# Characterization of Site-Specific N- and O-Glycopeptides from Recombinant Spike and ACE2 Glycoproteins Using LC-MS/MS Analysis

**DOI:** 10.3390/ijms252413649

**Published:** 2024-12-20

**Authors:** Ju Hwan Song, Sangeun Jang, Jin-Woong Choi, Seoyoung Hwang, Kyoung Heon Kim, Hye-Yeon Kim, Sun Cheol Park, Wonbin Lee, Ju Yeon Lee

**Affiliations:** 1 Digital Omics Research Center, Korea Basic Science Institute, Ochang 28119, Republic of Korea; sjhs5904@kbsi.re.kr (J.H.S.);; 2 Department of Biotechnology, Graduate School, Korea University, Seoul 02841, Republic of Korea; 3 Biopharmaceutical Research Center, Korea Basic Science Institute, Cheongju 28119, Republic of Korea; 4 Department of Bio-Analytical Science, University of Science and Technology, Daejeon 34113, Republic of Korea; 5 Critical Diseases Diagnostics Convergence Research Center, Korea Research Institute of Bioscience and Biotechnology, Daejeon 34141, Republic of Korea

**Keywords:** spike glycoprotein, reaction-binding domain, recombinant proteins, site-specific glycopeptides, modification of glycan, LC-MS/MS analysis

## Abstract

The COVID-19 pandemic, caused by severe acute respiratory syndrome coronavirus 2 (SARS-CoV-2), has resulted in hundreds of millions of infections and millions of deaths globally. Although vaccination campaigns are mitigating the pandemic, emerging viral variants continue to pose challenges. The spike (S) protein of SARS-CoV-2 plays a critical role in viral entry by binding to the angiotensin-converting enzyme 2 (ACE2) receptor, making both proteins essential targets for therapeutic and vaccine development. The glycosylation of these proteins influences their structure and function. This underscores the need for detailed site-specific glycoproteomic analysis. In this study, we characterized the N- or O-glycosylation profiles of the recombinant receptor-binding domain (RBD) of spike protein and ACE2 proteins expressed from Expi293F cells, as well as the S2 subunit of spike protein expressed in plant (*N. benthamiana*) cells. Using a high-resolution Orbitrap Eclipse Tribrid mass spectrometer equipped with the Ultimate 3000 RSLCnano and I-GPA (Integrated GlycoProteome Analyzer) developed in a previous study, 148 N- and 28 O-glycopeptides from RBD, 71 N-glycopeptides from the S2 subunit, and 139 N-glycopeptides from ACE2 were characterized. In addition, we report post-translational modifications (PTMs) of glycan, including mannose-6-phosphate (M6P) and GlcNAc-1-phosphate-6-O-mannose in N-glycan of RBD and ACE2, and O-acetylation in O-glycan of RBD, identified for the first time in these recombinant proteins. The relative abundance distribution according to glycosites and glycan types were analyzed by quantified site-specific N- and O (only from RBD)-glycopeptides from RBD, S2, and ACE2 using I-GPA. Asn331 for RBD, Asn1098 for S2, and Asn103 for ACE2 were majorly N-glycosylated, and dominant glycan-type was complex from RBD and ACE2 and high-mannose from S2. These findings will provide valuable insights into the glycosylation patterns that influence protein function and immunogenicity and offer new perspectives for the development of vaccines and antibody-based therapies against COVID-19.

## 1. Introduction

The COVID-19 pandemic, caused by severe acute respiratory syndrome coronavirus 2 (SARS-CoV-2), has led to a global health crisis with significant social and economic consequences. Since the initial outbreak, over 777 million infections and 7.1 million deaths have been reported according to the World Health Organization (WHO) as of November 2024. While vaccines are being deployed worldwide, the emergence of new variants with altered infectivity and immune evasion capabilities underscores the need for continued research into viral mechanisms and therapeutic strategies.

In the SARS-CoV-2 spike (S) protein and its interaction with the angiotensin-converting enzyme 2 (ACE2) receptor, which facilitates viral entry into host cells, one of the most important factors was glycosylation, which has been studied by many researchers [1,2,3]. Glycosylation, a form of post-translational modification (PTM), plays a key role in protein folding [4], stability [5], immune recognition, and host–pathogen interactions [6,7]. The spike protein of SARS-CoV-2 is glycosylated, and these glycan modifications are essential for modulating viral infectivity and immune evasion [8]. The ACE2 receptor, which serves as the viral entry point, is also glycosylated, and these modifications may impact its interaction with the virus [9,10]. The glycosylation of both RBD and ACE2 is very important for the binding of S protein and ACE2. In particular, the deletion of the glycosylation N331 and N343 of RBD drastically reduces the infectivity of SARS-CoV-2 [11,12]. Also, glycosylation at residues N53, N90, and N322 of ACE2 play a critical role in binding. For example, the deletion of N53 and N90 glycosylation led to increased susceptibility to infection [13]. The N53 and N90 glycosylation partly covers the binding interface of the RBD [14]. By contrast, the glycan at the N322 site strengthens RBD binding [13,14,15]. Understanding the site-specific glycosylation of the spike and ACE2 proteins can, therefore, offer critical insights into the design of vaccines, monoclonal antibodies, and therapeutic interventions.

Glycosylation sites are generally encoded by the gene sequence of the protein and, especially, N-glycosylation sites are conserved such as Asn-X -Ser/Thr (X for any amino acid except proline) without mutation. In contrast, O-glycosylation is the process of attaching a monosaccharide or mucin-type glycans to serine or threonine residues. Wang et al. [16] investigated 80 natural variants and 26 glycosylation spike mutants of SARS-CoV-2, including several mutations that critically affect the reactivity to neutralizing antibodies and virus infectivity. Although most N-glycosylation sites were conserved in SARS-CoV-2 variants, two novel potential glycosylation sites (N20 and N188) were formed in Gamma S, and N17 was eliminated in Delta S [16].

Many studies have used HEK 293 cells to produce SARS-CoV-2 spike protein [17,18,19], but other production systems have also been used to produce SARS-CoV-2 spike protein. For example, mammalian Chinese Hamster Ovary (CHO) cells [20], insect cells [21], the plant *Nicotiana benthamiana* (*N. benthamiana*) [15,22,23], and so on were used. However, different production systems from different cell types can change the glycosylation profiles of proteins, which may alter properties like stability and binding affinity. Specifically, the recombinant S protein expressed in CHO [20] increased in terminal sialyation as well as the presence of the N-glycosylneuraminic acid (NeuGc), which affects the potential immunogenicity in human body. In the case of the recombinant S protein expressed in the insect cells, the advantages include a low-cost and scalable production platform [24,25], but they present smaller and less processed glycans to be compared to mammalian cells [26]. Therefore, for the development of drugs produced in CHO, insect, and plant cells and so on, it should be accompanied by the additionally sophisticated quality control test. This plant production system can produce large amounts of recombinant proteins rapidly; *N. benthamiana* has been used as a production system of the recombinant proteins of the SARS-CoV-2 in several studies [15,22,23]. Song’s group [22] found that the plant-derived near-full-length S protein is highly immunogenic in mice and stimulates the production of antibodies that offer protection against SARS-CoV-2.

In this study, we performed a comprehensive site-specific glycosylation analysis of recombinant spike and ACE2 proteins using high-resolution mass spectrometry. Specifically, the receptor-binding domain (RBD) and ACE2 proteins were expressed in Expi293F cells, while the S2 subunit of the spike protein was produced in the plant *N. benthamiana* system because the S2 protein was poorly expressed in the mammalian cell expression system. In the previous study, we developed I-GPA (Integrated GlycoProteome Analyzer) [27]. The Orbitrap Eclipse Tribrid mass spectrometer and the Ultimate 3000 RSLCnano coupled with I-GPA were employed to identify N- and O-glycopeptides and to characterize their glycan compositions. This approach allowed us to uncover previously unreported PTMs, including mannose-6-phosphate (M6P) and GlcNAc-1-phosphate-6-O-mannose from N-glycans in RBD and ACE2 and O-acetylation from O-glycans in RBD protein. Also, the quantitative analysis of site-specific N- and O-glycopeptides from each RBD, S2, and ACE2 protein was processed according to glycosites and glycan types.

The findings of this study provide a deeper understanding of how glycosylation impacts the interaction between the SARS-CoV-2 spike protein and the ACE2 receptor, which is not only relevant for elucidating viral mechanisms, but also holds significant implications for the development of next-generation vaccines and antibody therapies to combat COVID-19 and its emerging variants.

## 2. Results and Discussion

### 2.1. Identification of Recombinant RBD and S2 Regions of Spike and ACE2 Glycoproteins

The RBD (receptor-binding domain) expressed in Expi293F cells contains two N-glycosylation and two O-glycosylation sites, and the S2 region of the spike protein expressed in plant (*N. benthamiana*) cells has nine N-glycosylation sites. The recombinant ACE2 protein expressed in Expi293F cells consists of six N-glycosylation sites (Supplementary Figure S1 and Supplementary Table S1). The RBD of the spike protein, treated with both trypsin and Glu-C, was prepared to generate peptides containing one N-glycosylation site and an appropriate O-glycopeptide length. For the S2 and ACE2 proteins, samples treated with trypsin only and samples treated with both trypsin and Glu-C were prepared to characterize site-specific glycopeptides. The glycopeptides from each digested sample were enriched using the ZIC-HILIC kit and analyzed by LC-MS/MS, like in the previous study [2,27]. Site-specific glycopeptides from enriched samples were automatically identified and quantified using I-GPA software version 3.55 [27].

**Figure 1:**
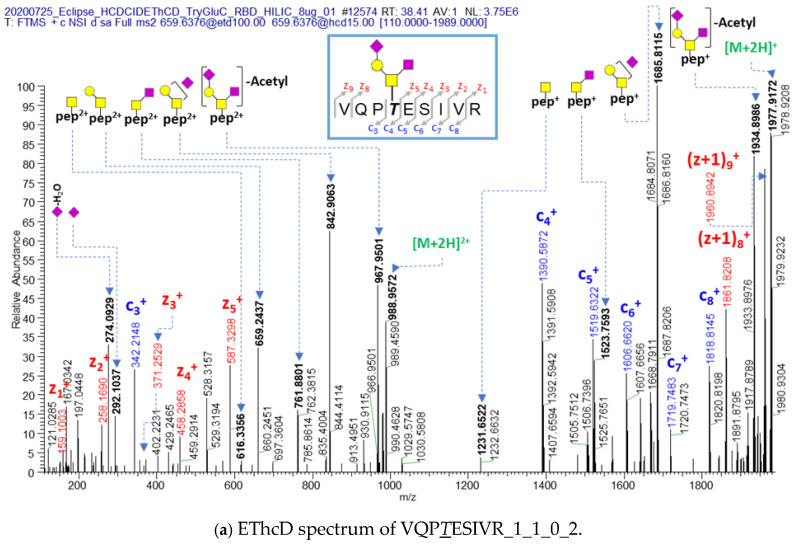

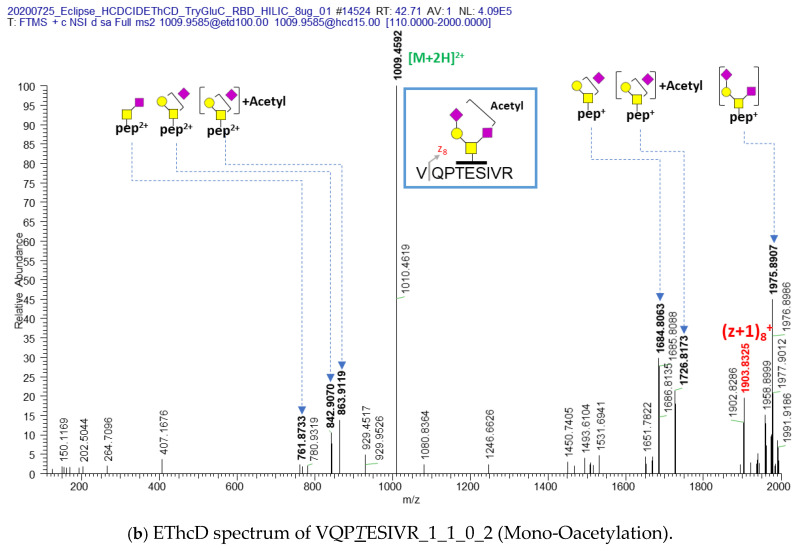
EThcD spectra of (**a**) non-acetylated and (**b**) acetylated VQP*T*ESIVR_1_1_0_2 O-glycopeptides from RBD of recombinant spike protein; 1_1_0_2 is the number of Hexose (Hex), N-acetylgalactosamine (GalNAc), fucose (Fuc), and N-acetyl neuraminic acid (NeuAc), respectively (the number of Hex_HexNac_Fuc_NeuAc). The italic and underlined T (*T*) or S (*S*) letters indicate the O-glycosylation site. The yellow circle indicates the galactose, the yellow square indicates the GalNAc, and the purple square indicates the NeuAc. The blue arrows indicate the match of theoretical *m*/*z* and actual *m*/*z*. The blue and the red colored letters indicate the c series and z (and z + 1) series ions of glycosylated peptide respectively. The green [M+2H] indicates the precursor ion.

The total number of identified O-glycopeptides/glycans from the RBD of spike glycoproteins was 28/23 (average 23.00 ± 0 glycopeptides) from VQP*T*ESIVR (24), IYQAGS*T*PCNGVE (1), and LLHAPA*T*VCGPK (3) (Table 1). The italic and underlined T (*T*) or S (*S*) indicates the O-glycosylation site. There are two possible O-glycosylation sites in the VQPTESIVR peptide of RBD, and 19 from 24 glycopeptides of VQPTESIVR were assigned to threonine as the O-glycosylation site (Figure 1a and Supplementary Figure S2). The oxonium ions, glycan-cleaved glycopeptide fragment ions (B/Y), and peptide fragment ions (c/z) are generally detected from HCD, CID, and EThcD, respectively [2,27]. In Figure 1a, the threonine glycosylation site is clearly characterized by c_3_ (342.2146 *m*/*z*, VQP fragment ion) and z_5_ (587.3298 *m*/*z*, ESIVR fragment ion) not containing glycans and c_4_ (1390.5782 *m*/*z*), (VQPT_1_1_0_2 = VQP (342.2146) + T (104.0477) + [1_1_0_2] (944.3159)), c_5_ (1519.6322 *m*/*z*), c_6_ (1606.6620 *m*/*z*), c_7_ (1719.7483 *m*/*z*), and c_8_ (1818.8145 *m*/*z*) ions containing glycans in the EThcD spectra. The threonine O-glycosite of many O-glycopeptides from VQP*T*ESIVR was manually assigned in EThcD spectra (Supplementary Figure S2). O-acetylation is a commonly known modification of sialic acid in biological and disease processes [28,29], and is related to the protein’s binding activity to the receptors [6,30]. The O-acetylation modification of VQP*T*ESIVR_1_1_0_2 was identified in this recombinant RBD protein and is clearly assigned in Figure 1. The glycan-cleaved O-acetylated glycopeptide fragment ions (863.9119 *m*/*z* (2+) and 1726.8173 *m*/*z* (1+) of VQP*T*ESIVR_1_1_0_1 (mono-acetylation)) were detected by only VQP*T*ESIVR_1_1_0_2 (mono-acetylation) O-glycopeptide (Figure 1b), but not by VQP*T*ESIVR_1_1_0_2 (Figure 1a). The spectra of HCD and CID for VQP*T*ESIVR_1_1_0_2 (mono-acetylation) O-glycopeptide are assigned in Supplementary Figure S2.

Potential O-glycopeptides containing multiple O-glycosylation sites were manually assigned based on the presence of more than two HexNAc glycans in VQPTESIVR. They were finally identified as single O-glycopeptides. The spectra of VQP*T*ESIVR_1_3_0_1, VQP*T*ESIVR_2_2_2_1, and VQP*T*ESIVR_3_3_0_1 O-glycopeptides were randomly selected and manually assigned for the verification of single-site glycosylated peptides (Supplementary Figure S2). The fragment ions (c_3_ or z_5_) that do not contain O-glycans, along with the glycan-containing fragment ions (c_4_ and z_6_), clearly identify the threonine O-glycosite of VQP*T*ESIVR glycopeptides. The information of 28 O-glycopeptides identified from RBD protein is listed with the information on glycan modification and the glycosylation site in Supplementary Table S2.

Yang’s group [31] reported the discovery of 30 uncertain O-glycosites and 11 clear O-glycosites from spike glycoproteins expressed in human cells using HCD and assigned by EThcD, respectively. Notably, they identified the IYQAGS*T*PCNGVE_1_1_0_1 glycopeptide with a Ser477 O-glycosite in their recombinant RBD protein. In contrast, in our recombinant RBD protein, Thr478 O-glycosite of IYQAGS*T*PCNGVE_1_1_0_2 glycopeptide, was distinctly identified from the EThcD spectrum with c_6_ (637.333 *m*/*z* from IYQAGS) and c_8_ (1782.758 *m*/*z* from IYQAGS*T*_1_1_0_2) fragment ions (Supplementary Figure S2). This Thr478 O-glycosylation has been reported in other research [7], but the O-glycosylation of LLHAPA*T*VCGPK (Thr523) is newly reported in this study. The spectra of HCD, CID, and EThcD for O-glycopeptides from IYQAGS*T*PCNGVE (1) and LLHAPA*T*VCGPK (3) are reported in Supplementary Figure S2.

The total number of identified N-glycopeptides/glycans from the RBD of spike glycoproteins was 148/114, with an average of 113.50 ± 13.44 glycopeptides (from FP***N***ITNLCPFGE (87) and VF***N***ATR (61)) across two replicate LC-MS/MS analyses (Table 1). Of the 87 FP*N*ITNLCPFGE and 61 VF*N*ATR N-glycopeptides, 92% and 77% were identified as complex type, while high-mannose and hybrid types represented 3% and 5% for FP*N*ITNLCPFGE and 11.48% for VF*N*ATR. The examples of glycan structure by glycan type are shown in Supplementary Figure S3.

**Figure 2:**
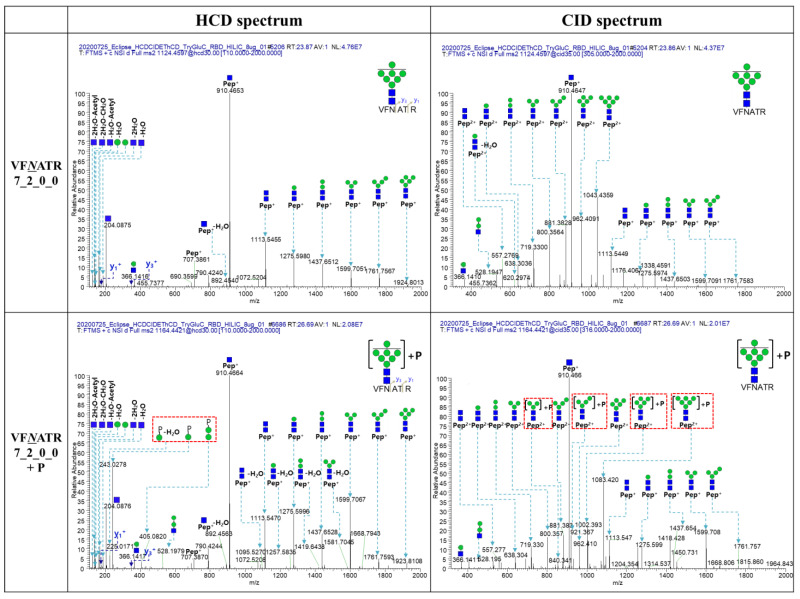
HCD and CID spectra of VF*N*ATR_7_2_0_0 and VF*N*ATR_7_2_0_0 (+Mannose-6-Phosphate). The italic and underlined N (*N*) letter indicates the N-glycosylation site of peptide. 7_2_0_2 is the number of Hexose (Hex), N-acetylglucosamine (GlcNAc), fucose (Fuc), and N-acetyl neuraminic acid (NeuAc), respectively (the number of Hex_HexNac_Fuc_NeuAc). The green circle and the blue square indicate the mannose and the GlcNAc respectively. The blue arrows indicate the match of theoretical *m*/*z* and actual *m*/*z*. The blue letters indicate the y series ions. The +P means that the glycopeptide contains the mannose-6-phosphate. The red dotted boxes are the fragment ions with M6P.

Several N-glycopeptides containing mannose-6-phosphate (M6P) and GlcNAc-1-phosphate, generated by the M6P biosynthetic pathway, were identified from this recombinant RBD. High-mannose N-glycopeptides with mannose-6-phosphate (M6P, 243.062) modification such as FP*N*ITNLCPFGE_5_2_0_0 (+M6P), FP*N*ITNLCPFGE_6_2_0_0 (+M6P), VF*N*ATR_6_2_0_0 (+M6P), and VF*N*ATR_7_2_0_0 (+M6P) were detected. The HCD and CID spectra of VF*N*ATR_7_2_0_0 and VF*N*ATR_7_2_0_0 (+M6P) were clearly characterized by the mannose-6-phosphated modification of VF*N*ATR_7_2_0_0 (+M6P) (Figure 2). Oxonium ions related to M6P such as *m*/*z* 243.028 (Hexose + M6P), 225.017 (Hexose + M6P-H_2_O), and 405.082 (2Hexose + M6P) were detected in the HCD of VFNATR_7_2_0_0 (+M6P) and the glycan-cleaved glycopeptide fragment ions such as *m*/*z* 840.341 (Hex3HexNAc2 + M6P, 2+), 921.367 (Hex4HexNAc2 + M6P, 2+), 1002.393 (Hex5HexNAc2 + M6P, 2+), and 1083.420 (Hex6HexNAc2 + M6P, 2+), which were detected in the CID spectrum of VF*N*ATR_7_2_0_0 (+M6P), but the HCD and CID spectra of VF*N*ATR_7_2_0_0 were not detected.

**Figure 3:**
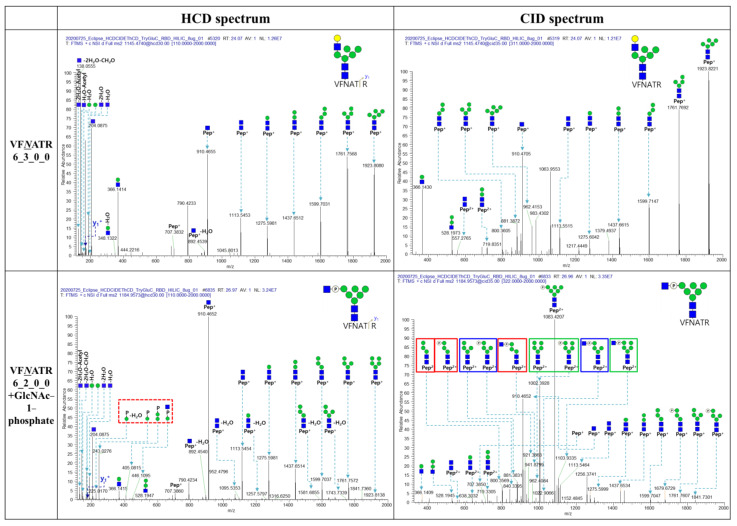
HCD and CID spectra of VF*N*ATR_6_3_0_0 and VF*N*ATR_6_2_0_0 (+GlcNAc-1-Phosphate). The italic and underlined N (*N*) letter indicates the N-glycosylation site of peptide. 6_2_0_0 is the number of Hexose (Hex), N-acetylglucosamine (GlcNAc), fucose (Fuc), and N-acetyl neuraminic acid (NeuAc), respectively (the number of Hex_HexNac_Fuc_NeuAc). The green circle, the blue square and the yellow circle indicates the mannose, the GlcNAc, and the galactose respectively. The blue arrows indicate the match of theoretical *m*/*z* and actual *m*/*z*. The blue letters indicate the y series ions. The P and circled P indicates the phosphate. The red dotted boxes are related to the fragment ions with Phosphate from GlcNAc-1-Phosphate or GlcNAc-1-Phosphate. The red, green and blue boxes are the pares of the fragment ions not containing P, containing P and GlcNAc-1-Phosphate.

GlcNAc-1-phosphate modified N-glycopeptides, including FP*N*ITNLCPFGE_5_2_0_0 (+GlcNAc-1-phosphate), FP*N*ITNLCPFGE_6_2_0_0 (+GlcNAc-1-phosphate), and VF*N*ATR_6_2_0_0 (+GlcNAc-1-phosphate), were identified in RBD. The HCD and CID spectra of VF*N*ATR_6_3_0_0 and VF*N*ATR_6_2_0_0 (+GlcNAc-1-phosphate) were compared to characterize this modification (Figure 3). The oxonium ions from GlcNAc-1-phosphate, *m*/*z* 243.028 (Hexose + M6P), 225.017 (Hexose + M6P-H_2_O), 405.082 (2Hexose + M6P), and 446.110 (Hexose + P + GlcNAc) were detected in the HCD of VF*N*ATR_6_2_0_0 (+GlcNAc-1-Phosphate), and the glycan-cleaved glycopeptide fragment ions, such as *m*/*z* 840.340 (Hex3HexNAc2 + M6P, 2+), 921.366 (Hex4HexNAc2 + M6P, 2+), 1002.393 (Hex5HexNAc2 + M6P, 2+), 1083.421 (Hex6HexNAc2 + M6P, 2+), 941.880 (Hex3HexNAc2 + GlcNAc-1-phosphate, 2+), 1022.907 (Hex4HexNAc2 + GlcNAc-1-phosphate, 2+), and 1103.934 (Hex5HexNAc2 + GlcNAc-1-phosphate, 2+), were detected in the CID spectrum of VF*N*ATR_6_2_0_0 (+GlcNAc-1-Phosphate), but the HCD and CID spectra of VF*N*ATR_6_3_0_0 were not detected. All other HCD, CID, and EThcD spectra of N-glycopeptides from RBD are represented in Supplementary Figures S4 and S5.

The lysosome, essential for recycling and digesting cellular materials, requires mannose-6-phosphate (M6P) residues as a signal for proper trafficking [32,33]. In this study, M6P biosynthetic pathways [34,35] were extensively studied because of their therapeutic relevance, such as in increasing M6P glycan content for the efficacy of treatments such as recombinant acid α-glucosidase for Pompe disease [32].

For the recombinant S2 region of the spike glycoprotein, 71/9 N-glycopeptides/glycans were identified from seven of nine N-glycosylation sites across two LC-MS/MS analyses (Table 1). The italic and underlined N (*N*) indicates the N-glycosylation site. The average number of glycopeptides was 28.00 ± 0.00 from trypsin-treated samples (S2-T) and 40.00 ± 1.41 from trypsin- and Glu-C-treated samples (S2-TG). Additionally, a total of 32 N-glycopeptides (NFS (Asn801): DFGGF*N*FSQILPDPSKPSK (5), DFGGF*N*FSQILPDPSKPSKR (3), and TPPIKDFGGF*N*FSQILPDPSKPSK (4); NGT (Asn1098): AHFPREGVFVS*N*GTHWFVTQR (1) and EGVFVS*N*GTHWFVTQR (8); NHT (Asn1158): *N*HTSPDVDLGDISGINASVVNIQK (6); NES (Asn1194): NL*N*ESLIDLQELGK (5)) and 46 N-glycopeptides (NFS (Ans801): DFGGF*N*FSQILPD (1) and FGGF*N*FSQILPDPSKPSK (7); NFT (Asn1074): *N*FTTAPAICHDGK (4) and *N*FTTAPAICHD (2); NGT (Asn423): GVFVS*N*GTHWFVTQR (9) and EGVFVS*N*GTHWFVTQR (8); NNT (Asn1134): NTFVSGNCDVVIGIV*N*NTVYD (3); NHT (Asn1173): *N*HTSPD (1) and *N*HTSPDVD (3); NAS (Asn1194): ISGI*N*ASVVNIQK (3) and LGDISGI*N*ASVVNIQK (5))) were identified from four N-glycosylation sites with an S2-T sample and from six N-glycosylation sites with an S2-TG sample, respectively. N-glycopeptides from *N*HTSPDVDLGDISGI*N*ASVVNIQK have two glycosylation sites such as Asn1158 and Ans1173, while the Asn1158 N-glycosylation site from this glycopeptide instead of the Asn1173 site was clearly characterized with fragment ions such as (z + 1)_9_ (975.536 *m*/*z* (1+), NASVVNIQK), (z + 1)_10_ (1070.622 *m*/*z* (1+), INASVVNIQK), (z + 1)_12_ (1214.676 *m*/*z* (1+), SGINASVVNIQK), (z + 1)_13_ (1442.790 *m*/*z* (1+), ISGINASVVNIQK), and (z + 1)_14_ (1442.790 *m*/*z* (1+), DISGINASVVNIQK) in the EThcD spectrum (Supplementary Figure S6). These five z fragment ions have Asn1173 amino acid but no glycans; therefore, this represents that the N-glycosite from *N*HTSPDVDLGDISGI*N*ASVVNIQK N-glycopeptides is Asn1158. All N-glycopeptide spectra of S2 are represented at Supplementary Figures S7–S10.

Another NSVAYS*N*NSIAIPTNFTISVTTEILPVSMTK N-glycopeptide containing two N-glycosylation sites was not detected in spite of both tryptic and Glu-C treatment in this study. The glycan types of all identified N-glycopeptides from the S2-T and S2-TG samples were only the high-mannose type such as 3_2_0_0, 4_2_0_0, 5_2_0_0, 6_2_0_0, 7_2_0_0, 8_2_0_0, 9_2_0_0, 10_2_0_0, or 11_2_0_0. The occupied glycoforms on the glycosylation sites from recombinant glycoproteins are different from the glycoforms of host cells [3]. The glycosite-specific occupancy among S proteins subunits expressed in human cells and insect cells was compared, and the glycoforms were characterized by different glycoforms based on host cells [3]. The major glycan type SARS-CoV-2 2 proteins expressed in human cells and in insect cells were complex glycoforms and high-mannose glycoforms, respectively.

For the recombinant ACE2 protein, a total of 139/79 N-glycopeptides/glycans from four of six N-glycosylation sites were identified across two replicate LC-MS/MS analyses (Table 1) with an average of 35.00 ± 2.83 glycopeptides from the trypsin-treated sample (ACE2-T) and 81.00 ± 9.90 glycopeptides from the trypsin- and Glu-C-treated sample (ACE2-TG). A total of 42 N-glycopeptides (NGS (Asn103): LQLQALQQ*N*GSSVLSEDK (28) and LQLQALQQ*N*GSSVLSEDKSK (1); NET (Asn432): SIGLLSPDFQED*N*ETEINFLLK (5); NST (Asn546): CDIS*N*STEAGQK (8)) and 97 N-glycopeptides (NLT (Asn90: IQ*N*LTVK (73); NGS (Asn103): LQLQALQQ*N*GSSVLSE (6); NST (Asn546): CDIS*N*STE (18))) were identified from three N-glycosylation sites with the ACE2-T sample and from three N-glycosylation sites with the ACE2-TG sample, respectively. In total, 72.15%, 12.66%, and 15.19% of 139 N-glycopeptides identified in ACE2 were complex, high-mannose, and hybrid-type, respectively.

The mannose-6-phosphate (M6P, 243.062) modification on high-mannose LQLQALQQ*N*GSSVLSEDK_7_2_0_0 N-glycopeptide and the GlcNAc-1-phosphate modification on N-glycan, such as LQLQALQQ*N*GSSVLSEDK_6_2_0_0 (+GlcNAc-1-phosphate), were identified from the recombinant ACE2 proteins like the RBD of spike glycoprotein. All N-glycopeptide spectra of ACE2 proteins are represented in Supplementary Figures S11–S14.

### 2.2. Quantification of Recombinant RBD and S2 Regions of Spike and ACE2 Glycoproteins

For the RBD domain of spike glycoprotein, a total of 28 O- and 148 N-glycopeptides from RBD, 71 N-glycopeptides from the S2 region and 139 N-glycopeptides from ACE2 proteins, were identified across two LC-MS/MS replicates using I-GPA, followed by a q-GPA (quantitated-GPA) [24] for label-free quantitative analysis. The abundances were calculated based on the sum of intensities of the top three isotope peaks at the three highest MS spectral points. If an N-glycopeptide was not identified in a given run, the corresponding N-glycopeptides within the mass tolerance (10 ppm) and the window of retention time (5 min) were extracted. The quantitative abundance of N- or O-glycopeptides identified at least once in two replicates and was extracted from the two replicates of data of each sample using q-GPA. The area intensities from the duplicates were averaged (glycopeptides with only one area intensity from two replicates were removed) and then quantitatively analyzed for site-specific peptides or glycan types. The medians of CV% measured from duplicate analyses were less than 25%, except the ACE2-T sample (median of CV%: 8.23%, 22.68%, 9.28%, 15.23%, 38.20%, and 24.36% from 16 O-glycopeptides of RBD, 119 N-glycopeptides of RBD, 28 N-glycopeptides of S2-T, 35 N-glycopeptides of S2-TG, 29 N-glycopeptides of ACE2-T, and 69 N-glycopeptiodes of ACE2-TG, respectively). These final data (Supplementary Table S3) were quantitatively analyzed according to the peptides with glycosites and glycan types.

**Figure 4:**
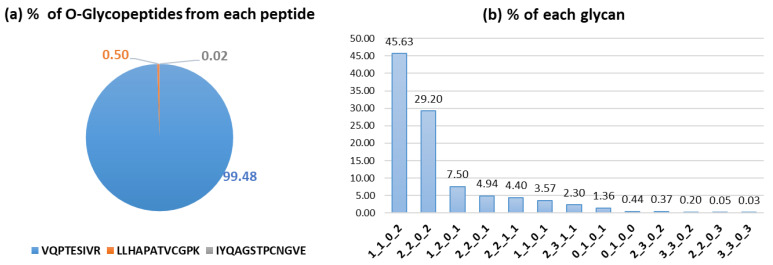
The quantitative percentage according to O-glycosites (**a**) and O-glycans (**b**) from RBD protein.

The quantitative distributions of O-glycopeptides in RBD were 99.48% from VQPTESIVR, 0.50% from LLHAPATVCGPK, and 0.02% from IYQAGSTPCNGVE (Figure 4a). The percentages from LLHAPATVCGPK and IYQAGSTPCNGVE were less than 1%, but O-glycopeptides from LLHAPATVCGPK were not previously identified in the recombinant RBD protein, and the O-glycosite in IYQAGSTPCNGVE was threonine (IYQAGS*T*PCNGVE) instead of serine (IYQAG*S*TPCNGVE) [31] in the RBD proteins. The quantitative analysis according to the O-glycan type is shown in Figure 4b. In total, 12 of the 13 O-glycopeptides were sialylated, representing about 99% of all quantified O-glycopeptides. This is similar to the finding by the O-glycopeptides from Yang’s group [31], where SARS-CoV-2 glycoproteins expressed in human cells were also predominantly identified as sialylated glycopeptides. In the RBD from our study, O-glycopeptides containing two sialic acids (1_1_0_2) were most abundant, while in Yang’s recombinant protein, O-glycopeptides containing 1_1_0_1 and 1_1_0_2 are most frequently identified.

**Figure 5:**
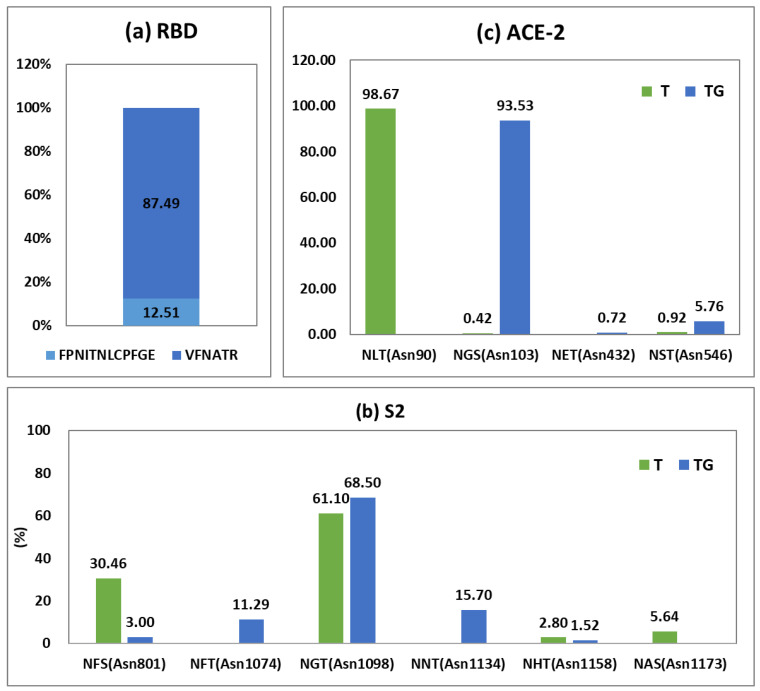
The quantitative distribution according to N-glycosites from (**a**) RBD, (**b**) S2, and (**c**) ACE-2 proteins. RBD sample was treated with trypsin and Glu-C. T: trypsin-treated S2 or ACE-2 samples. TG: trypsin- and Glu-C-treated S2 or ACE-2 samples.

The distribution of N-glycopeptides according to N-glycosylation sites from RBD, S2, and ACE2 proteins was relatively analyzed (Figure 5), while 76 and 43 N-glycopeptides were identified from FPNITNLCPFGE and VFNATR, respectively. FPNITNLCPFGE was dominant in quantitative distribution, with 88% and 18% quantification for FPNITNLCPFGE and VFNATR, respectively. In the S2 protein, the most relatively abundant N-glycosylation site was Asn1098 (NGT) in both S2-T and S2-TG samples, followed by Asn801 (NFS), Asn1134 (NNT), Asn1074 (NFT) or Asn1173 (NAS), and Asn1158 (NHT). N-glycopeptides identified from NHTSPDVDLGDISGINASVVNIQK contain two N-glycosylation sites (Ans1158 and Asn1173), with these glycosylation sites being clearly characterized by five z fragment ions ((z + 1)_9_, (z + 1)_10_, (z + 1)_12_, (z + 1)_13_, and (z + 1)_14_ ions) (Supplementary Figure S6). In ACE2-T and ACE2-TG samples, N-glycopeptides from Asn90 and Asn103 glycosites were quantitatively preponderant with 98.67% and 93.53%, respectively. Other N-glycopeptides from ACE2 recombinant proteins were less than 6%.

**Figure 6:**
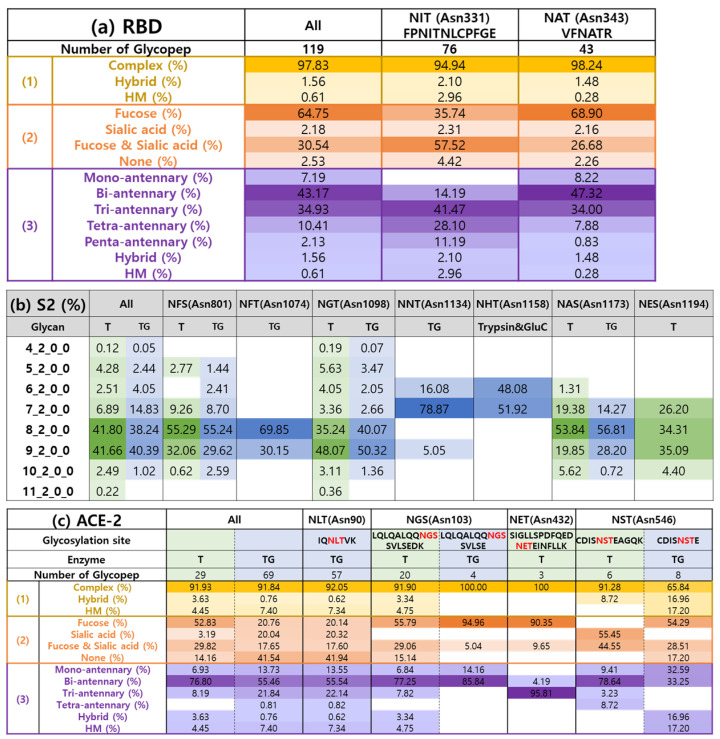
The quantitative distribution according to (**a**) glycan type (complex, hybrid, and high-mannose (HM)), (**b**) fucose (containing fucose but not sialic acid), sialic acid (containing sialic acid but not fucose), fucose and sialic acid (containing fucose and sialic acid), and none (glycopeptides without fucose or sialic acid), (**c**) the number of branches in N-glycans for all N-glycopeptides from RBD, S2, or ACE-2 protein. The RBD sample was treated with trypsin and Glu-C. T: trypsin-treated S2 or ACE-2 samples. TG: trypsin- and Glu-C-treated S2 or ACE-2 samples. A darker color indicates a higher percentage.

The quantitative distribution of N-glycopeptides according to the glycan type was examined in RBD, S2, and ACE2 proteins (Figure 6). Only high-mannose N-glycopeptides were identified from the recombinant S2 protein because it was expressed in plant (*N. benthamiana*) cells rather than human cells. The 8_2_0_0 high-mannose glycan type was the most abundant on the Asn801, Asn1074, and Asn1173 glycosylation sites of S2, while 9_2_0_0 and 7_2_0_0 glycan were mostly attached to the Asn1098 and Asn1134 N-glycosylation sites, respectively. The Asn1158 N-glycosylation site was characterized by two predominant high-mannose glycans, 6_2_0_0 and 7_2_0_0, and the Asn1194 site contained three major glycans: 7_2_0_0, 8_2_0_0, and 9_2_0_0. Recombinant RBD and ACE2 proteins expressed in human cells were quantitatively dominated by complex glycans, as for over 90% all N-glycosylation sites of RBD and ACE2, except Asn546 of ACE2 (65.84%) (Figure 6). In RBD, over 95% of FPNITNLCPFGE was glycosylated with fucose (35.74%), fucose and sialic acid (57.52%), or sialic acid (2.31%). The VFNATR peptide was mostly fucosylated (68.90%), with 26.68% containing both fucose and sialic acid, and 2.26% having sialic acid only. FPNITNLCPFGE glycopeptides were more branched (tri-antennary: 41.47%) than the VFNATR (bi-antennary: 47.32%). Recombinant ACE2 glycoproteins were dominantly fucosylated at the Asn103, Asn432, and Asn546. However, approximately 40% of the Asn90 N-sites were not modified fucose or sialic acid. Most of ACE2 N-glycosites were glycosylated by bi-antennary structures, but over 95% of Asn432 sites were glycosylated by tri-antennary structures.

The glycosylation of SARS-CoV-2 was broadly analyzed in many research papers [3]. The site-specific quantification of N-glycopeptides from S protein was analyzed within the top five N-glycans in Yang’s group, but the distributions according to the glycan types were analyzed using the number of identified N-glycopeptides, and the quantification of O-glycopeptides was not analyzed in Yang’s group. And, Watanabe and co-workers [8] discovered that, in Asn331 and Asn343 of S protein, the quantitatively dominant glycan type is complex glycan, and over 90% of N-glycopeptides contained fucose, as in our results. The several N-glycosites (Asn603, Asn709, Asn717, Asn801, and Asn1074 of S2 subunit) are dominantly occupied with the high-mannose glycan type, although this recombinant S protein was expressed in human HEK293 cells [8]. But, the identification and quantification of O-glycopeptides from S protein were not characterized in this reference. The major glycans at all N-glycosylation sites of ACE2 protein are complex-type, and the sialic acid linkages exist in the glycans [36,37]. The major antennary of complex glycan type is bi-antennary for both RBD and ACE2 proteins.

In this study, RBD and ACE2 recombinant proteins were produced in Expi293f cell, which is derived from human embryonic kidney 293 (HEK 293) cell. ACE2 has 7 N-glycosylation sites and SARS-CoV-2 S protein has 22 possible N-glycosylation sites. In 22 N-glycosylation sites, 2 are from RBD and 9 are from S2 subunit. We identified 4 N-glycosites of ACE2, 2 N-glycosites of RBD, and 7 N-glycosites of S2 subunit. Glycosylation at residues N53, N90, N322, and N546 of ACE2 and residues N331 and N343 of RBD are important in the binding of ACE2 and SARS-CoV-2 protein [13,14], as explained in the Introduction. Residues N90 and N546 of ACE2 were identified, and residues N331 and N343 were identified in this study. N165 of NTD is situated close to the receptor-binding site of the SARS-CoV-2 spike protein, and the key role of its glycosylation is reported as facilitating receptor binding and shielding epitopes [38,39,40]. Some researchers have demonstrated that mutations resulting in the deletion of glycosylation at N122 in the NTD, as well as N717 and N1158 in the S2 region, led to a significant reduction in viral infectivity [11,41,42]. Because these glycosylations play a critical role in viral infection, these sites can be possible drug targets for therapy [43,44]. The LC-MS/MS analysis approach provided us with the information of site-specific glycopeptides, but could not suggest the most important glycosylation sites of each protein. This proteomics technology could complement research [1,15,18,45] into the binding of S protein to ACE2 and the development of the target therapy.

Huang’s group [46] had reported the identification of mannose-6-phosphate (M6P) glycosylation on the N-terminal domain (NTD) of S protein using two-step enrichment processing involving Ti-IMAC and HILIC. Zhu’s group provided the comprehensive site-specific N-glycosylation of S protein using the structural and site-specific N-glycoproteomics sequencing algorithm, StrucGP [17]. Alongside the common N-glycans identified, they mapped several uncommon glycosylation structures, including LacdiNAc structures, Lewis structures, Mannose 6-phosphate (M6P) residues, and bisected core structures, across 20 glycosylation sites in the S protein trimer and protomer.

Several groups [16,18] studied variant S proteins (Alpha, Beta, Gamma, Delta, or Omicron [18]) and compared the N-glycosylation sites and the pattern of glycan types among them. The substitutions and deletions of the amino acid between variants affects the type and the abundance of glycans on the glycosylation sites of the spike protein.

In Antonopoules’s study [19], N- and O-glycan site-specific glycosylation from RBD were characterized using an integrated glycomic and glycoproteomic analytical strategy. They demonstrated the complex type of N-glycans with unusual fucosylated LacdiNAc at two N-glycosites (N331 and N431) and O-glycosite (T323). They identified many O-glycopeptides from T323, but there is no clear evidence of S325 sites.

In our study, RBD was prepared with trypsin and Glu-C treatment, and S2 and ACE2 were prepared with trypsin only (T) and trypsin combined with Glu-C (TG), and they were analyzed by LC-MS/MS with HCD, CID, and EThcD for MS/MS fragmentation for all precursors after one-step HILIC enrichment. The M6P modification of N-glycan was characterized in the RBD domain, where the involvement of the binding to ACE2 is known, as for NTD. Additionally, we present clear evidence of M6P modification on ACE2 with GlcNAc-1-phosphate-6-O-mannose in the N-glycan of RBD and ACE2. In the case of O-glycopeptide, the O-acetylation modification of RBD O-glycopeptide was clearly characterized from HCD, CID, and EThcD spectra. But, we did not characterize the other modification, such as LacdiNAc. We also covered the deep identification of O-glycopeptides, and 23 glycans from 28 O-glycopeptides were characterized in the T323 glycosite with site-specific information ions. Theses study will contribute to deeper understanding of the glycosylation function of both the S proteins and ACE2 in the context of viral infection.

## 3. Materials and Methods

### 3.1. Materials

1,4-Dithiothreitol (DTT), iodoacetamide (IAA), formic acid (FA), and ammonium bicarbonate (ABC) were purchased from Sigma-Aldrich (St. Louis, MO, USA). Trypsin and GluC were obtained from Promega (Madison, WI, USA). HPLC-grade acetonitrile (ACN) and water were obtained from Merck (Darmstadt, Germany). The AmiconUltra centrifugal filter (10 K) was from Millipore (Billerica, MA, USA).

### 3.2. Production of Recombinant Proteins

SARS-CoV2 spike receptor-binding domain (RBD, residues 318-541) and human ACE2 (residues 19-615) recombinant proteins were produced by using the Expi293F expression system (Thermo Fisher Scientific, Waltham, MA, USA). Expi293F cells are derived from human embryonic kidney (HEK) 293 cell line. The expression plasmids were transfected into Expi293F cells and, after 5 days, the supernatant containing secreted proteins was harvested and clarified by centrifugation and filtration. The supernatant was injected into the HisTrap HP column (Cytiva, Marlborough, MA, USA) with a solution containing 20 mM Tris pH 8.0, 300 mM NaCl. After the column wash step with a buffer of 20 mM Tris pH 8.0, 300 mM NaCl, and 50 mM imidazole, the RBD protein was eluted by using an elution buffer containing 20 mM Tris pH 8.0, 300 mM NaCl, and 250 mM imidazole. Proteins were further purified by size exclusion chromatography using a HiLoad Superdex 200 16/600 column (Cytiva, Marlborough, MA, USA) in 20 mM Tris pH 8.0 and 150 mM NaCl. SARS-CoV-2 spike S2 (residues 686-1213) recombinant protein expressed in *N. benthamiana* was purified as described previously [47] and obtained from BioApplications Inc. (Pohang, Republic of Korea).

### 3.3. Digestion of Samples

Recombinant RBD and ACE2 and S2 subunit glycoproteins were expressed from Expi293F and plant cells, respectively. Three recombinant glycoproteins were prepared in 50 mM ABC buffer for digestion after desalting by centrifugal filtration using a 10,000-Da membrane cutoff filter (Millipore, Billerica, MA, USA, product code UFC501096). The protein solution was reduced by 10 mM DTT at 60 °C for 1 h and was alkylated by 25 mM IAA in the dark at room temperature during a 1 h reaction time. Alkylated RBD, S2, and ACE2 samples were digested with trypsin at 37 °C overnight (16 h). S2 and ACE2 were equally divided into two. RBD and the other S2 and ACE2 samples were additionally digested with Glu-C at 37 °C overnight (16 h). All digest samples were quenched in a 3% final concentration of FA. The digested samples were dried using a SpeedVac concentrator for glycopeptide enrichment. For LC-MS/MS analysis, the solution was diluted by mobile phase A (99.9% water with 0.1% FA) of liquid chromatography.

### 3.4. Glycopeptide Enrichment

Glycopeptides from dried digested samples were enriched using the ZIC-HILIC kit according to the manufacturer’s instructions, with minor modifications [2]. Rehydrated digested samples were diluted with 50 μL ZIC binding buffer. This solution was mixed well with a ZIC glycocapture resin, and 50 μL was transferred to a new microcentrifuge tube. Then, the tube was centrifuged for 1 to 2 min at 2000–2500× *g*, and the supernatant was completely removed and discarded. The diluted sample was added to the ZIC glycocapture resin, mixed by pipetting 3–5 times, and incubated at 1200 rpm for 10–20 min. Then, the tube was centrifuged, and the supernatant was completely removed. Next, 150 μL of ZIC wash buffer was added to the ZIC glycocapture resin; it was mixed, incubated, and centrifuged, and the supernatant was removed. These steps were repeated three times. Then, 75–100 μL ZIC elution buffer was added to elute the glycopeptides, and the tube was mixed, incubated, and centrifuged. The supernatant was transferred to a new microcentrifuge tube, centrifuged for 2 min at 10,000× *g*, and transferred to a new microcentrifuge tube (avoiding the transfer of any resin particles). The supernatant was dried in a SpeedVac and rehydrated in 0.1% FA for LC-MS/MS analysis.

### 3.5. Nano LC-ESI-MSMS

Rehydrated samples by mobile phase A were analyzed using an LC-MS/MS system consisting of an UltiMate 3000 RSLCnano System and a Thermo Scientific Orbitrap Eclipse Tribrid Mass Spectrometer (Thermo Fisher Scientific, Bremen, Germany) equipped with a nano electrospray source. An autosampler was used to load the sample solutions into a C_18_ trap column (Acclaim PepMap^TM^ 100, 75 μm × 2 cm from Thermo Fisher Scientific (Vilnius, Lithuania)). The samples were desalted and concentrated on the trap column for 8 min at a flow rate of 3 μL/min. The trapped samples were then separated on a C_18_ analytical column (PepMap^TM^ RSLC C18, 2 μm, 100 Å, 75 μm × 50 cm from Thermo Fisher Scientific (Vilnius, Lithuania)). The mobile phases were composed of 99.9% water (A) and 99.9% ACN (B), and each contained 0.1% FA. The LC gradient started with 5% of B for 8 min, and was ramped to 20% of B for 77 min, 30% of B for 4 min, and 95% of B for 1 min; it was held at 95% of B for 10 min and then at 5% of B for another 0.5 min. The column was re-equilibrated with 5% of B for 9.5 min before the next run. The voltage applied to produce an electrospray was 2000 V. During the chromatographic separation, the Orbitrap Fusion Lumos (Thermo Fisher Scientific, San Jose, CA, USA) was operated in data-dependent mode, automatically switching between MS1 and MS2 with a 3 s cycle time. The MS data were acquired using the following parameters: Full-scan MS1 spectra (400–2000 *m*/*z*) were acquired in the Orbitrap for a maximum ion injection time of 100 ms at a resolution of 120,000 and an automatic gain control (AGC) target value of 4.0 × 10^5^. MS2 spectra were acquired in the Orbitrap mass analyzer at a resolution of 30,000 and with higher energy collision dissociation (HCD, 30% normalized collision energy, maximum ion injection time of 54 ms, automatic gain control (AGC) target value of 5.0 × 10^4^), collision-induced dissociation (CID, 35% normalized collision energy, maximum ion injection time of 54 ms, automatic gain control (AGC) target value of 5.0 × 10^4^), and electron–transfer/higher-energy collision dissociation (EThcD, 15% SA collision energy, maximum ion injection time of 250 ms, automatic gain control (AGC) target value of 1.5 × 10^5^). Previously fragmented ions were excluded for 30 s within 10 ppm tolerance.

### 3.6. Identification of Glycopeptides

The raw files were converted to ms1/ms2 using Raw Converter (1.1.0.22, 2014, Scripps Research Institute, La Jolla, CA, USA). Site-specific N-glycopeptides of recombinant RBD, S2, and ACE2 proteins were automatically identified by I-GPA (Integrated GlycoProteome Analyzer) [27], which our group developed for N-glycopeptide analysis. Briefly, N-glycopeptide spectra were first selected by 15 glycan-specific oxonium ions using tandem MS/MS spectra. Second, N-glycopeptide candidates were picked by matching their experimental MS isotope pattern to the theoretical ones of N-glycopeptides in the GAP-DB constructed by combining possible tryptic peptides including N-glycosite of AGP and 352 N-glycans (retrosynthetic glycans from Kronewitter, S.R. et al. [48] and glycans of penta and hexa polylactosamine series from Ozohanics, O. et al. [49]). Finally, N-glycopeptides were identified by Y-score from matching the experimental and theoretical fragment ions of *N-*glycopeptides. For Y-score, Y ions (partially glycan moiety fragmented ions), B ions (multi-mono-saccharide fragments of non-reducing end of glycans), and b and y ions (from the amino acid sequences of peptides) are considered.

O-glycopeptides of RBD protein were identified using O-GPA via the following procedures [50]. The O-glycopeptide database (O-GPADB) was generated considering 64 glycans [51,52], including Oglycans classified as core 1 (nine O-glycans), core 2 (22 Oglycans), extended core 1 (53 O-glycans), and extended core 2 (33 O-glycans) with up to 4 HexNAc. Detailed information about the 64 O-glycans is provided in Supporting Information (Excel document from Park’s study [50]). All MS/MS spectra were searched using N- and O-GPA with modification parameters set as carbamidomethyl cysteine (fixed for N- and O-glycopeptide) and mannose-6-phosphate (M6P, variable for N-glycopeptide) and O-acetylation (variable for O-linked glycopeptide), and a precursor mass tolerance of ±0.02 Da. MS/MS tolerances were set ±0.02 for HCD and ±0.05 Da for CID and EThcD. M- and S-score thresholds were 1.0 and 98.0. The Y-score was filtered to less than 1.0% of the estimated false discovery rate (FDR).

The nomenclature of the N- and O-glycopeptide is composed of the peptide sequence and the number of Hexose (Hex), N-acetylglucosamine (GlcNAc), fucose (Fuc), and N-acetyl neuraminic acid (NeuAc), respectively (#Hex_#HexNac_#Fuc_#NeuAc). The nomenclature of glycan is based on the reference [48] of retrosynthetic glycans database. For example, FP*N*ITNLCPFGE _5_4_1_2 N-glycopeptide is composed of FP*N*ITNLCPFGE for peptide sequence, five for the number of Hexose, four for the number of N-acetylglycosamine, one for the number of fucose, and two for the number of N-acetyl neuraminic acid. Italic and underlined N (*N*) is the N-glycosylation site.

### 3.7. Quantification of Glycopeptide

For the quantitative analysis of N- and O-glycopeptides, the cGPA, which sums the intensity of three ms1 points having three isotopic patterns of each precursor, was performed by I-GPA software version 3.55 [27]. Therefore, the intensity of all N-and O-glycopeptides from RBD, S2, and ACE2 were extracted by cGPA from two replicates and averaged for quantitative analyses for site-specific glycopeptides, glycan types, and antennary types.

## 4. Conclusions

This study provides an in-depth glycoproteomic analysis of SARS-CoV-2 spike (S) protein receptor-binding domain (RBD), the S2 subunit, and the ACE2 receptor. In total, 148 N-glycopeptides and 28 O-glycopeptides from RBD, 71 N-glycopeptides from the S2 subunit, and 139 N-glycopeptides from ACE2 were site-specifically characterized by LC-MS/MS analyses coupled with I-GPA platforms. Novel post-translational modifications (PTMs), mannose-6-phosphate (M6P), and GlcNAc-1-phosphate from RBD and ACE2 N-glycopeptides, and O-acetylation from RBD O-glycopeptide, were clearly characterized from HCD, CID, and EThcD spectra, and reported for the first time in this study. These novel glycosylation profiles may affect the proteins’ function, immunogenicity, receptor binding, and immune response.

The relative quantification according to glycosites and glycan type from each recombinant sample was performed: N-glycopeptides from Asn331 for RBD, Asn1098 for S2, and Asn103 for ACE2 were dominant, and the major glycan type of the recombinant RBD and ACE2 proteins expressed in Expi293F cells was a complex type, while the major glycan type of S2 subunit expressed in plant cells was a high-mannose type. Site-specific O-glycopeptides from RBD were also unambiguously characterized by LC-SM/MS analysis.

The characterization of novel glycan modification and comprehensive quantification in this study will contribute to the understanding of SARS-CoV-2 glycoprotein glycosylation research and will help drive the design of effective vaccines and antibody-based therapies.

## Figures and Tables

**Table 1:** List of identified glycopeptides from recombinant spike (RBD, S2) and ACE2 proteins.

Protein	Glycosylation Site	Repeat 1	Repeat 2	Average	STDEV	CV%	The Number of Identified Glycopeptide/Glycan
RBD (receptor-binding domain) P0DTC2-RBD: 2/2 sites	O from trypsin- and GluC-treated sample	23	23	23.00	0.00	0.00	28/23
	N from trypsin- and GluC-treated sample	104	123	113.50	13.44	11.84	148/114
S2 (spike SARS2 domain) P0DTC2-S2: 7/9 sites	N from trypsin-treated sample	28	28	28.00	0.00	0.00	71/9
	N from trypsin- and GluC-treated sample	41	39	40.00	1.41	3.54	
ACE2 (angiotensin-convertingenzyme2) Q9BYF1: 4/6 site	N from trypsin-treated sample	33	37	35.00	2.83	8.08	139/79
	N from trypsin- and GluC-treated sample	74	88	81.00	9.90	12.22	

## Data Availability

The raw data and search results are available at MassIVE: MSV000096403.

## Supplementary Materials

The following supporting information can be downloaded at: https://www.mdpi.com/article/10.3390/ijms252413649/s1.

## Abbreviations

SARS-CoV-2	severe acute respiratory syndrome coronavirus 2
ACE2	angiotensin-converting enzyme 2
RBD	receptor-binding domain
PTM	post-translational modification
M6P	mannose-6-phosphate
WHO	World Health Organization
rGAA	recombinant acid α-glucosidase
I-GPA	Integrated-GPA, Integrated GlycoProteome Analyzer
O-GPADB	O-glycopeptide database
q-GPA	quantitated-GPA, quantitated GlycoProteome Analyzer
DTT	1:4-dithiothreitol
IAA	iodoacetamide
FA	formic acid
ABC	ammonium bicarbonate
ACN	acetonitrile
HEK 293	human embryonic kidney 293
CHO cell	Chinese Hamster Ovary cell
AGC	automatic gain control
HCD	higher energy collision dissociation
CID	collision induced dissociation
EThcD	electron transfer/higher-energy collision dissociation
FDR	false discovery rate
Hex	Hexose
GlcNAc	N-acetylglucosamine
Fuc	fucose
NeuAc	N-acetyl neuraminic acid
