# Supplementary material for: Characterization of Site-Specific N- and O-Glycopeptides from Recombinant Spike and ACE2 Glycoproteins Using LC-MS/MS Analysis
Source: Int J Mol Sci. 2024 Dec 20;25(24):13649. doi: 10.3390/ijms252413649 (PMC11678118; doi:10.3390/ijms252413649)
Supplement: Supplementary file 1 [file ijms-25-13649-s001.zip › 3. Supplementary informations_Final_20241213.pdf]

Characterization of Site-Specific O- and N-Glycopeptides from Recombinant Spike and ACE2 Glycoproteins Using LC-MS/MS Analysis

Ju Hwan Song [1,2,†], Sangeun Jang[1,†], Jin-Woong Choi[1], Seoyoung Hwang[1], Kyoung Heon Kim[2], Hye-Yeon Kim[3], Sun Cheol Park[3], Wonbin Lee[3] and Ju Yeon Lee[1,4,5,*]

[1]Digital Omics Research Center, Korea Basic Science Institute, Ochang 28119, Republic of Korea

[2]Department of Biotechnology, Graduate School, Korea University, Seoul 02841, Republic of Korea

[3]Biopharmaceutical Research Center, Korea Basic Science Institute, Cheongju, 28119, Republic of Korea

[4]Department of Bio-Analytical Science, University of Science and Technology, Daejeon 34113, Republic of Korea

[5]Critical Diseases Diagnostics Convergence Research Center, Korea Research Institute of Bioscience and Biotechnology, Daejeon 34141, Republic of Korea

† Ju Hwan Song and Sangeun Jang have been contributed equally to this work

* **Corresponding authors**: jylee@kbsi.re.kr; Tel.:+82-43-240-5145

Supplementary Table S1. The information of RBD (receptor binding domain) and S2 of spike glycoprotein and ACE-2 protein

Protein	Domain	Glycosite*	Original glycosite	Peptide (Trypsin & GluC)*
Spike glycoprotein: P0DTC2	Receptor-binding domain (RBD)	Thr7	Thr323	VQP***T***ESIVR
		Ser9	Ser325	VQPTE***S***IVR
		Thr162**	Thr478	IYQAGS***T***PCNGVE
		Thr207**	Thr523	LLHAPA***T***VCGPK
		Asn15	Asn331	FP***N*IT**NLCPFGE
		Ans27	Asn343	VF***N*AT**R
	S2	Asn34 & Asn42	Asn709 & Asn717	NSVAYS***N*NS**IAIPT***N*FT**ISVTTE ILPVSMTK
		Asn126	Asn801	FGGF***N*FS**QILPD
		Asn399	Asn1074	***N*FT**TAPAICHD
		Asn423	Asn1098	GVFVS***N*GT**HWFVTQR
		Asn459	Asn1134	VVIGIV***N*NT**VYD
		Asn483	Asn1158	***N*HT**SPD
		Asn498	Asn1173	ISGI***N*AS**VVNIQK
		Asn519	Asn1194	NL***N*ES**LID (or NL**NE**)
Angiotensin-convertingenzyme2 (ACE): Q9BYF1		Asn35	Asn53	DLFYQSSLASWNYNT***N*IT**E
		Asn72	Asn90	IQ***N*LT**VK
		Asn85	Asn103	LQLQALQQ***N*GS**SVLSE
		Asn304	Asn322	FFVSVGLP***N*MT**QGFWE
		Asn414	Asn432	***N*ET**E (or NE)
		Asn528	Asn546	DIS***N*ST**E

*Italic bold underlined “Serine (***S***) & Threonine (***T***)”: O-linked glycosylation sites, Italic bold underlined “Asparagine (***N***)”: N-linked glycosylation site.

** O-glycosylation sites of RBD are firstly reported in this recombinant protein.

Supplementary Table S2. List of 28 O-glycopeptides identified from RBD protein

No.	O-glycopeptides of RBD	Glycan modification	Glycosylation site
1	VQPTESIVR_0_1_0_0		T
2	VQPTESIVR_0_1_0_1		T
3	VQPTESIVR_1_1_0_0		T
4	VQPTESIVR_1_1_0_1		T
5	VQPTESIVR_1_1_0_2		T
6	VQPTESIVR_1_1_0_2	Mono_O-acetylation	T
7	VQPTESIVR_1_1_1_0		T or S
8	VQPTESIVR_1_1_1_1		T or S
9	VQPTESIVR_1_2_0_0		T or S
10	VQPTESIVR_1_2_0_1		T
11	VQPTESIVR_1_3_0_1		T
12	VQPTESIVR_2_2_0_0		T or S
13	VQPTESIVR_2_2_0_1		T
14	VQPTESIVR_2_2_0_2		T
15	VQPTESIVR_2_2_0_3		T
16	VQPTESIVR_2_2_1_0		T or S
17	VQPTESIVR_2_2_1_1		T
18	VQPTESIVR_2_2_1_2		T
19	VQPTESIVR_2_2_2_1		T
20	VQPTESIVR_2_3_0_2		T
21	VQPTESIVR_2_3_1_1		T
22	VQPTESIVR_3_3_0_1		T
23	VQPTESIVR_3_3_0_2		T
24	VQPTESIVR_3_3_0_3		T
25	IYQAGSTPCNGVE_1_1_0_2		T
26	LLHAPATVCGPK_1_1_0_2		T
27	LLHAPATVCGPK_2_2_0_2		T
28	LLHAPATVCGPK_2_2_1_1		T

Supplementary Table S3 file is provided separately

Supplementary Figure S1. Sequence of RBD (receptor binding domain) and S2 of spike glycoprotein and ACE2 protein

1. Uniport >sp|P0DTC2|SPIKE_SARS2 Spike glycoprotein OS=Severe acute respiratory syndrome coronavirus2 OX=2697049 GN=S PE=1 SV=1
MFVFLVLLPLVSSQCVNLTTRTQLPPAYTNSFTRGVYYPDKVFRSSVLHSTQDLFLPFFSNVTWFHAIHVSGT
NGTKRFDNPVLPFNDGVYFASTEKSNIIRGWIFGTTLDSKTQSLLIVNNATNVVIKVCEFQFCNDPFLGVYYH
KNNKSWMESEFRVYSSANNCTFEYVSQPFLMDLEGKQGNFKNLREFVFKNIDGYFKIYSKHTPINLVRDLPQ
GFSALEPLVDLPIGINITRFQTLLALHRSYLTPGDSSSGWTAGAAAYYVGYLQPRTFLLKYNENGTITDAVDC
ALDPLSETKCTLKSFTVEKGIYQTSNFRVQP***T***E***S***IVRFP***N*****IT**NLCPFGEVF***N*****AT**RFASVYAWNRKRISNCVAD
YSVLYNSASFSTFKCYGVSPTKLNDLCFTNVYADSFVIRGDEVRQIAPGQTGKIADYNYKLPDDFTGCVIAW
NSNNLDSKVGGNYNYLYRLFRKSNLKPFERDISTEIYQAGSTPCNGVEGFNCYFPLQSYGFQPTNGVGYQPY
RVVVLSFELLHAPATVCGPKKSTNLVKNKCVNFNFNGLTGTGVLTESNKKFLPFQQFGRDIADTTDAVRDPQ
TLEILDITPCSFGGVSVITPGTNTSNQVAVLYQDVNCTEVPVAIHADQLTPTWRVYSTGSNVFQTRAGCLIGA
EHVNNSYECDIPIGAGICASYQTQTNSPRRARSVASQSIIAYTMSLGAENSVAYSNNSIAIPTNFTISVTTEILPV
SMTKTSVDCTMYICGDSTECSNLLLQYGSFCTQLNRALTGIAVEQDKNTQEVFAQVKQIYKTPPIKDFGGFNF
SQILPDPSKPSKRSFIEDLLFNKVTLADAGFIKQYGDCLGDIAARDLICAQKFNGLTVLPPLLTDEMIAQYTSA
LLAGTITSGWTFGAGAALQIPFAMQMAYRFNGIGVTQNVLYENQKLIANQFNSAIGKIQDSLSSTASALGKLQ
DVVNQNAQALNTLVKQLSSNFGAISSVLNDILSRLDKVEAEVQIDRLITGRLQSLQTYVTQQLIRAAEIRASA
NLAATKMSECVLGQSKRVDFCGKGYHLMSFPQSAPHGVVFLHVTYVPAQEKNFTTAPAICHDGKAHFPREG
VFVSNGTHWFVTQRNFYEPQIITTDNTFVSGNCDVVIGIVNNTVYDPLQPELDSFKEELDKYFKNHTSPDVDL
GDISGINASVVNIQKEIDRLNEVAKNLNESLIDLQELGKYEQYIKWPWYIWLGFIAGLIAIVMVTIMLCCMTSC
CSCLKGCCSCGSCCKFDEDDSEPVLKGVKLHYT

2. Recombinant protein >SARS-CoV2_RBD (Receptor-binding domain)
GSRVQP***T***E***S***IVR**FP*****N*****ITNLCPFGEVF*****N*****ATR**FASVYAWNRKRISNCVADYSVLYNSASFSTFKCYGVSPTKLN
DLCFTNVYADSFVIRGDEVRQIAPGQTGKIADYNYKLPDDFTGCVIAWNSNNLDSKVGGNYNYLYRLFRKS
NLKPFERDISTEIYQAGSTPCNGVEGFNCYFPLQSYGFQPTNGVGYQPYRVVVLSFELLHAPATVCGPKKSTN
LVKNKCVNFGTKHHHHHH

3. Uniport >sp|P0DTC2|SPIKE_SARS2 Spike glycoprotein OS=Severe acute respiratory syndrome coronavirus2 OX=2697049 GN=S PE=1 SV=1
MFVFLVLLPLVSSQCVNLTTRTQLPPAYTNSFTRGVYYPDKVFRSSVLHSTQDLFLPFFSNVTWFHAIHVSGT
NGTKRFDNPVLPFNDGVYFASTEKSNIIRGWIFGTTLDSKTQSLLIVNNATNVVIKVCEFQFCNDPFLGVYYH
KNNKSWMESEFRVYSSANNCTFEYVSQPFLMDLEGKQGNFKNLREFVFKNIDGYFKIYSKHTPINLVRDLPQ
GFSALEPLVDLPIGINITRFQTLLALHRSYLTPGDSSSGWTAGAAAYYVGYLQPRTFLLKYNENGTITDAVDC
ALDPLSETKCTLKSFTVEKGIYQTSNFRVQPTESIVRFPNITNLCPFGEVFNATRFASVYAWNRKRISNCVADY
SVLYNSASFSTFKCYGVSPTKLNDLCFTNVYADSFVIRGDEVRQIAPGQTGKIADYNYKLPDDFTGCVIAWNS
NNLDSKVGGNYNYLYRLFRKSNLKPFERDISTEIYQAGSTPCNGVEGFNCYFPLQSYGFQPTNGVGYQPYRV
VVLSFELLHAPATVCGPKKSTNLVKNKCVNFNFNGLTGTGVLTESNKKFLPFQQFGRDIADTTDAVRDPQTL
EILDITPCSFGGVSVITPGTNTSNQVAVLYQDVNCTEVPVAIHADQLTPTWRVYSTGSNVFQTRAGCLIGAEH
VNNSYECDIPIGAGICASYQTQTNSPRRARSVASQSIIAYTMSLGAENSVAYS***N*****NS**IAIPT***N*****FT**ISVTTEILPVSM
TKTSVDCTMYICGDSTECSNLLLQYGSFCTQLNRALTGIAVEQDKNTQEVFAQVKQIYKTPPIKDFGGF***N*****FS**Q
ILPDPSKPSKRSFIEDLLFNKVTLADAGFIKQYGDCLGDIAARDLICAQKFNGLTVLPPLLTDEMIAQYTSALL
AGTITSGWTFGAGAALQIPFAMQMAYRFNGIGVTQNVLYENQKLIANQFNSAIGKIQDSLSSTASALGKLQD
VVNQNAQALNTLVKQLSSNFGAISSVLNDILSRLDKVEAEVQIDRLITGRLQSLQTYVTQQLIRAAEIRASANL
AATKMSECVLGQSKRVDFCGKGYHLMSFPQSAPHGVVFLHVTYVPAQEK***N*****FT**TAPAICHDGKAHFPREGVF
VS***N*****GT**HWFVTQRNFYEPQIITTDNTFVSGNCDVVIGIV***N*****NT**VYDPLQPELDSFKEELDKYFK***N*****HT**SPDVDLG
DISGI***N*****AS**VVNIQKEIDRLNEVAKNL***N*****ES**LIDLQELGKYEQYIKWPWYIWLGFIAGLIAIVMVTIMLCCMTSCC
SCLKGCCSCGSCCKFDEDDSEPVLKGVKLHYT

4. Recombinant protein >SPIKE-SARS2 (S2) – 9 N-GLYCOSITE
IEEATKLRIQSVASQSIIAYTMSLGAENSVAYS***N*****NS**IAIPT***N*****FT**ISVTTEILPVSMTKTSVDCTMYICGDSTECSNL
LLQYGSFCTQLNRALTGIAVEQDKNTQEVFAQVKQIYKTPPIKDFGGF***N*****FS**QILPDPSKPSKRSFIEDLLFNKVT
LADAGFIKQYGDCLGDIAARDLICAQKFNGLTVLPPLLTDEMIAQYTSALLAGTITSGWTFGAGAALQIPFAM
QMAYRFNGIGVTQNVLYENQKLIANQFNSAIGKIQDSLSSTASALGKLQDVVNQNAQALNTLVKQLSSNFGA
ISSVLNDILSRLDKVEAEVQIDRLITGRLQSLQTYVTQQLIRAAEIRASANLAATKMSECVLGQSKRVDFCGKG
YHLMSFPQSAPHGVVFLHVTYVPAQEK***N*****FT**TAPAICHDGKAHFPREGVFVS***N*****GT**HWFVTQRNFYEPQIITTD
NTFVSGNCDVVIGIV***N*****NT**VYDPLQPELDSFKEELDKYFK***N*****HT**SPDVDLGDISGI***N*****AS**VVNIQKEIDRLNEVAK
NL***N*****ES**LIDLQELGKYEQYIKWPGGSGGSGGSGGSTSVSRLEEDVRNLNAIVQKLQERLDRLEETVQAKTRGH
HHHHHDEL

5. Uniport >sp|Q9BYF1|ACE2_HUMAN Angiotensin-converting enzyme 2 OS=Homo sapiens OX=9606 GN=ACE2 PE=1 SV=2

MSSSSWLLLSLVAVTAAQSTIEEQAKTFLDKFNHEAEDLFYQSSLASWNYNT**NIT**EENVQNMNNAGDKWSA
FLKEQSTLAQMYPLQEIQ**NLT**VKLQLQALQQ**NGS**SVLSEDKSKRLNTILNTMSTIYSTGKVCNPDNPQECLLL
EPGLNEIMANSLDYNERLWAWESWRSEVGKQLRPLYEEYVVLKNEMARANHYEDYGDYWRGDYEVNGV
DGYDYSRGQLIEDVEHTFEEIKPLYEHLHAYVRAKLMNAYPSYISPIGCLPAHLLGDMWGRFWTNLYSLTVP
FGQKPNIDVTDAMVDQAWDAQRIFKEAEKFFVSVGLP**NMT**QGFWENSMLTDPGNVQKAVCHPTAWDLGK
GDFRILMCTKVTMDDFLTAHHEMGHIQYDMAYAAQPFLLRNGANEGFHEAVGEIMSLSAATPKHLKSIGLL
SPDFQED**NET**EINFLLKQALTIVGTLPFTYMLEKWRWMVFKGEIPKDQWMKKWWEMKREIVGVVEPVPHD
ETYCDPASLFHVSNDYSFIRYYTRTLYQFQFQEALCQAAKHEGPLHKCDIS**NST**EAGQKLFNMLRLGKSEPW
TLALENVVGAKNMNVRPLLNYFEPLFTWLKDQNKNSFVGWSTDWSPYADQSIKVRISLKSALGDKAYEWN
DNEMYLFRSSVAYAMRQYFLKVKNQMILFGEEDVRVANLKPRISFNFFVTAPK**NVS**DIIPRTEVEKAIRMSRS
RINDAFRLNDNSLEFLGIQPTLGPPNQPPVSIWLIVFGVVMGVIVVGIVILIFTGIRDRKKKNKARSGENPYASI
DISKGENNPGFQNTDDVQTSF

6. Recombinant protein >humanACE-2(19-615residues)

STIEEQAKTFLDKFNHEAEDLFYQSSLASWNYNT**NIT**EENVQNMNNAGDKWSAFLKEQSTLAQMYPLQEIQ
NLTVKLQLQALQQ**NGS**SVLSEDKSKRLNTILNTMSTIYSTGKVCNPDNPQECLLLEPGLNEIMANSLDYNER
LWAWESWRSEVGKQLRPLYEEYVVLKNEMARANHYEDYGDYWRGDYEVNGVDGYDYSRGQLIEDVEHT
FEEIKPLYEHLHAYVRAKLMNAYPSYISPIGCLPAHLLGDMWGRFWTNLYSLTVPFGQKPNIDVTDAMVDQ
AWDAQRIFKEAEKFFVSVGLP**NMT**QGFWENSMLTDPGNVQKAVCHPTAWDLGKGDFRILMCTKVTMDDF
LTAHHEMGHIQYDMAYAAQPFLLRNGANEGFHEAVGEIMSLSAATPKHLKSIGLLSPDFQED**NET**EINFLLK
QALTIVGTLPFTYMLEKWRWMVFKGEIPKDQWMKKWWEMKREIVGVVEPVPHDETYCDPASLFHVSNDY
SFIRYYTRTLYQFQFQEALCQAAKHEGPLHKCDIS**NST**EAGQKLFNMLRLGKSEPWTLALENVVGAKNMNV
RPLLNYFEPLFTWLKDQNKNSFVGWSTDWSPYAD

Blue letters are common sequences between uniport and recombinant proteins. Italic bold red “Serine (S) & Threonine (T)”: O-linked glycosylation sites and “Asparagine (N)”: N-linked glycosylation site.

Supplementary Figure S2. Spectrum of 28 O-glycopeptides identified from RBD protein

Supplementary Figure S2 file is attached separately.

Supplementary Figure S3. The glycan structure examples according to glycan type.

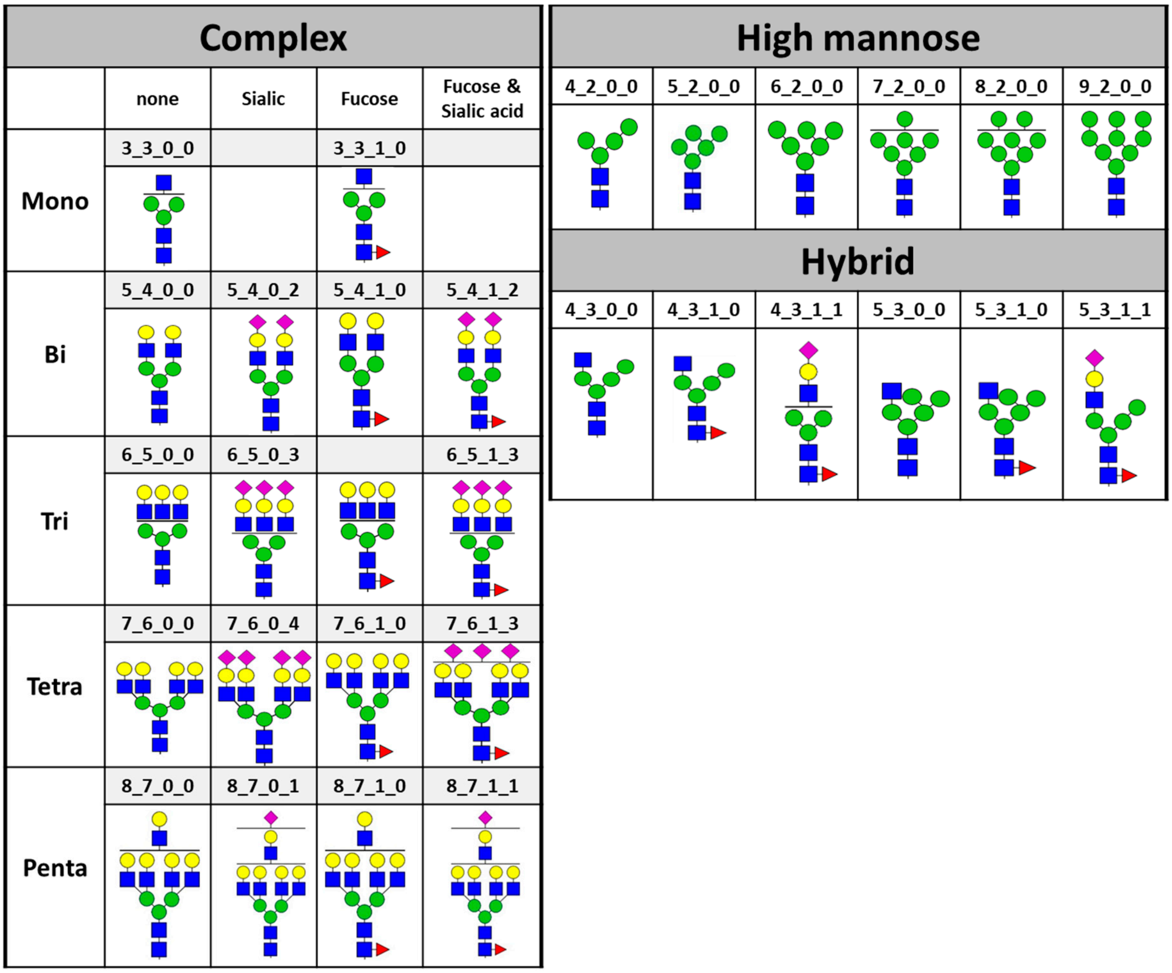

Supplementary Figure S4. HCD, CID and EThcD spectra of N-glycopeptides identified from RBD-T repeat 1

Supplementary Figure S5. HCD, CID and EThcD spectra of N-glycopeptides identified from RBD-T repeat 2

Supplementary Figures S4 and S5 files are provided separately.

Supplementary Figure S6. Spectrum of *N*HTSPDVDLGDISGINASVVNIQK_8_2_0_0 N-glycopeptide identified from S2 protein.

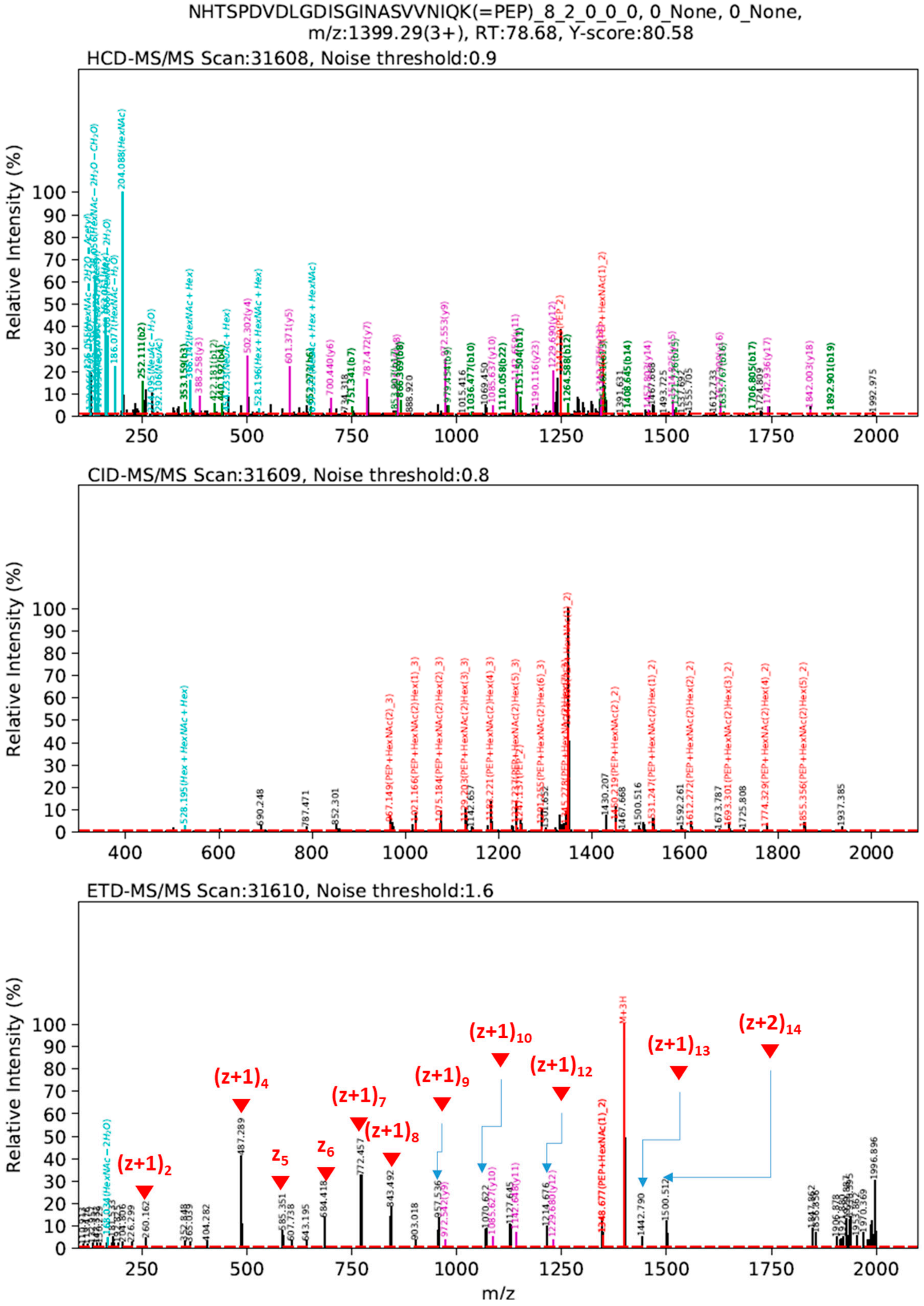

***N*HTSPDVDLGDISGINASVVNIQK_8_2_0_0**

=> *N*(Hex8HexNAc2)HTSPDVDLGDISGINASVVNIQK

MH^{+1}(mono)	MH^{+2}(mono)	MH^{+3}(mono)	MH^{+4}(mono)
4195.8393	2098.4233	1399.2846	1049.7153

b	b^{+2}	c	c^{+2}				y	y^{+2}	z	z^{+2}
---	---	1834.6581	---	1	N(HexNAc2Hex8)	24	---	---	---	---
1954.6904	977.8489	1971.717	986.3621	2	H	23	2379.215	1190.1112	2363.1963	1182.1018
2055.7381	1028.3727	2072.7647	1036.886	3	T	22	2242.1561	1121.5817	2226.1374	1113.5723
2142.7702	1071.8887	---	---	4	S	21	2141.1085	1071.0579	2125.0897	1063.0485
2239.8229	1120.4151	2256.8495	1128.9284	5	P	20	2054.0764	1027.5419	---	---
2354.8499	1177.9286	2371.8764	1186.4418	6	D	19	1957.0237	979.0155	1941.0049	971.0061
2453.9183	1227.4628	2470.9448	1235.9761	7	V	18	1841.9967	921.502	1825.978	913.4926
2568.9452	1284.9762	2585.9718	1293.4895	8	D	17	1742.9283	871.9678	1726.9096	863.9584
2682.0293	1341.5183	2699.0558	1350.0316	9	L	16	1627.9014	814.4543	1611.8826	806.445
2739.0507	1370.029	2756.0773	1378.5423	10	G	15	1514.8173	757.9123	1498.7986	749.9029
2854.0777	1427.5425	2871.1042	1436.0558	11	D	14	1457.7958	729.4016	1441.7771	721.3922
2967.1618	1484.0845	2984.1883	1492.5978	12	I	13	1342.7689	671.8881	1326.7502	663.8787
3054.1938	1527.6005	3071.2203	1536.1138	13	S	12	1229.6848	615.3461	1213.6661	607.3367
3111.2152	1556.1113	3128.2418	1564.6245	14	G	11	1142.6528	571.83	1126.6341	563.8207
3224.2993	1612.6533	3241.3259	1621.1666	15	I	10	1085.6313	543.3193	1069.6126	535.3099
3338.3422	1669.6748	3355.3688	1678.188	16	N	9	972.5473	486.7773	956.5285	478.7679
3409.3794	1705.1933	3426.4059	1713.7066	17	A	8	858.5043	429.7558	842.4856	421.7464
3496.4114	1748.7093	3513.4379	1757.2226	18	S	7	787.4672	394.2373	771.4485	386.2279
3595.4798	1798.2435	3612.5063	1806.7568	19	V	6	700.4352	350.7212	684.4165	342.7119
3694.5482	1847.7777	3711.5748	1856.291	20	V	5	601.3668	301.187	585.3481	293.1777
3808.5911	1904.7992	3825.6177	1913.3125	21	N	4	502.2984	251.6528	486.2796	243.6435
3921.6752	1961.3412	3938.7017	1969.8545	22	I	3	388.2554	194.6314	372.2367	186.622
4049.7338	2025.3705	4066.7603	2033.8838	23	Q	2	275.1714	138.0893	259.1527	130.08
---	---	---	---	24	K	1	147.1128	74.06	131.0941	66.0507

Supplementary Figure S7. HCD, CID and EThcD spectra of N-glycopeptides identified from S2-T repeat 1.

Supplementary Figure S8. HCD, CID and EThcD spectra of N-glycopeptides identified from S2-T repeat 2.

Supplementary Figure S9. HCD, CID and EThcD spectra of N-glycopeptides identified from S2-TG repeat 1.

Supplementary Figure S10. HCD, CID and EThcD spectra of N-glycopeptides identified from S2-TG repeat 2.

Supplementary Figure S11. HCD, CID and EThcD spectra of N-glycopeptides identified from ACE2-T repeat 1.

Supplementary Figure S12. HCD, CID and EThcD spectra of N-glycopeptides identified from ACE2-T repeat 2.

Supplementary Figure S13. HCD, CID and EThcD spectra of N-glycopeptides identified from ACE2-TG repeat 1.

Supplementary Figure S14. HCD, CID and EThcD spectra of N-glycopeptides identified from ACE2-TG repeat 2.

Supplementary Figures S7, S8, S9, S10, S11, S12, S13 and S14 files are provided separately.
