# Supplementary material for: Characterization of Site-Specific N- and O-Glycopeptides from Recombinant Spike and ACE2 Glycoproteins Using LC-MS/MS Analysis
Source: Int J Mol Sci. 2024 Dec 20;25(24):13649. doi: 10.3390/ijms252413649 (PMC11678118; doi:10.3390/ijms252413649)
Supplement: Supplementary file 1 [file ijms-25-13649-s001.zip › Supplementary Figure S10(S2_TG_N-glycopep_2).pdf]

EGVFVSNGTHWFVTQR(=PEP)_8_2_0_0_0, 0_None, 0_None,
m/z:1189.50(3+), RT:63.87, hcd-score:66.65

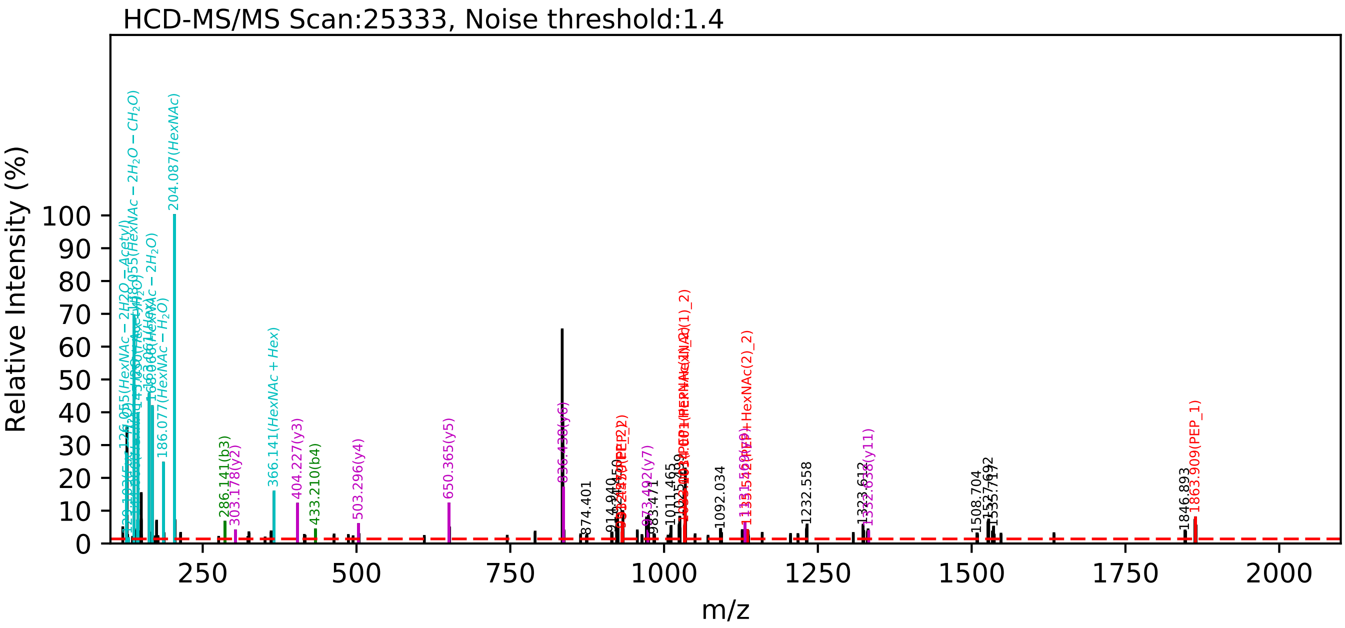

EGVFVSNGTHWFVTQR(=PEP)_8_2_0_0_0, 0_None, 0_None,
m/z:1189.50(3+), RT:63.87, hcd-score:66.65

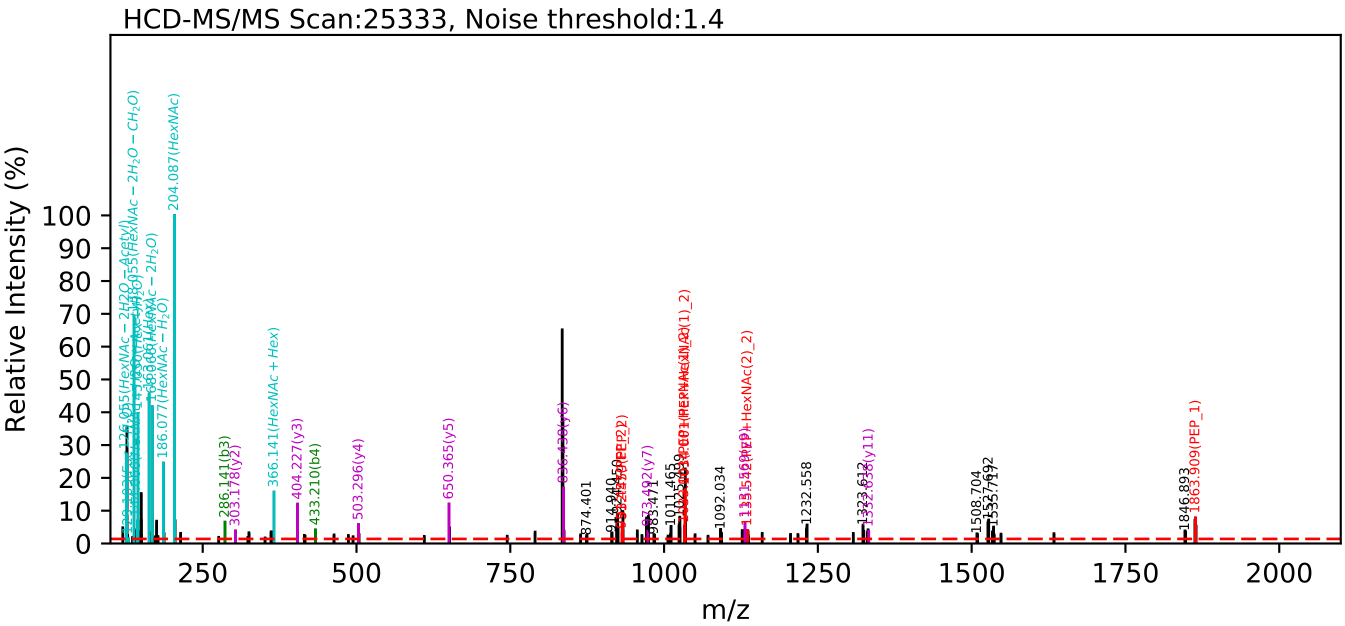

EGVFVSNGTHWFVTQR(=PEP)_10_2_0_0_0, 0_None, 0_None,
m/z:1297.54(2+), RT:64.39, hcd-score:71.34

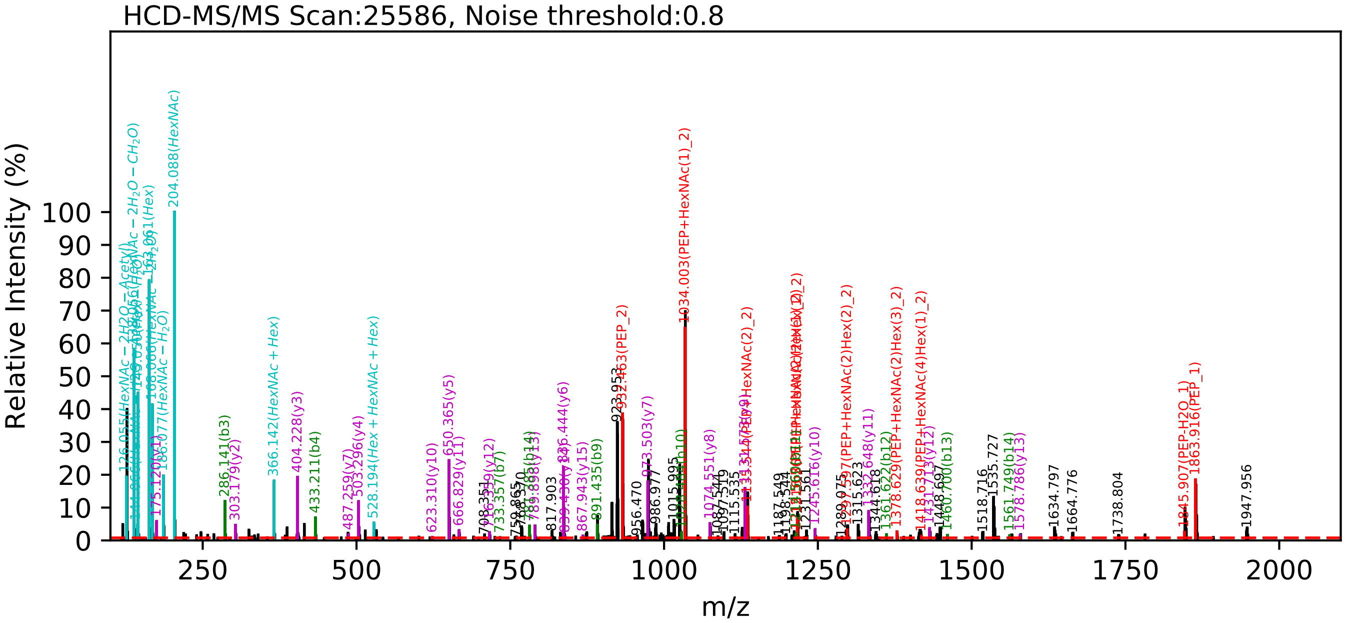

EGVFVSNGTHWFVTQR(=PEP)_10_2_0_0_0, 0_None, 0_None,
m/z:1297.54(2+), RT:64.39, hcd-score:71.34

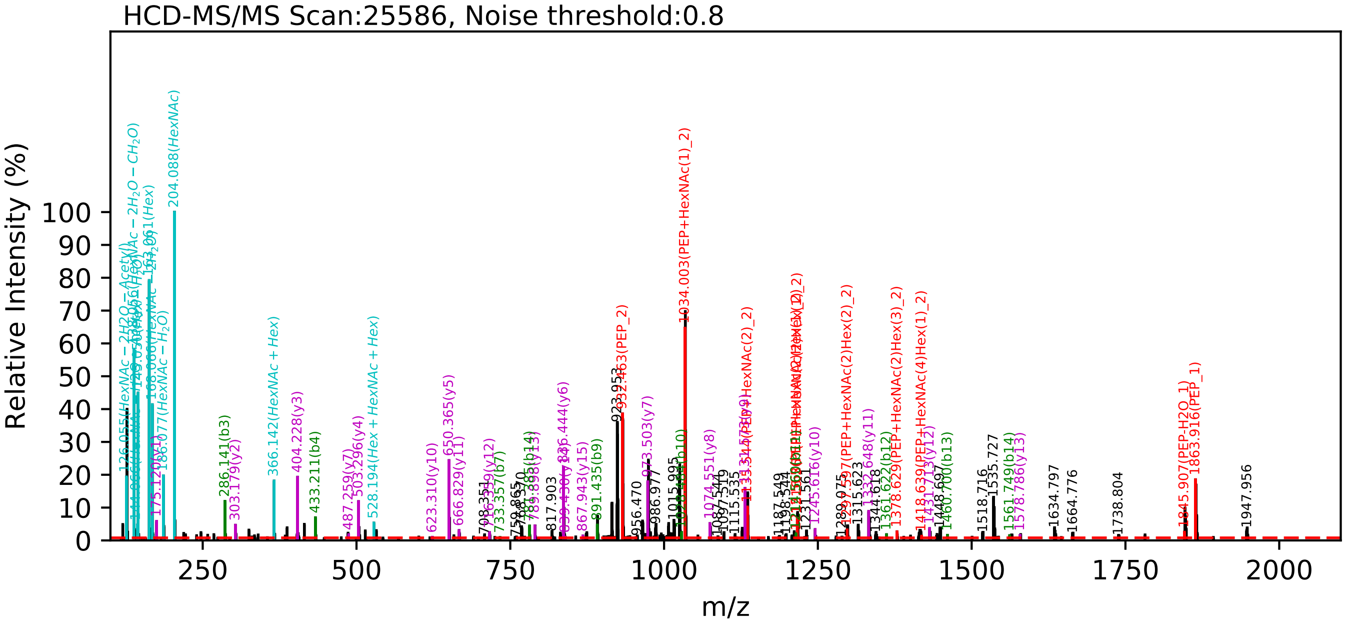

FGGFNFSQILPDPSKPSK(=PEP)_4_2_0_0_0, 0_None, 0_None,
m/z:1007.47(3+), RT:89.63, hcd-score:85.46

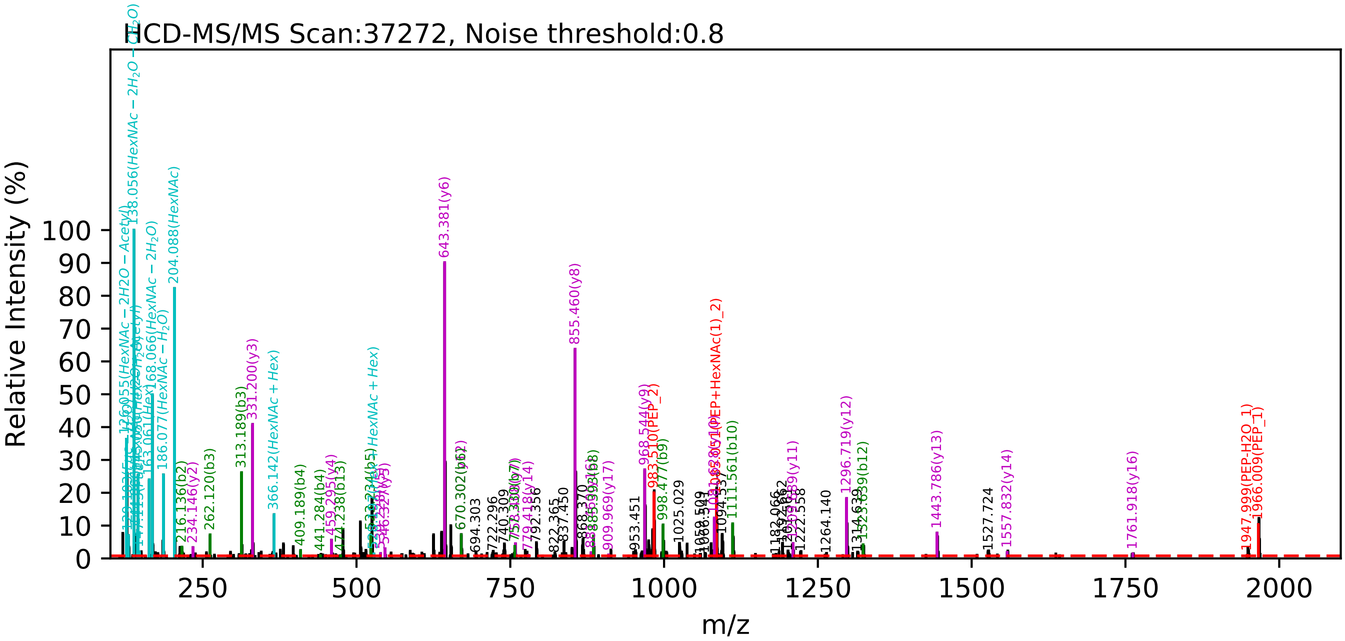

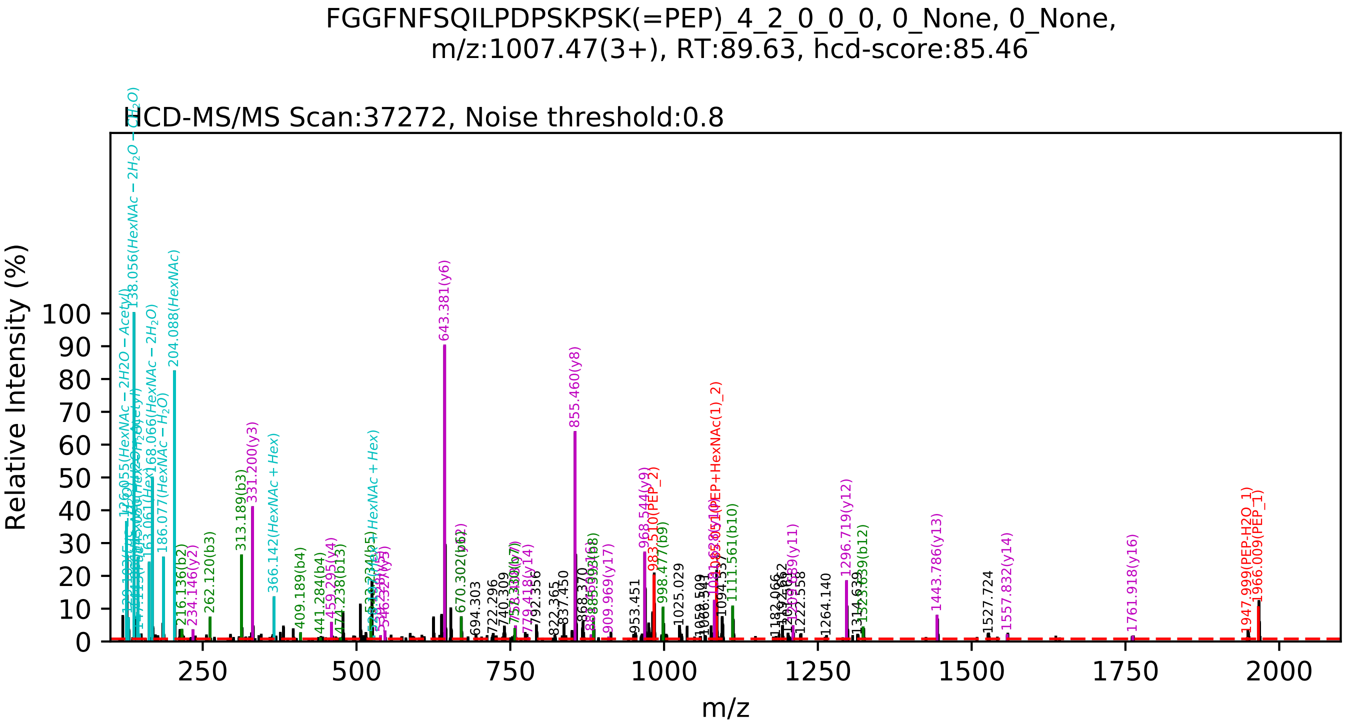
FGGFNFSQILPDPSKPSK(=PEP)_4_2_0_0_0, 0_None, 0_None,
m/z:1007.47(3+), RT:89.63, hcd-score:85.46
HCD-MS/MS Scan:37272, Noise threshold:0.8
Relative Intensity (%)
m/z
204.088(HexNAc)
186.077(HexNAc − H2O)
168.066(HexNAc − 2H2O)
216.136(b2)
234.146(y2)
262.120(b3)
313.189(b3)
331.200(y3)
366.142(HexNAc + Hex)
409.189(b4)
441.284(b4)
459.295(y4)
643.381(y6)
670.302(b6)
694.303
722.296
740.309
792.356
822.365
837.450
855.460(y8)
909.969(y17)
953.451
968.544(y9)
998.477(b9)
1025.029
1094.533
1111.561(b10)
1182.066
1222.558
1264.140
1296.719(y12)
1443.786(y13)
1527.724
1557.832(y14)
1761.918(y16)
1947.999(PEP-H2O_1)
1966.009(PEP_1)

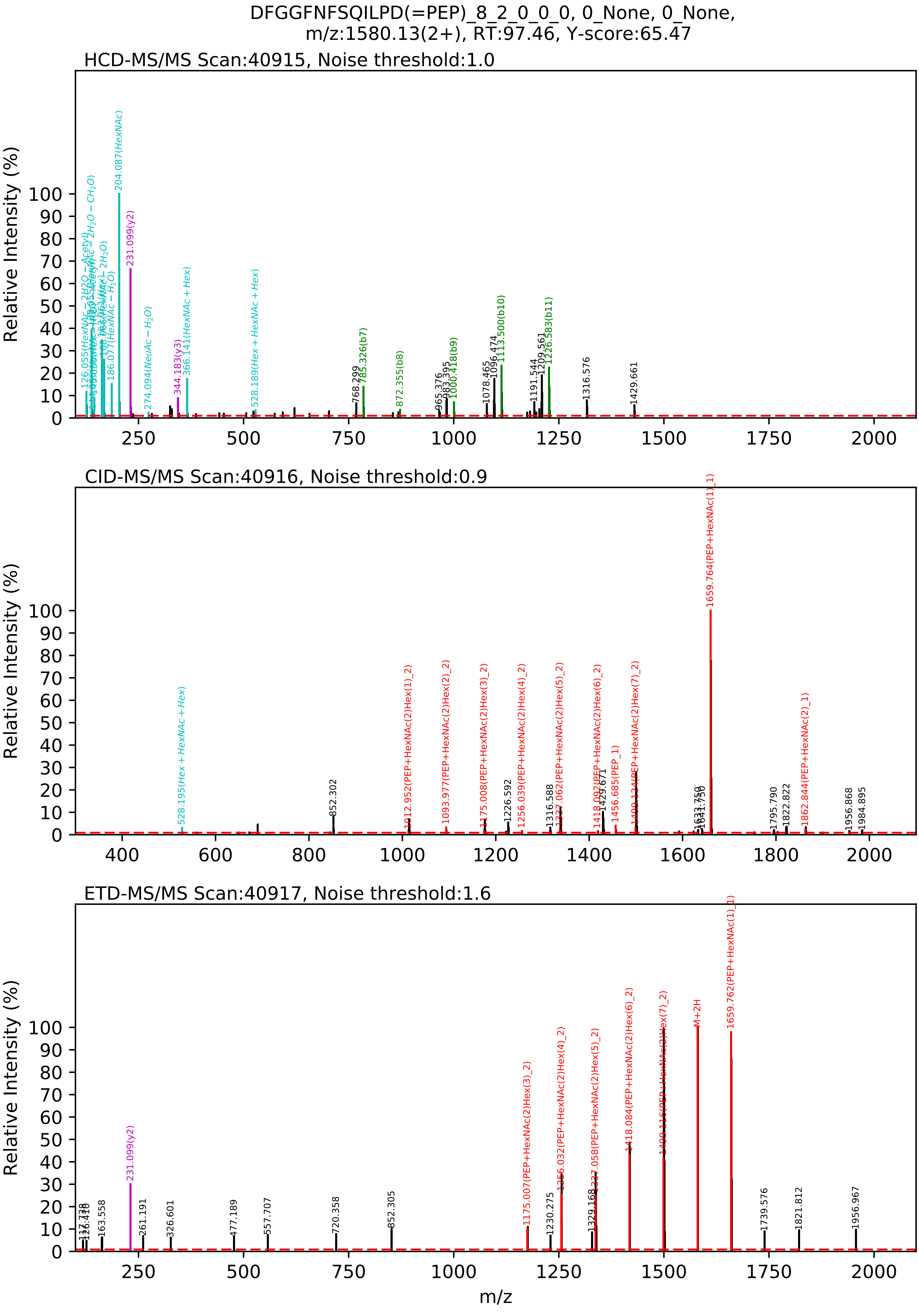

DFGGFNFSQILPD(=PEP)_8_2_0_0_0, 0_None, 0_None,
m/z:1580.13(2+), RT:97.46, Y-score:65.47
HCD-MS/MS Scan:40915, Noise threshold:1.0
Relative Intensity (%)
204.087(HexNAc)
231.099(y2)
344.183(y3)
366.141(HexNAc + Hex)
528.189(Hex + HexNAc + Hex)
785.326(b7)
872.355(b8)
1000.418(b9)
1113.500(b10)
1226.583(b11)
1316.576
1429.661
CID-MS/MS Scan:40916, Noise threshold:0.9
528.195(Hex + HexNAc + Hex)
852.302
1659.764(PEP+HexNAc(1)_1)
1862.844(PEP+HexNAc(2)_1)
1795.790
1822.822
1956.868
1984.895
ETD-MS/MS Scan:40917, Noise threshold:1.6
231.099(y2)
M+2H
1659.762(PEP+HexNAc(1)_1)
1739.576
1821.812
1956.967
m/z

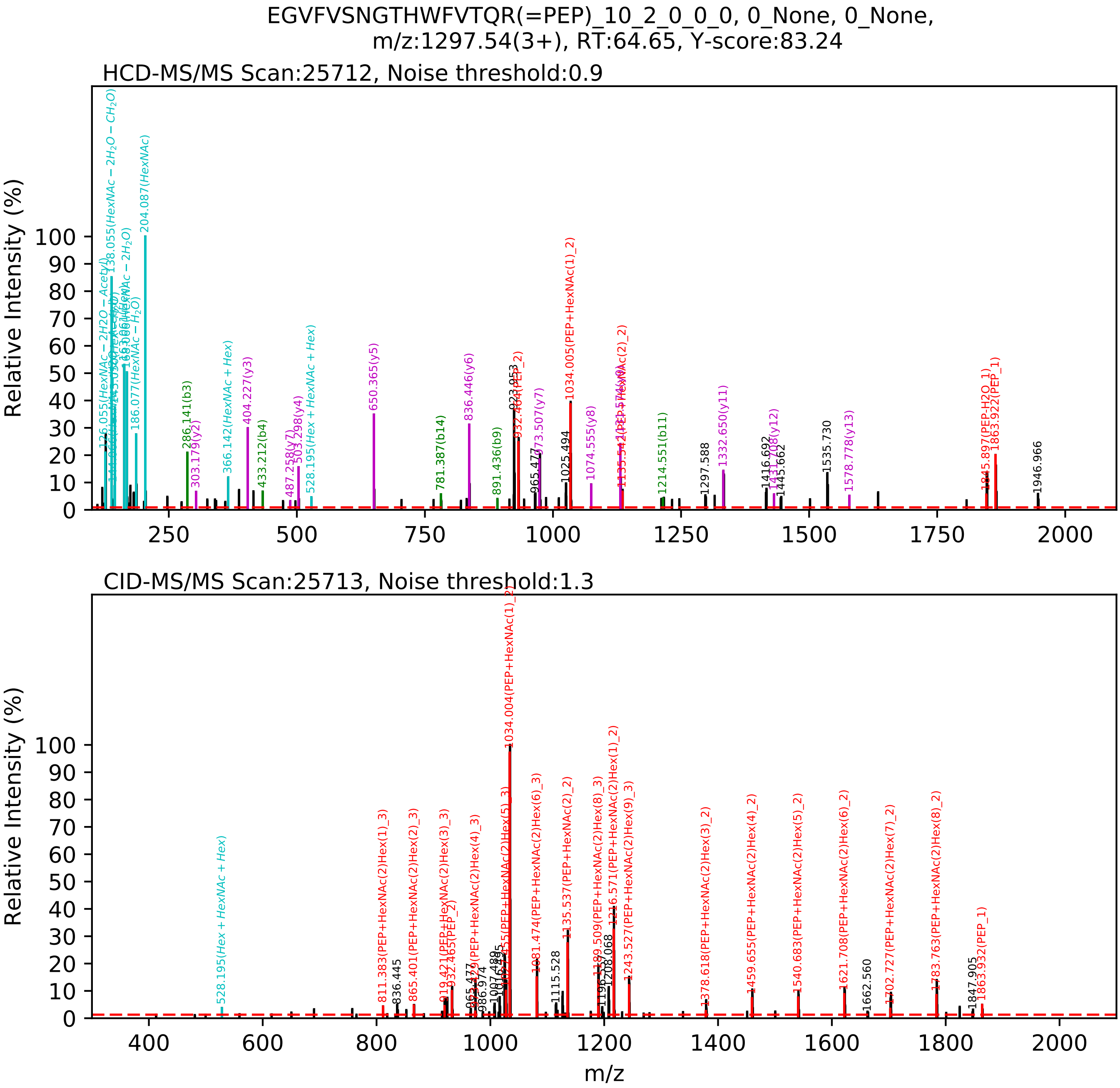
EGVFVSNGTHWFVTQR(=PEP)_10_2_0_0_0, 0_None, 0_None,
m/z:1297.54(3+), RT:64.65, Y-score:83.24
HCD-MS/MS Scan:25712, Noise threshold:0.9
Relative Intensity (%)
204.087(HexNAc)
286.141(b3)
303.179(y2)
366.142(HexNAc + Hex)
404.227(y3)
433.212(b4)
487.258(y7)
503.298(y4)
528.195(Hex + HexNAc + Hex)
650.365(y5)
781.387(b14)
836.446(y6)
891.436(b9)
923.953
965.477
973.507(y7)
1025.494
1034.005(PEP+HexNAc(1)_2)
1074.555(y8)
1214.551(b11)
1297.588
1332.650(y11)
1416.692
1445.662
1535.730
1578.778(y13)
1845.897(PEP-H2O_1)
1863.922(PEP_1)
1946.966
CID-MS/MS Scan:25713, Noise threshold:1.3
Relative Intensity (%)
528.195(Hex + HexNAc + Hex)
811.383(PEP+HexNAc(2)Hex(1)_3)
836.445
865.401(PEP+HexNAc(2)Hex(2)_3)
932.465(PEP_2)
965.477
986.974
1007.489
1034.004(PEP+HexNAc(1)_2)
1081.474(PEP+HexNAc(2)Hex(6)_3)
1115.528
1135.537(PEP+HexNAc(2)_2)
1189.509(PEP+HexNAc(2)Hex(8)_3)
1208.068
1216.571(PEP+HexNAc(2)Hex(1)_2)
1243.527(PEP+HexNAc(2)Hex(9)_3)
1378.618(PEP+HexNAc(2)Hex(3)_2)
1459.655(PEP+HexNAc(2)Hex(4)_2)
1540.683(PEP+HexNAc(2)Hex(5)_2)
1621.708(PEP+HexNAc(2)Hex(6)_2)
1662.560
1702.727(PEP+HexNAc(2)Hex(7)_2)
1783.763(PEP+HexNAc(2)Hex(8)_2)
1847.905
1863.932(PEP_1)
m/z

EGVFVSNGTHWFVTQR(=PEP)_11_2_0_0_0, 0_None, 0_None,
m/z:1351.55(3+), RT:64.27, Y-score:86.44

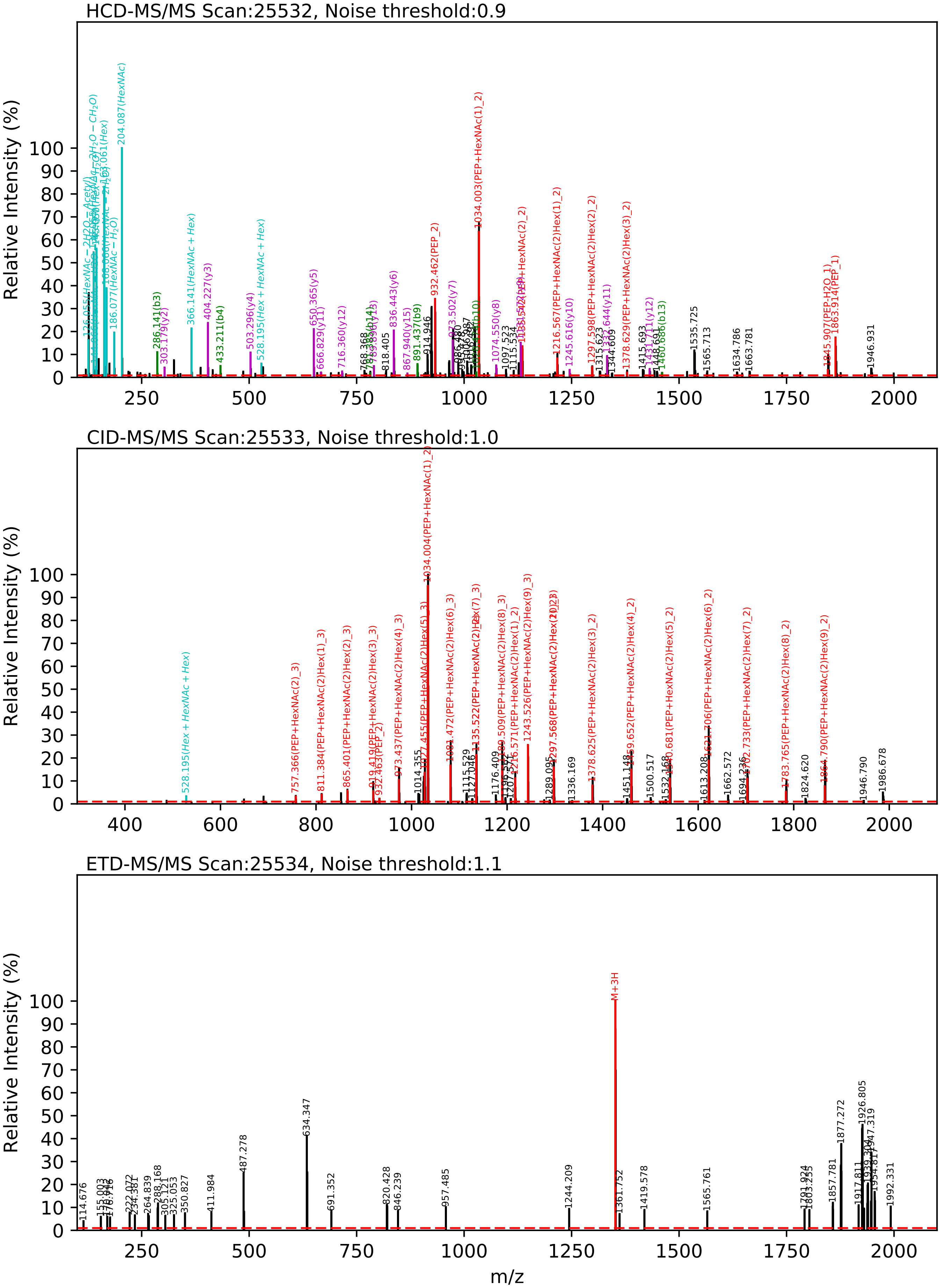

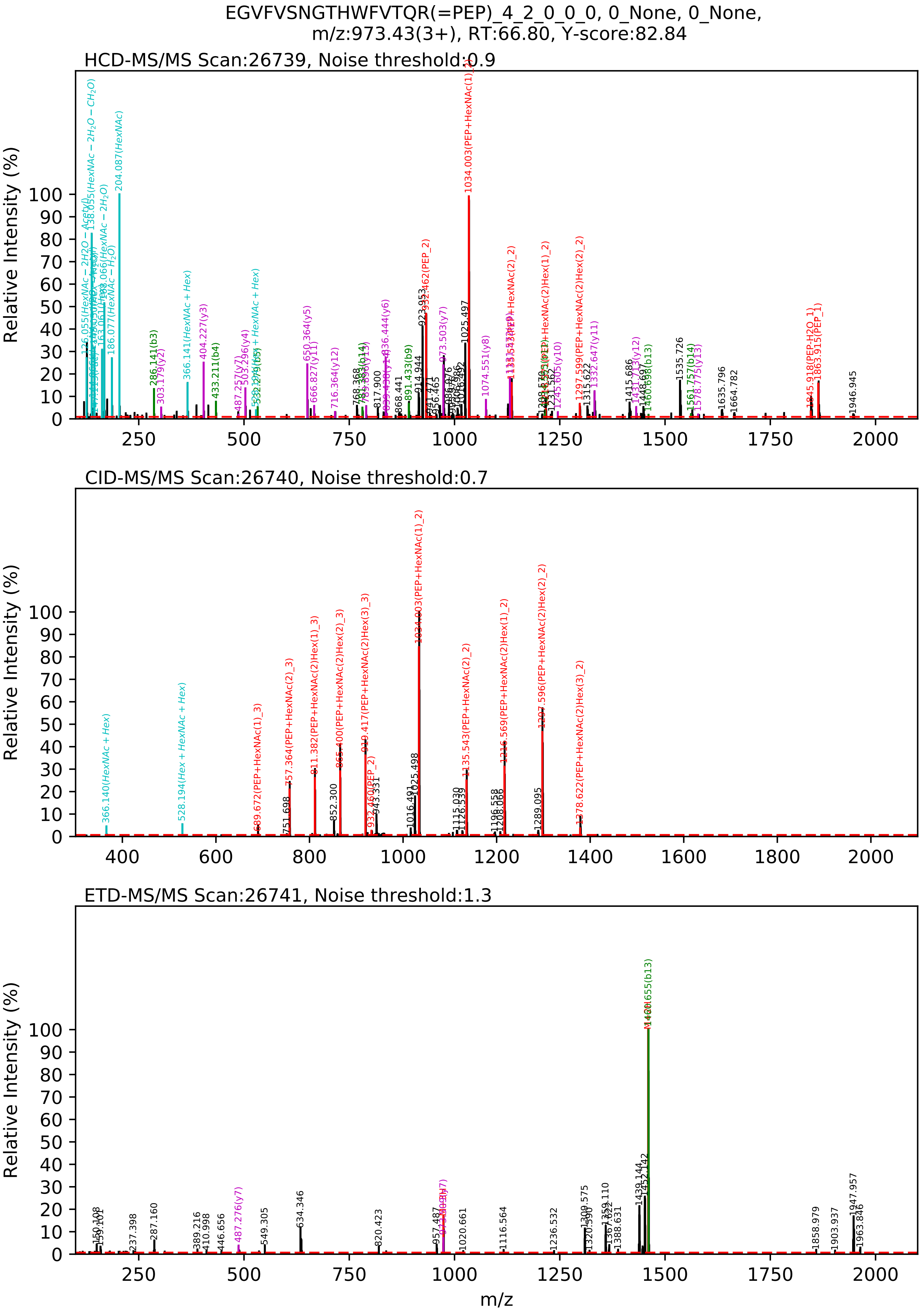

EGVFVSNGTHWFVTQR(=PEP)_4_2_0_0_0, 0_None, 0_None,
m/z:973.43(3+), RT:66.80, Y-score:82.84
HCD-MS/MS Scan:26739, Noise threshold:0.9
CID-MS/MS Scan:26740, Noise threshold:0.7
ETD-MS/MS Scan:26741, Noise threshold:1.3
Relative Intensity (%)
m/z

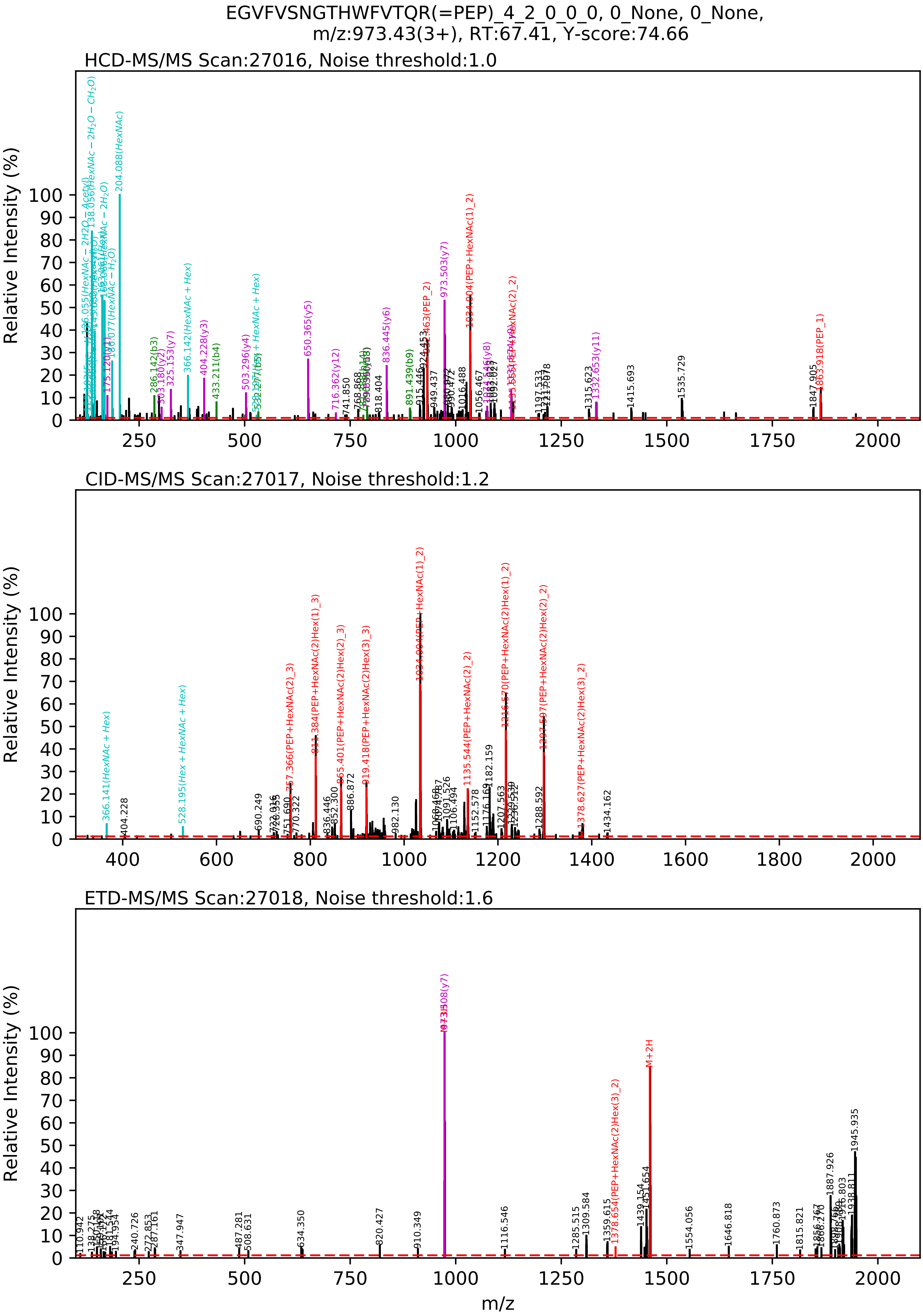

EGVFVSNGTHWFVTQR(=PEP)_4_2_0_0_0, 0_None, 0_None,
m/z:973.43(3+), RT:67.41, Y-score:74.66
HCD-MS/MS Scan:27016, Noise threshold:1.0
CID-MS/MS Scan:27017, Noise threshold:1.2
ETD-MS/MS Scan:27018, Noise threshold:1.6
Relative Intensity (%)
m/z
204.088(HexNAc)
973.503(y7)
1034.004(PEP+HexNAc(1)_2)
1863.918(PEP_1)
1332.653(y11)
650.365(y5)
836.445(y6)
1535.729
811.384(PEP+HexNAc(2)Hex(1)_3)
1216.570(PEP+HexNAc(2)Hex(1)_2)
1297.597(PEP+HexNAc(2)Hex(2)_2)
1135.544(PEP+HexNAc(2)_2)
1378.627(PEP+HexNAc(2)Hex(3)_2)
973.508(y7)
M+2H
1378.654(PEP+HexNAc(2)Hex(3)_2)
1945.935
1887.926

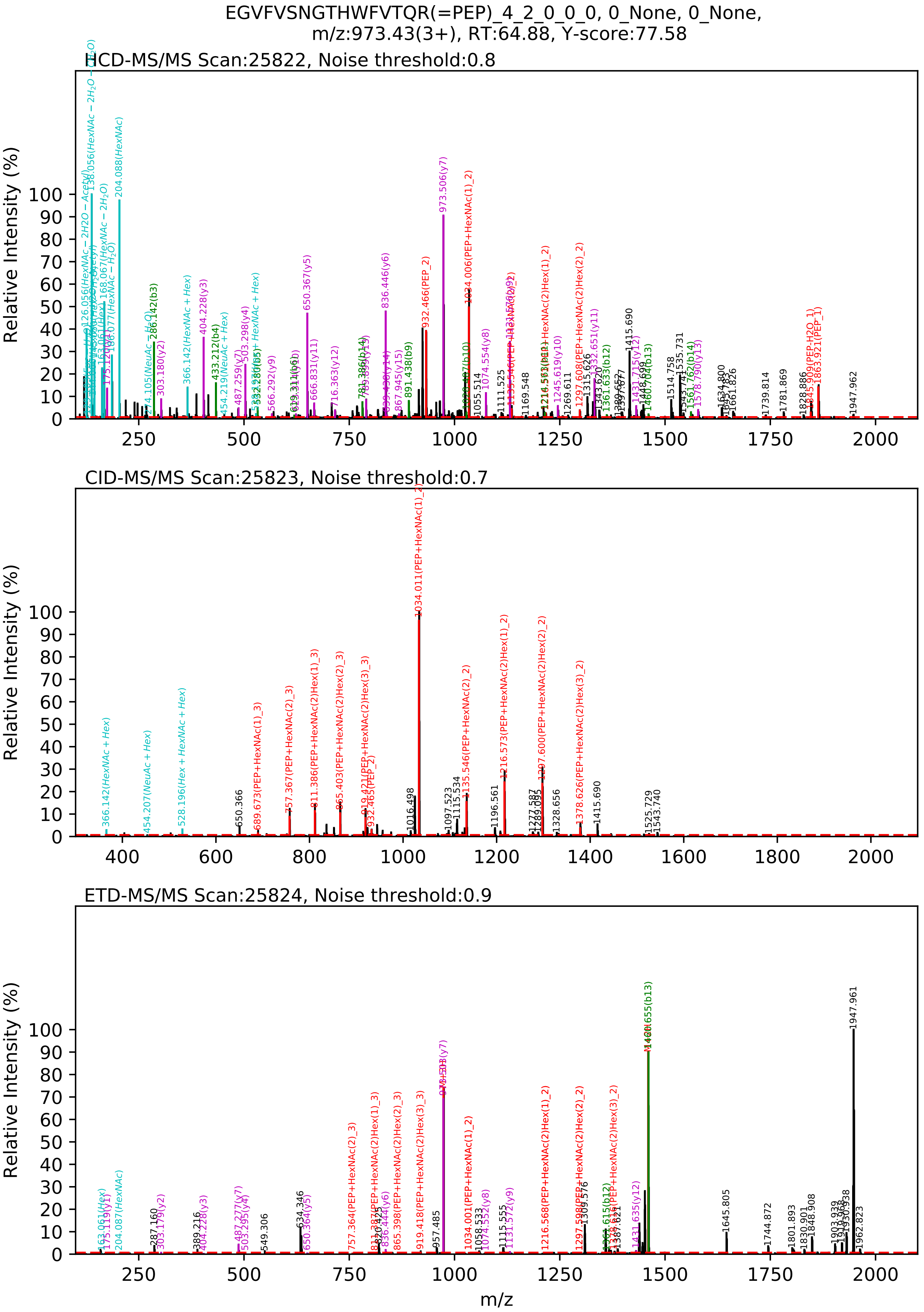
EGVFVSNGTHWFVTQR(=PEP)_4_2_0_0_0, 0_None, 0_None,
m/z:973.43(3+), RT:64.88, Y-score:77.58
HCD-MS/MS Scan:25822, Noise threshold:0.8
CID-MS/MS Scan:25823, Noise threshold:0.7
ETD-MS/MS Scan:25824, Noise threshold:0.9
Relative Intensity (%)
m/z

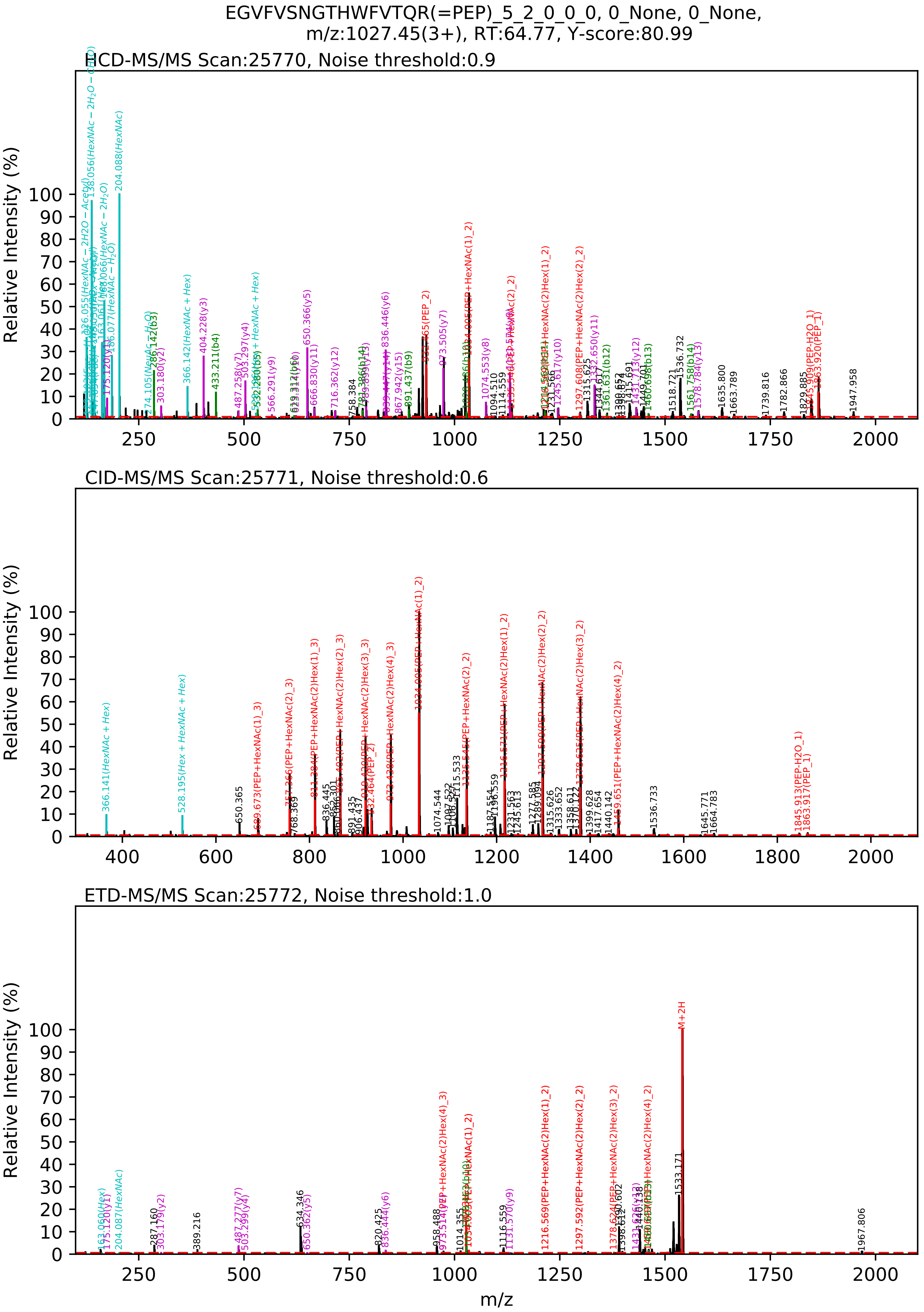

EGVFVSNGTHWFVTQR(=PEP)_5_2_0_0_0, 0_None, 0_None,
m/z:1027.45(3+), RT:64.77, Y-score:80.99
HCD-MS/MS Scan:25770, Noise threshold:0.9
CID-MS/MS Scan:25771, Noise threshold:0.6
ETD-MS/MS Scan:25772, Noise threshold:1.0
Relative Intensity (%)
m/z

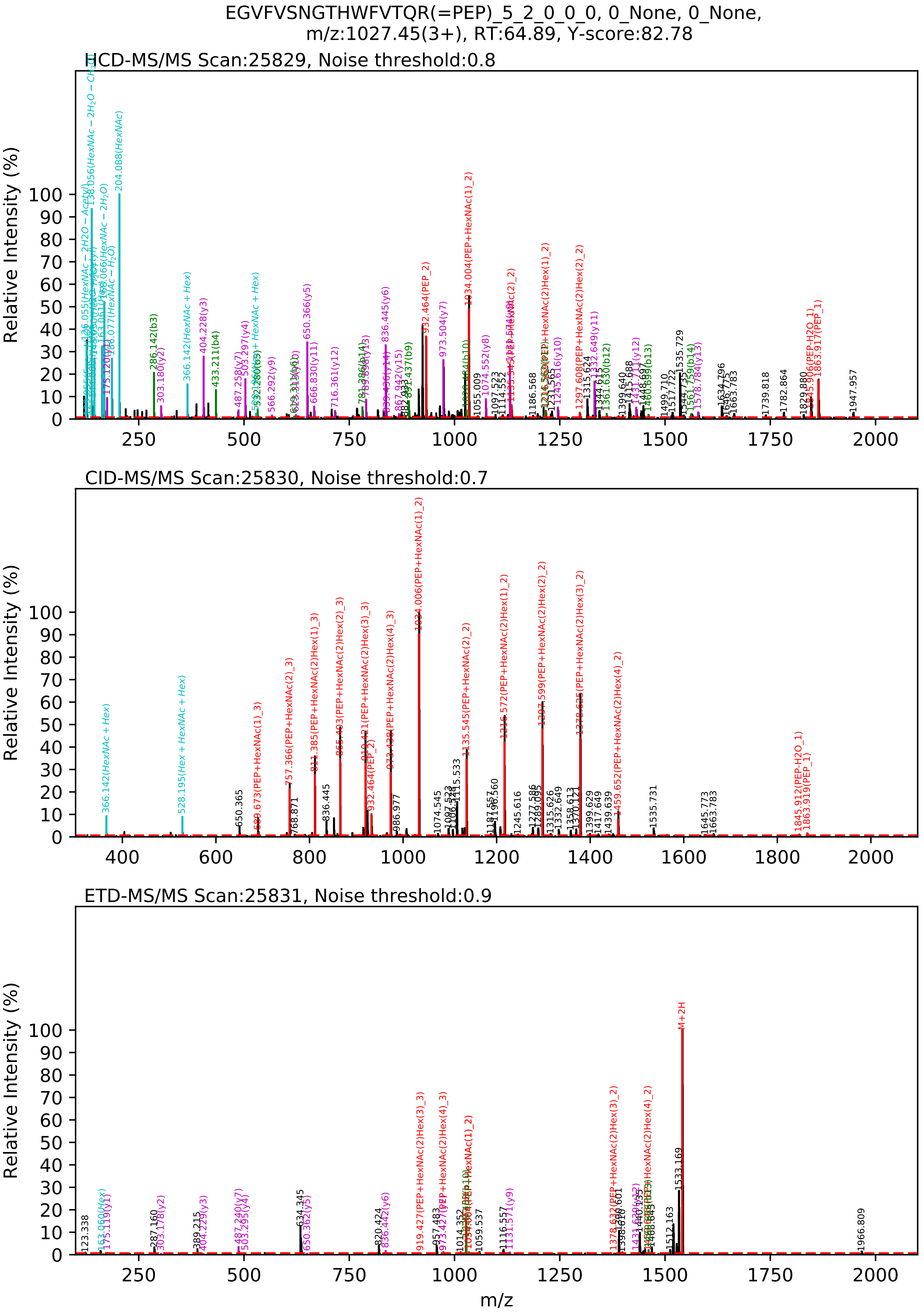
EGVFVSNGTHWFVTQR(=PEP)_5_2_0_0_0, 0_None, 0_None,
m/z:1027.45(3+), RT:64.89, Y-score:82.78
HCD-MS/MS Scan:25829, Noise threshold:0.8
CID-MS/MS Scan:25830, Noise threshold:0.7
ETD-MS/MS Scan:25831, Noise threshold:0.9
Relative Intensity (%)
m/z

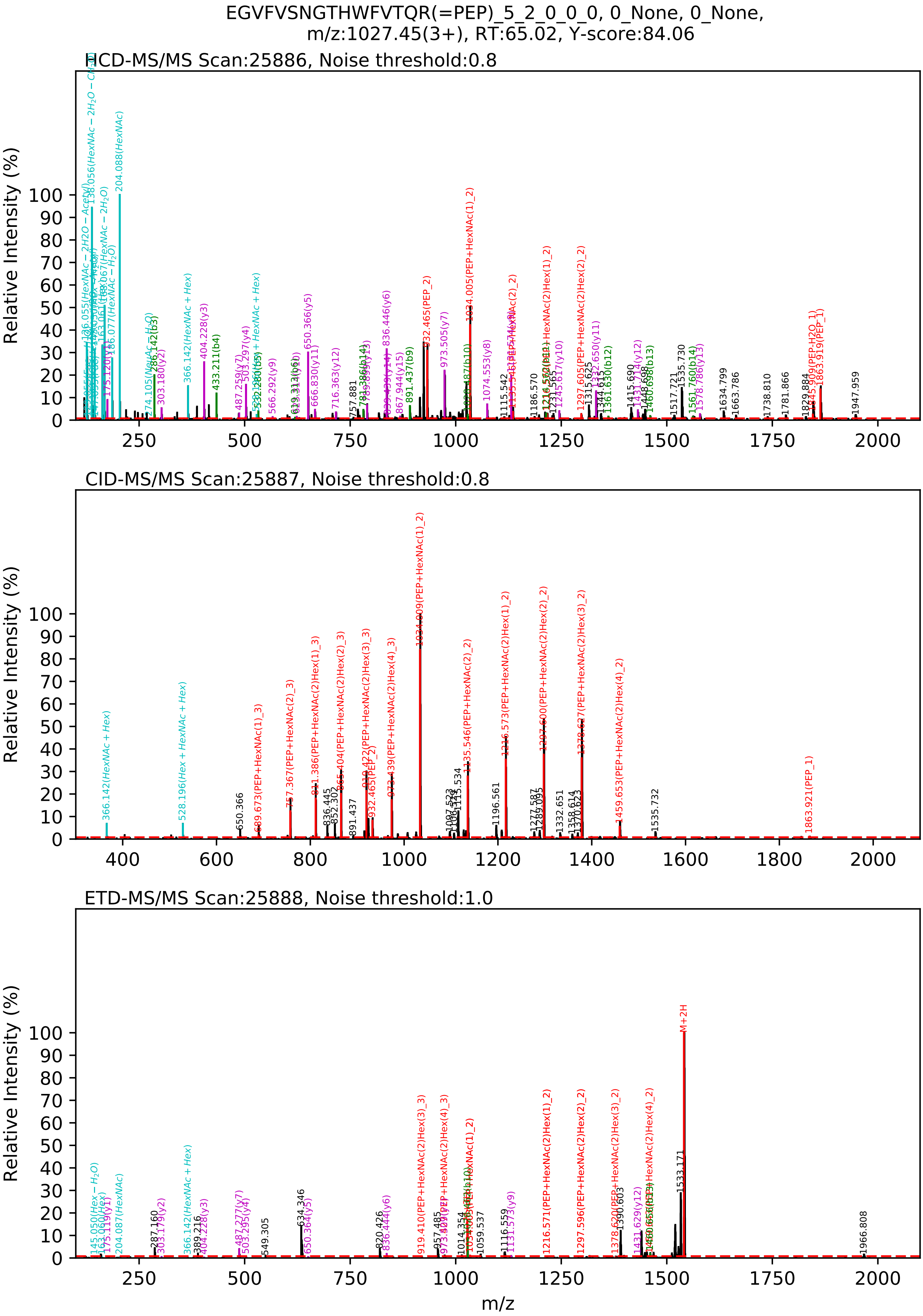

EGVFVSNGTHWFVTQR(=PEP)_5_2_0_0_0, 0_None, 0_None,
m/z:1027.45(3+), RT:65.02, Y-score:84.06
HCD-MS/MS Scan:25886, Noise threshold:0.8
Relative Intensity (%)
204.088(HexNAc)
1034.005(PEP+HexNAc(1)_2)
932.465(PEP_2)
973.505(y7)
836.446(y6)
650.366(y5)
404.228(y3)
433.211(b4)
366.142(HexNAc + Hex)
1863.919(PEP_1)
CID-MS/MS Scan:25887, Noise threshold:0.8
1034.009(PEP+HexNAc(1)_2)
1297.600(PEP+HexNAc(2)Hex(2)_2)
1378.627(PEP+HexNAc(2)Hex(3)_2)
1216.573(PEP+HexNAc(2)Hex(1)_2)
1135.546(PEP+HexNAc(2)_2)
1459.653(PEP+HexNAc(2)Hex(4)_2)
757.367(PEP+HexNAc(2)_3)
811.386(PEP+HexNAc(2)Hex(1)_3)
865.404(PEP+HexNAc(2)Hex(2)_3)
919.422(PEP+HexNAc(2)Hex(3)_3)
973.439(PEP+HexNAc(2)Hex(4)_3)
689.673(PEP+HexNAc(1)_3)
366.142(HexNAc + Hex)
528.196(Hex + HexNAc + Hex)
1863.921(PEP_1)
ETD-MS/MS Scan:25888, Noise threshold:1.0
M+2H
1533.171
1390.603
634.346
1966.808
1216.571(PEP+HexNAc(2)Hex(1)_2)
1297.596(PEP+HexNAc(2)Hex(2)_2)
1378.620(PEP+HexNAc(2)Hex(3)_2)
919.410(PEP+HexNAc(2)Hex(3)_3)
m/z

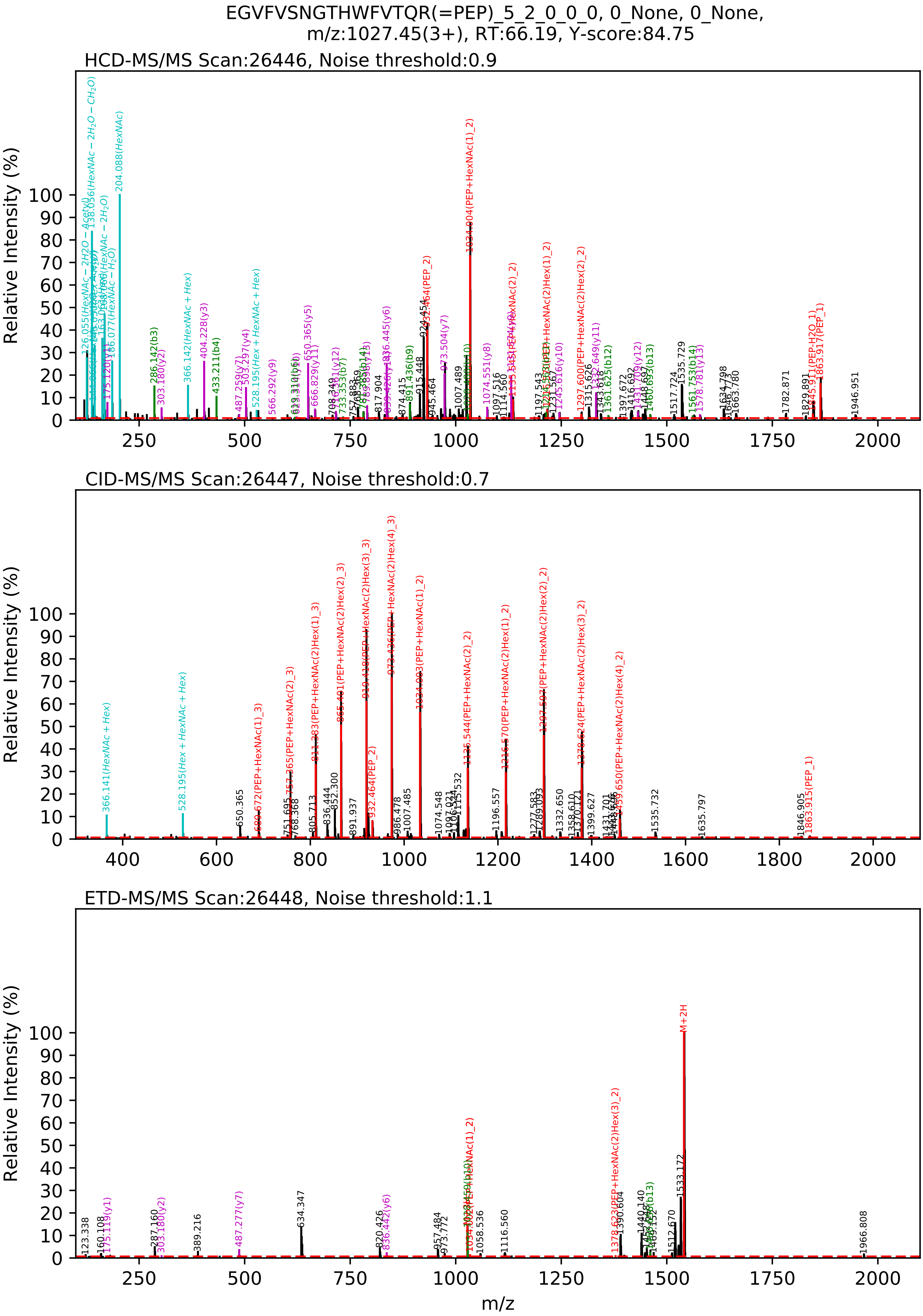
EGVFVSNGTHWFVTQR(=PEP)_5_2_0_0_0, 0_None, 0_None,
m/z:1027.45(3+), RT:66.19, Y-score:84.75
HCD-MS/MS Scan:26446, Noise threshold:0.9
CID-MS/MS Scan:26447, Noise threshold:0.7
ETD-MS/MS Scan:26448, Noise threshold:1.1
Relative Intensity (%)
m/z
M+2H

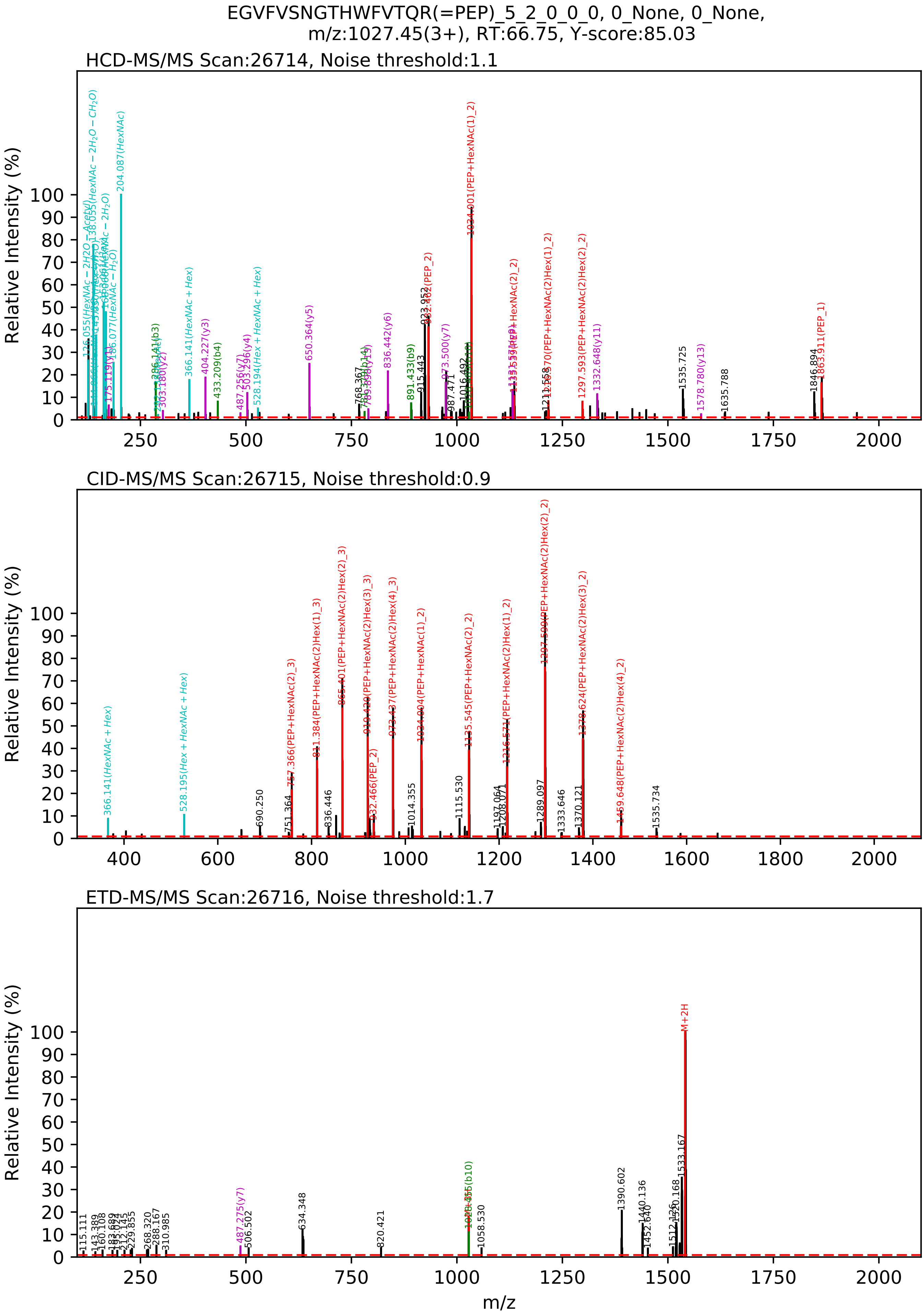

EGVFVSNGTHWFVTQR(=PEP)_5_2_0_0_0, 0_None, 0_None,
m/z:1027.45(3+), RT:66.75, Y-score:85.03
HCD-MS/MS Scan:26714, Noise threshold:1.1
Relative Intensity (%)
204.087(HexNAc)
1034.001(PEP+HexNAc(1)_2)
932.462(PEP_2)
923.952
650.364(y5)
836.442(y6)
891.433(b9)
973.500(y7)
1297.593(PEP+HexNAc(2)Hex(2)_2)
1332.648(y11)
1535.725
1578.780(y13)
1635.788
1846.894
1863.911(PEP_1)
366.141(HexNAc+Hex)
404.227(y3)
433.209(b4)
487.256(y7)
528.194(Hex+HexNAc+Hex)
175.119(y1)
286.141(b3)
303.180(y2)
768.367
915.443
987.471
1016.492
CID-MS/MS Scan:26715, Noise threshold:0.9
1297.590(PEP+HexNAc(2)Hex(2)_2)
865.401(PEP+HexNAc(2)Hex(2)_3)
919.420(PEP+HexNAc(2)Hex(3)_3)
973.437(PEP+HexNAc(2)Hex(4)_3)
1034.004(PEP+HexNAc(1)_2)
1135.545(PEP+HexNAc(2)_2)
1216.571(PEP+HexNAc(2)Hex(1)_2)
1378.624(PEP+HexNAc(2)Hex(3)_2)
1459.648(PEP+HexNAc(2)Hex(4)_2)
811.384(PEP+HexNAc(2)Hex(1)_3)
757.366(PEP+HexNAc(2)_3)
932.466(PEP_2)
366.141(HexNAc+Hex)
528.195(Hex+HexNAc+Hex)
690.250
751.364
836.446
1014.355
1115.530
1197.064
1289.097
1333.646
1370.121
1535.734
ETD-MS/MS Scan:26716, Noise threshold:1.7
M+2H
1533.167
1390.602
1440.136
1452.640
1512.126
1520.168
1058.530
820.421
634.348
487.275(y7)
506.502
115.111
143.389
160.108
268.320
288.167
310.985
m/z

EGVFVSNGTHWFVTQR(=PEP)_5_2_0_0_0, 0_None, 0_None,
m/z:1027.45(3+), RT:68.25, Y-score:86.63

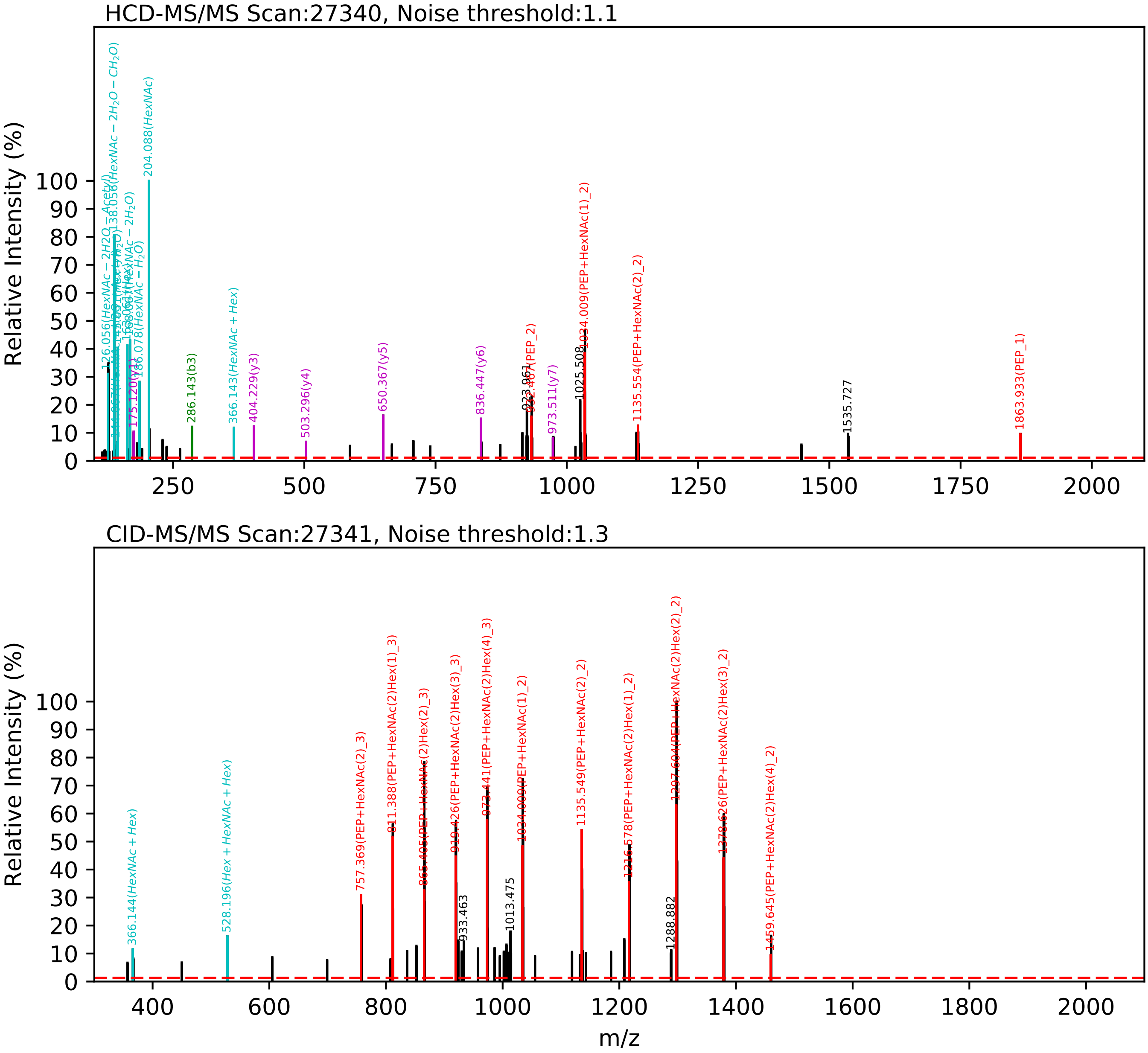

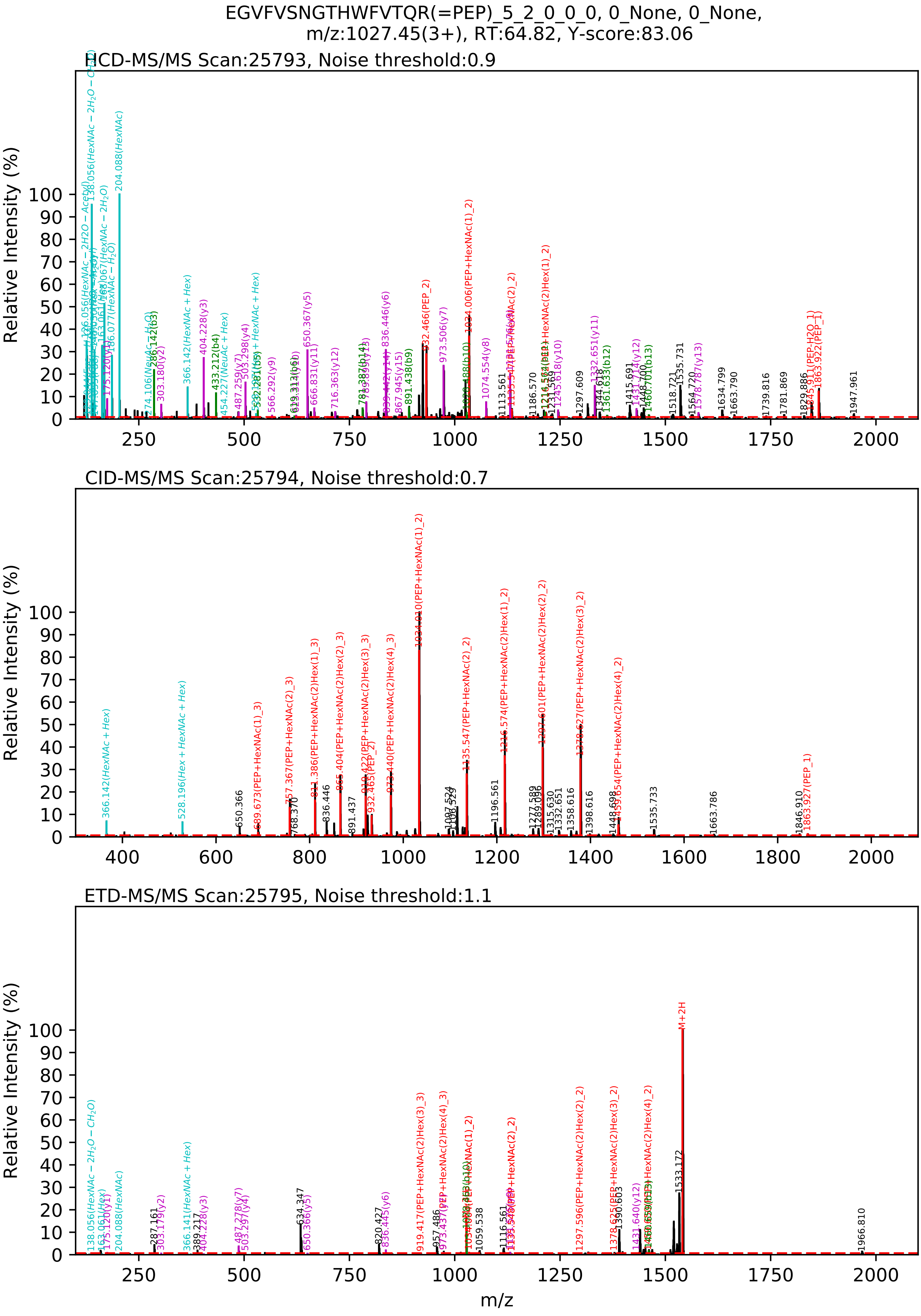

EGVFVSNGTHWFVTQR(=PEP)_5_2_0_0_0, 0_None, 0_None,
m/z:1027.45(3+), RT:64.82, Y-score:83.06
HCD-MS/MS Scan:25793, Noise threshold:0.9
CID-MS/MS Scan:25794, Noise threshold:0.7
ETD-MS/MS Scan:25795, Noise threshold:1.1
Relative Intensity (%)
m/z

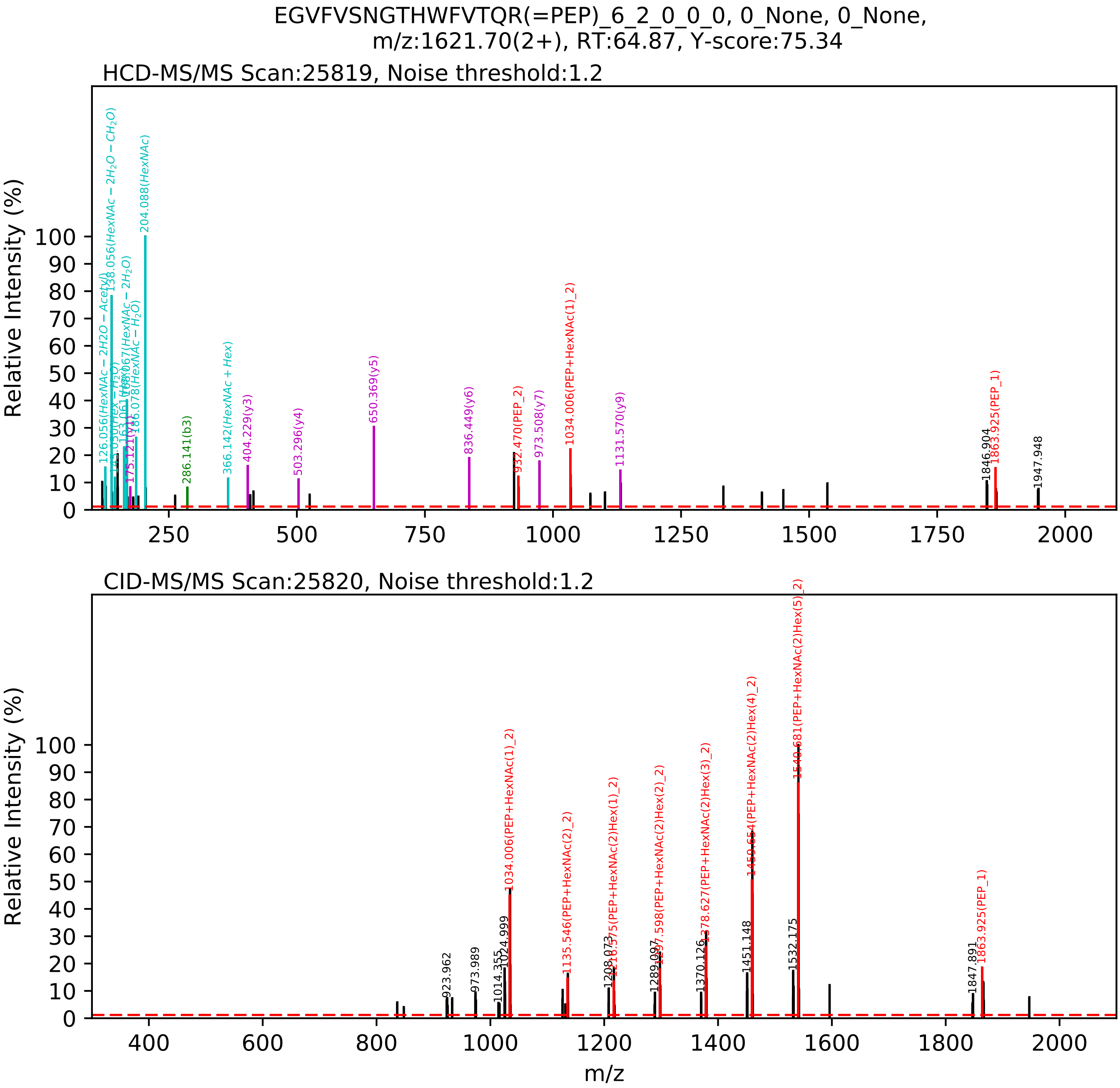

EGVFVSNGTHWFVTQR(=PEP)_6_2_0_0_0, 0_None, 0_None,
m/z:1621.70(2+), RT:64.87, Y-score:75.34
HCD-MS/MS Scan:25819, Noise threshold:1.2
Relative Intensity (%)
126.056(HexNAc − 2H2O − Acetyl)
138.056(HexNAc − 2H₂O − CH₂O)
186.078(HexNAc − H₂O)
204.088(HexNAc)
175.121(y1)
286.141(b3)
366.142(HexNAc + Hex)
404.229(y3)
503.296(y4)
650.369(y5)
836.449(y6)
932.470(PEP_2)
973.508(y7)
1034.006(PEP+HexNAc(1)_2)
1131.570(y9)
1846.904
1863.925(PEP_1)
1947.948
CID-MS/MS Scan:25820, Noise threshold:1.2
Relative Intensity (%)
923.962
973.989
1014.355
1024.999
1034.006(PEP+HexNAc(1)_2)
1135.546(PEP+HexNAc(2)_2)
1208.073
1216.575(PEP+HexNAc(2)Hex(1)_2)
1289.097
1297.598(PEP+HexNAc(2)Hex(2)_2)
1370.126
1378.627(PEP+HexNAc(2)Hex(3)_2)
1451.148
1459.654(PEP+HexNAc(2)Hex(4)_2)
1532.175
1540.681(PEP+HexNAc(2)Hex(5)_2)
1847.891
1863.925(PEP_1)
m/z

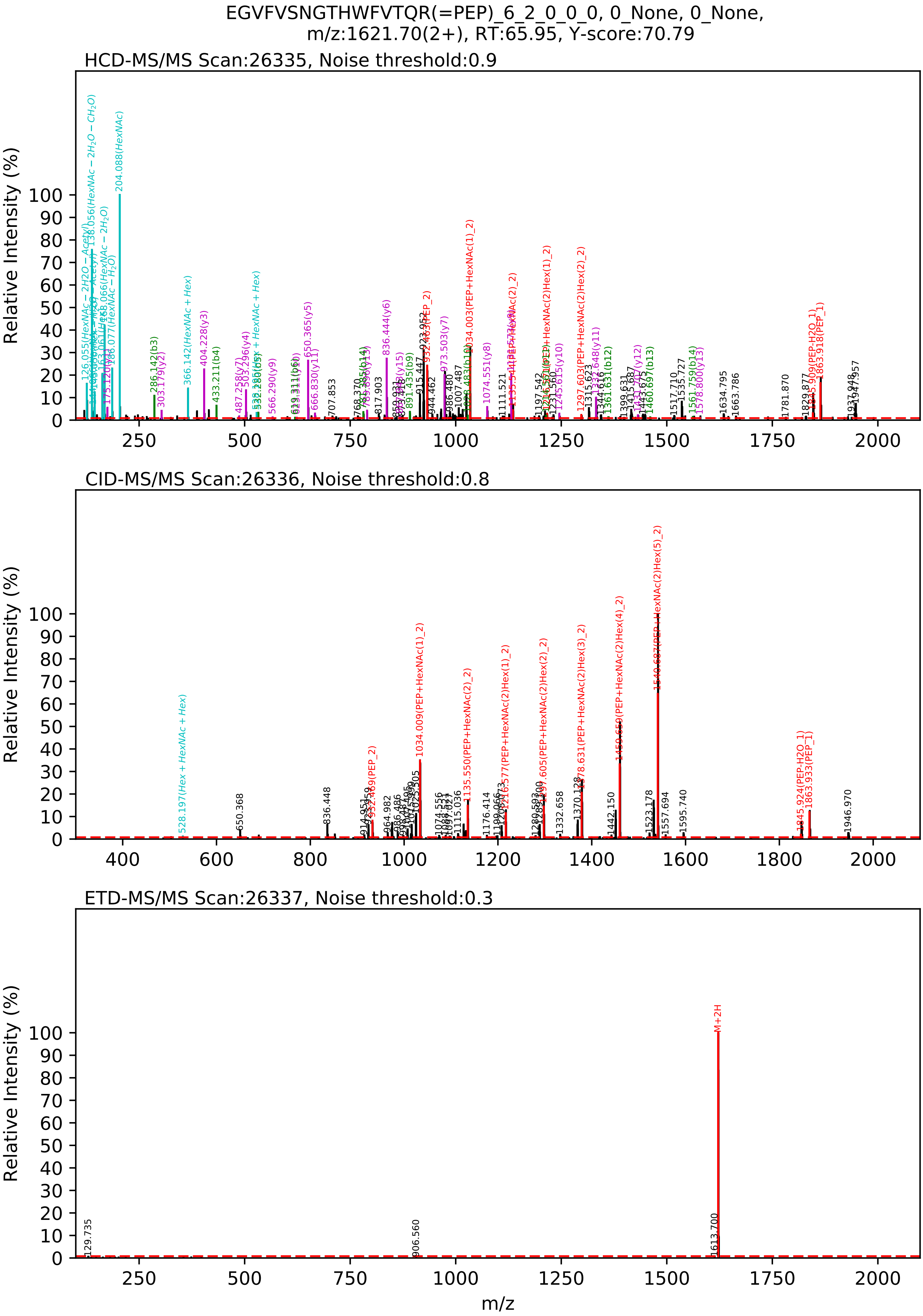
EGVFVSNGTHWFVTQR(=PEP)_6_2_0_0_0, 0_None, 0_None,
m/z:1621.70(2+), RT:65.95, Y-score:70.79
HCD-MS/MS Scan:26335, Noise threshold:0.9
Relative Intensity (%)
204.088(HexNAc)
366.142(HexNAc + Hex)
404.228(y3)
836.444(y6)
973.503(y7)
1034.003(PEP+HexNAc(1)_2)
1297.603(PEP+HexNAc(2)Hex(2)_2)
1863.918(PEP_1)
CID-MS/MS Scan:26336, Noise threshold:0.8
Relative Intensity (%)
528.197(Hex + HexNAc + Hex)
650.368
836.448
1034.009(PEP+HexNAc(1)_2)
1135.550(PEP+HexNAc(2)_2)
1216.577(PEP+HexNAc(2)Hex(1)_2)
1297.605(PEP+HexNAc(2)Hex(2)_2)
1378.631(PEP+HexNAc(2)Hex(3)_2)
1459.659(PEP+HexNAc(2)Hex(4)_2)
1845.924(PEP-H2O_1)
1863.933(PEP_1)
1946.970
ETD-MS/MS Scan:26337, Noise threshold:0.3
Relative Intensity (%)
M+2H
129.735
906.560
1613.700
m/z

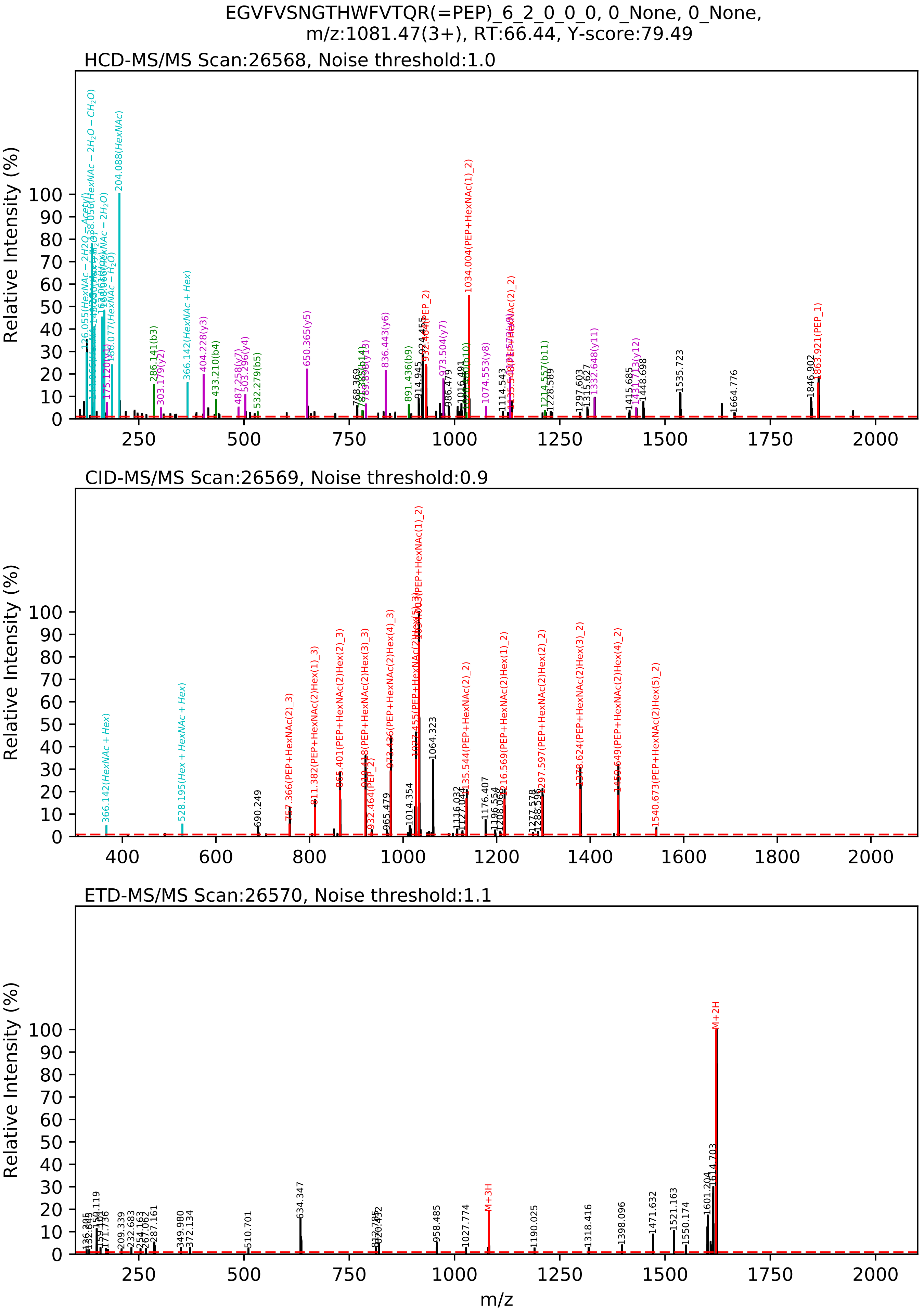

EGVFVSNGTHWFVTQR(=PEP)_6_2_0_0_0, 0_None, 0_None,
m/z:1081.47(3+), RT:66.44, Y-score:79.49
HCD-MS/MS Scan:26568, Noise threshold:1.0
CID-MS/MS Scan:26569, Noise threshold:0.9
ETD-MS/MS Scan:26570, Noise threshold:1.1
Relative Intensity (%)
m/z
204.088(HexNAc)
1034.004(PEP+HexNAc(1)_2)
1863.921(PEP_1)
650.365(y5)
836.443(y6)
973.504(y7)
1074.553(y8)
1332.648(y11)
404.228(y3)
366.142(HexNAc + Hex)
286.141(b3)
433.210(b4)
532.279(b5)
891.436(b9)
1214.557(b11)
1535.723
1846.902
1064.323
1378.624(PEP+HexNAc(2)Hex(3)_2)
1459.649(PEP+HexNAc(2)Hex(4)_2)
1540.673(PEP+HexNAc(2)Hex(5)_2)
1297.597(PEP+HexNAc(2)Hex(2)_2)
1216.569(PEP+HexNAc(2)Hex(1)_2)
1135.544(PEP+HexNAc(2)_2)
865.401(PEP+HexNAc(2)Hex(2)_3)
811.382(PEP+HexNAc(2)Hex(1)_3)
757.366(PEP+HexNAc(2)_3)
973.436(PEP+HexNAc(2)Hex(4)_3)
528.195(Hex + HexNAc + Hex)
M+2H
M+3H
634.347
1614.703
1601.204
1521.163
1471.632

EGVFVSNGTHWFVTQR(=PEP)_6_2_0_0_0, 0_None, 0_None,
m/z:1081.47(3+), RT:65.80, Y-score:83.09

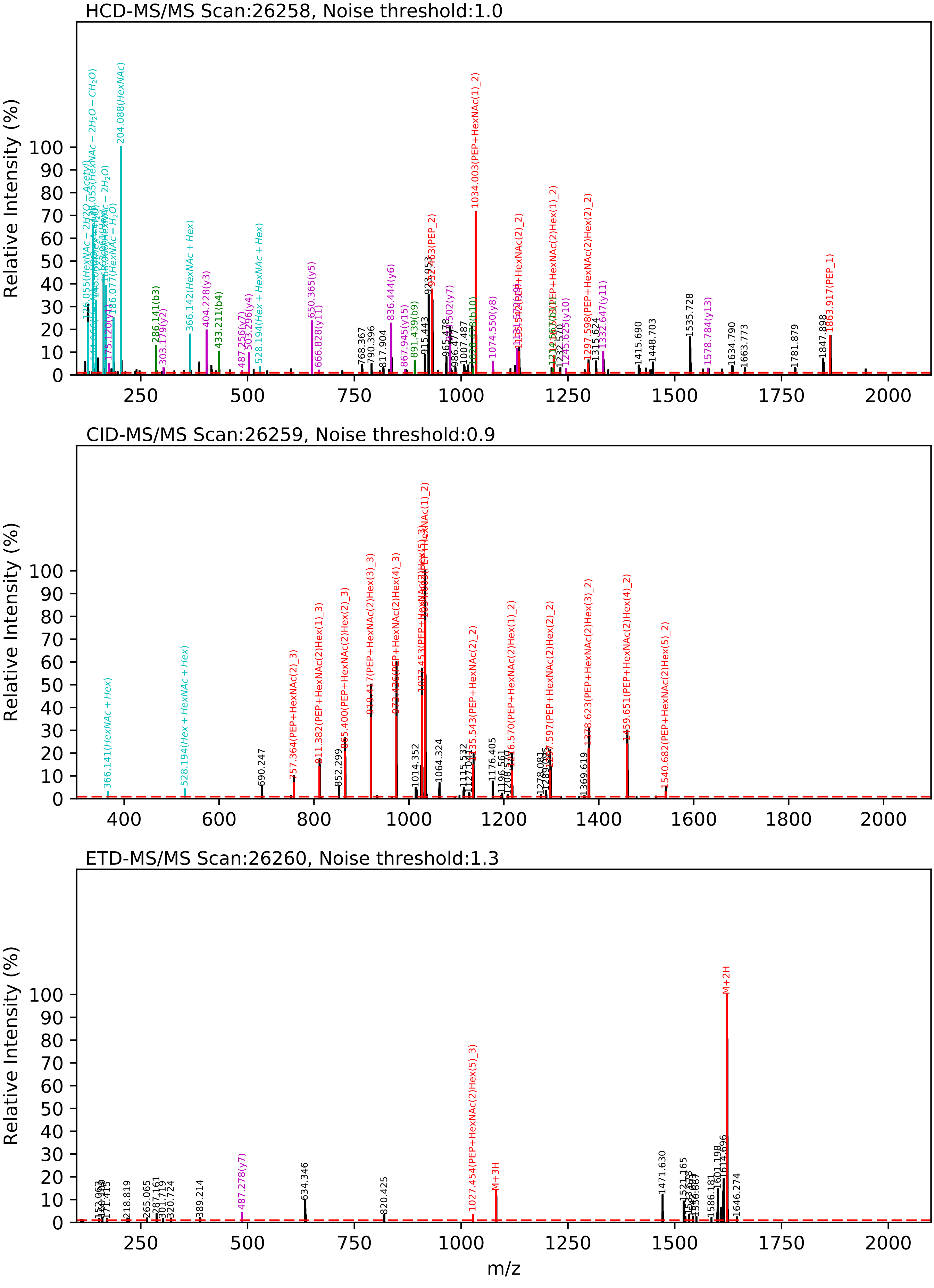

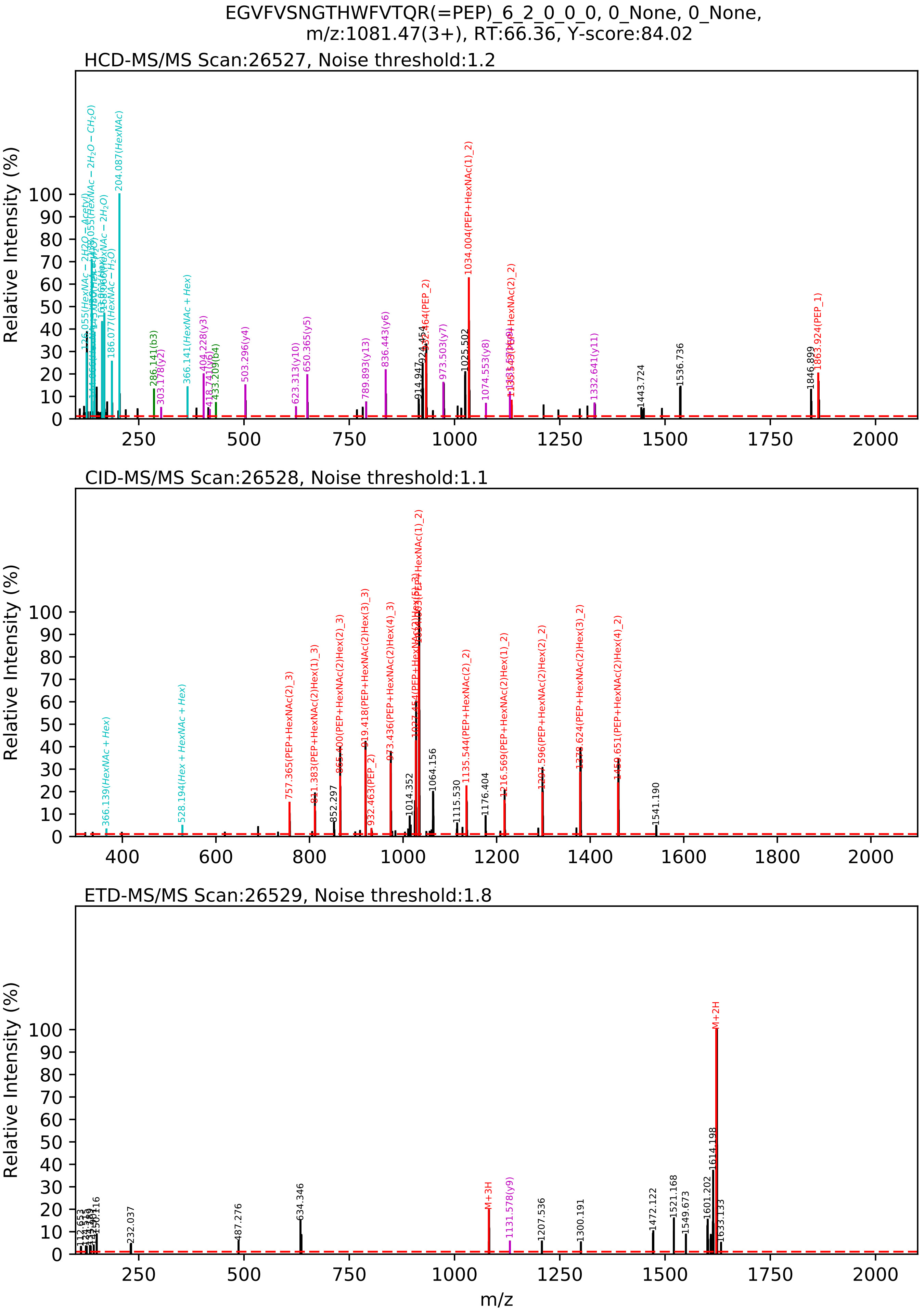
EGVFVSNGTHWFVTQR(=PEP)_6_2_0_0_0, 0_None, 0_None,
m/z:1081.47(3+), RT:66.36, Y-score:84.02
HCD-MS/MS Scan:26527, Noise threshold:1.2
CID-MS/MS Scan:26528, Noise threshold:1.1
ETD-MS/MS Scan:26529, Noise threshold:1.8
Relative Intensity (%)
m/z
204.087(HexNAc)
1034.004(PEP+HexNAc(1)_2)
1863.924(PEP_1)
1536.736
1846.899
1443.724
1332.641(y11)
1074.553(y8)
973.503(y7)
836.443(y6)
789.893(y13)
650.365(y5)
623.313(y10)
503.296(y4)
366.141(HexNAc+Hex)
286.141(b3)
303.178(y2)
1025.502
366.139(HexNAc+Hex)
528.194(Hex+HexNAc+Hex)
757.365(PEP+HexNAc(2)_3)
811.383(PEP+HexNAc(2)Hex(1)_3)
865.400(PEP+HexNAc(2)Hex(2)_3)
919.418(PEP+HexNAc(2)Hex(3)_3)
973.436(PEP+HexNAc(2)Hex(4)_3)
932.463(PEP_2)
852.297
1014.352
1064.156
1115.530
1135.544(PEP+HexNAc(2)_2)
1176.404
1216.569(PEP+HexNAc(2)Hex(1)_2)
1297.596(PEP+HexNAc(2)Hex(2)_2)
1378.624(PEP+HexNAc(2)Hex(3)_2)
1459.651(PEP+HexNAc(2)Hex(4)_2)
1541.190
M+2H
M+3H
1131.578(y9)
232.037
487.276
634.346
1207.536
1300.191
1472.122
1521.168
1549.673
1601.202
1614.198
1633.133

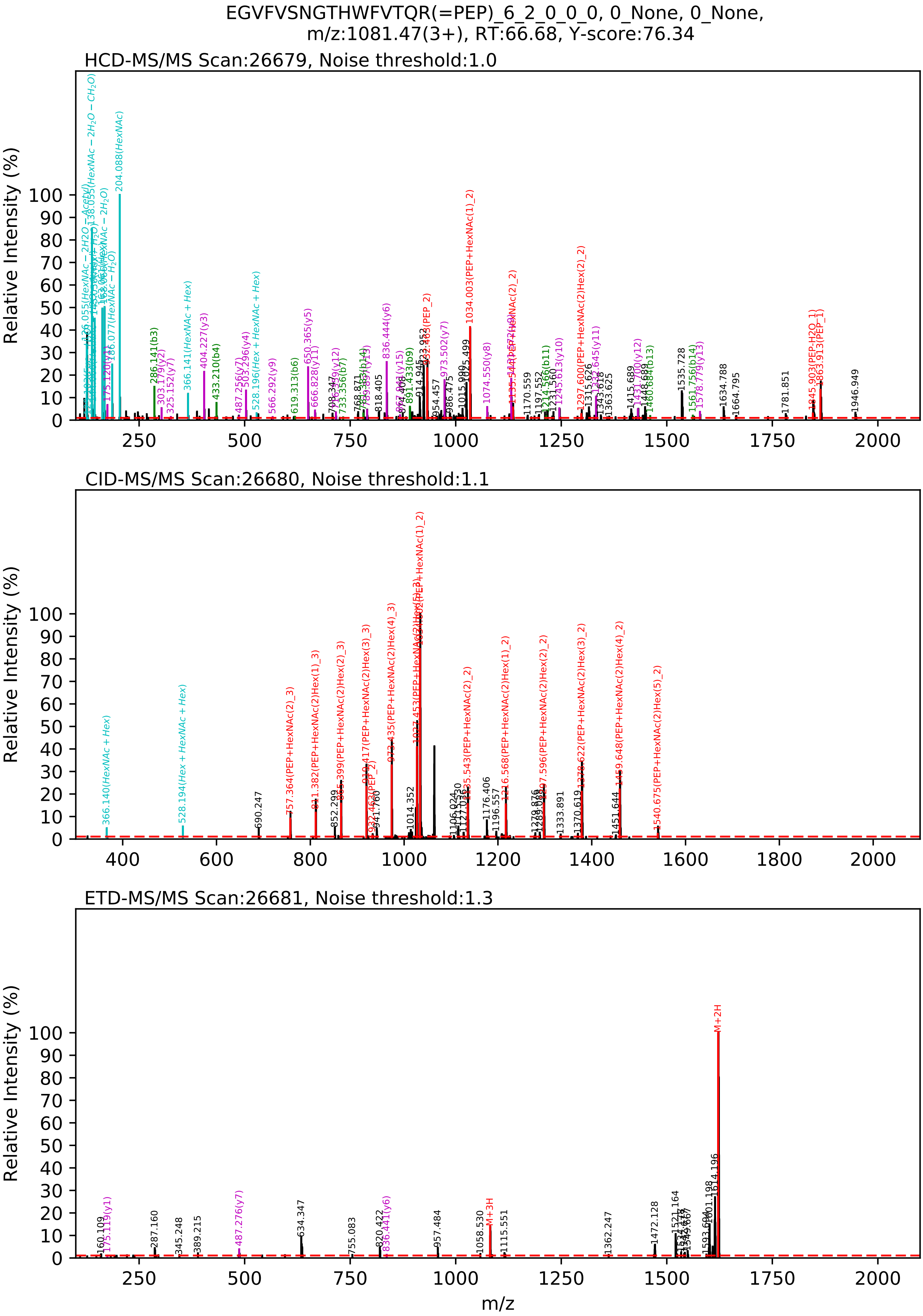

EGVFVSNGTHWFVTQR(=PEP)_6_2_0_0_0, 0_None, 0_None,
m/z:1081.47(3+), RT:66.68, Y-score:76.34
HCD-MS/MS Scan:26679, Noise threshold:1.0
Relative Intensity (%)
204.088(HexNAc)
286.141(b3)
303.179(y2)
325.152(y7)
366.141(HexNAc + Hex)
404.227(y3)
433.210(b4)
487.256(y7)
503.296(y4)
528.196(Hex + HexNAc + Hex)
566.292(y9)
619.313(b6)
650.365(y5)
666.828(y11)
733.356(b7)
768.871
818.405
836.444(y6)
891.433(b9)
923.953
954.457
973.502(y7)
986.475
1015.909
1025.499
1034.003(PEP+HexNAc(1)_2)
1074.550(y8)
1170.559
1197.552
1214.566(b11)
1231.560
1245.613(y10)
1297.600(PEP+HexNAc(2)Hex(2)_2)
1343.618
1363.625
1415.689
1460.684(b13)
1535.728
1561.756(b14)
1578.779(y13)
1634.788
1664.795
1781.851
1845.903(PEP-H2O_1)
1863.913(PEP_1)
1946.949
CID-MS/MS Scan:26680, Noise threshold:1.1
Relative Intensity (%)
366.140(HexNAc + Hex)
528.194(Hex + HexNAc + Hex)
690.247
757.364(PEP+HexNAc(2)_3)
811.382(PEP+HexNAc(2)Hex(1)_3)
852.299
865.399(PEP+HexNAc(2)Hex(2)_3)
919.417(PEP+HexNAc(2)Hex(3)_3)
932.462(PEP_2)
941.760
973.435(PEP+HexNAc(2)Hex(4)_3)
1014.352
1027.453(PEP+HexNAc(2)Hex(5)_3)
1106.024
1135.543(PEP+HexNAc(2)_2)
1176.406
1196.557
1216.568(PEP+HexNAc(2)Hex(1)_2)
1297.596(PEP+HexNAc(2)Hex(2)_2)
1333.891
1370.619
1378.622(PEP+HexNAc(2)Hex(3)_2)
1451.644
1459.648(PEP+HexNAc(2)Hex(4)_2)
1540.675(PEP+HexNAc(2)Hex(5)_2)
ETD-MS/MS Scan:26681, Noise threshold:1.3
Relative Intensity (%)
160.109
175.119(y1)
287.160
345.248
389.215
487.276(y7)
634.347
755.083
820.422
836.441(y6)
957.484
1058.530
M+3H
1115.551
1362.247
1472.128
1521.164
1601.198
1614.196
M+2H
m/z

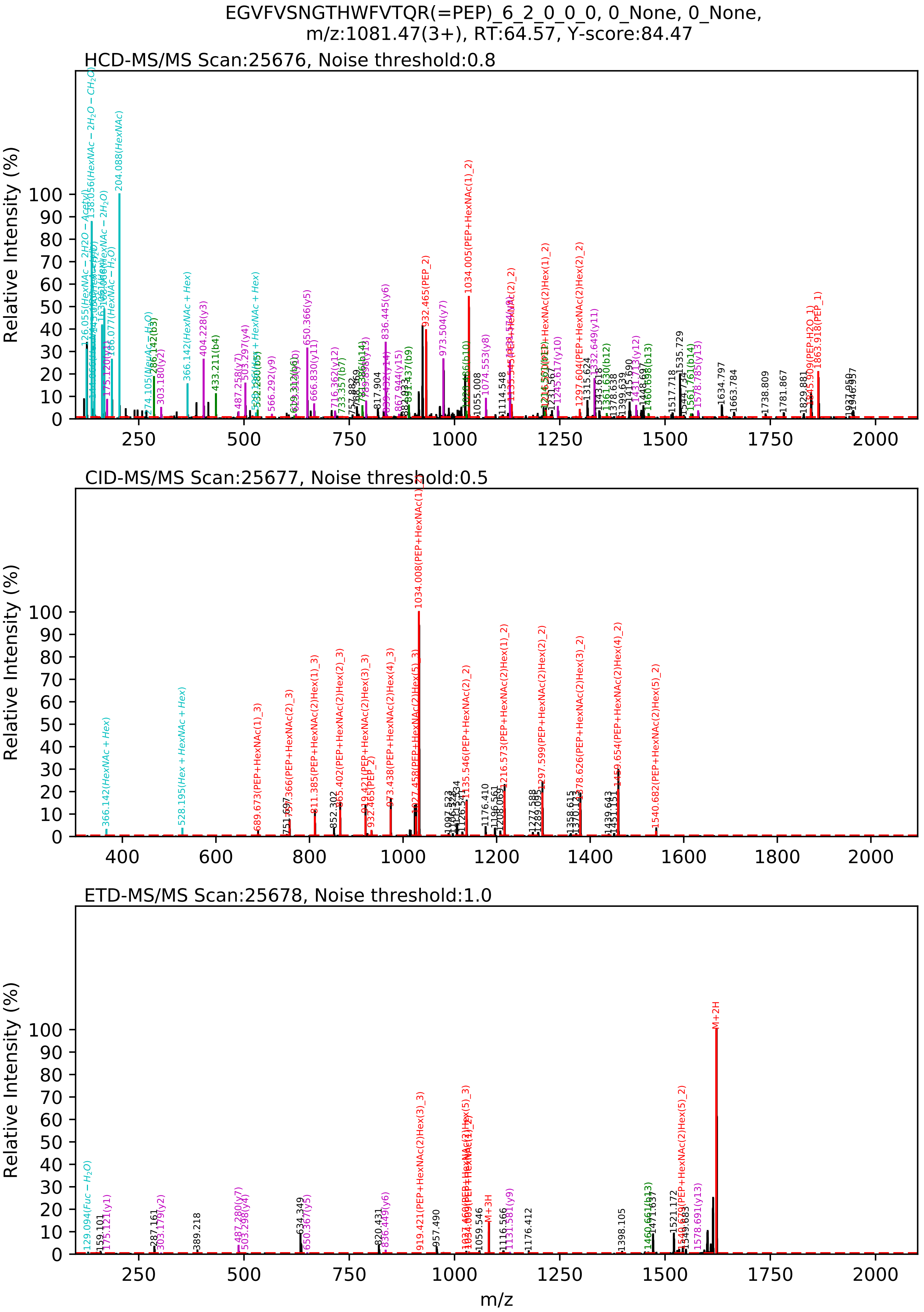

EGVFVSNGTHWFVTQR(=PEP)_6_2_0_0_0, 0_None, 0_None,
m/z:1081.47(3+), RT:64.57, Y-score:84.47
HCD-MS/MS Scan:25676, Noise threshold:0.8
Relative Intensity (%)
204.088(HexNAc)
1034.005(PEP+HexNAc(1)_2)
932.465(PEP_2)
973.504(y7)
836.445(y6)
650.366(y5)
404.228(y3)
366.142(HexNAc + Hex)
1863.918(PEP_1)
CID-MS/MS Scan:25677, Noise threshold:0.5
Relative Intensity (%)
1034.008(PEP+HexNAc(1)_2)
366.142(HexNAc + Hex)
528.195(Hex + HexNAc + Hex)
1540.682(PEP+HexNAc(2)Hex(5)_2)
ETD-MS/MS Scan:25678, Noise threshold:1.0
Relative Intensity (%)
M+2H
M+3H
m/z

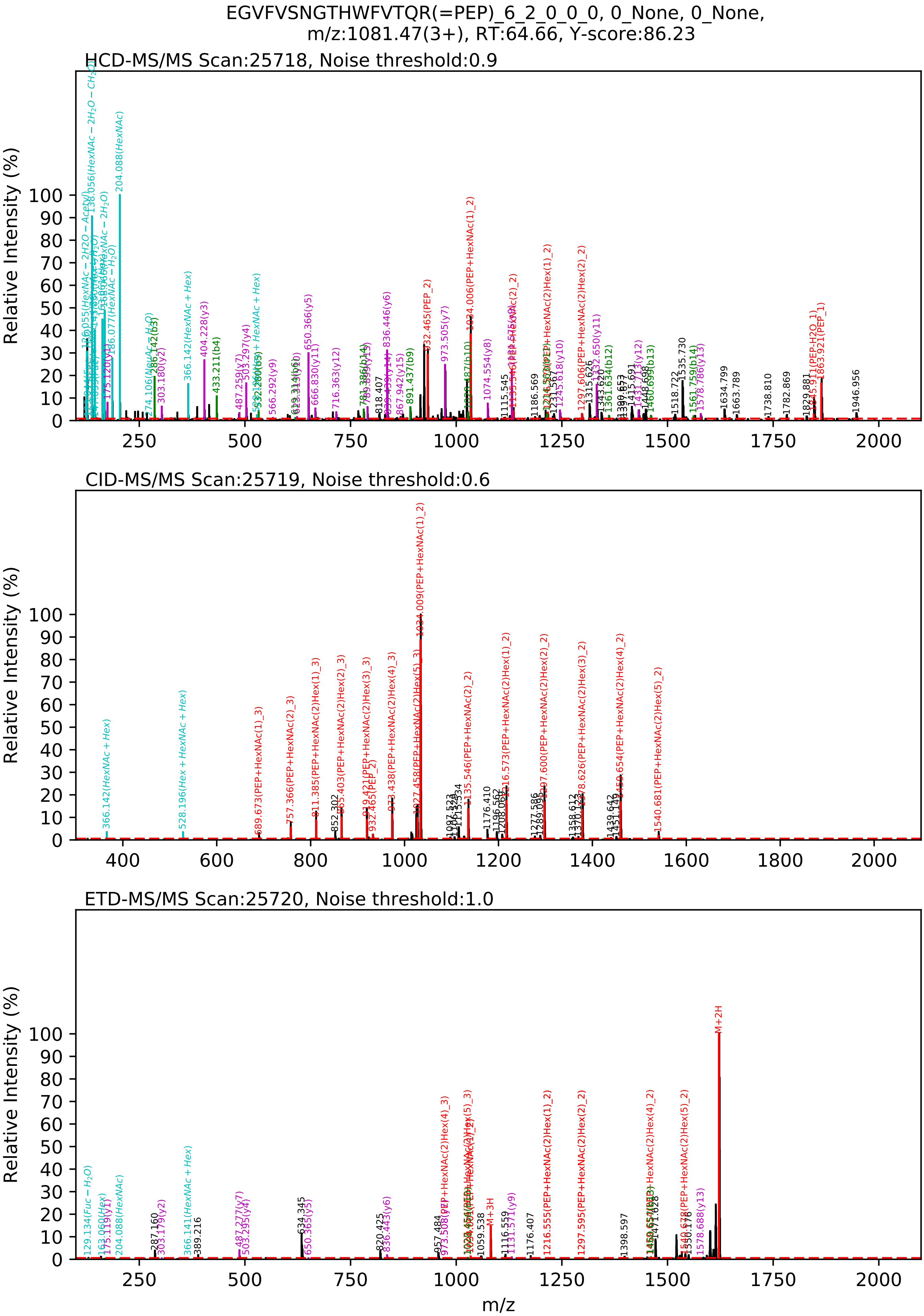
EGVFVSNGTHWFVTQR(=PEP)_6_2_0_0_0, 0_None, 0_None,
m/z:1081.47(3+), RT:64.66, Y-score:86.23
HCD-MS/MS Scan:25718, Noise threshold:0.9
CID-MS/MS Scan:25719, Noise threshold:0.6
ETD-MS/MS Scan:25720, Noise threshold:1.0
Relative Intensity (%)
m/z
204.088(HexNAc)
1034.006(PEP+HexNAc(1)_2)
932.465(PEP_2)
973.505(y7)
1863.921(PEP_1)
1034.009(PEP+HexNAc(1)_2)
1540.681(PEP+HexNAc(2)Hex(5)_2)
M+2H
M+3H

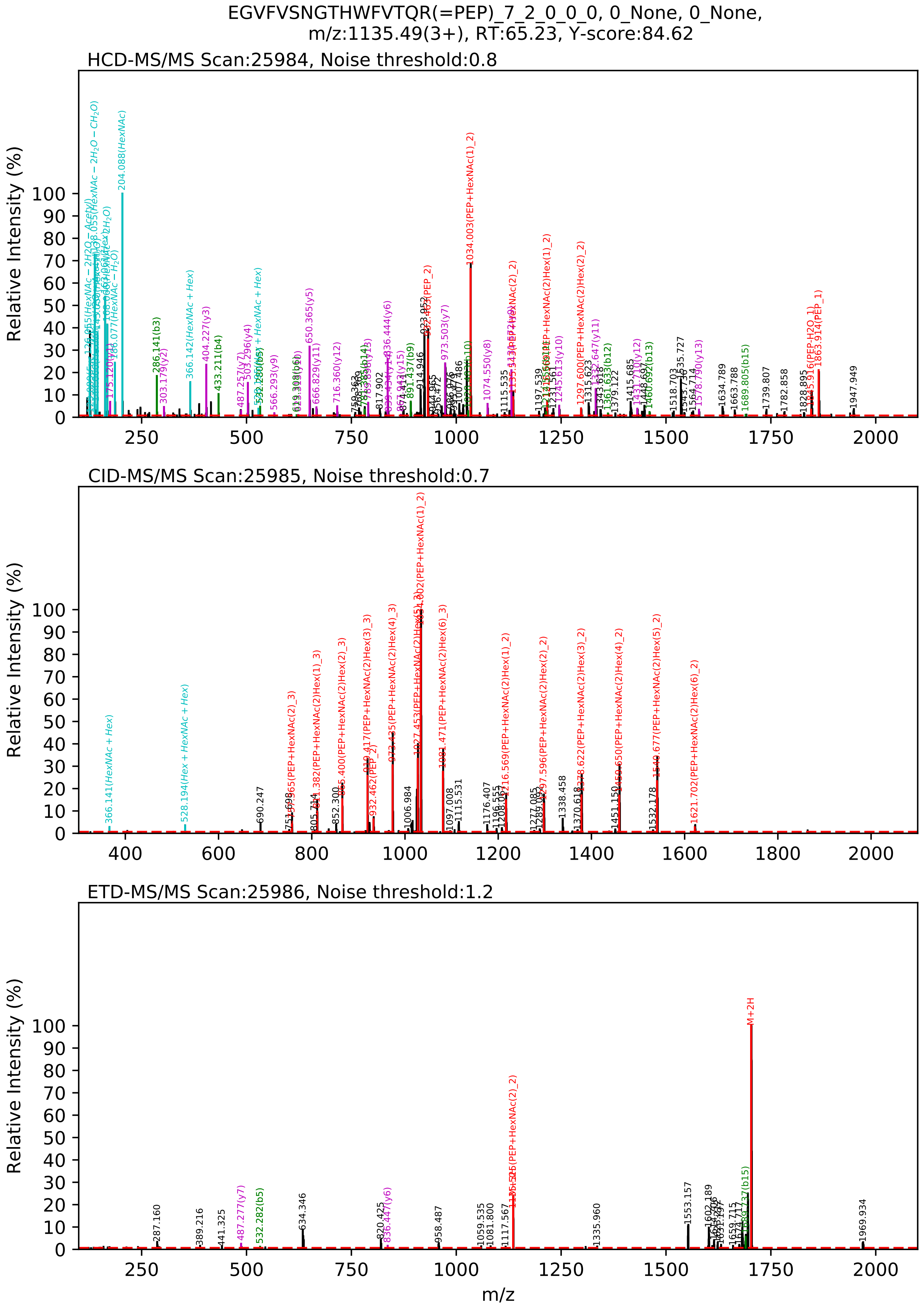

EGVFVSNGTHWFVTQR(=PEP)_7_2_0_0_0, 0_None, 0_None,
m/z:1135.49(3+), RT:65.23, Y-score:84.62
HCD-MS/MS Scan:25984, Noise threshold:0.8
CID-MS/MS Scan:25985, Noise threshold:0.7
ETD-MS/MS Scan:25986, Noise threshold:1.2
Relative Intensity (%)
m/z

EGVFVSNGTHWFVTQR(=PEP)_7_2_0_0_0, 0_None, 0_None,
m/z:1135.49(3+), RT:65.79, Y-score:84.29

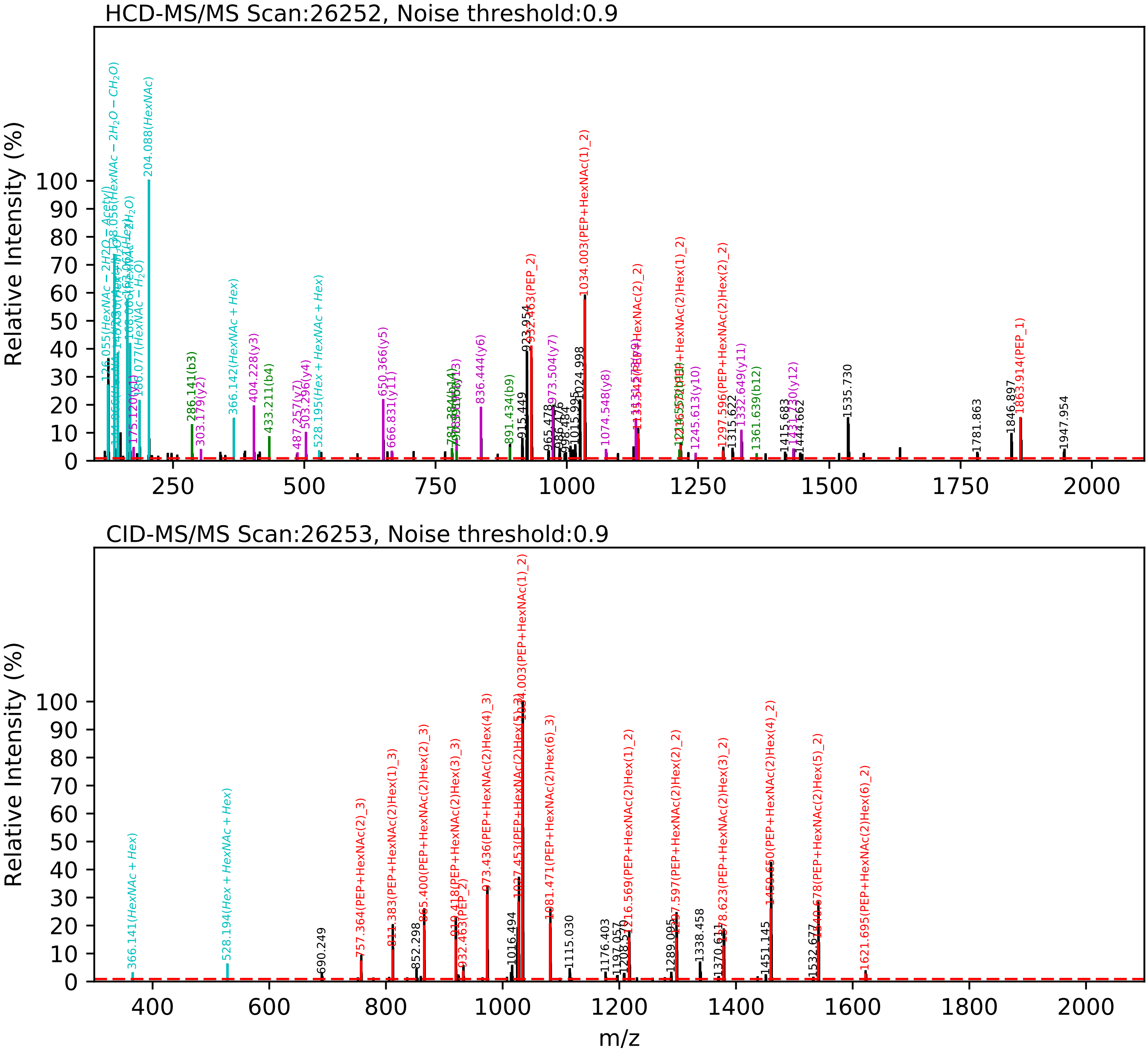

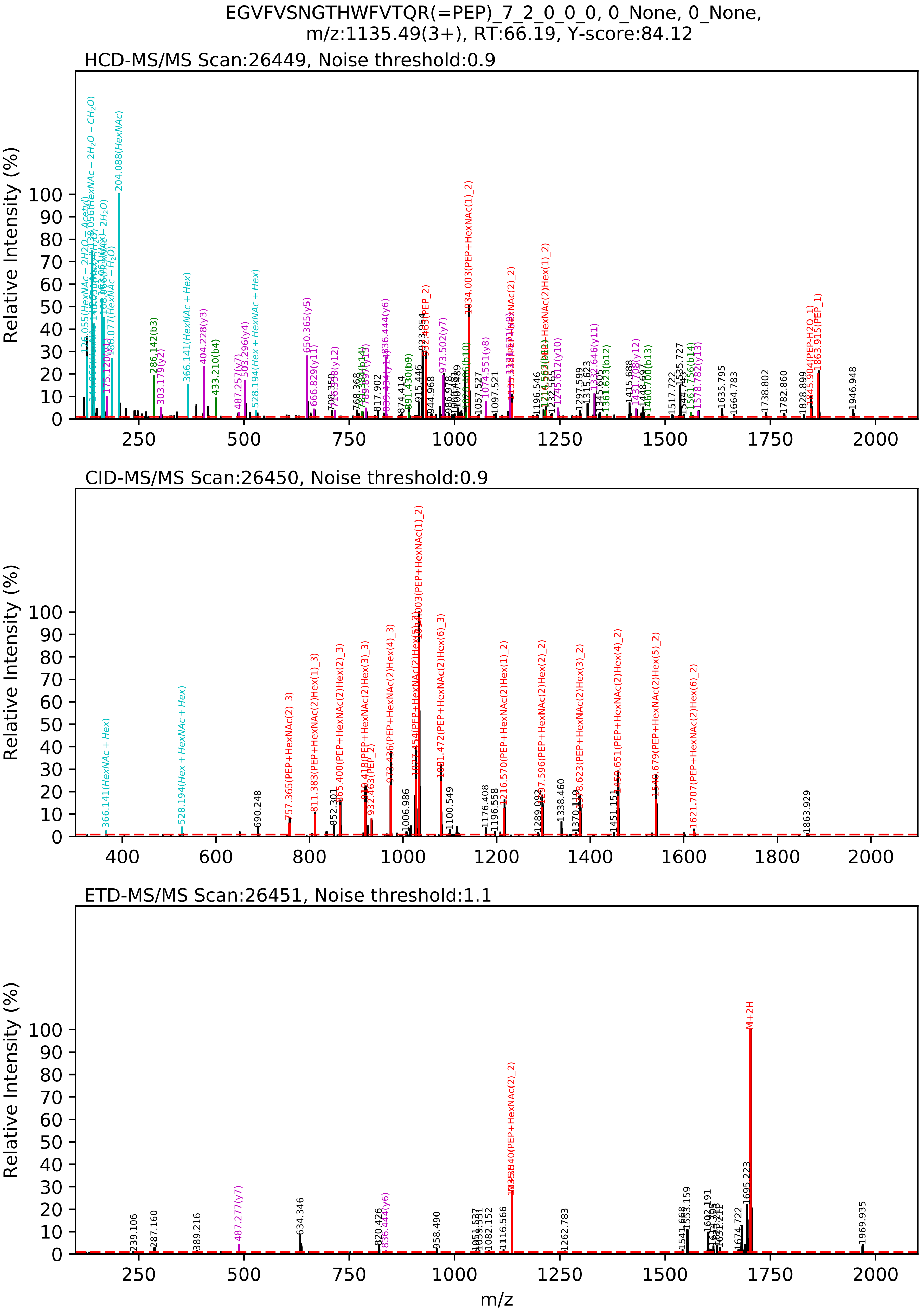
EGVFVSNGTHWFVTQR(=PEP)_7_2_0_0_0, 0_None, 0_None,
m/z:1135.49(3+), RT:66.19, Y-score:84.12
HCD-MS/MS Scan:26449, Noise threshold:0.9
Relative Intensity (%)
204.088(HexNAc)
286.142(b3)
303.179(y2)
366.141(HexNAc + Hex)
404.228(y3)
433.210(b4)
487.257(y7)
503.296(y4)
528.194(Hex + HexNAc + Hex)
650.365(y5)
666.829(y11)
708.350
768.368
817.902
874.414
891.430(b9)
915.446
923.954
944.968
973.502(y7)
1034.003(PEP+HexNAc(1)_2)
1057.527
1074.551(y8)
1097.521
1196.546
1232.565
1245.612(y10)
1297.599
1345.605
1361.623(b12)
1415.688
1460.700(b13)
1517.722
1535.727
1578.782(y13)
1635.795
1664.783
1738.802
1782.860
1828.899
1863.915(PEP_1)
1946.948
CID-MS/MS Scan:26450, Noise threshold:0.9
Relative Intensity (%)
366.141(HexNAc + Hex)
528.194(Hex + HexNAc + Hex)
690.248
757.365(PEP+HexNAc(2)_3)
811.383(PEP+HexNAc(2)Hex(1)_3)
852.301
865.400(PEP+HexNAc(2)Hex(2)_3)
973.436(PEP+HexNAc(2)Hex(4)_3)
1006.986
1081.472(PEP+HexNAc(2)Hex(6)_3)
1100.549
1176.408
1196.558
1216.570(PEP+HexNAc(2)Hex(1)_2)
1289.092
1338.460
1370.119
1451.151
1621.707(PEP+HexNAc(2)Hex(6)_2)
1863.929
ETD-MS/MS Scan:26451, Noise threshold:1.1
Relative Intensity (%)
M+2H
239.106
287.160
389.216
487.277(y7)
634.346
820.426
836.444(y6)
958.490
1082.152
1116.566
1262.783
1541.668
1553.159
1602.191
1674.722
1695.223
1969.935
m/z

EGVFVSNGTHWFVTQR(=PEP)_7_2_0_0_0, 0_None, 0_None,
m/z:1135.49(3+), RT:66.88, Y-score:74.99

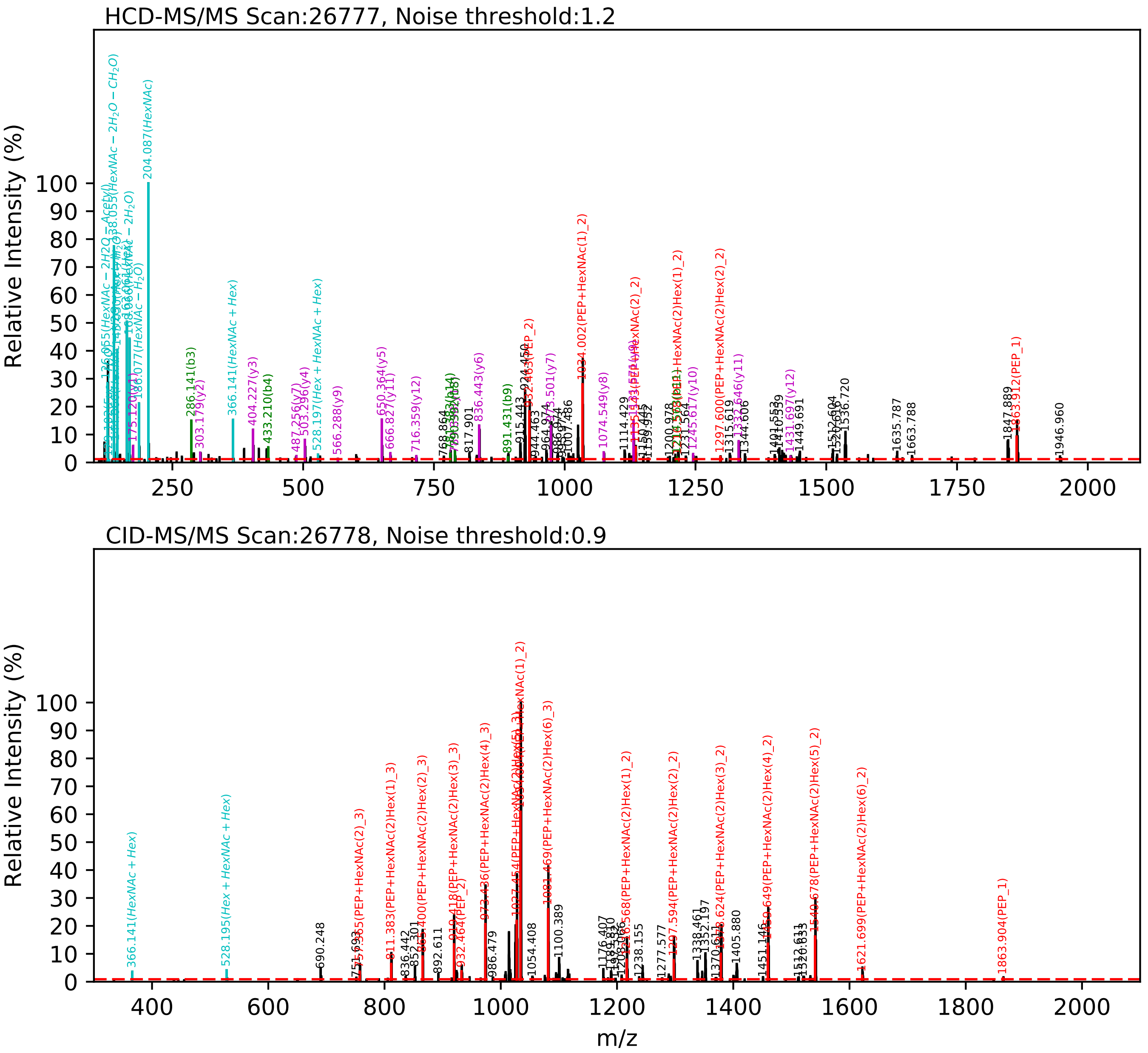

EGVFVSNGTHWFVTQR(=PEP)_7_2_0_0_0, 0_None, 0_None,
m/z:1135.49(3+), RT:67.01, Y-score:82.98

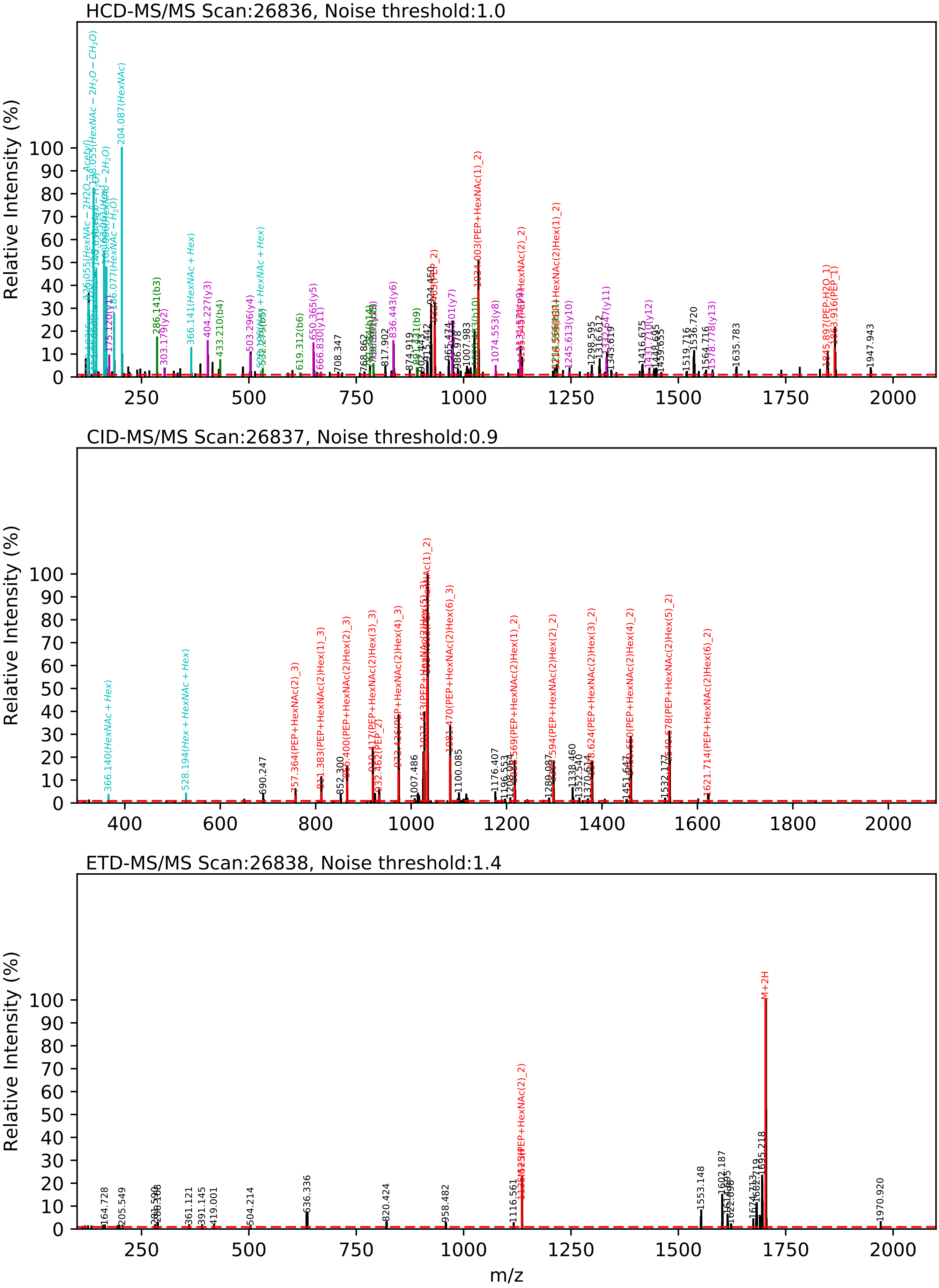

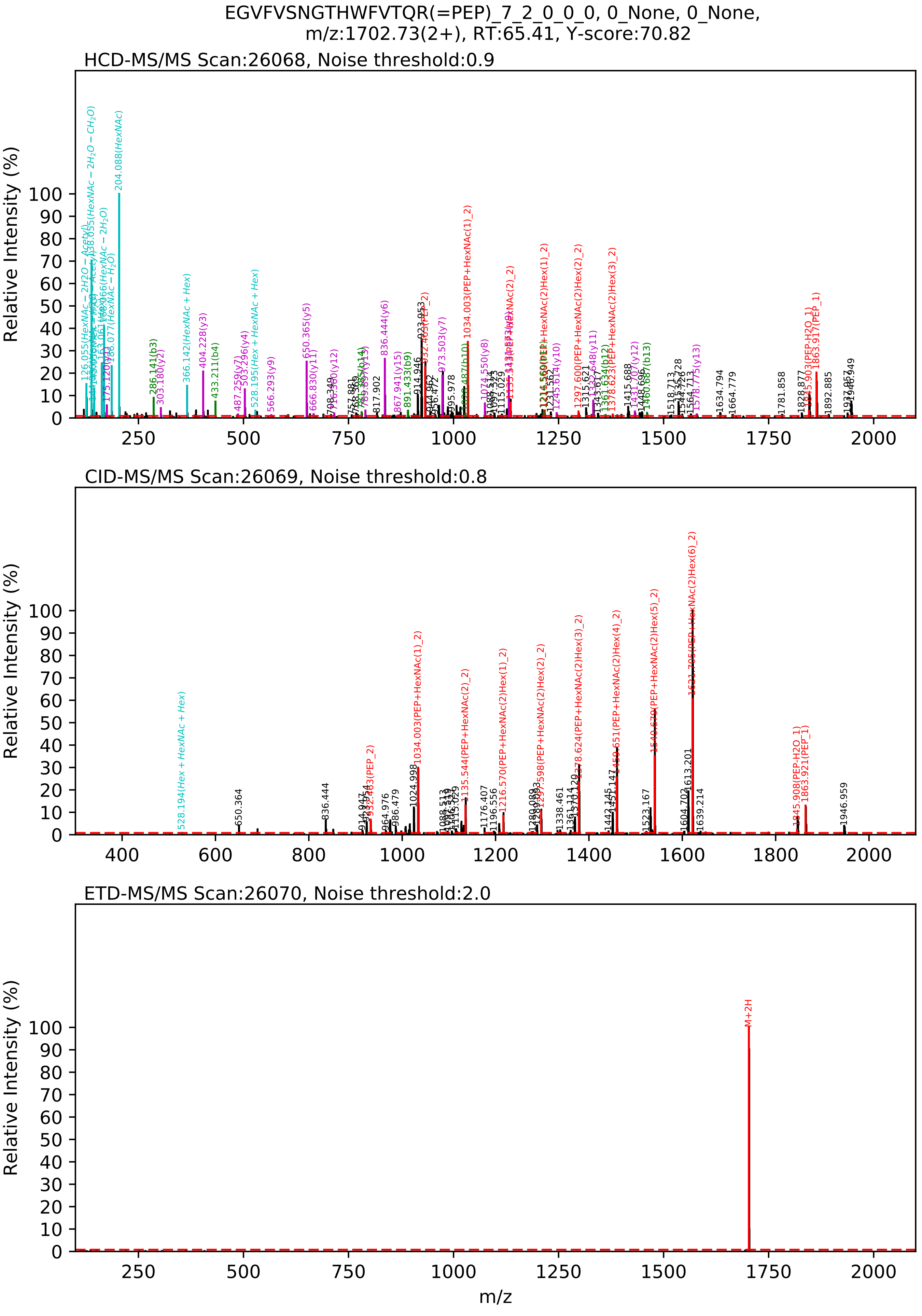

EGVFVSNGTHWFVTQR(=PEP)_7_2_0_0_0, 0_None, 0_None,
m/z:1702.73(2+), RT:65.41, Y-score:70.82
HCD-MS/MS Scan:26068, Noise threshold:0.9
CID-MS/MS Scan:26069, Noise threshold:0.8
ETD-MS/MS Scan:26070, Noise threshold:2.0
Relative Intensity (%)
m/z
204.088(HexNAc)
1034.003(PEP+HexNAc(1)_2)
1863.917(PEP_1)
1621.705(PEP+HexNAc(2)Hex(6)_2)
1540.670(PEP+HexNAc(2)Hex(5)_2)
1863.921(PEP_1)
M+2H

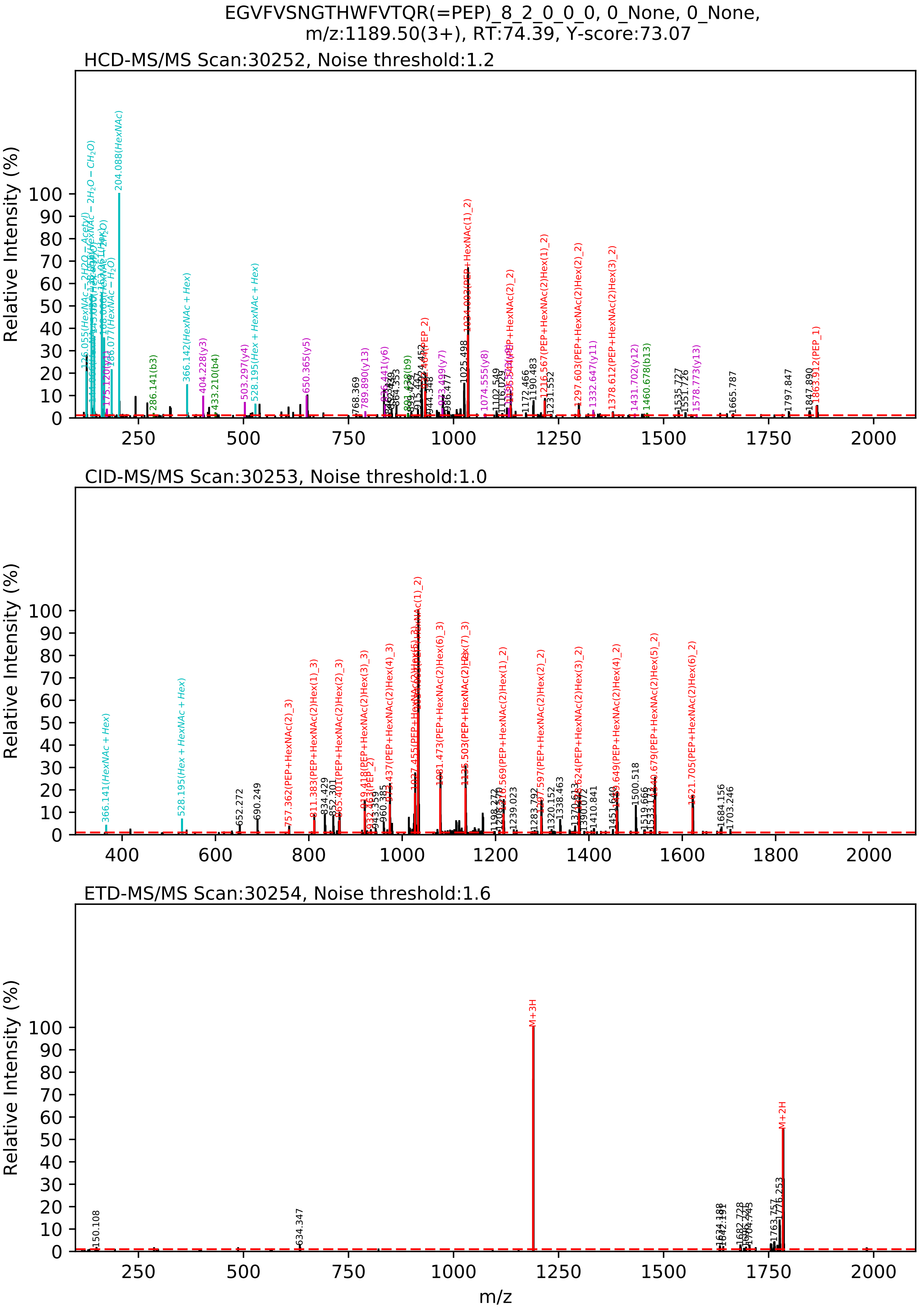

EGVFVSNGTHWFVTQR(=PEP)_8_2_0_0_0, 0_None, 0_None,
m/z:1189.50(3+), RT:74.39, Y-score:73.07
HCD-MS/MS Scan:30252, Noise threshold:1.2
Relative Intensity (%)
204.088(HexNAc)
366.142(HexNAc + Hex)
528.195(Hex + HexNAc + Hex)
286.141(b3)
404.228(y3)
433.210(b4)
503.297(y4)
650.365(y5)
768.369
789.890(y13)
1025.498
1034.003(PEP+HexNAc(1)_2)
1074.555(y8)
1172.466
1190.483
1216.567(PEP+HexNAc(2)Hex(1)_2)
1231.552
1297.603(PEP+HexNAc(2)Hex(2)_2)
1332.647(y11)
1378.612(PEP+HexNAc(2)Hex(3)_2)
1431.702(y12)
1460.678(b13)
1535.727
1551.726
1578.773(y13)
1665.787
1797.847
1847.890
1863.912(PEP_1)
CID-MS/MS Scan:30253, Noise threshold:1.0
366.141(HexNAc + Hex)
528.195(Hex + HexNAc + Hex)
652.272
690.249
757.362(PEP+HexNAc(2)_3)
811.383(PEP+HexNAc(2)Hex(1)_3)
834.429
865.401(PEP+HexNAc(2)Hex(2)_3)
960.385
1081.473(PEP+HexNAc(2)Hex(6)_3)
1135.503(PEP+HexNAc(2)Hex(7)_3)
1239.023
1283.792
1320.152
1338.463
1370.613
1410.841
1451.649
1500.518
1519.666
1621.705(PEP+HexNAc(2)Hex(6)_2)
1684.156
1703.246
ETD-MS/MS Scan:30254, Noise threshold:1.6
M+3H
M+2H
150.108
634.347
1682.728
1763.757
1776.253
m/z

EGVFVSNGTHWFVTQR(=PEP)_8_2_0_0_0, 0_None, 0_None,
m/z:1189.50(3+), RT:74.53, Y-score:66.39

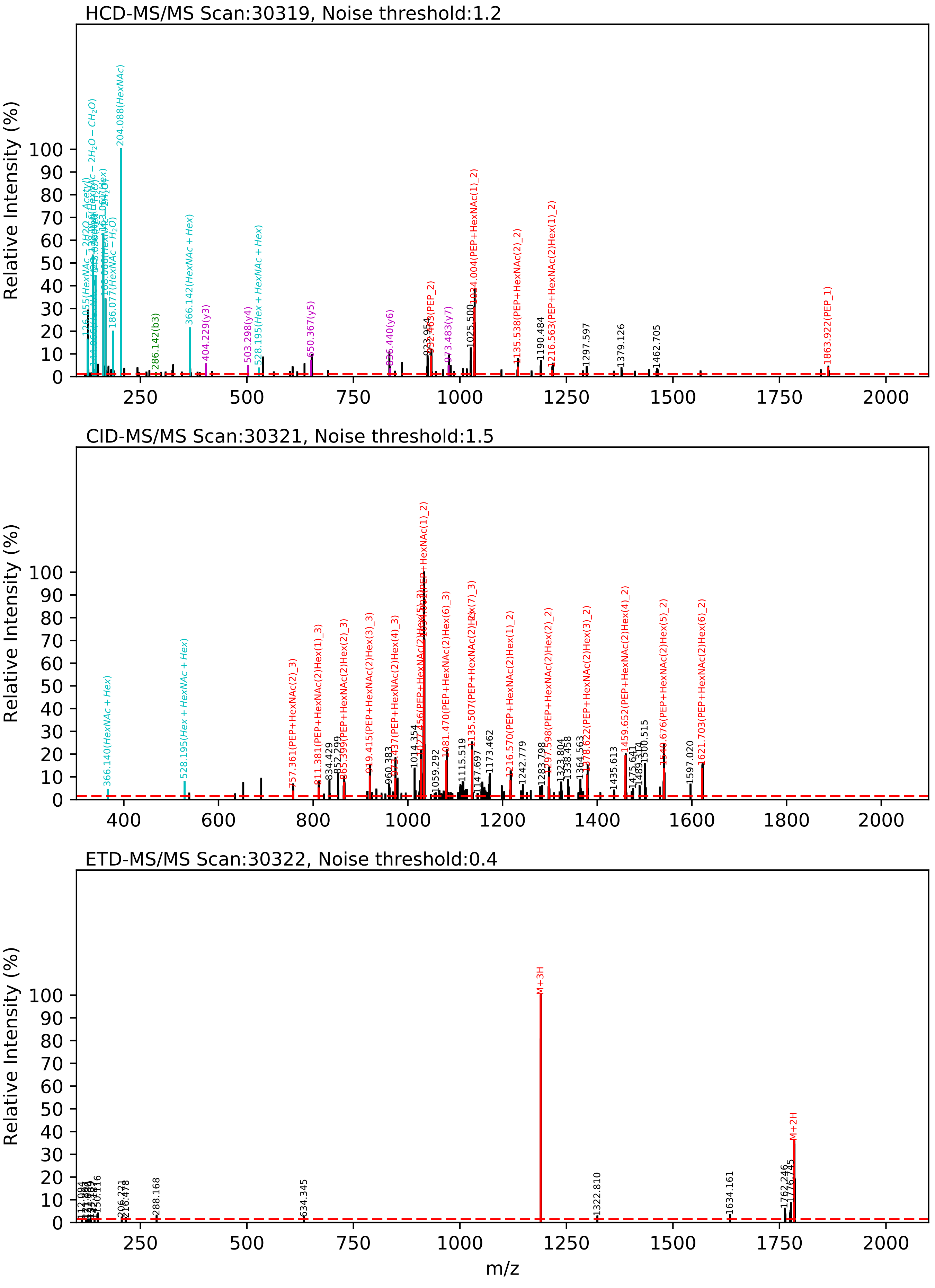

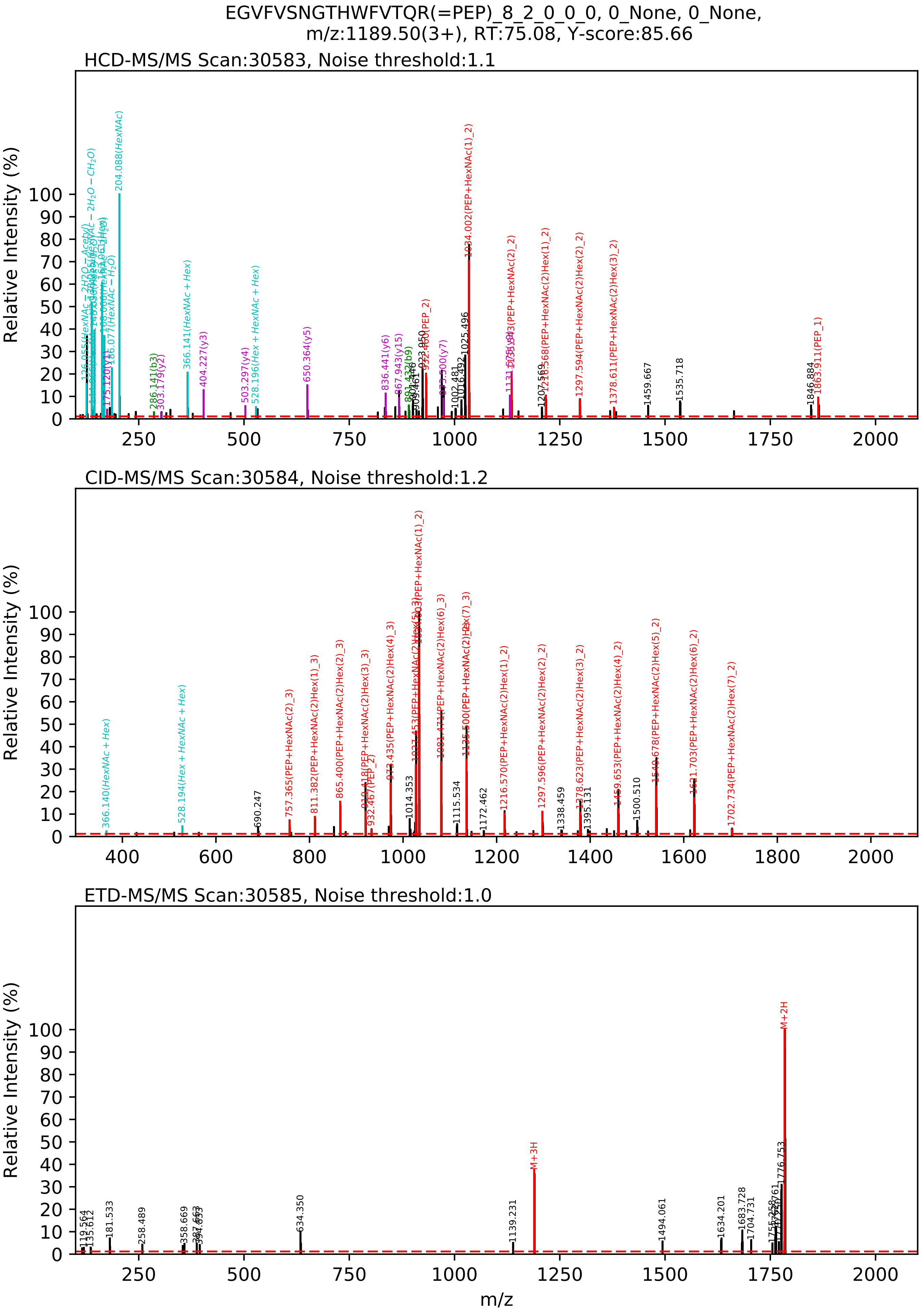
EGVFVSNGTHWFVTQR(=PEP)_8_2_0_0_0, 0_None, 0_None,
m/z:1189.50(3+), RT:75.08, Y-score:85.66
HCD-MS/MS Scan:30583, Noise threshold:1.1
Relative Intensity (%)
204.088(HexNAc)
366.141(HexNAc + Hex)
528.196(Hex + HexNAc + Hex)
286.141(b3)
303.179(y2)
404.227(y3)
503.297(y4)
650.364(y5)
836.441(y6)
867.943(y15)
891.432(b9)
923.950
932.460(PEP_2)
1002.481
1016.492
1025.496
1034.002(PEP+HexNAc(1)_2)
1135.093(PEP+HexNAc(2)_2)
1207.569
1216.568(PEP+HexNAc(2)Hex(1)_2)
1297.594(PEP+HexNAc(2)Hex(2)_2)
1378.611(PEP+HexNAc(2)Hex(3)_2)
1459.667
1535.718
1846.884
1863.911(PEP_1)
CID-MS/MS Scan:30584, Noise threshold:1.2
366.140(HexNAc + Hex)
528.194(Hex + HexNAc + Hex)
690.247
757.365(PEP+HexNAc(2)_3)
811.382(PEP+HexNAc(2)Hex(1)_3)
865.400(PEP+HexNAc(2)Hex(2)_3)
919.418(PEP+HexNAc(2)Hex(3)_3)
932.467(PEP_2)
973.435(PEP+HexNAc(2)Hex(4)_3)
1014.353
1034.003(PEP+HexNAc(1)_2)
1081.471(PEP+HexNAc(2)Hex(6)_3)
1115.534
1135.500(PEP+HexNAc(2)Hex(7)_3)
1172.462
1216.570(PEP+HexNAc(2)Hex(1)_2)
1297.596(PEP+HexNAc(2)Hex(2)_2)
1338.459
1378.623(PEP+HexNAc(2)Hex(3)_2)
1395.131
1459.653(PEP+HexNAc(2)Hex(4)_2)
1500.510
1540.678(PEP+HexNAc(2)Hex(5)_2)
1621.703(PEP+HexNAc(2)Hex(6)_2)
1702.734(PEP+HexNAc(2)Hex(7)_2)
ETD-MS/MS Scan:30585, Noise threshold:1.0
119.564
135.612
181.533
258.489
358.669
634.350
M+3H
1139.231
1494.061
1634.201
1683.728
1704.731
1776.753
M+2H
m/z

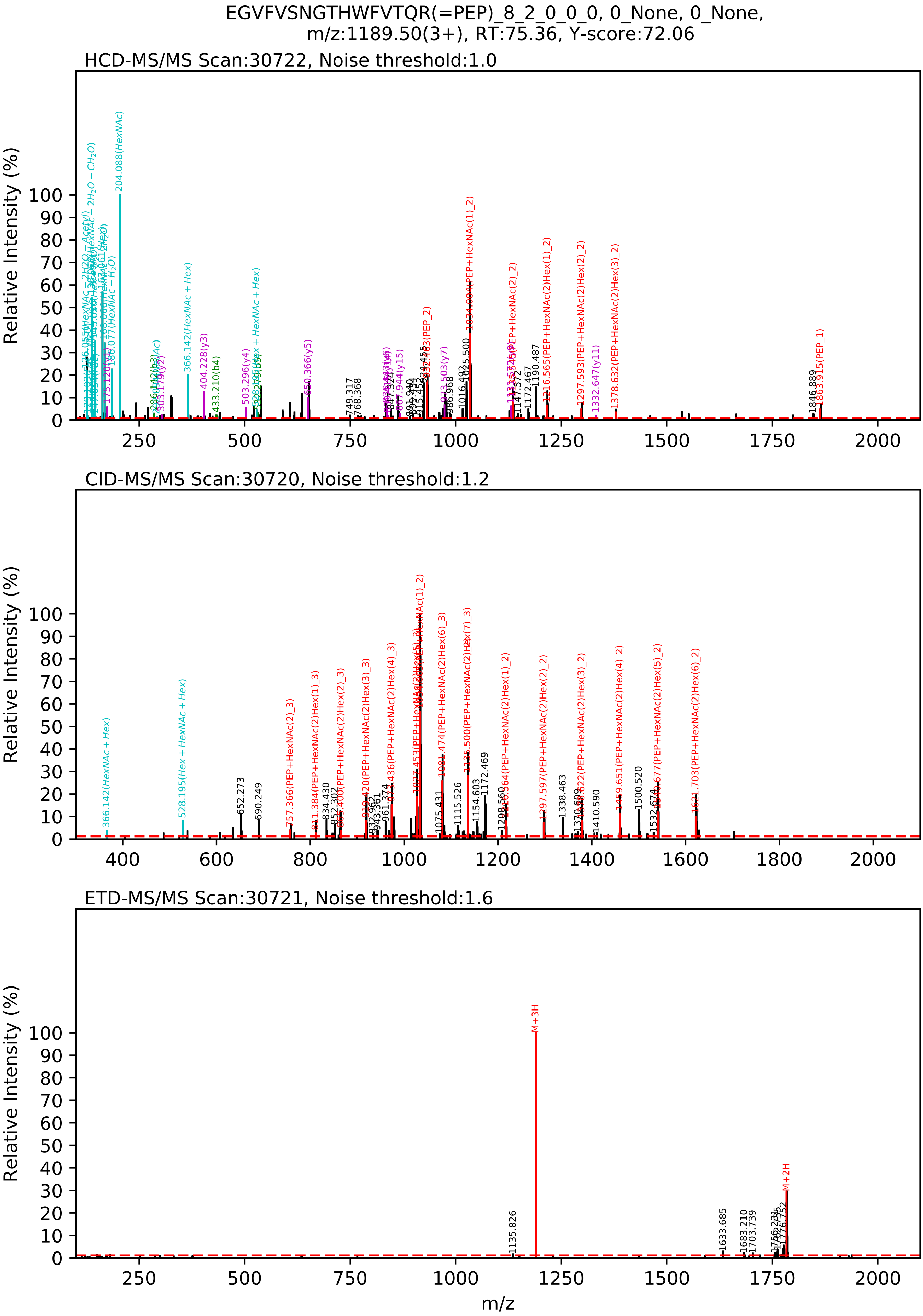
EGVFVSNGTHWFVTQR(=PEP)_8_2_0_0_0, 0_None, 0_None,
m/z:1189.50(3+), RT:75.36, Y-score:72.06
HCD-MS/MS Scan:30722, Noise threshold:1.0
Relative Intensity (%)
204.088(HexNAc)
366.142(HexNAc + Hex)
404.228(y3)
433.210(b4)
503.296(y4)
650.366(y5)
749.317
768.368
932.453(PEP_2)
973.503(y7)
986.968
1016.492
1025.500
1034.004(PEP+HexNAc(1)_2)
1147.572
1172.467
1190.487
1216.565(PEP+HexNAc(2)Hex(1)_2)
1297.593(PEP+HexNAc(2)Hex(2)_2)
1332.647(y11)
1378.632(PEP+HexNAc(2)Hex(3)_2)
1846.889
1863.915(PEP_1)
CID-MS/MS Scan:30720, Noise threshold:1.2
366.142(HexNAc + Hex)
528.195(Hex + HexNAc + Hex)
652.273
690.249
757.366(PEP+HexNAc(2)_3)
811.384(PEP+HexNAc(2)Hex(1)_3)
834.430
865.400(PEP+HexNAc(2)Hex(2)_3)
919.420(PEP+HexNAc(2)Hex(3)_3)
961.374
973.436(PEP+HexNAc(2)Hex(4)_3)
1075.431
1081.474(PEP+HexNAc(2)Hex(6)_3)
1115.526
1135.500(PEP+HexNAc(2)Hex(7)_3)
1154.603
1172.469
1208.560
1297.597(PEP+HexNAc(2)Hex(2)_2)
1338.463
1370.609
1410.590
1459.651(PEP+HexNAc(2)Hex(4)_2)
1500.520
1532.674
1540.677(PEP+HexNAc(2)Hex(5)_2)
1621.703(PEP+HexNAc(2)Hex(6)_2)
ETD-MS/MS Scan:30721, Noise threshold:1.6
M+3H
1135.826
1633.685
1683.210
1703.739
M+2H
m/z

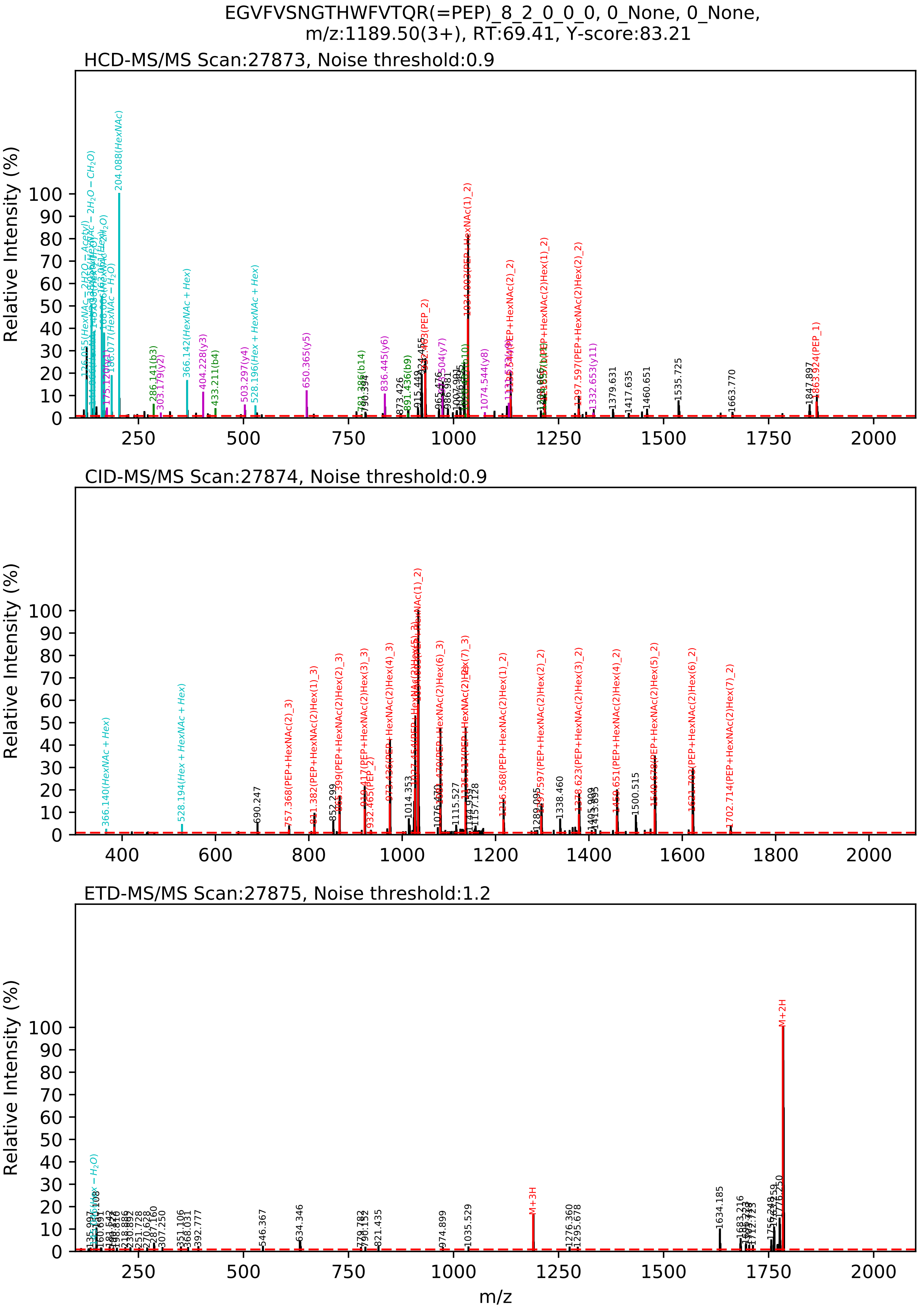
EGVFVSNGTHWFVTQR(=PEP)_8_2_0_0_0, 0_None, 0_None,
m/z:1189.50(3+), RT:69.41, Y-score:83.21
HCD-MS/MS Scan:27873, Noise threshold:0.9
CID-MS/MS Scan:27874, Noise threshold:0.9
ETD-MS/MS Scan:27875, Noise threshold:1.2
Relative Intensity (%)
m/z
204.088(HexNAc)
366.142(HexNAc + Hex)
528.196(Hex + HexNAc + Hex)
650.365(y5)
836.445(y6)
1034.903(PEP+HexNAc(1)_2)
1297.597(PEP+HexNAc(2)Hex(2)_2)
1863.924(PEP_1)
1535.725
M+2H
M+3H
1634.185

EGVFVSNGTHWFVTQR(=PEP)_8_2_0_0_0, 0_None, 0_None,
m/z:1189.50(3+), RT:69.73, Y-score:81.42

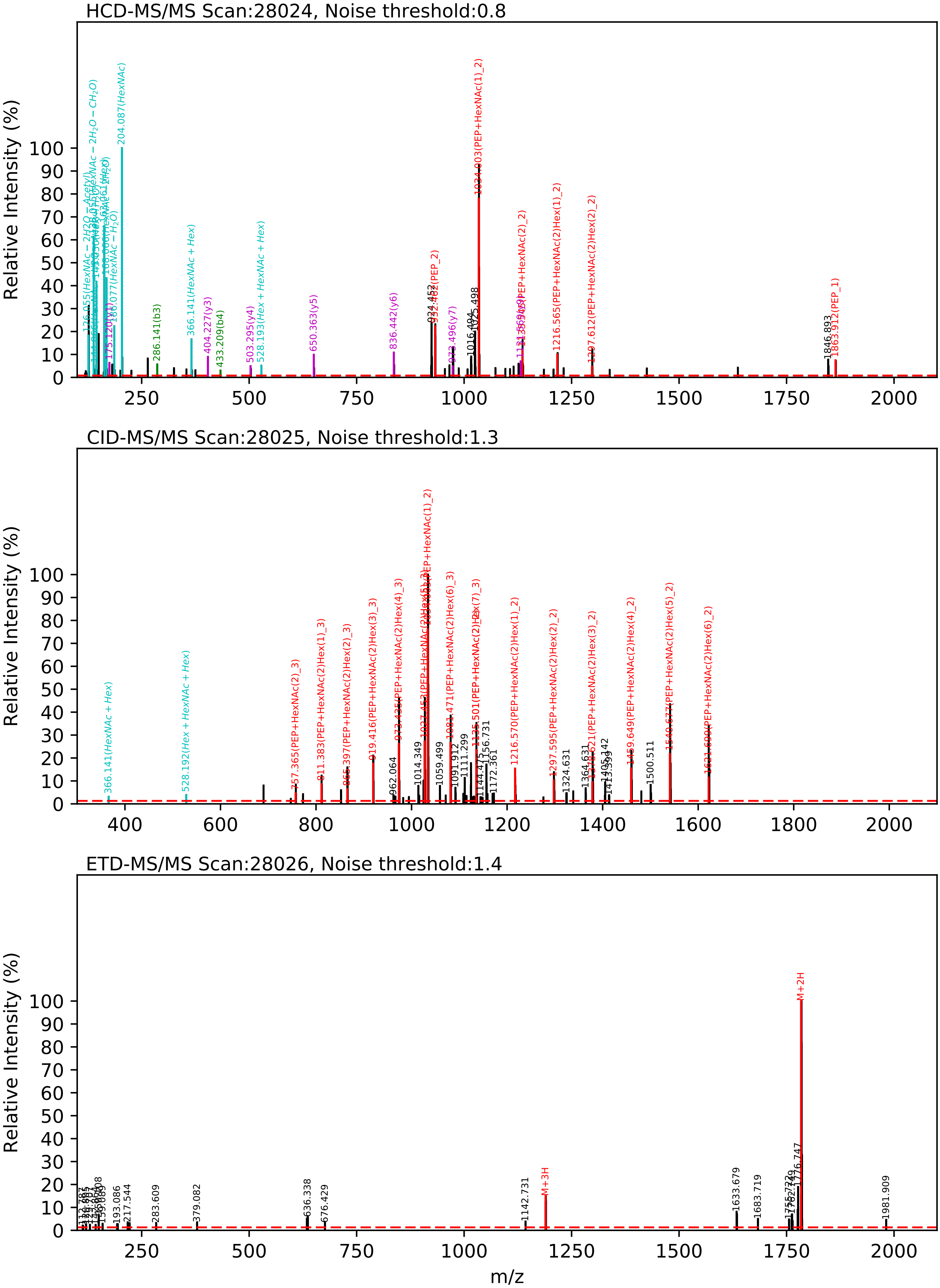

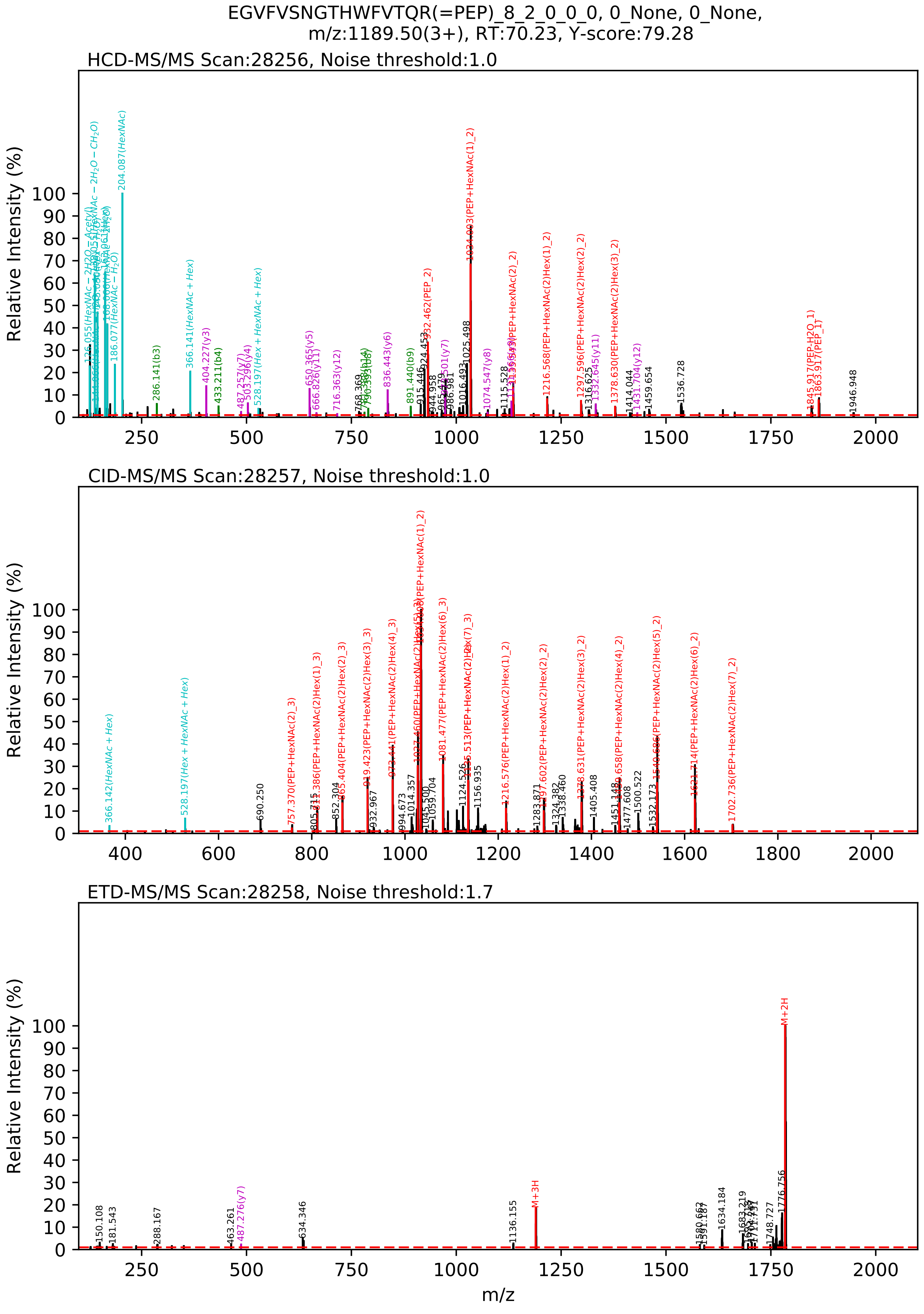

EGVFVSNGTHWFVTQR(=PEP)_8_2_0_0_0, 0_None, 0_None,
m/z:1189.50(3+), RT:70.23, Y-score:79.28
HCD-MS/MS Scan:28256, Noise threshold:1.0
CID-MS/MS Scan:28257, Noise threshold:1.0
ETD-MS/MS Scan:28258, Noise threshold:1.7
Relative Intensity (%)
m/z

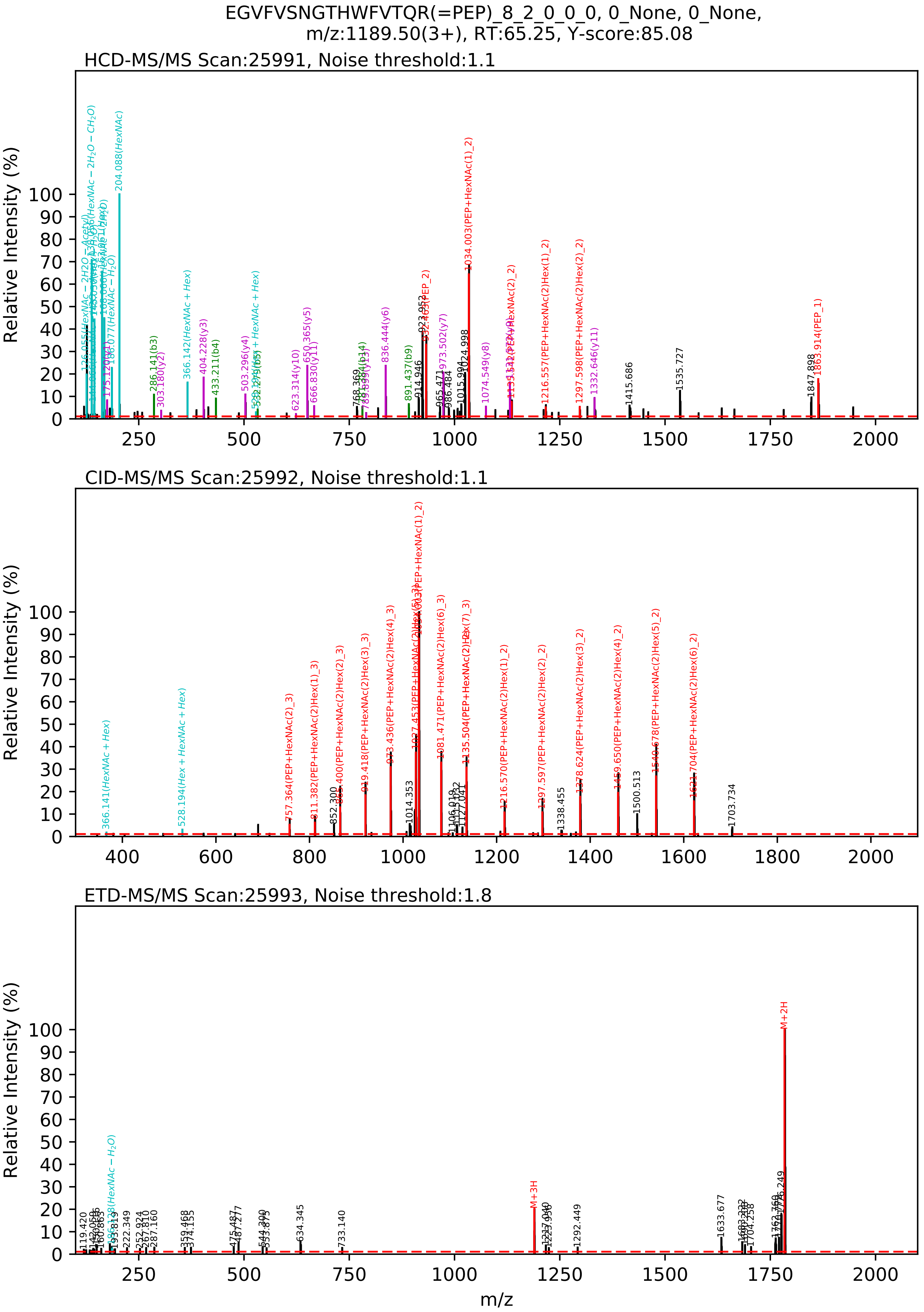

EGVFVSNGTHWFVTQR(=PEP)_8_2_0_0_0, 0_None, 0_None,
m/z:1189.50(3+), RT:65.25, Y-score:85.08
HCD-MS/MS Scan:25991, Noise threshold:1.1
CID-MS/MS Scan:25992, Noise threshold:1.1
ETD-MS/MS Scan:25993, Noise threshold:1.8
Relative Intensity (%)
m/z
204.088(HexNAc)
1034.003(PEP+HexNAc(1)_2)
1216.557(PEP+HexNAc(2)Hex(1)_2)
1297.598(PEP+HexNAc(2)Hex(2)_2)
1863.914(PEP_1)
1027.453(PEP+HexNAc(2)Hex(5)_3)
1081.471(PEP+HexNAc(2)Hex(6)_3)
1135.504(PEP+HexNAc(2)Hex(7)_3)
1378.624(PEP+HexNAc(2)Hex(3)_2)
1459.650(PEP+HexNAc(2)Hex(4)_2)
1540.678(PEP+HexNAc(2)Hex(5)_2)
1621.704(PEP+HexNAc(2)Hex(6)_2)
M+2H
M+3H

EGVFVSNGTHWFVTQR(=PEP)_8_2_0_0_0, 0_None, 0_None,
m/z:1189.50(3+), RT:65.80, Y-score:86.37

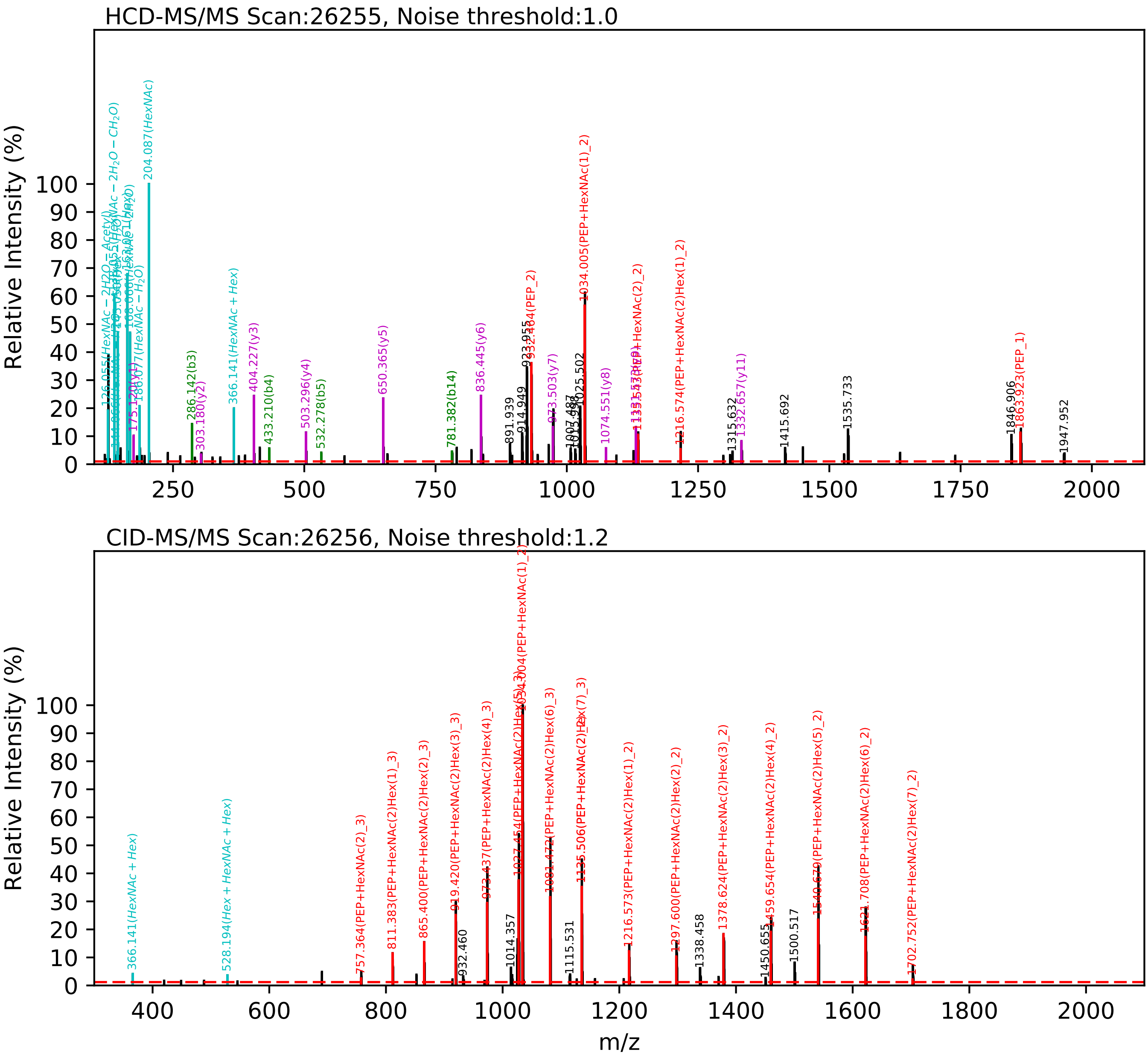

EGVFVSNGTHWFVTQR(=PEP)_8_2_0_0_0, 0_None, 0_None,
m/z:1189.50(3+), RT:66.45, Y-score:85.78

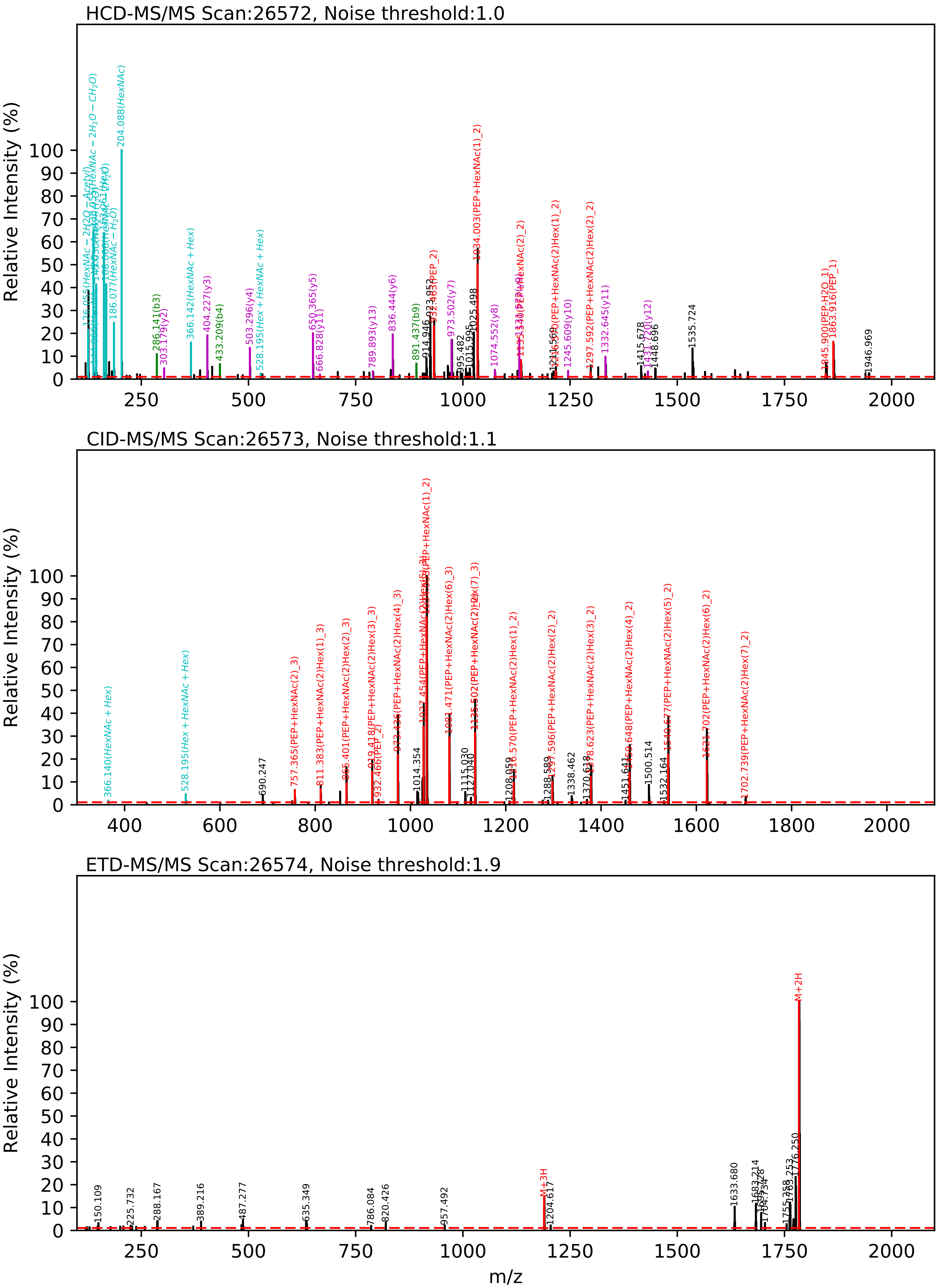

EGVFVSNGTHWFVTQR(=PEP)_8_2_0_0_0, 0_None, 0_None,
m/z:1189.50(3+), RT:67.05, Y-score:83.50

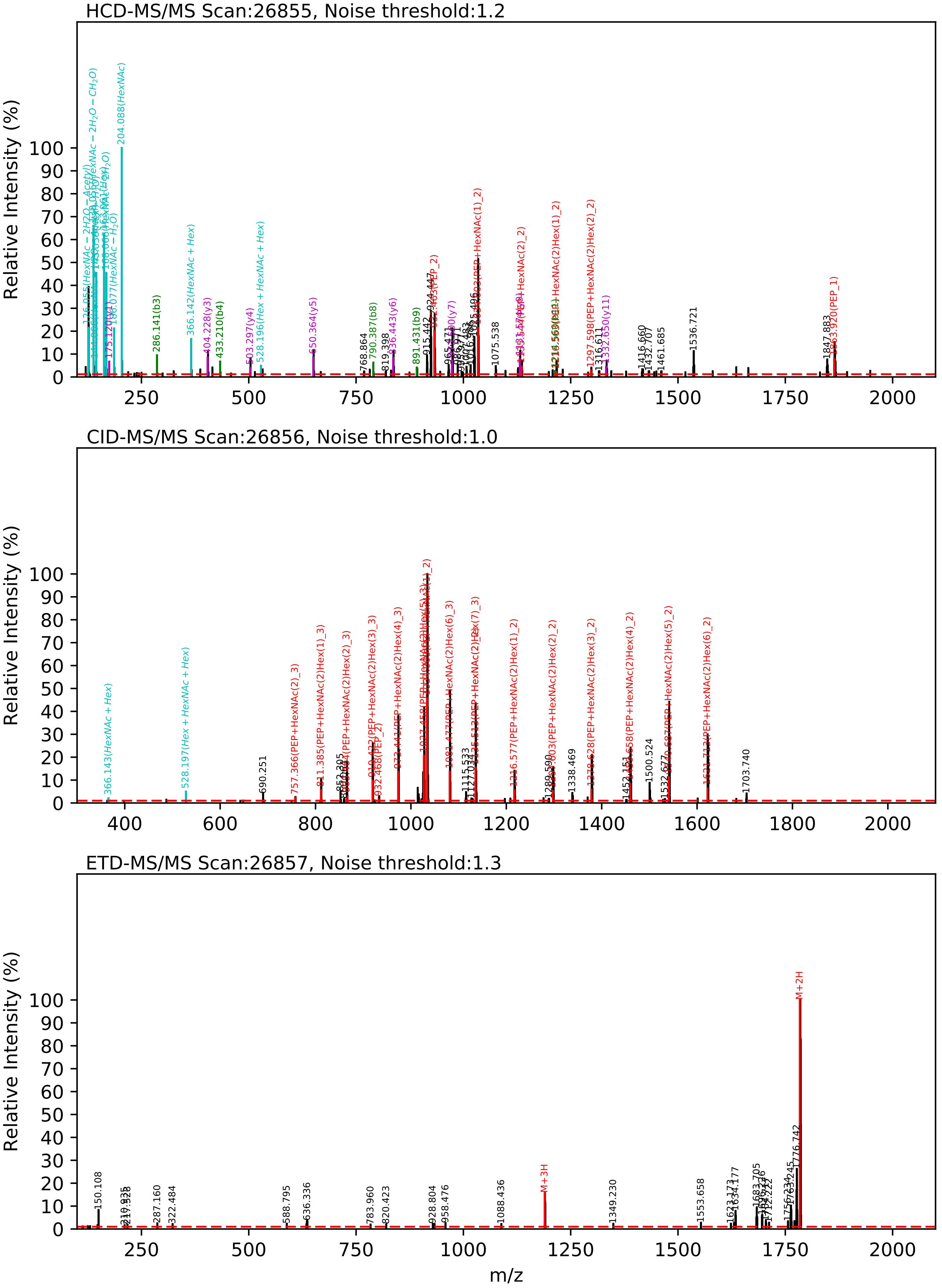

EGVFVSNGTHWFVTQR(=PEP)_8_2_0_0_0, 0_None, 0_None,
m/z:1189.50(3+), RT:68.26, Y-score:85.22

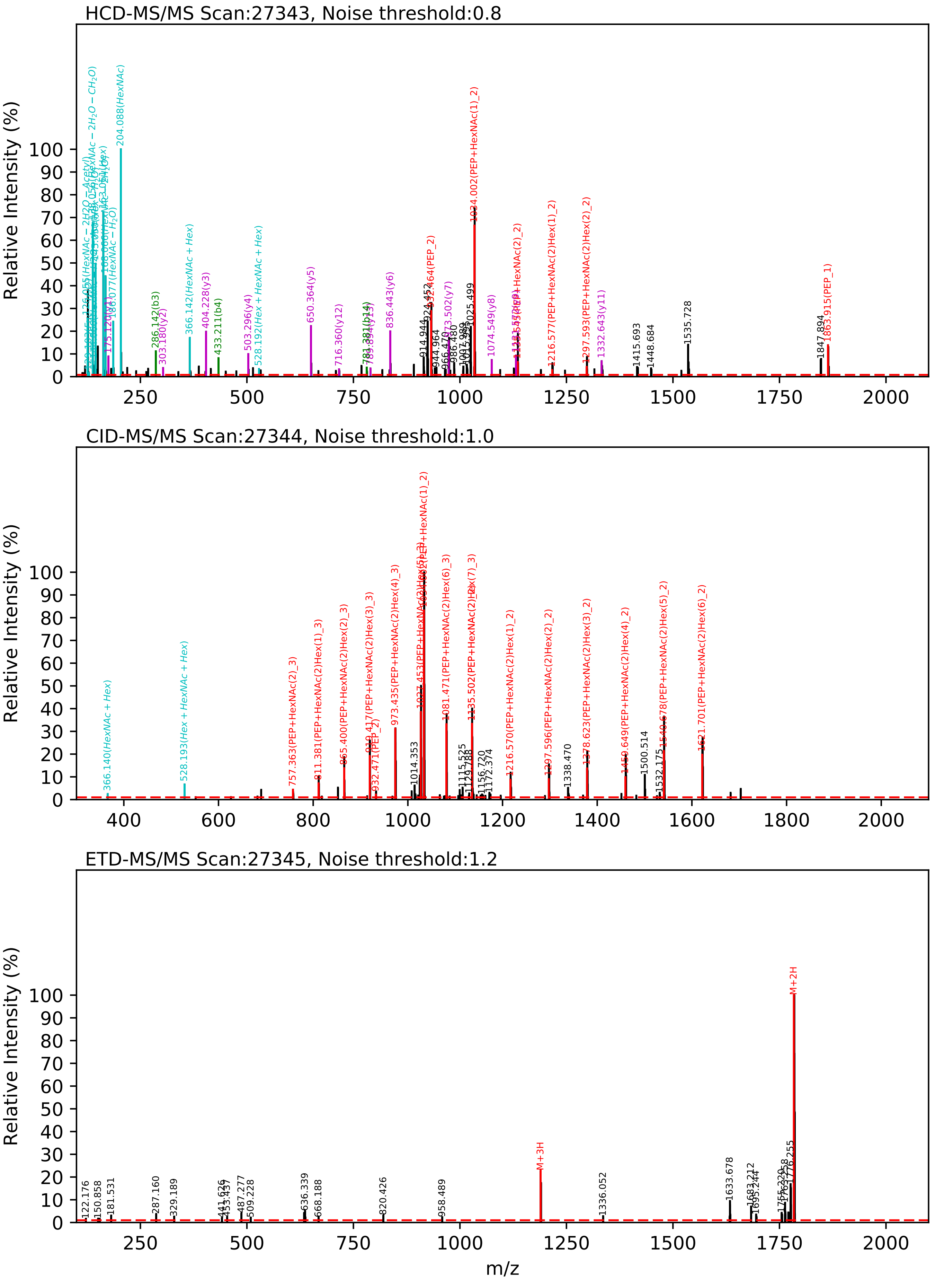

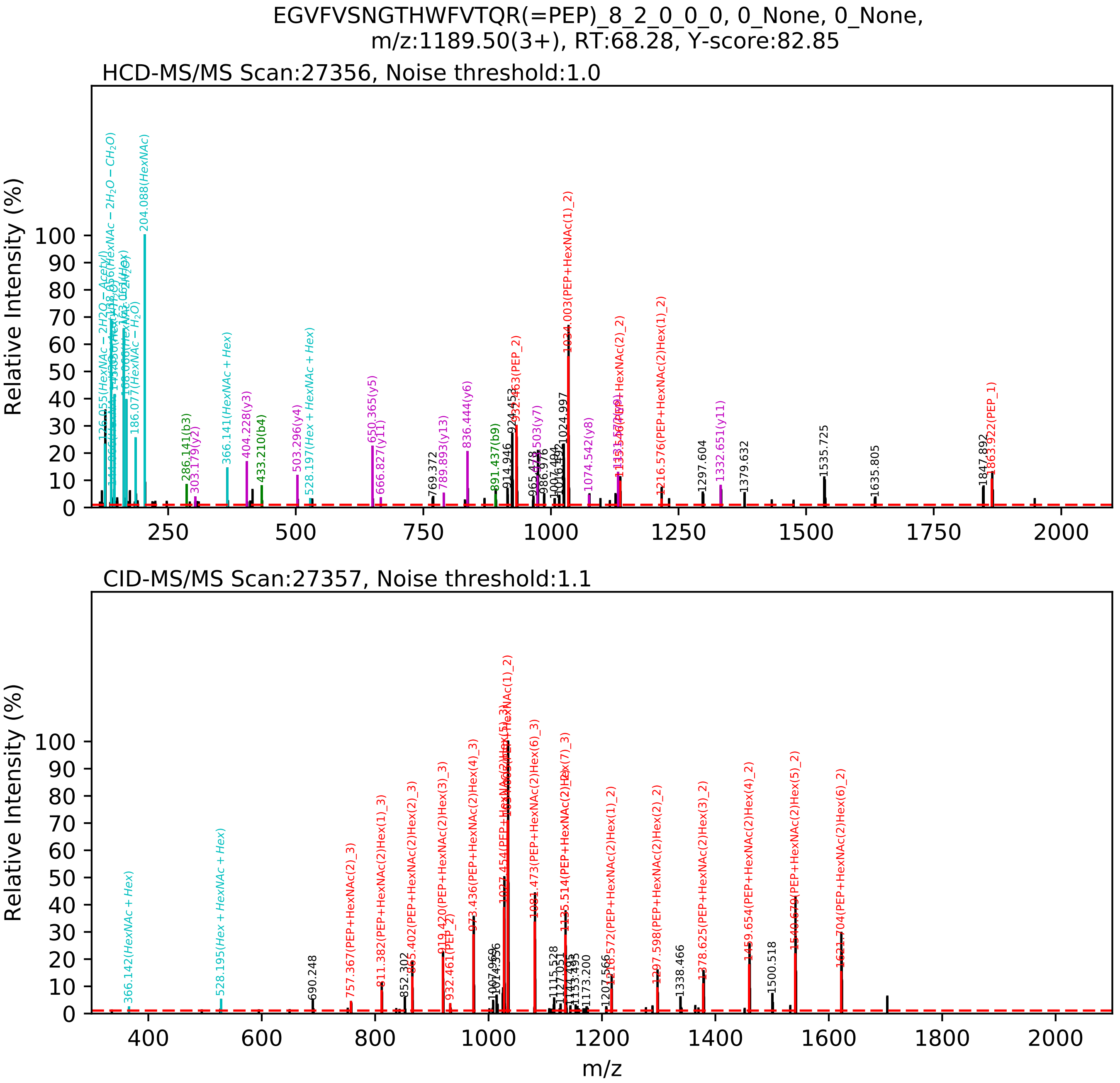

EGVFVSNGTHWFVTQR(=PEP)_8_2_0_0_0, 0_None, 0_None,
m/z:1189.50(3+), RT:68.28, Y-score:82.85
HCD-MS/MS Scan:27356, Noise threshold:1.0
Relative Intensity (%)
204.088(HexNAc)
1034.003(PEP+HexNAc(1)_2)
932.463(PEP_2)
1133.548(PEP+HexNAc(2)_2)
1216.576(PEP+HexNAc(2)Hex(1)_2)
1863.922(PEP_1)
366.141(HexNAc + Hex)
528.197(Hex + HexNAc + Hex)
650.365(y5)
666.827(y11)
836.444(y6)
789.893(y13)
404.228(y3)
503.296(y4)
303.179(y2)
286.141(b3)
433.210(b4)
891.437(b9)
1074.542(y8)
1332.651(y11)
186.077(HexNAc − H2O)
769.372
914.946
924.453
965.478
986.976
1024.997
1297.604
1379.632
1535.725
1635.805
1847.892
CID-MS/MS Scan:27357, Noise threshold:1.1
Relative Intensity (%)
366.142(HexNAc + Hex)
528.195(Hex + HexNAc + Hex)
690.248
757.367(PEP+HexNAc(2)_3)
811.382(PEP+HexNAc(2)Hex(1)_3)
852.302
865.402(PEP+HexNAc(2)Hex(2)_3)
919.420(PEP+HexNAc(2)Hex(3)_3)
932.461(PEP_2)
973.436(PEP+HexNAc(2)Hex(4)_3)
1007.969
1014.356
1027.454(PEP+HexNAc(2)Hex(5)_3)
1081.473(PEP+HexNAc(2)Hex(6)_3)
1115.528
1135.514(PEP+HexNAc(2)Hex(7)_3)
1173.200
1207.566
1216.572(PEP+HexNAc(2)Hex(1)_2)
1297.598(PEP+HexNAc(2)Hex(2)_2)
1338.466
1378.625(PEP+HexNAc(2)Hex(3)_2)
1459.654(PEP+HexNAc(2)Hex(4)_2)
1500.518
1540.679(PEP+HexNAc(2)Hex(5)_2)
1621.704(PEP+HexNAc(2)Hex(6)_2)
m/z

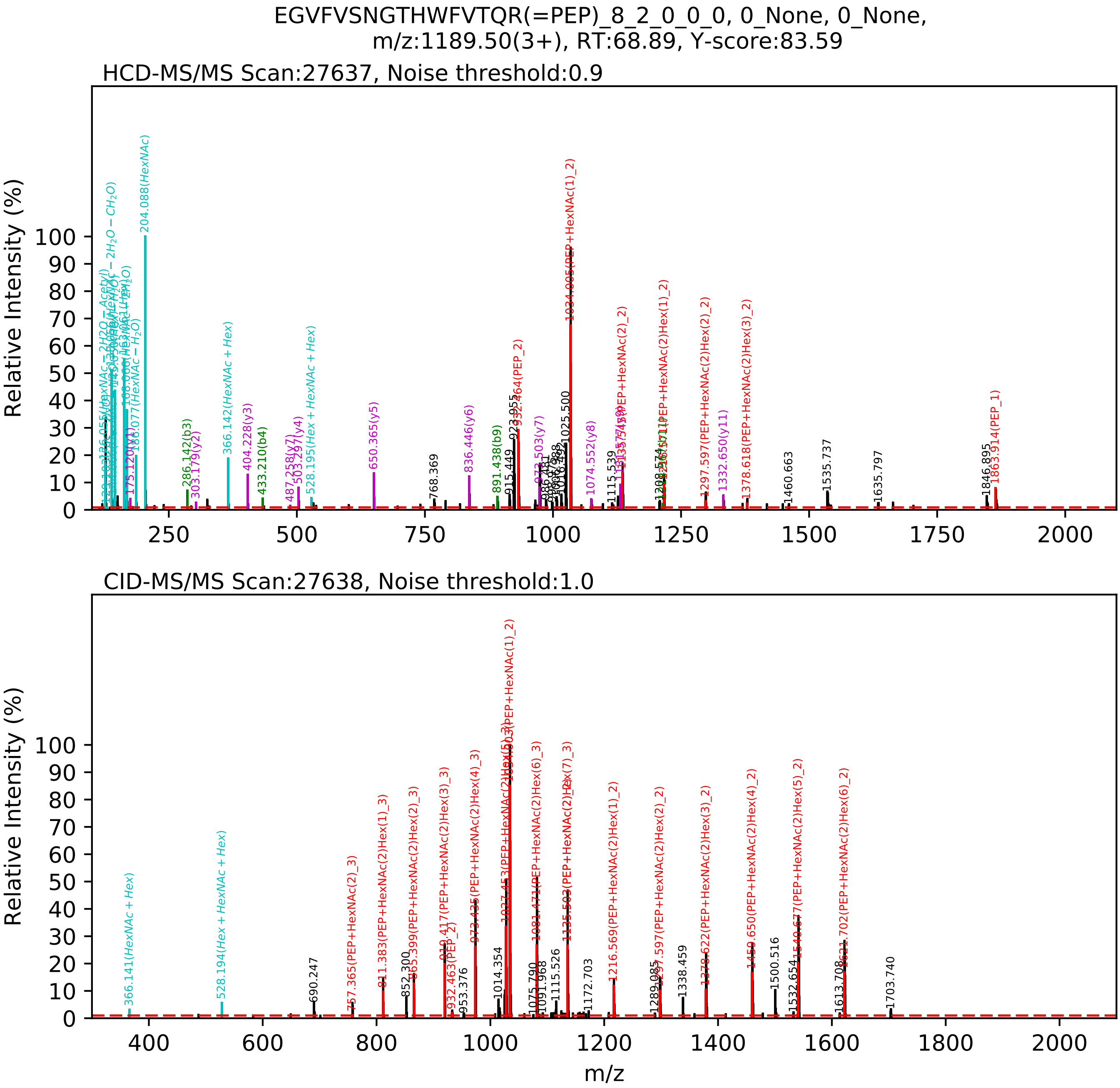
EGVFVSNGTHWFVTQR(=PEP)_8_2_0_0_0, 0_None, 0_None,
m/z:1189.50(3+), RT:68.89, Y-score:83.59
HCD-MS/MS Scan:27637, Noise threshold:0.9
Relative Intensity (%)
204.088(HexNAc)
1034.005(PEP+HexNAc(1)_2)
932.464(PEP_2)
923.955
1025.500
286.142(b3)
303.179(y2)
366.142(HexNAc+Hex)
404.228(y3)
433.210(b4)
487.258(y7)
528.195(Hex+HexNAc+Hex)
650.365(y5)
768.369
836.446(y6)
891.438(b9)
915.449
1074.552(y8)
1115.539
1208.574
1297.597(PEP+HexNAc(2)Hex(2)_2)
1332.650(y11)
1378.618(PEP+HexNAc(2)Hex(3)_2)
1460.663
1535.737
1635.797
1846.895
1863.914(PEP_1)
CID-MS/MS Scan:27638, Noise threshold:1.0
366.141(HexNAc+Hex)
528.194(Hex+HexNAc+Hex)
690.247
757.365(PEP+HexNAc(2)_3)
811.383(PEP+HexNAc(2)Hex(1)_3)
852.300
865.399(PEP+HexNAc(2)Hex(2)_3)
932.463(PEP_2)
953.376
973.435(PEP+HexNAc(2)Hex(4)_3)
1014.354
1075.790
1081.471(PEP+HexNAc(2)Hex(6)_3)
1091.968
1115.526
1135.503(PEP+HexNAc(2)Hex(7)_3)
1172.703
1216.569(PEP+HexNAc(2)Hex(1)_2)
1289.085
1338.459
1459.650(PEP+HexNAc(2)Hex(4)_2)
1500.516
1532.654
1613.708
1703.740
m/z

EGVFVSNGTHWFVTQR(=PEP)_8_2_0_0_0, 0_None, 0_None,
m/z:1189.50(3+), RT:68.96, Y-score:82.72

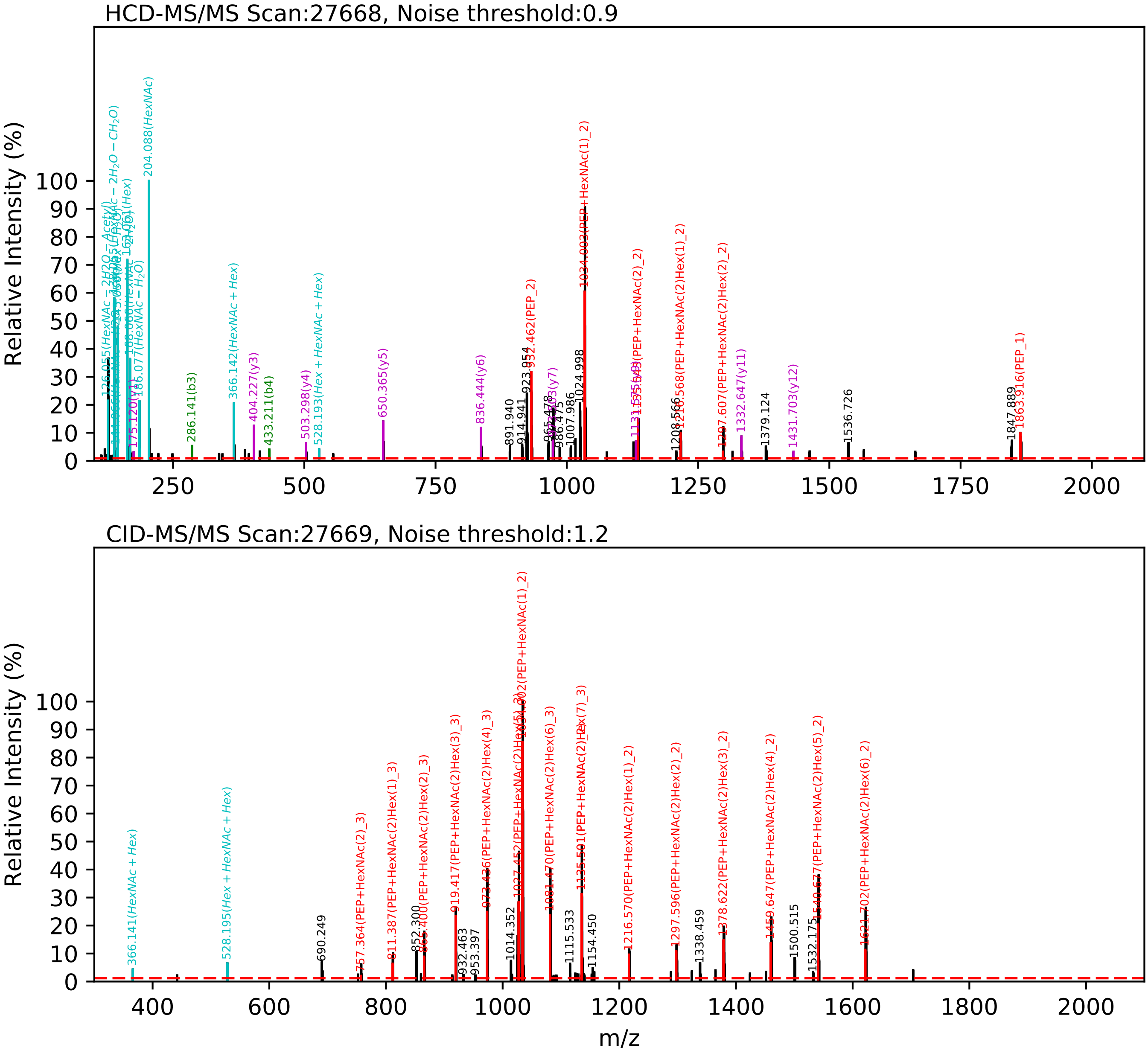

EGVFVSNGTHWFVTQR(=PEP)_8_2_0_0_0, 0_None, 0_None,
m/z:1189.50(3+), RT:61.62, Y-score:82.84

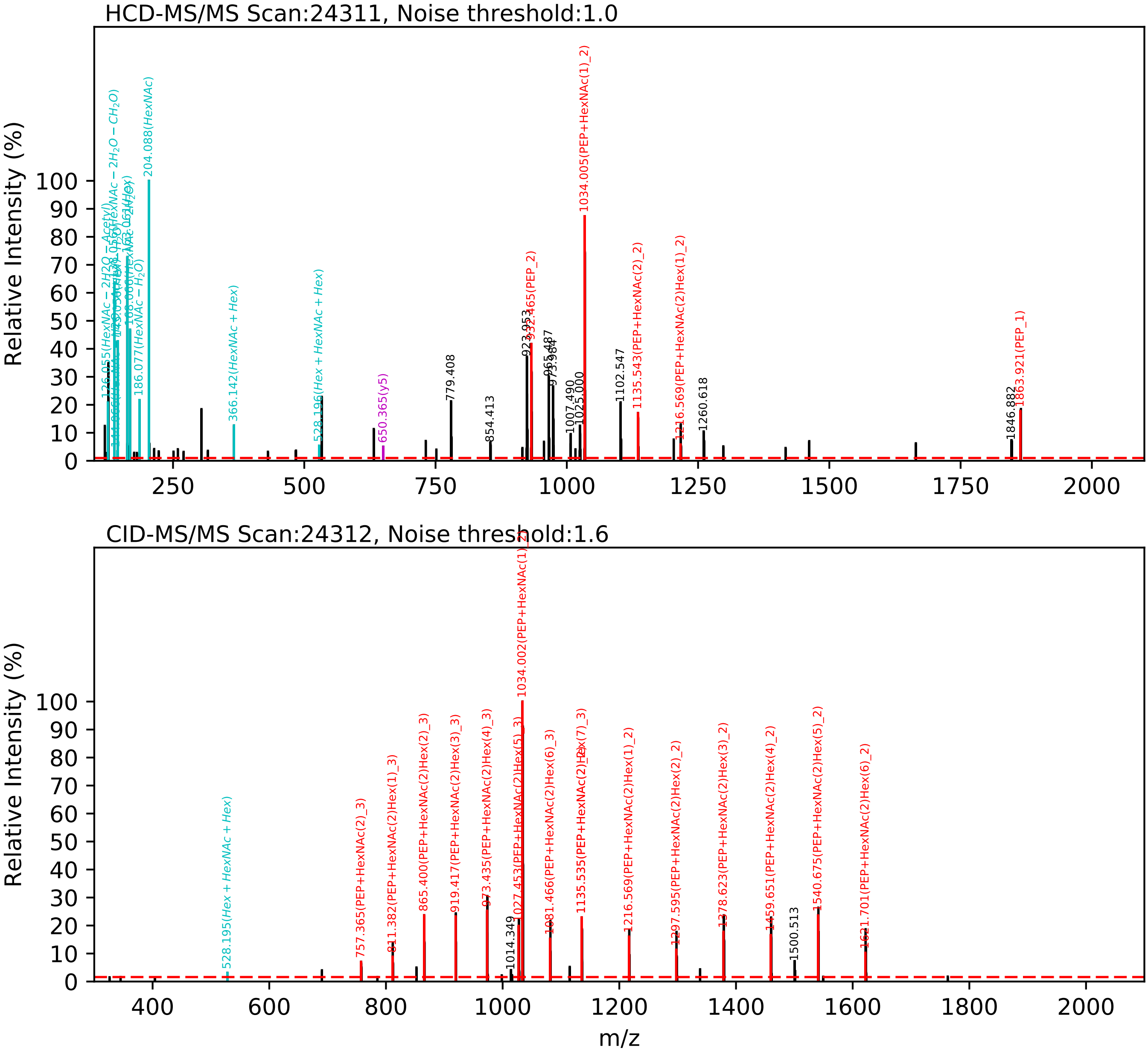

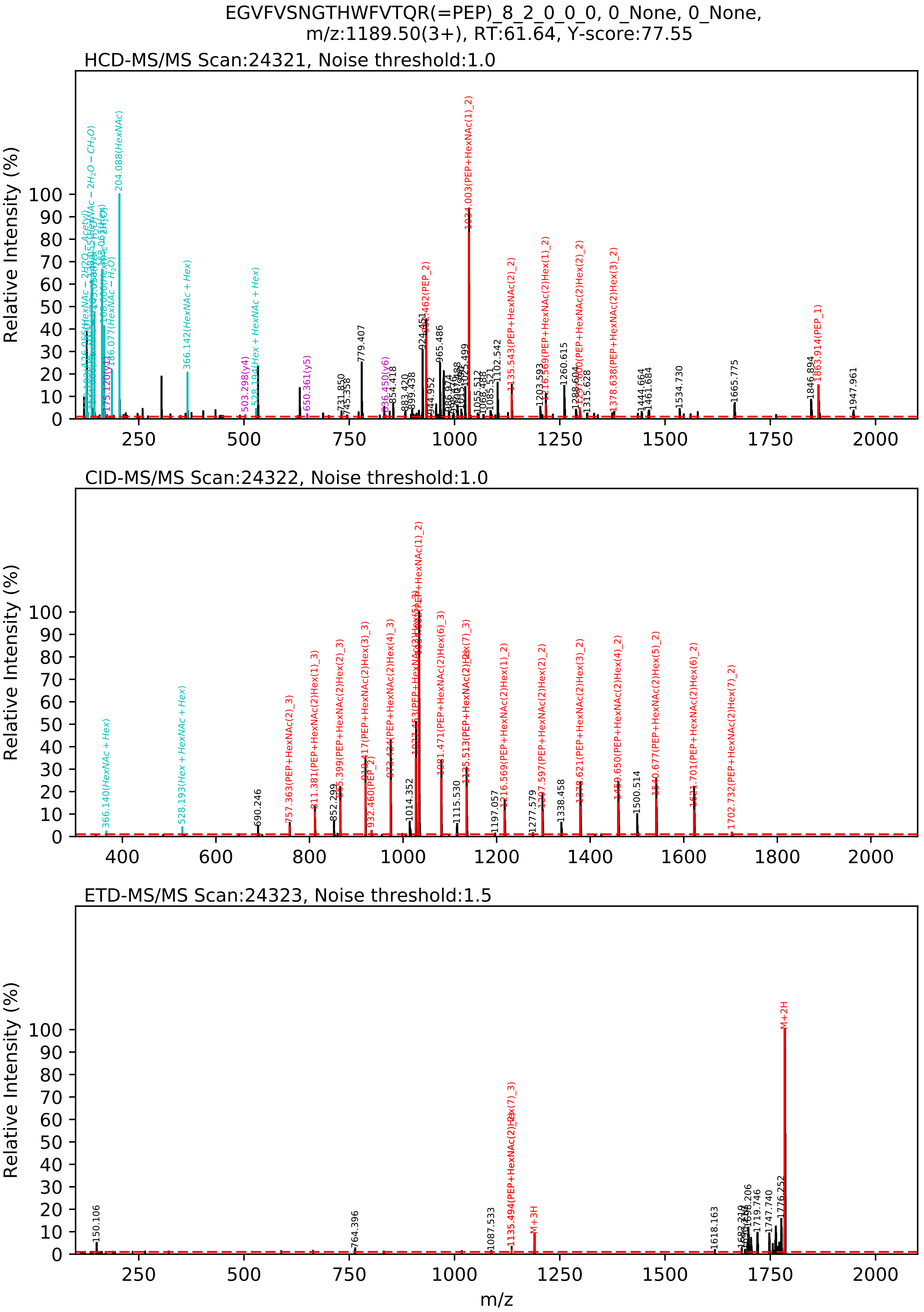
EGVFVSNGTHWFVTQR(=PEP)_8_2_0_0_0, 0_None, 0_None,
m/z:1189.50(3+), RT:61.64, Y-score:77.55
HCD-MS/MS Scan:24321, Noise threshold:1.0
CID-MS/MS Scan:24322, Noise threshold:1.0
ETD-MS/MS Scan:24323, Noise threshold:1.5
Relative Intensity (%)
m/z
204.088(HexNAc)
366.142(HexNAc+Hex)
1034.003(PEP+HexNAc(1)_2)
1135.543(PEP+HexNAc(2)_2)
1378.638(PEP+HexNAc(2)Hex(3)_2)
1863.914(PEP_1)
366.140(HexNAc+Hex)
528.193(Hex+HexNAc+Hex)
757.363(PEP+HexNAc(2)_3)
811.381(PEP+HexNAc(2)Hex(1)_3)
1081.471(PEP+HexNAc(2)Hex(6)_3)
1378.621(PEP+HexNAc(2)Hex(3)_2)
1459.650(PEP+HexNAc(2)Hex(4)_2)
1540.677(PEP+HexNAc(2)Hex(5)_2)
1621.701(PEP+HexNAc(2)Hex(6)_2)
1702.732(PEP+HexNAc(2)Hex(7)_2)
M+2H
M+3H
1135.494(PEP+HexNAc(2)Hex(7)_3)

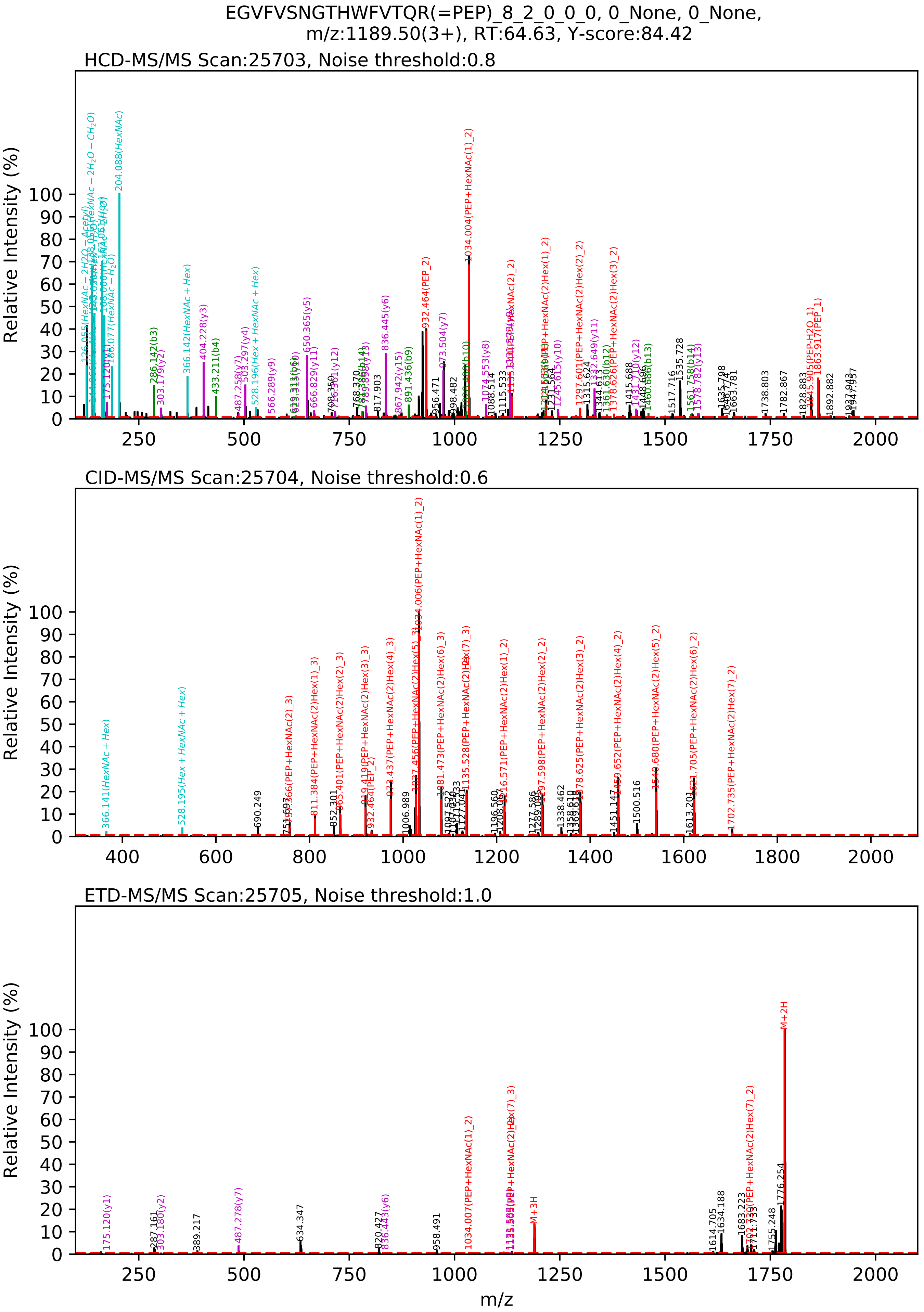

EGVFVSNGTHWFVTQR(=PEP)_8_2_0_0_0, 0_None, 0_None,
m/z:1189.50(3+), RT:64.63, Y-score:84.42
HCD-MS/MS Scan:25703, Noise threshold:0.8
Relative Intensity (%)
204.088(HexNAc)
1034.004(PEP+HexNAc(1)_2)
932.464(PEP_2)
836.445(y6)
650.365(y5)
404.228(y3)
366.142(HexNAc + Hex)
286.142(b3)
433.211(b4)
303.179(y2)
973.504(y7)
891.436(b9)
1297.601(PEP+HexNAc(2)Hex(2)_2)
1378.626(PEP+HexNAc(2)Hex(3)_2)
1863.917(PEP_1)
1535.728
1738.803
CID-MS/MS Scan:25704, Noise threshold:0.6
Relative Intensity (%)
1034.006(PEP+HexNAc(1)_2)
366.141(HexNAc + Hex)
528.195(Hex + HexNAc + Hex)
690.249
811.384(PEP+HexNAc(2)Hex(1)_3)
1081.473(PEP+HexNAc(2)Hex(6)_3)
1216.571(PEP+HexNAc(2)Hex(1)_2)
1297.598(PEP+HexNAc(2)Hex(2)_2)
1540.680(PEP+HexNAc(2)Hex(5)_2)
1702.735(PEP+HexNAc(2)Hex(7)_2)
1500.516
1613.201
ETD-MS/MS Scan:25705, Noise threshold:1.0
Relative Intensity (%)
M+2H
M+3H
175.120(y1)
303.180(y2)
487.278(y7)
836.443(y6)
287.161
389.217
634.347
820.427
958.491
1034.007(PEP+HexNAc(1)_2)
1614.705
1634.188
1683.223
1755.248
1776.254
m/z

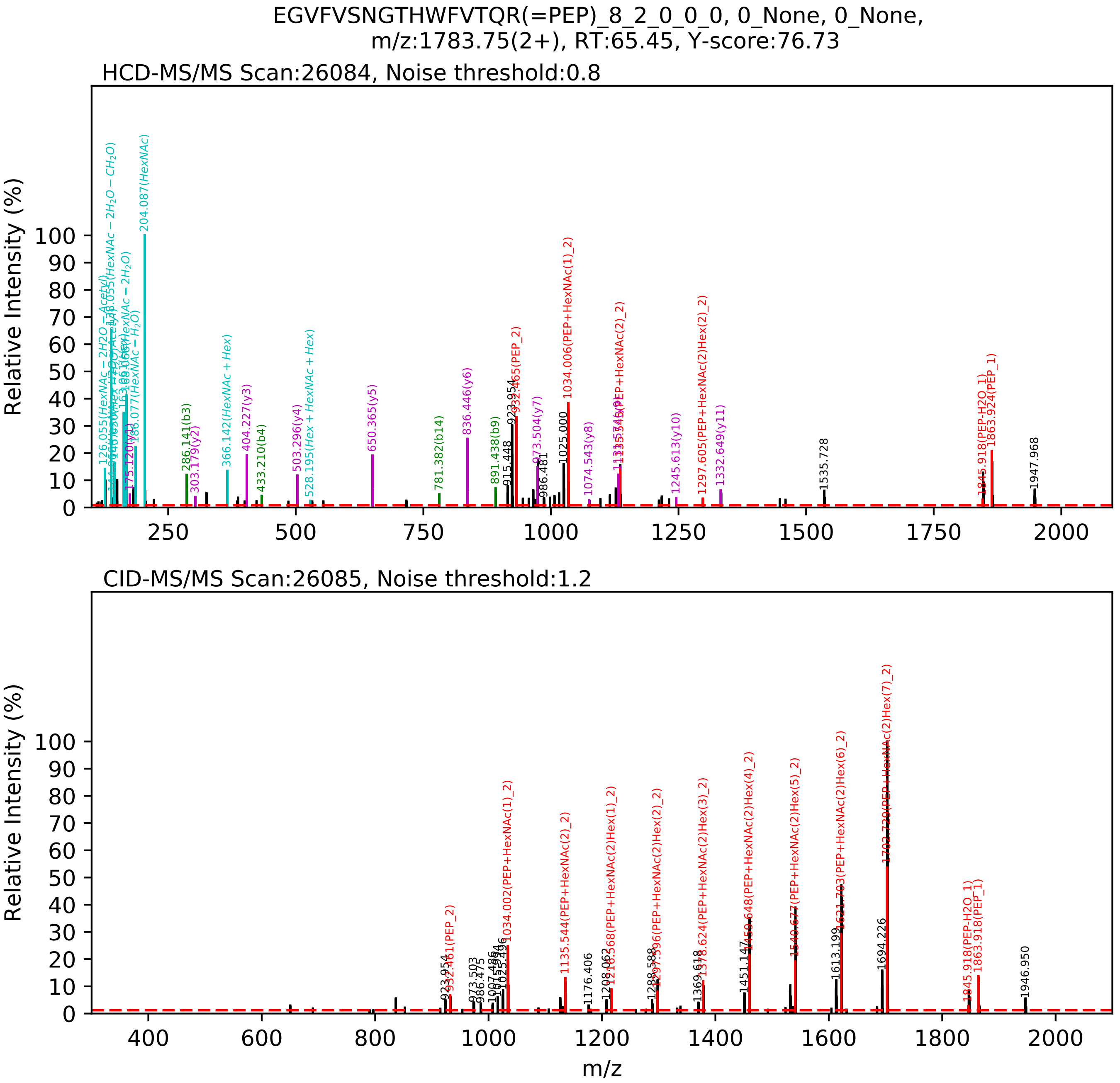

EGVFVSNGTHWFVTQR(=PEP)_8_2_0_0_0, 0_None, 0_None,
m/z:1783.75(2+), RT:65.45, Y-score:76.73
HCD-MS/MS Scan:26084, Noise threshold:0.8
Relative Intensity (%)
126.055(HexNAc − 2H2O − Acetyl)
138.055(HexNAc − 2H2O − CH2O)
168.066(HexNAc − 2H2O)
175.120(y1)
186.077(HexNAc − H2O)
204.087(HexNAc)
286.141(b3)
303.179(y2)
366.142(HexNAc + Hex)
404.227(y3)
433.210(b4)
503.296(y4)
528.195(Hex + HexNAc + Hex)
650.365(y5)
781.382(b14)
836.446(y6)
891.438(b9)
915.448
923.954
932.465(PEP_2)
973.504(y7)
986.481
1025.000
1034.006(PEP+HexNAc(1)_2)
1074.543(y8)
1135.545(PEP+HexNAc(2)_2)
1245.613(y10)
1297.605(PEP+HexNAc(2)Hex(2)_2)
1332.649(y11)
1535.728
1845.918(PEP-H2O_1)
1863.924(PEP_1)
1947.968
CID-MS/MS Scan:26085, Noise threshold:1.2
Relative Intensity (%)
923.954
932.461(PEP_2)
973.503
986.475
1007.486
1034.002(PEP+HexNAc(1)_2)
1135.544(PEP+HexNAc(2)_2)
1176.406
1208.062
1216.568(PEP+HexNAc(2)Hex(1)_2)
1288.588
1297.596(PEP+HexNAc(2)Hex(2)_2)
1369.618
1378.624(PEP+HexNAc(2)Hex(3)_2)
1451.147
1459.648(PEP+HexNAc(2)Hex(4)_2)
1540.677(PEP+HexNAc(2)Hex(5)_2)
1613.199
1621.703(PEP+HexNAc(2)Hex(6)_2)
1694.226
1702.729(PEP+HexNAc(2)Hex(7)_2)
1845.918(PEP-H2O_1)
1863.918(PEP_1)
1946.950
m/z

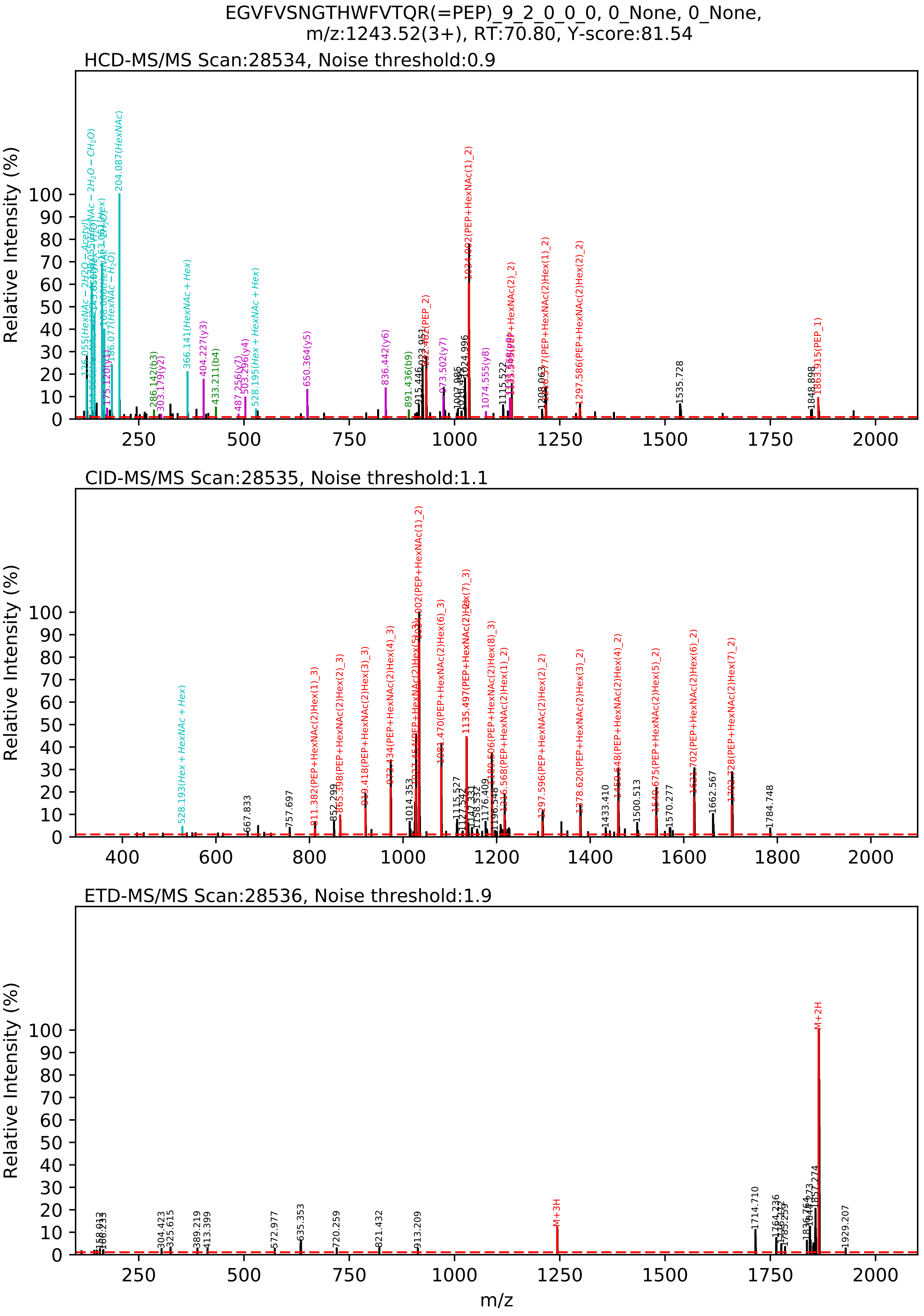

EGVFVSNGTHWFVTQR(=PEP)_9_2_0_0_0, 0_None, 0_None,
m/z:1243.52(3+), RT:70.80, Y-score:81.54
HCD-MS/MS Scan:28534, Noise threshold:0.9
Relative Intensity (%)
204.087(HexNAc)
366.141(HexNAc + Hex)
528.195(Hex + HexNAc + Hex)
286.142(b3)
303.179(y2)
404.227(y3)
433.211(b4)
487.256(y7)
503.296(y4)
650.364(y5)
836.442(y6)
891.436(b9)
915.446
923.951
932.482(PEP_2)
973.502(y7)
1007.995
1024.996
1034.002(PEP+HexNAc(1)_2)
1074.555(y8)
1115.522
1135.497(PEP+HexNAc(2)_2)
1208.063
1297.586(PEP+HexNAc(2)Hex(2)_2)
1535.728
1848.898
1863.915(PEP_1)
CID-MS/MS Scan:28535, Noise threshold:1.1
528.193(Hex + HexNAc + Hex)
667.833
757.697
811.382(PEP+HexNAc(2)Hex(1)_3)
852.299
865.398(PEP+HexNAc(2)Hex(2)_3)
919.418(PEP+HexNAc(2)Hex(3)_3)
973.434(PEP+HexNAc(2)Hex(4)_3)
1014.353
1034.002(PEP+HexNAc(1)_2)
1081.470(PEP+HexNAc(2)Hex(6)_3)
1115.527
1135.497(PEP+HexNAc(2)_2)
1176.409
1216.568(PEP+HexNAc(2)Hex(1)_2)
1297.596(PEP+HexNAc(2)Hex(2)_2)
1378.620(PEP+HexNAc(2)Hex(3)_2)
1433.410
1459.648(PEP+HexNAc(2)Hex(4)_2)
1500.513
1540.675(PEP+HexNAc(2)Hex(5)_2)
1570.277
1621.702(PEP+HexNAc(2)Hex(6)_2)
1662.567
1702.728(PEP+HexNAc(2)Hex(7)_2)
1784.748
ETD-MS/MS Scan:28536, Noise threshold:1.9
158.013
304.423
325.615
389.219
413.399
572.977
635.353
720.259
821.432
913.209
M+3H
1714.710
1764.236
1857.274
1929.207
M+2H
m/z

EGVFVSNGTHWFVTQR(=PEP)_9_2_0_0_0, 0_None, 0_None,
m/z:1243.52(3+), RT:74.15, Y-score:75.65

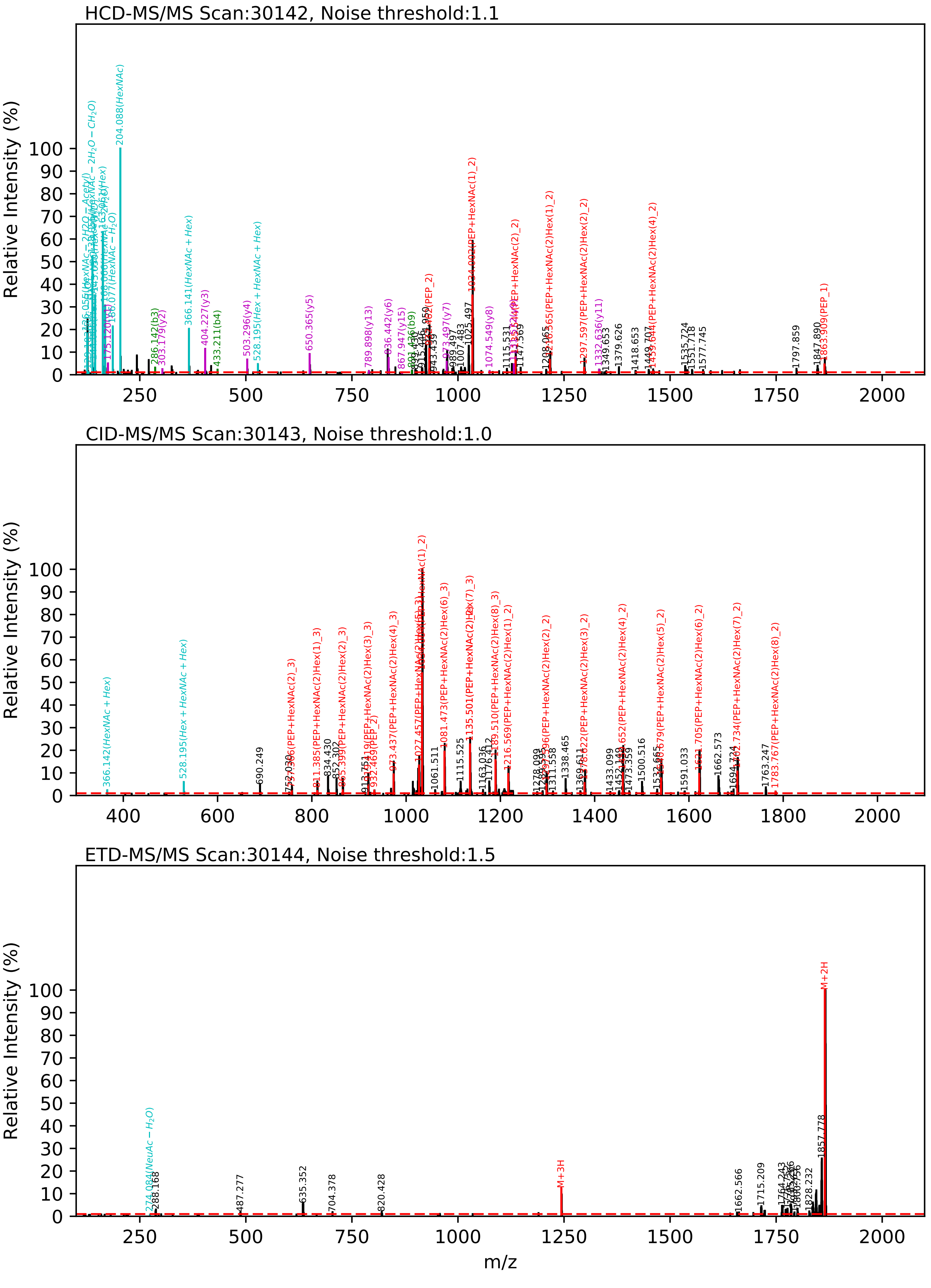

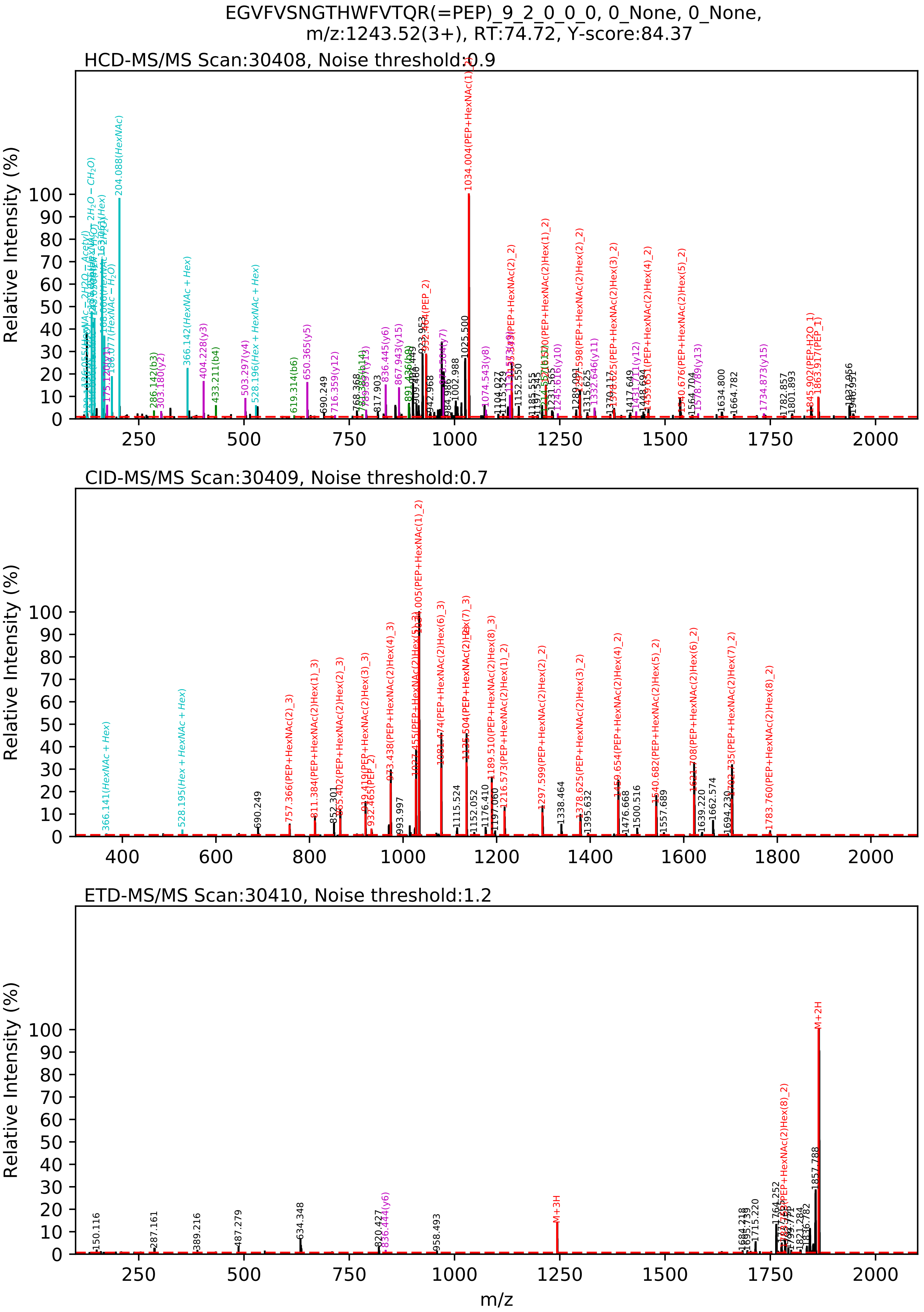
EGVFVSNGTHWFVTQR(=PEP)_9_2_0_0_0, 0_None, 0_None,
m/z:1243.52(3+), RT:74.72, Y-score:84.37
HCD-MS/MS Scan:30408, Noise threshold:0.9
CID-MS/MS Scan:30409, Noise threshold:0.7
ETD-MS/MS Scan:30410, Noise threshold:1.2
Relative Intensity (%)
m/z
1034.004(PEP+HexNAc(1)_2)
204.088(HexNAc)
M+2H
M+3H

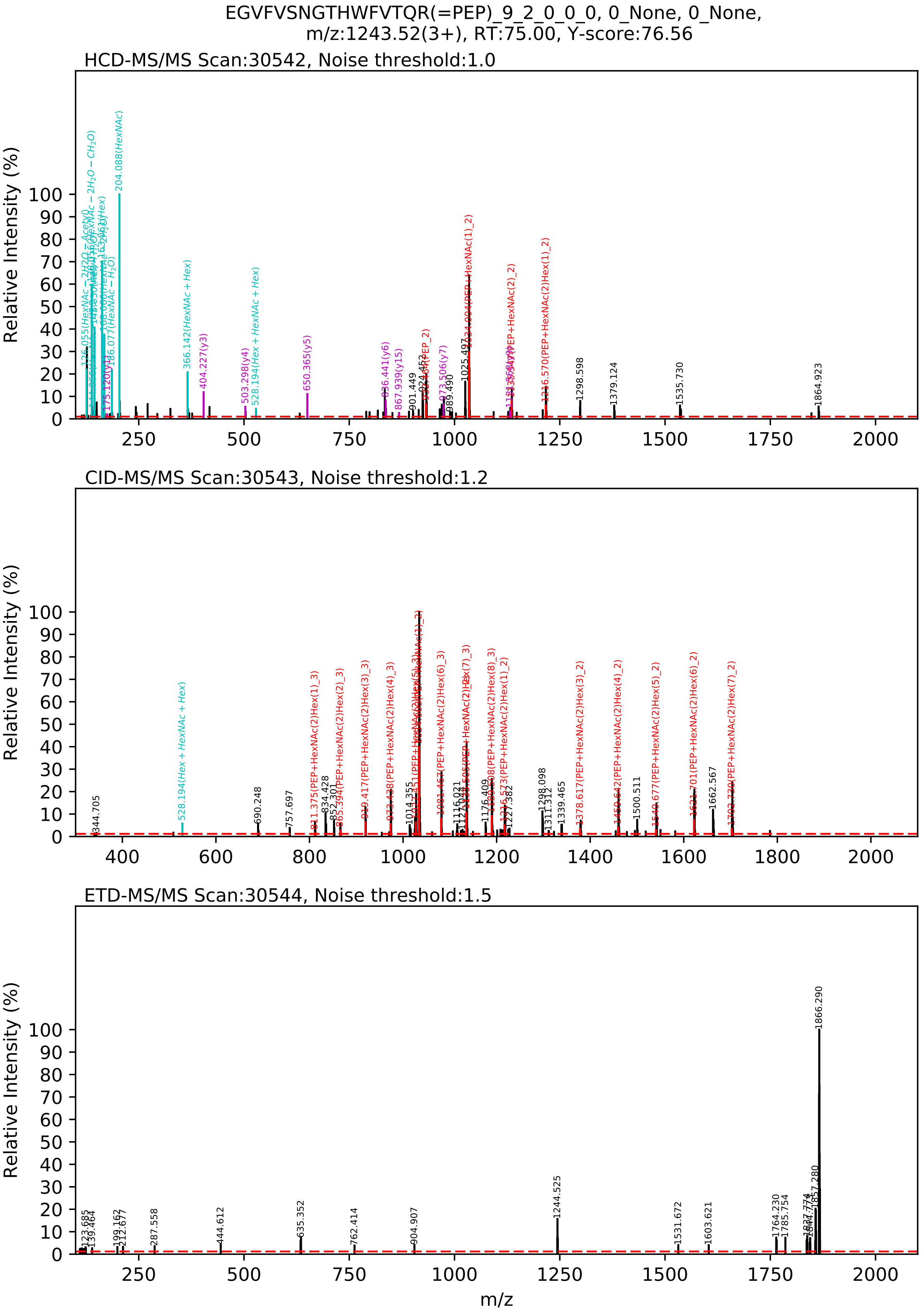

EGVFVSNGTHWFVTQR(=PEP)_9_2_0_0_0, 0_None, 0_None,
m/z:1243.52(3+), RT:75.00, Y-score:76.56
HCD-MS/MS Scan:30542, Noise threshold:1.0
Relative Intensity (%)
204.088(HexNAc)
366.142(HexNAc + Hex)
528.194(Hex + HexNAc + Hex)
404.227(y3)
503.298(y4)
650.365(y5)
836.441(y6)
867.939(y15)
973.506(y7)
1216.570(PEP+HexNAc(2)Hex(1)_2)
186.077(HexNAc − H2O)
175.120(y1)
901.449
924.452
989.490
1025.497
1298.598
1379.124
1535.730
1864.923
CID-MS/MS Scan:30543, Noise threshold:1.2
Relative Intensity (%)
344.705
528.194(Hex + HexNAc + Hex)
690.248
757.697
811.375(PEP+HexNAc(2)Hex(1)_3)
834.428
852.301
865.394(PEP+HexNAc(2)Hex(2)_3)
919.417(PEP+HexNAc(2)Hex(3)_3)
973.438(PEP+HexNAc(2)Hex(4)_3)
1014.355
1081.467(PEP+HexNAc(2)Hex(6)_3)
1116.021
1176.409
1216.573(PEP+HexNAc(2)Hex(1)_2)
1227.382
1298.098
1311.312
1339.465
1378.617(PEP+HexNAc(2)Hex(3)_2)
1459.642(PEP+HexNAc(2)Hex(4)_2)
1500.511
1540.677(PEP+HexNAc(2)Hex(5)_2)
1621.701(PEP+HexNAc(2)Hex(6)_2)
1662.567
1702.729(PEP+HexNAc(2)Hex(7)_2)
ETD-MS/MS Scan:30544, Noise threshold:1.5
Relative Intensity (%)
123.685
139.464
199.162
212.677
287.558
444.612
635.352
762.414
904.907
1244.525
1531.672
1603.621
1764.230
1785.754
1857.280
1866.290
m/z

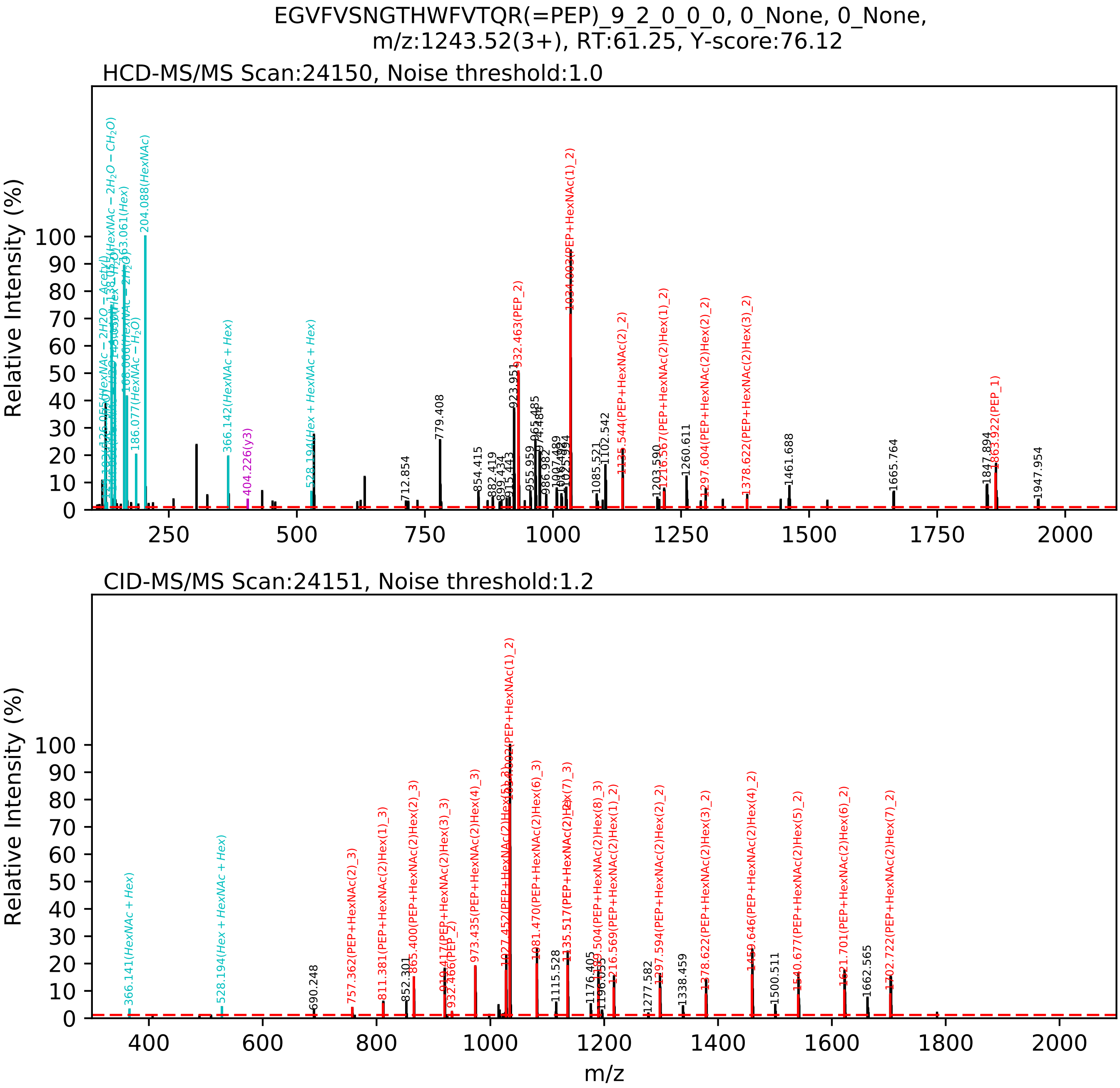

EGVFVSNGTHWFVTQR(=PEP)_9_2_0_0_0, 0_None, 0_None,
m/z:1243.52(3+), RT:61.25, Y-score:76.12
HCD-MS/MS Scan:24150, Noise threshold:1.0
CID-MS/MS Scan:24151, Noise threshold:1.2
Relative Intensity (%)
m/z

EGVFVSNGTHWFVTQR(=PEP)_9_2_0_0_0, 0_None, 0_None,
m/z:1243.52(3+), RT:61.31, Y-score:78.69

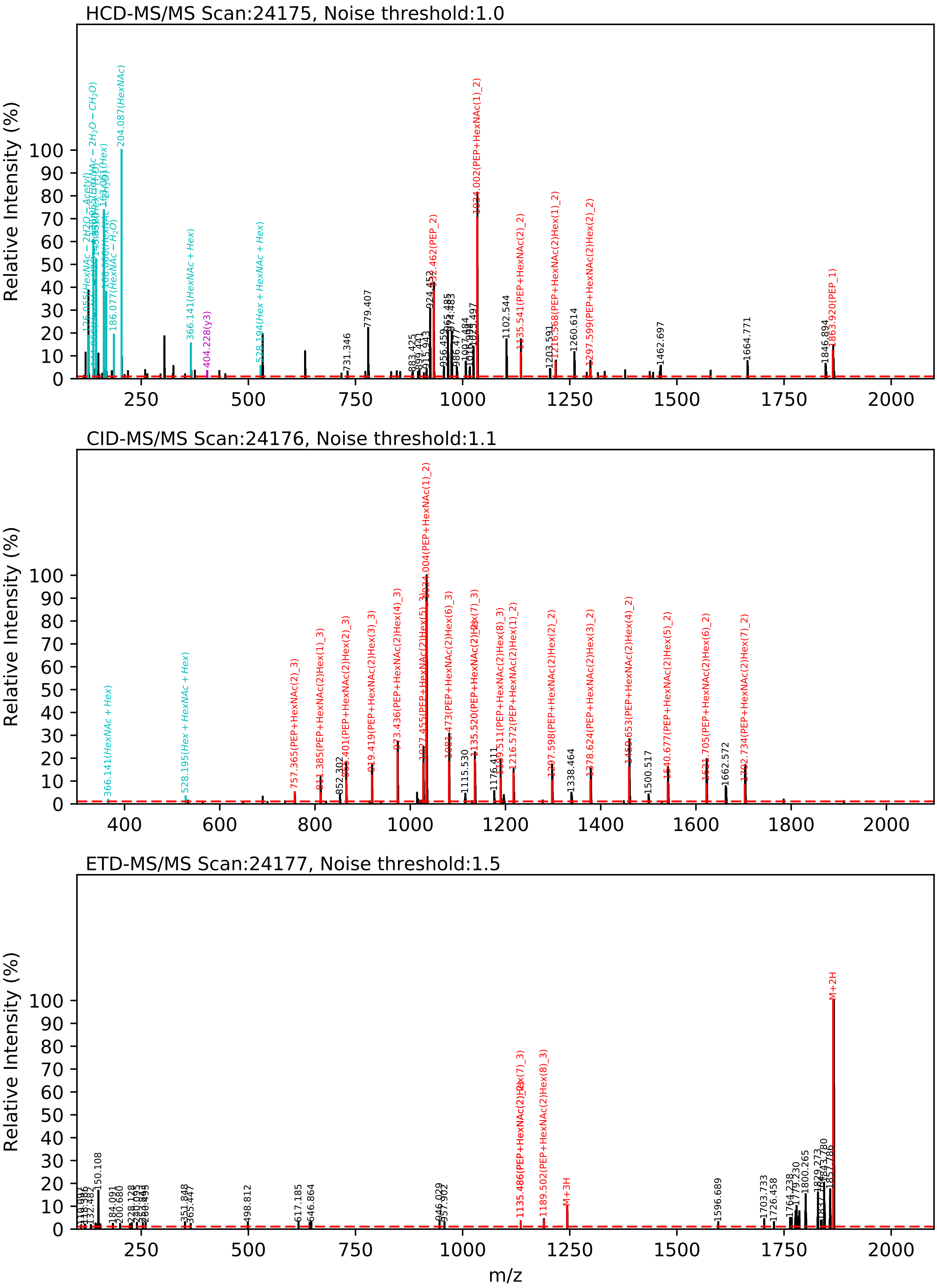

EGVFVSNGTHWFVTQR(=PEP)_9_2_0_0_0, 0_None, 0_None,
m/z:1243.52(3+), RT:64.48, Y-score:84.87

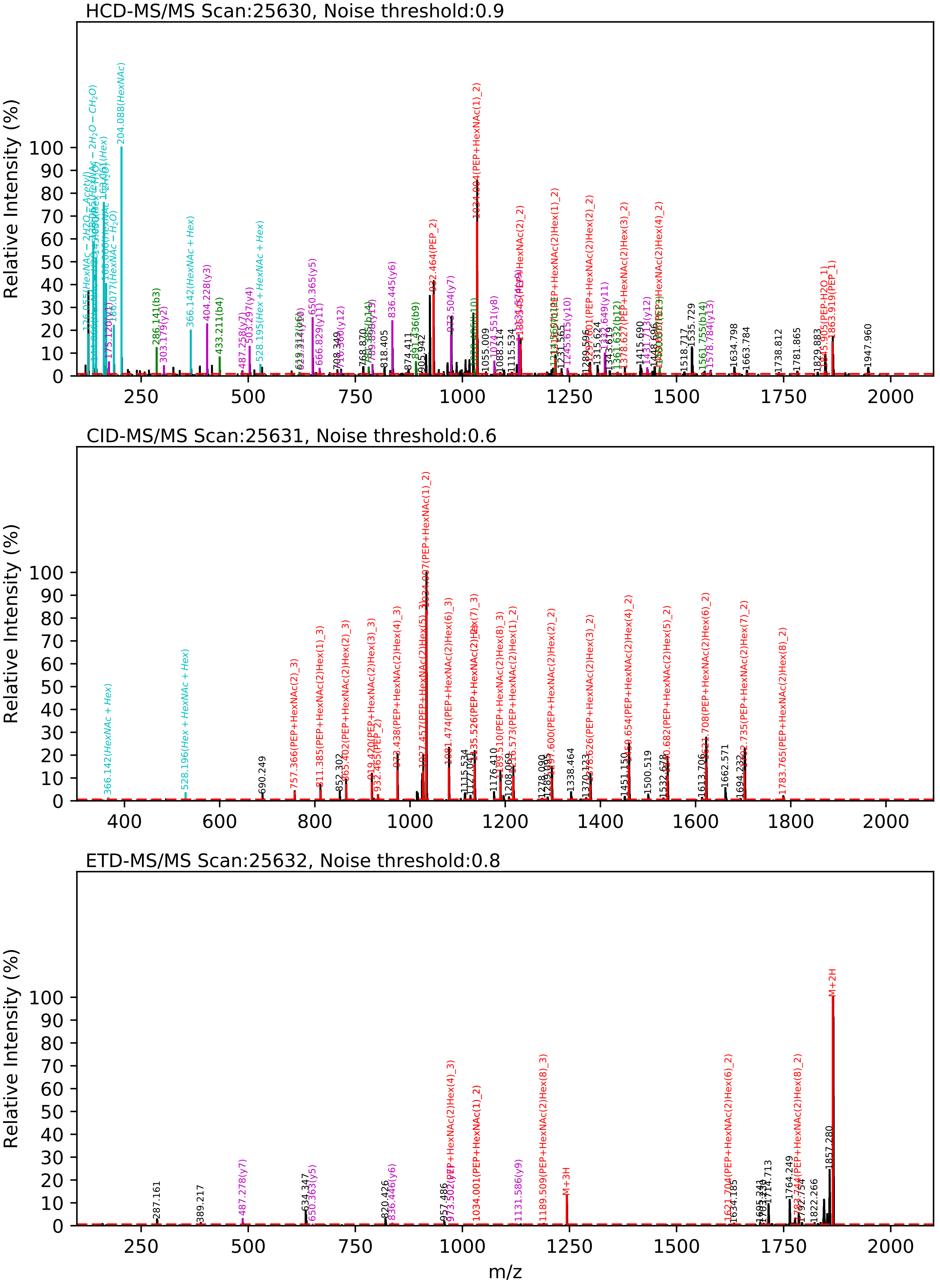

EGVFVSNGTHWFVTQR(=PEP)_9_2_0_0_0, 0_None, 0_None,
m/z:1243.52(3+), RT:64.93, Y-score:87.74

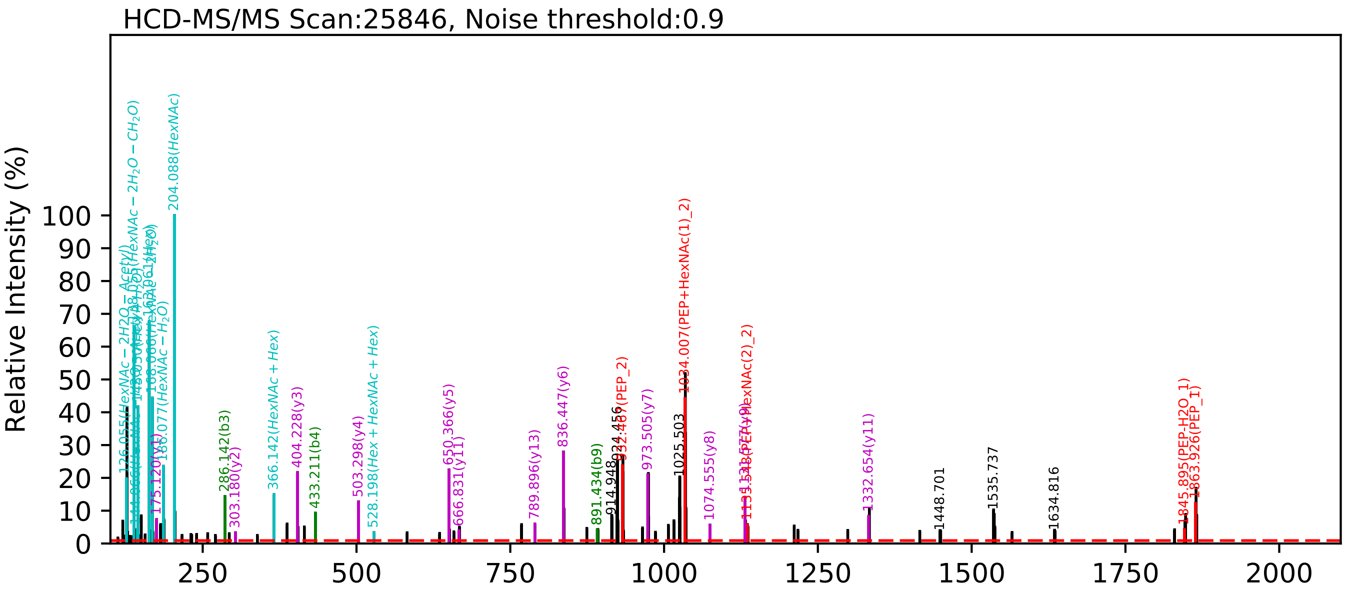

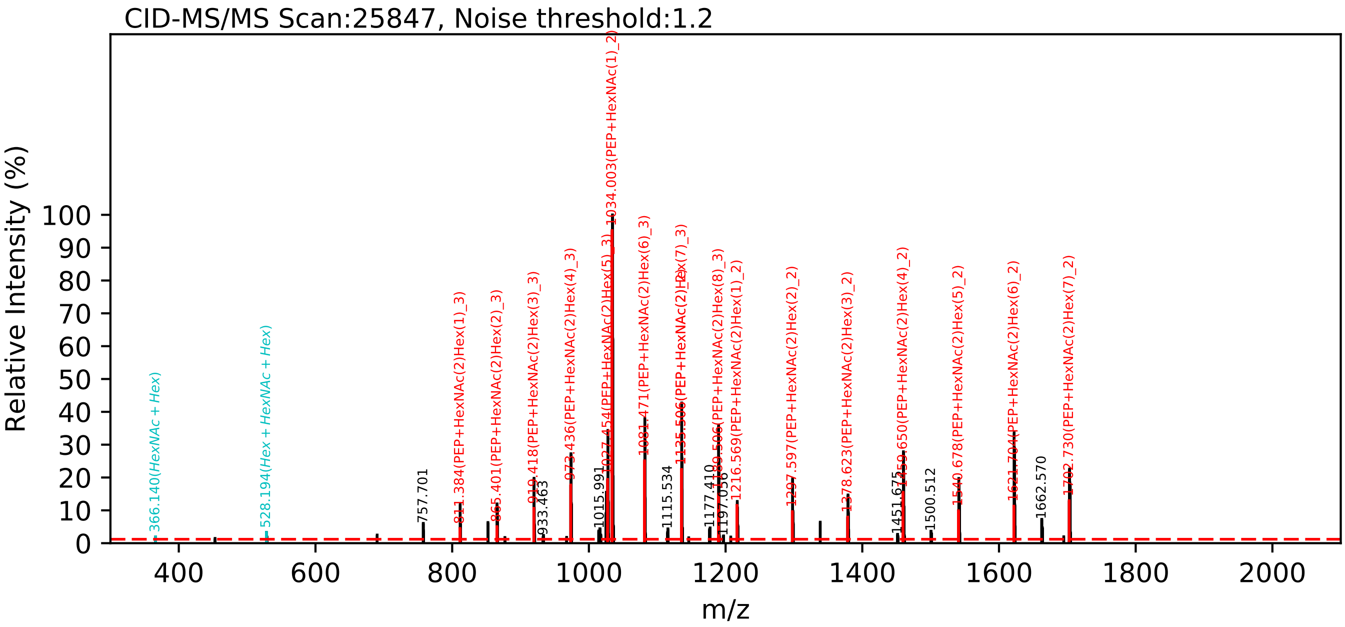

EGVFVSNGTHWFVTQR(=PEP)_9_2_0_0_0, 0_None, 0_None,
m/z:1243.52(3+), RT:65.06, Y-score:84.03

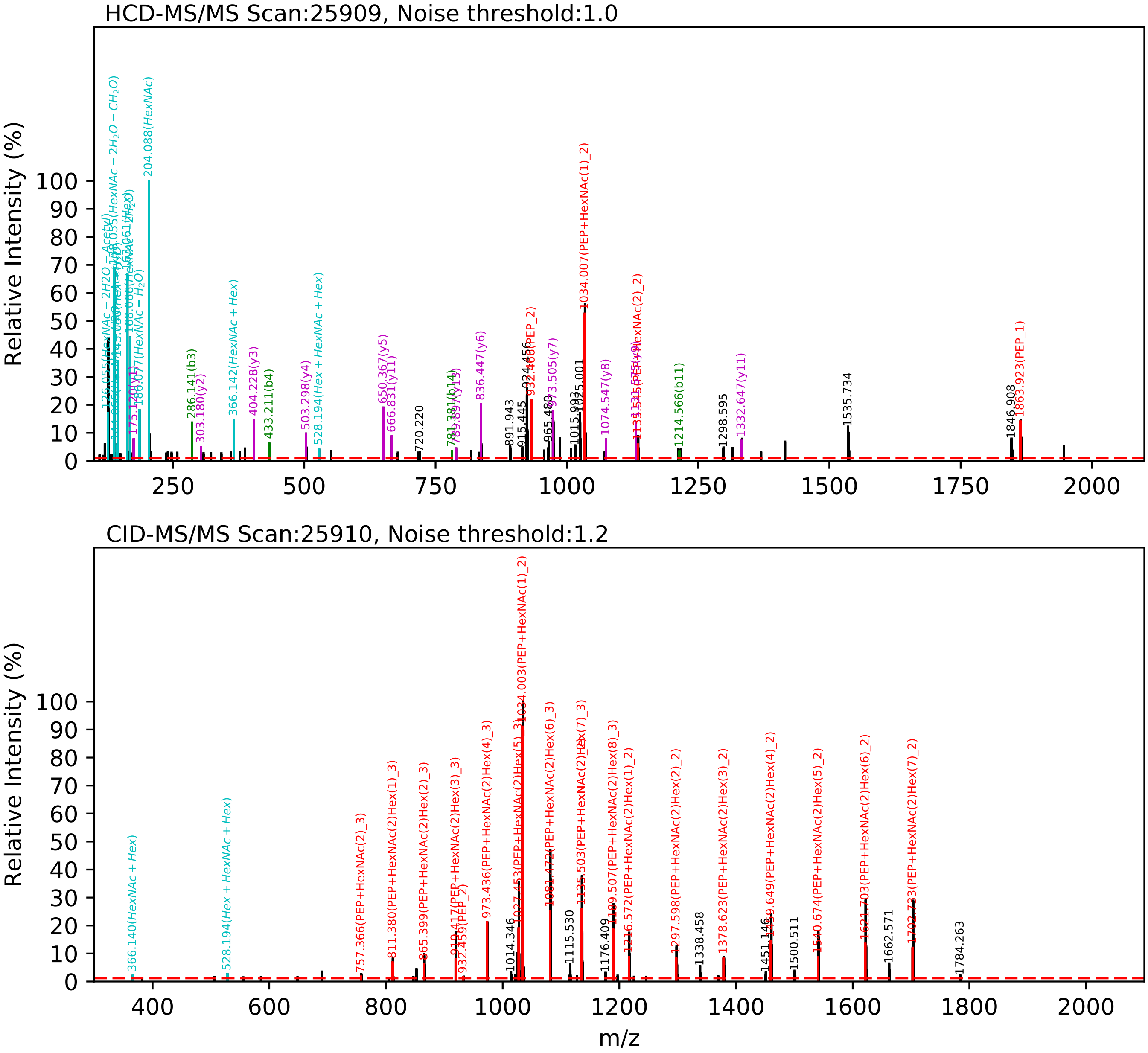

EGVFVSNGTHWFVTQR(=PEP)_9_2_0_0_0, 0_None, 0_None,
m/z:1243.52(3+), RT:65.77, Y-score:84.97

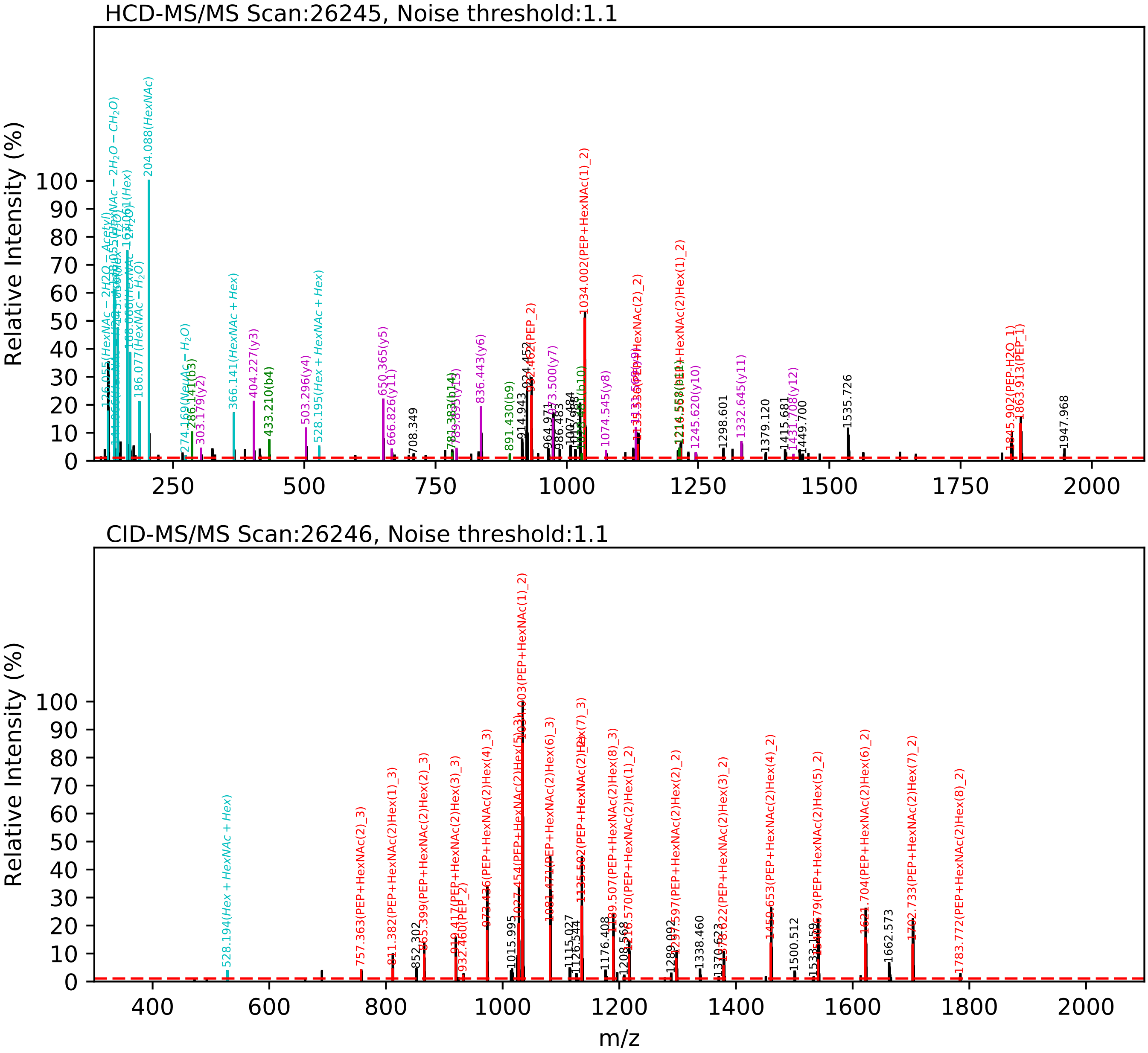

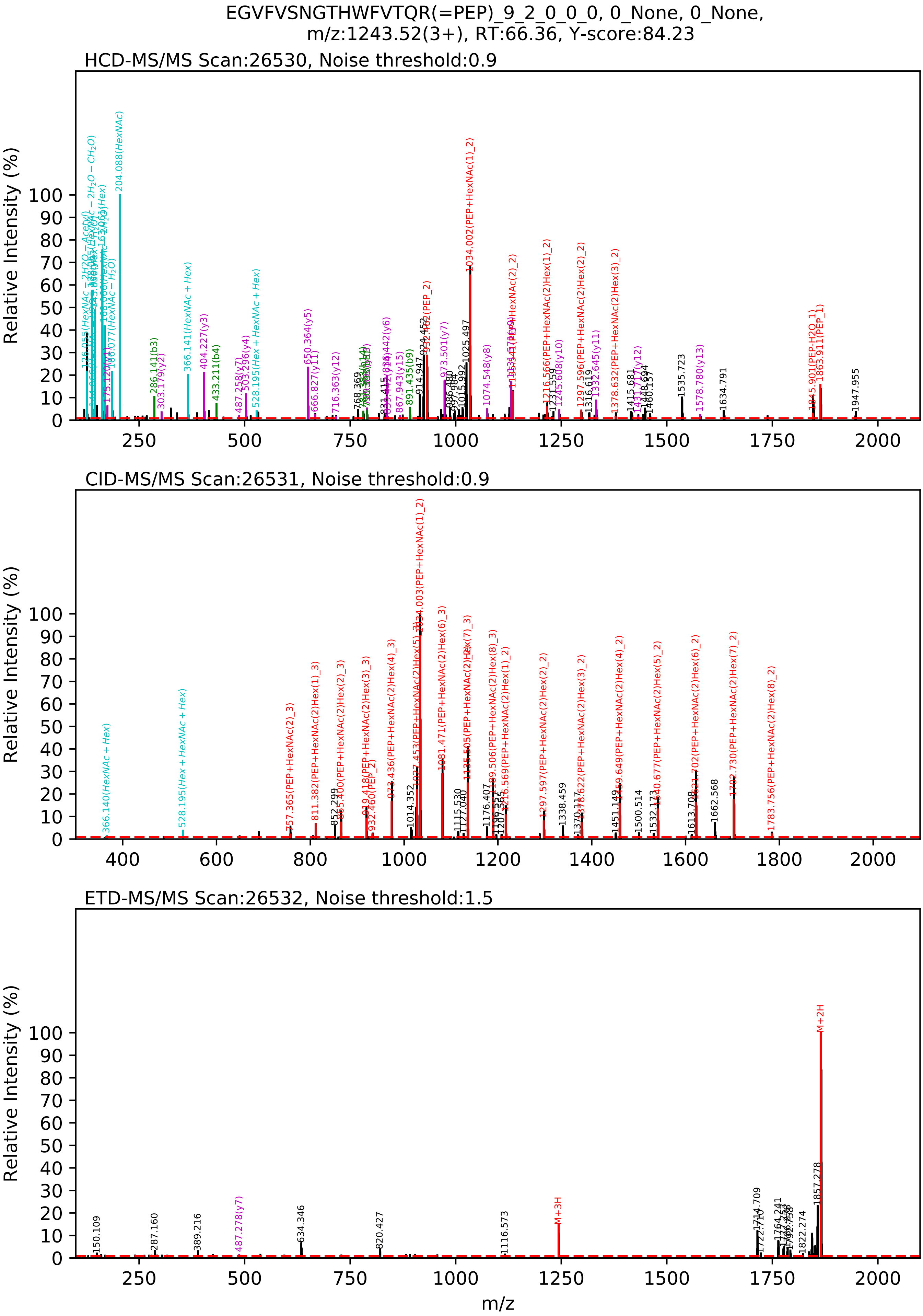

EGVFVSNGTHWFVTQR(=PEP)_9_2_0_0_0, 0_None, 0_None,
m/z:1243.52(3+), RT:66.36, Y-score:84.23
HCD-MS/MS Scan:26530, Noise threshold:0.9
CID-MS/MS Scan:26531, Noise threshold:0.9
ETD-MS/MS Scan:26532, Noise threshold:1.5
Relative Intensity (%)
m/z
204.088(HexNAc)
1034.002(PEP+HexNAc(1)_2)
1034.003(PEP+HexNAc(1)_2)
M+2H
M+3H
1857.278

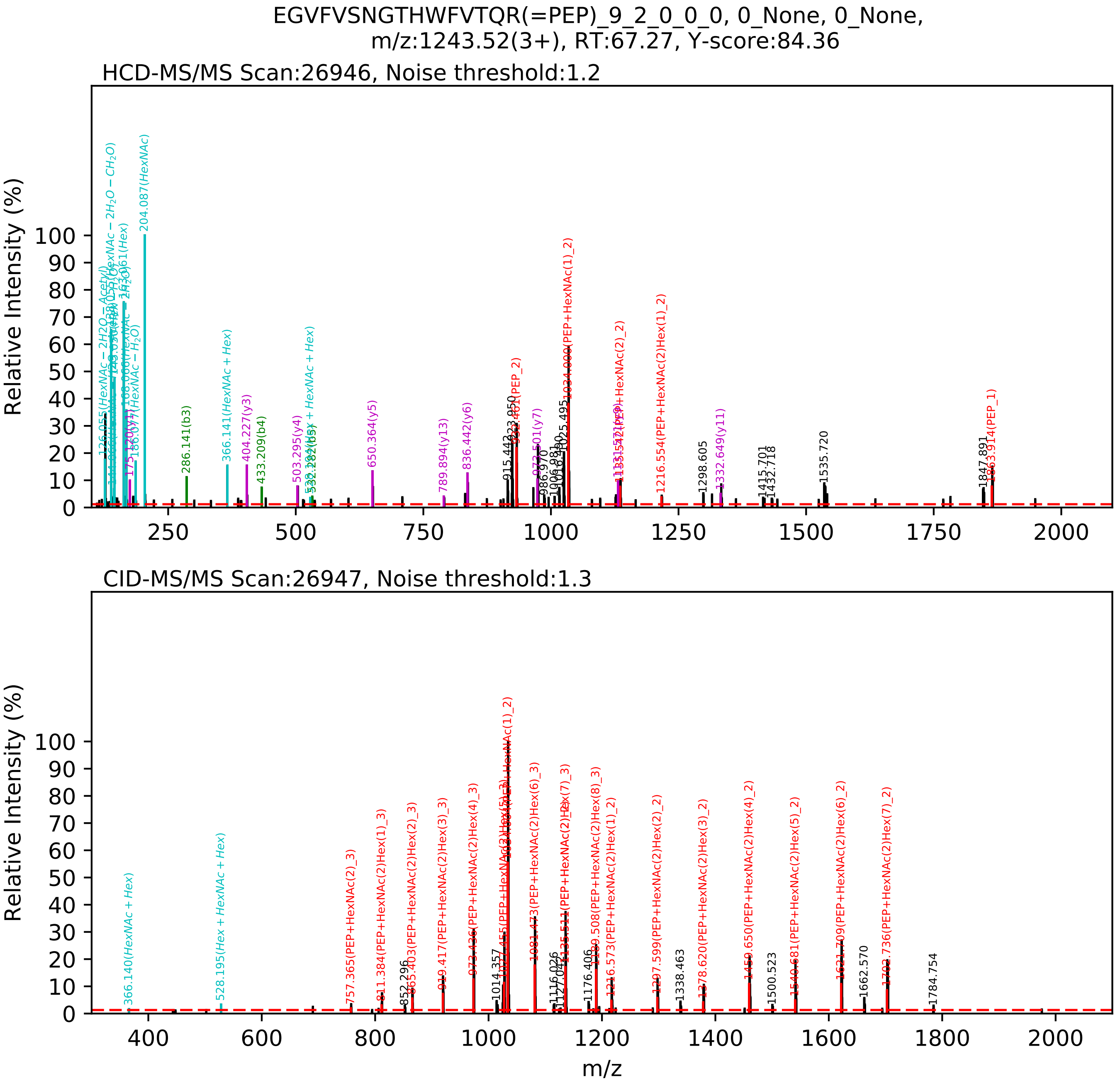

EGVFVSNGTHWFVTQR(=PEP)_9_2_0_0_0, 0_None, 0_None,
m/z:1243.52(3+), RT:67.27, Y-score:84.36
HCD-MS/MS Scan:26946, Noise threshold:1.2
Relative Intensity (%)
204.087(HexNAc)
286.141(b3)
366.141(HexNAc + Hex)
404.227(y3)
433.209(b4)
503.295(y4)
650.364(y5)
789.894(y13)
836.442(y6)
915.442
923.950
1034.000(PEP+HexNAc(1)_2)
1025.495
1216.554(PEP+HexNAc(2)Hex(1)_2)
1298.605
1332.649(y11)
1415.701
1432.718
1535.720
1847.891
1863.914(PEP_1)
CID-MS/MS Scan:26947, Noise threshold:1.3
Relative Intensity (%)
366.140(HexNAc + Hex)
528.195(Hex + HexNAc + Hex)
757.365(PEP+HexNAc(2)_3)
811.384(PEP+HexNAc(2)Hex(1)_3)
852.296
865.403(PEP+HexNAc(2)Hex(2)_3)
919.417(PEP+HexNAc(2)Hex(3)_3)
973.436(PEP+HexNAc(2)Hex(4)_3)
1014.357
1081.473(PEP+HexNAc(2)Hex(6)_3)
1116.026
1176.406
1216.573(PEP+HexNAc(2)Hex(1)_2)
1297.599(PEP+HexNAc(2)Hex(2)_2)
1338.463
1378.620(PEP+HexNAc(2)Hex(3)_2)
1459.650(PEP+HexNAc(2)Hex(4)_2)
1500.523
1540.681(PEP+HexNAc(2)Hex(5)_2)
1621.709(PEP+HexNAc(2)Hex(6)_2)
1662.570
1702.736(PEP+HexNAc(2)Hex(7)_2)
1784.754
m/z

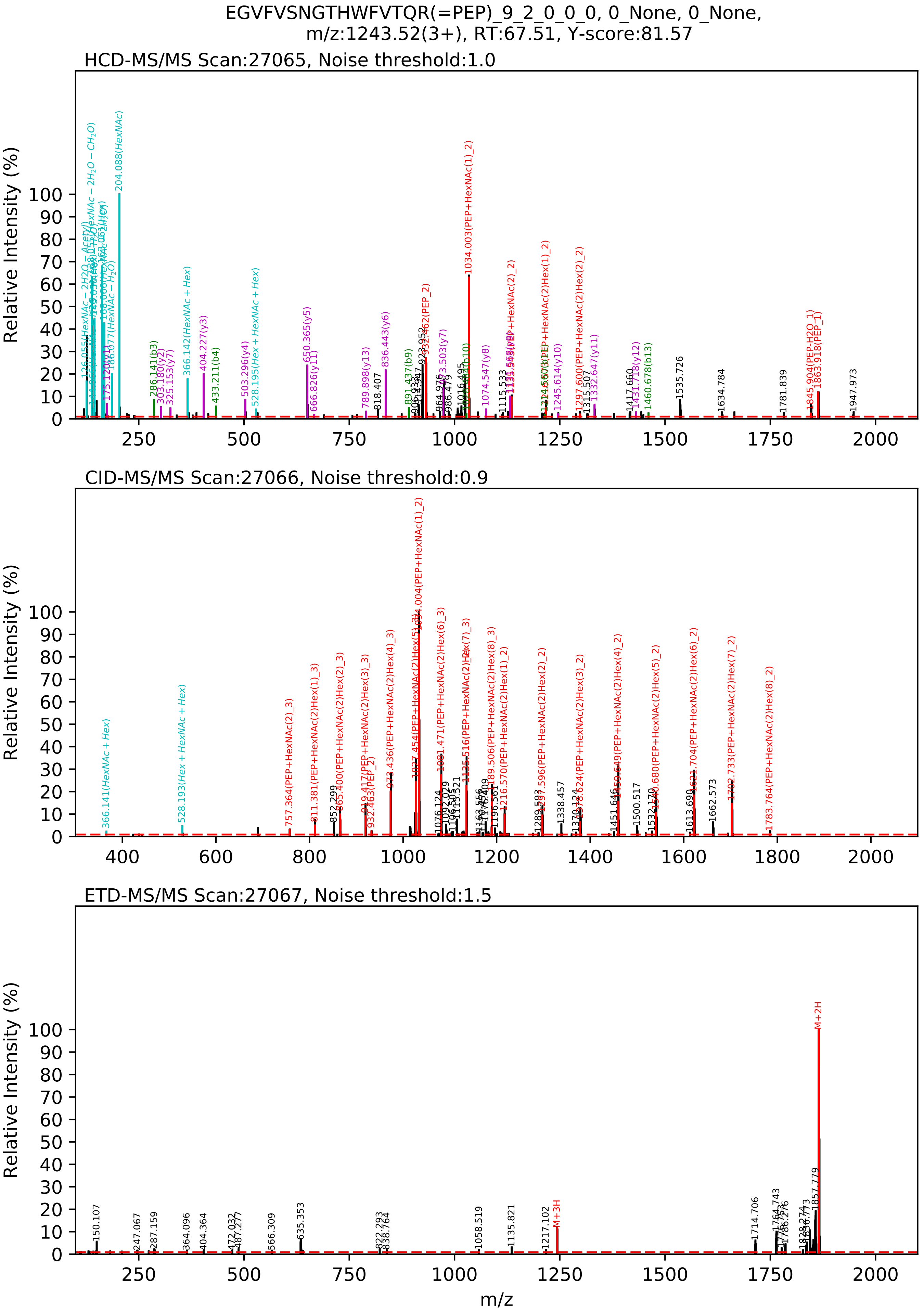

EGVFVSNGTHWFVTQR(=PEP)_9_2_0_0_0, 0_None, 0_None,
m/z:1243.52(3+), RT:67.51, Y-score:81.57
HCD-MS/MS Scan:27065, Noise threshold:1.0
Relative Intensity (%)
204.088(HexNAc)
1034.003(PEP+HexNAc(1)_2)
932.462(PEP_2)
650.365(y5)
836.443(y6)
404.227(y3)
366.142(HexNAc + Hex)
528.195(Hex + HexNAc + Hex)
286.141(b3)
433.211(b4)
503.296(y4)
1297.600(PEP+HexNAc(2)Hex(2)_2)
1845.904(PEP-H2O_1)
1863.918(PEP_1)
1535.726
1634.784
1781.839
1947.973
CID-MS/MS Scan:27066, Noise threshold:0.9
Relative Intensity (%)
366.141(HexNAc + Hex)
528.193(Hex + HexNAc + Hex)
757.364(PEP+HexNAc(2)_3)
811.381(PEP+HexNAc(2)Hex(1)_3)
972.436(PEP+HexNAc(2)Hex(4)_3)
1081.471(PEP+HexNAc(2)Hex(6)_3)
1216.570(PEP+HexNAc(2)Hex(1)_2)
1297.596(PEP+HexNAc(2)Hex(2)_2)
1378.624(PEP+HexNAc(2)Hex(3)_2)
1540.680(PEP+HexNAc(2)Hex(5)_2)
1621.704(PEP+HexNAc(2)Hex(6)_2)
1702.733(PEP+HexNAc(2)Hex(7)_2)
1783.764(PEP+HexNAc(2)Hex(8)_2)
ETD-MS/MS Scan:27067, Noise threshold:1.5
Relative Intensity (%)
M+2H
M+3H
150.107
635.353
1714.706
1857.779
m/z

EGVFVSNGTHWFVTQR(=PEP)_9_2_0_0_0, 0_None, 0_None,
m/z:1243.52(3+), RT:68.26, Y-score:81.42

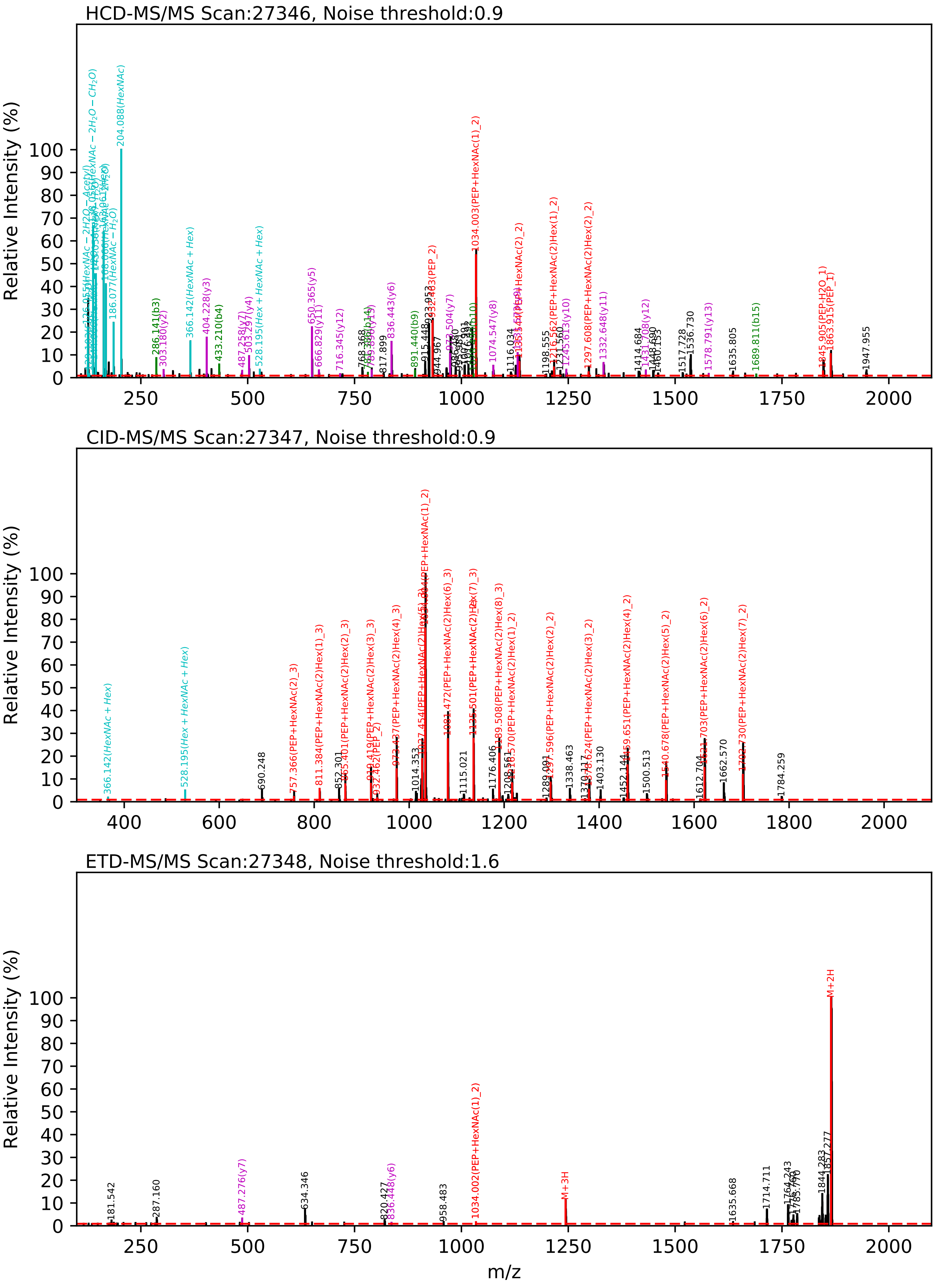

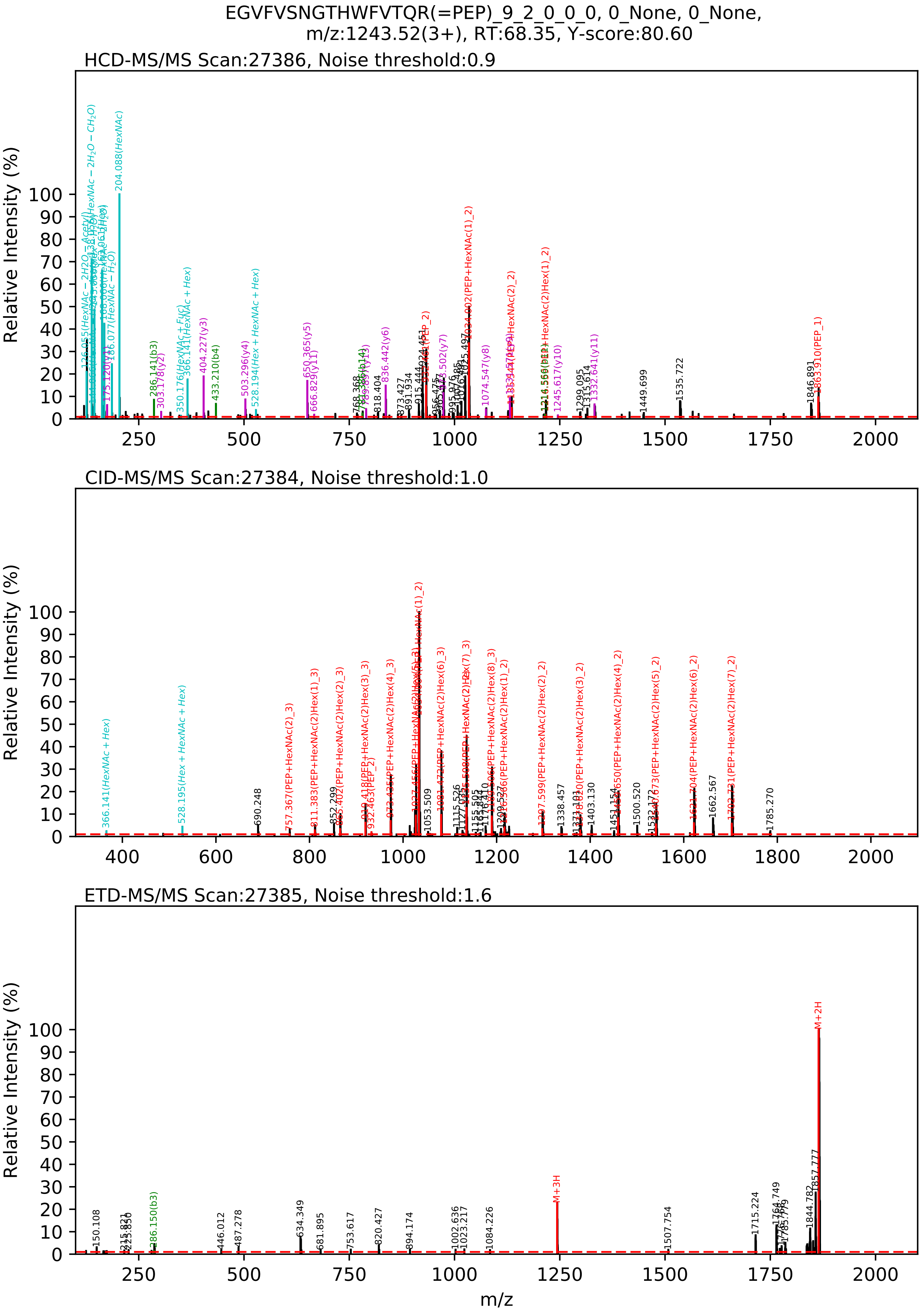
EGVFVSNGTHWFVTQR(=PEP)_9_2_0_0_0, 0_None, 0_None,
m/z:1243.52(3+), RT:68.35, Y-score:80.60
HCD-MS/MS Scan:27386, Noise threshold:0.9
CID-MS/MS Scan:27384, Noise threshold:1.0
ETD-MS/MS Scan:27385, Noise threshold:1.6
Relative Intensity (%)
m/z
204.088(HexNAc)
1034.002(PEP+HexNAc(1)_2)
1863.910(PEP_1)
M+2H
M+3H

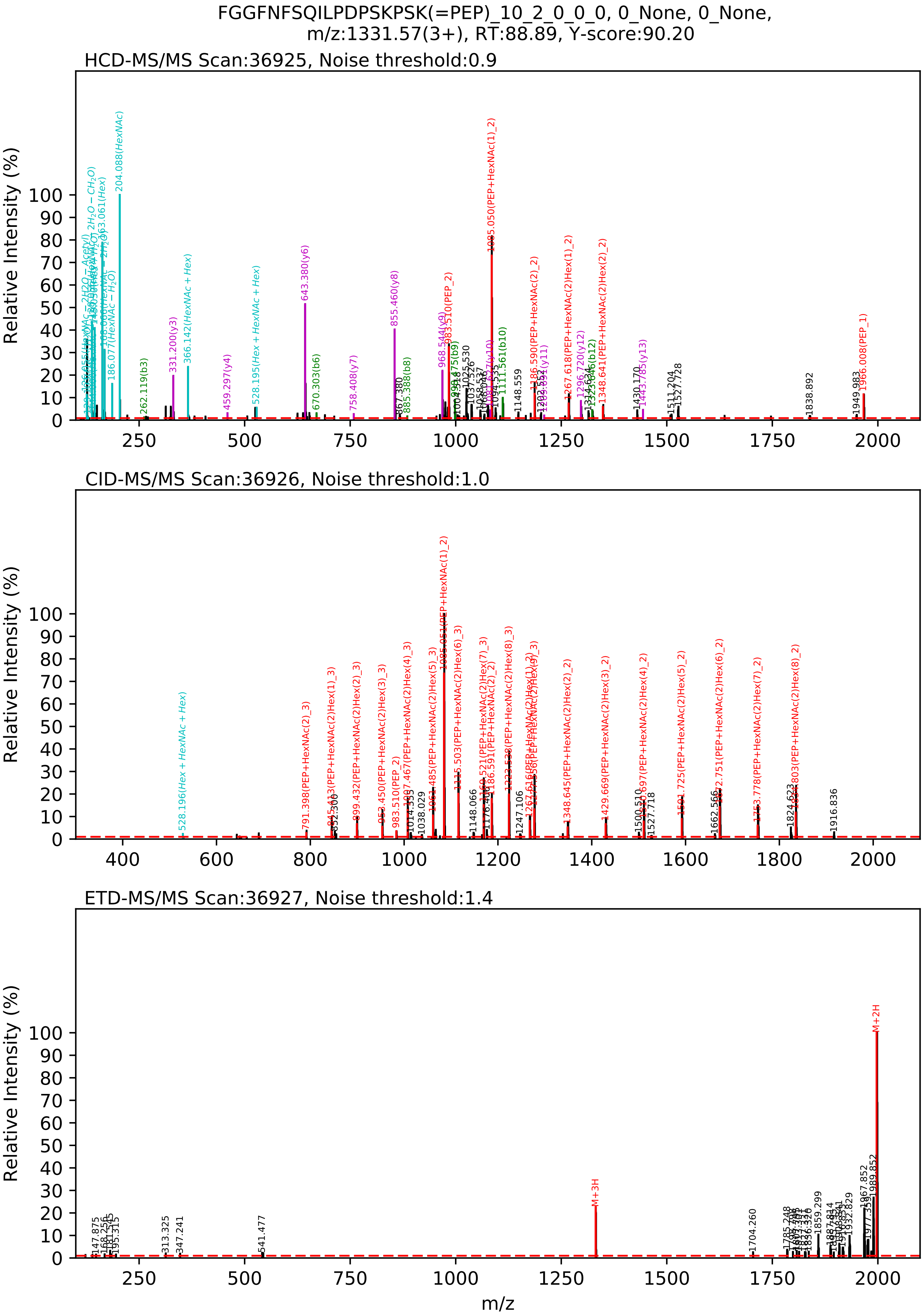

FGGFNFSQILPDPSKPSK(=PEP)_10_2_0_0_0, 0_None, 0_None,
m/z:1331.57(3+), RT:88.89, Y-score:90.20
HCD-MS/MS Scan:36925, Noise threshold:0.9
CID-MS/MS Scan:36926, Noise threshold:1.0
ETD-MS/MS Scan:36927, Noise threshold:1.4
Relative Intensity (%)
m/z

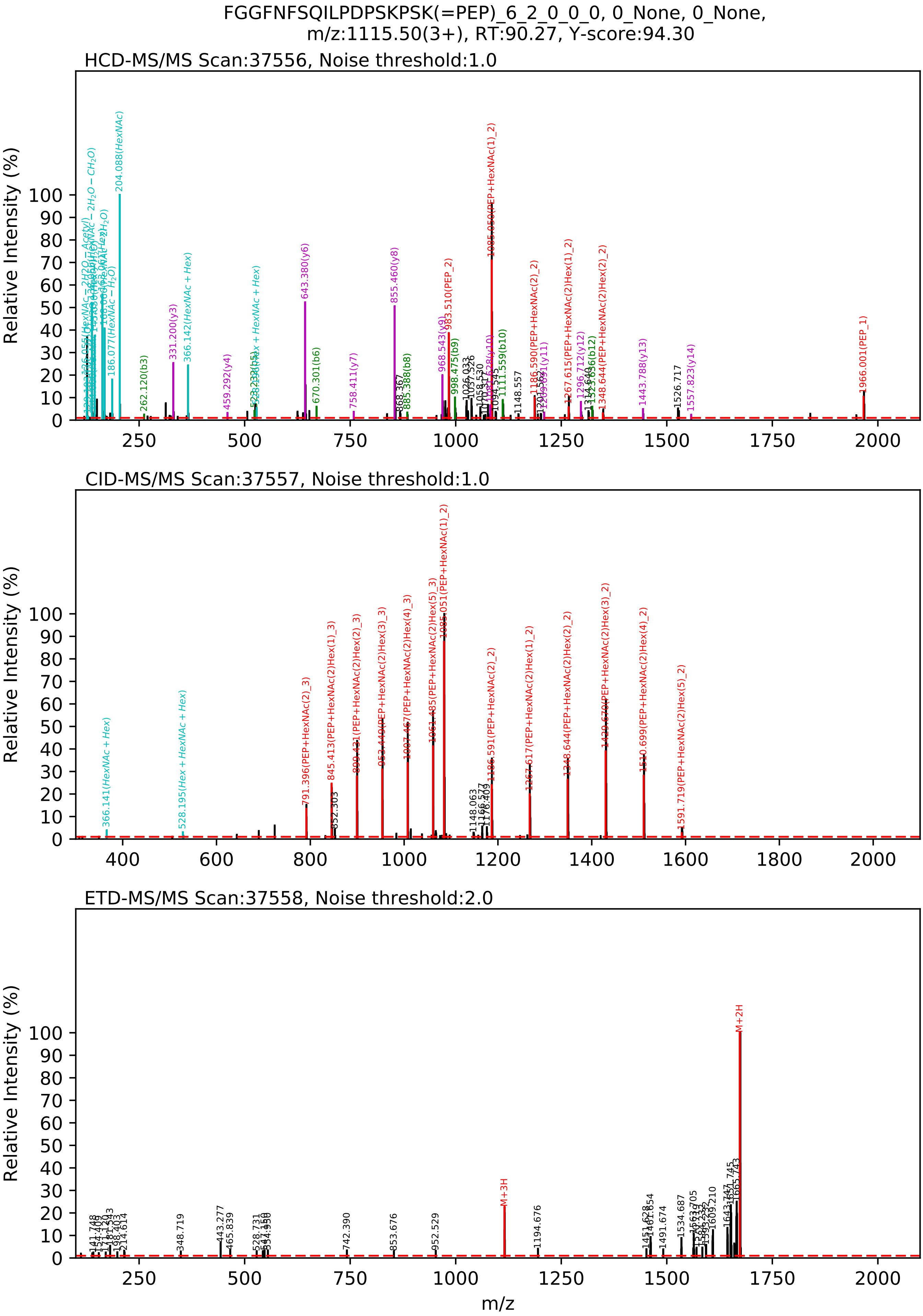

FGGFNFSQILPDPSKPSK(=PEP)_6_2_0_0_0, 0_None, 0_None,
m/z:1115.50(3+), RT:90.27, Y-score:94.30
HCD-MS/MS Scan:37556, Noise threshold:1.0
CID-MS/MS Scan:37557, Noise threshold:1.0
ETD-MS/MS Scan:37558, Noise threshold:2.0
Relative Intensity (%)
m/z

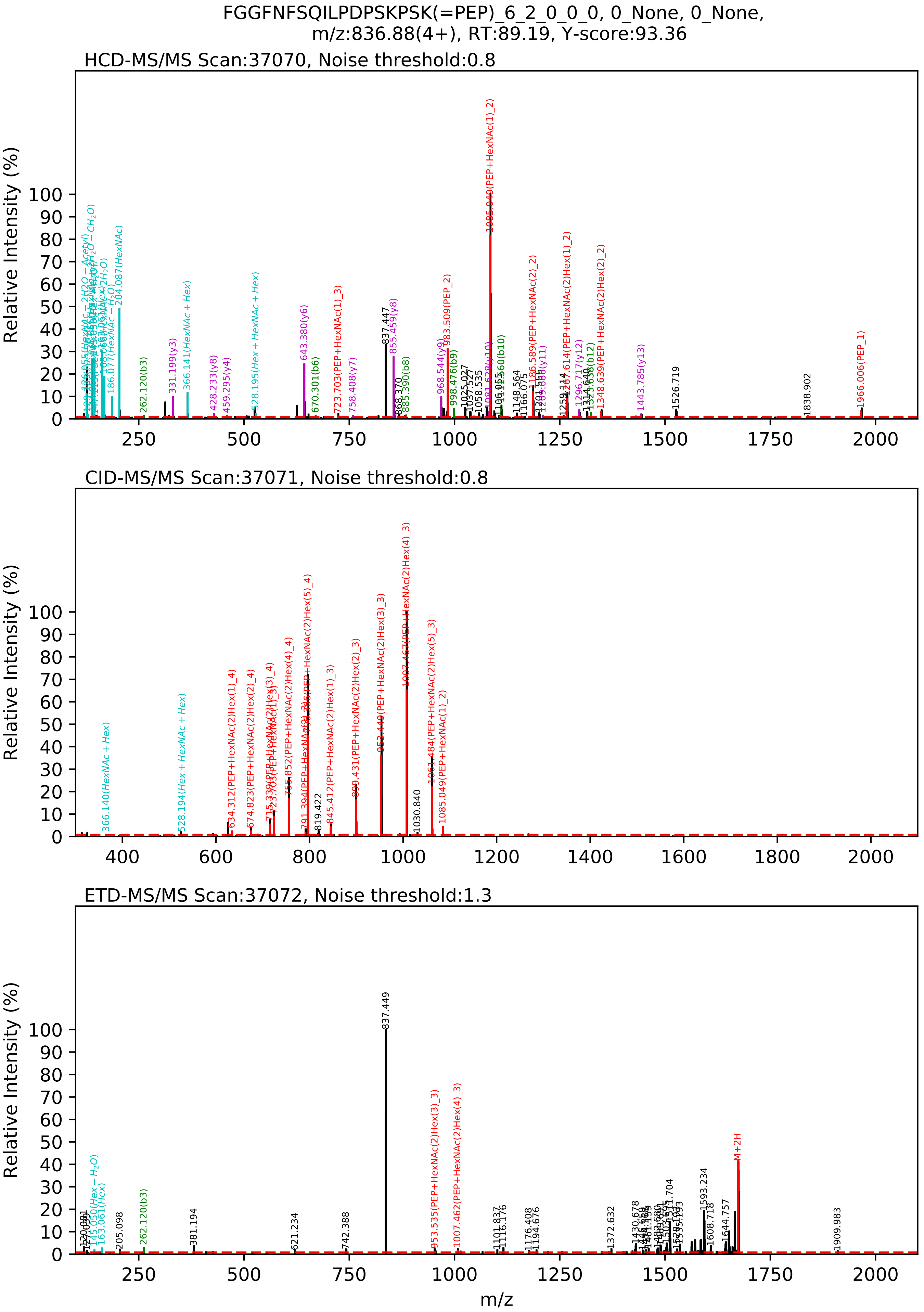

FGGFNFSQILPDPSKPSK(=PEP)_6_2_0_0_0, 0_None, 0_None,
m/z:836.88(4+), RT:89.19, Y-score:93.36
HCD-MS/MS Scan:37070, Noise threshold:0.8
Relative Intensity (%)
204.087(HexNAc)
262.120(b3)
331.199(y3)
366.141(HexNAc + Hex)
428.233(y8)
459.295(y4)
528.195(Hex + HexNAc + Hex)
643.380(y6)
670.301(b6)
723.703(PEP+HexNAc(1)_3)
758.408(y7)
837.447
855.459(y8)
868.370
885.390(b8)
968.544(y9)
983.509(PEP_2)
998.476(b9)
1085.049(PEP+HexNAc(1)_2)
1148.564
1166.075
1186.589(PEP+HexNAc(2)_2)
1267.614(PEP+HexNAc(2)Hex(1)_2)
1296.717(y12)
1348.639(PEP+HexNAc(2)Hex(2)_2)
1443.785(y13)
1526.719
1838.902
1966.006(PEP_1)
CID-MS/MS Scan:37071, Noise threshold:0.8
366.140(HexNAc + Hex)
528.194(Hex + HexNAc + Hex)
634.312(PEP+HexNAc(2)Hex(1)_4)
674.823(PEP+HexNAc(2)Hex(2)_4)
755.852(PEP+HexNAc(2)Hex(4)_4)
819.422
845.412(PEP+HexNAc(2)Hex(1)_3)
899.431(PEP+HexNAc(2)Hex(2)_3)
953.449(PEP+HexNAc(2)Hex(3)_3)
1007.467(PEP+HexNAc(2)Hex(4)_3)
1030.840
1061.484(PEP+HexNAc(2)Hex(5)_3)
1085.049(PEP+HexNAc(1)_2)
ETD-MS/MS Scan:37072, Noise threshold:1.3
145.050(Hex − H2O)
163.061(Hex)
205.098
262.120(b3)
381.194
621.234
742.388
837.449
953.535(PEP+HexNAc(2)Hex(3)_3)
1007.462(PEP+HexNAc(2)Hex(4)_3)
1101.837
1116.176
1176.408
1194.676
1372.632
1430.678
1593.234
1608.718
1644.757
M+2H
1909.983
m/z

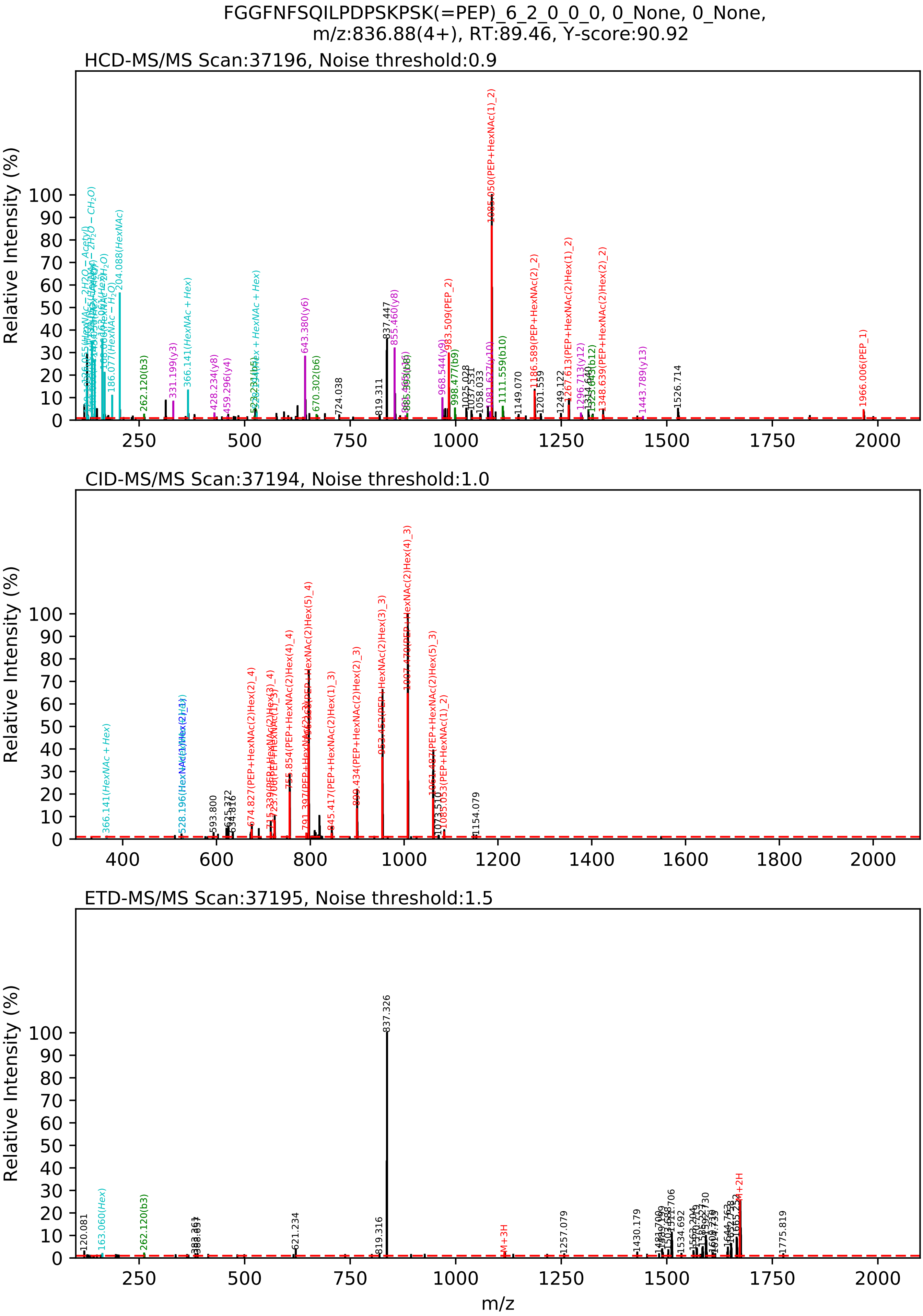

FGGFNFSQILPDPSKPSK(=PEP)_6_2_0_0_0, 0_None, 0_None,
m/z:836.88(4+), RT:89.46, Y-score:90.92
HCD-MS/MS Scan:37196, Noise threshold:0.9
CID-MS/MS Scan:37194, Noise threshold:1.0
ETD-MS/MS Scan:37195, Noise threshold:1.5
Relative Intensity (%)
m/z
1085.050(PEP+HexNAc(1)_2)
204.088(HexNAc)
186.077(HexNAc − H2O)
366.141(HexNAc + Hex)
643.380(y6)
855.460(y8)
837.447
983.509(PEP_2)
1186.589(PEP+HexNAc(2)_2)
1267.613(PEP+HexNAc(2)Hex(1)_2)
1348.639(PEP+HexNAc(2)Hex(2)_2)
1966.006(PEP_1)
1443.789(y13)
1526.714
1111.559(b10)
998.477(b9)
968.544(y9)
262.120(b3)
331.199(y3)
428.234(y8)
459.296(y4)
670.302(b6)
724.038
819.311
1149.070
1201.559
1249.122
1296.713(y12)
1007.470(PEP+HexNAc(2)Hex(4)_3)
953.452(PEP+HexNAc(2)Hex(3)_3)
899.434(PEP+HexNAc(2)Hex(2)_3)
845.417(PEP+HexNAc(2)Hex(1)_3)
755.854(PEP+HexNAc(2)Hex(4)_4)
674.827(PEP+HexNAc(2)Hex(2)_4)
1061.487(PEP+HexNAc(2)Hex(5)_3)
1085.053(PEP+HexNAc(1)_2)
1154.079
593.800
625.372
634.816
366.141(HexNAc + Hex)
837.326
M+2H
M+3H
163.060(Hex)
262.120(b3)
120.081
621.234
819.316
1257.079
1430.179
1534.692
1775.819

FGGFNFSQILPDPSKPSK(=PEP)_7_2_0_0_0, 0_None, 0_None,
m/z:1169.52(3+), RT:89.75, Y-score:93.29

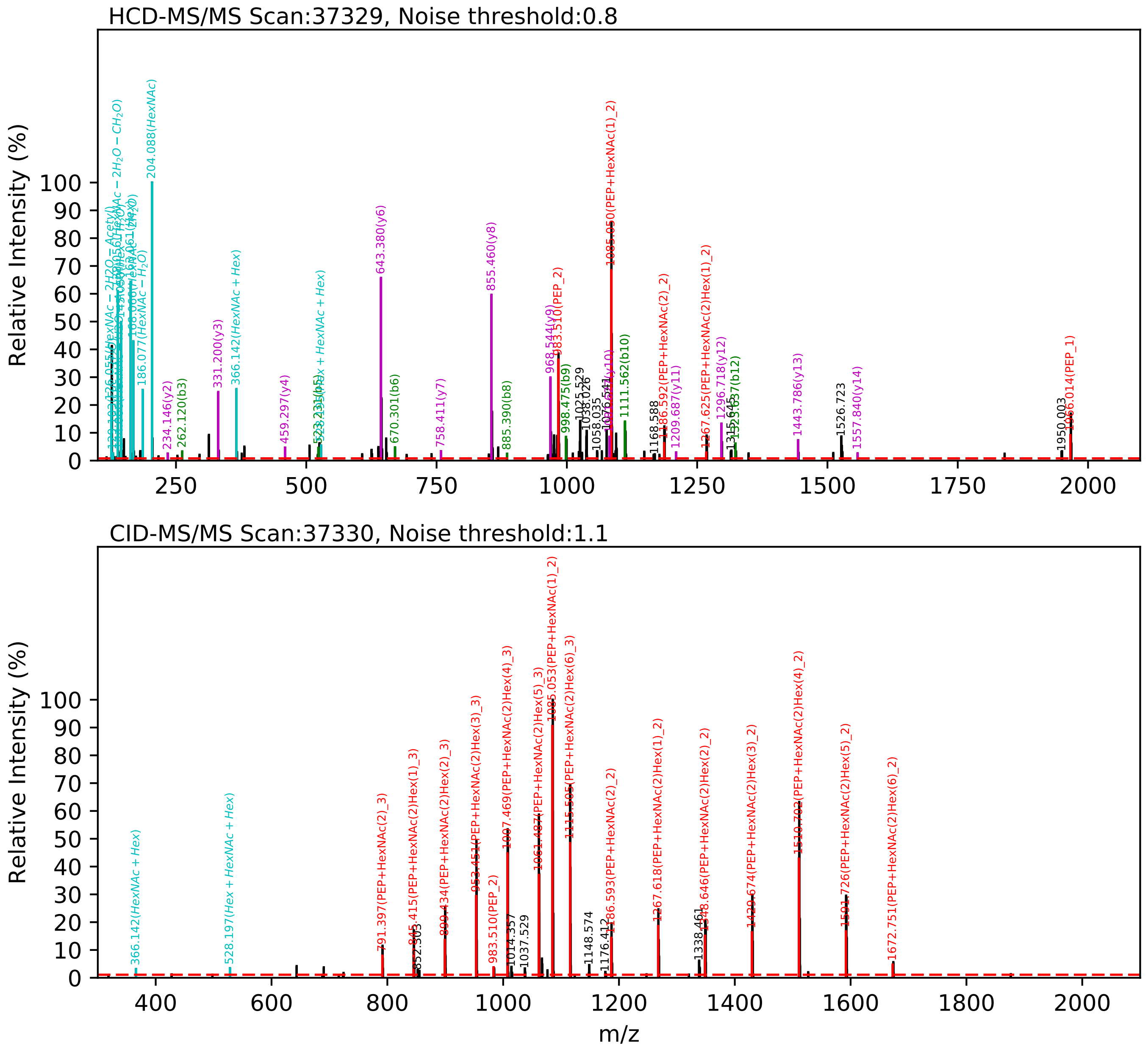

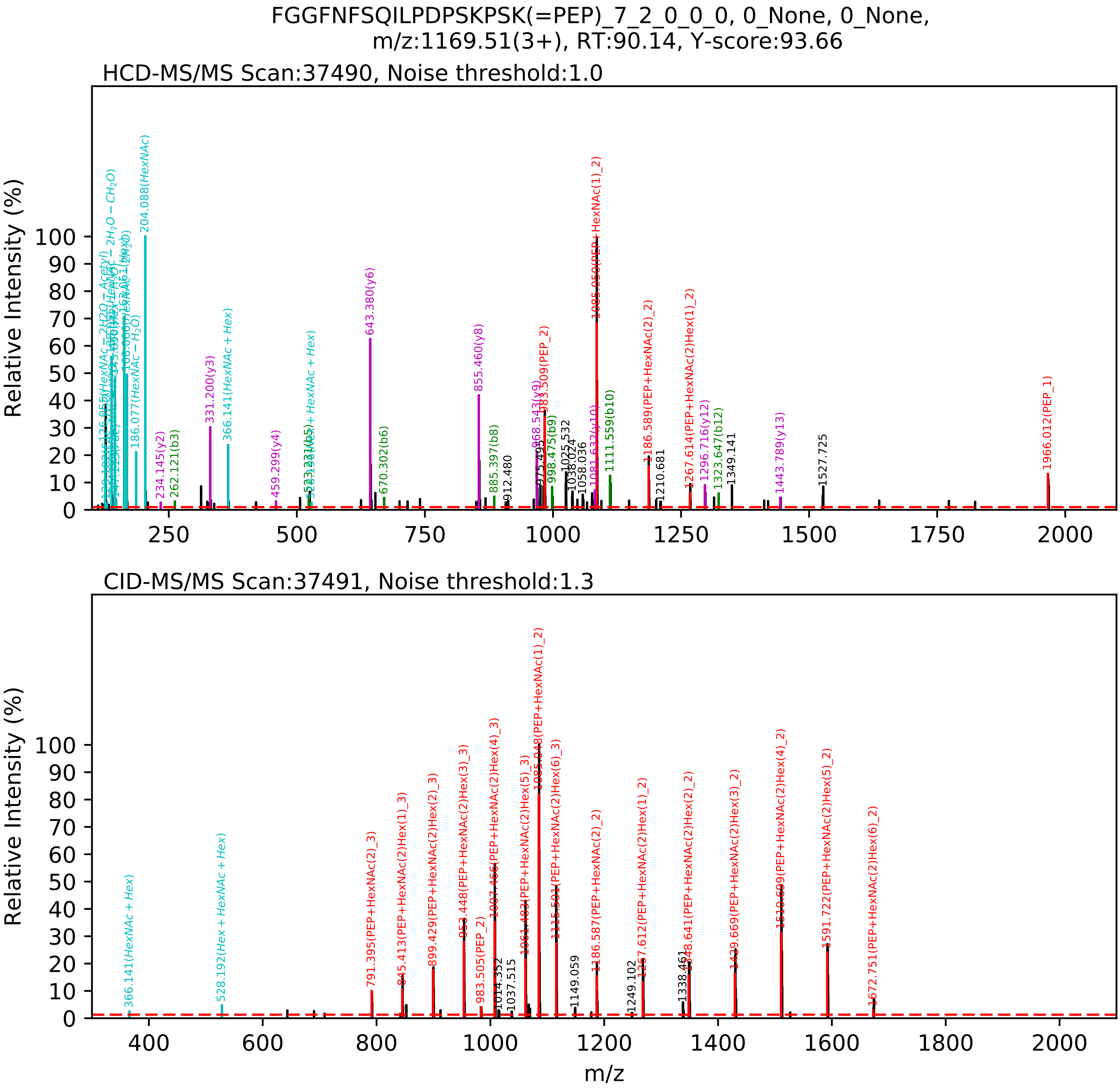
FGGFNFSQILPDPSKPSK(=PEP)_7_2_0_0_0, 0_None, 0_None,
m/z:1169.51(3+), RT:90.14, Y-score:93.66
HCD-MS/MS Scan:37490, Noise threshold:1.0
CID-MS/MS Scan:37491, Noise threshold:1.3
Relative Intensity (%)
m/z

FGGFNFSQILPDPSKPSK(=PEP)_8_2_0_0_0, 0_None, 0_None,
m/z:917.90(4+), RT:89.17, Y-score:92.94

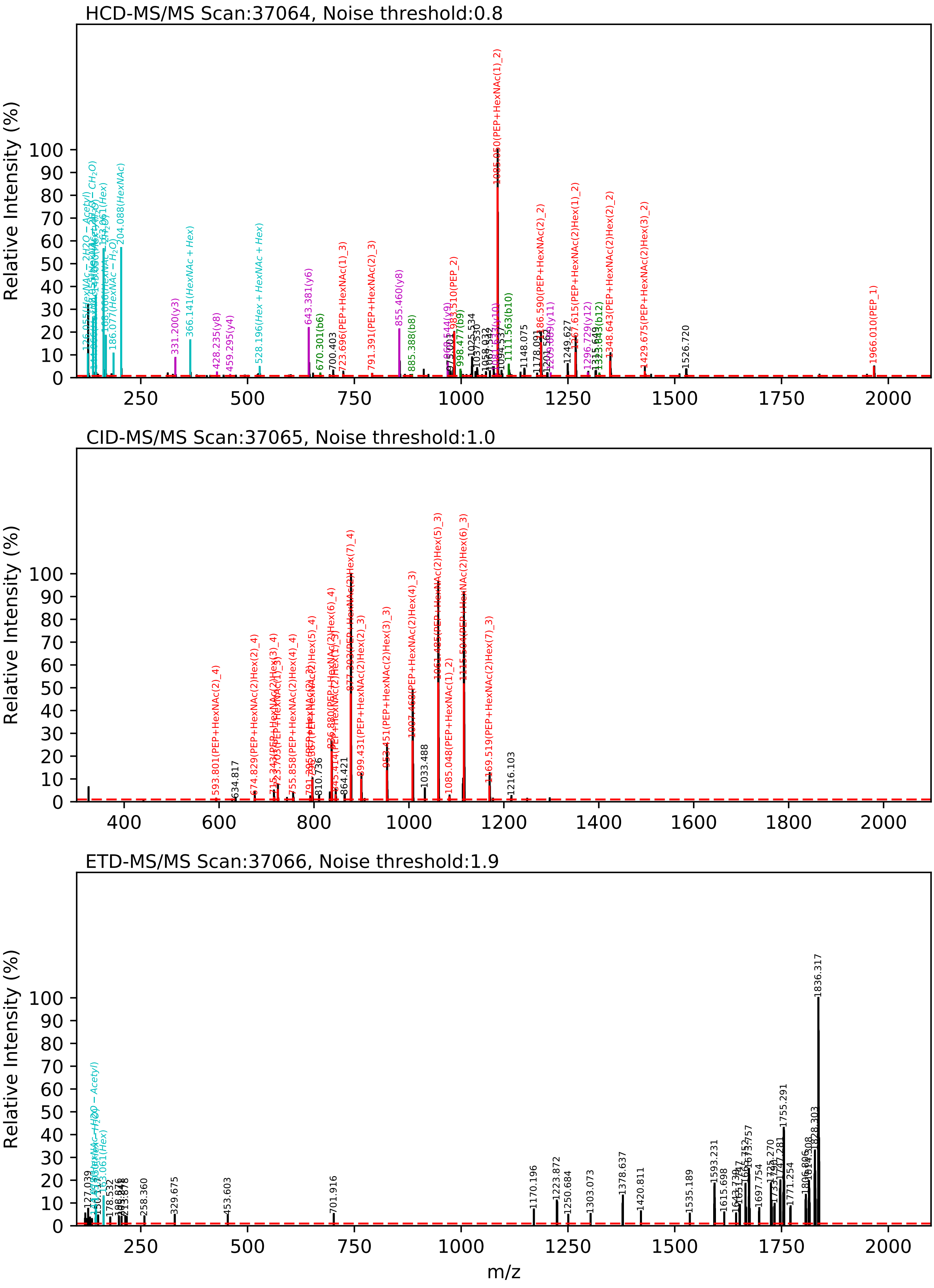

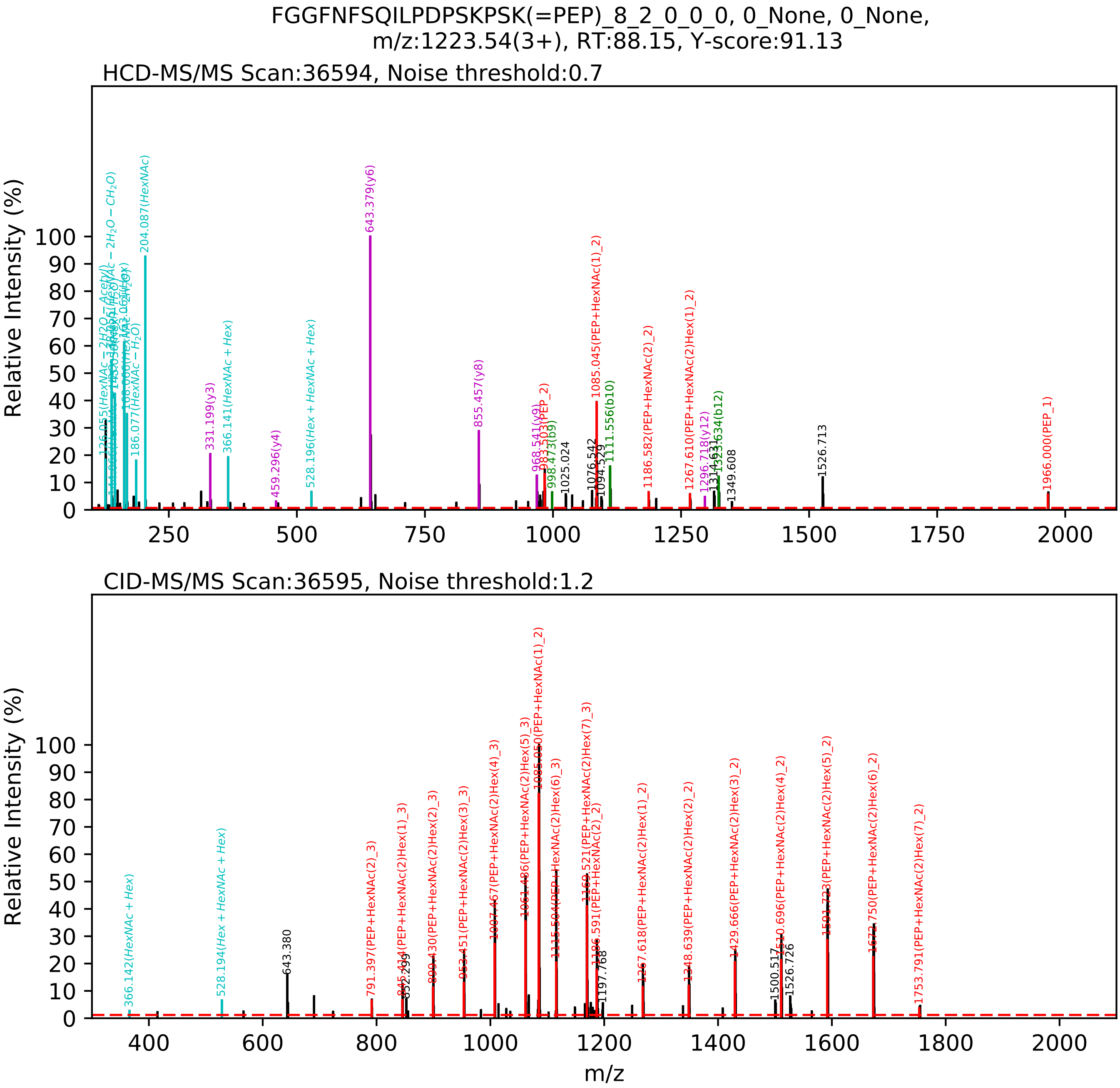
FGGFNFSQILPDPSKPSK(=PEP)_8_2_0_0_0, 0_None, 0_None,
m/z:1223.54(3+), RT:88.15, Y-score:91.13
HCD-MS/MS Scan:36594, Noise threshold:0.7
Relative Intensity (%)
204.087(HexNAc)
186.077(HexNAc − H2O)
331.199(y3)
366.141(HexNAc + Hex)
459.296(y4)
528.196(Hex + HexNAc + Hex)
643.379(y6)
855.457(y8)
968.541(y9)
998.473(b9)
1025.024
1076.542
1094.529
1085.045(PEP+HexNAc(1)_2)
1111.556(b10)
1186.582(PEP+HexNAc(2)_2)
1267.610(PEP+HexNAc(2)Hex(1)_2)
1296.718(y12)
1349.608
1526.713
1966.000(PEP_1)
CID-MS/MS Scan:36595, Noise threshold:1.2
366.142(HexNAc + Hex)
528.194(Hex + HexNAc + Hex)
643.380
791.397(PEP+HexNAc(2)_3)
852.299
899.430(PEP+HexNAc(2)Hex(2)_3)
953.451(PEP+HexNAc(2)Hex(3)_3)
1007.467(PEP+HexNAc(2)Hex(4)_3)
1061.486(PEP+HexNAc(2)Hex(5)_3)
1085.050(PEP+HexNAc(1)_2)
1115.504(PEP+HexNAc(2)Hex(6)_3)
1169.521(PEP+HexNAc(2)Hex(7)_3)
1186.591(PEP+HexNAc(2)_2)
1197.768
1267.618(PEP+HexNAc(2)Hex(1)_2)
1348.639(PEP+HexNAc(2)Hex(2)_2)
1429.666(PEP+HexNAc(2)Hex(3)_2)
1500.517
1526.726
1591.723(PEP+HexNAc(2)Hex(5)_2)
1672.750(PEP+HexNAc(2)Hex(6)_2)
1753.791(PEP+HexNAc(2)Hex(7)_2)
m/z

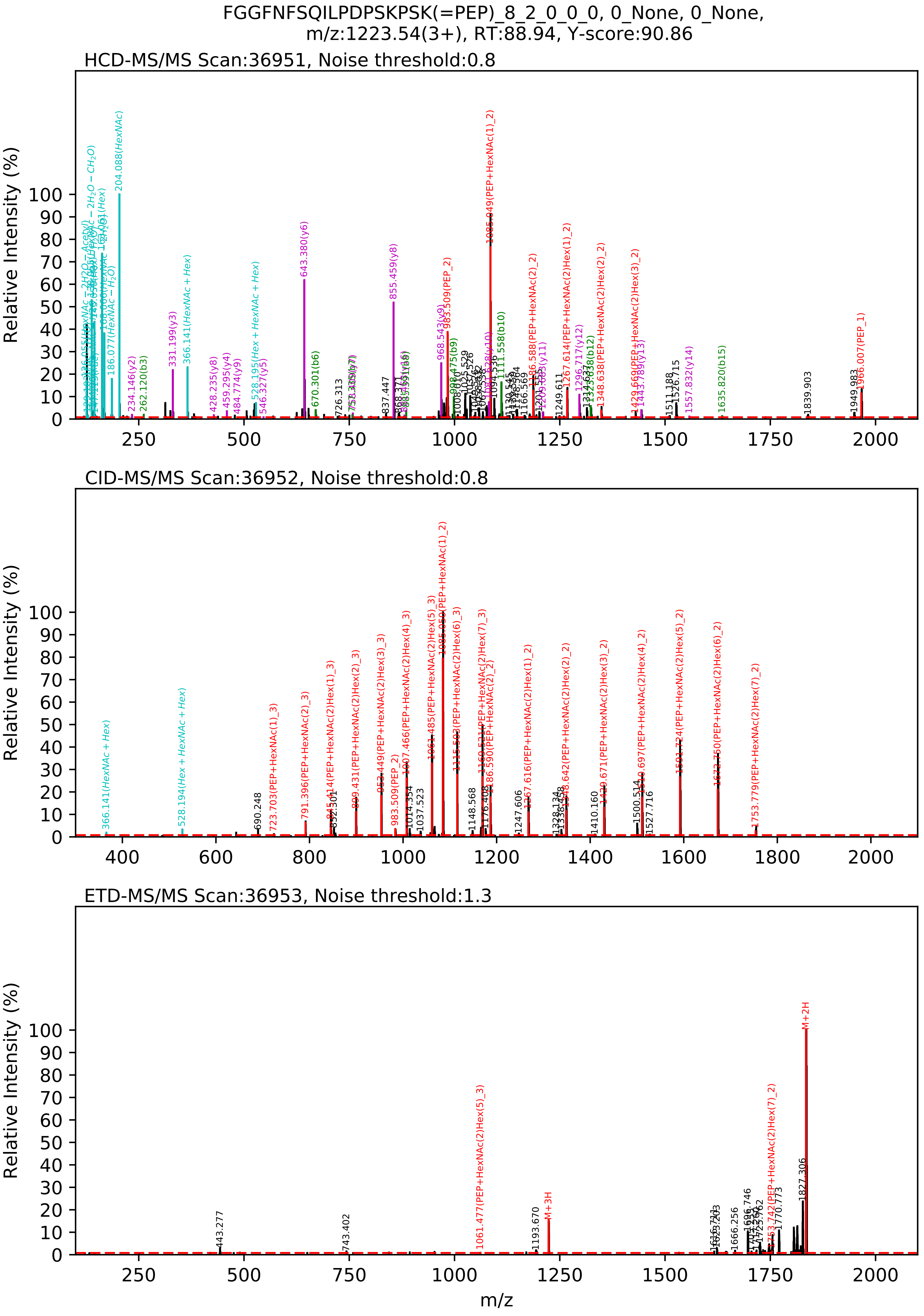

FGGFNFSQILPDPSKPSK(=PEP)_8_2_0_0_0, 0_None, 0_None,
m/z:1223.54(3+), RT:88.94, Y-score:90.86
HCD-MS/MS Scan:36951, Noise threshold:0.8
Relative Intensity (%)
204.088(HexNAc)
186.077(HexNAc − H2O)
262.120(b3)
234.146(y2)
331.199(y3)
366.141(HexNAc + Hex)
428.235(y8)
459.295(y4)
484.774(y9)
528.195(Hex + HexNAc + Hex)
546.327(y5)
643.380(y6)
670.301(b6)
726.313
837.447
855.459(y8)
868.371
968.543(y9)
983.509(PEP_2)
1085.049(PEP+HexNAc(1)_2)
1094.536
1111.558(b10)
1130.545
1166.569
1186.588(PEP+HexNAc(2)_2)
1249.611
1267.614(PEP+HexNAc(2)Hex(1)_2)
1296.717(y12)
1348.638(PEP+HexNAc(2)Hex(2)_2)
1429.660(PEP+HexNAc(2)Hex(3)_2)
1443.789(y13)
1511.188
1526.715
1557.832(y14)
1635.820(b15)
1839.903
1949.983
1966.007(PEP_1)
CID-MS/MS Scan:36952, Noise threshold:0.8
Relative Intensity (%)
366.141(HexNAc + Hex)
528.194(Hex + HexNAc + Hex)
690.248
723.703(PEP+HexNAc(1)_3)
791.396(PEP+HexNAc(2)_3)
852.301
899.431(PEP+HexNAc(2)Hex(2)_3)
983.509(PEP_2)
1007.466(PEP+HexNAc(2)Hex(4)_3)
1014.354
1037.523
1085.050(PEP+HexNAc(1)_2)
1115.503(PEP+HexNAc(2)Hex(6)_3)
1148.568
1176.408
1186.590(PEP+HexNAc(2)_2)
1247.606
1267.616(PEP+HexNAc(2)Hex(1)_2)
1328.134
1348.642(PEP+HexNAc(2)Hex(2)_2)
1410.160
1429.671(PEP+HexNAc(2)Hex(3)_2)
1500.514
1510.697(PEP+HexNAc(2)Hex(4)_2)
1527.716
1591.724(PEP+HexNAc(2)Hex(5)_2)
1672.750(PEP+HexNAc(2)Hex(6)_2)
1753.779(PEP+HexNAc(2)Hex(7)_2)
ETD-MS/MS Scan:36953, Noise threshold:1.3
Relative Intensity (%)
443.277
743.402
1061.477(PEP+HexNAc(2)Hex(5)_3)
1193.670
M+3H
1666.256
1696.746
1753.742(PEP+HexNAc(2)Hex(7)_2)
1770.773
1827.306
M+2H
m/z

FGGFNFSQILPDPSKPSK(=PEP)_8_2_0_0_0, 0_None, 0_None,
m/z:1223.54(3+), RT:89.52, Y-score:92.56

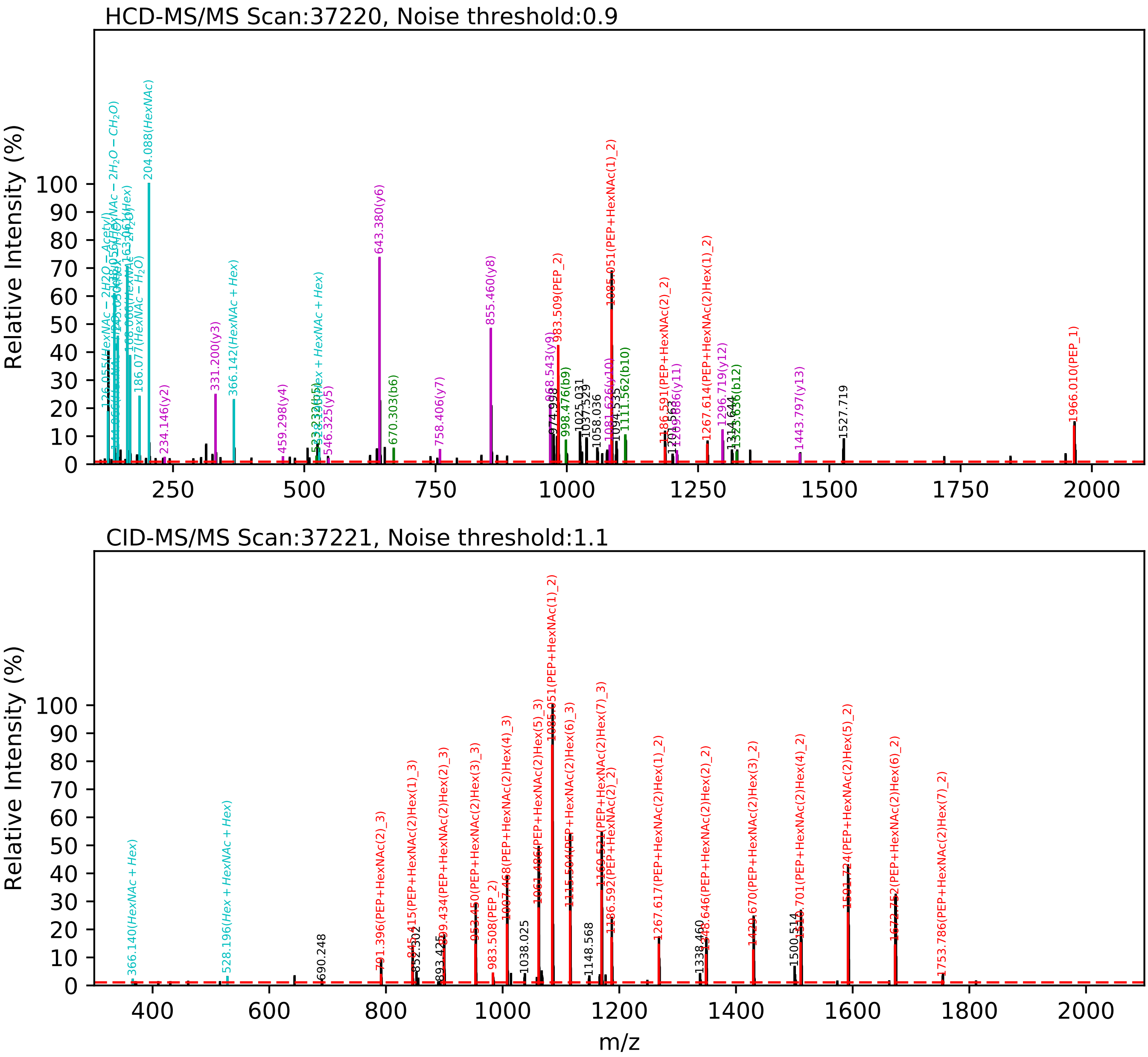

FGGFNFSQILPDPSKPSK(=PEP)_8_2_0_0_0, 0_None, 0_None,
m/z:1223.54(3+), RT:89.72, Y-score:93.86

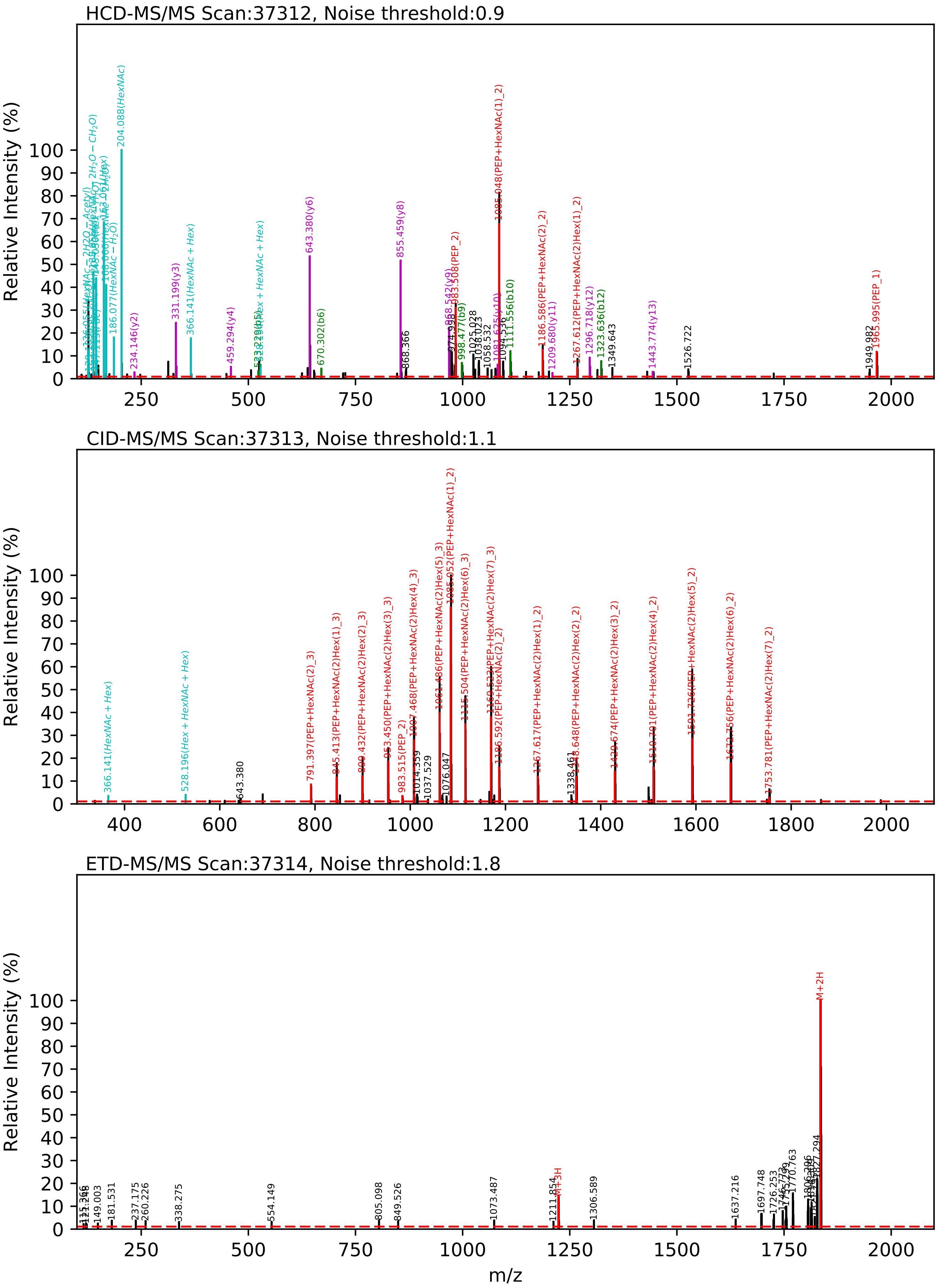

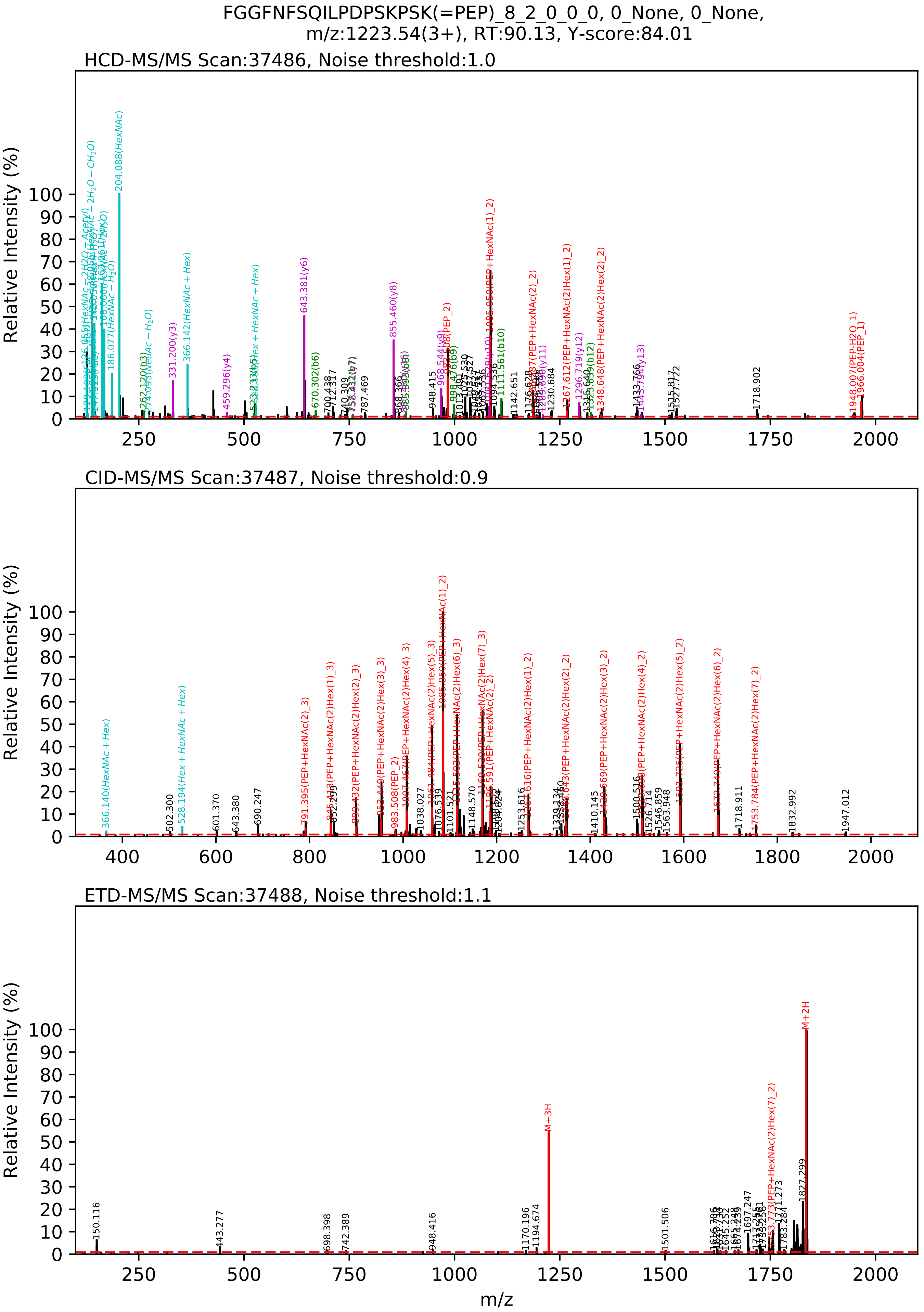
FGGFNFSQILPDPSKPSK(=PEP)_8_2_0_0_0, 0_None, 0_None,
m/z:1223.54(3+), RT:90.13, Y-score:84.01
HCD-MS/MS Scan:37486, Noise threshold:1.0
Relative Intensity (%)
204.088(HexNAc)
186.077(HexNAc − H2O)
262.120(b3)
331.200(y3)
366.142(HexNAc + Hex)
459.296(y4)
643.381(y6)
670.302(b6)
855.460(y8)
1085.050(PEP+HexNAc(1)_2)
1111.561(b10)
1267.612(PEP+HexNAc(2)Hex(1)_2)
1296.719(y12)
1348.648(PEP+HexNAc(2)Hex(2)_2)
1433.766
1515.817
1527.722
1718.902
1948.007(PEP-H2O_1)
1966.004(PEP_1)
CID-MS/MS Scan:37487, Noise threshold:0.9
366.140(HexNAc + Hex)
528.194(Hex + HexNAc + Hex)
502.300
601.370
643.380
690.247
791.395(PEP+HexNAc(2)_3)
900.432(PEP+HexNAc(2)Hex(2)_3)
953.440(PEP+HexNAc(2)Hex(3)_3)
983.508(PEP_2)
1038.027
1085.050(PEP+HexNAc(1)_2)
1076.539
1101.521
1148.570
1253.616
1329.134
1410.145
1500.516
1526.714
1546.859
1563.948
1591.725(PEP+HexNAc(2)Hex(5)_2)
1672.740(PEP+HexNAc(2)Hex(6)_2)
1753.784(PEP+HexNAc(2)Hex(7)_2)
1718.911
1832.992
1947.012
ETD-MS/MS Scan:37488, Noise threshold:1.1
M+3H
M+2H
150.116
443.277
698.398
742.389
948.416
1170.196
1194.674
1501.506
1697.247
1771.273
1783.284
1827.299
m/z

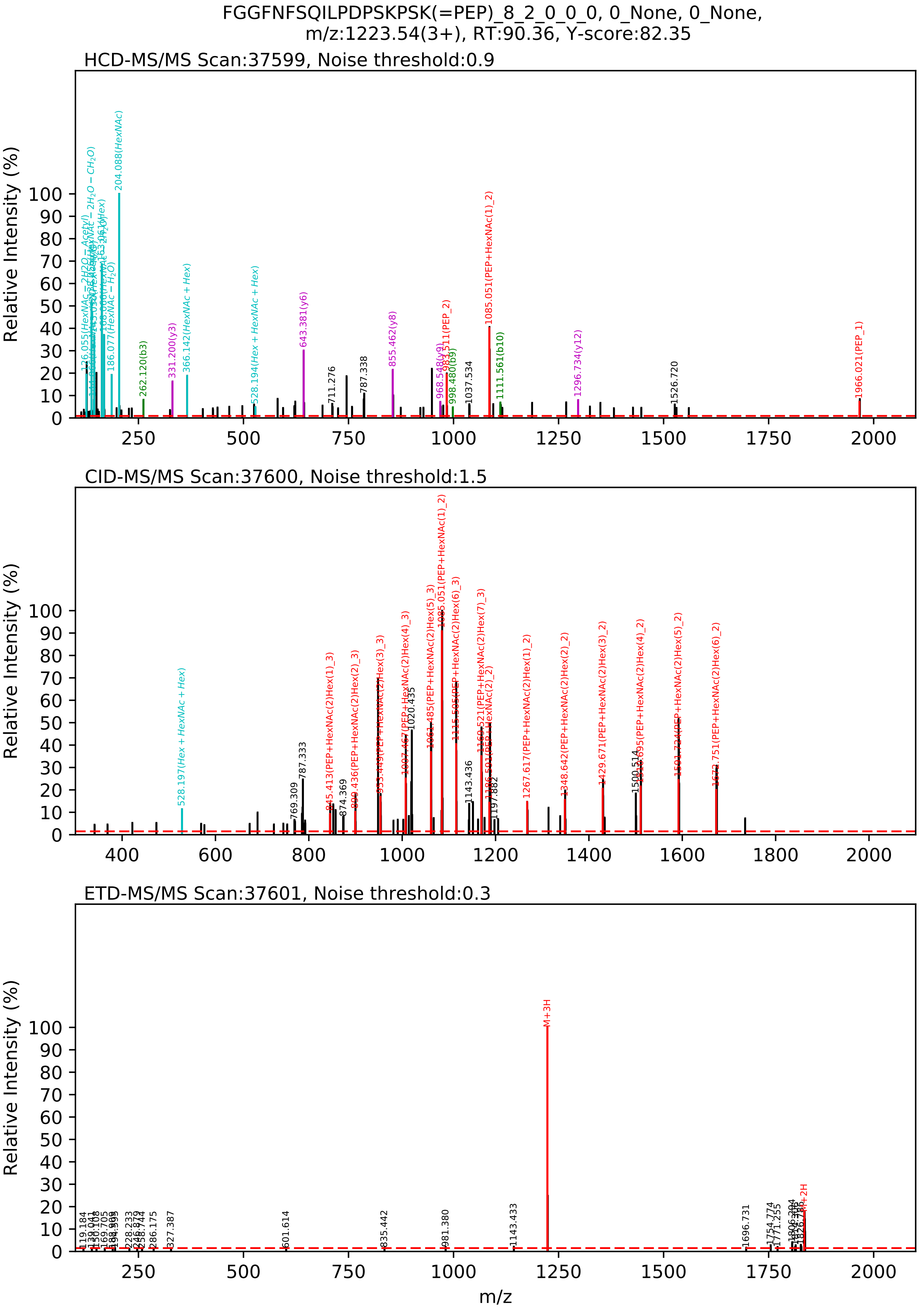

FGGFNFSQILPDPSKPSK(=PEP)_8_2_0_0_0, 0_None, 0_None,
m/z:1223.54(3+), RT:90.36, Y-score:82.35
HCD-MS/MS Scan:37599, Noise threshold:0.9
Relative Intensity (%)
204.088(HexNAc)
186.077(HexNAc − H2O)
262.120(b3)
331.200(y3)
366.142(HexNAc + Hex)
528.194(Hex + HexNAc + Hex)
643.381(y6)
711.276
787.338
855.462(y8)
968.548(y9)
998.480(b9)
1037.534
1085.051(PEP+HexNAc(1)_2)
1111.561(b10)
1296.734(y12)
1526.720
1966.021(PEP_1)
CID-MS/MS Scan:37600, Noise threshold:1.5
528.197(Hex + HexNAc + Hex)
769.309
787.333
845.413(PEP+HexNAc(2)Hex(1)_3)
874.369
899.436(PEP+HexNAc(2)Hex(2)_3)
1020.435
1085.051(PEP+HexNAc(1)_2)
1143.436
1197.882
1267.617(PEP+HexNAc(2)Hex(1)_2)
1348.642(PEP+HexNAc(2)Hex(2)_2)
1429.671(PEP+HexNAc(2)Hex(3)_2)
1500.514
1672.751(PEP+HexNAc(2)Hex(6)_2)
ETD-MS/MS Scan:37601, Noise threshold:0.3
M+3H
M+2H
119.184
286.175
327.387
601.614
835.442
981.380
1143.433
1696.731
1754.774
1771.255
m/z

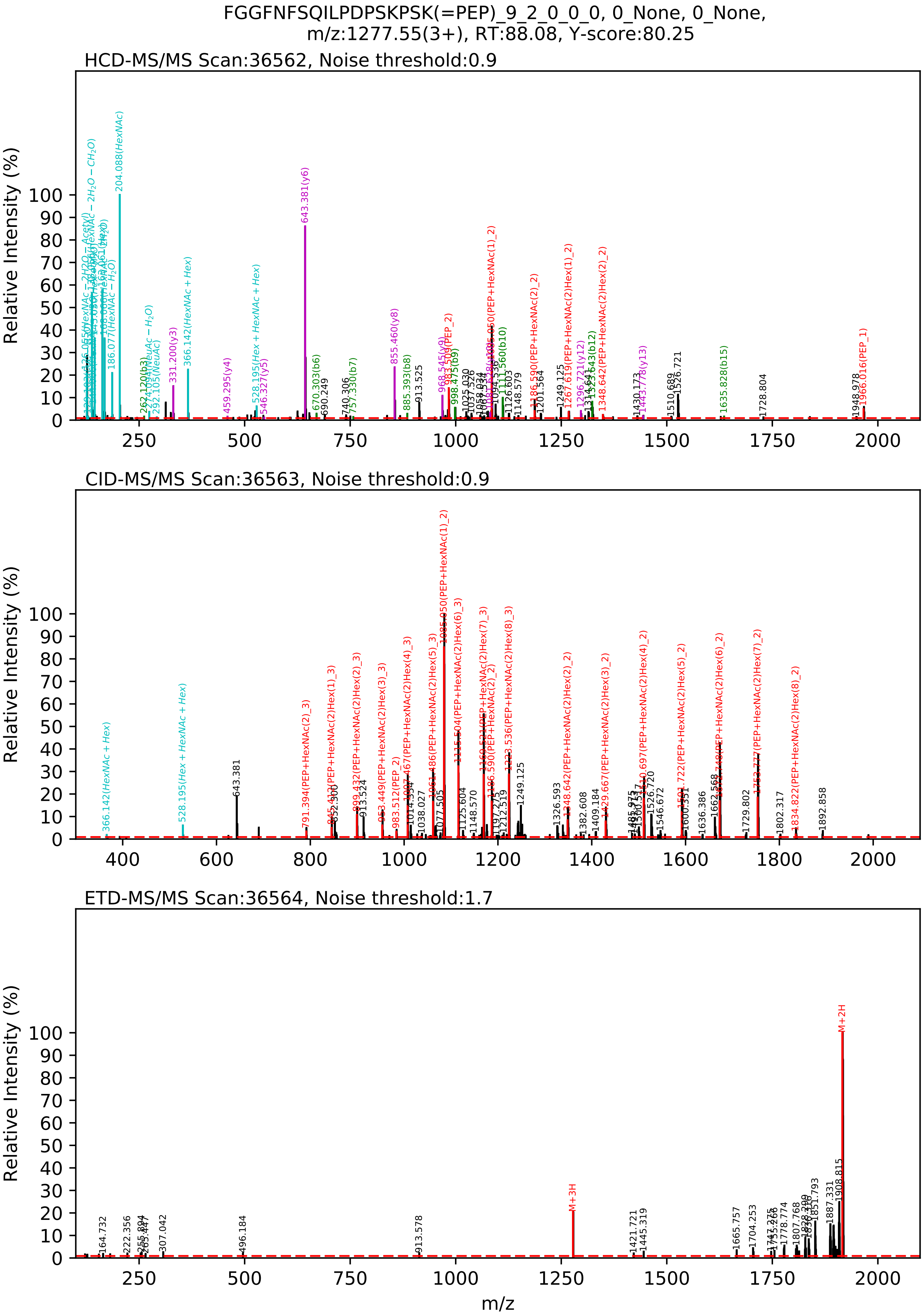
FGGFNFSQILPDPSKPSK(=PEP)_9_2_0_0_0, 0_None, 0_None,
m/z:1277.55(3+), RT:88.08, Y-score:80.25
HCD-MS/MS Scan:36562, Noise threshold:0.9
CID-MS/MS Scan:36563, Noise threshold:0.9
ETD-MS/MS Scan:36564, Noise threshold:1.7
Relative Intensity (%)
m/z
204.088(HexNAc)
643.381(y6)
855.460(y8)
366.142(HexNAc+Hex)
528.195(Hex+HexNAc+Hex)
331.200(y3)
1966.016(PEP_1)
1085.050(PEP+HexNAc(1)_2)
1348.642(PEP+HexNAc(2)Hex(2)_2)
1635.828(b15)
M+2H
M+3H

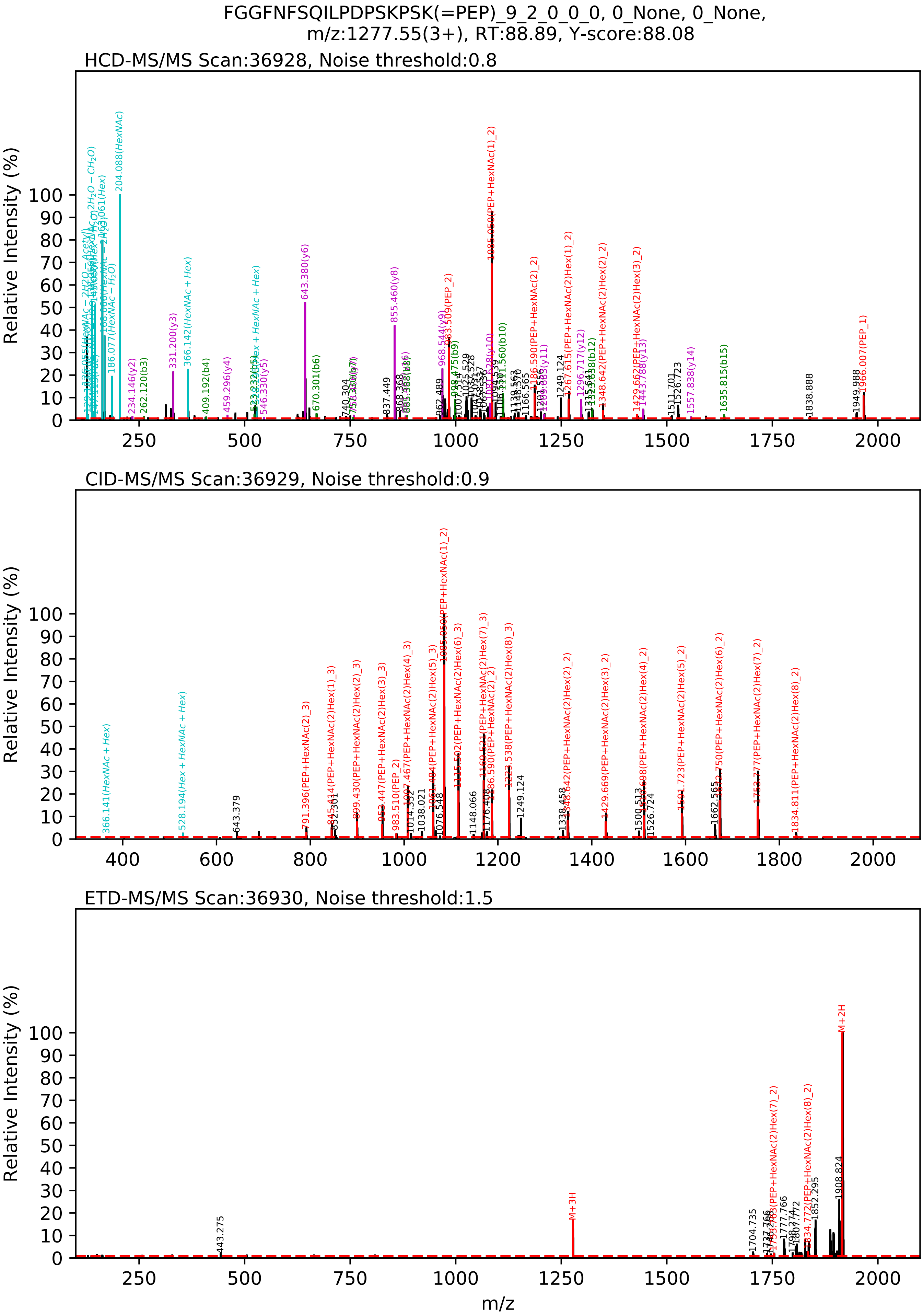
FGGFNFSQILPDPSKPSK(=PEP)_9_2_0_0_0, 0_None, 0_None,
m/z:1277.55(3+), RT:88.89, Y-score:88.08
HCD-MS/MS Scan:36928, Noise threshold:0.8
Relative Intensity (%)
204.088(HexNAc)
186.077(HexNAc − H2O)
234.146(y2)
262.120(b3)
331.200(y3)
366.142(HexNAc + Hex)
409.192(b4)
459.296(y4)
546.330(y5)
643.380(y6)
670.301(b6)
855.460(y8)
968.544(y9)
983.509(PEP_2)
1085.050(PEP+HexNAc(1)_2)
1186.590(PEP+HexNAc(2)_2)
1267.615(PEP+HexNAc(2)Hex(1)_2)
1296.717(y12)
1348.642(PEP+HexNAc(2)Hex(2)_2)
1429.662(PEP+HexNAc(2)Hex(3)_2)
1443.788(y13)
1557.838(y14)
1635.815(b15)
1838.888
1949.988
1966.007(PEP_1)
CID-MS/MS Scan:36929, Noise threshold:0.9
Relative Intensity (%)
366.141(HexNAc + Hex)
528.194(Hex + HexNAc + Hex)
643.379
791.396(PEP+HexNAc(2)_3)
899.430(PEP+HexNAc(2)Hex(2)_3)
953.447(PEP+HexNAc(2)Hex(3)_3)
983.510(PEP_2)
1014.332
1038.021
1076.548
1085.050(PEP+HexNAc(1)_2)
1115.502(PEP+HexNAc(2)Hex(6)_3)
1148.066
1186.590(PEP+HexNAc(2)_2)
1223.538(PEP+HexNAc(2)Hex(8)_3)
1249.124
1338.458
1429.669(PEP+HexNAc(2)Hex(3)_2)
1500.513
1526.724
1591.723(PEP+HexNAc(2)Hex(5)_2)
1662.565
1753.777(PEP+HexNAc(2)Hex(7)_2)
1834.811(PEP+HexNAc(2)Hex(8)_2)
ETD-MS/MS Scan:36930, Noise threshold:1.5
Relative Intensity (%)
443.275
M+3H
1704.735
1777.766
1834.772(PEP+HexNAc(2)Hex(8)_2)
1852.295
1908.824
M+2H
m/z

FGGFNFSQILPDPSKPSK(=PEP)_9_2_0_0_0, 0_None, 0_None,
m/z:1277.55(3+), RT:89.45, Y-score:86.95

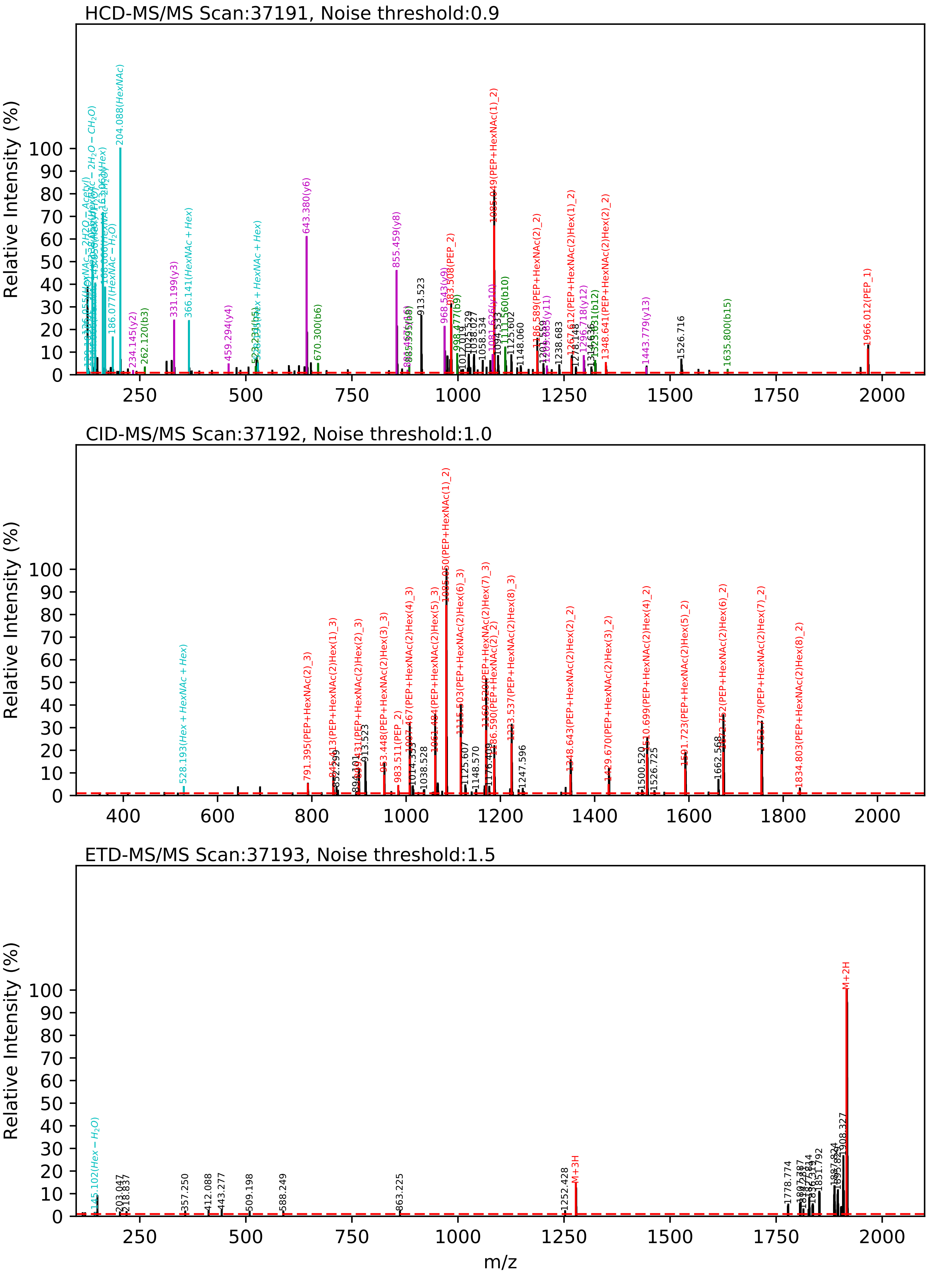

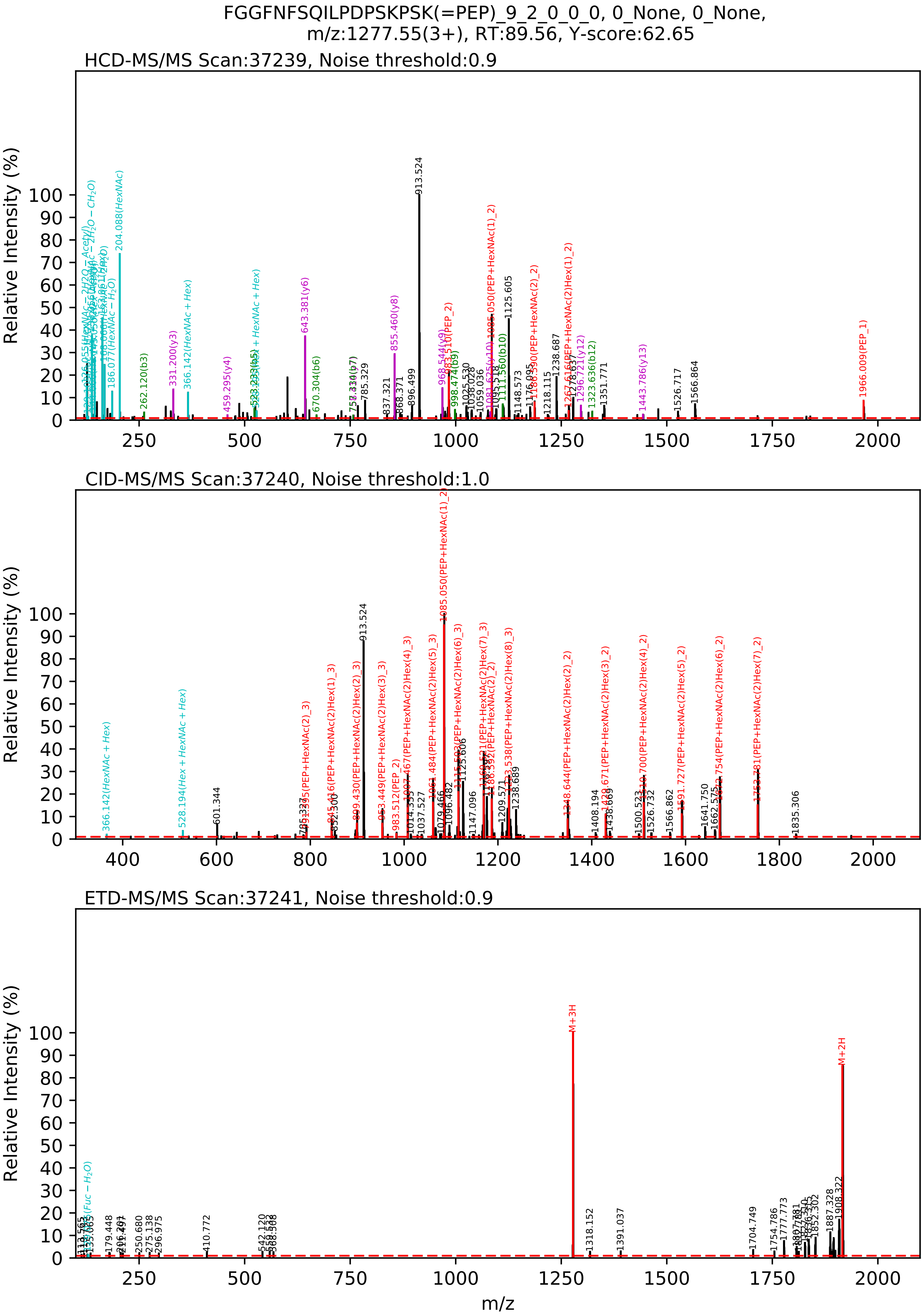

FGGFNFSQILPDPSKPSK(=PEP)_9_2_0_0_0, 0_None, 0_None,
m/z:1277.55(3+), RT:89.56, Y-score:62.65
HCD-MS/MS Scan:37239, Noise threshold:0.9
CID-MS/MS Scan:37240, Noise threshold:1.0
ETD-MS/MS Scan:37241, Noise threshold:0.9
Relative Intensity (%)
m/z
913.524
204.088(HexNAc)
643.381(y6)
855.460(y8)
1085.050(PEP+HexNAc(1)_2)
1125.605
1966.009(PEP_1)
1085.050(PEP+HexNAc(1)_2)
M+3H
M+2H

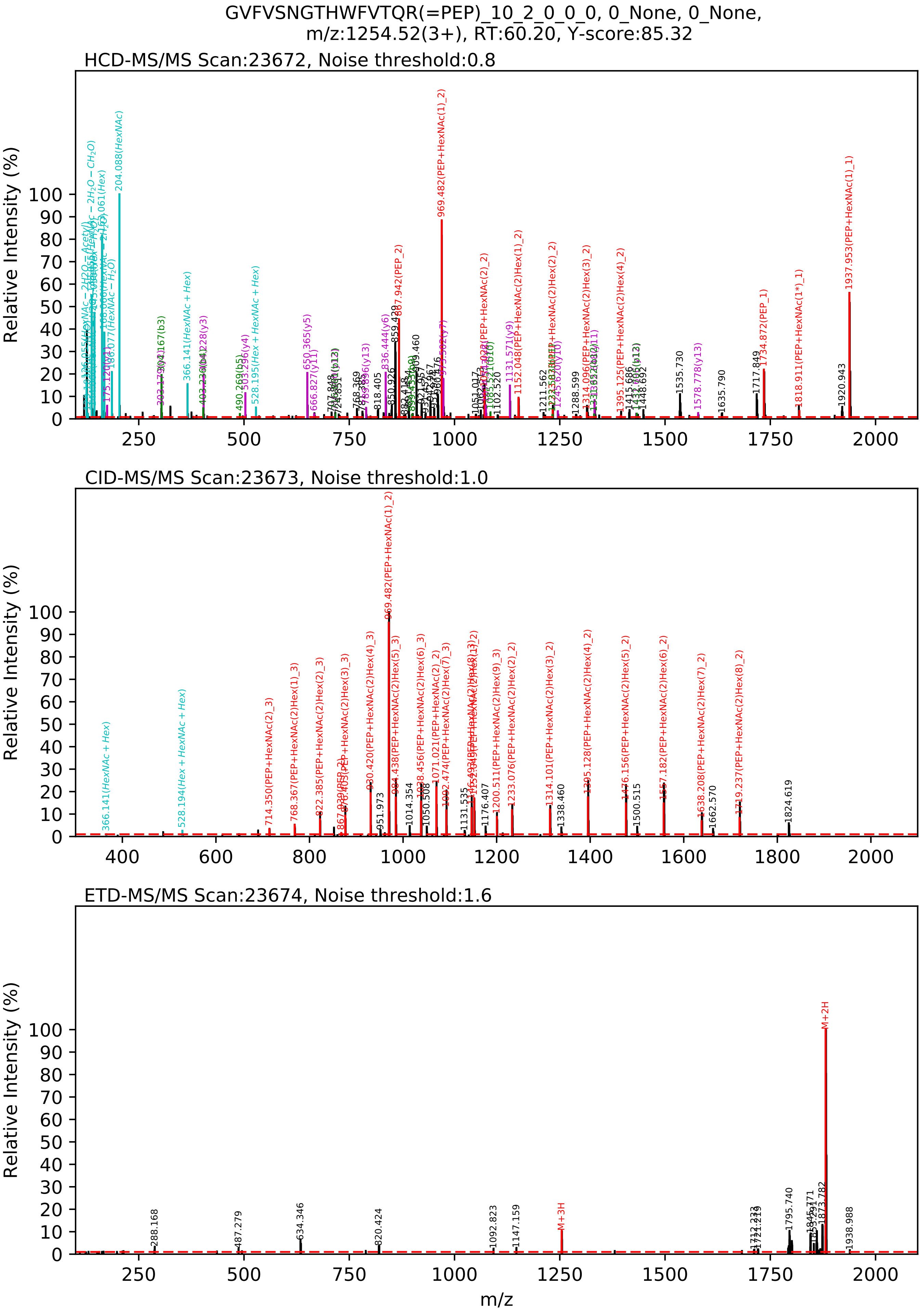

GVFVSNGTHWFVTQR(=PEP)_10_2_0_0_0, 0_None, 0_None,
m/z:1254.52(3+), RT:60.20, Y-score:85.32
HCD-MS/MS Scan:23672, Noise threshold:0.8
CID-MS/MS Scan:23673, Noise threshold:1.0
ETD-MS/MS Scan:23674, Noise threshold:1.6
Relative Intensity (%)
m/z
969.482(PEP+HexNAc(1)_2)
1937.953(PEP+HexNAc(1)_1)
204.088(HexNAc)
M+2H
M+3H

GVFVSNGTHWFVTQR(=PEP)_3_2_0_0_0, 0_None, 0_None,
m/z:876.40(3+), RT:60.67, Y-score:88.60

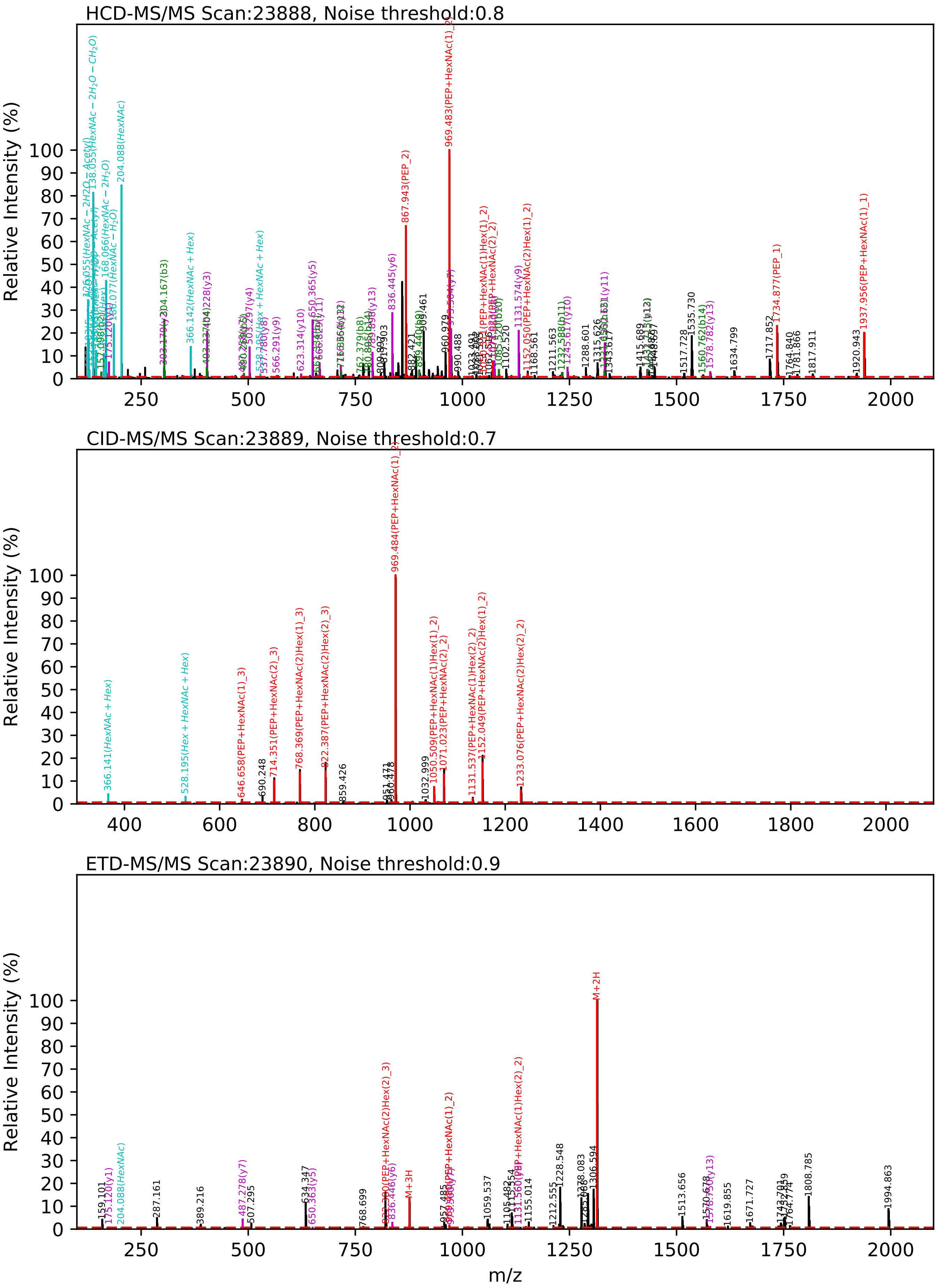

GVFVSNGTHWFVTQR(=PEP)_4_2_0_0_0, 0_None, 0_None,
m/z:930.42(3+), RT:62.58, Y-score:87.50

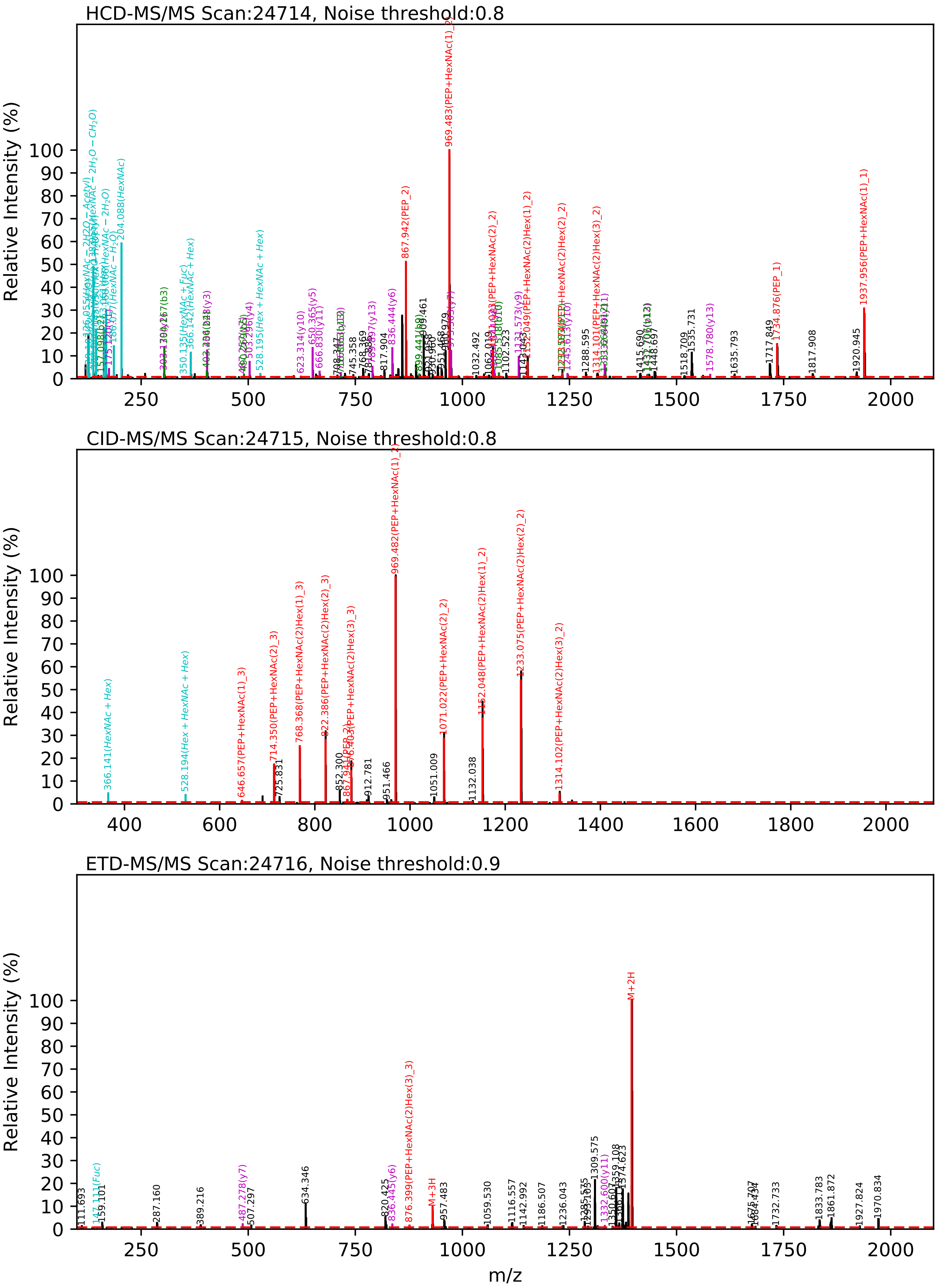

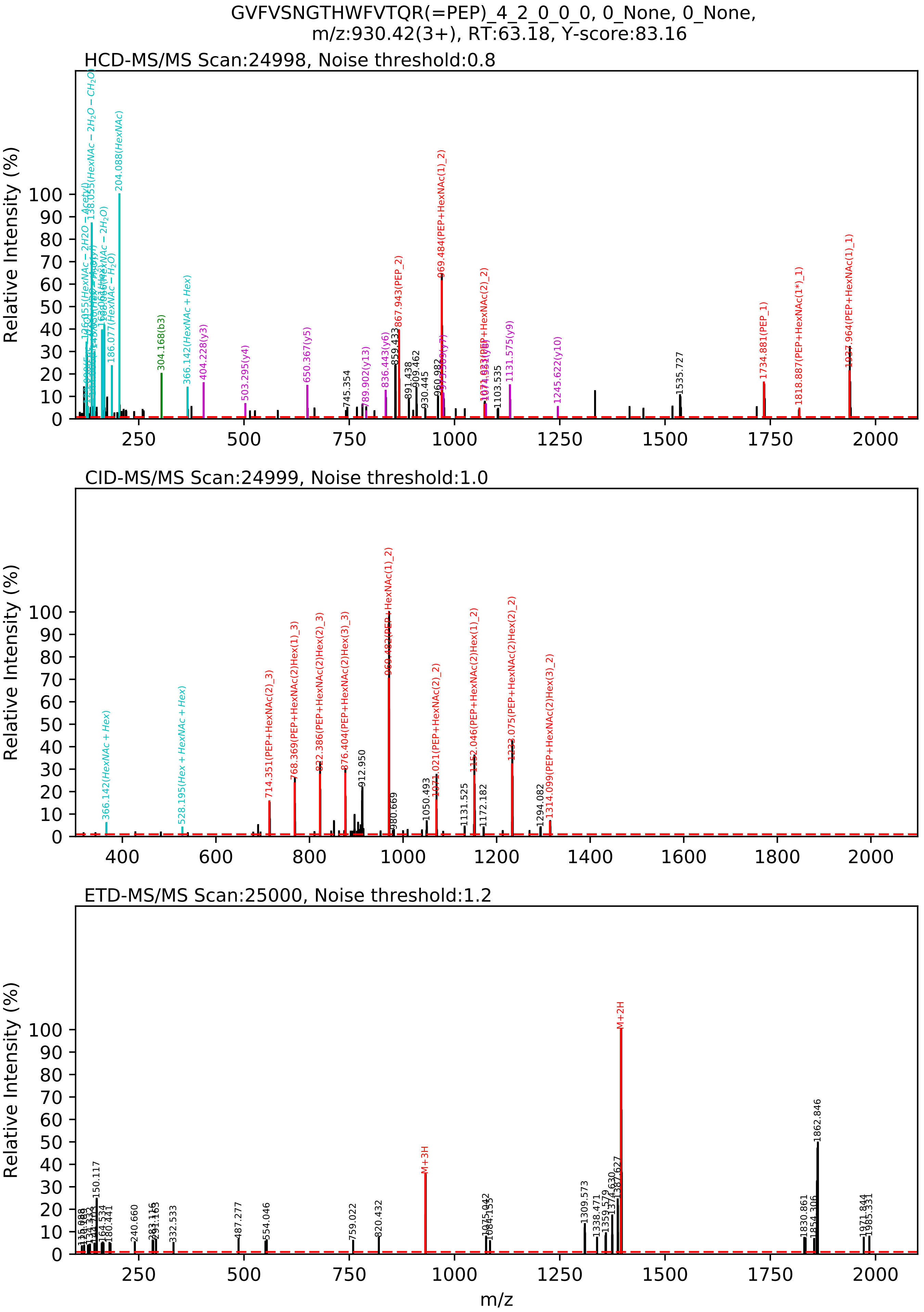

GVFVSNGTHWFVTQR(=PEP)_4_2_0_0_0, 0_None, 0_None,
m/z:930.42(3+), RT:63.18, Y-score:83.16
HCD-MS/MS Scan:24998, Noise threshold:0.8
CID-MS/MS Scan:24999, Noise threshold:1.0
ETD-MS/MS Scan:25000, Noise threshold:1.2
Relative Intensity (%)
m/z

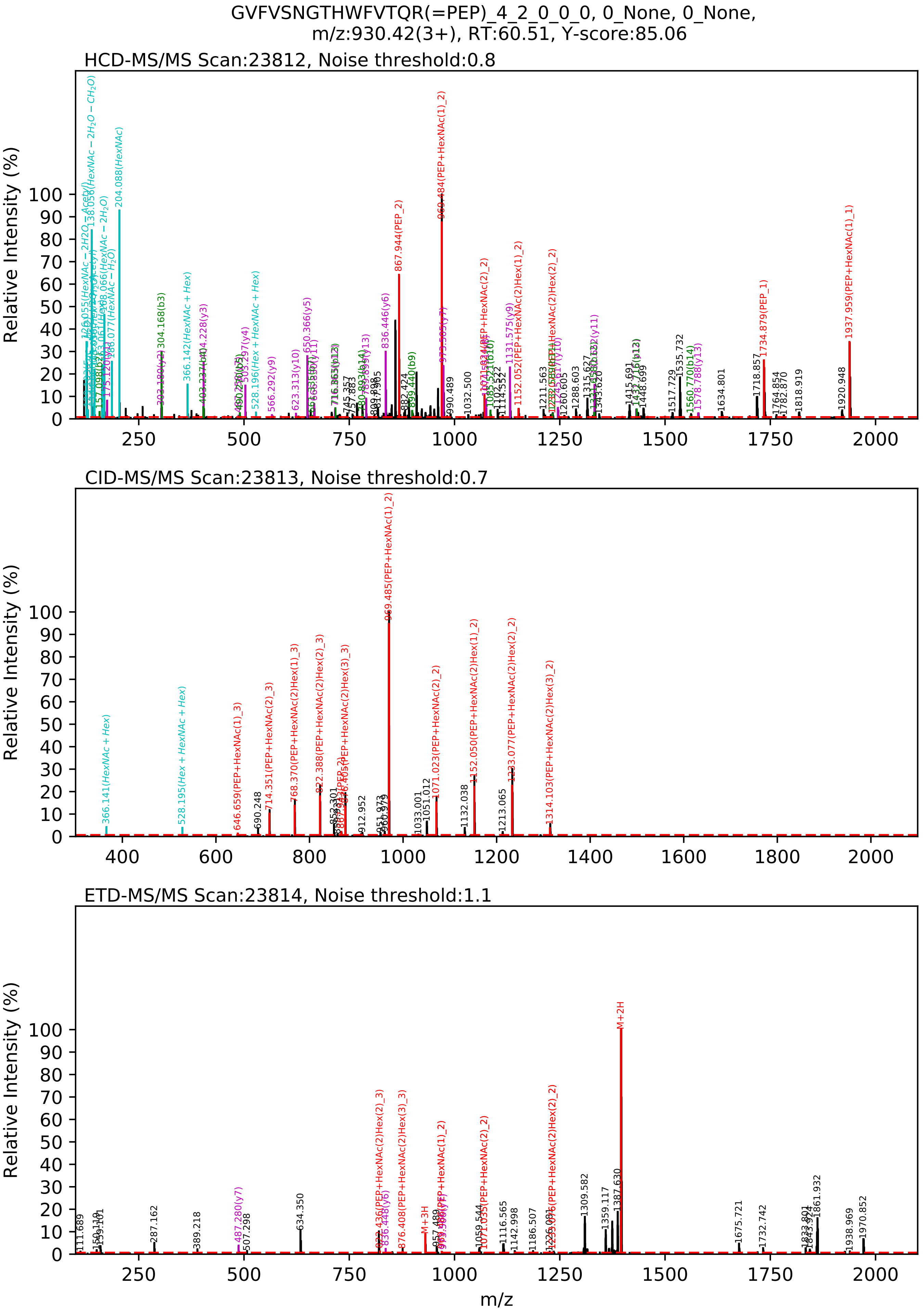

GVFVSNGTHWFVTQR(=PEP)_4_2_0_0_0, 0_None, 0_None,
m/z:930.42(3+), RT:60.51, Y-score:85.06
HCD-MS/MS Scan:23812, Noise threshold:0.8
Relative Intensity (%)
969.484(PEP+HexNAc(1)_2)
204.088(HexNAc)
867.944(PEP_2)
1937.959(PEP+HexNAc(1)_1)
1734.879(PEP_1)
836.446(y6)
650.366(y5)
404.228(y3)
366.142(HexNAc + Hex)
528.196(Hex + HexNAc + Hex)
304.168(b3)
1131.575(y9)
1152.052(PEP+HexNAc(2)Hex(1)_2)
1718.857
1535.732
1920.948
CID-MS/MS Scan:23813, Noise threshold:0.7
Relative Intensity (%)
969.485(PEP+HexNAc(1)_2)
366.141(HexNAc + Hex)
528.195(Hex + HexNAc + Hex)
646.659(PEP+HexNAc(1)_3)
714.351(PEP+HexNAc(2)_3)
768.370(PEP+HexNAc(2)Hex(1)_3)
822.388(PEP+HexNAc(2)Hex(2)_3)
876.405(PEP+HexNAc(2)Hex(3)_3)
1071.023(PEP+HexNAc(2)_2)
1152.050(PEP+HexNAc(2)Hex(1)_2)
1233.077(PEP+HexNAc(2)Hex(2)_2)
1314.103(PEP+HexNAc(2)Hex(3)_2)
690.248
1051.012
1132.038
1213.065
ETD-MS/MS Scan:23814, Noise threshold:1.1
Relative Intensity (%)
M+2H
M+3H
487.280(y7)
822.436(PEP+HexNAc(2)Hex(2)_3)
876.408(PEP+HexNAc(2)Hex(3)_3)
1071.035(PEP+HexNAc(2)_2)
1309.582
1359.117
1387.630
1861.932
1970.852
634.350
287.162
m/z

GVFVSNGTHWFVTQR(=PEP)_5_2_0_0_0, 0_None, 0_None,
m/z:1476.15(2+), RT:60.58, Y-score:85.18

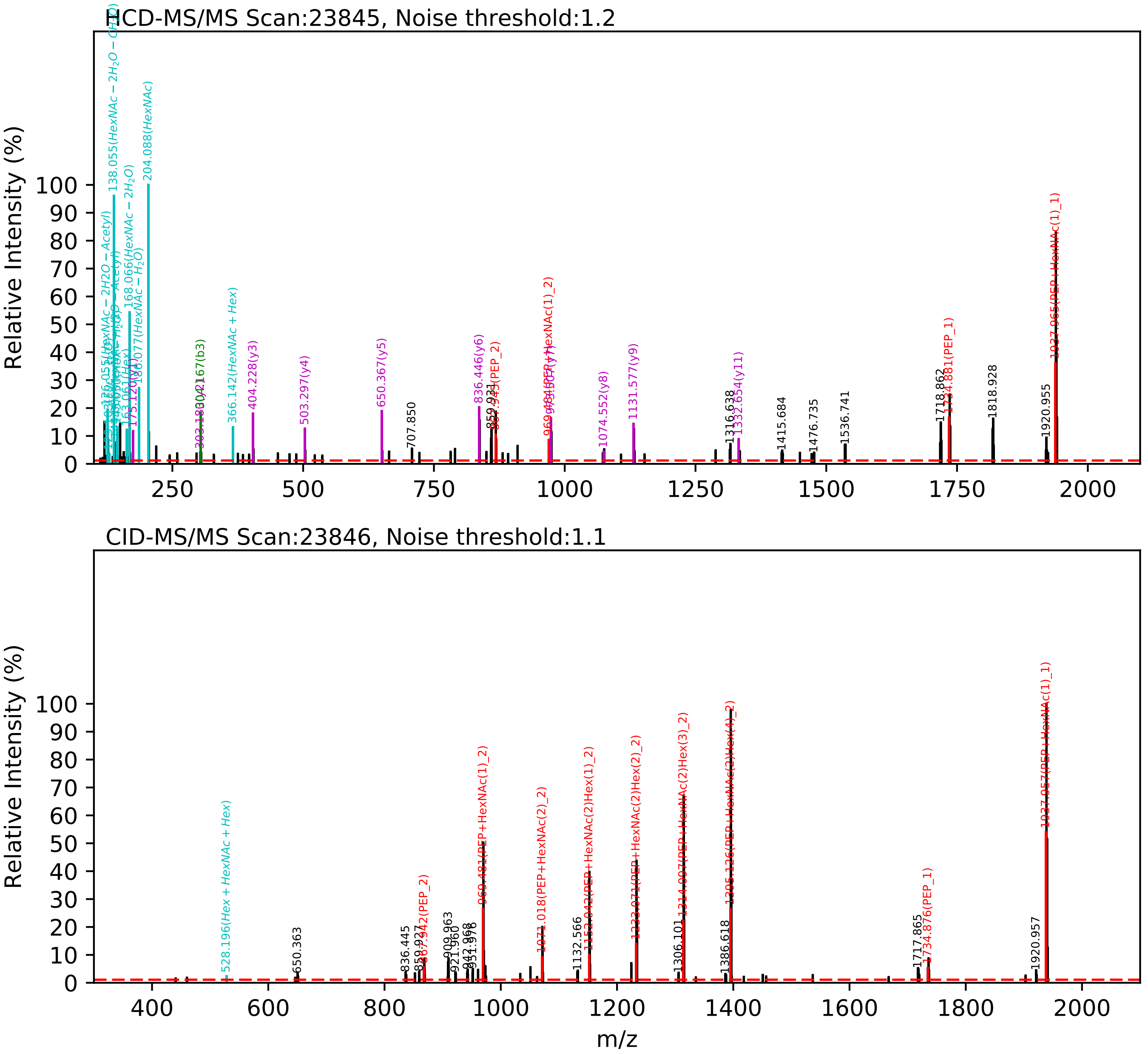

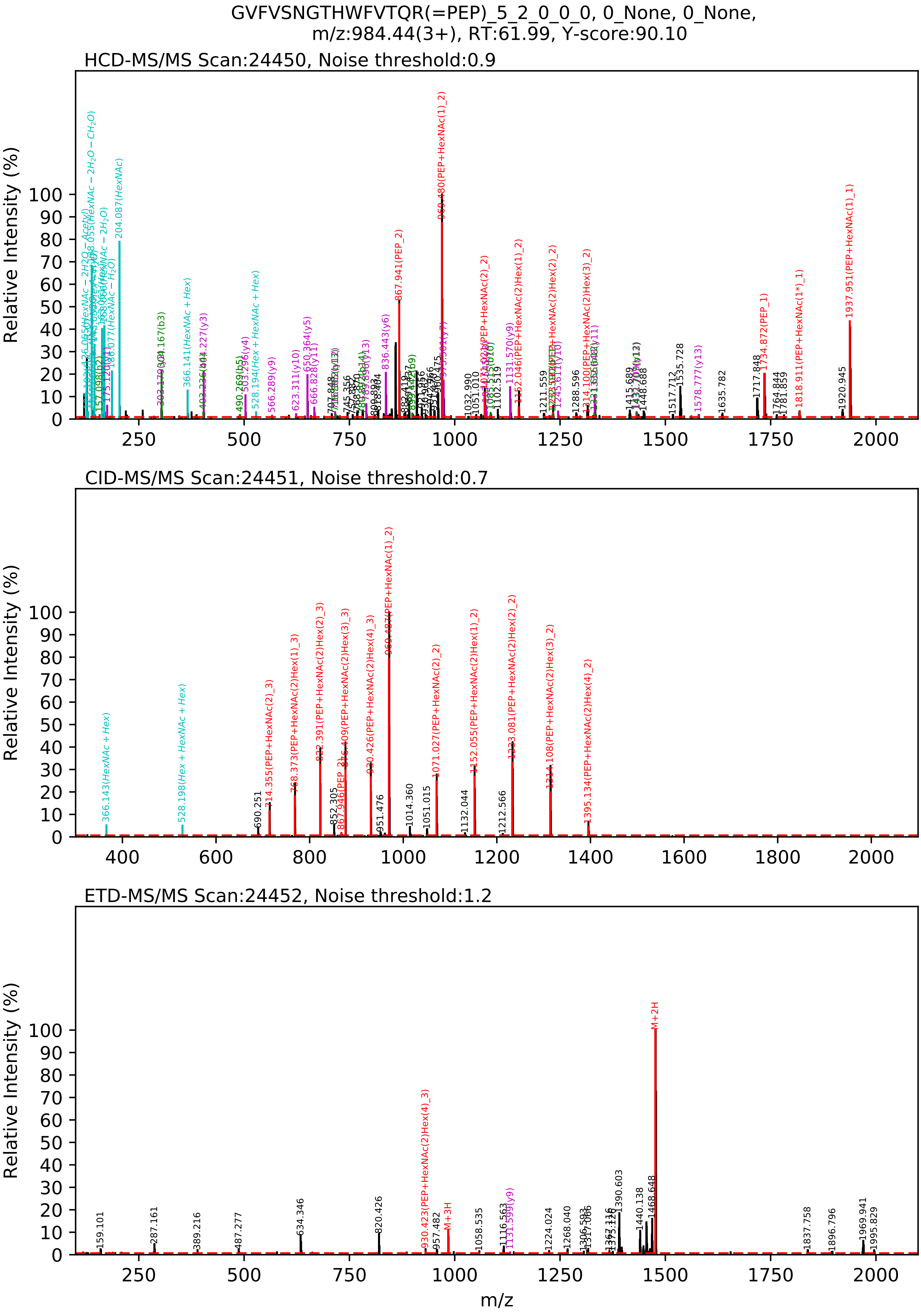
GVFVSNGTHWFVTQR(=PEP)_5_2_0_0_0, 0_None, 0_None,
m/z:984.44(3+), RT:61.99, Y-score:90.10
HCD-MS/MS Scan:24450, Noise threshold:0.9
Relative Intensity (%)
204.087(HexNAc)
366.141(HexNAc + Hex)
528.194(Hex + HexNAc + Hex)
867.941(PEP_2)
969.480(PEP+HexNAc(1)_2)
1152.046(PEP+HexNAc(2)Hex(1)_2)
1734.872(PEP_1)
1818.911(PEP+HexNAc(1*)_1)
1937.951(PEP+HexNAc(1)_1)
836.443(y6)
1131.570(y9)
1578.777(y13)
1535.728
1635.782
1717.848
1920.945
CID-MS/MS Scan:24451, Noise threshold:0.7
Relative Intensity (%)
366.143(HexNAc + Hex)
528.198(Hex + HexNAc + Hex)
714.355(PEP+HexNAc(2)_3)
768.373(PEP+HexNAc(2)Hex(1)_3)
822.391(PEP+HexNAc(2)Hex(2)_3)
876.409(PEP+HexNAc(2)Hex(3)_3)
930.426(PEP+HexNAc(2)Hex(4)_3)
969.487(PEP+HexNAc(1)_2)
1071.027(PEP+HexNAc(2)_2)
1152.055(PEP+HexNAc(2)Hex(1)_2)
1233.081(PEP+HexNAc(2)Hex(2)_2)
1314.108(PEP+HexNAc(2)Hex(3)_2)
1395.134(PEP+HexNAc(2)Hex(4)_2)
690.251
852.305
951.476
1014.360
1051.015
1132.044
1212.566
ETD-MS/MS Scan:24452, Noise threshold:1.2
Relative Intensity (%)
M+2H
M+3H
930.423(PEP+HexNAc(2)Hex(4)_3)
1131.599(y9)
159.101
287.161
389.216
487.277
634.346
820.426
957.482
1058.535
1116.563
1224.024
1268.040
1390.603
1440.138
1468.648
1837.758
1896.796
1969.941
1995.829
m/z

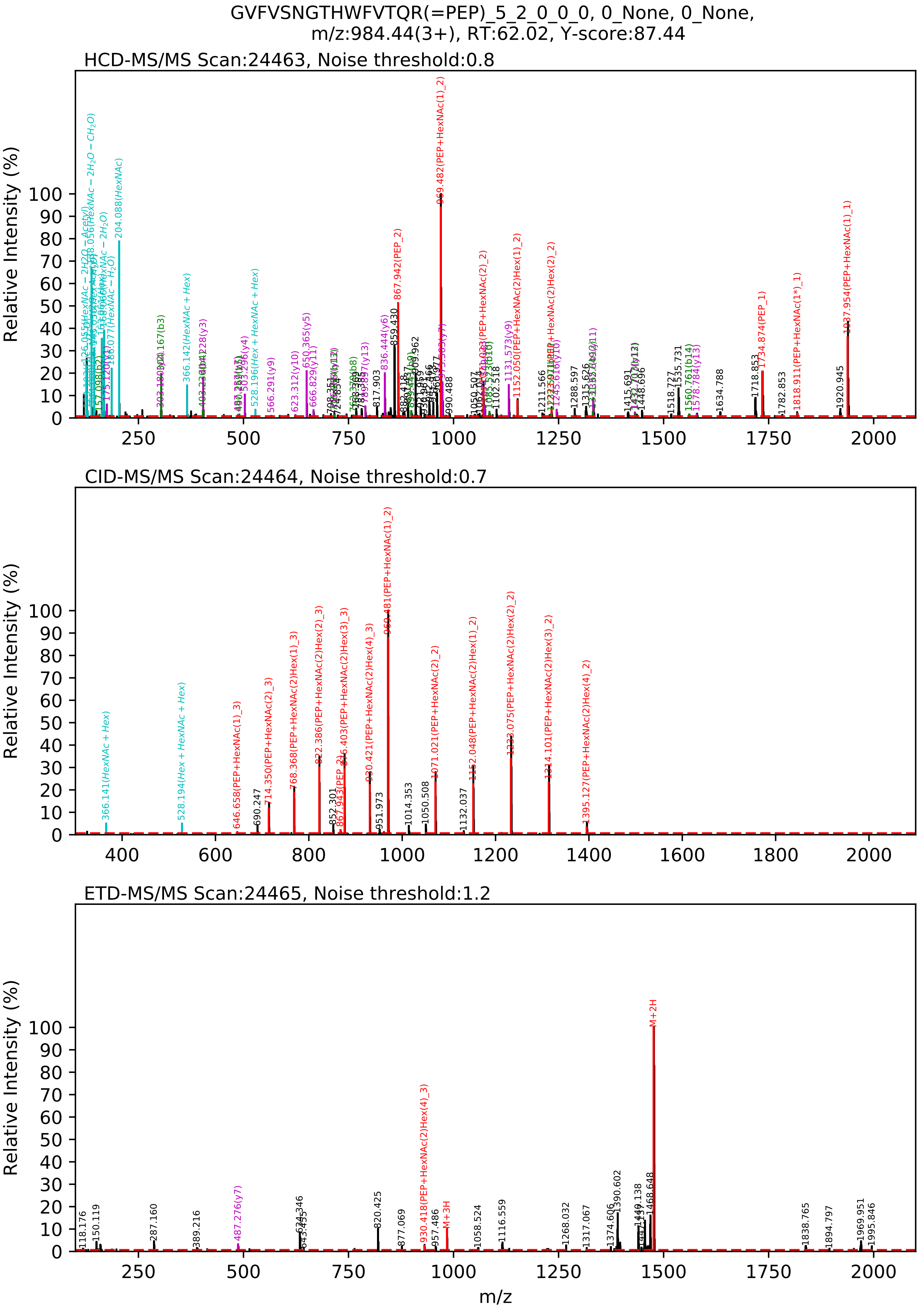

GVFVSNGTHWFVTQR(=PEP)_5_2_0_0_0, 0_None, 0_None,
m/z:984.44(3+), RT:62.02, Y-score:87.44
HCD-MS/MS Scan:24463, Noise threshold:0.8
Relative Intensity (%)
969.482(PEP+HexNAc(1)_2)
867.942(PEP_2)
1937.954(PEP+HexNAc(1)_1)
1734.874(PEP_1)
1818.911(PEP+HexNAc(1*)_1)
204.088(HexNAc)
366.142(HexNAc + Hex)
528.196(Hex + HexNAc + Hex)
1152.050(PEP+HexNAc(2)Hex(1)_2)
CID-MS/MS Scan:24464, Noise threshold:0.7
366.141(HexNAc + Hex)
528.194(Hex + HexNAc + Hex)
646.658(PEP+HexNAc(1)_3)
714.350(PEP+HexNAc(2)_3)
768.368(PEP+HexNAc(2)Hex(1)_3)
822.386(PEP+HexNAc(2)Hex(2)_3)
876.403(PEP+HexNAc(2)Hex(3)_3)
930.421(PEP+HexNAc(2)Hex(4)_3)
969.481(PEP+HexNAc(1)_2)
1071.021(PEP+HexNAc(2)_2)
1152.048(PEP+HexNAc(2)Hex(1)_2)
1233.075(PEP+HexNAc(2)Hex(2)_2)
1314.101(PEP+HexNAc(2)Hex(3)_2)
1395.127(PEP+HexNAc(2)Hex(4)_2)
ETD-MS/MS Scan:24465, Noise threshold:1.2
M+2H
M+3H
930.418(PEP+HexNAc(2)Hex(4)_3)
487.276(y7)
m/z

GVFVSNGTHWFVTQR(=PEP)_5_2_0_0_0, 0_None, 0_None,
m/z:984.44(3+), RT:62.60, Y-score:92.10

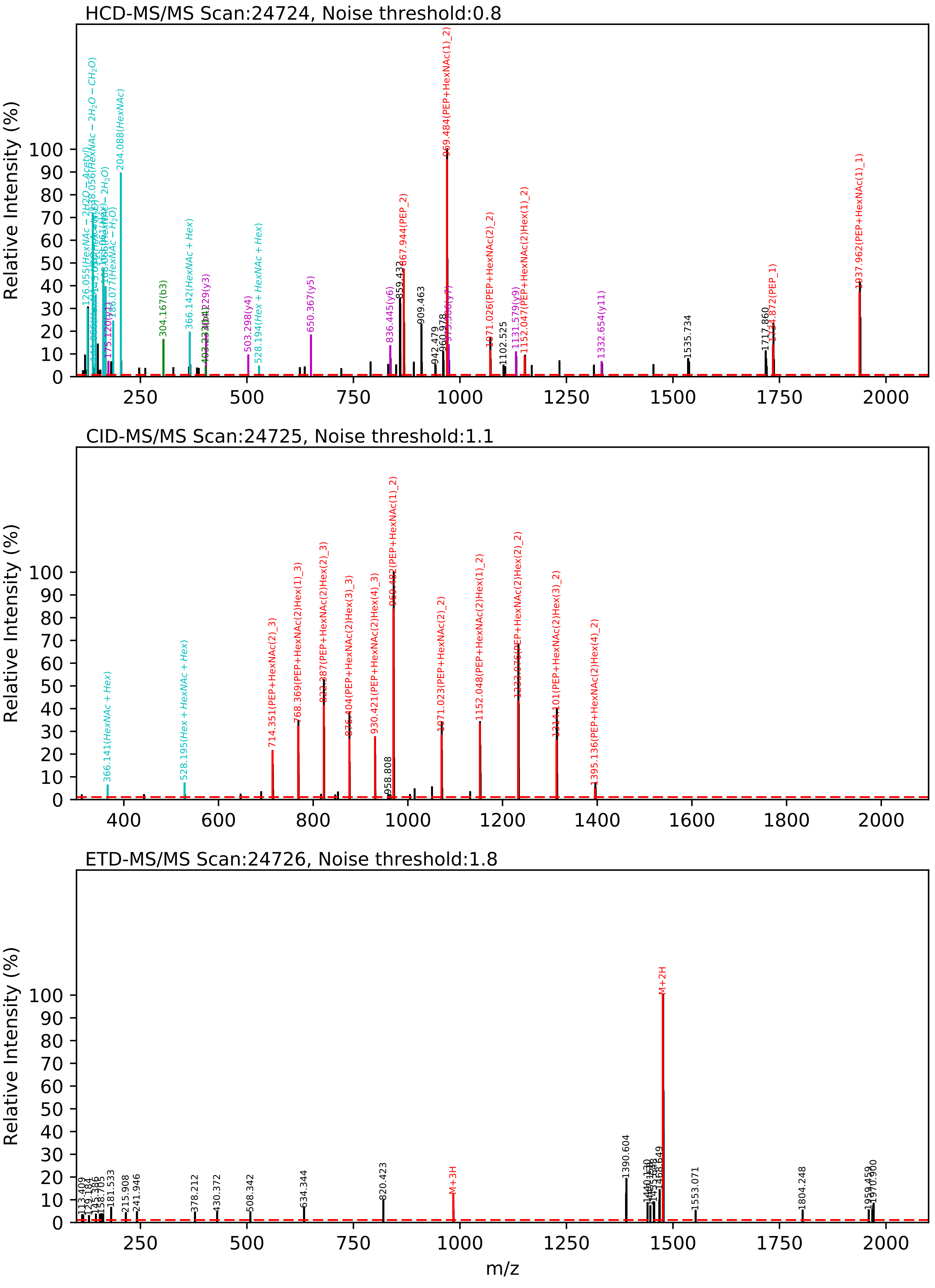

GVFVSNGTHWFVTQR(=PEP)_5_2_0_0_0, 0_None, 0_None,
m/z:984.44(3+), RT:62.74, Y-score:88.73

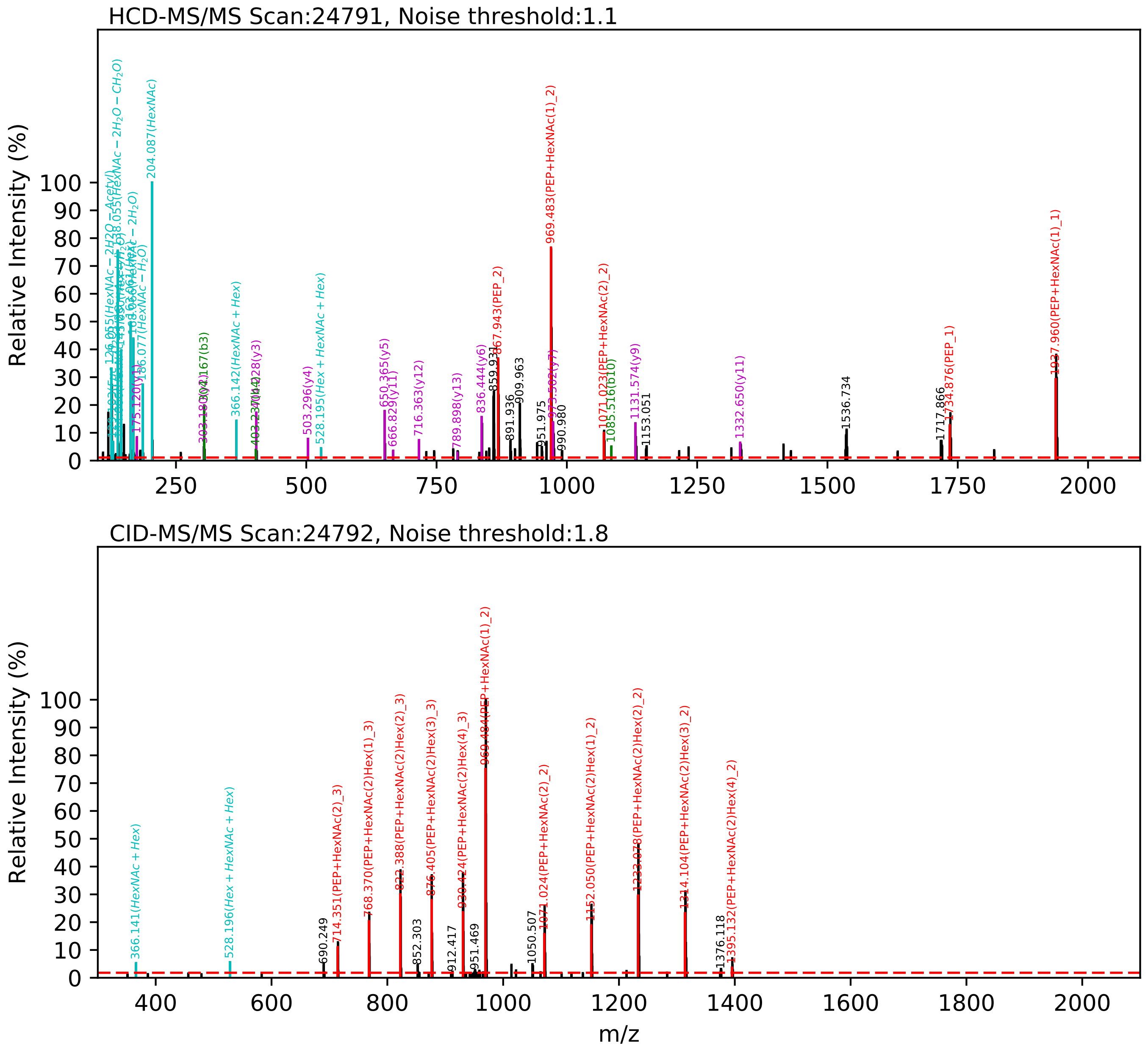

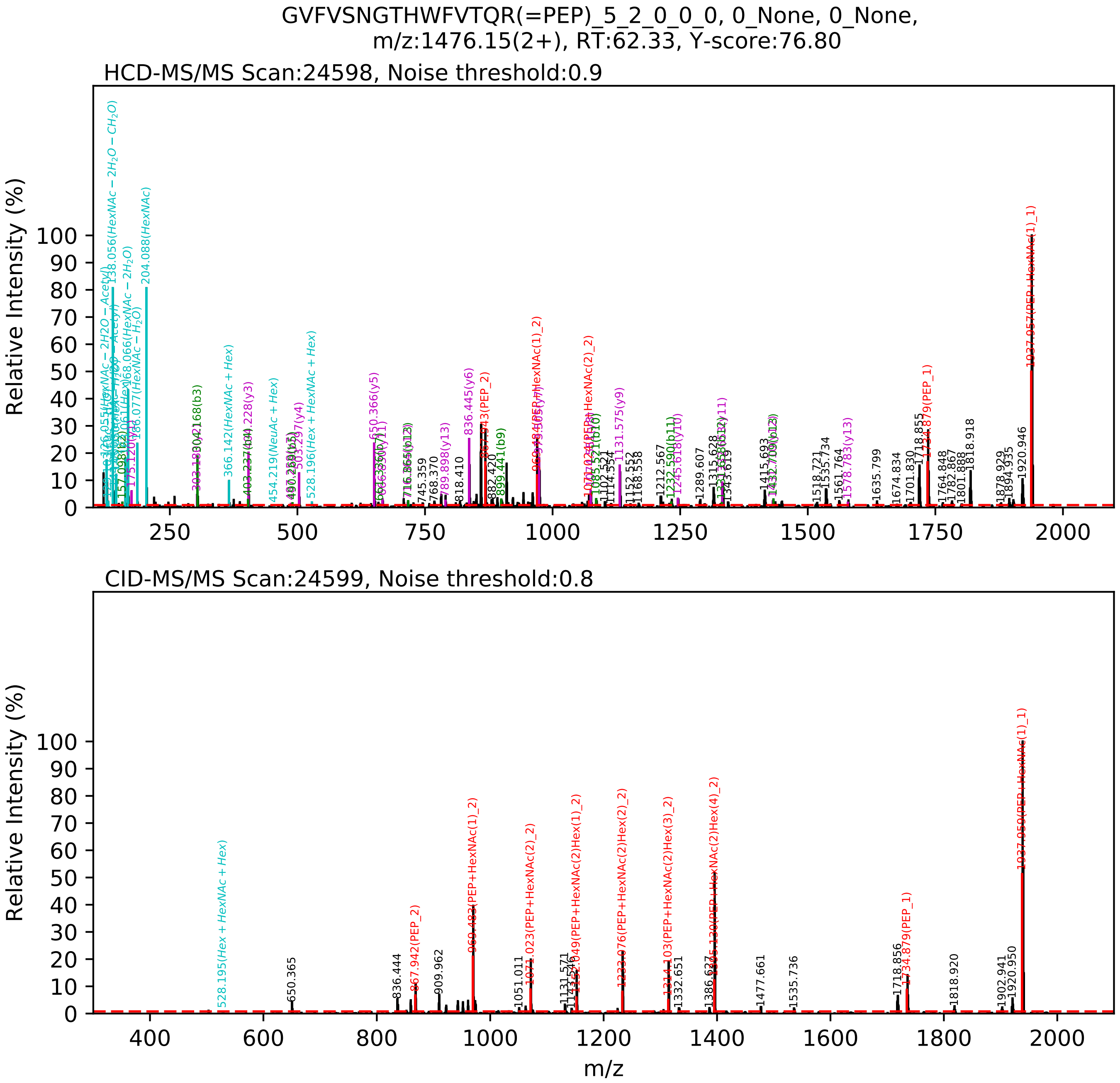
GVFVSNGTHWFVTQR(=PEP)_5_2_0_0_0, 0_None, 0_None,
m/z:1476.15(2+), RT:62.33, Y-score:76.80
HCD-MS/MS Scan:24598, Noise threshold:0.9
Relative Intensity (%)
204.088(HexNAc)
366.142(HexNAc + Hex)
528.196(Hex + HexNAc + Hex)
650.366(y5)
836.445(y6)
969.483(PEP+HexNAc(1)_2)
1131.575(y9)
1734.879(PEP_1)
1937.957(PEP+HexNAc(1)_1)
CID-MS/MS Scan:24599, Noise threshold:0.8
Relative Intensity (%)
528.195(Hex + HexNAc + Hex)
650.365
836.444
867.942(PEP_2)
909.962
969.482(PEP+HexNAc(1)_2)
1071.023(PEP+HexNAc(2)_2)
1152.049(PEP+HexNAc(2)Hex(1)_2)
1233.076(PEP+HexNAc(2)Hex(2)_2)
1314.103(PEP+HexNAc(2)Hex(3)_2)
1332.651
1386.627
1477.661
1535.736
1718.856
1734.879(PEP_1)
1818.920
1902.941
1920.950
1937.959(PEP+HexNAc(1)_1)
m/z

GVFVSNGTHWFVTQR(=PEP)_6_2_0_0_0, 0_None, 0_None,
m/z:1557.18(2+), RT:60.32, Y-score:76.89

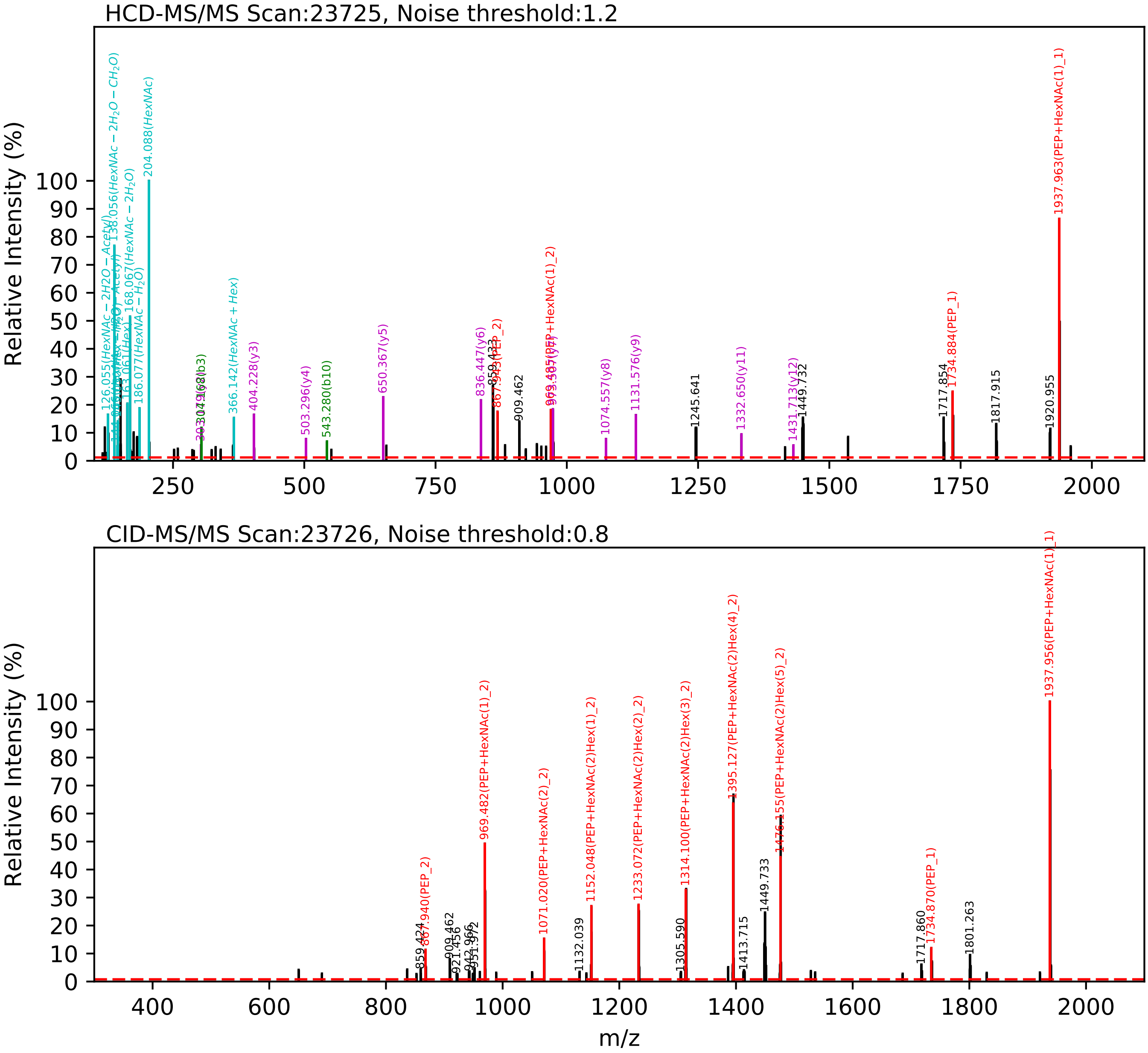

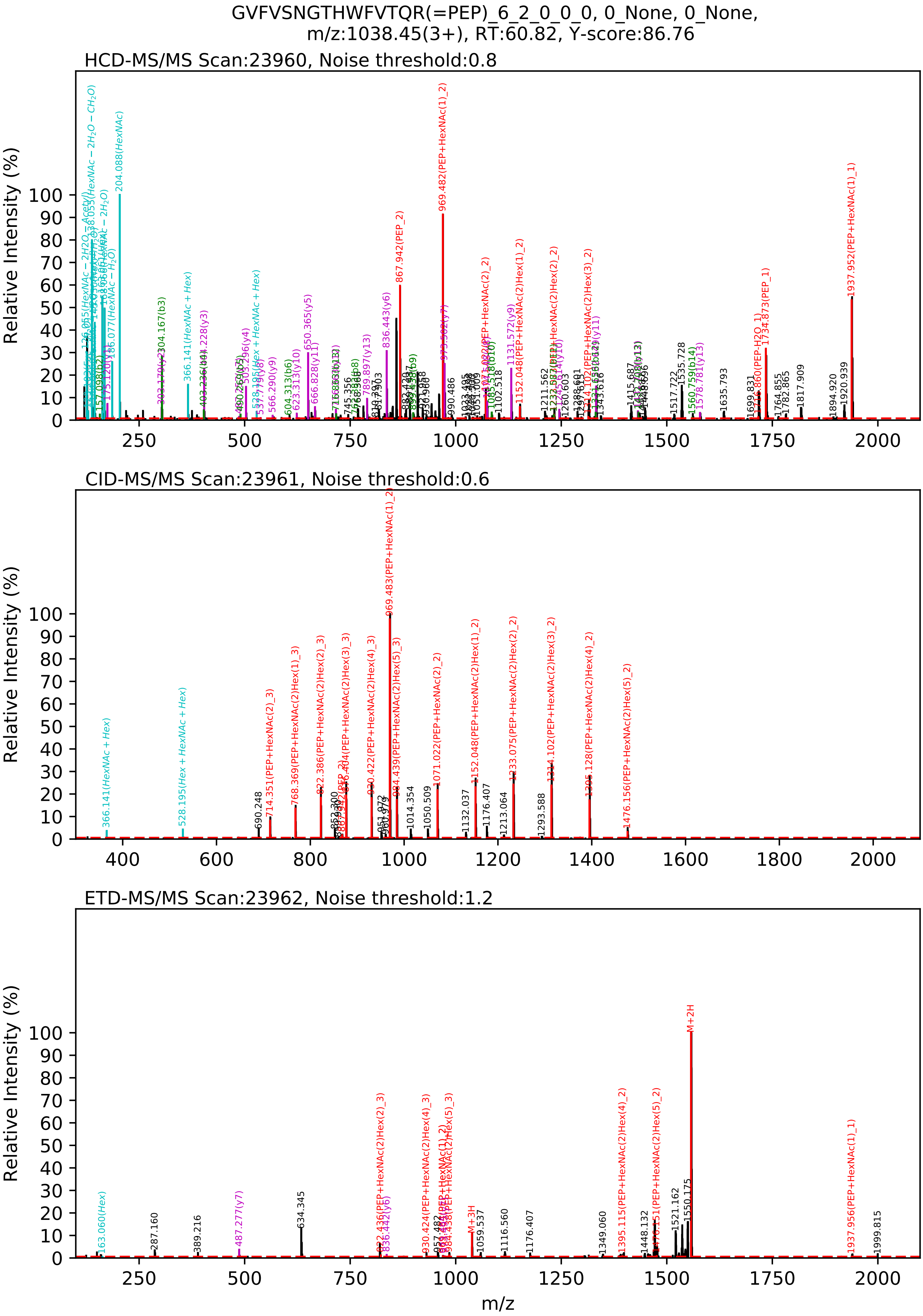

GVFVSNGTHWFVTQR(=PEP)_6_2_0_0_0, 0_None, 0_None,
m/z:1038.45(3+), RT:60.82, Y-score:86.76
HCD-MS/MS Scan:23960, Noise threshold:0.8
Relative Intensity (%)
204.088(HexNAc)
969.482(PEP+HexNAc(1)_2)
867.942(PEP_2)
1937.952(PEP+HexNAc(1)_1)
1734.873(PEP_1)
1152.048(PEP+HexNAc(2)Hex(1)_2)
1131.572(y9)
836.443(y6)
650.365(y5)
304.167(b3)
366.141(HexNAc + Hex)
528.195(Hex + HexNAc + Hex)
CID-MS/MS Scan:23961, Noise threshold:0.6
Relative Intensity (%)
969.483(PEP+HexNAc(1)_2)
366.141(HexNAc + Hex)
528.195(Hex + HexNAc + Hex)
714.351(PEP+HexNAc(2)_3)
768.369(PEP+HexNAc(2)Hex(1)_3)
822.386(PEP+HexNAc(2)Hex(2)_3)
930.422(PEP+HexNAc(2)Hex(4)_3)
984.439(PEP+HexNAc(2)Hex(5)_3)
1071.022(PEP+HexNAc(2)_2)
1152.048(PEP+HexNAc(2)Hex(1)_2)
1233.075(PEP+HexNAc(2)Hex(2)_2)
1314.102(PEP+HexNAc(2)Hex(3)_2)
1476.156(PEP+HexNAc(2)Hex(5)_2)
ETD-MS/MS Scan:23962, Noise threshold:1.2
Relative Intensity (%)
M+2H
M+3H
163.060(Hex)
287.160
389.216
487.277(y7)
634.345
1059.537
1116.560
1176.407
1349.060
1395.115(PEP+HexNAc(2)Hex(4)_2)
1448.132
1521.162
1550.175
1937.956(PEP+HexNAc(1)_1)
1999.815
m/z

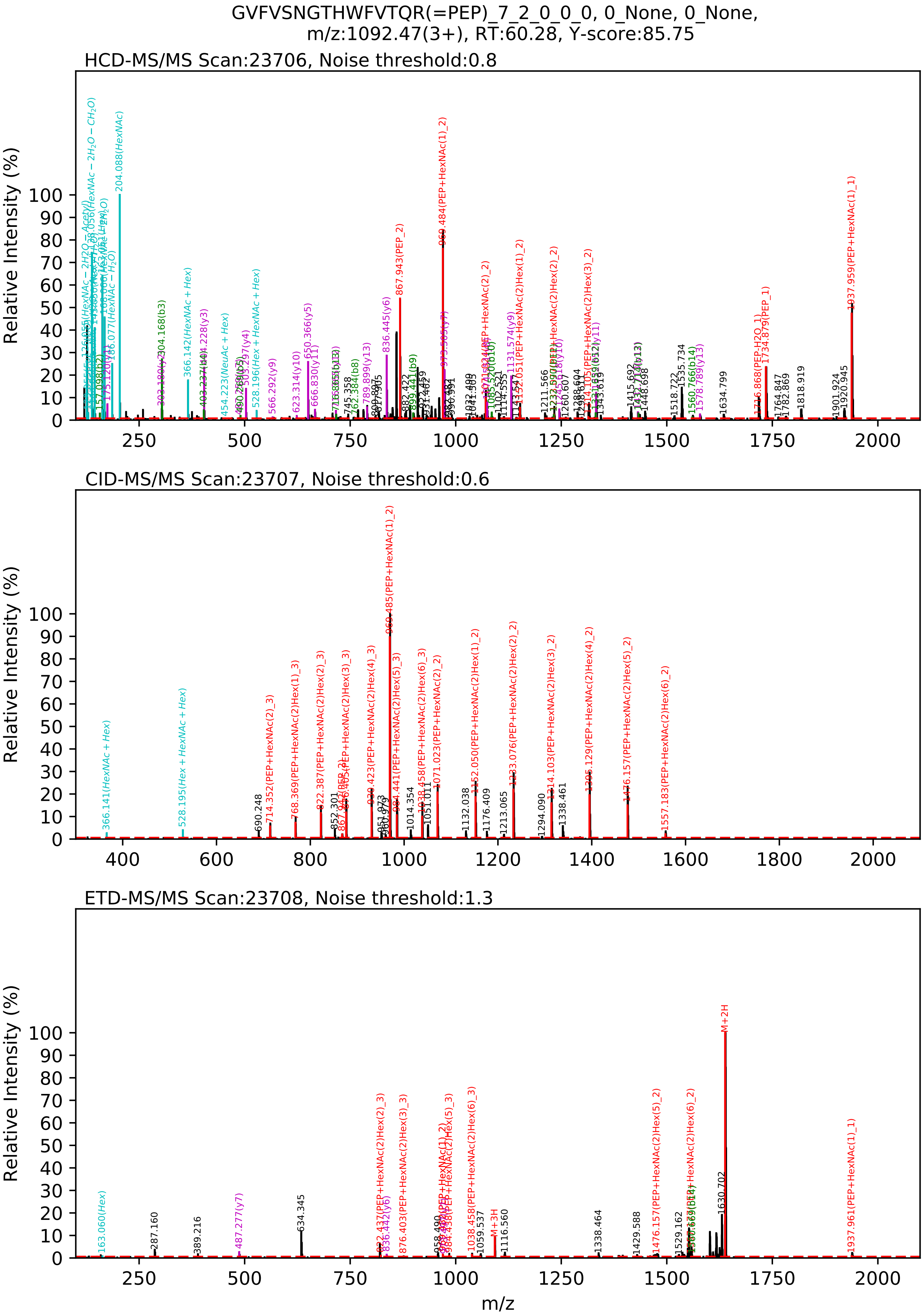

GVFVSNGTHWFVTQR(=PEP)_7_2_0_0_0, 0_None, 0_None,
m/z:1092.47(3+), RT:60.28, Y-score:85.75
HCD-MS/MS Scan:23706, Noise threshold:0.8
Relative Intensity (%)
204.088(HexNAc)
969.484(PEP+HexNAc(1)_2)
867.943(PEP_2)
1937.959(PEP+HexNAc(1)_1)
1734.879(PEP_1)
836.445(y6)
650.366(y5)
366.142(HexNAc + Hex)
304.168(b3)
CID-MS/MS Scan:23707, Noise threshold:0.6
969.485(PEP+HexNAc(1)_2)
1557.183(PEP+HexNAc(2)Hex(6)_2)
1476.157(PEP+HexNAc(2)Hex(5)_2)
1395.129(PEP+HexNAc(2)Hex(4)_2)
1314.103(PEP+HexNAc(2)Hex(3)_2)
1233.076(PEP+HexNAc(2)Hex(2)_2)
1152.050(PEP+HexNAc(2)Hex(1)_2)
1071.023(PEP+HexNAc(2)_2)
768.369(PEP+HexNAc(2)Hex(1)_3)
822.387(PEP+HexNAc(2)Hex(2)_3)
714.352(PEP+HexNAc(2)_3)
366.141(HexNAc + Hex)
528.195(Hex + HexNAc + Hex)
ETD-MS/MS Scan:23708, Noise threshold:1.3
M+2H
M+3H
1630.702
634.345
1937.961(PEP+HexNAc(1)_1)
1476.157(PEP+HexNAc(2)Hex(5)_2)
163.060(Hex)
487.277(y7)
m/z

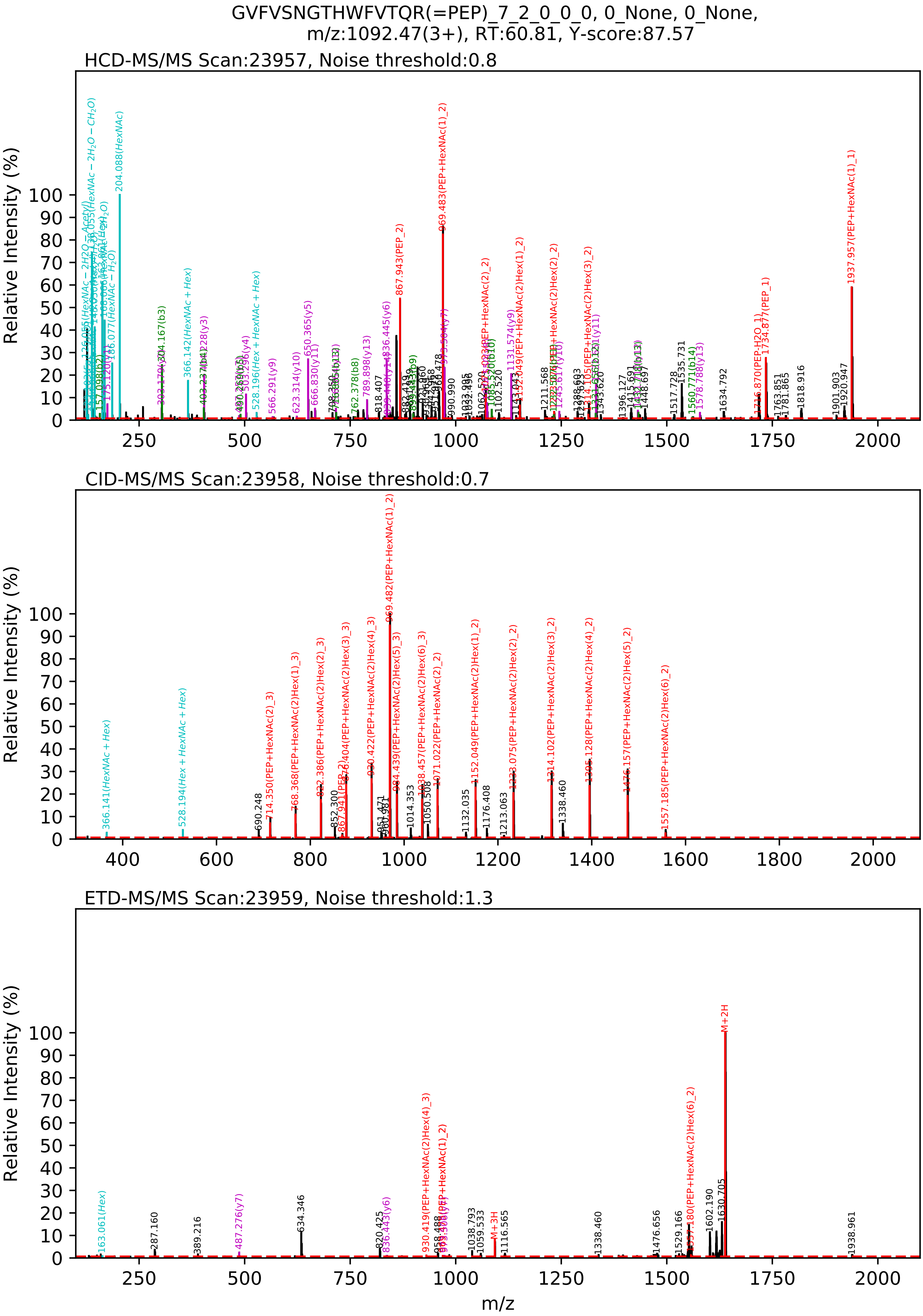
GVFVSNGTHWFVTQR(=PEP)_7_2_0_0_0, 0_None, 0_None,
m/z:1092.47(3+), RT:60.81, Y-score:87.57
HCD-MS/MS Scan:23957, Noise threshold:0.8
Relative Intensity (%)
204.088(HexNAc)
969.483(PEP+HexNAc(1)_2)
867.943(PEP_2)
1937.957(PEP+HexNAc(1)_1)
1734.877(PEP_1)
1716.870(PEP-H2O_1)
366.142(HexNAc+Hex)
528.196(Hex+HexNAc+Hex)
304.167(b3)
650.365(y5)
836.445(y6)
1131.574(y9)
CID-MS/MS Scan:23958, Noise threshold:0.7
969.482(PEP+HexNAc(1)_2)
366.141(HexNAc+Hex)
528.194(Hex+HexNAc+Hex)
714.350(PEP+HexNAc(2)_3)
768.368(PEP+HexNAc(2)Hex(1)_3)
822.386(PEP+HexNAc(2)Hex(2)_3)
876.404(PEP+HexNAc(2)Hex(3)_3)
930.422(PEP+HexNAc(2)Hex(4)_3)
984.439(PEP+HexNAc(2)Hex(5)_3)
1038.457(PEP+HexNAc(2)Hex(6)_3)
1071.022(PEP+HexNAc(2)_2)
1152.049(PEP+HexNAc(2)Hex(1)_2)
1233.075(PEP+HexNAc(2)Hex(2)_2)
1314.102(PEP+HexNAc(2)Hex(3)_2)
1395.128(PEP+HexNAc(2)Hex(4)_2)
1476.157(PEP+HexNAc(2)Hex(5)_2)
1557.185(PEP+HexNAc(2)Hex(6)_2)
ETD-MS/MS Scan:23959, Noise threshold:1.3
M+2H
M+3H
163.061(Hex)
487.276(y7)
836.443(y6)
930.419(PEP+HexNAc(2)Hex(4)_3)
1557.180(PEP+HexNAc(2)Hex(6)_2)
m/z

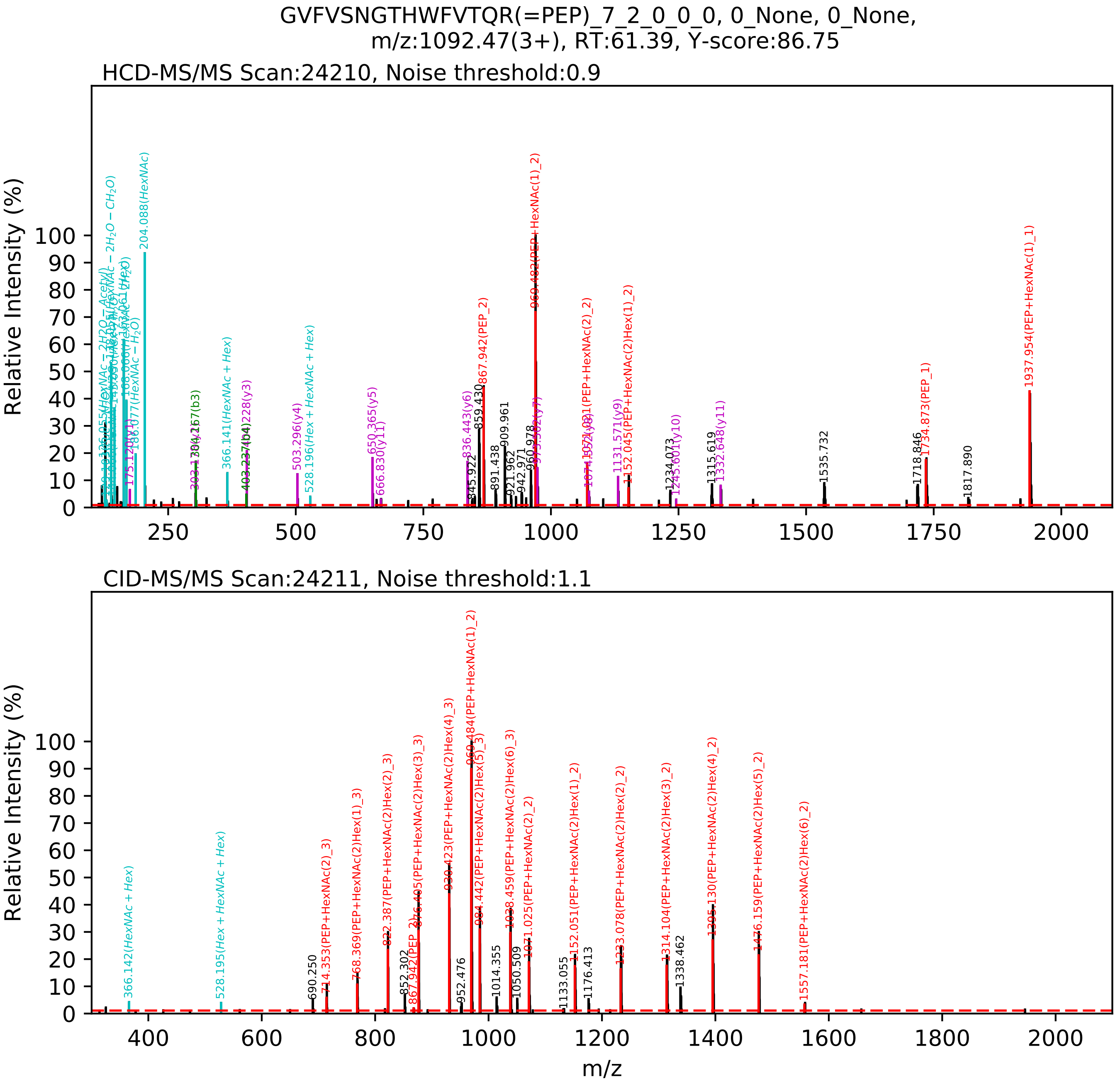
GVFVSNGTHWFVTQR(=PEP)_7_2_0_0_0, 0_None, 0_None,
m/z:1092.47(3+), RT:61.39, Y-score:86.75
HCD-MS/MS Scan:24210, Noise threshold:0.9
Relative Intensity (%)
204.088(HexNAc)
366.141(HexNAc + Hex)
528.196(Hex + HexNAc + Hex)
503.296(y4)
650.365(y5)
666.830(y11)
836.443(y6)
845.922
859.430
867.942(PEP_2)
891.438
909.961
921.962
942.971
960.978
969.482(PEP+HexNAc(1)_2)
1071.091(PEP+HexNAc(2)_2)
1131.571(y9)
1152.045(PEP+HexNAc(2)Hex(1)_2)
1234.073
1243.601(y10)
1315.619
1332.648(y11)
1535.732
1718.846
1734.873(PEP_1)
1817.890
1937.954(PEP+HexNAc(1)_1)
CID-MS/MS Scan:24211, Noise threshold:1.1
366.142(HexNAc + Hex)
528.195(Hex + HexNAc + Hex)
690.250
714.353(PEP+HexNAc(2)_3)
768.369(PEP+HexNAc(2)Hex(1)_3)
822.387(PEP+HexNAc(2)Hex(2)_3)
852.302
867.942(PEP_2)
876.405(PEP+HexNAc(2)Hex(3)_3)
930.423(PEP+HexNAc(2)Hex(4)_3)
952.476
969.484(PEP+HexNAc(1)_2)
984.442(PEP+HexNAc(2)Hex(5)_3)
1014.355
1038.459(PEP+HexNAc(2)Hex(6)_3)
1050.509
1071.025(PEP+HexNAc(2)_2)
1133.055
1152.051(PEP+HexNAc(2)Hex(1)_2)
1176.413
1233.078(PEP+HexNAc(2)Hex(2)_2)
1314.104(PEP+HexNAc(2)Hex(3)_2)
1338.462
1395.130(PEP+HexNAc(2)Hex(4)_2)
1476.159(PEP+HexNAc(2)Hex(5)_2)
1557.181(PEP+HexNAc(2)Hex(6)_2)
m/z

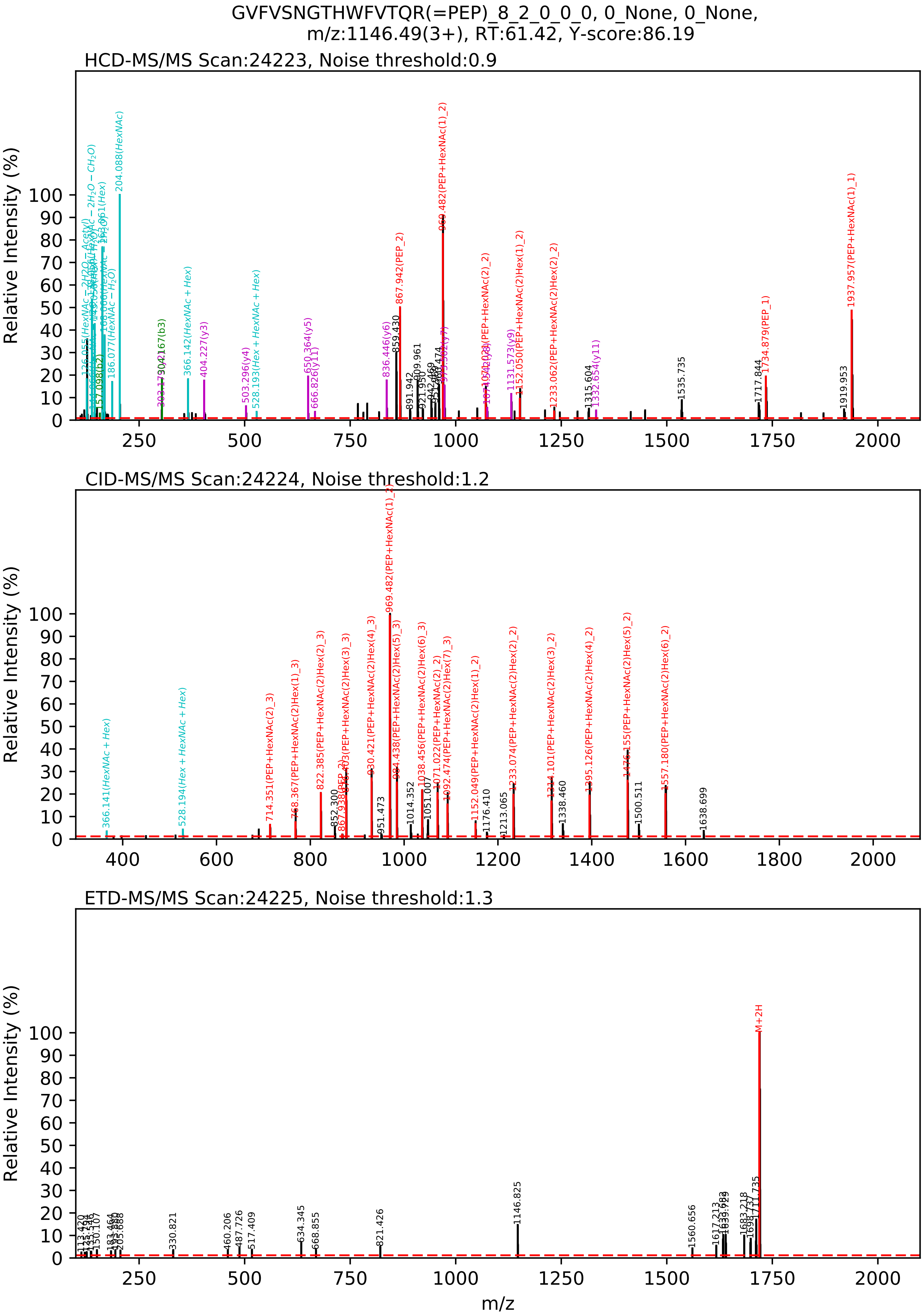
GVFVSNGTHWFVTQR(=PEP)_8_2_0_0_0, 0_None, 0_None,
m/z:1146.49(3+), RT:61.42, Y-score:86.19
HCD-MS/MS Scan:24223, Noise threshold:0.9
CID-MS/MS Scan:24224, Noise threshold:1.2
ETD-MS/MS Scan:24225, Noise threshold:1.3
Relative Intensity (%)
m/z

GVFVSNGTHWFVTQR(=PEP)_8_2_0_0_0, 0_None, 0_None,
m/z:1146.49(3+), RT:61.52, Y-score:85.33

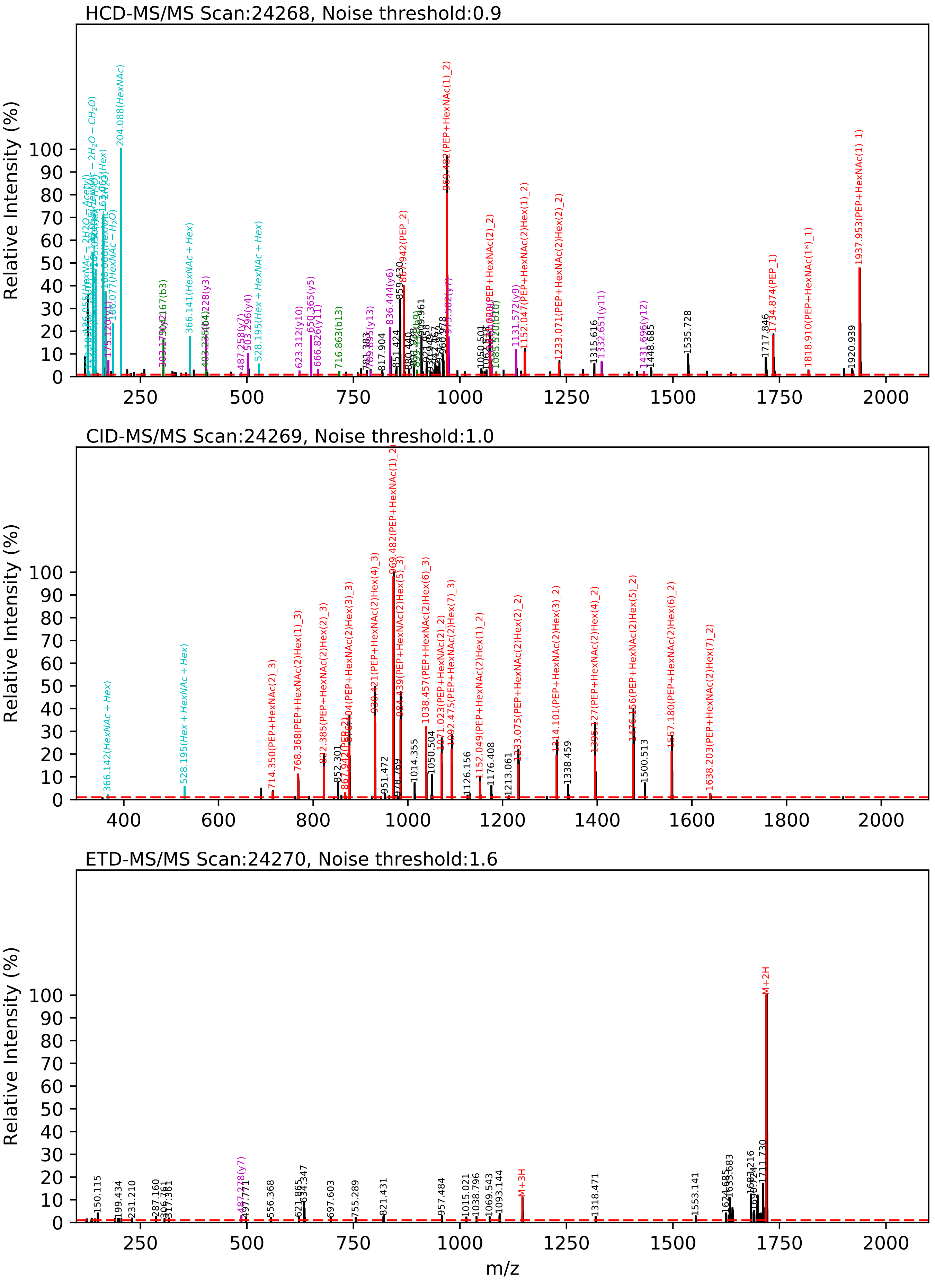

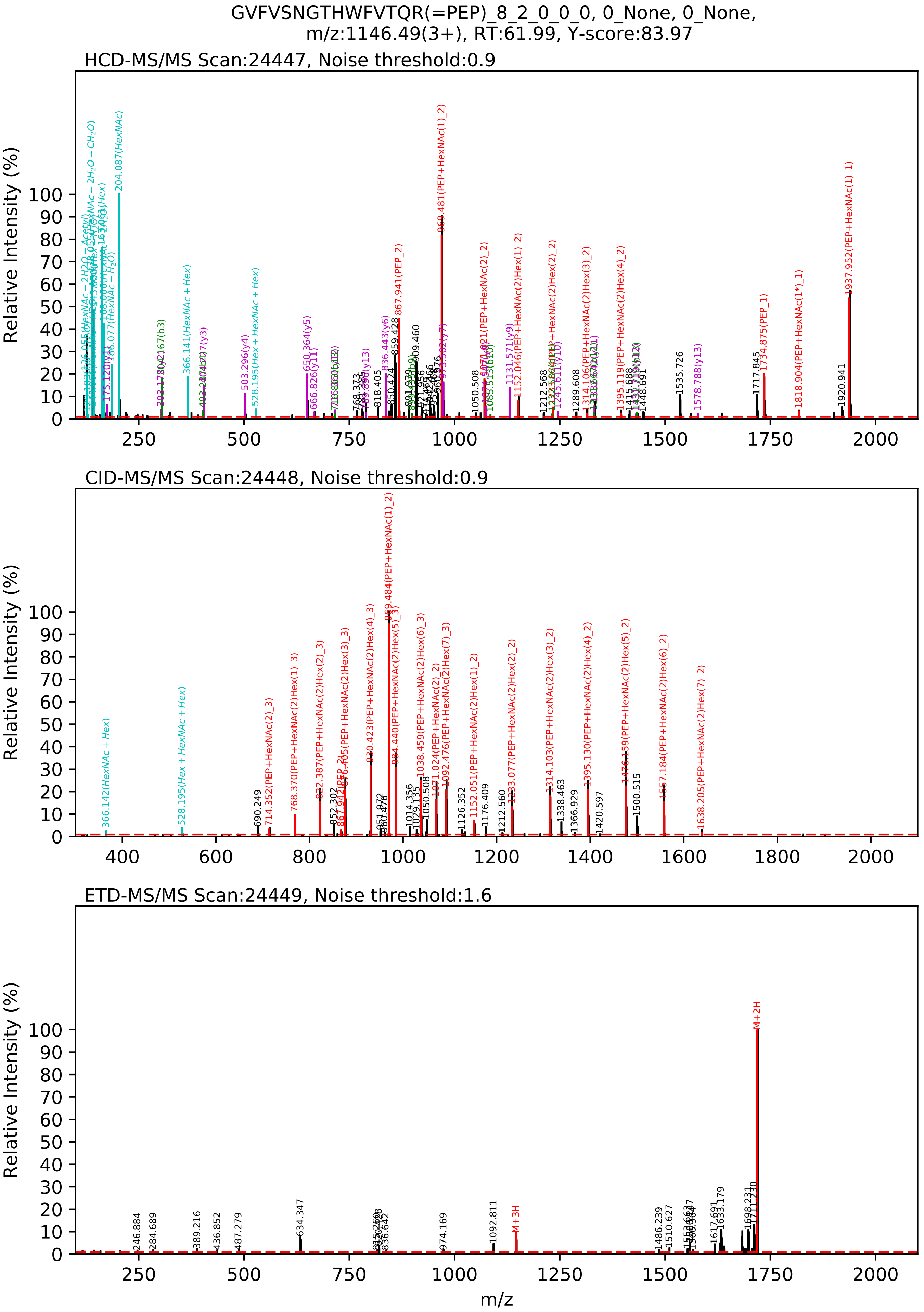
GVFVSNGTHWFVTQR(=PEP)_8_2_0_0_0, 0_None, 0_None,
m/z:1146.49(3+), RT:61.99, Y-score:83.97
HCD-MS/MS Scan:24447, Noise threshold:0.9
CID-MS/MS Scan:24448, Noise threshold:0.9
ETD-MS/MS Scan:24449, Noise threshold:1.6
Relative Intensity (%)
m/z
M+2H
M+3H

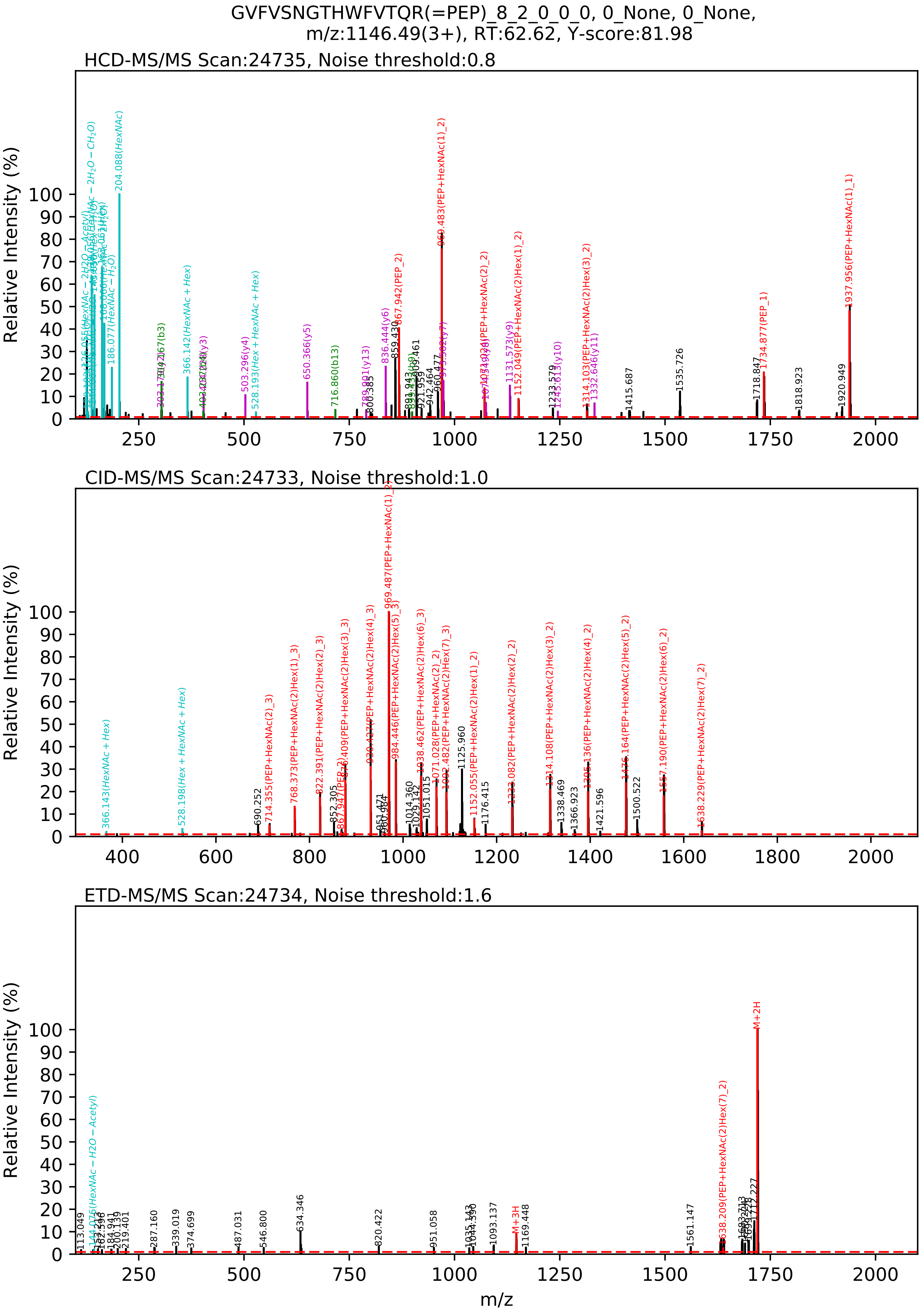

GVFVSNGTHWFVTQR(=PEP)_8_2_0_0_0, 0_None, 0_None,
m/z:1146.49(3+), RT:62.62, Y-score:81.98
HCD-MS/MS Scan:24735, Noise threshold:0.8
CID-MS/MS Scan:24733, Noise threshold:1.0
ETD-MS/MS Scan:24734, Noise threshold:1.6
Relative Intensity (%)
m/z

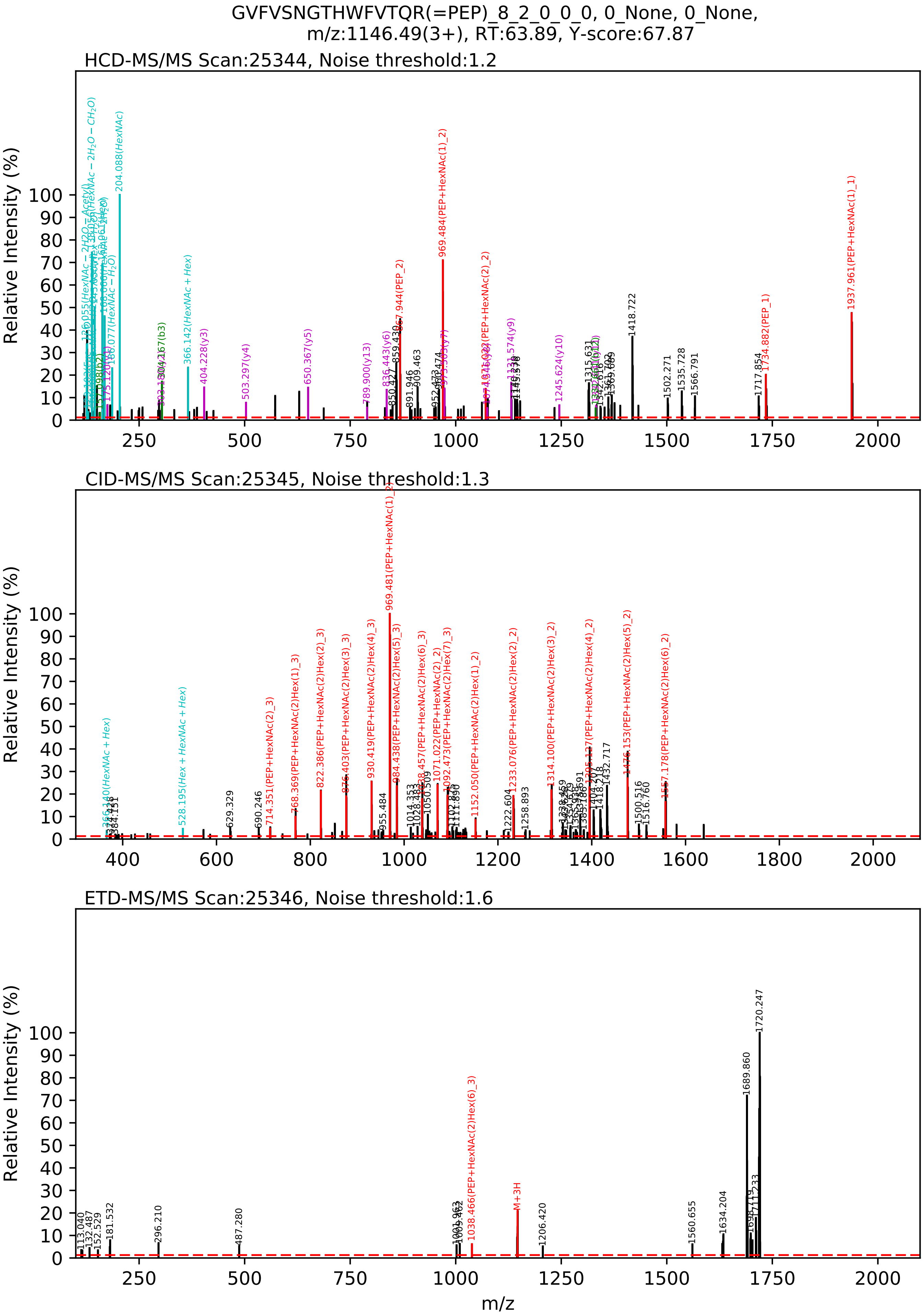

GVFVSNGTHWFVTQR(=PEP)_8_2_0_0_0, 0_None, 0_None,
m/z:1146.49(3+), RT:63.89, Y-score:67.87
HCD-MS/MS Scan:25344, Noise threshold:1.2
969.484(PEP+HexNAc(1)_2)
1937.961(PEP+HexNAc(1)_1)
1734.882(PEP_1)
867.944(PEP_2)
204.088(HexNAc)
366.142(HexNAc + Hex)
CID-MS/MS Scan:25345, Noise threshold:1.3
969.481(PEP+HexNAc(1)_2)
1476.153(PEP+HexNAc(2)Hex(5)_2)
1557.178(PEP+HexNAc(2)Hex(6)_2)
1314.100(PEP+HexNAc(2)Hex(3)_2)
1233.076(PEP+HexNAc(2)Hex(2)_2)
1152.050(PEP+HexNAc(2)Hex(1)_2)
528.195(Hex + HexNAc + Hex)
ETD-MS/MS Scan:25346, Noise threshold:1.6
1720.247
1689.860
1038.466(PEP+HexNAc(2)Hex(6)_3)
M+3H
Relative Intensity (%)
m/z

GVFVSNGTHWFVTQR(=PEP)_8_2_0_0_0, 0_None, 0_None,
m/z:1146.49(3+), RT:65.93, Y-score:74.15

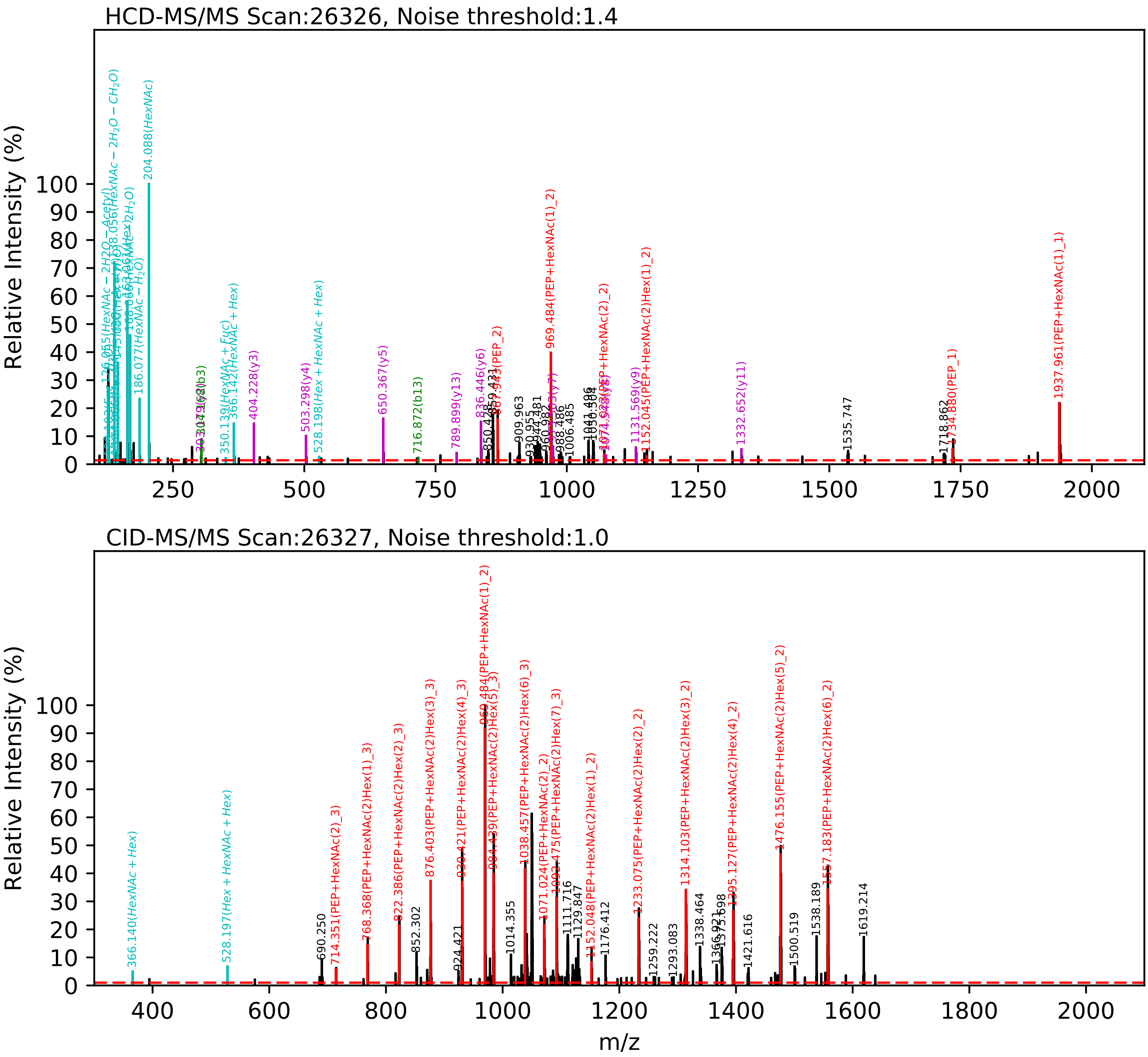

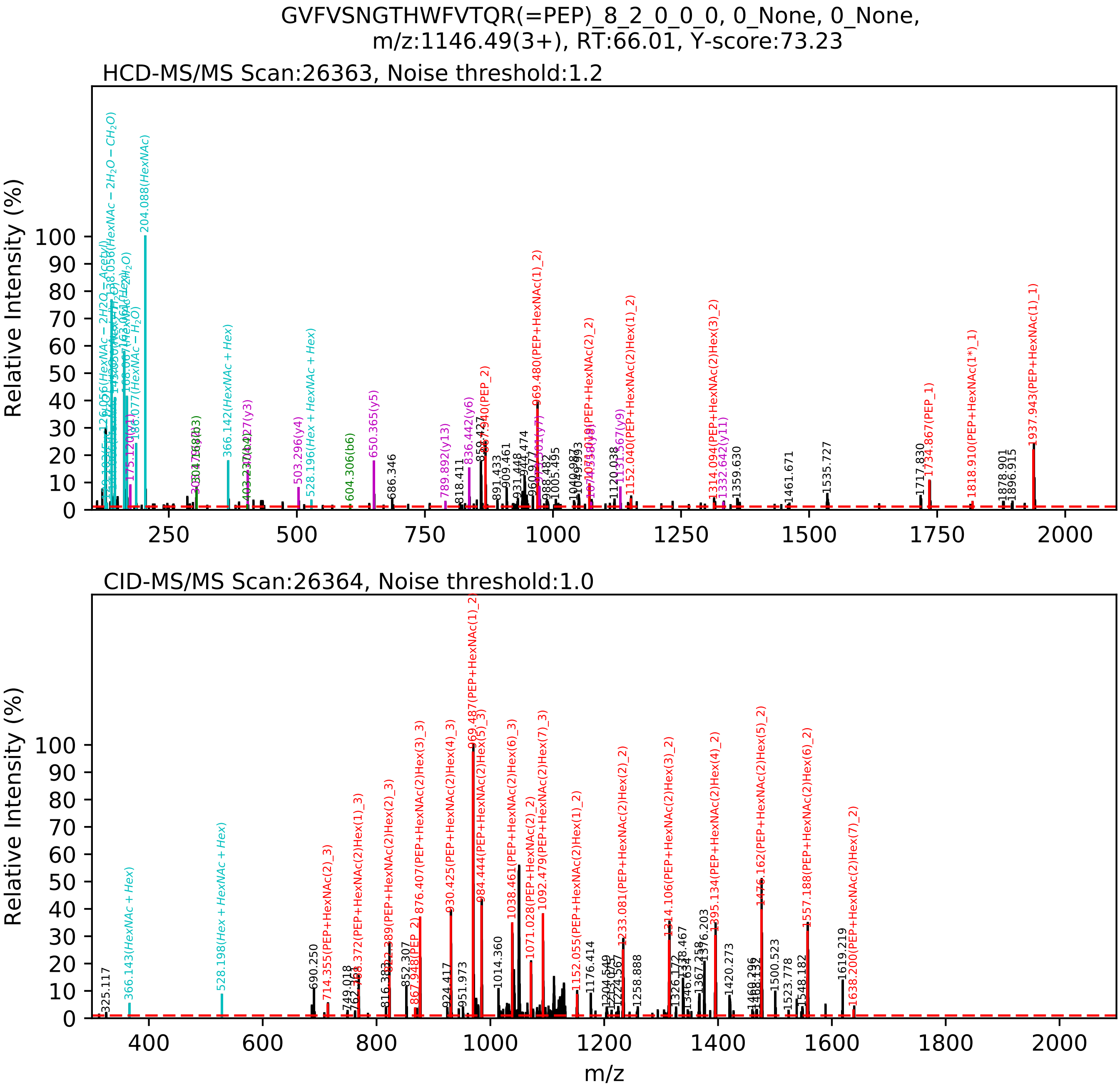
GVFVSNGTHWFVTQR(=PEP)_8_2_0_0_0, 0_None, 0_None,
m/z:1146.49(3+), RT:66.01, Y-score:73.23
HCD-MS/MS Scan:26363, Noise threshold:1.2
Relative Intensity (%)
204.088(HexNAc)
366.142(HexNAc + Hex)
528.196(Hex + HexNAc + Hex)
503.296(y4)
604.306(b6)
650.365(y5)
686.346
789.892(y13)
818.411
836.442(y6)
867.940(PEP_2)
969.480(PEP+HexNAc(1)_2)
1152.040(PEP+HexNAc(2)Hex(1)_2)
1314.094(PEP+HexNAc(2)Hex(3)_2)
1332.642(y11)
1359.630
1461.671
1535.727
1717.830
1734.867(PEP_1)
1818.910(PEP+HexNAc(1*)_1)
1878.901
1896.915
1937.943(PEP+HexNAc(1)_1)
CID-MS/MS Scan:26364, Noise threshold:1.0
325.117
366.143(HexNAc + Hex)
528.198(Hex + HexNAc + Hex)
690.250
714.355(PEP+HexNAc(2)_3)
768.372(PEP+HexNAc(2)Hex(1)_3)
822.389(PEP+HexNAc(2)Hex(2)_3)
852.307
867.948(PEP_2)
876.407(PEP+HexNAc(2)Hex(3)_3)
924.417
930.425(PEP+HexNAc(2)Hex(4)_3)
951.973
969.487(PEP+HexNAc(1)_2)
984.444(PEP+HexNAc(2)Hex(5)_3)
1014.360
1038.461(PEP+HexNAc(2)Hex(6)_3)
1071.028(PEP+HexNAc(2)_2)
1092.479(PEP+HexNAc(2)Hex(7)_3)
1152.055(PEP+HexNAc(2)Hex(1)_2)
1176.414
1233.081(PEP+HexNAc(2)Hex(2)_2)
1258.888
1314.106(PEP+HexNAc(2)Hex(3)_2)
1376.203
1395.134(PEP+HexNAc(2)Hex(4)_2)
1420.273
1476.162(PEP+HexNAc(2)Hex(5)_2)
1500.523
1523.778
1557.188(PEP+HexNAc(2)Hex(6)_2)
1619.219
1638.200(PEP+HexNAc(2)Hex(7)_2)
m/z

GVFVSNGTHWFVTQR(=PEP)_8_2_0_0_0, 0_None, 0_None,
m/z:1146.49(3+), RT:66.89, Y-score:70.40

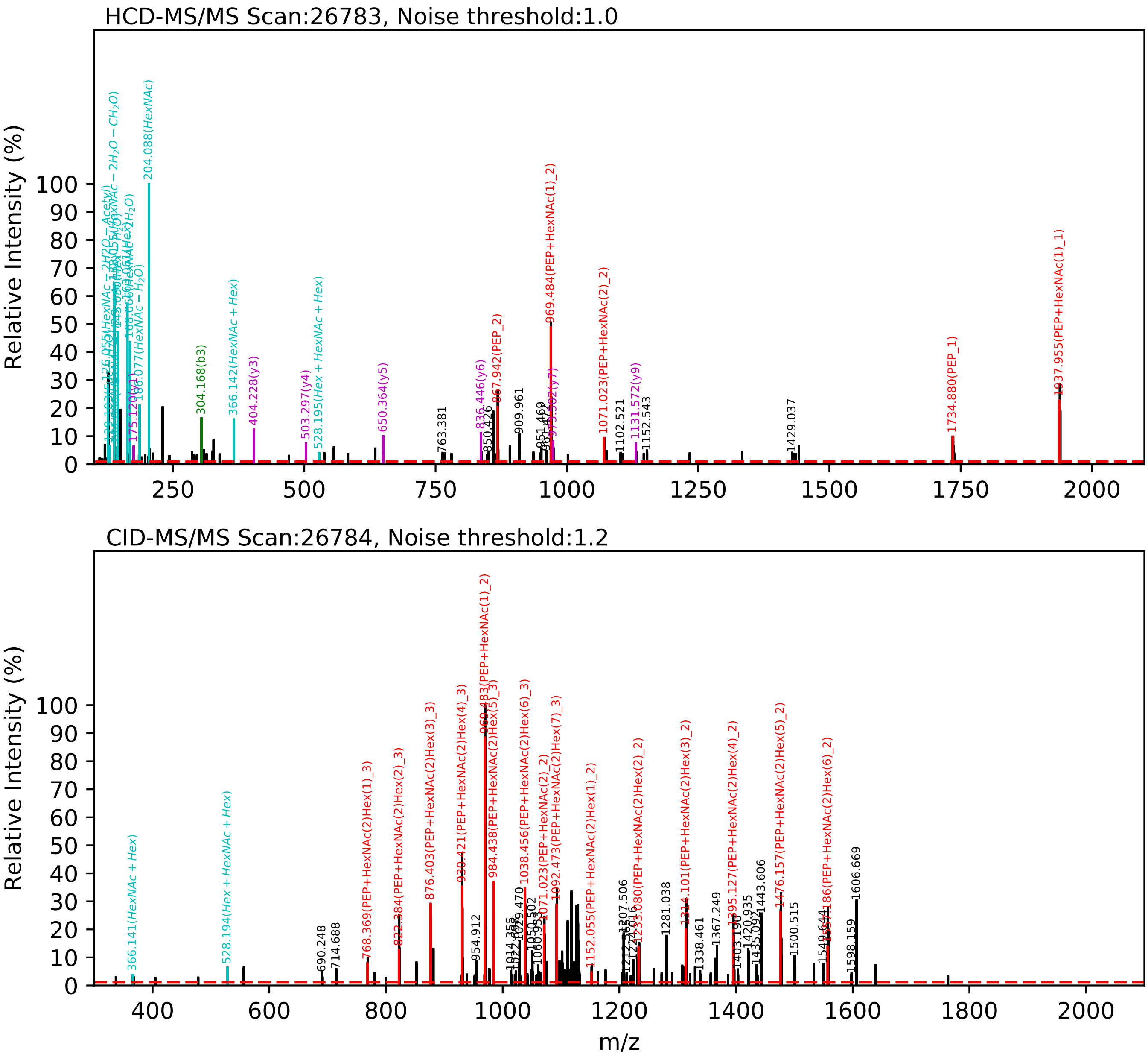

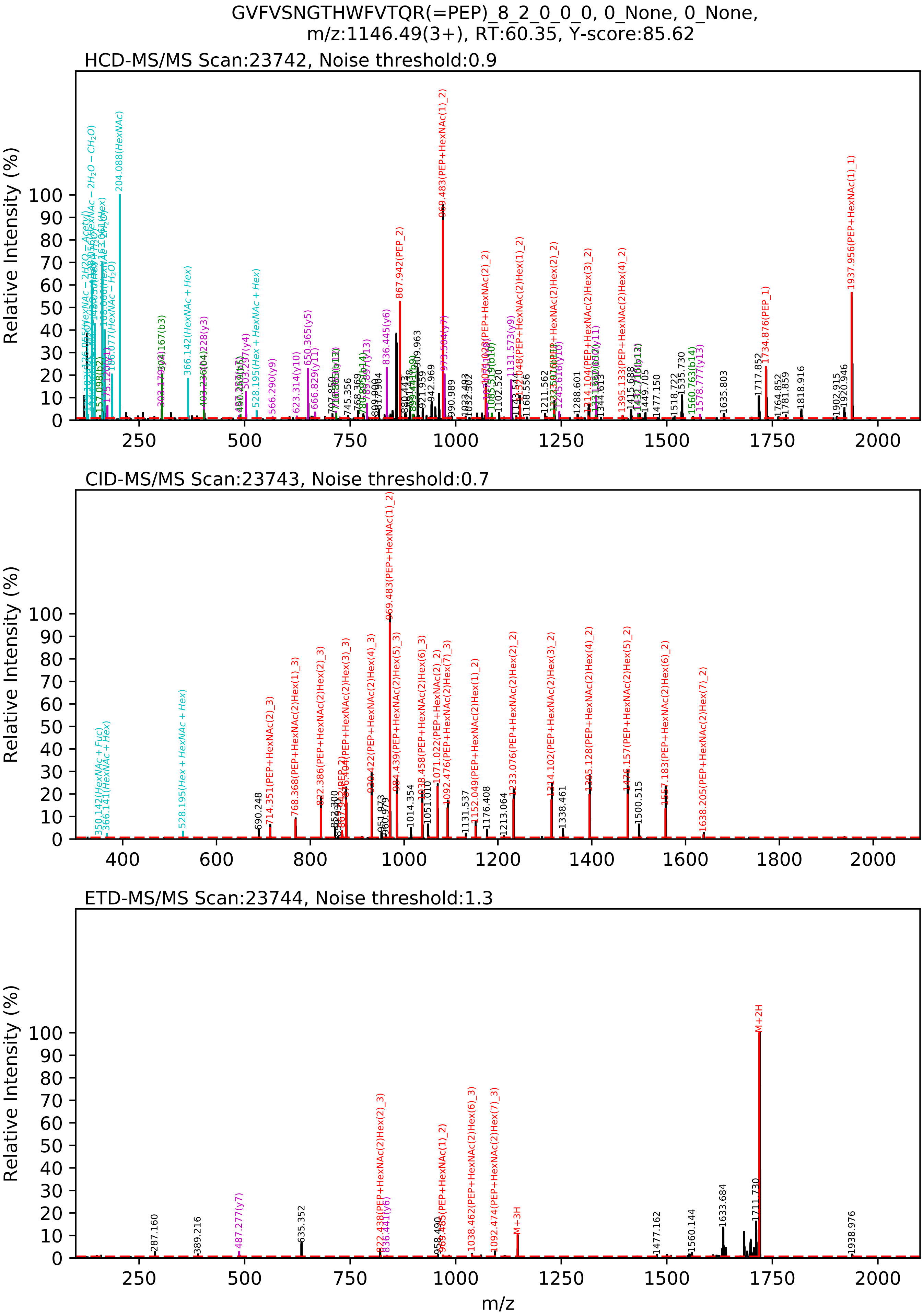

GVFVSNGTHWFVTQR(=PEP)_8_2_0_0_0, 0_None, 0_None,
m/z:1146.49(3+), RT:60.35, Y-score:85.62
HCD-MS/MS Scan:23742, Noise threshold:0.9
CID-MS/MS Scan:23743, Noise threshold:0.7
ETD-MS/MS Scan:23744, Noise threshold:1.3
Relative Intensity (%)
m/z

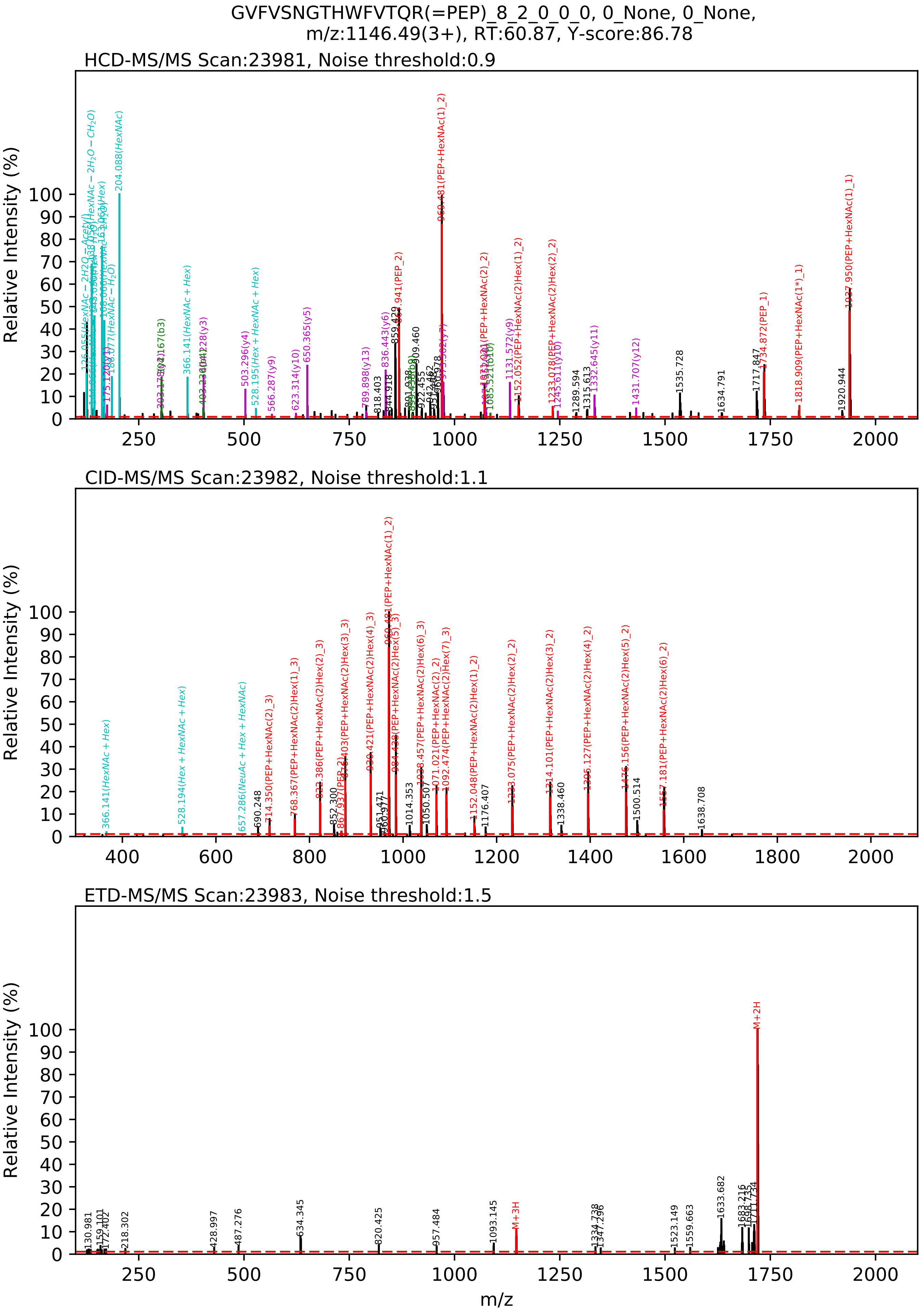
GVFVSNGTHWFVTQR(=PEP)_8_2_0_0_0, 0_None, 0_None,
m/z:1146.49(3+), RT:60.87, Y-score:86.78
HCD-MS/MS Scan:23981, Noise threshold:0.9
CID-MS/MS Scan:23982, Noise threshold:1.1
ETD-MS/MS Scan:23983, Noise threshold:1.5
Relative Intensity (%)
m/z
960.481(PEP+HexNAc(1)_2)
1937.950(PEP+HexNAc(1)_1)
1734.872(PEP_1)
1818.909(PEP+HexNAc(1*)_1)
204.088(HexNAc)
366.141(HexNAc+Hex)
528.195(Hex+HexNAc+Hex)
650.365(y5)
503.296(y4)
1535.728
1717.847
1920.944
1634.791
930.421(PEP+HexNAc(2)Hex(4)_3)
984.428(PEP+HexNAc(2)Hex(5)_3)
1233.075(PEP+HexNAc(2)Hex(2)_2)
1314.101(PEP+HexNAc(2)Hex(3)_2)
1395.127(PEP+HexNAc(2)Hex(4)_2)
1476.156(PEP+HexNAc(2)Hex(5)_2)
1557.181(PEP+HexNAc(2)Hex(6)_2)
1638.708
M+2H
M+3H
1633.682
634.345
1093.145

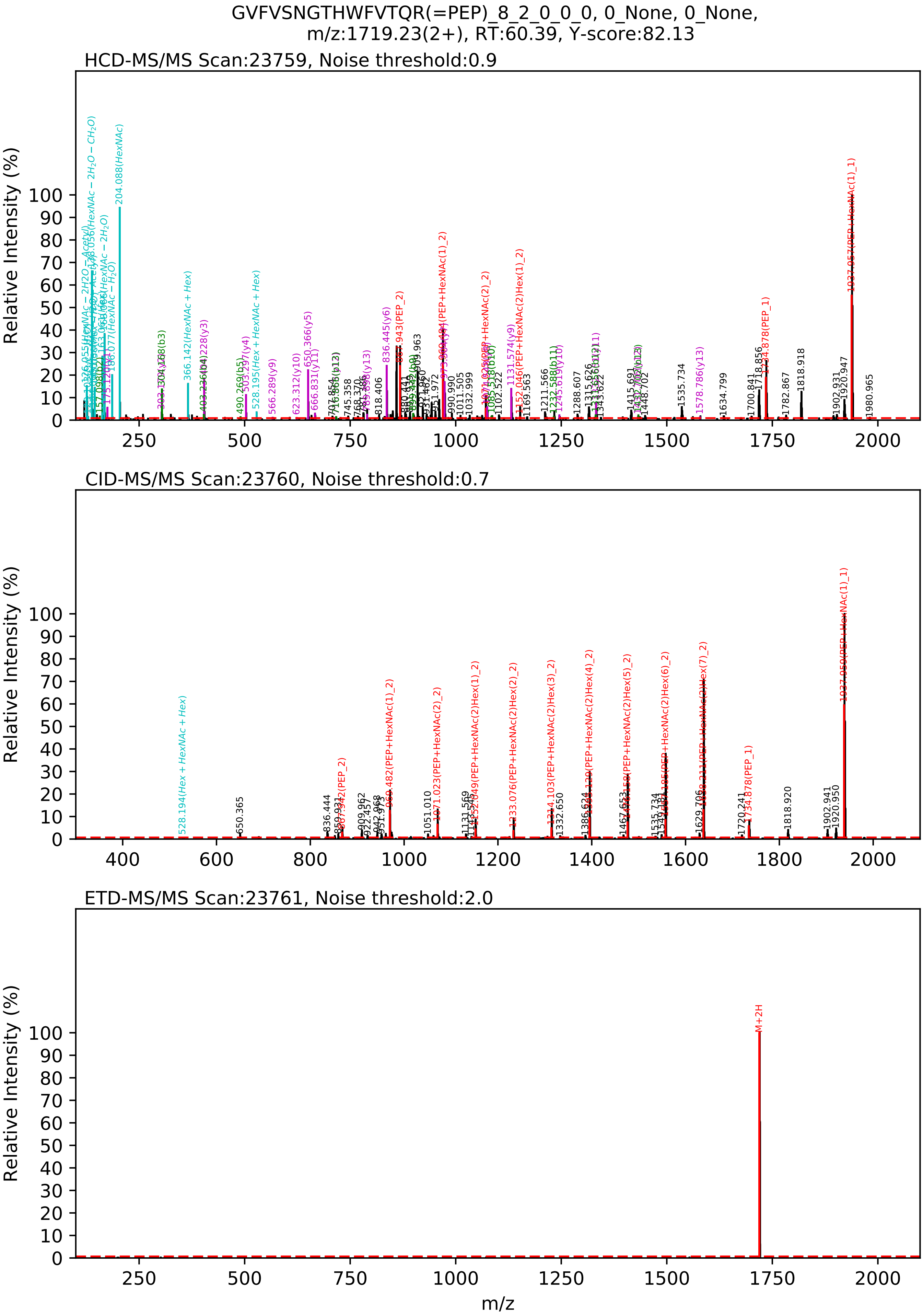

GVFVSNGTHWFVTQR(=PEP)_8_2_0_0_0, 0_None, 0_None,
m/z:1719.23(2+), RT:60.39, Y-score:82.13
HCD-MS/MS Scan:23759, Noise threshold:0.9
Relative Intensity (%)
204.088(HexNAc)
366.142(HexNAc + Hex)
528.195(Hex + HexNAc + Hex)
650.366(y5)
836.445(y6)
1131.574(y9)
1152.046(PEP+HexNAc(2)Hex(1)_2)
1578.786(y13)
1734.878(PEP_1)
1937.957(PEP+HexNAc(1)_1)
1535.734
1818.918
1902.931
1920.947
1980.965
CID-MS/MS Scan:23760, Noise threshold:0.7
528.194(Hex + HexNAc + Hex)
650.365
969.482(PEP+HexNAc(1)_2)
1071.023(PEP+HexNAc(2)_2)
1734.878(PEP_1)
1818.920
1937.950(PEP+HexNAc(1)_1)
ETD-MS/MS Scan:23761, Noise threshold:2.0
M+2H
m/z

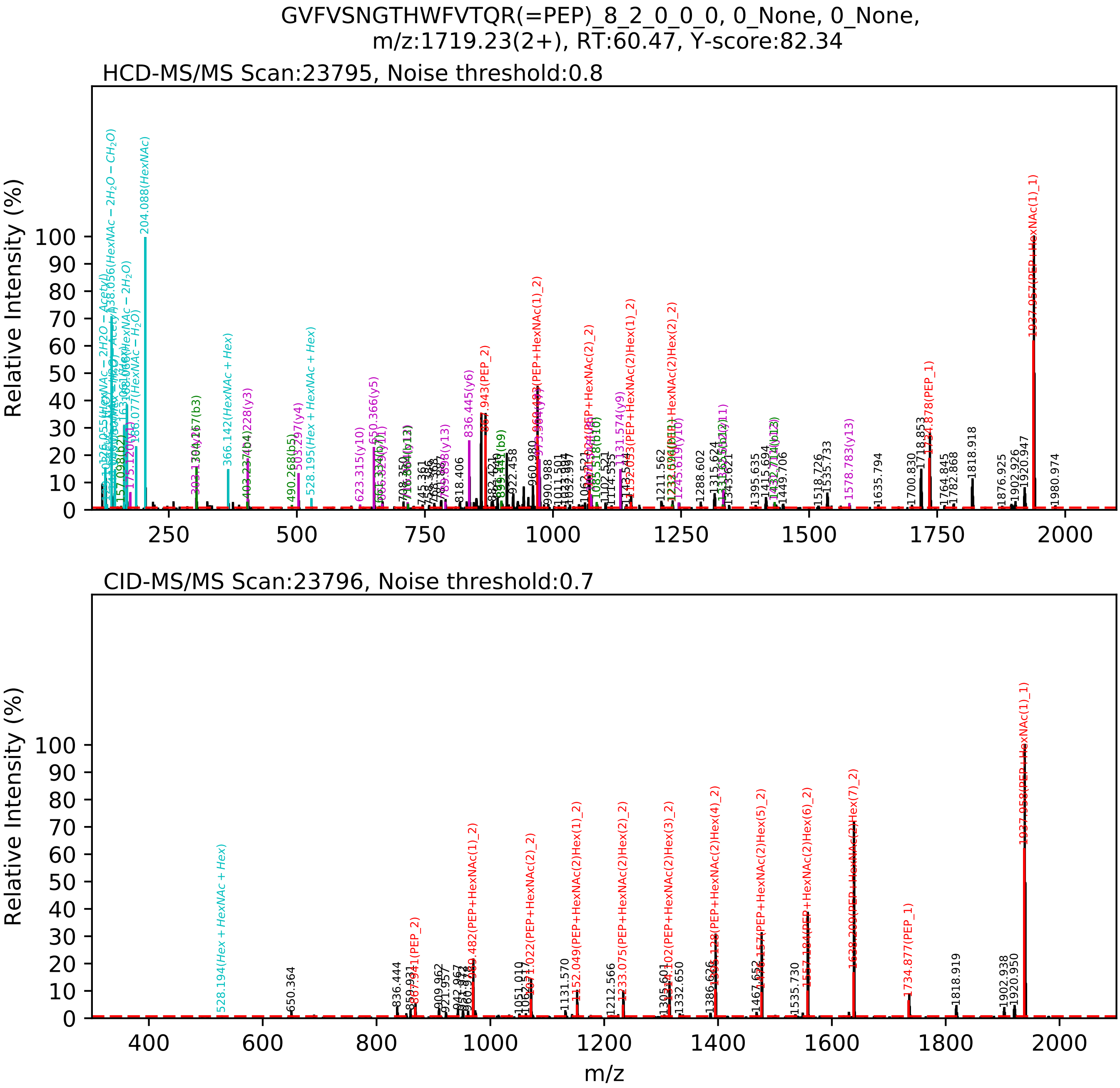

GVFVSNGTHWFVTQR(=PEP)_8_2_0_0_0, 0_None, 0_None,
m/z:1719.23(2+), RT:60.47, Y-score:82.34
HCD-MS/MS Scan:23795, Noise threshold:0.8
Relative Intensity (%)
204.088(HexNAc)
366.142(HexNAc + Hex)
528.195(Hex + HexNAc + Hex)
836.445(y6)
1937.957(PEP+HexNAc(1)_1)
CID-MS/MS Scan:23796, Noise threshold:0.7
Relative Intensity (%)
528.194(Hex + HexNAc + Hex)
1734.877(PEP_1)
1937.958(PEP+HexNAc(1)_1)
m/z

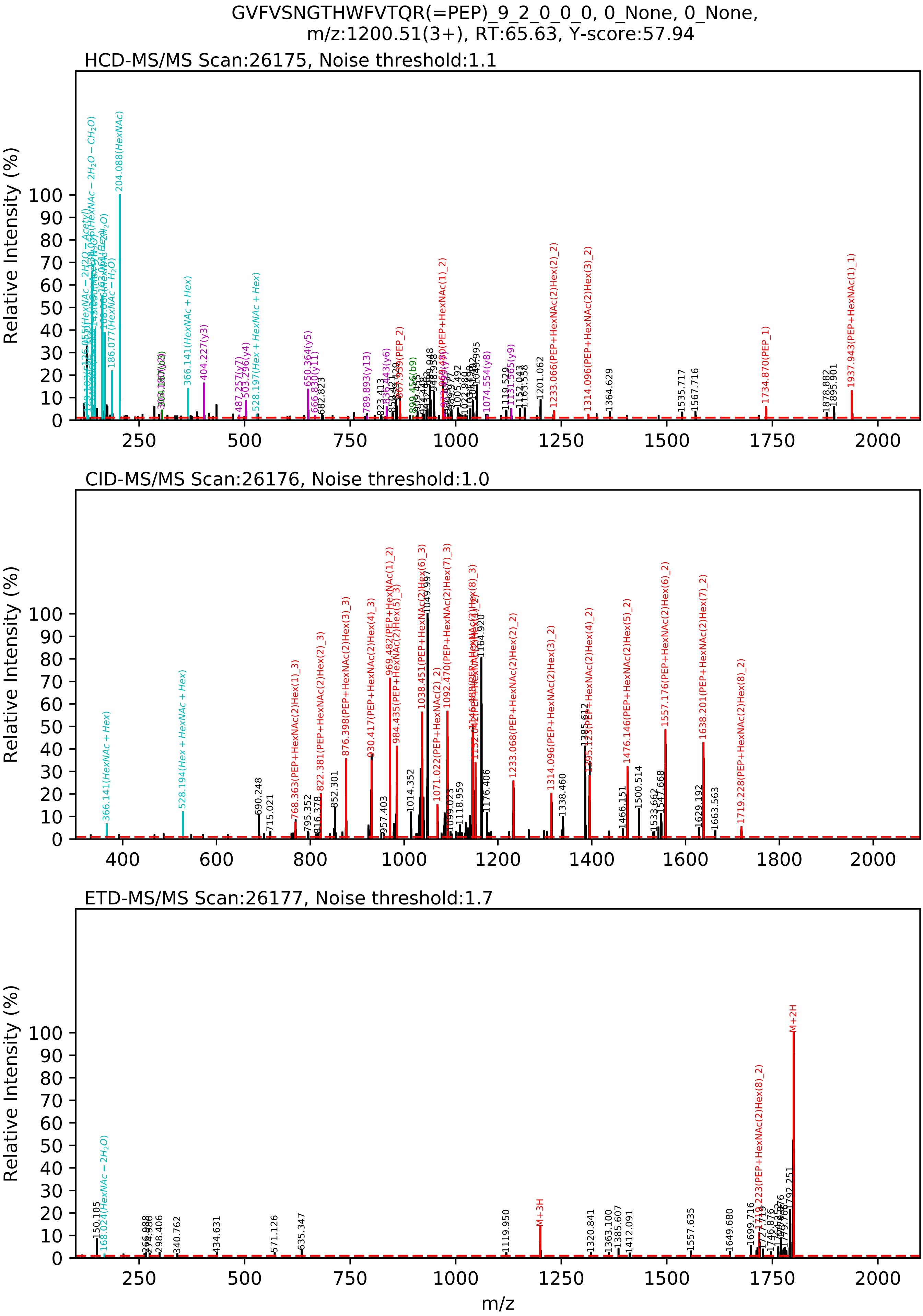

GVFVSNGTHWFVTQR(=PEP)_9_2_0_0_0, 0_None, 0_None,
m/z:1200.51(3+), RT:65.63, Y-score:57.94
HCD-MS/MS Scan:26175, Noise threshold:1.1
CID-MS/MS Scan:26176, Noise threshold:1.0
ETD-MS/MS Scan:26177, Noise threshold:1.7
Relative Intensity (%)
m/z

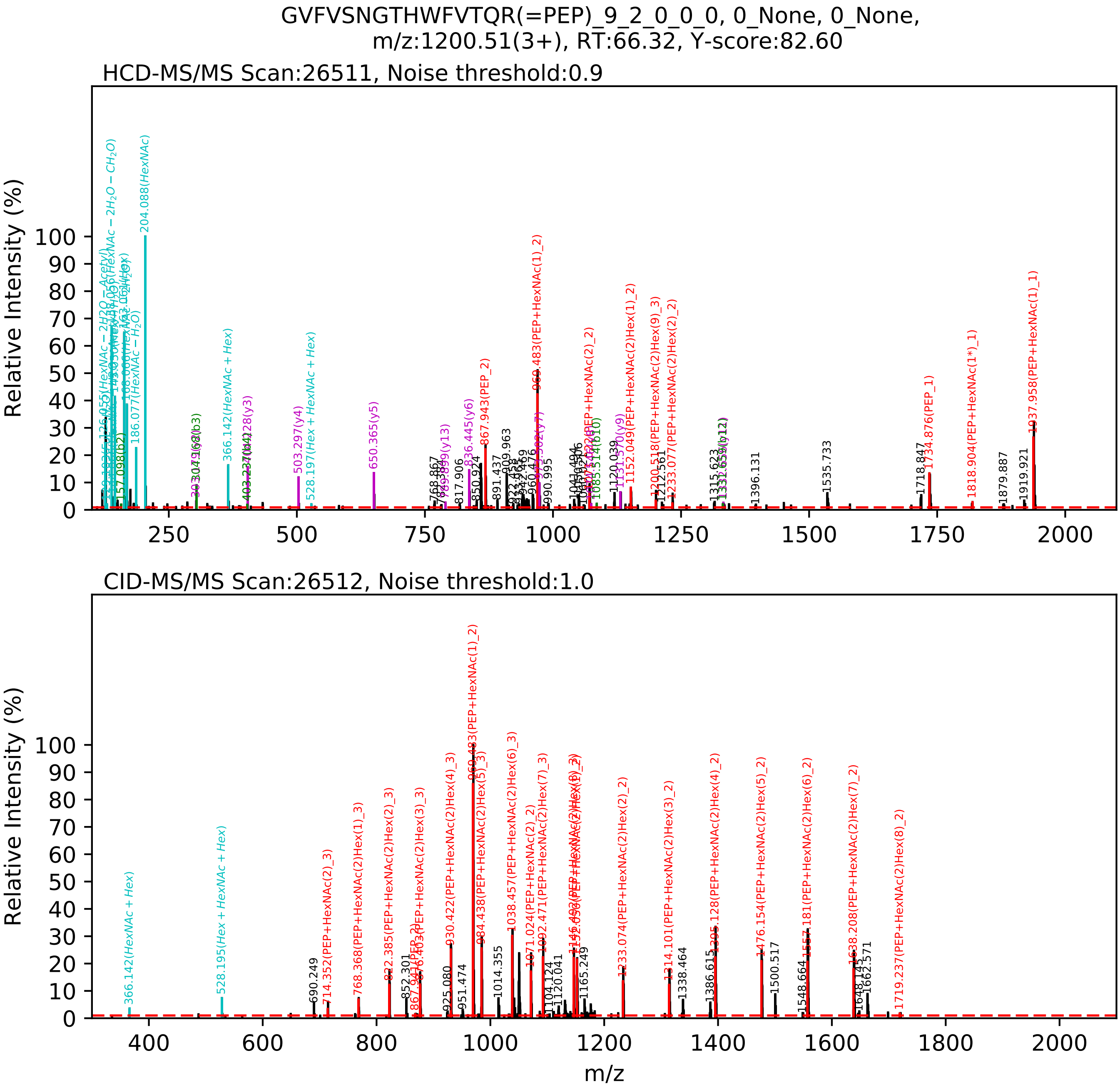
GVFVSNGTHWFVTQR(=PEP)_9_2_0_0_0, 0_None, 0_None,
m/z:1200.51(3+), RT:66.32, Y-score:82.60
HCD-MS/MS Scan:26511, Noise threshold:0.9
Relative Intensity (%)
CID-MS/MS Scan:26512, Noise threshold:1.0
Relative Intensity (%)
m/z

GVFVSNGTHWFVTQR(=PEP)_9_2_0_0_0, 0_None, 0_None,
m/z:1200.51(3+), RT:60.21, Y-score:85.13

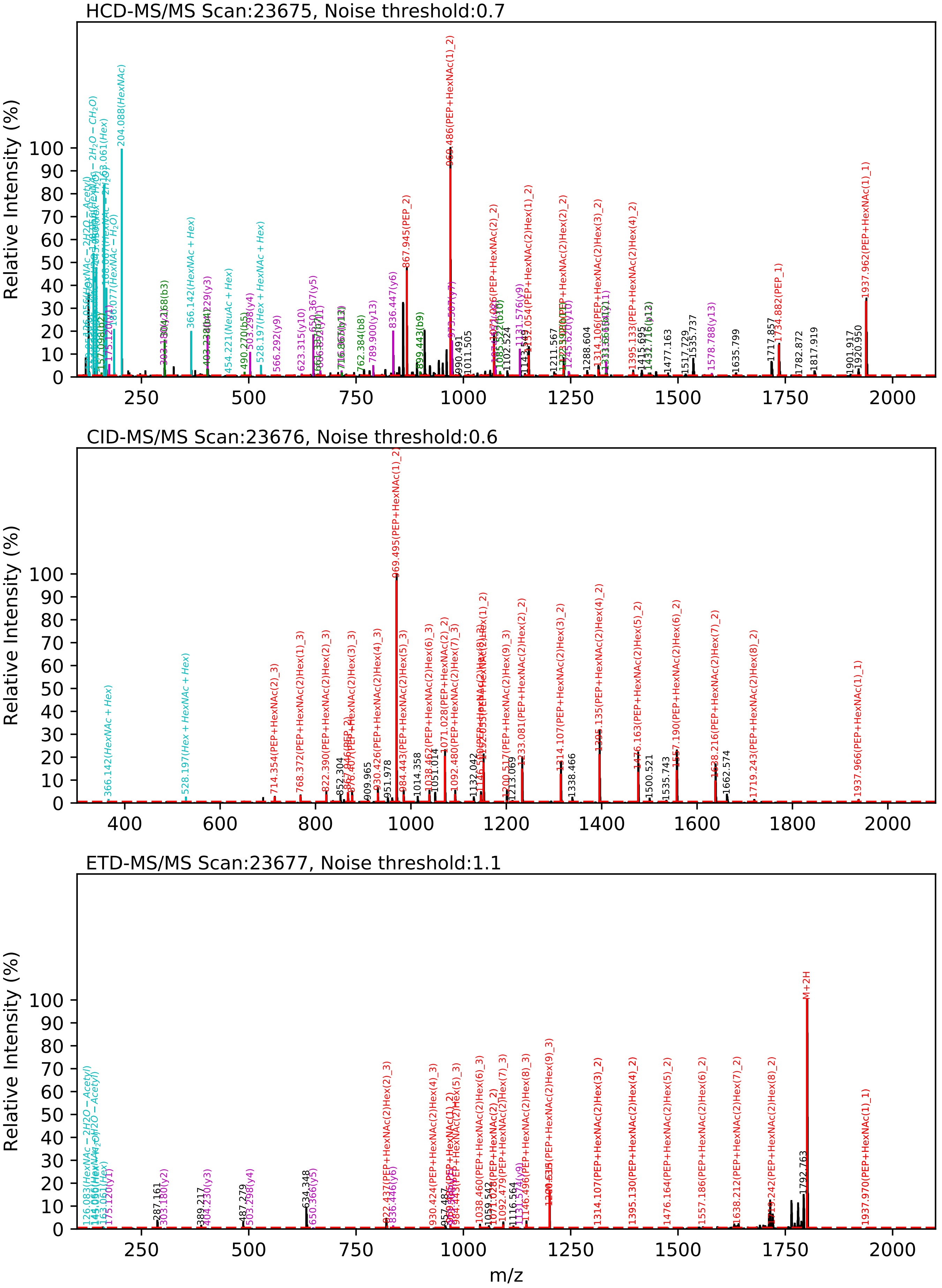

GVFVSNGTHWFVTQR(=PEP)_9_2_0_0_0, 0_None, 0_None,
m/z:1200.51(3+), RT:60.78, Y-score:89.29

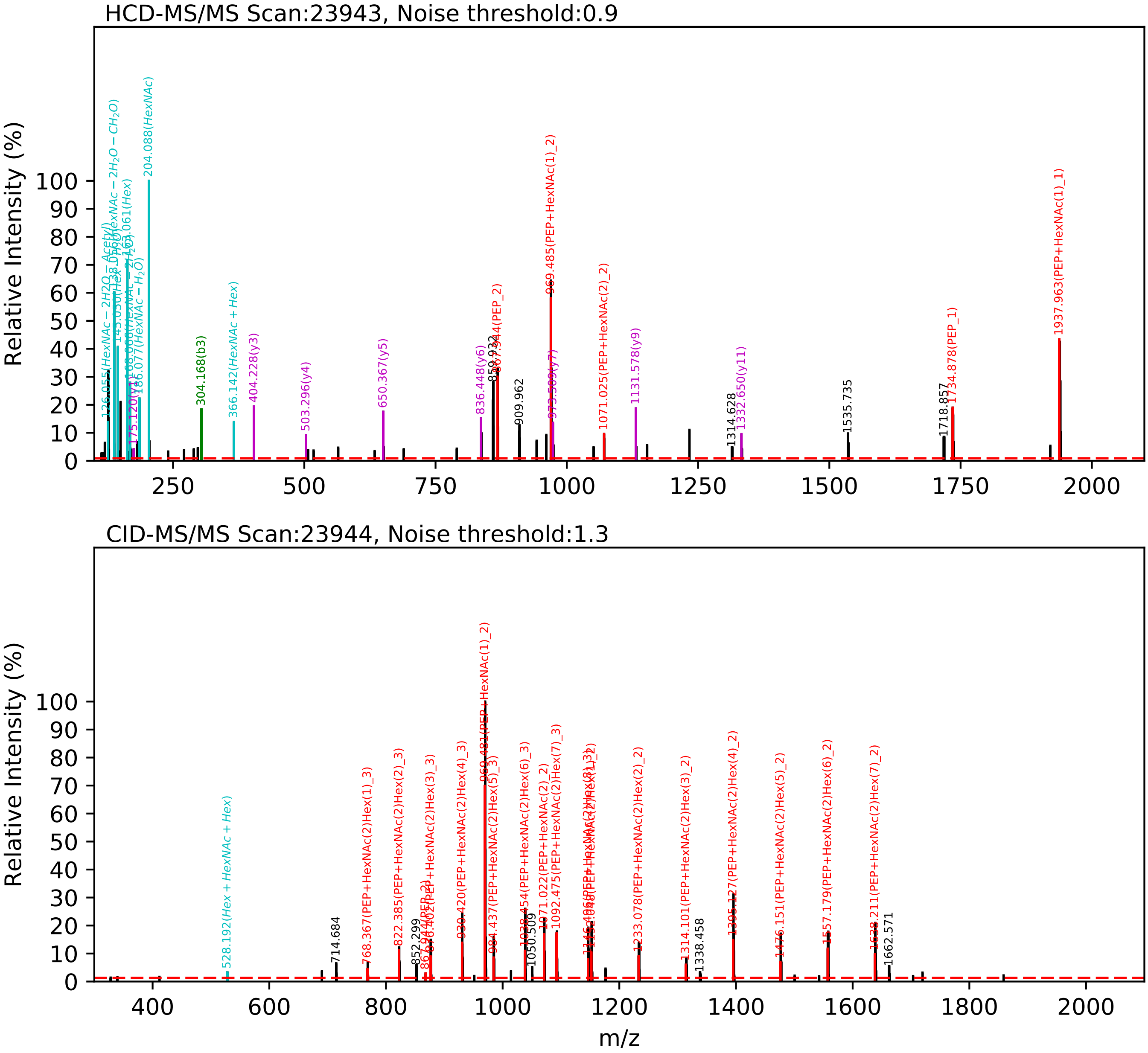

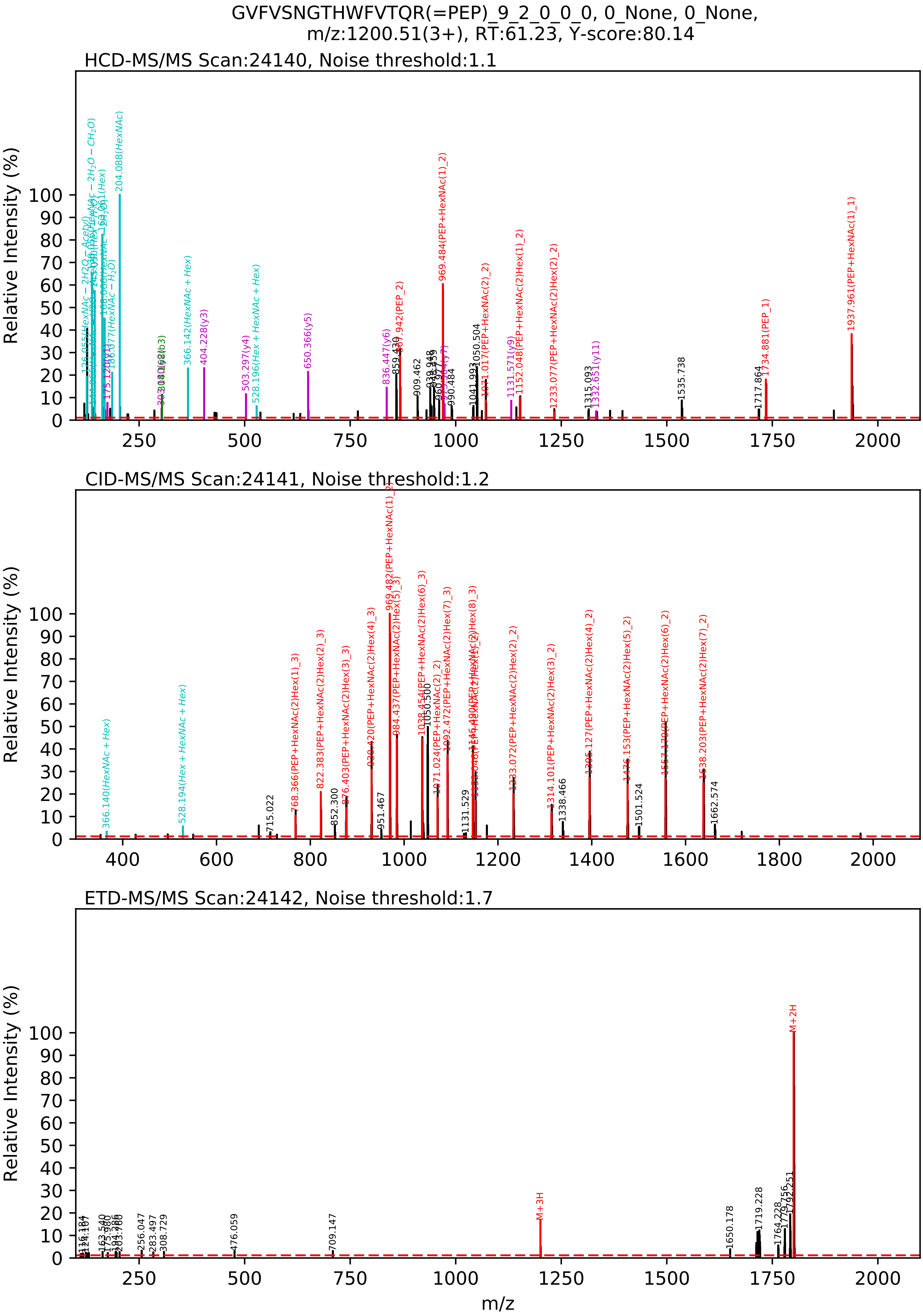

GVFVSNGTHWFVTQR(=PEP)_9_2_0_0_0, 0_None, 0_None,
m/z:1200.51(3+), RT:61.23, Y-score:80.14
HCD-MS/MS Scan:24140, Noise threshold:1.1
Relative Intensity (%)
204.088(HexNAc)
969.484(PEP+HexNAc(1)_2)
1937.961(PEP+HexNAc(1)_1)
1734.881(PEP_1)
867.942(PEP_2)
1071.017(PEP+HexNAc(2)_2)
1152.048(PEP+HexNAc(2)Hex(1)_2)
1233.077(PEP+HexNAc(2)Hex(2)_2)
366.142(HexNAc + Hex)
528.196(Hex + HexNAc + Hex)
404.228(y3)
503.297(y4)
650.366(y5)
836.447(y6)
1131.571(y9)
1332.651(y11)
1535.738
1717.864
1315.093
1050.504
CID-MS/MS Scan:24141, Noise threshold:1.2
969.482(PEP+HexNAc(1)_2)
984.437(PEP+HexNAc(2)Hex(5)_3)
1038.454(PEP+HexNAc(2)Hex(6)_3)
1092.472(PEP+HexNAc(2)Hex(7)_3)
1071.024(PEP+HexNAc(2)_2)
930.420(PEP+HexNAc(2)Hex(4)_3)
876.403(PEP+HexNAc(2)Hex(3)_3)
822.383(PEP+HexNAc(2)Hex(2)_3)
768.366(PEP+HexNAc(2)Hex(1)_3)
1233.072(PEP+HexNAc(2)Hex(2)_2)
1314.101(PEP+HexNAc(2)Hex(3)_2)
1395.127(PEP+HexNAc(2)Hex(4)_2)
1476.153(PEP+HexNAc(2)Hex(5)_2)
1557.179(PEP+HexNAc(2)Hex(6)_2)
1638.203(PEP+HexNAc(2)Hex(7)_2)
366.140(HexNAc + Hex)
528.194(Hex + HexNAc + Hex)
715.022
852.300
951.467
1050.500
1131.529
1338.466
1501.524
1662.574
ETD-MS/MS Scan:24142, Noise threshold:1.7
M+2H
M+3H
1650.178
1719.228
1764.228
1779.756
1792.251
476.059
709.147
256.047
283.497
308.729
m/z

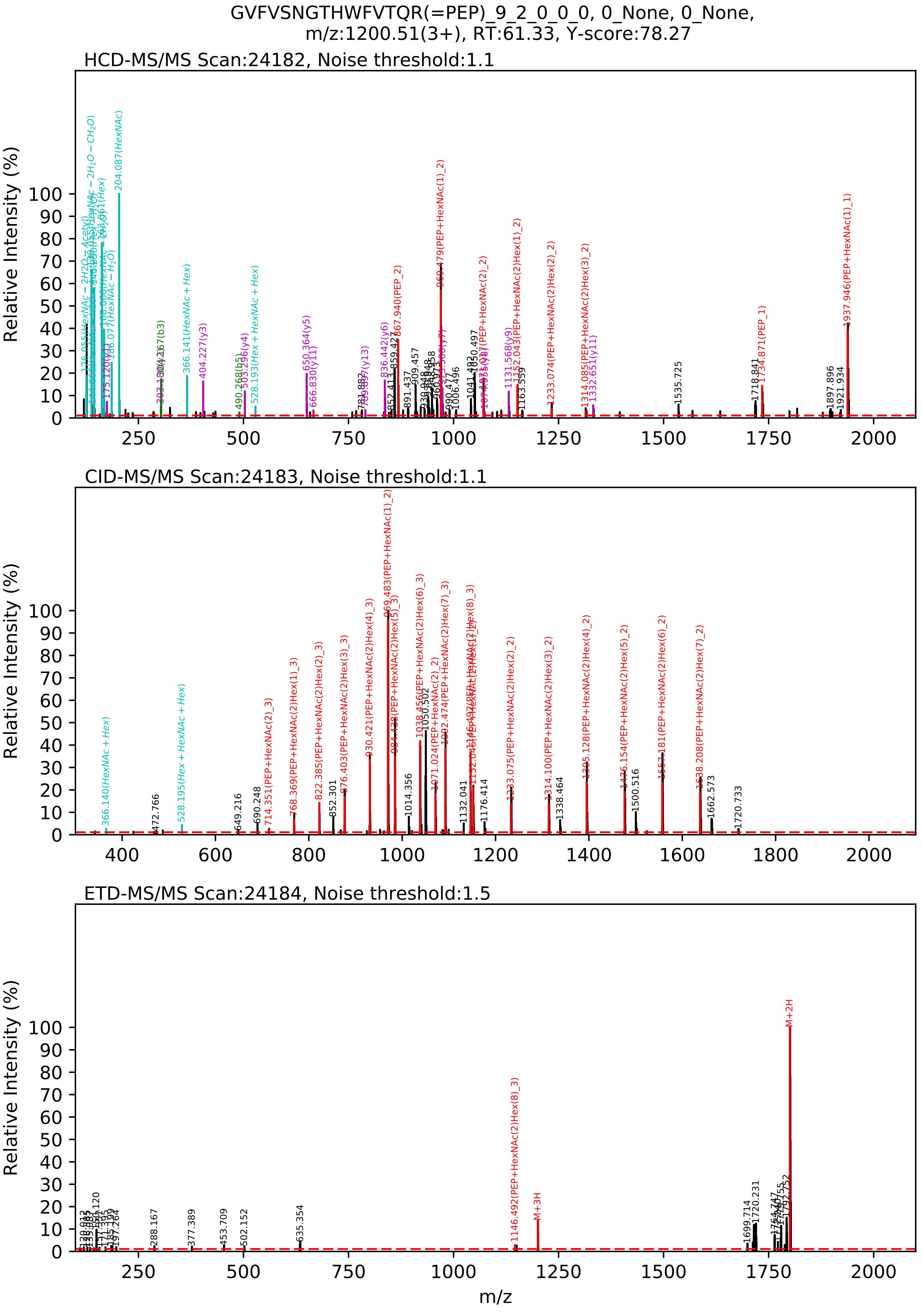

GVFVSNGTHWFVTQR(=PEP)_9_2_0_0_0, 0_None, 0_None,
m/z:1200.51(3+), RT:61.33, Y-score:78.27
HCD-MS/MS Scan:24182, Noise threshold:1.1
CID-MS/MS Scan:24183, Noise threshold:1.1
ETD-MS/MS Scan:24184, Noise threshold:1.5
Relative Intensity (%)
m/z

GVFVSNGTHWFVTQR(=PEP)_9_2_0_0_0, 0_None, 0_None,
m/z:1200.51(3+), RT:61.38, Y-score:82.18

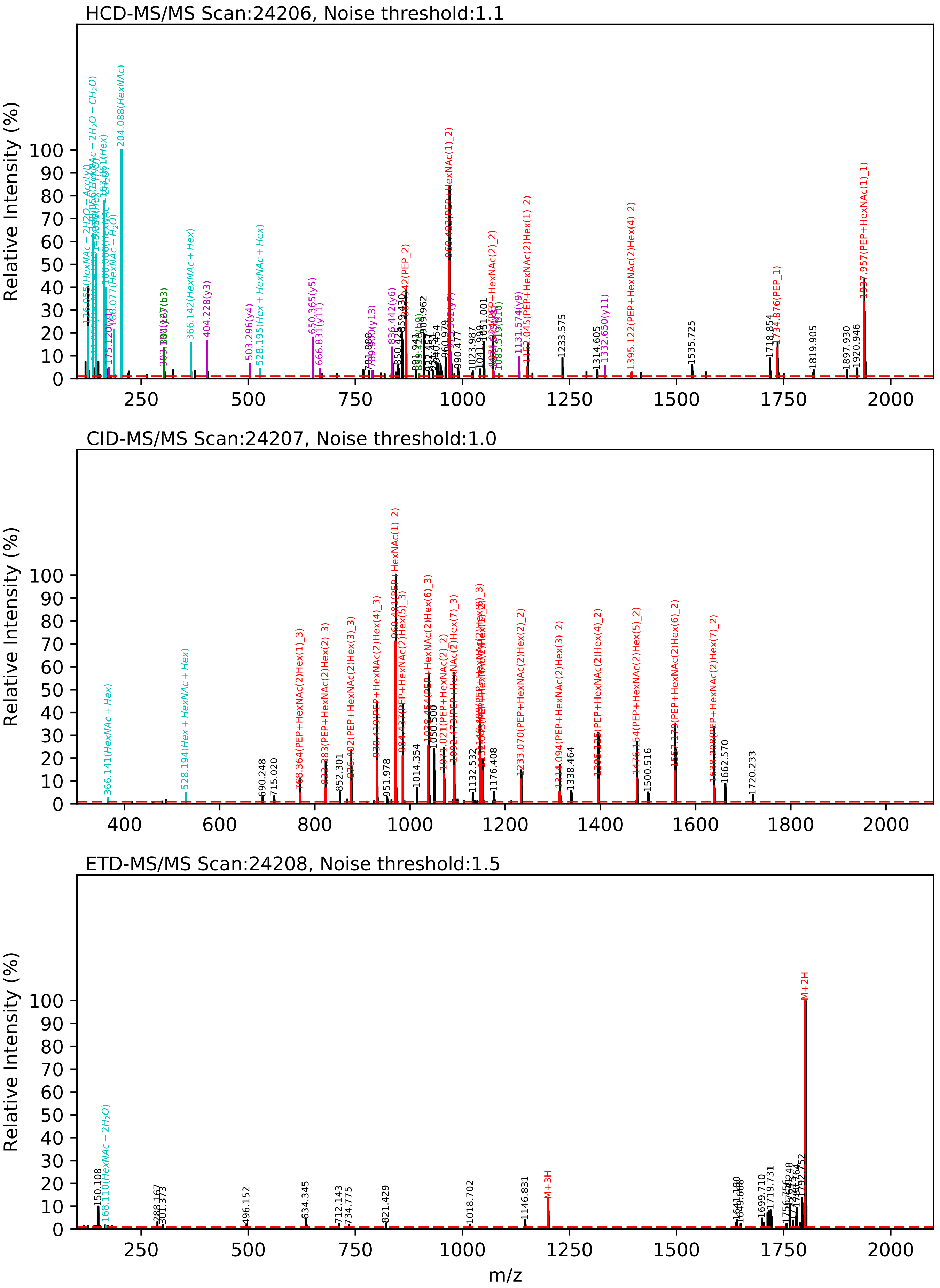

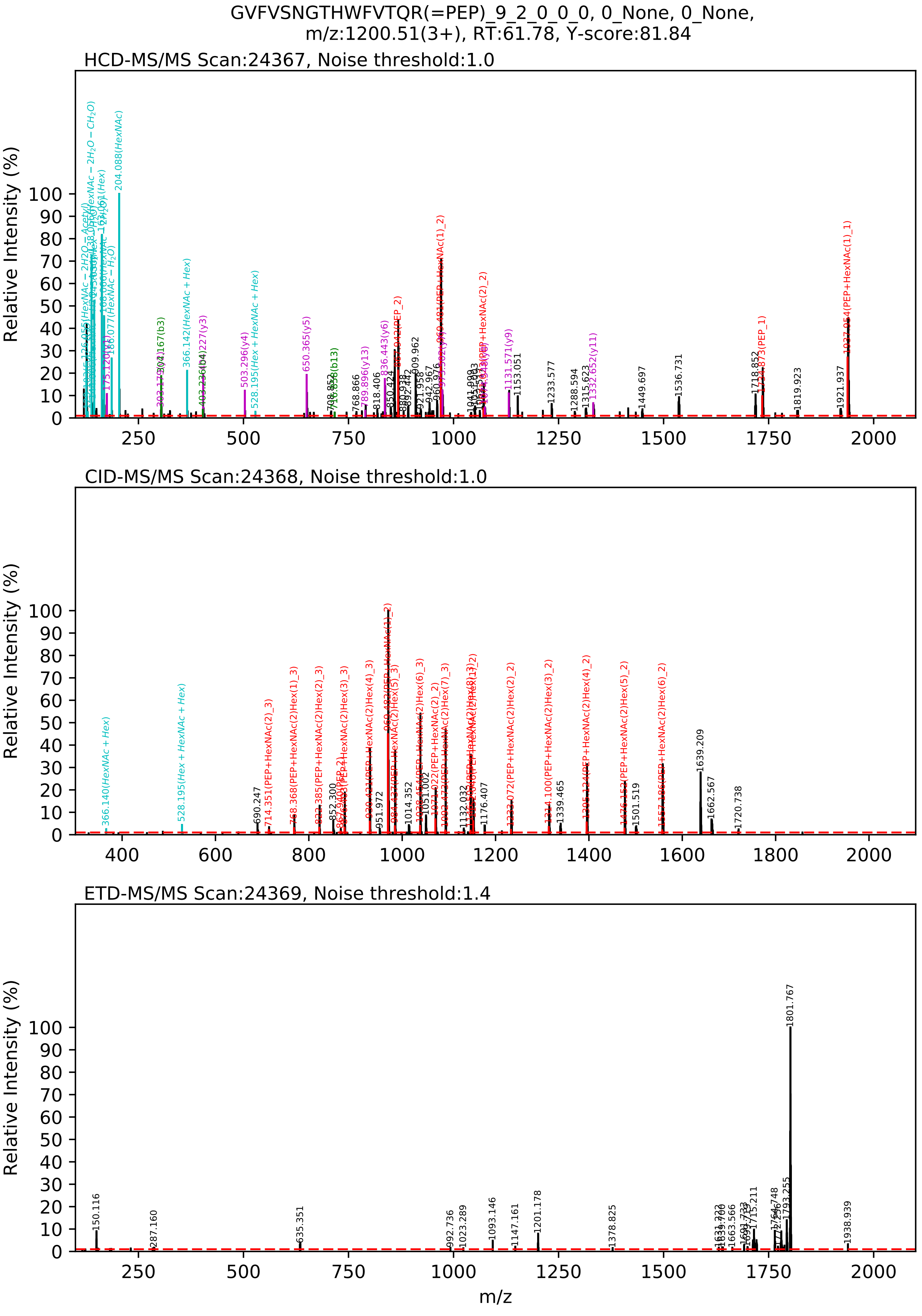

GVFVSNGTHWFVTQR(=PEP)_9_2_0_0_0, 0_None, 0_None,
m/z:1200.51(3+), RT:61.78, Y-score:81.84
HCD-MS/MS Scan:24367, Noise threshold:1.0
Relative Intensity (%)
204.088(HexNAc)
366.142(HexNAc + Hex)
528.195(Hex + HexNAc + Hex)
503.296(y4)
650.365(y5)
836.443(y6)
1131.571(y9)
1153.051
1233.577
1288.594
1315.623
1332.652(y11)
1449.697
1536.731
1718.852
1819.923
1921.937
1937.054(PEP+HexNAc(1)_1)
CID-MS/MS Scan:24368, Noise threshold:1.0
366.140(HexNAc + Hex)
528.195(Hex + HexNAc + Hex)
690.247
714.351(PEP+HexNAc(2)_3)
768.368(PEP+HexNAc(2)Hex(1)_3)
822.385(PEP+HexNAc(2)Hex(2)_3)
852.300
951.972
1014.352
1132.032
1176.407
1232.072(PEP+HexNAc(2)Hex(2)_2)
1314.100(PEP+HexNAc(2)Hex(3)_2)
1339.465
1395.124(PEP+HexNAc(2)Hex(4)_2)
1476.153(PEP+HexNAc(2)Hex(5)_2)
1501.519
1557.196(PEP+HexNAc(2)Hex(6)_2)
1639.209
1662.567
1720.738
ETD-MS/MS Scan:24369, Noise threshold:1.4
150.116
287.160
635.351
992.736
1023.289
1093.146
1147.161
1201.178
1378.825
1663.566
1715.211
1764.748
1793.255
1801.767
1938.939
m/z

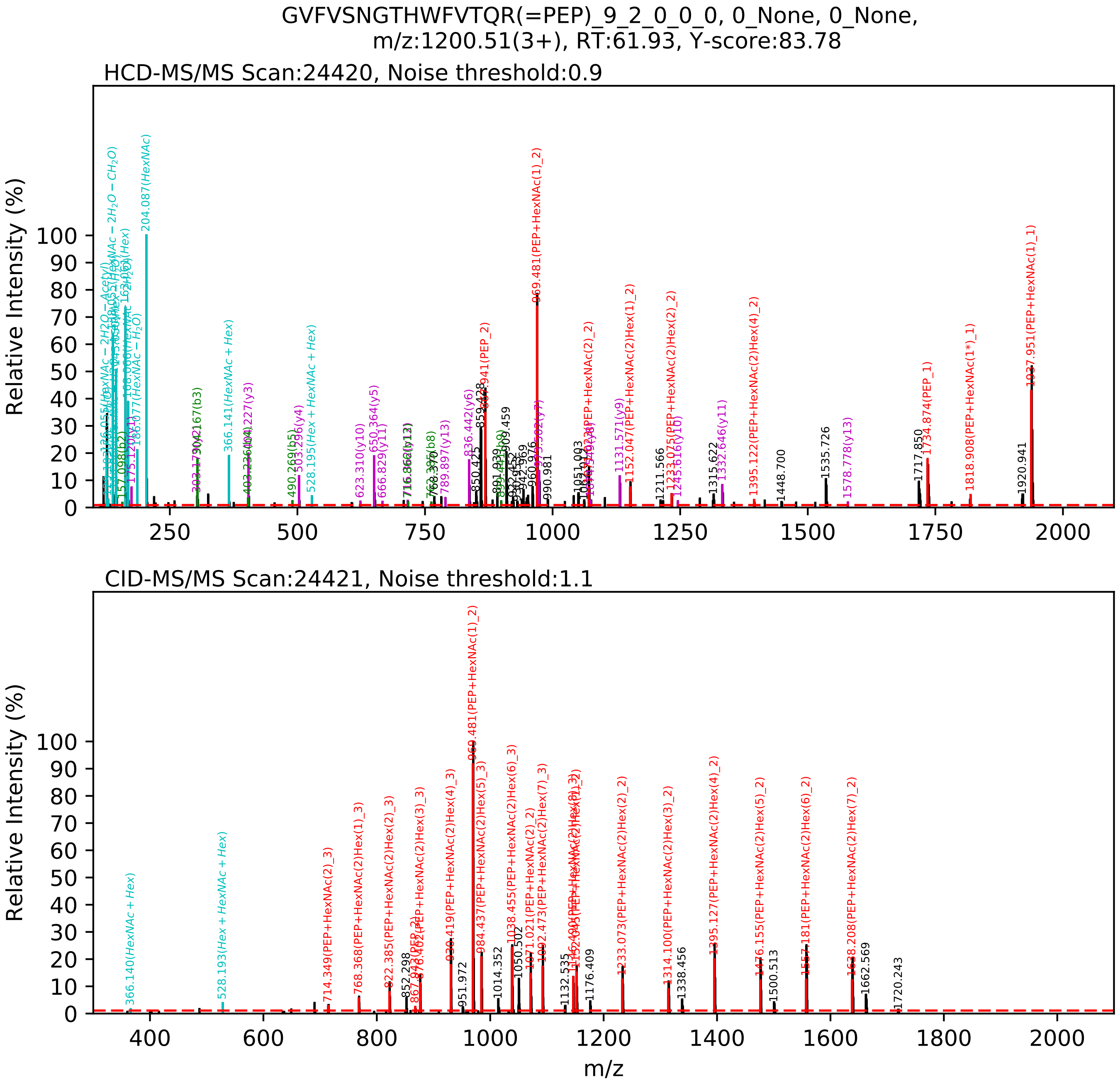
GVFVSNGTHWFVTQR(=PEP)_9_2_0_0_0, 0_None, 0_None,
m/z:1200.51(3+), RT:61.93, Y-score:83.78
HCD-MS/MS Scan:24420, Noise threshold:0.9
Relative Intensity (%)
204.087(HexNAc)
969.481(PEP+HexNAc(1)_2)
1937.951(PEP+HexNAc(1)_1)
1734.874(PEP_1)
1818.908(PEP+HexNAc(1*)_1)
1395.122(PEP+HexNAc(2)Hex(4)_2)
1233.075(PEP+HexNAc(2)Hex(2)_2)
1152.047(PEP+HexNAc(2)Hex(1)_2)
1131.571(y9)
1332.646(y11)
1578.778(y13)
1535.726
1717.850
1920.941
1448.700
1315.622
1211.566
366.141(HexNAc + Hex)
528.195(Hex + HexNAc + Hex)
490.269(b5)
503.296(y4)
623.310(y10)
650.364(y5)
666.829(y11)
789.897(y13)
836.442(y6)
CID-MS/MS Scan:24421, Noise threshold:1.1
Relative Intensity (%)
m/z
969.481(PEP+HexNAc(1)_2)
366.140(HexNAc + Hex)
528.193(Hex + HexNAc + Hex)
714.349(PEP+HexNAc(2)_3)
768.368(PEP+HexNAc(2)Hex(1)_3)
822.385(PEP+HexNAc(2)Hex(2)_3)
876.402(PEP+HexNAc(2)Hex(3)_3)
930.419(PEP+HexNAc(2)Hex(4)_3)
984.437(PEP+HexNAc(2)Hex(5)_3)
1038.455(PEP+HexNAc(2)Hex(6)_3)
1071.021(PEP+HexNAc(2)_2)
1092.473(PEP+HexNAc(2)Hex(7)_3)
1233.073(PEP+HexNAc(2)Hex(2)_2)
1314.100(PEP+HexNAc(2)Hex(3)_2)
1395.127(PEP+HexNAc(2)Hex(4)_2)
1476.155(PEP+HexNAc(2)Hex(5)_2)
1557.181(PEP+HexNAc(2)Hex(6)_2)
1638.208(PEP+HexNAc(2)Hex(7)_2)
852.298
951.972
1014.352
1050.502
1132.535
1176.409
1338.456
1500.513
1662.569
1720.243

GVFVSNGTHWFVTQR(=PEP)_9_2_0_0_0, 0_None, 0_None,
m/z:1200.51(3+), RT:62.46, Y-score:82.32

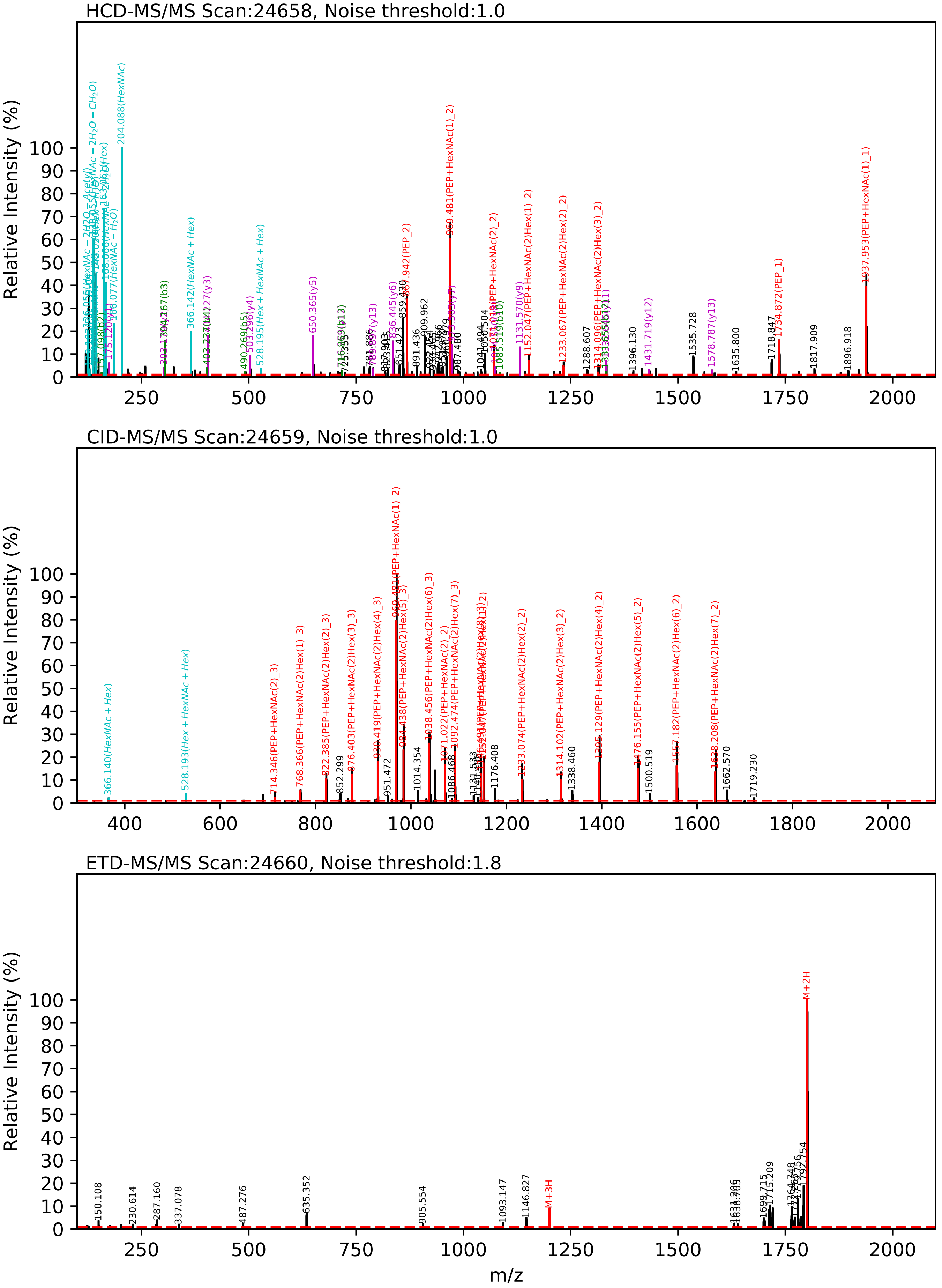

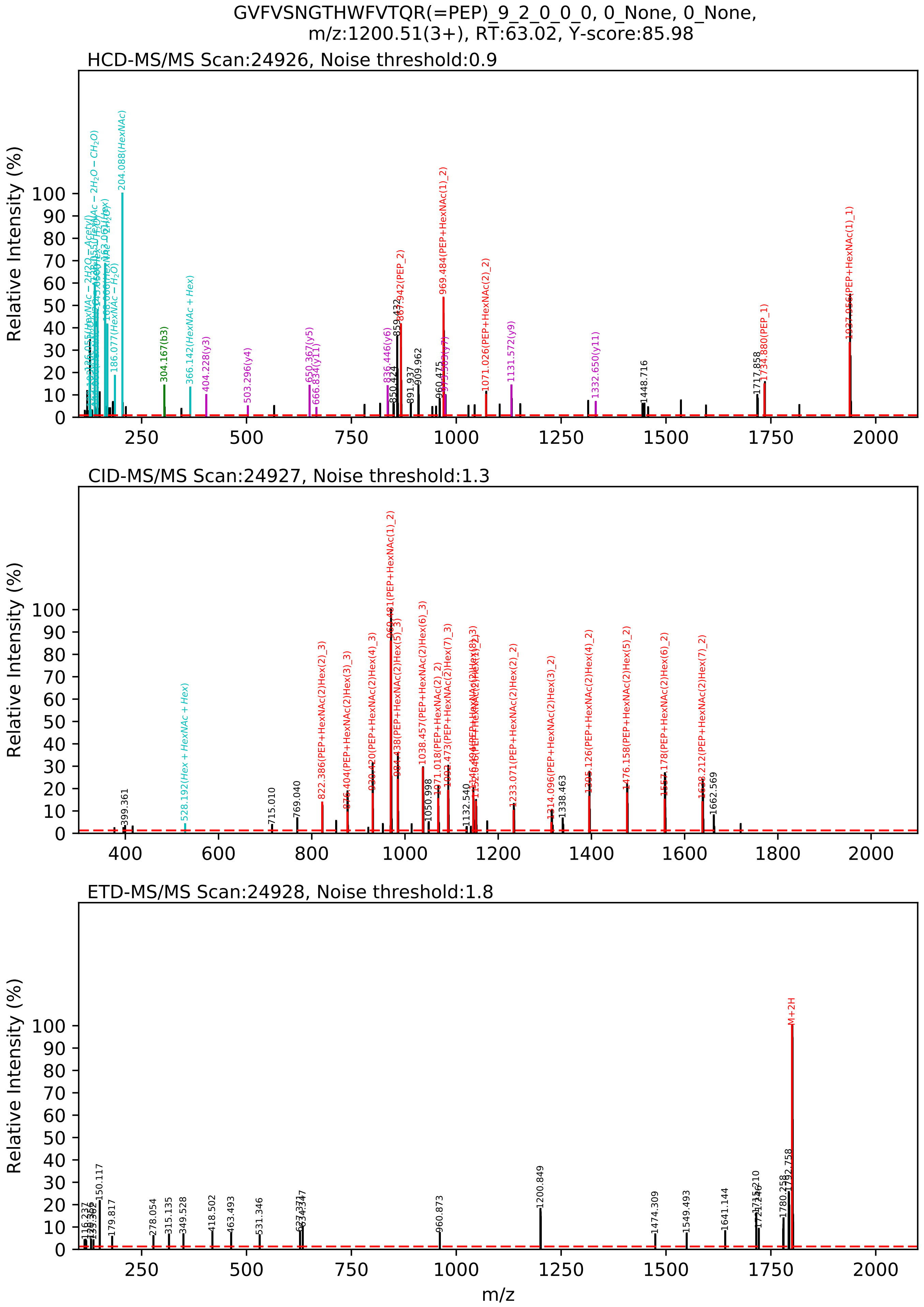
GVFVSNGTHWFVTQR(=PEP)_9_2_0_0_0, 0_None, 0_None,
m/z:1200.51(3+), RT:63.02, Y-score:85.98
HCD-MS/MS Scan:24926, Noise threshold:0.9
Relative Intensity (%)
204.088(HexNAc)
186.077(HexNAc − H2O)
304.167(b3)
366.142(HexNAc + Hex)
404.228(y3)
503.296(y4)
650.367(y5)
666.834(y11)
836.446(y6)
850.424
859.432
867.942(PEP_2)
891.937
909.962
960.475
969.484(PEP+HexNAc(1)_2)
1071.026(PEP+HexNAc(2)_2)
1131.572(y9)
1332.650(y11)
1448.716
1717.858
1734.880(PEP_1)
1937.956(PEP+HexNAc(1)_1)
CID-MS/MS Scan:24927, Noise threshold:1.3
Relative Intensity (%)
399.361
528.192(Hex + HexNAc + Hex)
715.010
769.040
822.386(PEP+HexNAc(2)Hex(2)_3)
876.404(PEP+HexNAc(2)Hex(3)_3)
930.420(PEP+HexNAc(2)Hex(4)_3)
969.481(PEP+HexNAc(1)_2)
984.438(PEP+HexNAc(2)Hex(5)_3)
1038.457(PEP+HexNAc(2)Hex(6)_3)
1050.998
1071.018(PEP+HexNAc(2)_2)
1092.473(PEP+HexNAc(2)Hex(7)_3)
1132.540
1233.071(PEP+HexNAc(2)Hex(2)_2)
1314.096(PEP+HexNAc(2)Hex(3)_2)
1338.463
1395.126(PEP+HexNAc(2)Hex(4)_2)
1476.158(PEP+HexNAc(2)Hex(5)_2)
1557.178(PEP+HexNAc(2)Hex(6)_2)
1638.212(PEP+HexNAc(2)Hex(7)_2)
1662.569
ETD-MS/MS Scan:24928, Noise threshold:1.8
Relative Intensity (%)
150.117
179.817
278.054
315.135
349.528
418.502
463.493
531.346
960.873
1200.849
1474.309
1549.493
1641.144
1780.258
1792.758
M+2H
m/z

ISGINASVVNIQK(=PEP)_7_2_0_0_0, 0_None, 0_None,
m/z:1442.15(2+), RT:56.27, Y-score:84.05

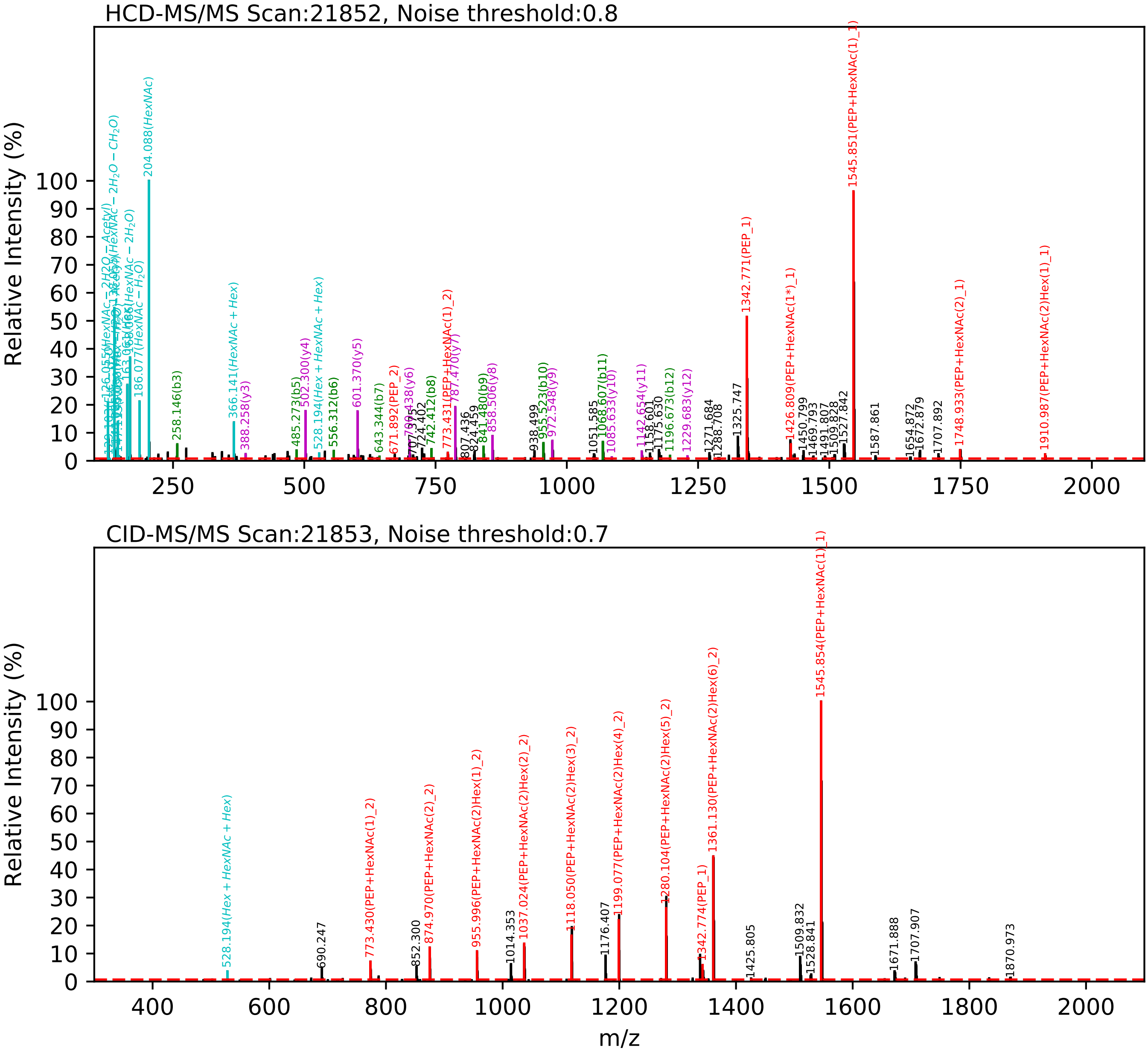

ISGINASVVNIQK(=PEP)_8_2_0_0_0, 0_None, 0_None,
m/z:1015.79(3+), RT:55.80, Y-score:93.21

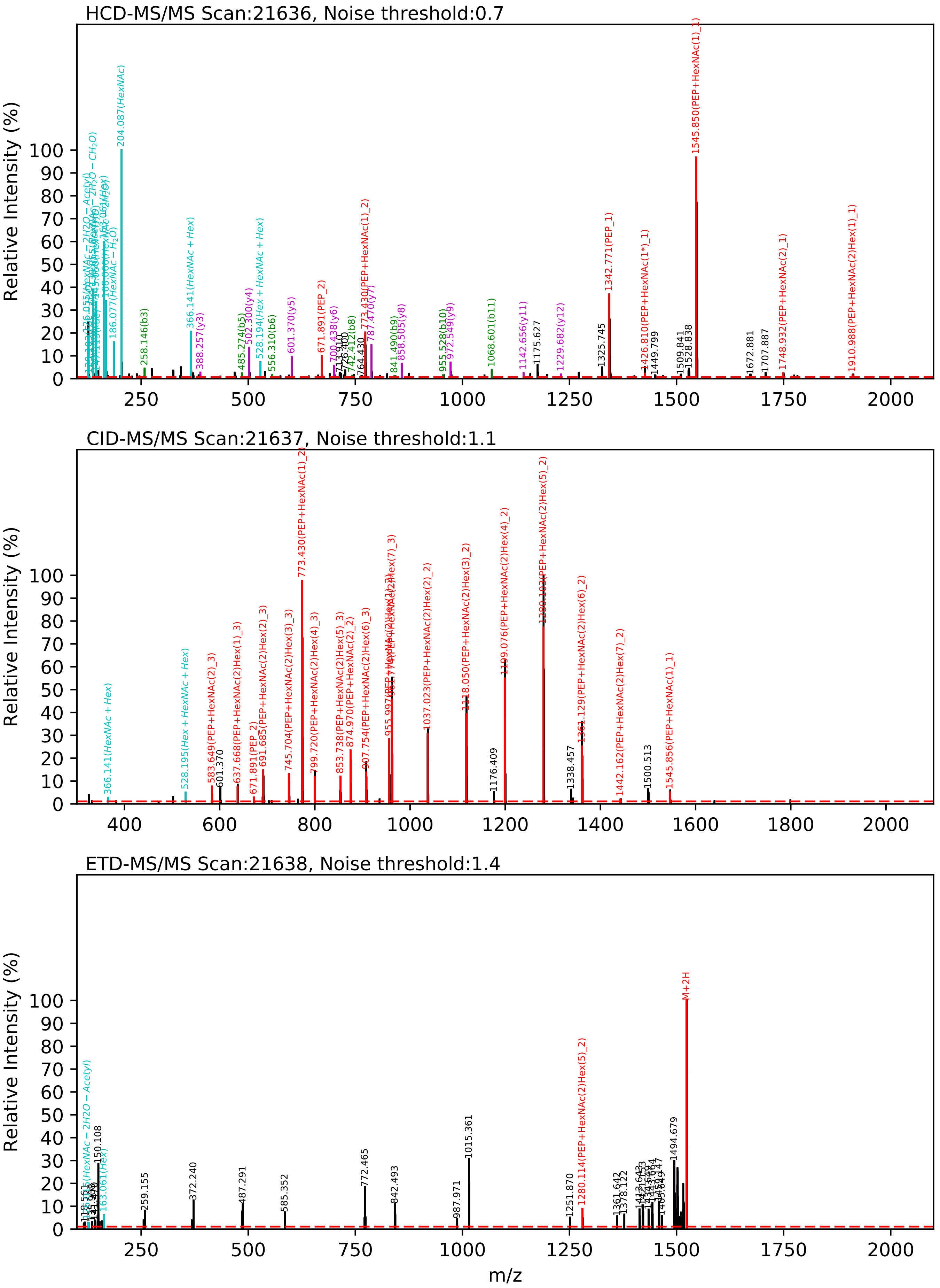

ISGINASVVNIQK(=PEP)_8_2_0_0_0, 0_None, 0_None,
m/z:1523.18(2+), RT:55.82, Y-score:82.24

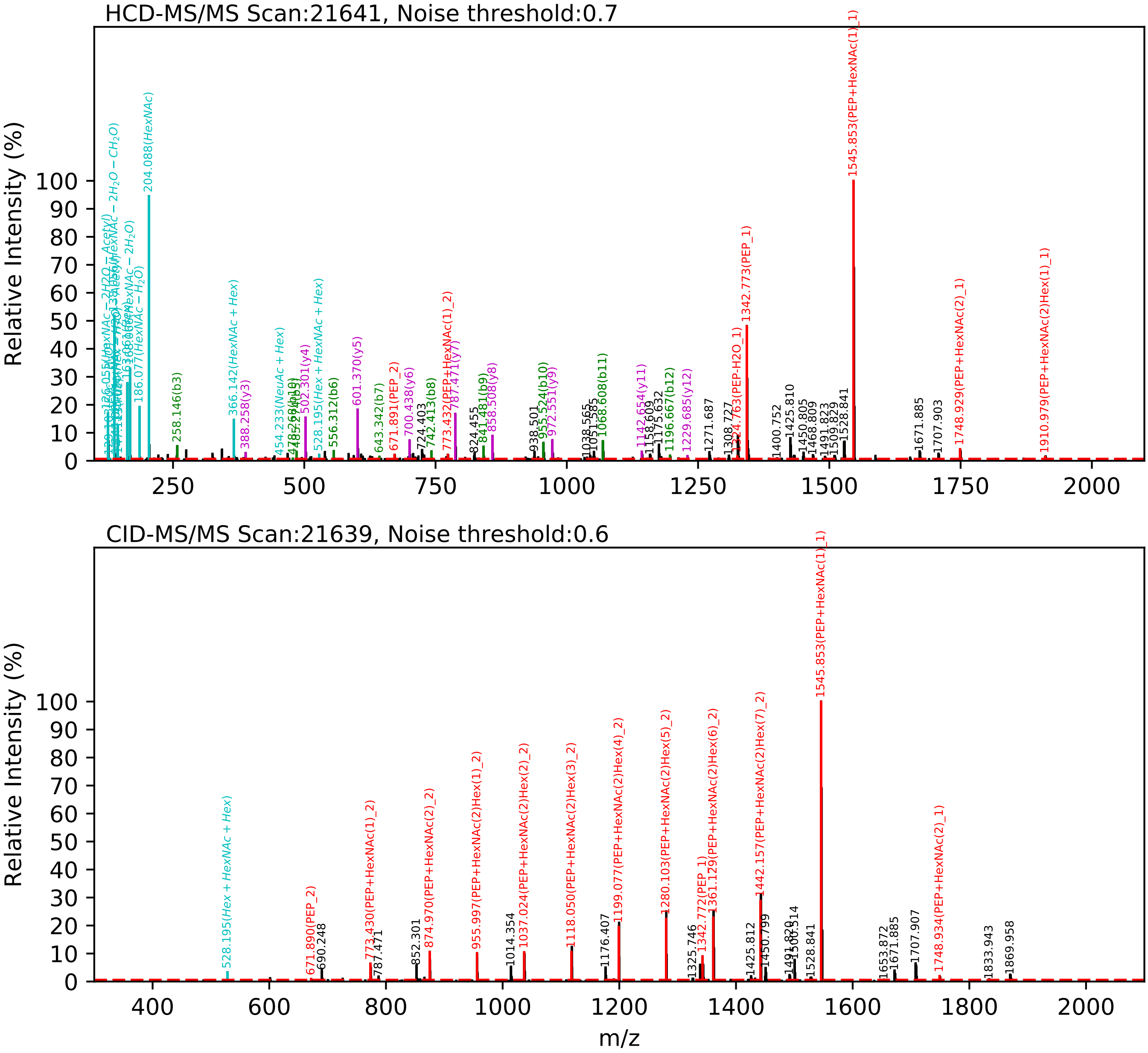

ISGINASVVNIQK(=PEP)_9_2_0_0_0, 0_None, 0_None,
m/z:1069.81(3+), RT:55.60, Y-score:92.85

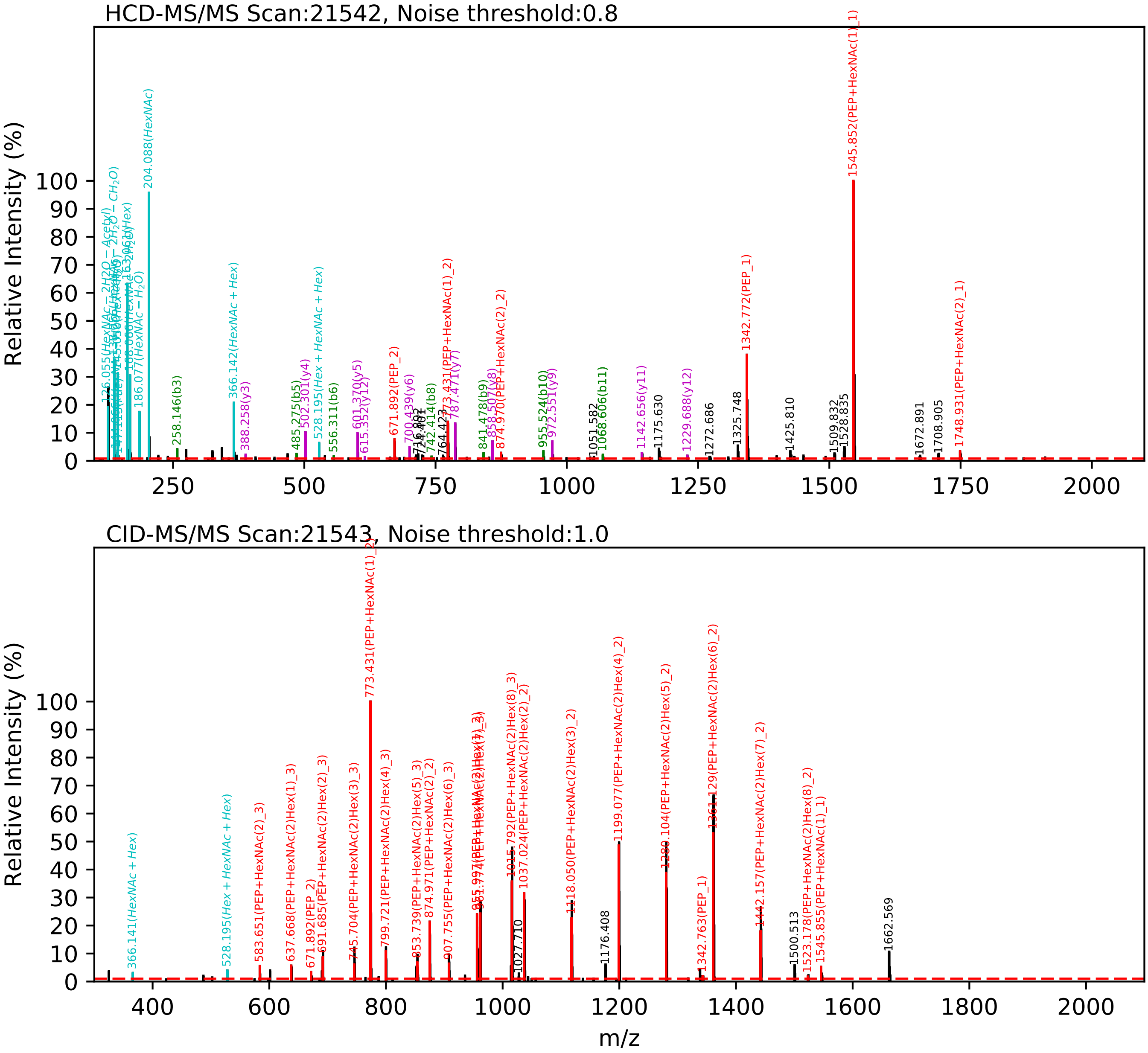

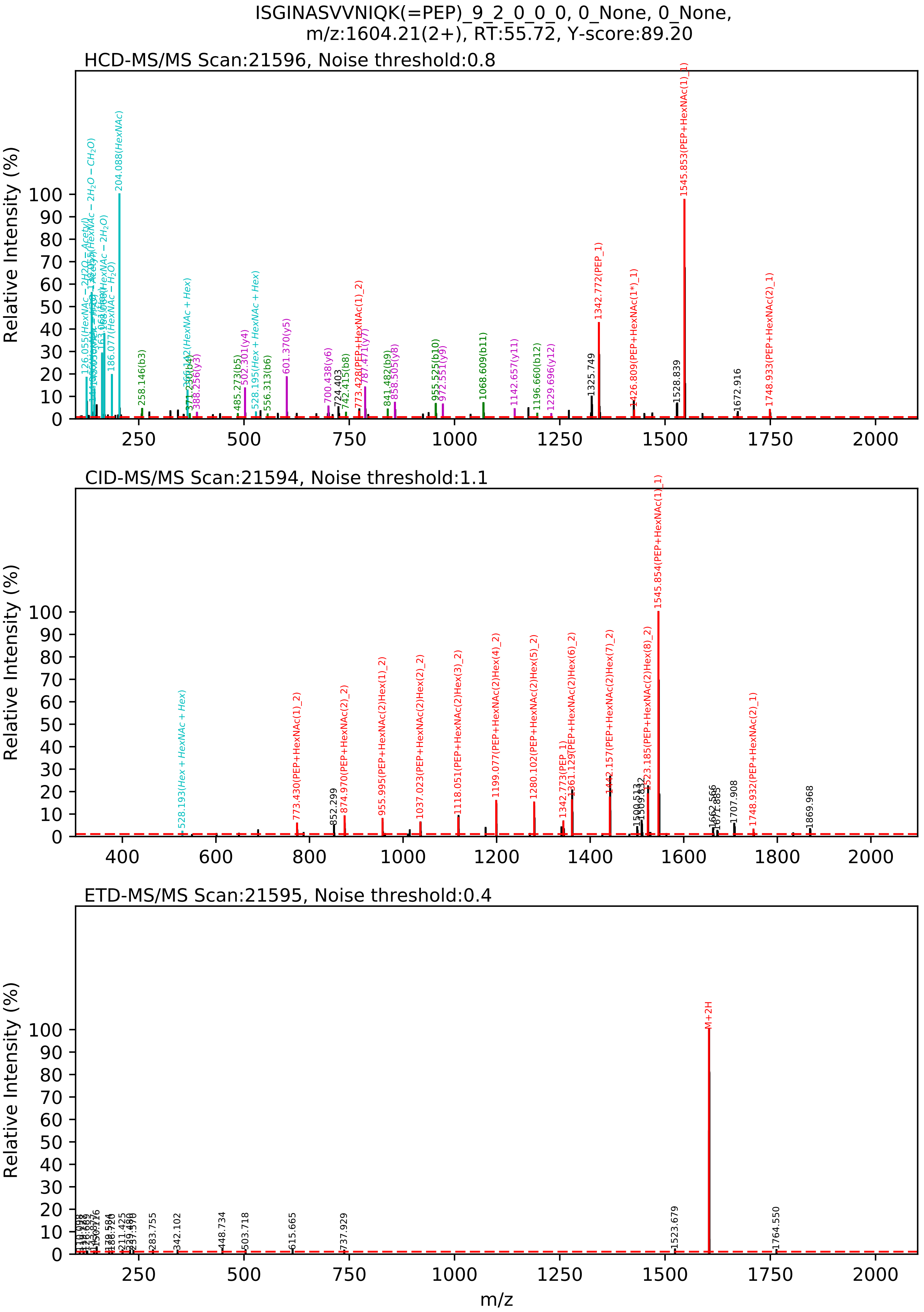

ISGINASVVNIQK(=PEP)_9_2_0_0_0, 0_None, 0_None,
m/z:1604.21(2+), RT:55.72, Y-score:89.20
HCD-MS/MS Scan:21596, Noise threshold:0.8
CID-MS/MS Scan:21594, Noise threshold:1.1
ETD-MS/MS Scan:21595, Noise threshold:0.4
Relative Intensity (%)
m/z
204.088(HexNAc)
1545.853(PEP+HexNAc(1)_1)
1342.772(PEP_1)
1426.809(PEP+HexNAc(1*)_1)
1748.933(PEP+HexNAc(2)_1)
773.428(PEP+HexNAc(1)_2)
601.370(y5)
1068.609(b11)
1545.854(PEP+HexNAc(1)_1)
528.193(Hex + HexNAc + Hex)
773.430(PEP+HexNAc(1)_2)
874.970(PEP+HexNAc(2)_2)
955.995(PEP+HexNAc(2)Hex(1)_2)
1037.023(PEP+HexNAc(2)Hex(2)_2)
1118.051(PEP+HexNAc(2)Hex(3)_2)
1199.077(PEP+HexNAc(2)Hex(4)_2)
1280.102(PEP+HexNAc(2)Hex(5)_2)
1361.129(PEP+HexNAc(2)Hex(6)_2)
1442.157(PEP+HexNAc(2)Hex(7)_2)
1523.185(PEP+HexNAc(2)Hex(8)_2)
1748.932(PEP+HexNAc(2)_1)
M+2H

LGDISGINASVVNIQK(=PEP)_5_2_0_0_0, 0_None, 0_None,
m/z:948.78(3+), RT:73.17, Y-score:82.96

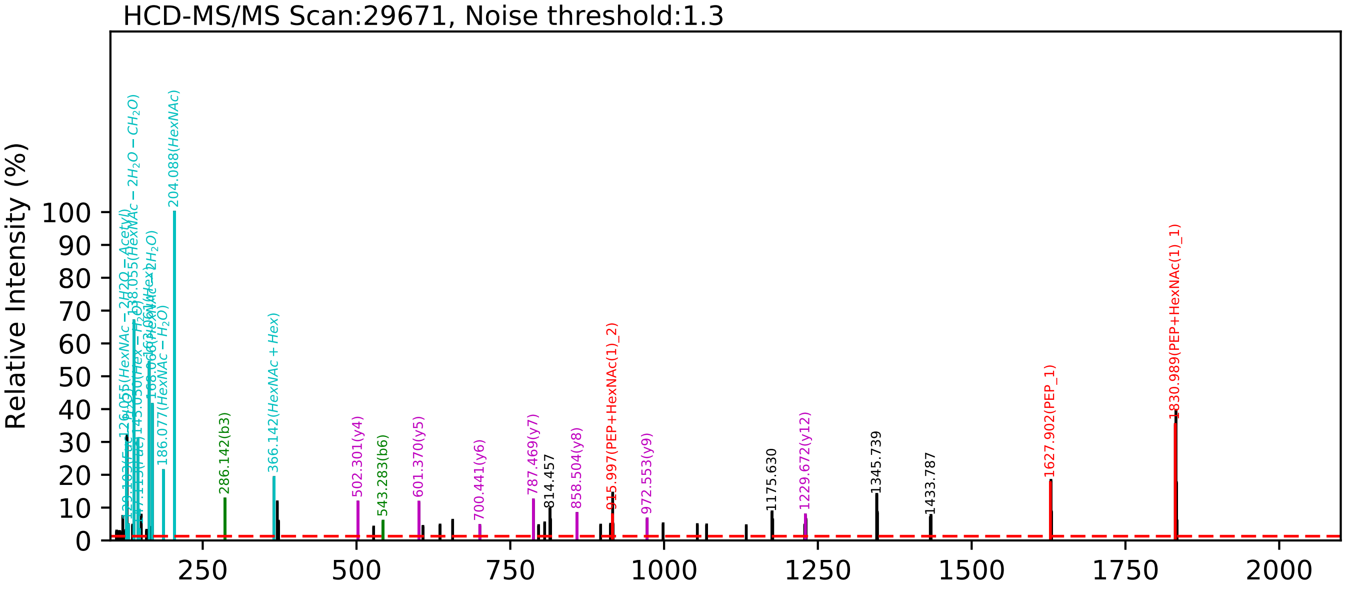

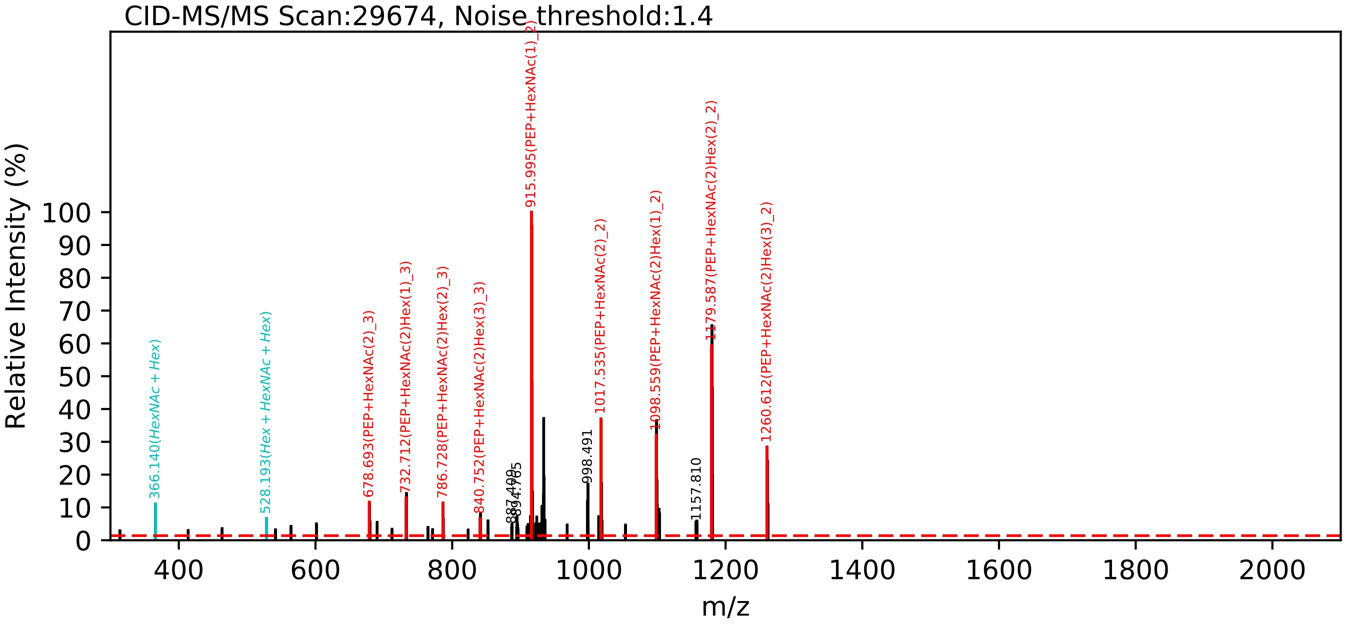

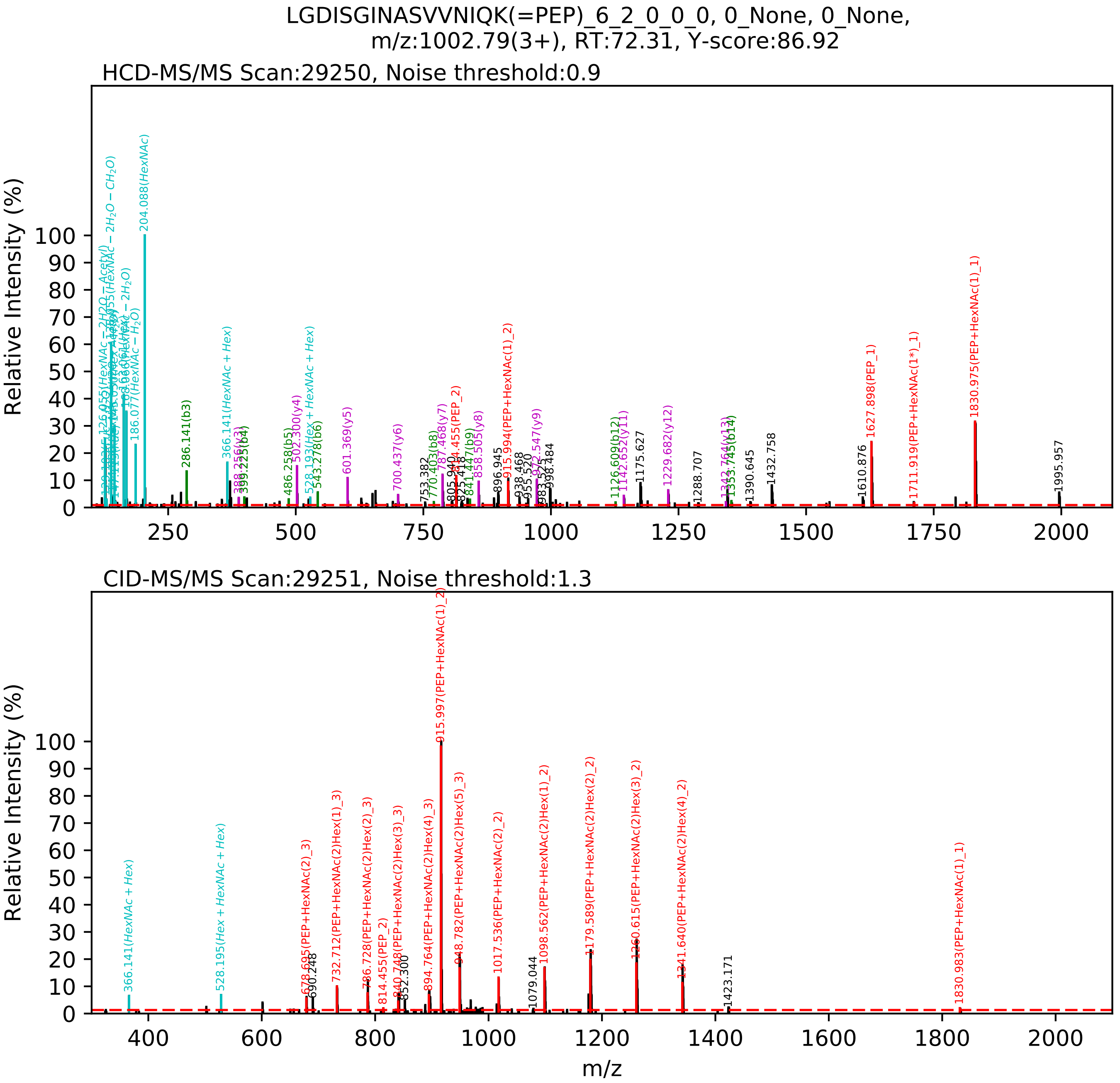

LGDISGINASVVNIQK(=PEP)_6_2_0_0_0, 0_None, 0_None,
m/z:1002.79(3+), RT:72.31, Y-score:86.92
HCD-MS/MS Scan:29250, Noise threshold:0.9
Relative Intensity (%)
204.088(HexNAc)
186.077(HexNAc − H2O)
286.141(b3)
366.141(HexNAc + Hex)
528.193(Hex + HexNAc + Hex)
543.278(b6)
486.258(b5)
502.300(y4)
601.369(y5)
700.437(y6)
753.382
770.403(b8)
787.468(y7)
804.455(PEP_2)
858.505(y8)
896.945
915.994(PEP+HexNAc(1)_2)
938.468
998.484
1126.609(b12)
1142.652(y11)
1175.627
1229.682(y12)
1288.707
1353.745(b14)
1390.645
1432.758
1610.876
1627.898(PEP_1)
1711.919(PEP+HexNAc(1*)_1)
1830.975(PEP+HexNAc(1)_1)
1995.957
CID-MS/MS Scan:29251, Noise threshold:1.3
Relative Intensity (%)
366.141(HexNAc + Hex)
528.195(Hex + HexNAc + Hex)
690.248
732.712(PEP+HexNAc(2)Hex(1)_3)
786.728(PEP+HexNAc(2)Hex(2)_3)
814.455(PEP_2)
840.748(PEP+HexNAc(2)Hex(3)_3)
852.300
894.764(PEP+HexNAc(2)Hex(4)_3)
915.997(PEP+HexNAc(1)_2)
948.782(PEP+HexNAc(2)Hex(5)_3)
1017.536(PEP+HexNAc(2)_2)
1079.044
1098.562(PEP+HexNAc(2)Hex(1)_2)
1179.589(PEP+HexNAc(2)Hex(2)_2)
1260.615(PEP+HexNAc(2)Hex(3)_2)
1341.640(PEP+HexNAc(2)Hex(4)_2)
1423.171
1830.983(PEP+HexNAc(1)_1)
m/z

LGDISGINASVVNIQK(=PEP)_7_2_0_0_0, 0_None, 0_None,
m/z:1056.81(3+), RT:72.00, Y-score:92.61

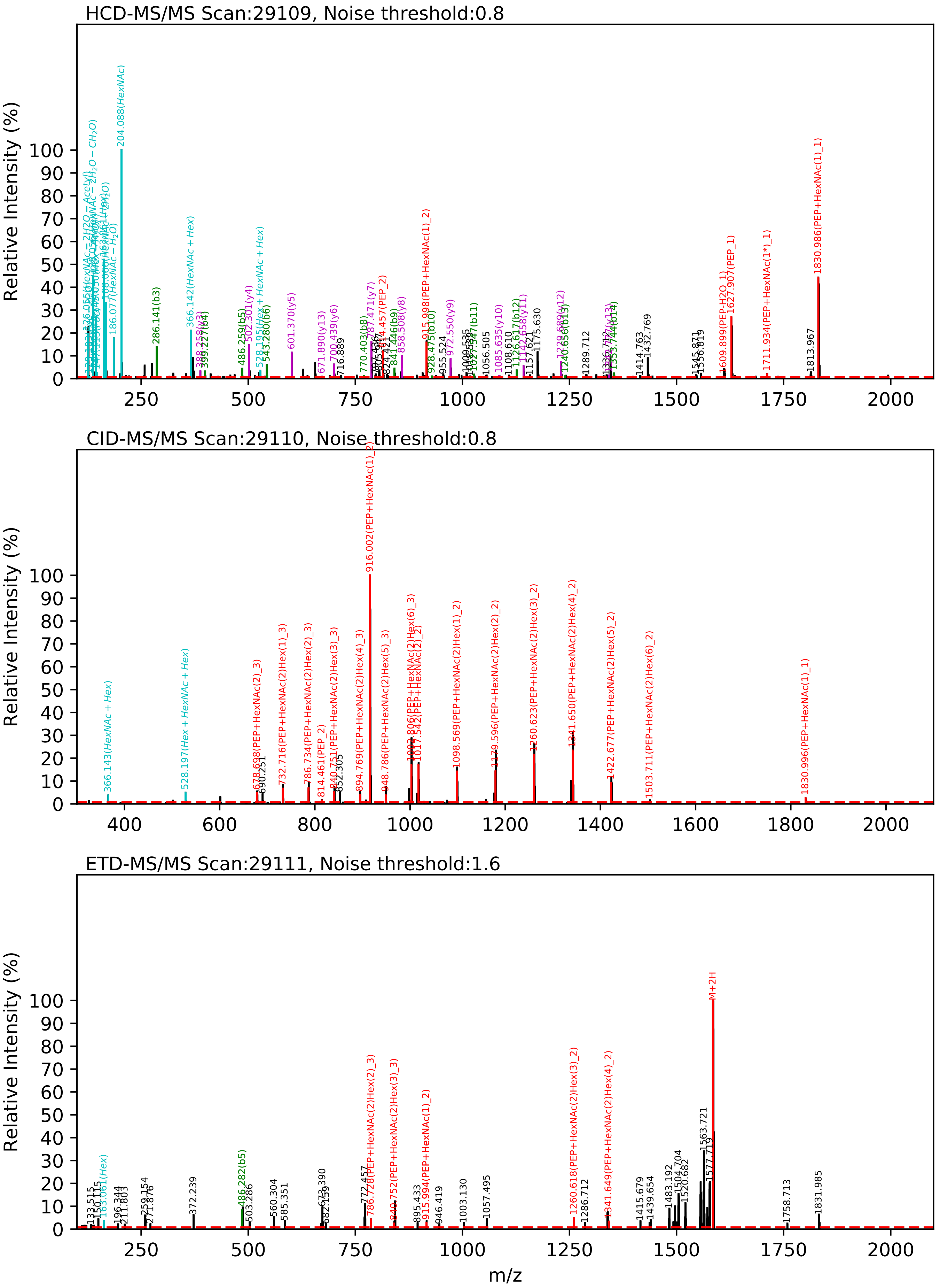

LGDISGINASVVNIQK(=PEP)_7_2_0_0_0, 0_None, 0_None,
m/z:1056.81(3+), RT:72.09, Y-score:90.85

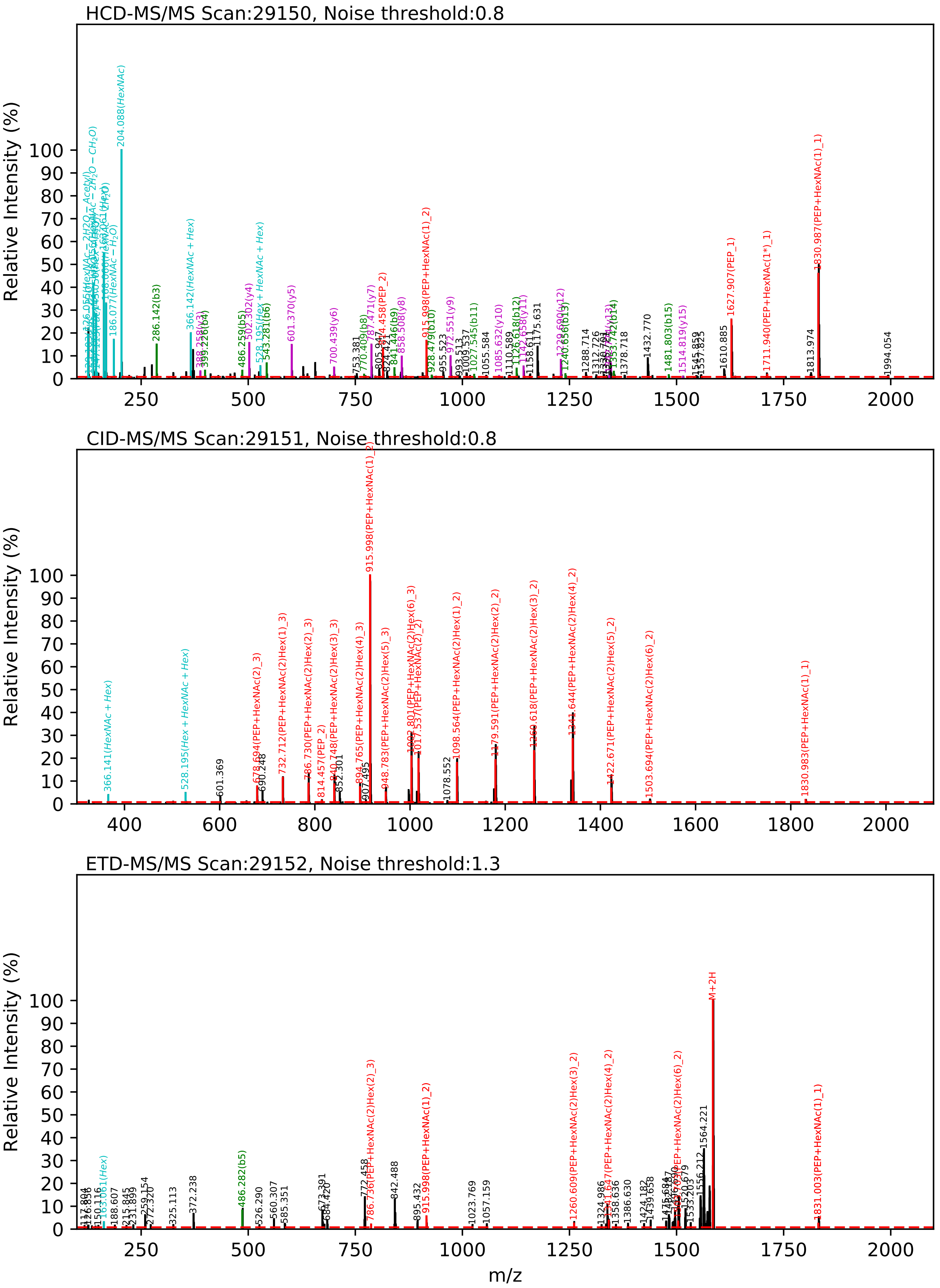

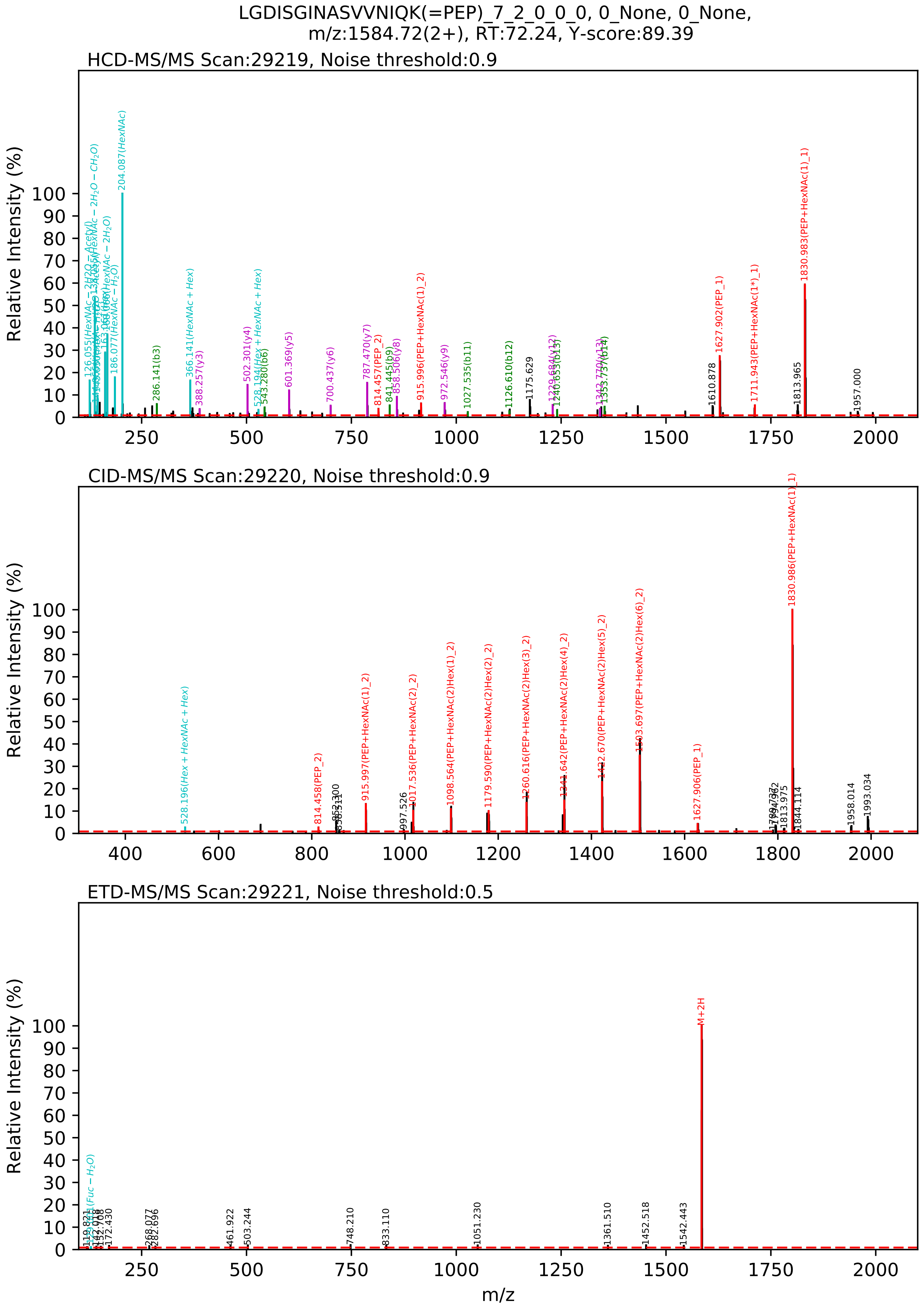

LGDISGINASVVNIQK(=PEP)_7_2_0_0_0, 0_None, 0_None,
m/z:1584.72(2+), RT:72.24, Y-score:89.39
HCD-MS/MS Scan:29219, Noise threshold:0.9
CID-MS/MS Scan:29220, Noise threshold:0.9
ETD-MS/MS Scan:29221, Noise threshold:0.5
Relative Intensity (%)
m/z
204.087(HexNAc)
366.141(HexNAc + Hex)
528.194(Hex + HexNAc + Hex)
286.141(b3)
388.257(y3)
502.301(y4)
543.280(b6)
601.369(y5)
700.437(y6)
787.470(y7)
814.457(PEP_2)
841.445(b9)
858.506(y8)
915.996(PEP+HexNAc(1)_2)
972.546(y9)
1027.535(b11)
1126.610(b12)
1175.629
1610.878
1627.902(PEP_1)
1711.943(PEP+HexNAc(1*)_1)
1813.965
1830.983(PEP+HexNAc(1)_1)
1957.000
528.196(Hex + HexNAc + Hex)
814.458(PEP_2)
852.300
915.997(PEP+HexNAc(1)_2)
997.526
1017.536(PEP+HexNAc(2)_2)
1098.564(PEP+HexNAc(2)Hex(1)_2)
1179.590(PEP+HexNAc(2)Hex(2)_2)
1260.616(PEP+HexNAc(2)Hex(3)_2)
1341.642(PEP+HexNAc(2)Hex(4)_2)
1422.670(PEP+HexNAc(2)Hex(5)_2)
1503.697(PEP+HexNAc(2)Hex(6)_2)
1627.906(PEP_1)
1813.975
1830.986(PEP+HexNAc(1)_1)
1844.114
1958.014
1993.034
M+2H
119.821
142.908
172.430
268.077
282.696
461.922
503.244
748.210
833.110
1051.230
1361.510
1452.518
1542.443

LGDISGINASVVNIQK(=PEP)_7_2_0_0_0, 0_None, 0_None,
m/z:1584.72(2+), RT:72.09, Y-score:86.91

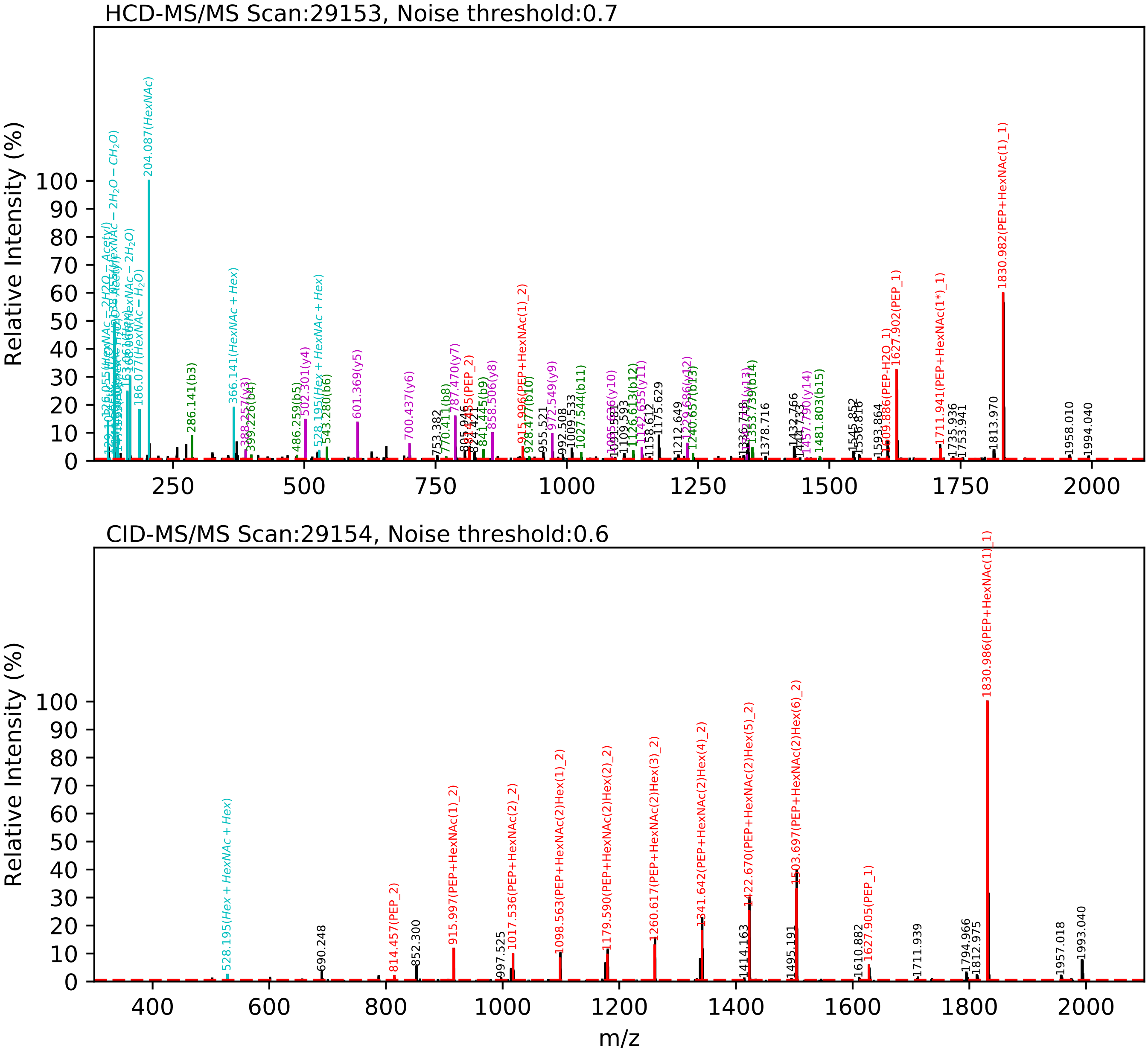

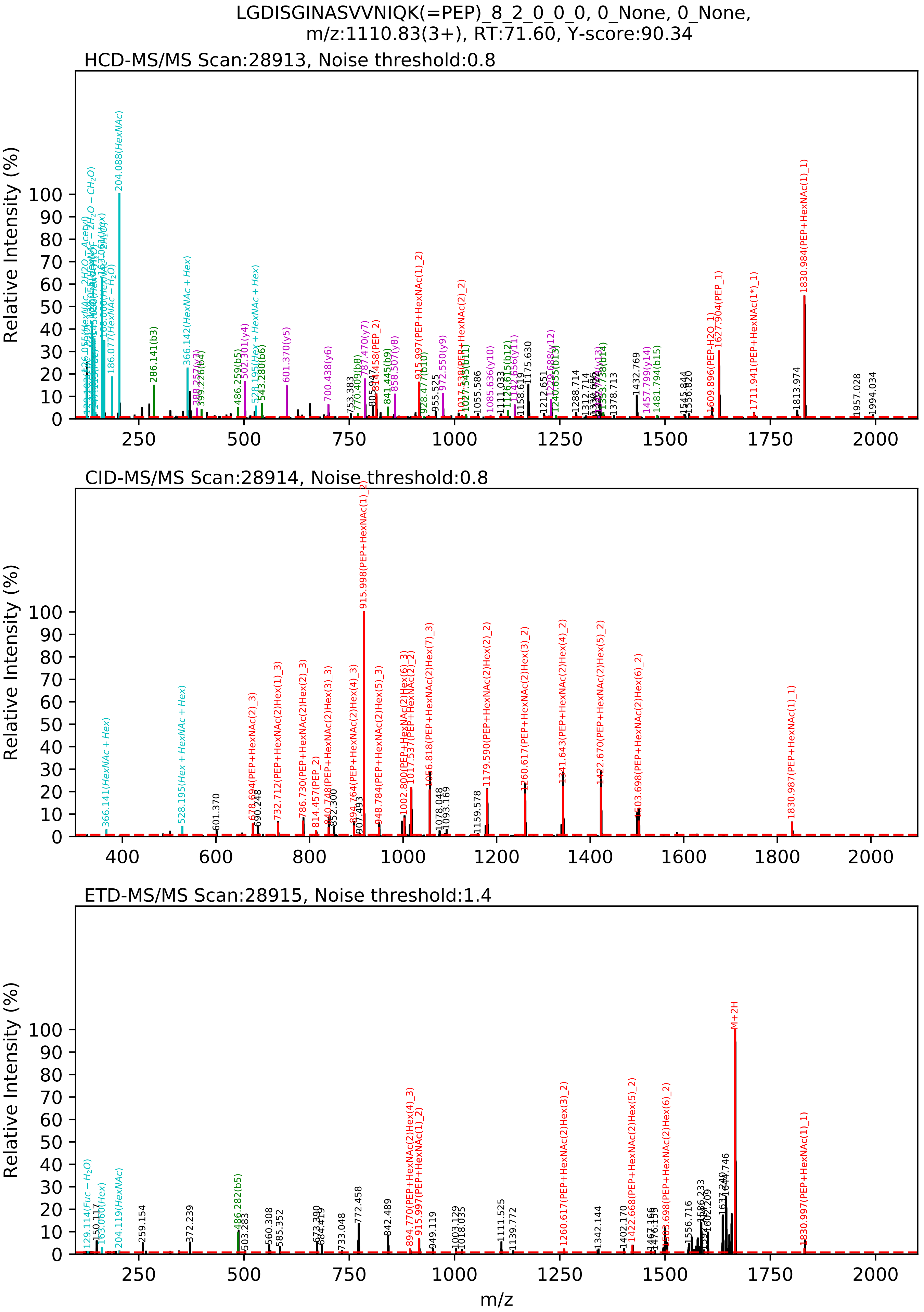

LGDISGINASVVNIQK(=PEP)_8_2_0_0_0, 0_None, 0_None,
m/z:1110.83(3+), RT:71.60, Y-score:90.34
HCD-MS/MS Scan:28913, Noise threshold:0.8
CID-MS/MS Scan:28914, Noise threshold:0.8
ETD-MS/MS Scan:28915, Noise threshold:1.4
Relative Intensity (%)
m/z
1830.984(PEP+HexNAc(1)_1)
915.998(PEP+HexNAc(1)_2)
M+2H

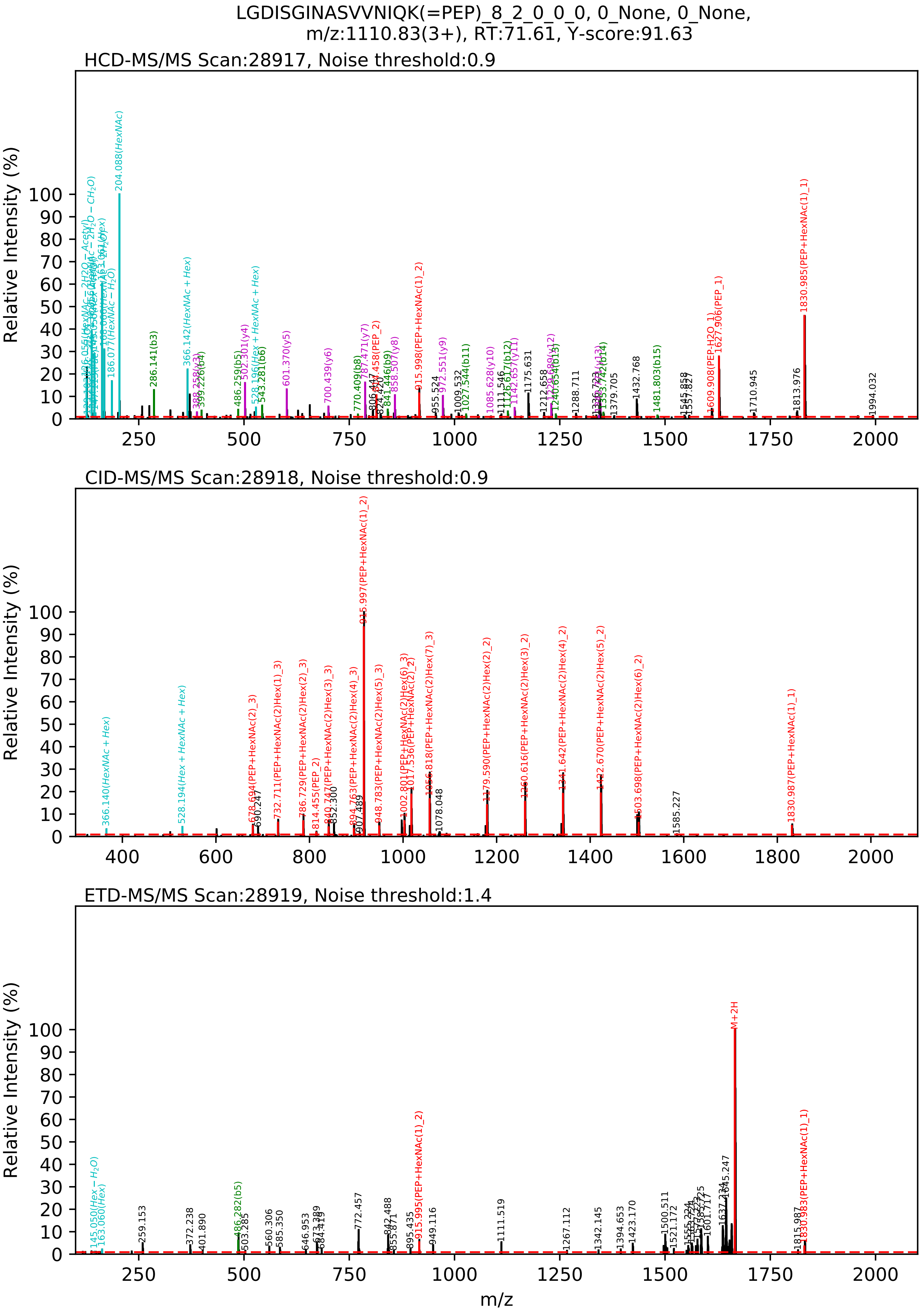

LGDISGINASVVNIQK(=PEP)_8_2_0_0_0, 0_None, 0_None,
m/z:1110.83(3+), RT:71.61, Y-score:91.63
HCD-MS/MS Scan:28917, Noise threshold:0.9
Relative Intensity (%)
204.088(HexNAc)
186.077(HexNAc − H2O)
168.066(HexNAc − 2H2O)
286.141(b3)
366.142(HexNAc + Hex)
388.258(y3)
399.226(b4)
486.259(b5)
502.301(y4)
528.196(Hex + HexNAc + Hex)
543.281(b6)
601.370(y5)
700.439(y6)
770.409(b8)
787.471(y7)
806.444
814.458(PEP_2)
841.446(b9)
858.507(y8)
915.998(PEP+HexNAc(1)_2)
955.524
972.551(y9)
1009.532
1027.544(b11)
1085.628(y10)
1111.546
1126.617(b12)
1142.657(y11)
1175.631
1212.658
1288.711
1336.723
1379.705
1432.768
1481.803(b15)
1545.858
1557.827
1609.908(PEP-H2O_1)
1627.906(PEP_1)
1710.945
1813.976
1830.985(PEP+HexNAc(1)_1)
1994.032
CID-MS/MS Scan:28918, Noise threshold:0.9
366.140(HexNAc + Hex)
528.194(Hex + HexNAc + Hex)
678.694(PEP+HexNAc(2)_3)
690.247
732.711(PEP+HexNAc(2)Hex(1)_3)
786.729(PEP+HexNAc(2)Hex(2)_3)
814.455(PEP_2)
840.747(PEP+HexNAc(2)Hex(3)_3)
852.300
894.763(PEP+HexNAc(2)Hex(4)_3)
907.489
915.997(PEP+HexNAc(1)_2)
948.783(PEP+HexNAc(2)Hex(5)_3)
1002.801(PEP+HexNAc(2)Hex(6)_3)
1017.536(PEP+HexNAc(2)_2)
1056.818(PEP+HexNAc(2)Hex(7)_3)
1078.048
1179.590(PEP+HexNAc(2)Hex(2)_2)
1260.616(PEP+HexNAc(2)Hex(3)_2)
1341.642(PEP+HexNAc(2)Hex(4)_2)
1422.670(PEP+HexNAc(2)Hex(5)_2)
1503.698(PEP+HexNAc(2)Hex(6)_2)
1585.227
1830.987(PEP+HexNAc(1)_1)
ETD-MS/MS Scan:28919, Noise threshold:1.4
145.050(Hex − H2O)
163.060(Hex)
259.153
372.238
401.890
486.282(b5)
503.285
560.306
585.350
646.953
673.389
684.419
772.457
842.488
855.871
895.435
915.995(PEP+HexNAc(1)_2)
949.116
1111.519
1267.112
1342.145
1394.653
1423.170
1500.511
1521.172
1555.224
1601.717
1637.234
1645.247
M+2H
1815.987
1830.983(PEP+HexNAc(1)_1)
m/z

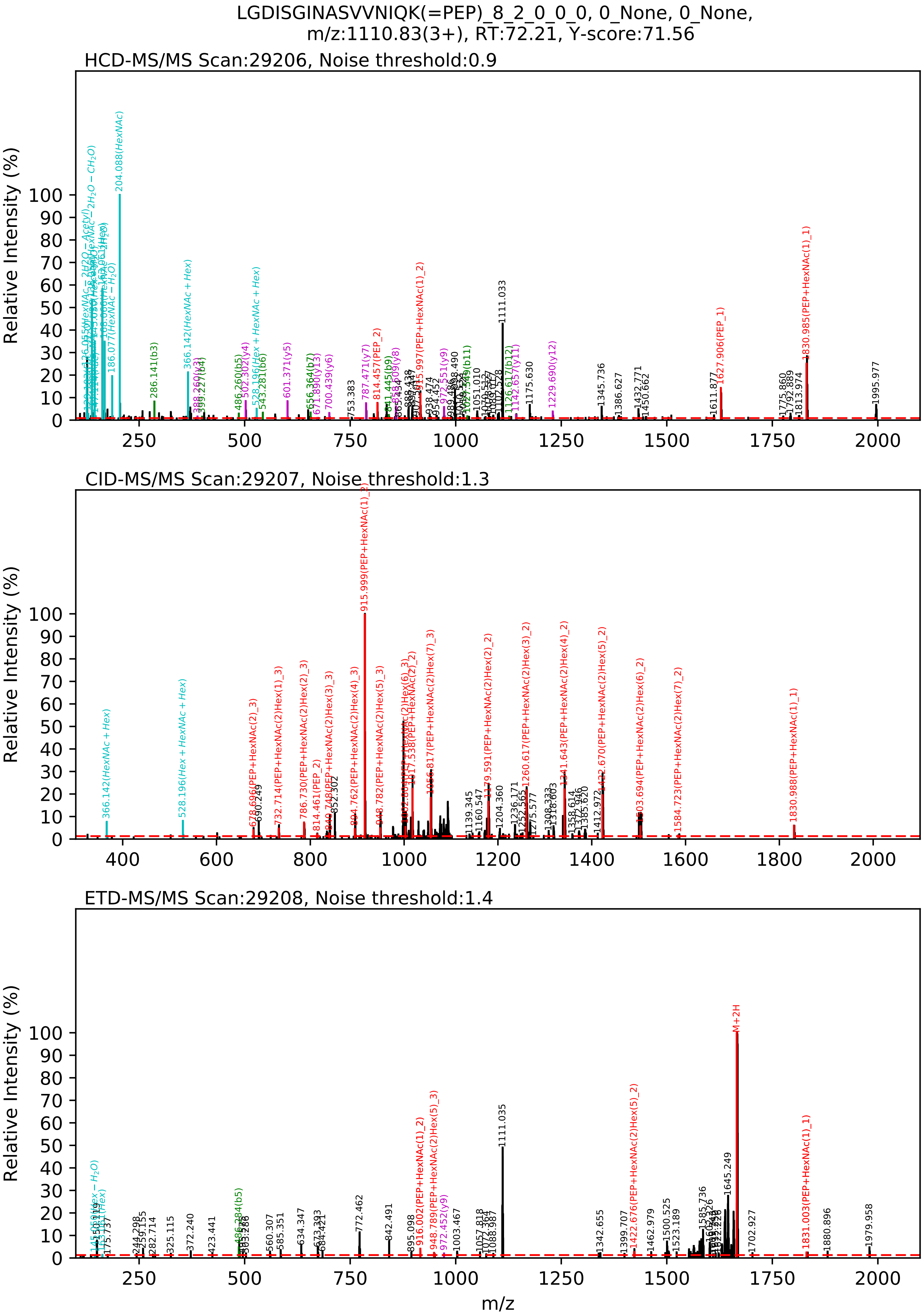
LGDISGINASVVNIQK(=PEP)_8_2_0_0_0, 0_None, 0_None,
m/z:1110.83(3+), RT:72.21, Y-score:71.56
HCD-MS/MS Scan:29206, Noise threshold:0.9
CID-MS/MS Scan:29207, Noise threshold:1.3
ETD-MS/MS Scan:29208, Noise threshold:1.4
Relative Intensity (%)
m/z
204.088(HexNAc)
1830.985(PEP+HexNAc(1)_1)
915.999(PEP+HexNAc(1)_2)
M+2H
1111.033
1111.035

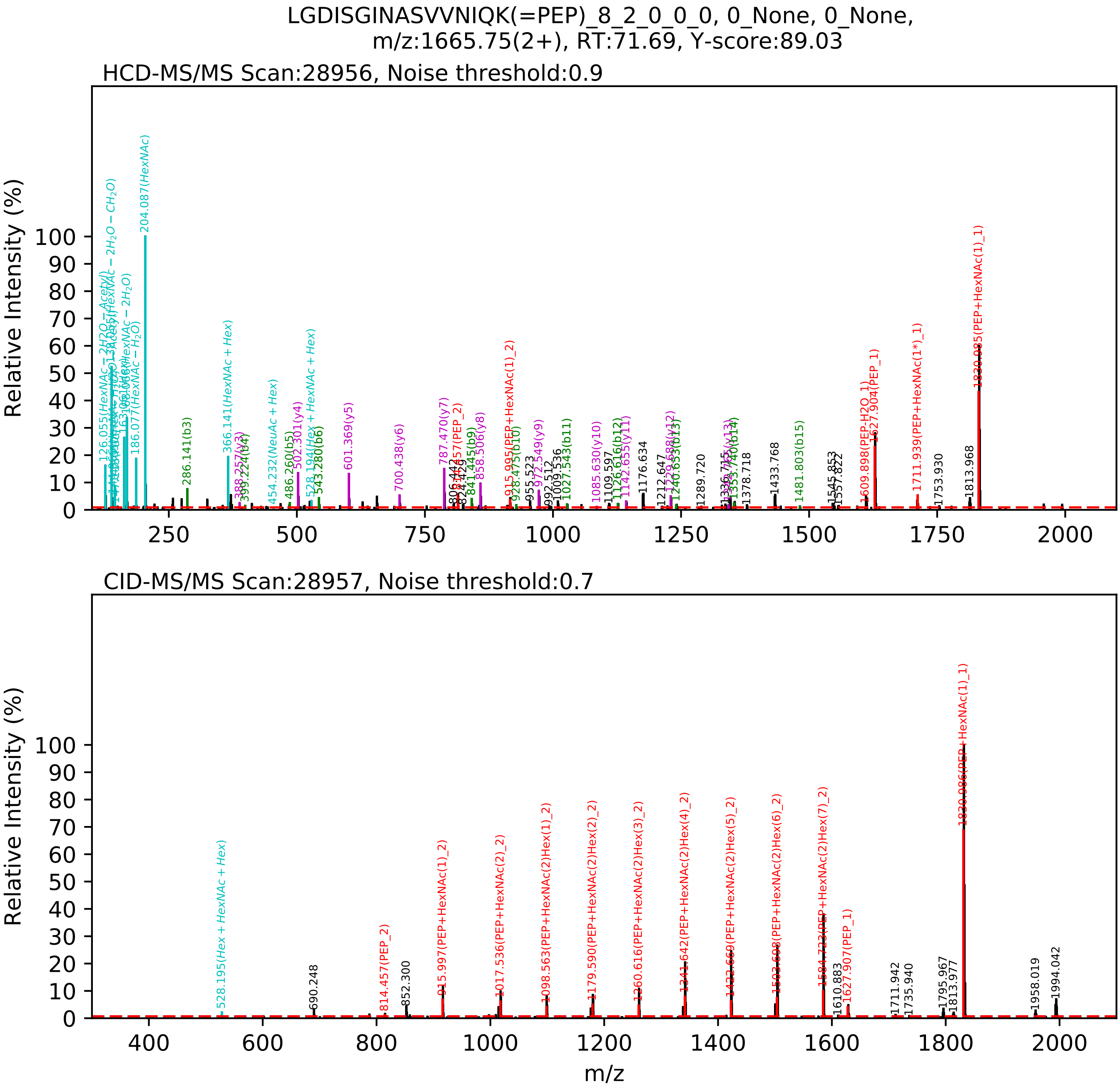

LGDISGINASVVNIQK(=PEP)_8_2_0_0_0, 0_None, 0_None,
m/z:1665.75(2+), RT:71.69, Y-score:89.03
HCD-MS/MS Scan:28956, Noise threshold:0.9
CID-MS/MS Scan:28957, Noise threshold:0.7
Relative Intensity (%)
m/z
204.087(HexNAc)
366.141(HexNAc + Hex)
528.194(Hex + HexNAc + Hex)
1830.985(PEP+HexNAc(1)_1)
1627.904(PEP_1)
1711.939(PEP+HexNAc(1*)_1)
915.995(PEP+HexNAc(1)_2)
1830.986(PEP+HexNAc(1)_1)
528.195(Hex + HexNAc + Hex)
814.457(PEP_2)
915.997(PEP+HexNAc(1)_2)
1017.536(PEP+HexNAc(2)_2)
1098.563(PEP+HexNAc(2)Hex(1)_2)
1179.590(PEP+HexNAc(2)Hex(2)_2)
1260.616(PEP+HexNAc(2)Hex(3)_2)
1341.642(PEP+HexNAc(2)Hex(4)_2)
1422.669(PEP+HexNAc(2)Hex(5)_2)
1503.698(PEP+HexNAc(2)Hex(6)_2)
1584.723(PEP+HexNAc(2)Hex(7)_2)
1627.907(PEP_1)

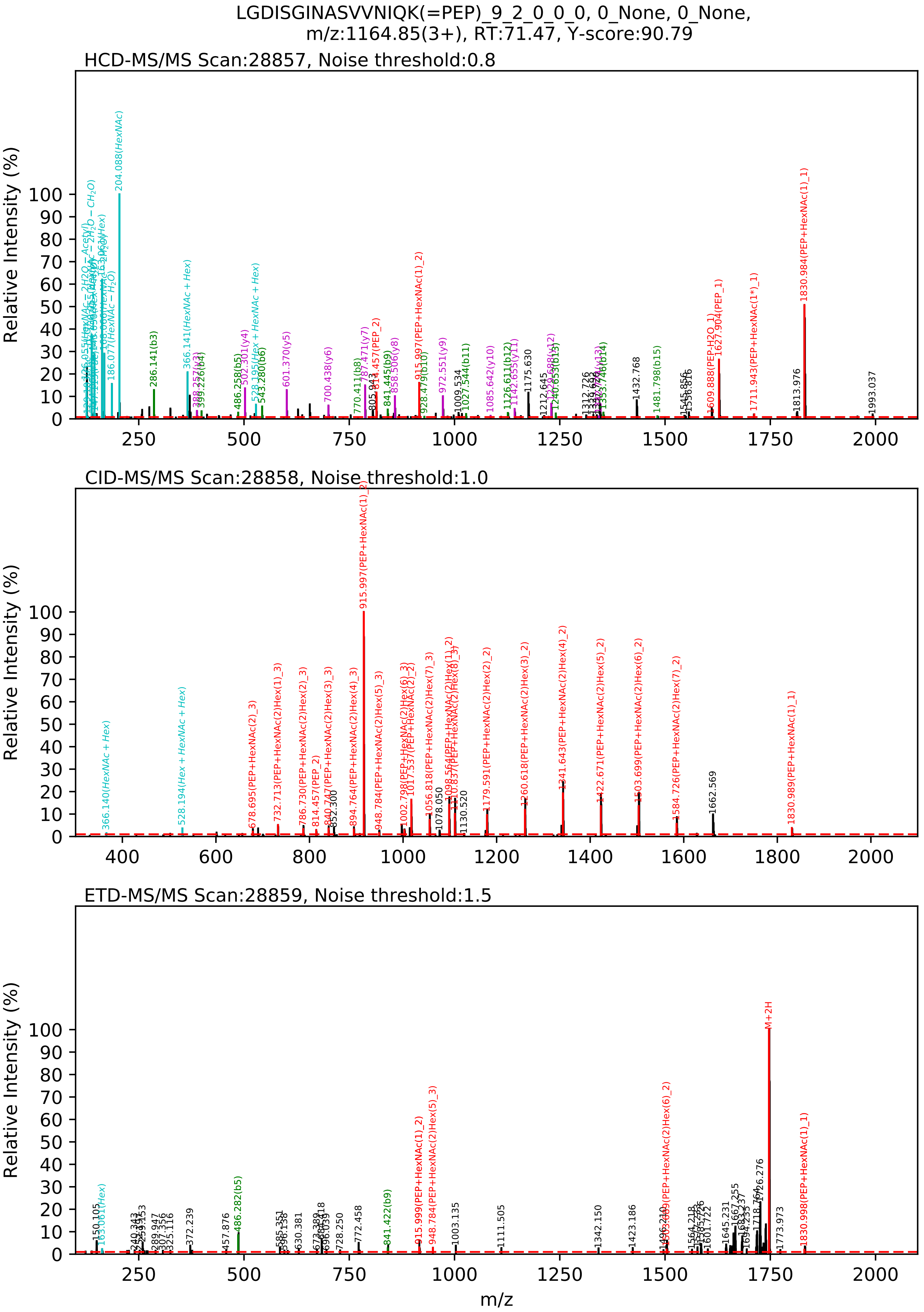
LGDISGINASVVNIQK(=PEP)_9_2_0_0_0, 0_None, 0_None,
m/z:1164.85(3+), RT:71.47, Y-score:90.79
HCD-MS/MS Scan:28857, Noise threshold:0.8
Relative Intensity (%)
204.088(HexNAc)
186.077(HexNAc − H2O)
286.141(b3)
366.141(HexNAc + Hex)
388.257(y3)
399.226(b4)
486.258(b5)
502.301(y4)
528.195(Hex + HexNAc + Hex)
543.280(b6)
601.370(y5)
700.438(y6)
770.411(b8)
787.471(y7)
805.913
814.457(PEP_2)
841.445(b9)
858.506(y8)
915.997(PEP+HexNAc(1)_2)
928.479(b10)
972.551(y9)
1009.534
1027.544(b11)
1085.642(y10)
1126.611(b12)
1142.655(y11)
1175.630
1212.645
1240.653(b13)
1312.726
1339.692
1353.740(b14)
1432.768
1481.798(b15)
1545.856
1556.816
1609.888(PEP-H2O_1)
1627.904(PEP_1)
1711.943(PEP+HexNAc(1*)_1)
1813.976
1830.984(PEP+HexNAc(1)_1)
1993.037
CID-MS/MS Scan:28858, Noise threshold:1.0
366.140(HexNAc + Hex)
528.194(Hex + HexNAc + Hex)
678.695(PEP+HexNAc(2)_3)
732.713(PEP+HexNAc(2)Hex(1)_3)
786.730(PEP+HexNAc(2)Hex(2)_3)
814.457(PEP_2)
840.747(PEP+HexNAc(2)Hex(3)_3)
852.300
894.764(PEP+HexNAc(2)Hex(4)_3)
915.997(PEP+HexNAc(1)_2)
948.784(PEP+HexNAc(2)Hex(5)_3)
1002.798(PEP+HexNAc(2)Hex(6)_3)
1017.537(PEP+HexNAc(2)_2)
1056.818(PEP+HexNAc(2)Hex(7)_3)
1078.050
1098.564(PEP+HexNAc(2)Hex(1)_2)
1110.837(PEP+HexNAc(2)Hex(8)_3)
1130.520
1179.591(PEP+HexNAc(2)Hex(2)_2)
1260.618(PEP+HexNAc(2)Hex(3)_2)
1341.643(PEP+HexNAc(2)Hex(4)_2)
1422.671(PEP+HexNAc(2)Hex(5)_2)
1503.699(PEP+HexNAc(2)Hex(6)_2)
1584.726(PEP+HexNAc(2)Hex(7)_2)
1662.569
1830.989(PEP+HexNAc(1)_1)
ETD-MS/MS Scan:28859, Noise threshold:1.5
150.105
163.061(Hex)
240.343
259.153
289.947
307.356
325.116
372.239
457.876
486.282(b5)
585.351
630.381
673.389
696.039
728.250
772.458
841.422(b9)
915.999(PEP+HexNAc(1)_2)
948.784(PEP+HexNAc(2)Hex(5)_3)
1003.135
1111.505
1342.150
1423.186
1496.210
1503.699(PEP+HexNAc(2)Hex(6)_2)
1564.218
1601.722
1645.231
1667.255
1683.237
1694.235
1718.764
1726.276
M+2H
1773.973
1830.998(PEP+HexNAc(1)_1)
m/z

NFTTAPAICHDGK(=PEP)_8_2_0_0_0, 0_None, 0_None,
m/z:1567.63(2+), RT:29.68, Y-score:74.95

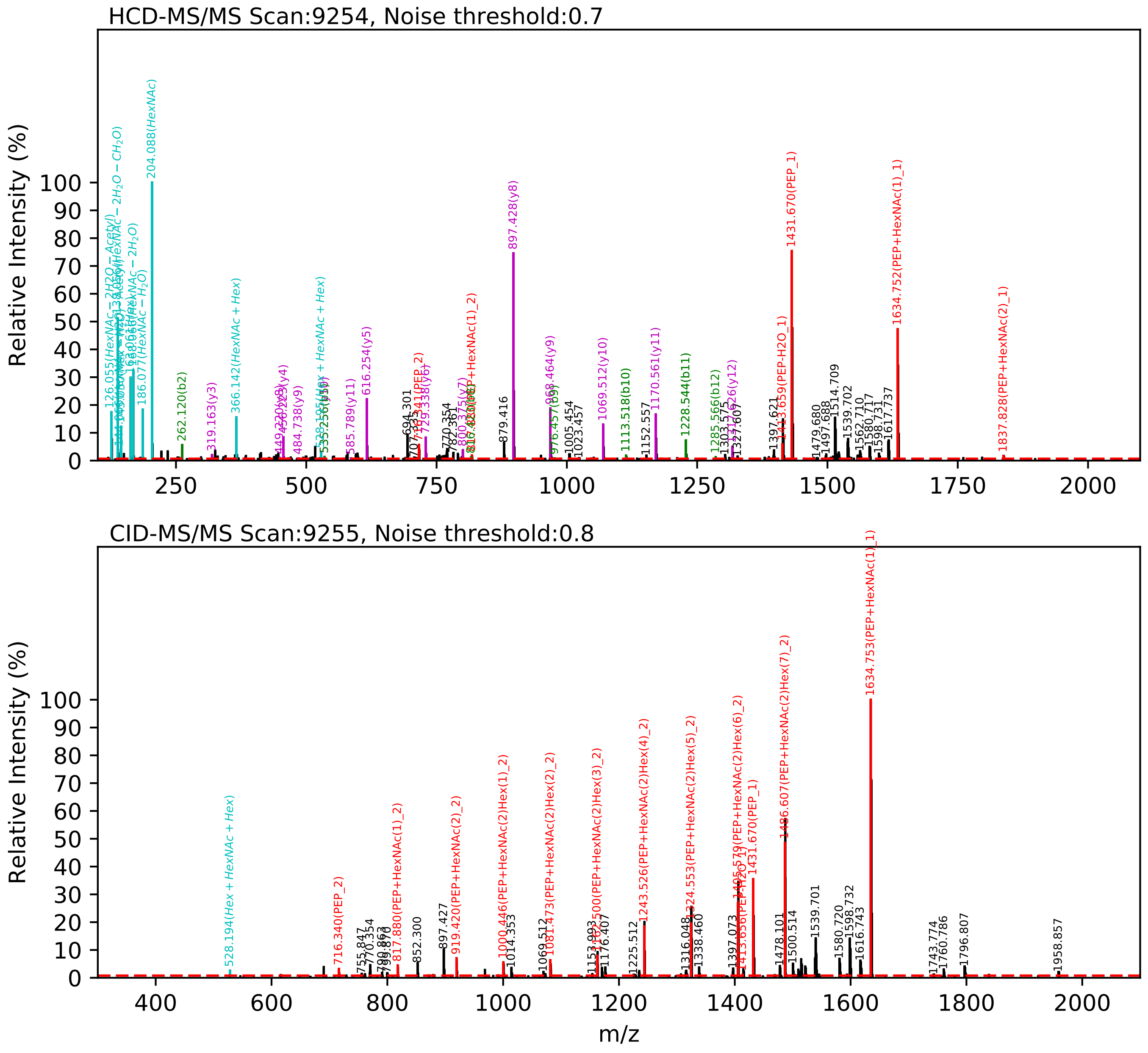

NFTTAPAICHDGK(=PEP)_8_2_0_0_0, 0_None, 0_None,
m/z:1567.63(2+), RT:30.07, Y-score:77.68

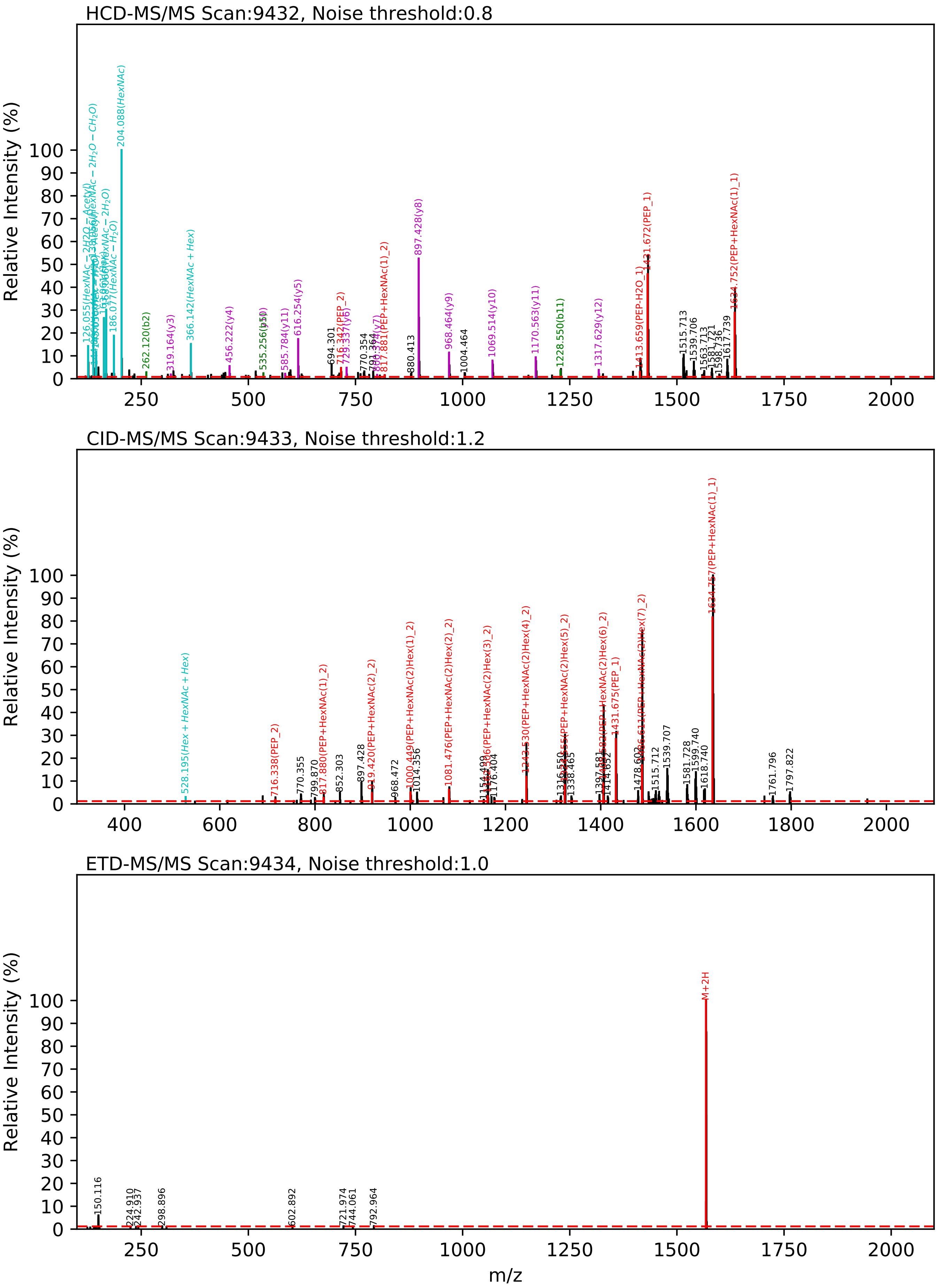

NFTTAPAICHDGK(=PEP)_8_2_0_0_0, 0_None, 0_None,
m/z:1567.63(2+), RT:32.36, Y-score:74.47

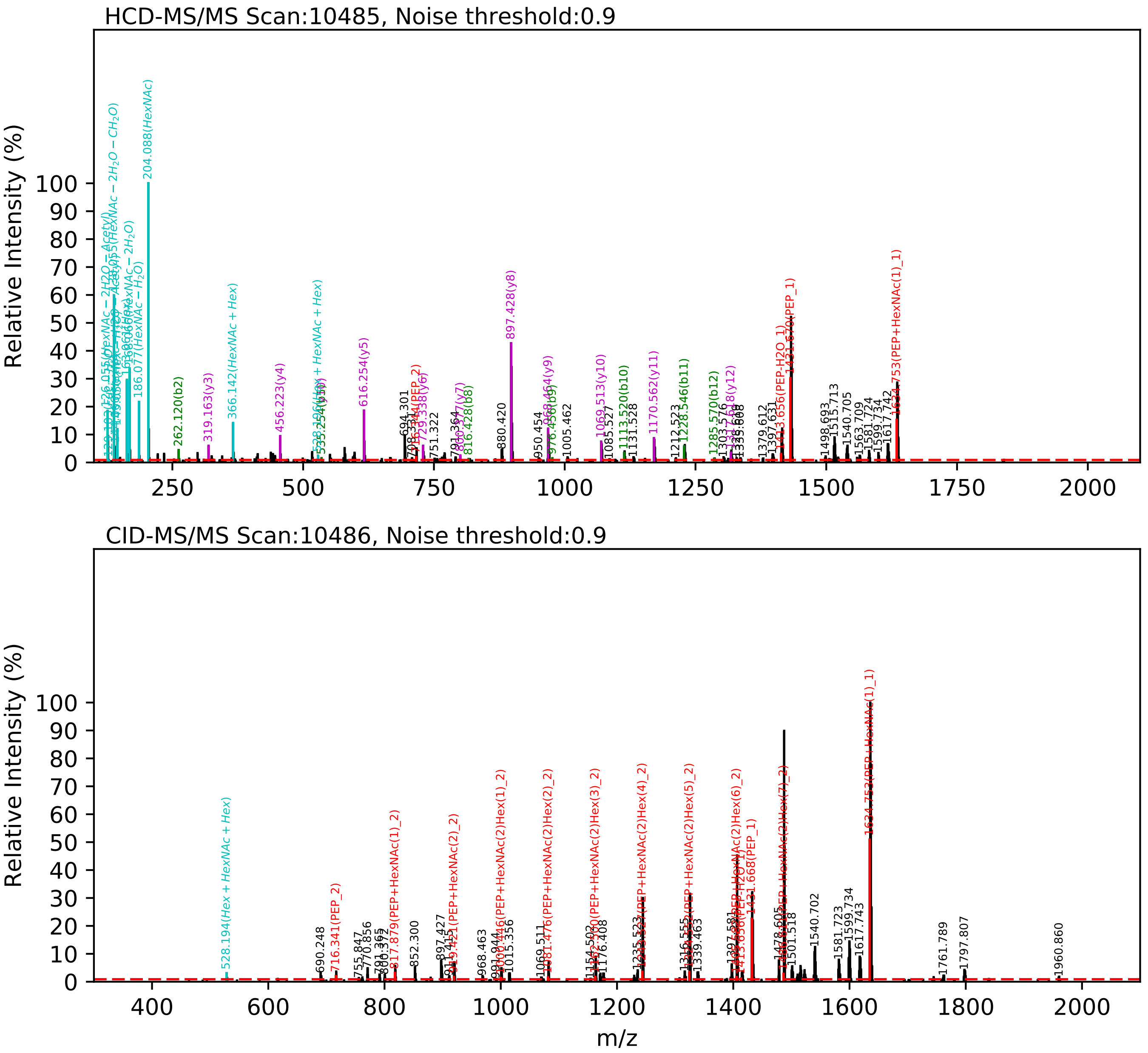

NFTTAPAICHDGK(=PEP)_8_2_0_0_0, 0_None, 0_None,
m/z:1045.42(3+), RT:27.97, Y-score:83.61

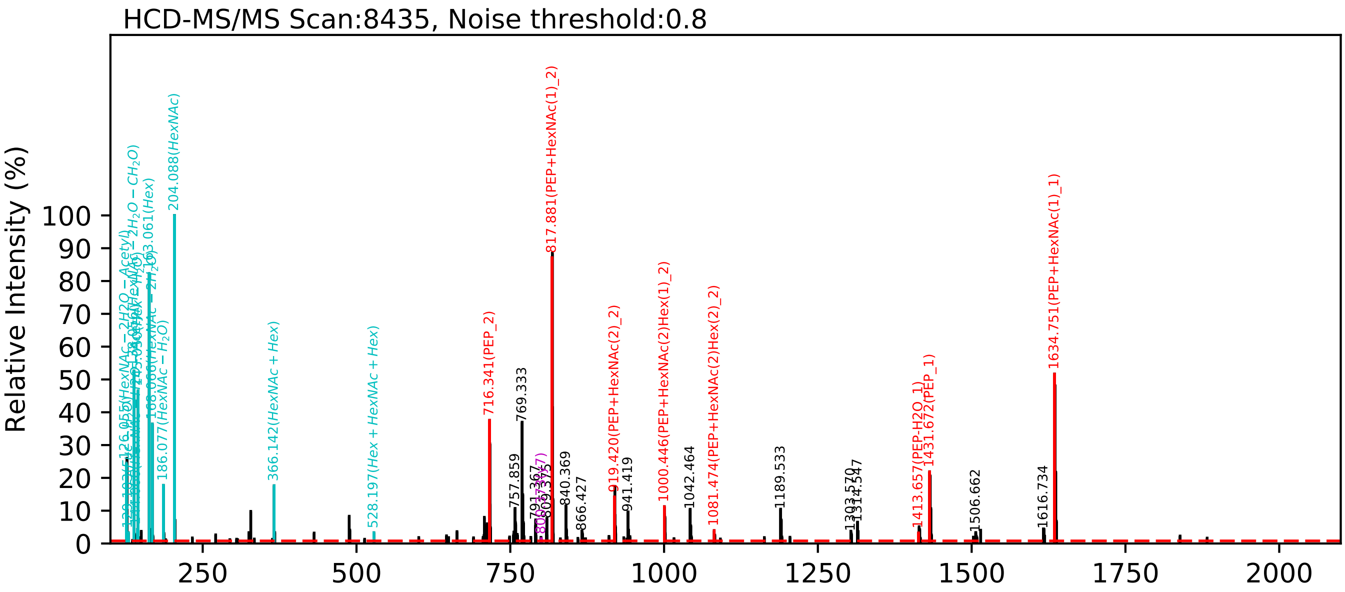

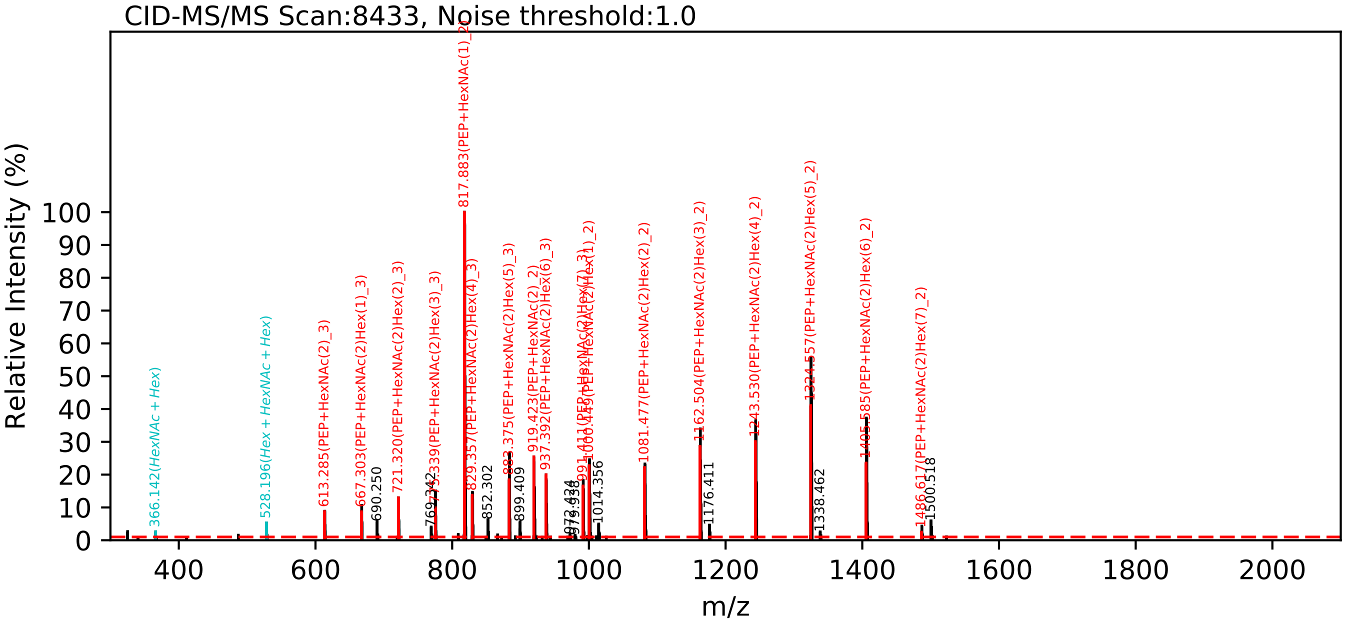

NFTTAPAICHDGK(=PEP)_9_2_0_0_0, 0_None, 0_None,
m/z:1099.44(3+), RT:27.95, Y-score:86.96

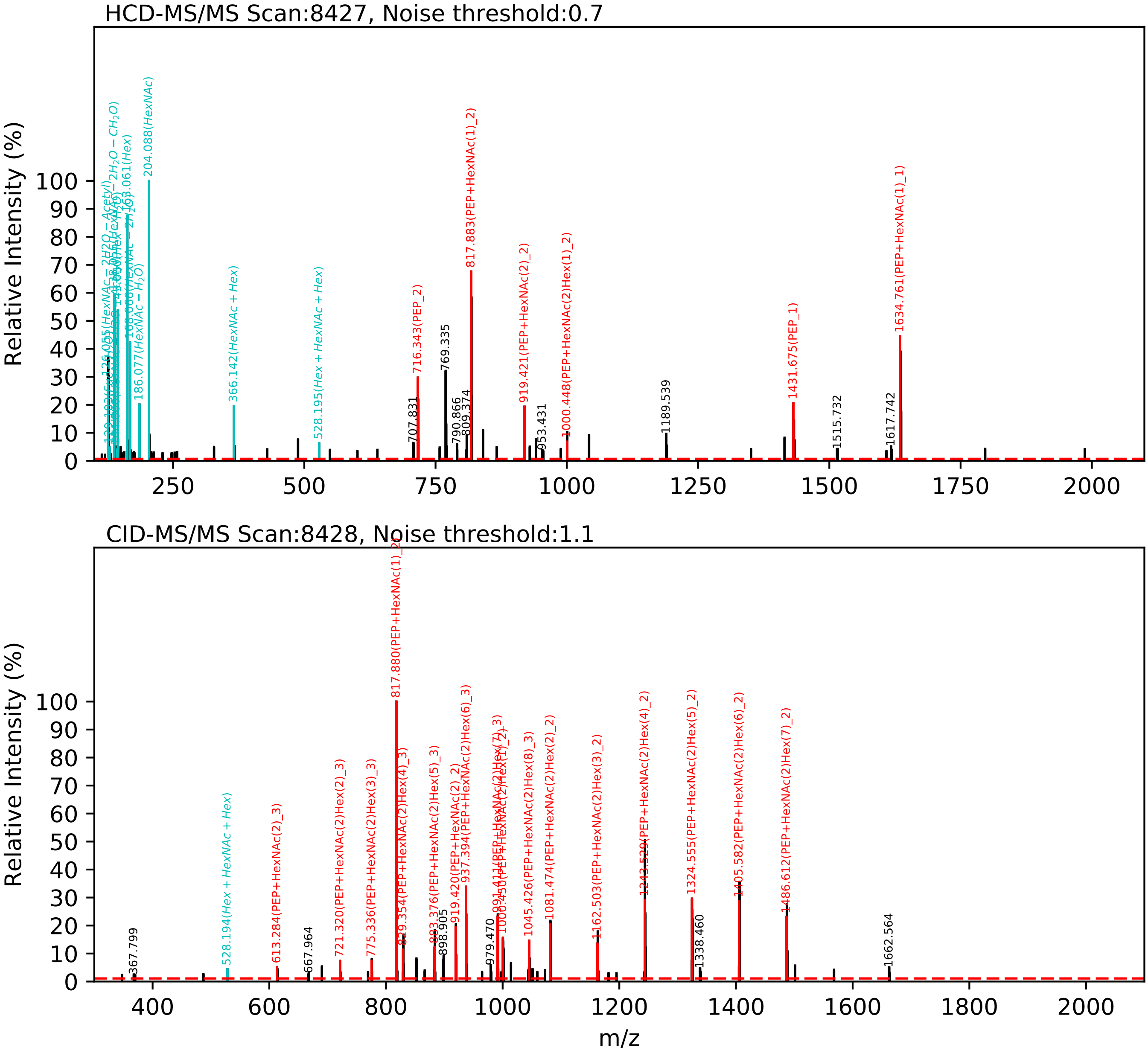

NFTTAPAICHDGK(=PEP)_9_2_0_0_0, 0_None, 0_None,
m/z:1099.44(3+), RT:30.10, Y-score:90.34

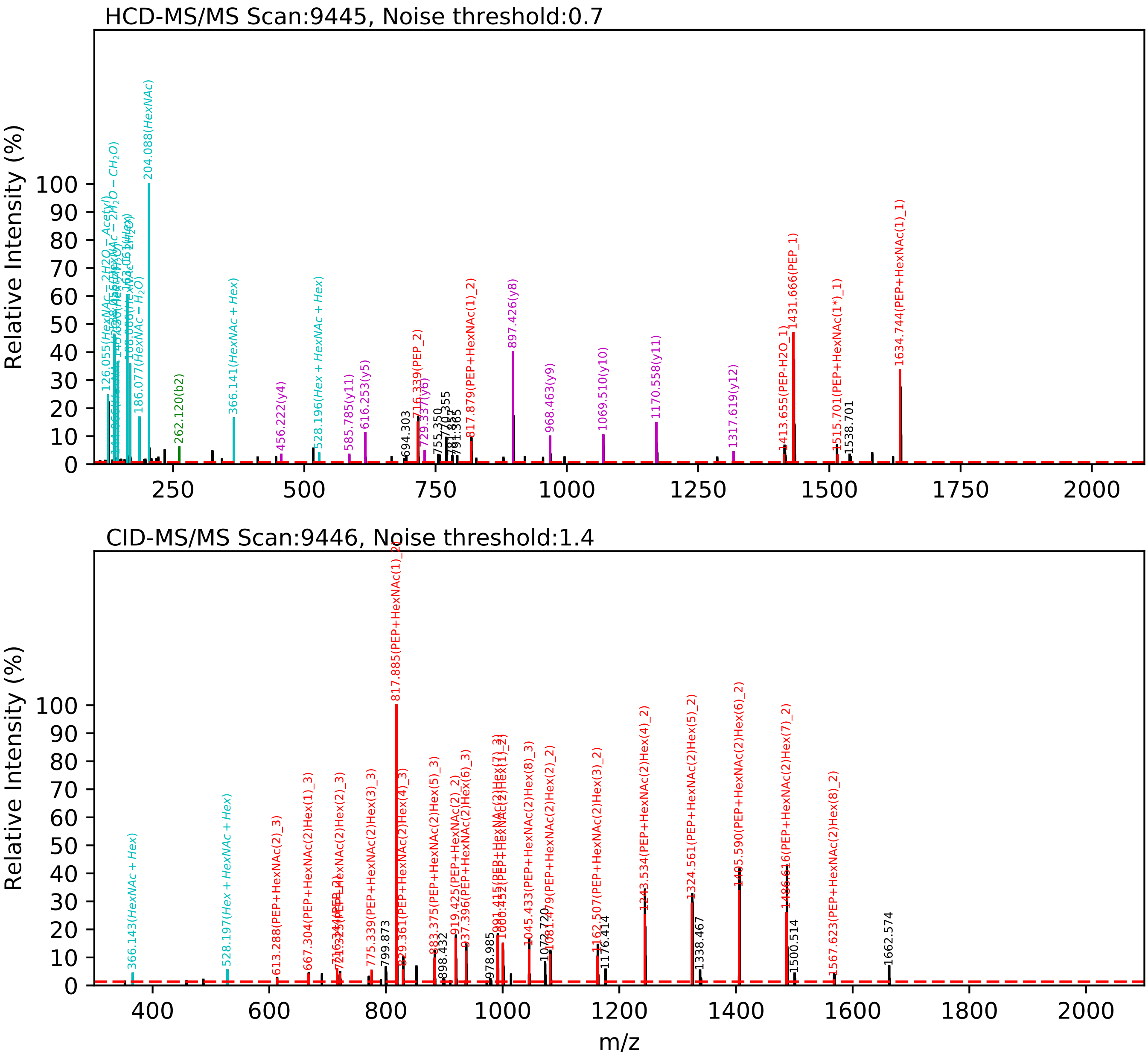

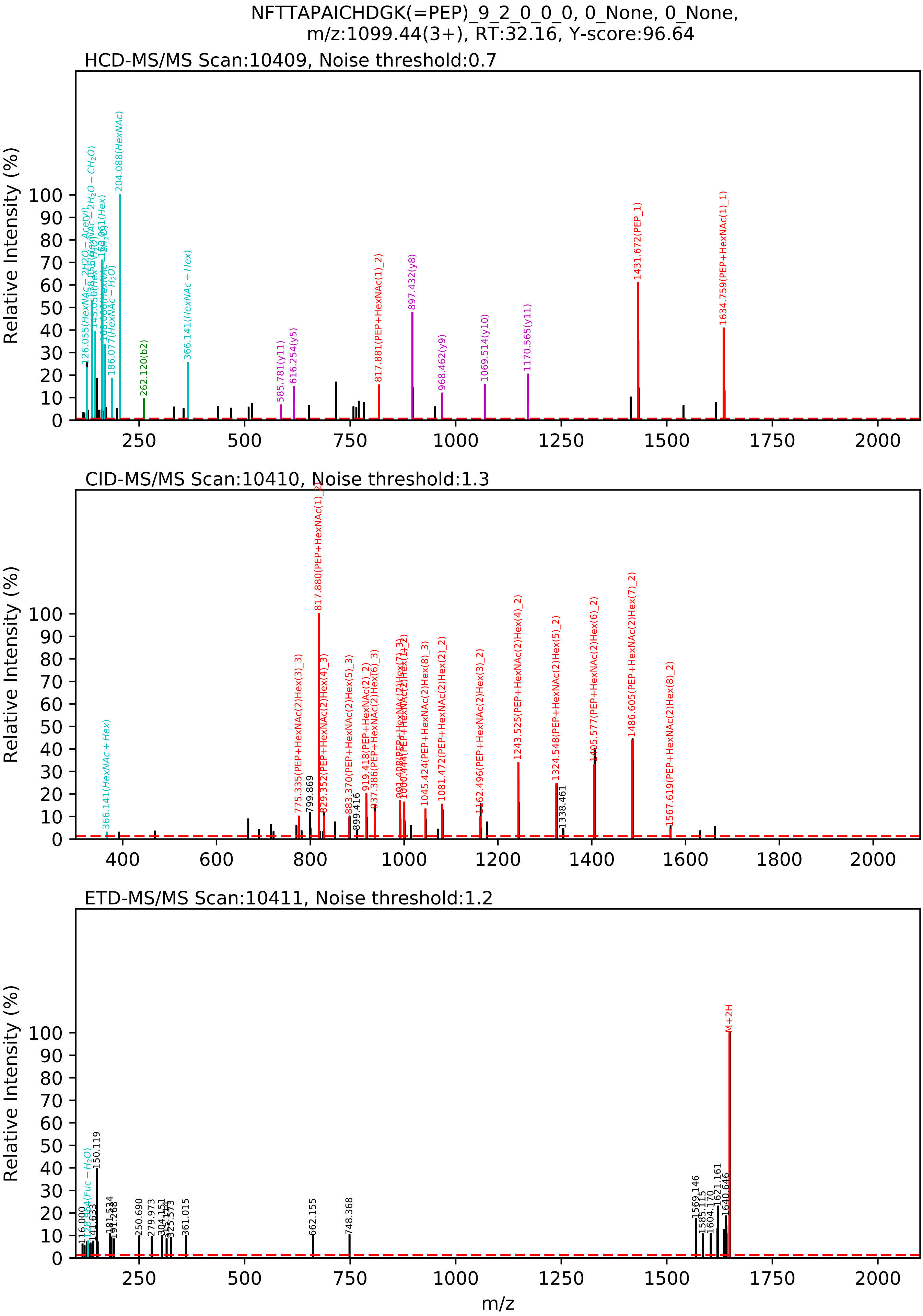

NFTTAPAICHDGK(=PEP)_9_2_0_0_0, 0_None, 0_None,
m/z:1099.44(3+), RT:32.16, Y-score:96.64
HCD-MS/MS Scan:10409, Noise threshold:0.7
Relative Intensity (%)
204.088(HexNAc)
366.141(HexNAc+Hex)
262.120(b2)
186.077(HexNAc−H2O)
126.055(HexNAc−2H2O−Acetyl)
585.781(y11)
616.254(y5)
817.881(PEP+HexNAc(1)_2)
897.432(y8)
968.462(y9)
1069.514(y10)
1170.565(y11)
1431.672(PEP_1)
1634.759(PEP+HexNAc(1)_1)
CID-MS/MS Scan:10410, Noise threshold:1.3
366.141(HexNAc+Hex)
775.335(PEP+HexNAc(2)Hex(3)_3)
799.869
817.880(PEP+HexNAc(1)_2)
829.352(PEP+HexNAc(2)Hex(4)_3)
883.370(PEP+HexNAc(2)Hex(5)_3)
899.416
919.418(PEP+HexNAc(2)_2)
937.386(PEP+HexNAc(2)Hex(6)_3)
1045.424(PEP+HexNAc(2)Hex(8)_3)
1081.472(PEP+HexNAc(2)Hex(2)_2)
1162.496(PEP+HexNAc(2)Hex(3)_2)
1243.525(PEP+HexNAc(2)Hex(4)_2)
1324.548(PEP+HexNAc(2)Hex(5)_2)
1338.461
1405.577(PEP+HexNAc(2)Hex(6)_2)
1486.605(PEP+HexNAc(2)Hex(7)_2)
1567.619(PEP+HexNAc(2)Hex(8)_2)
ETD-MS/MS Scan:10411, Noise threshold:1.2
M+2H
150.119
116.000
141.633
181.534
191.288
250.690
279.973
304.151
361.015
662.155
748.368
1569.146
1585.115
1604.170
1621.161
1640.646
m/z

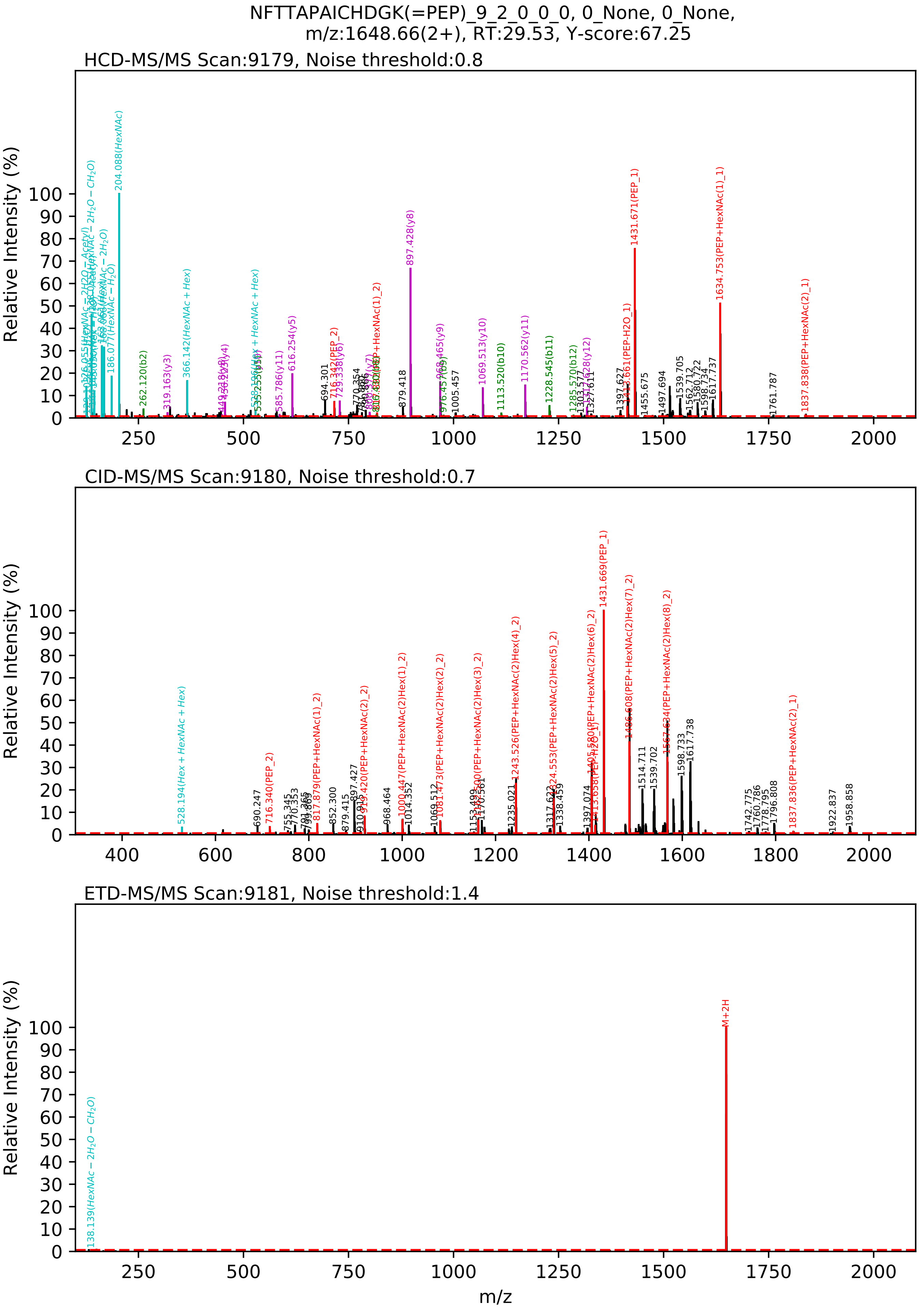

NFTTAPAICHDGK(=PEP)_9_2_0_0_0, 0_None, 0_None,
m/z:1648.66(2+), RT:29.53, Y-score:67.25
HCD-MS/MS Scan:9179, Noise threshold:0.8
Relative Intensity (%)
204.088(HexNAc)
186.077(HexNAc − H2O)
262.120(b2)
319.163(y3)
366.142(HexNAc + Hex)
585.786(y11)
616.254(y5)
694.301
716.342(PEP_2)
729.338(y6)
817.880(PEP+HexNAc(1)_2)
879.418
897.428(y8)
968.465(y9)
1005.457
1069.513(y10)
1113.520(b10)
1170.562(y11)
1228.545(b11)
1285.570(b12)
1303.577
1397.627
1431.671(PEP_1)
1455.675
1497.694
1539.705
1617.737
1634.753(PEP+HexNAc(1)_1)
1761.787
1837.838(PEP+HexNAc(2)_1)
CID-MS/MS Scan:9180, Noise threshold:0.7
528.194(Hex + HexNAc + Hex)
690.247
716.340(PEP_2)
755.345
770.353
817.879(PEP+HexNAc(1)_2)
852.300
879.415
897.427
919.420(PEP+HexNAc(2)_2)
968.464
1000.447(PEP+HexNAc(2)Hex(1)_2)
1014.352
1069.512
1081.473(PEP+HexNAc(2)Hex(2)_2)
1153.499
1170.561
1235.021
1243.526(PEP+HexNAc(2)Hex(4)_2)
1317.623
1338.459
1397.074
1431.669(PEP_1)
1514.711
1539.702
1598.733
1617.738
1742.775
1760.786
1778.795
1796.808
1837.836(PEP+HexNAc(2)_1)
1922.837
1958.858
ETD-MS/MS Scan:9181, Noise threshold:1.4
138.139(HexNAc − 2H2O − CH2O)
M+2H
m/z

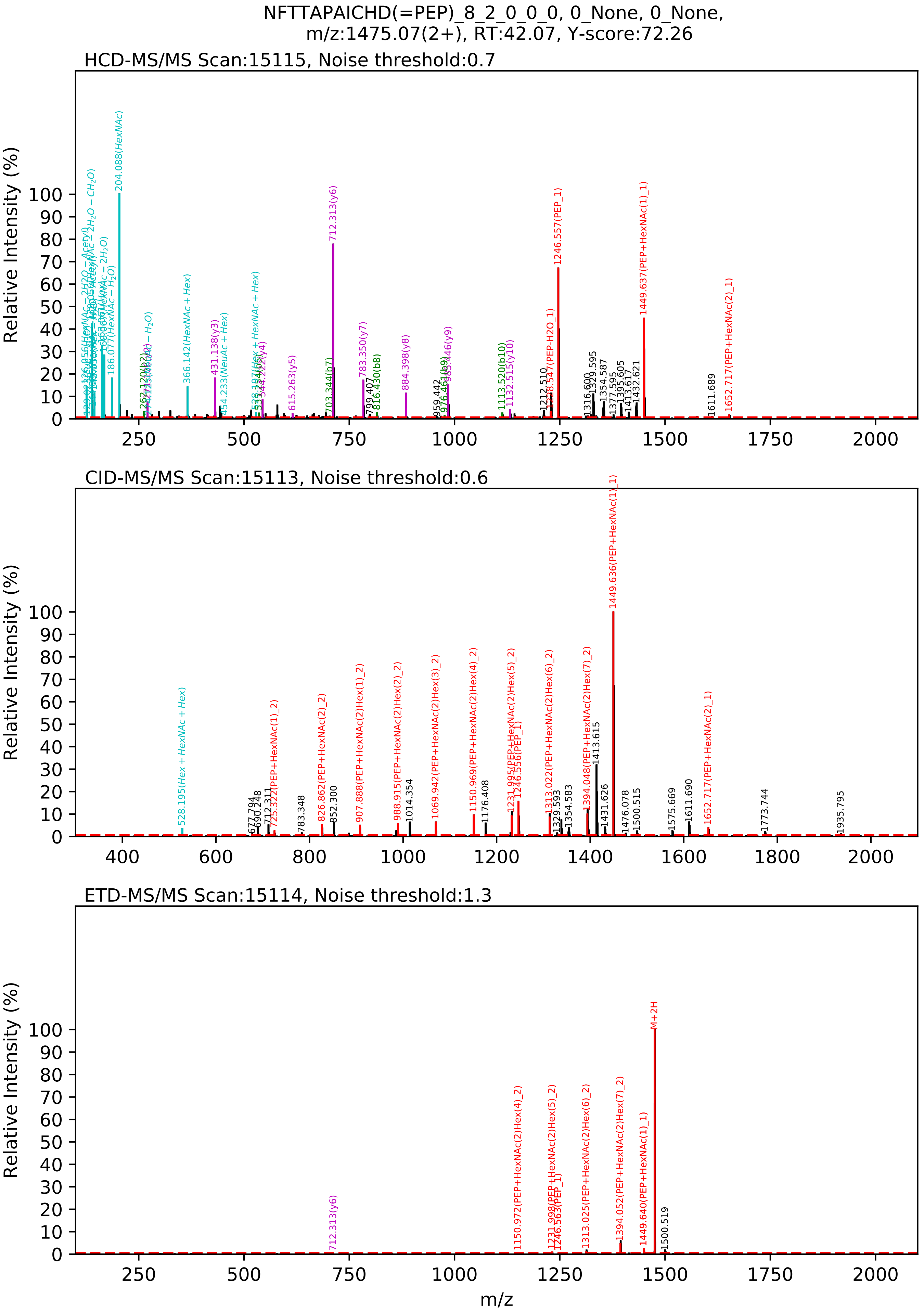
NFTTAPAICHD(=PEP)_8_2_0_0_0, 0_None, 0_None,
m/z:1475.07(2+), RT:42.07, Y-score:72.26
HCD-MS/MS Scan:15115, Noise threshold:0.7
Relative Intensity (%)
204.088(HexNAc)
186.077(HexNAc − H2O)
262.120(b2)
366.142(HexNAc + Hex)
431.138(y3)
454.233(NeuAc + Hex)
544.272(y4)
615.263(y5)
703.344(b7)
712.313(y6)
783.350(y7)
799.407
816.430(b8)
884.398(y8)
959.442
985.446(y9)
1113.520(b10)
1132.515(y10)
1212.510
1228.547(PEP-H2O_1)
1246.557(PEP_1)
1316.600
1329.595
1354.587
1377.595
1395.605
1413.617
1432.621
1449.637(PEP+HexNAc(1)_1)
1611.689
1652.717(PEP+HexNAc(2)_1)
CID-MS/MS Scan:15113, Noise threshold:0.6
Relative Intensity (%)
528.195(Hex + HexNAc + Hex)
677.794
690.248
712.311
725.322(PEP+HexNAc(1)_2)
783.348
826.862(PEP+HexNAc(2)_2)
852.300
907.888(PEP+HexNAc(2)Hex(1)_2)
988.915(PEP+HexNAc(2)Hex(2)_2)
1014.354
1069.942(PEP+HexNAc(2)Hex(3)_2)
1150.969(PEP+HexNAc(2)Hex(4)_2)
1176.408
1231.995(PEP+HexNAc(2)Hex(5)_2)
1246.556(PEP_1)
1313.022(PEP+HexNAc(2)Hex(6)_2)
1329.593
1354.583
1394.048(PEP+HexNAc(2)Hex(7)_2)
1413.615
1431.626
1449.636(PEP+HexNAc(1)_1)
1476.078
1500.515
1575.669
1611.690
1652.717(PEP+HexNAc(2)_1)
1773.744
1935.795
ETD-MS/MS Scan:15114, Noise threshold:1.3
Relative Intensity (%)
712.313(y6)
1150.972(PEP+HexNAc(2)Hex(4)_2)
1231.998(PEP+HexNAc(2)Hex(5)_2)
1246.563(PEP_1)
1313.025(PEP+HexNAc(2)Hex(6)_2)
1394.052(PEP+HexNAc(2)Hex(7)_2)
1449.640(PEP+HexNAc(1)_1)
M+2H
1500.519
m/z

NFTTAPAICHD(=PEP)_9_2_0_0_0, 0_None, 0_None,
m/z:1556.10(2+), RT:41.96, Y-score:73.99

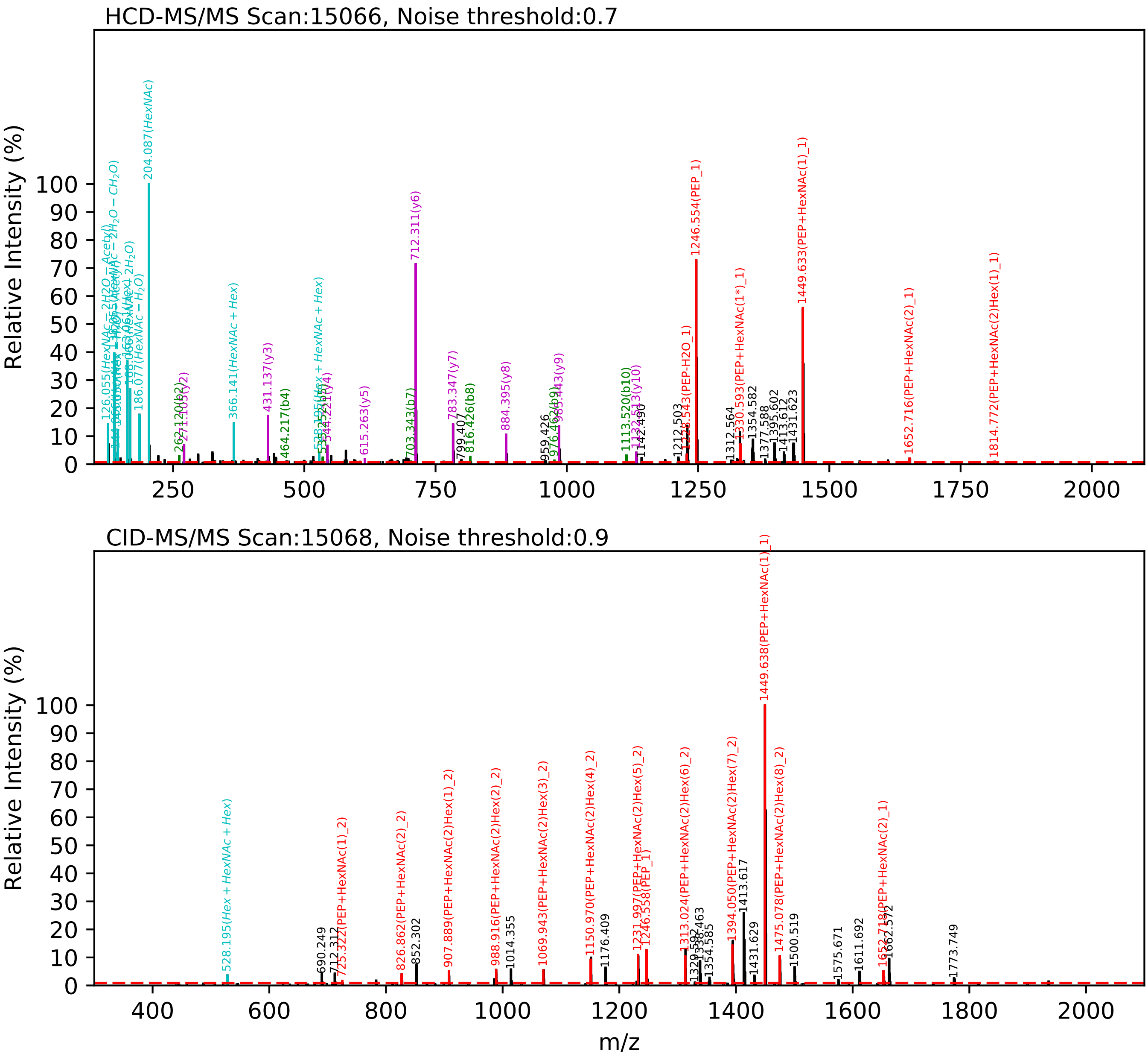

NHTSPDVD(=PEP)_7_2_0_0_0, 0_None, 0_None,
m/z:1212.95(2+), RT:13.93, Y-score:90.51

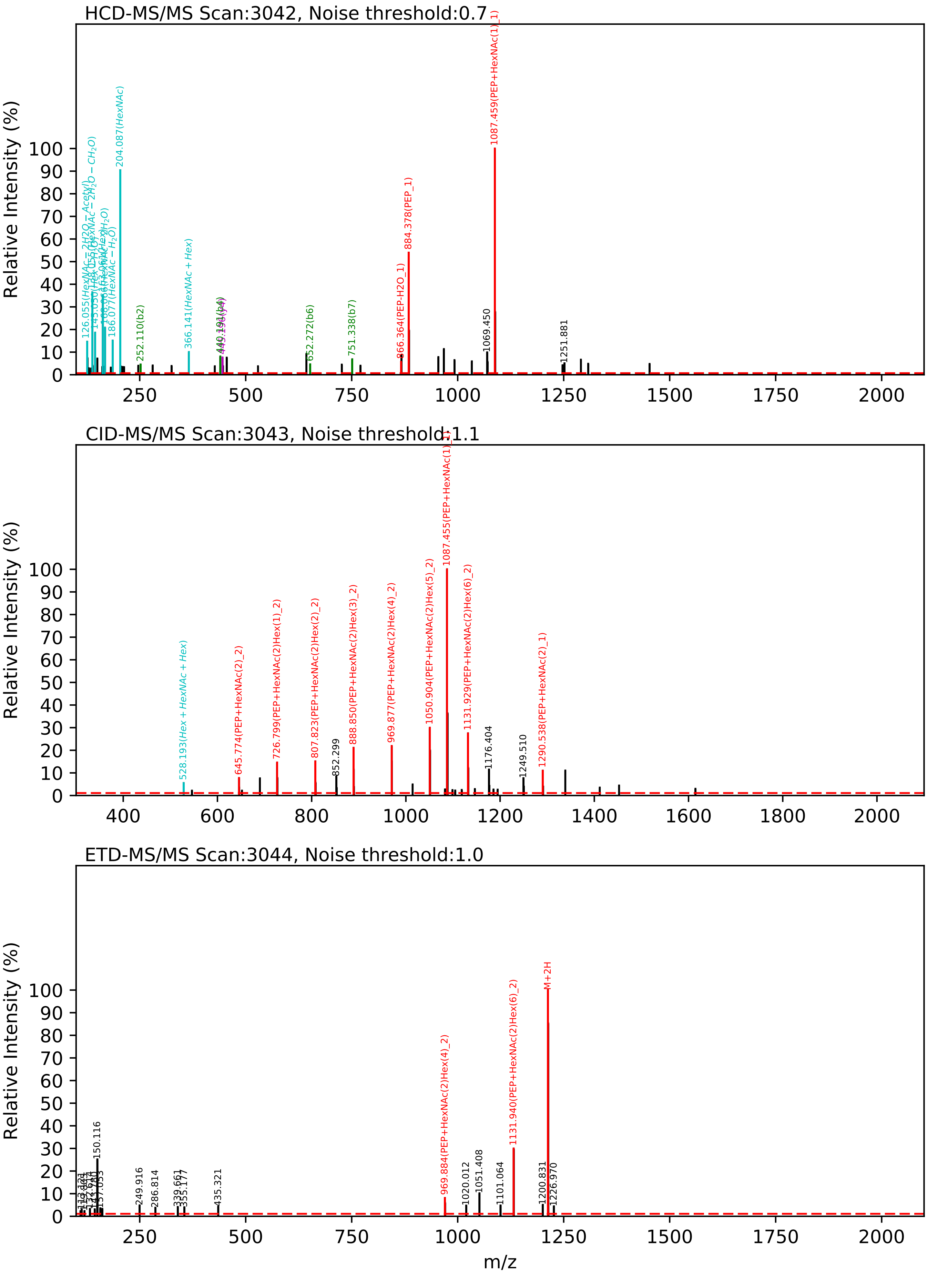

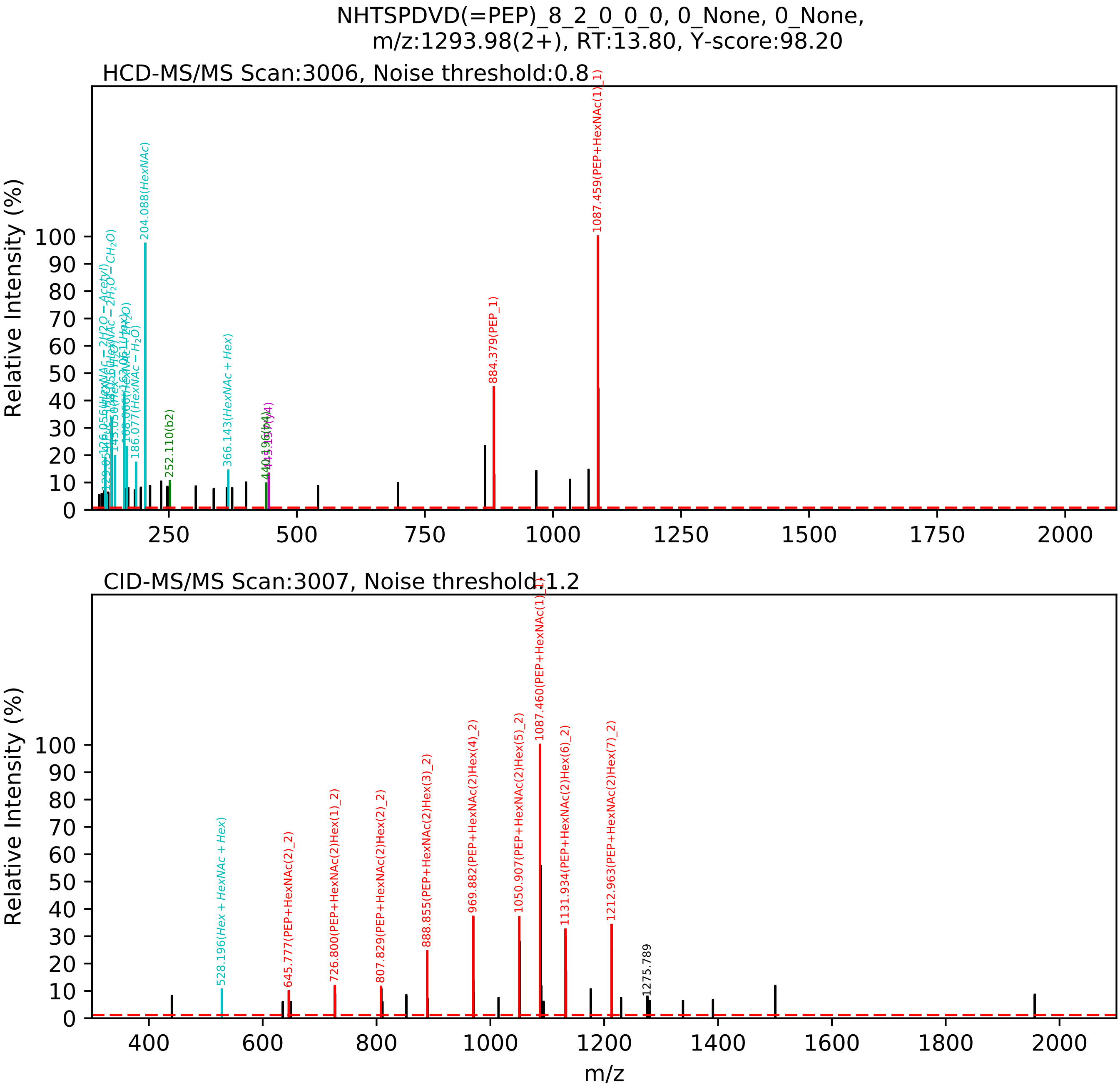

NHTSPDVD(=PEP)_8_2_0_0_0, 0_None, 0_None,
m/z:1293.98(2+), RT:13.80, Y-score:98.20
HCD-MS/MS Scan:3006, Noise threshold:0.8
Relative Intensity (%)
204.088(HexNAc)
1087.459(PEP+HexNAc(1)_1)
884.379(PEP_1)
366.143(HexNAc+Hex)
252.110(b2)
186.077(HexNAc−H2O)
CID-MS/MS Scan:3007, Noise threshold:1.2
528.196(Hex+HexNAc+Hex)
645.777(PEP+HexNAc(2)_2)
726.800(PEP+HexNAc(2)Hex(1)_2)
807.829(PEP+HexNAc(2)Hex(2)_2)
888.855(PEP+HexNAc(2)Hex(3)_2)
969.882(PEP+HexNAc(2)Hex(4)_2)
1050.907(PEP+HexNAc(2)Hex(5)_2)
1087.460(PEP+HexNAc(1)_1)
1131.934(PEP+HexNAc(2)Hex(6)_2)
1212.963(PEP+HexNAc(2)Hex(7)_2)
1275.789
m/z

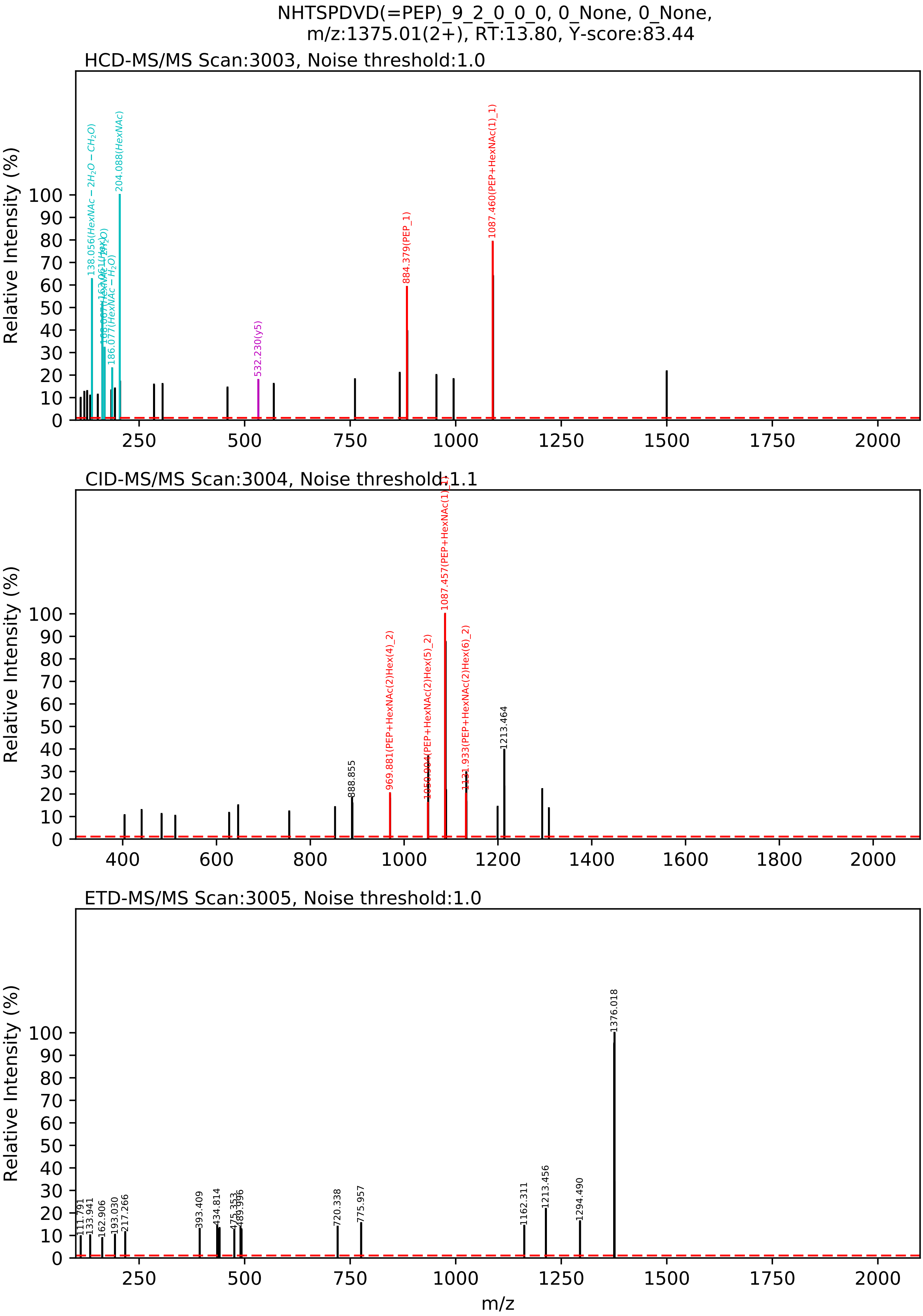

NHTSPDVD(=PEP)_9_2_0_0_0, 0_None, 0_None,
m/z:1375.01(2+), RT:13.80, Y-score:83.44
HCD-MS/MS Scan:3003, Noise threshold:1.0
204.088(HexNAc)
138.056(HexNAc − 2H2O − CH2O)
186.077(HexNAc − H2O)
532.230(y5)
884.379(PEP_1)
1087.460(PEP+HexNAc(1)_1)
CID-MS/MS Scan:3004, Noise threshold:1.1
888.855
969.881(PEP+HexNAc(2)Hex(4)_2)
1087.457(PEP+HexNAc(1)_1)
1131.933(PEP+HexNAc(2)Hex(6)_2)
1213.464
ETD-MS/MS Scan:3005, Noise threshold:1.0
111.791
133.941
162.906
193.030
217.266
393.409
434.814
489.996
720.338
775.957
1162.311
1213.456
1294.490
1376.018
Relative Intensity (%)
m/z

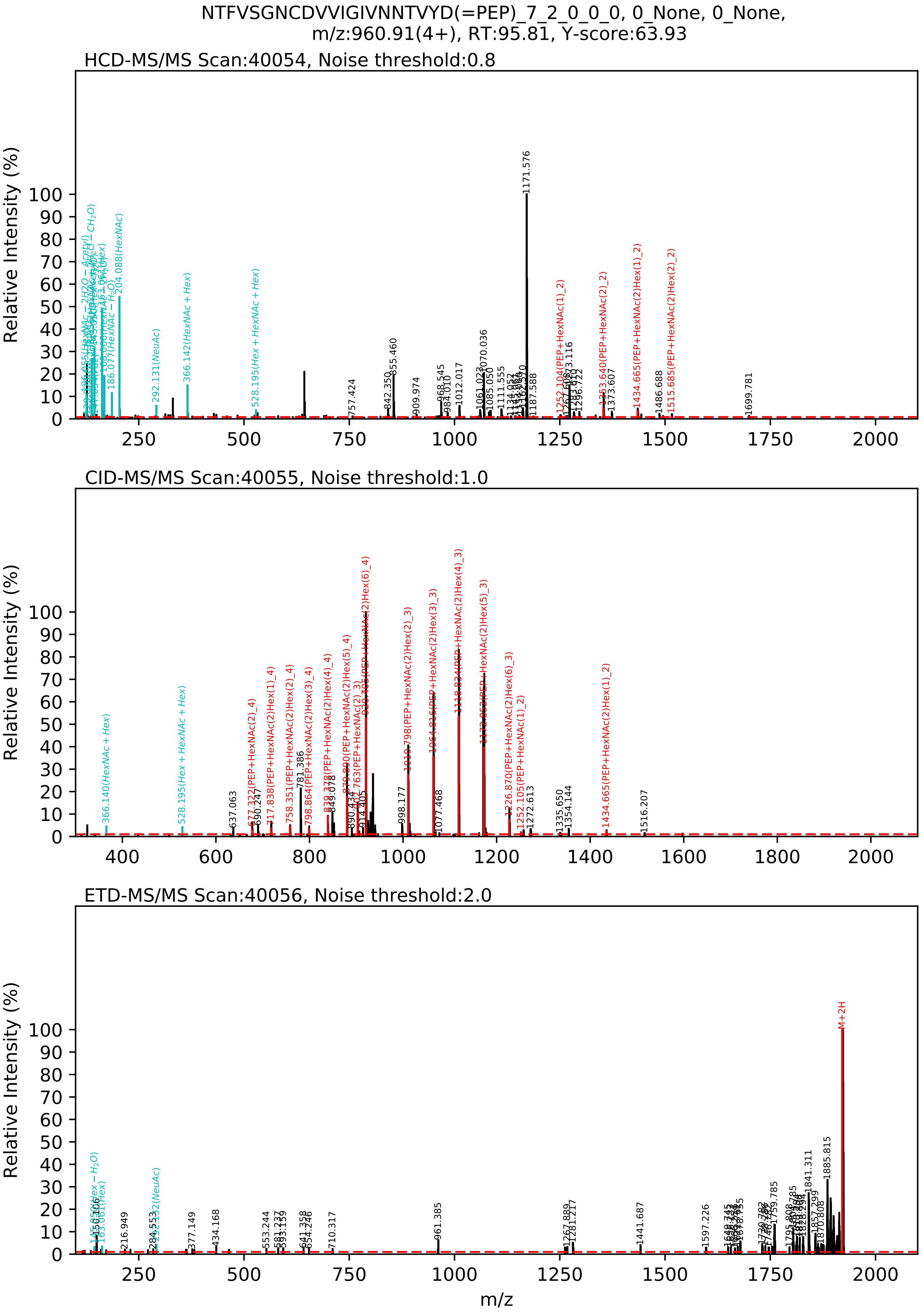
NTFVSGNCDVVIGIVNNTVYD(=PEP)_7_2_0_0_0, 0_None, 0_None,
m/z:960.91(4+), RT:95.81, Y-score:63.93
HCD-MS/MS Scan:40054, Noise threshold:0.8
CID-MS/MS Scan:40055, Noise threshold:1.0
ETD-MS/MS Scan:40056, Noise threshold:2.0
Relative Intensity (%)
m/z
1171.576
204.088(HexNAc)
366.142(HexNAc + Hex)
528.195(Hex + HexNAc + Hex)
292.131(NeuAc)
1252.104(PEP+HexNAc(1)_2)
1353.640(PEP+HexNAc(2)_2)
1434.665(PEP+HexNAc(2)Hex(1)_2)
1515.685(PEP+HexNAc(2)Hex(2)_2)
920.405(PEP+HexNAc(2)Hex(6)_4)
1010.798(PEP+HexNAc(2)Hex(2)_3)
1064.816(PEP+HexNAc(2)Hex(3)_3)
1118.834(PEP+HexNAc(2)Hex(4)_3)
1172.852(PEP+HexNAc(2)Hex(5)_3)
1226.870(PEP+HexNAc(2)Hex(6)_3)
1252.105(PEP+HexNAc(1)_2)
M+2H
1885.815
