# Supplementary material for: Characterization of Site-Specific N- and O-Glycopeptides from Recombinant Spike and ACE2 Glycoproteins Using LC-MS/MS Analysis
Source: Int J Mol Sci. 2024 Dec 20;25(24):13649. doi: 10.3390/ijms252413649 (PMC11678118; doi:10.3390/ijms252413649)
Supplement: Supplementary file 1 [file ijms-25-13649-s001.zip › Supplementary Figure S11(ACE2_T_N-glycopep_1).pdf]

CDISNSTEAGQK(=PEP)_3_3_1_0_0, 0_None, 0_None,
m/z:1276.01(3+), RT:23.50, hcd-score:73.13

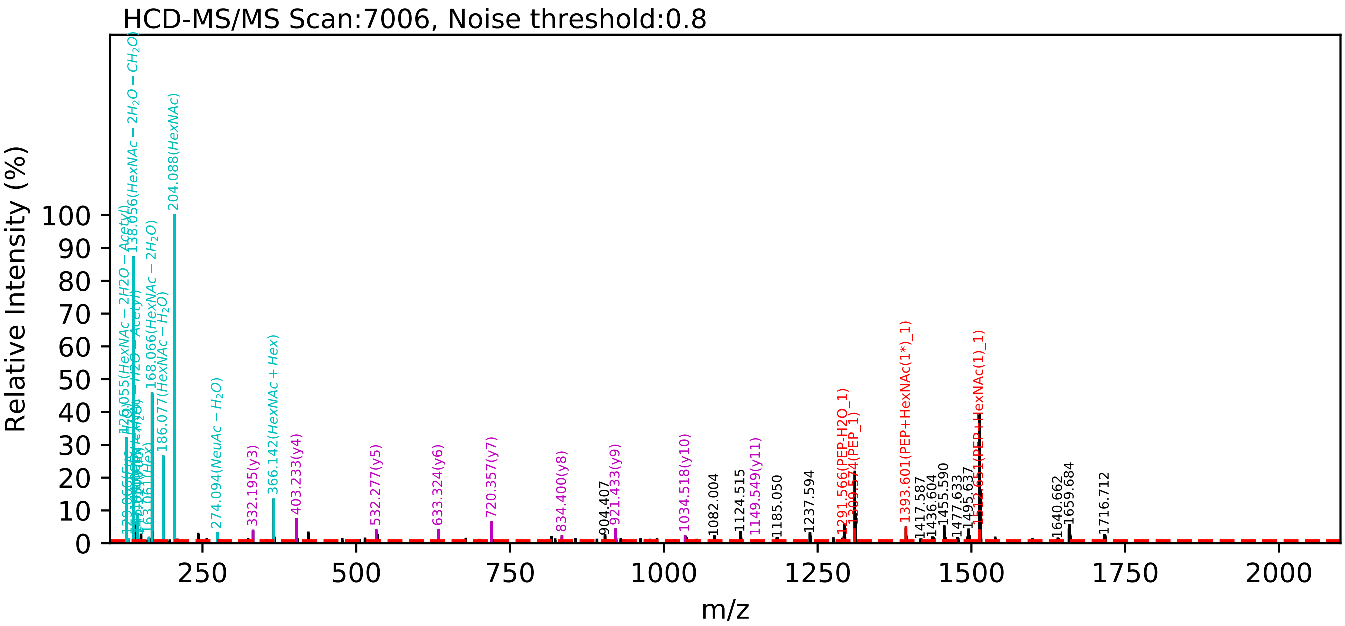

CDISNSTEAGQK(=PEP)_3_3_1_0_0, 0_None, 0_None,
m/z:1276.01(3+), RT:23.50, hcd-score:73.13

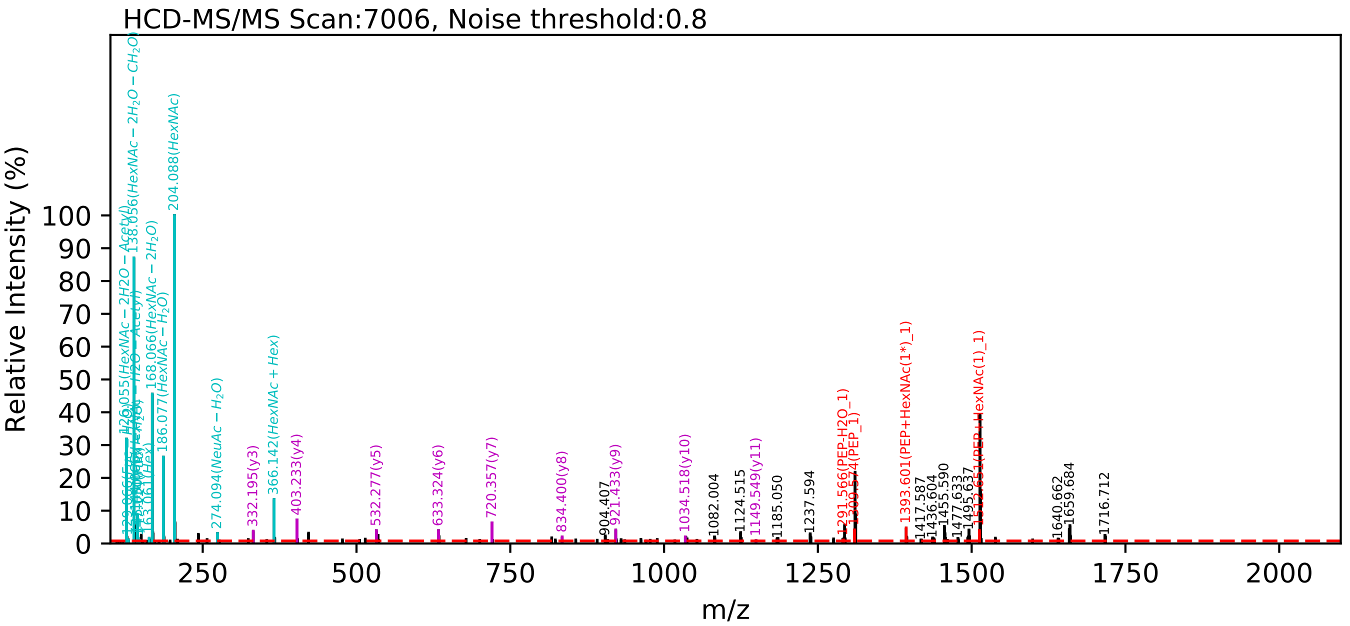

LQLQALQQNGSSVLSEDK(=PEP)_5_4_1_1_0, 0_None, 0_None,
m/z:1005.19(4+), RT:78.30, hcd-score:71.21

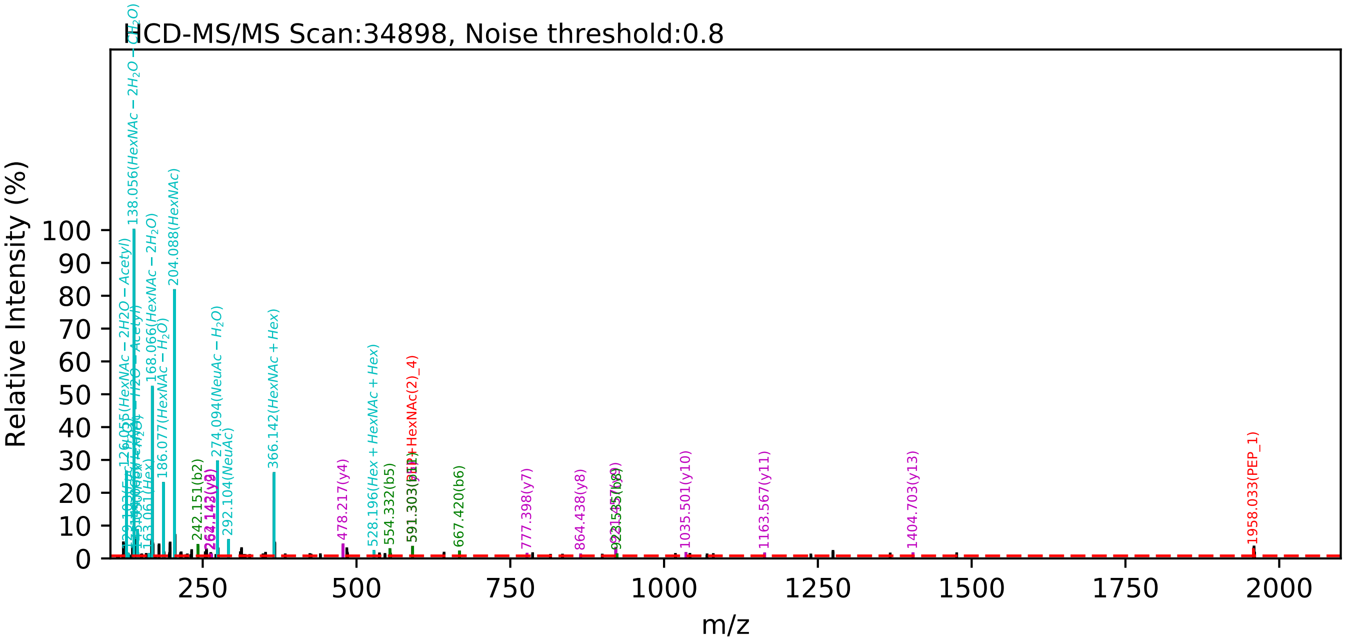

LQLQALQQNGSSVLSEDK(=PEP)_5_4_1_1_0, 0_None, 0_None,
m/z:1005.19(4+), RT:78.30, hcd-score:71.21

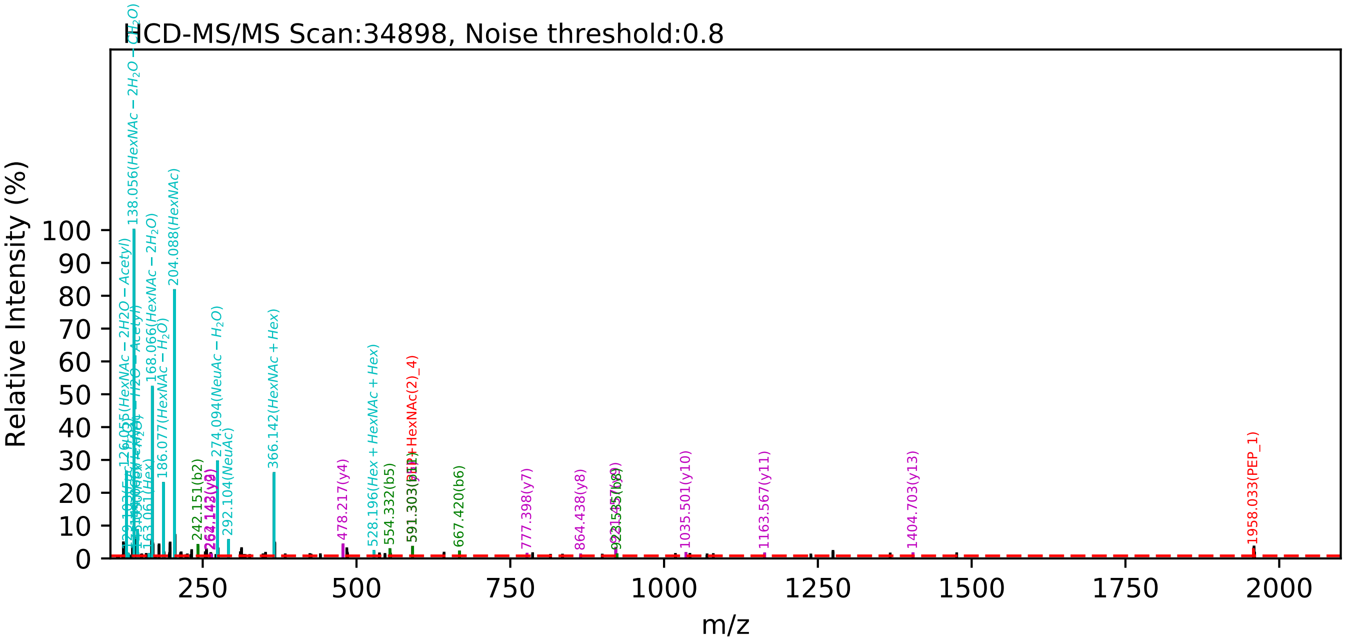

LQLQALQQNGSSVLSEDK(=PEP)_7_2_0_0_0, 0_None, 1_Hex_Phosphorylation,
m/z:1193.51(4+), RT:78.97, hcd-score:84.11

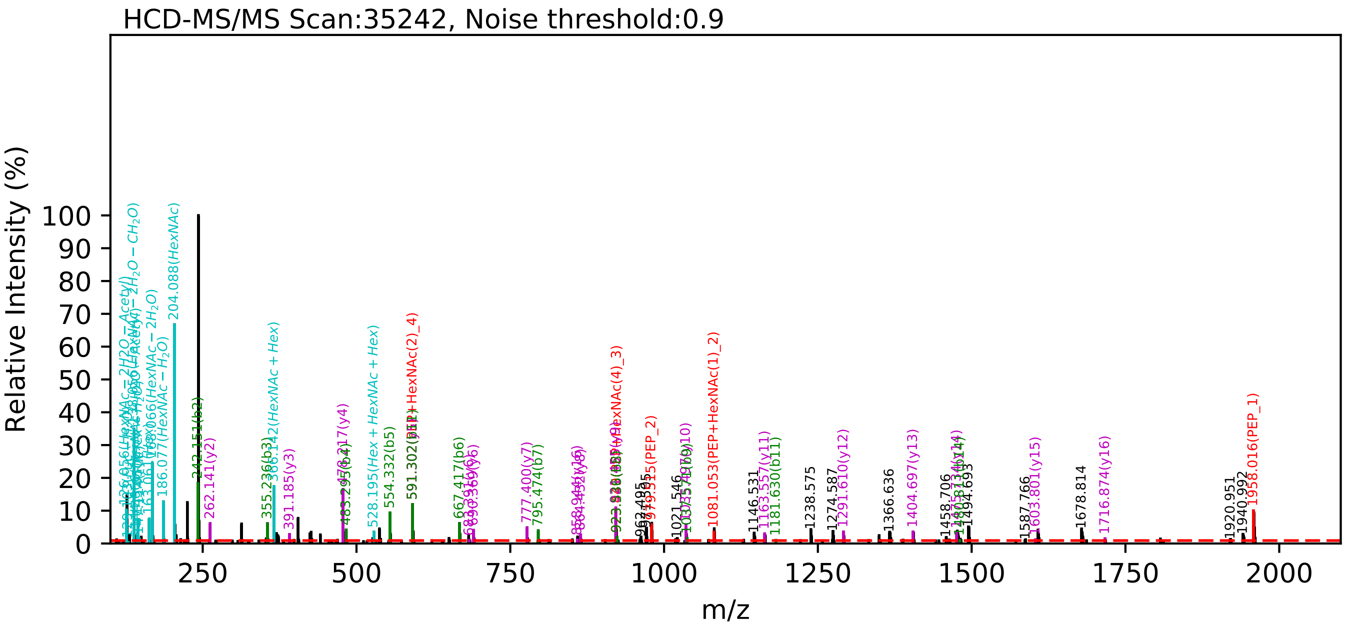

LQLQALQQNGSSVLSEDK(=PEP)_7_2_0_0_0, 0_None, 1_Hex_Phosphorylation,
m/z:1193.51(4+), RT:78.97, hcd-score:84.11

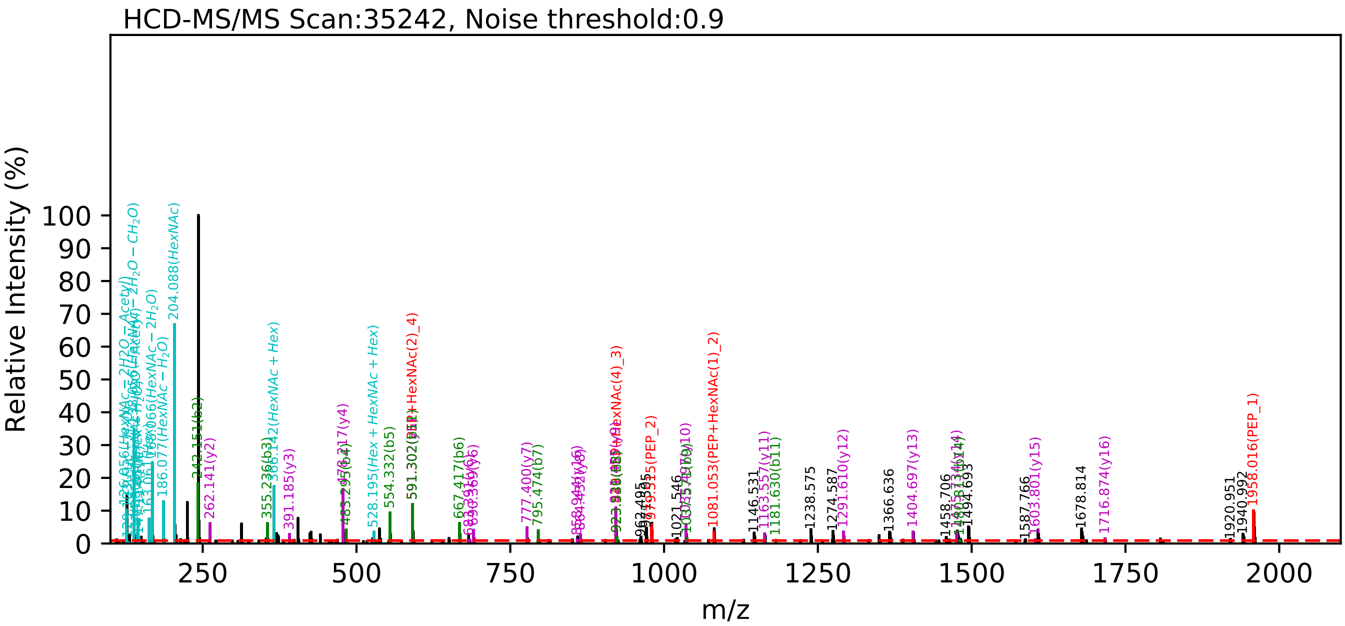

LQLQALQQNGSSVLSEDK(=PEP)_5_4_1_2_0, 0_None, 0_None,
m/z:1077.97(6+), RT:91.13, hcd-score:65.78

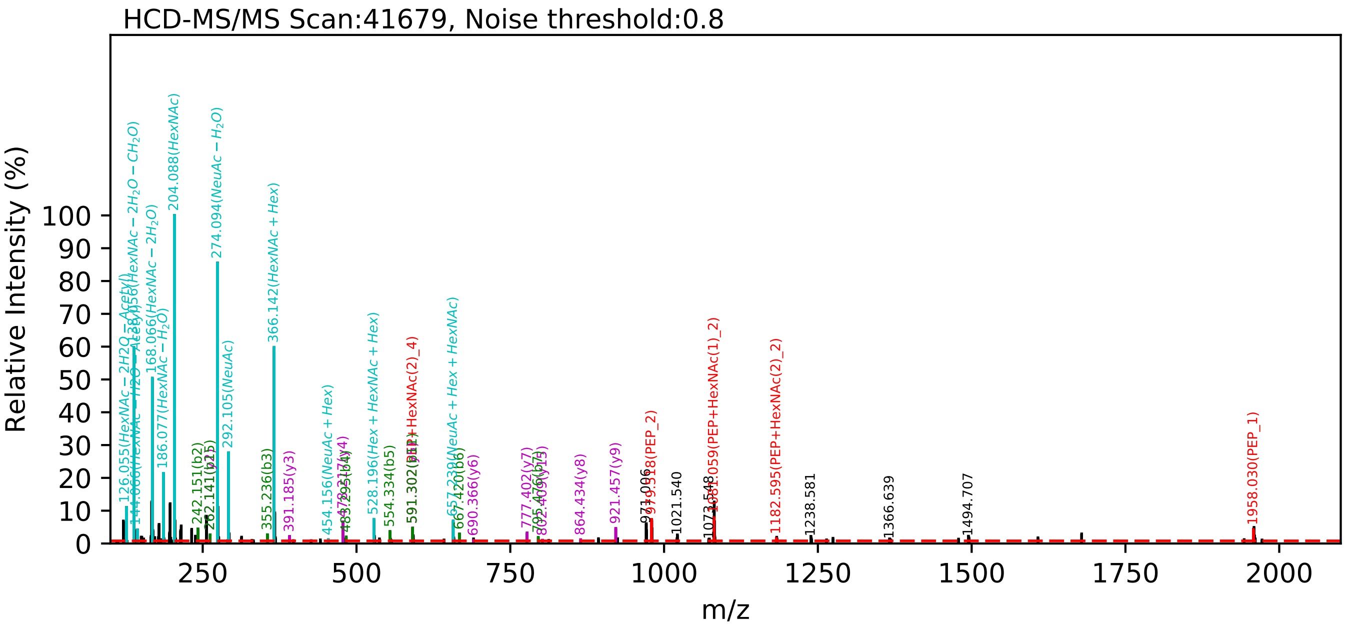

LQLQALQQNGSSVLSEDK(=PEP)_5_4_1_2_0, 0_None, 0_None,
m/z:1077.97(6+), RT:91.13, hcd-score:65.78

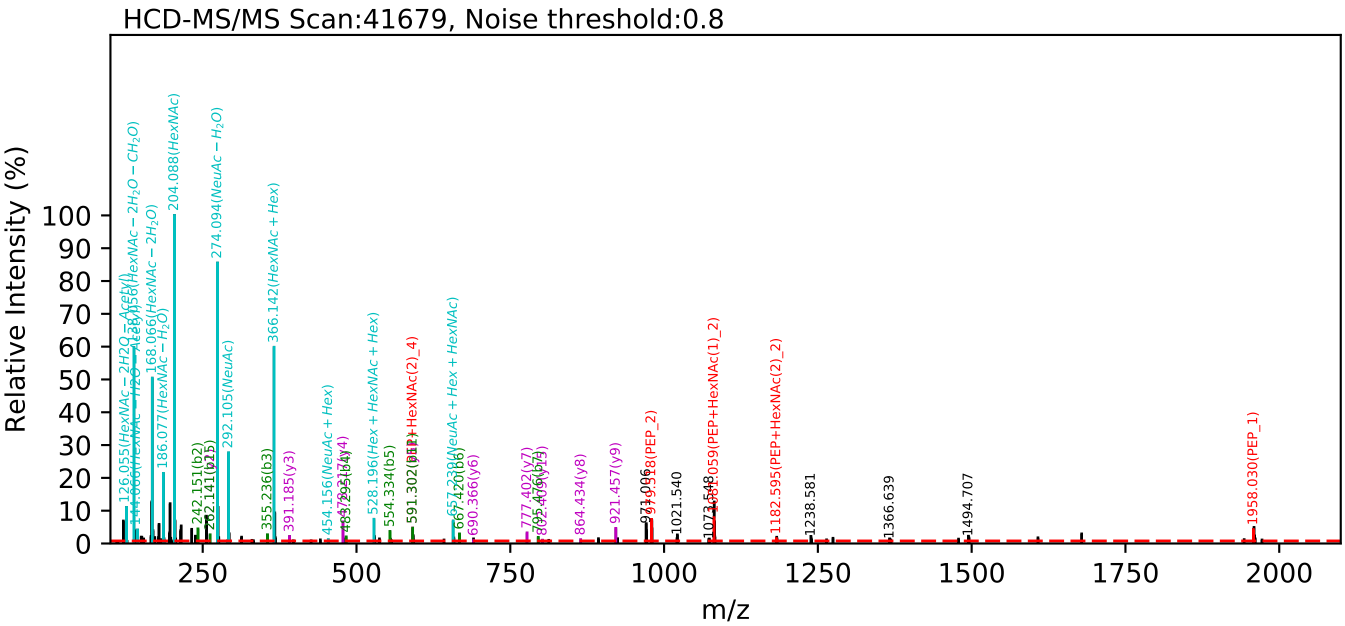

LQLQALQQNGSSVLSEDK(=PEP)_5_4_1_2_0, 0_None, 0_None,
m/z:1077.97(6+), RT:91.92, hcd-score:74.09

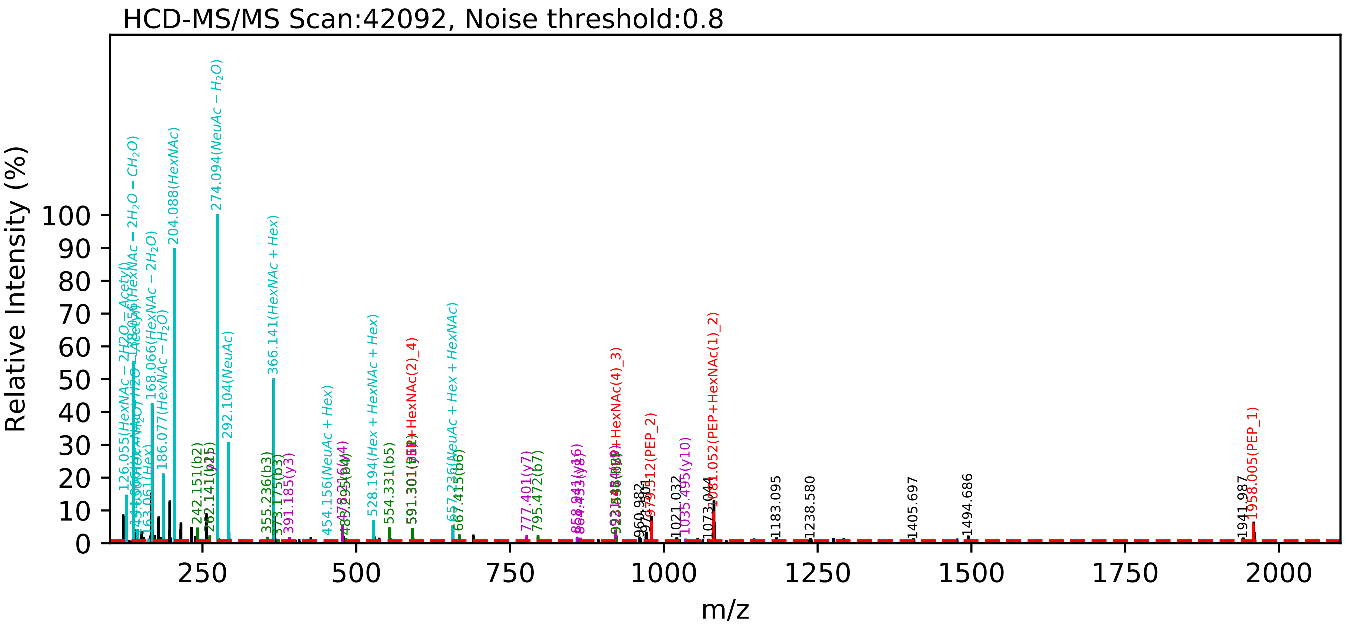

LQLQALQQNGSSVLSEDK(=PEP)_5_4_1_2_0, 0_None, 0_None,
m/z:1077.97(6+), RT:91.92, hcd-score:74.09

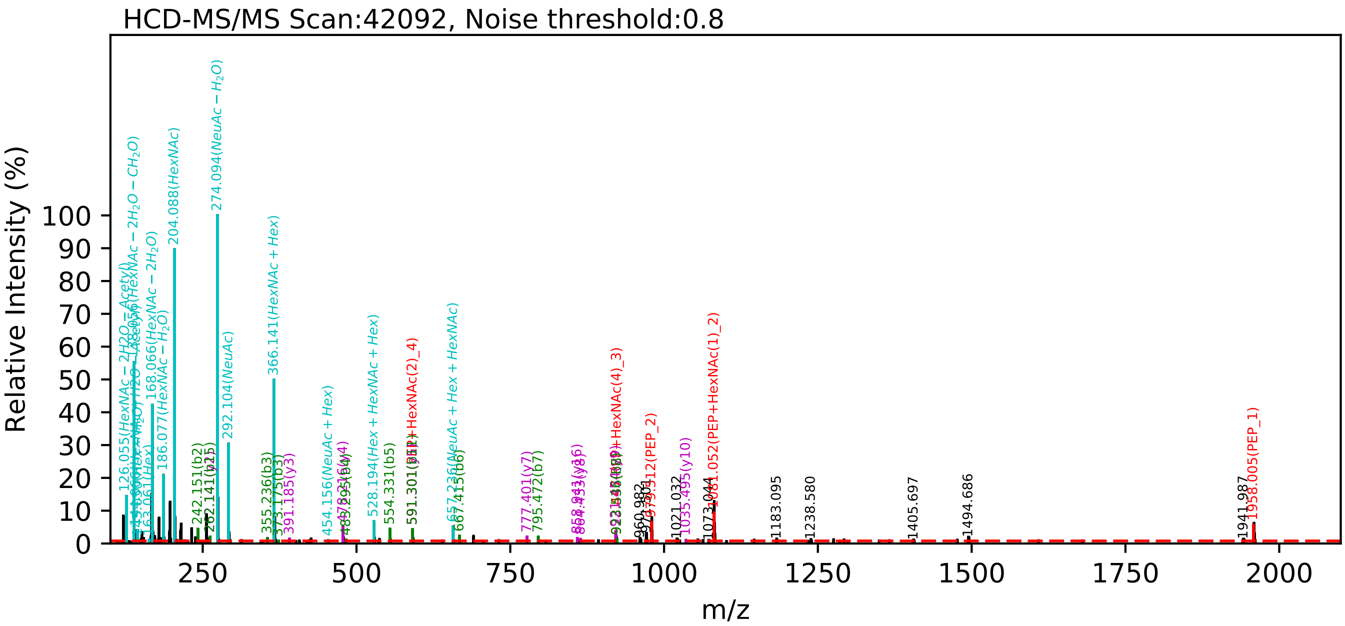

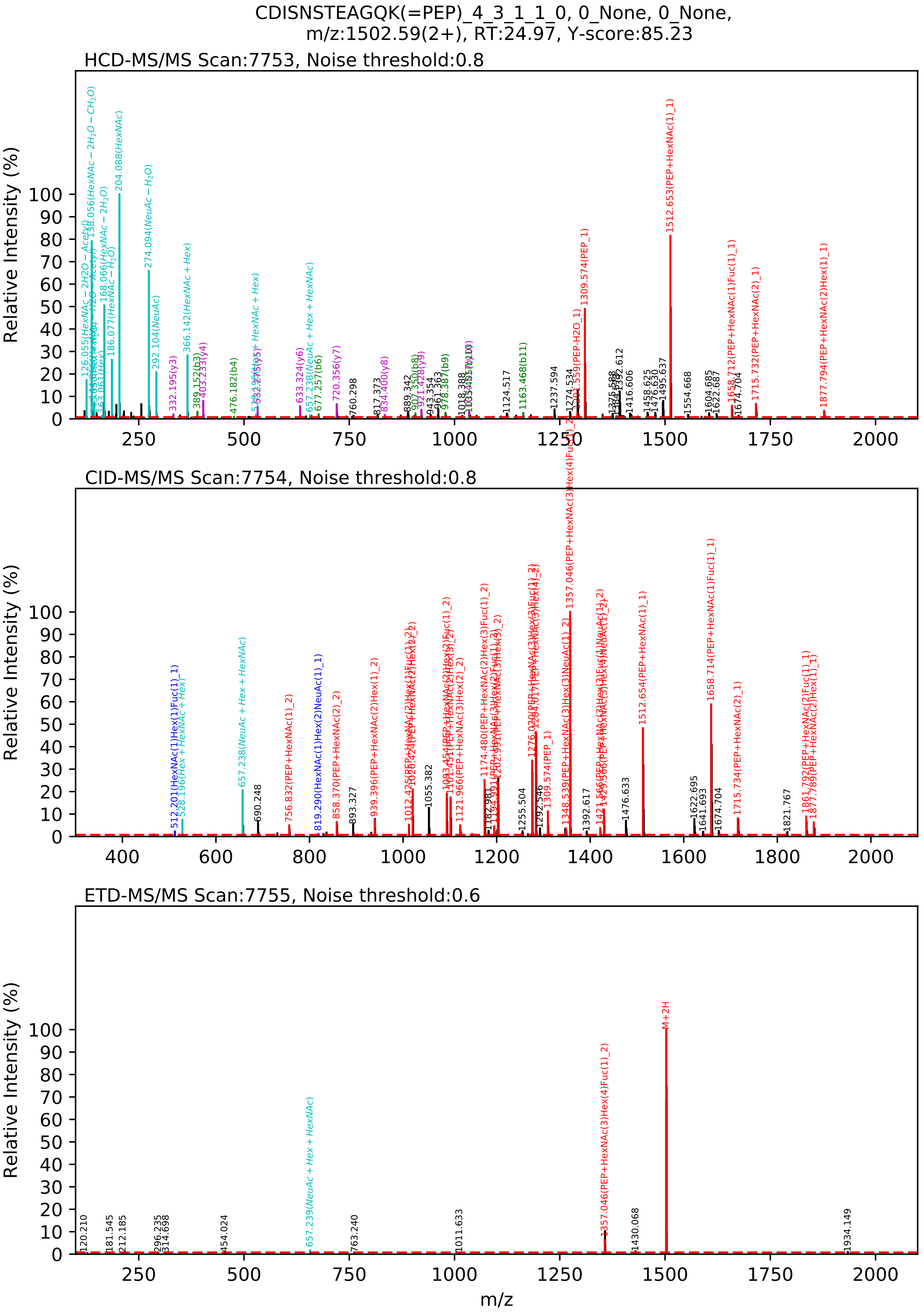

CDISNSTEAGQK(=PEP)_4_3_1_1_0, 0_None, 0_None,
m/z:1502.59(2+), RT:24.97, Y-score:85.23
HCD-MS/MS Scan:7753, Noise threshold:0.8
CID-MS/MS Scan:7754, Noise threshold:0.8
ETD-MS/MS Scan:7755, Noise threshold:0.6
Relative Intensity (%)
m/z
1512.653(PEP+HexNAc(1)_1)
1309.574(PEP_1)
204.088(HexNAc)
274.094(NeuAc − H2O)
1357.046(PEP+HexNAc(3)Hex(4)Fuc(1)_2)
1658.714(PEP+HexNAc(1)Fuc(1)_1)
1512.654(PEP+HexNAc(1)_1)
657.238(NeuAc + Hex + HexNAc)
M+2H

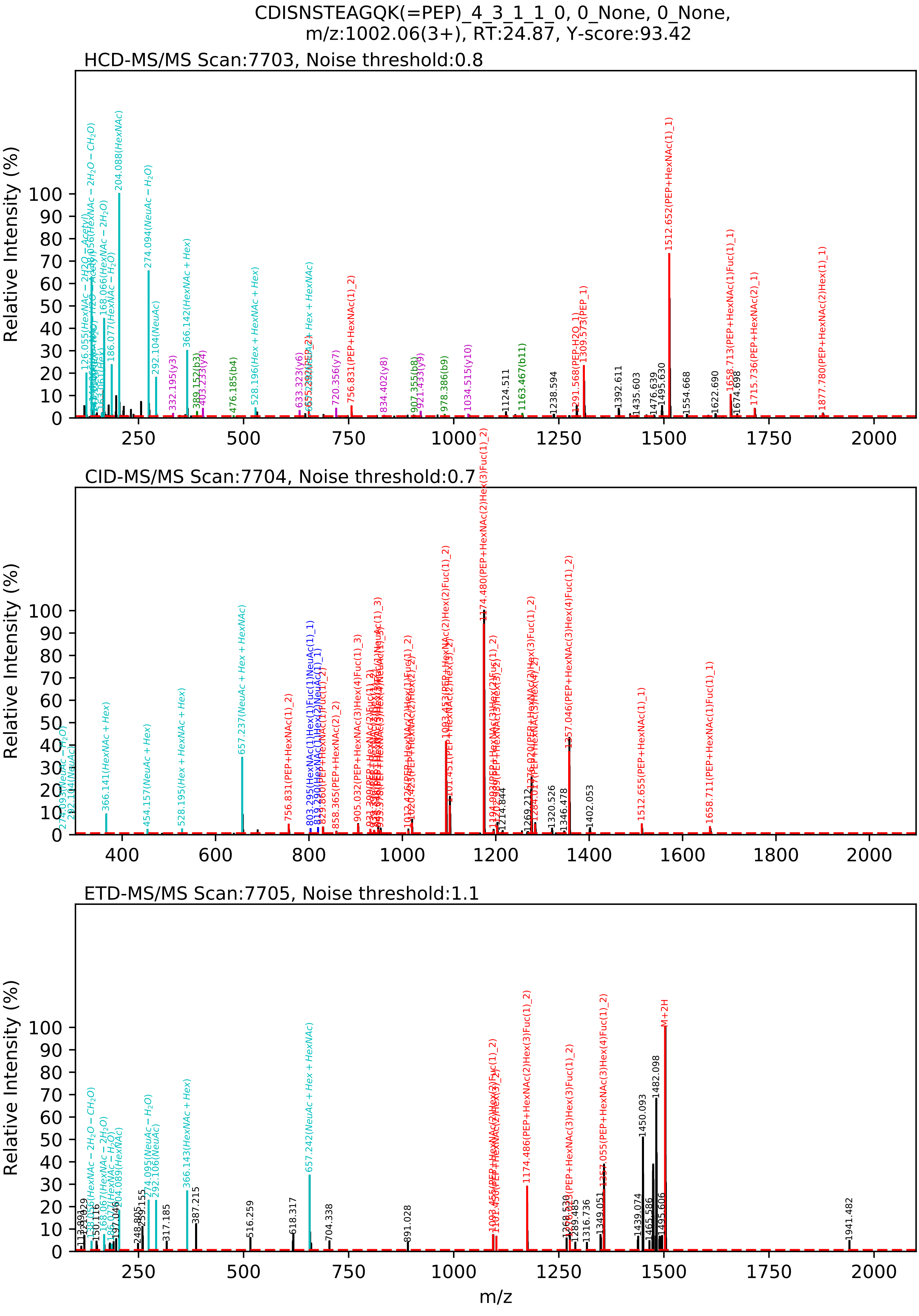

CDISNSTEAGQK(=PEP)_4_3_1_1_0, 0_None, 0_None,
m/z:1002.06(3+), RT:24.87, Y-score:93.42
HCD-MS/MS Scan:7703, Noise threshold:0.8
Relative Intensity (%)
204.088(HexNAc)
274.094(NeuAc − H2O)
292.104(NeuAc)
366.142(HexNAc + Hex)
528.196(Hex + HexNAc + Hex)
756.831(PEP+HexNAc(1)_2)
1309.573(PEP_1)
1512.652(PEP+HexNAc(1)_1)
1658.713(PEP+HexNAc(1)Fuc(1)_1)
1715.736(PEP+HexNAc(2)_1)
1877.780(PEP+HexNAc(2)Hex(1)_1)
CID-MS/MS Scan:7704, Noise threshold:0.7
Relative Intensity (%)
366.141(HexNAc + Hex)
454.157(NeuAc + Hex)
528.195(Hex + HexNAc + Hex)
657.237(NeuAc + Hex + HexNAc)
756.831(PEP+HexNAc(1)_2)
1174.480(PEP+HexNAc(2)Hex(3)Fuc(1)_2)
1357.046(PEP+HexNAc(3)Hex(4)Fuc(1)_2)
1512.655(PEP+HexNAc(1)_1)
1658.711(PEP+HexNAc(1)Fuc(1)_1)
ETD-MS/MS Scan:7705, Noise threshold:1.1
Relative Intensity (%)
366.143(HexNAc + Hex)
657.242(NeuAc + Hex + HexNAc)
1174.486(PEP+HexNAc(2)Hex(3)Fuc(1)_2)
1450.093
1482.098
M+2H
1941.482
m/z

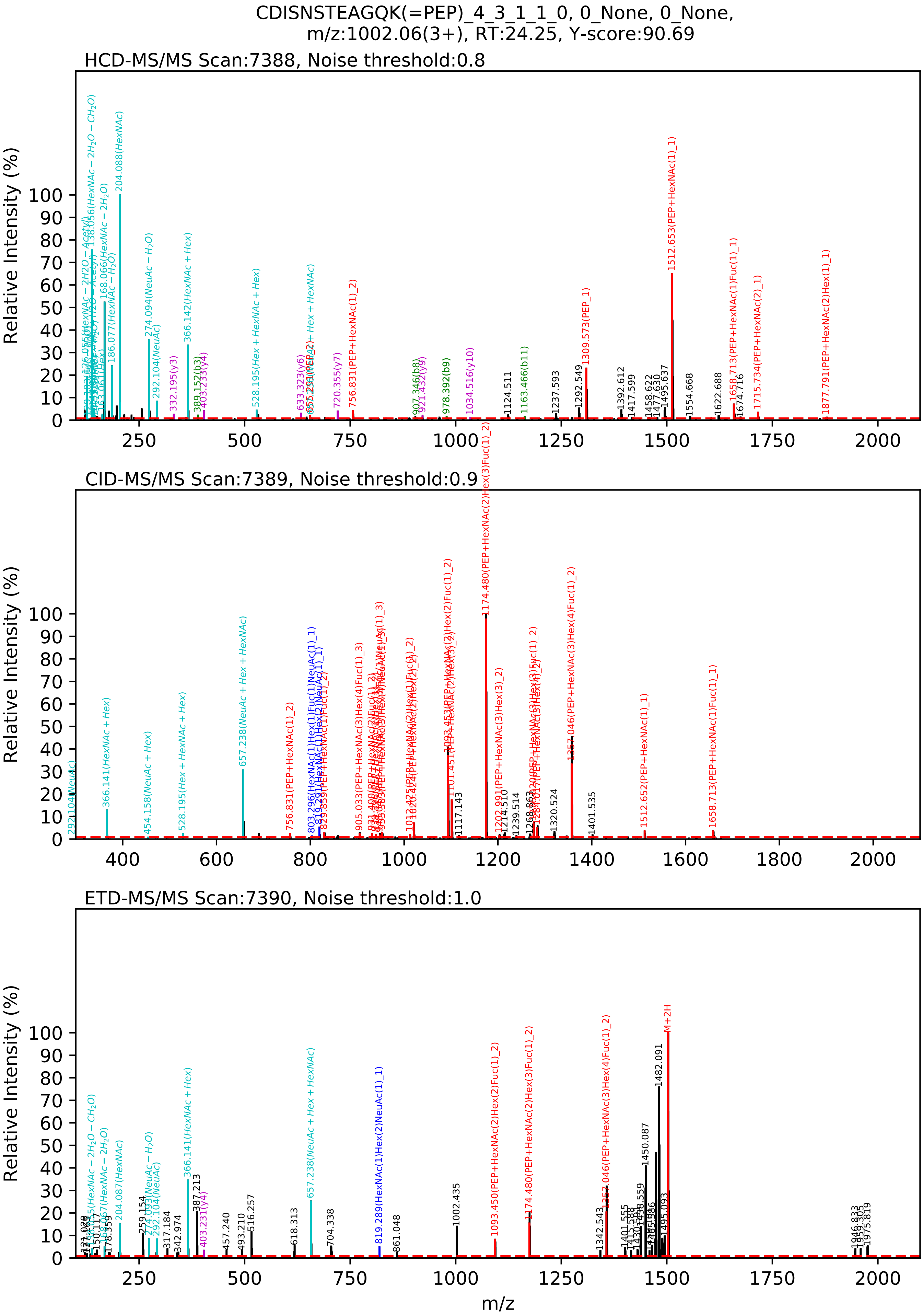
CDISNSTEAGQK(=PEP)_4_3_1_1_0, 0_None, 0_None,
m/z:1002.06(3+), RT:24.25, Y-score:90.69
HCD-MS/MS Scan:7388, Noise threshold:0.8
CID-MS/MS Scan:7389, Noise threshold:0.9
ETD-MS/MS Scan:7390, Noise threshold:1.0
Relative Intensity (%)
m/z
204.088(HexNAc)
1512.653(PEP+HexNAc(1)_1)
1174.480(PEP+HexNAc(2)Hex(3)Fuc(1)_2)
1357.046(PEP+HexNAc(3)Hex(4)Fuc(1)_2)
M+2H
1482.091
1450.087

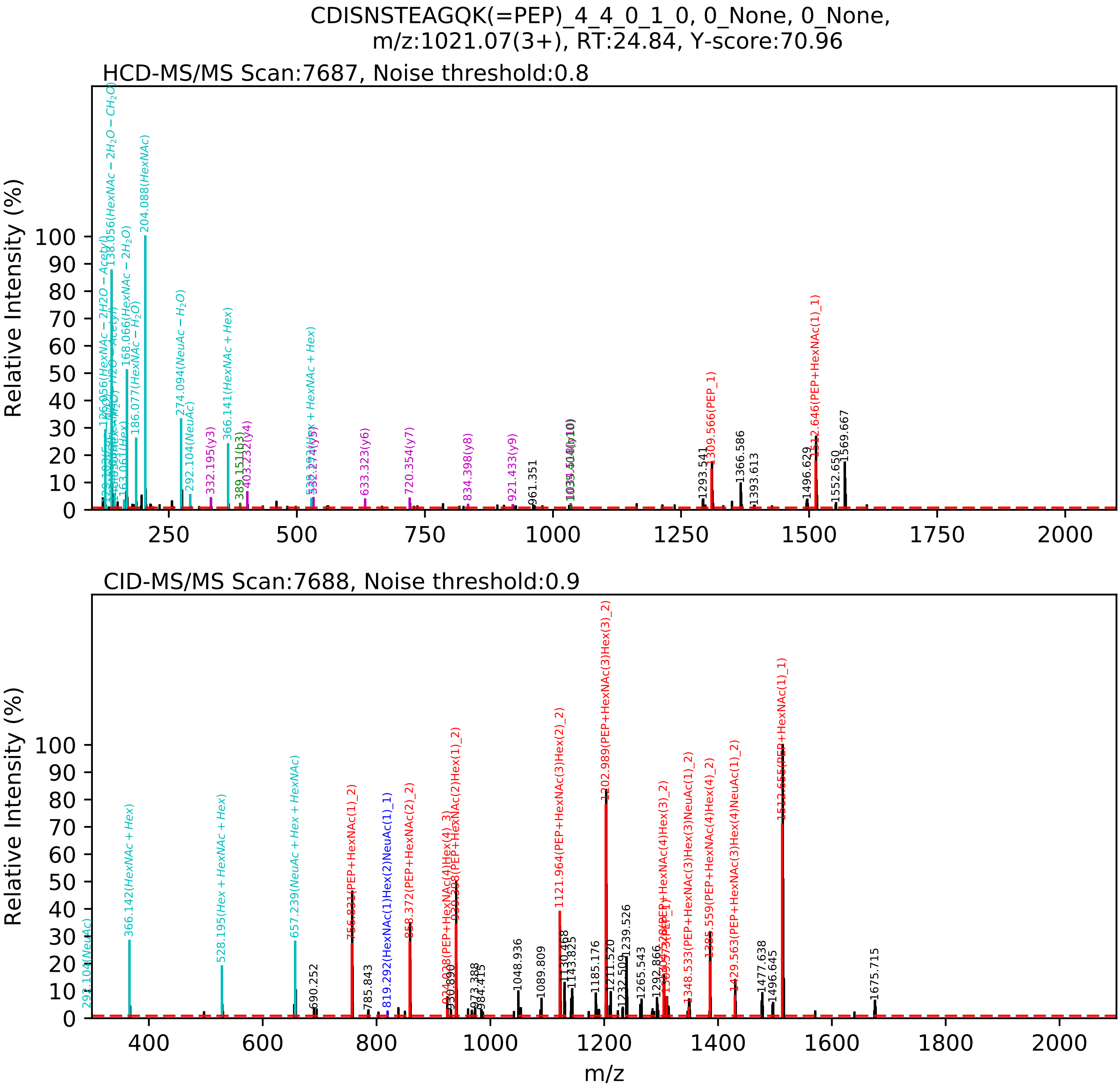

CDISNSTEAGQK(=PEP)_4_4_0_1_0, 0_None, 0_None,
m/z:1021.07(3+), RT:24.84, Y-score:70.96
HCD-MS/MS Scan:7687, Noise threshold:0.8
Relative Intensity (%)
204.088(HexNAc)
274.094(NeuAc − H2O)
292.104(NeuAc)
366.141(HexNAc + Hex)
332.195(y3)
389.151(b3)
403.232(y4)
633.323(y6)
720.354(y7)
834.398(y8)
921.433(y9)
961.351
1293.541
1309.566(PEP_1)
1366.586
1393.613
1496.629
1512.646(PEP+HexNAc(1)_1)
1552.650
1569.667
CID-MS/MS Scan:7688, Noise threshold:0.9
366.142(HexNAc + Hex)
528.195(Hex + HexNAc + Hex)
657.239(NeuAc + Hex + HexNAc)
690.252
785.843
819.292(HexNAc(1)Hex(2)NeuAc(1)_1)
858.372(PEP+HexNAc(2)_2)
1048.936
1089.809
1121.964(PEP+HexNAc(3)Hex(2)_2)
1130.468
1143.825
1185.176
1202.989(PEP+HexNAc(3)Hex(3)_2)
1211.520
1232.509
1239.526
1265.543
1292.866
1348.533(PEP+HexNAc(3)Hex(3)NeuAc(1)_2)
1385.559(PEP+HexNAc(4)Hex(4)_2)
1429.563(PEP+HexNAc(3)Hex(4)NeuAc(1)_2)
1477.638
1496.645
1675.715
m/z

CDISNSTEAGQK(=PEP)_4_4_1_1_0, 0_None, 0_None,
m/z:1069.76(3+), RT:24.20, Y-score:85.61

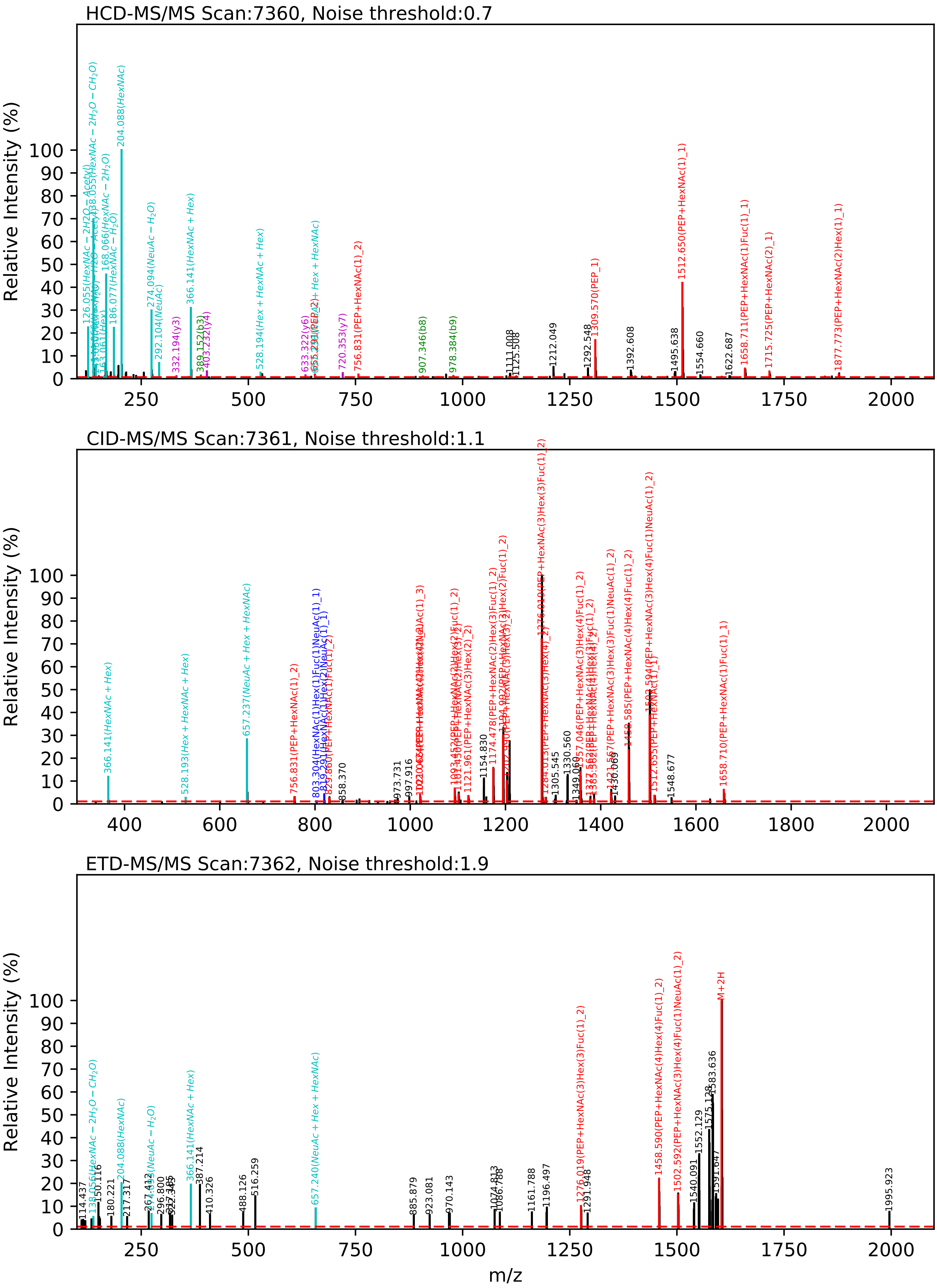

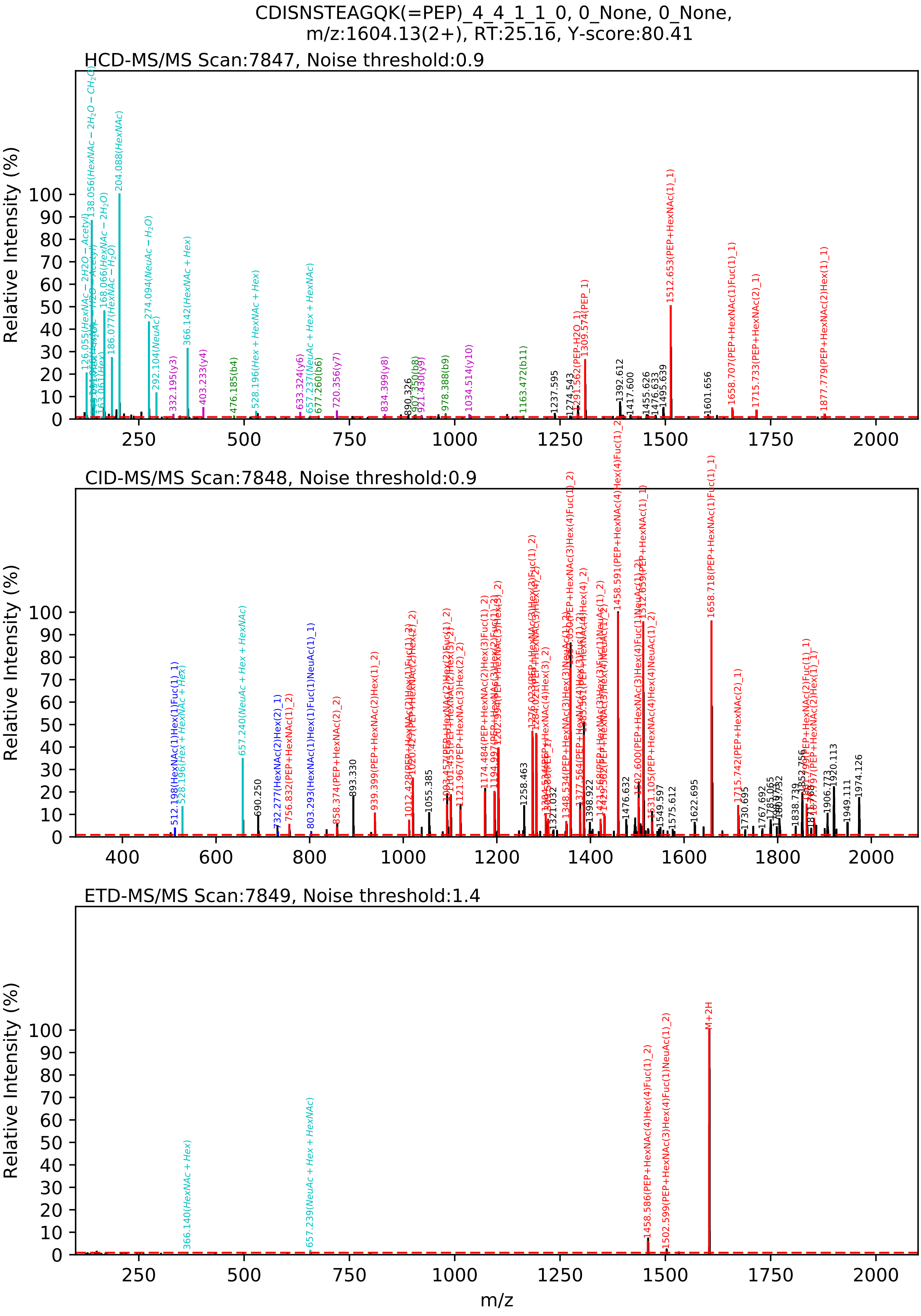
CDISNSTEAGQK(=PEP)_4_4_1_1_0, 0_None, 0_None,
m/z:1604.13(2+), RT:25.16, Y-score:80.41
HCD-MS/MS Scan:7847, Noise threshold:0.9
Relative Intensity (%)
204.088(HexNAc)
274.094(NeuAc − H2O)
292.104(NeuAc)
366.142(HexNAc + Hex)
332.195(y3)
403.233(y4)
476.185(b4)
528.196(Hex + HexNAc + Hex)
633.324(y6)
657.237(NeuAc + Hex + HexNAc)
677.260(b6)
720.356(y7)
834.399(y8)
890.326
907.350(b8)
921.430(y9)
978.388(b9)
1034.514(y10)
1163.472(b11)
1237.595
1274.543
1291.562(PEP-H2O_1)
1309.574(PEP_1)
1392.612
1417.600
1455.626
1476.633
1495.639
1512.653(PEP+HexNAc(1)_1)
1601.656
1658.707(PEP+HexNAc(1)Fuc(1)_1)
1715.733(PEP+HexNAc(2)_1)
1877.779(PEP+HexNAc(2)Hex(1)_1)
CID-MS/MS Scan:7848, Noise threshold:0.9
Relative Intensity (%)
512.198(HexNAc(1)Hex(1)Fuc(1)_1)
657.240(NeuAc + Hex + HexNAc)
690.250
732.277(HexNAc(2)Hex(2)_1)
756.832(PEP+HexNAc(1)_2)
803.293(HexNAc(1)Hex(1)Fuc(1)NeuAc(1)_1)
858.374(PEP+HexNAc(2)_2)
893.330
939.399(PEP+HexNAc(2)Hex(1)_2)
1055.385
1121.967(PEP+HexNAc(3)Hex(2)_2)
1174.484(PEP+HexNAc(2)Hex(3)Fuc(1)_2)
1258.463
1321.032
1348.534(PEP+HexNAc(3)Hex(3)NeuAc(1)_2)
1398.922
1458.591(PEP+HexNAc(4)Hex(4)Fuc(1)_2)
1476.632
1549.597
1575.612
1622.695
1658.718(PEP+HexNAc(1)Fuc(1)_1)
1715.742(PEP+HexNAc(2)_1)
1730.695
1767.692
1838.739
1906.773
1920.113
1949.111
1974.126
ETD-MS/MS Scan:7849, Noise threshold:1.4
Relative Intensity (%)
366.140(HexNAc + Hex)
657.239(NeuAc + Hex + HexNAc)
1458.586(PEP+HexNAc(4)Hex(4)Fuc(1)_2)
1502.599(PEP+HexNAc(3)Hex(4)Fuc(1)NeuAc(1)_2)
M+2H
m/z

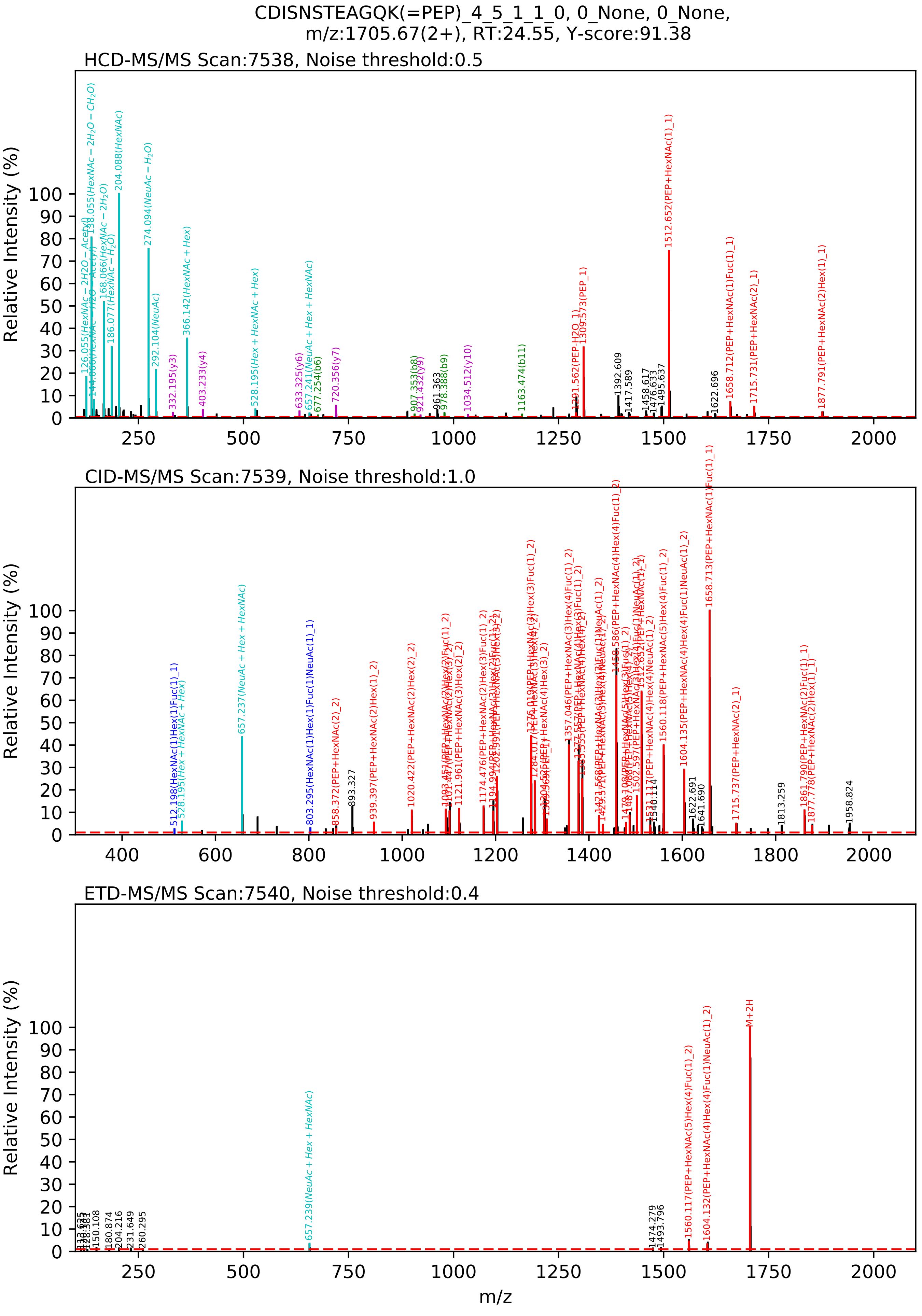

CDISNSTEAGQK(=PEP)_4_5_1_1_0, 0_None, 0_None,
m/z:1705.67(2+), RT:24.55, Y-score:91.38
HCD-MS/MS Scan:7538, Noise threshold:0.5
CID-MS/MS Scan:7539, Noise threshold:1.0
ETD-MS/MS Scan:7540, Noise threshold:0.4
Relative Intensity (%)
m/z
204.088(HexNAc)
274.094(NeuAc − H2O)
366.142(HexNAc + Hex)
1512.652(PEP+HexNAc(1)_1)
1309.573(PEP_1)
1658.712(PEP+HexNAc(1)Fuc(1)_1)
1715.731(PEP+HexNAc(2)_1)
1877.791(PEP+HexNAc(2)Hex(1)_1)
657.237(NeuAc + Hex + HexNAc)
1658.713(PEP+HexNAc(1)Fuc(1)_1)
1604.135(PEP+HexNAc(4)Hex(4)Fuc(1)NeuAc(1)_2)
1560.118(PEP+HexNAc(5)Hex(4)Fuc(1)_2)
M+2H
1560.117(PEP+HexNAc(5)Hex(4)Fuc(1)_2)
1604.132(PEP+HexNAc(4)Hex(4)Fuc(1)NeuAc(1)_2)
657.239(NeuAc + Hex + HexNAc)

CDISNSTEAGQK(=PEP)_5_3_1_1_0, 0_None, 0_None,
m/z:1056.08(3+), RT:24.62, Y-score:92.22

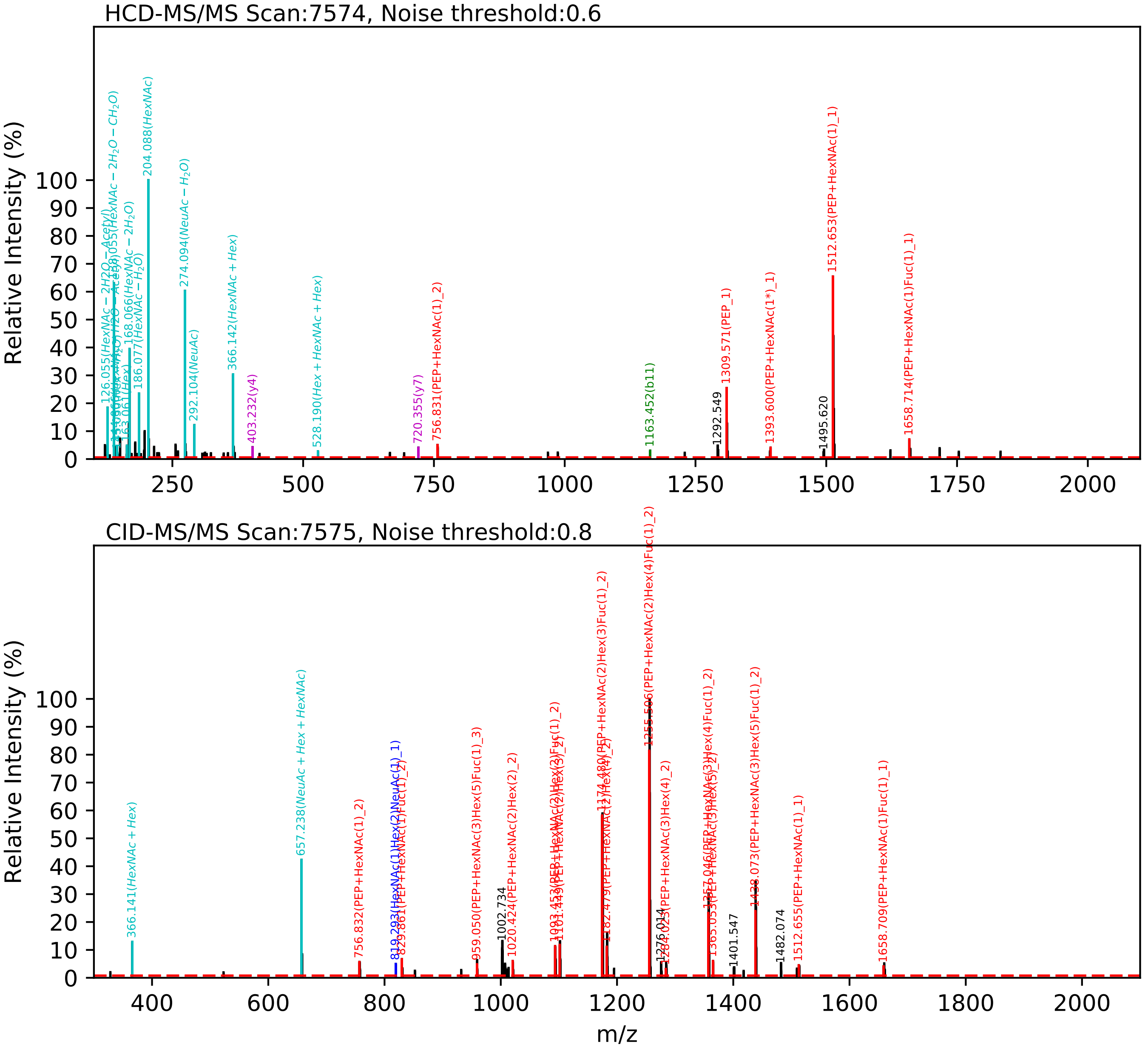

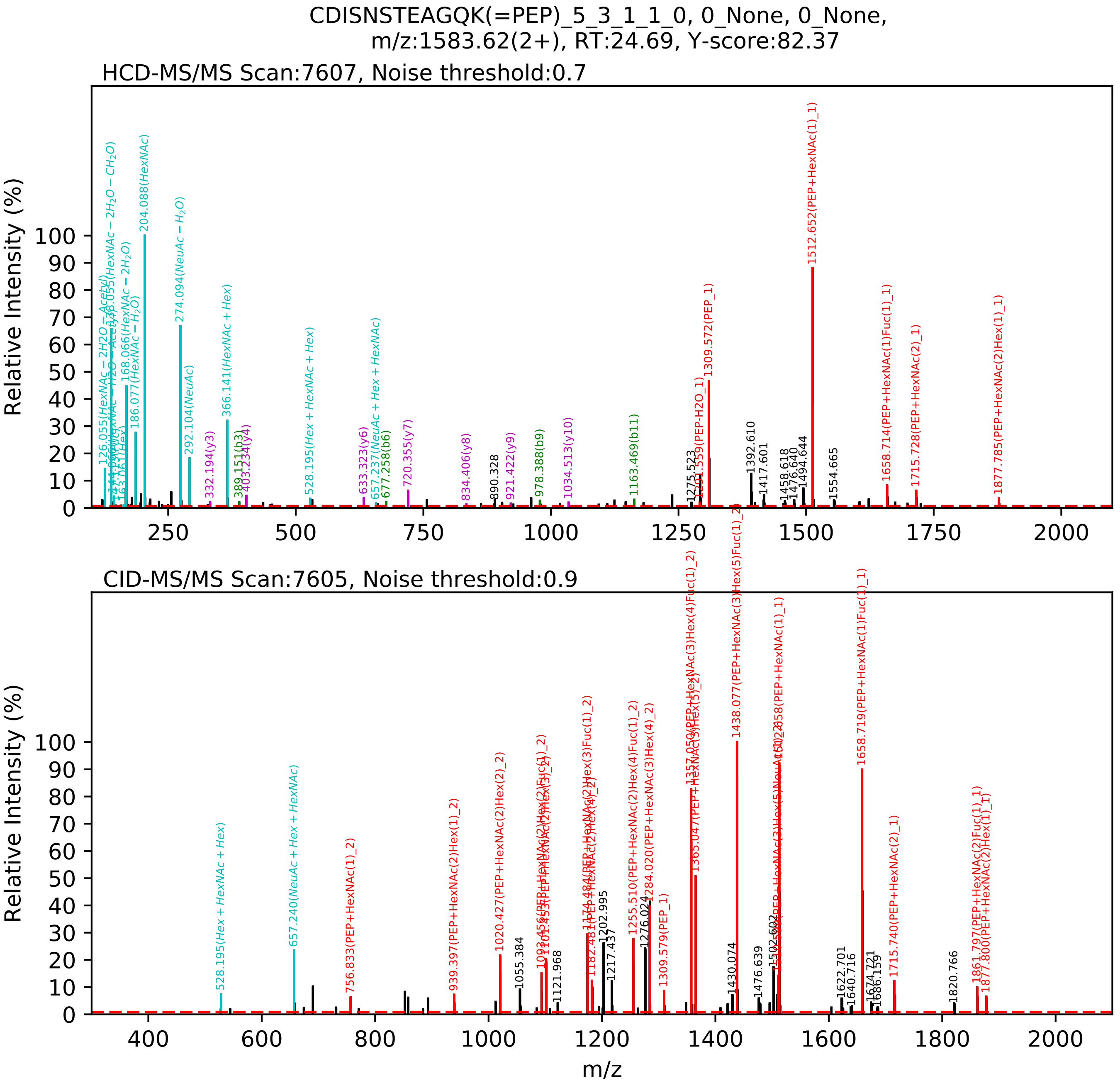

CDISNSTEAGQK(=PEP)_5_3_1_1_0, 0_None, 0_None,
m/z:1583.62(2+), RT:24.69, Y-score:82.37
HCD-MS/MS Scan:7607, Noise threshold:0.7
Relative Intensity (%)
204.088(HexNAc)
274.094(NeuAc − H2O)
292.104(NeuAc)
366.141(HexNAc + Hex)
332.194(y3)
389.151(b3)
403.234(y4)
528.195(Hex + HexNAc + Hex)
633.323(y6)
657.237(NeuAc + Hex + HexNAc)
677.258(b6)
720.355(y7)
834.406(y8)
890.328
921.422(y9)
978.388(b9)
1034.513(y10)
1163.469(b11)
1275.523
1309.572(PEP_1)
1392.610
1417.601
1458.618
1476.640
1494.644
1512.652(PEP+HexNAc(1)_1)
1554.665
1658.714(PEP+HexNAc(1)Fuc(1)_1)
1715.728(PEP+HexNAc(2)_1)
1877.785(PEP+HexNAc(2)Hex(1)_1)
CID-MS/MS Scan:7605, Noise threshold:0.9
Relative Intensity (%)
528.195(Hex + HexNAc + Hex)
657.240(NeuAc + Hex + HexNAc)
756.833(PEP+HexNAc(1)_2)
939.397(PEP+HexNAc(2)Hex(1)_2)
1020.427(PEP+HexNAc(2)Hex(2)_2)
1055.384
1121.968
1202.995
1217.437
1255.510(PEP+HexNAc(2)Hex(4)Fuc(1)_2)
1276.024
1284.020(PEP+HexNAc(3)Hex(4)_2)
1309.579(PEP_1)
1365.047
1430.074
1438.077(PEP+HexNAc(3)Hex(5)Fuc(1)_2)
1476.639
1502.602
1658.719(PEP+HexNAc(1)Fuc(1)_1)
1622.701
1640.716
1674.721
1686.159
1715.740(PEP+HexNAc(2)_1)
1820.766
1861.797(PEP+HexNAc(2)Fuc(1)_1)
1877.800(PEP+HexNAc(2)Hex(1)_1)
m/z

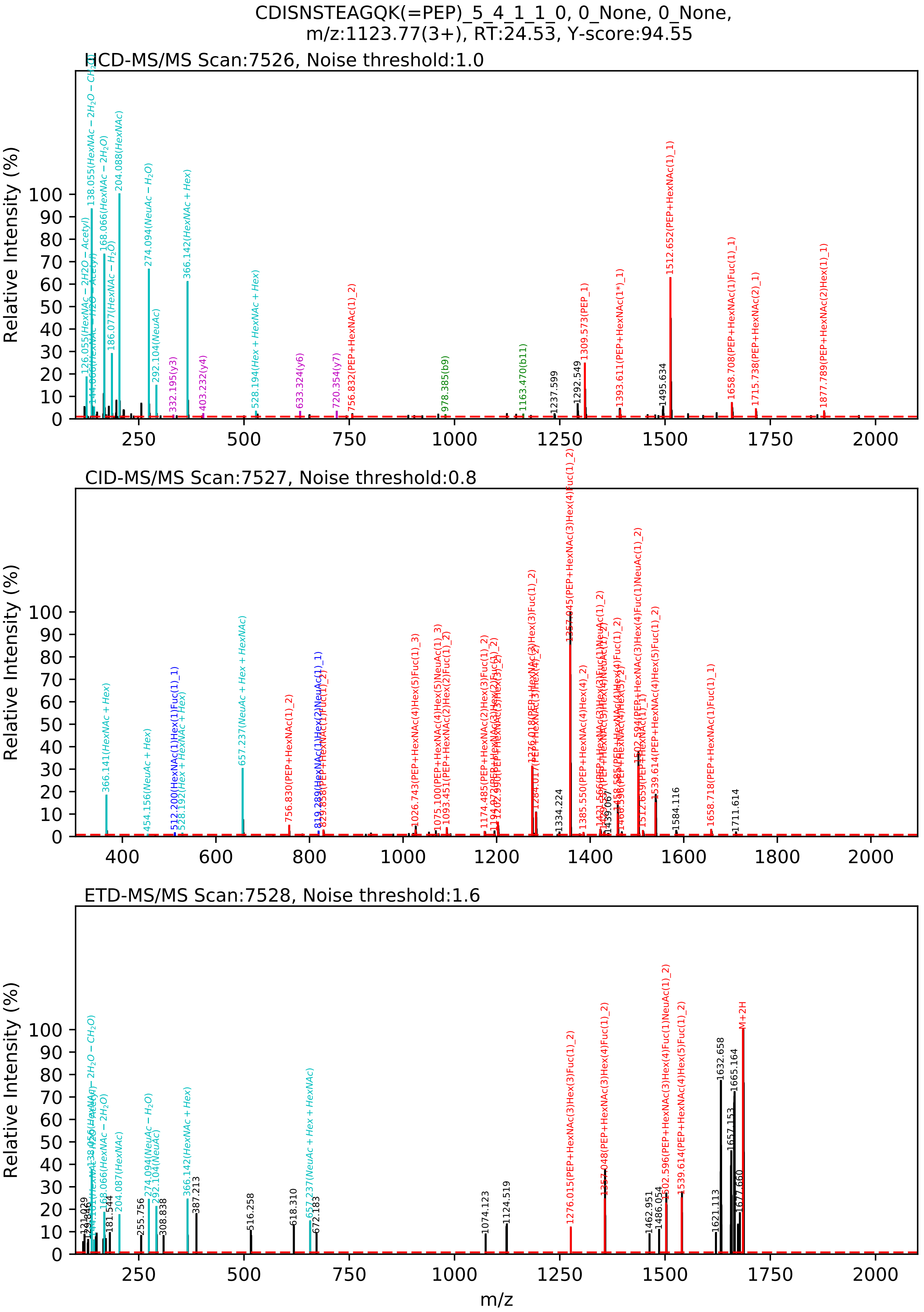

CDISNSTEAGQK(=PEP)_5_4_1_1_0, 0_None, 0_None,
m/z:1123.77(3+), RT:24.53, Y-score:94.55
HCD-MS/MS Scan:7526, Noise threshold:1.0
CID-MS/MS Scan:7527, Noise threshold:0.8
ETD-MS/MS Scan:7528, Noise threshold:1.6
Relative Intensity (%)
m/z

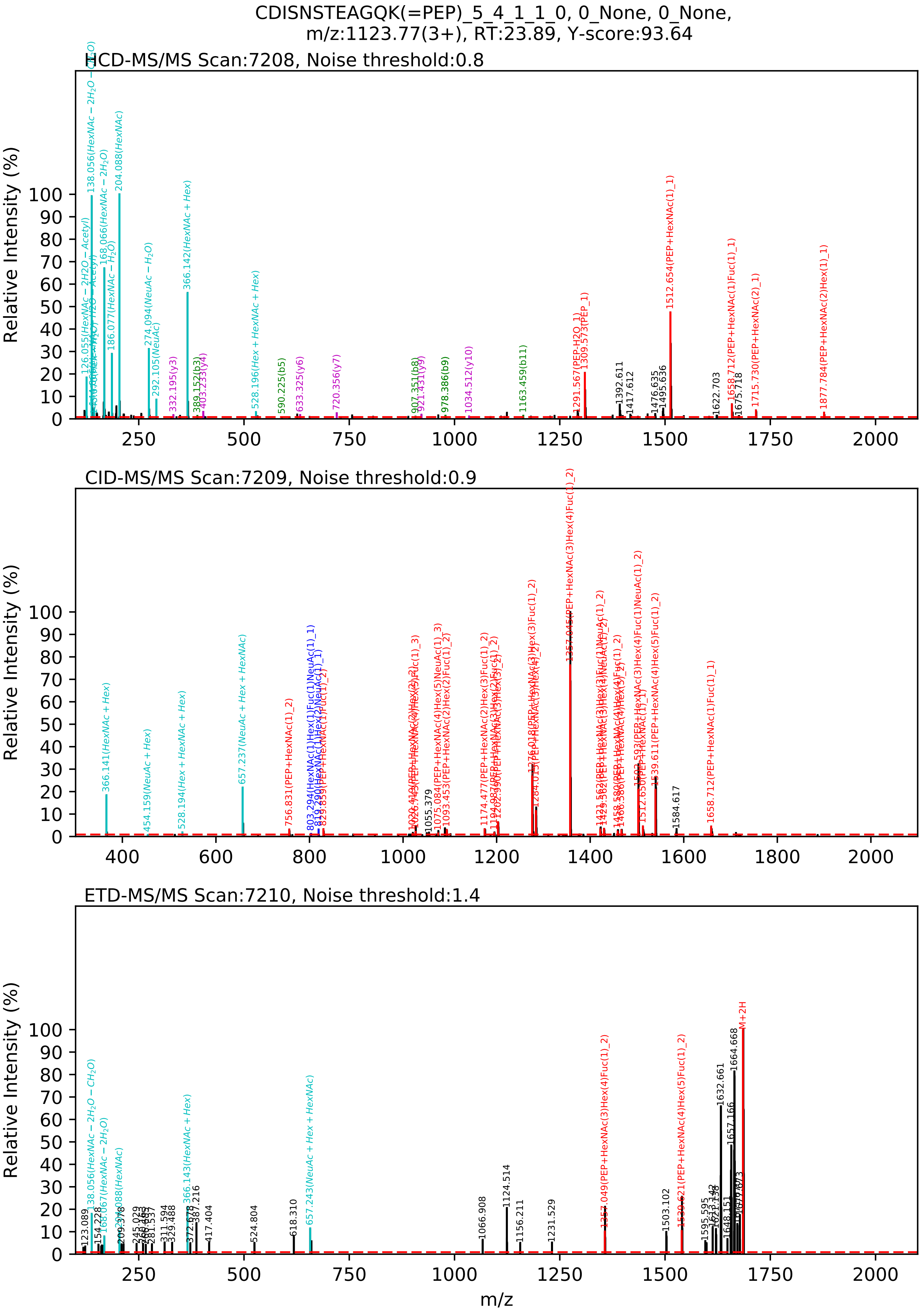
CDISNSTEAGQK(=PEP)_5_4_1_1_0, 0_None, 0_None,
m/z:1123.77(3+), RT:23.89, Y-score:93.64
HCD-MS/MS Scan:7208, Noise threshold:0.8
Relative Intensity (%)
138.056(HexNAc − 2H2O − CH2O)
204.088(HexNAc)
168.066(HexNAc − 2H2O)
186.077(HexNAc − H2O)
126.055(HexNAc − 2H2O − Acetyl)
274.094(NeuAc − H2O)
292.105(NeuAc)
366.142(HexNAc + Hex)
332.195(y3)
389.152(b3)
403.233(y4)
528.196(Hex + HexNAc + Hex)
590.225(b5)
633.325(y6)
720.356(y7)
907.351(b8)
921.431(y9)
978.386(b9)
1034.512(y10)
1163.459(b11)
1291.567(PEP-H2O_1)
1309.573(PEP_1)
1392.611
1417.612
1476.635
1495.636
1512.654(PEP+HexNAc(1)_1)
1622.703
1658.712(PEP+HexNAc(1)Fuc(1)_1)
1675.718
1715.730(PEP+HexNAc(2)_1)
1877.784(PEP+HexNAc(2)Hex(1)_1)
CID-MS/MS Scan:7209, Noise threshold:0.9
366.141(HexNAc + Hex)
454.159(NeuAc + Hex)
528.194(Hex + HexNAc + Hex)
657.237(NeuAc + Hex + HexNAc)
756.831(PEP+HexNAc(1)_2)
803.294(HexNAc(1)Hex(1)Fuc(1)NeuAc(1)_1)
1055.379
1075.084(PEP+HexNAc(4)Hex(5)NeuAc(1)_3)
1093.453(PEP+HexNAc(2)Fuc(1)_2)
1174.477(PEP+HexNAc(2)Hex(3)Fuc(1)_2)
1357.045(PEP+HexNAc(3)Hex(4)Fuc(1)_2)
1539.611(PEP+HexNAc(4)Hex(5)Fuc(1)_2)
1584.617
1658.712(PEP+HexNAc(1)Fuc(1)_1)
ETD-MS/MS Scan:7210, Noise threshold:1.4
123.089
138.056(HexNAc − 2H2O − CH2O)
154.228
168.067(HexNAc − 2H2O)
204.088(HexNAc)
209.378
245.029
281.537
311.594
329.488
366.143(HexNAc + Hex)
372.678
387.216
417.404
524.804
618.310
657.243(NeuAc + Hex + HexNAc)
1066.908
1124.514
1156.211
1231.529
1357.049(PEP+HexNAc(3)Hex(4)Fuc(1)_2)
1503.102
1539.621(PEP+HexNAc(4)Hex(5)Fuc(1)_2)
1595.595
1632.661
1648.151
1657.166
1664.668
M+2H
m/z

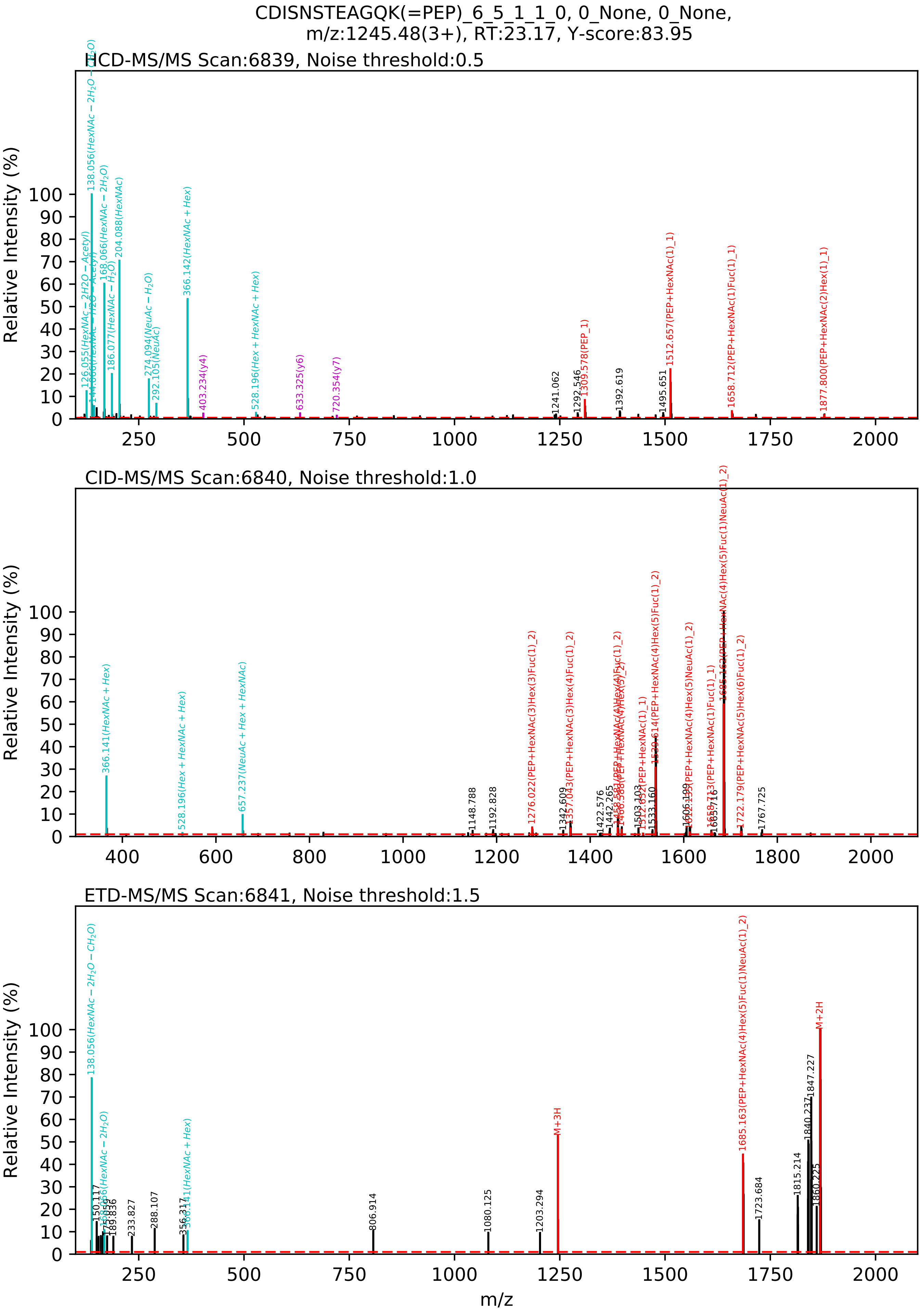

CDISNSTEAGQK(=PEP)_6_5_1_1_0, 0_None, 0_None,
m/z:1245.48(3+), RT:23.17, Y-score:83.95
HCD-MS/MS Scan:6839, Noise threshold:0.5
CID-MS/MS Scan:6840, Noise threshold:1.0
ETD-MS/MS Scan:6841, Noise threshold:1.5
Relative Intensity (%)
m/z
138.056(HexNAc − 2H2O − CH2O)
168.066(HexNAc − 2H2O)
204.088(HexNAc)
186.077(HexNAc − H2O)
274.094(NeuAc − H2O)
292.105(NeuAc)
366.142(HexNAc + Hex)
528.196(Hex + HexNAc + Hex)
403.234(y4)
633.325(y6)
720.354(y7)
1241.062
1292.546
1309.578(PEP_1)
1392.619
1495.651
1512.657(PEP+HexNAc(1)_1)
1658.712(PEP+HexNAc(1)Fuc(1)_1)
1877.800(PEP+HexNAc(2)Hex(1)_1)
366.141(HexNAc + Hex)
528.196(Hex + HexNAc + Hex)
657.237(NeuAc + Hex + HexNAc)
1148.788
1192.828
1276.022(PEP+HexNAc(3)Hex(3)Fuc(1)_2)
1342.609
1357.043(PEP+HexNAc(3)Hex(4)Fuc(1)_2)
1422.576
1442.265
1503.103
1533.160
1539.614(PEP+HexNAc(4)Hex(5)Fuc(1)_2)
1606.199
1612.193(PEP+HexNAc(4)Hex(5)NeuAc(1)_2)
1658.713(PEP+HexNAc(1)Fuc(1)_1)
1665.716
1685.163(PEP+HexNAc(4)Hex(5)Fuc(1)NeuAc(1)_2)
1722.179(PEP+HexNAc(5)Hex(6)Fuc(1)_2)
1767.725
150.117
189.836
233.827
288.107
356.317
366.141(HexNAc + Hex)
806.914
1080.125
1203.294
M+3H
1685.163(PEP+HexNAc(4)Hex(5)Fuc(1)NeuAc(1)_2)
1723.684
1815.214
1840.237
1847.227
1860.225
M+2H

CDISNSTEAGQK(=PEP)_7_6_1_1_0, 0_None, 0_None,
m/z:1367.19(3+), RT:23.49, Y-score:72.07

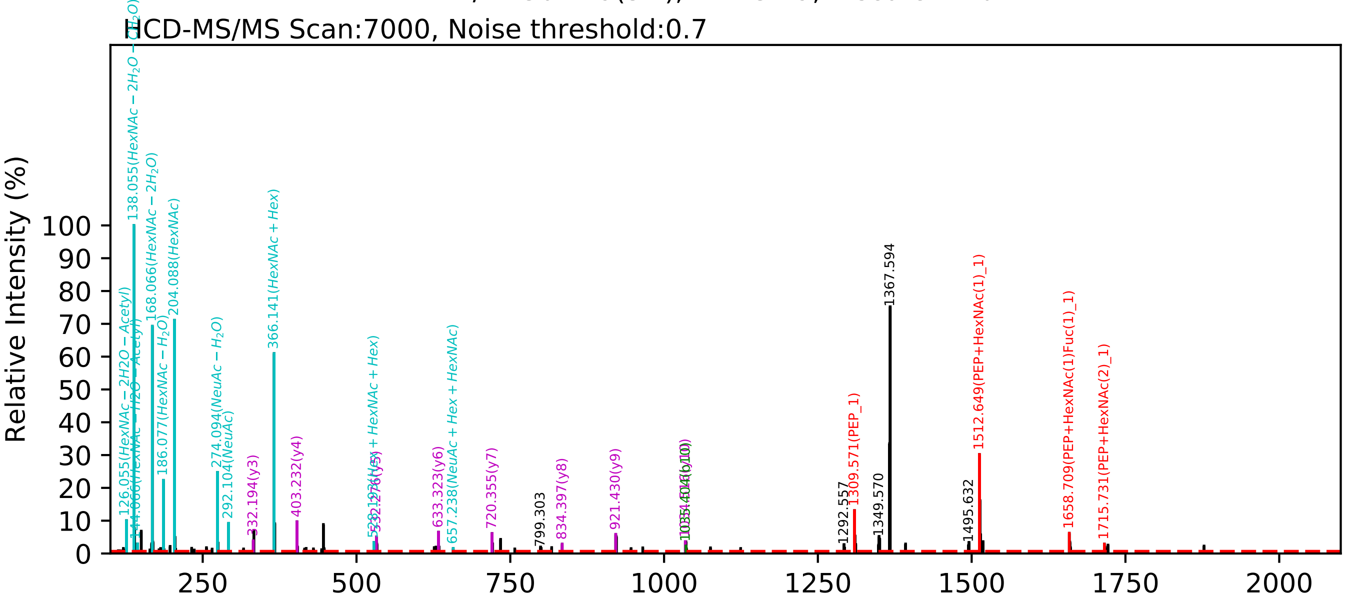

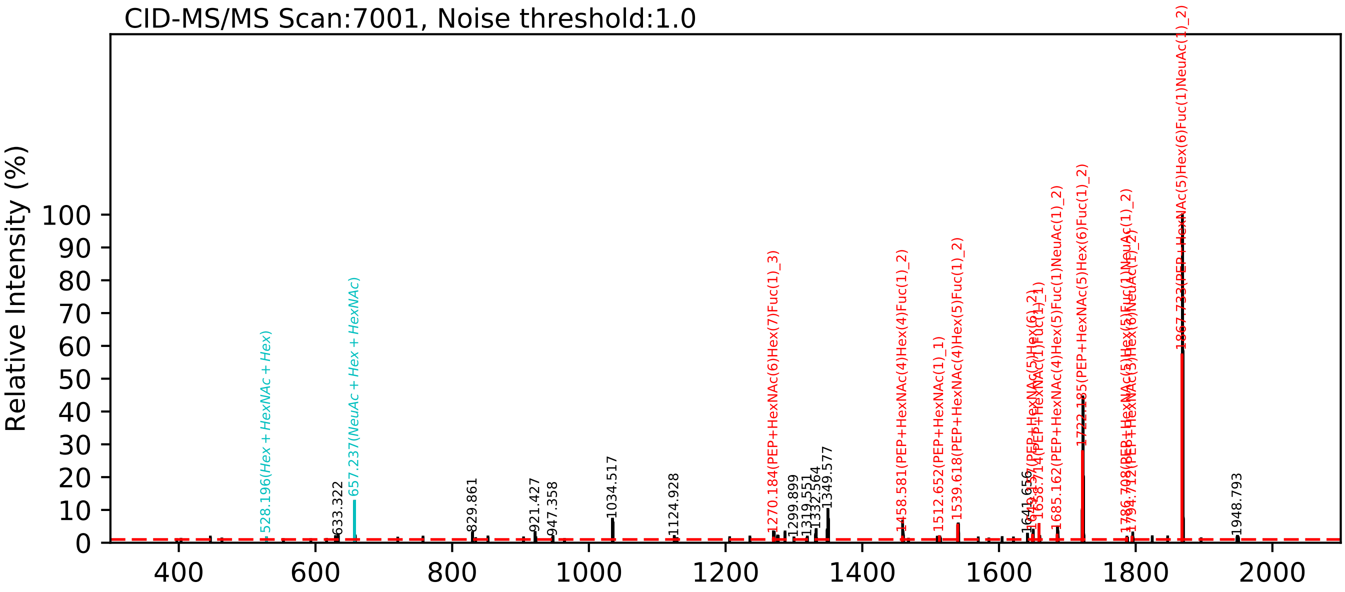

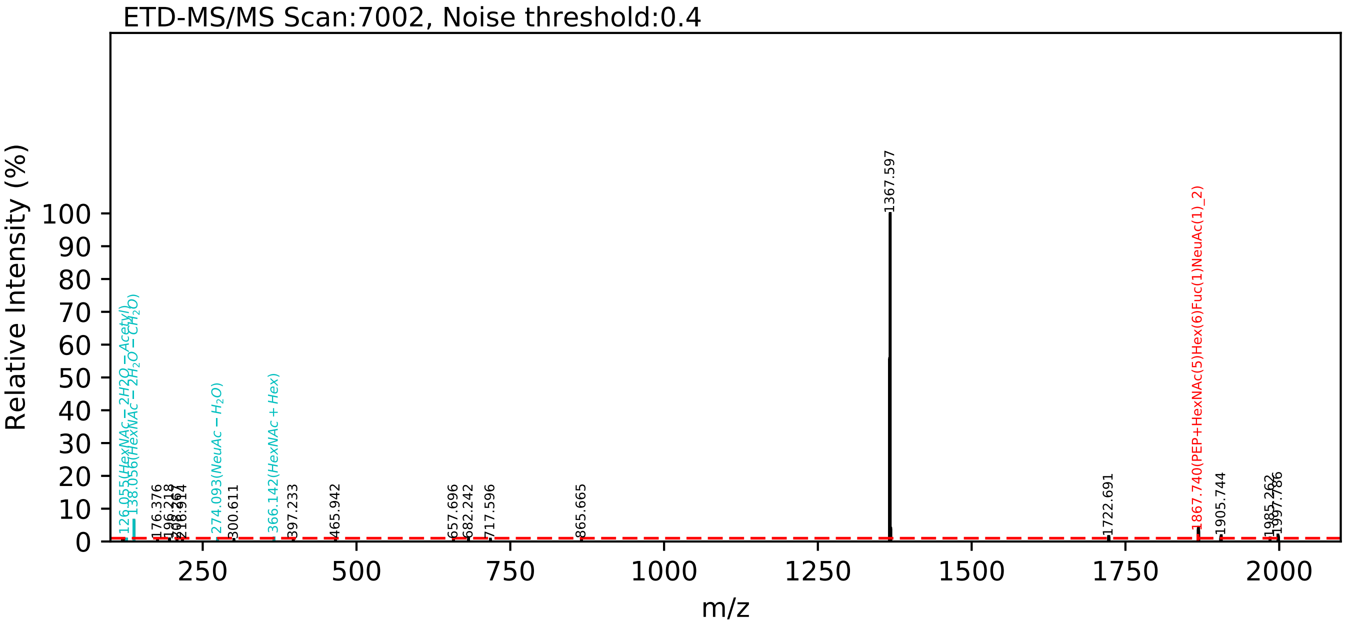

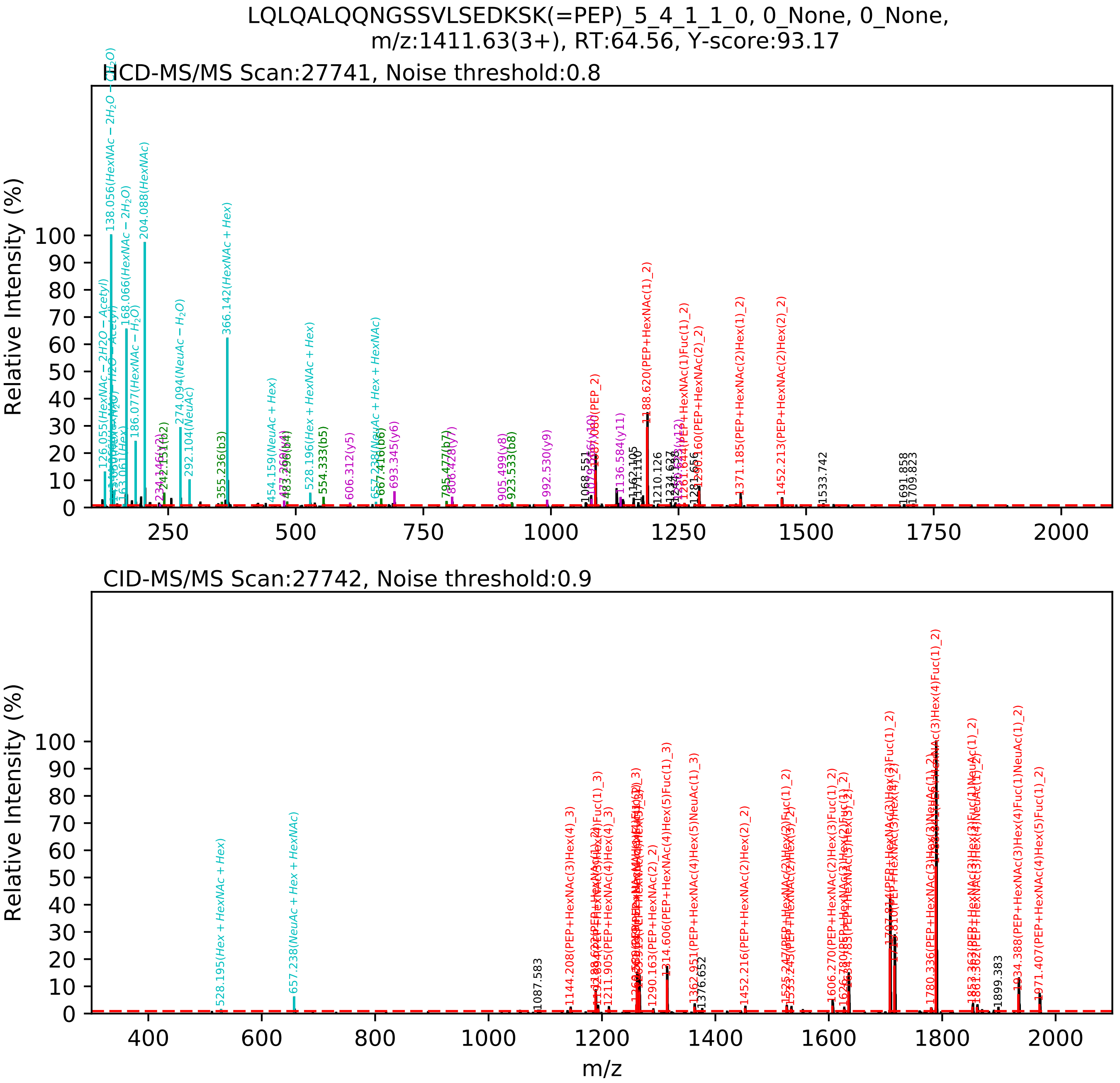

LQLQALQQNGSSVLSEDKSK(=PEP)_5_4_1_1_0, 0_None, 0_None,
m/z:1411.63(3+), RT:64.56, Y-score:93.17
HCD-MS/MS Scan:27741, Noise threshold:0.8
Relative Intensity (%)
138.056(HexNAc − 2H2O)
204.088(HexNAc)
168.066(HexNAc − 2H2O)
186.077(HexNAc − H2O)
366.142(HexNAc + Hex)
274.094(NeuAc − H2O)
292.104(NeuAc)
126.055(HexNAc − 2H2O − Acetyl)
163.061(Hex)
242.131(b2)
355.236(b3)
454.159(NeuAc + Hex)
528.196(Hex + HexNAc + Hex)
554.333(b5)
606.312(y5)
693.345(y6)
795.477(b7)
806.428(y7)
905.499(y8)
923.533(b8)
992.530(y9)
1068.551
1136.584(y11)
1188.620(PEP+HexNAc(1)_2)
1210.126
1234.632
1281.656
1290.160(PEP+HexNAc(2)_2)
1371.185(PEP+HexNAc(2)Hex(1)_2)
1452.213(PEP+HexNAc(2)Hex(2)_2)
1533.742
1691.858
1709.823
250
500
750
1000
1250
1500
1750
2000
CID-MS/MS Scan:27742, Noise threshold:0.9
528.195(Hex + HexNAc + Hex)
657.238(NeuAc + Hex + HexNAc)
1087.583
1144.208(PEP+HexNAc(3)Hex(4)_3)
1211.905(PEP+HexNAc(4)Hex(4)_3)
1290.163(PEP+HexNAc(2)_2)
1314.606(PEP+HexNAc(4)Hex(5)Fuc(1)_3)
1362.951(PEP+HexNAc(4)Hex(5)NeuAc(1)_3)
1376.652
1452.216(PEP+HexNAc(2)Hex(2)_2)
1606.270(PEP+HexNAc(2)Hex(3)Fuc(1)_2)
1780.336(PEP+HexNAc(3)Hex(4)Fuc(1)_2)
1899.383
1934.388(PEP+HexNAc(3)Hex(4)Fuc(1)NeuAc(1)_2)
1971.407(PEP+HexNAc(4)Hex(5)Fuc(1)_2)
400
600
800
1000
1200
1400
1600
1800
2000
m/z

LQLQALQQNGSSVLSEDK(=PEP)_3_2_1_0_0, 0_None, 0_None,
m/z:999.47(3+), RT:67.66, Y-score:75.83

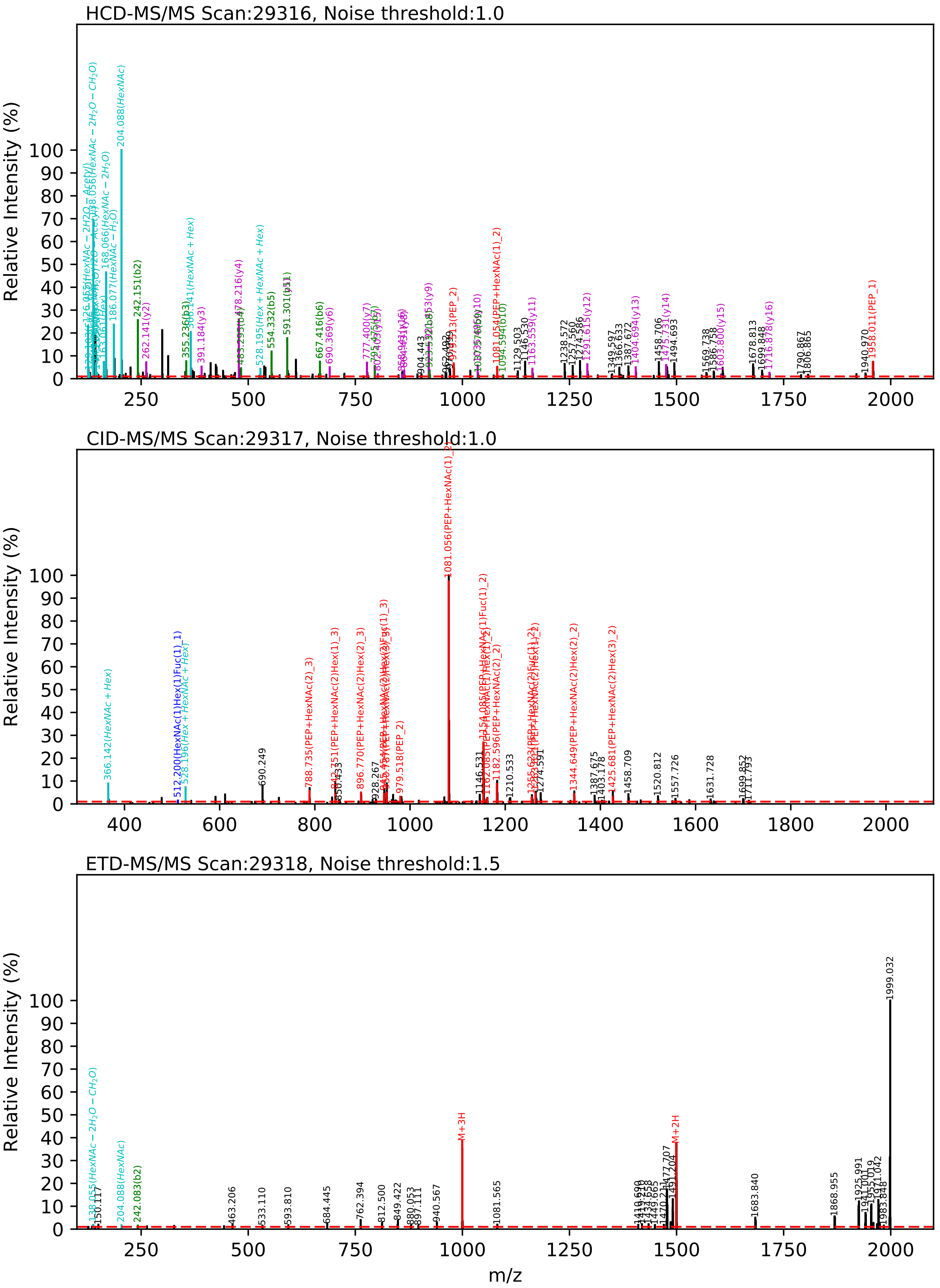

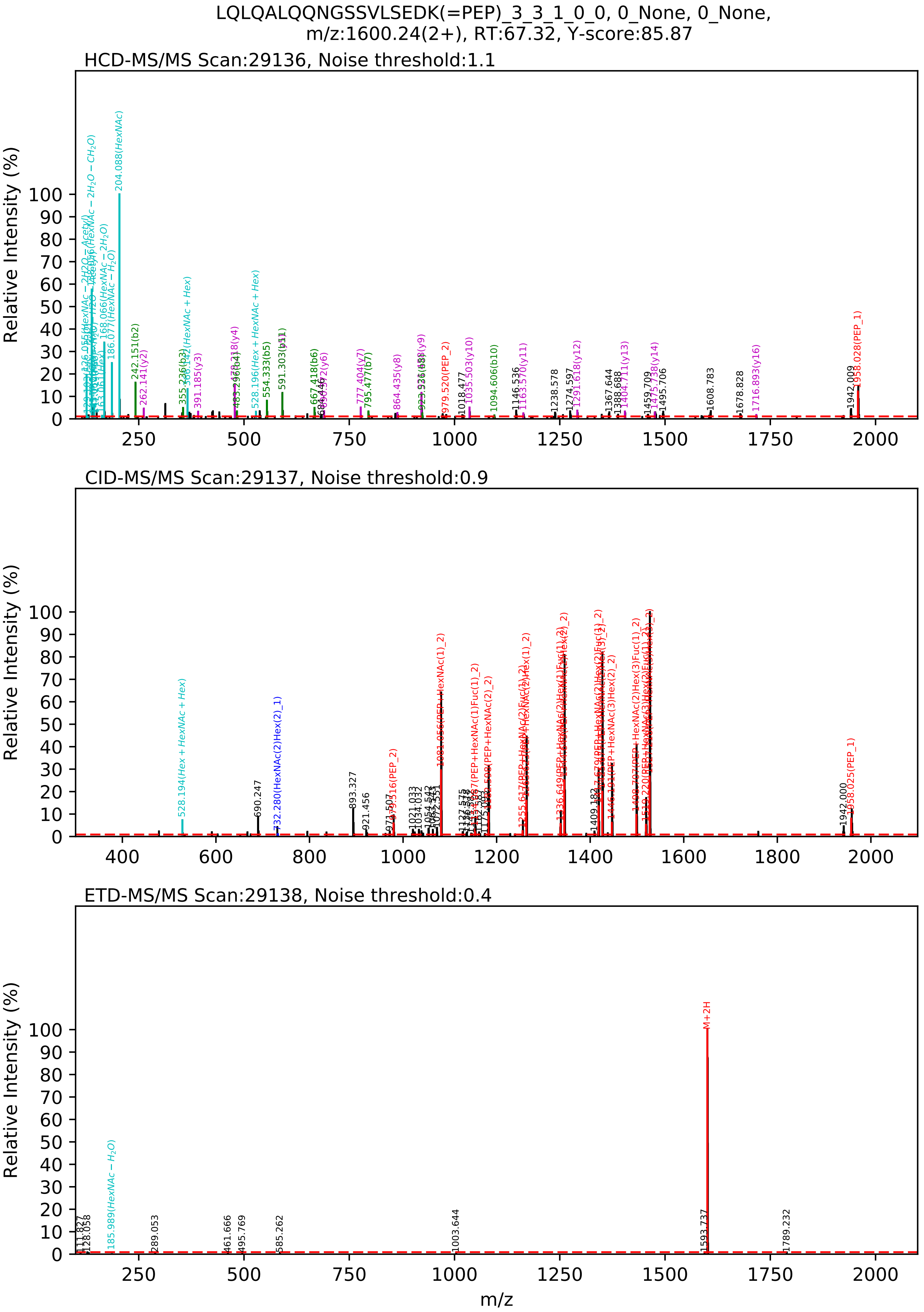
LQLQALQQNGSSVLSEDK(=PEP)_3_3_1_0_0, 0_None, 0_None,
m/z:1600.24(2+), RT:67.32, Y-score:85.87
HCD-MS/MS Scan:29136, Noise threshold:1.1
Relative Intensity (%)
204.088(HexNAc)
186.077(HexNAc − H2O)
168.066(HexNAc − 2H2O)
242.151(b2)
262.141(y2)
355.236(b3)
391.185(y3)
528.196(Hex + HexNAc + Hex)
554.333(b5)
591.303(y5)
667.418(b6)
777.404(y7)
795.477(b7)
864.435(y8)
979.520(PEP_2)
1018.477
1035.503(y10)
1094.606(b10)
1146.536
1163.570(y11)
1238.578
1274.597
1291.618(y12)
1367.644
1388.688
1404.711(y13)
1459.709
1475.738(y14)
1495.706
1608.783
1678.828
1716.893(y16)
1942.009
1958.028(PEP_1)
CID-MS/MS Scan:29137, Noise threshold:0.9
528.194(Hex + HexNAc + Hex)
690.247
732.280(HexNAc(2)Hex(2)_1)
893.327
921.456
971.507
979.516(PEP_2)
1021.033
1034.032
1072.551
1081.056(PEP+HexNAc(1)_2)
1127.575
1409.182
1942.000
1958.025(PEP_1)
ETD-MS/MS Scan:29138, Noise threshold:0.4
M+2H
111.827
128.058
185.989(HexNAc − H2O)
289.053
461.666
495.769
585.262
1003.644
1593.737
1789.232
m/z

LQLQALQQNGSSVLSEDK(=PEP)_3_3_1_0_0, 0_None, 0_None,
m/z:1600.24(2+), RT:67.45, Y-score:83.76

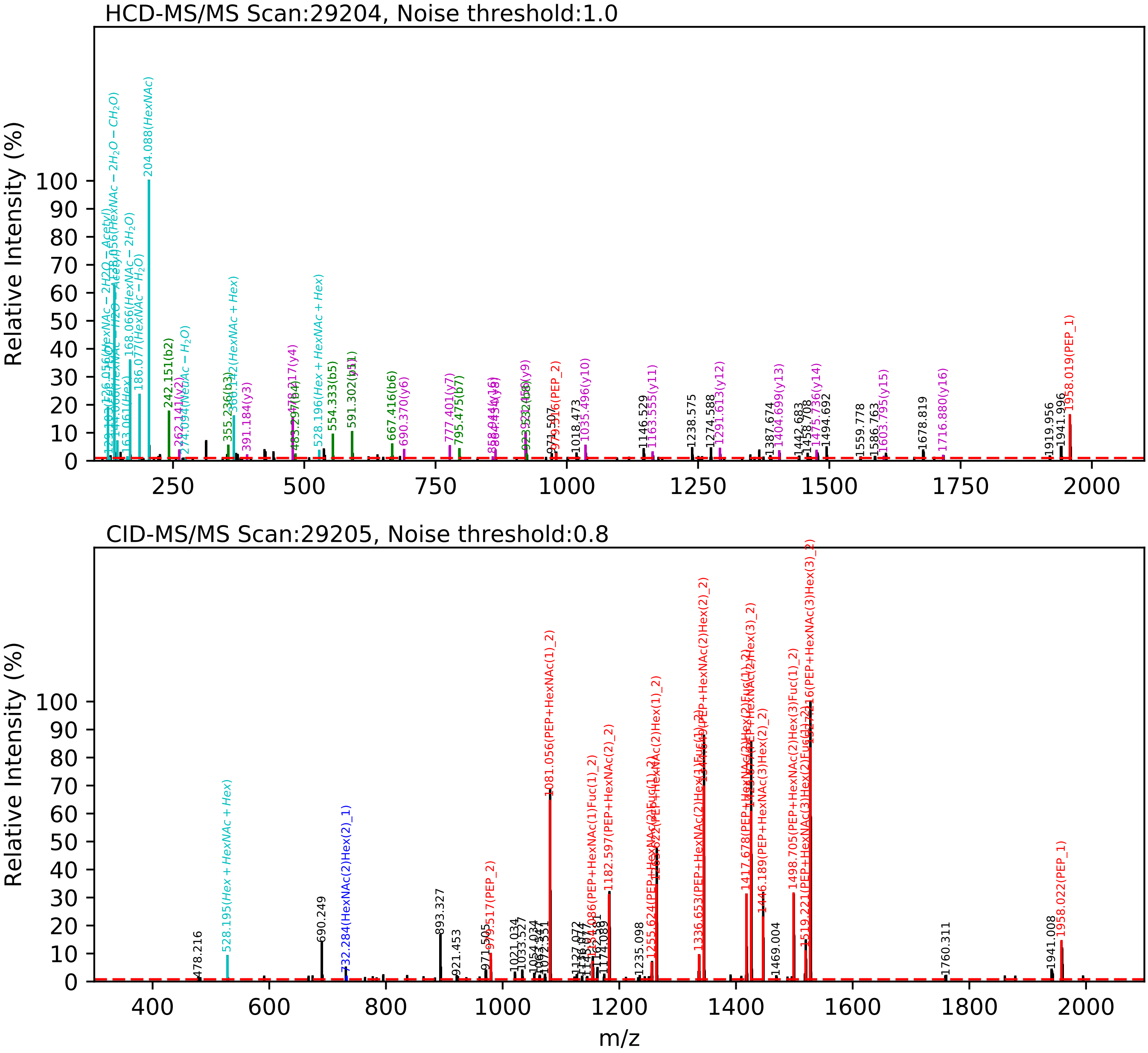

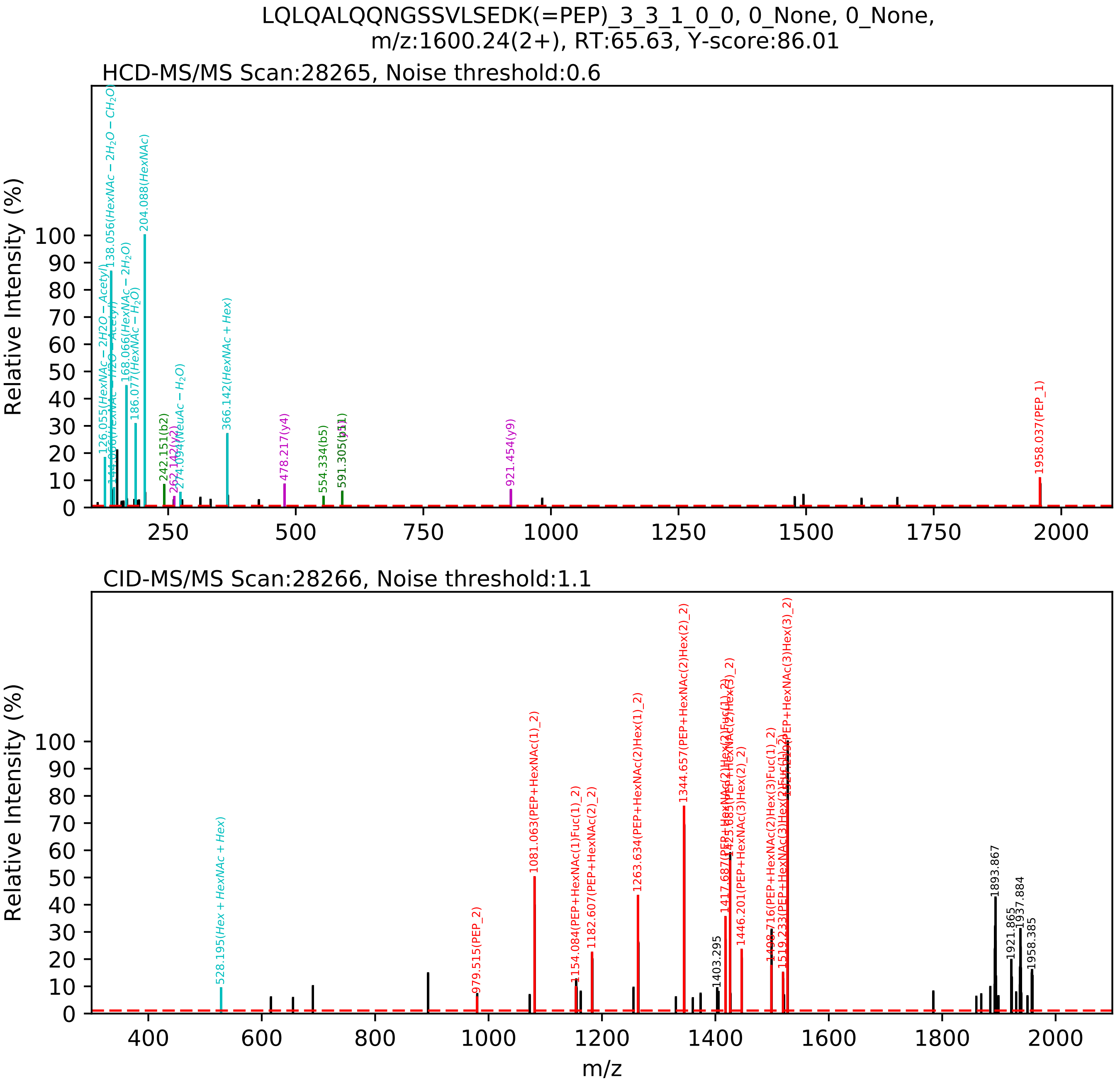
LQLQALQQNGSSVLSEDK(=PEP)_3_3_1_0_0, 0_None, 0_None,
m/z:1600.24(2+), RT:65.63, Y-score:86.01
HCD-MS/MS Scan:28265, Noise threshold:0.6
Relative Intensity (%)
204.088(HexNAc)
138.056(HexNAc − 2H2O − CH2O)
168.066(HexNAc − 2H2O)
186.077(HexNAc − H2O)
126.055(HexNAc − 2H2O − Acetyl)
366.142(HexNAc + Hex)
242.151(b2)
262.142(y2)
274.094(NeuAc − H2O)
478.217(y4)
554.334(b5)
591.305(b5)
921.454(y9)
1958.037(PEP_1)
CID-MS/MS Scan:28266, Noise threshold:1.1
528.195(Hex + HexNAc + Hex)
979.515(PEP_2)
1081.063(PEP+HexNAc(1)_2)
1154.084(PEP+HexNAc(1)Fuc(1)_2)
1182.607(PEP+HexNAc(2)_2)
1263.634(PEP+HexNAc(2)Hex(1)_2)
1344.657(PEP+HexNAc(2)Hex(2)_2)
1403.295
1417.687
1446.201(PEP+HexNAc(3)Hex(2)_2)
1498.716(PEP+HexNAc(2)Hex(3)Fuc(1)_2)
1519.233(PEP+HexNAc(3)Hex(2)Fuc(1)_2)
PEP+HexNAc(3)Hex(3)_2
1893.867
1921.865
1937.884
1958.385
m/z

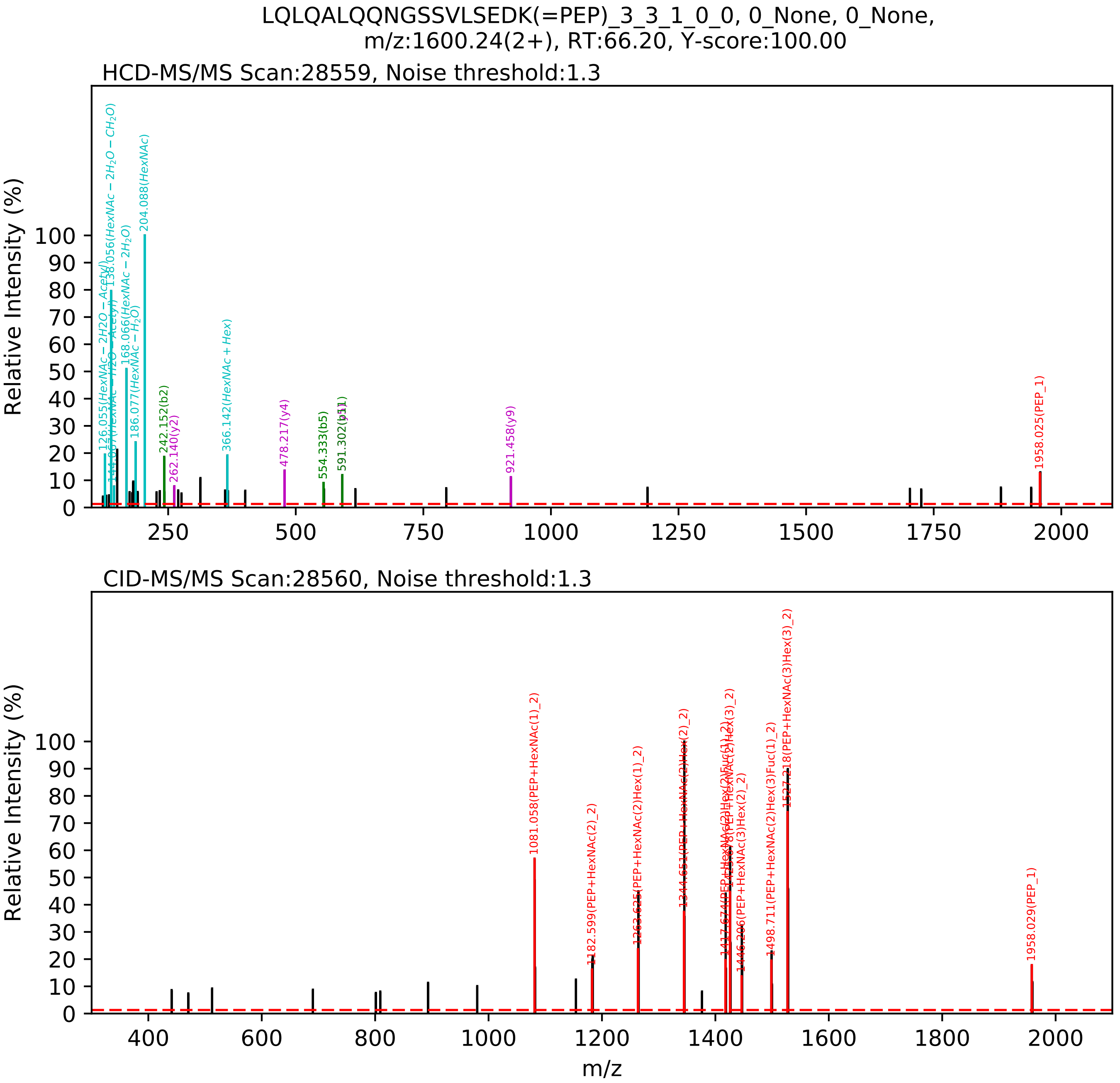
LQLQALQQNGSSVLSEDK(=PEP)_3_3_1_0_0, 0_None, 0_None,
m/z:1600.24(2+), RT:66.20, Y-score:100.00
HCD-MS/MS Scan:28559, Noise threshold:1.3
Relative Intensity (%)
126.055(HexNAc − 2H2O − Acetyl)
138.056(HexNAc − 2H2O − CH2O)
168.066(HexNAc − 2H2O)
186.077(HexNAc − H2O)
204.088(HexNAc)
242.152(b2)
262.140(y2)
366.142(HexNAc + Hex)
478.217(y4)
554.333(b5)
591.302(y5)
921.458(y9)
1958.025(PEP_1)
CID-MS/MS Scan:28560, Noise threshold:1.3
Relative Intensity (%)
1081.058(PEP+HexNAc(1)_2)
1182.599(PEP+HexNAc(2)_2)
1263.625(PEP+HexNAc(2)Hex(1)_2)
1344.651(PEP+HexNAc(2)Hex(2)_2)
1446.206(PEP+HexNAc(3)Hex(2)_2)
1498.711(PEP+HexNAc(2)Hex(3)Fuc(1)_2)
1527.218(PEP+HexNAc(3)Hex(3)_2)
1958.029(PEP_1)
m/z

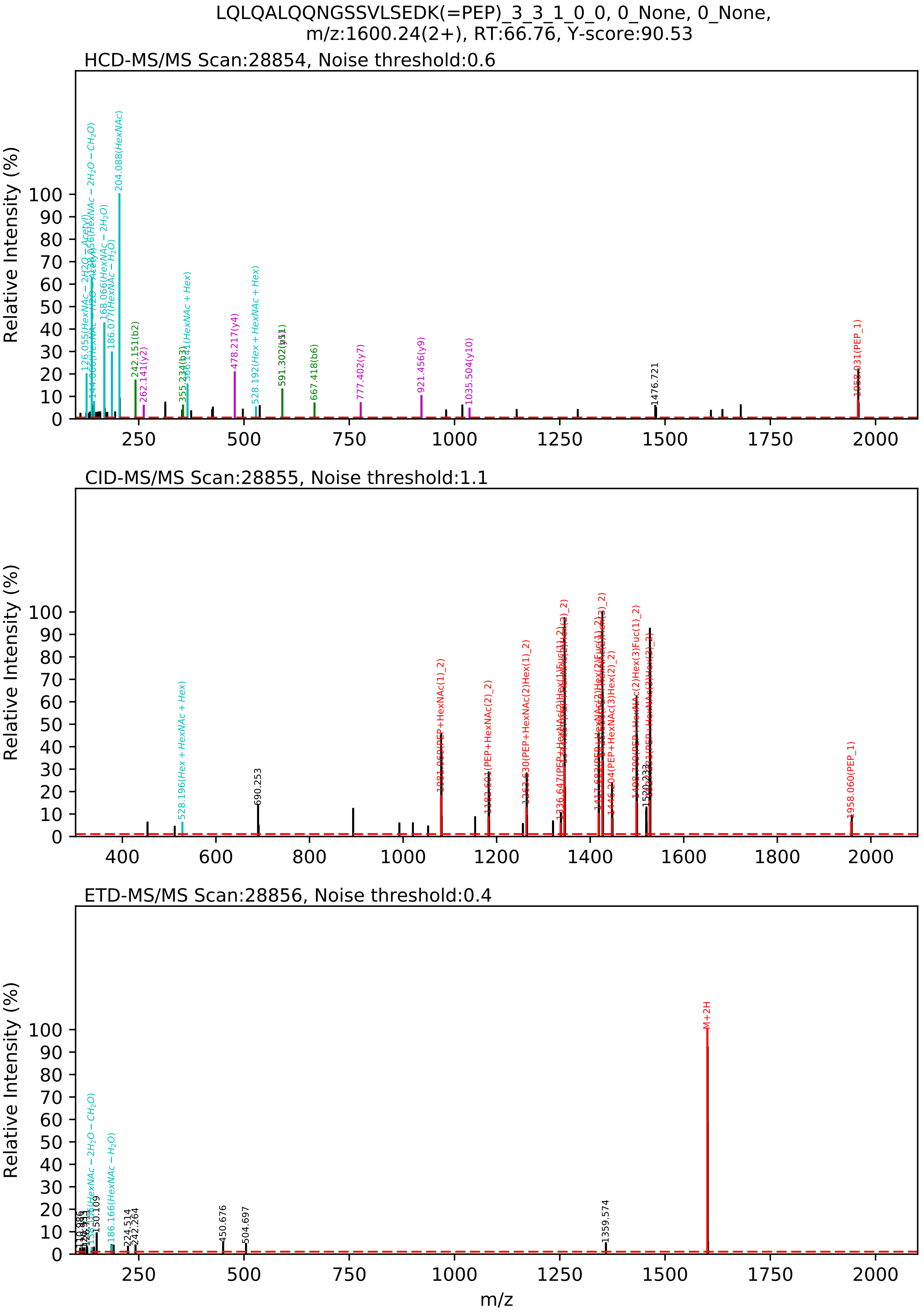

LQLQALQQNGSSVLSEDK(=PEP)_3_3_1_0_0, 0_None, 0_None,
m/z:1600.24(2+), RT:66.76, Y-score:90.53
HCD-MS/MS Scan:28854, Noise threshold:0.6
Relative Intensity (%)
204.088(HexNAc)
168.066(HexNAc − 2H2O)
186.077(HexNAc − H2O)
126.055(HexNAc − 2H2O − Acetyl)
242.151(b2)
262.141(y2)
355.234(b3)
478.217(y4)
528.192(Hex + HexNAc + Hex)
591.302(b5)
667.418(b6)
777.402(y7)
921.456(y9)
1035.504(y10)
1476.721
1958.031(PEP_1)
CID-MS/MS Scan:28855, Noise threshold:1.1
528.196(Hex + HexNAc + Hex)
690.253
1081.060(PEP+HexNAc(1)_2)
1182.603(PEP+HexNAc(2)_2)
1263.630(PEP+HexNAc(2)Hex(1)_2)
1446.204(PEP+HexNAc(3)Hex(2)_2)
1520.233
1958.060(PEP_1)
ETD-MS/MS Scan:28856, Noise threshold:0.4
M+2H
186.166(HexNAc − H2O)
150.109
224.514
242.264
450.676
504.697
1359.574
m/z

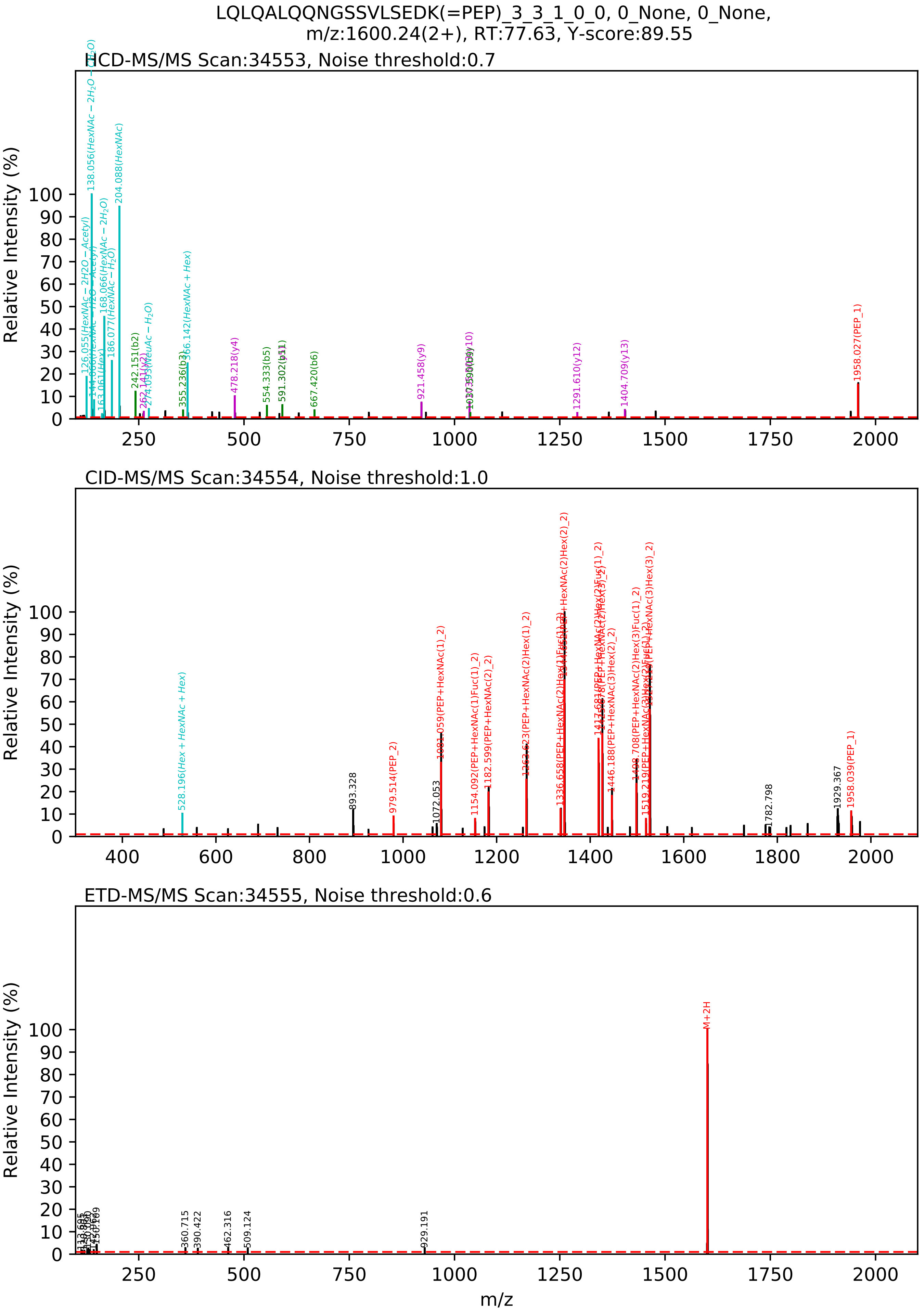

LQLQALQQNGSSVLSEDK(=PEP)_3_3_1_0_0, 0_None, 0_None,
m/z:1600.24(2+), RT:77.63, Y-score:89.55
HCD-MS/MS Scan:34553, Noise threshold:0.7
CID-MS/MS Scan:34554, Noise threshold:1.0
ETD-MS/MS Scan:34555, Noise threshold:0.6
Relative Intensity (%)
m/z
138.056(HexNAc − 2H2O − H2O)
204.088(HexNAc)
168.066(HexNAc − 2H2O)
186.077(HexNAc − H2O)
126.055(HexNAc − 2H2O − Acetyl)
366.142(HexNAc + Hex)
242.151(b2)
355.236(b3)
478.218(y4)
554.333(b5)
591.302(y5)
667.420(b6)
921.458(y9)
1291.610(y12)
1404.709(y13)
1958.027(PEP_1)
528.196(Hex + HexNAc + Hex)
893.328
979.514(PEP_2)
1072.053
1081.059(PEP+HexNAc(1)_2)
1154.092(PEP+HexNAc(1)Fuc(1)_2)
1182.599(PEP+HexNAc(2)_2)
1263.623(PEP+HexNAc(2)Hex(1)_2)
1336.658(PEP+HexNAc(2)Hex(1)Fuc(1)_2)
1417.681(PEP+HexNAc(2)Hex(2)Fuc(1)_2)
1446.188(PEP+HexNAc(3)Hex(2)_2)
1498.708(PEP+HexNAc(2)Hex(3)Fuc(1)_2)
1519.219(PEP+HexNAc(3)Hex(2)Fuc(1)_2)
1782.798
1929.367
1958.039(PEP_1)
M+2H
360.715
390.422
462.316
509.124
929.191

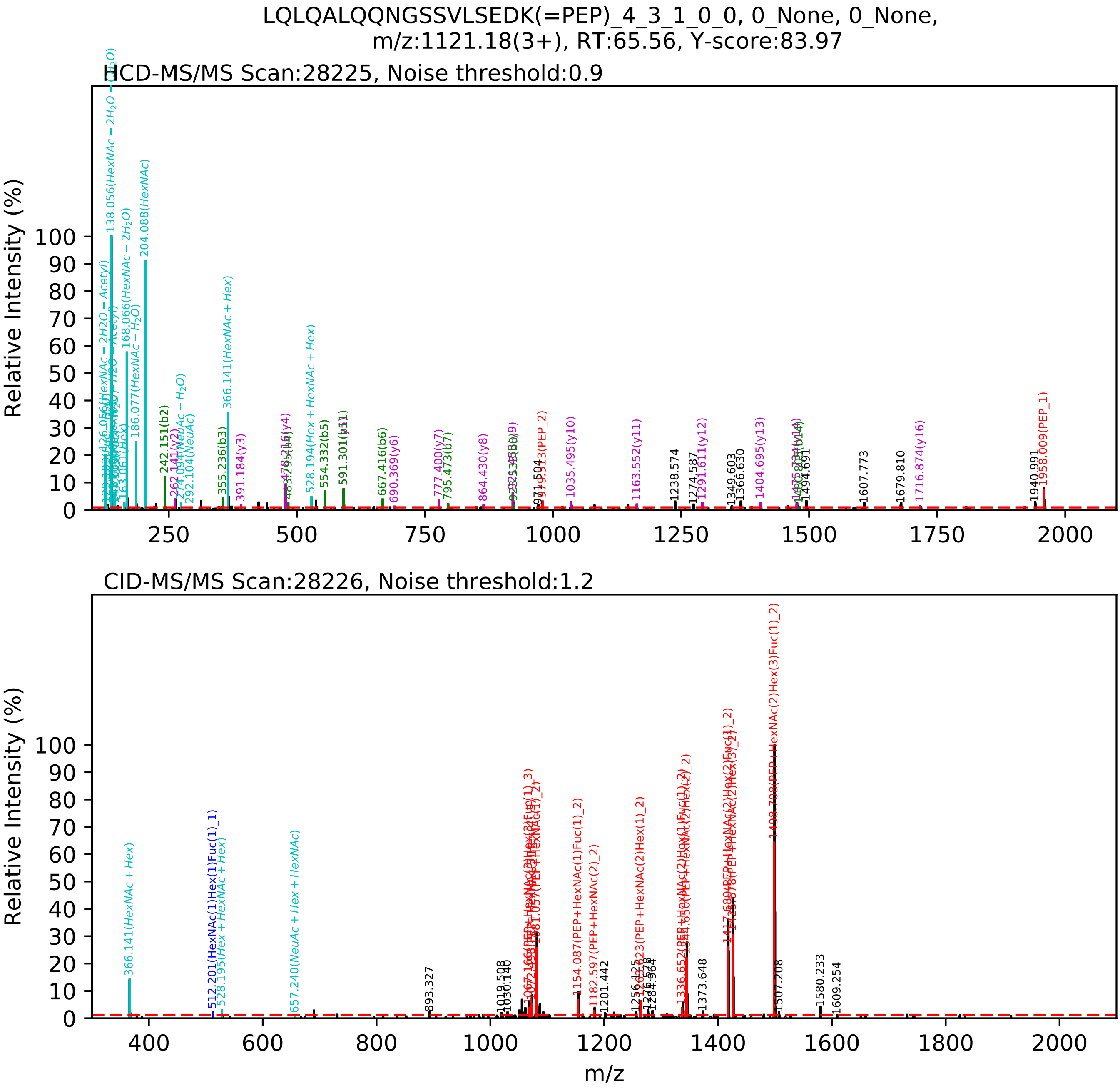

LQLQALQQNGSSVLSEDK(=PEP)_4_3_1_0_0, 0_None, 0_None,
m/z:1121.18(3+), RT:65.56, Y-score:83.97
HCD-MS/MS Scan:28225, Noise threshold:0.9
Relative Intensity (%)
138.056(HexNAc − 2H2O)
204.088(HexNAc)
168.066(HexNAc − 2H2O)
186.077(HexNAc − H2O)
366.141(HexNAc + Hex)
242.151(b2)
355.236(b3)
391.184(y3)
528.194(Hex + HexNAc + Hex)
554.332(b5)
591.301(b6)
667.416(b6)
690.369(y6)
777.400(y7)
795.473(b7)
864.430(y8)
1035.495(y10)
1163.552(y11)
1238.574
1274.587
1291.611(y12)
1349.603
1366.630
1404.695(y13)
1494.691
1607.773
1679.810
1716.874(y16)
1940.991
1958.009(PEP_1)
CID-MS/MS Scan:28226, Noise threshold:1.2
Relative Intensity (%)
366.141(HexNAc + Hex)
512.201(HexNAc(1)Hex(1)Fuc(1)_1)
657.240(NeuAc + Hex + HexNAc)
893.327
1154.087(PEP+HexNAc(1)Fuc(1)_2)
1182.597(PEP+HexNAc(2)_2)
1201.442
1373.648
1498.708(PEP+HexNAc(2)Hex(3)Fuc(1)_2)
1507.208
1580.233
1609.254
m/z

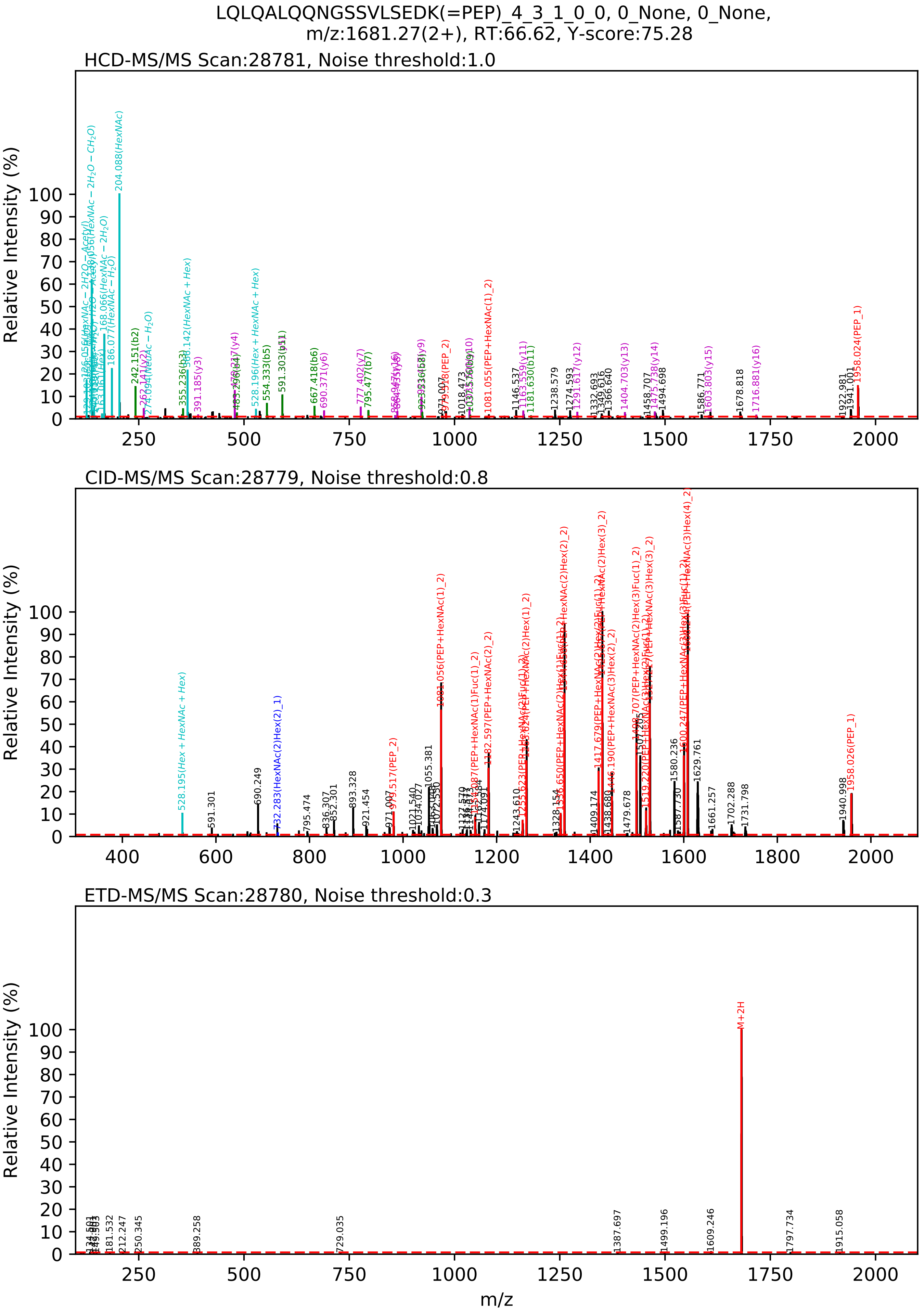

LQLQALQQNGSSVLSEDK(=PEP)_4_3_1_0_0, 0_None, 0_None,
m/z:1681.27(2+), RT:66.62, Y-score:75.28
HCD-MS/MS Scan:28781, Noise threshold:1.0
CID-MS/MS Scan:28779, Noise threshold:0.8
ETD-MS/MS Scan:28780, Noise threshold:0.3
Relative Intensity (%)
m/z

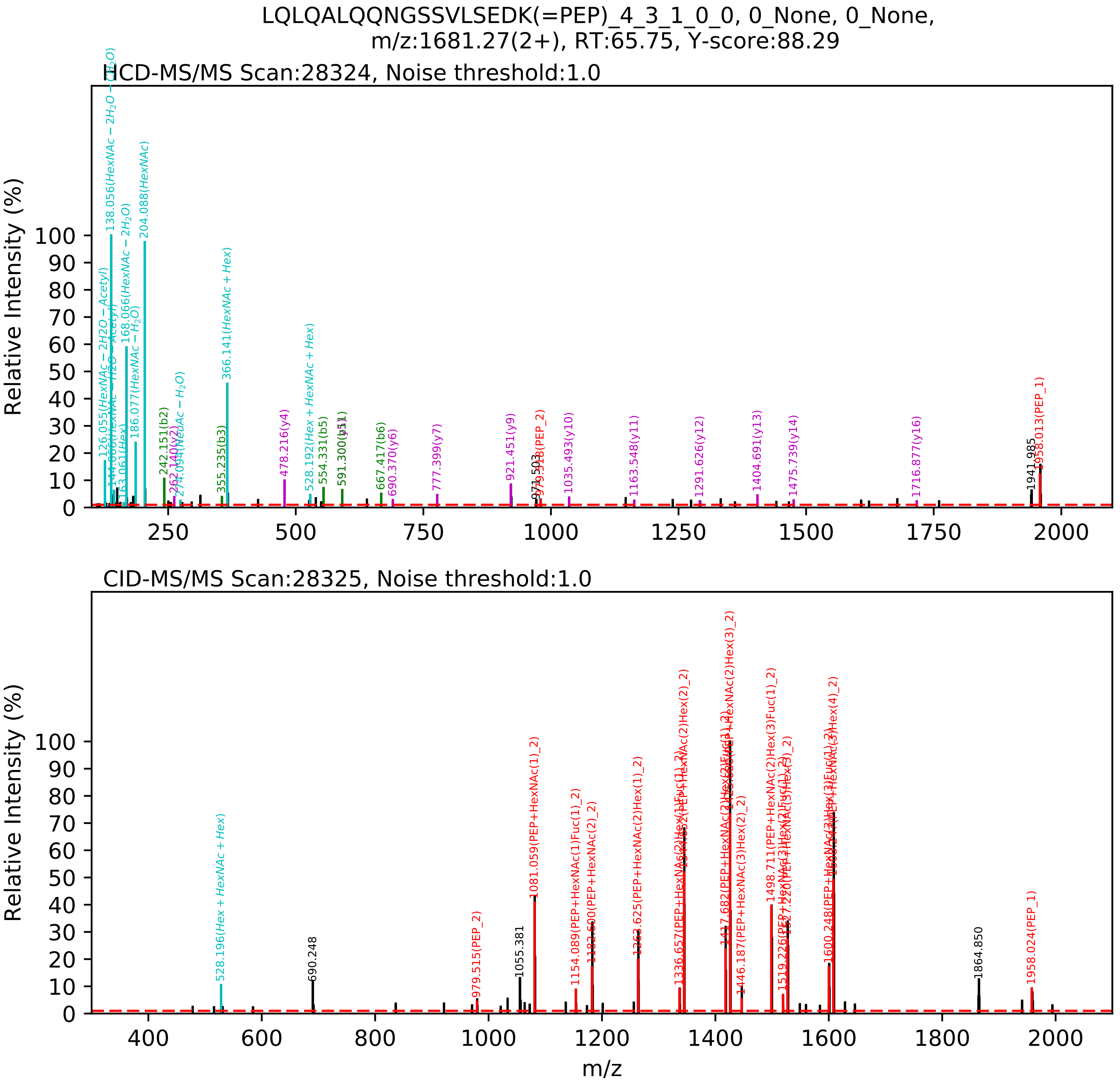

LQLQALQQNGSSVLSEDK(=PEP)_4_3_1_0_0, 0_None, 0_None,
m/z:1681.27(2+), RT:65.75, Y-score:88.29
HCD-MS/MS Scan:28324, Noise threshold:1.0
CID-MS/MS Scan:28325, Noise threshold:1.0
Relative Intensity (%)
m/z

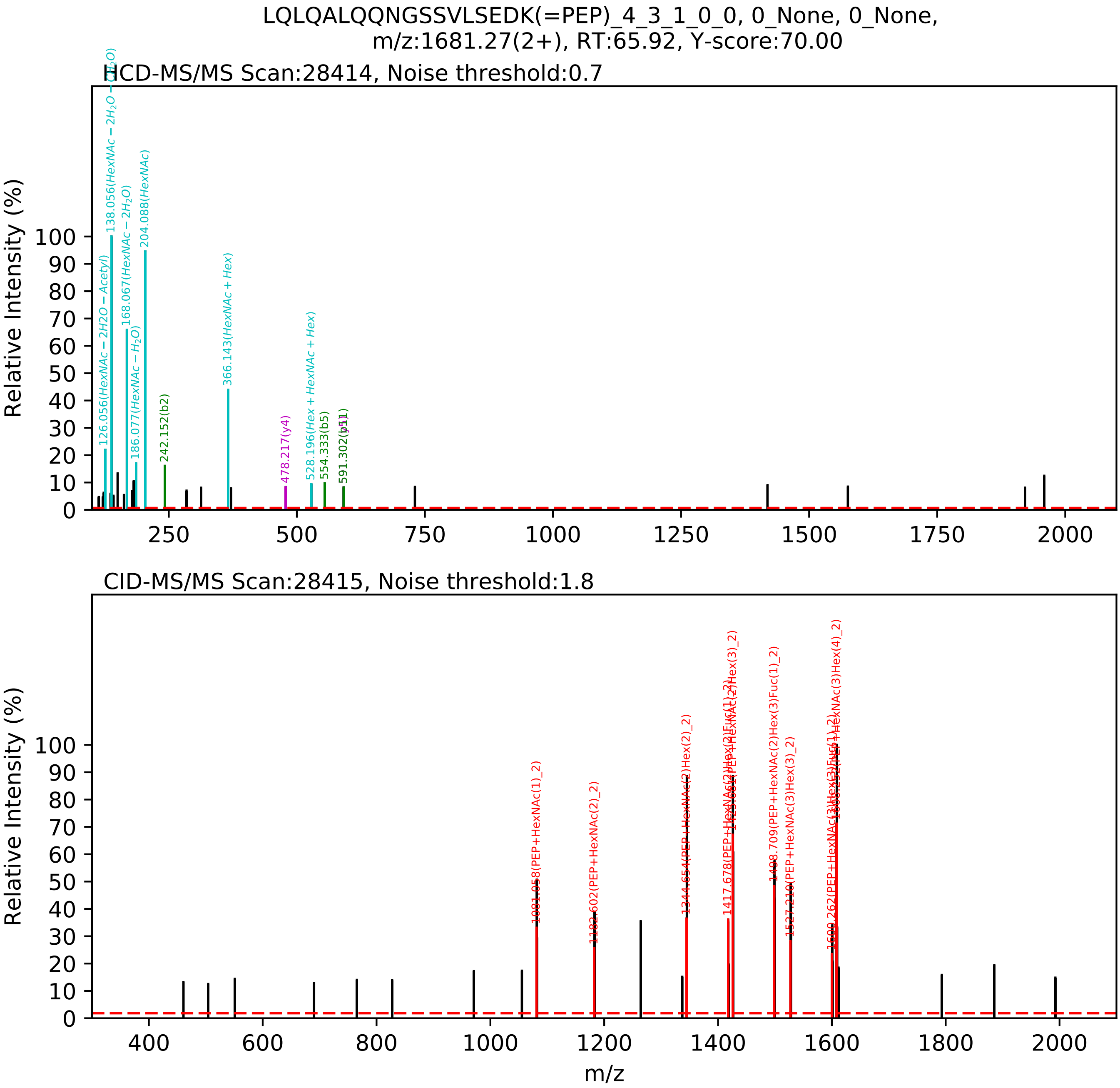

LQLQALQQNGSSVLSEDK(=PEP)_4_3_1_0_0, 0_None, 0_None,
m/z:1681.27(2+), RT:65.92, Y-score:70.00
HCD-MS/MS Scan:28414, Noise threshold:0.7
Relative Intensity (%)
126.056(HexNAc − 2H2O − Acetyl)
138.056(HexNAc − 2H2O − H2O)
168.067(HexNAc − 2H2O)
186.077(HexNAc − H2O)
204.088(HexNAc)
242.152(b2)
366.143(HexNAc + Hex)
478.217(y4)
528.196(Hex + HexNAc + Hex)
554.333(b5)
591.302(b11)
CID-MS/MS Scan:28415, Noise threshold:1.8
Relative Intensity (%)
1081.058(PEP+HexNAc(1)_2)
1182.602(PEP+HexNAc(2)_2)
1344.654(PEP+HexNAc(2)Hex(2)_2)
1498.709(PEP+HexNAc(2)Hex(3)Fuc(1)_2)
m/z

LQLQALQQNGSSVLSEDK(=PEP)_4_3_1_0_0, 0_None, 0_None,
m/z:1121.18(3+), RT:65.96, Y-score:89.07

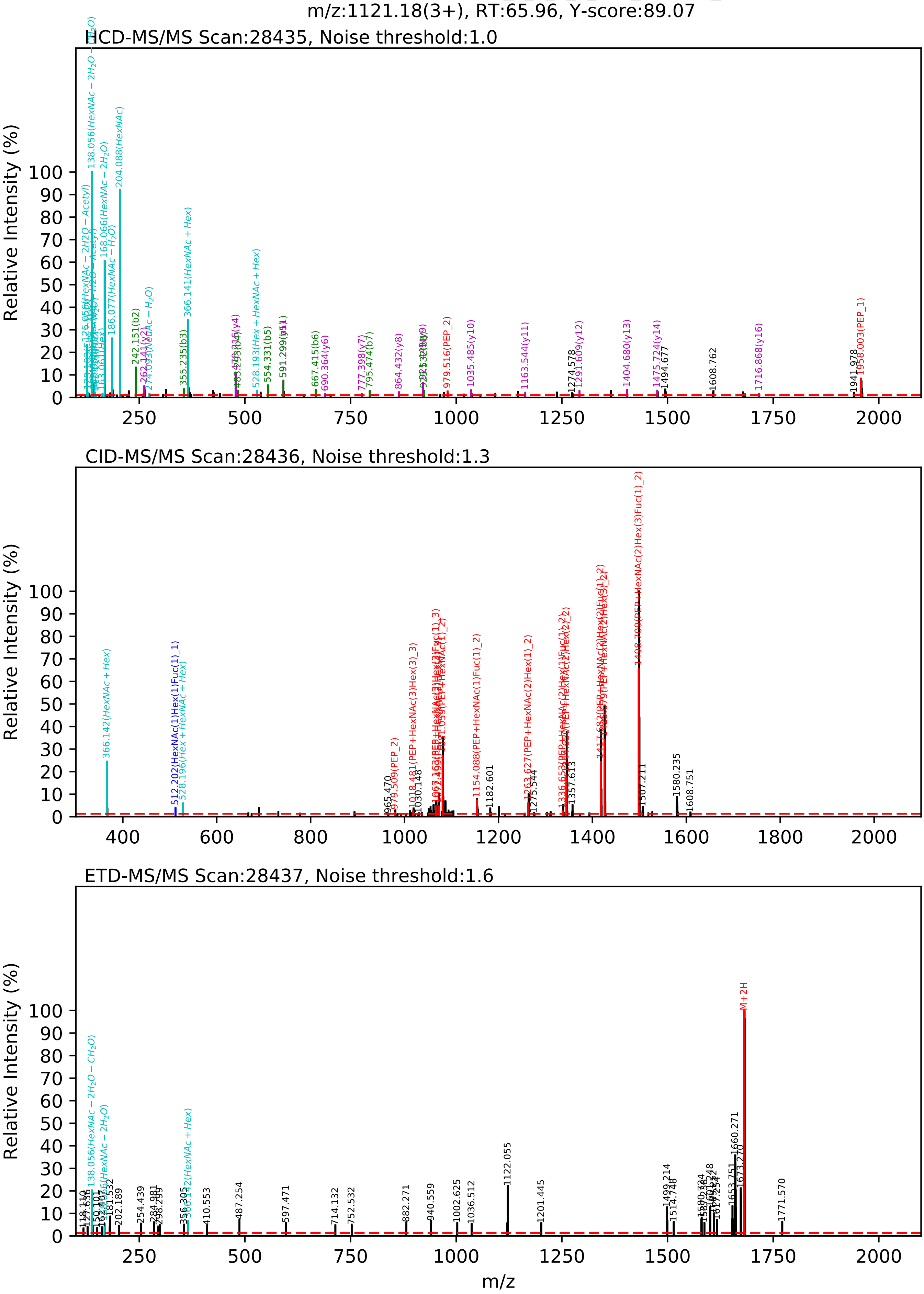

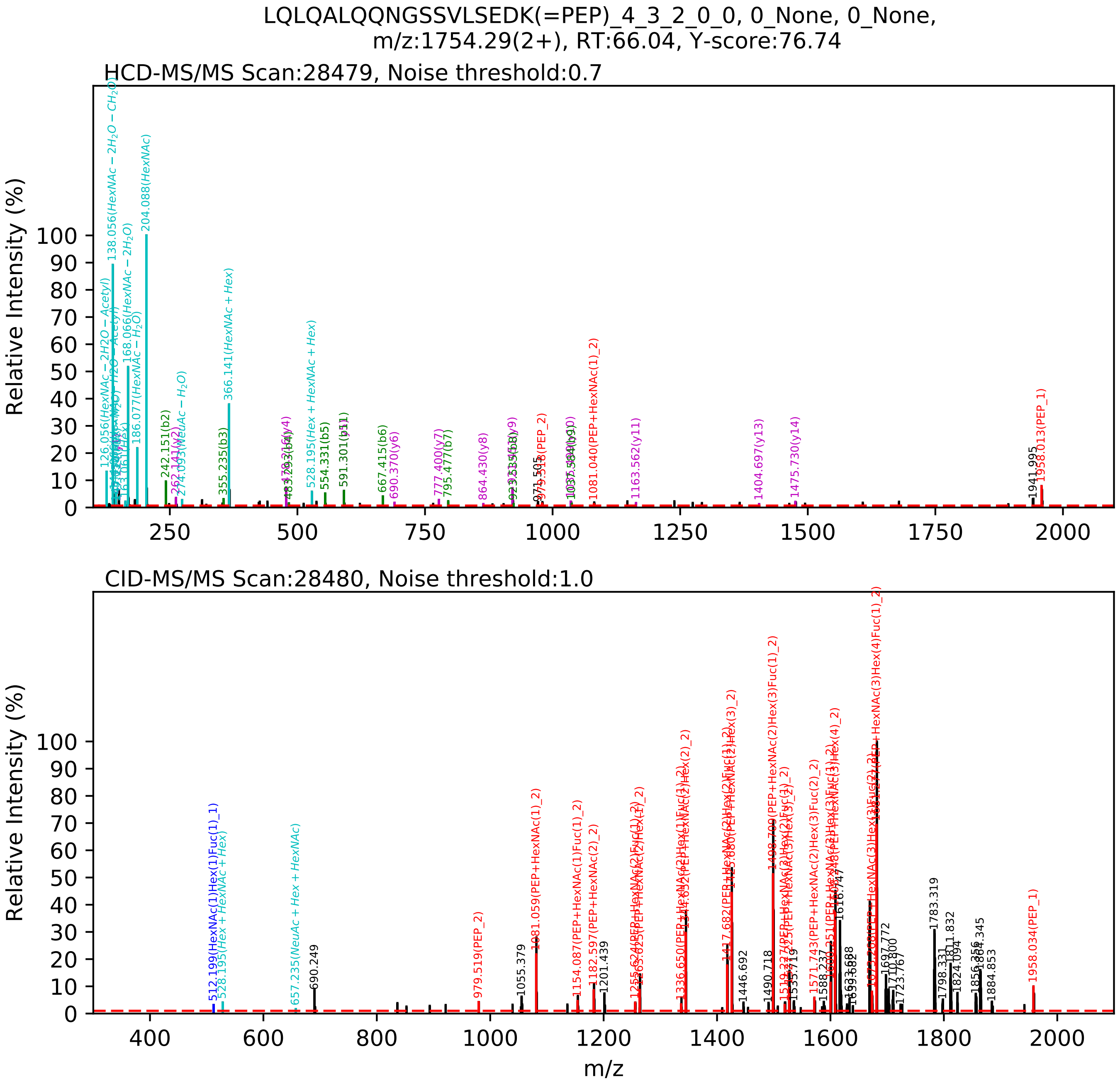

LQLQALQQNGSSVLSEDK(=PEP)_4_3_2_0_0, 0_None, 0_None,
m/z:1754.29(2+), RT:66.04, Y-score:76.74
HCD-MS/MS Scan:28479, Noise threshold:0.7
Relative Intensity (%)
204.088(HexNAc)
138.056(HexNAc − 2H2O − CH2O)
168.066(HexNAc − 2H2O)
186.077(HexNAc − H2O)
126.056(HexNAc − 2H2O − Acetyl)
366.141(HexNAc + Hex)
528.195(Hex + HexNAc + Hex)
242.151(b2)
355.235(b3)
554.331(b5)
591.301(y5)
667.415(b6)
690.370(y6)
777.400(y7)
795.477(b7)
864.430(y8)
979.518(PEP_2)
1081.040(PEP+HexNAc(1)_2)
1163.562(y11)
1404.697(y13)
1475.730(y14)
1941.995
1958.013(PEP_1)
CID-MS/MS Scan:28480, Noise threshold:1.0
Relative Intensity (%)
512.199(HexNAc(1)Hex(1)Fuc(1)_1)
528.195(Hex + HexNAc + Hex)
657.235(NeuAc + Hex + HexNAc)
690.249
979.519(PEP_2)
1055.379
1081.059(PEP+HexNAc(1)_2)
1154.087(PEP+HexNAc(1)Fuc(1)_2)
1182.597(PEP+HexNAc(2)_2)
1201.439
1336.650
1417.682
1446.692
1490.718
1535.719
1571.743(PEP+HexNAc(2)Hex(3)Fuc(2)_2)
1588.237
1616.747
1633.688
1783.319
1798.331
1811.832
1824.094
1856.356
1884.853
1958.034(PEP_1)
m/z

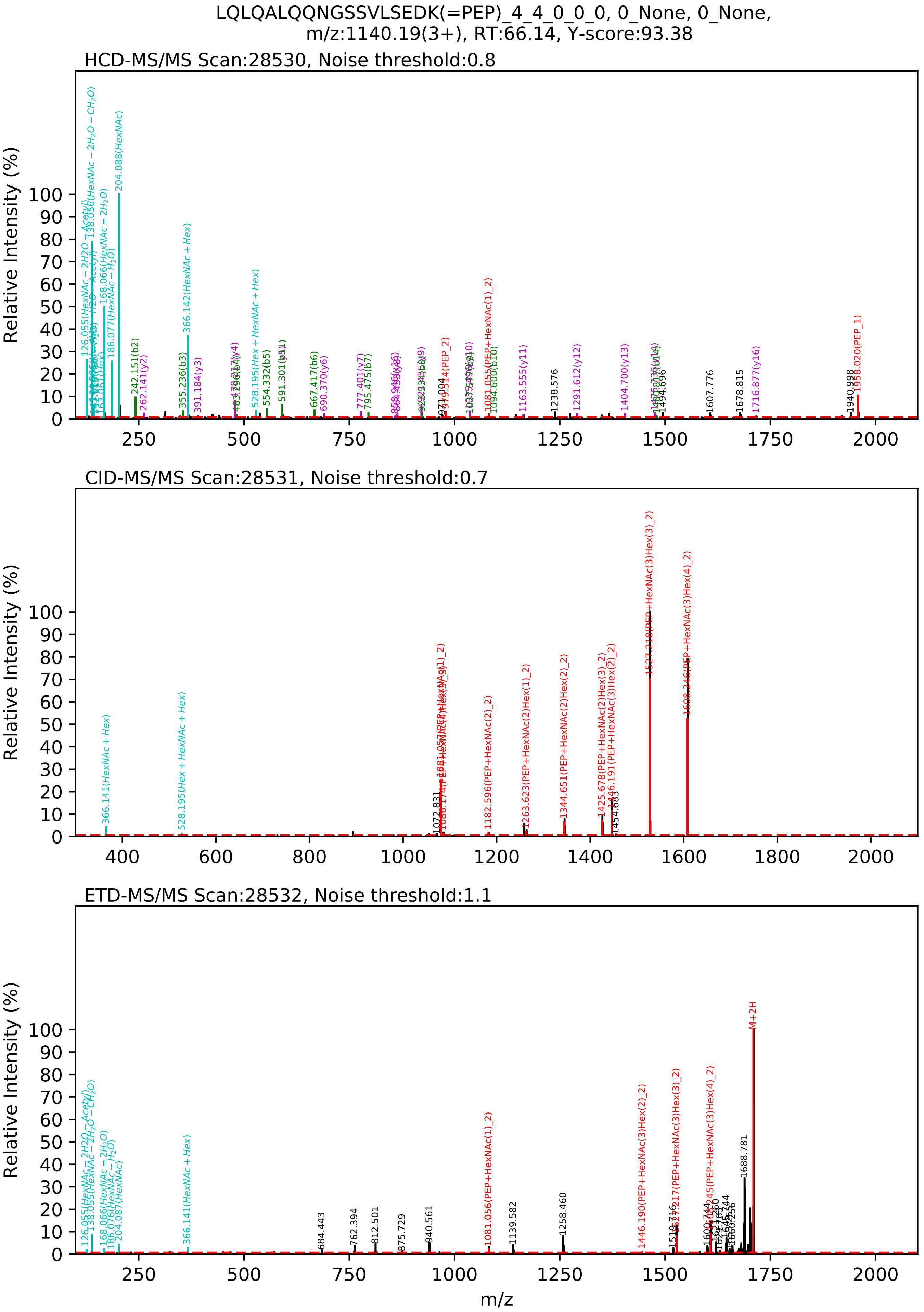

LQLQALQQNGSSVLSEDK(=PEP)_4_4_0_0_0, 0_None, 0_None,
m/z:1140.19(3+), RT:66.14, Y-score:93.38
HCD-MS/MS Scan:28530, Noise threshold:0.8
CID-MS/MS Scan:28531, Noise threshold:0.7
ETD-MS/MS Scan:28532, Noise threshold:1.1
Relative Intensity (%)
m/z

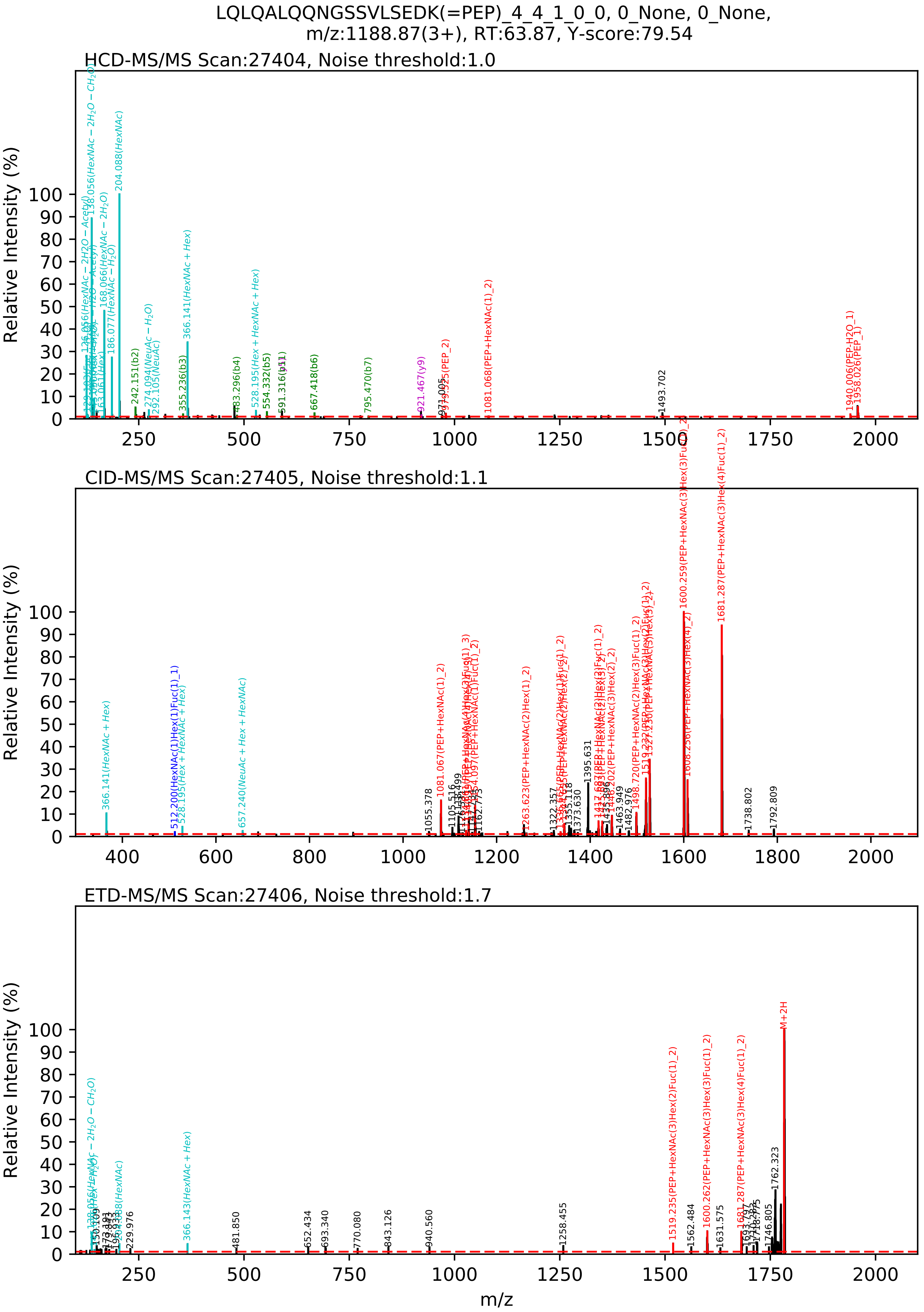

LQLQALQQNGSSVLSEDK(=PEP)_4_4_1_0_0, 0_None, 0_None,
m/z:1188.87(3+), RT:63.87, Y-score:79.54
HCD-MS/MS Scan:27404, Noise threshold:1.0
Relative Intensity (%)
204.088(HexNAc)
168.066(HexNAc − 2H2O)
186.077(HexNAc − H2O)
366.141(HexNAc + Hex)
242.151(b2)
274.094(NeuAc − H2O)
292.105(NeuAc)
355.236(b3)
528.195(Hex + HexNAc + Hex)
554.332(b5)
591.316(b11)
667.418(b6)
795.470(b7)
921.467(y9)
971.005
979.525(PEP_2)
1081.068(PEP+HexNAc(1)_2)
1493.702
1940.006(PEP-H2O_1)
1958.026(PEP_1)
CID-MS/MS Scan:27405, Noise threshold:1.1
366.141(HexNAc + Hex)
512.200(HexNAc(1)Hex(1)Fuc(1)_1)
528.195(Hex + HexNAc + Hex)
657.240(NeuAc + Hex + HexNAc)
1055.378
1081.067(PEP+HexNAc(1)_2)
1105.516
1263.623(PEP+HexNAc(2)Hex(1)_2)
1322.357
1335.118
1373.630
1395.631
1463.949
1482.976
1498.720(PEP+HexNAc(2)Hex(3)Fuc(1)_2)
1600.259(PEP+HexNAc(3)Hex(3)Fuc(1)_2)
1608.256(PEP+HexNAc(3)Hex(4)_2)
1681.287(PEP+HexNAc(3)Hex(4)Fuc(1)_2)
1738.802
1792.809
ETD-MS/MS Scan:27406, Noise threshold:1.7
204.088(HexNAc)
229.976
366.143(HexNAc + Hex)
481.850
652.434
693.340
770.080
843.126
940.560
1258.455
1519.235(PEP+HexNAc(3)Hex(2)Fuc(1)_2)
1562.484
1600.262(PEP+HexNAc(3)Hex(3)Fuc(1)_2)
1631.575
1681.287(PEP+HexNAc(3)Hex(4)Fuc(1)_2)
1718.775
1746.805
1762.323
M+2H
m/z

LQLQALQQNGSSVLSEDK(=PEP)_4_4_1_0_0, 0_None, 0_None,
m/z:1188.87(3+), RT:64.75, Y-score:88.81

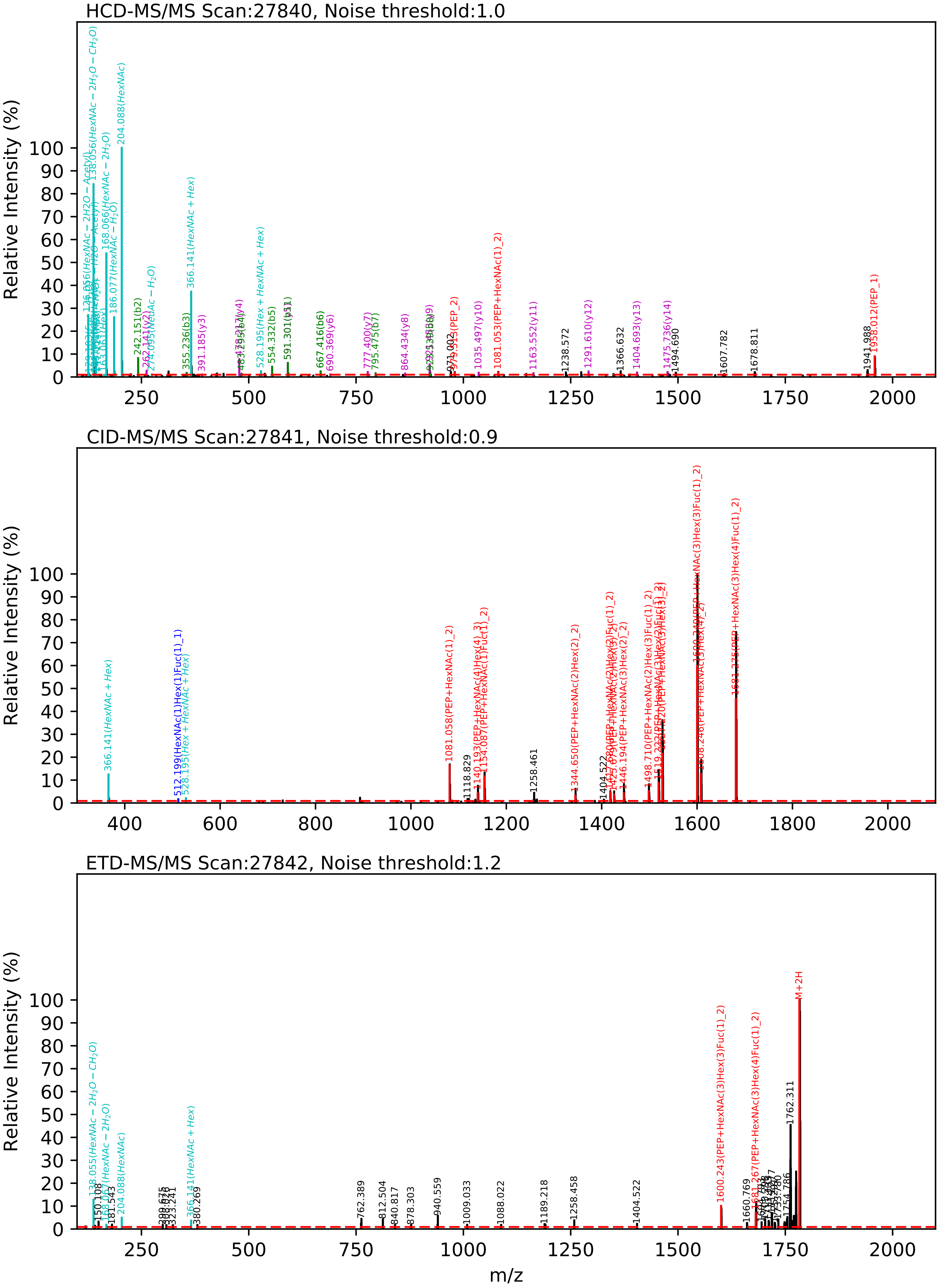

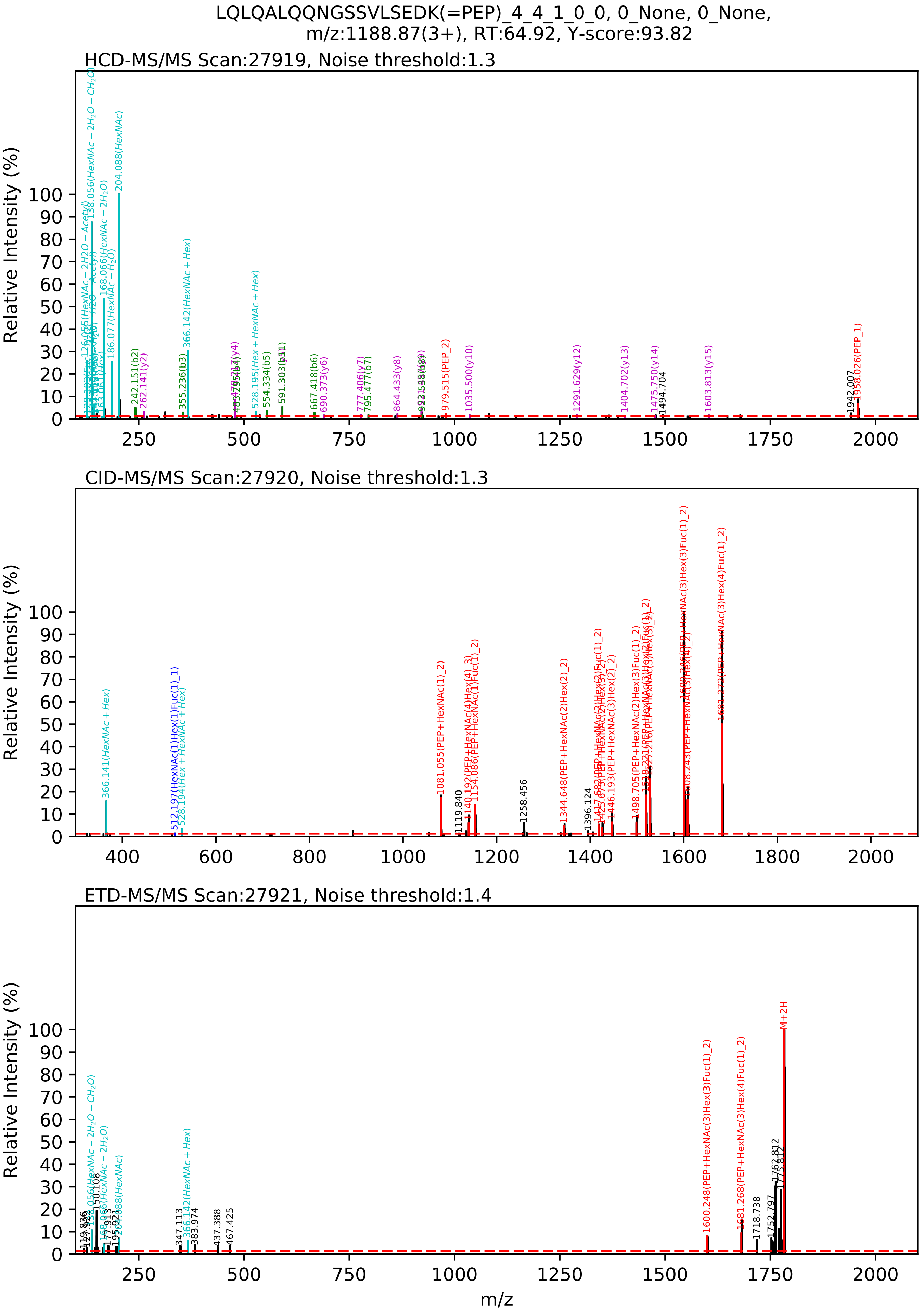

LQLQALQQNGSSVLSEDK(=PEP)_4_4_1_0_0, 0_None, 0_None,
m/z:1188.87(3+), RT:64.92, Y-score:93.82
HCD-MS/MS Scan:27919, Noise threshold:1.3
Relative Intensity (%)
204.088(HexNAc)
168.066(HexNAc − 2H2O)
186.077(HexNAc − H2O)
366.142(HexNAc + Hex)
528.195(Hex + HexNAc + Hex)
242.151(b2)
262.141(y2)
355.236(b3)
554.334(b5)
591.303(y5)
667.418(b6)
690.373(y6)
777.406(y7)
795.477(b7)
864.433(y8)
979.515(PEP_2)
1035.500(y10)
1291.629(y12)
1404.702(y13)
1475.750(y14)
1494.704
1603.813(y15)
1942.007
1958.026(PEP_1)
CID-MS/MS Scan:27920, Noise threshold:1.3
366.141(HexNAc + Hex)
512.197(HexNAc(1)Hex(1)Fuc(1)_1)
528.194(Hex + HexNAc + Hex)
1081.055(PEP+HexNAc(1)_2)
1119.840
1154.086(PEP+HexNAc(1)Fuc(1)_2)
1258.456
1344.648(PEP+HexNAc(2)Hex(2)_2)
1396.124
1446.193(PEP+HexNAc(3)Hex(2)_2)
1498.705(PEP+HexNAc(2)Hex(3)Fuc(1)_2)
1600.246(PEP+HexNAc(3)Hex(3)Fuc(1)_2)
1681.272(PEP+HexNAc(3)Hex(4)Fuc(1)_2)
ETD-MS/MS Scan:27921, Noise threshold:1.4
204.088(HexNAc)
366.142(HexNAc + Hex)
150.108
179.913
195.923
347.113
383.974
437.388
467.425
1600.248(PEP+HexNAc(3)Hex(3)Fuc(1)_2)
1681.268(PEP+HexNAc(3)Hex(4)Fuc(1)_2)
1718.738
1752.797
1763.812
1773.812
M+2H
m/z

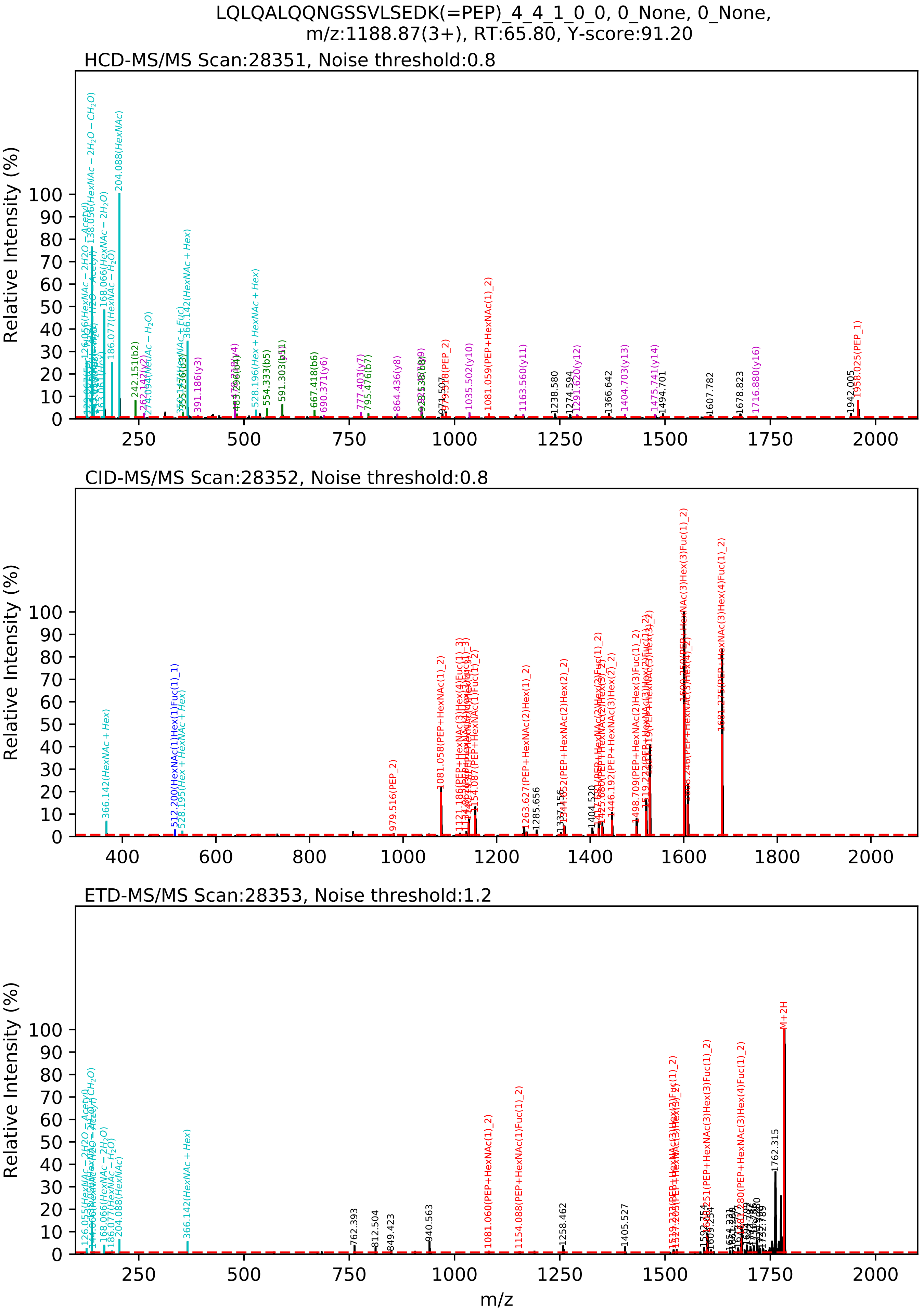

LQLQALQQNGSSVLSEDK(=PEP)_4_4_1_0_0, 0_None, 0_None,
m/z:1188.87(3+), RT:65.80, Y-score:91.20
HCD-MS/MS Scan:28351, Noise threshold:0.8
CID-MS/MS Scan:28352, Noise threshold:0.8
ETD-MS/MS Scan:28353, Noise threshold:1.2
Relative Intensity (%)
m/z

LQLQALQQNGSSVLSEDK(=PEP)_4_4_1_0_0, 0_None, 0_None,
m/z:1782.81(2+), RT:66.03, Y-score:84.83

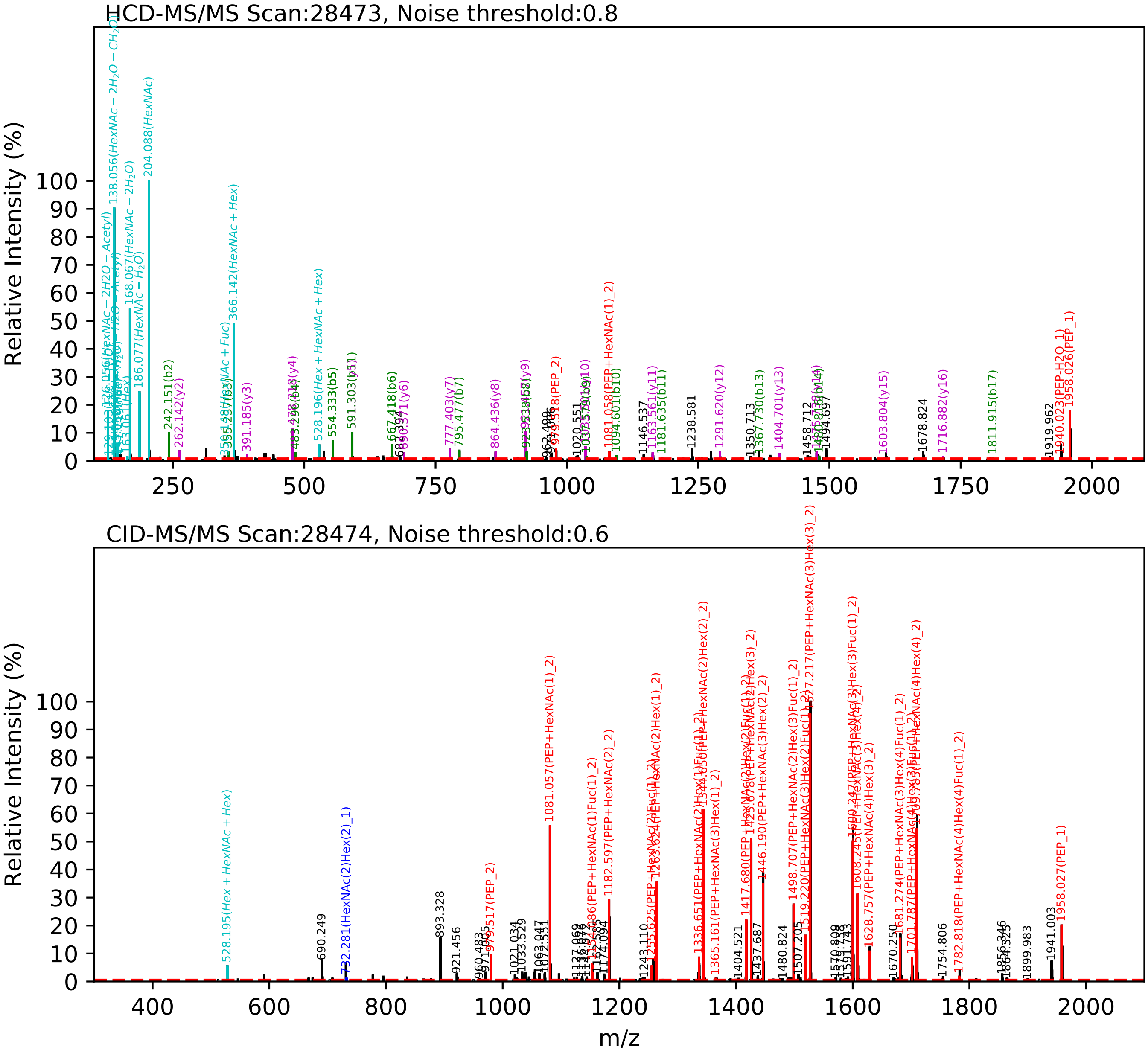

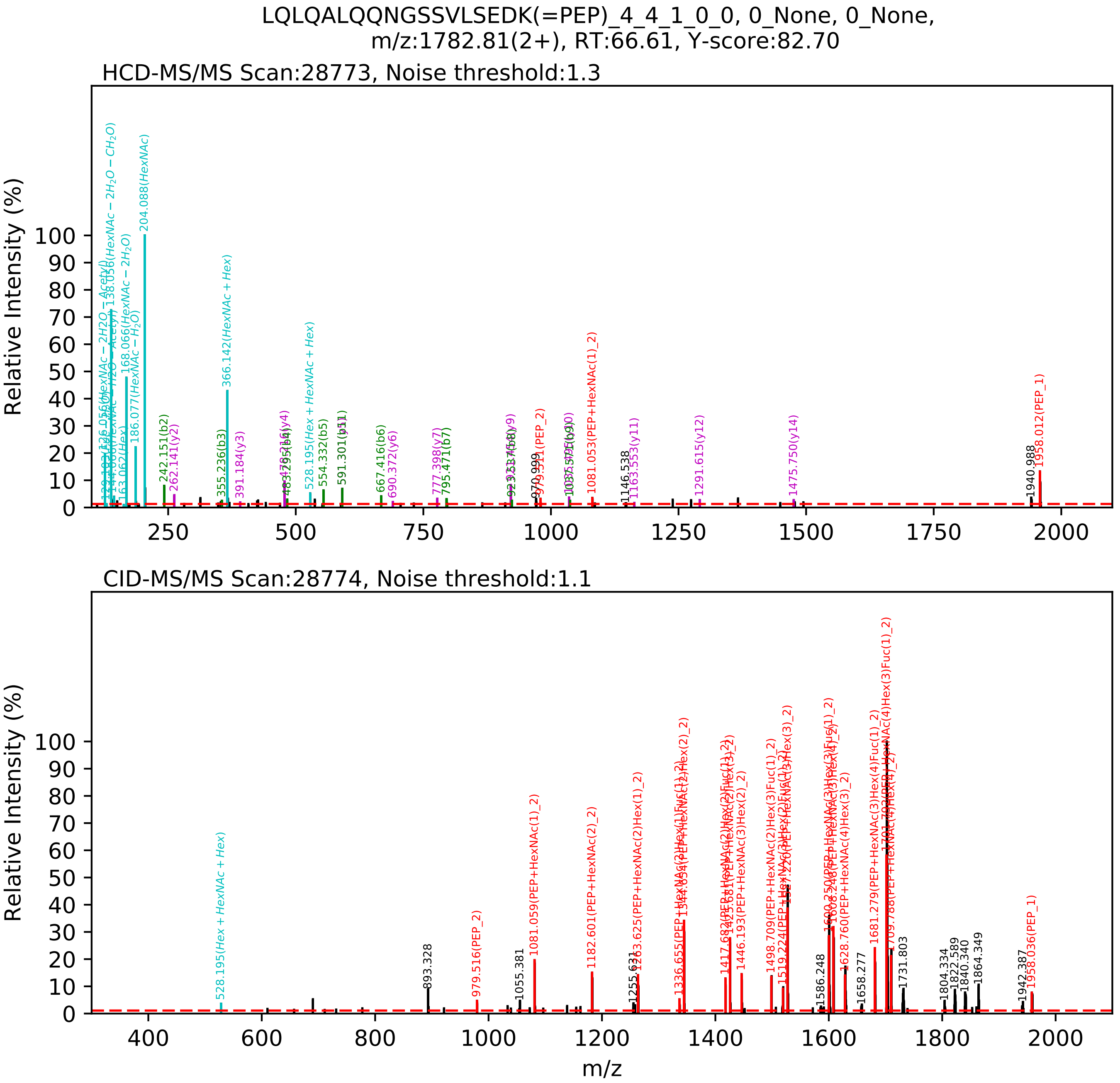

LQLQALQQNGSSVLSEDK(=PEP)_4_4_1_0_0, 0_None, 0_None,
m/z:1782.81(2+), RT:66.61, Y-score:82.70
HCD-MS/MS Scan:28773, Noise threshold:1.3
CID-MS/MS Scan:28774, Noise threshold:1.1
Relative Intensity (%)
m/z

LQLQALQQNGSSVLSEDK(=PEP)_4_4_1_0_0, 0_None, 0_None,
m/z:1188.87(3+), RT:66.52, Y-score:99.55

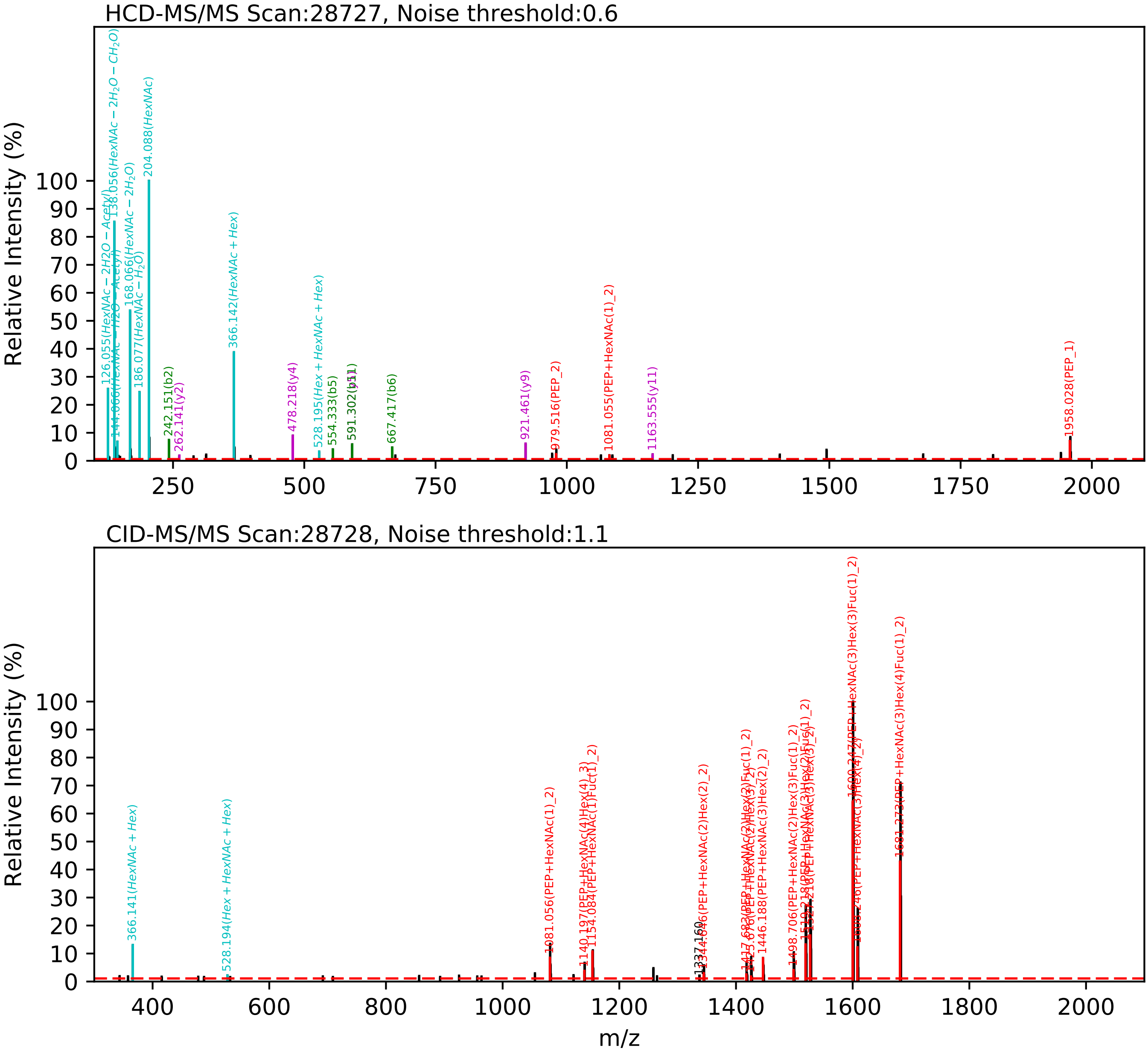

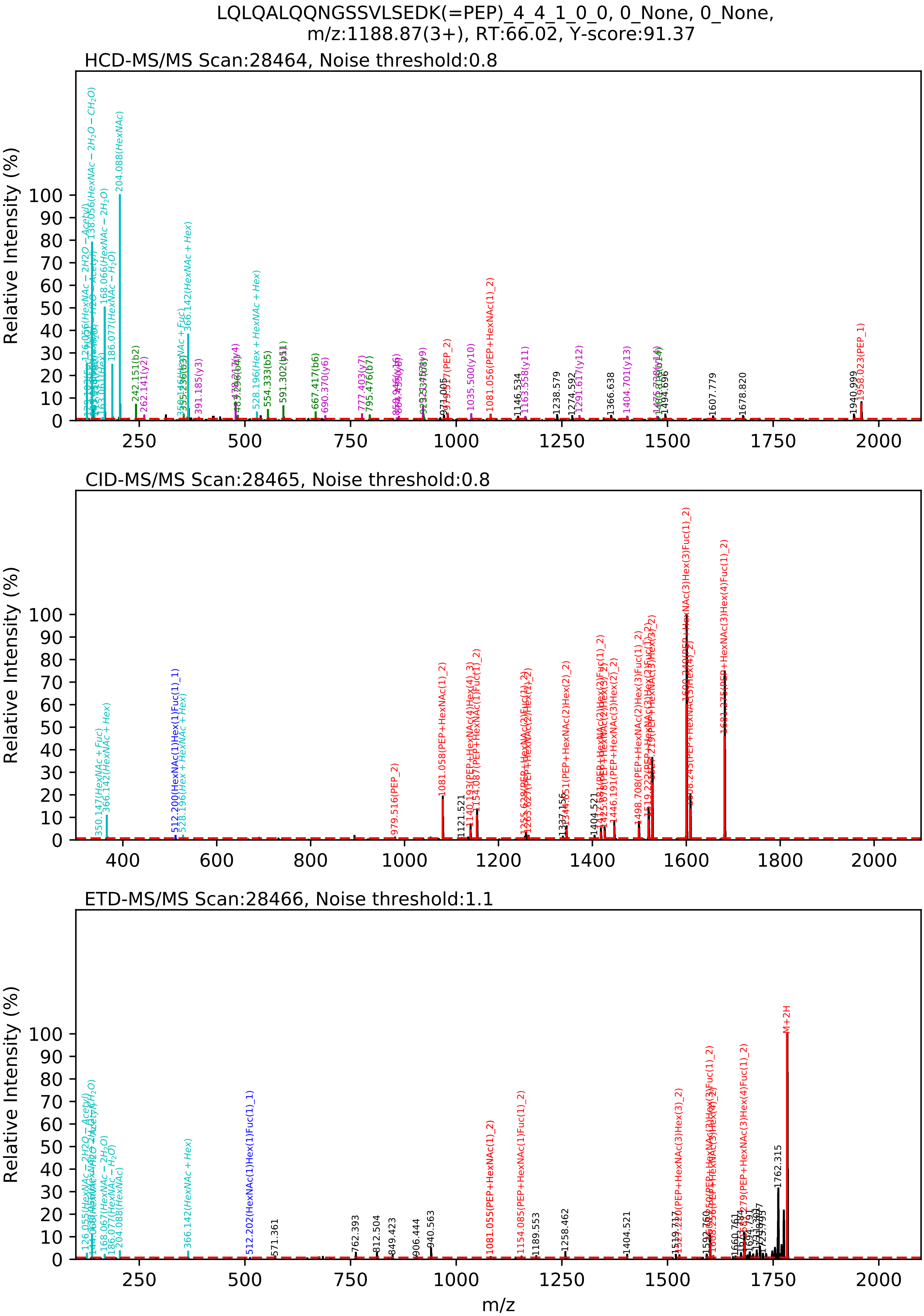
LQLQALQQNGSSVLSEDK(=PEP)_4_4_1_0_0, 0_None, 0_None,
m/z:1188.87(3+), RT:66.02, Y-score:91.37
HCD-MS/MS Scan:28464, Noise threshold:0.8
CID-MS/MS Scan:28465, Noise threshold:0.8
ETD-MS/MS Scan:28466, Noise threshold:1.1
Relative Intensity (%)
m/z
M+2H

LQLQALQQNGSSVLSEDK(=PEP)_4_4_1_0_0, 0_None, 0_None,
m/z:1188.87(3+), RT:66.95, Y-score:90.96

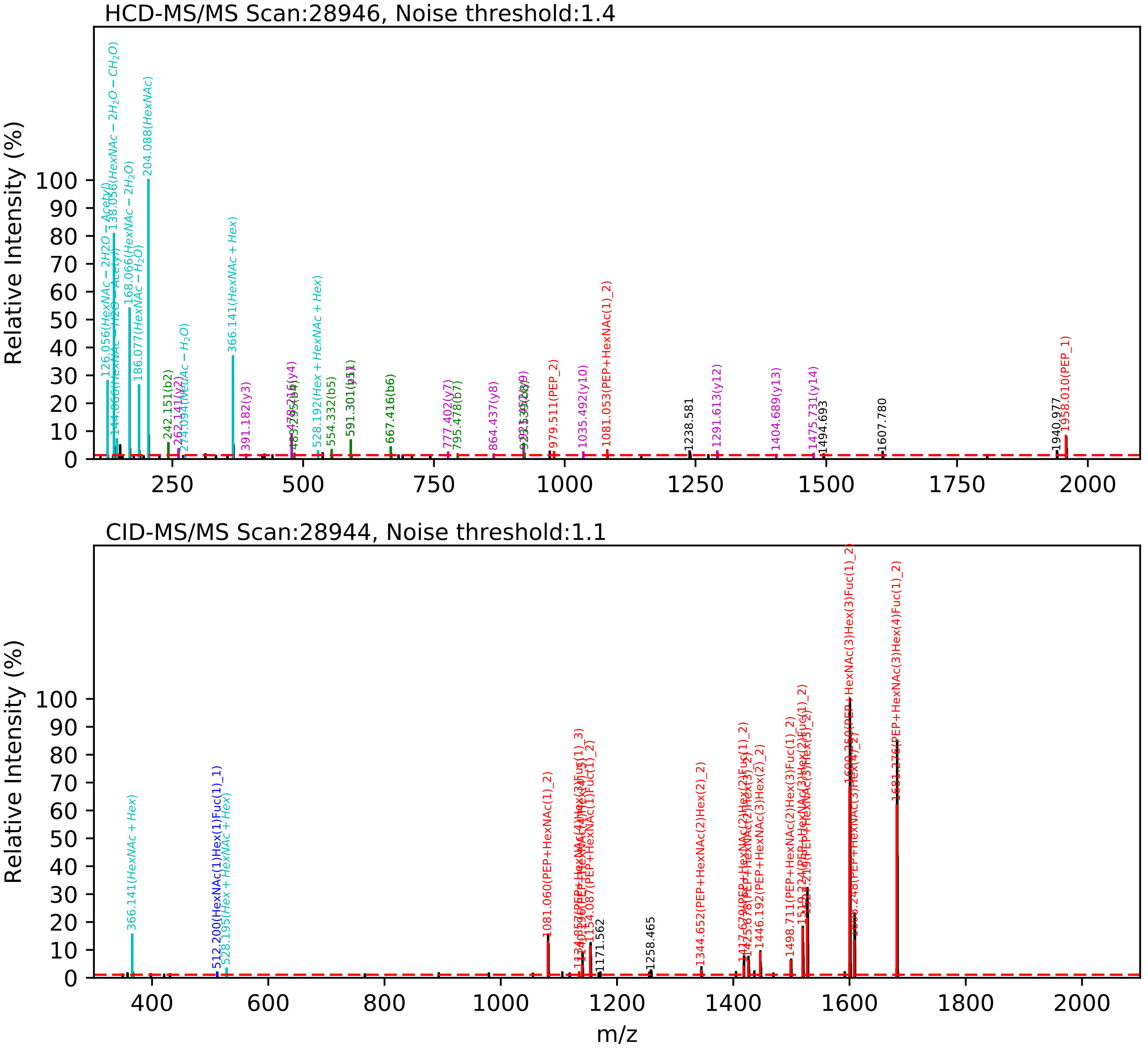

LQLQALQQNGSSVLSEDK(=PEP)_4_4_1_0_0, 0_None, 0_None,
m/z:1188.87(3+), RT:67.55, Y-score:79.69

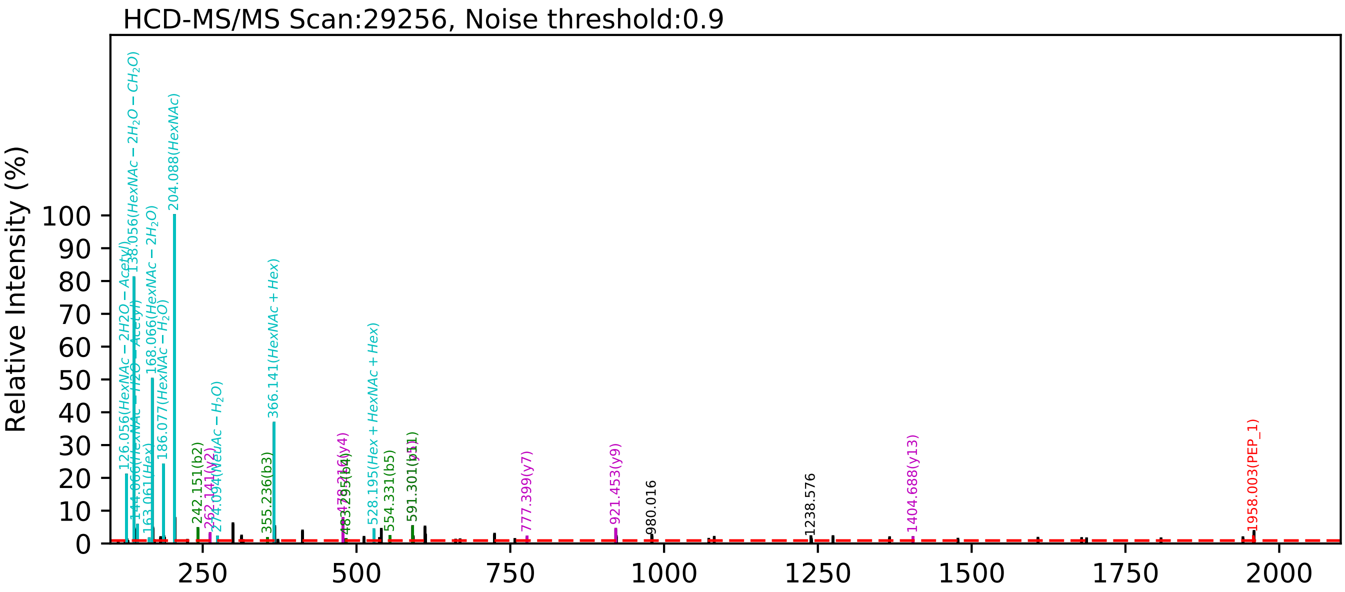

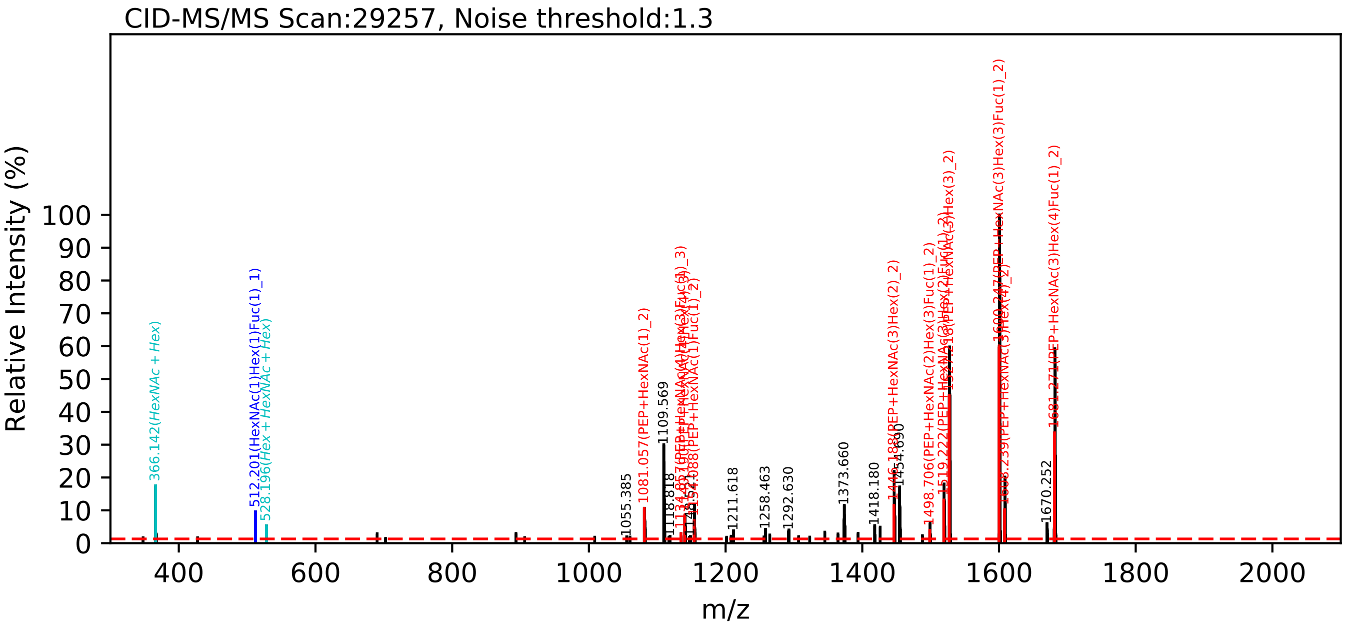

LQLQALQQNGSSVLSEDK(=PEP)_4_4_1_0_0, 0_None, 0_None,
m/z:1188.87(3+), RT:68.25, Y-score:80.37

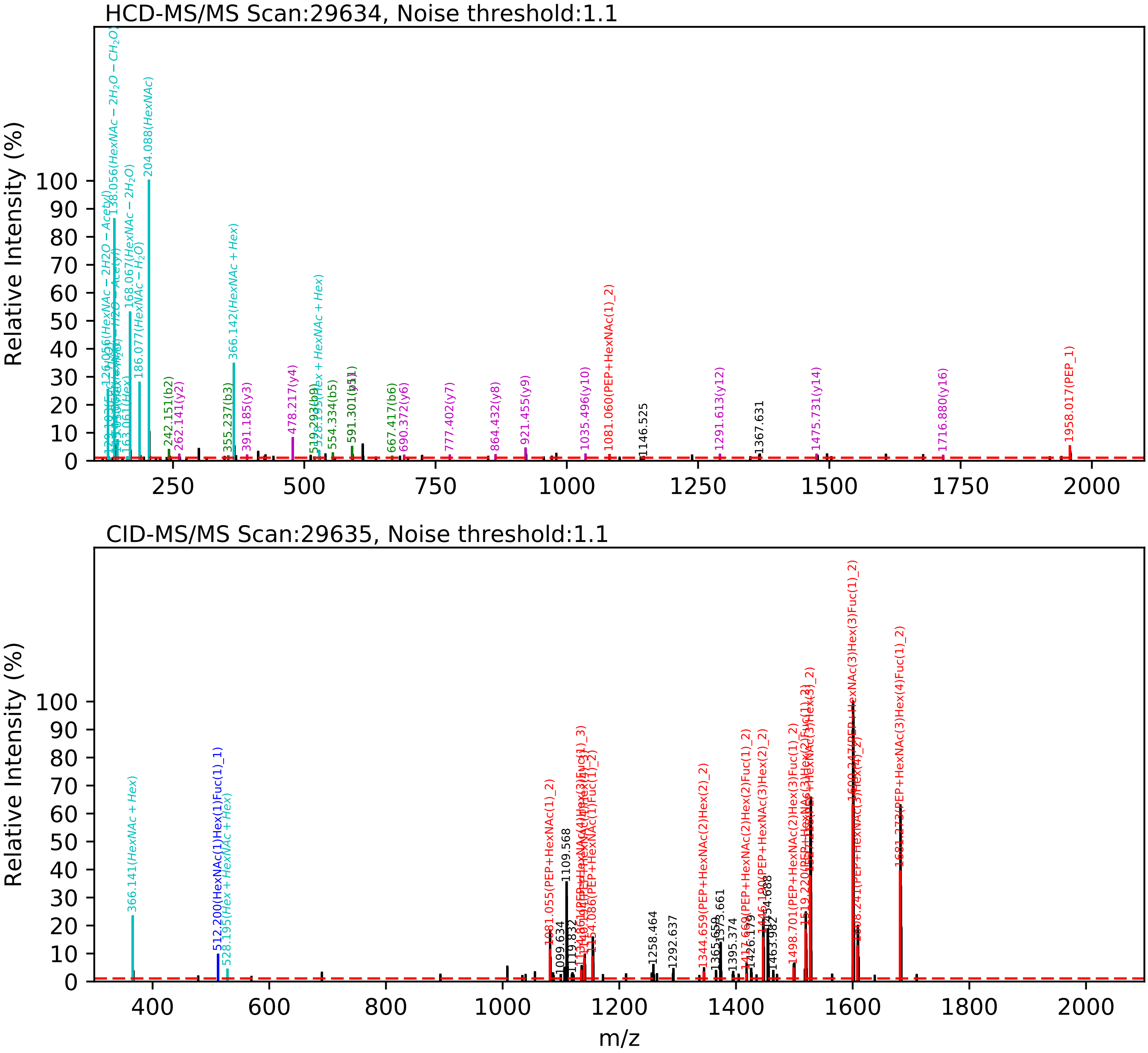

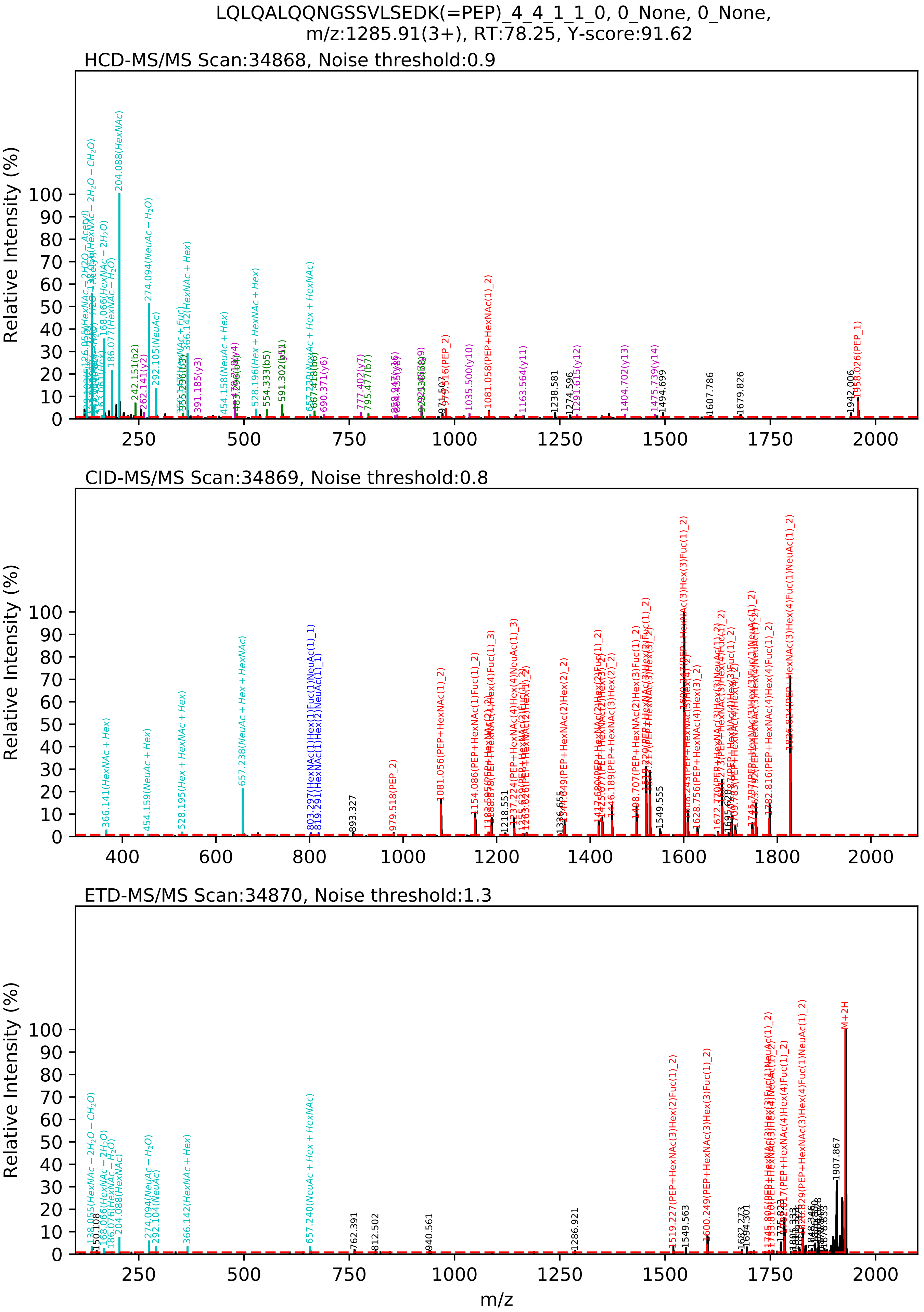

LQLQALQQNGSSVLSEDK(=PEP)_4_4_1_1_0, 0_None, 0_None,
m/z:1285.91(3+), RT:78.25, Y-score:91.62
HCD-MS/MS Scan:34868, Noise threshold:0.9
CID-MS/MS Scan:34869, Noise threshold:0.8
ETD-MS/MS Scan:34870, Noise threshold:1.3
Relative Intensity (%)
m/z
204.088(HexNAc)
274.094(NeuAc − H2O)
292.105(NeuAc)
366.142(HexNAc + Hex)
454.158(NeuAc + Hex)
528.196(Hex + HexNAc + Hex)
1081.058(PEP+HexNAc(1)_2)
1958.026(PEP_1)
657.238(NeuAc + Hex + HexNAc)
1081.056(PEP+HexNAc(1)_2)
979.518(PEP_2)
1826.824(PEP+HexNAc(3)Hex(4)Fuc(1)NeuAc(1)_2)
1519.227(PEP+HexNAc(3)Hex(2)Fuc(1)_2)
1600.249(PEP+HexNAc(3)Hex(3)Fuc(1)_2)
1907.867
M+2H

LQLQALQQNGSSVLSEDK(=PEP)_4_4_1_1_0, 0_None, 0_None,
m/z:1285.90(3+), RT:75.93, Y-score:89.15

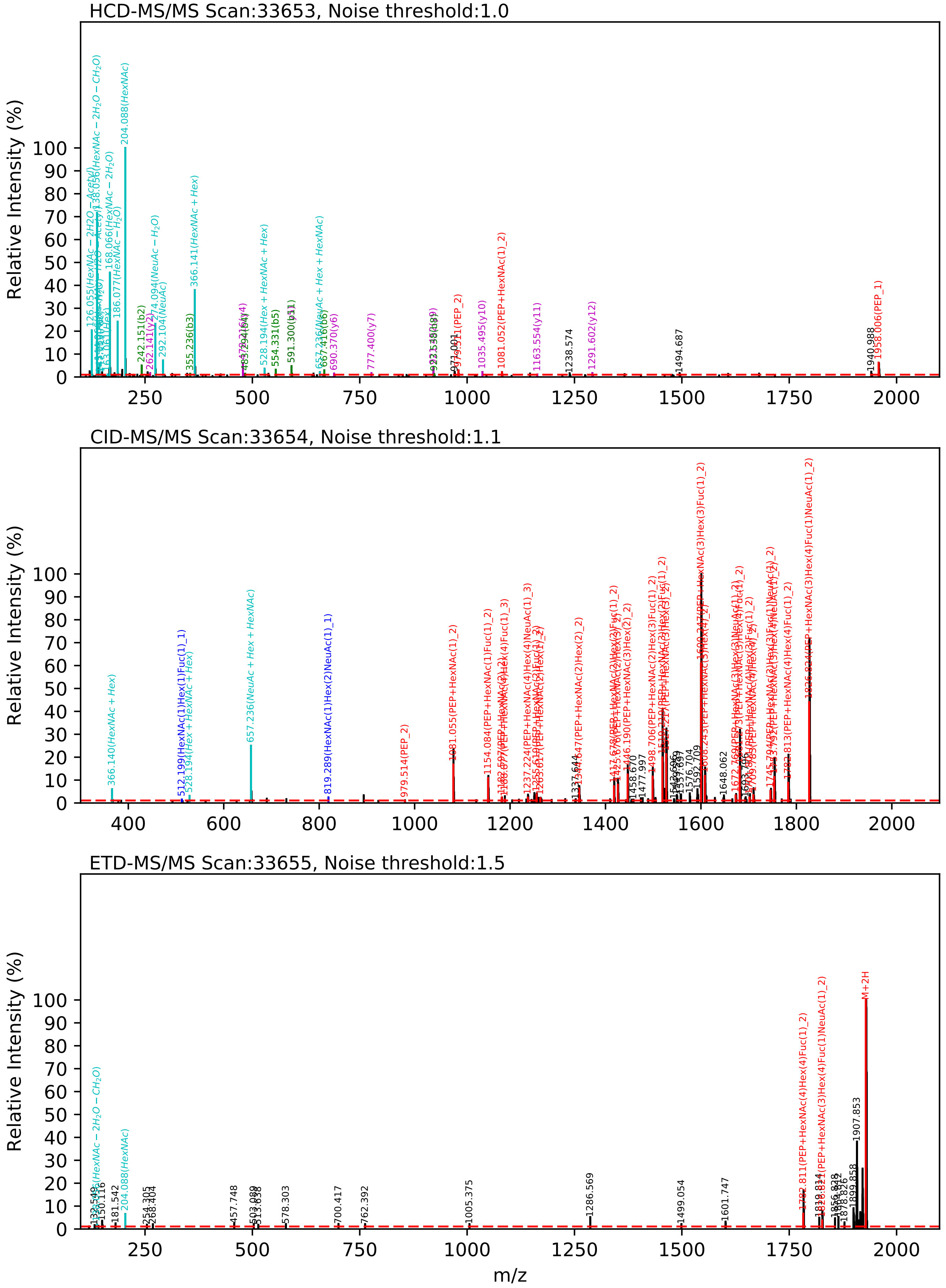

LQLQALQQNGSSVLSEDK(=PEP)_4_4_1_1_0, 0_None, 0_None,
m/z:1285.90(3+), RT:76.73, Y-score:73.10

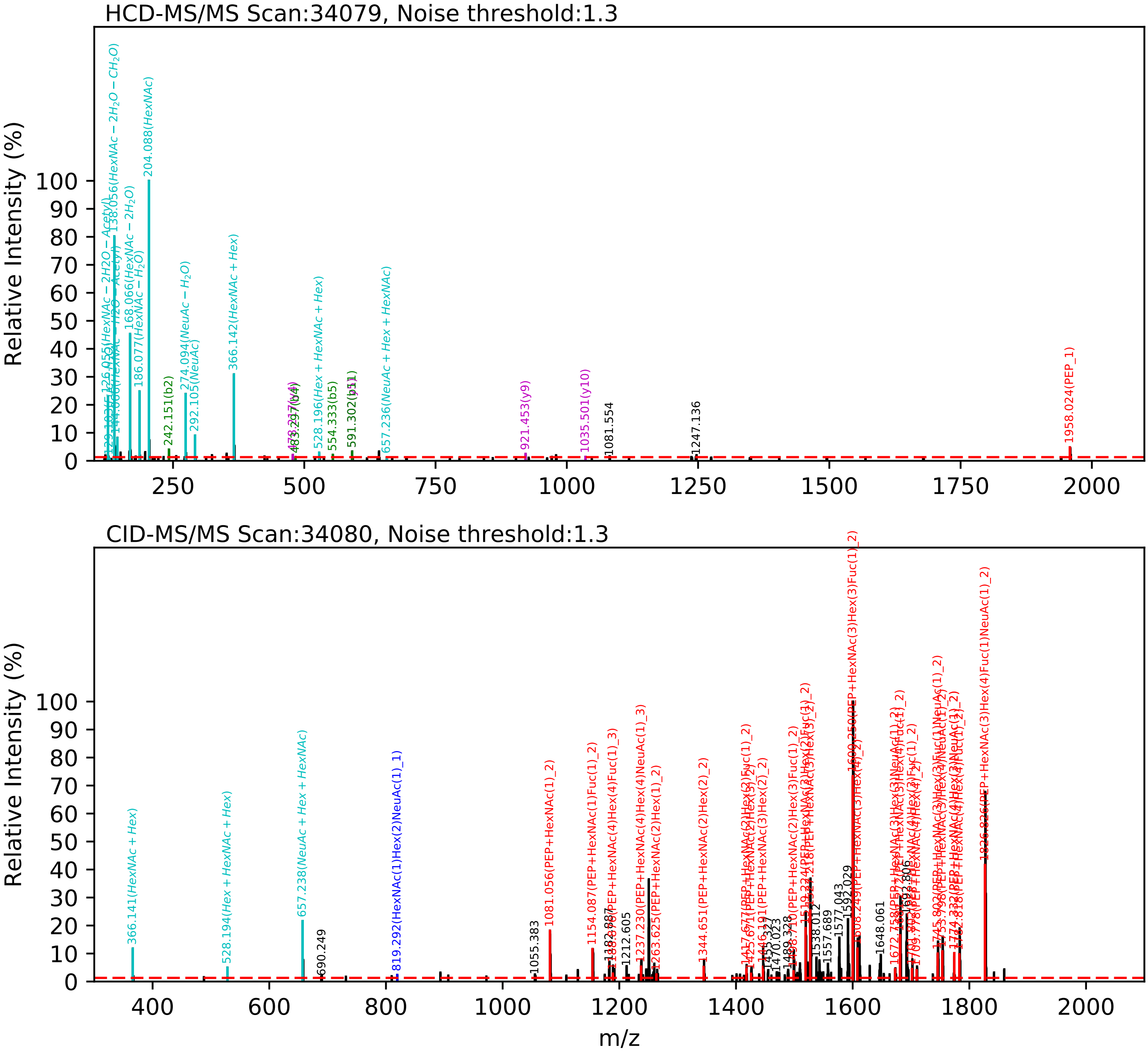

LQLQALQQNGSSVLSEDK(=PEP)_4_4_1_1_0, 0_None, 0_None,
m/z:1285.90(3+), RT:77.54, Y-score:90.96

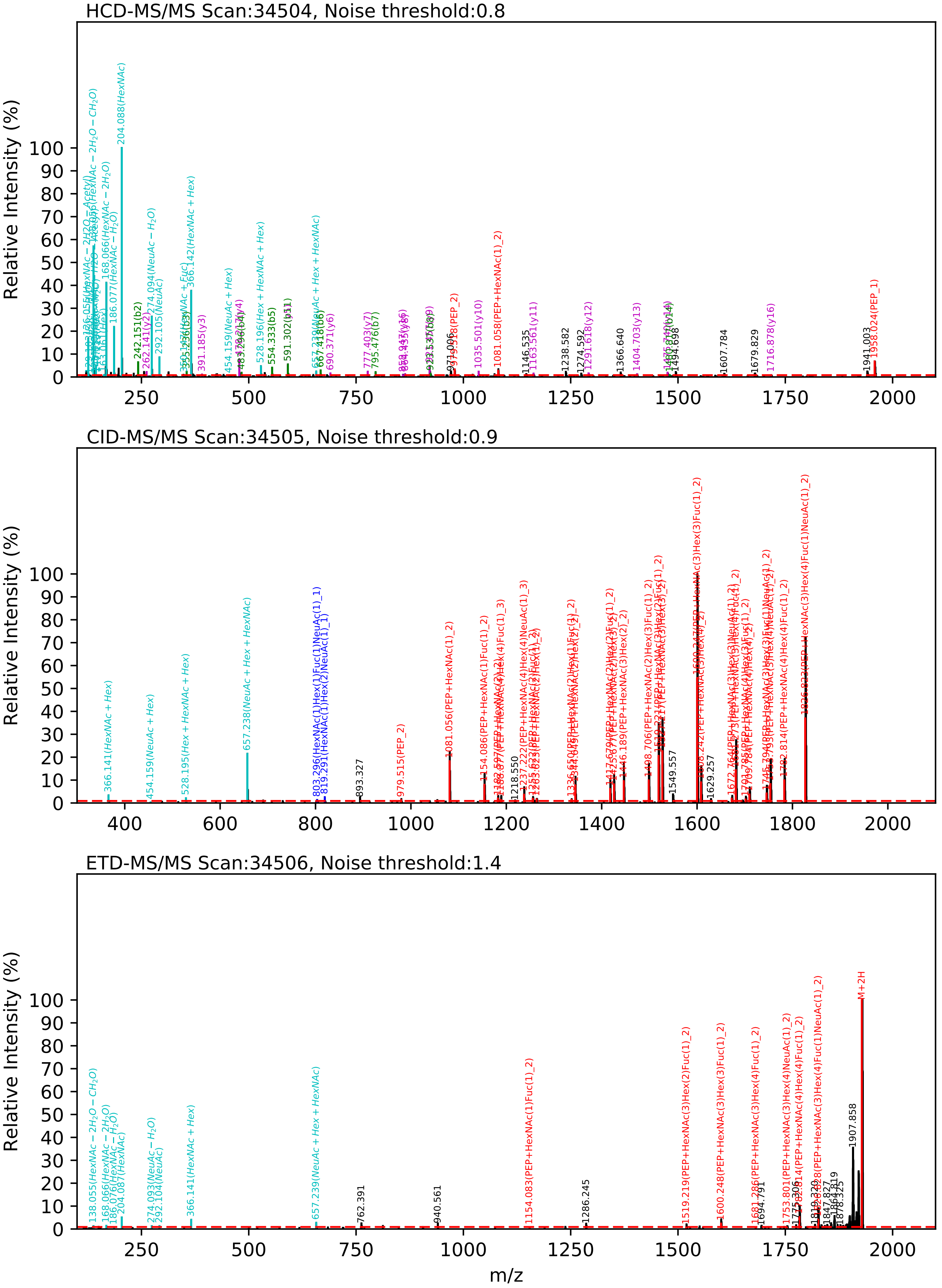

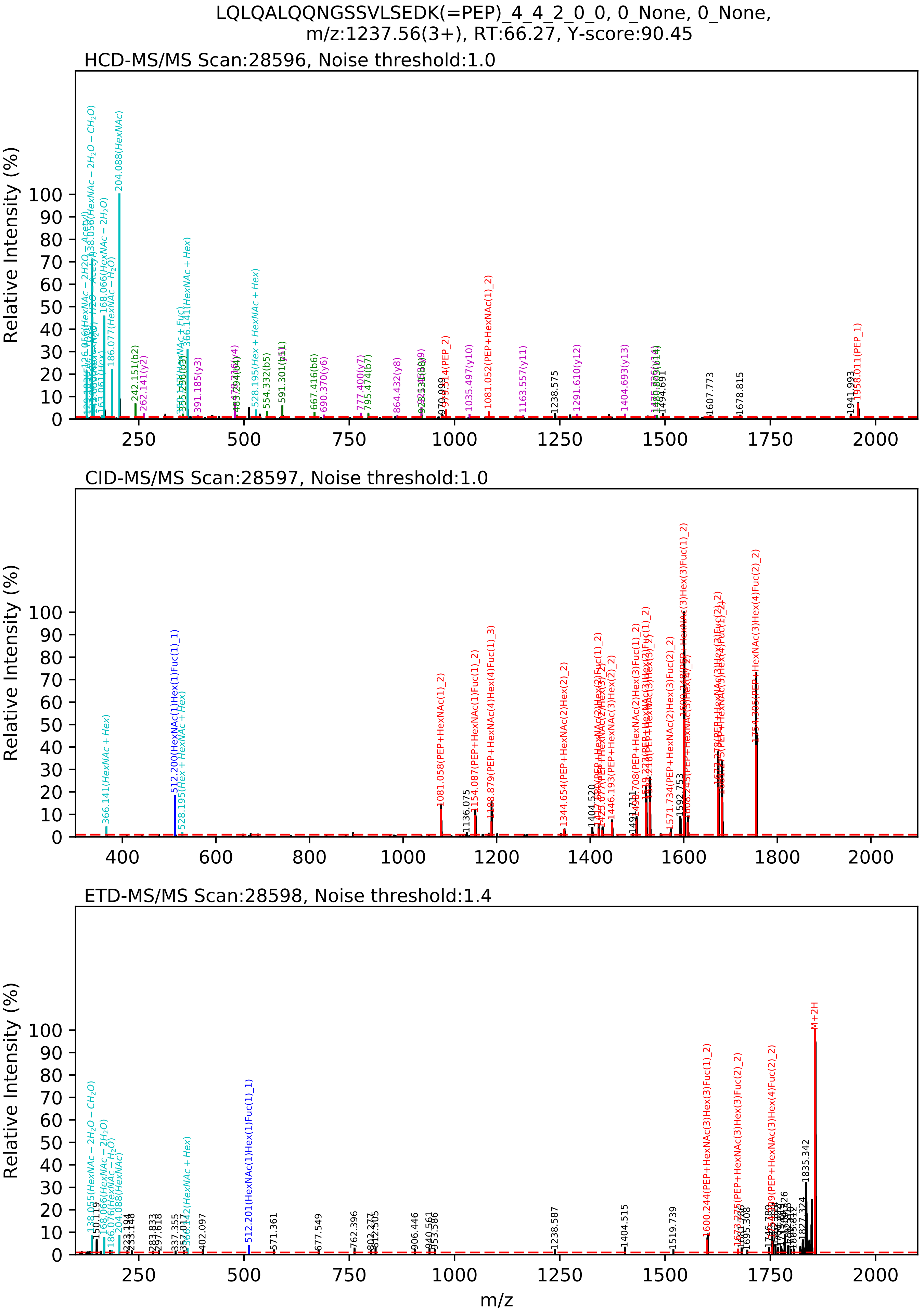
LQLQALQQNGSSVLSEDK(=PEP)_4_4_2_0_0, 0_None, 0_None,
m/z:1237.56(3+), RT:66.27, Y-score:90.45
HCD-MS/MS Scan:28596, Noise threshold:1.0
CID-MS/MS Scan:28597, Noise threshold:1.0
ETD-MS/MS Scan:28598, Noise threshold:1.4
Relative Intensity (%)
m/z

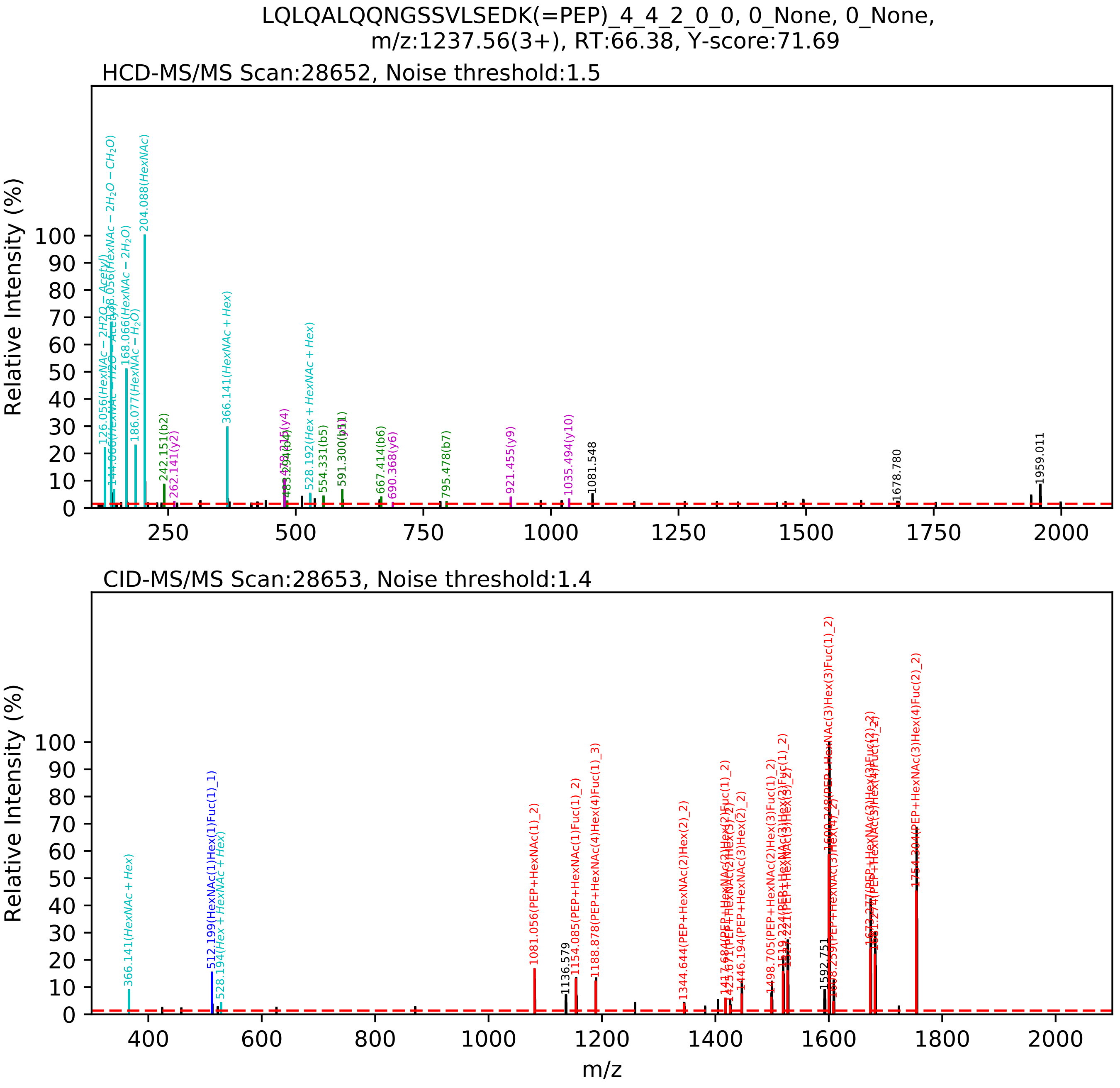

LQLQALQQNGSSVLSEDK(=PEP)_4_4_2_0_0, 0_None, 0_None,
m/z:1237.56(3+), RT:66.38, Y-score:71.69
HCD-MS/MS Scan:28652, Noise threshold:1.5
Relative Intensity (%)
204.088(HexNAc)
168.066(HexNAc − 2H2O)
186.077(HexNAc − H2O)
126.056(HexNAc − 2H2O − CH2O)
242.151(b2)
262.141(y2)
366.141(HexNAc + Hex)
528.192(Hex + HexNAc + Hex)
554.331(b5)
667.414(b6)
690.368(y6)
795.478(b7)
921.455(y9)
1035.494(y10)
1081.548
1678.780
1959.011
CID-MS/MS Scan:28653, Noise threshold:1.4
Relative Intensity (%)
366.141(HexNAc + Hex)
512.199(HexNAc(1)Hex(1)Fuc(1)_1)
528.194(Hex + HexNAc + Hex)
1081.056(PEP+HexNAc(1)_2)
1136.579
1154.085(PEP+HexNAc(1)Fuc(1)_2)
1188.878(PEP+HexNAc(4)Hex(4)Fuc(1)_3)
1344.644(PEP+HexNAc(2)Hex(2)_2)
1446.194(PEP+HexNAc(3)Hex(2)_2)
1498.705(PEP+HexNAc(2)Hex(3)Fuc(1)_2)
1592.751
1754.304(PEP+HexNAc(3)Hex(4)Fuc(2)_2)
m/z

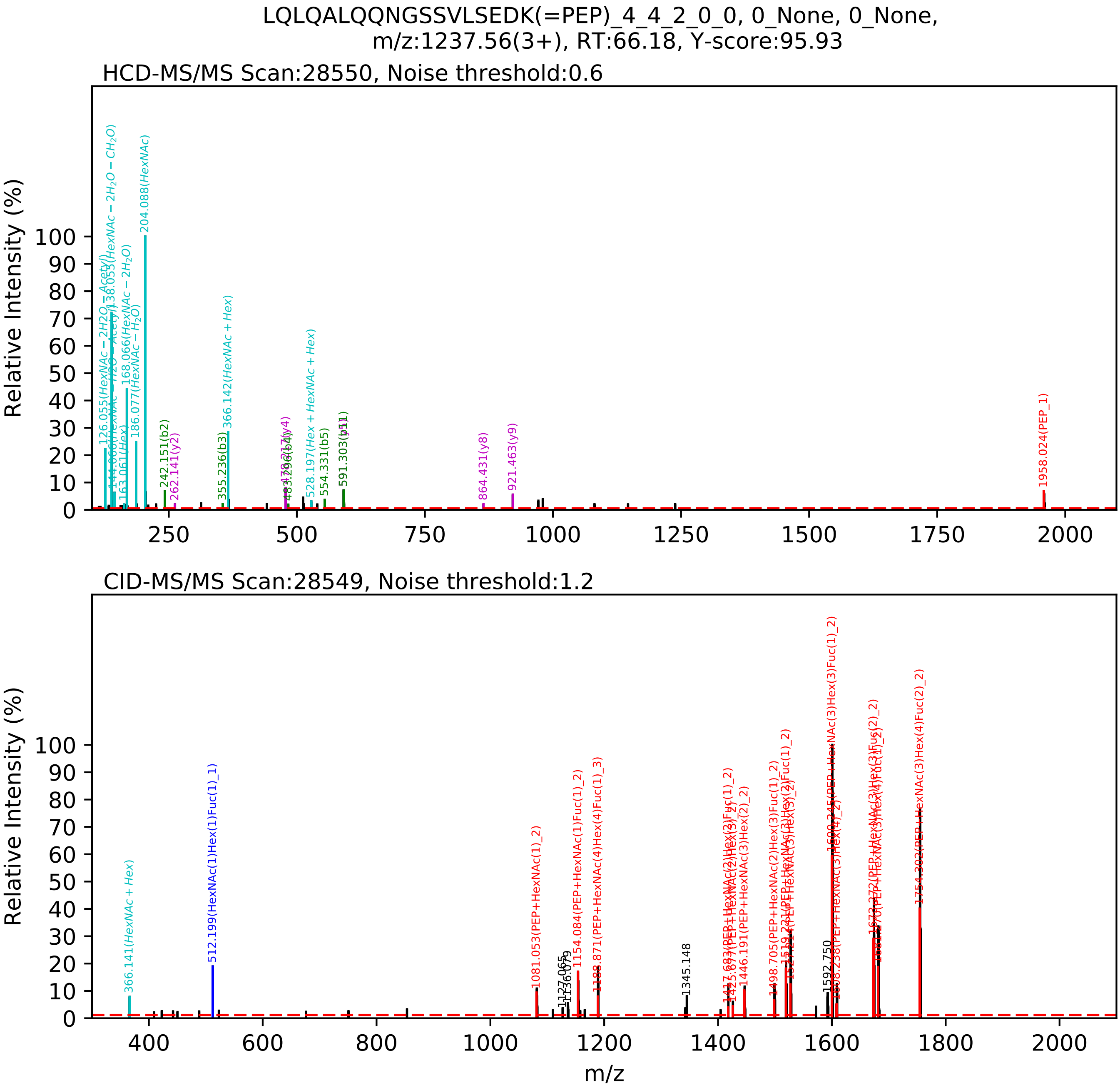

LQLQALQQNGSSVLSEDK(=PEP)_4_4_2_0_0, 0_None, 0_None,
m/z:1237.56(3+), RT:66.18, Y-score:95.93
HCD-MS/MS Scan:28550, Noise threshold:0.6
Relative Intensity (%)
204.088(HexNAc)
168.066(HexNAc − 2H2O)
186.077(HexNAc − H2O)
126.055(HexNAc − 2H2O − CH2O)
366.142(HexNAc + Hex)
528.197(Hex + HexNAc + Hex)
242.151(b2)
262.141(y2)
355.236(b3)
554.331(b5)
591.303(b11)
864.431(y8)
921.463(y9)
1958.024(PEP_1)
CID-MS/MS Scan:28549, Noise threshold:1.2
Relative Intensity (%)
366.141(HexNAc + Hex)
512.199(HexNAc(1)Hex(1)Fuc(1)_1)
1081.053(PEP+HexNAc(1)_2)
1127.065
1136.079
1154.084(PEP+HexNAc(1)Fuc(1)_2)
1188.871(PEP+HexNAc(4)Hex(4)Fuc(1)_3)
1345.148
1446.191(PEP+HexNAc(3)Hex(2)_2)
1498.705(PEP+HexNAc(2)Hex(3)Fuc(1)_2)
1592.750
1754.292(PEP+HexNAc(3)Hex(4)Fuc(2)_2)
m/z

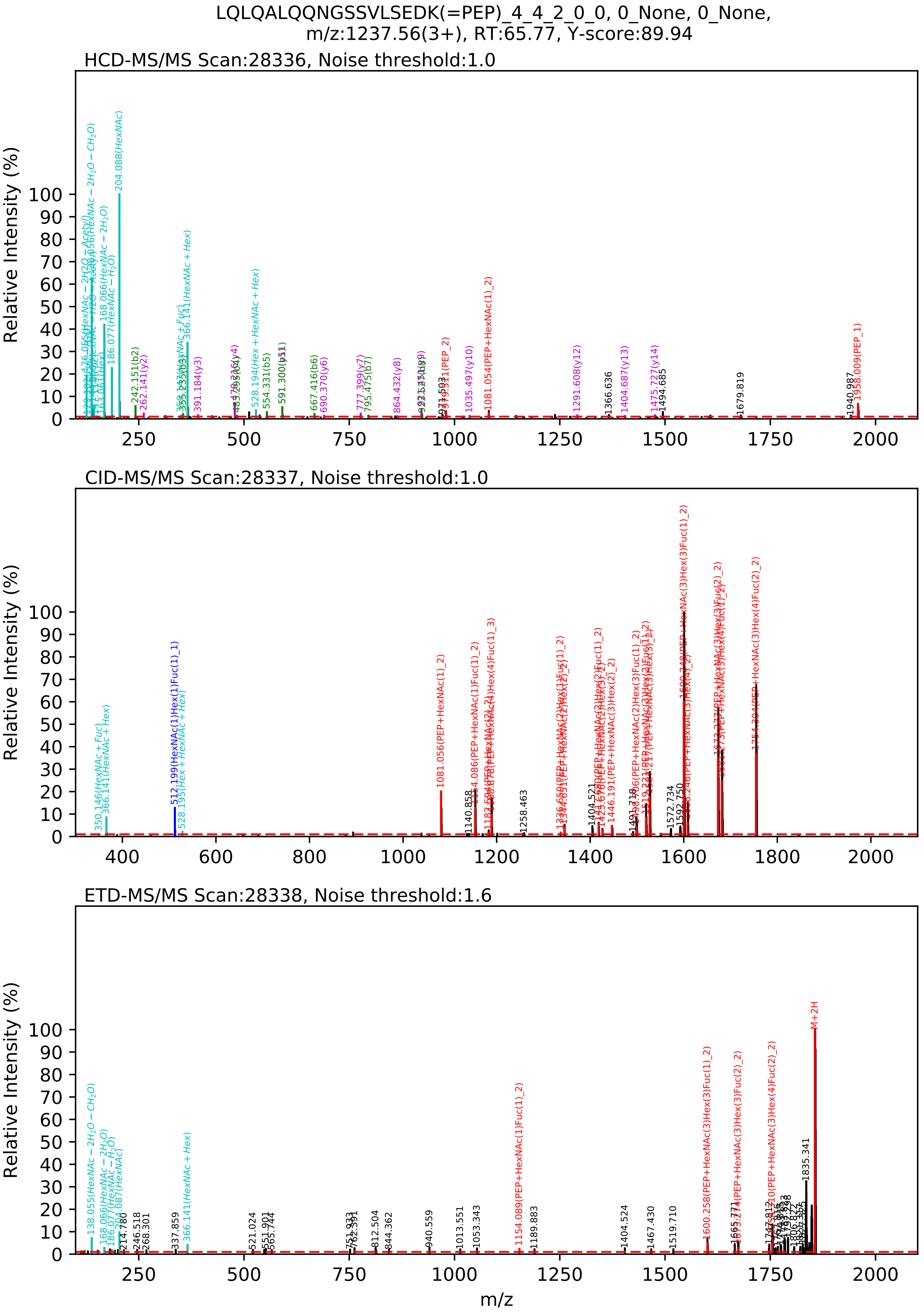

LQLQALQQNGSSVLSEDK(=PEP)_4_4_2_0_0, 0_None, 0_None,
m/z:1237.56(3+), RT:65.77, Y-score:89.94
HCD-MS/MS Scan:28336, Noise threshold:1.0
CID-MS/MS Scan:28337, Noise threshold:1.0
ETD-MS/MS Scan:28338, Noise threshold:1.6
Relative Intensity (%)
m/z
204.088(HexNAc)
366.141(HexNAc + Hex)
1081.054(PEP+HexNAc(1)_2)
1958.009(PEP_1)
512.199(HexNAc(1)Hex(1)Fuc(1)_1)
1081.056(PEP+HexNAc(1)_2)
1754.304(PEP+HexNAc(3)Hex(4)Fuc(2)_2)
1154.089(PEP+HexNAc(1)Fuc(1)_2)
1600.258(PEP+HexNAc(3)Hex(3)Fuc(1)_2)
M+2H
1835.341

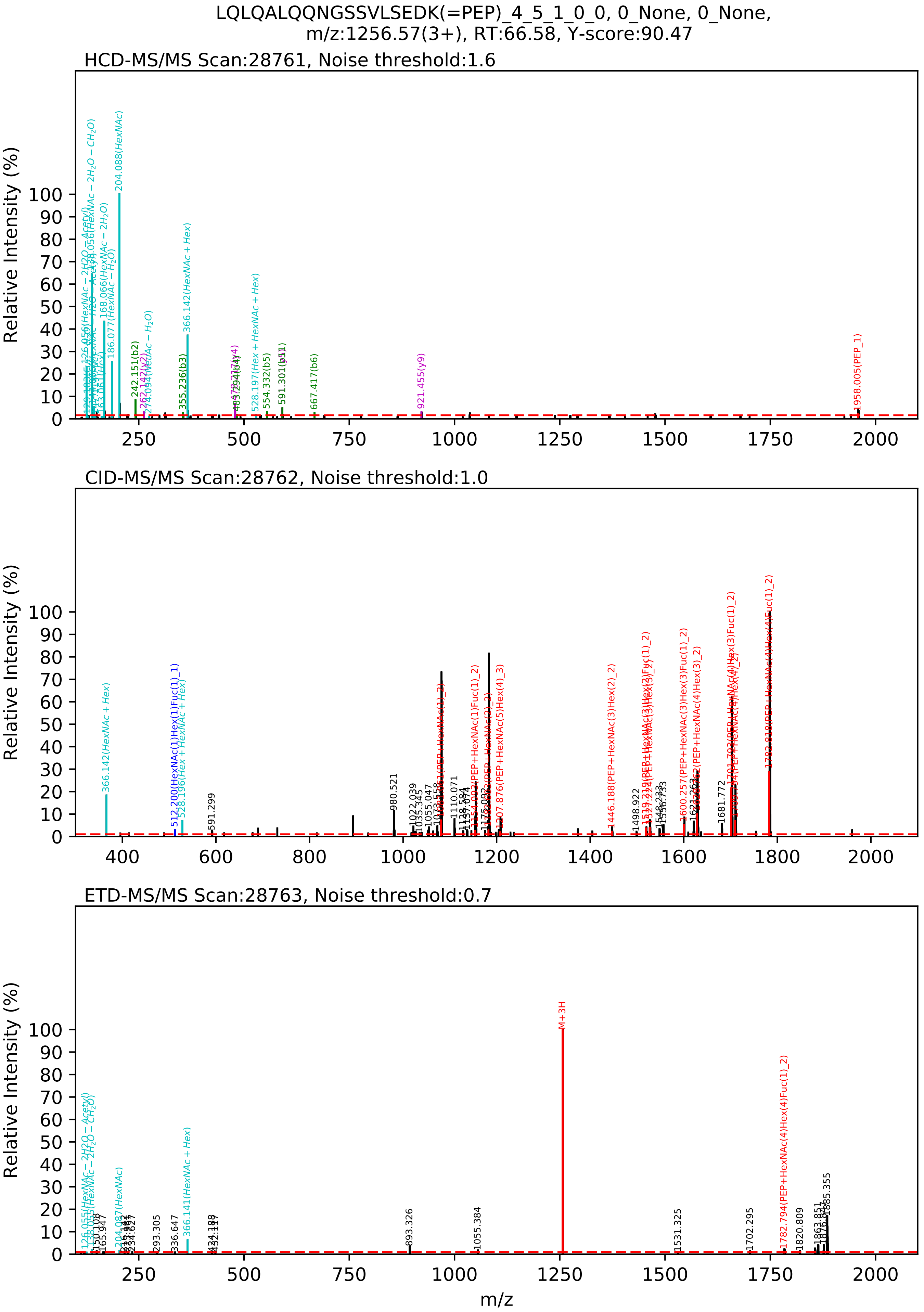

LQLQALQQNGSSVLSEDK(=PEP)_4_5_1_0_0, 0_None, 0_None,
m/z:1256.57(3+), RT:66.58, Y-score:90.47
HCD-MS/MS Scan:28761, Noise threshold:1.6
Relative Intensity (%)
204.088(HexNAc)
168.066(HexNAc − 2H2O)
186.077(HexNAc − H2O)
366.142(HexNAc + Hex)
242.151(b2)
262.142(y2)
355.236(b3)
528.197(Hex + HexNAc + Hex)
554.332(b5)
591.301(b5)
667.417(b6)
921.455(y9)
1958.005(PEP_1)
CID-MS/MS Scan:28762, Noise threshold:1.0
366.142(HexNAc + Hex)
512.200(HexNAc(1)Hex(1)Fuc(1)_1)
528.196(Hex + HexNAc + Hex)
591.299
980.521
1022.039
1035.345
1055.047
1110.071
1128.584
1137.074
1207.876(PEP+HexNAc(5)Hex(4)_3)
1446.188(PEP+HexNAc(3)Hex(2)_2)
1498.922
1548.233
1556.733
1600.257(PEP+HexNAc(3)Hex(3)Fuc(1)_2)
1681.772
1782.819(PEP+HexNAc(4)Hex(4)Fuc(1)_2)
ETD-MS/MS Scan:28763, Noise threshold:0.7
M+3H
150.103
165.947
204.087(HexNAc)
293.305
336.647
366.141(HexNAc + Hex)
434.188
893.326
1055.384
1531.325
1702.295
1782.794(PEP+HexNAc(4)Hex(4)Fuc(1)_2)
1820.809
1863.851
1885.355
m/z

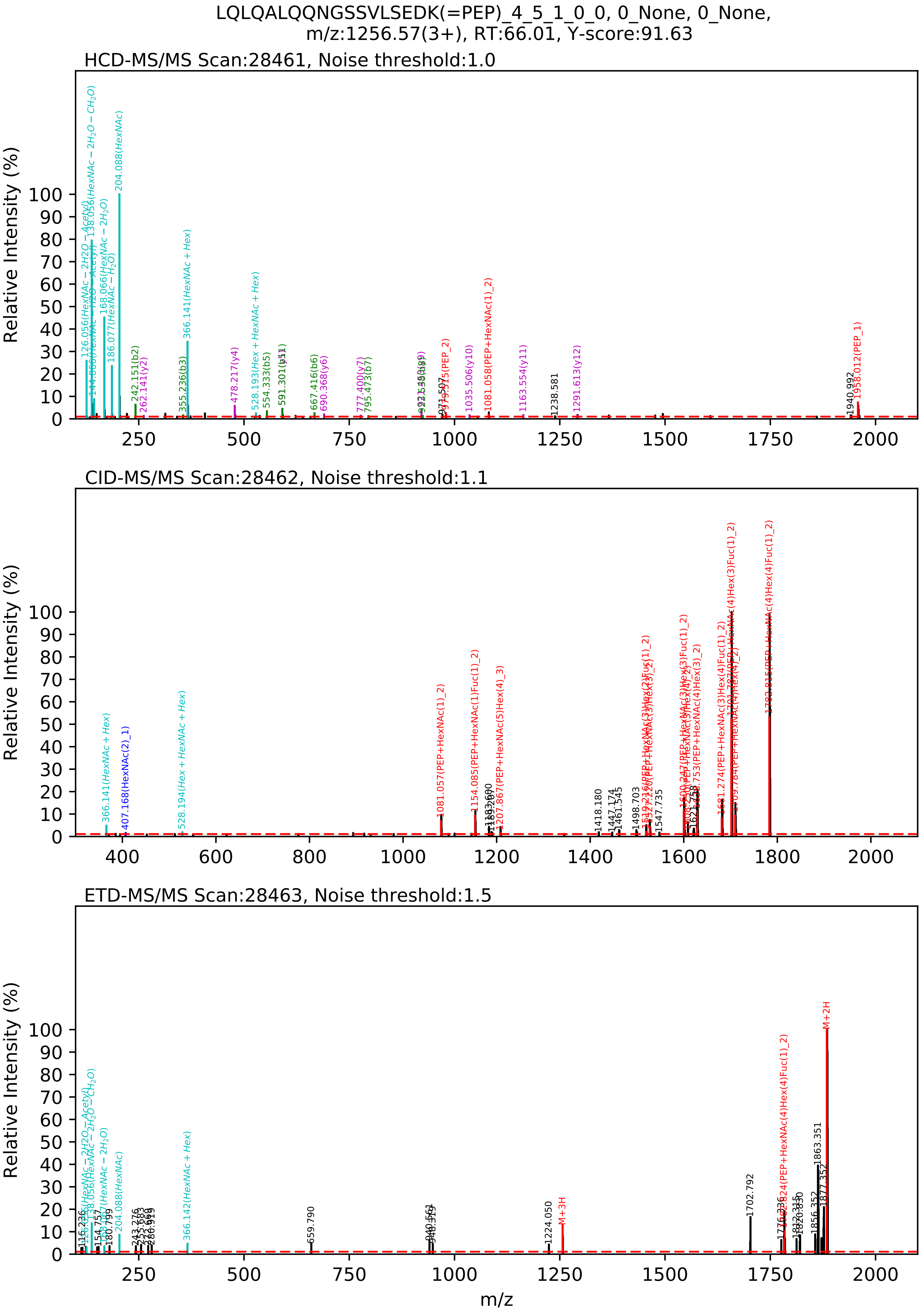
LQLQALQQNGSSVLSEDK(=PEP)_4_5_1_0_0, 0_None, 0_None,
m/z:1256.57(3+), RT:66.01, Y-score:91.63
HCD-MS/MS Scan:28461, Noise threshold:1.0
Relative Intensity (%)
204.088(HexNAc)
168.066(HexNAc − 2H2O)
186.077(HexNAc − H2O)
366.141(HexNAc + Hex)
242.151(b2)
262.141(y2)
355.236(b3)
478.217(y4)
528.193(Hex + HexNAc + Hex)
554.333(b5)
667.416(b6)
690.368(y6)
777.400(y7)
795.473(b7)
1035.506(y10)
1081.058(PEP+HexNAc(1)_2)
1163.554(y11)
1238.581
1291.613(y12)
1940.992
1958.012(PEP_1)
CID-MS/MS Scan:28462, Noise threshold:1.1
366.141(HexNAc + Hex)
407.168(HexNAc(2)_1)
528.194(Hex + HexNAc + Hex)
1081.057(PEP+HexNAc(1)_2)
1154.085(PEP+HexNAc(1)Fuc(1)_2)
1207.867(PEP+HexNAc(5)Hex(4)_3)
1418.180
1447.174
1461.545
1498.703
1547.735
1681.274(PEP+HexNAc(3)Hex(4)Fuc(1)_2)
1782.815(PEP+HexNAc(4)Hex(4)Fuc(1)_2)
ETD-MS/MS Scan:28463, Noise threshold:1.5
204.088(HexNAc)
366.142(HexNAc + Hex)
154.757
180.799
243.276
659.790
940.561
1224.050
M+3H
1702.792
1776.236
1812.315
1820.830
1856.352
1863.351
1877.352
M+2H
m/z

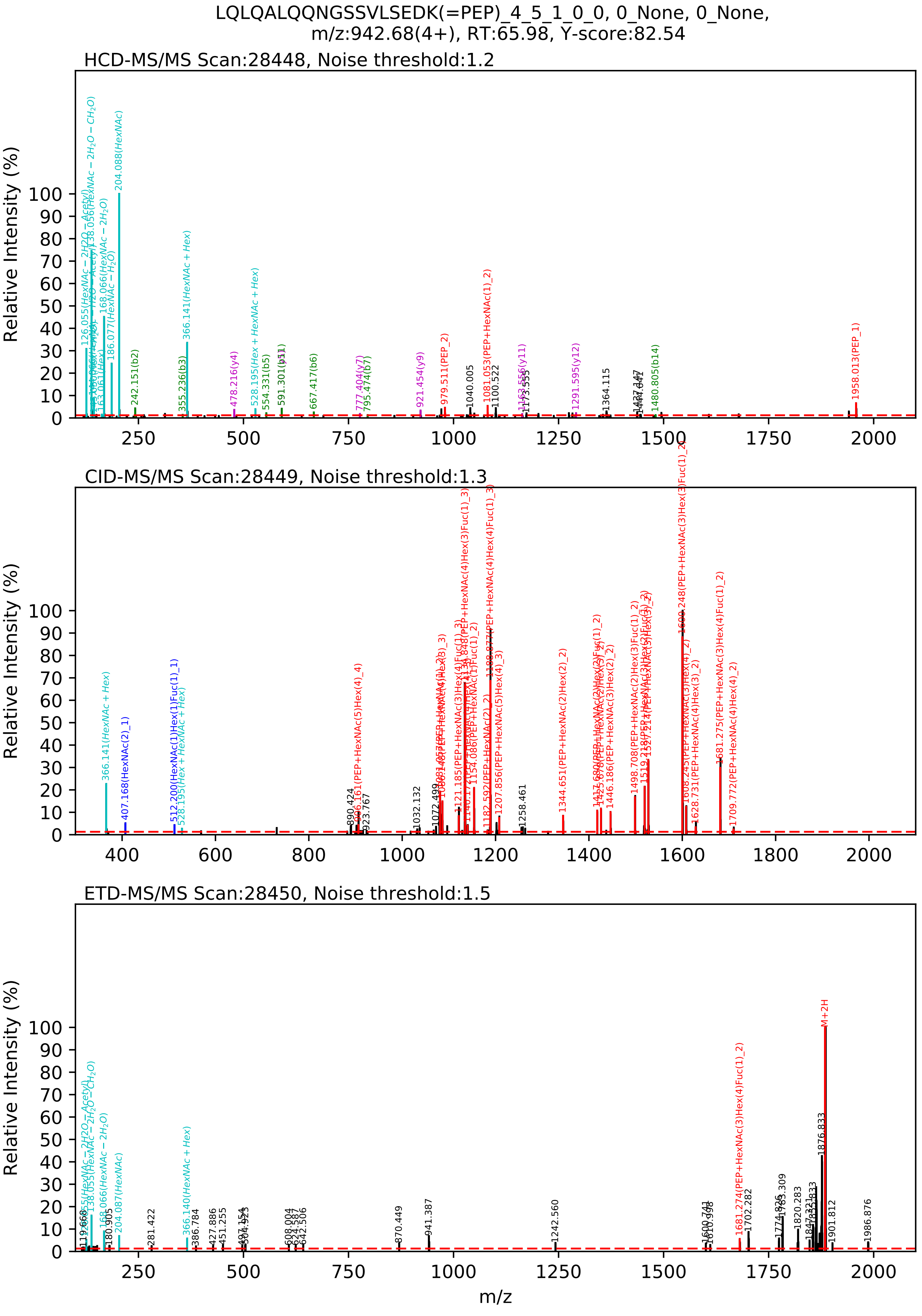
LQLQALQQNGSSVLSEDK(=PEP)_4_5_1_0_0, 0_None, 0_None,
m/z:942.68(4+), RT:65.98, Y-score:82.54
HCD-MS/MS Scan:28448, Noise threshold:1.2
CID-MS/MS Scan:28449, Noise threshold:1.3
ETD-MS/MS Scan:28450, Noise threshold:1.5
Relative Intensity (%)
m/z
204.088(HexNAc)
366.141(HexNAc + Hex)
1081.053(PEP+HexNAc(1)_2)
979.511(PEP_2)
1958.013(PEP_1)
1600.248(PEP+HexNAc(3)Hex(3)Fuc(1)_2)
1681.275(PEP+HexNAc(3)Hex(4)Fuc(1)_2)
1188.877(PEP+HexNAc(4)Hex(4)Fuc(1)_3)
M+2H
1876.833
1681.274(PEP+HexNAc(3)Hex(4)Fuc(1)_2)

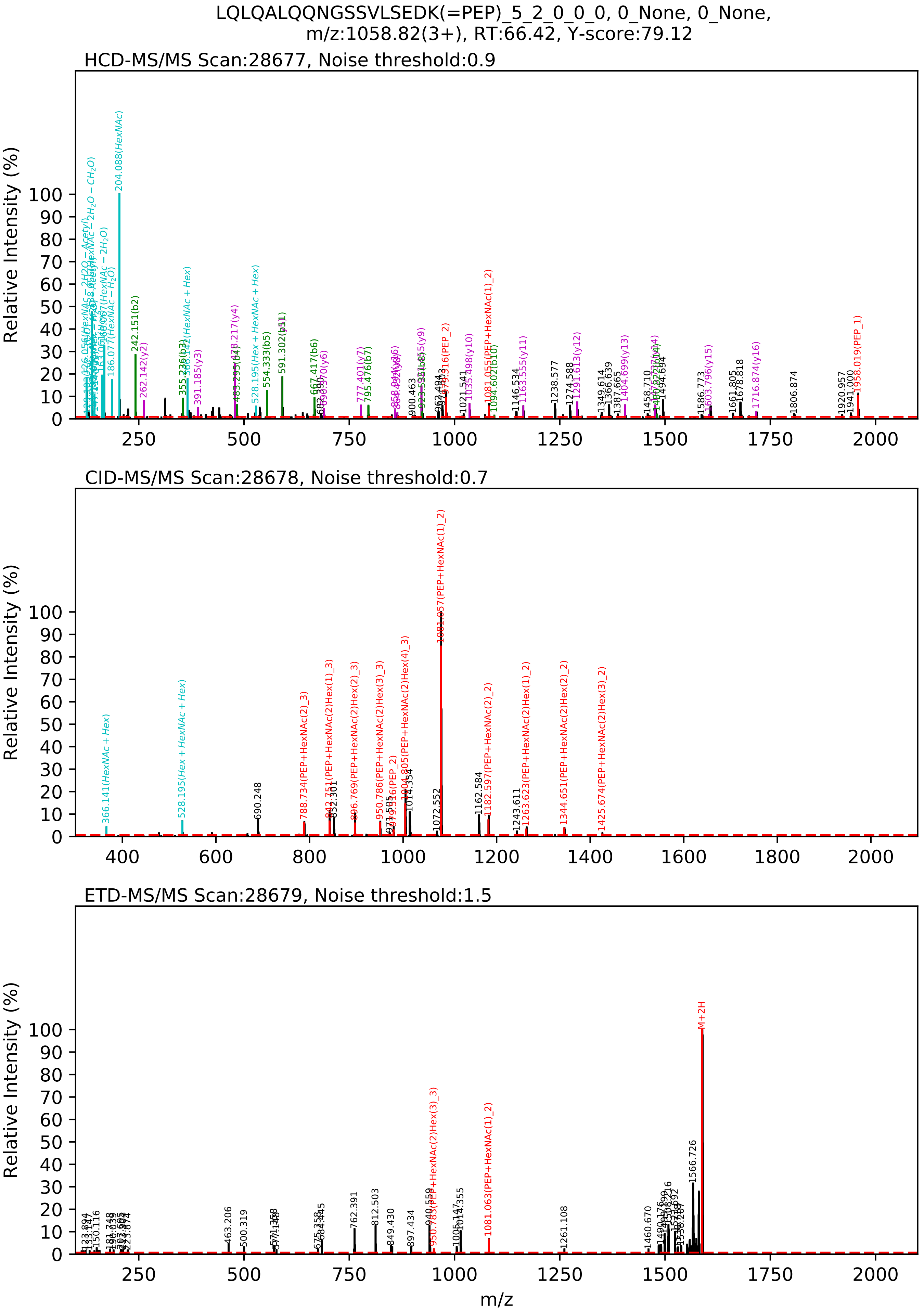
LQLQALQQNGSSVLSEDK(=PEP)_5_2_0_0_0, 0_None, 0_None,
m/z:1058.82(3+), RT:66.42, Y-score:79.12
HCD-MS/MS Scan:28677, Noise threshold:0.9
Relative Intensity (%)
204.088(HexNAc)
242.151(b2)
262.142(y2)
355.236(b3)
391.185(y3)
554.333(b5)
591.302(b5)
667.417(b6)
777.401(y7)
795.476(b7)
900.463
1021.541
1035.498(y10)
1081.055(PEP+HexNAc(1)_2)
1094.602(b10)
1146.534
1163.555(y11)
1238.577
1274.588
1291.613(y12)
1349.614
1366.639
1387.665
1404.699(y13)
1458.710
1494.694
1586.773
1603.796(y15)
1661.805
1678.818
1716.874(y16)
1806.874
1920.957
1941.000
1958.019(PEP_1)
CID-MS/MS Scan:28678, Noise threshold:0.7
366.141(HexNAc + Hex)
528.195(Hex + HexNAc + Hex)
690.248
788.734(PEP+HexNAc(2)_3)
852.301
896.769(PEP+HexNAc(2)Hex(2)_3)
950.786(PEP+HexNAc(2)Hex(3)_3)
1004.805(PEP+HexNAc(2)Hex(4)_3)
1014.354
1072.552
1081.057(PEP+HexNAc(1)_2)
1162.584
1182.597(PEP+HexNAc(2)_2)
1243.611
1263.623(PEP+HexNAc(2)Hex(1)_2)
1344.651(PEP+HexNAc(2)Hex(2)_2)
1425.674(PEP+HexNAc(2)Hex(3)_2)
ETD-MS/MS Scan:28679, Noise threshold:1.5
M+2H
1566.726
463.206
500.319
762.391
812.503
849.430
897.434
950.785(PEP+HexNAc(2)Hex(3)_3)
1014.355
1081.063(PEP+HexNAc(1)_2)
1261.108
1460.670
m/z

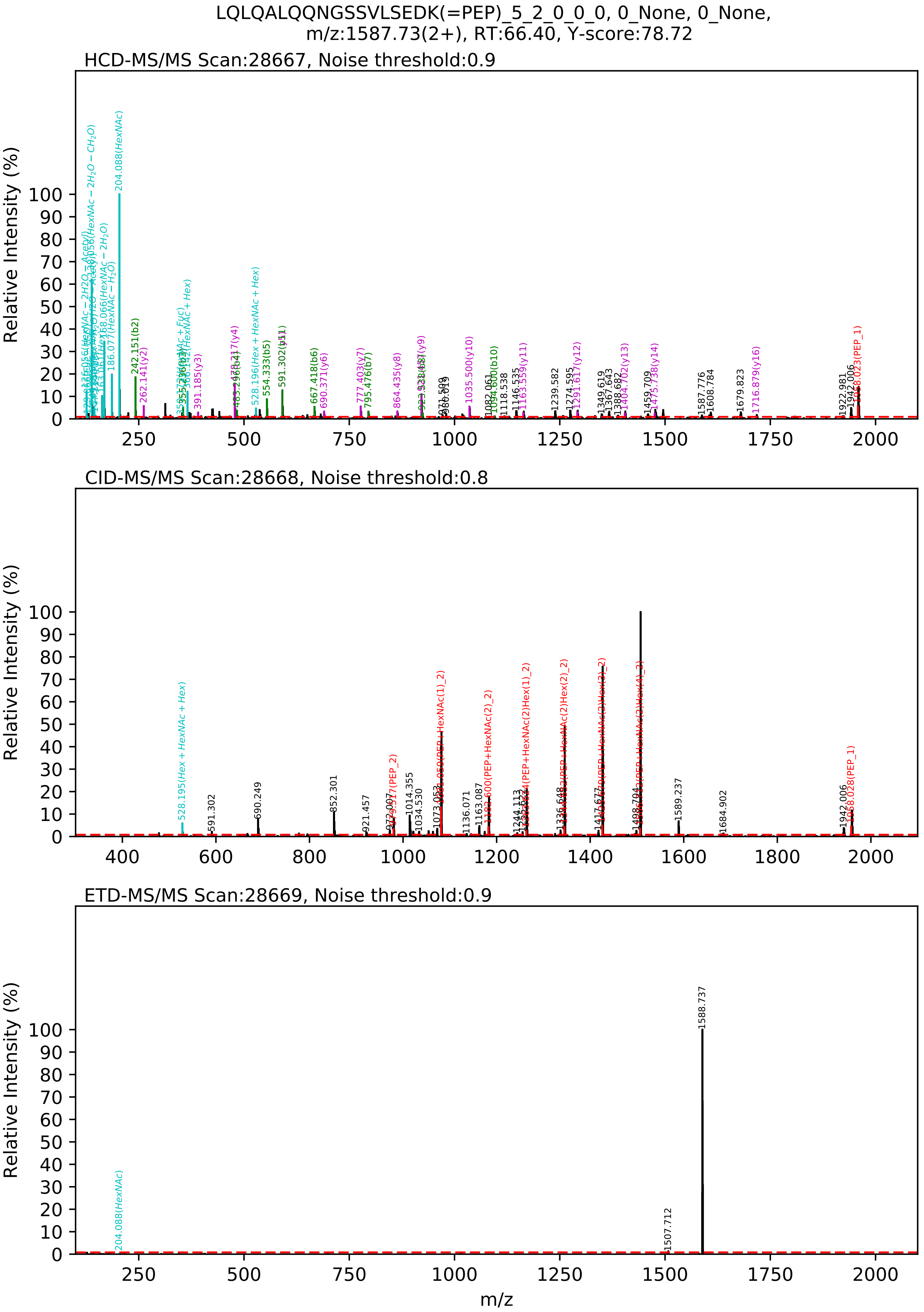

LQLQALQQNGSSVLSEDK(=PEP)_5_2_0_0_0, 0_None, 0_None,
m/z:1587.73(2+), RT:66.40, Y-score:78.72
HCD-MS/MS Scan:28667, Noise threshold:0.9
CID-MS/MS Scan:28668, Noise threshold:0.8
ETD-MS/MS Scan:28669, Noise threshold:0.9
Relative Intensity (%)
m/z

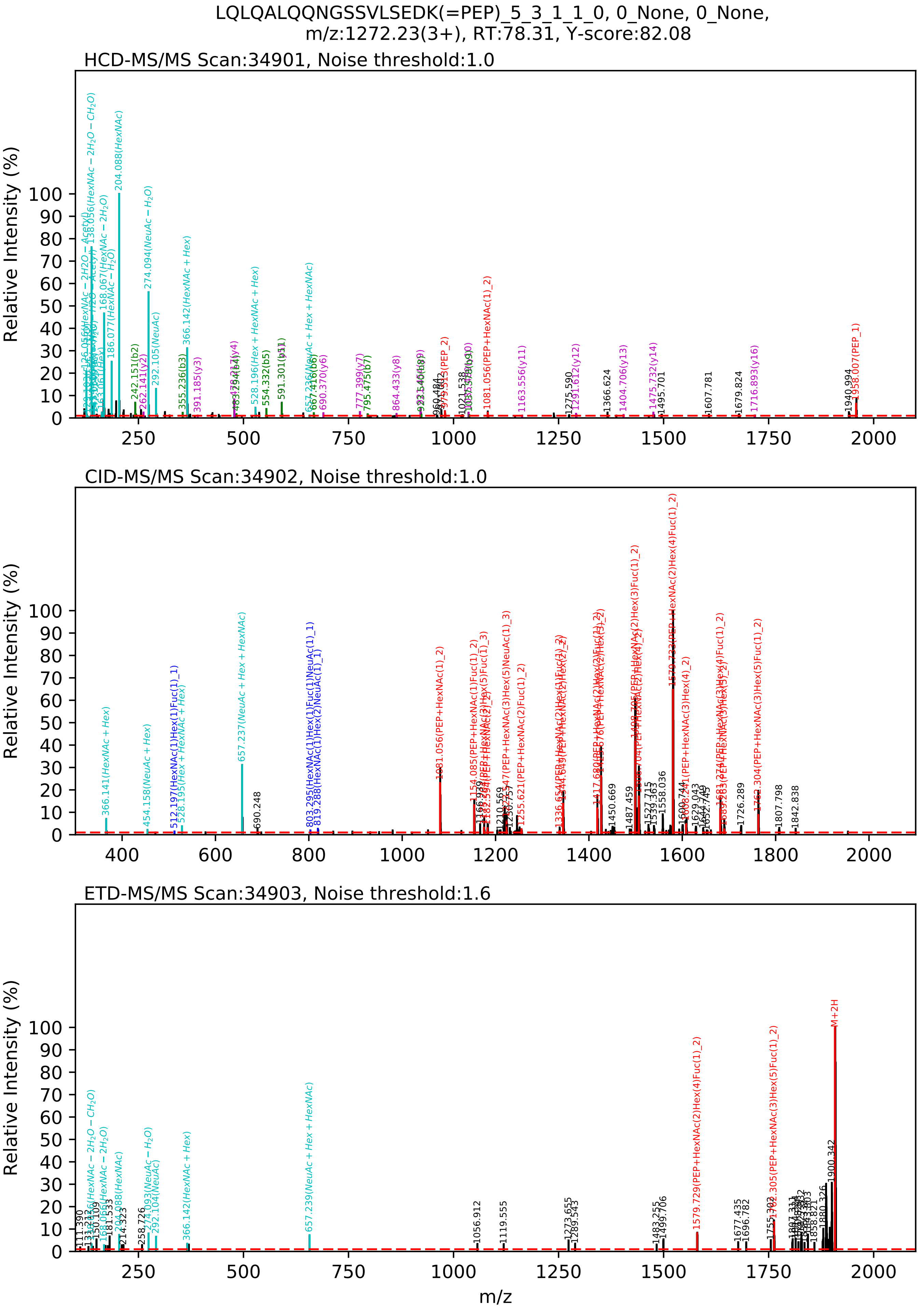

LQLQALQQNGSSVLSEDK(=PEP)_5_3_1_1_0, 0_None, 0_None,
m/z:1272.23(3+), RT:78.31, Y-score:82.08
HCD-MS/MS Scan:34901, Noise threshold:1.0
Relative Intensity (%)
204.088(HexNAc)
274.094(NeuAc − H2O)
366.142(HexNAc + Hex)
292.105(NeuAc)
528.196(Hex + HexNAc + Hex)
1081.056(PEP+HexNAc(1)_2)
1958.007(PEP_1)
CID-MS/MS Scan:34902, Noise threshold:1.0
657.237(NeuAc + Hex + HexNAc)
1081.056(PEP+HexNAc(1)_2)
1762.304(PEP+HexNAc(3)Hex(5)Fuc(1)_2)
ETD-MS/MS Scan:34903, Noise threshold:1.6
1579.729(PEP+HexNAc(2)Hex(4)Fuc(1)_2)
1762.305(PEP+HexNAc(3)Hex(5)Fuc(1)_2)
M+2H
1900.342
m/z

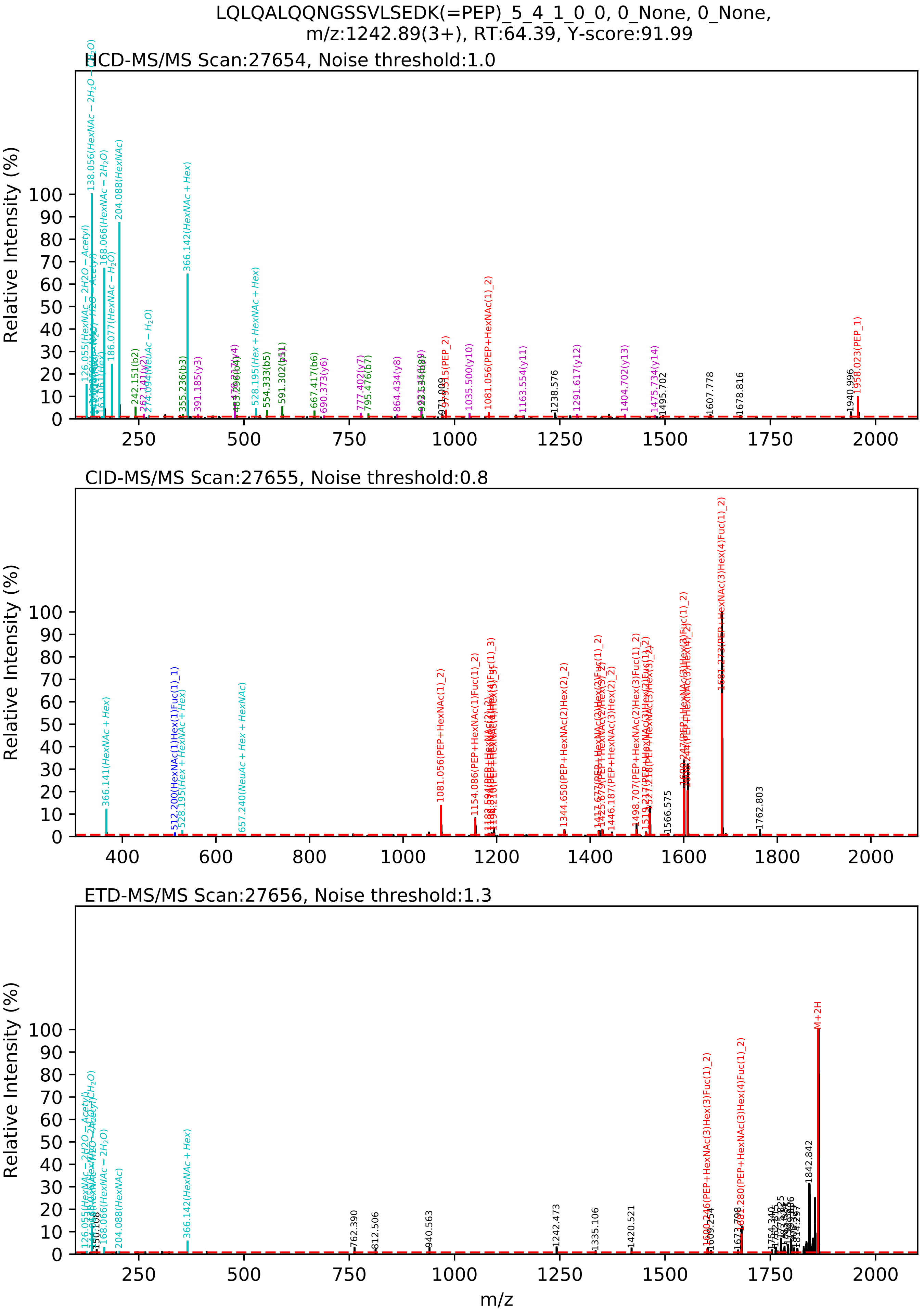

LQLQALQQNGSSVLSEDK(=PEP)_5_4_1_0_0, 0_None, 0_None,
m/z:1242.89(3+), RT:64.39, Y-score:91.99
HCD-MS/MS Scan:27654, Noise threshold:1.0
CID-MS/MS Scan:27655, Noise threshold:0.8
ETD-MS/MS Scan:27656, Noise threshold:1.3
Relative Intensity (%)
m/z

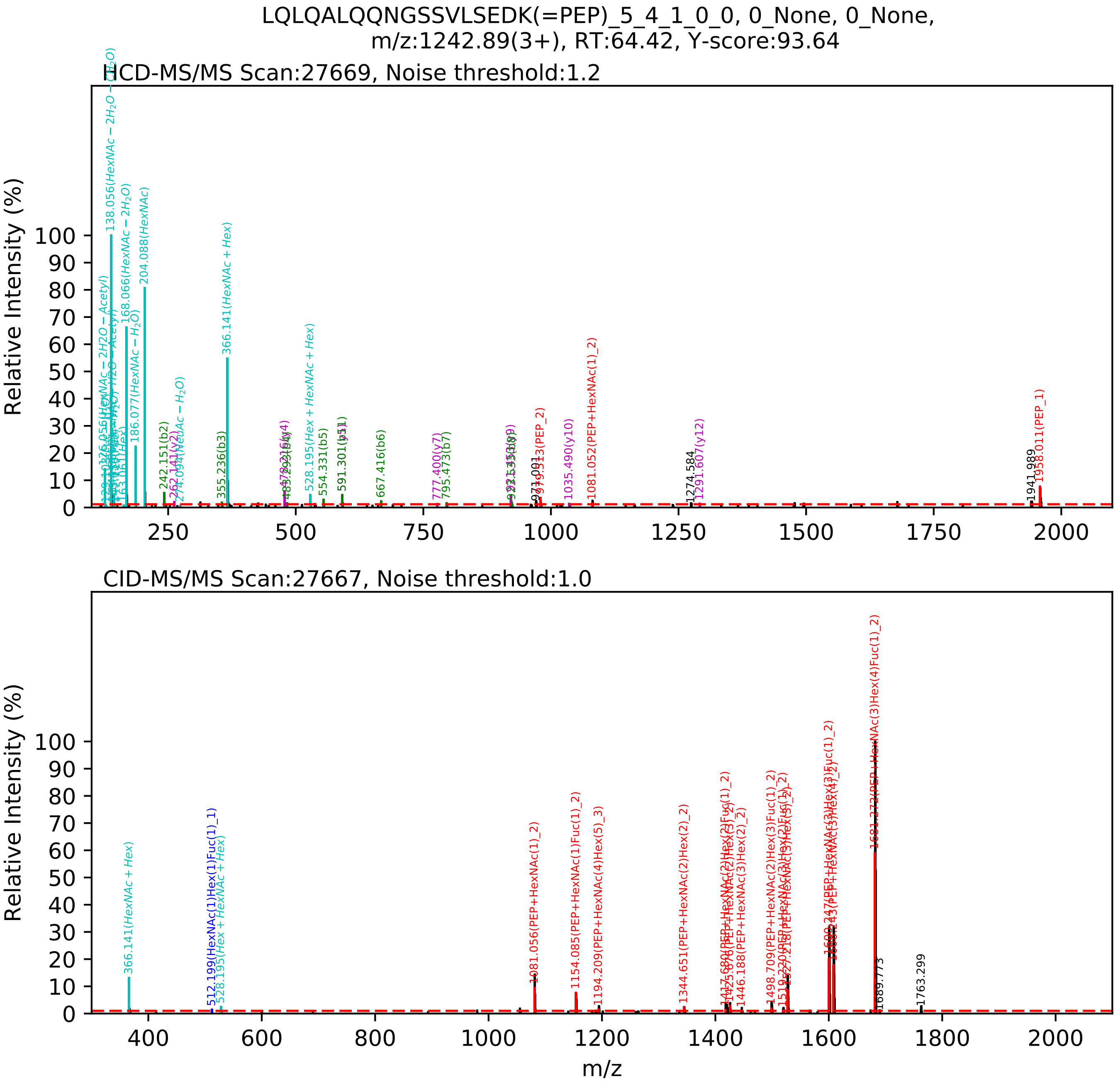

LQLQALQQNGSSVLSEDK(=PEP)_5_4_1_0_0, 0_None, 0_None,
m/z:1242.89(3+), RT:64.42, Y-score:93.64
HCD-MS/MS Scan:27669, Noise threshold:1.2
Relative Intensity (%)
138.056(HexNAc − 2H2O − H2O)
138.056(HexNAc − 2H2O)
168.066(HexNAc − 2H2O)
186.077(HexNAc − H2O)
204.088(HexNAc)
163.061(Hex)
366.141(HexNAc + Hex)
242.151(b2)
262.141(y2)
274.094(NeuAc − H2O)
355.236(b3)
528.195(Hex + HexNAc + Hex)
554.331(b5)
591.301(b11)
667.416(b6)
777.400(y7)
795.473(b7)
971.091
1035.490(y10)
1081.052(PEP+HexNAc(1)_2)
1274.584
1291.607(y12)
1941.989
1958.011(PEP_1)
250
500
750
1000
1250
1500
1750
2000
CID-MS/MS Scan:27667, Noise threshold:1.0
Relative Intensity (%)
366.141(HexNAc + Hex)
512.199(HexNAc(1)Hex(1)Fuc(1)_1)
528.195(Hex + HexNAc + Hex)
1081.056(PEP+HexNAc(1)_2)
1154.085(PEP+HexNAc(1)Fuc(1)_2)
1194.209(PEP+HexNAc(4)Hex(5)_3)
1344.651(PEP+HexNAc(2)Hex(2)_2)
1446.188(PEP+HexNAc(3)Hex(2)_2)
1498.709(PEP+HexNAc(2)Hex(3)Fuc(1)_2)
1681.272(PEP+HexNAc(3)Hex(4)Fuc(1)_2)
1689.773
1763.299
400
600
800
1000
1200
1400
1600
1800
2000
m/z

LQLQALQQNGSSVLSEDK(=PEP)_5_4_1_0_0, 0_None, 0_None,
m/z:1242.89(3+), RT:65.65, Y-score:88.32

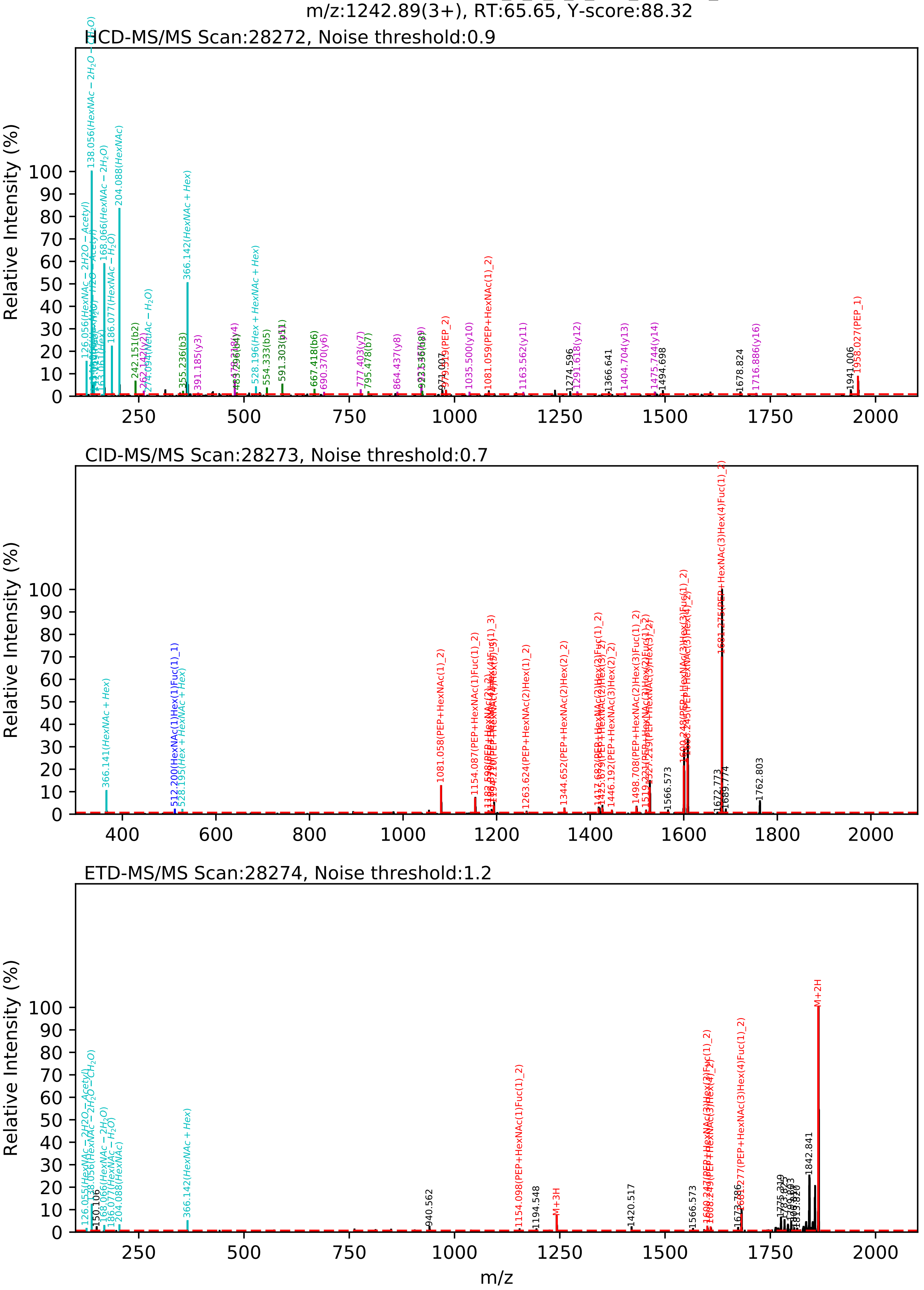

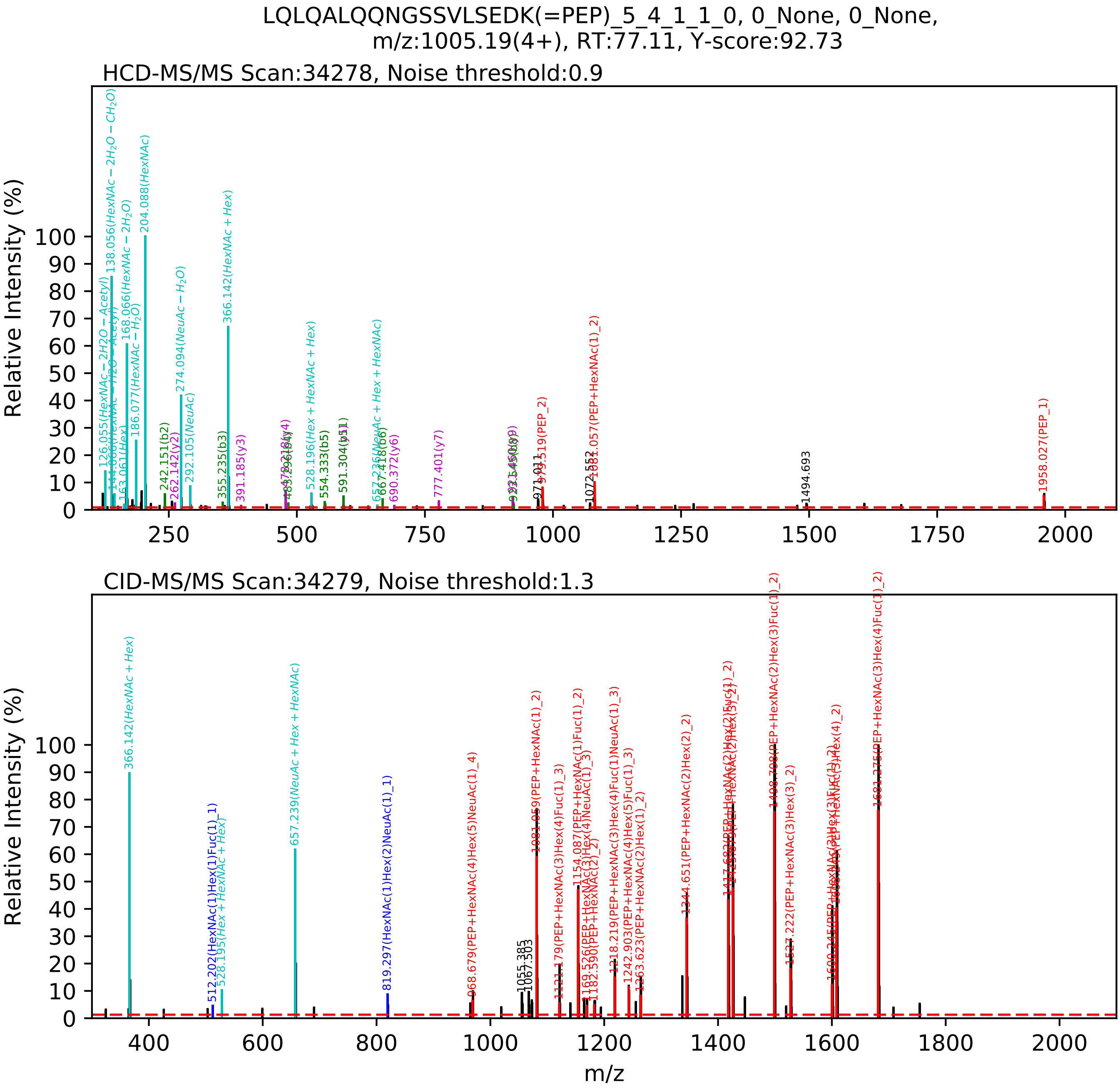

LQLQALQQNGSSVLSEDK(=PEP)_5_4_1_1_0, 0_None, 0_None,
m/z:1005.19(4+), RT:77.11, Y-score:92.73
HCD-MS/MS Scan:34278, Noise threshold:0.9
Relative Intensity (%)
204.088(HexNAc)
366.142(HexNAc + Hex)
274.094(NeuAc − H2O)
292.105(NeuAc)
186.077(HexNAc − H2O)
168.066(HexNAc − 2H2O)
242.151(b2)
262.142(y2)
355.235(b3)
391.185(y3)
528.196(Hex + HexNAc + Hex)
554.333(b5)
591.304(b5)
690.372(y6)
777.401(y7)
973.519(PEP_2)
1081.057(PEP+HexNAc(1)_2)
1072.552
1494.693
1958.027(PEP_1)
CID-MS/MS Scan:34279, Noise threshold:1.3
366.142(HexNAc + Hex)
657.239(NeuAc + Hex + HexNAc)
512.202(HexNAc(1)Hex(1)Fuc(1)_1)
528.195(Hex + HexNAc + Hex)
819.297(HexNAc(1)Hex(2)NeuAc(1)_1)
968.679(PEP+HexNAc(4)Hex(5)NeuAc(1)_4)
1055.385
1067.503
1081.059(PEP+HexNAc(1)_2)
1218.219(PEP+HexNAc(3)Hex(4)Fuc(1)NeuAc(1)_3)
1242.903(PEP+HexNAc(4)Hex(5)Fuc(1)_3)
1263.623(PEP+HexNAc(2)Hex(1)_2)
1344.651(PEP+HexNAc(2)Hex(2)_2)
1498.708(PEP+HexNAc(2)Hex(3)Fuc(1)_2)
1527.222(PEP+HexNAc(3)Hex(3)_2)
1681.275(PEP+HexNAc(3)Hex(4)Fuc(1)_2)
m/z

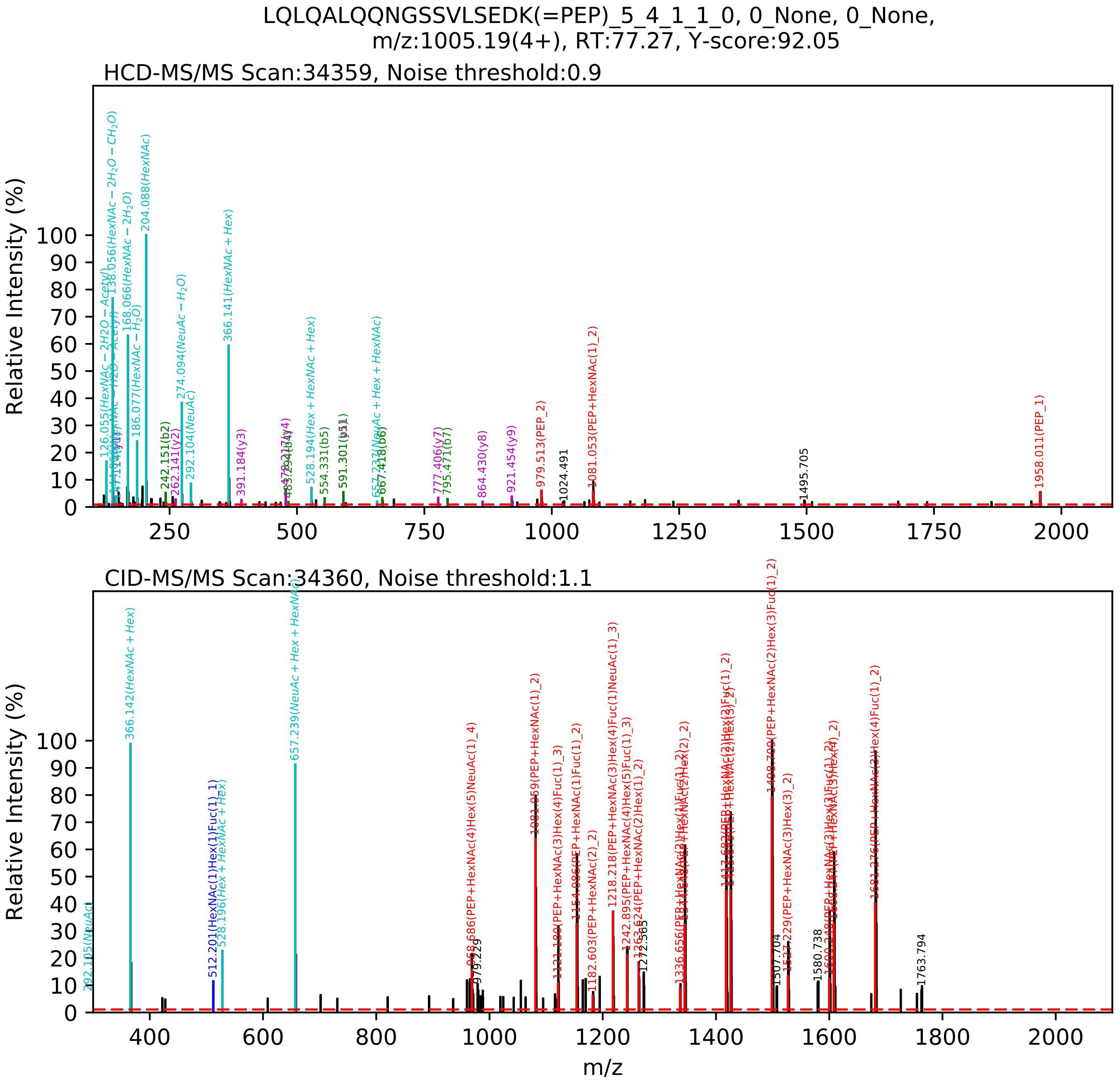

LQLQALQQNGSSVLSEDK(=PEP)_5_4_1_1_0, 0_None, 0_None,
m/z:1005.19(4+), RT:77.27, Y-score:92.05
HCD-MS/MS Scan:34359, Noise threshold:0.9
Relative Intensity (%)
204.088(HexNAc)
366.141(HexNAc + Hex)
274.094(NeuAc − H2O)
292.104(NeuAc)
186.077(HexNAc − H2O)
168.066(HexNAc − 2H2O)
126.055(HexNAc − 2H2O − Acetyl)
242.151(b2)
262.141(y2)
391.184(y3)
528.194(Hex + HexNAc + Hex)
554.331(b5)
591.301(b5)
667.418(b6)
777.406(y7)
795.471(b7)
864.430(y8)
921.454(y9)
979.513(PEP_2)
1024.491
1081.053(PEP+HexNAc(1)_2)
1495.705
1958.011(PEP_1)
CID-MS/MS Scan:34360, Noise threshold:1.1
366.142(HexNAc + Hex)
512.201(HexNAc(1)Hex(1)Fuc(1)_1)
528.196(Hex + HexNAc + Hex)
657.239(NeuAc + Hex + HexNAc)
292.105(NeuAc)
968.686(PEP+HexNAc(4)Hex(5)NeuAc(1)_4)
979.229
1081.059(PEP+HexNAc(1)_2)
1121.190(PEP+HexNAc(3)Hex(4)Fuc(1)_3)
1154.086(PEP+HexNAc(1)Fuc(1)_2)
1182.603(PEP+HexNAc(2)_2)
1218.218(PEP+HexNAc(3)Hex(4)Fuc(1)NeuAc(1)_3)
1242.895(PEP+HexNAc(4)Hex(5)Fuc(1)_3)
1263.624(PEP+HexNAc(2)Hex(1)_2)
1272.565
1336.656
1417.683
1498.709(PEP+HexNAc(2)Hex(3)Fuc(1)_2)
1507.704
1527.229(PEP+HexNAc(3)Hex(3)_2)
1580.738
1681.276
1763.794
m/z

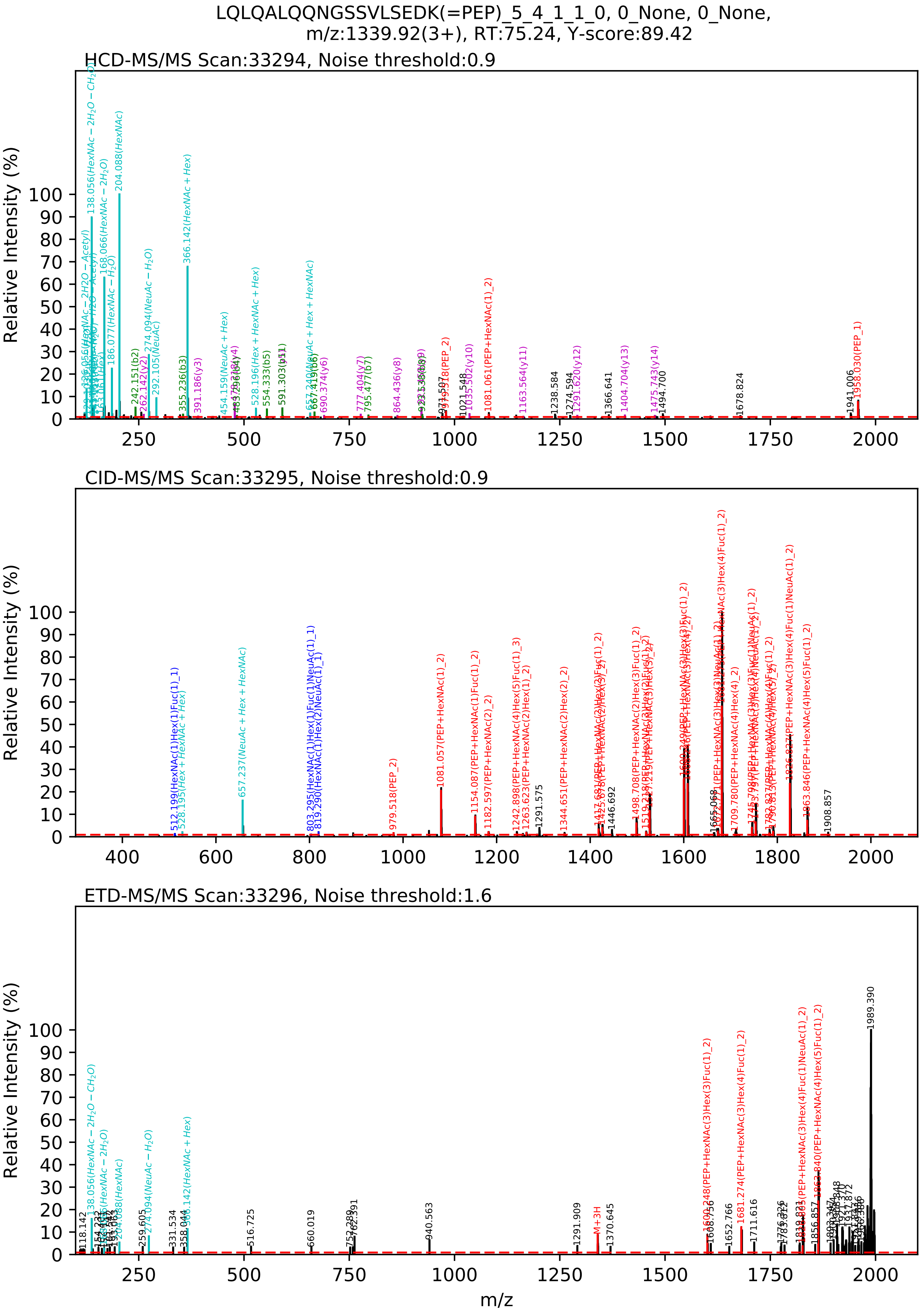

LQLQALQQNGSSVLSEDK(=PEP)_5_4_1_1_0, 0_None, 0_None,
m/z:1339.92(3+), RT:75.24, Y-score:89.42
HCD-MS/MS Scan:33294, Noise threshold:0.9
CID-MS/MS Scan:33295, Noise threshold:0.9
ETD-MS/MS Scan:33296, Noise threshold:1.6
Relative Intensity (%)
m/z

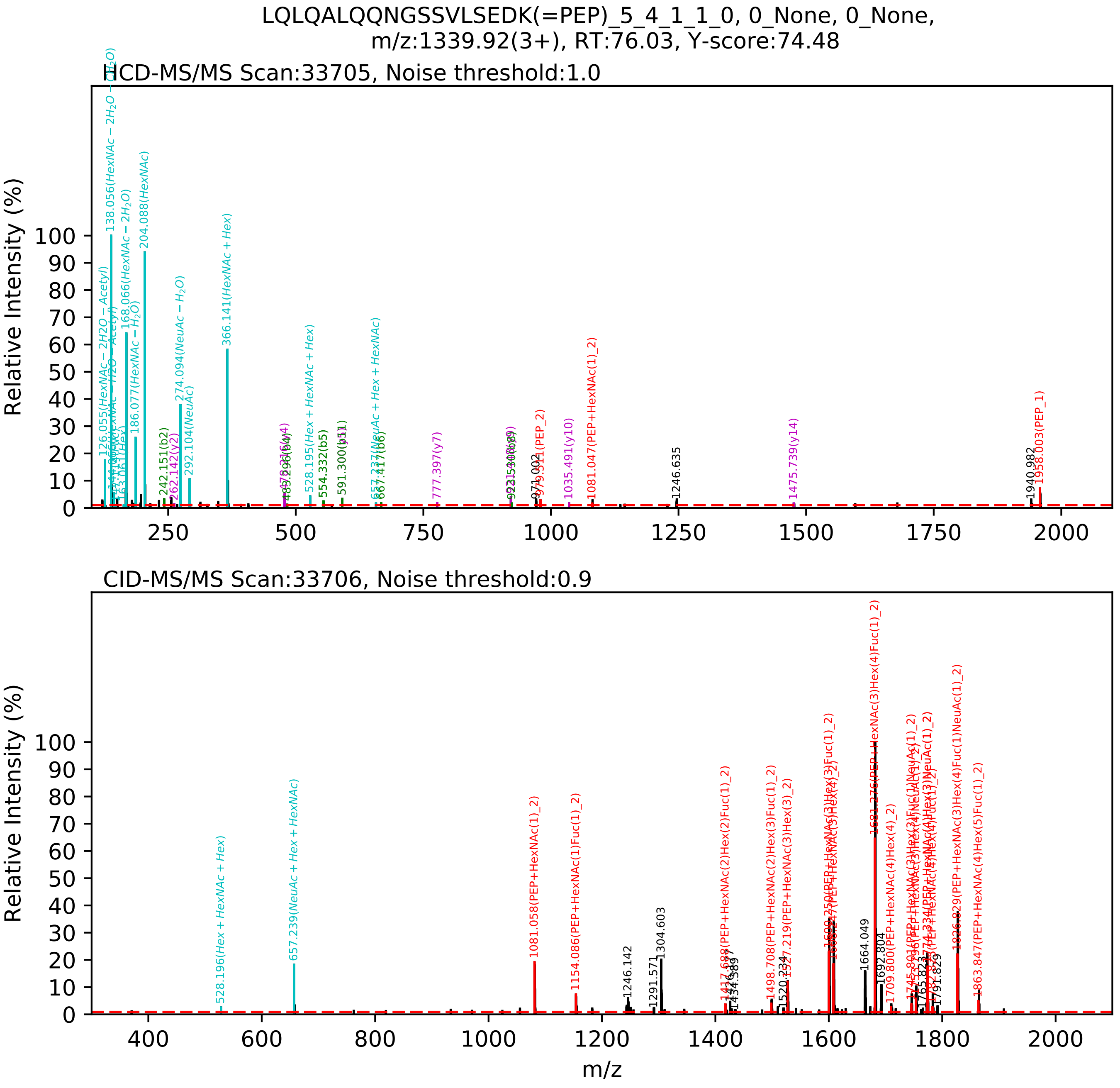

LQLQALQQNGSSVLSEDK(=PEP)_5_4_1_1_0, 0_None, 0_None,
m/z:1339.92(3+), RT:76.03, Y-score:74.48
HCD-MS/MS Scan:33705, Noise threshold:1.0
Relative Intensity (%)
138.056
204.088(HexNAc)
168.066
186.077
366.141(HexNAc + Hex)
274.094(NeuAc − H2O)
292.104(NeuAc)
242.151(b2)
262.142(y2)
528.195(Hex + HexNAc + Hex)
554.332(b5)
591.300(y5)
657.237(NeuAc + Hex + HexNAc)
667.417(b6)
777.397(y7)
971.002
1035.491(y10)
1081.047(PEP+HexNAc(1)_2)
1246.635
1475.739(y14)
1940.982
1958.003(PEP_1)
CID-MS/MS Scan:33706, Noise threshold:0.9
528.196(Hex + HexNAc + Hex)
657.239(NeuAc + Hex + HexNAc)
1081.058(PEP+HexNAc(1)_2)
1154.086(PEP+HexNAc(1)Fuc(1)_2)
1246.142
1291.571
1304.603
1417.688(PEP+HexNAc(2)Hex(2)Fuc(1)_2)
1434.389
1498.708(PEP+HexNAc(2)Hex(3)Fuc(1)_2)
1527.219(PEP+HexNAc(3)Hex(3)_2)
1664.049
1692.804
1709.800(PEP+HexNAc(4)Hex(4)_2)
1765.823
1791.829
1826.929(PEP+HexNAc(3)Hex(4)Fuc(1)NeuAc(1)_2)
1863.847(PEP+HexNAc(4)Hex(5)Fuc(1)_2)
m/z

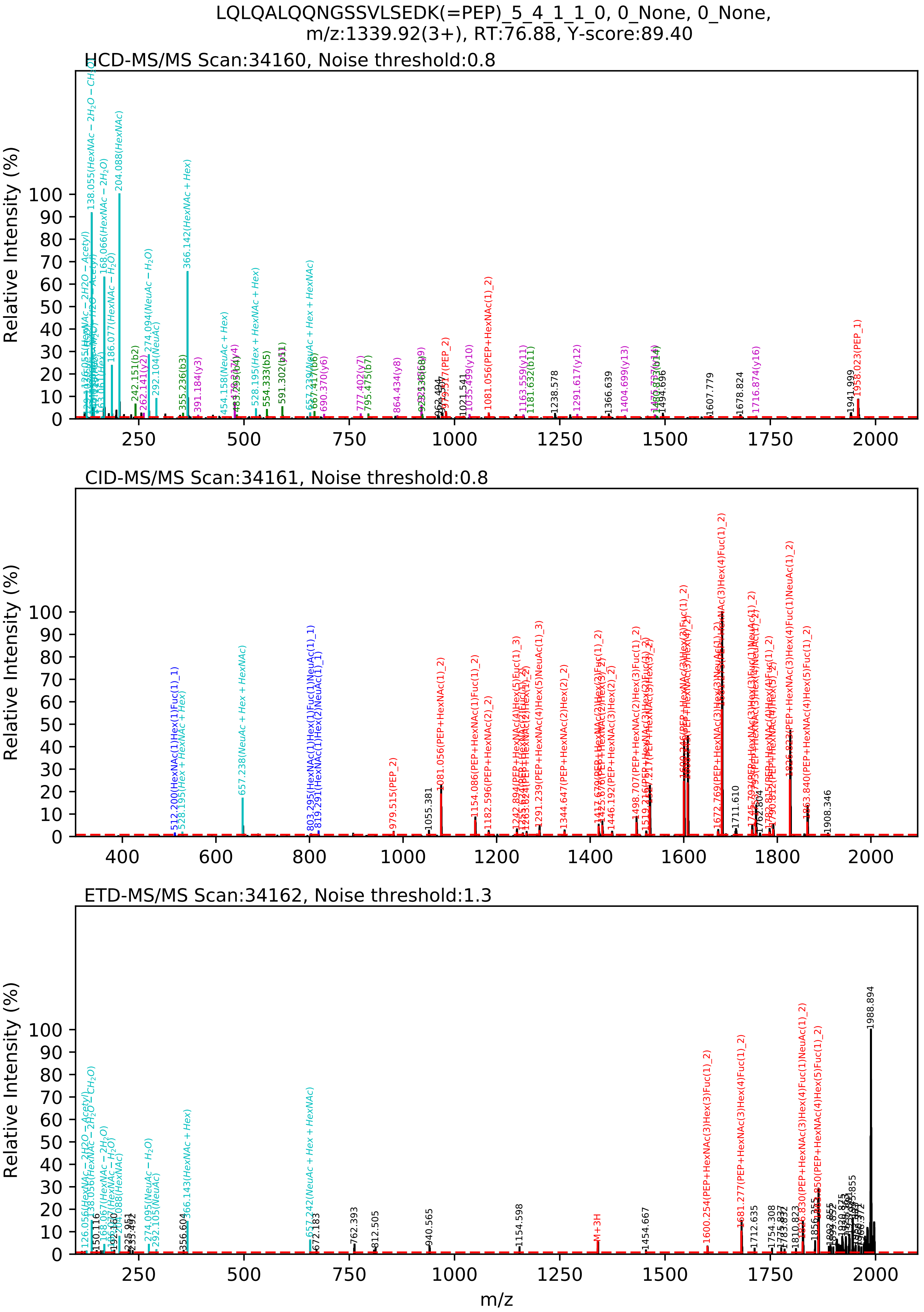
LQLQALQQNGSSVLSEDK(=PEP)_5_4_1_1_0, 0_None, 0_None,
m/z:1339.92(3+), RT:76.88, Y-score:89.40
HCD-MS/MS Scan:34160, Noise threshold:0.8
CID-MS/MS Scan:34161, Noise threshold:0.8
ETD-MS/MS Scan:34162, Noise threshold:1.3
Relative Intensity (%)
m/z
204.088(HexNAc)
138.055(HexNAc − 2H2O − CH2O)
168.066(HexNAc − 2H2O)
186.077(HexNAc − H2O)
366.142(HexNAc + Hex)
274.094(NeuAc − H2O)
292.104(NeuAc)
1958.023(PEP_1)
1081.056(PEP+HexNAc(1)_2)
979.517(PEP_2)
1678.824
1607.779
1941.999
1716.874(y16)
1291.617(y12)
1404.699(y13)
1163.559(y11)
1181.632(b11)
1238.578
1366.639
1494.696
1021.541
657.238(NeuAc + Hex + HexNAc)
512.200(HexNAc(1)Hex(1)Fuc(1)_1)
803.295(HexNAc(1)Hex(1)Fuc(1)NeuAc(1)_1)
819.291(HexNAc(1)Hex(2)NeuAc(1)_1)
979.515(PEP_2)
1055.381
1154.086(PEP+HexNAc(1)Fuc(1)_2)
1182.596(PEP+HexNAc(2)_2)
1291.239(PEP+HexNAc(4)Hex(5)NeuAc(1)_3)
1344.647(PEP+HexNAc(2)Hex(2)_2)
1446.192(PEP+HexNAc(3)Hex(2)_2)
1498.707(PEP+HexNAc(2)Hex(3)Fuc(1)_2)
1672.769(PEP+HexNAc(3)Hex(3)NeuAc(1)_2)
1711.610
1762.804
1826.822(PEP+HexNAc(3)Hex(4)Fuc(1)NeuAc(1)_2)
1863.840(PEP+HexNAc(4)Hex(5)Fuc(1)_2)
1908.346
1988.894
1600.254(PEP+HexNAc(3)Hex(3)Fuc(1)_2)
1681.277(PEP+HexNAc(3)Hex(4)Fuc(1)_2)
M+3H
1454.667
1154.598
940.565
812.505
762.393
672.183
356.604
1712.635
1754.308
1810.823

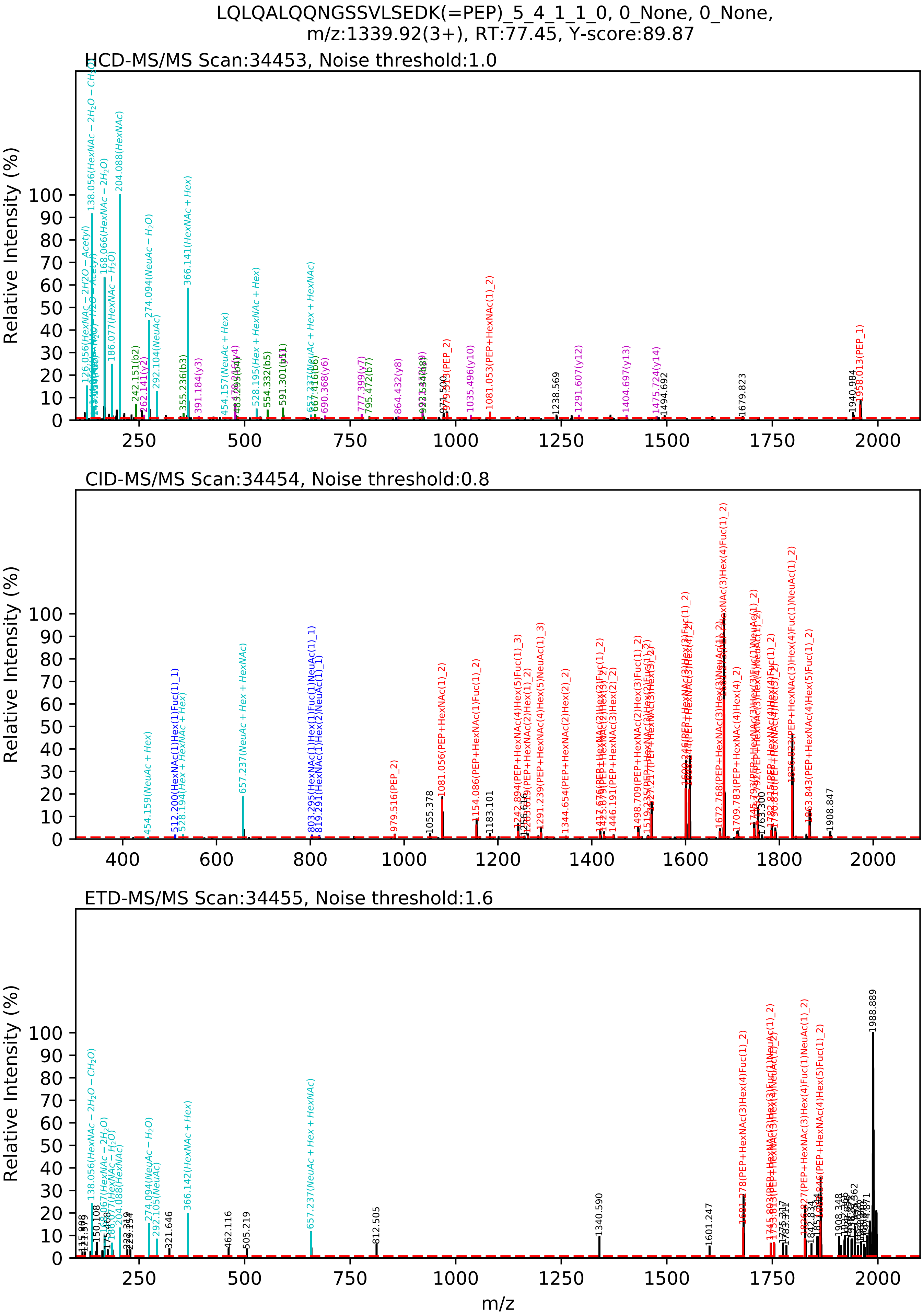

LQLQALQQNGSSVLSEDK(=PEP)_5_4_1_1_0, 0_None, 0_None,
m/z:1339.92(3+), RT:77.45, Y-score:89.87
HCD-MS/MS Scan:34453, Noise threshold:1.0
CID-MS/MS Scan:34454, Noise threshold:0.8
ETD-MS/MS Scan:34455, Noise threshold:1.6
Relative Intensity (%)
m/z

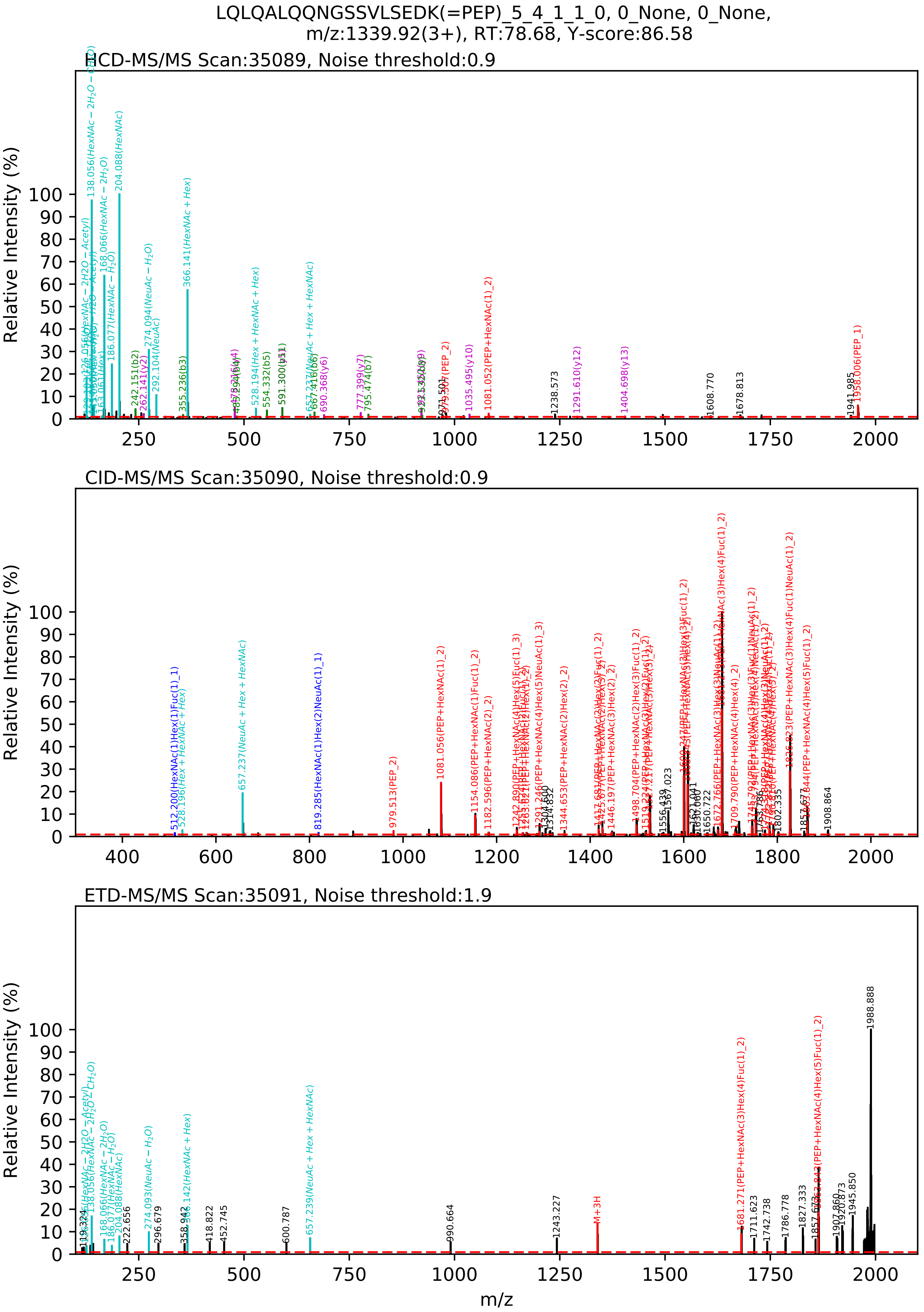
LQLQALQQNGSSVLSEDK(=PEP)_5_4_1_1_0, 0_None, 0_None,
m/z:1339.92(3+), RT:78.68, Y-score:86.58
HCD-MS/MS Scan:35089, Noise threshold:0.9
CID-MS/MS Scan:35090, Noise threshold:0.9
ETD-MS/MS Scan:35091, Noise threshold:1.9
Relative Intensity (%)
m/z

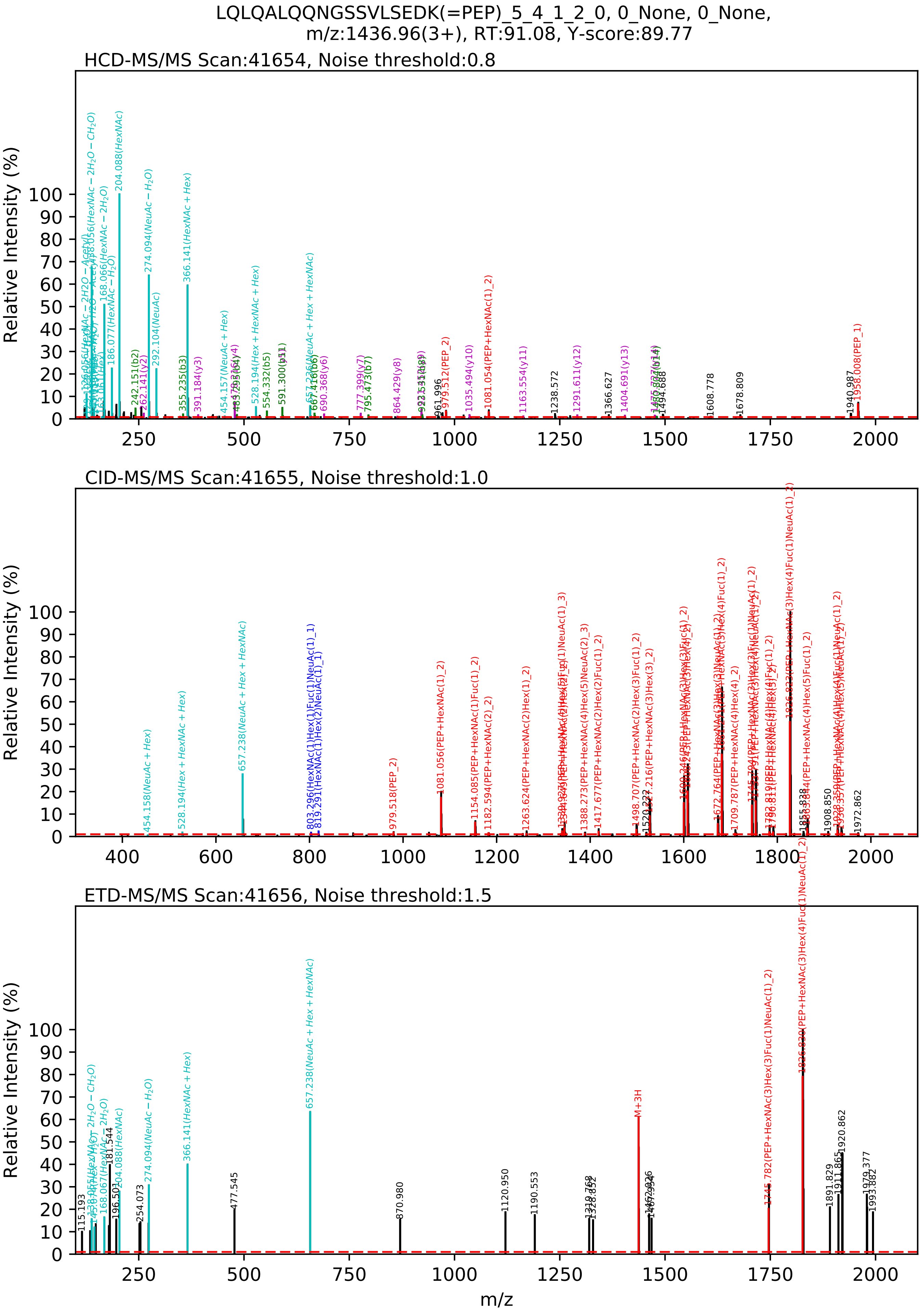
LQLQALQQNGSSVLSEDK(=PEP)_5_4_1_2_0, 0_None, 0_None,
m/z:1436.96(3+), RT:91.08, Y-score:89.77
HCD-MS/MS Scan:41654, Noise threshold:0.8
CID-MS/MS Scan:41655, Noise threshold:1.0
ETD-MS/MS Scan:41656, Noise threshold:1.5
Relative Intensity (%)
m/z
204.088(HexNAc)
274.094(NeuAc − H2O)
366.141(HexNAc + Hex)
292.104(NeuAc)
657.238(NeuAc + Hex + HexNAc)
1081.054(PEP+HexNAc(1)_2)
1958.008(PEP_1)
1826.833(PEP+HexNAc(3)Hex(4)Fuc(1)NeuAc(1)_2)
1745.782(PEP+HexNAc(3)Hex(3)Fuc(1)NeuAc(1)_2)
M+3H

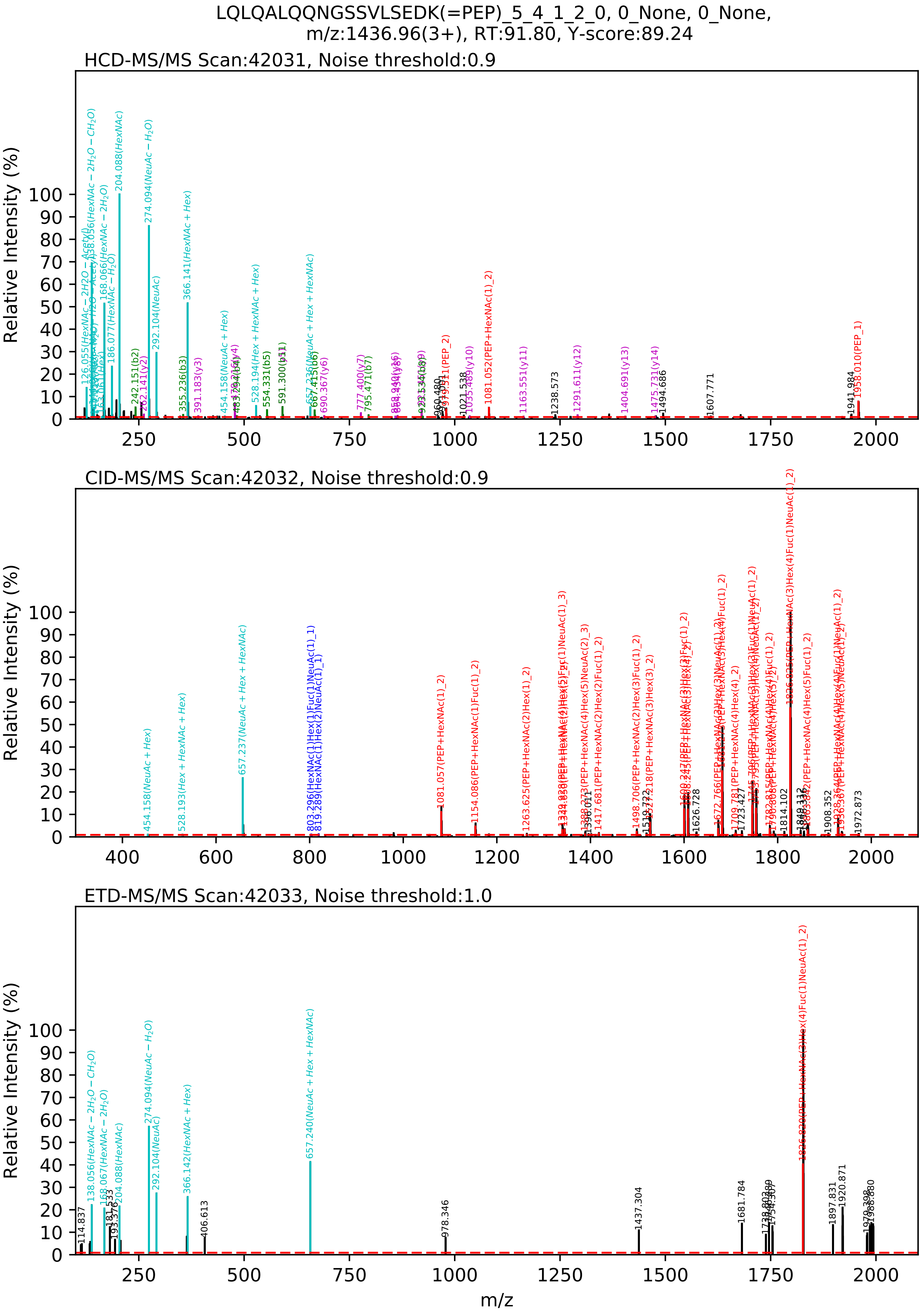
LQLQALQQNGSSVLSEDK(=PEP)_5_4_1_2_0, 0_None, 0_None,
m/z:1436.96(3+), RT:91.80, Y-score:89.24
HCD-MS/MS Scan:42031, Noise threshold:0.9
CID-MS/MS Scan:42032, Noise threshold:0.9
ETD-MS/MS Scan:42033, Noise threshold:1.0
Relative Intensity (%)
m/z

LQLQALQQNGSSVLSEDK(=PEP)_5_4_1_2_0, 0_None, 0_None,
m/z:1436.96(3+), RT:91.87, Y-score:90.79

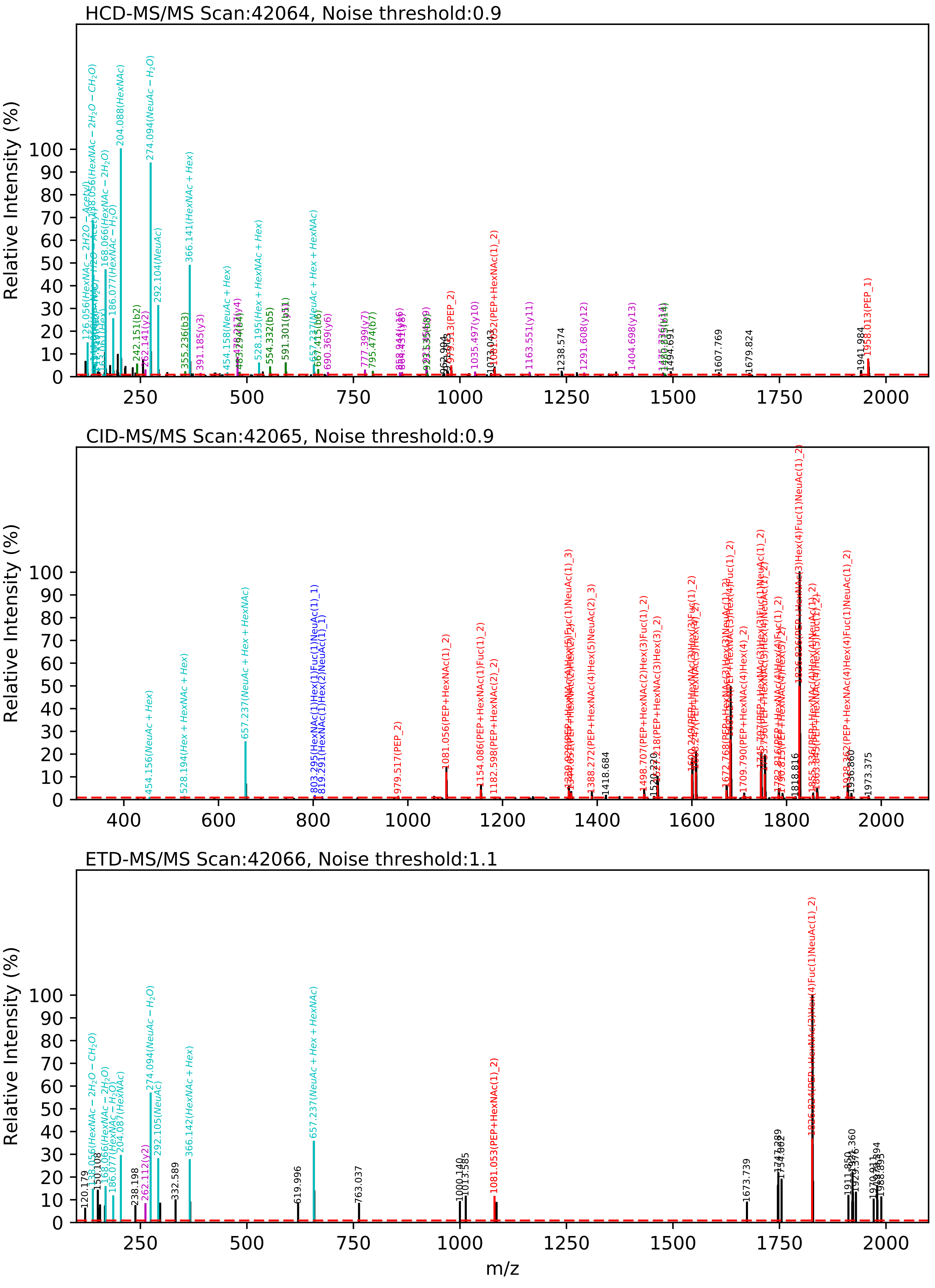

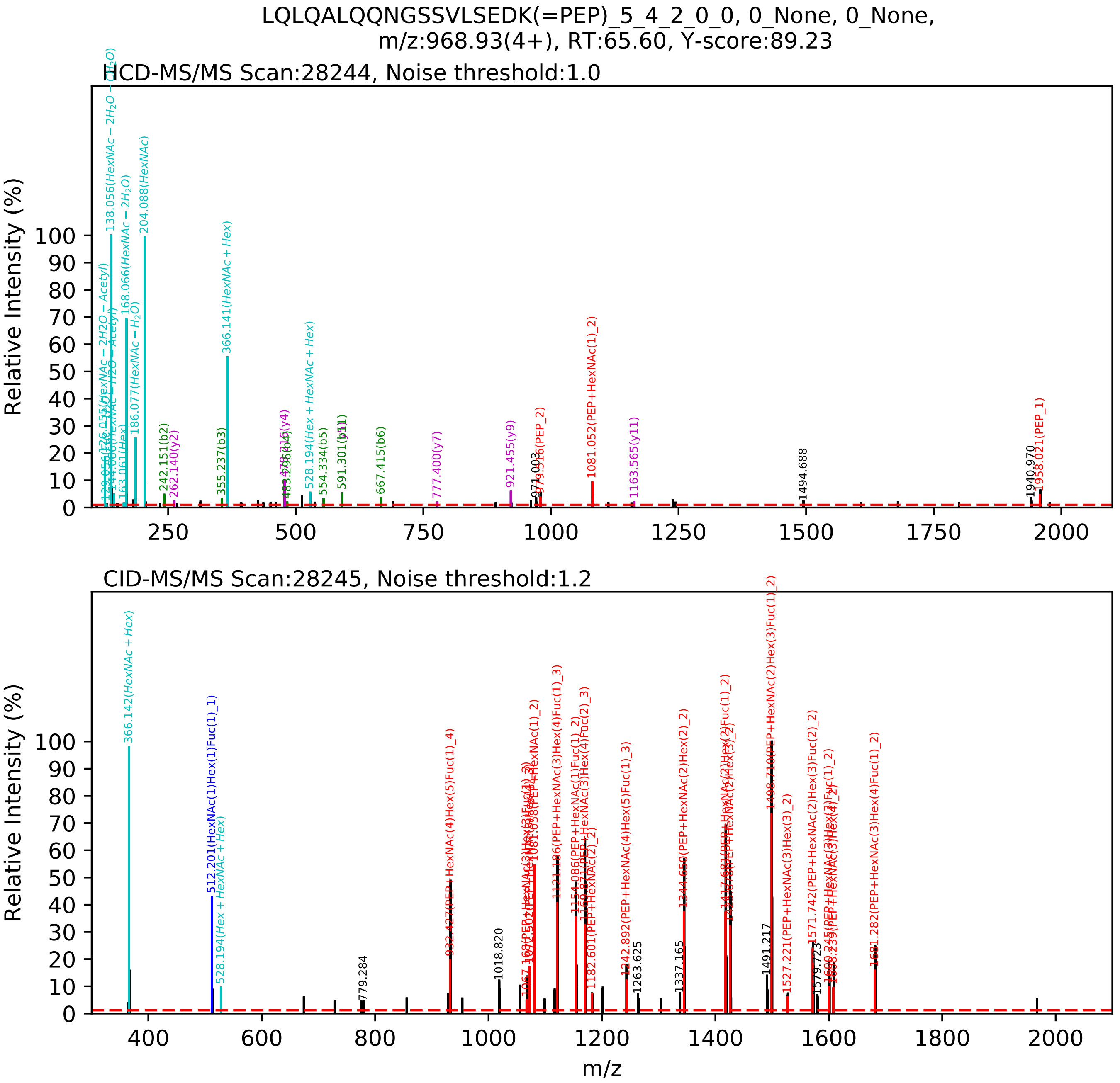
LQLQALQQNGSSVLSEDK(=PEP)_5_4_2_0_0, 0_None, 0_None,
m/z:968.93(4+), RT:65.60, Y-score:89.23
HCD-MS/MS Scan:28244, Noise threshold:1.0
Relative Intensity (%)
138.056(HexNAc − 2H2O − H2O)
204.088(HexNAc)
168.066(HexNAc − 2H2O)
186.077(HexNAc − H2O)
163.061(Hex)
366.141(HexNAc + Hex)
528.194(Hex + HexNAc + Hex)
242.151(b2)
262.140(y2)
355.237(b3)
554.334(b5)
591.301(b11)
667.415(b6)
777.400(y7)
921.455(y9)
1081.052(PEP+HexNAc(1)_2)
1163.565(y11)
1494.688
1940.970
1958.021(PEP_1)
CID-MS/MS Scan:28245, Noise threshold:1.2
366.142(HexNAc + Hex)
512.201(HexNAc(1)Hex(1)Fuc(1)_1)
528.194(Hex + HexNAc + Hex)
779.284
1018.820
1263.625
1337.165
1491.217
1579.723
1242.892(PEP+HexNAc(4)Hex(5)Fuc(1)_3)
1344.650(PEP+HexNAc(2)Hex(2)_2)
1498.710(PEP+HexNAc(2)Hex(3)Fuc(1)_2)
1527.221(PEP+HexNAc(3)Hex(3)_2)
1571.742(PEP+HexNAc(2)Hex(3)Fuc(2)_2)
1681.282(PEP+HexNAc(3)Hex(4)Fuc(1)_2)
1182.601(PEP+HexNAc(2)_2)
m/z

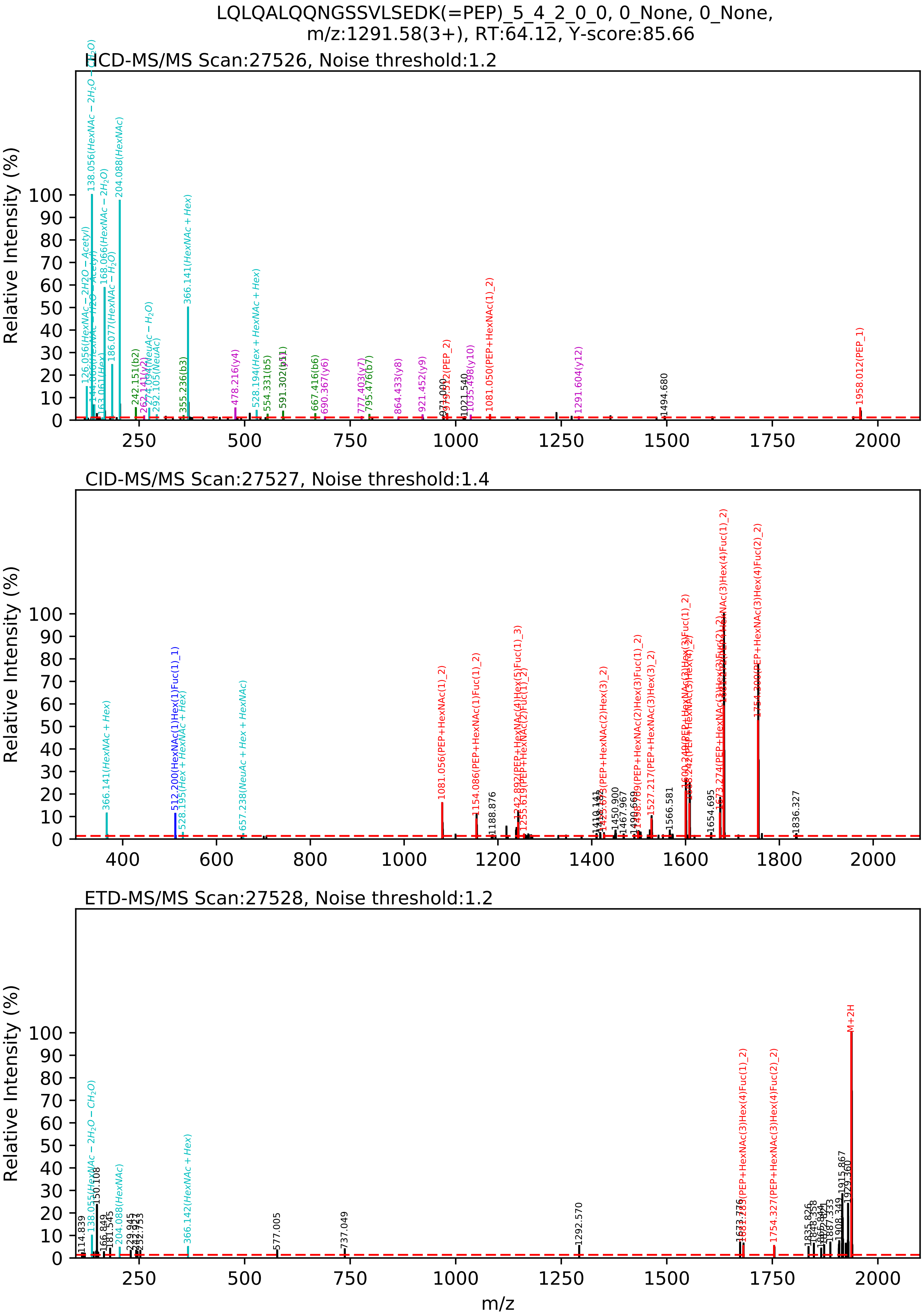
LQLQALQQNGSSVLSEDK(=PEP)_5_4_2_0_0, 0_None, 0_None,
m/z:1291.58(3+), RT:64.12, Y-score:85.66
HCD-MS/MS Scan:27526, Noise threshold:1.2
Relative Intensity (%)
138.056(HexNAc − 2H2O)
204.088(HexNAc)
168.066(HexNAc − 2H2O)
186.077(HexNAc − H2O)
366.141(HexNAc + Hex)
242.151(b2)
355.236(b3)
478.216(y4)
528.194(Hex + HexNAc + Hex)
554.331(b5)
667.416(b6)
690.367(y6)
777.403(y7)
795.476(b7)
864.433(y8)
921.452(y9)
1021.540
1035.498(y10)
1081.050(PEP+HexNAc(1)_2)
1291.604(y12)
1494.680
1958.012(PEP_1)
CID-MS/MS Scan:27527, Noise threshold:1.4
Relative Intensity (%)
366.141(HexNAc + Hex)
512.200(HexNAc(1)Hex(1)Fuc(1)_1)
528.195(Hex + HexNAc + Hex)
657.238(NeuAc + Hex + HexNAc)
1081.056(PEP+HexNAc(1)_2)
1154.086(PEP+HexNAc(1)Fuc(1)_2)
1188.876
1255.619(PEP+HexNAc(2)Fuc(1)_2)
1450.900
1467.967
1527.217(PEP+HexNAc(3)Hex(3)_2)
1566.581
1654.695
1754.209(PEP+HexNAc(3)Hex(4)Fuc(2)_2)
1836.327
ETD-MS/MS Scan:27528, Noise threshold:1.2
Relative Intensity (%)
114.839
150.108
166.849
181.545
204.088(HexNAc)
252.753
366.142(HexNAc + Hex)
577.005
737.049
1292.570
1673.776
1754.327(PEP+HexNAc(3)Hex(4)Fuc(2)_2)
1908.349
1915.867
1929.360
M+2H
m/z

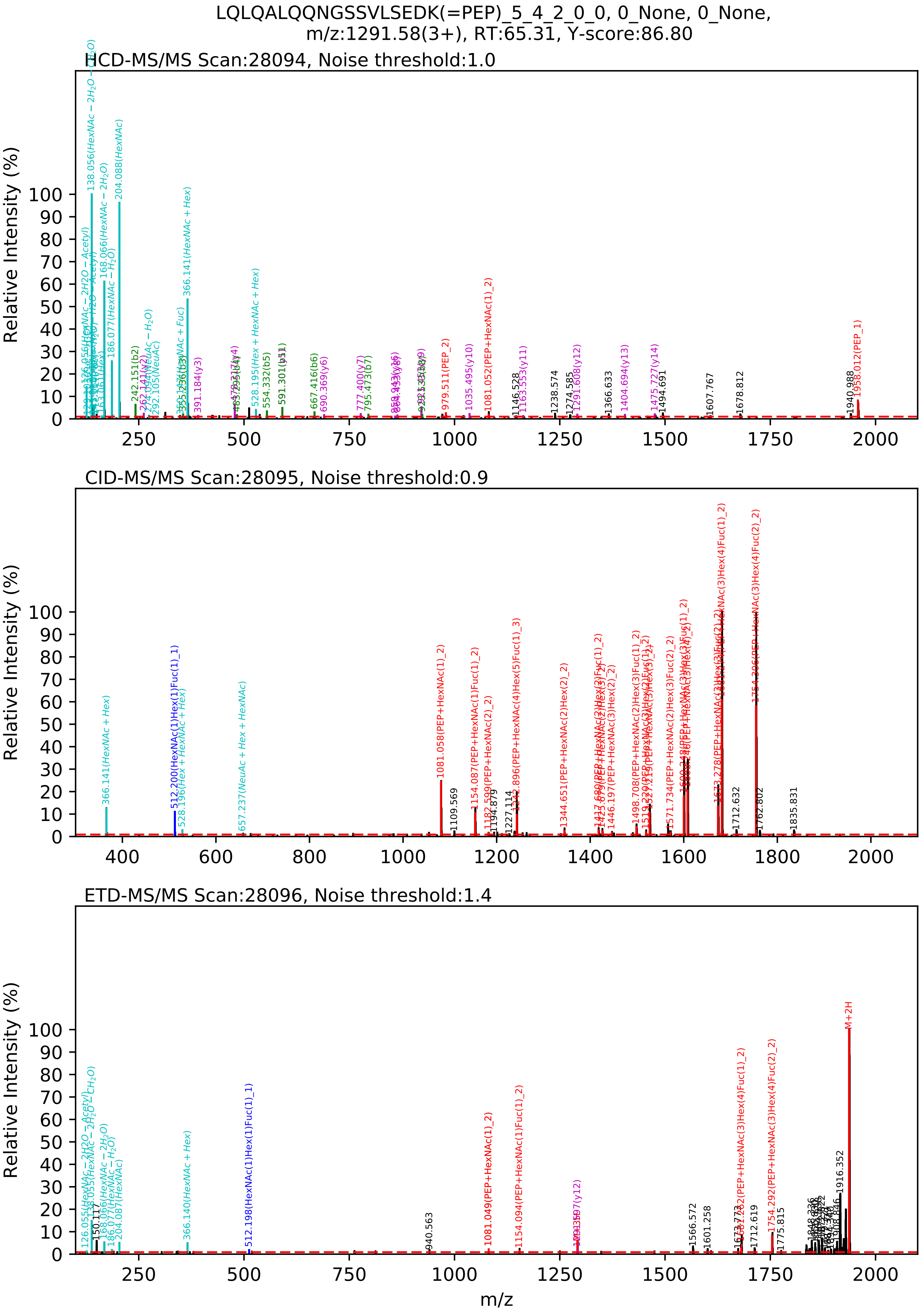

LQLQALQQNGSSVLSEDK(=PEP)_5_4_2_0_0, 0_None, 0_None,
m/z:1291.58(3+), RT:65.31, Y-score:86.80
HCD-MS/MS Scan:28094, Noise threshold:1.0
CID-MS/MS Scan:28095, Noise threshold:0.9
ETD-MS/MS Scan:28096, Noise threshold:1.4
Relative Intensity (%)
m/z

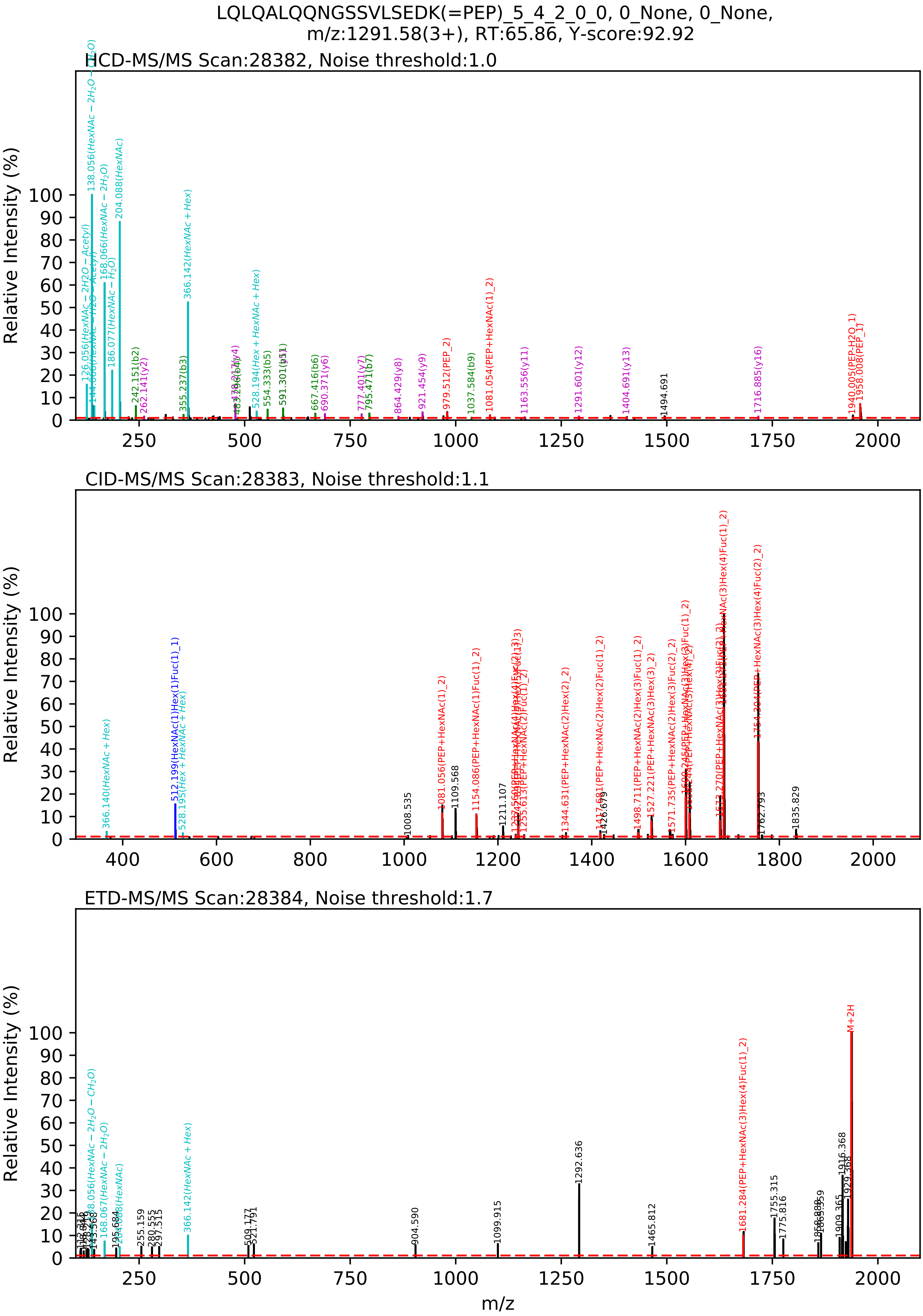

LQLQALQQNGSSVLSEDK(=PEP)_5_4_2_0_0, 0_None, 0_None,
m/z:1291.58(3+), RT:65.86, Y-score:92.92
HCD-MS/MS Scan:28382, Noise threshold:1.0
Relative Intensity (%)
138.056(HexNAc − 2H2O − CH2O)
204.088(HexNAc)
168.066(HexNAc − 2H2O)
186.077(HexNAc − H2O)
366.142(HexNAc + Hex)
528.194(Hex + HexNAc + Hex)
242.151(b2)
262.141(y2)
355.237(b3)
554.333(b5)
591.301(b11)
667.416(b6)
690.371(y6)
777.401(y7)
795.471(b7)
864.429(y8)
921.454(y9)
979.512(PEP_2)
1037.584(b9)
1081.054(PEP+HexNAc(1)_2)
1163.556(y11)
1291.601(y12)
1404.691(y13)
1494.691
1716.885(y16)
1940.005(PEP-H2O_1)
1958.008(PEP_1)
CID-MS/MS Scan:28383, Noise threshold:1.1
366.140(HexNAc + Hex)
512.199(HexNAc(1)Hex(1)Fuc(1)_1)
528.195(Hex + HexNAc + Hex)
1008.535
1081.056(PEP+HexNAc(1)_2)
1109.568
1154.086(PEP+HexNAc(1)Fuc(1)_2)
1211.107
1255.613(PEP+HexNAc(2)Fuc(1)_2)
1344.631(PEP+HexNAc(2)Hex(2)_2)
1417.681(PEP+HexNAc(2)Hex(2)Fuc(1)_2)
1426.679
1498.711(PEP+HexNAc(2)Hex(3)Fuc(1)_2)
1527.221(PEP+HexNAc(3)Hex(3)_2)
1571.735(PEP+HexNAc(2)Hex(3)Fuc(2)_2)
1673.270(PEP+HexNAc(3)Hex(3)Fuc(2)_2)
PEP+HexNAc(3)Hex(4)Fuc(1)_2
1754.294(PEP+HexNAc(3)Hex(4)Fuc(2)_2)
1762.793
1835.829
ETD-MS/MS Scan:28384, Noise threshold:1.7
138.056(HexNAc − 2H2O − CH2O)
168.067(HexNAc − 2H2O)
204.088(HexNAc)
143.568
195.684
255.159
280.555
297.515
366.142(HexNAc + Hex)
509.177
521.791
904.590
1099.915
1292.636
1465.812
1681.284(PEP+HexNAc(3)Hex(4)Fuc(1)_2)
1755.315
1775.816
1909.365
1916.368
1929.368
M+2H
m/z

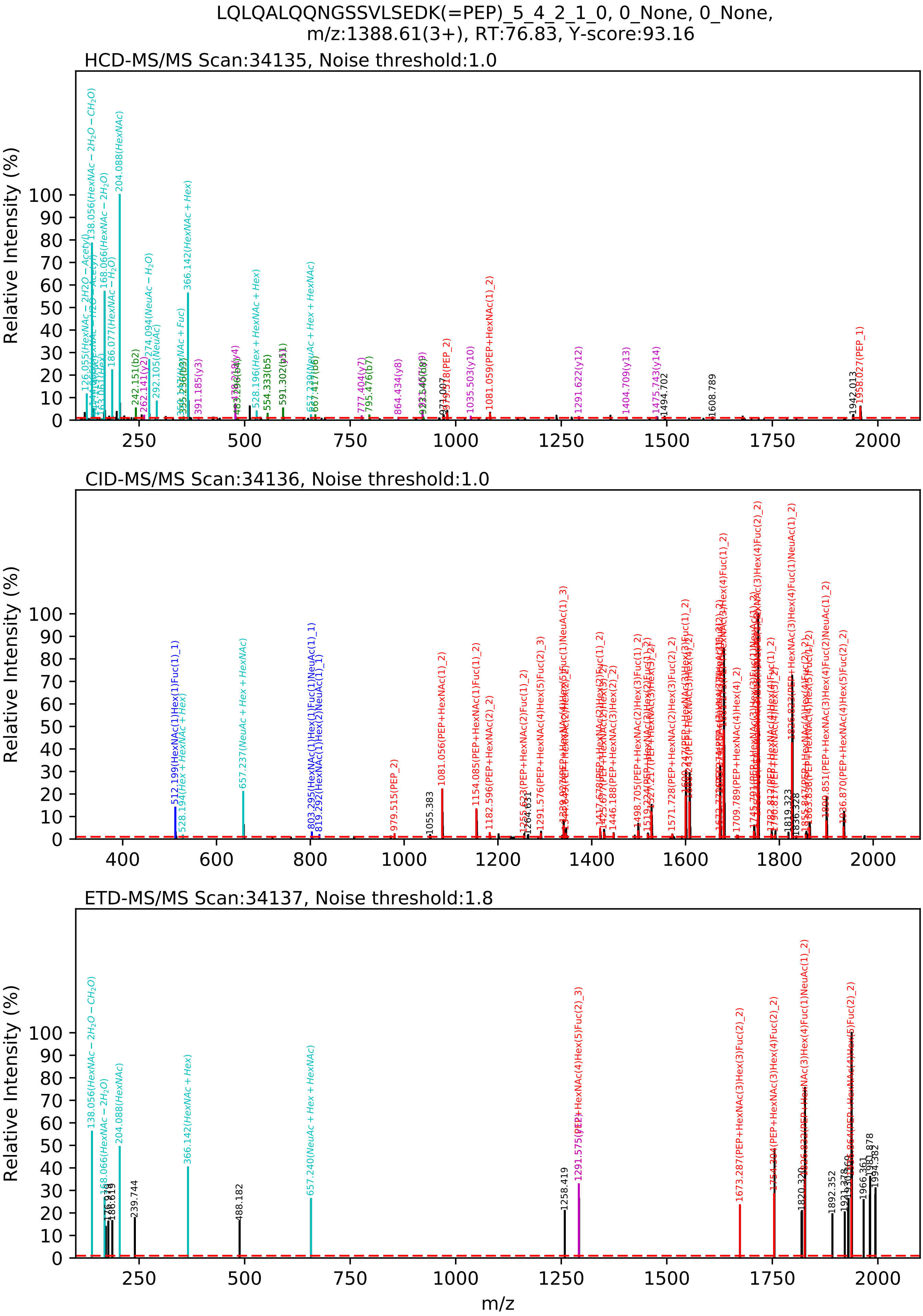
LQLQALQQNGSSVLSEDK(=PEP)_5_4_2_1_0, 0_None, 0_None,
m/z:1388.61(3+), RT:76.83, Y-score:93.16
HCD-MS/MS Scan:34135, Noise threshold:1.0
Relative Intensity (%)
204.088(HexNAc)
138.056(HexNAc − 2H2O)
168.066(HexNAc − H2O)
186.077(HexNAc − H2O)
366.142(HexNAc + Hex)
274.094(NeuAc − H2O)
292.105(NeuAc)
242.151(b2)
262.141(y2)
391.185(y3)
528.196(Hex + HexNAc + Hex)
554.333(b5)
591.302(b5)
657.239(NeuAc + Hex + HexNAc)
687.417(b6)
777.404(y7)
795.476(b7)
864.434(y8)
971.097
979.518(PEP_2)
1035.503(y10)
1081.059(PEP+HexNAc(1)_2)
1291.622(y12)
1404.709(y13)
1475.743(y14)
1494.702
1608.789
1942.013
1958.027(PEP_1)
CID-MS/MS Scan:34136, Noise threshold:1.0
512.199(HexNAc(1)Hex(1)Fuc(1)_1)
528.194(Hex + HexNAc + Hex)
657.237(NeuAc + Hex + HexNAc)
803.295(HexNAc(1)Hex(1)Fuc(1)NeuAc(1)_1)
819.292(HexNAc(1)Hex(2)NeuAc(1)_1)
979.515(PEP_2)
1055.383
1081.056(PEP+HexNAc(1)_2)
1154.085(PEP+HexNAc(1)Fuc(1)_2)
1182.596(PEP+HexNAc(2)_2)
1255.623(PEP+HexNAc(2)Fuc(1)_2)
1264.631
1291.576(PEP+HexNAc(4)Hex(5)Fuc(2)_3)
1446.188(PEP+HexNAc(3)Hex(2)_2)
1498.705(PEP+HexNAc(2)Hex(3)Fuc(1)_2)
1571.728(PEP+HexNAc(2)Hex(3)Fuc(2)_2)
1709.789(PEP+HexNAc(4)Hex(4)_2)
1819.323
1836.328
1826.823(PEP+HexNAc(3)Hex(4)Fuc(1)NeuAc(1)_2)
1899.851(PEP+HexNAc(3)Hex(4)Fuc(2)NeuAc(1)_2)
1936.870(PEP+HexNAc(4)Hex(5)Fuc(2)_2)
ETD-MS/MS Scan:34137, Noise threshold:1.8
138.056(HexNAc − 2H2O − CH2O)
168.066(HexNAc − 2H2O)
204.088(HexNAc)
239.744
366.142(HexNAc + Hex)
488.182
657.240(NeuAc + Hex + HexNAc)
1258.419
1291.575(PEP+HexNAc(4)Hex(5)Fuc(2)_3)
1673.287(PEP+HexNAc(3)Hex(3)Fuc(2)_2)
1754.204(PEP+HexNAc(3)Hex(4)Fuc(2)_2)
1820.320
1826.823(PEP+HexNAc(3)Hex(4)Fuc(1)NeuAc(1)_2)
1892.352
1966.361
1981.878
1994.382
m/z

LQLQALQQNGSSVLSEDK(=PEP)_5_4_2_1_0, 0_None, 0_None,
m/z:1388.61(3+), RT:77.95, Y-score:67.03

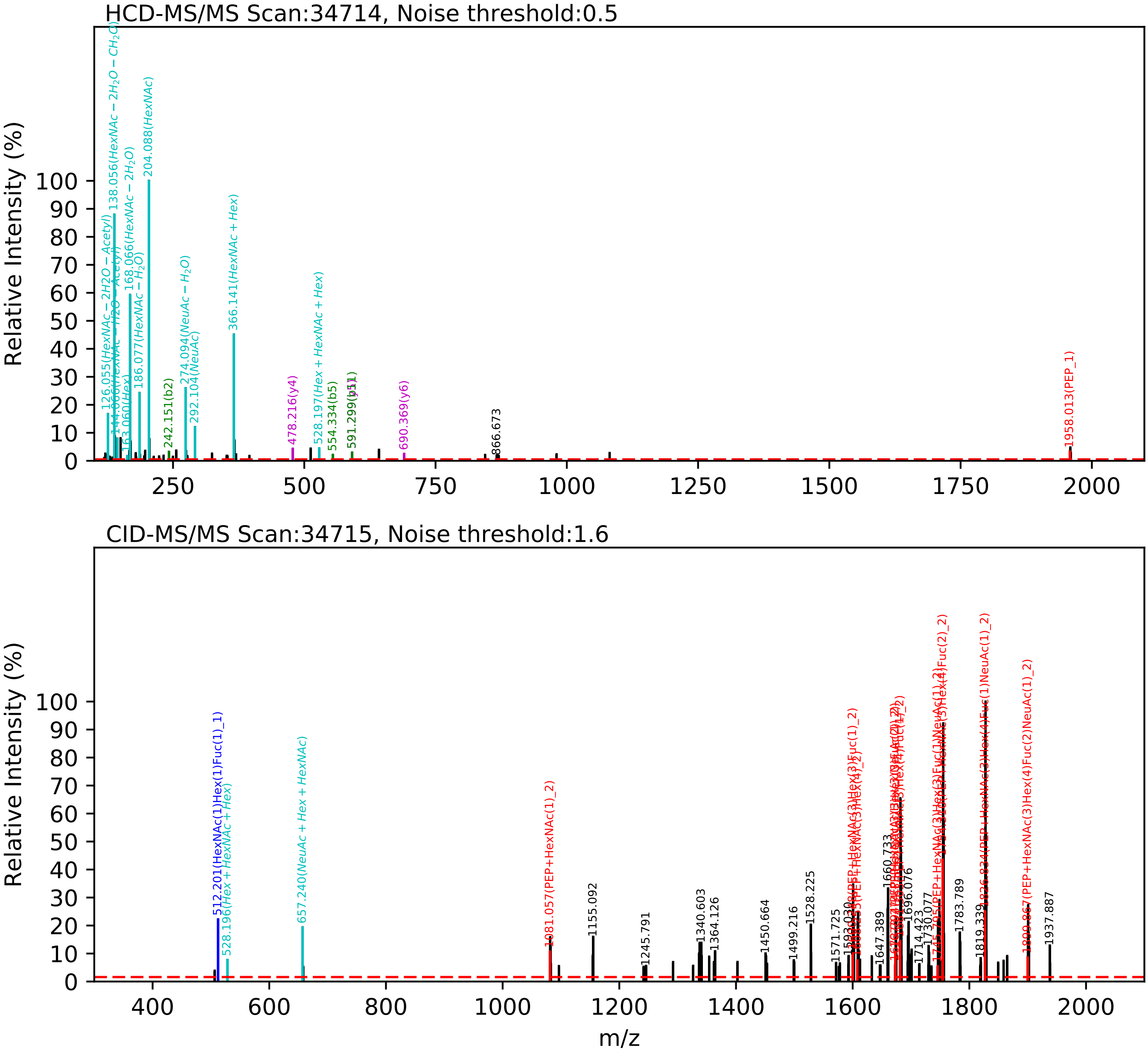

LQLQALQQNGSSVLSEDK(=PEP)_5_4_2_1_0, 0_None, 0_None,
m/z:1388.61(3+), RT:77.80, Y-score:88.13

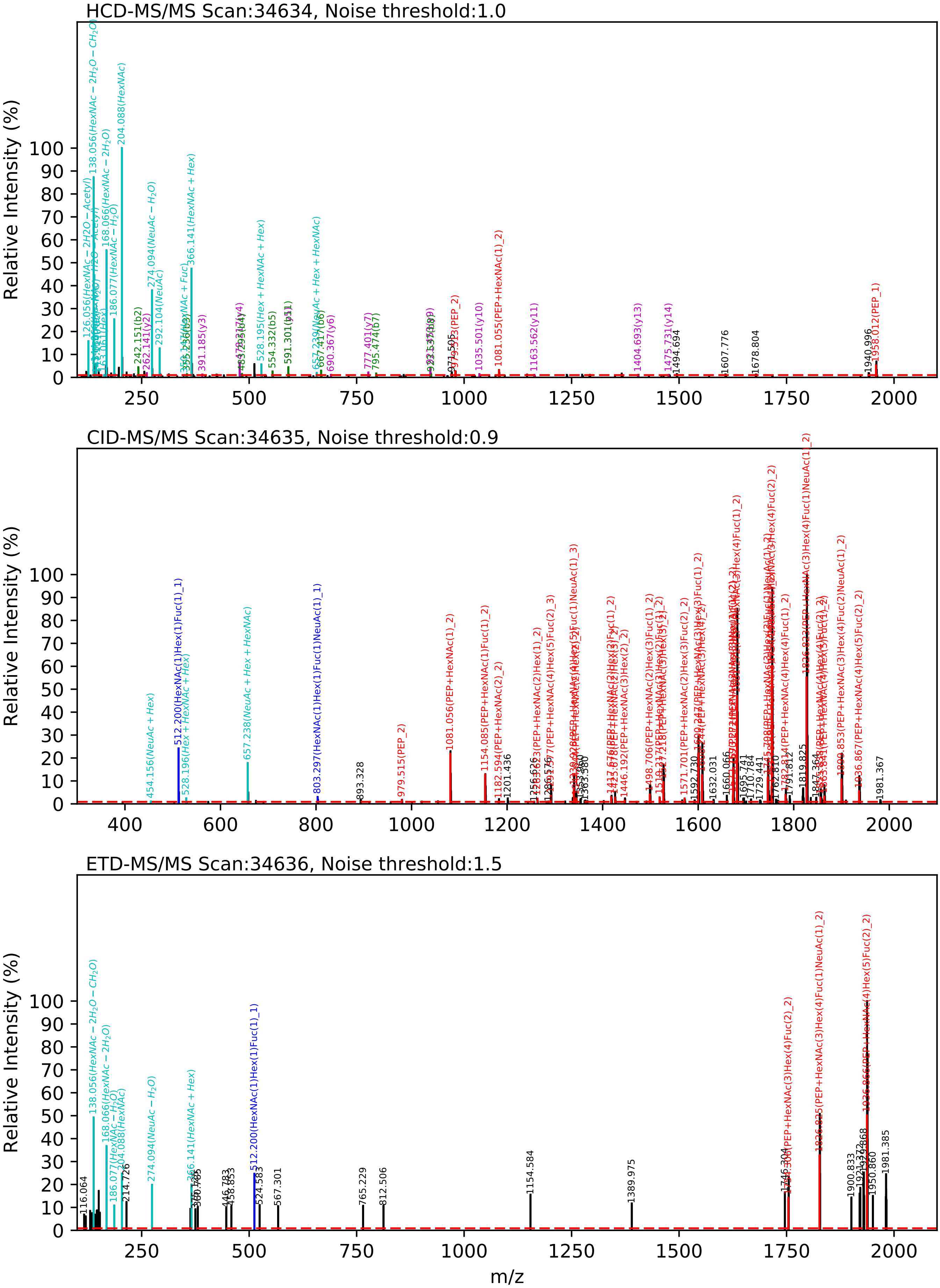

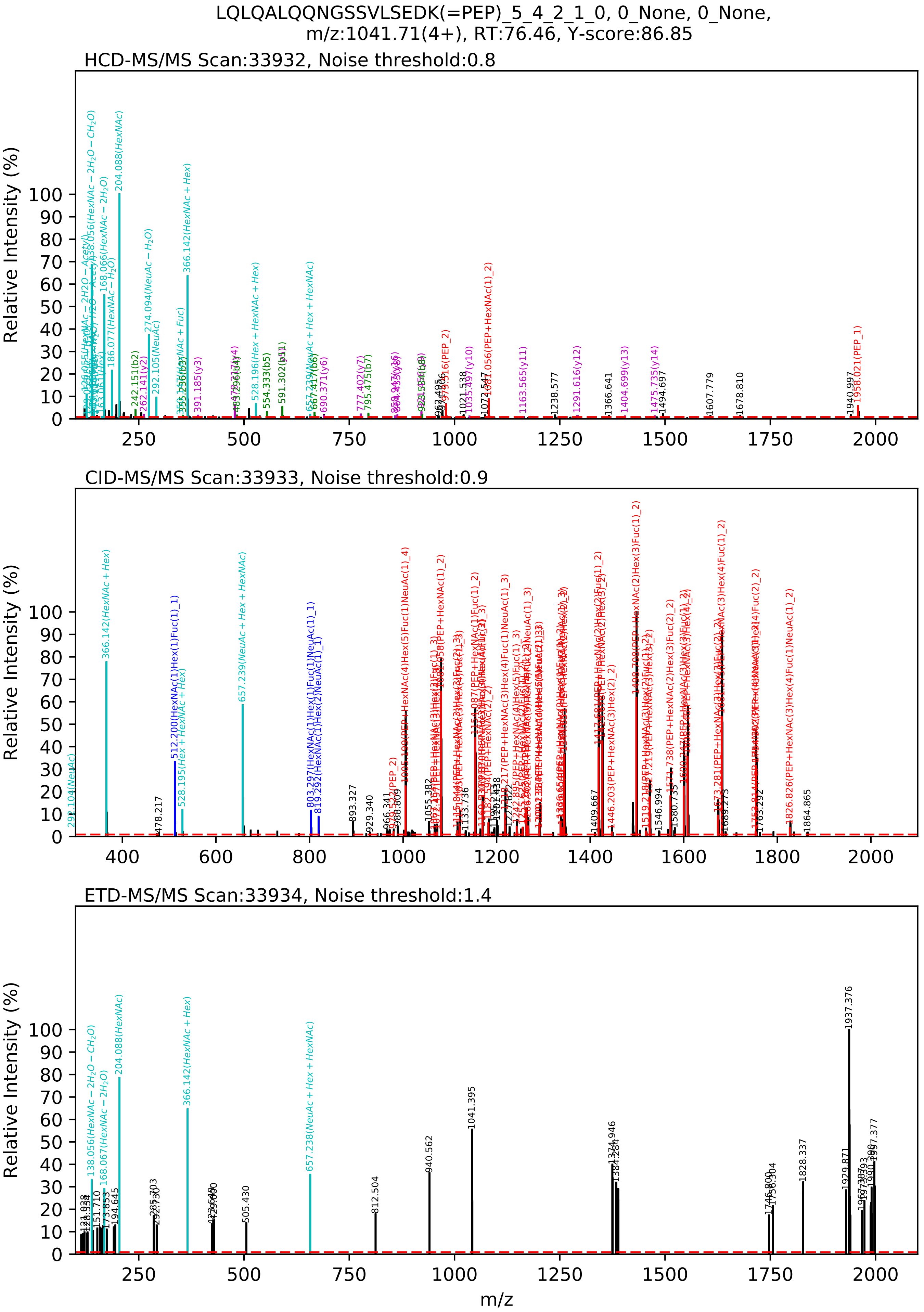

LQLQALQQNGSSVLSEDK(=PEP)_5_4_2_1_0, 0_None, 0_None,
m/z:1041.71(4+), RT:76.46, Y-score:86.85
HCD-MS/MS Scan:33932, Noise threshold:0.8
CID-MS/MS Scan:33933, Noise threshold:0.9
ETD-MS/MS Scan:33934, Noise threshold:1.4
Relative Intensity (%)
m/z

LQLQALQQNGSSVLSEDK(=PEP)_5_4_3_0_0, 0_None, 0_None,
m/z:1340.26(3+), RT:65.58, Y-score:72.70

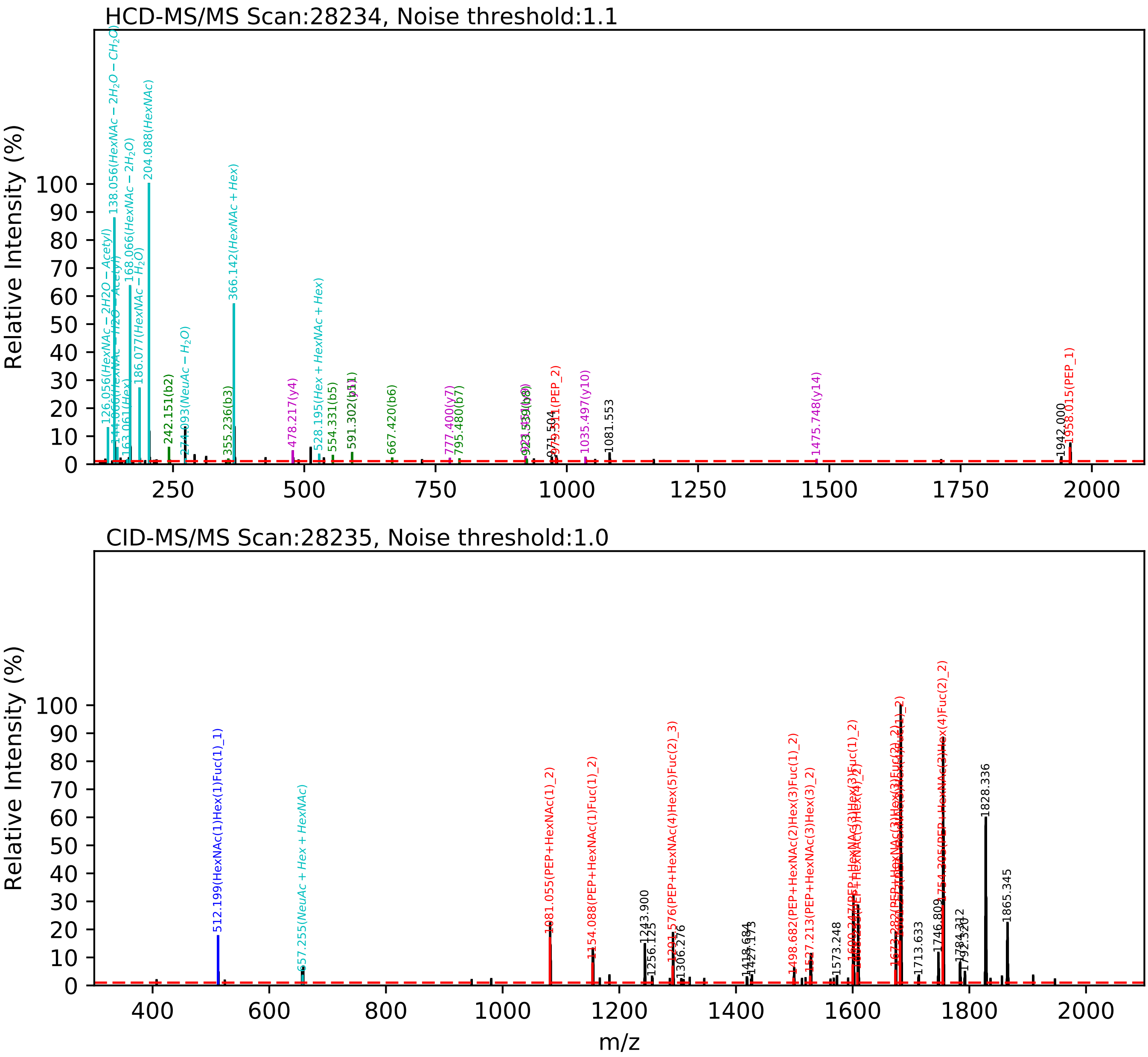

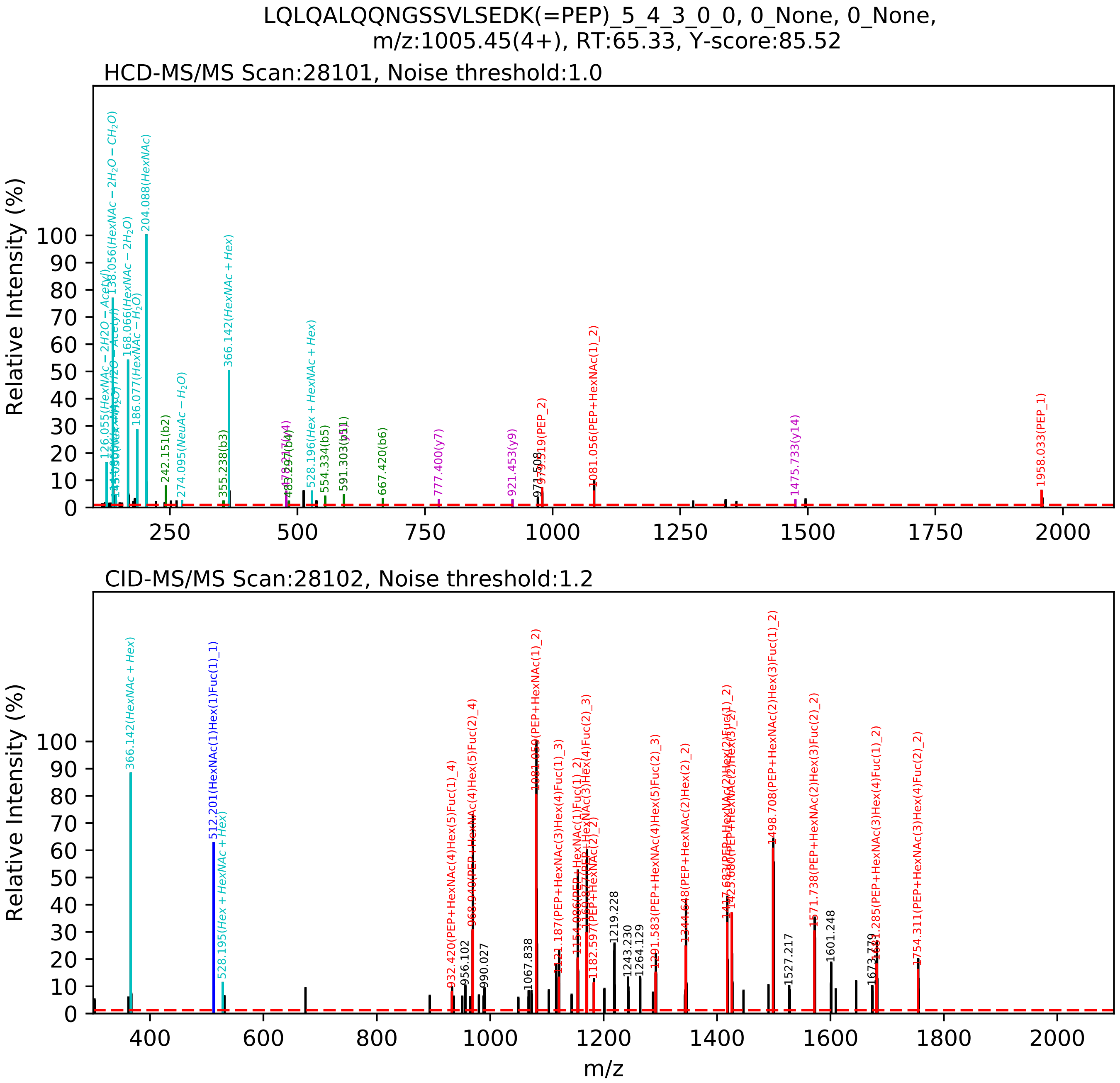
LQLQALQQNGSSVLSEDK(=PEP)_5_4_3_0_0, 0_None, 0_None,
m/z:1005.45(4+), RT:65.33, Y-score:85.52
HCD-MS/MS Scan:28101, Noise threshold:1.0
Relative Intensity (%)
204.088(HexNAc)
366.142(HexNAc + Hex)
168.066(HexNAc − 2H2O)
186.077(HexNAc − H2O)
242.151(b2)
274.095(NeuAc − H2O)
355.238(b3)
528.196(Hex + HexNAc + Hex)
554.334(b5)
591.303(b5)
667.420(b6)
777.400(y7)
921.453(y9)
971.508
975.519(PEP_2)
1081.056(PEP+HexNAc(1)_2)
1475.733(y14)
1958.033(PEP_1)
CID-MS/MS Scan:28102, Noise threshold:1.2
366.142(HexNAc + Hex)
512.201(HexNAc(1)Hex(1)Fuc(1)_1)
528.195(Hex + HexNAc + Hex)
932.420(PEP+HexNAc(4)Hex(5)Fuc(1)_4)
956.102
990.027
1067.838
1081.059(PEP+HexNAc(1)_2)
1219.228
1243.230
1264.129
1291.583(PEP+HexNAc(4)Hex(5)Fuc(2)_3)
1344.648(PEP+HexNAc(2)Hex(2)_2)
1498.708(PEP+HexNAc(2)Hex(3)Fuc(1)_2)
1527.217
1571.738(PEP+HexNAc(2)Hex(3)Fuc(2)_2)
1601.248
1673.779
1754.311(PEP+HexNAc(3)Hex(4)Fuc(2)_2)
m/z

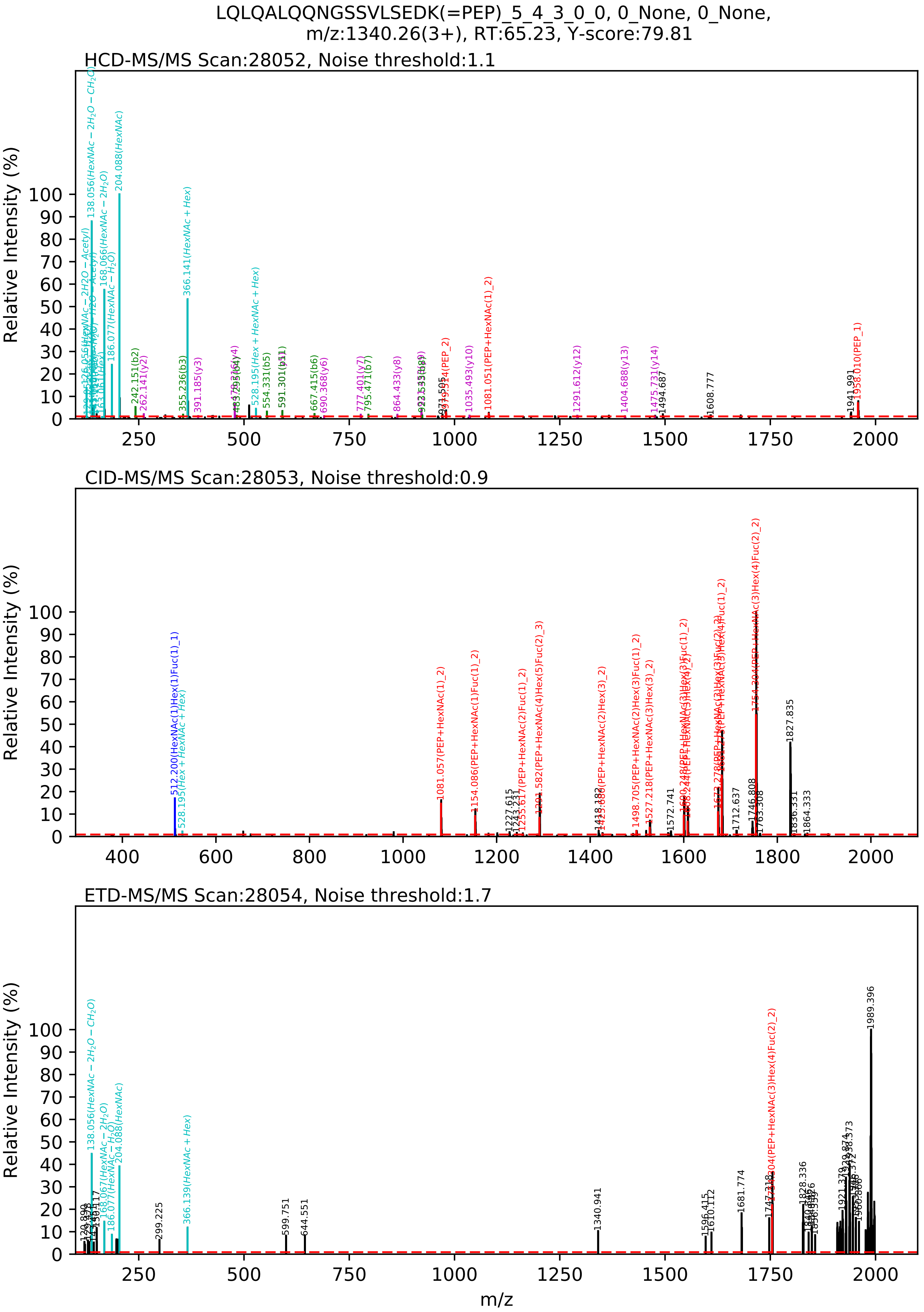
LQLQALQQNGSSVLSEDK(=PEP)_5_4_3_0_0, 0_None, 0_None,
m/z:1340.26(3+), RT:65.23, Y-score:79.81
HCD-MS/MS Scan:28052, Noise threshold:1.1
Relative Intensity (%)
204.088(HexNAc)
138.056(HexNAc − 2H2O − CH2O)
168.066(HexNAc − 2H2O)
186.077(HexNAc − H2O)
366.141(HexNAc + Hex)
528.195(Hex + HexNAc + Hex)
242.151(b2)
262.141(y2)
355.236(b3)
391.185(y3)
554.331(b5)
591.301(b5)
667.415(b6)
690.368(y6)
777.401(y7)
795.471(b7)
864.433(y8)
1035.493(y10)
1081.051(PEP+HexNAc(1)_2)
1291.612(y12)
1404.688(y13)
1475.731(y14)
1494.687
1608.777
1941.991
1958.010(PEP_1)
CID-MS/MS Scan:28053, Noise threshold:0.9
512.200(HexNAc(1)Hex(1)Fuc(1)_1)
528.195(Hex + HexNAc + Hex)
1081.057(PEP+HexNAc(1)_2)
1154.086(PEP+HexNAc(1)Fuc(1)_2)
1255.617(PEP+HexNAc(2)Fuc(1)_2)
1291.582(PEP+HexNAc(4)Hex(5)Fuc(2)_3)
1227.615
1243.231
1418.182
1498.705(PEP+HexNAc(2)Hex(3)Fuc(1)_2)
1527.218(PEP+HexNAc(3)Hex(3)_2)
1572.741
1712.637
1746.808
1763.308
1754.304(PEP+HexNAc(3)Hex(4)Fuc(2)_2)
1827.835
1836.331
1864.333
ETD-MS/MS Scan:28054, Noise threshold:1.7
138.056(HexNAc − 2H2O − CH2O)
168.067(HexNAc − 2H2O)
186.077(HexNAc − H2O)
204.088(HexNAc)
366.139(HexNAc + Hex)
299.225
599.751
644.551
1340.941
1596.415
1610.112
1681.774
1747.318
PEP+HexNAc(3)Hex(4)Fuc(2)_2
1828.336
1921.379
1938.373
1960.806
1989.396
m/z

LQLQALQQNGSSVLSEDK(=PEP)_5_4_3_1_0, 0_None, 0_None,
m/z:1437.29(3+), RT:76.57, Y-score:87.20

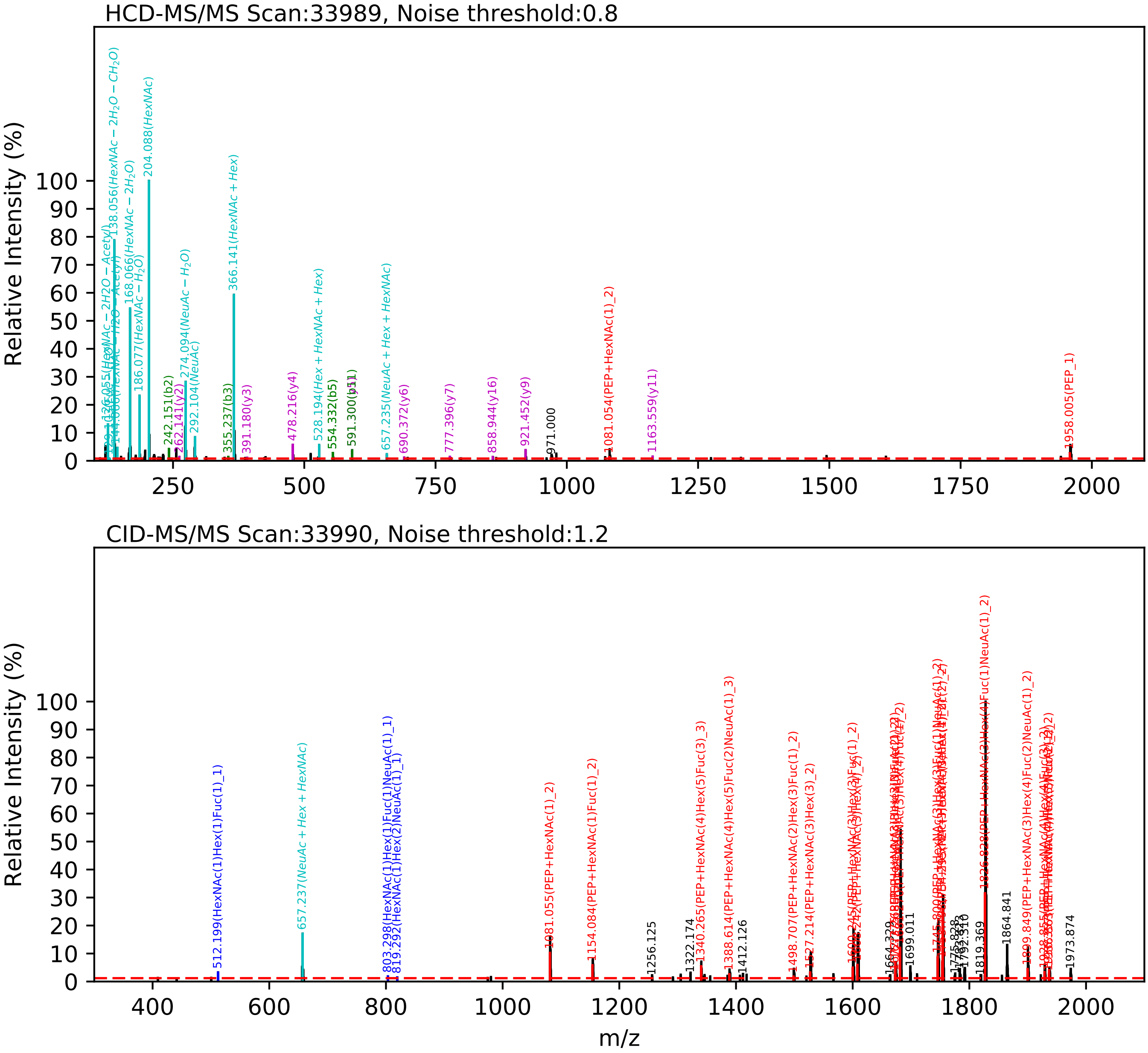

LQLQALQQNGSSVLSEDK(=PEP)_5_5_1_0_0, 0_None, 0_None,
m/z:983.19(4+), RT:65.37, Y-score:69.79

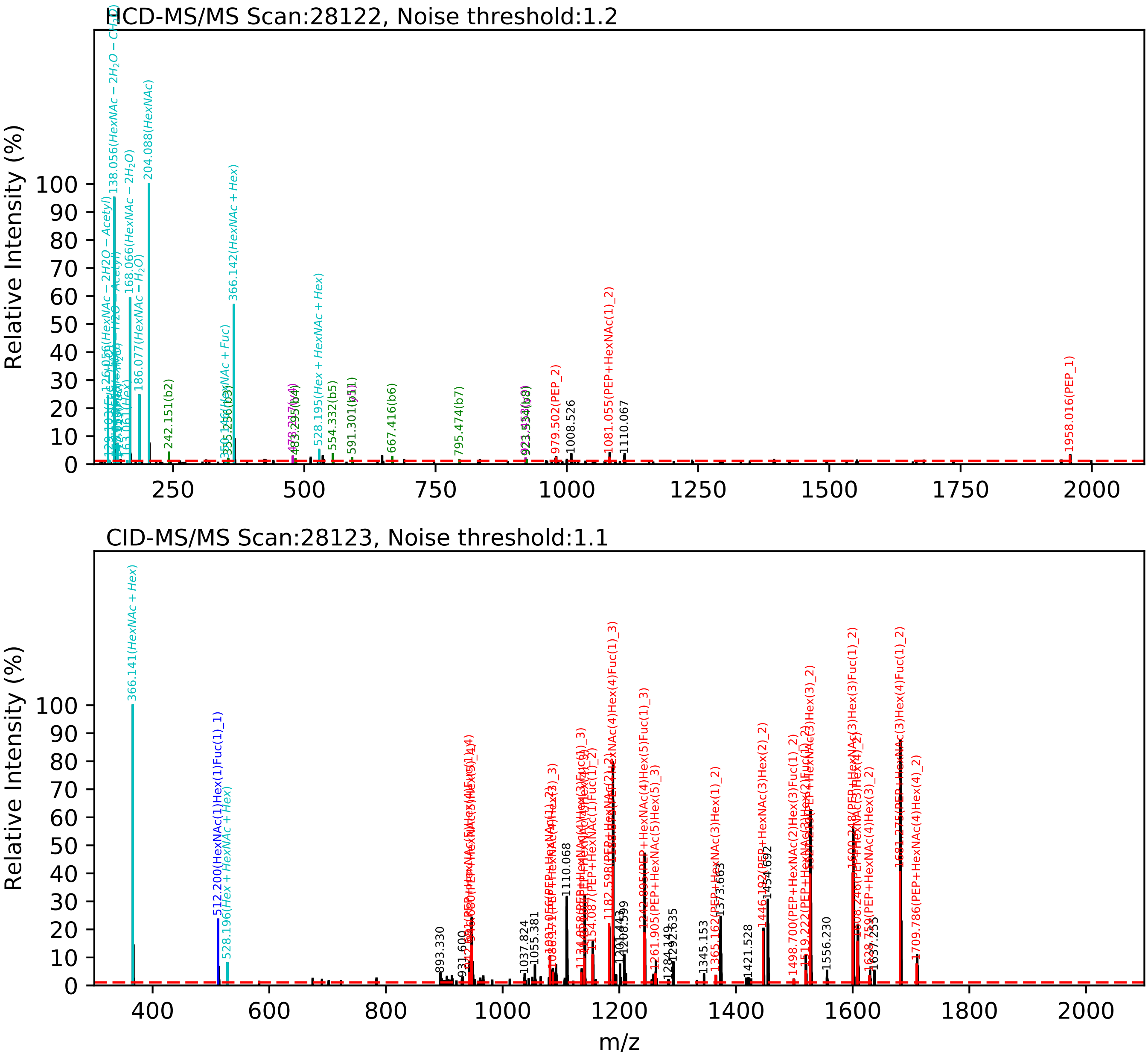

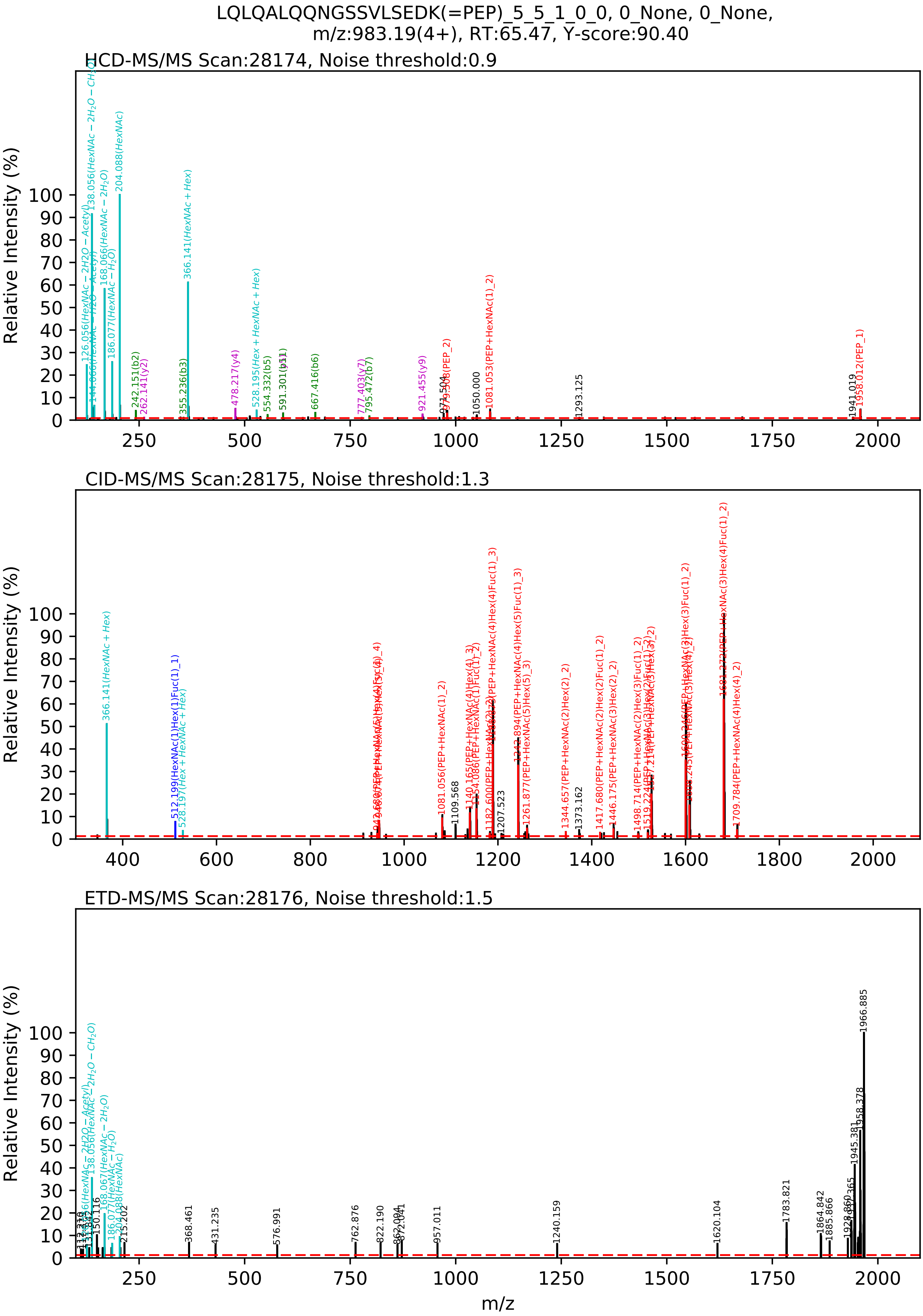

LQLQALQQNGSSVLSEDK(=PEP)_5_5_1_0_0, 0_None, 0_None,
m/z:983.19(4+), RT:65.47, Y-score:90.40
HCD-MS/MS Scan:28174, Noise threshold:0.9
Relative Intensity (%)
204.088(HexNAc)
366.141(HexNAc + Hex)
168.066(HexNAc − 2H₂O)
186.077(HexNAc − H₂O)
242.151(b2)
262.141(y2)
355.236(b3)
478.217(y4)
528.195(Hex + HexNAc + Hex)
554.332(b5)
667.416(b6)
777.403(y7)
795.472(b7)
921.455(y9)
1050.000
1081.053(PEP+HexNAc(1)_2)
1293.125
1941.019
1958.012(PEP_1)
CID-MS/MS Scan:28175, Noise threshold:1.3
366.141(HexNAc + Hex)
512.199(HexNAc(1)Hex(1)Fuc(1)_1)
528.197(Hex + HexNAc + Hex)
1081.056(PEP+HexNAc(1)_2)
1109.568
1207.523
1261.877(PEP+HexNAc(5)Hex(5)_3)
1344.657(PEP+HexNAc(2)Hex(2)_2)
1373.162
1417.680(PEP+HexNAc(2)Hex(2)Fuc(1)_2)
1446.175(PEP+HexNAc(3)Hex(2)_2)
1709.784(PEP+HexNAc(4)Hex(4)_2)
ETD-MS/MS Scan:28176, Noise threshold:1.5
150.116
215.202
368.461
431.235
576.991
762.876
822.190
872.041
957.011
1240.159
1620.104
1783.821
1864.842
1885.866
1945.381
1958.378
1966.885
m/z

LQLQALQQNGSSVLSEDK(=PEP)_5_5_1_1_0, 0_None, 0_None,
m/z:1407.62(3+), RT:77.41, Y-score:88.19

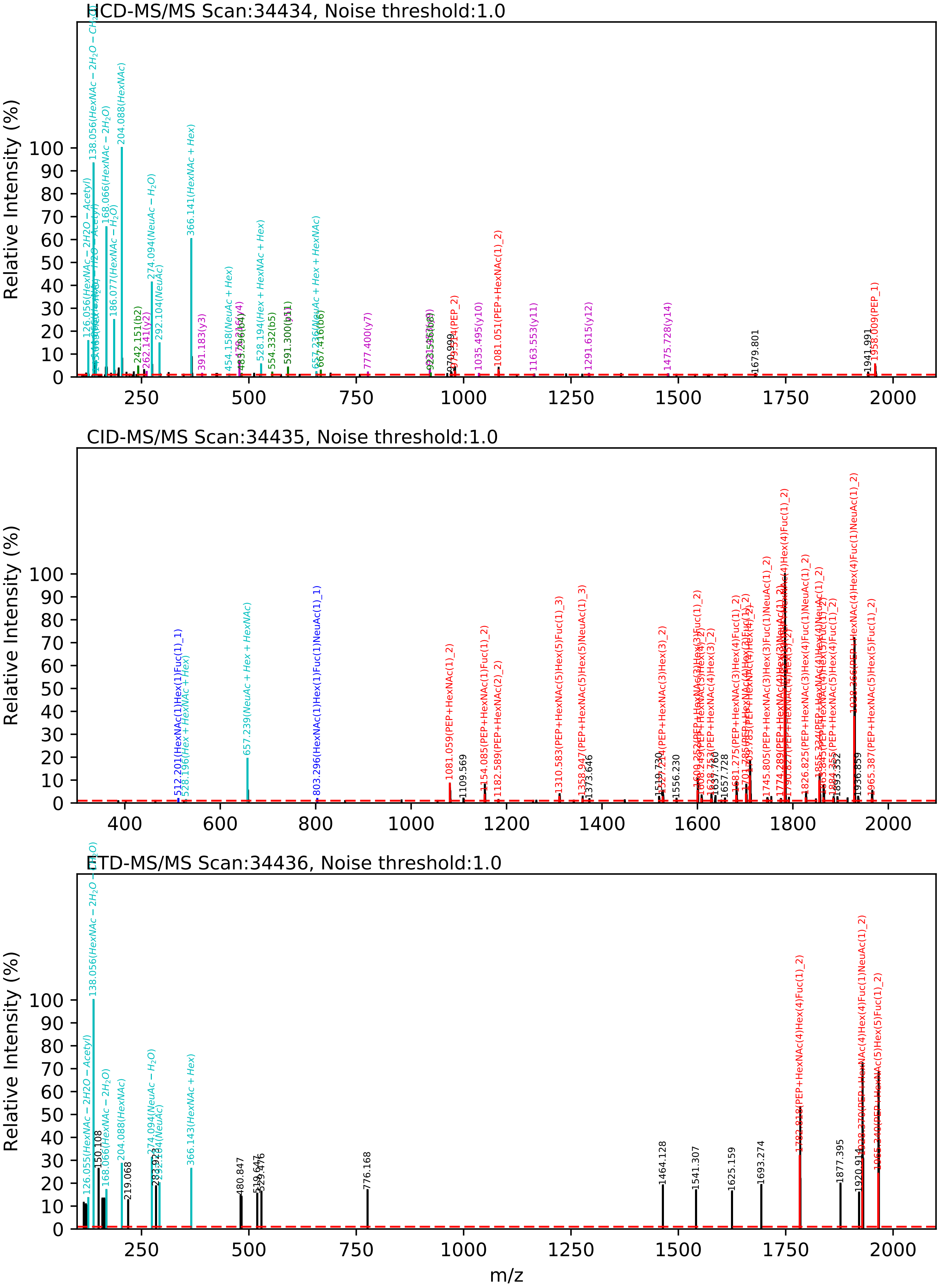

LQLQALQQNGSSVLSEDK(=PEP)_5_5_1_1_0, 0_None, 0_None,
m/z:1407.62(3+), RT:77.50, Y-score:84.03

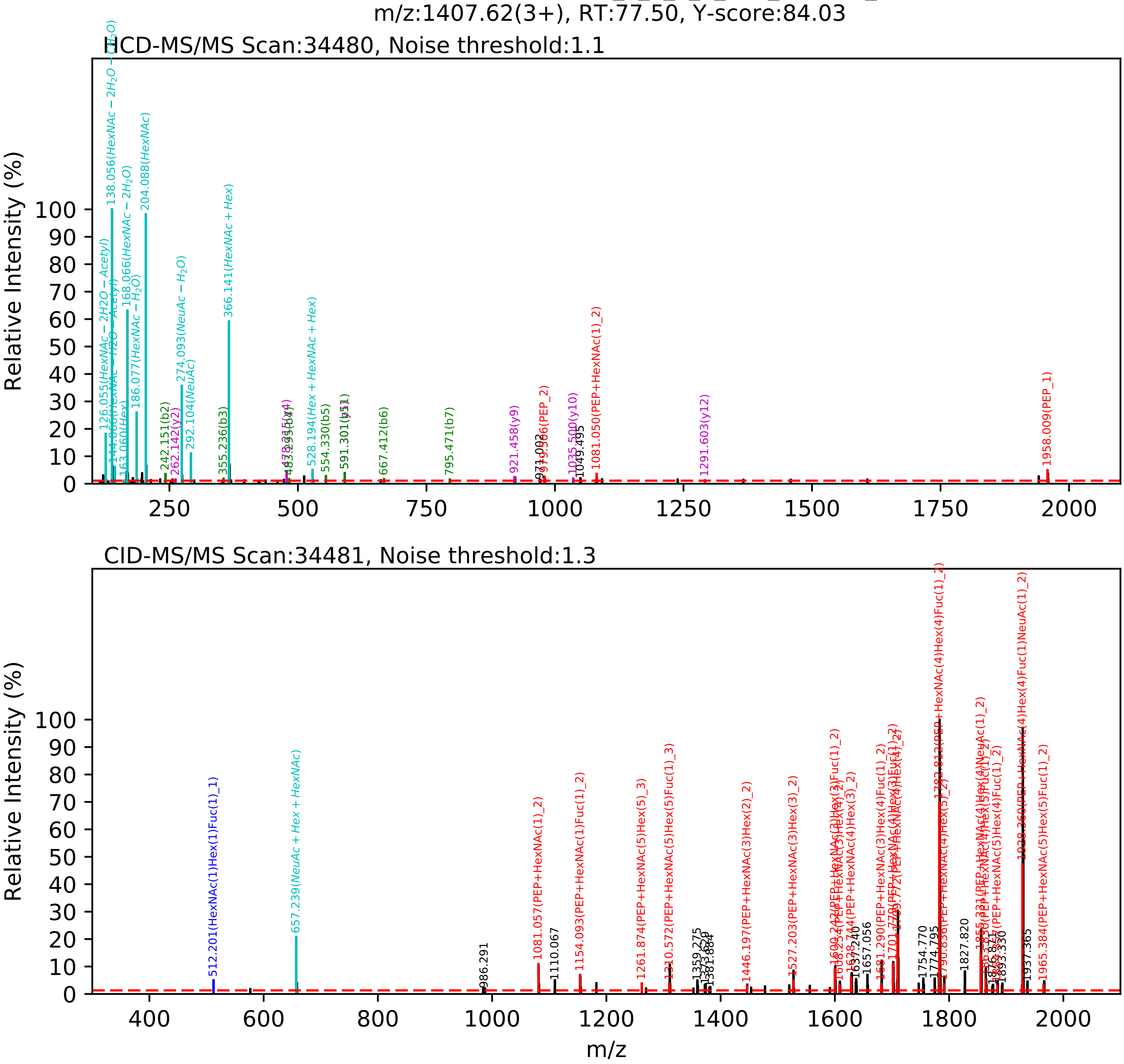

LQLQALQQNGSSVLSEDK(=PEP)_5_5_2_0_0, 0_None, 0_None,
m/z:1359.27(3+), RT:65.34, Y-score:85.13

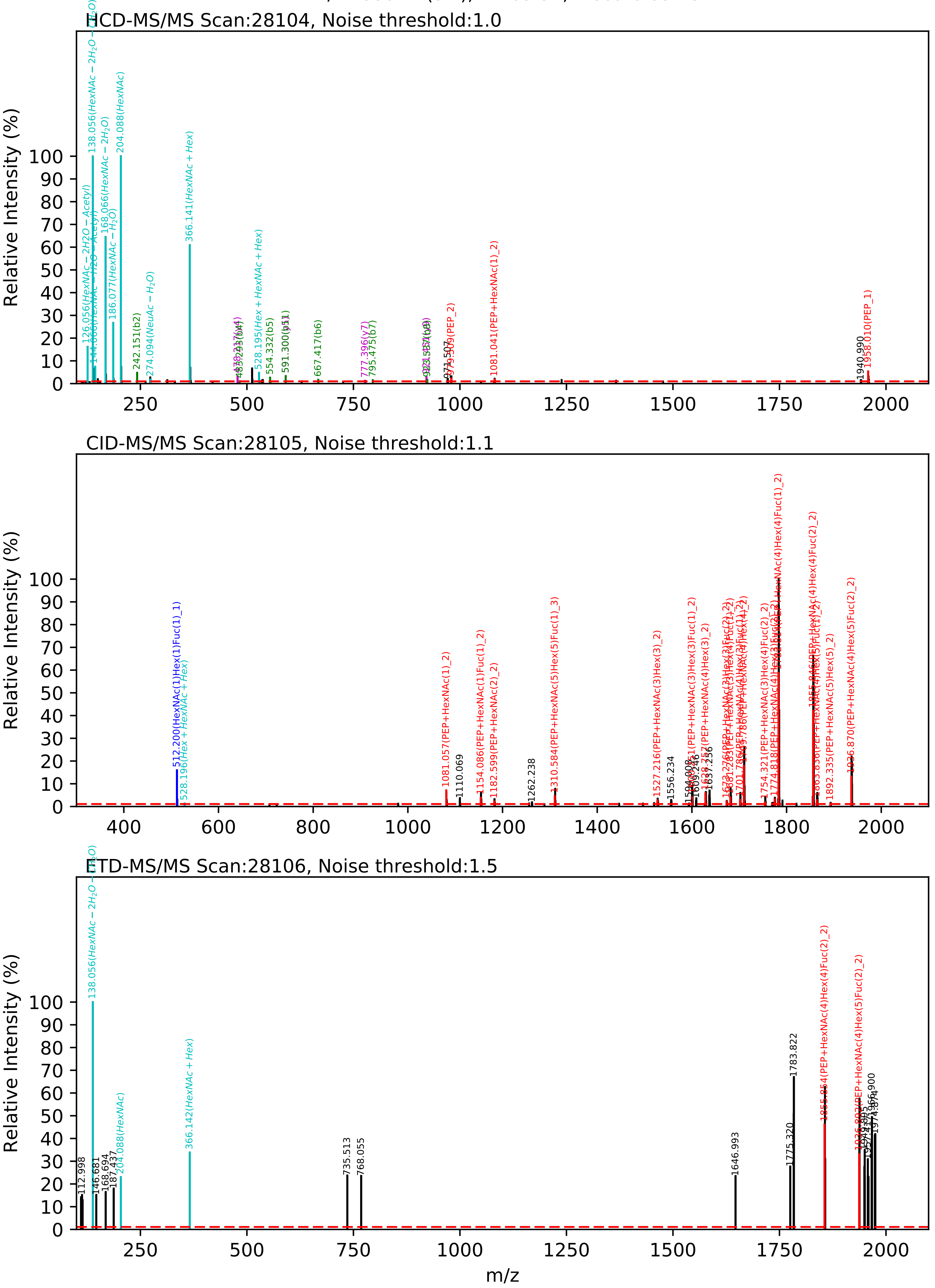

LQLQALQQNGSSVLSEDK(=PEP)_6_3_0_0_0, 0_None, 1_Hex_Phosphorylation, m/z:1207.19(3+), RT:79.82, Y-score:69.49

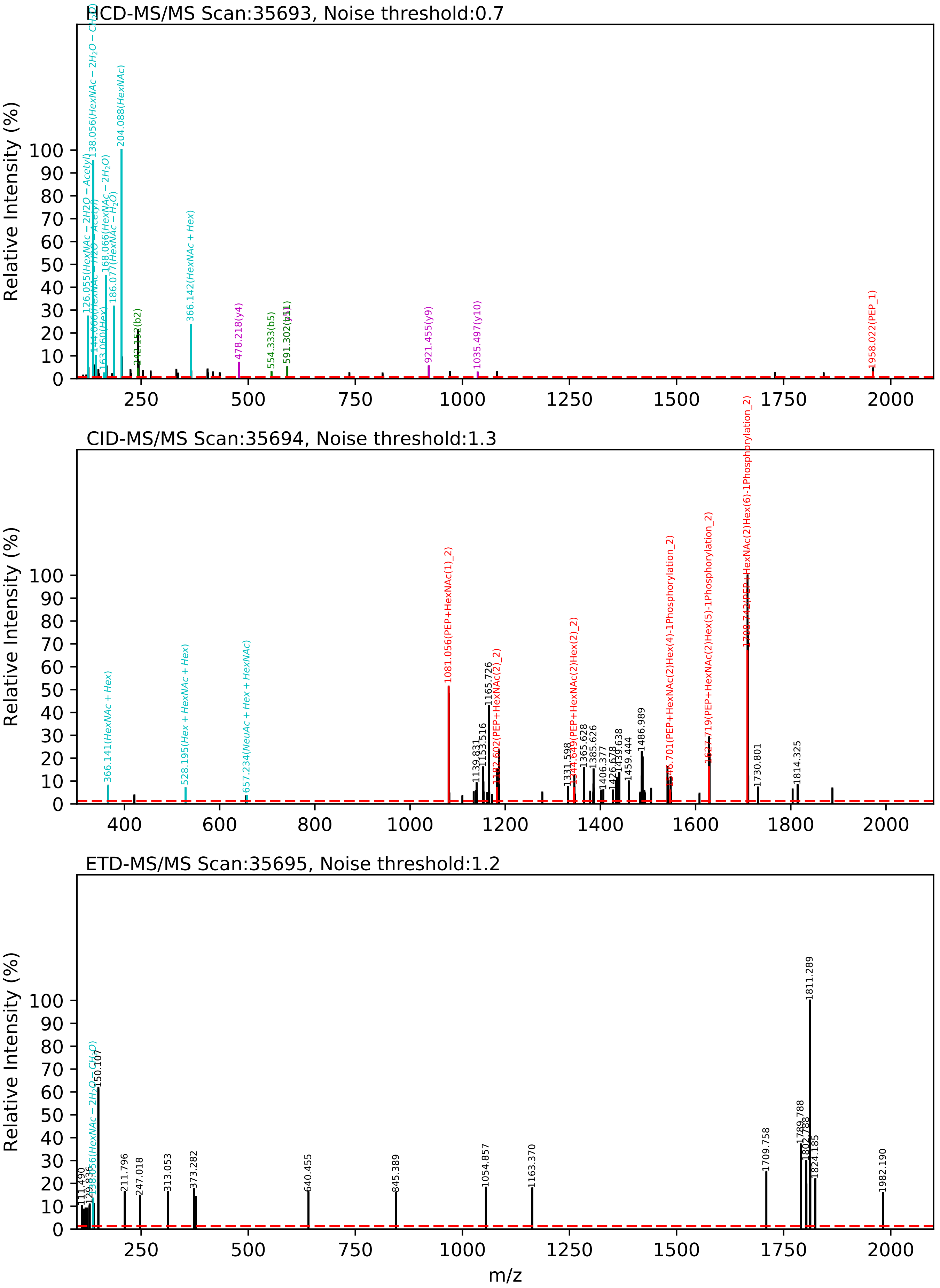

LQLQALQQNGSSVLSEDK(=PEP)_6_3_0_0_0, 0_None, 1_Hex_Phosphorylation,
m/z:1810.27(2+), RT:79.32, Y-score:68.67

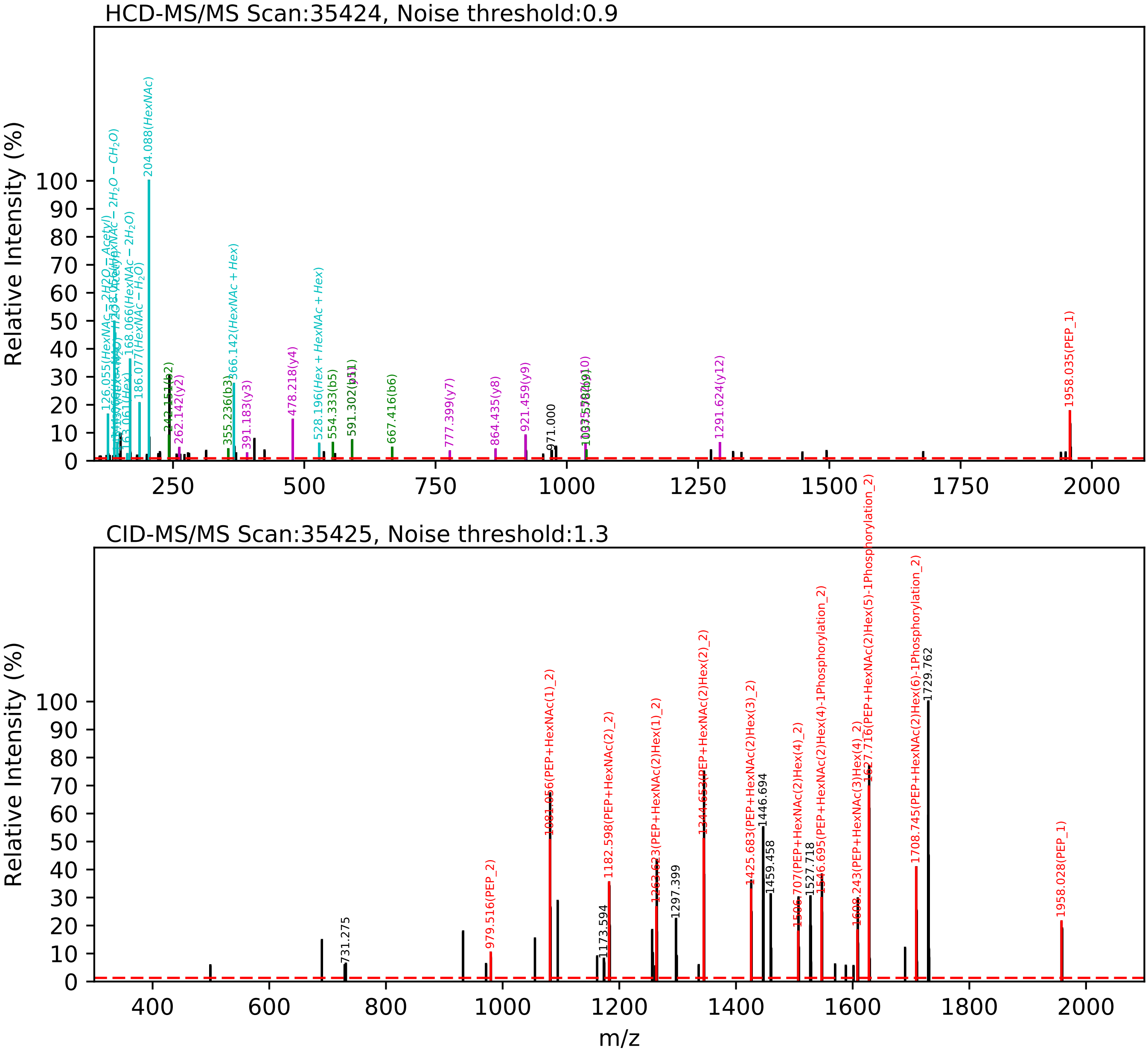

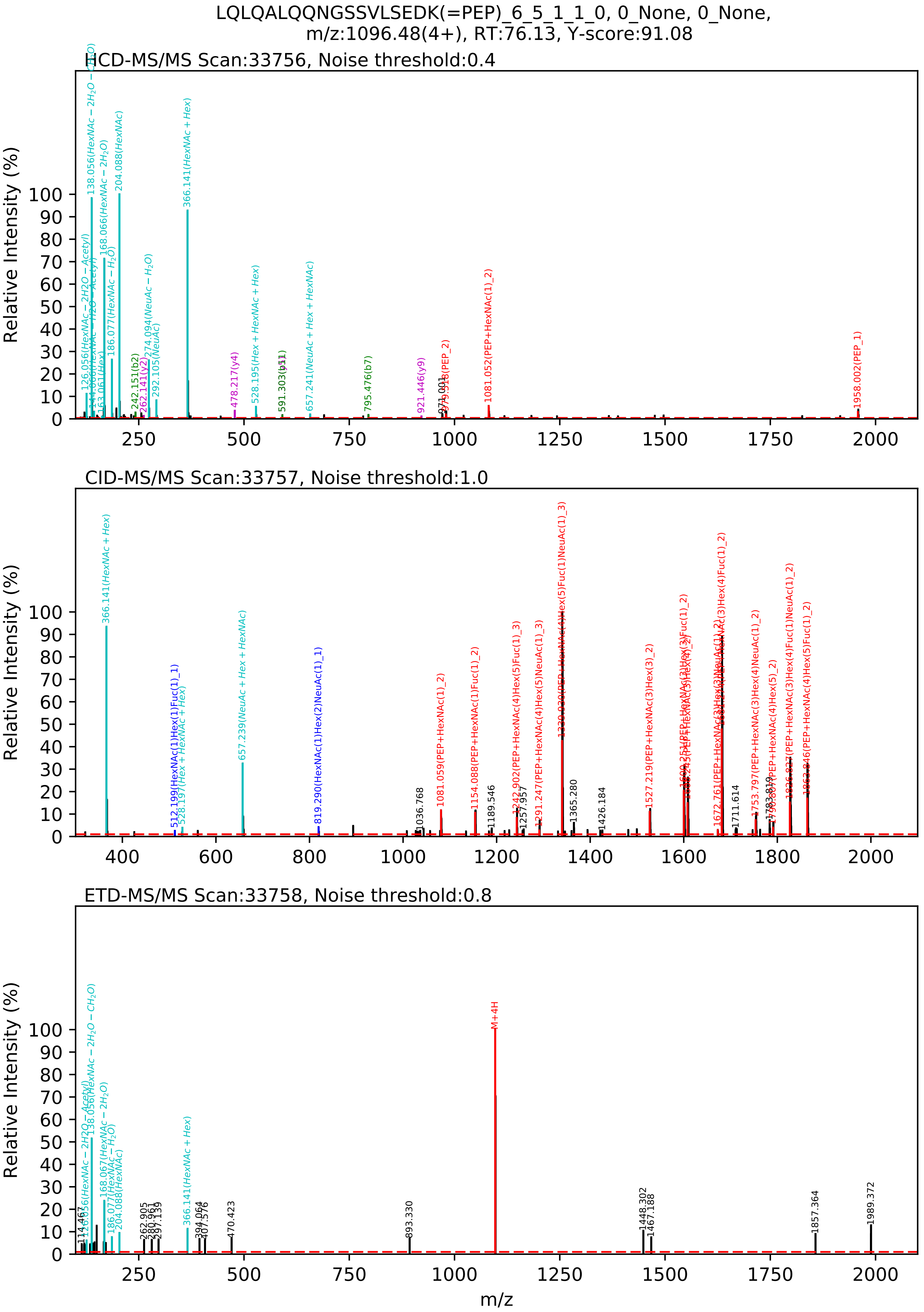

LQLQALQQNGSSVLSEDK(=PEP)_6_5_1_1_0, 0_None, 0_None,
m/z:1096.48(4+), RT:76.13, Y-score:91.08
HCD-MS/MS Scan:33756, Noise threshold:0.4
366.141(HexNAc + Hex)
204.088(HexNAc)
138.056(HexNAc − 2H2O − CH2O)
168.066(HexNAc − 2H2O)
186.077(HexNAc − H2O)
274.094(NeuAc − H2O)
292.105(NeuAc)
242.151(b2)
262.141(y2)
478.217(y4)
528.195(Hex + HexNAc + Hex)
591.303(b5)
657.241(NeuAc + Hex + HexNAc)
795.476(b7)
921.446(y9)
1081.052(PEP+HexNAc(1)_2)
1958.002(PEP_1)
CID-MS/MS Scan:33757, Noise threshold:1.0
366.141(HexNAc + Hex)
512.199(HexNAc(1)Hex(1)Fuc(1)_1)
528.197(Hex + HexNAc + Hex)
657.239(NeuAc + Hex + HexNAc)
819.290(HexNAc(1)Hex(2)NeuAc(1)_1)
1036.768
1081.059(PEP+HexNAc(1)_2)
1154.088(PEP+HexNAc(1)Fuc(1)_2)
1189.546
1242.902(PEP+HexNAc(4)Hex(5)Fuc(1)_3)
1257.957
1291.247(PEP+HexNAc(4)Hex(5)NeuAc(1)_3)
1365.280
1426.184
1527.219(PEP+HexNAc(3)Hex(3)_2)
1672.761(PEP+HexNAc(3)Hex(3)NeuAc(1)_2)
1711.614
1753.797(PEP+HexNAc(3)Hex(4)NeuAc(1)_2)
1826.827(PEP+HexNAc(3)Hex(4)Fuc(1)NeuAc(1)_2)
1863.846(PEP+HexNAc(4)Hex(5)Fuc(1)_2)
ETD-MS/MS Scan:33758, Noise threshold:0.8
M+4H
114.467
366.141(HexNAc + Hex)
204.088(HexNAc)
186.077(HexNAc − H2O)
168.067(HexNAc − 2H2O)
138.056(HexNAc − 2H2O − CH2O)
262.905
297.139
394.064
407.576
470.423
893.330
1448.302
1467.188
1857.364
1989.372
Relative Intensity (%)
m/z

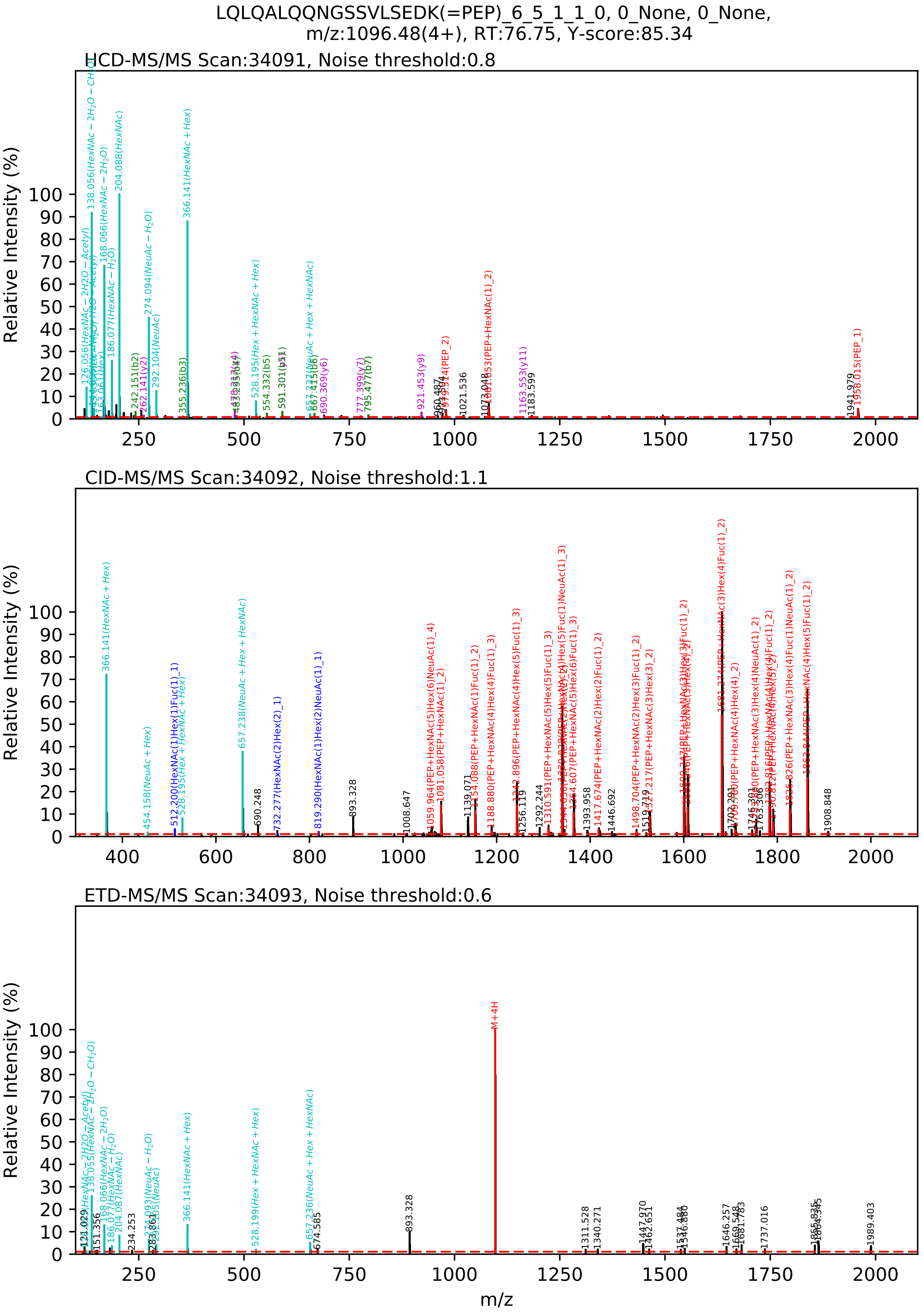

LQLQALQQNGSSVLSEDK(=PEP)_6_5_1_1_0, 0_None, 0_None,
m/z:1096.48(4+), RT:76.75, Y-score:85.34
HCD-MS/MS Scan:34091, Noise threshold:0.8
Relative Intensity (%)
204.088(HexNAc)
366.141(HexNAc + Hex)
274.094(NeuAc − H2O)
292.104(NeuAc)
528.195(Hex + HexNAc + Hex)
657.237(NeuAc + Hex + HexNAc)
1958.015(PEP_1)
CID-MS/MS Scan:34092, Noise threshold:1.1
366.141(HexNAc + Hex)
454.158(NeuAc + Hex)
657.238(NeuAc + Hex + HexNAc)
732.277(HexNAc(2)Hex(2)_1)
819.290(HexNAc(1)Hex(2)NeuAc(1)_1)
893.328
1908.848
ETD-MS/MS Scan:34093, Noise threshold:0.6
M+4H
893.328
1989.403
m/z

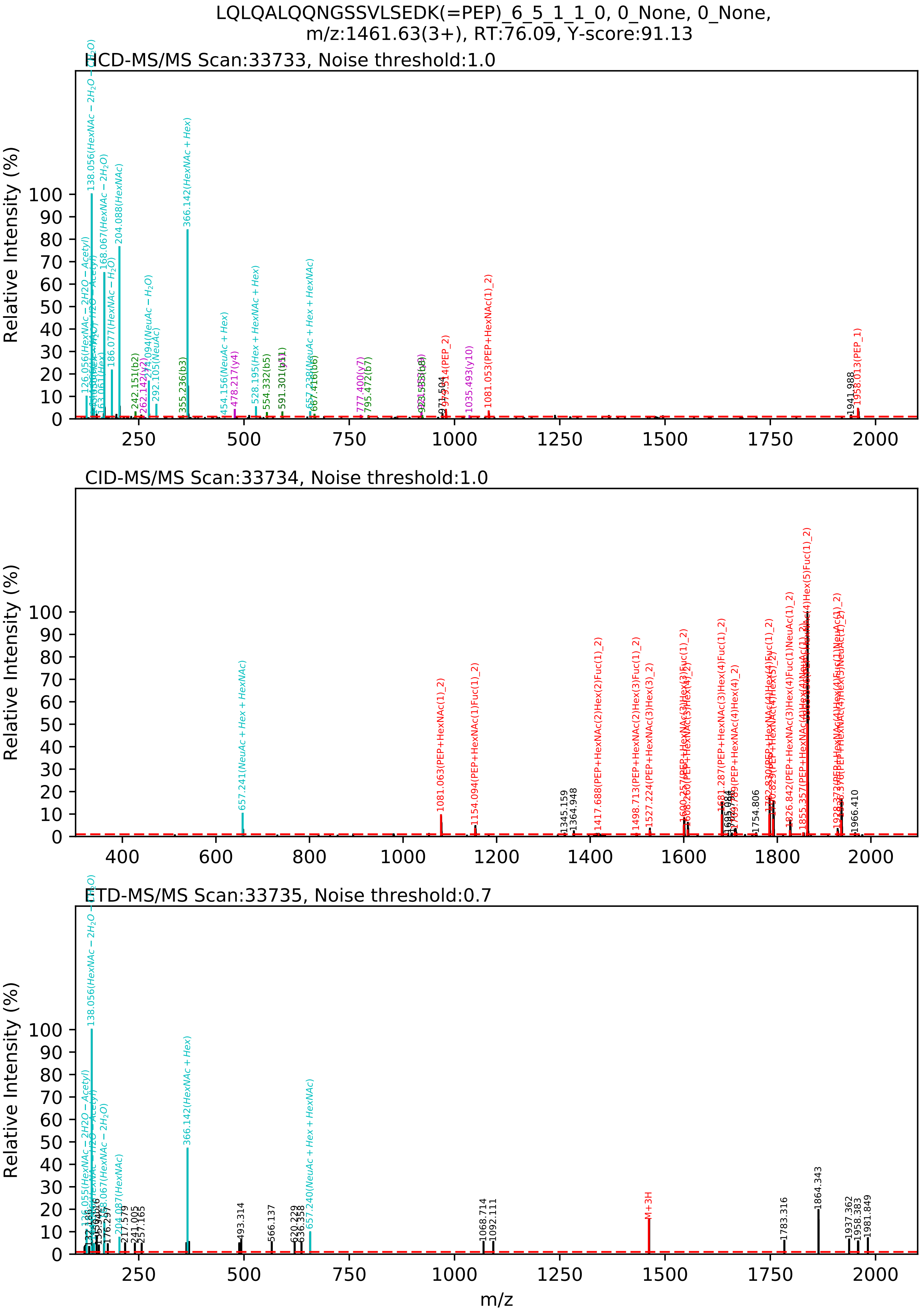
LQLQALQQNGSSVLSEDK(=PEP)_6_5_1_1_0, 0_None, 0_None,
m/z:1461.63(3+), RT:76.09, Y-score:91.13
HCD-MS/MS Scan:33733, Noise threshold:1.0
Relative Intensity (%)
138.056(HexNAc − 2H2O − H2O)
366.142(HexNAc + Hex)
204.088(HexNAc)
168.067(HexNAc − 2H2O)
186.077(HexNAc − H2O)
126.056(HexNAc − 2H2O − Acetyl)
274.094(NeuAc − H2O)
292.105(NeuAc)
242.151(b2)
262.142(y2)
355.236(b3)
454.156(NeuAc + Hex)
478.217(y4)
528.195(Hex + HexNAc + Hex)
554.332(b5)
657.238(NeuAc + Hex + HexNAc)
667.416(b6)
777.400(y7)
795.472(b7)
971.504
979.514(PEP_2)
1035.493(y10)
1081.053(PEP+HexNAc(1)_2)
1941.988
1958.013(PEP_1)
CID-MS/MS Scan:33734, Noise threshold:1.0
657.241(NeuAc + Hex + HexNAc)
1081.063(PEP+HexNAc(1)_2)
1154.094(PEP+HexNAc(1)Fuc(1)_2)
1345.159
1364.948
1417.688(PEP+HexNAc(2)Hex(2)Fuc(1)_2)
1498.713(PEP+HexNAc(2)Hex(3)Fuc(1)_2)
1527.224(PEP+HexNAc(3)Hex(3)_2)
1681.287(PEP+HexNAc(3)Hex(4)Fuc(1)_2)
1709.799(PEP+HexNAc(4)Hex(4)_2)
1754.806
1826.842(PEP+HexNAc(3)Hex(4)Fuc(1)NeuAc(1)_2)
1855.357(PEP+HexNAc(4)Hex(4)NeuAc(1)_2)
1966.410
ETD-MS/MS Scan:33735, Noise threshold:0.7
138.056(HexNAc − 2H2O − H2O)
366.142(HexNAc + Hex)
168.067(HexNAc − 2H2O)
204.087(HexNAc)
657.240(NeuAc + Hex + HexNAc)
217.579
241.005
257.165
493.314
566.137
620.229
636.358
1068.714
1092.111
M+3H
1783.316
1864.343
1937.362
1958.383
1981.849
m/z

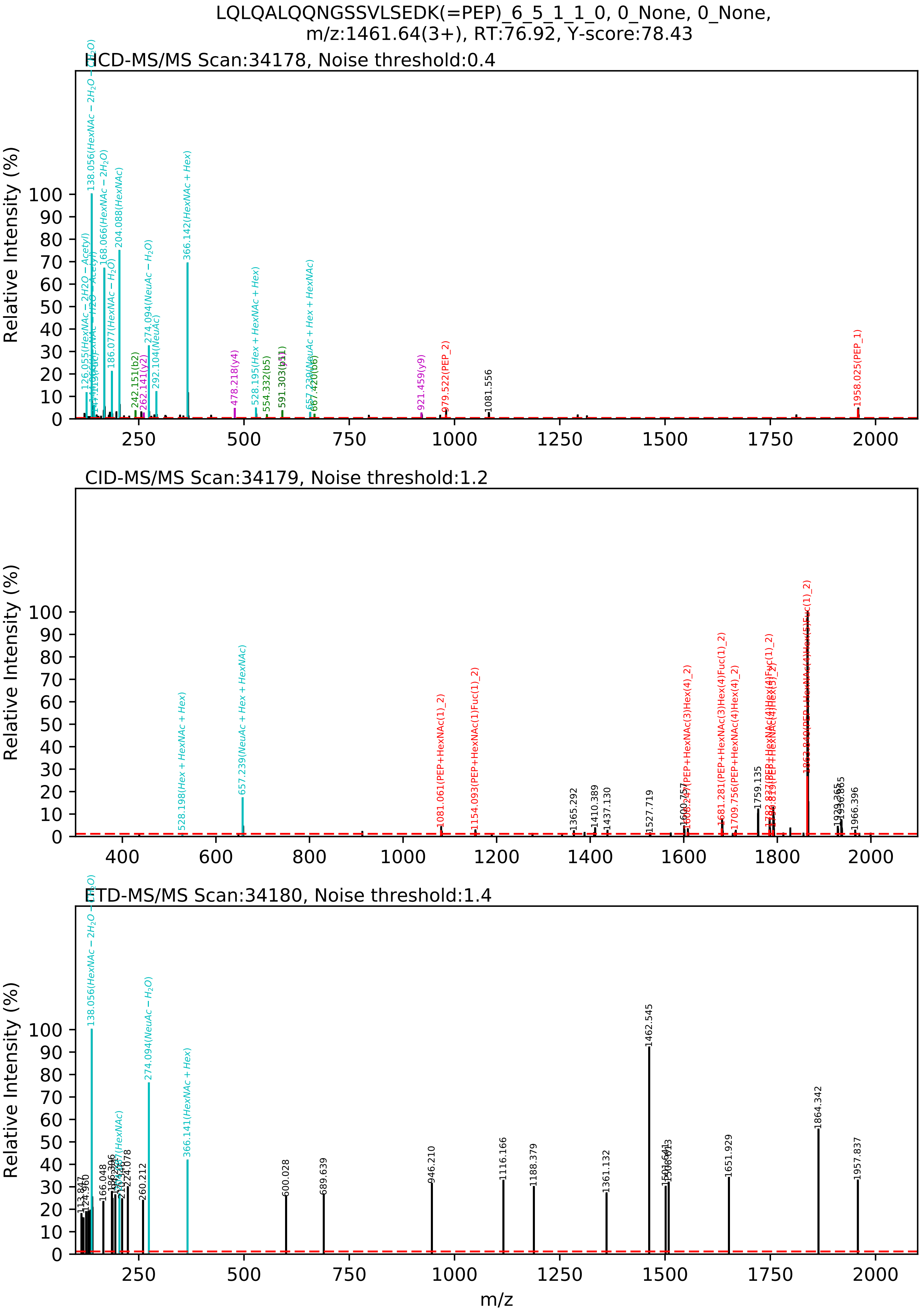

LQLQALQQNGSSVLSEDK(=PEP)_6_5_1_1_0, 0_None, 0_None,
m/z:1461.64(3+), RT:76.92, Y-score:78.43
HCD-MS/MS Scan:34178, Noise threshold:0.4
CID-MS/MS Scan:34179, Noise threshold:1.2
ETD-MS/MS Scan:34180, Noise threshold:1.4
Relative Intensity (%)
m/z
138.056(HexNAc − 2H2O − H2O)
168.066(HexNAc − 2H2O)
204.088(HexNAc)
186.077(HexNAc − H2O)
366.142(HexNAc + Hex)
274.094(NeuAc − H2O)
292.104(NeuAc)
242.151(b2)
262.141(y2)
478.218(y4)
528.195(Hex + HexNAc + Hex)
554.332(b5)
591.303(b11)
657.239(NeuAc + Hex + HexNAc)
667.420(b6)
921.459(y9)
979.522(PEP_2)
1081.556
1958.025(PEP_1)
528.198(Hex + HexNAc + Hex)
657.239(NeuAc + Hex + HexNAc)
1081.061(PEP+HexNAc(1)_2)
1154.093(PEP+HexNAc(1)Fuc(1)_2)
1365.292
1410.389
1437.130
1527.719
1681.281(PEP+HexNAc(3)Hex(4)Fuc(1)_2)
1709.756(PEP+HexNAc(4)Hex(4)_2)
1759.135
1966.396
1462.545
1864.342
1957.837
1651.929
1361.132
1188.379
1116.166
946.210
689.639
600.028
260.212
224.078
166.048
366.141(HexNAc + Hex)
274.094(NeuAc − H2O)

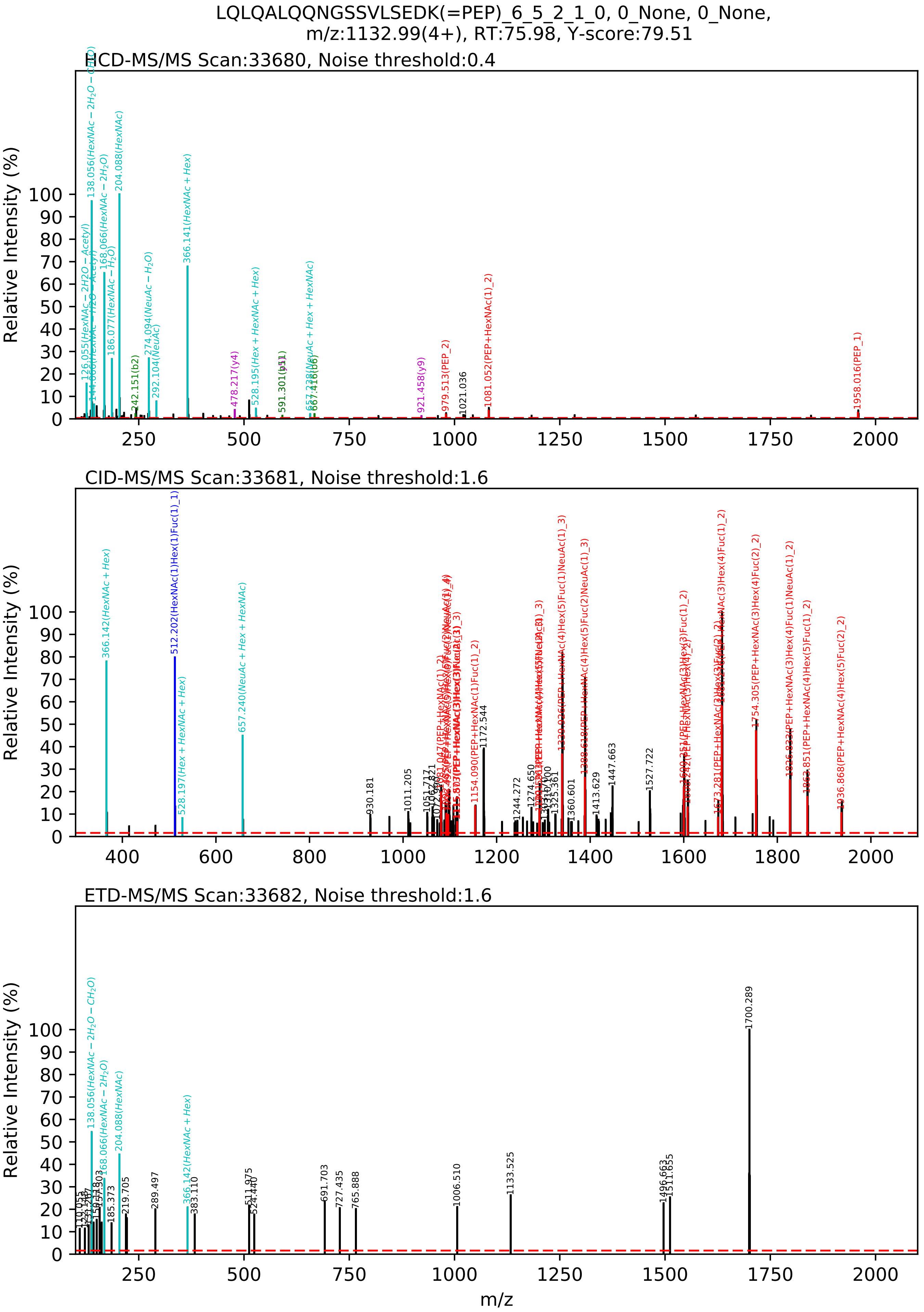

LQLQALQQNGSSVLSEDK(=PEP)_6_5_2_1_0, 0_None, 0_None,
m/z:1132.99(4+), RT:75.98, Y-score:79.51
HCD-MS/MS Scan:33680, Noise threshold:0.4
Relative Intensity (%)
204.088(HexNAc)
366.141(HexNAc + Hex)
528.195(Hex + HexNAc + Hex)
274.094(NeuAc − H2O)
292.104(NeuAc)
242.151(b2)
478.217(y4)
591.301(b5)
921.458(y9)
979.513(PEP_2)
1021.036
1081.052(PEP+HexNAc(1)_2)
1958.016(PEP_1)
CID-MS/MS Scan:33681, Noise threshold:1.6
366.142(HexNAc + Hex)
512.202(HexNAc(1)Hex(1)Fuc(1)_1)
528.197(Hex + HexNAc + Hex)
657.240(NeuAc + Hex + HexNAc)
930.181
1011.205
1154.090(PEP+HexNAc(1)Fuc(1)_2)
1172.544
1244.272
1274.650
1325.361
1360.601
1413.629
1447.663
1527.722
1754.305(PEP+HexNAc(3)Hex(4)Fuc(2)_2)
1826.822(PEP+HexNAc(3)Hex(4)Fuc(1)NeuAc(1)_2)
1862.851(PEP+HexNAc(4)Hex(5)Fuc(1)_2)
1936.868(PEP+HexNAc(4)Hex(5)Fuc(2)_2)
ETD-MS/MS Scan:33682, Noise threshold:1.6
138.056(HexNAc − 2H2O − CH2O)
168.066(HexNAc − 2H2O)
204.088(HexNAc)
366.142(HexNAc + Hex)
185.373
219.705
289.497
383.110
511.975
524.440
691.703
727.435
765.888
1006.510
1133.525
1496.663
1511.655
1700.289
m/z

LQLQALQQNGSSVLSEDK(=PEP)_7_2_0_0_0, 0_None, 0_None,
m/z:1166.85(3+), RT:65.70, Y-score:82.95

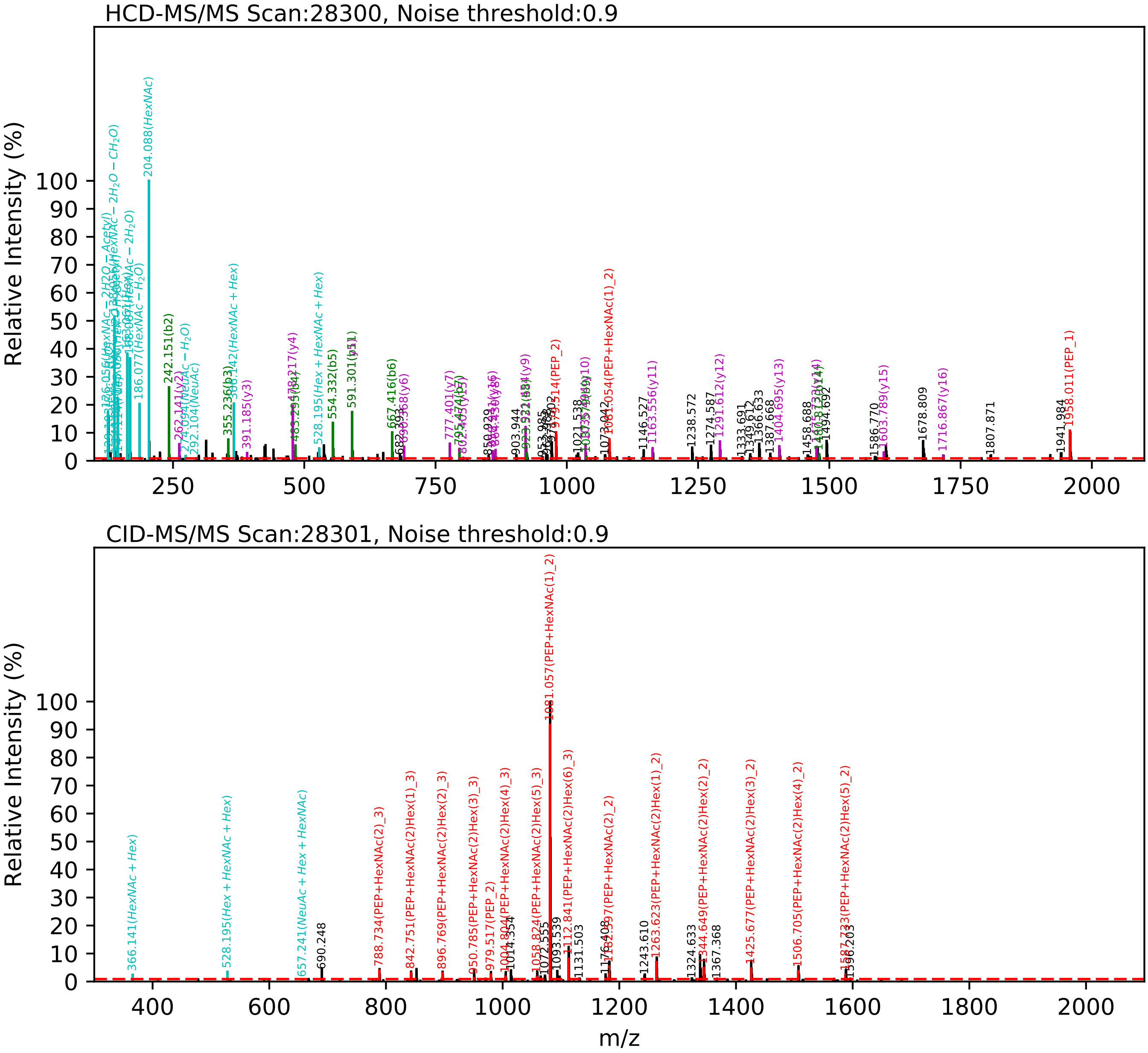

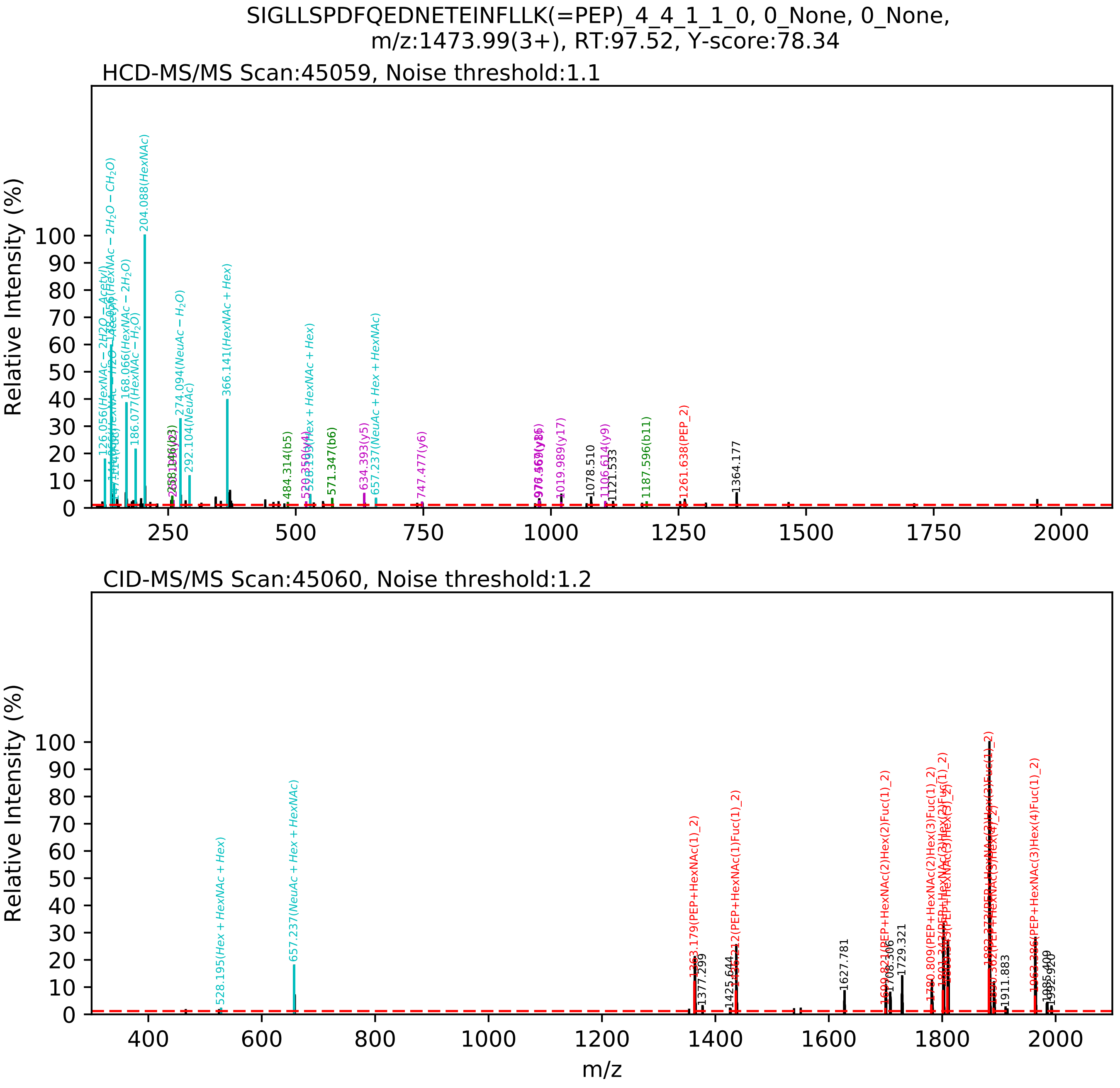
SIGLLSPDFQEDNETEINFLLK(=PEP)_4_4_1_1_0, 0_None, 0_None,
m/z:1473.99(3+), RT:97.52, Y-score:78.34
HCD-MS/MS Scan:45059, Noise threshold:1.1
Relative Intensity (%)
204.088(HexNAc)
366.141(HexNAc+Hex)
168.066(HexNAc−2H2O)
186.077(HexNAc−H2O)
126.056(HexNAc−2H2O−Acetyl)
274.094(NeuAc−H2O)
292.104(NeuAc)
484.314(b5)
571.347(b6)
634.393(y5)
657.237(NeuAc+Hex+HexNAc)
747.477(y6)
1019.989(y17)
1078.510
1121.533
1106.614(y9)
1187.596(b11)
1261.638(PEP_2)
1364.177
CID-MS/MS Scan:45060, Noise threshold:1.2
528.195(Hex+HexNAc+Hex)
657.237(NeuAc+Hex+HexNAc)
1377.299
1425.644
1627.781
1708.306
1729.321
1911.883
1963.296(PEP+HexNAc(3)Hex(4)Fuc(1)_2)
m/z

SIGLLSPDFQEDNETEINFLLK(=PEP)_4_4_1_1_0, 0_None, 0_None,
m/z:1105.74(4+), RT:97.61, Y-score:82.73

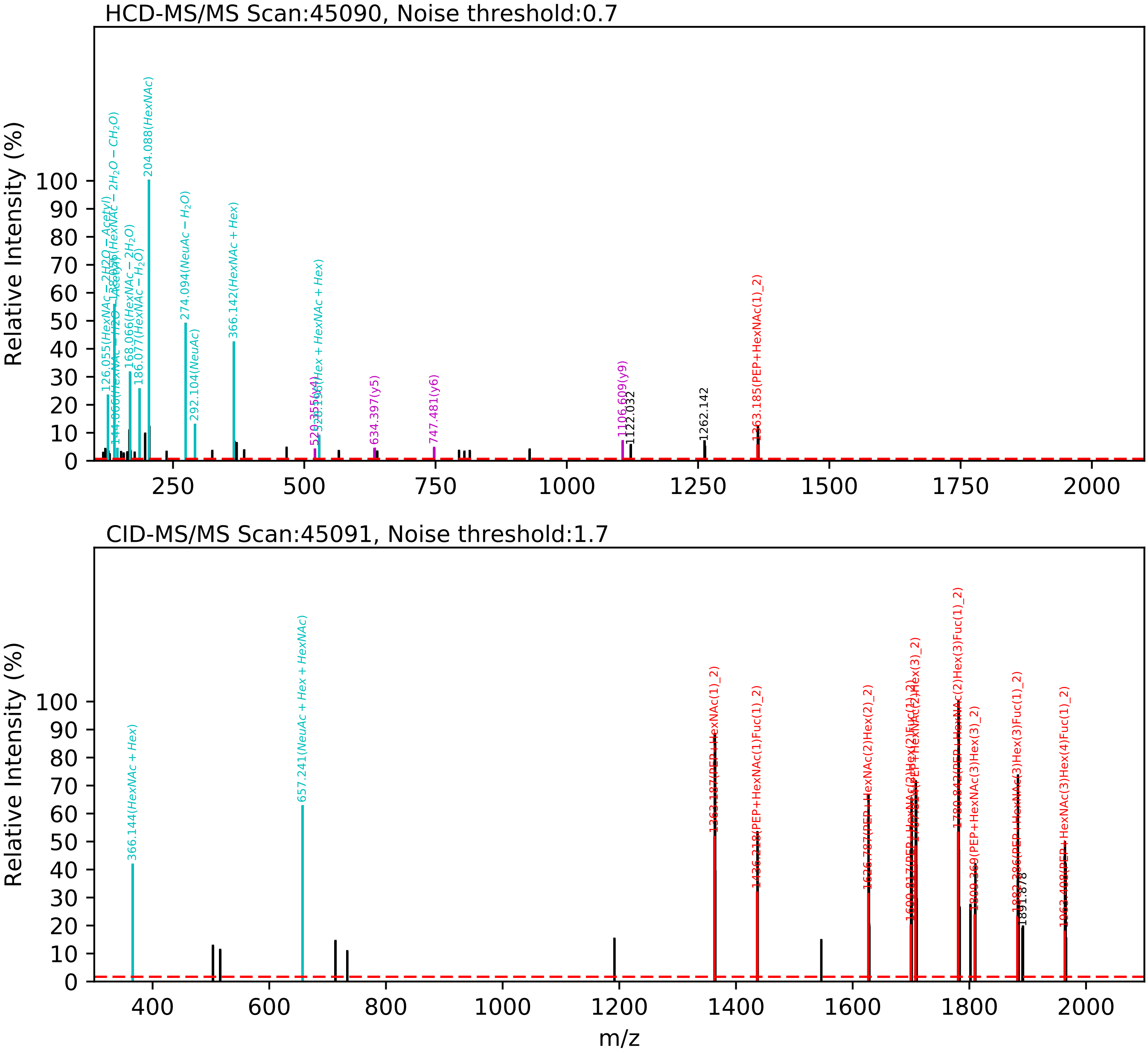

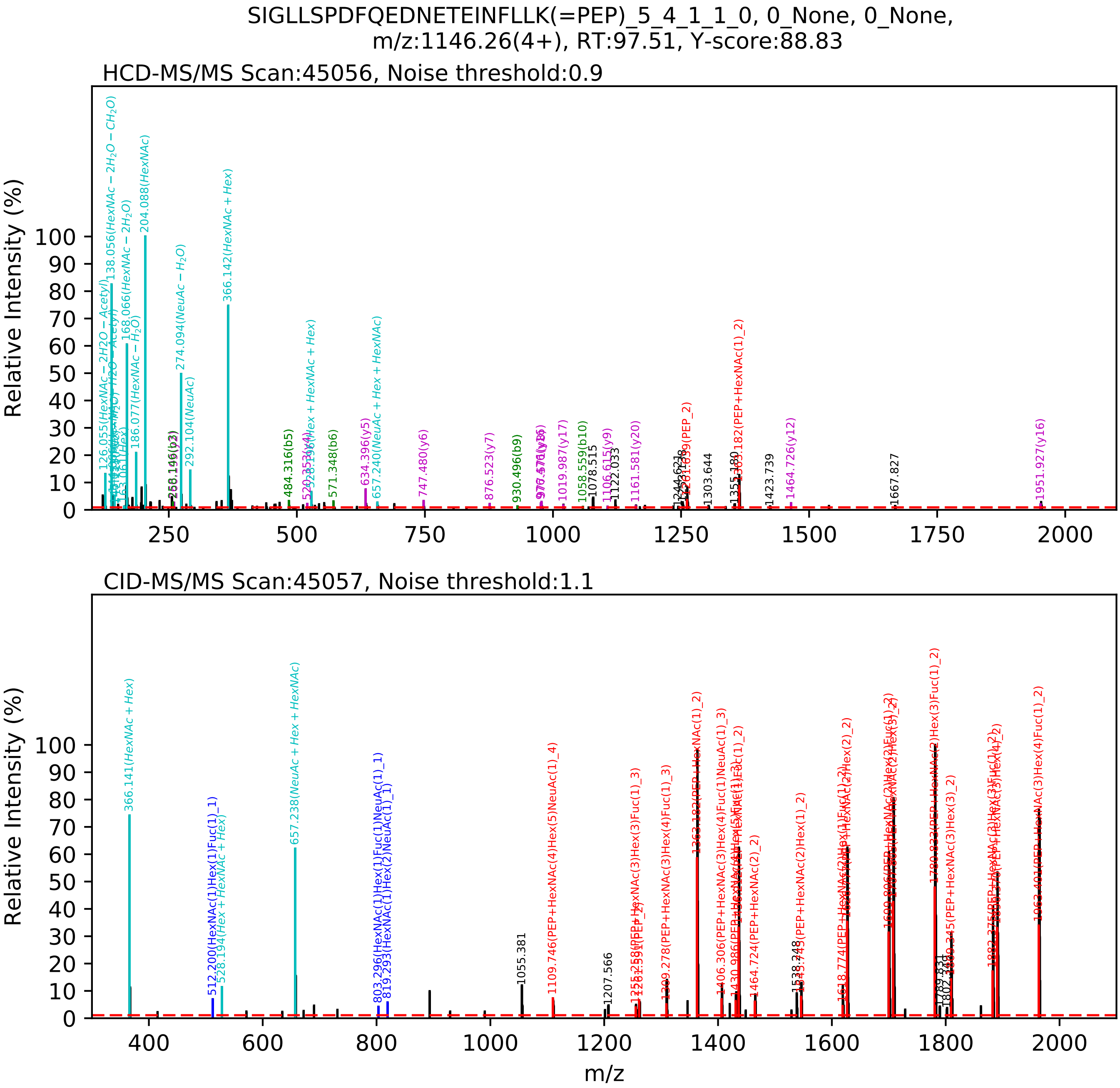
SIGLLSPDFQEDNETEINFLLK(=PEP)_5_4_1_1_0, 0_None, 0_None,
m/z:1146.26(4+), RT:97.51, Y-score:88.83
HCD-MS/MS Scan:45056, Noise threshold:0.9
Relative Intensity (%)
204.088(HexNAc)
366.142(HexNAc + Hex)
274.094(NeuAc − H2O)
292.104(NeuAc)
484.316(b5)
571.348(b6)
634.396(y5)
657.240(NeuAc + Hex + HexNAc)
747.480(y6)
876.523(y7)
930.496(b9)
1019.987(y17)
1058.559(b10)
1078.515
1122.033
1161.581(y20)
1303.644
1363.182(PEP+HexNAc(1)_2)
1423.739
1464.726(y12)
1667.827
1951.927(y16)
CID-MS/MS Scan:45057, Noise threshold:1.1
Relative Intensity (%)
366.141(HexNAc + Hex)
512.200(HexNAc(1)Hex(1)Fuc(1)_1)
528.194(Hex + HexNAc + Hex)
657.238(NeuAc + Hex + HexNAc)
803.296(HexNAc(1)Hex(1)Fuc(1)NeuAc(1)_1)
819.293(HexNAc(1)Hex(2)NeuAc(1)_1)
1055.381
1109.746(PEP+HexNAc(4)Hex(5)NeuAc(1)_4)
1207.566
1309.278(PEP+HexNAc(3)Hex(4)Fuc(1)_3)
1406.306(PEP+HexNAc(3)Hex(4)Fuc(1)NeuAc(1)_3)
1464.724(PEP+HexNAc(2)_2)
1538.748
m/z

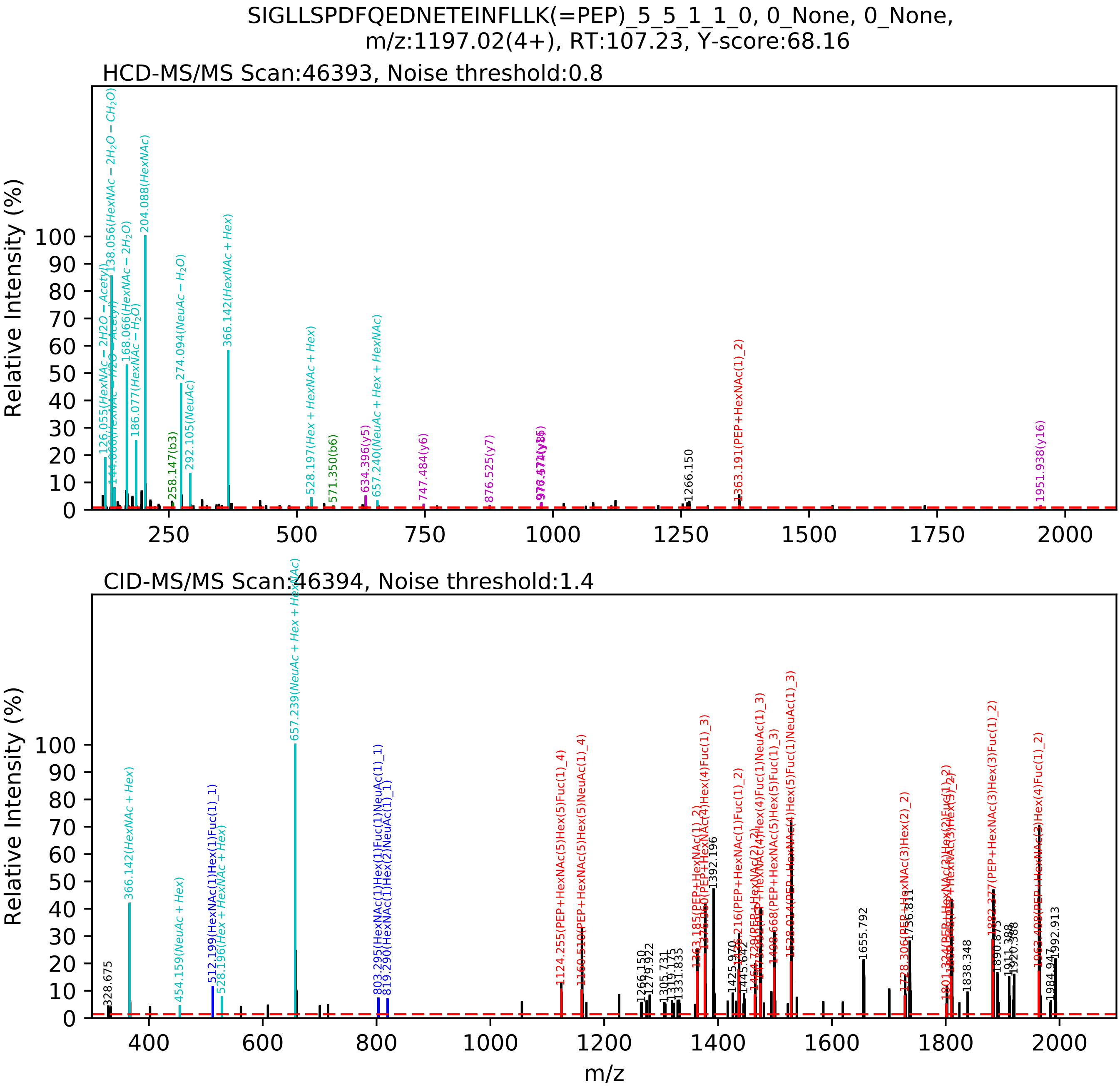

SIGLLSPDFQEDNETEINFLLK(=PEP)_5_5_1_1_0, 0_None, 0_None,
m/z:1197.02(4+), RT:107.23, Y-score:68.16
HCD-MS/MS Scan:46393, Noise threshold:0.8
Relative Intensity (%)
204.088(HexNAc)
138.056(HexNAc − 2H2O − CH2O)
168.066(HexNAc − 2H2O)
186.077(HexNAc − H2O)
126.055(HexNAc − 2H2O − Acetyl)
144.066(HexNAc − H2O − Acetyl)
274.094(NeuAc − H2O)
292.105(NeuAc)
258.147(b3)
366.142(HexNAc + Hex)
528.197(Hex + HexNAc + Hex)
571.350(b6)
634.396(y5)
657.240(NeuAc + Hex + HexNAc)
747.484(y6)
876.525(y7)
1266.150
1363.191(PEP+HexNAc(1)_2)
1951.938(y16)
CID-MS/MS Scan:46394, Noise threshold:1.4
657.239(NeuAc + Hex + HexNAc)
366.142(HexNAc + Hex)
454.159(NeuAc + Hex)
512.199(HexNAc(1)Hex(1)Fuc(1)_1)
528.196(Hex + HexNAc + Hex)
803.295(HexNAc(1)Hex(1)Fuc(1)NeuAc(1)_1)
819.290(HexNAc(1)Hex(2)NeuAc(1)_1)
328.675
1124.255(PEP+HexNAc(5)Hex(5)Fuc(1)_4)
1160.510(PEP+HexNAc(5)Hex(5)NeuAc(1)_4)
1266.150
1279.922
1305.731
1392.196
1425.970
1445.642
1655.792
1736.811
1838.348
1890.875
1911.388
1920.388
1984.947
1992.913
m/z

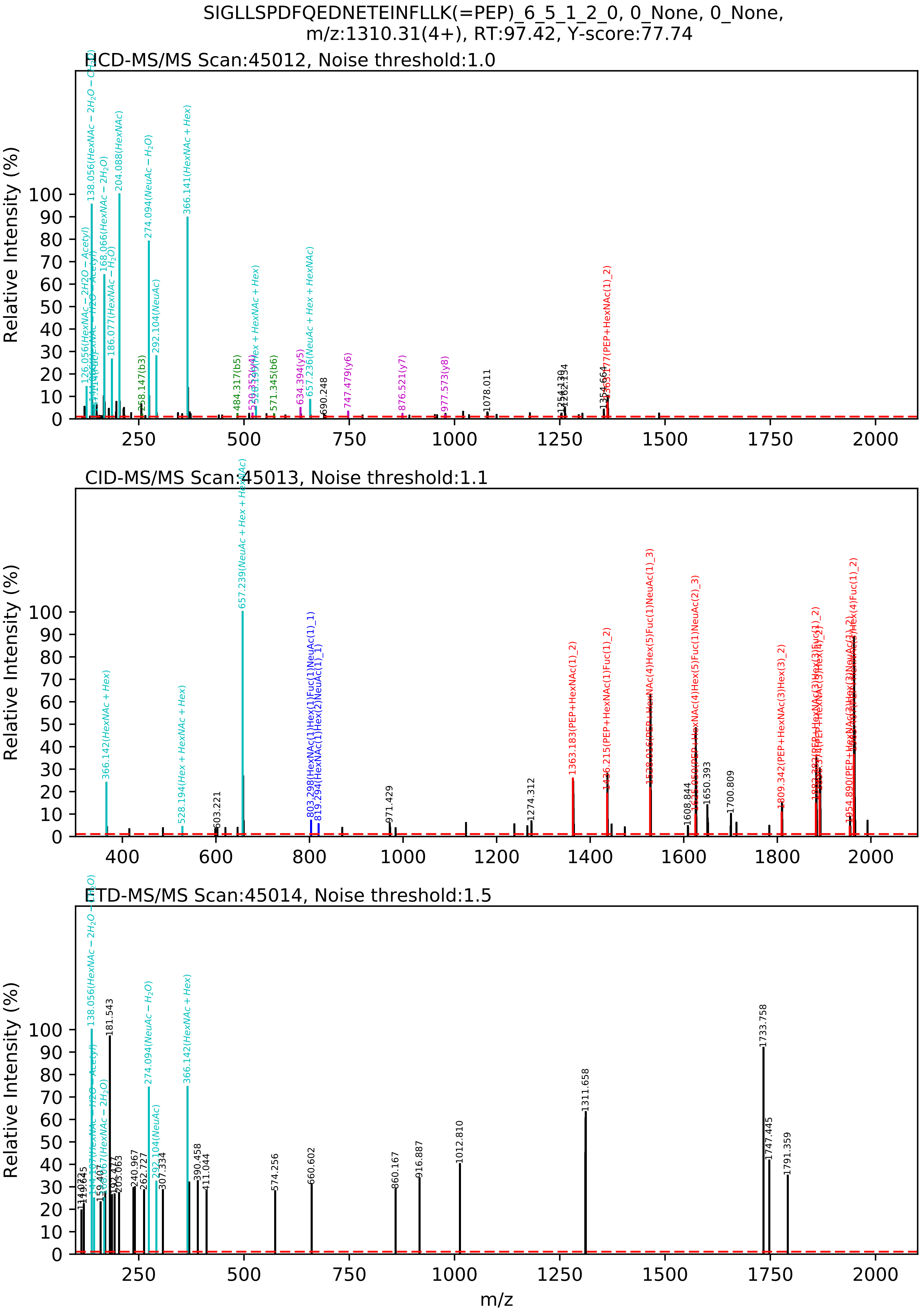
SIGLLSPDFQEDNETEINFLLK(=PEP)_6_5_1_2_0, 0_None, 0_None,
m/z:1310.31(4+), RT:97.42, Y-score:77.74
HCD-MS/MS Scan:45012, Noise threshold:1.0
Relative Intensity (%)
138.056(HexNAc − 2H₂O − CH₂O)
168.066(HexNAc − 2H₂O)
204.088(HexNAc)
186.077(HexNAc − H₂O)
274.094(NeuAc − H₂O)
292.104(NeuAc)
366.141(HexNAc + Hex)
258.147(b3)
484.317(b5)
571.345(b6)
634.394(y5)
657.236(NeuAc + Hex + HexNAc)
690.248
747.479(y6)
876.521(y7)
977.573(y8)
1078.011
1354.664
1363.177(PEP+HexNAc(1)_2)
CID-MS/MS Scan:45013, Noise threshold:1.1
366.142(HexNAc + Hex)
528.194(Hex + HexNAc + Hex)
603.221
657.239(NeuAc + Hex + HexNAc)
803.298(HexNAc(1)Hex(1)Fuc(1)NeuAc(1)_1)
819.294(HexNAc(1)Hex(2)NeuAc(1)_1)
971.429
1274.312
1363.183(PEP+HexNAc(1)_2)
1436.215(PEP+HexNAc(1)Fuc(1)_2)
1608.844
1650.393
1700.809
1809.342(PEP+HexNAc(3)Hex(3)_2)
ETD-MS/MS Scan:45014, Noise threshold:1.5
138.056(HexNAc − 2H₂O − CH₂O)
181.543
274.094(NeuAc − H₂O)
366.142(HexNAc + Hex)
240.967
262.727
292.104(NeuAc)
307.334
390.458
411.044
574.256
660.602
860.167
916.887
1012.810
1311.658
1733.758
1747.445
1791.359
m/z
