# Supplementary material for: Characterization of Site-Specific N- and O-Glycopeptides from Recombinant Spike and ACE2 Glycoproteins Using LC-MS/MS Analysis
Source: Int J Mol Sci. 2024 Dec 20;25(24):13649. doi: 10.3390/ijms252413649 (PMC11678118; doi:10.3390/ijms252413649)
Supplement: Supplementary file 1 [file ijms-25-13649-s001.zip › Supplementary Figure S12(ACE2_T_N-glycopep_2).pdf]

LQLQALQQNGSSVLSEDK(=PEP)_5_5_1_1_0, 0_None, 0_None,
m/z:1055.96(4+), RT:77.00, hcd-score:73.58

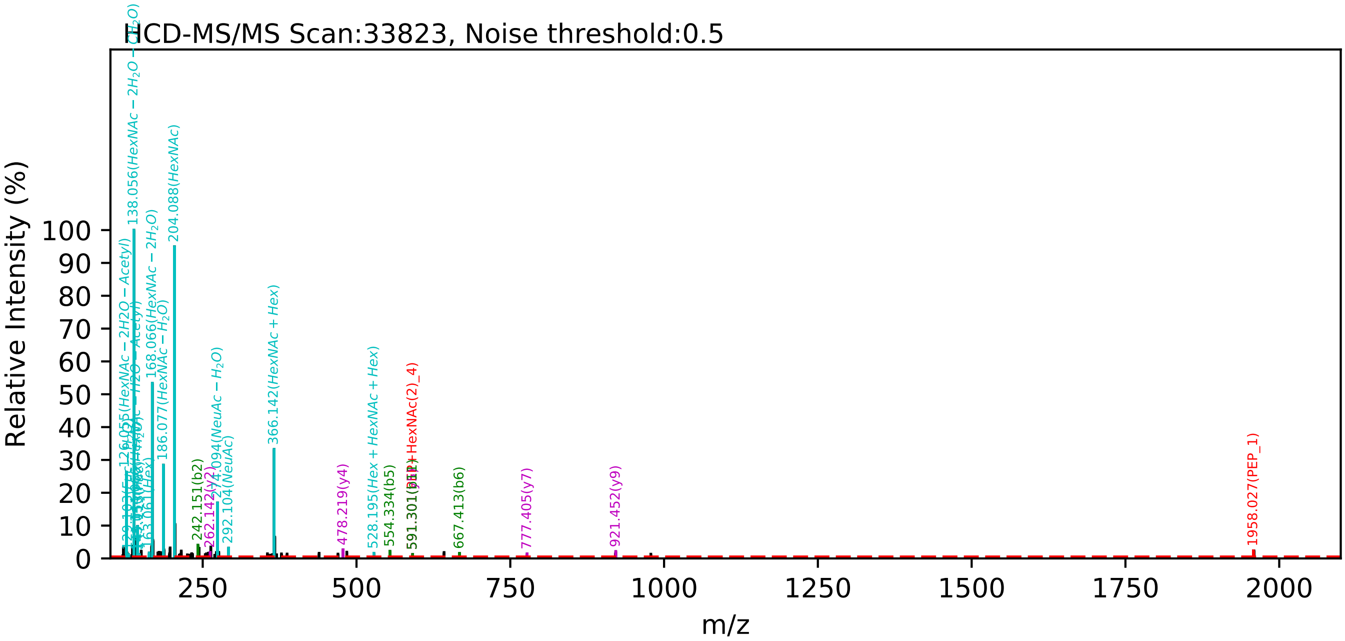

LQLQALQQNGSSVLSEDK(=PEP)_5_5_1_1_0, 0_None, 0_None,
m/z:1055.96(4+), RT:77.00, hcd-score:73.58

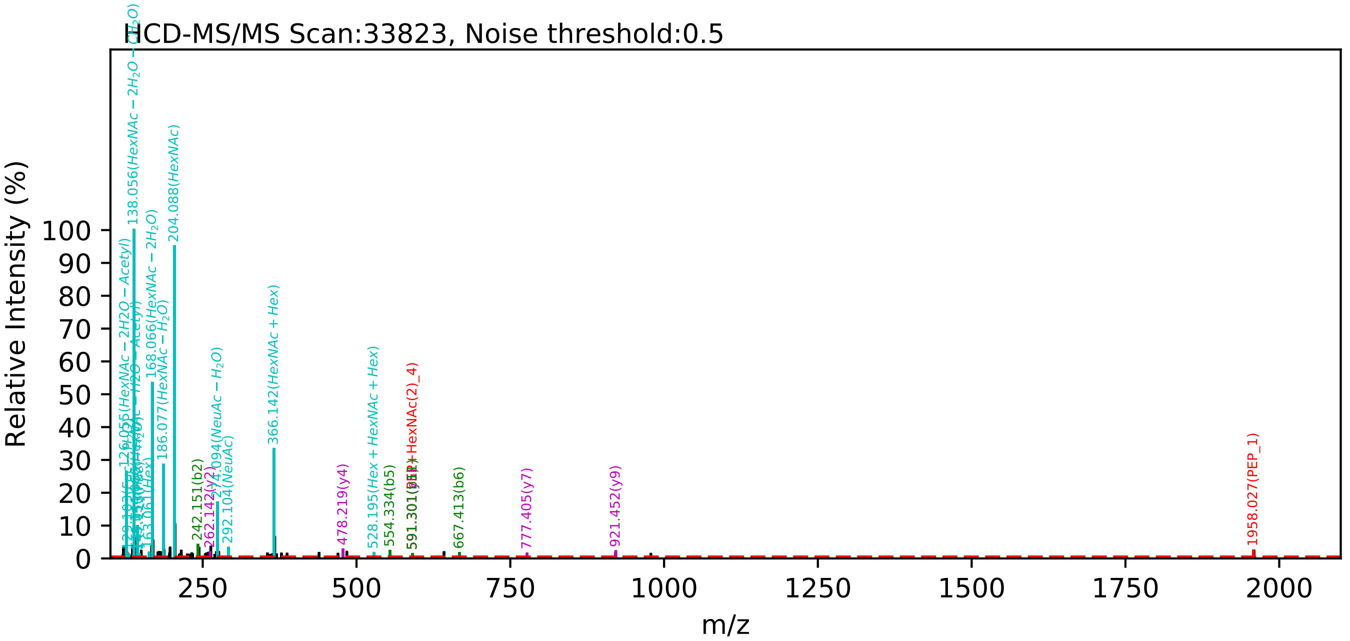

LQLQALQQNGSSVLSEDK(=PEP)_5_5_1_1_0, 0_None, 0_None,
m/z:1407.62(3+), RT:77.71, hcd-score:100.00

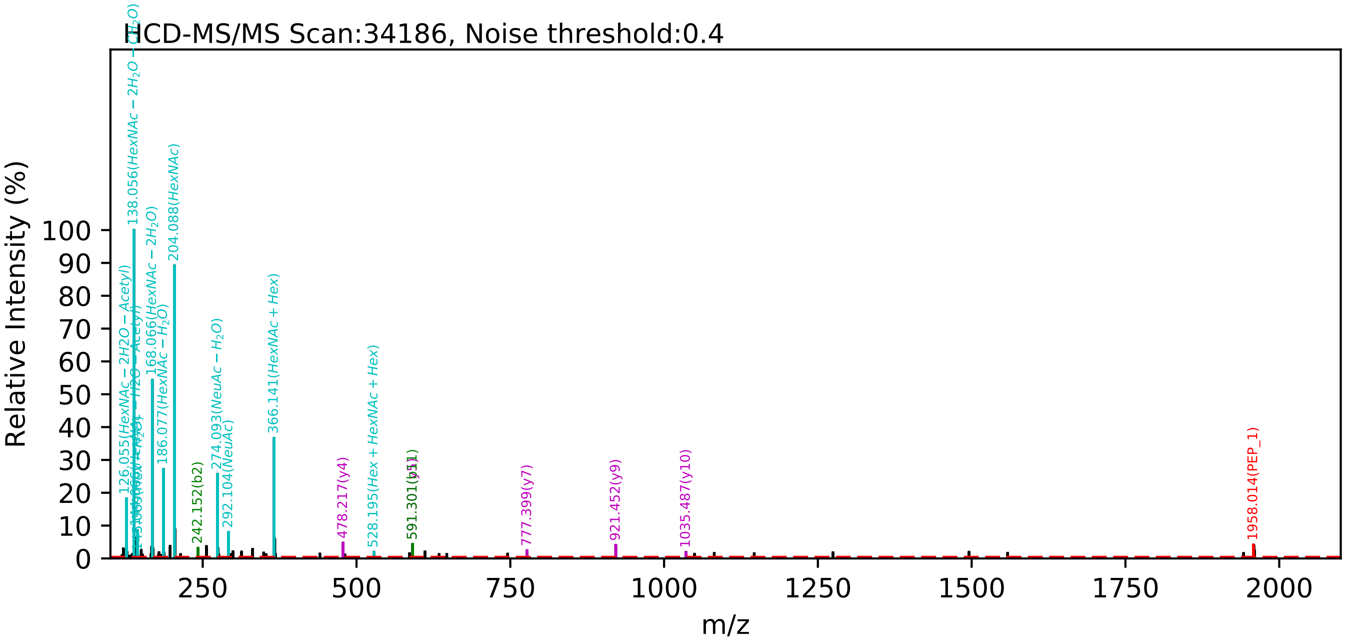

LQLQALQQNGSSVLSEDK(=PEP)_5_5_1_1_0, 0_None, 0_None,
m/z:1407.62(3+), RT:77.71, hcd-score:100.00

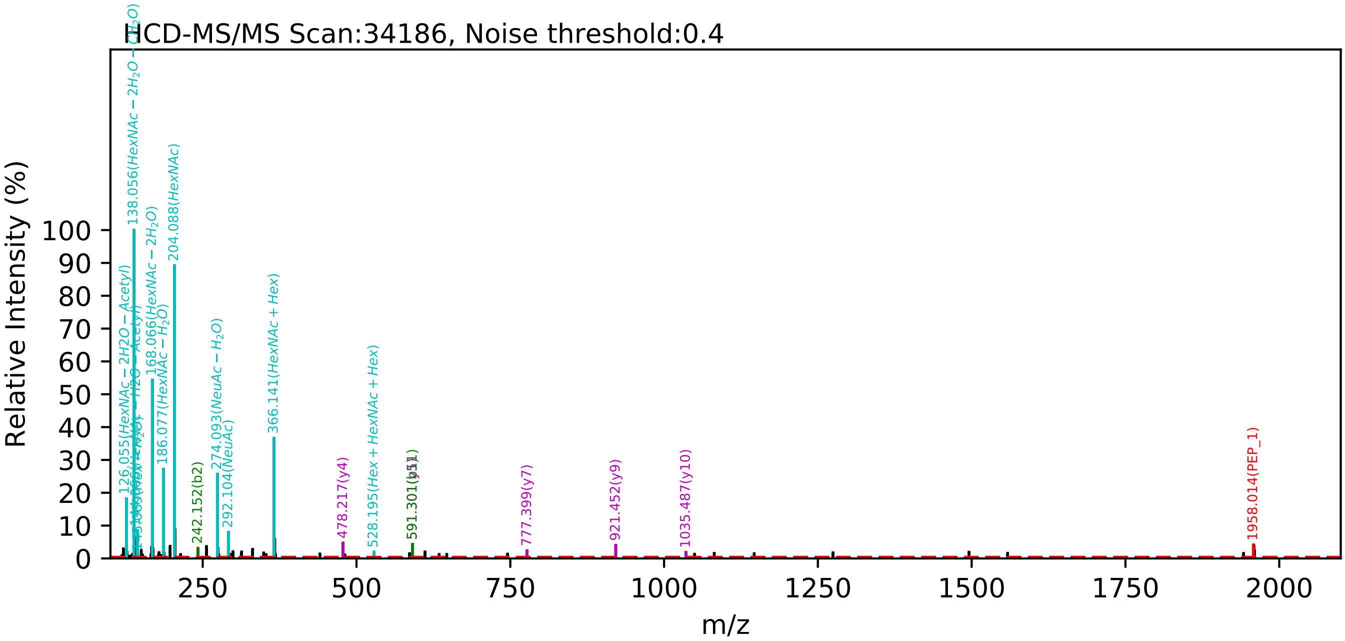

LQLQALQQNGSSVLSEDK(=PEP)_5_4_1_2_0, 0_None, 0_None,
m/z:1077.97(6+), RT:91.36, hcd-score:81.79

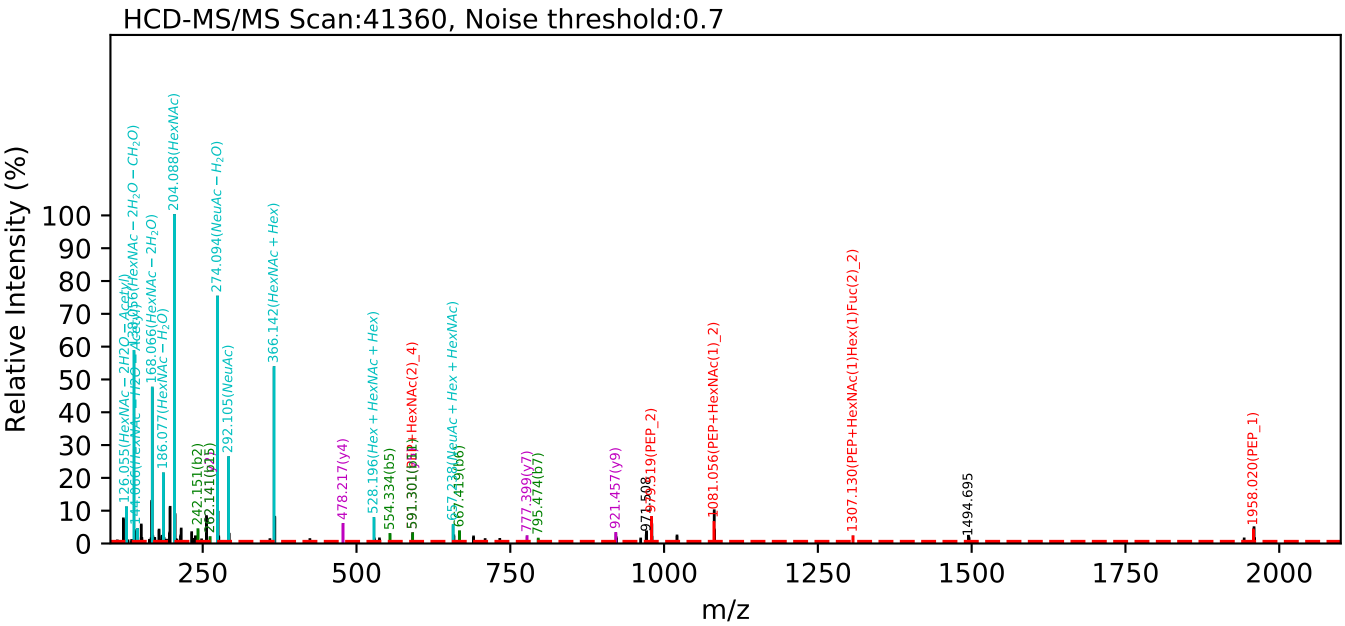

LQLQALQQNGSSVLSEDK(=PEP)_5_4_1_2_0, 0_None, 0_None,
m/z:1077.97(6+), RT:91.36, hcd-score:81.79

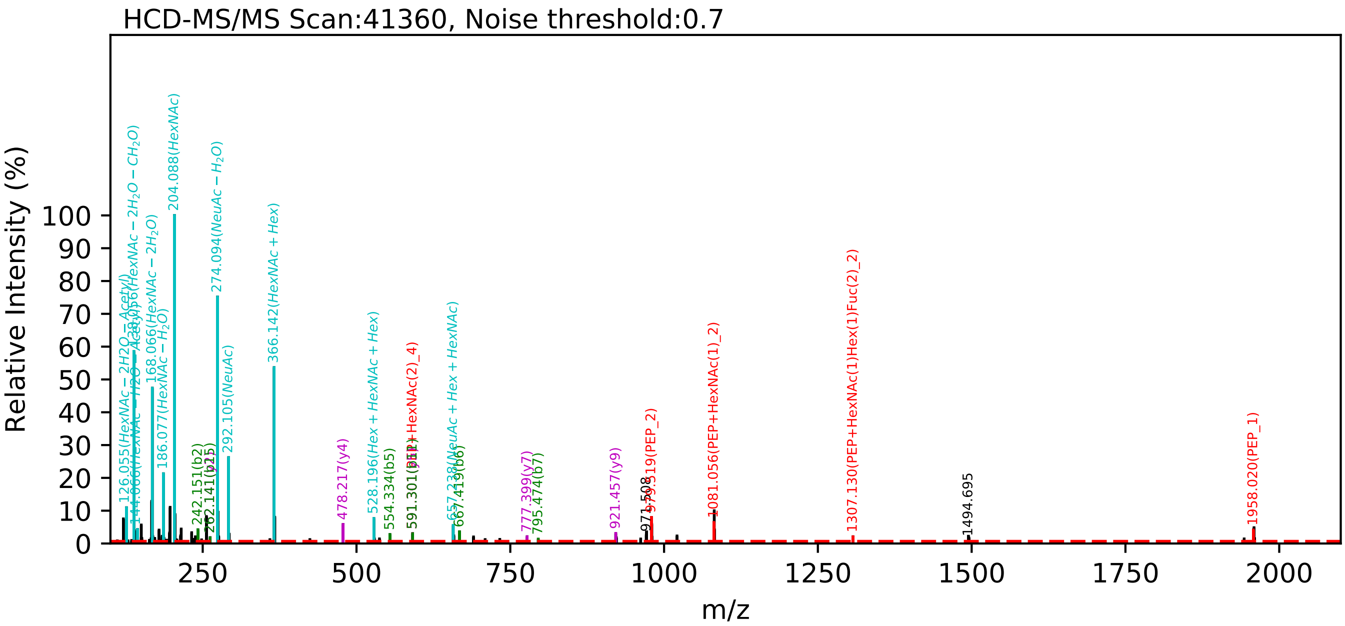

SIGLLSPDFQEDNETEINFLLK(=PEP)_5_4_1_1_0, 0_None, 0_None,
m/z:1146.25(4+), RT:107.30, hcd-score:100.00

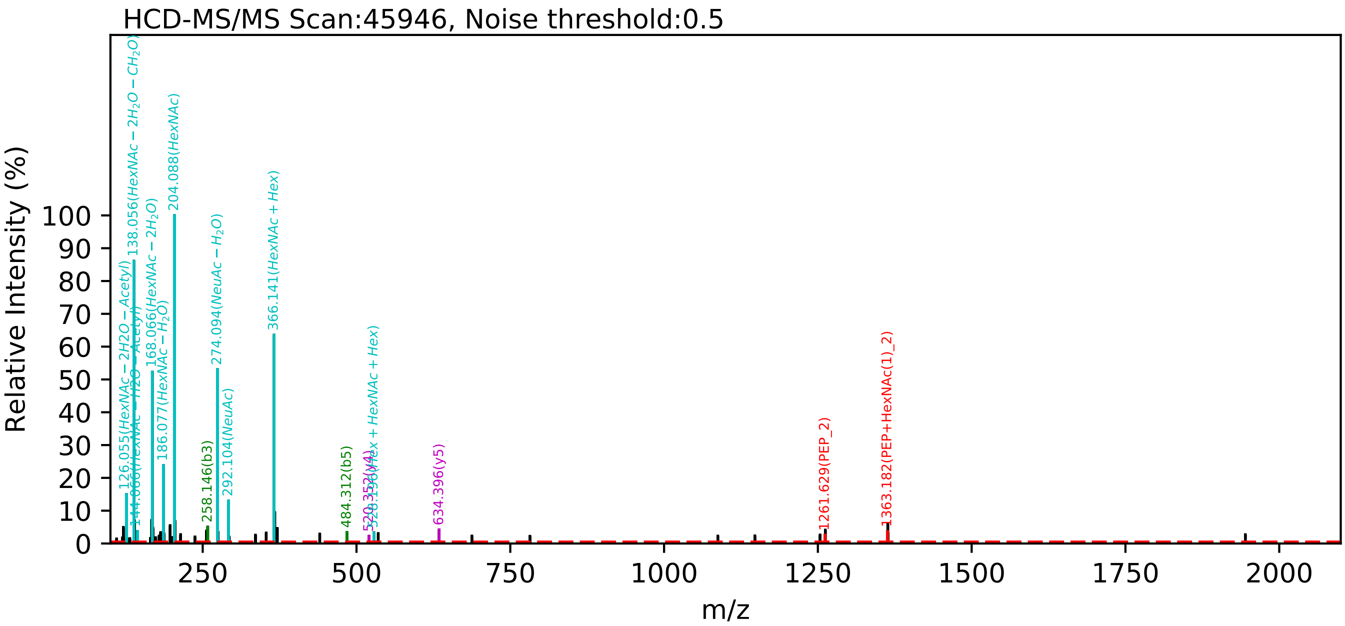

SIGLLSPDFQEDNETEINFLLK(=PEP)_5_4_1_1_0, 0_None, 0_None,
m/z:1146.25(4+), RT:107.30, hcd-score:100.00

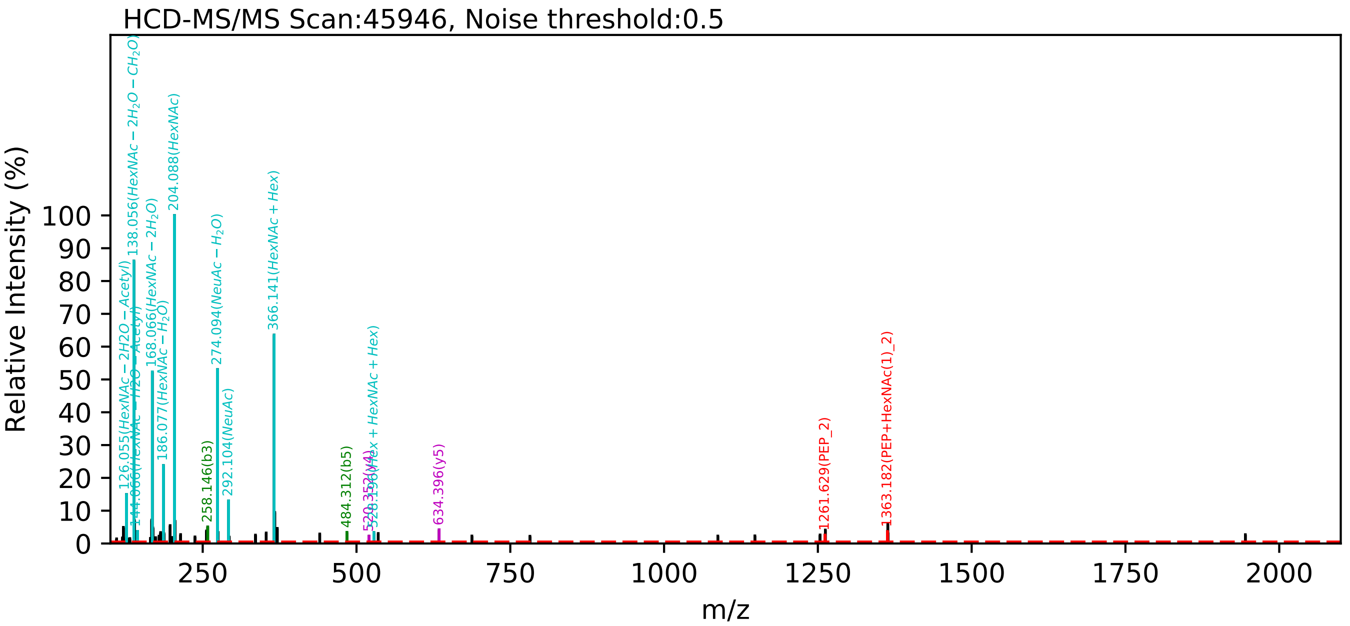

CDISNSTEAGQK(=PEP)_4_3_1_1_0, 0_None, 0_None,
m/z:1002.06(3+), RT:24.88, Y-score:93.09

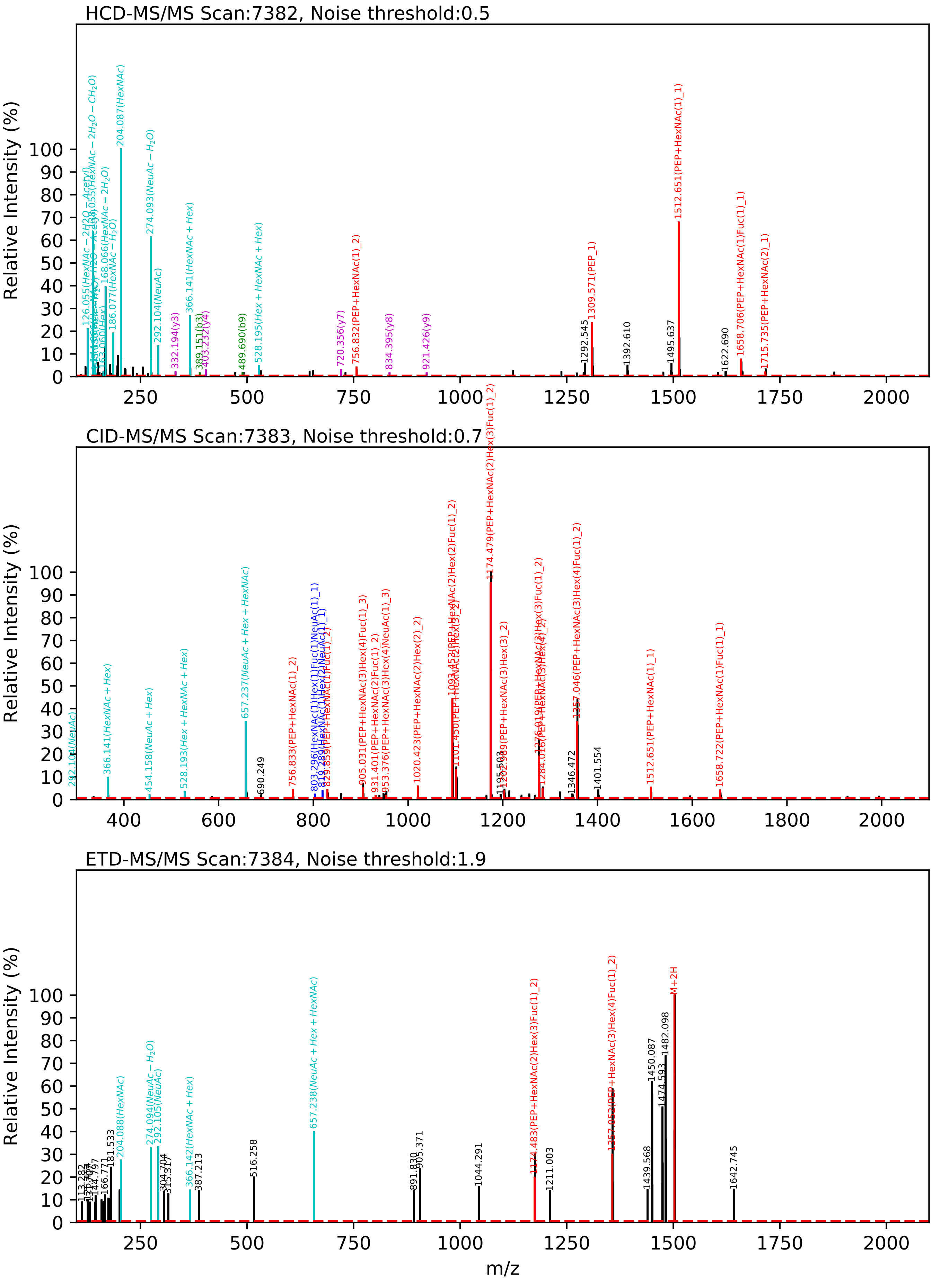

CDISNSTEAGQK(=PEP)_4_3_1_1_0, 0_None, 0_None,
m/z:1502.59(2+), RT:24.85, Y-score:85.75

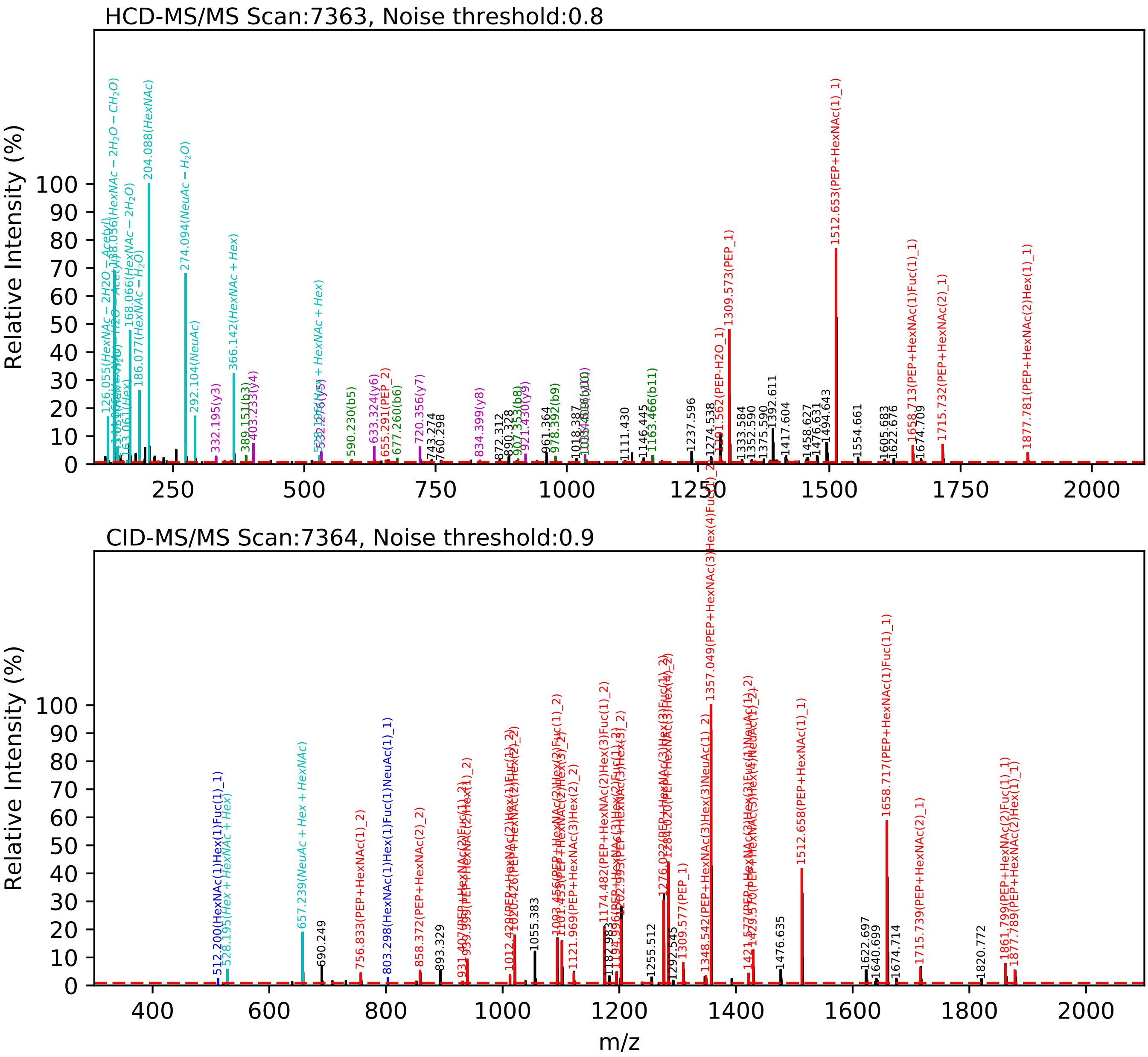

CDISNSTEAGQK(=PEP)_4_3_1_1_0, 0_None, 0_None,
m/z:1002.06(3+), RT:24.30, Y-score:90.36

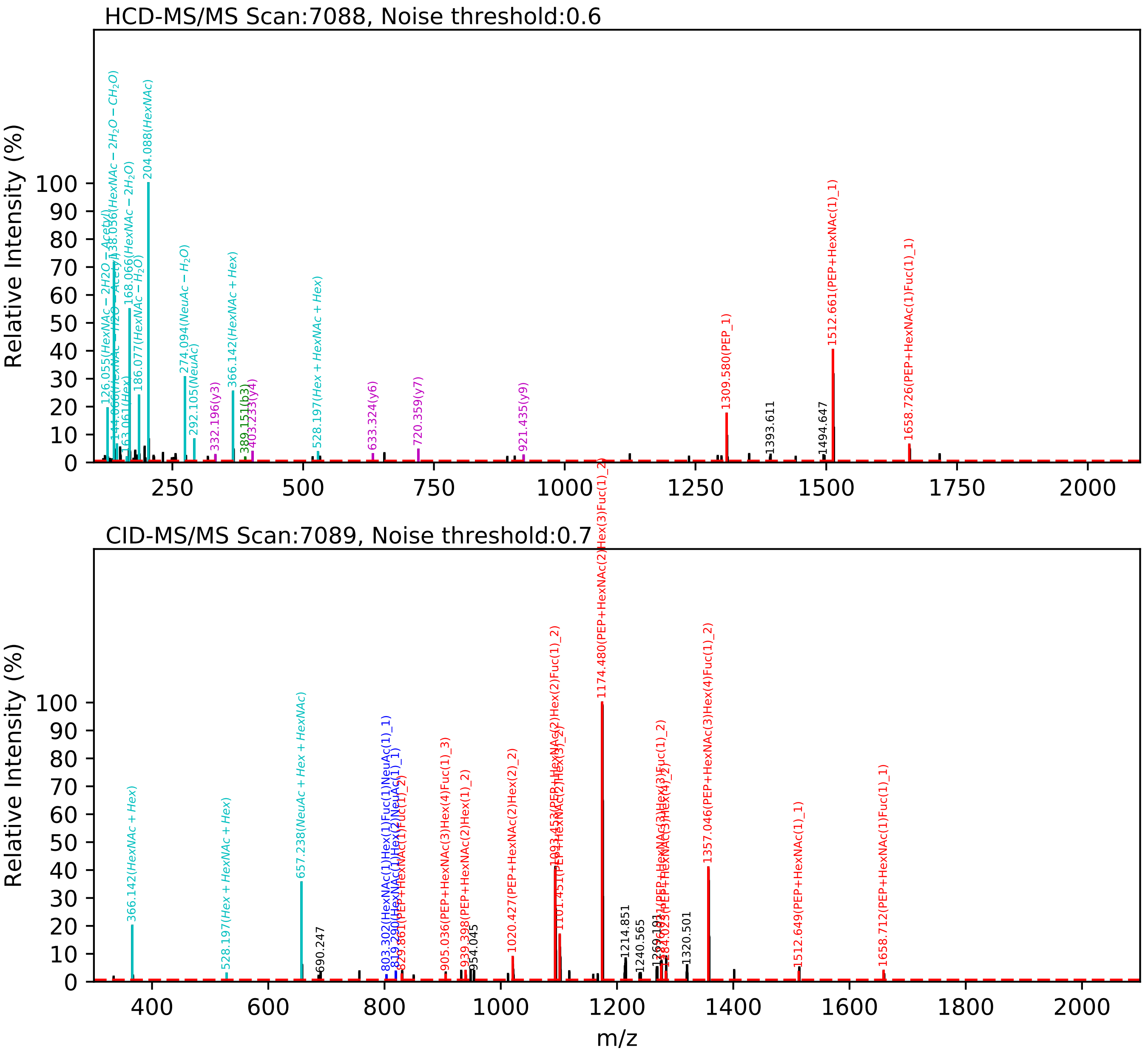

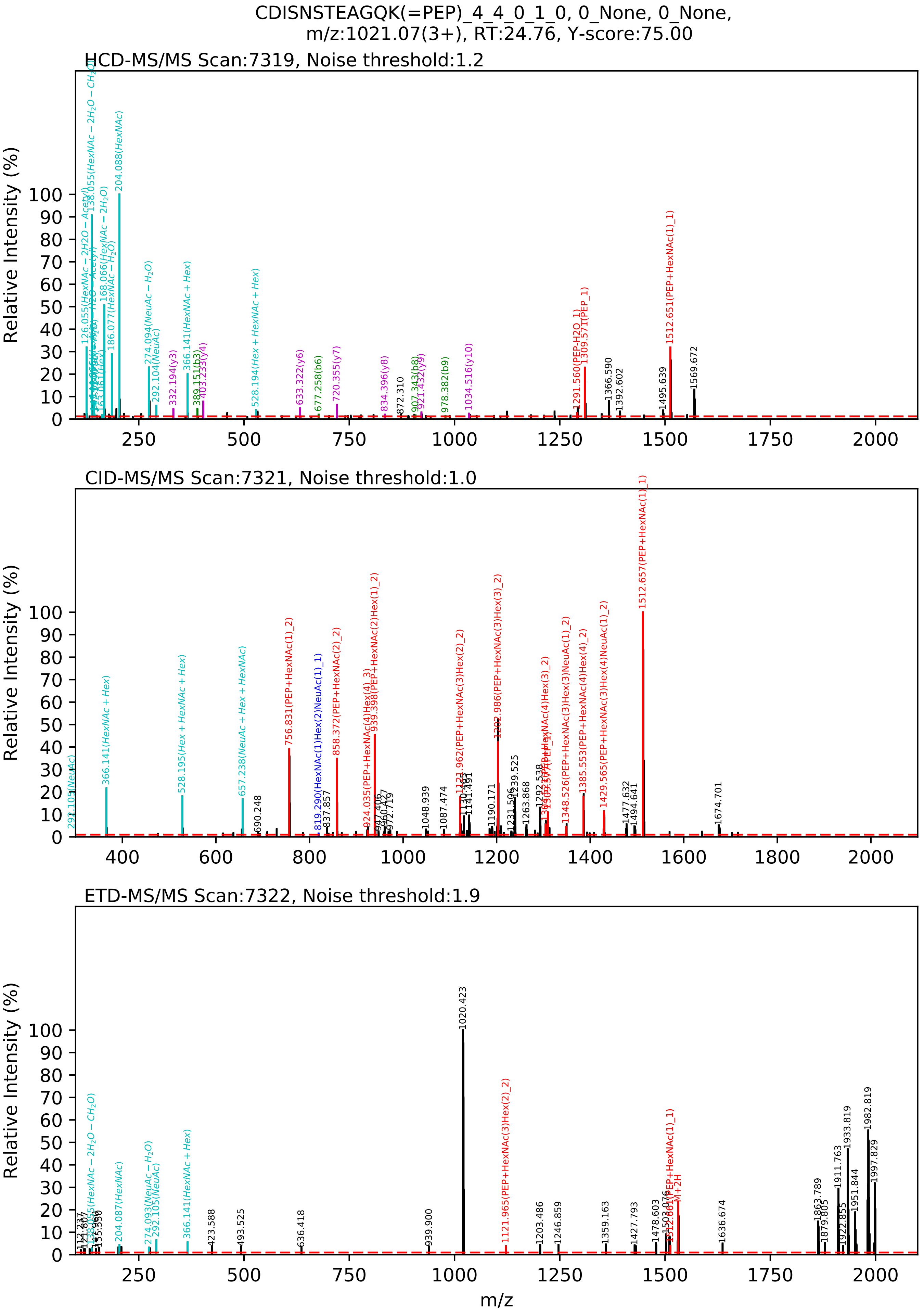

CDISNSTEAGQK(=PEP)_4_4_0_1_0, 0_None, 0_None,
m/z:1021.07(3+), RT:24.76, Y-score:75.00
HCD-MS/MS Scan:7319, Noise threshold:1.2
Relative Intensity (%)
204.088(HexNAc)
138.055(HexNAc − 2H2O − CH2O)
168.066(HexNAc − 2H2O)
186.077(HexNAc − H2O)
126.055(HexNAc − 2H2O − Acetyl)
274.094(NeuAc − H2O)
292.104(NeuAc)
366.141(HexNAc + Hex)
332.194(y3)
389.151(b3)
403.233(y4)
528.194(Hex + HexNAc + Hex)
633.322(y6)
677.258(b6)
720.355(y7)
834.396(y8)
872.310
907.343(b8)
921.432(y9)
978.382(b9)
1034.516(y10)
1291.560(PEP-H2O_1)
1309.571(PEP_1)
1366.590
1392.602
1495.639
1512.651(PEP+HexNAc(1)_1)
1569.672
CID-MS/MS Scan:7321, Noise threshold:1.0
Relative Intensity (%)
292.105(NeuAc)
366.141(HexNAc + Hex)
528.195(Hex + HexNAc + Hex)
657.238(NeuAc + Hex + HexNAc)
690.248
756.831(PEP+HexNAc(1)_2)
819.290(HexNAc(1)Hex(2)NeuAc(1)_1)
837.857
858.372(PEP+HexNAc(2)_2)
924.035(PEP+HexNAc(4)Hex(4)_3)
939.398(PEP+HexNAc(2)Hex(1)_2)
1048.939
1087.474
1121.962(PEP+HexNAc(3)Hex(2)_2)
1141.491
1190.171
1202.986(PEP+HexNAc(3)Hex(3)_2)
1231.506
1239.525
1263.868
1292.538
1348.526(PEP+HexNAc(3)Hex(3)NeuAc(1)_2)
1385.553(PEP+HexNAc(4)Hex(4)_2)
1429.565(PEP+HexNAc(3)Hex(4)NeuAc(1)_2)
1477.632
1494.641
1512.657(PEP+HexNAc(1)_1)
1674.701
ETD-MS/MS Scan:7322, Noise threshold:1.9
Relative Intensity (%)
204.087(HexNAc)
274.093(NeuAc − H2O)
292.105(NeuAc)
366.141(HexNAc + Hex)
423.588
493.525
636.418
939.900
1020.423
1121.965(PEP+HexNAc(3)Hex(2)_2)
1203.486
1246.859
1359.163
1427.793
1478.603
1512.651(PEP+HexNAc(1)_1)
1636.674
1863.789
1879.805
1911.763
1922.855
1933.819
1951.844
1982.819
1997.829
m/z

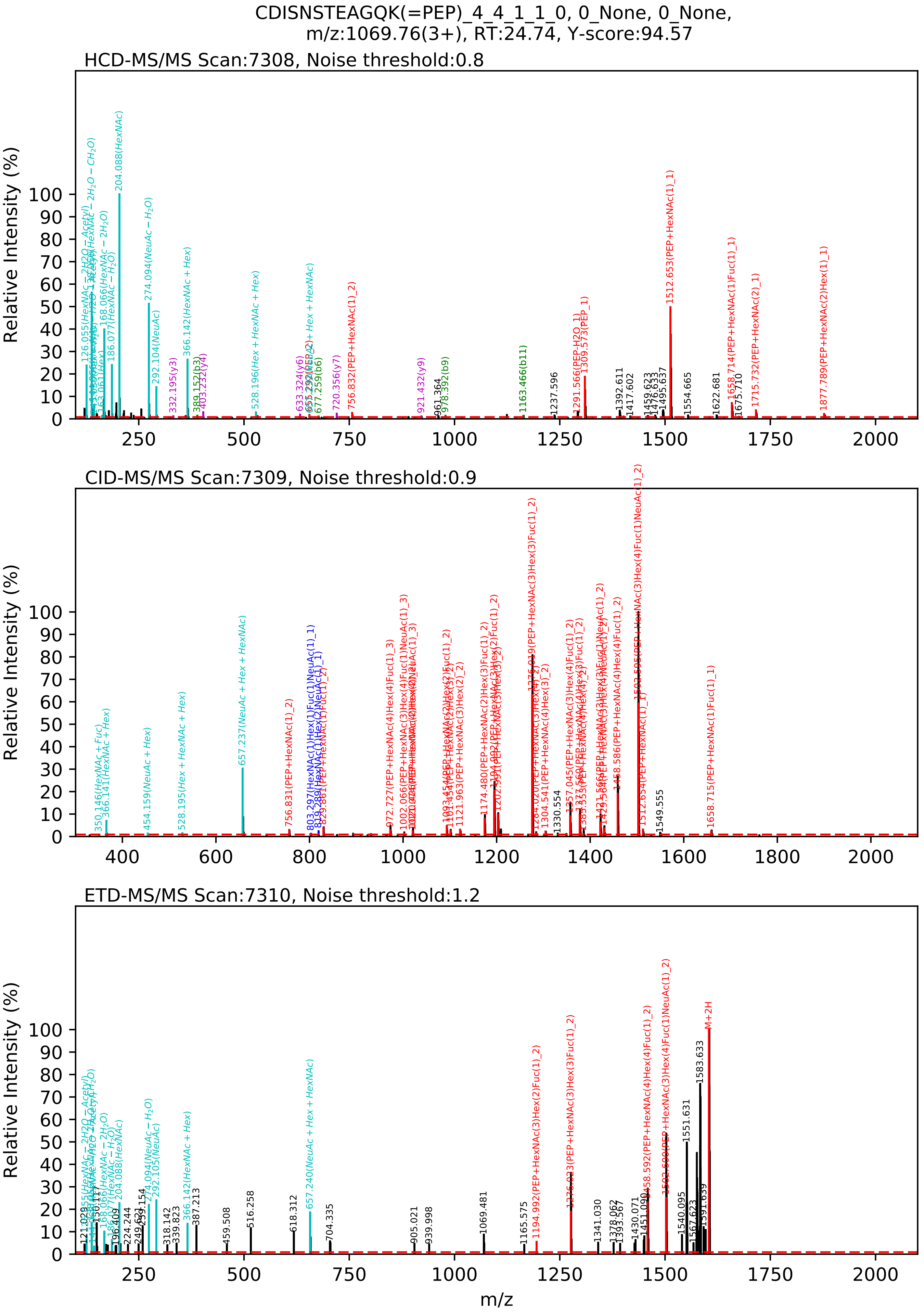
CDISNSTEAGQK(=PEP)_4_4_1_1_0, 0_None, 0_None,
m/z:1069.76(3+), RT:24.74, Y-score:94.57
HCD-MS/MS Scan:7308, Noise threshold:0.8
Relative Intensity (%)
204.088(HexNAc)
274.094(NeuAc − H2O)
292.104(NeuAc)
366.142(HexNAc + Hex)
332.195(y3)
389.152(b3)
403.232(y4)
528.196(Hex + HexNAc + Hex)
633.324(y6)
677.259(b6)
720.356(y7)
756.832(PEP+HexNAc(1)_2)
921.432(y9)
961.364
978.392(b9)
1163.466(b11)
1237.596
1291.566(PEP-H2O_1)
1309.573(PEP_1)
1392.611
1417.602
1459.623
1476.633
1495.637
1512.653(PEP+HexNAc(1)_1)
1554.665
1622.681
1658.714(PEP+HexNAc(1)Fuc(1)_1)
1675.710
1715.732(PEP+HexNAc(2)_1)
1877.789(PEP+HexNAc(2)Hex(1)_1)
CID-MS/MS Scan:7309, Noise threshold:0.9
Relative Intensity (%)
350.146(HexNAc + Fuc)
366.141(HexNAc + Hex)
454.159(NeuAc + Hex)
528.195(Hex + HexNAc + Hex)
657.237(NeuAc + Hex + HexNAc)
756.831(PEP+HexNAc(1)_2)
1121.963(PEP+HexNAc(3)Hex(2)_2)
1174.480(PEP+HexNAc(2)Hex(3)Fuc(1)_2)
1276.019(PEP+HexNAc(3)Hex(3)Fuc(1)_2)
1304.541(PEP+HexNAc(4)Hex(3)_2)
1330.554
1357.045(PEP+HexNAc(3)Hex(4)Fuc(1)_2)
1458.586(PEP+HexNAc(4)Hex(4)Fuc(1)_2)
1502.595(PEP+HexNAc(3)Hex(4)Fuc(1)NeuAc(1)_2)
1512.654(PEP+HexNAc(1)_1)
1549.555
1658.715(PEP+HexNAc(1)Fuc(1)_1)
ETD-MS/MS Scan:7310, Noise threshold:1.2
Relative Intensity (%)
204.088(HexNAc)
274.094(NeuAc − H2O)
292.105(NeuAc)
366.142(HexNAc + Hex)
387.213
459.508
516.258
618.312
657.240(NeuAc + Hex + HexNAc)
704.335
905.021
939.998
1069.481
1165.575
1194.992(PEP+HexNAc(3)Hex(2)Fuc(1)_2)
1276.023(PEP+HexNAc(3)Hex(3)Fuc(1)_2)
1341.030
1378.062
1393.567
1430.071
1451.090
1458.592(PEP+HexNAc(4)Hex(4)Fuc(1)_2)
1502.600(PEP+HexNAc(3)Hex(4)Fuc(1)NeuAc(1)_2)
1540.095
1551.631
1567.623
1583.633
1591.639
M+2H
m/z

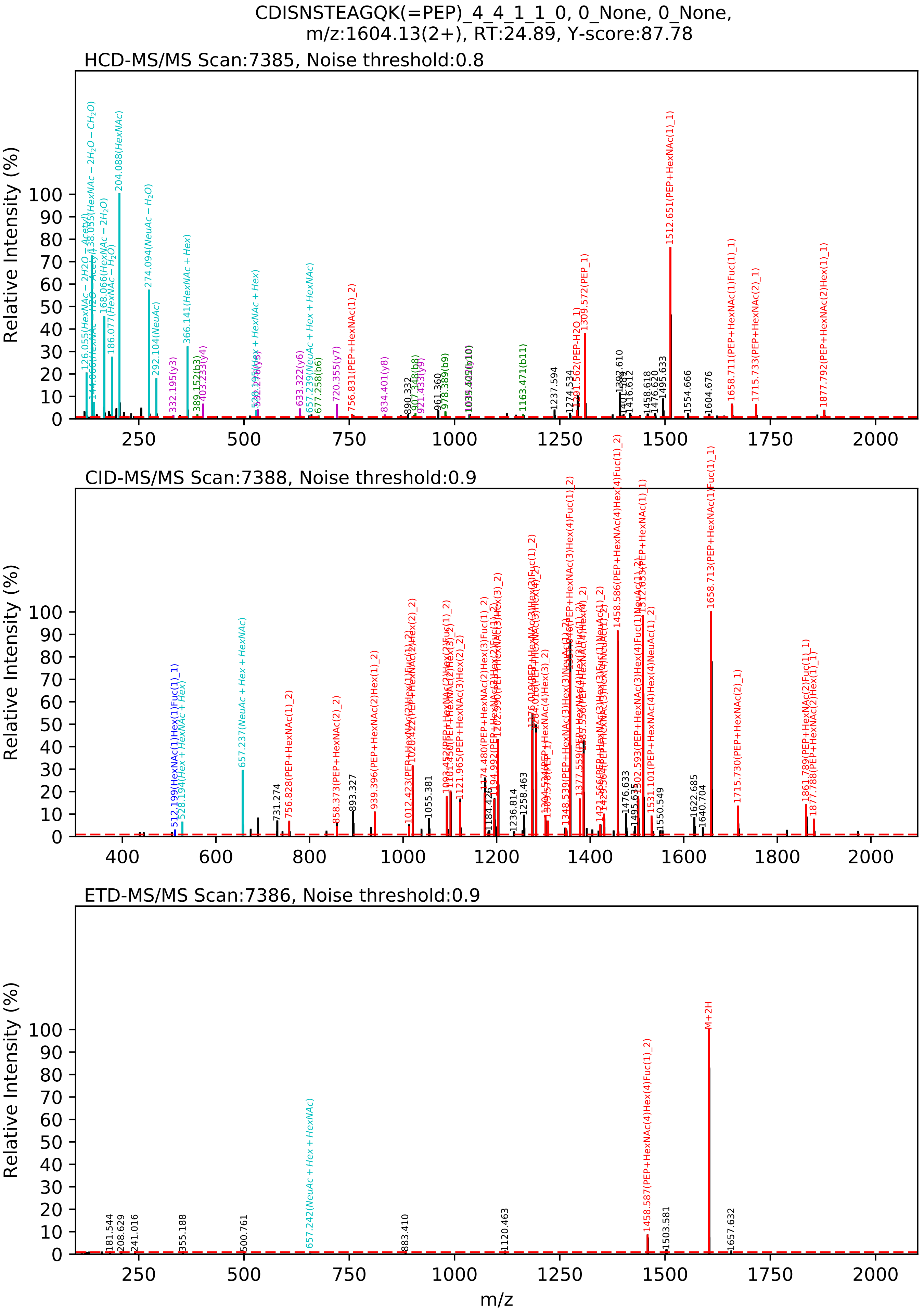
CDISNSTEAGQK(=PEP)_4_4_1_1_0, 0_None, 0_None,
m/z:1604.13(2+), RT:24.89, Y-score:87.78
HCD-MS/MS Scan:7385, Noise threshold:0.8
Relative Intensity (%)
204.088(HexNAc)
274.094(NeuAc − H2O)
366.141(HexNAc + Hex)
292.104(NeuAc)
186.077(HexNAc − H2O)
168.066(HexNAc − 2H2O)
1512.651(PEP+HexNAc(1)_1)
1309.572(PEP_1)
1658.711(PEP+HexNAc(1)Fuc(1)_1)
1715.733(PEP+HexNAc(2)_1)
1877.792(PEP+HexNAc(2)Hex(1)_1)
756.831(PEP+HexNAc(1)_2)
1163.471(b11)
978.389(b9)
720.355(y7)
633.322(y6)
834.401(y8)
332.195(y3)
403.233(y4)
389.152(b3)
677.258(b6)
1237.594
1274.534
1554.666
1604.676
1495.633
m/z
CID-MS/MS Scan:7388, Noise threshold:0.9
1658.713(PEP+HexNAc(1)Fuc(1)_1)
1458.586(PEP+HexNAc(4)Hex(4)Fuc(1)_2)
657.237(NeuAc + Hex + HexNAc)
512.199(HexNAc(1)Hex(1)Fuc(1)_1)
756.828(PEP+HexNAc(1)_2)
858.373(PEP+HexNAc(2)_2)
939.396(PEP+HexNAc(2)Hex(1)_2)
1715.730(PEP+HexNAc(2)_1)
1861.789(PEP+HexNAc(2)Fuc(1)_1)
1877.788(PEP+HexNAc(2)Hex(1)_1)
731.274
893.327
1055.381
1236.814
1258.463
1476.633
1550.549
1622.685
1640.704
ETD-MS/MS Scan:7386, Noise threshold:0.9
M+2H
1458.587(PEP+HexNAc(4)Hex(4)Fuc(1)_2)
657.242(NeuAc + Hex + HexNAc)
181.544
208.629
241.016
355.188
500.761
883.410
1120.463
1503.581
1657.632

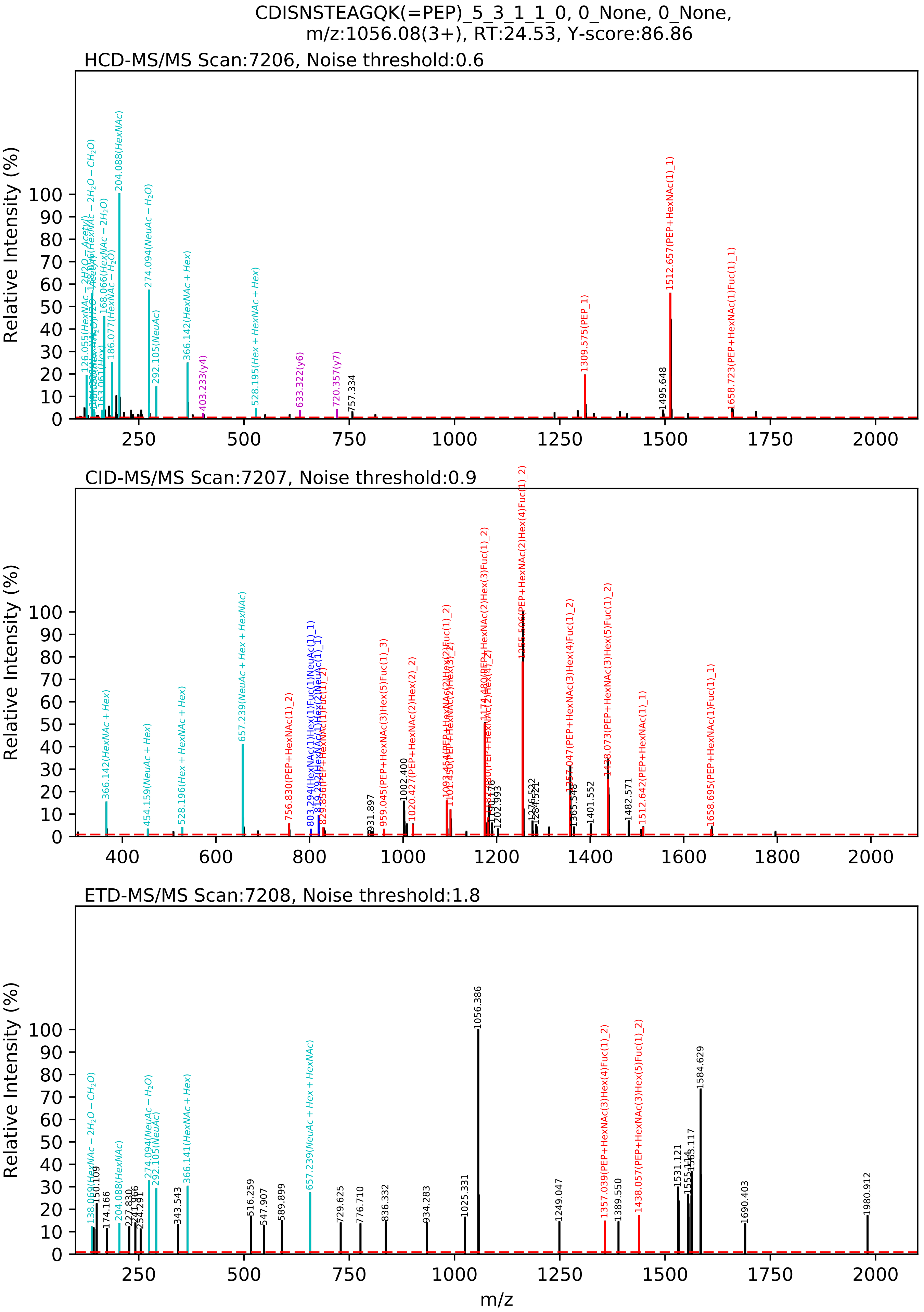
CDISNSTEAGQK(=PEP)_5_3_1_1_0, 0_None, 0_None,
m/z:1056.08(3+), RT:24.53, Y-score:86.86
HCD-MS/MS Scan:7206, Noise threshold:0.6
Relative Intensity (%)
204.088(HexNAc)
274.094(NeuAc − H2O)
366.142(HexNAc + Hex)
292.105(NeuAc)
528.195(Hex + HexNAc + Hex)
403.233(y4)
633.322(y6)
720.357(y7)
757.334
1309.575(PEP_1)
1512.657(PEP+HexNAc(1)_1)
1495.648
1658.723(PEP+HexNAc(1)Fuc(1)_1)
186.077(HexNAc − H2O)
168.066(HexNAc − 2H2O)
126.055(HexNAc − 2H2O − CH2O)
CID-MS/MS Scan:7207, Noise threshold:0.9
366.142(HexNAc + Hex)
454.159(NeuAc + Hex)
528.196(Hex + HexNAc + Hex)
657.239(NeuAc + Hex + HexNAc)
756.830(PEP+HexNAc(1)_2)
803.294(HexNAc(1)Hex(1)Fuc(1)NeuAc(1)_1)
931.897
959.045(PEP+HexNAc(3)Hex(5)Fuc(1)_3)
1002.400
1020.427(PEP+HexNAc(2)Hex(2)_2)
1174.480(PEP+HexNAc(2)Hex(3)Fuc(1)_2)
1202.993
1255.506(PEP+HexNAc(2)Hex(4)Fuc(1)_2)
1276.532
1357.047(PEP+HexNAc(3)Hex(4)Fuc(1)_2)
1365.548
1401.552
1438.073(PEP+HexNAc(3)Hex(5)Fuc(1)_2)
1482.571
1512.642(PEP+HexNAc(1)_1)
1658.695(PEP+HexNAc(1)Fuc(1)_1)
ETD-MS/MS Scan:7208, Noise threshold:1.8
138.060(HexNAc − 2H2O − CH2O)
150.109
174.166
204.088(HexNAc)
274.094(NeuAc − H2O)
292.105(NeuAc)
366.141(HexNAc + Hex)
343.543
516.259
547.907
589.899
657.239(NeuAc + Hex + HexNAc)
729.625
776.710
836.332
934.283
1025.331
1056.386
1249.047
1357.039(PEP+HexNAc(3)Hex(4)Fuc(1)_2)
1389.550
1438.057(PEP+HexNAc(3)Hex(5)Fuc(1)_2)
1531.121
1563.117
1584.629
1690.403
1980.912
m/z

CDISNSTEAGQK(=PEP)_5_3_1_1_0, 0_None, 0_None,
m/z:1583.62(2+), RT:24.49, Y-score:79.40

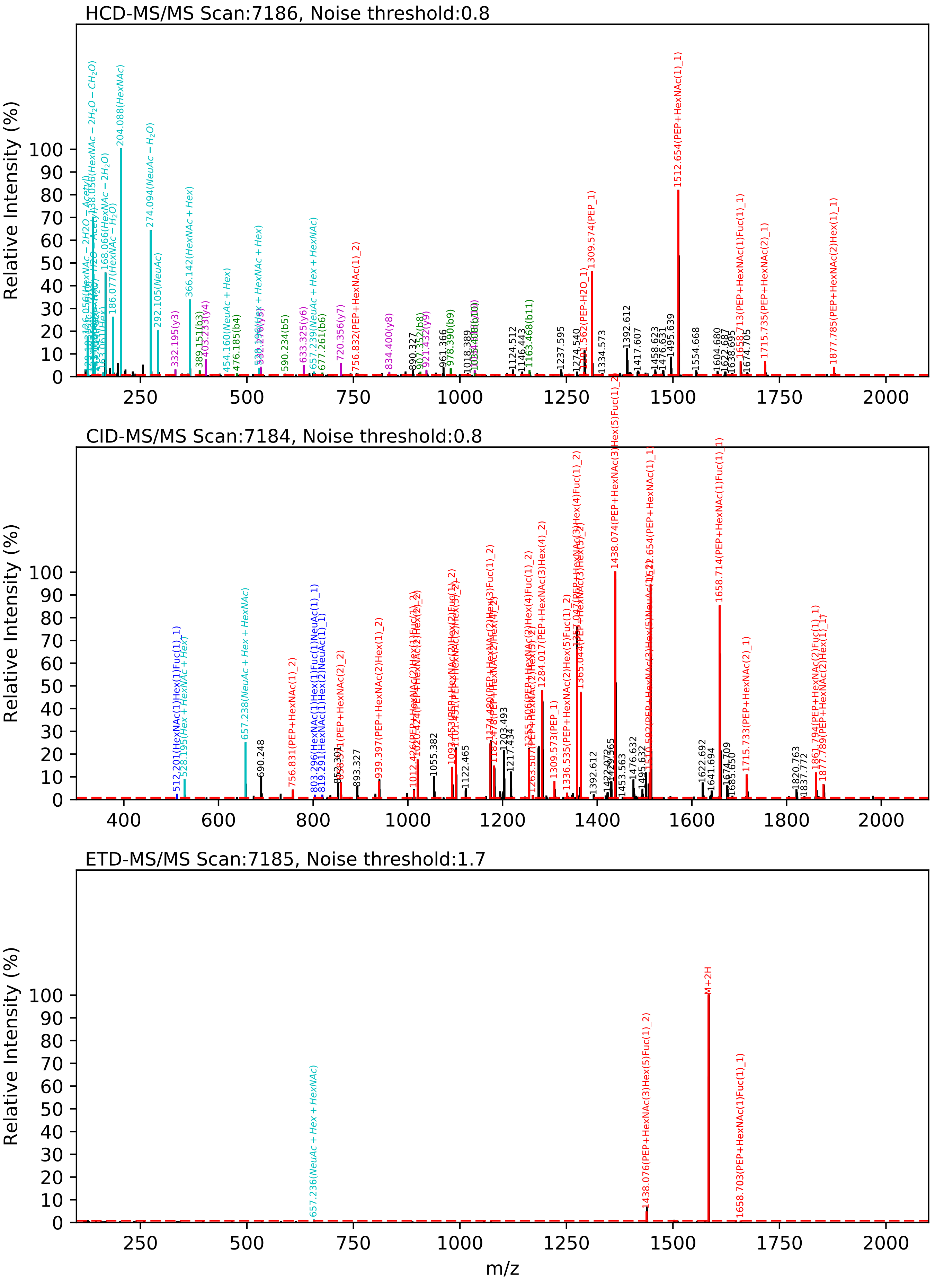

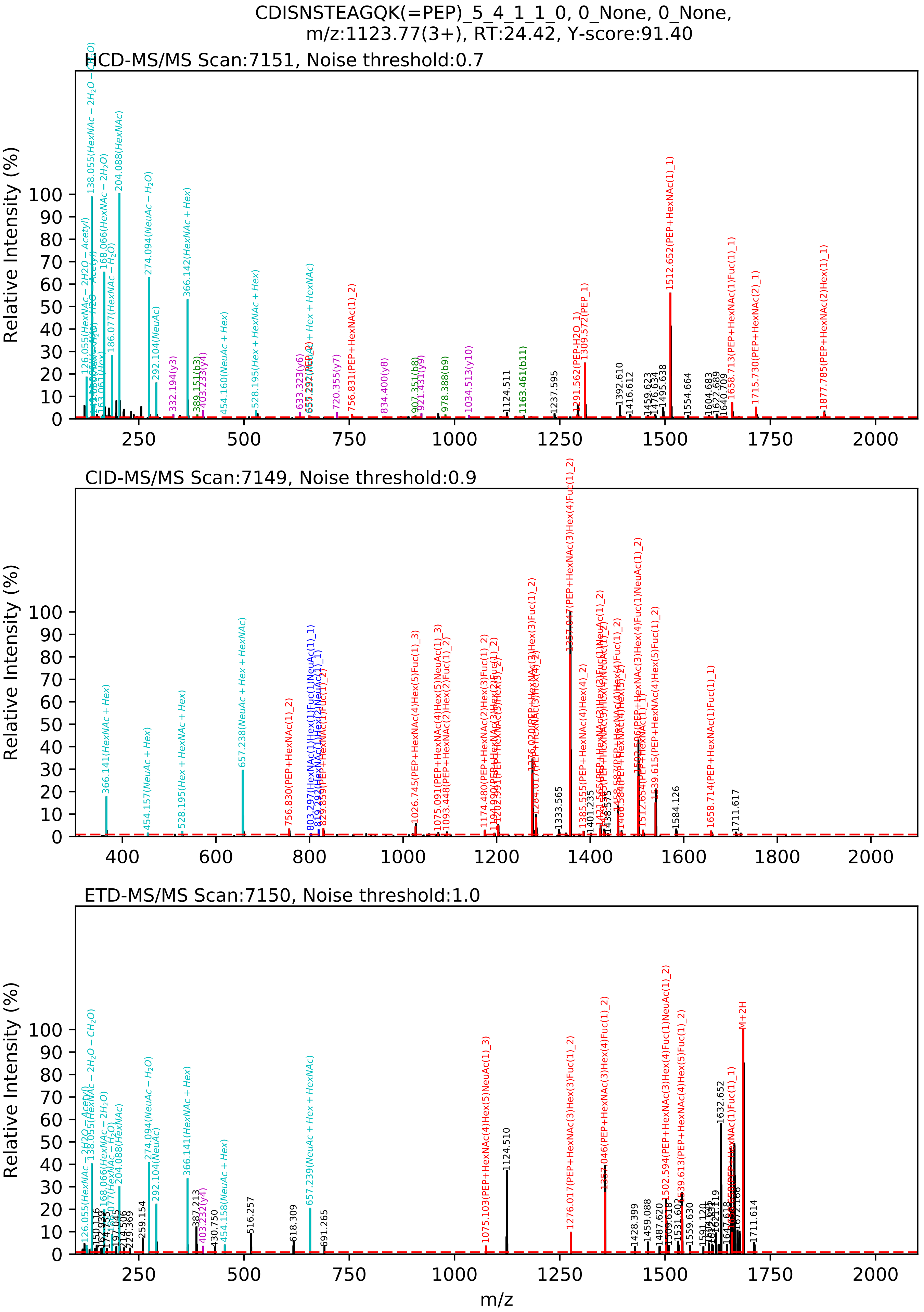
CDISNSTEAGQK(=PEP)_5_4_1_1_0, 0_None, 0_None,
m/z:1123.77(3+), RT:24.42, Y-score:91.40
HCD-MS/MS Scan:7151, Noise threshold:0.7
CID-MS/MS Scan:7149, Noise threshold:0.9
ETD-MS/MS Scan:7150, Noise threshold:1.0
Relative Intensity (%)
m/z
138.055(HexNAc − 2H2O − CH2O)
204.088(HexNAc)
168.066(HexNAc − 2H2O)
186.077(HexNAc − H2O)
274.094(NeuAc − H2O)
292.104(NeuAc)
366.142(HexNAc + Hex)
454.160(NeuAc + Hex)
528.195(Hex + HexNAc + Hex)
657.292(NeuAc + Hex + HexNAc)
332.194(y3)
403.233(y4)
389.151(b3)
633.323(y6)
720.355(y7)
756.831(PEP+HexNAc(1)_2)
834.400(y8)
907.351(b8)
921.431(y9)
978.388(b9)
1034.513(y10)
1124.511
1163.461(b11)
1237.595
1291.562(PEP-H2O_1)
1309.572(PEP_1)
1392.610
1416.612
1459.623
1476.634
1495.638
1512.652(PEP+HexNAc(1)_1)
1554.664
1604.683
1622.689
1640.709
1658.713(PEP+HexNAc(1)Fuc(1)_1)
1715.730(PEP+HexNAc(2)_1)
1877.785(PEP+HexNAc(2)Hex(1)_1)
366.141(HexNAc + Hex)
454.157(NeuAc + Hex)
528.195(Hex + HexNAc + Hex)
657.238(NeuAc + Hex + HexNAc)
756.830(PEP+HexNAc(1)_2)
803.297(HexNAc(1)Hex(1)Fuc(1)NeuAc(1)_1)
1026.745(PEP+HexNAc(4)Hex(5)Fuc(1)_3)
1075.091(PEP+HexNAc(4)Hex(5)NeuAc(1)_3)
1093.448(PEP+HexNAc(2)Hex(2)Fuc(1)_2)
1174.480(PEP+HexNAc(2)Hex(3)Fuc(1)_2)
1276.020(PEP+HexNAc(3)Hex(3)Fuc(1)_2)
1357.047(PEP+HexNAc(3)Hex(4)Fuc(1)_2)
1333.565
1385.555(PEP+HexNAc(4)Hex(4)_2)
1401.235
1438.575
1502.596(PEP+HexNAc(3)Hex(4)Fuc(1)NeuAc(1)_2)
1512.654(PEP+HexNAc(1)_1)
1539.615(PEP+HexNAc(4)Hex(5)Fuc(1)_2)
1584.126
1658.714(PEP+HexNAc(1)Fuc(1)_1)
1711.617
126.055(HexNAc − 2H2O − Acetyl)
138.055(HexNAc − 2H2O − CH2O)
168.066(HexNAc − 2H2O)
204.088(HexNAc)
274.094(NeuAc − H2O)
292.104(NeuAc)
366.141(HexNAc + Hex)
403.232(y4)
454.158(NeuAc + Hex)
657.239(NeuAc + Hex + HexNAc)
150.116
197.045
214.506
229.369
259.154
387.213
430.750
516.257
618.309
691.265
1075.103(PEP+HexNAc(4)Hex(5)NeuAc(1)_3)
1124.510
1276.017(PEP+HexNAc(3)Hex(3)Fuc(1)_2)
1357.046(PEP+HexNAc(3)Hex(4)Fuc(1)_2)
1428.399
1459.088
1487.620
1502.594(PEP+HexNAc(3)Hex(4)Fuc(1)NeuAc(1)_2)
1531.602
1539.613(PEP+HexNAc(4)Hex(5)Fuc(1)_2)
1559.630
1632.652
1658.716(PEP+HexNAc(1)Fuc(1)_1)
1672.166
1711.614
M+2H

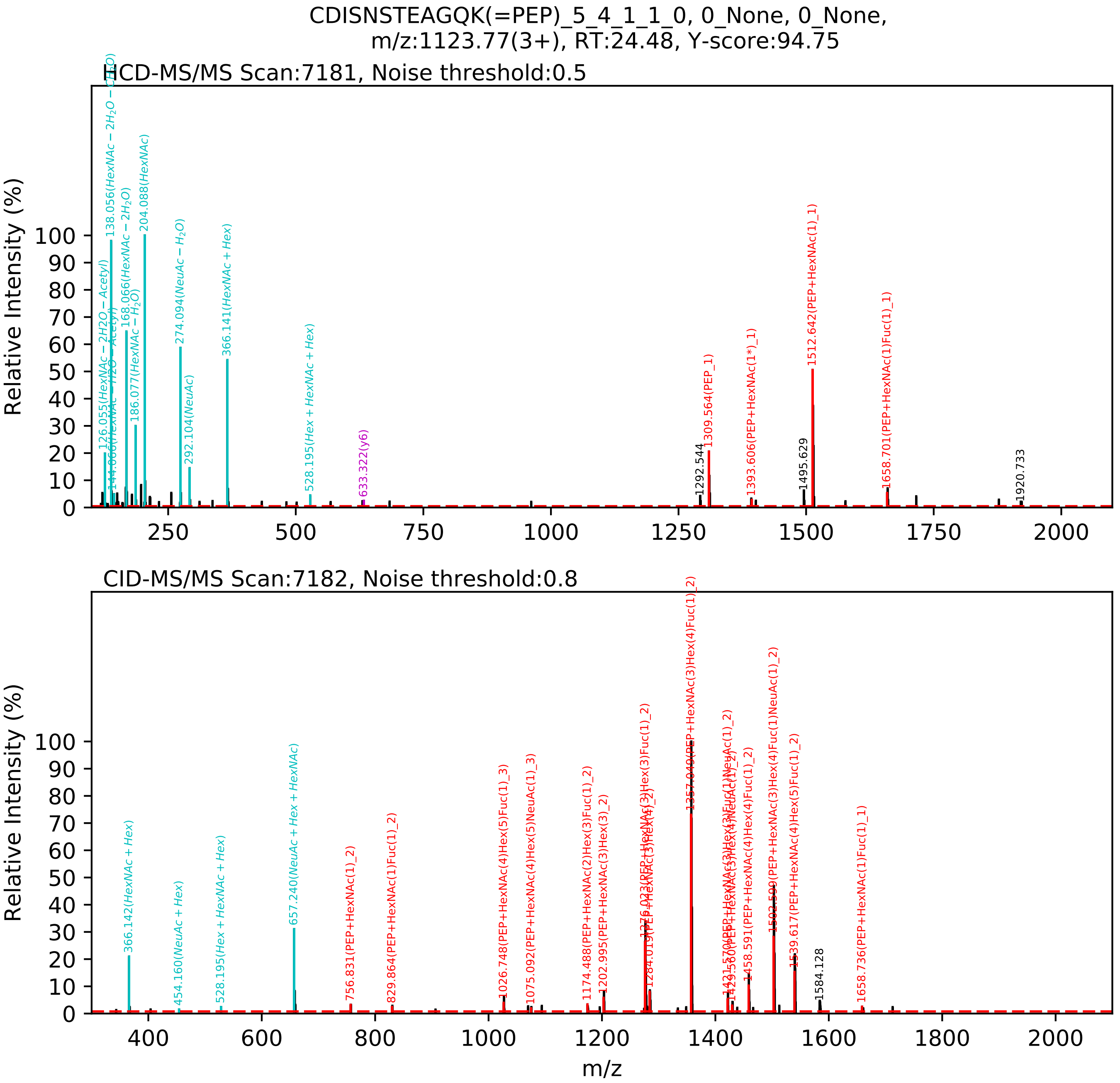

CDISNSTEAGQK(=PEP)_5_4_1_1_0, 0_None, 0_None,
m/z:1123.77(3+), RT:24.48, Y-score:94.75
HCD-MS/MS Scan:7181, Noise threshold:0.5
Relative Intensity (%)
126.055(HexNAc − 2H2O − Acetyl)
138.056(HexNAc − 2H2O − C2H2O)
168.066(HexNAc − 2H2O)
186.077(HexNAc − H2O)
204.088(HexNAc)
274.094(NeuAc − H2O)
292.104(NeuAc)
366.141(HexNAc + Hex)
528.195(Hex + HexNAc + Hex)
633.322(y6)
1292.544
1309.564(PEP_1)
1393.606(PEP+HexNAc(1*)_1)
1495.629
1512.642(PEP+HexNAc(1)_1)
1658.701(PEP+HexNAc(1)Fuc(1)_1)
1920.733
CID-MS/MS Scan:7182, Noise threshold:0.8
Relative Intensity (%)
366.142(HexNAc + Hex)
454.160(NeuAc + Hex)
528.195(Hex + HexNAc + Hex)
657.240(NeuAc + Hex + HexNAc)
756.831(PEP+HexNAc(1)_2)
829.864(PEP+HexNAc(1)Fuc(1)_2)
1026.748(PEP+HexNAc(4)Hex(5)Fuc(1)_3)
1075.092(PEP+HexNAc(4)Hex(5)NeuAc(1)_3)
1174.488(PEP+HexNAc(2)Hex(3)Fuc(1)_2)
1202.995(PEP+HexNAc(3)Hex(3)_2)
1276.023(PEP+HexNAc(3)Hex(3)Fuc(1)_2)
1357.040(PEP+HexNAc(3)Hex(4)Fuc(1)_2)
1458.591(PEP+HexNAc(4)Hex(4)Fuc(1)_2)
1503.599(PEP+HexNAc(3)Hex(4)Fuc(1)NeuAc(1)_2)
1539.617(PEP+HexNAc(4)Hex(5)Fuc(1)_2)
1584.128
1658.736(PEP+HexNAc(1)Fuc(1)_1)
m/z

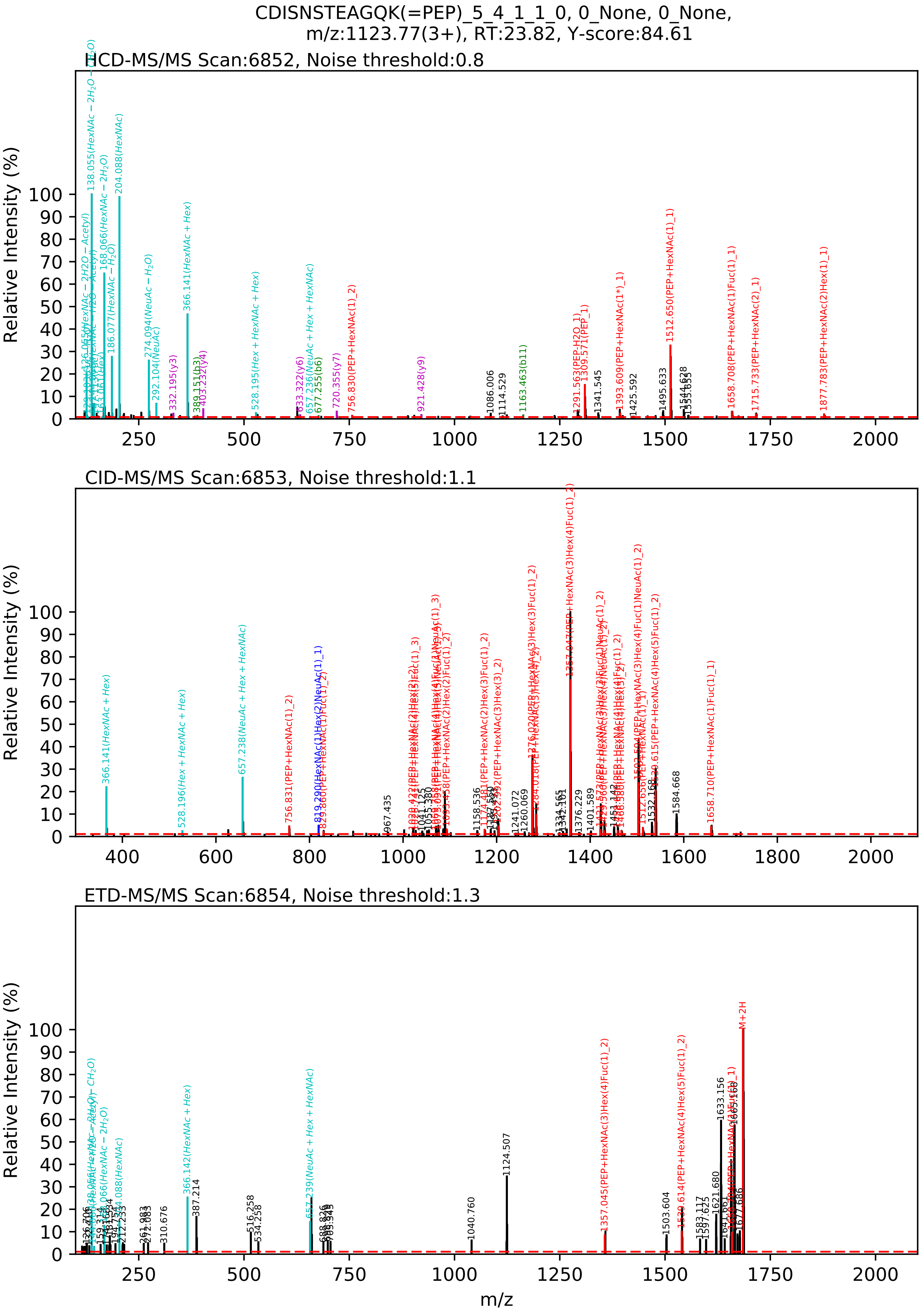

CDISNSTEAGQK(=PEP)_5_4_1_1_0, 0_None, 0_None,
m/z:1123.77(3+), RT:23.82, Y-score:84.61
HCD-MS/MS Scan:6852, Noise threshold:0.8
Relative Intensity (%)
204.088(HexNAc)
168.066(HexNAc − 2H2O)
186.077(HexNAc − H2O)
274.094(NeuAc − H2O)
292.104(NeuAc)
366.141(HexNAc + Hex)
332.195(y3)
389.151(b3)
403.232(y4)
528.195(Hex + HexNAc + Hex)
633.322(y6)
657.236(NeuAc + Hex + HexNAc)
677.255(b6)
720.355(y7)
756.830(PEP+HexNAc(1)_2)
921.428(y9)
1086.006
1114.529
1163.463(b11)
1291.563(PEP-H2O_1)
1309.571(PEP_1)
1341.545
1393.609(PEP+HexNAc(1*)_1)
1425.592
1495.633
1512.650(PEP+HexNAc(1)_1)
1658.708(PEP+HexNAc(1)Fuc(1)_1)
1715.733(PEP+HexNAc(2)_1)
1877.783(PEP+HexNAc(2)Hex(1)_1)
CID-MS/MS Scan:6853, Noise threshold:1.1
Relative Intensity (%)
366.141(HexNAc + Hex)
528.196(Hex + HexNAc + Hex)
657.238(NeuAc + Hex + HexNAc)
756.831(PEP+HexNAc(1)_2)
967.435
1158.536
1241.072
1260.069
1276.020(PEP+HexNAc(3)Hex(3)Fuc(1)_2)
1357.047(PEP+HexNAc(3)Hex(4)Fuc(1)_2)
1376.229
1401.589
1451.142
1502.594(PEP+HexNAc(3)Hex(4)Fuc(1)NeuAc(1)_2)
1532.168
1584.668
1658.710(PEP+HexNAc(1)Fuc(1)_1)
ETD-MS/MS Scan:6854, Noise threshold:1.3
Relative Intensity (%)
366.142(HexNAc + Hex)
387.214
516.258
534.258
657.239(NeuAc + Hex + HexNAc)
1040.760
1124.507
1357.045(PEP+HexNAc(3)Hex(4)Fuc(1)_2)
1503.604
1539.614(PEP+HexNAc(4)Hex(5)Fuc(1)_2)
1583.117
1621.680
1633.156
1677.686
M+2H
m/z

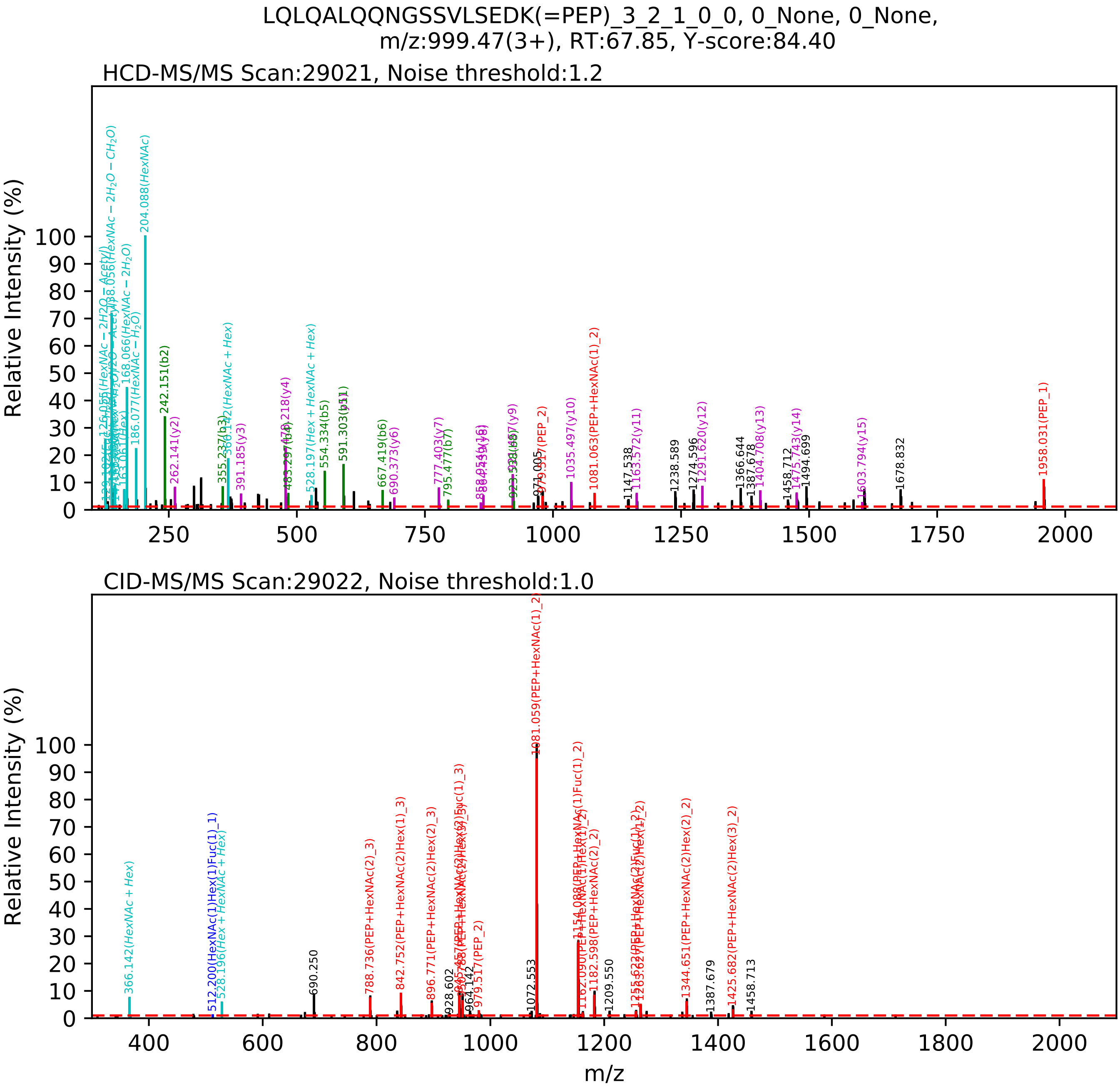
LQLQALQQNGSSVLSEDK(=PEP)_3_2_1_0_0, 0_None, 0_None,
m/z:999.47(3+), RT:67.85, Y-score:84.40
HCD-MS/MS Scan:29021, Noise threshold:1.2
Relative Intensity (%)
204.088(HexNAc)
242.151(b2)
262.141(y2)
355.237(b3)
391.185(y3)
528.197(Hex + HexNAc + Hex)
554.334(b5)
667.419(b6)
690.373(y6)
777.403(y7)
795.477(b7)
971.005
979.517(PEP_2)
1035.497(y10)
1081.063(PEP+HexNAc(1)_2)
1147.538
1163.572(y11)
1238.589
1274.596
1291.620(y12)
1366.644
1387.678
1404.708(y13)
1458.712
1494.699
1603.794(y15)
1678.832
1958.031(PEP_1)
CID-MS/MS Scan:29022, Noise threshold:1.0
Relative Intensity (%)
366.142(HexNAc + Hex)
512.200(HexNAc(1)Hex(1)Fuc(1)_1)
690.250
788.736(PEP+HexNAc(2)_3)
842.752(PEP+HexNAc(2)Hex(1)_3)
896.771(PEP+HexNAc(2)Hex(2)_3)
928.602
964.142
979.517(PEP_2)
1072.553
1081.059(PEP+HexNAc(1)_2
1182.598(PEP+HexNAc(2)_2)
1209.550
1344.651(PEP+HexNAc(2)Hex(2)_2)
1387.679
1425.682(PEP+HexNAc(2)Hex(3)_2)
1458.713
m/z

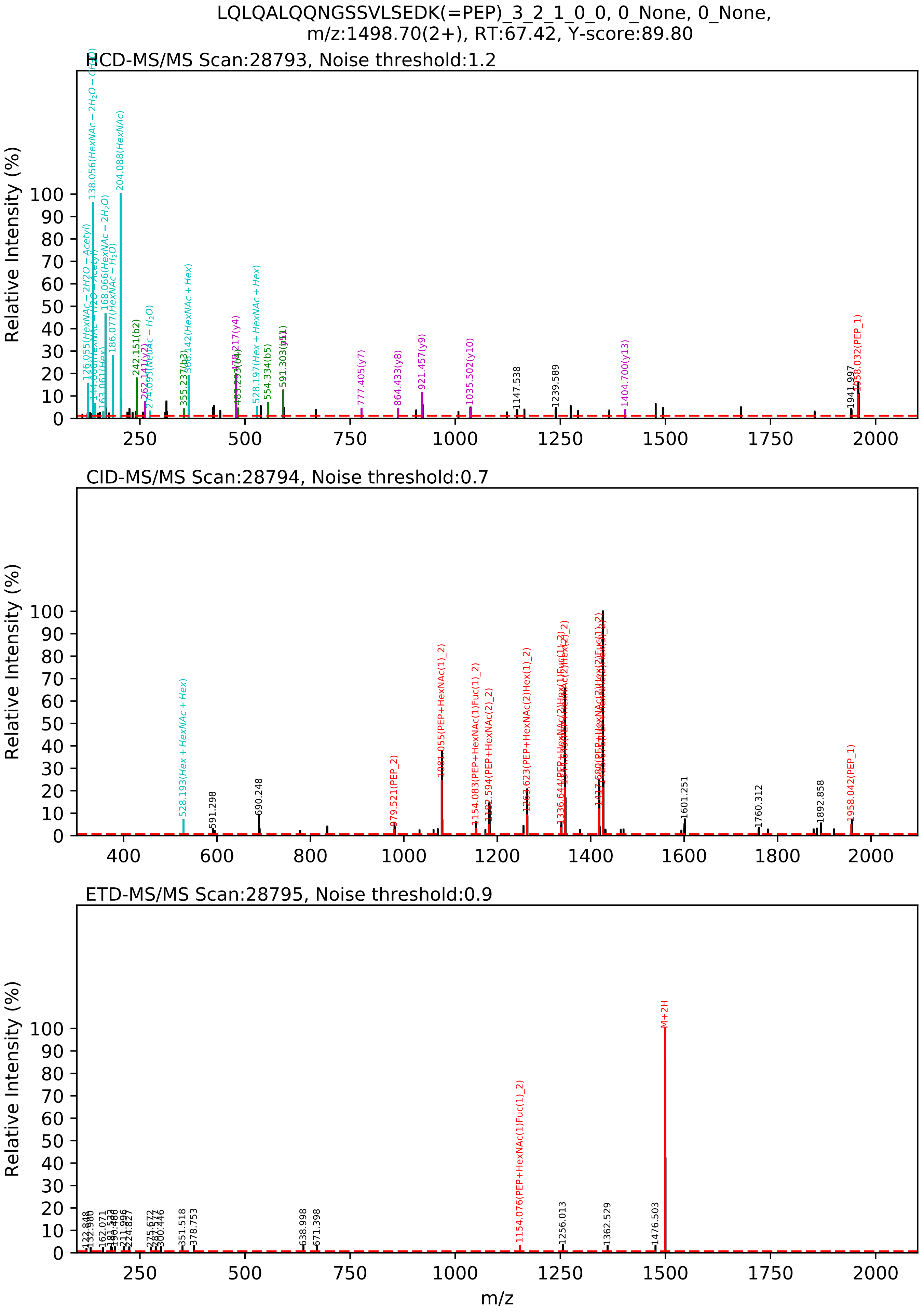
LQLQALQQNGSSVLSEDK(=PEP)_3_2_1_0_0, 0_None, 0_None,
m/z:1498.70(2+), RT:67.42, Y-score:89.80
HCD-MS/MS Scan:28793, Noise threshold:1.2
Relative Intensity (%)
204.088(HexNAc)
138.056(HexNAc − 2H2O − CH2O)
168.066(HexNAc − 2H2O)
186.077(HexNAc − H2O)
126.055(HexNAc − 2H2O − Acetyl)
163.061(Hex)
242.151(b2)
262.141(y2)
355.237(b3)
366.142(HexNAc + Hex)
483.293(b4)
528.197(Hex + HexNAc + Hex)
554.334(b5)
591.303(b6)
777.405(y7)
864.433(y8)
921.457(y9)
1035.502(y10)
1147.538
1239.589
1404.700(y13)
1941.997
1958.032(PEP_1)
CID-MS/MS Scan:28794, Noise threshold:0.7
Relative Intensity (%)
528.193(Hex + HexNAc + Hex)
591.298
690.248
979.521(PEP_2)
1154.083(PEP+HexNAc(1)Fuc(1)_2)
1182.594(PEP+HexNAc(2)_2)
1263.623(PEP+HexNAc(2)Hex(1)_2)
1601.251
1760.312
1892.858
1958.042(PEP_1)
ETD-MS/MS Scan:28795, Noise threshold:0.9
Relative Intensity (%)
M+2H
1154.076(PEP+HexNAc(1)Fuc(1)_2)
351.518
378.753
638.998
671.398
1256.013
1362.529
1476.503
m/z

LQLQALQQNGSSVLSEDK(=PEP)_3_3_1_0_0, 0_None, 0_None,
m/z:1600.24(2+), RT:67.16, Y-score:84.34

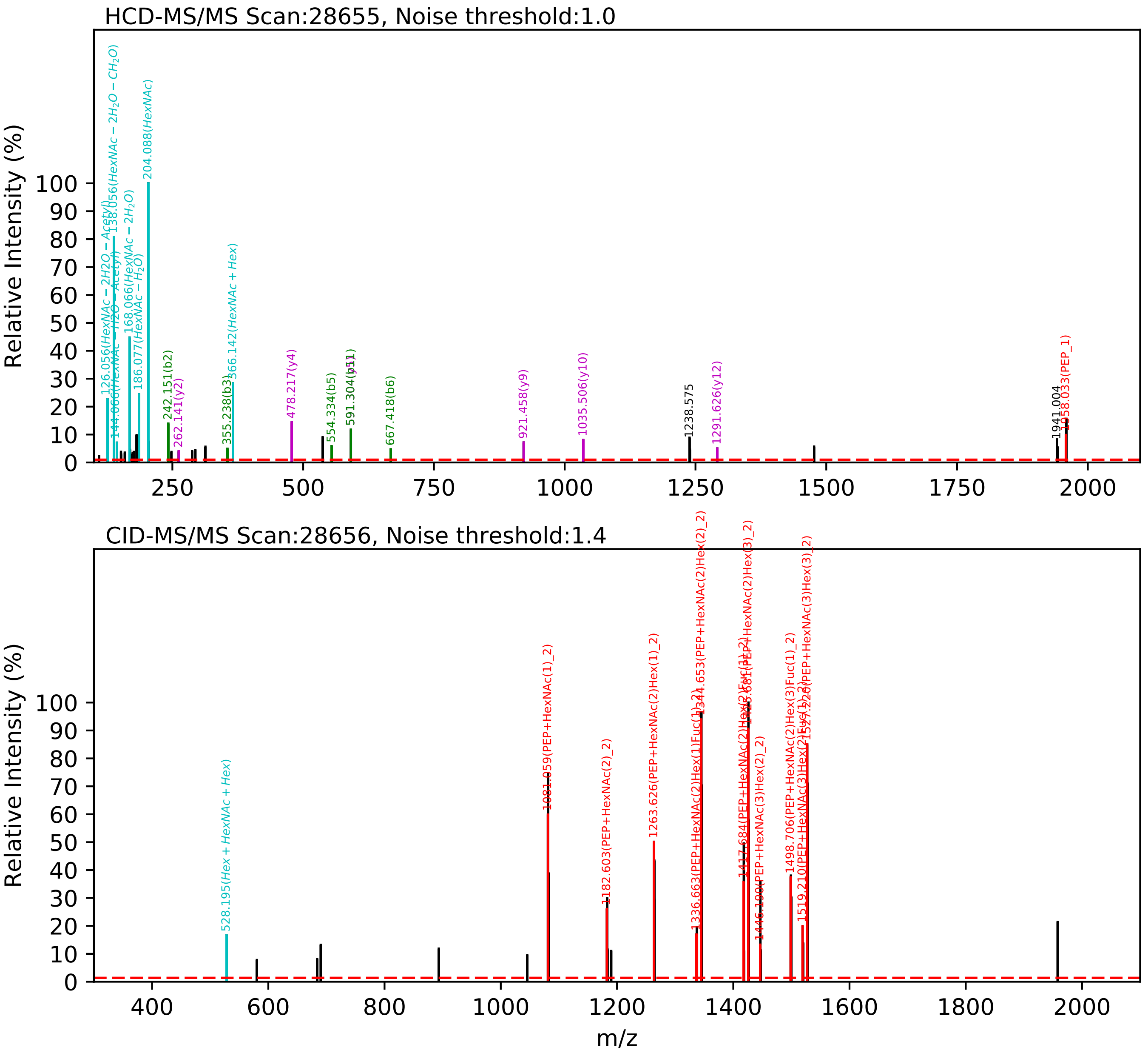

LQLQALQQNGSSVLSEDK(=PEP)_3_3_1_0_0, 0_None, 0_None,
m/z:1600.24(2+), RT:67.78, Y-score:82.99

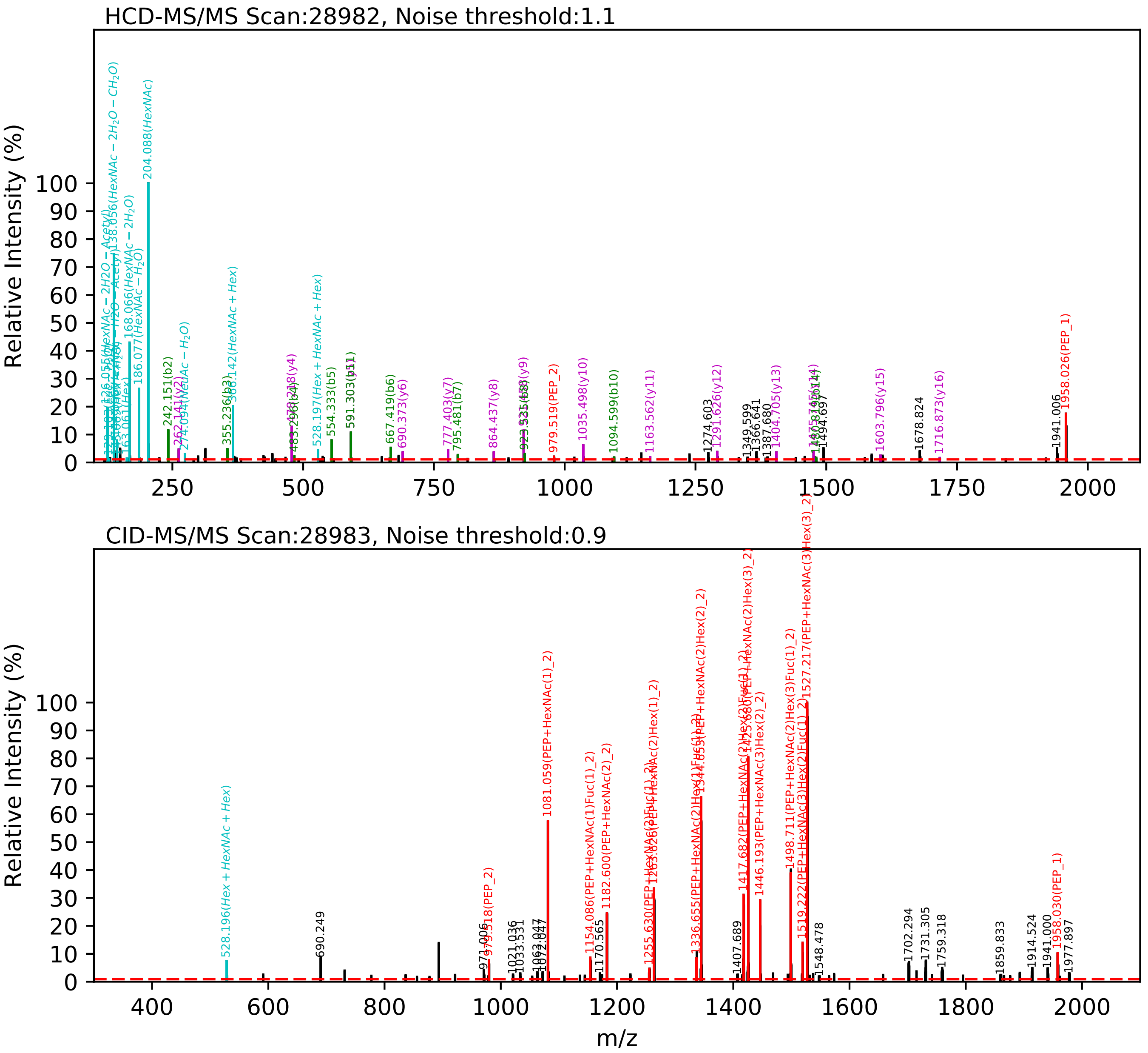

LQLQALQQNGSSVLSEDK(=PEP)_3_3_1_0_0, 0_None, 0_None,
m/z:1600.24(2+), RT:77.79, Y-score:79.78

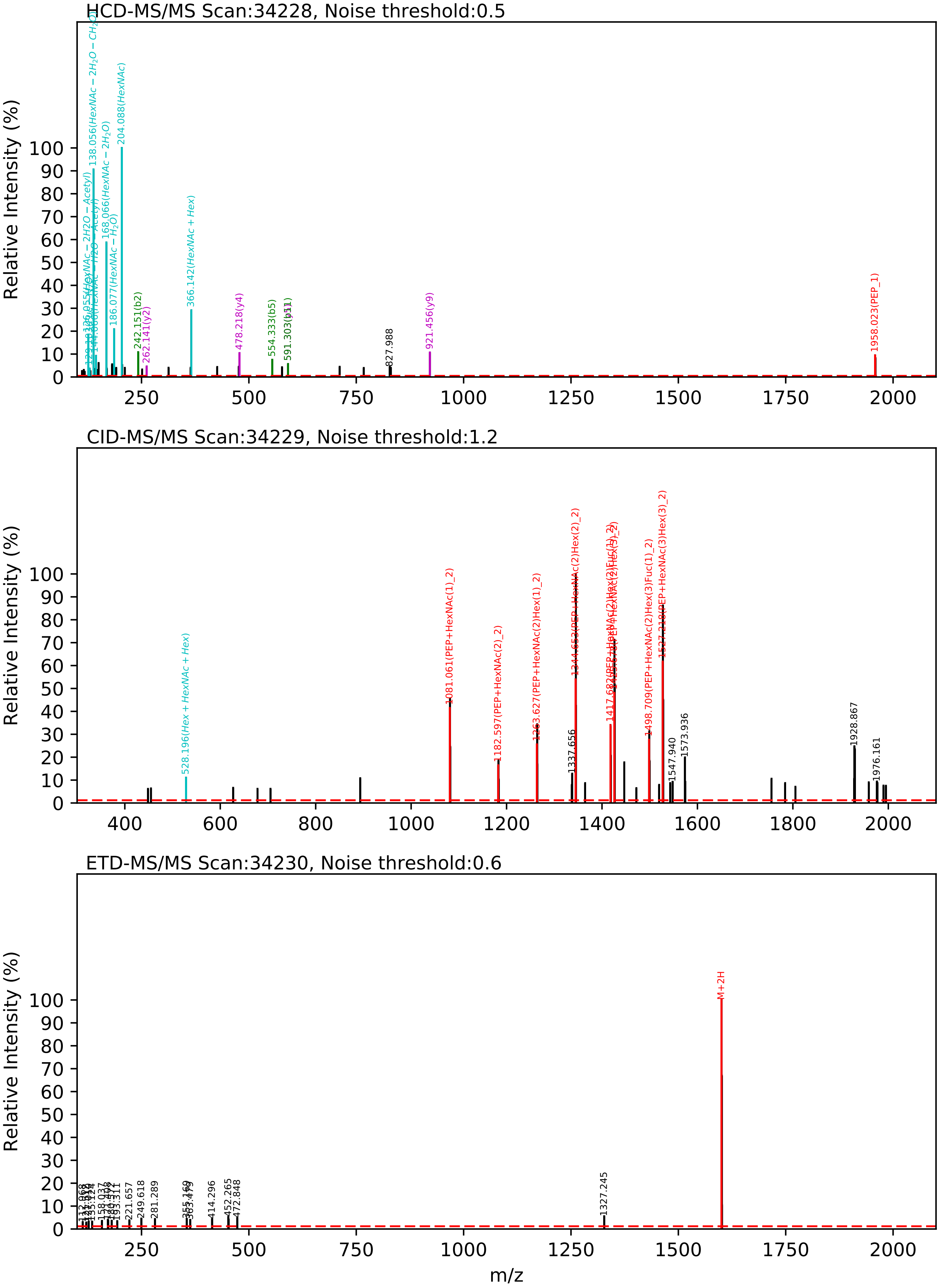

LQLQALQQNGSSVLSEDK(=PEP)_3_3_1_0_0, 0_None, 0_None,
m/z:1600.24(2+), RT:66.58, Y-score:91.74

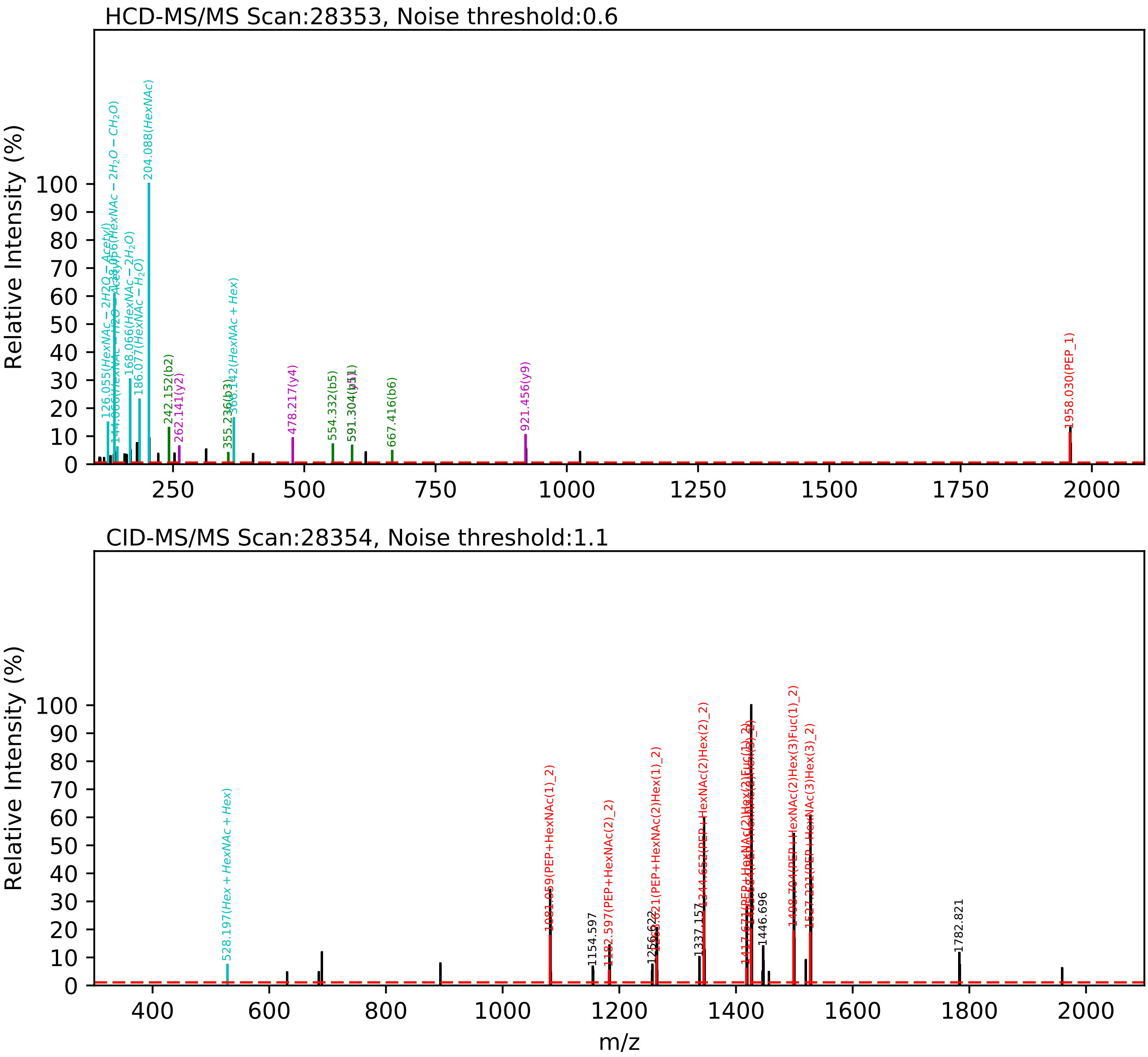

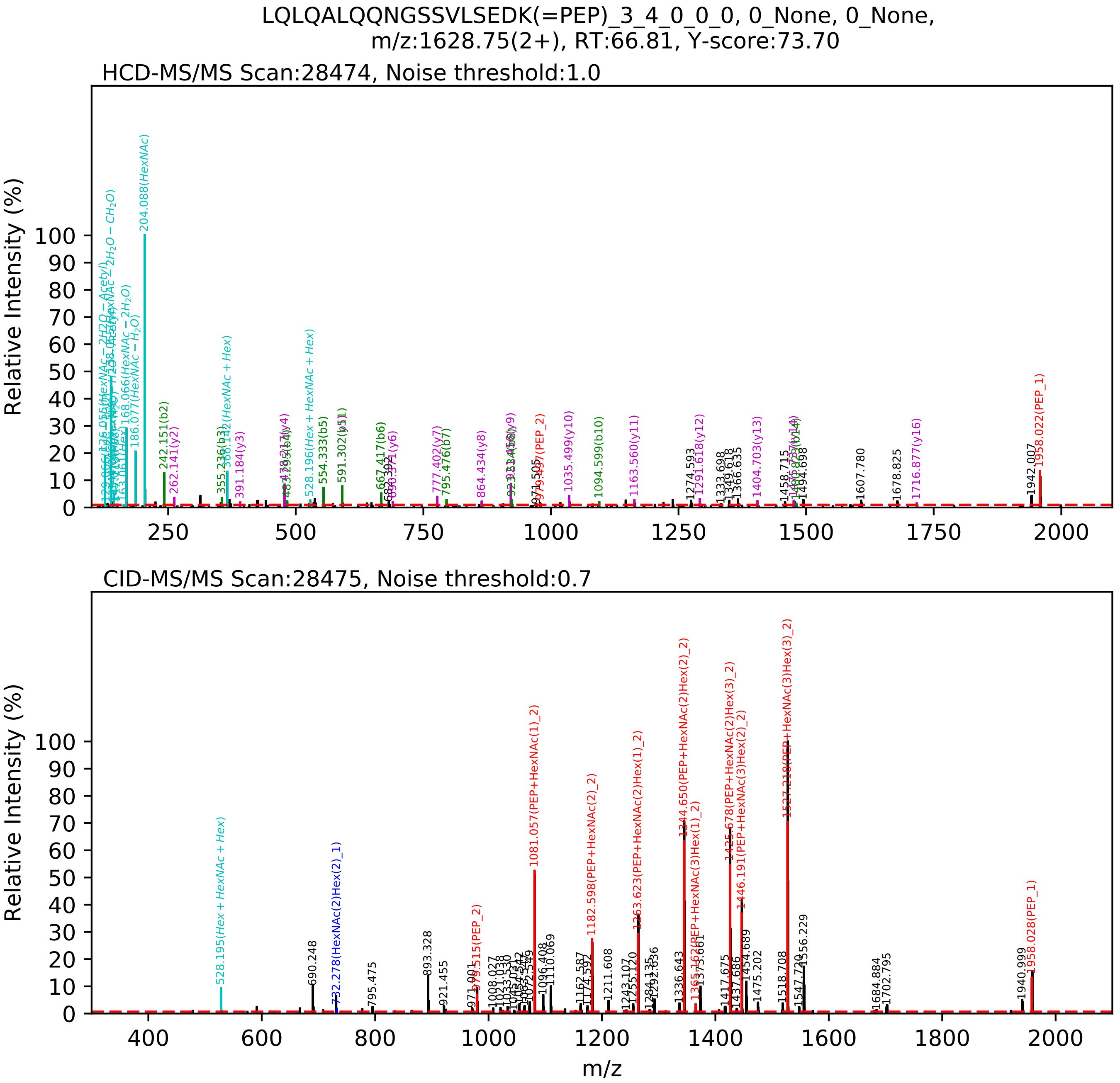

LQLQALQQNGSSVLSEDK(=PEP)_3_4_0_0_0, 0_None, 0_None,
m/z:1628.75(2+), RT:66.81, Y-score:73.70
HCD-MS/MS Scan:28474, Noise threshold:1.0
Relative Intensity (%)
204.088(HexNAc)
186.077(HexNAc − H2O)
168.066(HexNAc − 2H2O)
242.151(b2)
262.141(y2)
355.236(b3)
366.142(HexNAc + Hex)
391.184(y3)
528.196(Hex + HexNAc + Hex)
554.333(b5)
591.302(b11)
667.417(b6)
777.402(y7)
795.476(b7)
864.434(y8)
979.497(PEP_2)
1035.499(y10)
1094.599(b10)
1163.560(y11)
1274.593
1291.618(y12)
1333.698
1349.618
1366.635
1404.703(y13)
1458.715
1494.698
1607.780
1678.825
1716.877(y16)
1942.007
1958.022(PEP_1)
CID-MS/MS Scan:28475, Noise threshold:0.7
Relative Intensity (%)
m/z
528.195(Hex + HexNAc + Hex)
690.248
732.278(HexNAc(2)Hex(2)_1)
795.475
893.328
921.455
979.515(PEP_2)
1081.057(PEP+HexNAc(1)_2)
1110.069
1182.598(PEP+HexNAc(2)_2)
1211.608
1263.623(PEP+HexNAc(2)Hex(1)_2)
1344.650(PEP+HexNAc(2)Hex(2)_2)
1373.661
1425.678(PEP+HexNAc(2)Hex(3)_2)
1446.191(PEP+HexNAc(3)Hex(2)_2)
1454.689
1475.202
1518.708
1527.218(PEP+HexNAc(3)Hex(3)_2)
1556.229
1684.884
1702.795
1940.999
1958.028(PEP_1)

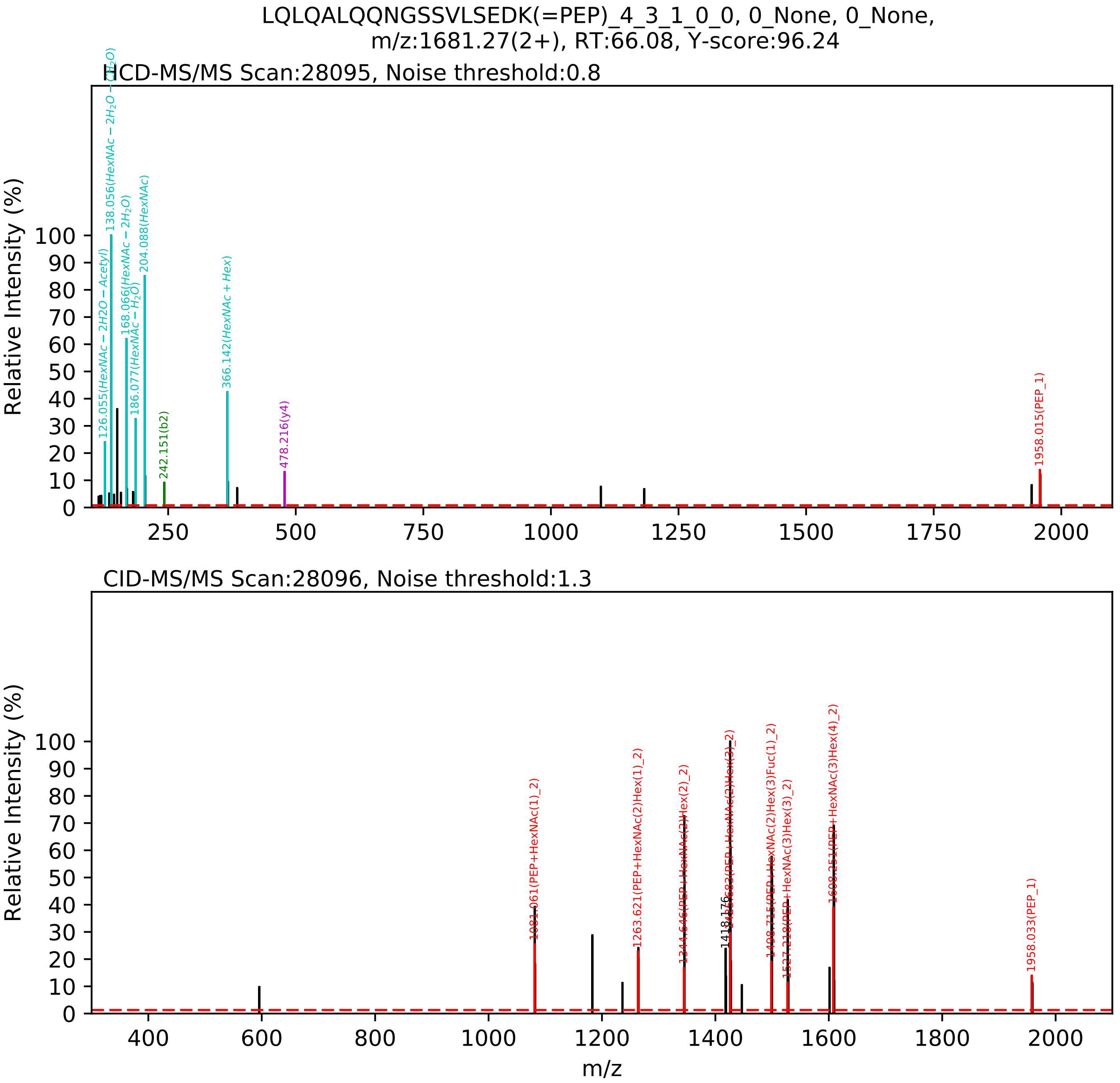

LQLQALQQNGSSVLSEDK(=PEP)_4_3_1_0_0, 0_None, 0_None,
m/z:1681.27(2+), RT:66.08, Y-score:96.24
HCD-MS/MS Scan:28095, Noise threshold:0.8
Relative Intensity (%)
126.055(HexNAc − 2H2O − Acetyl)
138.056(HexNAc − 2H2O − H2O)
168.066(HexNAc − 2H2O)
186.077(HexNAc − H2O)
204.088(HexNAc)
242.151(b2)
366.142(HexNAc + Hex)
478.216(y4)
1958.015(PEP_1)
CID-MS/MS Scan:28096, Noise threshold:1.3
Relative Intensity (%)
1081.061(PEP+HexNAc(1)_2)
1263.621(PEP+HexNAc(2)Hex(1)_2)
1418.176
1498.715(PEP+HexNAc(2)Hex(3)Fuc(1)_2)
1527.218(PEP+HexNAc(3)Hex(3)_2)
1608.251(PEP+HexNAc(3)Hex(4)_2)
1958.033(PEP_1)
m/z

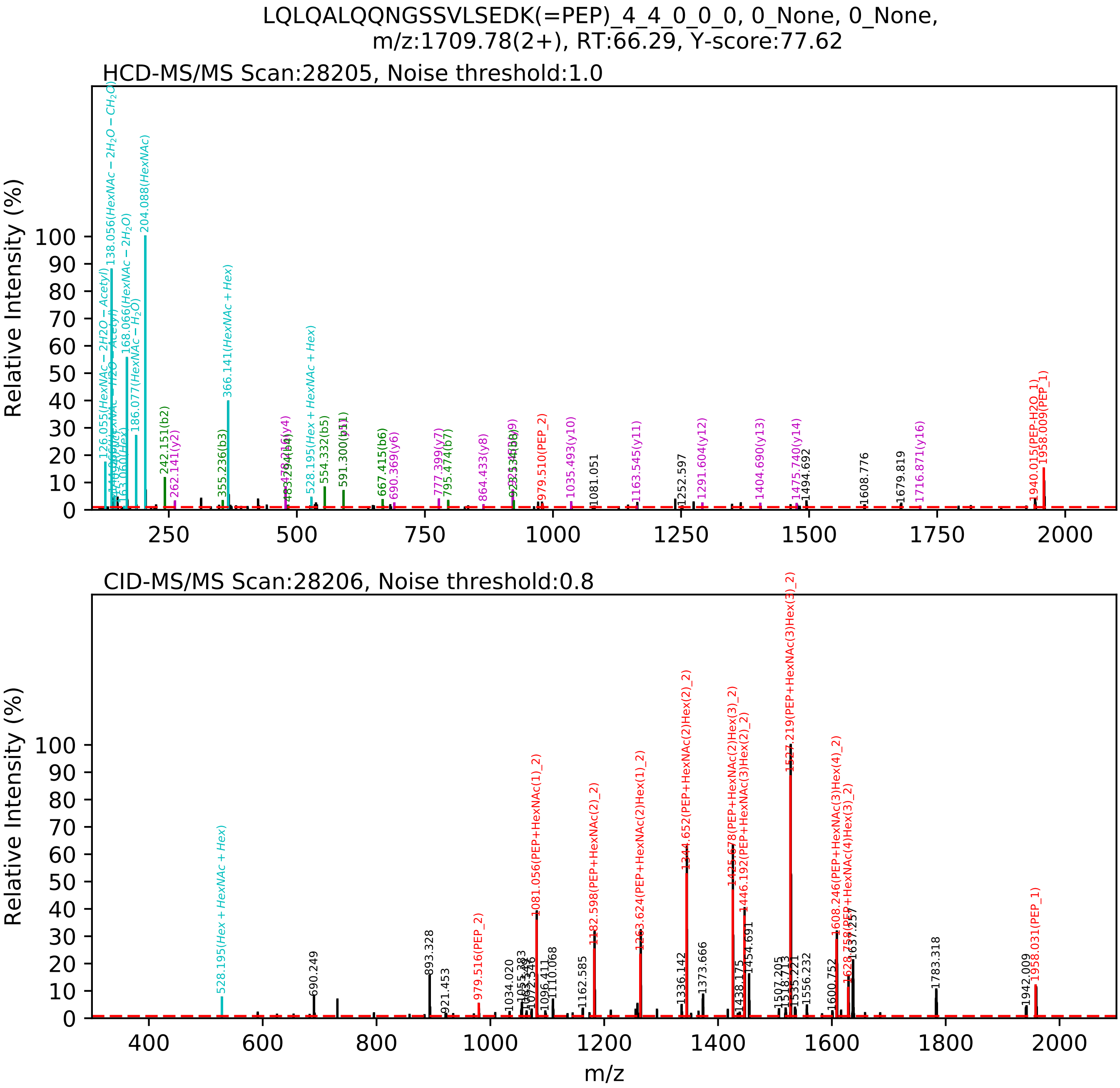
LQLQALQQNGSSVLSEDK(=PEP)_4_4_0_0_0, 0_None, 0_None,
m/z:1709.78(2+), RT:66.29, Y-score:77.62
HCD-MS/MS Scan:28205, Noise threshold:1.0
Relative Intensity (%)
204.088(HexNAc)
138.056(HexNAc − 2H2O − CH2O)
366.141(HexNAc + Hex)
168.066(HexNAc − 2H2O)
186.077(HexNAc − H2O)
126.055(HexNAc − 2H2O − Acetyl)
528.195(Hex + HexNAc + Hex)
242.151(b2)
262.141(y2)
355.236(b3)
554.332(b5)
591.300(y5)
667.415(b6)
690.369(y6)
777.399(y7)
795.474(b7)
864.433(y8)
979.510(PEP_2)
1035.493(y10)
1081.051
1163.545(y11)
1252.597
1291.604(y12)
1404.690(y13)
1475.740(y14)
1494.692
1608.776
1679.819
1716.871(y16)
1940.015(PEP-H2O_1)
1958.009(PEP_1)
CID-MS/MS Scan:28206, Noise threshold:0.8
Relative Intensity (%)
528.195(Hex + HexNAc + Hex)
690.249
893.328
921.453
979.516(PEP_2)
1034.020
1081.056(PEP+HexNAc(1)_2)
1096.411
1110.068
1162.585
1182.598(PEP+HexNAc(2)_2)
1263.624(PEP+HexNAc(2)Hex(1)_2)
1336.142
1344.652(PEP+HexNAc(2)Hex(2)_2)
1373.666
1425.678(PEP+HexNAc(2)Hex(3)_2)
1438.175
1446.192(PEP+HexNAc(3)Hex(2)_2)
1454.691
1507.205
1518.713
1527.219(PEP+HexNAc(3)Hex(3)_2)
1535.221
1556.232
1600.752
1608.246(PEP+HexNAc(3)Hex(4)_2)
1628.759(PEP+HexNAc(4)Hex(3)_2)
1637.257
1783.318
1942.009
1958.031(PEP_1)
m/z

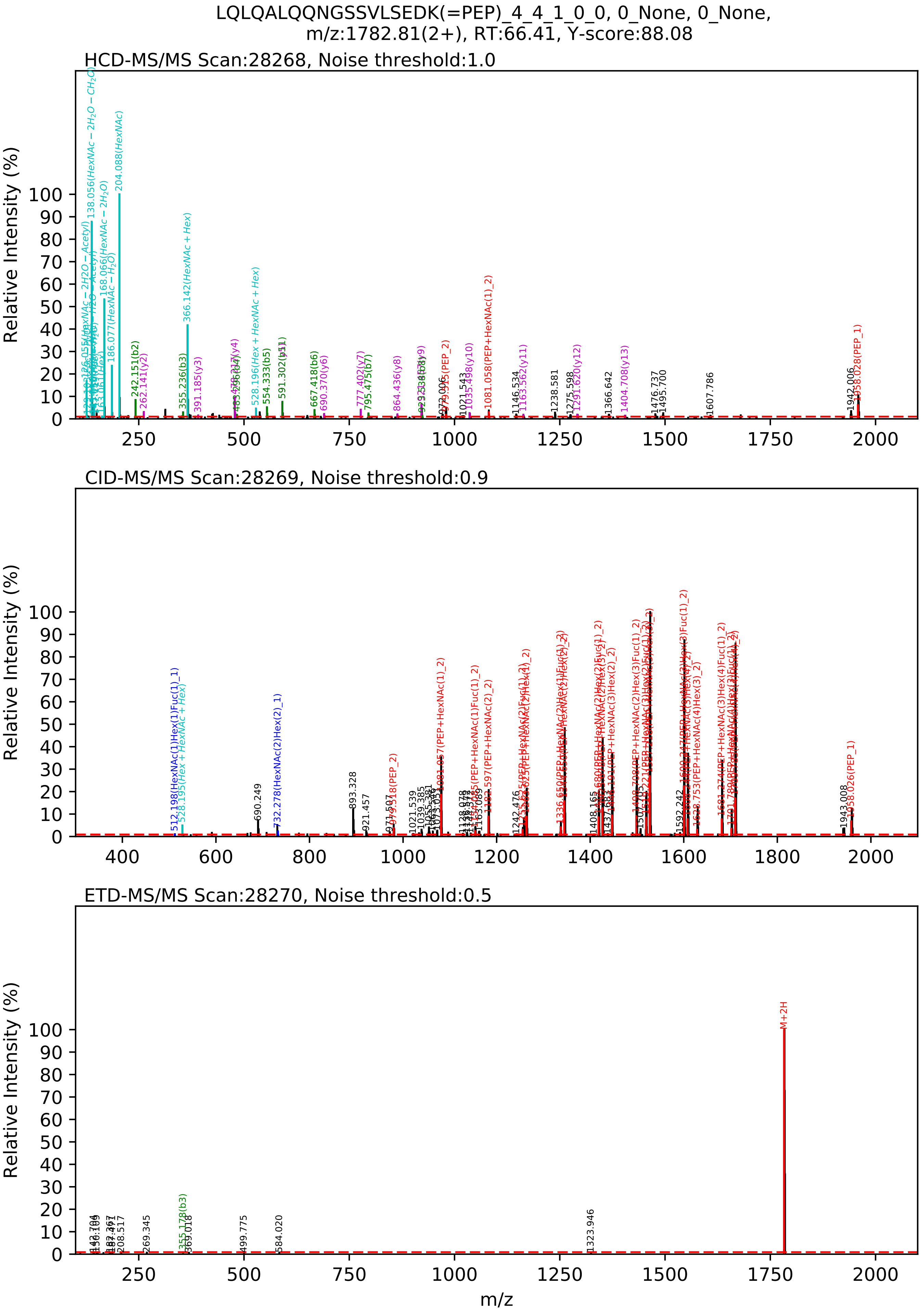
LQLQALQQNGSSVLSEDK(=PEP)_4_4_1_0_0, 0_None, 0_None,
m/z:1782.81(2+), RT:66.41, Y-score:88.08
HCD-MS/MS Scan:28268, Noise threshold:1.0
CID-MS/MS Scan:28269, Noise threshold:0.9
ETD-MS/MS Scan:28270, Noise threshold:0.5
Relative Intensity (%)
m/z
204.088(HexNAc)
366.142(HexNAc + Hex)
1081.058(PEP+HexNAc(1)_2)
1958.028(PEP_1)
1958.026(PEP_1)
M+2H

LQLQALQQNGSSVLSEDK(=PEP)_4_4_1_0_0, 0_None, 0_None,
m/z:1782.81(2+), RT:66.57, Y-score:85.74

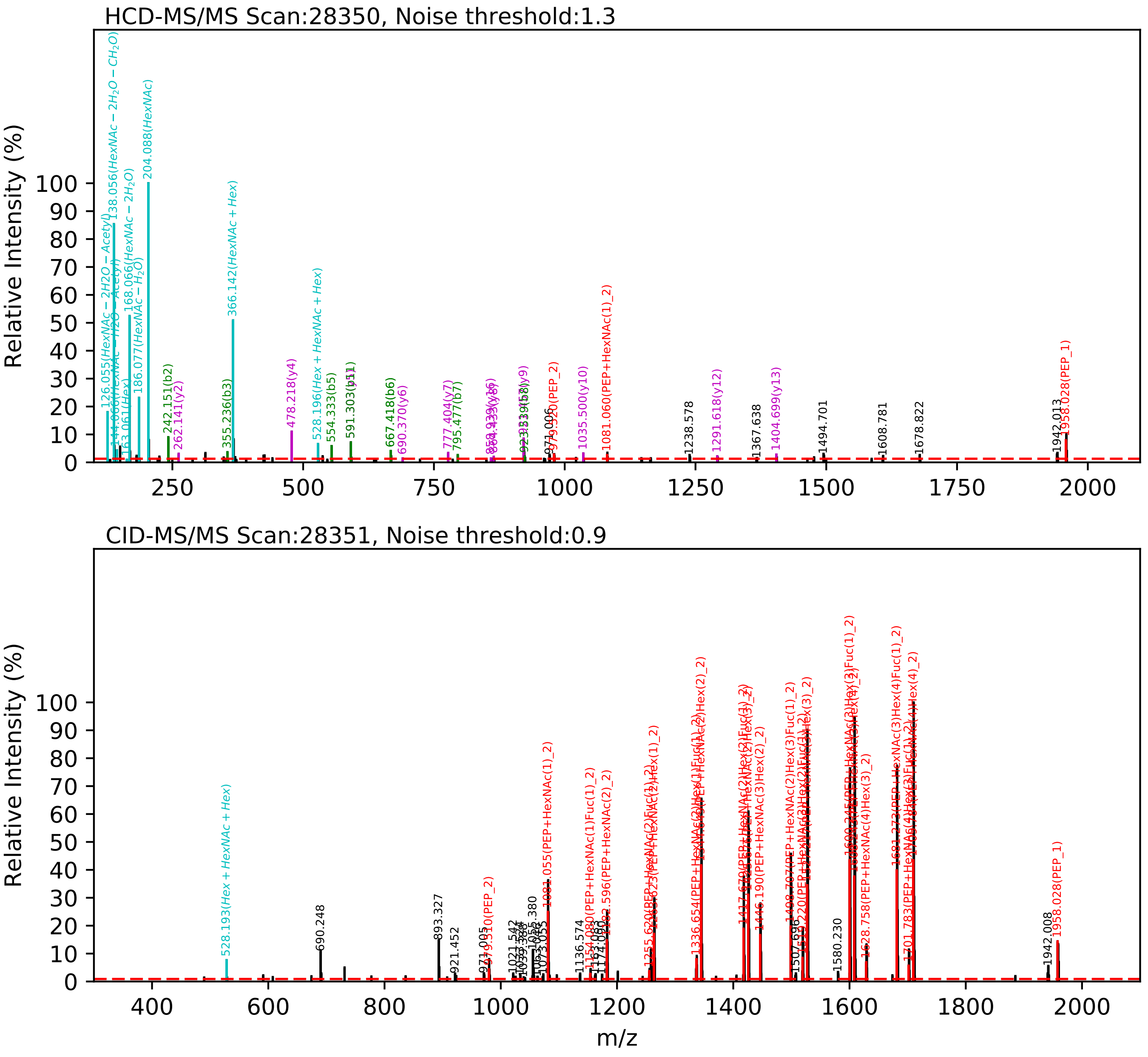

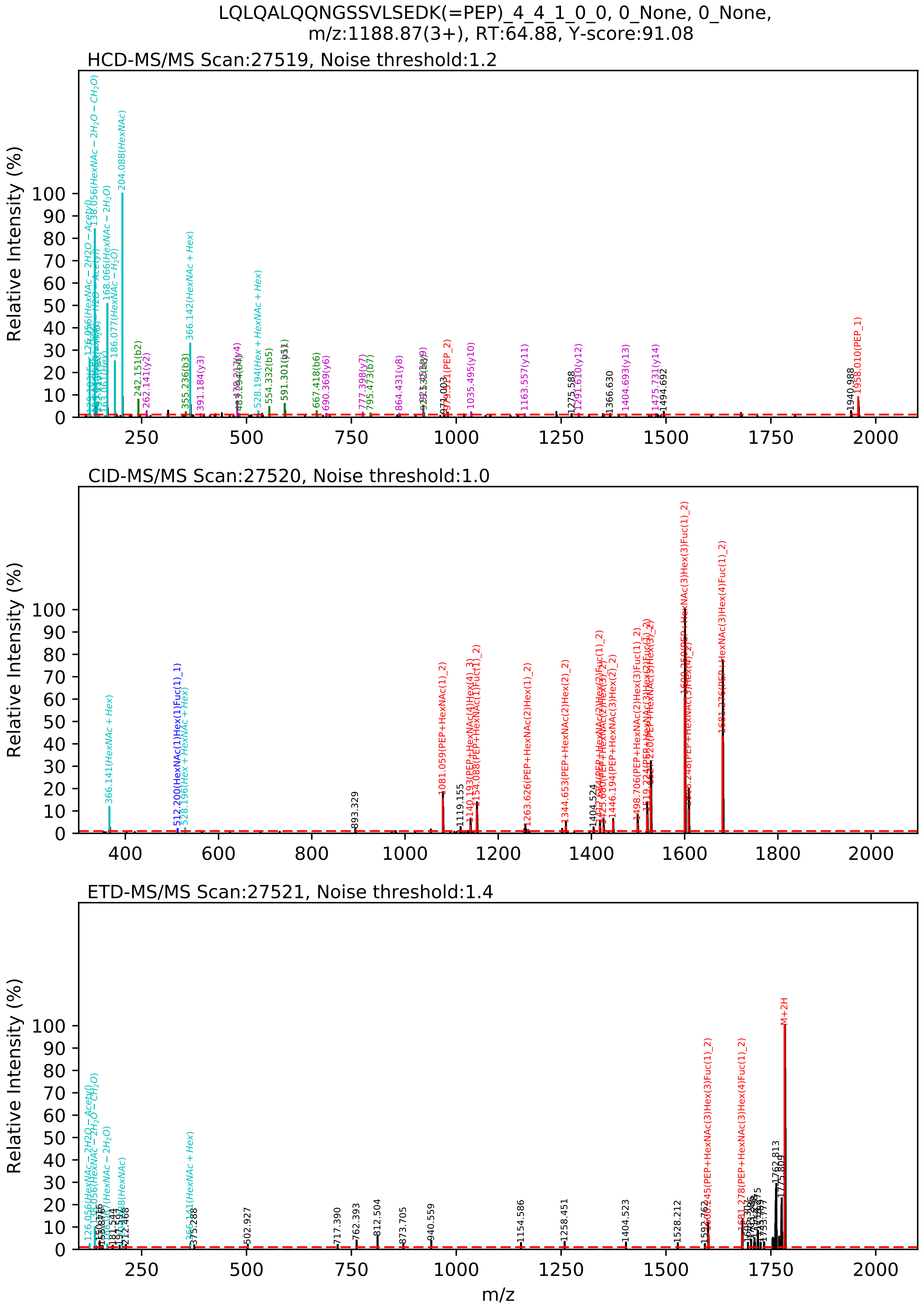

LQLQALQQNGSSVLSEDK(=PEP)_4_4_1_0_0, 0_None, 0_None,
m/z:1188.87(3+), RT:64.88, Y-score:91.08
HCD-MS/MS Scan:27519, Noise threshold:1.2
CID-MS/MS Scan:27520, Noise threshold:1.0
ETD-MS/MS Scan:27521, Noise threshold:1.4
Relative Intensity (%)
m/z
204.088(HexNAc)
366.142(HexNAc + Hex)
1958.010(PEP_1)
1600.250(PEP+HexNAc(3)Hex(3)Fuc(1)_2)
1681.276(PEP+HexNAc(3)Hex(4)Fuc(1)_2)
1081.059(PEP+HexNAc(1)_2)
512.200(HexNAc(1)Hex(1)Fuc(1)_1)
M+2H

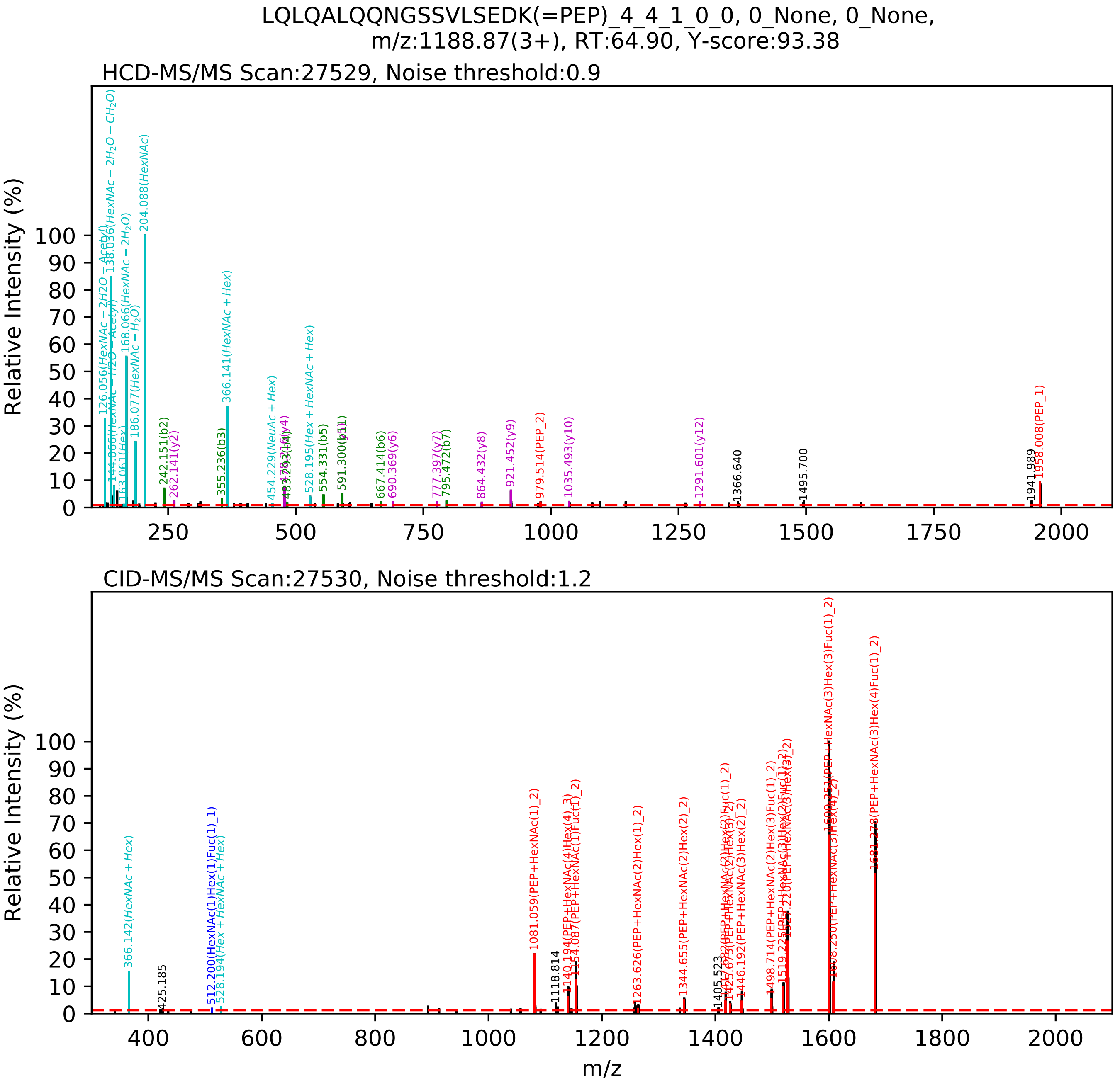
LQLQALQQNGSSVLSEDK(=PEP)_4_4_1_0_0, 0_None, 0_None,
m/z:1188.87(3+), RT:64.90, Y-score:93.38
HCD-MS/MS Scan:27529, Noise threshold:0.9
Relative Intensity (%)
204.088(HexNAc)
366.141(HexNAc + Hex)
242.151(b2)
262.141(y2)
355.236(b3)
554.331(b5)
667.414(b6)
690.369(y6)
777.397(y7)
795.472(b7)
864.432(y8)
921.452(y9)
979.514(PEP_2)
1035.493(y10)
1291.601(y12)
1366.640
1495.700
1941.989
1958.008(PEP_1)
CID-MS/MS Scan:27530, Noise threshold:1.2
Relative Intensity (%)
366.142(HexNAc + Hex)
425.185
512.200(HexNAc(1)Hex(1)Fuc(1)_1)
528.194(Hex + HexNAc + Hex)
1081.059(PEP+HexNAc(1)_2)
1118.814
1263.626(PEP+HexNAc(2)Hex(1)_2)
1344.655(PEP+HexNAc(2)Hex(2)_2)
1405.523
1446.192(PEP+HexNAc(3)Hex(2)_2)
1681.278(PEP+HexNAc(3)Hex(4)Fuc(1)_2)
m/z

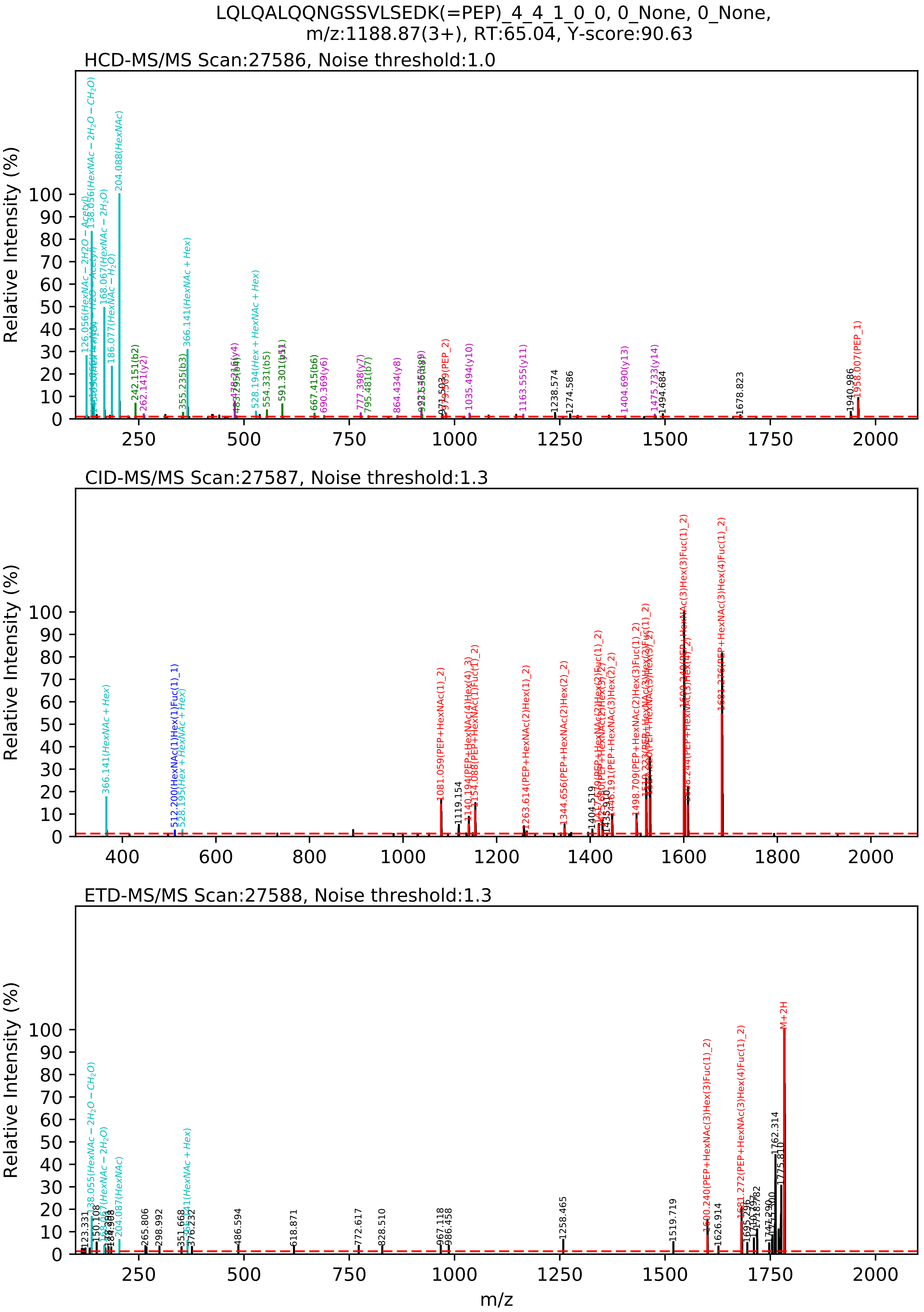

LQLQALQQNGSSVLSEDK(=PEP)_4_4_1_0_0, 0_None, 0_None,
m/z:1188.87(3+), RT:65.04, Y-score:90.63
HCD-MS/MS Scan:27586, Noise threshold:1.0
CID-MS/MS Scan:27587, Noise threshold:1.3
ETD-MS/MS Scan:27588, Noise threshold:1.3
Relative Intensity (%)
m/z
204.088(HexNAc)
366.141(HexNAc + Hex)
1958.007(PEP_1)
1600.240(PEP+HexNAc(3)Hex(3)Fuc(1)_2)
1681.272(PEP+HexNAc(3)Hex(4)Fuc(1)_2)
M+2H

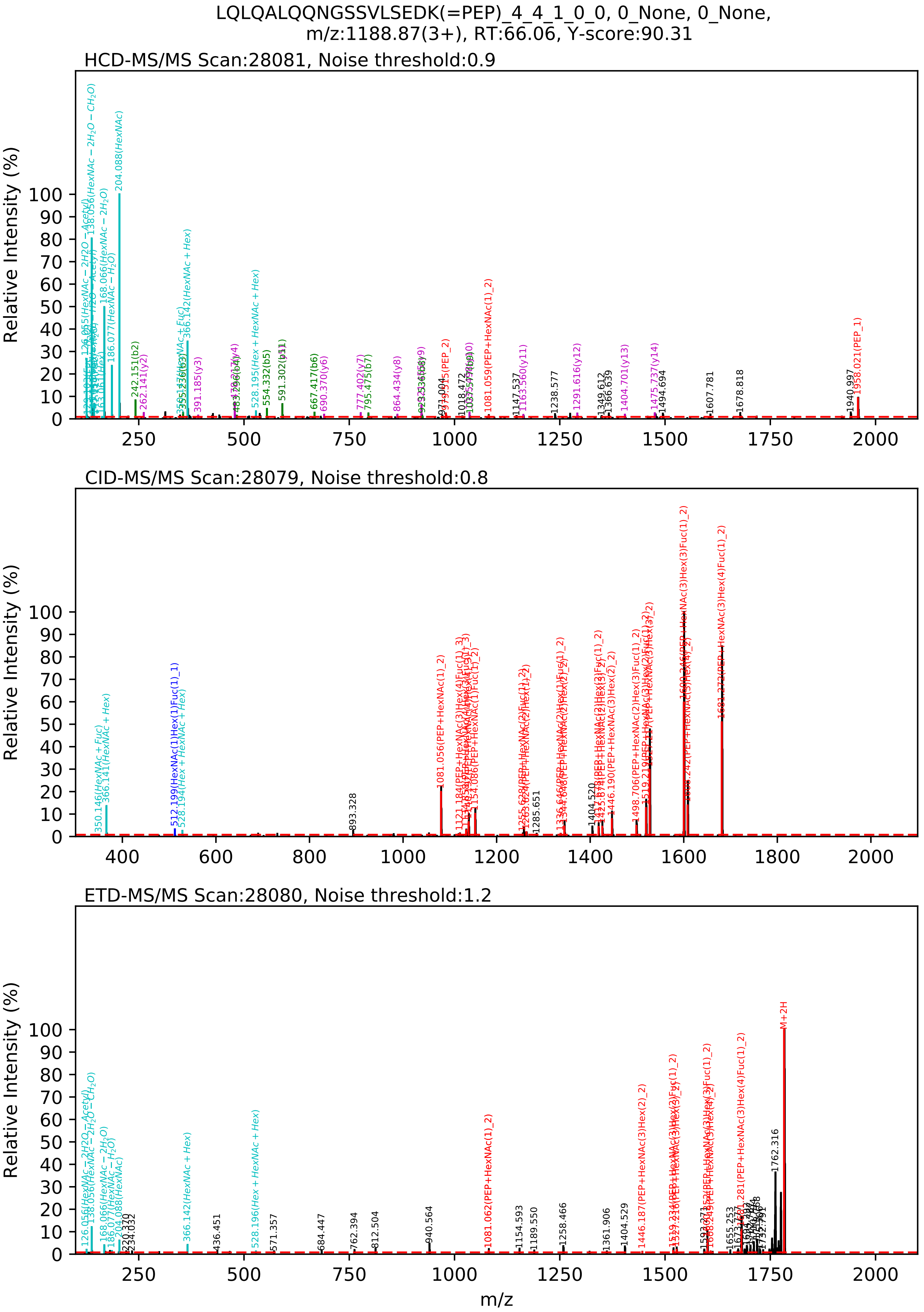

LQLQALQQNGSSVLSEDK(=PEP)_4_4_1_0_0, 0_None, 0_None,
m/z:1188.87(3+), RT:66.06, Y-score:90.31
HCD-MS/MS Scan:28081, Noise threshold:0.9
Relative Intensity (%)
204.088(HexNAc)
366.142(HexNAc + Hex)
528.195(Hex + HexNAc + Hex)
1081.059(PEP+HexNAc(1)_2)
1958.021(PEP_1)
CID-MS/MS Scan:28079, Noise threshold:0.8
Relative Intensity (%)
366.141(HexNAc + Hex)
512.199(HexNAc(1)Hex(1)Fuc(1)_1)
1081.056(PEP+HexNAc(1)_2)
1681.272(PEP+HexNAc(3)Hex(4)Fuc(1)_2)
ETD-MS/MS Scan:28080, Noise threshold:1.2
Relative Intensity (%)
M+2H
1762.316
1081.062(PEP+HexNAc(1)_2)
1446.187(PEP+HexNAc(3)Hex(2)_2)
m/z

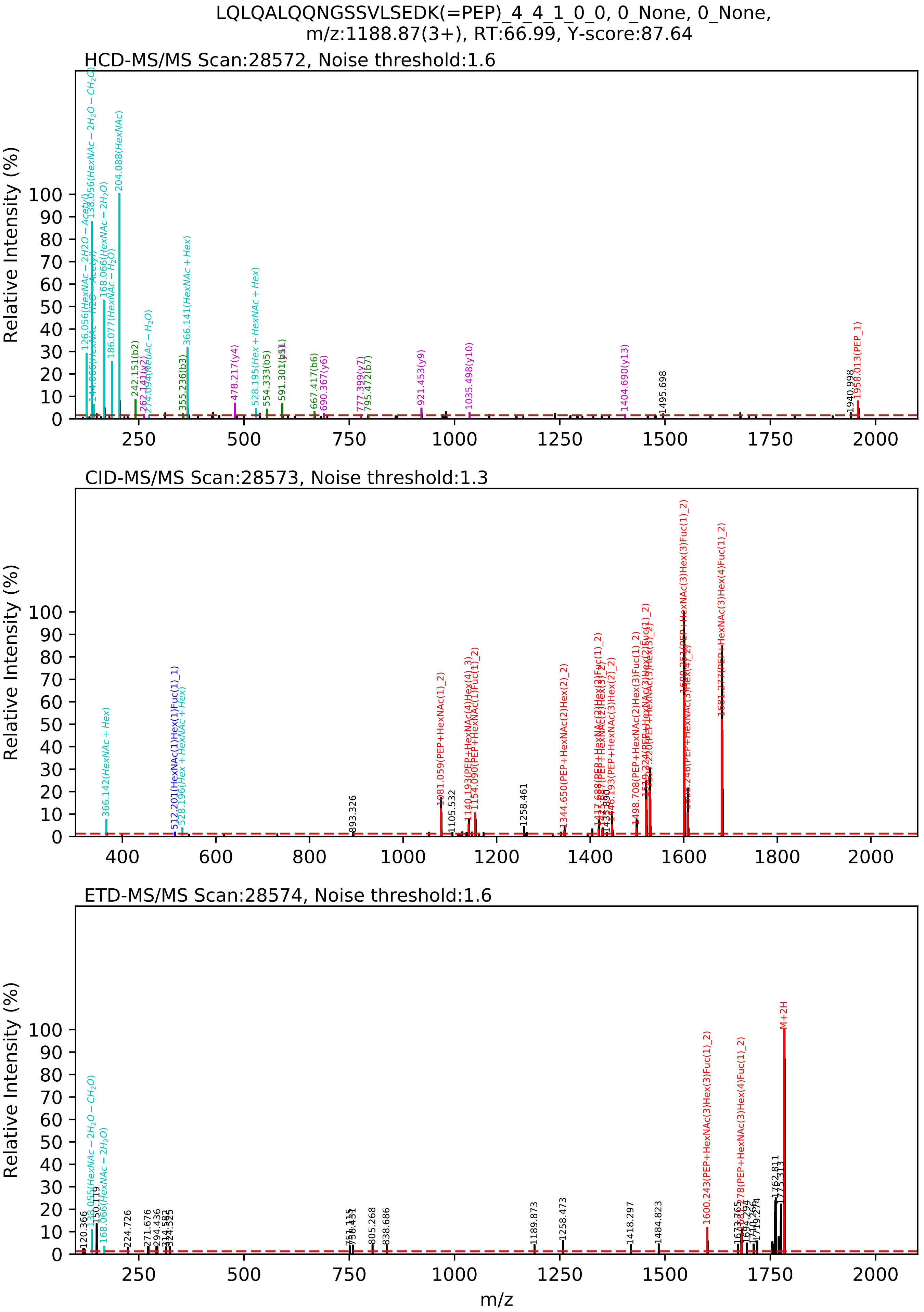

LQLQALQQNGSSVLSEDK(=PEP)_4_4_1_0_0, 0_None, 0_None,
m/z:1188.87(3+), RT:66.99, Y-score:87.64
HCD-MS/MS Scan:28572, Noise threshold:1.6
CID-MS/MS Scan:28573, Noise threshold:1.3
ETD-MS/MS Scan:28574, Noise threshold:1.6
Relative Intensity (%)
m/z
204.088(HexNAc)
366.141(HexNAc + Hex)
1958.013(PEP_1)
1600.251(PEP+HexNAc(3)Hex(3)Fuc(1)_2)
1681.277(PEP+HexNAc(3)Hex(4)Fuc(1)_2)
1081.059(PEP+HexNAc(1)_2)
1344.650(PEP+HexNAc(2)Hex(2)_2)
1600.243(PEP+HexNAc(3)Hex(3)Fuc(1)_2)
M+2H

LQLQALQQNGSSVLSEDK(=PEP)_4_4_1_0_0, 0_None, 0_None,
m/z:1188.87(3+), RT:66.75, Y-score:100.00

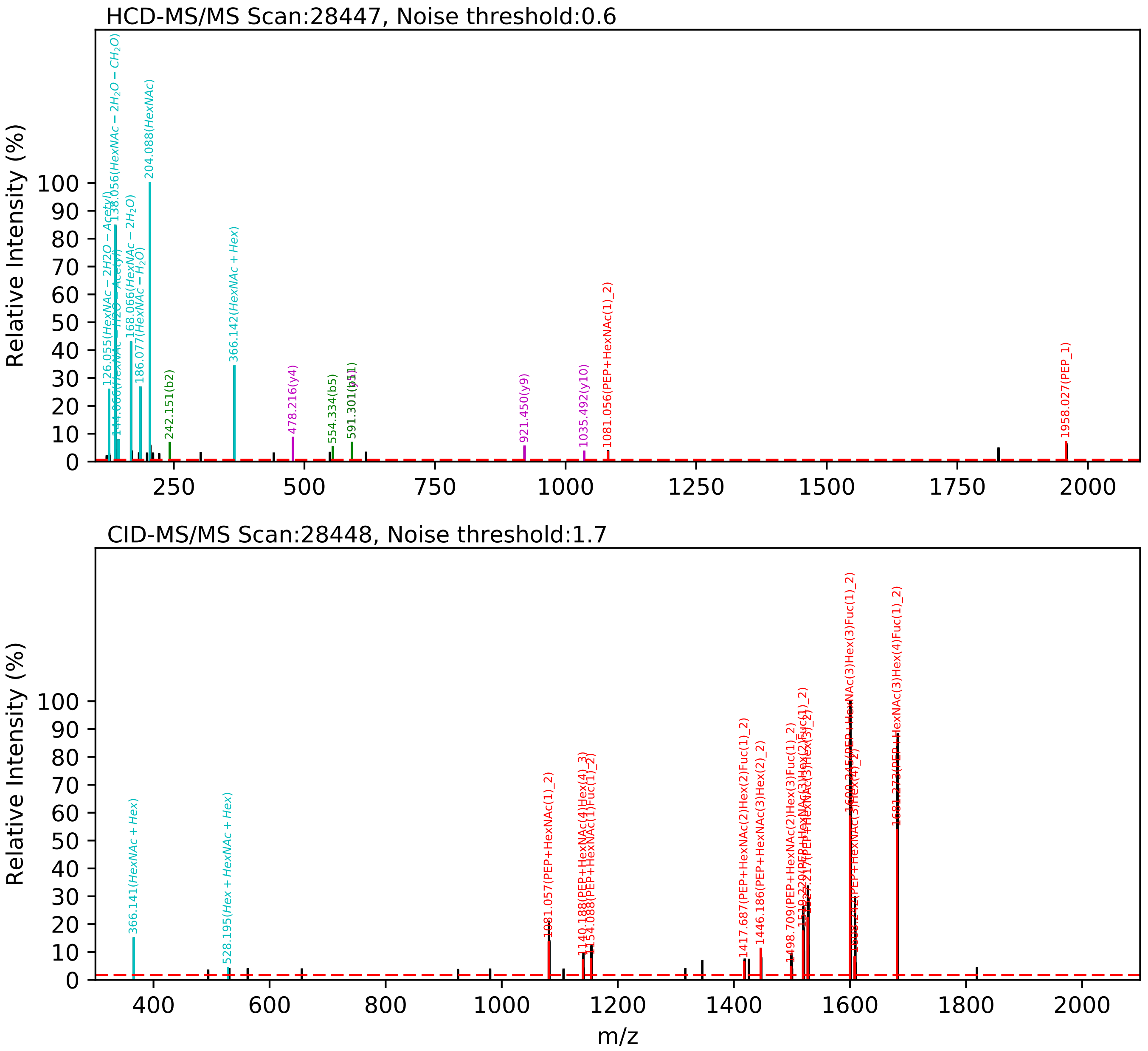

LQLQALQQNGSSVLSEDK(=PEP)_4_4_1_0_0, 0_None, 0_None,
m/z:1188.87(3+), RT:68.01, Y-score:85.25

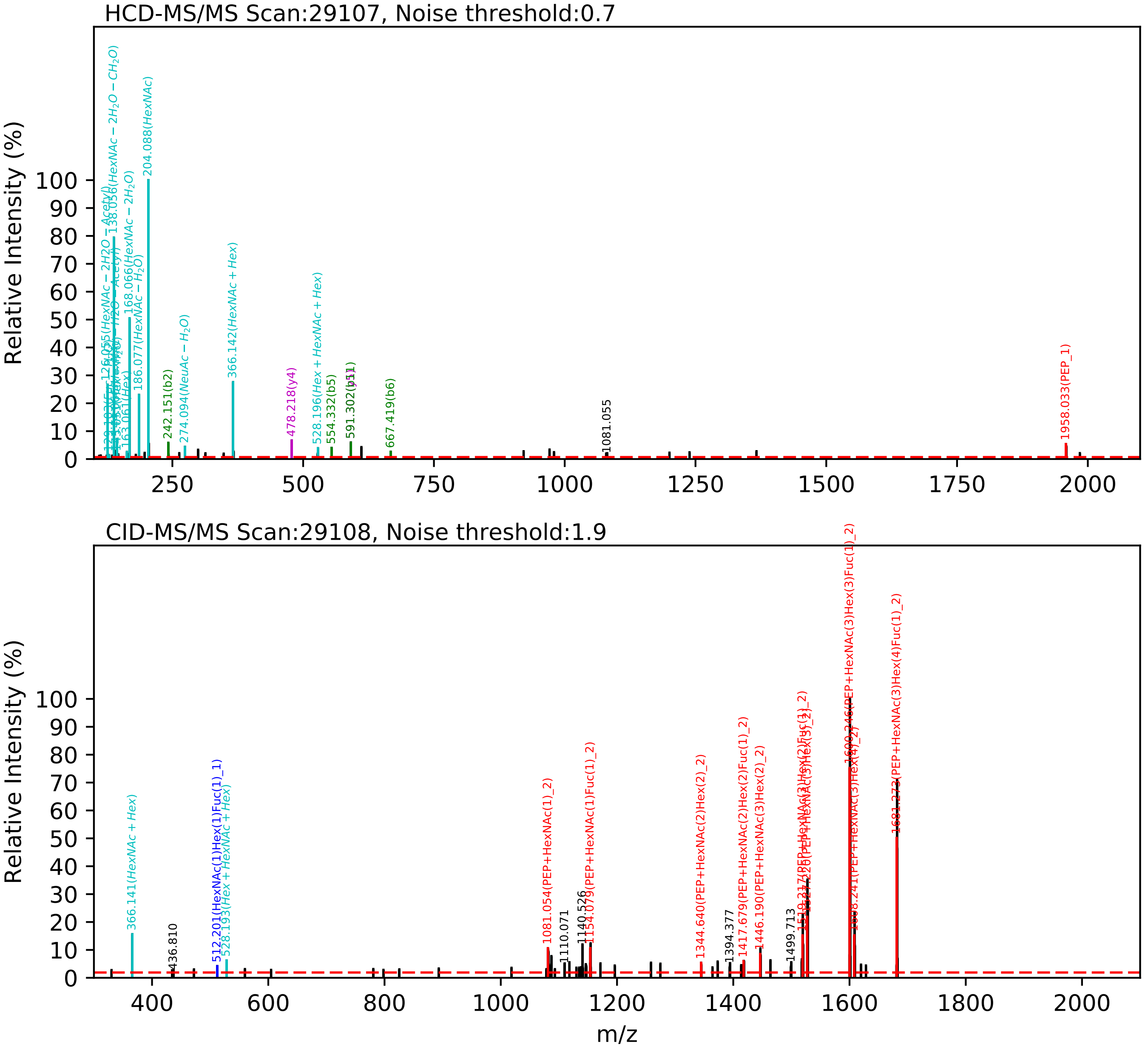

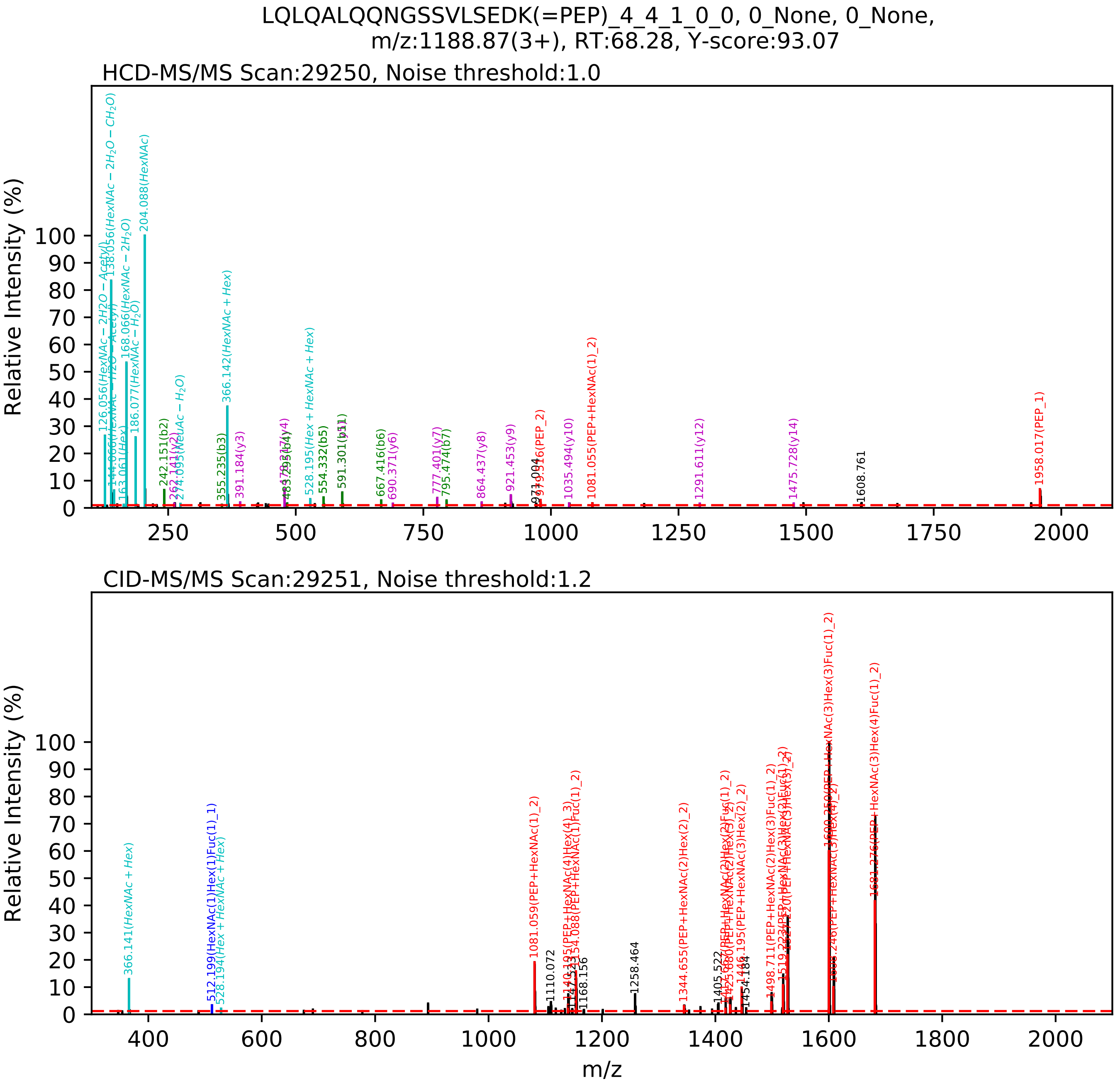

LQLQALQQNGSSVLSEDK(=PEP)_4_4_1_0_0, 0_None, 0_None,
m/z:1188.87(3+), RT:68.28, Y-score:93.07
HCD-MS/MS Scan:29250, Noise threshold:1.0
CID-MS/MS Scan:29251, Noise threshold:1.2
Relative Intensity (%)
m/z
204.088(HexNAc)
366.142(HexNAc+Hex)
1958.017(PEP_1)
1081.055(PEP+HexNAc(1)_2)
1081.059(PEP+HexNAc(1)_2)
1600.250(PEP+HexNAc(3)Hex(3)Fuc(1)_2)
1681.276(PEP+HexNAc(3)Hex(4)Fuc(1)_2)

LQLQALQQNGSSVLSEDK(=PEP)_4_4_1_0_0, 0_None, 0_None,
m/z:891.91(4+), RT:66.63, Y-score:89.97

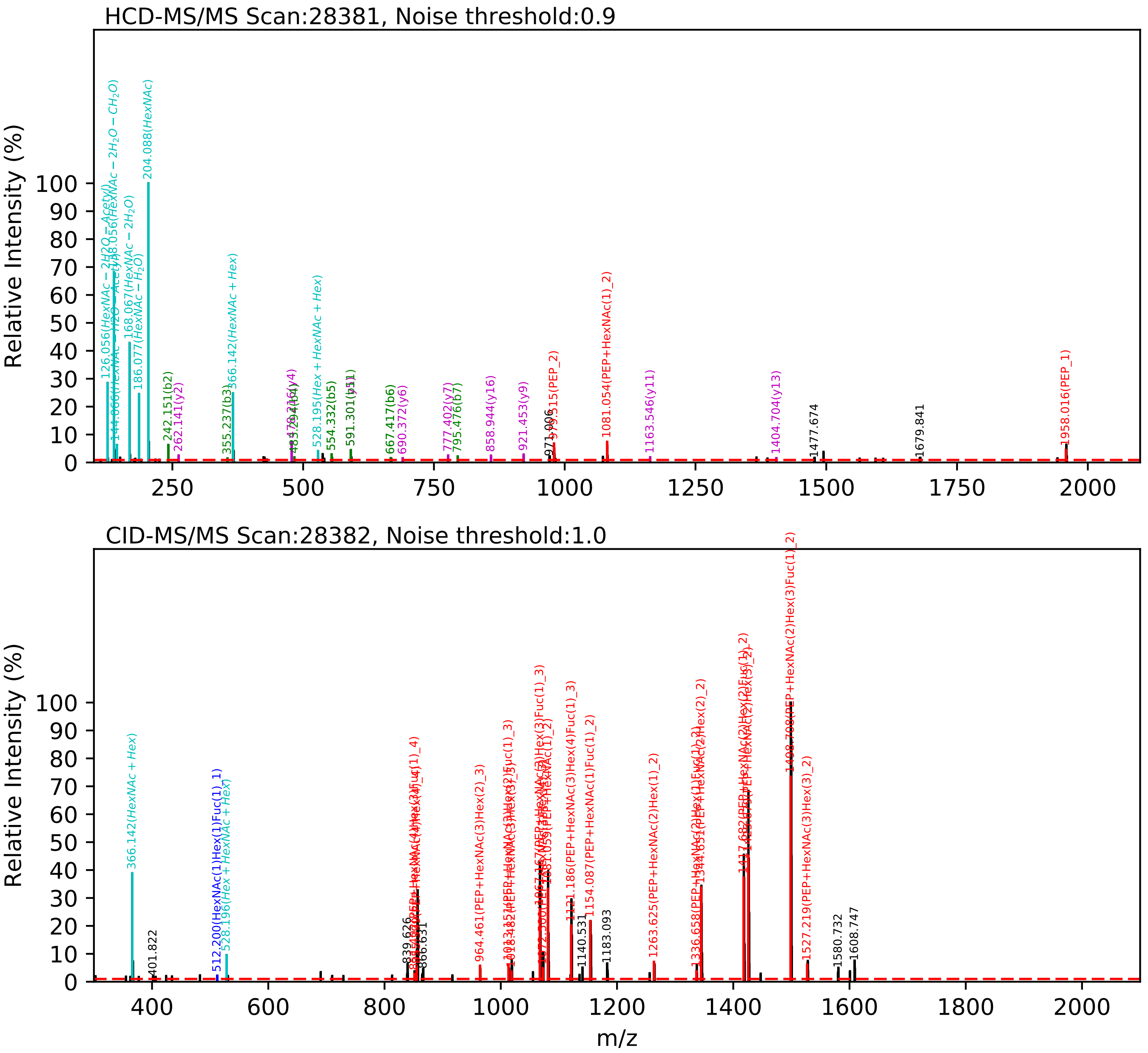

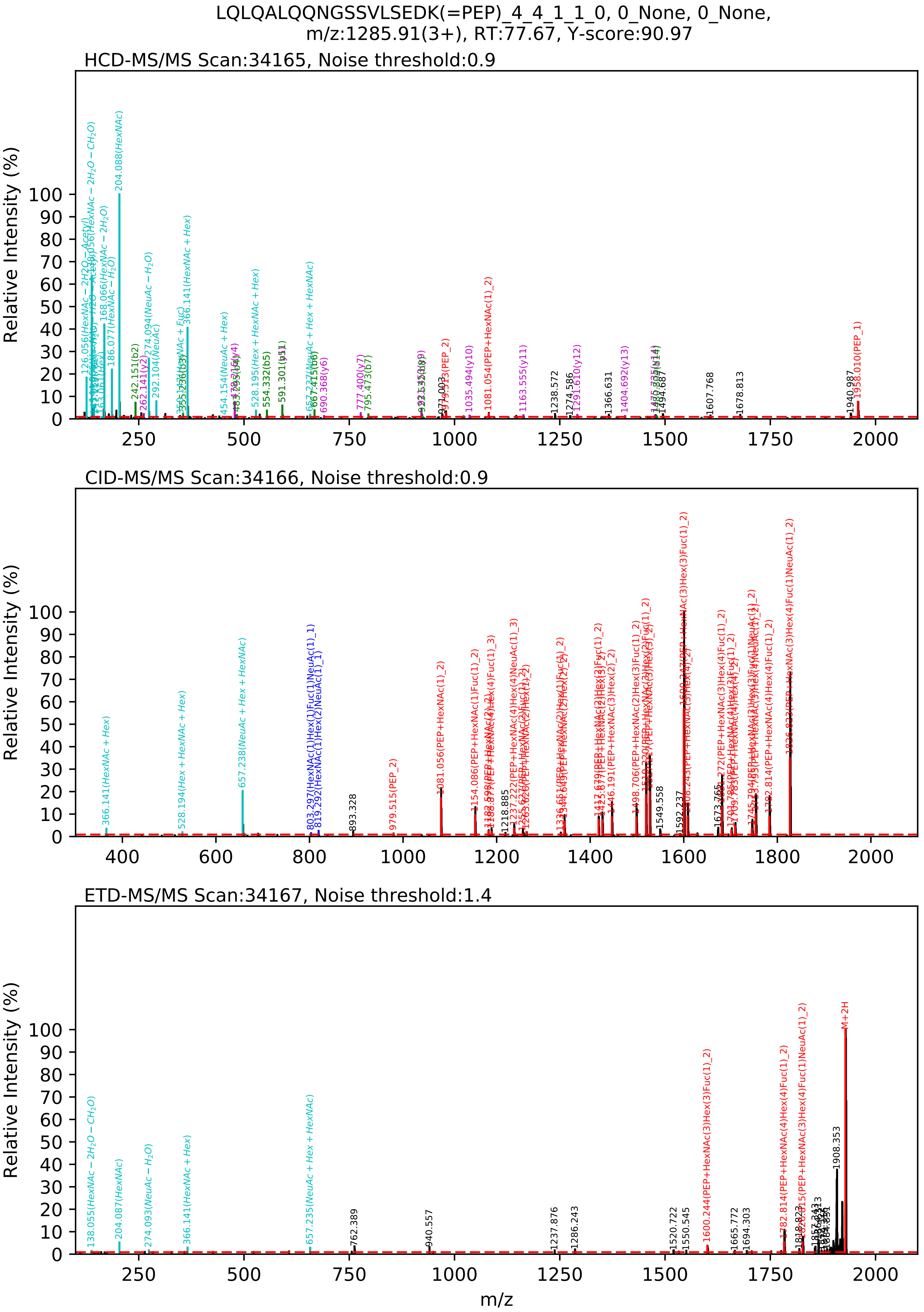

LQLQALQQNGSSVLSEDK(=PEP)_4_4_1_1_0, 0_None, 0_None,
m/z:1285.91(3+), RT:77.67, Y-score:90.97
HCD-MS/MS Scan:34165, Noise threshold:0.9
Relative Intensity (%)
204.088(HexNAc)
366.141(HexNAc + Hex)
274.094(NeuAc − H2O)
292.104(NeuAc)
168.066(HexNAc − 2H2O)
186.077(HexNAc − H2O)
242.151(b2)
262.141(y2)
454.154(NeuAc + Hex)
528.195(Hex + HexNAc + Hex)
554.332(b5)
591.301(y5)
690.368(y6)
777.400(y7)
795.473(b7)
971.003
979.513(PEP_2)
1035.494(y10)
1081.054(PEP+HexNAc(1)_2)
1163.555(y11)
1238.572
1274.586
1291.610(y12)
1366.631
1404.692(y13)
1494.687
1607.768
1678.813
1940.987
1958.010(PEP_1)
CID-MS/MS Scan:34166, Noise threshold:0.9
Relative Intensity (%)
366.141(HexNAc + Hex)
528.194(Hex + HexNAc + Hex)
657.238(NeuAc + Hex + HexNAc)
803.297(HexNAc(1)Hex(1)Fuc(1)NeuAc(1)_1)
819.292(HexNAc(1)Hex(2)NeuAc(1)_1)
893.328
979.515(PEP_2)
1081.056(PEP+HexNAc(1)_2)
1154.086(PEP+HexNAc(1)Fuc(1)_2)
1218.885
1237.222(PEP+HexNAc(4)Hex(4)NeuAc(1)_3)
1446.191(PEP+HexNAc(3)Hex(2)_2)
1498.706(PEP+HexNAc(2)Hex(3)Fuc(1)_2)
1549.558
1592.237
1600.247(PEP+HexNAc(3)Hex(3)Fuc(1)_2)
1673.765
1782.814(PEP+HexNAc(4)Hex(4)Fuc(1)_2)
1826.823(PEP+HexNAc(3)Hex(4)Fuc(1)NeuAc(1)_2)
ETD-MS/MS Scan:34167, Noise threshold:1.4
Relative Intensity (%)
138.055(HexNAc − 2H2O − CH2O)
204.087(HexNAc)
274.093(NeuAc − H2O)
366.141(HexNAc + Hex)
657.235(NeuAc + Hex + HexNAc)
762.389
940.557
1237.876
1286.243
1520.722
1550.545
1600.244(PEP+HexNAc(3)Hex(3)Fuc(1)_2)
1665.772
1694.303
1782.814(PEP+HexNAc(4)Hex(4)Fuc(1)_2)
1818.823
1908.353
M+2H
m/z

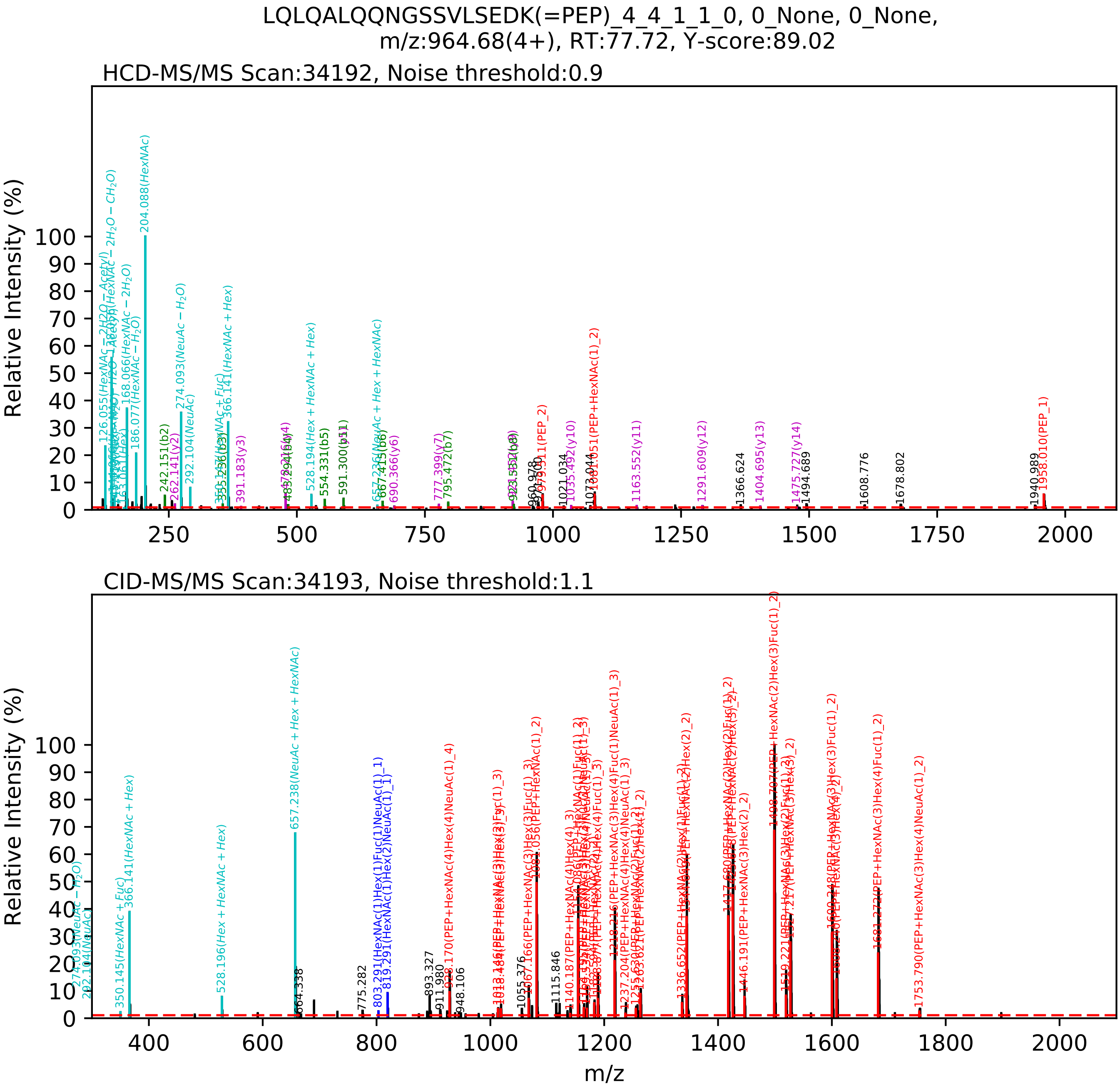
LQLQALQQNGSSVLSEDK(=PEP)_4_4_1_1_0, 0_None, 0_None,
m/z:964.68(4+), RT:77.72, Y-score:89.02
HCD-MS/MS Scan:34192, Noise threshold:0.9
Relative Intensity (%)
204.088(HexNAc)
1958.010(PEP_1)
1081.051(PEP+HexNAc(1)_2)
CID-MS/MS Scan:34193, Noise threshold:1.1
Relative Intensity (%)
657.238(NeuAc + Hex + HexNAc)
366.141(HexNAc + Hex)
1498.707(PEP+HexNAc(2)Hex(3)Fuc(1)_2)
m/z

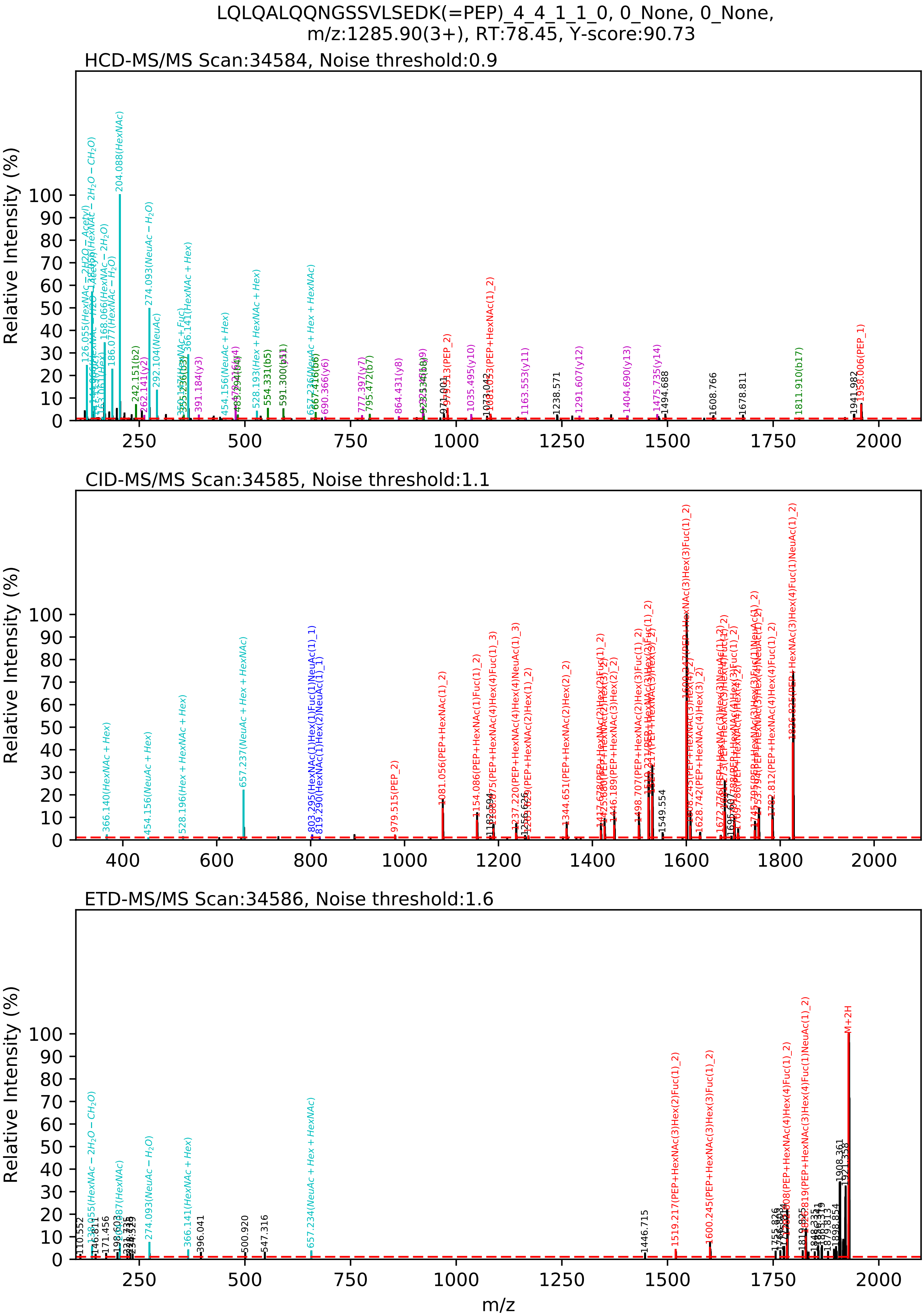
LQLQALQQNGSSVLSEDK(=PEP)_4_4_1_1_0, 0_None, 0_None,
m/z:1285.90(3+), RT:78.45, Y-score:90.73
HCD-MS/MS Scan:34584, Noise threshold:0.9
CID-MS/MS Scan:34585, Noise threshold:1.1
ETD-MS/MS Scan:34586, Noise threshold:1.6
Relative Intensity (%)
m/z
204.088(HexNAc)
274.093(NeuAc − H2O)
292.104(NeuAc)
366.141(HexNAc + Hex)
528.193(Hex + HexNAc + Hex)
657.237(NeuAc + Hex + HexNAc)
1958.006(PEP_1)
1081.053(PEP+HexNAc(1)_2)
1811.910(b17)
1475.735(y14)
1600.247(PEP+HexNAc(3)Hex(3)Fuc(1)_2)
1826.925(PEP+HexNAc(3)Hex(4)Fuc(1)NeuAc(1)_2)
1081.056(PEP+HexNAc(1)_2)
979.515(PEP_2)
803.295(HexNAc(1)Hex(1)Fuc(1)NeuAc(1)_1)
819.290(HexNAc(1)Hex(2)NeuAc(1)_1)
M+2H
1519.217(PEP+HexNAc(3)Hex(2)Fuc(1)_2)
1600.245(PEP+HexNAc(3)Hex(3)Fuc(1)_2)
1908.361
1921.358

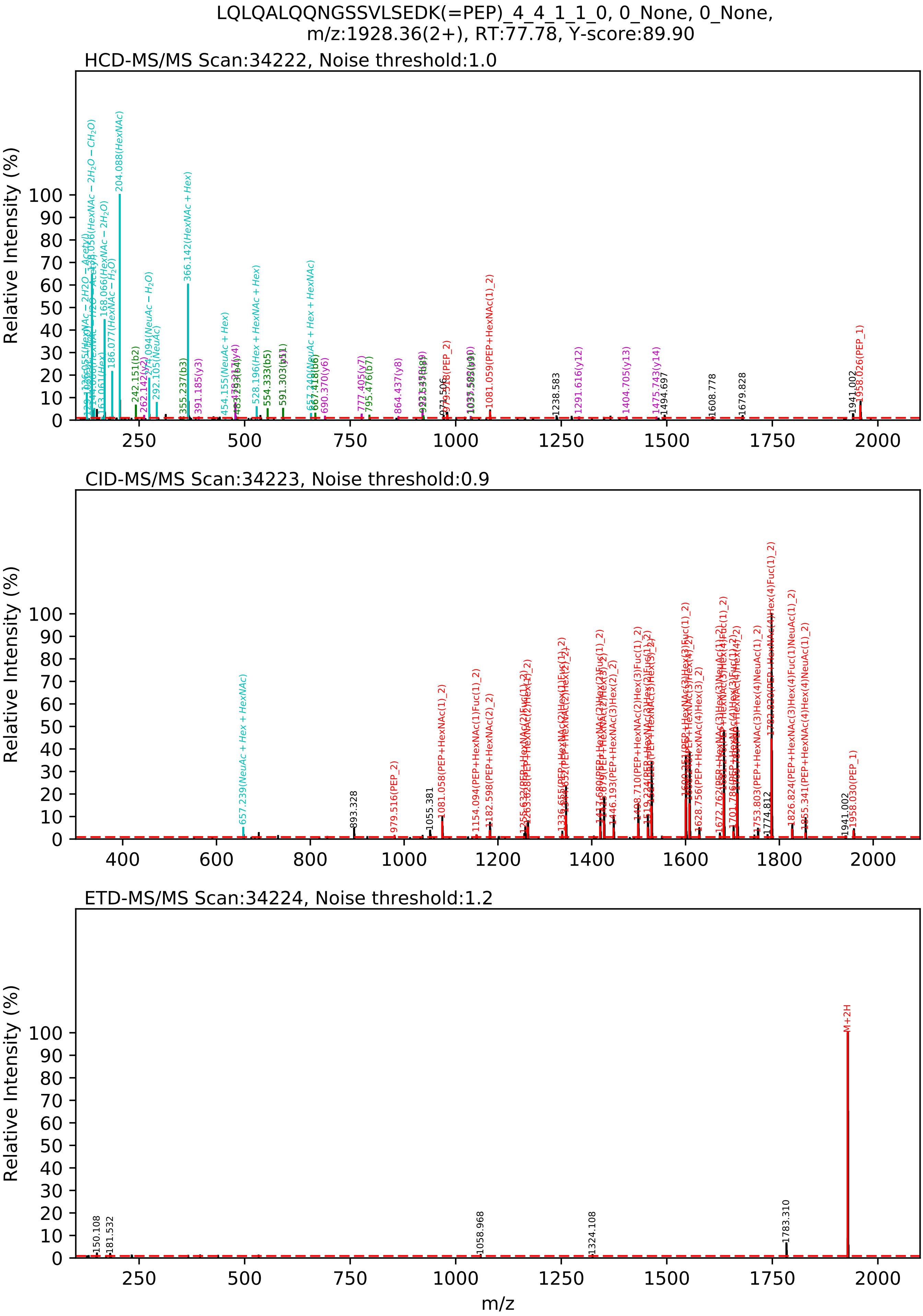
LQLQALQQNGSSVLSEDK(=PEP)_4_4_1_1_0, 0_None, 0_None,
m/z:1928.36(2+), RT:77.78, Y-score:89.90
HCD-MS/MS Scan:34222, Noise threshold:1.0
CID-MS/MS Scan:34223, Noise threshold:0.9
ETD-MS/MS Scan:34224, Noise threshold:1.2
Relative Intensity (%)
m/z

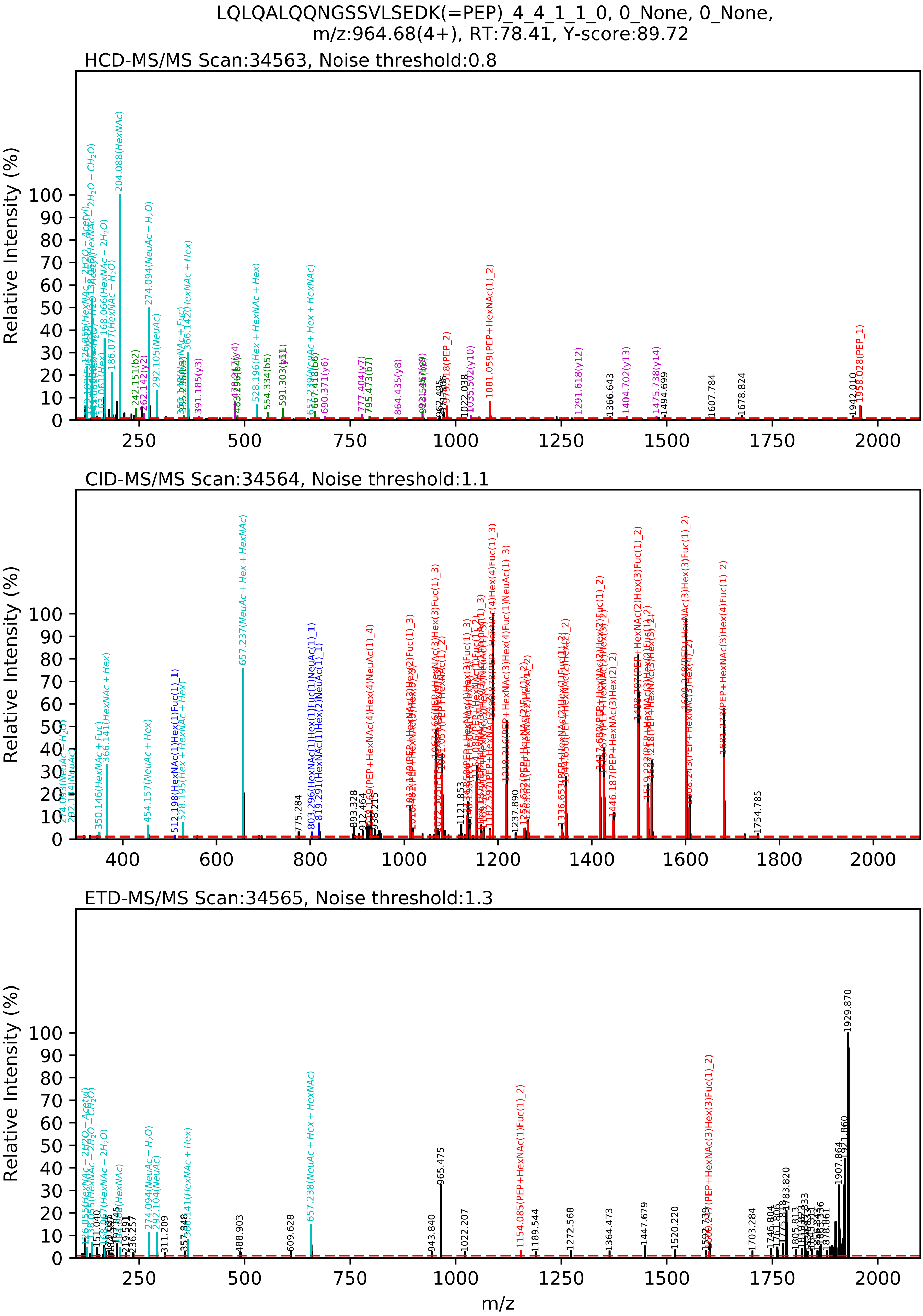

LQLQALQQNGSSVLSEDK(=PEP)_4_4_1_1_0, 0_None, 0_None,
m/z:964.68(4+), RT:78.41, Y-score:89.72
HCD-MS/MS Scan:34563, Noise threshold:0.8
CID-MS/MS Scan:34564, Noise threshold:1.1
ETD-MS/MS Scan:34565, Noise threshold:1.3
Relative Intensity (%)
m/z

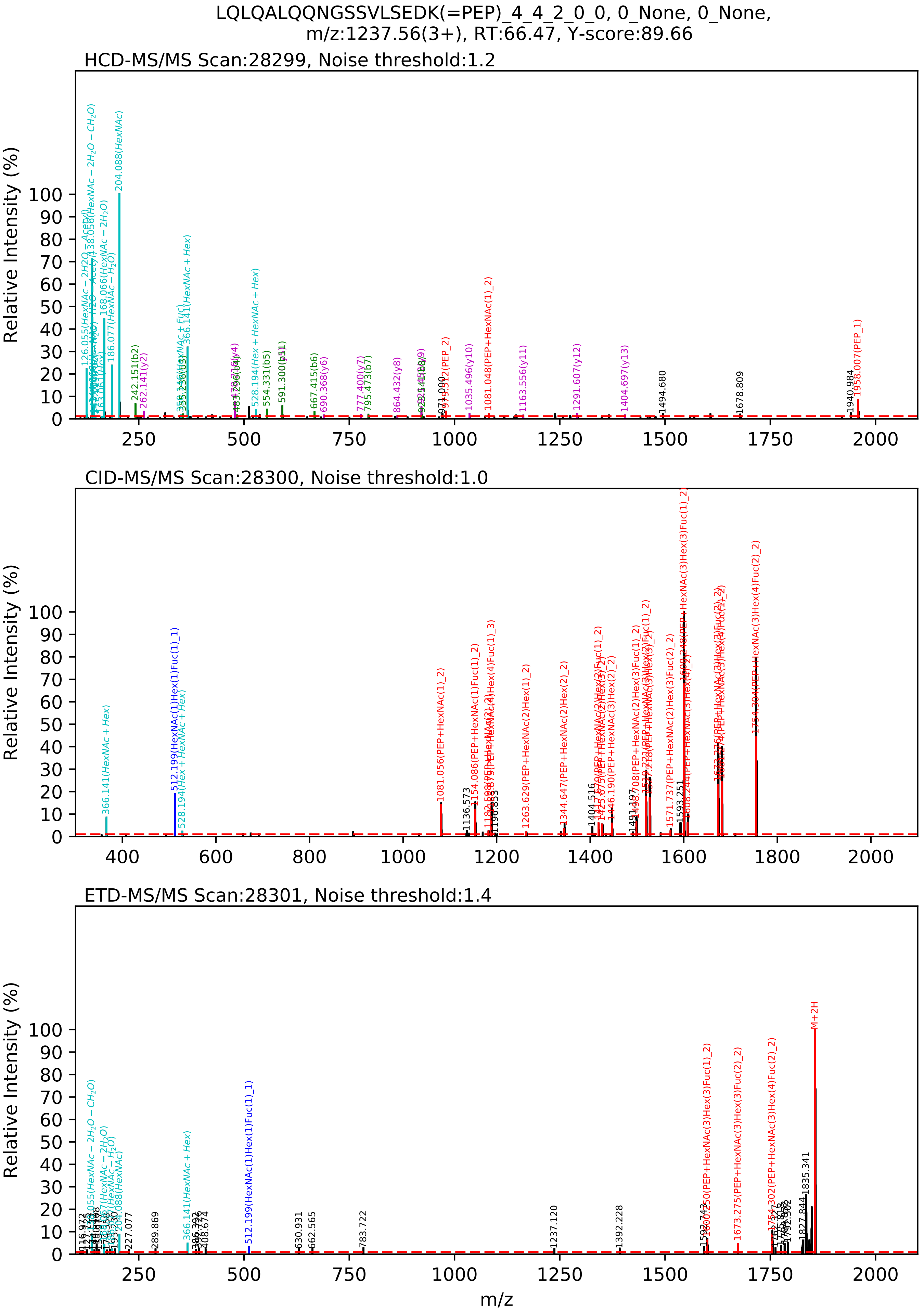
LQLQALQQNGSSVLSEDK(=PEP)_4_4_2_0_0, 0_None, 0_None,
m/z:1237.56(3+), RT:66.47, Y-score:89.66
HCD-MS/MS Scan:28299, Noise threshold:1.2
CID-MS/MS Scan:28300, Noise threshold:1.0
ETD-MS/MS Scan:28301, Noise threshold:1.4
Relative Intensity (%)
m/z

LQLQALQQNGSSVLSEDK(=PEP)_4_4_2_0_0, 0_None, 0_None,
m/z:1237.56(3+), RT:66.60, Y-score:98.15

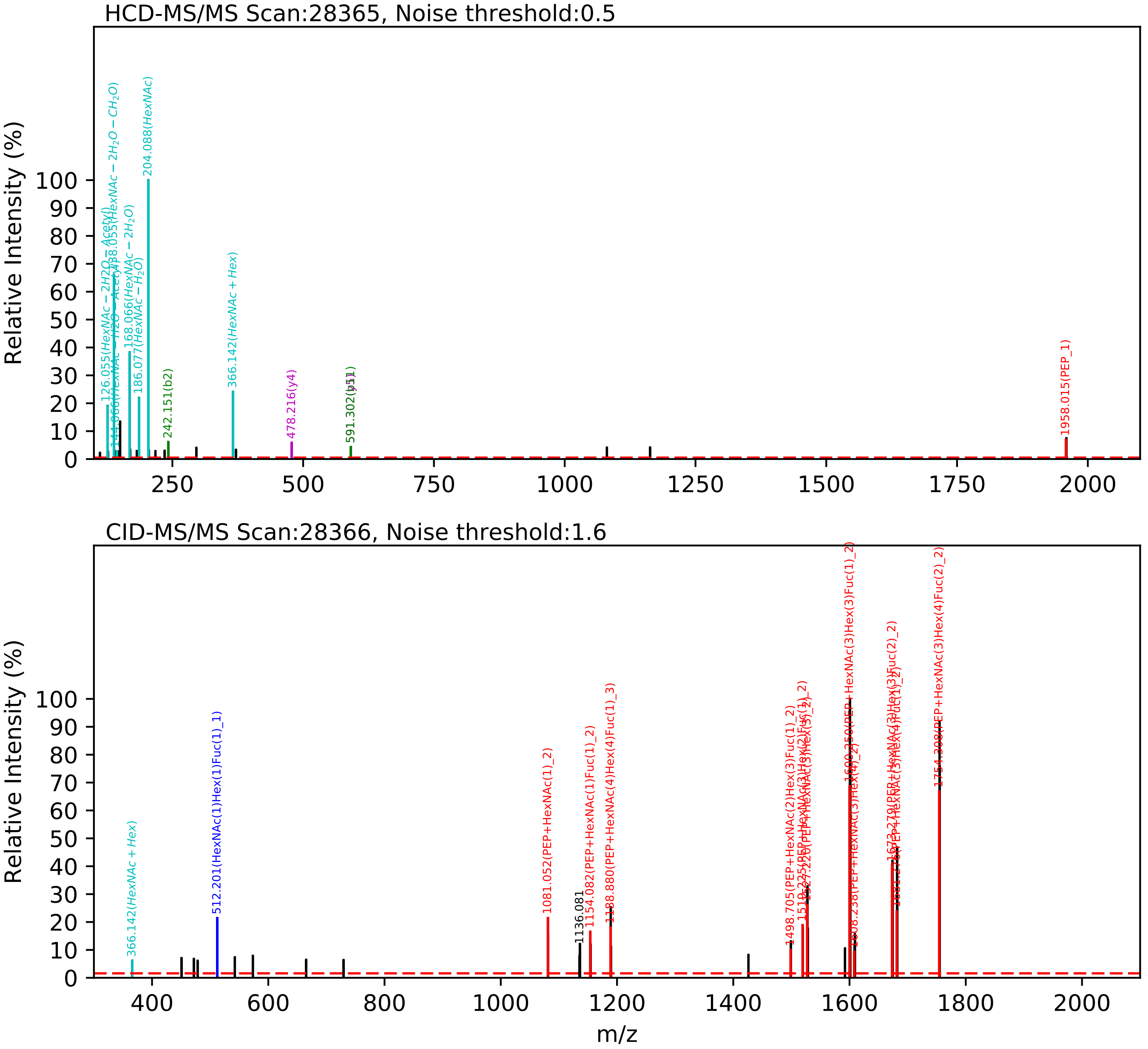

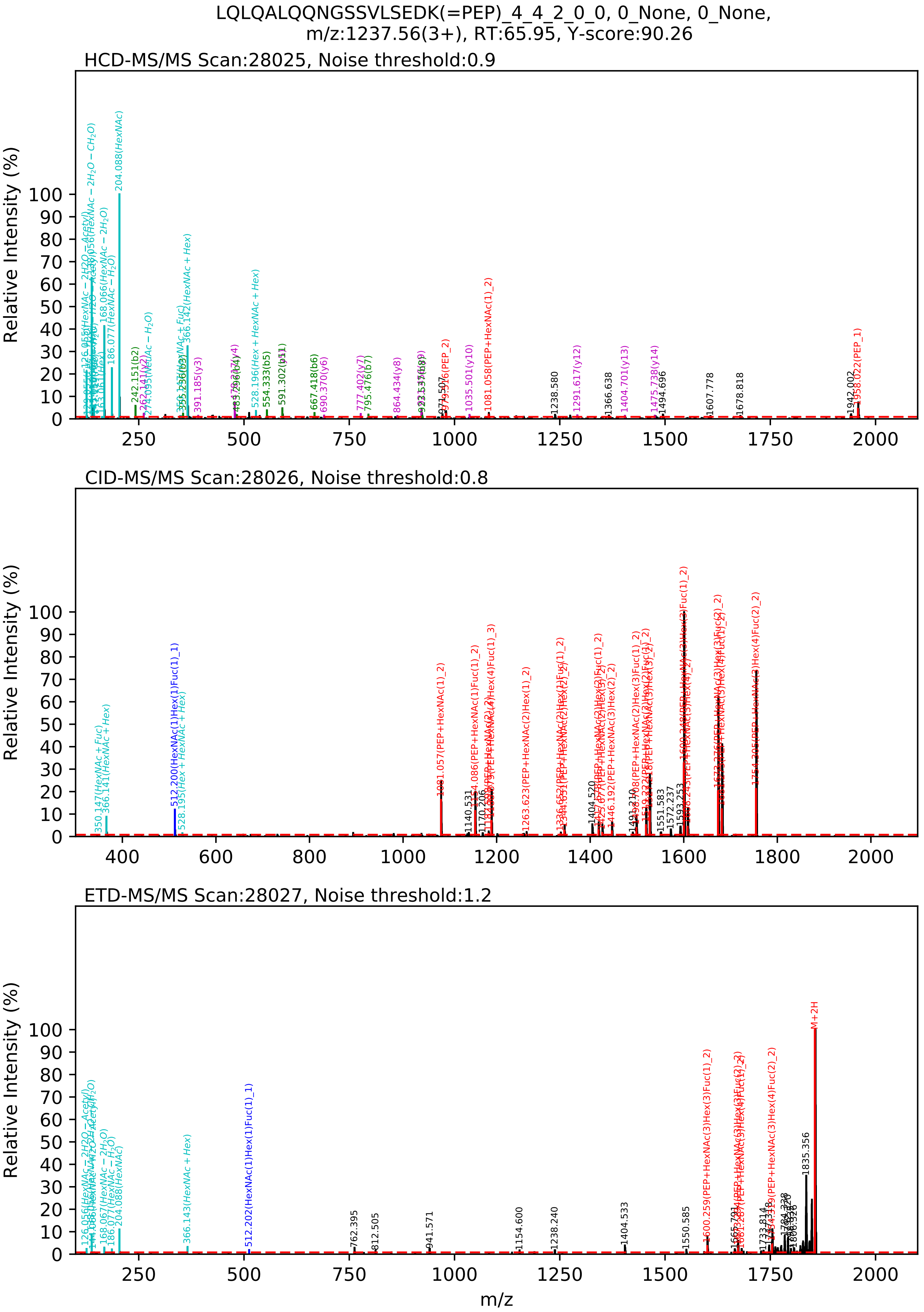

LQLQALQQNGSSVLSEDK(=PEP)_4_4_2_0_0, 0_None, 0_None,
m/z:1237.56(3+), RT:65.95, Y-score:90.26
HCD-MS/MS Scan:28025, Noise threshold:0.9
CID-MS/MS Scan:28026, Noise threshold:0.8
ETD-MS/MS Scan:28027, Noise threshold:1.2
Relative Intensity (%)
m/z

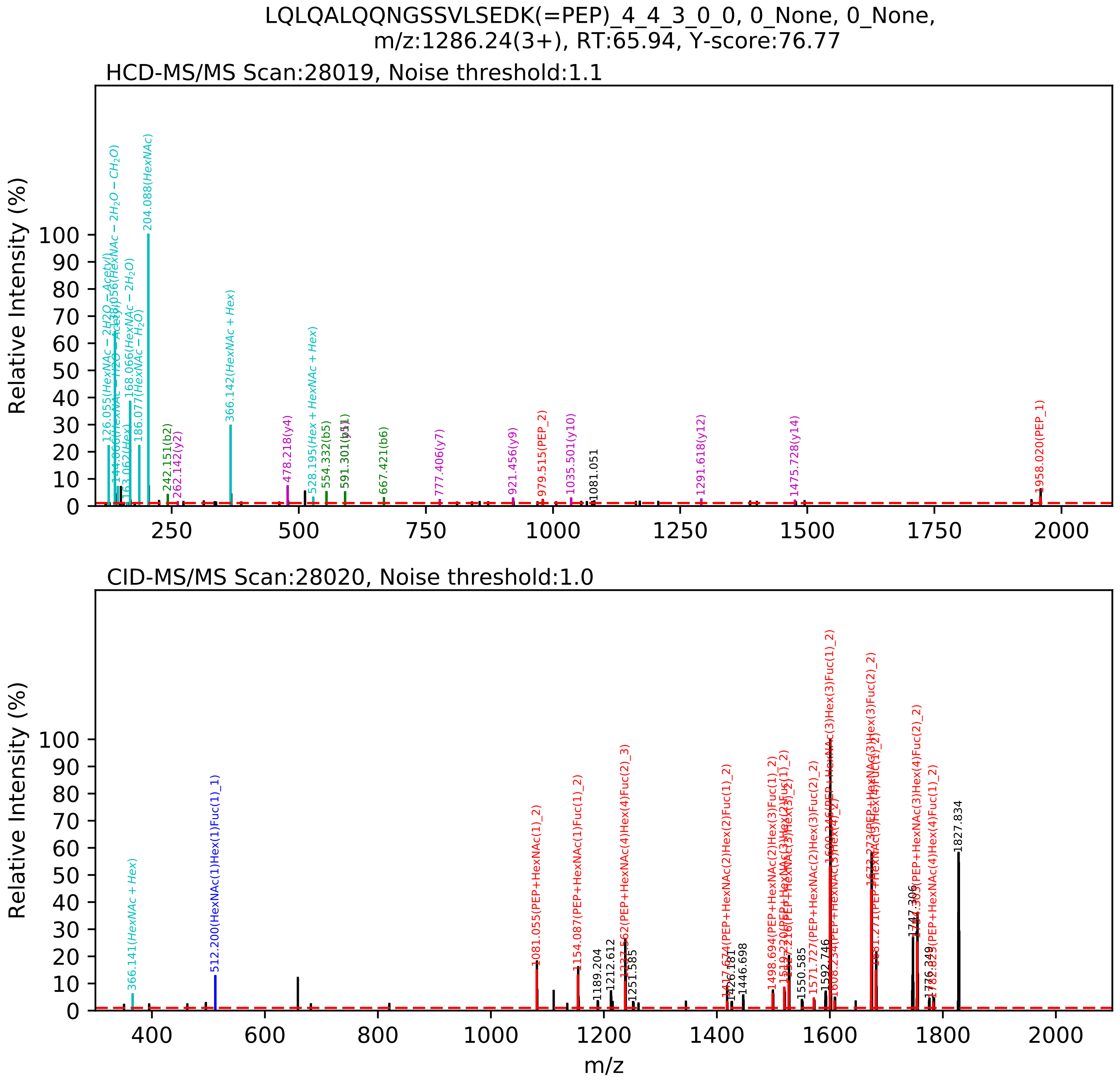
LQLQALQQNGSSVLSEDK(=PEP)_4_4_3_0_0, 0_None, 0_None,
m/z:1286.24(3+), RT:65.94, Y-score:76.77
HCD-MS/MS Scan:28019, Noise threshold:1.1
Relative Intensity (%)
204.088(HexNAc)
366.142(HexNAc + Hex)
478.218(y4)
554.332(b5)
667.421(b6)
777.406(y7)
921.456(y9)
979.515(PEP_2)
1035.501(y10)
1081.051
1291.618(y12)
1475.728(y14)
1958.020(PEP_1)
CID-MS/MS Scan:28020, Noise threshold:1.0
366.141(HexNAc + Hex)
512.200(HexNAc(1)Hex(1)Fuc(1)_1)
1081.055(PEP+HexNAc(1)_2)
1154.087(PEP+HexNAc(1)Fuc(1)_2)
1189.204
1212.612
1251.585
1426.181
1446.698
1550.585
1592.746
1747.306
1776.349
1827.834
m/z

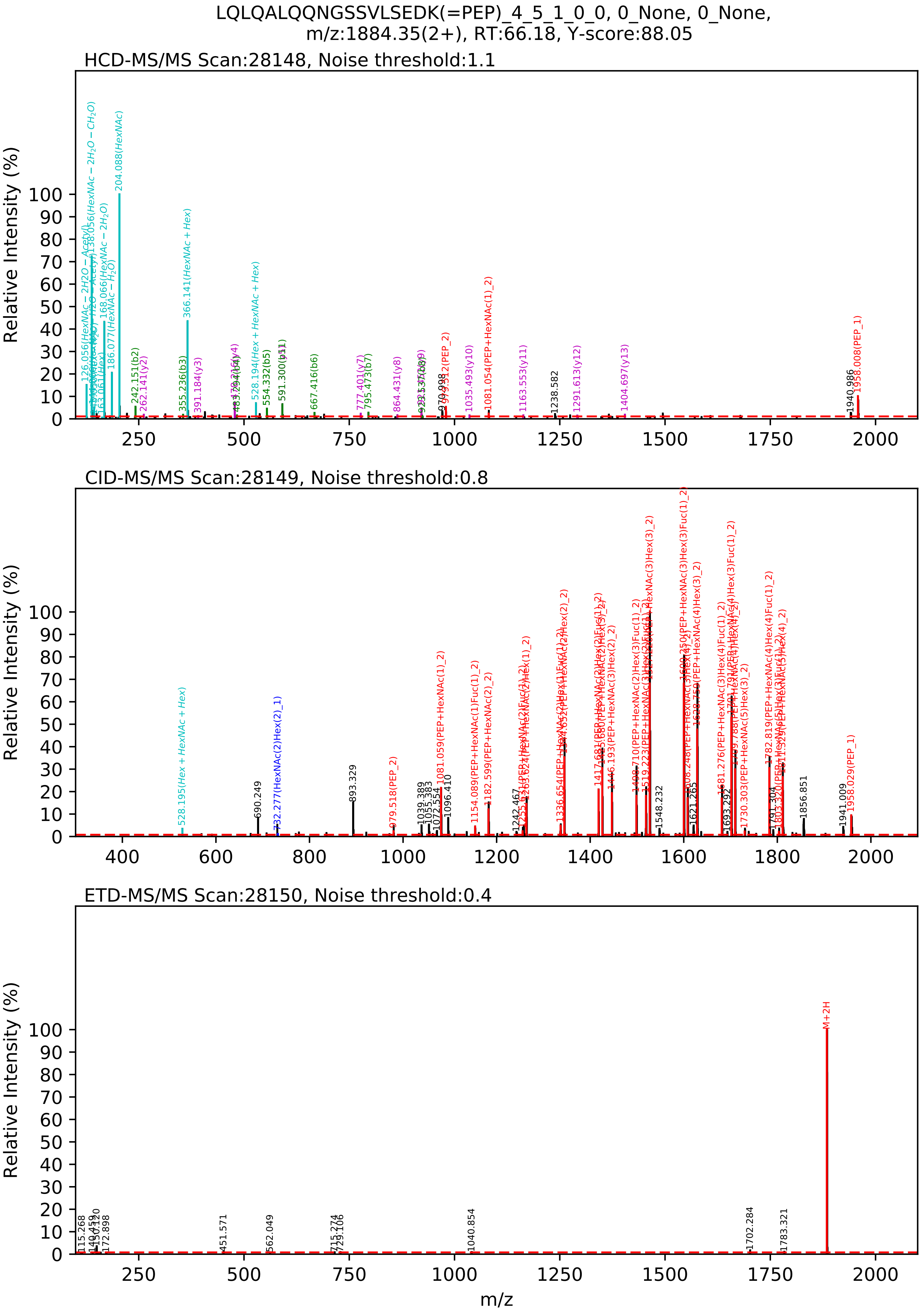
LQLQALQQNGSSVLSEDK(=PEP)_4_5_1_0_0, 0_None, 0_None,
m/z:1884.35(2+), RT:66.18, Y-score:88.05
HCD-MS/MS Scan:28148, Noise threshold:1.1
Relative Intensity (%)
204.088(HexNAc)
366.141(HexNAc + Hex)
168.066(HexNAc − 2H2O)
186.077(HexNAc − H2O)
242.151(b2)
262.141(y2)
355.236(b3)
391.184(y3)
528.194(Hex + HexNAc + Hex)
554.332(b5)
591.300(b6)
667.416(b6)
777.401(y7)
795.473(b7)
864.431(y8)
979.512(PEP_2)
1035.493(y10)
1081.054(PEP+HexNAc(1)_2)
1163.553(y11)
1238.582
1291.613(y12)
1404.697(y13)
1940.986
1958.008(PEP_1)
CID-MS/MS Scan:28149, Noise threshold:0.8
528.195(Hex + HexNAc + Hex)
690.249
732.277(HexNAc(2)Hex(2)_1)
893.329
979.518(PEP_2)
1039.389
1055.383
1072.554
1081.059(PEP+HexNAc(1)_2)
1096.410
1154.089(PEP+HexNAc(1)Fuc(1)_2)
1182.599(PEP+HexNAc(2)_2)
1242.467
1336.654
1417.681
1446.193(PEP+HexNAc(3)Hex(2)_2)
1498.710(PEP+HexNAc(2)Hex(3)Fuc(1)_2)
1519.223(PEP+HexNAc(3)Hex(2)Fuc(1)_2)
1548.232
1600.250(PEP+HexNAc(3)Hex(3)Fuc(1)_2)
1621.265
1628.759(PEP+HexNAc(4)Hex(3)_2)
1681.276(PEP+HexNAc(3)Hex(4)Fuc(1)_2)
1693.292
1730.303(PEP+HexNAc(5)Hex(3)_2)
1782.819(PEP+HexNAc(4)Hex(4)Fuc(1)_2)
1791.304
1856.851
1941.009
1958.029(PEP_1)
ETD-MS/MS Scan:28150, Noise threshold:0.4
M+2H
115.268
172.898
451.571
562.049
715.274
729.106
1040.854
1702.284
1783.321
m/z

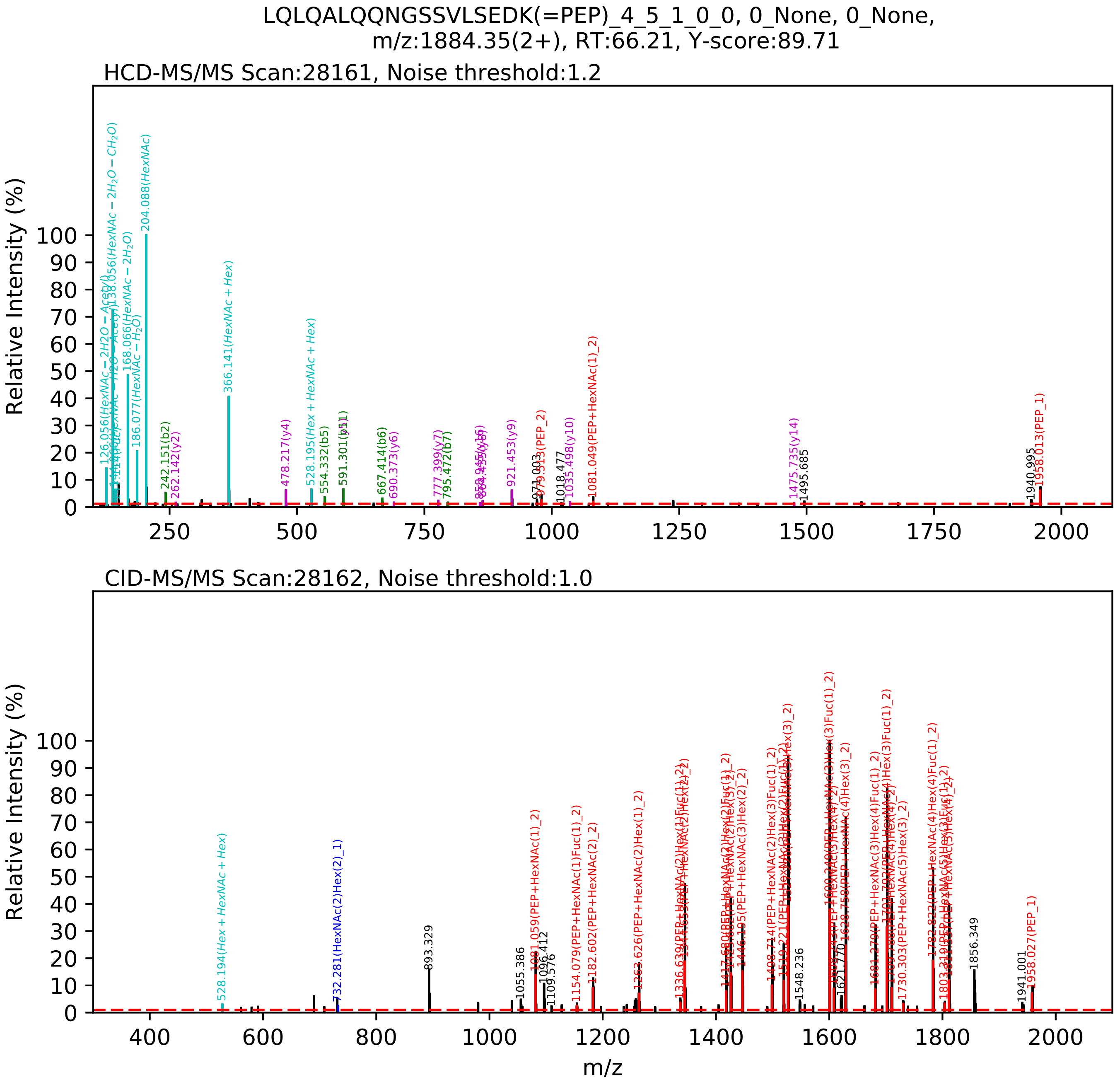
LQLQALQQNGSSVLSEDK(=PEP)_4_5_1_0_0, 0_None, 0_None,
m/z:1884.35(2+), RT:66.21, Y-score:89.71
HCD-MS/MS Scan:28161, Noise threshold:1.2
Relative Intensity (%)
204.088(HexNAc)
366.141(HexNAc + Hex)
242.151(b2)
262.142(y2)
478.217(y4)
528.195(Hex + HexNAc + Hex)
554.332(b5)
667.414(b6)
690.373(y6)
777.399(y7)
795.472(b7)
921.453(y9)
1018.477
1035.498(y10)
1081.049(PEP+HexNAc(1)_2)
1475.735(y14)
1495.685
1940.995
1958.013(PEP_1)
CID-MS/MS Scan:28162, Noise threshold:1.0
Relative Intensity (%)
528.194(Hex + HexNAc + Hex)
732.281(HexNAc(2)Hex(2)_1)
893.329
1055.386
1081.059(PEP+HexNAc(1)_2)
1154.079(PEP+HexNAc(1)Fuc(1)_2)
1182.602(PEP+HexNAc(2)_2)
1263.626(PEP+HexNAc(2)Hex(1)_2)
1446.195(PEP+HexNAc(3)Hex(2)_2)
1498.714(PEP+HexNAc(2)Hex(3)Fuc(1)_2)
1548.236
1621.770
1681.270(PEP+HexNAc(3)Hex(4)Fuc(1)_2)
1730.303(PEP+HexNAc(5)Hex(3)_2)
1856.349
1941.001
1958.027(PEP_1)
m/z

LQLQALQQNGSSVLSEDK(=PEP)_4_5_1_0_0, 0_None, 0_None,
m/z:1256.57(3+), RT:66.40, Y-score:90.16

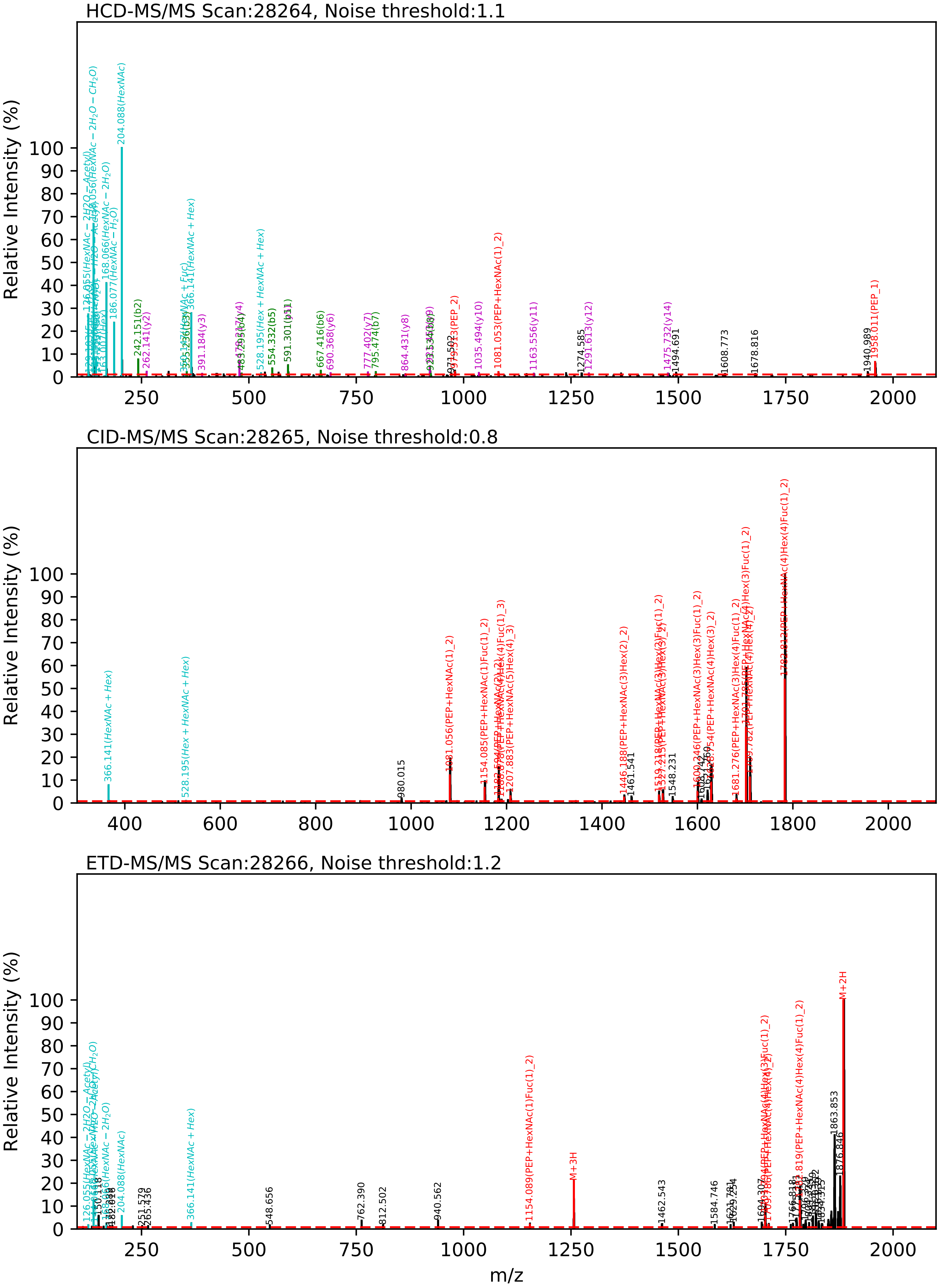

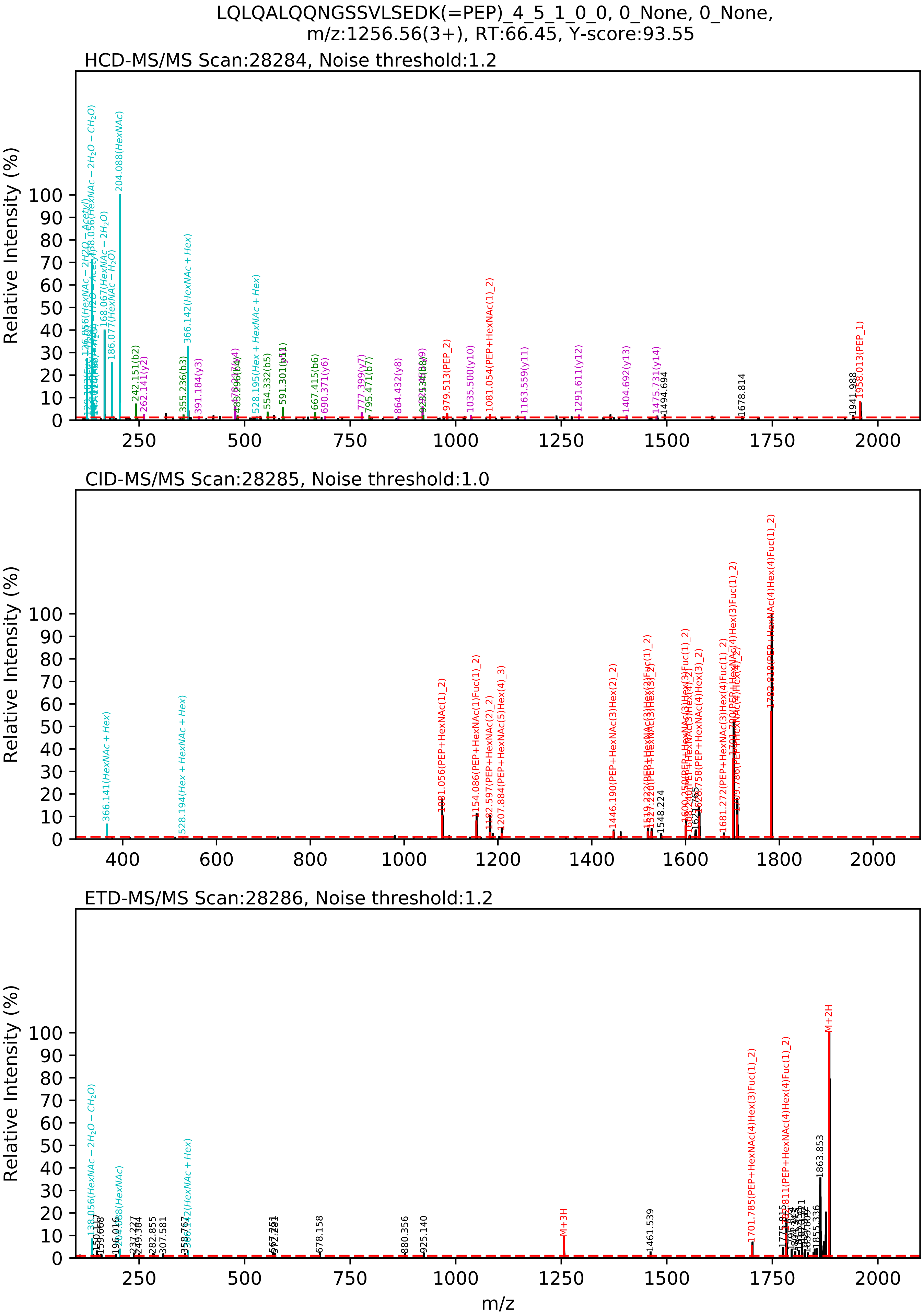
LQLQALQQNGSSVLSEDK(=PEP)_4_5_1_0_0, 0_None, 0_None,
m/z:1256.56(3+), RT:66.45, Y-score:93.55
HCD-MS/MS Scan:28284, Noise threshold:1.2
Relative Intensity (%)
204.088(HexNAc)
366.142(HexNAc + Hex)
168.067(HexNAc − 2H2O)
186.077(HexNAc − H2O)
242.151(b2)
262.141(y2)
355.236(b3)
391.184(y3)
528.195(Hex + HexNAc + Hex)
554.332(b5)
667.415(b6)
690.371(y6)
777.399(y7)
795.471(b7)
864.432(y8)
979.513(PEP_2)
1035.500(y10)
1081.054(PEP+HexNAc(1)_2)
1163.559(y11)
1291.611(y12)
1404.692(y13)
1475.731(y14)
1494.694
1678.814
1941.988
1958.013(PEP_1)
CID-MS/MS Scan:28285, Noise threshold:1.0
366.141(HexNAc + Hex)
528.194(Hex + HexNAc + Hex)
1081.056(PEP+HexNAc(1)_2)
1154.086(PEP+HexNAc(1)Fuc(1)_2)
1182.597(PEP+HexNAc(2)_2)
1207.884(PEP+HexNAc(5)Hex(4)_3)
1446.190(PEP+HexNAc(3)Hex(2)_2)
1548.224
1628.758(PEP+HexNAc(4)Hex(3)_2)
1681.272(PEP+HexNAc(3)Hex(4)Fuc(1)_2)
1782.818(PEP+HexNAc(4)Hex(4)Fuc(1)_2)
ETD-MS/MS Scan:28286, Noise threshold:1.2
138.056(HexNAc − 2H2O − CH2O)
204.088(HexNAc)
366.142(HexNAc + Hex)
196.016
237.227
249.384
282.855
307.581
358.767
678.158
880.356
925.140
M+3H
1461.539
1701.785(PEP+HexNAc(4)Hex(3)Fuc(1)_2)
1863.853
M+2H
m/z

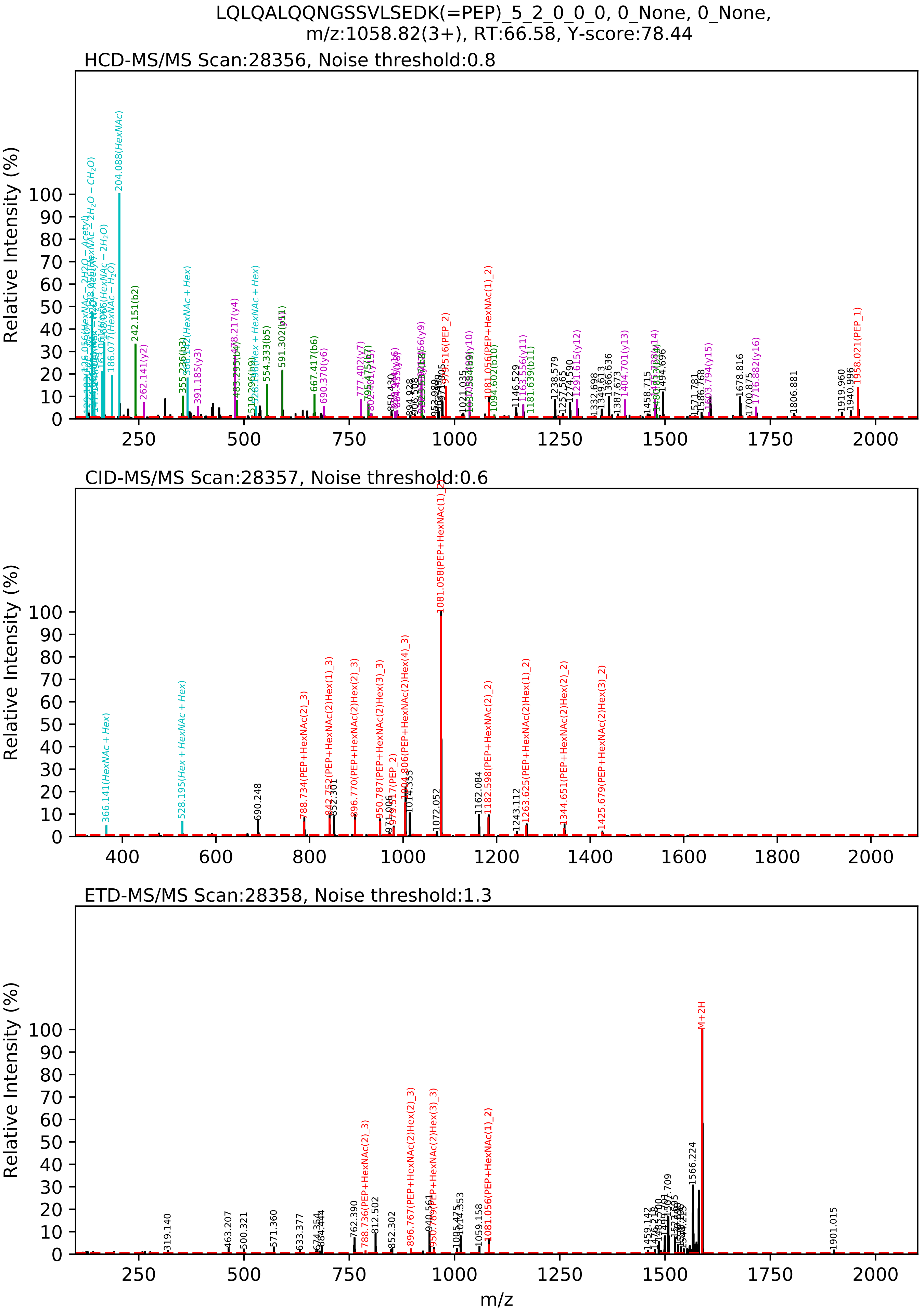
LQLQALQQNGSSVLSEDK(=PEP)_5_2_0_0_0, 0_None, 0_None,
m/z:1058.82(3+), RT:66.58, Y-score:78.44
HCD-MS/MS Scan:28356, Noise threshold:0.8
CID-MS/MS Scan:28357, Noise threshold:0.6
ETD-MS/MS Scan:28358, Noise threshold:1.3
Relative Intensity (%)
m/z

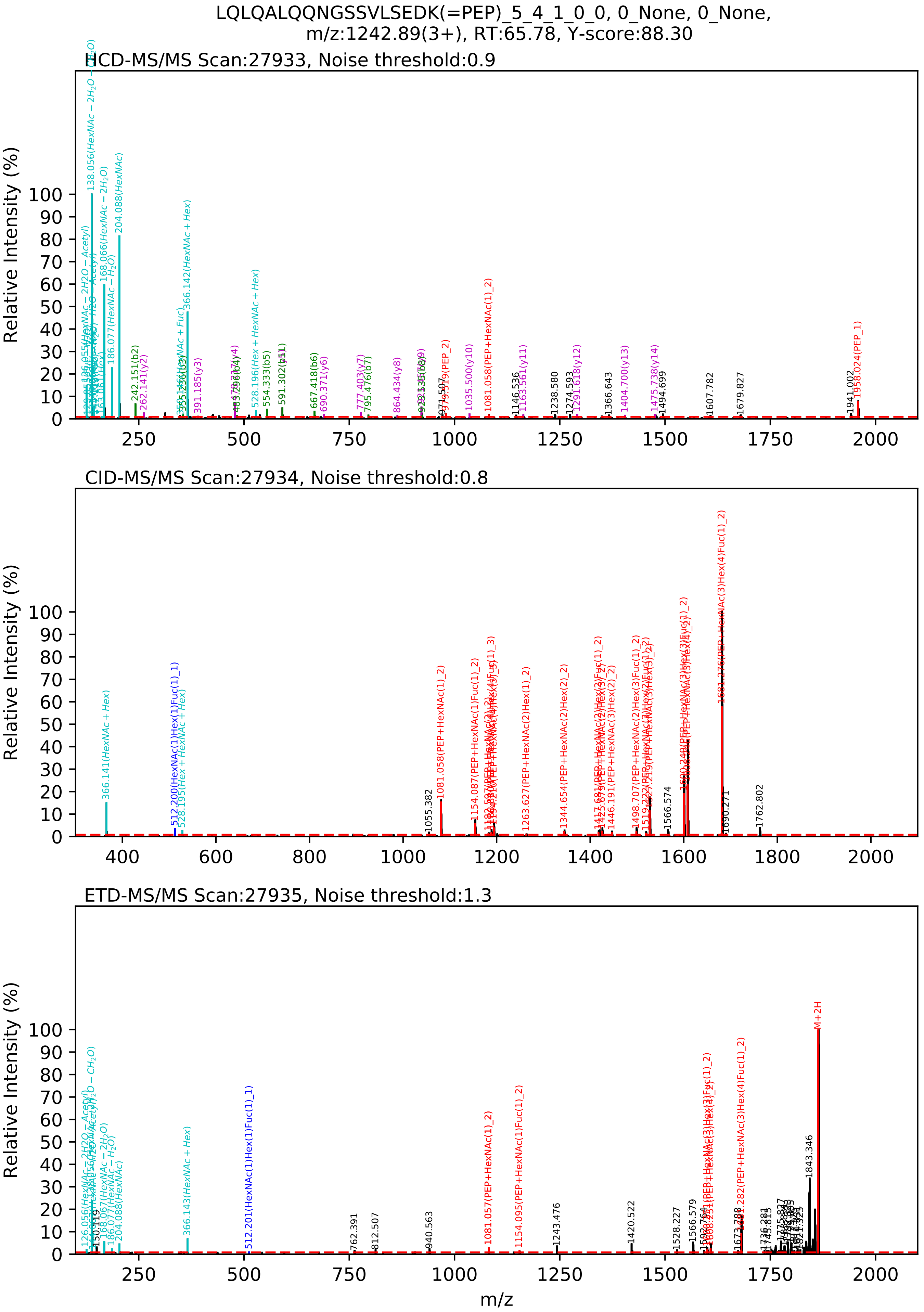
LQLQALQQNGSSVLSEDK(=PEP)_5_4_1_0_0, 0_None, 0_None,
m/z:1242.89(3+), RT:65.78, Y-score:88.30
HCD-MS/MS Scan:27933, Noise threshold:0.9
CID-MS/MS Scan:27934, Noise threshold:0.8
ETD-MS/MS Scan:27935, Noise threshold:1.3
Relative Intensity (%)
m/z
138.056(HexNAc − 2H2O − CH2O)
204.088(HexNAc)
366.142(HexNAc + Hex)
528.196(HexNAc + Hex + Hex)
1081.058(PEP+HexNAc(1)_2)
1958.024(PEP_1)
1681.276(PEP+HexNAc(3)Hex(4)Fuc(1)_2)
512.200(HexNAc(1)Hex(1)Fuc(1)_1)
M+2H
1843.346

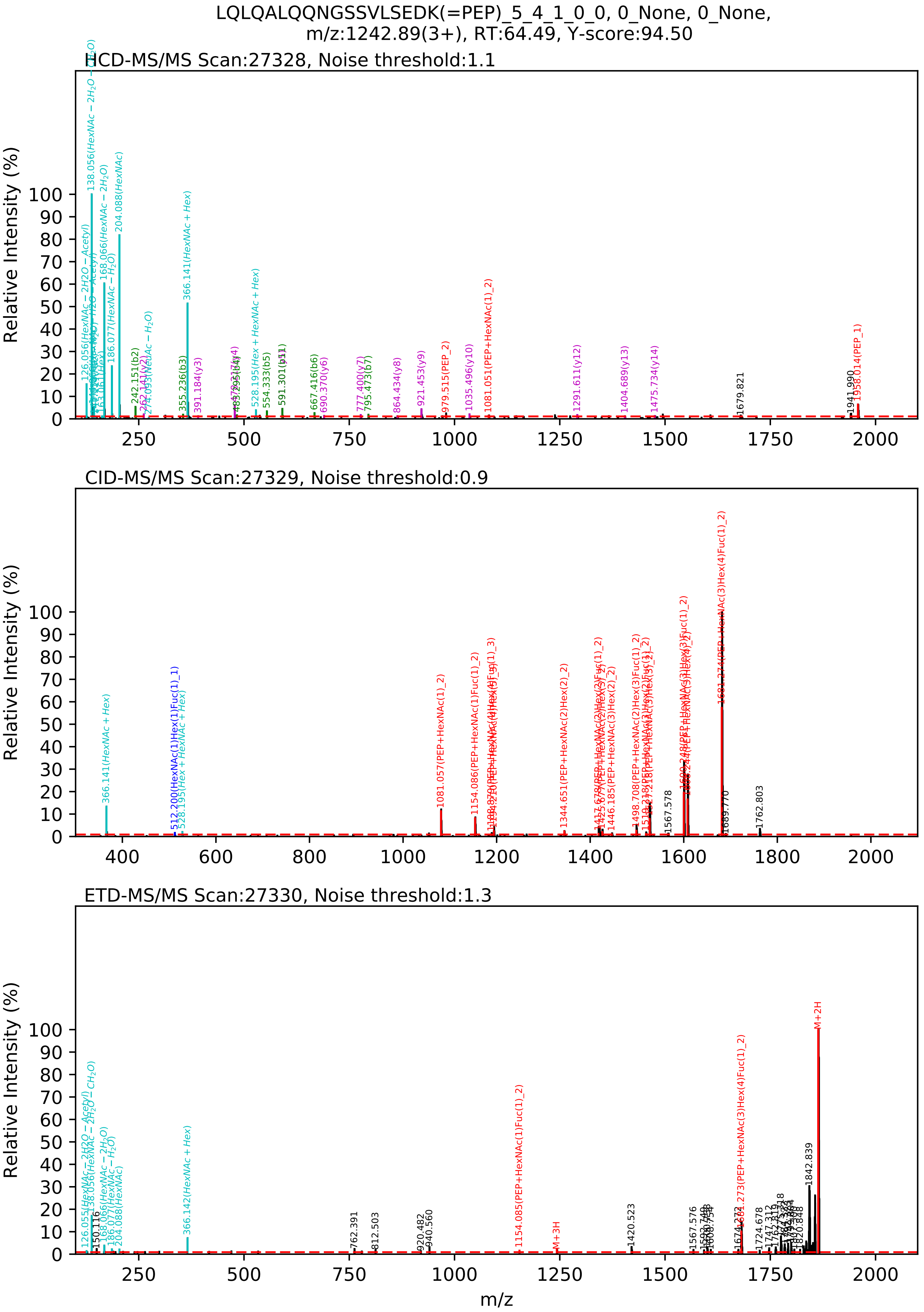
LQLQALQQNGSSVLSEDK(=PEP)_5_4_1_0_0, 0_None, 0_None,
m/z:1242.89(3+), RT:64.49, Y-score:94.50
HCD-MS/MS Scan:27328, Noise threshold:1.1
Relative Intensity (%)
138.056(HexNAc − 2H2O − CH2O)
204.088(HexNAc)
168.066(HexNAc − 2H2O)
186.077(HexNAc − H2O)
366.141(HexNAc + Hex)
242.151(b2)
355.236(b3)
391.184(y3)
528.195(Hex + HexNAc + Hex)
554.333(b5)
591.301(b6)
667.416(b6)
690.370(y6)
777.400(y7)
795.473(b7)
864.434(y8)
921.453(y9)
979.515(PEP_2)
1035.496(y10)
1081.051(PEP+HexNAc(1)_2)
1291.611(y12)
1404.689(y13)
1475.734(y14)
1679.821
1941.990
1958.014(PEP_1)
CID-MS/MS Scan:27329, Noise threshold:0.9
Relative Intensity (%)
366.141(HexNAc + Hex)
512.200(HexNAc(1)Hex(1)Fuc(1)_1)
528.195(Hex + HexNAc + Hex)
1081.057(PEP+HexNAc(1)_2)
1154.086(PEP+HexNAc(1)Fuc(1)_2)
1344.651(PEP+HexNAc(2)Hex(2)_2)
1446.185(PEP+HexNAc(3)Hex(2)_2)
1567.578
1681.274(PEP+HexNAc(3)Hex(4)Fuc(1)_2)
1689.770
1762.803
ETD-MS/MS Scan:27330, Noise threshold:1.3
Relative Intensity (%)
150.116
204.088(HexNAc)
366.142(HexNAc + Hex)
762.391
812.503
920.482
940.560
1154.085(PEP+HexNAc(1)Fuc(1)_2)
M+3H
1420.523
1567.576
1608.754
1674.273
1724.678
1747.312
1842.839
M+2H
m/z

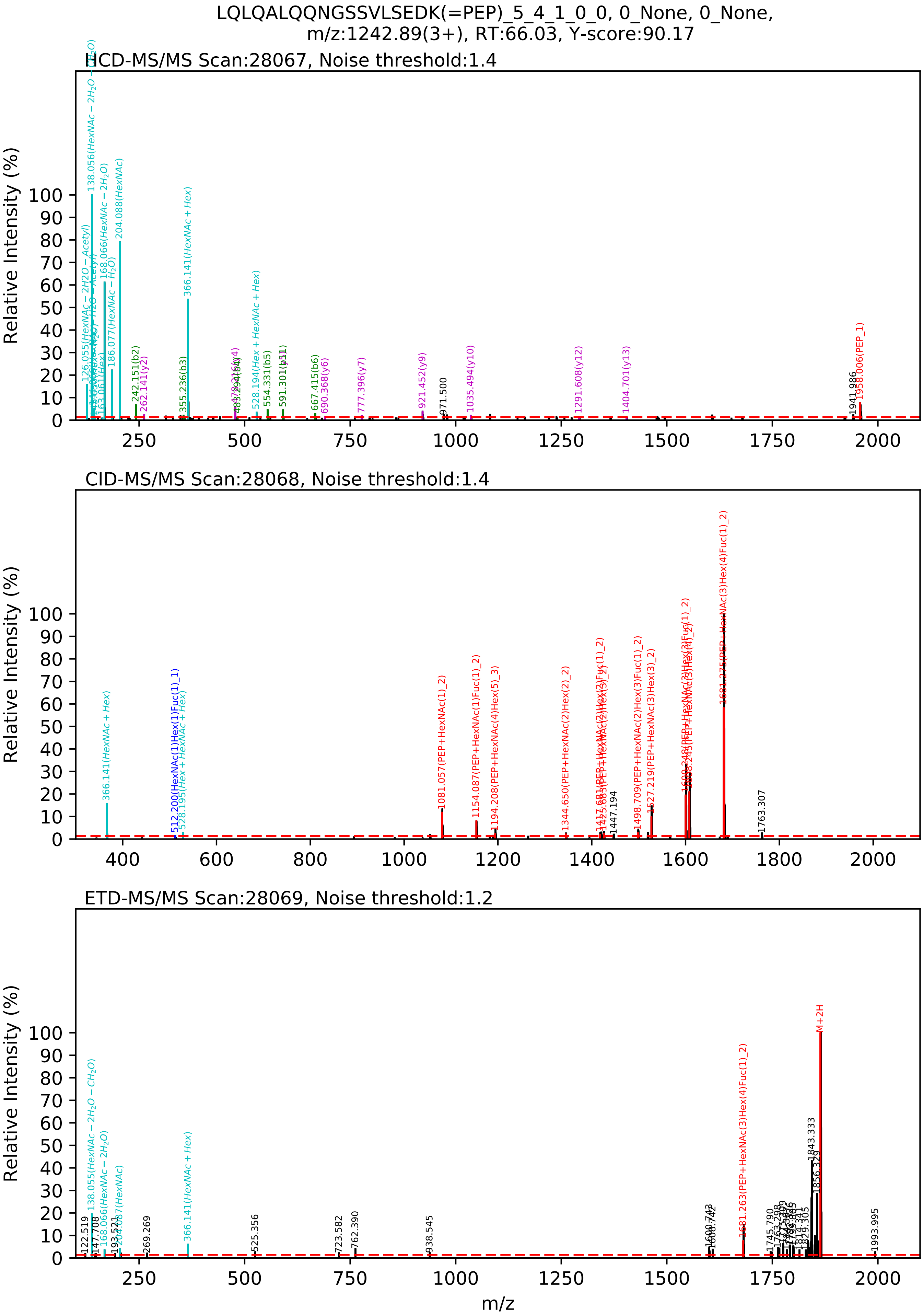
LQLQALQQNGSSVLSEDK(=PEP)_5_4_1_0_0, 0_None, 0_None,
m/z:1242.89(3+), RT:66.03, Y-score:90.17
HCD-MS/MS Scan:28067, Noise threshold:1.4
CID-MS/MS Scan:28068, Noise threshold:1.4
ETD-MS/MS Scan:28069, Noise threshold:1.2
Relative Intensity (%)
m/z

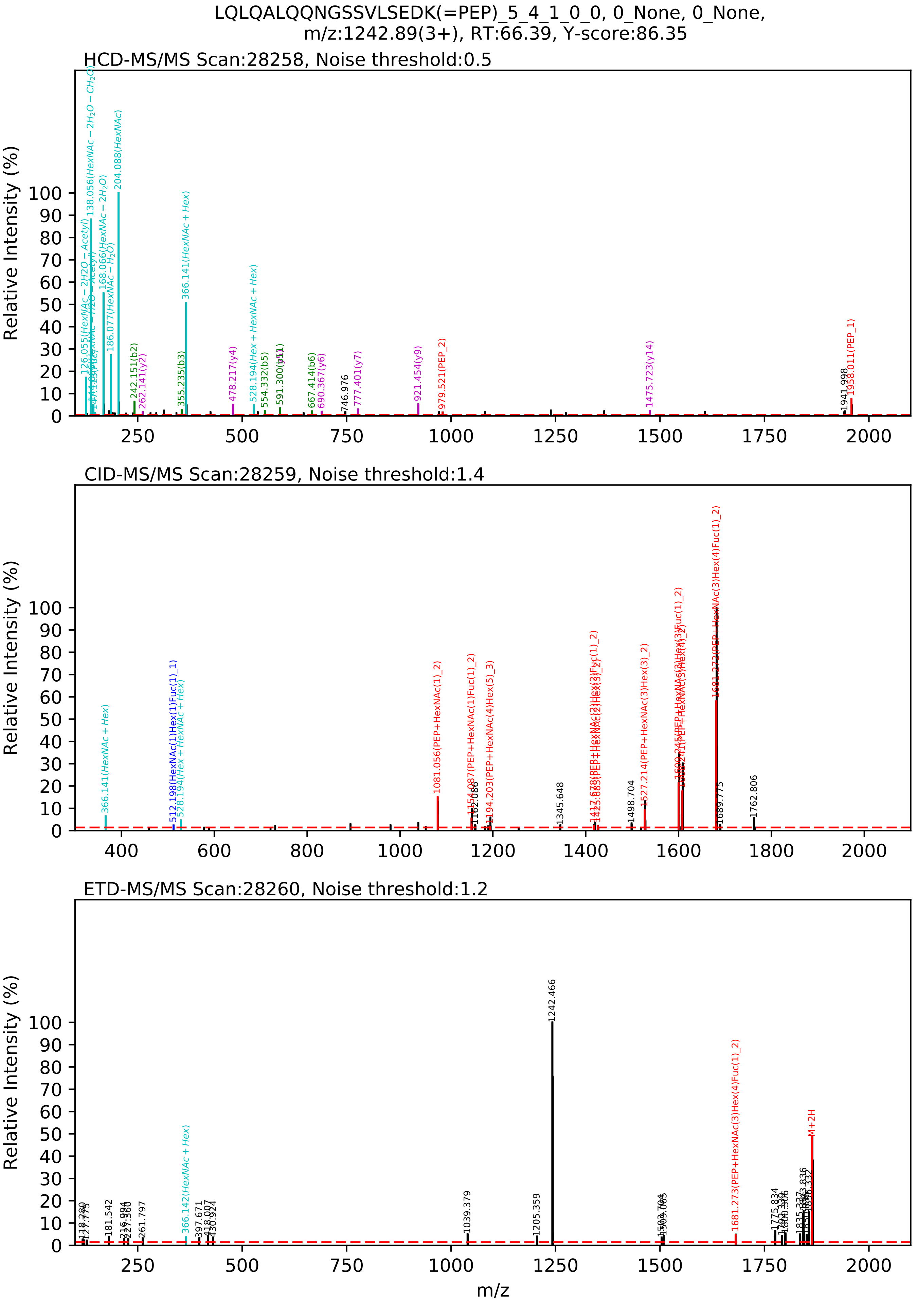

LQLQALQQNGSSVLSEDK(=PEP)_5_4_1_0_0, 0_None, 0_None,
m/z:1242.89(3+), RT:66.39, Y-score:86.35
HCD-MS/MS Scan:28258, Noise threshold:0.5
CID-MS/MS Scan:28259, Noise threshold:1.4
ETD-MS/MS Scan:28260, Noise threshold:1.2
Relative Intensity (%)
m/z
204.088(HexNAc)
366.141(HexNAc+Hex)
528.194(Hex+HexNAc+Hex)
1958.011(PEP_1)
1681.273(PEP+HexNAc(3)Hex(4)Fuc(1)_2)
1081.056(PEP+HexNAc(1)_2)
1242.466
M+2H

LQLQALQQNGSSVLSEDK(=PEP)_5_4_1_0_0, 0_None, 0_None,
m/z:932.42(4+), RT:66.08, Y-score:86.48

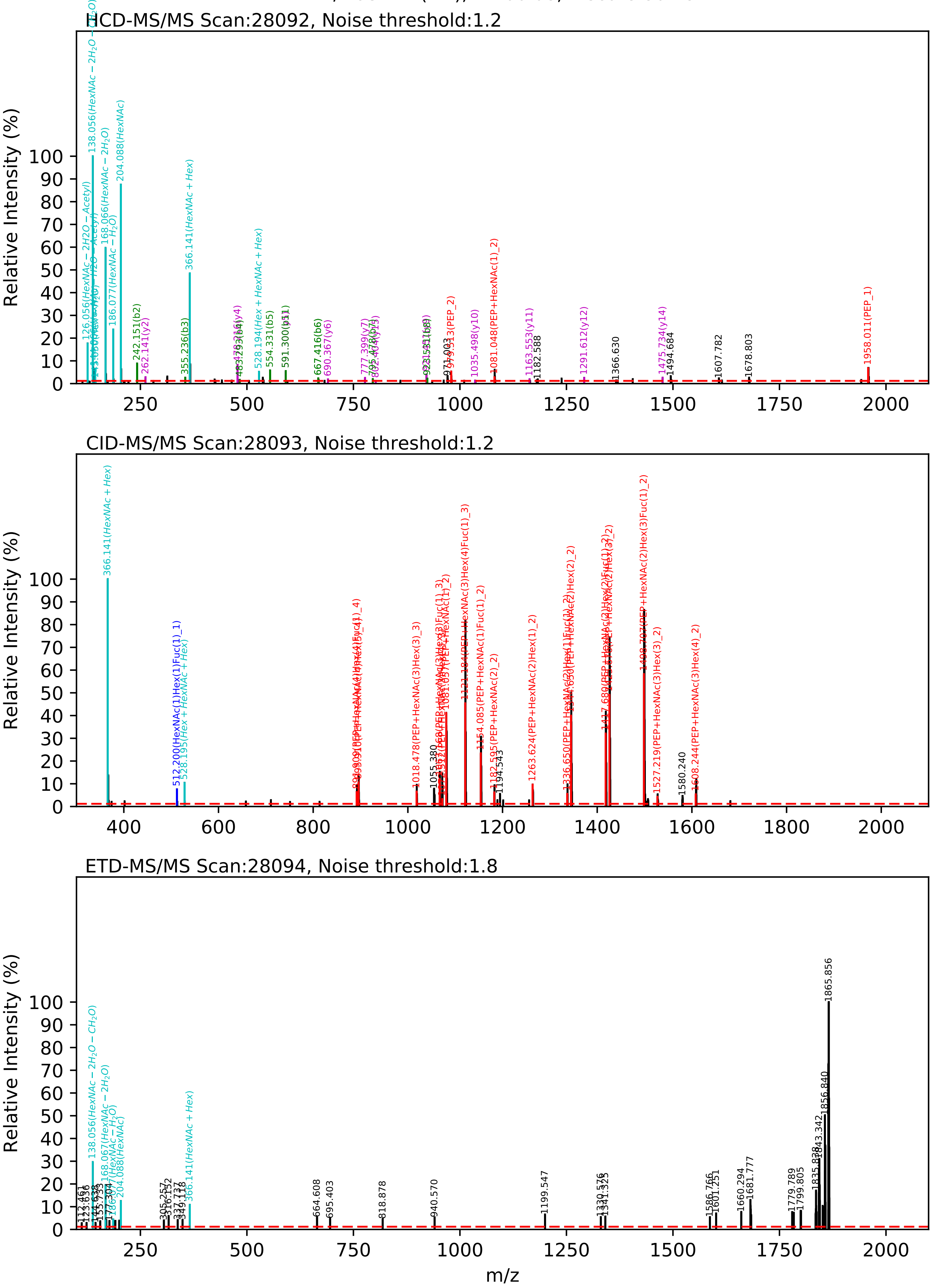

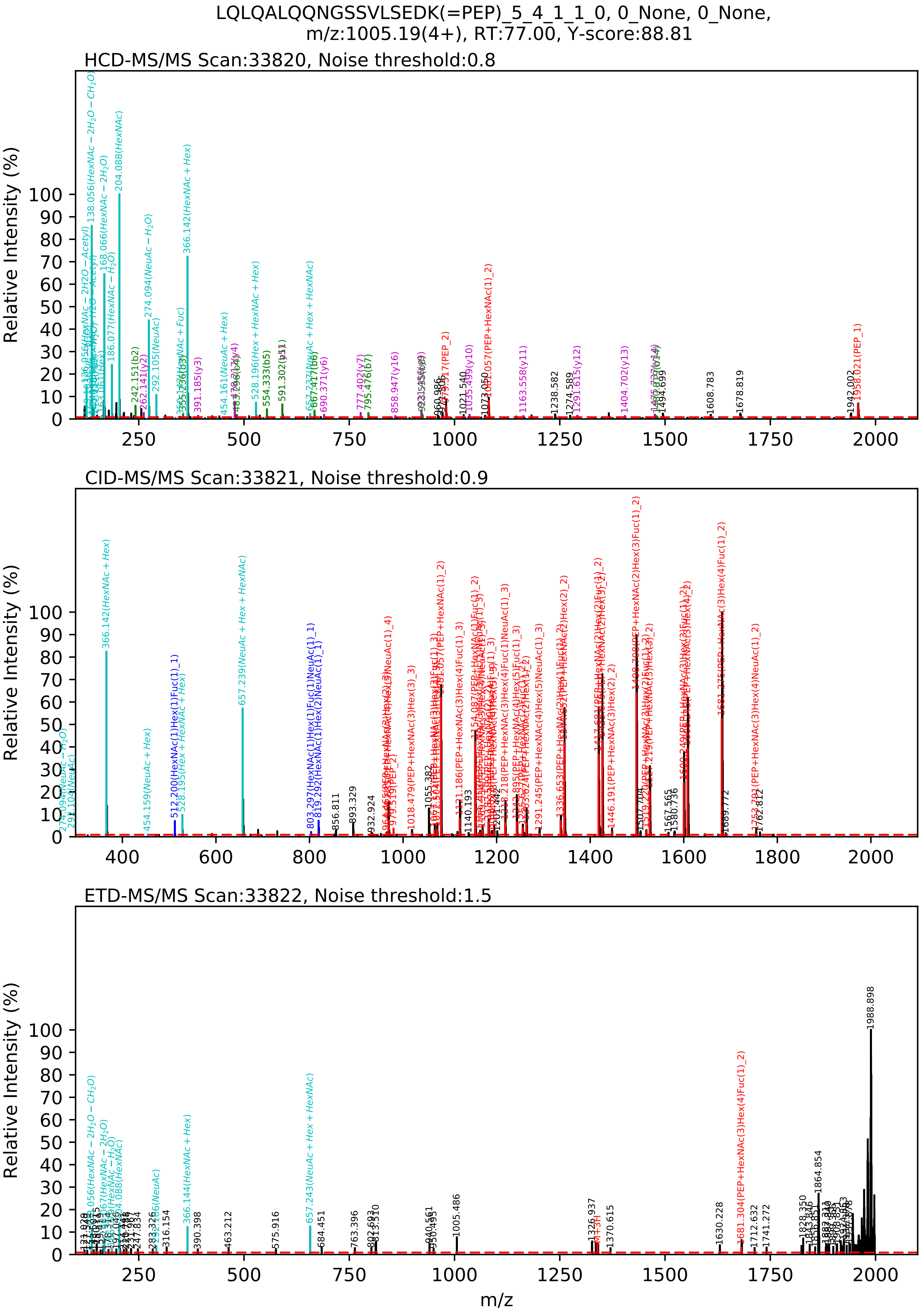
LQLQALQQNGSSVLSEDK(=PEP)_5_4_1_1_0, 0_None, 0_None,
m/z:1005.19(4+), RT:77.00, Y-score:88.81
HCD-MS/MS Scan:33820, Noise threshold:0.8
Relative Intensity (%)
204.088(HexNAc)
138.056(HexNAc − 2H2O − CH2O)
168.066(HexNAc − 2H2O)
186.077(HexNAc − H2O)
366.142(HexNAc + Hex)
274.094(NeuAc − H2O)
292.105(NeuAc)
242.151(b2)
262.141(y2)
391.185(y3)
454.161(NeuAc + Hex)
528.196(Hex + HexNAc + Hex)
554.333(b5)
591.302(y5)
657.239(NeuAc + Hex + HexNAc)
690.371(y6)
777.402(y7)
795.476(b7)
858.947(y16)
960.986
979.517(PEP_2)
1021.540
1035.499(y10)
1073.050
1081.057(PEP+HexNAc(1)_2)
1163.558(y11)
1238.582
1274.589
1291.615(y12)
1404.702(y13)
1494.699
1608.783
1678.819
1942.002
1958.021(PEP_1)
CID-MS/MS Scan:33821, Noise threshold:0.9
366.142(HexNAc + Hex)
454.159(NeuAc + Hex)
512.200(HexNAc(1)Hex(1)Fuc(1)_1)
528.195(Hex + HexNAc + Hex)
657.239(NeuAc + Hex + HexNAc)
803.297(HexNAc(1)Hex(1)Fuc(1)NeuAc(1)_1)
819.292(HexNAc(1)Hex(2)NeuAc(1)_1)
856.811
893.329
932.924
979.519(PEP_2)
1018.479(PEP+HexNAc(3)Hex(3)_3)
1055.382
1081.057(PEP+HexNAc(1)_2)
1121.186(PEP+HexNAc(3)Hex(4)Fuc(1)_3)
1140.193
1201.442
1218.218(PEP+HexNAc(3)Hex(4)Fuc(1)NeuAc(1)_3)
1291.245(PEP+HexNAc(4)Hex(5)NeuAc(1)_3)
1336.653(PEP+HexNAc(2)Hex(2)_2)
1417.681
1446.191(PEP+HexNAc(3)Hex(2)_2)
1499.708(PEP+HexNAc(2)Hex(3)Fuc(1)_2)
1567.565
1580.736
1600.240
1681.275(PEP+HexNAc(3)Hex(4)Fuc(1)_2)
1689.772
1753.791(PEP+HexNAc(3)Hex(4)NeuAc(1)_2)
1762.812
ETD-MS/MS Scan:33822, Noise threshold:1.5
138.056(HexNAc − 2H2O − CH2O)
204.088(HexNAc)
283.326
292.106(NeuAc)
316.154
366.144(HexNAc + Hex)
390.398
463.212
575.916
657.243(NeuAc + Hex + HexNAc)
684.451
763.396
802.693
813.510
940.561
1005.486
1326.937
M+3H
1370.615
1630.228
1681.304(PEP+HexNAc(3)Hex(4)Fuc(1)_2)
1712.632
1741.272
1828.350
1864.854
1988.898
m/z

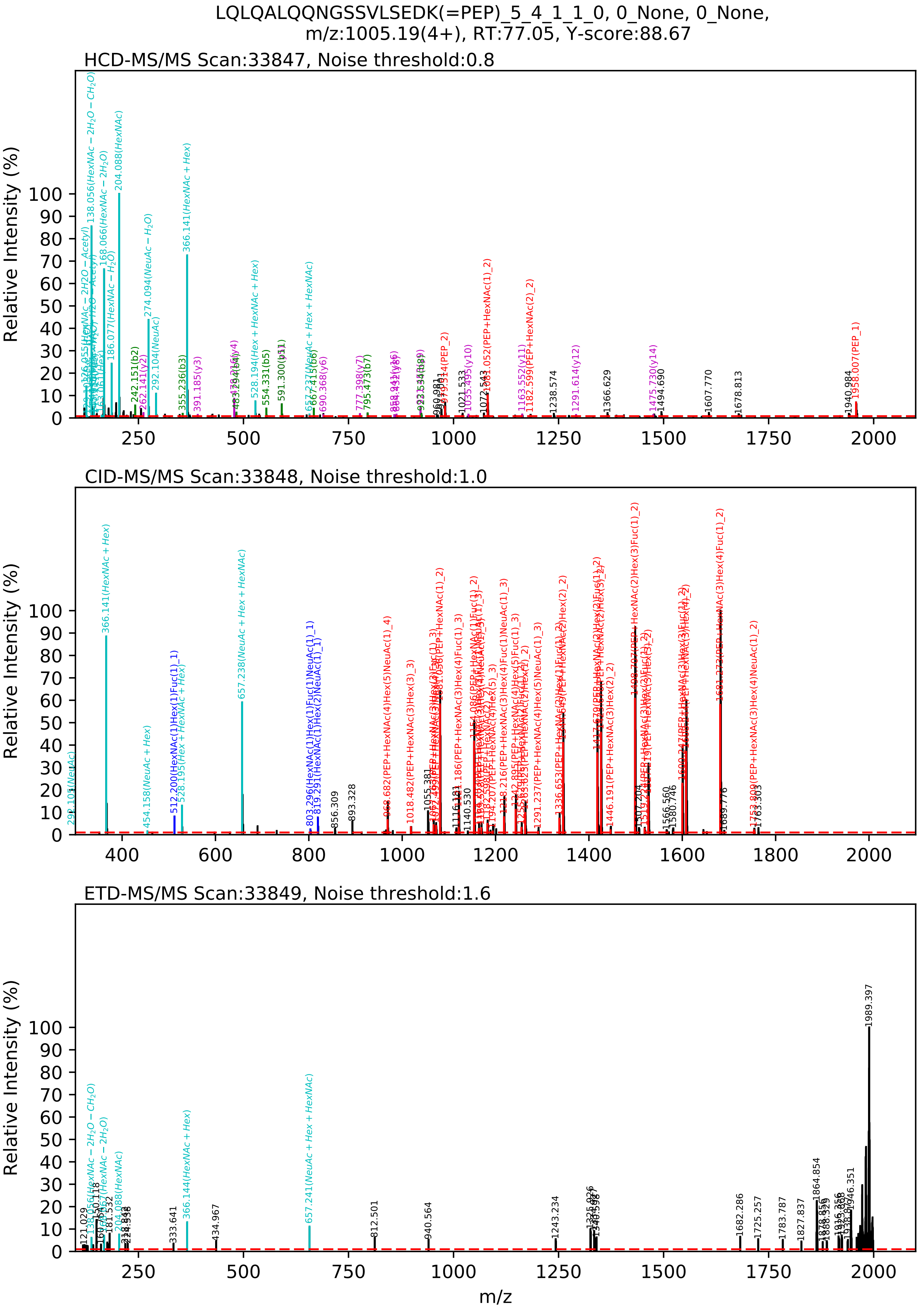
LQLQALQQNGSSVLSEDK(=PEP)_5_4_1_1_0, 0_None, 0_None,
m/z:1005.19(4+), RT:77.05, Y-score:88.67
HCD-MS/MS Scan:33847, Noise threshold:0.8
CID-MS/MS Scan:33848, Noise threshold:1.0
ETD-MS/MS Scan:33849, Noise threshold:1.6
Relative Intensity (%)
m/z

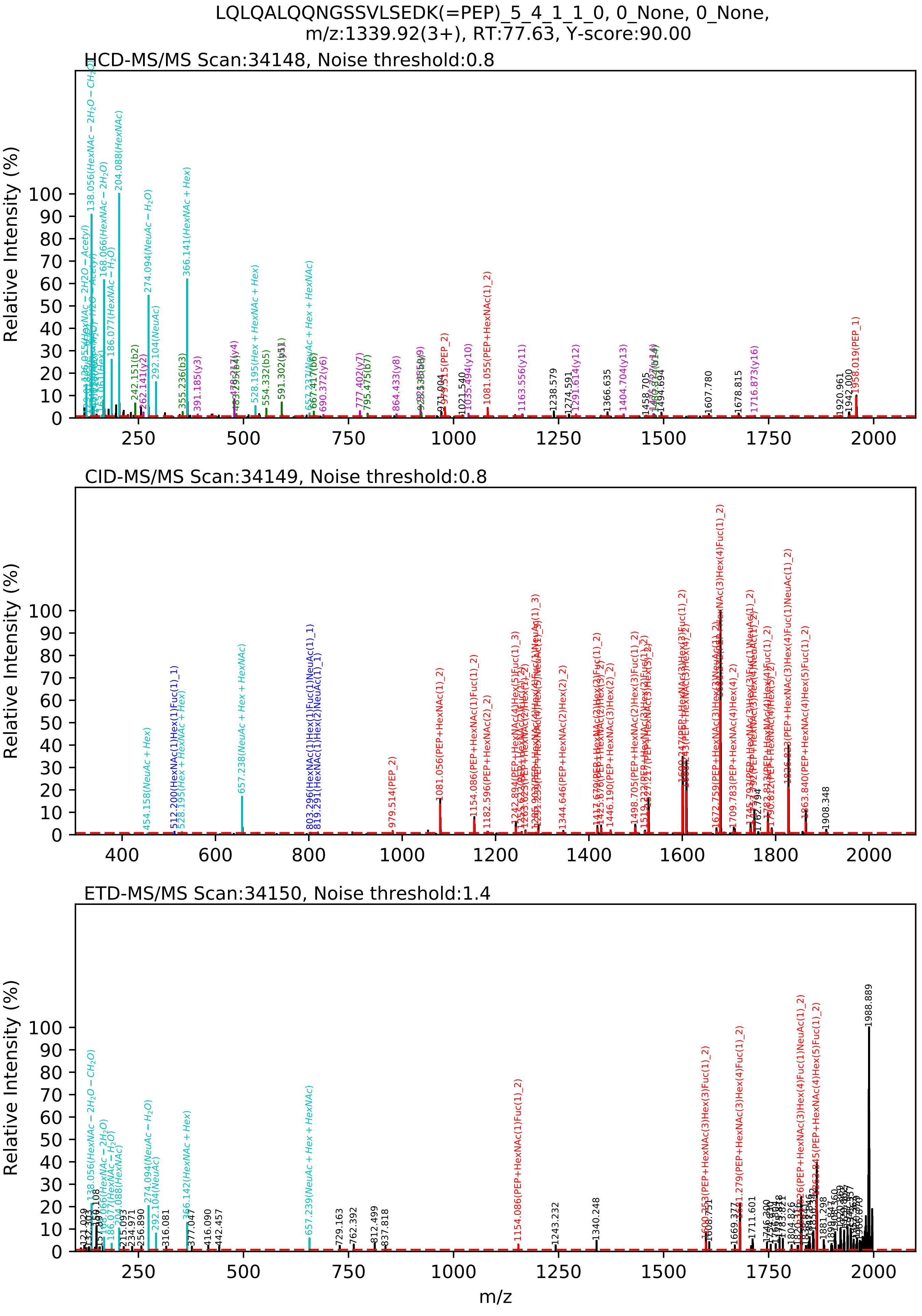
LQLQALQQNGSSVLSEDK(=PEP)_5_4_1_1_0, 0_None, 0_None,
m/z:1339.92(3+), RT:77.63, Y-score:90.00
HCD-MS/MS Scan:34148, Noise threshold:0.8
CID-MS/MS Scan:34149, Noise threshold:0.8
ETD-MS/MS Scan:34150, Noise threshold:1.4
Relative Intensity (%)
m/z

LQLQALQQNGSSVLSEDK(=PEP)_5_4_1_1_0, 0_None, 0_None,
m/z:1005.19(4+), RT:77.73, Y-score:88.64

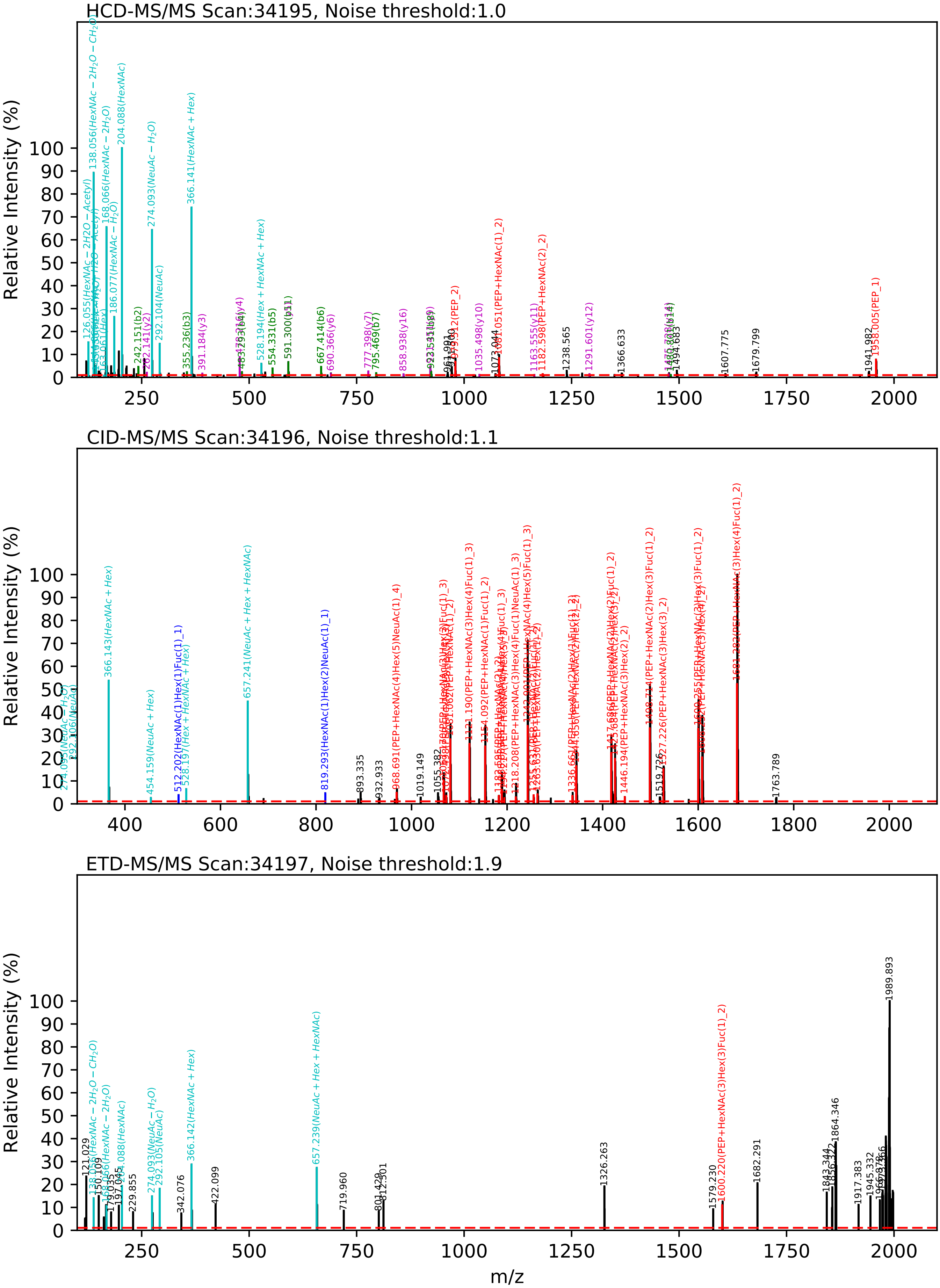

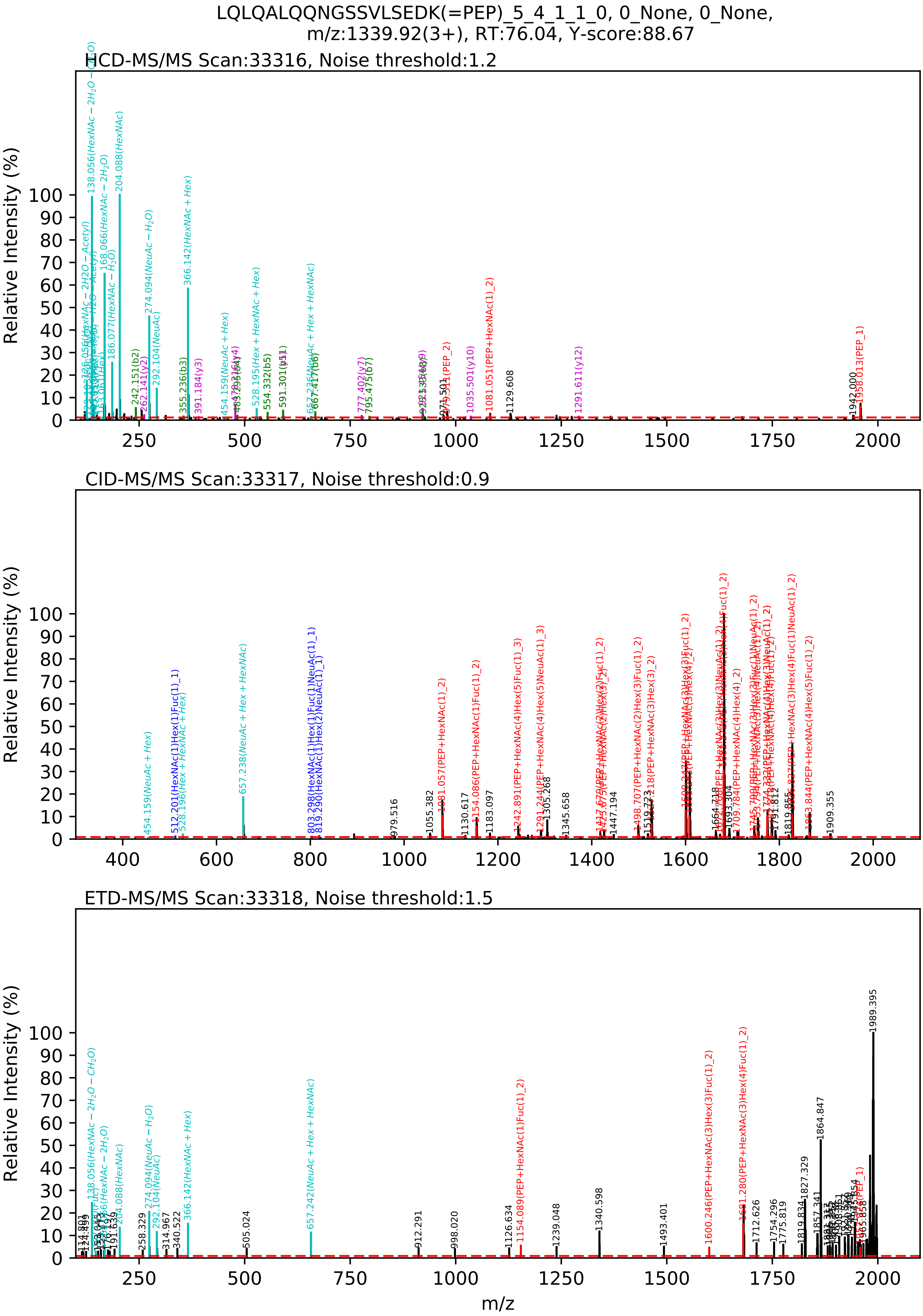
LQLQALQQNGSSVLSEDK(=PEP)_5_4_1_1_0, 0_None, 0_None,
m/z:1339.92(3+), RT:76.04, Y-score:88.67
HCD-MS/MS Scan:33316, Noise threshold:1.2
CID-MS/MS Scan:33317, Noise threshold:0.9
ETD-MS/MS Scan:33318, Noise threshold:1.5
Relative Intensity (%)
m/z

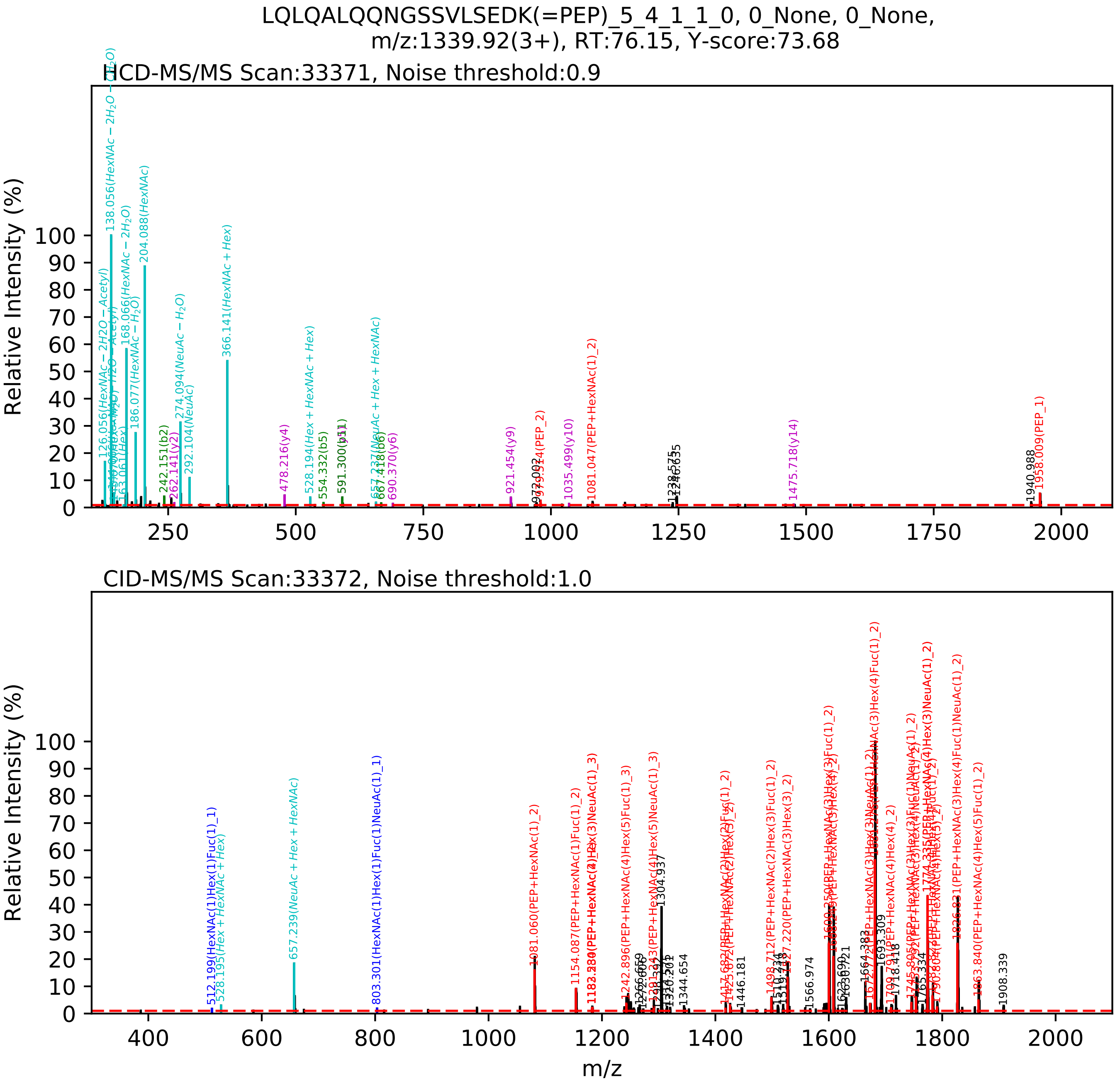
LQLQALQQNGSSVLSEDK(=PEP)_5_4_1_1_0, 0_None, 0_None,
m/z:1339.92(3+), RT:76.15, Y-score:73.68
HCD-MS/MS Scan:33371, Noise threshold:0.9
Relative Intensity (%)
CID-MS/MS Scan:33372, Noise threshold:1.0
Relative Intensity (%)
m/z

LQLQALQQNGSSVLSEDK(=PEP)_5_4_1_1_0, 0_None, 0_None,
m/z:1339.92(3+), RT:77.04, Y-score:91.26

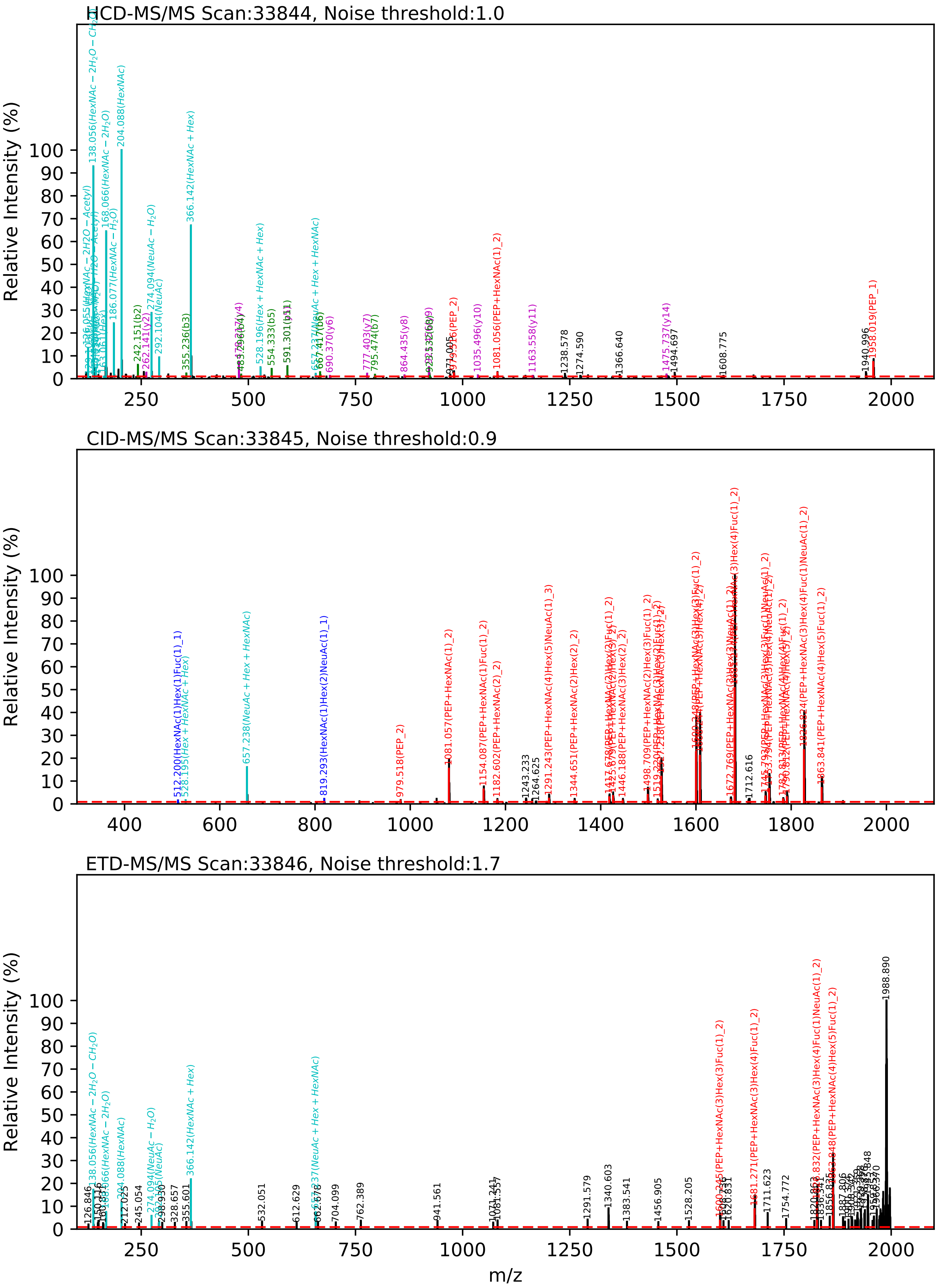

LQLQALQQNGSSVLSEDK(=PEP)_5_4_1_1_0, 0_None, 0_None,
m/z:1339.92(3+), RT:77.40, Y-score:91.11

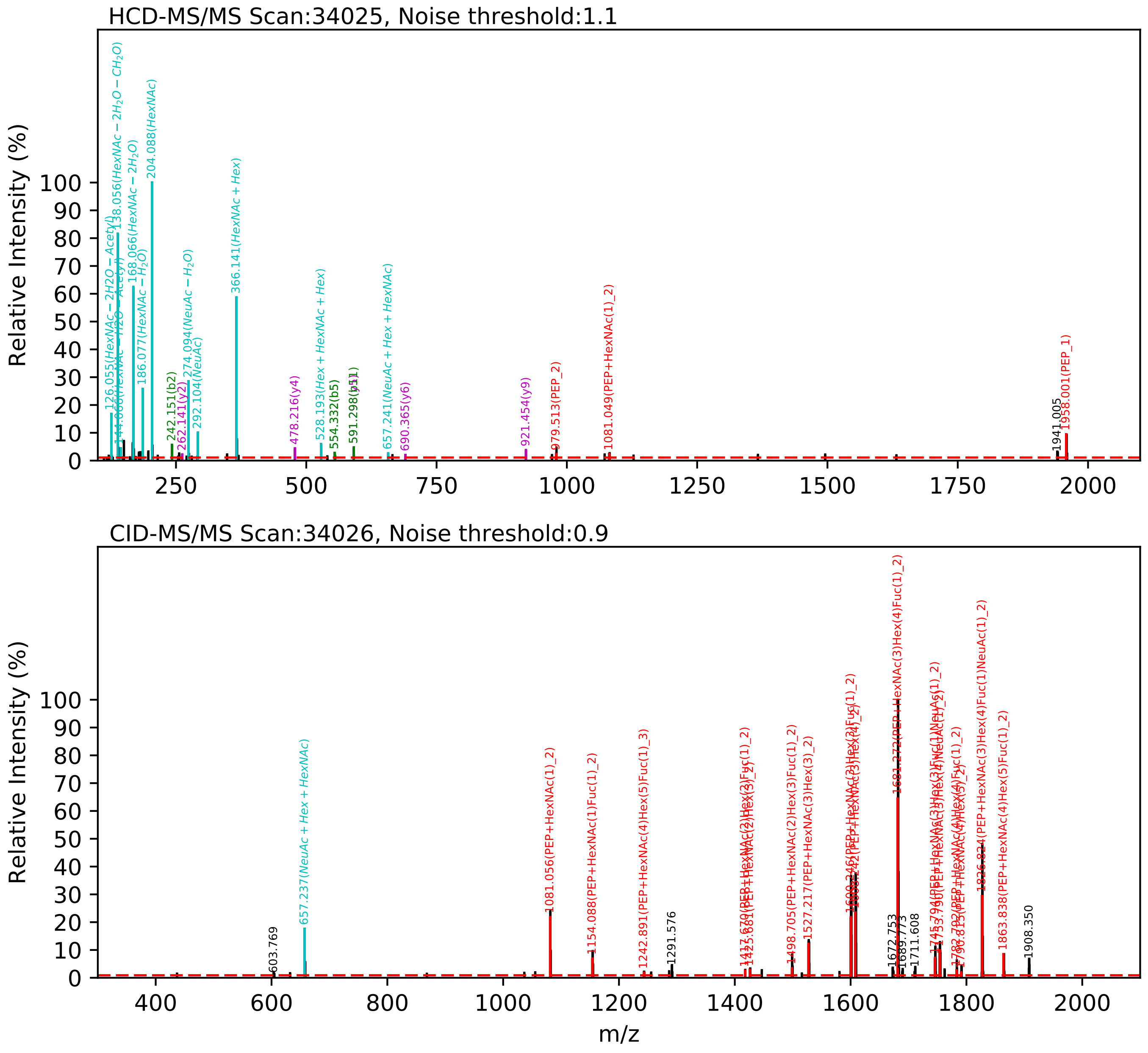

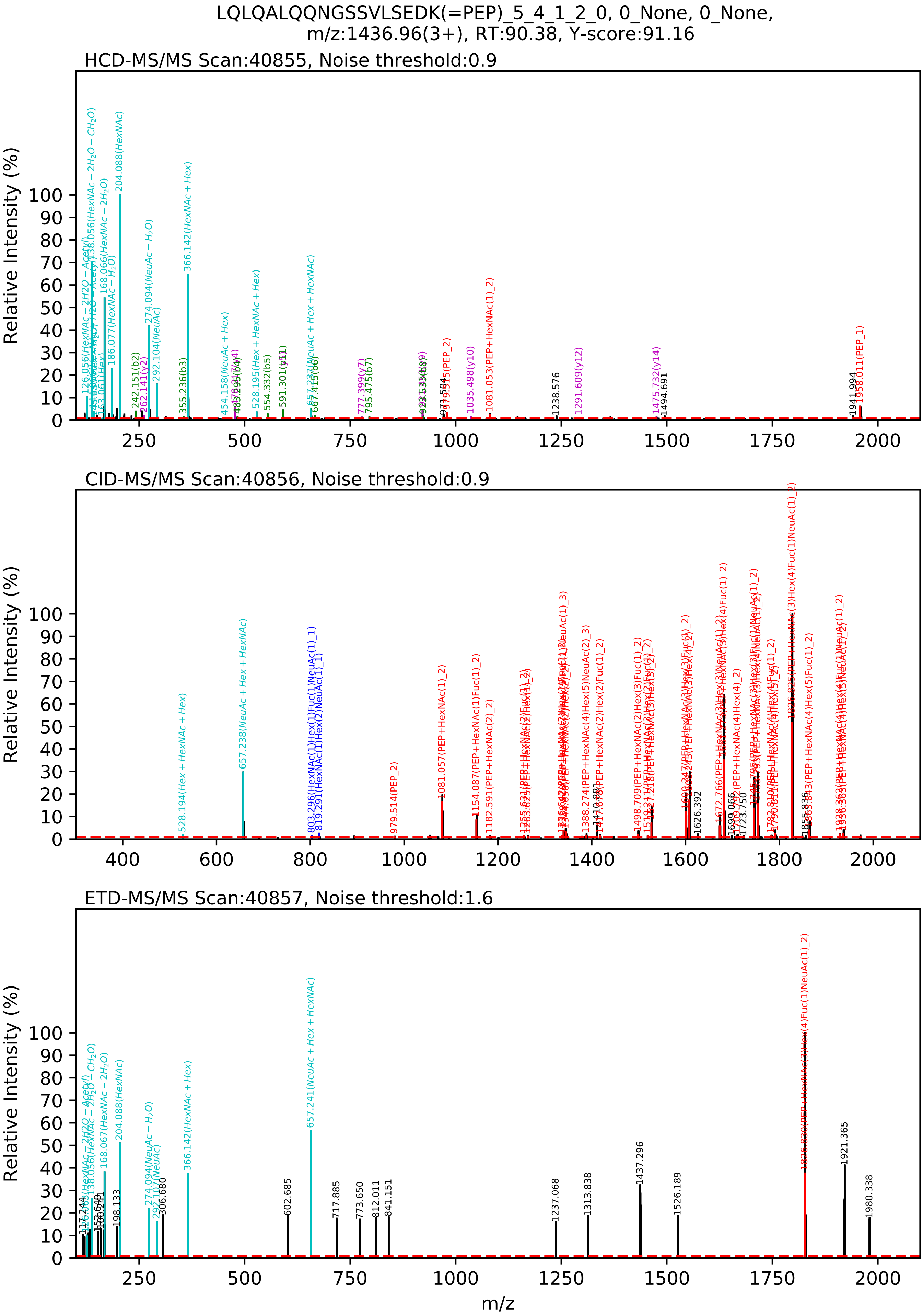

LQLQALQQNGSSVLSEDK(=PEP)_5_4_1_2_0, 0_None, 0_None,
m/z:1436.96(3+), RT:90.38, Y-score:91.16
HCD-MS/MS Scan:40855, Noise threshold:0.9
Relative Intensity (%)
204.088(HexNAc)
366.142(HexNAc + Hex)
274.094(NeuAc − H2O)
292.104(NeuAc)
186.077(HexNAc − H2O)
168.066(HexNAc − 2H2O)
126.056(HexNAc − 2H2O − CH2O)
242.151(b2)
262.141(y2)
355.236(b3)
454.158(NeuAc + Hex)
528.195(Hex + HexNAc + Hex)
554.332(b5)
591.301(y5)
657.237(NeuAc + Hex + HexNAc)
667.413(b6)
777.399(y7)
795.475(b7)
979.515(PEP_2)
1035.498(y10)
1081.053(PEP+HexNAc(1)_2)
1238.576
1291.609(y12)
1475.732(y14)
1494.691
1941.994
1958.011(PEP_1)
CID-MS/MS Scan:40856, Noise threshold:0.9
528.194(Hex + HexNAc + Hex)
657.238(NeuAc + Hex + HexNAc)
803.296(HexNAc(1)Hex(1)Fuc(1)NeuAc(1)_1)
819.291(HexNAc(1)Hex(2)NeuAc(1)_1)
979.514(PEP_2)
1081.057(PEP+HexNAc(1)_2)
1154.087(PEP+HexNAc(1)Fuc(1)_2)
1182.591(PEP+HexNAc(2)_2)
1388.274(PEP+HexNAc(4)Hex(5)NeuAc(2)_3)
1410.881
1626.392
1699.066
1723.750
1826.825(PEP+HexNAc(3)Hex(4)Fuc(1)NeuAc(1)_2)
1855.836
ETD-MS/MS Scan:40857, Noise threshold:1.6
117.244
198.133
204.088(HexNAc)
168.067(HexNAc − 2H2O)
274.094(NeuAc − H2O)
306.680
366.142(HexNAc + Hex)
602.685
657.241(NeuAc + Hex + HexNAc)
717.885
773.650
812.011
841.151
1237.068
1313.838
1437.296
1526.189
1826.820(PEP+HexNAc(3)Hex(4)Fuc(1)NeuAc(1)_2)
1921.365
1980.338
m/z

LQLQALQQNGSSVLSEDK(=PEP)_5_4_1_2_0, 0_None, 0_None,
m/z:1436.96(3+), RT:90.45, Y-score:90.50

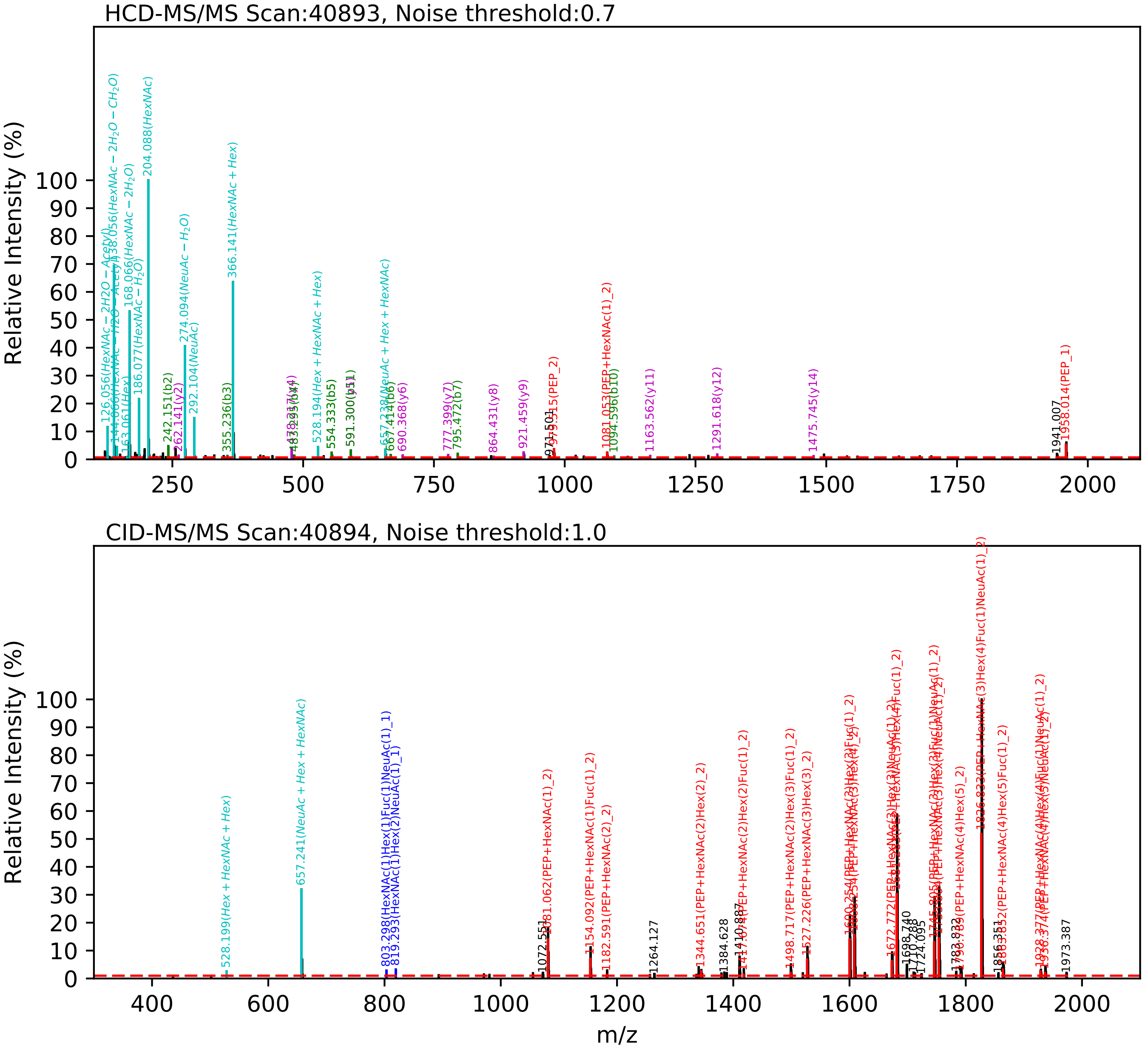

LQLQALQQNGSSVLSEDK(=PEP)_5_4_1_2_0, 0_None, 0_None,
m/z:1436.96(3+), RT:91.48, Y-score:77.71

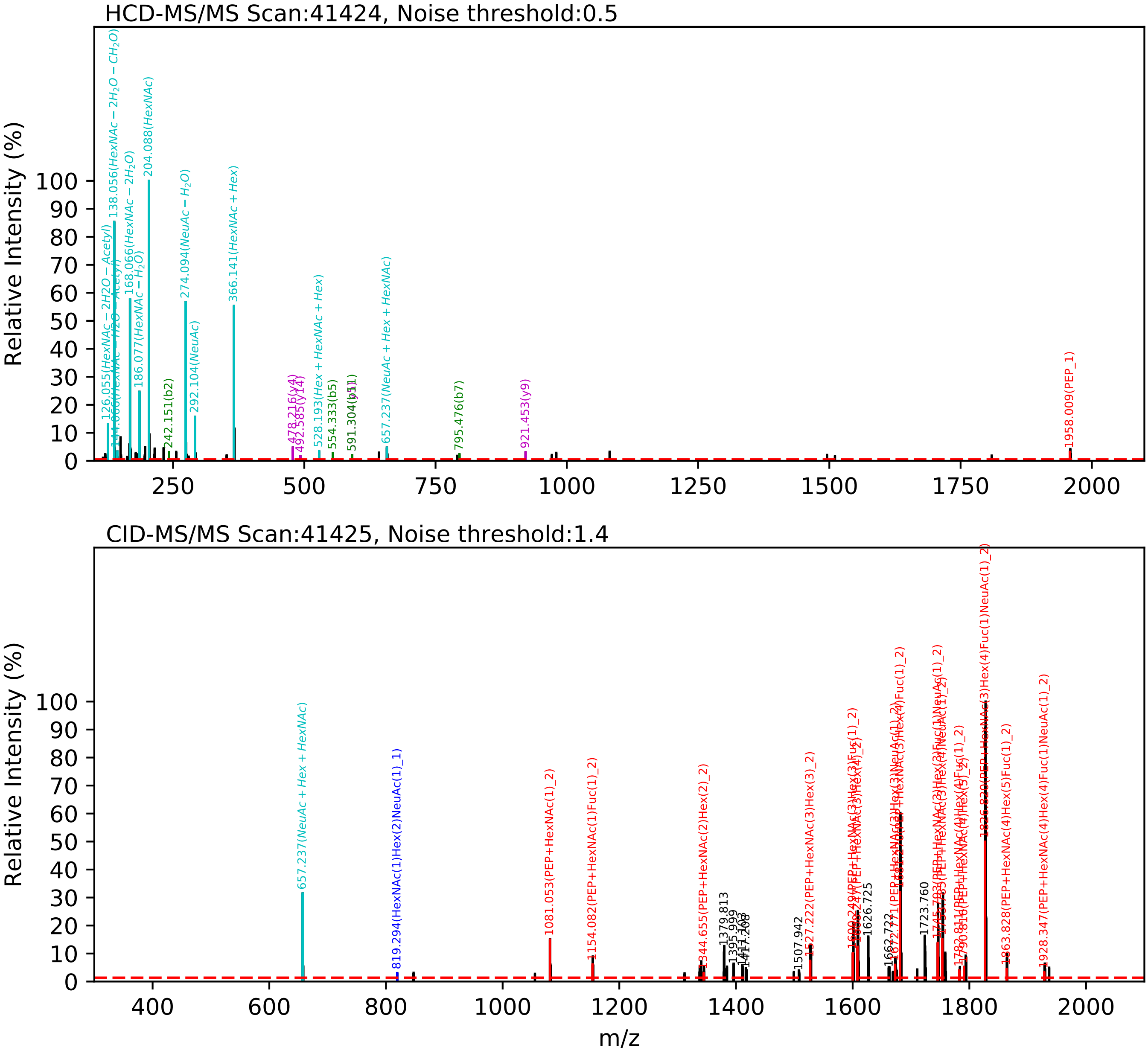

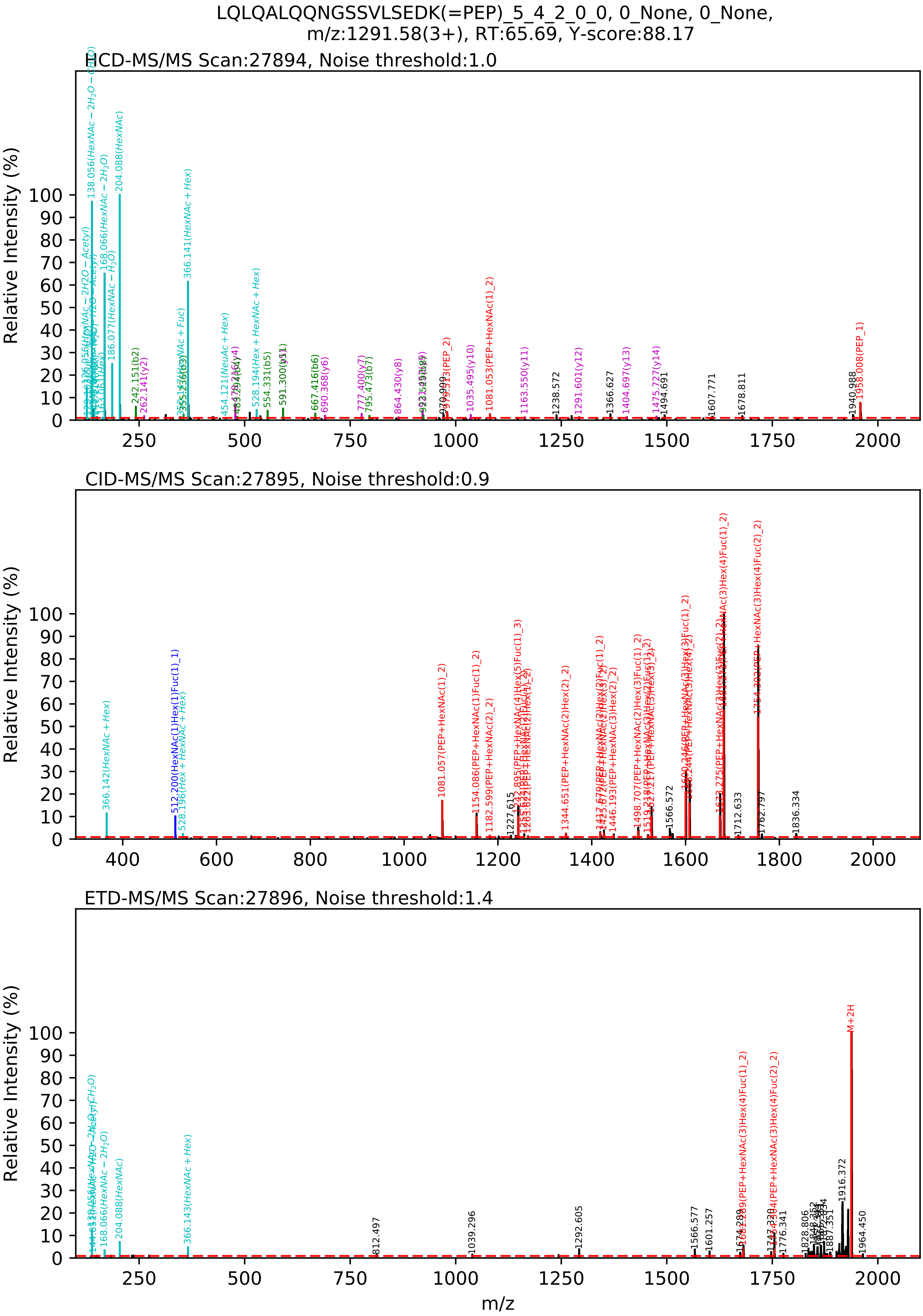

LQLQALQQNGSSVLSEDK(=PEP)_5_4_2_0_0, 0_None, 0_None,
m/z:1291.58(3+), RT:65.69, Y-score:88.17
HCD-MS/MS Scan:27894, Noise threshold:1.0
Relative Intensity (%)
138.056(HexNAc − 2H2O − CH2O)
204.088(HexNAc)
168.066(HexNAc − 2H2O)
186.077(HexNAc − H2O)
366.141(HexNAc + Hex)
528.194(Hex + HexNAc + Hex)
454.121(NeuAc + Hex)
242.151(b2)
262.141(y2)
554.331(b5)
591.300(y5)
667.416(b6)
690.368(y6)
777.400(y7)
795.473(b7)
864.430(y8)
975.513(PEP_2)
1035.495(y10)
1081.053(PEP+HexNAc(1)_2)
1163.550(y11)
1238.572
1291.601(y12)
1366.627
1404.697(y13)
1475.727(y14)
1494.691
1607.771
1678.811
1940.988
1958.008(PEP_1)
CID-MS/MS Scan:27895, Noise threshold:0.9
Relative Intensity (%)
366.142(HexNAc + Hex)
512.200(HexNAc(1)Hex(1)Fuc(1)_1)
528.196(Hex + HexNAc + Hex)
1081.057(PEP+HexNAc(1)_2)
1154.086(PEP+HexNAc(1)Fuc(1)_2)
1182.599(PEP+HexNAc(2)_2)
1227.615
1344.651(PEP+HexNAc(2)Hex(2)_2)
1446.193(PEP+HexNAc(3)Hex(2)_2)
1498.707(PEP+HexNAc(2)Hex(3)Fuc(1)_2)
1566.572
1673.275(PEP+HexNAc(3)Hex(4)Fuc(1)_2)
1712.633
1754.302(PEP+HexNAc(3)Hex(4)Fuc(2)_2)
1762.797
1836.334
ETD-MS/MS Scan:27896, Noise threshold:1.4
Relative Intensity (%)
168.066(HexNAc − 2H2O)
204.088(HexNAc)
366.143(HexNAc + Hex)
812.497
1039.296
1292.605
1566.577
1601.257
1674.289
1747.320
1776.341
1828.806
1916.372
1964.450
M+2H
m/z

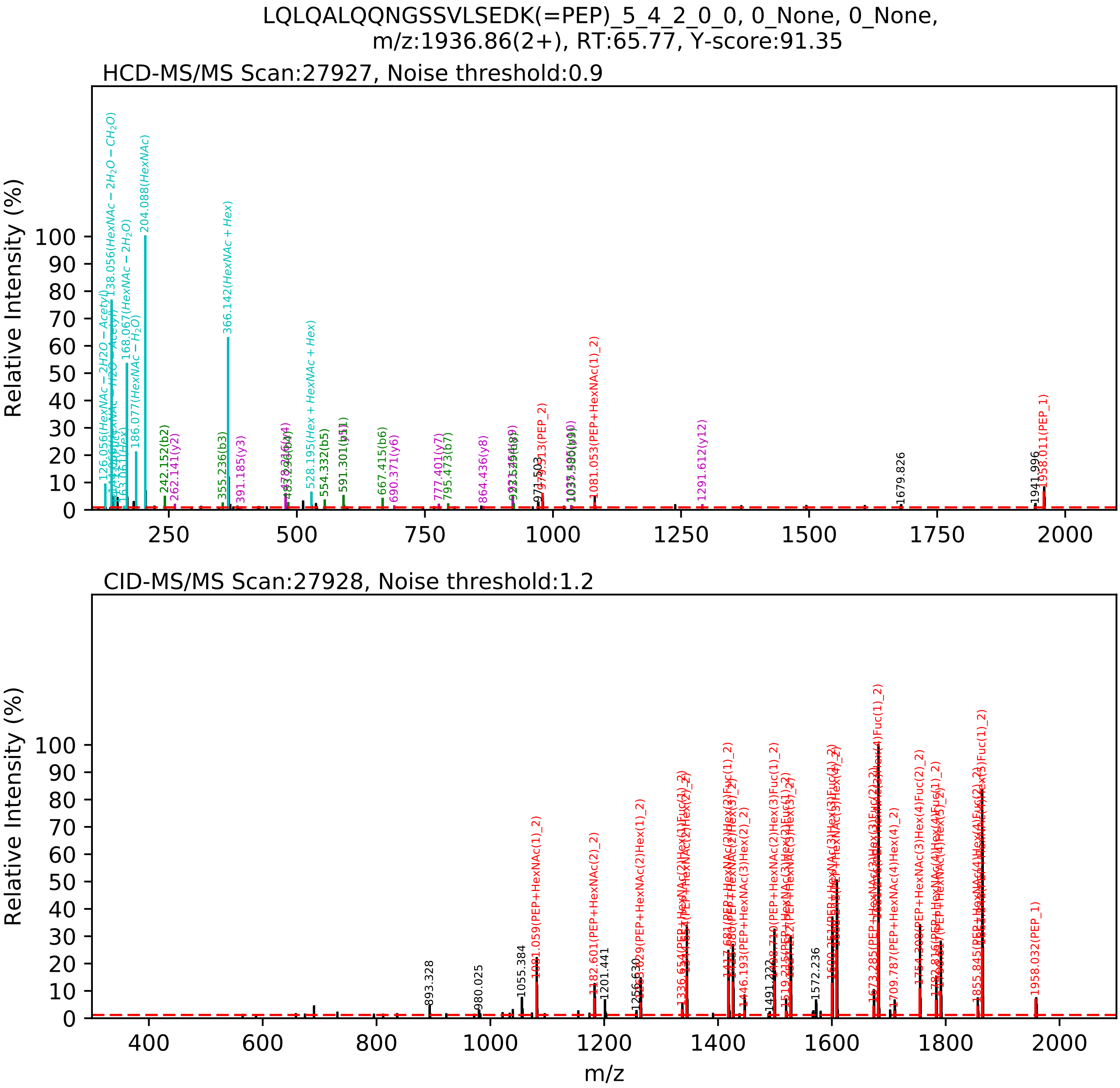
LQLQALQQNGSSVLSEDK(=PEP)_5_4_2_0_0, 0_None, 0_None,
m/z:1936.86(2+), RT:65.77, Y-score:91.35
HCD-MS/MS Scan:27927, Noise threshold:0.9
Relative Intensity (%)
204.088(HexNAc)
366.142(HexNAc + Hex)
168.067(HexNAc − 2H2O)
186.077(HexNAc − H2O)
126.056(HexNAc − 2H2O − Acetyl)
528.195(Hex + HexNAc + Hex)
242.152(b2)
262.141(y2)
355.236(b3)
391.185(y3)
554.332(b5)
591.301(b5)
667.415(b6)
690.371(y6)
777.401(y7)
795.473(b7)
864.436(y8)
971.503
979.513(PEP_2)
1081.053(PEP+HexNAc(1)_2)
1291.612(y12)
1679.826
1941.996
1958.011(PEP_1)
CID-MS/MS Scan:27928, Noise threshold:1.2
Relative Intensity (%)
893.328
980.025
1055.384
1081.059(PEP+HexNAc(1)_2)
1182.601(PEP+HexNAc(2)_2)
1201.441
1256.630
1336.654
1446.193(PEP+HexNAc(3)Hex(2)_2)
1491.222
1572.236
1709.787(PEP+HexNAc(4)Hex(4)_2)
1754.308(PEP+HexNAc(3)Hex(4)Fuc(2)_2)
1855.845
1958.032(PEP_1)
m/z

LQLQALQQNGSSVLSEDK(=PEP)_5_4_2_0_0, 0_None, 0_None,
m/z:1936.86(2+), RT:65.78, Y-score:90.82

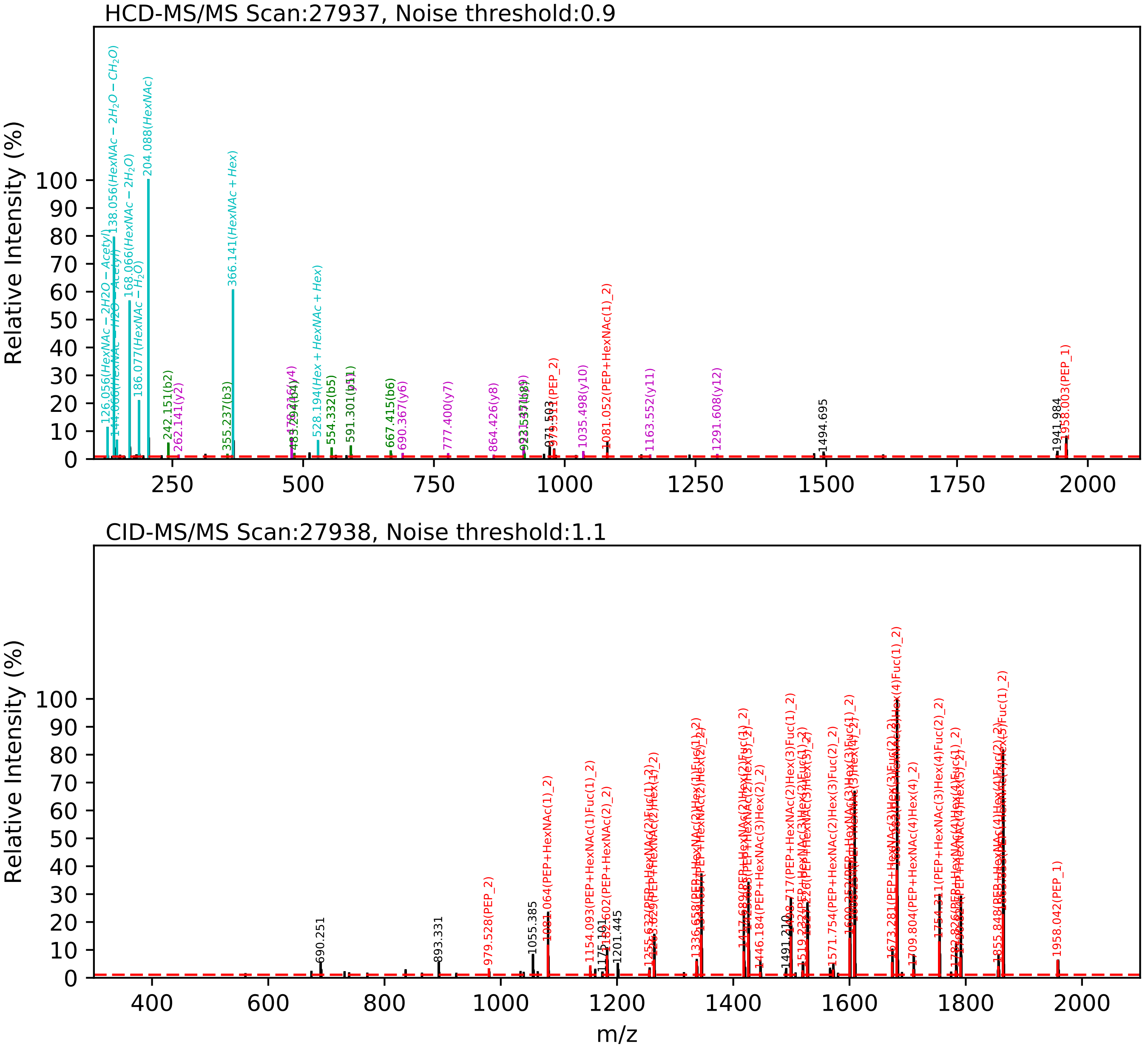

LQLQALQQNGSSVLSEDK(=PEP)_5_4_2_1_0, 0_None, 0_None,
m/z:1388.61(3+), RT:76.95, Y-score:90.95

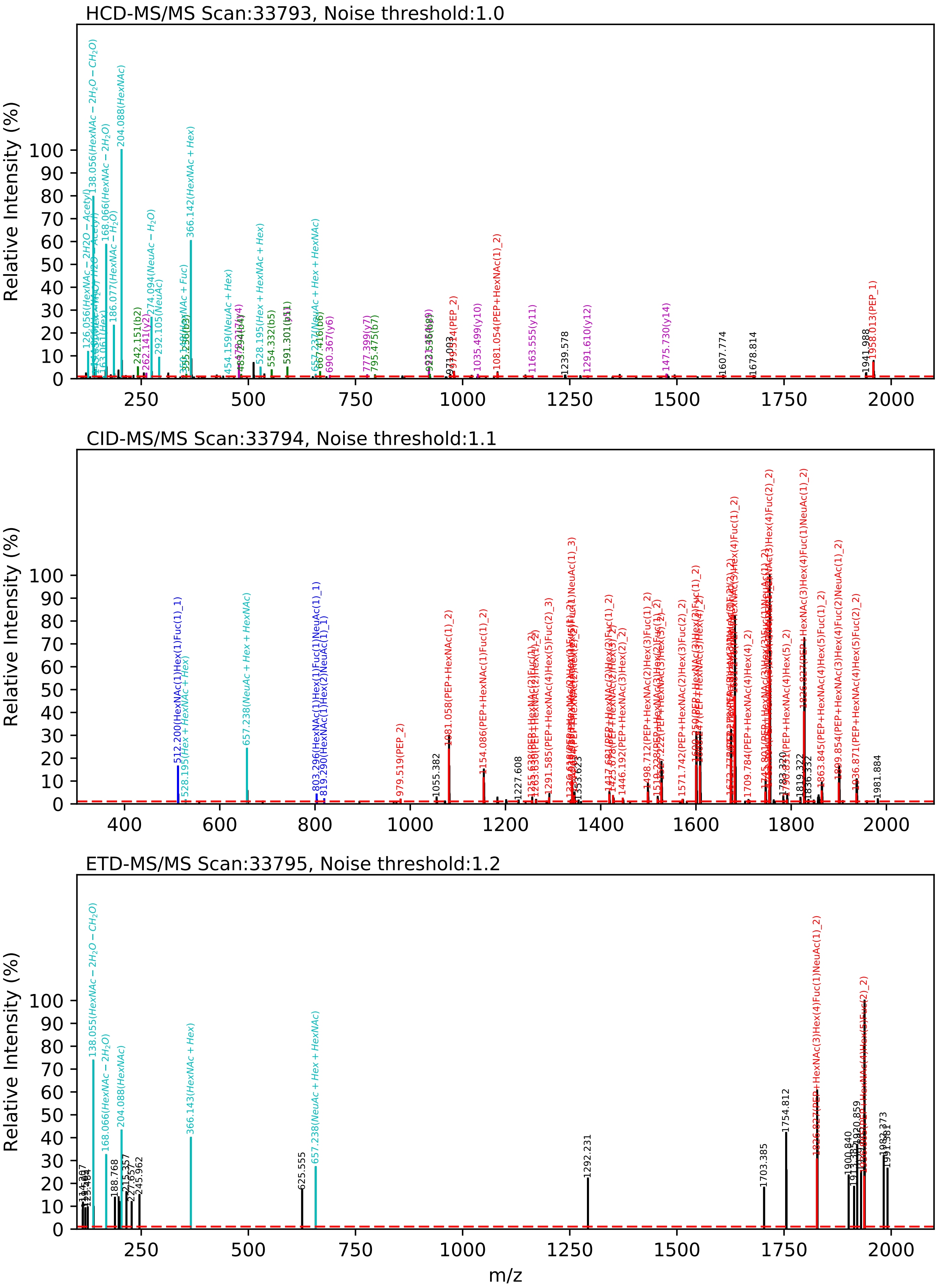

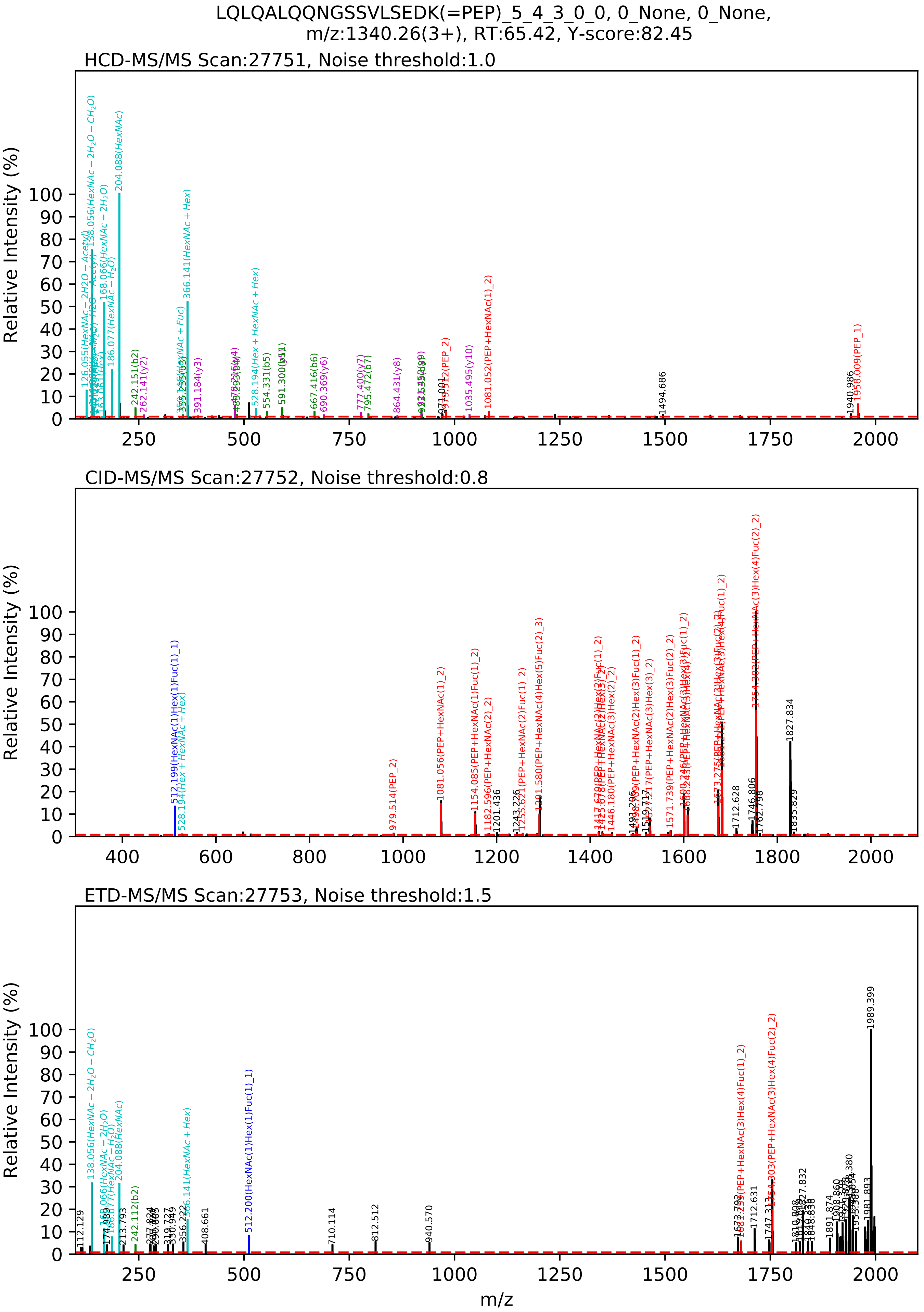
LQLQALQQNGSSVLSEDK(=PEP)_5_4_3_0_0, 0_None, 0_None,
m/z:1340.26(3+), RT:65.42, Y-score:82.45
HCD-MS/MS Scan:27751, Noise threshold:1.0
CID-MS/MS Scan:27752, Noise threshold:0.8
ETD-MS/MS Scan:27753, Noise threshold:1.5
Relative Intensity (%)
m/z
204.088(HexNAc)
366.141(HexNAc + Hex)
1081.052(PEP+HexNAc(1)_2)
1958.009(PEP_1)
1494.686
1940.986
512.199(HexNAc(1)Hex(1)Fuc(1)_1)
1754.303(PEP+HexNAc(3)Hex(4)Fuc(2)_2)
1827.834
1989.399
512.200(HexNAc(1)Hex(1)Fuc(1)_1)

LQLQALQQNGSSVLSEDK(=PEP)_5_4_3_0_0, 0_None, 0_None,
m/z:1340.26(3+), RT:65.45, Y-score:79.67

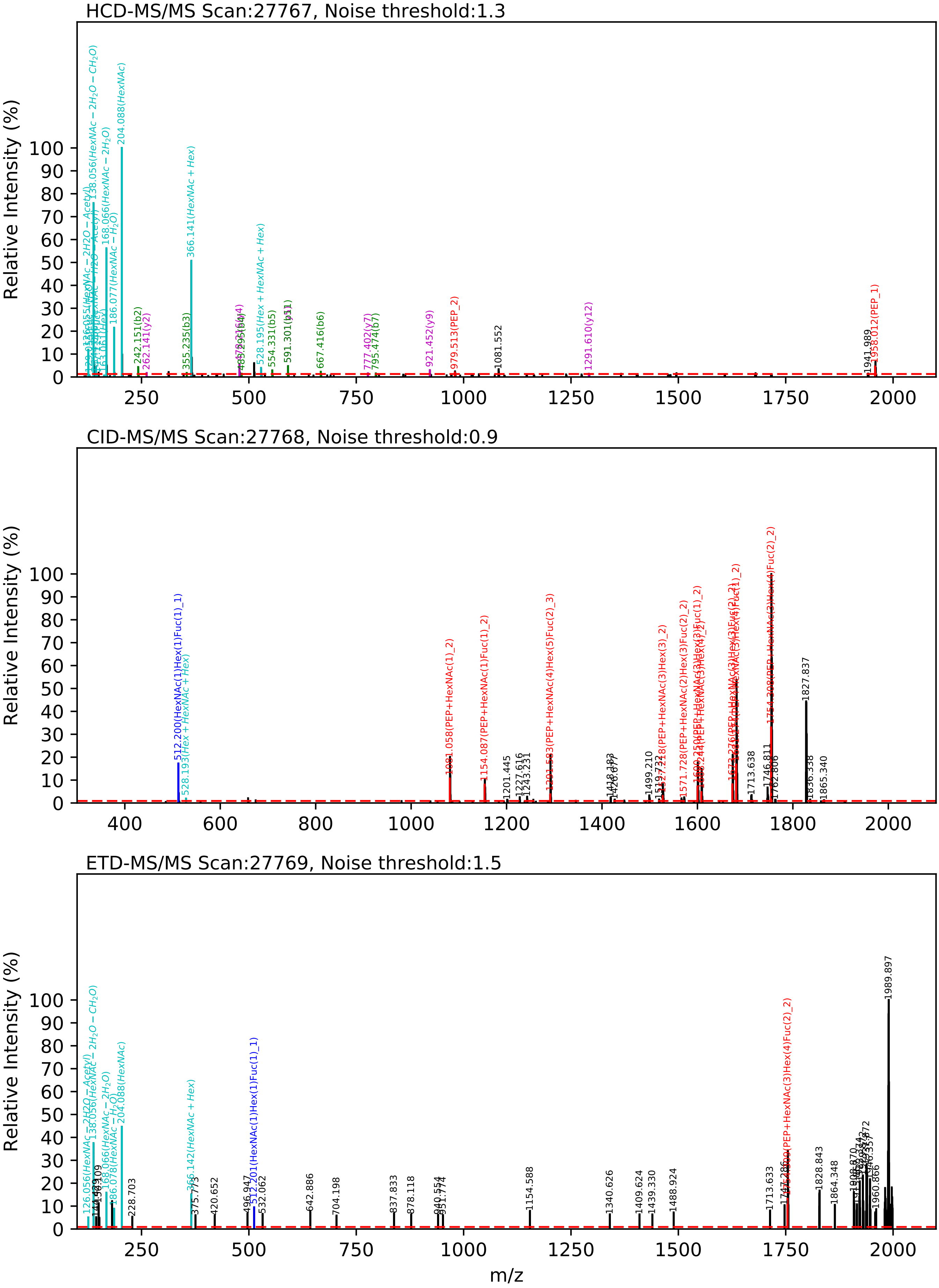

LQLQALQQNGSSVLSEDK(=PEP)_5_4_3_1_0, 0_None, 0_None,
m/z:1437.30(3+), RT:76.57, Y-score:89.98

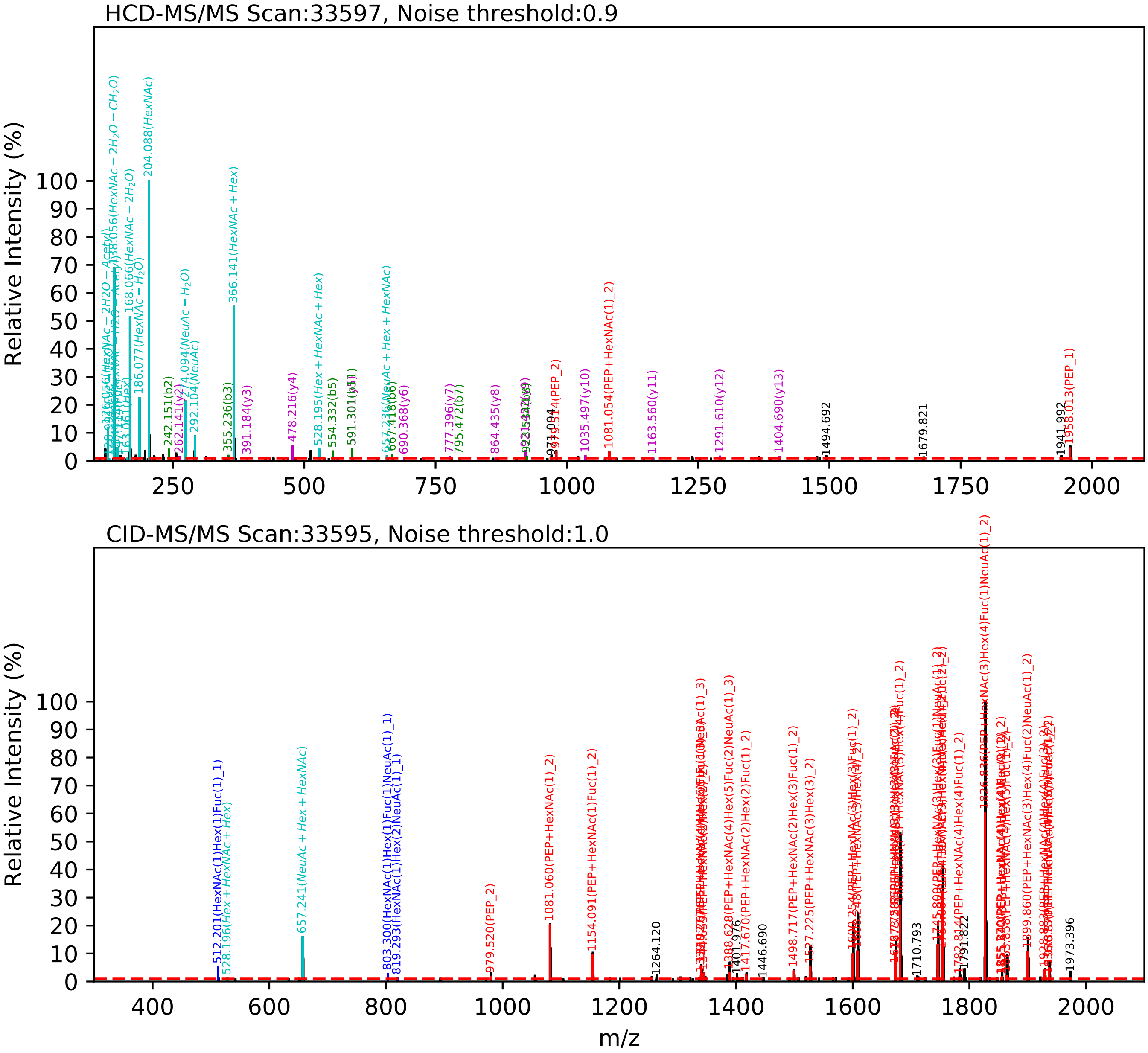

LQLQALQQNGSSVLSEDK(=PEP)_5_4_3_1_0, 0_None, 0_None,
m/z:1078.22(4+), RT:76.50, Y-score:84.63

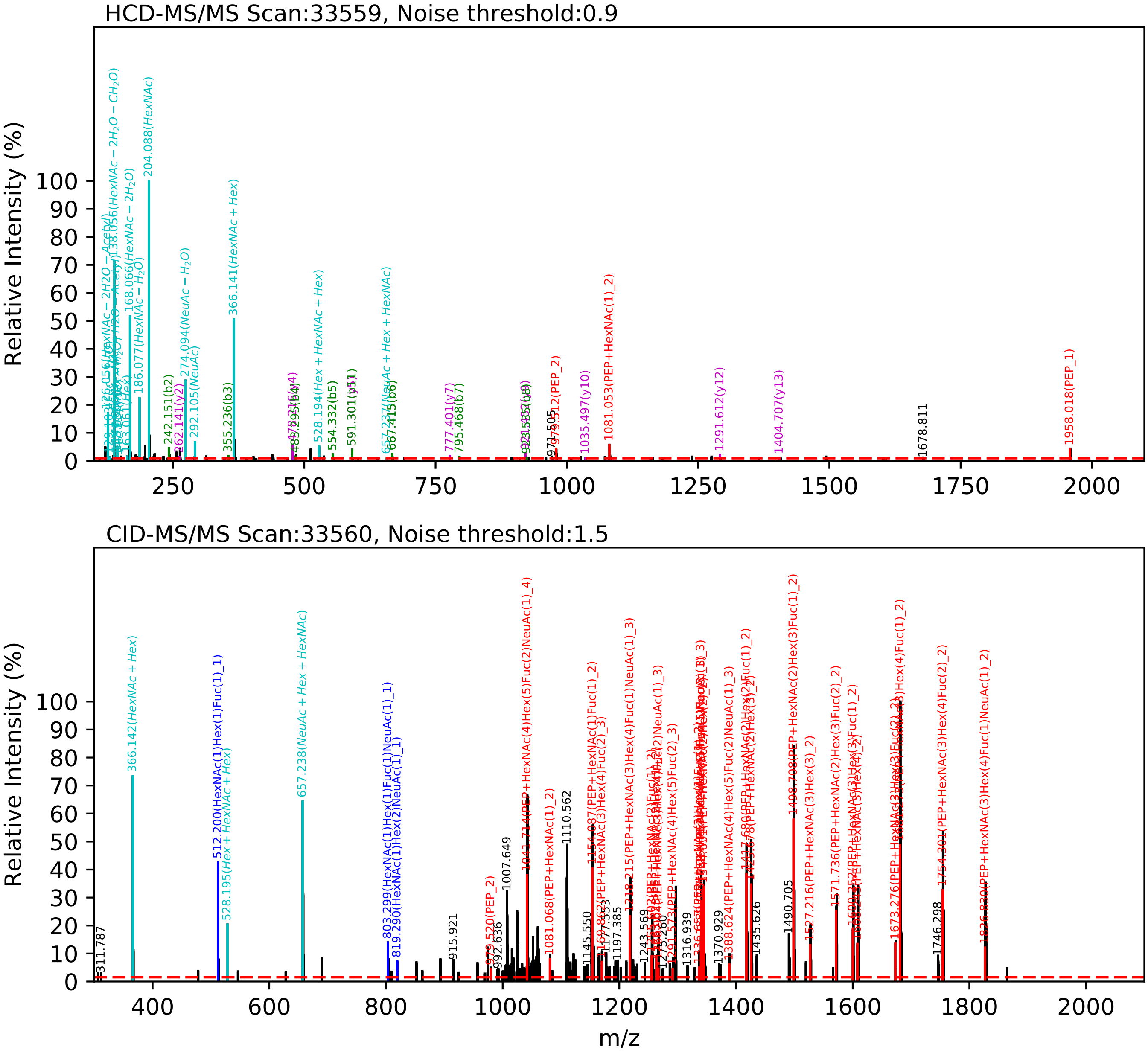

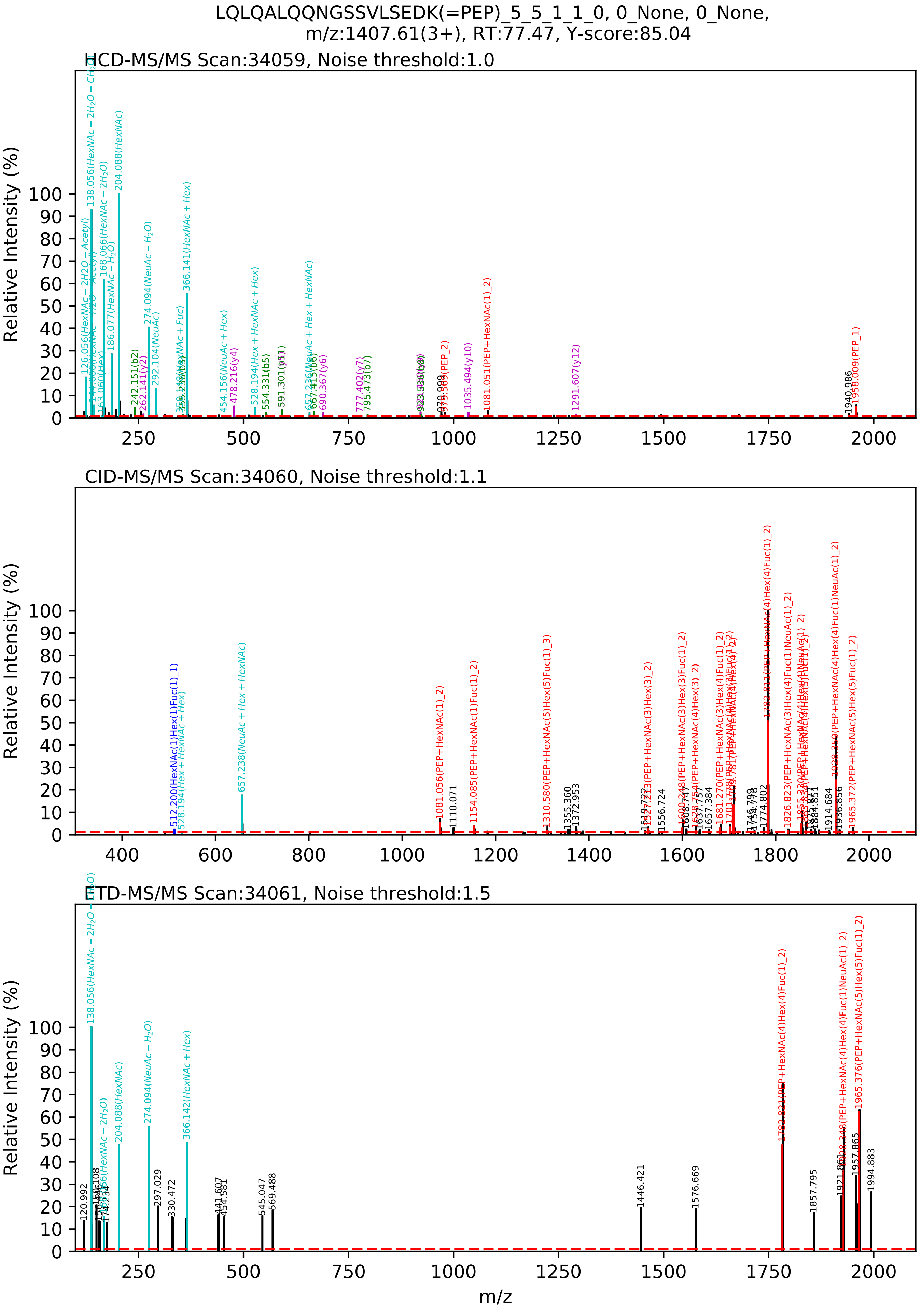
LQLQALQQNGSSVLSEDK(=PEP)_5_5_1_1_0, 0_None, 0_None,
m/z:1407.61(3+), RT:77.47, Y-score:85.04
HCD-MS/MS Scan:34059, Noise threshold:1.0
CID-MS/MS Scan:34060, Noise threshold:1.1
ETD-MS/MS Scan:34061, Noise threshold:1.5
Relative Intensity (%)
m/z

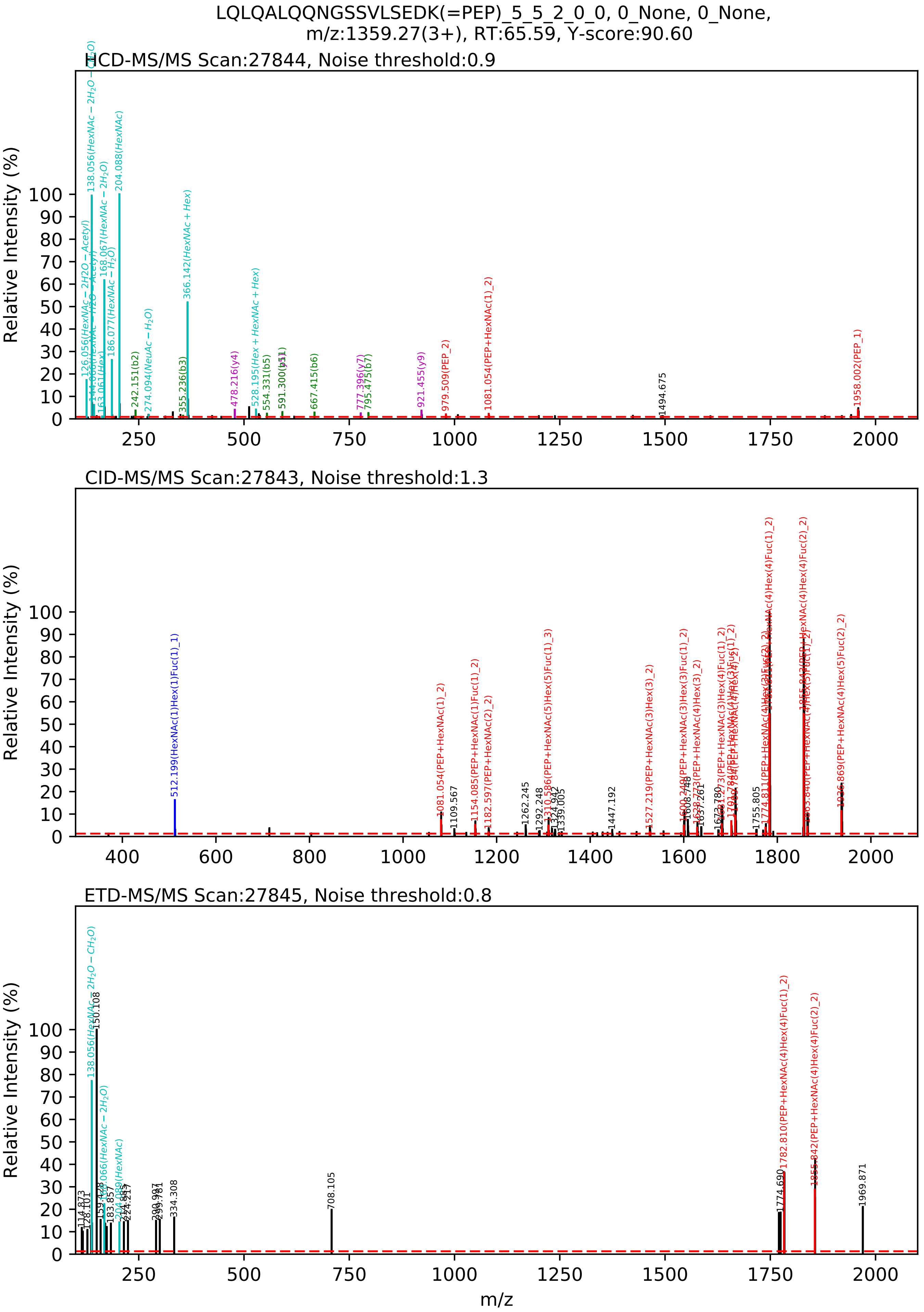
LQLQALQQNGSSVLSEDK(=PEP)_5_5_2_0_0, 0_None, 0_None,
m/z:1359.27(3+), RT:65.59, Y-score:90.60
HCD-MS/MS Scan:27844, Noise threshold:0.9
Relative Intensity (%)
138.056(HexNAc)
168.067(HexNAc − 2H2O)
186.077(HexNAc − H2O)
204.088(HexNAc)
366.142(HexNAc + Hex)
242.151(b2)
274.094(NeuAc − H2O)
355.236(b3)
478.216(y4)
528.195(Hex + HexNAc + Hex)
554.331(b5)
667.415(b6)
777.396(y7)
795.475(b7)
921.455(y9)
979.509(PEP_2)
1081.054(PEP+HexNAc(1)_2)
1494.675
1958.002(PEP_1)
CID-MS/MS Scan:27843, Noise threshold:1.3
Relative Intensity (%)
512.199(HexNAc(1)Hex(1)Fuc(1)_1)
1081.054(PEP+HexNAc(1)_2)
1109.567
1154.085(PEP+HexNAc(1)Fuc(1)_2)
1182.597(PEP+HexNAc(2)_2)
1262.245
1292.248
1310.586(PEP+HexNAc(5)Hex(5)Fuc(1)_3)
1324.942
1339.005
1447.192
1527.219(PEP+HexNAc(3)Hex(3)_2)
1600.249(PEP+HexNAc(3)Hex(3)Fuc(1)_2)
1608.748
1628.773(PEP+HexNAc(4)Hex(3)_2)
1637.261
1673.780
1755.805
1774.811(PEP+HexNAc(4)Hex(3)Fuc(2)_2)
1782.810(PEP+HexNAc(4)Hex(4)Fuc(1)_2)
1855.842(PEP+HexNAc(4)Hex(4)Fuc(2)_2)
1936.869(PEP+HexNAc(4)Hex(5)Fuc(2)_2)
ETD-MS/MS Scan:27845, Noise threshold:0.8
Relative Intensity (%)
138.056(HexNAc − 2H2O − CH2O)
150.108
168.066(HexNAc − 2H2O)
204.089(HexNAc)
114.873
128.101
159.428
183.857
214.965
224.217
290.997
299.781
334.308
708.105
1774.690
1782.810(PEP+HexNAc(4)Hex(4)Fuc(1)_2)
1855.842(PEP+HexNAc(4)Hex(4)Fuc(2)_2)
1969.871
m/z

LQLQALQQNGSSVLSEDK(=PEP)_6_2_0_0_0, 0_None, 0_None,
m/z:1112.83(3+), RT:66.31, Y-score:82.46

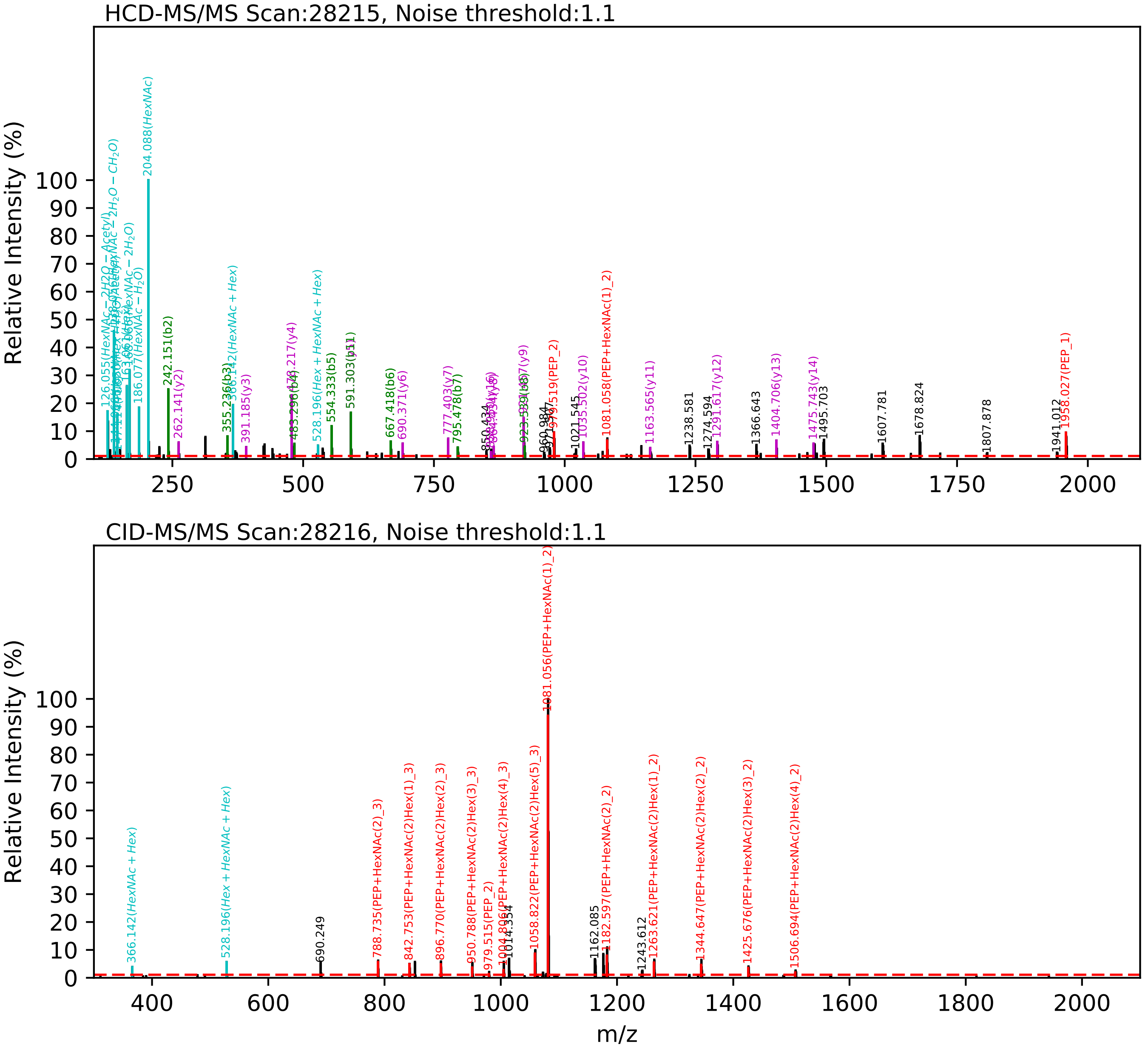

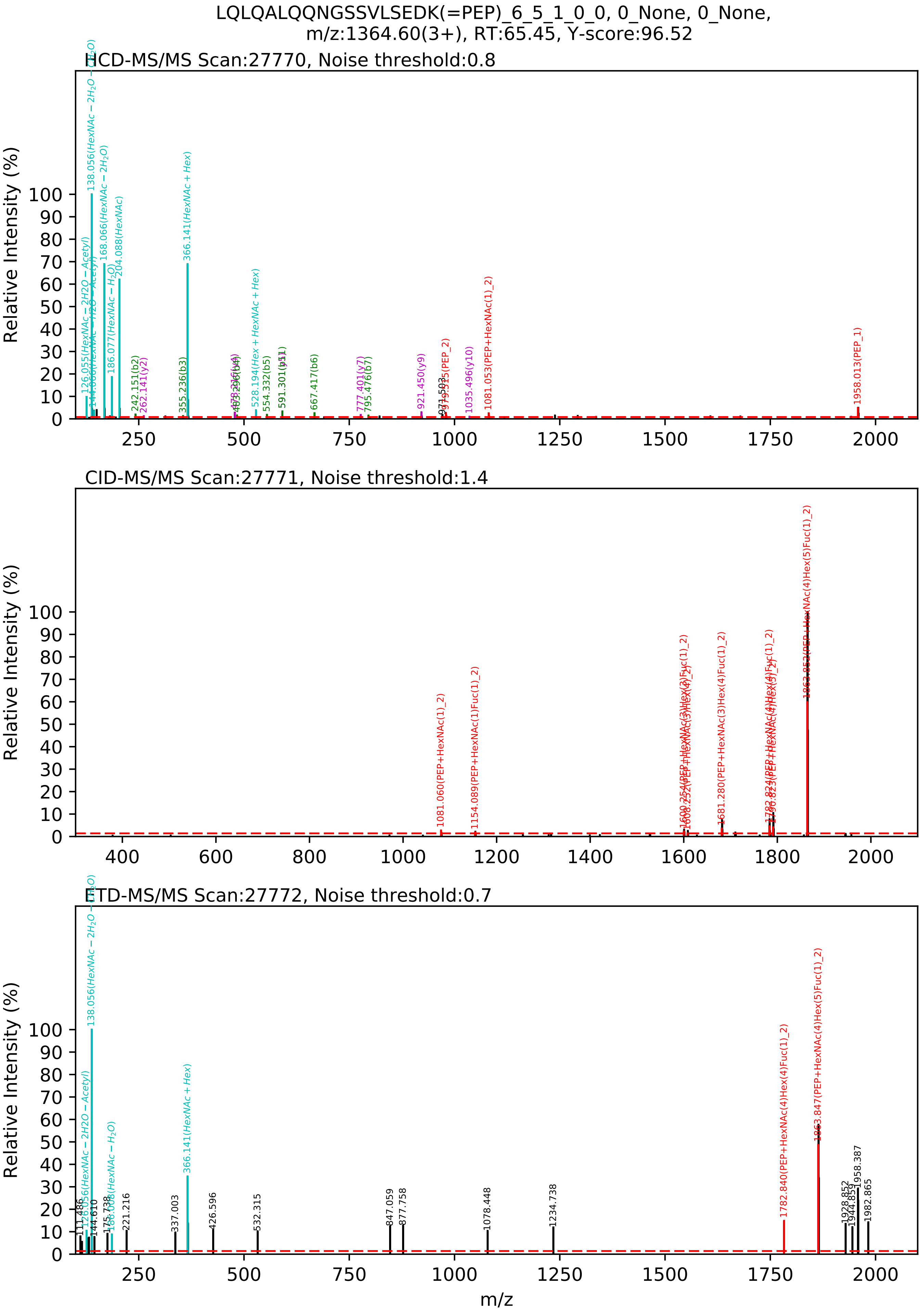

LQLQALQQNGSSVLSEDK(=PEP)_6_5_1_0_0, 0_None, 0_None,
m/z:1364.60(3+), RT:65.45, Y-score:96.52
HCD-MS/MS Scan:27770, Noise threshold:0.8
138.056(HexNAc − 2H2O − H2O)
168.066(HexNAc − 2H2O)
204.088(HexNAc)
186.077(HexNAc − H2O)
366.141(HexNAc + Hex)
528.194(Hex + HexNAc + Hex)
242.151(b2)
262.141(y2)
355.236(b3)
554.332(b5)
591.301(b11)
667.417(b6)
777.401(y7)
795.476(b7)
921.450(y9)
971.503
1035.496(y10)
1081.053(PEP+HexNAc(1)_2)
1958.013(PEP_1)
CID-MS/MS Scan:27771, Noise threshold:1.4
1081.060(PEP+HexNAc(1)_2)
1154.089(PEP+HexNAc(1)Fuc(1)_2)
1681.280(PEP+HexNAc(3)Hex(4)Fuc(1)_2)
1863.852(PEP+HexNAc(4)Hex(5)Fuc(1)_2)
ETD-MS/MS Scan:27772, Noise threshold:0.7
138.056(HexNAc − 2H2O − H2O)
366.141(HexNAc + Hex)
111.486
144.610
175.738
221.216
337.003
426.596
532.315
847.059
877.758
1078.448
1234.738
1782.840(PEP+HexNAc(4)Hex(4)Fuc(1)_2)
1863.847(PEP+HexNAc(4)Hex(5)Fuc(1)_2)
1928.852
1944.859
1958.387
1982.865
Relative Intensity (%)
m/z

LQLQALQQNGSSVLSEDK(=PEP)_6_5_1_1_0, 0_None, 0_None,
m/z:1461.63(3+), RT:76.28, Y-score:83.23

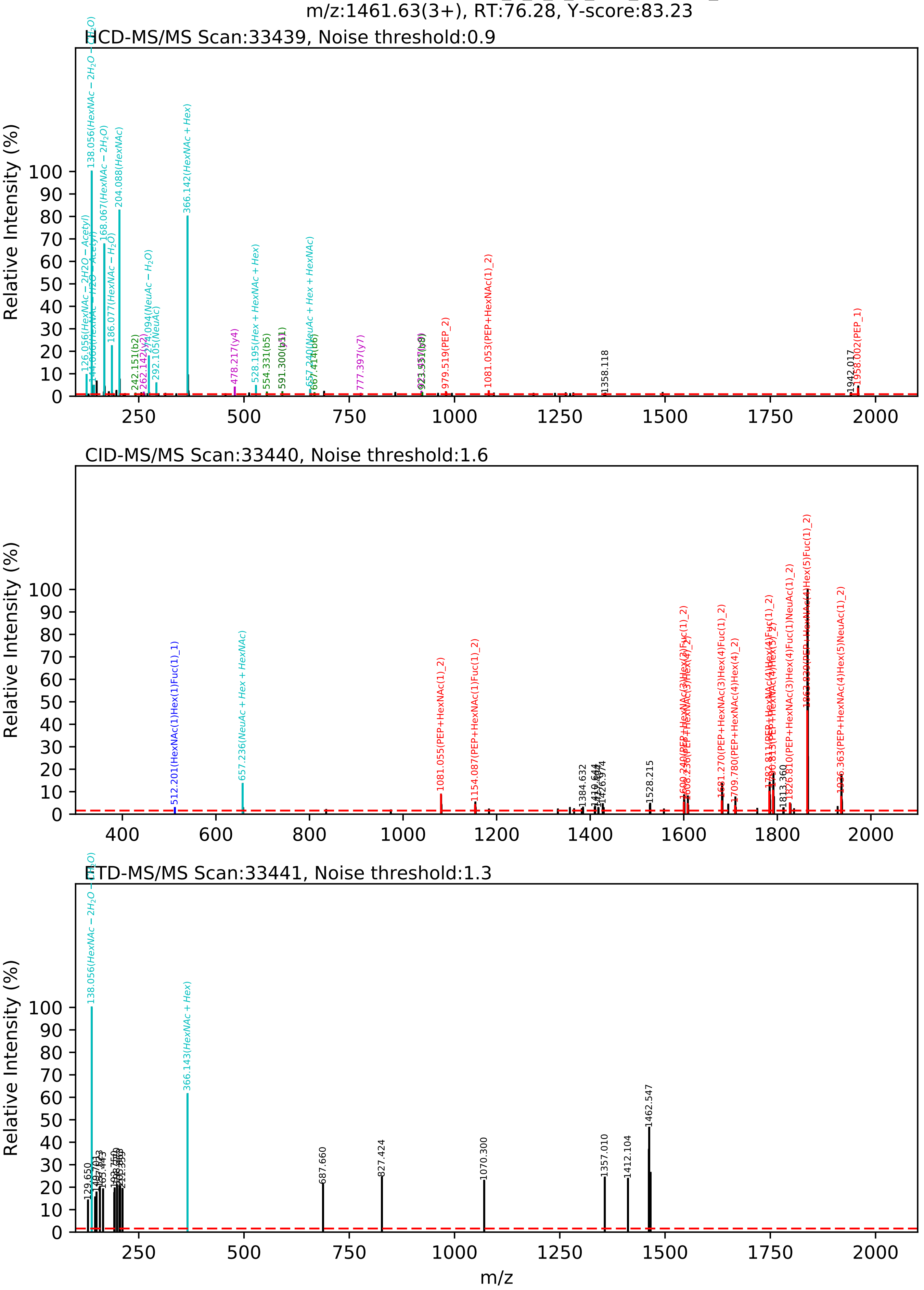

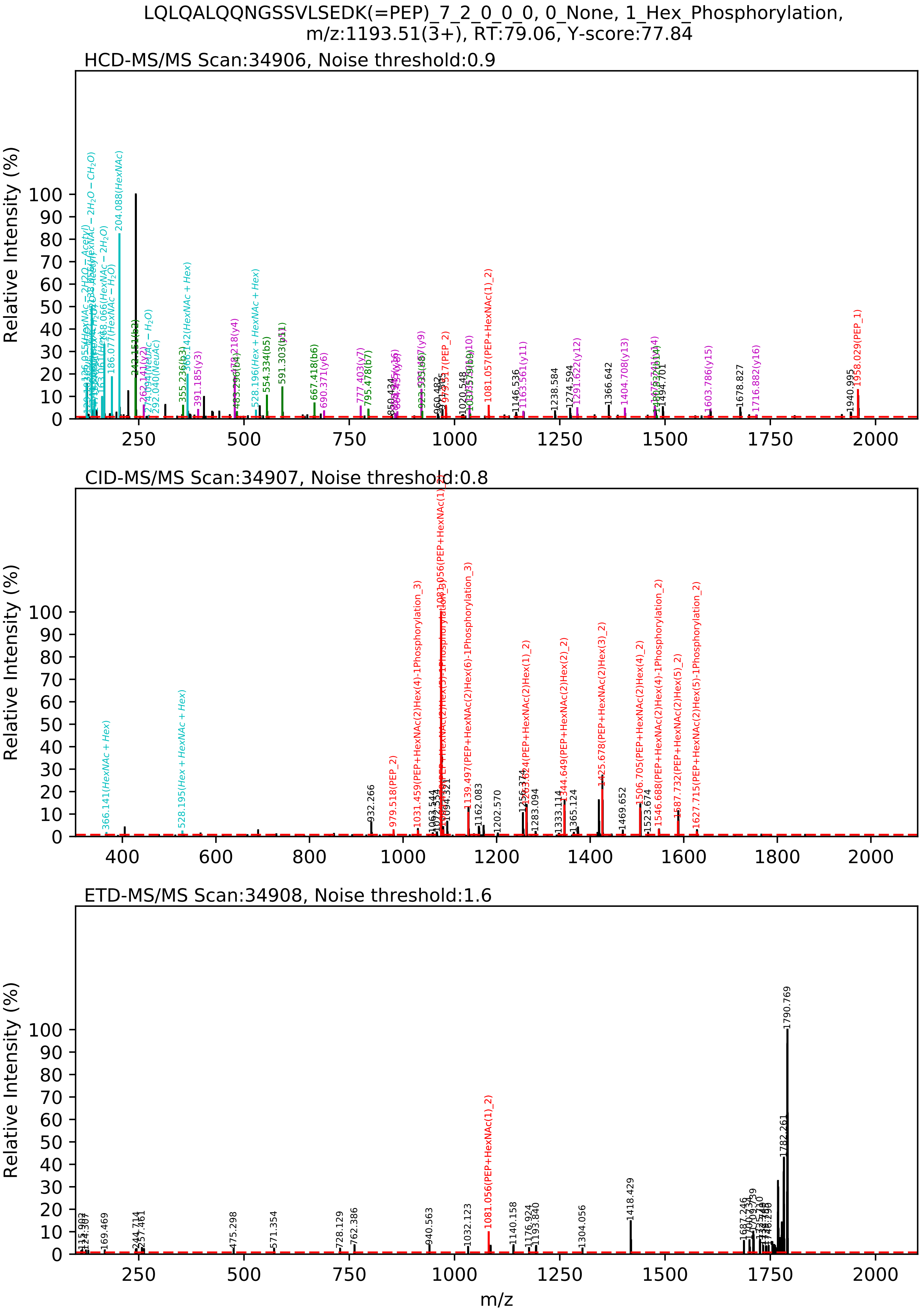

LQLQALQQNGSSVLSEDK(=PEP)_7_2_0_0_0, 0_None, 1_Hex_Phosphorylation,
m/z:1193.51(3+), RT:79.06, Y-score:77.84
HCD-MS/MS Scan:34906, Noise threshold:0.9
204.088(HexNAc)
1081.057(PEP+HexNAc(1)_2)
1958.029(PEP_1)
CID-MS/MS Scan:34907, Noise threshold:0.8
1425.678(PEP+HexNAc(2)Hex(3)_2)
1139.497(PEP+HexNAc(2)Hex(6)-1Phosphorylation_3)
ETD-MS/MS Scan:34908, Noise threshold:1.6
1790.769
1782.261
1418.429
1081.056(PEP+HexNAc(1)_2)
Relative Intensity (%)
m/z

LQLQALQQNGSSVLSEDK(=PEP)_7_2_0_0_0, 0_None, 0_None,
m/z:1166.85(3+), RT:65.94, Y-score:81.77

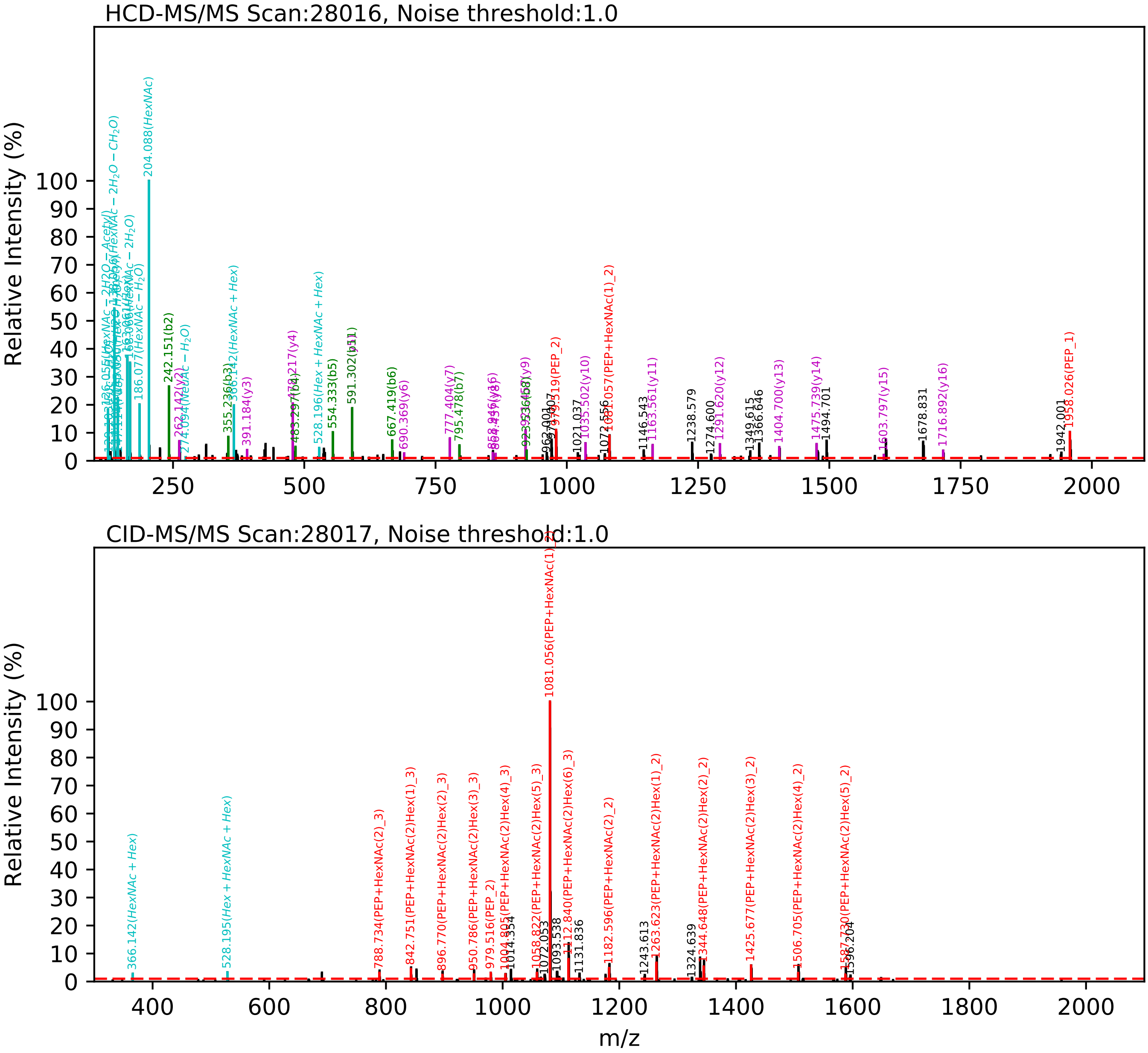

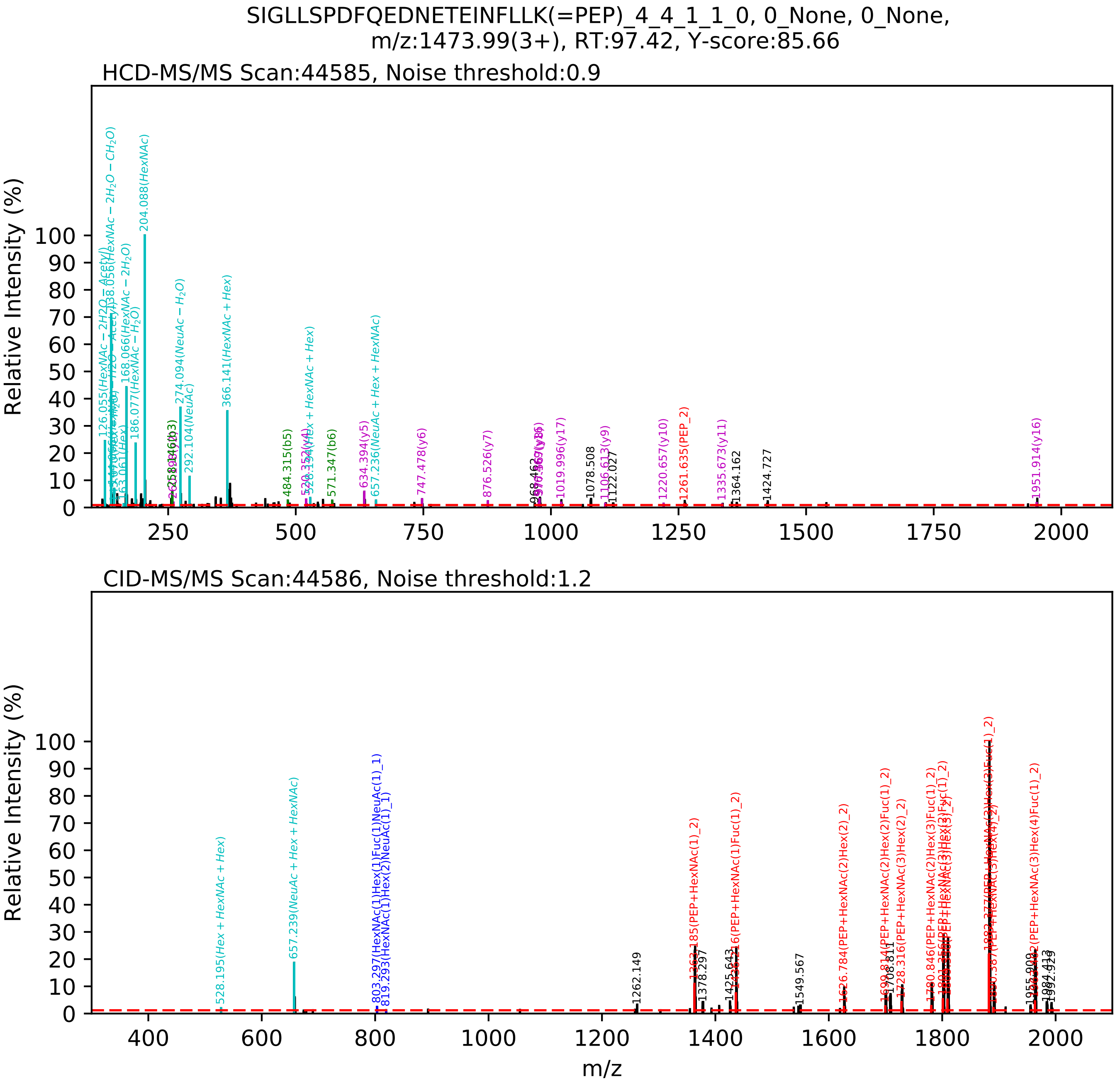
SIGLLSPDFQEDNETEINFLLK(=PEP)_4_4_1_1_0, 0_None, 0_None,
m/z:1473.99(3+), RT:97.42, Y-score:85.66
HCD-MS/MS Scan:44585, Noise threshold:0.9
Relative Intensity (%)
204.088(HexNAc)
366.141(HexNAc + Hex)
274.094(NeuAc − H2O)
292.104(NeuAc)
186.077(HexNAc − H2O)
168.066(HexNAc − 2H2O)
126.055(HexNAc − 2H2O − CH2O)
484.315(b5)
571.347(b6)
634.394(y5)
657.236(NeuAc + Hex + HexNAc)
747.478(y6)
876.526(y7)
1019.996(y17)
1078.508
1122.027
1106.613(y9)
1220.657(y10)
1261.635(PEP_2)
1335.673(y11)
1364.162
1424.727
1951.914(y16)
CID-MS/MS Scan:44586, Noise threshold:1.2
528.195(Hex + HexNAc + Hex)
657.239(NeuAc + Hex + HexNAc)
803.297(HexNAc(1)Hex(1)Fuc(1)NeuAc(1)_1)
819.293(HexNAc(1)Hex(2)NeuAc(1)_1)
1262.149
1378.297
1425.643
1549.567
1626.784(PEP+HexNAc(2)Hex(2)_2)
1728.316(PEP+HexNAc(3)Hex(2)_2)
1992.929
m/z

SIGLLSPDFQEDNETEINFLLK(=PEP)_4_5_1_0_0, 0_None, 0_None,
m/z:1444.65(3+), RT:96.87, Y-score:85.01

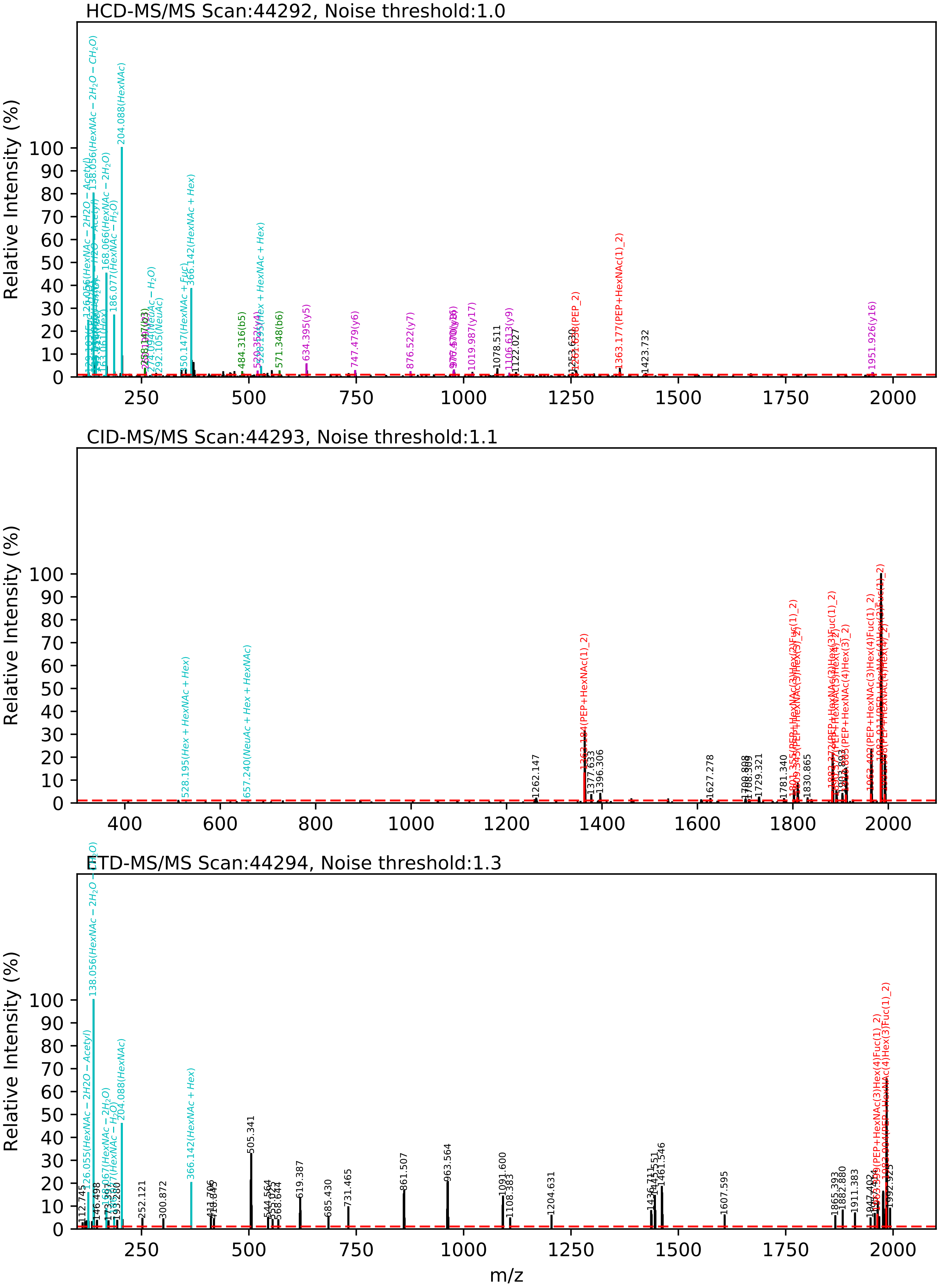

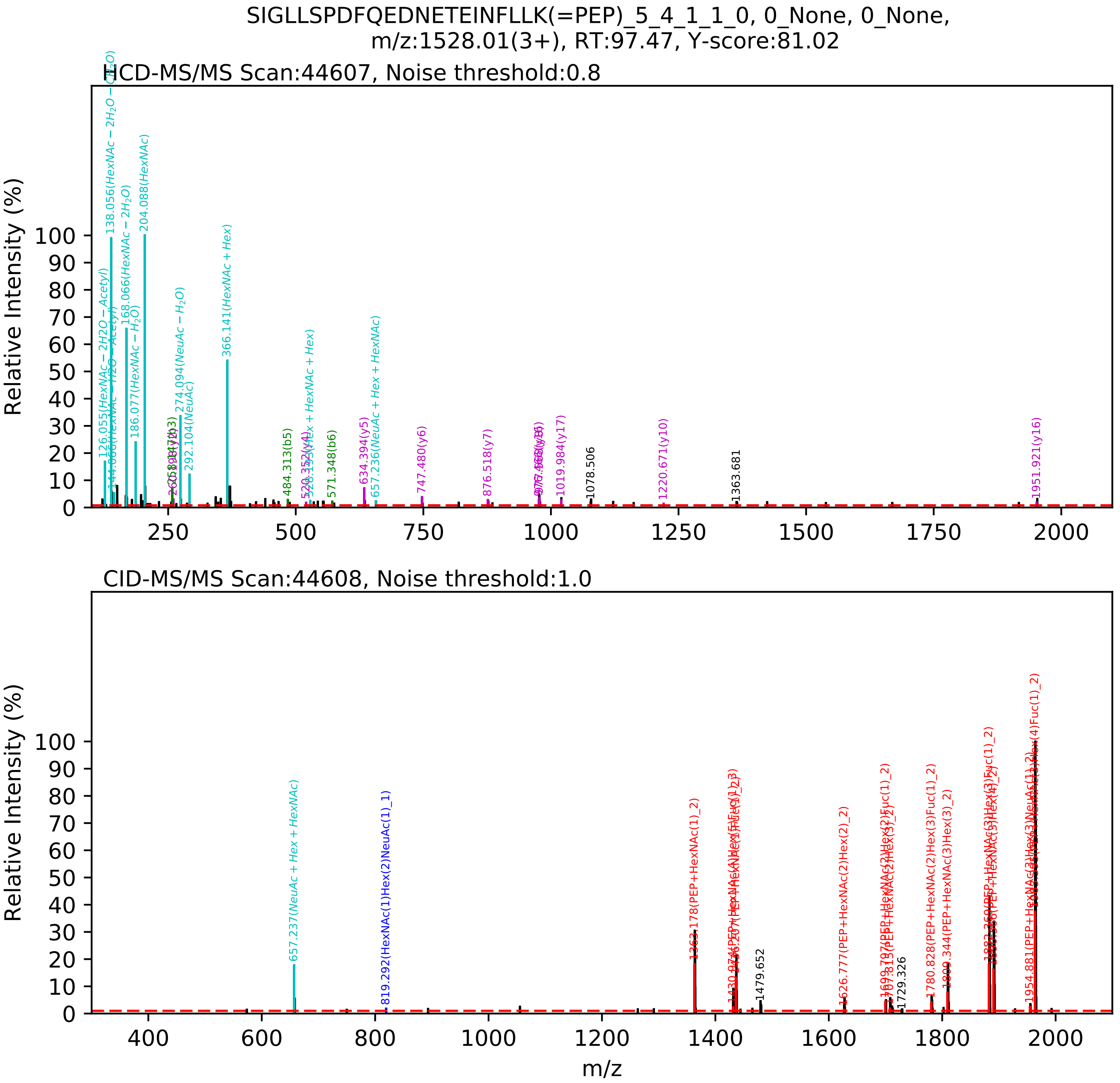
SIGLLSPDFQEDNETEINFLLK(=PEP)_5_4_1_1_0, 0_None, 0_None,
m/z:1528.01(3+), RT:97.47, Y-score:81.02
HCD-MS/MS Scan:44607, Noise threshold:0.8
Relative Intensity (%)
138.056(HexNAc − 2H2O − C2H4O)
204.088(HexNAc)
168.066(HexNAc − 2H2O)
186.077(HexNAc − H2O)
126.055(HexNAc − 2H2O − Acetyl)
274.094(NeuAc − H2O)
292.104(NeuAc)
366.141(HexNAc + Hex)
484.313(b5)
571.348(b6)
634.394(y5)
657.236(NeuAc + Hex + HexNAc)
747.480(y6)
876.518(y7)
1019.984(y17)
1078.506
1220.671(y10)
1363.681
1951.921(y16)
CID-MS/MS Scan:44608, Noise threshold:1.0
Relative Intensity (%)
657.237(NeuAc + Hex + HexNAc)
819.292(HexNAc(1)Hex(2)NeuAc(1)_1)
1363.178(PEP+HexNAc(1)_2)
1479.652
1626.777(PEP+HexNAc(2)Hex(2)_2)
1729.326
1780.828(PEP+HexNAc(2)Hex(3)Fuc(1)_2)
1809.344(PEP+HexNAc(3)Hex(3)_2)
1954.881(PEP+HexNAc(3)Hex(3)NeuAc(1)_2)
m/z
