# Supplementary material for: Characterization of Site-Specific N- and O-Glycopeptides from Recombinant Spike and ACE2 Glycoproteins Using LC-MS/MS Analysis
Source: Int J Mol Sci. 2024 Dec 20;25(24):13649. doi: 10.3390/ijms252413649 (PMC11678118; doi:10.3390/ijms252413649)

IQNLTVK(=PEP)_5_4_1_0_0, 0_None, 0_None,
m/z:1292.57(4+), RT:26.03, hcd-score:87.50

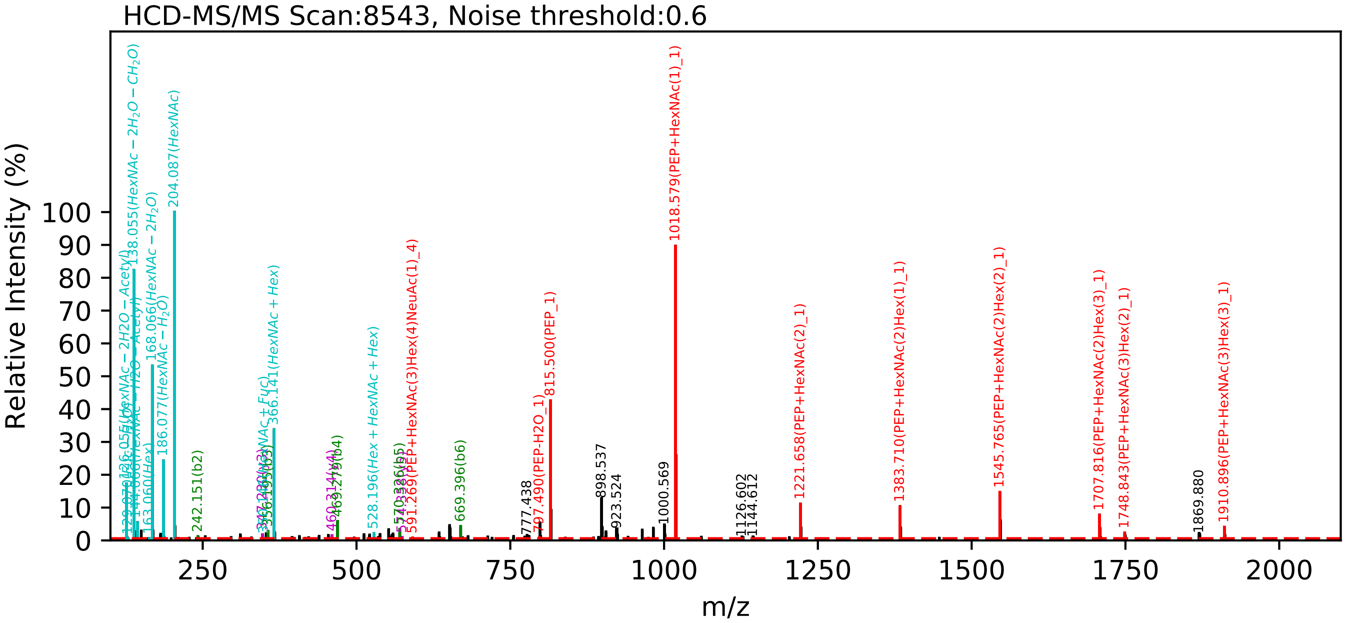

IQNLTVK(=PEP)_5_4_1_0_0, 0_None, 0_None,
m/z:1292.57(4+), RT:26.03, hcd-score:87.50

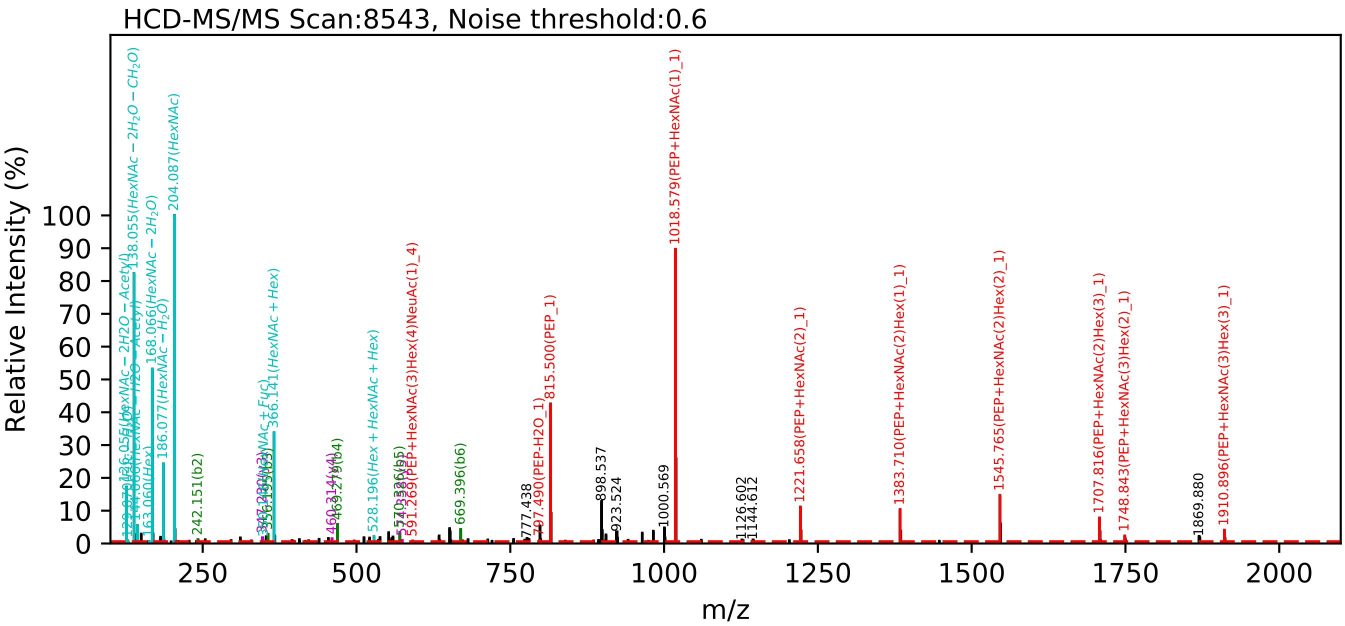

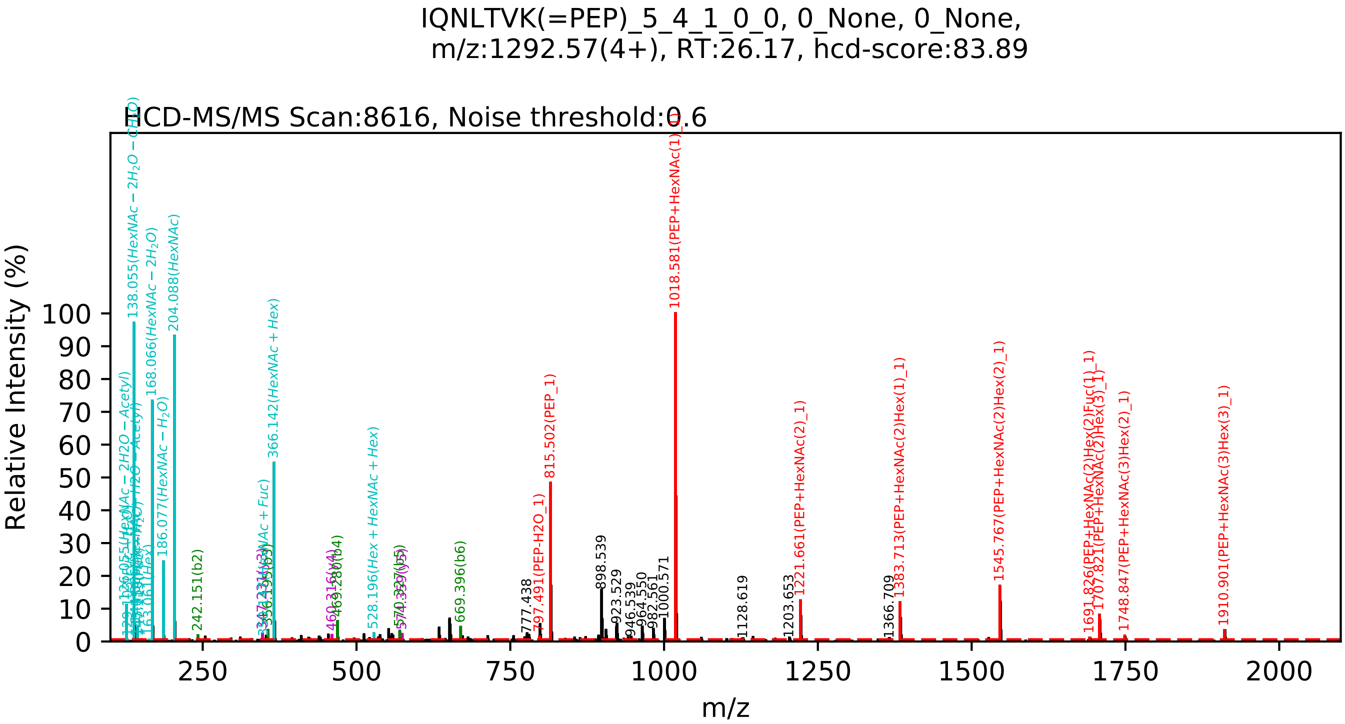

IQNLTVK(=PEP)_5_4_1_0_0, 0_None, 0_None,
m/z:1292.57(4+), RT:26.17, hcd-score:83.89
HCD-MS/MS Scan:8616, Noise threshold:0.6
Relative Intensity (%)
m/z
138.055(HexNAc − 2H2O − CH2O)
168.066(HexNAc − 2H2O)
186.077(HexNAc − H2O)
204.088(HexNAc)
366.142(HexNAc + Hex)
242.151(b2)
469.280(b4)
528.196(Hex + HexNAc + Hex)
669.396(b6)
815.502(PEP_1)
797.491(PEP-H2O_1)
777.438
898.539
923.529
946.539
964.550
982.561
1000.571
1018.581(PEP+HexNAc(1)_1)
1128.619
1203.653
1221.661(PEP+HexNAc(2)_1)
1366.709
1383.713(PEP+HexNAc(2)Hex(1)_1)
1545.767(PEP+HexNAc(2)Hex(2)_1)
1691.826(PEP+HexNAc(2)Hex(2)Fuc(1)_1)
1707.821(PEP+HexNAc(2)Hex(3)_1)
1748.847(PEP+HexNAc(3)Hex(2)_1)
1910.901(PEP+HexNAc(3)Hex(3)_1)

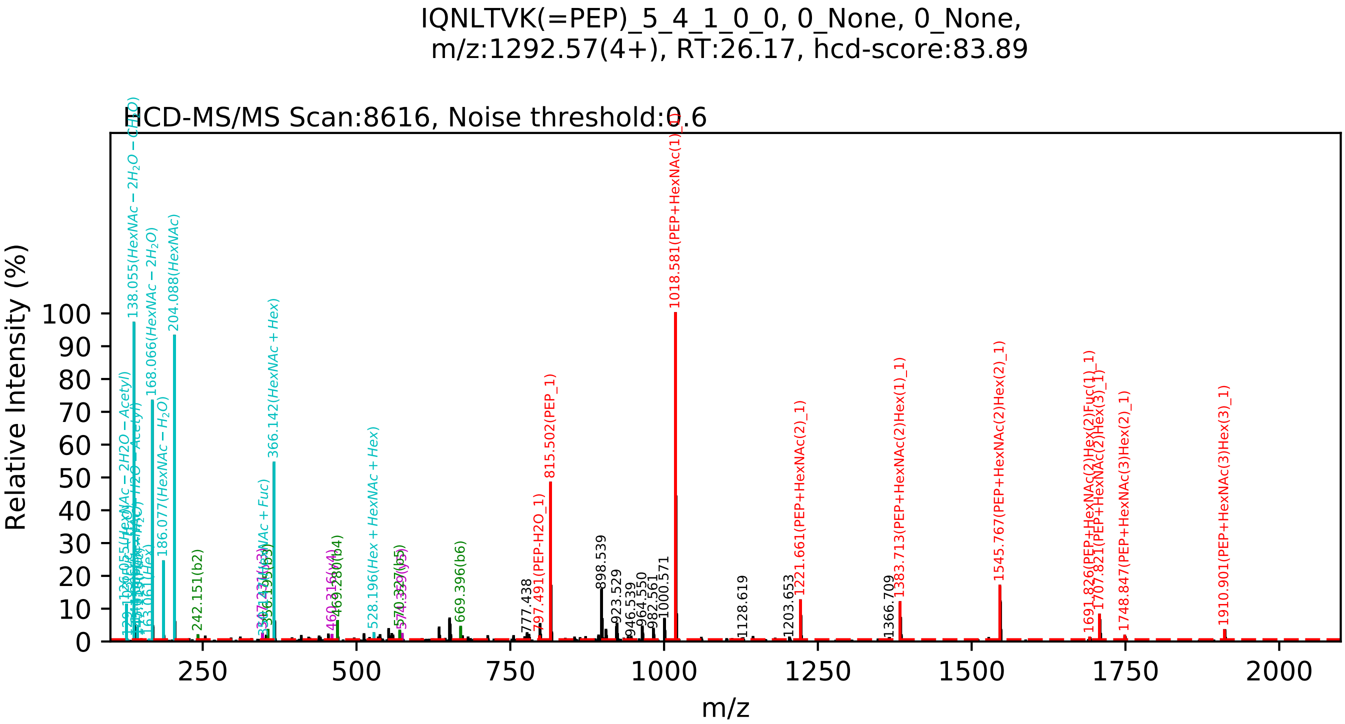
IQNLTVK(=PEP)_5_4_1_0_0, 0_None, 0_None,
m/z:1292.57(4+), RT:26.17, hcd-score:83.89
HCD-MS/MS Scan:8616, Noise threshold:0.6
Relative Intensity (%)
m/z
138.055(HexNAc − 2H2O − CH2O)
168.066(HexNAc − 2H2O)
186.077(HexNAc − H2O)
204.088(HexNAc)
366.142(HexNAc + Hex)
528.196(Hex + HexNAc + Hex)
242.151(b2)
469.280(b4)
669.396(b6)
815.502(PEP_1)
797.491(PEP-H2O_1)
1018.581(PEP+HexNAc(1)_1)
1221.661(PEP+HexNAc(2)_1)
1383.713(PEP+HexNAc(2)Hex(1)_1)
1545.767(PEP+HexNAc(2)Hex(2)_1)
1691.826(PEP+HexNAc(2)Hex(2)Fuc(1)_1)
1707.821(PEP+HexNAc(2)Hex(3)_1)
1748.847(PEP+HexNAc(3)Hex(2)_1)
1910.901(PEP+HexNAc(3)Hex(3)_1)
777.438
898.539
923.529
946.539
964.550
982.561
1000.571
1128.619
1203.653
1366.709

IQNLTVK(=PEP)_6_3_0_0_0, 0_None, 0_None,
m/z:1199.03(2+), RT:26.31, hcd-score:83.07

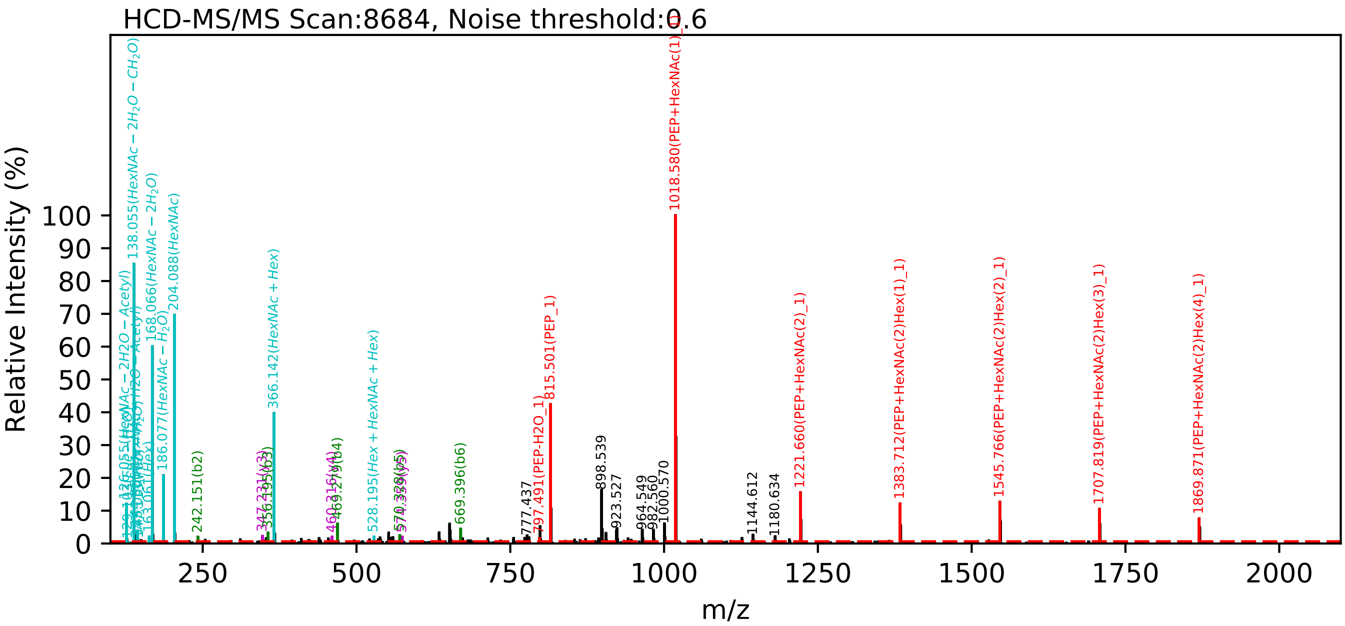

IQNLTVK(=PEP)_6_3_0_0_0, 0_None, 0_None,
m/z:1199.03(2+), RT:26.31, hcd-score:83.07

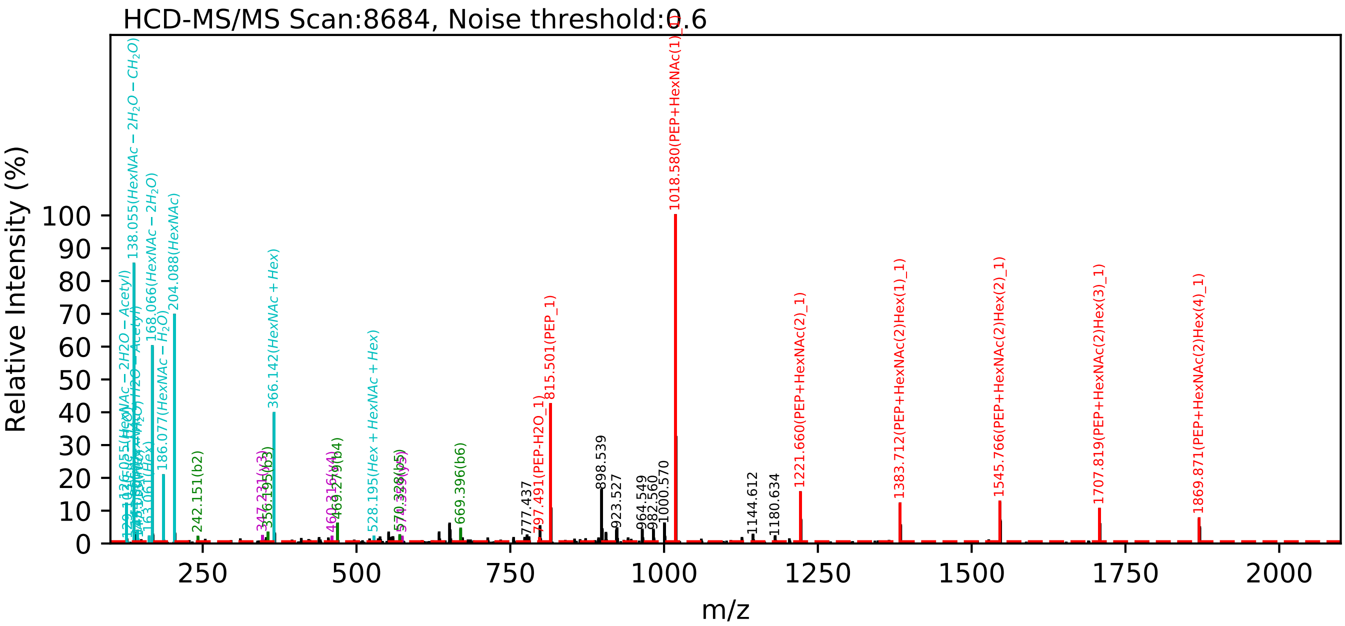

IQNLTVK(=PEP)_5_4_1_0_0, 0_None, 0_None,
m/z:1292.57(4+), RT:26.60, hcd-score:77.18

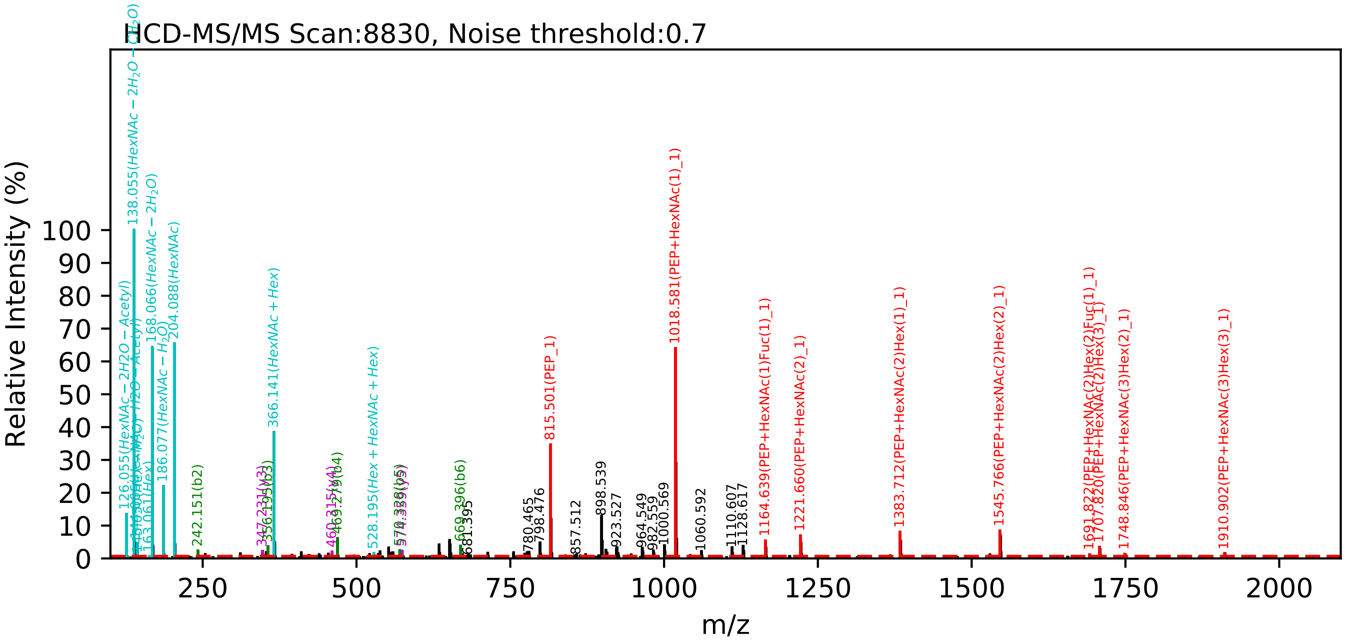

IQNLTVK(=PEP)_5_4_1_0_0, 0_None, 0_None,
m/z:1292.57(4+), RT:26.60, hcd-score:77.18

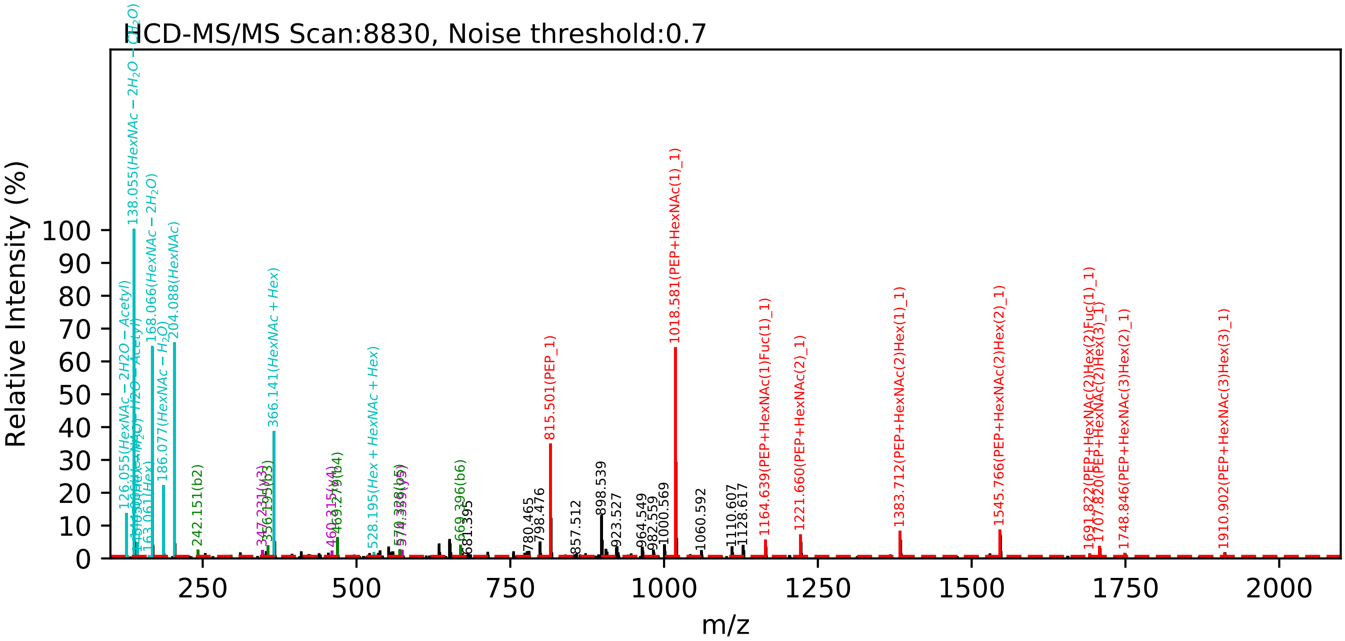

IQNLTVK(=PEP)_5_4_1_0_0, 0_None, 0_None,
m/z:1292.57(4+), RT:27.20, hcd-score:83.15

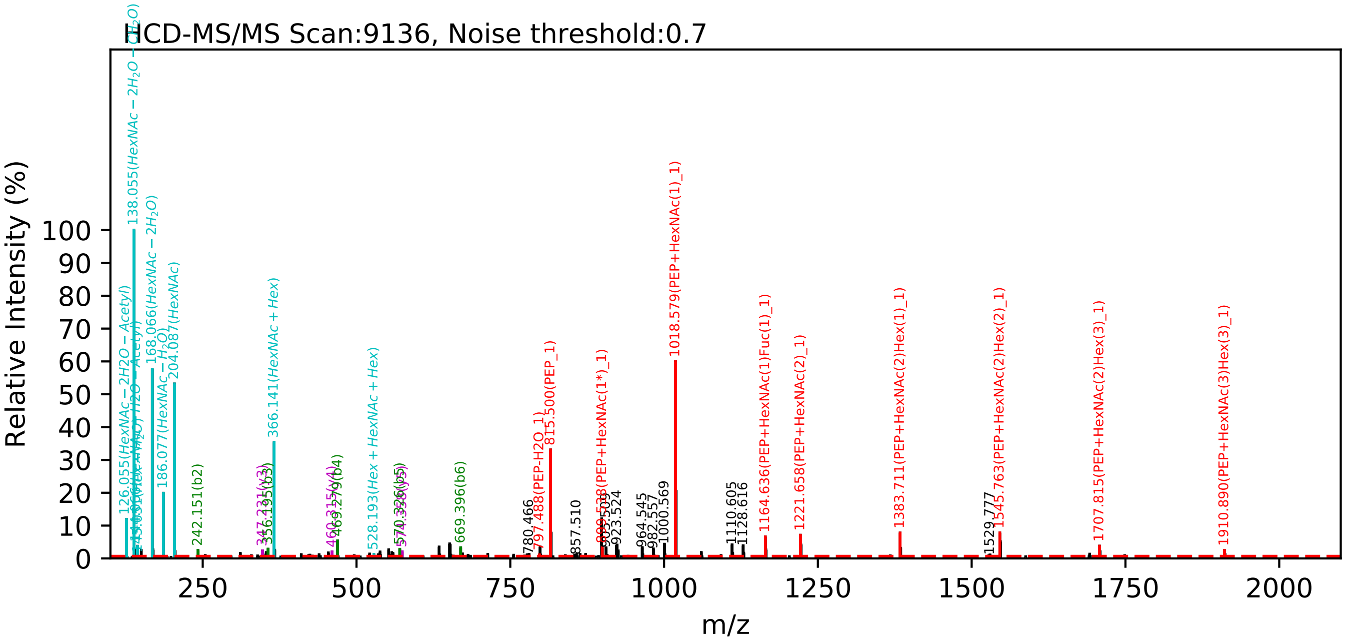

IQNLTVK(=PEP)_5_4_1_0_0, 0_None, 0_None,
m/z:1292.57(4+), RT:27.20, hcd-score:83.15

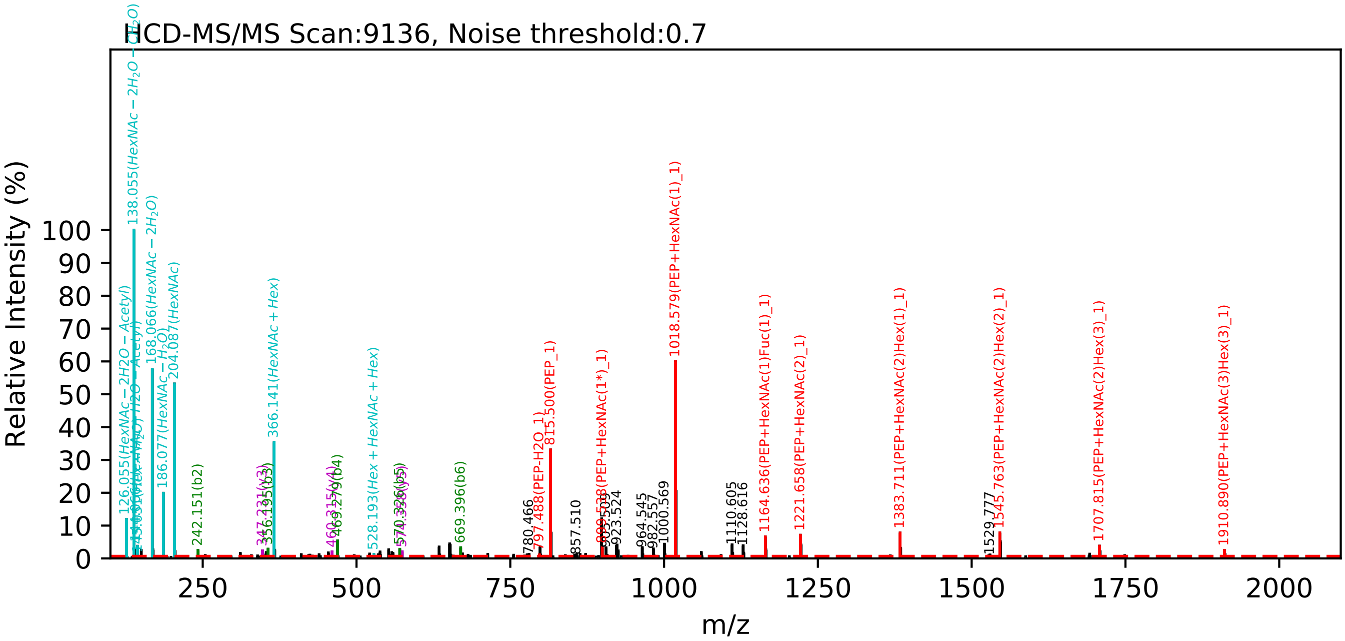

IQNLTVK(=PEP)_5_4_1_0_0, 0_None, 0_None,
m/z:1292.57(4+), RT:27.76, hcd-score:81.01

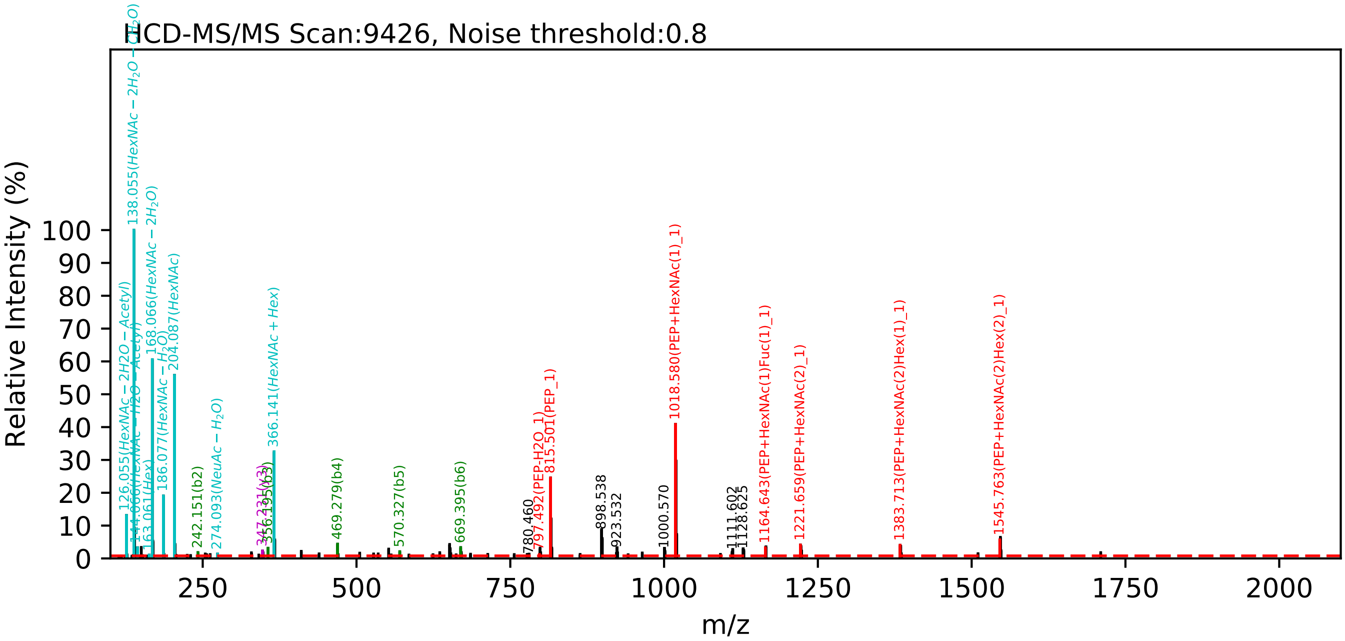

IQNLTVK(=PEP)_5_4_1_0_0, 0_None, 0_None,
m/z:1292.57(4+), RT:27.76, hcd-score:81.01

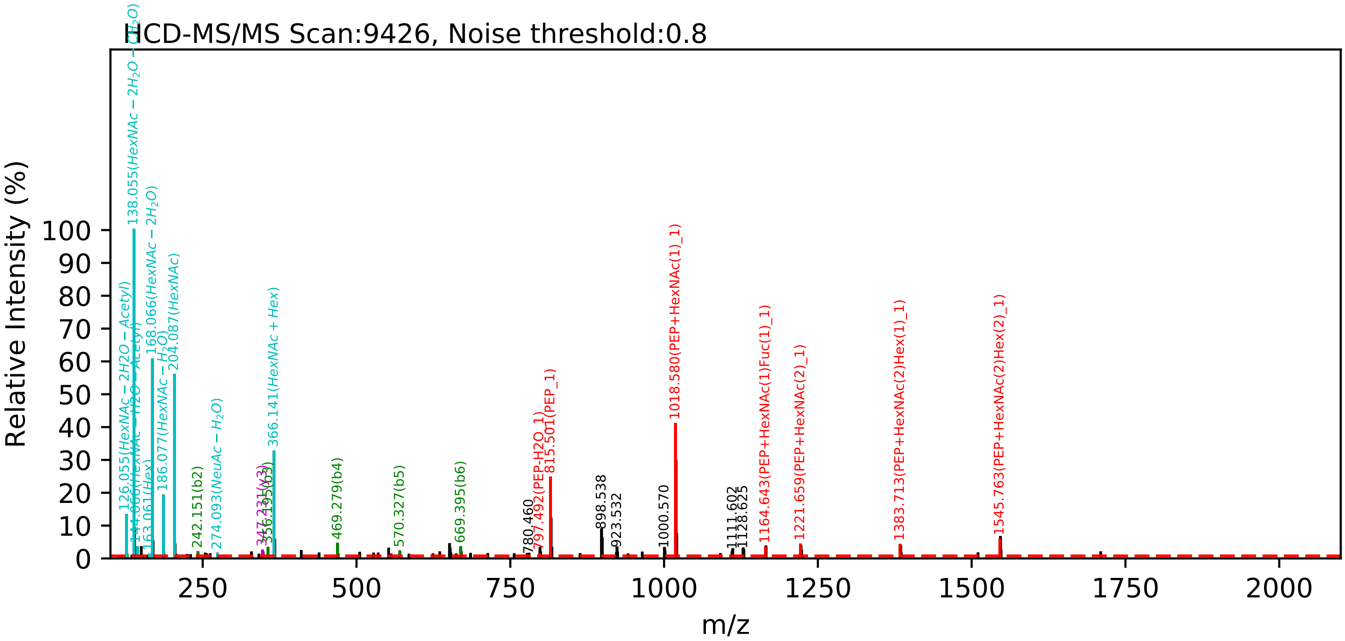

IQNLTVK(=PEP)_6_5_1_2_0, 0_None, 0_None,
m/z:883.62(4+), RT:47.40, hcd-score:88.13

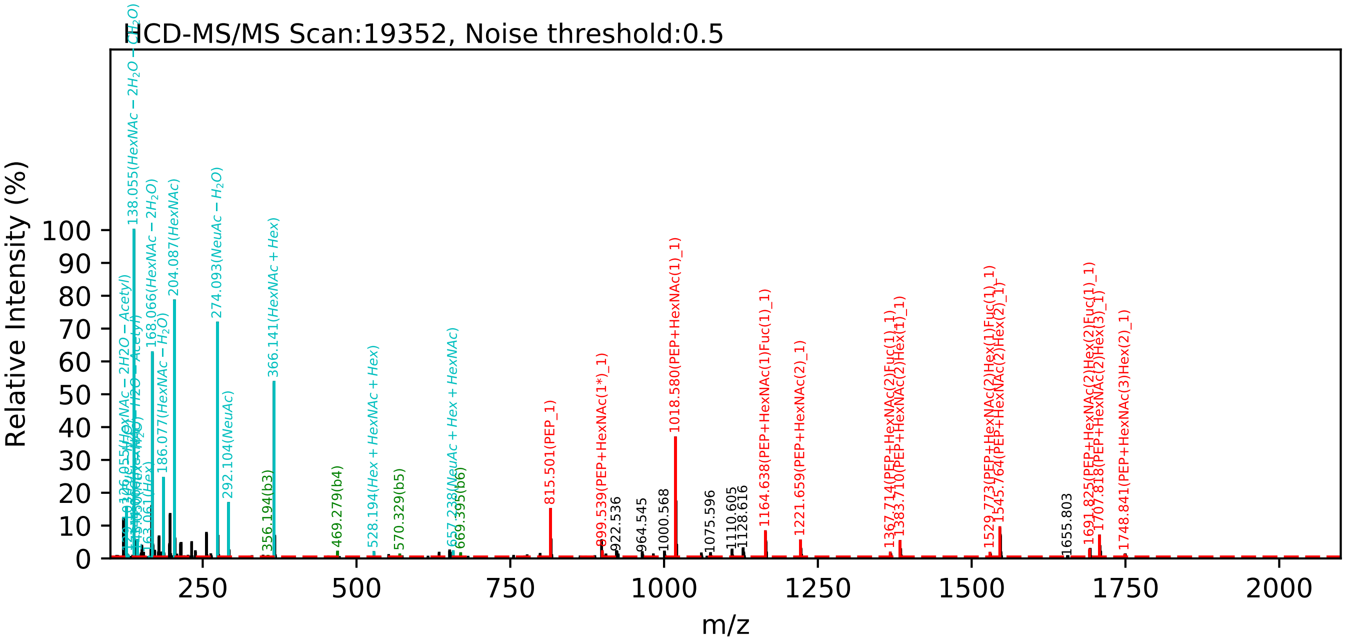

IQNLTVK(=PEP)_6_5_1_2_0, 0_None, 0_None,
m/z:883.62(4+), RT:47.40, hcd-score:88.13

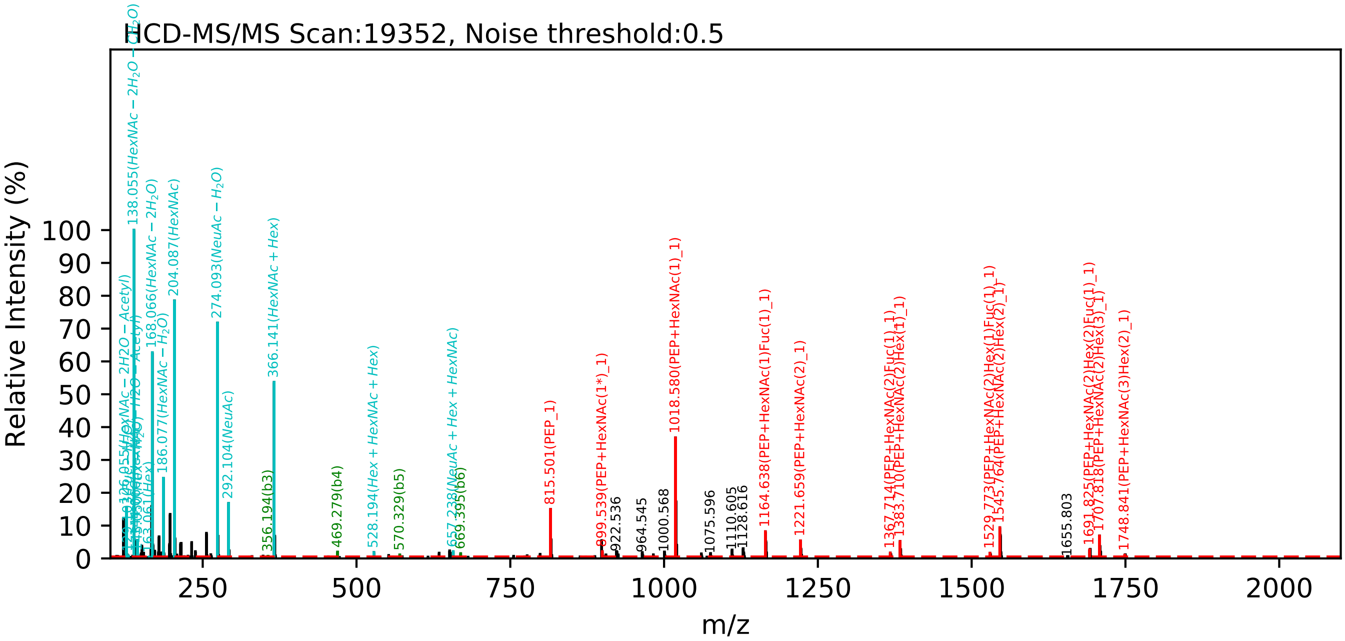

IQNLTVK(=PEP)_5_4_0_2_0, 0_None, 0_None,
m/z:1007.43(3+), RT:49.95, hcd-score:90.54

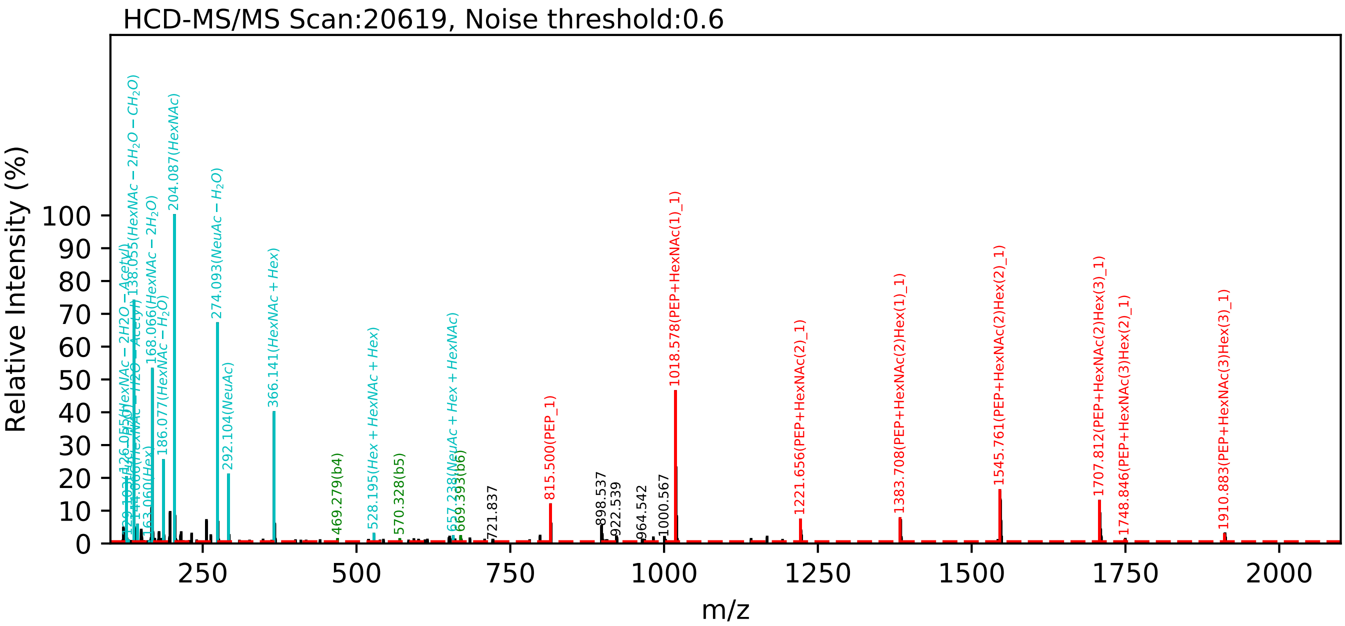

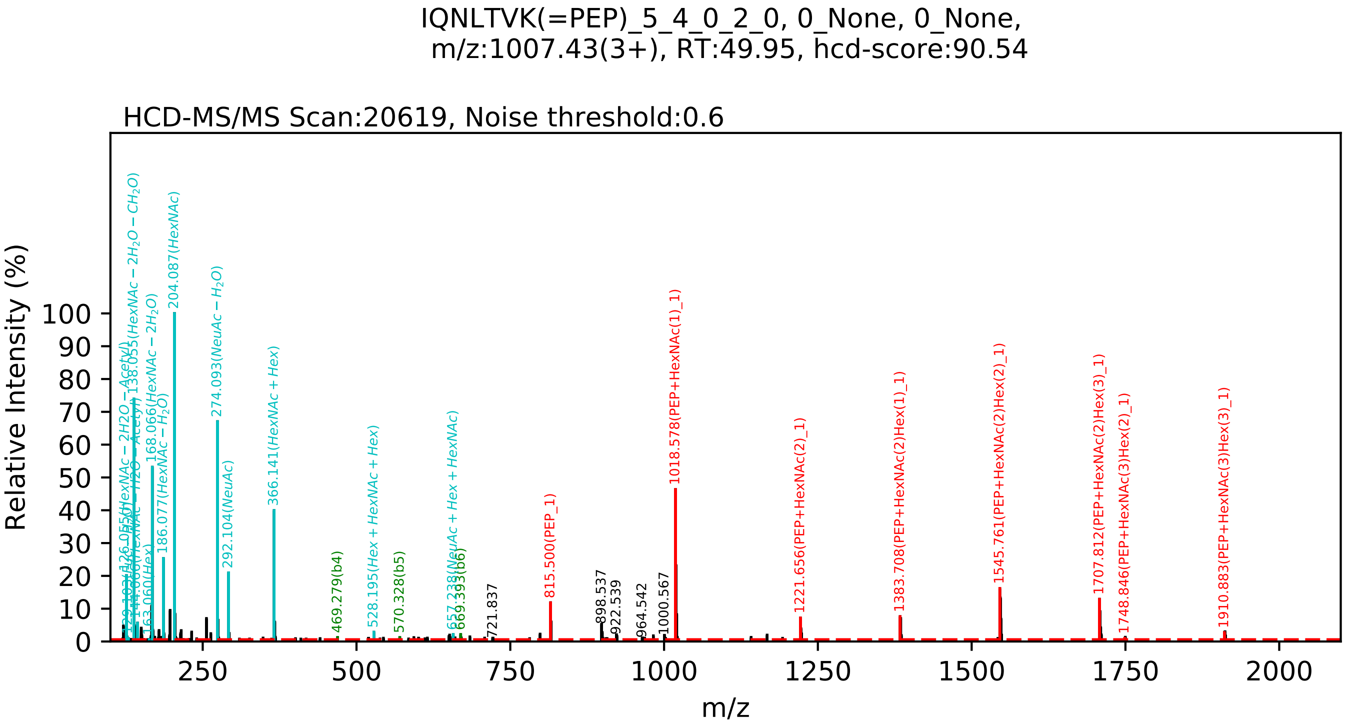

IQNLTVK(=PEP)_5_4_0_2_0, 0_None, 0_None,
m/z:1007.43(3+), RT:49.95, hcd-score:90.54
HCD-MS/MS Scan:20619, Noise threshold:0.6
Relative Intensity (%)
m/z
204.087(HexNAc)
274.093(NeuAc − H2O)
292.104(NeuAc)
366.141(HexNAc + Hex)
186.077(HexNAc − H2O)
168.066(HexNAc − 2H2O)
163.060(Hex)
469.279(b4)
528.195(Hex + HexNAc + Hex)
570.328(b5)
669.393(b6)
721.837
815.500(PEP_1)
898.537
922.539
964.542
1000.567
1018.578(PEP+HexNAc(1)_1)
1221.656(PEP+HexNAc(2)_1)
1383.708(PEP+HexNAc(2)Hex(1)_1)
1545.761(PEP+HexNAc(2)Hex(2)_1)
1707.812(PEP+HexNAc(2)Hex(3)_1)
1748.846(PEP+HexNAc(3)Hex(2)_1)
1910.883(PEP+HexNAc(3)Hex(3)_1)

CDISNSTE(=PEP)_4_2_0_0_0, 0_None, 0_None,
m/z:990.37(2+), RT:22.86, Y-score:78.89

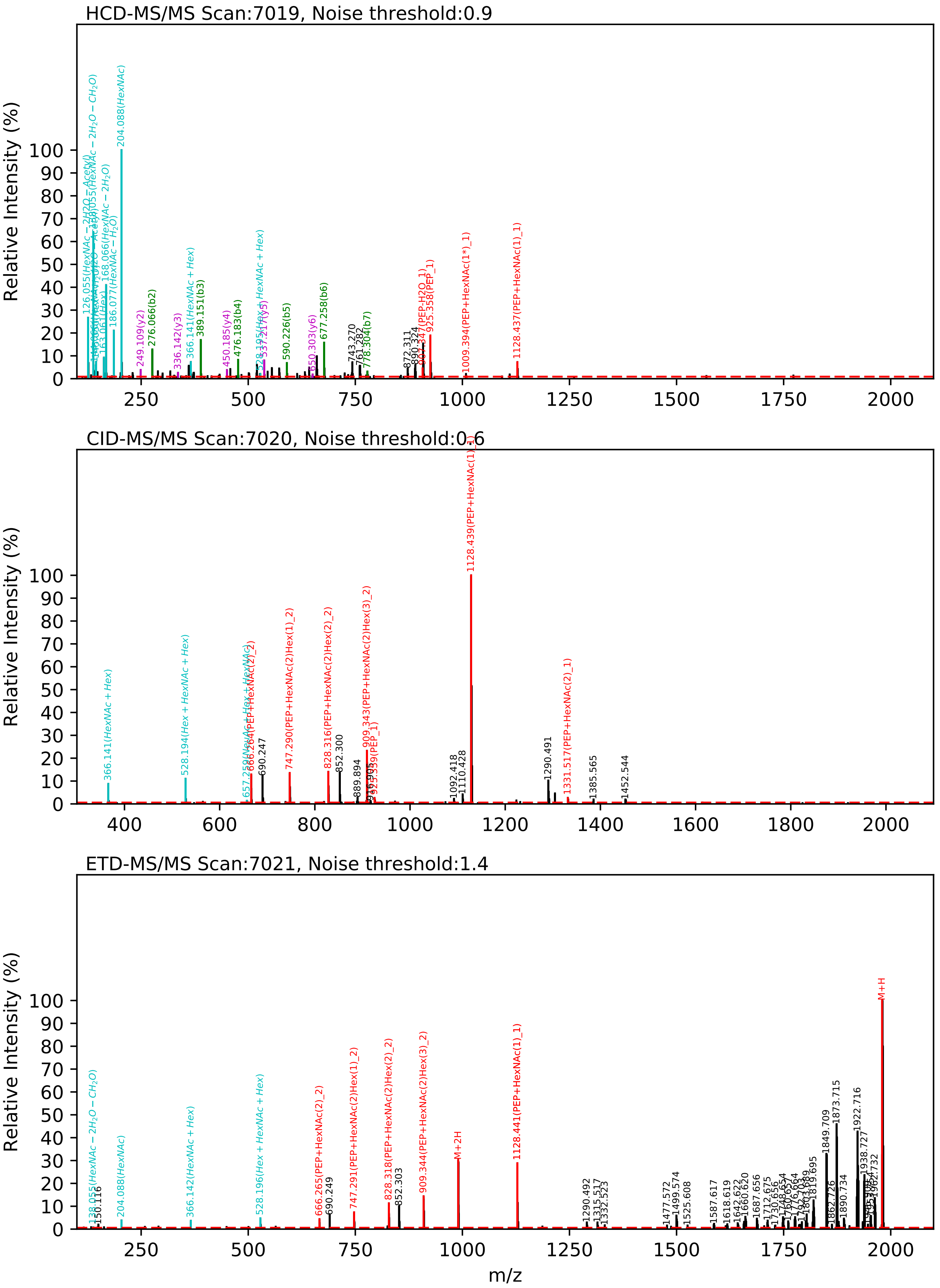

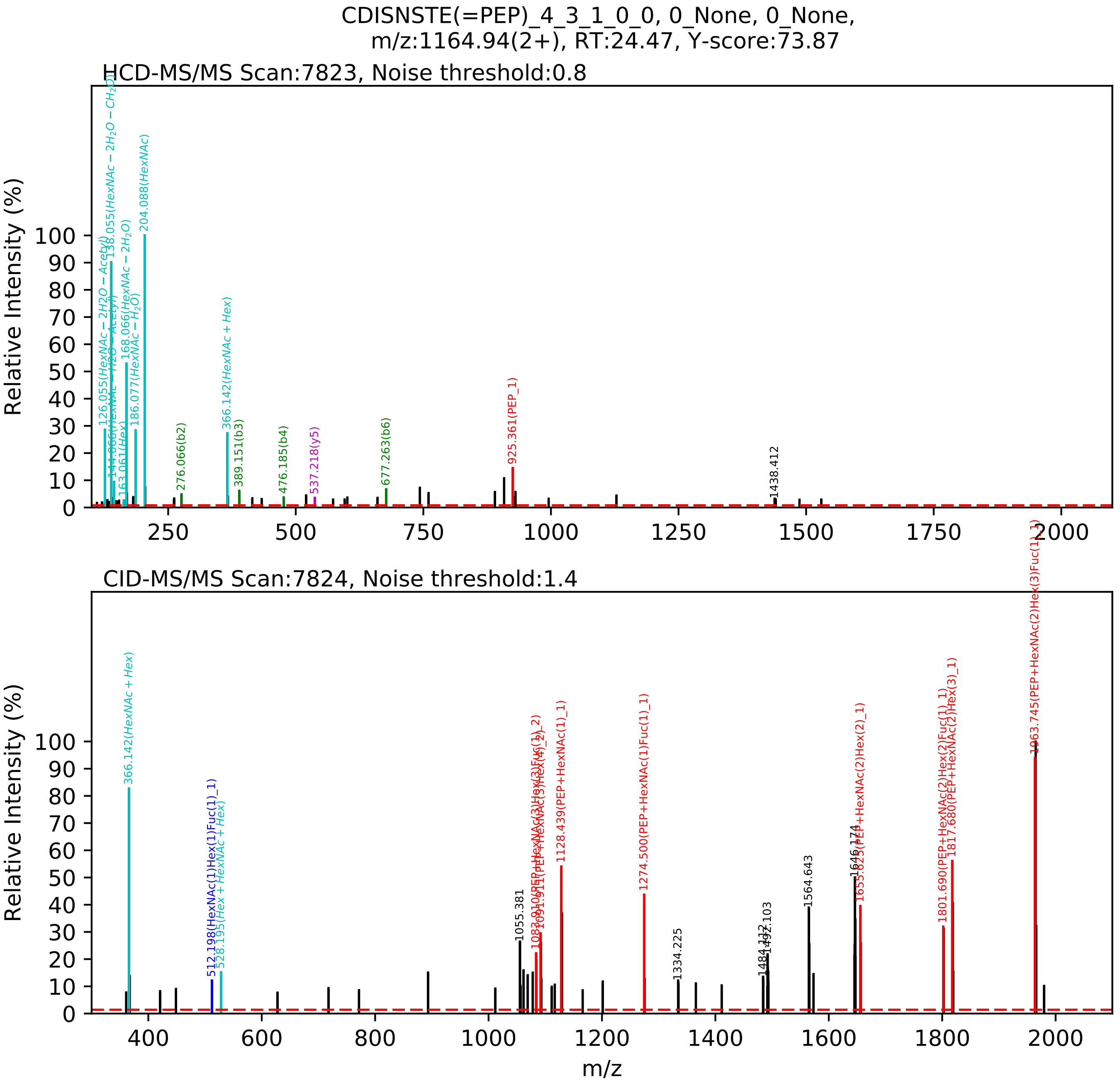

CDISNSTE(=PEP)_4_3_1_0_0, 0_None, 0_None,
m/z:1164.94(2+), RT:24.47, Y-score:73.87
HCD-MS/MS Scan:7823, Noise threshold:0.8
Relative Intensity (%)
204.088(HexNAc)
138.055(HexNAc − 2H2O − CH2O)
126.055(HexNAc − 2H2O − Acetyl)
168.066(HexNAc − 2H2O)
186.077(HexNAc − H2O)
366.142(HexNAc + Hex)
276.066(b2)
389.151(b3)
476.185(b4)
537.218(y5)
677.263(b6)
925.361(PEP_1)
1438.412
CID-MS/MS Scan:7824, Noise threshold:1.4
366.142(HexNAc + Hex)
512.198(HexNAc(1)Hex(1)Fuc(1)_1)
528.195(Hex + HexNAc + Hex)
1055.381
1128.439(PEP+HexNAc(1)_1)
1274.500(PEP+HexNAc(1)Fuc(1)_1)
1334.225
1492.103
1564.643
1646.174
1655.825(PEP+HexNAc(2)Hex(2)_1)
1801.690(PEP+HexNAc(2)Hex(2)Fuc(1)_1)
1817.680(PEP+HexNAc(2)Hex(3)_1)
1963.745(PEP+HexNAc(2)Hex(3)Fuc(1)_1)
m/z

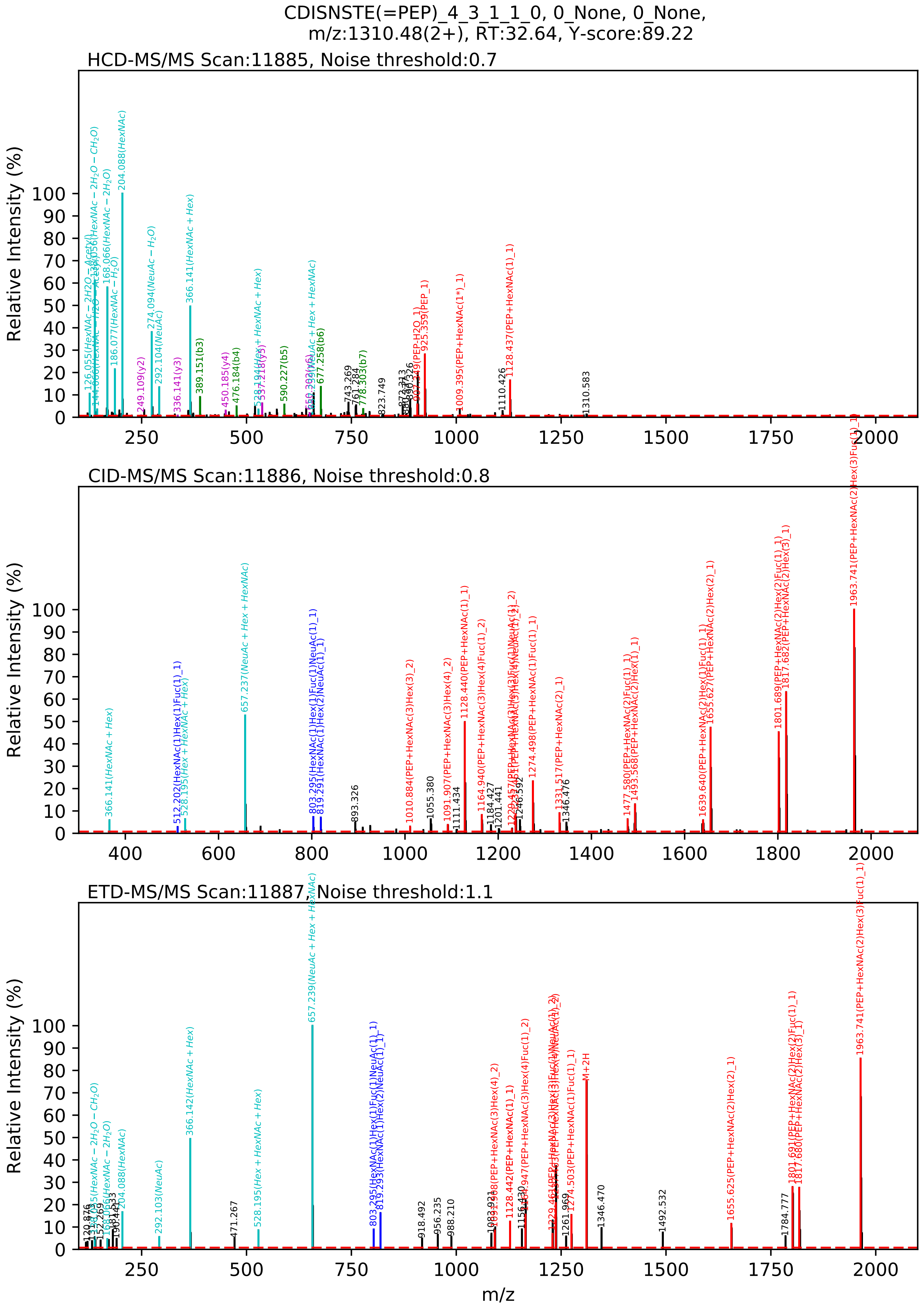

CDISNSTE(=PEP)_4_3_1_1_0, 0_None, 0_None,
m/z:1310.48(2+), RT:32.64, Y-score:89.22
HCD-MS/MS Scan:11885, Noise threshold:0.7
CID-MS/MS Scan:11886, Noise threshold:0.8
ETD-MS/MS Scan:11887, Noise threshold:1.1
Relative Intensity (%)
m/z
204.088(HexNAc)
366.141(HexNAc + Hex)
168.066(HexNAc − 2H2O)
186.077(HexNAc − H2O)
274.094(NeuAc − H2O)
292.104(NeuAc)
1128.437(PEP+HexNAc(1)_1)
1009.395(PEP+HexNAc(1*)_1)
925.359(PEP_1)
677.258(b6)
590.227(b5)
389.151(b3)
476.184(b4)
249.109(y2)
336.141(y3)
450.185(y4)
537.218(y5)
778.303(b7)
1110.426
1310.583
657.237(NeuAc + Hex + HexNAc)
1963.741(PEP+HexNAc(2)Hex(3)Fuc(1)_1)
1801.689(PEP+HexNAc(2)Hex(2)Fuc(1)_1)
1817.682(PEP+HexNAc(2)Hex(3)_1)
1655.627(PEP+HexNAc(2)Hex(2)_1)
1639.640(PEP+HexNAc(2)Hex(1)Fuc(1)_1)
1477.580(PEP+HexNAc(2)Fuc(1)_1)
1493.568(PEP+HexNAc(2)Hex(1)_1)
1331.517(PEP+HexNAc(2)_1)
1274.498(PEP+HexNAc(1)Fuc(1)_1)
1128.440(PEP+HexNAc(1)_1)
1164.940(PEP+HexNAc(3)Hex(4)Fuc(1)_2)
1091.907(PEP+HexNAc(3)Hex(4)_2)
1010.884(PEP+HexNAc(3)Hex(3)_2)
803.295(HexNAc(1)Hex(1)Fuc(1)NeuAc(1)_1)
819.291(HexNAc(1)Hex(2)NeuAc(1)_1)
512.202(HexNAc(1)Hex(1)Fuc(1)_1)
528.195(Hex + HexNAc + Hex)
657.239(NeuAc + Hex + HexNAc)
M+2H
1274.503(PEP+HexNAc(1)Fuc(1)_1)
1655.625(PEP+HexNAc(2)Hex(2)_1)
1801.691(PEP+HexNAc(2)Hex(2)Fuc(1)_1)
1817.680(PEP+HexNAc(2)Hex(3)_1)

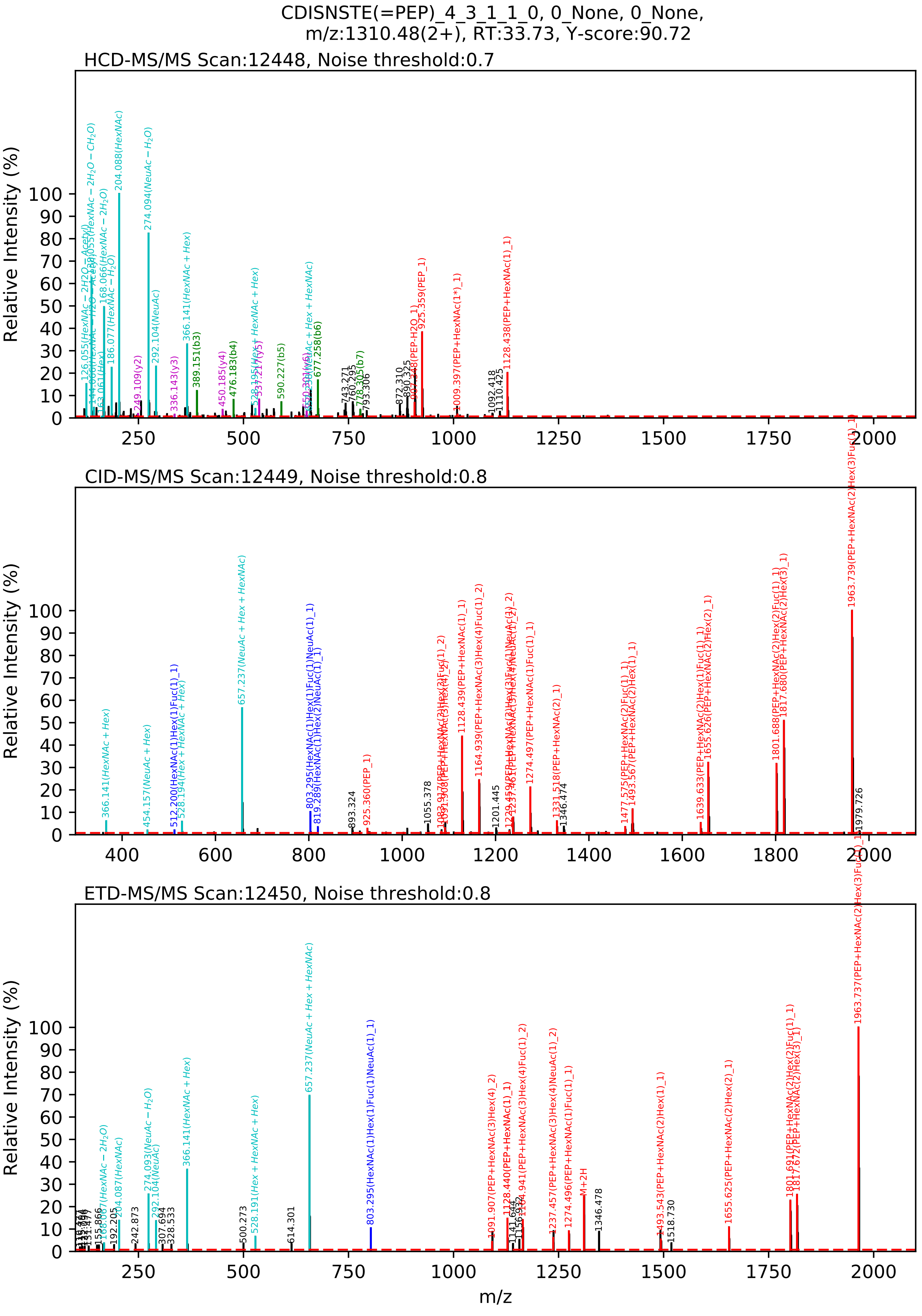
CDISNSTE(=PEP)_4_3_1_1_0, 0_None, 0_None,
m/z:1310.48(2+), RT:33.73, Y-score:90.72
HCD-MS/MS Scan:12448, Noise threshold:0.7
CID-MS/MS Scan:12449, Noise threshold:0.8
ETD-MS/MS Scan:12450, Noise threshold:0.8
Relative Intensity (%)
m/z

CDISNSTE(=PEP)_4_3_1_1_0, 0_None, 0_None,
m/z:873.99(3+), RT:32.63, Y-score:62.59

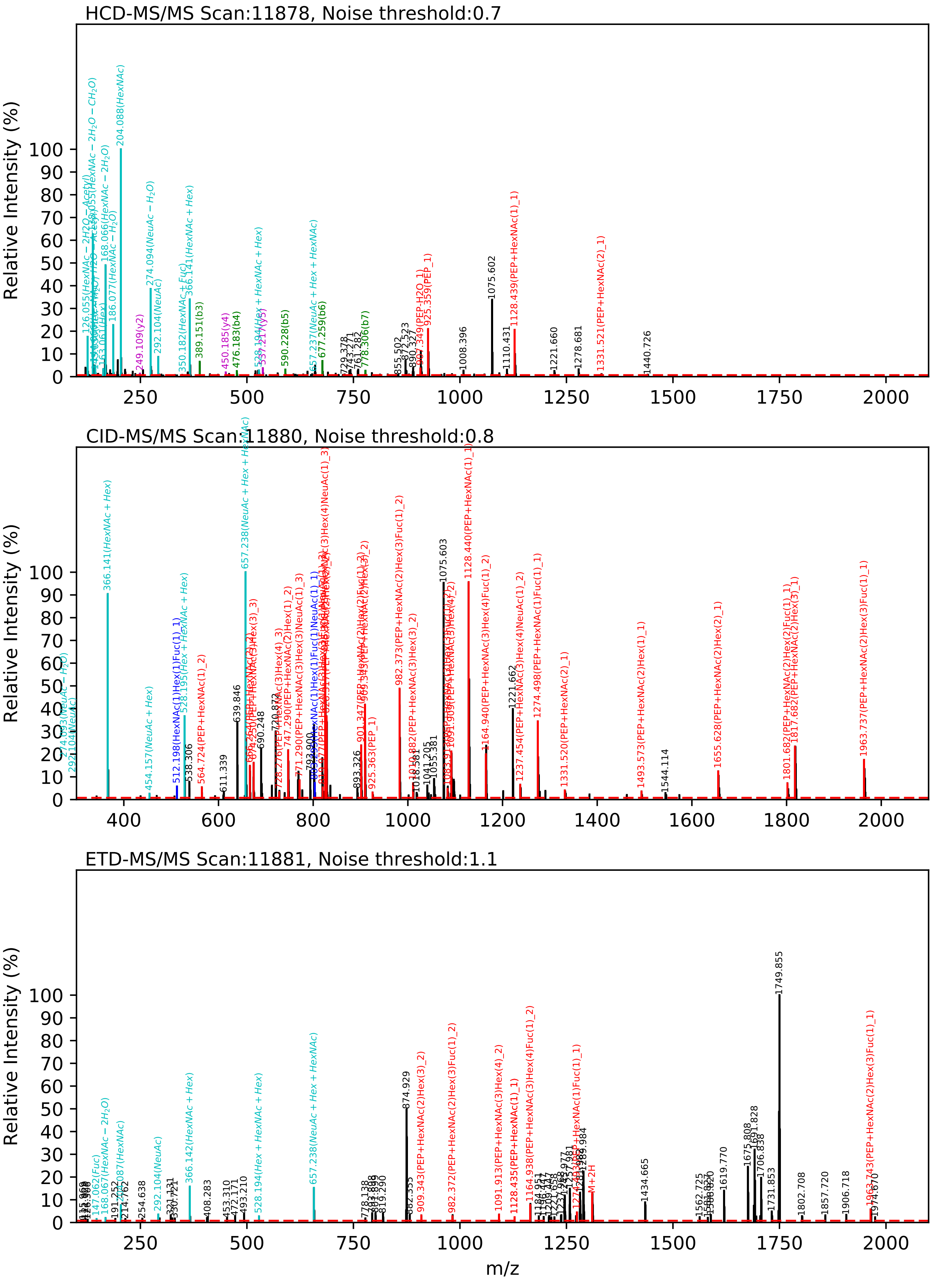

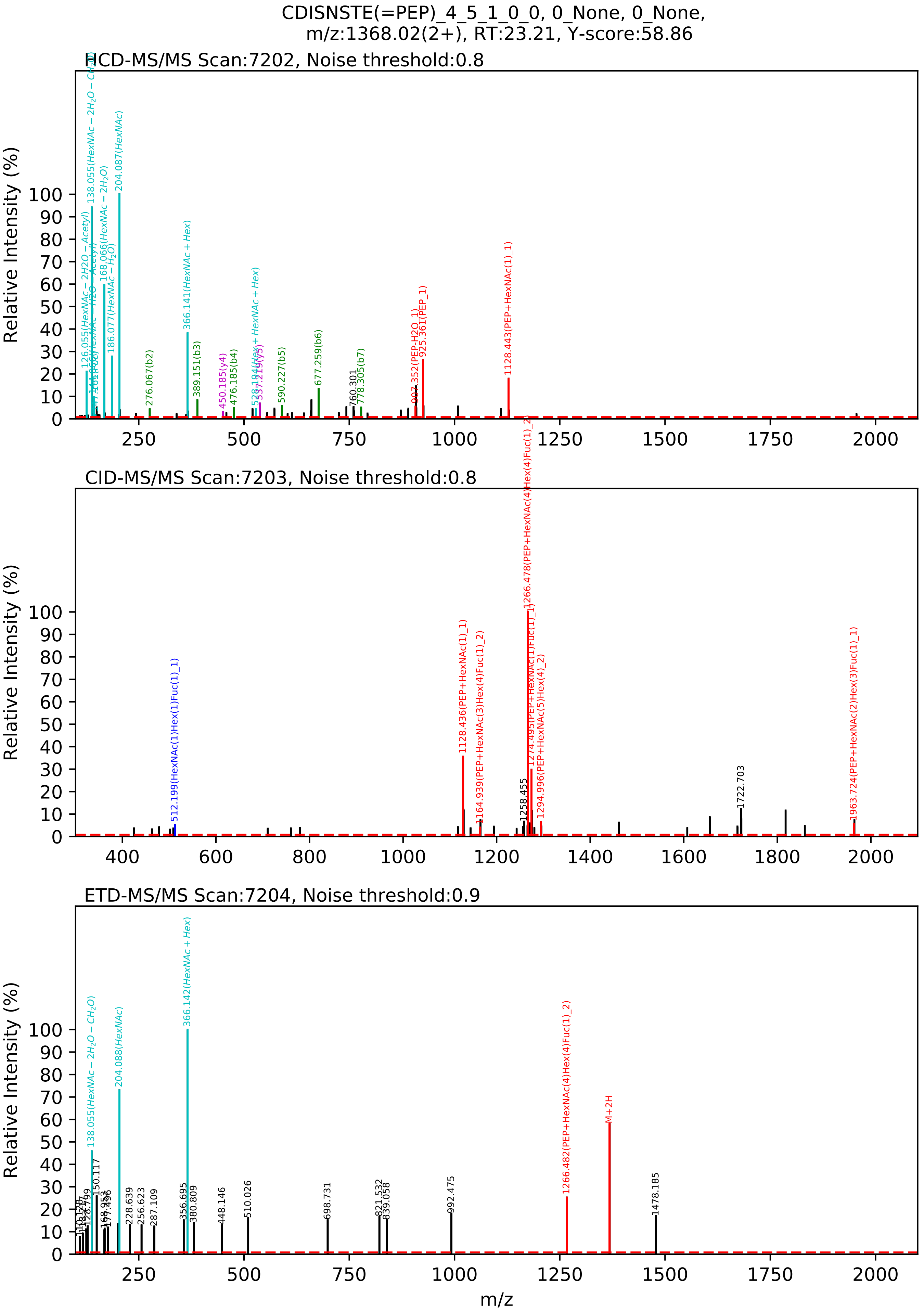
CDISNSTE(=PEP)_4_5_1_0_0, 0_None, 0_None,
m/z:1368.02(2+), RT:23.21, Y-score:58.86
HCD-MS/MS Scan:7202, Noise threshold:0.8
CID-MS/MS Scan:7203, Noise threshold:0.8
ETD-MS/MS Scan:7204, Noise threshold:0.9
Relative Intensity (%)
m/z
204.087(HexNAc)
366.141(HexNAc+Hex)
1128.443(PEP+HexNAc(1)_1)
925.361(PEP_1)
677.259(b6)
512.199(HexNAc(1)Hex(1)Fuc(1)_1)
1128.436(PEP+HexNAc(1)_1)
1266.478(PEP+HexNAc(4)Hex(4)Fuc(1)_2)
1963.724(PEP+HexNAc(2)Hex(3)Fuc(1)_1)
1722.703
366.142(HexNAc+Hex)
204.088(HexNAc)
1266.482(PEP+HexNAc(4)Hex(4)Fuc(1)_2)
M+2H
1478.185

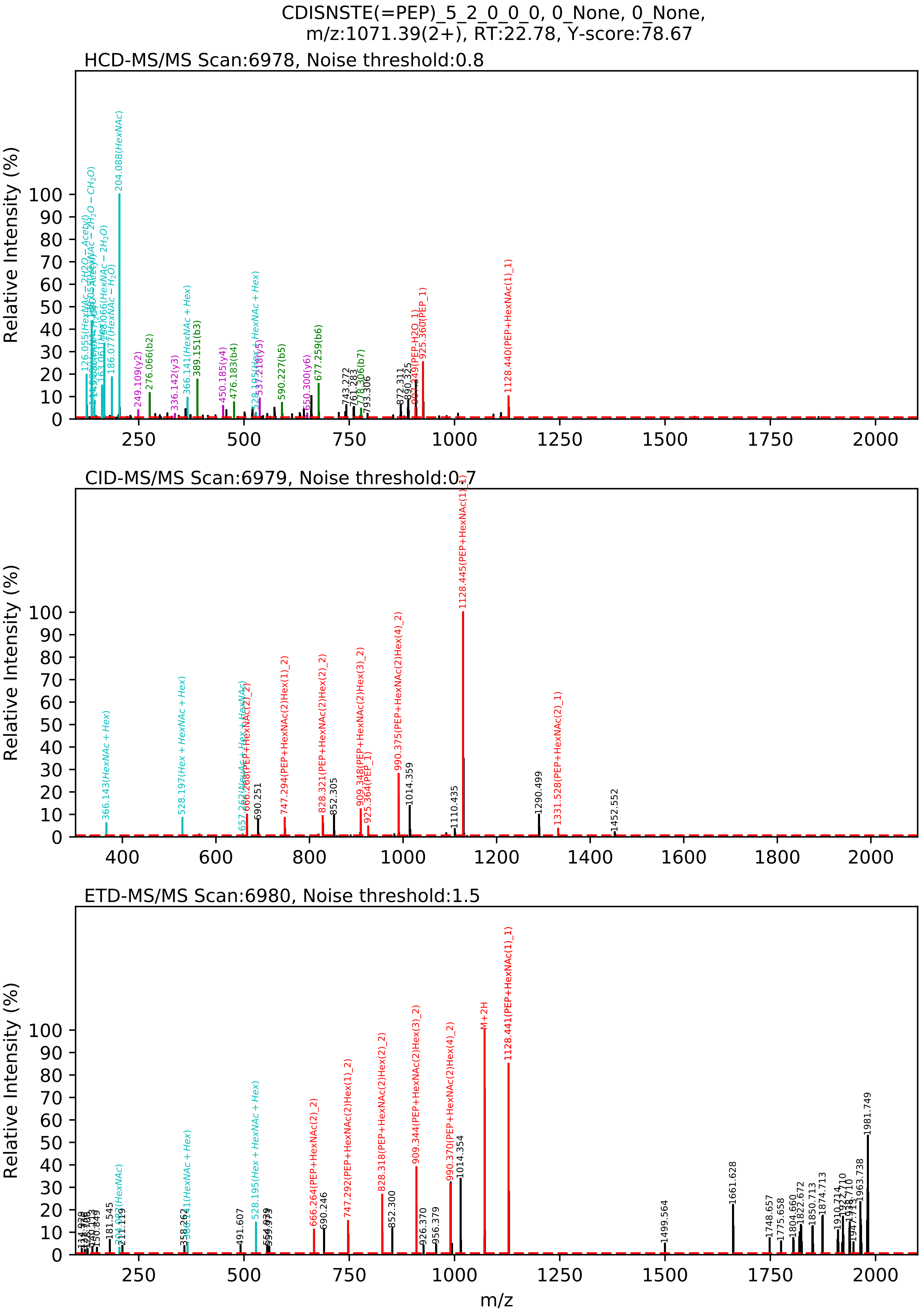

CDISNSTE(=PEP)_5_2_0_0_0, 0_None, 0_None,
m/z:1071.39(2+), RT:22.78, Y-score:78.67
HCD-MS/MS Scan:6978, Noise threshold:0.8
Relative Intensity (%)
204.088(HexNAc)
186.077(HexNAc − H2O)
126.055(HexNAc − 2H2O − Acetyl)
249.109(y2)
276.066(b2)
336.142(y3)
366.141(HexNAc + Hex)
389.151(b3)
450.185(y4)
476.183(b4)
528.195(Hex + HexNAc + Hex)
537.218(y5)
590.227(b5)
650.300(y6)
677.259(b6)
743.272
761.283
778.306(b7)
793.306
872.311
890.325
907.349(PEP-H2O_1)
925.360(PEP_1)
1128.440(PEP+HexNAc(1)_1)
CID-MS/MS Scan:6979, Noise threshold:0.7
366.143(HexNAc + Hex)
528.197(Hex + HexNAc + Hex)
690.251
747.294(PEP+HexNAc(2)Hex(1)_2)
828.321(PEP+HexNAc(2)Hex(2)_2)
852.305
909.348(PEP+HexNAc(2)Hex(3)_2)
925.364(PEP_1)
990.375(PEP+HexNAc(2)Hex(4)_2)
1014.359
1110.435
1128.445(PEP+HexNAc(1)_1)
1290.499
1331.528(PEP+HexNAc(2)_1)
1452.552
ETD-MS/MS Scan:6980, Noise threshold:1.5
181.545
358.262
491.607
528.195(Hex + HexNAc + Hex)
666.264(PEP+HexNAc(2)_2)
690.246
747.292(PEP+HexNAc(2)Hex(1)_2)
828.318(PEP+HexNAc(2)Hex(2)_2)
852.300
909.344(PEP+HexNAc(2)Hex(3)_2)
926.370
956.379
990.370(PEP+HexNAc(2)Hex(4)_2)
1014.354
M+2H
1128.441(PEP+HexNAc(1)_1)
1499.564
1661.628
1748.657
1775.658
1804.660
1822.672
1850.713
1874.713
1963.738
1981.749
m/z

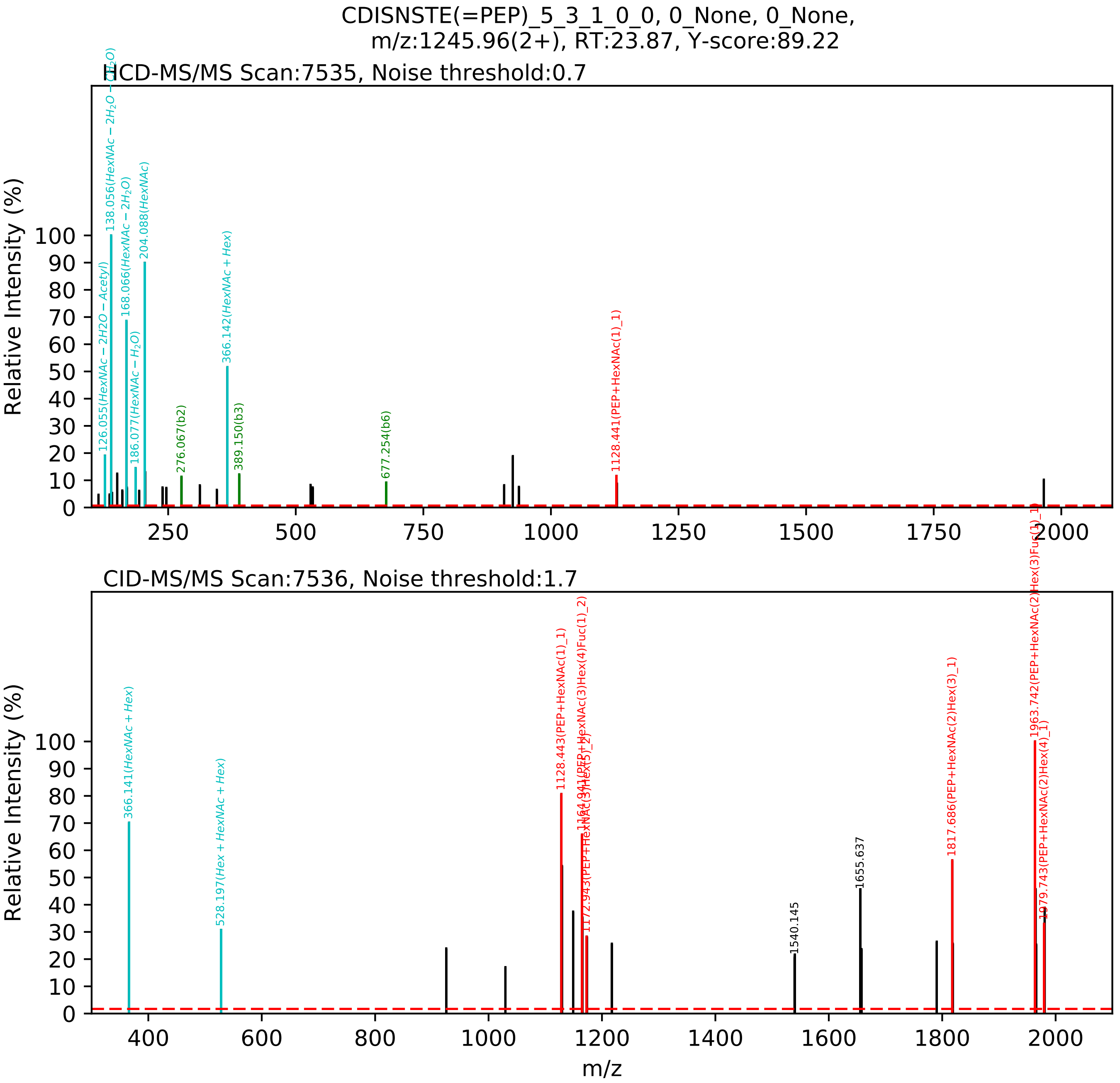

CDISNSTE(=PEP)_5_3_1_0_0, 0_None, 0_None,
m/z:1245.96(2+), RT:23.87, Y-score:89.22
HCD-MS/MS Scan:7535, Noise threshold:0.7
Relative Intensity (%)
126.055(HexNAc − 2H2O − Acetyl)
138.056(HexNAc − 2H2O − H2O)
168.066(HexNAc − 2H2O)
186.077(HexNAc − H2O)
204.088(HexNAc)
276.067(b2)
366.142(HexNAc + Hex)
389.150(b3)
677.254(b6)
1128.441(PEP+HexNAc(1)_1)
CID-MS/MS Scan:7536, Noise threshold:1.7
366.141(HexNAc + Hex)
528.197(Hex + HexNAc + Hex)
1128.443(PEP+HexNAc(1)_1)
1164.941(PEP+HexNAc(3)Hex(4)Fuc(1)_2)
1172.943(PEP+HexNAc(3)Hex(5)_2)
1540.145
1655.637
1817.686(PEP+HexNAc(2)Hex(3)_1)
1963.742(PEP+HexNAc(2)Hex(3)Fuc(1)_1)
1979.743(PEP+HexNAc(2)Hex(4)_1)
m/z

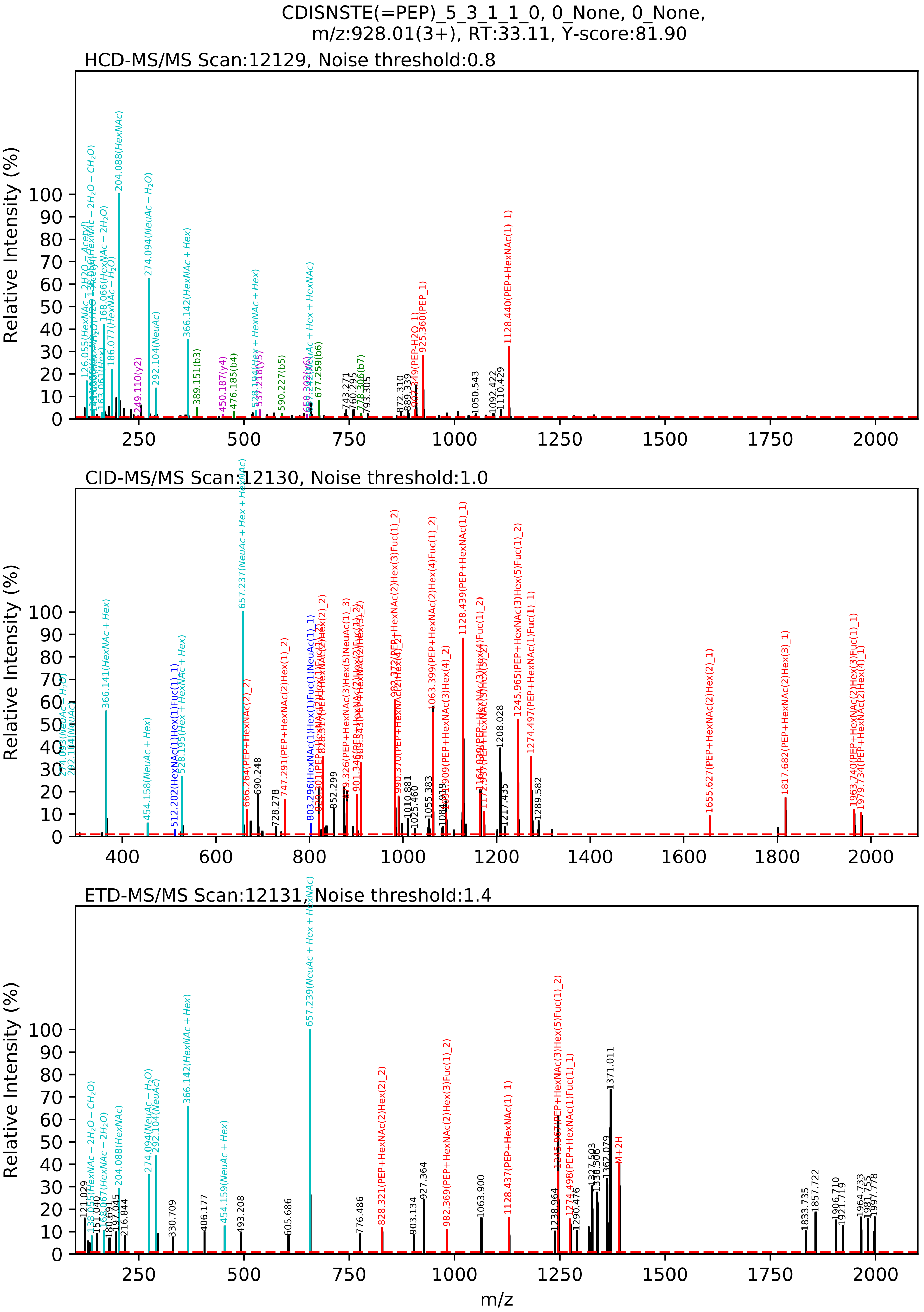

CDISNSTE(=PEP)_5_3_1_1_0, 0_None, 0_None,
m/z:928.01(3+), RT:33.11, Y-score:81.90
HCD-MS/MS Scan:12129, Noise threshold:0.8
CID-MS/MS Scan:12130, Noise threshold:1.0
ETD-MS/MS Scan:12131, Noise threshold:1.4
Relative Intensity (%)
m/z
204.088(HexNAc)
274.094(NeuAc − H2O)
366.142(HexNAc + Hex)
1128.440(PEP+HexNAc(1)_1)
925.360(PEP_1)
657.237(NeuAc + Hex + HexNAc)
1128.439(PEP+HexNAc(1)_1)
1063.399(PEP+HexNAc(2)Hex(4)Fuc(1)_2)
1245.965(PEP+HexNAc(3)Hex(5)Fuc(1)_2)
1274.497(PEP+HexNAc(1)Fuc(1)_1)
1655.627(PEP+HexNAc(2)Hex(2)_1)
1817.682(PEP+HexNAc(2)Hex(3)_1)
657.239(NeuAc + Hex + HexNAc)
1371.011
M+2H

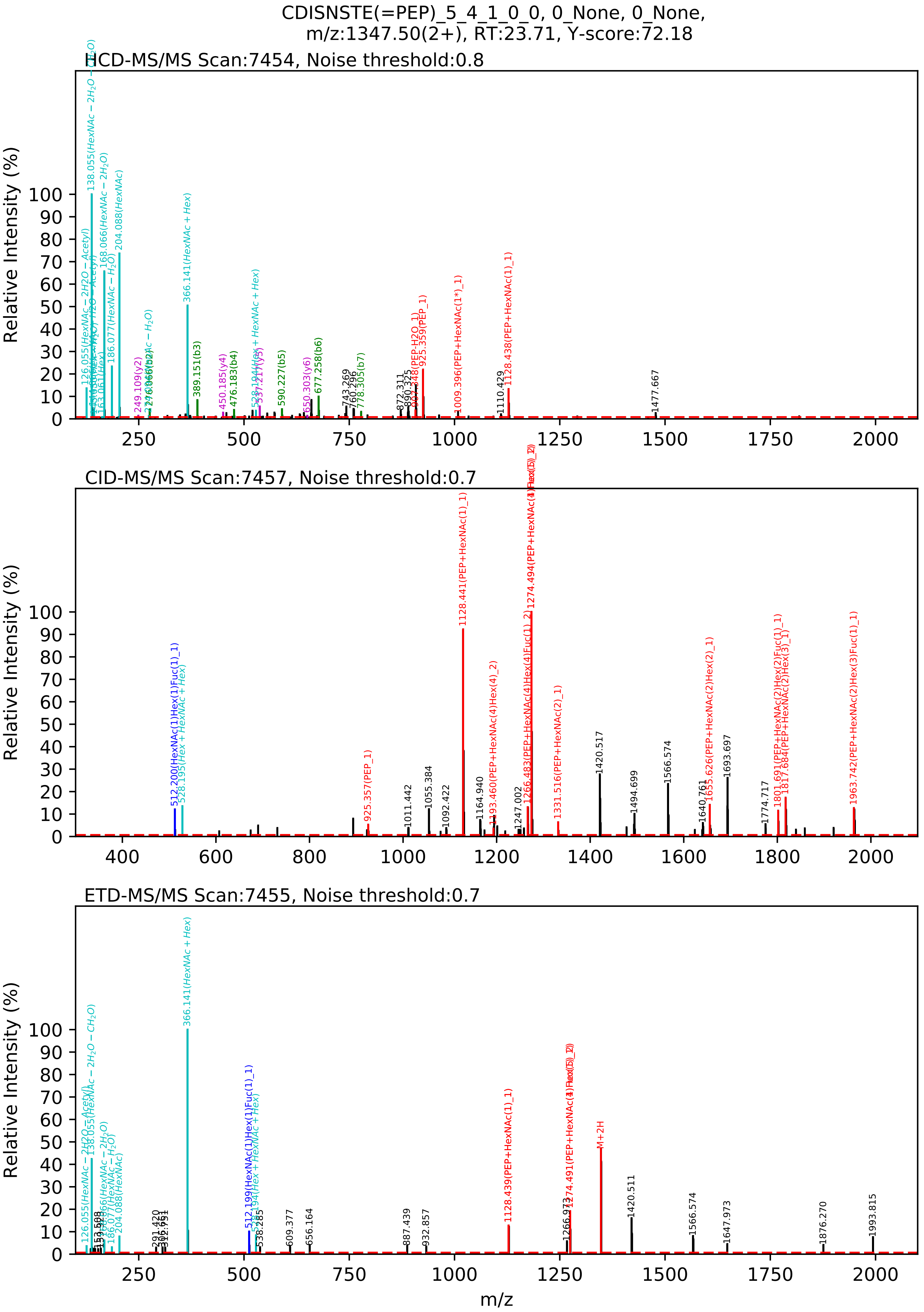
CDISNSTE(=PEP)_5_4_1_0_0, 0_None, 0_None,
m/z:1347.50(2+), RT:23.71, Y-score:72.18
HCD-MS/MS Scan:7454, Noise threshold:0.8
Relative Intensity (%)
138.055
168.066
204.088(HexNAc)
366.141(HexNAc + Hex)
389.151(b3)
476.183(b4)
590.227(b5)
677.258(b6)
778.305(b7)
925.359(PEP_1)
1009.396(PEP+HexNAc(1*)_1)
1128.438(PEP+HexNAc(1)_1)
1110.429
1477.667
CID-MS/MS Scan:7457, Noise threshold:0.7
512.200(HexNAc(1)Hex(1)Fuc(1)_1)
528.195(Hex + HexNAc + Hex)
925.357(PEP_1)
1128.441(PEP+HexNAc(1)_1)
1193.460(PEP+HexNAc(4)Hex(4)_2)
1274.494(PEP+HexNAc(4)Hex(4)Fuc(1)_2)
1331.516(PEP+HexNAc(2)_1)
1420.517
1566.574
1655.626(PEP+HexNAc(2)Hex(2)_1)
1693.697
1801.691(PEP+HexNAc(2)Hex(2)Fuc(1)_1)
1817.684(PEP+HexNAc(2)Hex(3)_1)
1963.742(PEP+HexNAc(2)Hex(3)Fuc(1)_1)
ETD-MS/MS Scan:7455, Noise threshold:0.7
366.141(HexNAc + Hex)
512.199(HexNAc(1)Hex(1)Fuc(1)_1)
1128.439(PEP+HexNAc(1)_1)
M+2H
1420.511
1566.574
1647.973
1876.270
1993.815
m/z

CDISNSTE(=PEP)_5_4_1_0_0, 0_None, 0_None,
m/z:898.67(3+), RT:23.70, Y-score:87.43

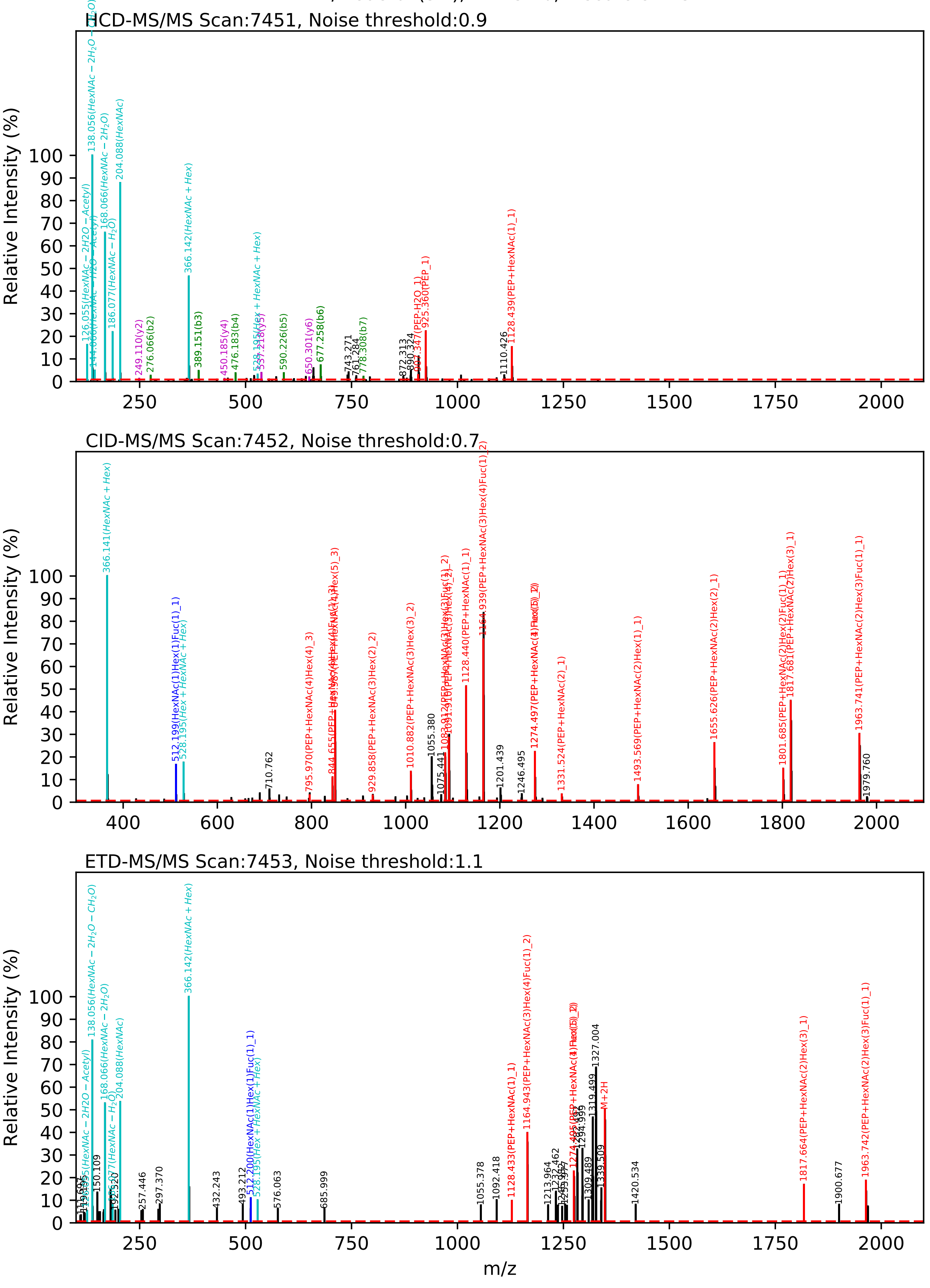

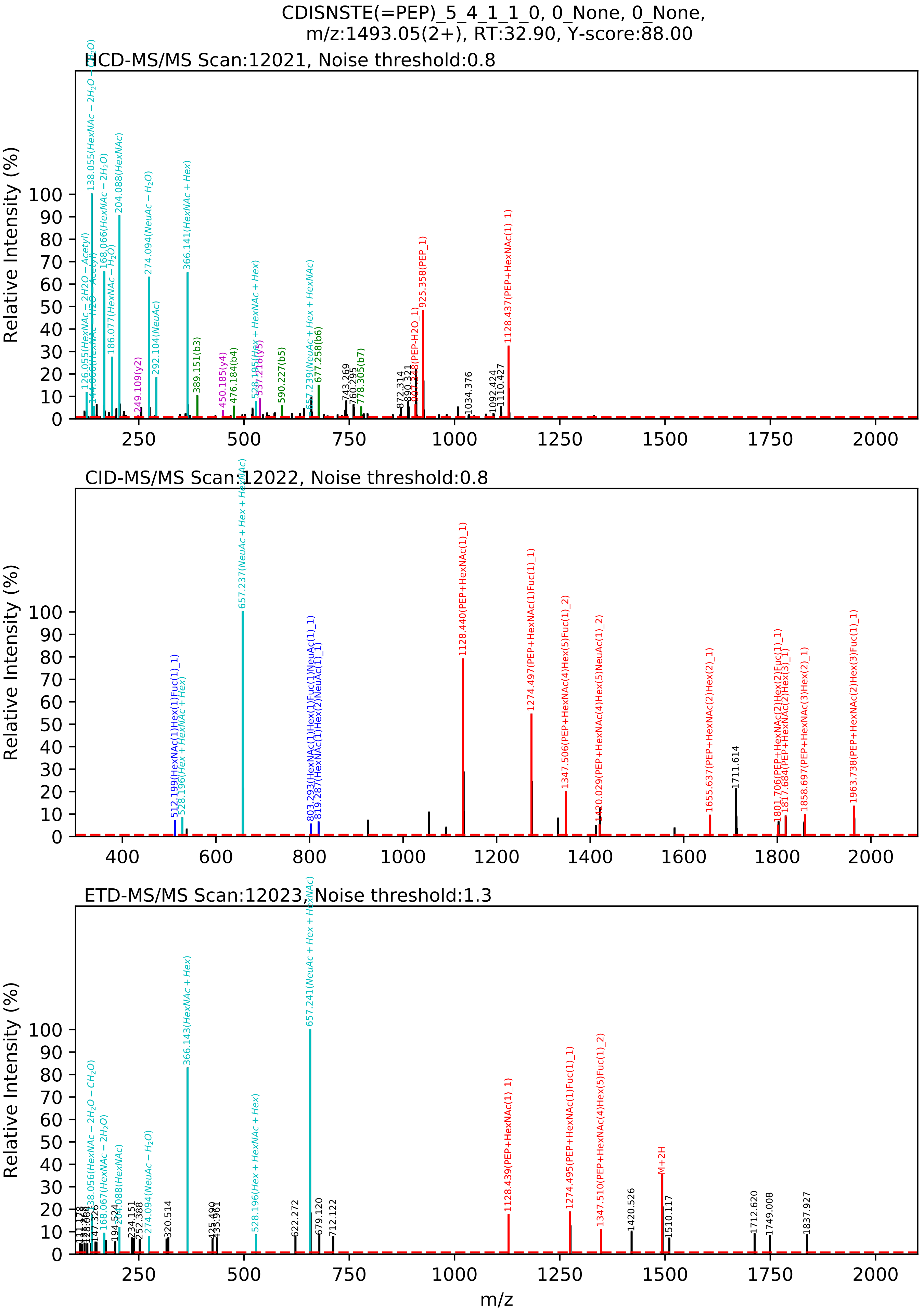
CDISNSTE(=PEP)_5_4_1_1_0, 0_None, 0_None,
m/z:1493.05(2+), RT:32.90, Y-score:88.00
HCD-MS/MS Scan:12021, Noise threshold:0.8
CID-MS/MS Scan:12022, Noise threshold:0.8
ETD-MS/MS Scan:12023, Noise threshold:1.3
Relative Intensity (%)
m/z
138.055(HexNAc − 2H2O − CH2O)
204.088(HexNAc)
274.094(NeuAc − H2O)
366.141(HexNAc + Hex)
292.104(NeuAc)
389.151(b3)
476.184(b4)
450.185(y4)
249.109(y2)
590.227(b5)
677.258(b6)
778.305(b7)
925.358(PEP_1)
1128.437(PEP+HexNAc(1)_1)
1034.376
1092.424
1110.427
657.237(NeuAc + Hex + HexNAc)
512.199(HexNAc(1)Hex(1)Fuc(1)_1)
528.196(Hex + HexNAc + Hex)
803.293(HexNAc(1)Hex(1)Fuc(1)NeuAc(1)_1)
819.287(HexNAc(1)Hex(2)NeuAc(1)_1)
1128.440(PEP+HexNAc(1)_1)
1274.497(PEP+HexNAc(1)Fuc(1)_1)
1347.506(PEP+HexNAc(4)Hex(5)Fuc(1)_2)
1420.029(PEP+HexNAc(4)Hex(5)NeuAc(1)_2)
1655.637(PEP+HexNAc(2)Hex(2)_1)
1711.614
1801.706(PEP+HexNAc(2)Hex(2)Fuc(1)_1)
1817.684(PEP+HexNAc(2)Hex(3)_1)
1858.697(PEP+HexNAc(3)Hex(2)_1)
1963.738(PEP+HexNAc(2)Hex(3)Fuc(1)_1)
366.143(HexNAc + Hex)
657.241(NeuAc + Hex + HexNAc)
528.196(Hex + HexNAc + Hex)
1128.439(PEP+HexNAc(1)_1)
1274.495(PEP+HexNAc(1)Fuc(1)_1)
1347.510(PEP+HexNAc(4)Hex(5)Fuc(1)_2)
M+2H
1420.526
1510.117
1712.620
1749.008
1837.927
622.272
679.120
712.122
320.514
234.151
252.388
194.524
147.326

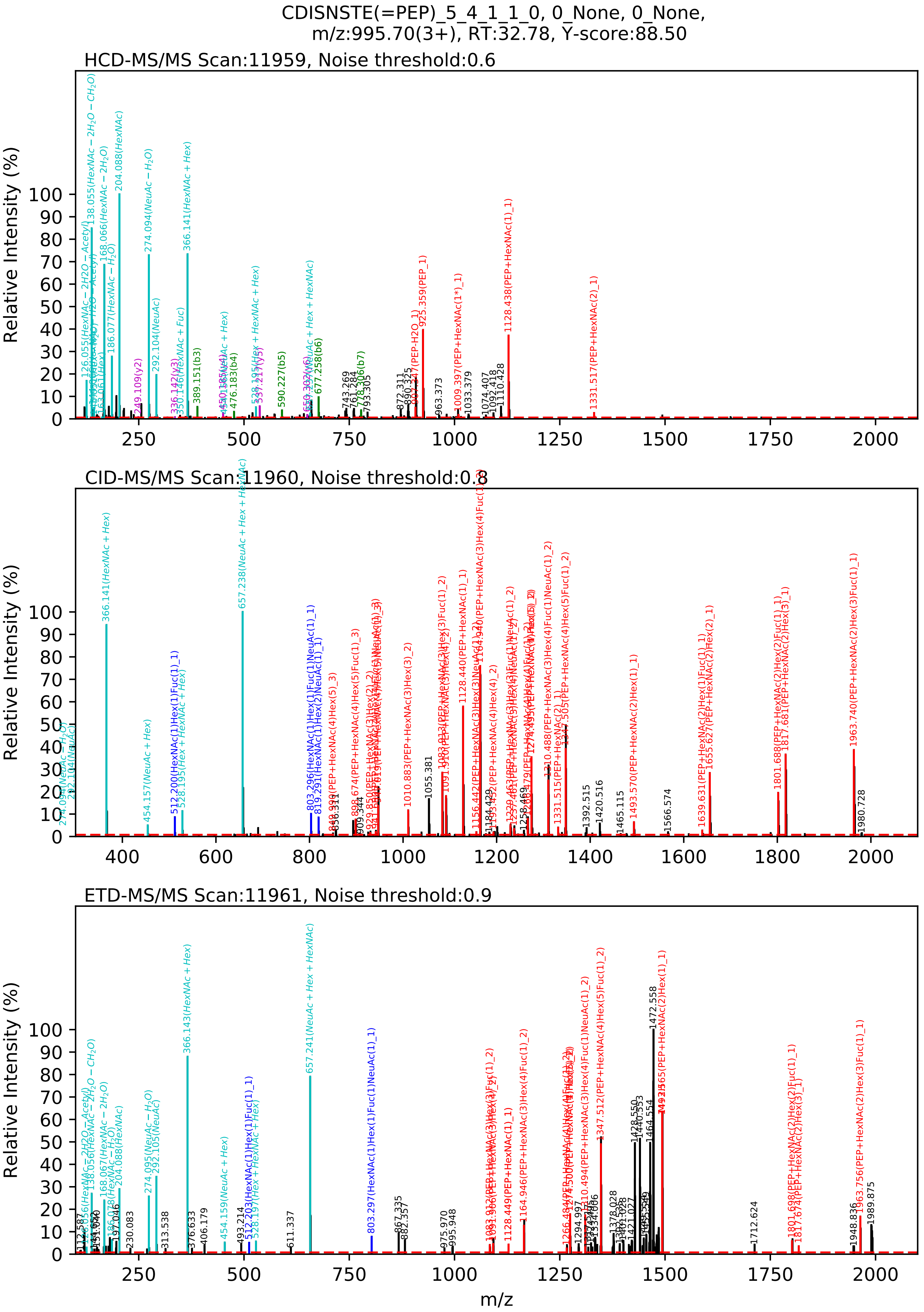
CDISNSTE(=PEP)_5_4_1_1_0, 0_None, 0_None,
m/z:995.70(3+), RT:32.78, Y-score:88.50
HCD-MS/MS Scan:11959, Noise threshold:0.6
CID-MS/MS Scan:11960, Noise threshold:0.8
ETD-MS/MS Scan:11961, Noise threshold:0.9
Relative Intensity (%)
m/z

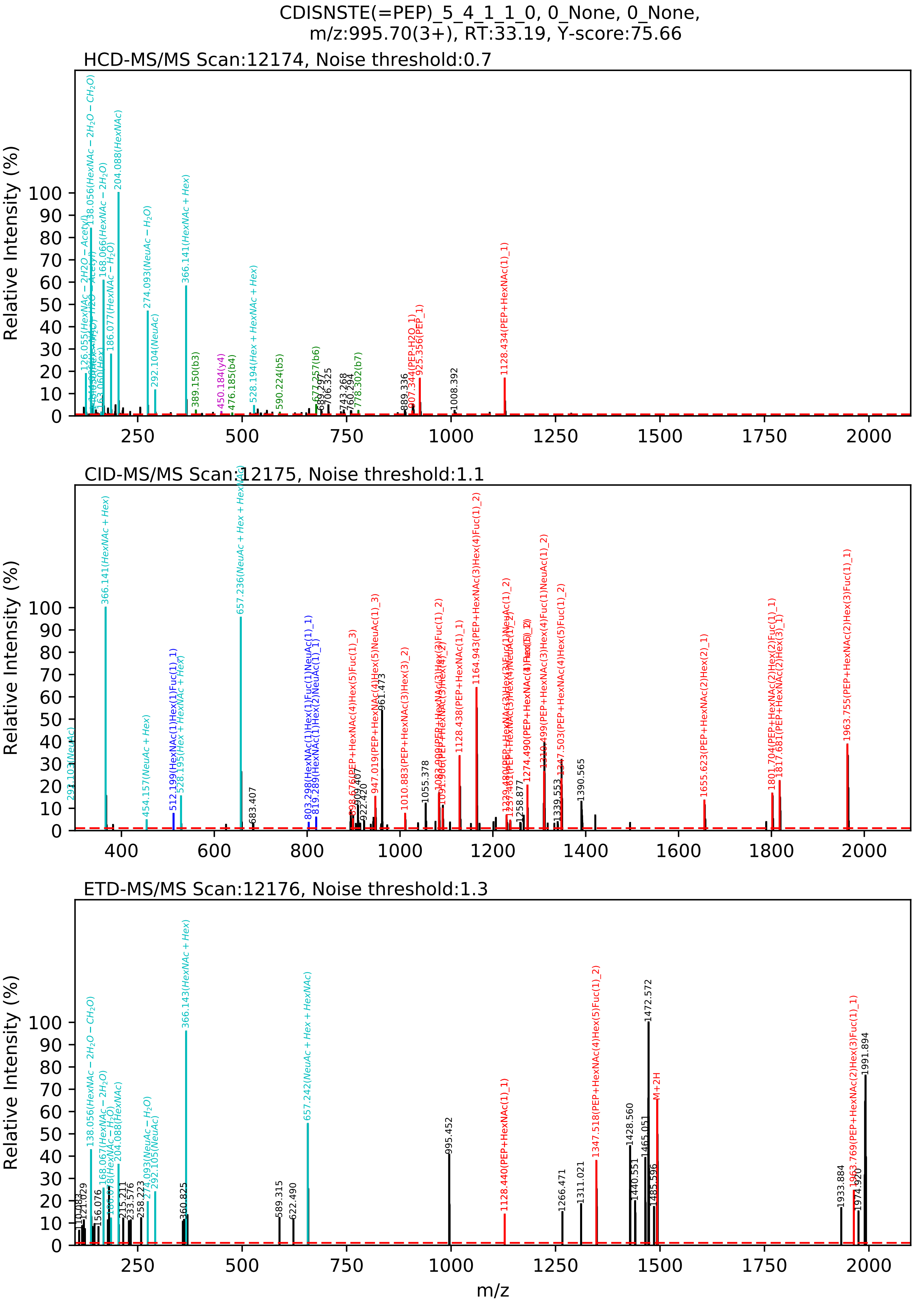

CDISNSTE(=PEP)_5_4_1_1_0, 0_None, 0_None,
m/z:995.70(3+), RT:33.19, Y-score:75.66
HCD-MS/MS Scan:12174, Noise threshold:0.7
CID-MS/MS Scan:12175, Noise threshold:1.1
ETD-MS/MS Scan:12176, Noise threshold:1.3
Relative Intensity (%)
m/z

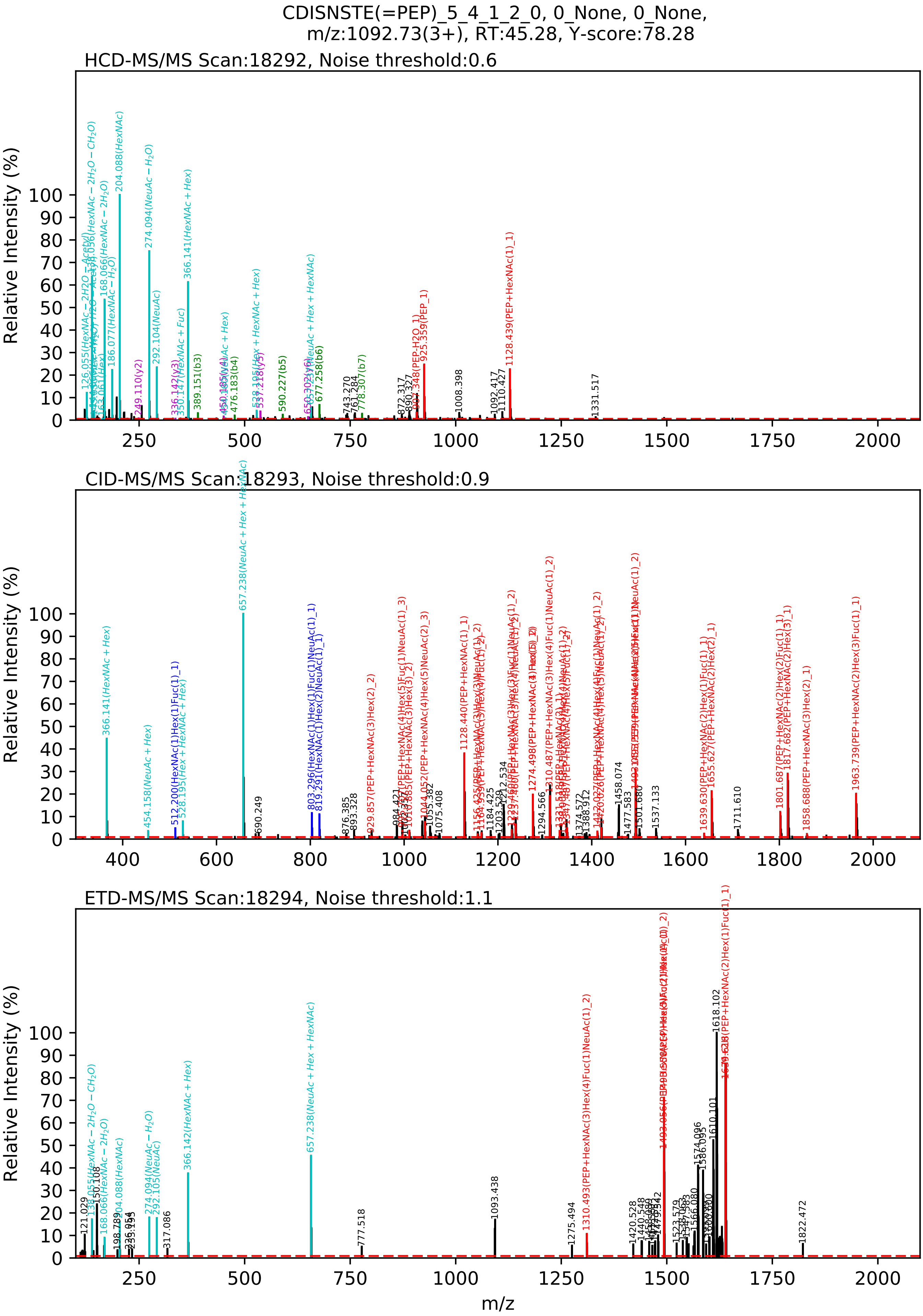
CDISNSTE(=PEP)_5_4_1_2_0, 0_None, 0_None,
m/z:1092.73(3+), RT:45.28, Y-score:78.28
HCD-MS/MS Scan:18292, Noise threshold:0.6
Relative Intensity (%)
CID-MS/MS Scan:18293, Noise threshold:0.9
Relative Intensity (%)
ETD-MS/MS Scan:18294, Noise threshold:1.1
Relative Intensity (%)
m/z

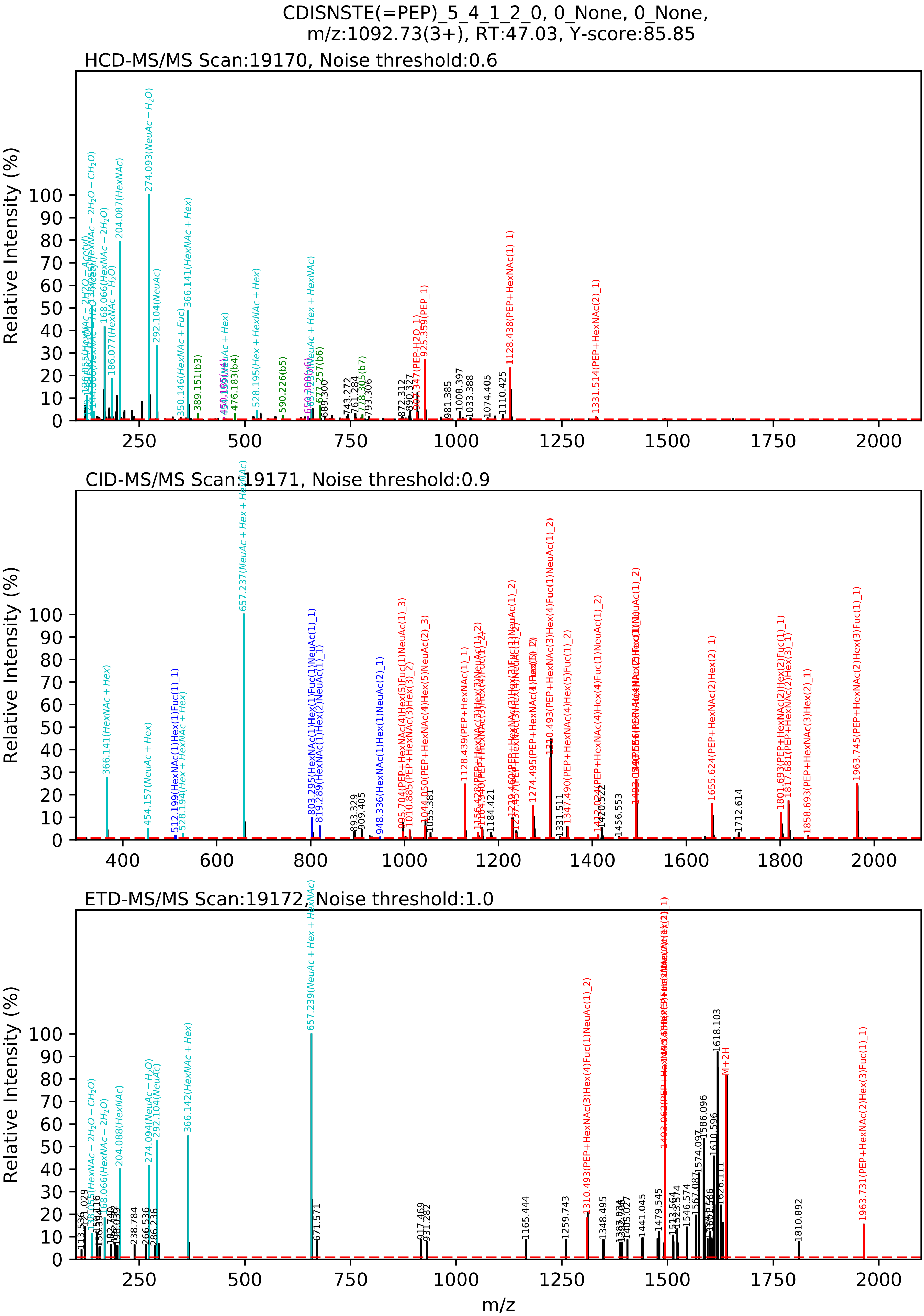
CDISNSTE(=PEP)_5_4_1_2_0, 0_None, 0_None,
m/z:1092.73(3+), RT:47.03, Y-score:85.85
HCD-MS/MS Scan:19170, Noise threshold:0.6
Relative Intensity (%)
274.093(NeuAc − H2O)
204.087(HexNAc)
168.066(HexNAc − 2H2O)
186.077(HexNAc − H2O)
292.104(NeuAc)
366.141(HexNAc + Hex)
350.146(HexNAc + Fuc)
389.151(b3)
476.183(b4)
528.195(Hex + HexNAc + Hex)
590.226(b5)
677.257(b6)
689.300
743.272
761.284
793.306
872.312
890.327
925.359(PEP_1)
981.385
1008.397
1033.388
1074.405
1110.425
1128.438(PEP+HexNAc(1)_1)
1331.514(PEP+HexNAc(2)_1)
CID-MS/MS Scan:19171, Noise threshold:0.9
657.237(NeuAc + Hex + HexNAc)
366.141(HexNAc + Hex)
454.157(NeuAc + Hex)
512.199(HexNAc(1)Hex(1)Fuc(1)_1)
528.194(Hex + HexNAc + Hex)
803.295(HexNAc(1)Hex(1)Fuc(1)NeuAc(1)_1)
819.289(HexNAc(1)Hex(2)NeuAc(1)_1)
893.329
909.405
948.336(HexNAc(1)Hex(1)NeuAc(2)_1)
1044.050(PEP+HexNAc(4)Hex(5)NeuAc(2)_3)
1055.381
1128.439(PEP+HexNAc(1)_1)
1184.421
1274.495(PEP+HexNAc(4)Hex(5)_2)
1310.493(PEP+HexNAc(3)Hex(4)Fuc(1)NeuAc(1)_2)
1331.511
1347.490(PEP+HexNAc(4)Hex(5)Fuc(1)_2)
1420.522
1422.024(PEP+HexNAc(4)Hex(4)Fuc(1)NeuAc(1)_2)
1456.553
1655.624(PEP+HexNAc(2)Hex(2)_1)
1712.614
1801.693(PEP+HexNAc(2)Hex(2)Fuc(1)_1)
1817.681(PEP+HexNAc(2)Hex(3)_1)
1858.693(PEP+HexNAc(3)Hex(2)_1)
1963.745(PEP+HexNAc(2)Hex(3)Fuc(1)_1)
ETD-MS/MS Scan:19172, Noise threshold:1.0
657.239(NeuAc + Hex + HexNAc)
168.066(HexNAc − 2H2O)
204.088(HexNAc)
274.094(NeuAc − H2O)
292.104(NeuAc)
366.142(HexNAc + Hex)
238.784
266.536
286.236
671.571
917.469
931.282
1165.444
1259.743
1310.493(PEP+HexNAc(3)Hex(4)Fuc(1)NeuAc(1)_2)
1348.495
1405.027
1441.045
1479.545
1513.564
1523.574
1546.574
1574.097
1586.096
1610.596
1618.103
1626.111
M+2H
1810.892
1963.731(PEP+HexNAc(2)Hex(3)Fuc(1)_1)
m/z

CDISNSTE(=PEP)_5_5_1_0_0, 0_None, 0_None,
m/z:966.36(3+), RT:23.18, Y-score:86.29

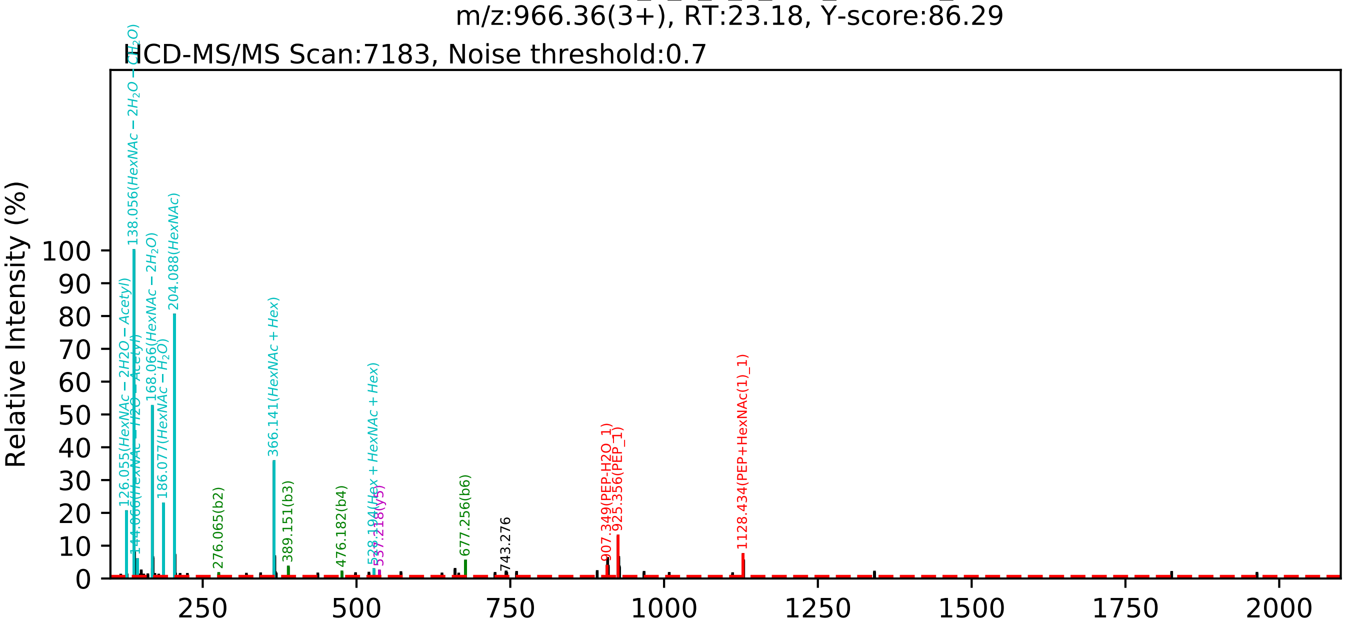

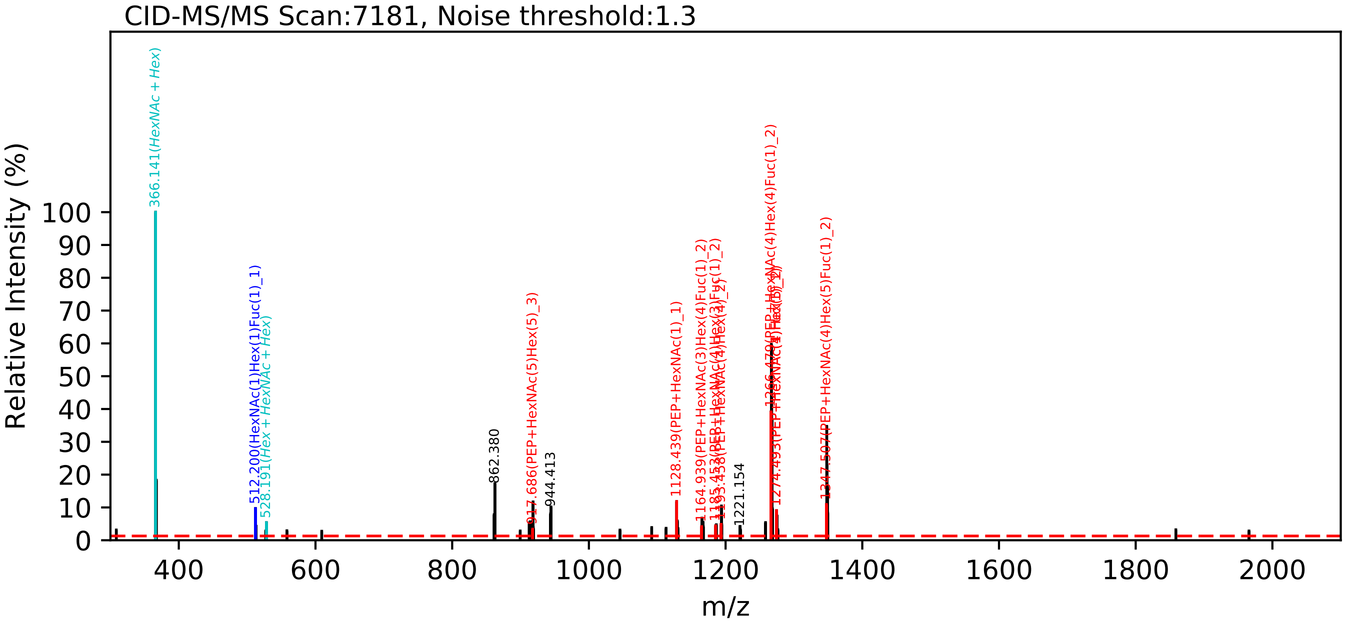

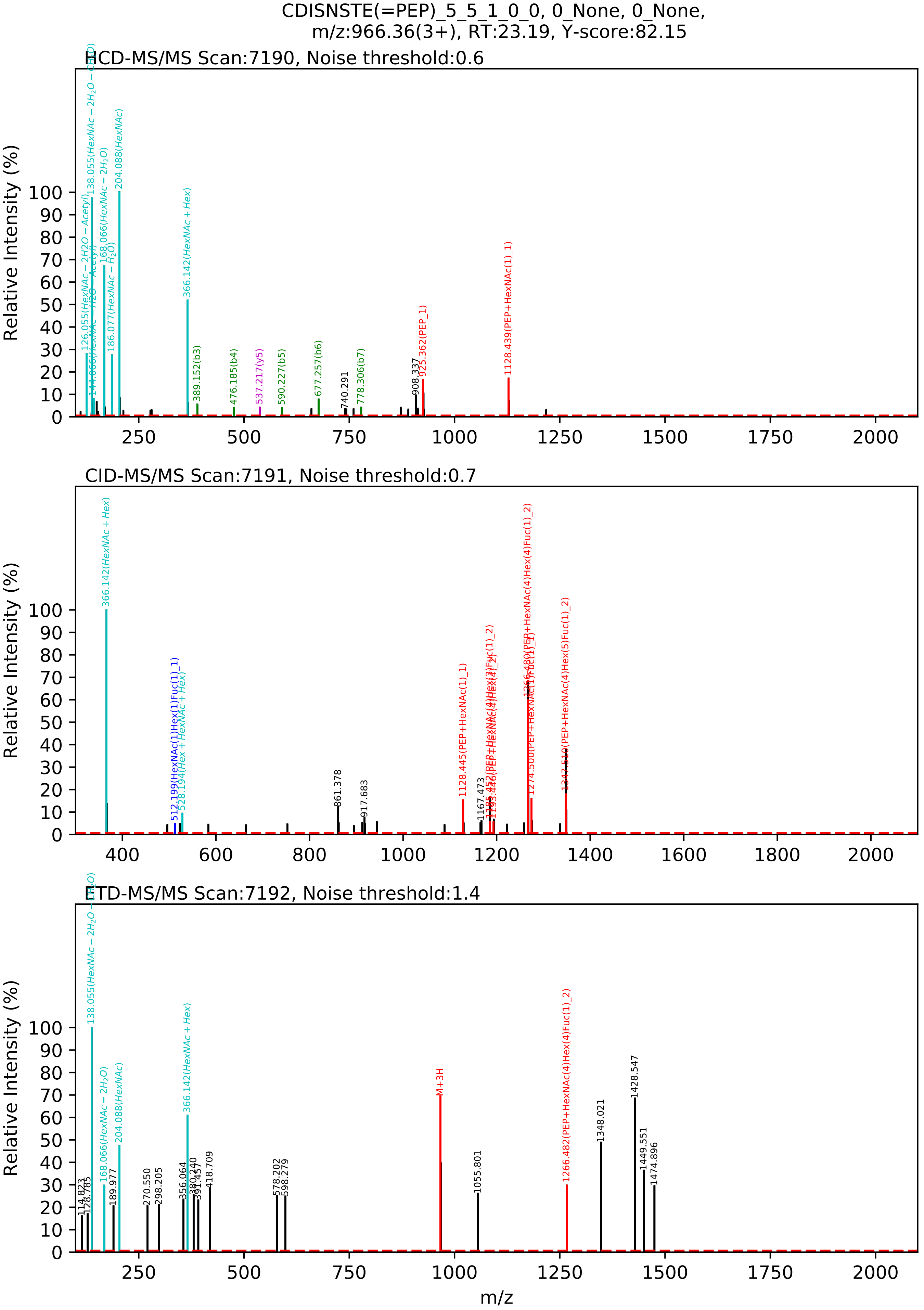

CDISNSTE(=PEP)_5_5_1_0_0, 0_None, 0_None,
m/z:966.36(3+), RT:23.19, Y-score:82.15
HCD-MS/MS Scan:7190, Noise threshold:0.6
Relative Intensity (%)
126.055(HexNAc − 2H2O − Acetyl)
138.055(HexNAc − 2H2O − CH2O)
144.066(HexNAc − H2O − Acetyl)
168.066(HexNAc − 2H2O)
186.077(HexNAc − H2O)
204.088(HexNAc)
366.142(HexNAc + Hex)
389.152(b3)
476.185(b4)
537.217(y5)
590.227(b5)
677.257(b6)
740.291
778.306(b7)
908.337
925.362(PEP_1)
1128.439(PEP+HexNAc(1)_1)
CID-MS/MS Scan:7191, Noise threshold:0.7
366.142(HexNAc + Hex)
512.199(HexNAc(1)Hex(1)Fuc(1)_1)
528.194(Hex + HexNAc + Hex)
861.378
917.683
1128.445(PEP+HexNAc(1)_1)
1167.473
1185.452(PEP+HexNAc(4)Hex(3)Fuc(1)_2)
1193.446(PEP+HexNAc(4)Hex(4)_2)
1266.480(PEP+HexNAc(4)Hex(4)Fuc(1)_2)
1274.500(PEP+HexNAc(1)Fuc(1)_1)
1347.510(PEP+HexNAc(4)Hex(5)Fuc(1)_2)
ETD-MS/MS Scan:7192, Noise threshold:1.4
114.823
128.785
138.055(HexNAc − 2H2O − CH2O)
168.066(HexNAc − 2H2O)
189.977
204.088(HexNAc)
270.550
298.205
356.064
366.142(HexNAc + Hex)
380.240
391.451
418.709
578.202
598.279
M+3H
1055.801
1266.482(PEP+HexNAc(4)Hex(4)Fuc(1)_2)
1348.021
1428.547
1449.551
1474.896
m/z

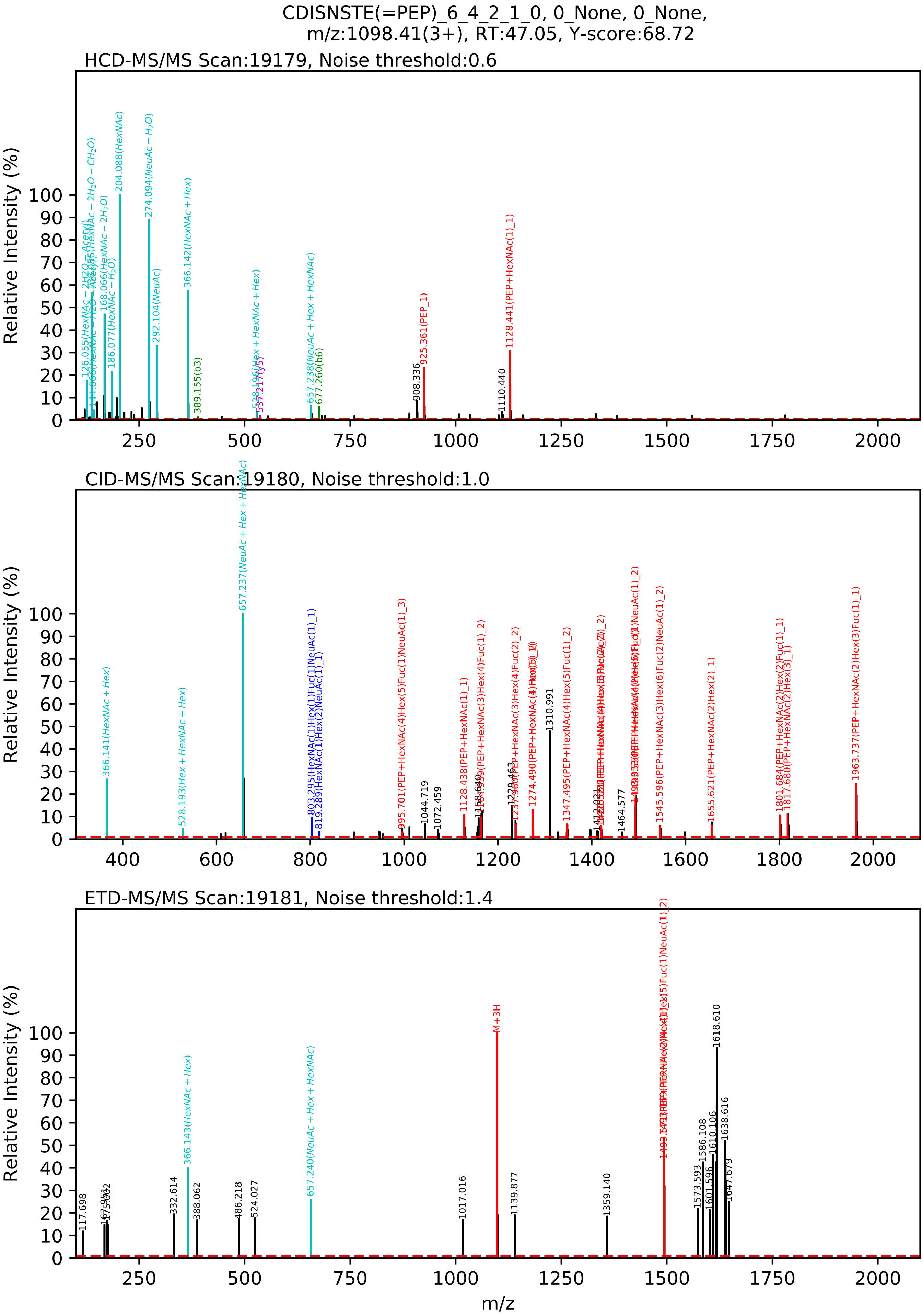
CDISNSTE(=PEP)_6_4_2_1_0, 0_None, 0_None,
m/z:1098.41(3+), RT:47.05, Y-score:68.72
HCD-MS/MS Scan:19179, Noise threshold:0.6
Relative Intensity (%)
204.088(HexNAc)
274.094(NeuAc − H2O)
366.142(HexNAc + Hex)
292.104(NeuAc)
168.066(HexNAc − 2H2O)
186.077(HexNAc − H2O)
126.055(HexNAc − 2H2O − Acetyl)
389.155(b3)
537.217(y5)
657.238(NeuAc + Hex + HexNAc)
677.260(b6)
908.336
925.361(PEP_1)
1110.440
1128.441(PEP+HexNAc(1)_1)
CID-MS/MS Scan:19180, Noise threshold:1.0
657.237(NeuAc + Hex + HexNAc)
366.141(HexNAc + Hex)
528.193(Hex + HexNAc + Hex)
803.295(HexNAc(1)Hex(1)Fuc(1)NeuAc(1)_1)
819.289(HexNAc(1)Hex(2)NeuAc(1)_1)
995.701(PEP+HexNAc(4)Hex(5)Fuc(1)NeuAc(1)_3)
1044.719
1072.459
1128.438(PEP+HexNAc(1)_1)
1229.463
1274.490(PEP+HexNAc(1)Hex(1)_1)
1310.991
1347.495(PEP+HexNAc(4)Hex(5)Fuc(1)_2)
1412.021
1464.577
1545.596(PEP+HexNAc(3)Hex(6)Fuc(2)NeuAc(1)_2)
1655.621(PEP+HexNAc(2)Hex(2)_1)
1801.684(PEP+HexNAc(2)Hex(2)Fuc(1)_1)
1817.680(PEP+HexNAc(2)Hex(3)_1)
1963.737(PEP+HexNAc(2)Hex(3)Fuc(1)_1)
ETD-MS/MS Scan:19181, Noise threshold:1.4
M+3H
1618.610
1638.616
1586.108
1610.106
1573.593
1601.596
1647.679
366.143(HexNAc + Hex)
657.240(NeuAc + Hex + HexNAc)
117.698
332.614
388.062
486.218
524.027
1017.016
1139.877
1359.140
m/z

CDISNSTE(=PEP)_6_5_1_0_0, 0_None, 0_None,
m/z:1020.38(3+), RT:22.73, Y-score:73.94

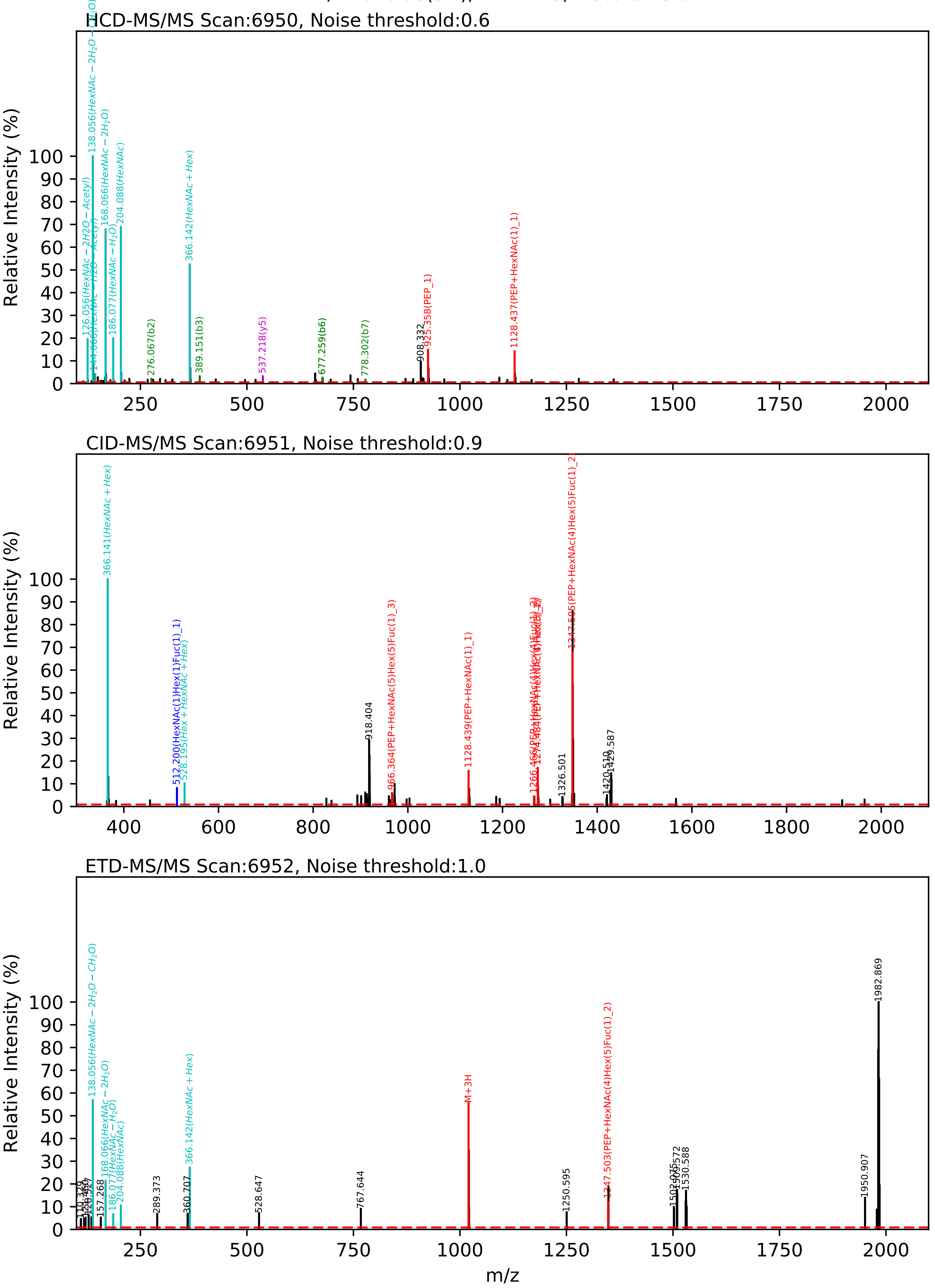

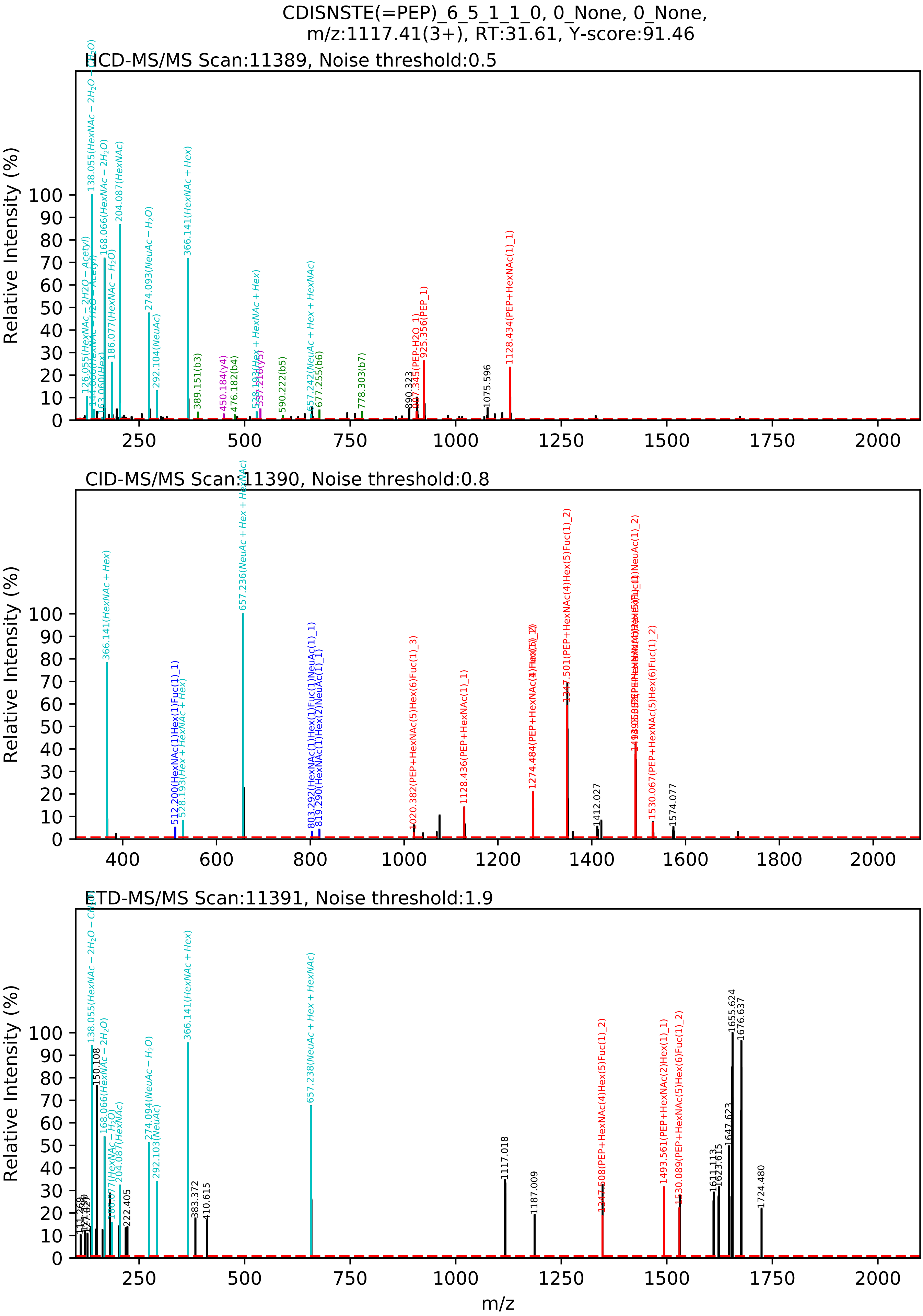
CDISNSTE(=PEP)_6_5_1_1_0, 0_None, 0_None,
m/z:1117.41(3+), RT:31.61, Y-score:91.46
HCD-MS/MS Scan:11389, Noise threshold:0.5
CID-MS/MS Scan:11390, Noise threshold:0.8
ETD-MS/MS Scan:11391, Noise threshold:1.9
Relative Intensity (%)
m/z
138.055(HexNAc − 2H2O − C2H4O)
168.066(HexNAc − 2H2O)
204.087(HexNAc)
186.077(HexNAc − H2O)
274.093(NeuAc − H2O)
292.104(NeuAc)
366.141(HexNAc + Hex)
389.151(b3)
450.184(y4)
476.182(b4)
537.216(y5)
590.222(b5)
657.242(NeuAc + Hex + HexNAc)
677.255(b6)
778.303(b7)
890.323
925.356(PEP_1)
1075.596
1128.434(PEP+HexNAc(1)_1)
657.236(NeuAc + Hex + HexNAc)
512.200(HexNAc(1)Hex(1)Fuc(1)_1)
528.193(Hex + HexNAc + Hex)
1020.382(PEP+HexNAc(5)Hex(6)Fuc(1)_3)
1128.436(PEP+HexNAc(1)_1)
1274.484(PEP+HexNAc(1)Fuc(1)_1)
1347.501(PEP+HexNAc(4)Hex(5)Fuc(1)_2)
1412.027
1530.067(PEP+HexNAc(5)Hex(6)Fuc(1)_2)
1574.077
150.108
222.405
383.372
410.615
657.238(NeuAc + Hex + HexNAc)
1117.018
1187.009
1347.508(PEP+HexNAc(4)Hex(5)Fuc(1)_2)
1493.561(PEP+HexNAc(2)Hex(1)_1)
1530.089(PEP+HexNAc(5)Hex(6)Fuc(1)_2)
1611.113
1623.615
1647.623
1655.624
1676.637
1724.480

CDISNSTE(=PEP)_6_5_1_2_0, 0_None, 0_None,
m/z:1214.44(3+), RT:44.75, Y-score:85.60

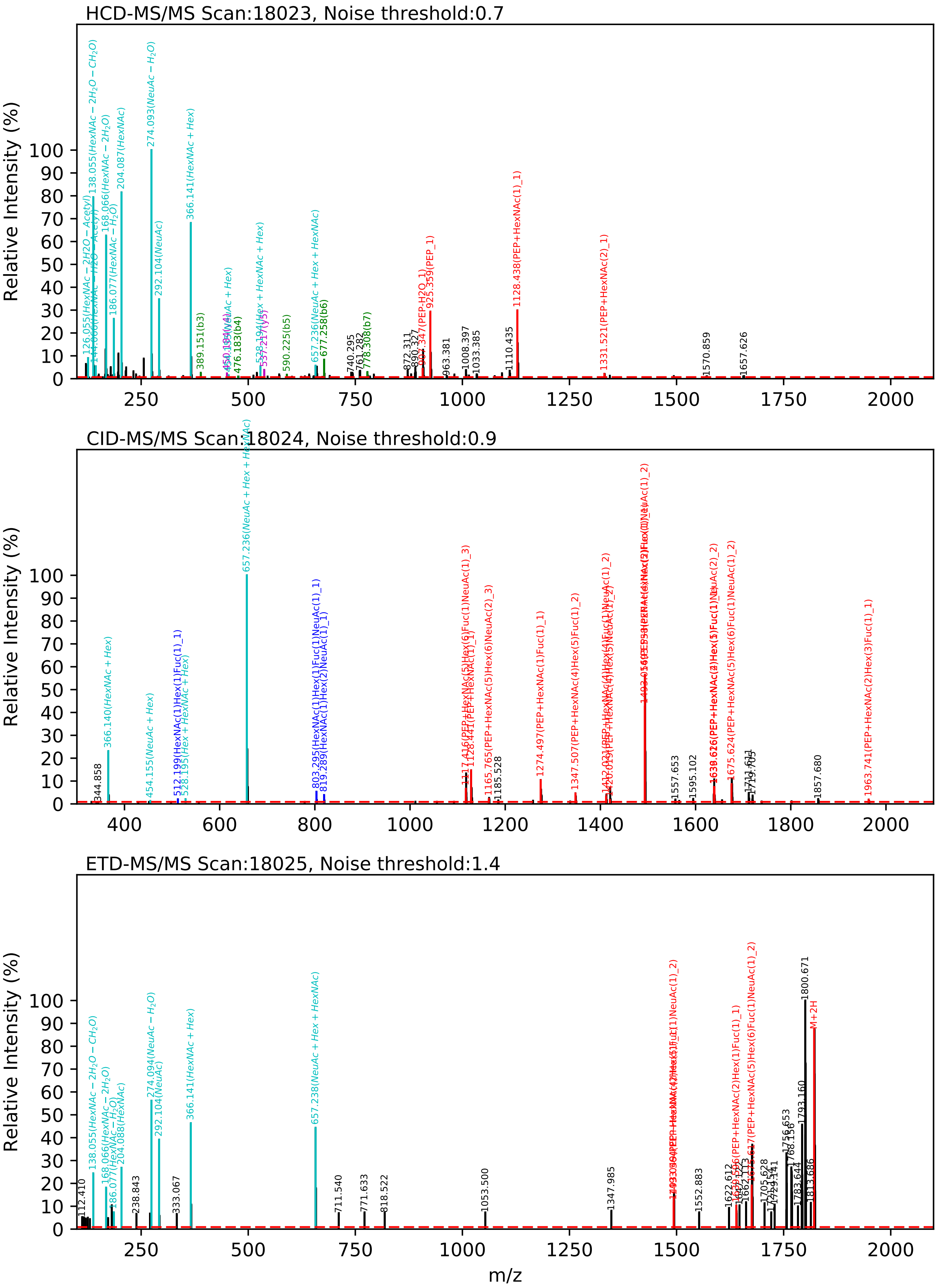

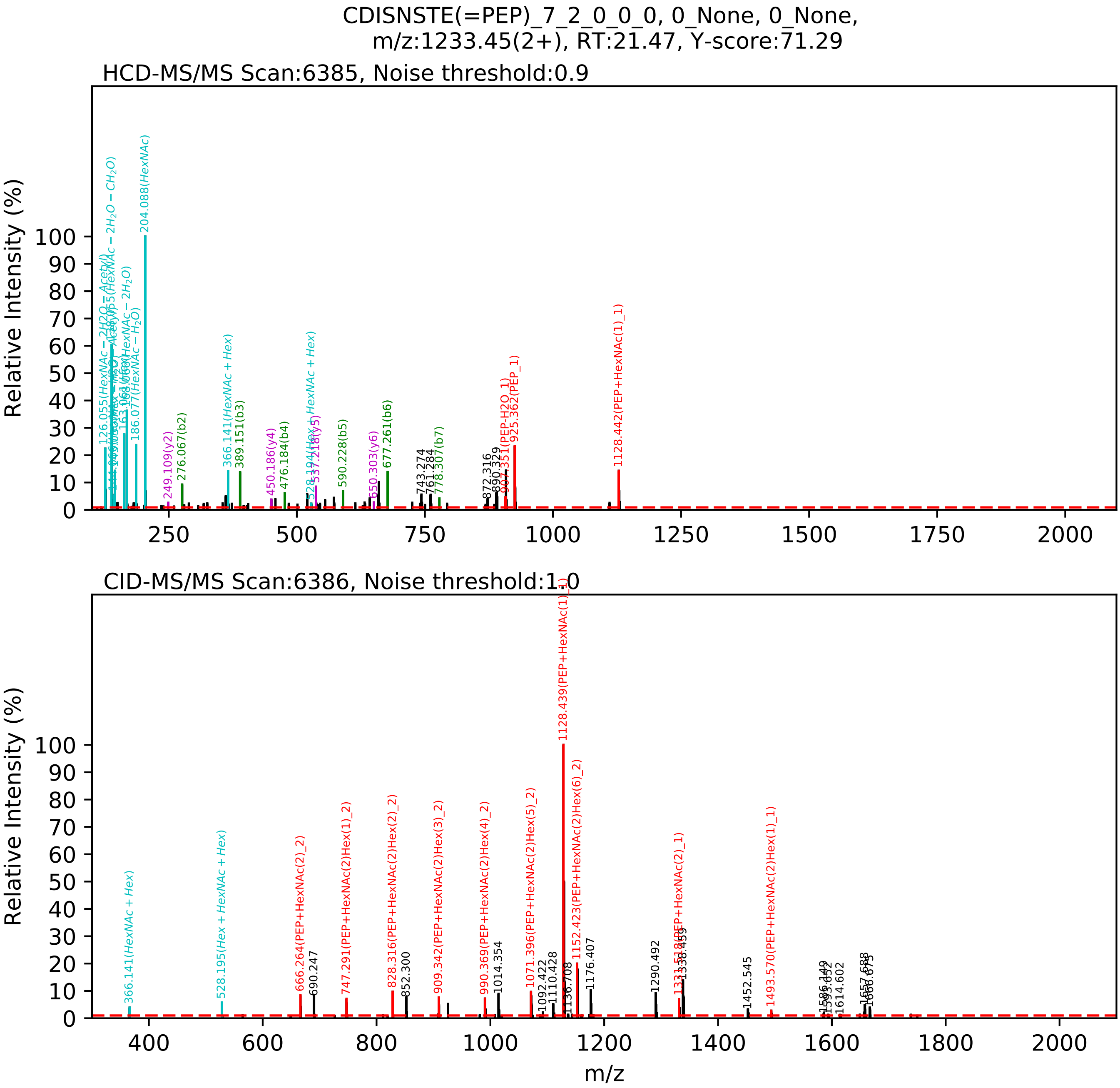
CDISNSTE(=PEP)_7_2_0_0_0, 0_None, 0_None,
m/z:1233.45(2+), RT:21.47, Y-score:71.29
HCD-MS/MS Scan:6385, Noise threshold:0.9
Relative Intensity (%)
204.088(HexNAc)
186.077(HexNAc − H2O)
126.055(HexNAc − 2H2O − Acetyl)
276.067(b2)
249.109(y2)
366.141(HexNAc + Hex)
389.151(b3)
450.186(y4)
476.184(b4)
537.218(y5)
590.228(b5)
650.303(y6)
677.261(b6)
743.274
761.284
778.307(b7)
872.316
890.329
907.351(PEP-H2O_1)
925.362(PEP_1)
1128.442(PEP+HexNAc(1)_1)
CID-MS/MS Scan:6386, Noise threshold:1.0
Relative Intensity (%)
366.141(HexNAc + Hex)
528.195(Hex + HexNAc + Hex)
666.264(PEP+HexNAc(2)_2)
690.247
747.291(PEP+HexNAc(2)Hex(1)_2)
828.316(PEP+HexNAc(2)Hex(2)_2)
852.300
909.342(PEP+HexNAc(2)Hex(3)_2)
990.369(PEP+HexNAc(2)Hex(4)_2)
1014.354
1071.396(PEP+HexNAc(2)Hex(5)_2)
1092.422
1110.428
1136.708
1128.439(PEP+HexNAc(1)_1)
1152.423(PEP+HexNAc(2)Hex(6)_2)
1176.407
1290.492
1338.459
1452.545
1493.570(PEP+HexNAc(2)Hex(1)_1)
1614.602
1657.688
m/z

IQNLTVK(=PEP)_3_2_0_0_0, 0_None, 0_None,
m/z:854.41(2+), RT:26.51, Y-score:95.20

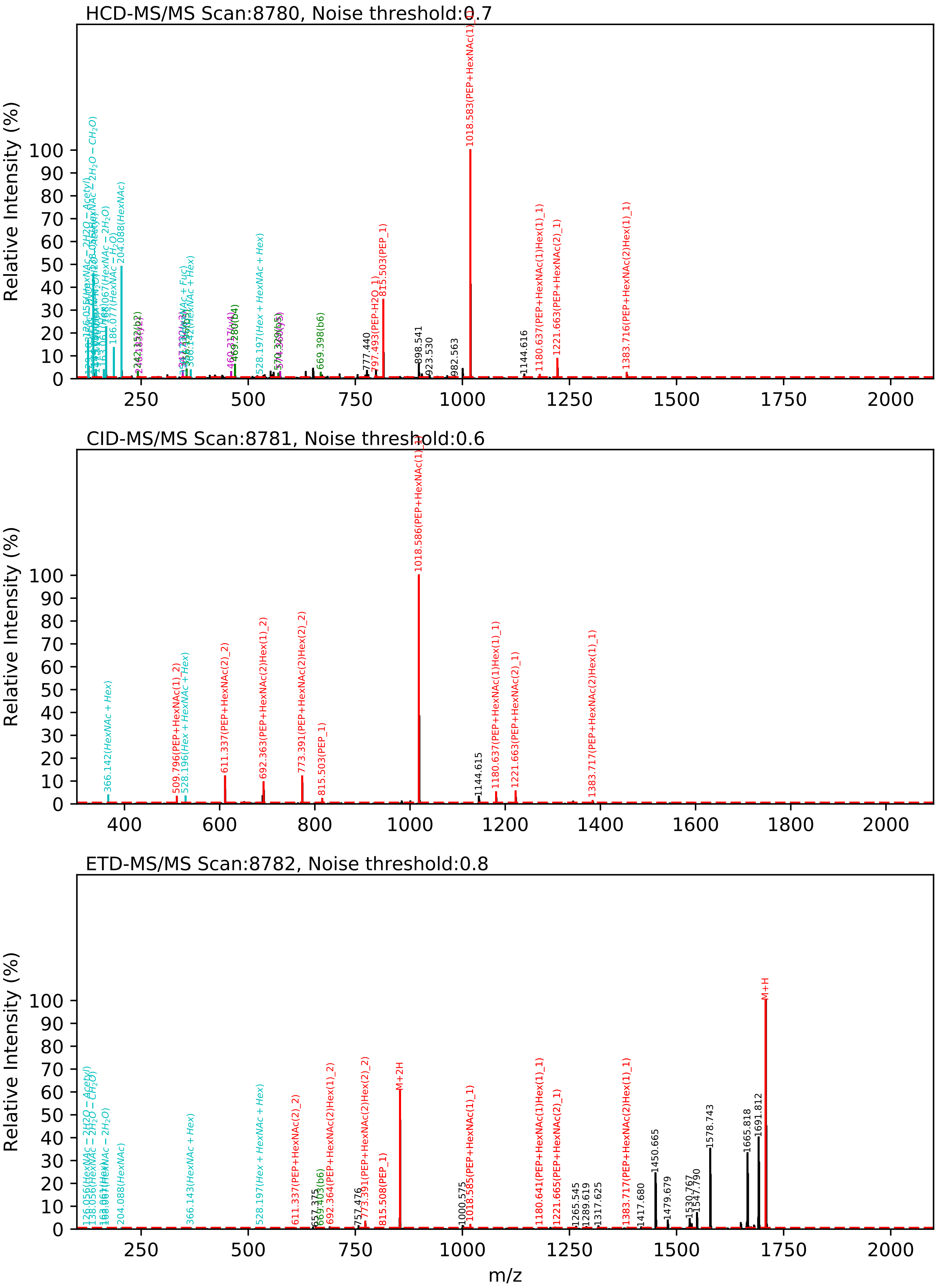

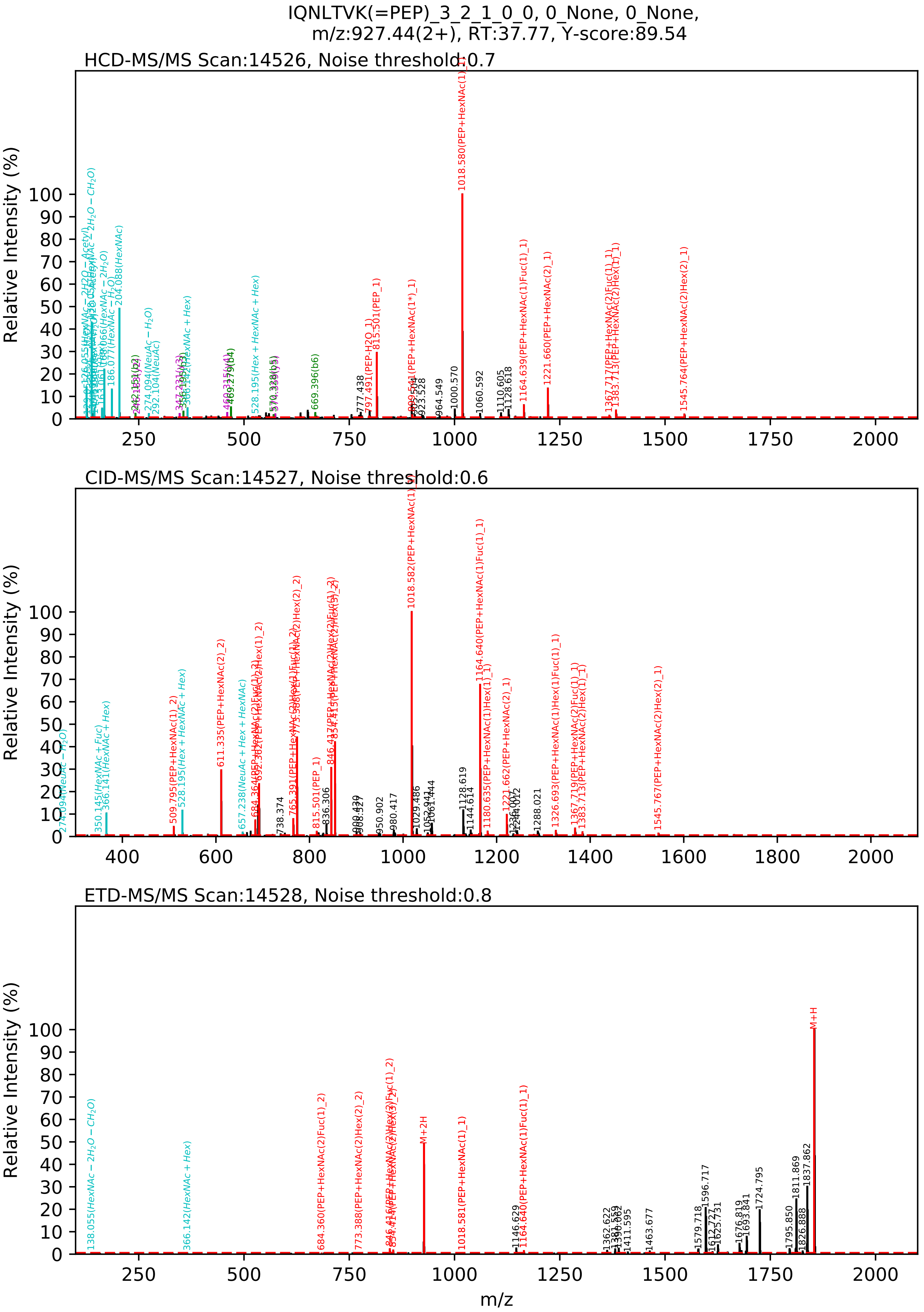

IQNLTVK(=PEP)_3_2_1_0_0, 0_None, 0_None,
m/z:927.44(2+), RT:37.77, Y-score:89.54
HCD-MS/MS Scan:14526, Noise threshold:0.7
CID-MS/MS Scan:14527, Noise threshold:0.6
ETD-MS/MS Scan:14528, Noise threshold:0.8
Relative Intensity (%)
m/z
1018.580(PEP+HexNAc(1)_1)
815.501(PEP_1)
1164.639(PEP+HexNAc(1)Fuc(1)_1)
1221.660(PEP+HexNAc(2)_1)
1545.764(PEP+HexNAc(2)Hex(2)_1)
204.088(HexNAc)
1018.582(PEP+HexNAc(1)_1)
1164.640(PEP+HexNAc(1)Fuc(1)_1)
611.335(PEP+HexNAc(2)_2)
1545.767(PEP+HexNAc(2)Hex(2)_1)
M+H
M+2H
1837.862
1811.869
1724.795
1596.717

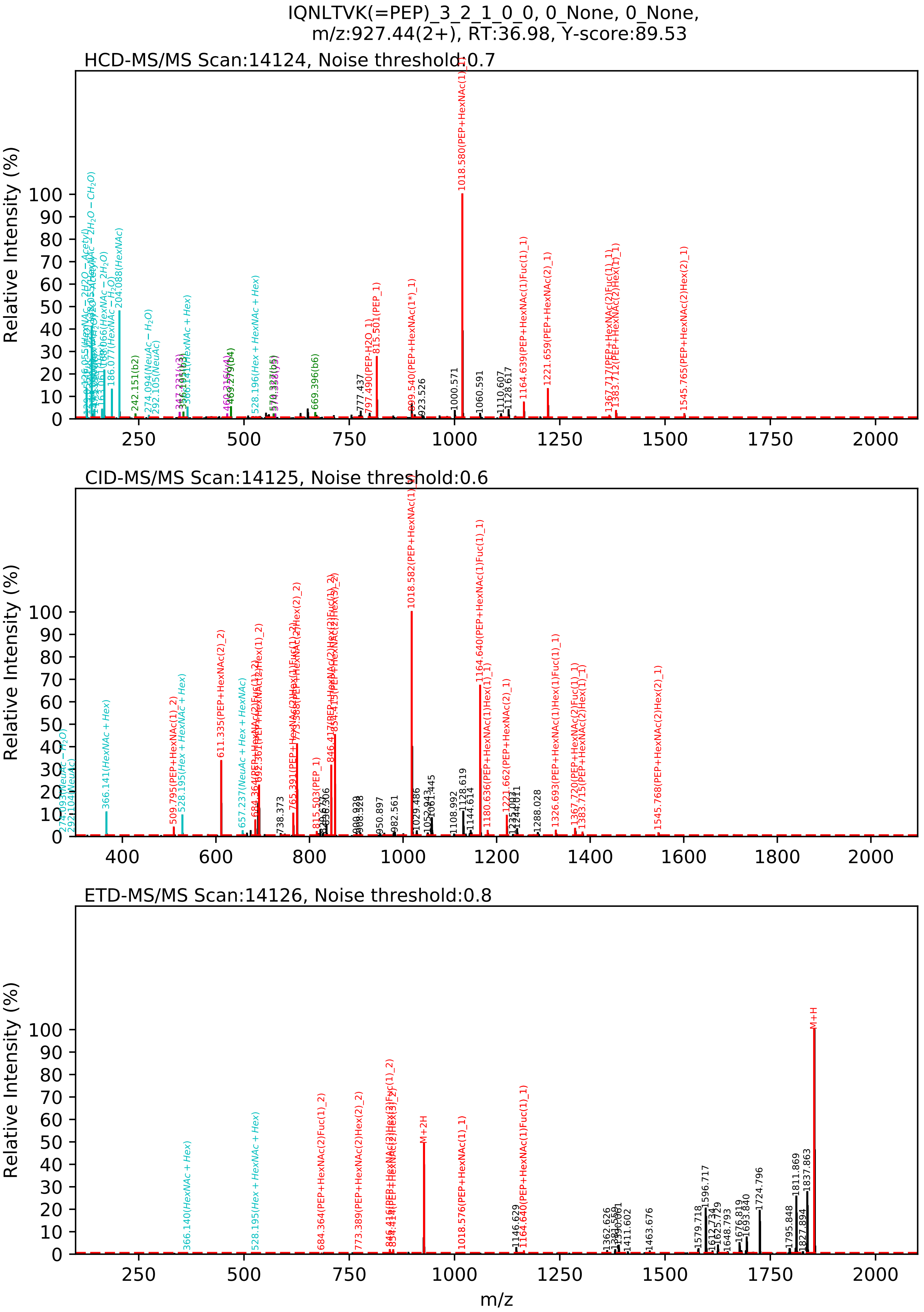

IQNLTVK(=PEP)_3_2_1_0_0, 0_None, 0_None,
m/z:927.44(2+), RT:36.98, Y-score:89.53
HCD-MS/MS Scan:14124, Noise threshold:0.7
CID-MS/MS Scan:14125, Noise threshold:0.6
ETD-MS/MS Scan:14126, Noise threshold:0.8
Relative Intensity (%)
m/z
1018.580(PEP+HexNAc(1)_1)
815.501(PEP_1)
1164.639(PEP+HexNAc(1)Fuc(1)_1)
1221.659(PEP+HexNAc(2)_1)
1545.765(PEP+HexNAc(2)Hex(2)_1)
204.088(HexNAc)
1018.582(PEP+HexNAc(1)_1)
1164.640(PEP+HexNAc(1)Fuc(1)_1)
611.335(PEP+HexNAc(2)_2)
366.141(HexNAc+Hex)
M+H
M+2H
1837.863
1811.869
1724.796
1596.717

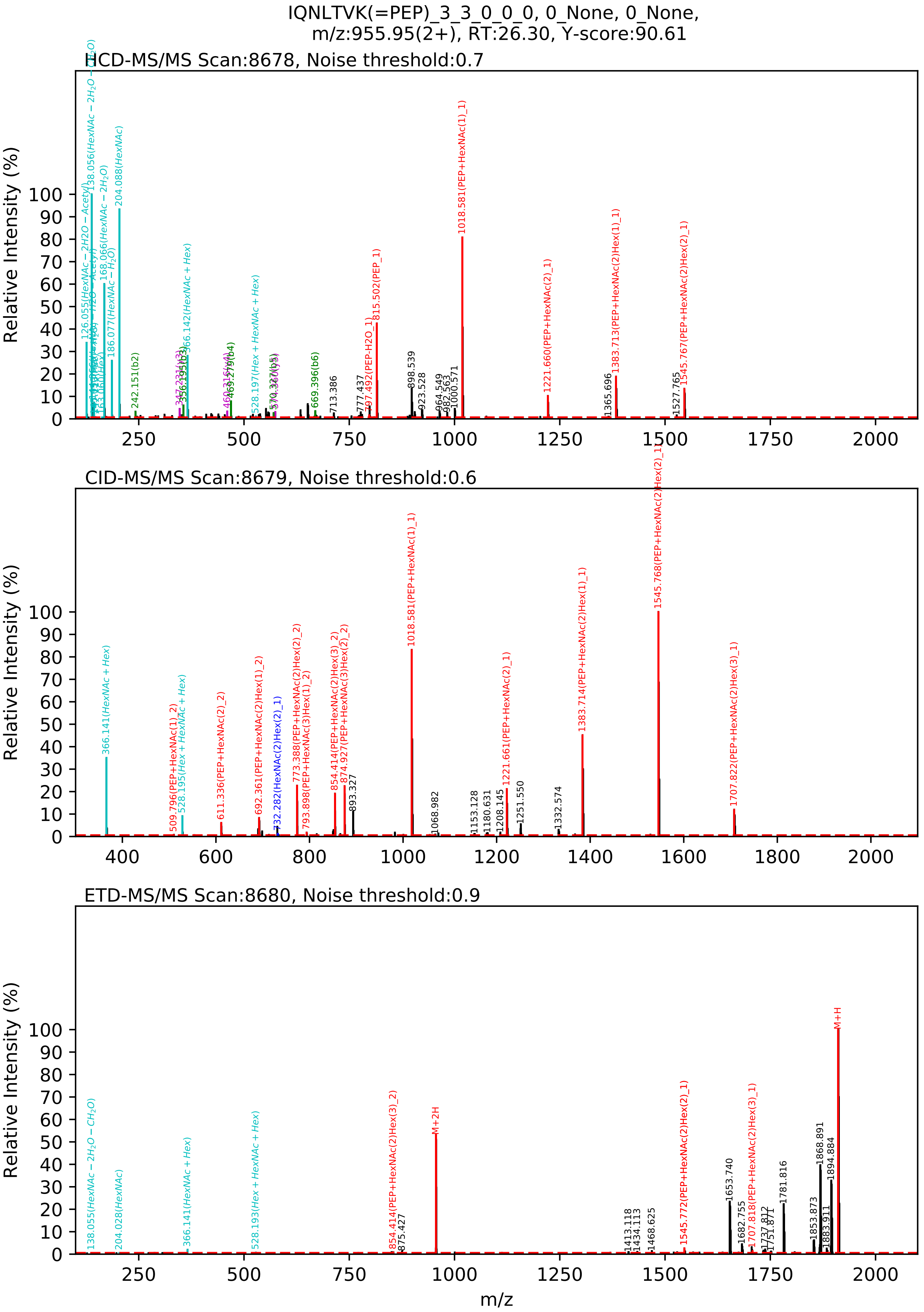
IQNLTVK(=PEP)_3_3_0_0_0, 0_None, 0_None,
m/z:955.95(2+), RT:26.30, Y-score:90.61
HCD-MS/MS Scan:8678, Noise threshold:0.7
CID-MS/MS Scan:8679, Noise threshold:0.6
ETD-MS/MS Scan:8680, Noise threshold:0.9
Relative Intensity (%)
m/z
1018.581(PEP+HexNAc(1)_1)
1221.660(PEP+HexNAc(2)_1)
1383.713(PEP+HexNAc(2)Hex(1)_1)
1545.767(PEP+HexNAc(2)Hex(2)_1)
815.502(PEP_1)
204.088(HexNAc)
366.142(HexNAc+Hex)
528.197(Hex+HexNAc+Hex)
1545.768(PEP+HexNAc(2)Hex(2)_1)
1383.714(PEP+HexNAc(2)Hex(1)_1)
1707.822(PEP+HexNAc(2)Hex(3)_1)
1221.661(PEP+HexNAc(2)_1)
366.141(HexNAc+Hex)
M+H
M+2H
854.414(PEP+HexNAc(2)Hex(3)_2)
1545.772(PEP+HexNAc(2)Hex(2)_1)
1707.818(PEP+HexNAc(2)Hex(3)_1)

IQNLTVK(=PEP)_3_3_0_0_0, 0_None, 0_None,
m/z:955.95(2+), RT:26.85, Y-score:92.29

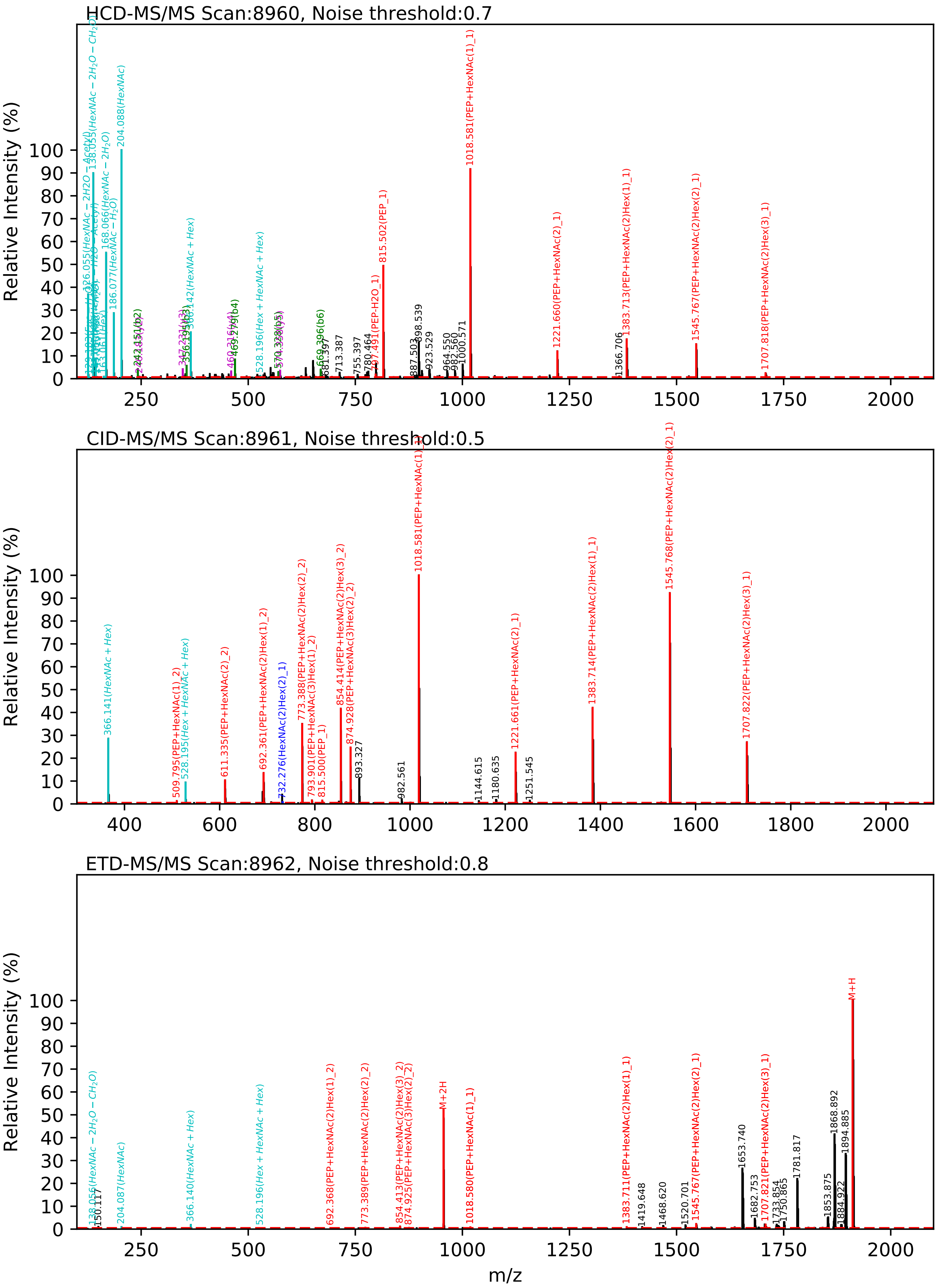

IQNLTVK(=PEP)_3_3_0_0_0, 0_None, 0_None,
m/z:955.95(2+), RT:48.73, Y-score:92.00

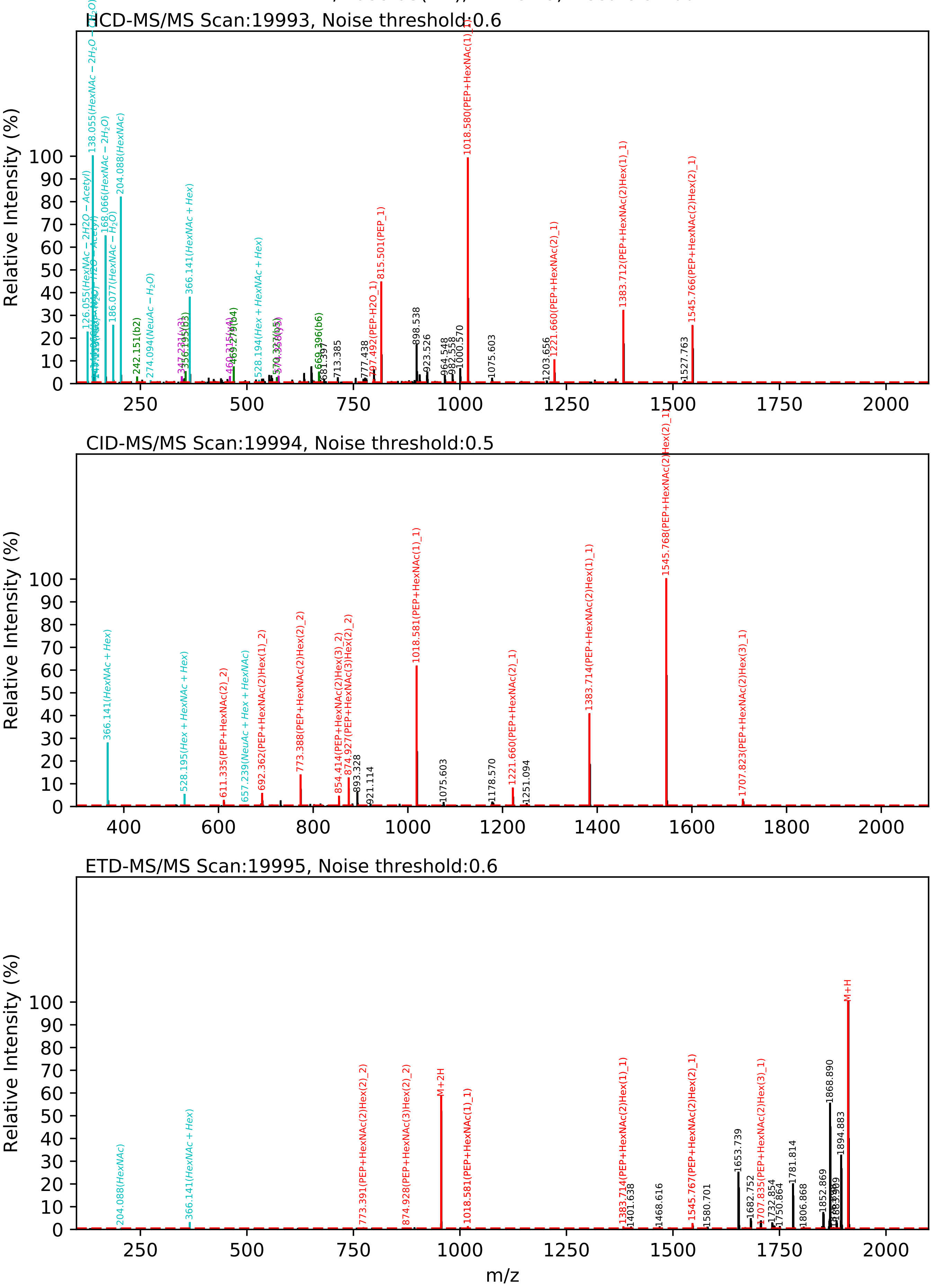

IQNLTVK(=PEP)_3_3_0_0_0, 0_None, 0_None,
m/z:955.95(2+), RT:35.78, Y-score:90.33

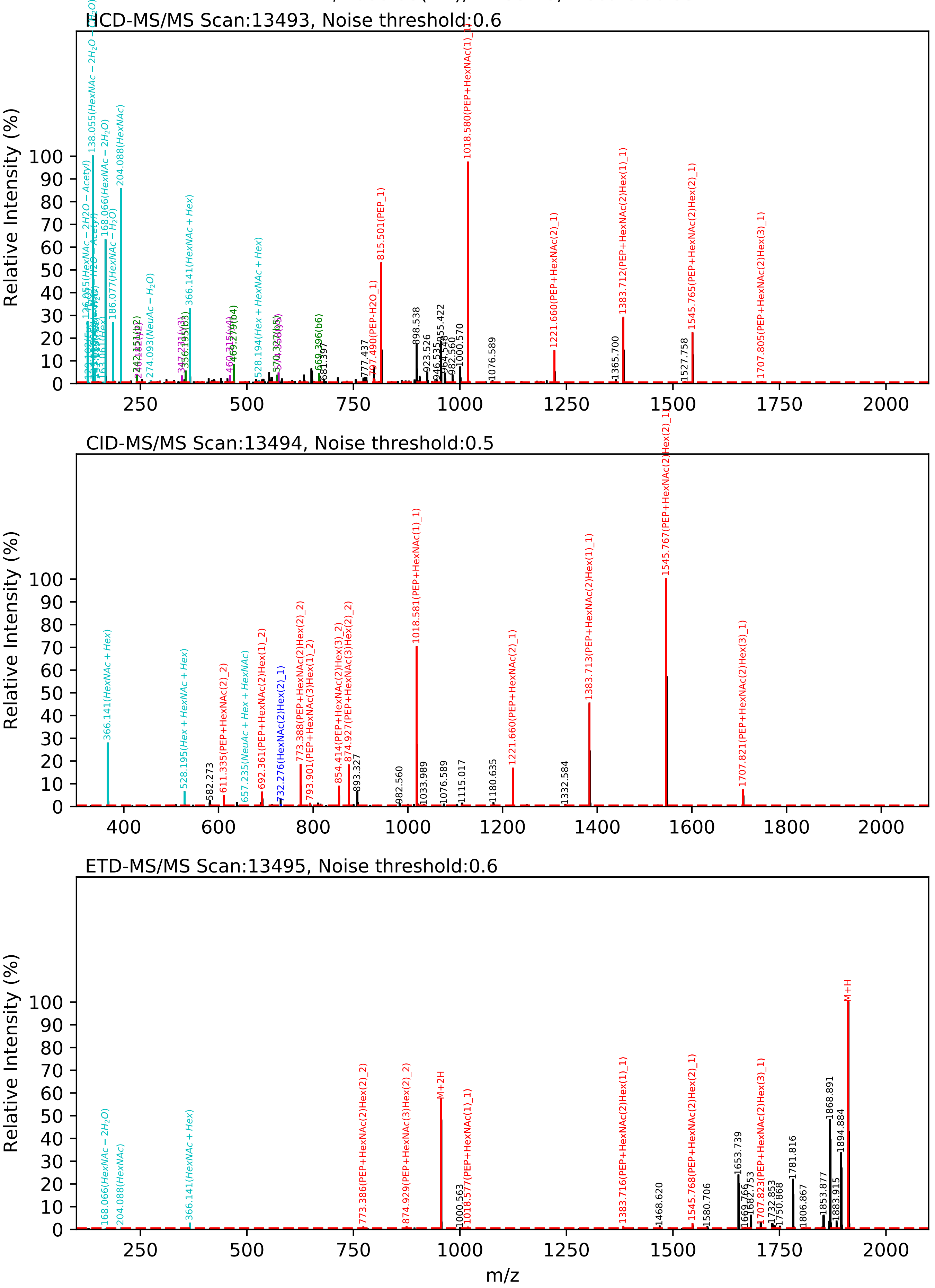

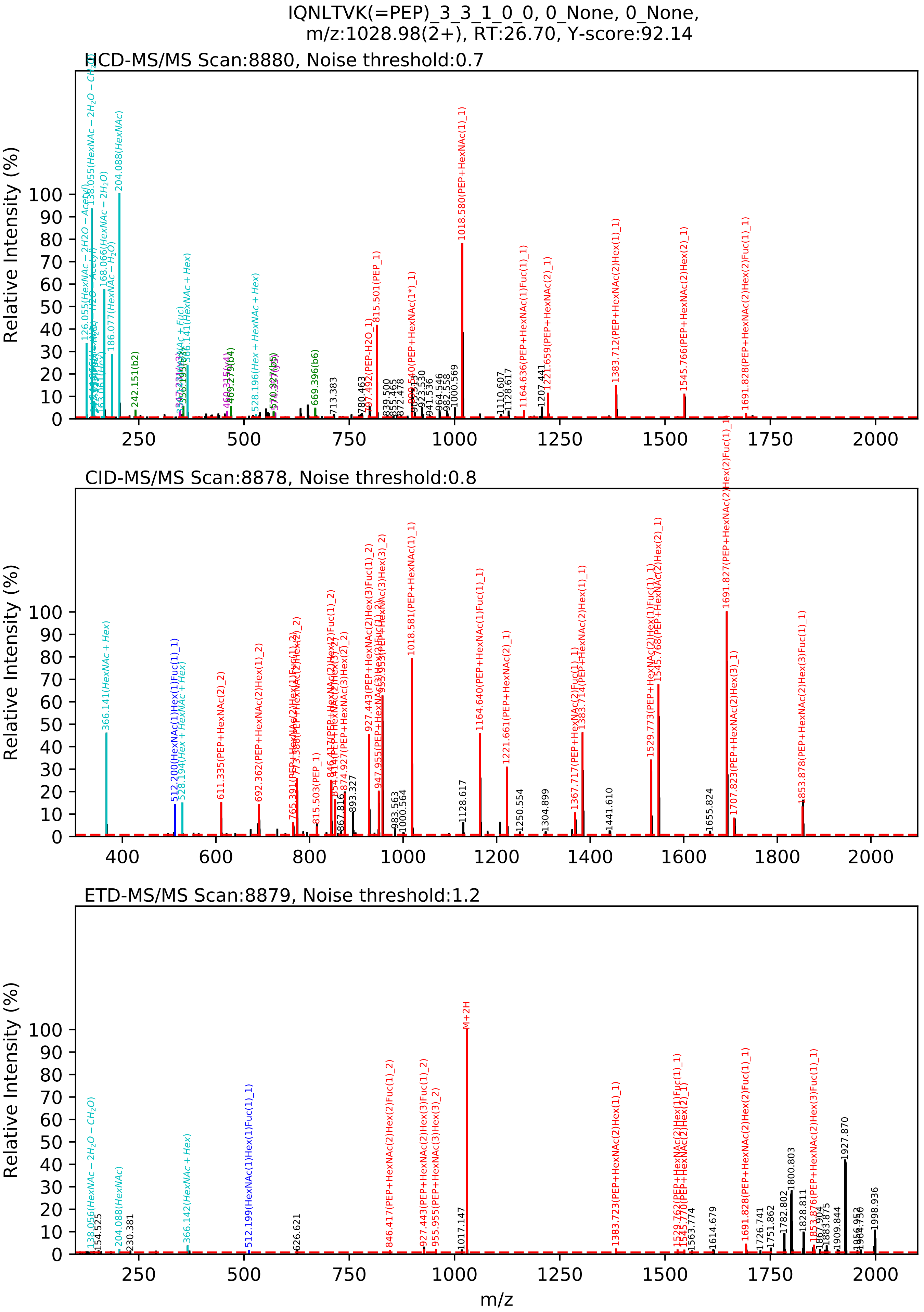

IQNLTVK(=PEP)_3_3_1_0_0, 0_None, 0_None,
m/z:1028.98(2+), RT:26.70, Y-score:92.14
HCD-MS/MS Scan:8880, Noise threshold:0.7
Relative Intensity (%)
204.088(HexNAc)
1018.580(PEP+HexNAc(1)_1)
815.501(PEP_1)
1383.712(PEP+HexNAc(2)Hex(1)_1)
1545.766(PEP+HexNAc(2)Hex(2)_1)
1691.828(PEP+HexNAc(2)Hex(2)Fuc(1)_1)
1164.636(PEP+HexNAc(1)Fuc(1)_1)
1221.659(PEP+HexNAc(2)_1)
669.396(b6)
242.151(b2)
CID-MS/MS Scan:8878, Noise threshold:0.8
366.141(HexNAc + Hex)
512.200(HexNAc(1)Hex(1)Fuc(1)_1)
611.335(PEP+HexNAc(2)_2)
692.362(PEP+HexNAc(2)Hex(1)_2)
1018.581(PEP+HexNAc(1)_1)
1164.640(PEP+HexNAc(1)Fuc(1)_1)
1221.661(PEP+HexNAc(2)_1)
1691.827(PEP+HexNAc(2)Hex(2)Fuc(1)_1)
1707.823(PEP+HexNAc(2)Hex(3)_1)
1853.878(PEP+HexNAc(2)Hex(3)Fuc(1)_1)
ETD-MS/MS Scan:8879, Noise threshold:1.2
M+2H
1927.870
1800.803
1998.936
m/z

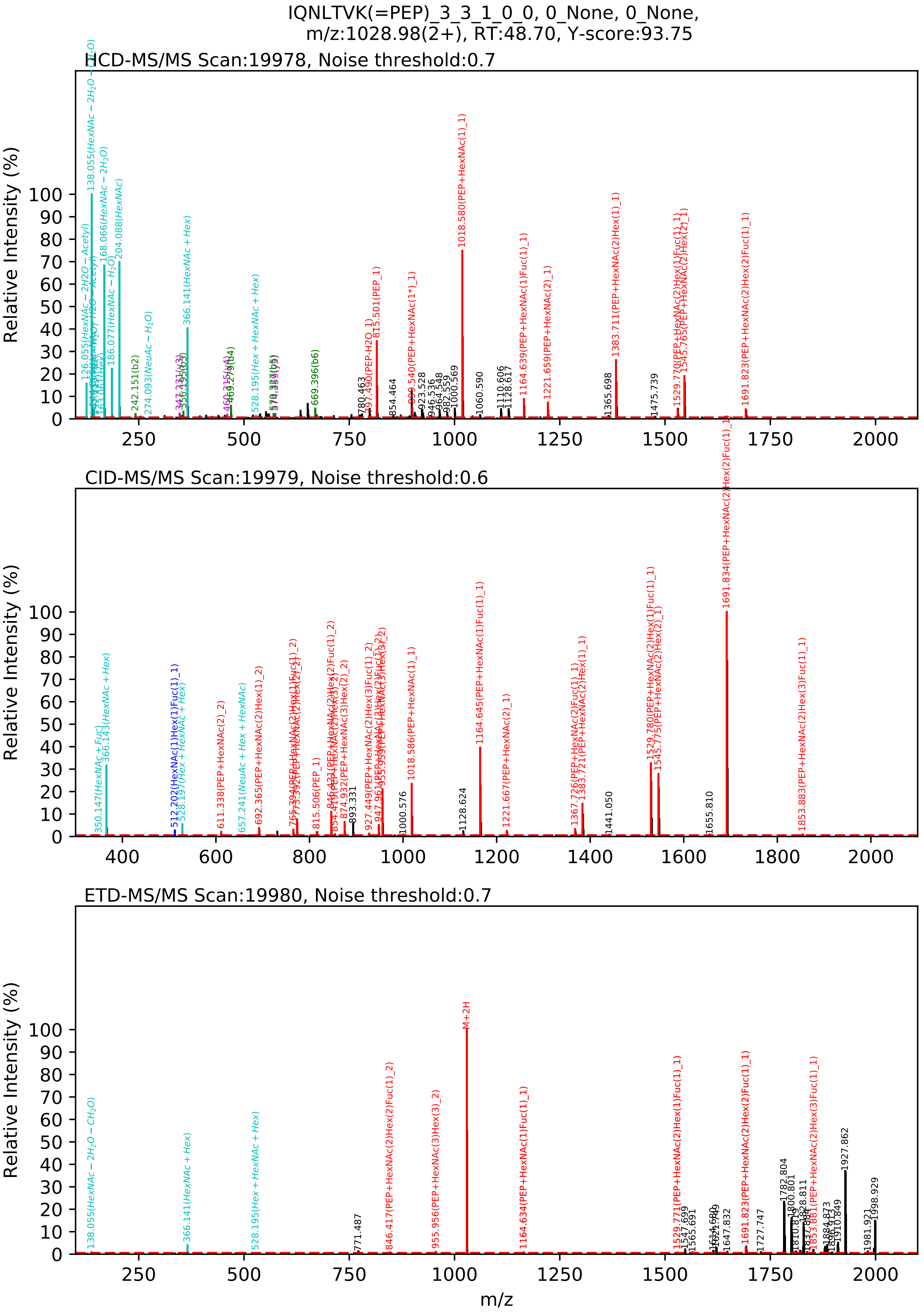
IQNLTVK(=PEP)_3_3_1_0_0, 0_None, 0_None,
m/z:1028.98(2+), RT:48.70, Y-score:93.75
HCD-MS/MS Scan:19978, Noise threshold:0.7
CID-MS/MS Scan:19979, Noise threshold:0.6
ETD-MS/MS Scan:19980, Noise threshold:0.7
Relative Intensity (%)
m/z

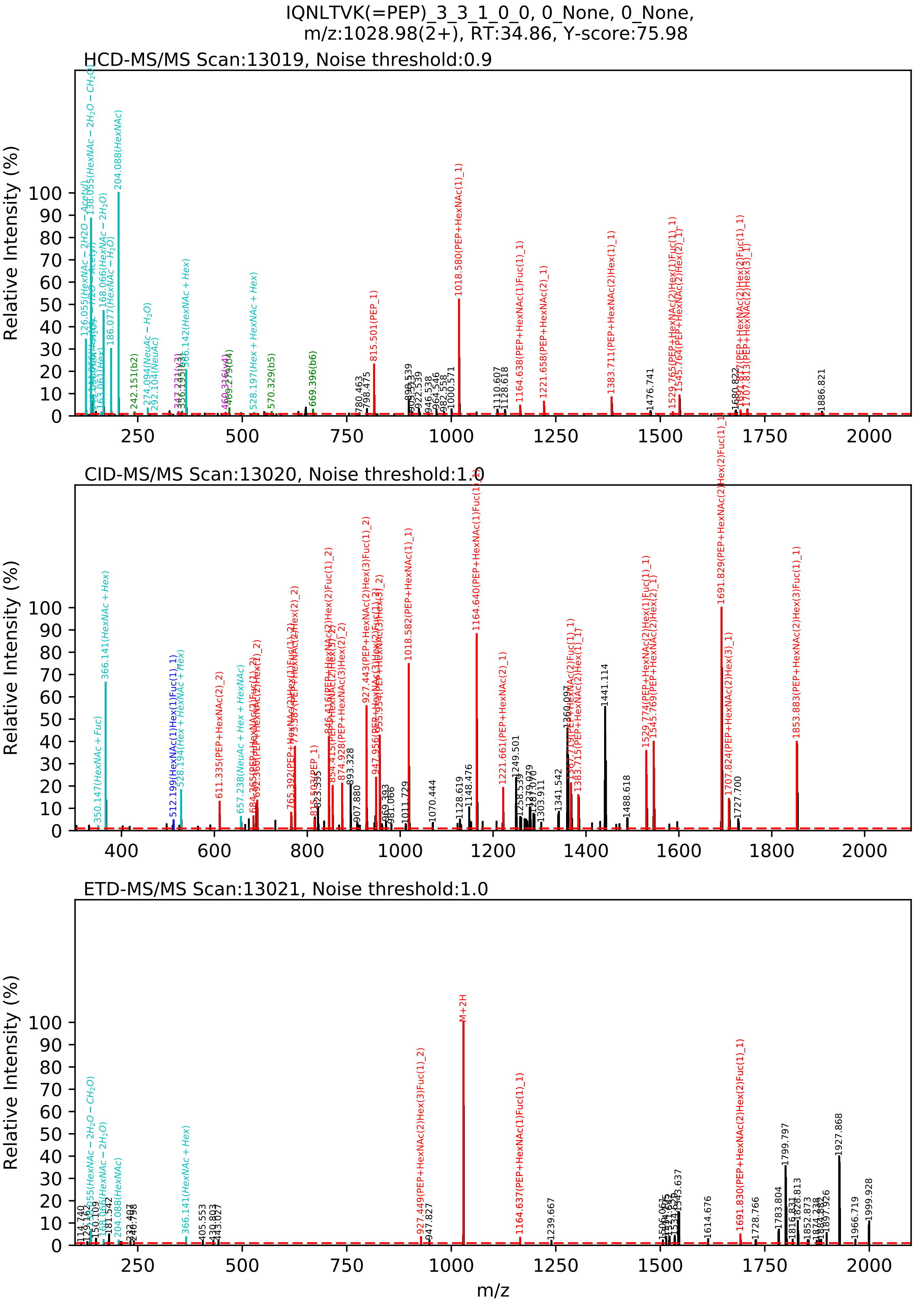
IQNLTVK(=PEP)_3_3_1_0_0, 0_None, 0_None,
m/z:1028.98(2+), RT:34.86, Y-score:75.98
HCD-MS/MS Scan:13019, Noise threshold:0.9
CID-MS/MS Scan:13020, Noise threshold:1.0
ETD-MS/MS Scan:13021, Noise threshold:1.0
Relative Intensity (%)
m/z
204.088(HexNAc)
1018.580(PEP+HexNAc(1)_1)
815.501(PEP_1)
1164.638(PEP+HexNAc(1)Fuc(1)_1)
1221.658(PEP+HexNAc(2)_1)
1383.711(PEP+HexNAc(2)Hex(1)_1)
366.141(HexNAc + Hex)
1691.829(PEP+HexNAc(2)Hex(2)Fuc(1)_1)
1853.883(PEP+HexNAc(2)Hex(3)Fuc(1)_1)
1164.640(PEP+HexNAc(1)Fuc(1)_1)
1018.582(PEP+HexNAc(1)_1)
1441.114
M+2H
927.449(PEP+HexNAc(2)Hex(3)Fuc(1)_2)
1164.637(PEP+HexNAc(1)Fuc(1)_1)
1691.830(PEP+HexNAc(2)Hex(2)Fuc(1)_1)
1927.868
1799.797

IQNLTVK(=PEP)_3_3_1_0_0, 0_None, 0_None,
m/z:1028.98(2+), RT:36.27, Y-score:79.99

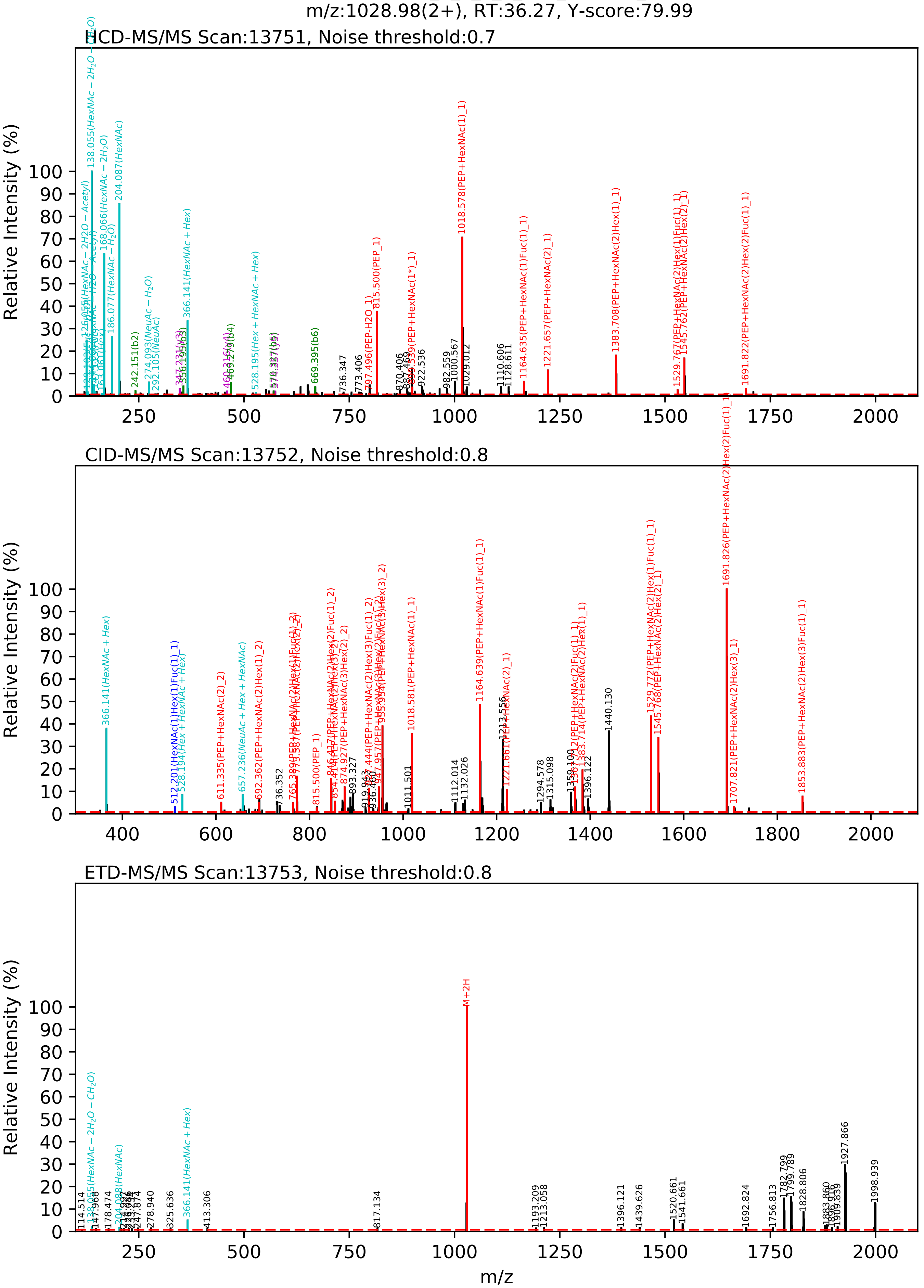

IQNLTVK(=PEP)_3_3_1_0_0, 0_None, 0_None,
m/z:1028.98(2+), RT:36.89, Y-score:91.98

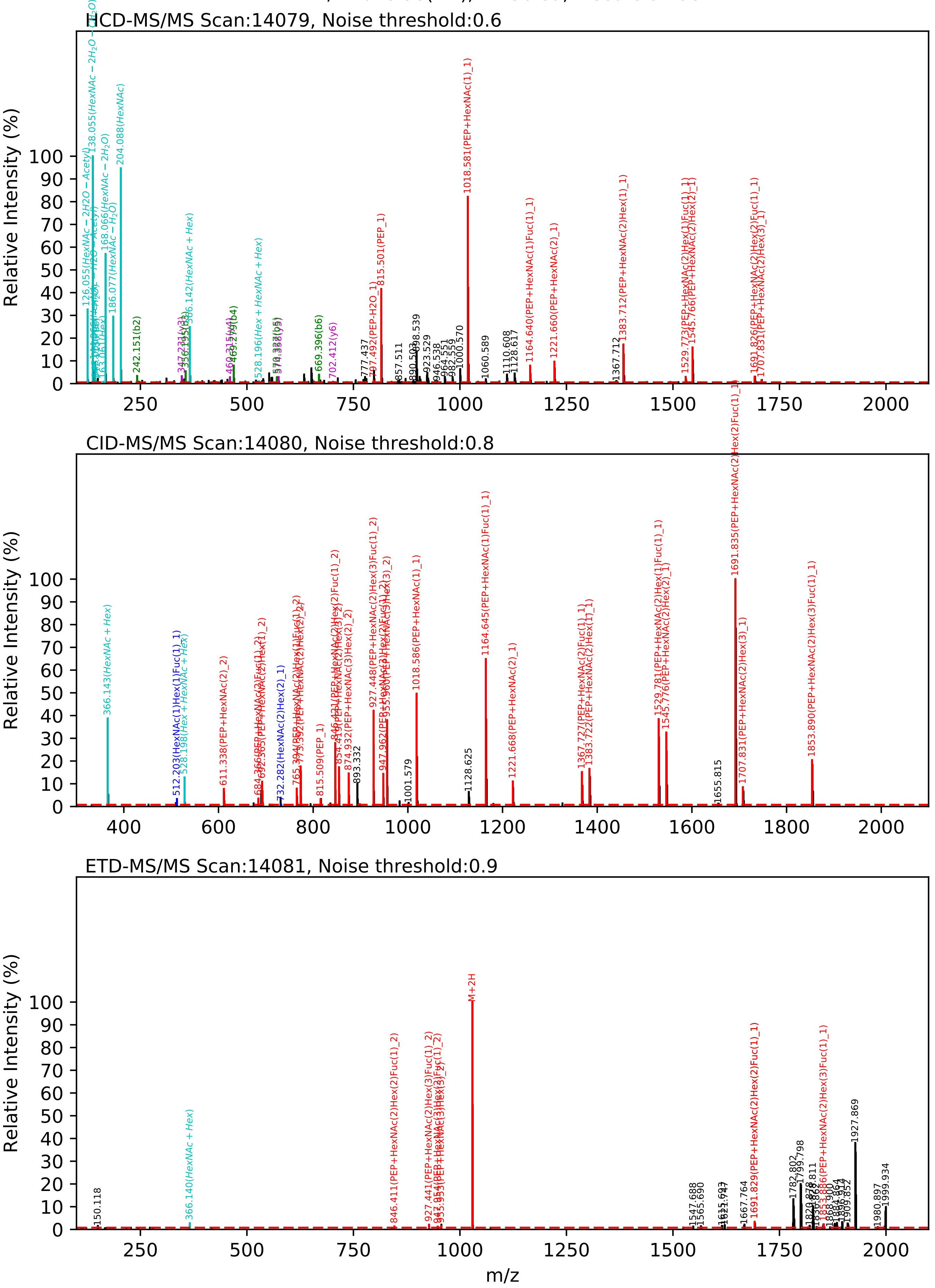

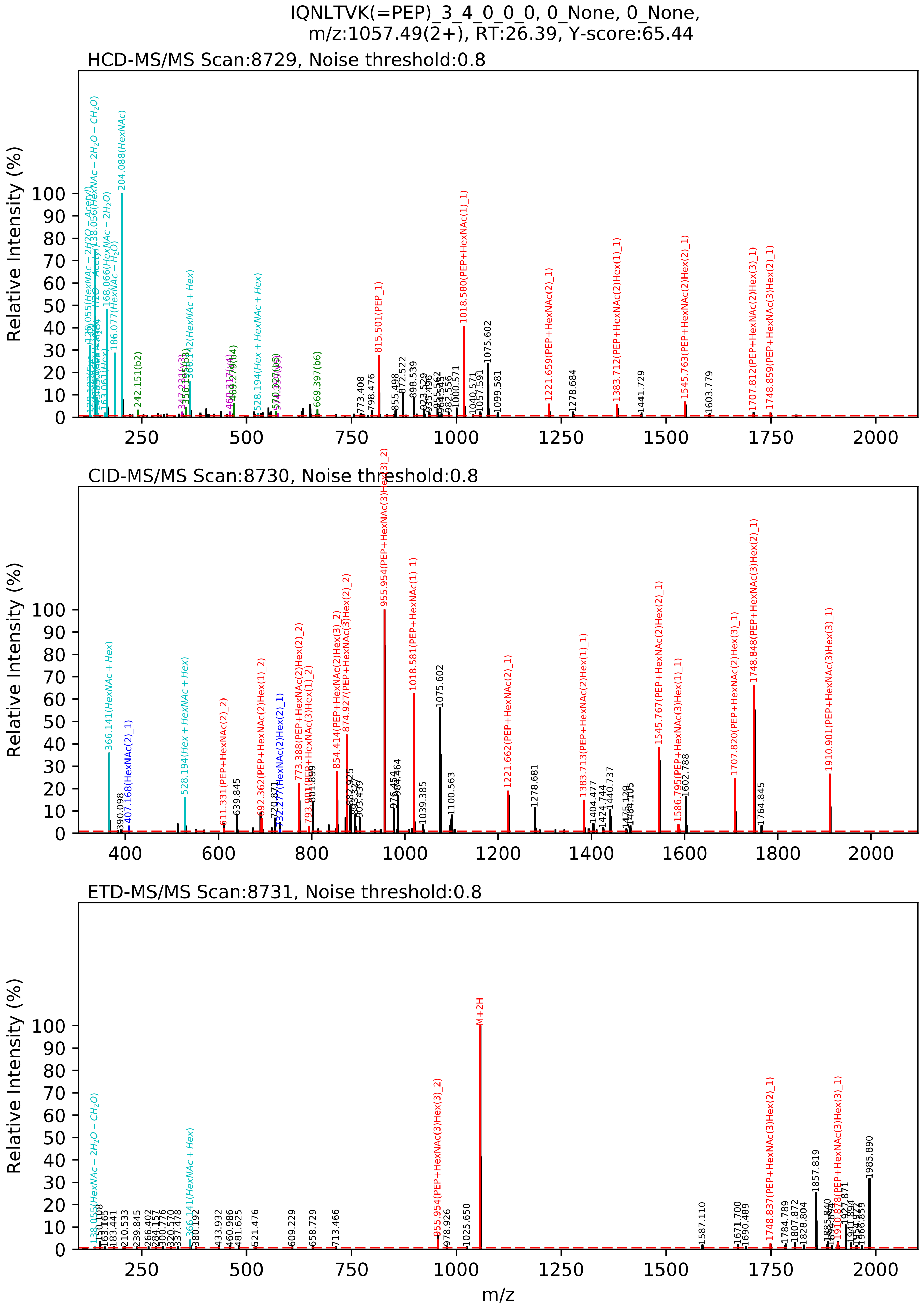
IQNLTVK(=PEP)_3_4_0_0_0, 0_None, 0_None,
m/z:1057.49(2+), RT:26.39, Y-score:65.44
HCD-MS/MS Scan:8729, Noise threshold:0.8
CID-MS/MS Scan:8730, Noise threshold:0.8
ETD-MS/MS Scan:8731, Noise threshold:0.8
Relative Intensity (%)
m/z
204.088(HexNAc)
815.501(PEP_1)
1018.580(PEP+HexNAc(1)_1)
1075.602
1221.659(PEP+HexNAc(2)_1)
1383.712(PEP+HexNAc(2)Hex(1)_1)
1545.763(PEP+HexNAc(2)Hex(2)_1)
1707.812(PEP+HexNAc(2)Hex(3)_1)
1748.859(PEP+HexNAc(3)Hex(2)_1)
366.141(HexNAc+Hex)
528.194(Hex+HexNAc+Hex)
955.954(PEP+HexNAc(3)Hex(3)_2)
1018.581(PEP+HexNAc(1)_1)
1221.662(PEP+HexNAc(2)_1)
1383.713(PEP+HexNAc(2)Hex(1)_1)
1545.767(PEP+HexNAc(2)Hex(2)_1)
1707.820(PEP+HexNAc(2)Hex(3)_1)
1748.848(PEP+HexNAc(3)Hex(2)_1)
1910.901(PEP+HexNAc(3)Hex(3)_1)
M+2H
955.954(PEP+HexNAc(3)Hex(3)_2)
1748.837(PEP+HexNAc(3)Hex(2)_1)
1857.819
1985.890

IQNLTVK(=PEP)_4_2_0_0_0, 0_None, 0_None,
m/z:935.44(2+), RT:25.63, Y-score:92.56

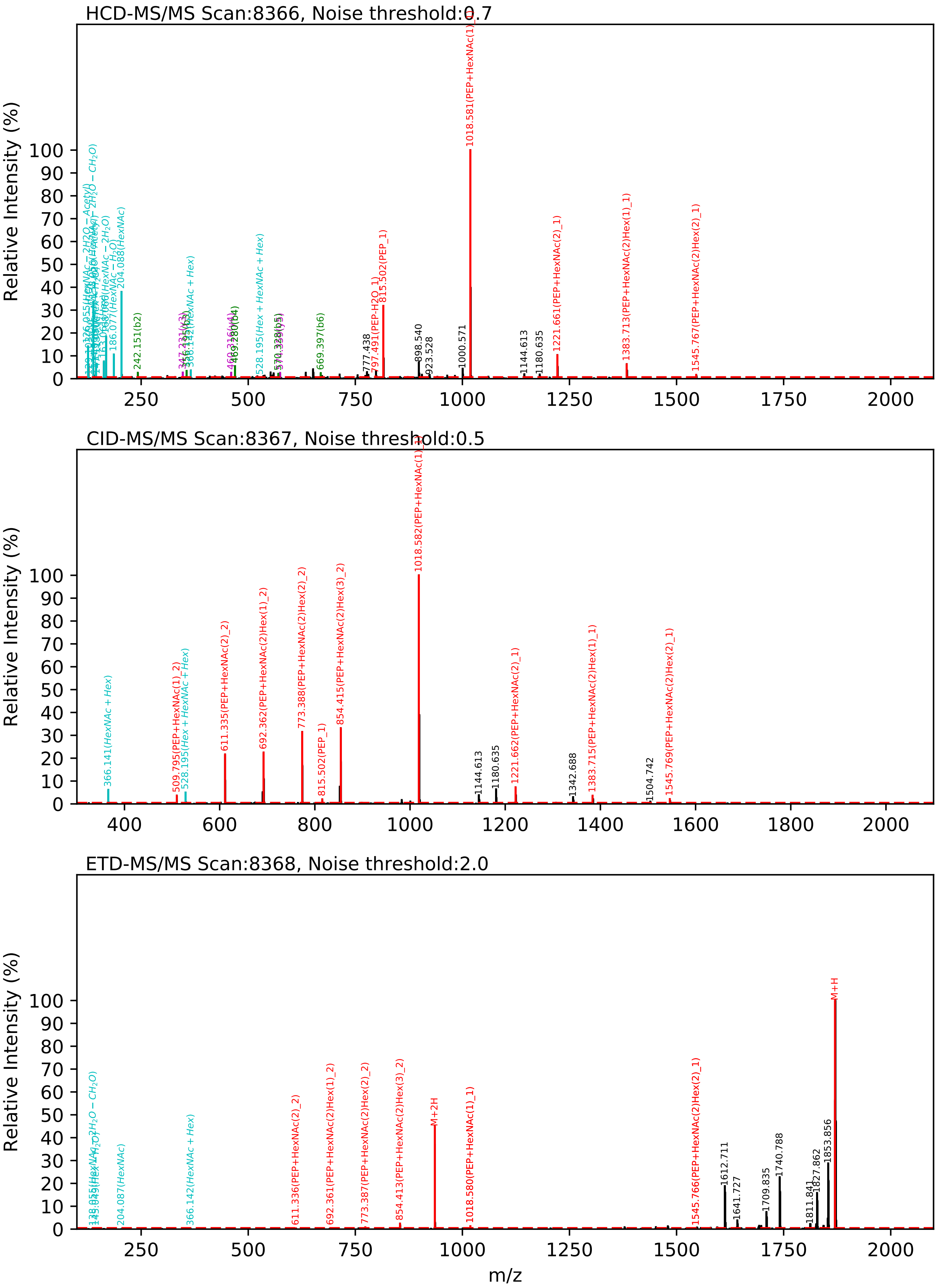

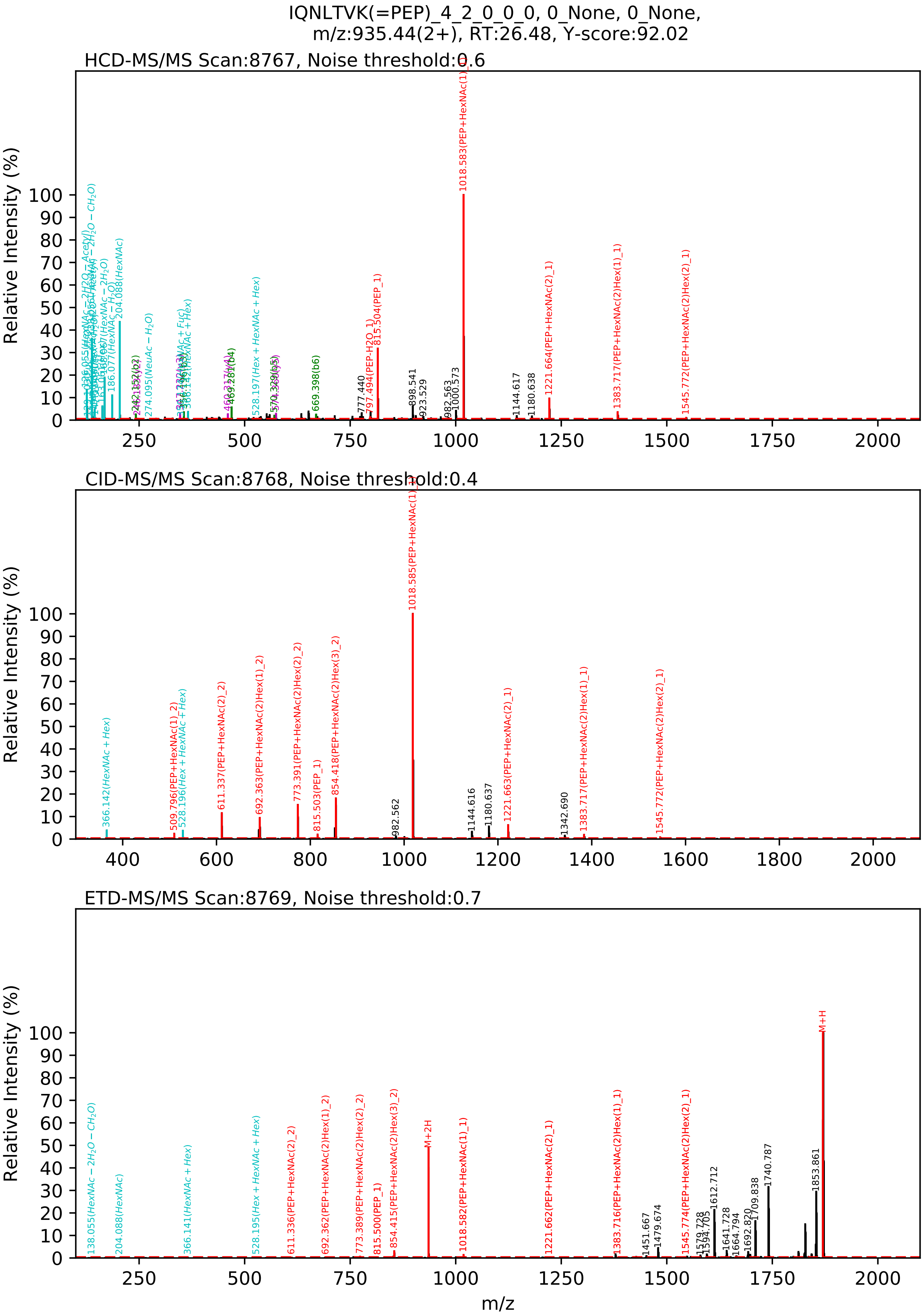
IQNLTVK(=PEP)_4_2_0_0_0, 0_None, 0_None,
m/z:935.44(2+), RT:26.48, Y-score:92.02
HCD-MS/MS Scan:8767, Noise threshold:0.6
Relative Intensity (%)
1018.583(PEP+HexNAc(1)_1)
815.504(PEP_1)
797.494(PEP-H2O_1)
1221.664(PEP+HexNAc(2)_1)
1383.717(PEP+HexNAc(2)Hex(1)_1)
1545.772(PEP+HexNAc(2)Hex(2)_1)
204.088(HexNAc)
186.077(HexNAc − H2O)
274.095(NeuAc − H2O)
366.142(HexNAc + Hex)
528.197(Hex + HexNAc + Hex)
669.398(b6)
469.281(b4)
898.541
923.529
982.563
1000.573
1144.617
1180.638
777.440
CID-MS/MS Scan:8768, Noise threshold:0.4
Relative Intensity (%)
1018.585(PEP+HexNAc(1)_1)
366.142(HexNAc + Hex)
509.796(PEP+HexNAc(1)_2)
528.196(Hex + HexNAc + Hex)
611.337(PEP+HexNAc(2)_2)
692.363(PEP+HexNAc(2)Hex(1)_2)
773.391(PEP+HexNAc(2)Hex(2)_2)
815.503(PEP_1)
854.418(PEP+HexNAc(2)Hex(3)_2)
982.562
1144.616
1180.637
1221.663(PEP+HexNAc(2)_1)
1342.690
1383.717(PEP+HexNAc(2)Hex(1)_1)
1545.772(PEP+HexNAc(2)Hex(2)_1)
ETD-MS/MS Scan:8769, Noise threshold:0.7
Relative Intensity (%)
138.055(HexNAc − 2H2O − CH2O)
204.088(HexNAc)
366.141(HexNAc + Hex)
528.195(Hex + HexNAc + Hex)
611.336(PEP+HexNAc(2)_2)
692.362(PEP+HexNAc(2)Hex(1)_2)
773.389(PEP+HexNAc(2)Hex(2)_2)
815.500(PEP_1)
854.415(PEP+HexNAc(2)Hex(3)_2)
M+2H
1018.582(PEP+HexNAc(1)_1)
1221.662(PEP+HexNAc(2)_1)
1383.716(PEP+HexNAc(2)Hex(1)_1)
1451.667
1479.674
1545.774(PEP+HexNAc(2)Hex(2)_1)
1579.728
1594.705
1612.712
1641.728
1664.794
1692.820
1709.838
1740.787
1853.861
M+H
m/z

IQNLTVK(=PEP)_4_2_0_0_0, 0_None, 0_None,
m/z:935.44(2+), RT:27.09, Y-score:94.63

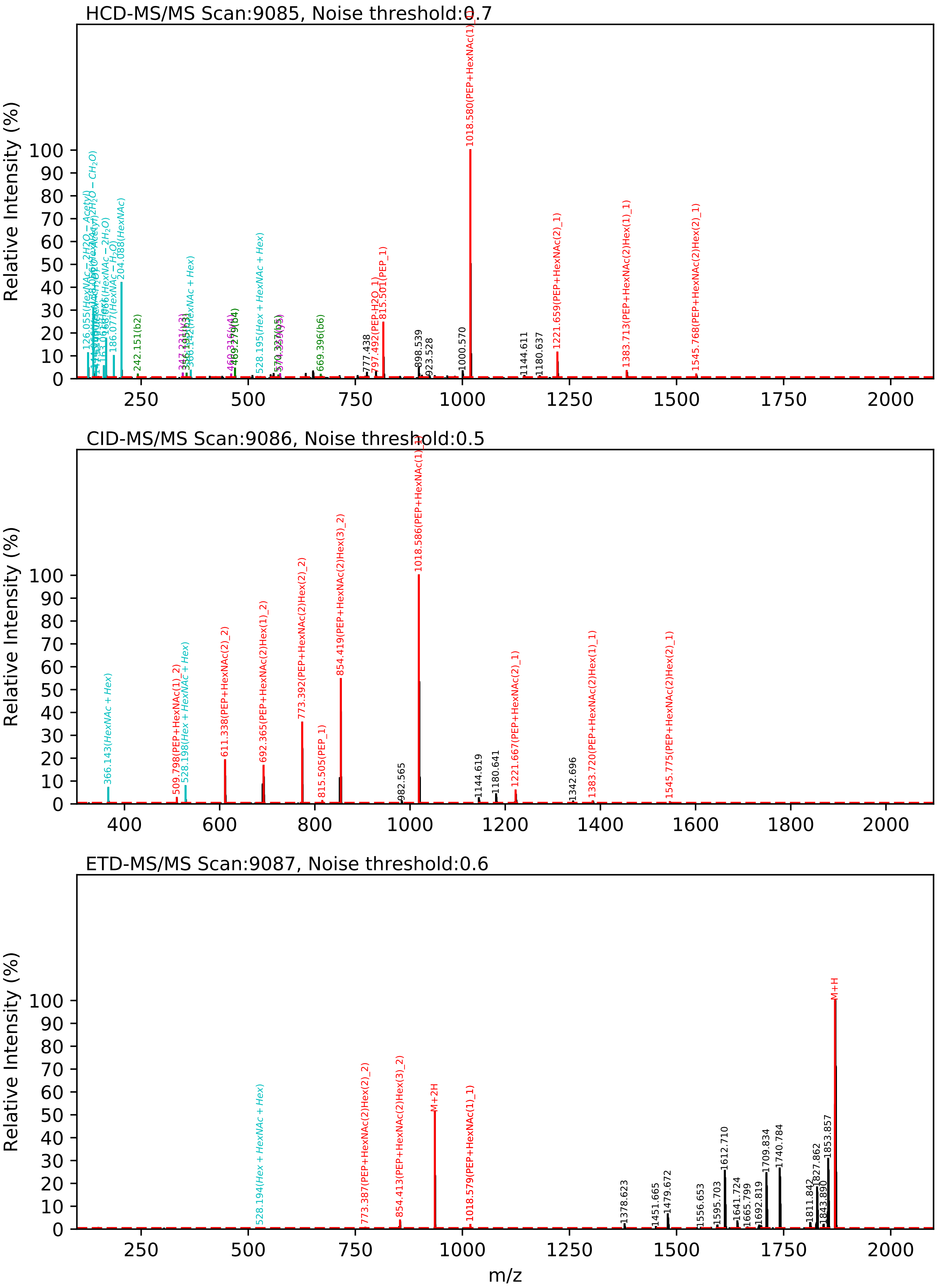

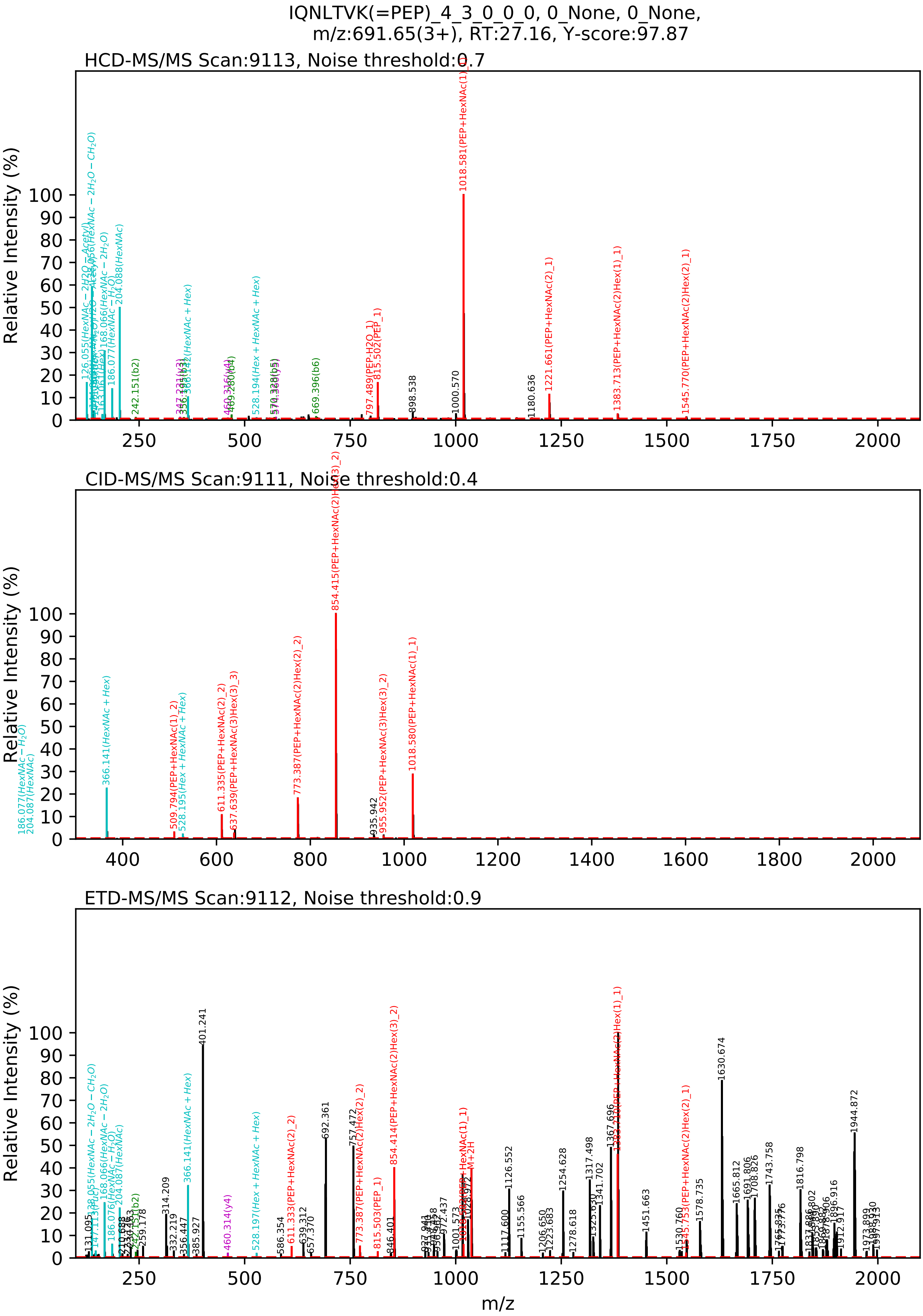

IQNLTVK(=PEP)_4_3_0_0_0, 0_None, 0_None,
m/z:691.65(3+), RT:27.16, Y-score:97.87
HCD-MS/MS Scan:9113, Noise threshold:0.7
CID-MS/MS Scan:9111, Noise threshold:0.4
ETD-MS/MS Scan:9112, Noise threshold:0.9
Relative Intensity (%)
m/z
1018.581(PEP+HexNAc(1)_1)
1221.661(PEP+HexNAc(2)_1)
1383.713(PEP+HexNAc(2)Hex(1)_1)
1545.770(PEP+HexNAc(2)Hex(2)_1)
815.502(PEP_1)
204.088(HexNAc)
186.077(HexNAc − H2O)
242.151(b2)
669.396(b6)
898.538
1000.570
1180.636
854.415(PEP+HexNAc(2)Hex(3)_2)
773.387(PEP+HexNAc(2)Hex(2)_2)
611.335(PEP+HexNAc(2)_2)
637.639(PEP+HexNAc(3)Hex(3)_3)
509.794(PEP+HexNAc(1)_2)
528.195(Hex + HexNAc + Hex)
366.141(HexNAc + Hex)
955.952(PEP+HexNAc(3)Hex(3)_2)
1018.580(PEP+HexNAc(1)_1)
935.942
401.241
692.361
757.472
1630.674
1944.872
1367.696
1317.498
1254.628
1126.552
1743.758
1816.798
1578.735
1451.663
314.209
460.314(y4)

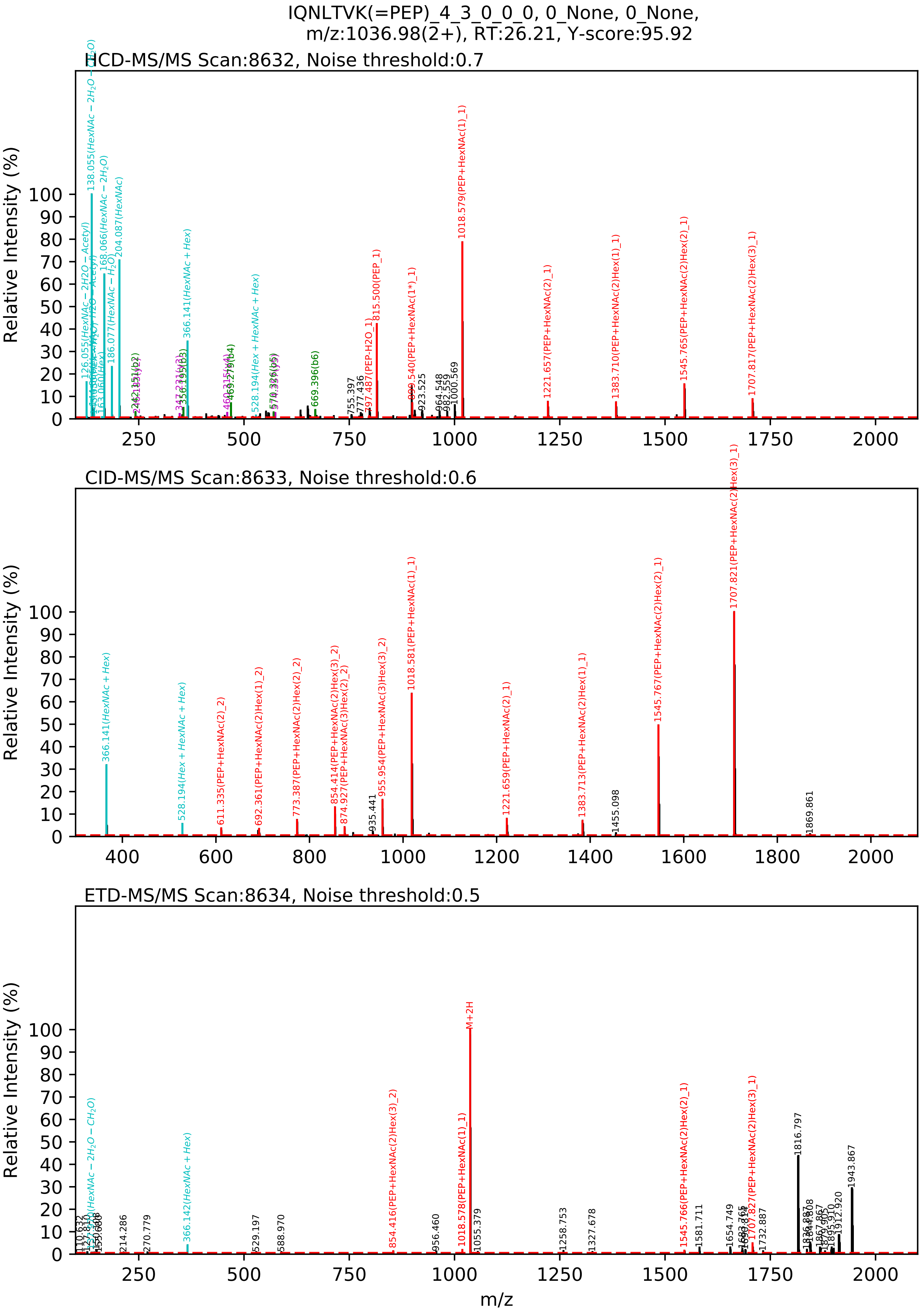
IQNLTVK(=PEP)_4_3_0_0_0, 0_None, 0_None,
m/z:1036.98(2+), RT:26.21, Y-score:95.92
HCD-MS/MS Scan:8632, Noise threshold:0.7
CID-MS/MS Scan:8633, Noise threshold:0.6
ETD-MS/MS Scan:8634, Noise threshold:0.5
Relative Intensity (%)
m/z
138.055(HexNAc − 2H2O − CH2O)
204.087(HexNAc)
366.141(HexNAc + Hex)
528.194(Hex + HexNAc + Hex)
669.396(b6)
815.500(PEP_1)
1018.579(PEP+HexNAc(1)_1)
1221.657(PEP+HexNAc(2)_1)
1383.710(PEP+HexNAc(2)Hex(1)_1)
1545.765(PEP+HexNAc(2)Hex(2)_1)
1707.817(PEP+HexNAc(2)Hex(3)_1)
611.335(PEP+HexNAc(2)_2)
692.361(PEP+HexNAc(2)Hex(1)_2)
773.387(PEP+HexNAc(2)Hex(2)_2)
854.414(PEP+HexNAc(2)Hex(3)_2)
874.927(PEP+HexNAc(3)Hex(2)_2)
955.954(PEP+HexNAc(3)Hex(3)_2)
1018.581(PEP+HexNAc(1)_1)
1221.659(PEP+HexNAc(2)_1)
1383.713(PEP+HexNAc(2)Hex(1)_1)
1545.767(PEP+HexNAc(2)Hex(2)_1)
1707.821(PEP+HexNAc(2)Hex(3)_1)
1455.098
1869.861
M+2H
1816.797
1943.867
1912.920
1581.711
1654.749
1732.887
1327.678
1258.753
1055.379
956.460
529.197
588.970
214.286
270.779

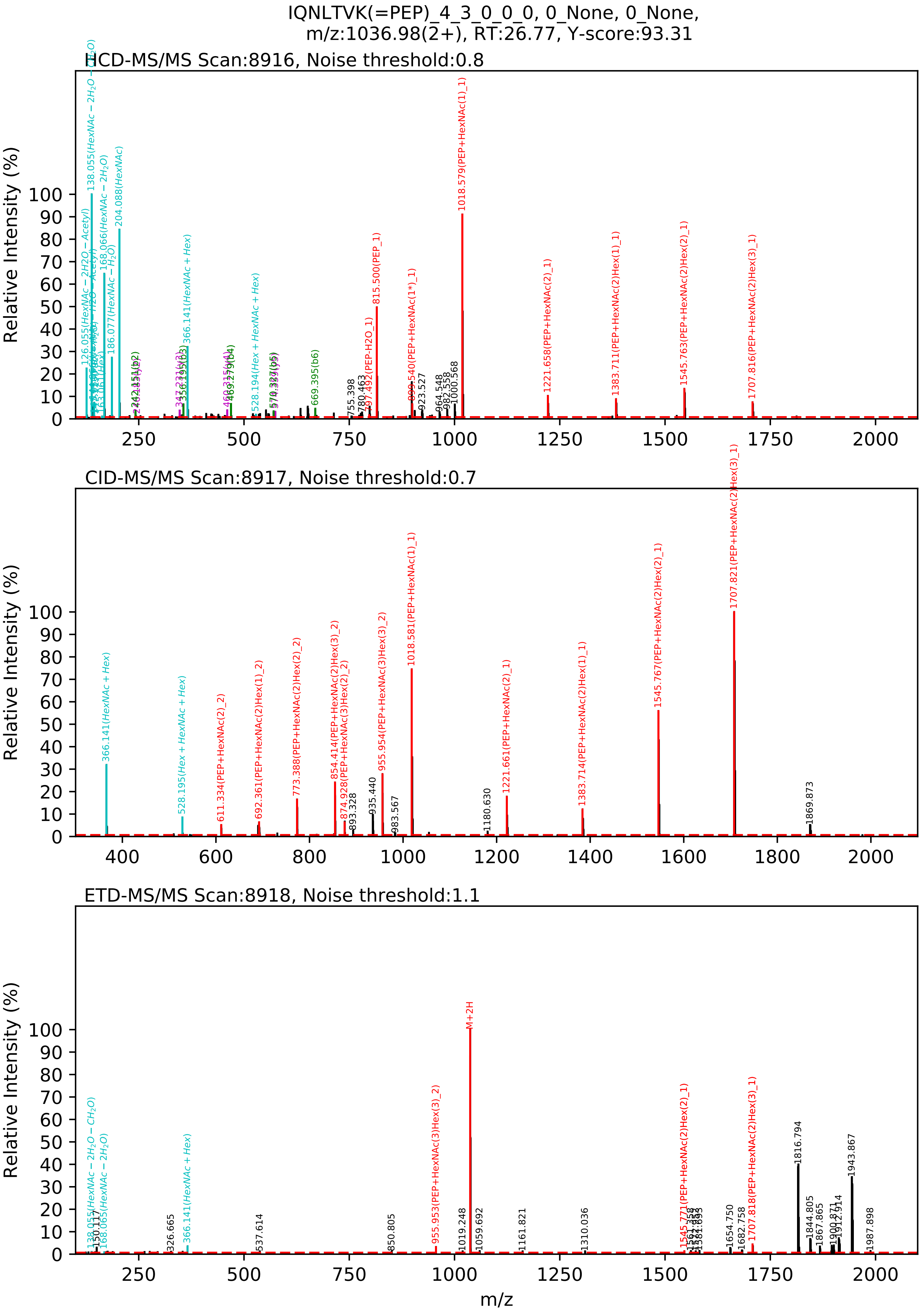
IQNLTVK(=PEP)_4_3_0_0_0, 0_None, 0_None,
m/z:1036.98(2+), RT:26.77, Y-score:93.31
HCD-MS/MS Scan:8916, Noise threshold:0.8
CID-MS/MS Scan:8917, Noise threshold:0.7
ETD-MS/MS Scan:8918, Noise threshold:1.1
Relative Intensity (%)
m/z
1018.579(PEP+HexNAc(1)_1)
815.500(PEP_1)
1221.658(PEP+HexNAc(2)_1)
1383.711(PEP+HexNAc(2)Hex(1)_1)
1545.763(PEP+HexNAc(2)Hex(2)_1)
1707.816(PEP+HexNAc(2)Hex(3)_1)
1707.821(PEP+HexNAc(2)Hex(3)_1)
1545.767(PEP+HexNAc(2)Hex(2)_1)
1018.581(PEP+HexNAc(1)_1)
M+2H

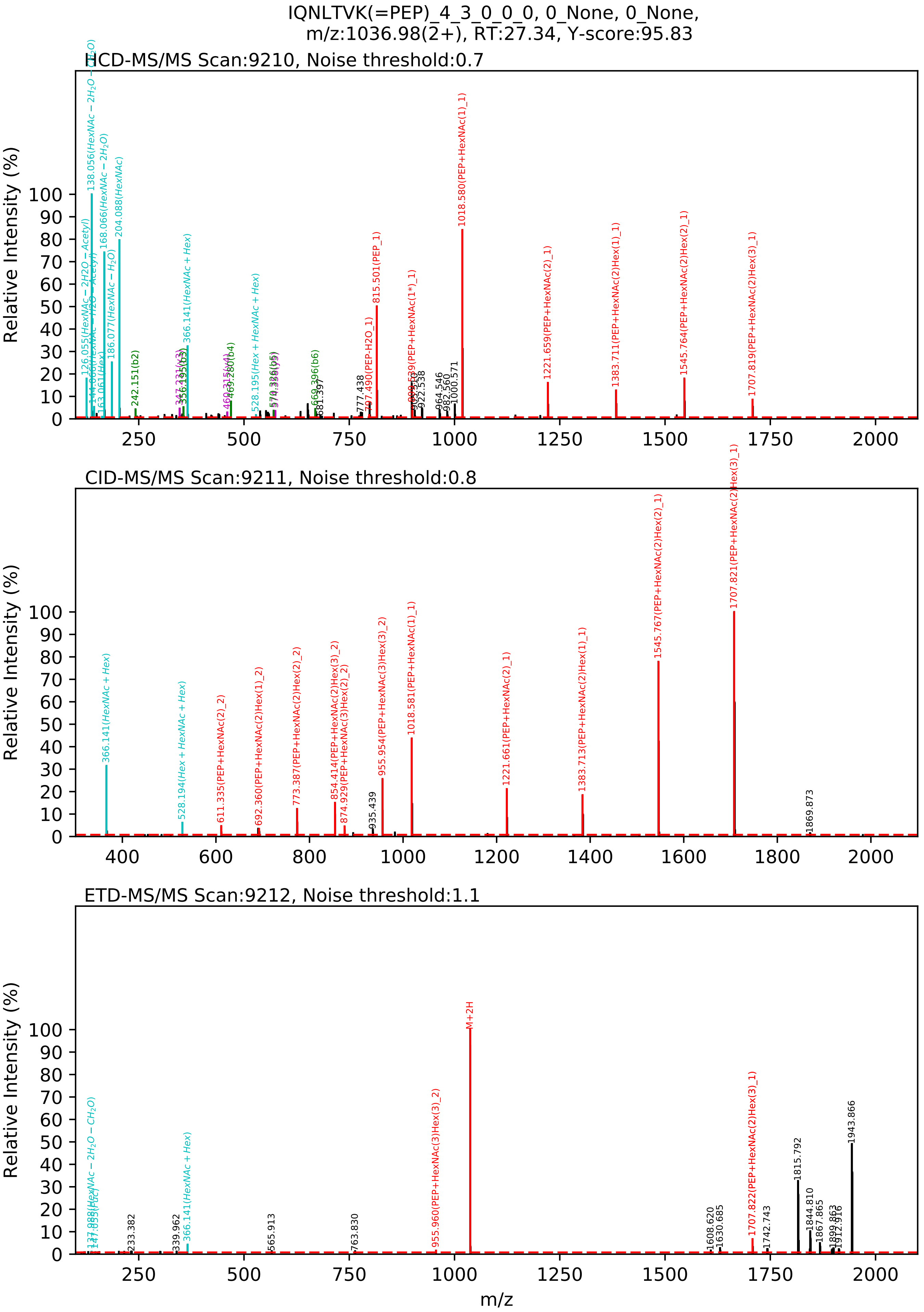
IQNLTVK(=PEP)_4_3_0_0_0, 0_None, 0_None,
m/z:1036.98(2+), RT:27.34, Y-score:95.83
HCD-MS/MS Scan:9210, Noise threshold:0.7
CID-MS/MS Scan:9211, Noise threshold:0.8
ETD-MS/MS Scan:9212, Noise threshold:1.1
Relative Intensity (%)
m/z
1018.580(PEP+HexNAc(1)_1)
815.501(PEP_1)
1221.659(PEP+HexNAc(2)_1)
1383.711(PEP+HexNAc(2)Hex(1)_1)
1545.764(PEP+HexNAc(2)Hex(2)_1)
1707.819(PEP+HexNAc(2)Hex(3)_1)
366.141(HexNAc + Hex)
204.088(HexNAc)
1707.821(PEP+HexNAc(2)Hex(3)_1)
1545.767(PEP+HexNAc(2)Hex(2)_1)
1383.713(PEP+HexNAc(2)Hex(1)_1)
1221.661(PEP+HexNAc(2)_1)
1018.581(PEP+HexNAc(1)_1)
955.954(PEP+HexNAc(3)Hex(3)_2)
773.387(PEP+HexNAc(2)Hex(2)_2)
692.360(PEP+HexNAc(2)Hex(1)_2)
611.335(PEP+HexNAc(2)_2)
528.194(Hex + HexNAc + Hex)
1869.873
M+2H
955.960(PEP+HexNAc(3)Hex(3)_2)
1707.822(PEP+HexNAc(2)Hex(3)_1)
1943.866
1815.792
1844.810
1867.865

IQNLTVK(=PEP)_4_3_0_0_0, 0_None, 0_None,
m/z:1036.98(2+), RT:35.74, Y-score:93.16

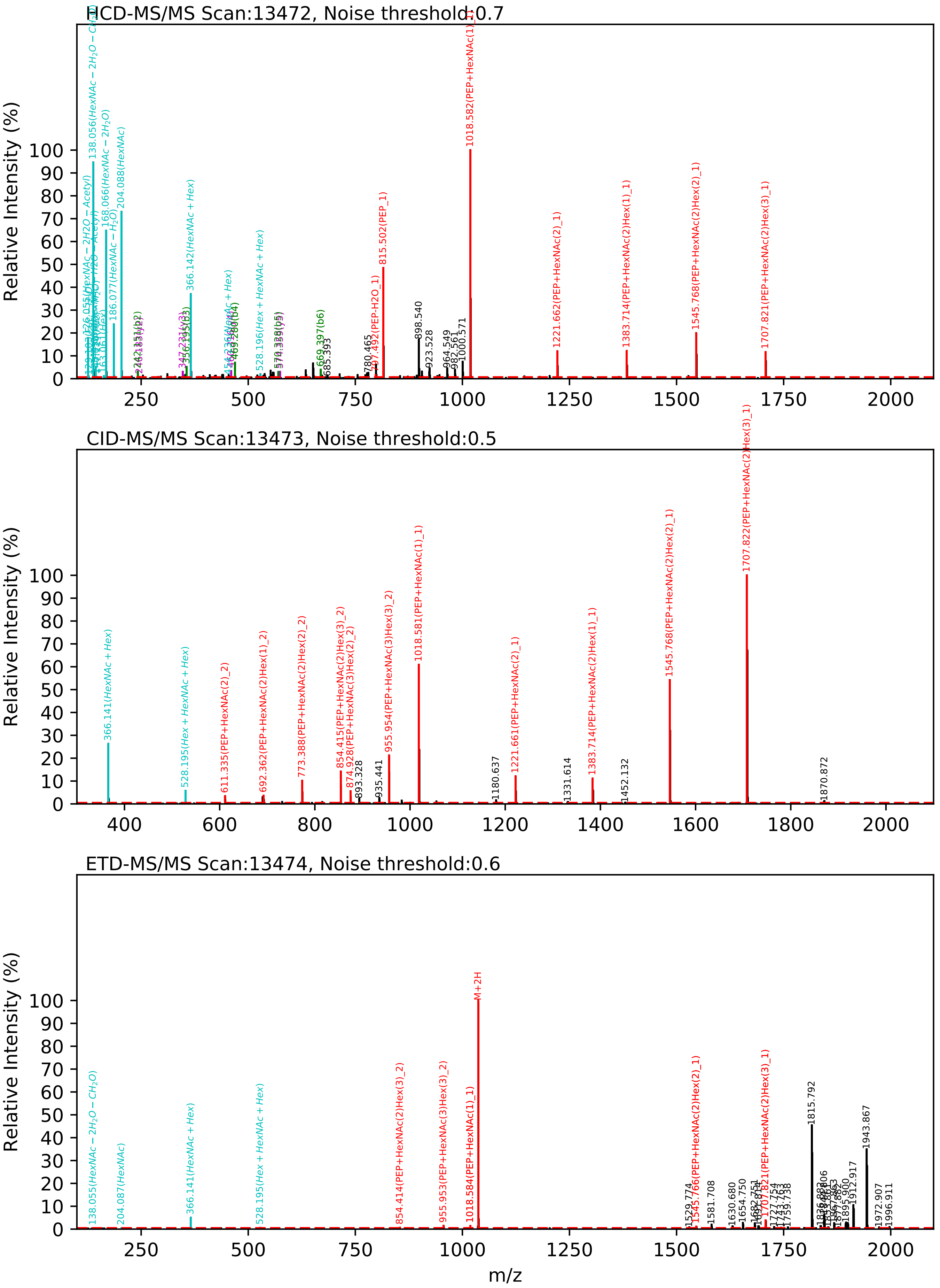

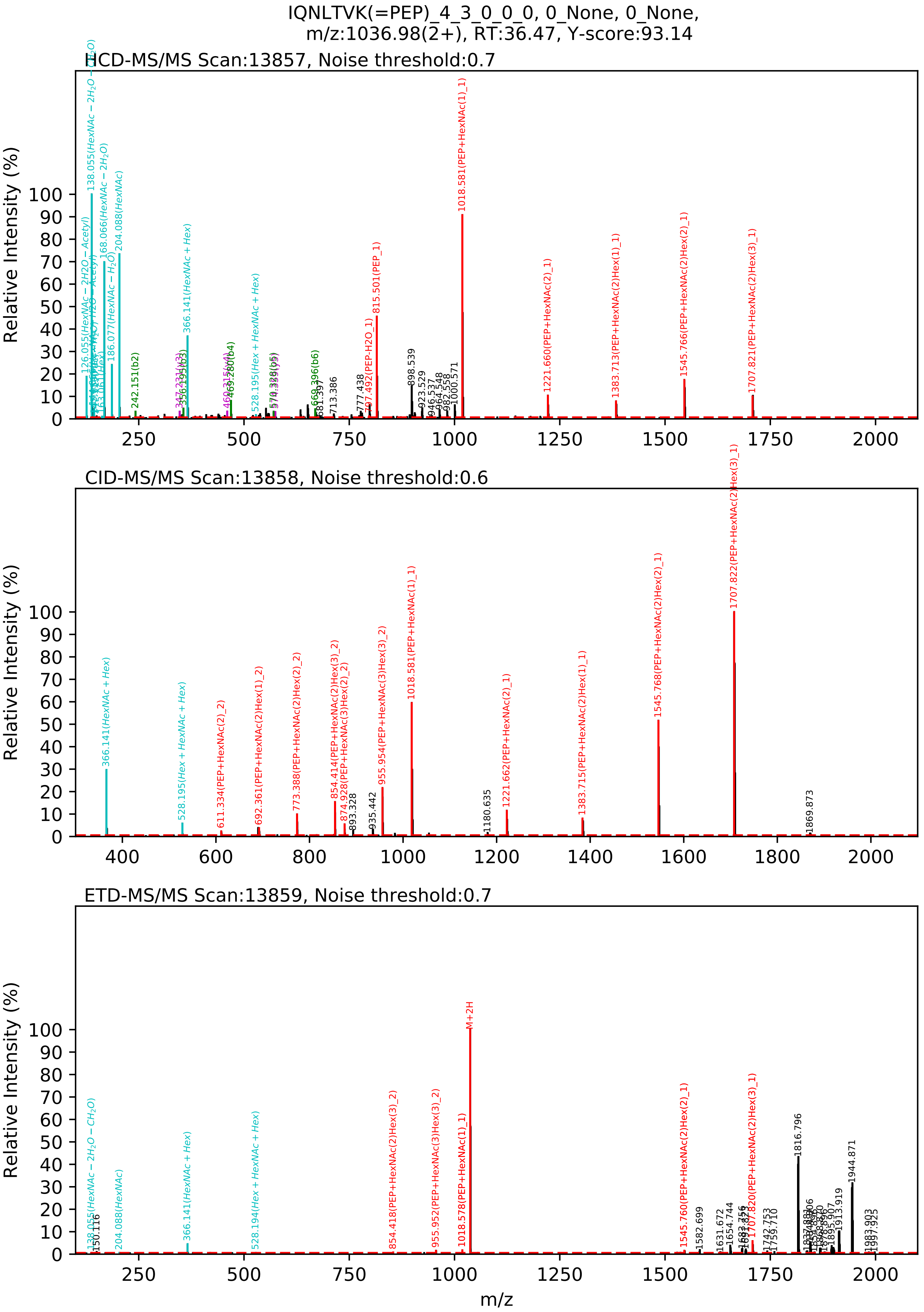
IQNLTVK(=PEP)_4_3_0_0_0, 0_None, 0_None,
m/z:1036.98(2+), RT:36.47, Y-score:93.14
HCD-MS/MS Scan:13857, Noise threshold:0.7
CID-MS/MS Scan:13858, Noise threshold:0.6
ETD-MS/MS Scan:13859, Noise threshold:0.7
Relative Intensity (%)
m/z
1018.581(PEP+HexNAc(1)_1)
1221.660(PEP+HexNAc(2)_1)
1383.713(PEP+HexNAc(2)Hex(1)_1)
1545.766(PEP+HexNAc(2)Hex(2)_1)
1707.821(PEP+HexNAc(2)Hex(3)_1)
815.501(PEP_1)
366.141(HexNAc + Hex)
528.195(Hex + HexNAc + Hex)
204.088(HexNAc)
1707.822(PEP+HexNAc(2)Hex(3)_1)
1545.768(PEP+HexNAc(2)Hex(2)_1)
1383.715(PEP+HexNAc(2)Hex(1)_1)
1221.662(PEP+HexNAc(2)_1)
955.954(PEP+HexNAc(3)Hex(3)_2)
854.414(PEP+HexNAc(2)Hex(3)_2)
773.388(PEP+HexNAc(2)Hex(2)_2)
692.361(PEP+HexNAc(2)Hex(1)_2)
611.334(PEP+HexNAc(2)_2)
M+2H
1816.796
1944.871

IQNLTVK(=PEP)_4_3_0_0_0, 0_None, 0_None,
m/z:1036.98(2+), RT:37.19, Y-score:91.00

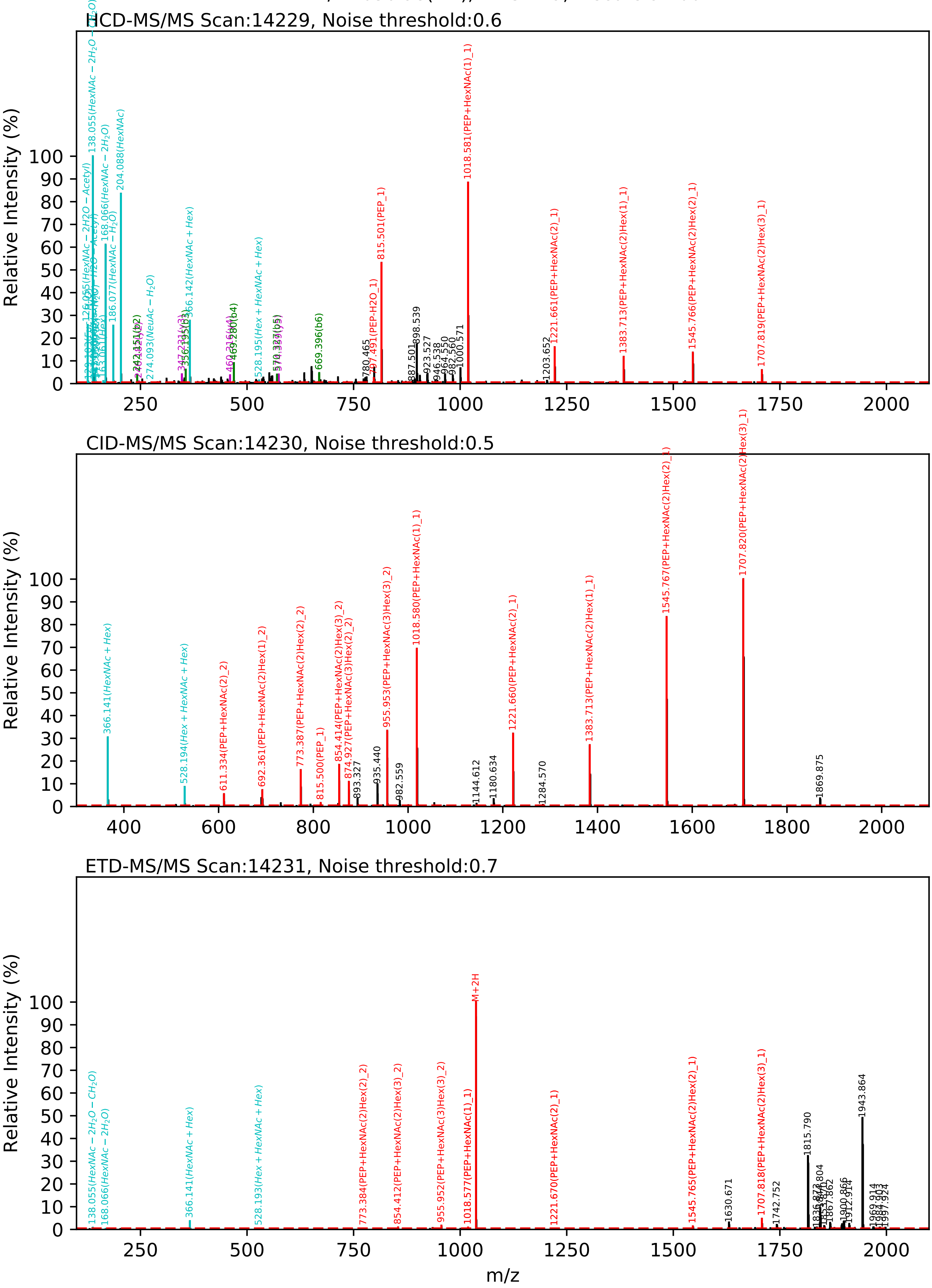

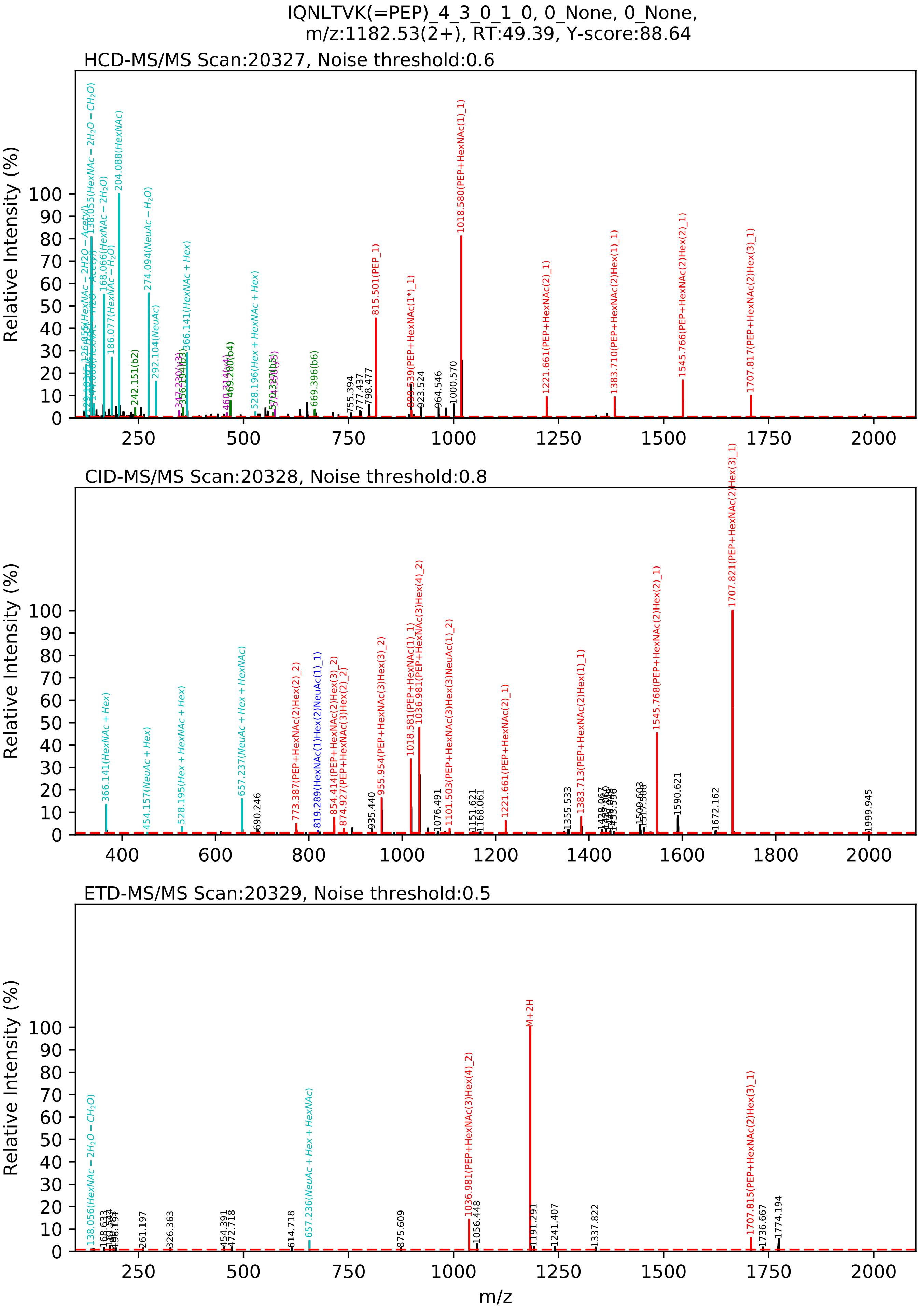

IQNLTVK(=PEP)_4_3_0_1_0, 0_None, 0_None,
m/z:1182.53(2+), RT:49.39, Y-score:88.64
HCD-MS/MS Scan:20327, Noise threshold:0.6
Relative Intensity (%)
204.088(HexNAc)
168.066(HexNAc − 2H2O)
186.077(HexNAc − H2O)
274.094(NeuAc − H2O)
292.104(NeuAc)
242.151(b2)
366.141(HexNAc + Hex)
528.196(Hex + HexNAc + Hex)
669.396(b6)
755.394
777.437
798.477
815.501(PEP_1)
899.539(PEP+HexNAc(1*)_1)
923.524
964.546
1000.570
1018.580(PEP+HexNAc(1)_1)
1221.661(PEP+HexNAc(2)_1)
1383.710(PEP+HexNAc(2)Hex(1)_1)
1545.766(PEP+HexNAc(2)Hex(2)_1)
1707.817(PEP+HexNAc(2)Hex(3)_1)
CID-MS/MS Scan:20328, Noise threshold:0.8
366.141(HexNAc + Hex)
454.157(NeuAc + Hex)
528.195(Hex + HexNAc + Hex)
657.237(NeuAc + Hex + HexNAc)
690.246
773.387(PEP+HexNAc(2)Hex(2)_2)
819.289(HexNAc(1)Hex(2)NeuAc(1)_1)
854.414(PEP+HexNAc(2)Hex(3)_2)
874.927(PEP+HexNAc(3)Hex(2)_2)
935.440
955.954(PEP+HexNAc(3)Hex(3)_2)
1018.581(PEP+HexNAc(1)_1)
1036.981(PEP+HexNAc(3)Hex(4)_2)
1076.491
1101.503(PEP+HexNAc(3)Hex(3)NeuAc(1)_2)
1151.621
1168.061
1221.661(PEP+HexNAc(2)_1)
1355.533
1383.713(PEP+HexNAc(2)Hex(1)_1)
1545.768(PEP+HexNAc(2)Hex(2)_1)
1590.621
1672.162
1707.821(PEP+HexNAc(2)Hex(3)_1)
1999.945
ETD-MS/MS Scan:20329, Noise threshold:0.5
138.056(HexNAc − 2H2O − CH2O)
261.197
326.363
454.391
472.718
614.718
657.236(NeuAc + Hex + HexNAc)
875.609
1036.981(PEP+HexNAc(3)Hex(4)_2)
1056.448
M+2H
1191.291
1241.407
1337.822
1707.815(PEP+HexNAc(2)Hex(3)_1)
1736.667
1774.194
m/z

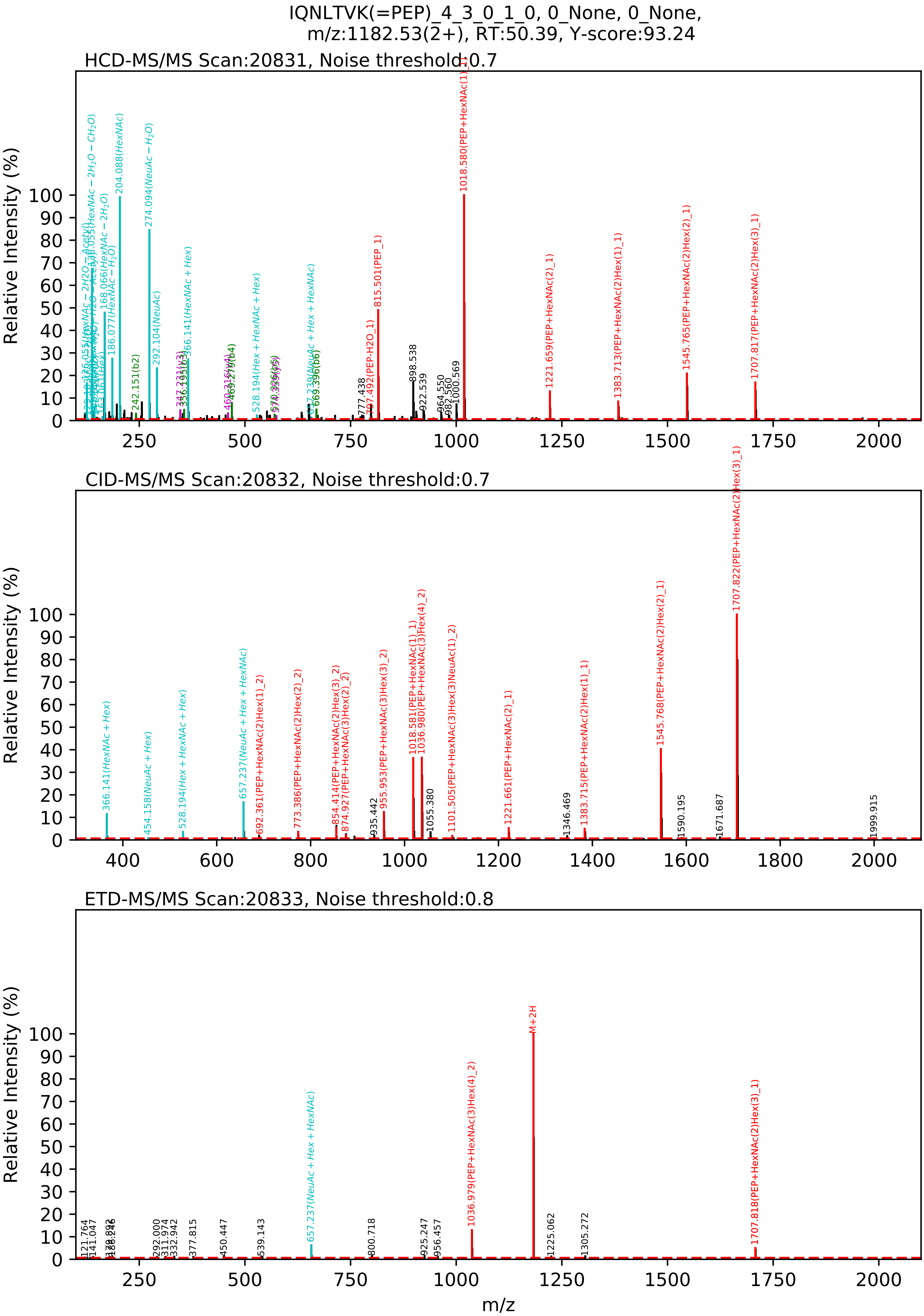
IQNLTVK(=PEP)_4_3_0_1_0, 0_None, 0_None,
m/z:1182.53(2+), RT:50.39, Y-score:93.24
HCD-MS/MS Scan:20831, Noise threshold:0.7
Relative Intensity (%)
1018.580(PEP+HexNAc(1)_1)
204.088(HexNAc)
274.094(NeuAc − H2O)
292.104(NeuAc)
186.077(HexNAc − H2O)
168.066(HexNAc − 2H2O)
366.141(HexNAc + Hex)
528.194(Hex + HexNAc + Hex)
657.237(NeuAc + Hex + HexNAc)
242.151(b2)
469.279(b4)
669.396(b6)
815.501(PEP_1)
797.492(PEP-H2O_1)
898.538
922.539
777.438
964.550
982.560
1000.569
1221.659(PEP+HexNAc(2)_1)
1383.713(PEP+HexNAc(2)Hex(1)_1)
1545.765(PEP+HexNAc(2)Hex(2)_1)
1707.817(PEP+HexNAc(2)Hex(3)_1)
CID-MS/MS Scan:20832, Noise threshold:0.7
366.141(HexNAc + Hex)
454.158(NeuAc + Hex)
528.194(Hex + HexNAc + Hex)
657.237(NeuAc + Hex + HexNAc)
692.361(PEP+HexNAc(2)Hex(1)_2)
773.386(PEP+HexNAc(2)Hex(2)_2)
854.414(PEP+HexNAc(2)Hex(3)_2)
874.927(PEP+HexNAc(3)Hex(2)_2)
935.442
955.953(PEP+HexNAc(3)Hex(3)_2)
1018.581(PEP+HexNAc(1)_1)
1036.980(PEP+HexNAc(3)Hex(4)_2)
1055.380
1101.505(PEP+HexNAc(3)Hex(3)NeuAc(1)_2)
1221.661(PEP+HexNAc(2)_1)
1346.469
1383.715(PEP+HexNAc(2)Hex(1)_1)
1545.768(PEP+HexNAc(2)Hex(2)_1)
1590.195
1671.687
1707.822(PEP+HexNAc(2)Hex(3)_1)
1999.915
ETD-MS/MS Scan:20833, Noise threshold:0.8
M+2H
121.764
141.047
292.000
311.974
332.942
377.815
450.447
539.143
657.237(NeuAc + Hex + HexNAc)
800.718
925.247
956.457
1036.979(PEP+HexNAc(3)Hex(4)_2)
1225.062
1305.272
1707.818(PEP+HexNAc(2)Hex(3)_1)
m/z

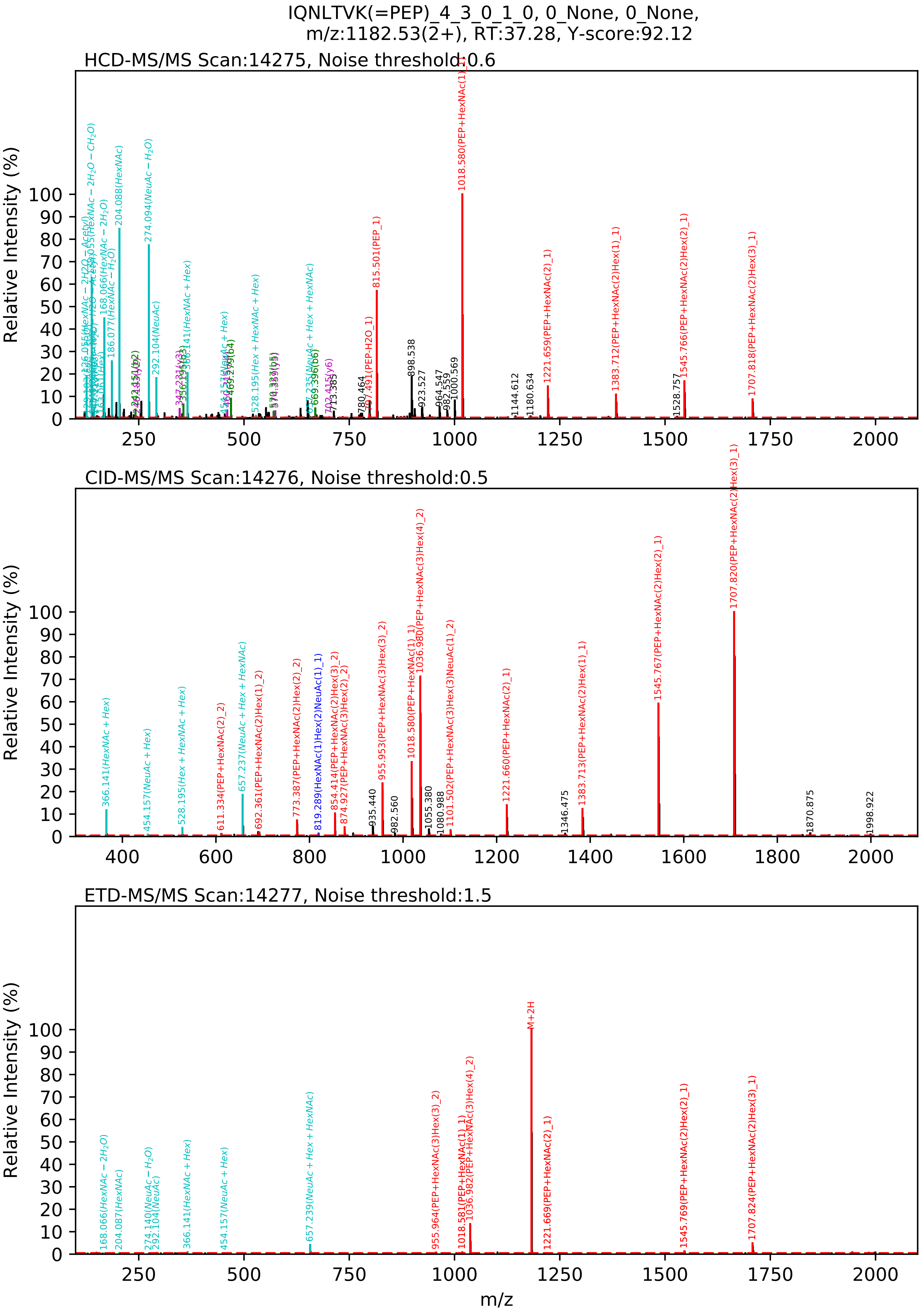
IQNLTVK(=PEP)_4_3_0_1_0, 0_None, 0_None,
m/z:1182.53(2+), RT:37.28, Y-score:92.12
HCD-MS/MS Scan:14275, Noise threshold:0.6
CID-MS/MS Scan:14276, Noise threshold:0.5
ETD-MS/MS Scan:14277, Noise threshold:1.5
Relative Intensity (%)
m/z
1018.580(PEP+HexNAc(1)_1)
815.501(PEP_1)
1221.659(PEP+HexNAc(2)_1)
1383.712(PEP+HexNAc(2)Hex(1)_1)
1545.766(PEP+HexNAc(2)Hex(2)_1)
1707.818(PEP+HexNAc(2)Hex(3)_1)
204.088(HexNAc)
274.094(NeuAc − H2O)
292.104(NeuAc)
1707.820(PEP+HexNAc(2)Hex(3)_1)
1545.767(PEP+HexNAc(2)Hex(2)_1)
1383.713(PEP+HexNAc(2)Hex(1)_1)
1221.660(PEP+HexNAc(2)_1)
1036.980(PEP+HexNAc(3)Hex(4)_2)
1018.580(PEP+HexNAc(1)_1)
955.953(PEP+HexNAc(3)Hex(3)_2)
M+2H
1036.982(PEP+HexNAc(3)Hex(4)_2)
1221.669(PEP+HexNAc(2)_1)
1545.769(PEP+HexNAc(2)Hex(2)_1)
1707.824(PEP+HexNAc(2)Hex(3)_1)

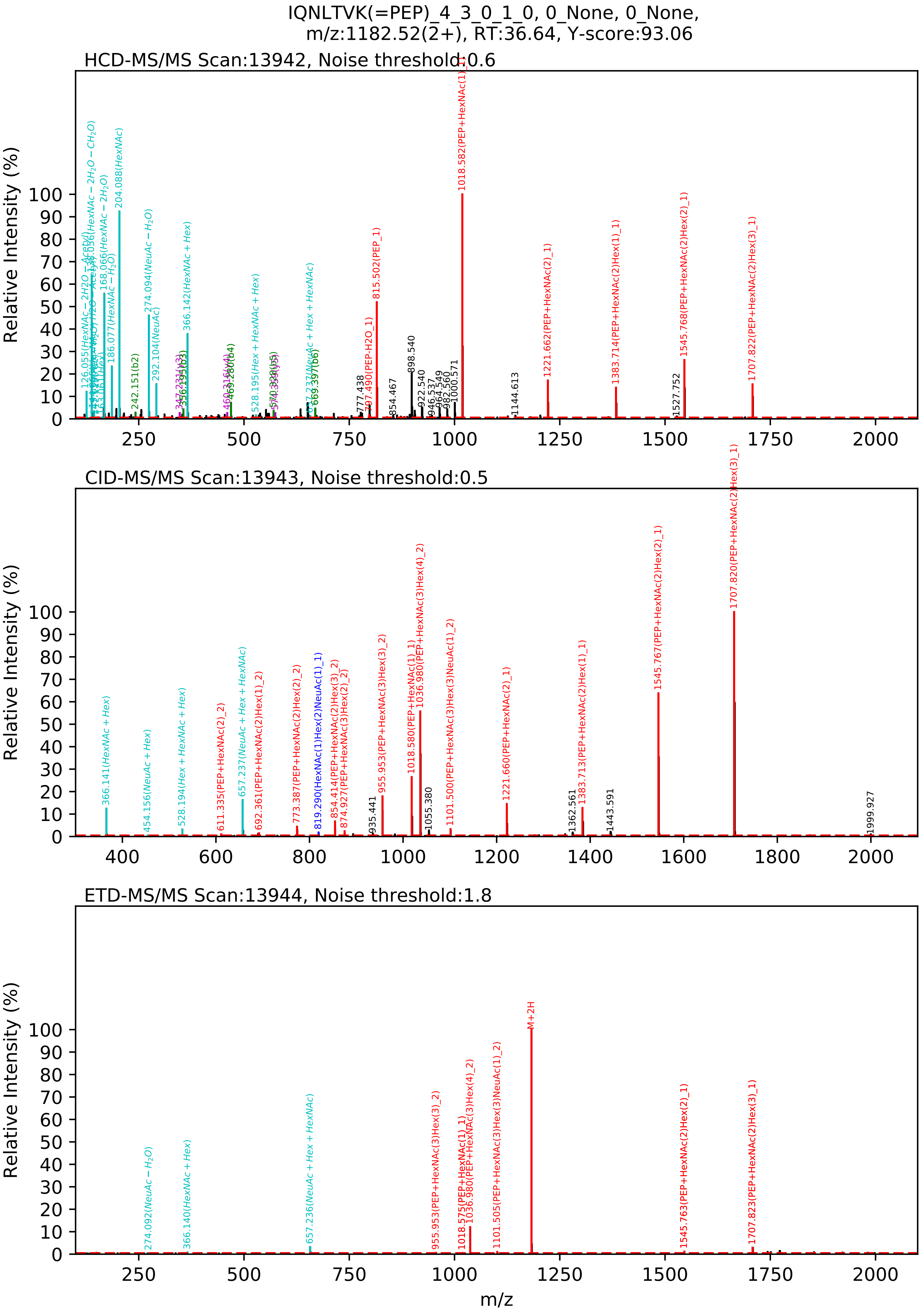
IQNLTVK(=PEP)_4_3_0_1_0, 0_None, 0_None,
m/z:1182.52(2+), RT:36.64, Y-score:93.06
HCD-MS/MS Scan:13942, Noise threshold:0.6
Relative Intensity (%)
1018.582(PEP+HexNAc(1)_1)
815.502(PEP_1)
1221.662(PEP+HexNAc(2)_1)
1383.714(PEP+HexNAc(2)Hex(1)_1)
1545.768(PEP+HexNAc(2)Hex(2)_1)
1707.822(PEP+HexNAc(2)Hex(3)_1)
204.088(HexNAc)
186.077(HexNAc − H2O)
168.066(HexNAc − 2H2O)
274.094(NeuAc − H2O)
292.104(NeuAc)
366.142(HexNAc + Hex)
242.151(b2)
469.280(b4)
528.195(Hex + HexNAc + Hex)
669.397(b6)
898.540
922.540
1144.613
1527.752
CID-MS/MS Scan:13943, Noise threshold:0.5
Relative Intensity (%)
1707.820(PEP+HexNAc(2)Hex(3)_1)
1545.767(PEP+HexNAc(2)Hex(2)_1)
1383.713(PEP+HexNAc(2)Hex(1)_1)
1221.660(PEP+HexNAc(2)_1)
1101.500(PEP+HexNAc(3)Hex(3)NeuAc(1)_2)
1036.980(PEP+HexNAc(3)Hex(4)_2)
1018.580(PEP+HexNAc(1)_1)
955.953(PEP+HexNAc(3)Hex(3)_2)
874.927(PEP+HexNAc(3)Hex(2)_2)
854.414(PEP+HexNAc(2)Hex(3)_2)
819.290(HexNAc(1)Hex(2)NeuAc(1)_1)
773.387(PEP+HexNAc(2)Hex(2)_2)
692.361(PEP+HexNAc(2)Hex(1)_2)
657.237(NeuAc + Hex + HexNAc)
611.335(PEP+HexNAc(2)_2)
528.194(Hex + HexNAc + Hex)
454.156(NeuAc + Hex)
366.141(HexNAc + Hex)
935.441
1055.380
1362.561
1443.591
1999.927
ETD-MS/MS Scan:13944, Noise threshold:1.8
Relative Intensity (%)
M+2H
274.092(NeuAc − H2O)
366.140(HexNAc + Hex)
657.236(NeuAc + Hex + HexNAc)
955.953(PEP+HexNAc(3)Hex(3)_2)
1018.575(PEP+HexNAc(1)_1)
1036.980(PEP+HexNAc(3)Hex(4)_2)
1101.505(PEP+HexNAc(3)Hex(3)NeuAc(1)_2)
1545.763(PEP+HexNAc(2)Hex(2)_1)
1707.823(PEP+HexNAc(2)Hex(3)_1)
m/z

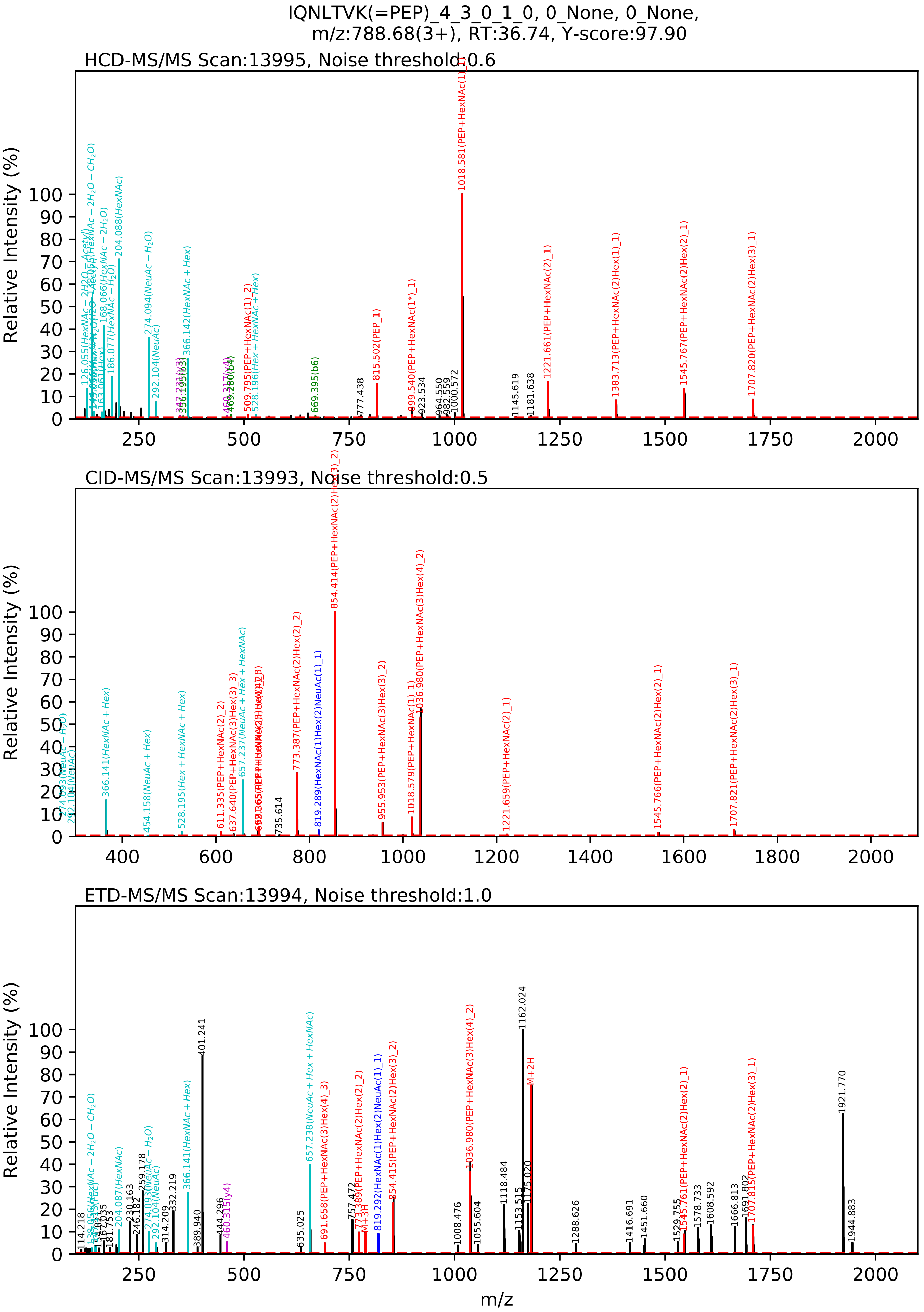
IQNLTVK(=PEP)_4_3_0_1_0, 0_None, 0_None,
m/z:788.68(3+), RT:36.74, Y-score:97.90
HCD-MS/MS Scan:13995, Noise threshold:0.6
CID-MS/MS Scan:13993, Noise threshold:0.5
ETD-MS/MS Scan:13994, Noise threshold:1.0
Relative Intensity (%)
m/z

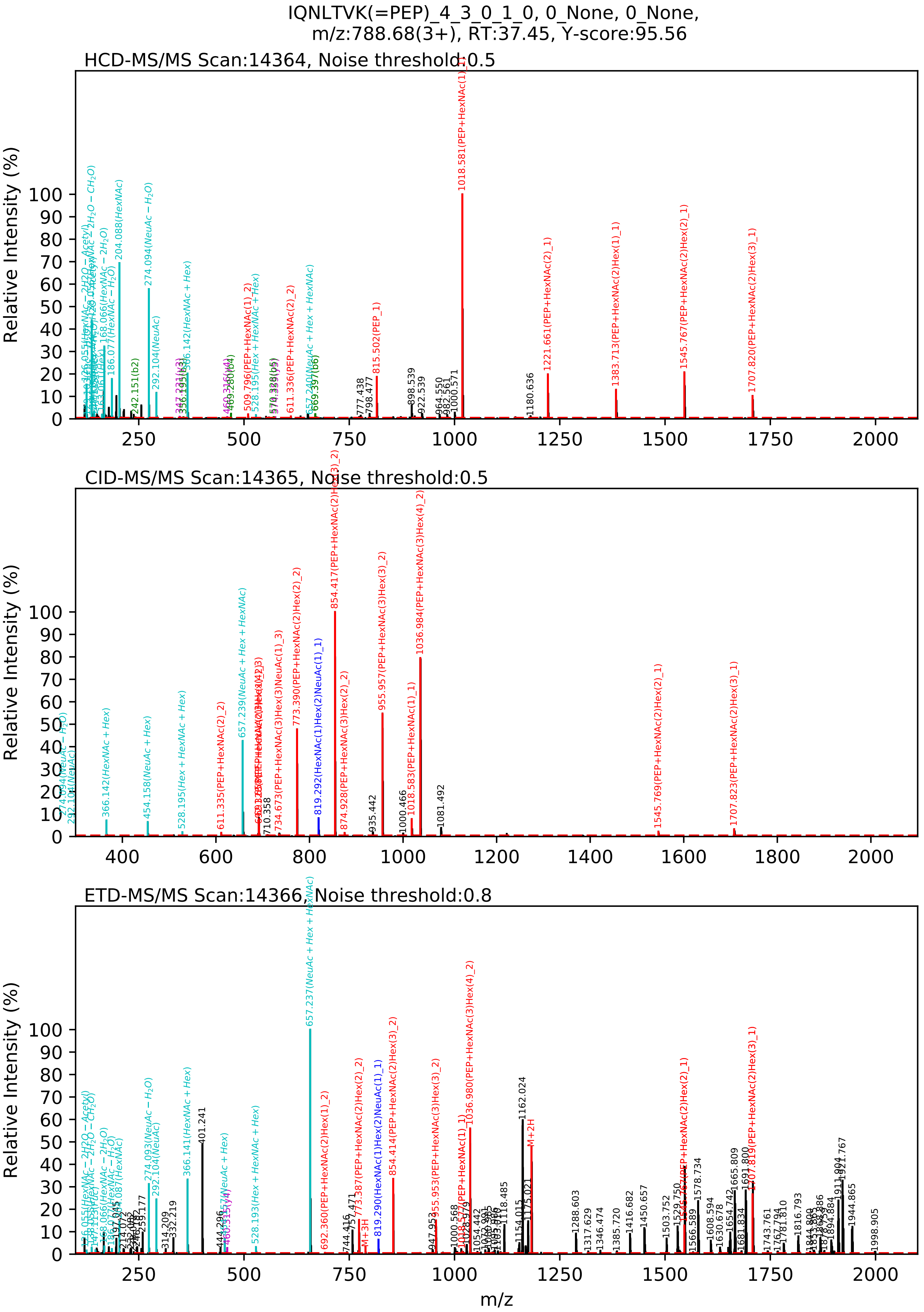
IQNLTVK(=PEP)_4_3_0_1_0, 0_None, 0_None,
m/z:788.68(3+), RT:37.45, Y-score:95.56
HCD-MS/MS Scan:14364, Noise threshold:0.5
CID-MS/MS Scan:14365, Noise threshold:0.5
ETD-MS/MS Scan:14366, Noise threshold:0.8
Relative Intensity (%)
m/z
1018.581(PEP+HexNAc(1)_1)
1221.661(PEP+HexNAc(2)_1)
1383.713(PEP+HexNAc(2)Hex(1)_1)
1545.767(PEP+HexNAc(2)Hex(2)_1)
1707.820(PEP+HexNAc(2)Hex(3)_1)
815.502(PEP_1)
204.088(HexNAc)
274.094(NeuAc − H2O)
292.104(NeuAc)
366.142(HexNAc + Hex)
854.417(PEP+HexNAc(2)Hex(3)_2)
1036.984(PEP+HexNAc(3)Hex(4)_2)
955.957(PEP+HexNAc(3)Hex(3)_2)
773.390(PEP+HexNAc(2)Hex(2)_2)
819.292(HexNAc(1)Hex(2)NeuAc(1)_1)
657.239(NeuAc + Hex + HexNAc)
1545.769(PEP+HexNAc(2)Hex(2)_1)
1707.823(PEP+HexNAc(2)Hex(3)_1)
657.237(NeuAc + Hex + HexNAc)
1036.980(PEP+HexNAc(3)Hex(4)_2)
854.414(PEP+HexNAc(2)Hex(3)_2)
1162.024
M+2H
M+3H
401.241
1921.767

IQNLTVK(=PEP)_4_3_1_0_0, 0_None, 0_None,
m/z:1110.01(2+), RT:27.92, Y-score:92.52

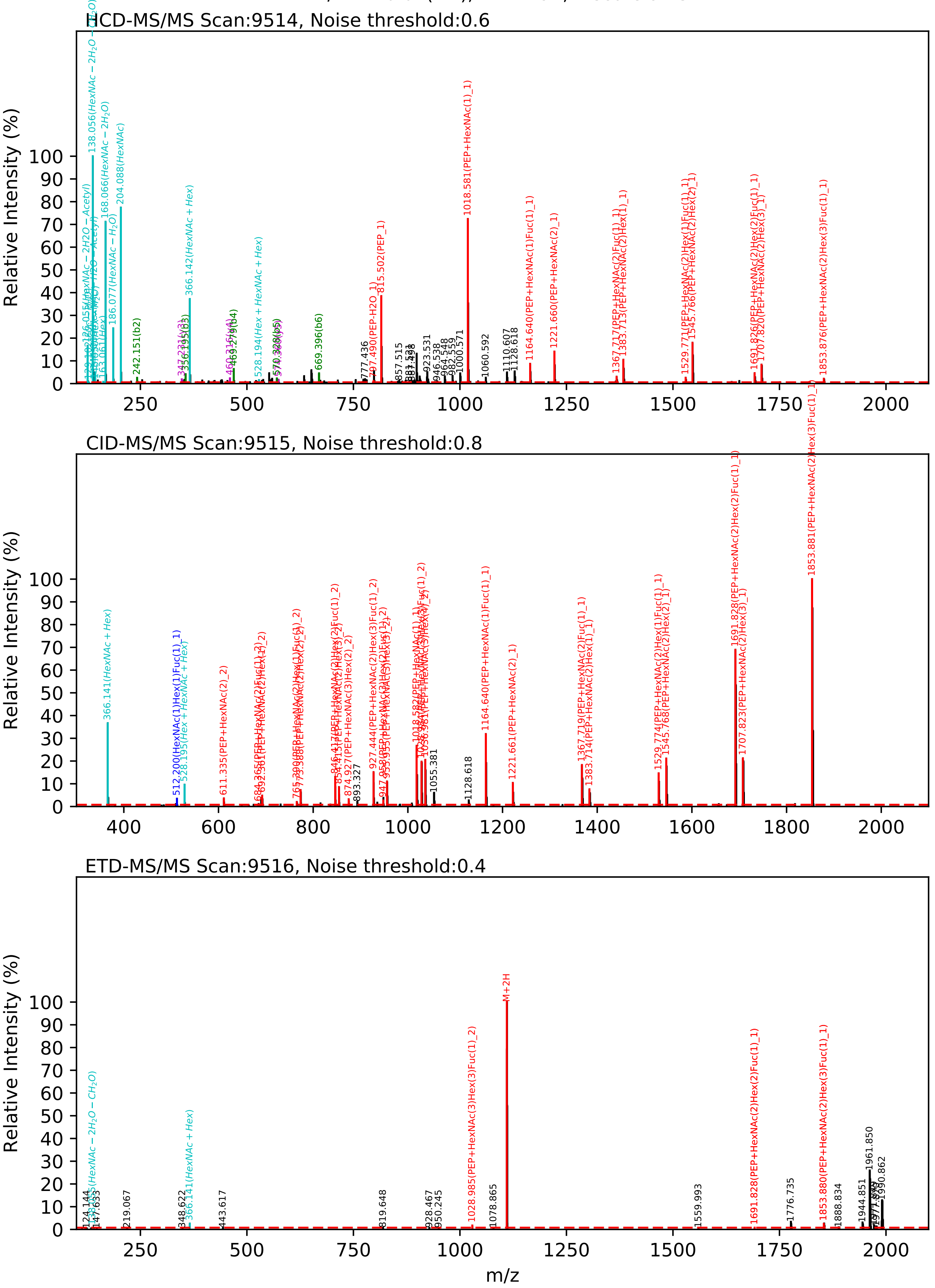

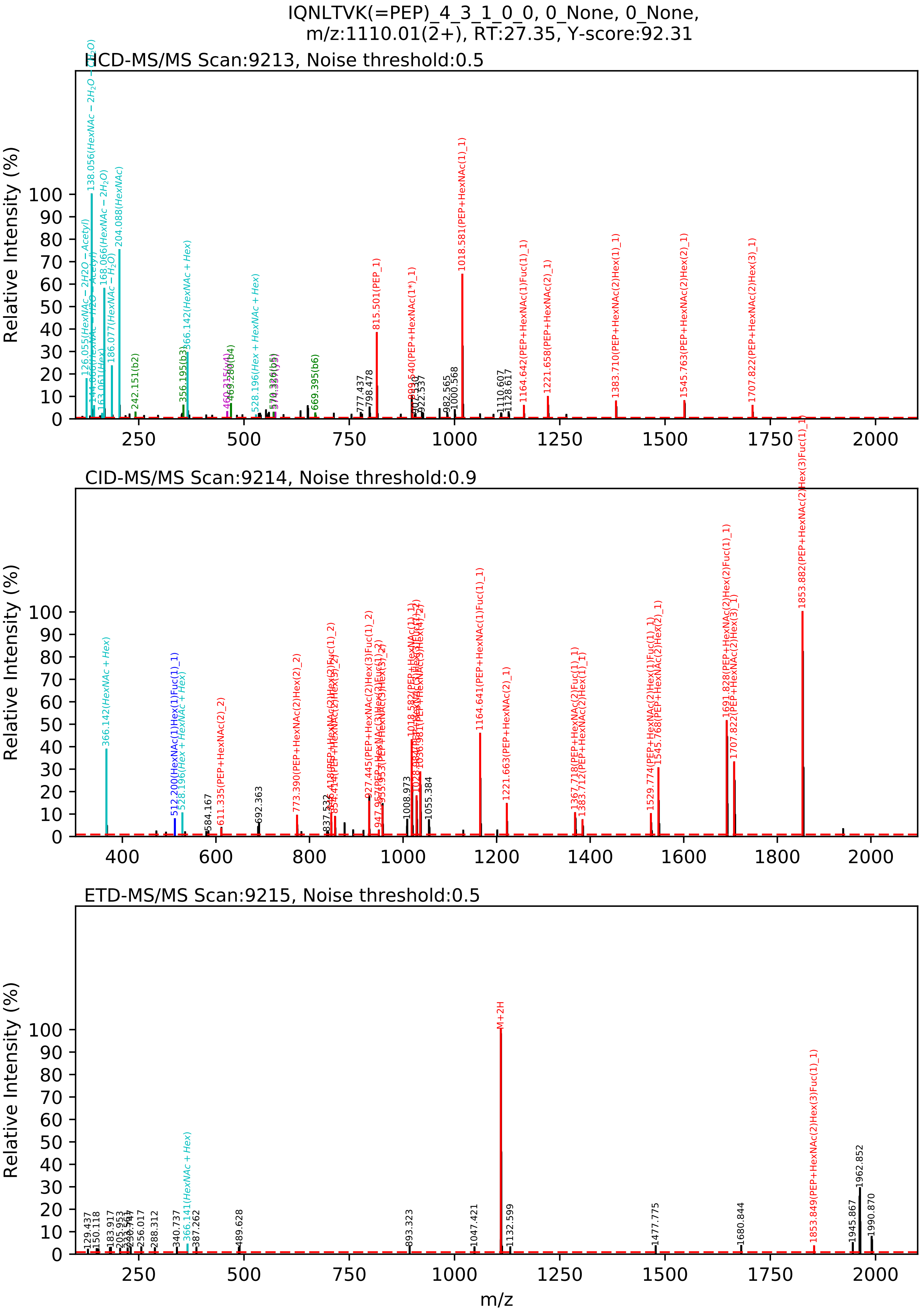

IQNLTVK(=PEP)_4_3_1_0_0, 0_None, 0_None,
m/z:1110.01(2+), RT:27.35, Y-score:92.31
HCD-MS/MS Scan:9213, Noise threshold:0.5
CID-MS/MS Scan:9214, Noise threshold:0.9
ETD-MS/MS Scan:9215, Noise threshold:0.5
Relative Intensity (%)
m/z
138.056(HexNAc − 2H2O − H2O)
204.088(HexNAc)
168.066(HexNAc − 2H2O)
186.077(HexNAc − H2O)
366.142(HexNAc + Hex)
242.151(b2)
356.195(b3)
469.280(b4)
528.196(Hex + HexNAc + Hex)
669.395(b6)
777.437
798.478
815.501(PEP_1)
899.540(PEP+HexNAc(1*)_1)
922.537
982.565
1000.568
1018.581(PEP+HexNAc(1)_1)
1110.607
1128.617
1164.642(PEP+HexNAc(1)Fuc(1)_1)
1221.658(PEP+HexNAc(2)_1)
1383.710(PEP+HexNAc(2)Hex(1)_1)
1545.763(PEP+HexNAc(2)Hex(2)_1)
1707.822(PEP+HexNAc(2)Hex(3)_1)
1853.882(PEP+HexNAc(2)Hex(3)Fuc(1)_1)
512.200(HexNAc(1)Hex(1)Fuc(1)_1)
584.167
611.335(PEP+HexNAc(2)_2)
692.363
773.390(PEP+HexNAc(2)Hex(2)_2)
837.532
927.445(PEP+HexNAc(2)Hex(3)Fuc(1)_2)
1008.973
1055.384
1164.641(PEP+HexNAc(1)Fuc(1)_1)
1221.663(PEP+HexNAc(2)_1)
1367.718(PEP+HexNAc(2)Fuc(1)_1)
1383.712(PEP+HexNAc(2)Hex(1)_1)
1529.774(PEP+HexNAc(2)Hex(1)Fuc(1)_1)
1545.768(PEP+HexNAc(2)Hex(2)_1)
1691.828(PEP+HexNAc(2)Hex(2)Fuc(1)_1)
1707.822(PEP+HexNAc(2)Hex(3)_1)
M+2H
129.437
150.118
183.917
256.017
288.312
340.737
366.141(HexNAc + Hex)
387.262
489.628
893.323
1047.421
1132.599
1477.775
1680.844
1853.849(PEP+HexNAc(2)Hex(3)Fuc(1)_1)
1945.867
1962.852
1990.870

IQNLTVK(=PEP)_4_3_1_0_0, 0_None, 0_None,
m/z:1110.01(2+), RT:26.22, Y-score:92.79

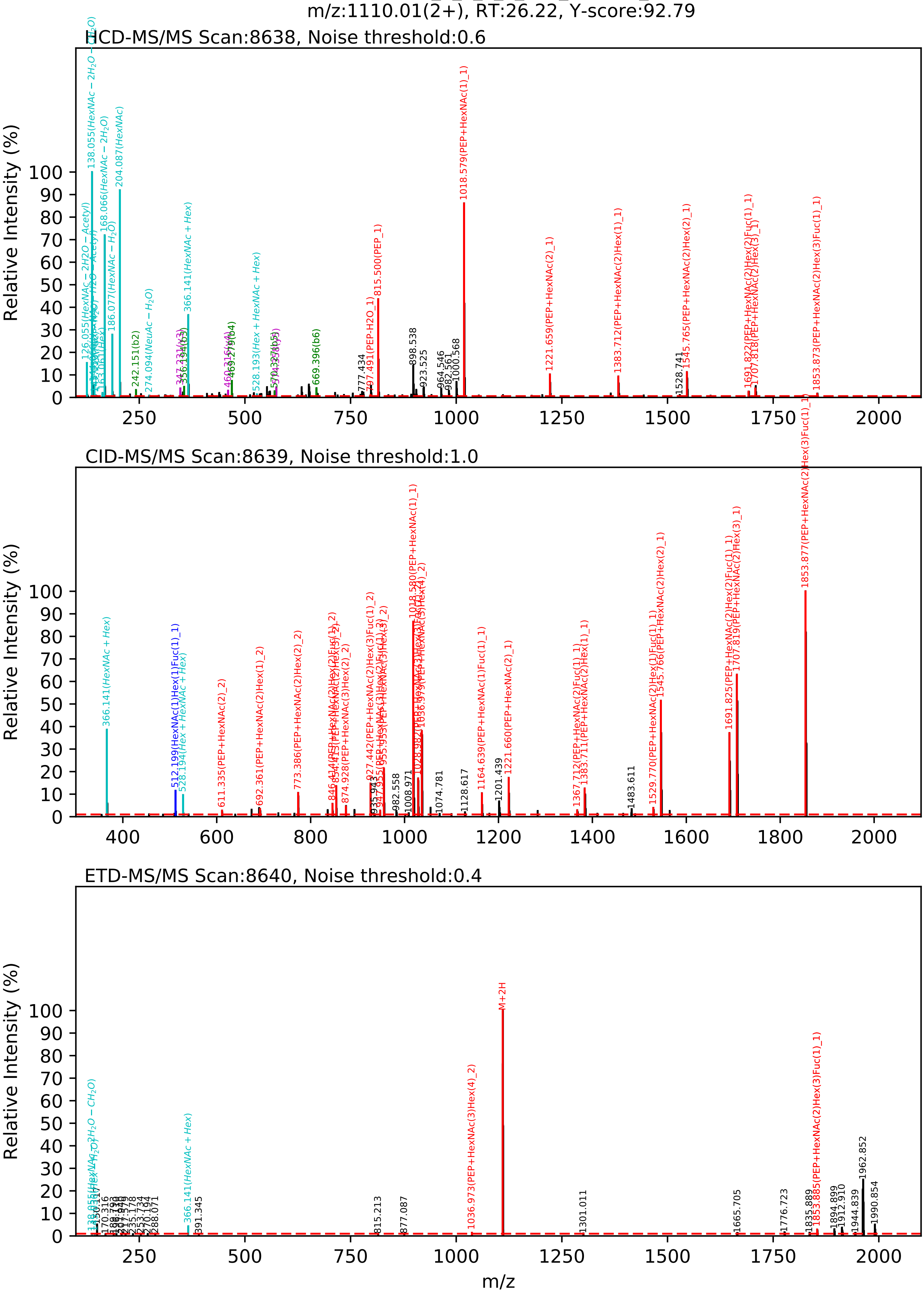

IQNLTVK(=PEP)_4_3_1_0_0, 0_None, 0_None,
m/z:1110.01(2+), RT:26.80, Y-score:93.96

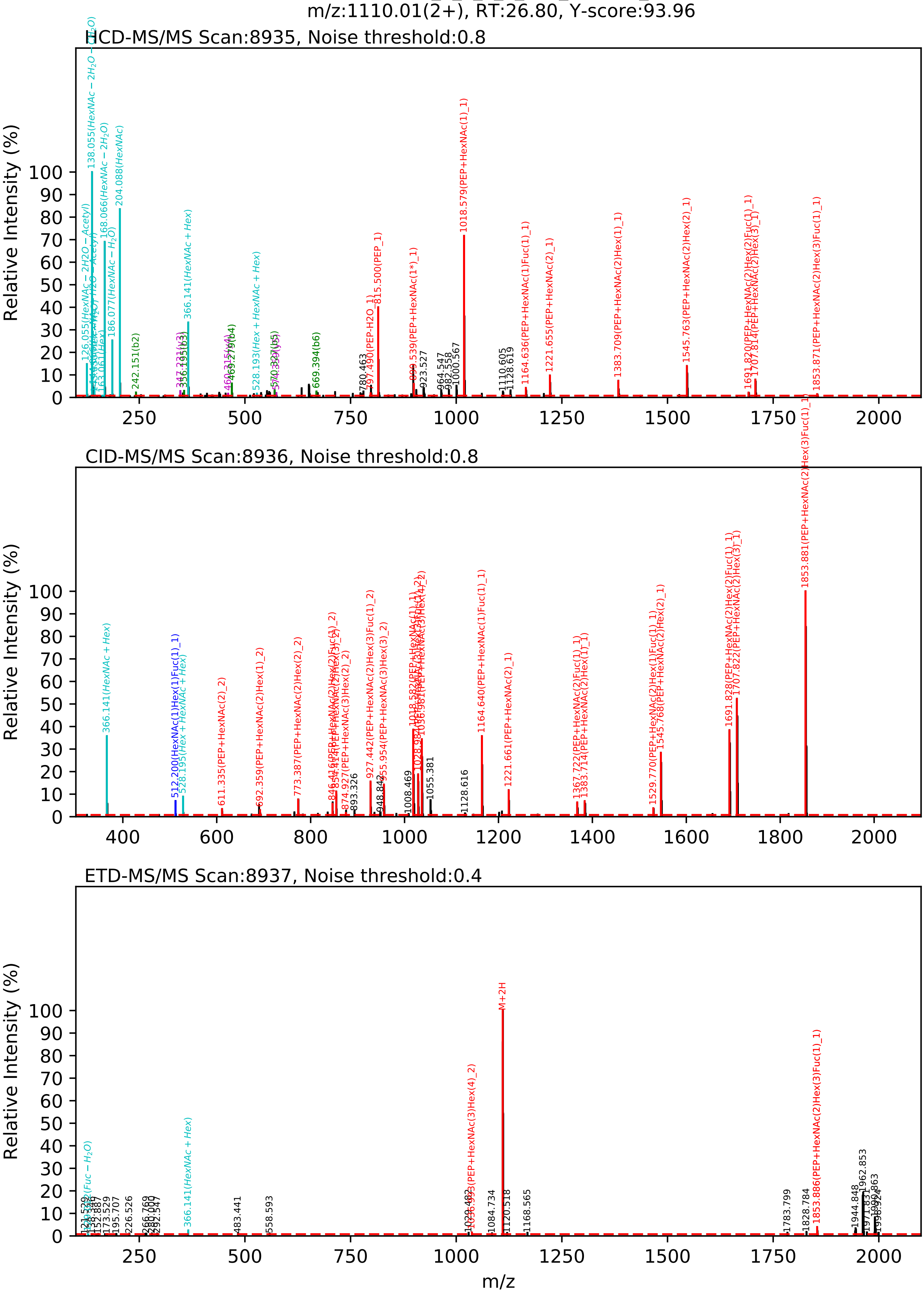

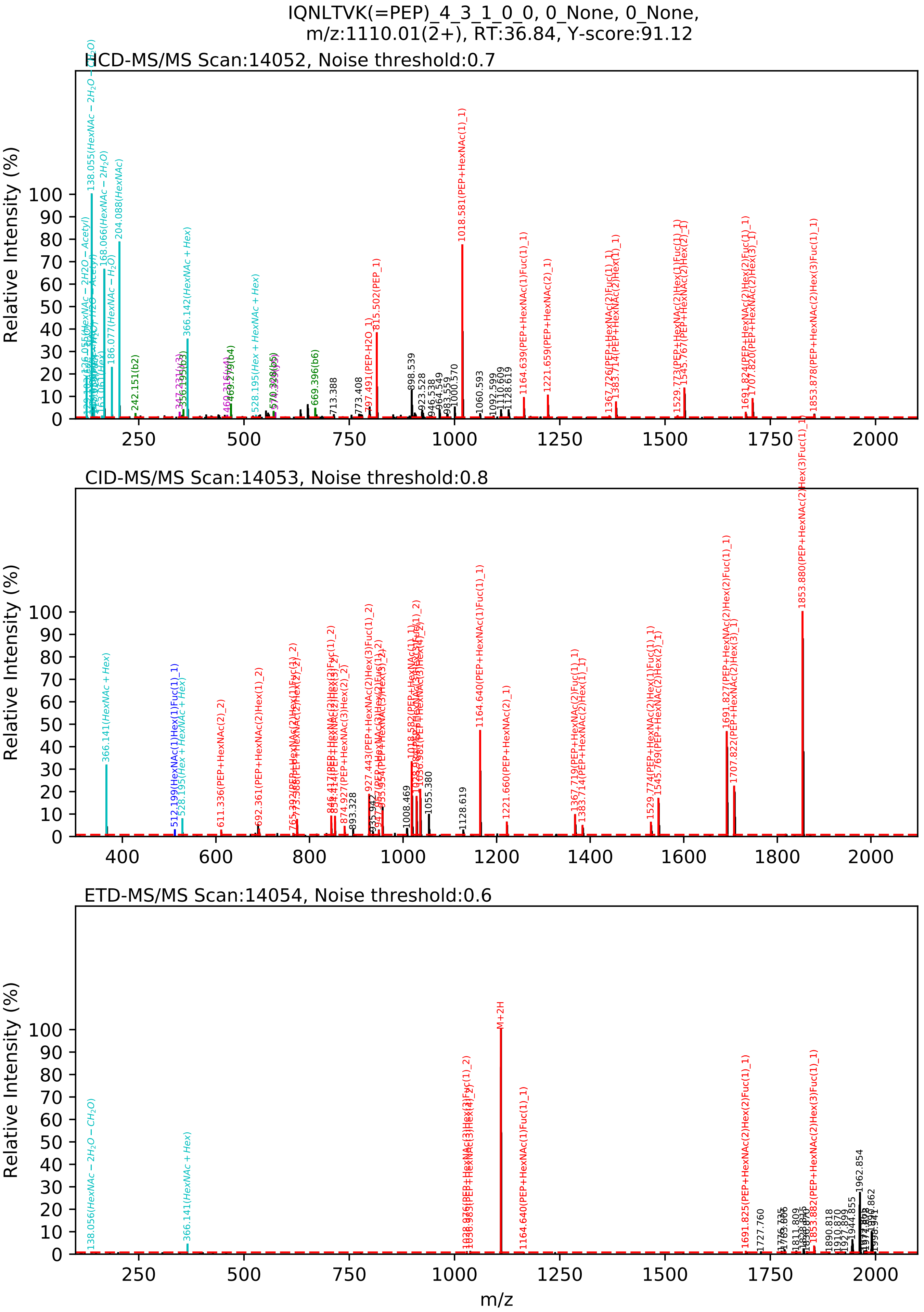
IQNLTVK(=PEP)_4_3_1_0_0, 0_None, 0_None,
m/z:1110.01(2+), RT:36.84, Y-score:91.12
HCD-MS/MS Scan:14052, Noise threshold:0.7
CID-MS/MS Scan:14053, Noise threshold:0.8
ETD-MS/MS Scan:14054, Noise threshold:0.6
Relative Intensity (%)
m/z

IQNLTVK(=PEP)_4_3_1_0_0, 0_None, 0_None,
m/z:1110.01(2+), RT:37.77, Y-score:89.50

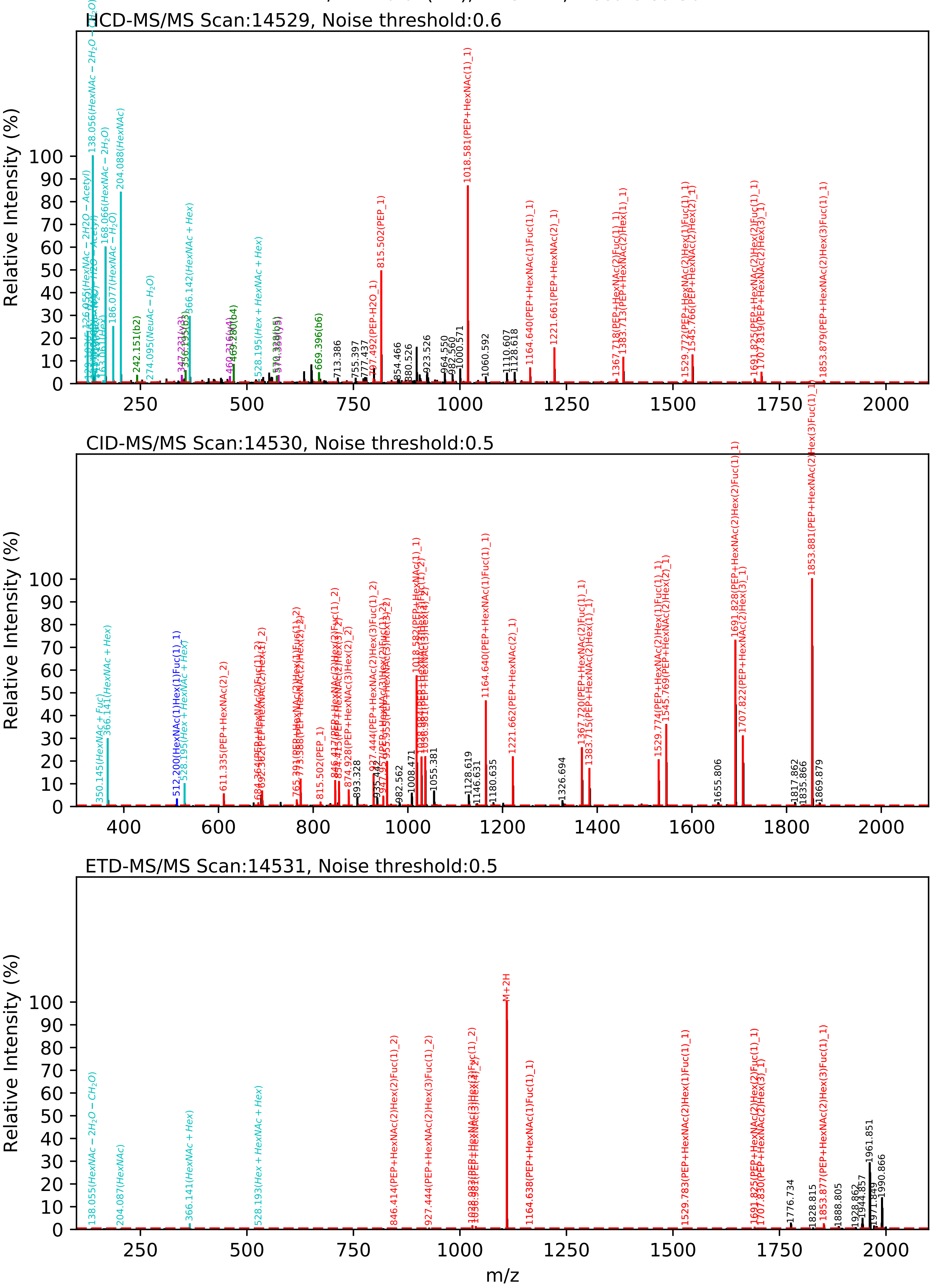

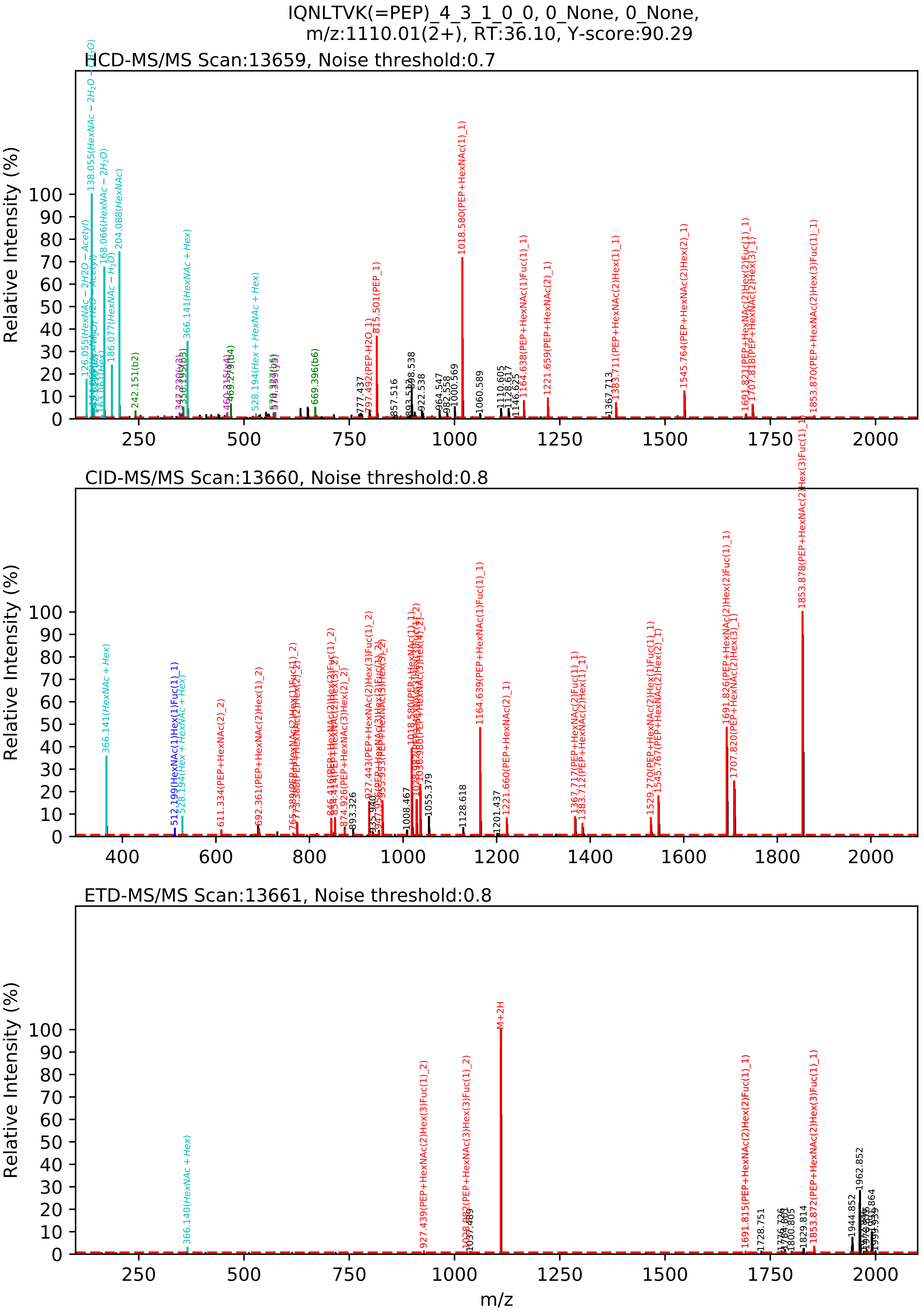
IQNLTVK(=PEP)_4_3_1_0_0, 0_None, 0_None,
m/z:1110.01(2+), RT:36.10, Y-score:90.29
HCD-MS/MS Scan:13659, Noise threshold:0.7
CID-MS/MS Scan:13660, Noise threshold:0.8
ETD-MS/MS Scan:13661, Noise threshold:0.8
Relative Intensity (%)
m/z
1018.580(PEP+HexNAc(1)_1)
815.501(PEP_1)
1853.878(PEP+HexNAc(2)Hex(3)Fuc(1)_1)
1691.826(PEP+HexNAc(2)Hex(2)Fuc(1)_1)
1164.639(PEP+HexNAc(1)Fuc(1)_1)
M+2H
1962.852

IQNLTVK(=PEP)_4_3_1_1_0, 0_None, 0_None,
m/z:1255.55(2+), RT:50.18, Y-score:95.62

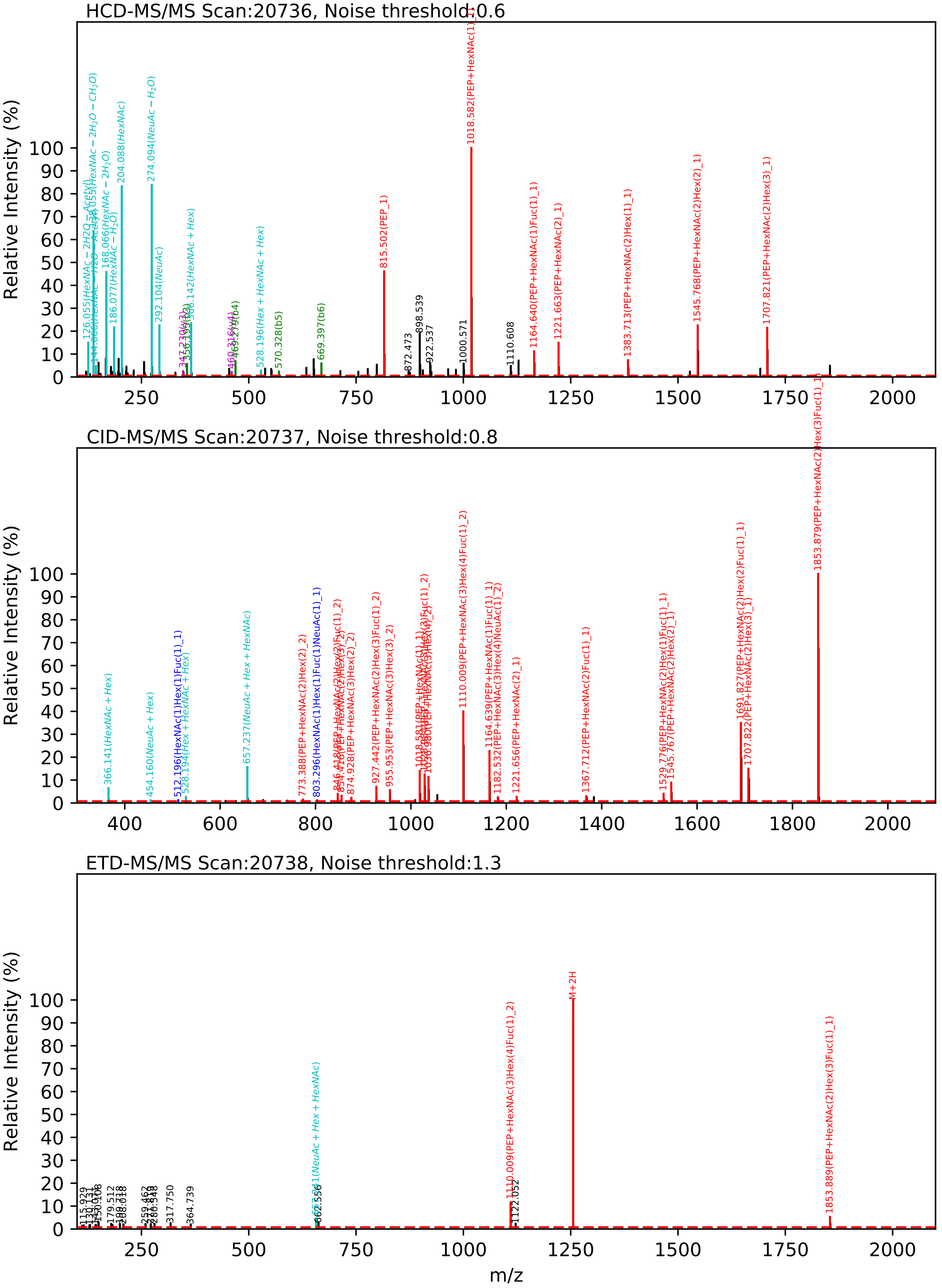

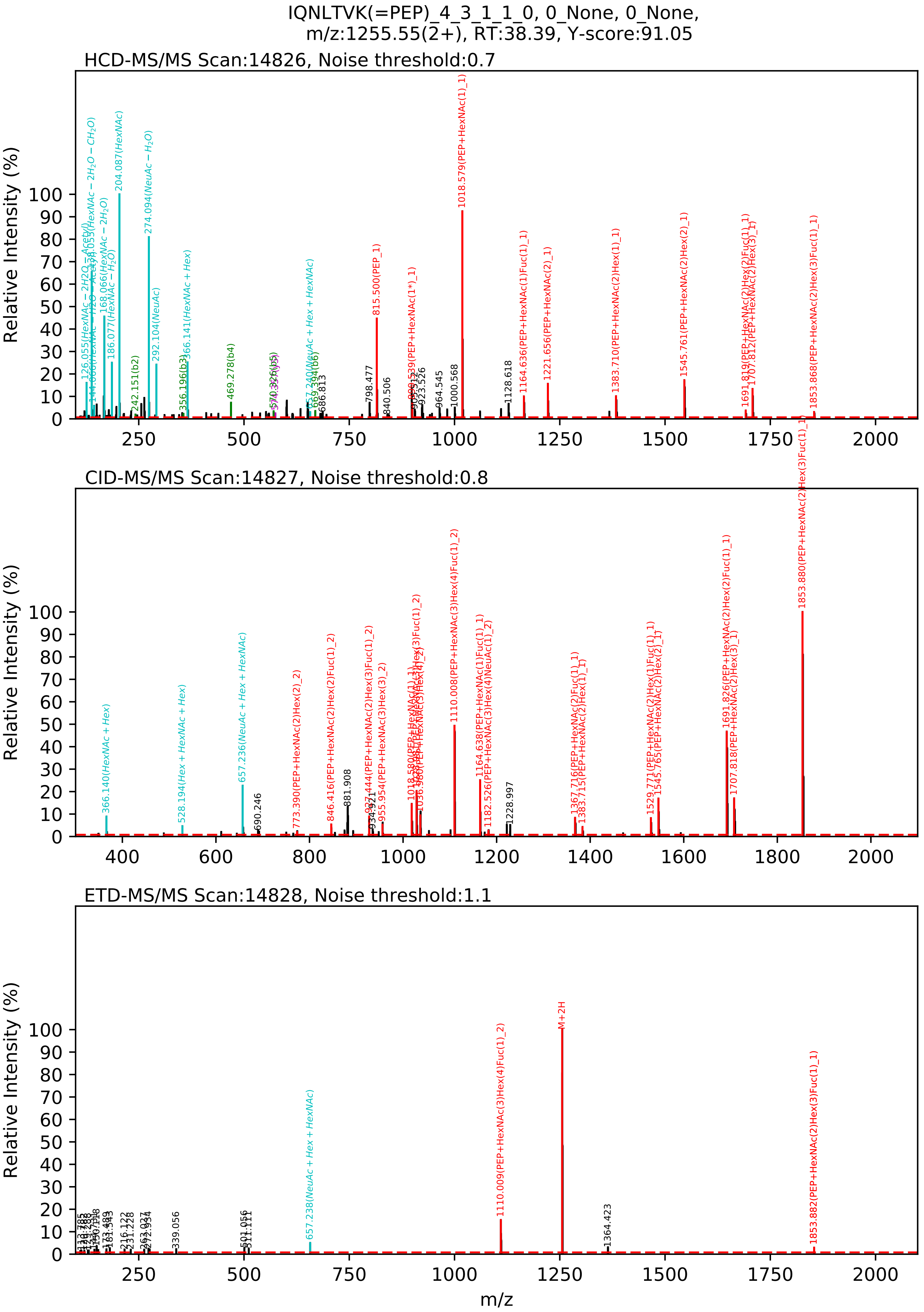
IQNLTVK(=PEP)_4_3_1_1_0, 0_None, 0_None,
m/z:1255.55(2+), RT:38.39, Y-score:91.05
HCD-MS/MS Scan:14826, Noise threshold:0.7
Relative Intensity (%)
204.087(HexNAc)
274.094(NeuAc − H2O)
292.104(NeuAc)
366.141(HexNAc + Hex)
242.151(b2)
356.196(b3)
469.278(b4)
815.500(PEP_1)
1018.579(PEP+HexNAc(1)_1)
1164.636(PEP+HexNAc(1)Fuc(1)_1)
1221.656(PEP+HexNAc(2)_1)
1383.710(PEP+HexNAc(2)Hex(1)_1)
1545.761(PEP+HexNAc(2)Hex(2)_1)
1853.868(PEP+HexNAc(2)Hex(3)Fuc(1)_1)
CID-MS/MS Scan:14827, Noise threshold:0.8
Relative Intensity (%)
366.140(HexNAc + Hex)
528.194(Hex + HexNAc + Hex)
657.236(NeuAc + Hex + HexNAc)
773.390(PEP+HexNAc(2)Hex(2)_2)
846.416(PEP+HexNAc(2)Hex(2)Fuc(1)_2)
1110.008(PEP+HexNAc(3)Hex(4)Fuc(1)_2)
1691.826(PEP+HexNAc(2)Hex(2)Fuc(1)_1)
1853.880(PEP+HexNAc(2)Hex(3)Fuc(1)_1)
ETD-MS/MS Scan:14828, Noise threshold:1.1
Relative Intensity (%)
657.238(NeuAc + Hex + HexNAc)
1110.009(PEP+HexNAc(3)Hex(4)Fuc(1)_2)
M+2H
1364.423
1853.882(PEP+HexNAc(2)Hex(3)Fuc(1)_1)
m/z

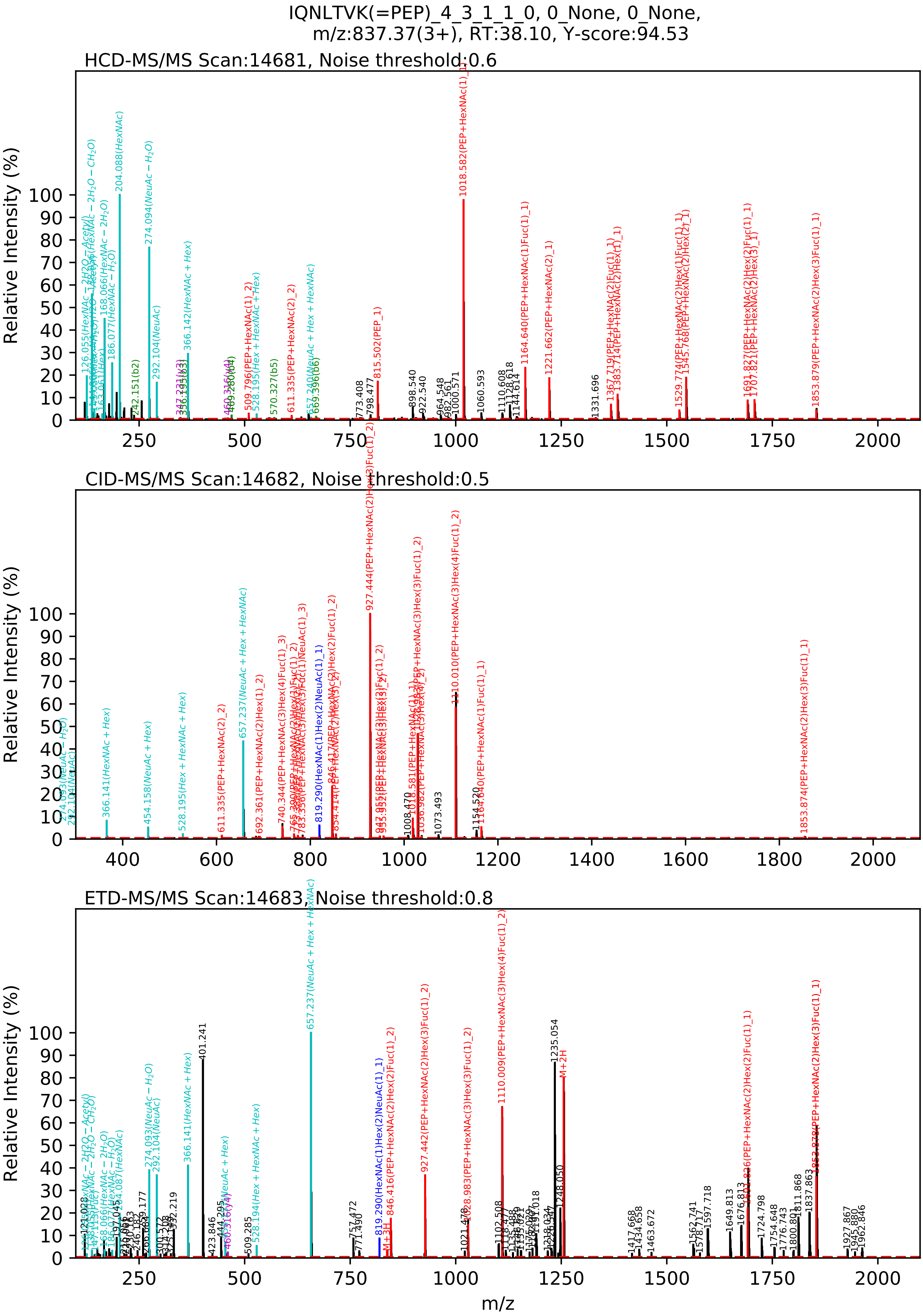
IQNLTVK(=PEP)_4_3_1_1_0, 0_None, 0_None,
m/z:837.37(3+), RT:38.10, Y-score:94.53
HCD-MS/MS Scan:14681, Noise threshold:0.6
CID-MS/MS Scan:14682, Noise threshold:0.5
ETD-MS/MS Scan:14683, Noise threshold:0.8
Relative Intensity (%)
m/z
1018.582(PEP+HexNAc(1)_1)
927.444(PEP+HexNAc(2)Hex(3)Fuc(1)_2)
1110.010(PEP+HexNAc(3)Hex(4)Fuc(1)_2)
657.237(NeuAc + Hex + HexNAc)
1235.054
401.241
M+2H

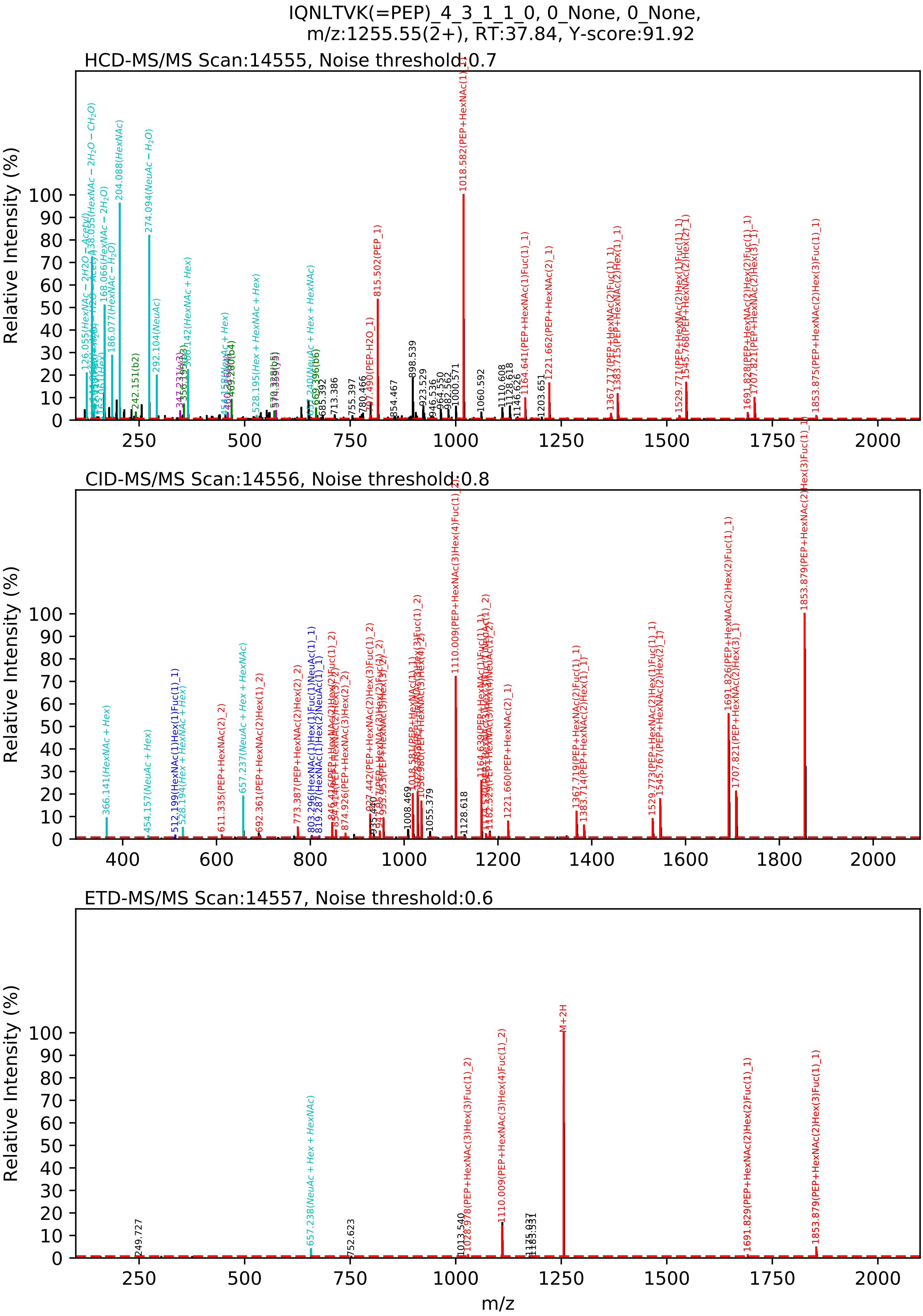
IQNLTVK(=PEP)_4_3_1_1_0, 0_None, 0_None,
m/z:1255.55(2+), RT:37.84, Y-score:91.92
HCD-MS/MS Scan:14555, Noise threshold:0.7
CID-MS/MS Scan:14556, Noise threshold:0.8
ETD-MS/MS Scan:14557, Noise threshold:0.6
Relative Intensity (%)
m/z
1018.582(PEP+HexNAc(1)_1)
815.502(PEP_1)
1853.879(PEP+HexNAc(2)Hex(3)Fuc(1)_1)
1691.826(PEP+HexNAc(2)Hex(2)Fuc(1)_1)
1110.009(PEP+HexNAc(3)Hex(4)Fuc(1)_2)
M+2H

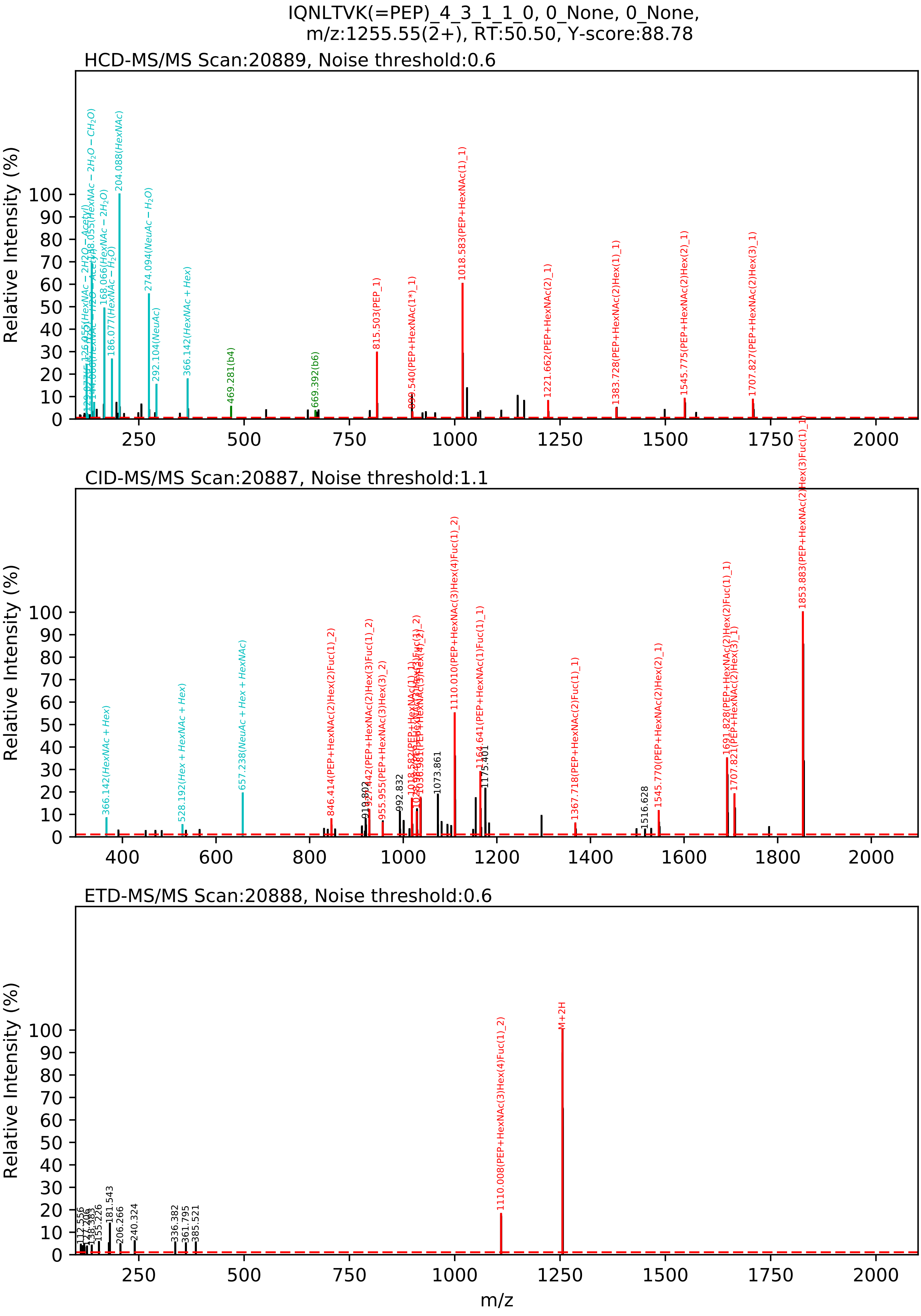
IQNLTVK(=PEP)_4_3_1_1_0, 0_None, 0_None,
m/z:1255.55(2+), RT:50.50, Y-score:88.78
HCD-MS/MS Scan:20889, Noise threshold:0.6
Relative Intensity (%)
204.088(HexNAc)
186.077(HexNAc − H2O)
168.066(HexNAc − 2H2O)
274.094(NeuAc − H2O)
292.104(NeuAc)
366.142(HexNAc + Hex)
469.281(b4)
669.392(b6)
815.503(PEP_1)
899.540(PEP+HexNAc(1*)_1)
1018.583(PEP+HexNAc(1)_1)
1221.662(PEP+HexNAc(2)_1)
1383.728(PEP+HexNAc(2)Hex(1)_1)
1545.775(PEP+HexNAc(2)Hex(2)_1)
1707.827(PEP+HexNAc(2)Hex(3)_1)
1853.883(PEP+HexNAc(2)Hex(3)Fuc(1)_1)
CID-MS/MS Scan:20887, Noise threshold:1.1
366.142(HexNAc + Hex)
528.192(Hex + HexNAc + Hex)
657.238(NeuAc + Hex + HexNAc)
846.414(PEP+HexNAc(2)Hex(2)Fuc(1)_2)
919.803
955.955(PEP+HexNAc(3)Hex(3)_2)
992.832
1036.981(PEP+HexNAc(3)Hex(4)_2)
1073.861
1110.010(PEP+HexNAc(3)Hex(4)Fuc(1)_2)
1164.641(PEP+HexNAc(1)Fuc(1)_1)
1175.401
1367.718(PEP+HexNAc(2)Fuc(1)_1)
1516.628
1545.770(PEP+HexNAc(2)Hex(2)_1)
1691.828(PEP+HexNAc(2)Hex(2)Fuc(1)_1)
1707.821(PEP+HexNAc(2)Hex(3)_1)
1853.883(PEP+HexNAc(2)Hex(3)Fuc(1)_1)
ETD-MS/MS Scan:20888, Noise threshold:0.6
112.556
138.385
155.226
181.543
206.266
240.324
336.382
361.795
385.521
1110.008(PEP+HexNAc(3)Hex(4)Fuc(1)_2)
M+2H
m/z

IQNLTVK(=PEP)_4_3_1_1_0, 0_None, 0_None,
m/z:1255.55(2+), RT:36.20, Y-score:92.64

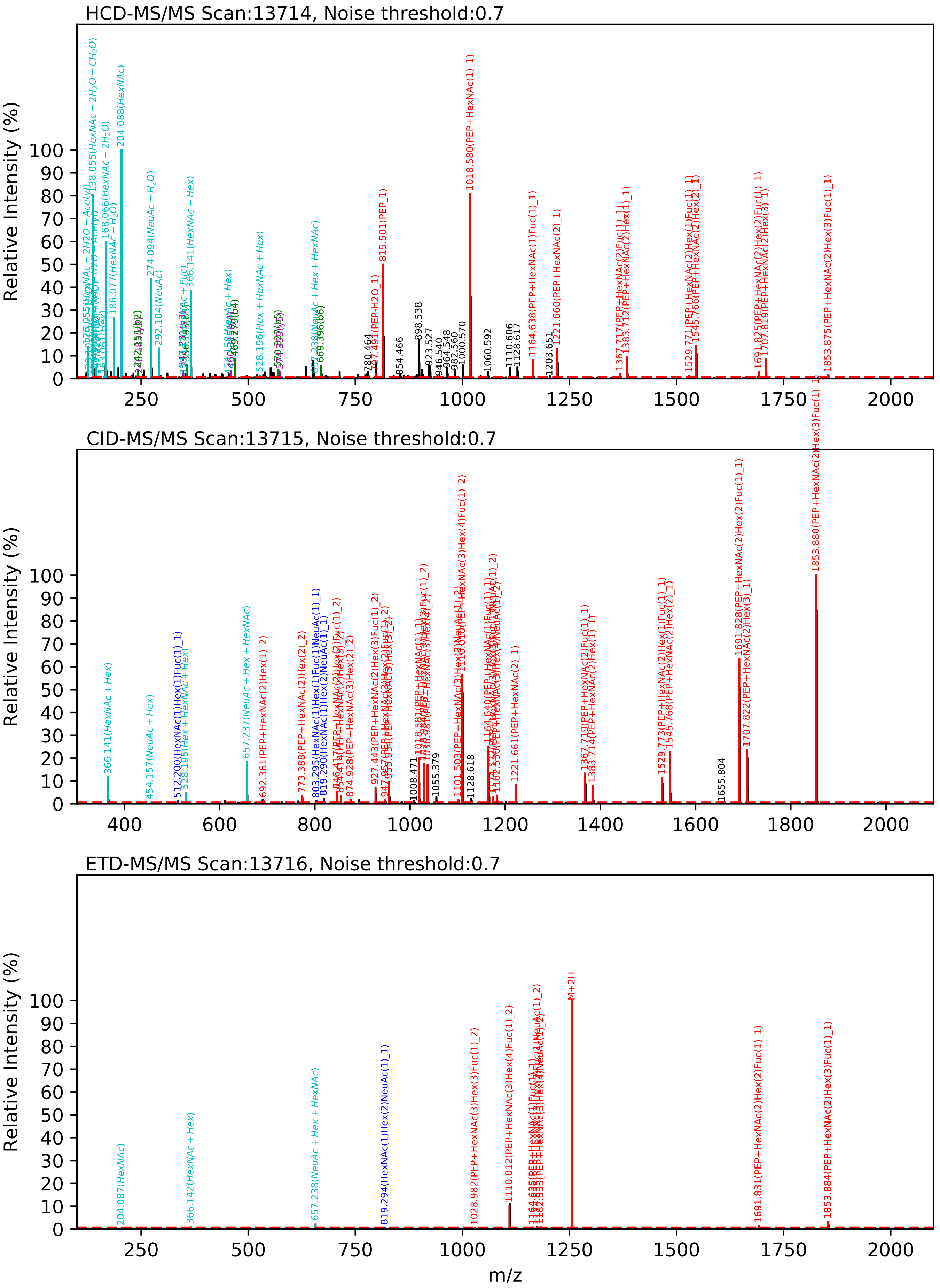

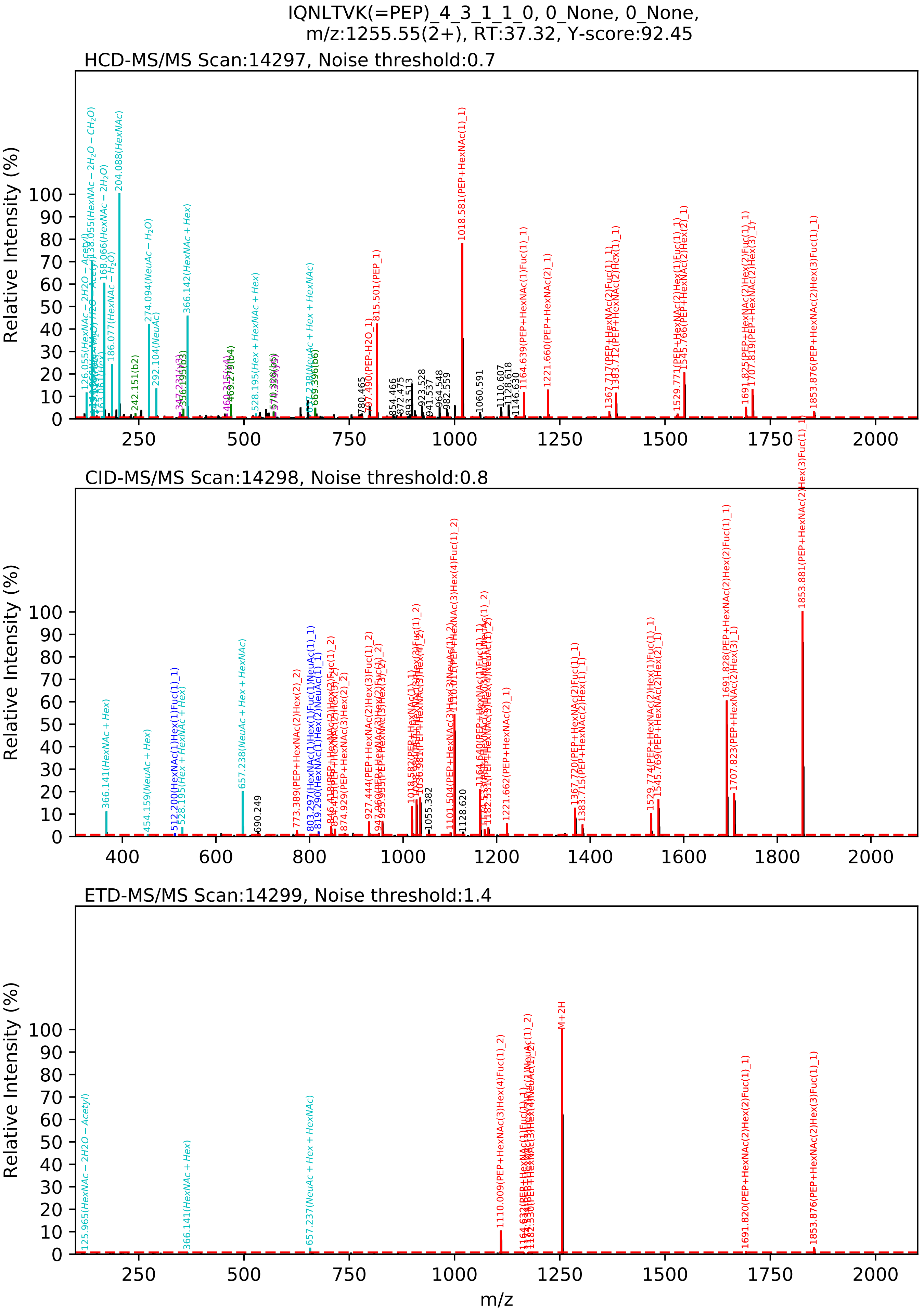
IQNLTVK(=PEP)_4_3_1_1_0, 0_None, 0_None,
m/z:1255.55(2+), RT:37.32, Y-score:92.45
HCD-MS/MS Scan:14297, Noise threshold:0.7
CID-MS/MS Scan:14298, Noise threshold:0.8
ETD-MS/MS Scan:14299, Noise threshold:1.4
Relative Intensity (%)
m/z

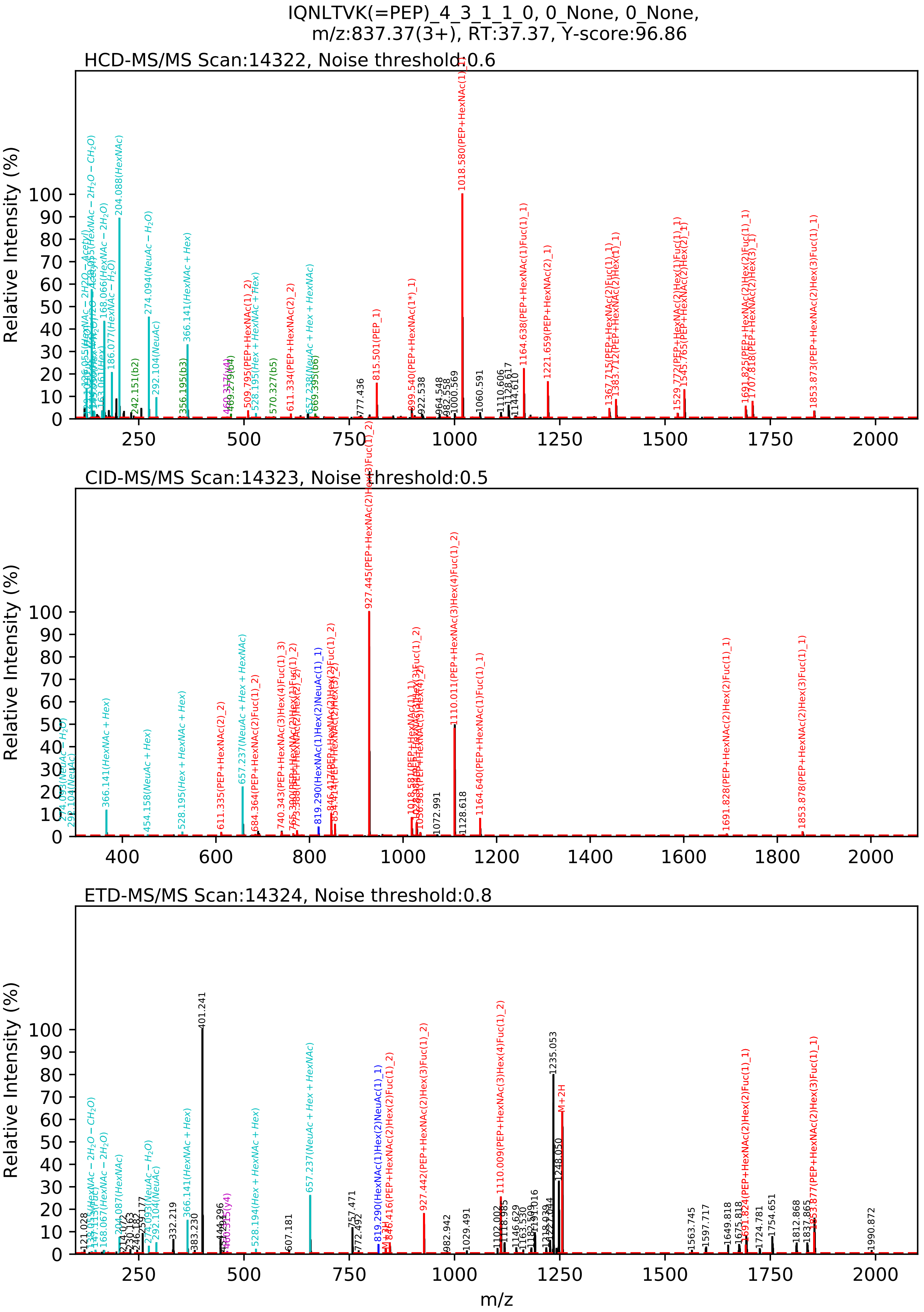
IQNLTVK(=PEP)_4_3_1_1_0, 0_None, 0_None,
m/z:837.37(3+), RT:37.37, Y-score:96.86
HCD-MS/MS Scan:14322, Noise threshold:0.6
CID-MS/MS Scan:14323, Noise threshold:0.5
ETD-MS/MS Scan:14324, Noise threshold:0.8
Relative Intensity (%)
m/z
1018.580(PEP+HexNAc(1)_1)
1164.638(PEP+HexNAc(1)Fuc(1)_1)
1221.659(PEP+HexNAc(2)_1)
815.501(PEP_1)
204.088(HexNAc)
274.094(NeuAc − H2O)
366.141(HexNAc + Hex)
927.445(PEP+HexNAc(2)Hex(3)Fuc(1)_2)
1110.011(PEP+HexNAc(3)Hex(4)Fuc(1)_2)
1164.640(PEP+HexNAc(1)Fuc(1)_1)
1691.828(PEP+HexNAc(2)Hex(2)Fuc(1)_1)
1853.878(PEP+HexNAc(2)Hex(3)Fuc(1)_1)
657.237(NeuAc + Hex + HexNAc)
401.241
1235.053
1248.050
M+2H
927.442(PEP+HexNAc(2)Hex(3)Fuc(1)_2)
1110.009(PEP+HexNAc(3)Hex(4)Fuc(1)_2)
1853.877(PEP+HexNAc(2)Hex(3)Fuc(1)_1)
1691.824(PEP+HexNAc(2)Hex(2)Fuc(1)_1)
819.290(HexNAc(1)Hex(2)NeuAc(1)_1)

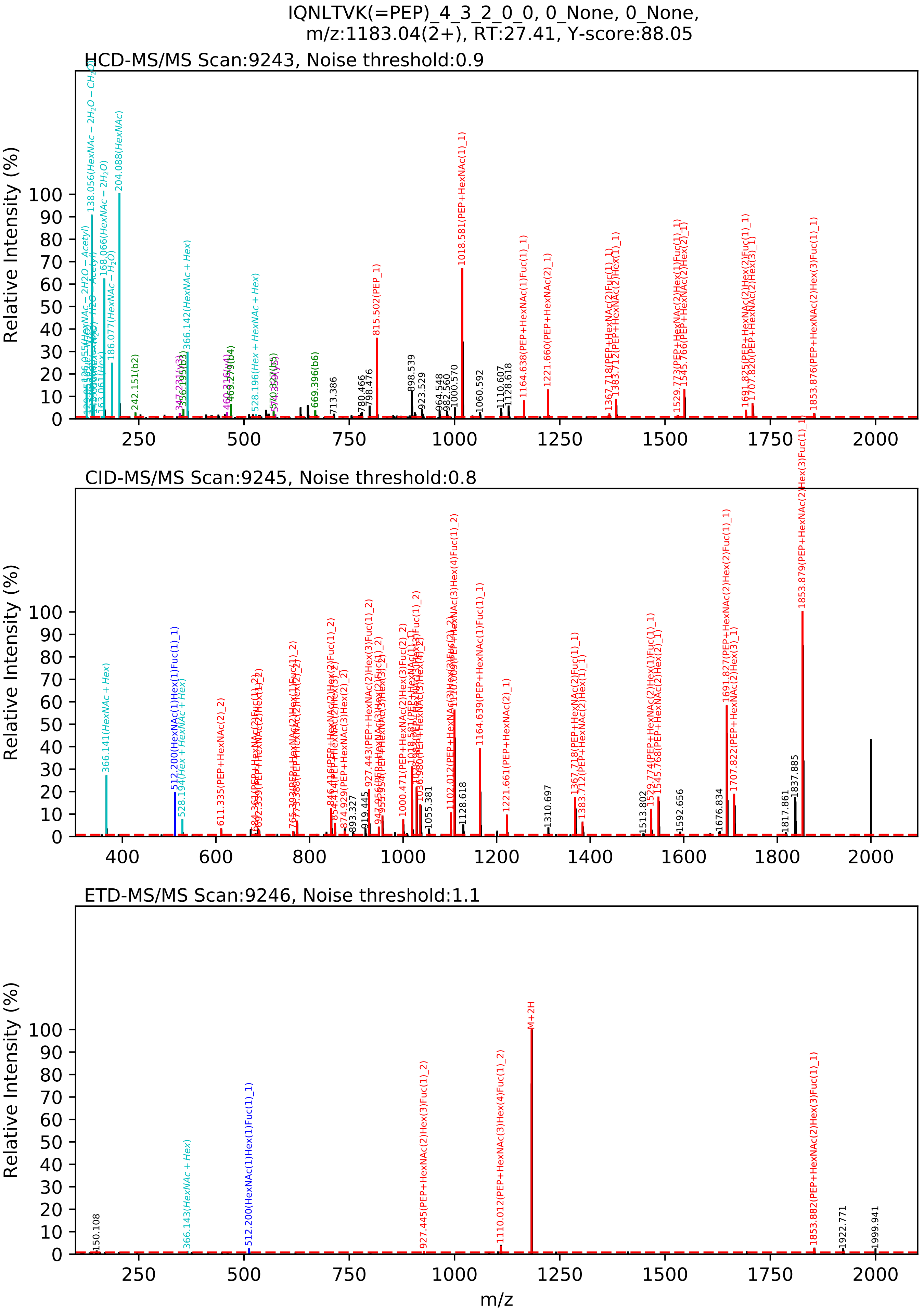
IQNLTVK(=PEP)_4_3_2_0_0, 0_None, 0_None,
m/z:1183.04(2+), RT:27.41, Y-score:88.05
HCD-MS/MS Scan:9243, Noise threshold:0.9
Relative Intensity (%)
204.088(HexNAc)
138.056
168.066
186.077
366.142(HexNAc + Hex)
242.151(b2)
528.196(Hex + HexNAc + Hex)
669.396(b6)
815.502(PEP_1)
1018.581(PEP+HexNAc(1)_1)
1164.638(PEP+HexNAc(1)Fuc(1)_1)
1221.660(PEP+HexNAc(2)_1)
1853.876(PEP+HexNAc(2)Hex(3)Fuc(1)_1)
713.386
898.539
923.529
1060.592
1110.607
1128.618
CID-MS/MS Scan:9245, Noise threshold:0.8
Relative Intensity (%)
366.141(HexNAc + Hex)
512.200(HexNAc(1)Hex(1)Fuc(1)_1)
611.335(PEP+HexNAc(2)_2)
1000.471(PEP+HexNAc(2)Hex(3)Fuc(2)_2)
1164.639(PEP+HexNAc(1)Fuc(1)_1)
1221.661(PEP+HexNAc(2)_1)
1691.827(PEP+HexNAc(2)Hex(2)Fuc(1)_1)
1707.822(PEP+HexNAc(2)Hex(3)_1)
1853.879(PEP+HexNAc(2)Hex(3)Fuc(1)_1)
1837.885
1128.618
1310.697
1592.656
1676.834
ETD-MS/MS Scan:9246, Noise threshold:1.1
Relative Intensity (%)
M+2H
150.108
366.143(HexNAc + Hex)
512.200(HexNAc(1)Hex(1)Fuc(1)_1)
927.445(PEP+HexNAc(2)Hex(3)Fuc(1)_2)
1110.012(PEP+HexNAc(3)Hex(4)Fuc(1)_2)
1853.882(PEP+HexNAc(2)Hex(3)Fuc(1)_1)
1922.771
1999.941
m/z

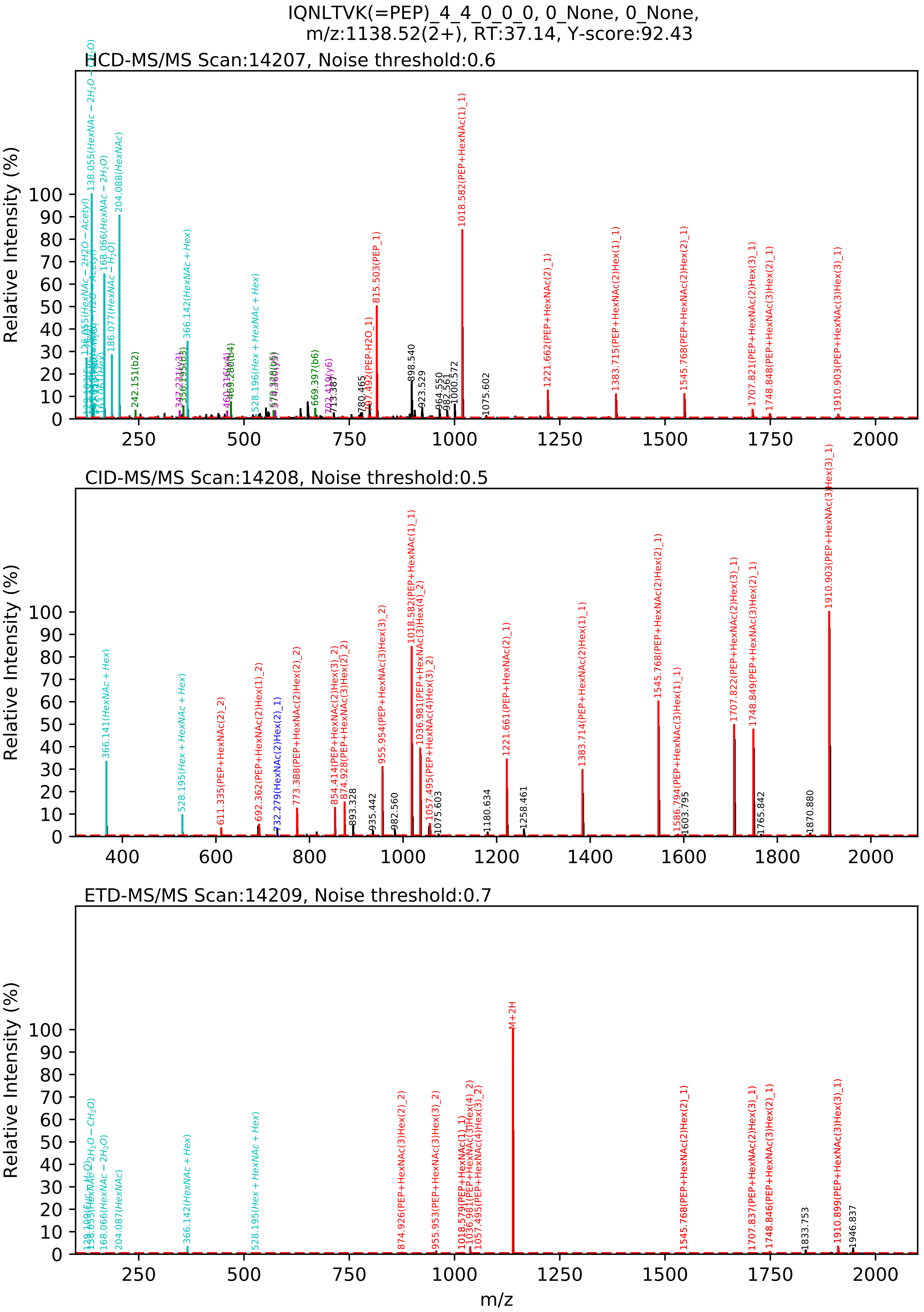
IQNLTVK(=PEP)_4_4_0_0_0, 0_None, 0_None,
m/z:1138.52(2+), RT:37.14, Y-score:92.43
HCD-MS/MS Scan:14207, Noise threshold:0.6
CID-MS/MS Scan:14208, Noise threshold:0.5
ETD-MS/MS Scan:14209, Noise threshold:0.7
Relative Intensity (%)
m/z
1018.582(PEP+HexNAc(1)_1)
815.503(PEP_1)
1221.662(PEP+HexNAc(2)_1)
1383.715(PEP+HexNAc(2)Hex(1)_1)
1545.768(PEP+HexNAc(2)Hex(2)_1)
1707.821(PEP+HexNAc(2)Hex(3)_1)
1748.848(PEP+HexNAc(3)Hex(2)_1)
1910.903(PEP+HexNAc(3)Hex(3)_1)
M+2H

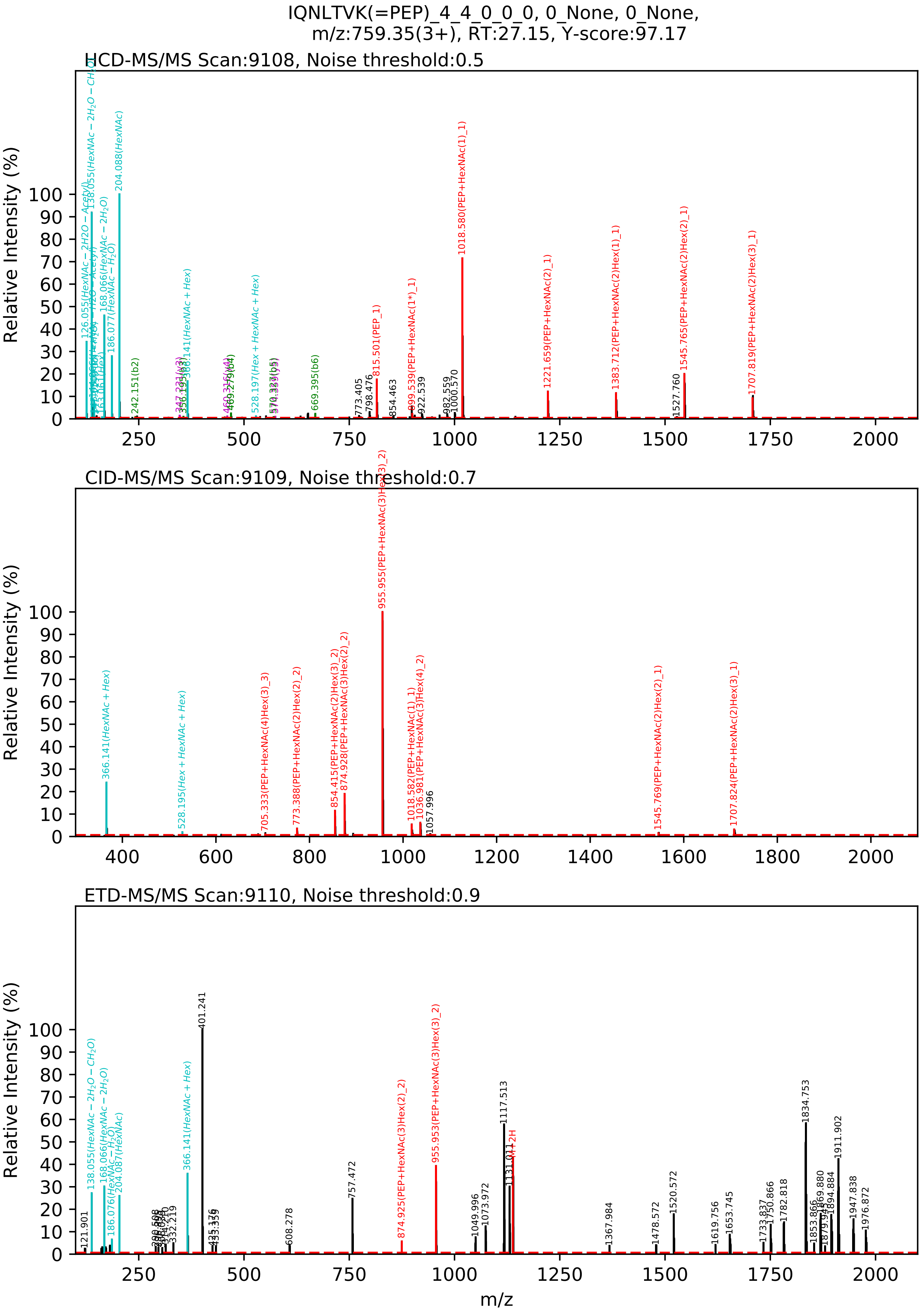
IQNLTVK(=PEP)_4_4_0_0_0, 0_None, 0_None,
m/z:759.35(3+), RT:27.15, Y-score:97.17
HCD-MS/MS Scan:9108, Noise threshold:0.5
Relative Intensity (%)
1018.580(PEP+HexNAc(1)_1)
1221.659(PEP+HexNAc(2)_1)
1383.712(PEP+HexNAc(2)Hex(1)_1)
1545.765(PEP+HexNAc(2)Hex(2)_1)
1707.819(PEP+HexNAc(2)Hex(3)_1)
815.501(PEP_1)
899.539(PEP+HexNAc(1*)_1)
204.088(HexNAc)
186.077(HexNAc − H2O)
168.066(HexNAc − 2H2O)
366.141(HexNAc + Hex)
528.197(Hex + HexNAc + Hex)
242.151(b2)
669.395(b6)
773.405
798.476
854.463
922.539
982.559
1000.570
1527.760
CID-MS/MS Scan:9109, Noise threshold:0.7
955.955(PEP+HexNAc(3)Hex(3)_2)
366.141(HexNAc + Hex)
528.195(Hex + HexNAc + Hex)
705.333(PEP+HexNAc(4)Hex(3)_3)
773.388(PEP+HexNAc(2)Hex(2)_2)
854.415(PEP+HexNAc(2)Hex(3)_2)
874.928(PEP+HexNAc(3)Hex(2)_2)
1018.582(PEP+HexNAc(1)_1)
1036.981(PEP+HexNAc(3)Hex(4)_2)
1057.996
1545.769(PEP+HexNAc(2)Hex(2)_1)
1707.824(PEP+HexNAc(2)Hex(3)_1)
ETD-MS/MS Scan:9110, Noise threshold:0.9
401.241
138.055(HexNAc − 2H2O − CH2O)
168.066(HexNAc − 2H2O)
186.076(HexNAc − H2O)
204.087(HexNAc)
366.141(HexNAc + Hex)
121.901
332.219
608.278
757.472
874.925(PEP+HexNAc(3)Hex(2)_2)
955.953(PEP+HexNAc(3)Hex(3)_2)
1049.996
1073.972
1117.513
1131.011
1367.984
1478.572
1520.572
1619.756
1653.745
1733.837
1750.866
1782.818
1834.753
1853.866
1869.880
1894.884
1911.902
1947.838
1976.872
m/z

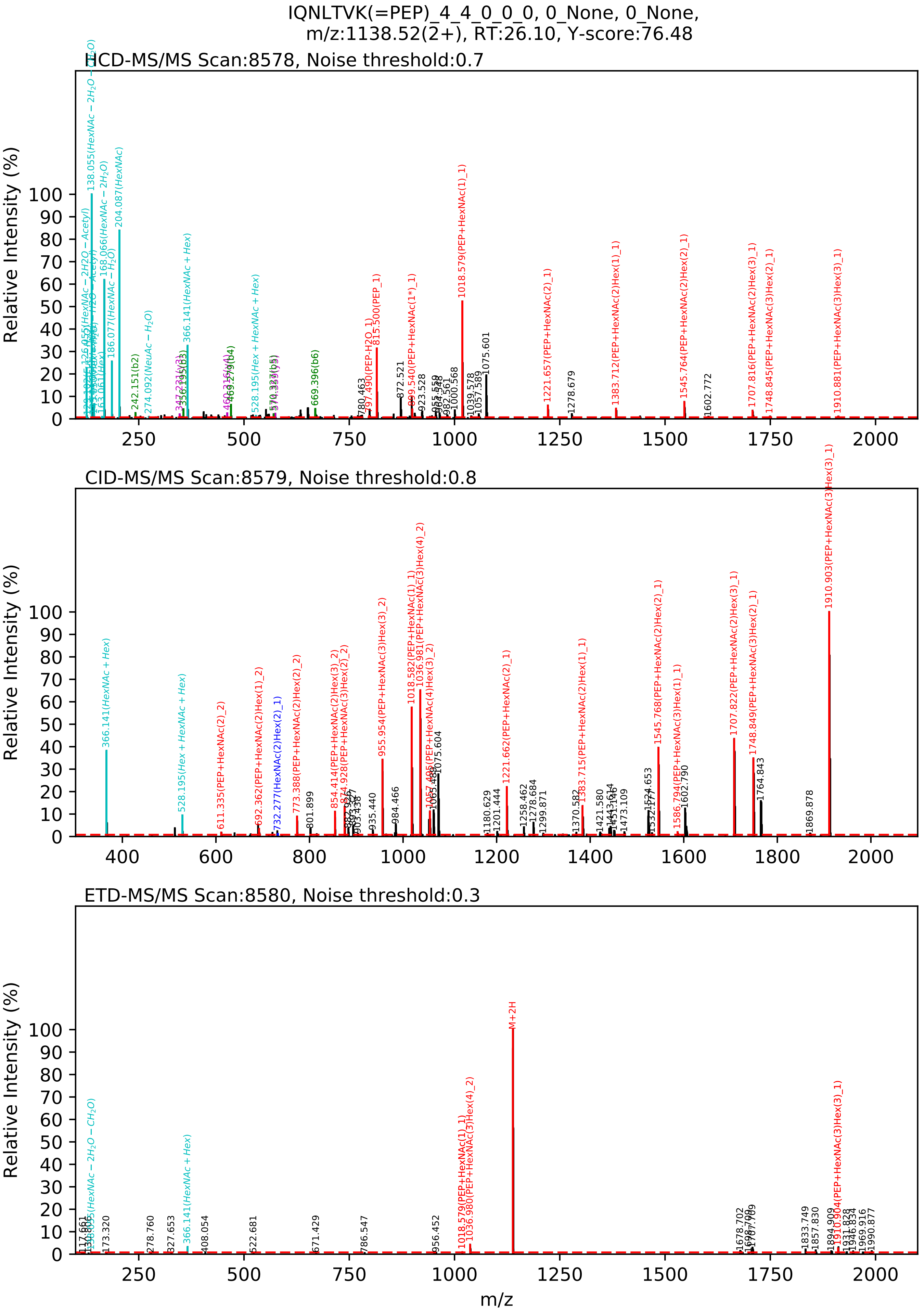
IQNLTVK(=PEP)_4_4_0_0_0, 0_None, 0_None,
m/z:1138.52(2+), RT:26.10, Y-score:76.48
HCD-MS/MS Scan:8578, Noise threshold:0.7
Relative Intensity (%)
138.055(HexNAc − 2H2O − H2O)
204.087(HexNAc)
168.066(HexNAc − 2H2O)
186.077(HexNAc − H2O)
366.141(HexNAc + Hex)
274.092(NeuAc − H2O)
242.151(b2)
528.195(Hex + HexNAc + Hex)
669.396(b6)
815.500(PEP_1)
797.490(PEP-H2O_1)
872.521
899.540(PEP+HexNAc(1*)_1)
923.528
1018.579(PEP+HexNAc(1)_1)
1075.601
1221.657(PEP+HexNAc(2)_1)
1278.679
1383.712(PEP+HexNAc(2)Hex(1)_1)
1545.764(PEP+HexNAc(2)Hex(2)_1)
1602.772
1707.816(PEP+HexNAc(2)Hex(3)_1)
1748.845(PEP+HexNAc(3)Hex(2)_1)
1910.881(PEP+HexNAc(3)Hex(3)_1)
CID-MS/MS Scan:8579, Noise threshold:0.8
Relative Intensity (%)
366.141(HexNAc + Hex)
528.195(Hex + HexNAc + Hex)
611.335(PEP+HexNAc(2)_2)
692.362(PEP+HexNAc(2)Hex(1)_2)
732.277(HexNAc(2)Hex(2)_1)
773.388(PEP+HexNAc(2)Hex(2)_2)
801.899
854.414(PEP+HexNAc(2)Hex(3)_2)
874.928(PEP+HexNAc(3)Hex(2)_2)
935.440
955.954(PEP+HexNAc(3)Hex(3)_2)
984.466
1018.582(PEP+HexNAc(1)_1)
1036.981(PEP+HexNAc(3)Hex(4)_2)
1057.495(PEP+HexNAc(4)Hex(3)_2)
1075.604
1180.629
1201.444
1221.662(PEP+HexNAc(2)_1)
1258.462
1278.684
1299.871
1370.582
1383.715(PEP+HexNAc(2)Hex(1)_1)
1421.580
1473.109
1524.653
1545.768(PEP+HexNAc(2)Hex(2)_1)
1586.794(PEP+HexNAc(3)Hex(1)_1)
1602.790
1707.822(PEP+HexNAc(2)Hex(3)_1)
1748.849(PEP+HexNAc(3)Hex(2)_1)
1764.843
1869.878
1910.903(PEP+HexNAc(3)Hex(3)_1)
ETD-MS/MS Scan:8580, Noise threshold:0.3
Relative Intensity (%)
138.055(HexNAc − 2H2O − CH2O)
173.320
278.760
327.653
366.141(HexNAc + Hex)
408.054
522.681
671.429
786.547
956.452
1018.579(PEP+HexNAc(1)_1)
1036.980(PEP+HexNAc(3)Hex(4)_2)
M+2H
1678.702
1833.749
1857.830
1894.909
1910.904(PEP+HexNAc(3)Hex(3)_1)
1931.828
1946.834
1969.916
1990.877
m/z

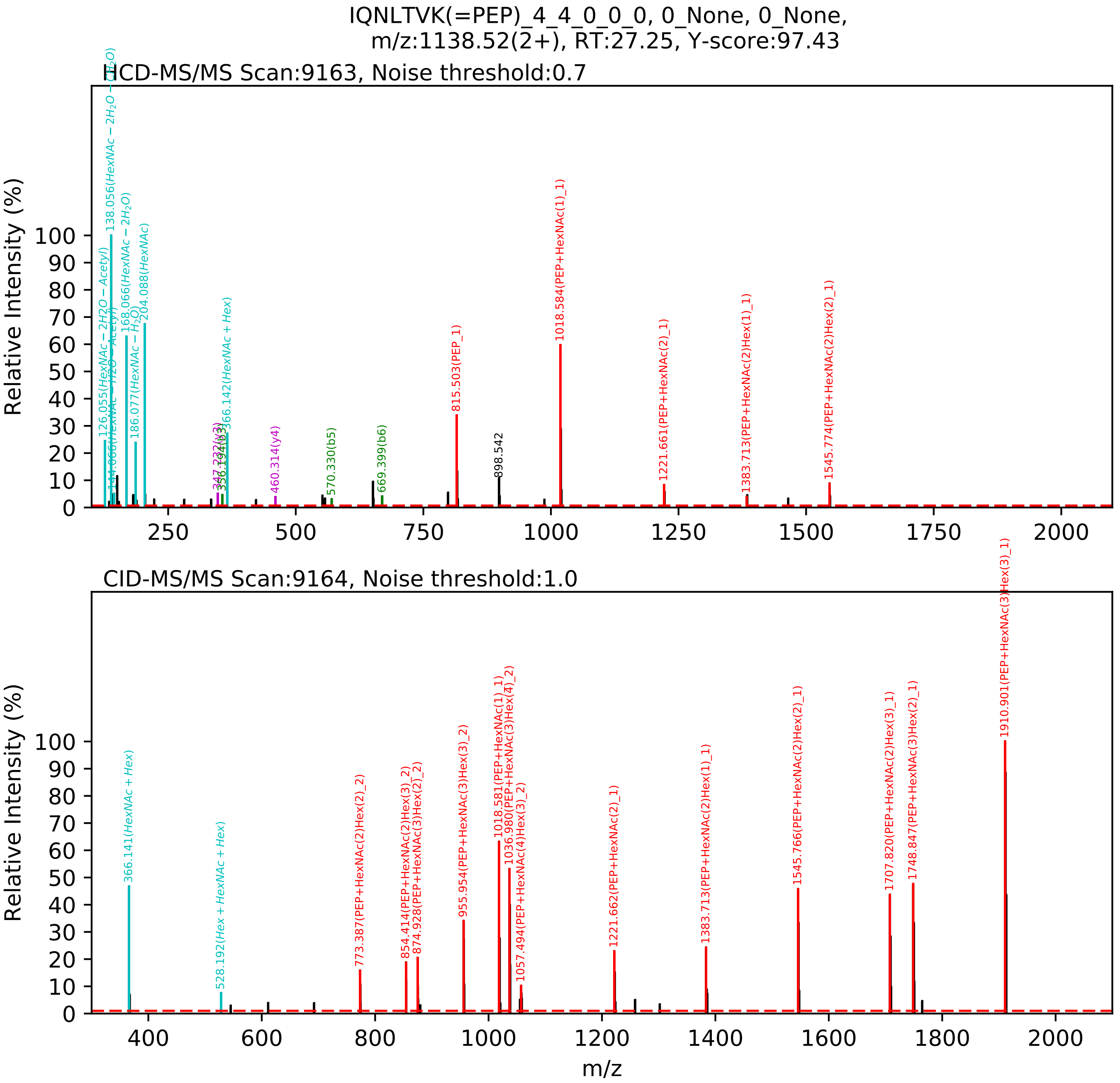
IQNLTVK(=PEP)_4_4_0_0_0, 0_None, 0_None,
m/z:1138.52(2+), RT:27.25, Y-score:97.43
HCD-MS/MS Scan:9163, Noise threshold:0.7
Relative Intensity (%)
126.055(HexNAc − 2H2O − Acetyl)
138.056(HexNAc − 2H2O)
168.066(HexNAc − 2H2O)
186.077(HexNAc − H2O)
204.088(HexNAc)
366.142(HexNAc + Hex)
460.314(y4)
570.330(b5)
669.399(b6)
815.503(PEP_1)
898.542
1018.584(PEP+HexNAc(1)_1)
1221.661(PEP+HexNAc(2)_1)
1383.713(PEP+HexNAc(2)Hex(1)_1)
1545.774(PEP+HexNAc(2)Hex(2)_1)
CID-MS/MS Scan:9164, Noise threshold:1.0
Relative Intensity (%)
366.141(HexNAc + Hex)
528.192(Hex + HexNAc + Hex)
773.387(PEP+HexNAc(2)Hex(2)_2)
854.414(PEP+HexNAc(2)Hex(3)_2)
874.928(PEP+HexNAc(3)Hex(2)_2)
955.954(PEP+HexNAc(3)Hex(3)_2)
1018.581(PEP+HexNAc(1)_1)
1036.980(PEP+HexNAc(3)Hex(4)_2)
1057.494(PEP+HexNAc(4)Hex(3)_2)
1221.662(PEP+HexNAc(2)_1)
1383.713(PEP+HexNAc(2)Hex(1)_1)
1545.766(PEP+HexNAc(2)Hex(2)_1)
1707.820(PEP+HexNAc(2)Hex(3)_1)
1748.847(PEP+HexNAc(3)Hex(2)_1)
1910.901(PEP+HexNAc(3)Hex(3)_1)
m/z

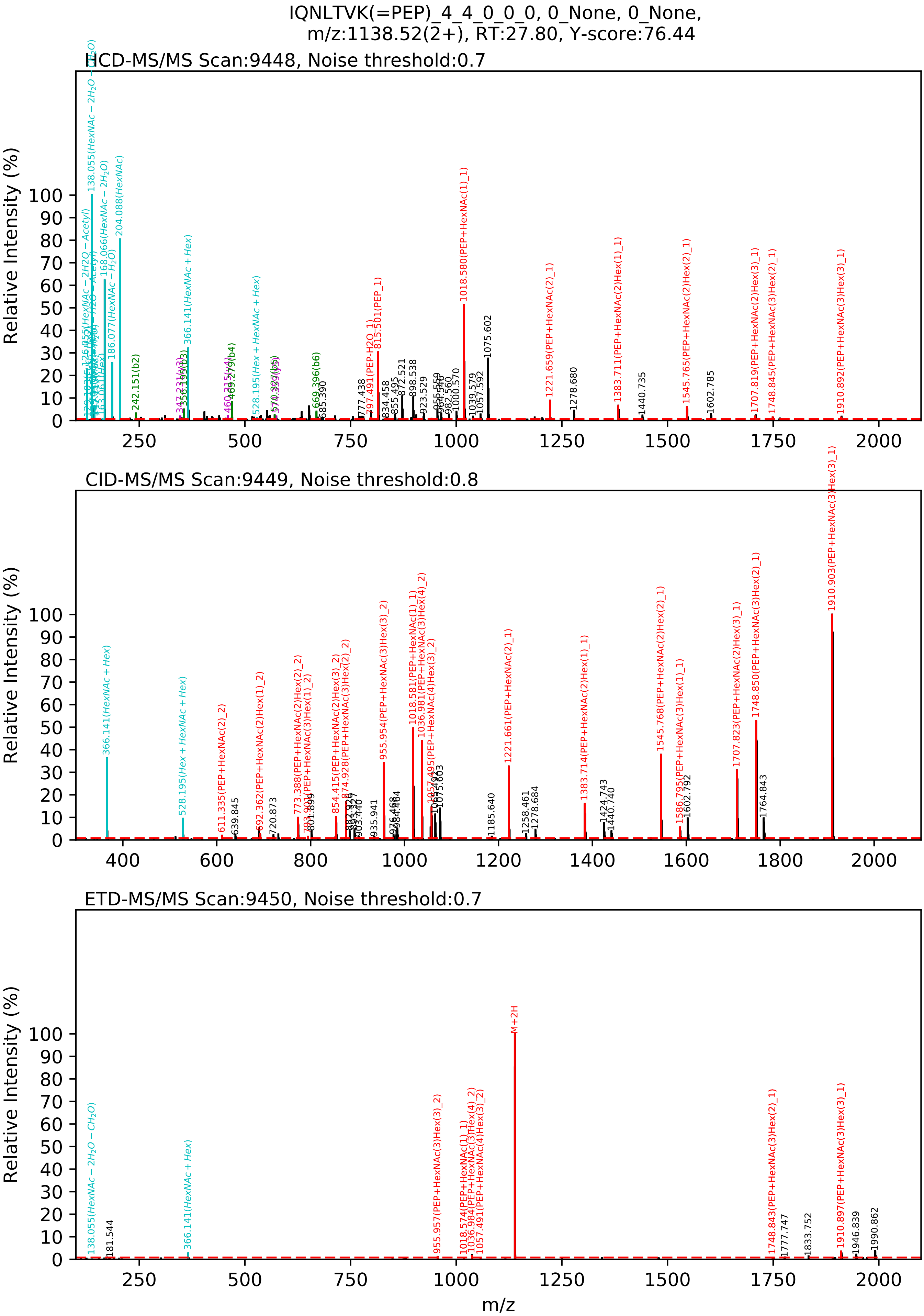
IQNLTVK(=PEP)_4_4_0_0_0, 0_None, 0_None,
m/z:1138.52(2+), RT:27.80, Y-score:76.44
HCD-MS/MS Scan:9448, Noise threshold:0.7
CID-MS/MS Scan:9449, Noise threshold:0.8
ETD-MS/MS Scan:9450, Noise threshold:0.7
Relative Intensity (%)
m/z

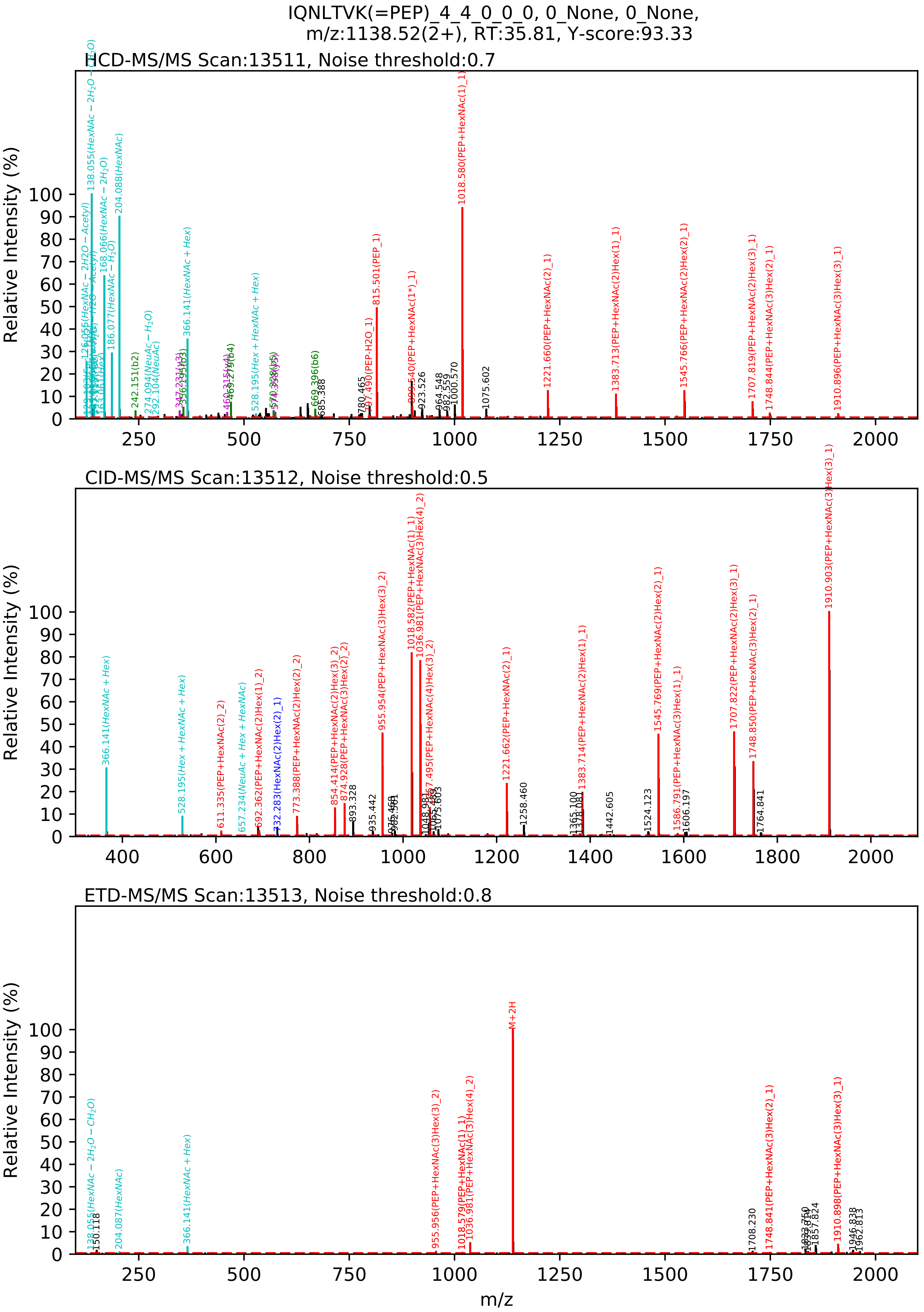
IQNLTVK(=PEP)_4_4_0_0_0, 0_None, 0_None,
m/z:1138.52(2+), RT:35.81, Y-score:93.33
HCD-MS/MS Scan:13511, Noise threshold:0.7
CID-MS/MS Scan:13512, Noise threshold:0.5
ETD-MS/MS Scan:13513, Noise threshold:0.8
Relative Intensity (%)
m/z
1018.580(PEP+HexNAc(1)_1)
815.501(PEP_1)
1221.660(PEP+HexNAc(2)_1)
1383.713(PEP+HexNAc(2)Hex(1)_1)
1545.766(PEP+HexNAc(2)Hex(2)_1)
1707.819(PEP+HexNAc(2)Hex(3)_1)
1748.844(PEP+HexNAc(3)Hex(2)_1)
1910.896(PEP+HexNAc(3)Hex(3)_1)
138.055(HexNAc − 2H2O − CH2O)
204.088(HexNAc)
366.141(HexNAc + Hex)
528.195(Hex + HexNAc + Hex)
1910.903(PEP+HexNAc(3)Hex(3)_1)
1018.582(PEP+HexNAc(1)_1)
1036.981(PEP+HexNAc(3)Hex(4)_2)
955.954(PEP+HexNAc(3)Hex(3)_2)
1221.662(PEP+HexNAc(2)_1)
1383.714(PEP+HexNAc(2)Hex(1)_1)
1545.769(PEP+HexNAc(2)Hex(2)_1)
1707.822(PEP+HexNAc(2)Hex(3)_1)
1748.850(PEP+HexNAc(3)Hex(2)_1)
M+2H
955.956(PEP+HexNAc(3)Hex(3)_2)
1018.579(PEP+HexNAc(1)_1)
1036.981(PEP+HexNAc(3)Hex(4)_2)
1748.841(PEP+HexNAc(3)Hex(2)_1)
1910.898(PEP+HexNAc(3)Hex(3)_1)

IQNLTVK(=PEP)_4_4_0_0_0, 0_None, 0_None,
m/z:1138.52(2+), RT:33.95, Y-score:91.17

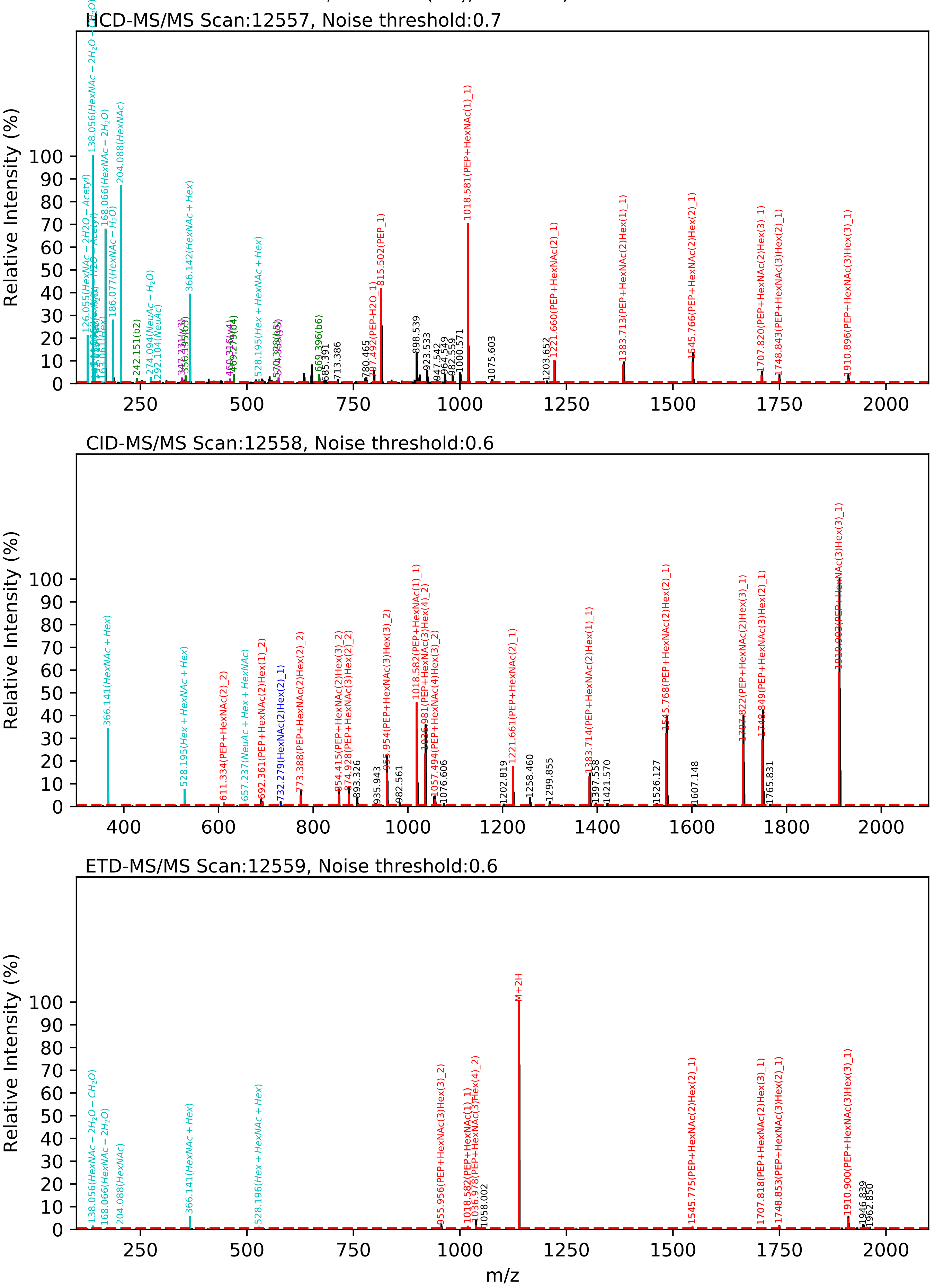

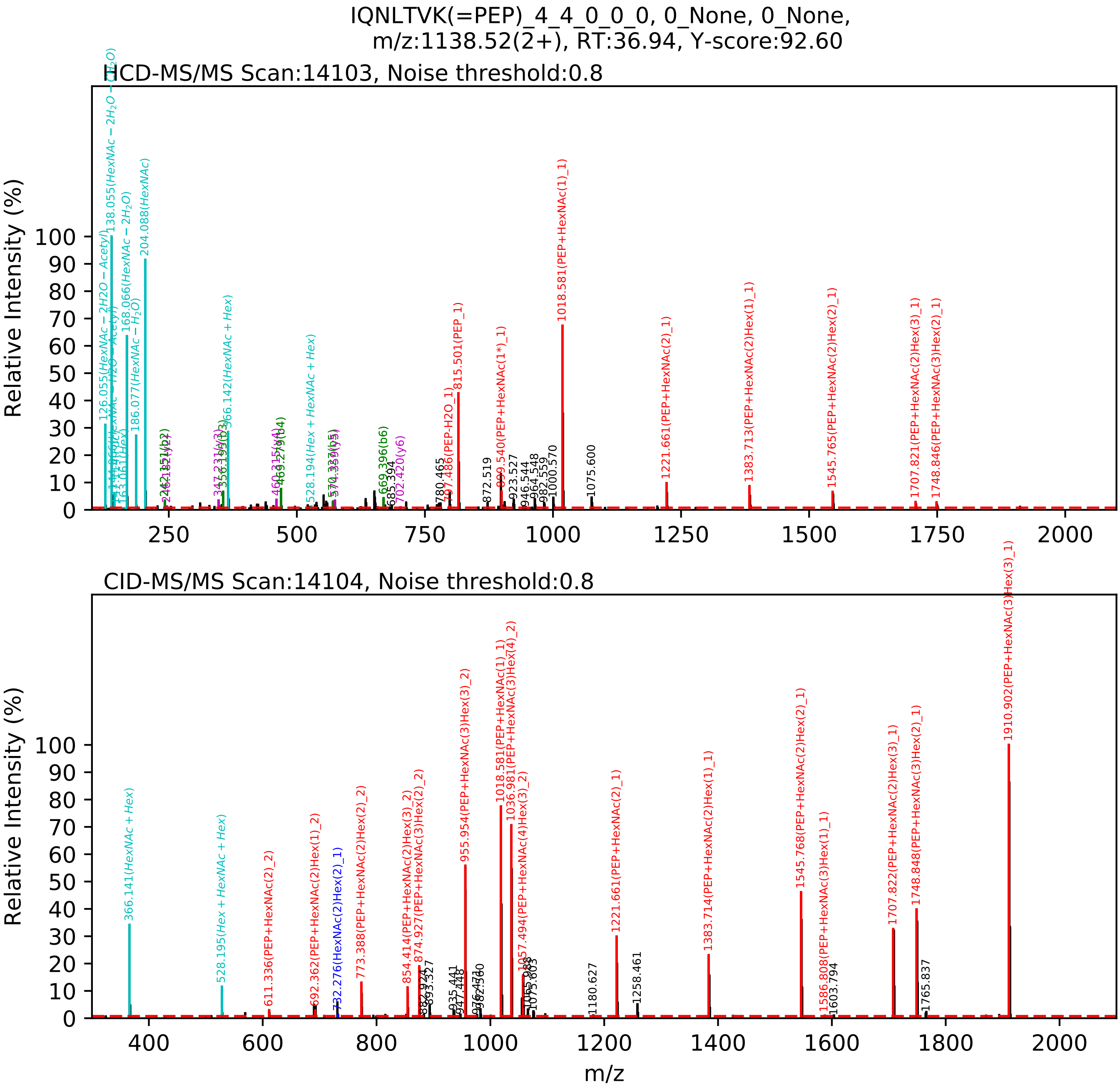

IQNLTVK(=PEP)_4_4_0_0_0, 0_None, 0_None,
m/z:1138.52(2+), RT:36.94, Y-score:92.60
HCD-MS/MS Scan:14103, Noise threshold:0.8
Relative Intensity (%)
138.055(HexNAc − 2H2O − Acetyl)
204.088(HexNAc)
168.066(HexNAc − 2H2O)
186.077(HexNAc − H2O)
126.055(HexNAc − 2H2O − Acetyl)
366.142(HexNAc + Hex)
528.194(Hex + HexNAc + Hex)
815.501(PEP_1)
797.486(PEP-H2O_1)
899.540(PEP+HexNAc(1*)_1)
1018.581(PEP+HexNAc(1)_1)
1221.661(PEP+HexNAc(2)_1)
1383.713(PEP+HexNAc(2)Hex(1)_1)
1545.765(PEP+HexNAc(2)Hex(2)_1)
1707.821(PEP+HexNAc(2)Hex(3)_1)
1748.846(PEP+HexNAc(3)Hex(2)_1)
469.279(b4)
669.396(b6)
702.420(y6)
570.337(b5)
780.465
872.519
923.527
946.544
964.548
1000.570
1075.600
CID-MS/MS Scan:14104, Noise threshold:0.8
Relative Intensity (%)
366.141(HexNAc + Hex)
528.195(Hex + HexNAc + Hex)
611.336(PEP+HexNAc(2)_2)
692.362(PEP+HexNAc(2)Hex(1)_2)
732.276(HexNAc(2)Hex(2)_1)
773.388(PEP+HexNAc(2)Hex(2)_2)
854.414(PEP+HexNAc(2)Hex(3)_2)
874.927(PEP+HexNAc(3)Hex(2)_2)
893.327
955.954(PEP+HexNAc(3)Hex(3)_2)
1018.581(PEP+HexNAc(1)_1)
1036.981(PEP+HexNAc(3)Hex(4)_2)
1057.494(PEP+HexNAc(4)Hex(3)_2)
1075.603
1180.627
1221.661(PEP+HexNAc(2)_1)
1258.461
1383.714(PEP+HexNAc(2)Hex(1)_1)
1545.768(PEP+HexNAc(2)Hex(2)_1)
1586.808(PEP+HexNAc(3)Hex(1)_1)
1603.794
1707.822(PEP+HexNAc(2)Hex(3)_1)
1748.848(PEP+HexNAc(3)Hex(2)_1)
1765.837
1910.902(PEP+HexNAc(3)Hex(3)_1)
m/z

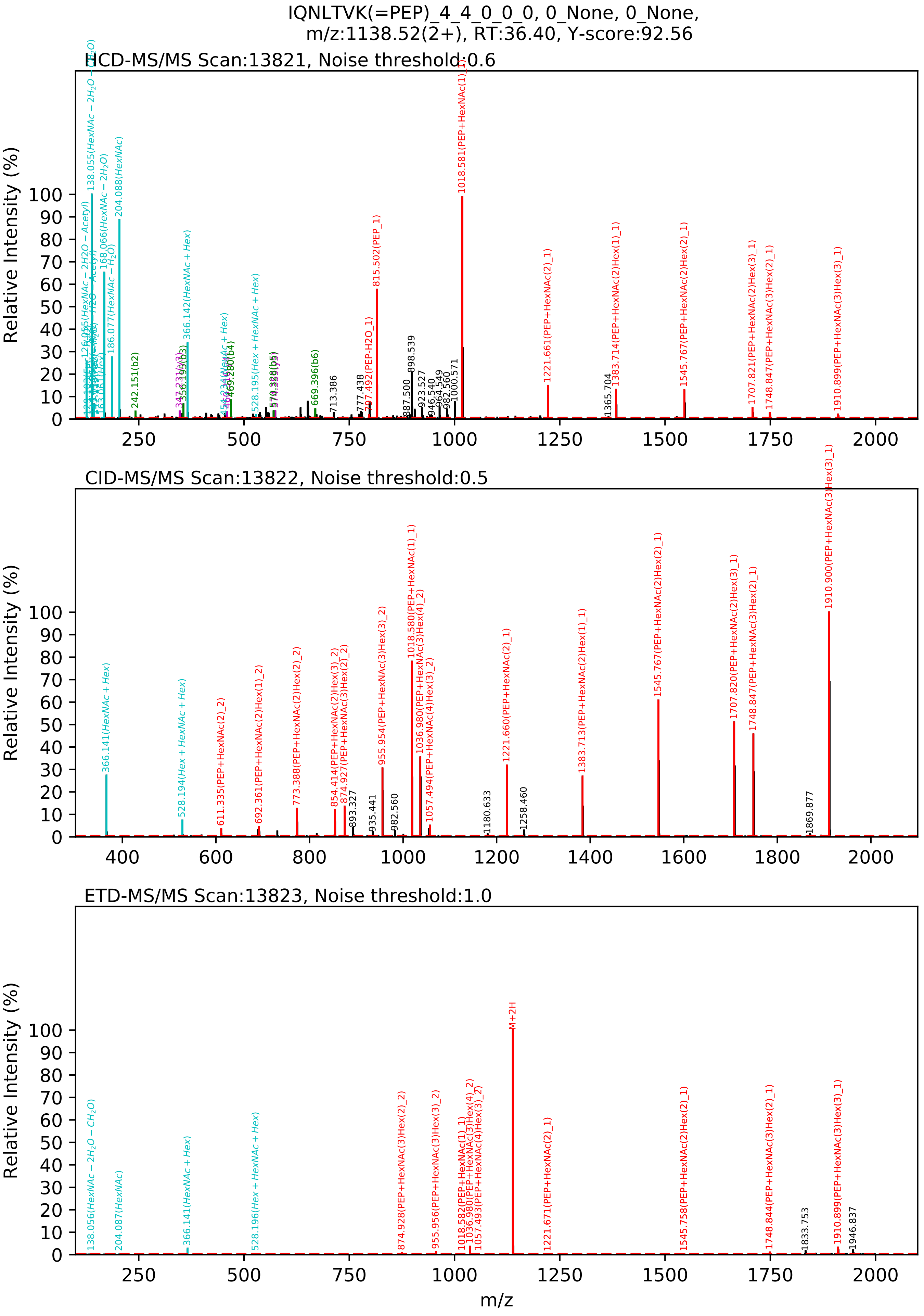
IQNLTVK(=PEP)_4_4_0_0_0, 0_None, 0_None,
m/z:1138.52(2+), RT:36.40, Y-score:92.56
HCD-MS/MS Scan:13821, Noise threshold:0.6
CID-MS/MS Scan:13822, Noise threshold:0.5
ETD-MS/MS Scan:13823, Noise threshold:1.0
Relative Intensity (%)
m/z
1018.581(PEP+HexNAc(1)_1)
815.502(PEP_1)
1221.661(PEP+HexNAc(2)_1)
1383.714(PEP+HexNAc(2)Hex(1)_1)
1545.767(PEP+HexNAc(2)Hex(2)_1)
1707.821(PEP+HexNAc(2)Hex(3)_1)
1748.847(PEP+HexNAc(3)Hex(2)_1)
1910.899(PEP+HexNAc(3)Hex(3)_1)
204.088(HexNAc)
366.142(HexNAc+Hex)
1910.900(PEP+HexNAc(3)Hex(3)_1)
1545.767(PEP+HexNAc(2)Hex(2)_1)
1707.820(PEP+HexNAc(2)Hex(3)_1)
1748.847(PEP+HexNAc(3)Hex(2)_1)
1018.580(PEP+HexNAc(1)_1)
955.954(PEP+HexNAc(3)Hex(3)_2)
M+2H

IQNLTVK(=PEP)_4_4_0_1_0, 0_None, 0_None,
m/z:1284.07(2+), RT:44.75, Y-score:94.32

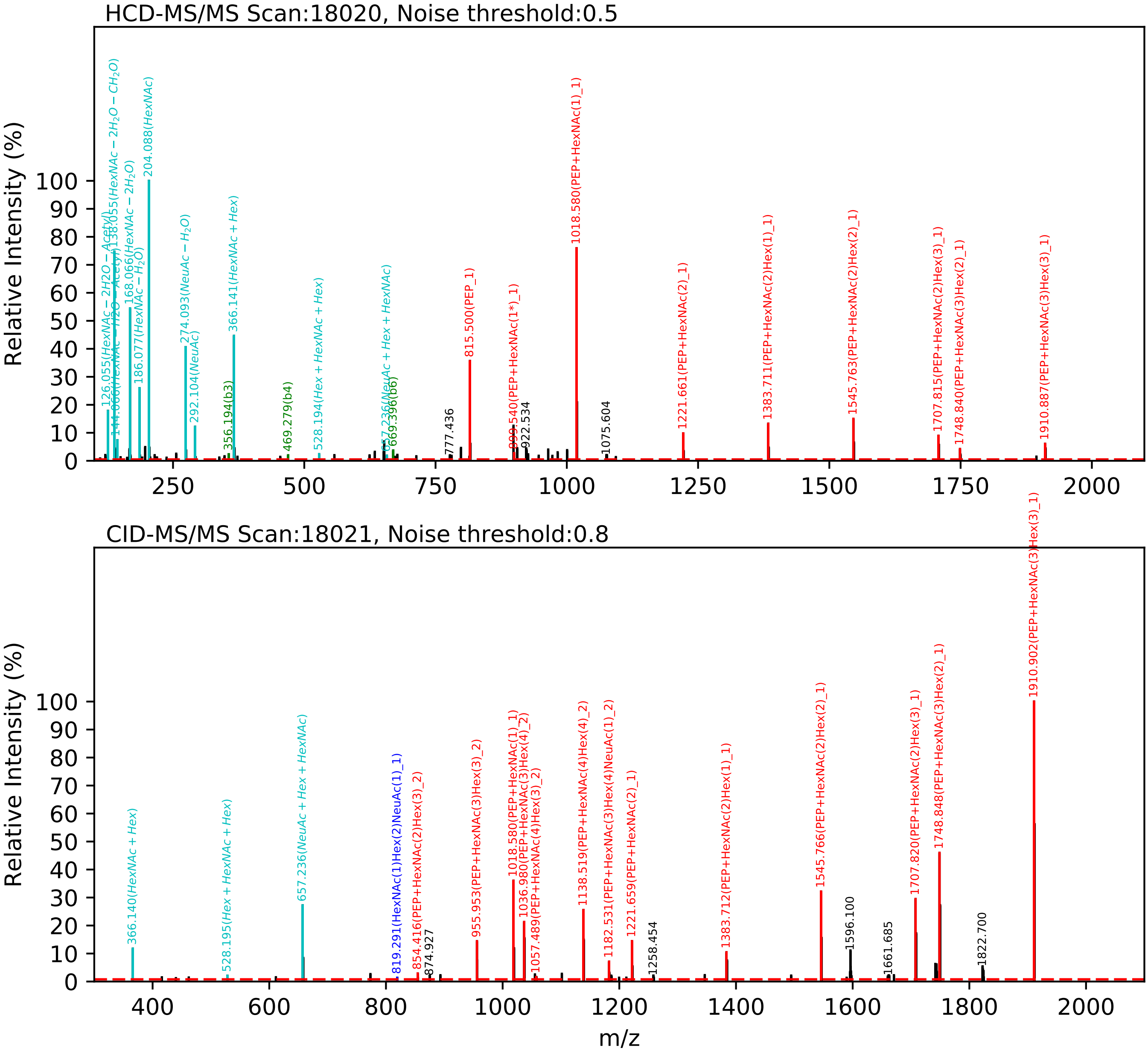

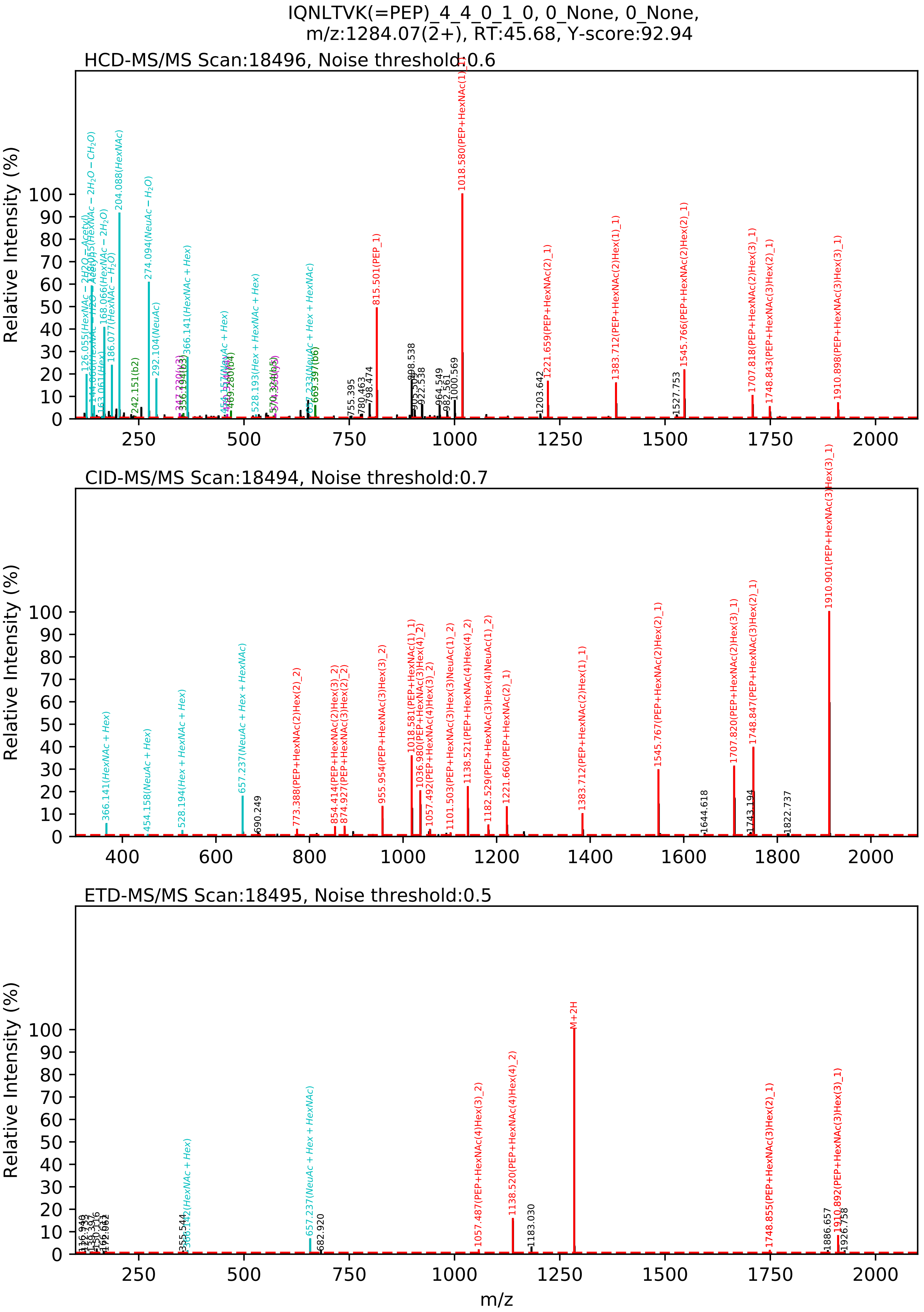
IQNLTVK(=PEP)_4_4_0_1_0, 0_None, 0_None,
m/z:1284.07(2+), RT:45.68, Y-score:92.94
HCD-MS/MS Scan:18496, Noise threshold:0.6
Relative Intensity (%)
1018.580(PEP+HexNAc(1)_1)
815.501(PEP_1)
1221.659(PEP+HexNAc(2)_1)
1383.712(PEP+HexNAc(2)Hex(1)_1)
1545.766(PEP+HexNAc(2)Hex(2)_1)
1707.818(PEP+HexNAc(2)Hex(3)_1)
1748.843(PEP+HexNAc(3)Hex(2)_1)
1910.898(PEP+HexNAc(3)Hex(3)_1)
204.088(HexNAc)
274.094(NeuAc − H2O)
292.104(NeuAc)
366.141(HexNAc + Hex)
528.193(Hex + HexNAc + Hex)
669.397(b6)
242.151(b2)
186.077(HexNAc − H2O)
168.066(HexNAc − 2H2O)
163.061(Hex)
126.055(HexNAc − 2H2O − CH2O)
898.538
922.538
1000.569
964.549
755.395
780.463
798.474
1203.642
1527.753
CID-MS/MS Scan:18494, Noise threshold:0.7
1910.901(PEP+HexNAc(3)Hex(3)_1)
366.141(HexNAc + Hex)
454.158(NeuAc + Hex)
528.194(Hex + HexNAc + Hex)
657.237(NeuAc + Hex + HexNAc)
690.249
773.388(PEP+HexNAc(2)Hex(2)_2)
854.414(PEP+HexNAc(2)Hex(3)_2)
874.927(PEP+HexNAc(3)Hex(2)_2)
955.954(PEP+HexNAc(3)Hex(3)_2)
1018.581(PEP+HexNAc(1)_1)
1036.980(PEP+HexNAc(3)Hex(4)_2)
1057.492(PEP+HexNAc(4)Hex(3)_2)
1101.503(PEP+HexNAc(3)Hex(3)NeuAc(1)_2)
1138.521(PEP+HexNAc(4)Hex(4)_2)
1182.529(PEP+HexNAc(3)Hex(4)NeuAc(1)_2)
1221.660(PEP+HexNAc(2)_1)
1383.712(PEP+HexNAc(2)Hex(1)_1)
1545.767(PEP+HexNAc(2)Hex(2)_1)
1644.618
1707.820(PEP+HexNAc(2)Hex(3)_1)
1748.847(PEP+HexNAc(3)Hex(2)_1)
1743.194
1822.737
ETD-MS/MS Scan:18495, Noise threshold:0.5
M+2H
366.142(HexNAc + Hex)
355.544
657.237(NeuAc + Hex + HexNAc)
682.920
1057.487(PEP+HexNAc(4)Hex(3)_2)
1138.520(PEP+HexNAc(4)Hex(4)_2)
1183.030
1748.855(PEP+HexNAc(3)Hex(2)_1)
1886.657
1910.892(PEP+HexNAc(3)Hex(3)_1)
1926.758
m/z

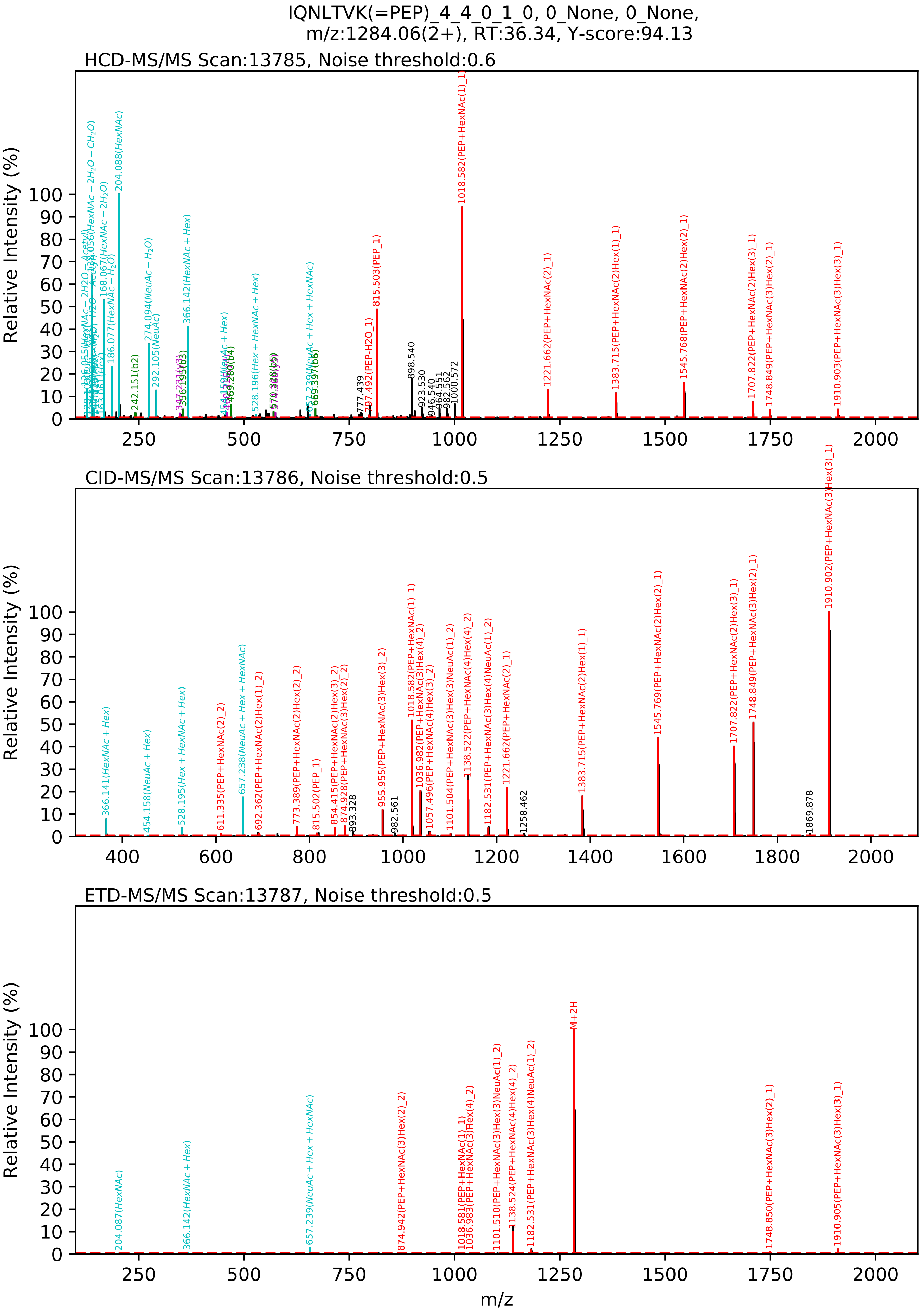

IQNLTVK(=PEP)_4_4_0_1_0, 0_None, 0_None,
m/z:1284.06(2+), RT:36.34, Y-score:94.13
HCD-MS/MS Scan:13785, Noise threshold:0.6
Relative Intensity (%)
204.088(HexNAc)
366.142(HexNAc + Hex)
274.094(NeuAc − H2O)
292.105(NeuAc)
168.067(HexNAc − 2H2O)
186.077(HexNAc − H2O)
242.151(b2)
528.196(Hex + HexNAc + Hex)
815.503(PEP_1)
898.540
923.530
1000.572
1018.582(PEP+HexNAc(1)_1)
1221.662(PEP+HexNAc(2)_1)
1383.715(PEP+HexNAc(2)Hex(1)_1)
1545.768(PEP+HexNAc(2)Hex(2)_1)
1707.822(PEP+HexNAc(2)Hex(3)_1)
1748.849(PEP+HexNAc(3)Hex(2)_1)
1910.903(PEP+HexNAc(3)Hex(3)_1)
CID-MS/MS Scan:13786, Noise threshold:0.5
366.141(HexNAc + Hex)
454.158(NeuAc + Hex)
528.195(Hex + HexNAc + Hex)
611.335(PEP+HexNAc(2)_2)
657.238(NeuAc + Hex + HexNAc)
692.362(PEP+HexNAc(2)Hex(1)_2)
773.389(PEP+HexNAc(2)Hex(2)_2)
815.502(PEP_1)
854.415(PEP+HexNAc(2)Hex(3)_2)
874.928(PEP+HexNAc(3)Hex(2)_2)
893.328
955.955(PEP+HexNAc(3)Hex(3)_2)
982.561
1018.582(PEP+HexNAc(1)_1)
1036.982(PEP+HexNAc(3)Hex(4)_2)
1057.496(PEP+HexNAc(4)Hex(3)_2)
1101.504(PEP+HexNAc(3)Hex(3)NeuAc(1)_2)
1138.522(PEP+HexNAc(4)Hex(4)_2)
1182.531(PEP+HexNAc(3)Hex(4)NeuAc(1)_2)
1221.662(PEP+HexNAc(2)_1)
1258.462
1383.715(PEP+HexNAc(2)Hex(1)_1)
1545.769(PEP+HexNAc(2)Hex(2)_1)
1707.822(PEP+HexNAc(2)Hex(3)_1)
1748.849(PEP+HexNAc(3)Hex(2)_1)
1869.878
1910.902(PEP+HexNAc(3)Hex(3)_1)
ETD-MS/MS Scan:13787, Noise threshold:0.5
204.087(HexNAc)
366.142(HexNAc + Hex)
657.239(NeuAc + Hex + HexNAc)
874.942(PEP+HexNAc(3)Hex(2)_2)
1018.581(PEP+HexNAc(1)_1)
1036.983(PEP+HexNAc(3)Hex(4)_2)
1101.510(PEP+HexNAc(3)Hex(3)NeuAc(1)_2)
1138.524(PEP+HexNAc(4)Hex(4)_2)
1182.531(PEP+HexNAc(3)Hex(4)NeuAc(1)_2)
M+2H
1748.850(PEP+HexNAc(3)Hex(2)_1)
1910.905(PEP+HexNAc(3)Hex(3)_1)
m/z

IQNLTVK(=PEP)_4_4_0_1_0, 0_None, 0_None,
m/z:1284.06(2+), RT:36.38, Y-score:94.32

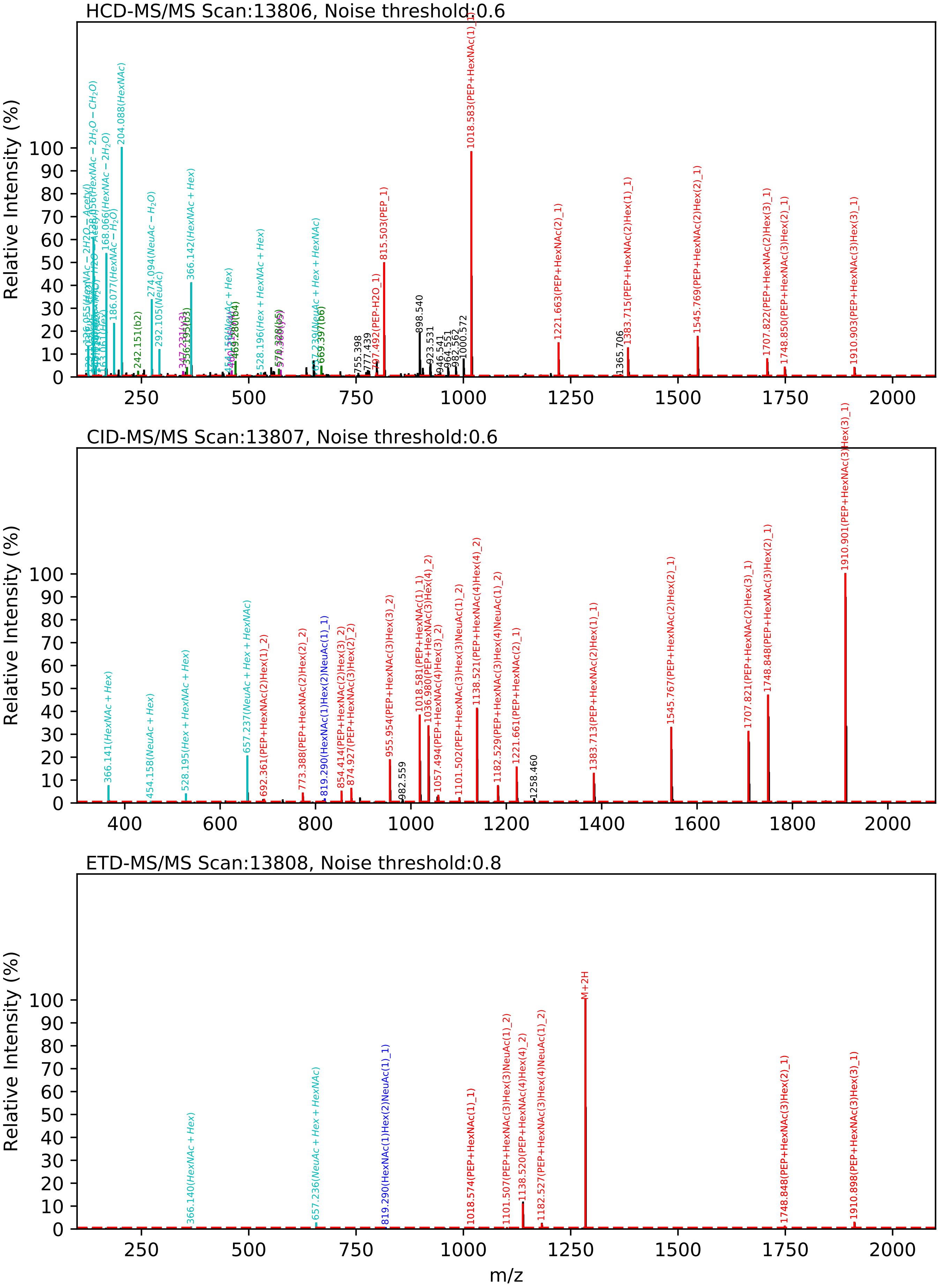

IQNLTVK(=PEP)_4_4_0_1_0, 0_None, 0_None,
m/z:856.38(3+), RT:40.72, Y-score:92.95

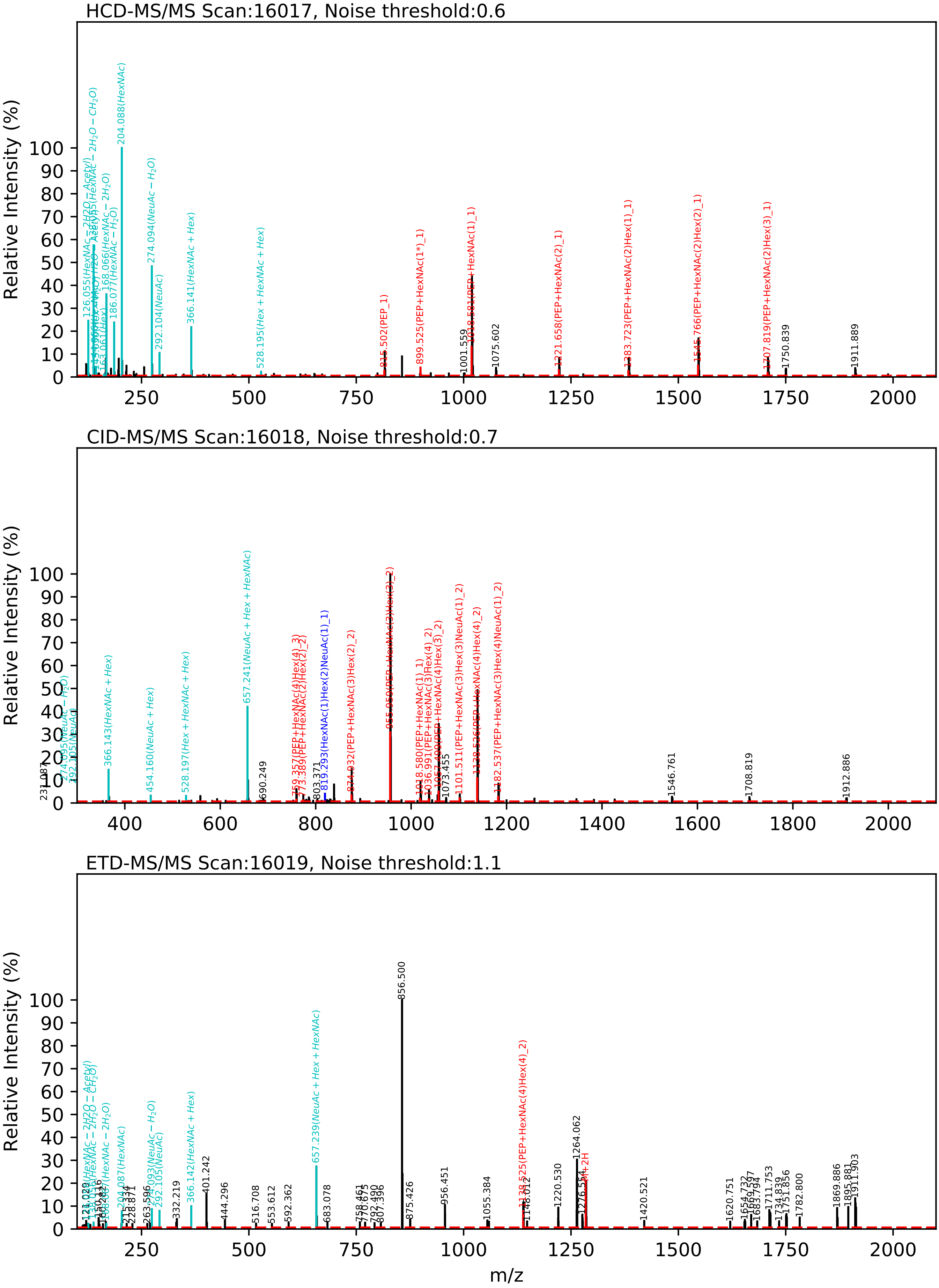

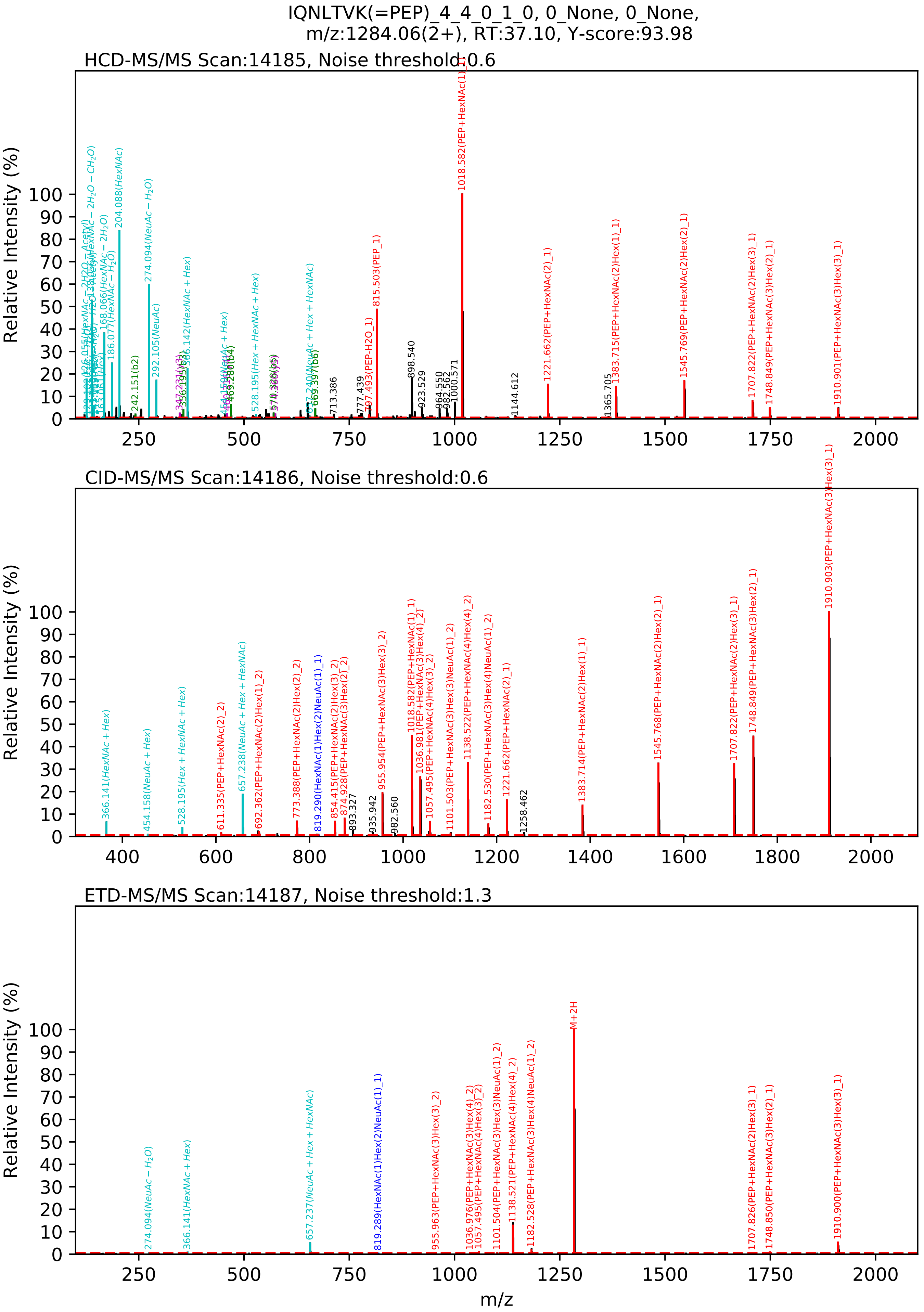

IQNLTVK(=PEP)_4_4_0_1_0, 0_None, 0_None,
m/z:1284.06(2+), RT:37.10, Y-score:93.98
HCD-MS/MS Scan:14185, Noise threshold:0.6
Relative Intensity (%)
204.088(HexNAc)
274.094(NeuAc − H2O)
292.105(NeuAc)
186.077(HexNAc − H2O)
168.066(HexNAc − 2H2O)
366.142(HexNAc + Hex)
242.151(b2)
469.280(b4)
528.195(Hex + HexNAc + Hex)
669.397(b6)
713.386
777.439
815.503(PEP_1)
898.540
923.529
964.550
1000.571
1018.582(PEP+HexNAc(1)_1)
1144.612
1221.662(PEP+HexNAc(2)_1)
1365.705
1383.715(PEP+HexNAc(2)Hex(1)_1)
1545.769(PEP+HexNAc(2)Hex(2)_1)
1707.822(PEP+HexNAc(2)Hex(3)_1)
1748.849(PEP+HexNAc(3)Hex(2)_1)
1910.901(PEP+HexNAc(3)Hex(3)_1)
CID-MS/MS Scan:14186, Noise threshold:0.6
366.141(HexNAc + Hex)
454.158(NeuAc + Hex)
528.195(Hex + HexNAc + Hex)
611.335(PEP+HexNAc(2)_2)
657.238(NeuAc + Hex + HexNAc)
692.362(PEP+HexNAc(2)Hex(1)_2)
773.388(PEP+HexNAc(2)Hex(2)_2)
819.290(HexNAc(1)Hex(2)NeuAc(1)_1)
854.415(PEP+HexNAc(2)Hex(3)_2)
874.928(PEP+HexNAc(3)Hex(2)_2)
893.327
935.942
955.954(PEP+HexNAc(3)Hex(3)_2)
982.560
1018.582(PEP+HexNAc(1)_1)
1036.981(PEP+HexNAc(3)Hex(4)_2)
1057.495(PEP+HexNAc(4)Hex(3)_2)
1101.503(PEP+HexNAc(3)Hex(3)NeuAc(1)_2)
1138.522(PEP+HexNAc(4)Hex(4)_2)
1182.530(PEP+HexNAc(3)Hex(4)NeuAc(1)_2)
1221.662(PEP+HexNAc(2)_1)
1258.462
1383.714(PEP+HexNAc(2)Hex(1)_1)
1545.768(PEP+HexNAc(2)Hex(2)_1)
1707.822(PEP+HexNAc(2)Hex(3)_1)
1748.849(PEP+HexNAc(3)Hex(2)_1)
1910.903(PEP+HexNAc(3)Hex(3)_1)
ETD-MS/MS Scan:14187, Noise threshold:1.3
274.094(NeuAc − H2O)
366.141(HexNAc + Hex)
657.237(NeuAc + Hex + HexNAc)
819.289(HexNAc(1)Hex(2)NeuAc(1)_1)
955.963(PEP+HexNAc(3)Hex(3)_2)
1036.976(PEP+HexNAc(3)Hex(4)_2)
1057.495(PEP+HexNAc(4)Hex(3)_2)
1101.504(PEP+HexNAc(3)Hex(3)NeuAc(1)_2)
1138.521(PEP+HexNAc(4)Hex(4)_2)
1182.528(PEP+HexNAc(3)Hex(4)NeuAc(1)_2)
M+2H
1707.826(PEP+HexNAc(2)Hex(3)_1)
1748.850(PEP+HexNAc(3)Hex(2)_1)
1910.900(PEP+HexNAc(3)Hex(3)_1)
m/z

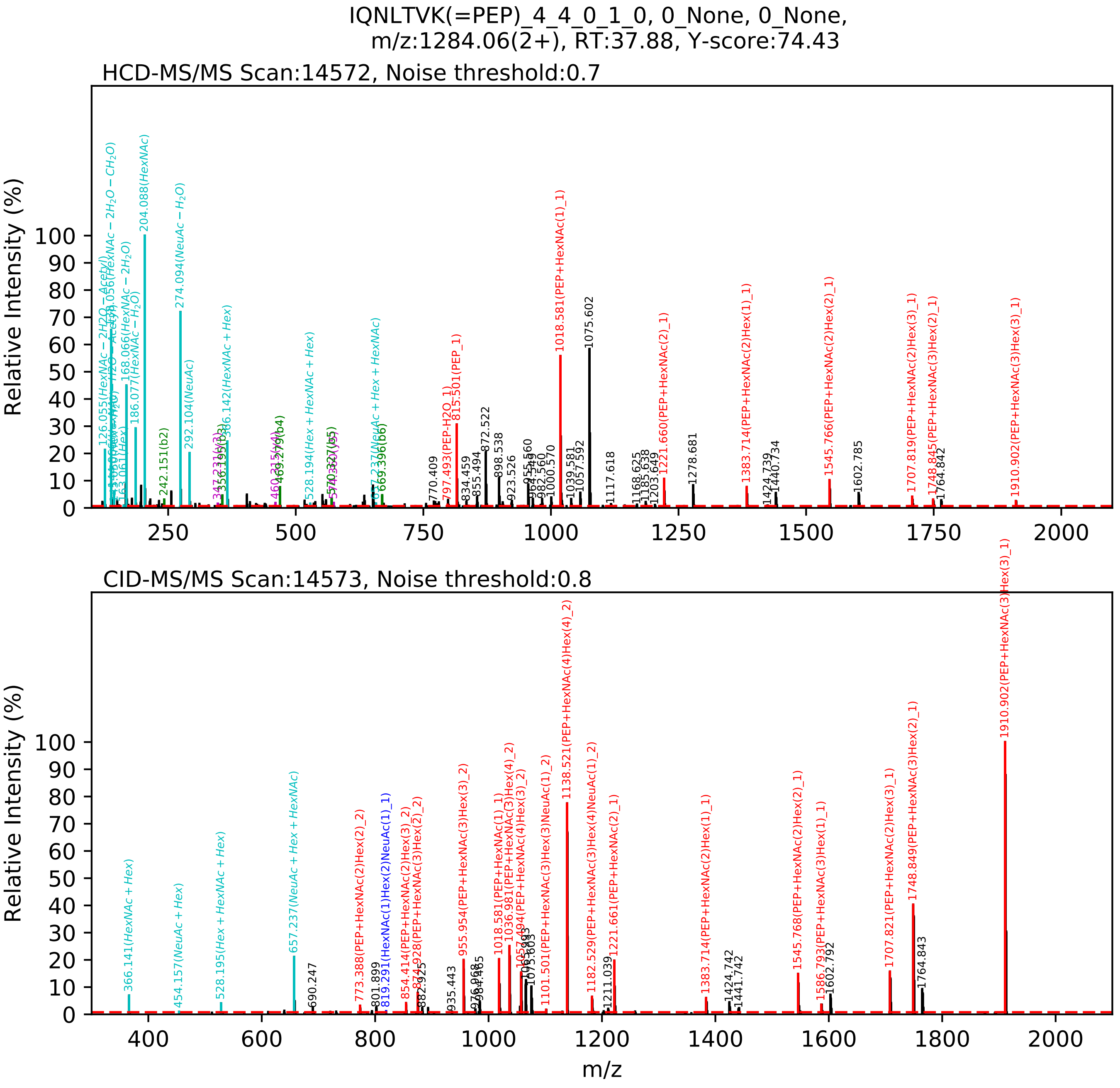
IQNLTVK(=PEP)_4_4_0_1_0, 0_None, 0_None,
m/z:1284.06(2+), RT:37.88, Y-score:74.43
HCD-MS/MS Scan:14572, Noise threshold:0.7
Relative Intensity (%)
204.088(HexNAc)
274.094(NeuAc − H2O)
1018.581(PEP+HexNAc(1)_1)
1075.602
815.501(PEP_1)
1221.660(PEP+HexNAc(2)_1)
1383.714(PEP+HexNAc(2)Hex(1)_1)
1545.766(PEP+HexNAc(2)Hex(2)_1)
1707.819(PEP+HexNAc(2)Hex(3)_1)
1748.845(PEP+HexNAc(3)Hex(2)_1)
1910.902(PEP+HexNAc(3)Hex(3)_1)
CID-MS/MS Scan:14573, Noise threshold:0.8
Relative Intensity (%)
1910.902(PEP+HexNAc(3)Hex(3)_1)
1138.521(PEP+HexNAc(4)Hex(4)_2)
1748.849(PEP+HexNAc(3)Hex(2)_1)
1707.821(PEP+HexNAc(2)Hex(3)_1)
1545.768(PEP+HexNAc(2)Hex(2)_1)
1221.661(PEP+HexNAc(2)_1)
1018.581(PEP+HexNAc(1)_1)
1036.981(PEP+HexNAc(3)Hex(4)_2)
657.237(NeuAc + Hex + HexNAc)
m/z

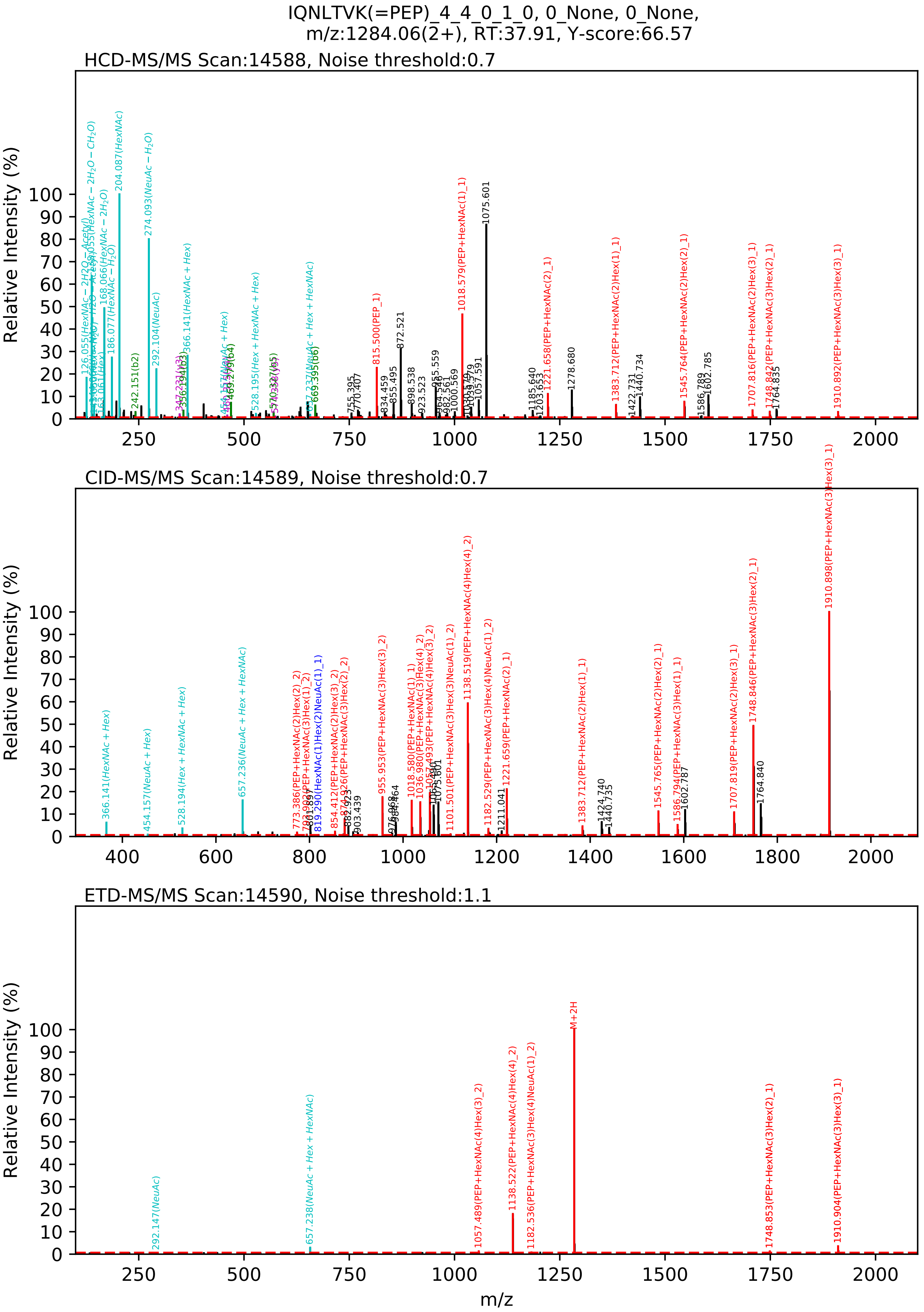

IQNLTVK(=PEP)_4_4_0_1_0, 0_None, 0_None,
m/z:1284.06(2+), RT:37.91, Y-score:66.57
HCD-MS/MS Scan:14588, Noise threshold:0.7
CID-MS/MS Scan:14589, Noise threshold:0.7
ETD-MS/MS Scan:14590, Noise threshold:1.1
Relative Intensity (%)
m/z
1018.579(PEP+HexNAc(1)_1)
1075.601
815.500(PEP_1)
872.521
1221.658(PEP+HexNAc(2)_1)
1383.712(PEP+HexNAc(2)Hex(1)_1)
1545.764(PEP+HexNAc(2)Hex(2)_1)
1707.816(PEP+HexNAc(2)Hex(3)_1)
1748.842(PEP+HexNAc(3)Hex(2)_1)
1910.892(PEP+HexNAc(3)Hex(3)_1)
204.087(HexNAc)
274.093(NeuAc − H2O)
292.104(NeuAc)
366.141(HexNAc + Hex)
1910.898(PEP+HexNAc(3)Hex(3)_1)
1138.519(PEP+HexNAc(4)Hex(4)_2)
1748.846(PEP+HexNAc(3)Hex(2)_1)
955.953(PEP+HexNAc(3)Hex(3)_2)
657.236(NeuAc + Hex + HexNAc)
M+2H
1138.522(PEP+HexNAc(4)Hex(4)_2)
1182.536(PEP+HexNAc(3)Hex(4)NeuAc(1)_2)
1057.489(PEP+HexNAc(4)Hex(3)_2)
1748.853(PEP+HexNAc(3)Hex(2)_1)
1910.904(PEP+HexNAc(3)Hex(3)_1)
292.147(NeuAc)
657.238(NeuAc + Hex + HexNAc)

IQNLTVK(=PEP)_4_4_0_1_0, 0_None, 0_None,
m/z:1284.06(2+), RT:40.60, Y-score:56.15

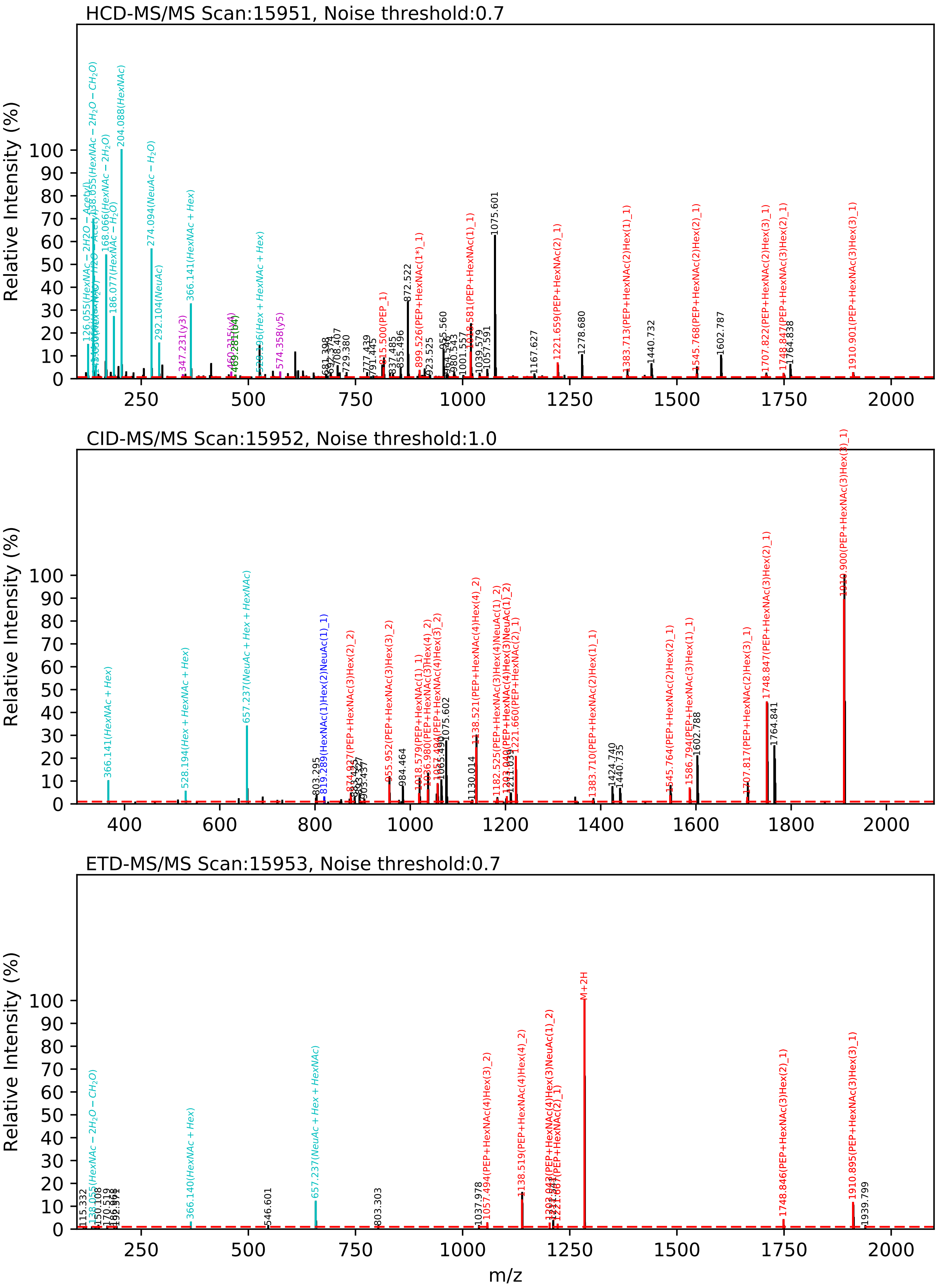

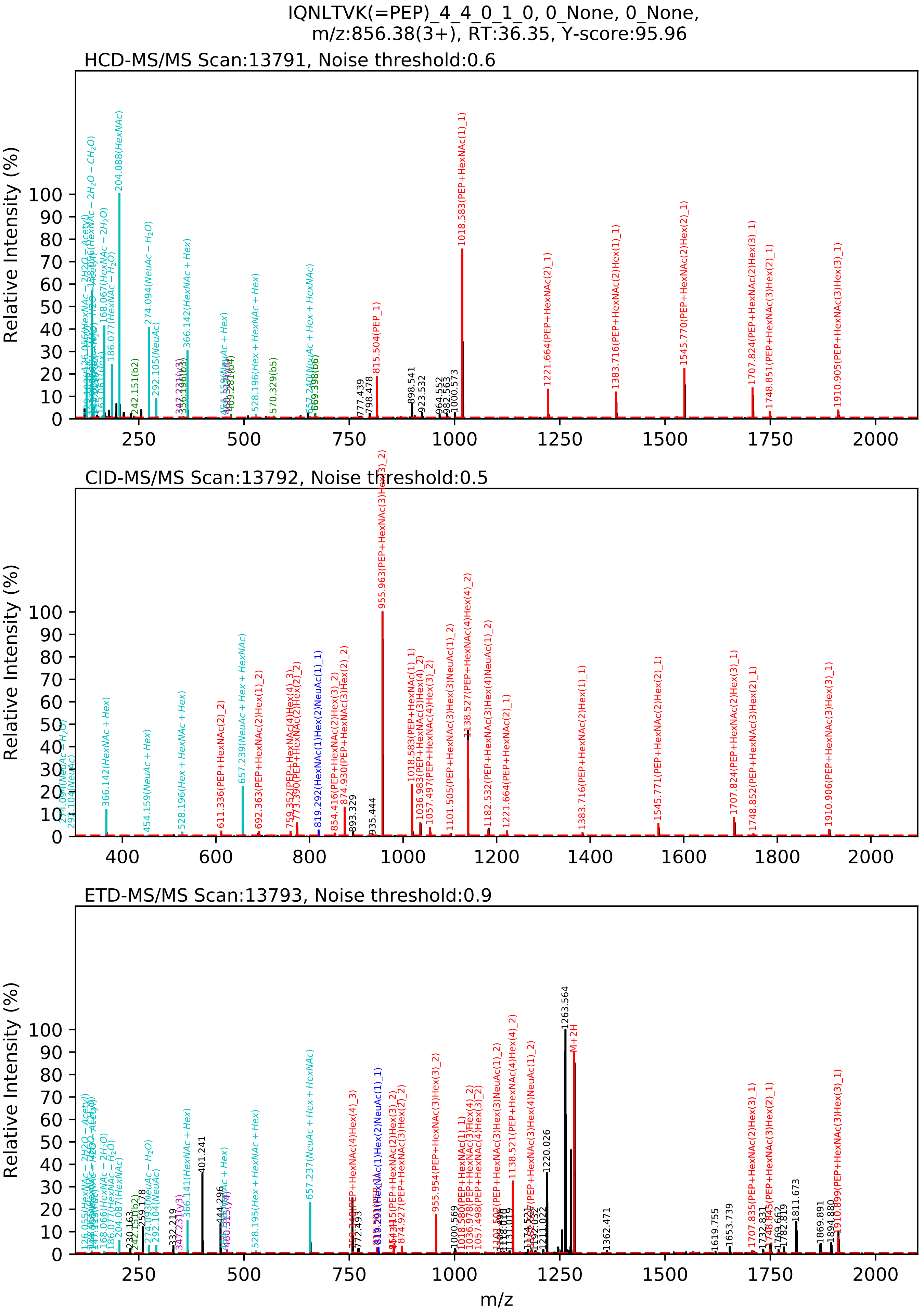

IQNLTVK(=PEP)_4_4_0_1_0, 0_None, 0_None,
m/z:856.38(3+), RT:36.35, Y-score:95.96
HCD-MS/MS Scan:13791, Noise threshold:0.6
CID-MS/MS Scan:13792, Noise threshold:0.5
ETD-MS/MS Scan:13793, Noise threshold:0.9
Relative Intensity (%)
m/z
204.088(HexNAc)
274.094(NeuAc − H2O)
366.142(HexNAc + Hex)
815.504(PEP_1)
1018.583(PEP+HexNAc(1)_1)
1221.664(PEP+HexNAc(2)_1)
1383.716(PEP+HexNAc(2)Hex(1)_1)
1545.770(PEP+HexNAc(2)Hex(2)_1)
1707.824(PEP+HexNAc(2)Hex(3)_1)
1748.851(PEP+HexNAc(3)Hex(2)_1)
1910.905(PEP+HexNAc(3)Hex(3)_1)
955.963(PEP+HexNAc(3)Hex(3)_2)
1138.527(PEP+HexNAc(4)Hex(4)_2)
657.239(NeuAc + Hex + HexNAc)
1263.564
M+2H
1220.026
401.241
1811.673

IQNLTVK(=PEP)_4_4_0_1_0, 0_None, 0_None,
m/z:856.38(3+), RT:37.11, Y-score:96.38

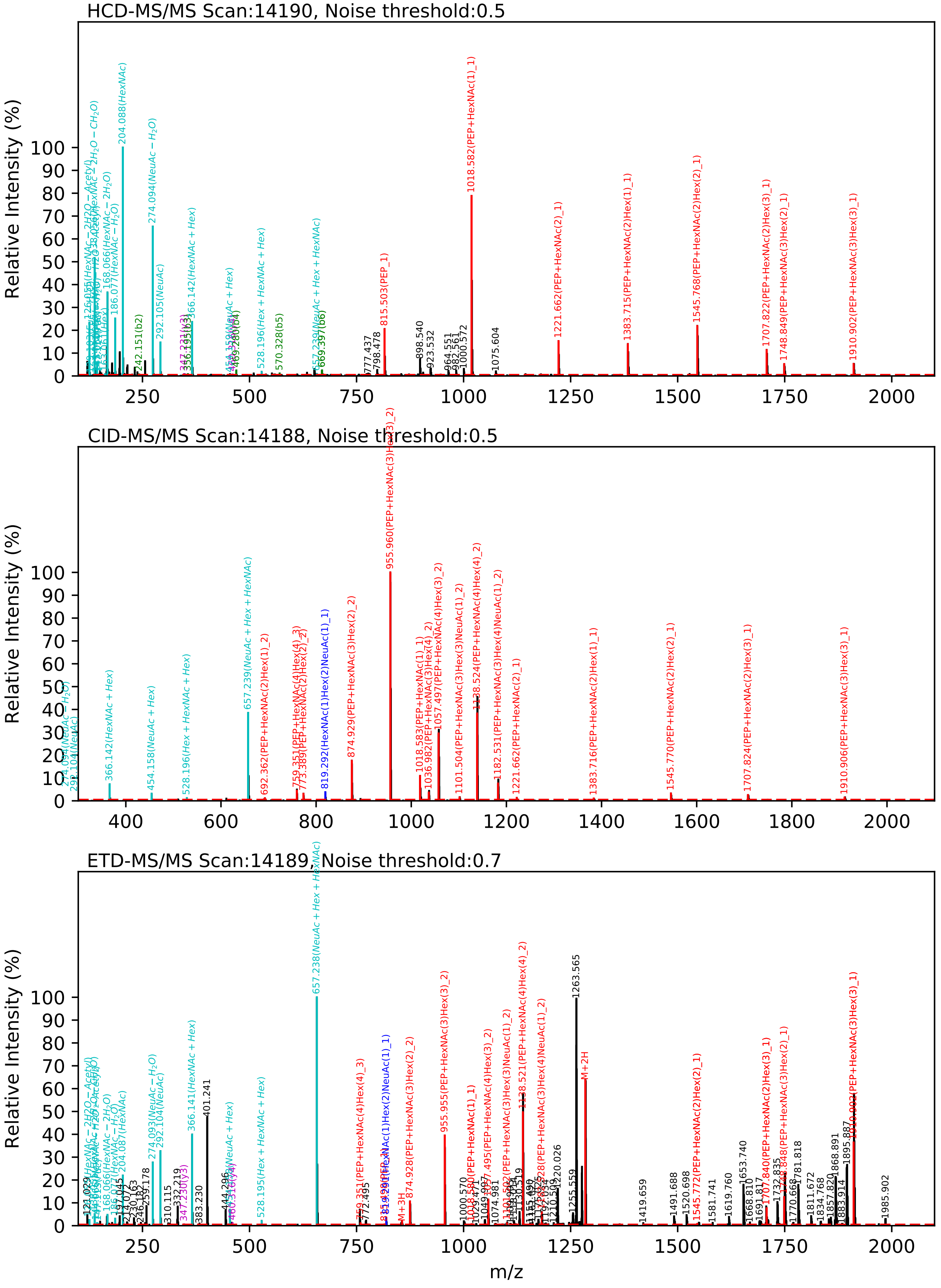

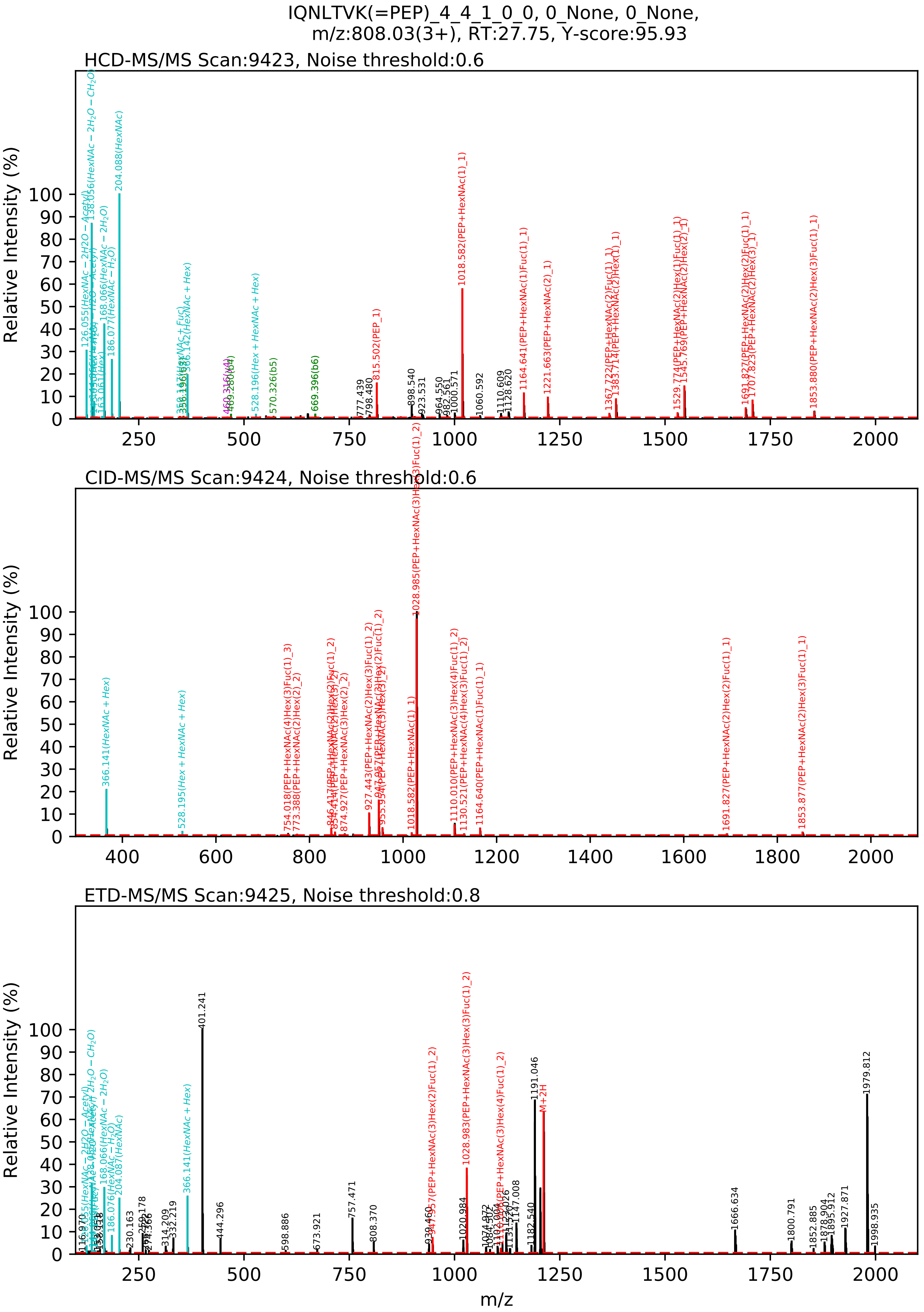
IQNLTVK(=PEP)_4_4_1_0_0, 0_None, 0_None,
m/z:808.03(3+), RT:27.75, Y-score:95.93
HCD-MS/MS Scan:9423, Noise threshold:0.6
CID-MS/MS Scan:9424, Noise threshold:0.6
ETD-MS/MS Scan:9425, Noise threshold:0.8
Relative Intensity (%)
m/z
204.088(HexNAc)
168.066(HexNAc − 2H2O)
186.077(HexNAc − H2O)
126.055(HexNAc − 2H2O − Acetyl)
366.142(HexNAc + Hex)
528.196(Hex + HexNAc + Hex)
570.326(b5)
669.396(b6)
815.502(PEP_1)
1018.582(PEP+HexNAc(1)_1)
1164.641(PEP+HexNAc(1)Fuc(1)_1)
1221.663(PEP+HexNAc(2)_1)
1383.714(PEP+HexNAc(2)Hex(1)_1)
1545.769(PEP+HexNAc(2)Hex(2)_1)
1853.880(PEP+HexNAc(2)Hex(3)Fuc(1)_1)
1028.985(PEP+HexNAc(3)Hex(3)Fuc(1)_2)
366.141(HexNAc + Hex)
528.195(Hex + HexNAc + Hex)
754.018(PEP+HexNAc(4)Hex(3)Fuc(1)_3)
773.388(PEP+HexNAc(2)Hex(2)_2)
874.927(PEP+HexNAc(3)Hex(2)_2)
927.443(PEP+HexNAc(2)Hex(3)Fuc(1)_2)
1018.582(PEP+HexNAc(1)_1)
1110.010(PEP+HexNAc(3)Hex(4)Fuc(1)_2)
1130.521(PEP+HexNAc(4)Hex(3)Fuc(1)_2)
1164.640(PEP+HexNAc(1)Fuc(1)_1)
1691.827(PEP+HexNAc(2)Hex(2)Fuc(1)_1)
1853.877(PEP+HexNAc(2)Hex(3)Fuc(1)_1)
401.241
1028.983(PEP+HexNAc(3)Hex(3)Fuc(1)_2)
1191.046
M+2H
1979.812
1666.634
1800.791
1927.871
757.471

IQNLTVK(=PEP)_4_4_1_0_0, 0_None, 0_None, m/z:808.03(3+), RT:30.81, Y-score:57.66

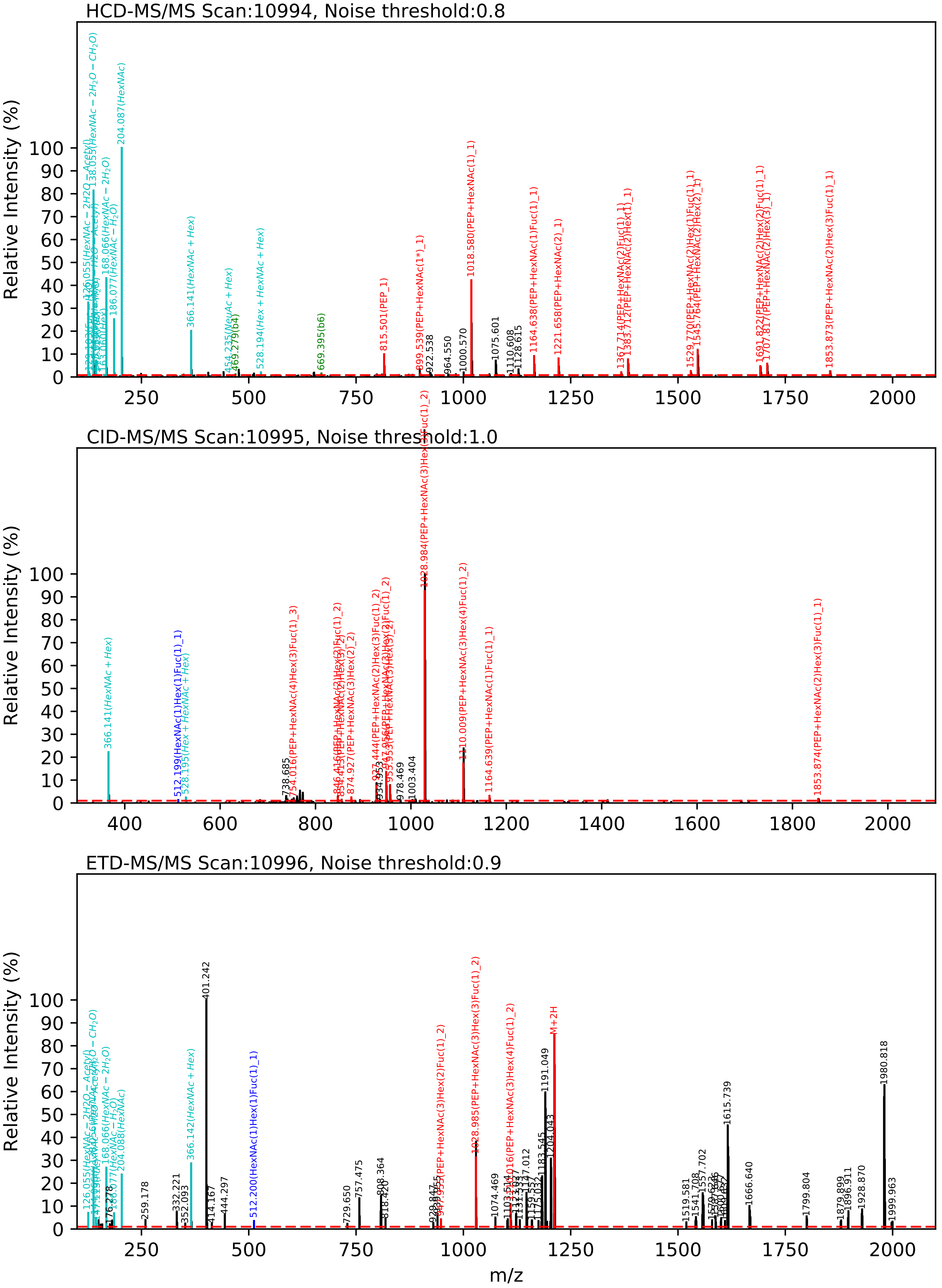

IQNLTVK(=PEP)_4_4_1_0_0, 0_None, 0_None,
m/z:808.03(3+), RT:26.55, Y-score:96.78

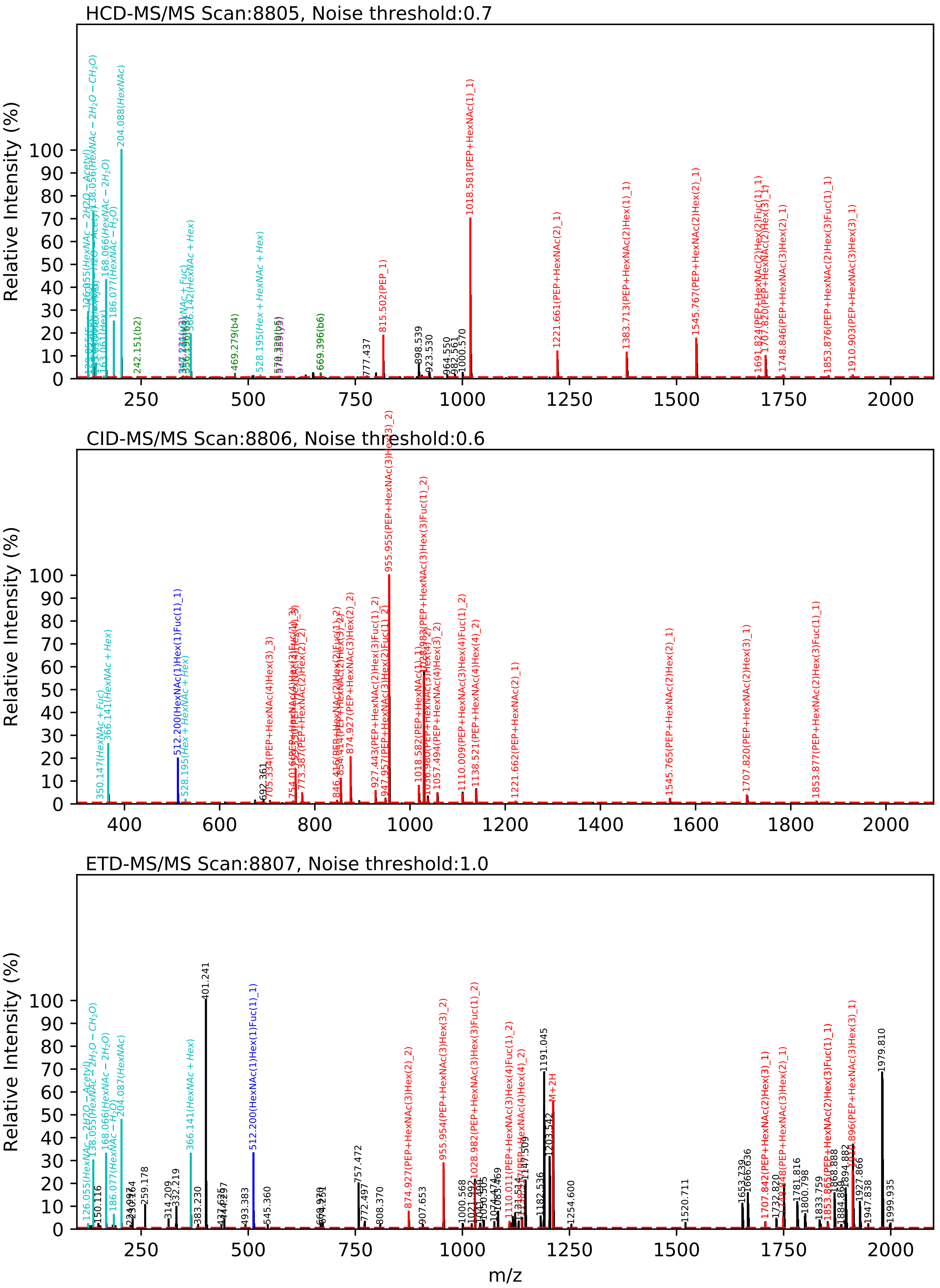

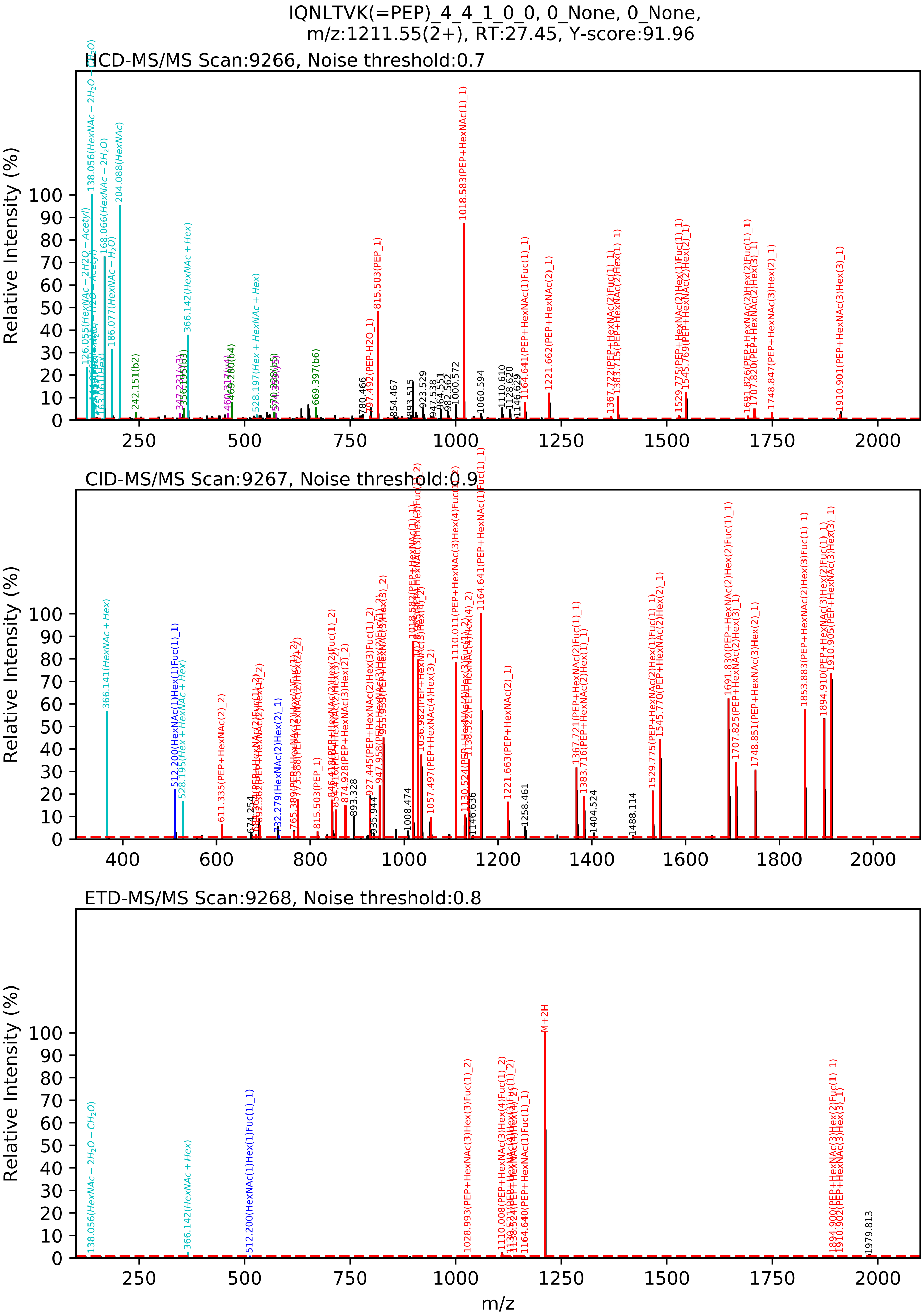
IQNLTVK(=PEP)_4_4_1_0_0, 0_None, 0_None,
m/z:1211.55(2+), RT:27.45, Y-score:91.96
HCD-MS/MS Scan:9266, Noise threshold:0.7
Relative Intensity (%)
138.056(HexNAc − 2H2O − CH2O)
204.088(HexNAc)
168.066(HexNAc − 2H2O)
186.077(HexNAc − H2O)
366.142(HexNAc + Hex)
528.197(Hex + HexNAc + Hex)
1018.583(PEP+HexNAc(1)_1)
815.503(PEP_1)
797.492(PEP-H2O_1)
1164.641(PEP+HexNAc(1)Fuc(1)_1)
1221.662(PEP+HexNAc(2)_1)
1383.715(PEP+HexNAc(2)Hex(1)_1)
1545.769(PEP+HexNAc(2)Hex(2)_1)
1748.847(PEP+HexNAc(3)Hex(2)_1)
1910.901(PEP+HexNAc(3)Hex(3)_1)
CID-MS/MS Scan:9267, Noise threshold:0.9
366.141(HexNAc + Hex)
512.200(HexNAc(1)Hex(1)Fuc(1)_1)
611.335(PEP+HexNAc(2)_2)
732.279(HexNAc(2)Hex(2)_1)
1164.641(PEP+HexNAc(1)Fuc(1)_1)
1221.663(PEP+HexNAc(2)_1)
1691.830(PEP+HexNAc(2)Hex(2)Fuc(1)_1)
1748.851(PEP+HexNAc(3)Hex(2)_1)
1853.883(PEP+HexNAc(2)Hex(3)Fuc(1)_1)
ETD-MS/MS Scan:9268, Noise threshold:0.8
M+2H
138.056(HexNAc − 2H2O − CH2O)
366.142(HexNAc + Hex)
512.200(HexNAc(1)Hex(1)Fuc(1)_1)
1028.993(PEP+HexNAc(3)Hex(3)Fuc(1)_2)
1164.640(PEP+HexNAc(1)Fuc(1)_1)
1979.813
m/z

IQNLTVK(=PEP)_4_4_1_0_0, 0_None, 0_None,
m/z:1211.55(2+), RT:28.00, Y-score:92.83

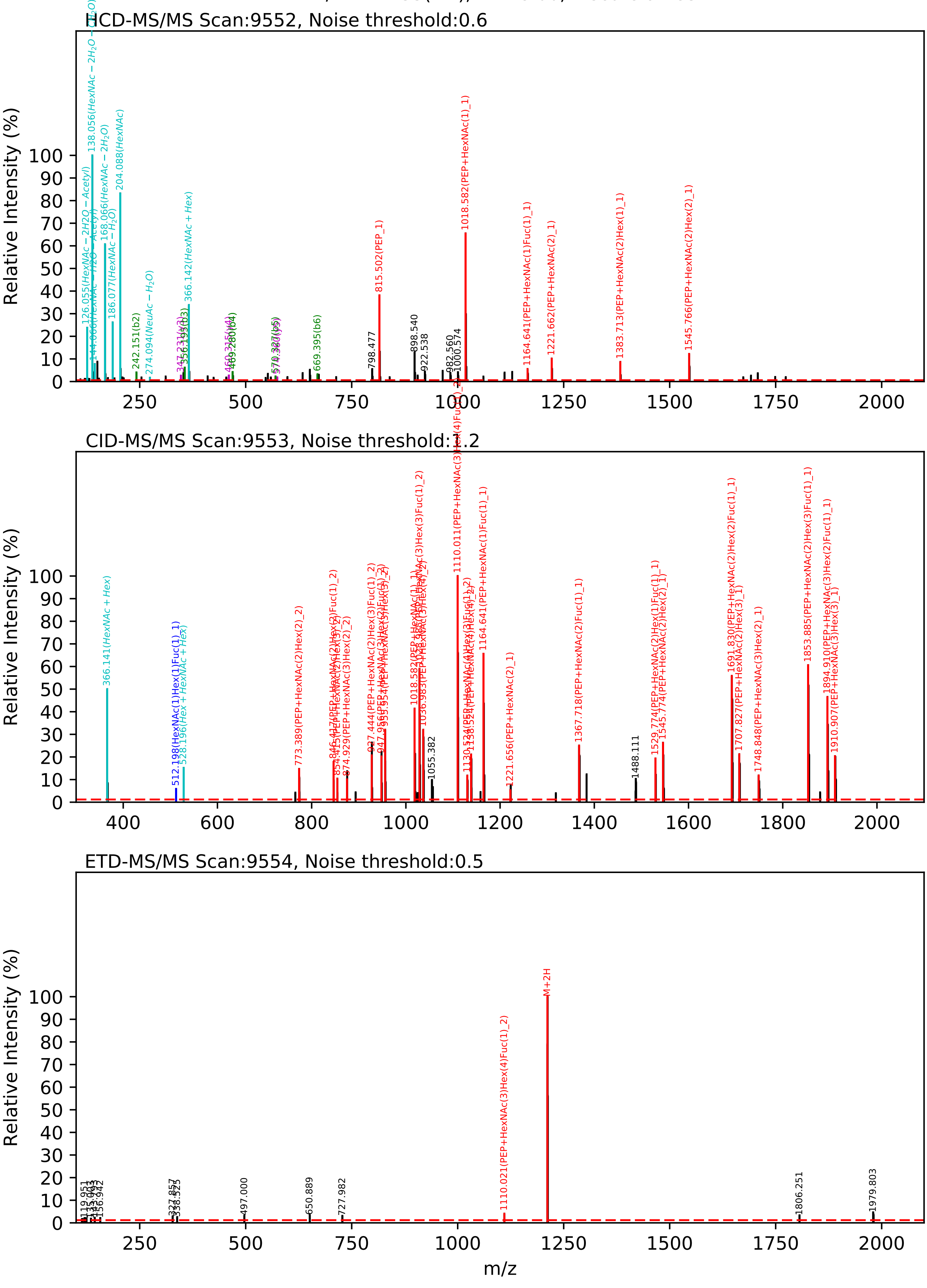

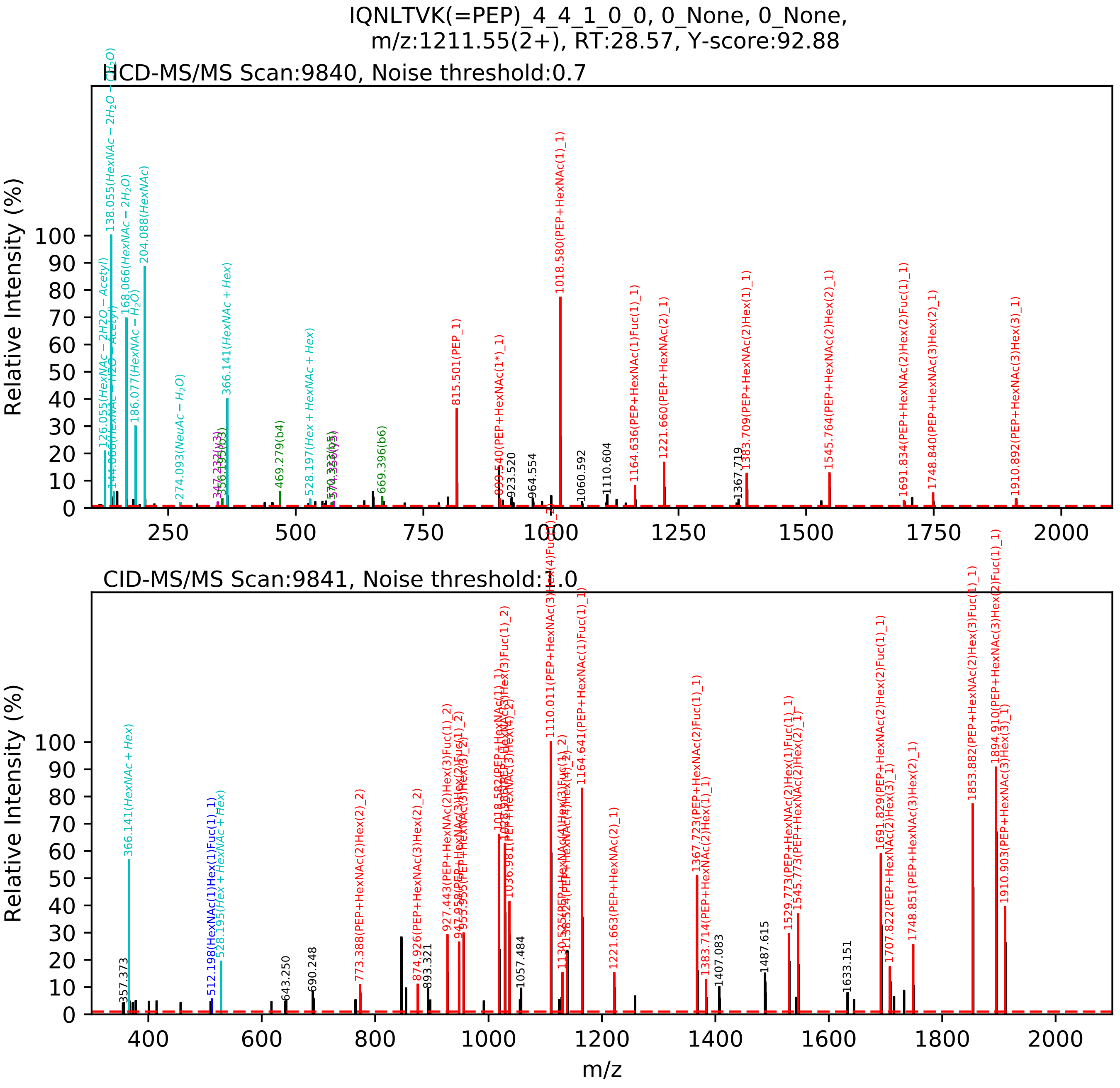
IQNLTVK(=PEP)_4_4_1_0_0, 0_None, 0_None,
m/z:1211.55(2+), RT:28.57, Y-score:92.88
HCD-MS/MS Scan:9840, Noise threshold:0.7
Relative Intensity (%)
138.055(HexNAc)
204.088(HexNAc)
126.055(HexNAc − 2H2O − Acetyl)
168.066(HexNAc − 2H2O)
186.077(HexNAc − H2O)
274.093(NeuAc − H2O)
366.141(HexNAc + Hex)
469.279(b4)
528.197(Hex + HexNAc + Hex)
669.396(b6)
815.501(PEP_1)
923.520
964.554
1018.580(PEP+HexNAc(1)_1)
1060.592
1110.604
1164.636(PEP+HexNAc(1)Fuc(1)_1)
1221.660(PEP+HexNAc(2)_1)
1367.719
1383.709(PEP+HexNAc(2)Hex(1)_1)
1545.764(PEP+HexNAc(2)Hex(2)_1)
1691.834(PEP+HexNAc(2)Hex(2)Fuc(1)_1)
1748.840(PEP+HexNAc(3)Hex(2)_1)
1910.892(PEP+HexNAc(3)Hex(3)_1)
CID-MS/MS Scan:9841, Noise threshold:1.0
366.141(HexNAc + Hex)
357.373
512.198(HexNAc(1)Hex(1)Fuc(1)_1)
528.195(Hex + HexNAc + Hex)
643.250
690.248
773.388(PEP+HexNAc(2)Hex(2)_2)
874.926(PEP+HexNAc(3)Hex(2)_2)
893.321
927.443(PEP+HexNAc(2)Hex(3)Fuc(1)_2)
1036.981(PEP+HexNAc(3)Hex(4)_2)
1057.484
1110.011(PEP+HexNAc(3)Hex(4)Fuc(1)_2)
1164.641(PEP+HexNAc(1)Fuc(1)_1)
1221.663(PEP+HexNAc(2)_1)
1367.723(PEP+HexNAc(2)Fuc(1)_1)
1383.714(PEP+HexNAc(2)Hex(1)_1)
1407.083
1487.615
1529.773(PEP+HexNAc(2)Hex(1)Fuc(1)_1)
1545.773(PEP+HexNAc(2)Hex(2)_1)
1633.151
1691.829(PEP+HexNAc(2)Hex(2)Fuc(1)_1)
1707.822(PEP+HexNAc(2)Hex(3)_1)
1748.851(PEP+HexNAc(3)Hex(2)_1)
1853.882(PEP+HexNAc(2)Hex(3)Fuc(1)_1)
1894.910(PEP+HexNAc(3)Hex(2)Fuc(1)_1)
1910.903(PEP+HexNAc(3)Hex(3)_1)
m/z

IQNLTVK(=PEP)_4_4_1_0_0, 0_None, 0_None, m/z:1211.55(2+), RT:29.64, Y-score:80.07

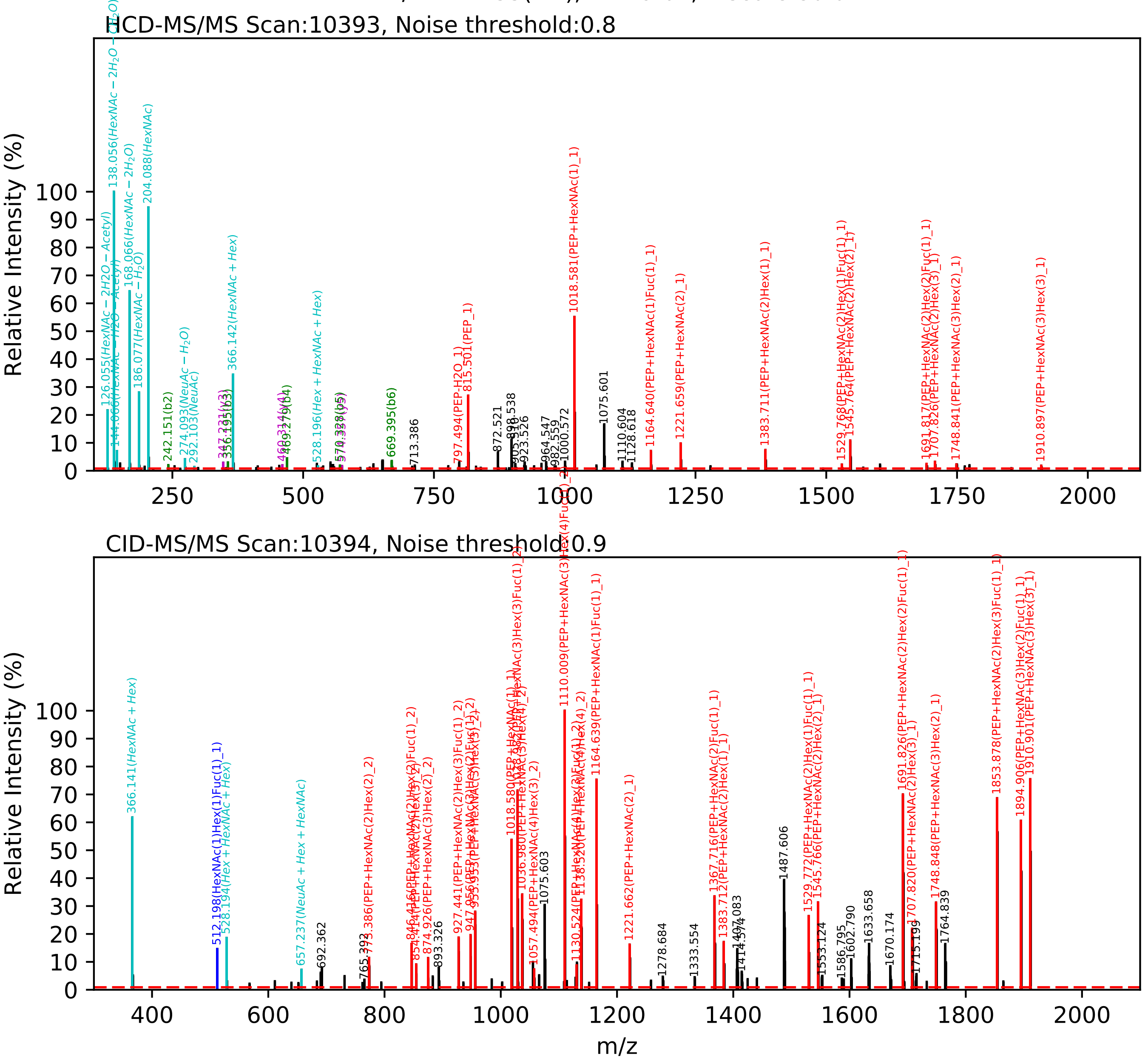

IQNLTVK(=PEP)_4_4_1_0_0, 0_None, 0_None,
m/z:1211.55(2+), RT:30.61, Y-score:80.78

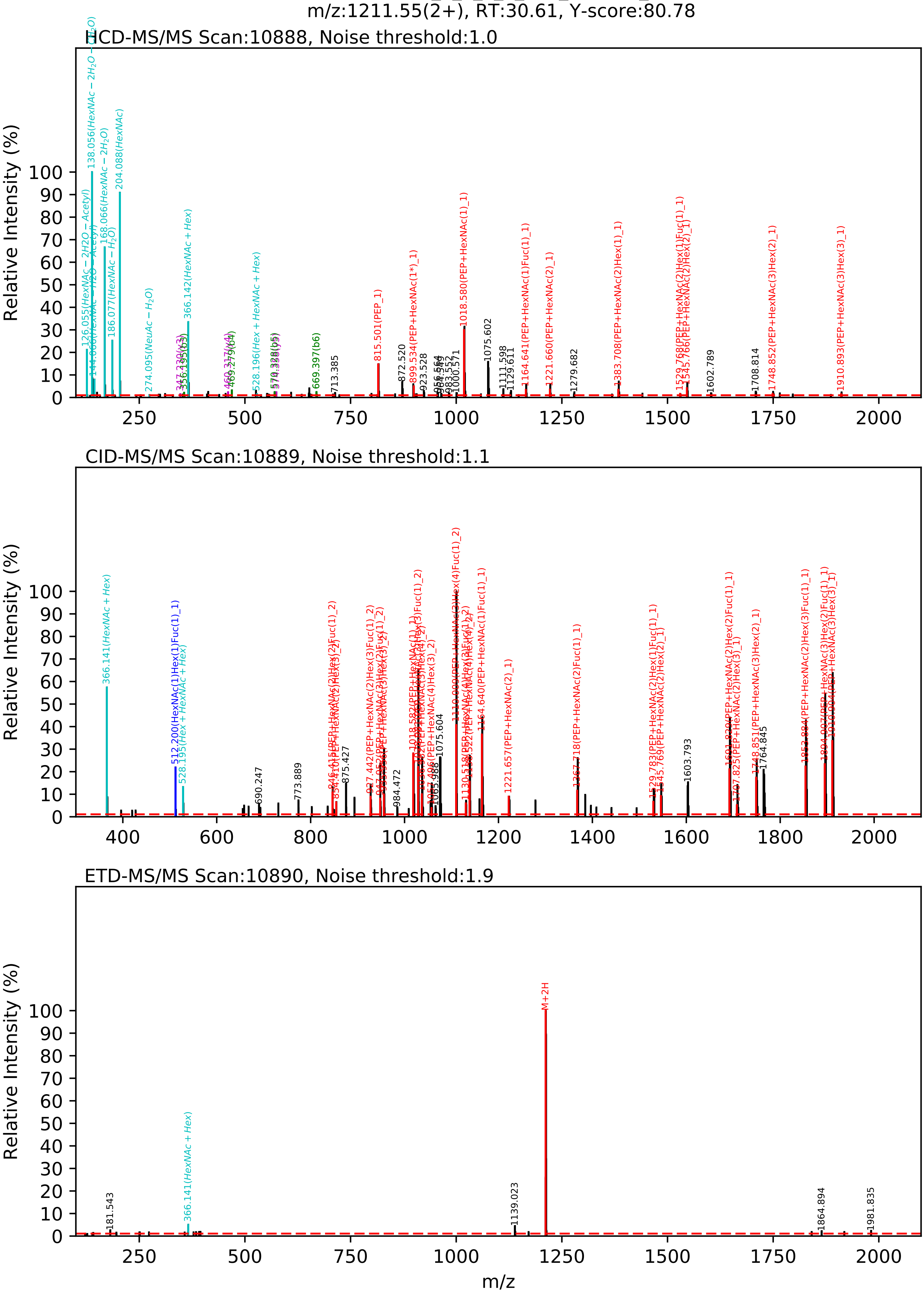

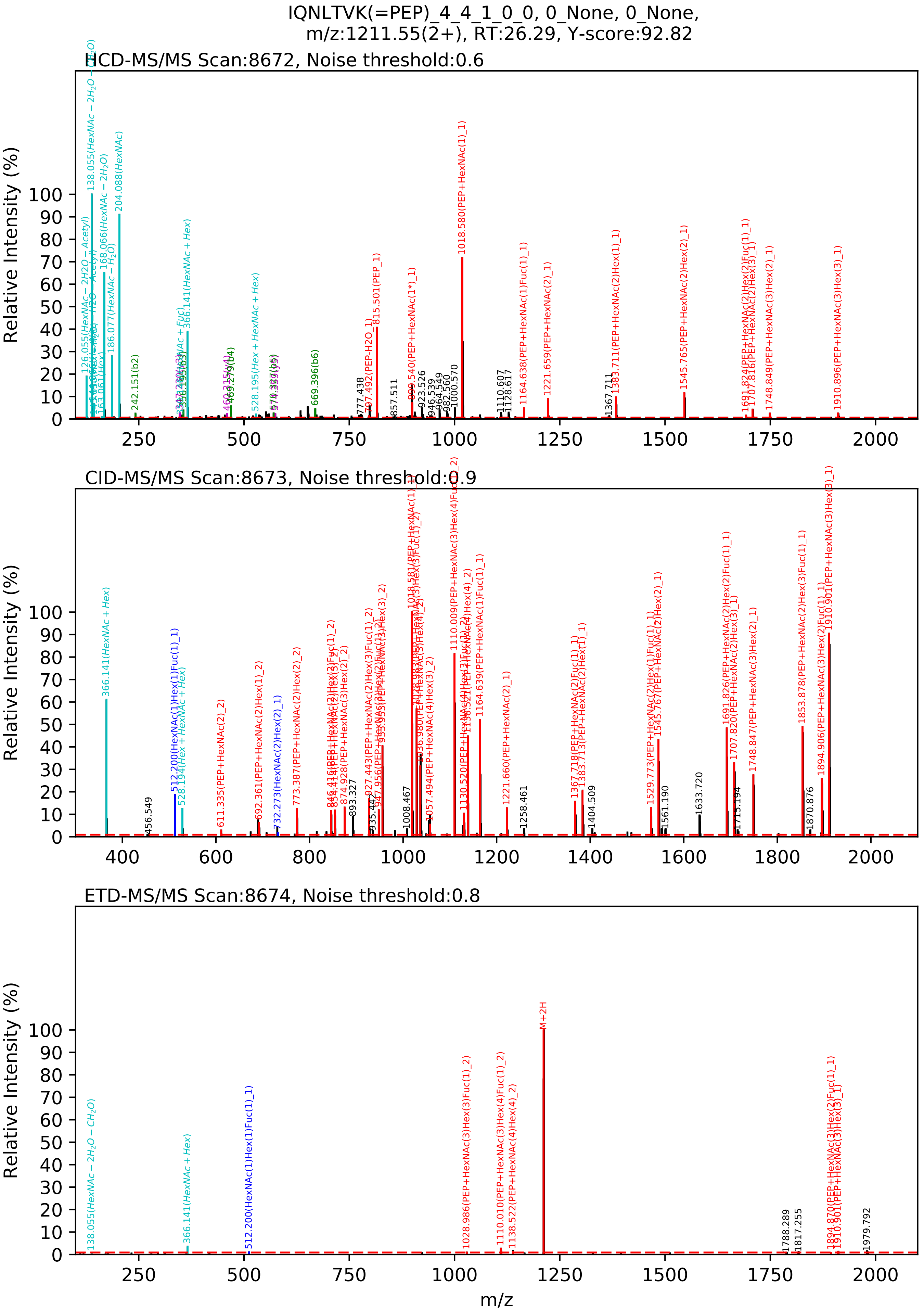
IQNLTVK(=PEP)_4_4_1_0_0, 0_None, 0_None,
m/z:1211.55(2+), RT:26.29, Y-score:92.82
HCD-MS/MS Scan:8672, Noise threshold:0.6
CID-MS/MS Scan:8673, Noise threshold:0.9
ETD-MS/MS Scan:8674, Noise threshold:0.8
Relative Intensity (%)
m/z
1018.580(PEP+HexNAc(1)_1)
815.501(PEP_1)
204.088(HexNAc)
138.055(HexNAc − 2H2O − CH2O)
366.141(HexNAc + Hex)
1545.765(PEP+HexNAc(2)Hex(2)_1)
1383.711(PEP+HexNAc(2)Hex(1)_1)
1910.901(PEP+HexNAc(3)Hex(3)_1)
1853.878(PEP+HexNAc(2)Hex(3)Fuc(1)_1)
1691.826(PEP+HexNAc(2)Hex(2)Fuc(1)_1)
1110.009(PEP+HexNAc(3)Hex(4)Fuc(1)_2)
512.200(HexNAc(1)Hex(1)Fuc(1)_1)
M+2H

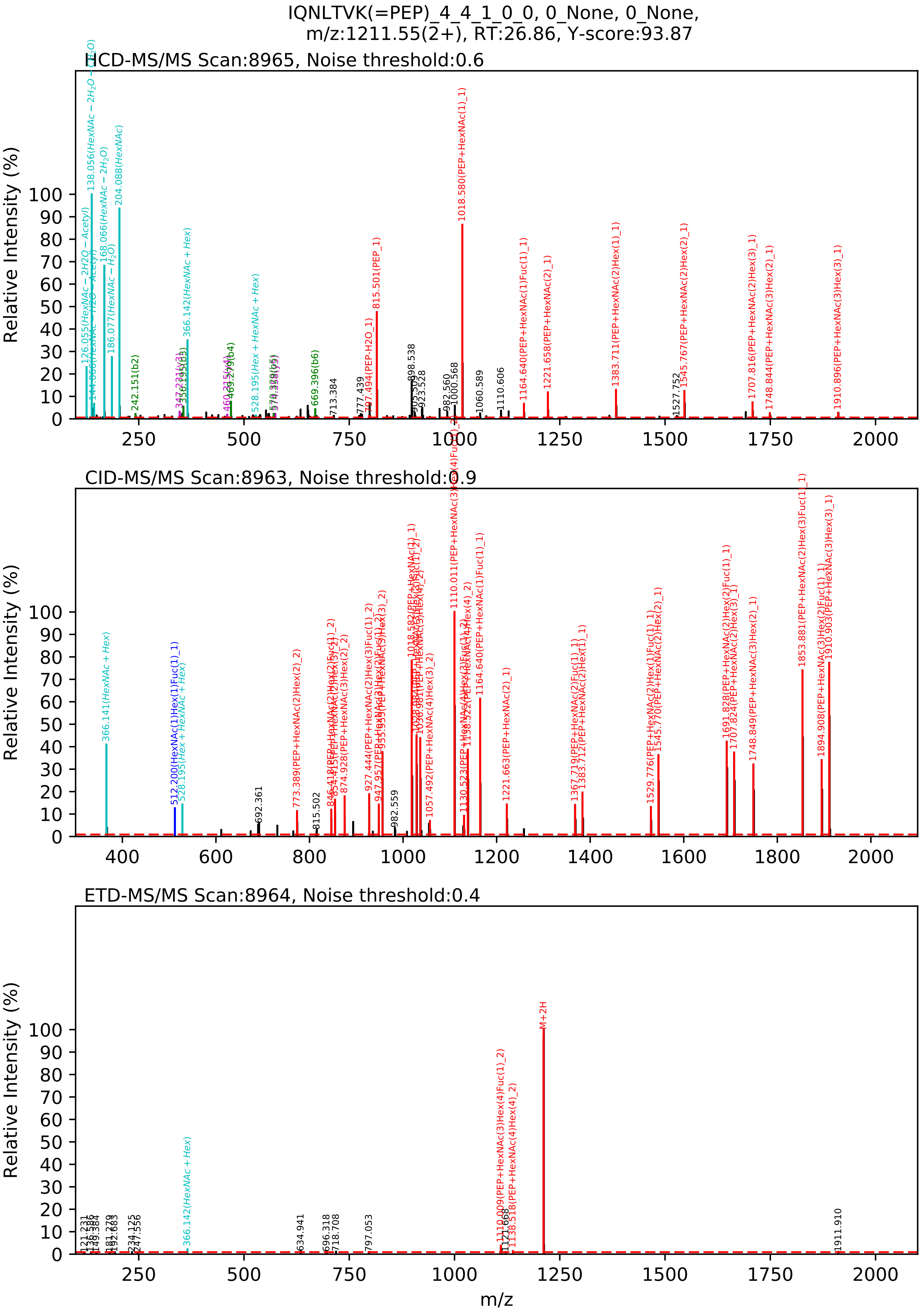
IQNLTVK(=PEP)_4_4_1_0_0, 0_None, 0_None,
m/z:1211.55(2+), RT:26.86, Y-score:93.87
HCD-MS/MS Scan:8965, Noise threshold:0.6
CID-MS/MS Scan:8963, Noise threshold:0.9
ETD-MS/MS Scan:8964, Noise threshold:0.4
Relative Intensity (%)
m/z
138.056(HexNAc − 2H2O − H2O)
204.088(HexNAc)
168.066(HexNAc − 2H2O)
186.077(HexNAc − H2O)
366.142(HexNAc + Hex)
815.501(PEP_1)
1018.580(PEP+HexNAc(1)_1)
1164.640(PEP+HexNAc(1)Fuc(1)_1)
1221.658(PEP+HexNAc(2)_1)
1383.711(PEP+HexNAc(2)Hex(1)_1)
1545.767(PEP+HexNAc(2)Hex(2)_1)
1707.816(PEP+HexNAc(2)Hex(3)_1)
1748.844(PEP+HexNAc(3)Hex(2)_1)
1910.896(PEP+HexNAc(3)Hex(3)_1)
242.151(b2)
669.396(b6)
366.141(HexNAc + Hex)
512.200(HexNAc(1)Hex(1)Fuc(1)_1)
773.389(PEP+HexNAc(2)Hex(2)_2)
1110.011(PEP+HexNAc(3)Hex(4)Fuc(1)_2)
1164.640(PEP+HexNAc(1)Fuc(1)_1)
1221.663(PEP+HexNAc(2)_1)
1853.881(PEP+HexNAc(2)Hex(3)Fuc(1)_1)
1691.828(PEP+HexNAc(2)Hex(2)Fuc(1)_1)
M+2H
1110.009(PEP+HexNAc(3)Hex(4)Fuc(1)_2)
1138.518(PEP+HexNAc(4)Hex(4)_2)
1911.910

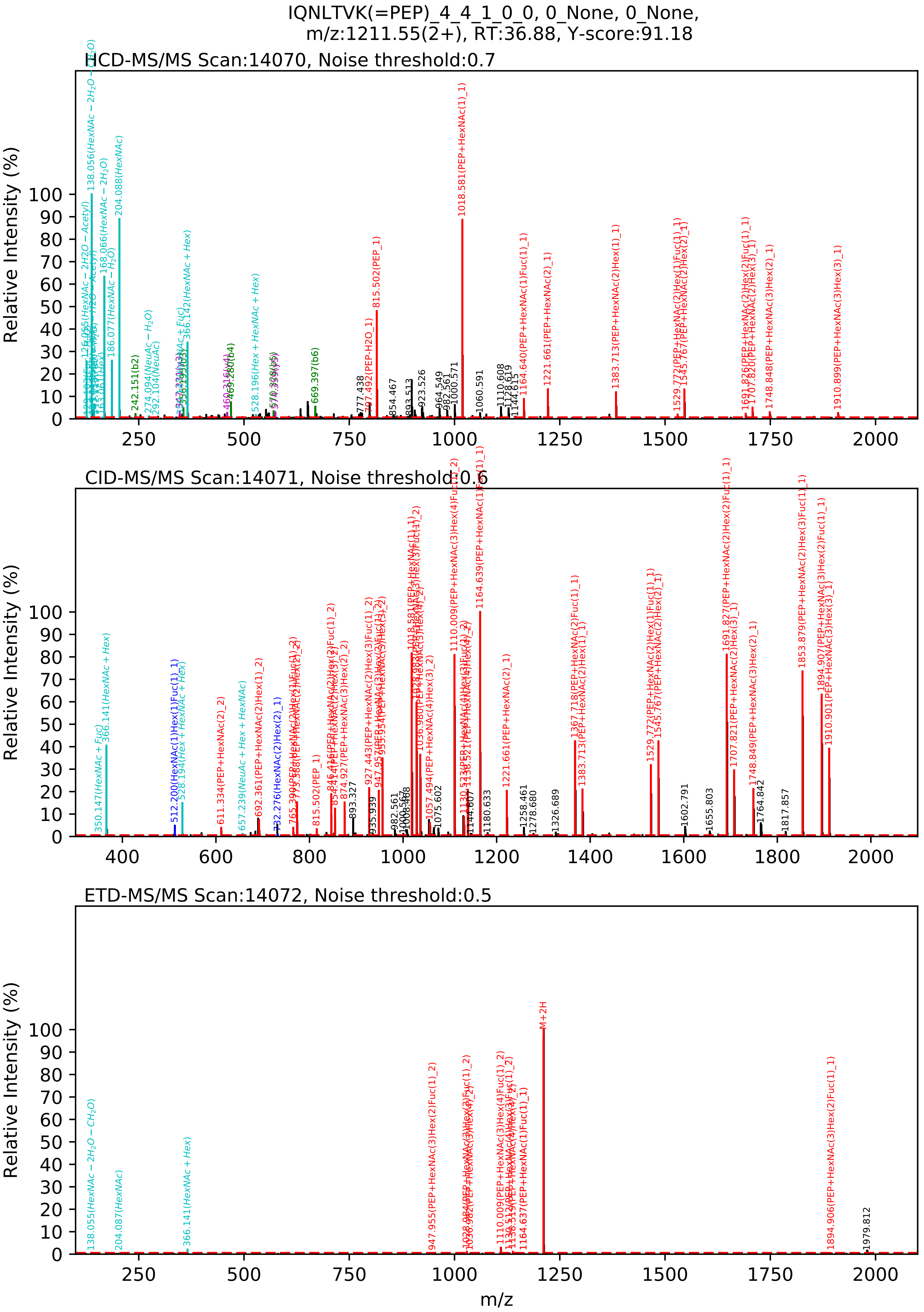
IQNLTVK(=PEP)_4_4_1_0_0, 0_None, 0_None,
m/z:1211.55(2+), RT:36.88, Y-score:91.18
HCD-MS/MS Scan:14070, Noise threshold:0.7
Relative Intensity (%)
138.056(HexNAc − 2H2O)
204.088(HexNAc)
168.066(HexNAc − 2H2O)
186.077(HexNAc − H2O)
366.142(HexNAc + Hex)
242.151(b2)
274.094(NeuAc − H2O)
292.104(NeuAc)
469.280(b4)
528.196(Hex + HexNAc + Hex)
669.397(b6)
777.438
797.492(PEP-H2O_1)
815.502(PEP_1)
854.467
893.513
923.526
964.549
1000.571
1018.581(PEP+HexNAc(1)_1)
1060.591
1110.608
1128.619
1144.615
1164.640(PEP+HexNAc(1)Fuc(1)_1)
1221.661(PEP+HexNAc(2)_1)
1383.713(PEP+HexNAc(2)Hex(1)_1)
1529.772(PEP+HexNAc(2)Hex(1)Fuc(1)_1)
1545.767(PEP+HexNAc(2)Hex(2)_1)
1691.826(PEP+HexNAc(2)Hex(2)Fuc(1)_1)
1707.820(PEP+HexNAc(2)Hex(3)_1)
1748.848(PEP+HexNAc(3)Hex(2)_1)
1910.899(PEP+HexNAc(3)Hex(3)_1)
CID-MS/MS Scan:14071, Noise threshold:0.6
350.147(HexNAc + Fuc)
366.141(HexNAc + Hex)
512.200(HexNAc(1)Hex(1)Fuc(1)_1)
528.194(Hex + HexNAc + Hex)
611.334(PEP+HexNAc(2)_2)
657.239(NeuAc + Hex + HexNAc)
692.361(PEP+HexNAc(2)Hex(1)_2)
732.276(HexNAc(2)Hex(2)_1)
815.502(PEP_1)
874.927(PEP+HexNAc(3)Hex(2)_2)
893.327
927.443(PEP+HexNAc(2)Hex(3)Fuc(1)_2)
1036.980(PEP+HexNAc(4)Hex(3)_2)
1057.494(PEP+HexNAc(4)Hex(3)_2)
1075.602
1110.009(PEP+HexNAc(3)Hex(4)Fuc(1)_2)
1164.639(PEP+HexNAc(1)Fuc(1)_1)
1180.633
1221.661(PEP+HexNAc(2)_1)
1258.461
1278.680
1326.689
1367.718(PEP+HexNAc(2)Fuc(1)_1)
1383.713(PEP+HexNAc(2)Hex(1)_1)
1529.772(PEP+HexNAc(2)Hex(1)Fuc(1)_1)
1545.767(PEP+HexNAc(2)Hex(2)_1)
1602.791
1655.803
1691.827(PEP+HexNAc(2)Hex(2)Fuc(1)_1)
1707.821(PEP+HexNAc(2)Hex(3)_1)
1748.849(PEP+HexNAc(3)Hex(2)_1)
1764.842
1817.857
1853.879(PEP+HexNAc(2)Hex(3)Fuc(1)_1)
1894.907(PEP+HexNAc(3)Hex(2)Fuc(1)_1)
1910.901(PEP+HexNAc(3)Hex(3)_1)
ETD-MS/MS Scan:14072, Noise threshold:0.5
138.055(HexNAc − 2H2O − CH2O)
204.087(HexNAc)
366.141(HexNAc + Hex)
947.955(PEP+HexNAc(3)Hex(2)Fuc(1)_2)
1110.009(PEP+HexNAc(3)Hex(4)Fuc(1)_2)
1164.637(PEP+HexNAc(1)Fuc(1)_1)
M+2H
1894.906(PEP+HexNAc(3)Hex(2)Fuc(1)_1)
1979.812
m/z

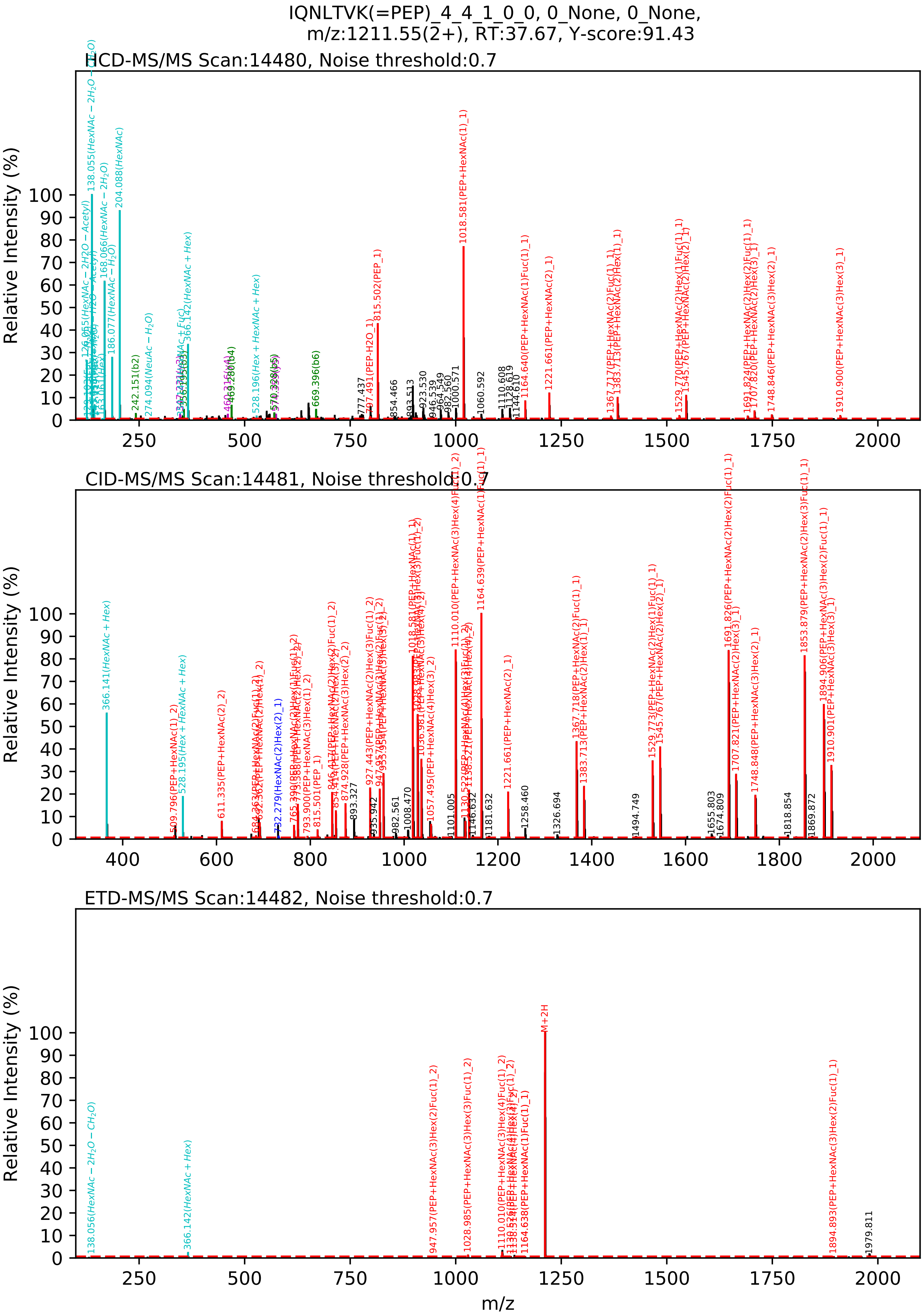

IQNLTVK(=PEP)_4_4_1_0_0, 0_None, 0_None,
m/z:1211.55(2+), RT:37.67, Y-score:91.43
HCD-MS/MS Scan:14480, Noise threshold:0.7
CID-MS/MS Scan:14481, Noise threshold:0.7
ETD-MS/MS Scan:14482, Noise threshold:0.7
Relative Intensity (%)
m/z
M+2H

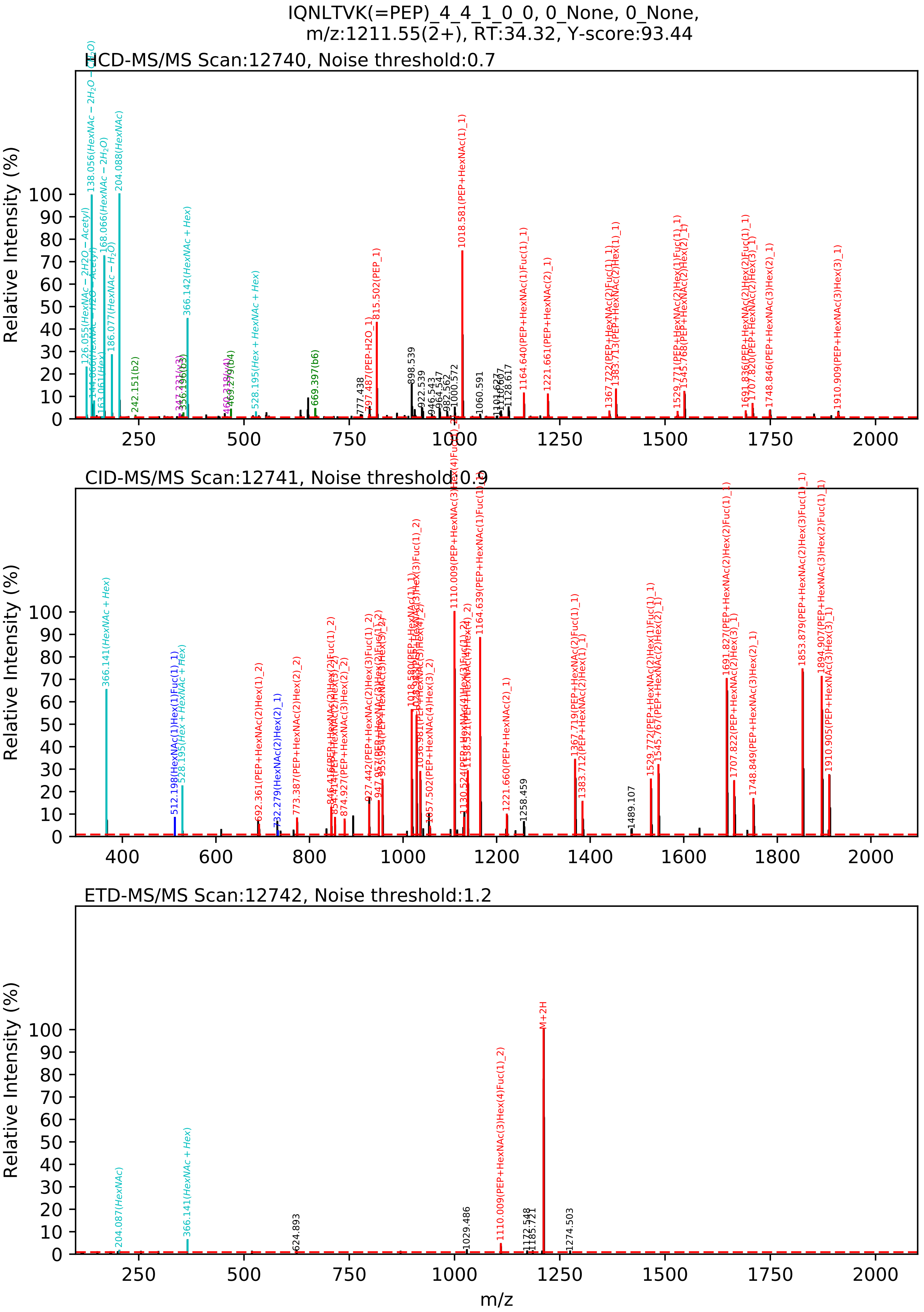
IQNLTVK(=PEP)_4_4_1_0_0, 0_None, 0_None,
m/z:1211.55(2+), RT:34.32, Y-score:93.44
HCD-MS/MS Scan:12740, Noise threshold:0.7
CID-MS/MS Scan:12741, Noise threshold:0.9
ETD-MS/MS Scan:12742, Noise threshold:1.2
Relative Intensity (%)
m/z

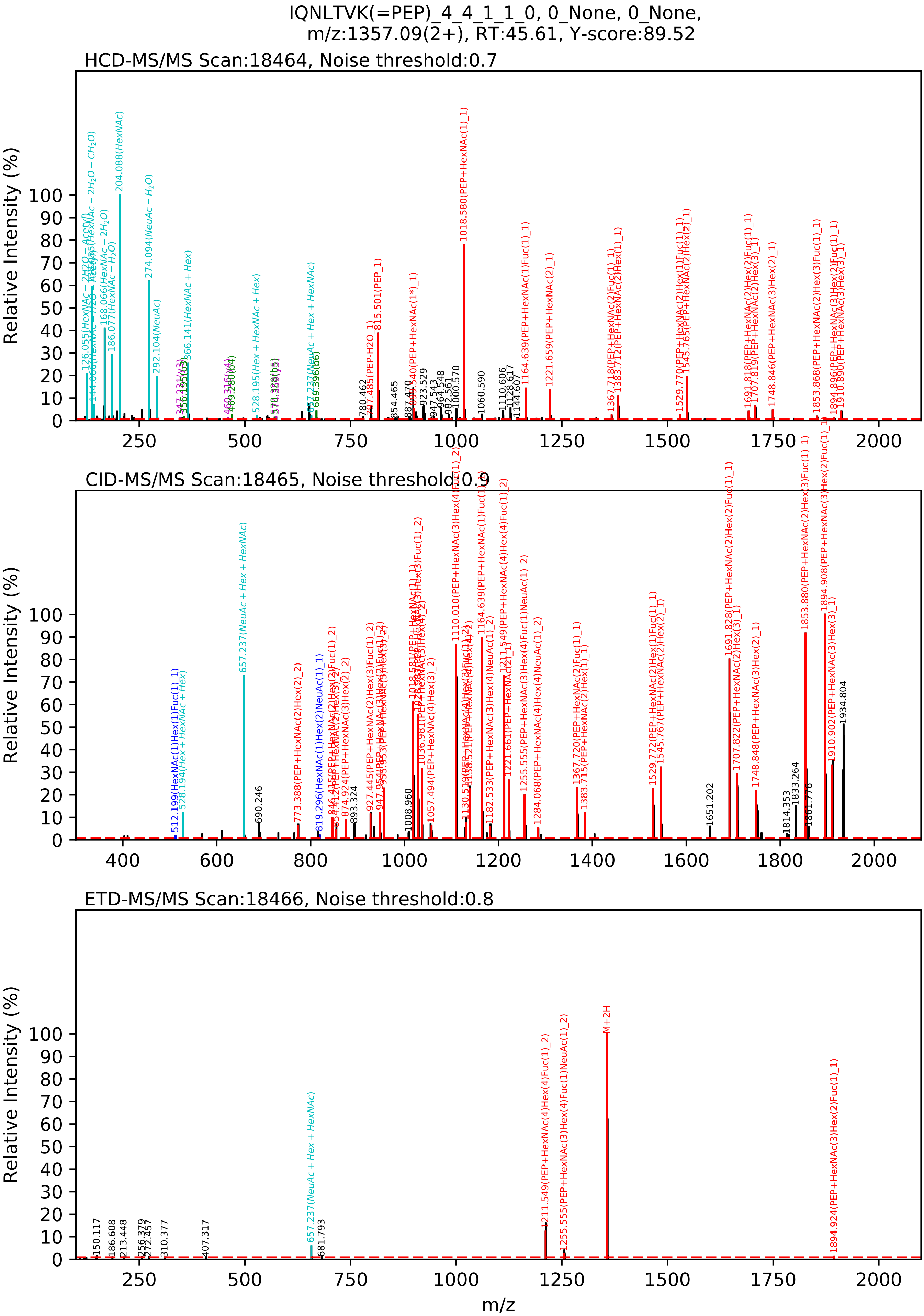
IQNLTVK(=PEP)_4_4_1_1_0, 0_None, 0_None,
m/z:1357.09(2+), RT:45.61, Y-score:89.52
HCD-MS/MS Scan:18464, Noise threshold:0.7
CID-MS/MS Scan:18465, Noise threshold:0.9
ETD-MS/MS Scan:18466, Noise threshold:0.8
Relative Intensity (%)
m/z
M+2H

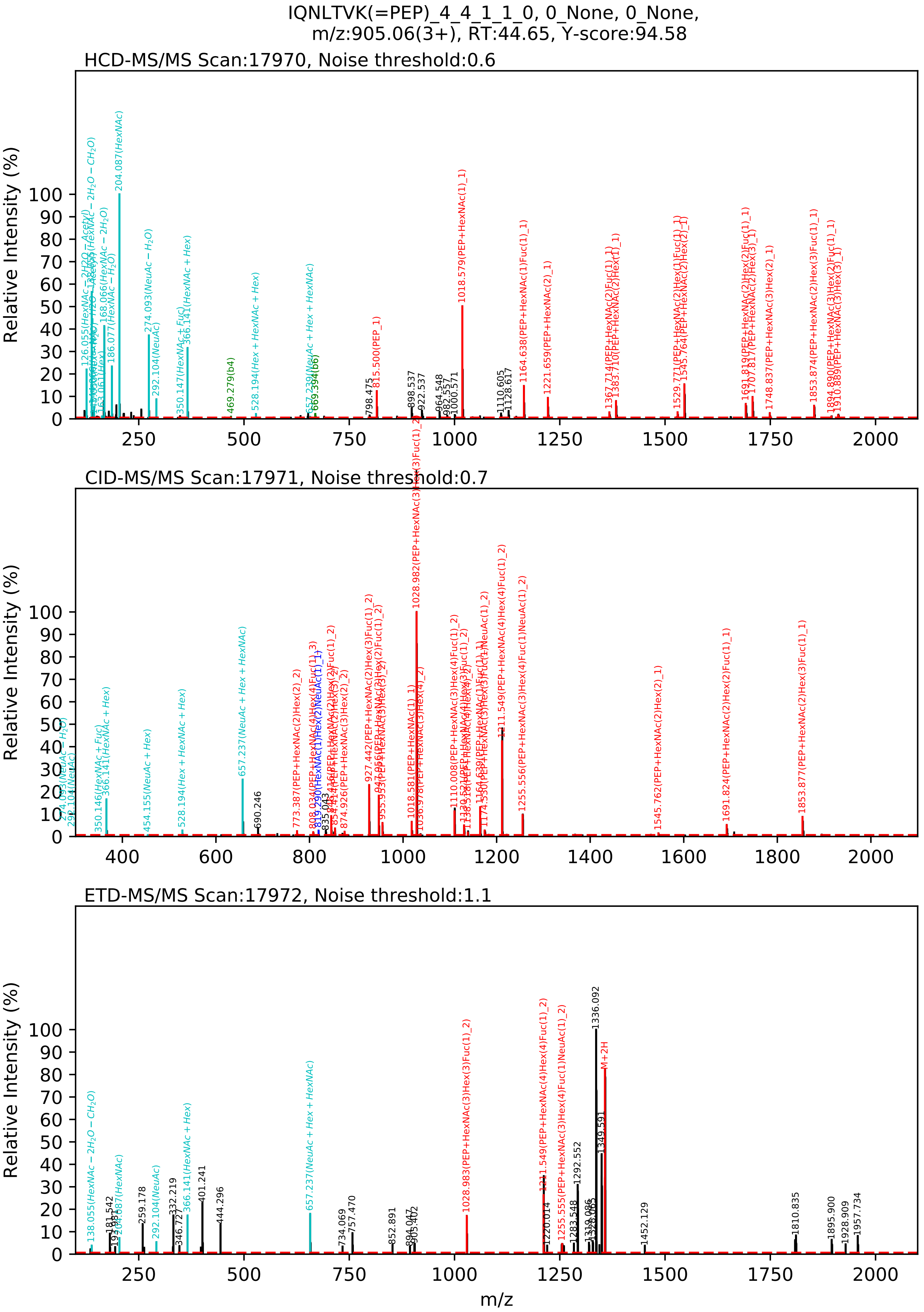

IQNLTVK(=PEP)_4_4_1_1_0, 0_None, 0_None,
m/z:905.06(3+), RT:44.65, Y-score:94.58
HCD-MS/MS Scan:17970, Noise threshold:0.6
CID-MS/MS Scan:17971, Noise threshold:0.7
ETD-MS/MS Scan:17972, Noise threshold:1.1
Relative Intensity (%)
m/z
204.087(HexNAc)
274.093(NeuAc − H2O)
366.141(HexNAc + Hex)
1018.579(PEP+HexNAc(1)_1)
1164.638(PEP+HexNAc(1)Fuc(1)_1)
1221.659(PEP+HexNAc(2)_1)
815.500(PEP_1)
1028.982(PEP+HexNAc(3)Hex(3)Fuc(1)_2)
1211.549(PEP+HexNAc(4)Hex(4)Fuc(1)_2)
1255.556(PEP+HexNAc(3)Hex(4)Fuc(1)NeuAc(1)_2)
657.237(NeuAc + Hex + HexNAc)
1853.877(PEP+HexNAc(2)Hex(3)Fuc(1)_1)
1691.824(PEP+HexNAc(2)Hex(2)Fuc(1)_1)
1545.762(PEP+HexNAc(2)Hex(2)_1)
1336.092
M+2H
1349.591
1292.552
401.241
444.296
1028.983(PEP+HexNAc(3)Hex(3)Fuc(1)_2)
1211.549(PEP+HexNAc(4)Hex(4)Fuc(1)_2)
1255.555(PEP+HexNAc(3)Hex(4)Fuc(1)NeuAc(1)_2)
1810.835
1895.900
1928.909
1957.734

IQNLTVK(=PEP)_4_4_1_1_0, 0_None, 0_None,
m/z:1357.09(2+), RT:36.60, Y-score:93.53

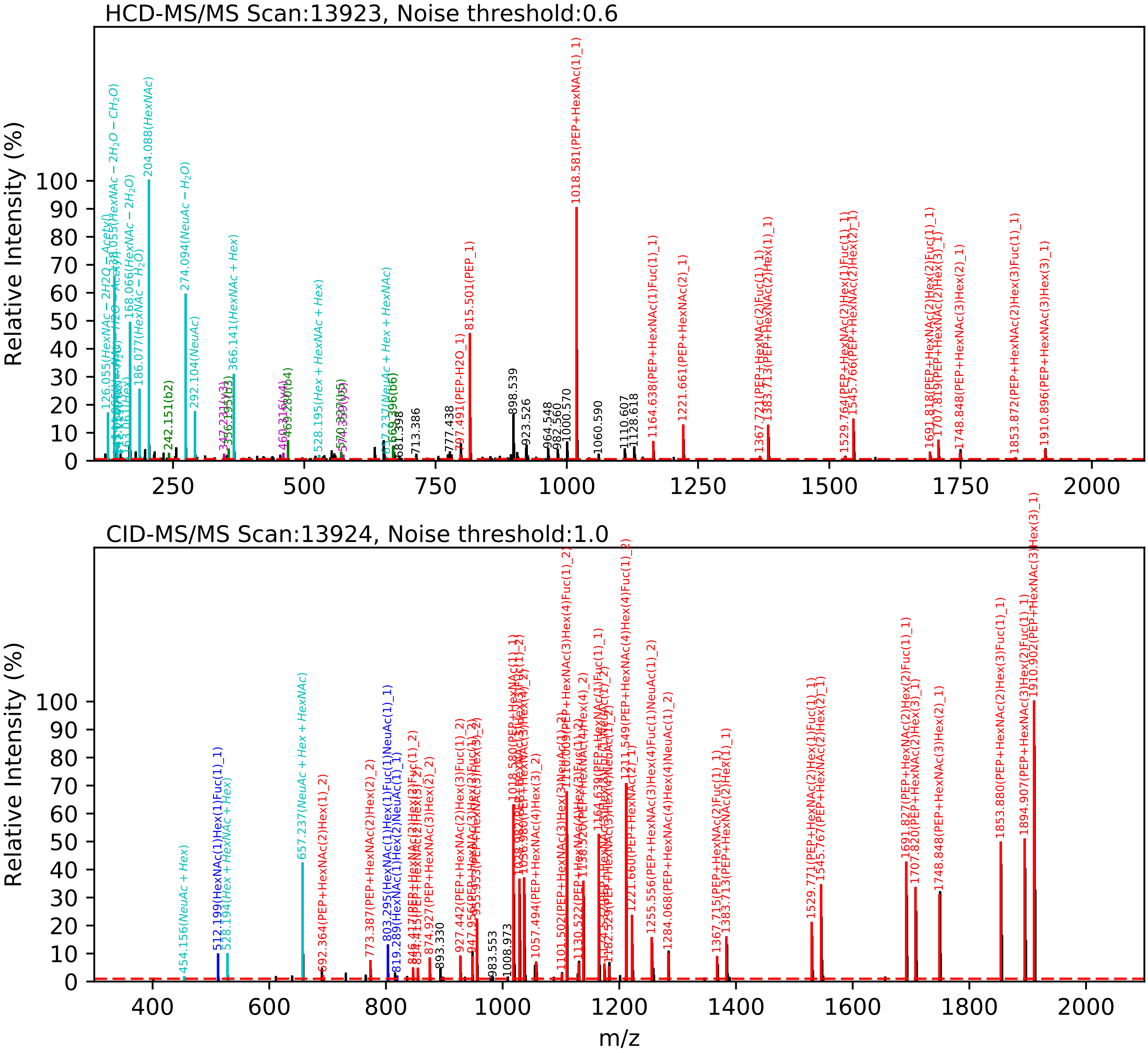

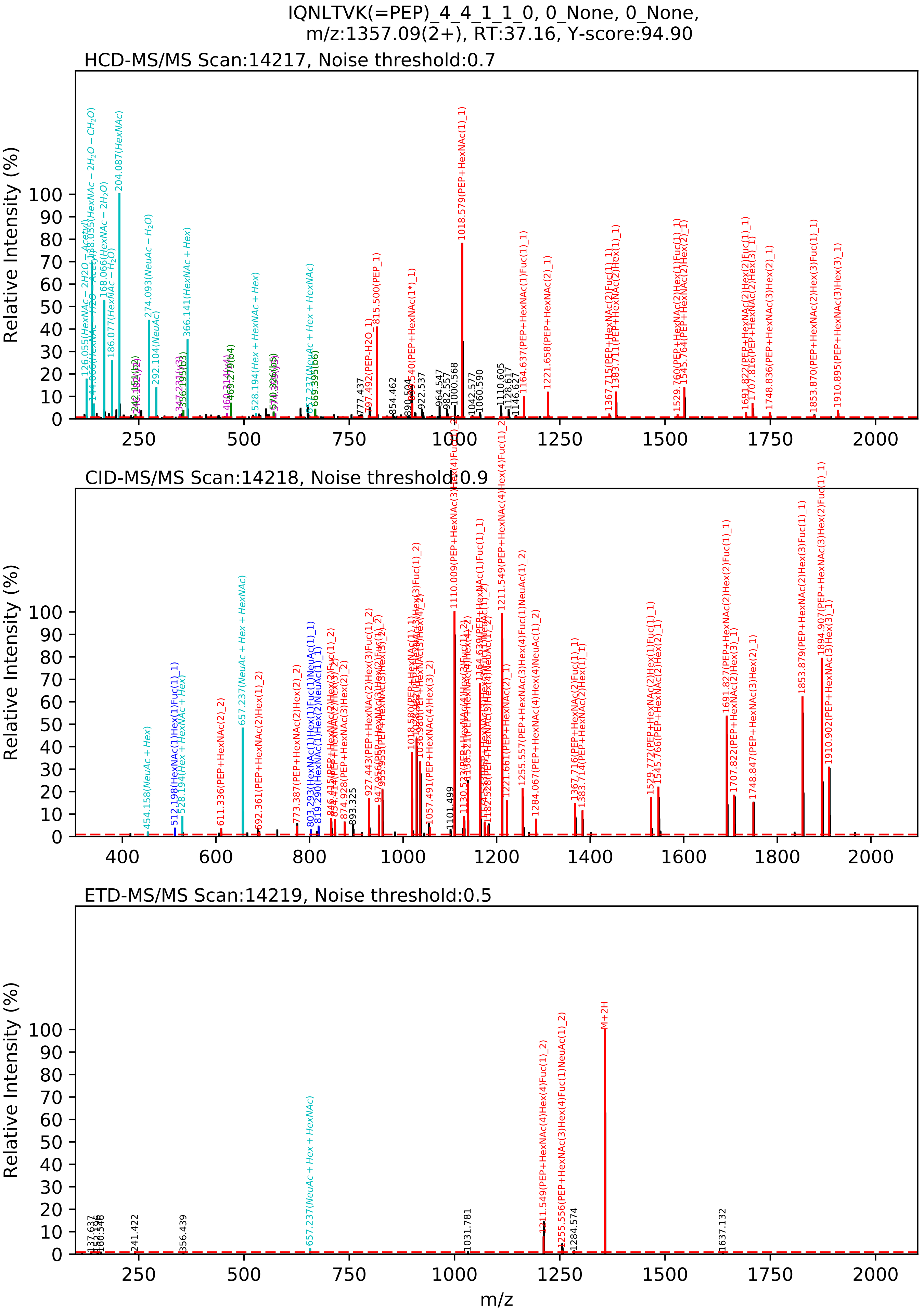

IQNLTVK(=PEP)_4_4_1_1_0, 0_None, 0_None,
m/z:1357.09(2+), RT:37.16, Y-score:94.90
HCD-MS/MS Scan:14217, Noise threshold:0.7
CID-MS/MS Scan:14218, Noise threshold:0.9
ETD-MS/MS Scan:14219, Noise threshold:0.5
Relative Intensity (%)
m/z

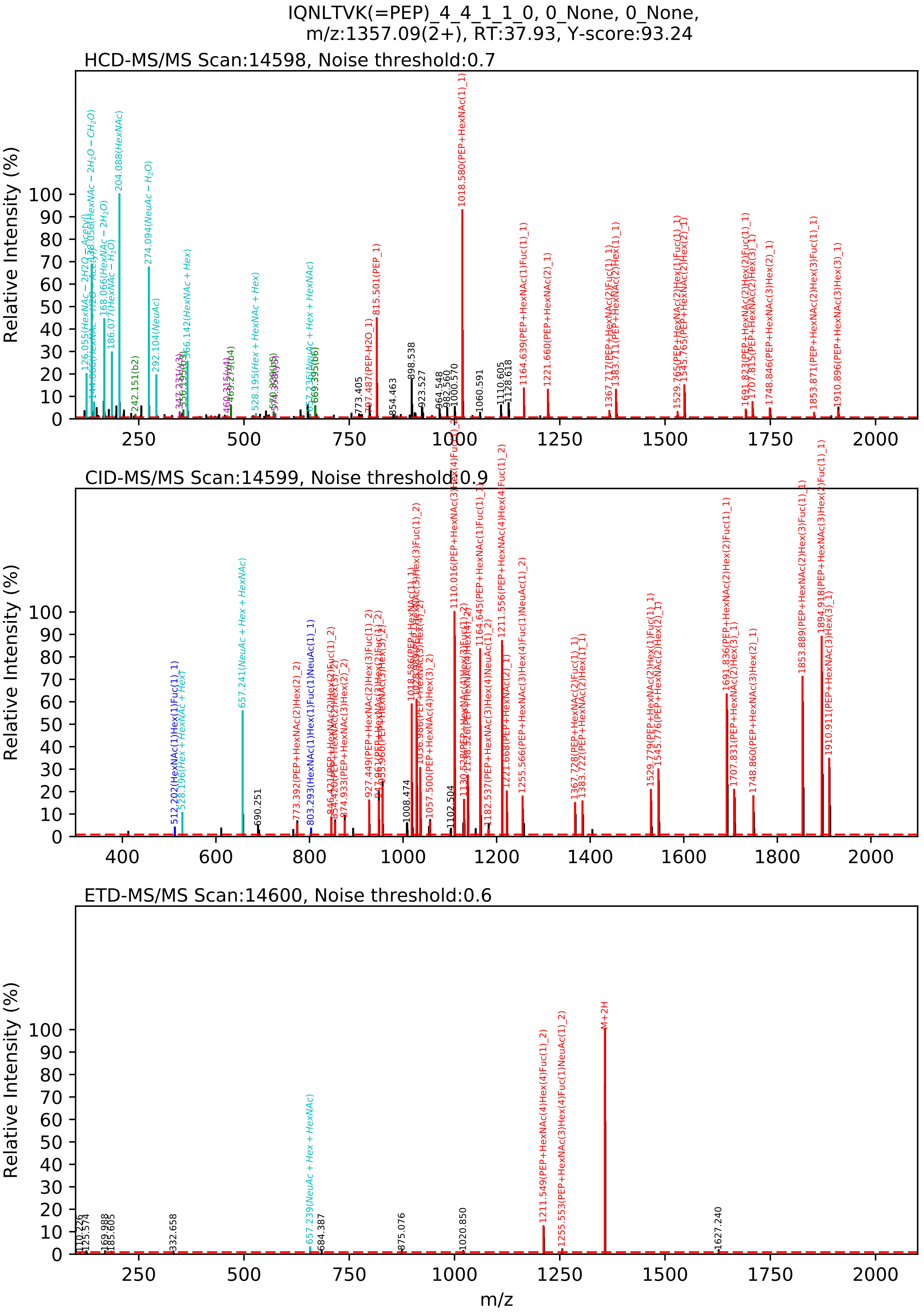
IQNLTVK(=PEP)_4_4_1_1_0, 0_None, 0_None,
m/z:1357.09(2+), RT:37.93, Y-score:93.24
HCD-MS/MS Scan:14598, Noise threshold:0.7
CID-MS/MS Scan:14599, Noise threshold:0.9
ETD-MS/MS Scan:14600, Noise threshold:0.6
Relative Intensity (%)
m/z
1018.580(PEP+HexNAc(1)_1)
204.088(HexNAc)
274.094(NeuAc − H2O)
815.501(PEP_1)
1110.016(PEP+HexNAc(3)Hex(4)Fuc(1)_2)
1164.645(PEP+HexNAc(1)Fuc(1)_1)
1211.556(PEP+HexNAc(4)Hex(4)Fuc(1)_2)
1894.918(PEP+HexNAc(3)Hex(2)Fuc(1)_1)
1853.889(PEP+HexNAc(2)Hex(3)Fuc(1)_1)
657.241(NeuAc + Hex + HexNAc)
M+2H
1211.549(PEP+HexNAc(4)Hex(4)Fuc(1)_2)
1255.553(PEP+HexNAc(3)Hex(4)Fuc(1)NeuAc(1)_2)

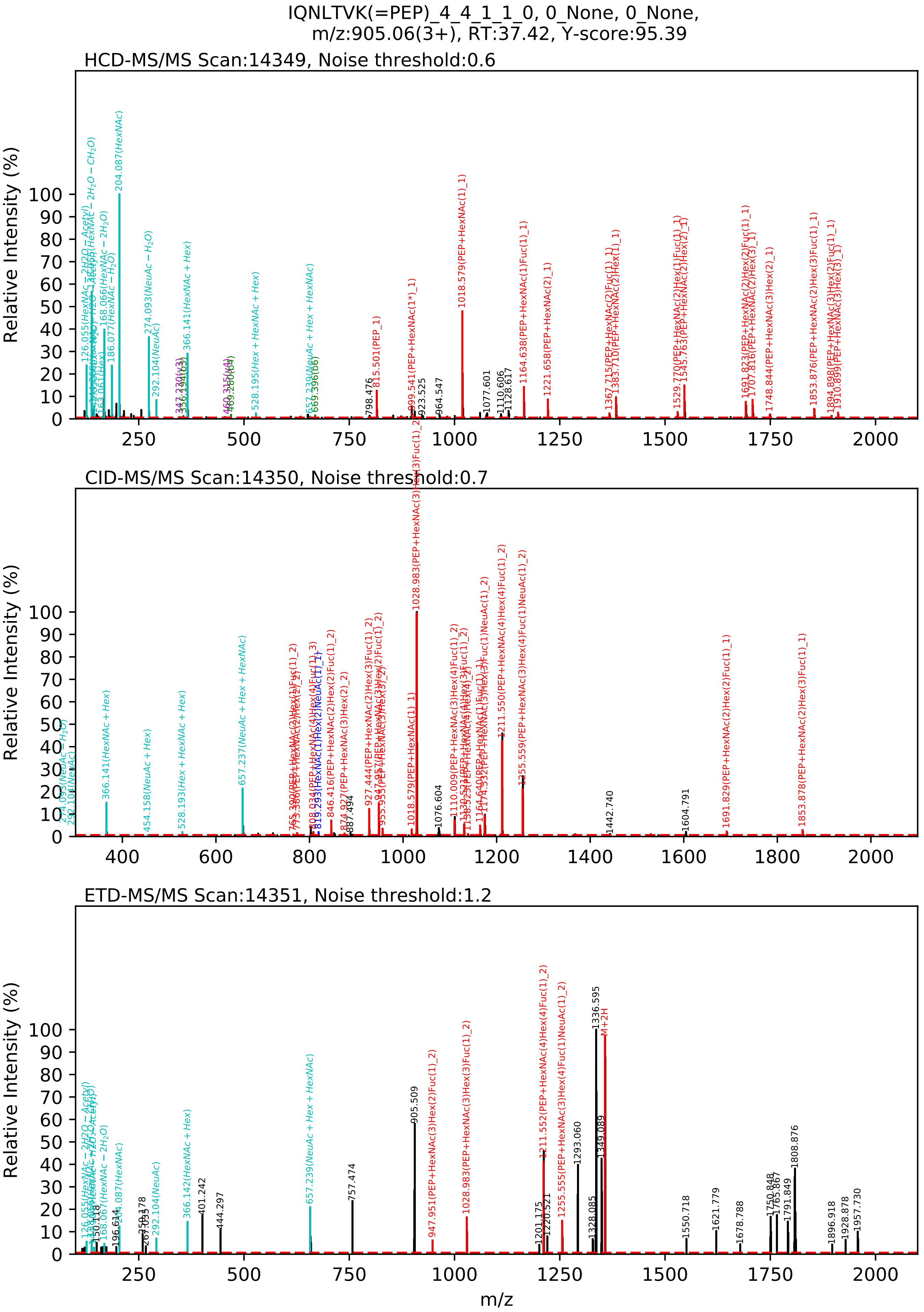
IQNLTVK(=PEP)_4_4_1_1_0, 0_None, 0_None,
m/z:905.06(3+), RT:37.42, Y-score:95.39
HCD-MS/MS Scan:14349, Noise threshold:0.6
Relative Intensity (%)
204.087(HexNAc)
168.066(HexNAc − 2H2O)
186.077(HexNAc − H2O)
274.093(NeuAc − H2O)
292.104(NeuAc)
366.141(HexNAc + Hex)
528.195(Hex + HexNAc + Hex)
657.239(NeuAc + Hex + HexNAc)
669.396(b6)
798.476
815.501(PEP_1)
999.541(PEP+HexNAc(1*)_1)
923.525
964.547
1018.579(PEP+HexNAc(1)_1)
1077.601
1110.606
1128.617
1164.638(PEP+HexNAc(1)Fuc(1)_1)
1221.658(PEP+HexNAc(2)_1)
1383.710(PEP+HexNAc(2)Hex(1)_1)
1545.763(PEP+HexNAc(2)Hex(2)_1)
1691.823(PEP+HexNAc(2)Hex(2)Fuc(1)_1)
1748.844(PEP+HexNAc(3)Hex(2)_1)
1853.876(PEP+HexNAc(2)Hex(3)Fuc(1)_1)
CID-MS/MS Scan:14350, Noise threshold:0.7
366.141(HexNAc + Hex)
454.158(NeuAc + Hex)
528.193(Hex + HexNAc + Hex)
657.237(NeuAc + Hex + HexNAc)
846.416(PEP+HexNAc(2)Hex(2)Fuc(1)_2)
874.927(PEP+HexNAc(3)Hex(2)_2)
887.494
927.444(PEP+HexNAc(2)Hex(3)Fuc(1)_2)
1018.579(PEP+HexNAc(1)_1)
1028.983(PEP+HexNAc(3)Hex(3)Fuc(1)_2)
1076.604
1110.009(PEP+HexNAc(3)Hex(4)Fuc(1)_2)
1211.550(PEP+HexNAc(4)Hex(4)Fuc(1)_2)
1255.559(PEP+HexNAc(3)Hex(4)Fuc(1)NeuAc(1)_2)
1442.740
1604.791
1691.829(PEP+HexNAc(2)Hex(2)Fuc(1)_1)
1853.878(PEP+HexNAc(2)Hex(3)Fuc(1)_1)
ETD-MS/MS Scan:14351, Noise threshold:1.2
204.087(HexNAc)
292.104(NeuAc)
366.142(HexNAc + Hex)
401.242
444.297
657.239(NeuAc + Hex + HexNAc)
757.474
905.509
947.951(PEP+HexNAc(3)Hex(2)Fuc(1)_2)
1028.983(PEP+HexNAc(3)Hex(3)Fuc(1)_2)
1201.175
1211.552(PEP+HexNAc(4)Hex(4)Fuc(1)_2)
1220.521
1255.555(PEP+HexNAc(3)Hex(4)Fuc(1)NeuAc(1)_2)
1293.060
1328.085
1336.595
M+2H
1349.089
1550.718
1621.779
1678.788
1750.848
1765.867
1791.849
1808.876
1896.918
1928.878
1957.730
m/z

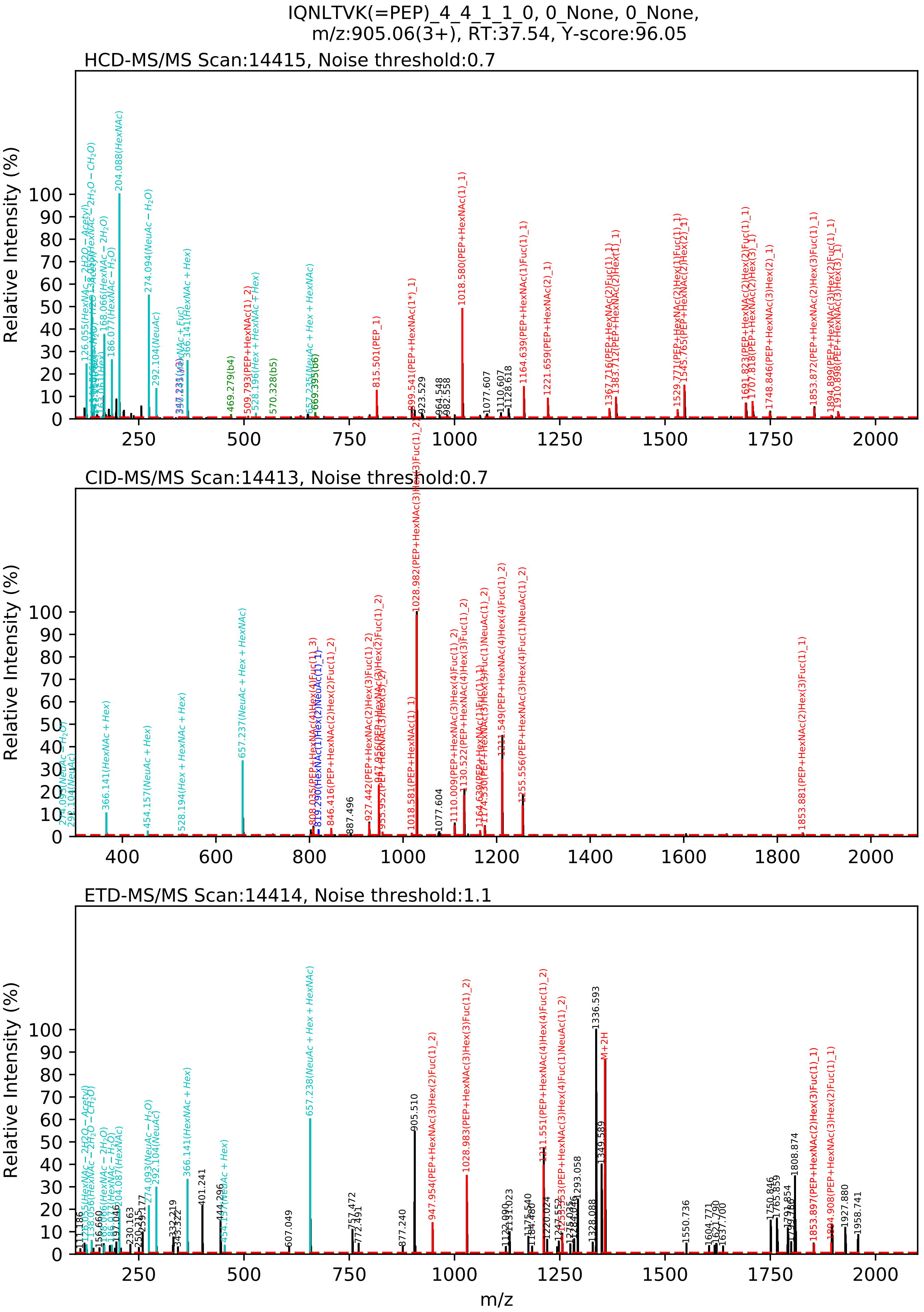
IQNLTVK(=PEP)_4_4_1_1_0, 0_None, 0_None,
m/z:905.06(3+), RT:37.54, Y-score:96.05
HCD-MS/MS Scan:14415, Noise threshold:0.7
CID-MS/MS Scan:14413, Noise threshold:0.7
ETD-MS/MS Scan:14414, Noise threshold:1.1
Relative Intensity (%)
m/z

IQNLTVK(=PEP)_4_4_1_1_0, 0_None, 0_None,
m/z:905.06(3+), RT:36.80, Y-score:97.22

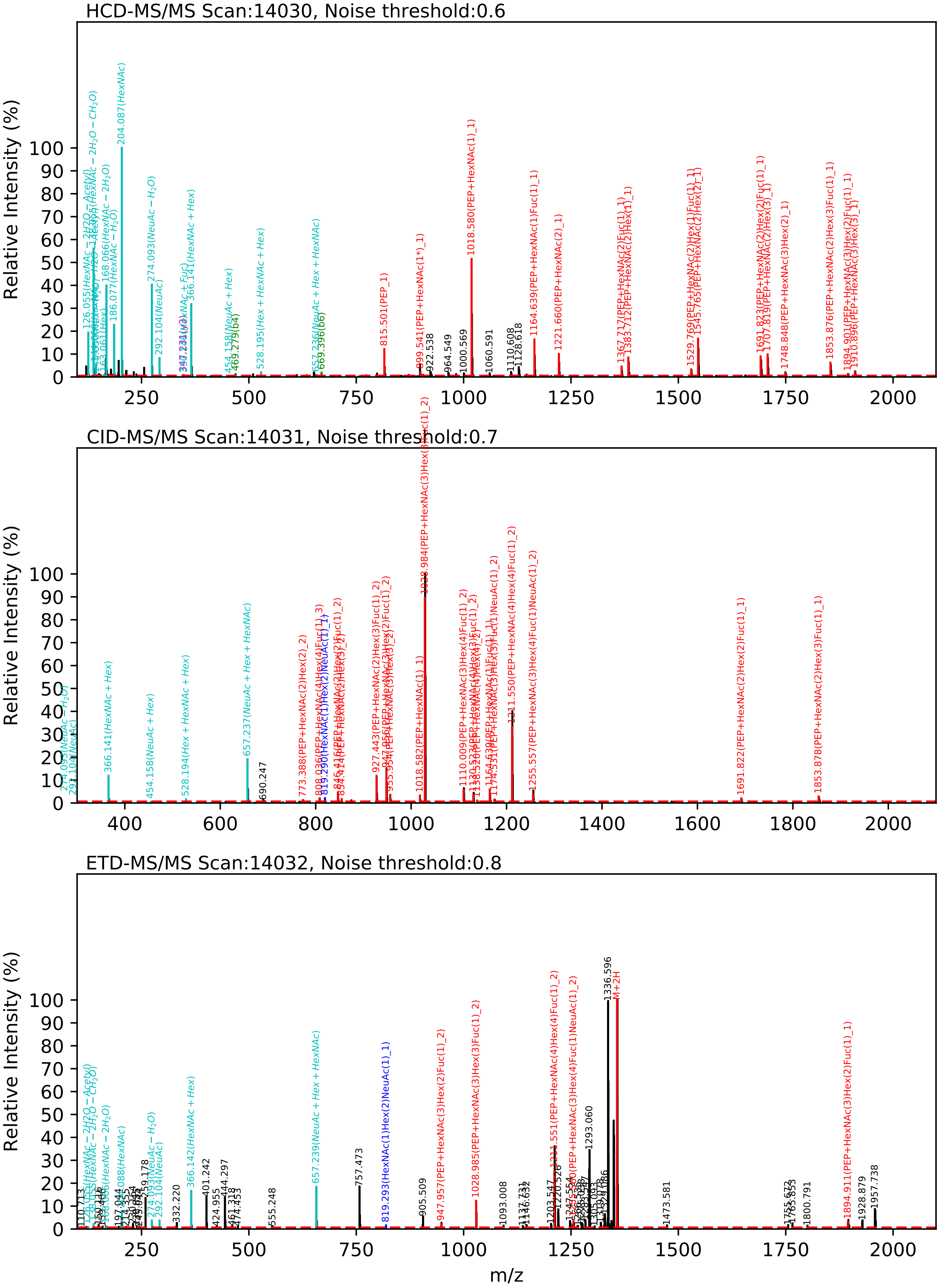

IQNLTVK(=PEP)_4_4_2_0_0, 0_None, 0_None,
m/z:856.72(3+), RT:27.13, Y-score:95.17

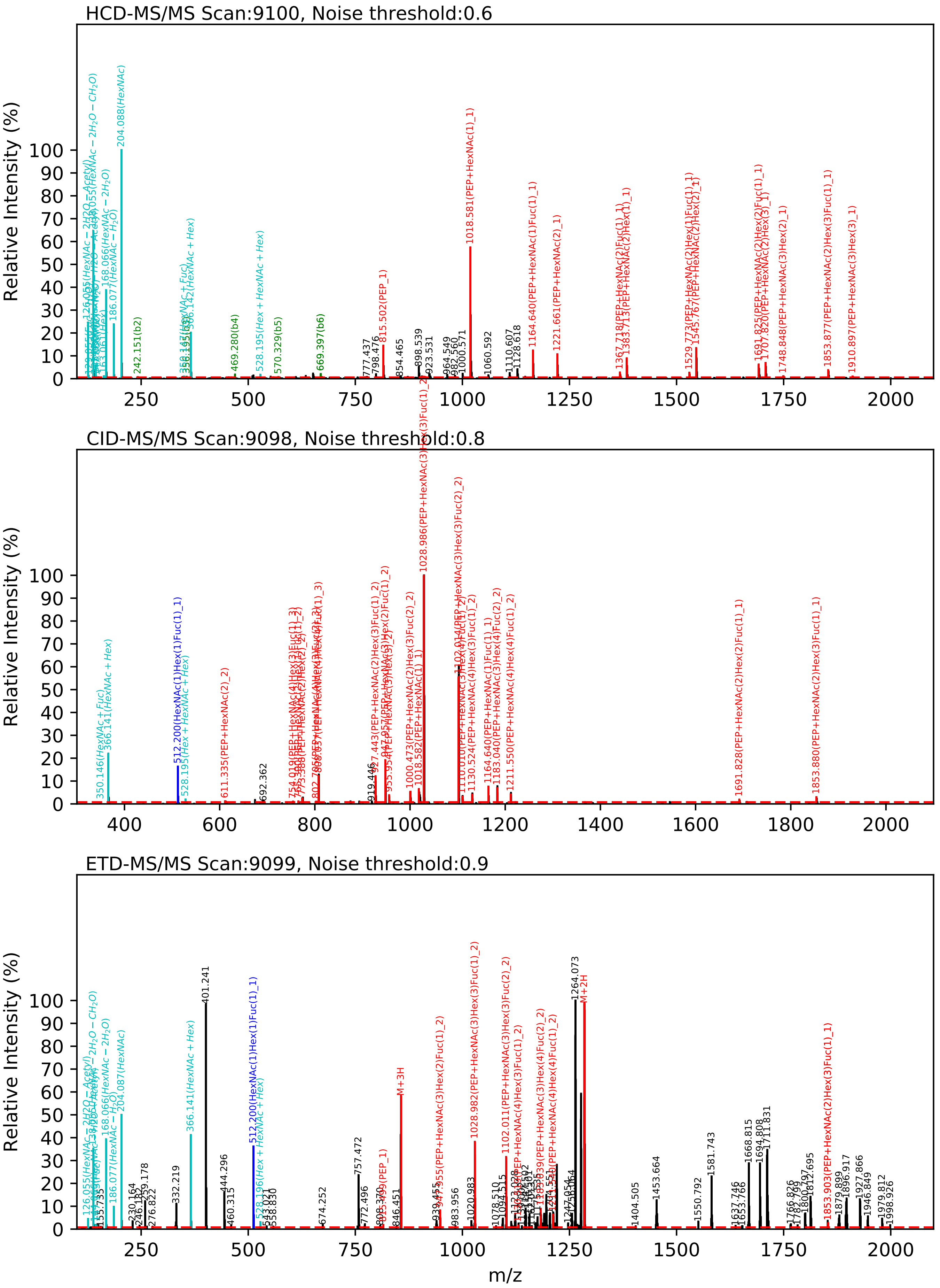

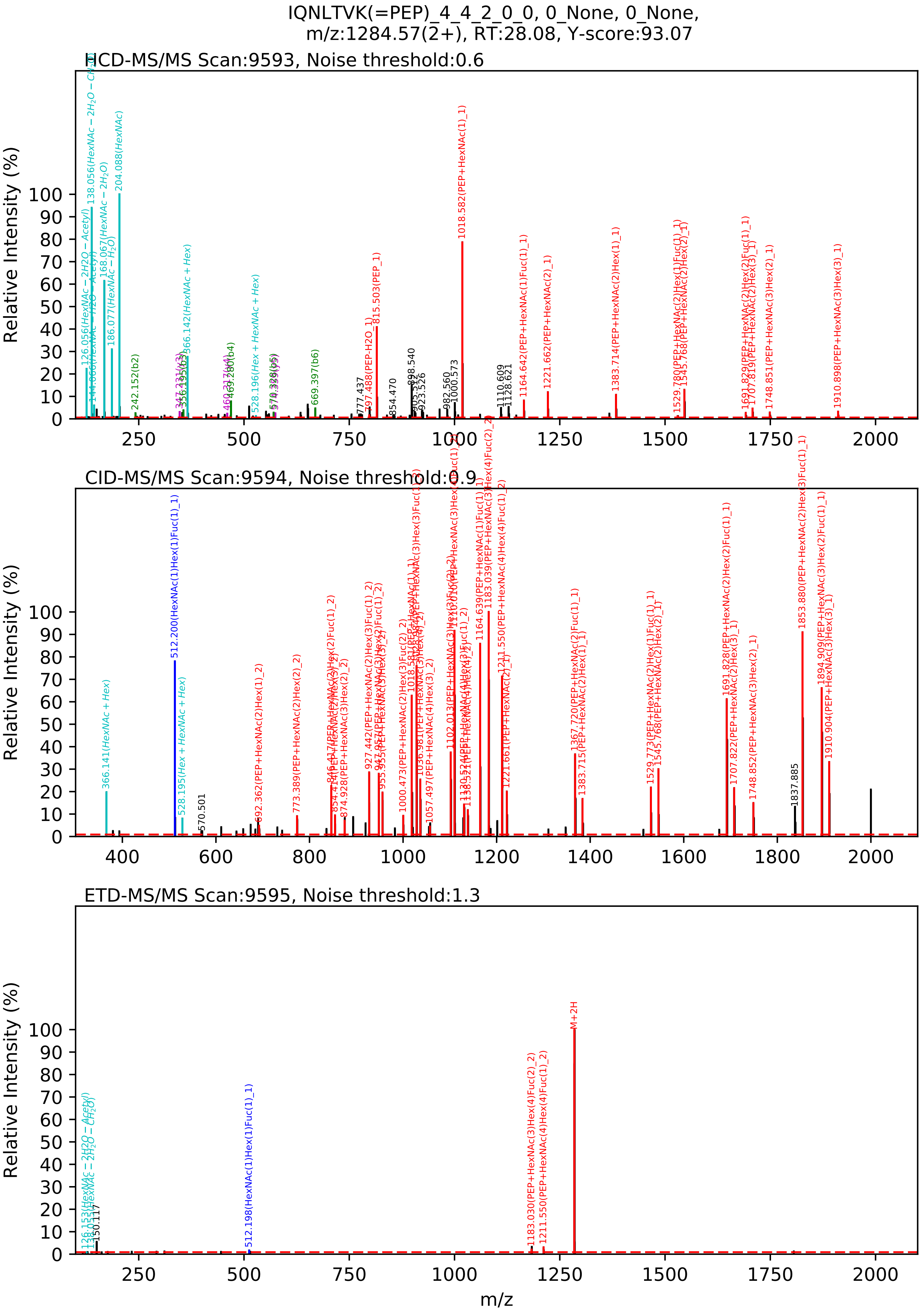
IQNLTVK(=PEP)_4_4_2_0_0, 0_None, 0_None,
m/z:1284.57(2+), RT:28.08, Y-score:93.07
HCD-MS/MS Scan:9593, Noise threshold:0.6
Relative Intensity (%)
126.056(HexNAc − 2H2O − Acetyl)
138.056(HexNAc − 2H2O − CH4O)
168.067(HexNAc − 2H2O)
186.077(HexNAc − H2O)
204.088(HexNAc)
242.152(b2)
366.142(HexNAc + Hex)
469.280(b4)
528.196(Hex + HexNAc + Hex)
669.397(b6)
777.437
797.488(PEP-H2O_1)
815.503(PEP_1)
854.470
898.540
923.526
982.560
1000.573
1018.582(PEP+HexNAc(1)_1)
1110.609
1128.621
1164.642(PEP+HexNAc(1)Fuc(1)_1)
1221.662(PEP+HexNAc(2)_1)
1383.714(PEP+HexNAc(2)Hex(1)_1)
1529.780(PEP+HexNAc(2)Hex(1)Fuc(1)_1)
1545.768(PEP+HexNAc(2)Hex(2)_1)
1691.829(PEP+HexNAc(2)Hex(2)Fuc(1)_1)
1707.819(PEP+HexNAc(2)Hex(3)_1)
1748.851(PEP+HexNAc(3)Hex(2)_1)
1910.898(PEP+HexNAc(3)Hex(3)_1)
CID-MS/MS Scan:9594, Noise threshold:0.9
Relative Intensity (%)
366.141(HexNAc + Hex)
512.200(HexNAc(1)Hex(1)Fuc(1)_1)
528.195(Hex + HexNAc + Hex)
570.501
692.362(PEP+HexNAc(2)Hex(1)_2)
773.389(PEP+HexNAc(2)Hex(2)_2)
874.928(PEP+HexNAc(3)Hex(2)_2)
927.442(PEP+HexNAc(2)Hex(3)Fuc(1)_2)
1000.473(PEP+HexNAc(2)Hex(3)Fuc(2)_2)
1036.981(PEP+HexNAc(3)Hex(3)Fuc(1)_2)
1057.497(PEP+HexNAc(4)Hex(3)_2)
1102.013(PEP+HexNAc(3)Hex(4)Fuc(1)_2)
1164.639(PEP+HexNAc(1)Fuc(1)_1)
1183.039(PEP+HexNAc(3)Hex(4)Fuc(2)_2)
1211.550(PEP+HexNAc(4)Hex(4)Fuc(1)_2)
1221.661(PEP+HexNAc(2)_1)
1367.720(PEP+HexNAc(2)Fuc(1)_1)
1383.715(PEP+HexNAc(2)Hex(1)_1)
1529.773(PEP+HexNAc(2)Hex(1)Fuc(1)_1)
1545.768(PEP+HexNAc(2)Hex(2)_1)
1691.828(PEP+HexNAc(2)Hex(2)Fuc(1)_1)
1707.822(PEP+HexNAc(2)Hex(3)_1)
1748.852(PEP+HexNAc(3)Hex(2)_1)
1837.885
1853.880(PEP+HexNAc(2)Hex(3)Fuc(1)_1)
1894.909(PEP+HexNAc(3)Hex(2)Fuc(1)_1)
1910.904(PEP+HexNAc(3)Hex(3)_1)
ETD-MS/MS Scan:9595, Noise threshold:1.3
Relative Intensity (%)
150.117
512.198(HexNAc(1)Hex(1)Fuc(1)_1)
1183.030(PEP+HexNAc(3)Hex(4)Fuc(2)_2)
1211.550(PEP+HexNAc(4)Hex(4)Fuc(1)_2)
M+2H
m/z

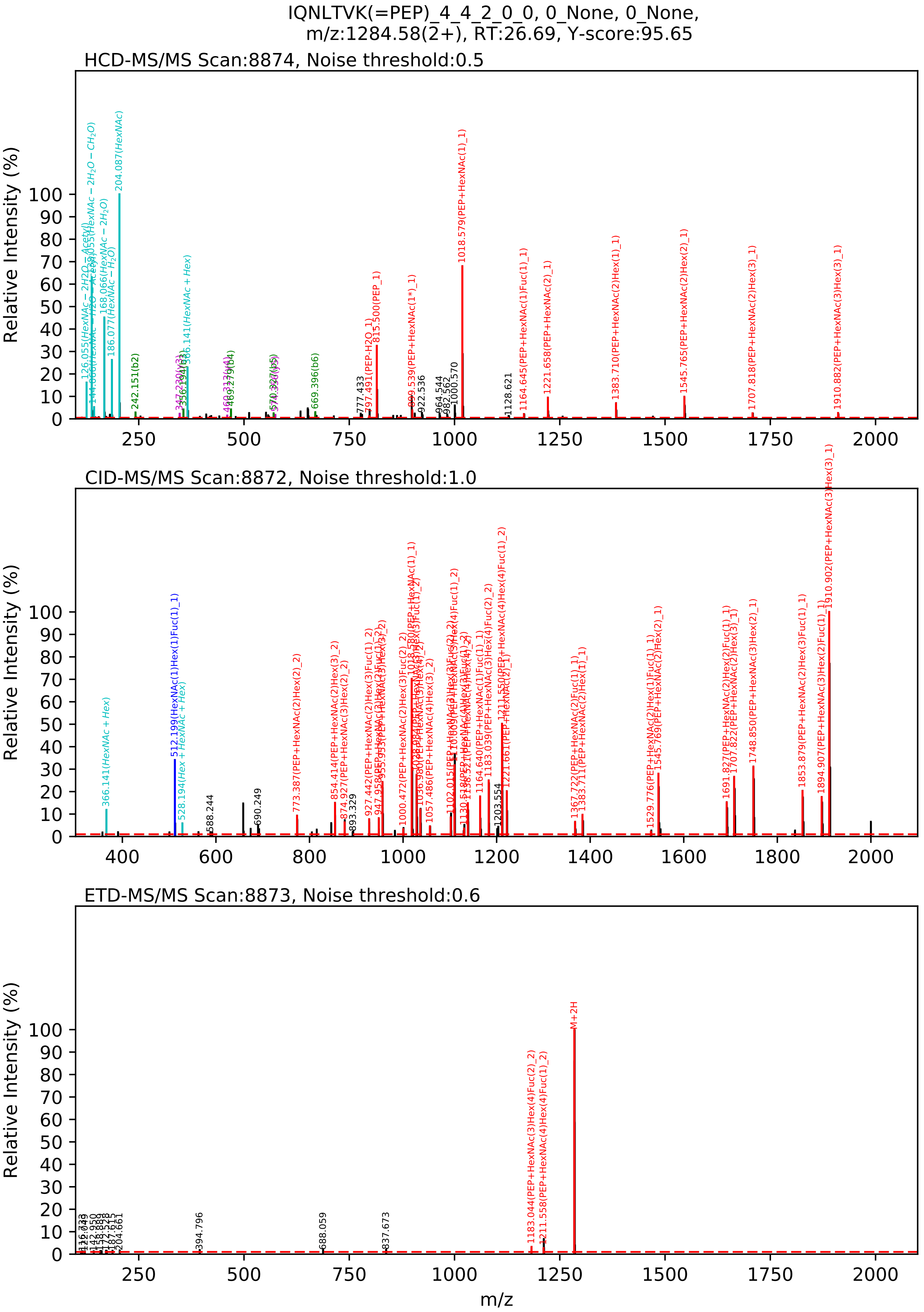
IQNLTVK(=PEP)_4_4_2_0_0, 0_None, 0_None,
m/z:1284.58(2+), RT:26.69, Y-score:95.65
HCD-MS/MS Scan:8874, Noise threshold:0.5
CID-MS/MS Scan:8872, Noise threshold:1.0
ETD-MS/MS Scan:8873, Noise threshold:0.6
Relative Intensity (%)
m/z
204.087(HexNAc)
1018.579(PEP+HexNAc(1)_1)
815.500(PEP_1)
1910.902(PEP+HexNAc(3)Hex(3)_1)
M+2H

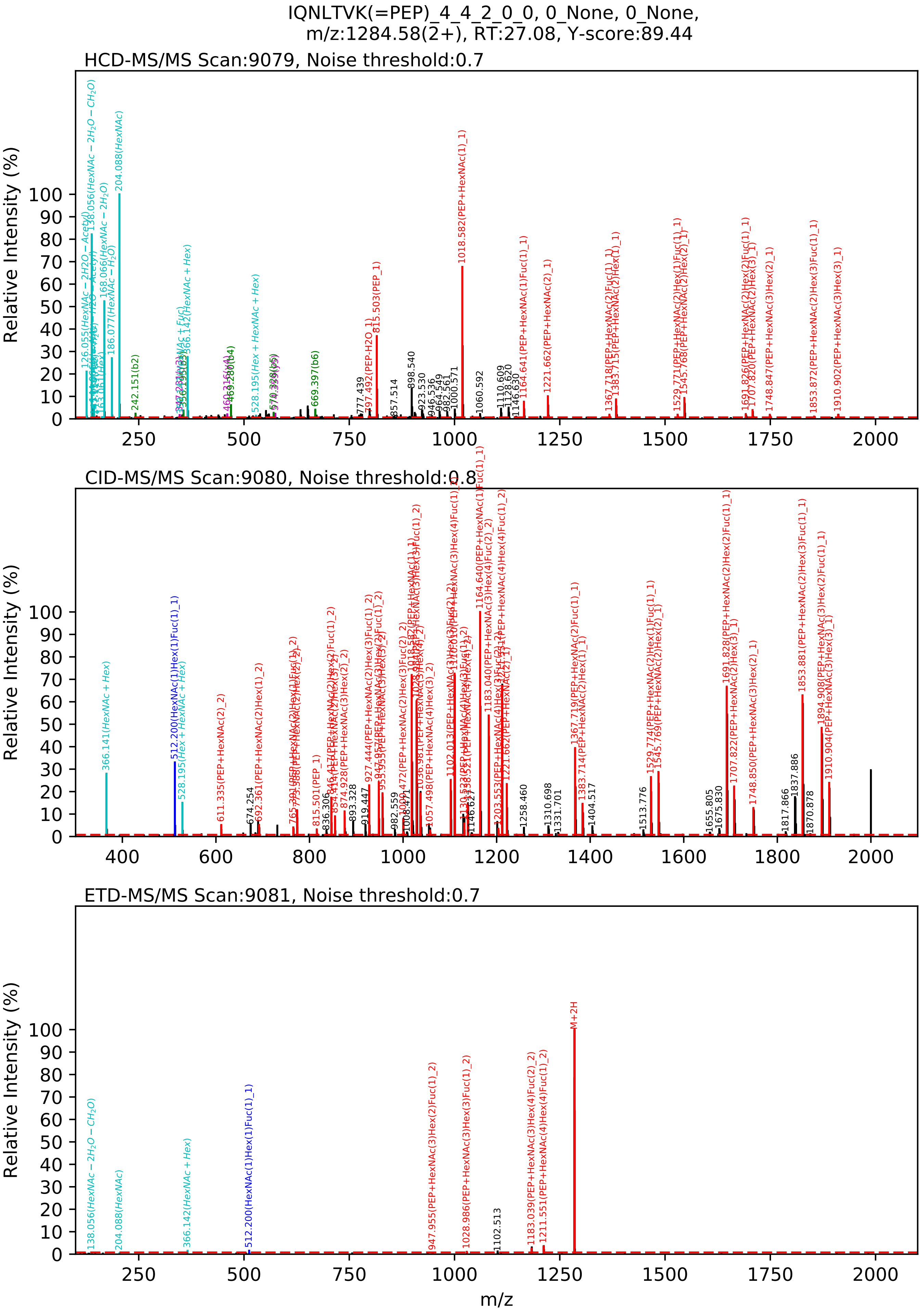
IQNLTVK(=PEP)_4_4_2_0_0, 0_None, 0_None,
m/z:1284.58(2+), RT:27.08, Y-score:89.44
HCD-MS/MS Scan:9079, Noise threshold:0.7
Relative Intensity (%)
204.088(HexNAc)
1018.582(PEP+HexNAc(1)_1)
815.503(PEP_1)
797.492(PEP-H2O_1)
1164.641(PEP+HexNAc(1)Fuc(1)_1)
1221.662(PEP+HexNAc(2)_1)
1383.715(PEP+HexNAc(2)Hex(1)_1)
1545.768(PEP+HexNAc(2)Hex(2)_1)
1707.820(PEP+HexNAc(2)Hex(3)_1)
1748.847(PEP+HexNAc(3)Hex(2)_1)
1853.872(PEP+HexNAc(2)Hex(3)Fuc(1)_1)
1910.902(PEP+HexNAc(3)Hex(3)_1)
669.397(b6)
242.151(b2)
CID-MS/MS Scan:9080, Noise threshold:0.8
366.141(HexNAc + Hex)
512.200(HexNAc(1)Hex(1)Fuc(1)_1)
1164.640(PEP+HexNAc(1)Fuc(1)_1)
1183.040(PEP+HexNAc(3)Hex(4)Fuc(2)_2)
1691.828(PEP+HexNAc(2)Hex(2)Fuc(1)_1)
1853.881(PEP+HexNAc(2)Hex(3)Fuc(1)_1)
ETD-MS/MS Scan:9081, Noise threshold:0.7
M+2H
947.955(PEP+HexNAc(3)Hex(2)Fuc(1)_2)
1028.986(PEP+HexNAc(3)Hex(3)Fuc(1)_2)
1102.513
1183.039(PEP+HexNAc(3)Hex(4)Fuc(2)_2)
1211.551(PEP+HexNAc(4)Hex(4)Fuc(1)_2)
m/z

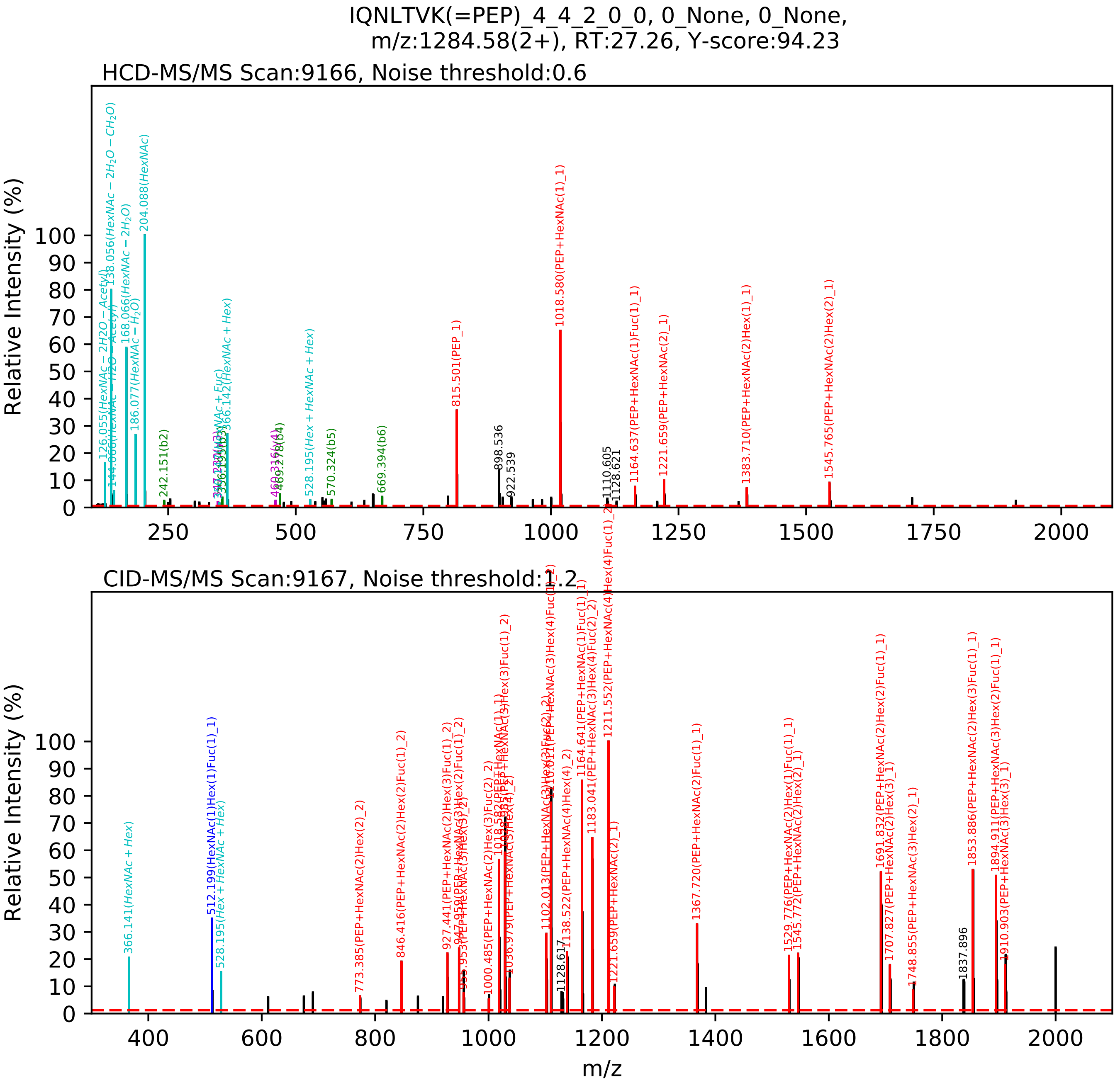
IQNLTVK(=PEP)_4_4_2_0_0, 0_None, 0_None,
m/z:1284.58(2+), RT:27.26, Y-score:94.23
HCD-MS/MS Scan:9166, Noise threshold:0.6
Relative Intensity (%)
204.088(HexNAc)
1018.580(PEP+HexNAc(1)_1)
815.501(PEP_1)
1164.637(PEP+HexNAc(1)Fuc(1)_1)
1221.659(PEP+HexNAc(2)_1)
1383.710(PEP+HexNAc(2)Hex(1)_1)
1545.765(PEP+HexNAc(2)Hex(2)_1)
242.151(b2)
570.324(b5)
669.394(b6)
898.536
922.539
1110.605
1128.621
CID-MS/MS Scan:9167, Noise threshold:1.2
366.141(HexNAc+Hex)
512.199(HexNAc(1)Hex(1)Fuc(1)_1)
528.195(Hex+HexNAc+Hex)
773.385(PEP+HexNAc(2)Hex(2)_2)
846.416(PEP+HexNAc(2)Hex(2)Fuc(1)_2)
1211.552(PEP+HexNAc(4)Hex(4)Fuc(1)_2)
1367.720(PEP+HexNAc(2)Fuc(1)_1)
1691.832(PEP+HexNAc(2)Hex(2)Fuc(1)_1)
1853.886(PEP+HexNAc(2)Hex(3)Fuc(1)_1)
1837.896
1128.617
m/z

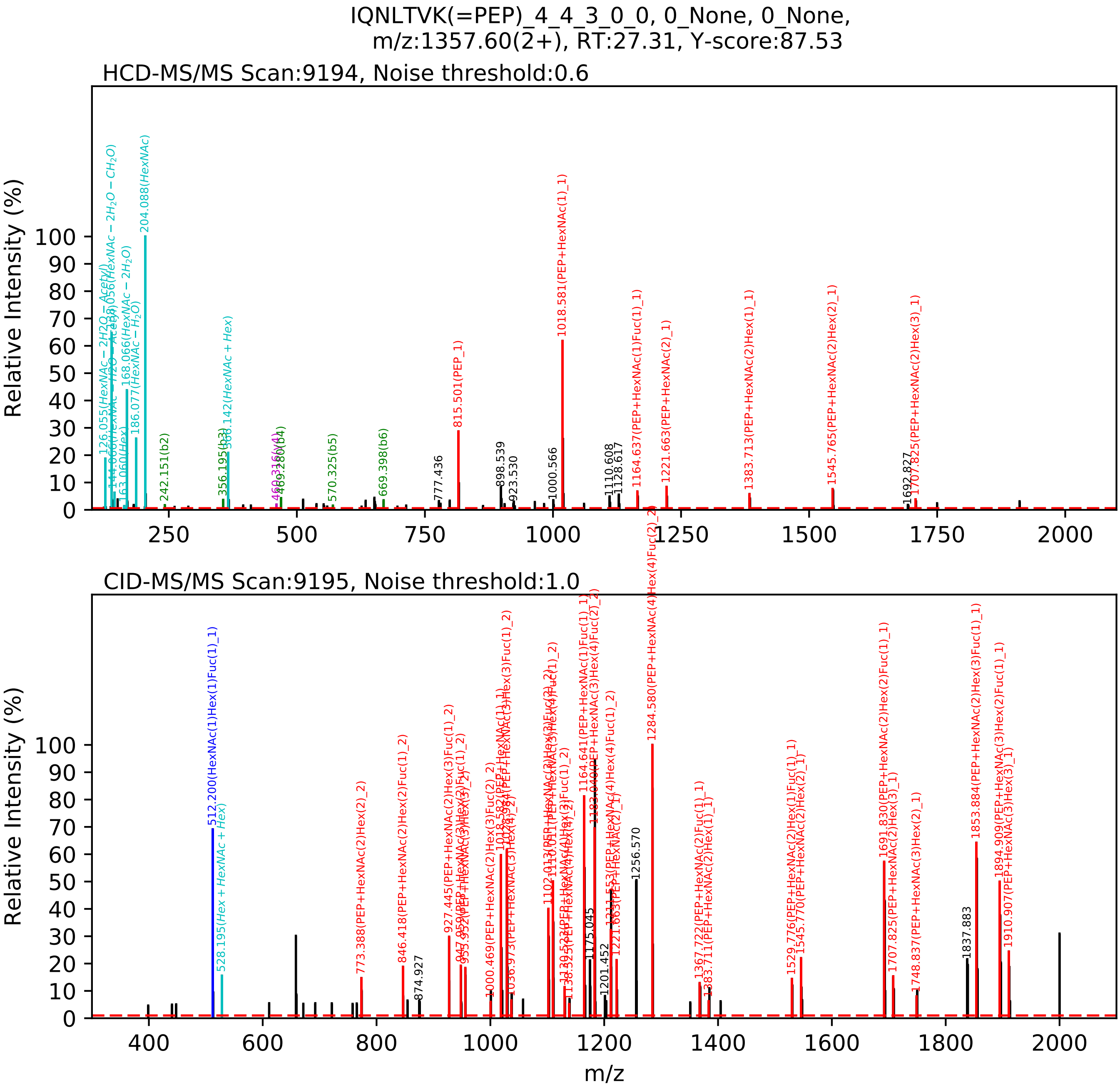
IQNLTVK(=PEP)_4_4_3_0_0, 0_None, 0_None,
m/z:1357.60(2+), RT:27.31, Y-score:87.53
HCD-MS/MS Scan:9194, Noise threshold:0.6
Relative Intensity (%)
204.088(HexNAc)
1018.581(PEP+HexNAc(1)_1)
815.501(PEP_1)
1164.637(PEP+HexNAc(1)Fuc(1)_1)
1221.663(PEP+HexNAc(2)_1)
1383.713(PEP+HexNAc(2)Hex(1)_1)
1545.765(PEP+HexNAc(2)Hex(2)_1)
1707.825(PEP+HexNAc(2)Hex(3)_1)
242.151(b2)
356.195(b3)
570.325(b5)
669.398(b6)
CID-MS/MS Scan:9195, Noise threshold:1.0
512.200(HexNAc(1)Hex(1)Fuc(1)_1)
528.195(Hex + HexNAc + Hex)
773.388(PEP+HexNAc(2)Hex(2)_2)
846.418(PEP+HexNAc(2)Hex(2)Fuc(1)_2)
874.927
1284.580(PEP+HexNAc(4)Hex(4)Fuc(1)_2)
1256.570
1164.641(PEP+HexNAc(1)Fuc(1)_1)
1175.045
1201.452
1367.722(PEP+HexNAc(2)Fuc(1)_1)
1529.776(PEP+HexNAc(2)Hex(1)Fuc(1)_1)
1545.770(PEP+HexNAc(2)Hex(2)_1)
1691.830(PEP+HexNAc(2)Hex(2)Fuc(1)_1)
1707.825(PEP+HexNAc(2)Hex(3)_1)
1748.837(PEP+HexNAc(3)Hex(2)_1)
1837.883
1853.884(PEP+HexNAc(2)Hex(3)Fuc(1)_1)
1894.909(PEP+HexNAc(3)Hex(2)Fuc(1)_1)
1910.907(PEP+HexNAc(3)Hex(3)_1)
m/z

IQNLTVK(=PEP)_4_5_0_0_0, 0_None, 0_None,
m/z:1240.06(2+), RT:26.24, Y-score:92.44

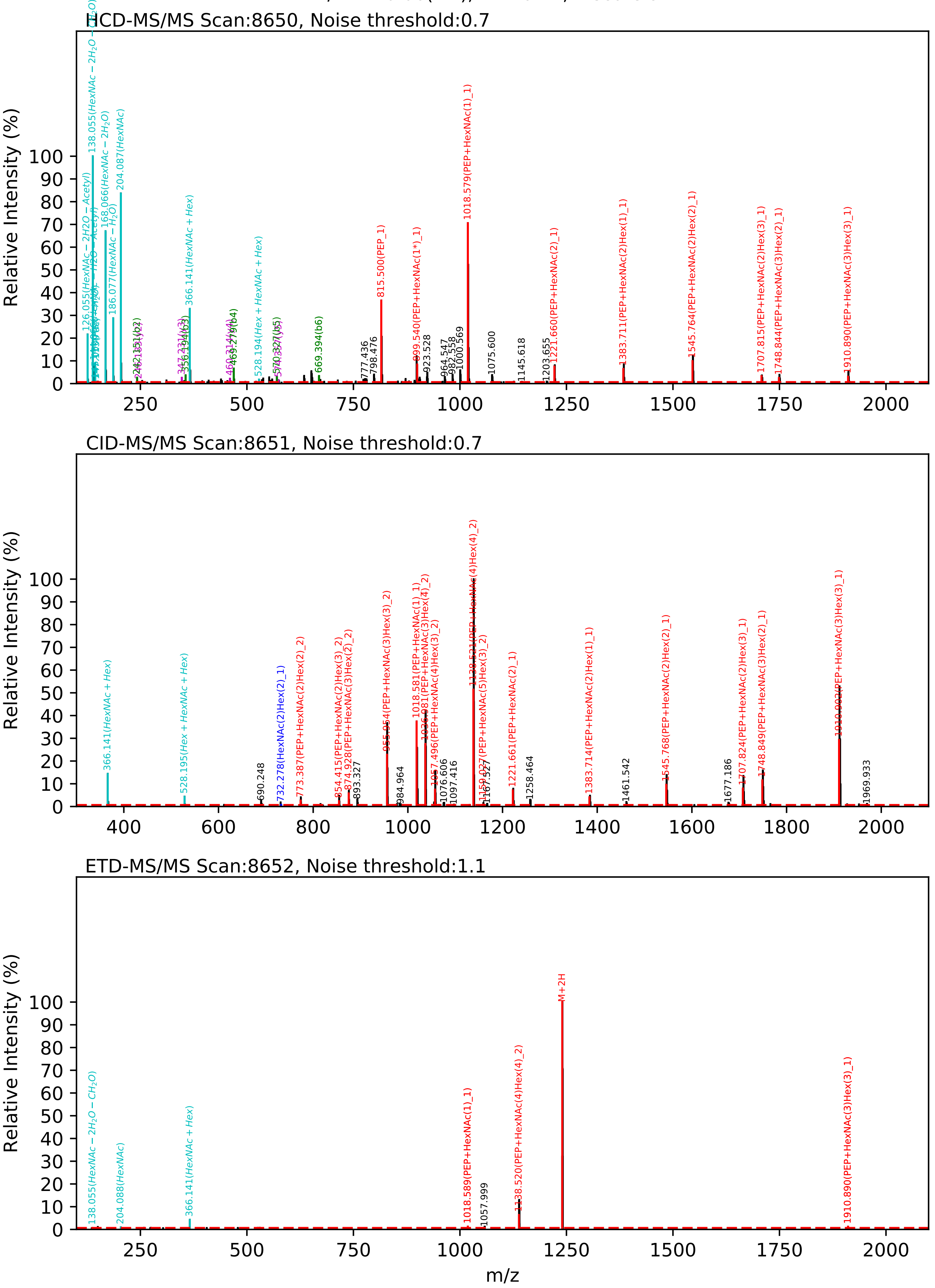

IQNLTVK(=PEP)_4_5_0_0_0, 0_None, 0_None,
m/z:1240.06(2+), RT:26.26, Y-score:92.00

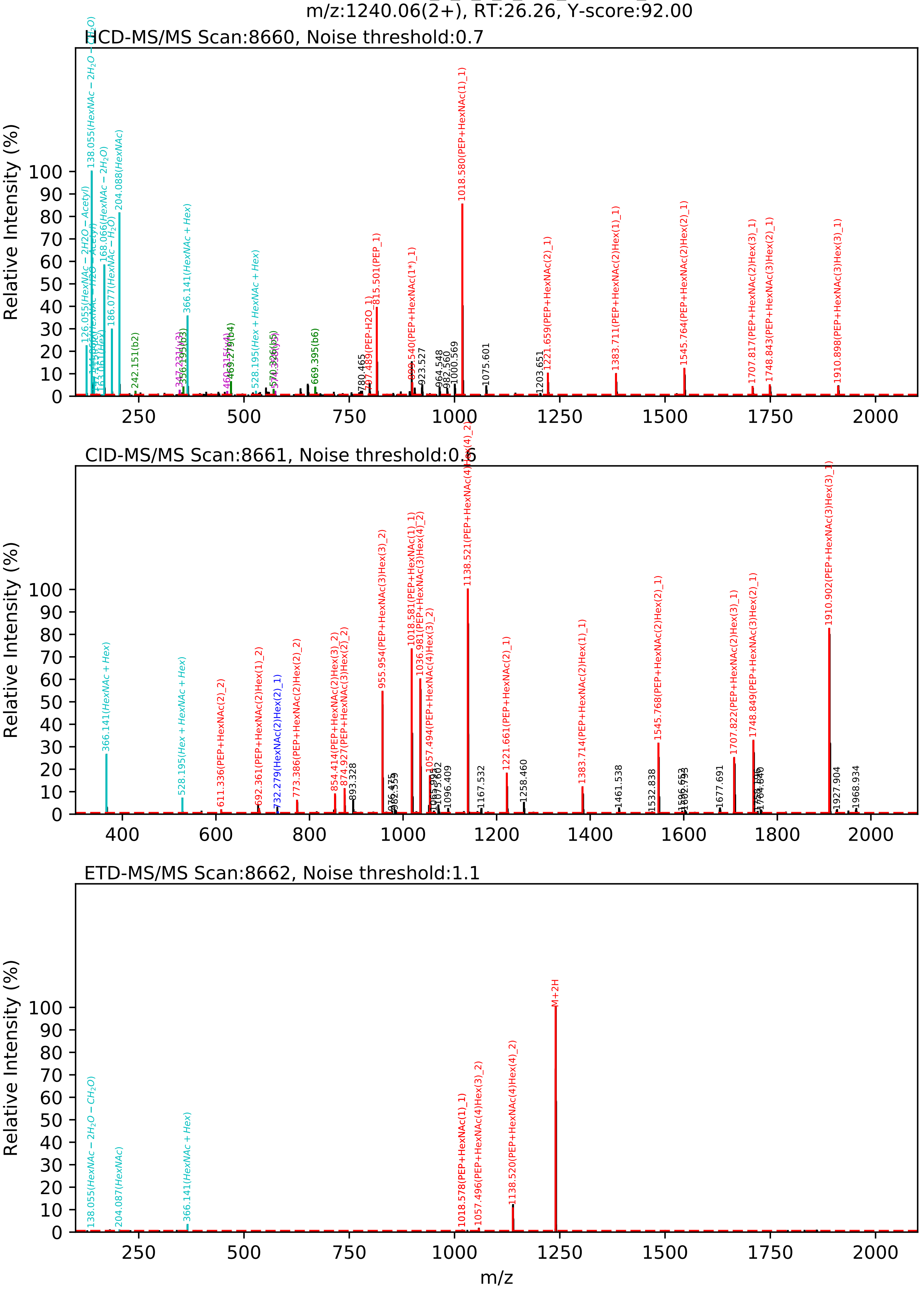

IQNLTVK(=PEP)_4_5_0_0_0, 0_None, 0_None,
m/z:1240.06(2+), RT:27.96, Y-score:71.27

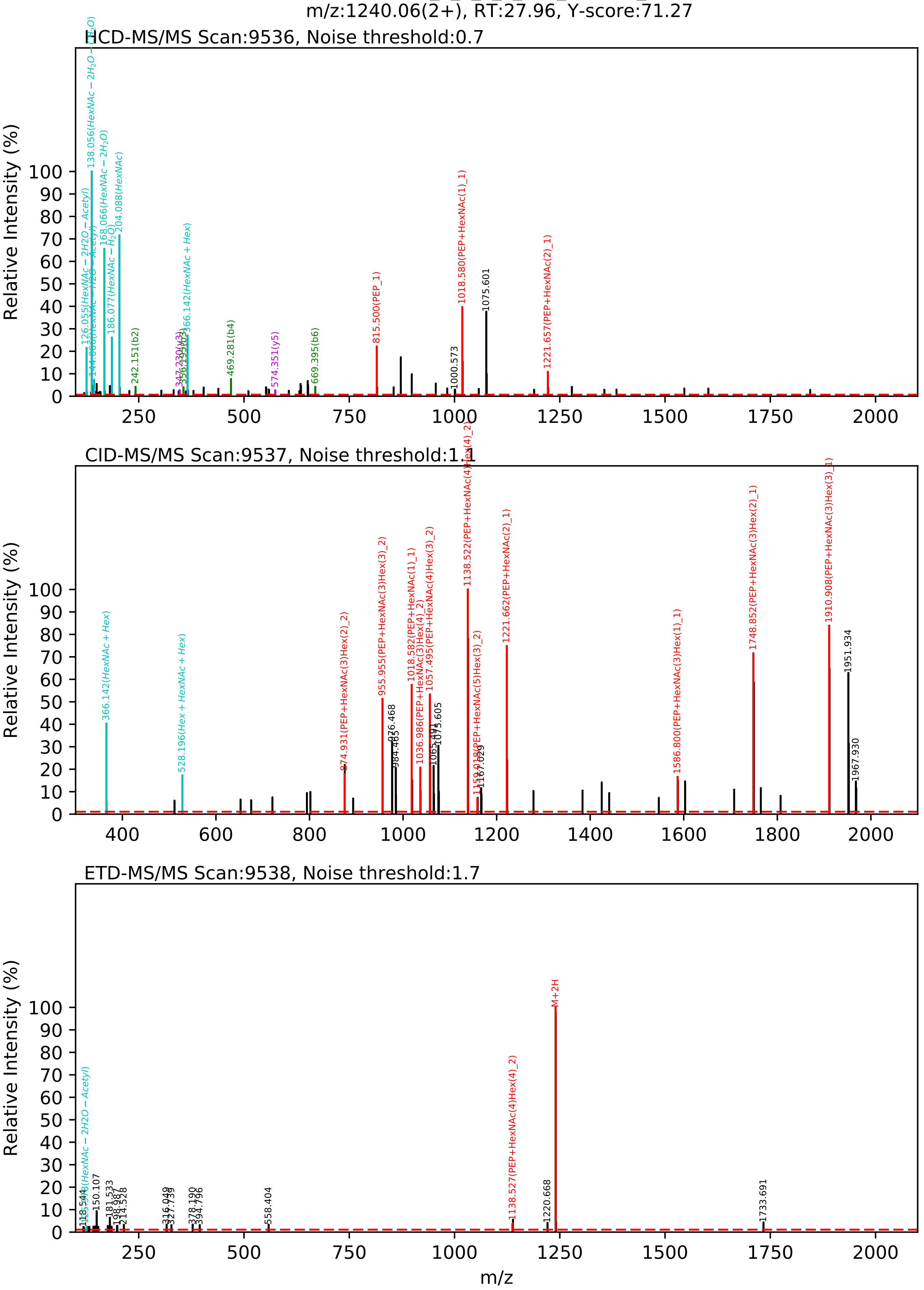

IQNLTVK(=PEP)_4_5_0_0_0, 0_None, 0_None,
m/z:827.04(3+), RT:28.02, Y-score:84.00

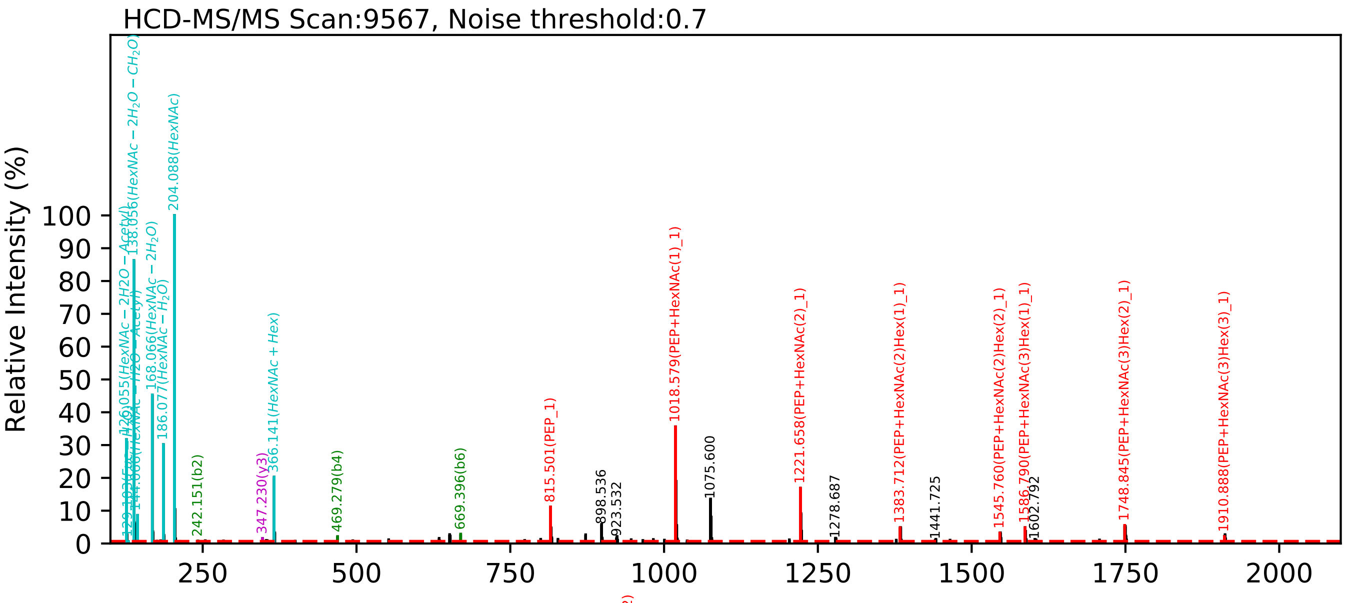

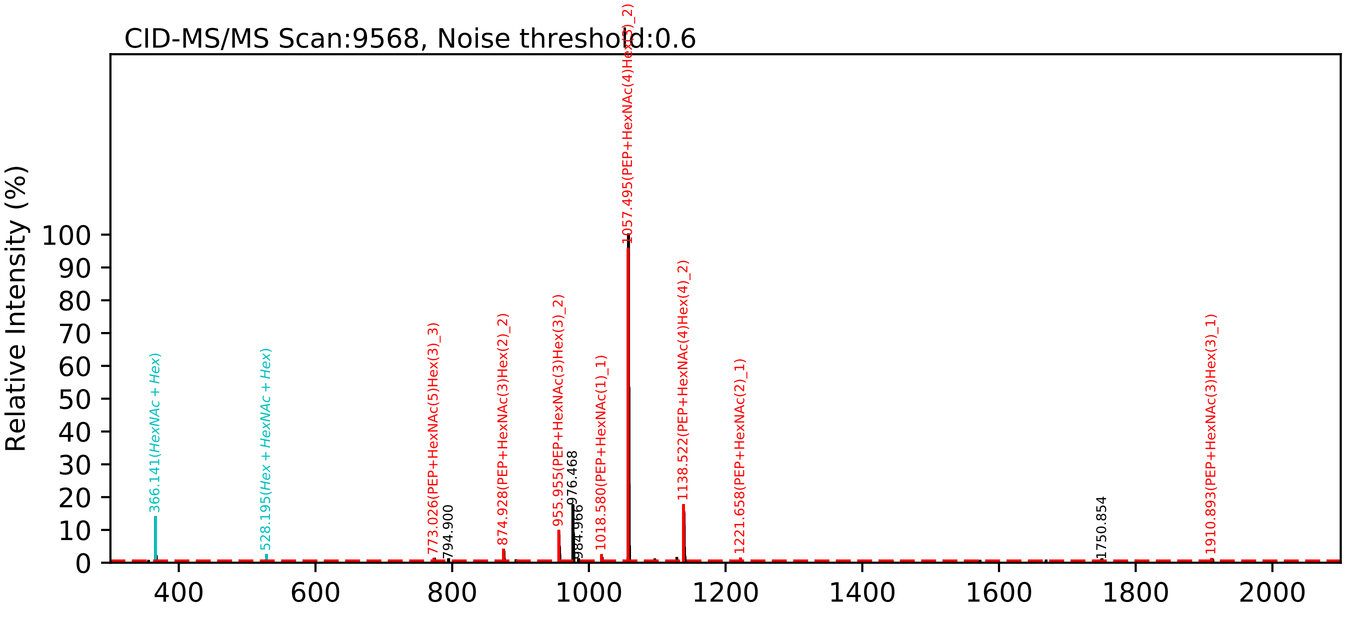

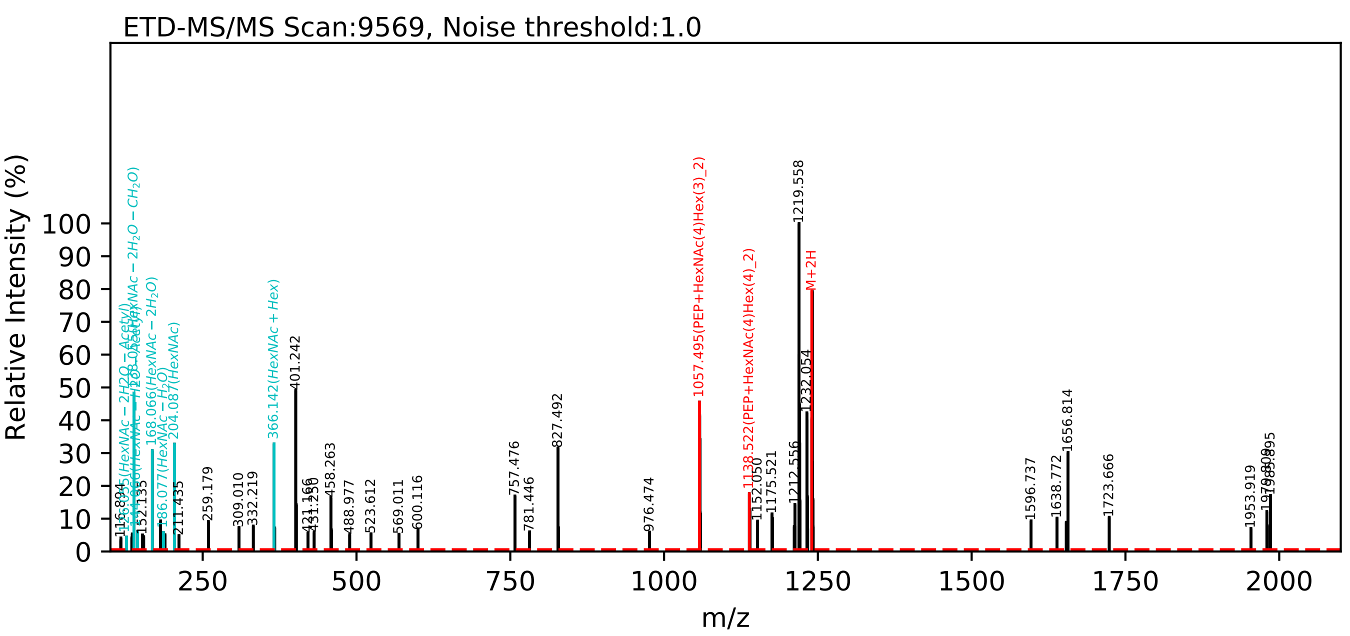

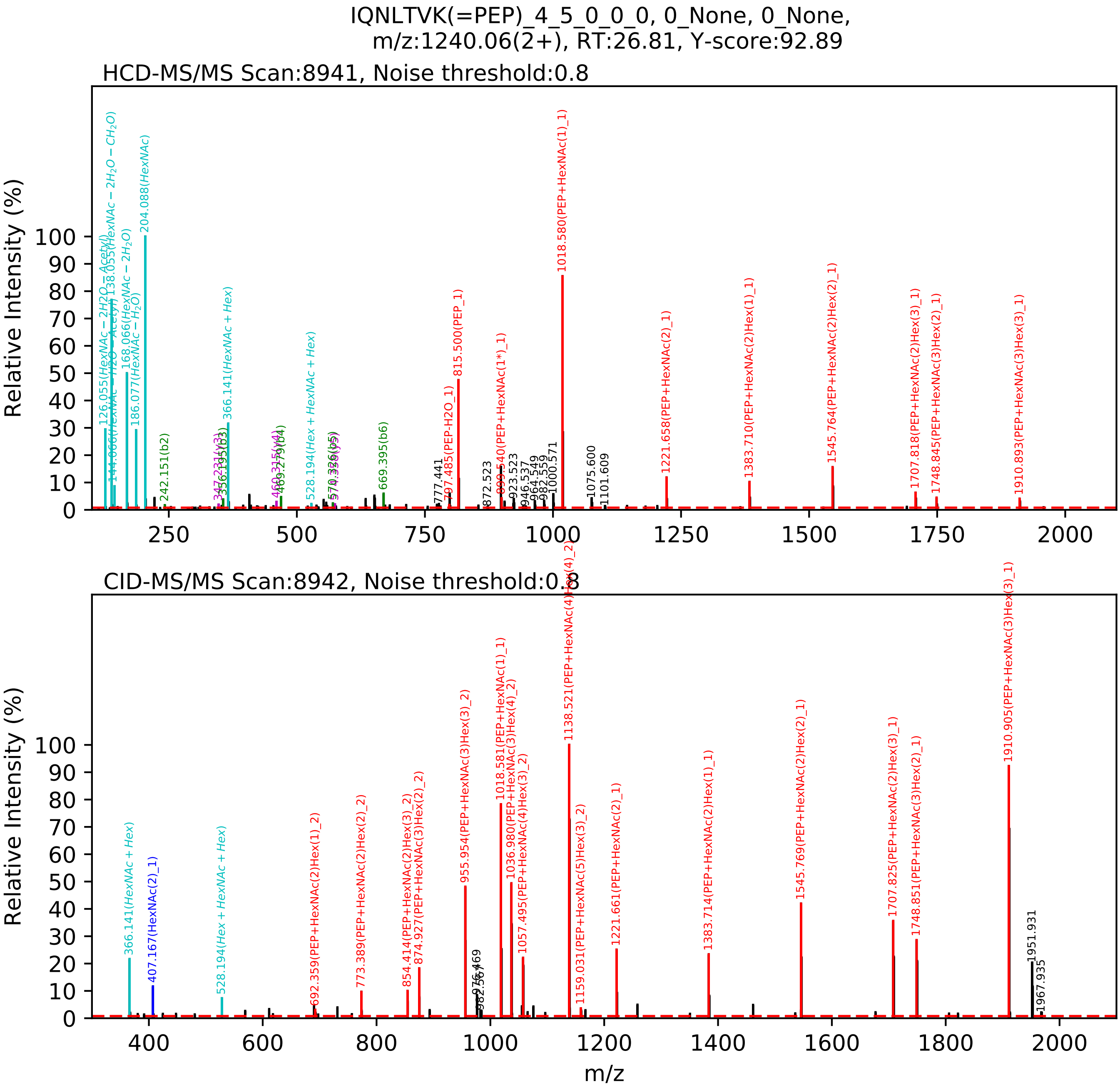
IQNLTVK(=PEP)_4_5_0_0_0, 0_None, 0_None,
m/z:1240.06(2+), RT:26.81, Y-score:92.89
HCD-MS/MS Scan:8941, Noise threshold:0.8
Relative Intensity (%)
204.088(HexNAc)
1018.580(PEP+HexNAc(1)_1)
815.500(PEP_1)
366.141(HexNAc+Hex)
1221.658(PEP+HexNAc(2)_1)
1383.710(PEP+HexNAc(2)Hex(1)_1)
1545.764(PEP+HexNAc(2)Hex(2)_1)
1707.818(PEP+HexNAc(2)Hex(3)_1)
1748.845(PEP+HexNAc(3)Hex(2)_1)
1910.893(PEP+HexNAc(3)Hex(3)_1)
242.151(b2)
669.395(b6)
528.194(Hex+HexNAc+Hex)
1075.600
1101.609
CID-MS/MS Scan:8942, Noise threshold:0.8
Relative Intensity (%)
366.141(HexNAc+Hex)
407.167(HexNAc(2)_1)
528.194(Hex+HexNAc+Hex)
692.359(PEP+HexNAc(2)Hex(1)_2)
773.389(PEP+HexNAc(2)Hex(2)_2)
854.414(PEP+HexNAc(2)Hex(3)_2)
874.927(PEP+HexNAc(3)Hex(2)_2)
955.954(PEP+HexNAc(3)Hex(3)_2)
1018.581(PEP+HexNAc(1)_1)
1036.980(PEP+HexNAc(3)Hex(4)_2)
1057.495(PEP+HexNAc(4)Hex(3)_2)
1138.521(PEP+HexNAc(4)Hex(4)_2)
1159.031(PEP+HexNAc(5)Hex(3)_2)
1221.661(PEP+HexNAc(2)_1)
1383.714(PEP+HexNAc(2)Hex(1)_1)
1545.769(PEP+HexNAc(2)Hex(2)_1)
1707.825(PEP+HexNAc(2)Hex(3)_1)
1748.851(PEP+HexNAc(3)Hex(2)_1)
1910.905(PEP+HexNAc(3)Hex(3)_1)
1951.931
1967.935
m/z

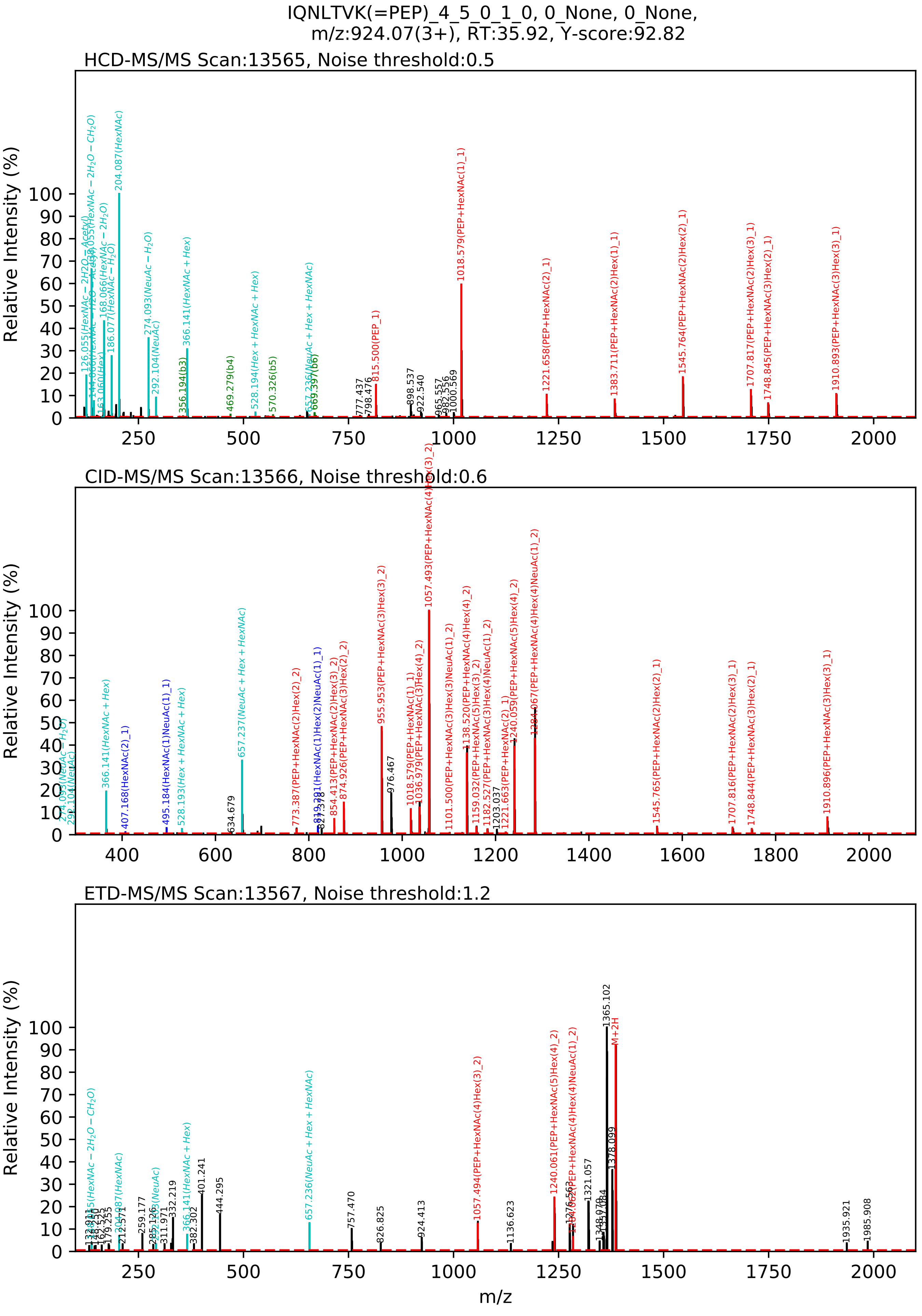

IQNLTVK(=PEP)_4_5_0_1_0, 0_None, 0_None,
m/z:924.07(3+), RT:35.92, Y-score:92.82
HCD-MS/MS Scan:13565, Noise threshold:0.5
CID-MS/MS Scan:13566, Noise threshold:0.6
ETD-MS/MS Scan:13567, Noise threshold:1.2
Relative Intensity (%)
m/z
1018.579(PEP+HexNAc(1)_1)
1057.493(PEP+HexNAc(4)Hex(3)_2)
1365.102
M+2H

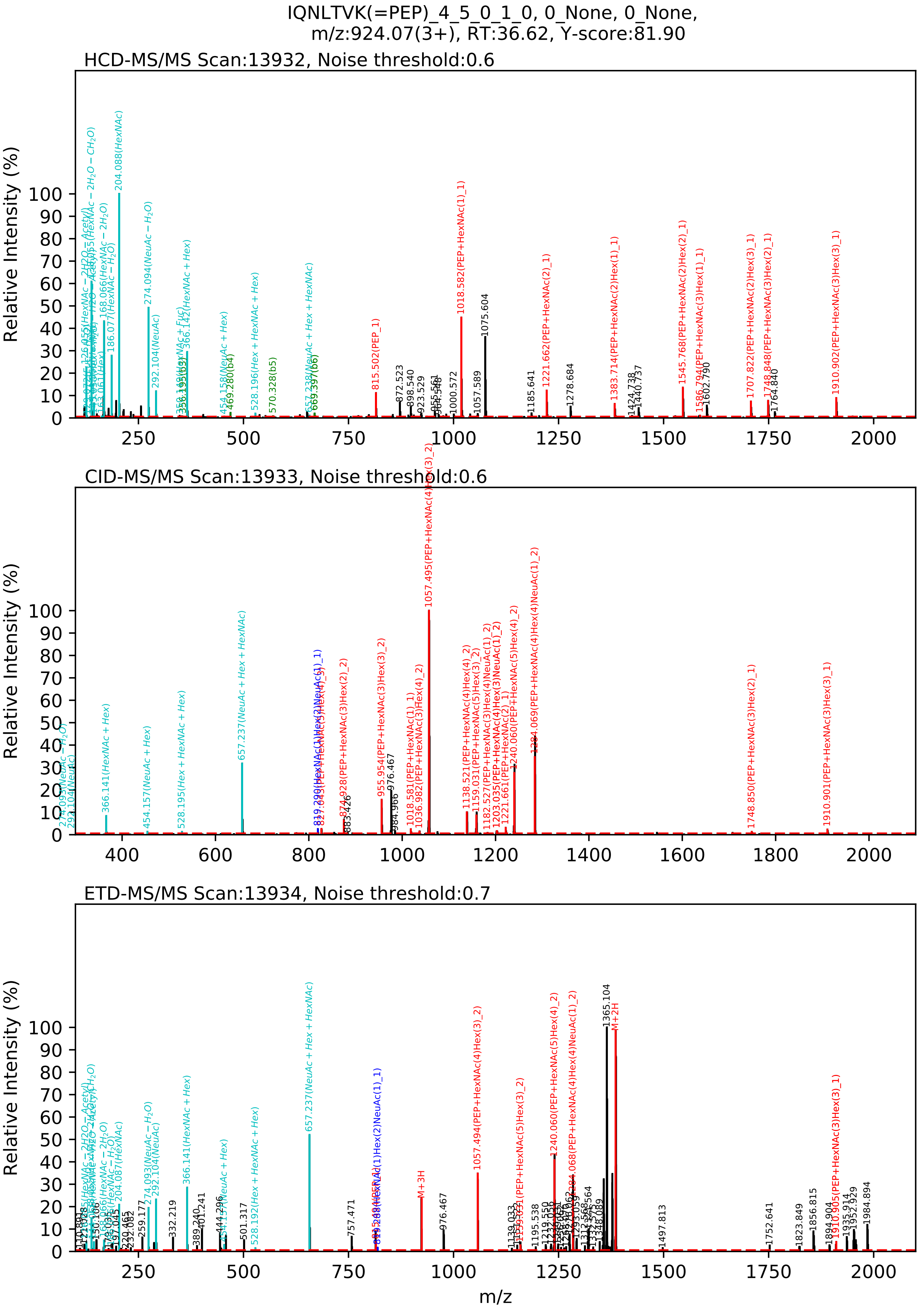
IQNLTVK(=PEP)_4_5_0_1_0, 0_None, 0_None,
m/z:924.07(3+), RT:36.62, Y-score:81.90
HCD-MS/MS Scan:13932, Noise threshold:0.6
CID-MS/MS Scan:13933, Noise threshold:0.6
ETD-MS/MS Scan:13934, Noise threshold:0.7
Relative Intensity (%)
m/z
204.088(HexNAc)
274.094(NeuAc − H2O)
292.104(NeuAc)
366.142(HexNAc + Hex)
454.158(NeuAc + Hex)
528.196(Hex + HexNAc + Hex)
657.238(NeuAc + Hex + HexNAc)
815.502(PEP_1)
1018.582(PEP+HexNAc(1)_1)
1075.604
1221.662(PEP+HexNAc(2)_1)
1383.714(PEP+HexNAc(2)Hex(1)_1)
1545.768(PEP+HexNAc(2)Hex(2)_1)
1586.794(PEP+HexNAc(3)Hex(1)_1)
1707.822(PEP+HexNAc(2)Hex(3)_1)
1748.848(PEP+HexNAc(3)Hex(2)_1)
1910.902(PEP+HexNAc(3)Hex(3)_1)
657.237(NeuAc + Hex + HexNAc)
874.928(PEP+HexNAc(3)Hex(2)_2)
955.954(PEP+HexNAc(3)Hex(3)_2)
1057.495(PEP+HexNAc(4)Hex(3)_2)
1138.521(PEP+HexNAc(4)Hex(4)_2)
1240.060(PEP+HexNAc(5)Hex(4)_2)
1284.069(PEP+HexNAc(4)Hex(4)NeuAc(1)_2)
1748.850(PEP+HexNAc(3)Hex(2)_1)
1910.901(PEP+HexNAc(3)Hex(3)_1)
1057.494(PEP+HexNAc(4)Hex(3)_2)
1365.104
M+2H
M+3H
1984.894

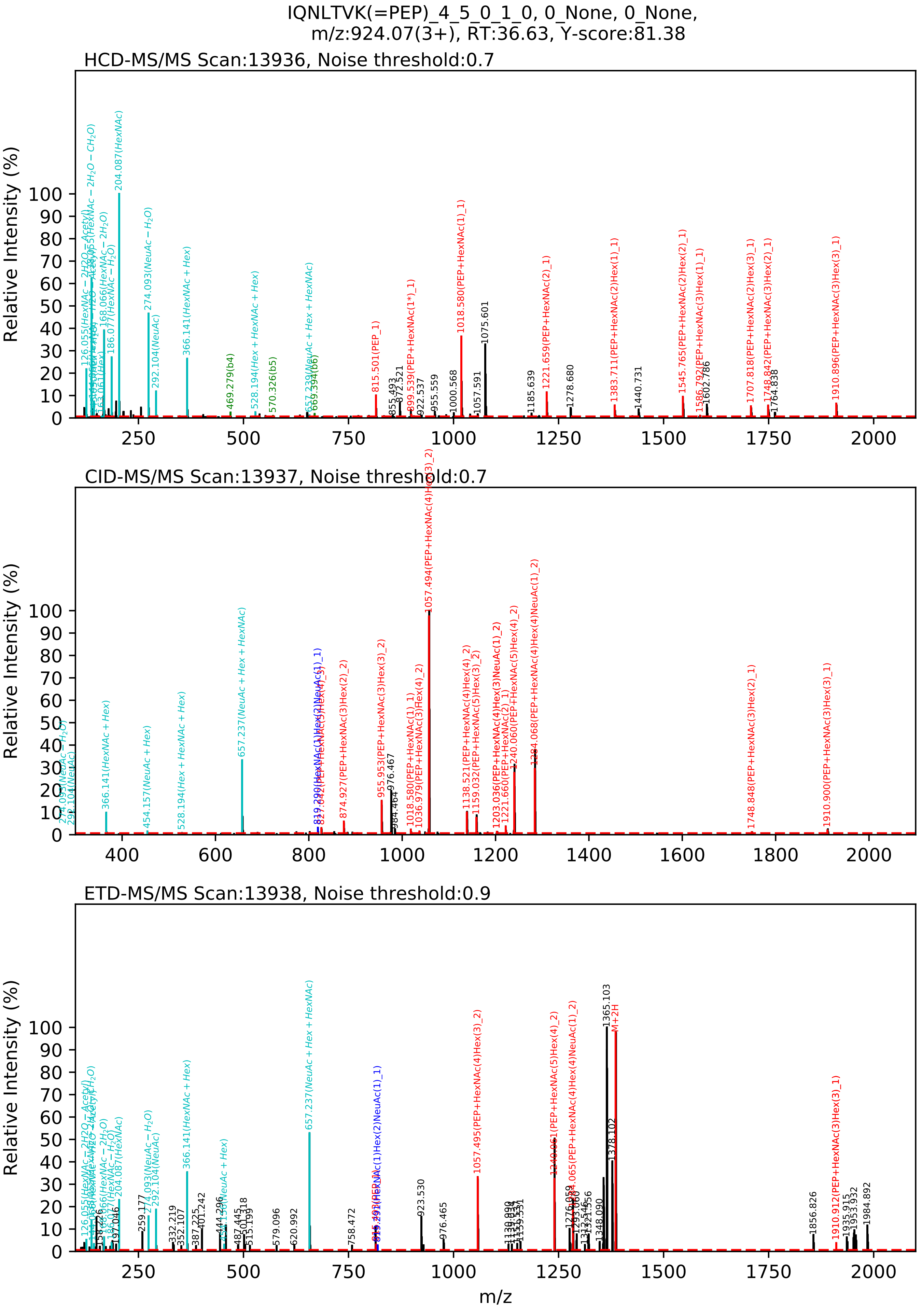
IQNLTVK(=PEP)_4_5_0_1_0, 0_None, 0_None,
m/z:924.07(3+), RT:36.63, Y-score:81.38
HCD-MS/MS Scan:13936, Noise threshold:0.7
Relative Intensity (%)
204.087(HexNAc)
274.093(NeuAc − H2O)
292.104(NeuAc)
366.141(HexNAc + Hex)
469.279(b4)
528.194(Hex + HexNAc + Hex)
570.326(b5)
669.394(b6)
815.501(PEP_1)
855.493
872.521
899.539(PEP+HexNAc(1*)_1)
922.537
955.559
1000.568
1018.580(PEP+HexNAc(1)_1)
1057.591
1075.601
1185.639
1221.659(PEP+HexNAc(2)_1)
1278.680
1383.711(PEP+HexNAc(2)Hex(1)_1)
1440.731
1545.765(PEP+HexNAc(2)Hex(2)_1)
1586.792(PEP+HexNAc(3)Hex(1)_1)
1602.786
1707.818(PEP+HexNAc(2)Hex(3)_1)
1748.842(PEP+HexNAc(3)Hex(2)_1)
1764.838
1910.896(PEP+HexNAc(3)Hex(3)_1)
CID-MS/MS Scan:13937, Noise threshold:0.7
Relative Intensity (%)
366.141(HexNAc + Hex)
454.157(NeuAc + Hex)
528.194(Hex + HexNAc + Hex)
657.237(NeuAc + Hex + HexNAc)
874.927(PEP+HexNAc(3)Hex(2)_2)
955.953(PEP+HexNAc(3)Hex(3)_2)
976.467
984.464
1018.580(PEP+HexNAc(1)_1)
1036.979(PEP+HexNAc(3)Hex(4)_2)
1057.494(PEP+HexNAc(4)Hex(3)_2)
1138.521(PEP+HexNAc(4)Hex(4)_2)
1159.032(PEP+HexNAc(5)Hex(3)_2)
1203.036(PEP+HexNAc(4)Hex(3)NeuAc(1)_2)
1221.660(PEP+HexNAc(2)_1)
1240.060(PEP+HexNAc(5)Hex(4)_2)
1284.068(PEP+HexNAc(4)Hex(4)NeuAc(1)_2)
1748.848(PEP+HexNAc(3)Hex(2)_1)
1910.900(PEP+HexNAc(3)Hex(3)_1)
ETD-MS/MS Scan:13938, Noise threshold:0.9
Relative Intensity (%)
204.087(HexNAc)
259.177
274.093(NeuAc − H2O)
292.104(NeuAc)
332.219
352.107
366.141(HexNAc + Hex)
387.225
401.242
444.296
487.445
501.318
515.199
579.096
620.992
657.237(NeuAc + Hex + HexNAc)
758.472
923.530
976.465
1057.495(PEP+HexNAc(4)Hex(3)_2)
1284.065(PEP+HexNAc(4)Hex(4)NeuAc(1)_2)
1348.090
1365.103
M+2H
1378.102
1856.826
1910.912(PEP+HexNAc(3)Hex(3)_1)
1935.915
1953.932
1984.892
m/z

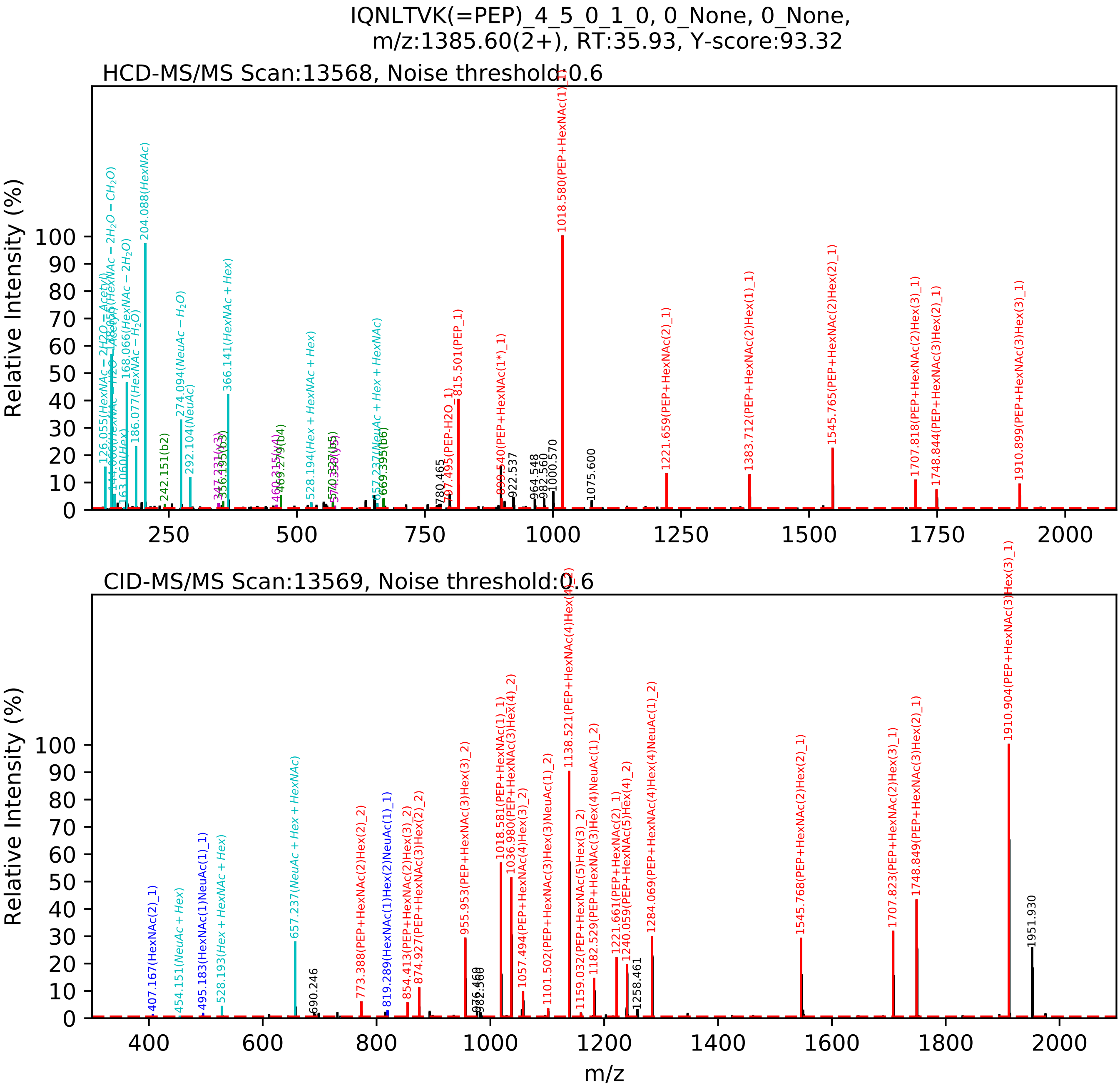
IQNLTVK(=PEP)_4_5_0_1_0, 0_None, 0_None,
m/z:1385.60(2+), RT:35.93, Y-score:93.32
HCD-MS/MS Scan:13568, Noise threshold:0.6
Relative Intensity (%)
204.088(HexNAc)
366.141(HexNAc + Hex)
274.094(NeuAc − H2O)
292.104(NeuAc)
168.066(HexNAc − 2H2O)
186.077(HexNAc − H2O)
242.151(b2)
528.194(Hex + HexNAc + Hex)
657.237(NeuAc + Hex + HexNAc)
669.395(b6)
815.501(PEP_1)
797.495(PEP-H2O_1)
899.540(PEP+HexNAc(1*)_1)
922.537
964.548
1000.570
1075.600
1018.580(PEP+HexNAc(1)_1)
1221.659(PEP+HexNAc(2)_1)
1383.712(PEP+HexNAc(2)Hex(1)_1)
1545.765(PEP+HexNAc(2)Hex(2)_1)
1707.818(PEP+HexNAc(2)Hex(3)_1)
1748.844(PEP+HexNAc(3)Hex(2)_1)
1910.899(PEP+HexNAc(3)Hex(3)_1)
CID-MS/MS Scan:13569, Noise threshold:0.6
Relative Intensity (%)
407.167(HexNAc(2)_1)
454.151(NeuAc + Hex)
495.183(HexNAc(1)NeuAc(1)_1)
528.193(Hex + HexNAc + Hex)
657.237(NeuAc + Hex + HexNAc)
690.246
773.388(PEP+HexNAc(2)Hex(2)_2)
819.289(HexNAc(1)Hex(2)NeuAc(1)_1)
854.413(PEP+HexNAc(2)Hex(3)_2)
874.927(PEP+HexNAc(3)Hex(2)_2)
955.953(PEP+HexNAc(3)Hex(3)_2)
1018.581(PEP+HexNAc(1)_1)
1036.980(PEP+HexNAc(3)Hex(4)_2)
1057.494(PEP+HexNAc(4)Hex(3)_2)
1101.502(PEP+HexNAc(3)Hex(3)NeuAc(1)_2)
1138.521(PEP+HexNAc(4)Hex(4)_2)
1159.032(PEP+HexNAc(5)Hex(3)_2)
1182.529(PEP+HexNAc(3)Hex(4)NeuAc(1)_2)
1221.661(PEP+HexNAc(2)_1)
1240.059(PEP+HexNAc(5)Hex(4)_2)
1258.461
1284.069(PEP+HexNAc(4)Hex(4)NeuAc(1)_2)
1545.768(PEP+HexNAc(2)Hex(2)_1)
1707.823(PEP+HexNAc(2)Hex(3)_1)
1748.849(PEP+HexNAc(3)Hex(2)_1)
1910.904(PEP+HexNAc(3)Hex(3)_1)
1951.930
m/z

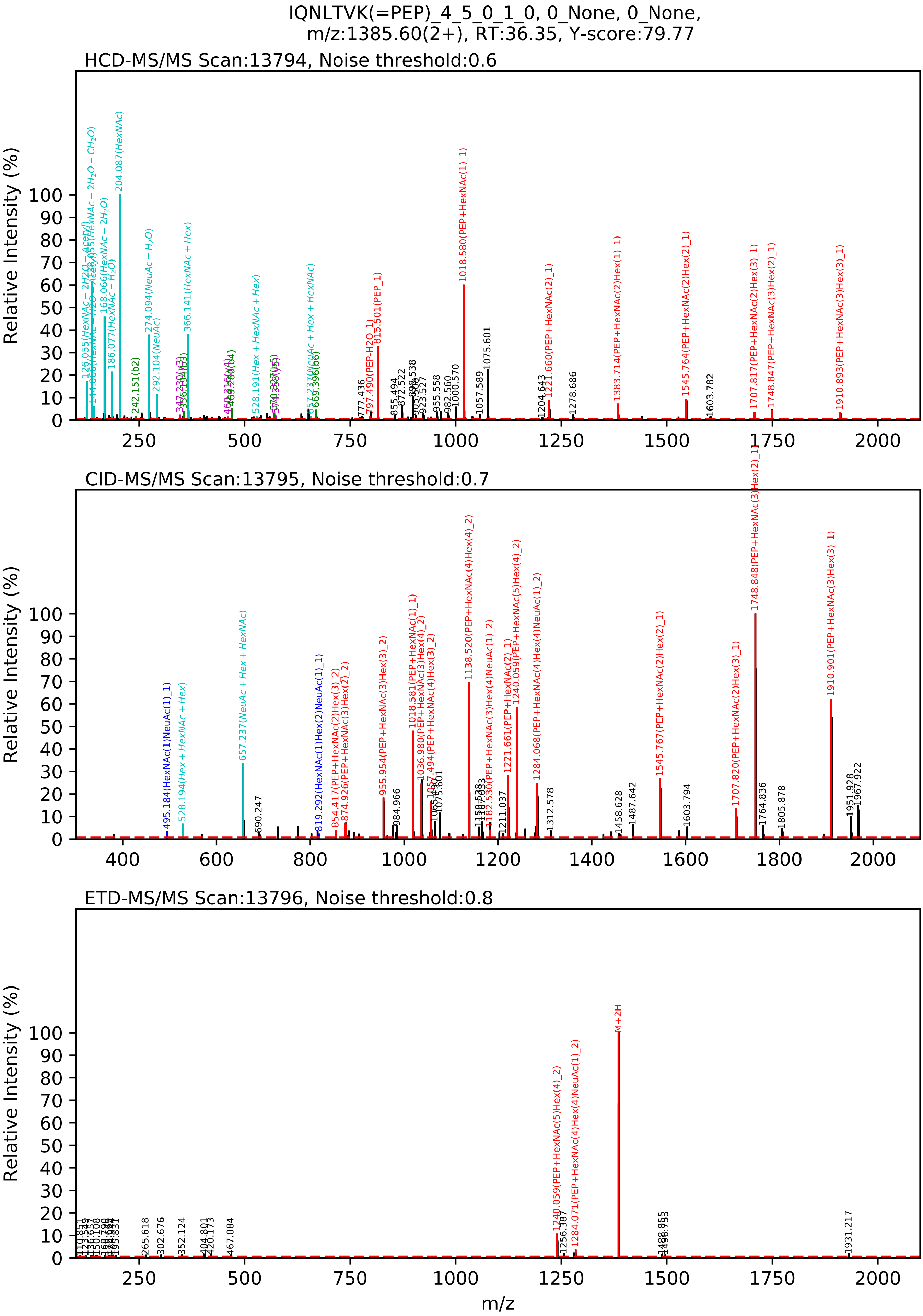
IQNLTVK(=PEP)_4_5_0_1_0, 0_None, 0_None,
m/z:1385.60(2+), RT:36.35, Y-score:79.77
HCD-MS/MS Scan:13794, Noise threshold:0.6
CID-MS/MS Scan:13795, Noise threshold:0.7
ETD-MS/MS Scan:13796, Noise threshold:0.8
Relative Intensity (%)
m/z
204.087(HexNAc)
366.141(HexNAc + Hex)
274.094(NeuAc − H2O)
292.104(NeuAc)
1018.580(PEP+HexNAc(1)_1)
815.501(PEP_1)
797.490(PEP-H2O_1)
1221.660(PEP+HexNAc(2)_1)
1383.714(PEP+HexNAc(2)Hex(1)_1)
1545.764(PEP+HexNAc(2)Hex(2)_1)
1707.817(PEP+HexNAc(2)Hex(3)_1)
1748.847(PEP+HexNAc(3)Hex(2)_1)
1910.893(PEP+HexNAc(3)Hex(3)_1)
1075.601
1748.848(PEP+HexNAc(3)Hex(2)_1)
1910.901(PEP+HexNAc(3)Hex(3)_1)
1138.520(PEP+HexNAc(4)Hex(4)_2)
1240.059(PEP+HexNAc(5)Hex(4)_2)
1284.068(PEP+HexNAc(4)Hex(4)NeuAc(1)_2)
1545.767(PEP+HexNAc(2)Hex(2)_1)
1707.820(PEP+HexNAc(2)Hex(3)_1)
1221.661(PEP+HexNAc(2)_1)
955.954(PEP+HexNAc(3)Hex(3)_2)
657.237(NeuAc + Hex + HexNAc)
495.184(HexNAc(1)NeuAc(1)_1)
528.194(Hex + HexNAc + Hex)
819.292(HexNAc(1)Hex(2)NeuAc(1)_1)
M+2H
1240.059(PEP+HexNAc(5)Hex(4)_2)
1284.071(PEP+HexNAc(4)Hex(4)NeuAc(1)_2)

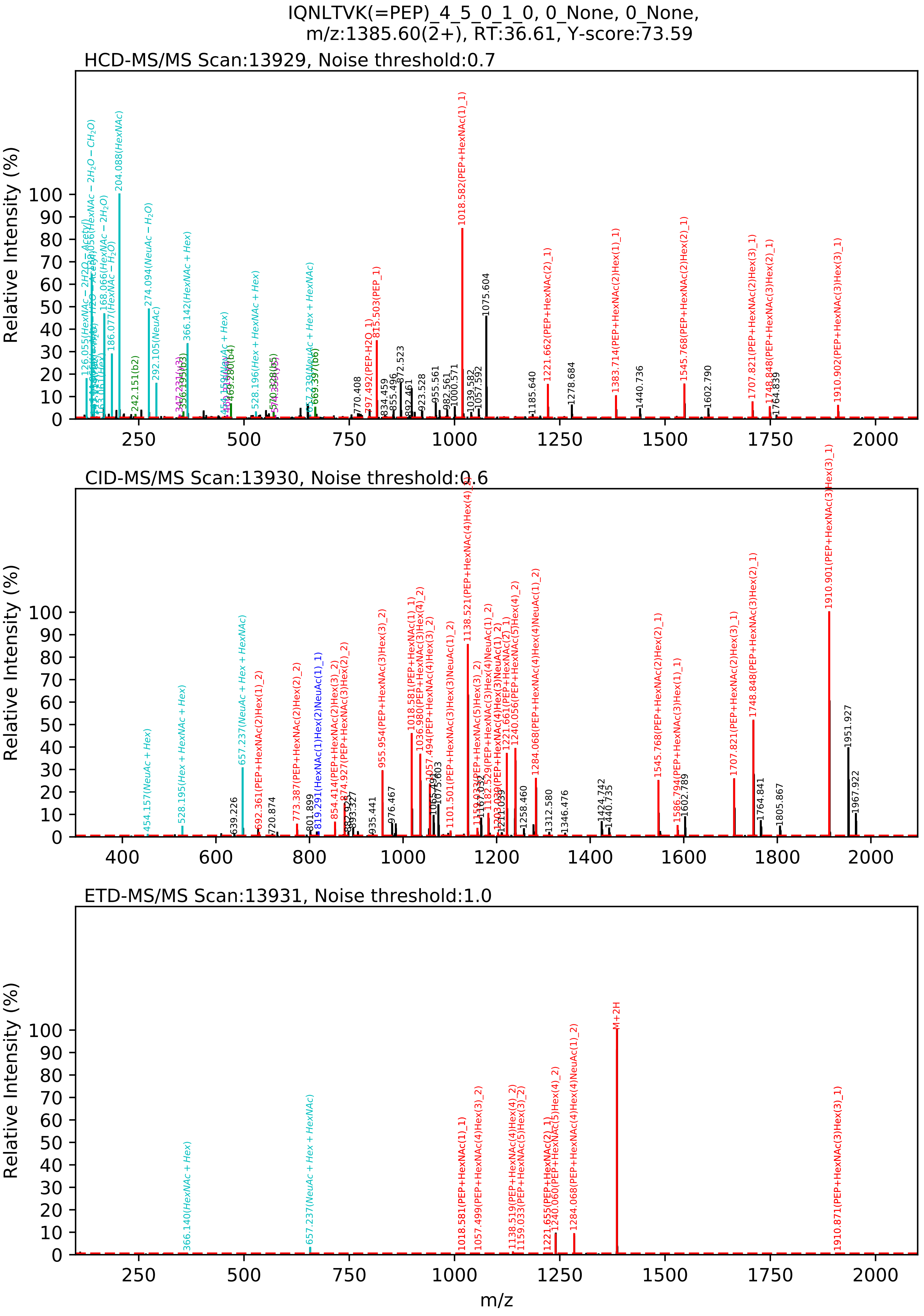
IQNLTVK(=PEP)_4_5_0_1_0, 0_None, 0_None,
m/z:1385.60(2+), RT:36.61, Y-score:73.59
HCD-MS/MS Scan:13929, Noise threshold:0.7
Relative Intensity (%)
204.088(HexNAc)
274.094(NeuAc − H2O)
292.105(NeuAc)
366.142(HexNAc + Hex)
186.077(HexNAc − H2O)
168.066(HexNAc − 2H2O)
242.151(b2)
469.280(b4)
528.196(Hex + HexNAc + Hex)
570.328(b5)
669.397(b6)
797.492(PEP-H2O_1)
815.503(PEP_1)
770.408
834.459
855.496
872.523
892.461
923.528
955.561
982.561
1000.571
1018.582(PEP+HexNAc(1)_1)
1039.582
1057.592
1075.604
1185.640
1221.662(PEP+HexNAc(2)_1)
1278.684
1383.714(PEP+HexNAc(2)Hex(1)_1)
1440.736
1545.768(PEP+HexNAc(2)Hex(2)_1)
1602.790
1707.821(PEP+HexNAc(2)Hex(3)_1)
1748.848(PEP+HexNAc(3)Hex(2)_1)
1764.839
1910.902(PEP+HexNAc(3)Hex(3)_1)
CID-MS/MS Scan:13930, Noise threshold:0.6
454.157(NeuAc + Hex)
528.195(Hex + HexNAc + Hex)
639.226
657.237(NeuAc + Hex + HexNAc)
692.361(PEP+HexNAc(2)Hex(1)_2)
720.874
773.387(PEP+HexNAc(2)Hex(2)_2)
801.899
819.291(HexNAc(1)Hex(2)NeuAc(1)_1)
854.414(PEP+HexNAc(2)Hex(3)_2)
874.927(PEP+HexNAc(3)Hex(2)_2)
882.928
893.327
935.441
955.954(PEP+HexNAc(3)Hex(3)_2)
976.467
1018.581(PEP+HexNAc(1)_1)
1036.980(PEP+HexNAc(3)Hex(4)_2)
1057.494(PEP+HexNAc(4)Hex(3)_2)
1075.603
1101.501(PEP+HexNAc(3)Hex(3)NeuAc(1)_2)
1138.521(PEP+HexNAc(4)Hex(4)_2)
1159.033(PEP+HexNAc(5)Hex(3)_2)
1182.529(PEP+HexNAc(3)Hex(4)NeuAc(1)_2)
1203.039(PEP+HexNAc(4)Hex(3)NeuAc(1)_2)
1221.661(PEP+HexNAc(2)_1)
1240.056(PEP+HexNAc(5)Hex(4)_2)
1258.460
1284.068(PEP+HexNAc(4)Hex(4)NeuAc(1)_2)
1312.580
1346.476
1424.742
1440.735
1545.768(PEP+HexNAc(2)Hex(2)_1)
1586.794(PEP+HexNAc(3)Hex(1)_1)
1602.789
1707.821(PEP+HexNAc(2)Hex(3)_1)
1748.848(PEP+HexNAc(3)Hex(2)_1)
1764.841
1805.867
1910.901(PEP+HexNAc(3)Hex(3)_1)
1951.927
1967.922
ETD-MS/MS Scan:13931, Noise threshold:1.0
366.140(HexNAc + Hex)
657.237(NeuAc + Hex + HexNAc)
1018.581(PEP+HexNAc(1)_1)
1057.499(PEP+HexNAc(4)Hex(3)_2)
1138.519(PEP+HexNAc(4)Hex(4)_2)
1159.033(PEP+HexNAc(5)Hex(3)_2)
1221.655(PEP+HexNAc(2)_1)
1240.060(PEP+HexNAc(5)Hex(4)_2)
1284.068(PEP+HexNAc(4)Hex(4)NeuAc(1)_2)
M+2H
1910.871(PEP+HexNAc(3)Hex(3)_1)
m/z

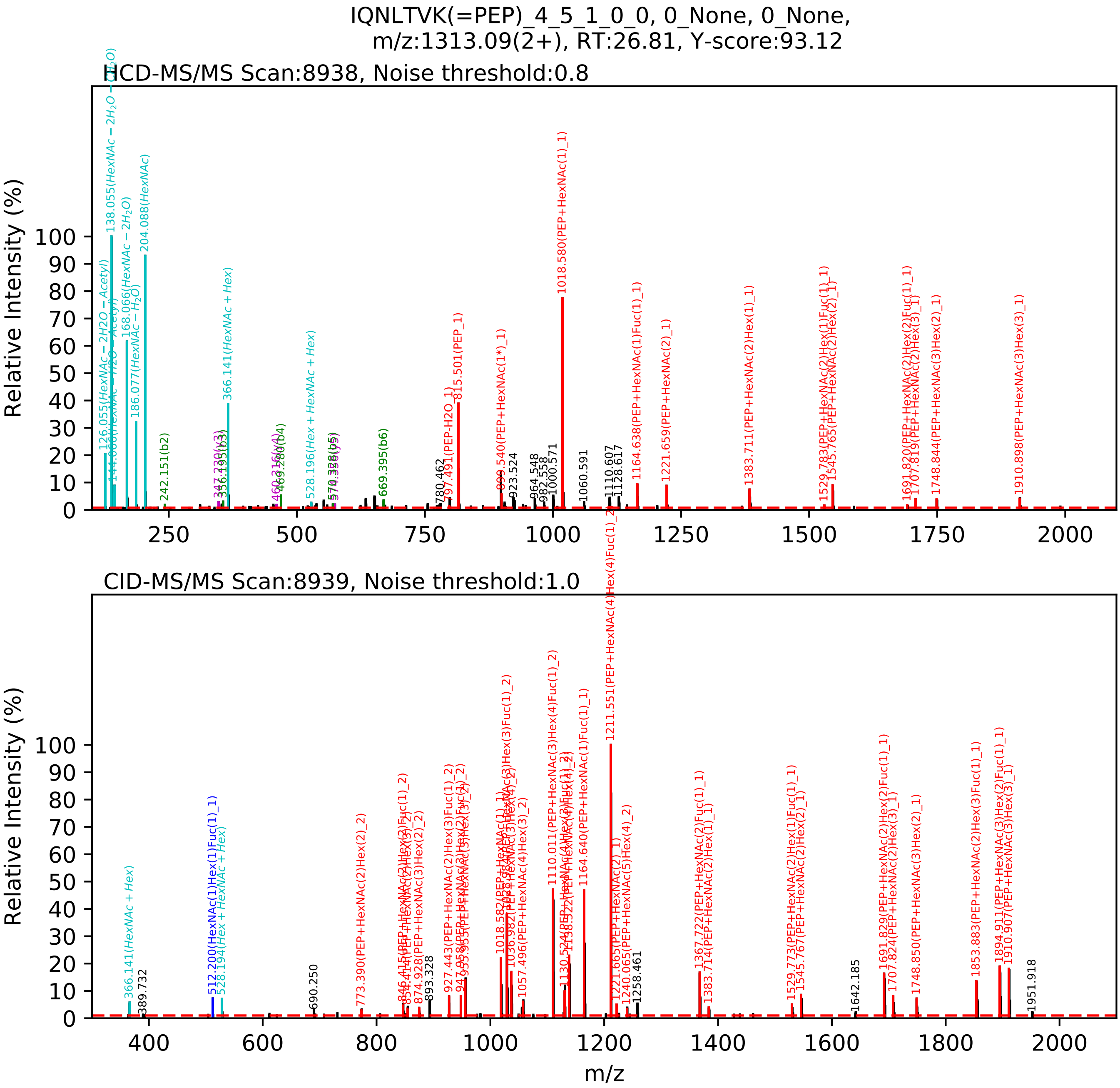
IQNLTVK(=PEP)_4_5_1_0_0, 0_None, 0_None,
m/z:1313.09(2+), RT:26.81, Y-score:93.12
HCD-MS/MS Scan:8938, Noise threshold:0.8
Relative Intensity (%)
126.055(HexNAc − 2H2O − Acetyl)
138.055(HexNAc − 2H2O − H2O)
168.066(HexNAc − 2H2O)
186.077(HexNAc − H2O)
204.088(HexNAc)
242.151(b2)
366.141(HexNAc + Hex)
469.260(b4)
528.196(Hex + HexNAc + Hex)
669.395(b6)
780.462
797.491(PEP-H2O_1)
815.501(PEP_1)
899.540(PEP+HexNAc(1*)_1)
923.524
964.548
1000.571
1018.580(PEP+HexNAc(1)_1)
1060.591
1110.607
1128.617
1164.638(PEP+HexNAc(1)Fuc(1)_1)
1221.659(PEP+HexNAc(2)_1)
1383.711(PEP+HexNAc(2)Hex(1)_1)
1529.783(PEP+HexNAc(2)Hex(1)Fuc(1)_1)
1545.765(PEP+HexNAc(2)Hex(2)_1)
1691.820(PEP+HexNAc(2)Hex(2)Fuc(1)_1)
1707.819(PEP+HexNAc(2)Hex(3)_1)
1748.844(PEP+HexNAc(3)Hex(2)_1)
1910.898(PEP+HexNAc(3)Hex(3)_1)
CID-MS/MS Scan:8939, Noise threshold:1.0
Relative Intensity (%)
366.141(HexNAc + Hex)
389.732
512.200(HexNAc(1)Hex(1)Fuc(1)_1)
528.194(Hex + HexNAc + Hex)
690.250
773.390(PEP+HexNAc(2)Hex(2)_2)
874.928(PEP+HexNAc(3)Hex(2)_2)
893.328
927.443(PEP+HexNAc(2)Hex(3)Fuc(1)_2)
1057.496(PEP+HexNAc(4)Hex(3)_2)
1110.011(PEP+HexNAc(3)Hex(4)Fuc(1)_2)
1164.640(PEP+HexNAc(1)Fuc(1)_1)
1211.551(PEP+HexNAc(4)Hex(4)Fuc(1)_2)
1221.665(PEP+HexNAc(2)_1)
1240.065(PEP+HexNAc(5)Hex(4)_2)
1258.461
1367.722(PEP+HexNAc(2)Fuc(1)_1)
1383.714(PEP+HexNAc(2)Hex(1)_1)
1529.773(PEP+HexNAc(2)Hex(1)Fuc(1)_1)
1545.767(PEP+HexNAc(2)Hex(2)_1)
1642.185
1691.829(PEP+HexNAc(2)Hex(2)Fuc(1)_1)
1707.824(PEP+HexNAc(2)Hex(3)_1)
1748.850(PEP+HexNAc(3)Hex(2)_1)
1853.883(PEP+HexNAc(2)Hex(3)Fuc(1)_1)
1894.911(PEP+HexNAc(3)Hex(2)Fuc(1)_1)
1910.907(PEP+HexNAc(3)Hex(3)_1)
1951.918
m/z

IQNLTVK(=PEP)_4_5_1_0_0, 0_None, 0_None,
m/z:1313.09(2+), RT:27.40, Y-score:92.73

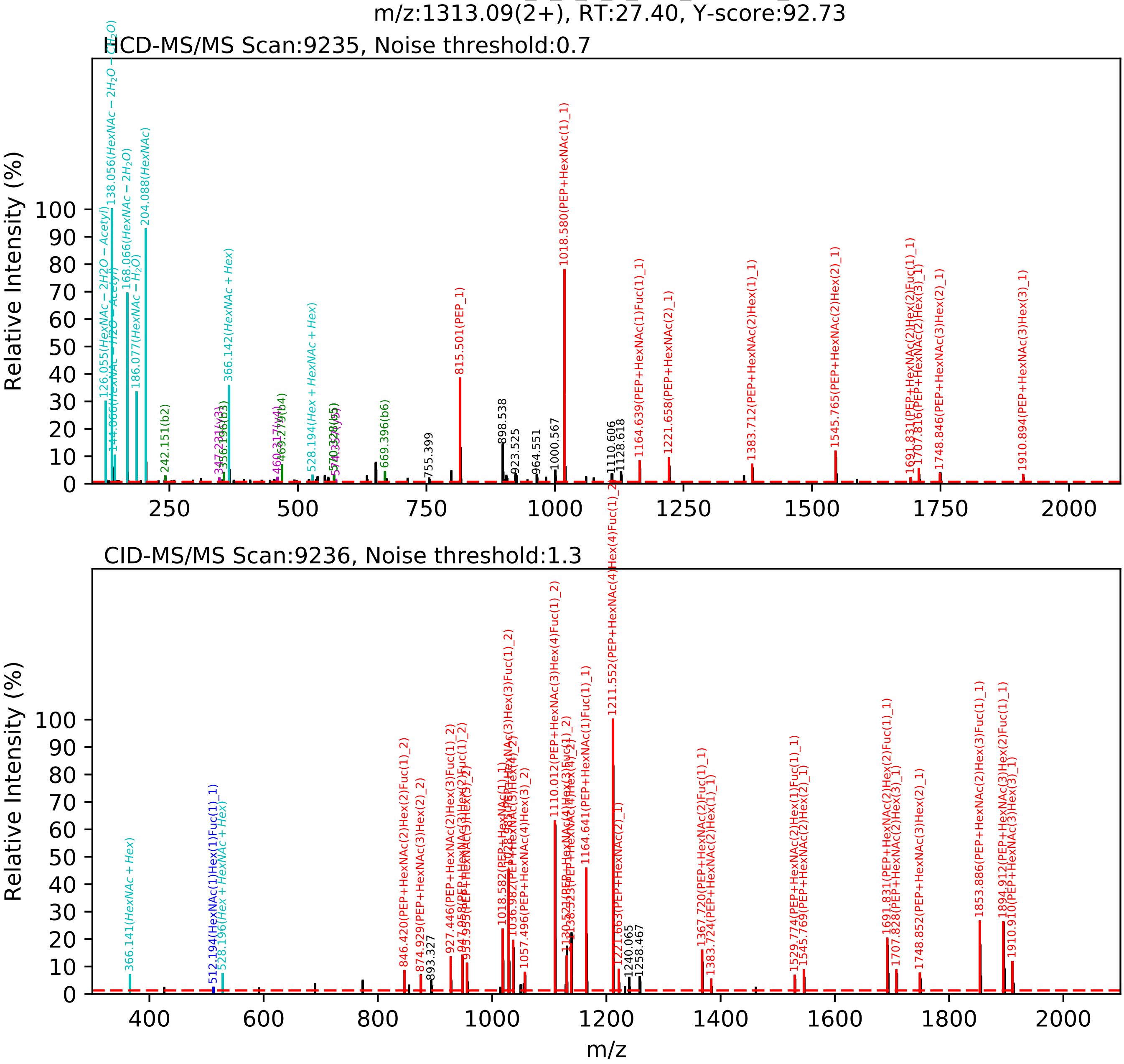

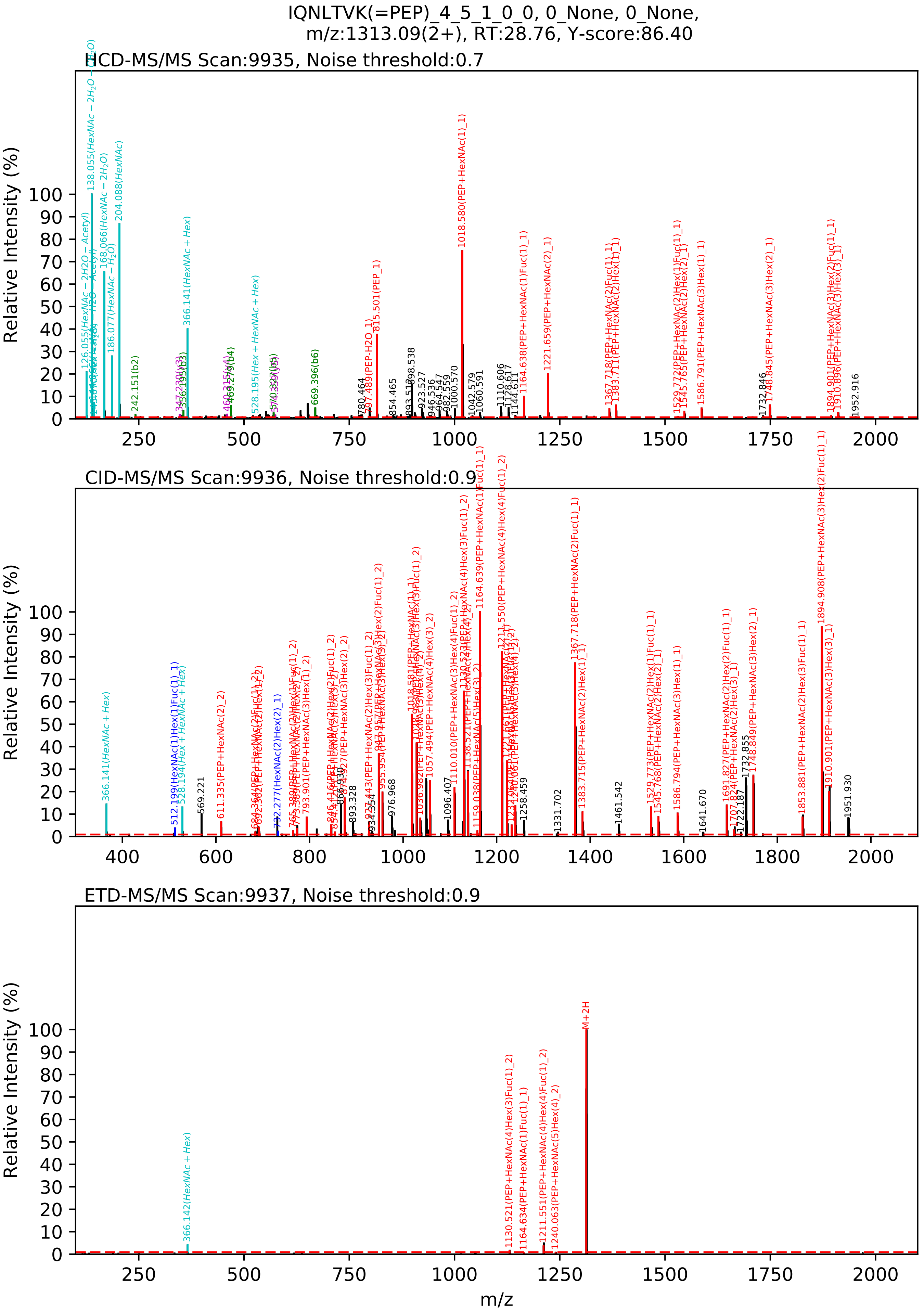
IQNLTVK(=PEP)_4_5_1_0_0, 0_None, 0_None,
m/z:1313.09(2+), RT:28.76, Y-score:86.40
HCD-MS/MS Scan:9935, Noise threshold:0.7
CID-MS/MS Scan:9936, Noise threshold:0.9
ETD-MS/MS Scan:9937, Noise threshold:0.9
Relative Intensity (%)
m/z
1018.580(PEP+HexNAc(1)_1)
815.501(PEP_1)
1221.659(PEP+HexNAc(2)_1)
1164.638(PEP+HexNAc(1)Fuc(1)_1)
204.088(HexNAc)
366.141(HexNAc + Hex)
1894.908(PEP+HexNAc(3)Hex(2)Fuc(1)_1)
1367.718(PEP+HexNAc(2)Fuc(1)_1)
1164.639(PEP+HexNAc(1)Fuc(1)_1)
M+2H
366.142(HexNAc + Hex)

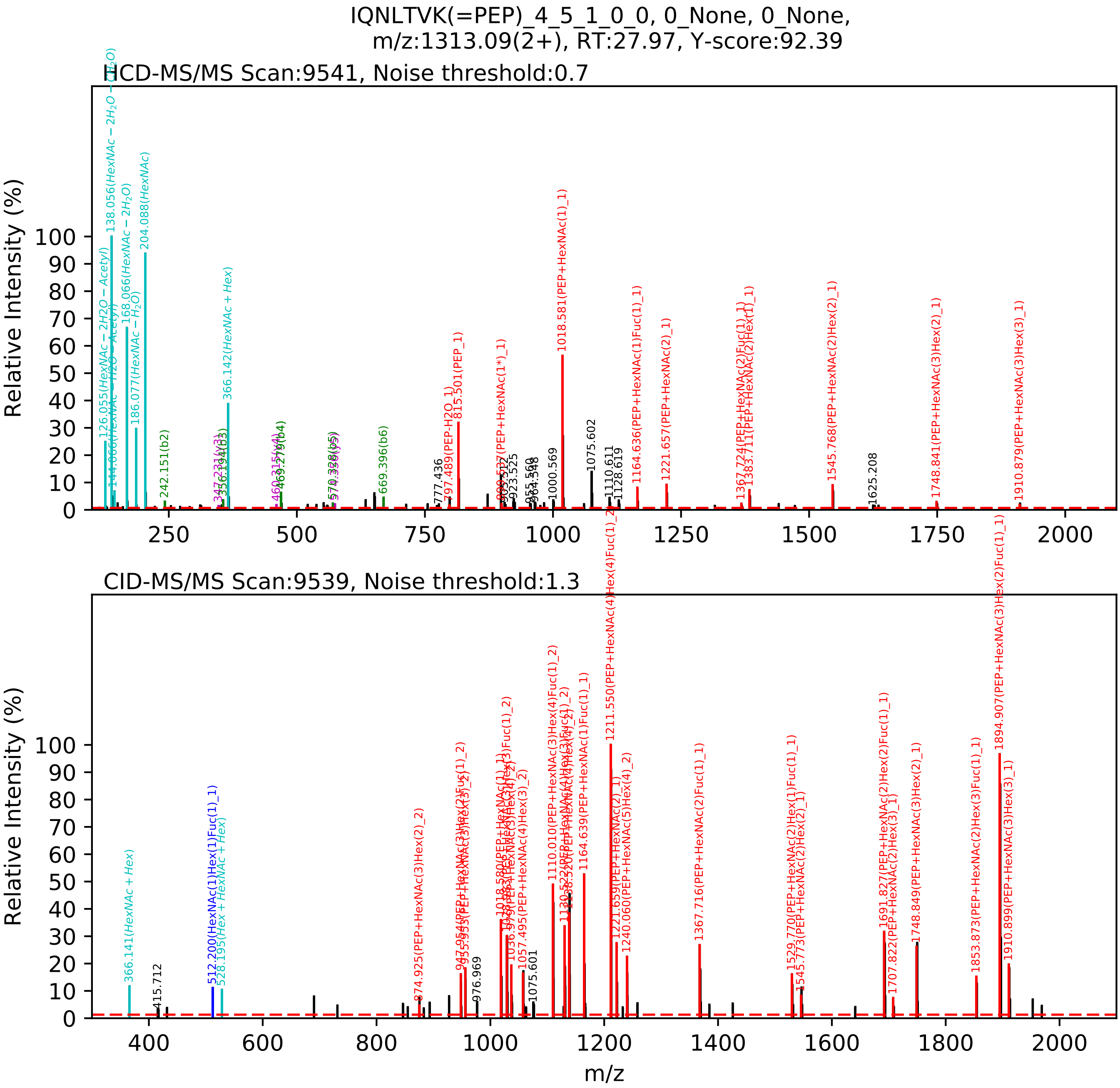
IQNLTVK(=PEP)_4_5_1_0_0, 0_None, 0_None,
m/z:1313.09(2+), RT:27.97, Y-score:92.39
HCD-MS/MS Scan:9541, Noise threshold:0.7
Relative Intensity (%)
138.056(HexNAc)
204.088(HexNAc)
168.066(HexNAc − 2H2O)
186.077(HexNAc − H2O)
366.142(HexNAc + Hex)
242.151(b2)
669.396(b6)
777.436
797.489(PEP-H2O_1)
815.501(PEP_1)
923.525
1000.569
1018.581(PEP+HexNAc(1)_1)
1075.602
1110.611
1128.619
1164.636(PEP+HexNAc(1)Fuc(1)_1)
1221.657(PEP+HexNAc(2)_1)
1545.768(PEP+HexNAc(2)Hex(2)_1)
1625.208
1748.841(PEP+HexNAc(3)Hex(2)_1)
1910.879(PEP+HexNAc(3)Hex(3)_1)
CID-MS/MS Scan:9539, Noise threshold:1.3
Relative Intensity (%)
366.141(HexNAc + Hex)
415.712
512.200(HexNAc(1)Hex(1)Fuc(1)_1)
528.195(Hex + HexNAc + Hex)
874.925(PEP+HexNAc(3)Hex(2)_2)
976.969
1057.495(PEP+HexNAc(4)Hex(3)_2)
1075.601
1110.010(PEP+HexNAc(3)Hex(4)Fuc(1)_2)
1164.639(PEP+HexNAc(1)Fuc(1)_1)
1211.550(PEP+HexNAc(4)Hex(4)Fuc(1)_2)
1221.659(PEP+HexNAc(2)_1)
1240.060(PEP+HexNAc(5)Hex(4)_2)
1367.716(PEP+HexNAc(2)Fuc(1)_1)
1529.770(PEP+HexNAc(2)Hex(1)Fuc(1)_1)
1545.773(PEP+HexNAc(2)Hex(2)_1)
1691.827(PEP+HexNAc(2)Hex(2)Fuc(1)_1)
1707.822(PEP+HexNAc(2)Hex(3)_1)
1748.849(PEP+HexNAc(3)Hex(2)_1)
1853.873(PEP+HexNAc(2)Hex(3)Fuc(1)_1)
1894.907(PEP+HexNAc(3)Hex(2)Fuc(1)_1)
1910.899(PEP+HexNAc(3)Hex(3)_1)
m/z

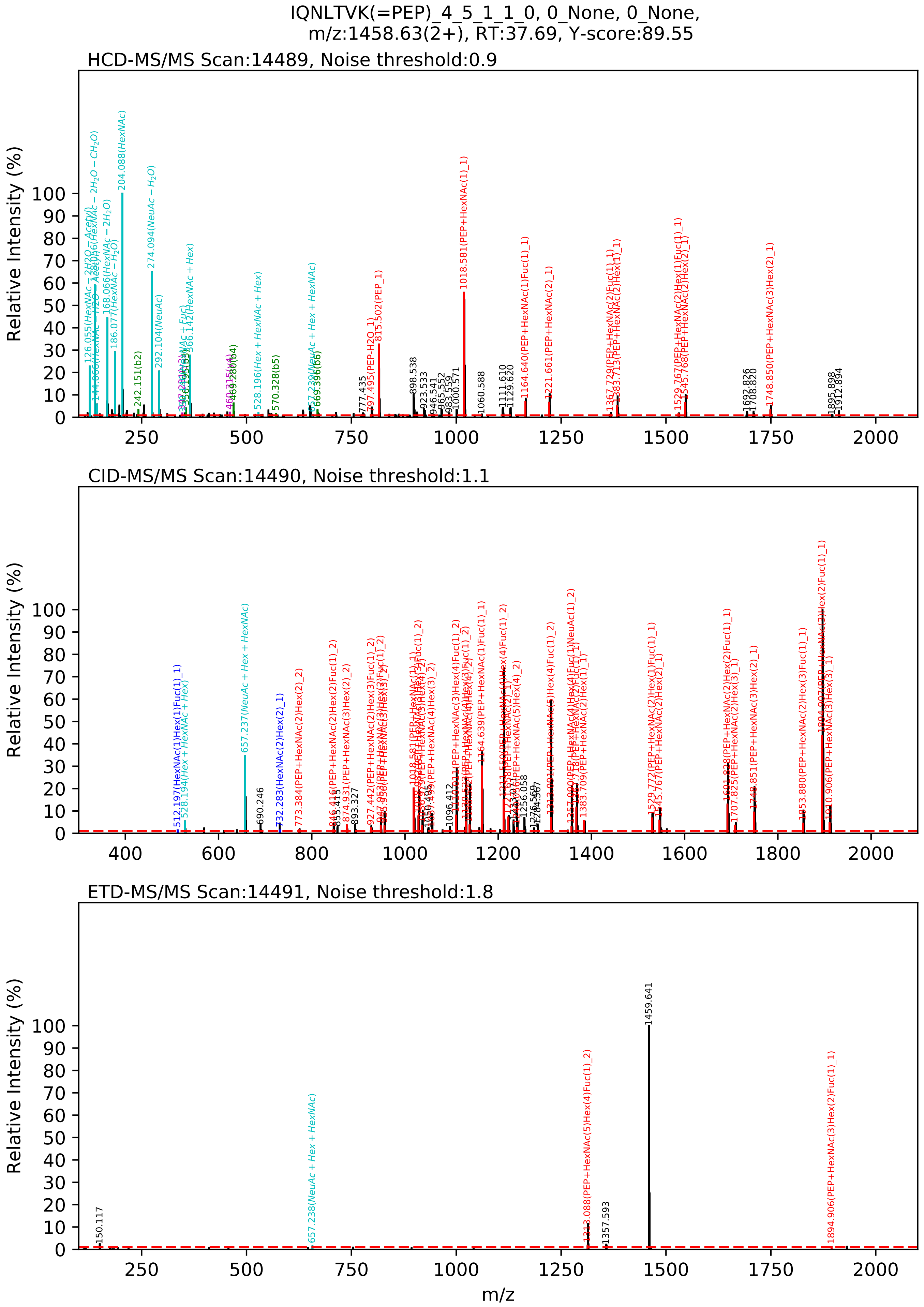

IQNLTVK(=PEP)_4_5_1_1_0, 0_None, 0_None,
m/z:1458.63(2+), RT:37.69, Y-score:89.55
HCD-MS/MS Scan:14489, Noise threshold:0.9
Relative Intensity (%)
204.088(HexNAc)
274.094(NeuAc − H2O)
292.104(NeuAc)
168.066(HexNAc − 2H2O)
186.077(HexNAc − H2O)
126.055(HexNAc − 2H2O − CH2O)
242.151(b2)
366.142(HexNAc + Hex)
463.280(b4)
528.196(Hex + HexNAc + Hex)
570.328(b5)
669.396(b6)
777.435
797.495(PEP-H2O_1)
815.502(PEP_1)
898.538
923.533
946.541
965.552
983.559
1000.571
1018.581(PEP+HexNAc(1)_1)
1060.588
1111.610
1129.620
1164.640(PEP+HexNAc(1)Fuc(1)_1)
1221.661(PEP+HexNAc(2)_1)
1367.729(PEP+HexNAc(2)Fuc(1)_1)
1383.713(PEP+HexNAc(2)Hex(1)_1)
1529.767(PEP+HexNAc(2)Hex(1)Fuc(1)_1)
1545.768(PEP+HexNAc(2)Hex(2)_1)
1692.826
1708.820
1748.850(PEP+HexNAc(3)Hex(2)_1)
1895.898
1912.894
CID-MS/MS Scan:14490, Noise threshold:1.1
Relative Intensity (%)
512.197(HexNAc(1)Hex(1)Fuc(1)_1)
528.194(Hex + HexNAc + Hex)
657.237(NeuAc + Hex + HexNAc)
690.246
732.283(HexNAc(2)Hex(2)_1)
773.384(PEP+HexNAc(2)Hex(2)_2)
846.416(PEP+HexNAc(2)Hex(2)Fuc(1)_2)
855.415
874.931(PEP+HexNAc(3)Hex(2)_2)
893.327
927.442(PEP+HexNAc(2)Hex(3)Fuc(1)_2)
1096.412
1164.639(PEP+HexNAc(1)Fuc(1)_1)
1256.058
1529.772(PEP+HexNAc(2)Hex(1)Fuc(1)_1)
1545.767(PEP+HexNAc(2)Hex(2)_1)
1691.828(PEP+HexNAc(2)Hex(2)Fuc(1)_1)
1707.825(PEP+HexNAc(2)Hex(3)_1)
1748.851(PEP+HexNAc(3)Hex(2)_1)
1853.880(PEP+HexNAc(2)Hex(3)Fuc(1)_1)
1910.906(PEP+HexNAc(3)Hex(3)_1)
ETD-MS/MS Scan:14491, Noise threshold:1.8
Relative Intensity (%)
150.117
657.238(NeuAc + Hex + HexNAc)
1313.088(PEP+HexNAc(5)Hex(4)Fuc(1)_2)
1357.593
1459.641
1894.906(PEP+HexNAc(3)Hex(2)Fuc(1)_1)
m/z

IQNLTVK(=PEP)_4_5_1_1_0, 0_None, 0_None,
m/z:1458.63(2+), RT:37.89, Y-score:94.65

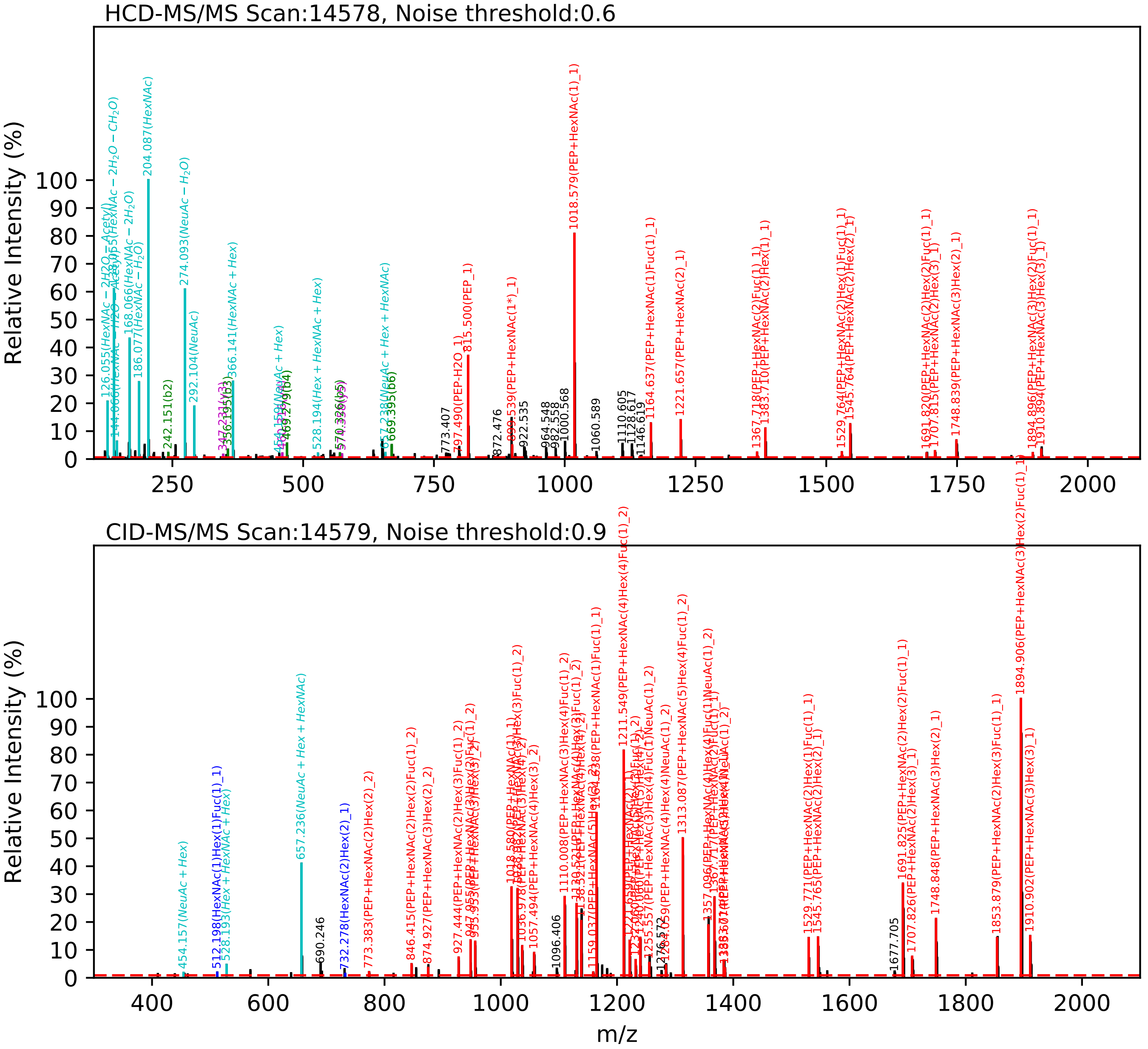

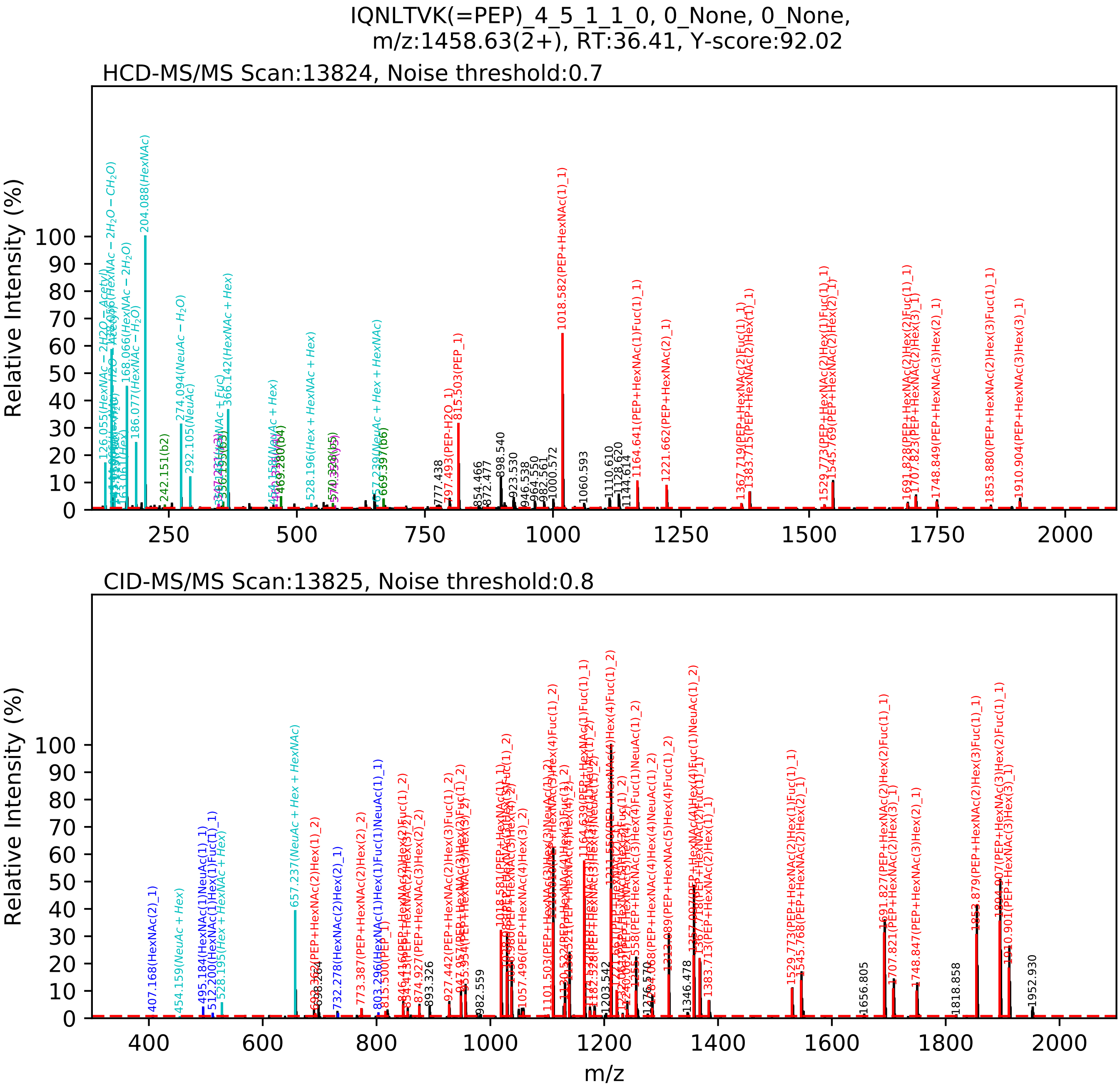
IQNLTVK(=PEP)_4_5_1_1_0, 0_None, 0_None,
m/z:1458.63(2+), RT:36.41, Y-score:92.02
HCD-MS/MS Scan:13824, Noise threshold:0.7
Relative Intensity (%)
1018.582(PEP+HexNAc(1)_1)
204.088(HexNAc)
815.503(PEP_1)
1164.641(PEP+HexNAc(1)Fuc(1)_1)
1221.662(PEP+HexNAc(2)_1)
366.142(HexNAc + Hex)
274.094(NeuAc − H2O)
292.105(NeuAc)
CID-MS/MS Scan:13825, Noise threshold:0.8
Relative Intensity (%)
657.237(NeuAc + Hex + HexNAc)
1691.827(PEP+HexNAc(2)Hex(2)Fuc(1)_1)
1853.879(PEP+HexNAc(2)Hex(3)Fuc(1)_1)
1894.907(PEP+HexNAc(3)Hex(2)Fuc(1)_1)
m/z

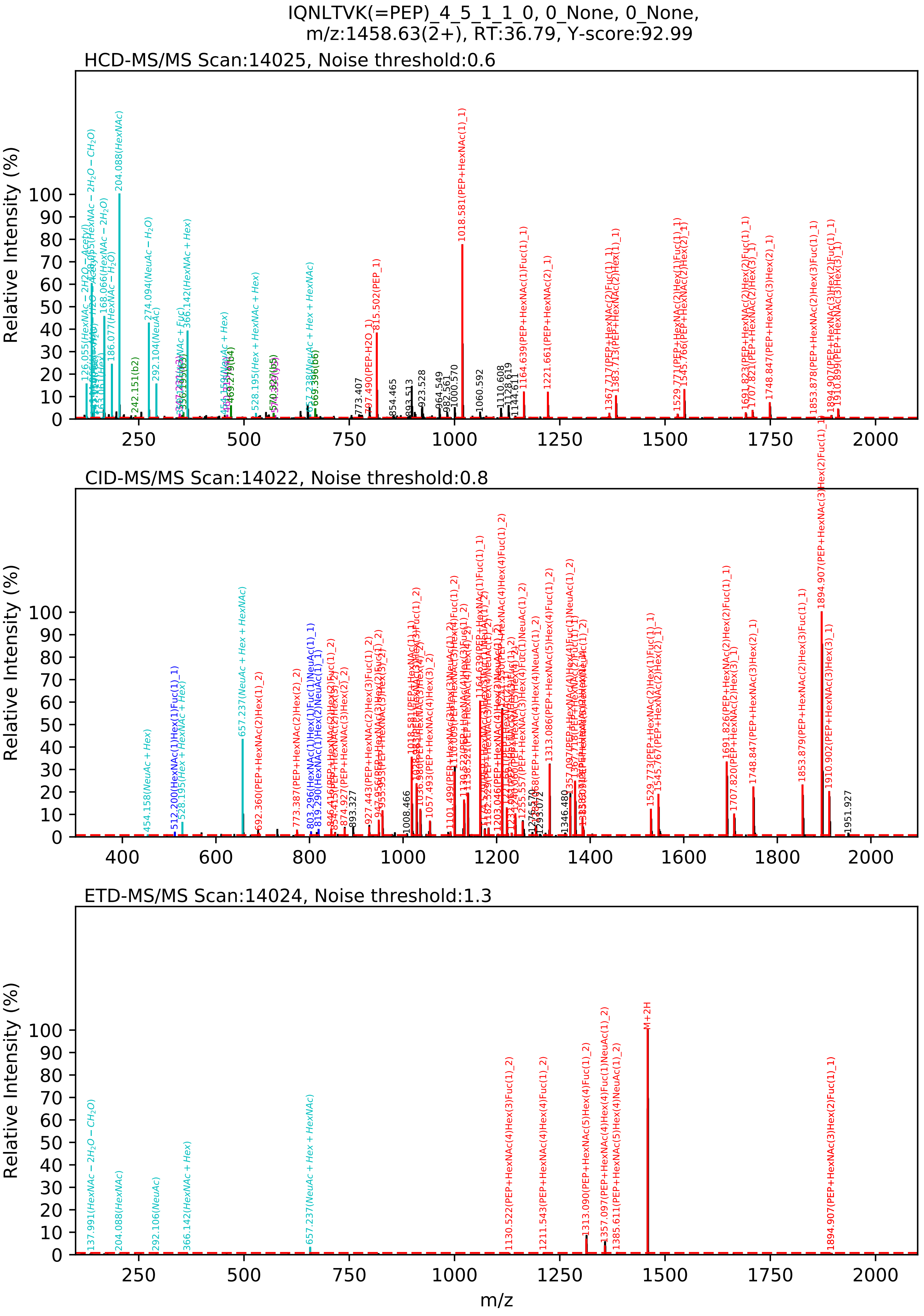
IQNLTVK(=PEP)_4_5_1_1_0, 0_None, 0_None,
m/z:1458.63(2+), RT:36.79, Y-score:92.99
HCD-MS/MS Scan:14025, Noise threshold:0.6
Relative Intensity (%)
204.088(HexNAc)
1018.581(PEP+HexNAc(1)_1)
815.502(PEP_1)
1164.639(PEP+HexNAc(1)Fuc(1)_1)
1221.661(PEP+HexNAc(2)_1)
1748.847(PEP+HexNAc(3)Hex(2)_1)
1853.878(PEP+HexNAc(2)Hex(3)Fuc(1)_1)
366.142(HexNAc + Hex)
274.094(NeuAc − H2O)
292.104(NeuAc)
186.077(HexNAc − H2O)
242.151(b2)
528.195(Hex + HexNAc + Hex)
923.528
854.465
773.407
1060.592
1110.608
1128.619
1144.611
CID-MS/MS Scan:14022, Noise threshold:0.8
1894.907(PEP+HexNAc(3)Hex(2)Fuc(1)_1)
1910.902(PEP+HexNAc(3)Hex(3)_1)
1853.879(PEP+HexNAc(2)Hex(3)Fuc(1)_1)
1748.847(PEP+HexNAc(3)Hex(2)_1)
1707.820(PEP+HexNAc(2)Hex(3)_1)
1691.826(PEP+HexNAc(2)Hex(2)Fuc(1)_1)
1545.767(PEP+HexNAc(2)Hex(2)_1)
1529.773(PEP+HexNAc(2)Hex(1)Fuc(1)_1)
1313.086(PEP+HexNAc(5)Hex(4)Fuc(1)_2)
657.237(NeuAc + Hex + HexNAc)
454.158(NeuAc + Hex)
692.360(PEP+HexNAc(2)Hex(1)_2)
773.387(PEP+HexNAc(2)Hex(2)_2)
1057.493(PEP+HexNAc(4)Hex(3)_2)
1008.466
1346.480
1951.927
ETD-MS/MS Scan:14024, Noise threshold:1.3
M+2H
137.991(HexNAc − 2H2O − CH2O)
204.088(HexNAc)
292.106(NeuAc)
366.142(HexNAc + Hex)
657.237(NeuAc + Hex + HexNAc)
1130.522(PEP+HexNAc(4)Hex(3)Fuc(1)_2)
1211.543(PEP+HexNAc(4)Hex(4)Fuc(1)_2)
1313.090(PEP+HexNAc(5)Hex(4)Fuc(1)_2)
1357.097(PEP+HexNAc(4)Hex(4)Fuc(1)NeuAc(1)_2)
1385.611(PEP+HexNAc(5)Hex(4)NeuAc(1)_2)
1894.907(PEP+HexNAc(3)Hex(2)Fuc(1)_1)
m/z

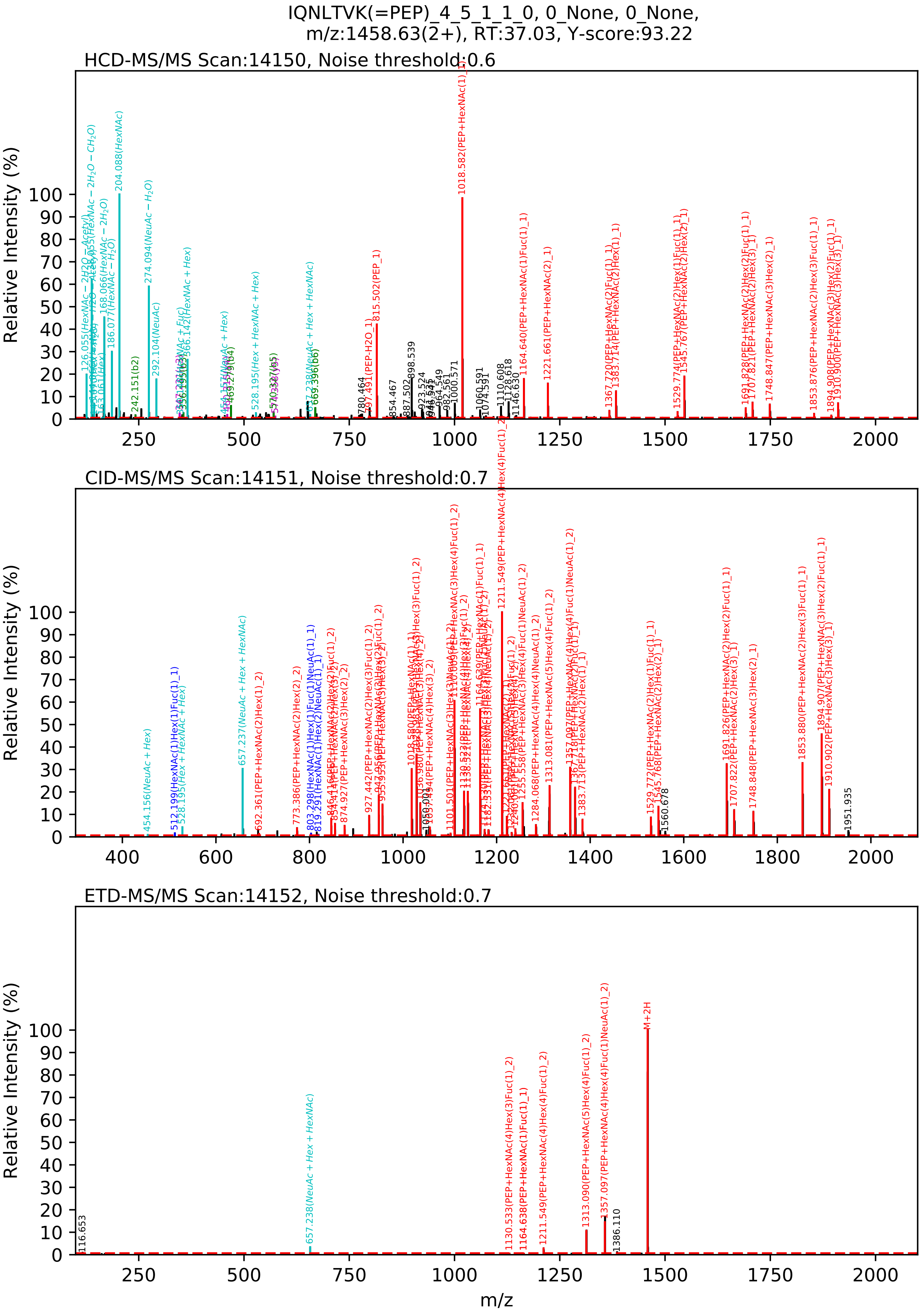

IQNLTVK(=PEP)_4_5_1_1_0, 0_None, 0_None,
m/z:1458.63(2+), RT:37.03, Y-score:93.22
HCD-MS/MS Scan:14150, Noise threshold:0.6
Relative Intensity (%)
1018.582(PEP+HexNAc(1)_1)
204.088(HexNAc)
274.094(NeuAc − H2O)
292.104(NeuAc)
168.066(HexNAc − 2H2O)
186.077(HexNAc − H2O)
126.055(HexNAc − 2H2O − CH2O)
366.142(HexNAc + Hex)
528.195(Hex + HexNAc + Hex)
657.238(NeuAc + Hex + HexNAc)
669.396(b6)
242.151(b2)
815.502(PEP_1)
797.491(PEP-H2O_1)
898.539
1000.571
1110.608
1128.618
1146.630
1164.640(PEP+HexNAc(1)Fuc(1)_1)
1221.661(PEP+HexNAc(2)_1)
1367.720(PEP+HexNAc(2)Fuc(1)_1)
1383.714(PEP+HexNAc(2)Hex(1)_1)
1529.774(PEP+HexNAc(2)Hex(1)Fuc(1)_1)
1545.767(PEP+HexNAc(2)Hex(2)_1)
1691.828(PEP+HexNAc(2)Hex(2)Fuc(1)_1)
1707.821(PEP+HexNAc(2)Hex(3)_1)
1748.847(PEP+HexNAc(3)Hex(2)_1)
1853.876(PEP+HexNAc(2)Hex(3)Fuc(1)_1)
1894.908(PEP+HexNAc(3)Hex(2)Fuc(1)_1)
1910.900(PEP+HexNAc(3)Hex(3)_1)
CID-MS/MS Scan:14151, Noise threshold:0.7
1211.549(PEP+HexNAc(4)Hex(4)Fuc(1)_2)
454.156(NeuAc + Hex)
512.199(HexNAc(1)Hex(1)Fuc(1)_1)
528.195(Hex + HexNAc + Hex)
657.237(NeuAc + Hex + HexNAc)
692.361(PEP+HexNAc(2)Hex(1)_2)
773.386(PEP+HexNAc(2)Hex(2)_2)
803.298(HexNAc(1)Hex(1)Fuc(1)NeuAc(1)_1)
819.291(HexNAc(1)Hex(2)NeuAc(1)_1)
927.442(PEP+HexNAc(2)Hex(3)Fuc(1)_2)
1101.501
1130.523
1164.639
1255.558(PEP+HexNAc(3)Hex(4)Fuc(1)NeuAc(1)_2)
1284.068(PEP+HexNAc(4)Hex(4)NeuAc(1)_2)
1313.081(PEP+HexNAc(5)Hex(4)Fuc(1)_2)
1357.097(PEP+HexNAc(4)Hex(4)Fuc(1)NeuAc(1)_2)
1383.713(PEP+HexNAc(2)Hex(1)_1)
1529.772(PEP+HexNAc(2)Hex(1)Fuc(1)_1)
1545.768(PEP+HexNAc(2)Hex(2)_1)
1560.678
1691.826(PEP+HexNAc(2)Hex(2)Fuc(1)_1)
1707.822(PEP+HexNAc(2)Hex(3)_1)
1748.848(PEP+HexNAc(3)Hex(2)_1)
1853.880(PEP+HexNAc(2)Hex(3)Fuc(1)_1)
1894.907(PEP+HexNAc(3)Hex(2)Fuc(1)_1)
1910.902(PEP+HexNAc(3)Hex(3)_1)
1951.935
ETD-MS/MS Scan:14152, Noise threshold:0.7
116.653
657.238(NeuAc + Hex + HexNAc)
1130.533(PEP+HexNAc(4)Hex(3)Fuc(1)_2)
1164.638(PEP+HexNAc(1)Fuc(1)_1)
1211.549(PEP+HexNAc(4)Hex(4)Fuc(1)_2)
1313.090(PEP+HexNAc(5)Hex(4)Fuc(1)_2)
1357.097(PEP+HexNAc(4)Hex(4)Fuc(1)NeuAc(1)_2)
1386.110
M+2H
m/z

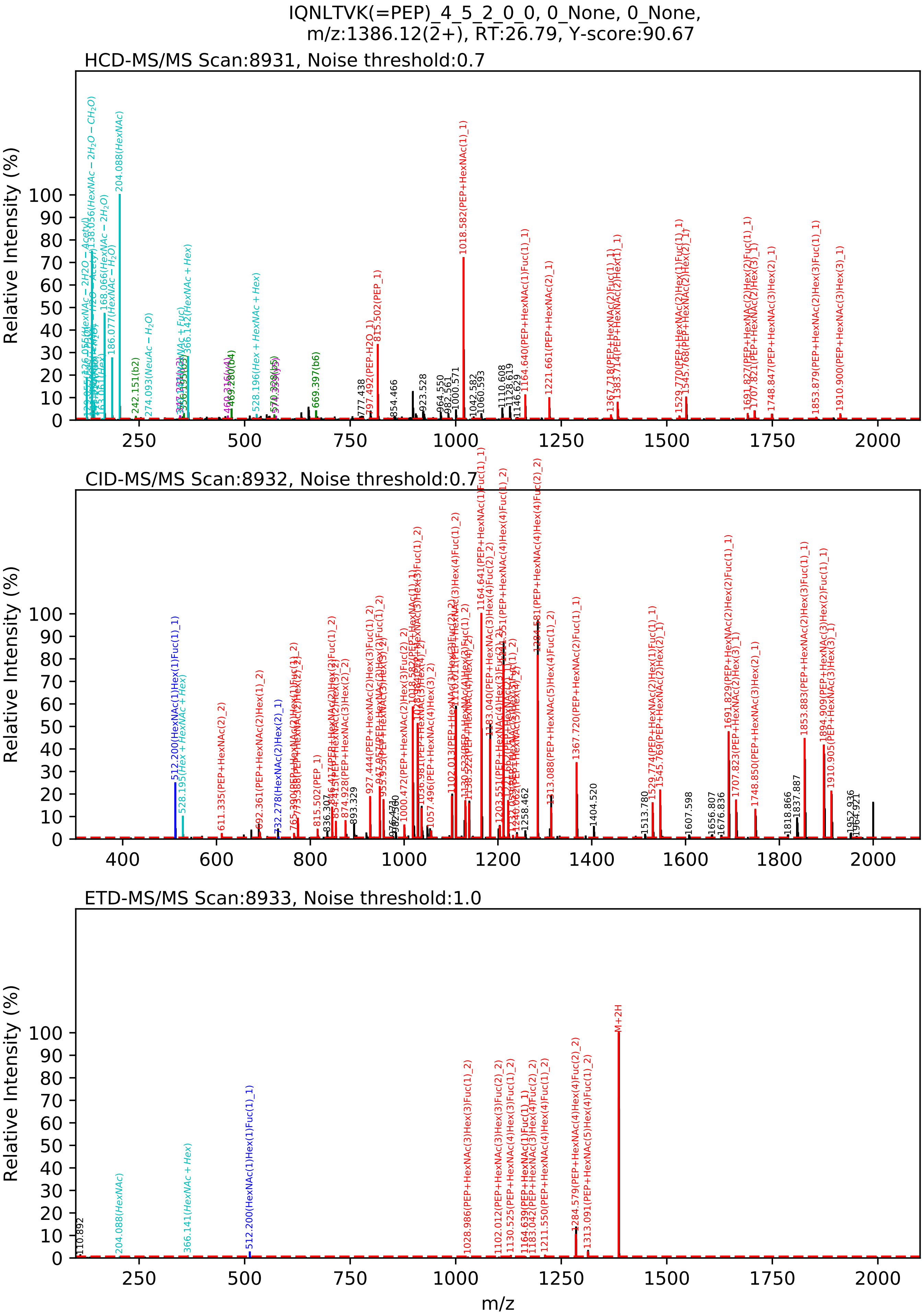
IQNLTVK(=PEP)_4_5_2_0_0, 0_None, 0_None,
m/z:1386.12(2+), RT:26.79, Y-score:90.67
HCD-MS/MS Scan:8931, Noise threshold:0.7
CID-MS/MS Scan:8932, Noise threshold:0.7
ETD-MS/MS Scan:8933, Noise threshold:1.0
Relative Intensity (%)
m/z

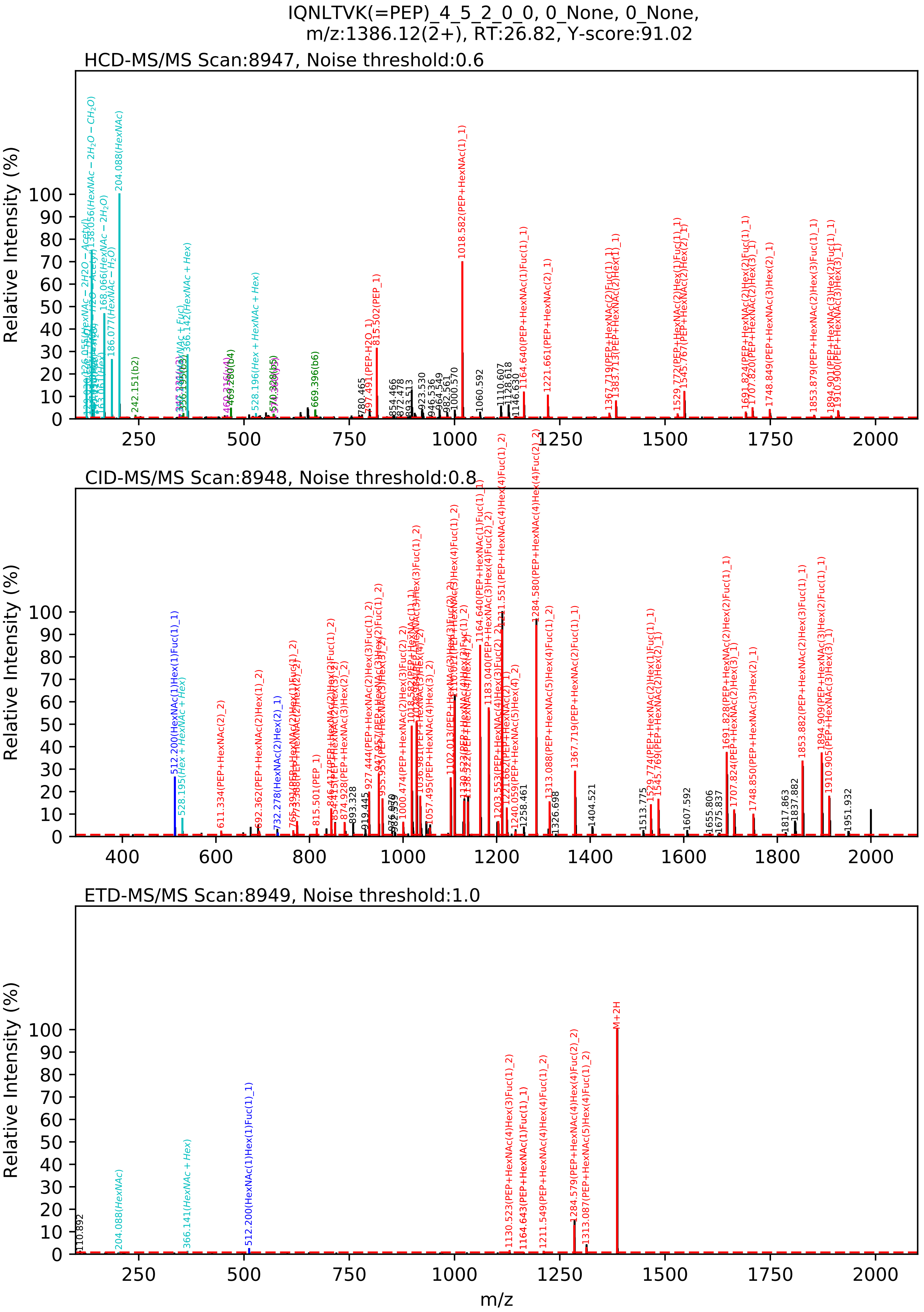
IQNLTVK(=PEP)_4_5_2_0_0, 0_None, 0_None,
m/z:1386.12(2+), RT:26.82, Y-score:91.02
HCD-MS/MS Scan:8947, Noise threshold:0.6
CID-MS/MS Scan:8948, Noise threshold:0.8
ETD-MS/MS Scan:8949, Noise threshold:1.0
Relative Intensity (%)
m/z
1018.582(PEP+HexNAc(1)_1)
204.088(HexNAc)
815.502(PEP_1)
1284.580(PEP+HexNAc(4)Hex(4)Fuc(2)_2)
512.200(HexNAc(1)Hex(1)Fuc(1)_1)
M+2H

IQNLTVK(=PEP)_4_6_0_0_0, 0_None, 0_None,
m/z:1341.60(2+), RT:26.67, Y-score:69.54

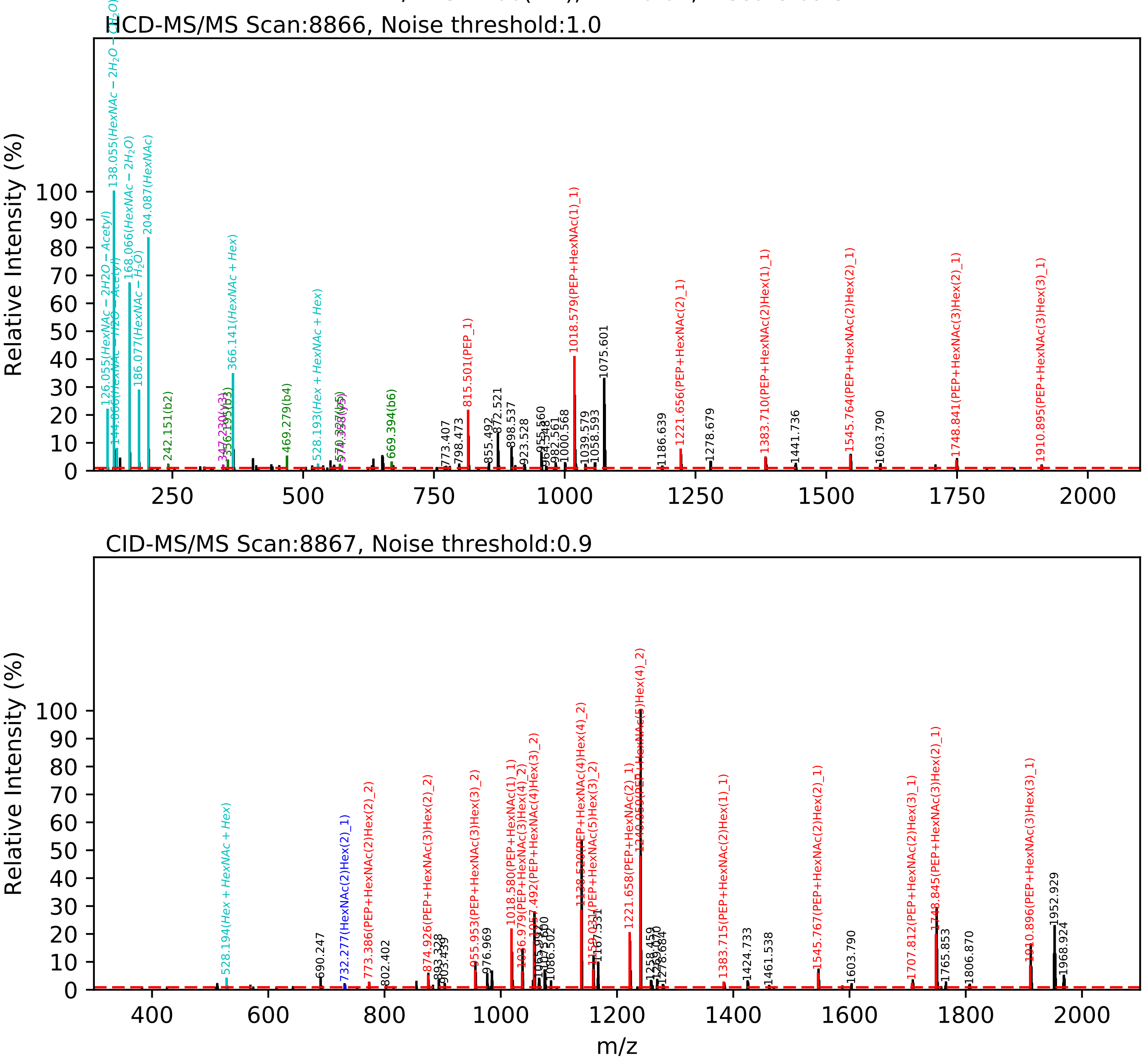

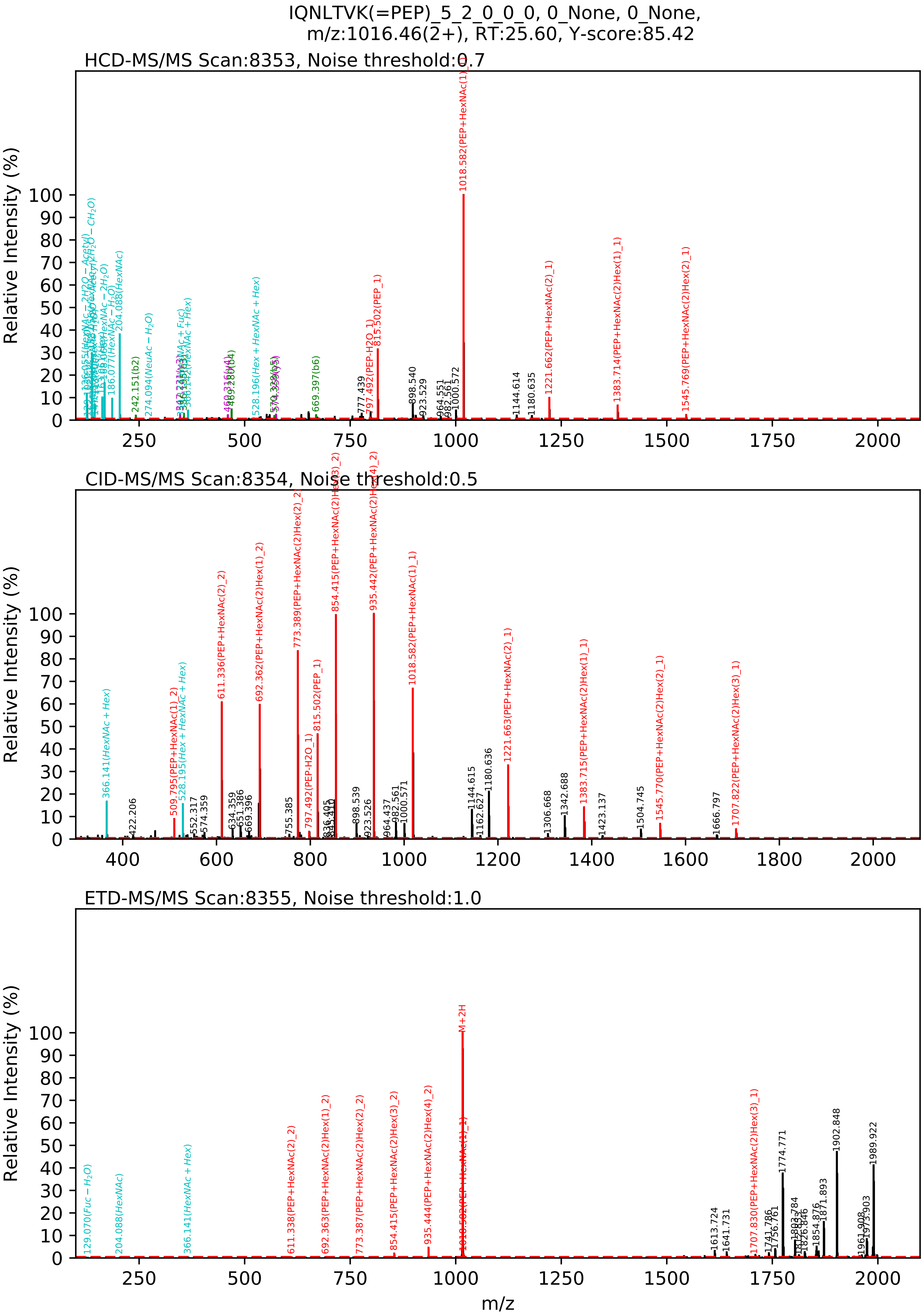
IQNLTVK(=PEP)_5_2_0_0_0, 0_None, 0_None,
m/z:1016.46(2+), RT:25.60, Y-score:85.42
HCD-MS/MS Scan:8353, Noise threshold:0.7
CID-MS/MS Scan:8354, Noise threshold:0.5
ETD-MS/MS Scan:8355, Noise threshold:1.0
Relative Intensity (%)
m/z
1018.582(PEP+HexNAc(1)_1)
1221.662(PEP+HexNAc(2)_1)
1383.714(PEP+HexNAc(2)Hex(1)_1)
1545.769(PEP+HexNAc(2)Hex(2)_1)
815.502(PEP_1)
797.492(PEP-H2O_1)
854.415(PEP+HexNAc(2)Hex(3)_2)
935.442(PEP+HexNAc(2)Hex(4)_2)
773.389(PEP+HexNAc(2)Hex(2)_2)
692.362(PEP+HexNAc(2)Hex(1)_2)
611.336(PEP+HexNAc(2)_2)
1707.822(PEP+HexNAc(2)Hex(3)_1)
M+2H

IQNLTVK(=PEP)_5_2_0_0_0, 0_None, 0_None,
m/z:1016.46(2+), RT:26.43, Y-score:89.06

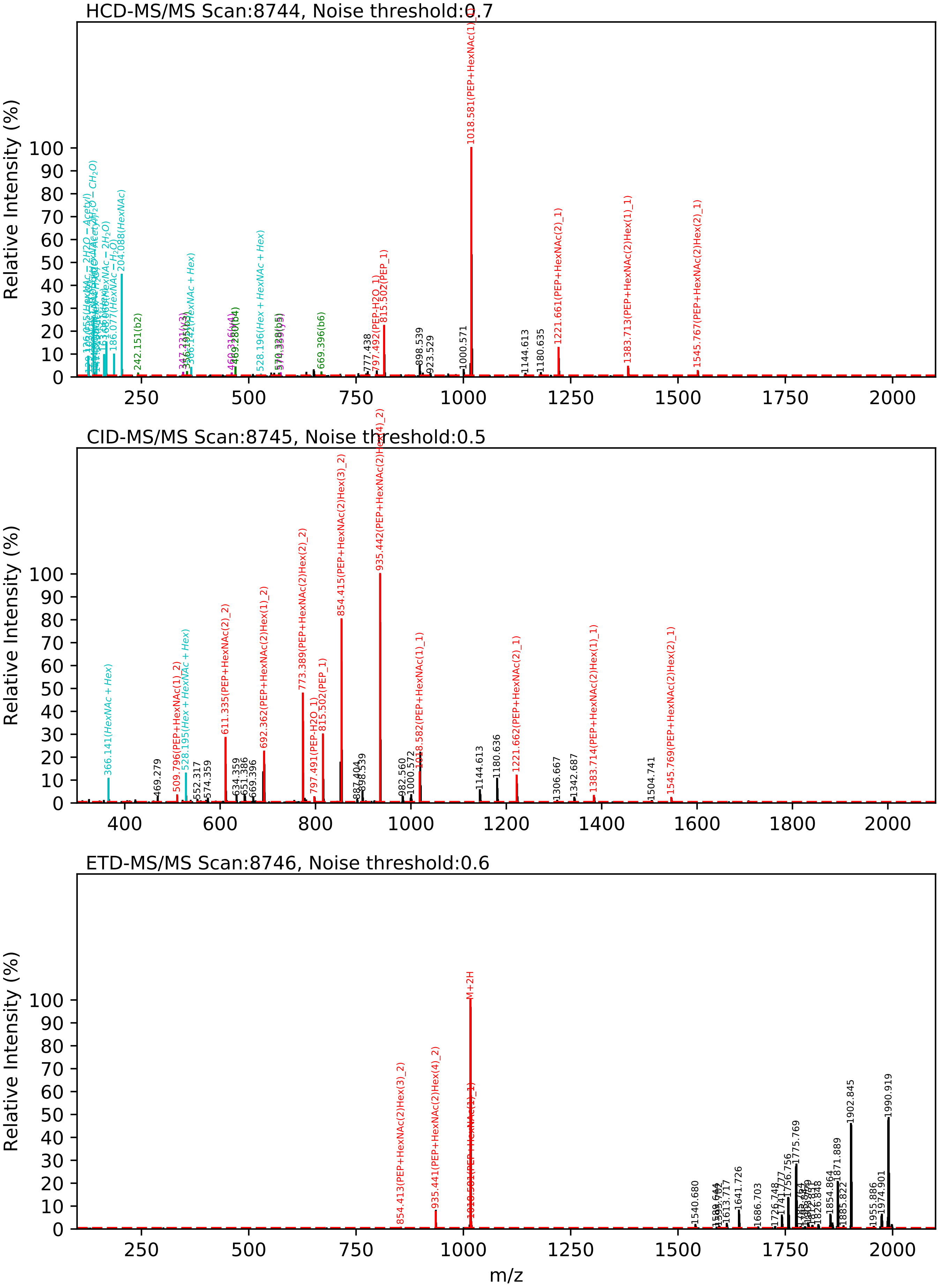

IQNLTVK(=PEP)_5_2_0_0_0, 0_None, 0_None,
m/z:1016.46(2+), RT:27.02, Y-score:83.31

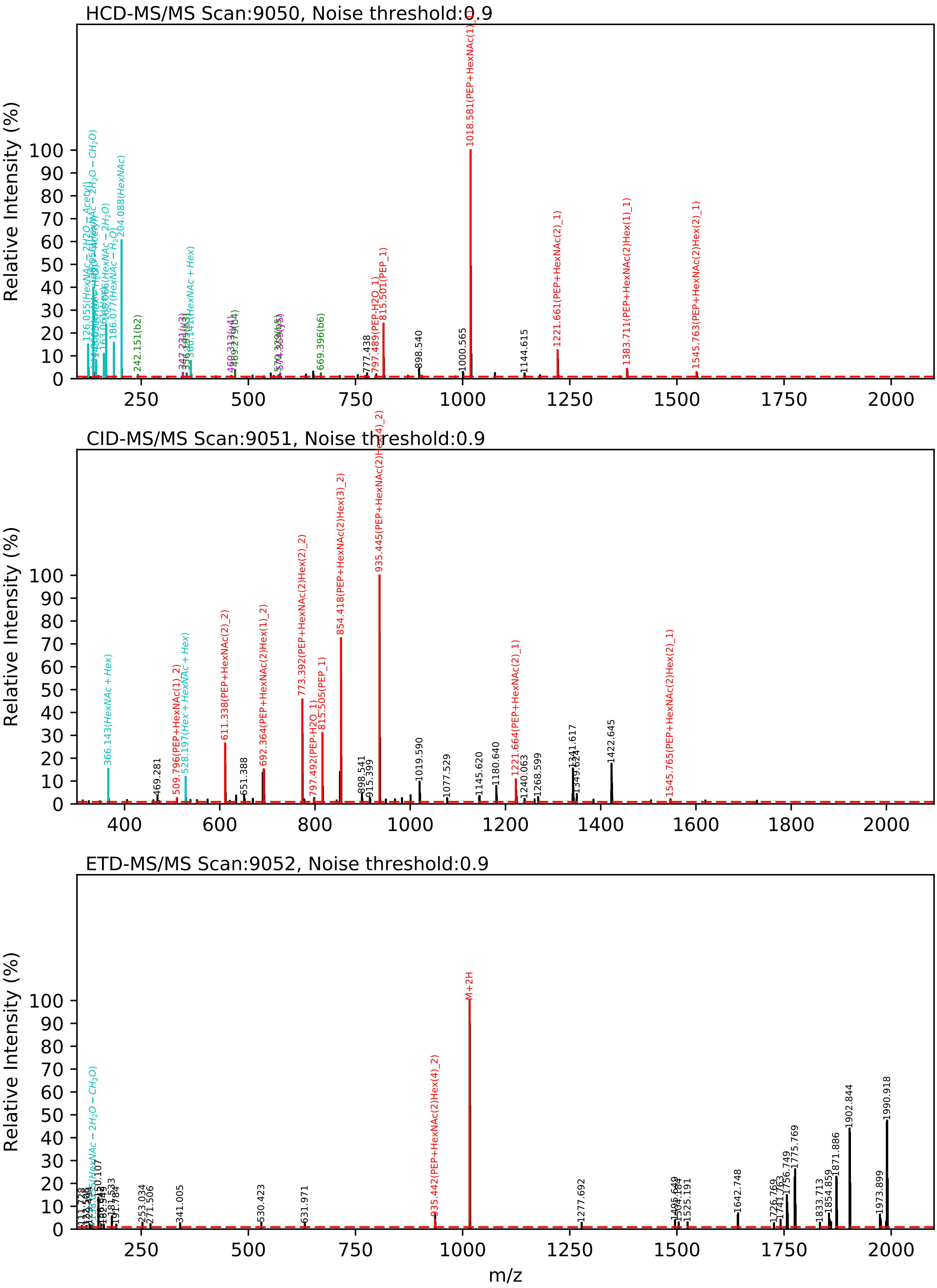

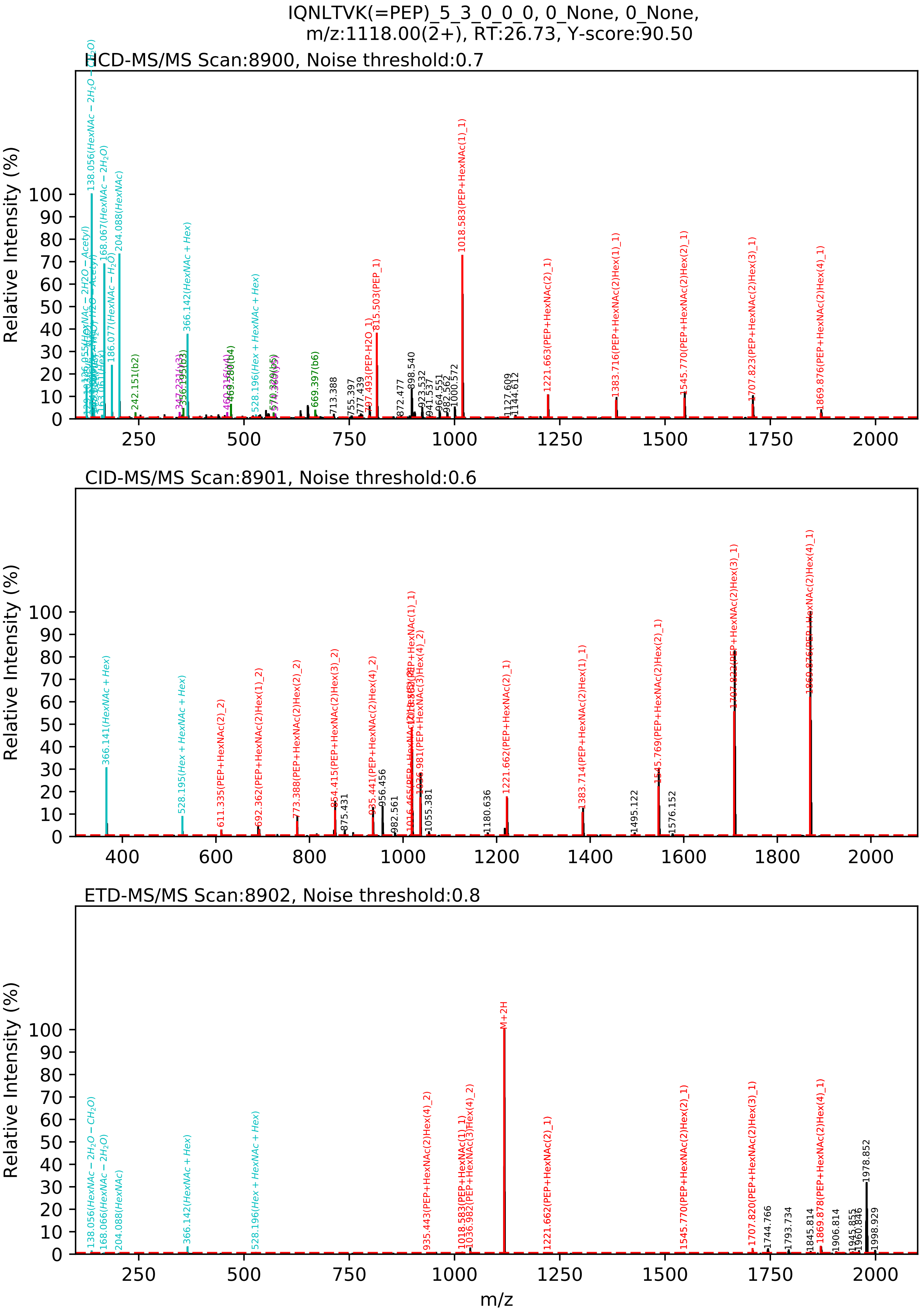
IQNLTVK(=PEP)_5_3_0_0_0, 0_None, 0_None,
m/z:1118.00(2+), RT:26.73, Y-score:90.50
HCD-MS/MS Scan:8900, Noise threshold:0.7
CID-MS/MS Scan:8901, Noise threshold:0.6
ETD-MS/MS Scan:8902, Noise threshold:0.8
Relative Intensity (%)
m/z
138.056(HexNAc − 2H2O − CH2O)
168.067(HexNAc − 2H2O)
186.077(HexNAc − H2O)
204.088(HexNAc)
242.151(b2)
366.142(HexNAc + Hex)
528.196(Hex + HexNAc + Hex)
669.397(b6)
815.503(PEP_1)
1018.583(PEP+HexNAc(1)_1)
1221.663(PEP+HexNAc(2)_1)
1383.716(PEP+HexNAc(2)Hex(1)_1)
1545.770(PEP+HexNAc(2)Hex(2)_1)
1707.823(PEP+HexNAc(2)Hex(3)_1)
1869.876(PEP+HexNAc(2)Hex(4)_1)
366.141(HexNAc + Hex)
528.195(Hex + HexNAc + Hex)
611.335(PEP+HexNAc(2)_2)
692.362(PEP+HexNAc(2)Hex(1)_2)
773.388(PEP+HexNAc(2)Hex(2)_2)
854.415(PEP+HexNAc(2)Hex(3)_2)
935.441(PEP+HexNAc(2)Hex(4)_2)
1221.662(PEP+HexNAc(2)_1)
1383.714(PEP+HexNAc(2)Hex(1)_1)
1545.769(PEP+HexNAc(2)Hex(2)_1)
M+2H
935.443(PEP+HexNAc(2)Hex(4)_2)
1018.583(PEP+HexNAc(1)_1)
1036.982(PEP+HexNAc(3)Hex(4)_2)
1221.662(PEP+HexNAc(2)_1)
1545.770(PEP+HexNAc(2)Hex(2)_1)
1707.820(PEP+HexNAc(2)Hex(3)_1)
1869.878(PEP+HexNAc(2)Hex(4)_1)
1978.852

IQNLTVK(=PEP)_5_3_0_0_0, 0_None, 0_None,
m/z:1118.00(2+), RT:35.75, Y-score:83.97

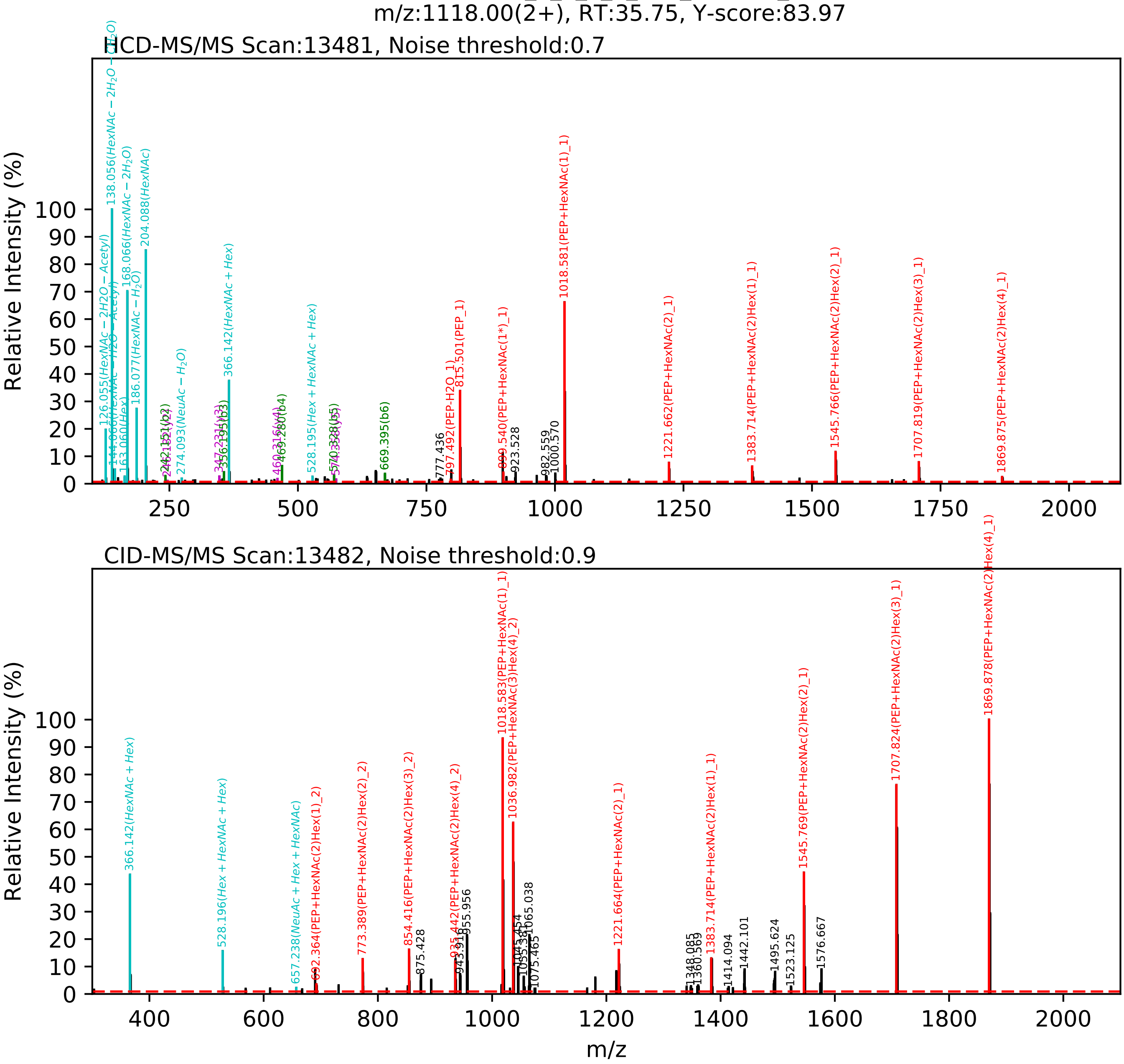

IQNLTVK(=PEP)_5_3_1_0_0, 0_None, 0_None,
m/z:1191.03(2+), RT:27.53, Y-score:89.47

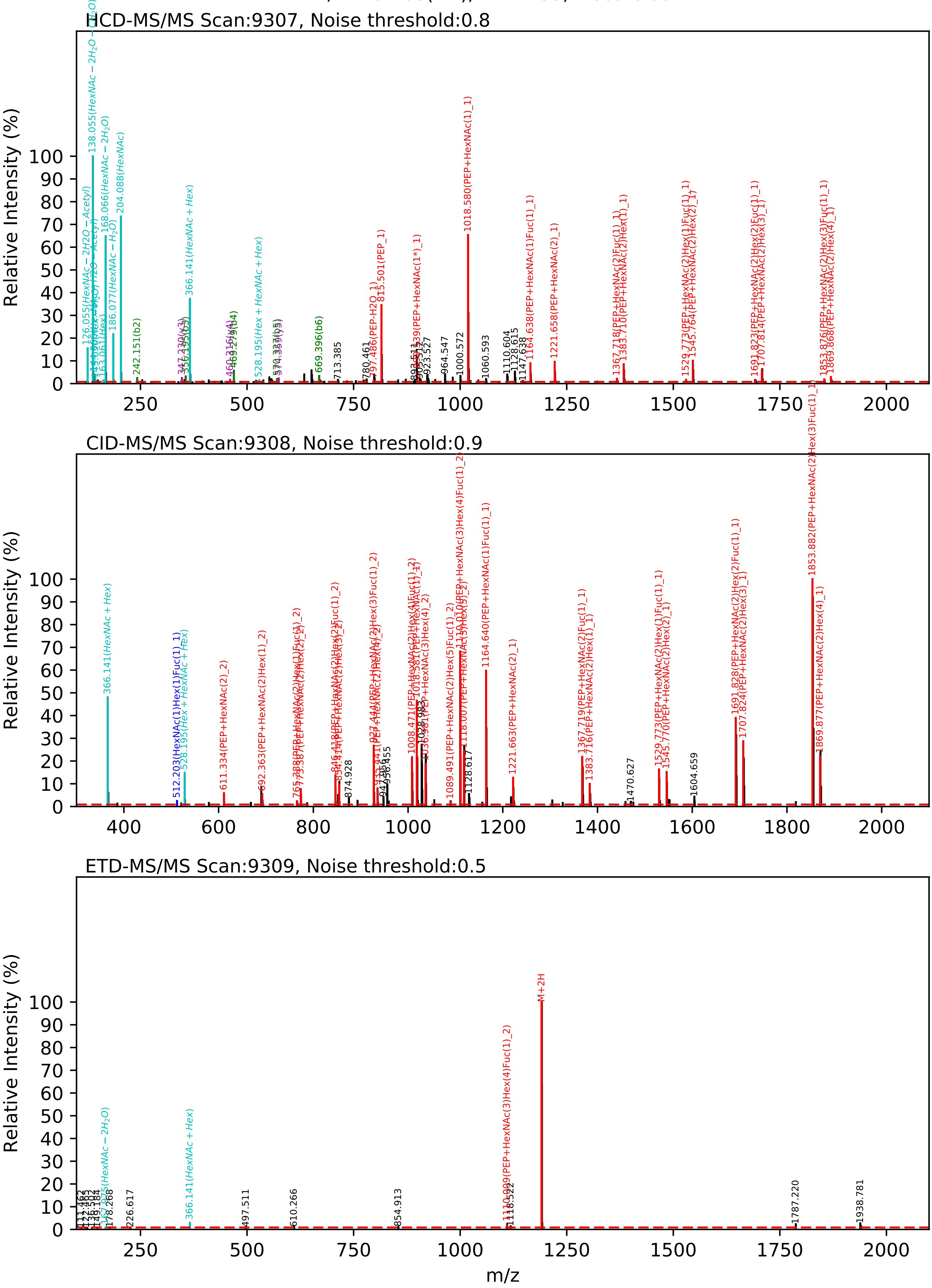

IQNLTVK(=PEP)_5_3_1_0_0, 0_None, 0_None,
m/z:1191.03(2+), RT:26.23, Y-score:91.67

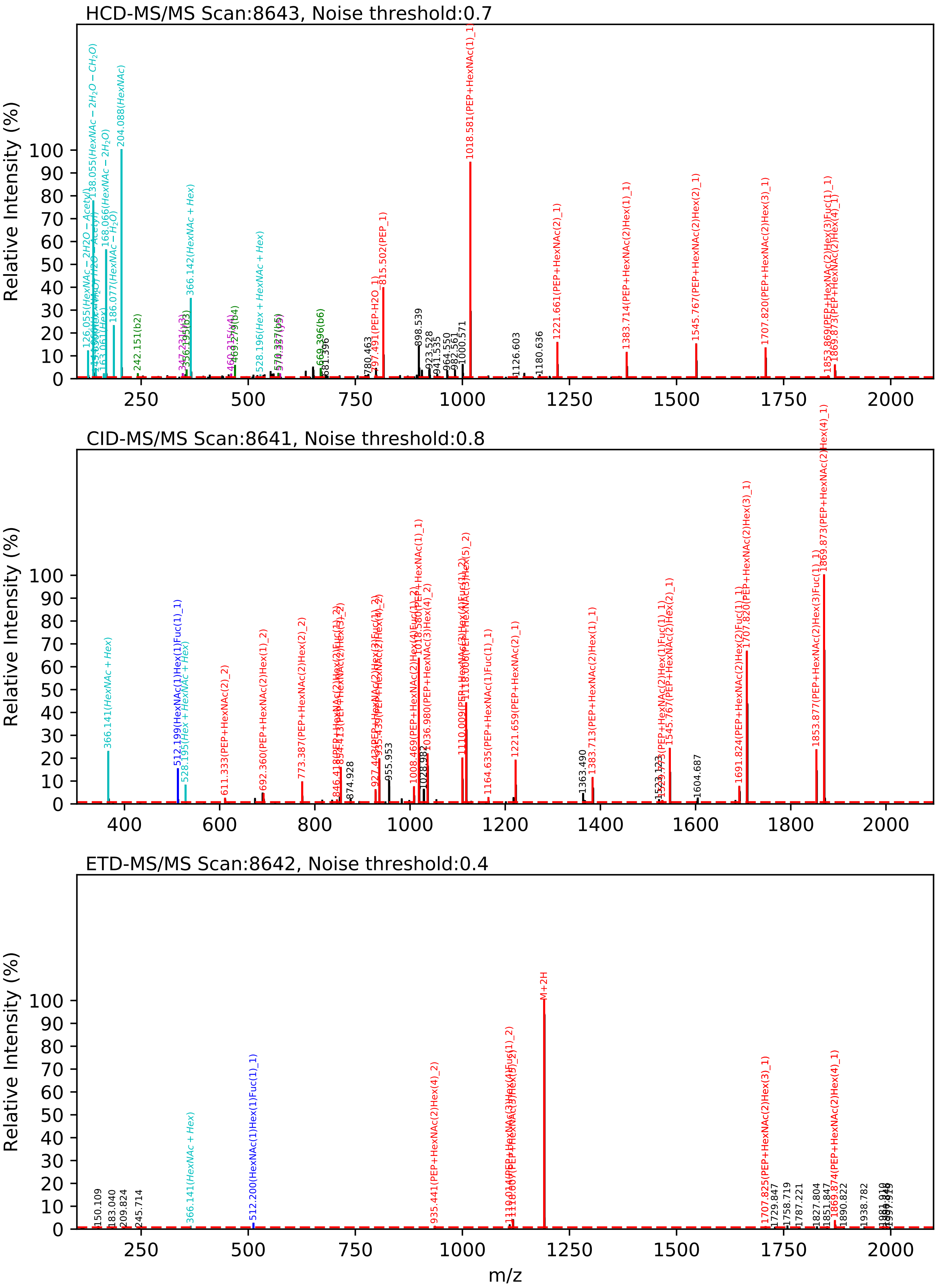

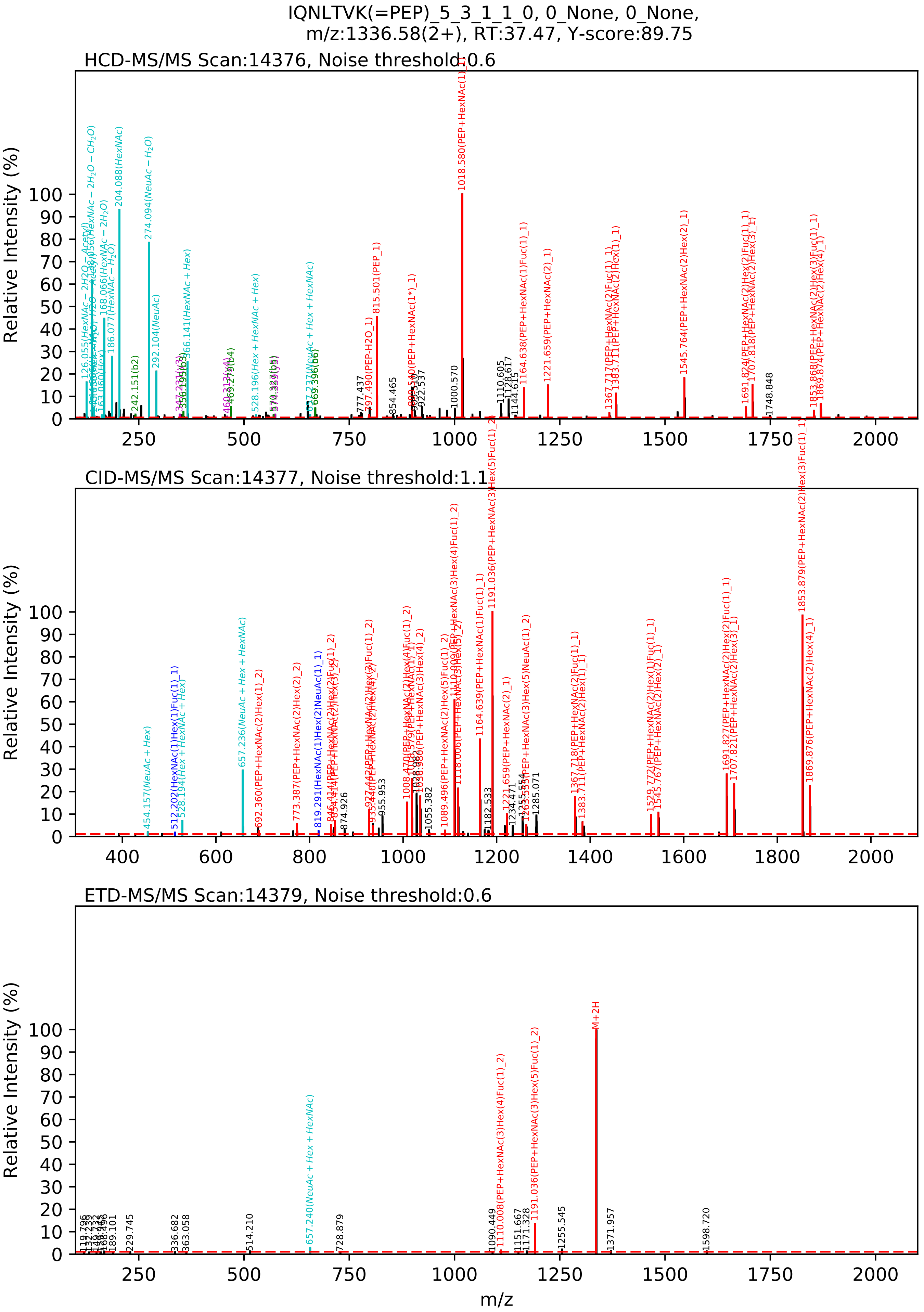
IQNLTVK(=PEP)_5_3_1_1_0, 0_None, 0_None,
m/z:1336.58(2+), RT:37.47, Y-score:89.75
HCD-MS/MS Scan:14376, Noise threshold:0.6
CID-MS/MS Scan:14377, Noise threshold:1.1
ETD-MS/MS Scan:14379, Noise threshold:0.6
Relative Intensity (%)
m/z

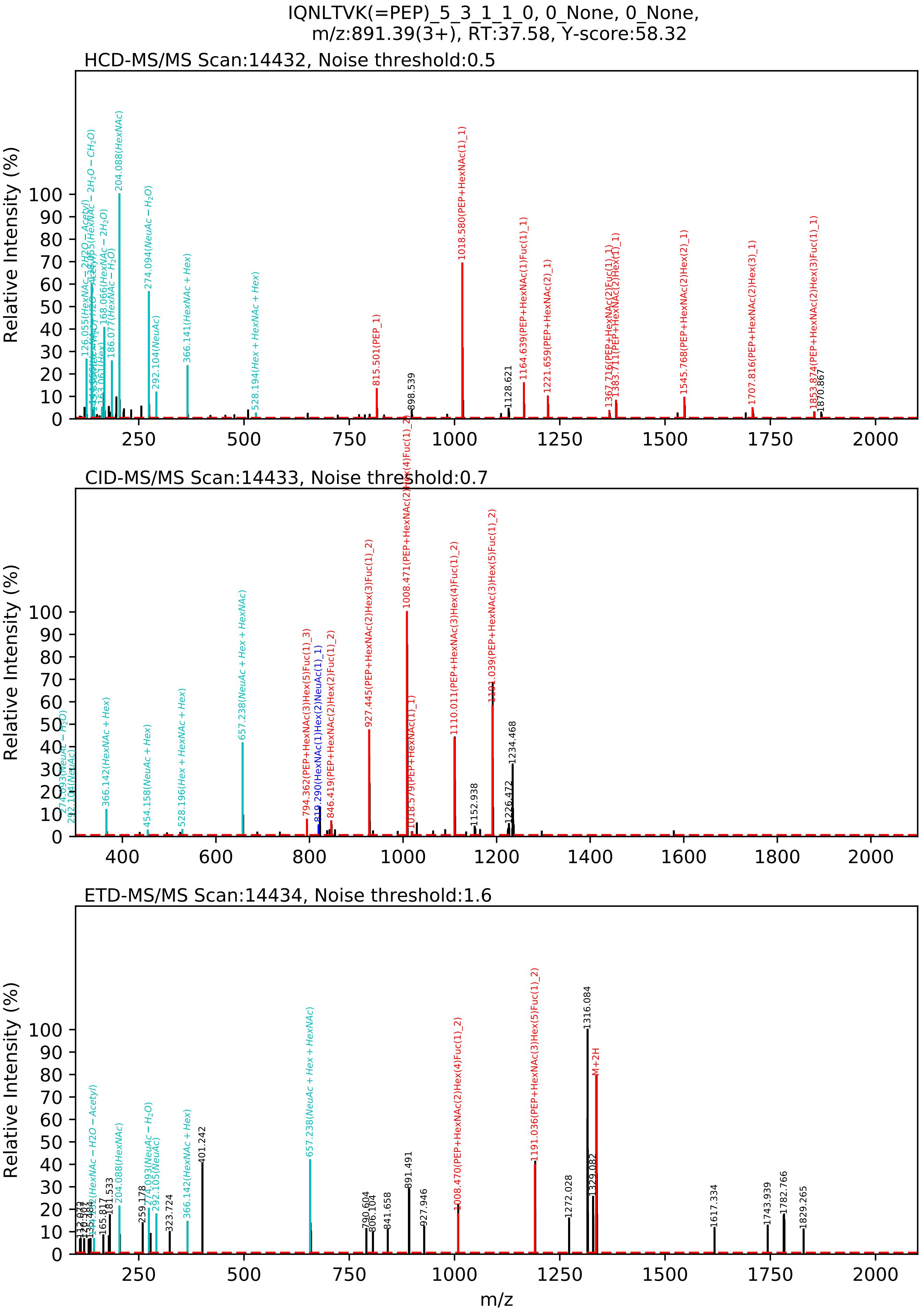

IQNLTVK(=PEP)_5_3_1_1_0, 0_None, 0_None,
m/z:891.39(3+), RT:37.58, Y-score:58.32
HCD-MS/MS Scan:14432, Noise threshold:0.5
CID-MS/MS Scan:14433, Noise threshold:0.7
ETD-MS/MS Scan:14434, Noise threshold:1.6
Relative Intensity (%)
m/z
204.088(HexNAc)
274.094(NeuAc − H2O)
292.104(NeuAc)
366.141(HexNAc + Hex)
528.194(Hex + HexNAc + Hex)
815.501(PEP_1)
898.539
1018.580(PEP+HexNAc(1)_1)
1128.621
1164.639(PEP+HexNAc(1)Fuc(1)_1)
1221.659(PEP+HexNAc(2)_1)
1545.768(PEP+HexNAc(2)Hex(2)_1)
1707.816(PEP+HexNAc(2)Hex(3)_1)
1853.874(PEP+HexNAc(2)Hex(3)Fuc(1)_1)
1870.867
366.142(HexNAc + Hex)
454.158(NeuAc + Hex)
528.196(Hex + HexNAc + Hex)
657.238(NeuAc + Hex + HexNAc)
794.362(PEP+HexNAc(3)Hex(5)Fuc(1)_3)
819.290(HexNAc(1)Hex(2)NeuAc(1)_1)
846.419(PEP+HexNAc(2)Hex(2)Fuc(1)_2)
927.445(PEP+HexNAc(2)Hex(3)Fuc(1)_2)
1008.471(PEP+HexNAc(2)Hex(4)Fuc(1)_2)
1018.579(PEP+HexNAc(1)_1)
1110.011(PEP+HexNAc(3)Hex(4)Fuc(1)_2)
1152.938
1191.039(PEP+HexNAc(3)Hex(5)Fuc(1)_2)
1226.472
1234.468
165.817
181.533
204.088(HexNAc)
259.178
274.093(NeuAc − H2O)
292.105(NeuAc)
323.724
366.142(HexNAc + Hex)
401.242
657.238(NeuAc + Hex + HexNAc)
790.604
806.104
841.658
891.491
927.946
1008.470(PEP+HexNAc(2)Hex(4)Fuc(1)_2)
1191.036(PEP+HexNAc(3)Hex(5)Fuc(1)_2)
1272.028
1316.084
1329.082
M+2H
1617.334
1743.939
1782.766
1829.265

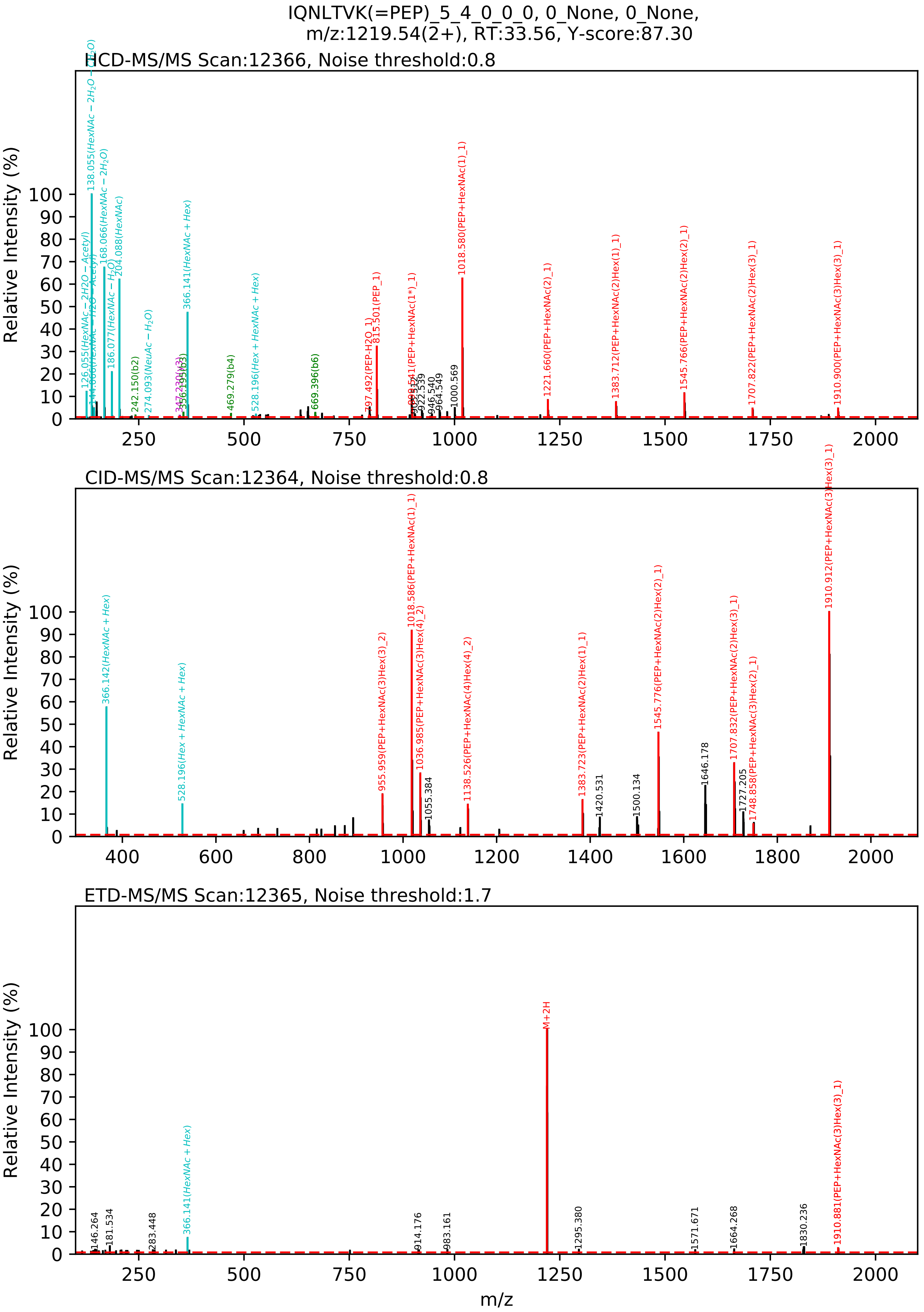
IQNLTVK(=PEP)_5_4_0_0_0, 0_None, 0_None,
m/z:1219.54(2+), RT:33.56, Y-score:87.30
HCD-MS/MS Scan:12366, Noise threshold:0.8
Relative Intensity (%)
126.055(HexNAc − 2H2O − Acetyl)
138.055(HexNAc − 2H2O − H2O)
168.066(HexNAc − 2H2O)
186.077(HexNAc − H2O)
204.088(HexNAc)
242.150(b2)
274.093(NeuAc − H2O)
356.193(b3)
366.141(HexNAc + Hex)
469.279(b4)
528.196(Hex + HexNAc + Hex)
669.396(b6)
797.492(PEP-H2O_1)
815.501(PEP_1)
922.539
946.540
964.549
1000.569
918.541(PEP+HexNAc(1*)_1)
1018.580(PEP+HexNAc(1)_1)
1221.660(PEP+HexNAc(2)_1)
1383.712(PEP+HexNAc(2)Hex(1)_1)
1545.766(PEP+HexNAc(2)Hex(2)_1)
1707.822(PEP+HexNAc(2)Hex(3)_1)
1910.900(PEP+HexNAc(3)Hex(3)_1)
CID-MS/MS Scan:12364, Noise threshold:0.8
Relative Intensity (%)
366.142(HexNAc + Hex)
528.196(Hex + HexNAc + Hex)
955.959(PEP+HexNAc(3)Hex(3)_2)
1018.586(PEP+HexNAc(1)_1)
1036.985(PEP+HexNAc(3)Hex(4)_2)
1055.384
1138.526(PEP+HexNAc(4)Hex(4)_2)
1383.723(PEP+HexNAc(2)Hex(1)_1)
1420.531
1500.134
1545.776(PEP+HexNAc(2)Hex(2)_1)
1646.178
1707.832(PEP+HexNAc(2)Hex(3)_1)
1727.205
1748.858(PEP+HexNAc(3)Hex(2)_1)
1910.912(PEP+HexNAc(3)Hex(3)_1)
ETD-MS/MS Scan:12365, Noise threshold:1.7
Relative Intensity (%)
146.264
181.534
283.448
366.141(HexNAc + Hex)
914.176
983.161
M+2H
1295.380
1571.671
1664.268
1830.236
1910.881(PEP+HexNAc(3)Hex(3)_1)
m/z

IQNLTVK(=PEP)_5_4_0_0_0, 0_None, 0_None,
m/z:1219.54(2+), RT:25.79, Y-score:83.01

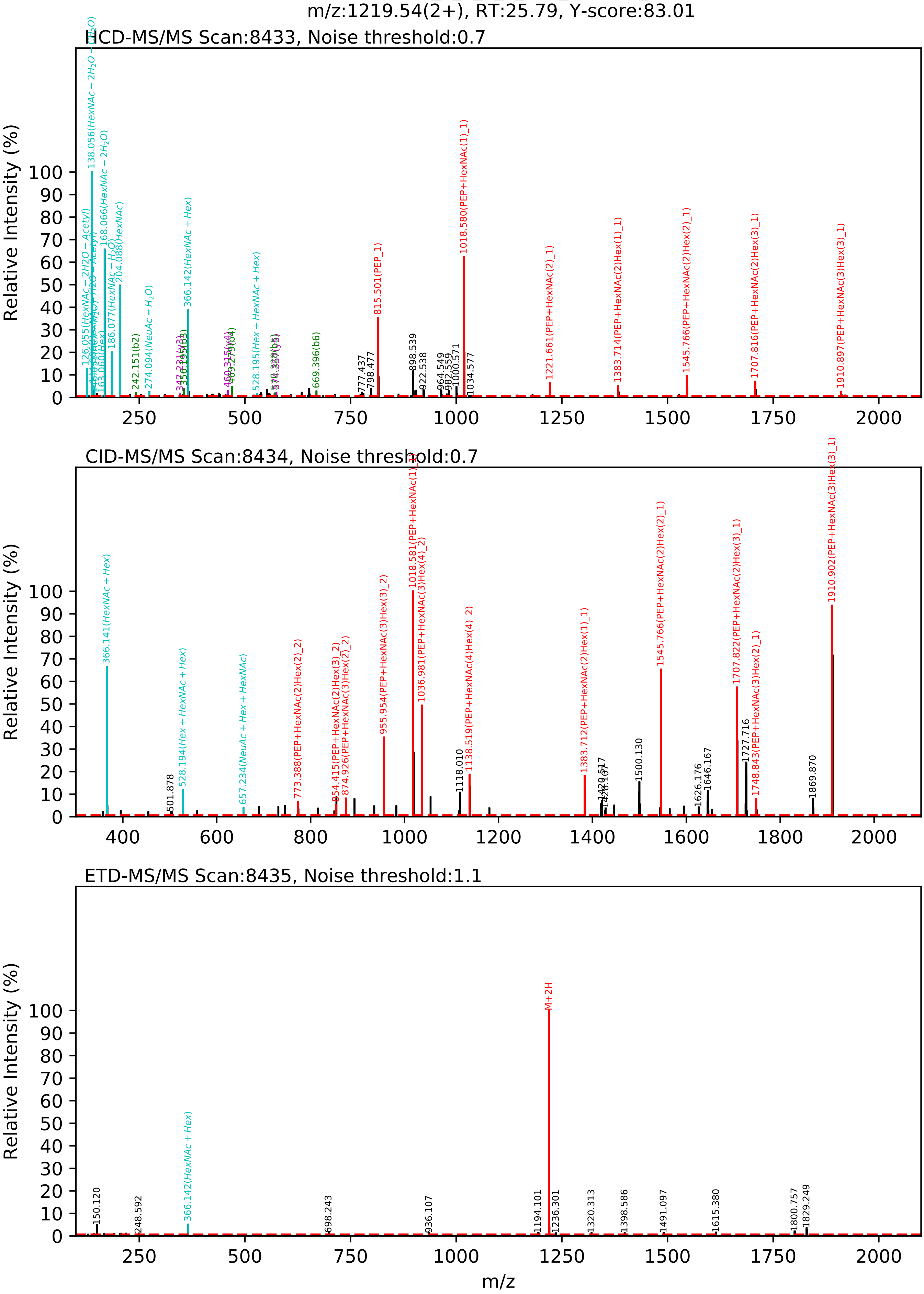

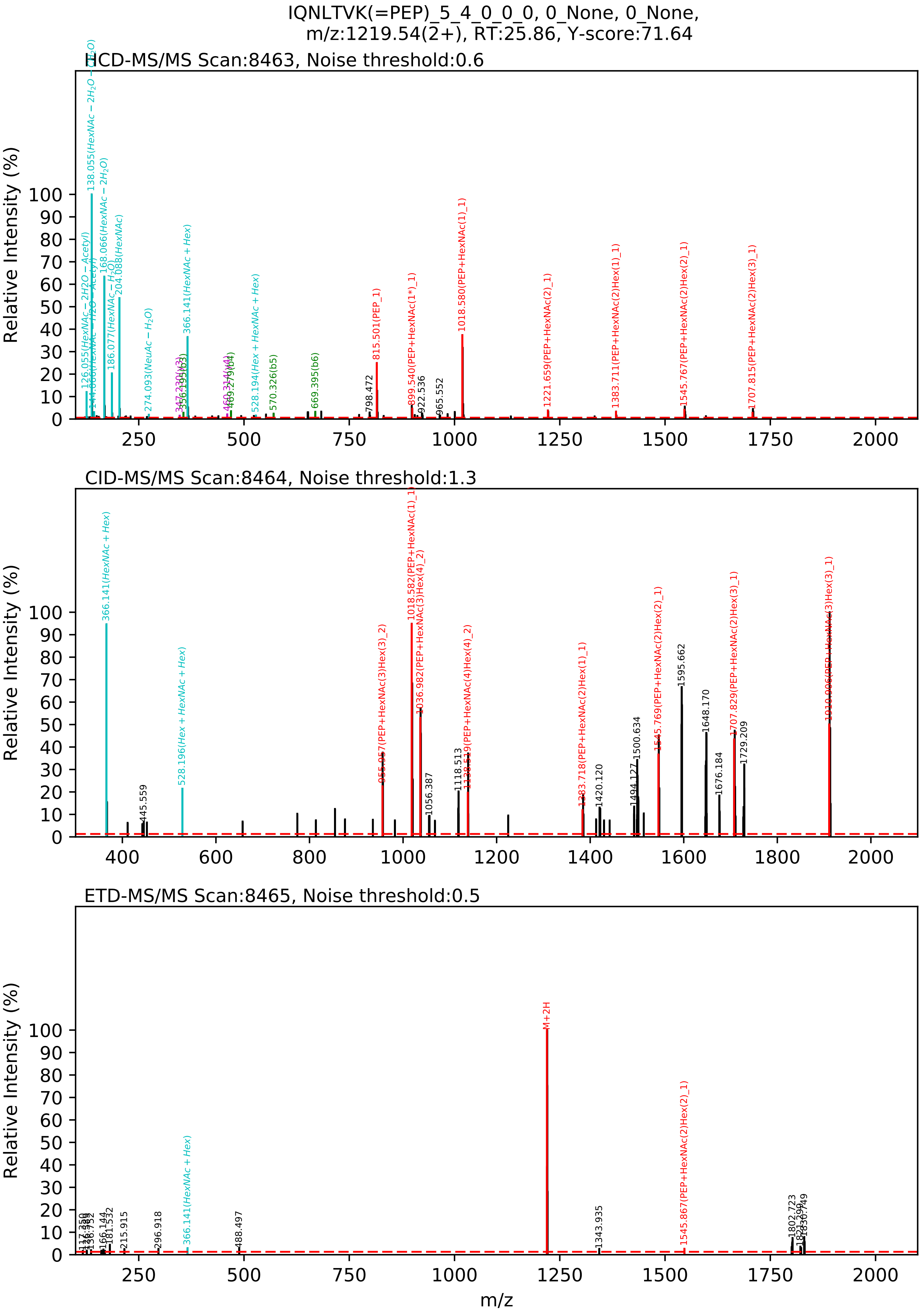
IQNLTVK(=PEP)_5_4_0_0_0, 0_None, 0_None,
m/z:1219.54(2+), RT:25.86, Y-score:71.64
HCD-MS/MS Scan:8463, Noise threshold:0.6
Relative Intensity (%)
138.055(HexNAc − 2H2O − H2O)
168.066(HexNAc − 2H2O)
204.088(HexNAc)
186.077(HexNAc − H2O)
366.141(HexNAc + Hex)
274.093(NeuAc − H2O)
528.194(Hex + HexNAc + Hex)
570.326(b5)
669.395(b6)
798.472
815.501(PEP_1)
899.540(PEP+HexNAc(1*)_1)
922.536
965.552
1018.580(PEP+HexNAc(1)_1)
1221.659(PEP+HexNAc(2)_1)
1383.711(PEP+HexNAc(2)Hex(1)_1)
1545.767(PEP+HexNAc(2)Hex(2)_1)
1707.815(PEP+HexNAc(2)Hex(3)_1)
CID-MS/MS Scan:8464, Noise threshold:1.3
366.141(HexNAc + Hex)
528.196(Hex + HexNAc + Hex)
445.559
955.957(PEP+HexNAc(3)Hex(3)_2)
1018.582(PEP+HexNAc(1)_1)
1036.982(PEP+HexNAc(3)Hex(4)_2)
1056.387
1118.513
1138.519(PEP+HexNAc(4)Hex(4)_2)
1383.718(PEP+HexNAc(2)Hex(1)_1)
1420.120
1494.127
1500.634
1545.769(PEP+HexNAc(2)Hex(2)_1)
1595.662
1648.170
1676.184
1707.829(PEP+HexNAc(2)Hex(3)_1)
1729.209
1910.906(PEP+HexNAc(3)Hex(3)_1)
ETD-MS/MS Scan:8465, Noise threshold:0.5
M+2H
366.141(HexNAc + Hex)
1545.867(PEP+HexNAc(2)Hex(2)_1)
166.144
181.532
215.915
296.918
488.497
1343.935
1802.723
1830.749
m/z

IQNLTVK(=PEP)_5_4_0_0_0, 0_None, 0_None,
m/z:1219.54(2+), RT:26.45, Y-score:82.31

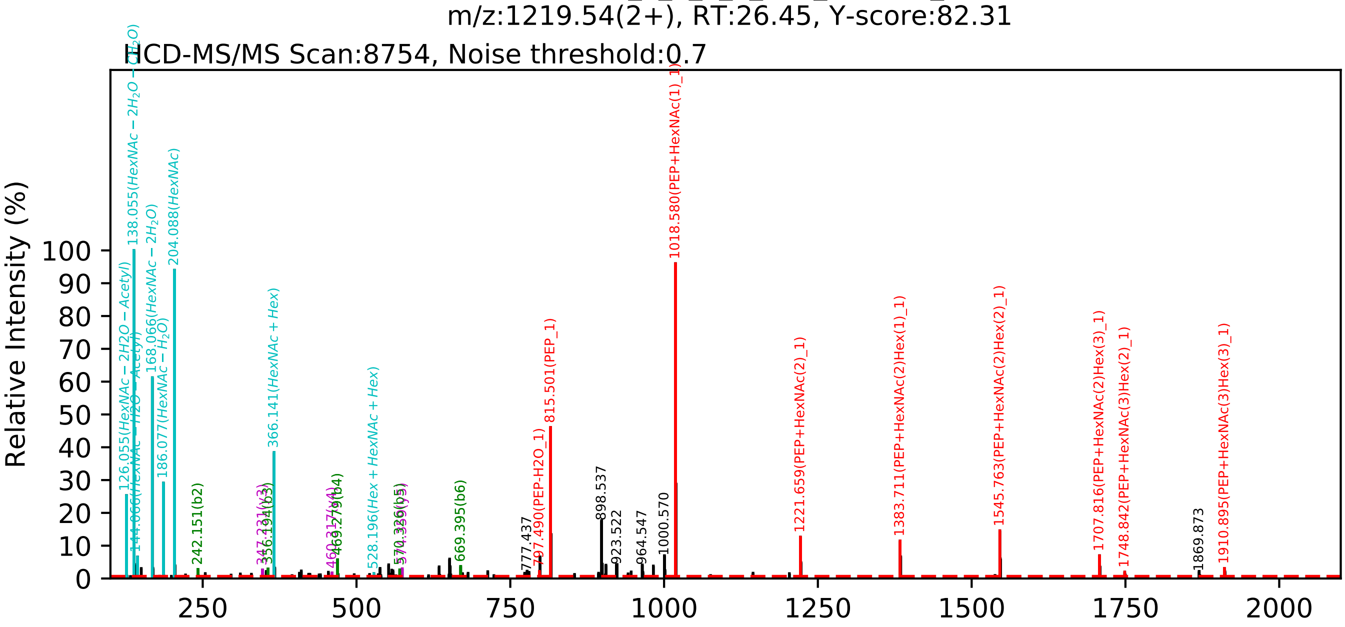

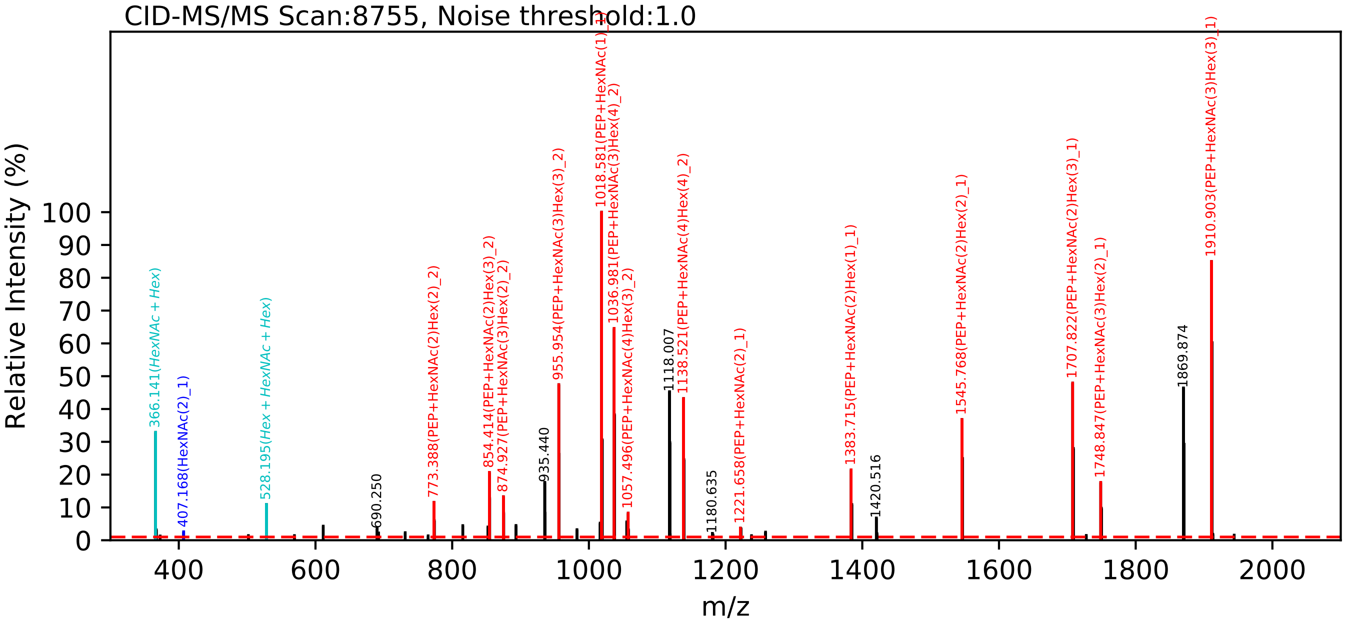

IQNLTVK(=PEP)_5_4_0_0_0, 0_None, 0_None,
m/z:1219.54(2+), RT:27.00, Y-score:94.85

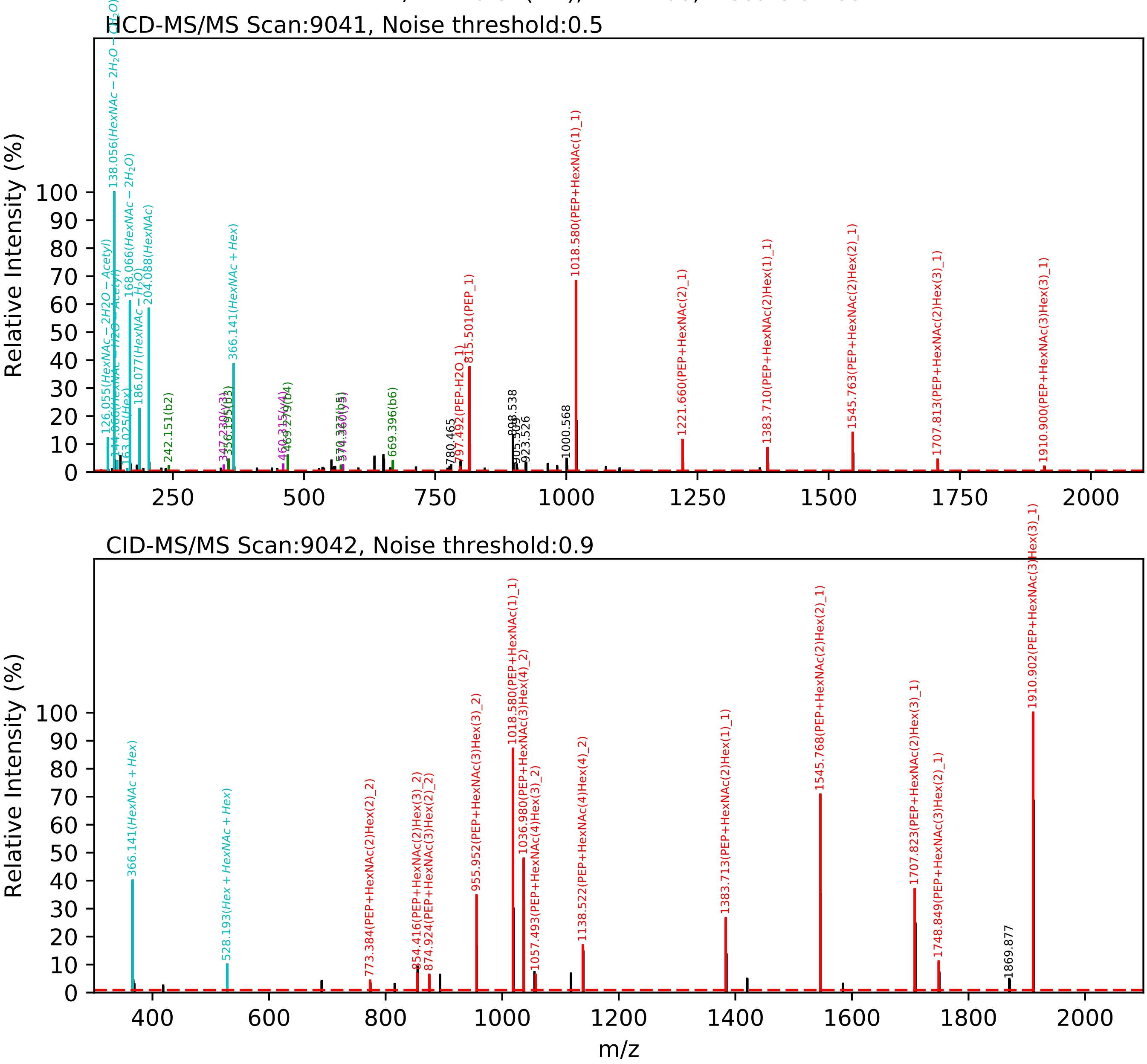

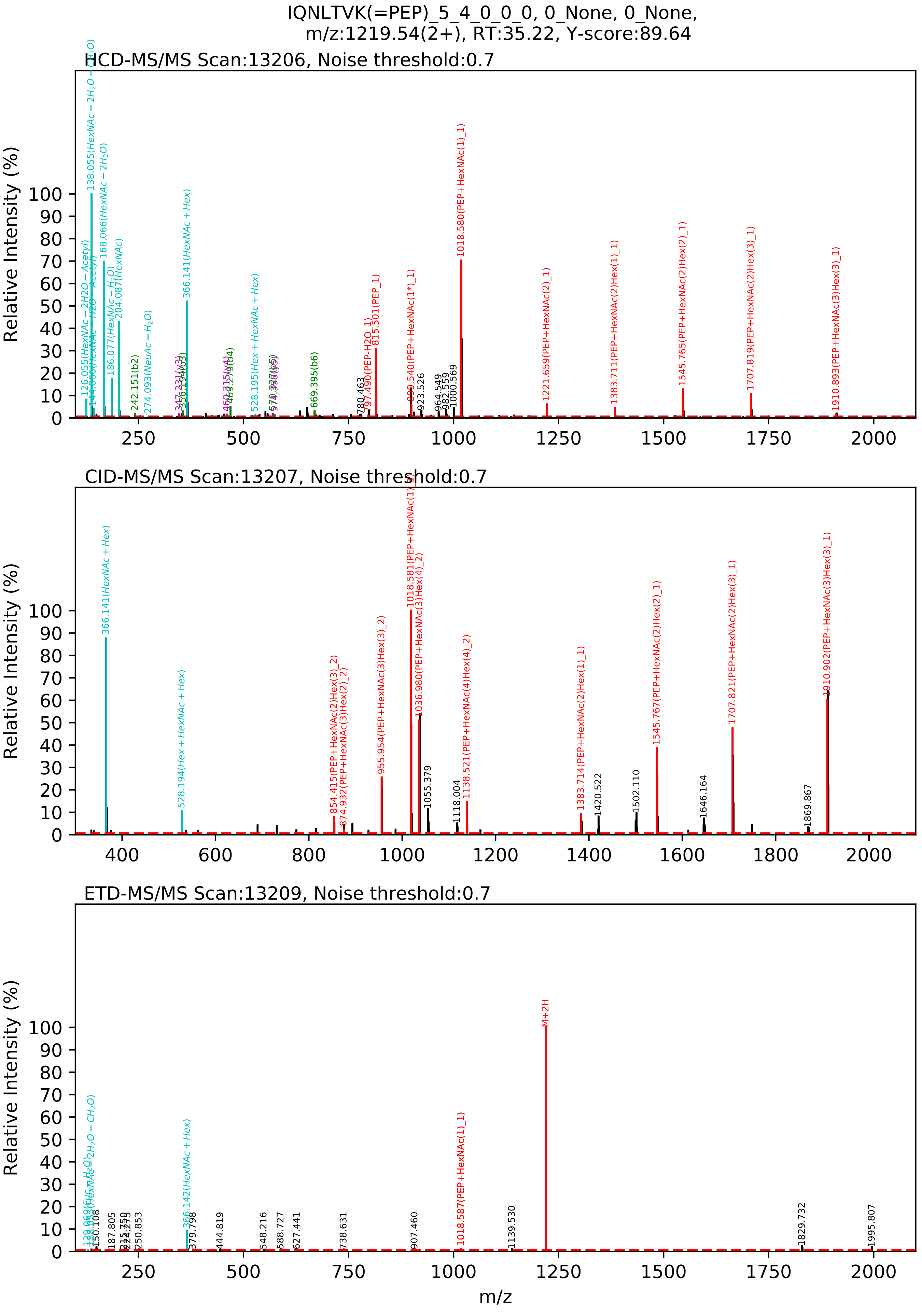

IQNLTVK(=PEP)_5_4_0_0_0, 0_None, 0_None,
m/z:1219.54(2+), RT:35.22, Y-score:89.64
HCD-MS/MS Scan:13206, Noise threshold:0.7
CID-MS/MS Scan:13207, Noise threshold:0.7
ETD-MS/MS Scan:13209, Noise threshold:0.7
Relative Intensity (%)
m/z
138.055(HexNAc − 2H2O − CH2O)
168.066(HexNAc − 2H2O)
204.087(HexNAc)
366.141(HexNAc + Hex)
528.195(Hex + HexNAc + Hex)
669.395(b6)
815.501(PEP_1)
1018.580(PEP+HexNAc(1)_1)
1221.659(PEP+HexNAc(2)_1)
1383.711(PEP+HexNAc(2)Hex(1)_1)
1545.765(PEP+HexNAc(2)Hex(2)_1)
1707.819(PEP+HexNAc(2)Hex(3)_1)
1910.893(PEP+HexNAc(3)Hex(3)_1)
366.141(HexNAc + Hex)
528.194(Hex + HexNAc + Hex)
854.415(PEP+HexNAc(2)Hex(3)_2)
874.932(PEP+HexNAc(3)Hex(2)_2)
955.954(PEP+HexNAc(3)Hex(3)_2)
1018.581(PEP+HexNAc(1)_1)
1036.980(PEP+HexNAc(3)Hex(4)_2)
1055.379
1118.004
1138.521(PEP+HexNAc(4)Hex(4)_2)
1383.714(PEP+HexNAc(2)Hex(1)_1)
1420.522
1502.110
1545.767(PEP+HexNAc(2)Hex(2)_1)
1646.164
1707.821(PEP+HexNAc(2)Hex(3)_1)
1869.867
1910.902(PEP+HexNAc(3)Hex(3)_1)
M+2H
366.142(HexNAc + Hex)
1018.587(PEP+HexNAc(1)_1)
1139.530
1829.732
1995.807

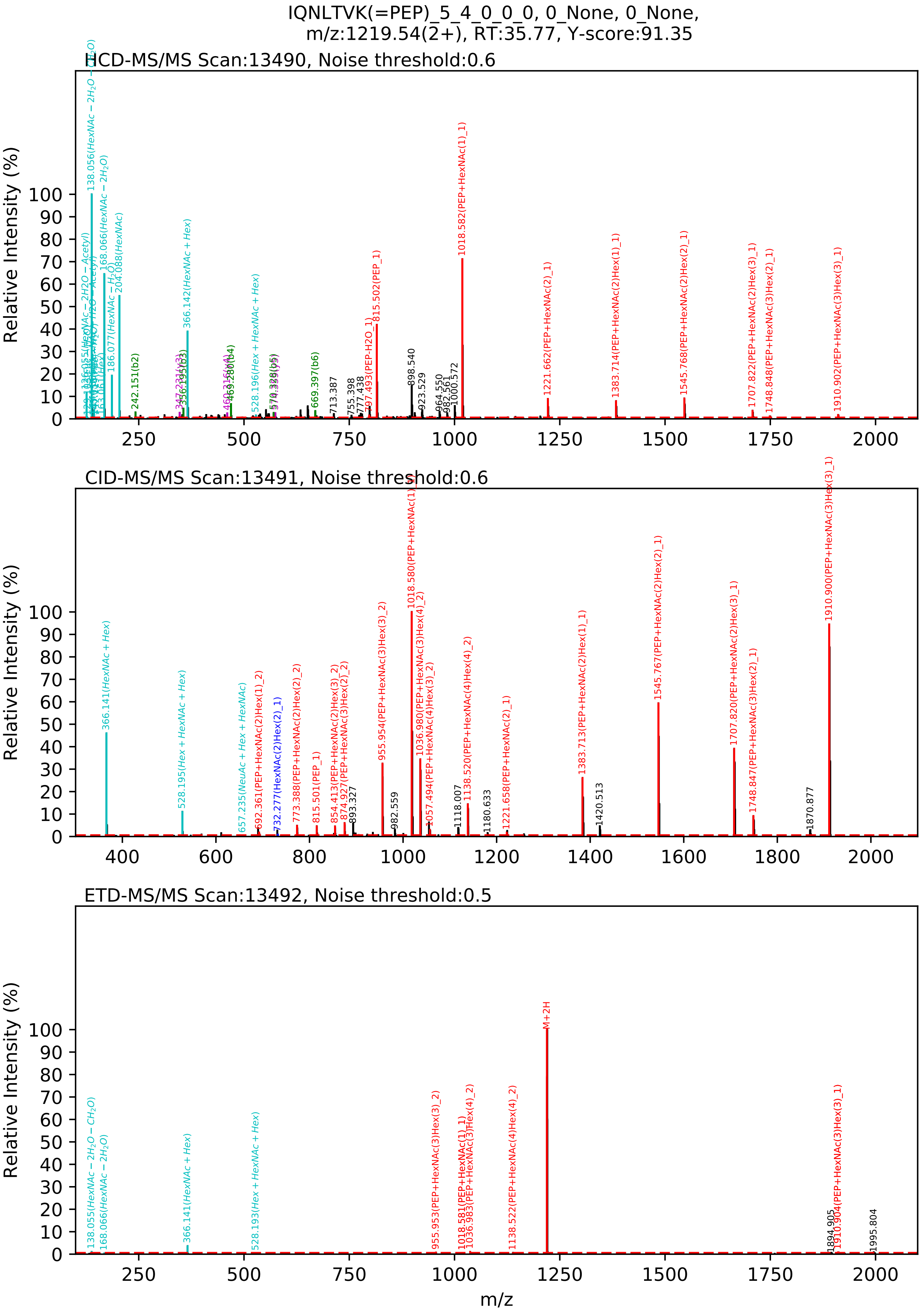

IQNLTVK(=PEP)_5_4_0_0_0, 0_None, 0_None,
m/z:1219.54(2+), RT:35.77, Y-score:91.35
HCD-MS/MS Scan:13490, Noise threshold:0.6
CID-MS/MS Scan:13491, Noise threshold:0.6
ETD-MS/MS Scan:13492, Noise threshold:0.5
Relative Intensity (%)
m/z
1018.582(PEP+HexNAc(1)_1)
815.502(PEP_1)
1221.662(PEP+HexNAc(2)_1)
1383.714(PEP+HexNAc(2)Hex(1)_1)
1545.768(PEP+HexNAc(2)Hex(2)_1)
1707.822(PEP+HexNAc(2)Hex(3)_1)
1748.848(PEP+HexNAc(3)Hex(2)_1)
1910.902(PEP+HexNAc(3)Hex(3)_1)
1018.580(PEP+HexNAc(1)_1)
1910.900(PEP+HexNAc(3)Hex(3)_1)
1545.767(PEP+HexNAc(2)Hex(2)_1)
1707.820(PEP+HexNAc(2)Hex(3)_1)
1383.713(PEP+HexNAc(2)Hex(1)_1)
955.954(PEP+HexNAc(3)Hex(3)_2)
1036.980(PEP+HexNAc(3)Hex(4)_2)
1138.520(PEP+HexNAc(4)Hex(4)_2)
366.141(HexNAc+Hex)
528.195(Hex+HexNAc+Hex)
M+2H

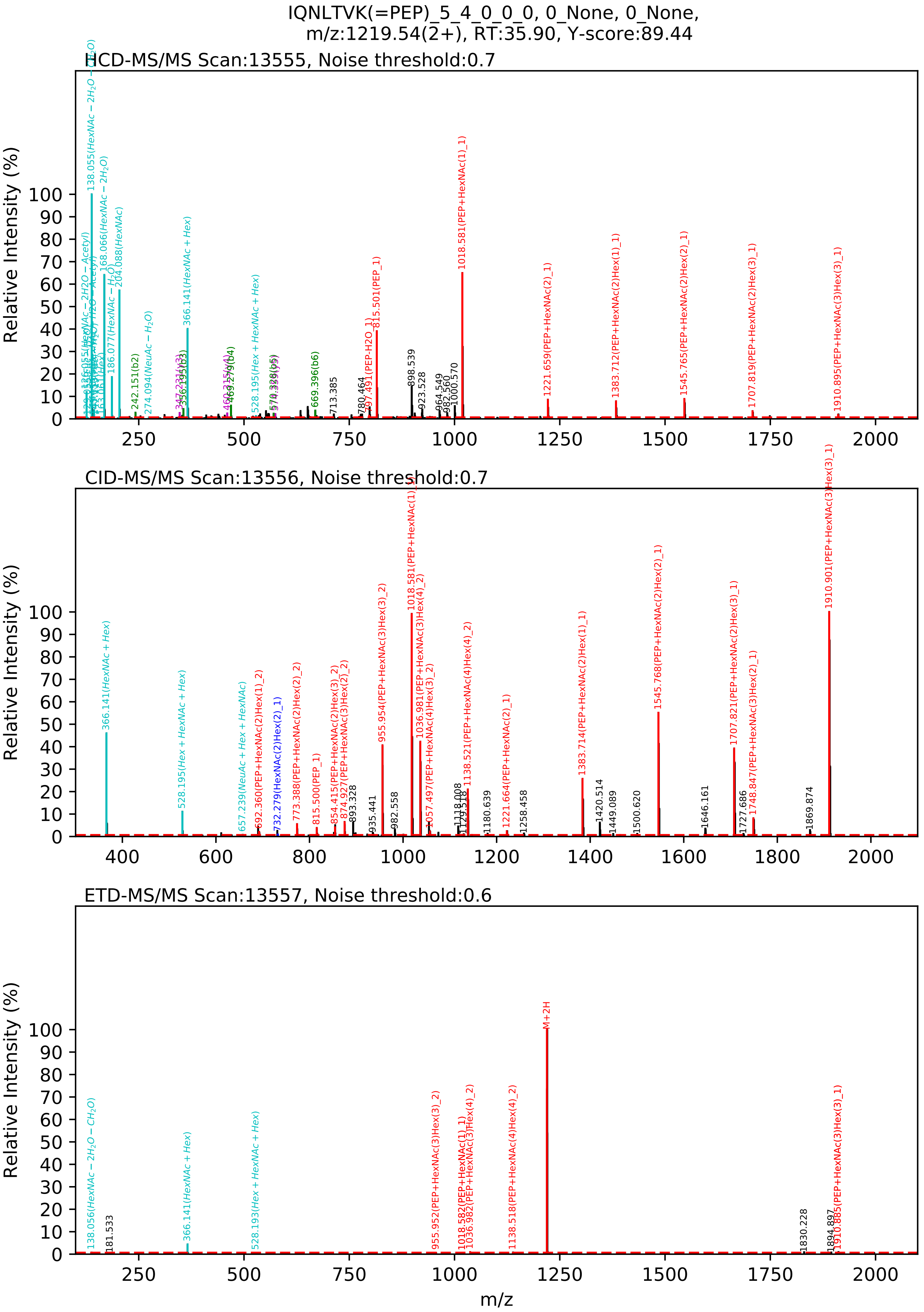
IQNLTVK(=PEP)_5_4_0_0_0, 0_None, 0_None,
m/z:1219.54(2+), RT:35.90, Y-score:89.44
HCD-MS/MS Scan:13555, Noise threshold:0.7
CID-MS/MS Scan:13556, Noise threshold:0.7
ETD-MS/MS Scan:13557, Noise threshold:0.6
Relative Intensity (%)
m/z
1018.581(PEP+HexNAc(1)_1)
815.501(PEP_1)
1221.659(PEP+HexNAc(2)_1)
1383.712(PEP+HexNAc(2)Hex(1)_1)
1545.765(PEP+HexNAc(2)Hex(2)_1)
1707.819(PEP+HexNAc(2)Hex(3)_1)
1910.895(PEP+HexNAc(3)Hex(3)_1)
138.055(HexNAc − 2H2O − CH2O)
168.066(HexNAc − 2H2O)
204.088(HexNAc)
186.077(HexNAc − H2O)
366.141(HexNAc + Hex)
528.195(Hex + HexNAc + Hex)
669.396(b6)
242.151(b2)
1910.901(PEP+HexNAc(3)Hex(3)_1)
1545.768(PEP+HexNAc(2)Hex(2)_1)
1383.714(PEP+HexNAc(2)Hex(1)_1)
1707.821(PEP+HexNAc(2)Hex(3)_1)
1748.847(PEP+HexNAc(3)Hex(2)_1)
1138.521(PEP+HexNAc(4)Hex(4)_2)
1221.664(PEP+HexNAc(2)_1)
955.954(PEP+HexNAc(3)Hex(3)_2)
1036.981(PEP+HexNAc(3)Hex(4)_2)
773.388(PEP+HexNAc(2)Hex(2)_2)
692.360(PEP+HexNAc(2)Hex(1)_2)
732.279(HexNAc(2)Hex(2)_1)
657.239(NeuAc + Hex + HexNAc)
M+2H
181.533
1830.228
1894.897
1910.885(PEP+HexNAc(3)Hex(3)_1)
1138.518(PEP+HexNAc(4)Hex(4)_2)
955.952(PEP+HexNAc(3)Hex(3)_2)
528.193(Hex + HexNAc + Hex)
138.056(HexNAc − 2H2O − CH2O)

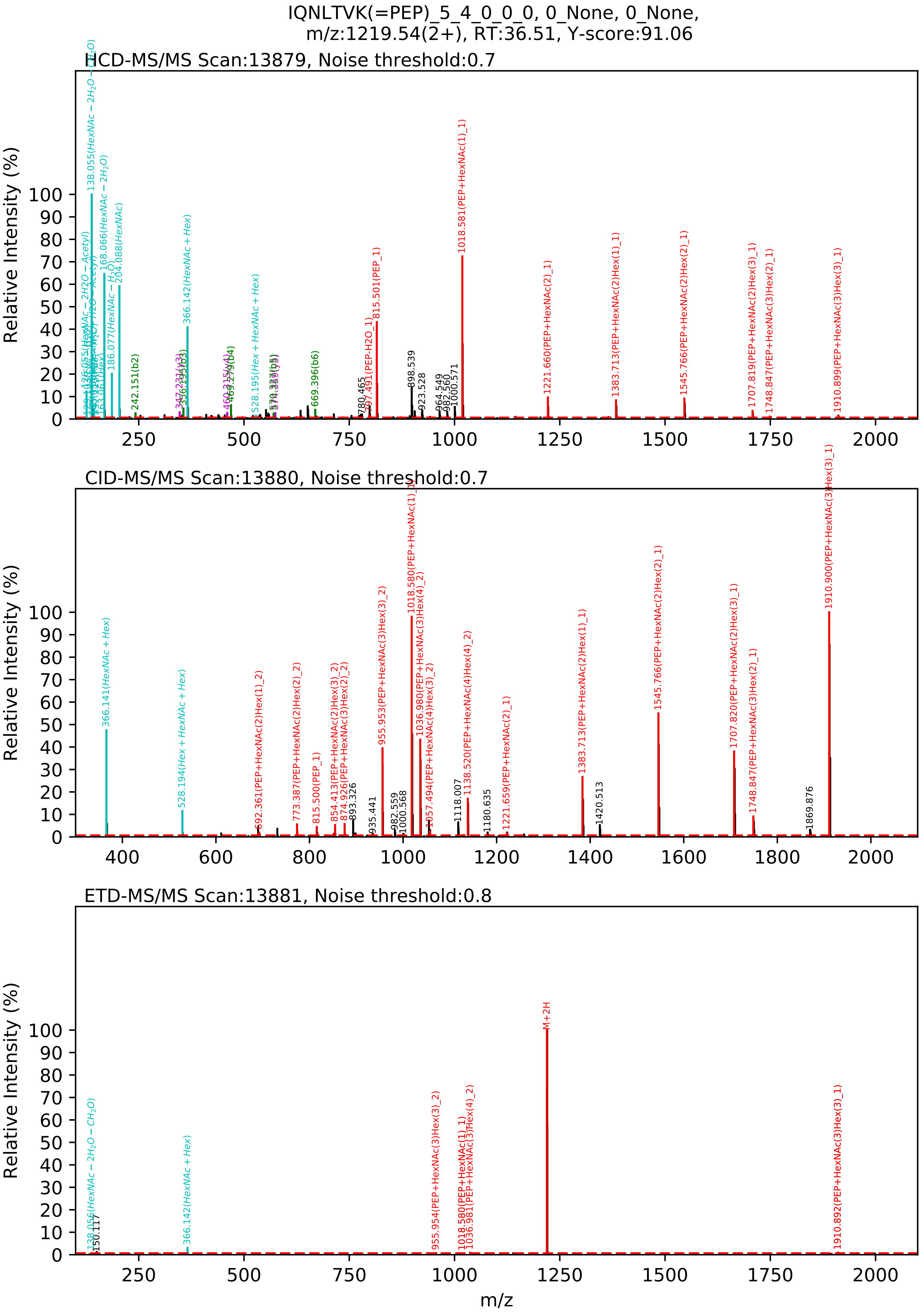
IQNLTVK(=PEP)_5_4_0_0_0, 0_None, 0_None,
m/z:1219.54(2+), RT:36.51, Y-score:91.06
HCD-MS/MS Scan:13879, Noise threshold:0.7
Relative Intensity (%)
138.055(HexNAc − 2H2O − Acetyl)
168.066(HexNAc − 2H2O)
186.077(HexNAc − H2O)
204.088(HexNAc)
366.142(HexNAc + Hex)
528.195(Hex + HexNAc + Hex)
242.151(b2)
356.195(b3)
469.279(b4)
570.327(b5)
669.396(b6)
780.465
797.491(PEP-H2O_1)
815.501(PEP_1)
898.539
923.528
964.549
982.560
1000.571
1018.581(PEP+HexNAc(1)_1)
1221.660(PEP+HexNAc(2)_1)
1383.713(PEP+HexNAc(2)Hex(1)_1)
1545.766(PEP+HexNAc(2)Hex(2)_1)
1707.819(PEP+HexNAc(2)Hex(3)_1)
1748.847(PEP+HexNAc(3)Hex(2)_1)
1910.899(PEP+HexNAc(3)Hex(3)_1)
CID-MS/MS Scan:13880, Noise threshold:0.7
366.141(HexNAc + Hex)
528.194(Hex + HexNAc + Hex)
692.361(PEP+HexNAc(2)Hex(1)_2)
773.387(PEP+HexNAc(2)Hex(2)_2)
815.500(PEP_1)
854.413(PEP+HexNAc(2)Hex(3)_2)
874.926(PEP+HexNAc(3)Hex(2)_2)
893.326
935.441
955.953(PEP+HexNAc(3)Hex(3)_2)
982.559
1000.568
1018.580(PEP+HexNAc(1)_1)
1036.980(PEP+HexNAc(3)Hex(4)_2)
1057.494(PEP+HexNAc(4)Hex(3)_2)
1118.007
1138.520(PEP+HexNAc(4)Hex(4)_2)
1180.635
1221.659(PEP+HexNAc(2)_1)
1383.713(PEP+HexNAc(2)Hex(1)_1)
1420.513
1545.766(PEP+HexNAc(2)Hex(2)_1)
1707.820(PEP+HexNAc(2)Hex(3)_1)
1748.847(PEP+HexNAc(3)Hex(2)_1)
1869.876
1910.900(PEP+HexNAc(3)Hex(3)_1)
ETD-MS/MS Scan:13881, Noise threshold:0.8
138.056(HexNAc − 2H2O − CH2O)
150.117
366.142(HexNAc + Hex)
955.954(PEP+HexNAc(3)Hex(3)_2)
1018.580(PEP+HexNAc(1)_1)
1036.981(PEP+HexNAc(3)Hex(4)_2)
M+2H
1910.892(PEP+HexNAc(3)Hex(3)_1)
m/z

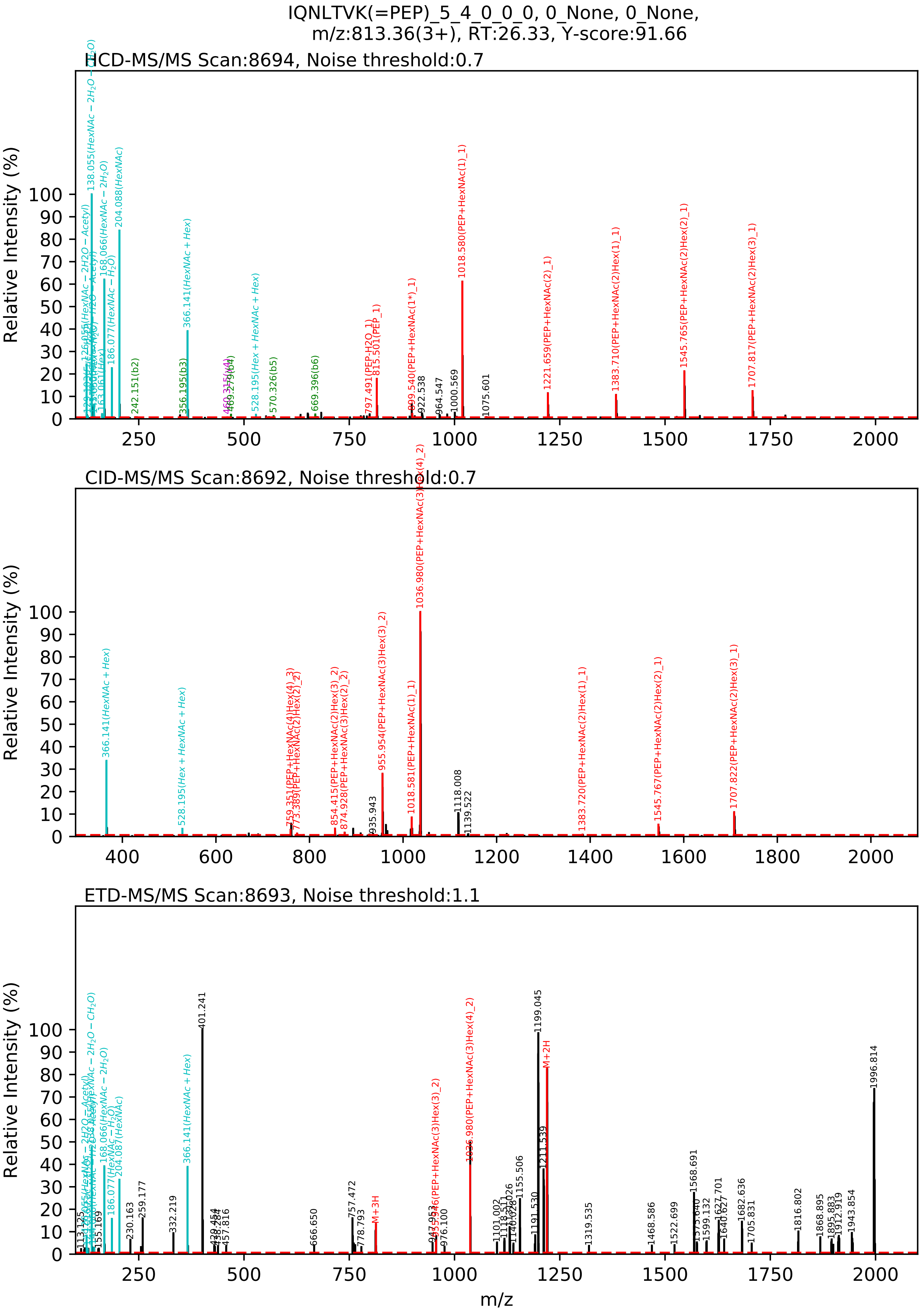

IQNLTVK(=PEP)_5_4_0_0_0, 0_None, 0_None,
m/z:813.36(3+), RT:26.33, Y-score:91.66
HCD-MS/MS Scan:8694, Noise threshold:0.7
138.055(HexNAc − 2H2O − H2O)
204.088(HexNAc)
168.066(HexNAc − 2H2O)
186.077(HexNAc − H2O)
366.141(HexNAc + Hex)
163.061(Hex)
242.151(b2)
356.195(b3)
460.315(y4)
469.279(b4)
528.195(Hex + HexNAc + Hex)
570.326(b5)
669.396(b6)
797.491(PEP-H2O_1)
815.501(PEP_1)
909.540(PEP+HexNAc(1*)_1)
922.538
964.547
1000.569
1018.580(PEP+HexNAc(1)_1)
1075.601
1221.659(PEP+HexNAc(2)_1)
1383.710(PEP+HexNAc(2)Hex(1)_1)
1545.765(PEP+HexNAc(2)Hex(2)_1)
1707.817(PEP+HexNAc(2)Hex(3)_1)
CID-MS/MS Scan:8692, Noise threshold:0.7
366.141(HexNAc + Hex)
528.195(Hex + HexNAc + Hex)
759.351(PEP+HexNAc(4)Hex(4)_3)
773.389(PEP+HexNAc(2)Hex(2)_2)
854.415(PEP+HexNAc(2)Hex(3)_2)
874.928(PEP+HexNAc(3)Hex(2)_2)
935.943
955.954(PEP+HexNAc(3)Hex(3)_2)
1018.581(PEP+HexNAc(1)_1)
1036.980(PEP+HexNAc(3)Hex(4)_2)
1118.008
1139.522
1383.720(PEP+HexNAc(2)Hex(1)_1)
1545.767(PEP+HexNAc(2)Hex(2)_1)
1707.822(PEP+HexNAc(2)Hex(3)_1)
ETD-MS/MS Scan:8693, Noise threshold:1.1
113.125
155.169
168.066(HexNAc − 2H2O)
186.077(HexNAc − H2O)
204.087(HexNAc)
230.163
259.177
332.219
366.141(HexNAc + Hex)
401.241
428.454
438.284
457.816
666.650
757.472
778.793
M+3H
947.953
976.100
1036.980(PEP+HexNAc(3)Hex(4)_2)
1101.002
1140.028
1155.506
1191.530
1199.045
M+2H
1211.539
1319.535
1468.586
1522.699
1568.691
1599.132
1627.701
1640.627
1682.636
1705.831
1816.802
1868.895
1895.883
1912.919
1943.854
1996.814
Relative Intensity (%)
m/z

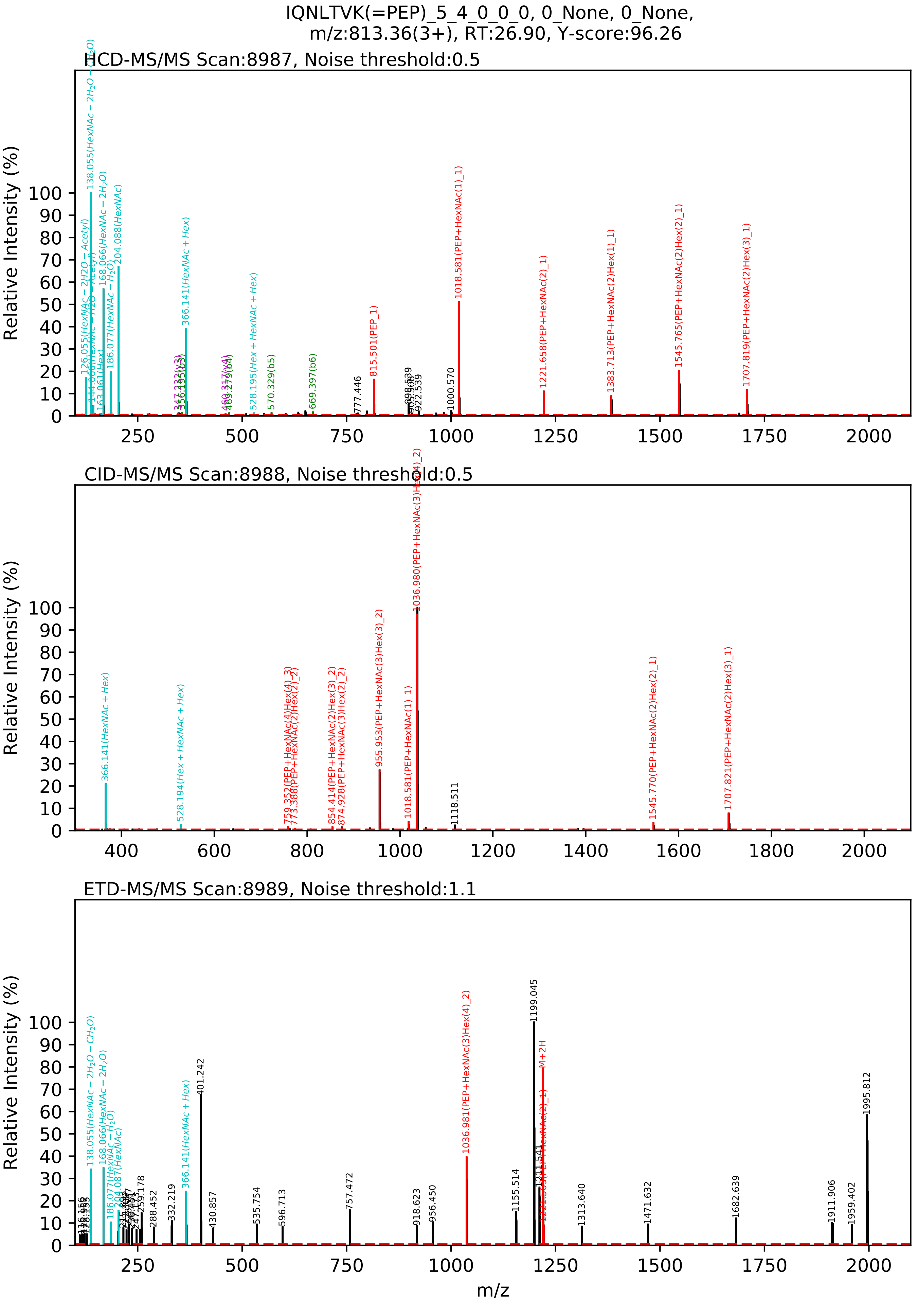

IQNLTVK(=PEP)_5_4_0_0_0, 0_None, 0_None,
m/z:813.36(3+), RT:26.90, Y-score:96.26
HCD-MS/MS Scan:8987, Noise threshold:0.5
CID-MS/MS Scan:8988, Noise threshold:0.5
ETD-MS/MS Scan:8989, Noise threshold:1.1
Relative Intensity (%)
m/z
138.055(HexNAc − 2H2O − CH2O)
168.066(HexNAc − 2H2O)
204.088(HexNAc)
366.141(HexNAc + Hex)
528.195(Hex + HexNAc + Hex)
570.329(b5)
669.397(b6)
815.501(PEP_1)
1018.581(PEP+HexNAc(1)_1)
1221.658(PEP+HexNAc(2)_1)
1383.713(PEP+HexNAc(2)Hex(1)_1)
1545.765(PEP+HexNAc(2)Hex(2)_1)
1707.819(PEP+HexNAc(2)Hex(3)_1)
1036.980(PEP+HexNAc(3)Hex(4)_2)
955.953(PEP+HexNAc(3)Hex(3)_2)
1545.770(PEP+HexNAc(2)Hex(2)_1)
1707.821(PEP+HexNAc(2)Hex(3)_1)
1118.511
1036.981(PEP+HexNAc(3)Hex(4)_2)
1199.045
M+2H
401.242
1995.812

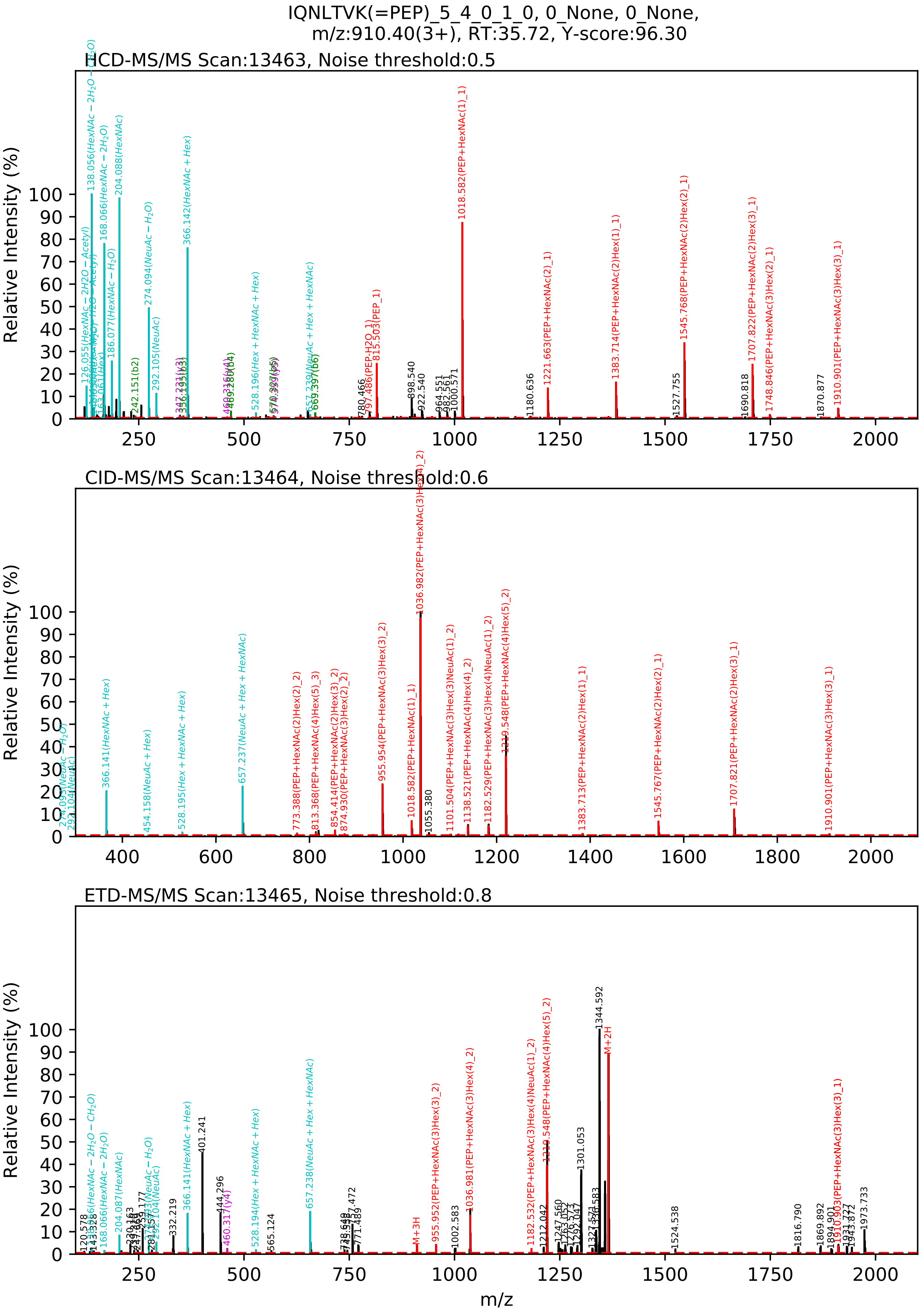
IQNLTVK(=PEP)_5_4_0_1_0, 0_None, 0_None,
m/z:910.40(3+), RT:35.72, Y-score:96.30
HCD-MS/MS Scan:13463, Noise threshold:0.5
CID-MS/MS Scan:13464, Noise threshold:0.6
ETD-MS/MS Scan:13465, Noise threshold:0.8
Relative Intensity (%)
m/z
1018.582(PEP+HexNAc(1)_1)
1221.663(PEP+HexNAc(2)_1)
1383.714(PEP+HexNAc(2)Hex(1)_1)
1545.768(PEP+HexNAc(2)Hex(2)_1)
1707.822(PEP+HexNAc(2)Hex(3)_1)
1748.846(PEP+HexNAc(3)Hex(2)_1)
1910.901(PEP+HexNAc(3)Hex(3)_1)
1036.982(PEP+HexNAc(3)Hex(4)_2)
1219.548(PEP+HexNAc(4)Hex(5)_2)
955.954(PEP+HexNAc(3)Hex(3)_2)
1344.592
M+2H
M+3H

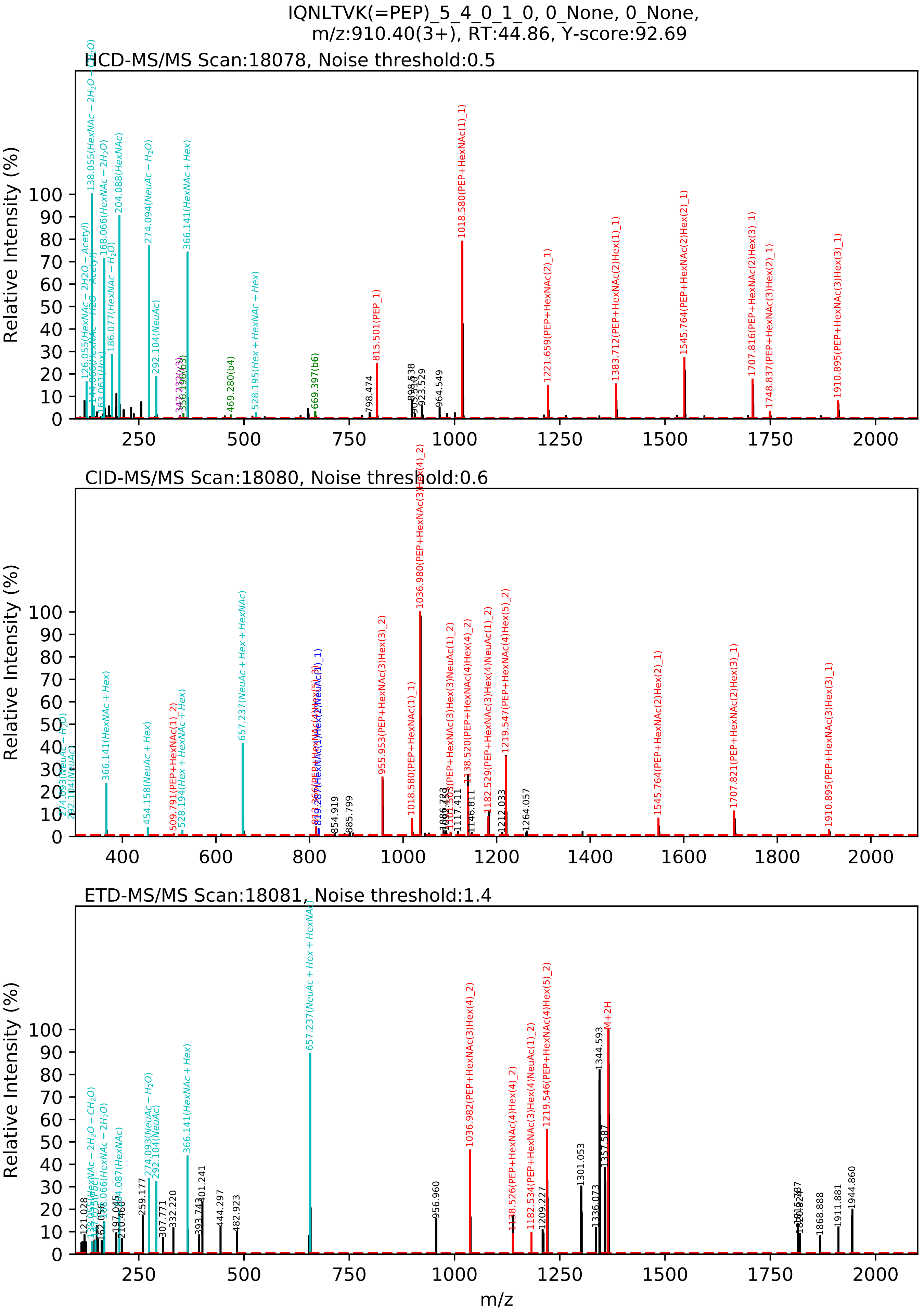
IQNLTVK(=PEP)_5_4_0_1_0, 0_None, 0_None,
m/z:910.40(3+), RT:44.86, Y-score:92.69
HCD-MS/MS Scan:18078, Noise threshold:0.5
Relative Intensity (%)
138.055(HexNAc − 2H2O − CH2O)
126.055(HexNAc − 2H2O − Acetyl)
144.066(HexNAc − H2O − Acetyl)
163.061(Hex)
168.066(HexNAc − 2H2O)
186.077(HexNAc − H2O)
204.088(HexNAc)
274.094(NeuAc − H2O)
292.104(NeuAc)
366.141(HexNAc + Hex)
469.280(b4)
528.195(Hex + HexNAc + Hex)
669.397(b6)
798.474
815.501(PEP_1)
898.538
923.529
964.549
1018.580(PEP+HexNAc(1)_1)
1221.659(PEP+HexNAc(2)_1)
1383.712(PEP+HexNAc(2)Hex(1)_1)
1545.764(PEP+HexNAc(2)Hex(2)_1)
1707.816(PEP+HexNAc(2)Hex(3)_1)
1748.837(PEP+HexNAc(3)Hex(2)_1)
1910.895(PEP+HexNAc(3)Hex(3)_1)
CID-MS/MS Scan:18080, Noise threshold:0.6
Relative Intensity (%)
366.141(HexNAc + Hex)
454.158(NeuAc + Hex)
509.791(PEP+HexNAc(1)_2)
528.194(Hex + HexNAc + Hex)
657.237(NeuAc + Hex + HexNAc)
854.919
885.799
955.953(PEP+HexNAc(3)Hex(3)_2)
1018.580(PEP+HexNAc(1)_1)
1036.980(PEP+HexNAc(3)Hex(4)_2)
1117.411
1146.811
1138.520(PEP+HexNAc(4)Hex(4)_2)
1182.529(PEP+HexNAc(3)Hex(4)NeuAc(1)_2)
1212.033
1219.547(PEP+HexNAc(4)Hex(5)_2)
1264.057
1545.764(PEP+HexNAc(2)Hex(2)_1)
1707.821(PEP+HexNAc(2)Hex(3)_1)
1910.895(PEP+HexNAc(3)Hex(3)_1)
ETD-MS/MS Scan:18081, Noise threshold:1.4
Relative Intensity (%)
121.028
168.066(HexNAc − 2H2O)
197.045
204.087(HexNAc)
210.460
259.177
274.093(NeuAc − H2O)
292.104(NeuAc)
307.771
332.220
366.141(HexNAc + Hex)
393.743
401.241
444.297
482.923
657.237(NeuAc + Hex + HexNAc)
956.960
1036.982(PEP+HexNAc(3)Hex(4)_2)
1138.526(PEP+HexNAc(4)Hex(4)_2)
1182.534(PEP+HexNAc(3)Hex(4)NeuAc(1)_2)
1209.227
1219.546(PEP+HexNAc(4)Hex(5)_2)
1301.053
1336.073
1344.593
1357.587
M+2H
1868.888
1911.881
1944.860
m/z

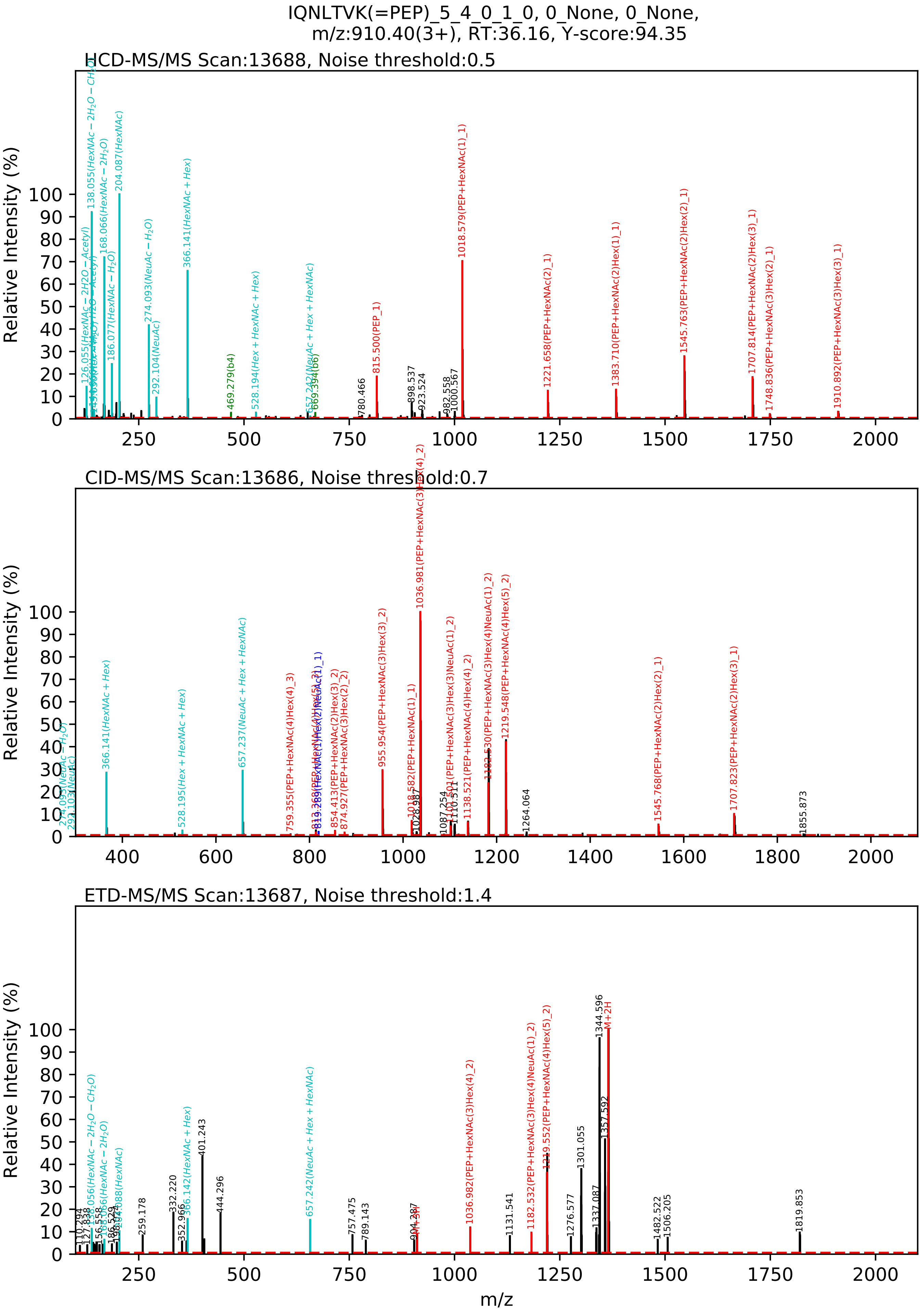

IQNLTVK(=PEP)_5_4_0_1_0, 0_None, 0_None,
m/z:910.40(3+), RT:36.16, Y-score:94.35
HCD-MS/MS Scan:13688, Noise threshold:0.5
Relative Intensity (%)
138.055(HexNAc − 2H2O − CH2O)
204.087(HexNAc)
168.066(HexNAc − 2H2O)
186.077(HexNAc − H2O)
274.093(NeuAc − H2O)
292.104(NeuAc)
366.141(HexNAc + Hex)
469.279(b4)
528.194(Hex + HexNAc + Hex)
657.242(NeuAc + Hex + HexNAc)
669.394(b6)
780.466
815.500(PEP_1)
898.537
923.524
982.558
1000.567
1018.579(PEP+HexNAc(1)_1)
1221.658(PEP+HexNAc(2)_1)
1383.710(PEP+HexNAc(2)Hex(1)_1)
1545.763(PEP+HexNAc(2)Hex(2)_1)
1707.814(PEP+HexNAc(2)Hex(3)_1)
1748.836(PEP+HexNAc(3)Hex(2)_1)
1910.892(PEP+HexNAc(3)Hex(3)_1)
CID-MS/MS Scan:13686, Noise threshold:0.7
366.141(HexNAc + Hex)
528.195(Hex + HexNAc + Hex)
657.237(NeuAc + Hex + HexNAc)
759.355(PEP+HexNAc(4)Hex(4)_3)
854.413(PEP+HexNAc(2)Hex(3)_2)
874.927(PEP+HexNAc(3)Hex(2)_2)
955.954(PEP+HexNAc(3)Hex(3)_2)
1018.582(PEP+HexNAc(1)_1)
1028.987
1036.981(PEP+HexNAc(3)Hex(4)_2)
1087.254
1110.511
1138.521(PEP+HexNAc(4)Hex(4)_2)
1219.548(PEP+HexNAc(4)Hex(5)_2)
1264.064
1545.768(PEP+HexNAc(2)Hex(2)_1)
1707.823(PEP+HexNAc(2)Hex(3)_1)
1855.873
ETD-MS/MS Scan:13687, Noise threshold:1.4
259.178
332.220
352.966
366.142(HexNAc + Hex)
401.243
444.296
657.242(NeuAc + Hex + HexNAc)
757.475
789.143
1036.982(PEP+HexNAc(3)Hex(4)_2)
1131.541
1182.532(PEP+HexNAc(3)Hex(4)NeuAc(1)_2)
1219.552(PEP+HexNAc(4)Hex(5)_2)
1276.577
1301.055
1337.087
1344.596
1357.592
M+2H
1482.522
1506.205
1819.853
m/z

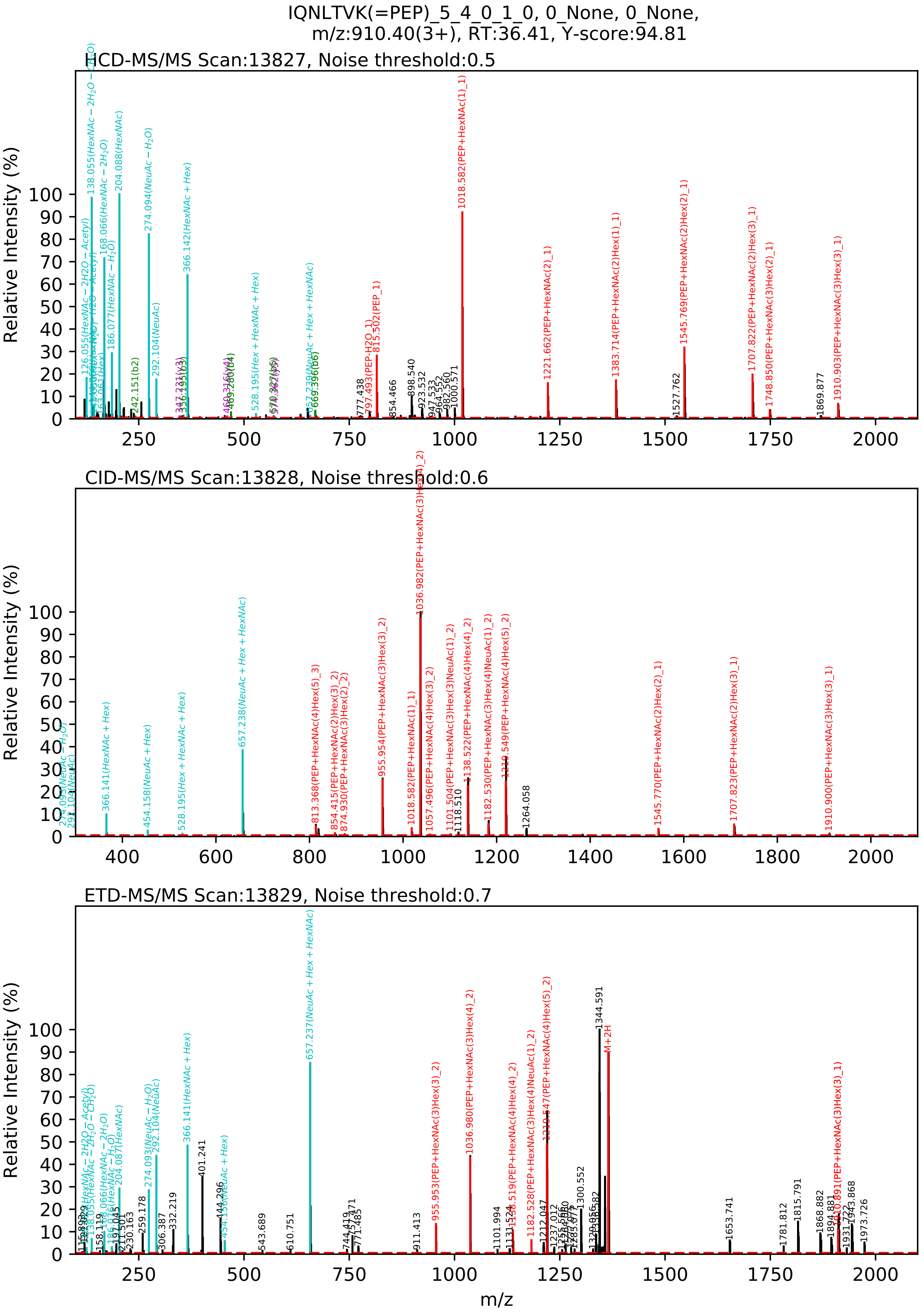

IQNLTVK(=PEP)_5_4_0_1_0, 0_None, 0_None,
m/z:910.40(3+), RT:36.41, Y-score:94.81
HCD-MS/MS Scan:13827, Noise threshold:0.5
Relative Intensity (%)
1018.582(PEP+HexNAc(1)_1)
1221.662(PEP+HexNAc(2)_1)
1383.714(PEP+HexNAc(2)Hex(1)_1)
1545.769(PEP+HexNAc(2)Hex(2)_1)
1707.822(PEP+HexNAc(2)Hex(3)_1)
1748.850(PEP+HexNAc(3)Hex(2)_1)
1910.903(PEP+HexNAc(3)Hex(3)_1)
815.502(PEP_1)
797.493(PEP-H2O_1)
204.088(HexNAc)
274.094(NeuAc − H2O)
366.142(HexNAc + Hex)
292.104(NeuAc)
186.077(HexNAc − H2O)
168.066(HexNAc − 2H2O)
138.055(HexNAc − 2H2O − CH2O)
528.195(Hex + HexNAc + Hex)
657.239(NeuAc + Hex + HexNAc)
CID-MS/MS Scan:13828, Noise threshold:0.6
1036.982(PEP+HexNAc(3)Hex(4)_2)
955.954(PEP+HexNAc(3)Hex(3)_2)
1219.549(PEP+HexNAc(4)Hex(5)_2)
1138.522(PEP+HexNAc(4)Hex(4)_2)
1182.530(PEP+HexNAc(3)Hex(4)NeuAc(1)_2)
1101.504(PEP+HexNAc(3)Hex(3)NeuAc(1)_2)
1057.496(PEP+HexNAc(4)Hex(3)_2)
1018.582(PEP+HexNAc(1)_1)
813.368(PEP+HexNAc(4)Hex(5)_3)
854.415(PEP+HexNAc(2)Hex(3)_2)
874.930(PEP+HexNAc(3)Hex(2)_2)
1545.770(PEP+HexNAc(2)Hex(2)_1)
1707.823(PEP+HexNAc(2)Hex(3)_1)
1910.900(PEP+HexNAc(3)Hex(3)_1)
657.238(NeuAc + Hex + HexNAc)
366.141(HexNAc + Hex)
454.158(NeuAc + Hex)
528.195(Hex + HexNAc + Hex)
1118.510
1264.058
ETD-MS/MS Scan:13829, Noise threshold:0.7
1344.591
M+2H
657.237(NeuAc + Hex + HexNAc)
1036.980(PEP+HexNAc(3)Hex(4)_2)
955.953(PEP+HexNAc(3)Hex(3)_2)
1219.547(PEP+HexNAc(4)Hex(5)_2)
1182.528(PEP+HexNAc(3)Hex(4)NeuAc(1)_2)
1910.891(PEP+HexNAc(3)Hex(3)_1)
366.141(HexNAc + Hex)
292.104(NeuAc)
274.093(NeuAc − H2O)
401.241
332.219
259.178
1300.552
1653.741
1815.791
1781.812
1868.882
1894.881
1943.868
1973.726
911.413
1101.994
1237.012
610.751
543.689
771.485
m/z

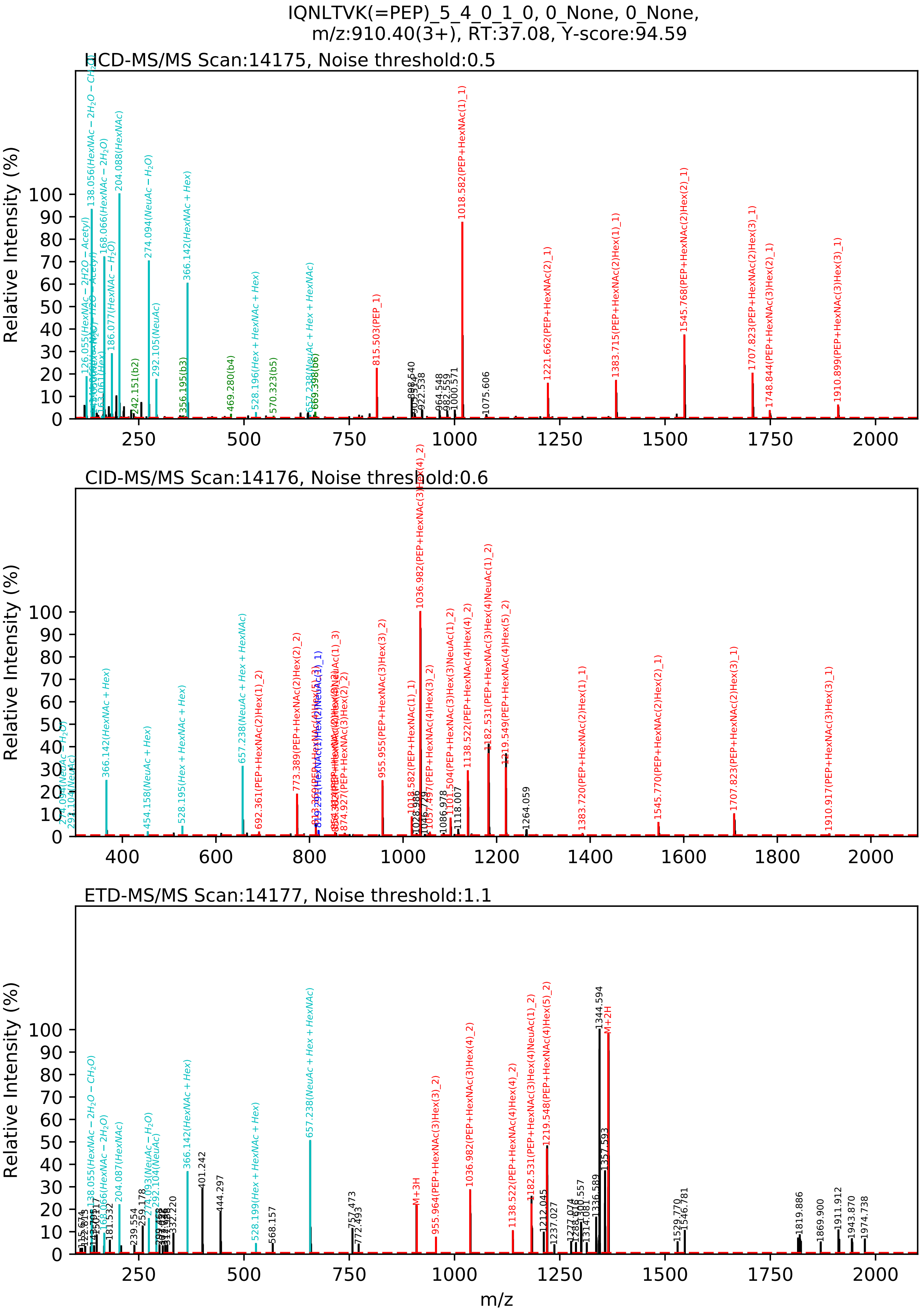
IQNLTVK(=PEP)_5_4_0_1_0, 0_None, 0_None,
m/z:910.40(3+), RT:37.08, Y-score:94.59
HCD-MS/MS Scan:14175, Noise threshold:0.5
CID-MS/MS Scan:14176, Noise threshold:0.6
ETD-MS/MS Scan:14177, Noise threshold:1.1
Relative Intensity (%)
m/z
1018.582(PEP+HexNAc(1)_1)
1221.662(PEP+HexNAc(2)_1)
1383.715(PEP+HexNAc(2)Hex(1)_1)
1545.768(PEP+HexNAc(2)Hex(2)_1)
1707.823(PEP+HexNAc(2)Hex(3)_1)
1748.844(PEP+HexNAc(3)Hex(2)_1)
1910.899(PEP+HexNAc(3)Hex(3)_1)
815.503(PEP_1)
204.088(HexNAc)
274.094(NeuAc − H2O)
292.105(NeuAc)
366.142(HexNAc + Hex)
1036.982(PEP+HexNAc(3)Hex(4)_2)
1182.531(PEP+HexNAc(3)Hex(4)NeuAc(1)_2)
1219.549(PEP+HexNAc(4)Hex(5)_2)
1138.522(PEP+HexNAc(4)Hex(4)_2)
955.955(PEP+HexNAc(3)Hex(3)_2)
657.238(NeuAc + Hex + HexNAc)
1344.594
M+2H
M+3H
1357.593

IQNLTVK(=PEP)_5_4_0_1_0, 0_None, 0_None,
m/z:1365.09(2+), RT:50.51, Y-score:90.59

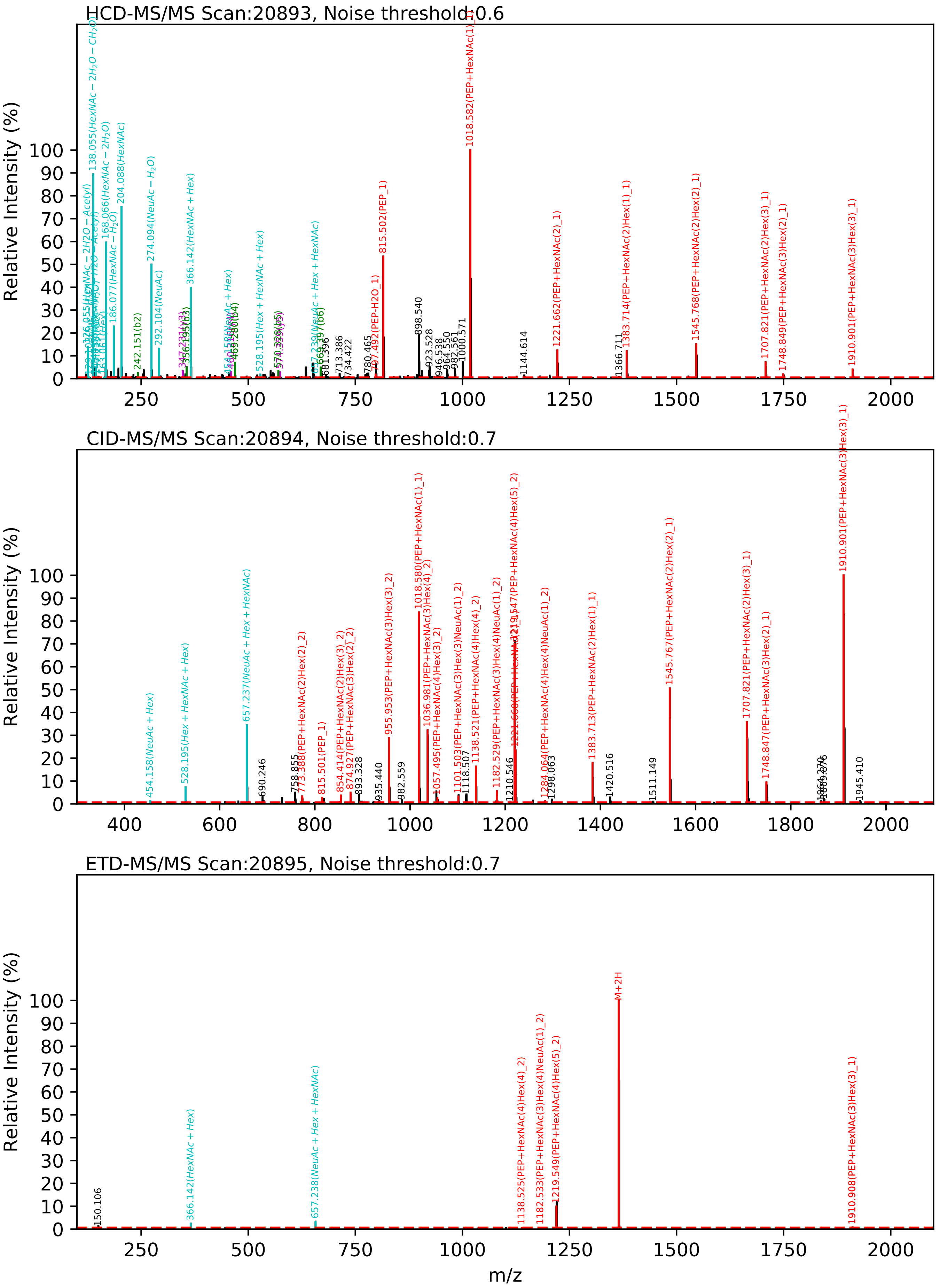

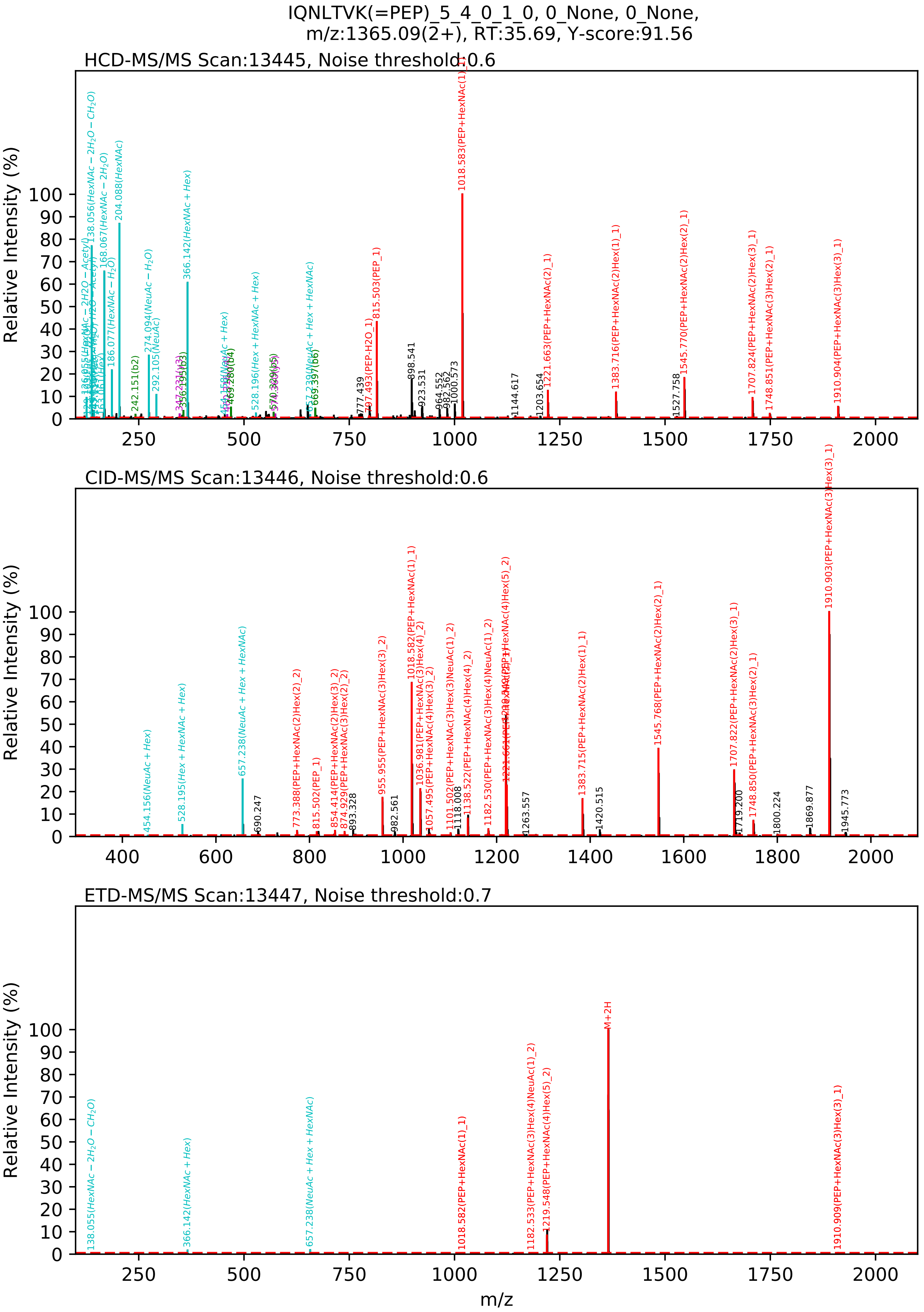
IQNLTVK(=PEP)_5_4_0_1_0, 0_None, 0_None,
m/z:1365.09(2+), RT:35.69, Y-score:91.56
HCD-MS/MS Scan:13445, Noise threshold:0.6
CID-MS/MS Scan:13446, Noise threshold:0.6
ETD-MS/MS Scan:13447, Noise threshold:0.7
Relative Intensity (%)
m/z
1018.583(PEP+HexNAc(1)_1)
815.503(PEP_1)
1221.663(PEP+HexNAc(2)_1)
1383.716(PEP+HexNAc(2)Hex(1)_1)
1545.770(PEP+HexNAc(2)Hex(2)_1)
1707.824(PEP+HexNAc(2)Hex(3)_1)
1748.851(PEP+HexNAc(3)Hex(2)_1)
1910.904(PEP+HexNAc(3)Hex(3)_1)
204.088(HexNAc)
366.142(HexNAc + Hex)
274.094(NeuAc − H2O)
292.105(NeuAc)
528.196(Hex + HexNAc + Hex)
1910.903(PEP+HexNAc(3)Hex(3)_1)
1018.582(PEP+HexNAc(1)_1)
1545.768(PEP+HexNAc(2)Hex(2)_1)
1707.822(PEP+HexNAc(2)Hex(3)_1)
1383.715(PEP+HexNAc(2)Hex(1)_1)
955.955(PEP+HexNAc(3)Hex(3)_2)
657.238(NeuAc + Hex + HexNAc)
454.156(NeuAc + Hex)
M+2H
1219.548(PEP+HexNAc(4)Hex(5)_2)
1182.533(PEP+HexNAc(3)Hex(4)NeuAc(1)_2)
1910.909(PEP+HexNAc(3)Hex(3)_1)
138.055(HexNAc − 2H2O − CH2O)

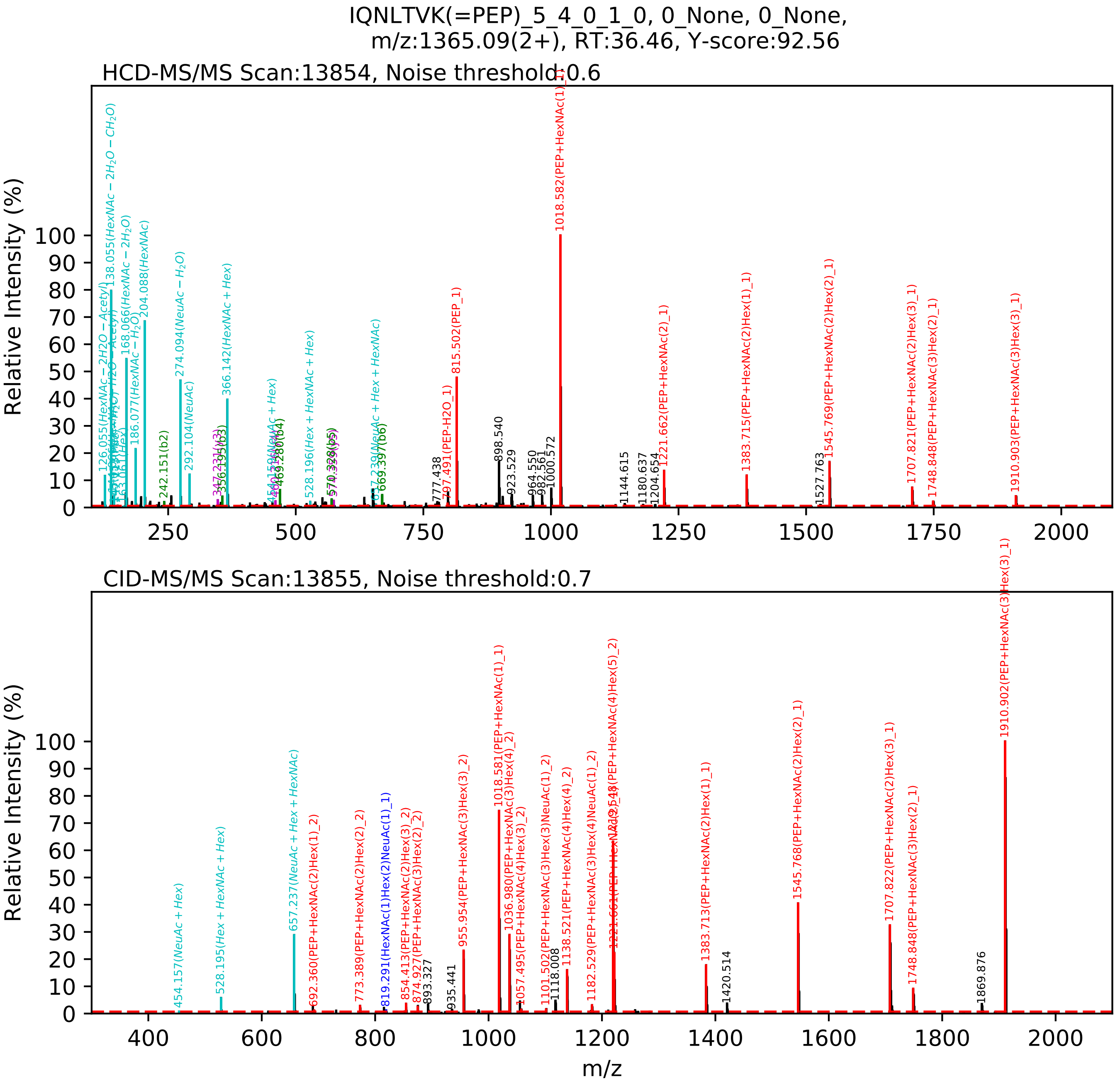
IQNLTVK(=PEP)_5_4_0_1_0, 0_None, 0_None,
m/z:1365.09(2+), RT:36.46, Y-score:92.56
HCD-MS/MS Scan:13854, Noise threshold:0.6
Relative Intensity (%)
126.055(HexNAc − 2H2O − Acetyl)
138.055(HexNAc − 2H2O − CH2O)
168.066(HexNAc − 2H2O)
186.077(HexNAc − H2O)
204.088(HexNAc)
163.061(Hex)
242.151(b2)
274.094(NeuAc − H2O)
292.104(NeuAc)
366.142(HexNAc + Hex)
356.193(b3)
469.280(b4)
528.196(Hex + HexNAc + Hex)
570.328(b5)
669.397(b6)
NeuAc + Hex + HexNAc
815.502(PEP_1)
797.491(PEP-H2O_1)
777.438
898.540
923.529
964.550
1000.572
1018.582(PEP+HexNAc(1)_1)
1144.615
1180.637
1204.654
1221.662(PEP+HexNAc(2)_1)
1383.715(PEP+HexNAc(2)Hex(1)_1)
1527.763
1545.769(PEP+HexNAc(2)Hex(2)_1)
1707.821(PEP+HexNAc(2)Hex(3)_1)
1748.848(PEP+HexNAc(3)Hex(2)_1)
1910.903(PEP+HexNAc(3)Hex(3)_1)
CID-MS/MS Scan:13855, Noise threshold:0.7
454.157(NeuAc + Hex)
528.195(Hex + HexNAc + Hex)
657.237(NeuAc + Hex + HexNAc)
692.360(PEP+HexNAc(2)Hex(1)_2)
773.389(PEP+HexNAc(2)Hex(2)_2)
819.291(HexNAc(1)Hex(2)NeuAc(1)_1)
854.413(PEP+HexNAc(2)Hex(3)_2)
874.927(PEP+HexNAc(3)Hex(2)_2)
893.327
935.441
955.954(PEP+HexNAc(3)Hex(3)_2)
1018.581(PEP+HexNAc(1)_1)
1036.980(PEP+HexNAc(3)Hex(4)_2)
1057.495(PEP+HexNAc(4)Hex(3)_2)
1101.502(PEP+HexNAc(3)Hex(3)NeuAc(1)_2)
1118.008
1138.521(PEP+HexNAc(4)Hex(4)_2)
1182.529(PEP+HexNAc(3)Hex(4)NeuAc(1)_2)
1221.661
(PEP+HexNAc(4)Hex(5)_2)
1383.713(PEP+HexNAc(2)Hex(1)_1)
1420.514
1545.768(PEP+HexNAc(2)Hex(2)_1)
1707.822(PEP+HexNAc(2)Hex(3)_1)
1748.848(PEP+HexNAc(3)Hex(2)_1)
1869.876
1910.902(PEP+HexNAc(3)Hex(3)_1)
m/z

IQNLTVK(=PEP)_5_4_0_1_0, 0_None, 0_None,
m/z:1365.09(2+), RT:37.21, Y-score:89.02

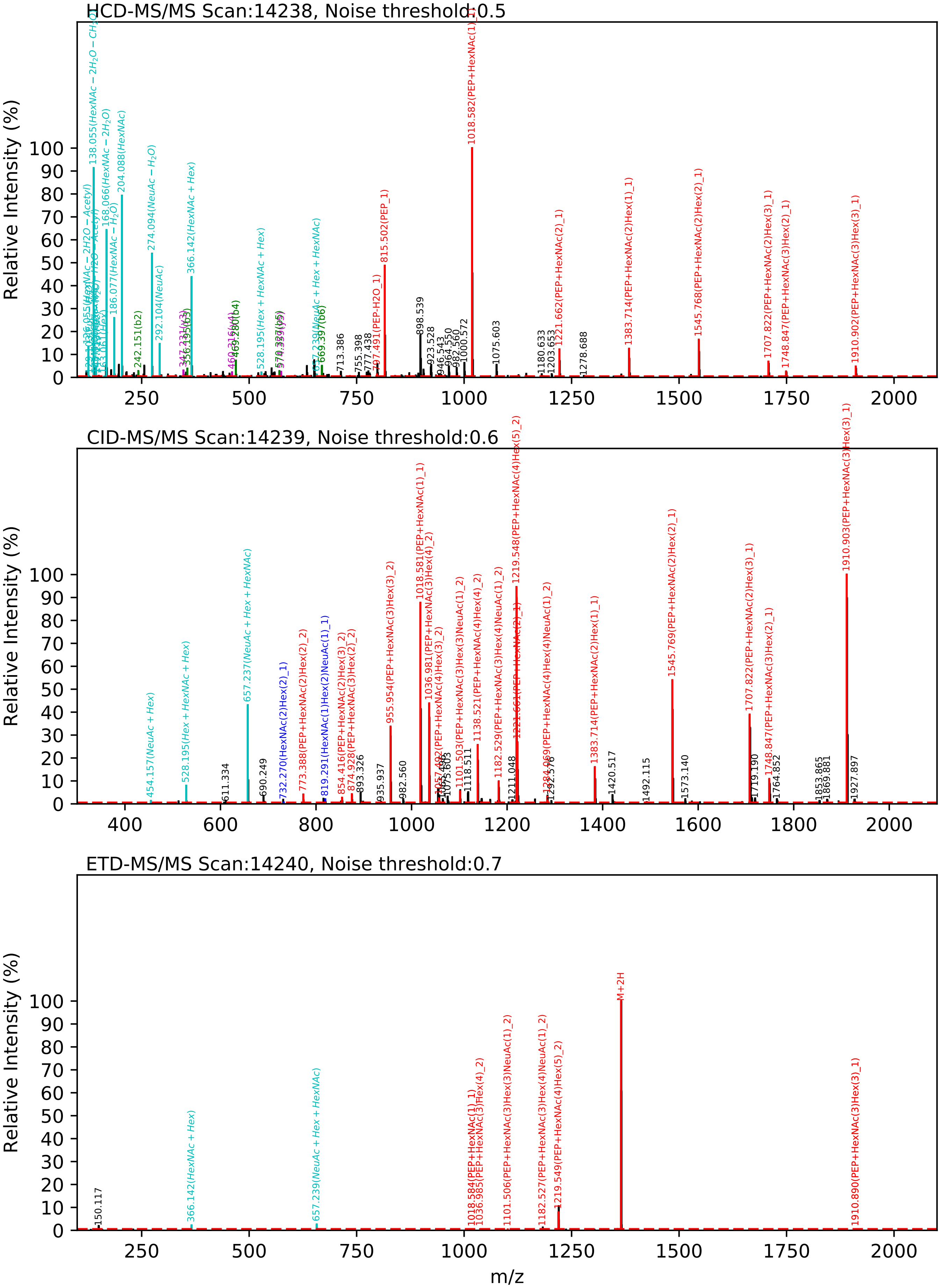

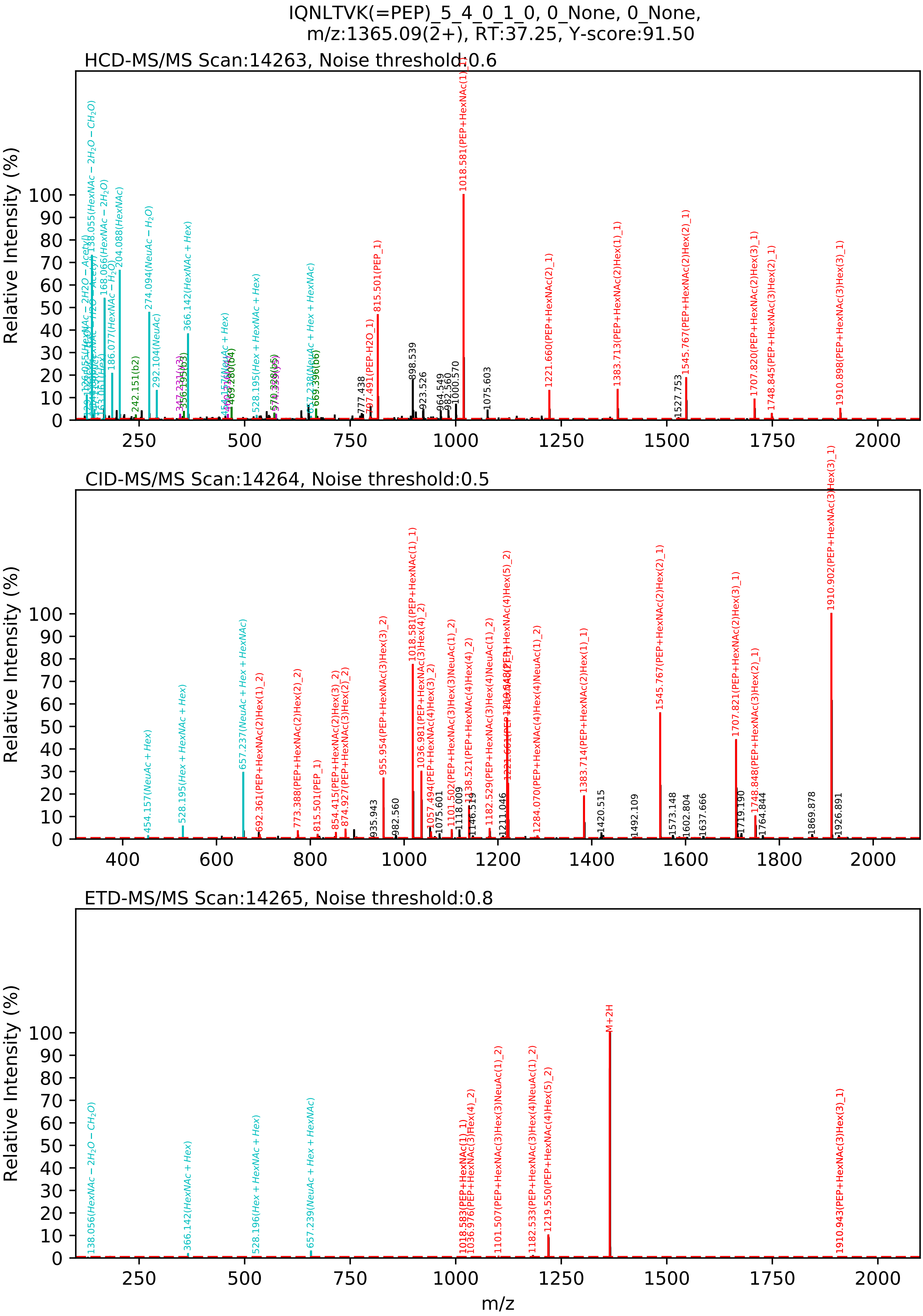
IQNLTVK(=PEP)_5_4_0_1_0, 0_None, 0_None,
m/z:1365.09(2+), RT:37.25, Y-score:91.50
HCD-MS/MS Scan:14263, Noise threshold:0.6
CID-MS/MS Scan:14264, Noise threshold:0.5
ETD-MS/MS Scan:14265, Noise threshold:0.8
Relative Intensity (%)
m/z
1018.581(PEP+HexNAc(1)_1)
815.501(PEP_1)
1221.660(PEP+HexNAc(2)_1)
1383.713(PEP+HexNAc(2)Hex(1)_1)
1545.767(PEP+HexNAc(2)Hex(2)_1)
1707.820(PEP+HexNAc(2)Hex(3)_1)
1748.845(PEP+HexNAc(3)Hex(2)_1)
1910.898(PEP+HexNAc(3)Hex(3)_1)
204.088(HexNAc)
366.142(HexNAc+Hex)
274.094(NeuAc−H2O)
292.104(NeuAc)
1910.902(PEP+HexNAc(3)Hex(3)_1)
1018.581(PEP+HexNAc(1)_1)
955.954(PEP+HexNAc(3)Hex(3)_2)
657.237(NeuAc+Hex+HexNAc)
M+2H
1219.550(PEP+HexNAc(4)Hex(5)_2)
1910.943(PEP+HexNAc(3)Hex(3)_1)

IQNLTVK(=PEP)_5_4_0_2_0, 0_None, 0_None,
m/z:1007.43(3+), RT:48.22, Y-score:93.26

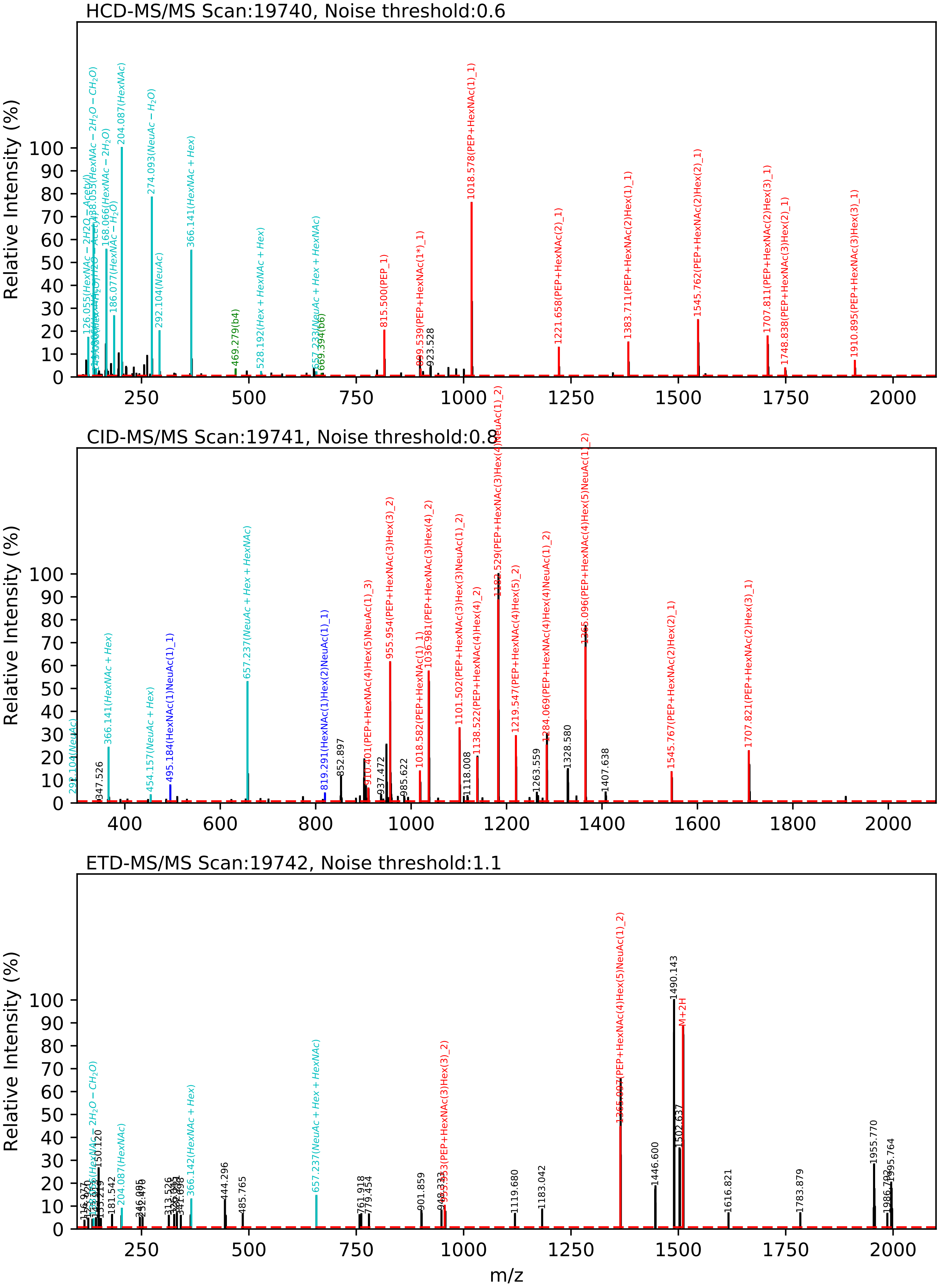

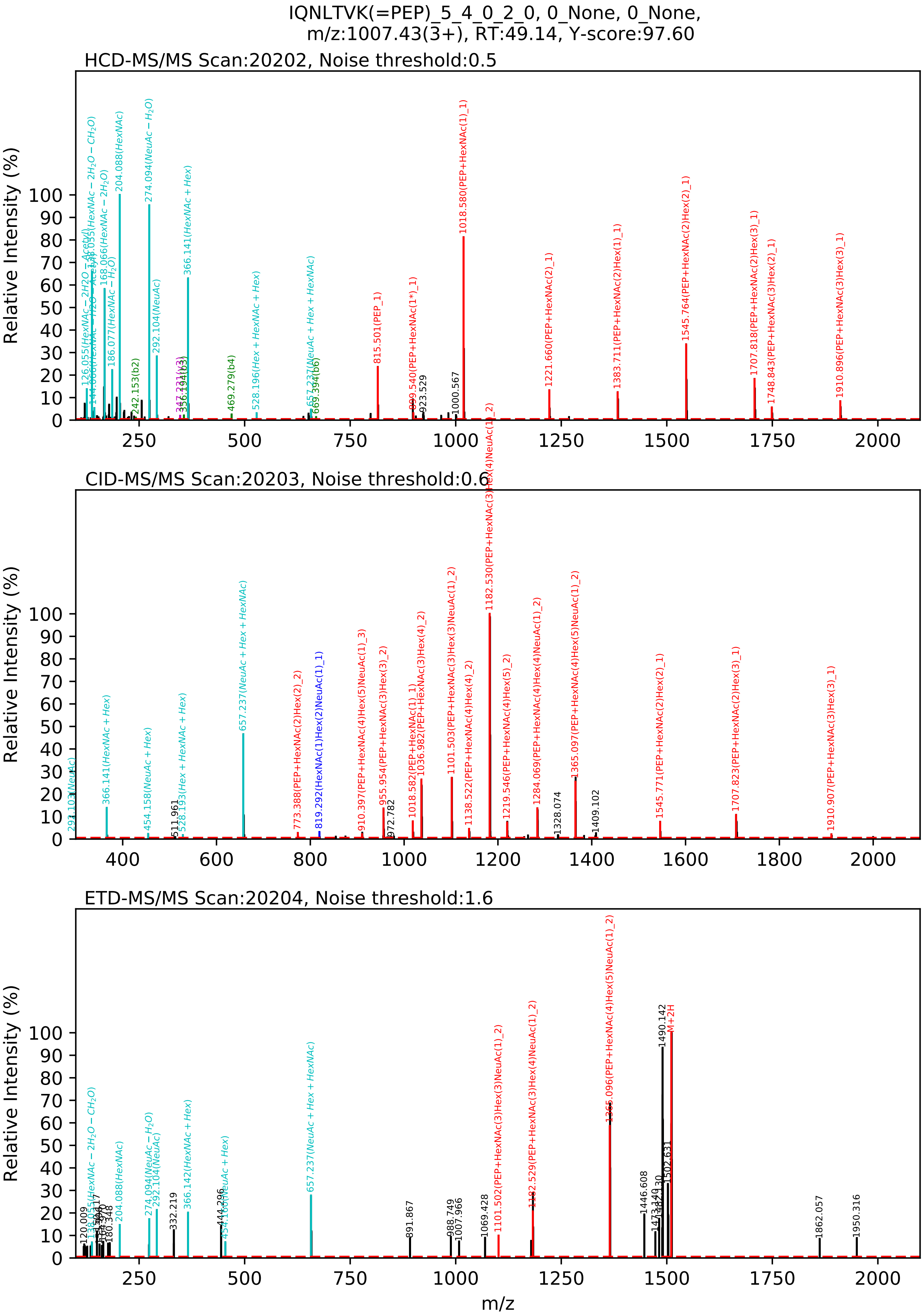
IQNLTVK(=PEP)_5_4_0_2_0, 0_None, 0_None,
m/z:1007.43(3+), RT:49.14, Y-score:97.60
HCD-MS/MS Scan:20202, Noise threshold:0.5
Relative Intensity (%)
126.055(HexNAc − 2H2O − CH2O)
138.055(HexNAc − 2H2O)
144.066(HexNAc − H2O − Acetyl)
168.066(HexNAc − 2H2O)
186.077(HexNAc − H2O)
204.088(HexNAc)
242.153(b2)
274.094(NeuAc − H2O)
292.104(NeuAc)
336.134(b3)
347.231(y3)
366.141(HexNAc + Hex)
469.279(b4)
528.196(Hex + HexNAc + Hex)
657.237(NeuAc + Hex + HexNAc)
669.394(b6)
815.501(PEP_1)
899.540(PEP+HexNAc(1*)_1)
923.529
1000.567
1018.580(PEP+HexNAc(1)_1)
1221.660(PEP+HexNAc(2)_1)
1383.711(PEP+HexNAc(2)Hex(1)_1)
1545.764(PEP+HexNAc(2)Hex(2)_1)
1707.818(PEP+HexNAc(2)Hex(3)_1)
1748.843(PEP+HexNAc(3)Hex(2)_1)
1910.896(PEP+HexNAc(3)Hex(3)_1)
CID-MS/MS Scan:20203, Noise threshold:0.6
Relative Intensity (%)
292.103(NeuAc)
366.141(HexNAc + Hex)
454.158(NeuAc + Hex)
511.961
528.193(Hex + HexNAc + Hex)
657.237(NeuAc + Hex + HexNAc)
773.388(PEP+HexNAc(2)Hex(2)_2)
819.292(HexNAc(1)Hex(2)NeuAc(1)_1)
910.397(PEP+HexNAc(4)Hex(5)NeuAc(1)_3)
955.954(PEP+HexNAc(3)Hex(3)_2)
972.782
1018.582(PEP+HexNAc(1)_1)
1036.982(PEP+HexNAc(3)Hex(4)_2)
1101.503(PEP+HexNAc(3)Hex(3)NeuAc(1)_2)
1138.522(PEP+HexNAc(4)Hex(4)_2)
1182.530(PEP+HexNAc(3)Hex(4)NeuAc(1)_2)
1219.546(PEP+HexNAc(4)Hex(5)_2)
1284.069(PEP+HexNAc(4)Hex(4)NeuAc(1)_2)
1328.074
1365.097(PEP+HexNAc(4)Hex(5)NeuAc(1)_2)
1409.102
1545.771(PEP+HexNAc(2)Hex(2)_1)
1707.823(PEP+HexNAc(2)Hex(3)_1)
1910.907(PEP+HexNAc(3)Hex(3)_1)
ETD-MS/MS Scan:20204, Noise threshold:1.6
Relative Intensity (%)
120.009
126.055(HexNAc − 2H2O − CH2O)
204.088(HexNAc)
274.094(NeuAc − H2O)
292.104(NeuAc)
332.219
366.142(HexNAc + Hex)
444.296
454.160(NeuAc + Hex)
657.237(NeuAc + Hex + HexNAc)
891.867
988.749
1007.966
1069.428
1101.502(PEP+HexNAc(3)Hex(3)NeuAc(1)_2)
1182.529(PEP+HexNAc(3)Hex(4)NeuAc(1)_2)
1365.096(PEP+HexNAc(4)Hex(5)NeuAc(1)_2)
1446.608
1490.142
1502.631
M+2H
1862.057
1950.316
m/z

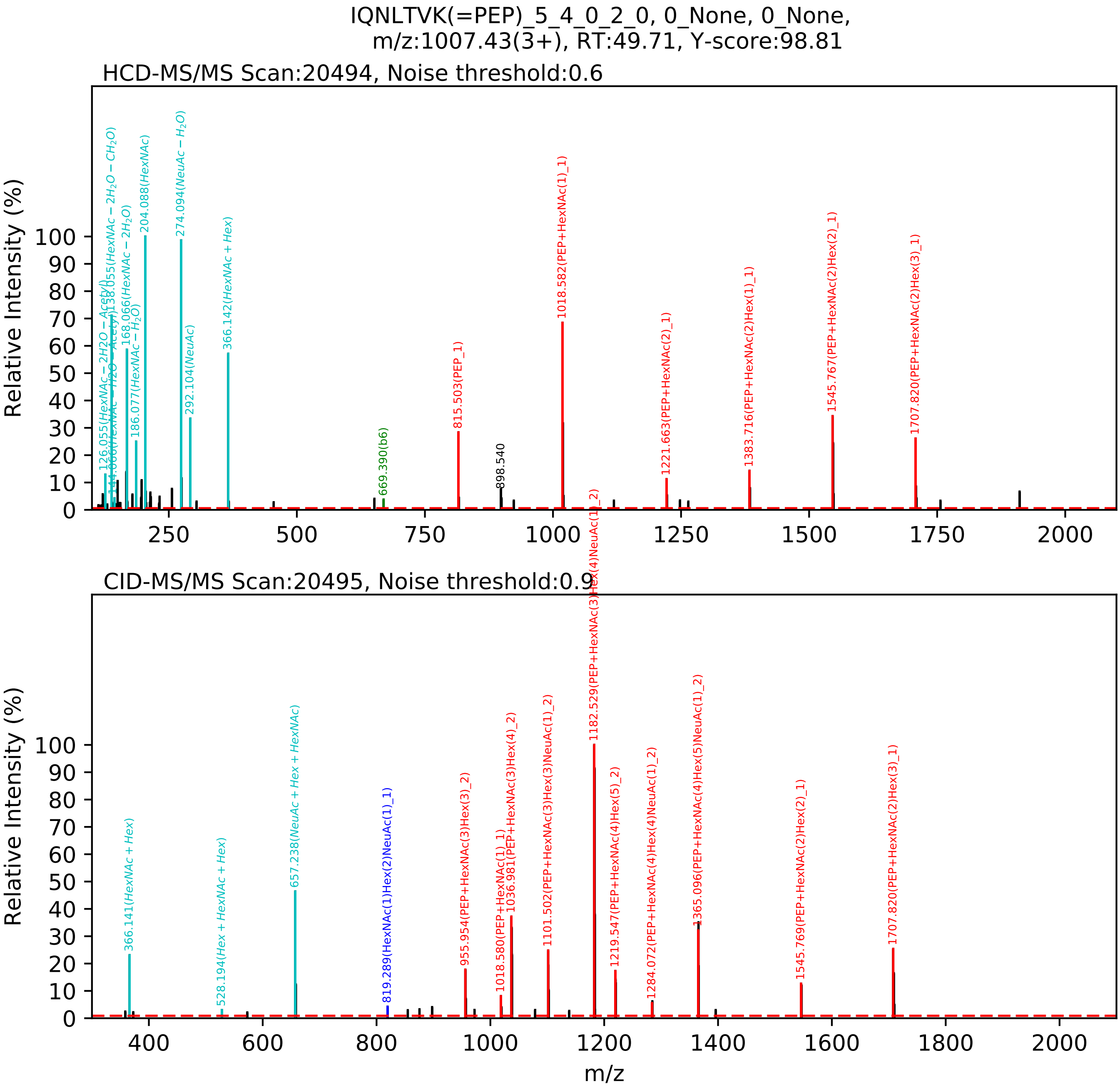
IQNLTVK(=PEP)_5_4_0_2_0, 0_None, 0_None,
m/z:1007.43(3+), RT:49.71, Y-score:98.81
HCD-MS/MS Scan:20494, Noise threshold:0.6
CID-MS/MS Scan:20495, Noise threshold:0.9
Relative Intensity (%)
m/z
1018.582(PEP+HexNAc(1)_1)
815.503(PEP_1)
1221.663(PEP+HexNAc(2)_1)
1383.716(PEP+HexNAc(2)Hex(1)_1)
1545.767(PEP+HexNAc(2)Hex(2)_1)
1707.820(PEP+HexNAc(2)Hex(3)_1)
204.088(HexNAc)
274.094(NeuAc − H2O)
292.104(NeuAc)
366.142(HexNAc + Hex)
669.390(b6)
898.540
1182.529(PEP+HexNAc(3)Hex(4)NeuAc(1)_2)
1365.096(PEP+HexNAc(4)Hex(5)NeuAc(1)_2)
1101.502(PEP+HexNAc(3)Hex(3)NeuAc(1)_2)
1036.981(PEP+HexNAc(3)Hex(4)_2)
955.954(PEP+HexNAc(3)Hex(3)_2)
1018.580(PEP+HexNAc(1)_1)
1219.547(PEP+HexNAc(4)Hex(5)_2)
1284.072(PEP+HexNAc(4)Hex(4)NeuAc(1)_2)
1545.769(PEP+HexNAc(2)Hex(2)_1)
1707.820(PEP+HexNAc(2)Hex(3)_1)
819.289(HexNAc(1)Hex(2)NeuAc(1)_1)
657.238(NeuAc + Hex + HexNAc)
528.194(Hex + HexNAc + Hex)
366.141(HexNAc + Hex)

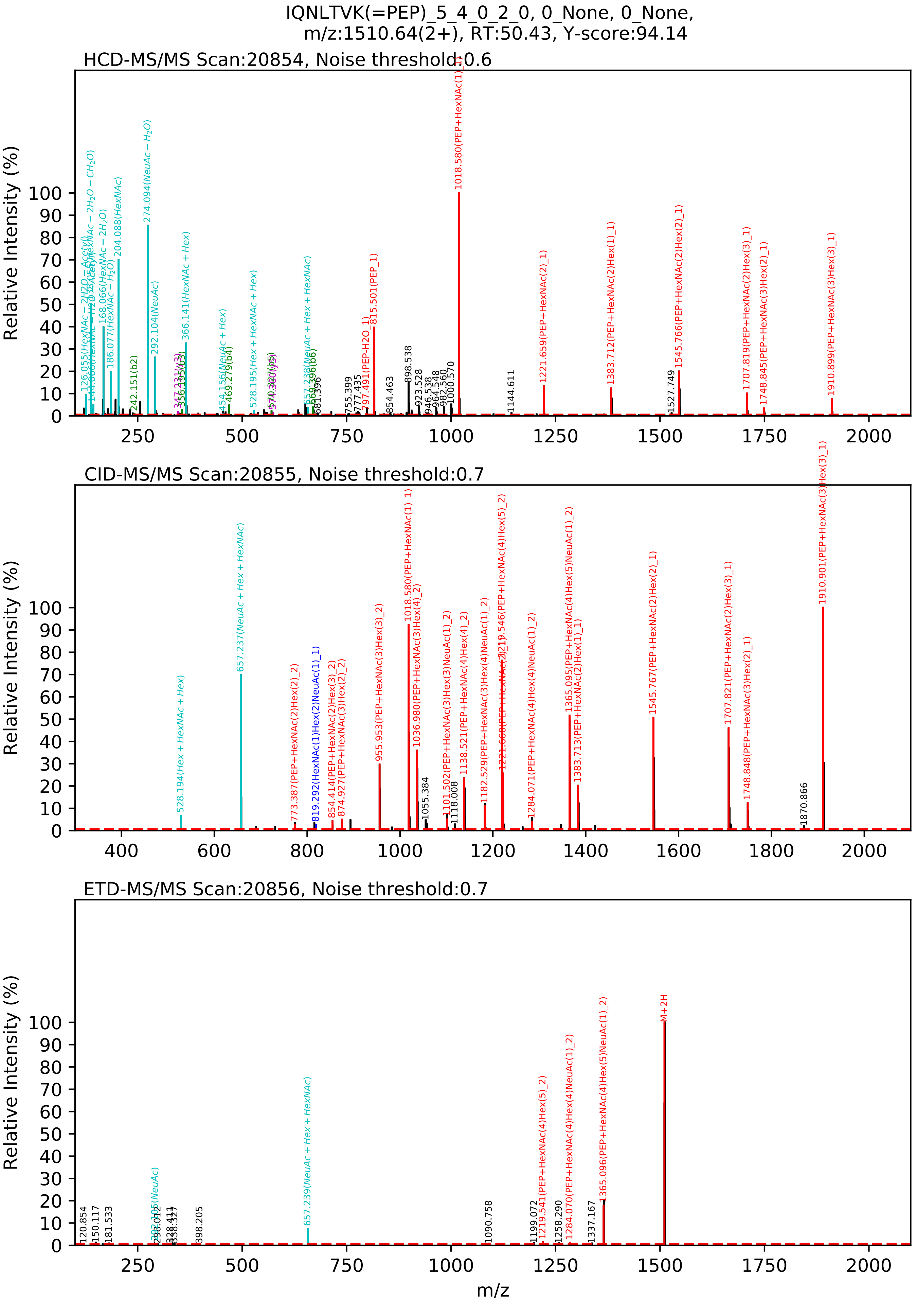
IQNLTVK(=PEP)_5_4_0_2_0, 0_None, 0_None,
m/z:1510.64(2+), RT:50.43, Y-score:94.14
HCD-MS/MS Scan:20854, Noise threshold:0.6
CID-MS/MS Scan:20855, Noise threshold:0.7
ETD-MS/MS Scan:20856, Noise threshold:0.7
Relative Intensity (%)
m/z
1018.580(PEP+HexNAc(1)_1)
274.094(NeuAc − H2O)
204.088(HexNAc)
815.501(PEP_1)
1221.659(PEP+HexNAc(2)_1)
1383.712(PEP+HexNAc(2)Hex(1)_1)
1545.766(PEP+HexNAc(2)Hex(2)_1)
1707.819(PEP+HexNAc(2)Hex(3)_1)
1748.845(PEP+HexNAc(3)Hex(2)_1)
1910.899(PEP+HexNAc(3)Hex(3)_1)
1910.901(PEP+HexNAc(3)Hex(3)_1)
657.237(NeuAc + Hex + HexNAc)
1365.095(PEP+HexNAc(4)Hex(5)NeuAc(1)_2)
1545.767(PEP+HexNAc(2)Hex(2)_1)
1707.821(PEP+HexNAc(2)Hex(3)_1)
M+2H
1365.096(PEP+HexNAc(4)Hex(5)NeuAc(1)_2)
657.239(NeuAc + Hex + HexNAc)

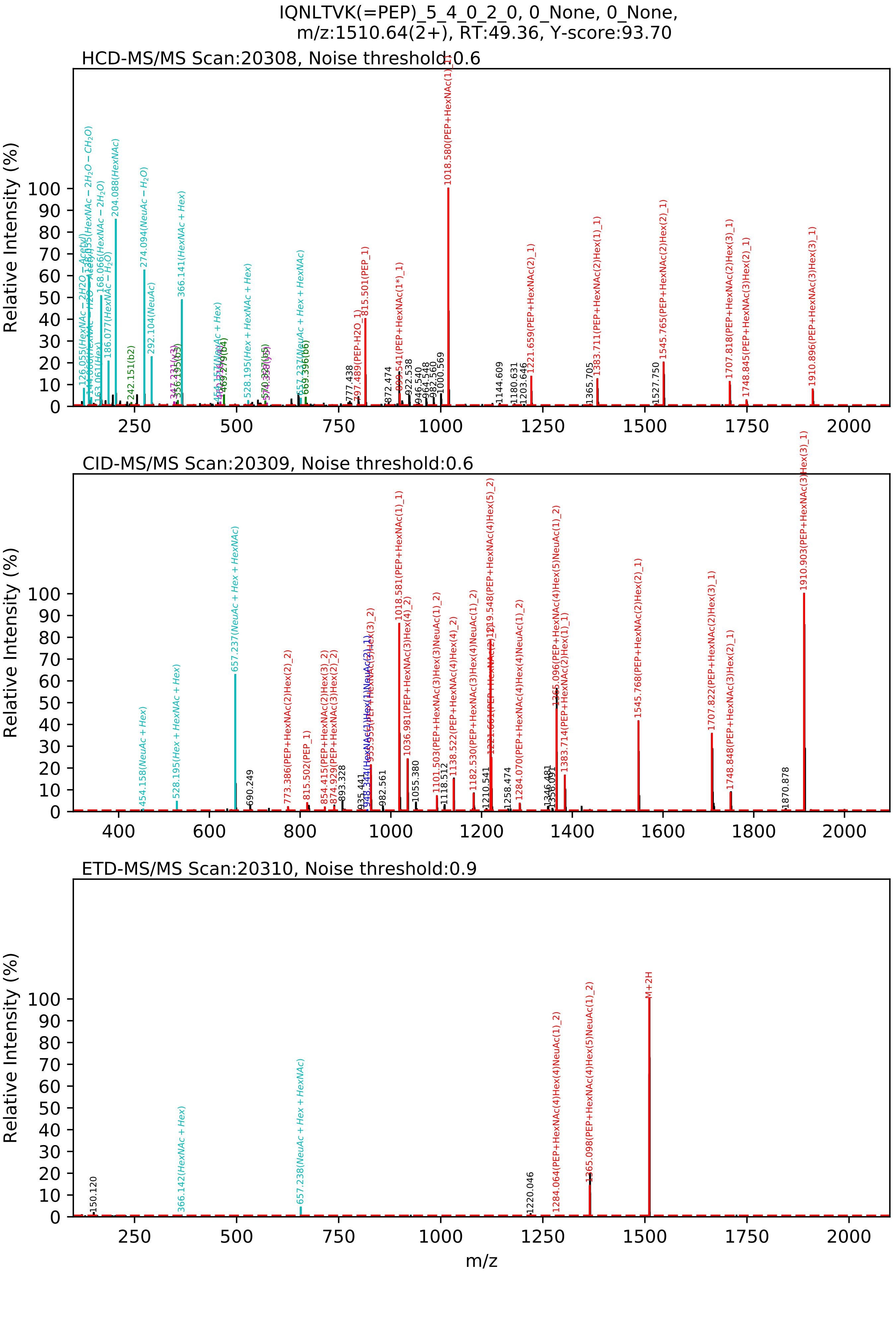
IQNLTVK(=PEP)_5_4_0_2_0, 0_None, 0_None,
m/z:1510.64(2+), RT:49.36, Y-score:93.70
HCD-MS/MS Scan:20308, Noise threshold:0.6
CID-MS/MS Scan:20309, Noise threshold:0.6
ETD-MS/MS Scan:20310, Noise threshold:0.9
Relative Intensity (%)
m/z
1018.580(PEP+HexNAc(1)_1)
815.501(PEP_1)
1221.659(PEP+HexNAc(2)_1)
1383.711(PEP+HexNAc(2)Hex(1)_1)
1545.765(PEP+HexNAc(2)Hex(2)_1)
1707.818(PEP+HexNAc(2)Hex(3)_1)
1748.845(PEP+HexNAc(3)Hex(2)_1)
1910.896(PEP+HexNAc(3)Hex(3)_1)
204.088(HexNAc)
274.094(NeuAc − H2O)
292.104(NeuAc)
366.141(HexNAc + Hex)
528.195(Hex + HexNAc + Hex)
657.237(NeuAc + Hex + HexNAc)
1910.903(PEP+HexNAc(3)Hex(3)_1)
1018.581(PEP+HexNAc(1)_1)
1036.981(PEP+HexNAc(3)Hex(4)_2)
1219.548(PEP+HexNAc(4)Hex(5)_2)
1365.096(PEP+HexNAc(4)Hex(5)NeuAc(1)_2)
1383.714(PEP+HexNAc(2)Hex(1)_1)
1545.768(PEP+HexNAc(2)Hex(2)_1)
1707.822(PEP+HexNAc(2)Hex(3)_1)
1748.848(PEP+HexNAc(3)Hex(2)_1)
454.158(NeuAc + Hex)
M+2H
1365.098(PEP+HexNAc(4)Hex(5)NeuAc(1)_2)
1284.064(PEP+HexNAc(4)Hex(4)NeuAc(1)_2)
366.142(HexNAc + Hex)
657.238(NeuAc + Hex + HexNAc)
150.120
1220.046

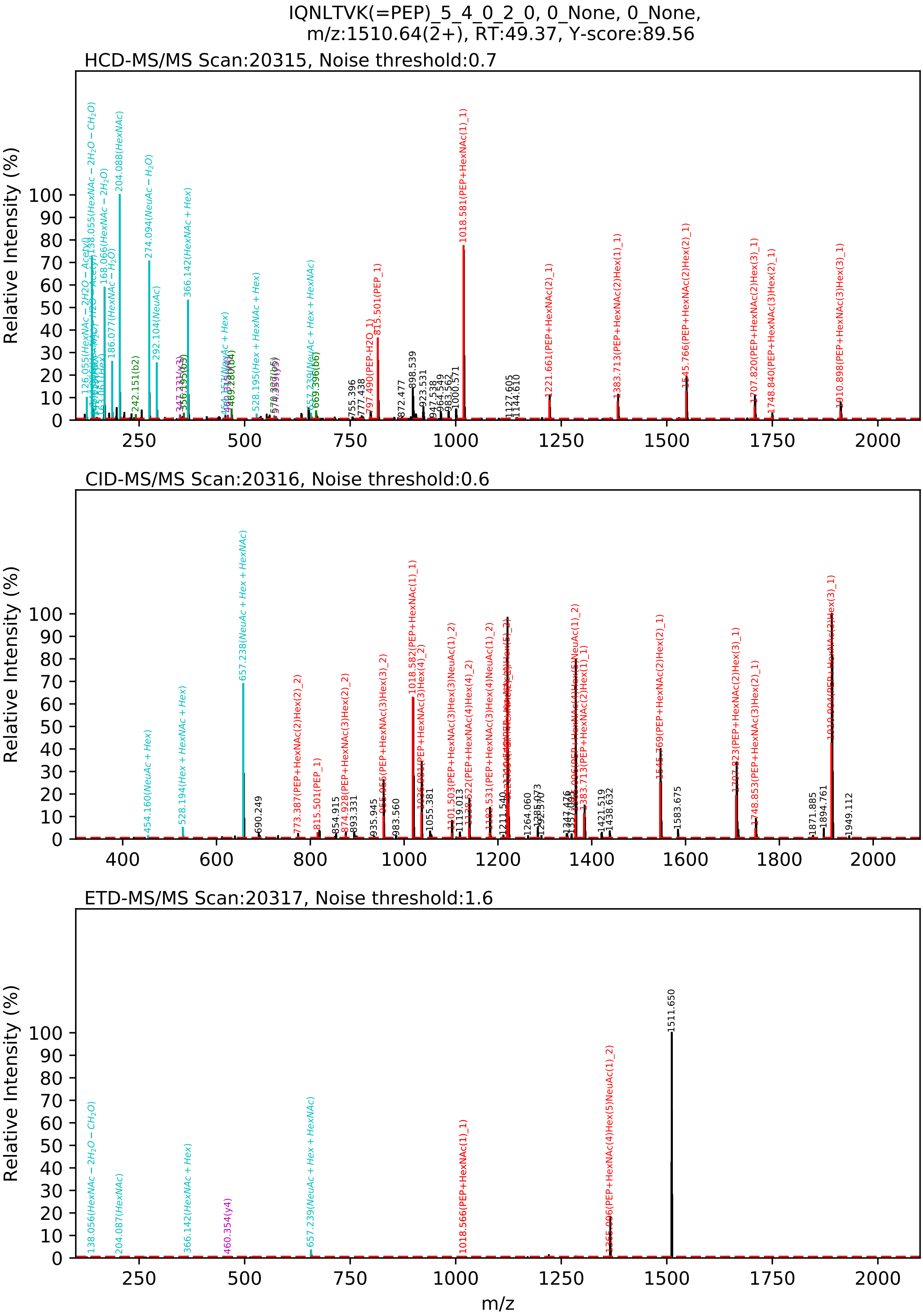

IQNLTVK(=PEP)_5_4_0_2_0, 0_None, 0_None,
m/z:1510.64(2+), RT:49.37, Y-score:89.56
HCD-MS/MS Scan:20315, Noise threshold:0.7
Relative Intensity (%)
138.055(HexNAc − 2H2O − CH2O)
204.088(HexNAc)
168.066(HexNAc − 2H2O)
186.077(HexNAc − H2O)
274.094(NeuAc − H2O)
292.104(NeuAc)
366.142(HexNAc + Hex)
242.151(b2)
528.195(Hex + HexNAc + Hex)
657.239(NeuAc + Hex + HexNAc)
669.396(b6)
797.490(PEP-H2O_1)
815.501(PEP_1)
898.539
923.531
1018.581(PEP+HexNAc(1)_1)
1127.605
1144.610
1221.661(PEP+HexNAc(2)_1)
1383.713(PEP+HexNAc(2)Hex(1)_1)
1545.766(PEP+HexNAc(2)Hex(2)_1)
1707.820(PEP+HexNAc(2)Hex(3)_1)
1748.840(PEP+HexNAc(3)Hex(2)_1)
1910.898(PEP+HexNAc(3)Hex(3)_1)
CID-MS/MS Scan:20316, Noise threshold:0.6
Relative Intensity (%)
454.160(NeuAc + Hex)
528.194(Hex + HexNAc + Hex)
657.238(NeuAc + Hex + HexNAc)
690.249
773.387(PEP+HexNAc(2)Hex(2)_2)
815.501(PEP_1)
854.915
874.928(PEP+HexNAc(3)Hex(2)_2)
893.331
935.945
983.560
1018.582(PEP+HexNAc(1)_1)
1055.381
1119.013
1211.540
1264.060
1292.570
1383.713(PEP+HexNAc(2)Hex(1)_1)
1421.519
1438.632
1583.675
1748.853(PEP+HexNAc(3)Hex(2)_1)
1871.885
1894.761
1949.112
ETD-MS/MS Scan:20317, Noise threshold:1.6
Relative Intensity (%)
138.056(HexNAc − 2H2O − CH2O)
204.087(HexNAc)
366.142(HexNAc + Hex)
460.354(y4)
657.239(NeuAc + Hex + HexNAc)
1018.566(PEP+HexNAc(1)_1)
1511.650
m/z

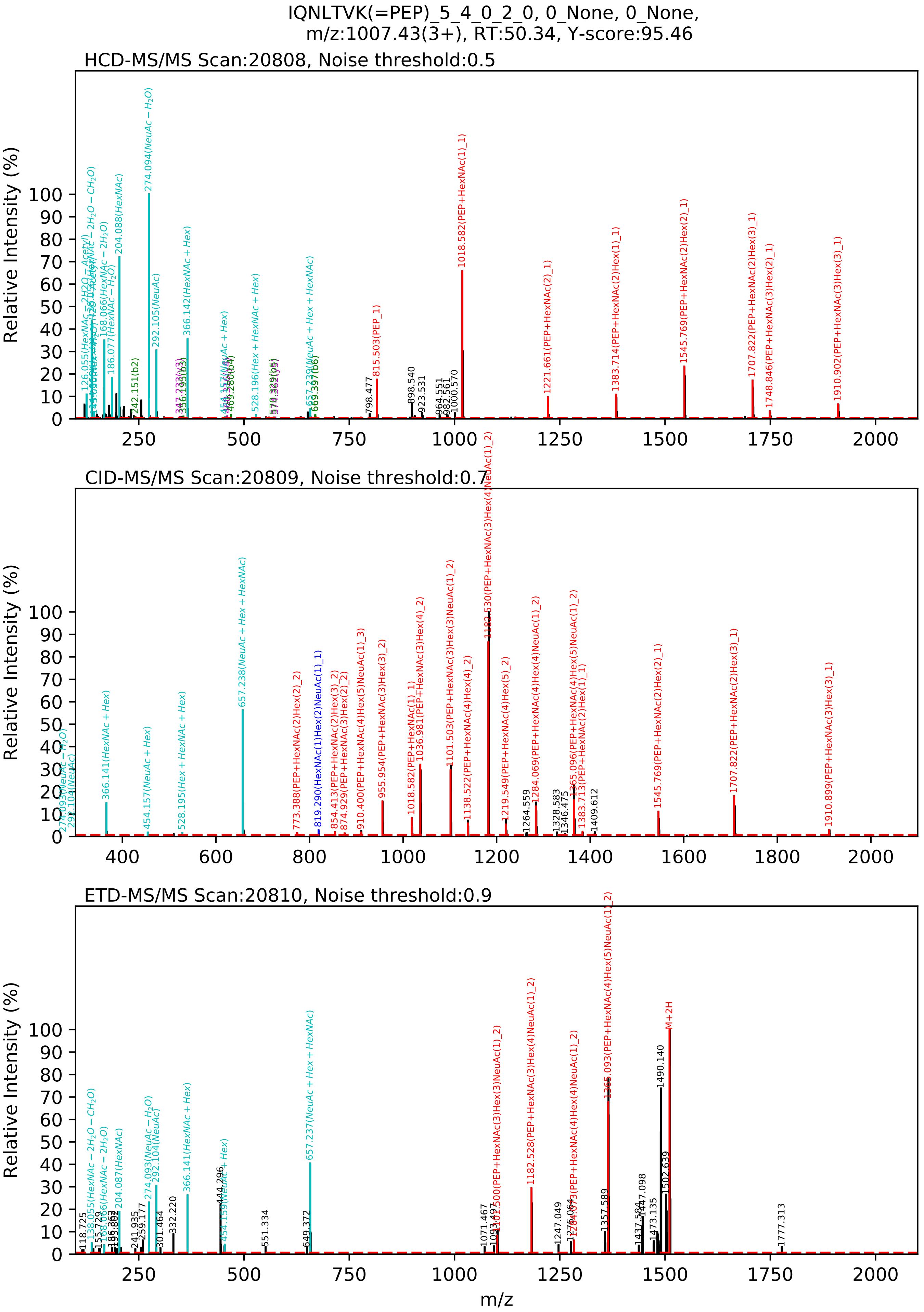
IQNLTVK(=PEP)_5_4_0_2_0, 0_None, 0_None,
m/z:1007.43(3+), RT:50.34, Y-score:95.46
HCD-MS/MS Scan:20808, Noise threshold:0.5
Relative Intensity (%)
274.094(NeuAc − H2O)
204.088(HexNAc)
292.105(NeuAc)
366.142(HexNAc + Hex)
168.066(HexNAc − 2H2O)
186.077(HexNAc − H2O)
242.151(b2)
528.196(Hex + HexNAc + Hex)
815.503(PEP_1)
798.477
898.540
923.531
964.551
1000.570
1018.582(PEP+HexNAc(1)_1)
1221.661(PEP+HexNAc(2)_1)
1383.714(PEP+HexNAc(2)Hex(1)_1)
1545.769(PEP+HexNAc(2)Hex(2)_1)
1707.822(PEP+HexNAc(2)Hex(3)_1)
1748.846(PEP+HexNAc(3)Hex(2)_1)
1910.902(PEP+HexNAc(3)Hex(3)_1)
CID-MS/MS Scan:20809, Noise threshold:0.7
366.141(HexNAc + Hex)
454.157(NeuAc + Hex)
528.195(Hex + HexNAc + Hex)
657.238(NeuAc + Hex + HexNAc)
773.388(PEP+HexNAc(2)Hex(2)_2)
819.290(HexNAc(1)Hex(2)NeuAc(1)_1)
854.413(PEP+HexNAc(2)Hex(3)_2)
874.929(PEP+HexNAc(3)Hex(2)_2)
910.400(PEP+HexNAc(4)Hex(5)NeuAc(1)_3)
955.954(PEP+HexNAc(3)Hex(3)_2)
1018.582(PEP+HexNAc(1)_1)
1036.981(PEP+HexNAc(3)Hex(4)_2)
1101.503(PEP+HexNAc(3)Hex(3)NeuAc(1)_2)
1138.522(PEP+HexNAc(4)Hex(4)_2)
1182.530(PEP+HexNAc(3)Hex(4)NeuAc(1)_2)
1219.549(PEP+HexNAc(4)Hex(5)_2)
1264.559
1284.069(PEP+HexNAc(4)Hex(4)NeuAc(1)_2)
1328.583
1346.475
1365.096(PEP+HexNAc(4)Hex(5)NeuAc(1)_2)
1383.713(PEP+HexNAc(2)Hex(1)_1)
1409.612
1545.769(PEP+HexNAc(2)Hex(2)_1)
1707.822(PEP+HexNAc(2)Hex(3)_1)
1910.899(PEP+HexNAc(3)Hex(3)_1)
ETD-MS/MS Scan:20810, Noise threshold:0.9
118.725
204.087(HexNAc)
241.935
259.177
274.093(NeuAc − H2O)
292.104(NeuAc)
301.464
332.220
366.141(HexNAc + Hex)
444.296
551.334
649.372
657.237(NeuAc + Hex + HexNAc)
1071.467
1182.528(PEP+HexNAc(3)Hex(4)NeuAc(1)_2)
1247.049
1365.093(PEP+HexNAc(4)Hex(5)NeuAc(1)_2)
1357.589
1437.584
1447.098
1473.135
1490.140
1502.639
M+2H
1777.313
m/z

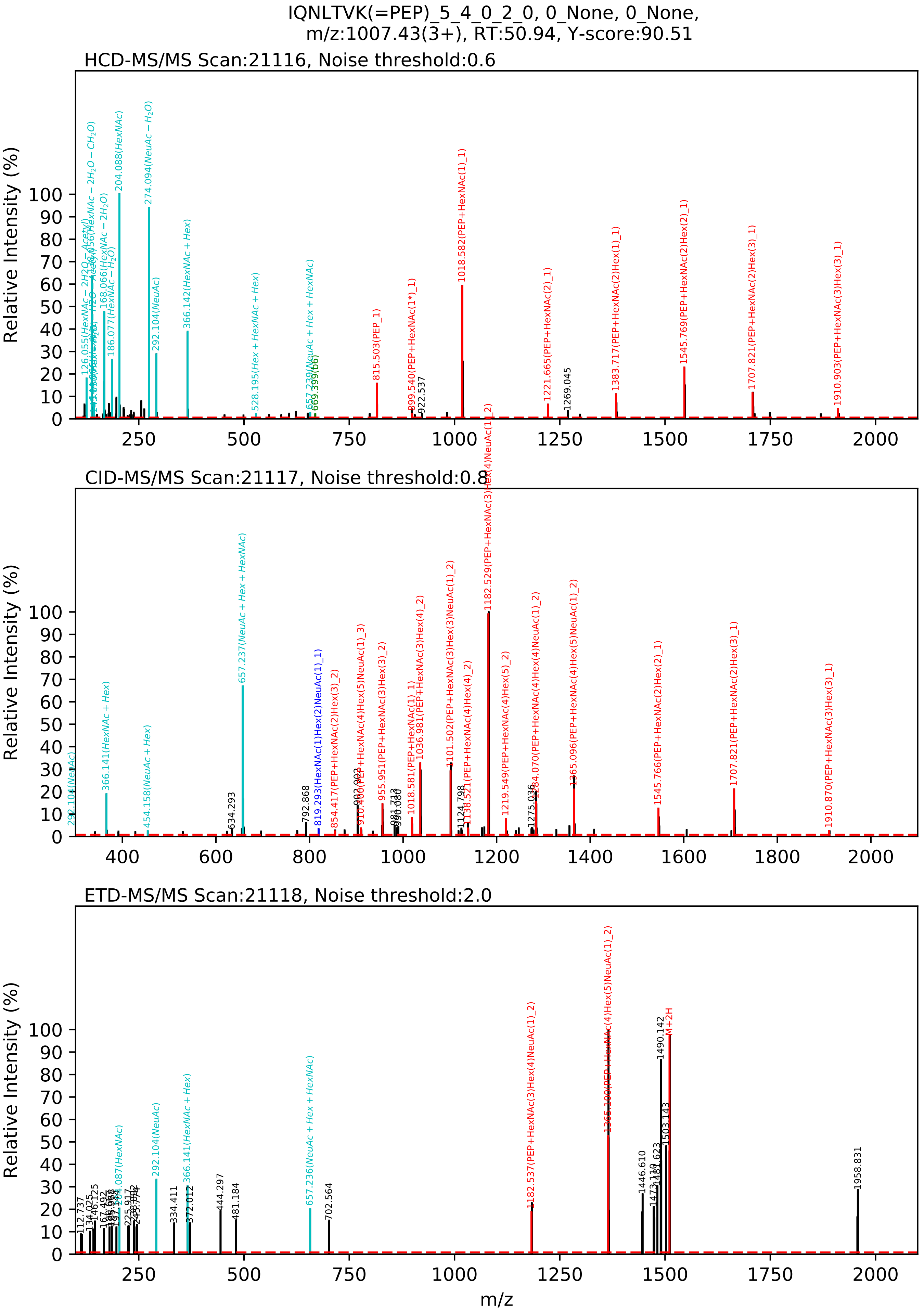
IQNLTVK(=PEP)_5_4_0_2_0, 0_None, 0_None,
m/z:1007.43(3+), RT:50.94, Y-score:90.51
HCD-MS/MS Scan:21116, Noise threshold:0.6
Relative Intensity (%)
204.088(HexNAc)
274.094(NeuAc − H2O)
292.104(NeuAc)
366.142(HexNAc + Hex)
528.195(Hex + HexNAc + Hex)
657.239(NeuAc + Hex + HexNAc)
669.399(b6)
815.503(PEP_1)
899.540(PEP+HexNAc(1*)_1)
922.537
1018.582(PEP+HexNAc(1)_1)
1221.665(PEP+HexNAc(2)_1)
1269.045
1383.717(PEP+HexNAc(2)Hex(1)_1)
1545.769(PEP+HexNAc(2)Hex(2)_1)
1707.821(PEP+HexNAc(2)Hex(3)_1)
1910.903(PEP+HexNAc(3)Hex(3)_1)
CID-MS/MS Scan:21117, Noise threshold:0.8
366.141(HexNAc + Hex)
454.158(NeuAc + Hex)
634.293
657.237(NeuAc + Hex + HexNAc)
792.868
819.293(HexNAc(1)Hex(2)NeuAc(1)_1)
854.417(PEP+HexNAc(2)Hex(3)_2)
902.902
955.951(PEP+HexNAc(3)Hex(3)_2)
1018.581(PEP+HexNAc(1)_1)
1036.981(PEP+HexNAc(3)Hex(4)_2)
1101.502(PEP+HexNAc(3)Hex(3)NeuAc(1)_2)
1124.798
1138.521(PEP+HexNAc(4)Hex(4)_2)
1182.529(PEP+HexNAc(3)Hex(4)NeuAc(1)_2)
1219.549(PEP+HexNAc(4)Hex(5)_2)
1275.036
1365.096(PEP+HexNAc(4)Hex(5)NeuAc(1)_2)
1545.766(PEP+HexNAc(2)Hex(2)_1)
1707.821(PEP+HexNAc(2)Hex(3)_1)
1910.870(PEP+HexNAc(3)Hex(3)_1)
ETD-MS/MS Scan:21118, Noise threshold:2.0
112.737
146.125
292.104(NeuAc)
334.411
366.141(HexNAc + Hex)
372.012
444.297
481.184
657.236(NeuAc + Hex + HexNAc)
702.564
1182.537(PEP+HexNAc(3)Hex(4)NeuAc(1)_2)
1365.100(PEP+HexNAc(4)Hex(5)NeuAc(1)_2)
1446.610
1490.142
1503.143
M+2H
1958.831
m/z

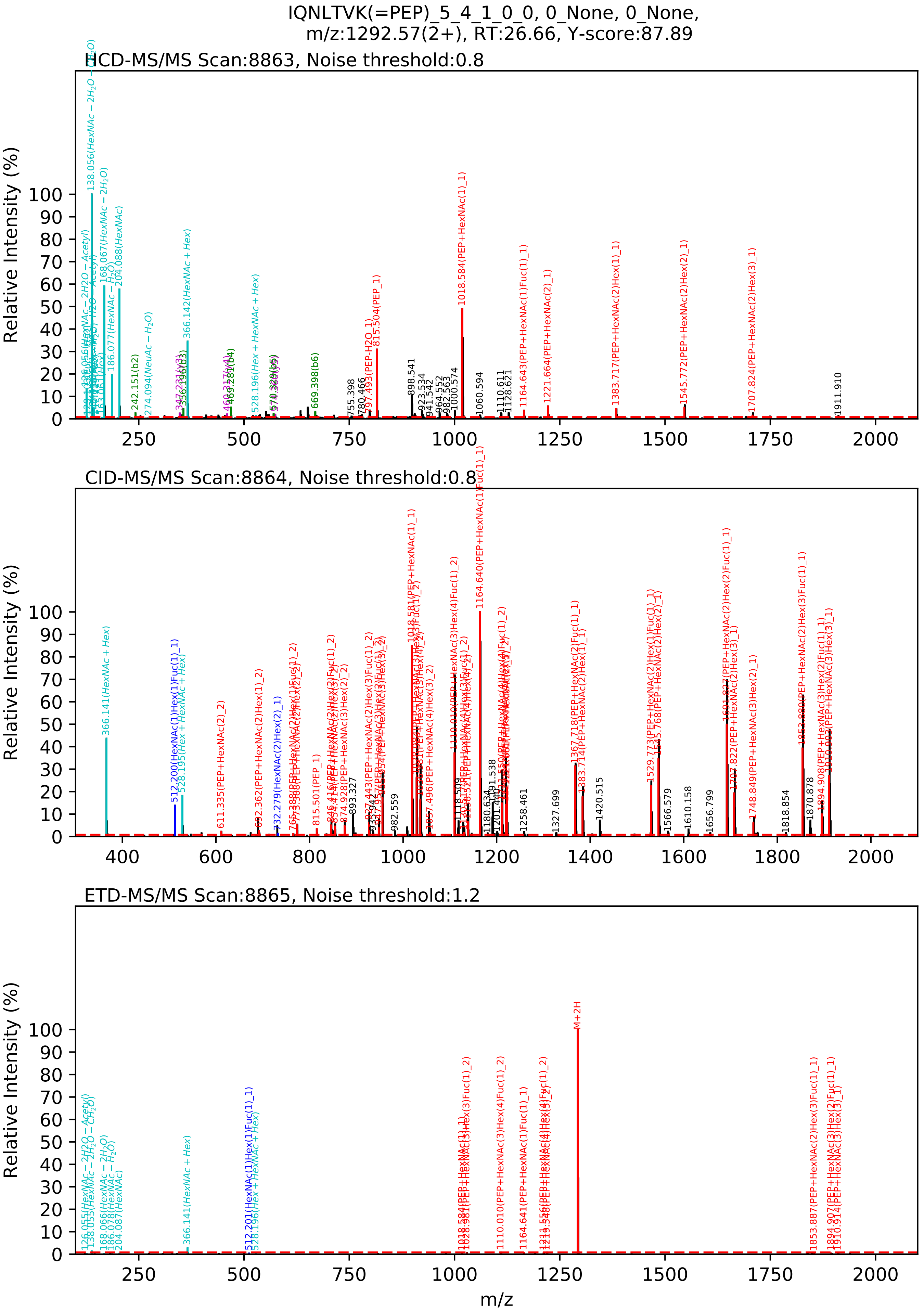
IQNLTVK(=PEP)_5_4_1_0_0, 0_None, 0_None,
m/z:1292.57(2+), RT:26.66, Y-score:87.89
HCD-MS/MS Scan:8863, Noise threshold:0.8
CID-MS/MS Scan:8864, Noise threshold:0.8
ETD-MS/MS Scan:8865, Noise threshold:1.2
Relative Intensity (%)
m/z
1018.584(PEP+HexNAc(1)_1)
815.504(PEP_1)
1164.643(PEP+HexNAc(1)Fuc(1)_1)
1221.664(PEP+HexNAc(2)_1)
1383.717(PEP+HexNAc(2)Hex(1)_1)
1545.772(PEP+HexNAc(2)Hex(2)_1)
1707.824(PEP+HexNAc(2)Hex(3)_1)
366.142(HexNAc+Hex)
204.088(HexNAc)
1164.640(PEP+HexNAc(1)Fuc(1)_1)
M+2H

IQNLTVK(=PEP)_5_4_1_0_0, 0_None, 0_None,
m/z:862.05(3+), RT:27.39, Y-score:96.03

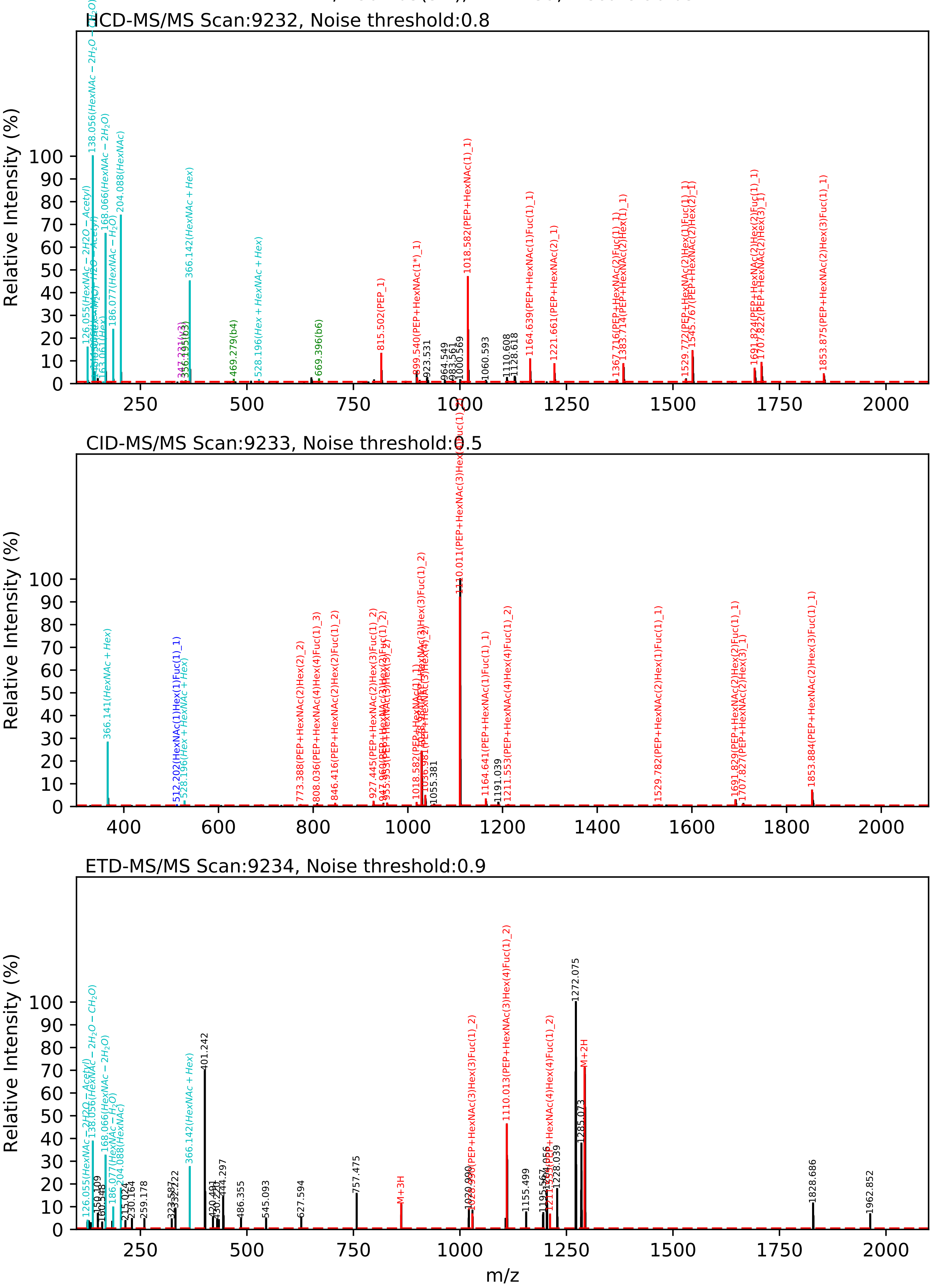

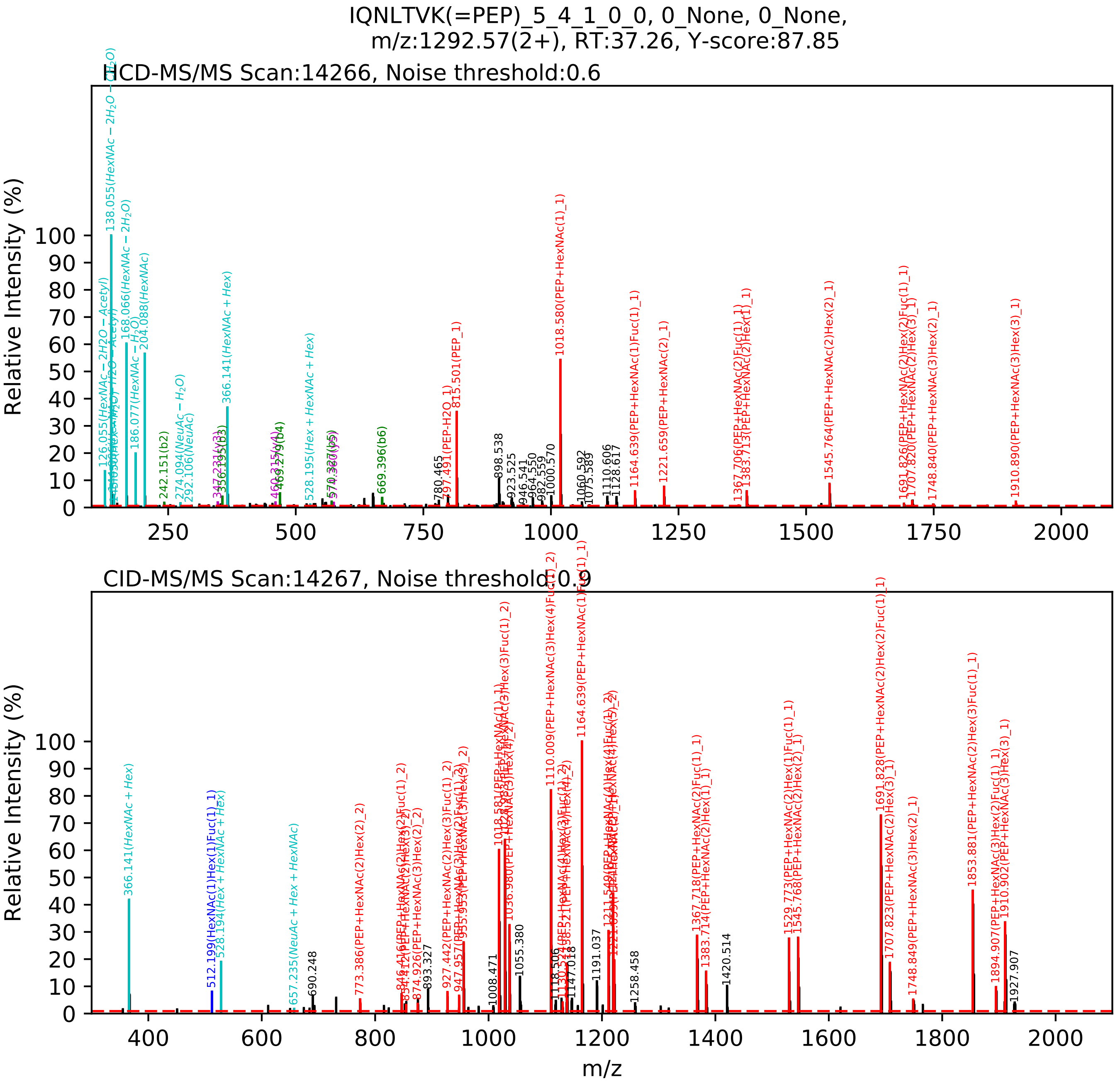
IQNLTVK(=PEP)_5_4_1_0_0, 0_None, 0_None,
m/z:1292.57(2+), RT:37.26, Y-score:87.85
HCD-MS/MS Scan:14266, Noise threshold:0.6
CID-MS/MS Scan:14267, Noise threshold:0.9
Relative Intensity (%)
m/z

IQNLTVK(=PEP)_5_4_1_0_0, 0_None, 0_None,
m/z:862.05(3+), RT:26.83, Y-score:94.47

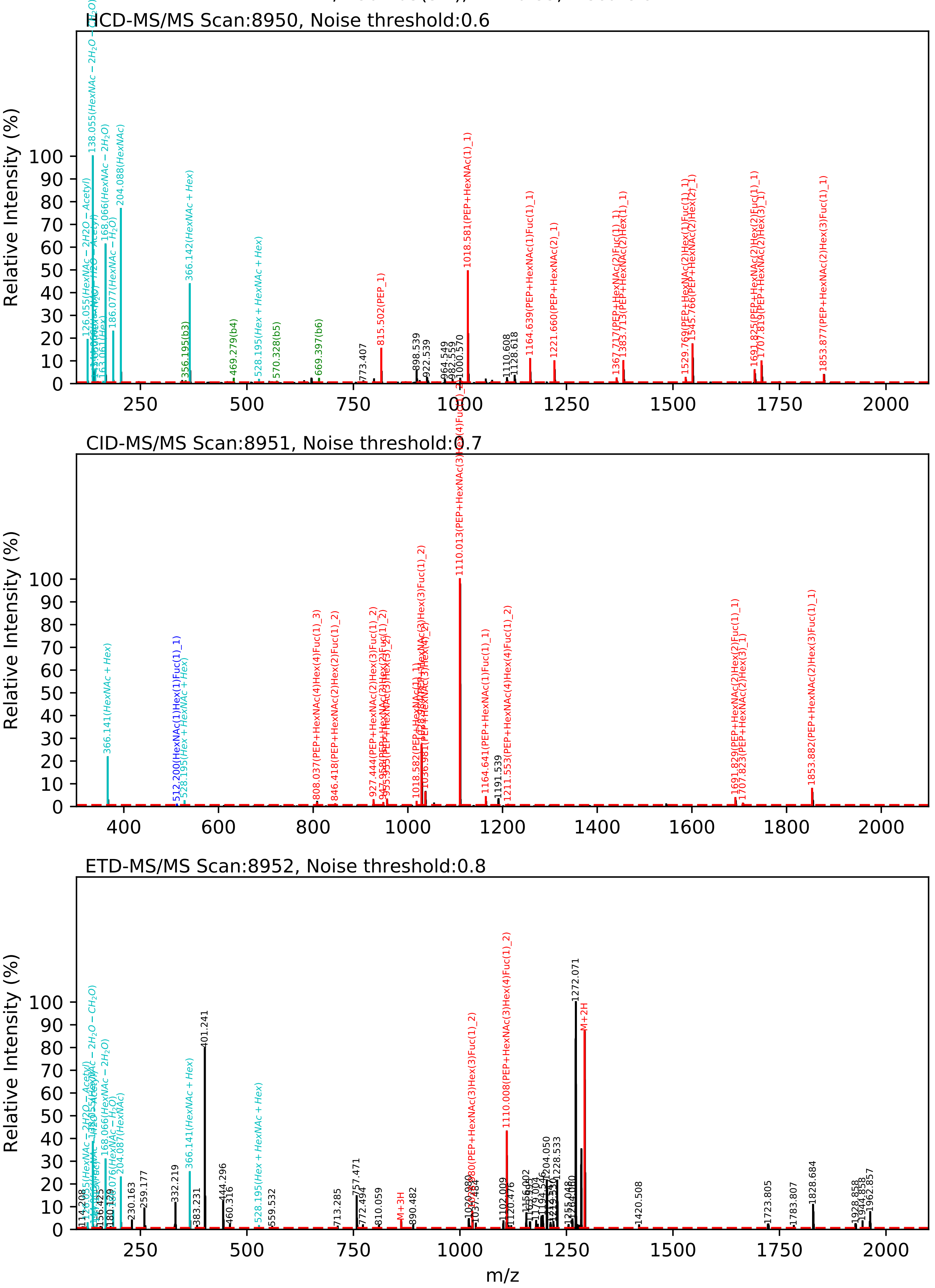

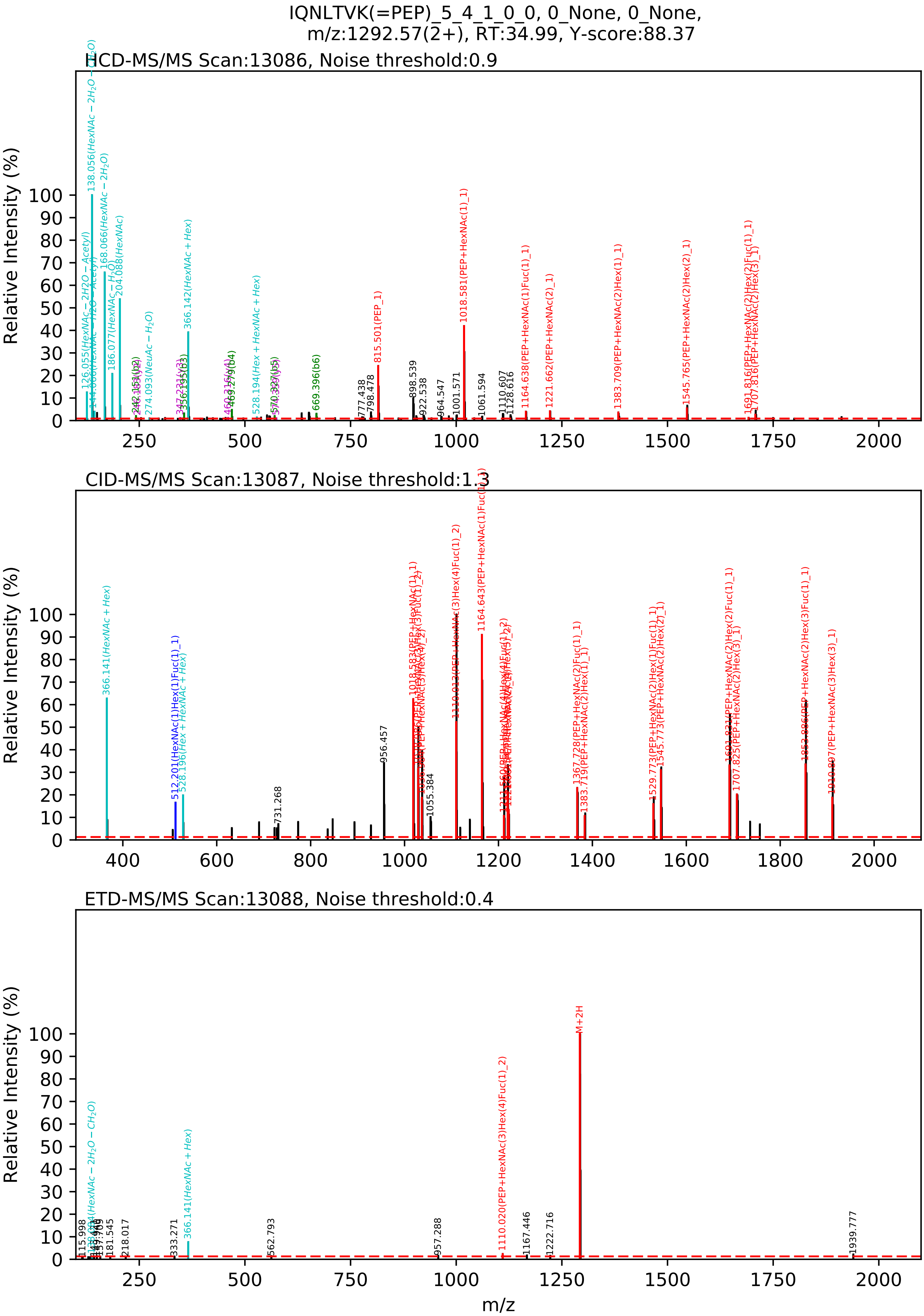
IQNLTVK(=PEP)_5_4_1_0_0, 0_None, 0_None,
m/z:1292.57(2+), RT:34.99, Y-score:88.37
HCD-MS/MS Scan:13086, Noise threshold:0.9
Relative Intensity (%)
138.056(HexNAc − 2H2O)
168.066(HexNAc − 2H2O)
204.088(HexNAc)
186.077(HexNAc − H2O)
126.055(HexNAc − 2H2O − Acetyl)
366.142(HexNAc + Hex)
528.194(Hex + HexNAc + Hex)
274.093(NeuAc − H2O)
669.396(b6)
815.501(PEP_1)
1018.581(PEP+HexNAc(1)_1)
1164.638(PEP+HexNAc(1)Fuc(1)_1)
1221.662(PEP+HexNAc(2)_1)
1383.709(PEP+HexNAc(2)Hex(1)_1)
1545.765(PEP+HexNAc(2)Hex(2)_1)
1691.816(PEP+HexNAc(2)Hex(2)Fuc(1)_1)
1707.816(PEP+HexNAc(2)Hex(3)_1)
777.438
798.478
898.539
922.538
964.547
1001.571
1061.594
1110.607
1128.616
CID-MS/MS Scan:13087, Noise threshold:1.3
366.141(HexNAc + Hex)
512.201(HexNAc(1)Hex(1)Fuc(1)_1)
528.196(Hex + HexNAc + Hex)
731.268
956.457
1055.384
1110.012(PEP+HexNAc(3)Hex(4)Fuc(1)_2)
1164.643(PEP+HexNAc(1)Fuc(1)_1)
1367.728(PEP+HexNAc(2)Fuc(1)_1)
1383.719(PEP+HexNAc(2)Hex(1)_1)
1529.773(PEP+HexNAc(2)Hex(1)Fuc(1)_1)
1545.773(PEP+HexNAc(2)Hex(2)_1)
1691.824(PEP+HexNAc(2)Hex(2)Fuc(1)_1)
1707.825(PEP+HexNAc(2)Hex(3)_1)
1853.886(PEP+HexNAc(2)Hex(3)Fuc(1)_1)
1910.907(PEP+HexNAc(3)Hex(3)_1)
ETD-MS/MS Scan:13088, Noise threshold:0.4
M+2H
1110.020(PEP+HexNAc(3)Hex(4)Fuc(1)_2)
366.141(HexNAc + Hex)
115.998
137.709
181.545
218.017
333.271
562.793
957.288
1167.446
1222.716
1939.777
m/z

IQNLTVK(=PEP)_5_4_1_0_0, 0_None, 0_None,
m/z:1292.57(2+), RT:35.03, Y-score:92.72

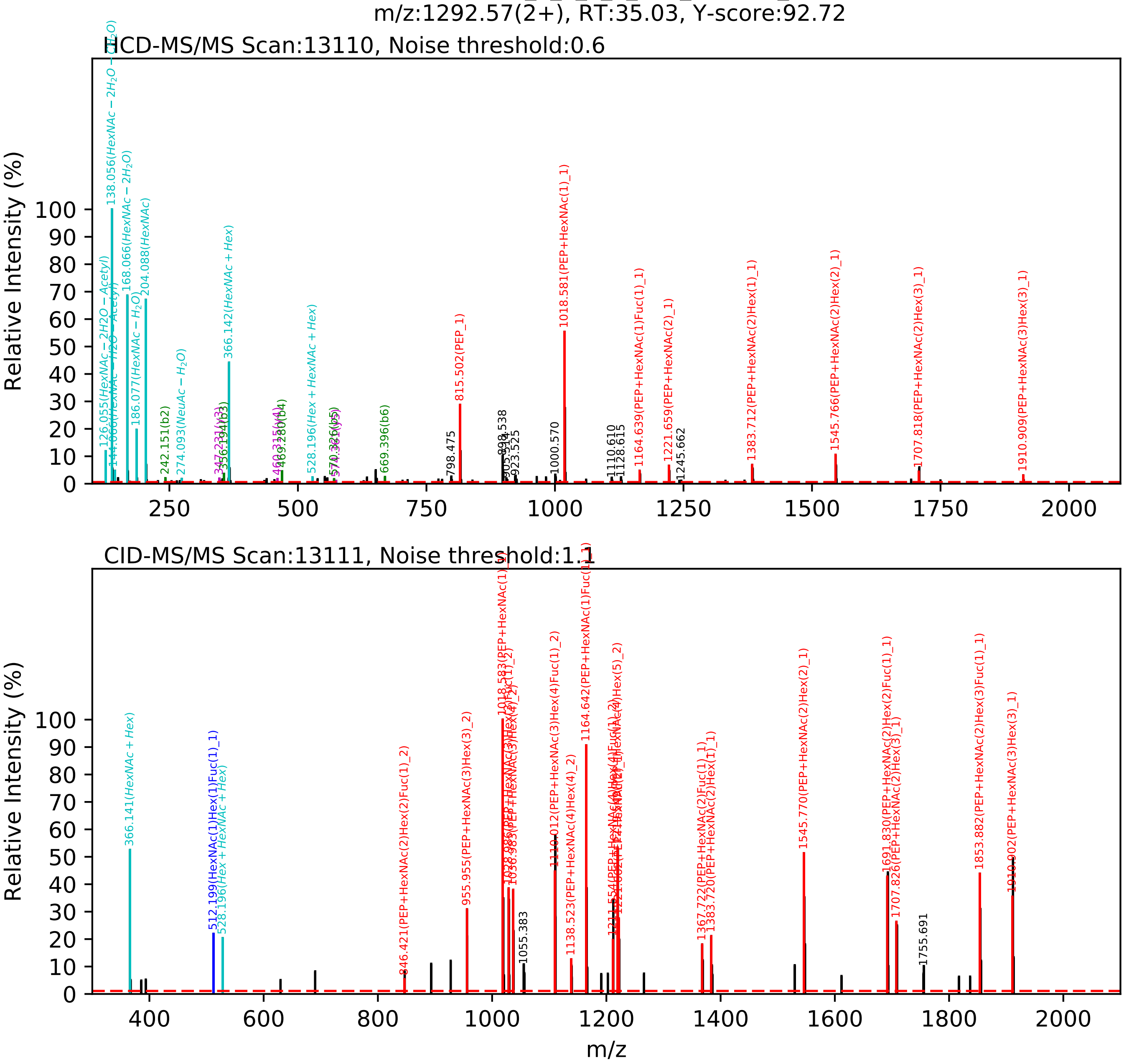

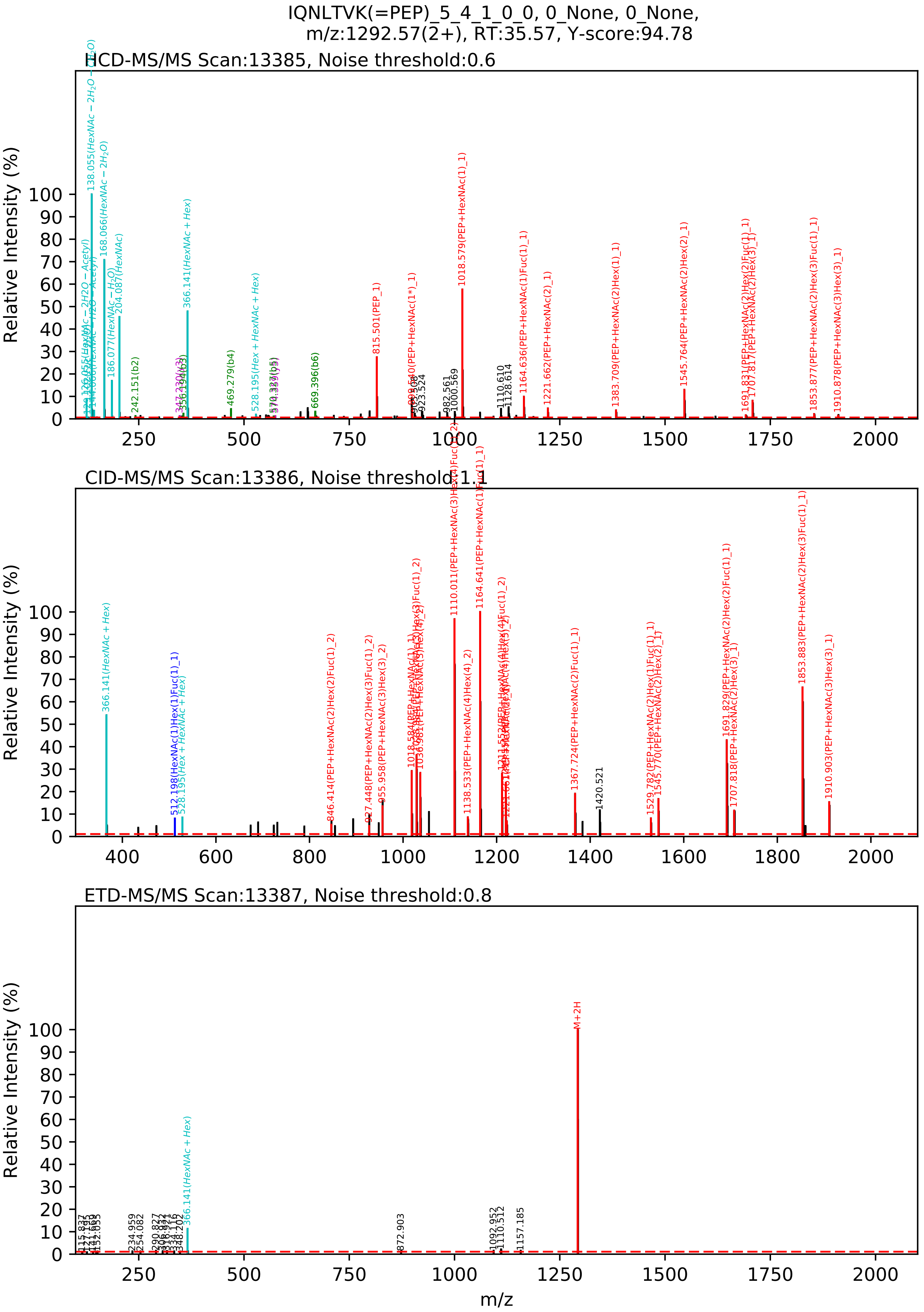
IQNLTVK(=PEP)_5_4_1_0_0, 0_None, 0_None,
m/z:1292.57(2+), RT:35.57, Y-score:94.78
HCD-MS/MS Scan:13385, Noise threshold:0.6
Relative Intensity (%)
138.055(HexNAc − 2H2O)
168.066(HexNAc − 2H2O)
186.077(HexNAc − H2O)
204.087(HexNAc)
366.141(HexNAc + Hex)
242.151(b2)
469.279(b4)
528.195(Hex + HexNAc + Hex)
669.396(b6)
815.501(PEP_1)
1018.579(PEP+HexNAc(1)_1)
923.524
982.561
1000.569
1110.610
1128.614
1164.636(PEP+HexNAc(1)Fuc(1)_1)
1221.662(PEP+HexNAc(2)_1)
1383.709(PEP+HexNAc(2)Hex(1)_1)
1545.764(PEP+HexNAc(2)Hex(2)_1)
1691.831(PEP+HexNAc(2)Hex(2)Fuc(1)_1)
1707.817(PEP+HexNAc(2)Hex(3)_1)
1853.877(PEP+HexNAc(2)Hex(3)Fuc(1)_1)
1910.878(PEP+HexNAc(3)Hex(3)_1)
CID-MS/MS Scan:13386, Noise threshold:1.1
366.141(HexNAc + Hex)
512.198(HexNAc(1)Hex(1)Fuc(1)_1)
528.195(Hex + HexNAc + Hex)
846.414(PEP+HexNAc(2)Hex(2)Fuc(1)_2)
927.448(PEP+HexNAc(2)Hex(3)Fuc(1)_2)
955.958(PEP+HexNAc(3)Hex(3)_2)
1110.011(PEP+HexNAc(3)Hex(3)Fuc(1)_2)
1164.641(PEP+HexNAc(1)Fuc(1)_1)
1138.533(PEP+HexNAc(4)Hex(4)_2)
1367.724(PEP+HexNAc(2)Fuc(1)_1)
1420.521
1529.782(PEP+HexNAc(2)Hex(1)Fuc(1)_1)
1545.770(PEP+HexNAc(2)Hex(2)_1)
1691.829(PEP+HexNAc(2)Hex(2)Fuc(1)_1)
1707.818(PEP+HexNAc(2)Hex(3)_1)
1853.883(PEP+HexNAc(2)Hex(3)Fuc(1)_1)
1910.903(PEP+HexNAc(3)Hex(3)_1)
ETD-MS/MS Scan:13387, Noise threshold:0.8
M+2H
366.141(HexNAc + Hex)
234.959
254.082
872.903
1092.952
1110.512
1157.185
m/z

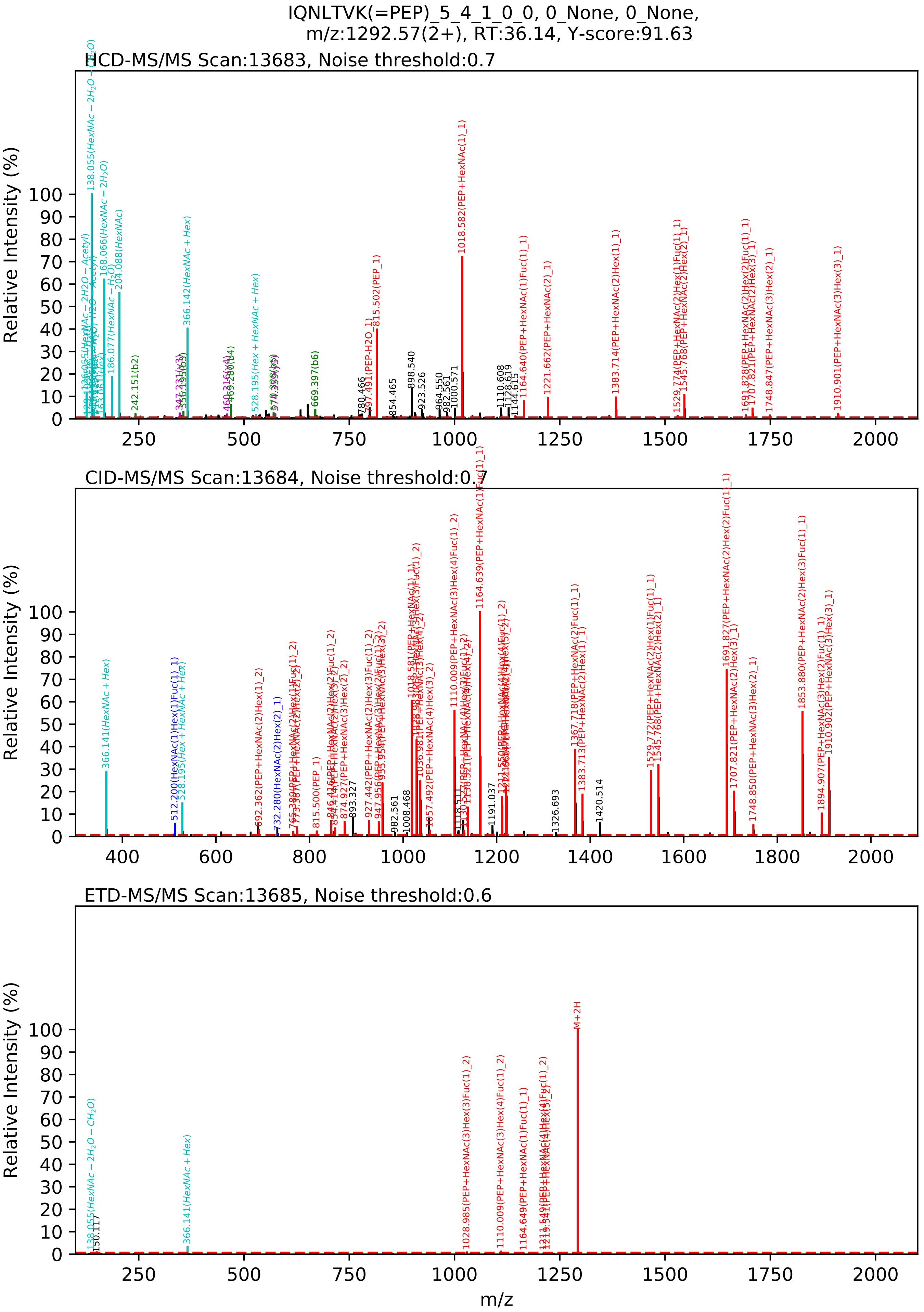

IQNLTVK(=PEP)_5_4_1_0_0, 0_None, 0_None,
m/z:1292.57(2+), RT:36.14, Y-score:91.63
HCD-MS/MS Scan:13683, Noise threshold:0.7
CID-MS/MS Scan:13684, Noise threshold:0.7
ETD-MS/MS Scan:13685, Noise threshold:0.6
Relative Intensity (%)
m/z
1018.582(PEP+HexNAc(1)_1)
1164.639(PEP+HexNAc(1)Fuc(1)_1)
1691.827(PEP+HexNAc(2)Hex(2)Fuc(1)_1)
1853.880(PEP+HexNAc(2)Hex(3)Fuc(1)_1)
M+2H

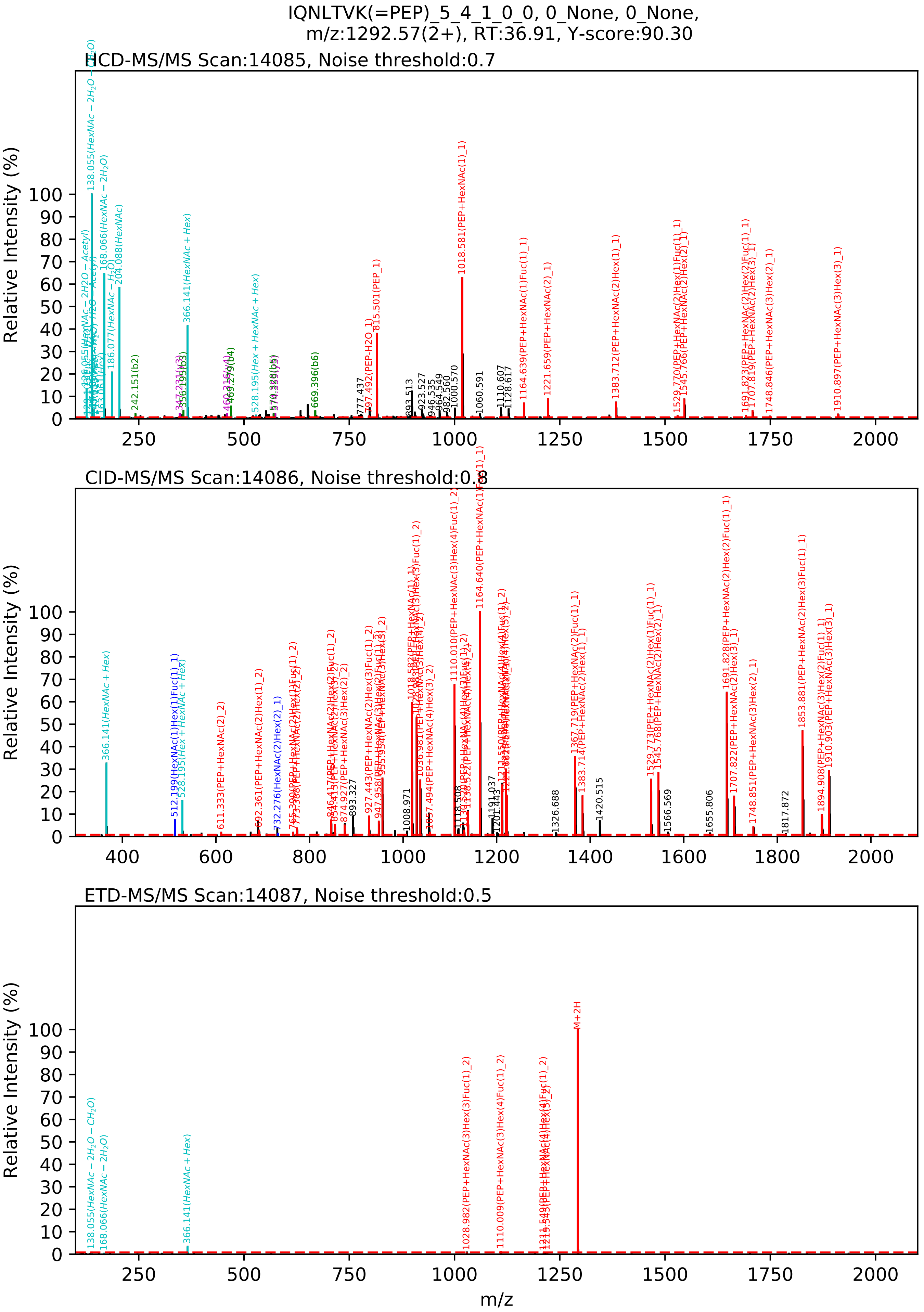
IQNLTVK(=PEP)_5_4_1_0_0, 0_None, 0_None,
m/z:1292.57(2+), RT:36.91, Y-score:90.30
HCD-MS/MS Scan:14085, Noise threshold:0.7
CID-MS/MS Scan:14086, Noise threshold:0.8
ETD-MS/MS Scan:14087, Noise threshold:0.5
Relative Intensity (%)
m/z
1018.581(PEP+HexNAc(1)_1)
815.501(PEP_1)
797.492(PEP-H2O_1)
1164.639(PEP+HexNAc(1)Fuc(1)_1)
1221.659(PEP+HexNAc(2)_1)
1383.712(PEP+HexNAc(2)Hex(1)_1)
1545.766(PEP+HexNAc(2)Hex(2)_1)
1748.846(PEP+HexNAc(3)Hex(2)_1)
1910.897(PEP+HexNAc(3)Hex(3)_1)
366.141(HexNAc+Hex)
528.195(Hex+HexNAc+Hex)
204.088(HexNAc)
242.151(b2)
669.396(b6)
1164.640(PEP+HexNAc(1)Fuc(1)_1)
1110.010(PEP+HexNAc(3)Hex(4)Fuc(1)_2)
1367.719(PEP+HexNAc(2)Fuc(1)_1)
1529.773(PEP+HexNAc(2)Hex(1)Fuc(1)_1)
1691.828(PEP+HexNAc(2)Hex(2)Fuc(1)_1)
1707.822(PEP+HexNAc(2)Hex(3)_1)
1853.881(PEP+HexNAc(2)Hex(3)Fuc(1)_1)
512.199(HexNAc(1)Hex(1)Fuc(1)_1)
732.276(HexNAc(2)Hex(2)_1)
M+2H
1028.982(PEP+HexNAc(3)Hex(3)Fuc(1)_2)
1110.009(PEP+HexNAc(3)Hex(4)Fuc(1)_2)
138.055(HexNAc − 2H2O − CH2O)
168.066(HexNAc − 2H2O)

IQNLTVK(=PEP)_5_4_1_0_0, 0_None, 0_None,
m/z:1292.57(2+), RT:27.82, Y-score:85.29

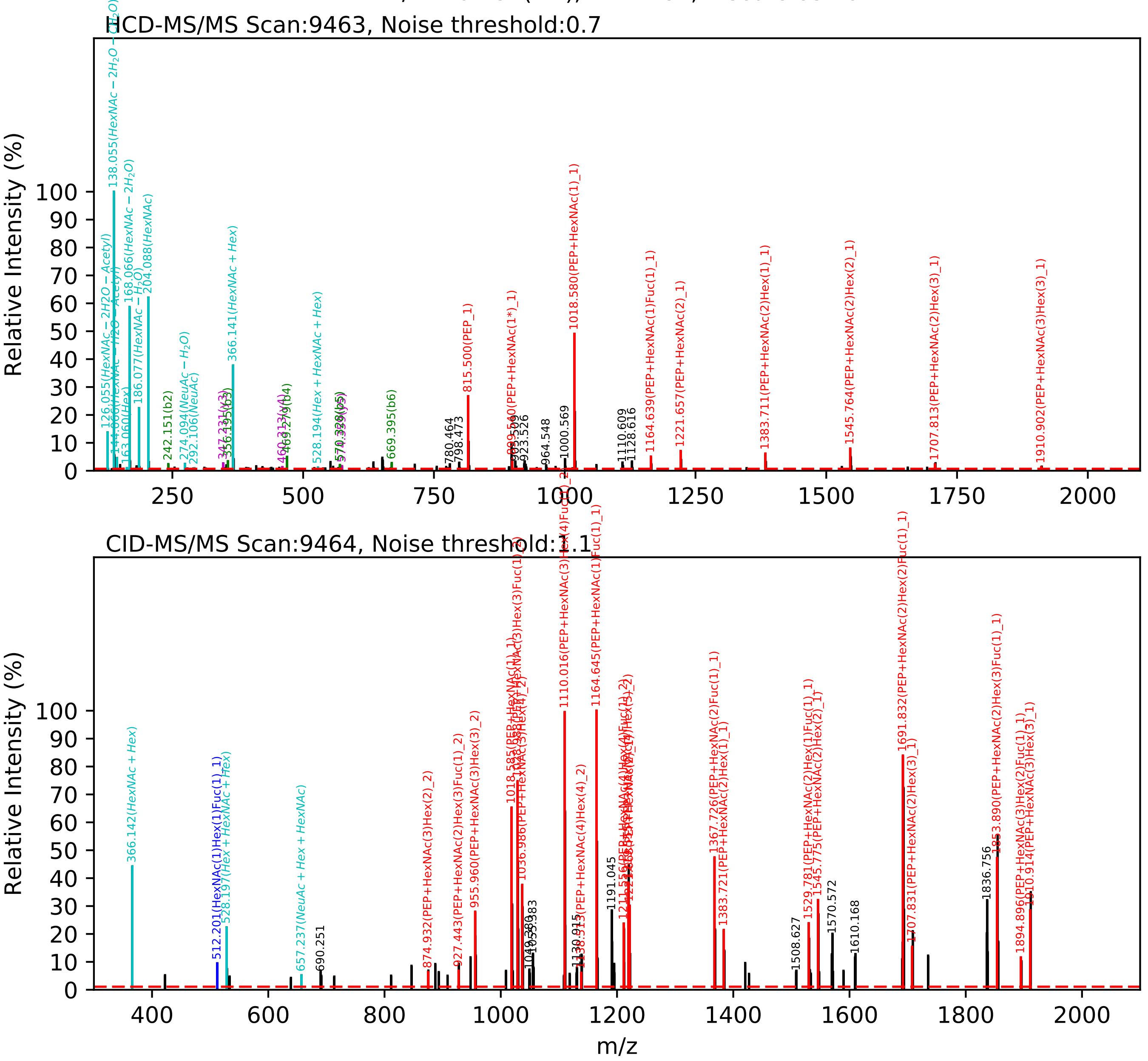

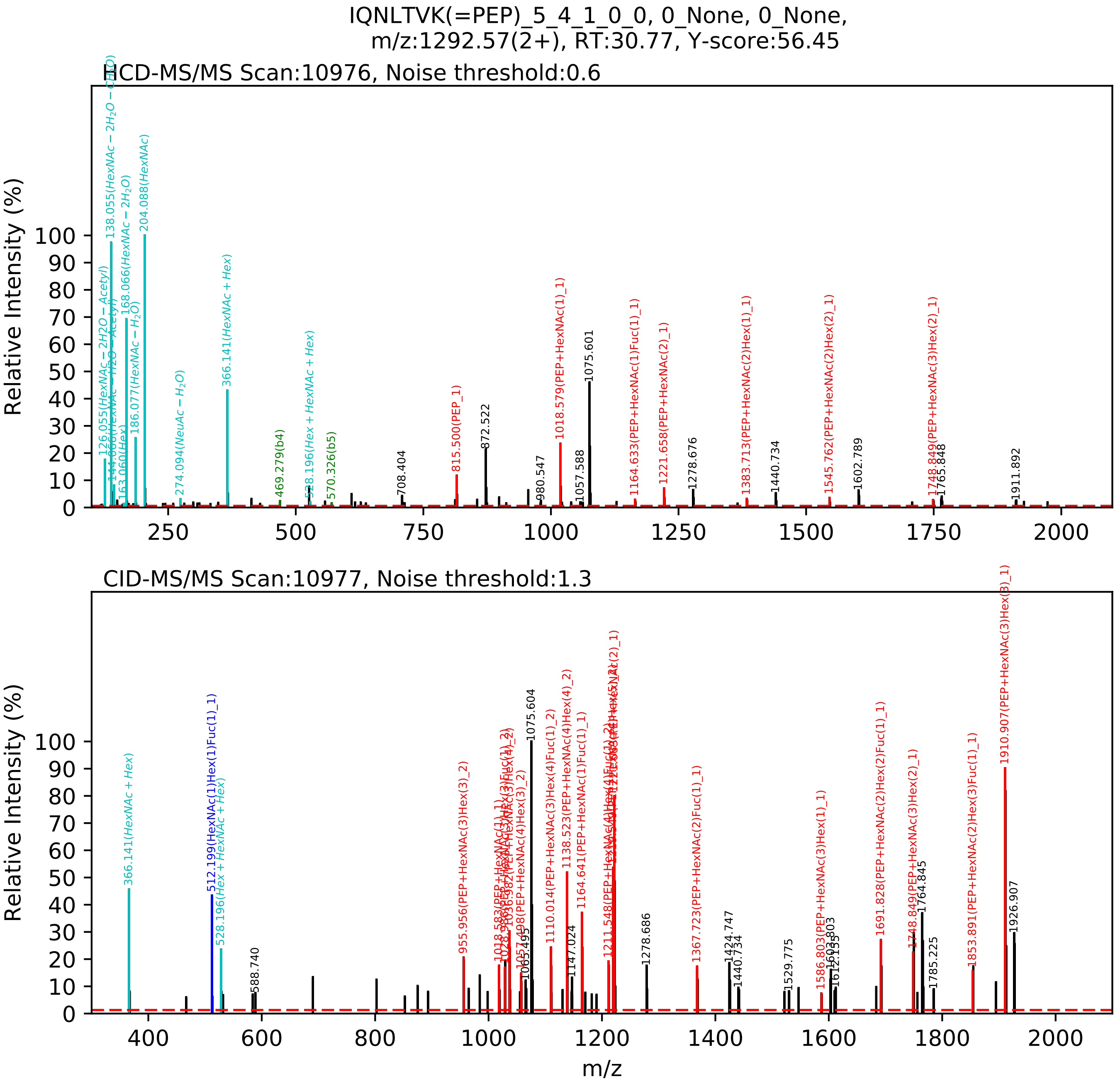
IQNLTVK(=PEP)_5_4_1_0_0, 0_None, 0_None,
m/z:1292.57(2+), RT:30.77, Y-score:56.45
HCD-MS/MS Scan:10976, Noise threshold:0.6
Relative Intensity (%)
126.055(HexNAc − 2H2O − Acetyl)
138.055(HexNAc − 2H2O − C2H4O)
168.066(HexNAc − 2H2O)
186.077(HexNAc − H2O)
204.088(HexNAc)
163.060(Hex)
274.094(NeuAc − H2O)
366.141(HexNAc + Hex)
469.279(b4)
528.196(Hex + HexNAc + Hex)
570.326(b5)
708.404
815.500(PEP_1)
872.522
980.547
1018.579(PEP+HexNAc(1)_1)
1057.588
1075.601
1164.633(PEP+HexNAc(1)Fuc(1)_1)
1221.658(PEP+HexNAc(2)_1)
1278.676
1383.713(PEP+HexNAc(2)Hex(1)_1)
1440.734
1545.762(PEP+HexNAc(2)Hex(2)_1)
1602.789
1748.849(PEP+HexNAc(3)Hex(2)_1)
1765.848
1911.892
CID-MS/MS Scan:10977, Noise threshold:1.3
366.141(HexNAc + Hex)
512.199(HexNAc(1)Hex(1)Fuc(1)_1)
528.196(Hex + HexNAc + Hex)
588.740
955.956(PEP+HexNAc(3)Hex(3)_2)
1057.498(PEP+HexNAc(4)Hex(3)_2)
1065.495
1075.604
1110.014(PEP+HexNAc(3)Hex(4)Fuc(1)_2)
1138.523(PEP+HexNAc(4)Hex(4)_2)
1147.024
1164.641(PEP+HexNAc(1)Fuc(1)_1)
1211.548
1278.686
1367.723(PEP+HexNAc(2)Fuc(1)_1)
1424.747
1440.734
1529.775
1586.803(PEP+HexNAc(3)Hex(1)_1)
1603.803
1612.155
1691.828(PEP+HexNAc(2)Hex(2)Fuc(1)_1)
1748.849(PEP+HexNAc(3)Hex(2)_1)
1764.845
1785.225
1853.891(PEP+HexNAc(2)Hex(3)Fuc(1)_1)
1910.907(PEP+HexNAc(3)Hex(3)_1)
1926.907
m/z

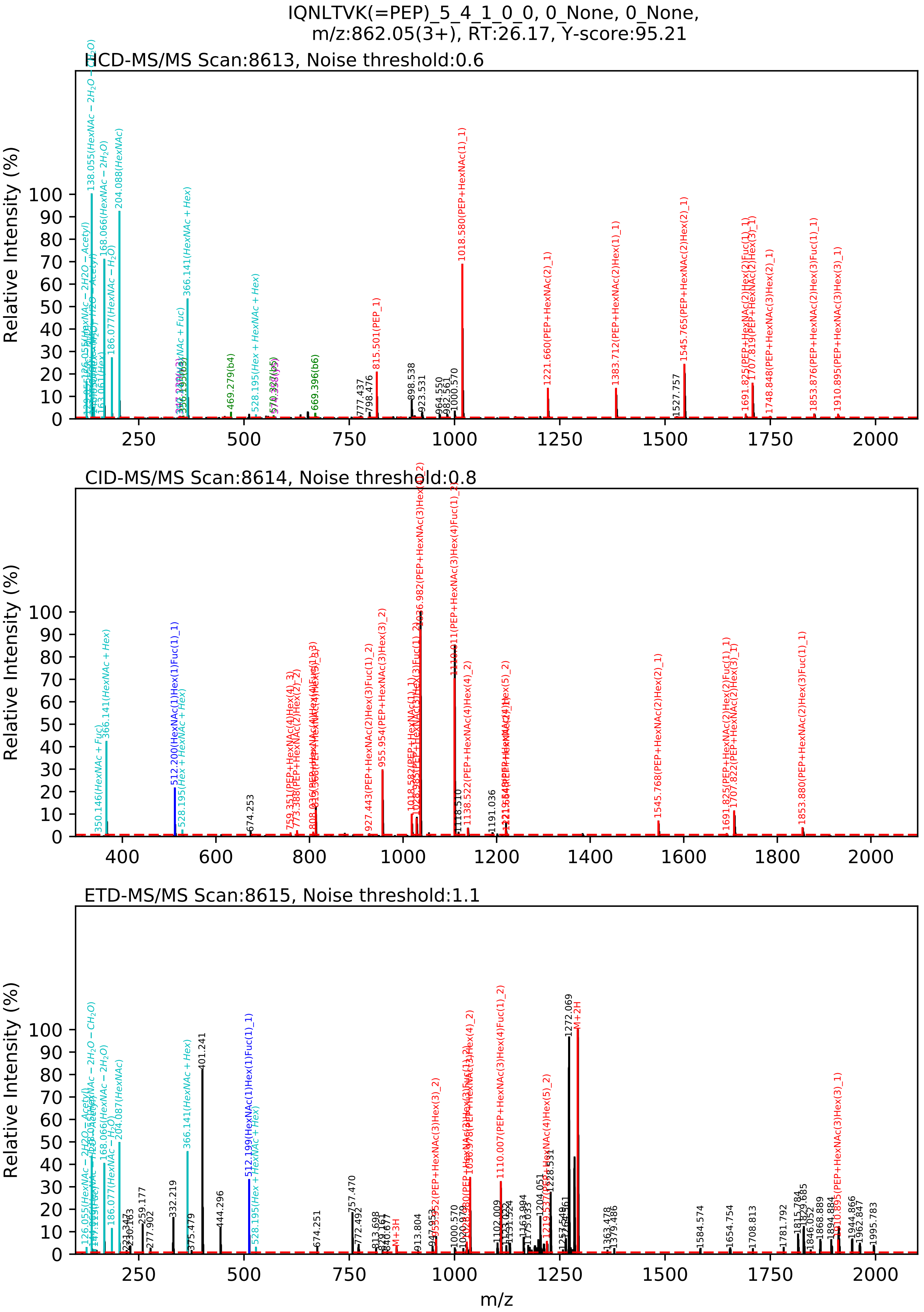
IQNLTVK(=PEP)_5_4_1_0_0, 0_None, 0_None,
m/z:862.05(3+), RT:26.17, Y-score:95.21
HCD-MS/MS Scan:8613, Noise threshold:0.6
CID-MS/MS Scan:8614, Noise threshold:0.8
ETD-MS/MS Scan:8615, Noise threshold:1.1
Relative Intensity (%)
m/z
1018.580(PEP+HexNAc(1)_1)
1221.660(PEP+HexNAc(2)_1)
1383.712(PEP+HexNAc(2)Hex(1)_1)
1545.765(PEP+HexNAc(2)Hex(2)_1)
1748.848(PEP+HexNAc(3)Hex(2)_1)
1853.876(PEP+HexNAc(2)Hex(3)Fuc(1)_1)
1910.895(PEP+HexNAc(3)Hex(3)_1)
815.501(PEP_1)
204.088(HexNAc)
366.141(HexNAc+Hex)
1110.011(PEP+HexNAc(3)Hex(4)Fuc(1)_2)
955.954(PEP+HexNAc(3)Hex(3)_2)
1545.768(PEP+HexNAc(2)Hex(2)_1)
1853.880(PEP+HexNAc(2)Hex(3)Fuc(1)_1)
512.200(HexNAc(1)Hex(1)Fuc(1)_1)
1272.069
M+2H
401.241
1110.007(PEP+HexNAc(3)Hex(4)Fuc(1)_2)

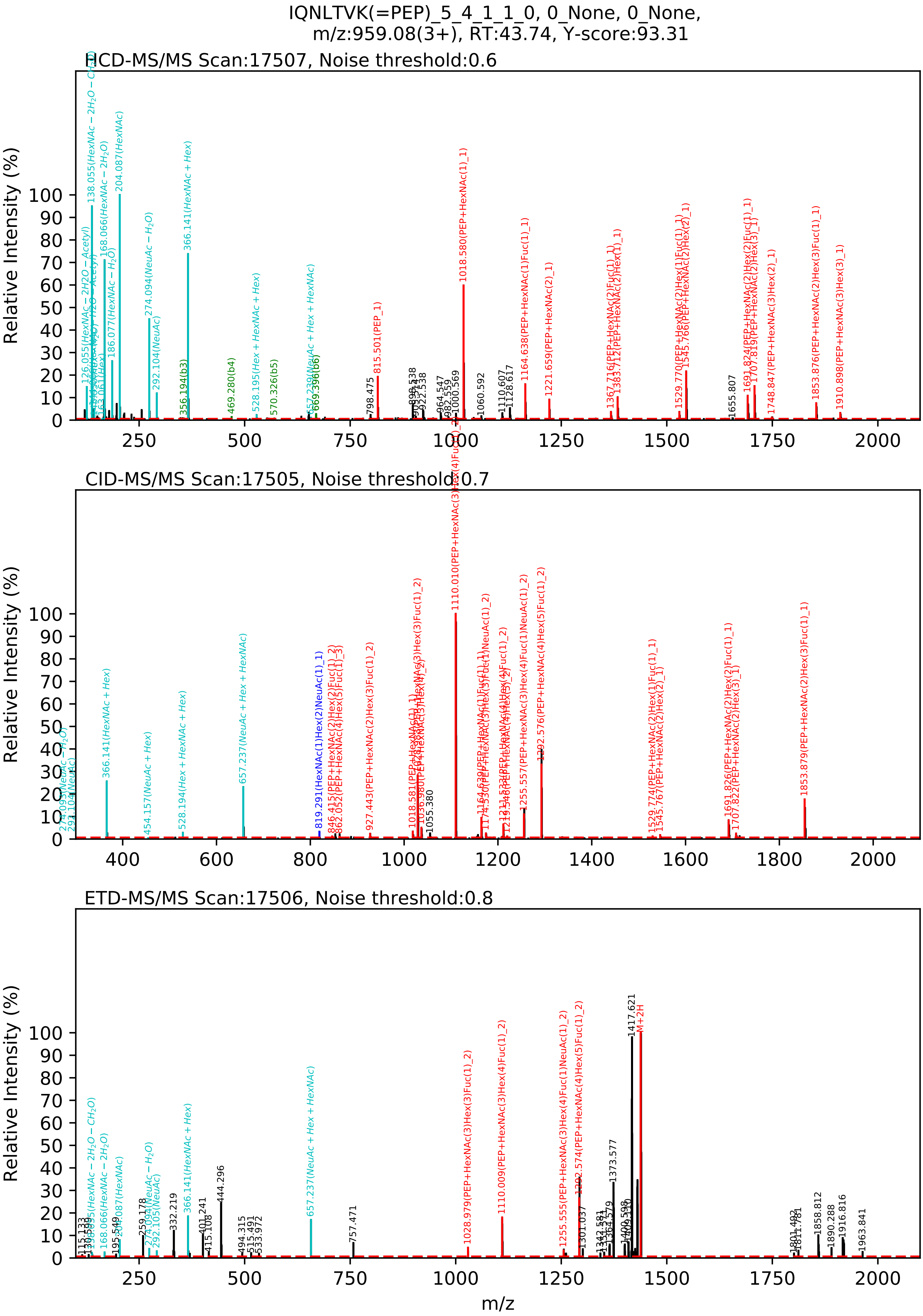
IQNLTVK(=PEP)_5_4_1_1_0, 0_None, 0_None,
m/z:959.08(3+), RT:43.74, Y-score:93.31
HCD-MS/MS Scan:17507, Noise threshold:0.6
CID-MS/MS Scan:17505, Noise threshold:0.7
ETD-MS/MS Scan:17506, Noise threshold:0.8
Relative Intensity (%)
m/z

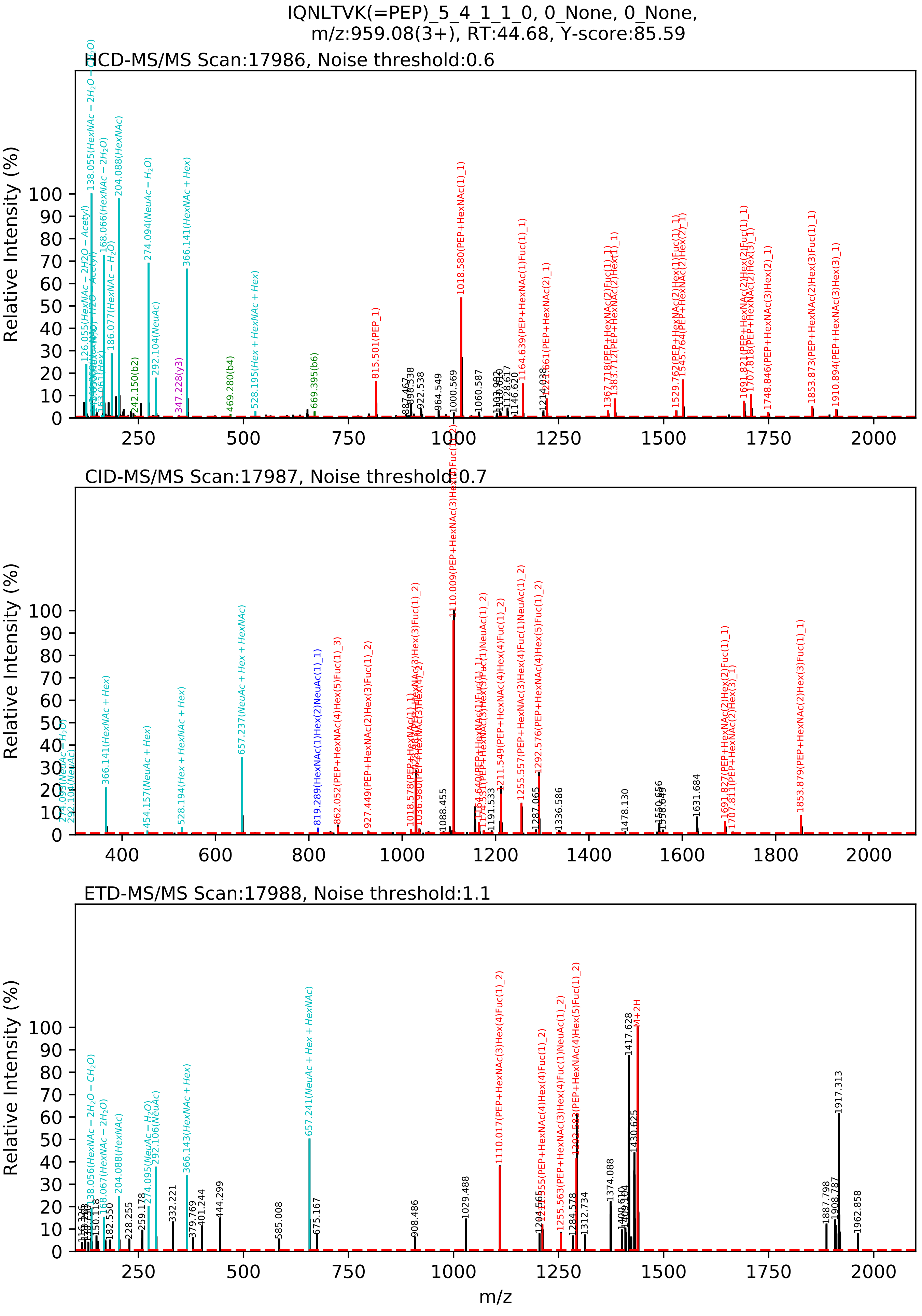
IQNLTVK(=PEP)_5_4_1_1_0, 0_None, 0_None,
m/z:959.08(3+), RT:44.68, Y-score:85.59
HCD-MS/MS Scan:17986, Noise threshold:0.6
CID-MS/MS Scan:17987, Noise threshold:0.7
ETD-MS/MS Scan:17988, Noise threshold:1.1
Relative Intensity (%)
m/z
1018.580(PEP+HexNAc(1)_1)
815.501(PEP_1)
1164.639(PEP+HexNAc(1)Fuc(1)_1)
1221.661(PEP+HexNAc(2)_1)
1853.873(PEP+HexNAc(2)Hex(3)Fuc(1)_1)
1910.894(PEP+HexNAc(3)Hex(3)_1)
1748.846(PEP+HexNAc(3)Hex(2)_1)
366.141(HexNAc + Hex)
274.094(NeuAc − H2O)
292.104(NeuAc)
204.088(HexNAc)
657.237(NeuAc + Hex + HexNAc)
819.289(HexNAc(1)Hex(2)NeuAc(1)_1)
862.052(PEP+HexNAc(4)Hex(5)Fuc(1)_3)
927.449(PEP+HexNAc(2)Hex(3)Fuc(1)_2)
1211.549(PEP+HexNAc(4)Hex(4)Fuc(1)_2)
1255.557(PEP+HexNAc(3)Hex(4)Fuc(1)NeuAc(1)_2)
1292.576(PEP+HexNAc(4)Hex(5)Fuc(1)_2)
1110.017(PEP+HexNAc(3)Hex(4)Fuc(1)_2)
1417.628
1430.625
1917.313
M+2H

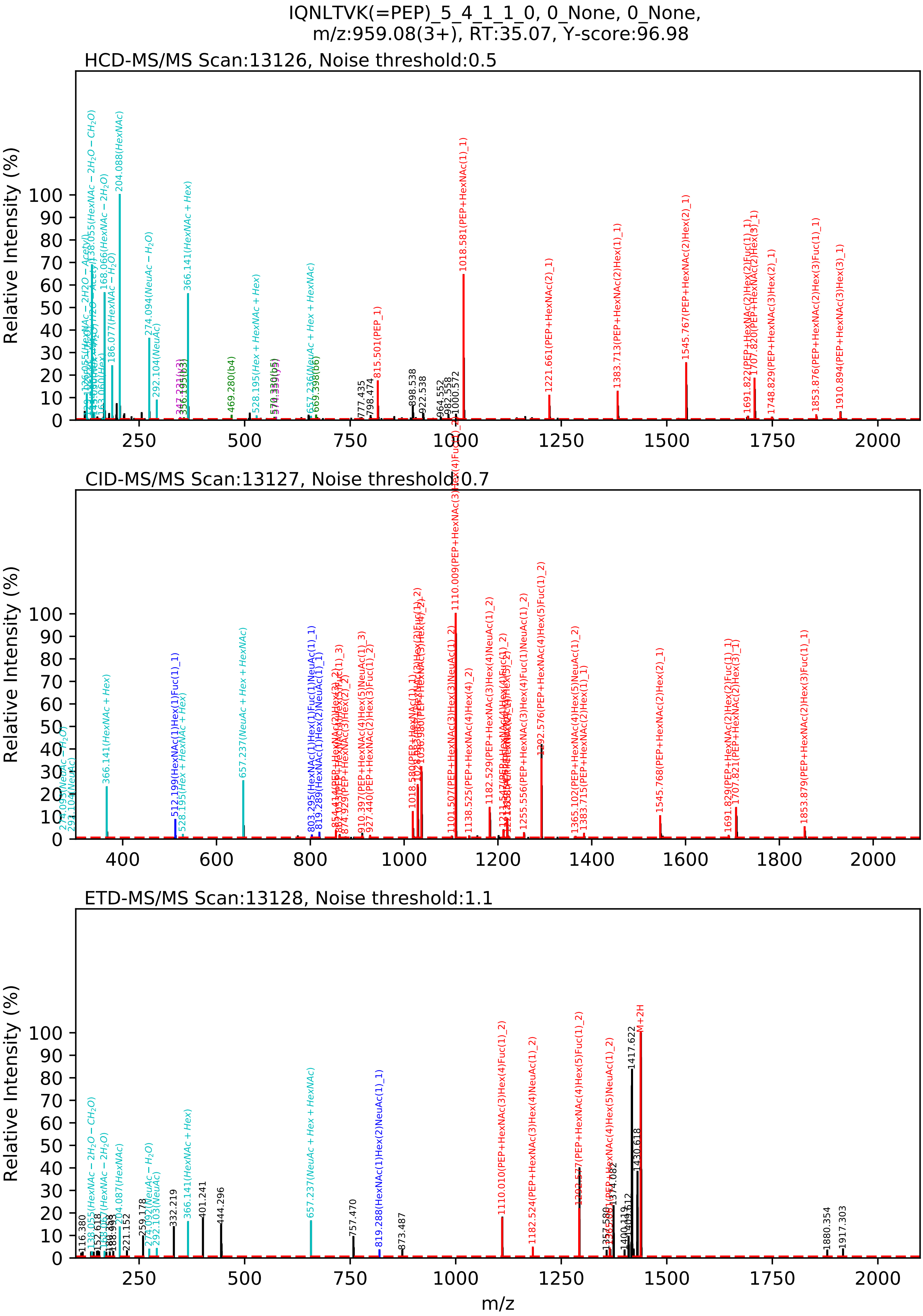

IQNLTVK(=PEP)_5_4_1_1_0, 0_None, 0_None,
m/z:959.08(3+), RT:35.07, Y-score:96.98
HCD-MS/MS Scan:13126, Noise threshold:0.5
CID-MS/MS Scan:13127, Noise threshold:0.7
ETD-MS/MS Scan:13128, Noise threshold:1.1
Relative Intensity (%)
m/z
204.088(HexNAc)
366.141(HexNAc+Hex)
274.094(NeuAc−H2O)
292.104(NeuAc)
186.077(HexNAc−H2O)
168.066(HexNAc−2H2O)
469.280(b4)
528.195(Hex+HexNAc+Hex)
657.236(NeuAc+Hex+HexNAc)
669.398(b6)
777.435
798.474
815.501(PEP_1)
898.538
922.538
964.552
1000.572
1018.581(PEP+HexNAc(1)_1)
1221.661(PEP+HexNAc(2)_1)
1383.713(PEP+HexNAc(2)Hex(1)_1)
1545.767(PEP+HexNAc(2)Hex(2)_1)
1691.822(PEP+HexNAc(2)Hex(2)Fuc(1)_1)
1707.820(PEP+HexNAc(2)Hex(3)_1)
1748.829(PEP+HexNAc(3)Hex(2)_1)
1853.876(PEP+HexNAc(2)Hex(3)Fuc(1)_1)
1910.894(PEP+HexNAc(3)Hex(3)_1)
366.141(HexNAc+Hex)
512.199(HexNAc(1)Hex(1)Fuc(1)_1)
528.195(Hex+HexNAc+Hex)
657.237(NeuAc+Hex+HexNAc)
803.295(HexNAc(1)Hex(1)Fuc(1)NeuAc(1)_1)
819.289(HexNAc(1)Hex(2)NeuAc(1)_1)
910.397(PEP+HexNAc(4)Hex(5)NeuAc(1)_3)
927.440(PEP+HexNAc(2)Hex(3)Fuc(1)_2)
1110.009(PEP+HexNAc(3)Hex(4)Fuc(1)_2)
1101.507(PEP+HexNAc(3)Hex(3)NeuAc(1)_2)
1138.525(PEP+HexNAc(4)Hex(4)_2)
1182.529(PEP+HexNAc(3)Hex(4)NeuAc(1)_2)
1255.556(PEP+HexNAc(3)Hex(4)Fuc(1)NeuAc(1)_2)
1292.576(PEP+HexNAc(4)Hex(5)Fuc(1)_2)
1365.102(PEP+HexNAc(4)Hex(5)NeuAc(1)_2)
1383.715(PEP+HexNAc(2)Hex(1)_1)
1545.768(PEP+HexNAc(2)Hex(2)_1)
1691.829(PEP+HexNAc(2)Hex(2)Fuc(1)_1)
1707.821(PEP+HexNAc(2)Hex(3)_1)
1853.879(PEP+HexNAc(2)Hex(3)Fuc(1)_1)
116.380
221.152
259.178
332.219
401.241
444.296
657.237(NeuAc+Hex+HexNAc)
757.470
819.288(HexNAc(1)Hex(2)NeuAc(1)_1)
873.487
1110.010(PEP+HexNAc(3)Hex(4)Fuc(1)_2)
1182.524(PEP+HexNAc(3)Hex(4)NeuAc(1)_2)
1292.577(PEP+HexNAc(4)Hex(5)Fuc(1)_2)
1374.082
1417.622
1430.618
M+2H
1880.354
1917.303

IQNLTVK(=PEP)_5_4_1_1_0, 0_None, 0_None, m/z:959.08(3+), RT:35.80, Y-score:96.95

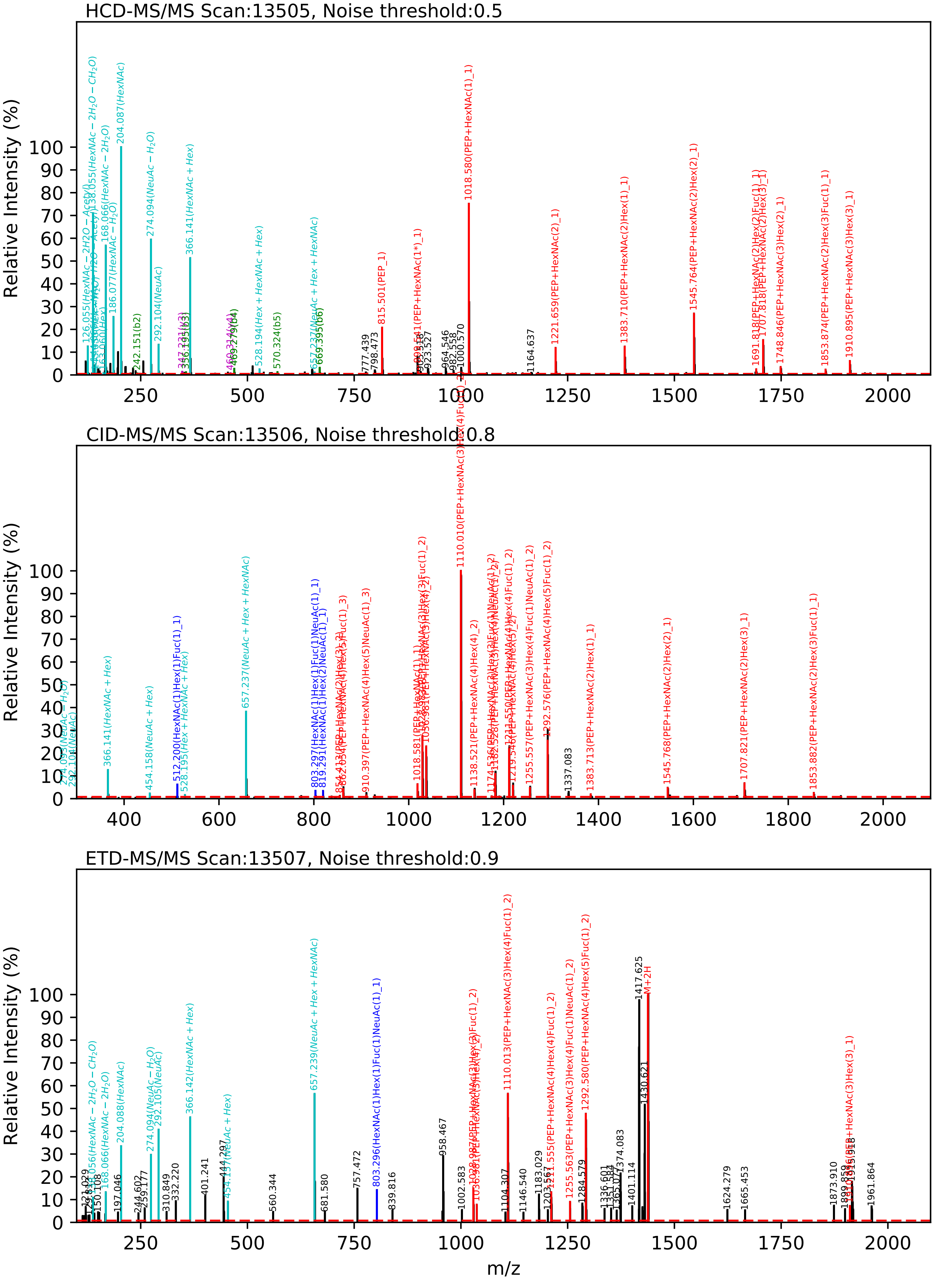

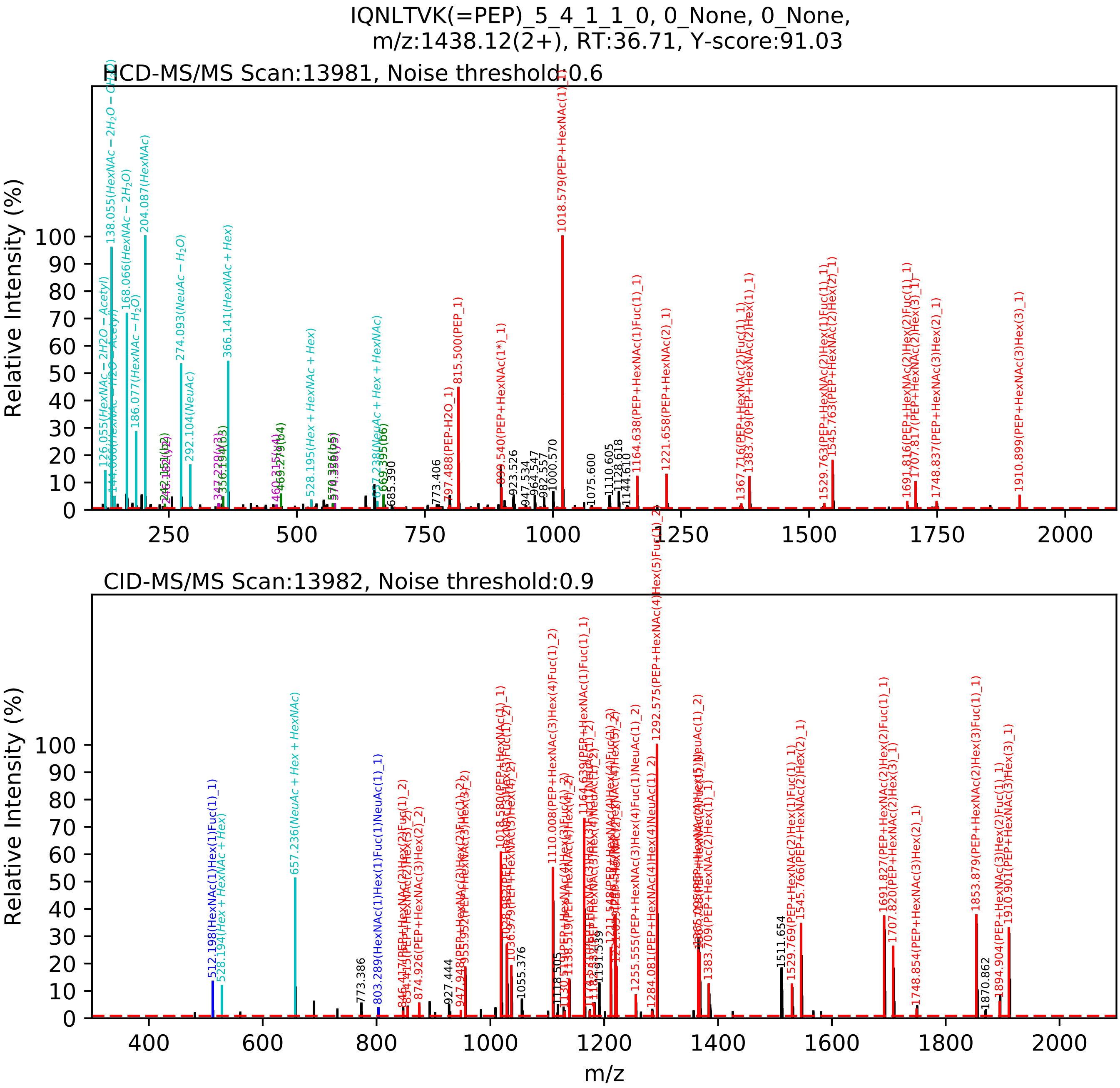

IQNLTVK(=PEP)_5_4_1_1_0, 0_None, 0_None,
m/z:1438.12(2+), RT:36.71, Y-score:91.03
HCD-MS/MS Scan:13981, Noise threshold:0.6
CID-MS/MS Scan:13982, Noise threshold:0.9
Relative Intensity (%)
m/z
1018.579(PEP+HexNAc(1)_1)
815.500(PEP_1)
1292.575(PEP+HexNAc(4)Hex(5)Fuc(1)_2)
657.236(NeuAc + Hex + HexNAc)

IQNLTVK(=PEP)_5_4_1_1_0, 0_None, 0_None,
m/z:1438.12(2+), RT:36.94, Y-score:95.24

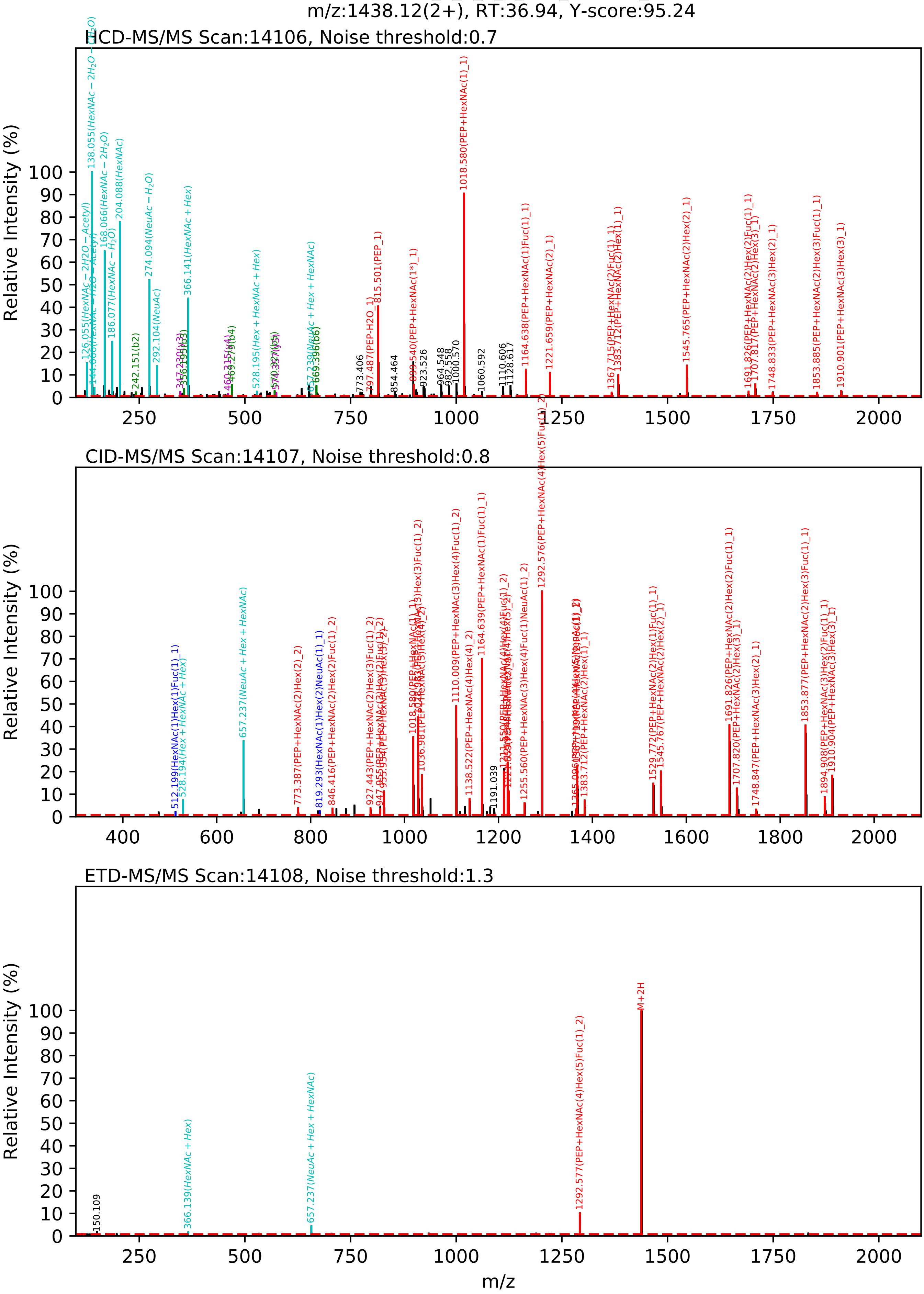

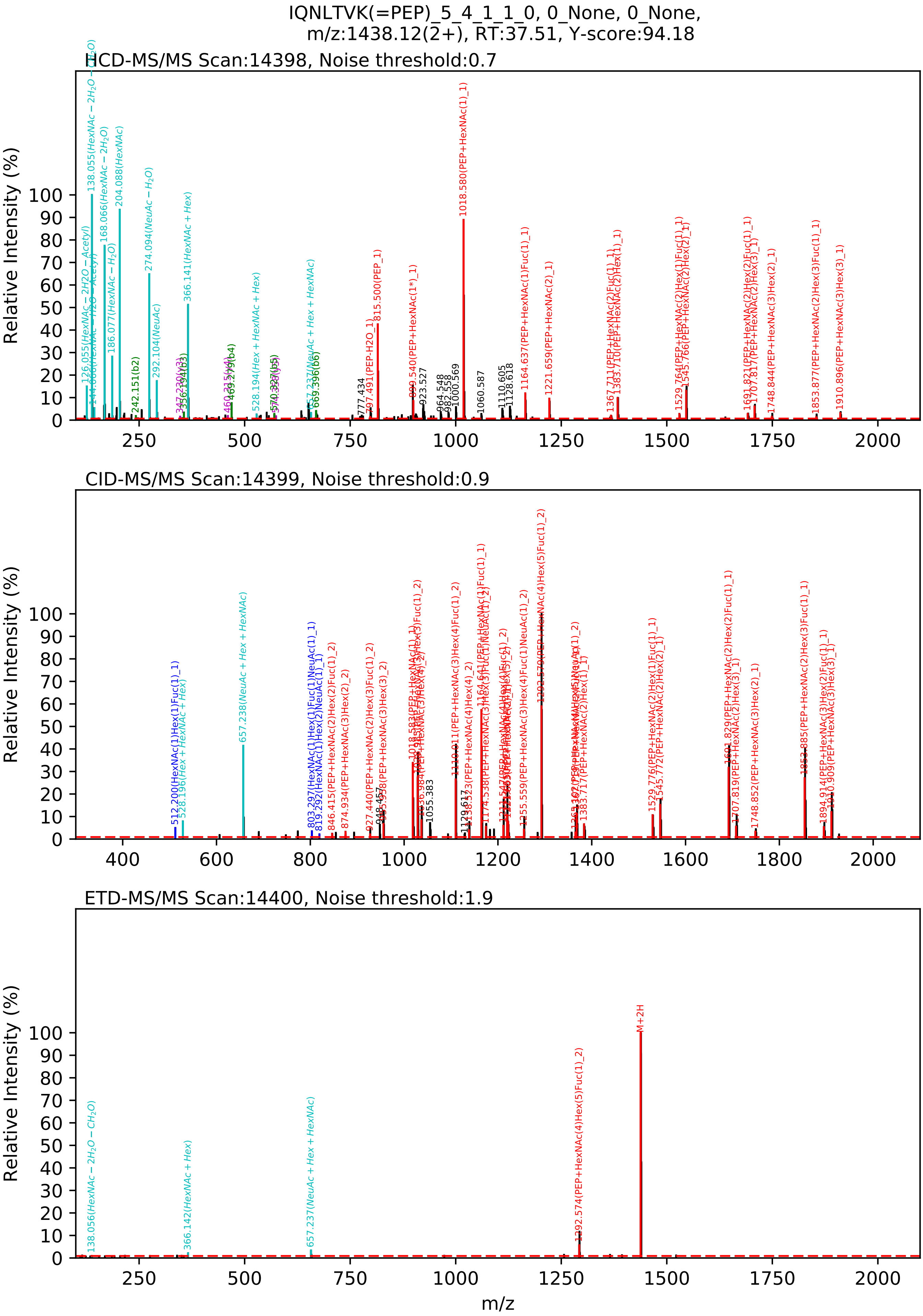

IQNLTVK(=PEP)_5_4_1_1_0, 0_None, 0_None,
m/z:1438.12(2+), RT:37.51, Y-score:94.18
HCD-MS/MS Scan:14398, Noise threshold:0.7
CID-MS/MS Scan:14399, Noise threshold:0.9
ETD-MS/MS Scan:14400, Noise threshold:1.9
Relative Intensity (%)
m/z
1018.580(PEP+HexNAc(1)_1)
815.500(PEP_1)
1292.574(PEP+HexNAc(4)Hex(5)Fuc(1)_2)
M+2H

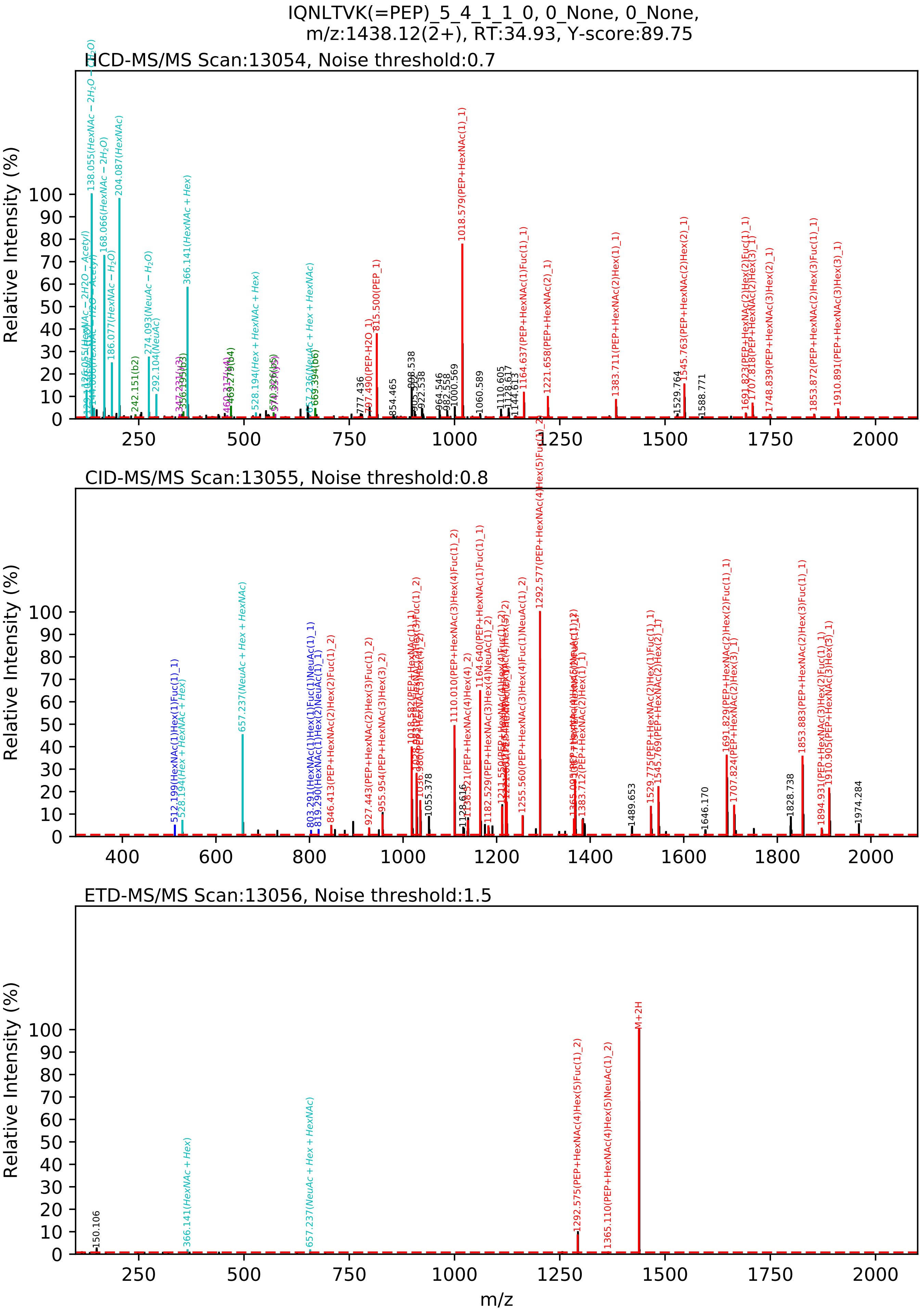

IQNLTVK(=PEP)_5_4_1_1_0, 0_None, 0_None,
m/z:1438.12(2+), RT:34.93, Y-score:89.75
HCD-MS/MS Scan:13054, Noise threshold:0.7
CID-MS/MS Scan:13055, Noise threshold:0.8
ETD-MS/MS Scan:13056, Noise threshold:1.5
Relative Intensity (%)
m/z
138.055(HexNAc − 2H2O)
168.066(HexNAc − 2H2O)
186.077(HexNAc − H2O)
204.087(HexNAc)
242.151(b2)
274.093(NeuAc − H2O)
292.104(NeuAc)
366.141(HexNAc + Hex)
528.194(Hex + HexNAc + Hex)
657.236(NeuAc + Hex + HexNAc)
815.500(PEP_1)
1018.579(PEP+HexNAc(1)_1)
1164.637(PEP+HexNAc(1)Fuc(1)_1)
1221.658(PEP+HexNAc(2)_1)
1383.711(PEP+HexNAc(2)Hex(1)_1)
1545.763(PEP+HexNAc(2)Hex(2)_1)
1748.839(PEP+HexNAc(3)Hex(2)_1)
1853.872(PEP+HexNAc(2)Hex(3)Fuc(1)_1)
1910.891(PEP+HexNAc(3)Hex(3)_1)
512.199(HexNAc(1)Hex(1)Fuc(1)_1)
657.237(NeuAc + Hex + HexNAc)
803.291(HexNAc(1)Hex(1)Fuc(1)NeuAc(1)_1)
819.290(HexNAc(1)Hex(2)NeuAc(1)_1)
846.413(PEP+HexNAc(2)Hex(2)Fuc(1)_2)
927.443(PEP+HexNAc(2)Hex(3)Fuc(1)_2)
955.954(PEP+HexNAc(3)Hex(3)_2)
1110.010(PEP+HexNAc(3)Hex(4)Fuc(1)_2)
1164.640(PEP+HexNAc(1)Fuc(1)_1)
1255.560(PEP+HexNAc(3)Hex(4)Fuc(1)NeuAc(1)_2)
1292.577(PEP+HexNAc(4)Hex(5)Fuc(1)_2)
1691.829(PEP+HexNAc(2)Hex(2)Fuc(1)_1)
1707.824(PEP+HexNAc(2)Hex(3)_1)
1853.883(PEP+HexNAc(2)Hex(3)Fuc(1)_1)
1910.905(PEP+HexNAc(3)Hex(3)_1)
1292.575(PEP+HexNAc(4)Hex(5)Fuc(1)_2)
1365.110(PEP+HexNAc(4)Hex(5)NeuAc(1)_2)
M+2H

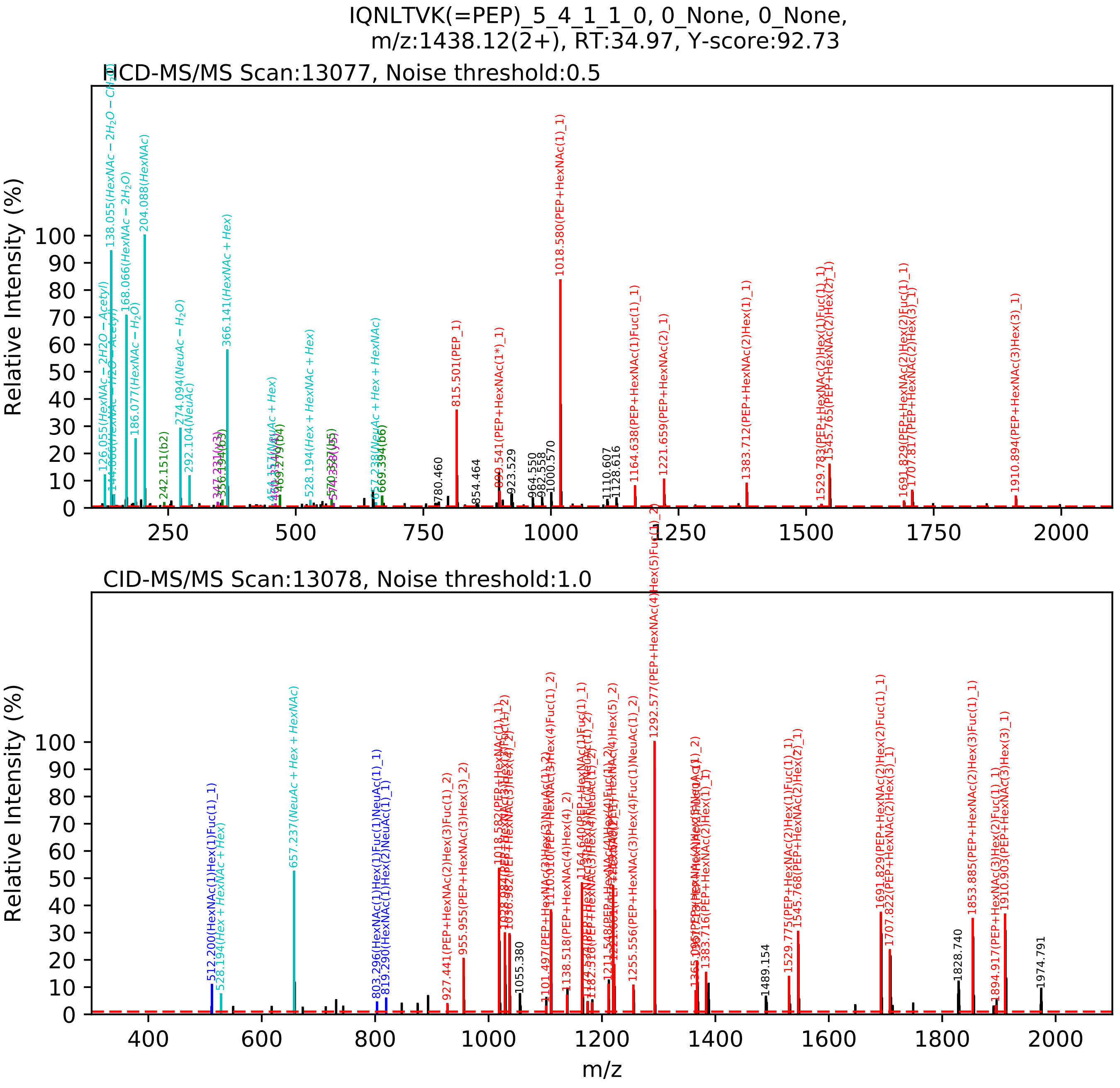
IQNLTVK(=PEP)_5_4_1_1_0, 0_None, 0_None,
m/z:1438.12(2+), RT:34.97, Y-score:92.73
HCD-MS/MS Scan:13077, Noise threshold:0.5
CID-MS/MS Scan:13078, Noise threshold:1.0
Relative Intensity (%)
m/z

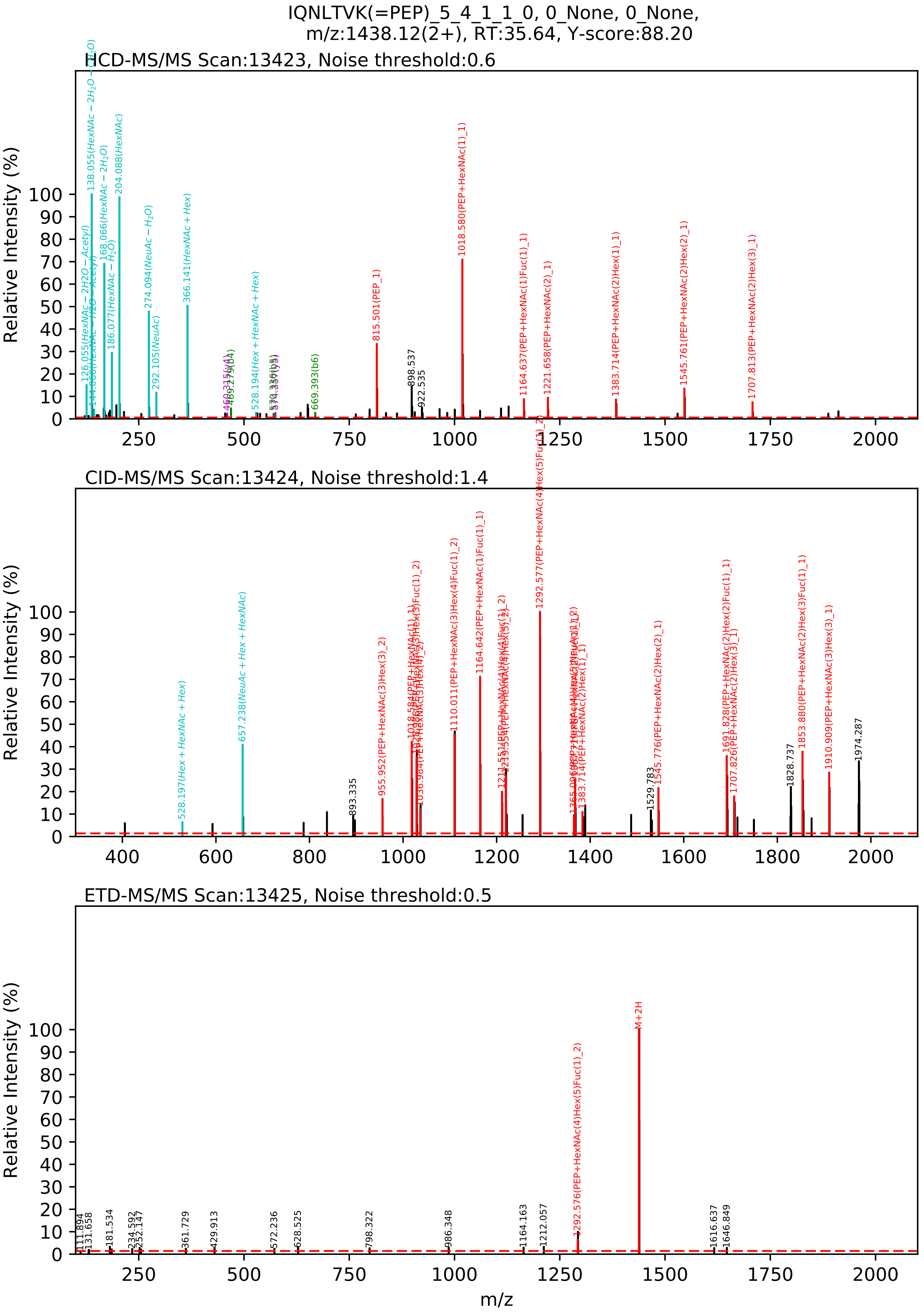
IQNLTVK(=PEP)_5_4_1_1_0, 0_None, 0_None,
m/z:1438.12(2+), RT:35.64, Y-score:88.20
HCD-MS/MS Scan:13423, Noise threshold:0.6
Relative Intensity (%)
138.055(HexNAc − 2H2O − H2O)
204.088(HexNAc)
168.066(HexNAc − 2H2O)
186.077(HexNAc − H2O)
126.055(HexNAc − 2H2O − Acetyl)
274.094(NeuAc − H2O)
292.105(NeuAc)
366.141(HexNAc + Hex)
469.275(b4)
528.194(Hex + HexNAc + Hex)
570.336(b5)
669.393(b6)
815.501(PEP_1)
898.537
922.535
1018.580(PEP+HexNAc(1)_1)
1164.637(PEP+HexNAc(1)Fuc(1)_1)
1221.658(PEP+HexNAc(2)_1)
1383.714(PEP+HexNAc(2)Hex(1)_1)
1545.761(PEP+HexNAc(2)Hex(2)_1)
1707.813(PEP+HexNAc(2)Hex(3)_1)
CID-MS/MS Scan:13424, Noise threshold:1.4
528.197(Hex + HexNAc + Hex)
657.238(NeuAc + Hex + HexNAc)
893.335
955.952(PEP+HexNAc(3)Hex(3)_2)
1110.011(PEP+HexNAc(3)Hex(4)Fuc(1)_2)
1164.642(PEP+HexNAc(1)Fuc(1)_1)
1292.577(PEP+HexNAc(4)Hex(5)Fuc(1)_2)
1383.714(PEP+HexNAc(2)Hex(1)_1)
1529.783
1545.776(PEP+HexNAc(2)Hex(2)_1)
1691.828(PEP+HexNAc(2)Hex(2)Fuc(1)_1)
1707.826(PEP+HexNAc(2)Hex(3)_1)
1828.737
1853.880(PEP+HexNAc(2)Hex(3)Fuc(1)_1)
1910.909(PEP+HexNAc(3)Hex(3)_1)
1974.287
ETD-MS/MS Scan:13425, Noise threshold:0.5
M+2H
1292.576(PEP+HexNAc(4)Hex(5)Fuc(1)_2)
111.894
131.658
181.534
234.592
252.147
361.729
429.913
572.236
628.525
798.322
986.348
1164.163
1212.057
1616.637
1646.849
m/z

IQNLTVK(=PEP)_5_4_1_1_0, 0_None, 0_None,
m/z:1438.12(2+), RT:35.80, Y-score:92.45

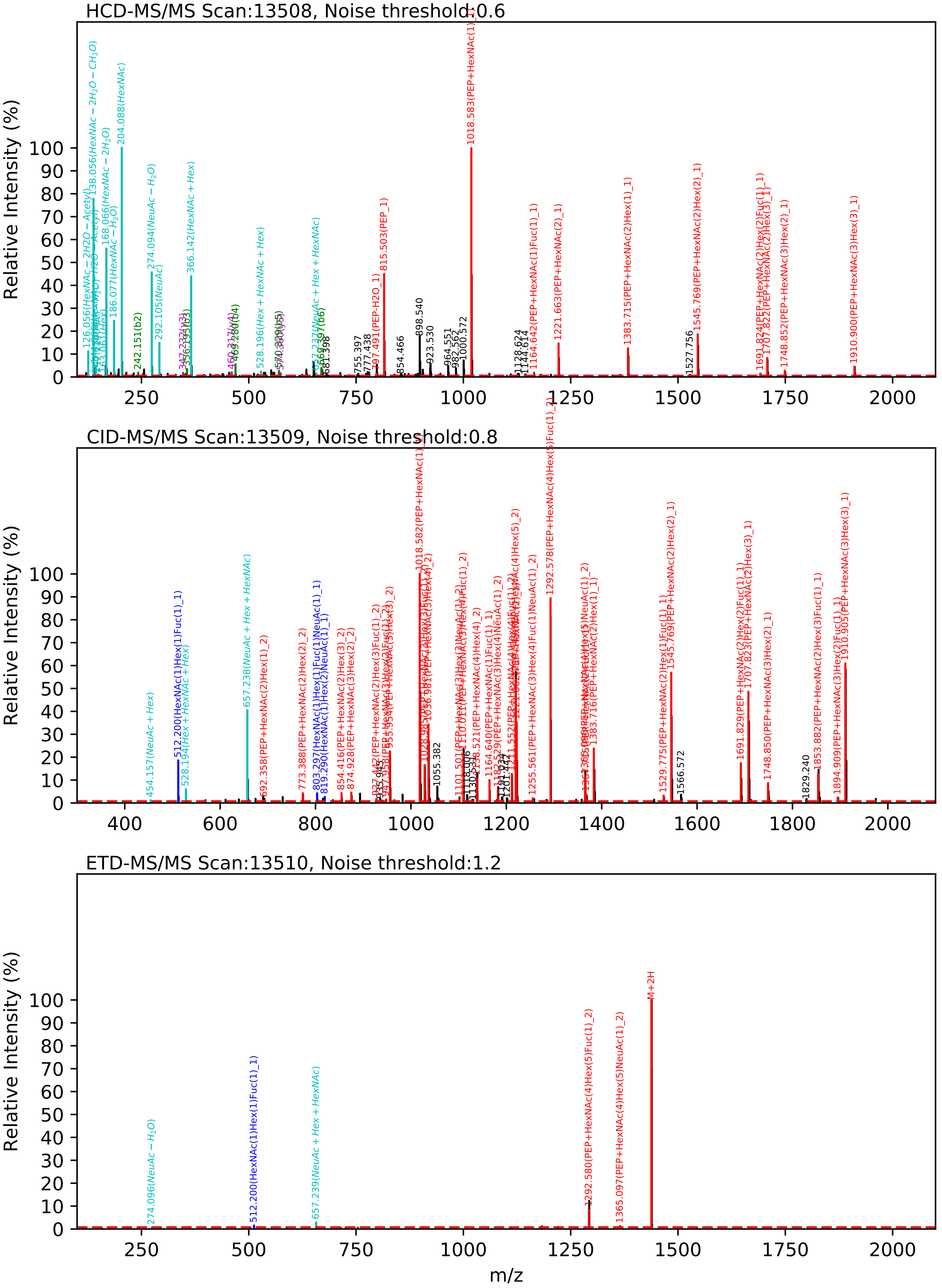

IQNLTVK(=PEP)_5_4_1_1_0, 0_None, 0_None,
m/z:1438.12(2+), RT:36.37, Y-score:93.63

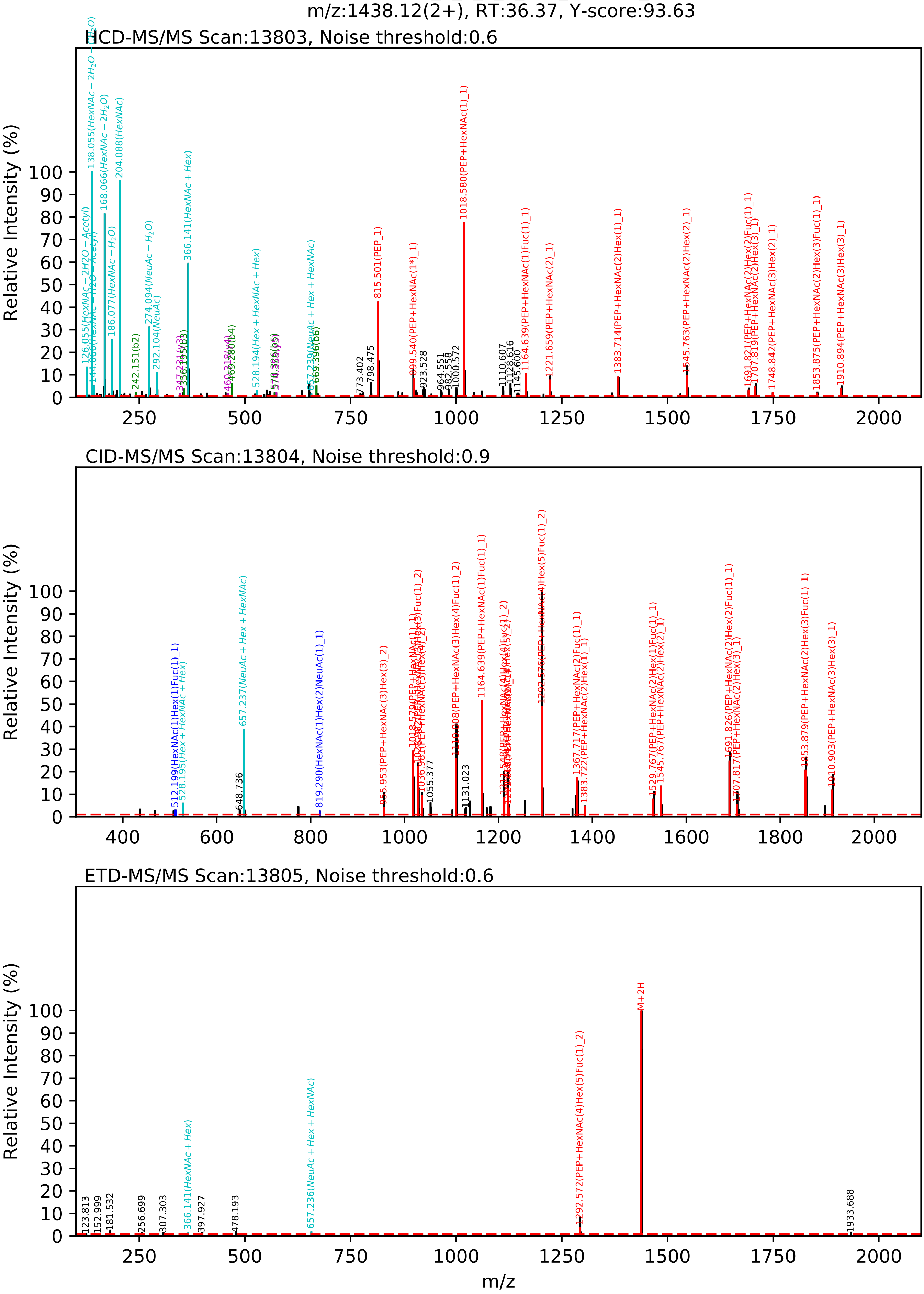

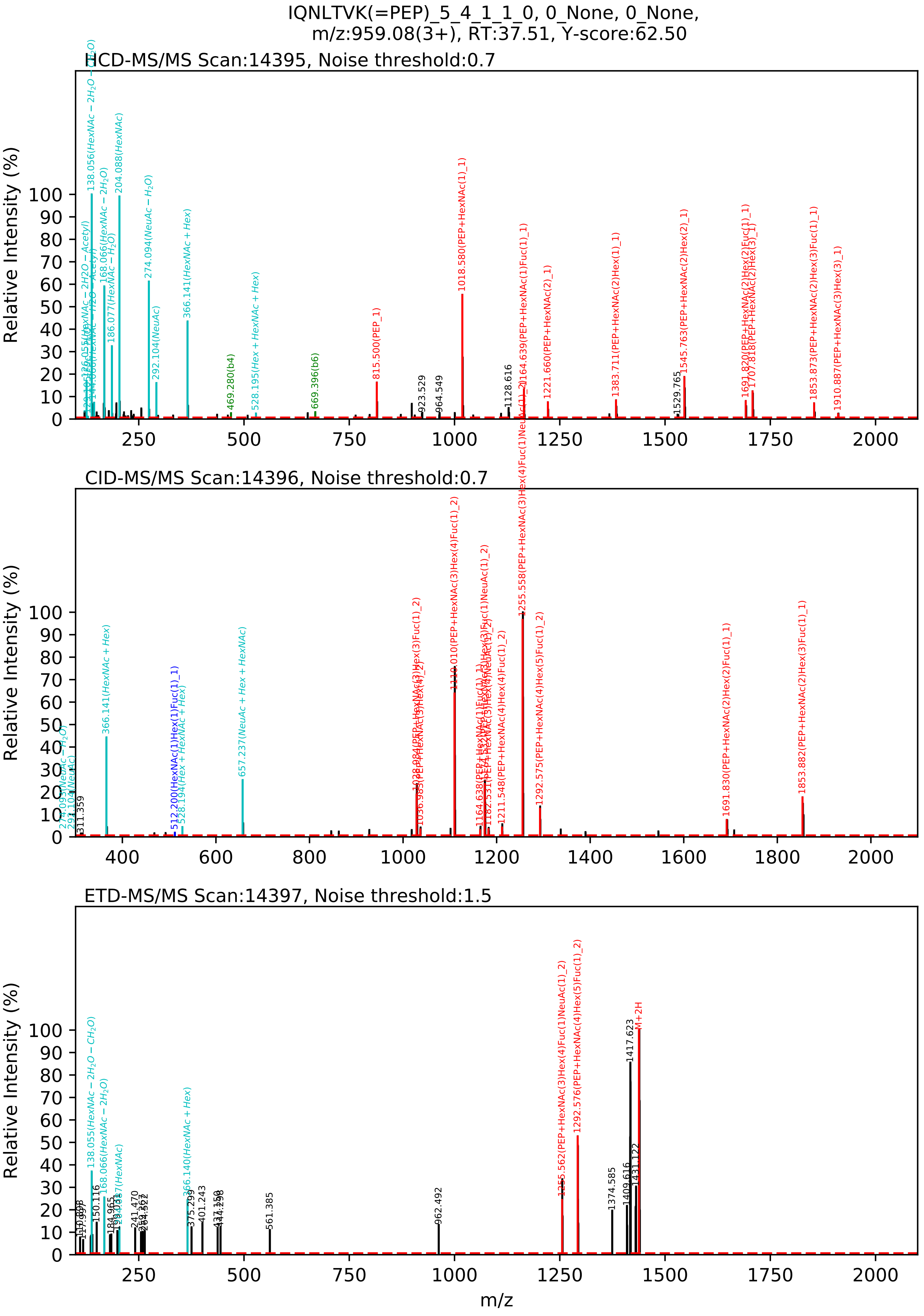
IQNLTVK(=PEP)_5_4_1_1_0, 0_None, 0_None,
m/z:959.08(3+), RT:37.51, Y-score:62.50
HCD-MS/MS Scan:14395, Noise threshold:0.7
Relative Intensity (%)
138.056(HexNAc − 2H2O − CH2O)
204.088(HexNAc)
168.066(HexNAc − 2H2O)
186.077(HexNAc − H2O)
274.094(NeuAc − H2O)
292.104(NeuAc)
366.141(HexNAc + Hex)
469.280(b4)
528.195(Hex + HexNAc + Hex)
669.396(b6)
815.500(PEP_1)
923.529
964.549
1018.580(PEP+HexNAc(1)_1)
1128.616
1164.639(PEP+HexNAc(1)Fuc(1)_1)
1221.660(PEP+HexNAc(2)_1)
1383.711(PEP+HexNAc(2)Hex(1)_1)
1529.765
1545.763(PEP+HexNAc(2)Hex(2)_1)
1691.820(PEP+HexNAc(2)Hex(2)Fuc(1)_1)
1707.818(PEP+HexNAc(2)Hex(3)_1)
1853.873(PEP+HexNAc(2)Hex(3)Fuc(1)_1)
1910.887(PEP+HexNAc(3)Hex(3)_1)
CID-MS/MS Scan:14396, Noise threshold:0.7
311.359
366.141(HexNAc + Hex)
512.200(HexNAc(1)Hex(1)Fuc(1)_1)
528.194(Hex + HexNAc + Hex)
657.237(NeuAc + Hex + HexNAc)
1028.984(PEP+HexNAc(3)Hex(3)Fuc(1)_2)
1110.010(PEP+HexNAc(3)Hex(4)Fuc(1)_2)
1164.638(PEP+HexNAc(1)Fuc(1)_1)
1211.548(PEP+HexNAc(4)Hex(4)Fuc(1)_2)
1255.558(PEP+HexNAc(3)Hex(4)Fuc(1)NeuAc(1)_2)
1292.575(PEP+HexNAc(4)Hex(5)Fuc(1)_2)
1691.830(PEP+HexNAc(2)Hex(2)Fuc(1)_1)
1853.882(PEP+HexNAc(2)Hex(3)Fuc(1)_1)
ETD-MS/MS Scan:14397, Noise threshold:1.5
138.055(HexNAc − 2H2O − CH2O)
168.066(HexNAc − 2H2O)
150.116
184.965
366.140(HexNAc + Hex)
375.299
401.243
561.385
962.492
1255.562(PEP+HexNAc(3)Hex(4)Fuc(1)NeuAc(1)_2)
1292.576(PEP+HexNAc(4)Hex(5)Fuc(1)_2)
1374.585
1409.616
1417.623
1431.122
M+2H
m/z

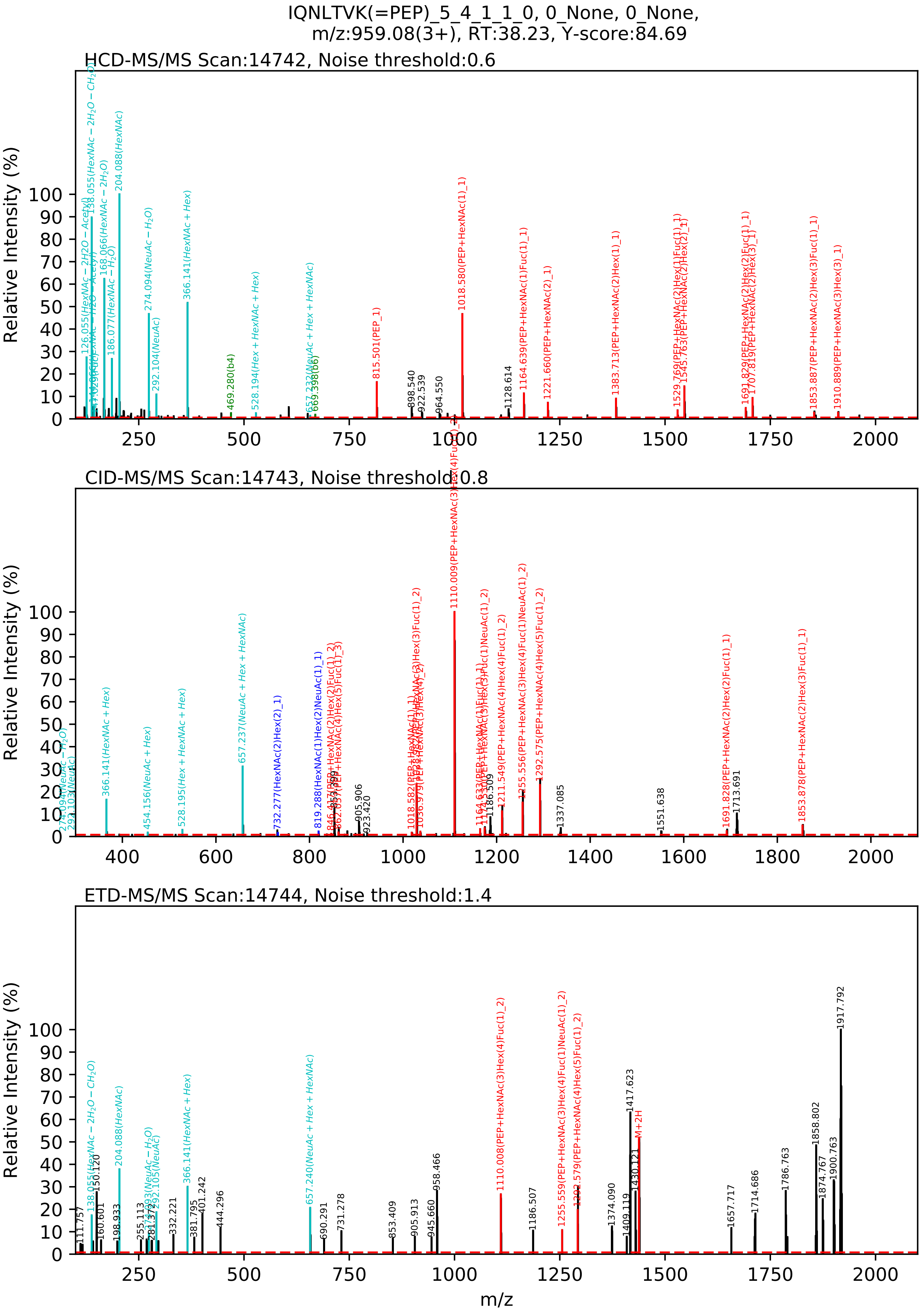
IQNLTVK(=PEP)_5_4_1_1_0, 0_None, 0_None,
m/z:959.08(3+), RT:38.23, Y-score:84.69
HCD-MS/MS Scan:14742, Noise threshold:0.6
Relative Intensity (%)
204.088(HexNAc)
274.094(NeuAc − H2O)
292.104(NeuAc)
366.141(HexNAc + Hex)
469.280(b4)
528.194(Hex + HexNAc + Hex)
657.232(NeuAc + Hex + HexNAc)
669.398(b6)
815.501(PEP_1)
898.540
922.539
964.550
1018.580(PEP+HexNAc(1)_1)
1128.614
1164.639(PEP+HexNAc(1)Fuc(1)_1)
1221.660(PEP+HexNAc(2)_1)
1383.713(PEP+HexNAc(2)Hex(1)_1)
1545.763(PEP+HexNAc(2)Hex(2)_1)
1707.819(PEP+HexNAc(2)Hex(3)_1)
1853.887(PEP+HexNAc(2)Hex(3)Fuc(1)_1)
1910.889(PEP+HexNAc(3)Hex(3)_1)
CID-MS/MS Scan:14743, Noise threshold:0.8
Relative Intensity (%)
366.141(HexNAc + Hex)
454.156(NeuAc + Hex)
528.195(Hex + HexNAc + Hex)
657.237(NeuAc + Hex + HexNAc)
732.277(HexNAc(2)Hex(2)_1)
819.288(HexNAc(1)Hex(2)NeuAc(1)_1)
905.906
923.420
1110.009(PEP+HexNAc(3)Hex(4)Fuc(1)_2)
1186.509
1211.549(PEP+HexNAc(4)Hex(4)Fuc(1)_2)
1255.556(PEP+HexNAc(3)Hex(4)Fuc(1)NeuAc(1)_2)
1292.575(PEP+HexNAc(4)Hex(5)Fuc(1)_2)
1337.085
1551.638
1691.828(PEP+HexNAc(2)Hex(2)Fuc(1)_1)
1713.691
1853.878(PEP+HexNAc(2)Hex(3)Fuc(1)_1)
ETD-MS/MS Scan:14744, Noise threshold:1.4
Relative Intensity (%)
111.757
150.120
160.601
198.933
204.088(HexNAc)
255.113
292.105(NeuAc)
332.221
366.141(HexNAc + Hex)
381.795
401.242
444.296
657.240(NeuAc + Hex + HexNAc)
690.291
731.278
853.409
905.913
945.660
958.466
1110.008(PEP+HexNAc(3)Hex(4)Fuc(1)_2)
1186.507
1255.559(PEP+HexNAc(3)Hex(4)Fuc(1)NeuAc(1)_2)
1292.579(PEP+HexNAc(4)Hex(5)Fuc(1)_2)
1374.090
1409.119
1417.623
1430.121
M+2H
1657.717
1714.686
1786.763
1858.802
1874.767
1900.763
1917.792
m/z

IQNLTVK(=PEP)_5_4_1_1_0, 0_None, 0_None,
m/z:1438.12(2+), RT:49.25, Y-score:92.95

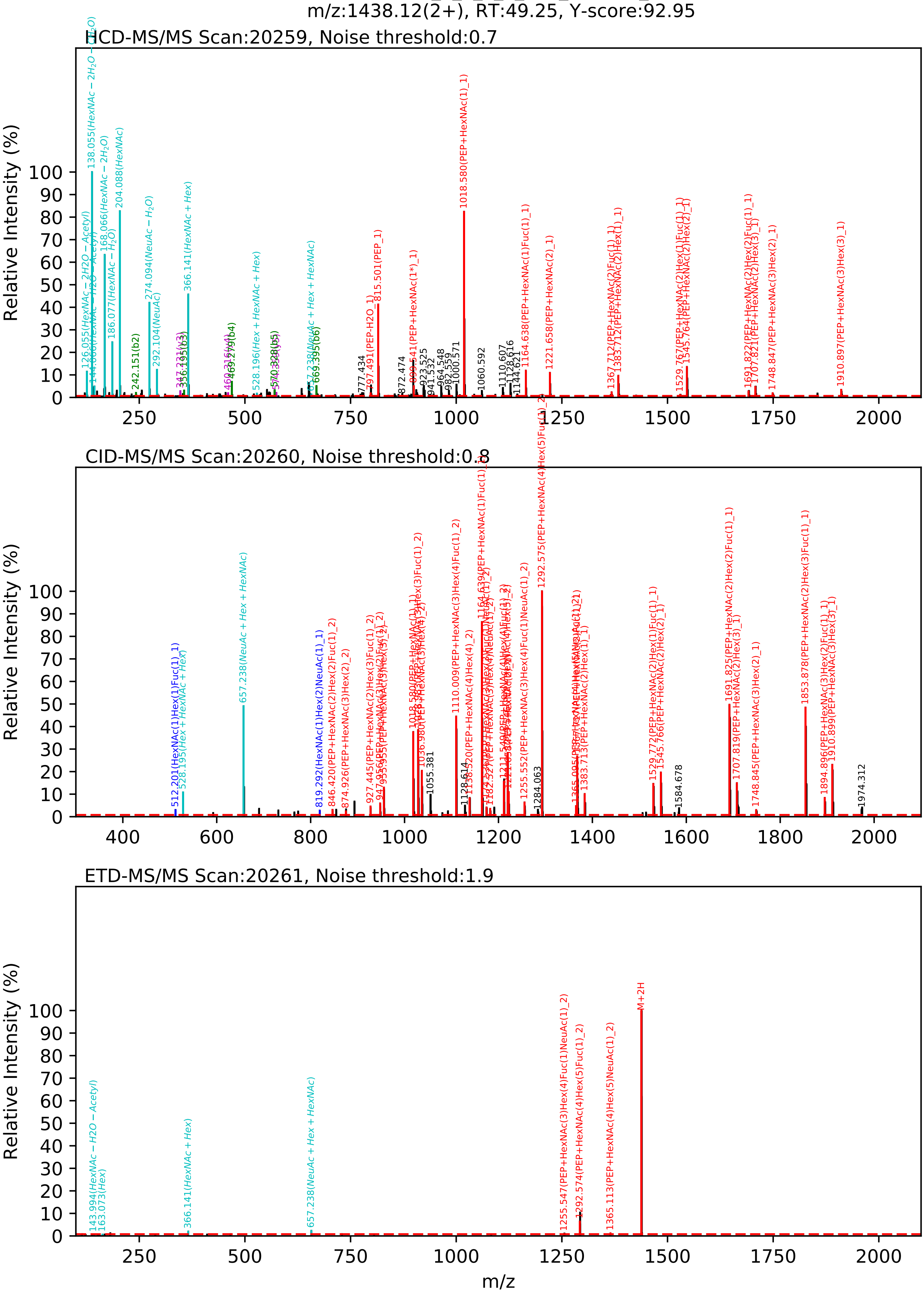

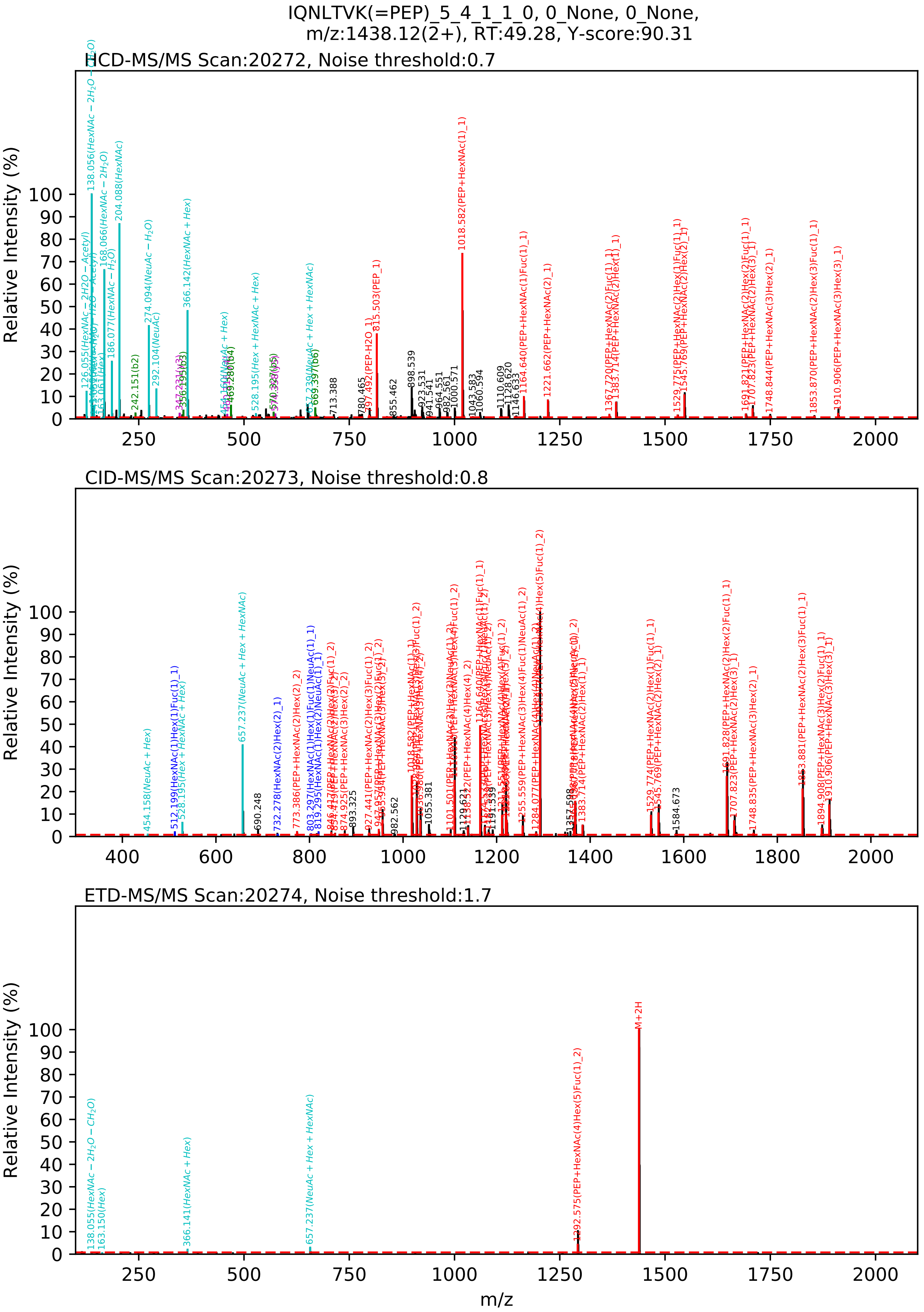

IQNLTVK(=PEP)_5_4_1_1_0, 0_None, 0_None,
m/z:1438.12(2+), RT:49.28, Y-score:90.31
HCD-MS/MS Scan:20272, Noise threshold:0.7
CID-MS/MS Scan:20273, Noise threshold:0.8
ETD-MS/MS Scan:20274, Noise threshold:1.7
Relative Intensity (%)
m/z
1018.582(PEP+HexNAc(1)_1)
815.503(PEP_1)
366.142(HexNAc + Hex)
204.088(HexNAc)
138.056(HexNAc − 2H2O − CH2O)
657.237(NeuAc + Hex + HexNAc)
1292.575(PEP+HexNAc(4)Hex(5)Fuc(1)_2)
M+2H

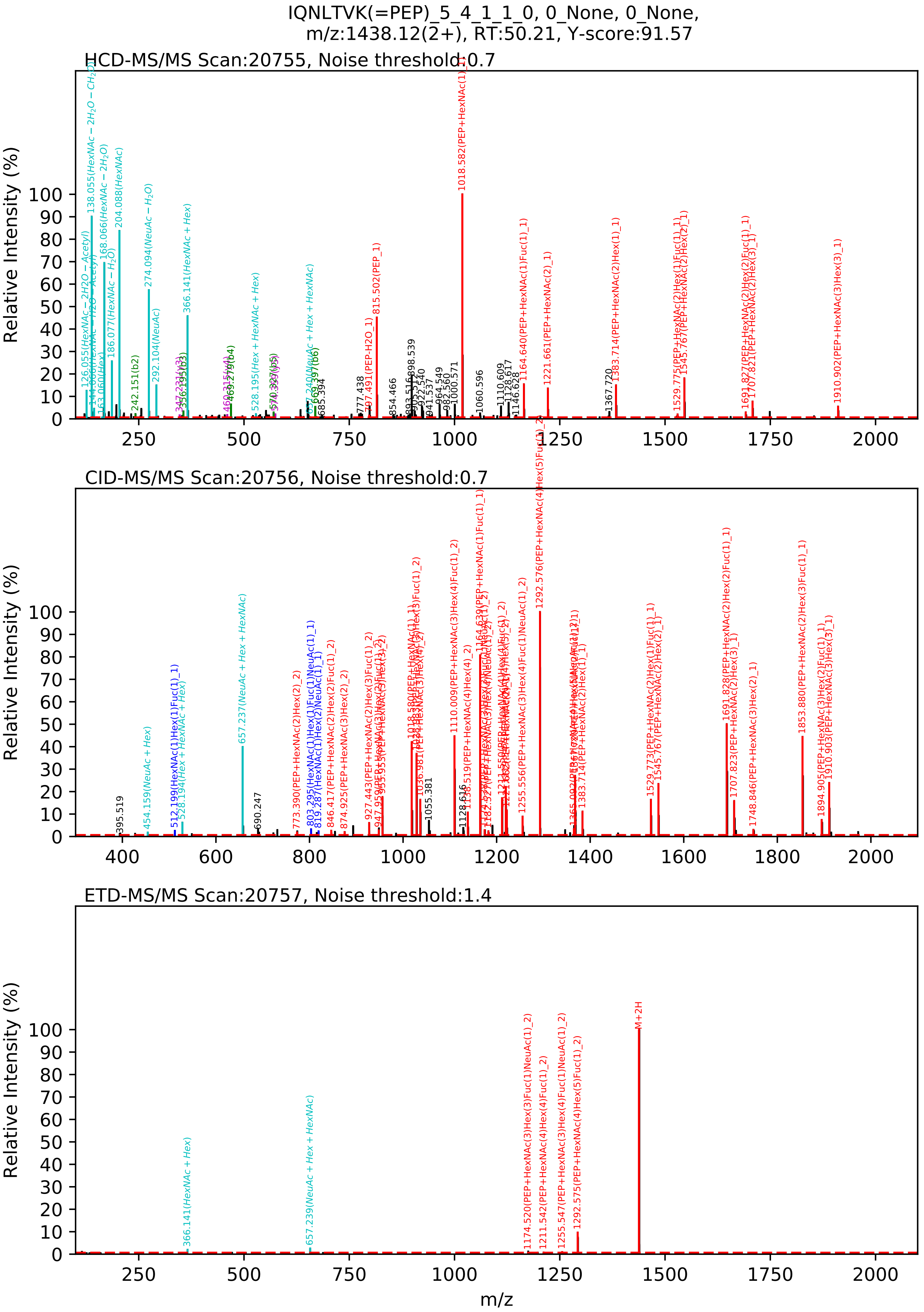
IQNLTVK(=PEP)_5_4_1_1_0, 0_None, 0_None,
m/z:1438.12(2+), RT:50.21, Y-score:91.57
HCD-MS/MS Scan:20755, Noise threshold:0.7
CID-MS/MS Scan:20756, Noise threshold:0.7
ETD-MS/MS Scan:20757, Noise threshold:1.4
Relative Intensity (%)
m/z
1018.582(PEP+HexNAc(1)_1)
815.502(PEP_1)
1164.640(PEP+HexNAc(1)Fuc(1)_1)
1221.661(PEP+HexNAc(2)_1)
1383.714(PEP+HexNAc(2)Hex(1)_1)
1910.902(PEP+HexNAc(3)Hex(3)_1)
204.088(HexNAc)
274.094(NeuAc − H2O)
366.141(HexNAc + Hex)
292.104(NeuAc)
1292.576(PEP+HexNAc(4)Hex(5)Fuc(1)_2)
1110.009(PEP+HexNAc(3)Hex(4)Fuc(1)_2)
1691.828(PEP+HexNAc(2)Hex(2)Fuc(1)_1)
1853.880(PEP+HexNAc(2)Hex(3)Fuc(1)_1)
657.237(NeuAc + Hex + HexNAc)
1255.556(PEP+HexNAc(3)Hex(4)Fuc(1)NeuAc(1)_2)
1748.846(PEP+HexNAc(3)Hex(2)_1)
M+2H
1174.520(PEP+HexNAc(3)Hex(3)Fuc(1)NeuAc(1)_2)
1211.542(PEP+HexNAc(4)Hex(4)Fuc(1)_2)
1255.547(PEP+HexNAc(3)Hex(4)Fuc(1)NeuAc(1)_2)
1292.575(PEP+HexNAc(4)Hex(5)Fuc(1)_2)
366.141(HexNAc + Hex)
657.239(NeuAc + Hex + HexNAc)

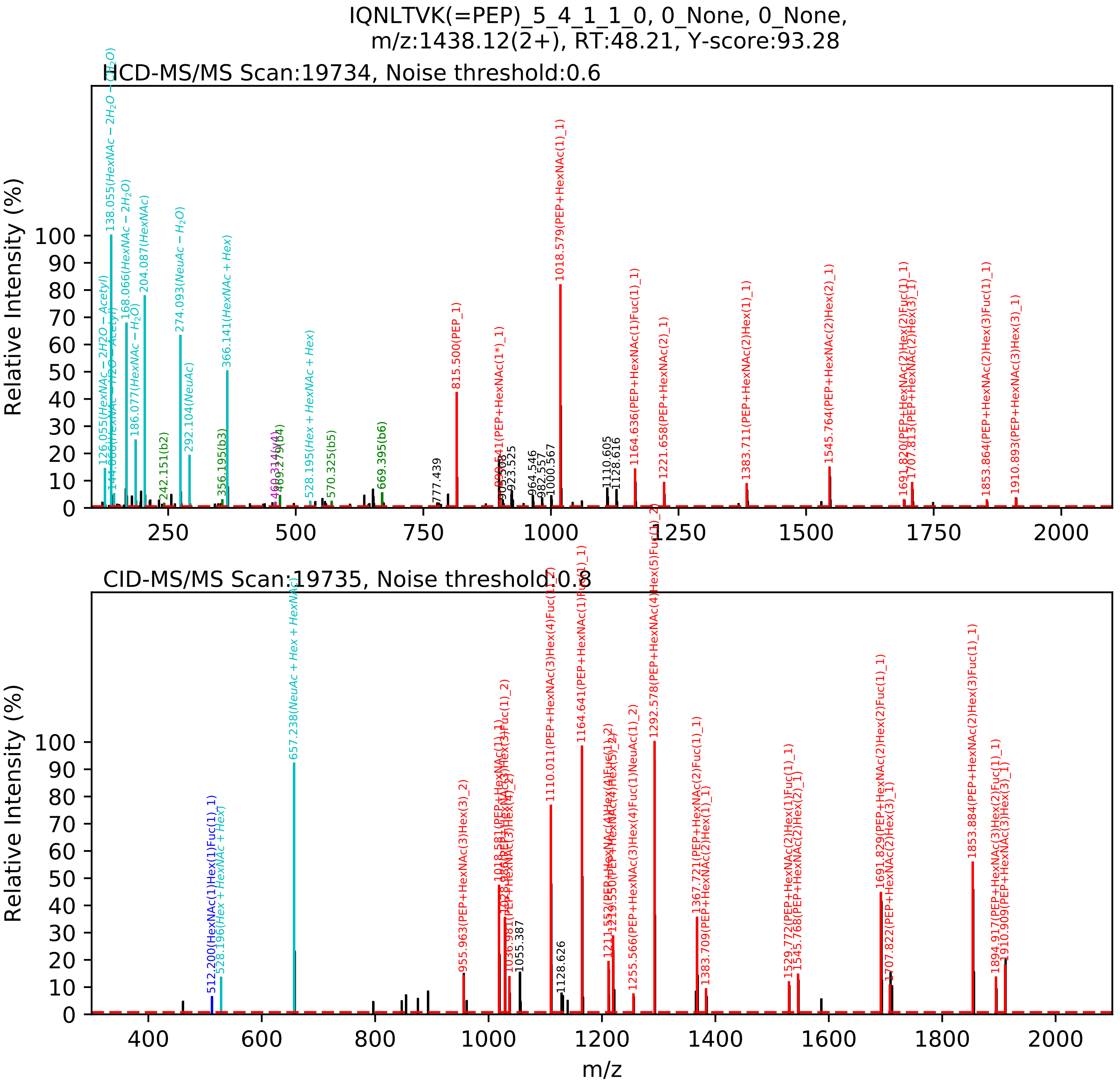

IQNLTVK(=PEP)_5_4_1_1_0, 0_None, 0_None,
m/z:1438.12(2+), RT:48.21, Y-score:93.28
HCD-MS/MS Scan:19734, Noise threshold:0.6
Relative Intensity (%)
138.055(HexNAc − 2H2O − H2O)
126.055(HexNAc − 2H2O − Acetyl)
168.066(HexNAc − 2H2O)
186.077(HexNAc − H2O)
204.087(HexNAc)
274.093(NeuAc − H2O)
292.104(NeuAc)
366.141(HexNAc + Hex)
242.151(b2)
356.195(b3)
469.279(b4)
528.195(Hex + HexNAc + Hex)
570.325(b5)
669.395(b6)
777.439
815.500(PEP_1)
923.525
964.546
1000.567
1018.579(PEP+HexNAc(1)_1)
1110.605
1128.616
1164.636(PEP+HexNAc(1)Fuc(1)_1)
1221.658(PEP+HexNAc(2)_1)
1383.711(PEP+HexNAc(2)Hex(1)_1)
1545.764(PEP+HexNAc(2)Hex(2)_1)
1691.820(PEP+HexNAc(2)Hex(2)Fuc(1)_1)
1707.813(PEP+HexNAc(2)Hex(3)_1)
1853.864(PEP+HexNAc(2)Hex(3)Fuc(1)_1)
1910.893(PEP+HexNAc(3)Hex(3)_1)
CID-MS/MS Scan:19735, Noise threshold:0.8
512.200(HexNAc(1)Hex(1)Fuc(1)_1)
528.196(Hex + HexNAc + Hex)
657.238(NeuAc + Hex + HexNAc)
955.963(PEP+HexNAc(3)Hex(3)_2)
1055.387
1110.011(PEP+HexNAc(3)Hex(4)Fuc(1)_2)
1128.626
1164.641(PEP+HexNAc(1)Fuc(1)_1)
1255.566(PEP+HexNAc(3)Hex(4)Fuc(1)NeuAc(1)_2)
1292.578(PEP+HexNAc(4)Hex(5)Fuc(1)_2)
1367.721(PEP+HexNAc(2)Fuc(1)_1)
1383.709(PEP+HexNAc(2)Hex(1)_1)
1529.772(PEP+HexNAc(2)Hex(1)Fuc(1)_1)
1545.768(PEP+HexNAc(2)Hex(2)_1)
1691.829(PEP+HexNAc(2)Hex(2)Fuc(1)_1)
1707.822(PEP+HexNAc(2)Hex(3)_1)
1853.884(PEP+HexNAc(2)Hex(3)Fuc(1)_1)
1894.917(PEP+HexNAc(3)Hex(2)Fuc(1)_1)
1910.909(PEP+HexNAc(3)Hex(3)_1)
m/z

IQNLTVK(=PEP)_5_4_1_1_0, 0_None, 0_None,
m/z:1438.12(2+), RT:48.63, Y-score:95.90

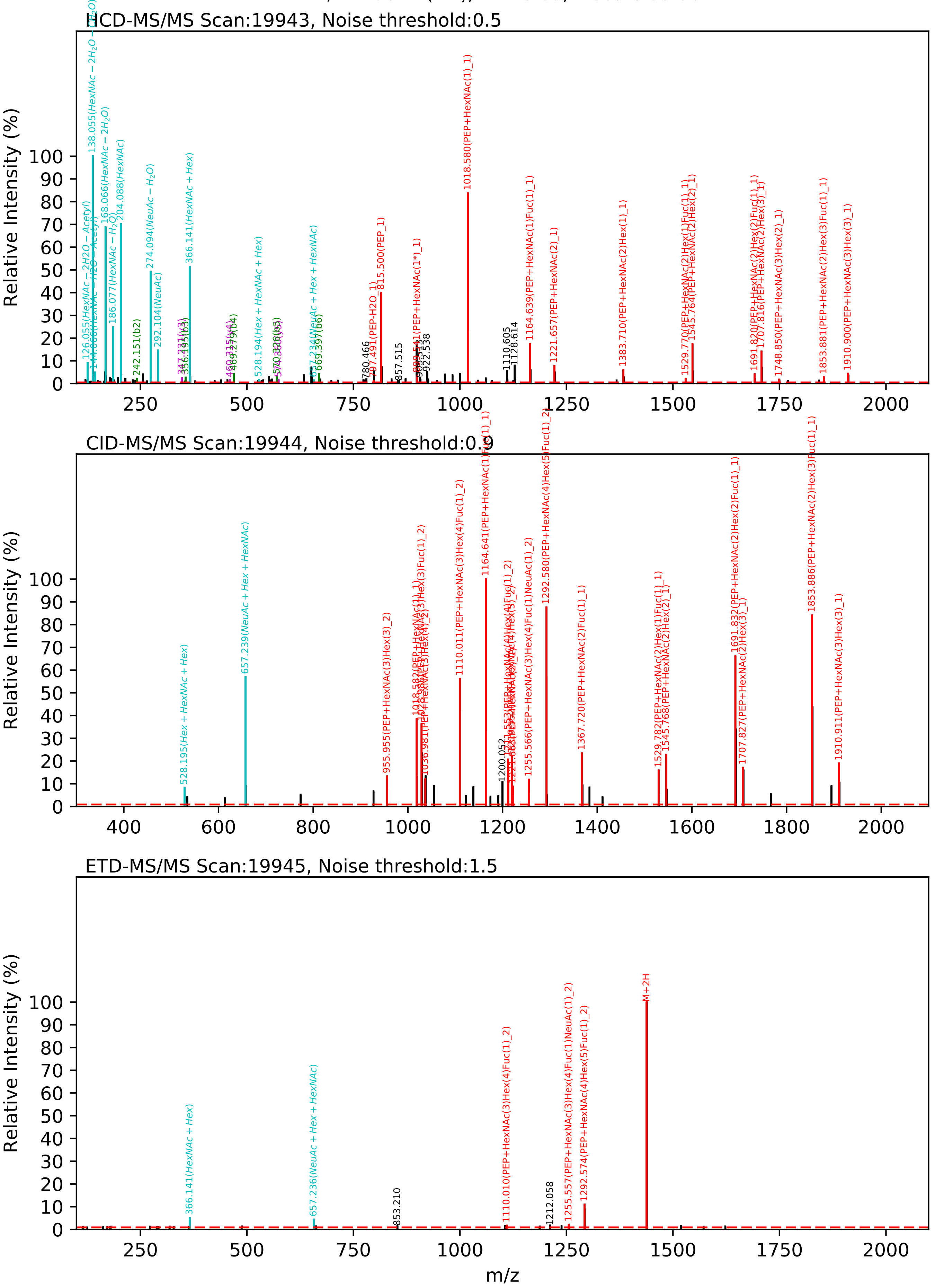

IQNLTVK(=PEP)_5_4_1_1_0, 0_None, 0_None,
m/z:1438.12(2+), RT:37.71, Y-score:93.52

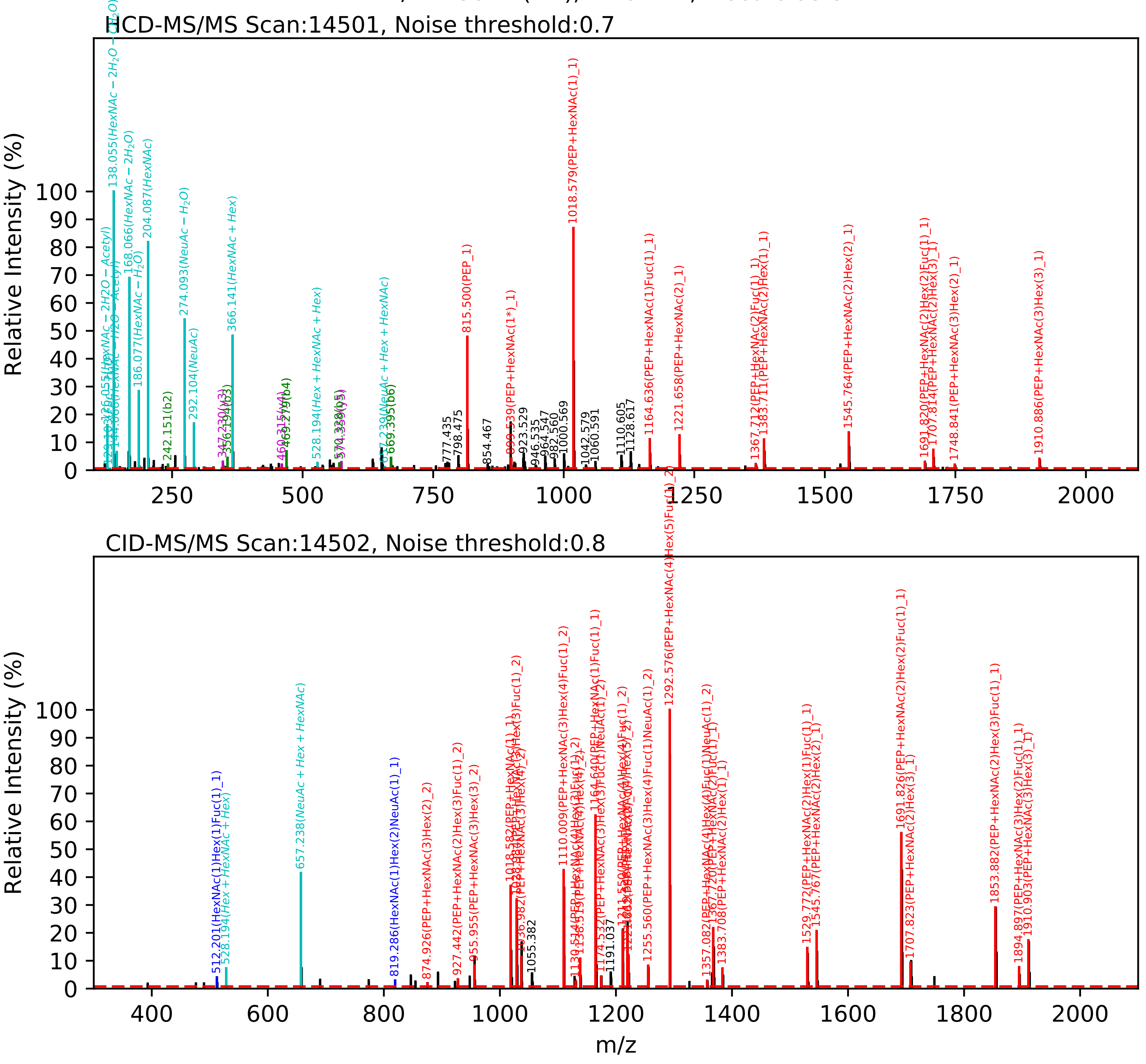

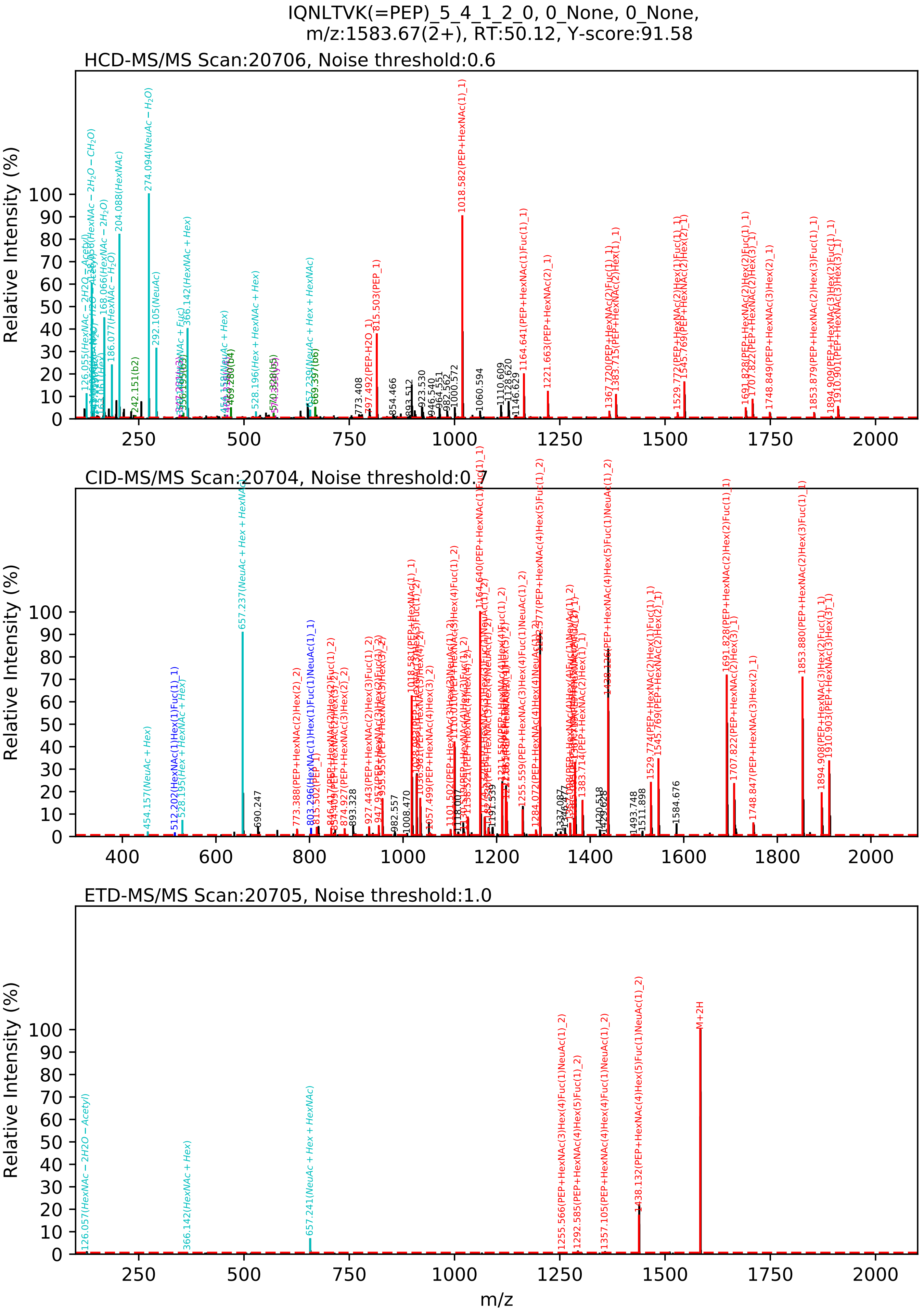
IQNLTVK(=PEP)_5_4_1_2_0, 0_None, 0_None,
m/z:1583.67(2+), RT:50.12, Y-score:91.58
HCD-MS/MS Scan:20706, Noise threshold:0.6
CID-MS/MS Scan:20704, Noise threshold:0.7
ETD-MS/MS Scan:20705, Noise threshold:1.0
Relative Intensity (%)
m/z
M+2H

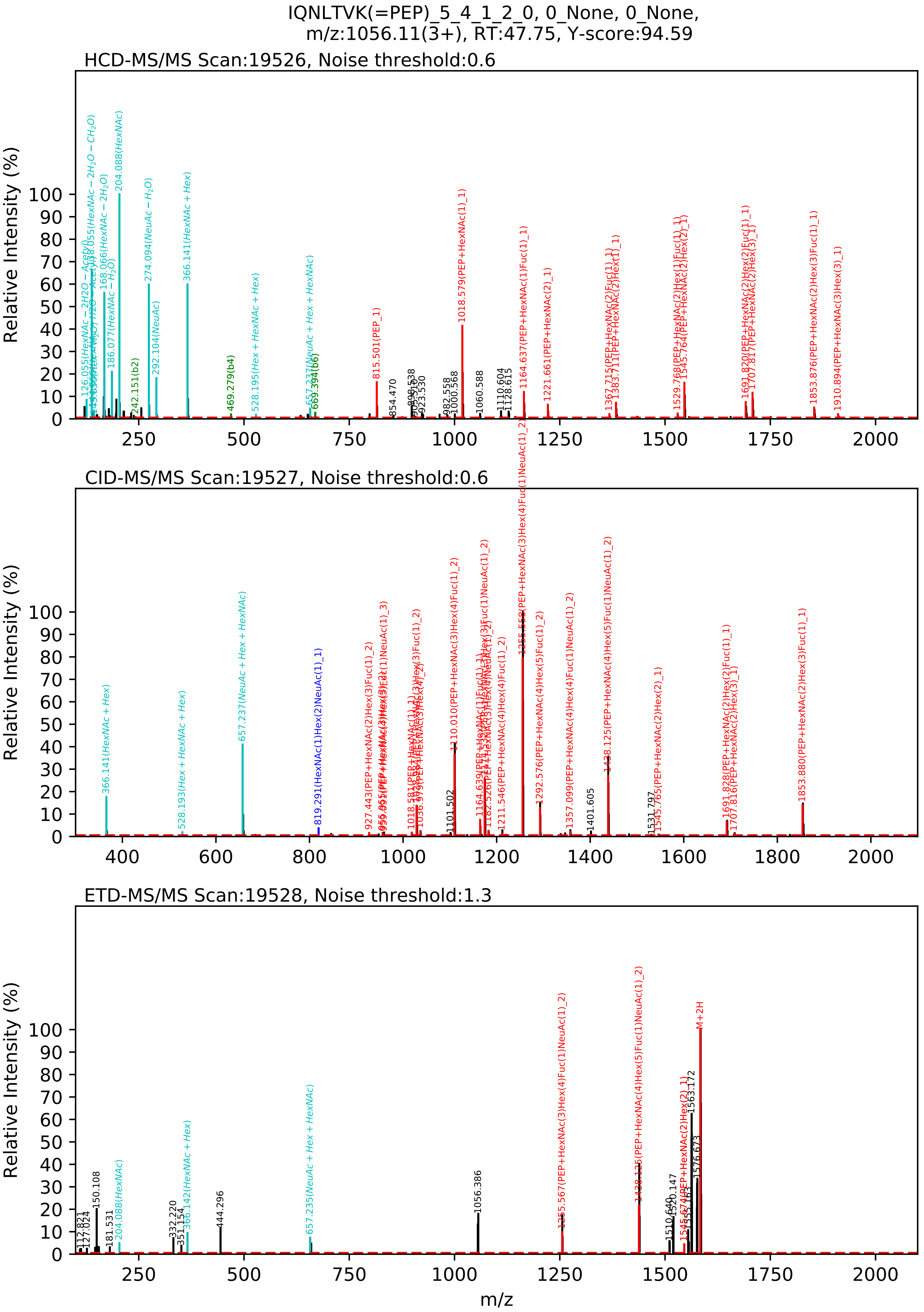
IQNLTVK(=PEP)_5_4_1_2_0, 0_None, 0_None,
m/z:1056.11(3+), RT:47.75, Y-score:94.59
HCD-MS/MS Scan:19526, Noise threshold:0.6
Relative Intensity (%)
204.088(HexNAc)
366.141(HexNAc + Hex)
274.094(NeuAc − H2O)
292.104(NeuAc)
186.077(HexNAc − H2O)
168.066(HexNAc − 2H2O)
242.151(b2)
469.279(b4)
669.394(b6)
528.195(Hex + HexNAc + Hex)
657.237(NeuAc + Hex + HexNAc)
815.501(PEP_1)
1018.579(PEP+HexNAc(1)_1)
1164.637(PEP+HexNAc(1)Fuc(1)_1)
1221.661(PEP+HexNAc(2)_1)
1383.711(PEP+HexNAc(2)Hex(1)_1)
1545.764(PEP+HexNAc(2)Hex(2)_1)
1691.820(PEP+HexNAc(2)Hex(2)Fuc(1)_1)
1707.817(PEP+HexNAc(2)Hex(3)_1)
1853.876(PEP+HexNAc(2)Hex(3)Fuc(1)_1)
1910.894(PEP+HexNAc(3)Hex(3)_1)
854.470
923.530
982.558
1000.568
1060.588
1110.604
1128.615
CID-MS/MS Scan:19527, Noise threshold:0.6
366.141(HexNAc + Hex)
528.193(Hex + HexNAc + Hex)
657.237(NeuAc + Hex + HexNAc)
819.291(HexNAc(1)Hex(2)NeuAc(1)_1)
927.443(PEP+HexNAc(2)Hex(3)Fuc(1)_2)
1110.010(PEP+HexNAc(3)Hex(4)Fuc(1)_2)
1255.558(PEP+HexNAc(3)Hex(4)Fuc(1)NeuAc(1)_2)
1292.576(PEP+HexNAc(4)Hex(5)Fuc(1)_2)
1357.099(PEP+HexNAc(4)Hex(4)Fuc(1)NeuAc(1)_2)
1438.125(PEP+HexNAc(4)Hex(5)Fuc(1)NeuAc(1)_2)
1545.765(PEP+HexNAc(2)Hex(2)_1)
1691.828(PEP+HexNAc(2)Hex(2)Fuc(1)_1)
1707.816(PEP+HexNAc(2)Hex(3)_1)
1853.880(PEP+HexNAc(2)Hex(3)Fuc(1)_1)
1211.546(PEP+HexNAc(4)Hex(4)Fuc(1)_2)
1101.502
1401.605
1531.797
ETD-MS/MS Scan:19528, Noise threshold:1.3
M+2H
1255.567(PEP+HexNAc(3)Hex(4)Fuc(1)NeuAc(1)_2)
1438.135(PEP+HexNAc(4)Hex(5)Fuc(1)NeuAc(1)_2)
204.088(HexNAc)
366.142(HexNAc + Hex)
657.235(NeuAc + Hex + HexNAc)
150.108
181.531
332.220
351.154
444.296
1056.386
1510.640
1520.147
1563.172
1576.673
m/z

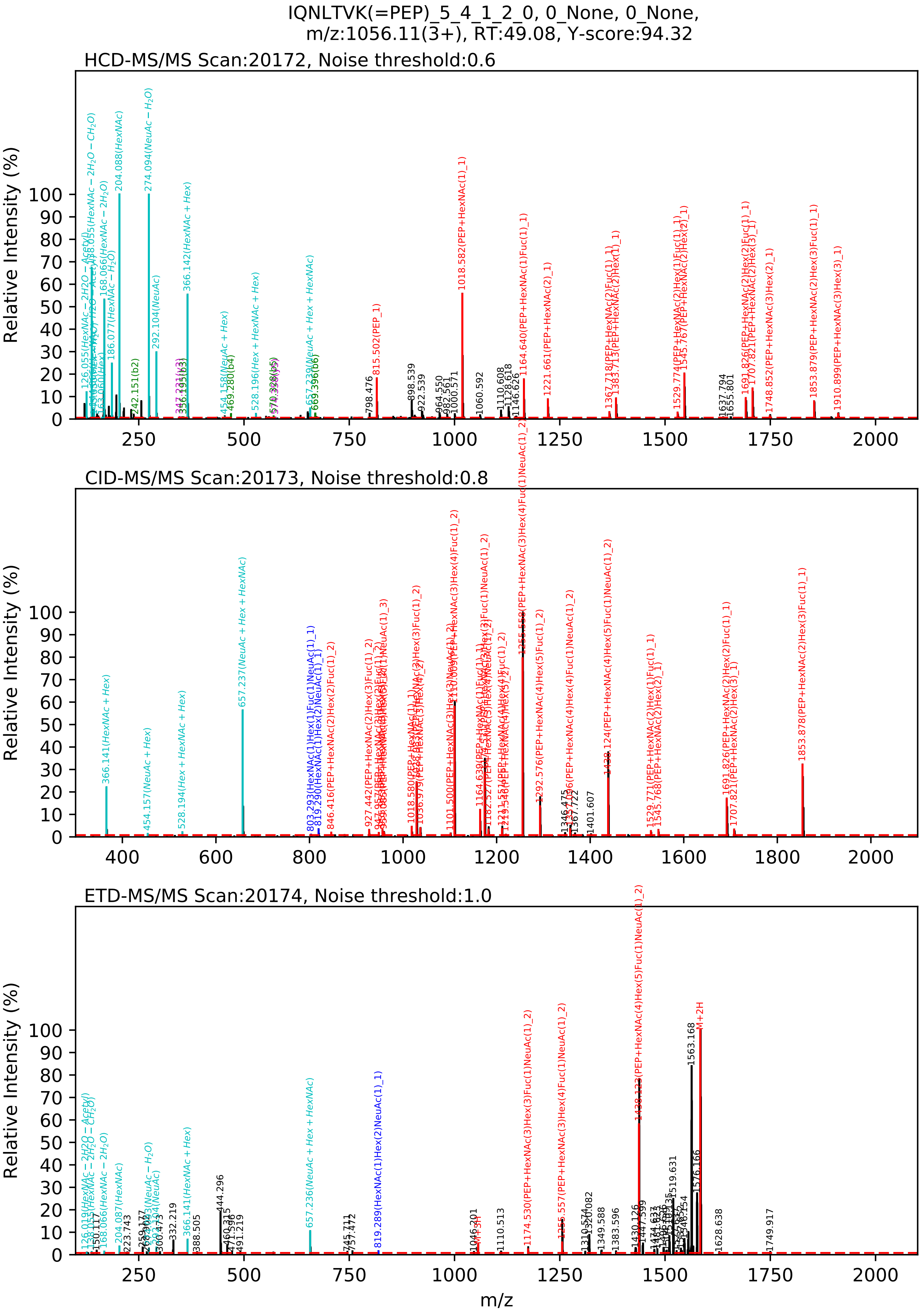
IQNLTVK(=PEP)_5_4_1_2_0, 0_None, 0_None,
m/z:1056.11(3+), RT:49.08, Y-score:94.32
HCD-MS/MS Scan:20172, Noise threshold:0.6
CID-MS/MS Scan:20173, Noise threshold:0.8
ETD-MS/MS Scan:20174, Noise threshold:1.0
Relative Intensity (%)
m/z
1018.582(PEP+HexNAc(1)_1)
815.502(PEP_1)
1164.640(PEP+HexNAc(1)Fuc(1)_1)
1221.661(PEP+HexNAc(2)_1)
1853.879(PEP+HexNAc(2)Hex(3)Fuc(1)_1)
1910.899(PEP+HexNAc(3)Hex(3)_1)
1748.852(PEP+HexNAc(3)Hex(2)_1)
204.088(HexNAc)
366.142(HexNAc + Hex)
292.104(NeuAc)
657.237(NeuAc + Hex + HexNAc)
1853.878(PEP+HexNAc(2)Hex(3)Fuc(1)_1)
1438.124(PEP+HexNAc(4)Hex(5)Fuc(1)NeuAc(1)_2)
1292.576(PEP+HexNAc(4)Hex(5)Fuc(1)_2)
1174.530(PEP+HexNAc(3)Hex(3)Fuc(1)NeuAc(1)_2)
1255.557(PEP+HexNAc(3)Hex(4)Fuc(1)NeuAc(1)_2)
1438.123(PEP+HexNAc(4)Hex(5)Fuc(1)NeuAc(1)_2)
1563.168
1576.166
M+2H
819.289(HexNAc(1)Hex(2)NeuAc(1)_1)

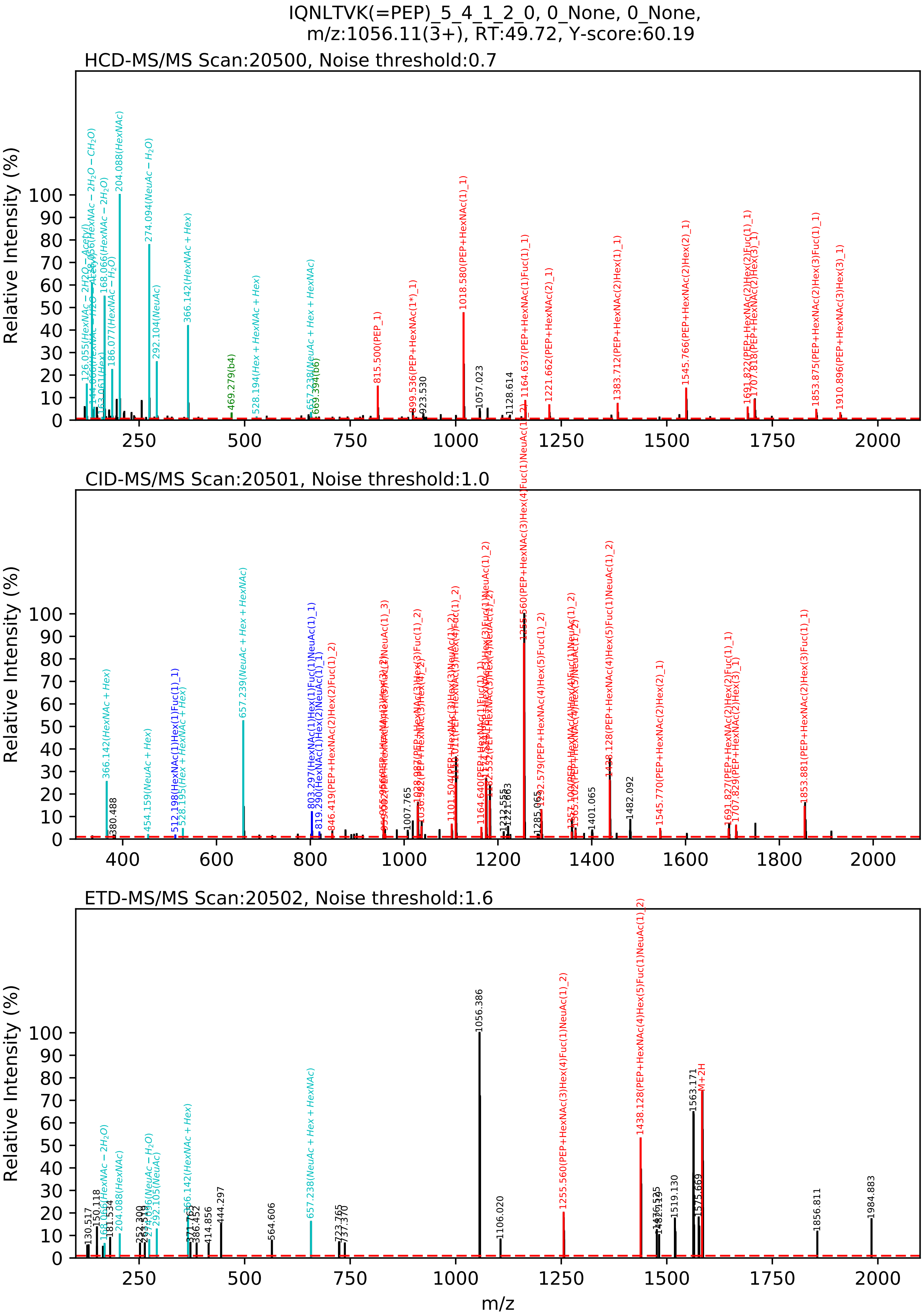
IQNLTVK(=PEP)_5_4_1_2_0, 0_None, 0_None,
m/z:1056.11(3+), RT:49.72, Y-score:60.19
HCD-MS/MS Scan:20500, Noise threshold:0.7
Relative Intensity (%)
204.088(HexNAc)
274.094(NeuAc − H2O)
292.104(NeuAc)
366.142(HexNAc + Hex)
469.279(b4)
528.194(Hex + HexNAc + Hex)
815.500(PEP_1)
1018.580(PEP+HexNAc(1)_1)
1057.023
1128.614
1164.637(PEP+HexNAc(1)Fuc(1)_1)
1221.662(PEP+HexNAc(2)_1)
1383.712(PEP+HexNAc(2)Hex(1)_1)
1545.766(PEP+HexNAc(2)Hex(2)_1)
1853.875(PEP+HexNAc(2)Hex(3)Fuc(1)_1)
1910.896(PEP+HexNAc(3)Hex(3)_1)
CID-MS/MS Scan:20501, Noise threshold:1.0
366.142(HexNAc + Hex)
454.159(NeuAc + Hex)
657.239(NeuAc + Hex + HexNAc)
846.419(PEP+HexNAc(2)Hex(2)Fuc(1)_2)
1007.765
1255.560(PEP+HexNAc(3)Hex(4)Fuc(1)NeuAc(1)_2)
1292.579(PEP+HexNAc(4)Hex(5)Fuc(1)_2)
1401.065
1438.128(PEP+HexNAc(4)Hex(5)Fuc(1)NeuAc(1)_2)
1482.092
1545.770(PEP+HexNAc(2)Hex(2)_1)
1853.881(PEP+HexNAc(2)Hex(3)Fuc(1)_1)
ETD-MS/MS Scan:20502, Noise threshold:1.6
1056.386
1106.020
1255.560(PEP+HexNAc(3)Hex(4)Fuc(1)NeuAc(1)_2)
1438.128(PEP+HexNAc(4)Hex(5)Fuc(1)NeuAc(1)_2)
1519.130
1563.171
M+2H
1856.811
1984.883
m/z

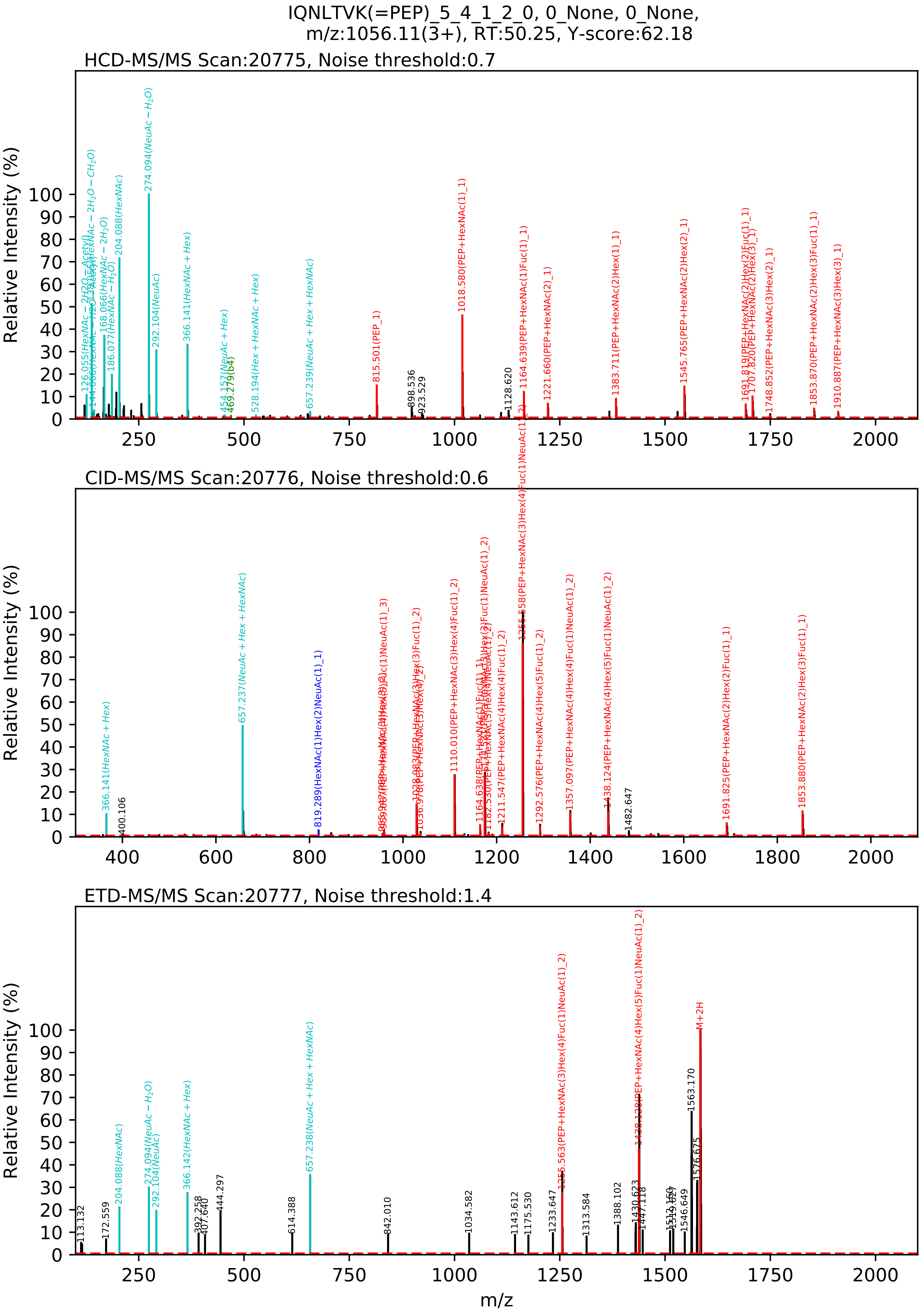

IQNLTVK(=PEP)_5_4_1_2_0, 0_None, 0_None,
m/z:1056.11(3+), RT:50.25, Y-score:62.18
HCD-MS/MS Scan:20775, Noise threshold:0.7
Relative Intensity (%)
274.094(NeuAc − H2O)
204.088(HexNAc)
168.066(HexNAc − 2H2O)
186.077(HexNAc − H2O)
126.055(HexNAc − 2H2O − Acetyl)
292.104(NeuAc)
366.141(HexNAc + Hex)
454.157(NeuAc + Hex)
469.279(b4)
528.194(Hex + HexNAc + Hex)
657.239(NeuAc + Hex + HexNAc)
815.501(PEP_1)
898.536
923.529
1018.580(PEP+HexNAc(1)_1)
1128.620
1164.639(PEP+HexNAc(1)Fuc(1)_1)
1221.660(PEP+HexNAc(2)_1)
1383.711(PEP+HexNAc(2)Hex(1)_1)
1545.765(PEP+HexNAc(2)Hex(2)_1)
1691.819(PEP+HexNAc(2)Hex(2)Fuc(1)_1)
1707.820(PEP+HexNAc(2)Hex(3)_1)
1748.852(PEP+HexNAc(3)Hex(2)_1)
1853.870(PEP+HexNAc(2)Hex(3)Fuc(1)_1)
1910.887(PEP+HexNAc(3)Hex(3)_1)
CID-MS/MS Scan:20776, Noise threshold:0.6
366.141(HexNAc + Hex)
400.106
657.237(NeuAc + Hex + HexNAc)
819.289(HexNAc(1)Hex(2)NeuAc(1)_1)
1028.983(PEP+HexNAc(3)Hex(3)Fuc(1)_2)
1110.010(PEP+HexNAc(3)Hex(4)Fuc(1)_2)
1211.547(PEP+HexNAc(4)Hex(4)Fuc(1)_2)
1255.558(PEP+HexNAc(3)Hex(4)Fuc(1)NeuAc(1)_2)
1292.576(PEP+HexNAc(4)Hex(5)Fuc(1)_2)
1357.097(PEP+HexNAc(4)Hex(4)Fuc(1)NeuAc(1)_2)
1438.124(PEP+HexNAc(4)Hex(5)Fuc(1)NeuAc(1)_2)
1482.647
1691.825(PEP+HexNAc(2)Hex(2)Fuc(1)_1)
1853.880(PEP+HexNAc(2)Hex(3)Fuc(1)_1)
ETD-MS/MS Scan:20777, Noise threshold:1.4
113.132
172.559
204.088(HexNAc)
274.094(NeuAc − H2O)
292.104(NeuAc)
366.142(HexNAc + Hex)
392.258
407.640
444.297
614.388
657.238(NeuAc + Hex + HexNAc)
842.010
1034.582
1143.612
1175.530
1233.647
1255.563(PEP+HexNAc(3)Hex(4)Fuc(1)NeuAc(1)_2)
1313.584
1388.102
1430.623
1438.128(PEP+HexNAc(4)Hex(5)Fuc(1)NeuAc(1)_2)
1447.118
1512.169
1519.627
1546.649
1563.170
1576.675
M+2H
m/z

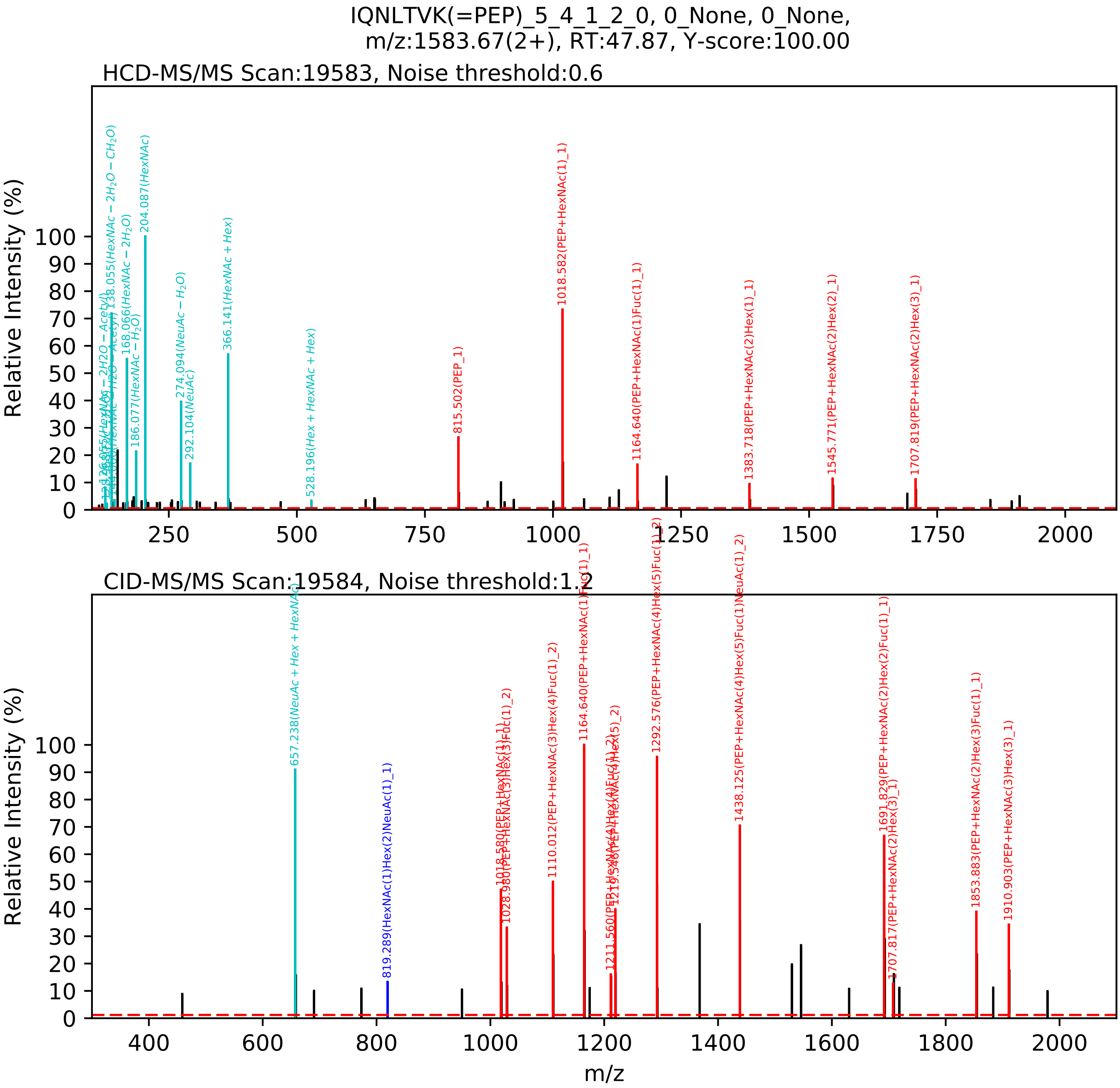

IQNLTVK(=PEP)_5_4_1_2_0, 0_None, 0_None,
m/z:1583.67(2+), RT:47.87, Y-score:100.00
HCD-MS/MS Scan:19583, Noise threshold:0.6
Relative Intensity (%)
204.087(HexNAc)
366.141(HexNAc + Hex)
274.094(NeuAc − H2O)
292.104(NeuAc)
168.066(HexNAc − 2H2O)
186.077(HexNAc − H2O)
138.055(HexNAc − 2H2O − CH2O)
528.196(Hex + HexNAc + Hex)
815.502(PEP_1)
1018.582(PEP+HexNAc(1)_1)
1164.640(PEP+HexNAc(1)Fuc(1)_1)
1383.718(PEP+HexNAc(2)Hex(1)_1)
1545.771(PEP+HexNAc(2)Hex(2)_1)
1707.819(PEP+HexNAc(2)Hex(3)_1)
CID-MS/MS Scan:19584, Noise threshold:1.2
Relative Intensity (%)
657.238(NeuAc + Hex + HexNAc)
819.289(HexNAc(1)Hex(2)NeuAc(1)_1)
1018.580(PEP+HexNAc(1)_1)
1028.980(PEP+HexNAc(3)Hex(3)Fuc(1)_2)
1110.012(PEP+HexNAc(3)Hex(4)Fuc(1)_2)
1164.640(PEP+HexNAc(1)Fuc(1)_1)
1211.560(PEP+HexNAc(4)Hex(4)Fuc(1)_2)
1219.540(PEP+HexNAc(4)Hex(5)_2)
1292.576(PEP+HexNAc(4)Hex(5)Fuc(1)_2)
1438.125(PEP+HexNAc(4)Hex(5)Fuc(1)NeuAc(1)_2)
1691.829(PEP+HexNAc(2)Hex(2)Fuc(1)_1)
1707.817(PEP+HexNAc(2)Hex(3)_1)
1853.883(PEP+HexNAc(2)Hex(3)Fuc(1)_1)
1910.903(PEP+HexNAc(3)Hex(3)_1)
m/z

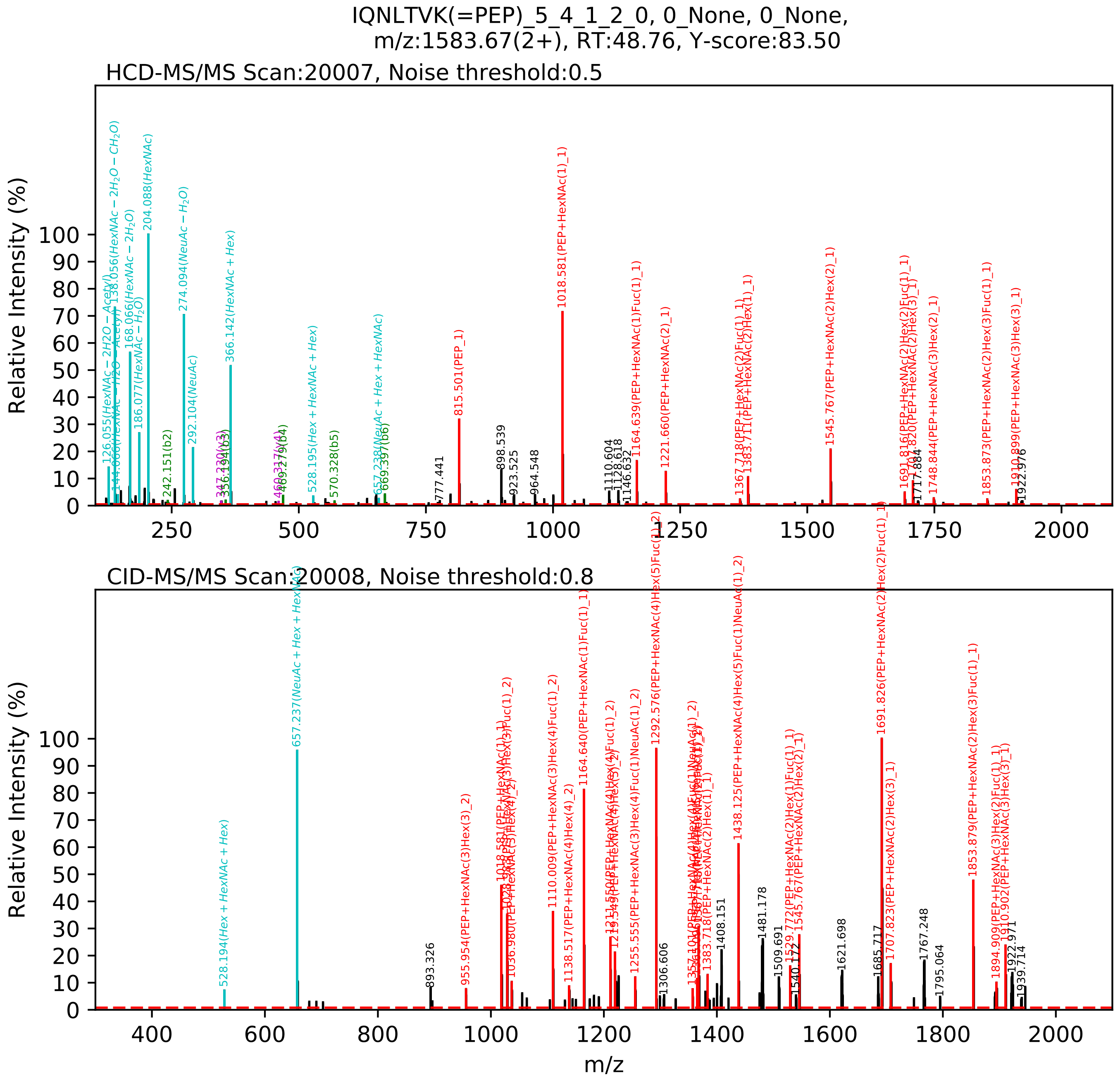

IQNLTVK(=PEP)_5_4_1_2_0, 0_None, 0_None,
m/z:1583.67(2+), RT:48.76, Y-score:83.50
HCD-MS/MS Scan:20007, Noise threshold:0.5
Relative Intensity (%)
204.088(HexNAc)
1018.581(PEP+HexNAc(1)_1)
274.094(NeuAc − H2O)
366.142(HexNAc + Hex)
815.501(PEP_1)
1164.639(PEP+HexNAc(1)Fuc(1)_1)
1221.660(PEP+HexNAc(2)_1)
1545.767(PEP+HexNAc(2)Hex(2)_1)
1383.711(PEP+HexNAc(2)Hex(1)_1)
1748.844(PEP+HexNAc(3)Hex(2)_1)
1853.873(PEP+HexNAc(2)Hex(3)Fuc(1)_1)
1910.899(PEP+HexNAc(3)Hex(3)_1)
898.539
923.525
964.548
777.441
1110.604
1128.618
1146.632
1717.884
1922.976
CID-MS/MS Scan:20008, Noise threshold:0.8
657.237(NeuAc + Hex + HexNAc)
528.194(Hex + HexNAc + Hex)
1691.826(PEP+HexNAc(2)Hex(2)Fuc(1)_1)
1292.576(PEP+HexNAc(4)Hex(5)Fuc(1)_2)
1164.640(PEP+HexNAc(1)Fuc(1)_1)
1438.125(PEP+HexNAc(4)Hex(5)Fuc(1)NeuAc(1)_2)
1853.879(PEP+HexNAc(2)Hex(3)Fuc(1)_1)
1110.009(PEP+HexNAc(3)Hex(4)Fuc(1)_2)
955.954(PEP+HexNAc(3)Hex(3)_2)
1707.823(PEP+HexNAc(2)Hex(3)_1)
1255.555(PEP+HexNAc(3)Hex(4)Fuc(1)NeuAc(1)_2)
1138.517(PEP+HexNAc(4)Hex(4)_2)
893.326
1306.606
1408.151
1481.178
1509.691
1621.698
1685.717
1767.248
1795.064
1922.971
1939.714
m/z

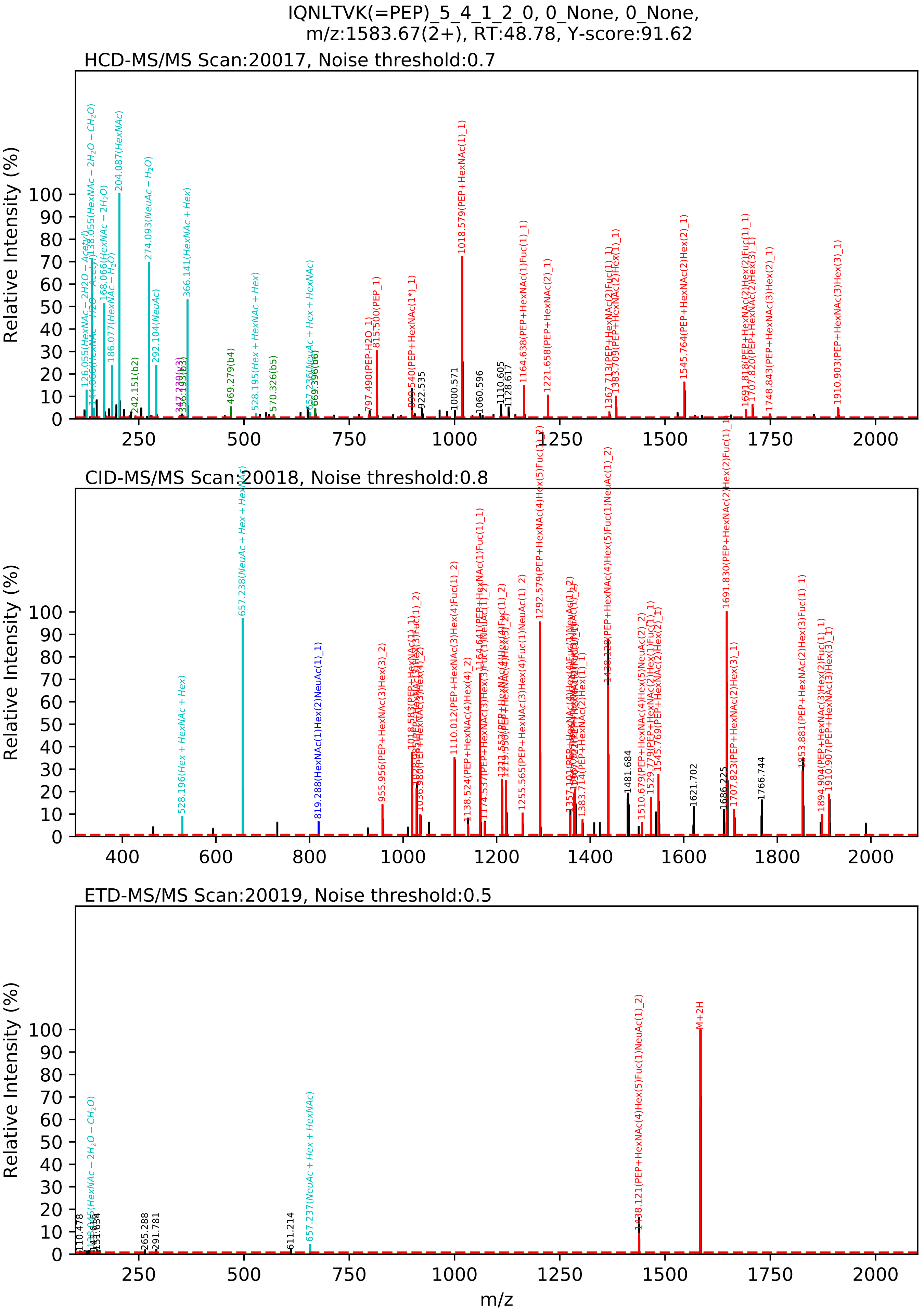
IQNLTVK(=PEP)_5_4_1_2_0, 0_None, 0_None,
m/z:1583.67(2+), RT:48.78, Y-score:91.62
HCD-MS/MS Scan:20017, Noise threshold:0.7
Relative Intensity (%)
204.087(HexNAc)
274.093(NeuAc − H2O)
366.141(HexNAc + Hex)
168.066(HexNAc − 2H2O)
186.077(HexNAc − H2O)
292.104(NeuAc)
242.151(b2)
469.279(b4)
528.195(Hex + HexNAc + Hex)
570.326(b5)
669.396(b6)
797.490(PEP-H2O_1)
815.500(PEP_1)
899.540(PEP+HexNAc(1*)_1)
922.535
1000.571
1018.579(PEP+HexNAc(1)_1)
1060.596
1110.605
1128.617
1164.638(PEP+HexNAc(1)Fuc(1)_1)
1221.658(PEP+HexNAc(2)_1)
1383.709(PEP+HexNAc(2)Hex(1)_1)
1545.764(PEP+HexNAc(2)Hex(2)_1)
1691.818(PEP+HexNAc(2)Hex(2)Fuc(1)_1)
1707.820(PEP+HexNAc(2)Hex(3)_1)
1748.843(PEP+HexNAc(3)Hex(2)_1)
1910.903(PEP+HexNAc(3)Hex(3)_1)
CID-MS/MS Scan:20018, Noise threshold:0.8
528.196(Hex + HexNAc + Hex)
657.238(NeuAc + Hex + HexNAc)
819.288(HexNAc(1)Hex(2)NeuAc(1)_1)
955.956(PEP+HexNAc(3)Hex(3)_2)
1110.012(PEP+HexNAc(3)Hex(4)Fuc(1)_2)
1138.524(PEP+HexNAc(4)Hex(4)_2)
1255.565(PEP+HexNAc(3)Hex(4)Fuc(1)NeuAc(1)_2)
1292.579(PEP+HexNAc(4)Hex(5)Fuc(1)_2)
1383.714(PEP+HexNAc(2)Hex(1)_1)
1438.128(PEP+HexNAc(4)Hex(5)Fuc(1)NeuAc(1)_2)
1481.684
1621.702
1686.225
1691.830(PEP+HexNAc(2)Hex(2)Fuc(1)_1)
1707.823(PEP+HexNAc(2)Hex(3)_1)
1766.744
1853.881(PEP+HexNAc(2)Hex(3)Fuc(1)_1)
ETD-MS/MS Scan:20019, Noise threshold:0.5
110.478
265.288
291.781
611.214
657.237(NeuAc + Hex + HexNAc)
1438.121(PEP+HexNAc(4)Hex(5)Fuc(1)NeuAc(1)_2)
M+2H
m/z

IQNLTVK(=PEP)_5_4_1_2_0, 0_None, 0_None,
m/z:1583.67(2+), RT:49.29, Y-score:93.90

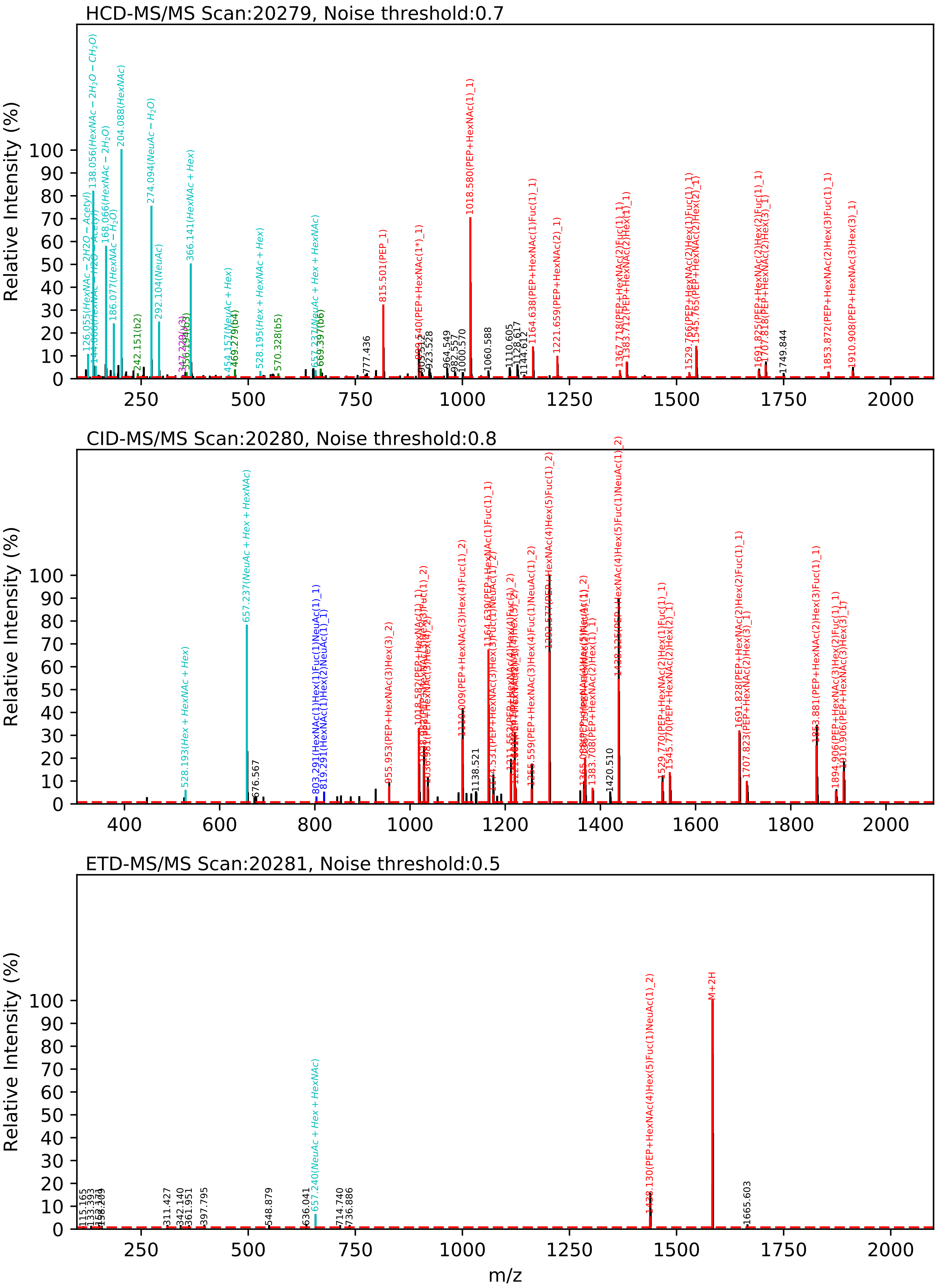

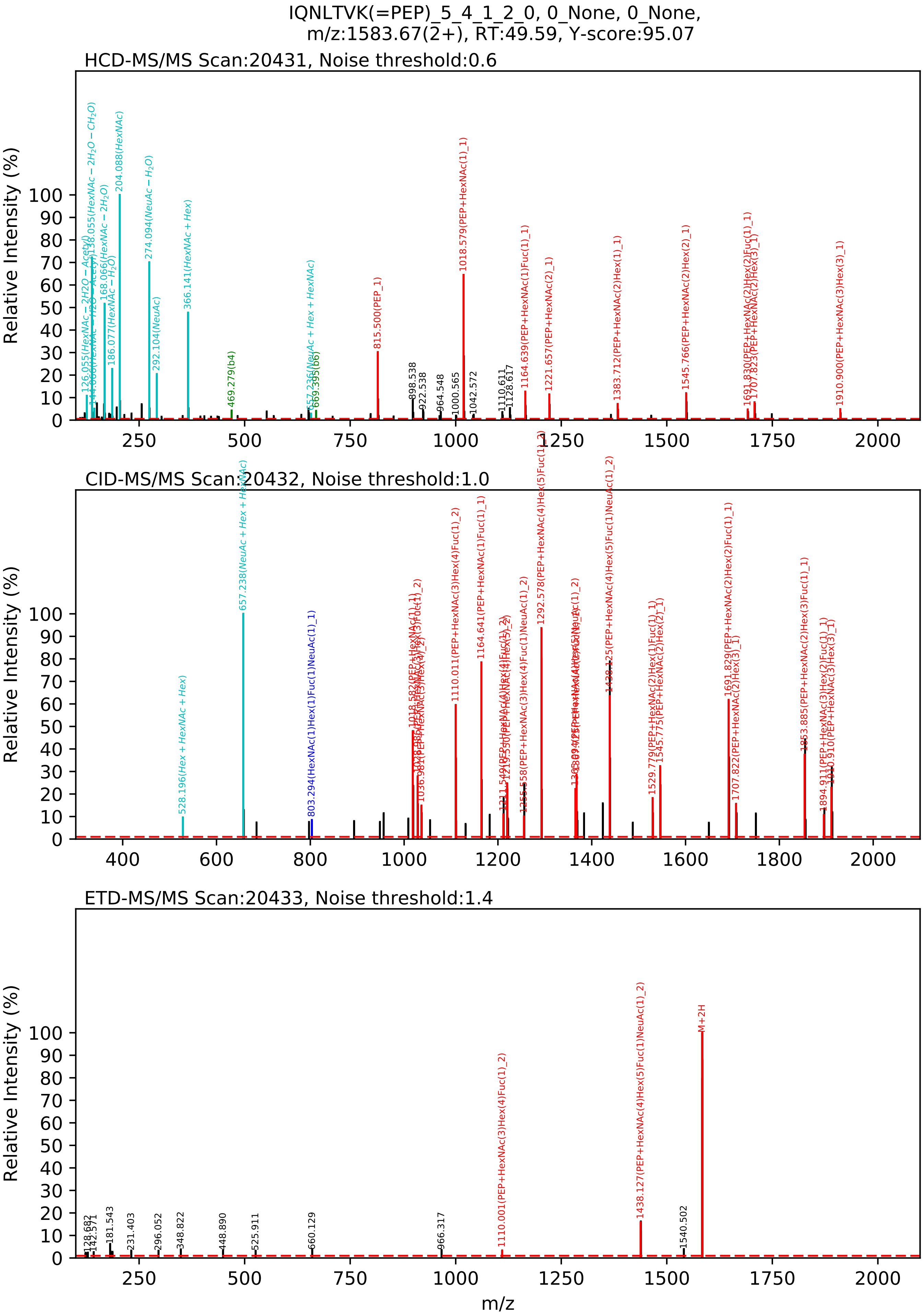
IQNLTVK(=PEP)_5_4_1_2_0, 0_None, 0_None,
m/z:1583.67(2+), RT:49.59, Y-score:95.07
HCD-MS/MS Scan:20431, Noise threshold:0.6
Relative Intensity (%)
204.088(HexNAc)
274.094(NeuAc − H2O)
366.141(HexNAc + Hex)
292.104(NeuAc)
186.077(HexNAc − H2O)
168.066(HexNAc − 2H2O)
469.279(b4)
669.395(b6)
815.500(PEP_1)
898.538
922.538
964.548
1000.565
1042.572
1110.611
1128.617
1018.579(PEP+HexNAc(1)_1)
1164.639(PEP+HexNAc(1)Fuc(1)_1)
1221.657(PEP+HexNAc(2)_1)
1383.712(PEP+HexNAc(2)Hex(1)_1)
1545.766(PEP+HexNAc(2)Hex(2)_1)
1910.900(PEP+HexNAc(3)Hex(3)_1)
CID-MS/MS Scan:20432, Noise threshold:1.0
657.238(NeuAc + Hex + HexNAc)
528.196(Hex + HexNAc + Hex)
803.294(HexNAc(1)Hex(1)Fuc(1)NeuAc(1)_1)
1110.011(PEP+HexNAc(3)Hex(4)Fuc(1)_2)
1164.641(PEP+HexNAc(1)Fuc(1)_1)
1255.558(PEP+HexNAc(3)Hex(4)Fuc(1)NeuAc(1)_2)
1292.578(PEP+HexNAc(4)Hex(5)Fuc(1)_2)
1438.125(PEP+HexNAc(4)Hex(5)Fuc(1)NeuAc(1)_2)
1529.779(PEP+HexNAc(2)Hex(1)Fuc(1)_1)
1545.775(PEP+HexNAc(2)Hex(2)_1)
1691.829(PEP+HexNAc(2)Hex(2)Fuc(1)_1)
1707.822(PEP+HexNAc(2)Hex(3)_1)
1853.885(PEP+HexNAc(2)Hex(3)Fuc(1)_1)
ETD-MS/MS Scan:20433, Noise threshold:1.4
128.682
142.571
181.543
231.403
296.052
348.822
448.890
525.911
660.129
966.317
1110.001(PEP+HexNAc(3)Hex(4)Fuc(1)_2)
1438.127(PEP+HexNAc(4)Hex(5)Fuc(1)NeuAc(1)_2)
1540.502
M+2H
m/z

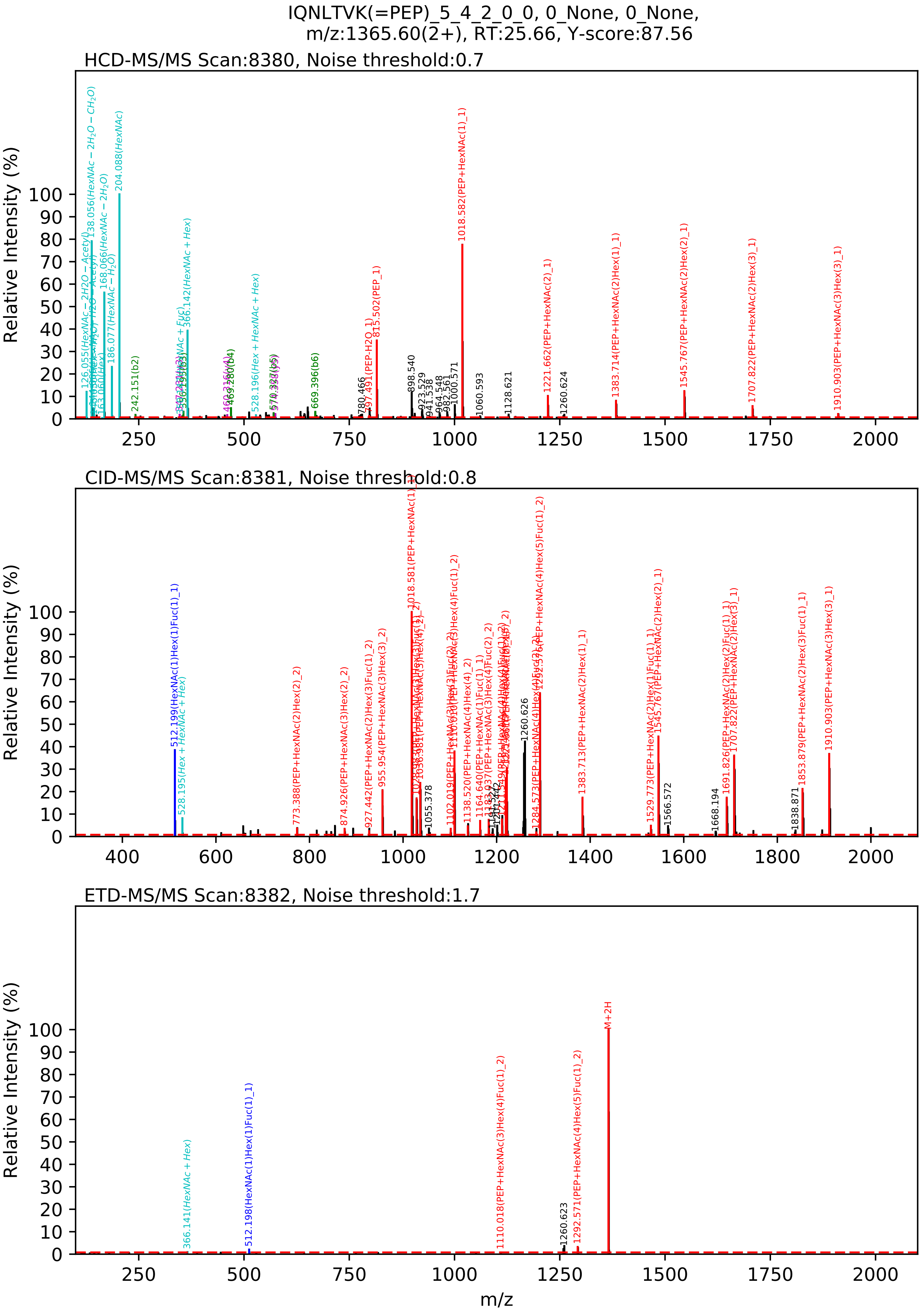
IQNLTVK(=PEP)_5_4_2_0_0, 0_None, 0_None,
m/z:1365.60(2+), RT:25.66, Y-score:87.56
HCD-MS/MS Scan:8380, Noise threshold:0.7
CID-MS/MS Scan:8381, Noise threshold:0.8
ETD-MS/MS Scan:8382, Noise threshold:1.7
Relative Intensity (%)
m/z
204.088(HexNAc)
366.142(HexNAc + Hex)
1018.582(PEP+HexNAc(1)_1)
815.502(PEP_1)
1221.662(PEP+HexNAc(2)_1)
1383.714(PEP+HexNAc(2)Hex(1)_1)
1545.767(PEP+HexNAc(2)Hex(2)_1)
1707.822(PEP+HexNAc(2)Hex(3)_1)
1910.903(PEP+HexNAc(3)Hex(3)_1)
512.199(HexNAc(1)Hex(1)Fuc(1)_1)
1018.581(PEP+HexNAc(1)_1)
1292.576(PEP+HexNAc(4)Hex(5)Fuc(1)_2)
1383.713(PEP+HexNAc(2)Hex(1)_1)
1545.767(PEP+HexNAc(2)Hex(2)_1)
1853.879(PEP+HexNAc(2)Hex(3)Fuc(1)_1)
1910.903(PEP+HexNAc(3)Hex(3)_1)
M+2H
512.198(HexNAc(1)Hex(1)Fuc(1)_1)
366.141(HexNAc + Hex)
1110.018(PEP+HexNAc(3)Hex(4)Fuc(1)_2)
1292.571(PEP+HexNAc(4)Hex(5)Fuc(1)_2)
1260.623

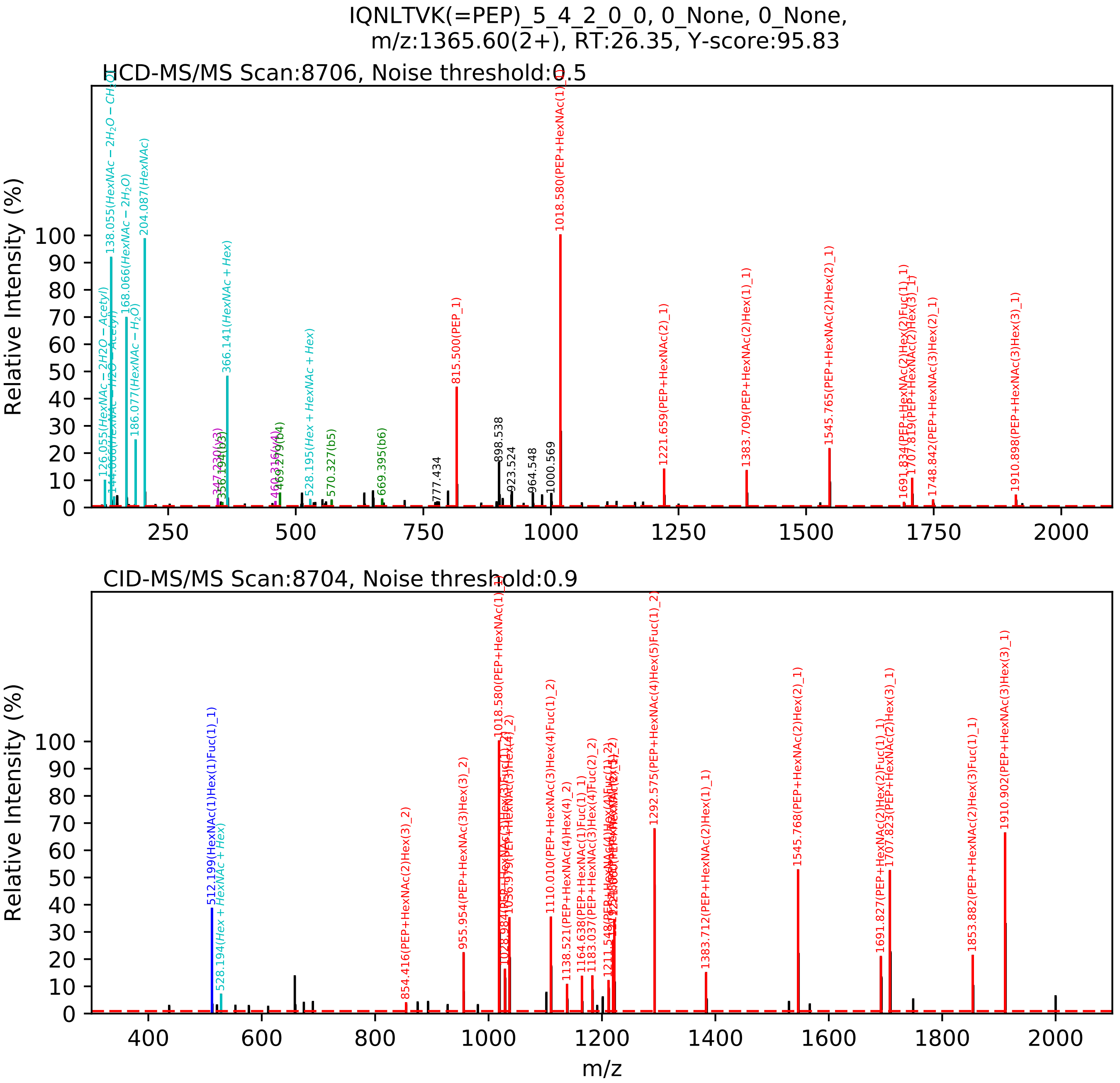
IQNLTVK(=PEP)_5_4_2_0_0, 0_None, 0_None,
m/z:1365.60(2+), RT:26.35, Y-score:95.83
HCD-MS/MS Scan:8706, Noise threshold:0.5
Relative Intensity (%)
126.055(HexNAc − 2H2O − Acetyl)
138.055(HexNAc − 2H2O − CH4O)
168.066(HexNAc − 2H2O)
186.077(HexNAc − H2O)
204.087(HexNAc)
366.141(HexNAc + Hex)
356.194(b3)
460.316(y4)
469.279(b4)
528.195(Hex + HexNAc + Hex)
570.327(b5)
669.395(b6)
777.434
815.500(PEP_1)
898.538
923.524
964.548
1000.569
1018.580(PEP+HexNAc(1)_1)
1221.659(PEP+HexNAc(2)_1)
1383.709(PEP+HexNAc(2)Hex(1)_1)
1545.765(PEP+HexNAc(2)Hex(2)_1)
1691.834(PEP+HexNAc(2)Hex(2)Fuc(1)_1)
1707.819(PEP+HexNAc(2)Hex(3)_1)
1748.842(PEP+HexNAc(3)Hex(2)_1)
1910.898(PEP+HexNAc(3)Hex(3)_1)
CID-MS/MS Scan:8704, Noise threshold:0.9
512.199(HexNAc(1)Hex(1)Fuc(1)_1)
528.194(Hex + HexNAc + Hex)
854.416(PEP+HexNAc(2)Hex(3)_2)
955.954(PEP+HexNAc(3)Hex(3)_2)
1018.580(PEP+HexNAc(1)_1)
1110.010(PEP+HexNAc(3)Hex(4)Fuc(1)_2)
1138.521(PEP+HexNAc(4)Hex(4)_2)
1164.638(PEP+HexNAc(1)Fuc(1)_1)
1183.037(PEP+HexNAc(3)Hex(4)Fuc(2)_2)
1292.575(PEP+HexNAc(4)Hex(5)Fuc(1)_2)
1383.712(PEP+HexNAc(2)Hex(1)_1)
1545.768(PEP+HexNAc(2)Hex(2)_1)
1691.827(PEP+HexNAc(2)Hex(2)Fuc(1)_1)
1707.823(PEP+HexNAc(2)Hex(3)_1)
1853.882(PEP+HexNAc(2)Hex(3)Fuc(1)_1)
1910.902(PEP+HexNAc(3)Hex(3)_1)
m/z

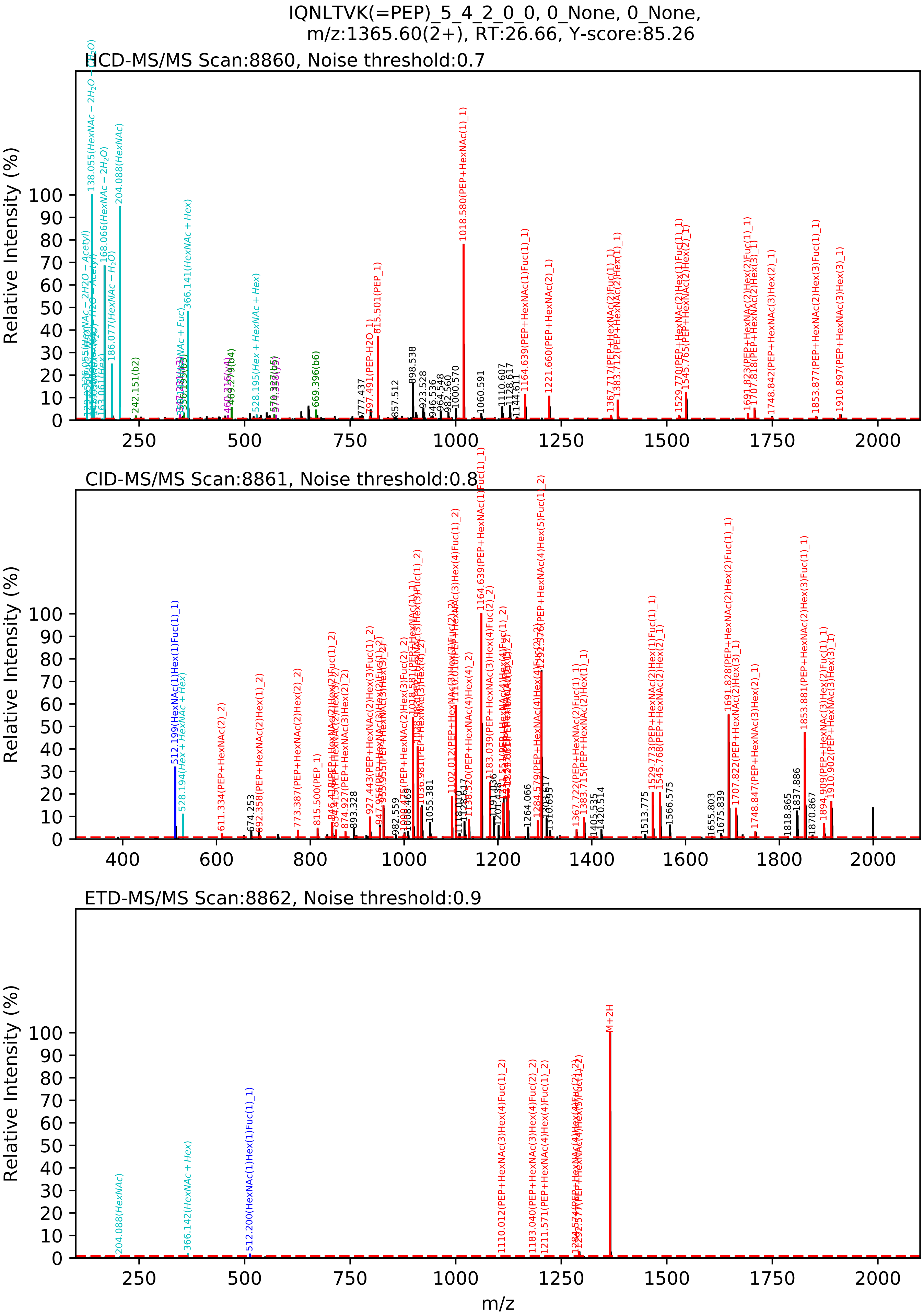
IQNLTVK(=PEP)_5_4_2_0_0, 0_None, 0_None,
m/z:1365.60(2+), RT:26.66, Y-score:85.26
HCD-MS/MS Scan:8860, Noise threshold:0.7
CID-MS/MS Scan:8861, Noise threshold:0.8
ETD-MS/MS Scan:8862, Noise threshold:0.9
Relative Intensity (%)
m/z
1018.580(PEP+HexNAc(1)_1)
815.501(PEP_1)
1164.639(PEP+HexNAc(1)Fuc(1)_1)
1221.660(PEP+HexNAc(2)_1)
1383.712(PEP+HexNAc(2)Hex(1)_1)
1545.765(PEP+HexNAc(2)Hex(2)_1)
1748.842(PEP+HexNAc(3)Hex(2)_1)
1853.877(PEP+HexNAc(2)Hex(3)Fuc(1)_1)
1910.897(PEP+HexNAc(3)Hex(3)_1)
204.088(HexNAc)
366.141(HexNAc+Hex)
512.199(HexNAc(1)Hex(1)Fuc(1)_1)
1164.639(PEP+HexNAc(1)Fuc(1)_1)
1691.828(PEP+HexNAc(2)Hex(2)Fuc(1)_1)
1853.881(PEP+HexNAc(2)Hex(3)Fuc(1)_1)
M+2H
204.088(HexNAc)
366.142(HexNAc+Hex)
512.200(HexNAc(1)Hex(1)Fuc(1)_1)
1110.012(PEP+HexNAc(3)Hex(4)Fuc(1)_2)
1183.040(PEP+HexNAc(3)Hex(4)Fuc(2)_2)
1211.571(PEP+HexNAc(4)Hex(4)Fuc(1)_2)

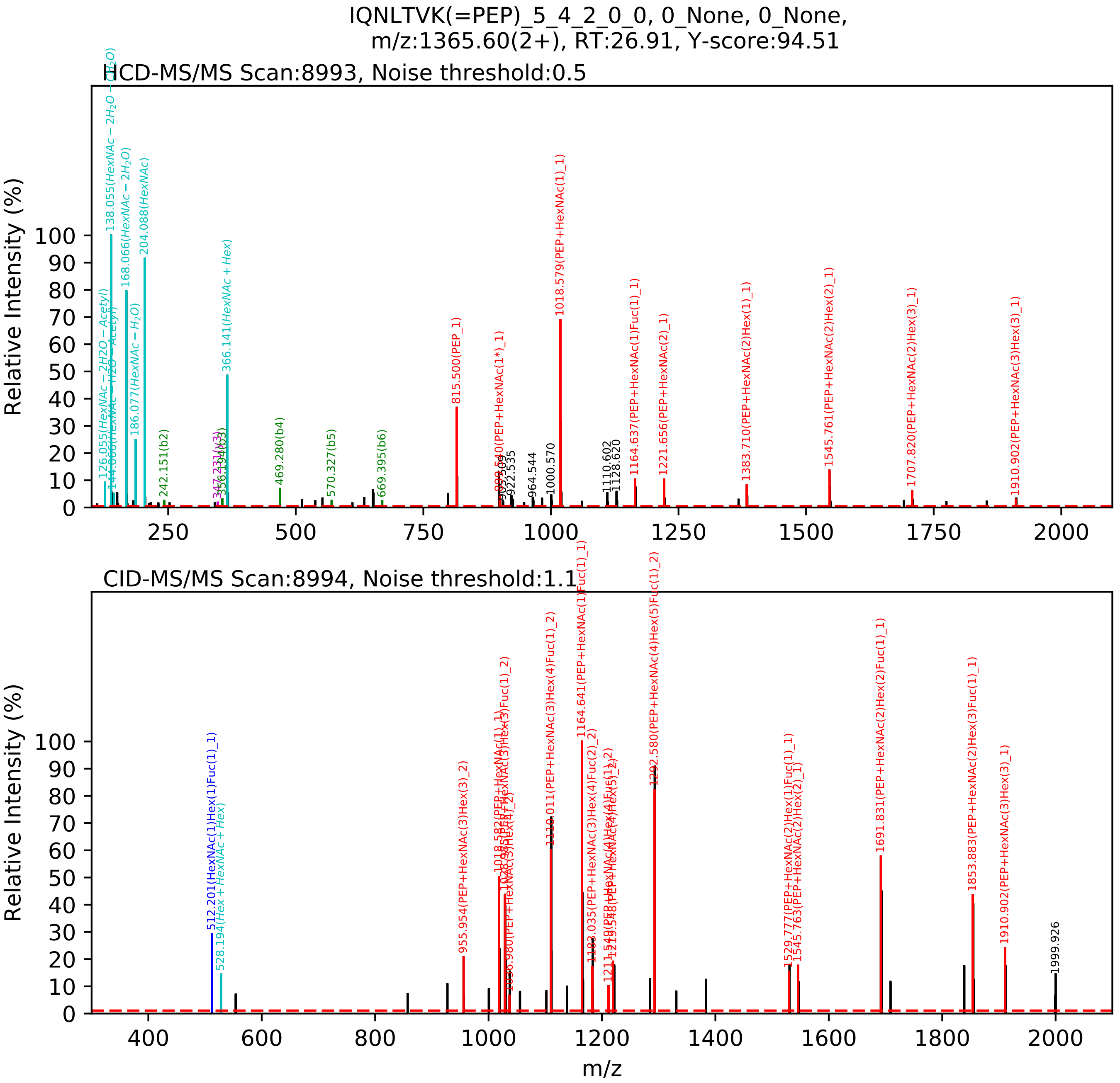
IQNLTVK(=PEP)_5_4_2_0_0, 0_None, 0_None,
m/z:1365.60(2+), RT:26.91, Y-score:94.51
HCD-MS/MS Scan:8993, Noise threshold:0.5
Relative Intensity (%)
138.055
168.066
204.088(HexNAc)
186.077
126.055
366.141(HexNAc + Hex)
242.151(b2)
469.280(b4)
570.327(b5)
669.395(b6)
815.500(PEP_1)
1018.579(PEP+HexNAc(1)_1)
964.544
1000.570
922.535
1110.602
1128.620
1164.637(PEP+HexNAc(1)Fuc(1)_1)
1221.656(PEP+HexNAc(2)_1)
1383.710(PEP+HexNAc(2)Hex(1)_1)
1545.761(PEP+HexNAc(2)Hex(2)_1)
1707.820(PEP+HexNAc(2)Hex(3)_1)
1910.902(PEP+HexNAc(3)Hex(3)_1)
CID-MS/MS Scan:8994, Noise threshold:1.1
512.201(HexNAc(1)Hex(1)Fuc(1)_1)
528.194(Hex + HexNAc + Hex)
955.954(PEP+HexNAc(3)Hex(3)_2)
1164.641(PEP+HexNAc(1)Fuc(1)_1)
1292.580(PEP+HexNAc(4)Hex(5)Fuc(1)_2)
1529.777(PEP+HexNAc(2)Hex(1)Fuc(1)_1)
1545.763(PEP+HexNAc(2)Hex(2)_1)
1691.831(PEP+HexNAc(2)Hex(2)Fuc(1)_1)
1853.883(PEP+HexNAc(2)Hex(3)Fuc(1)_1)
1910.902(PEP+HexNAc(3)Hex(3)_1)
1999.926
m/z

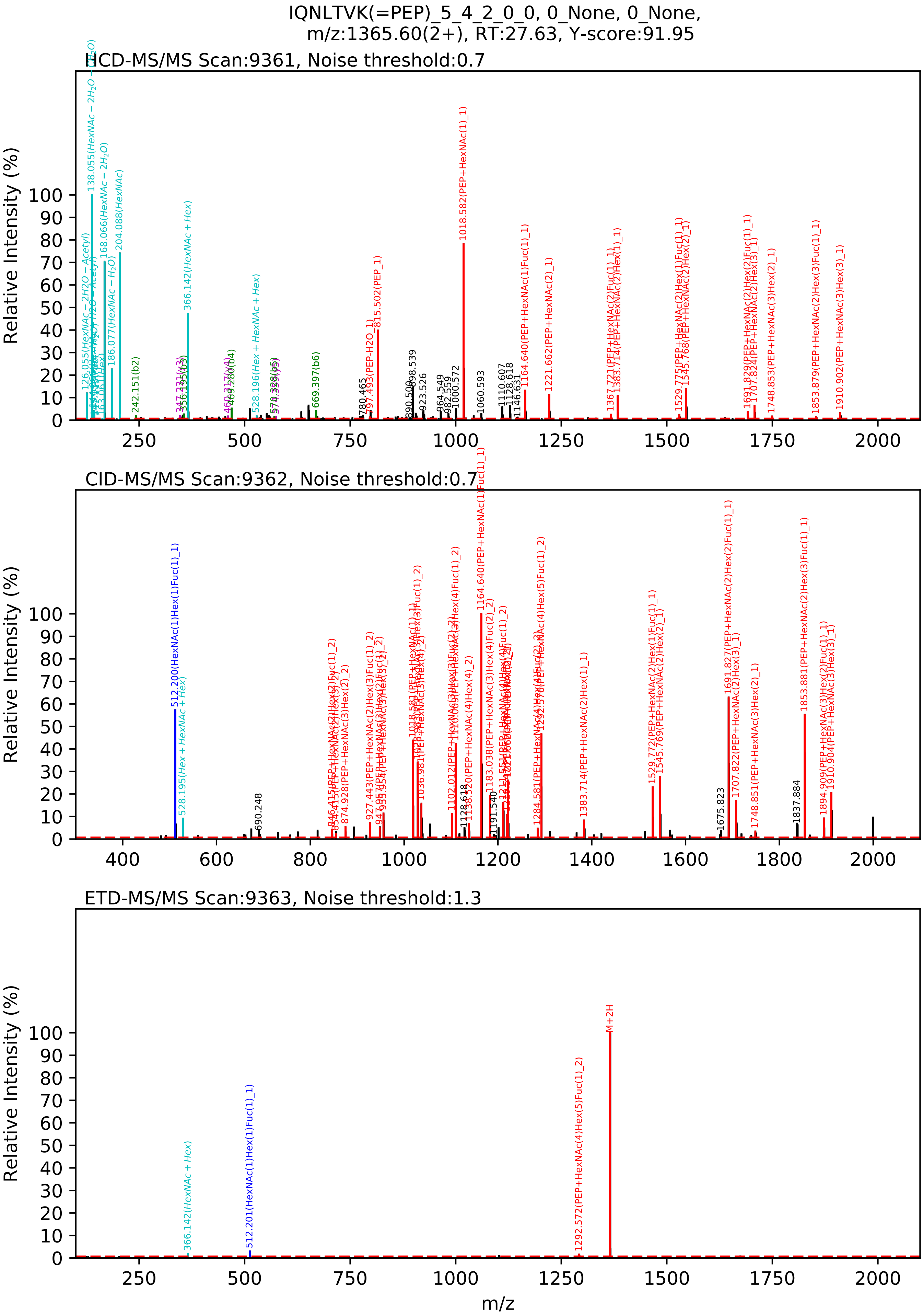
IQNLTVK(=PEP)_5_4_2_0_0, 0_None, 0_None,
m/z:1365.60(2+), RT:27.63, Y-score:91.95
HCD-MS/MS Scan:9361, Noise threshold:0.7
Relative Intensity (%)
138.055(HexNAc − 2H2O − Acetyl)
168.066(HexNAc − 2H2O)
186.077(HexNAc − H2O)
204.088(HexNAc)
366.142(HexNAc + Hex)
242.151(b2)
469.260(b4)
669.397(b6)
815.502(PEP_1)
797.493(PEP-H2O_1)
1018.582(PEP+HexNAc(1)_1)
1164.640(PEP+HexNAc(1)Fuc(1)_1)
1221.662(PEP+HexNAc(2)_1)
1545.768(PEP+HexNAc(2)Hex(2)_1)
1748.853(PEP+HexNAc(3)Hex(2)_1)
1853.879(PEP+HexNAc(2)Hex(3)Fuc(1)_1)
1910.902(PEP+HexNAc(3)Hex(3)_1)
CID-MS/MS Scan:9362, Noise threshold:0.7
512.200(HexNAc(1)Hex(1)Fuc(1)_1)
528.195(Hex + HexNAc + Hex)
690.248
874.928(PEP+HexNAc(3)Hex(2)_2)
1164.640(PEP+HexNAc(1)Fuc(1)_1)
1383.714(PEP+HexNAc(2)Hex(1)_1)
1545.769(PEP+HexNAc(2)Hex(2)_1)
1675.823
1691.827(PEP+HexNAc(2)Hex(2)Fuc(1)_1)
1707.822(PEP+HexNAc(2)Hex(3)_1)
1748.851(PEP+HexNAc(3)Hex(2)_1)
1837.884
1853.881(PEP+HexNAc(2)Hex(3)Fuc(1)_1)
ETD-MS/MS Scan:9363, Noise threshold:1.3
366.142(HexNAc + Hex)
512.201(HexNAc(1)Hex(1)Fuc(1)_1)
1292.572(PEP+HexNAc(4)Hex(5)Fuc(1)_2)
M+2H
m/z

IQNLTVK(=PEP)_5_4_2_0_0, 0_None, 0_None,
m/z:910.74(3+), RT:25.66, Y-score:96.65

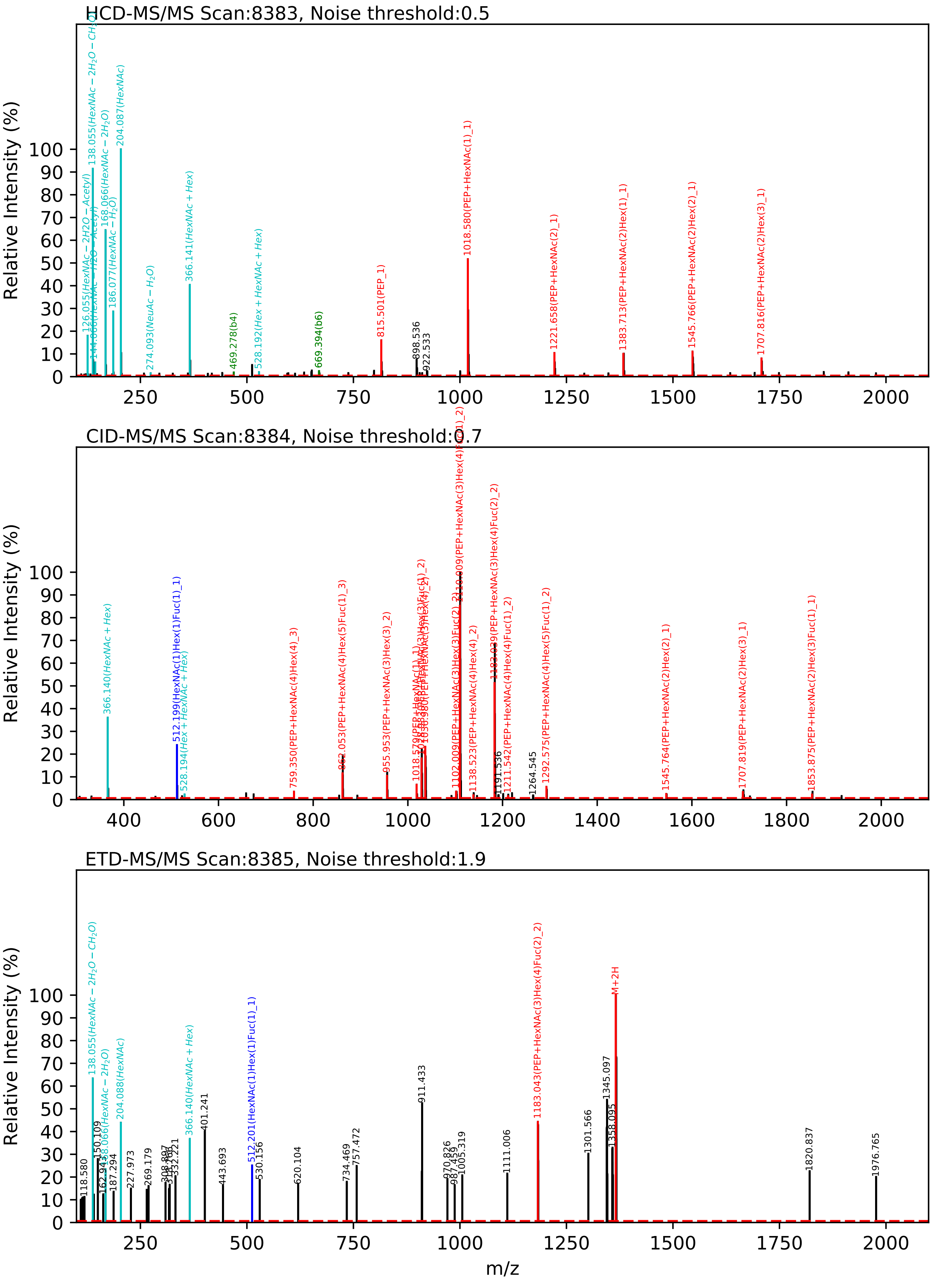

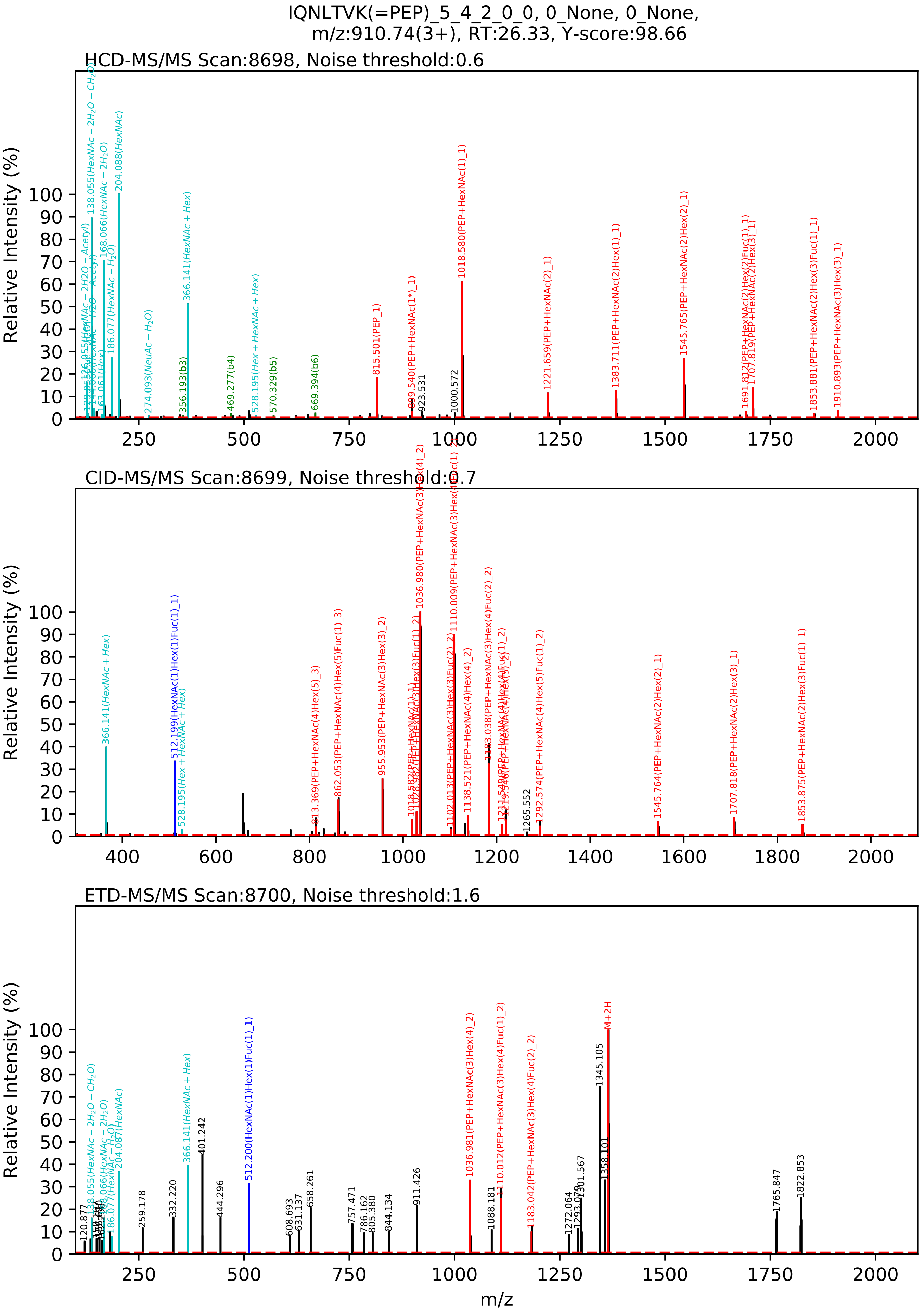

IQNLTVK(=PEP)_5_4_2_0_0, 0_None, 0_None,
m/z:910.74(3+), RT:26.33, Y-score:98.66
HCD-MS/MS Scan:8698, Noise threshold:0.6
Relative Intensity (%)
204.088(HexNAc)
366.141(HexNAc+Hex)
815.501(PEP_1)
1018.580(PEP+HexNAc(1)_1)
1221.659(PEP+HexNAc(2)_1)
1383.711(PEP+HexNAc(2)Hex(1)_1)
1545.765(PEP+HexNAc(2)Hex(2)_1)
1853.881(PEP+HexNAc(2)Hex(3)Fuc(1)_1)
1910.893(PEP+HexNAc(3)Hex(3)_1)
CID-MS/MS Scan:8699, Noise threshold:0.7
366.141(HexNAc+Hex)
512.199(HexNAc(1)Hex(1)Fuc(1)_1)
862.053(PEP+HexNAc(4)Hex(5)Fuc(1)_3)
955.953(PEP+HexNAc(3)Hex(3)_2)
1036.980(PEP+HexNAc(3)Hex(4)_2)
1545.764(PEP+HexNAc(2)Hex(2)_1)
1707.818(PEP+HexNAc(2)Hex(3)_1)
1853.875(PEP+HexNAc(2)Hex(3)Fuc(1)_1)
ETD-MS/MS Scan:8700, Noise threshold:1.6
512.200(HexNAc(1)Hex(1)Fuc(1)_1)
1036.981(PEP+HexNAc(3)Hex(4)_2)
1110.012(PEP+HexNAc(3)Hex(4)Fuc(1)_2)
1183.042(PEP+HexNAc(3)Hex(4)Fuc(2)_2)
M+2H
1345.105
1765.847
1822.853
m/z

IQNLTVK(=PEP)_5_4_2_0_0, 0_None, 0_None,
m/z:910.74(3+), RT:26.91, Y-score:63.38

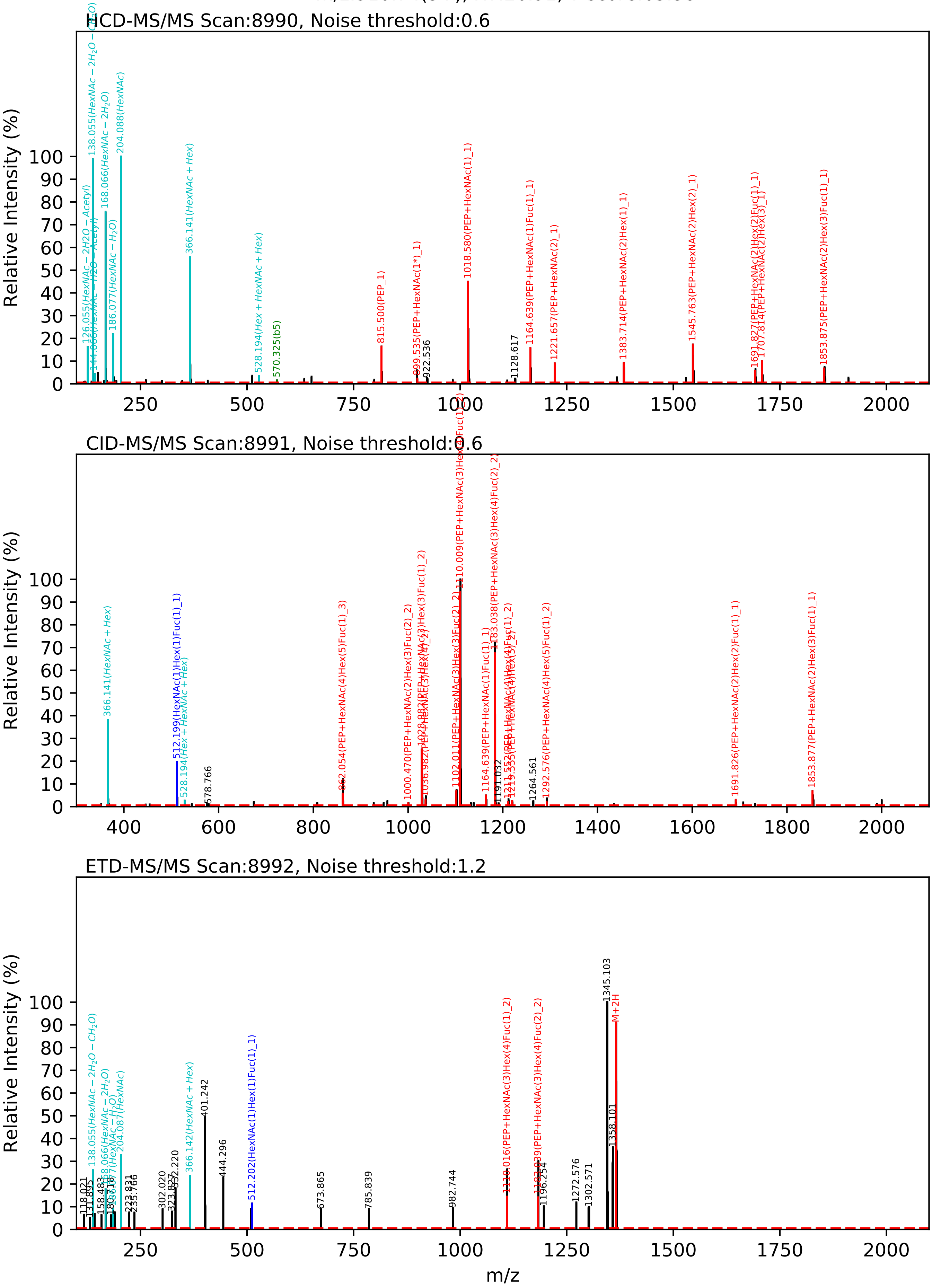

IQNLTVK(=PEP)_5_4_2_0_0, 0_None, 0_None,
m/z:910.74(3+), RT:27.71, Y-score:60.03

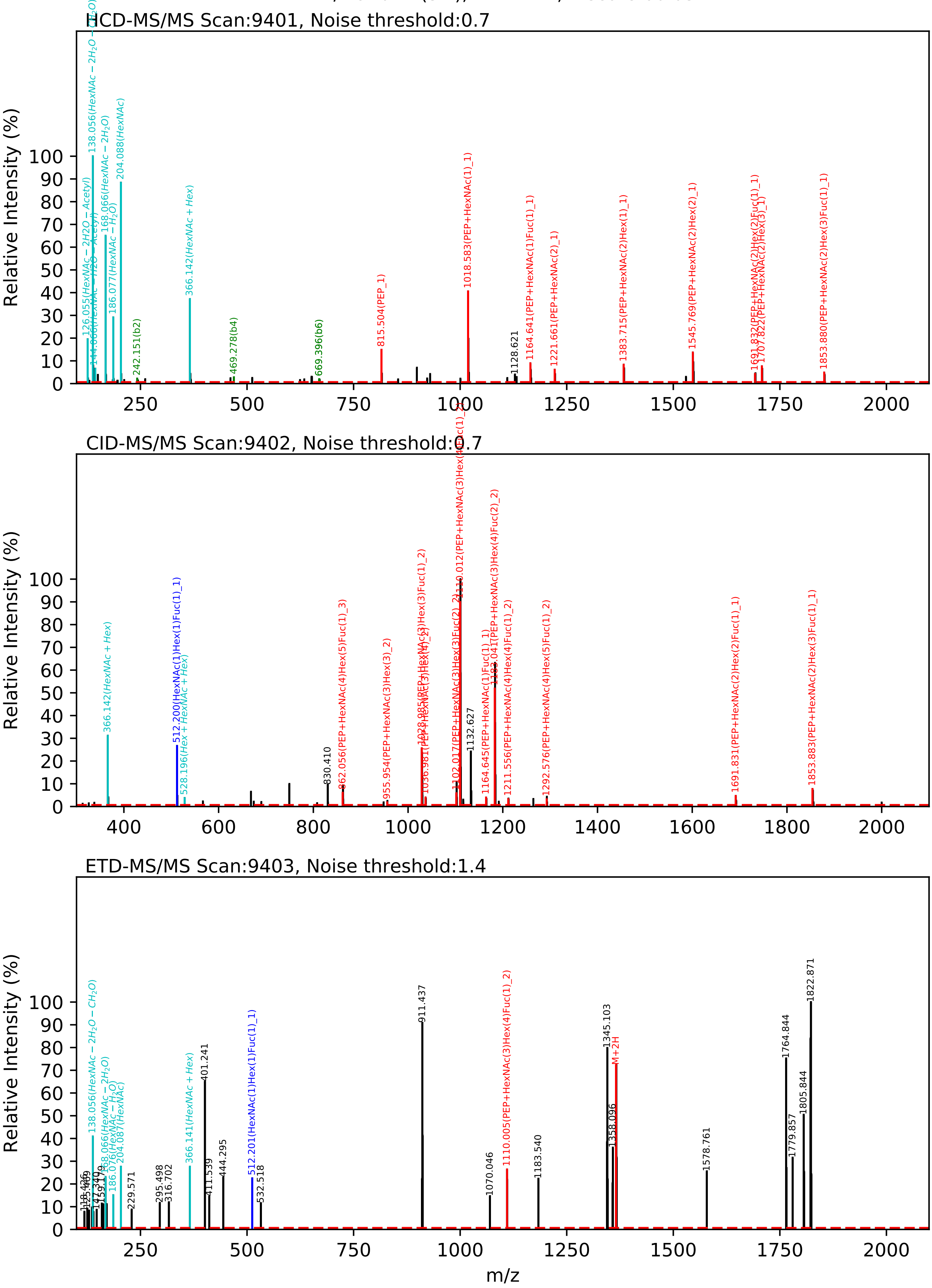

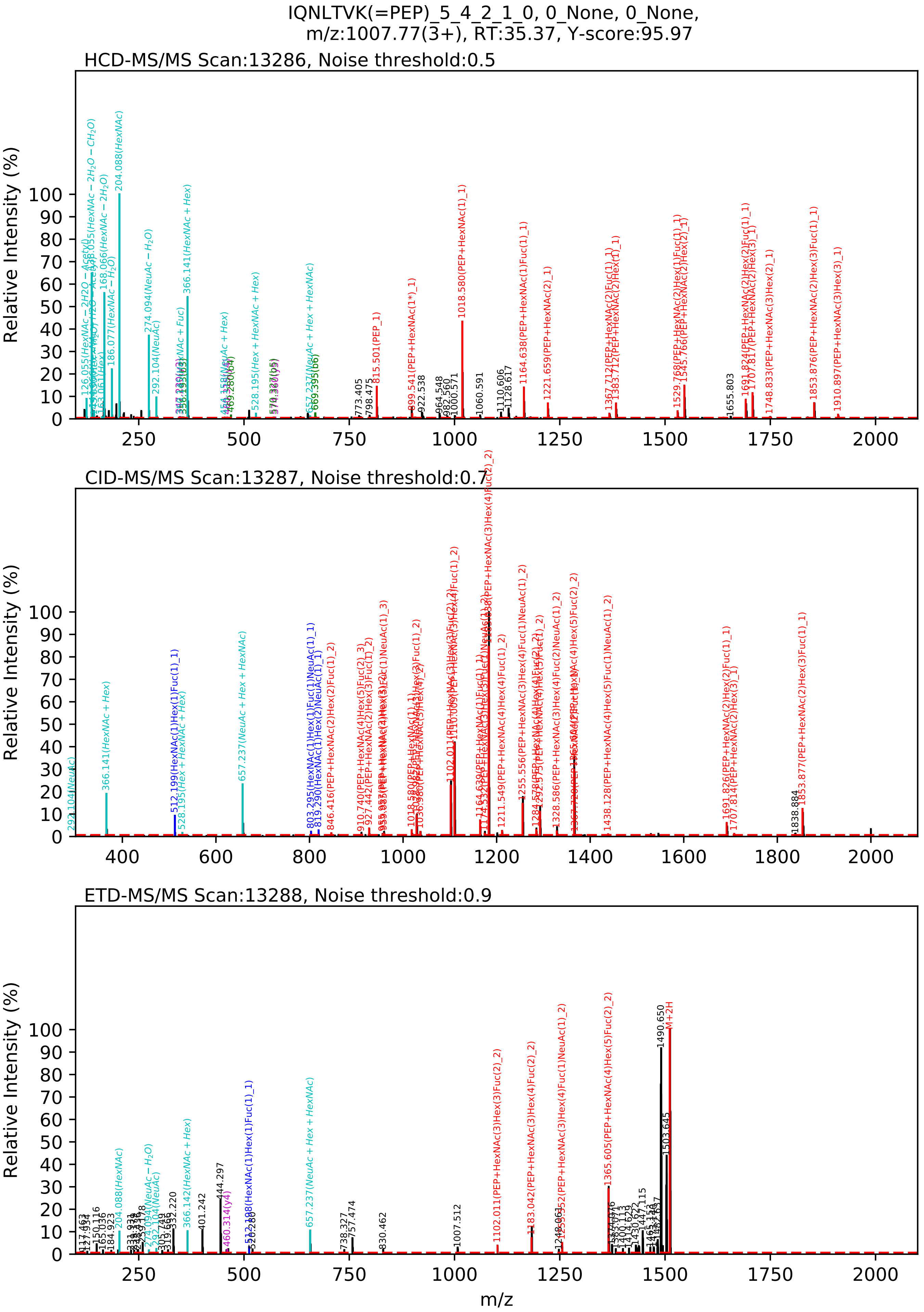

IQNLTVK(=PEP)_5_4_2_1_0, 0_None, 0_None,
m/z:1007.77(3+), RT:35.37, Y-score:95.97
HCD-MS/MS Scan:13286, Noise threshold:0.5
CID-MS/MS Scan:13287, Noise threshold:0.7
ETD-MS/MS Scan:13288, Noise threshold:0.9
Relative Intensity (%)
m/z
204.088(HexNAc)
366.141(HexNAc+Hex)
274.094(NeuAc−H2O)
292.104(NeuAc)
168.066(HexNAc−H2O)
186.077(HexNAc−H2O)
528.195(Hex+HexNAc+Hex)
657.237(NeuAc+Hex+HexNAc)
815.501(PEP_1)
899.541(PEP+HexNAc(1*)_1)
1018.580(PEP+HexNAc(1)_1)
1164.638(PEP+HexNAc(1)Fuc(1)_1)
1221.659(PEP+HexNAc(2)_1)
1748.833(PEP+HexNAc(3)Hex(2)_1)
1853.876(PEP+HexNAc(2)Hex(3)Fuc(1)_1)
1910.897(PEP+HexNAc(3)Hex(3)_1)
512.199(HexNAc(1)Hex(1)Fuc(1)_1)
1438.128(PEP+HexNAc(4)Hex(5)Fuc(1)NeuAc(1)_2)
1691.826(PEP+HexNAc(2)Hex(2)Fuc(1)_1)
1853.877(PEP+HexNAc(2)Hex(3)Fuc(1)_1)
1102.011(PEP+HexNAc(3)Hex(3)Fuc(2)_2)
1183.042(PEP+HexNAc(3)Hex(4)Fuc(2)_2)
1365.605(PEP+HexNAc(4)Hex(5)Fuc(2)_2)
1490.650
1503.645
M+2H

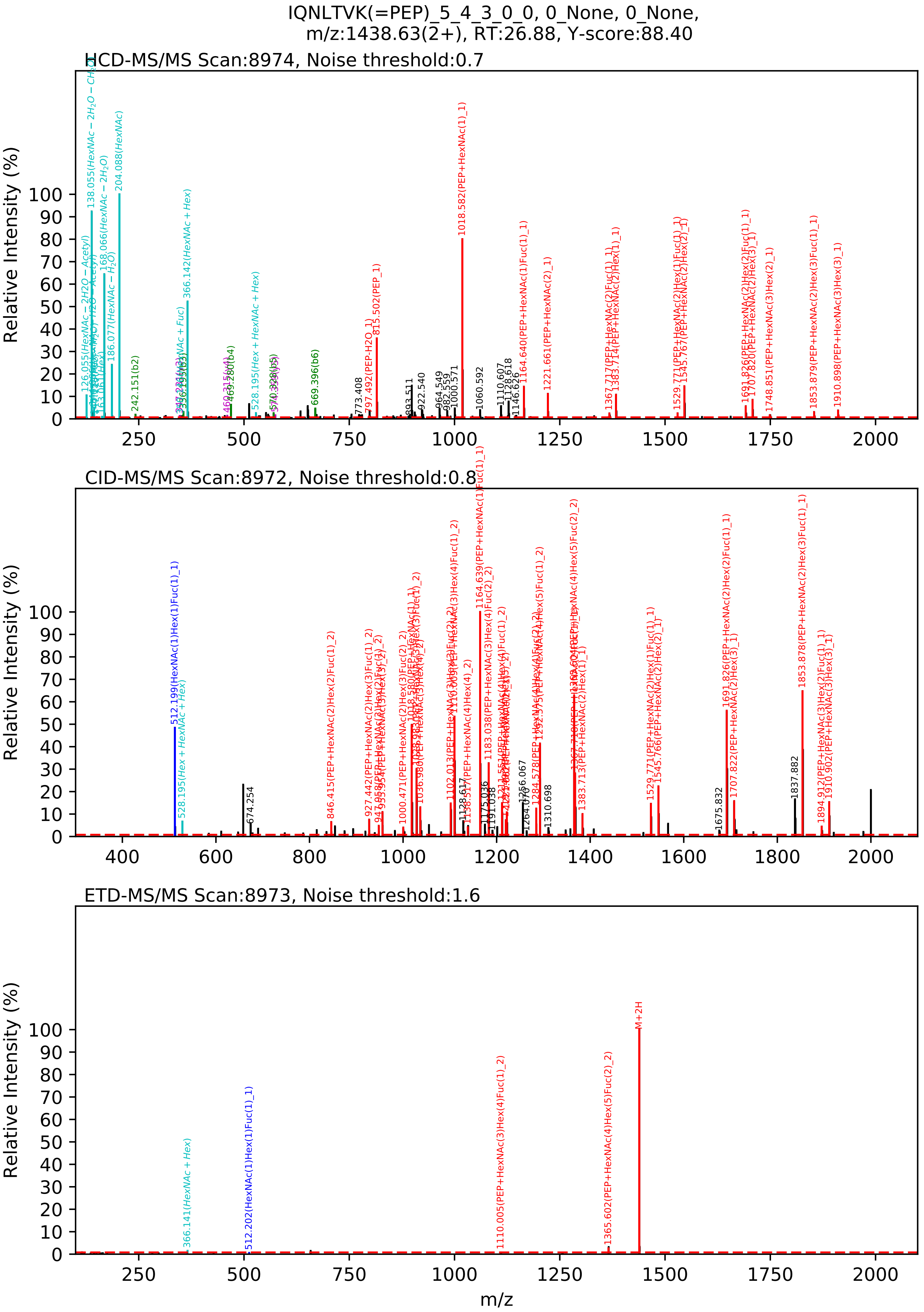
IQNLTVK(=PEP)_5_4_3_0_0, 0_None, 0_None,
m/z:1438.63(2+), RT:26.88, Y-score:88.40
HCD-MS/MS Scan:8974, Noise threshold:0.7
Relative Intensity (%)
204.088(HexNAc)
138.055(HexNAc − 2H2O − CH3)
366.142(HexNAc + Hex)
1018.582(PEP+HexNAc(1)_1)
815.502(PEP_1)
797.492(PEP-H2O_1)
1164.640(PEP+HexNAc(1)Fuc(1)_1)
1221.661(PEP+HexNAc(2)_1)
242.151(b2)
469.280(b4)
669.396(b6)
CID-MS/MS Scan:8972, Noise threshold:0.8
512.199(HexNAc(1)Hex(1)Fuc(1)_1)
528.195(Hex + HexNAc + Hex)
674.254
846.415(PEP+HexNAc(2)Hex(2)Fuc(1)_2)
1164.639(PEP+HexNAc(1)Fuc(1)_1)
1183.038(PEP+HexNAc(3)Hex(4)Fuc(1)_2)
1691.826(PEP+HexNAc(2)Hex(2)Fuc(1)_1)
1707.822(PEP+HexNAc(2)Hex(3)_1)
1853.878(PEP+HexNAc(2)Hex(3)Fuc(1)_1)
1837.882
1675.832
ETD-MS/MS Scan:8973, Noise threshold:1.6
366.141(HexNAc + Hex)
512.202(HexNAc(1)Hex(1)Fuc(1)_1)
1110.005(PEP+HexNAc(3)Hex(4)Fuc(1)_2)
1365.602(PEP+HexNAc(4)Hex(5)Fuc(2)_2)
M+2H
m/z

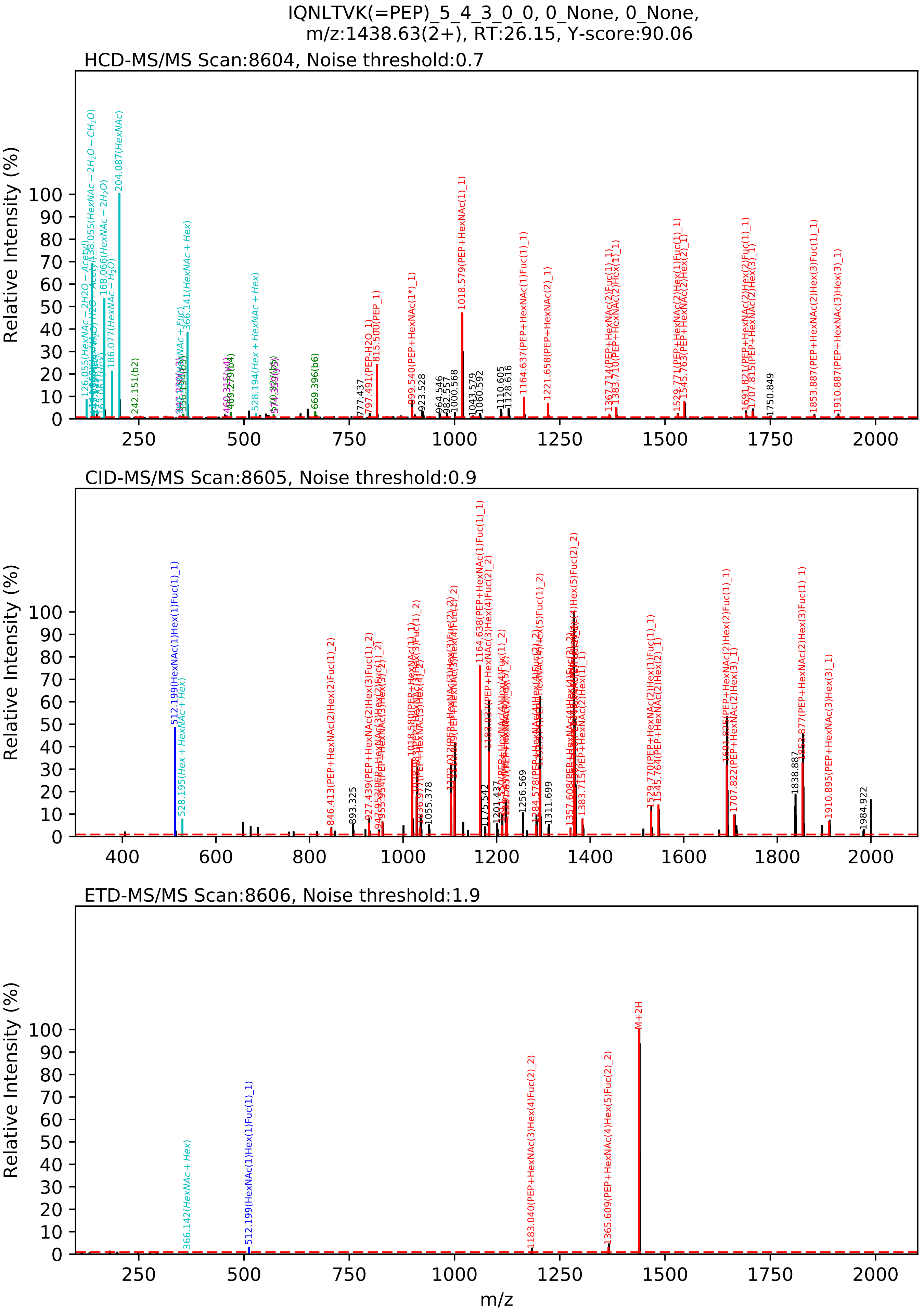
IQNLTVK(=PEP)_5_4_3_0_0, 0_None, 0_None,
m/z:1438.63(2+), RT:26.15, Y-score:90.06
HCD-MS/MS Scan:8604, Noise threshold:0.7
Relative Intensity (%)
204.087(HexNAc)
168.066(HexNAc − 2H2O)
186.077(HexNAc − H2O)
366.141(HexNAc + Hex)
242.151(b2)
469.279(b4)
528.194(Hex + HexNAc + Hex)
669.396(b6)
777.437
797.491(PEP-H2O_1)
815.500(PEP_1)
899.540(PEP+HexNAc(1*)_1)
923.528
964.546
1000.568
1018.579(PEP+HexNAc(1)_1)
1043.579
1060.592
1110.605
1128.616
1164.637(PEP+HexNAc(1)Fuc(1)_1)
1221.658(PEP+HexNAc(2)_1)
1383.710(PEP+HexNAc(2)Hex(1)_1)
1529.771(PEP+HexNAc(2)Hex(1)Fuc(1)_1)
1545.763(PEP+HexNAc(2)Hex(2)_1)
1691.821(PEP+HexNAc(2)Hex(2)Fuc(1)_1)
1707.815(PEP+HexNAc(2)Hex(3)_1)
1750.849
1853.887(PEP+HexNAc(2)Hex(3)Fuc(1)_1)
1910.887(PEP+HexNAc(3)Hex(3)_1)
CID-MS/MS Scan:8605, Noise threshold:0.9
512.199(HexNAc(1)Hex(1)Fuc(1)_1)
528.195(Hex + HexNAc + Hex)
846.413(PEP+HexNAc(2)Hex(2)Fuc(1)_2)
893.325
1055.378
1164.638(PEP+HexNAc(1)Fuc(1)_1)
1201.437
1256.569
1311.699
1383.715(PEP+HexNAc(2)Hex(1)_1)
1529.770(PEP+HexNAc(2)Hex(1)Fuc(1)_1)
1545.764(PEP+HexNAc(2)Hex(2)_1)
1707.822(PEP+HexNAc(2)Hex(3)_1)
1838.887
1910.895(PEP+HexNAc(3)Hex(3)_1)
1984.922
ETD-MS/MS Scan:8606, Noise threshold:1.9
366.142(HexNAc + Hex)
512.199(HexNAc(1)Hex(1)Fuc(1)_1)
1183.040(PEP+HexNAc(3)Hex(4)Fuc(2)_2)
1365.609(PEP+HexNAc(4)Hex(5)Fuc(2)_2)
M+2H
m/z

IQNLTVK(=PEP)_5_5_0_0_0, 0_None, 0_None,
m/z:1321.08(2+), RT:26.88, Y-score:59.38

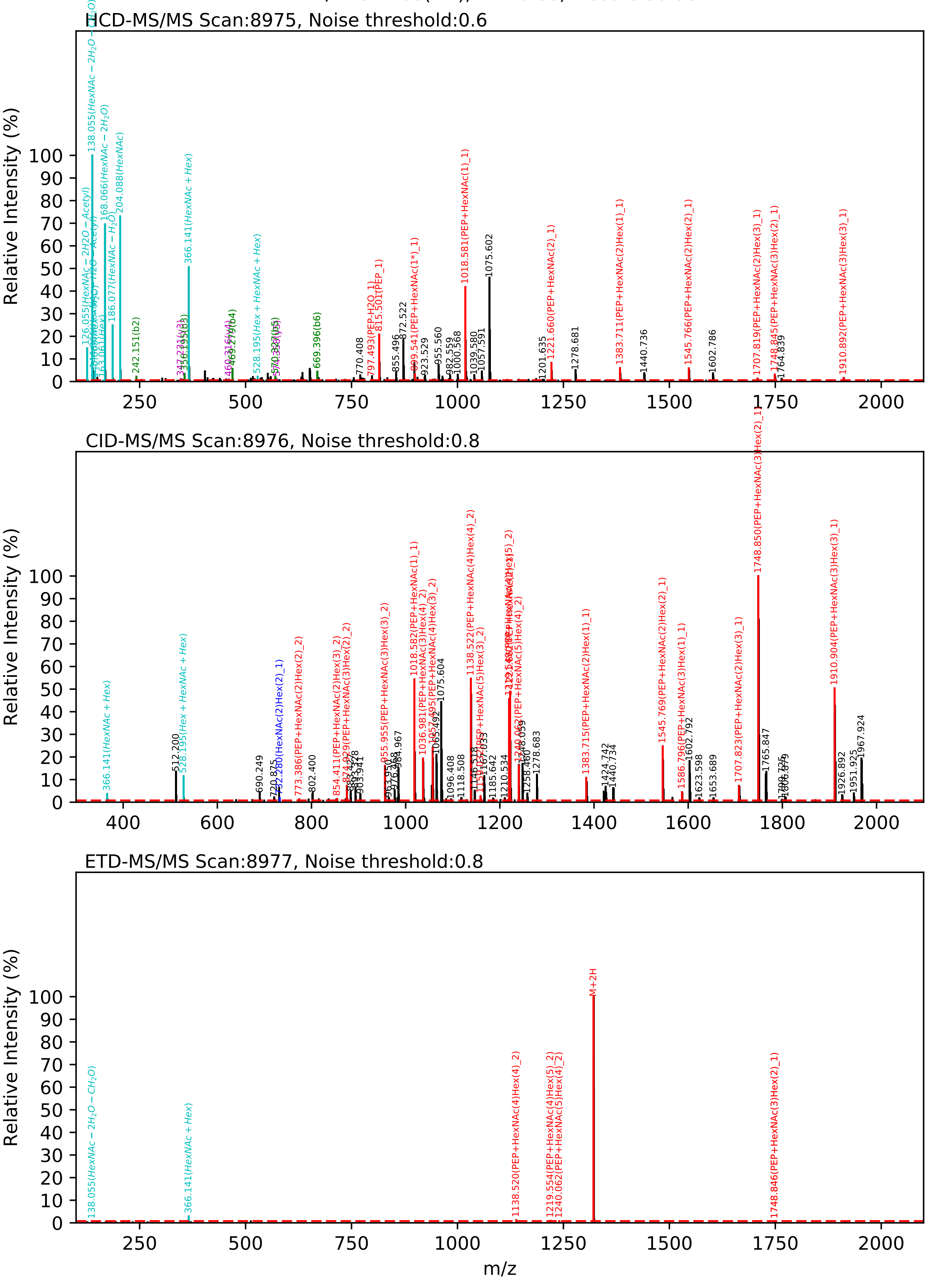

IQNLTVK(=PEP)_5_5_0_0_0, 0_None, 0_None,
m/z:1321.08(2+), RT:27.77, Y-score:89.48

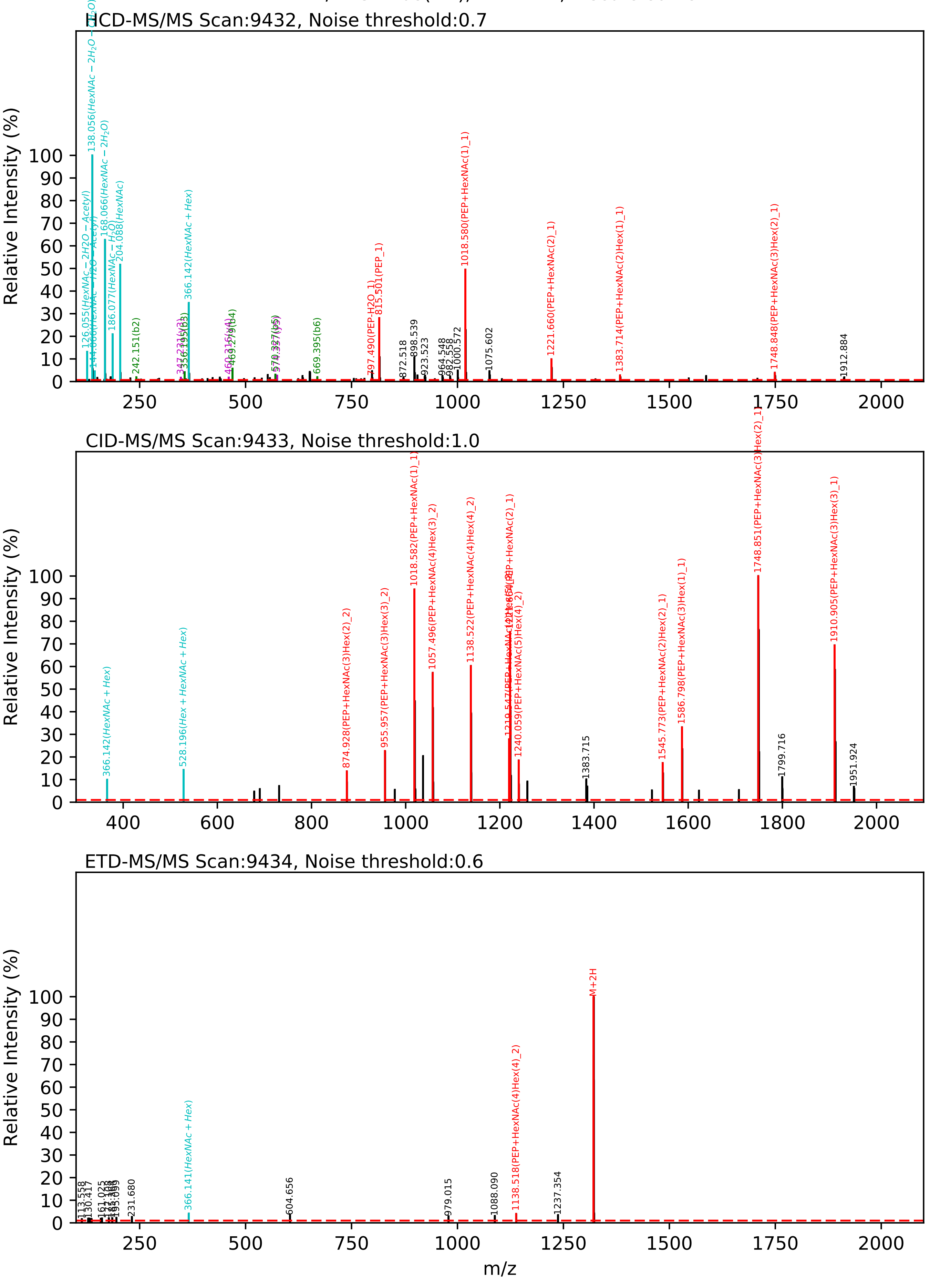

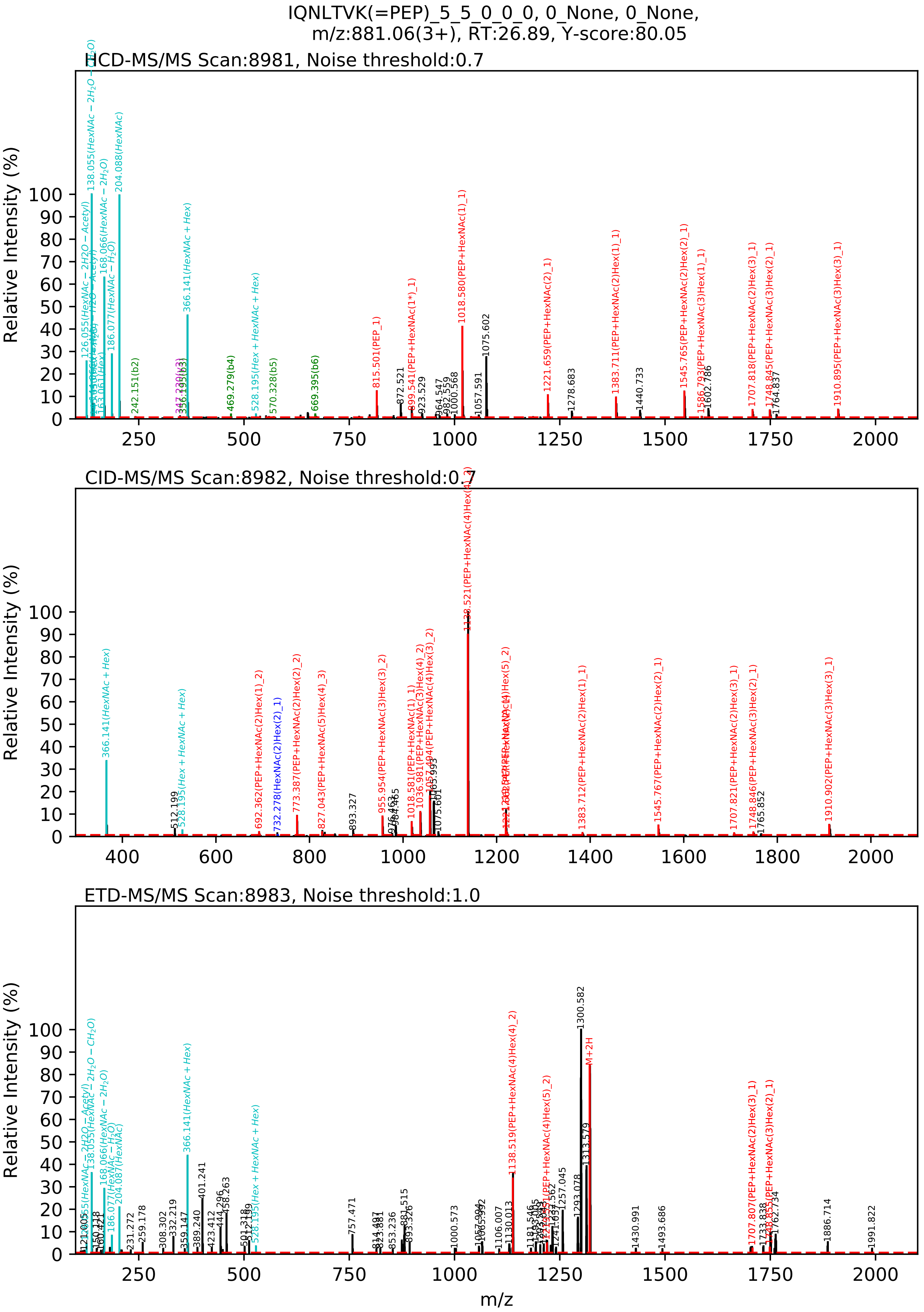

IQNLTVK(=PEP)_5_5_0_0_0, 0_None, 0_None,
m/z:881.06(3+), RT:26.89, Y-score:80.05
HCD-MS/MS Scan:8981, Noise threshold:0.7
CID-MS/MS Scan:8982, Noise threshold:0.7
ETD-MS/MS Scan:8983, Noise threshold:1.0
Relative Intensity (%)
m/z
138.055(HexNAc − 2H2O − CH2O)
204.088(HexNAc)
168.066(HexNAc − 2H2O)
186.077(HexNAc − H2O)
366.141(HexNAc + Hex)
528.195(Hex + HexNAc + Hex)
242.151(b2)
469.279(b4)
570.328(b5)
669.395(b6)
815.501(PEP_1)
899.541(PEP+HexNAc(1*)_1)
1018.580(PEP+HexNAc(1)_1)
1075.602
1221.659(PEP+HexNAc(2)_1)
1383.711(PEP+HexNAc(2)Hex(1)_1)
1545.765(PEP+HexNAc(2)Hex(2)_1)
1586.793(PEP+HexNAc(3)Hex(1)_1)
1707.818(PEP+HexNAc(2)Hex(3)_1)
1748.845(PEP+HexNAc(3)Hex(2)_1)
1910.895(PEP+HexNAc(3)Hex(3)_1)
692.362(PEP+HexNAc(2)Hex(1)_2)
732.278(HexNAc(2)Hex(2)_1)
773.387(PEP+HexNAc(2)Hex(2)_2)
827.043(PEP+HexNAc(5)Hex(4)_3)
955.954(PEP+HexNAc(3)Hex(3)_2)
1138.521(PEP+HexNAc(4)Hex(4)_2)
1300.582
M+2H
1313.579

IQNLTVK(=PEP)_5_5_0_0_0, 0_None, 0_None, m/z:881.06(3+), RT:26.98, Y-score:88.40

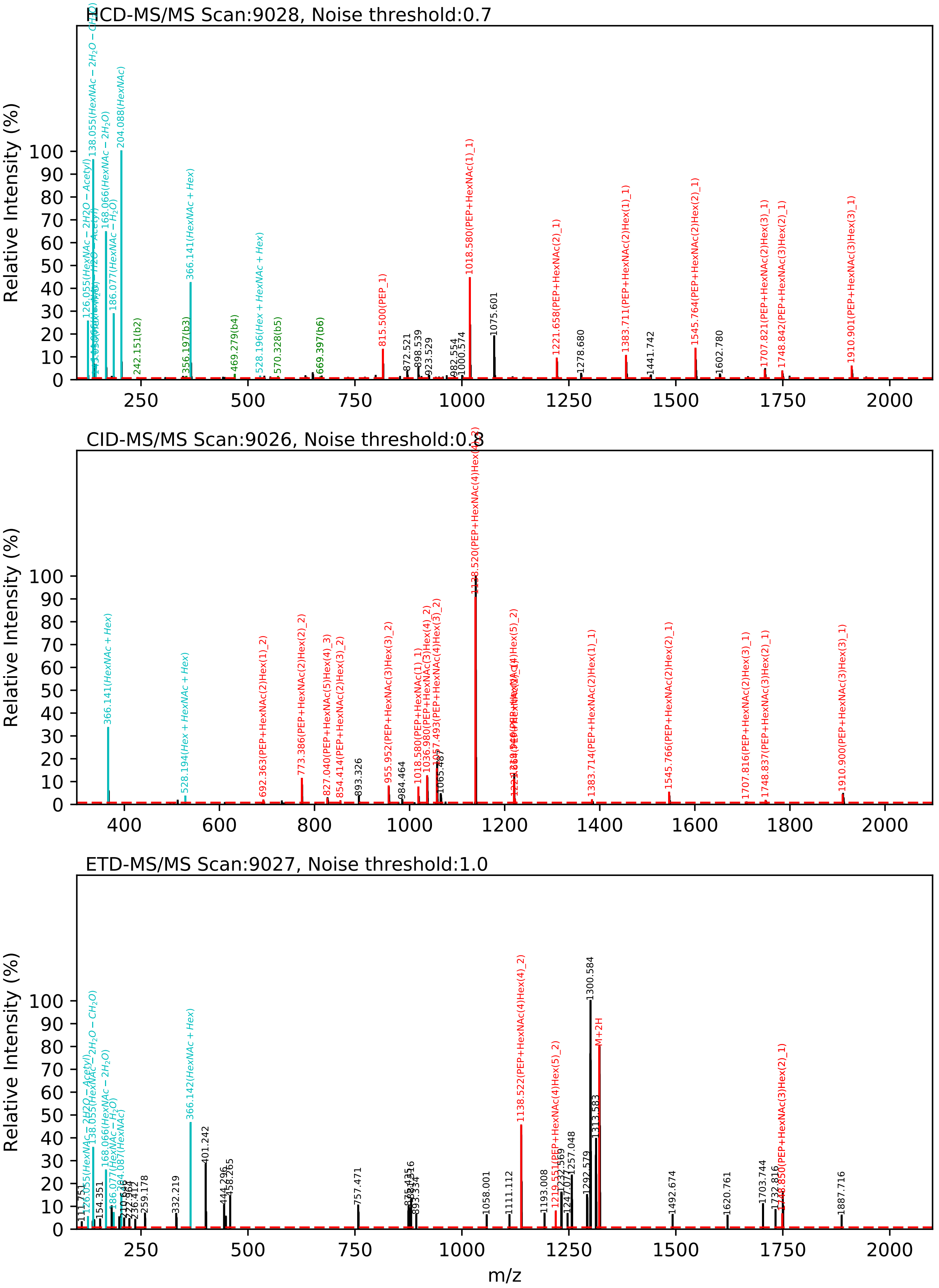

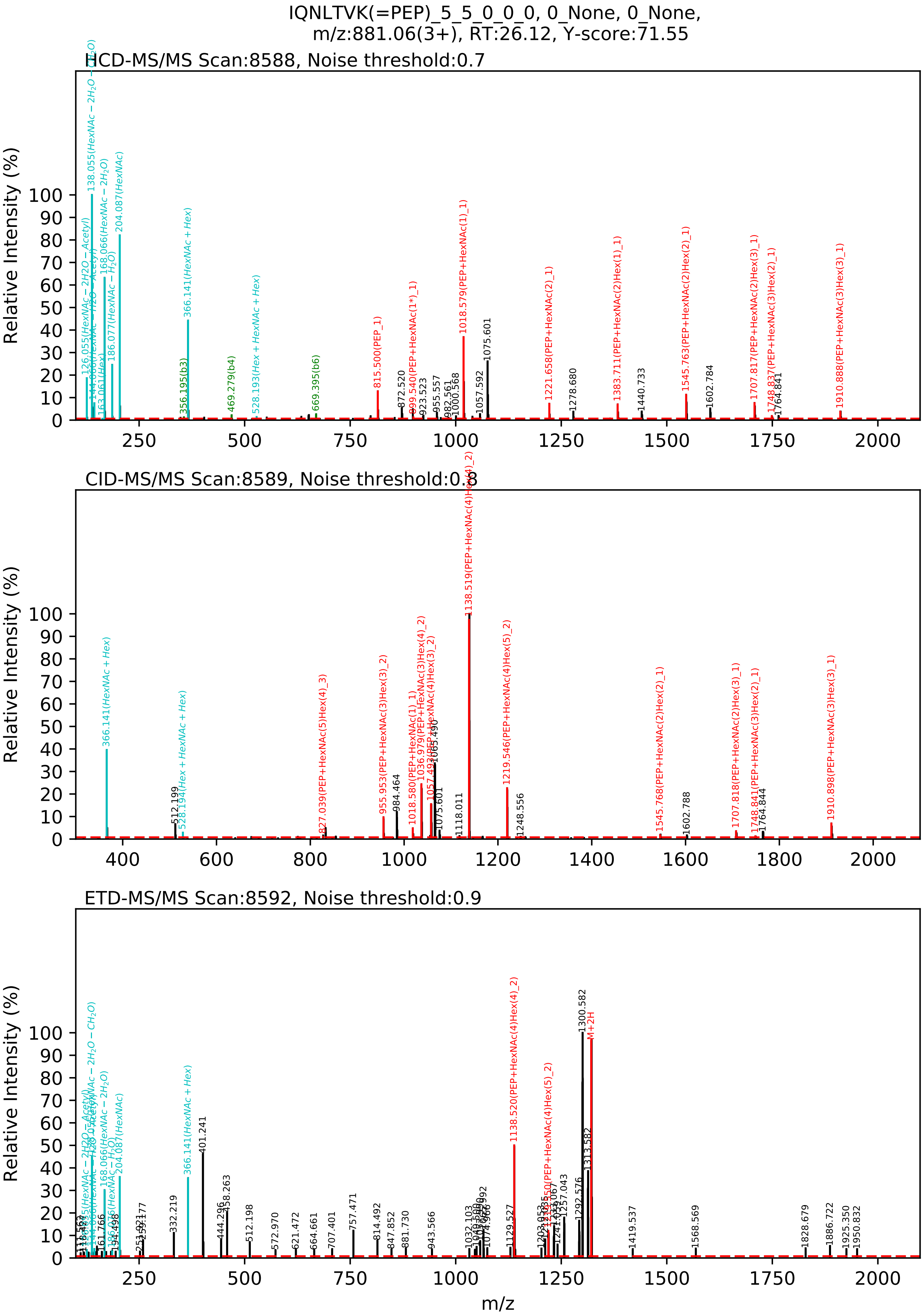

IQNLTVK(=PEP)_5_5_0_0_0, 0_None, 0_None,
m/z:881.06(3+), RT:26.12, Y-score:71.55
HCD-MS/MS Scan:8588, Noise threshold:0.7
CID-MS/MS Scan:8589, Noise threshold:0.8
ETD-MS/MS Scan:8592, Noise threshold:0.9
Relative Intensity (%)
m/z
815.500(PEP_1)
1018.579(PEP+HexNAc(1)_1)
1138.519(PEP+HexNAc(4)Hex(4)_2)
1138.520(PEP+HexNAc(4)Hex(4)_2)
1219.546(PEP+HexNAc(4)Hex(5)_2)
1910.888(PEP+HexNAc(3)Hex(3)_1)
M+2H

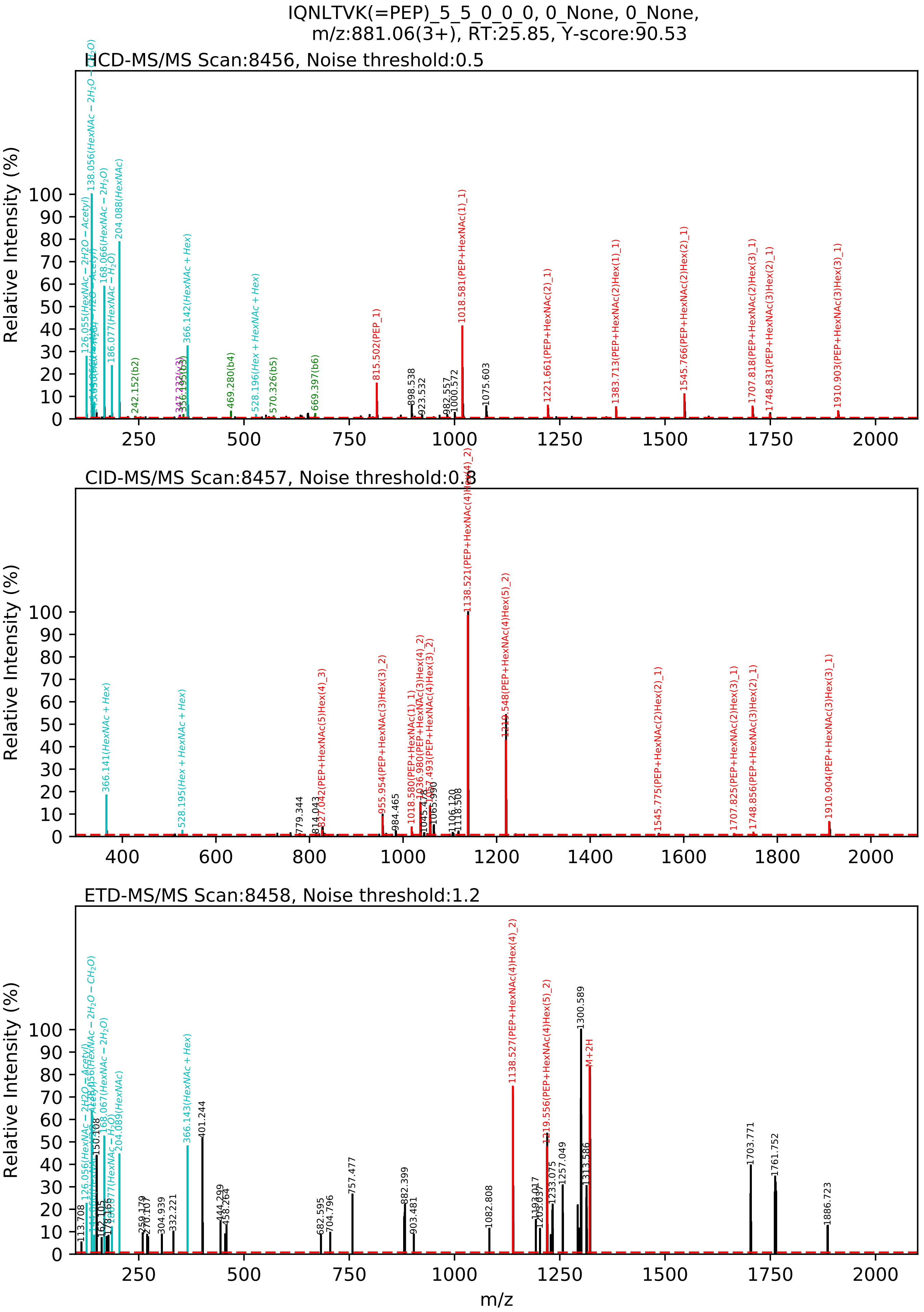
IQNLTVK(=PEP)_5_5_0_0_0, 0_None, 0_None,
m/z:881.06(3+), RT:25.85, Y-score:90.53
HCD-MS/MS Scan:8456, Noise threshold:0.5
CID-MS/MS Scan:8457, Noise threshold:0.8
ETD-MS/MS Scan:8458, Noise threshold:1.2
Relative Intensity (%)
m/z
815.502(PEP_1)
1018.581(PEP+HexNAc(1)_1)
1221.661(PEP+HexNAc(2)_1)
1383.713(PEP+HexNAc(2)Hex(1)_1)
1545.766(PEP+HexNAc(2)Hex(2)_1)
1707.818(PEP+HexNAc(2)Hex(3)_1)
1748.831(PEP+HexNAc(3)Hex(2)_1)
1910.903(PEP+HexNAc(3)Hex(3)_1)
1138.521(PEP+HexNAc(4)Hex(4)_2)
1219.548(PEP+HexNAc(4)Hex(5)_2)
1138.527(PEP+HexNAc(4)Hex(4)_2)
1219.556(PEP+HexNAc(4)Hex(5)_2)
1300.589
M+2H

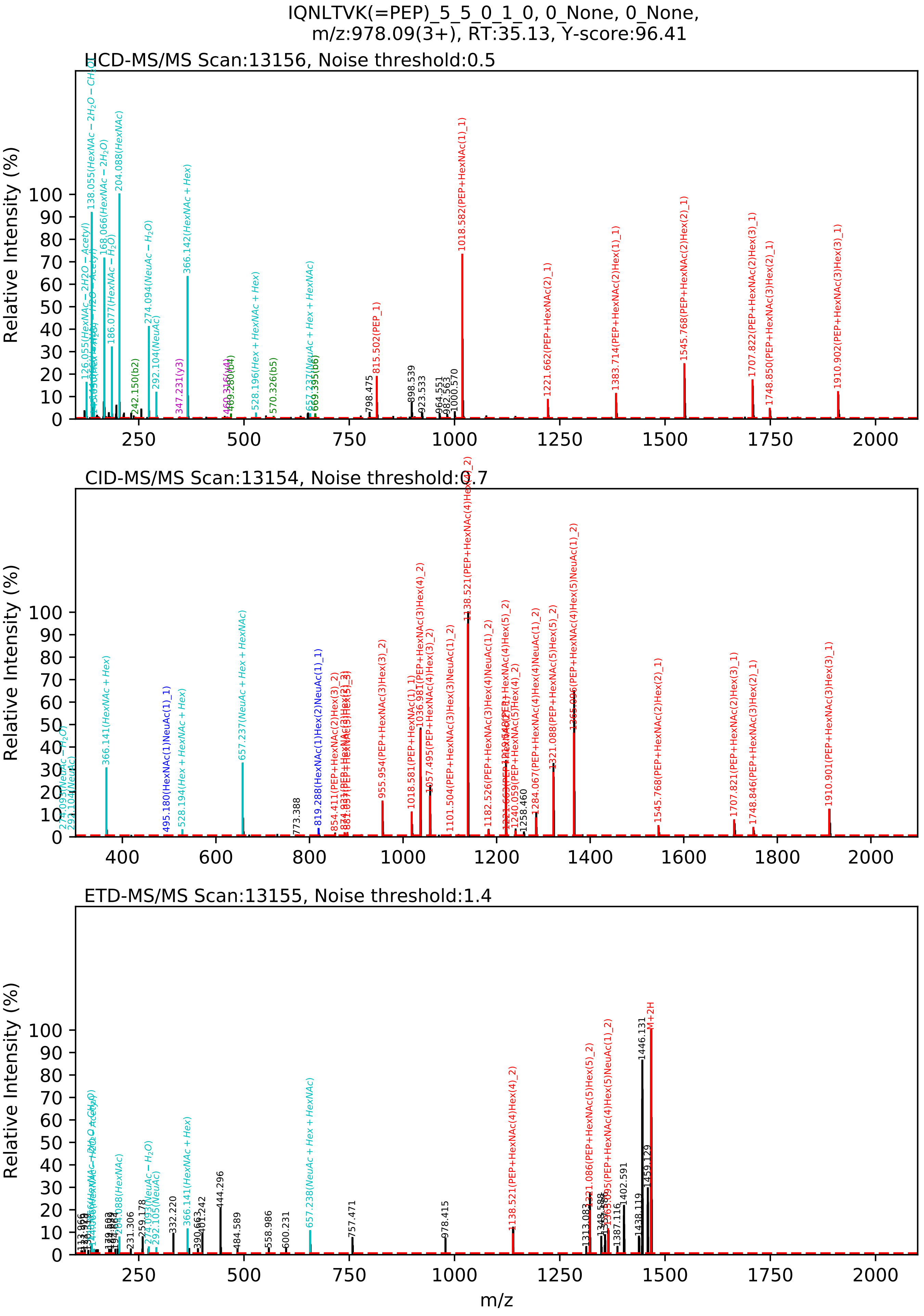
IQNLTVK(=PEP)_5_5_0_1_0, 0_None, 0_None,
m/z:978.09(3+), RT:35.13, Y-score:96.41
HCD-MS/MS Scan:13156, Noise threshold:0.5
Relative Intensity (%)
138.055(HexNAc − 2H2O − CH4O)
204.088(HexNAc)
168.066(HexNAc − 2H2O)
186.077(HexNAc − H2O)
126.055(HexNAc − 2H2O − Acetyl)
274.094(NeuAc − H2O)
292.104(NeuAc)
366.142(HexNAc + Hex)
242.150(b2)
347.231(y3)
528.196(Hex + HexNAc + Hex)
570.326(b5)
669.395(b6)
815.502(PEP_1)
798.475
898.539
923.533
964.551
1000.570
1018.582(PEP+HexNAc(1)_1)
1221.662(PEP+HexNAc(2)_1)
1383.714(PEP+HexNAc(2)Hex(1)_1)
1545.768(PEP+HexNAc(2)Hex(2)_1)
1707.822(PEP+HexNAc(2)Hex(3)_1)
1748.850(PEP+HexNAc(3)Hex(2)_1)
1910.902(PEP+HexNAc(3)Hex(3)_1)
CID-MS/MS Scan:13154, Noise threshold:0.7
366.141(HexNAc + Hex)
495.180(HexNAc(1)NeuAc(1)_1)
528.194(Hex + HexNAc + Hex)
657.237(NeuAc + Hex + HexNAc)
773.388
819.288(HexNAc(1)Hex(2)NeuAc(1)_1)
955.954(PEP+HexNAc(3)Hex(3)_2)
1018.581(PEP+HexNAc(1)_1)
1036.981(PEP+HexNAc(3)Hex(4)_2)
1057.495(PEP+HexNAc(4)Hex(3)_2)
1101.504(PEP+HexNAc(3)Hex(3)NeuAc(1)_2)
1138.521(PEP+HexNAc(4)Hex(4)_2)
1182.526(PEP+HexNAc(3)Hex(4)NeuAc(1)_2)
1240.059(PEP+HexNAc(5)Hex(4)_2)
1258.460
1284.067(PEP+HexNAc(4)Hex(4)NeuAc(1)_2)
1321.088(PEP+HexNAc(5)Hex(5)_2)
1365.096(PEP+HexNAc(4)Hex(5)NeuAc(1)_2)
1545.768(PEP+HexNAc(2)Hex(2)_1)
1707.821(PEP+HexNAc(2)Hex(3)_1)
1748.846(PEP+HexNAc(3)Hex(2)_1)
1910.901(PEP+HexNAc(3)Hex(3)_1)
ETD-MS/MS Scan:13155, Noise threshold:1.4
204.088(HexNAc)
274.093(NeuAc − H2O)
292.105(NeuAc)
231.306
259.178
332.220
366.141(HexNAc + Hex)
390.663
401.242
444.296
484.589
558.986
600.231
657.238(NeuAc + Hex + HexNAc)
757.471
978.415
1138.521(PEP+HexNAc(4)Hex(4)_2)
1313.083
1321.086(PEP+HexNAc(5)Hex(5)_2)
1365.095(PEP+HexNAc(4)Hex(5)NeuAc(1)_2)
1387.116
1402.591
1438.119
1446.131
1459.129
M+2H
m/z

IQNLTVK(=PEP)_5_5_0_1_0, 0_None, 0_None,
m/z:1466.63(2+), RT:37.38, Y-score:68.51

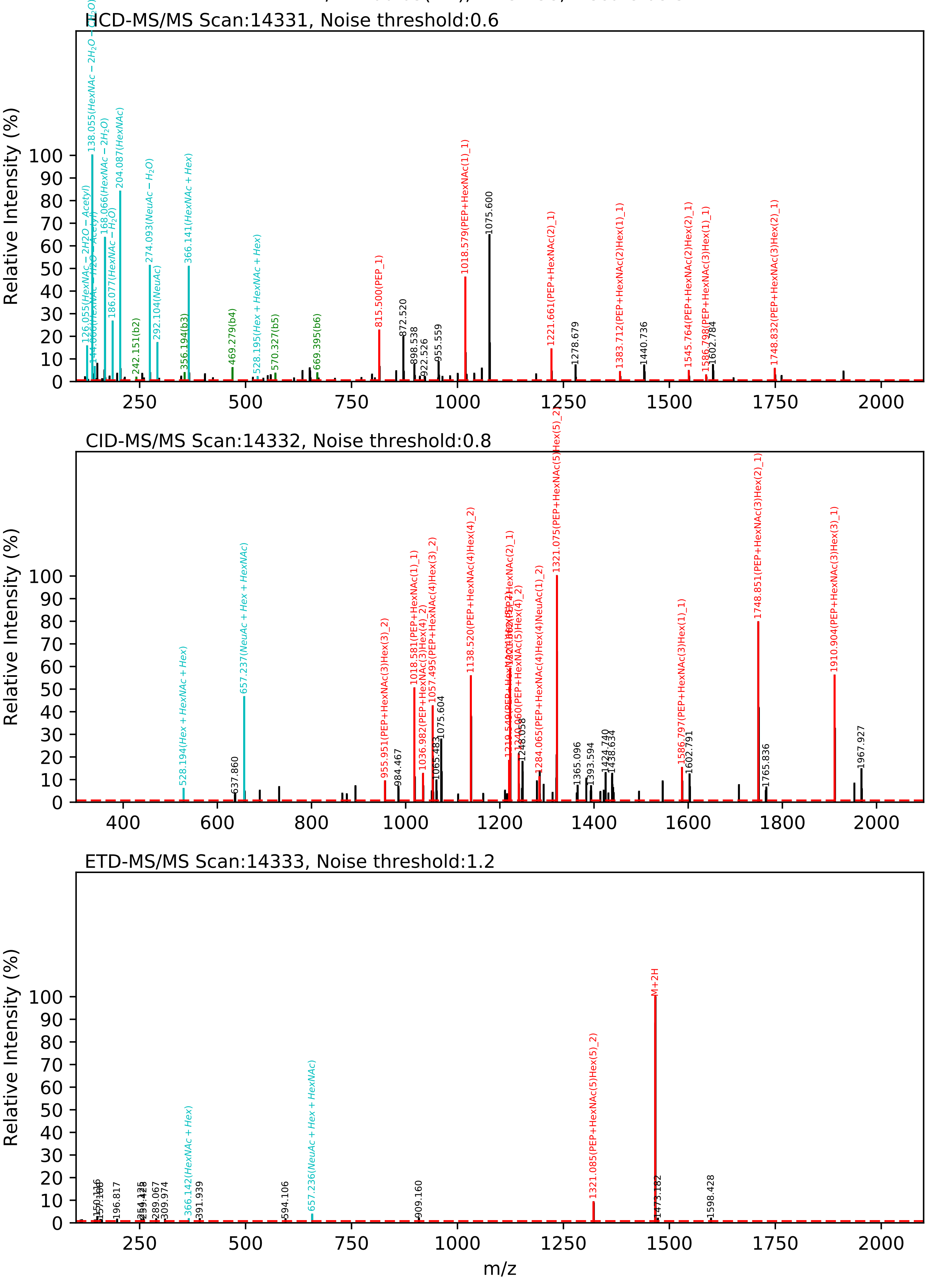

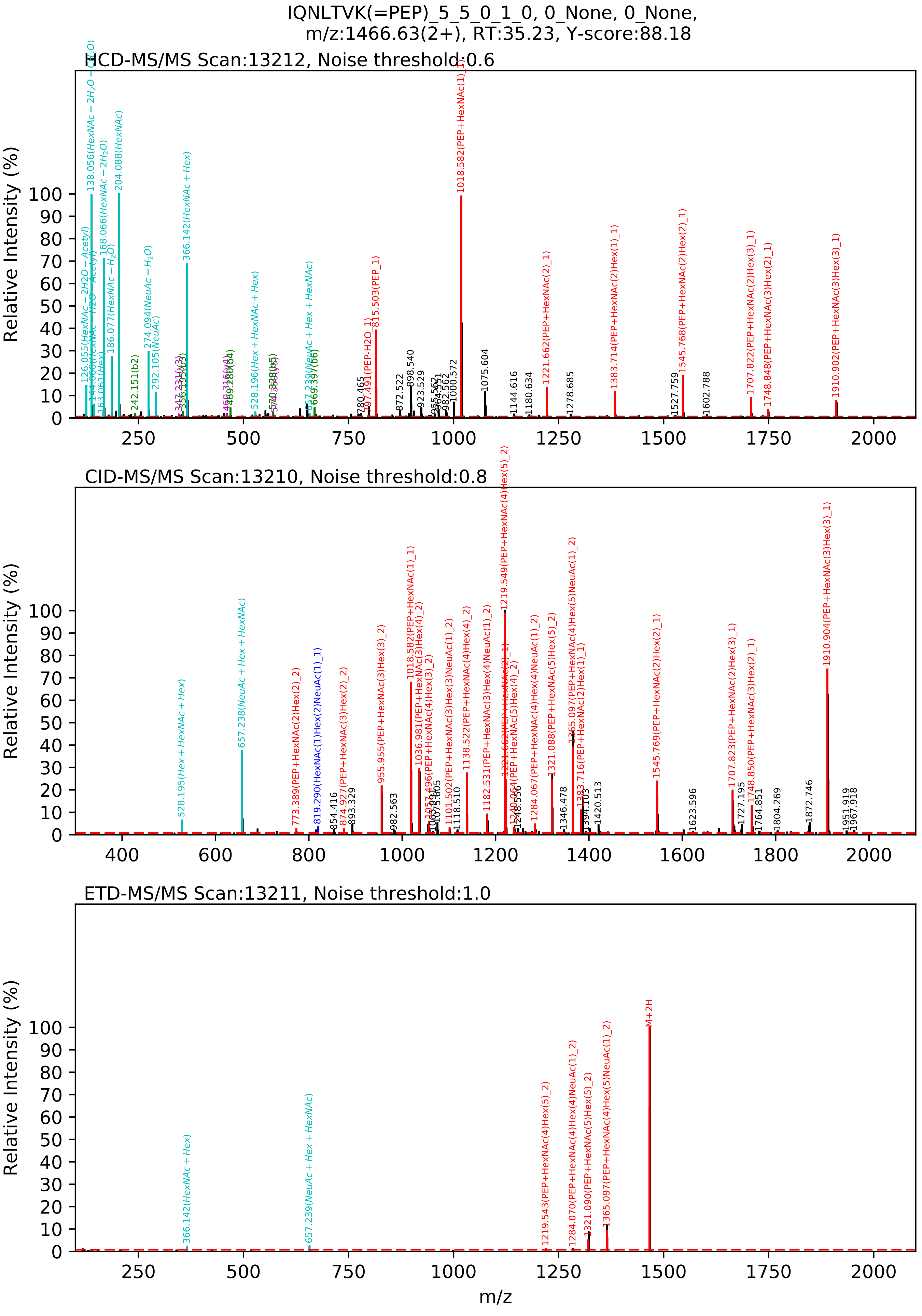
IQNLTVK(=PEP)_5_5_0_1_0, 0_None, 0_None,
m/z:1466.63(2+), RT:35.23, Y-score:88.18
HCD-MS/MS Scan:13212, Noise threshold:0.6
Relative Intensity (%)
138.056(HexNAc − 2H2O − Acetyl)
204.088(HexNAc)
168.066(HexNAc − 2H2O)
186.077(HexNAc − H2O)
126.055(HexNAc − 2H2O − Acetyl)
163.061(Hex)
366.142(HexNAc + Hex)
274.094(NeuAc − H2O)
292.105(NeuAc)
242.151(b2)
469.280(b4)
528.196(Hex + HexNAc + Hex)
657.239(NeuAc + Hex + HexNAc)
669.397(b6)
1018.582(PEP+HexNAc(1)_1)
815.503(PEP_1)
797.491(PEP-H2O_1)
780.465
872.522
898.540
923.529
1000.572
1075.604
1144.616
1180.634
1221.662(PEP+HexNAc(2)_1)
1278.685
1383.714(PEP+HexNAc(2)Hex(1)_1)
1545.768(PEP+HexNAc(2)Hex(2)_1)
1527.759
1602.788
1707.822(PEP+HexNAc(2)Hex(3)_1)
1748.848(PEP+HexNAc(3)Hex(2)_1)
1910.902(PEP+HexNAc(3)Hex(3)_1)
CID-MS/MS Scan:13210, Noise threshold:0.8
528.195(Hex + HexNAc + Hex)
657.238(NeuAc + Hex + HexNAc)
773.389(PEP+HexNAc(2)Hex(2)_2)
819.290(HexNAc(1)Hex(2)NeuAc(1)_1)
874.927(PEP+HexNAc(3)Hex(2)_2)
854.416
893.329
955.955(PEP+HexNAc(3)Hex(3)_2)
982.563
1018.582(PEP+HexNAc(1)_1)
1036.981(PEP+HexNAc(3)Hex(4)_2)
1057.496(PEP+HexNAc(4)Hex(3)_2)
1075.605
1101.502(PEP+HexNAc(3)Hex(3)NeuAc(1)_2)
1118.510
1138.522(PEP+HexNAc(4)Hex(4)_2)
1182.531(PEP+HexNAc(3)Hex(4)NeuAc(1)_2)
1219.549(PEP+HexNAc(4)Hex(5)_2)
1221.662(PEP+HexNAc(2)_1)
1240.064(PEP+HexNAc(5)Hex(4)_2)
1248.556
1284.067(PEP+HexNAc(4)Hex(4)NeuAc(1)_2)
1321.088(PEP+HexNAc(5)Hex(5)_2)
1346.478
1365.097(PEP+HexNAc(4)Hex(5)NeuAc(1)_2)
1383.716(PEP+HexNAc(2)Hex(1)_1)
1394.103
1420.513
1545.769(PEP+HexNAc(2)Hex(2)_1)
1623.596
1707.823(PEP+HexNAc(2)Hex(3)_1)
1727.195
1748.850(PEP+HexNAc(3)Hex(2)_1)
1764.851
1804.269
1872.746
1910.904(PEP+HexNAc(3)Hex(3)_1)
1951.919
1967.918
ETD-MS/MS Scan:13211, Noise threshold:1.0
366.142(HexNAc + Hex)
657.239(NeuAc + Hex + HexNAc)
1219.543(PEP+HexNAc(4)Hex(5)_2)
1284.070(PEP+HexNAc(4)Hex(4)NeuAc(1)_2)
1321.090(PEP+HexNAc(5)Hex(5)_2)
1365.097(PEP+HexNAc(4)Hex(5)NeuAc(1)_2)
M+2H
m/z

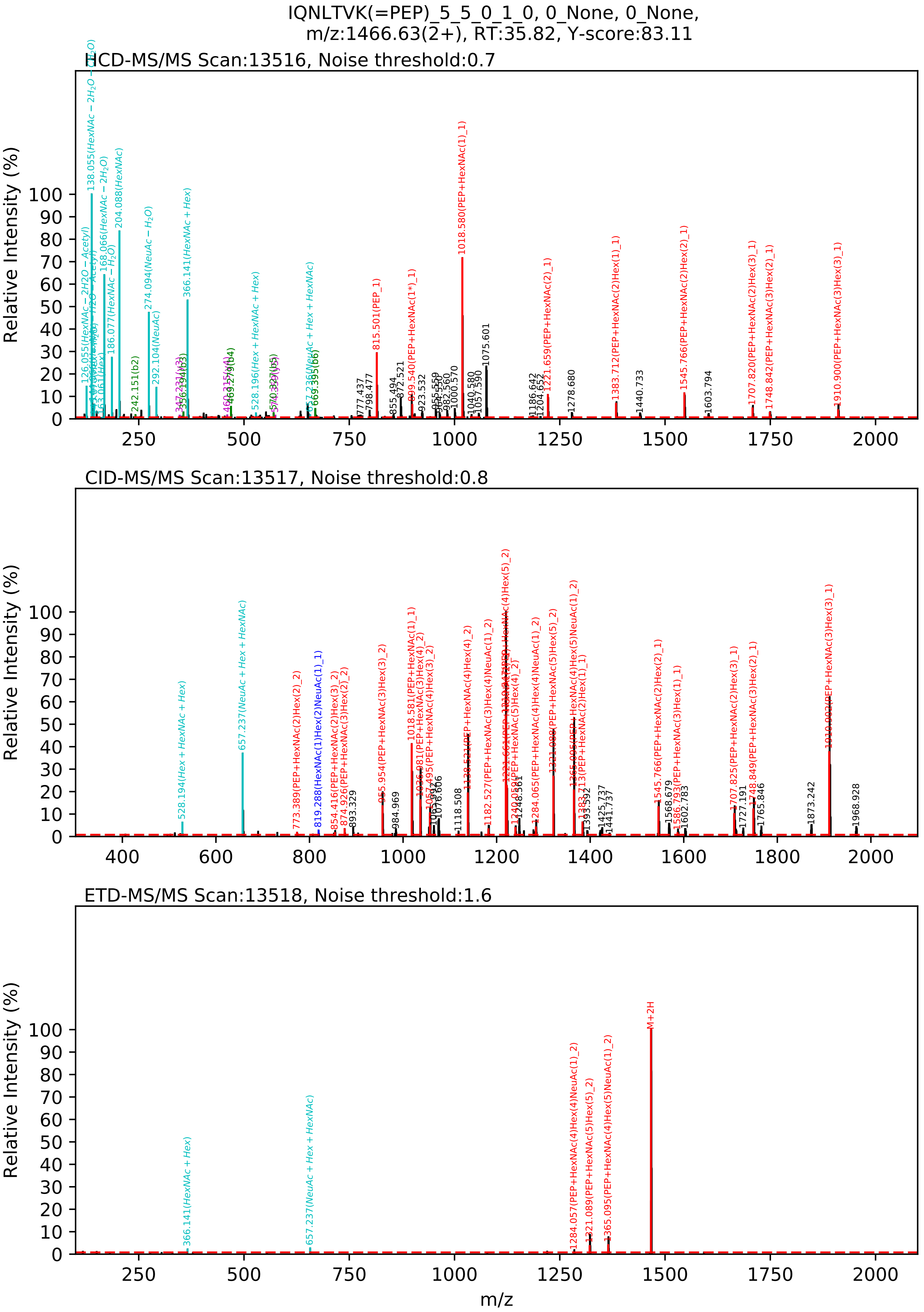
IQNLTVK(=PEP)_5_5_0_1_0, 0_None, 0_None,
m/z:1466.63(2+), RT:35.82, Y-score:83.11
HCD-MS/MS Scan:13516, Noise threshold:0.7
CID-MS/MS Scan:13517, Noise threshold:0.8
ETD-MS/MS Scan:13518, Noise threshold:1.6
Relative Intensity (%)
m/z
1018.580(PEP+HexNAc(1)_1)
815.501(PEP_1)
1221.659(PEP+HexNAc(2)_1)
1383.712(PEP+HexNAc(2)Hex(1)_1)
1545.766(PEP+HexNAc(2)Hex(2)_1)
1707.820(PEP+HexNAc(2)Hex(3)_1)
1748.842(PEP+HexNAc(3)Hex(2)_1)
1910.900(PEP+HexNAc(3)Hex(3)_1)
138.055(HexNAc − 2H2O − Acetyl)
204.088(HexNAc)
274.094(NeuAc − H2O)
292.104(NeuAc)
366.141(HexNAc + Hex)
528.196(Hex + HexNAc + Hex)
657.237(NeuAc + Hex + HexNAc)
1910.902(PEP+HexNAc(3)Hex(3)_1)
1321.089(PEP+HexNAc(5)Hex(5)_2)
1365.095(PEP+HexNAc(4)Hex(5)NeuAc(1)_2)
1284.057(PEP+HexNAc(4)Hex(4)NeuAc(1)_2)
M+2H

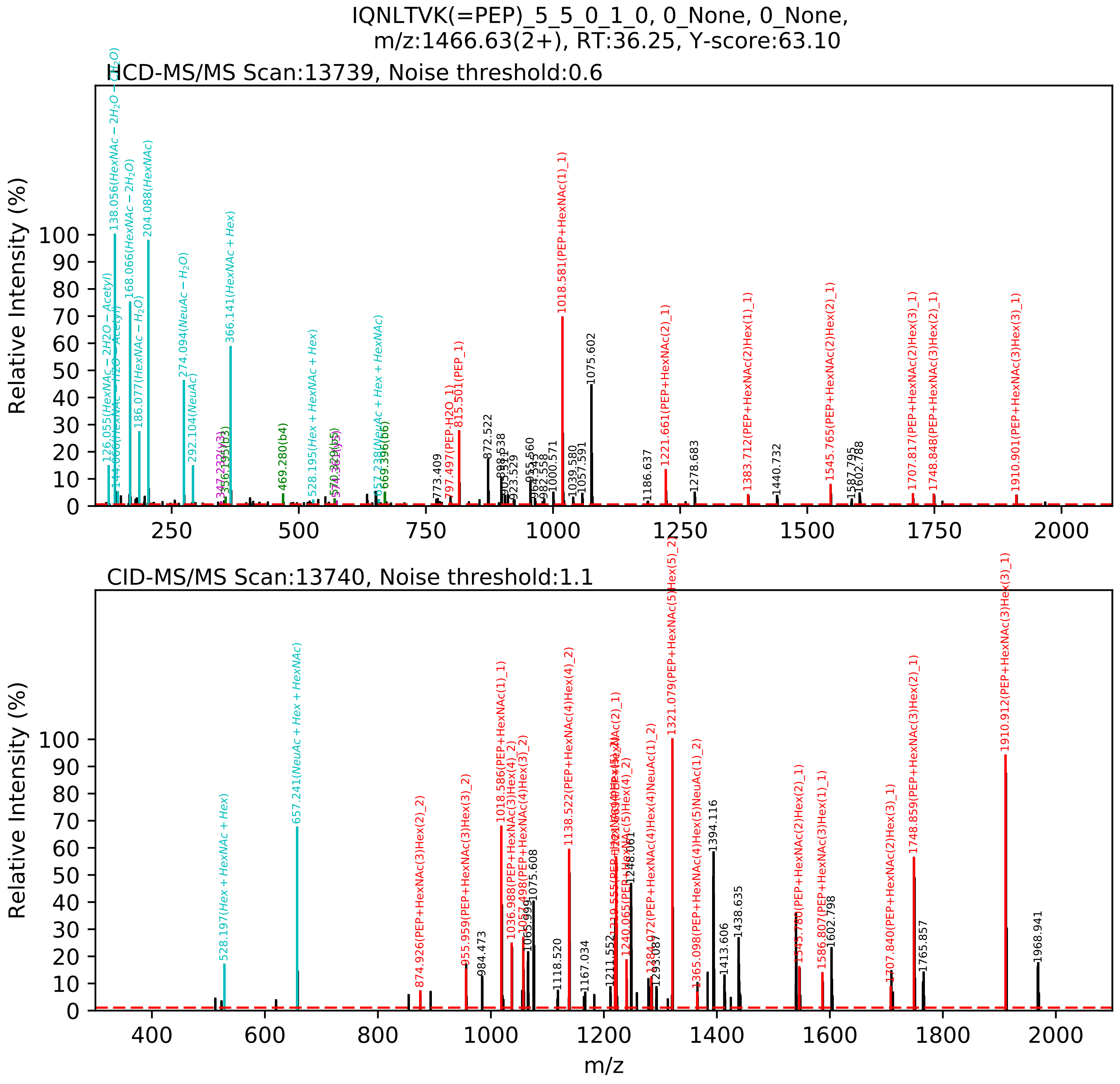
IQNLTVK(=PEP)_5_5_0_1_0, 0_None, 0_None,
m/z:1466.63(2+), RT:36.25, Y-score:63.10
HCD-MS/MS Scan:13739, Noise threshold:0.6
Relative Intensity (%)
1018.581(PEP+HexNAc(1)_1)
1075.602
1221.661(PEP+HexNAc(2)_1)
1383.712(PEP+HexNAc(2)Hex(1)_1)
1545.765(PEP+HexNAc(2)Hex(2)_1)
1707.817(PEP+HexNAc(2)Hex(3)_1)
1748.848(PEP+HexNAc(3)Hex(2)_1)
1910.901(PEP+HexNAc(3)Hex(3)_1)
815.501(PEP_1)
797.497(PEP-H2O_1)
204.088(HexNAc)
138.056(HexNAc − 2H2O − H2O)
168.066(HexNAc − 2H2O)
186.077(HexNAc − H2O)
274.094(NeuAc − H2O)
292.104(NeuAc)
366.141(HexNAc + Hex)
469.280(b4)
528.195(Hex + HexNAc + Hex)
CID-MS/MS Scan:13740, Noise threshold:1.1
Relative Intensity (%)
528.197(Hex + HexNAc + Hex)
657.241(NeuAc + Hex + HexNAc)
874.926(PEP+HexNAc(3)Hex(2)_2)
955.959(PEP+HexNAc(3)Hex(3)_2)
1018.586(PEP+HexNAc(1)_1)
1036.988(PEP+HexNAc(3)Hex(4)_2)
1138.522(PEP+HexNAc(4)Hex(4)_2)
1321.079(PEP+HexNAc(5)Hex(5)_2)
1365.098(PEP+HexNAc(4)Hex(5)NeuAc(1)_2)
1394.116
1545.780(PEP+HexNAc(2)Hex(2)_1)
1586.807(PEP+HexNAc(3)Hex(1)_1)
1707.840(PEP+HexNAc(2)Hex(3)_1)
1748.859(PEP+HexNAc(3)Hex(2)_1)
1910.912(PEP+HexNAc(3)Hex(3)_1)
1968.941
m/z

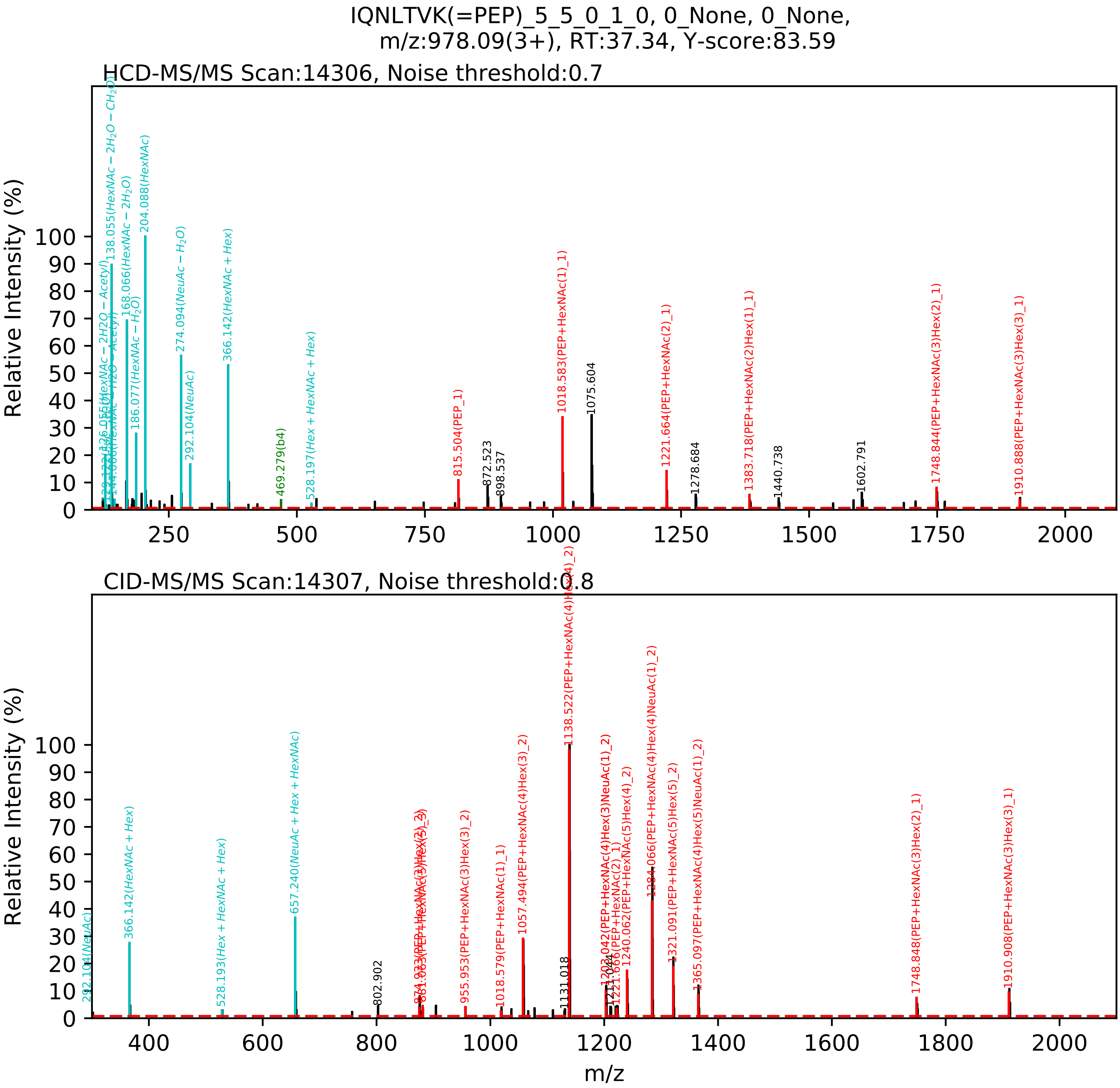
IQNLTVK(=PEP)_5_5_0_1_0, 0_None, 0_None,
m/z:978.09(3+), RT:37.34, Y-score:83.59
HCD-MS/MS Scan:14306, Noise threshold:0.7
Relative Intensity (%)
138.055(HexNAc − 2H2O − CH2O)
204.088(HexNAc)
168.066(HexNAc − 2H2O)
186.077(HexNAc − H2O)
274.094(NeuAc − H2O)
292.104(NeuAc)
366.142(HexNAc + Hex)
469.279(b4)
528.197(Hex + HexNAc + Hex)
815.504(PEP_1)
872.523
898.537
1018.583(PEP+HexNAc(1)_1)
1075.604
1221.664(PEP+HexNAc(2)_1)
1278.684
1383.718(PEP+HexNAc(2)Hex(1)_1)
1440.738
1602.791
1748.844(PEP+HexNAc(3)Hex(2)_1)
1910.888(PEP+HexNAc(3)Hex(3)_1)
CID-MS/MS Scan:14307, Noise threshold:0.8
Relative Intensity (%)
366.142(HexNAc + Hex)
528.193(Hex + HexNAc + Hex)
657.240(NeuAc + Hex + HexNAc)
802.902
955.953(PEP+HexNAc(3)Hex(3)_2)
1018.579(PEP+HexNAc(1)_1)
1057.494(PEP+HexNAc(4)Hex(3)_2)
1131.018
1138.522(PEP+HexNAc(4)Hex(4)_2)
1240.062(PEP+HexNAc(5)Hex(4)_2)
1284.066(PEP+HexNAc(4)Hex(4)NeuAc(1)_2)
1321.091(PEP+HexNAc(5)Hex(5)_2)
1365.097(PEP+HexNAc(4)Hex(5)NeuAc(1)_2)
1748.848(PEP+HexNAc(3)Hex(2)_1)
1910.908(PEP+HexNAc(3)Hex(3)_1)
m/z

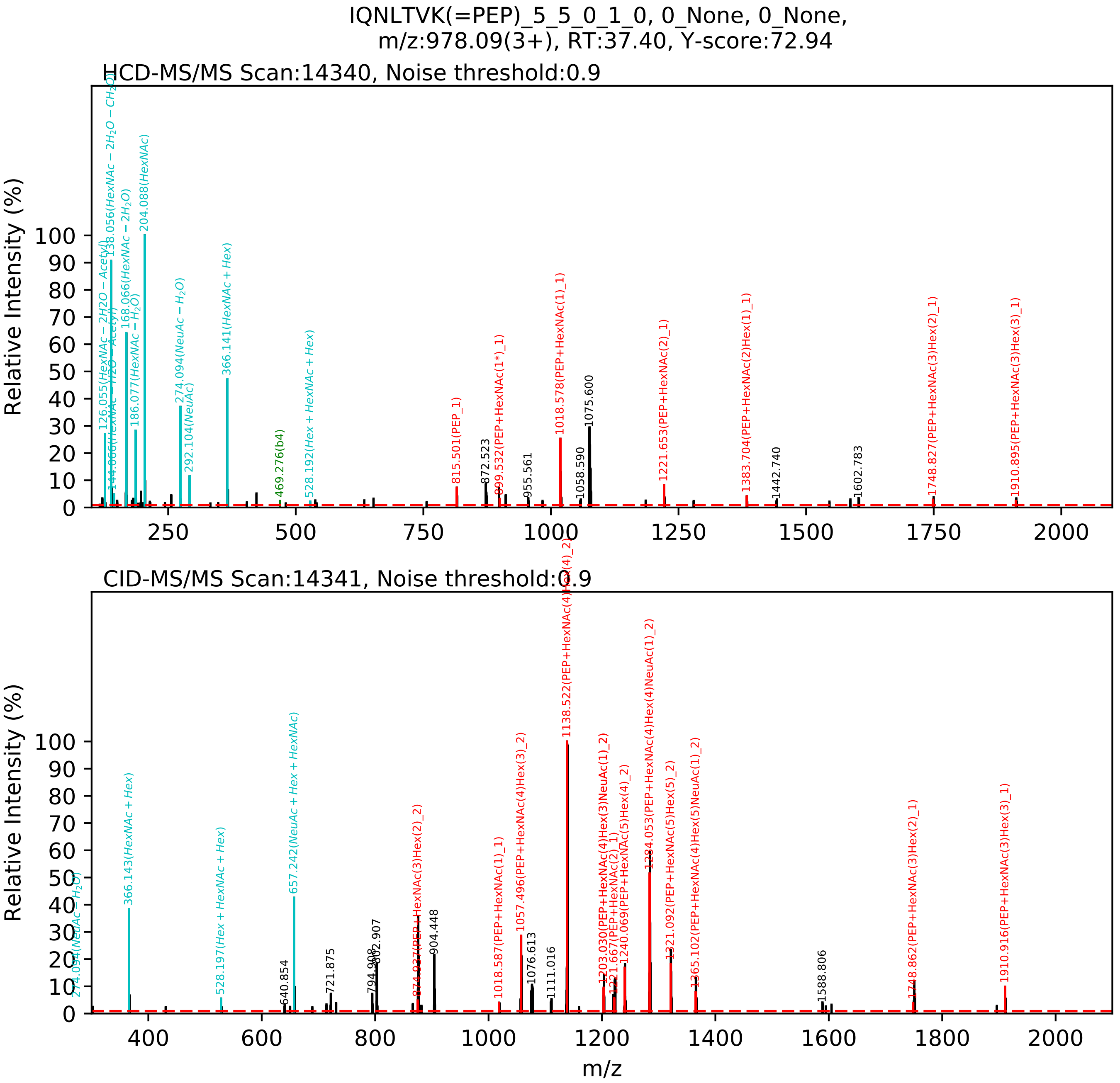
IQNLTVK(=PEP)_5_5_0_1_0, 0_None, 0_None,
m/z:978.09(3+), RT:37.40, Y-score:72.94
HCD-MS/MS Scan:14340, Noise threshold:0.9
Relative Intensity (%)
126.055(HexNAc − 2H2O − Acetyl)
138.056(HexNAc − 2H2O − CH2O)
144.066(HexNAc − H2O − Acetyl)
168.066(HexNAc − 2H2O)
186.077(HexNAc − H2O)
204.088(HexNAc)
274.094(NeuAc − H2O)
292.104(NeuAc)
366.141(HexNAc + Hex)
469.276(b4)
528.192(Hex + HexNAc + Hex)
815.501(PEP_1)
872.523
899.532(PEP+HexNAc(1*)_1)
955.561
1018.578(PEP+HexNAc(1)_1)
1058.590
1075.600
1221.653(PEP+HexNAc(2)_1)
1383.704(PEP+HexNAc(2)Hex(1)_1)
1442.740
1602.783
1748.827(PEP+HexNAc(3)Hex(2)_1)
1910.895(PEP+HexNAc(3)Hex(3)_1)
CID-MS/MS Scan:14341, Noise threshold:0.9
Relative Intensity (%)
274.094(NeuAc − H2O)
366.143(HexNAc + Hex)
528.197(Hex + HexNAc + Hex)
657.242(NeuAc + Hex + HexNAc)
640.854
721.875
794.908
802.907
874.037(PEP+HexNAc(3)Hex(2)_2)
904.448
1018.587(PEP+HexNAc(1)_1)
1057.496(PEP+HexNAc(4)Hex(3)_2)
1076.613
1111.016
1138.522(PEP+HexNAc(4)Hex(4)_2)
1203.030(PEP+HexNAc(4)Hex(3)NeuAc(1)_2)
1221.667(PEP+HexNAc(2)_1)
1240.069(PEP+HexNAc(5)Hex(4)_2)
1284.053(PEP+HexNAc(4)Hex(4)NeuAc(1)_2)
1321.092(PEP+HexNAc(5)Hex(5)_2)
1365.102(PEP+HexNAc(4)Hex(5)NeuAc(1)_2)
1588.806
1748.862(PEP+HexNAc(3)Hex(2)_1)
1910.916(PEP+HexNAc(3)Hex(3)_1)
m/z

IQNLTVK(=PEP)_5_5_0_1_0, 0_None, 0_None,
m/z:978.09(3+), RT:35.63, Y-score:86.90

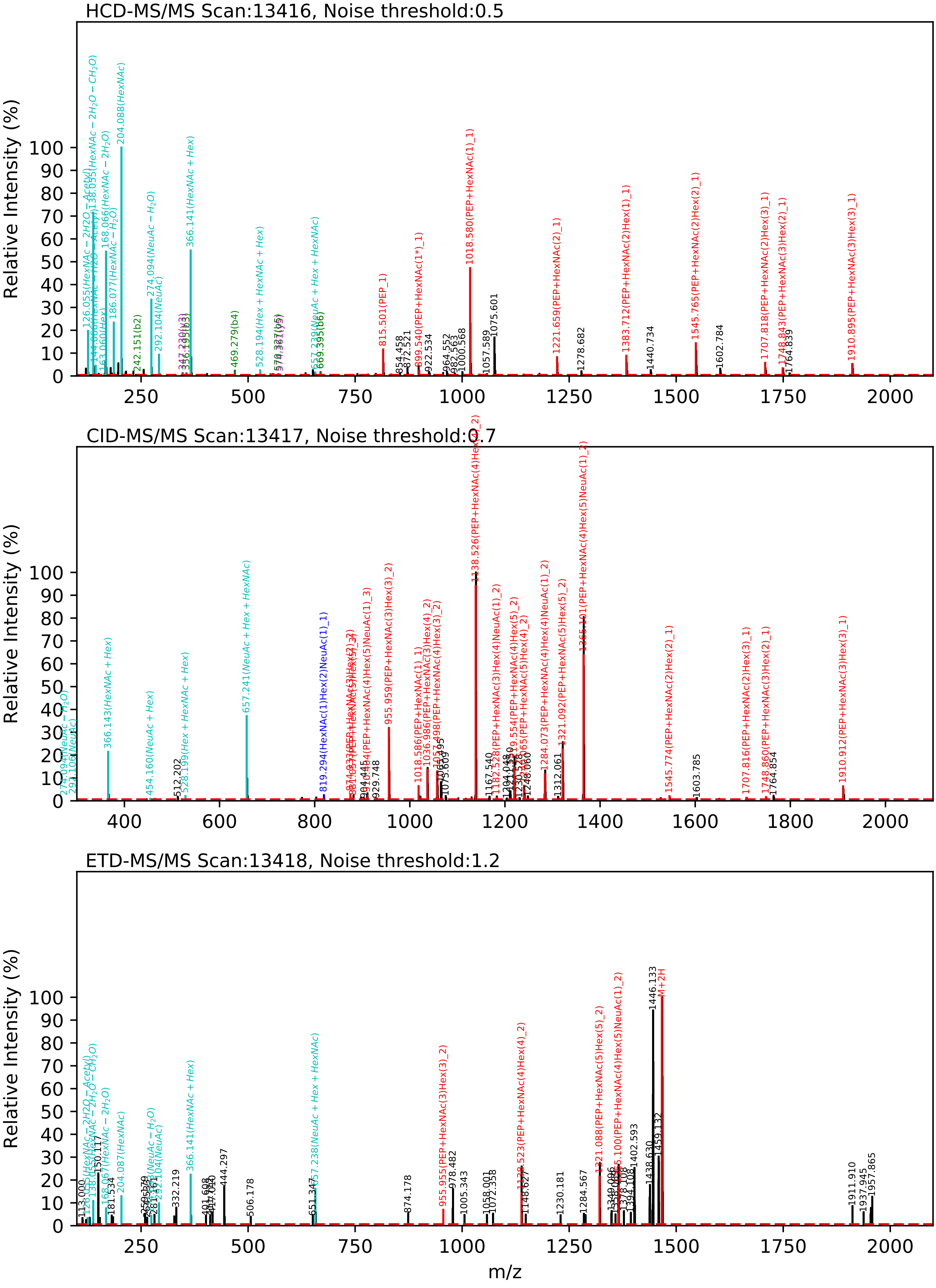

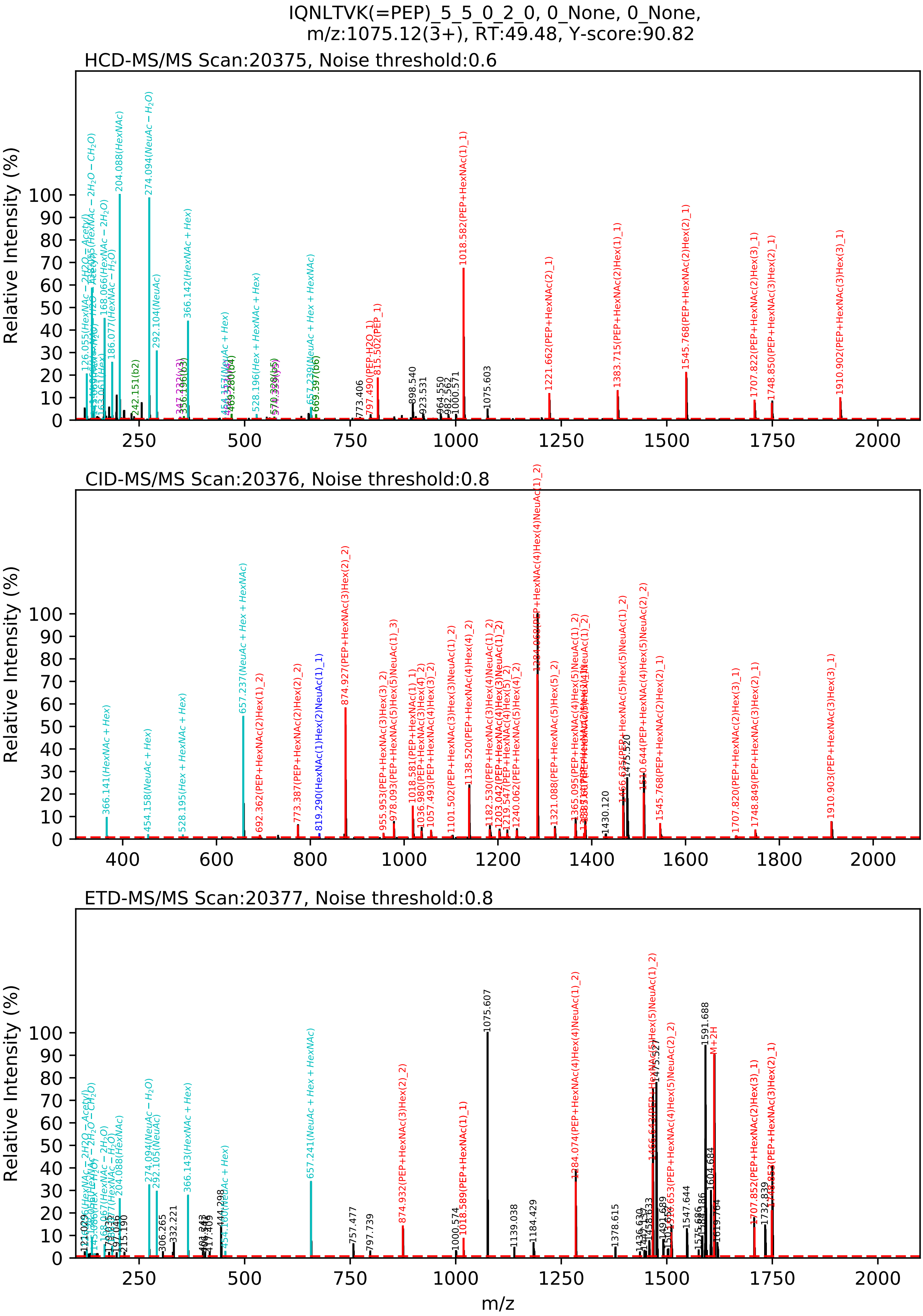
IQNLTVK(=PEP)_5_5_0_2_0, 0_None, 0_None,
m/z:1075.12(3+), RT:49.48, Y-score:90.82
HCD-MS/MS Scan:20375, Noise threshold:0.6
Relative Intensity (%)
204.088(HexNAc)
274.094(NeuAc − H2O)
292.104(NeuAc)
366.142(HexNAc + Hex)
168.066(HexNAc − 2H2O)
186.077(HexNAc − H2O)
528.196(Hex + HexNAc + Hex)
657.239(NeuAc + Hex + HexNAc)
242.151(b2)
469.280(b4)
669.397(b6)
797.490(PEP-H2O_1)
815.502(PEP_1)
773.406
898.540
923.531
964.550
1000.571
1075.603
1018.582(PEP+HexNAc(1)_1)
1221.662(PEP+HexNAc(2)_1)
1383.715(PEP+HexNAc(2)Hex(1)_1)
1545.768(PEP+HexNAc(2)Hex(2)_1)
1707.822(PEP+HexNAc(2)Hex(3)_1)
1748.850(PEP+HexNAc(3)Hex(2)_1)
1910.902(PEP+HexNAc(3)Hex(3)_1)
CID-MS/MS Scan:20376, Noise threshold:0.8
366.141(HexNAc + Hex)
454.158(NeuAc + Hex)
528.195(Hex + HexNAc + Hex)
657.237(NeuAc + Hex + HexNAc)
692.362(PEP+HexNAc(2)Hex(1)_2)
773.387(PEP+HexNAc(2)Hex(2)_2)
819.290(HexNAc(1)Hex(2)NeuAc(1)_1)
874.927(PEP+HexNAc(3)Hex(2)_2)
955.953(PEP+HexNAc(3)Hex(3)_2)
978.093(PEP+HexNAc(5)Hex(5)NeuAc(1)_3)
1018.581(PEP+HexNAc(1)_1)
1036.980(PEP+HexNAc(3)Hex(4)_2)
1057.493(PEP+HexNAc(4)Hex(3)_2)
1101.502(PEP+HexNAc(3)Hex(3)NeuAc(1)_2)
1138.520(PEP+HexNAc(4)Hex(4)_2)
1182.530(PEP+HexNAc(3)Hex(4)NeuAc(1)_2)
1203.042(PEP+HexNAc(4)Hex(3)NeuAc(1)_2)
1219.547(PEP+HexNAc(4)Hex(5)_2)
1240.062(PEP+HexNAc(5)Hex(4)_2)
1284.068(PEP+HexNAc(4)Hex(4)NeuAc(1)_2)
1321.088(PEP+HexNAc(5)Hex(5)_2)
1365.095(PEP+HexNAc(4)Hex(5)NeuAc(1)_2)
1430.120
1475.520
1510.644(PEP+HexNAc(4)Hex(5)NeuAc(2)_2)
1545.768(PEP+HexNAc(2)Hex(2)_1)
1707.820(PEP+HexNAc(2)Hex(3)_1)
1748.849(PEP+HexNAc(3)Hex(2)_1)
1910.903(PEP+HexNAc(3)Hex(3)_1)
ETD-MS/MS Scan:20377, Noise threshold:0.8
204.088(HexNAc)
274.094(NeuAc − H2O)
292.105(NeuAc)
366.143(HexNAc + Hex)
657.241(NeuAc + Hex + HexNAc)
215.190
306.265
332.221
444.298
757.477
797.739
874.932(PEP+HexNAc(3)Hex(2)_2)
1000.574
1018.589(PEP+HexNAc(1)_1)
1075.607
1139.038
1184.429
1284.074(PEP+HexNAc(4)Hex(4)NeuAc(1)_2)
1378.615
1475.527
1491.689
1547.644
1591.688
M+2H
1604.684
1619.764
1707.852(PEP+HexNAc(2)Hex(3)_1)
1732.839
m/z

IQNLTVK(=PEP)_5_5_0_2_0, 0_None, 0_None,
m/z:1075.12(3+), RT:49.55, Y-score:90.44

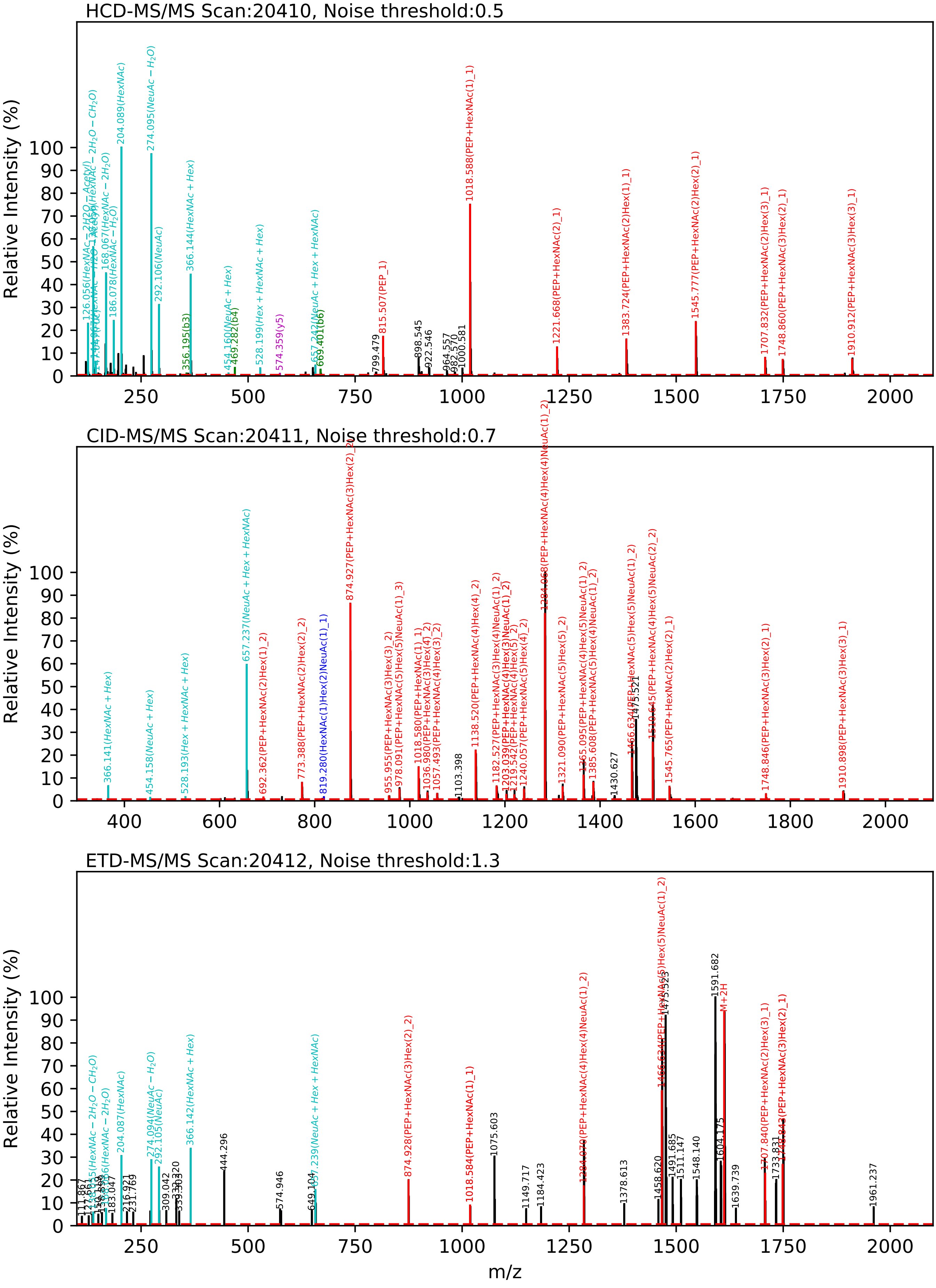

IQNLTVK(=PEP)_5_5_0_2_0, 0_None, 0_None,
m/z:1612.18(2+), RT:49.55, Y-score:94.73

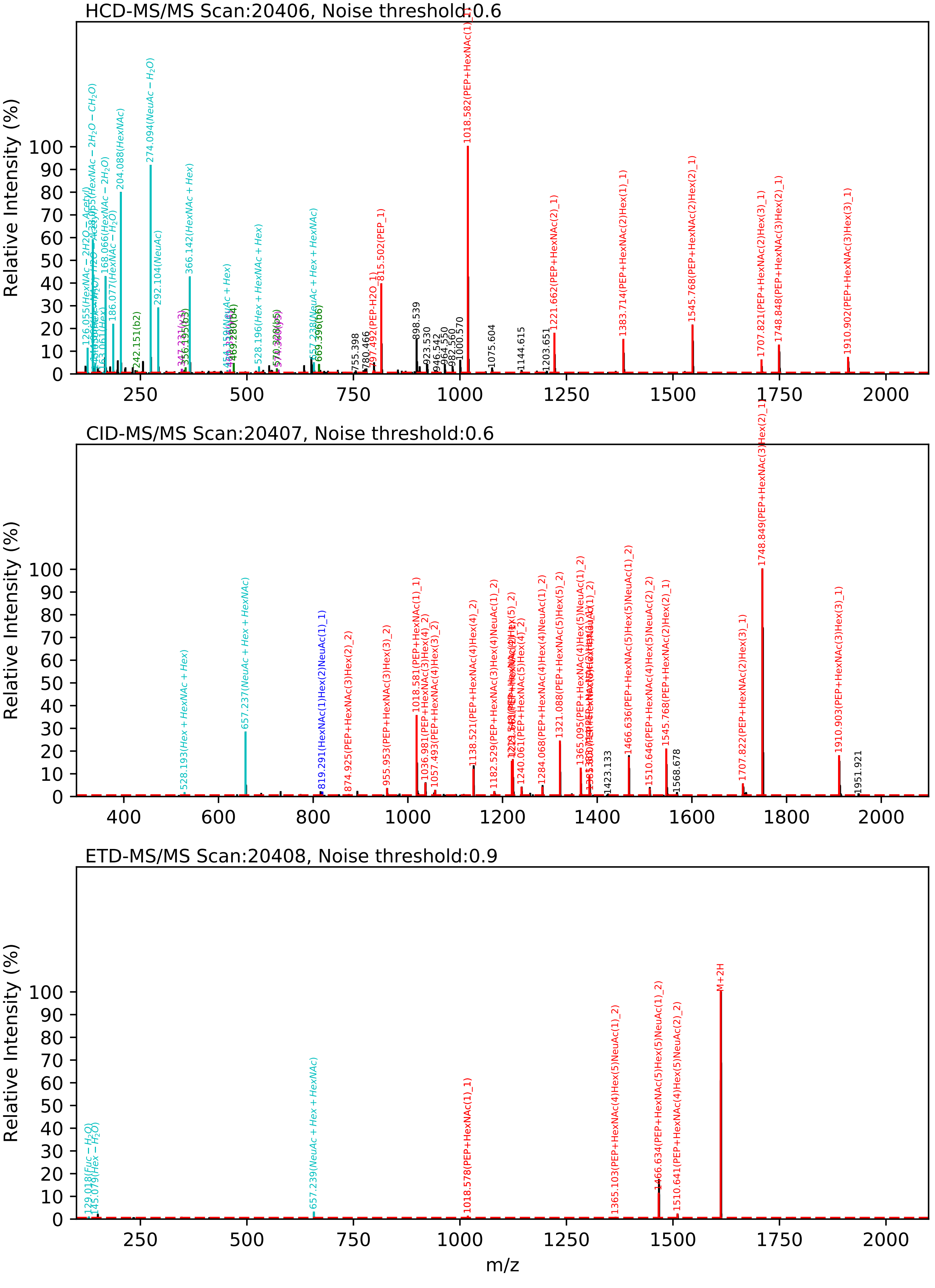

IQNLTVK(=PEP)_5_5_0_2_0, 0_None, 0_None,
m/z:1612.18(2+), RT:49.66, Y-score:94.94

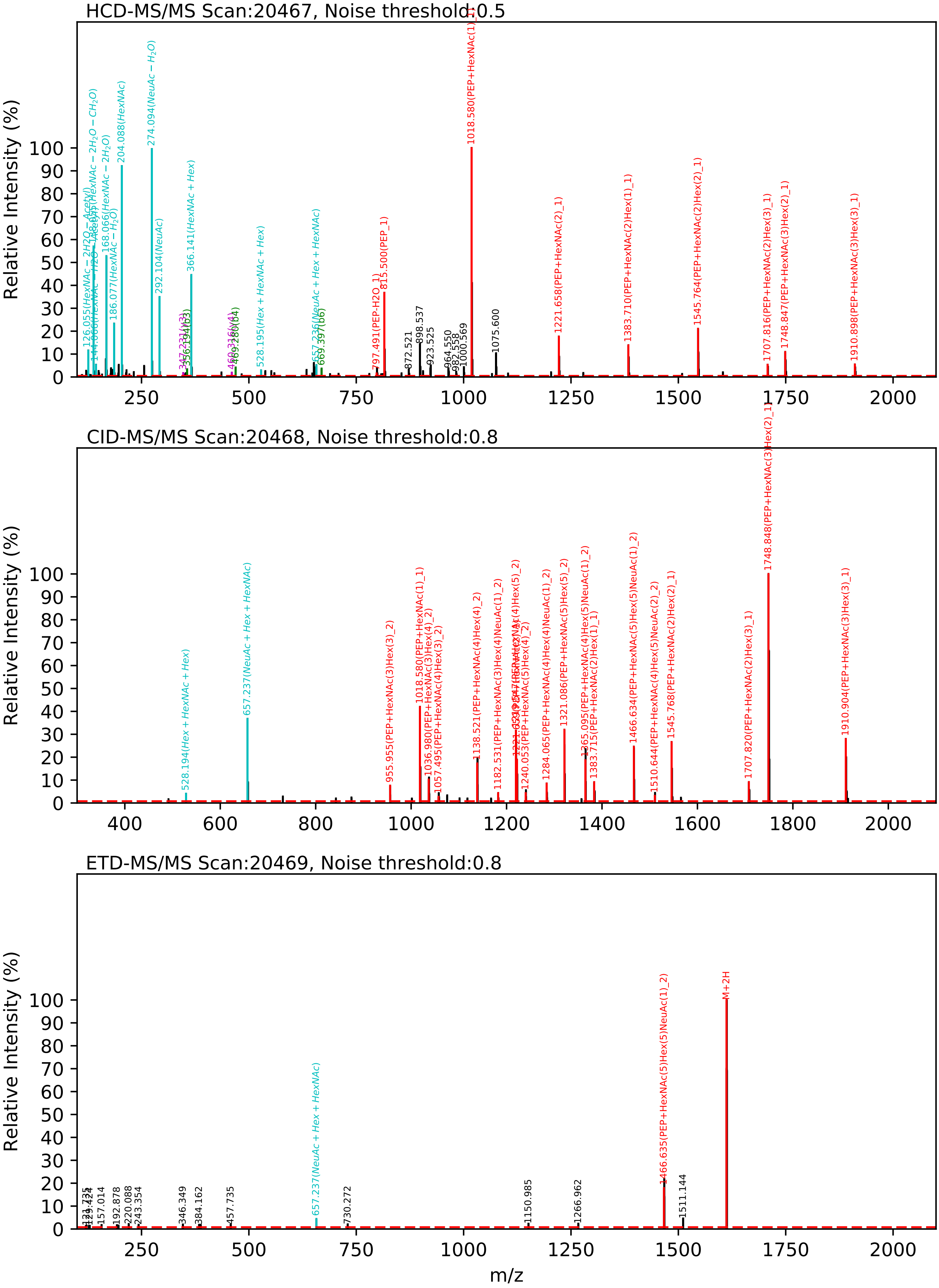

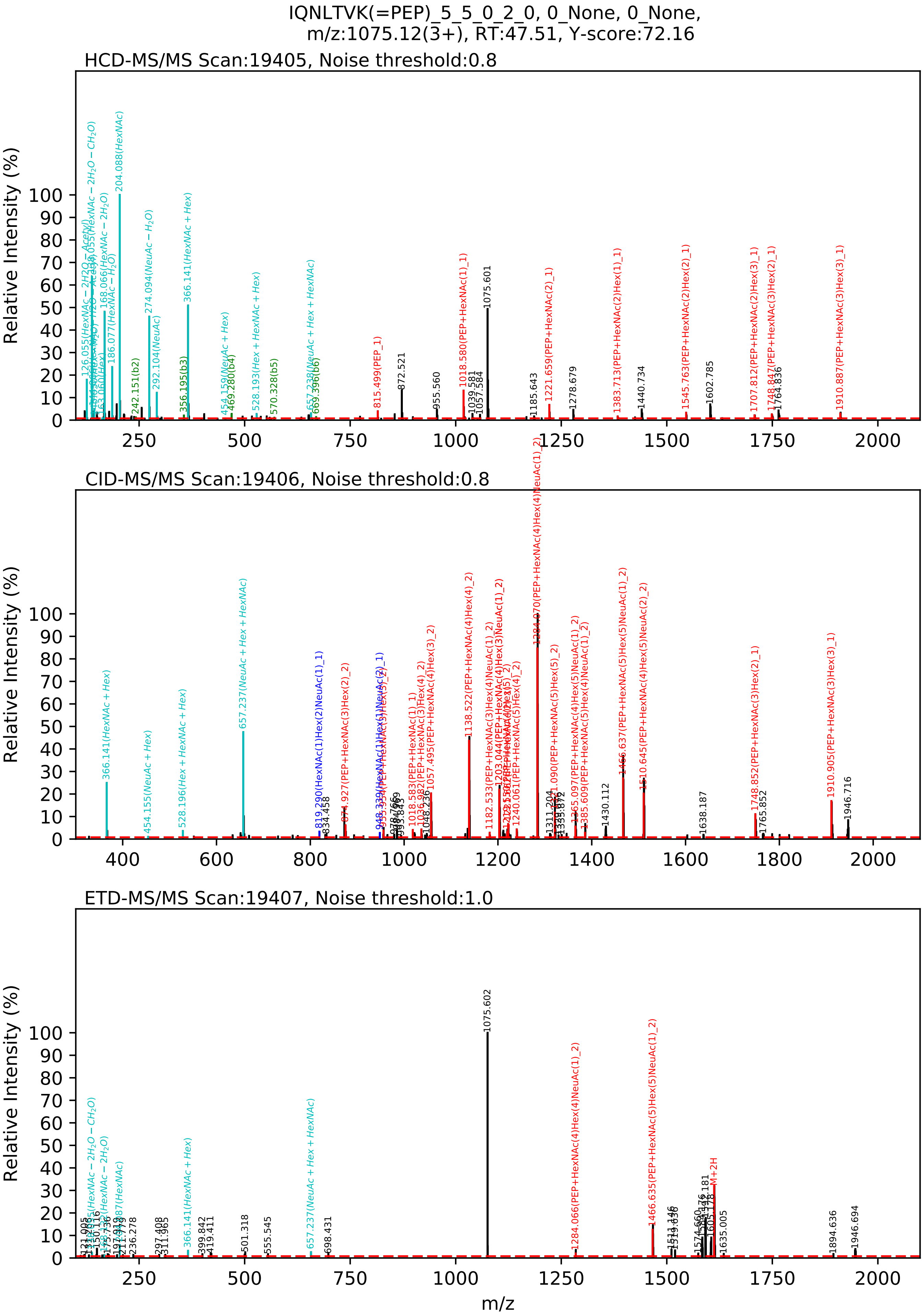
IQNLTVK(=PEP)_5_5_0_2_0, 0_None, 0_None,
m/z:1075.12(3+), RT:47.51, Y-score:72.16
HCD-MS/MS Scan:19405, Noise threshold:0.8
CID-MS/MS Scan:19406, Noise threshold:0.8
ETD-MS/MS Scan:19407, Noise threshold:1.0
Relative Intensity (%)
m/z
204.088(HexNAc)
366.141(HexNAc+Hex)
274.094(NeuAc−H2O)
292.104(NeuAc)
657.237(NeuAc+Hex+HexNAc)
1018.580(PEP+HexNAc(1)_1)
1075.601
1221.659(PEP+HexNAc(2)_1)
1383.713(PEP+HexNAc(2)Hex(1)_1)
1545.763(PEP+HexNAc(2)Hex(2)_1)
1707.812(PEP+HexNAc(2)Hex(3)_1)
1910.887(PEP+HexNAc(3)Hex(3)_1)
1284.070(PEP+HexNAc(4)Hex(4)NeuAc(1)_2)
1138.522(PEP+HexNAc(4)Hex(4)_2)
1466.637(PEP+HexNAc(5)Hex(5)NeuAc(1)_2)
1510.645(PEP+HexNAc(4)Hex(5)NeuAc(2)_2)
1748.852(PEP+HexNAc(3)Hex(2)_1)
1910.905(PEP+HexNAc(3)Hex(3)_1)
1946.716
1075.602
1284.066(PEP+HexNAc(4)Hex(4)NeuAc(1)_2)
1466.635(PEP+HexNAc(5)Hex(5)NeuAc(1)_2)
M+2H

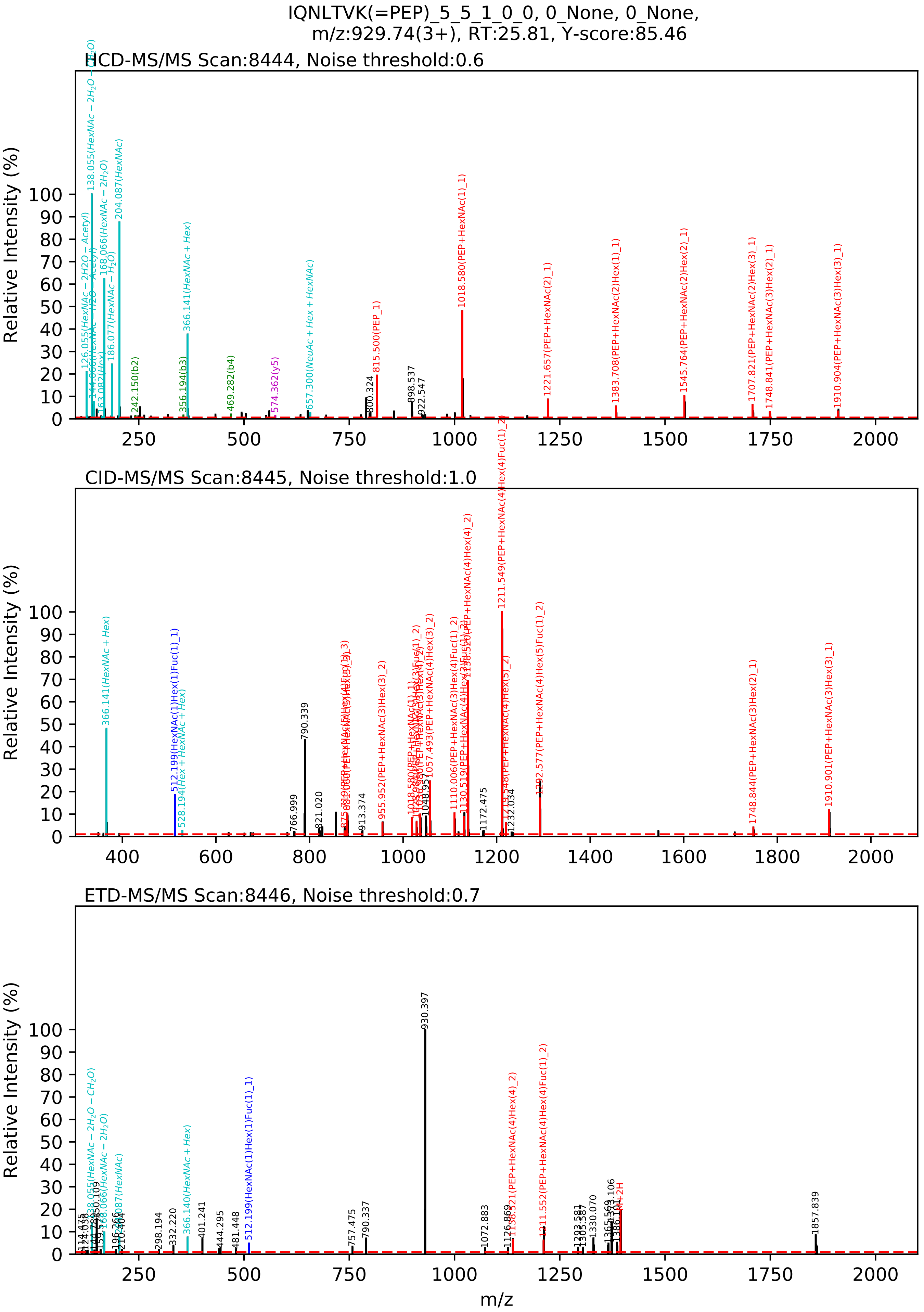

IQNLTVK(=PEP)_5_5_1_0_0, 0_None, 0_None,
m/z:929.74(3+), RT:25.81, Y-score:85.46
HCD-MS/MS Scan:8444, Noise threshold:0.6
CID-MS/MS Scan:8445, Noise threshold:1.0
ETD-MS/MS Scan:8446, Noise threshold:0.7
Relative Intensity (%)
m/z
138.055(HexNAc − 2H2O − CH2O)
204.087(HexNAc)
168.066(HexNAc − 2H2O)
186.077(HexNAc − H2O)
366.141(HexNAc + Hex)
242.150(b2)
356.194(b3)
469.282(b4)
574.362(y5)
657.300(NeuAc + Hex + HexNAc)
815.500(PEP_1)
800.324
898.537
922.547
1018.580(PEP+HexNAc(1)_1)
1221.657(PEP+HexNAc(2)_1)
1383.708(PEP+HexNAc(2)Hex(1)_1)
1545.764(PEP+HexNAc(2)Hex(2)_1)
1707.821(PEP+HexNAc(2)Hex(3)_1)
1748.841(PEP+HexNAc(3)Hex(2)_1)
1910.904(PEP+HexNAc(3)Hex(3)_1)
512.199(HexNAc(1)Hex(1)Fuc(1)_1)
528.194(Hex + HexNAc + Hex)
790.339
766.999
821.020
913.374
955.952(PEP+HexNAc(3)Hex(3)_2)
1057.493(PEP+HexNAc(4)Hex(3)_2)
1110.006(PEP+HexNAc(3)Hex(4)Fuc(1)_2)
1211.549(PEP+HexNAc(4)Hex(4)Fuc(1)_2)
1172.475
1232.034
1292.577(PEP+HexNAc(4)Hex(5)Fuc(1)_2)
1748.844(PEP+HexNAc(3)Hex(2)_1)
1910.901(PEP+HexNAc(3)Hex(3)_1)
930.397
150.109
196.266
210.404
298.194
332.220
366.140(HexNAc + Hex)
401.241
444.295
481.448
512.199(HexNAc(1)Hex(1)Fuc(1)_1)
757.475
790.337
1072.883
1126.869
1138.521(PEP+HexNAc(4)Hex(4)_2)
1211.552(PEP+HexNAc(4)Hex(4)Fuc(1)_2)
1293.581
1305.587
1330.070
1371.106
1857.839

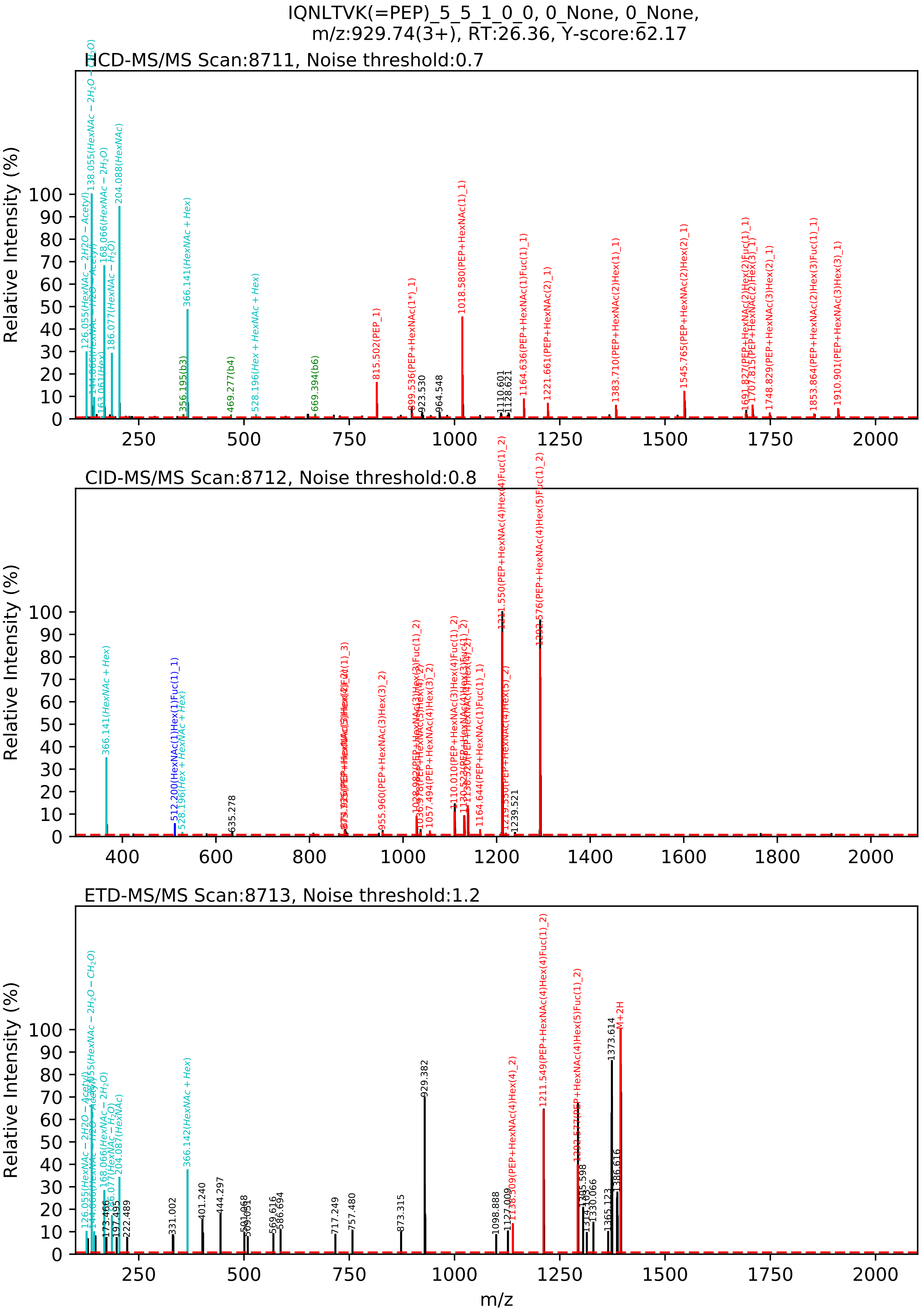
IQNLTVK(=PEP)_5_5_1_0_0, 0_None, 0_None,
m/z:929.74(3+), RT:26.36, Y-score:62.17
HCD-MS/MS Scan:8711, Noise threshold:0.7
Relative Intensity (%)
126.055(HexNAc − 2H2O − Acetyl)
138.055(HexNAc − 2H2O − CH2O)
168.066(HexNAc − 2H2O)
186.077(HexNAc − H2O)
204.088(HexNAc)
356.195(b3)
366.141(HexNAc + Hex)
469.277(b4)
528.196(Hex + HexNAc + Hex)
669.394(b6)
815.502(PEP_1)
899.536(PEP+HexNAc(1*)_1)
923.530
964.548
1018.580(PEP+HexNAc(1)_1)
1110.601
1128.621
1164.636(PEP+HexNAc(1)Fuc(1)_1)
1221.661(PEP+HexNAc(2)_1)
1383.710(PEP+HexNAc(2)Hex(1)_1)
1545.765(PEP+HexNAc(2)Hex(2)_1)
1748.829(PEP+HexNAc(3)Hex(2)_1)
1853.864(PEP+HexNAc(2)Hex(3)Fuc(1)_1)
1910.901(PEP+HexNAc(3)Hex(3)_1)
CID-MS/MS Scan:8712, Noise threshold:0.8
366.141(HexNAc + Hex)
512.200(HexNAc(1)Hex(1)Fuc(1)_1)
635.278
955.960(PEP+HexNAc(3)Hex(3)_2)
1057.494(PEP+HexNAc(4)Hex(3)_2)
1164.644(PEP+HexNAc(1)Fuc(1)_1)
1211.550(PEP+HexNAc(4)Hex(4)Fuc(1)_2)
1239.521
1292.576(PEP+HexNAc(4)Hex(5)Fuc(1)_2)
ETD-MS/MS Scan:8713, Noise threshold:1.2
366.142(HexNAc + Hex)
222.489
331.002
401.240
444.297
569.616
586.694
717.249
757.480
873.315
929.382
1098.888
1138.509(PEP+HexNAc(4)Hex(4)_2)
1211.549(PEP+HexNAc(4)Hex(4)Fuc(1)_2)
1292.577(PEP+HexNAc(4)Hex(5)Fuc(1)_2)
1330.066
1365.123
1373.614
1386.616
M+2H
m/z

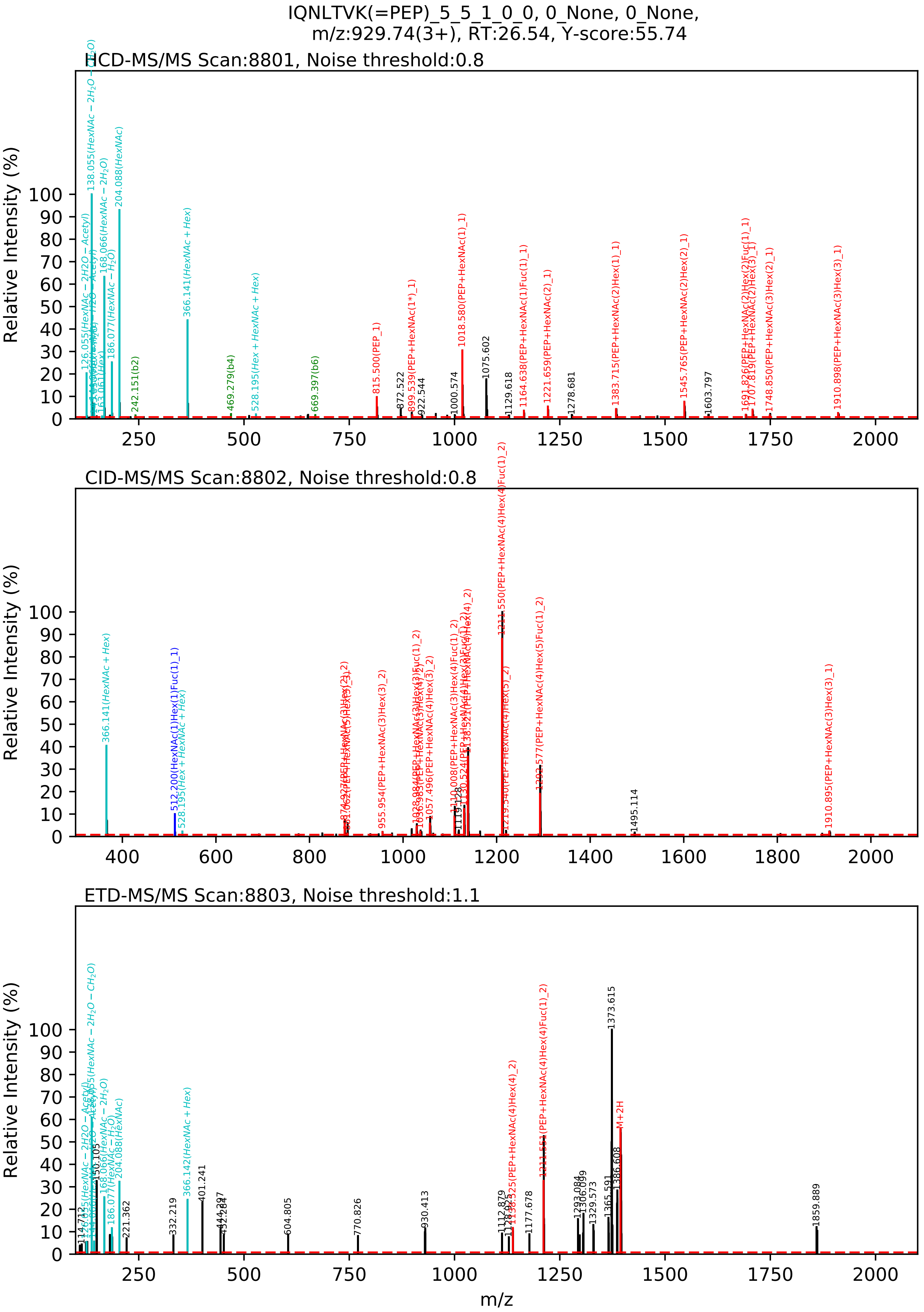

IQNLTVK(=PEP)_5_5_1_0_0, 0_None, 0_None,
m/z:929.74(3+), RT:26.54, Y-score:55.74
HCD-MS/MS Scan:8801, Noise threshold:0.8
CID-MS/MS Scan:8802, Noise threshold:0.8
ETD-MS/MS Scan:8803, Noise threshold:1.1
Relative Intensity (%)
m/z

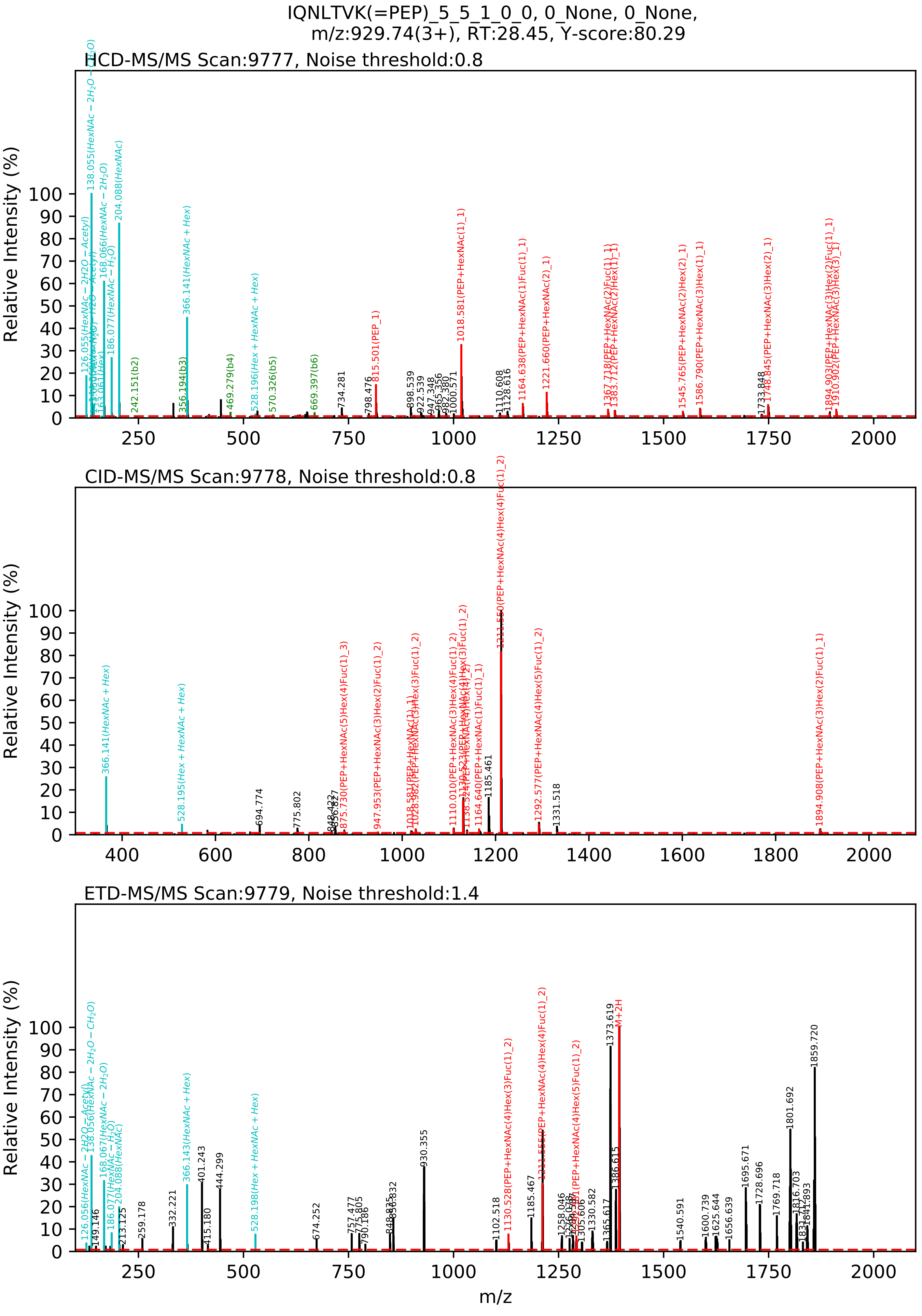

IQNLTVK(=PEP)_5_5_1_0_0, 0_None, 0_None,
m/z:929.74(3+), RT:28.45, Y-score:80.29
HCD-MS/MS Scan:9777, Noise threshold:0.8
CID-MS/MS Scan:9778, Noise threshold:0.8
ETD-MS/MS Scan:9779, Noise threshold:1.4
Relative Intensity (%)
m/z

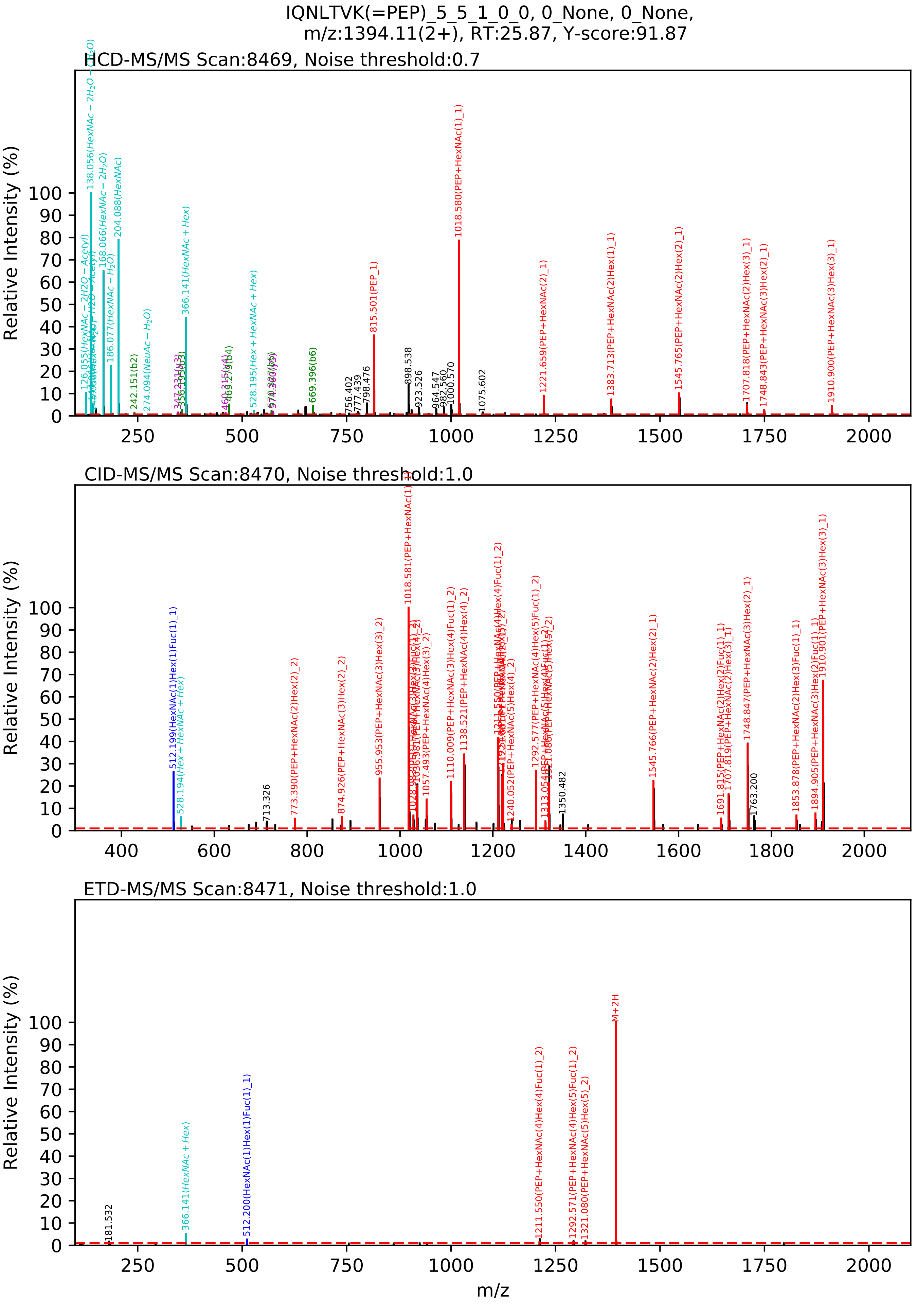
IQNLTVK(=PEP)_5_5_1_0_0, 0_None, 0_None,
m/z:1394.11(2+), RT:25.87, Y-score:91.87
HCD-MS/MS Scan:8469, Noise threshold:0.7
Relative Intensity (%)
138.056(HexNAc − 2H2O − H2O)
168.066(HexNAc − 2H2O)
204.088(HexNAc)
186.077(HexNAc − H2O)
126.055(HexNAc − 2H2O − Acetyl)
242.151(b2)
274.094(NeuAc − H2O)
366.141(HexNAc + Hex)
469.279(b4)
528.195(Hex + HexNAc + Hex)
669.396(b6)
815.501(PEP_1)
756.402
777.439
798.476
898.538
923.526
964.547
982.560
1000.570
1018.580(PEP+HexNAc(1)_1)
1075.602
1221.659(PEP+HexNAc(2)_1)
1383.713(PEP+HexNAc(2)Hex(1)_1)
1545.765(PEP+HexNAc(2)Hex(2)_1)
1707.818(PEP+HexNAc(2)Hex(3)_1)
1748.843(PEP+HexNAc(3)Hex(2)_1)
1910.900(PEP+HexNAc(3)Hex(3)_1)
CID-MS/MS Scan:8470, Noise threshold:1.0
512.199(HexNAc(1)Hex(1)Fuc(1)_1)
528.194(Hex + HexNAc + Hex)
713.326
773.390(PEP+HexNAc(2)Hex(2)_2)
874.926(PEP+HexNAc(3)Hex(2)_2)
955.953(PEP+HexNAc(3)Hex(3)_2)
1018.581(PEP+HexNAc(1)_1)
1057.493(PEP+HexNAc(4)Hex(3)_2)
1110.009(PEP+HexNAc(3)Hex(4)Fuc(1)_2)
1138.521(PEP+HexNAc(4)Hex(4)_2)
1211.550(PEP+HexNAc(4)Hex(4)Fuc(1)_2)
1240.052(PEP+HexNAc(5)Hex(4)_2)
1292.577(PEP+HexNAc(4)Hex(5)Fuc(1)_2)
1350.482
1545.766(PEP+HexNAc(2)Hex(2)_1)
1691.815(PEP+HexNAc(2)Hex(2)Fuc(1)_1)
1707.819(PEP+HexNAc(2)Hex(3)_1)
1748.847(PEP+HexNAc(3)Hex(2)_1)
1763.200
1853.878(PEP+HexNAc(2)Hex(3)Fuc(1)_1)
1894.905(PEP+HexNAc(3)Hex(2)Fuc(1)_1)
1910.901(PEP+HexNAc(3)Hex(3)_1)
ETD-MS/MS Scan:8471, Noise threshold:1.0
181.532
366.141(HexNAc + Hex)
512.200(HexNAc(1)Hex(1)Fuc(1)_1)
1211.550(PEP+HexNAc(4)Hex(4)Fuc(1)_2)
1292.571(PEP+HexNAc(4)Hex(5)Fuc(1)_2)
1321.080(PEP+HexNAc(5)Hex(5)_2)
M+2H
m/z

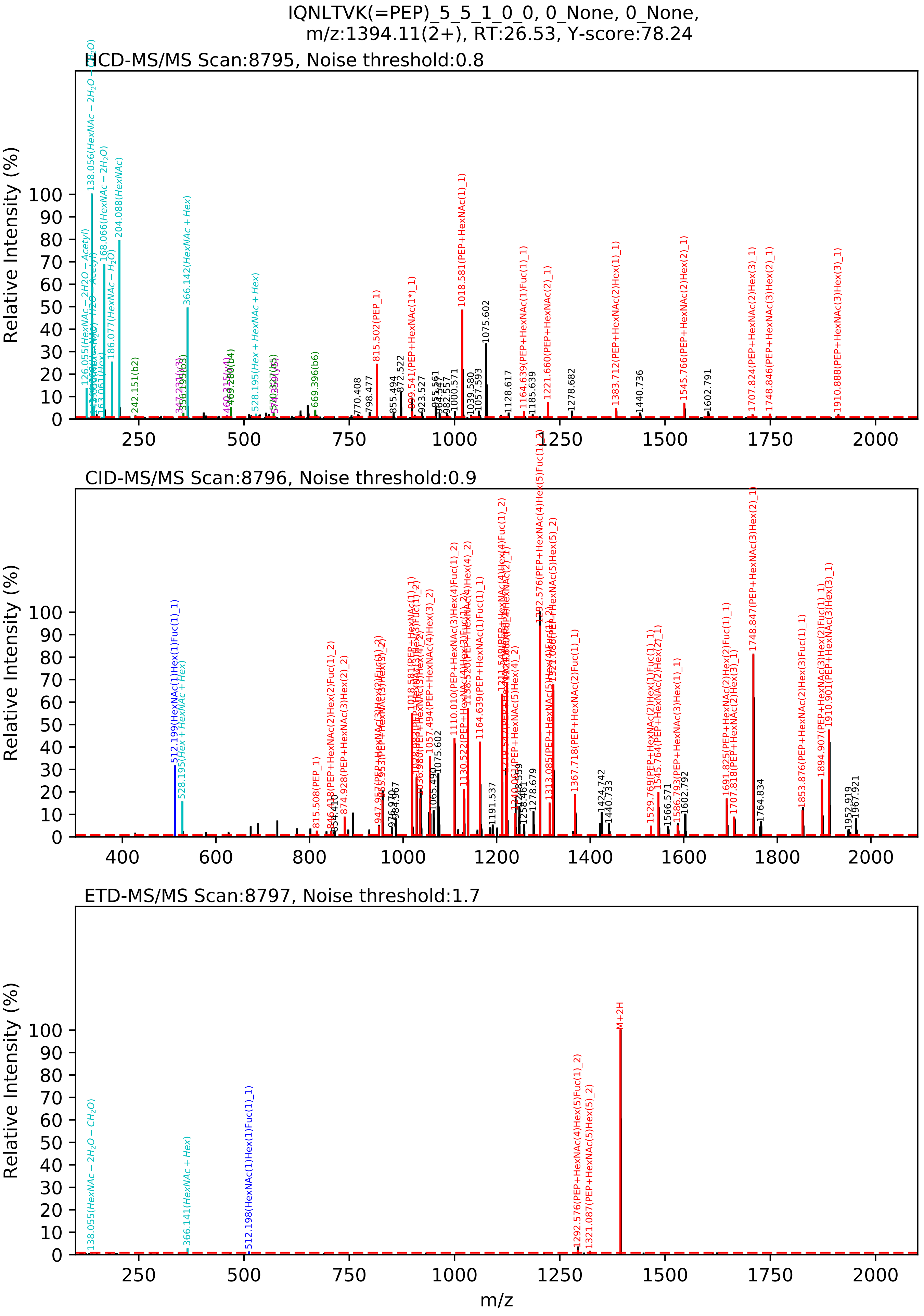

IQNLTVK(=PEP)_5_5_1_0_0, 0_None, 0_None,
m/z:1394.11(2+), RT:26.53, Y-score:78.24
HCD-MS/MS Scan:8795, Noise threshold:0.8
Relative Intensity (%)
138.056(HexNAc − 2H2O − CH2O)
204.088(HexNAc)
168.066(HexNAc − 2H2O)
186.077(HexNAc − H2O)
366.142(HexNAc + Hex)
528.195(Hex + HexNAc + Hex)
242.151(b2)
669.396(b6)
815.502(PEP_1)
1018.581(PEP+HexNAc(1)_1)
1075.602
1164.639(PEP+HexNAc(1)Fuc(1)_1)
1221.660(PEP+HexNAc(2)_1)
1278.682
1383.712(PEP+HexNAc(2)Hex(1)_1)
1440.736
1545.766(PEP+HexNAc(2)Hex(2)_1)
1602.791
1707.824(PEP+HexNAc(2)Hex(3)_1)
1748.846(PEP+HexNAc(3)Hex(2)_1)
1910.888(PEP+HexNAc(3)Hex(3)_1)
CID-MS/MS Scan:8796, Noise threshold:0.9
512.199(HexNAc(1)Hex(1)Fuc(1)_1)
528.195(Hex + HexNAc + Hex)
815.508(PEP_1)
874.928(PEP+HexNAc(3)Hex(2)_2)
1110.010(PEP+HexNAc(3)Hex(4)Fuc(1)_2)
1164.639(PEP+HexNAc(1)Fuc(1)_1)
1292.576(PEP+HexNAc(4)Hex(5)Fuc(1)_2)
1313.085(PEP+HexNAc(5)Hex(5)_2)
1367.718(PEP+HexNAc(2)Fuc(1)_1)
1691.825(PEP+HexNAc(2)Hex(2)Fuc(1)_1)
1748.847(PEP+HexNAc(3)Hex(2)_1)
1853.876(PEP+HexNAc(2)Hex(3)Fuc(1)_1)
1894.907(PEP+HexNAc(3)Hex(2)Fuc(1)_1)
1910.901(PEP+HexNAc(3)Hex(3)_1)
ETD-MS/MS Scan:8797, Noise threshold:1.7
138.055(HexNAc − 2H2O − CH2O)
366.141(HexNAc + Hex)
512.198(HexNAc(1)Hex(1)Fuc(1)_1)
1292.576(PEP+HexNAc(4)Hex(5)Fuc(1)_2)
1321.087(PEP+HexNAc(5)Hex(5)_2)
M+2H
m/z

IQNLTVK(=PEP)_5_5_1_0_0, 0_None, 0_None,
m/z:1394.11(2+), RT:26.57, Y-score:79.40

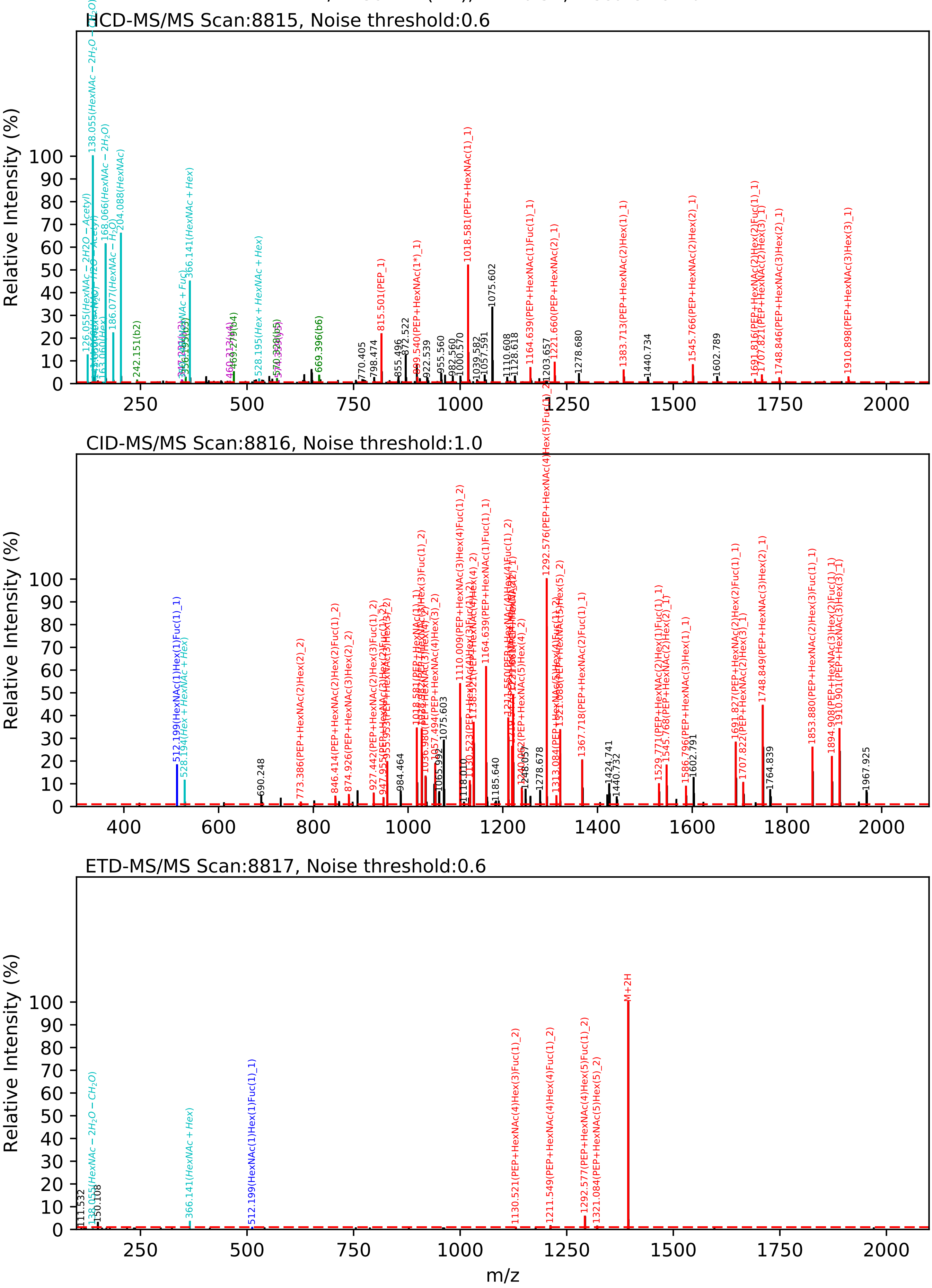

IQNLTVK(=PEP)_5_5_1_0_0, 0_None, 0_None,
m/z:929.74(3+), RT:26.95, Y-score:98.87

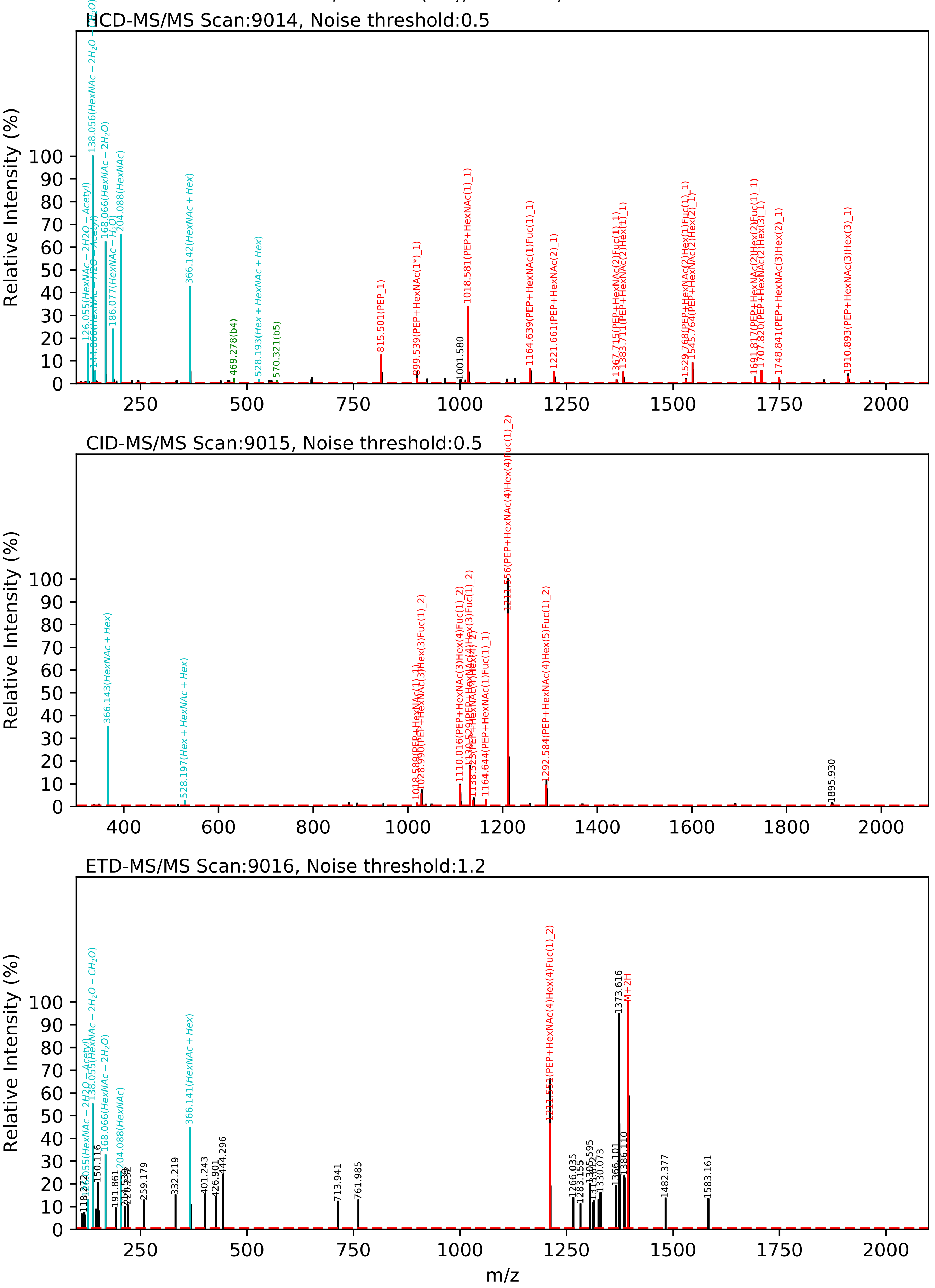

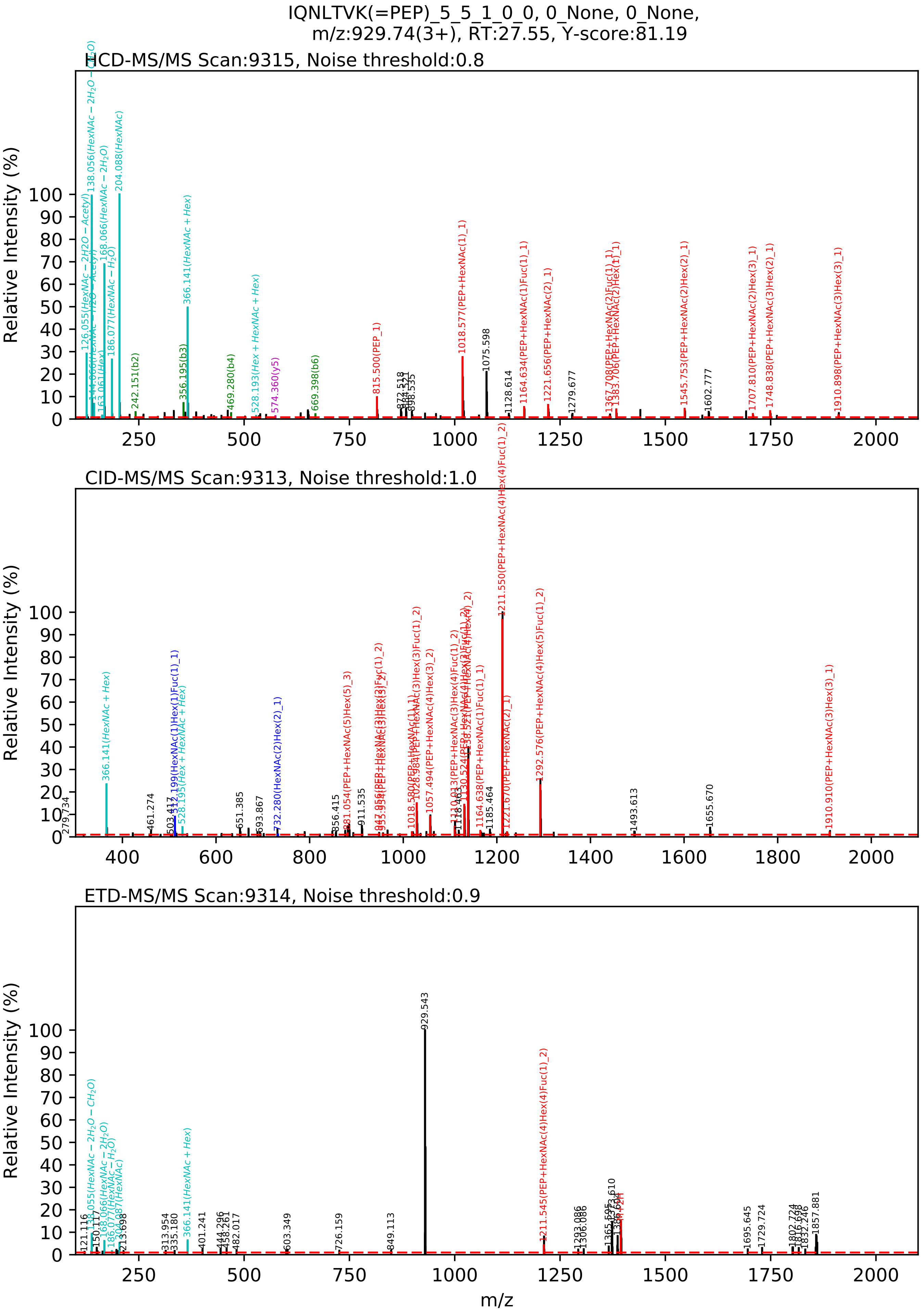

IQNLTVK(=PEP)_5_5_1_0_0, 0_None, 0_None,
m/z:929.74(3+), RT:27.55, Y-score:81.19
HCD-MS/MS Scan:9315, Noise threshold:0.8
Relative Intensity (%)
138.056(HexNAc − 2H2O − CH2O)
204.088(HexNAc)
168.066(HexNAc − 2H2O)
186.077(HexNAc − H2O)
126.055(HexNAc − 2H2O − Acetyl)
366.141(HexNAc + Hex)
528.193(Hex + HexNAc + Hex)
242.151(b2)
356.195(b3)
469.280(b4)
574.360(y5)
669.398(b6)
815.500(PEP_1)
1018.577(PEP+HexNAc(1)_1)
1075.598
1128.614
1164.634(PEP+HexNAc(1)Fuc(1)_1)
1221.656(PEP+HexNAc(2)_1)
1279.677
1545.753(PEP+HexNAc(2)Hex(2)_1)
1602.777
1707.810(PEP+HexNAc(2)Hex(3)_1)
1748.838(PEP+HexNAc(3)Hex(2)_1)
1910.898(PEP+HexNAc(3)Hex(3)_1)
CID-MS/MS Scan:9313, Noise threshold:1.0
1211.550(PEP+HexNAc(4)Hex(4)Fuc(1)_2)
1292.576(PEP+HexNAc(4)Hex(5)Fuc(1)_2)
1910.910(PEP+HexNAc(3)Hex(3)_1)
732.280(HexNAc(2)Hex(2)_1)
881.054(PEP+HexNAc(5)Hex(5)_3)
1057.494(PEP+HexNAc(4)Hex(3)_2)
1164.638(PEP+HexNAc(1)Fuc(1)_1)
1221.670(PEP+HexNAc(2)_1)
279.734
461.274
651.385
693.867
856.415
911.535
1185.464
1493.613
1655.670
ETD-MS/MS Scan:9314, Noise threshold:0.9
929.543
1211.545(PEP+HexNAc(4)Hex(4)Fuc(1)_2)
121.116
150.117
213.098
313.954
335.180
401.241
444.296
458.261
482.017
603.349
726.159
849.113
1293.086
1306.086
1695.645
1729.724
1802.724
1832.246
1857.881
m/z

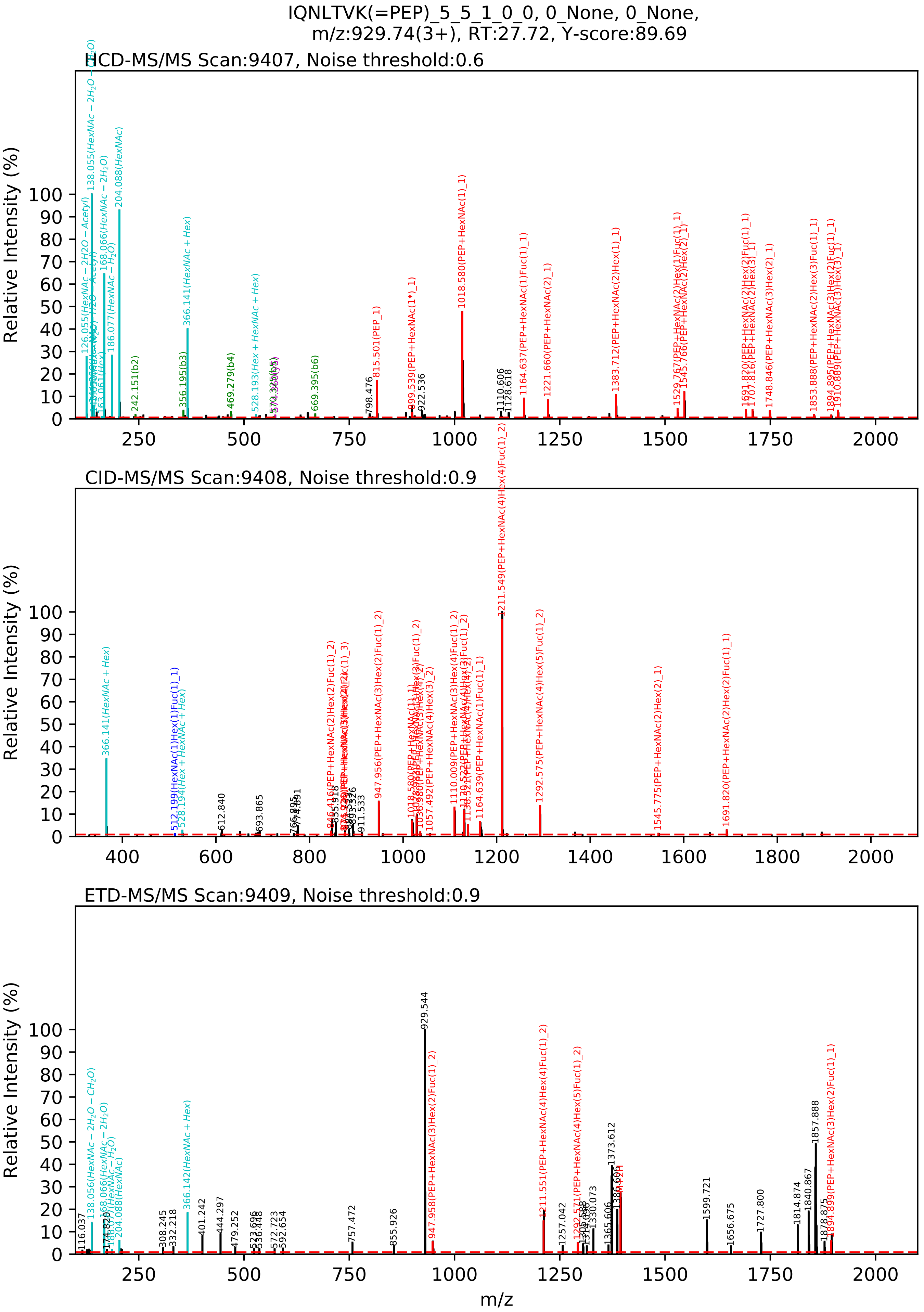

IQNLTVK(=PEP)_5_5_1_0_0, 0_None, 0_None,
m/z:929.74(3+), RT:27.72, Y-score:89.69
HCD-MS/MS Scan:9407, Noise threshold:0.6
CID-MS/MS Scan:9408, Noise threshold:0.9
ETD-MS/MS Scan:9409, Noise threshold:0.9
Relative Intensity (%)
m/z

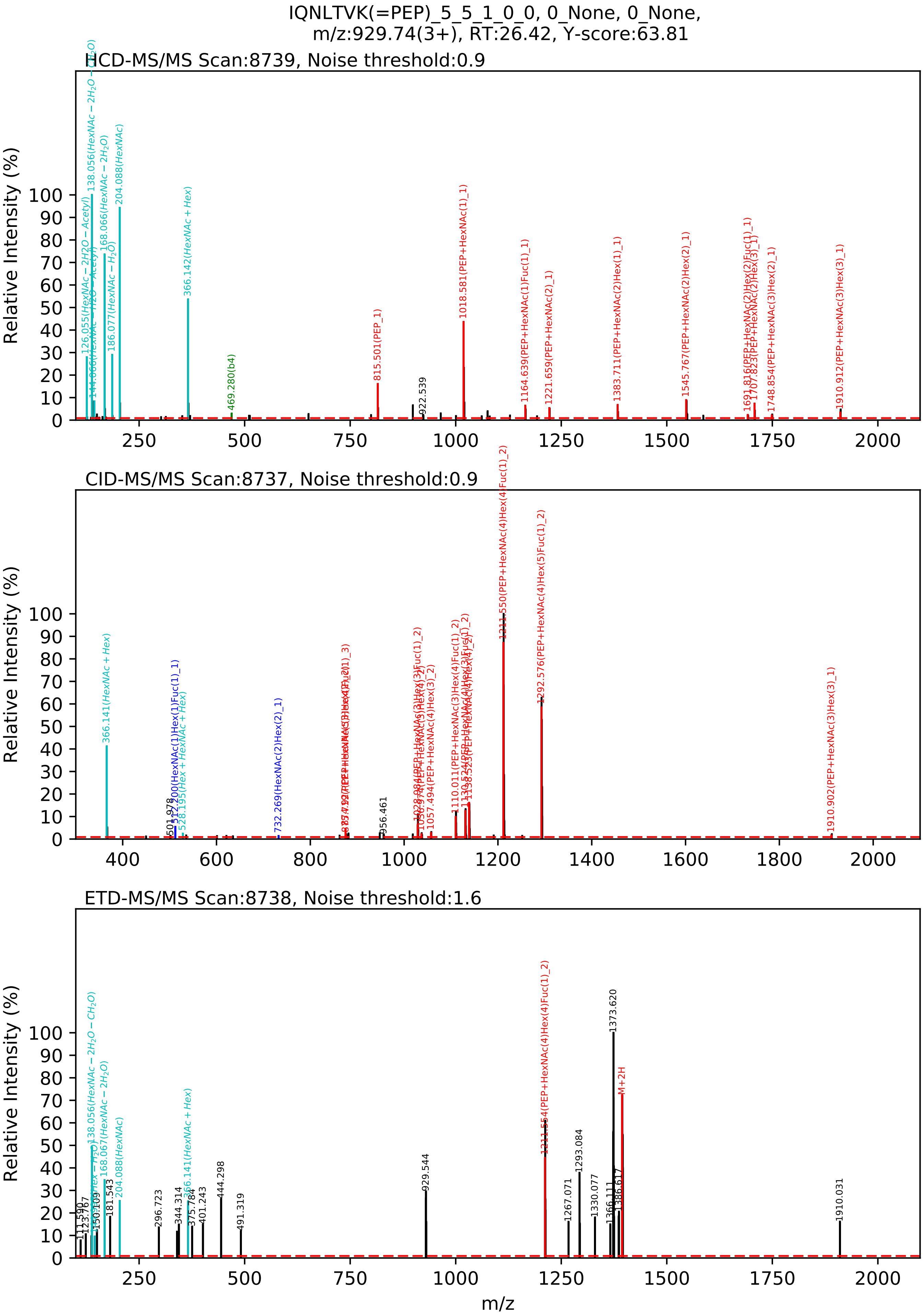
IQNLTVK(=PEP)_5_5_1_0_0, 0_None, 0_None,
m/z:929.74(3+), RT:26.42, Y-score:63.81
HCD-MS/MS Scan:8739, Noise threshold:0.9
Relative Intensity (%)
126.055
138.056
168.066
186.077
204.088(HexNAc)
366.142(HexNAc + Hex)
469.280(b4)
815.501(PEP_1)
922.539
1018.581(PEP+HexNAc(1)_1)
1164.639(PEP+HexNAc(1)Fuc(1)_1)
1221.659(PEP+HexNAc(2)_1)
1383.711(PEP+HexNAc(2)Hex(1)_1)
1545.767(PEP+HexNAc(2)Hex(2)_1)
1748.854(PEP+HexNAc(3)Hex(2)_1)
1910.912(PEP+HexNAc(3)Hex(3)_1)
CID-MS/MS Scan:8737, Noise threshold:0.9
Relative Intensity (%)
366.141(HexNAc + Hex)
732.269(HexNAc(2)Hex(2)_1)
956.461
1211.550(PEP+HexNAc(4)Hex(4)Fuc(1)_2)
1292.576(PEP+HexNAc(4)Hex(5)Fuc(1)_2)
1910.902(PEP+HexNAc(3)Hex(3)_1)
ETD-MS/MS Scan:8738, Noise threshold:1.6
Relative Intensity (%)
138.056
168.067
204.088(HexNAc)
366.141(HexNAc + Hex)
181.543
296.723
344.314
375.784
401.243
444.298
491.319
929.544
1211.554(PEP+HexNAc(4)Hex(4)Fuc(1)_2)
1267.071
1293.084
1330.077
1373.620
M+2H
1910.031
m/z

IQNLTVK(=PEP)_5_5_1_0_0, 0_None, 0_None,
m/z:1394.11(2+), RT:27.24, Y-score:97.72

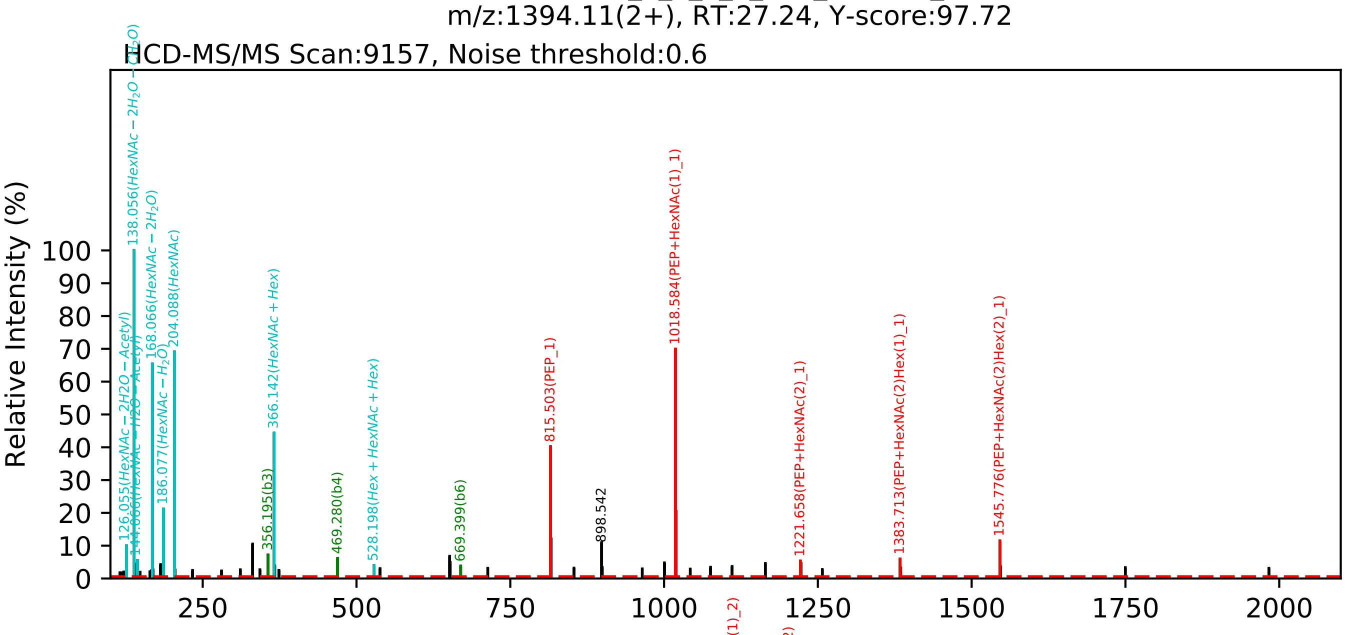

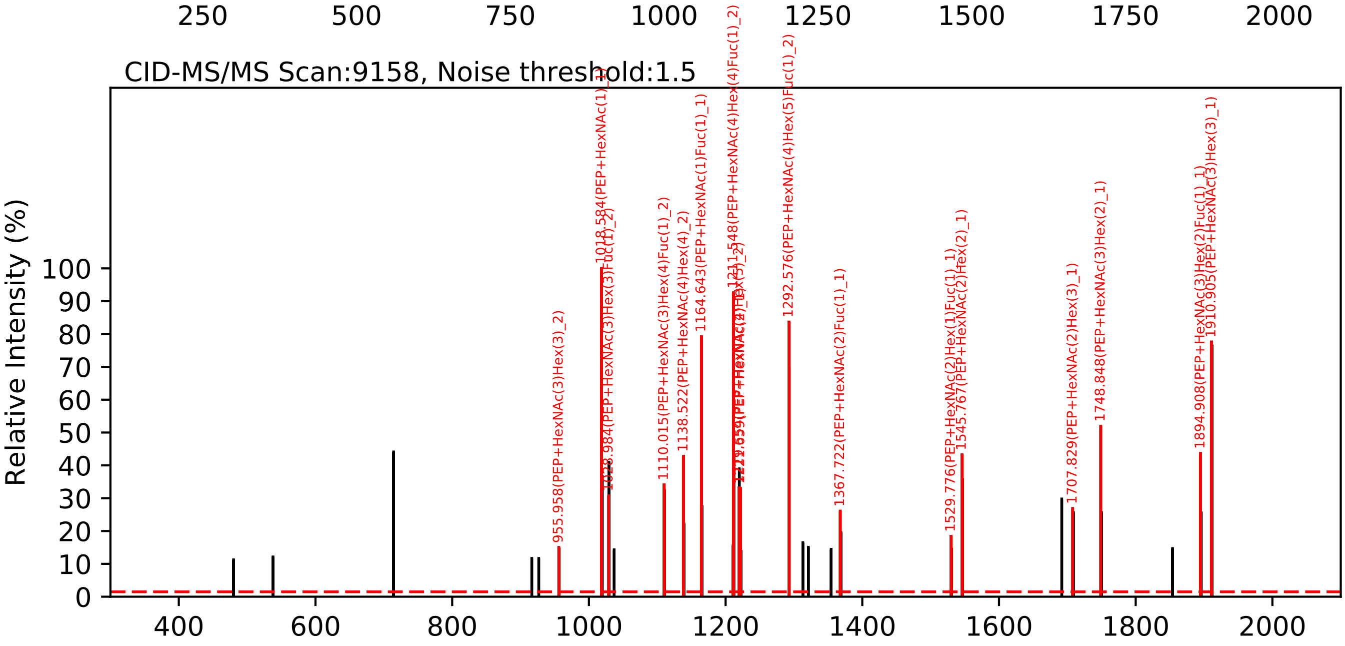

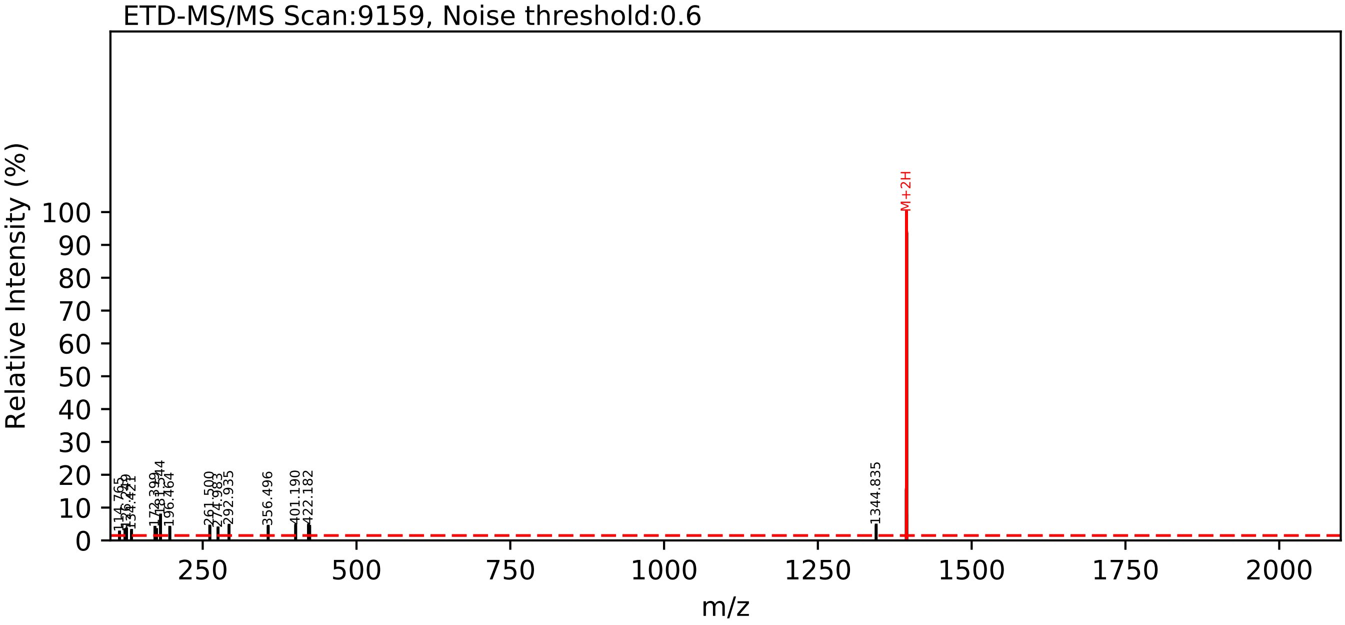

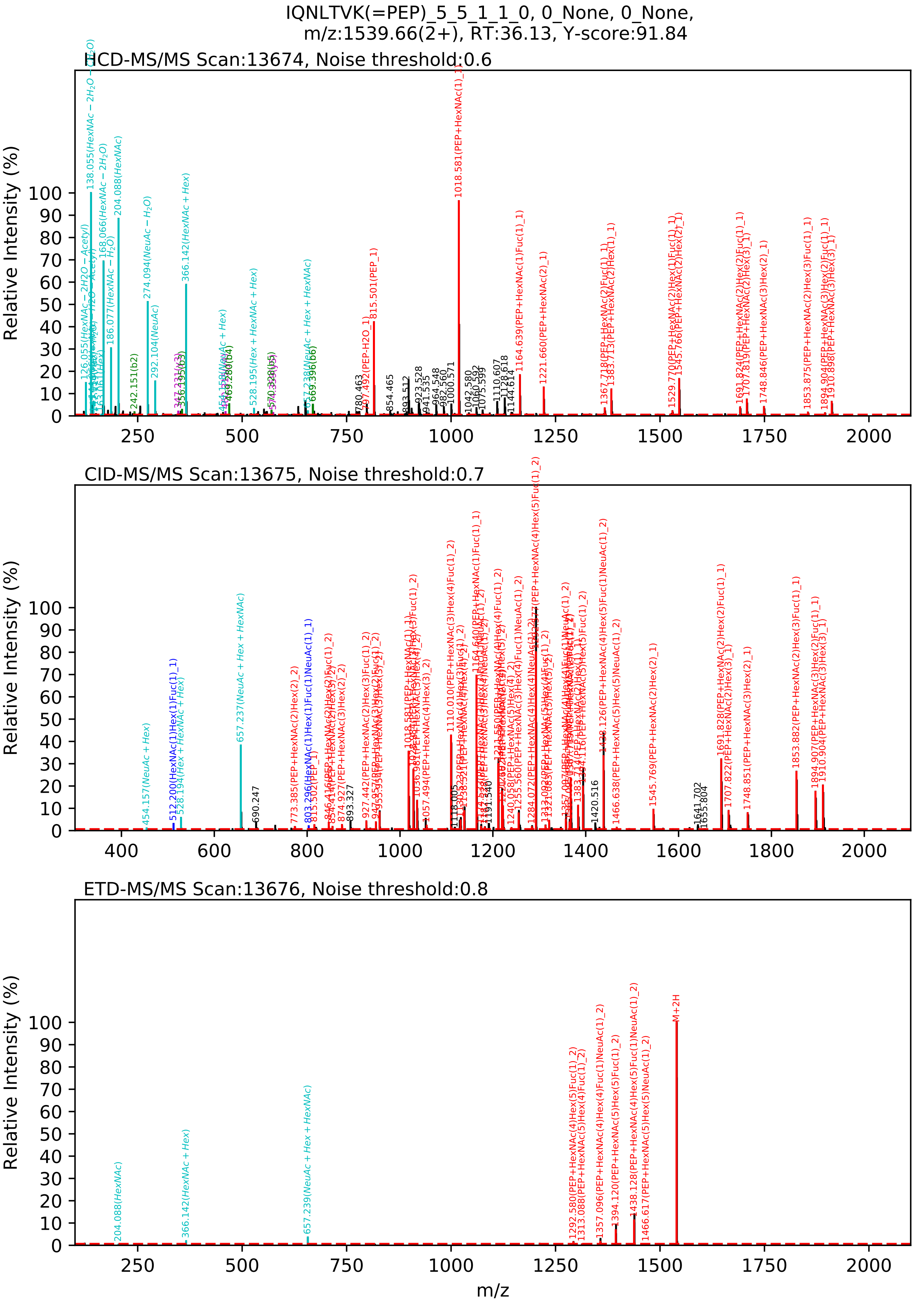
IQNLTVK(=PEP)_5_5_1_1_0, 0_None, 0_None,
m/z:1539.66(2+), RT:36.13, Y-score:91.84
HCD-MS/MS Scan:13674, Noise threshold:0.6
CID-MS/MS Scan:13675, Noise threshold:0.7
ETD-MS/MS Scan:13676, Noise threshold:0.8
Relative Intensity (%)
m/z

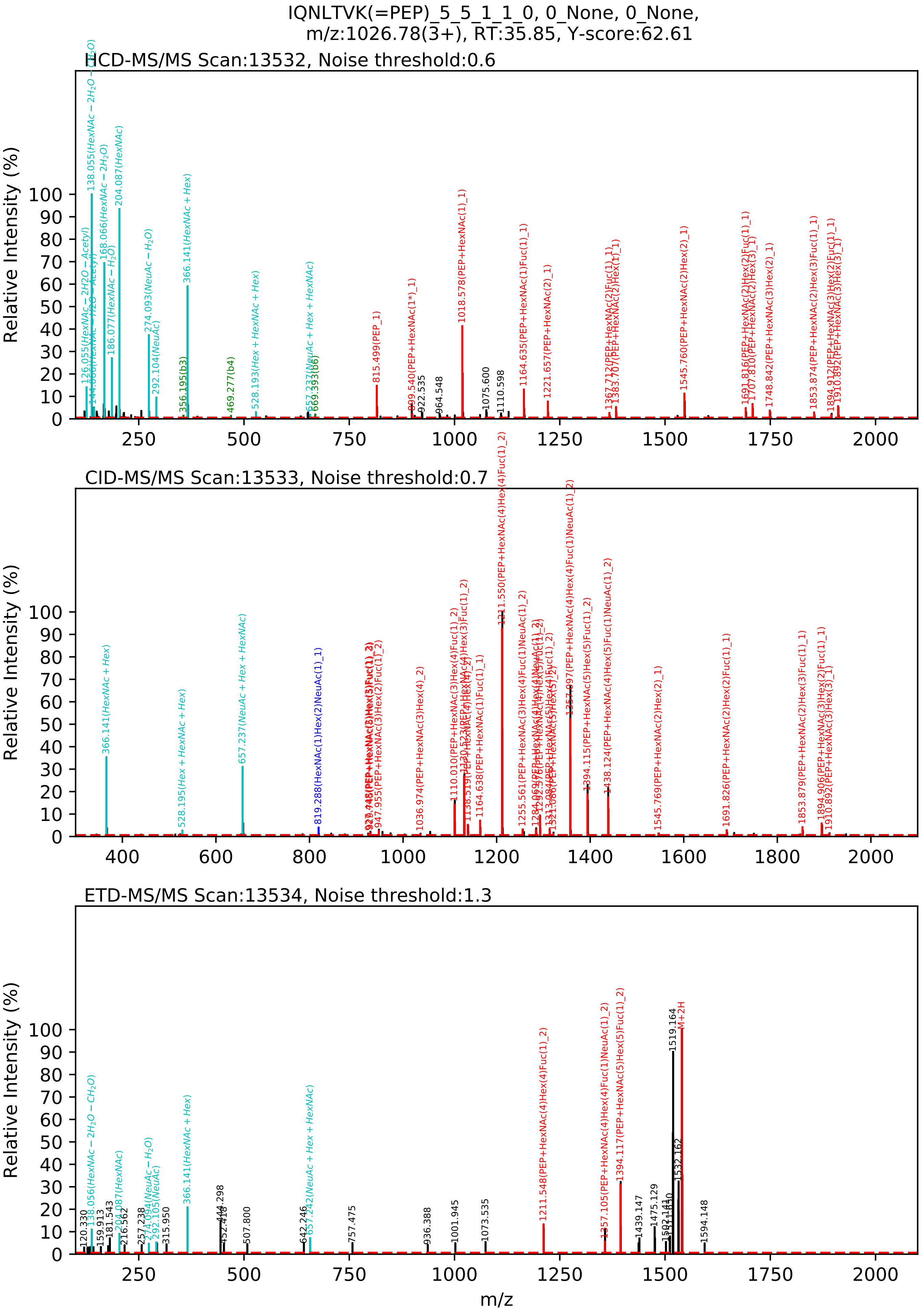

IQNLTVK(=PEP)_5_5_1_1_0, 0_None, 0_None,
m/z:1026.78(3+), RT:35.85, Y-score:62.61
HCD-MS/MS Scan:13532, Noise threshold:0.6
Relative Intensity (%)
138.055(HexNAc − 2H2O − CH2O)
204.087(HexNAc)
168.066(HexNAc − 2H2O)
186.077(HexNAc − H2O)
274.093(NeuAc − H2O)
292.104(NeuAc)
366.141(HexNAc + Hex)
356.195(b3)
469.277(b4)
528.193(Hex + HexNAc + Hex)
657.237(NeuAc + Hex + HexNAc)
669.393(b6)
815.499(PEP_1)
899.540(PEP+HexNAc(1*)_1)
922.535
964.548
1018.578(PEP+HexNAc(1)_1)
1075.600
1110.598
1164.635(PEP+HexNAc(1)Fuc(1)_1)
1221.657(PEP+HexNAc(2)_1)
1545.760(PEP+HexNAc(2)Hex(2)_1)
1748.842(PEP+HexNAc(3)Hex(2)_1)
1853.874(PEP+HexNAc(2)Hex(3)Fuc(1)_1)
CID-MS/MS Scan:13533, Noise threshold:0.7
366.141(HexNAc + Hex)
528.195(Hex + HexNAc + Hex)
657.237(NeuAc + Hex + HexNAc)
819.288(HexNAc(1)Hex(2)NeuAc(1)_1)
947.955(PEP+HexNAc(3)Hex(2)Fuc(1)_2)
1036.974(PEP+HexNAc(3)Hex(4)_2)
1110.010(PEP+HexNAc(3)Hex(4)Fuc(1)_2)
1164.638(PEP+HexNAc(1)Fuc(1)_1)
1211.550(PEP+HexNAc(4)Hex(4)Fuc(1)_2)
1255.561(PEP+HexNAc(3)Hex(4)Fuc(1)NeuAc(1)_2)
1357.097(PEP+HexNAc(4)Hex(4)Fuc(1)NeuAc(1)_2)
1394.115(PEP+HexNAc(5)Hex(5)Fuc(1)_2)
1438.124(PEP+HexNAc(4)Hex(5)Fuc(1)NeuAc(1)_2)
1545.769(PEP+HexNAc(2)Hex(2)_1)
1691.826(PEP+HexNAc(2)Hex(2)Fuc(1)_1)
1853.879(PEP+HexNAc(2)Hex(3)Fuc(1)_1)
ETD-MS/MS Scan:13534, Noise threshold:1.3
1211.548(PEP+HexNAc(4)Hex(4)Fuc(1)_2)
1357.105(PEP+HexNAc(4)Hex(4)Fuc(1)NeuAc(1)_2)
1394.117(PEP+HexNAc(5)Hex(5)Fuc(1)_2)
1519.164
1532.162
M+2H
1594.148
m/z

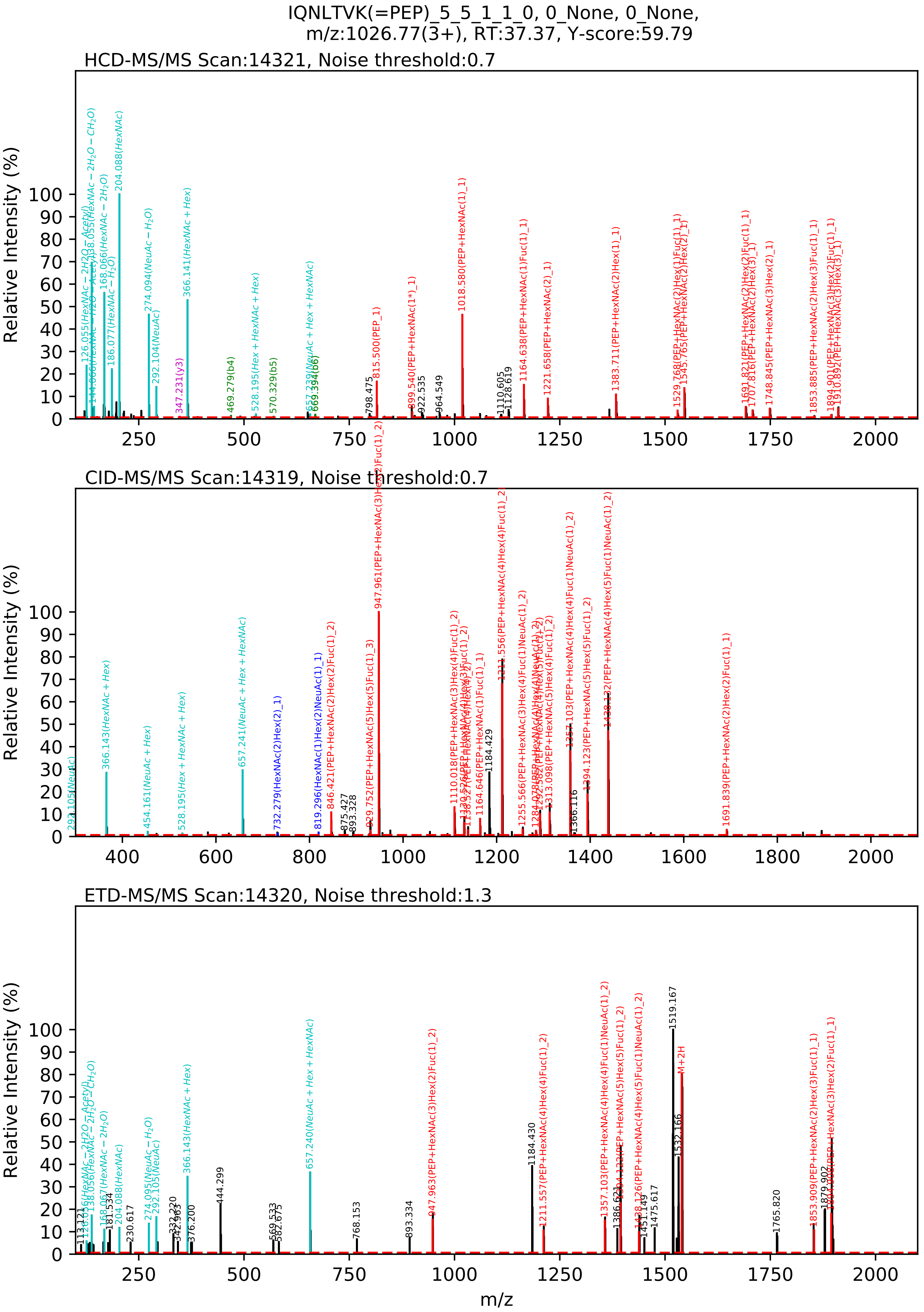
IQNLTVK(=PEP)_5_5_1_1_0, 0_None, 0_None,
m/z:1026.77(3+), RT:37.37, Y-score:59.79
HCD-MS/MS Scan:14321, Noise threshold:0.7
CID-MS/MS Scan:14319, Noise threshold:0.7
ETD-MS/MS Scan:14320, Noise threshold:1.3
Relative Intensity (%)
m/z

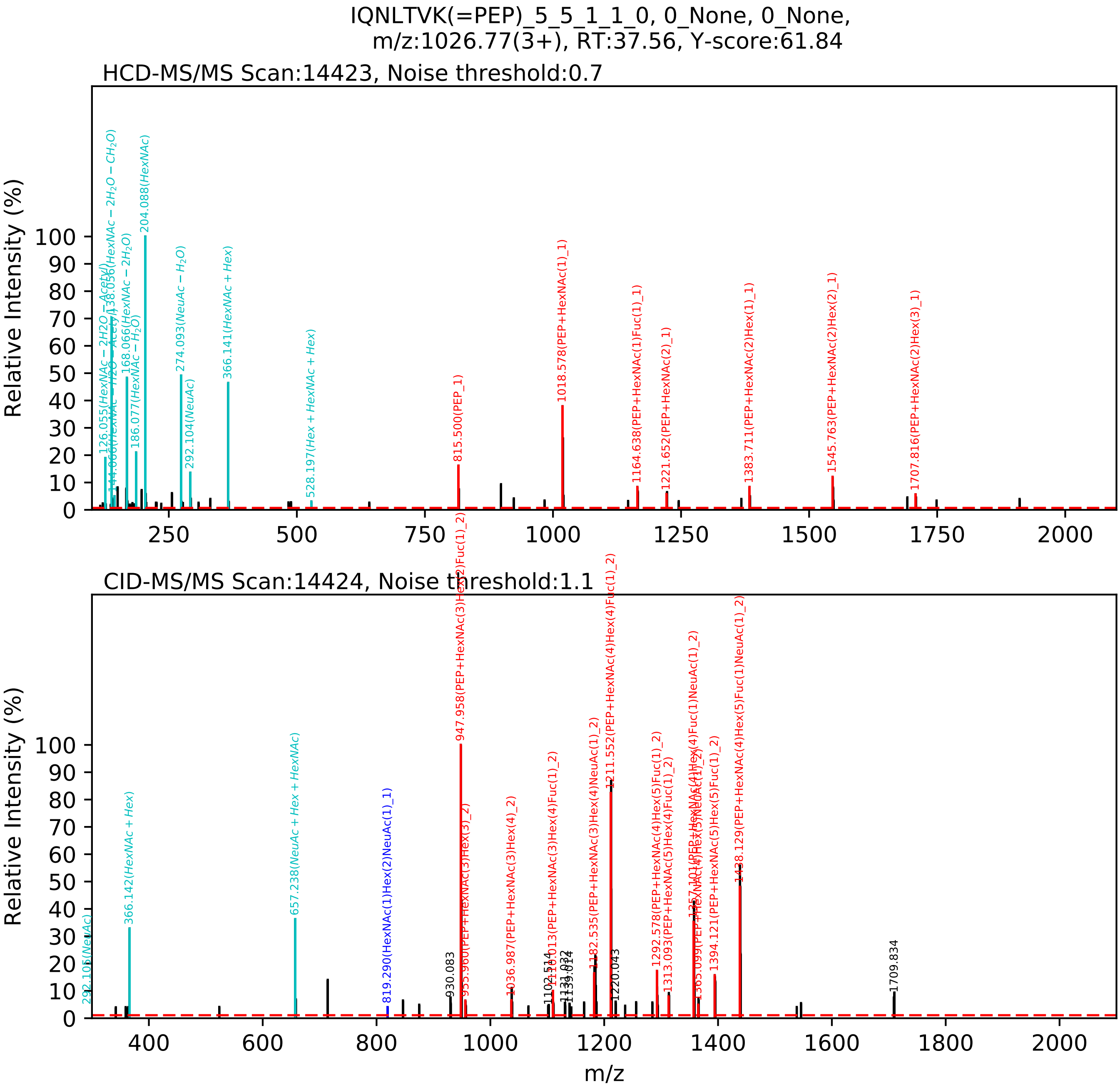

IQNLTVK(=PEP)_5_5_1_1_0, 0_None, 0_None,
m/z:1026.77(3+), RT:37.56, Y-score:61.84
HCD-MS/MS Scan:14423, Noise threshold:0.7
Relative Intensity (%)
204.088(HexNAc)
366.141(HexNAc+Hex)
274.093(NeuAc−H2O)
292.104(NeuAc)
168.066(HexNAc−2H2O)
186.077(HexNAc−H2O)
126.055(HexNAc−2H2O−Acetyl)
528.197(Hex+HexNAc+Hex)
815.500(PEP_1)
1018.578(PEP+HexNAc(1)_1)
1164.638(PEP+HexNAc(1)Fuc(1)_1)
1221.652(PEP+HexNAc(2)_1)
1383.711(PEP+HexNAc(2)Hex(1)_1)
1545.763(PEP+HexNAc(2)Hex(2)_1)
1707.816(PEP+HexNAc(2)Hex(3)_1)
CID-MS/MS Scan:14424, Noise threshold:1.1
Relative Intensity (%)
366.142(HexNAc+Hex)
657.238(NeuAc+Hex+HexNAc)
819.290(HexNAc(1)Hex(2)NeuAc(1)_1)
930.083
947.958(PEP+HexNAc(3)Hex(2)Fuc(1)_2)
1036.987(PEP+HexNAc(3)Hex(4)_2)
1182.535(PEP+HexNAc(3)Hex(4)NeuAc(1)_2)
1211.552(PEP+HexNAc(4)Hex(4)Fuc(1)_2)
1220.043
1292.578(PEP+HexNAc(4)Hex(5)Fuc(1)_2)
1313.093(PEP+HexNAc(5)Hex(4)Fuc(1)_2)
1394.121(PEP+HexNAc(5)Hex(5)Fuc(1)_2)
1438.129(PEP+HexNAc(4)Hex(5)Fuc(1)NeuAc(1)_2)
1709.834
m/z

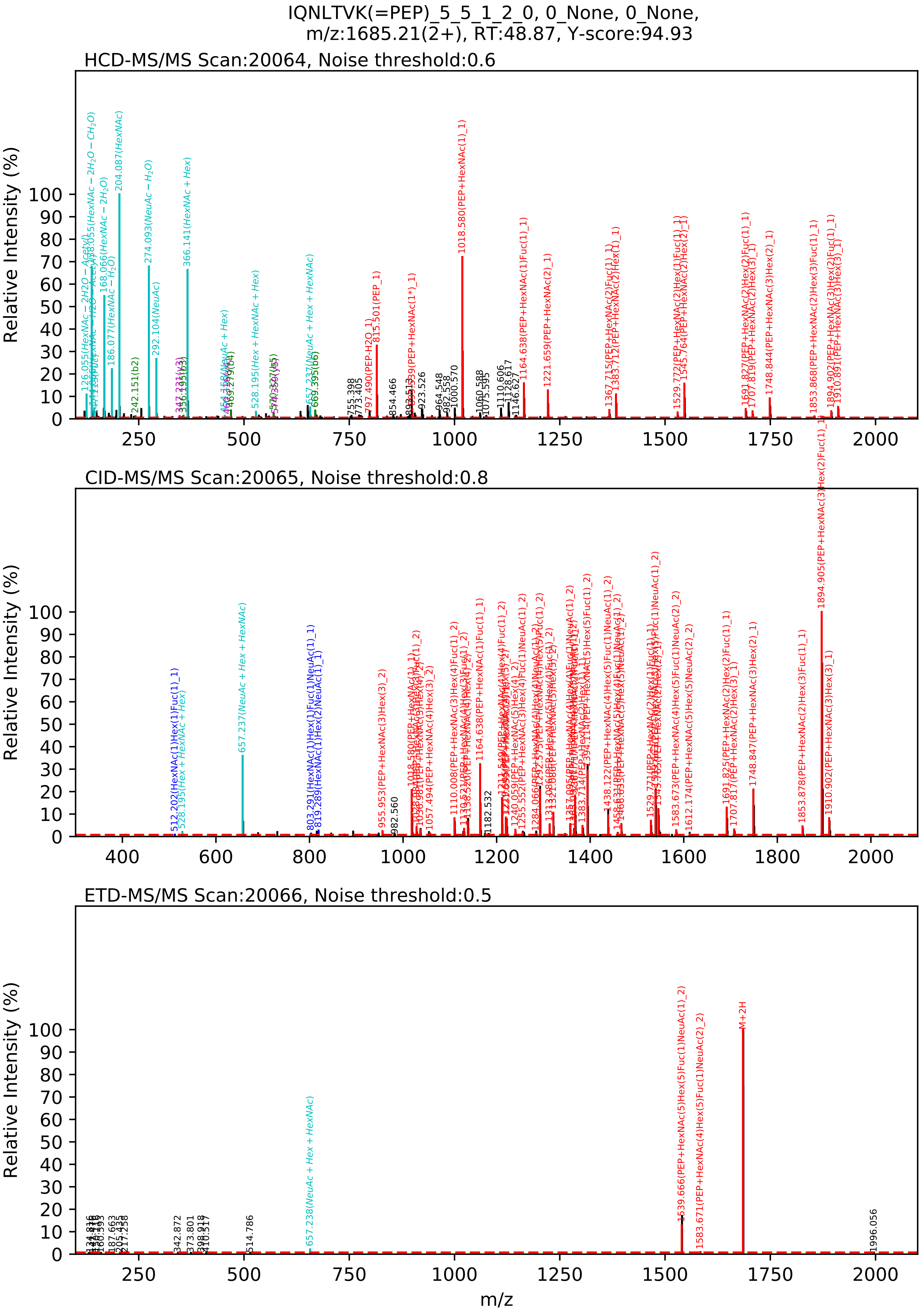
IQNLTVK(=PEP)_5_5_1_2_0, 0_None, 0_None,
m/z:1685.21(2+), RT:48.87, Y-score:94.93
HCD-MS/MS Scan:20064, Noise threshold:0.6
Relative Intensity (%)
204.087(HexNAc)
274.093(NeuAc − H2O)
366.141(HexNAc + Hex)
292.104(NeuAc)
168.066(HexNAc − 2H2O)
186.077(HexNAc − H2O)
126.055(HexNAc − 2H2O − CH2O)
242.151(b2)
1018.580(PEP+HexNAc(1)_1)
815.501(PEP_1)
797.490(PEP-H2O_1)
1164.638(PEP+HexNAc(1)Fuc(1)_1)
1221.659(PEP+HexNAc(2)_1)
1383.712(PEP+HexNAc(2)Hex(1)_1)
1545.764(PEP+HexNAc(2)Hex(2)_1)
1748.844(PEP+HexNAc(3)Hex(2)_1)
1910.891(PEP+HexNAc(3)Hex(3)_1)
CID-MS/MS Scan:20065, Noise threshold:0.8
1894.905(PEP+HexNAc(3)Hex(2)Fuc(1)_1)
657.237(NeuAc + Hex + HexNAc)
1164.638(PEP+HexNAc(1)Fuc(1)_1)
1748.847(PEP+HexNAc(3)Hex(2)_1)
1691.825(PEP+HexNAc(2)Hex(2)Fuc(1)_1)
1910.902(PEP+HexNAc(3)Hex(3)_1)
1853.878(PEP+HexNAc(2)Hex(3)Fuc(1)_1)
955.953(PEP+HexNAc(3)Hex(3)_2)
ETD-MS/MS Scan:20066, Noise threshold:0.5
M+2H
1539.666(PEP+HexNAc(5)Hex(5)Fuc(1)NeuAc(1)_2)
1583.671(PEP+HexNAc(4)Hex(5)Fuc(1)NeuAc(2)_2)
657.238(NeuAc + Hex + HexNAc)
1996.056
m/z

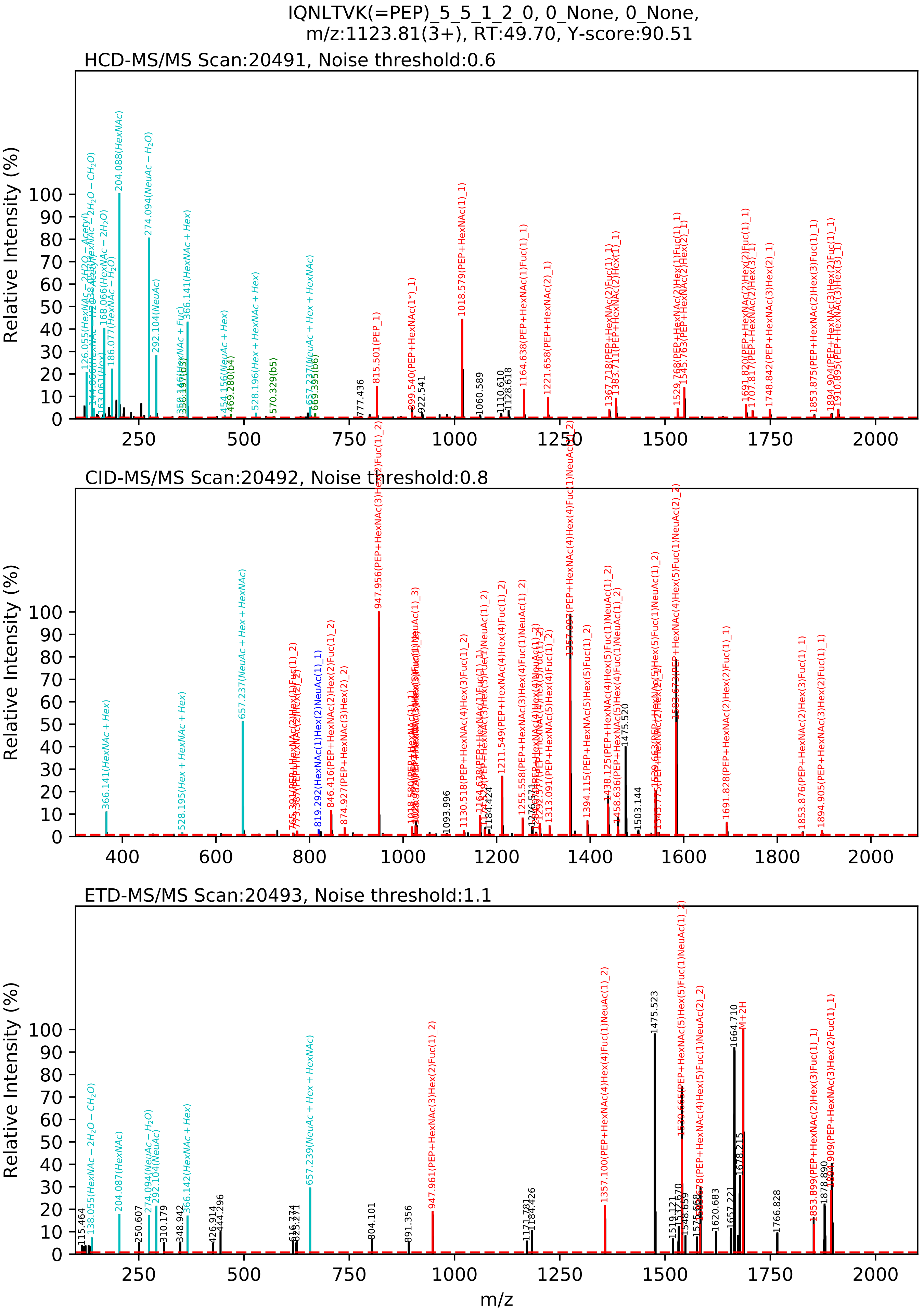
IQNLTVK(=PEP)_5_5_1_2_0, 0_None, 0_None,
m/z:1123.81(3+), RT:49.70, Y-score:90.51
HCD-MS/MS Scan:20491, Noise threshold:0.6
CID-MS/MS Scan:20492, Noise threshold:0.8
ETD-MS/MS Scan:20493, Noise threshold:1.1
Relative Intensity (%)
m/z

IQNLTVK(=PEP)_5_5_1_2_0, 0_None, 0_None,
m/z:1123.81(3+), RT:49.04, Y-score:58.92

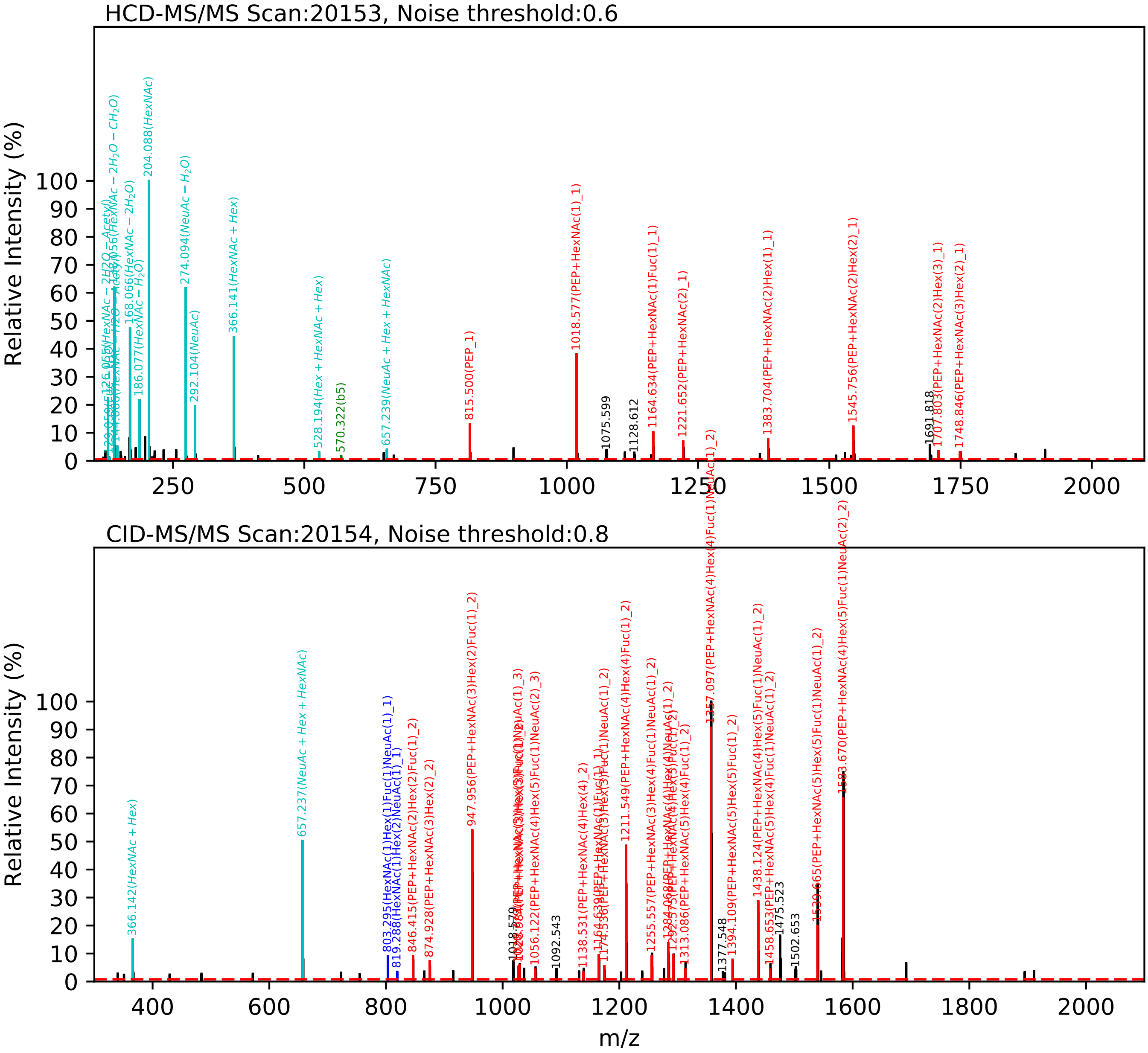

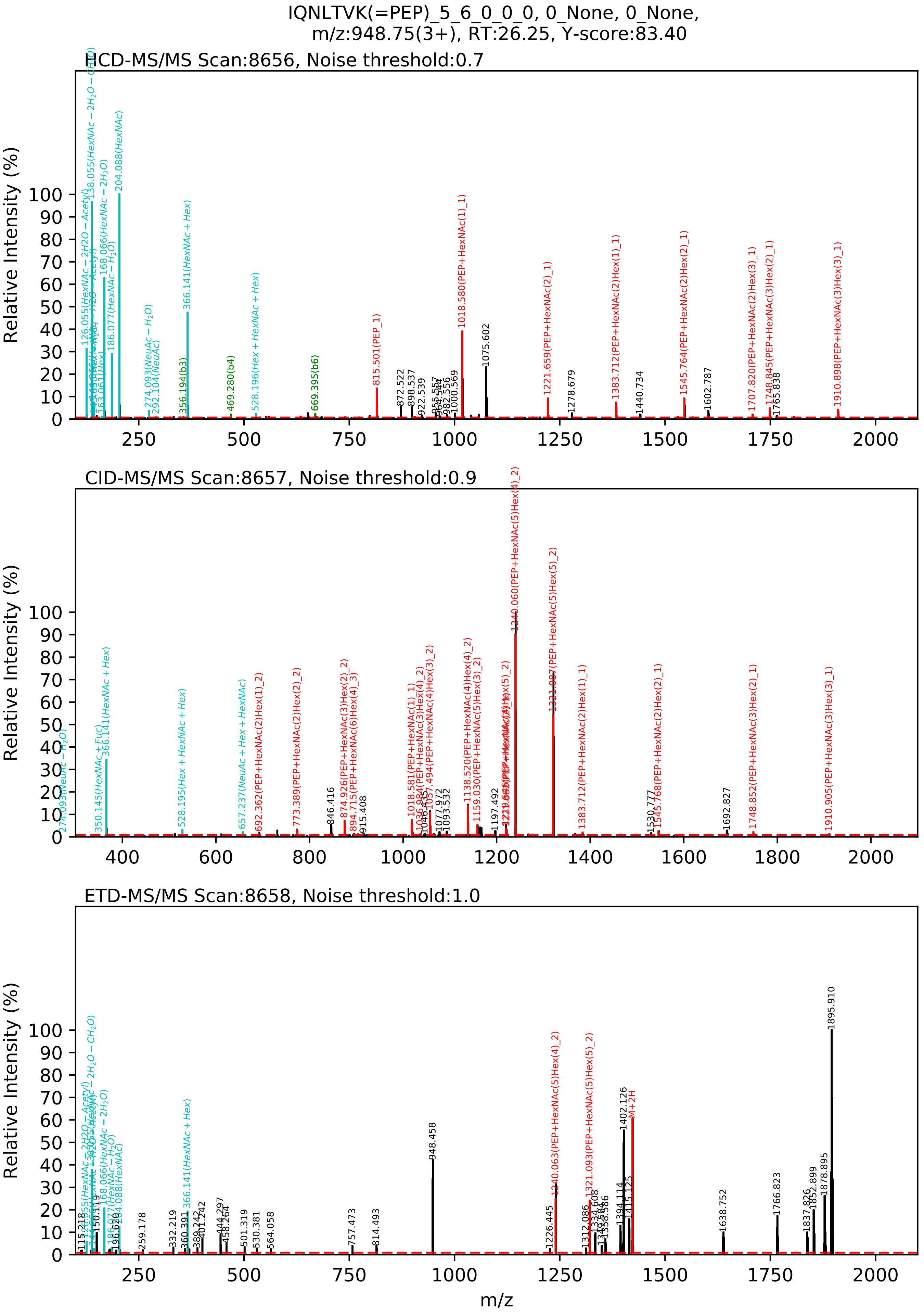
IQNLTVK(=PEP)_5_6_0_0_0, 0_None, 0_None,
m/z:948.75(3+), RT:26.25, Y-score:83.40
HCD-MS/MS Scan:8656, Noise threshold:0.7
1018.580(PEP+HexNAc(1)_1)
1221.659(PEP+HexNAc(2)_1)
1383.712(PEP+HexNAc(2)Hex(1)_1)
1545.764(PEP+HexNAc(2)Hex(2)_1)
1707.820(PEP+HexNAc(2)Hex(3)_1)
1748.845(PEP+HexNAc(3)Hex(2)_1)
1910.898(PEP+HexNAc(3)Hex(3)_1)
815.501(PEP_1)
CID-MS/MS Scan:8657, Noise threshold:0.9
1240.060(PEP+HexNAc(5)Hex(4)_2)
1321.087(PEP+HexNAc(5)Hex(5)_2)
ETD-MS/MS Scan:8658, Noise threshold:1.0
1895.910
M+2H
Relative Intensity (%)
m/z

IQNLTVK(=PEP)_5_6_1_0_0, 0_None, 0_None,
m/z:997.44(3+), RT:26.94, Y-score:59.16

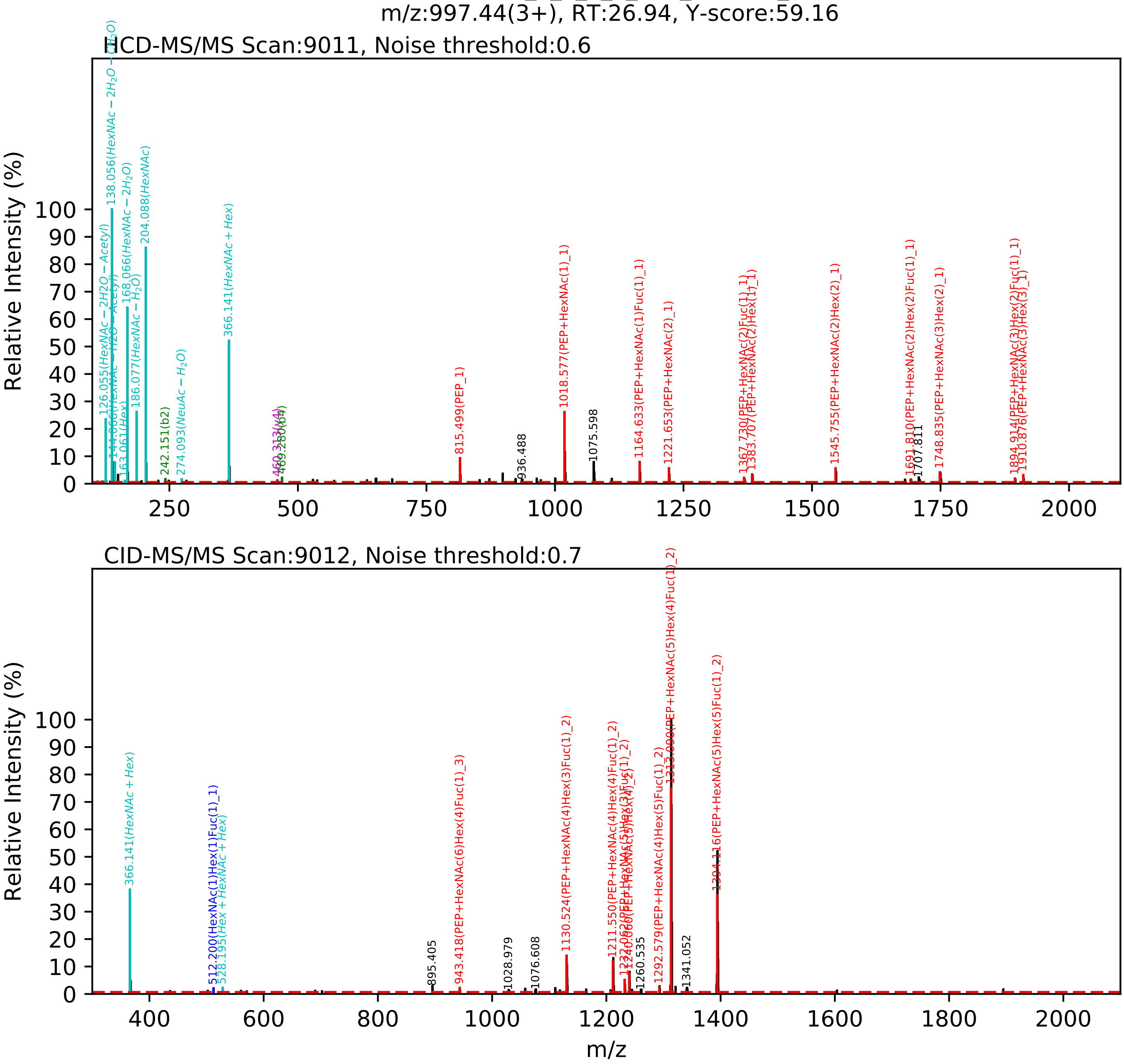

IQNLTVK(=PEP)_6_2_0_0_0, 0_None, 0_None,
m/z:1097.49(2+), RT:26.72, Y-score:93.48

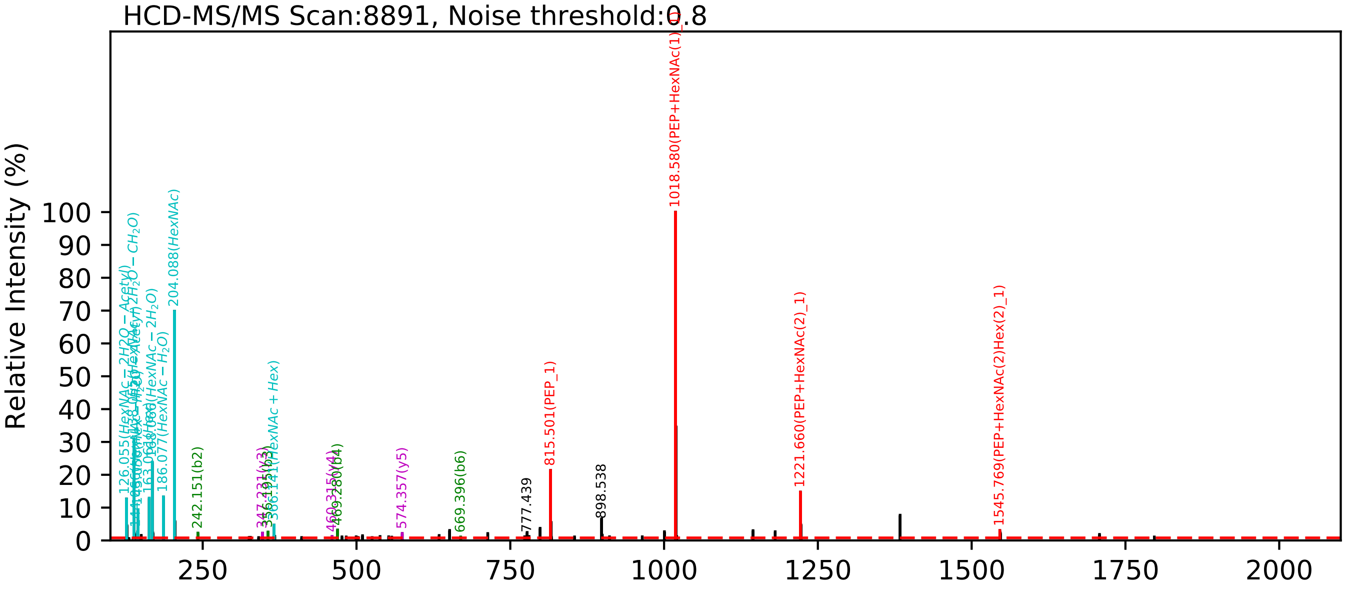

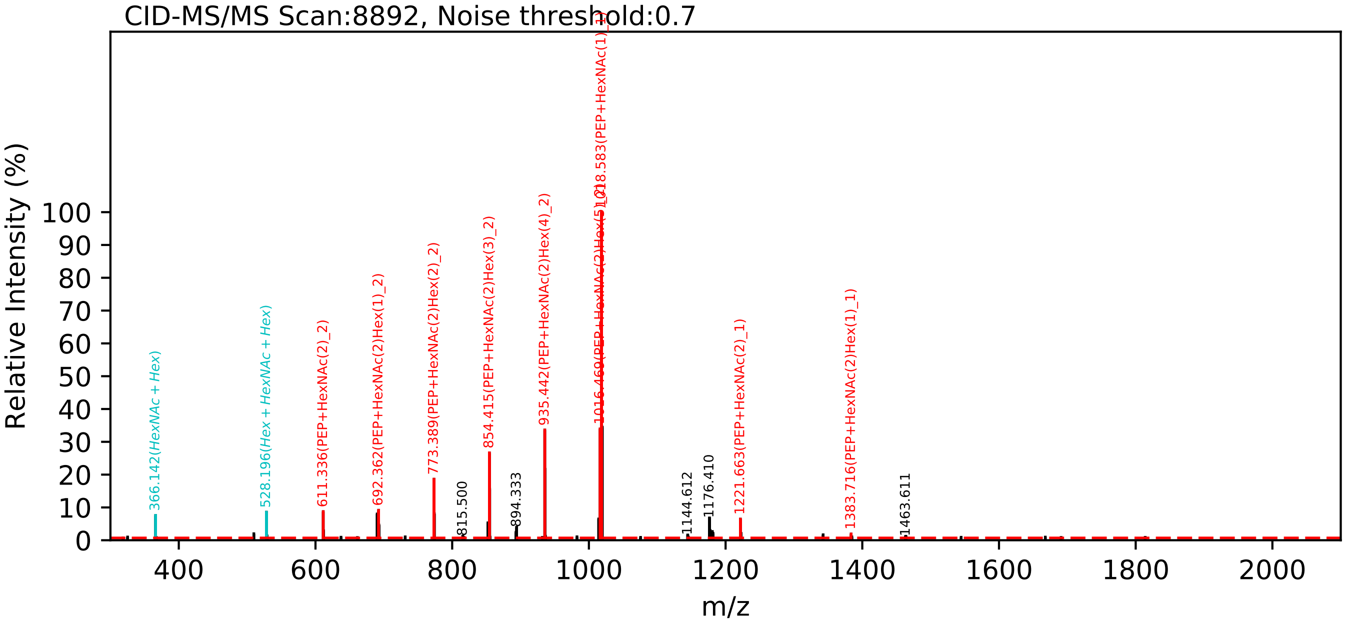

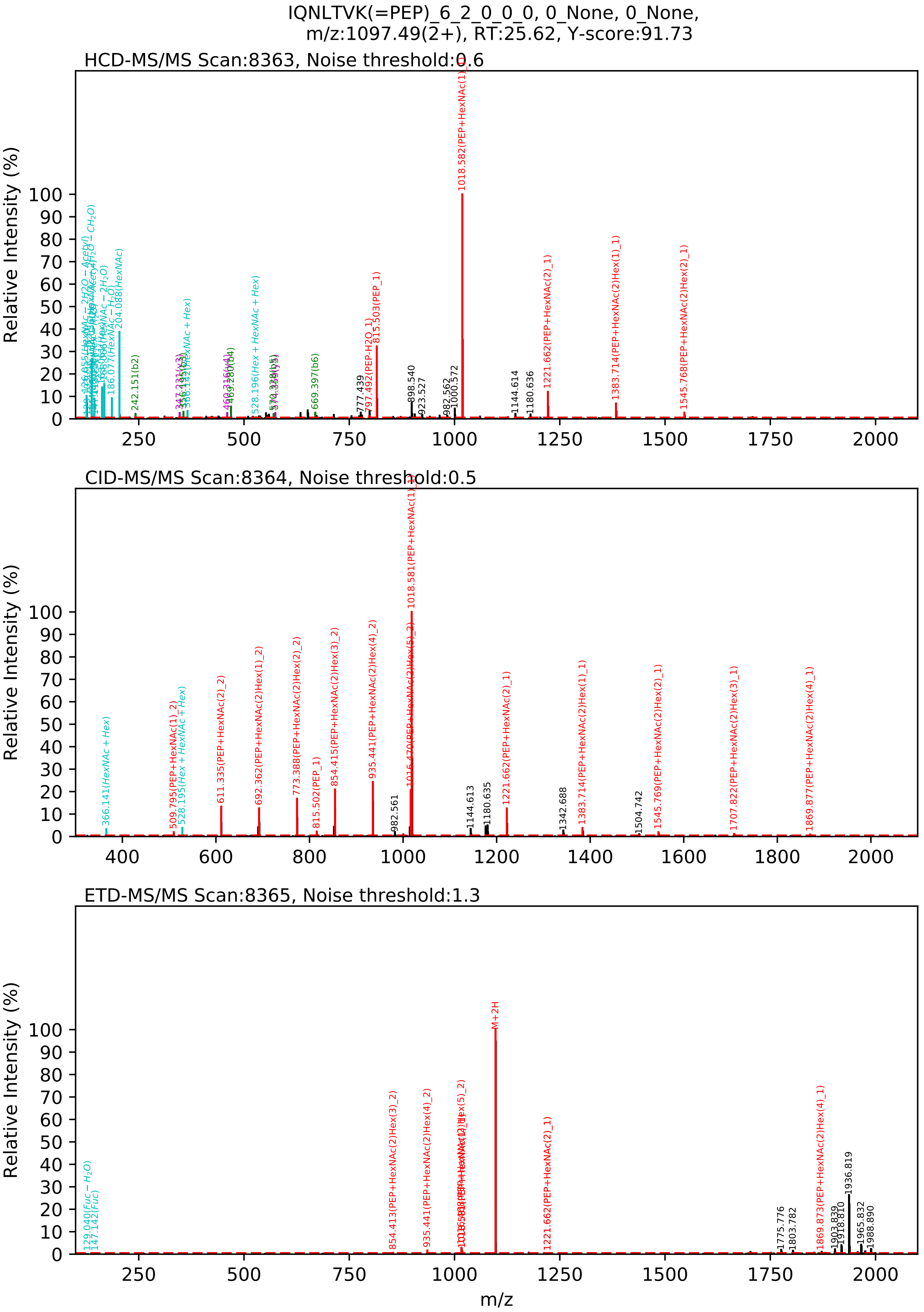
IQNLTVK(=PEP)_6_2_0_0_0, 0_None, 0_None,
m/z:1097.49(2+), RT:25.62, Y-score:91.73
HCD-MS/MS Scan:8363, Noise threshold:0.6
Relative Intensity (%)
1018.582(PEP+HexNAc(1)_1)
1221.662(PEP+HexNAc(2)_1)
1383.714(PEP+HexNAc(2)Hex(1)_1)
1545.768(PEP+HexNAc(2)Hex(2)_1)
815.503(PEP_1)
797.492(PEP-H2O_1)
204.088(HexNAc)
186.077(HexNAc − H2O)
242.151(b2)
669.397(b6)
528.196(Hex + HexNAc + Hex)
898.540
923.527
982.562
1000.572
1144.614
1180.636
777.439
CID-MS/MS Scan:8364, Noise threshold:0.5
366.141(HexNAc + Hex)
509.795(PEP+HexNAc(1)_2)
528.195(Hex + HexNAc + Hex)
611.335(PEP+HexNAc(2)_2)
692.362(PEP+HexNAc(2)Hex(1)_2)
773.388(PEP+HexNAc(2)Hex(2)_2)
815.502(PEP_1)
854.415(PEP+HexNAc(2)Hex(3)_2)
935.441(PEP+HexNAc(2)Hex(4)_2)
982.561
1016.470(PEP+HexNAc(2)Hex(5)_2)
1018.581(PEP+HexNAc(1)_1)
1144.613
1180.635
1221.662(PEP+HexNAc(2)_1)
1342.688
1383.714(PEP+HexNAc(2)Hex(1)_1)
1504.742
1545.769(PEP+HexNAc(2)Hex(2)_1)
1707.822(PEP+HexNAc(2)Hex(3)_1)
1869.877(PEP+HexNAc(2)Hex(4)_1)
ETD-MS/MS Scan:8365, Noise threshold:1.3
129.040(Fuc − H2O)
147.142(Fuc)
854.413(PEP+HexNAc(2)Hex(3)_2)
935.441(PEP+HexNAc(2)Hex(4)_2)
M+2H
1221.662(PEP+HexNAc(2)_1)
1775.776
1803.782
1869.873(PEP+HexNAc(2)Hex(4)_1)
1903.839
1918.810
1936.819
1965.832
1988.890
m/z

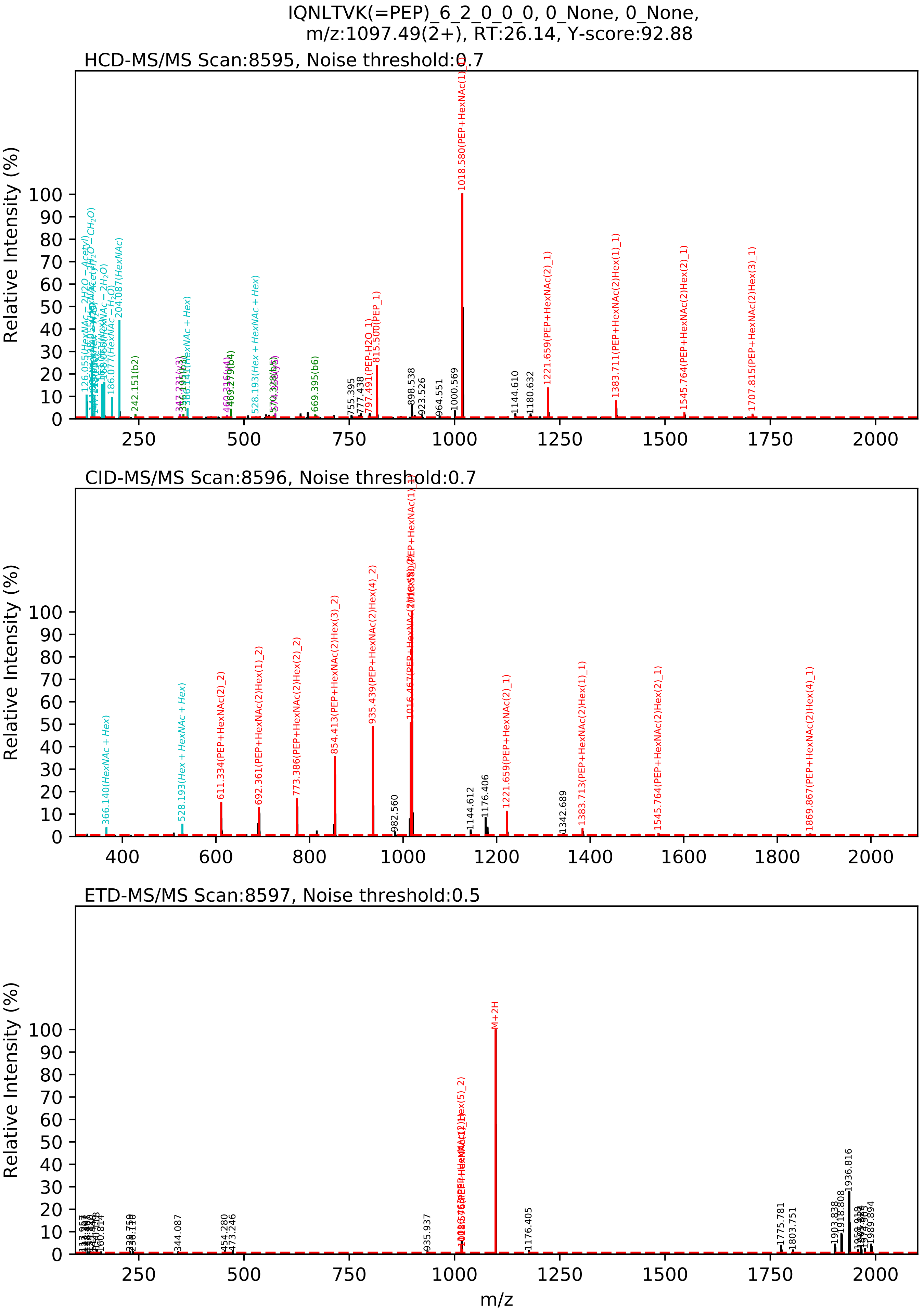
IQNLTVK(=PEP)_6_2_0_0_0, 0_None, 0_None,
m/z:1097.49(2+), RT:26.14, Y-score:92.88
HCD-MS/MS Scan:8595, Noise threshold:0.7
CID-MS/MS Scan:8596, Noise threshold:0.7
ETD-MS/MS Scan:8597, Noise threshold:0.5
Relative Intensity (%)
m/z

IQNLTVK(=PEP)_6_3_0_0_0, 0_None, 0_None,
m/z:799.69(3+), RT:26.33, Y-score:96.30

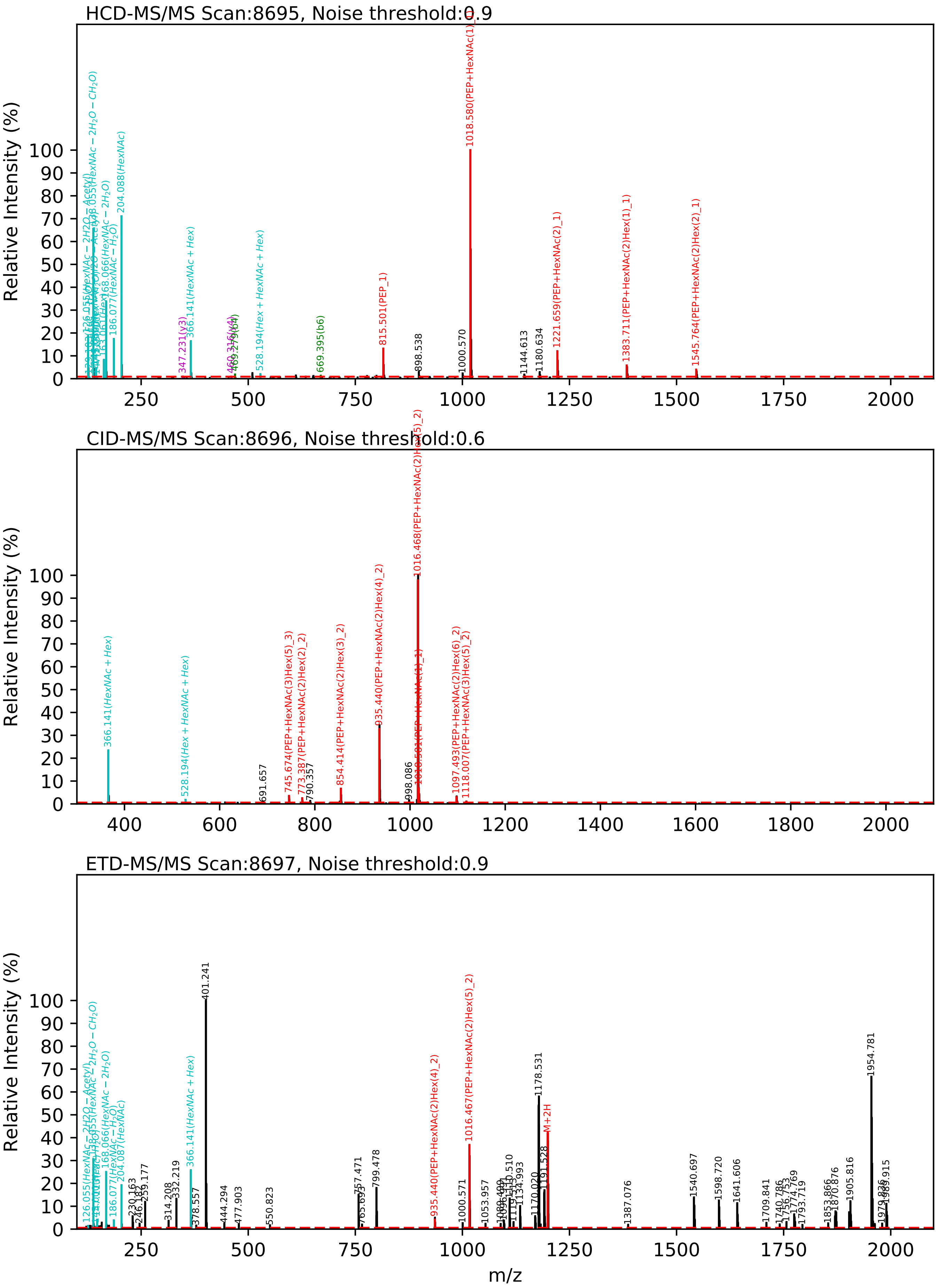

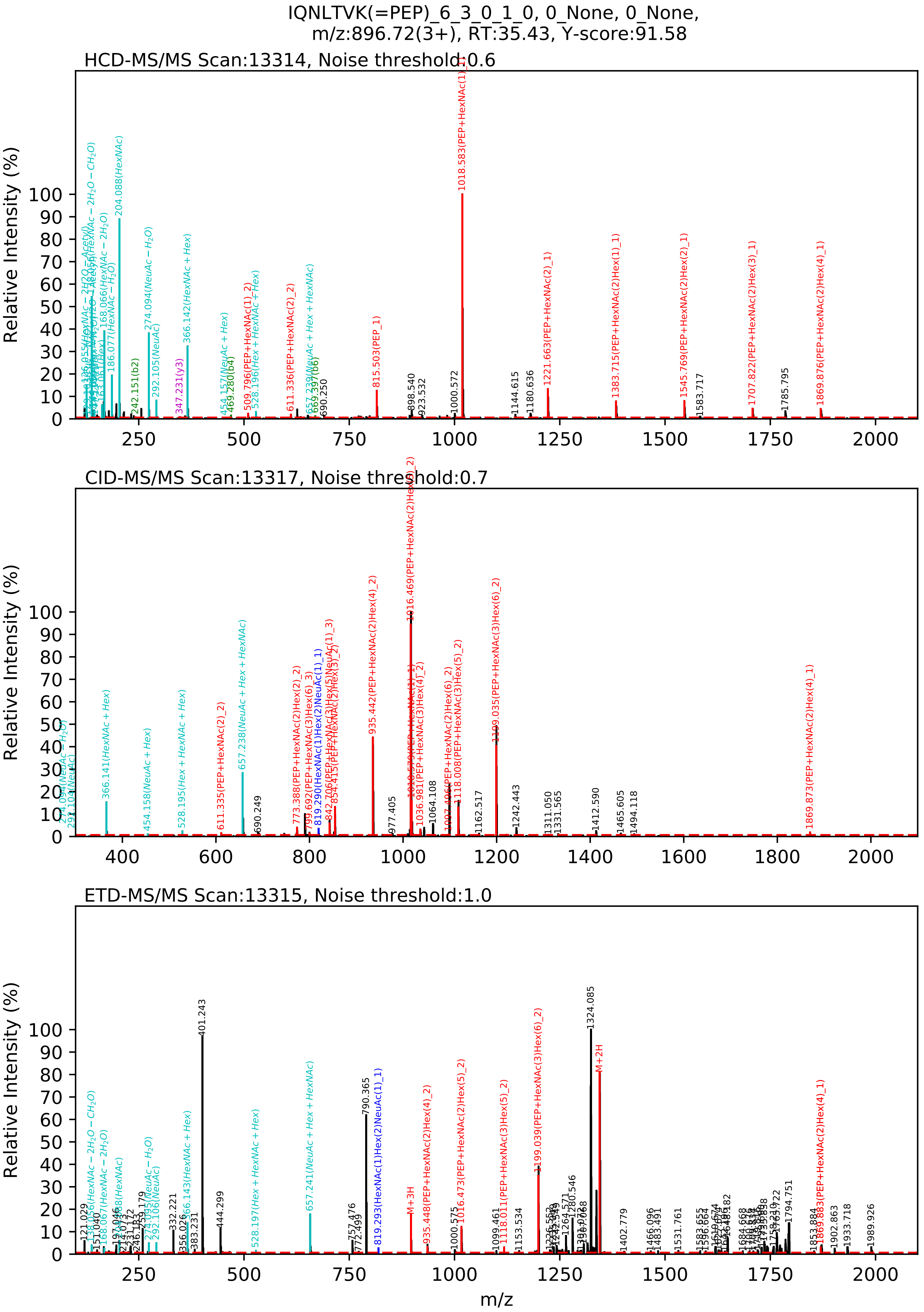
IQNLTVK(=PEP)_6_3_0_1_0, 0_None, 0_None,
m/z:896.72(3+), RT:35.43, Y-score:91.58
HCD-MS/MS Scan:13314, Noise threshold:0.6
CID-MS/MS Scan:13317, Noise threshold:0.7
ETD-MS/MS Scan:13315, Noise threshold:1.0
Relative Intensity (%)
m/z
1018.583(PEP+HexNAc(1)_1)
1016.469(PEP+HexNAc(2)Hex(4)_2)
1199.035(PEP+HexNAc(3)Hex(6)_2)
935.442(PEP+HexNAc(2)Hex(4)_2)
1869.873(PEP+HexNAc(2)Hex(4)_1)
401.243
1324.085
M+2H
M+3H
790.365
819.293(HexNAc(1)Hex(2)NeuAc(1)_1)

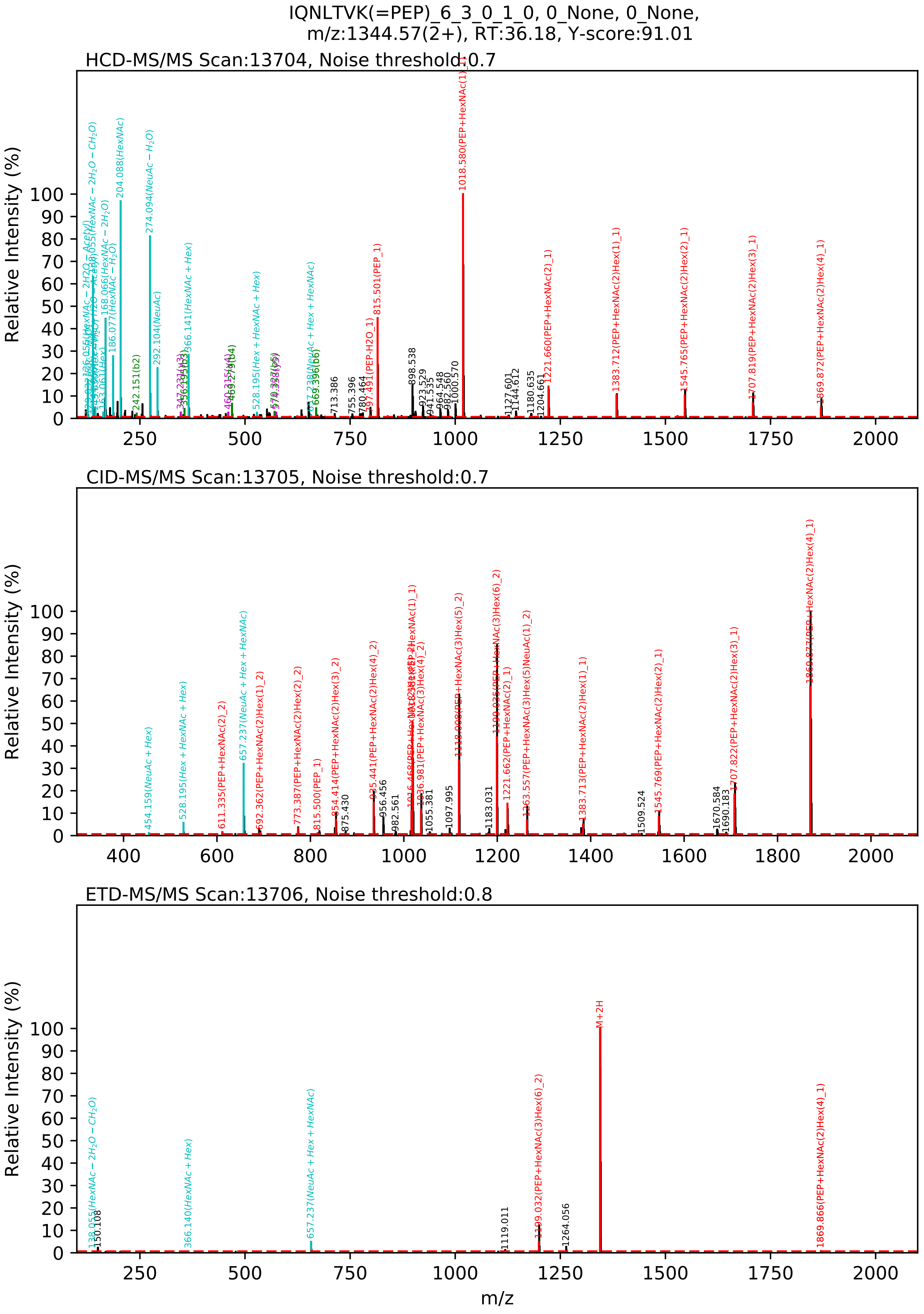
IQNLTVK(=PEP)_6_3_0_1_0, 0_None, 0_None,
m/z:1344.57(2+), RT:36.18, Y-score:91.01
HCD-MS/MS Scan:13704, Noise threshold:0.7
Relative Intensity (%)
1018.580(PEP+HexNAc(1)_1)
204.088(HexNAc)
274.094(NeuAc − H2O)
292.104(NeuAc)
366.141(HexNAc + Hex)
528.195(Hex + HexNAc + Hex)
815.501(PEP_1)
797.491(PEP-H2O_1)
242.151(b2)
469.279(b4)
669.396(b6)
898.538
1221.660(PEP+HexNAc(2)_1)
1383.712(PEP+HexNAc(2)Hex(1)_1)
1545.765(PEP+HexNAc(2)Hex(2)_1)
1707.819(PEP+HexNAc(2)Hex(3)_1)
1869.872(PEP+HexNAc(2)Hex(4)_1)
CID-MS/MS Scan:13705, Noise threshold:0.7
Relative Intensity (%)
454.159(NeuAc + Hex)
528.195(Hex + HexNAc + Hex)
657.237(NeuAc + Hex + HexNAc)
611.335(PEP+HexNAc(2)_2)
692.362(PEP+HexNAc(2)Hex(1)_2)
773.387(PEP+HexNAc(2)Hex(2)_2)
815.500(PEP_1)
854.414(PEP+HexNAc(2)Hex(3)_2)
935.441(PEP+HexNAc(2)Hex(4)_2)
1118.008(PEP+HexNAc(3)Hex(5)_2)
1199.035(PEP+HexNAc(3)Hex(6)_2)
1221.662(PEP+HexNAc(2)_1)
1263.557(PEP+HexNAc(3)Hex(5)NeuAc(1)_2)
1383.713(PEP+HexNAc(2)Hex(1)_1)
1545.769(PEP+HexNAc(2)Hex(2)_1)
1707.822(PEP+HexNAc(2)Hex(3)_1)
1869.877(PEP+HexNAc(2)Hex(4)_1)
ETD-MS/MS Scan:13706, Noise threshold:0.8
Relative Intensity (%)
138.055(HexNAc − 2H2O − CH2O)
150.108
366.140(HexNAc + Hex)
657.237(NeuAc + Hex + HexNAc)
1119.011
1199.032(PEP+HexNAc(3)Hex(6)_2)
1264.056
M+2H
1869.866(PEP+HexNAc(2)Hex(4)_1)
m/z

IQNLTVK(=PEP)_6_4_0_0_0, 0_None, 0_None,
m/z:1300.57(2+), RT:26.09, Y-score:80.97

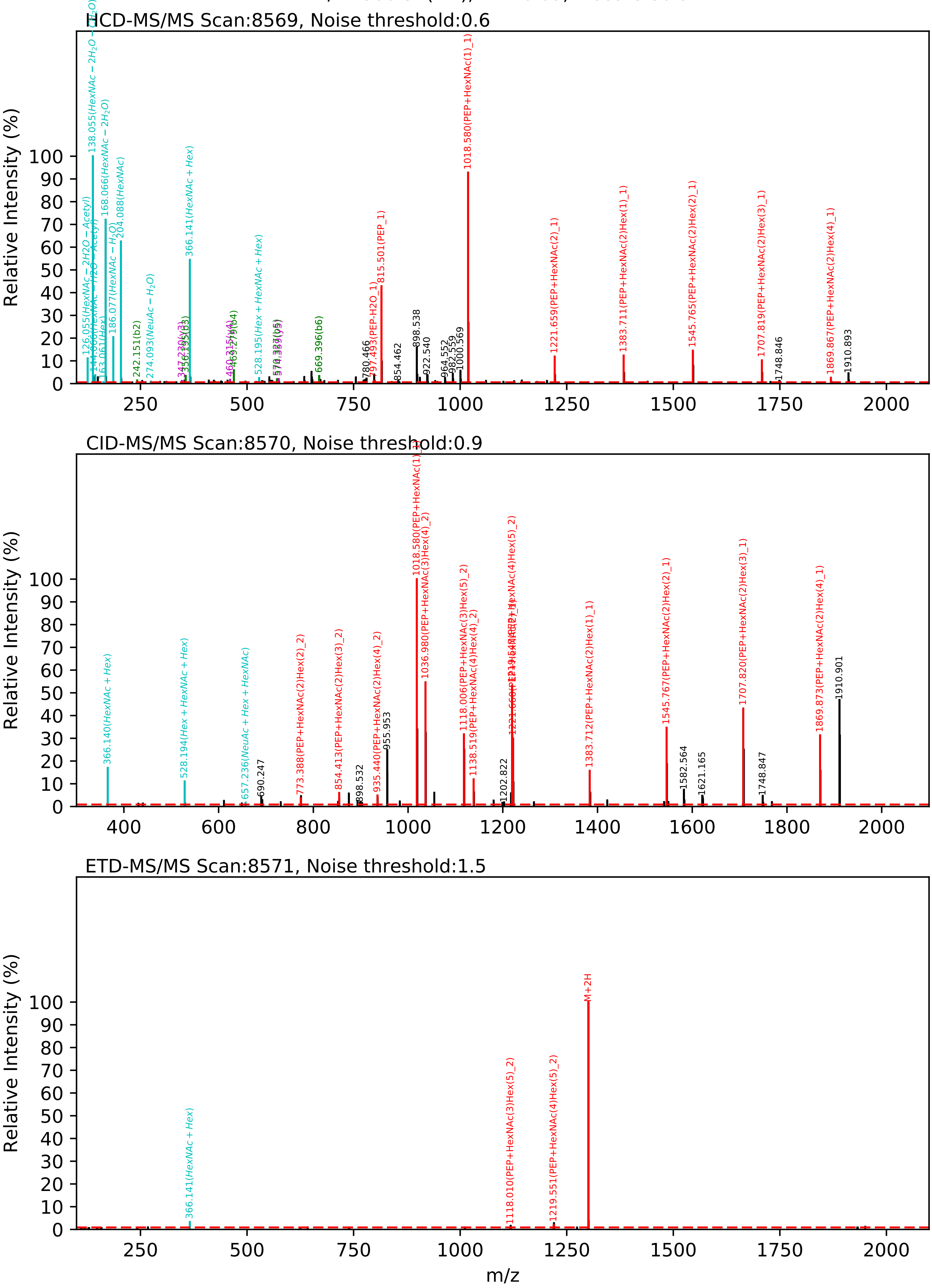

IQNLTVK(=PEP)_6_4_0_1_0, 0_None, 0_None,
m/z:1446.12(2+), RT:35.09, Y-score:91.30

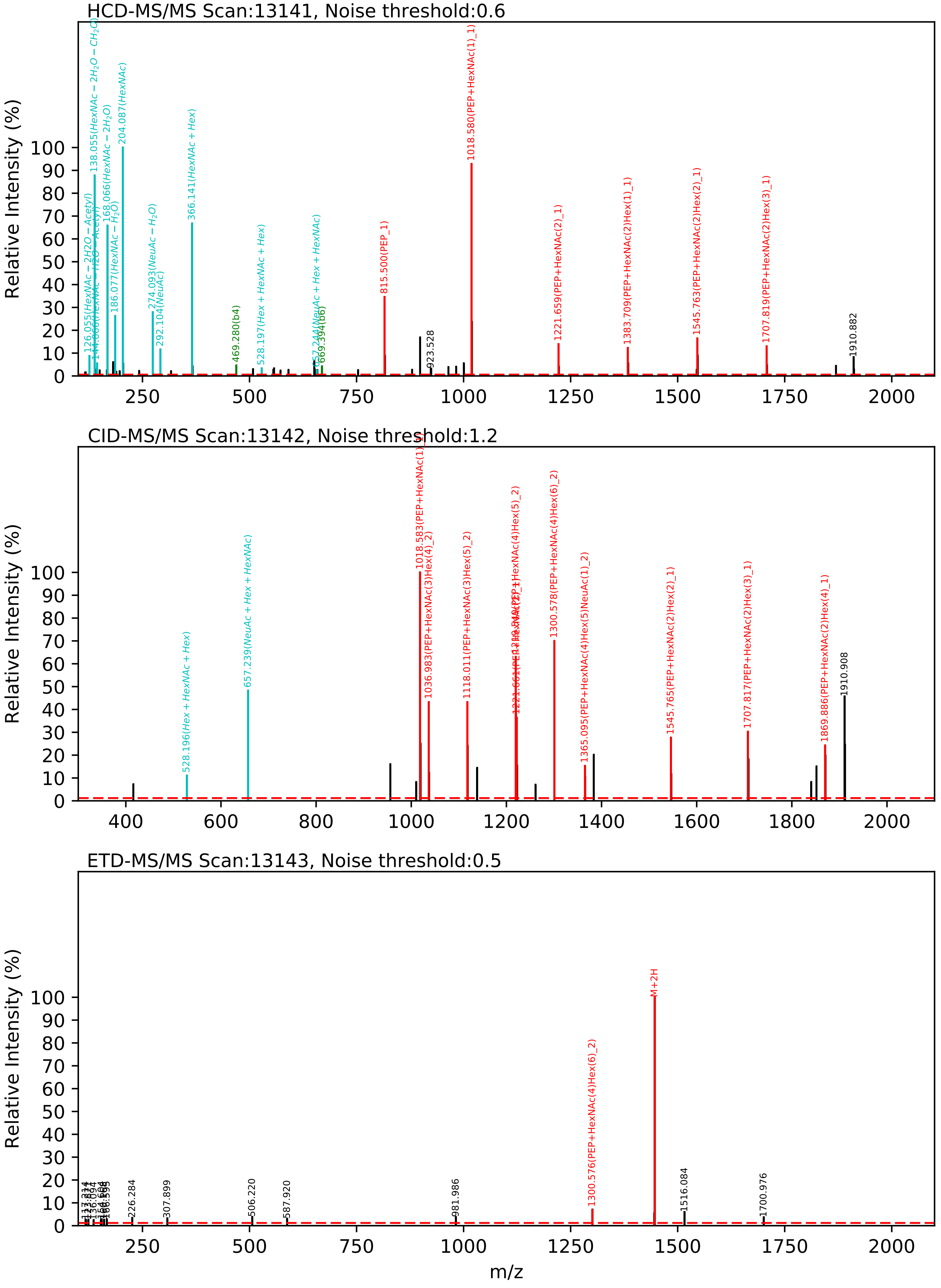

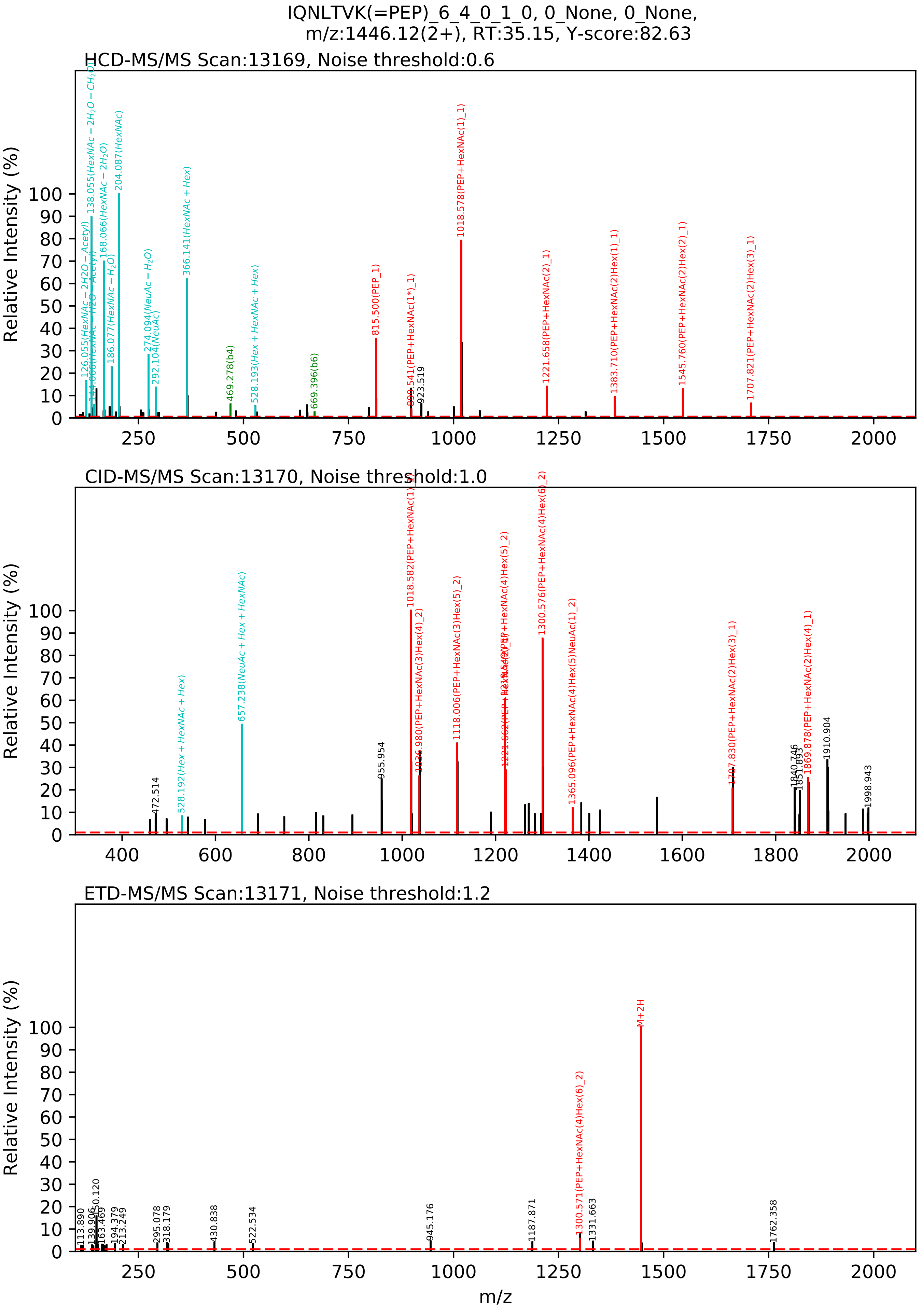
IQNLTVK(=PEP)_6_4_0_1_0, 0_None, 0_None,
m/z:1446.12(2+), RT:35.15, Y-score:82.63
HCD-MS/MS Scan:13169, Noise threshold:0.6
CID-MS/MS Scan:13170, Noise threshold:1.0
ETD-MS/MS Scan:13171, Noise threshold:1.2
Relative Intensity (%)
m/z
204.087(HexNAc)
366.141(HexNAc+Hex)
469.278(b4)
528.193(Hex+HexNAc+Hex)
669.396(b6)
815.500(PEP_1)
923.519
1018.578(PEP+HexNAc(1)_1)
1221.658(PEP+HexNAc(2)_1)
1383.710(PEP+HexNAc(2)Hex(1)_1)
1545.760(PEP+HexNAc(2)Hex(2)_1)
1707.821(PEP+HexNAc(2)Hex(3)_1)
472.514
528.192(Hex+HexNAc+Hex)
657.238(NeuAc+Hex+HexNAc)
955.954
1118.006(PEP+HexNAc(3)Hex(5)_2)
1300.576(PEP+HexNAc(4)Hex(6)_2)
1365.096(PEP+HexNAc(4)Hex(5)NeuAc(1)_2)
1707.830(PEP+HexNAc(2)Hex(3)_1)
1840.746
1869.878(PEP+HexNAc(2)Hex(4)_1)
1910.904
1998.943
M+2H
1300.571(PEP+HexNAc(4)Hex(6)_2)
1331.663
1762.358
1187.871
945.176
522.534
430.838

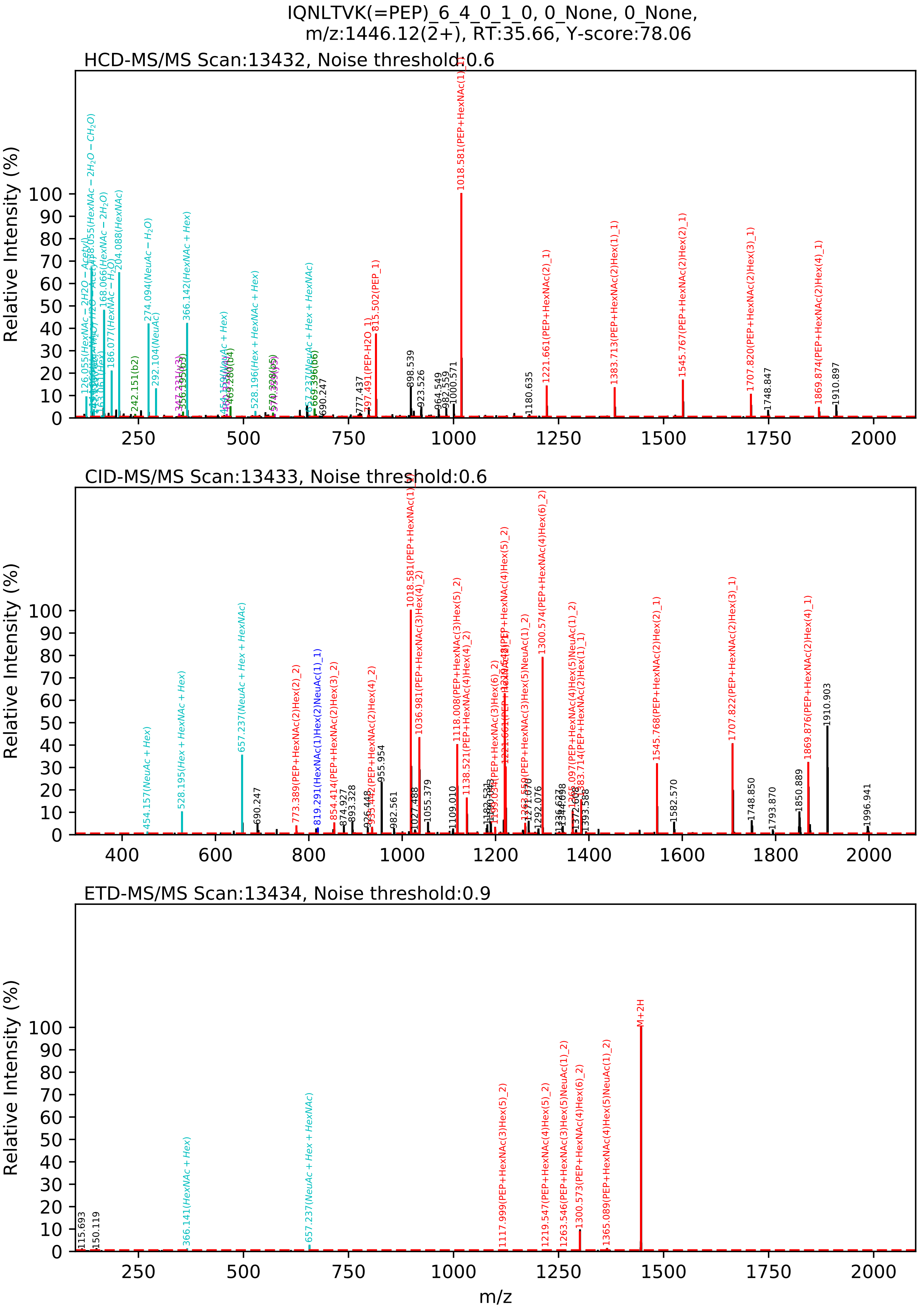

IQNLTVK(=PEP)_6_4_0_1_0, 0_None, 0_None,
m/z:1446.12(2+), RT:35.66, Y-score:78.06
HCD-MS/MS Scan:13432, Noise threshold:0.6
CID-MS/MS Scan:13433, Noise threshold:0.6
ETD-MS/MS Scan:13434, Noise threshold:0.9
Relative Intensity (%)
m/z
1018.581(PEP+HexNAc(1)_1)
815.502(PEP_1)
1221.661(PEP+HexNAc(2)_1)
1383.713(PEP+HexNAc(2)Hex(1)_1)
1545.767(PEP+HexNAc(2)Hex(2)_1)
1707.820(PEP+HexNAc(2)Hex(3)_1)
1869.874(PEP+HexNAc(2)Hex(4)_1)
204.088(HexNAc)
274.094(NeuAc − H2O)
292.104(NeuAc)
366.142(HexNAc + Hex)
528.196(Hex + HexNAc + Hex)
657.237(NeuAc + Hex + HexNAc)
1300.574(PEP+HexNAc(4)Hex(6)_2)
1910.903
M+2H

IQNLTVK(=PEP)_6_4_0_2_0, 0_None, 0_None,
m/z:1061.44(3+), RT:49.65, Y-score:82.10

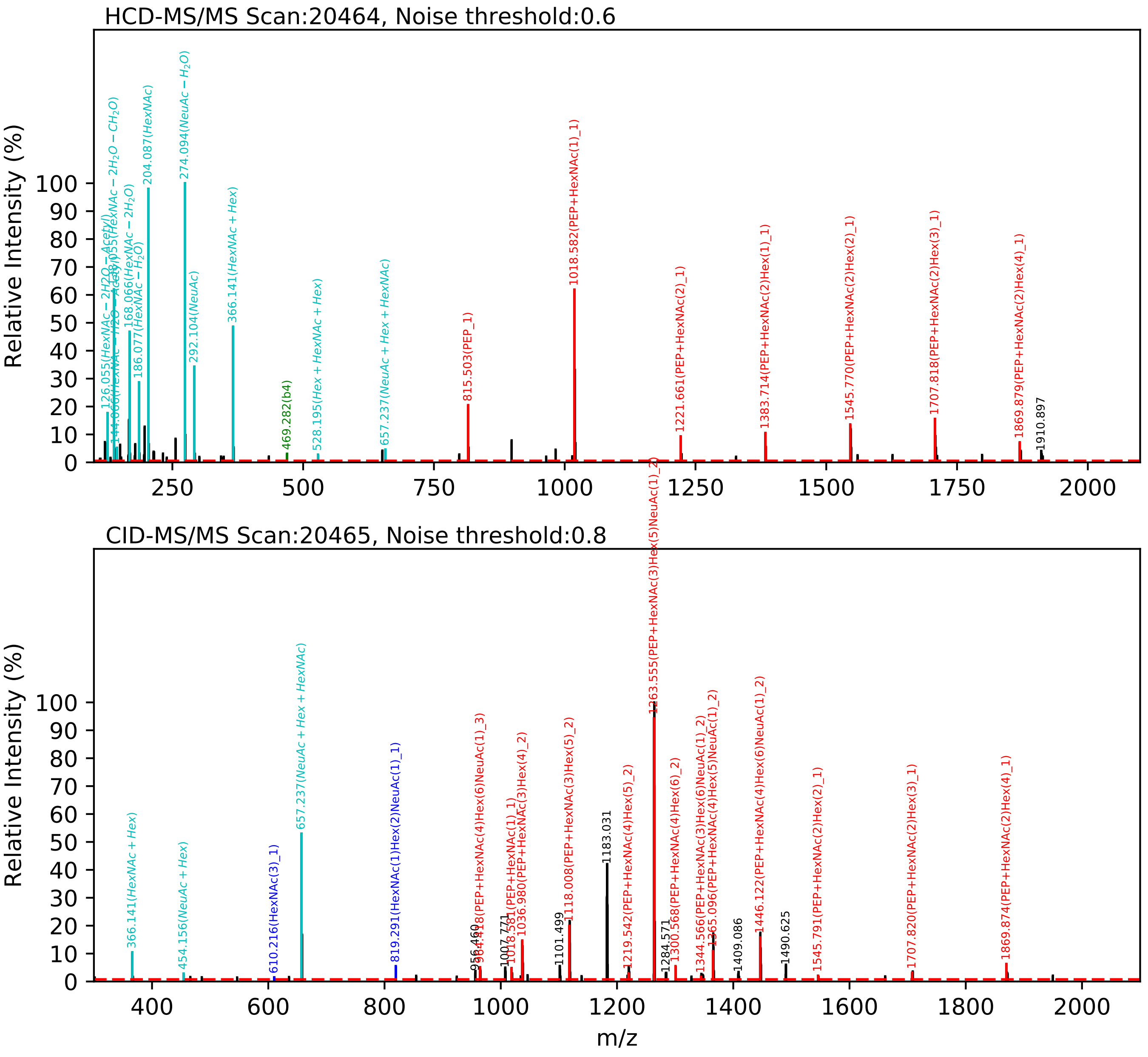

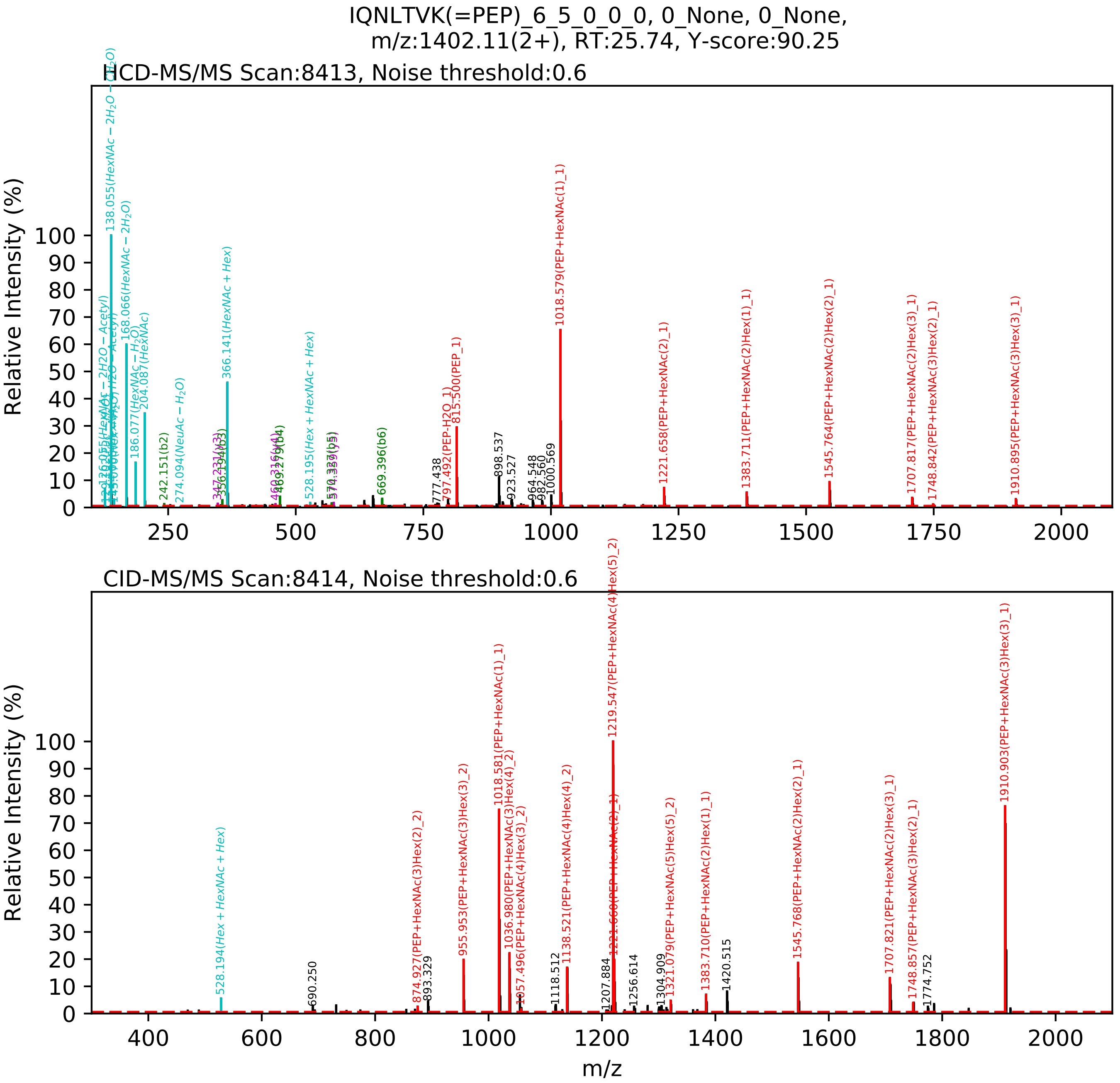

IQNLTVK(=PEP)_6_5_0_0_0, 0_None, 0_None,
m/z:1402.11(2+), RT:25.74, Y-score:90.25
HCD-MS/MS Scan:8413, Noise threshold:0.6
Relative Intensity (%)
138.055(HexNAc − 2H2O − H2O)
168.066(HexNAc − 2H2O)
186.077(HexNAc − H2O)
204.087(HexNAc)
242.151(b2)
274.094(NeuAc − H2O)
366.141(HexNAc + Hex)
469.279(b4)
528.195(Hex + HexNAc + Hex)
669.396(b6)
777.438
797.492(PEP-H2O_1)
815.500(PEP_1)
898.537
923.527
964.548
1000.569
1018.579(PEP+HexNAc(1)_1)
1221.658(PEP+HexNAc(2)_1)
1383.711(PEP+HexNAc(2)Hex(1)_1)
1545.764(PEP+HexNAc(2)Hex(2)_1)
1707.817(PEP+HexNAc(2)Hex(3)_1)
1748.842(PEP+HexNAc(3)Hex(2)_1)
1910.895(PEP+HexNAc(3)Hex(3)_1)
CID-MS/MS Scan:8414, Noise threshold:0.6
528.194(Hex + HexNAc + Hex)
690.250
874.927(PEP+HexNAc(3)Hex(2)_2)
893.329
955.953(PEP+HexNAc(3)Hex(3)_2)
1018.581(PEP+HexNAc(1)_1)
1036.980(PEP+HexNAc(3)Hex(4)_2)
1057.496(PEP+HexNAc(4)Hex(3)_2)
1118.512
1138.521(PEP+HexNAc(4)Hex(4)_2)
1207.884
1219.547(PEP+HexNAc(4)Hex(5)_2)
1256.614
1304.909
1321.079(PEP+HexNAc(5)Hex(5)_2)
1383.710(PEP+HexNAc(2)Hex(1)_1)
1420.515
1545.768(PEP+HexNAc(2)Hex(2)_1)
1707.821(PEP+HexNAc(2)Hex(3)_1)
1748.857(PEP+HexNAc(3)Hex(2)_1)
1774.752
1910.903(PEP+HexNAc(3)Hex(3)_1)
m/z

IQNLTVK(=PEP)_6_5_0_0_0, 0_None, 0_None,
m/z:1402.11(2+), RT:25.78, Y-score:91.65

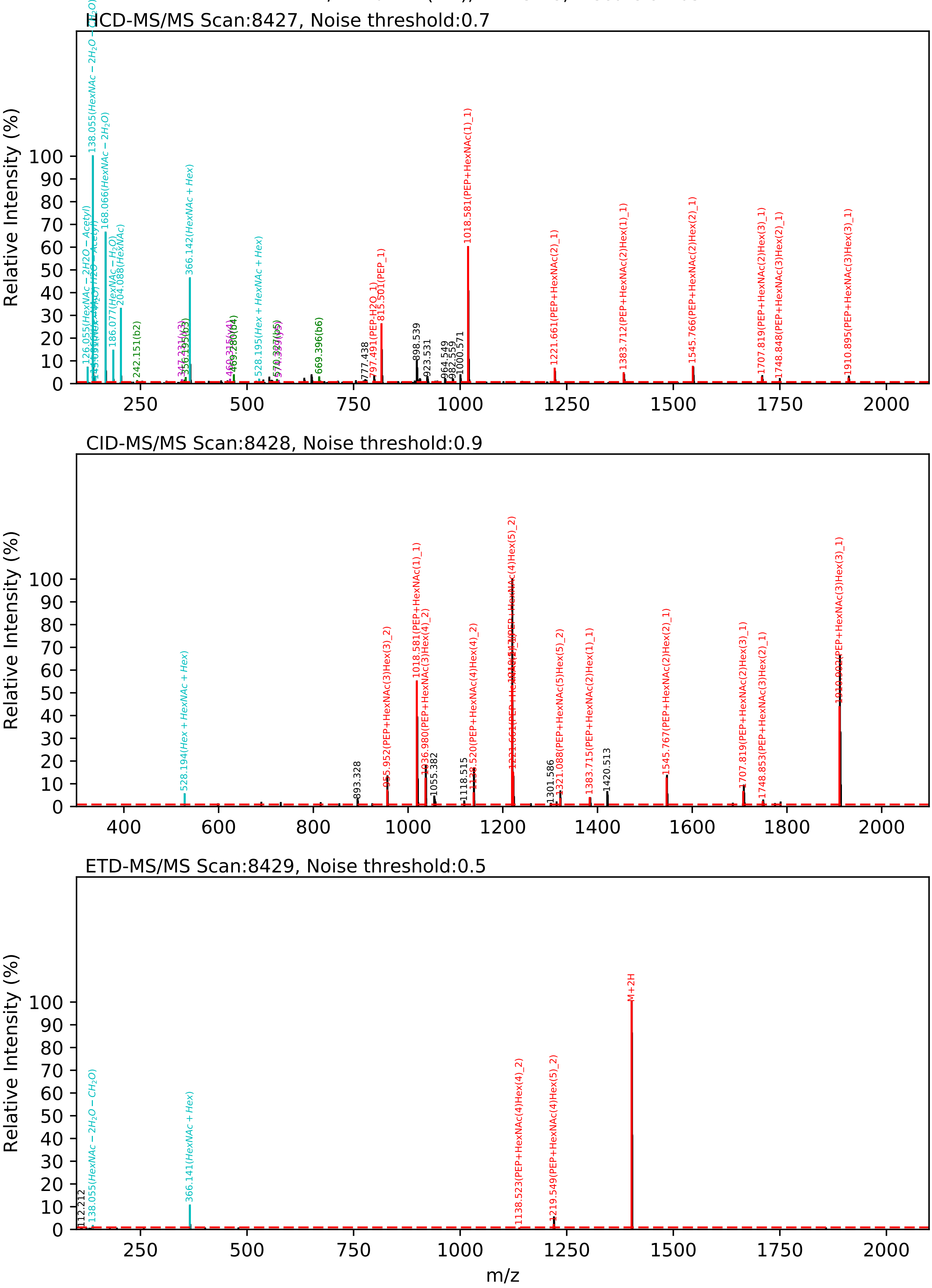

IQNLTVK(=PEP)_6_5_0_0_0, 0_None, 0_None,
m/z:935.08(3+), RT:25.80, Y-score:96.37

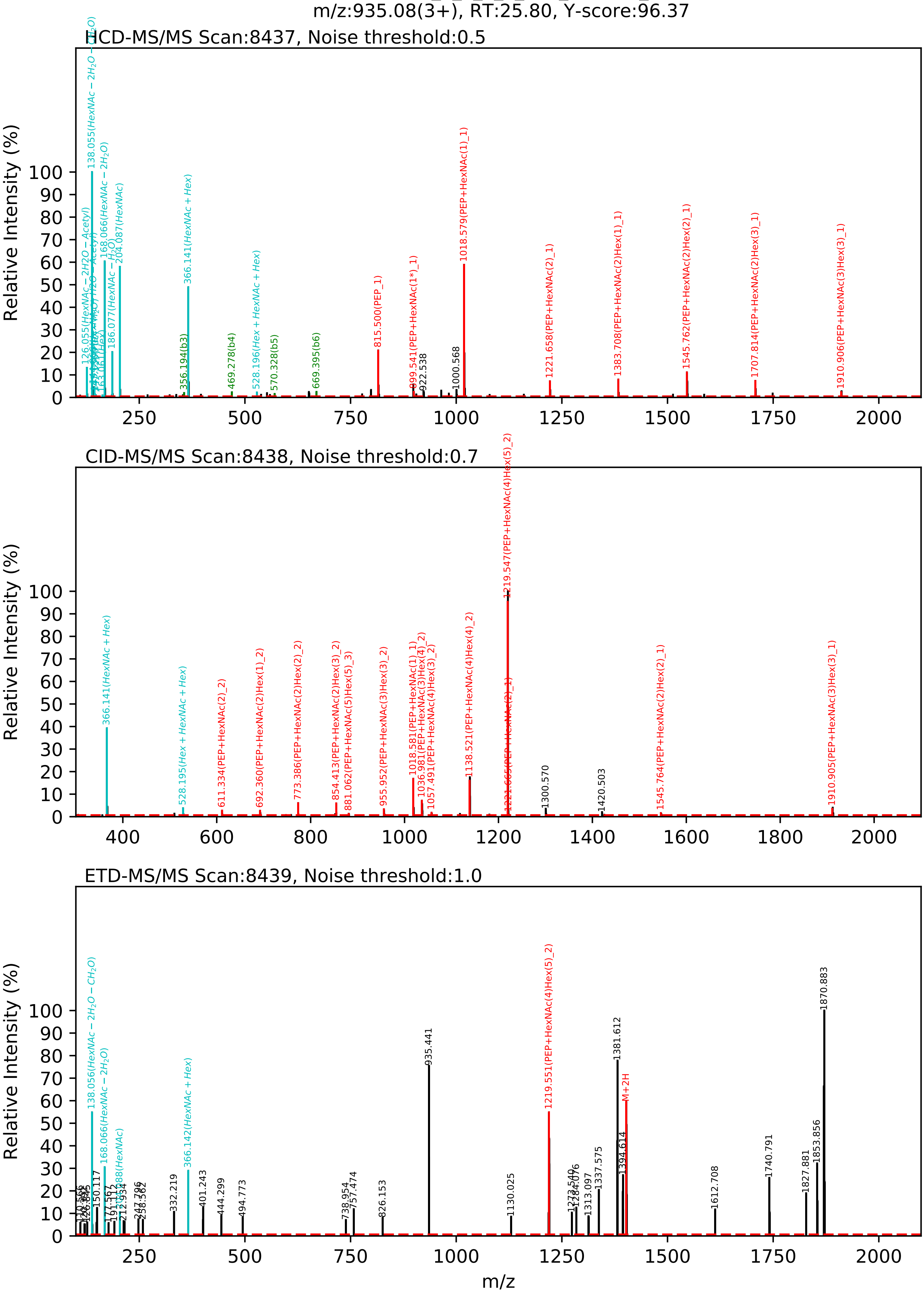

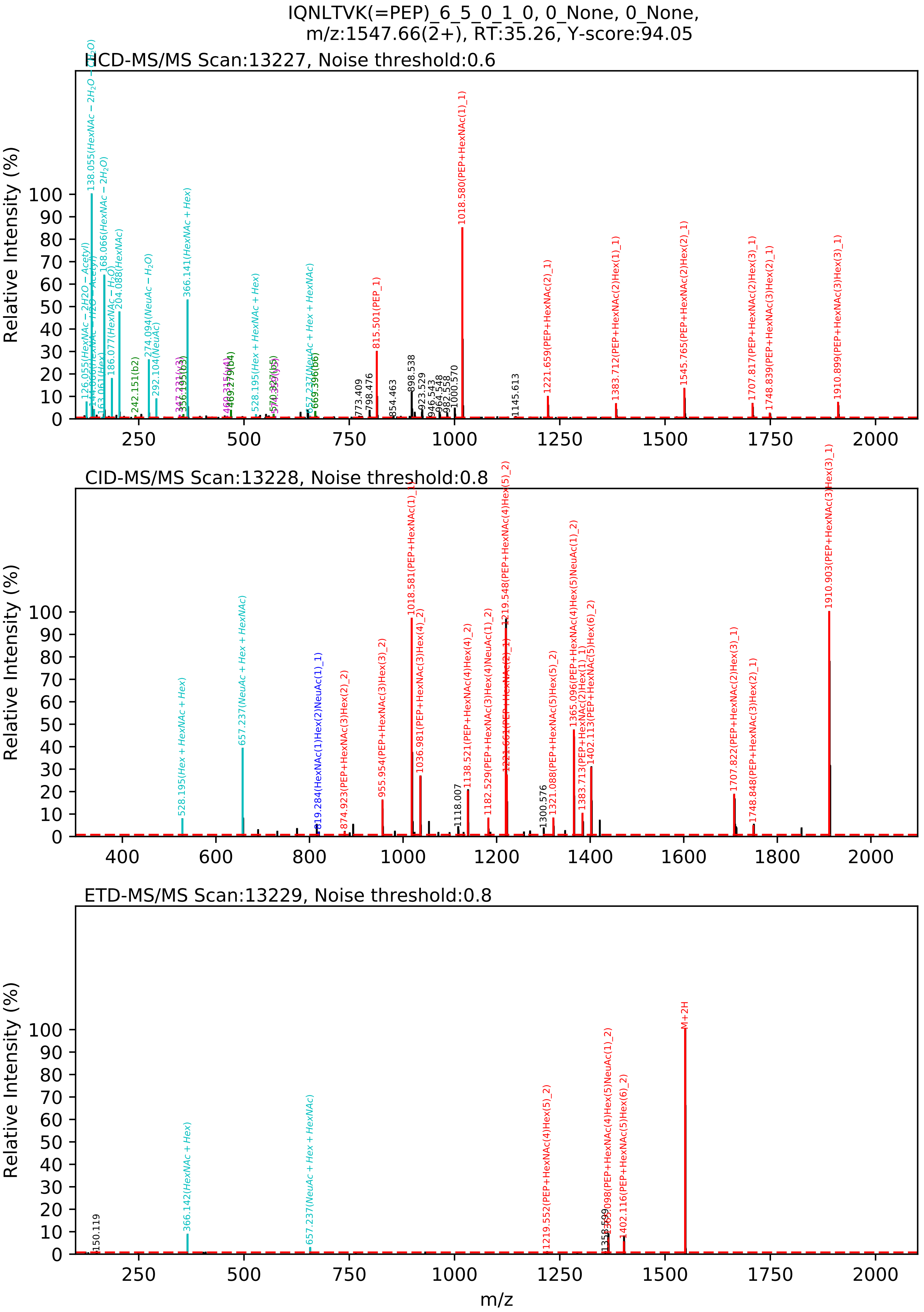

IQNLTVK(=PEP)_6_5_0_1_0, 0_None, 0_None,
m/z:1547.66(2+), RT:35.26, Y-score:94.05
HCD-MS/MS Scan:13227, Noise threshold:0.6
Relative Intensity (%)
138.055(HexNAc − 2H2O − C2H4O)
168.066(HexNAc − 2H2O)
186.077(HexNAc − H2O)
204.088(HexNAc)
126.055(HexNAc − 2H2O − Acetyl)
163.061(Hex)
242.151(b2)
274.094(NeuAc − H2O)
292.104(NeuAc)
366.141(HexNAc + Hex)
356.195(b3)
469.279(b4)
528.195(Hex + HexNAc + Hex)
657.237(NeuAc + Hex + HexNAc)
669.396(b6)
815.501(PEP_1)
773.409
798.476
854.463
898.538
923.529
946.543
964.548
1000.570
1018.580(PEP+HexNAc(1)_1)
1145.613
1221.659(PEP+HexNAc(2)_1)
1383.712(PEP+HexNAc(2)Hex(1)_1)
1545.765(PEP+HexNAc(2)Hex(2)_1)
1707.817(PEP+HexNAc(2)Hex(3)_1)
1748.839(PEP+HexNAc(3)Hex(2)_1)
1910.899(PEP+HexNAc(3)Hex(3)_1)
CID-MS/MS Scan:13228, Noise threshold:0.8
Relative Intensity (%)
528.195(Hex + HexNAc + Hex)
657.237(NeuAc + Hex + HexNAc)
819.284(HexNAc(1)Hex(2)NeuAc(1)_1)
874.923(PEP+HexNAc(3)Hex(2)_2)
955.954(PEP+HexNAc(3)Hex(3)_2)
1018.581(PEP+HexNAc(1)_1)
1036.981(PEP+HexNAc(3)Hex(4)_2)
1118.007
1138.521(PEP+HexNAc(4)Hex(4)_2)
1182.529(PEP+HexNAc(3)Hex(4)NeuAc(1)_2)
1219.548(PEP+HexNAc(4)Hex(5)_2)
1221.661(PEP+HexNAc(2)_1)
1300.576
1321.088(PEP+HexNAc(5)Hex(5)_2)
1365.096(PEP+HexNAc(4)Hex(5)NeuAc(1)_2)
1383.713(PEP+HexNAc(2)Hex(1)_1)
1402.113(PEP+HexNAc(5)Hex(6)_2)
1707.822(PEP+HexNAc(2)Hex(3)_1)
1748.848(PEP+HexNAc(3)Hex(2)_1)
1910.903(PEP+HexNAc(3)Hex(3)_1)
ETD-MS/MS Scan:13229, Noise threshold:0.8
Relative Intensity (%)
150.119
366.142(HexNAc + Hex)
657.237(NeuAc + Hex + HexNAc)
1219.552(PEP+HexNAc(4)Hex(5)_2)
1358.599
1365.098(PEP+HexNAc(4)Hex(5)NeuAc(1)_2)
1402.116(PEP+HexNAc(5)Hex(6)_2)
M+2H
m/z

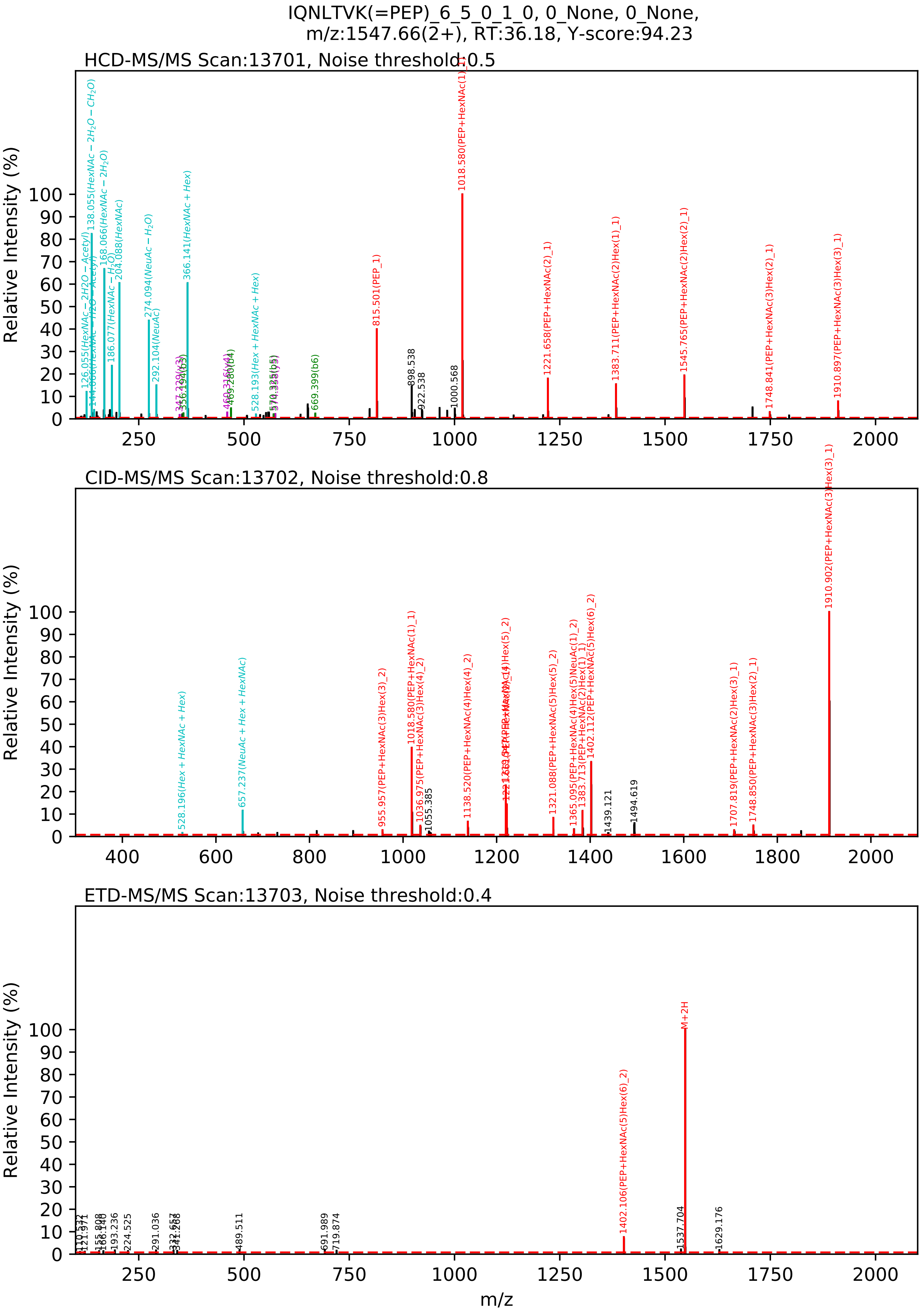

IQNLTVK(=PEP)_6_5_0_1_0, 0_None, 0_None,
m/z:1547.66(2+), RT:36.18, Y-score:94.23
HCD-MS/MS Scan:13701, Noise threshold:0.5
CID-MS/MS Scan:13702, Noise threshold:0.8
ETD-MS/MS Scan:13703, Noise threshold:0.4
Relative Intensity (%)
m/z
1018.580(PEP+HexNAc(1)_1)
815.501(PEP_1)
1221.658(PEP+HexNAc(2)_1)
1383.711(PEP+HexNAc(2)Hex(1)_1)
1545.765(PEP+HexNAc(2)Hex(2)_1)
1748.841(PEP+HexNAc(3)Hex(2)_1)
1910.897(PEP+HexNAc(3)Hex(3)_1)
366.141(HexNAc + Hex)
204.088(HexNAc)
274.094(NeuAc − H2O)
292.104(NeuAc)
1910.902(PEP+HexNAc(3)Hex(3)_1)
1402.112(PEP+HexNAc(5)Hex(6)_2)
1018.580(PEP+HexNAc(1)_1)
657.237(NeuAc + Hex + HexNAc)
528.196(Hex + HexNAc + Hex)
1402.106(PEP+HexNAc(5)Hex(6)_2)
M+2H

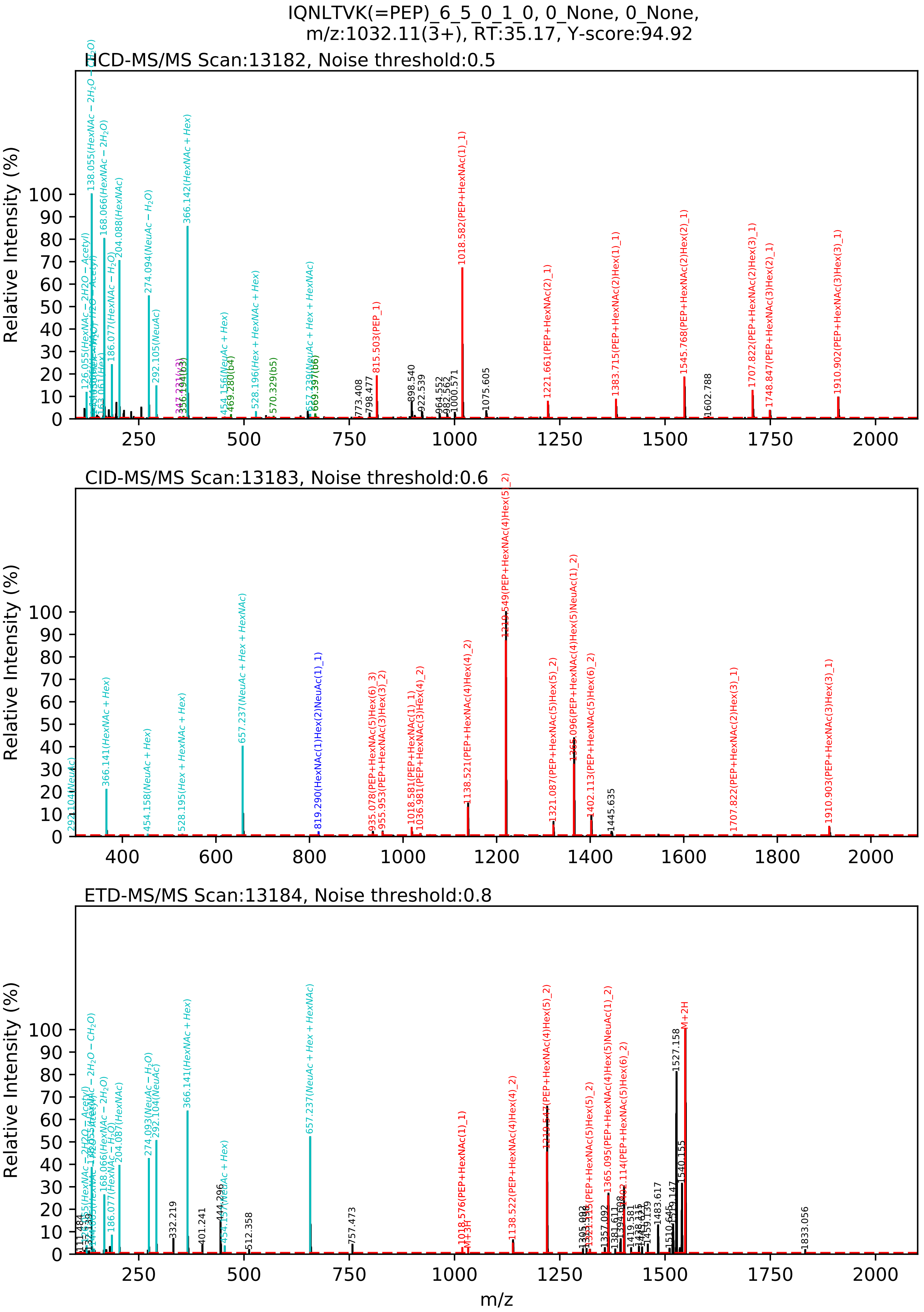
IQNLTVK(=PEP)_6_5_0_1_0, 0_None, 0_None,
m/z:1032.11(3+), RT:35.17, Y-score:94.92
HCD-MS/MS Scan:13182, Noise threshold:0.5
Relative Intensity (%)
138.055(HexNAc − 2H2O − CH2O)
168.066(HexNAc − 2H2O)
204.088(HexNAc)
186.077(HexNAc − H2O)
274.094(NeuAc − H2O)
292.105(NeuAc)
366.142(HexNAc + Hex)
454.156(NeuAc + Hex)
528.196(Hex + HexNAc + Hex)
570.329(b5)
469.280(b4)
657.239(NeuAc + Hex + HexNAc)
669.397(b6)
773.408
798.477
815.503(PEP_1)
898.540
922.539
964.552
1000.571
1018.582(PEP+HexNAc(1)_1)
1075.605
1221.661(PEP+HexNAc(2)_1)
1383.715(PEP+HexNAc(2)Hex(1)_1)
1545.768(PEP+HexNAc(2)Hex(2)_1)
1602.788
1707.822(PEP+HexNAc(2)Hex(3)_1)
1748.847(PEP+HexNAc(3)Hex(2)_1)
1910.902(PEP+HexNAc(3)Hex(3)_1)
CID-MS/MS Scan:13183, Noise threshold:0.6
366.141(HexNAc + Hex)
454.158(NeuAc + Hex)
528.195(Hex + HexNAc + Hex)
657.237(NeuAc + Hex + HexNAc)
819.290(HexNAc(1)Hex(2)NeuAc(1)_1)
935.078(PEP+HexNAc(5)Hex(6)_3)
955.953(PEP+HexNAc(3)Hex(3)_2)
1018.581(PEP+HexNAc(1)_1)
1036.981(PEP+HexNAc(3)Hex(4)_2)
1138.521(PEP+HexNAc(4)Hex(4)_2)
1219.549(PEP+HexNAc(4)Hex(5)_2)
1321.087(PEP+HexNAc(5)Hex(5)_2)
1365.096(PEP+HexNAc(4)Hex(5)NeuAc(1)_2)
1402.113(PEP+HexNAc(5)Hex(6)_2)
1445.635
1707.822(PEP+HexNAc(2)Hex(3)_1)
1910.903(PEP+HexNAc(3)Hex(3)_1)
ETD-MS/MS Scan:13184, Noise threshold:0.8
168.066(HexNAc − 2H2O)
186.077(HexNAc − H2O)
204.087(HexNAc)
274.093(NeuAc − H2O)
292.104(NeuAc)
332.219
366.141(HexNAc + Hex)
401.241
444.296
512.358
657.237(NeuAc + Hex + HexNAc)
757.473
1018.576(PEP+HexNAc(1)_1)
M+3H
1138.522(PEP+HexNAc(4)Hex(4)_2)
1219.547(PEP+HexNAc(4)Hex(5)_2)
1365.095(PEP+HexNAc(4)Hex(5)NeuAc(1)_2)
1357.092
1419.581
1459.139
1483.617
1527.158
M+2H
1833.056
m/z

IQNLTVK(=PEP)_6_5_0_1_0, 0_None, 0_None, m/z:1032.11(3+), RT:34.55, Y-score:95.28

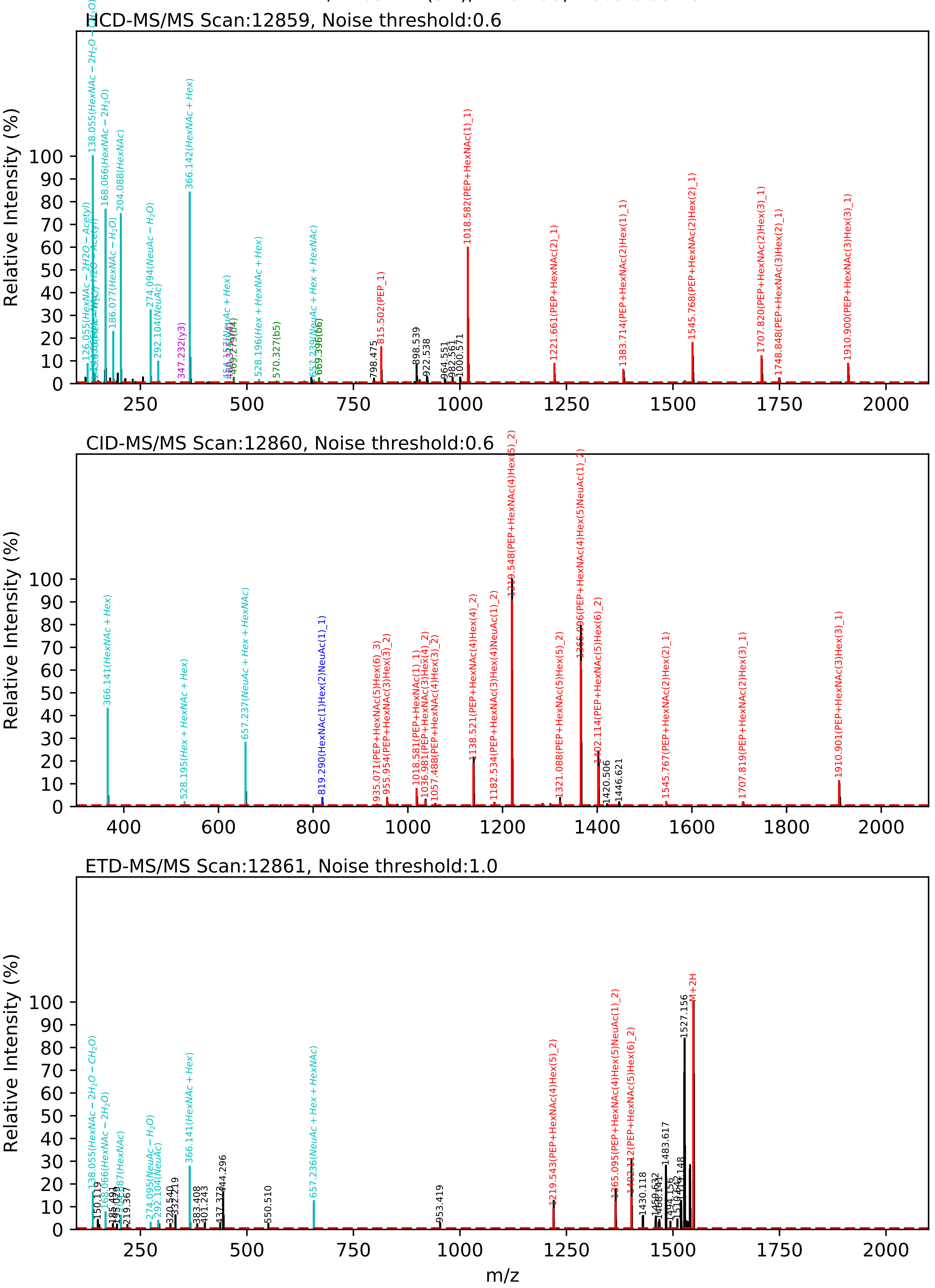

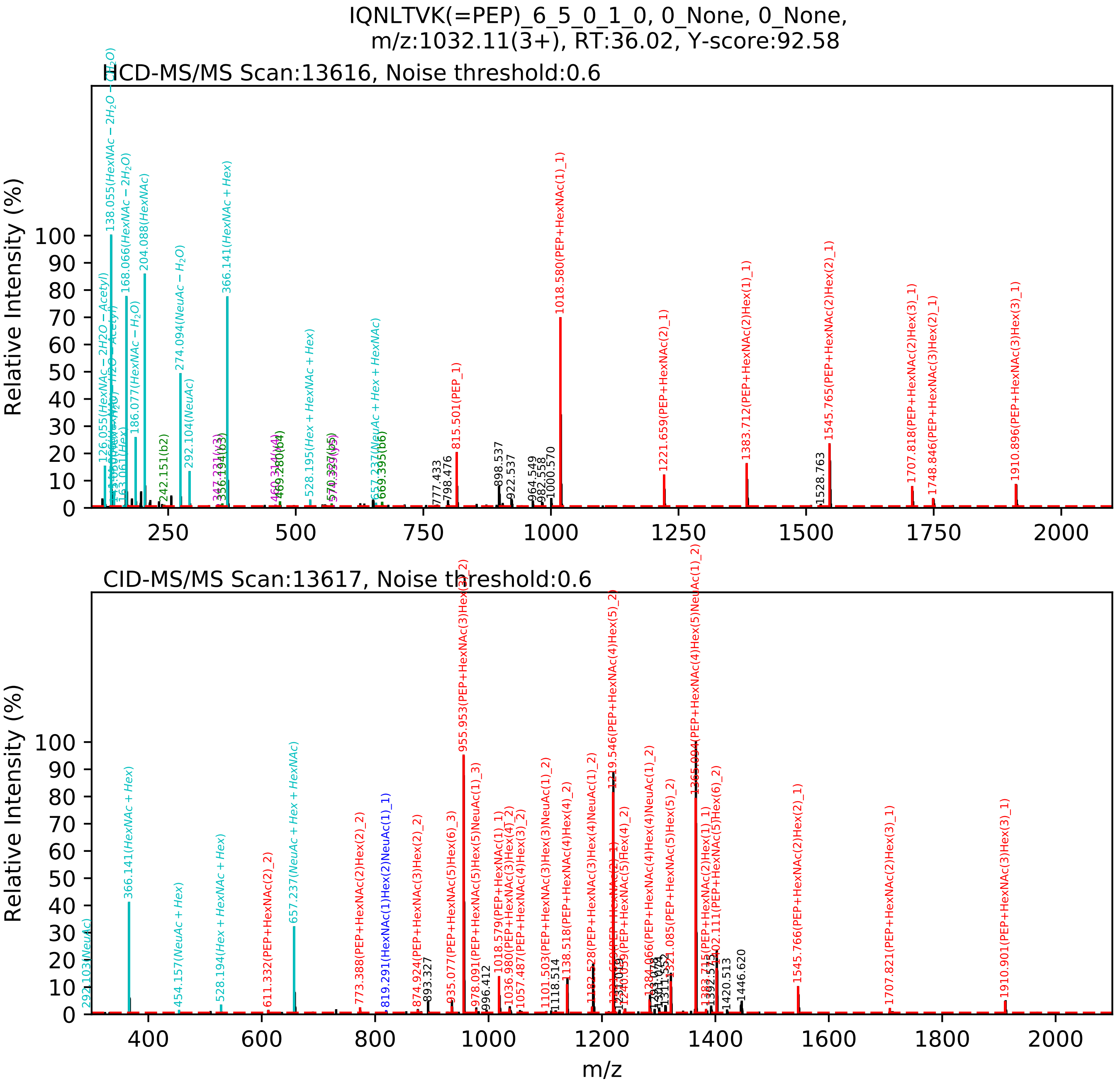
IQNLTVK(=PEP)_6_5_0_1_0, 0_None, 0_None,
m/z:1032.11(3+), RT:36.02, Y-score:92.58
HCD-MS/MS Scan:13616, Noise threshold:0.6
CID-MS/MS Scan:13617, Noise threshold:0.6
Relative Intensity (%)
m/z
1018.580(PEP+HexNAc(1)_1)
1221.659(PEP+HexNAc(2)_1)
1383.712(PEP+HexNAc(2)Hex(1)_1)
1545.765(PEP+HexNAc(2)Hex(2)_1)
1707.818(PEP+HexNAc(2)Hex(3)_1)
1748.846(PEP+HexNAc(3)Hex(2)_1)
1910.896(PEP+HexNAc(3)Hex(3)_1)
815.501(PEP_1)
366.141(HexNAc + Hex)
204.088(HexNAc)
955.953(PEP+HexNAc(3)Hex(2)_2)
1219.546(PEP+HexNAc(4)Hex(5)_2)
1365.094(PEP+HexNAc(4)Hex(5)NeuAc(1)_2)
1545.766(PEP+HexNAc(2)Hex(2)_1)
1707.821(PEP+HexNAc(2)Hex(3)_1)
1910.901(PEP+HexNAc(3)Hex(3)_1)
657.237(NeuAc + Hex + HexNAc)

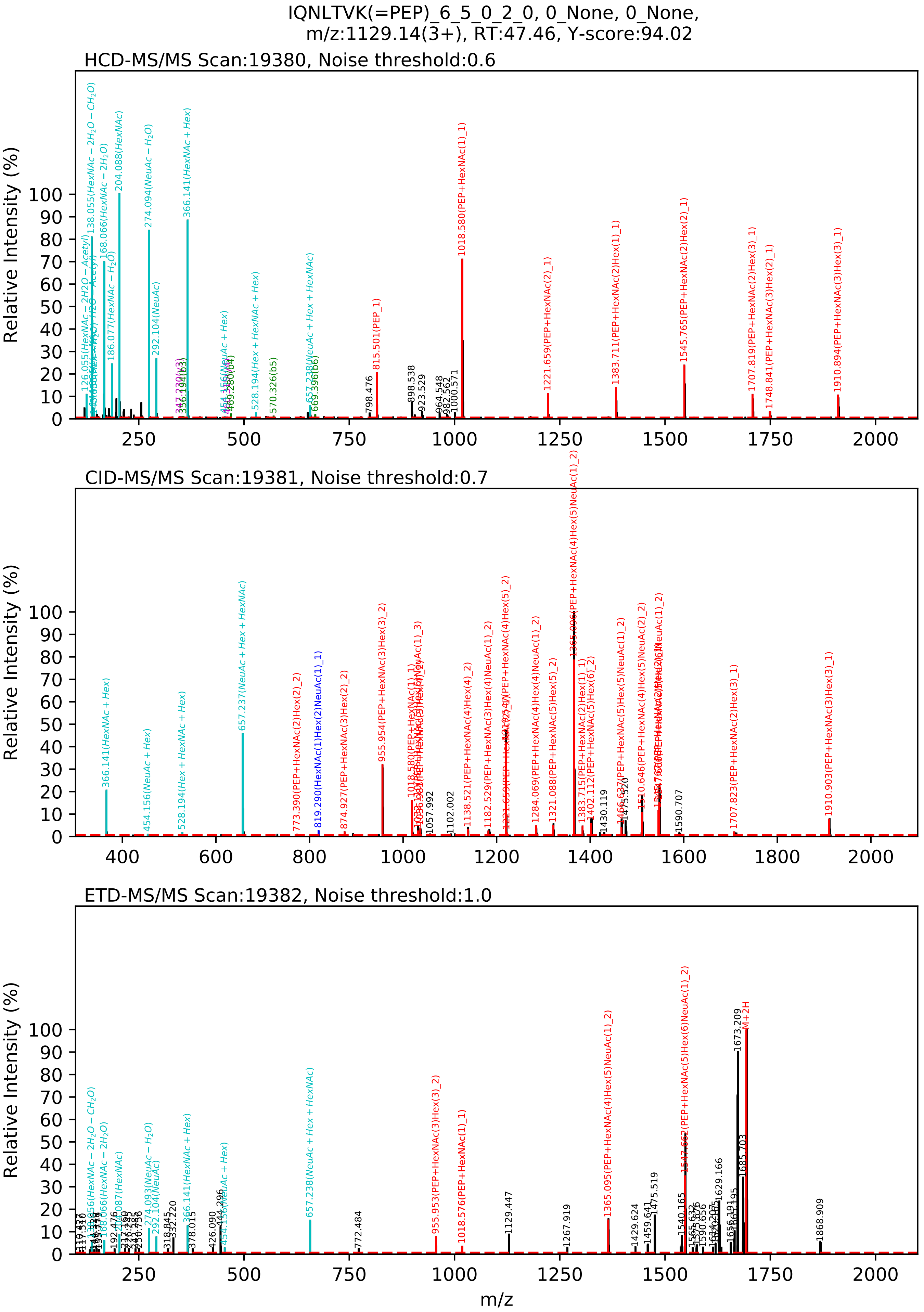

IQNLTVK(=PEP)_6_5_0_2_0, 0_None, 0_None,
m/z:1129.14(3+), RT:47.46, Y-score:94.02
HCD-MS/MS Scan:19380, Noise threshold:0.6
Relative Intensity (%)
204.088(HexNAc)
366.141(HexNAc + Hex)
274.094(NeuAc − H2O)
168.066(HexNAc − 2H2O)
292.104(NeuAc)
186.077(HexNAc − H2O)
1018.580(PEP+HexNAc(1)_1)
815.501(PEP_1)
1221.659(PEP+HexNAc(2)_1)
1383.711(PEP+HexNAc(2)Hex(1)_1)
1545.765(PEP+HexNAc(2)Hex(2)_1)
1707.819(PEP+HexNAc(2)Hex(3)_1)
1748.841(PEP+HexNAc(3)Hex(2)_1)
1910.894(PEP+HexNAc(3)Hex(3)_1)
528.194(Hex + HexNAc + Hex)
570.326(b5)
669.396(b6)
657.238(NeuAc + Hex + HexNAc)
798.476
898.538
923.529
964.548
1000.571
CID-MS/MS Scan:19381, Noise threshold:0.7
Relative Intensity (%)
366.141(HexNAc + Hex)
454.156(NeuAc + Hex)
528.194(Hex + HexNAc + Hex)
657.237(NeuAc + Hex + HexNAc)
773.390(PEP+HexNAc(2)Hex(2)_2)
819.290(HexNAc(1)Hex(2)NeuAc(1)_1)
874.927(PEP+HexNAc(3)Hex(2)_2)
955.954(PEP+HexNAc(3)Hex(3)_2)
1057.992
1102.002
1138.521(PEP+HexNAc(4)Hex(4)_2)
1182.529(PEP+HexNAc(3)Hex(4)NeuAc(1)_2)
1284.069(PEP+HexNAc(4)Hex(4)NeuAc(1)_2)
1321.088(PEP+HexNAc(5)Hex(5)_2)
1365.096(PEP+HexNAc(4)Hex(5)NeuAc(1)_2)
1383.715(PEP+HexNAc(2)Hex(1)_1)
1402.112(PEP+HexNAc(5)Hex(6)_2)
1430.119
1475.520
1510.646(PEP+HexNAc(4)Hex(5)NeuAc(2)_2)
1590.707
1707.823(PEP+HexNAc(2)Hex(3)_1)
1910.903(PEP+HexNAc(3)Hex(3)_1)
ETD-MS/MS Scan:19382, Noise threshold:1.0
Relative Intensity (%)
657.238(NeuAc + Hex + HexNAc)
772.484
955.953(PEP+HexNAc(3)Hex(3)_2)
1018.576(PEP+HexNAc(1)_1)
1129.447
1267.919
1365.095(PEP+HexNAc(4)Hex(5)NeuAc(1)_2)
1429.624
1459.641
1475.519
1540.165
1547.662(PEP+HexNAc(5)Hex(6)NeuAc(1)_2)
1590.656
1629.166
1673.209
1685.703
M+2H
1868.909
m/z

IQNLTVK(=PEP)_6_5_0_2_0, 0_None, 0_None,
m/z:1129.14(3+), RT:48.49, Y-score:87.35

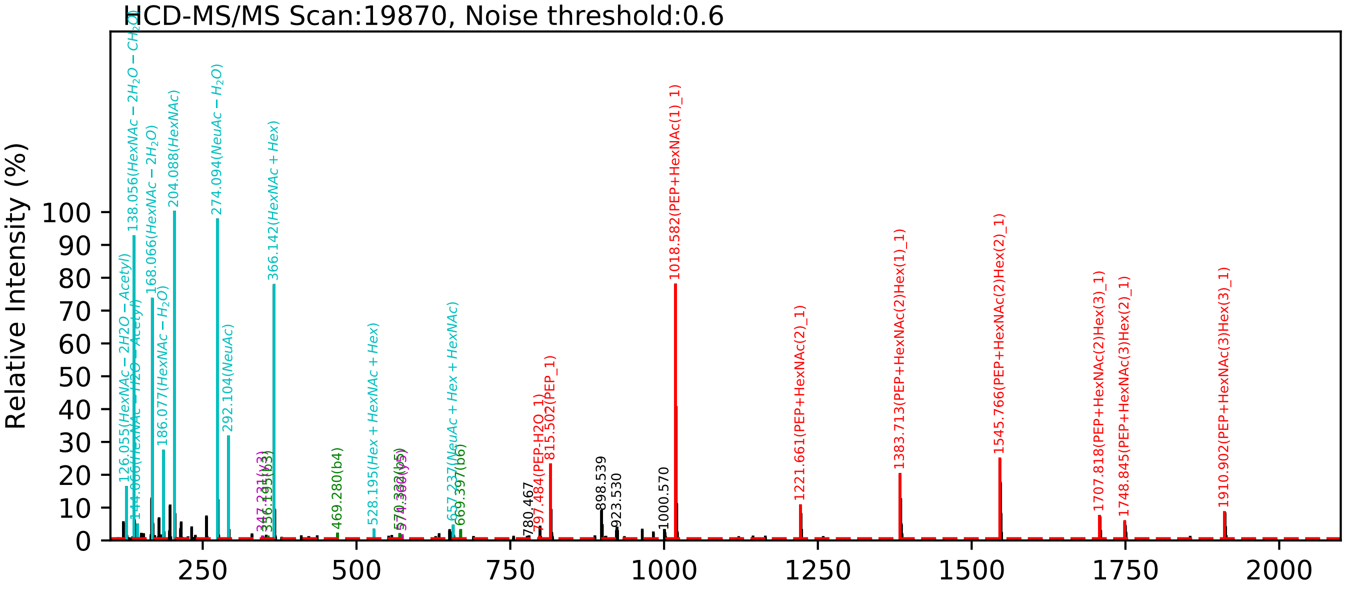

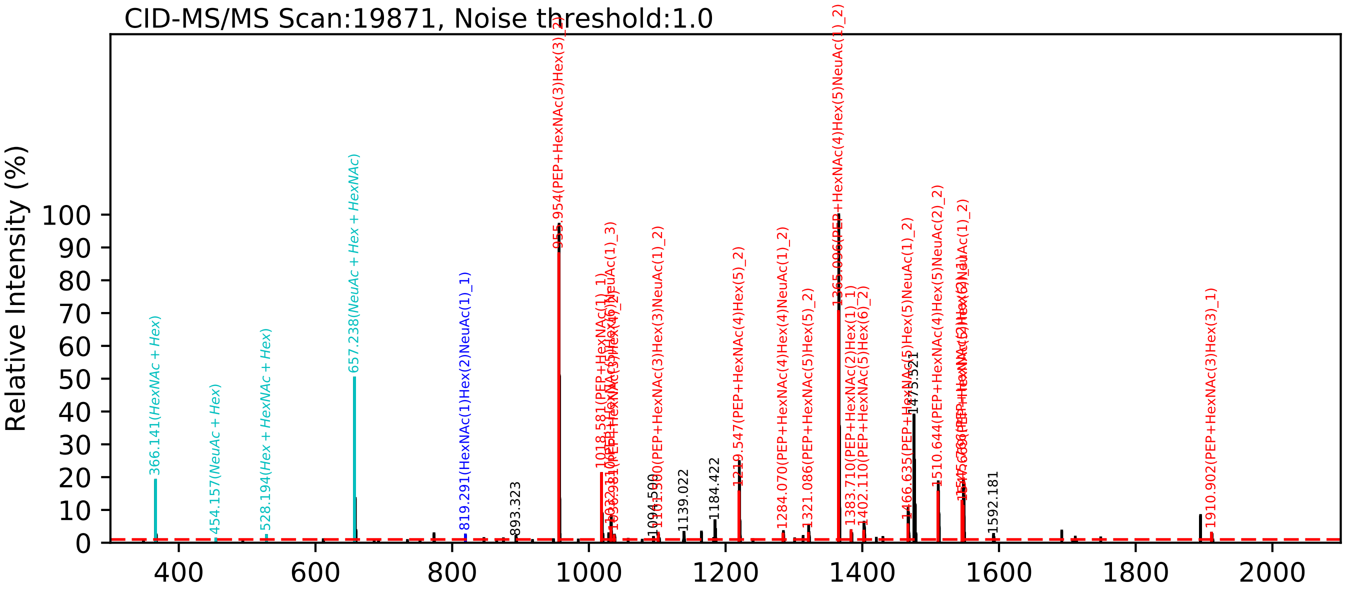

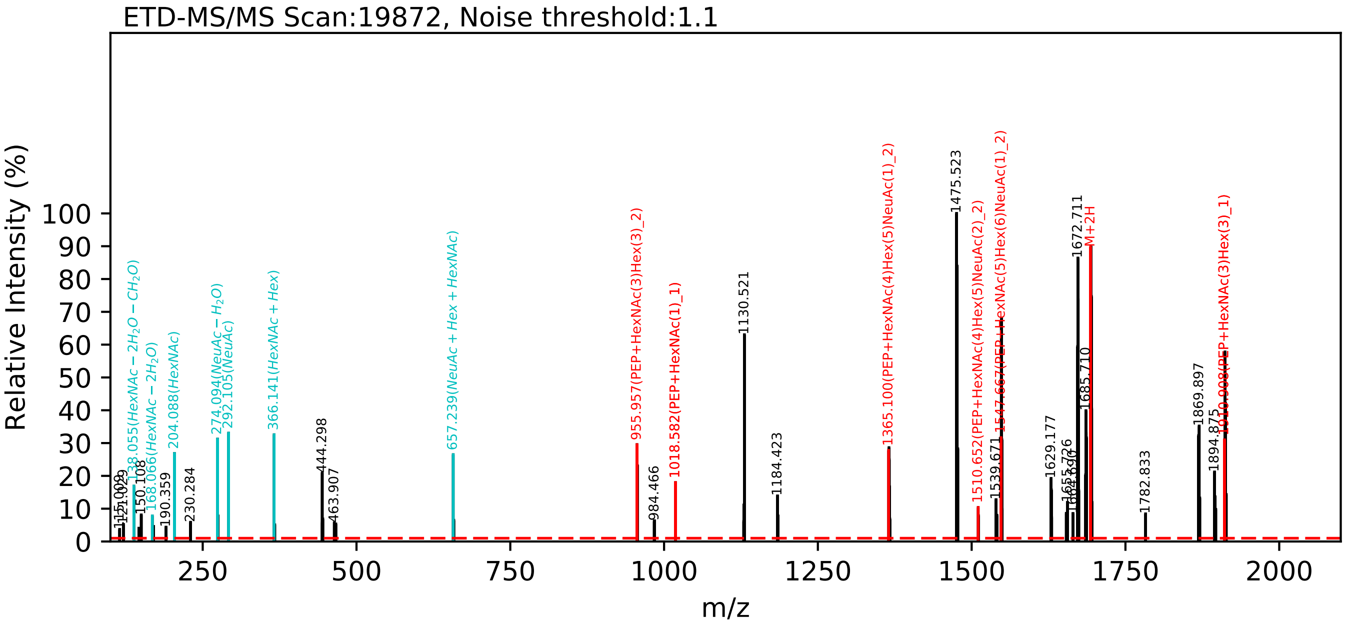

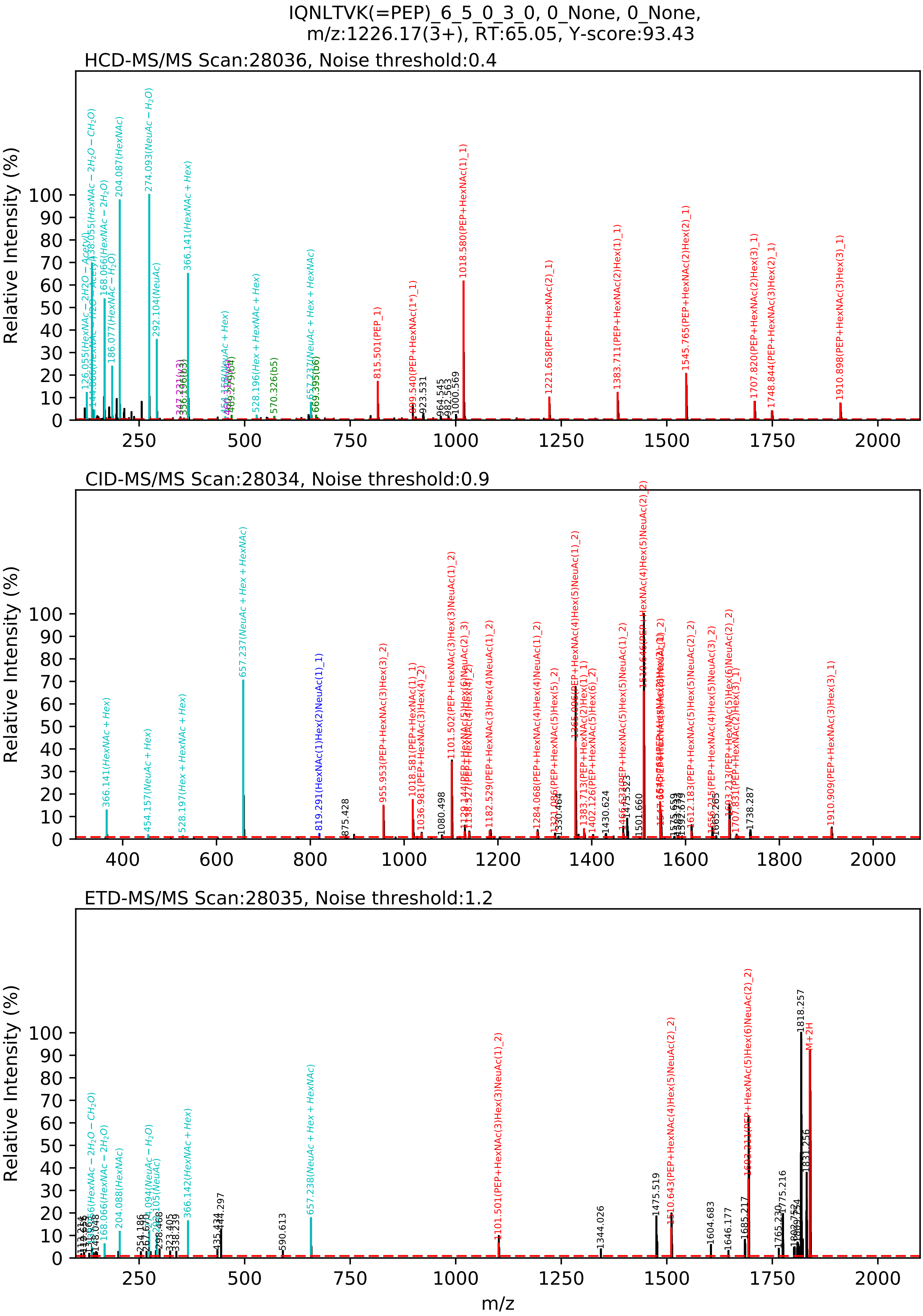

IQNLTVK(=PEP)_6_5_0_3_0, 0_None, 0_None,
m/z:1226.17(3+), RT:65.05, Y-score:93.43
HCD-MS/MS Scan:28036, Noise threshold:0.4
Relative Intensity (%)
204.087(HexNAc)
274.093(NeuAc − H2O)
292.104(NeuAc)
366.141(HexNAc + Hex)
168.066(HexNAc − 2H2O)
186.077(HexNAc − H2O)
528.196(Hex + HexNAc + Hex)
570.326(b5)
657.237(NeuAc + Hex + HexNAc)
669.395(b6)
815.501(PEP_1)
899.540(PEP+HexNAc(1*)_1)
923.531
964.545
1000.569
1018.580(PEP+HexNAc(1)_1)
1221.658(PEP+HexNAc(2)_1)
1383.711(PEP+HexNAc(2)Hex(1)_1)
1545.765(PEP+HexNAc(2)Hex(2)_1)
1707.820(PEP+HexNAc(2)Hex(3)_1)
1748.844(PEP+HexNAc(3)Hex(2)_1)
1910.898(PEP+HexNAc(3)Hex(3)_1)
CID-MS/MS Scan:28034, Noise threshold:0.9
366.141(HexNAc + Hex)
454.157(NeuAc + Hex)
528.197(Hex + HexNAc + Hex)
657.237(NeuAc + Hex + HexNAc)
819.291(HexNAc(1)Hex(2)NeuAc(1)_1)
875.428
955.953(PEP+HexNAc(3)Hex(3)_2)
1018.581(PEP+HexNAc(1)_1)
1036.981(PEP+HexNAc(3)Hex(4)_2)
1080.498
1101.502(PEP+HexNAc(3)Hex(3)NeuAc(1)_2)
1182.529(PEP+HexNAc(3)Hex(4)NeuAc(1)_2)
1284.068(PEP+HexNAc(4)Hex(4)NeuAc(1)_2)
1330.464
1365.096(PEP+HexNAc(4)Hex(5)NeuAc(1)_2)
1430.624
1475.523
1501.660
1510.646(PEP+HexNAc(4)Hex(5)NeuAc(2)_2)
1575.659
1612.183(PEP+HexNAc(5)Hex(5)NeuAc(2)_2)
1665.265
1738.287
1910.909(PEP+HexNAc(3)Hex(3)_1)
ETD-MS/MS Scan:28035, Noise threshold:1.2
168.066(HexNAc − 2H2O)
204.088(HexNAc)
366.142(HexNAc + Hex)
444.297
590.613
657.238(NeuAc + Hex + HexNAc)
1101.501(PEP+HexNAc(3)Hex(3)NeuAc(1)_2)
1344.026
1475.519
1510.643(PEP+HexNAc(4)Hex(5)NeuAc(2)_2)
1604.683
1646.177
1685.217
1693.211(PEP+HexNAc(5)Hex(6)NeuAc(2)_2)
1775.216
1818.257
1831.256
M+2H
m/z

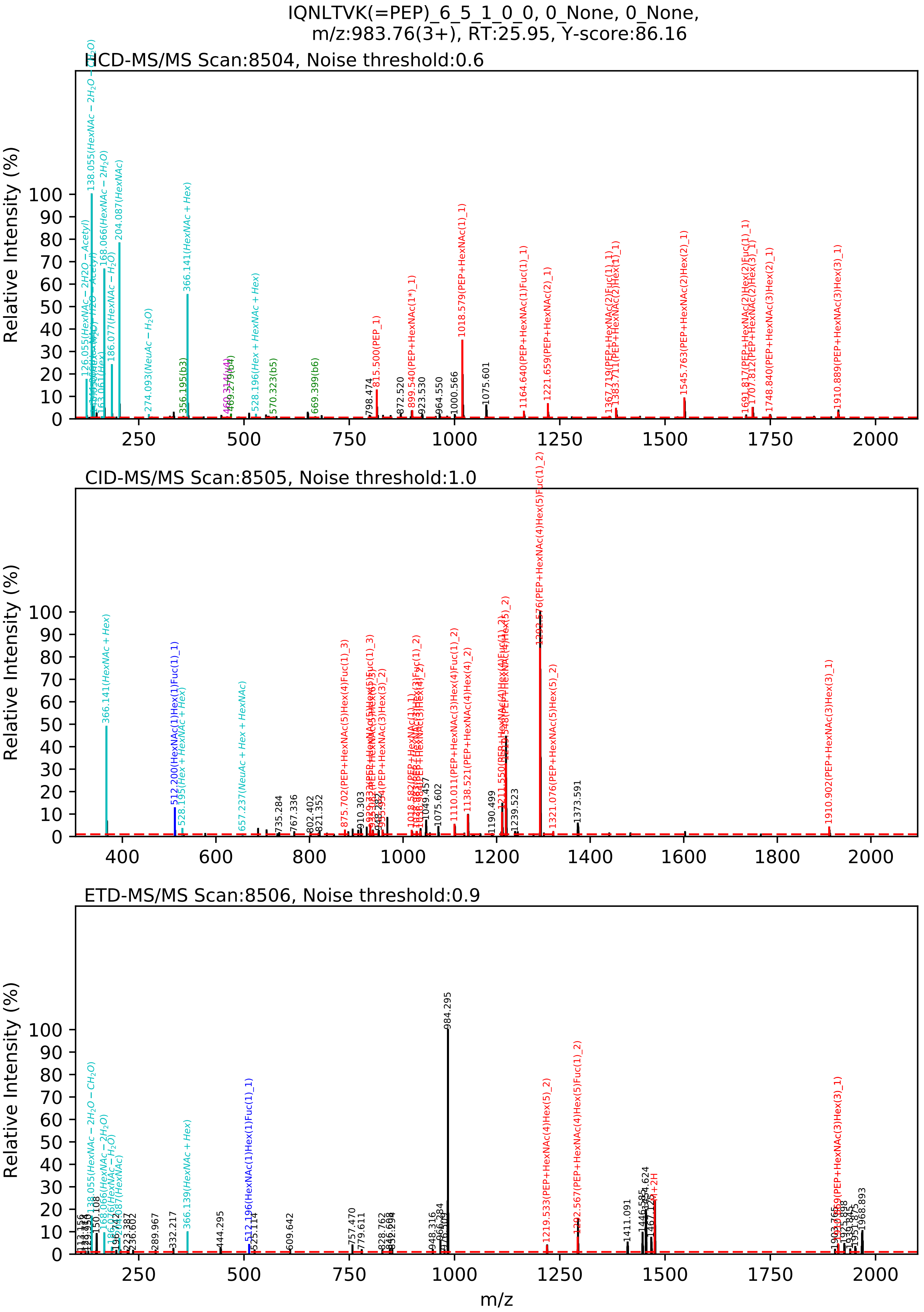
IQNLTVK(=PEP)_6_5_1_0_0, 0_None, 0_None,
m/z:983.76(3+), RT:25.95, Y-score:86.16
HCD-MS/MS Scan:8504, Noise threshold:0.6
Relative Intensity (%)
138.055(HexNAc − 2H2O − CH2O)
168.066(HexNAc − 2H2O)
204.087(HexNAc)
186.077(HexNAc − H2O)
126.055(HexNAc − 2H2O − Acetyl)
163.061(Hex)
274.093(NeuAc − H2O)
356.195(b3)
366.141(HexNAc + Hex)
469.279(b4)
528.196(Hex + HexNAc + Hex)
570.323(b5)
669.399(b6)
798.474
815.500(PEP_1)
872.520
899.540(PEP+HexNAc(1*)_1)
923.530
964.550
1000.566
1018.579(PEP+HexNAc(1)_1)
1075.601
1164.640(PEP+HexNAc(1)Fuc(1)_1)
1221.659(PEP+HexNAc(2)_1)
1383.711(PEP+HexNAc(2)Hex(1)_1)
1545.763(PEP+HexNAc(2)Hex(2)_1)
1748.840(PEP+HexNAc(3)Hex(2)_1)
1910.889(PEP+HexNAc(3)Hex(3)_1)
CID-MS/MS Scan:8505, Noise threshold:1.0
366.141(HexNAc + Hex)
512.200(HexNAc(1)Hex(1)Fuc(1)_1)
528.195(Hex + HexNAc + Hex)
657.237(NeuAc + Hex + HexNAc)
735.284
767.336
802.402
821.352
875.702(PEP+HexNAc(5)Hex(4)Fuc(1)_3)
910.303
955.374(PEP+HexNAc(3)Hex(3)_2)
1049.457
1075.602
1110.011(PEP+HexNAc(3)Hex(4)Fuc(1)_2)
1138.521(PEP+HexNAc(4)Hex(4)_2)
1190.499
1211.550
1239.523
1292.576(PEP+HexNAc(4)Hex(5)Fuc(1)_2)
1321.076(PEP+HexNAc(5)Hex(5)_2)
1373.591
1910.902(PEP+HexNAc(3)Hex(3)_1)
ETD-MS/MS Scan:8506, Noise threshold:0.9
138.055(HexNAc − 2H2O − CH2O)
168.066(HexNAc − 2H2O)
204.087(HexNAc)
150.108
289.967
332.217
366.139(HexNAc + Hex)
444.295
512.196(HexNAc(1)Hex(1)Fuc(1)_1)
525.114
609.642
757.470
779.611
828.762
948.316
984.295
1219.533(PEP+HexNAc(4)Hex(5)_2)
1292.567(PEP+HexNAc(4)Hex(5)Fuc(1)_2)
1411.091
1446.585
1474.624
1903.769(PEP+HexNAc(3)Hex(3)_1)
1968.893
m/z

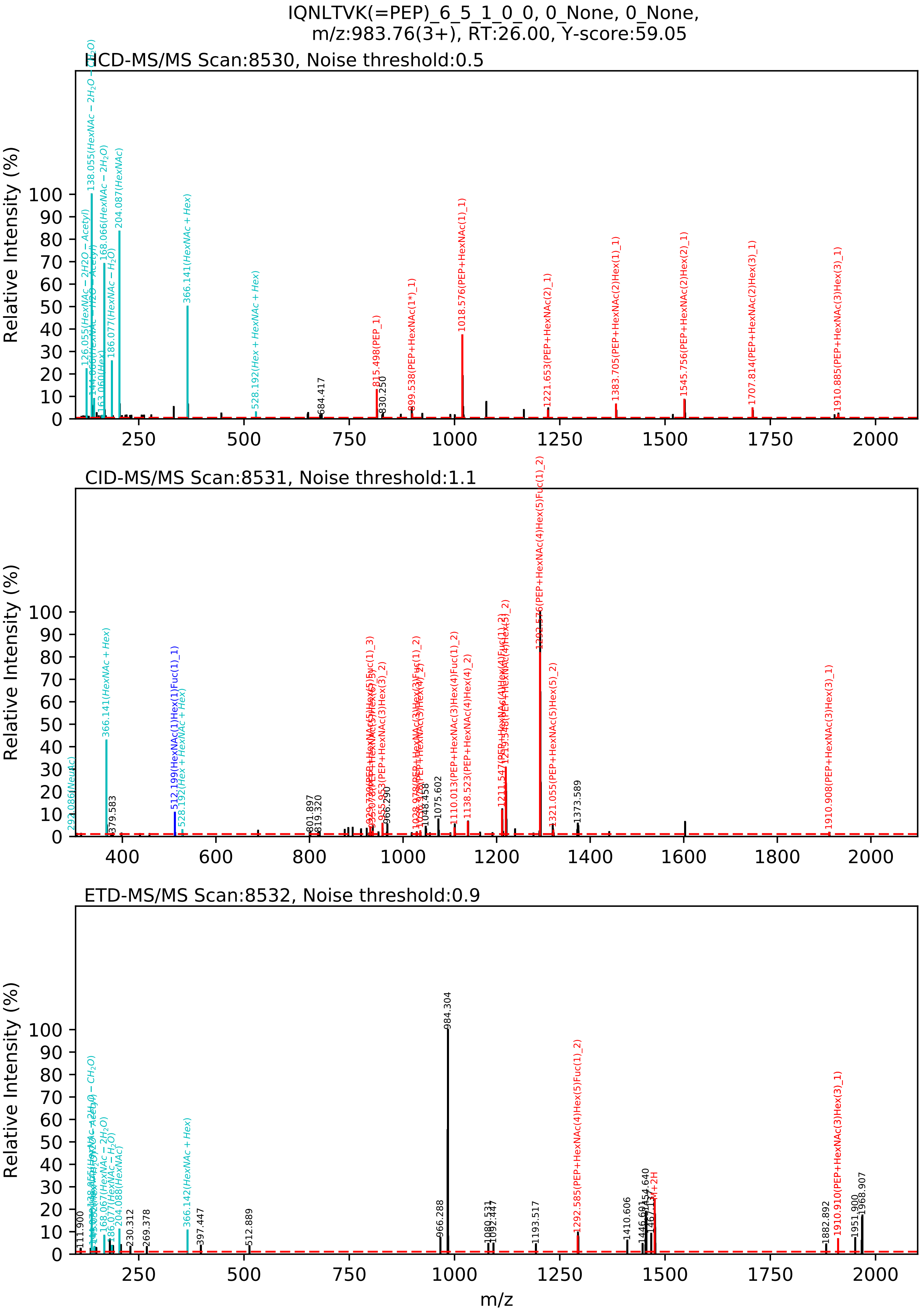
IQNLTVK(=PEP)_6_5_1_0_0, 0_None, 0_None,
m/z:983.76(3+), RT:26.00, Y-score:59.05
HCD-MS/MS Scan:8530, Noise threshold:0.5
CID-MS/MS Scan:8531, Noise threshold:1.1
ETD-MS/MS Scan:8532, Noise threshold:0.9
Relative Intensity (%)
m/z

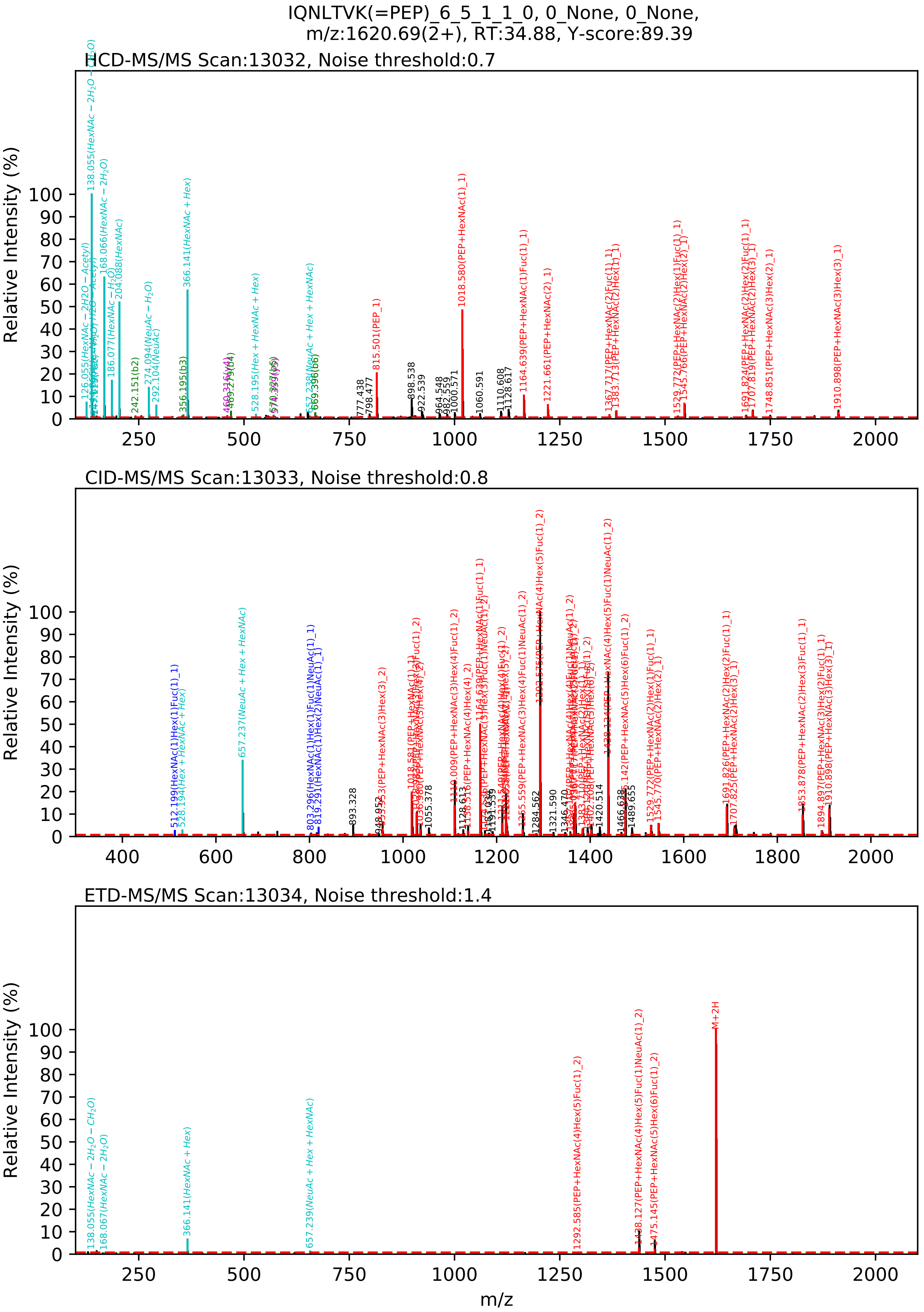
IQNLTVK(=PEP)_6_5_1_1_0, 0_None, 0_None,
m/z:1620.69(2+), RT:34.88, Y-score:89.39
HCD-MS/MS Scan:13032, Noise threshold:0.7
CID-MS/MS Scan:13033, Noise threshold:0.8
ETD-MS/MS Scan:13034, Noise threshold:1.4
Relative Intensity (%)
m/z
1018.580(PEP+HexNAc(1)_1)
815.501(PEP_1)
366.141(HexNAc + Hex)
1164.639(PEP+HexNAc(1)Fuc(1)_1)
1221.661(PEP+HexNAc(2)_1)
1910.898(PEP+HexNAc(3)Hex(3)_1)
1748.851(PEP+HexNAc(3)Hex(2)_1)
657.237(NeuAc + Hex + HexNAc)
1292.575(PEP+HexNAc(4)Hex(5)Fuc(1)_2)
1438.124(PEP+HexNAc(4)Hex(5)Fuc(1)NeuAc(1)_2)
1691.826(PEP+HexNAc(2)Hex(2)Fuc(1)_1)
1853.878(PEP+HexNAc(3)Hex(3)Fuc(1)_1)
M+2H
1292.585(PEP+HexNAc(4)Hex(5)Fuc(1)_2)
1438.127(PEP+HexNAc(4)Hex(5)Fuc(1)NeuAc(1)_2)
1475.145(PEP+HexNAc(5)Hex(6)Fuc(1)_2)

IQNLTVK(=PEP)_6_5_1_1_0, 0_None, 0_None,
m/z:1620.69(2+), RT:35.57, Y-score:91.84

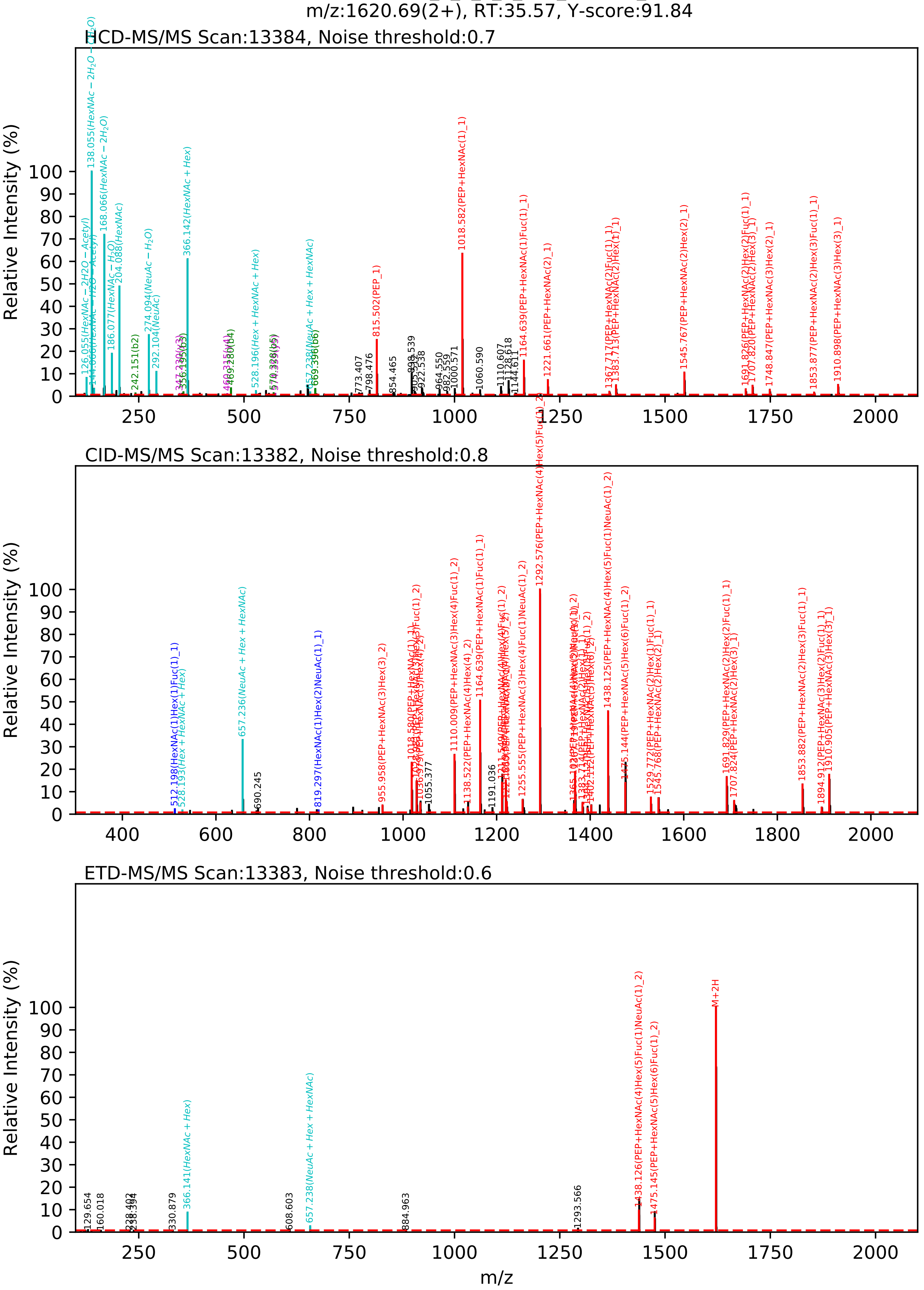

IQNLTVK(=PEP)_6_5_1_1_0, 0_None, 0_None,
m/z:1620.69(2+), RT:35.79, Y-score:93.34

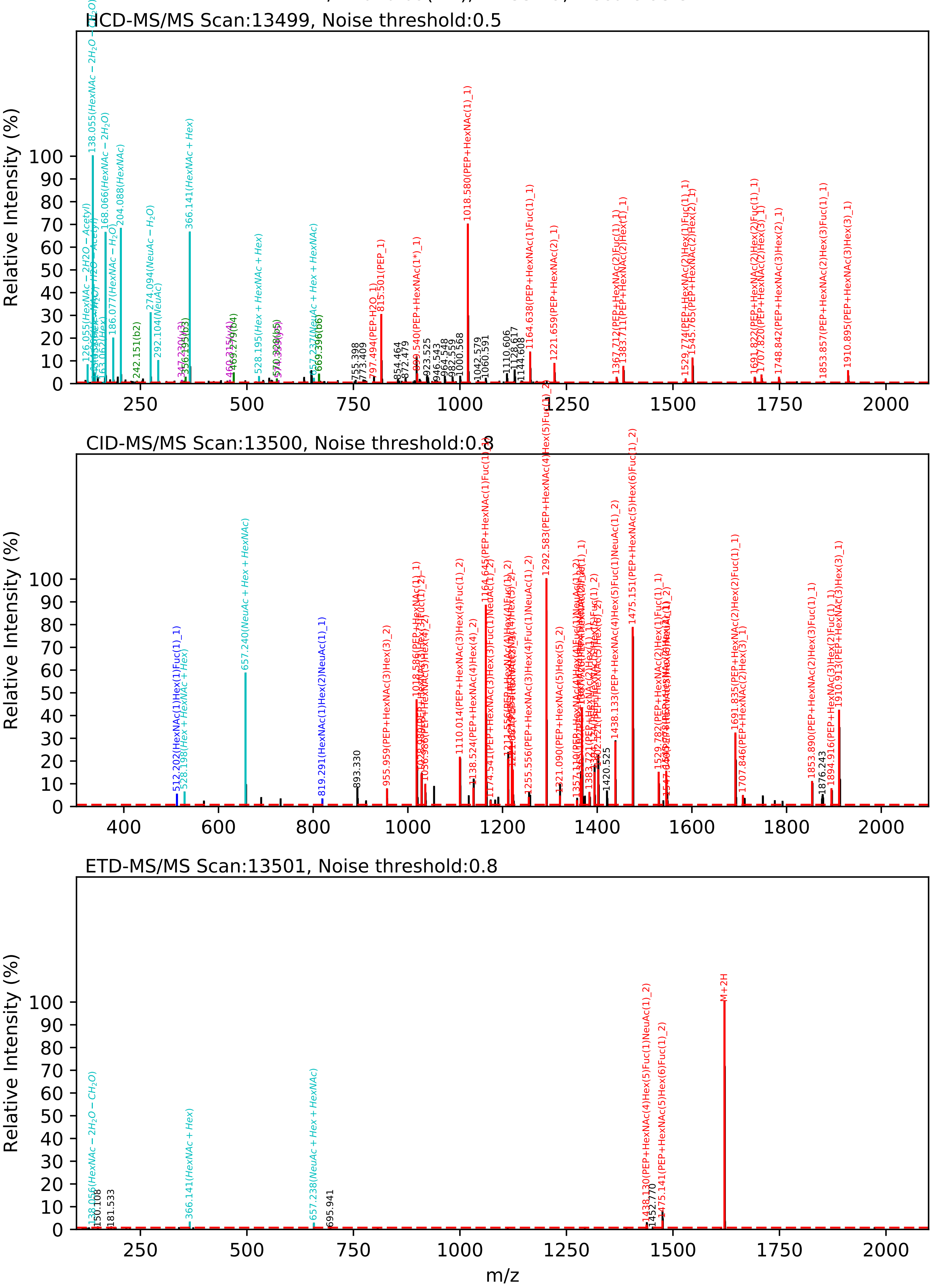

IQNLTVK(=PEP)_6_5_1_1_0, 0_None, 0_None,
m/z:1620.69(2+), RT:36.69, Y-score:93.80

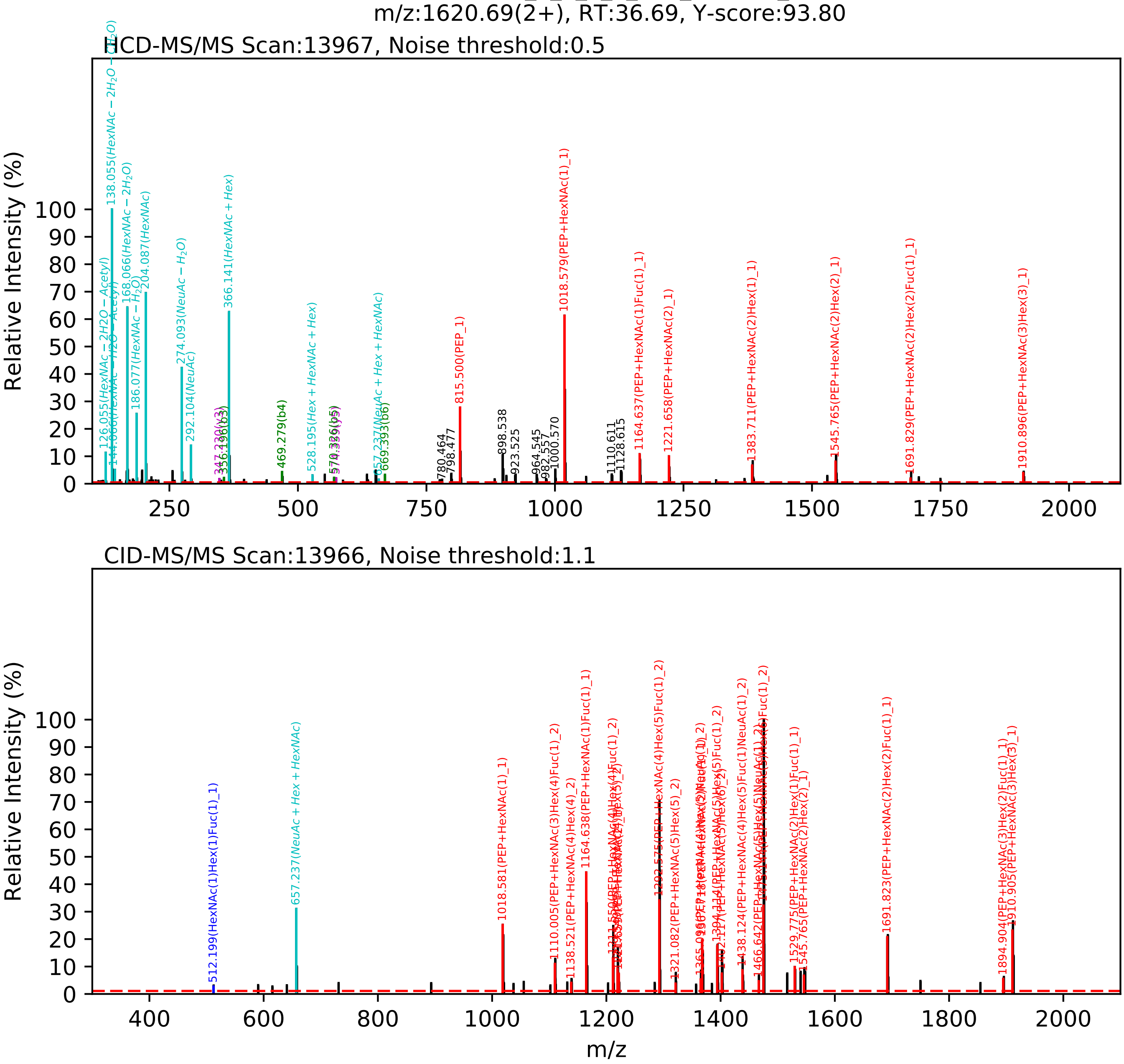

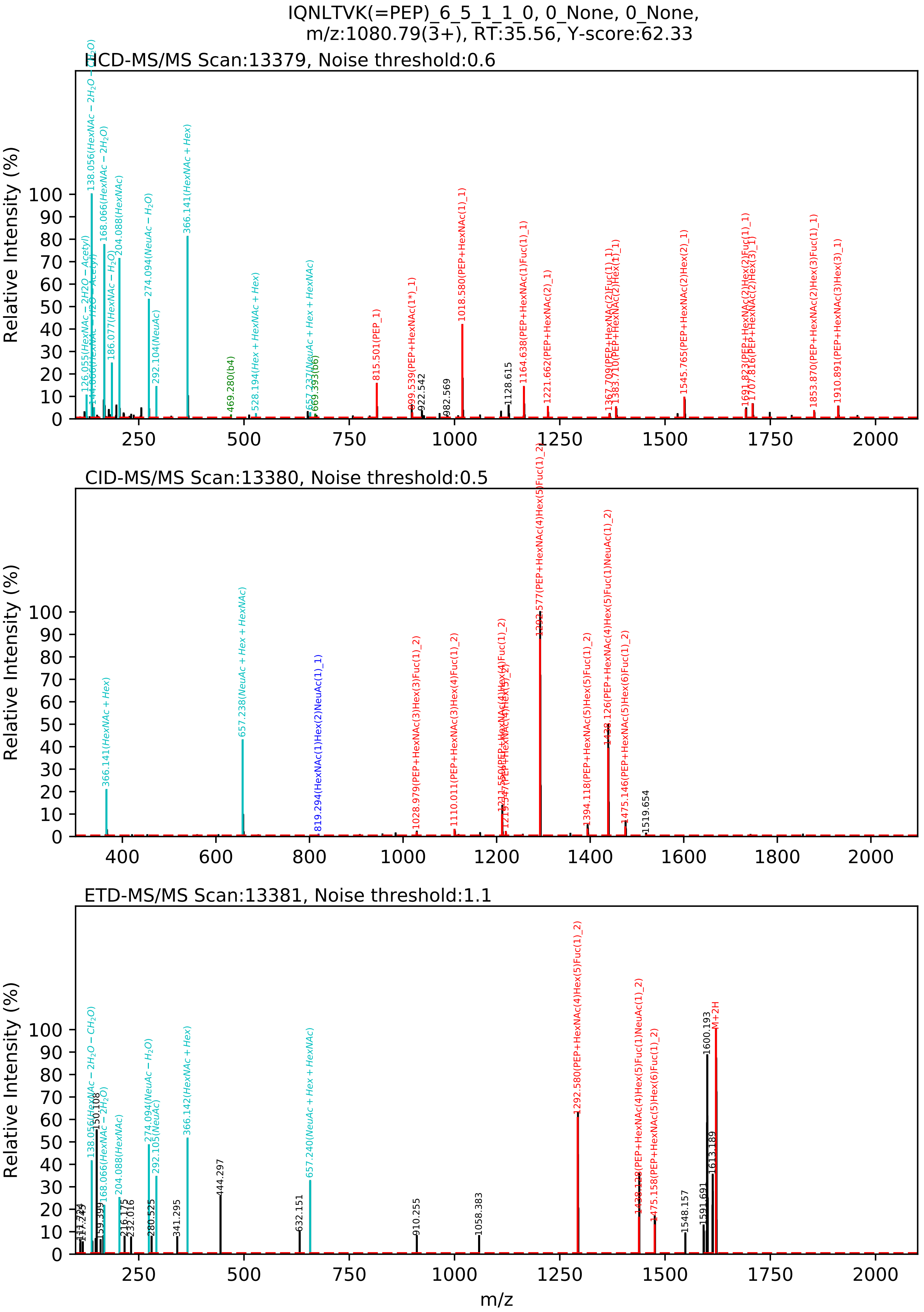
IQNLTVK(=PEP)_6_5_1_1_0, 0_None, 0_None,
m/z:1080.79(3+), RT:35.56, Y-score:62.33
HCD-MS/MS Scan:13379, Noise threshold:0.6
CID-MS/MS Scan:13380, Noise threshold:0.5
ETD-MS/MS Scan:13381, Noise threshold:1.1
Relative Intensity (%)
m/z
1018.580(PEP+HexNAc(1)_1)
815.501(PEP_1)
1293.577(PEP+HexNAc(4)Hex(5)Fuc(1)_2)
1438.126(PEP+HexNAc(4)Hex(5)Fuc(1)NeuAc(1)_2)
657.238(NeuAc+Hex+HexNAc)
366.141(HexNAc+Hex)
1292.580(PEP+HexNAc(4)Hex(5)Fuc(1)_2)
1600.193
M+2H

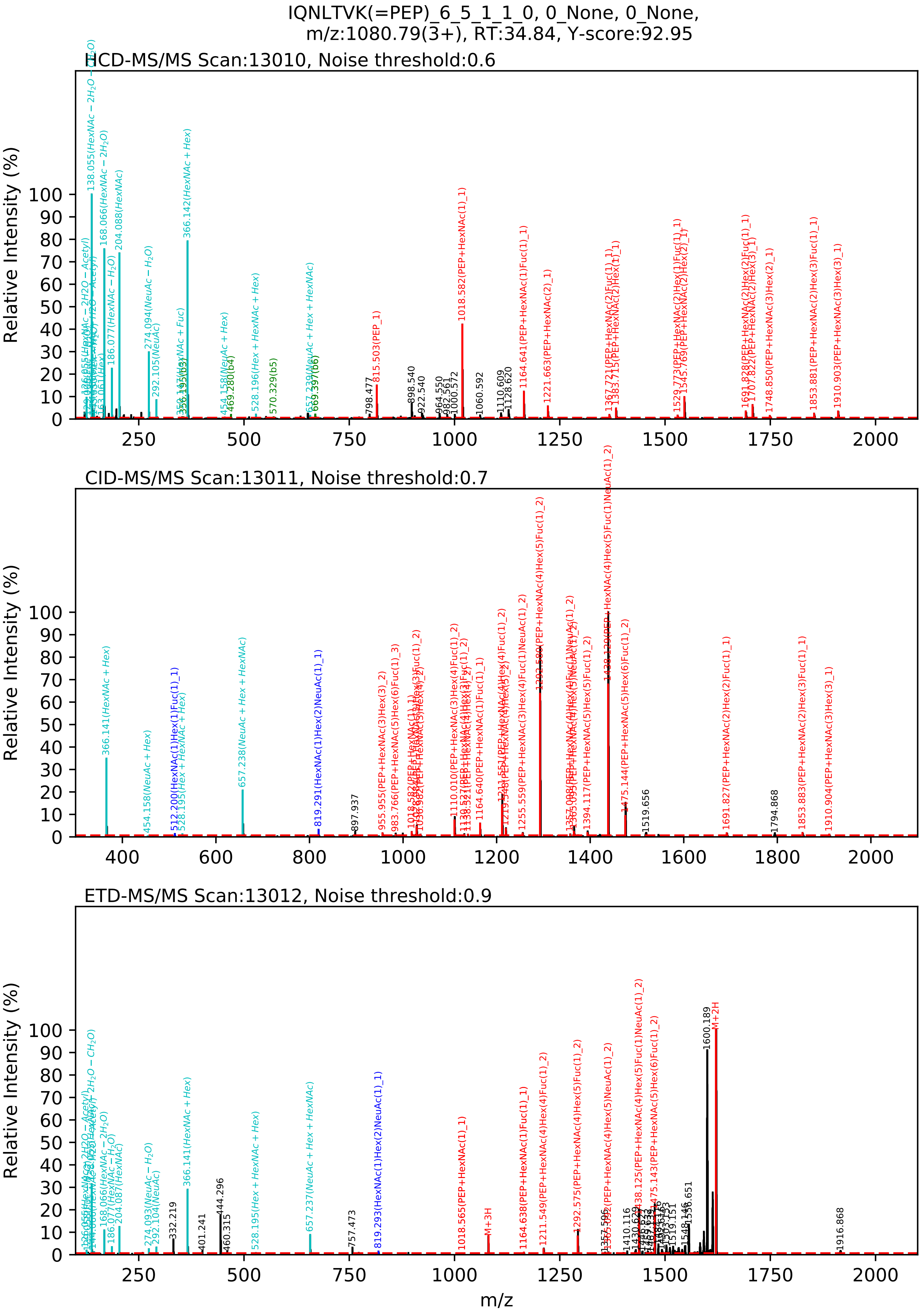

IQNLTVK(=PEP)_6_5_1_1_0, 0_None, 0_None,
m/z:1080.79(3+), RT:34.84, Y-score:92.95
HCD-MS/MS Scan:13010, Noise threshold:0.6
CID-MS/MS Scan:13011, Noise threshold:0.7
ETD-MS/MS Scan:13012, Noise threshold:0.9
Relative Intensity (%)
m/z

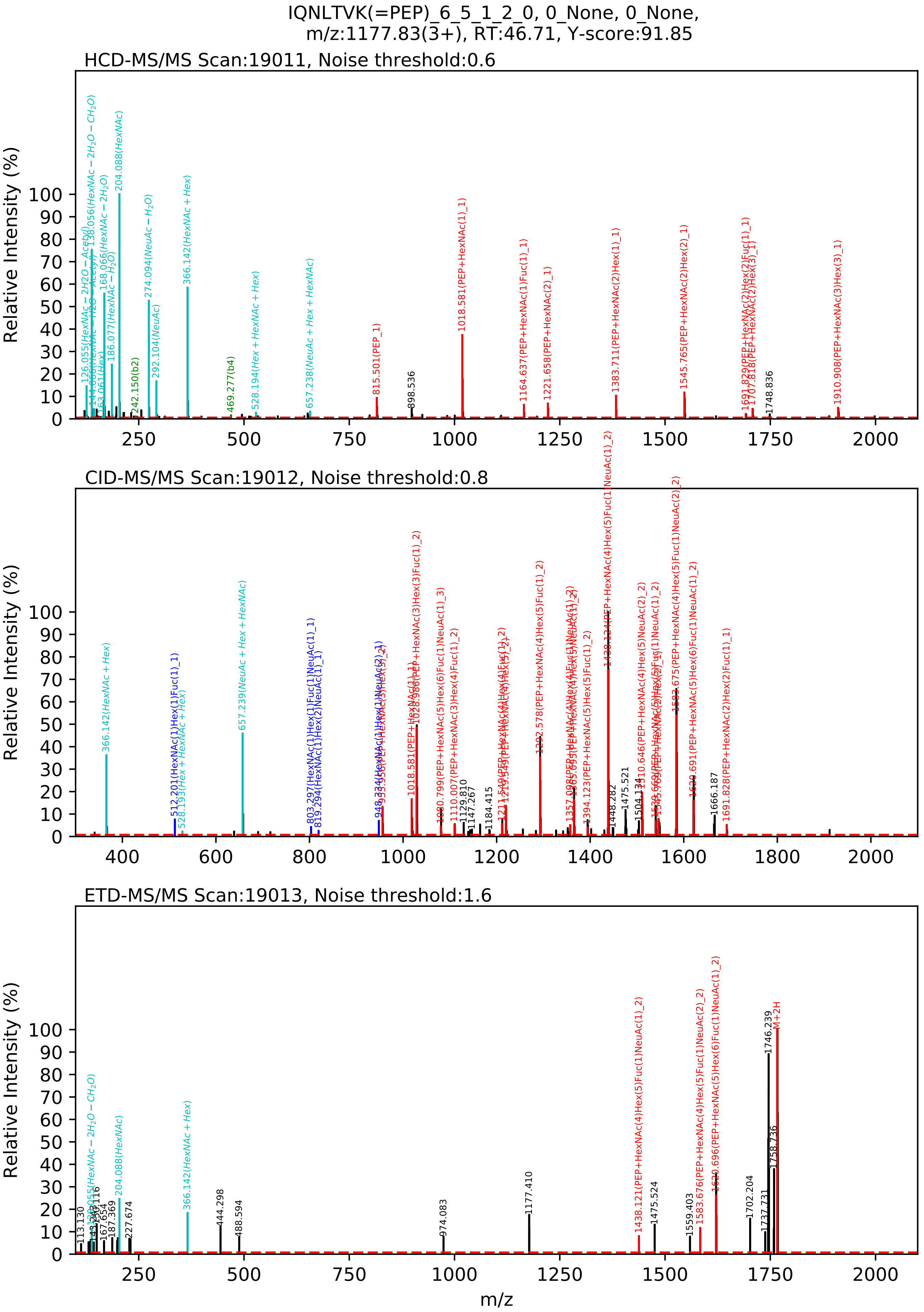
IQNLTVK(=PEP)_6_5_1_2_0, 0_None, 0_None,
m/z:1177.83(3+), RT:46.71, Y-score:91.85
HCD-MS/MS Scan:19011, Noise threshold:0.6
Relative Intensity (%)
204.088(HexNAc)
168.066(HexNAc − H2O)
186.077(HexNAc)
274.094(NeuAc − H2O)
292.104(NeuAc)
366.142(HexNAc + Hex)
242.150(b2)
469.277(b4)
528.194(Hex + HexNAc + Hex)
657.238(NeuAc + Hex + HexNAc)
815.501(PEP_1)
898.536
1018.581(PEP+HexNAc(1)_1)
1164.637(PEP+HexNAc(1)Fuc(1)_1)
1221.658(PEP+HexNAc(2)_1)
1383.711(PEP+HexNAc(2)Hex(1)_1)
1545.765(PEP+HexNAc(2)Hex(2)_1)
1748.836
1910.908(PEP+HexNAc(3)Hex(3)_1)
CID-MS/MS Scan:19012, Noise threshold:0.8
Relative Intensity (%)
366.142(HexNAc + Hex)
512.201(HexNAc(1)Hex(1)Fuc(1)_1)
528.193(Hex + HexNAc + Hex)
657.239(NeuAc + Hex + HexNAc)
803.297(HexNAc(1)Hex(1)Fuc(1)NeuAc(1)_1)
819.294(HexNAc(1)Hex(2)NeuAc(1)_1)
1018.581(PEP+HexNAc(1)_1)
1080.799(PEP+HexNAc(5)Hex(6)Fuc(1)NeuAc(1)_3)
1110.007(PEP+HexNAc(3)Hex(4)Fuc(1)_2)
1129.810
1147.267
1184.415
1292.578(PEP+HexNAc(4)Hex(5)Fuc(1)_2)
1394.123(PEP+HexNAc(5)Hex(5)Fuc(1)_2)
1438.12(PEP+HexNAc(4)Hex(5)Fuc(1)NeuAc(1)_2)
1448.282
1475.521
1504.134
1510.646(PEP+HexNAc(4)Hex(5)NeuAc(2)_2)
1583.675(PEP+HexNAc(4)Hex(5)Fuc(1)NeuAc(2)_2)
1620.691(PEP+HexNAc(5)Hex(6)Fuc(1)NeuAc(1)_2)
1666.187
1691.828(PEP+HexNAc(2)Hex(2)Fuc(1)_1)
ETD-MS/MS Scan:19013, Noise threshold:1.6
Relative Intensity (%)
113.130
167.654
187.369
204.088(HexNAc)
227.674
366.142(HexNAc + Hex)
444.298
488.594
974.083
1177.410
1438.121(PEP+HexNAc(4)Hex(5)Fuc(1)NeuAc(1)_2)
1475.524
1559.403
1583.676(PEP+HexNAc(4)Hex(5)Fuc(1)NeuAc(2)_2)
1620.696(PEP+HexNAc(5)Hex(6)Fuc(1)NeuAc(1)_2)
1702.204
1737.731
1746.239
1758.736
M+2H
m/z

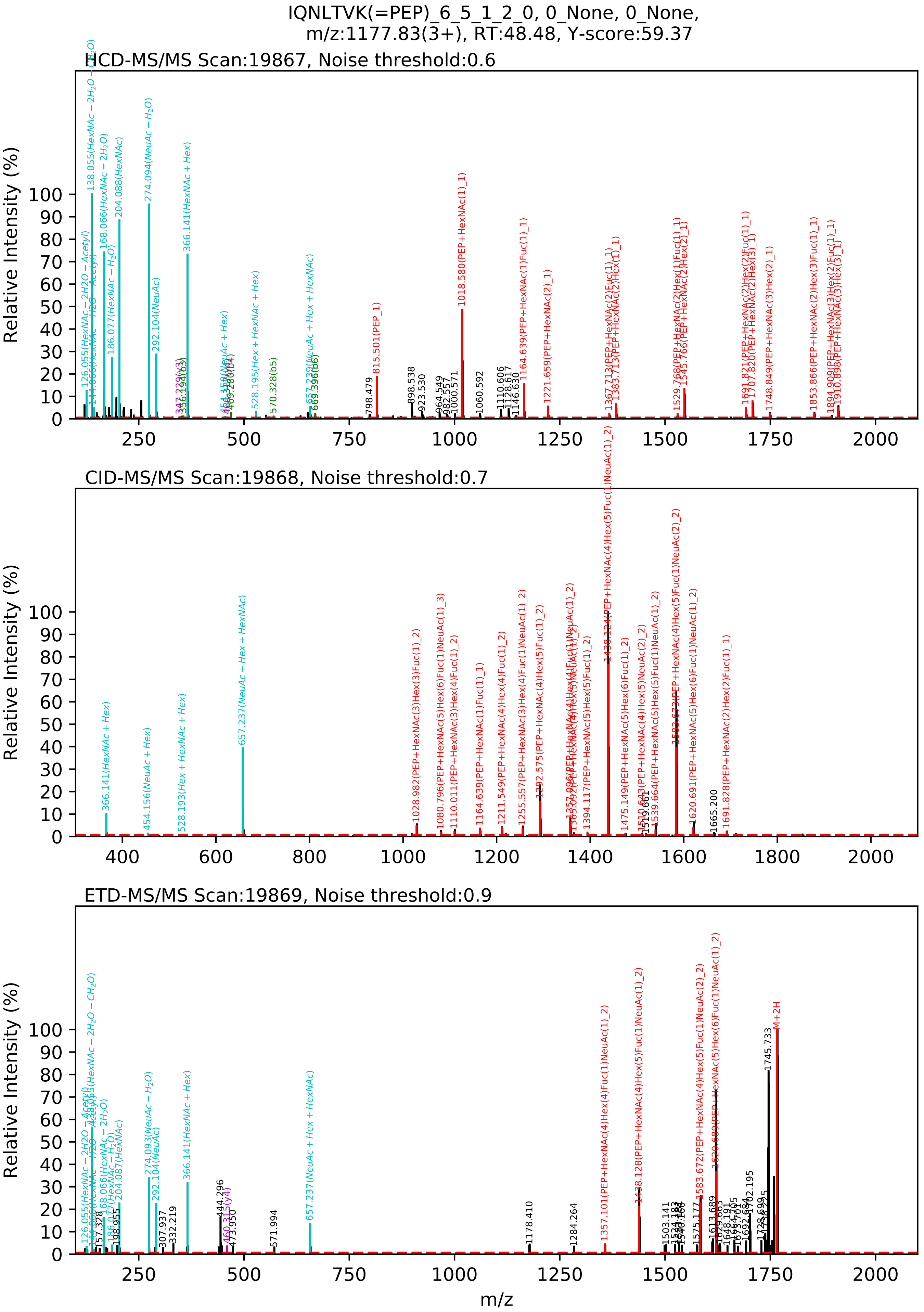
IQNLTVK(=PEP)_6_5_1_2_0, 0_None, 0_None,
m/z:1177.83(3+), RT:48.48, Y-score:59.37
HCD-MS/MS Scan:19867, Noise threshold:0.6
CID-MS/MS Scan:19868, Noise threshold:0.7
ETD-MS/MS Scan:19869, Noise threshold:0.9
Relative Intensity (%)
m/z

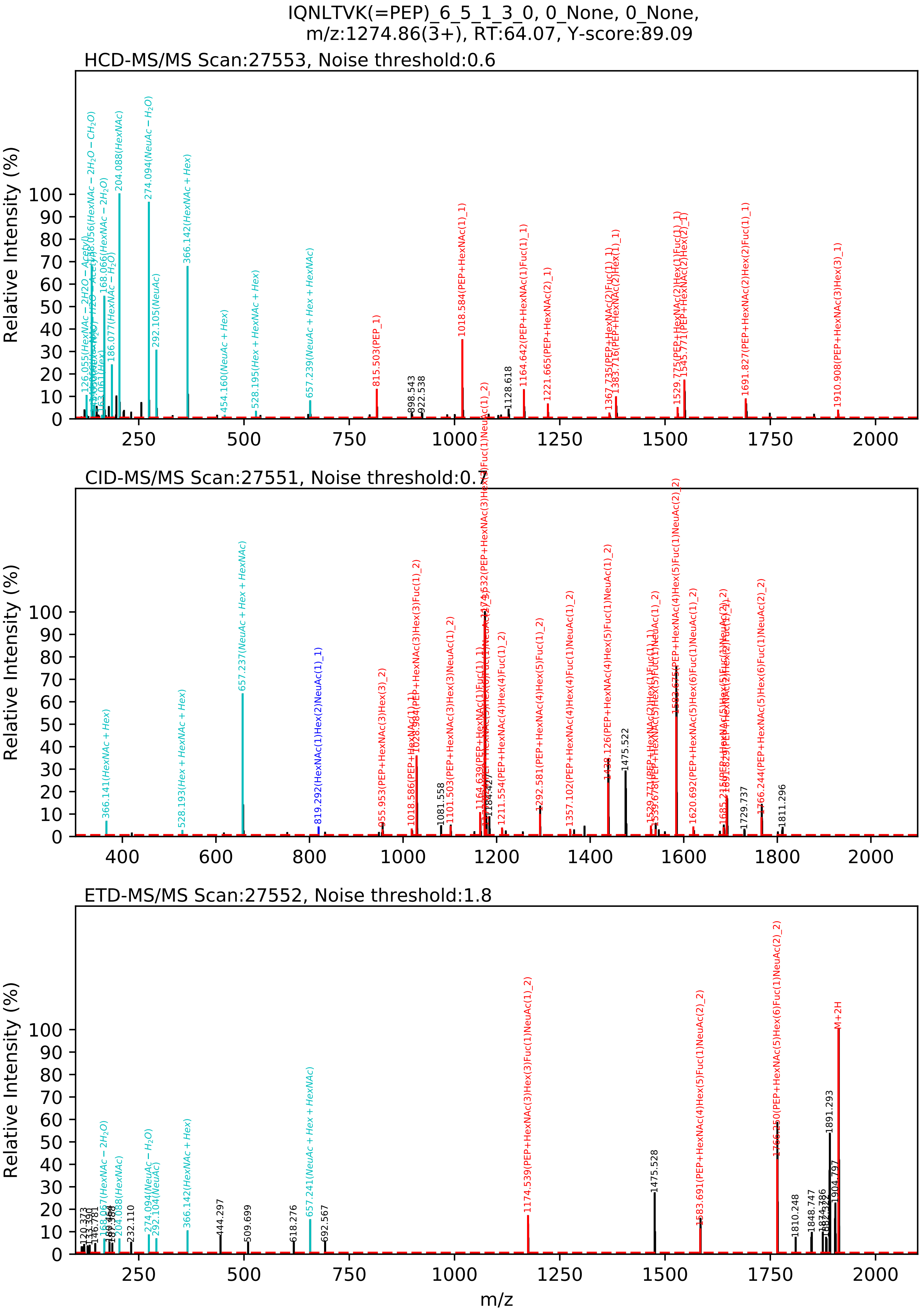

IQNLTVK(=PEP)_6_5_1_3_0, 0_None, 0_None,
m/z:1274.86(3+), RT:64.07, Y-score:89.09
HCD-MS/MS Scan:27553, Noise threshold:0.6
CID-MS/MS Scan:27551, Noise threshold:0.7
ETD-MS/MS Scan:27552, Noise threshold:1.8
Relative Intensity (%)
m/z

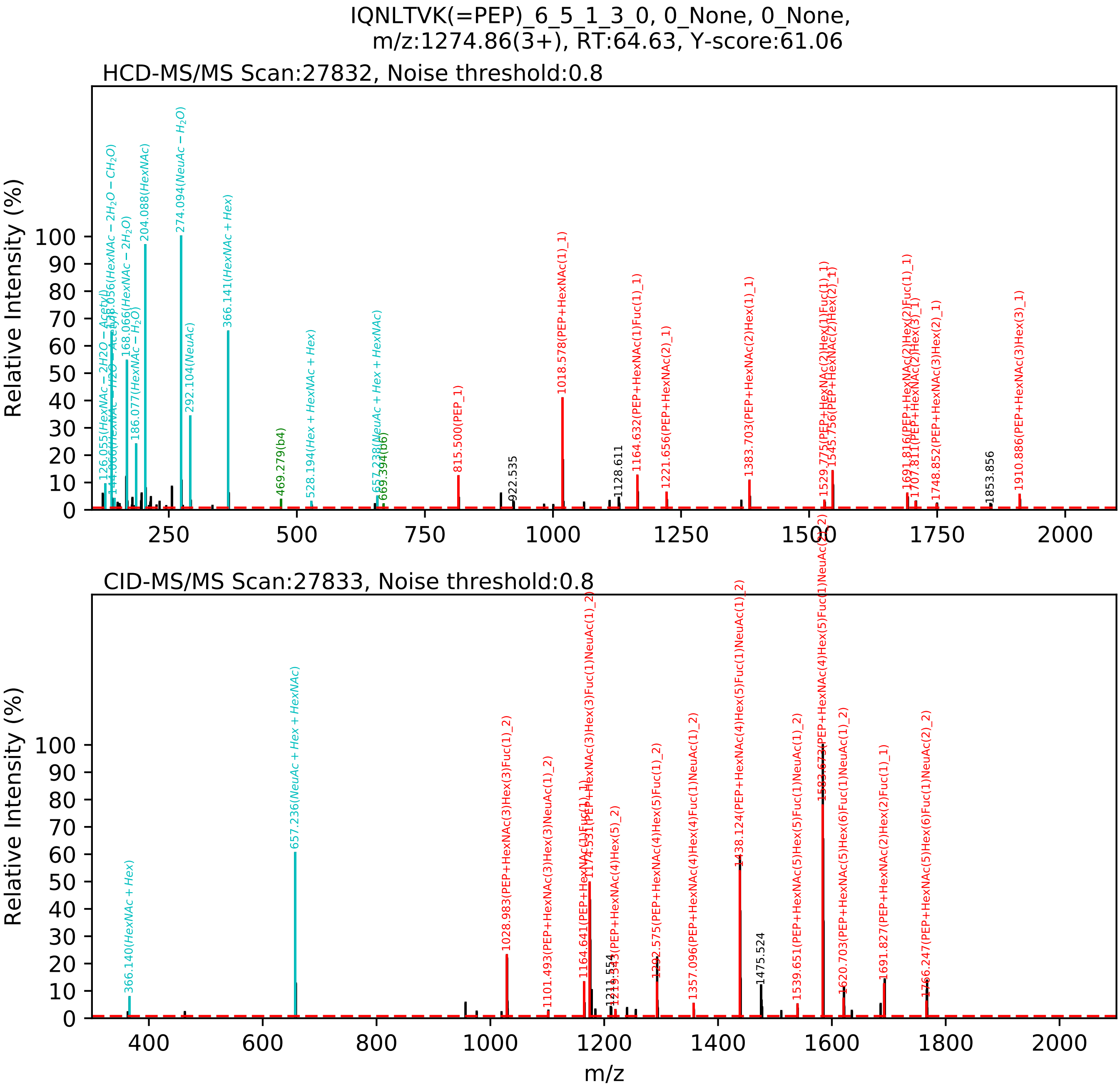
IQNLTVK(=PEP)_6_5_1_3_0, 0_None, 0_None,
m/z:1274.86(3+), RT:64.63, Y-score:61.06
HCD-MS/MS Scan:27832, Noise threshold:0.8
Relative Intensity (%)
274.094(NeuAc − H2O)
204.088(HexNAc)
168.066(HexNAc − 2H2O)
186.077(HexNAc − H2O)
366.141(HexNAc + Hex)
292.104(NeuAc)
469.279(b4)
528.194(Hex + HexNAc + Hex)
669.394(b6)
815.500(PEP_1)
922.535
1018.578(PEP+HexNAc(1)_1)
1128.611
1164.632(PEP+HexNAc(1)Fuc(1)_1)
1221.656(PEP+HexNAc(2)_1)
1383.703(PEP+HexNAc(2)Hex(1)_1)
1545.756(PEP+HexNAc(2)Hex(2)_1)
1748.852(PEP+HexNAc(3)Hex(2)_1)
1853.856
1910.886(PEP+HexNAc(3)Hex(3)_1)
CID-MS/MS Scan:27833, Noise threshold:0.8
Relative Intensity (%)
366.140(HexNAc + Hex)
657.236(NeuAc + Hex + HexNAc)
1028.983(PEP+HexNAc(3)Hex(3)Fuc(1)_2)
1101.493(PEP+HexNAc(3)Hex(3)NeuAc(1)_2)
1211.554
1292.575(PEP+HexNAc(4)Hex(5)Fuc(1)_2)
1357.096(PEP+HexNAc(4)Hex(4)Fuc(1)NeuAc(1)_2)
1438.124(PEP+HexNAc(4)Hex(5)Fuc(1)NeuAc(1)_2)
1475.524
1539.651(PEP+HexNAc(5)Hex(5)Fuc(1)NeuAc(1)_2)
1620.703(PEP+HexNAc(5)Hex(6)Fuc(1)NeuAc(1)_2)
1691.827(PEP+HexNAc(2)Hex(2)Fuc(1)_1)
1766.247(PEP+HexNAc(5)Hex(6)Fuc(1)NeuAc(2)_2)
m/z

IQNLTVK(=PEP)_6_5_2_0_0, 0_None, 0_None,
m/z:1548.16(2+), RT:25.83, Y-score:91.35

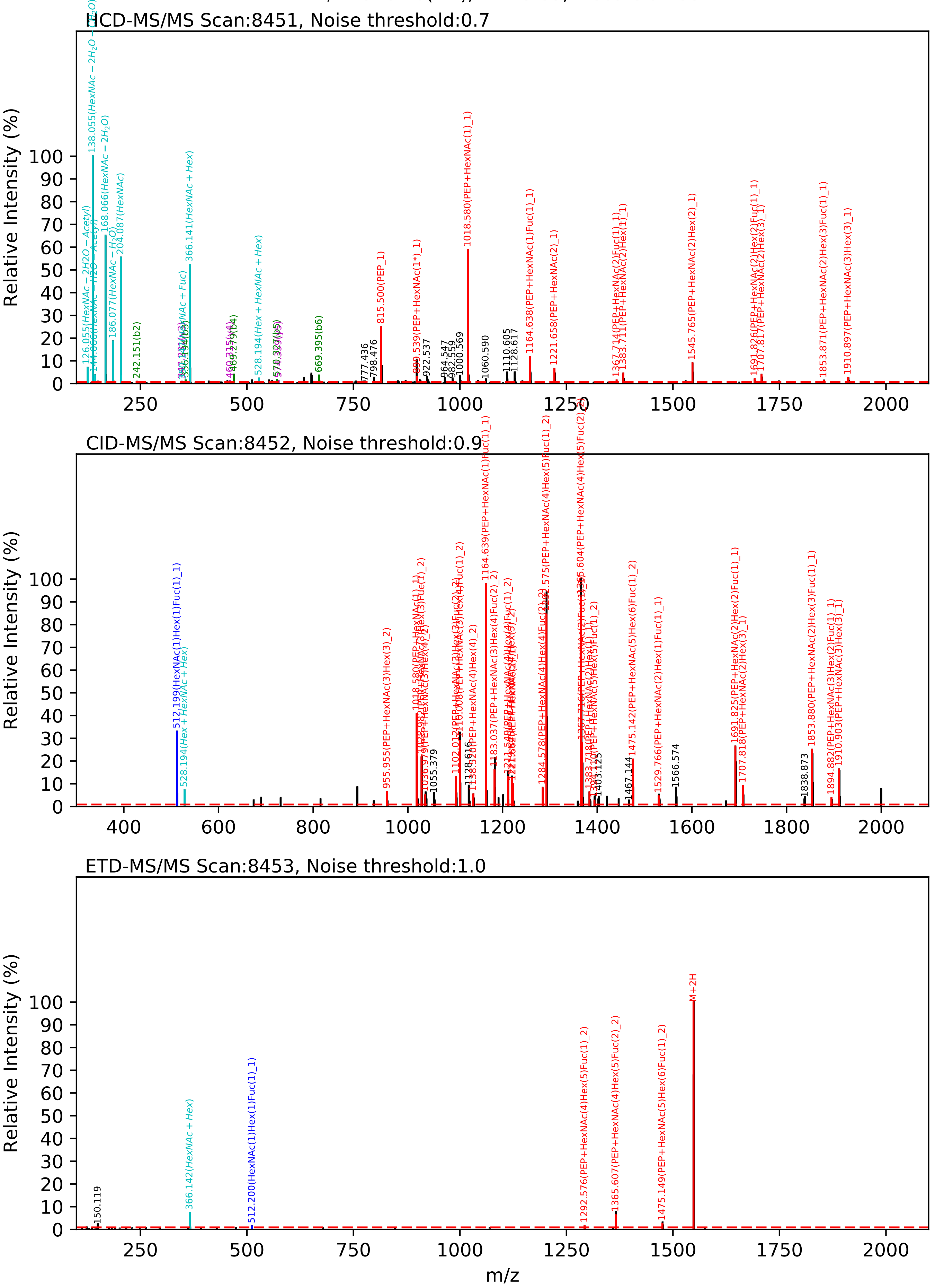

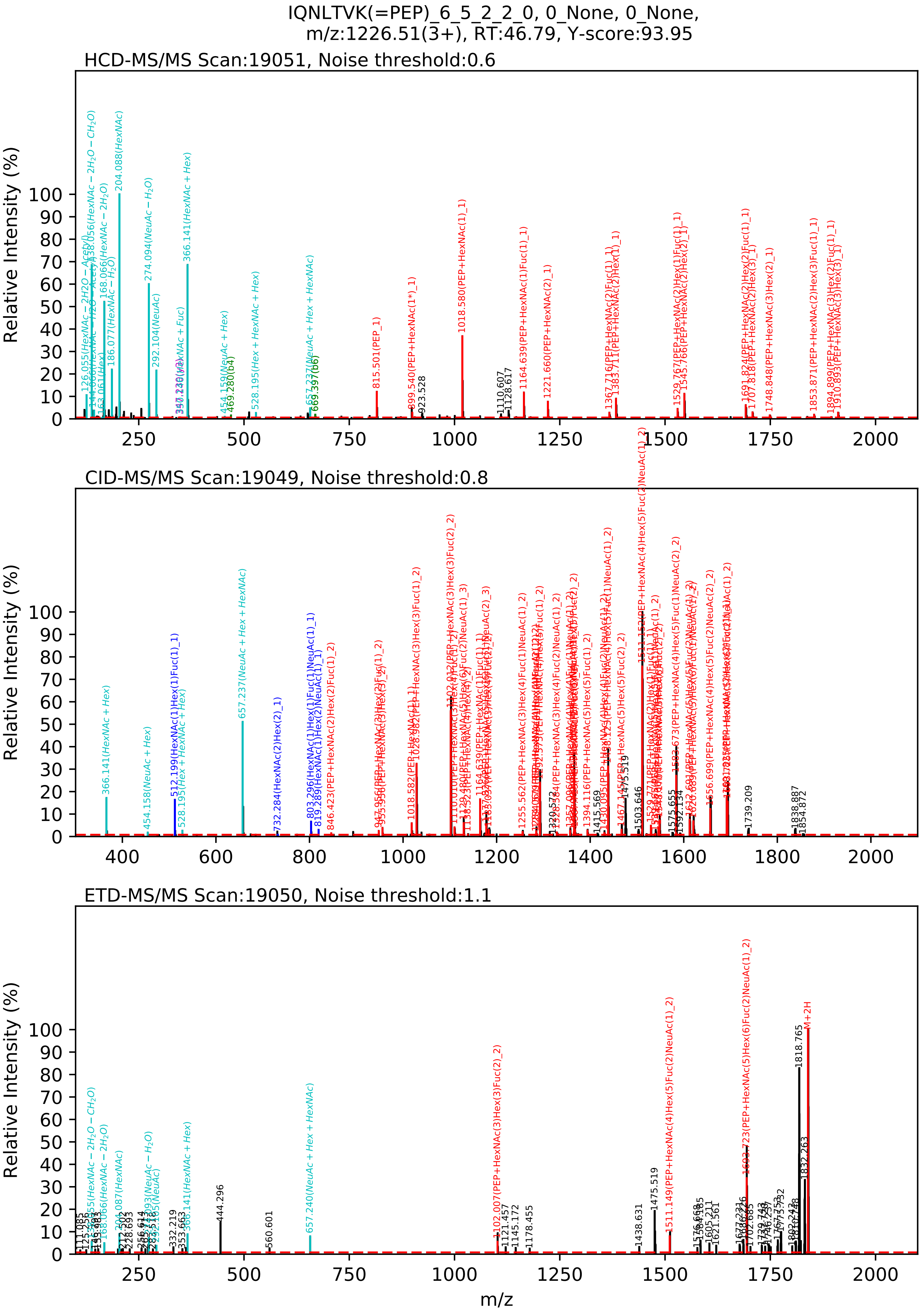
IQNLTVK(=PEP)_6_5_2_2_0, 0_None, 0_None,
m/z:1226.51(3+), RT:46.79, Y-score:93.95
HCD-MS/MS Scan:19051, Noise threshold:0.6
Relative Intensity (%)
204.088(HexNAc)
366.141(HexNAc + Hex)
274.094(NeuAc − H2O)
292.104(NeuAc)
528.195(Hex + HexNAc + Hex)
657.237(NeuAc + Hex + HexNAc)
815.501(PEP_1)
899.540(PEP+HexNAc(1*)_1)
923.528
1018.580(PEP+HexNAc(1)_1)
1110.607
1128.617
1164.639(PEP+HexNAc(1)Fuc(1)_1)
1221.660(PEP+HexNAc(2)_1)
1748.848(PEP+HexNAc(3)Hex(2)_1)
1853.871(PEP+HexNAc(2)Hex(3)Fuc(1)_1)
CID-MS/MS Scan:19049, Noise threshold:0.8
366.141(HexNAc + Hex)
454.158(NeuAc + Hex)
512.199(HexNAc(1)Hex(1)Fuc(1)_1)
657.237(NeuAc + Hex + HexNAc)
732.284(HexNAc(2)Hex(2)_1)
846.423(PEP+HexNAc(2)Hex(2)Fuc(1)_2)
1028.982(PEP+HexNAc(3)Hex(3)Fuc(1)_2)
1475.519
1503.646
1575.655
1656.699(PEP+HexNAc(4)Hex(5)Fuc(2)NeuAc(2)_2)
1739.209
1838.887
1854.872
ETD-MS/MS Scan:19050, Noise threshold:1.1
204.087(HexNAc)
366.141(HexNAc + Hex)
444.296
560.601
657.240(NeuAc + Hex + HexNAc)
1102.007(PEP+HexNAc(3)Hex(3)Fuc(2)_2)
1121.457
1145.172
1178.455
1438.631
1475.519
1511.149(PEP+HexNAc(4)Hex(5)Fuc(2)NeuAc(1)_2)
1692.723(PEP+HexNAc(5)Hex(6)Fuc(2)NeuAc(1)_2)
1818.765
1832.263
M+2H
m/z

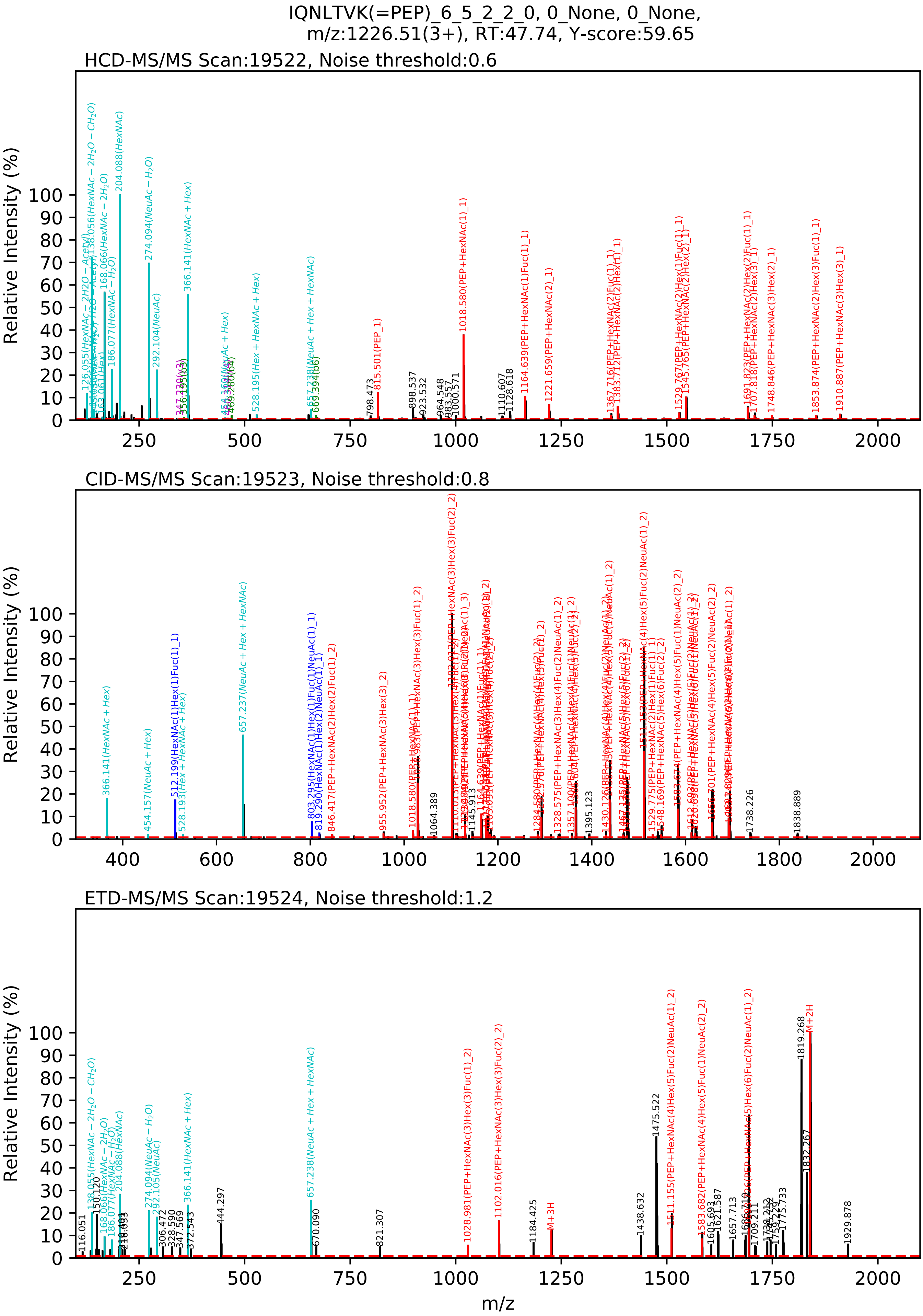
IQNLTVK(=PEP)_6_5_2_2_0, 0_None, 0_None,
m/z:1226.51(3+), RT:47.74, Y-score:59.65
HCD-MS/MS Scan:19522, Noise threshold:0.6
CID-MS/MS Scan:19523, Noise threshold:0.8
ETD-MS/MS Scan:19524, Noise threshold:1.2
Relative Intensity (%)
m/z
204.088(HexNAc)
274.094(NeuAc − H2O)
366.141(HexNAc + Hex)
292.104(NeuAc)
528.195(Hex + HexNAc + Hex)
657.238(NeuAc + Hex + HexNAc)
815.501(PEP_1)
1018.580(PEP+HexNAc(1)_1)
1164.639(PEP+HexNAc(1)Fuc(1)_1)
1221.659(PEP+HexNAc(2)_1)
1748.846(PEP+HexNAc(3)Hex(2)_1)
1853.874(PEP+HexNAc(2)Hex(3)Fuc(1)_1)
1910.887(PEP+HexNAc(3)Hex(3)_1)
366.141(HexNAc + Hex)
454.157(NeuAc + Hex)
512.199(HexNAc(1)Hex(1)Fuc(1)_1)
657.237(NeuAc + Hex + HexNAc)
846.417(PEP+HexNAc(2)Hex(2)Fuc(1)_2)
955.952(PEP+HexNAc(3)Hex(3)_2)
1738.226
1838.889
1028.981(PEP+HexNAc(3)Hex(3)Fuc(1)_2)
1102.016(PEP+HexNAc(3)Hex(3)Fuc(2)_2)
1511.155(PEP+HexNAc(4)Hex(5)Fuc(2)NeuAc(1)_2)
1583.682(PEP+HexNAc(4)Hex(5)Fuc(1)NeuAc(2)_2)
1475.522
1819.268
1832.267
1929.878
M+2H
M+3H

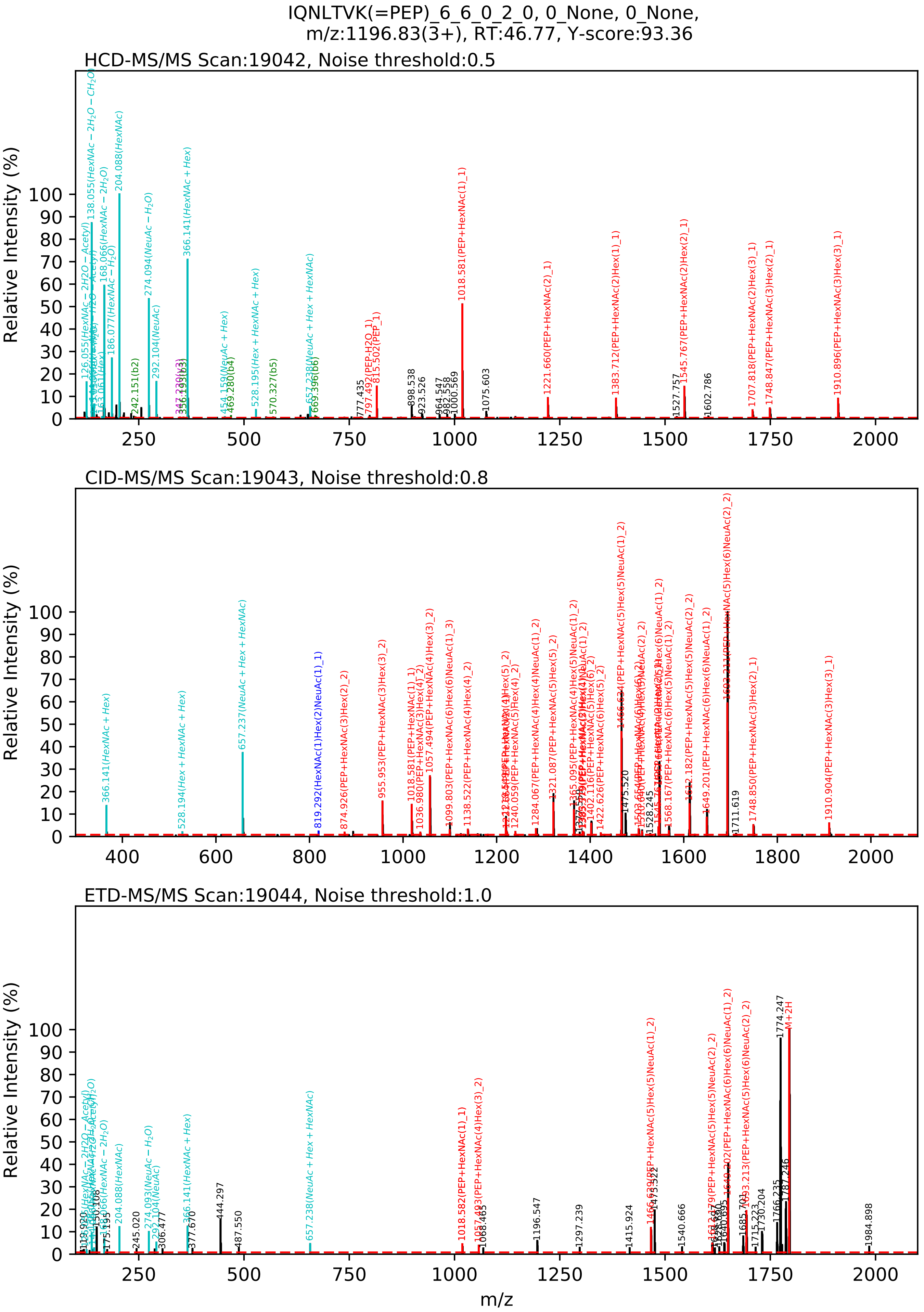

IQNLTVK(=PEP)_6_6_0_2_0, 0_None, 0_None,
m/z:1196.83(3+), RT:46.77, Y-score:93.36
HCD-MS/MS Scan:19042, Noise threshold:0.5
CID-MS/MS Scan:19043, Noise threshold:0.8
ETD-MS/MS Scan:19044, Noise threshold:1.0
Relative Intensity (%)
m/z

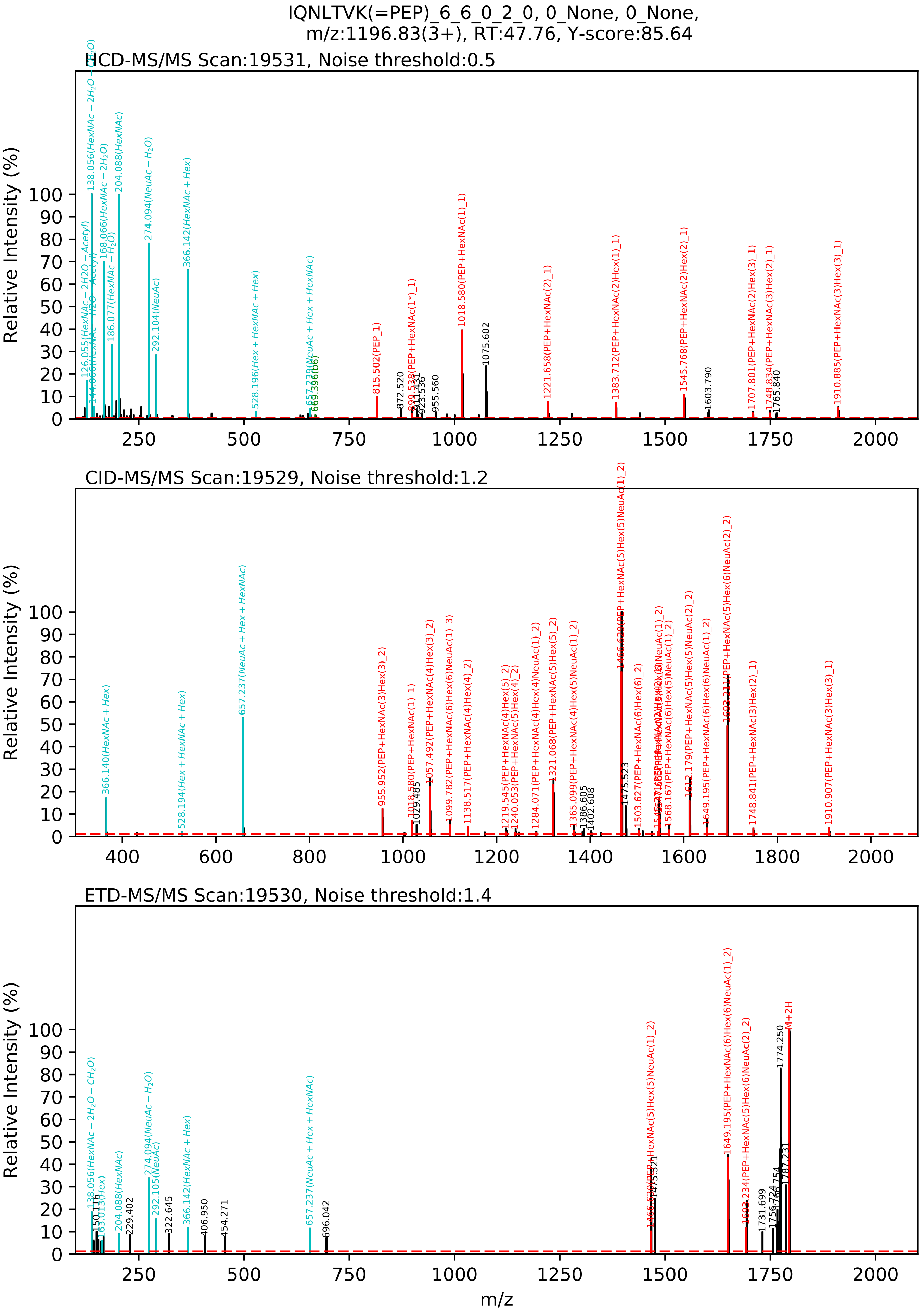
IQNLTVK(=PEP)_6_6_0_2_0, 0_None, 0_None,
m/z:1196.83(3+), RT:47.76, Y-score:85.64
HCD-MS/MS Scan:19531, Noise threshold:0.5
CID-MS/MS Scan:19529, Noise threshold:1.2
ETD-MS/MS Scan:19530, Noise threshold:1.4
Relative Intensity (%)
m/z
815.502(PEP_1)
1018.580(PEP+HexNAc(1)_1)
1221.658(PEP+HexNAc(2)_1)
1383.712(PEP+HexNAc(2)Hex(1)_1)
1545.768(PEP+HexNAc(2)Hex(2)_1)
1707.801(PEP+HexNAc(2)Hex(3)_1)
1748.834(PEP+HexNAc(3)Hex(2)_1)
1910.885(PEP+HexNAc(3)Hex(3)_1)
366.142(HexNAc+Hex)
292.104(NeuAc)
204.088(HexNAc)
657.237(NeuAc+Hex+HexNAc)
1466.620(PEP+HexNAc(5)Hex(5)NeuAc(1)_2)
1693.211(PEP+HexNAc(5)Hex(6)NeuAc(2)_2)
1649.195(PEP+HexNAc(6)Hex(6)NeuAc(1)_2)
1321.068(PEP+HexNAc(5)Hex(5)_2)
1057.492(PEP+HexNAc(4)Hex(3)_2)
955.952(PEP+HexNAc(3)Hex(3)_2)
M+2H

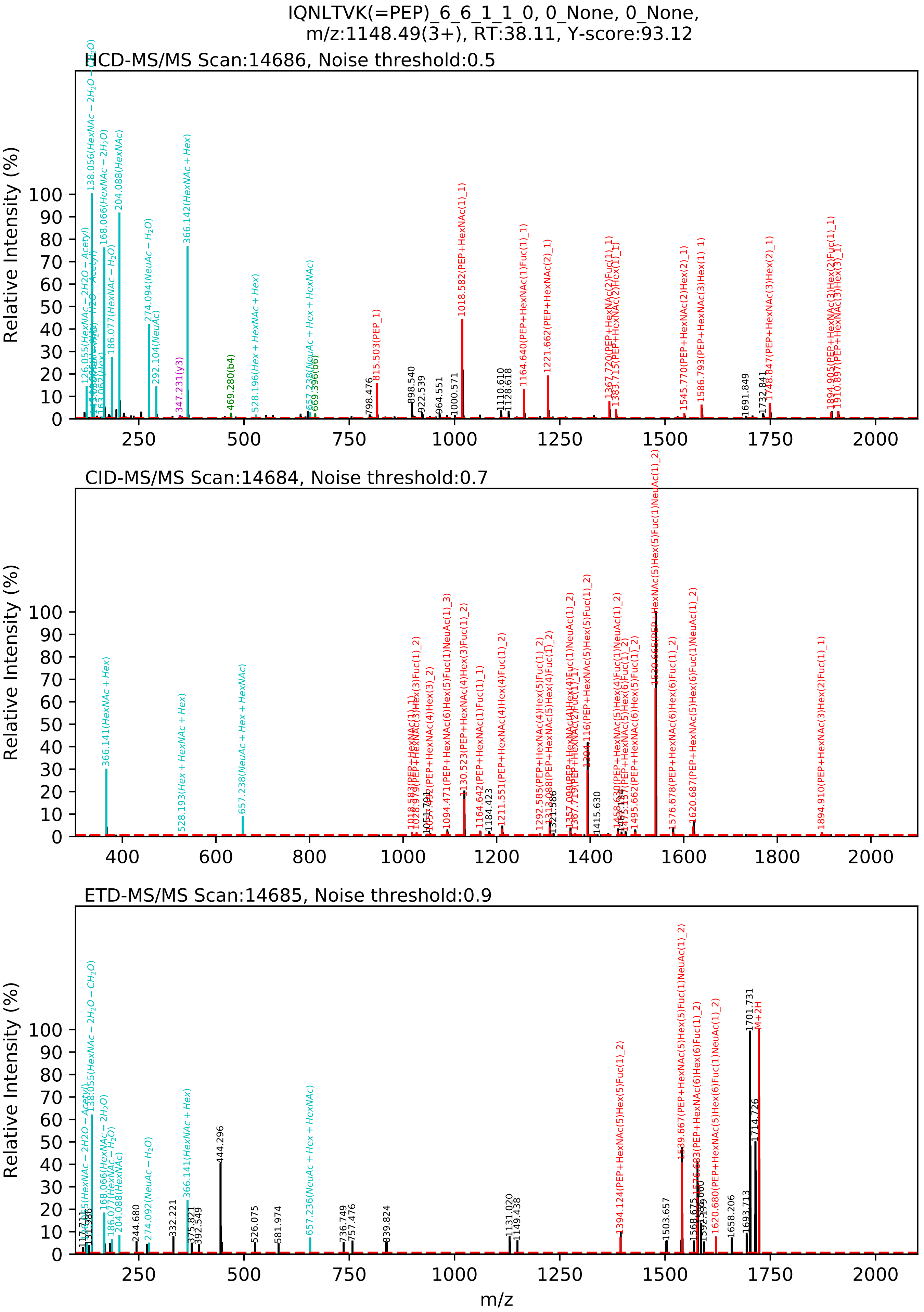
IQNLTVK(=PEP)_6_6_1_1_0, 0_None, 0_None,
m/z:1148.49(3+), RT:38.11, Y-score:93.12
HCD-MS/MS Scan:14686, Noise threshold:0.5
CID-MS/MS Scan:14684, Noise threshold:0.7
ETD-MS/MS Scan:14685, Noise threshold:0.9
Relative Intensity (%)
m/z
1018.582(PEP+HexNAc(1)_1)
1539.667(PEP+HexNAc(5)Hex(5)Fuc(1)NeuAc(1)_2)
1701.731
M+2H

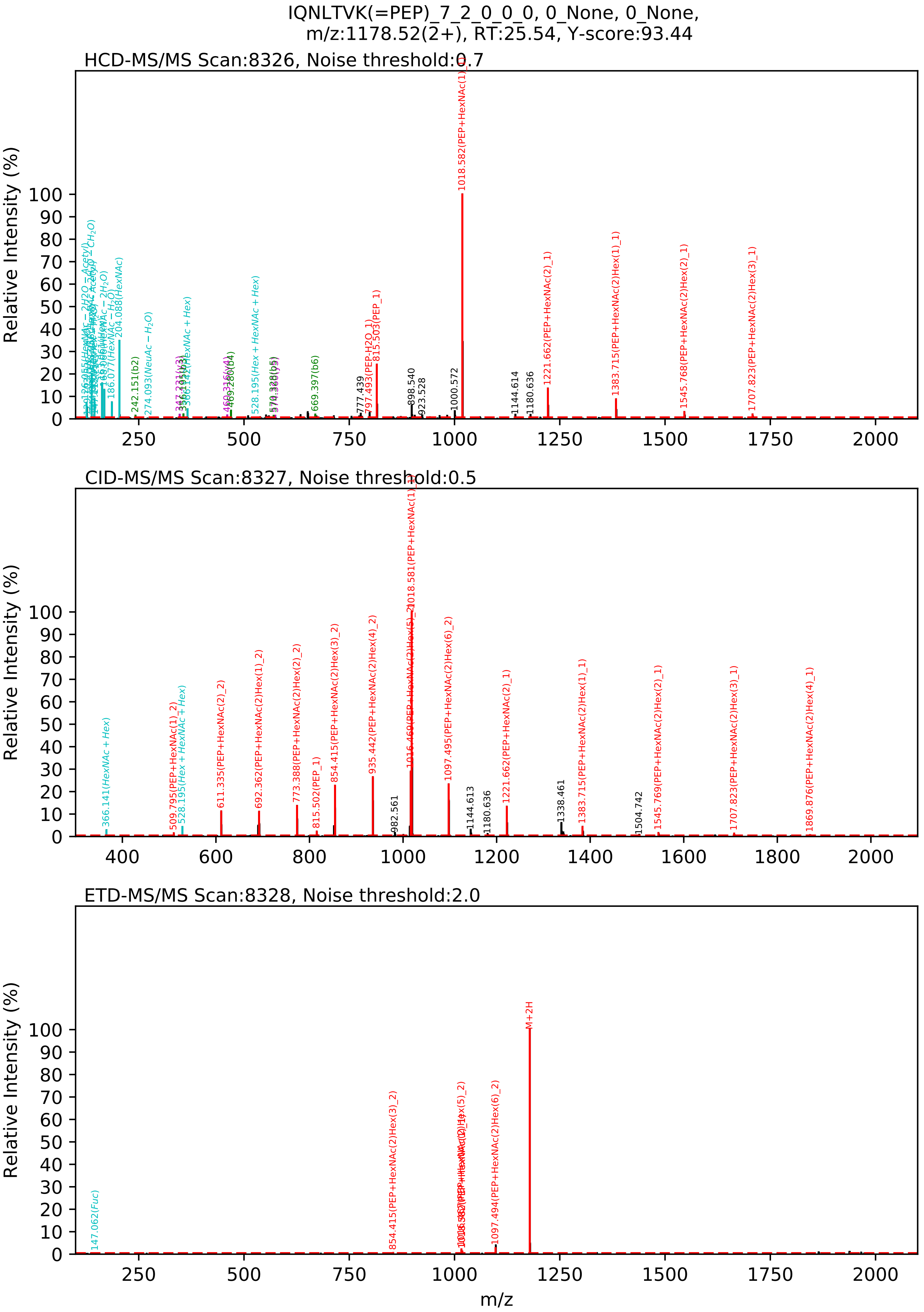
IQNLTVK(=PEP)_7_2_0_0_0, 0_None, 0_None,
m/z:1178.52(2+), RT:25.54, Y-score:93.44
HCD-MS/MS Scan:8326, Noise threshold:0.7
1018.582(PEP+HexNAc(1)_1)
1221.662(PEP+HexNAc(2)_1)
1383.715(PEP+HexNAc(2)Hex(1)_1)
1545.768(PEP+HexNAc(2)Hex(2)_1)
1707.823(PEP+HexNAc(2)Hex(3)_1)
815.503(PEP_1)
797.493(PEP-H2O_1)
204.088(HexNAc)
186.077(HexNAc − H2O)
242.151(b2)
274.093(NeuAc − H2O)
528.195(Hex + HexNAc + Hex)
669.397(b6)
777.439
898.540
923.528
1000.572
1144.614
1180.636
CID-MS/MS Scan:8327, Noise threshold:0.5
366.141(HexNAc + Hex)
509.795(PEP+HexNAc(1)_2)
528.195(Hex + HexNAc + Hex)
611.335(PEP+HexNAc(2)_2)
692.362(PEP+HexNAc(2)Hex(1)_2)
773.388(PEP+HexNAc(2)Hex(2)_2)
815.502(PEP_1)
854.415(PEP+HexNAc(2)Hex(3)_2)
935.442(PEP+HexNAc(2)Hex(4)_2)
982.561
1018.581(PEP+HexNAc(1)_1)
1097.495(PEP+HexNAc(2)Hex(6)_2)
1144.613
1180.636
1221.662(PEP+HexNAc(2)_1)
1338.461
1383.715(PEP+HexNAc(2)Hex(1)_1)
1504.742
1545.769(PEP+HexNAc(2)Hex(2)_1)
1707.823(PEP+HexNAc(2)Hex(3)_1)
1869.876(PEP+HexNAc(2)Hex(4)_1)
ETD-MS/MS Scan:8328, Noise threshold:2.0
147.062(Fuc)
854.415(PEP+HexNAc(2)Hex(3)_2)
1097.494(PEP+HexNAc(2)Hex(6)_2)
M+2H
Relative Intensity (%)
m/z

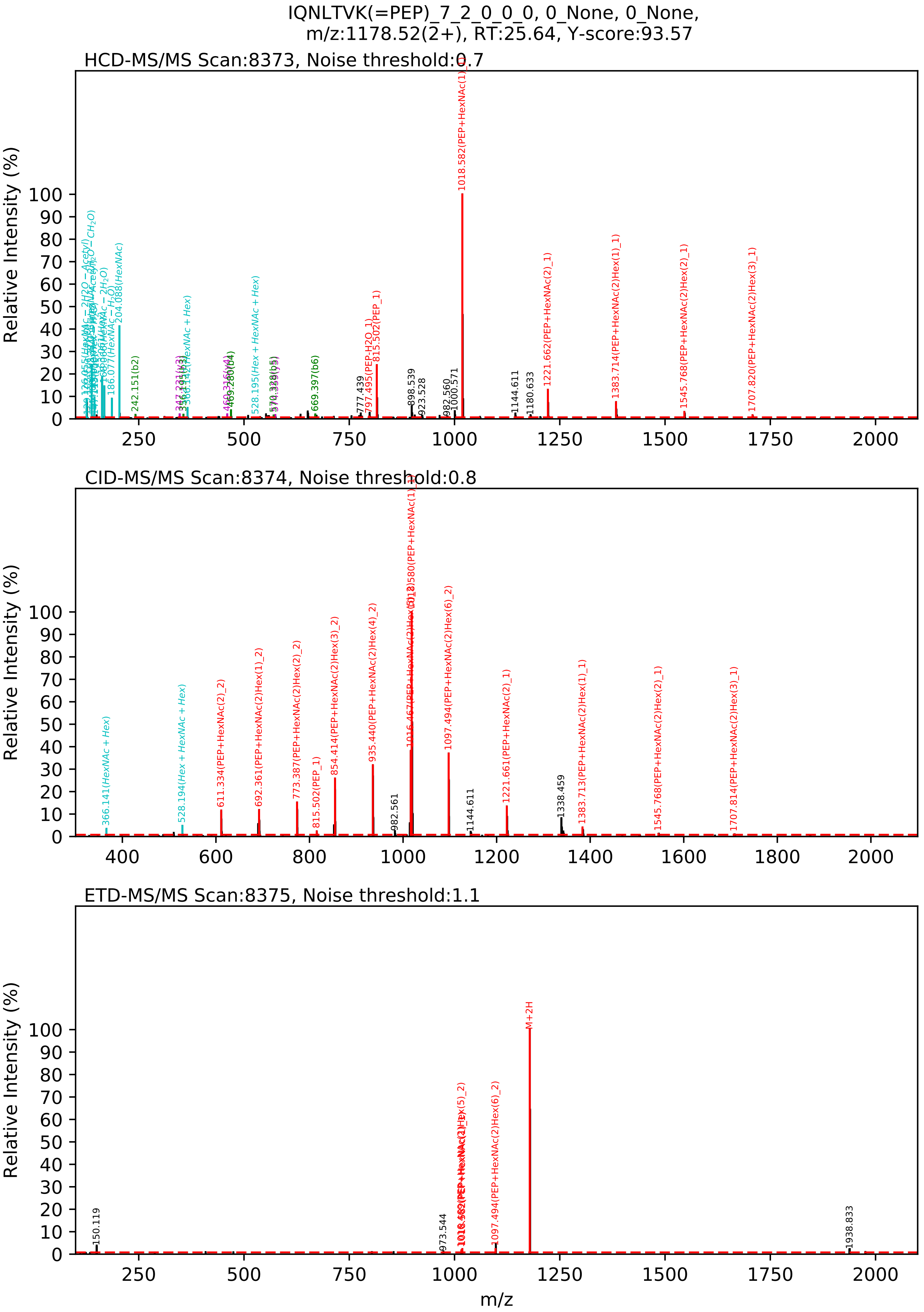

IQNLTVK(=PEP)_7_2_0_0_0, 0_None, 0_None,
m/z:1178.52(2+), RT:25.64, Y-score:93.57
HCD-MS/MS Scan:8373, Noise threshold:0.7
CID-MS/MS Scan:8374, Noise threshold:0.8
ETD-MS/MS Scan:8375, Noise threshold:1.1
Relative Intensity (%)
m/z
1018.582(PEP+HexNAc(1)_1)
1221.662(PEP+HexNAc(2)_1)
1383.714(PEP+HexNAc(2)Hex(1)_1)
1545.768(PEP+HexNAc(2)Hex(2)_1)
1707.820(PEP+HexNAc(2)Hex(3)_1)
815.502(PEP_1)
204.088(HexNAc)
242.151(b2)
669.397(b6)
528.195(Hex+HexNAc+Hex)
611.334(PEP+HexNAc(2)_2)
692.361(PEP+HexNAc(2)Hex(1)_2)
773.387(PEP+HexNAc(2)Hex(2)_2)
854.414(PEP+HexNAc(2)Hex(3)_2)
935.440(PEP+HexNAc(2)Hex(4)_2)
1097.494(PEP+HexNAc(2)Hex(6)_2)
1221.661(PEP+HexNAc(2)_1)
1383.713(PEP+HexNAc(2)Hex(1)_1)
1545.768(PEP+HexNAc(2)Hex(2)_1)
1707.814(PEP+HexNAc(2)Hex(3)_1)
366.141(HexNAc+Hex)
528.194(Hex+HexNAc+Hex)
1338.459
1144.611
982.561
M+2H
150.119
973.544
1938.833

IQNLTVK(=PEP)_7_2_0_0_0, 0_None, 0_None,
m/z:1178.52(2+), RT:26.08, Y-score:93.25

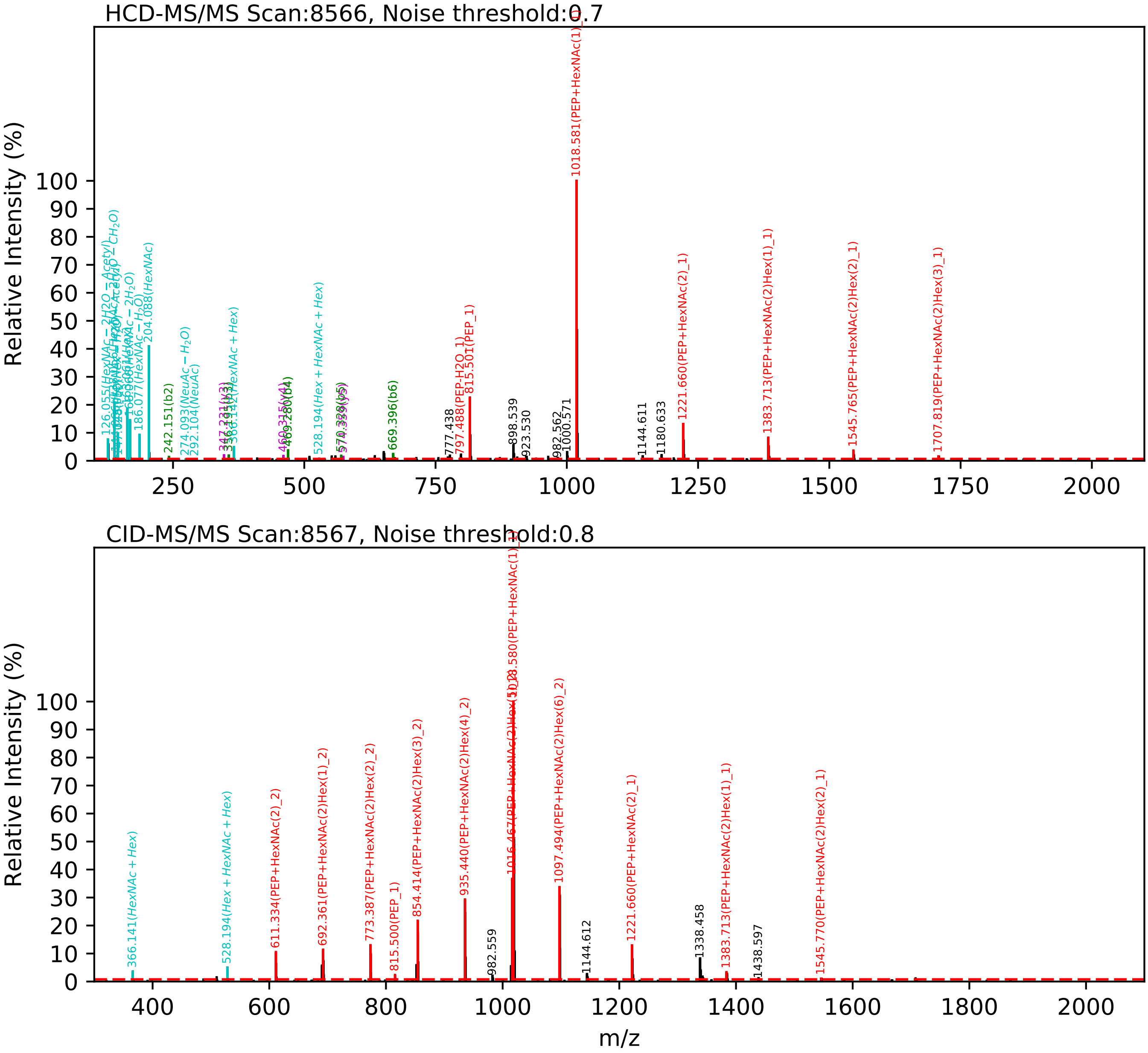

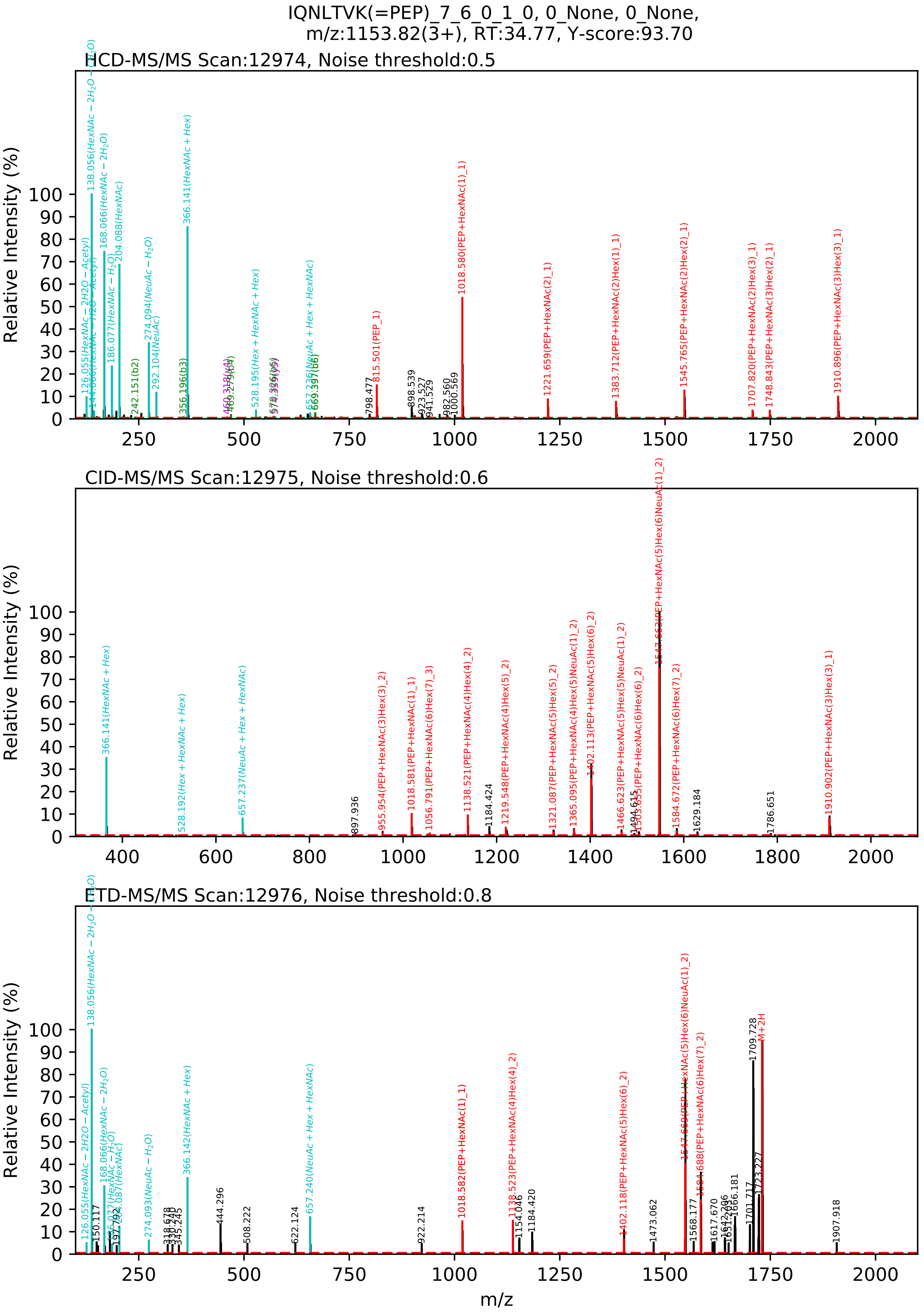
IQNLTVK(=PEP)_7_6_0_1_0, 0_None, 0_None,
m/z:1153.82(3+), RT:34.77, Y-score:93.70
HCD-MS/MS Scan:12974, Noise threshold:0.5
CID-MS/MS Scan:12975, Noise threshold:0.6
ETD-MS/MS Scan:12976, Noise threshold:0.8
Relative Intensity (%)
m/z
1018.580(PEP+HexNAc(1)_1)
815.501(PEP_1)
1221.659(PEP+HexNAc(2)_1)
1383.712(PEP+HexNAc(2)Hex(1)_1)
1545.765(PEP+HexNAc(2)Hex(2)_1)
1707.820(PEP+HexNAc(2)Hex(3)_1)
1748.843(PEP+HexNAc(3)Hex(2)_1)
1910.896(PEP+HexNAc(3)Hex(3)_1)
366.141(HexNAc + Hex)
138.056(HexNAc − 2H2O − CH2O)
168.066(HexNAc − 2H2O)
204.088(HexNAc)
274.094(NeuAc − H2O)
292.104(NeuAc)
1547.663(PEP+HexNAc(5)Hex(6)NeuAc(1)_2)
1402.113(PEP+HexNAc(5)Hex(6)_2)
1138.521(PEP+HexNAc(4)Hex(4)_2)
1910.902(PEP+HexNAc(3)Hex(3)_1)
1709.728
M+2H

IQNLTVK(=PEP)_7_6_0_2_0, 0_None, 0_None,
m/z:938.39(4+), RT:46.99, Y-score:95.61

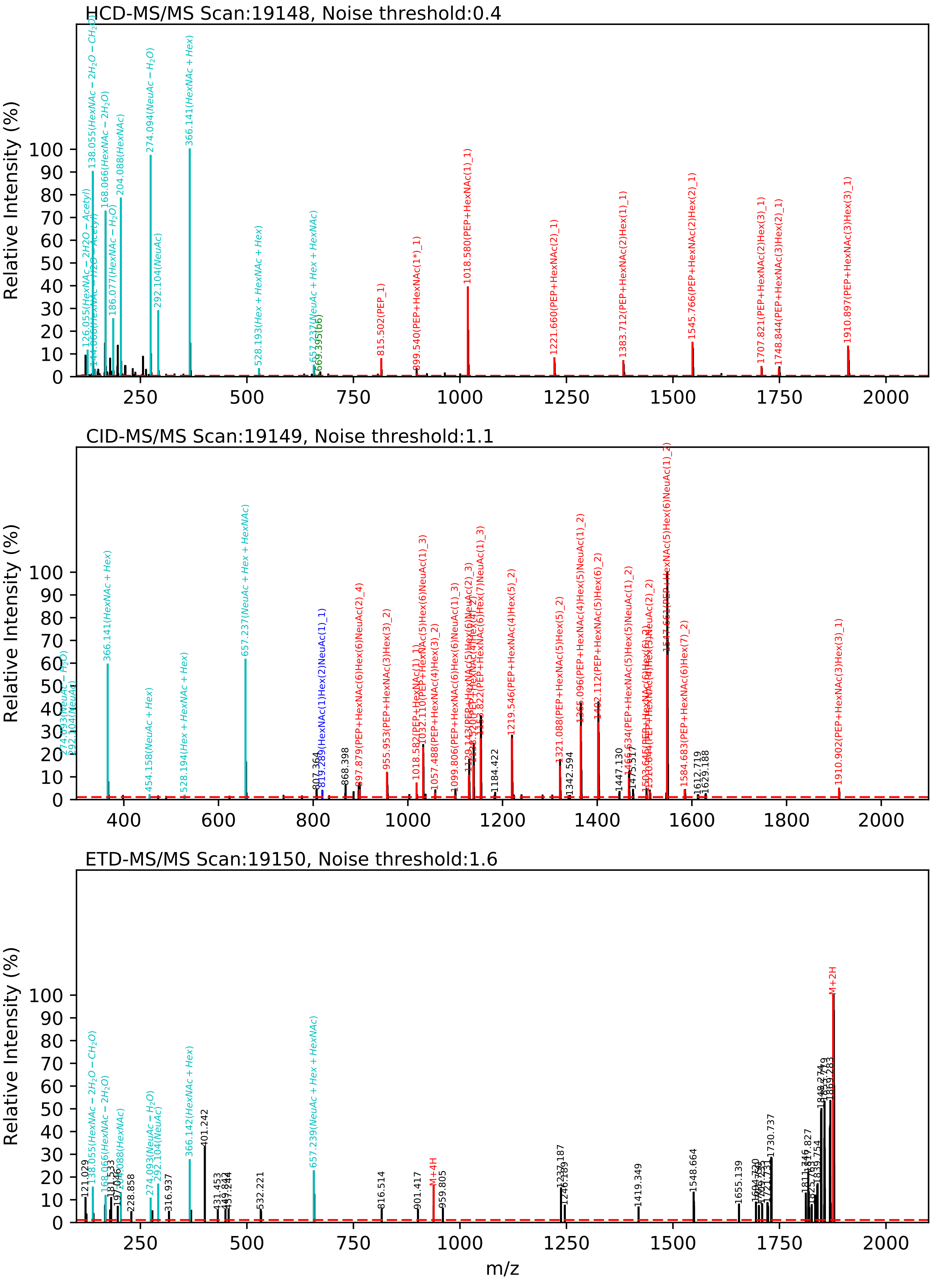

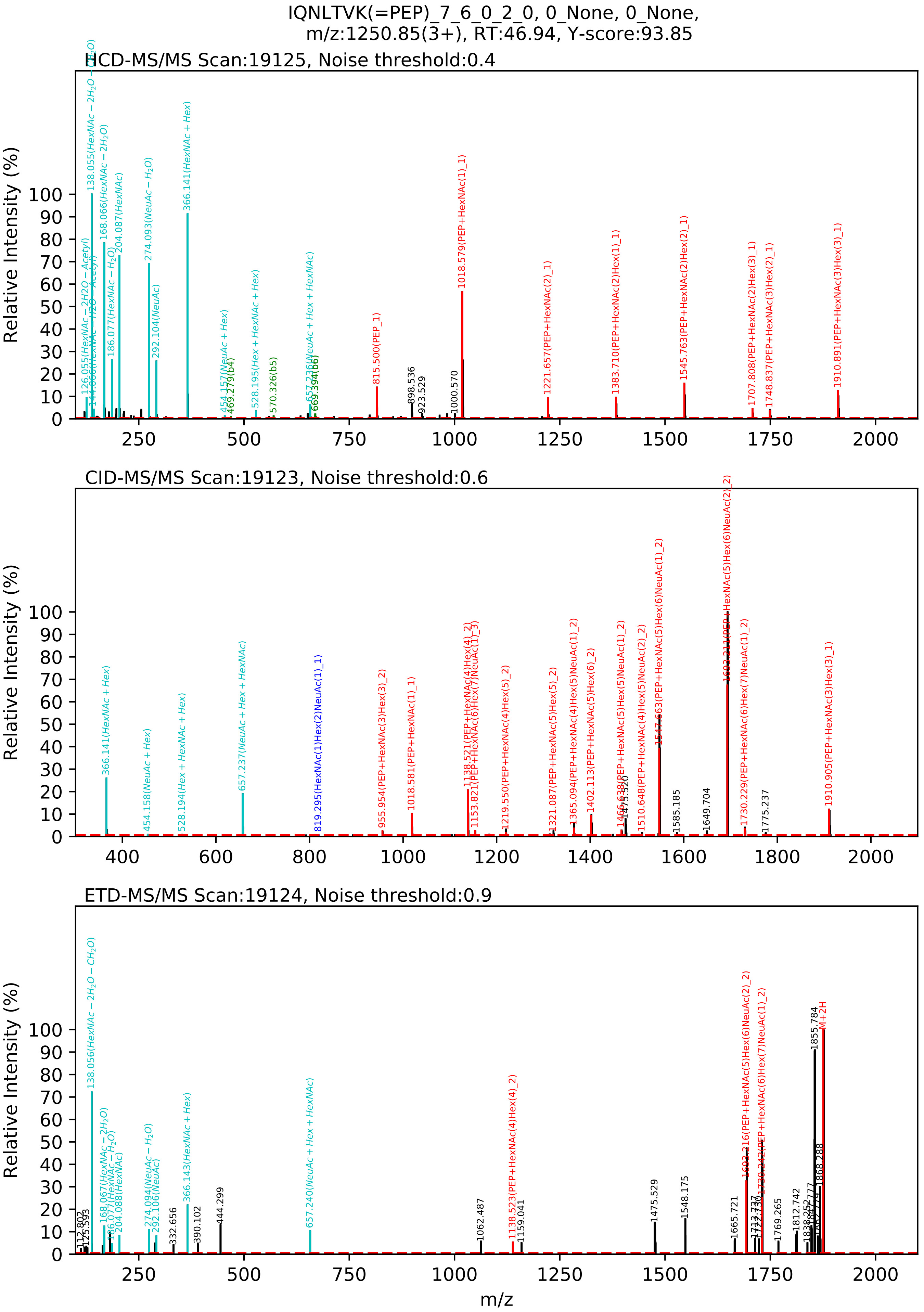

IQNLTVK(=PEP)_7_6_0_2_0, 0_None, 0_None,
m/z:1250.85(3+), RT:46.94, Y-score:93.85
HCD-MS/MS Scan:19125, Noise threshold:0.4
Relative Intensity (%)
126.055(HexNAc − 2H2O − Acetyl)
144.066(HexNAc − H2O − Acetyl)
138.055(HexNAc − 2H2O − CH2O)
168.066(HexNAc − 2H2O)
186.077(HexNAc − H2O)
204.087(HexNAc)
274.093(NeuAc − H2O)
292.104(NeuAc)
366.141(HexNAc + Hex)
454.157(NeuAc + Hex)
469.279(b4)
528.195(Hex + HexNAc + Hex)
570.326(b5)
657.236(NeuAc + Hex + HexNAc)
669.394(b6)
815.500(PEP_1)
898.536
923.529
1000.570
1018.579(PEP+HexNAc(1)_1)
1221.657(PEP+HexNAc(2)_1)
1383.710(PEP+HexNAc(2)Hex(1)_1)
1545.763(PEP+HexNAc(2)Hex(2)_1)
1707.808(PEP+HexNAc(2)Hex(3)_1)
1748.837(PEP+HexNAc(3)Hex(2)_1)
1910.891(PEP+HexNAc(3)Hex(3)_1)
CID-MS/MS Scan:19123, Noise threshold:0.6
Relative Intensity (%)
366.141(HexNAc + Hex)
454.158(NeuAc + Hex)
528.194(Hex + HexNAc + Hex)
657.237(NeuAc + Hex + HexNAc)
819.295(HexNAc(1)Hex(2)NeuAc(1)_1)
955.954(PEP+HexNAc(3)Hex(3)_2)
1018.581(PEP+HexNAc(1)_1)
1138.521(PEP+HexNAc(4)Hex(4)_2)
1153.821(PEP+HexNAc(6)Hex(7)NeuAc(1)_3)
1219.550(PEP+HexNAc(4)Hex(5)_2)
1321.087(PEP+HexNAc(5)Hex(5)_2)
1365.094(PEP+HexNAc(4)Hex(5)NeuAc(1)_2)
1402.113(PEP+HexNAc(5)Hex(6)_2)
1466.638(PEP+HexNAc(5)Hex(5)NeuAc(1)_2)
1475.520
1510.648(PEP+HexNAc(4)Hex(5)NeuAc(2)_2)
1547.663(PEP+HexNAc(5)Hex(6)NeuAc(1)_2)
1585.185
1649.704
1692.211(PEP+HexNAc(5)Hex(6)NeuAc(2)_2)
1730.229(PEP+HexNAc(6)Hex(7)NeuAc(1)_2)
1775.237
1910.905(PEP+HexNAc(3)Hex(3)_1)
ETD-MS/MS Scan:19124, Noise threshold:0.9
Relative Intensity (%)
112.802
125.593
138.056(HexNAc − 2H2O − CH2O)
168.067(HexNAc − 2H2O)
186.077(HexNAc − H2O)
204.088(HexNAc)
274.094(NeuAc − H2O)
292.106(NeuAc)
332.656
366.143(HexNAc + Hex)
390.102
444.299
657.240(NeuAc + Hex + HexNAc)
1062.487
1138.523(PEP+HexNAc(4)Hex(4)_2)
1159.041
1475.529
1548.175
1665.721
1692.216(PEP+HexNAc(5)Hex(6)NeuAc(2)_2)
1713.737
1722.730
1730.242(PEP+HexNAc(6)Hex(7)NeuAc(1)_2)
1769.265
1812.742
1838.252
1855.784
1868.288
M+2H
m/z

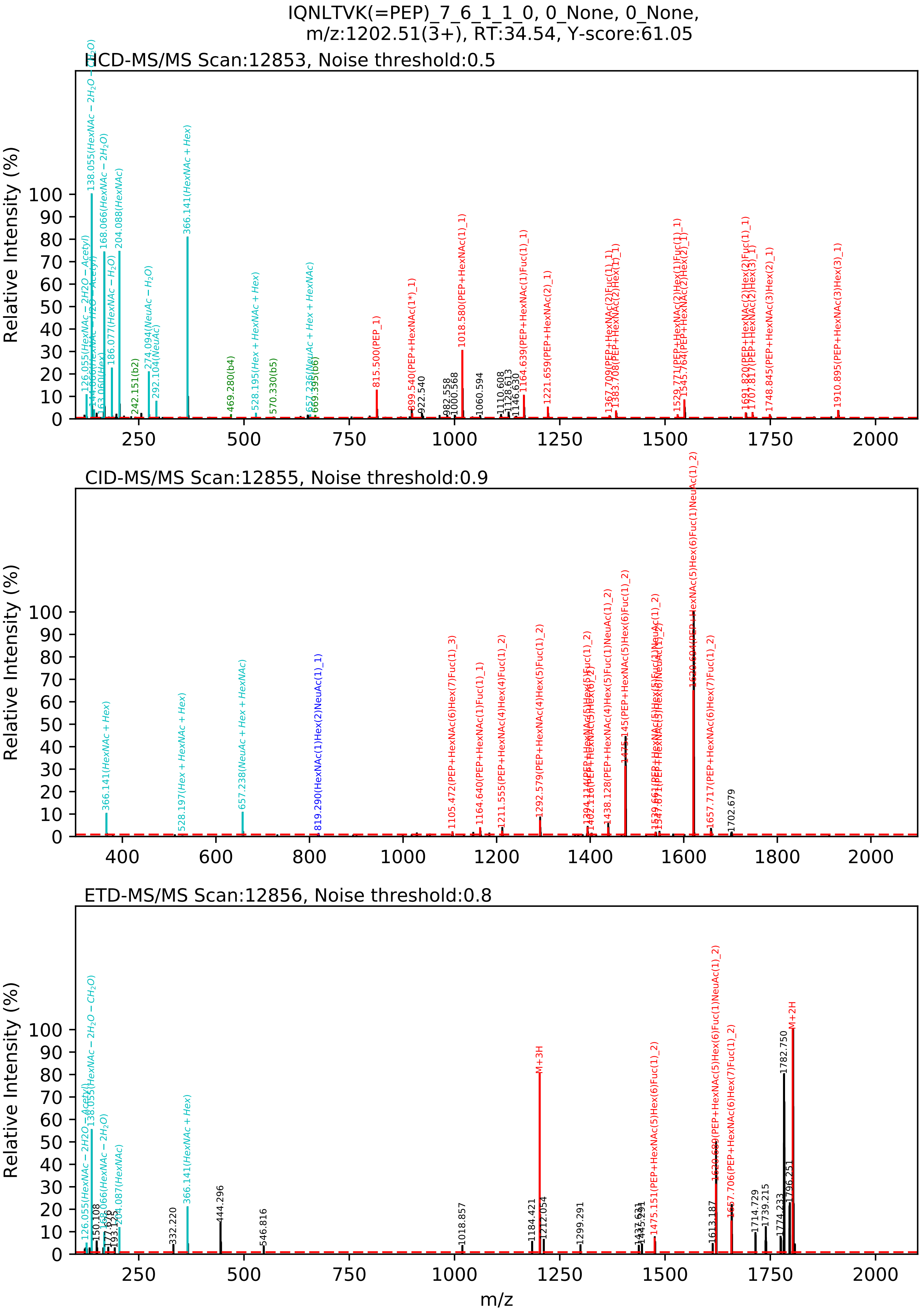

IQNLTVK(=PEP)_7_6_1_1_0, 0_None, 0_None,
m/z:1202.51(3+), RT:34.54, Y-score:61.05
HCD-MS/MS Scan:12853, Noise threshold:0.5
CID-MS/MS Scan:12855, Noise threshold:0.9
ETD-MS/MS Scan:12856, Noise threshold:0.8
Relative Intensity (%)
m/z

IQNLTVK(=PEP)_7_6_1_2_0, 0_None, 0_None,
m/z:1299.54(3+), RT:46.92, Y-score:62.92

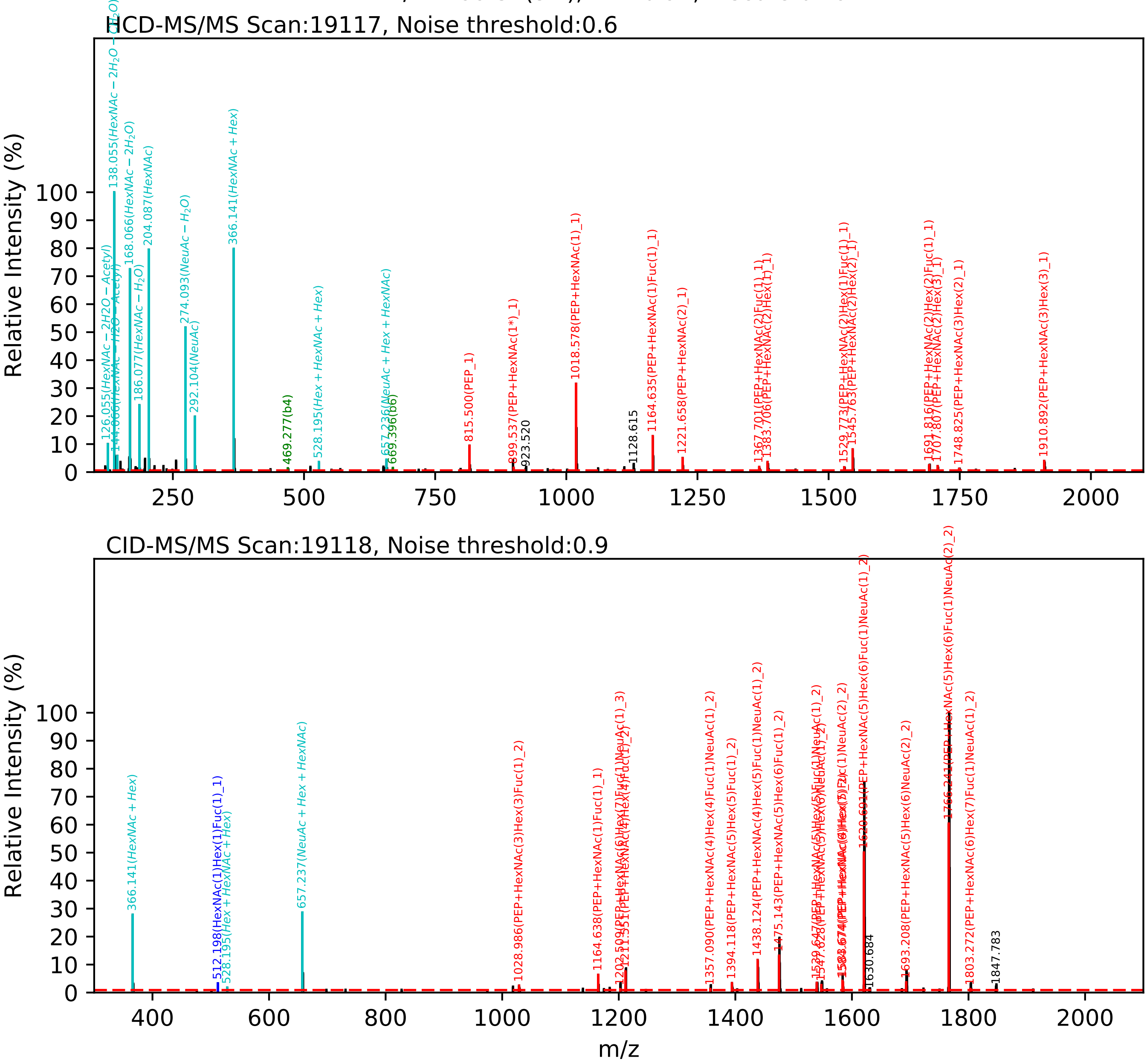

IQNLTVK(=PEP)_8_2_0_0_0, 0_None, 0_None,
m/z:1259.54(2+), RT:25.51, Y-score:95.22

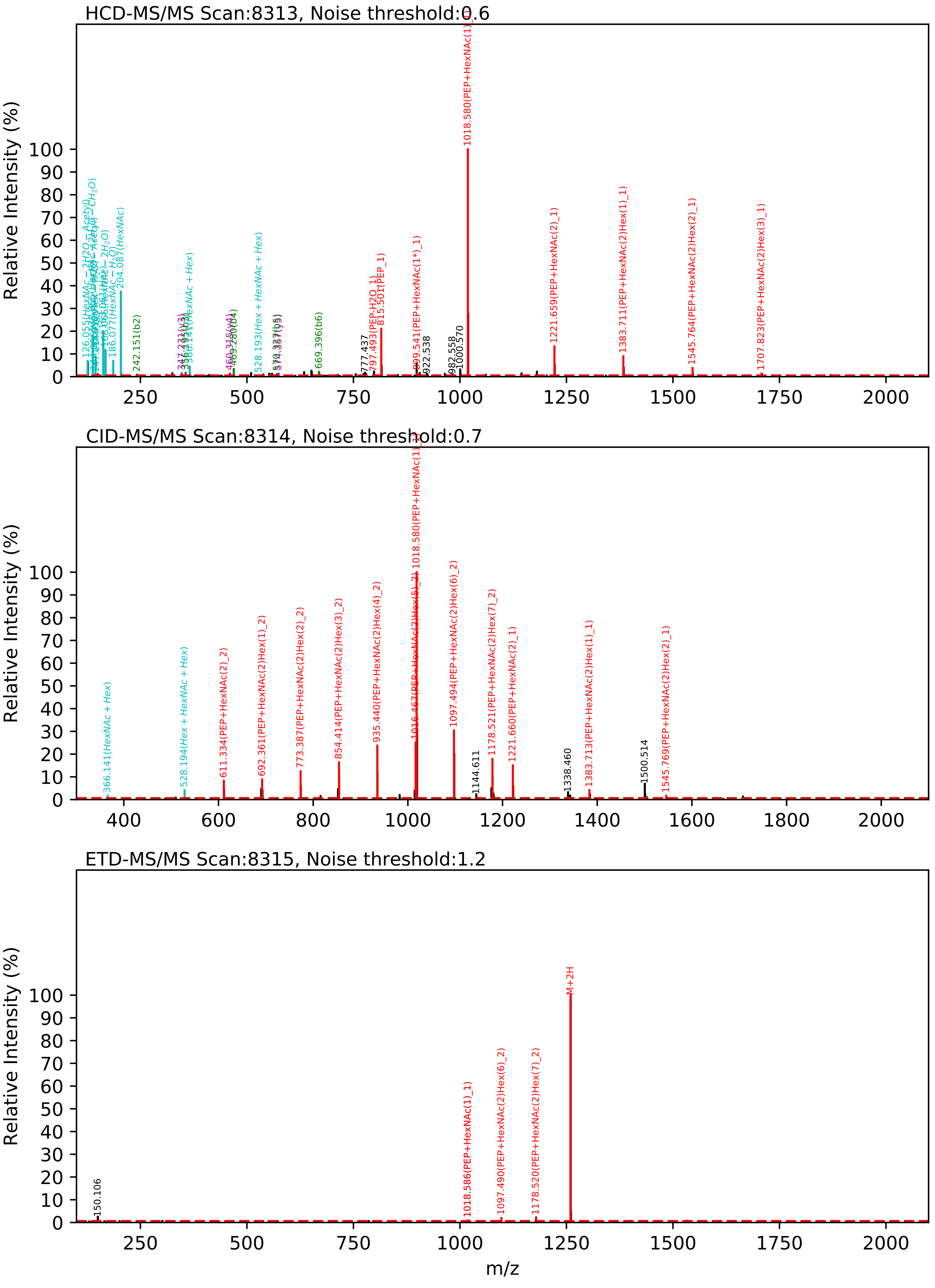

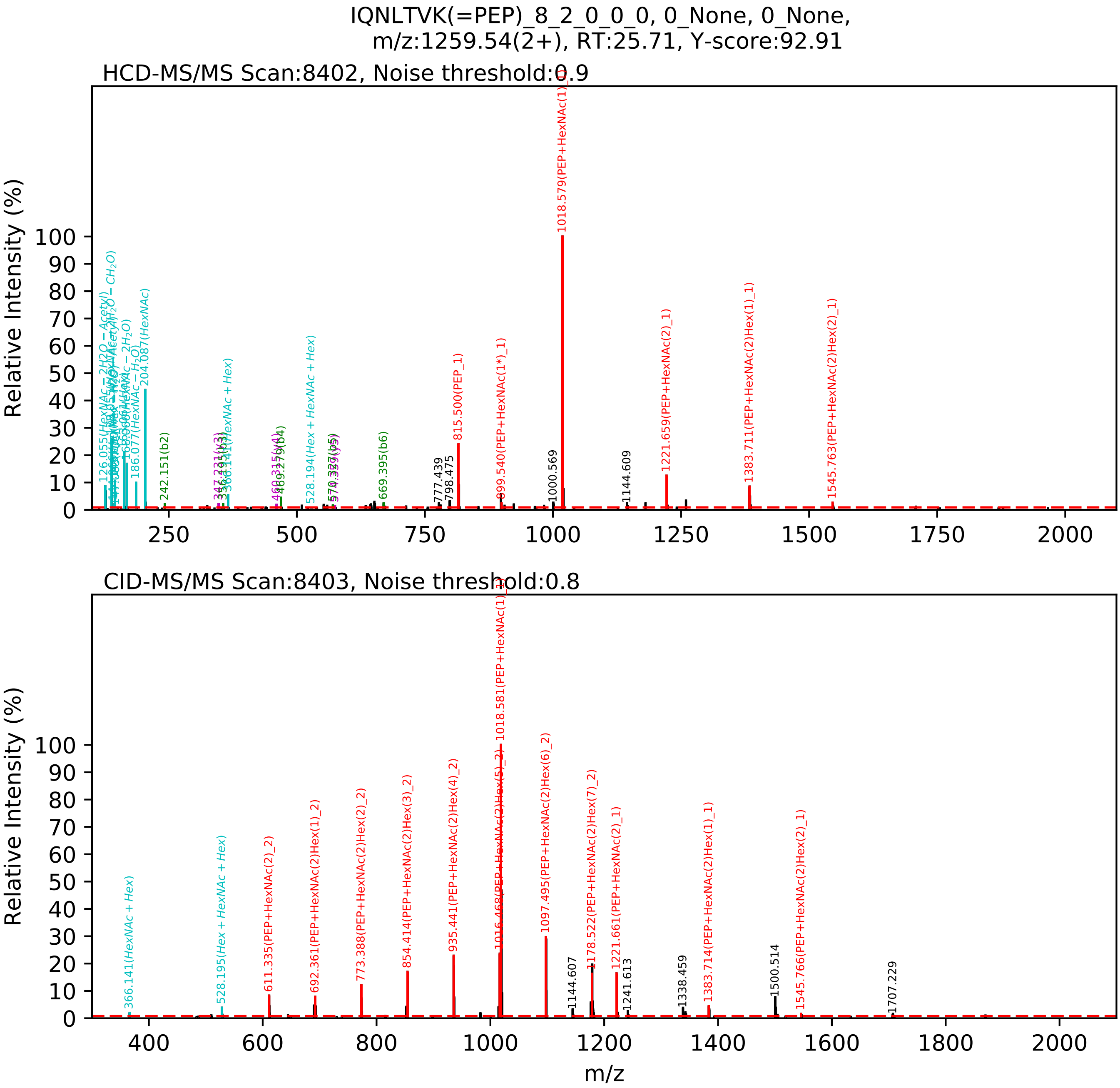

IQNLTVK(=PEP)_8_2_0_0_0, 0_None, 0_None,
m/z:1259.54(2+), RT:25.71, Y-score:92.91
HCD-MS/MS Scan:8402, Noise threshold:0.9
Relative Intensity (%)
1018.579(PEP+HexNAc(1)_1)
1221.659(PEP+HexNAc(2)_1)
1383.711(PEP+HexNAc(2)Hex(1)_1)
1545.763(PEP+HexNAc(2)Hex(2)_1)
815.500(PEP_1)
899.540(PEP+HexNAc(1*)_1)
1000.569
1144.609
777.439
798.475
669.395(b6)
528.194(Hex+HexNAc+Hex)
469.279(b4)
366.141(HexNAc+Hex)
242.151(b2)
204.087(HexNAc)
186.077(HexNAc−H2O)
126.055(HexNAc−2H2O−Acetyl)
CID-MS/MS Scan:8403, Noise threshold:0.8
1018.581(PEP+HexNAc(1)_1)
1097.495(PEP+HexNAc(2)Hex(6)_2)
1178.522(PEP+HexNAc(2)Hex(7)_2)
1221.661(PEP+HexNAc(2)_1)
1383.714(PEP+HexNAc(2)Hex(1)_1)
1545.766(PEP+HexNAc(2)Hex(2)_1)
935.441(PEP+HexNAc(2)Hex(4)_2)
854.414(PEP+HexNAc(2)Hex(3)_2)
773.388(PEP+HexNAc(2)Hex(2)_2)
692.361(PEP+HexNAc(2)Hex(1)_2)
611.335(PEP+HexNAc(2)_2)
528.195(Hex+HexNAc+Hex)
366.141(HexNAc+Hex)
1144.607
1241.613
1338.459
1500.514
1707.229
m/z

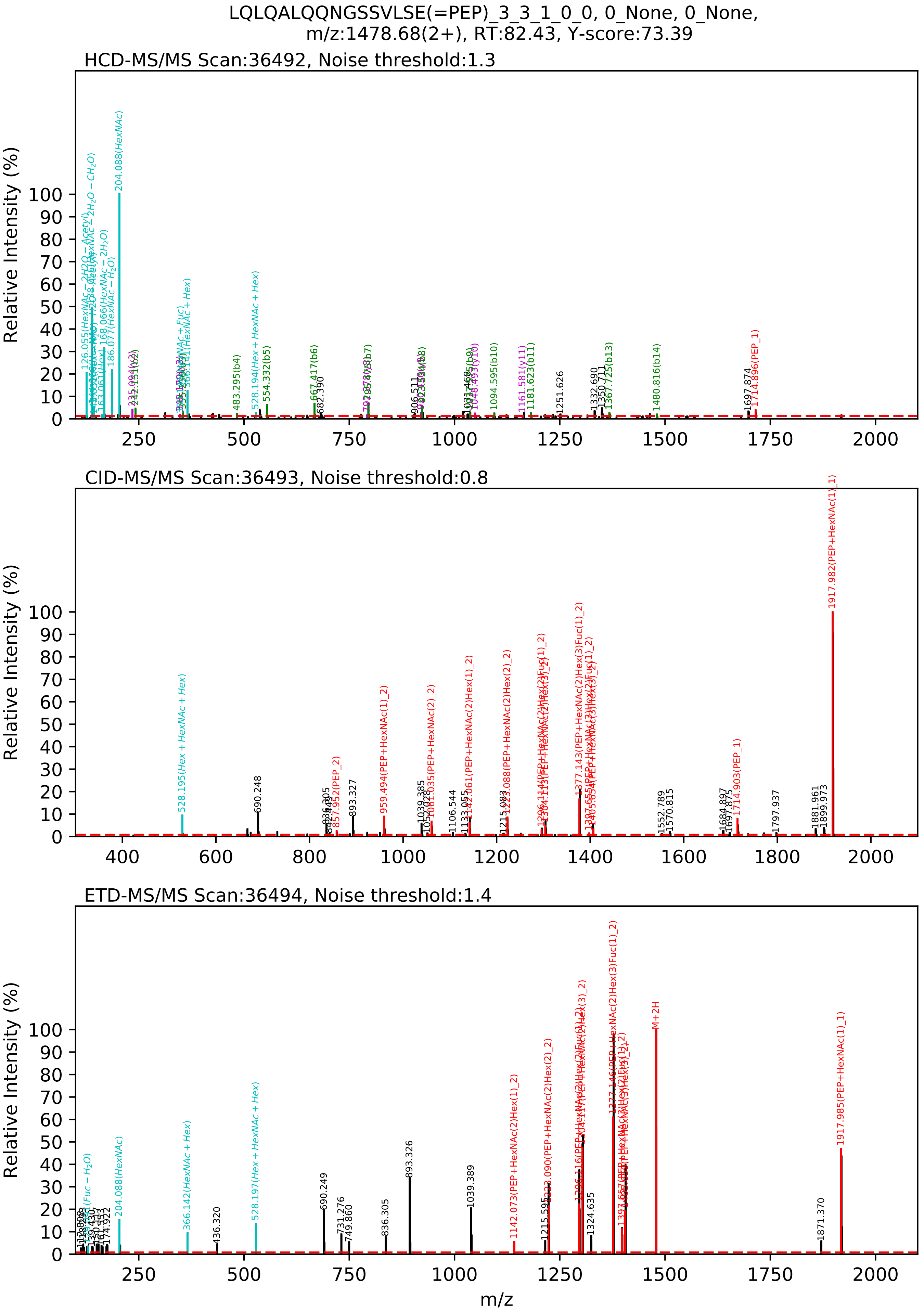

LQLQALQQNGSSVLSE(=PEP)_3_3_1_0_0, 0_None, 0_None,
m/z:1478.68(2+), RT:82.43, Y-score:73.39
HCD-MS/MS Scan:36492, Noise threshold:1.3
CID-MS/MS Scan:36493, Noise threshold:0.8
ETD-MS/MS Scan:36494, Noise threshold:1.4
Relative Intensity (%)
m/z

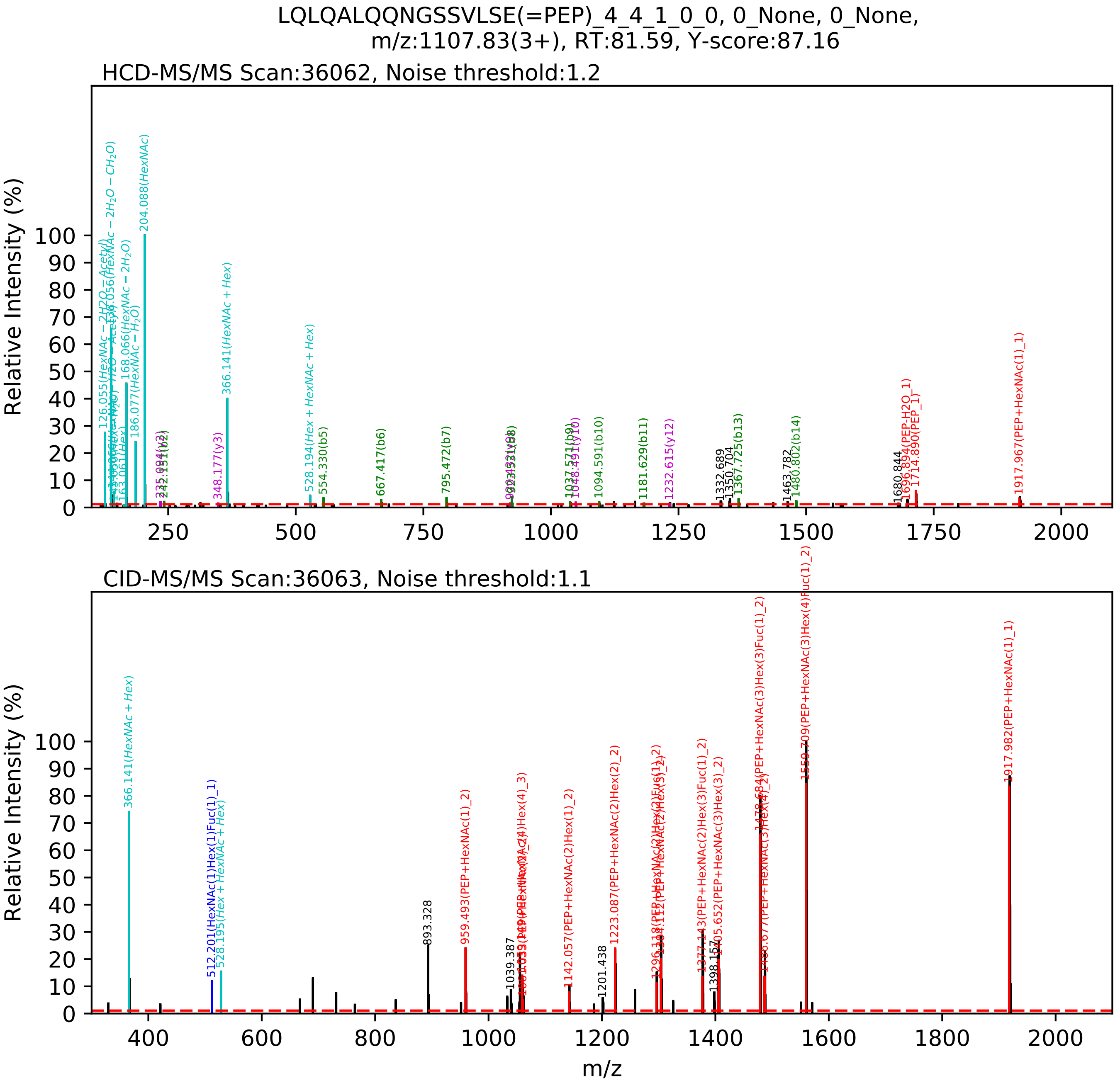
LQLQALQQNGSSVLSE(=PEP)_4_4_1_0_0, 0_None, 0_None,
m/z:1107.83(3+), RT:81.59, Y-score:87.16
HCD-MS/MS Scan:36062, Noise threshold:1.2
Relative Intensity (%)
204.088(HexNAc)
366.141(HexNAc + Hex)
186.077(HexNAc − H2O)
168.066(HexNAc − 2H2O)
126.055(HexNAc − 2H2O − Acetyl)
528.194(Hex + HexNAc + Hex)
554.330(b5)
667.417(b6)
795.472(b7)
348.177(y3)
242.151(b2)
1094.591(b10)
1181.629(b11)
1232.615(y12)
1332.689
1350.704
1367.725(b13)
1463.782
1480.802(b14)
1680.844
1696.894(PEP-H2O_1)
1714.890(PEP_1)
1917.967(PEP+HexNAc(1)_1)
CID-MS/MS Scan:36063, Noise threshold:1.1
Relative Intensity (%)
366.141(HexNAc + Hex)
512.201(HexNAc(1)Hex(1)Fuc(1)_1)
528.195(Hex + HexNAc + Hex)
893.328
959.493(PEP+HexNAc(1)_2)
1039.387
1142.057(PEP+HexNAc(2)Hex(1)_2)
1201.438
1223.087(PEP+HexNAc(2)Hex(2)_2)
1277.143(PEP+HexNAc(2)Hex(3)Fuc(1)_2)
1305.652(PEP+HexNAc(3)Hex(3)_2)
1398.157
1478.684(PEP+HexNAc(3)Hex(3)Fuc(1)_2)
1559.709(PEP+HexNAc(3)Hex(4)Fuc(1)_2)
1917.982(PEP+HexNAc(1)_1)
m/z

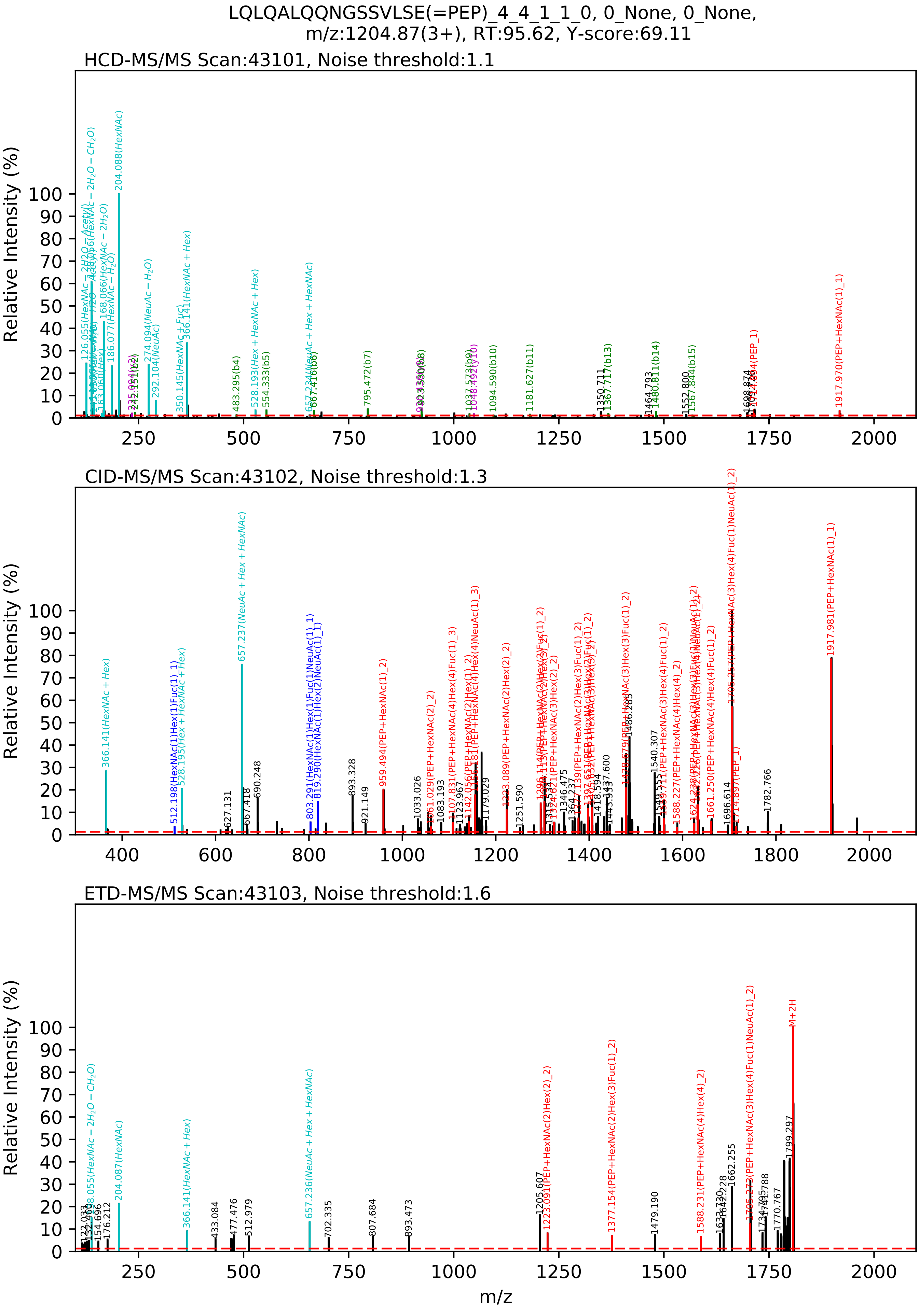
LQLQALQQNGSSVLSE(=PEP)_4_4_1_1_0, 0_None, 0_None,
m/z:1204.87(3+), RT:95.62, Y-score:69.11
HCD-MS/MS Scan:43101, Noise threshold:1.1
Relative Intensity (%)
204.088(HexNAc)
168.066(HexNAc − 2H2O)
186.077(HexNAc − H2O)
274.094(NeuAc − H2O)
292.104(NeuAc)
366.141(HexNAc + Hex)
350.145(HexNAc + Fuc)
483.295(b4)
528.193(Hex + HexNAc + Hex)
554.333(b5)
667.416(b6)
795.472(b7)
1094.590(b10)
1181.627(b11)
1350.711
1367.717(b13)
1464.793
1480.811(b14)
1552.800
1567.844(b15)
1917.970(PEP+HexNAc(1)_1)
CID-MS/MS Scan:43102, Noise threshold:1.3
366.141(HexNAc + Hex)
512.198(HexNAc(1)Hex(1)Fuc(1)_1)
657.237(NeuAc + Hex + HexNAc)
627.131
667.418
690.248
803.291(HexNAc(1)Hex(1)Fuc(1)NeuAc(1)_1)
819.290(HexNAc(1)Hex(2)NeuAc(1)_1)
893.328
921.149
959.494(PEP+HexNAc(1)_2)
1033.026
1061.029(PEP+HexNAc(2)_2)
1083.193
1123.967
1179.029
1223.089(PEP+HexNAc(2)Hex(2)_2)
1251.590
1346.475
1364.237
1418.594
1486.285
1540.307
1588.227(PEP+HexNAc(4)Hex(4)_2)
1661.250(PEP+HexNAc(4)Fuc(1)_2)
1696.614
1705.257(PEP+HexNAc(3)Hex(4)Fuc(1)NeuAc(1)_2)
1714.897(PEP_1)
1782.766
1917.981(PEP+HexNAc(1)_1)
ETD-MS/MS Scan:43103, Noise threshold:1.6
204.087(HexNAc)
366.141(HexNAc + Hex)
433.084
477.476
512.979
657.236(NeuAc + Hex + HexNAc)
702.335
807.684
893.473
1205.607
1223.091(PEP+HexNAc(2)Hex(2)_2)
1377.154(PEP+HexNAc(2)Hex(3)Fuc(1)_2)
1479.190
1588.231(PEP+HexNAc(4)Hex(4)_2)
1662.255
1705.273(PEP+HexNAc(3)Hex(4)Fuc(1)NeuAc(1)_2)
1770.767
1799.297
M+2H
m/z

LQLQALQQNGSSVLSE(=PEP)_4_5_1_0_0, 0_None, 0_None,
m/z:1175.52(3+), RT:81.20, Y-score:79.48

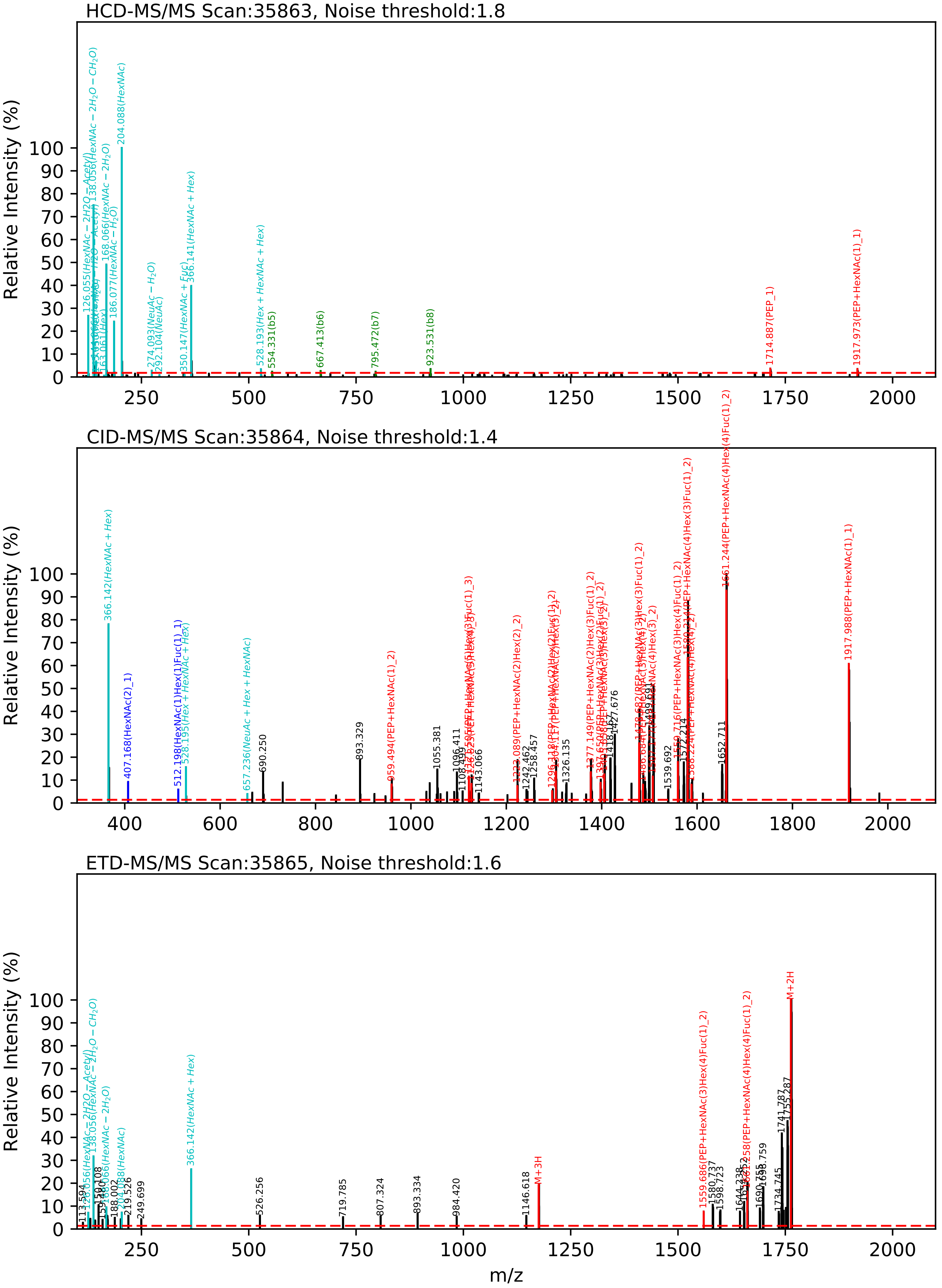

Supplement: Supplementary file 1 [file ijms-25-13649-s001.zip › Supplementary Figure S13(ACE2_TG_N-glycopep_1).pdf]
