# Supplementary material for: Characterization of Site-Specific N- and O-Glycopeptides from Recombinant Spike and ACE2 Glycoproteins Using LC-MS/MS Analysis
Source: Int J Mol Sci. 2024 Dec 20;25(24):13649. doi: 10.3390/ijms252413649 (PMC11678118; doi:10.3390/ijms252413649)
Supplement: Supplementary file 1 [file ijms-25-13649-s001.zip › Supplementary Figure S14(ACE2_TG_N-glycopep_2).pdf]

CDISNSTE(=PEP)_6_3_1_0_0, 0_None, 0_None,
m/z:1326.99(2+), RT:22.05, hcd-score:72.83

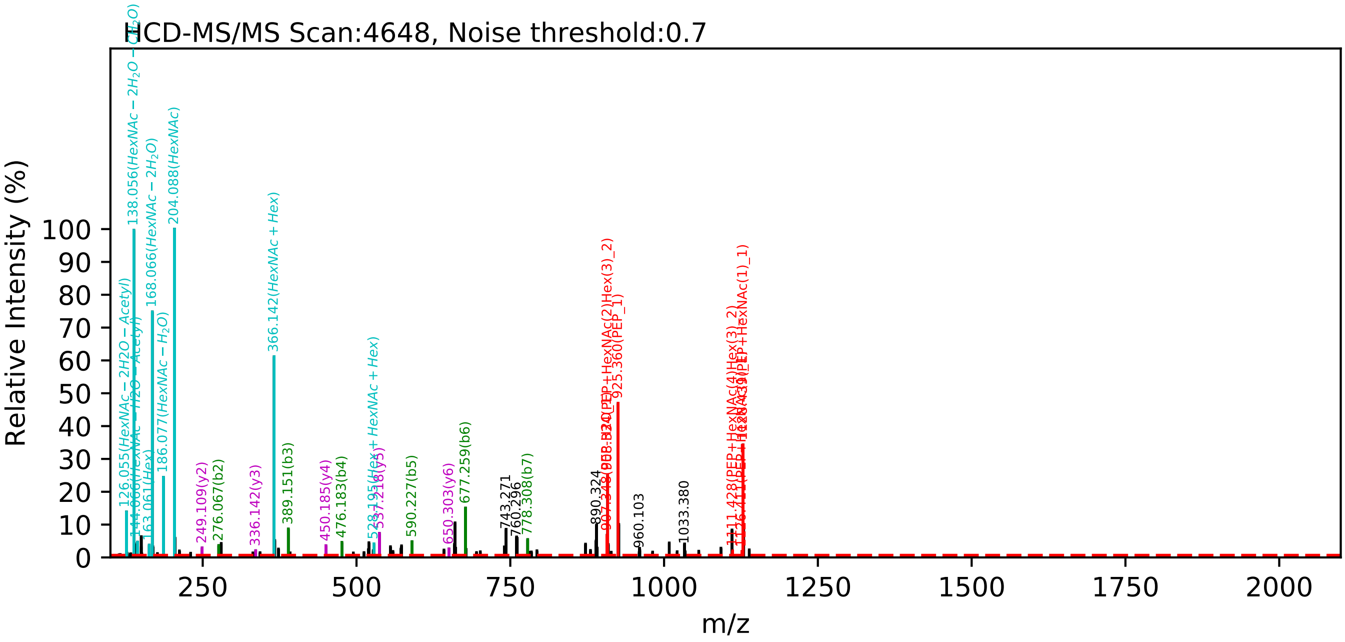

CDISNSTE(=PEP)_6_3_1_0_0, 0_None, 0_None,
m/z:1326.99(2+), RT:22.05, hcd-score:72.83

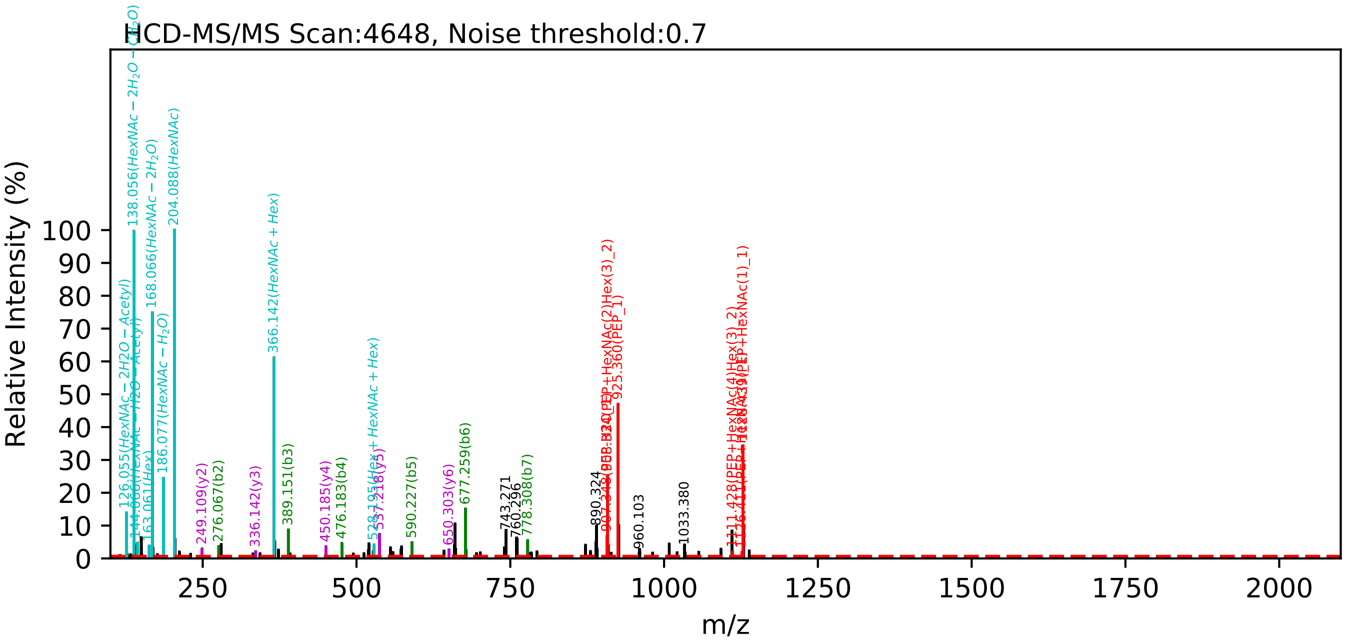

IQNLTVK(=PEP)_6_3_1_0_0, 0_None, 0_None,
m/z:1272.06(4+), RT:25.59, hcd-score:88.78

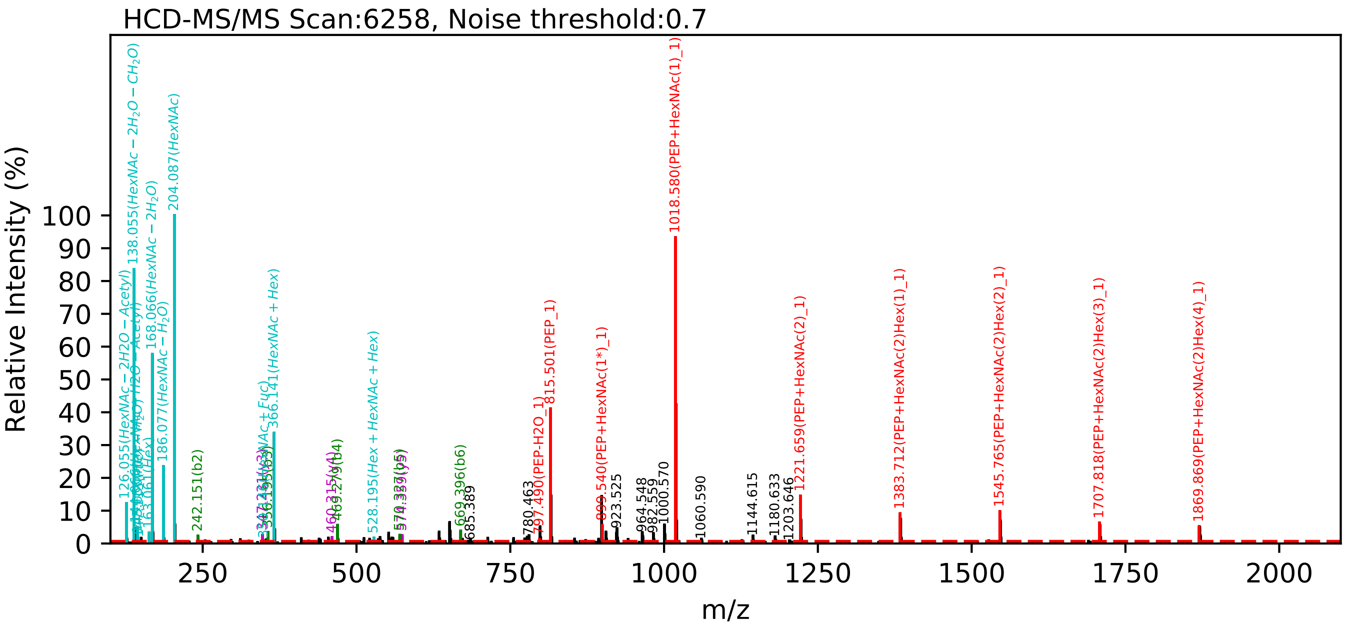

IQNLTVK(=PEP)_6_3_1_0_0, 0_None, 0_None,
m/z:1272.06(4+), RT:25.59, hcd-score:88.78

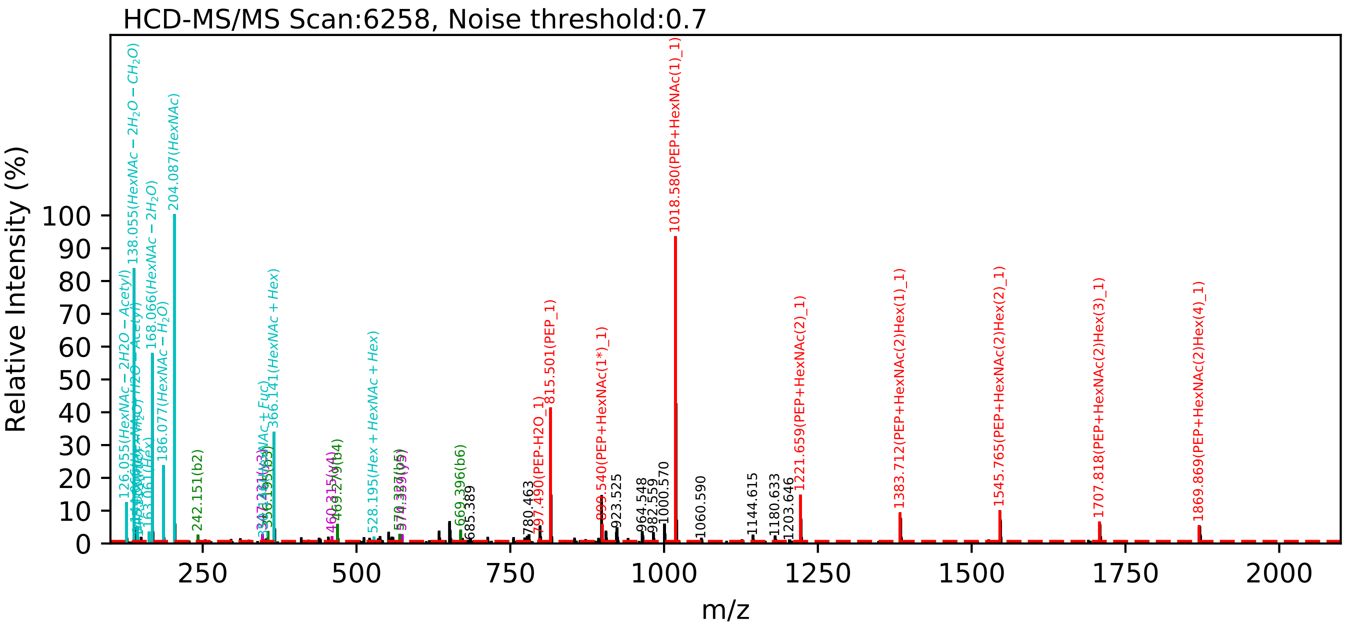

IQNLTVK(=PEP)_6_3_1_0_0, 0_None, 0_None,
m/z:1272.06(4+), RT:25.66, hcd-score:84.91

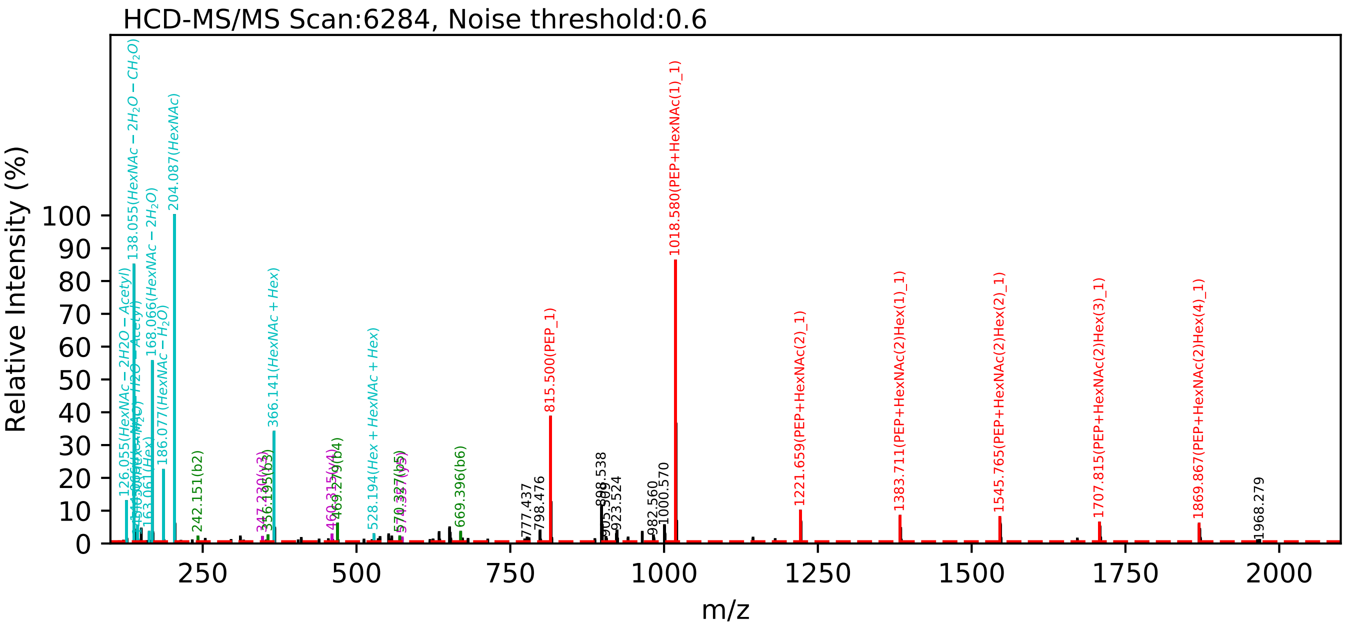

IQNLTVK(=PEP)_6_3_1_0_0, 0_None, 0_None,
m/z:1272.06(4+), RT:25.66, hcd-score:84.91

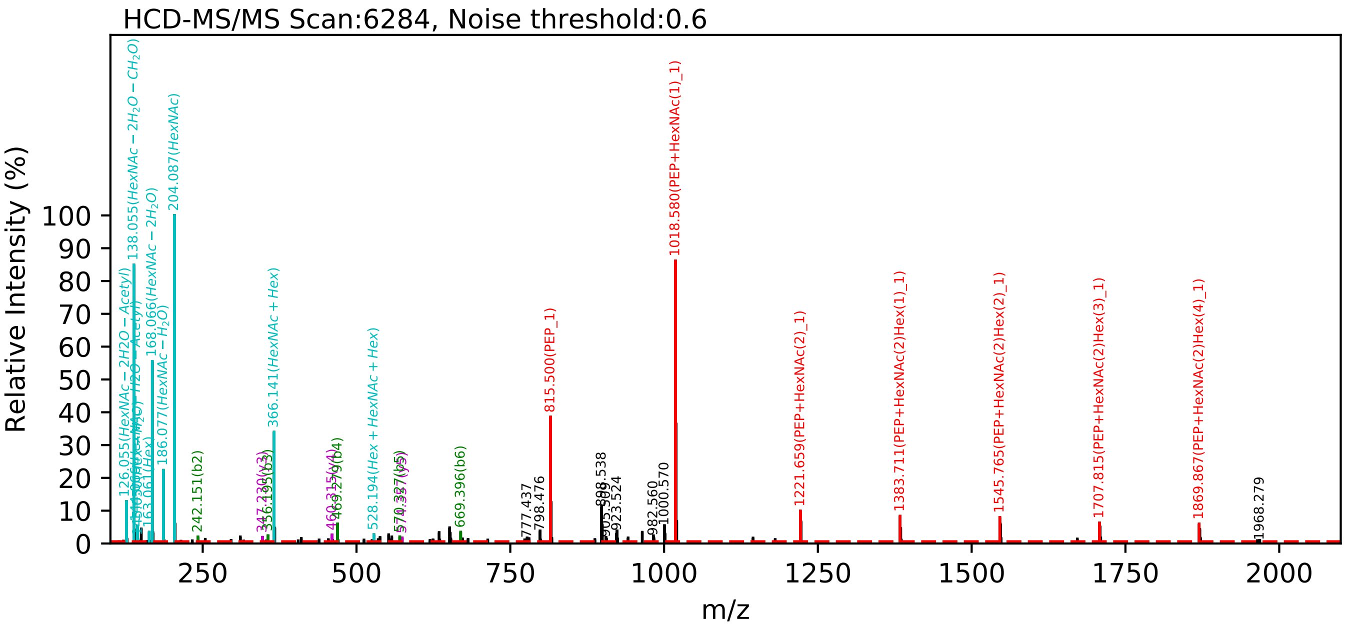

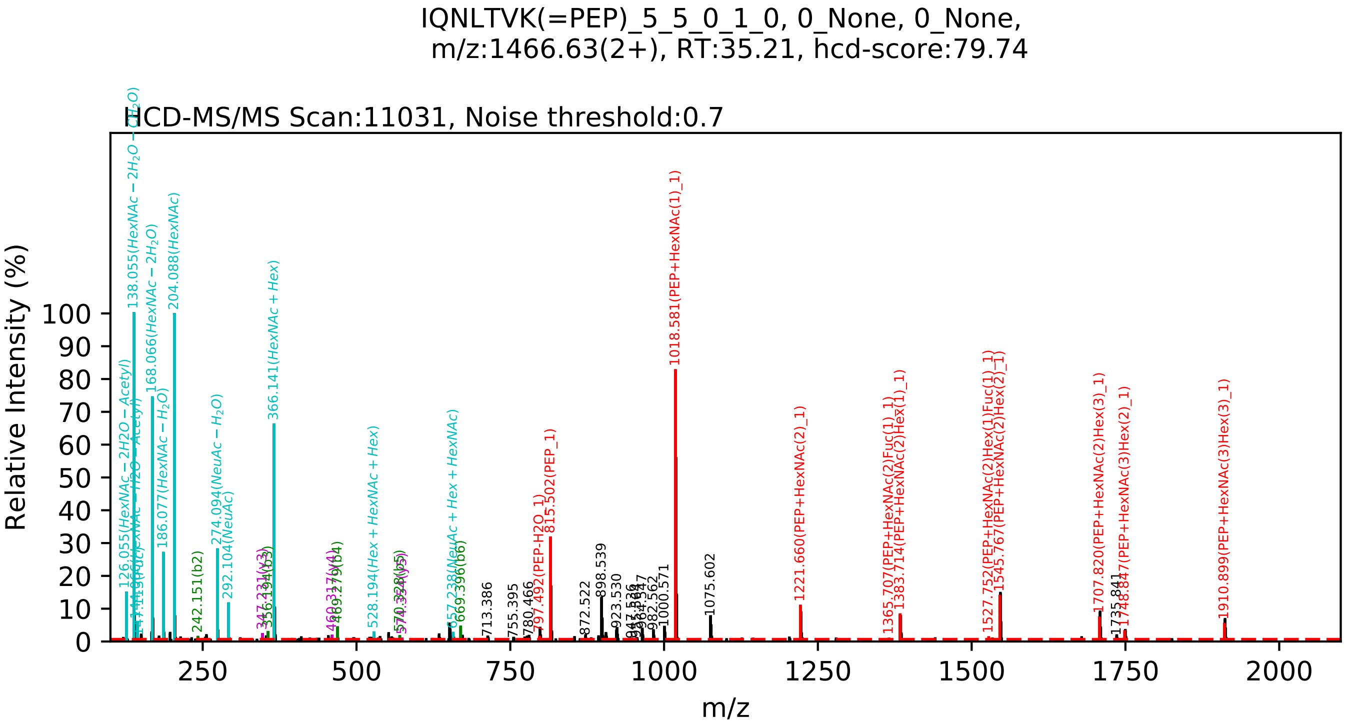
IQNLTVK(=PEP)_5_5_0_1_0, 0_None, 0_None,
m/z:1466.63(2+), RT:35.21, hcd-score:79.74
HCD-MS/MS Scan:11031, Noise threshold:0.7
Relative Intensity (%)
m/z
138.055(HexNAc − 2H2O − H2O)
204.088(HexNAc)
168.066(HexNAc − 2H2O)
186.077(HexNAc − H2O)
366.141(HexNAc + Hex)
274.094(NeuAc − H2O)
292.104(NeuAc)
126.055(HexNAc − 2H2O − Acetyl)
242.151(b2)
528.194(Hex + HexNAc + Hex)
657.238(NeuAc + Hex + HexNAc)
669.396(b6)
713.386
755.395
780.466
797.492(PEP-H2O_1)
815.502(PEP_1)
872.522
898.539
923.530
1000.571
1018.581(PEP+HexNAc(1)_1)
1075.602
1221.660(PEP+HexNAc(2)_1)
1365.707(PEP+HexNAc(2)Fuc(1)_1)
1383.714(PEP+HexNAc(2)Hex(1)_1)
1527.752(PEP+HexNAc(2)Hex(1)Fuc(1)_1)
1545.767(PEP+HexNAc(2)Hex(2)_1)
1707.820(PEP+HexNAc(2)Hex(3)_1)
1735.841
1748.847(PEP+HexNAc(3)Hex(2)_1)
1910.899(PEP+HexNAc(3)Hex(3)_1)

IQNLTVK(=PEP)_5_5_0_1_0, 0_None, 0_None,
m/z:1466.63(2+), RT:35.21, hcd-score:79.74

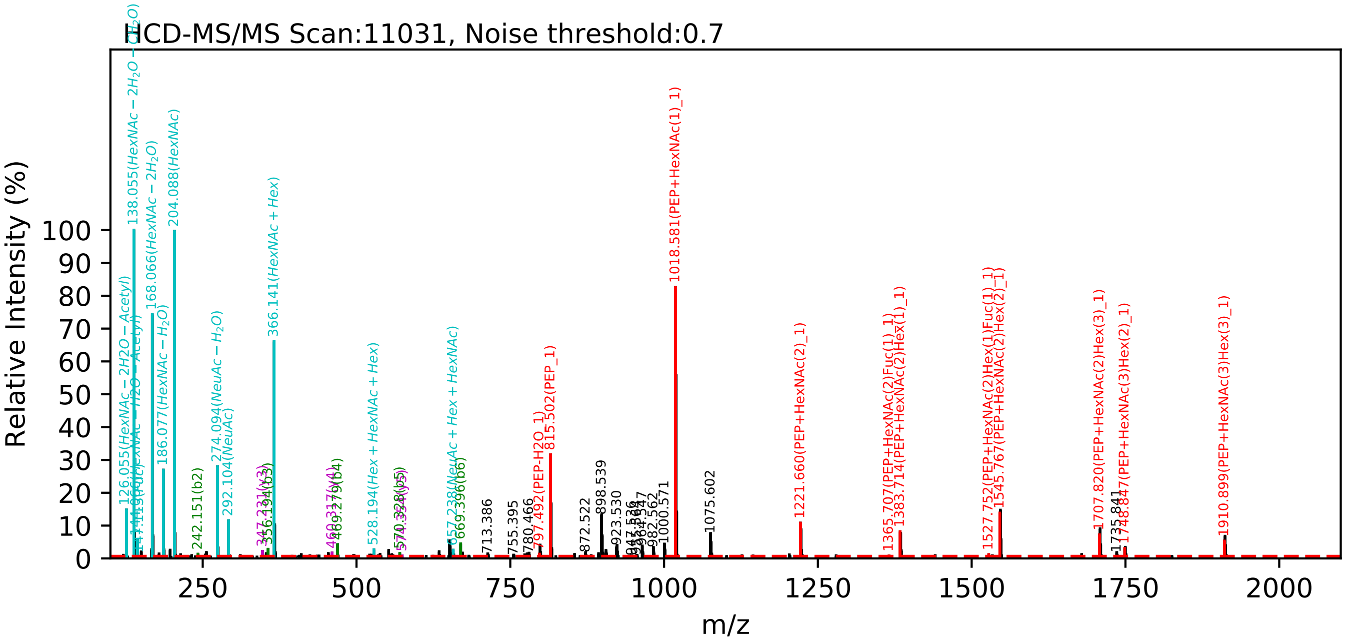

IQNLTVK(=PEP)_5_4_1_1_0, 0_None, 0_None,
m/z:959.08(2+), RT:35.79, hcd-score:96.32

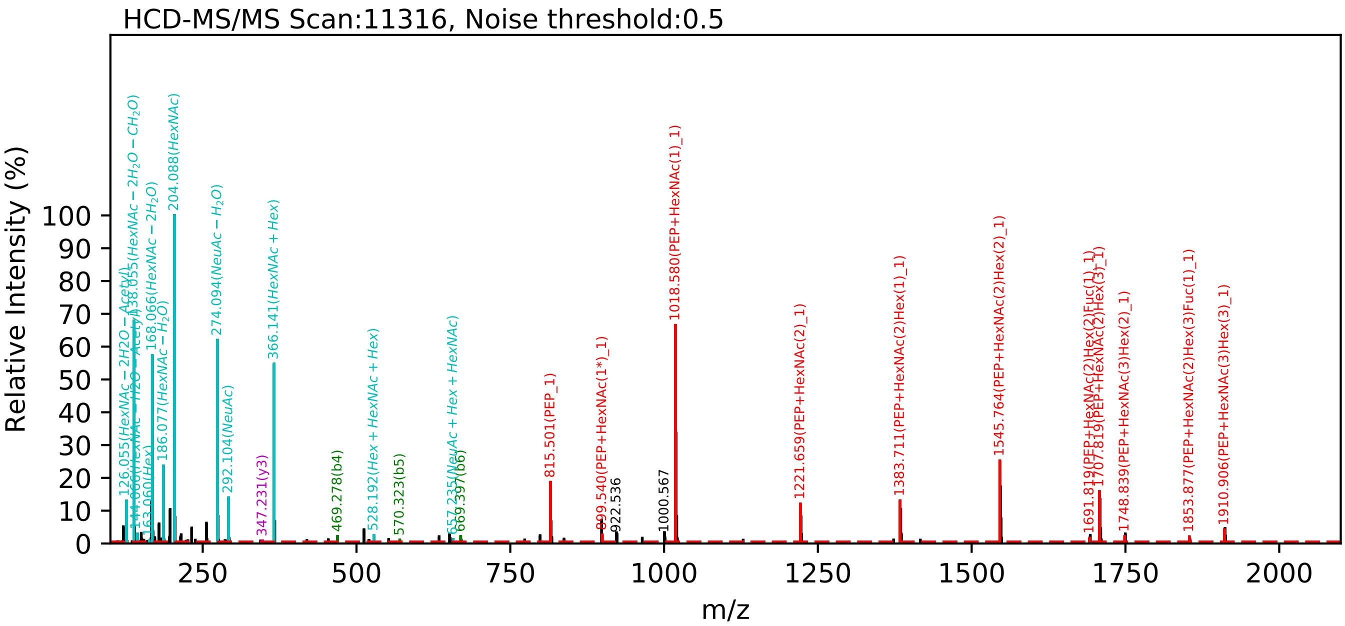

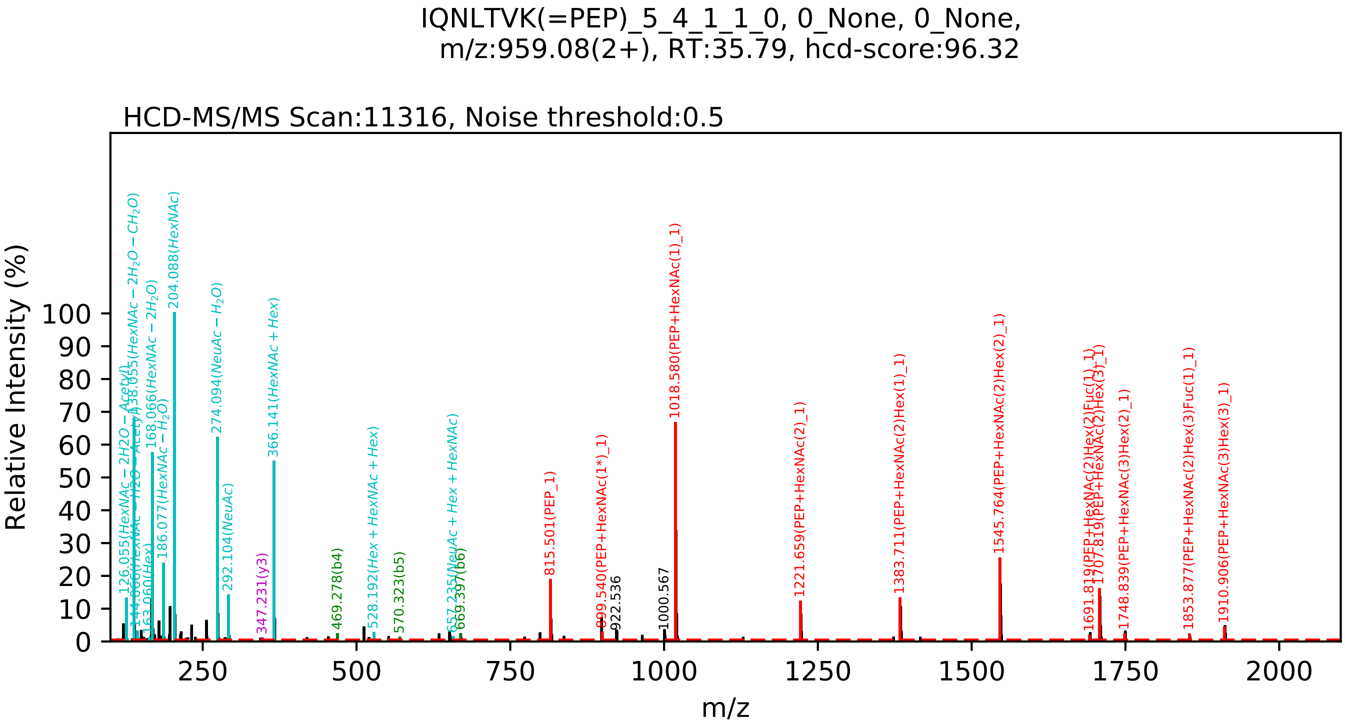
IQNLTVK(=PEP)_5_4_1_1_0, 0_None, 0_None,
m/z:959.08(2+), RT:35.79, hcd-score:96.32
HCD-MS/MS Scan:11316, Noise threshold:0.5
Relative Intensity (%)
m/z
126.055(HexNAc − 2H2O − Acetyl)
138.055(HexNAc − 2H2O − CH2O)
144.066(HexNAc − H2O − Acetyl)
163.060(Hex)
168.066(HexNAc − 2H2O)
186.077(HexNAc − H2O)
204.088(HexNAc)
274.094(NeuAc − H2O)
292.104(NeuAc)
347.231(y3)
366.141(HexNAc + Hex)
469.278(b4)
528.192(Hex + HexNAc + Hex)
570.323(b5)
657.235(NeuAc + Hex + HexNAc)
669.397(b6)
815.501(PEP_1)
899.540(PEP+HexNAc(1*)_1)
922.536
1000.567
1018.580(PEP+HexNAc(1)_1)
1221.659(PEP+HexNAc(2)_1)
1383.711(PEP+HexNAc(2)Hex(1)_1)
1545.764(PEP+HexNAc(2)Hex(2)_1)
1691.819(PEP+HexNAc(2)Hex(2)Fuc(1)_1)
1707.819(PEP+HexNAc(2)Hex(3)_1)
1748.839(PEP+HexNAc(3)Hex(2)_1)
1853.877(PEP+HexNAc(2)Hex(3)Fuc(1)_1)
1910.906(PEP+HexNAc(3)Hex(3)_1)

IQNLTVK(=PEP)_5_4_1_1_0, 0_None, 0_None,
m/z:1438.12(2+), RT:36.02, hcd-score:80.79

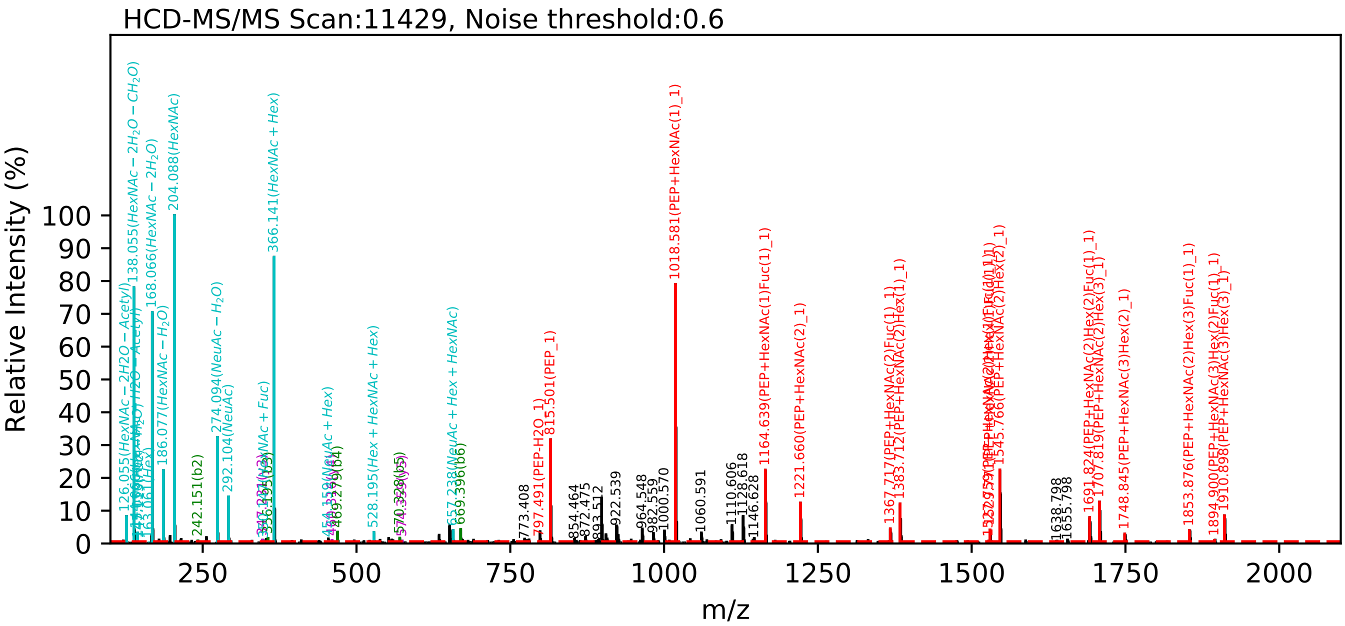

IQNLTVK(=PEP)_5_4_1_1_0, 0_None, 0_None,
m/z:1438.12(2+), RT:36.02, hcd-score:80.79

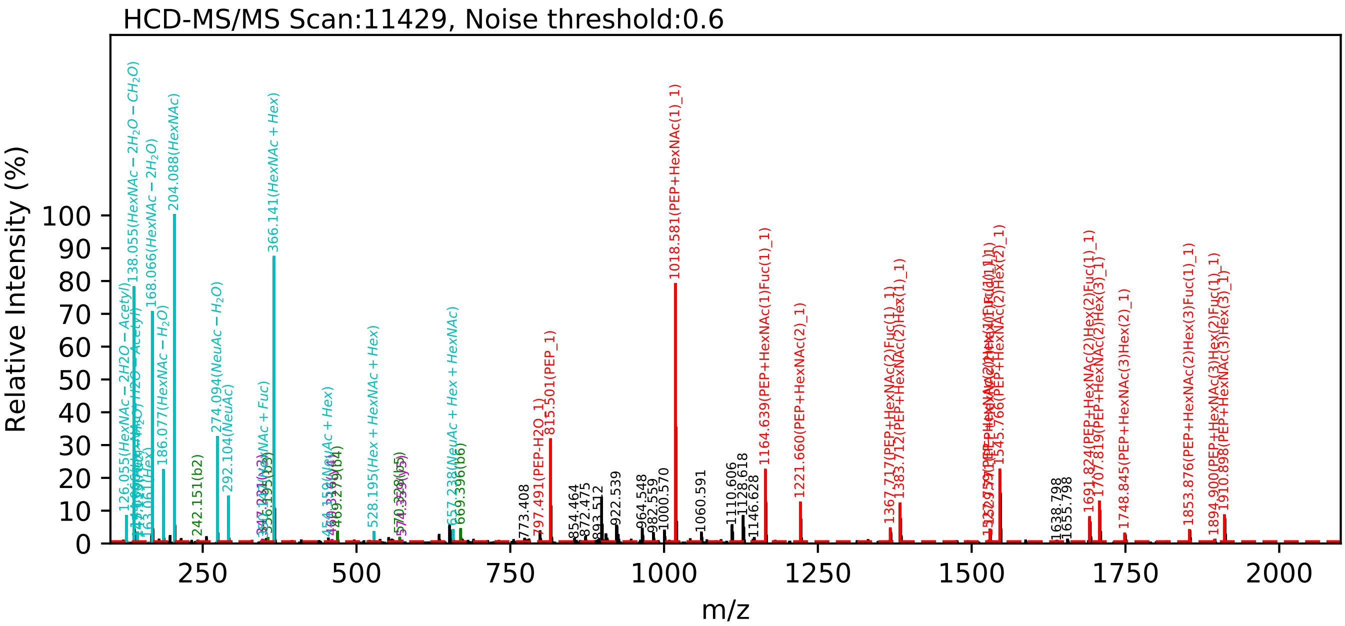

IQNLTVK(=PEP)_4_3_0_1_0, 0_None, 0_None,
m/z:1182.52(3+), RT:50.57, hcd-score:82.68

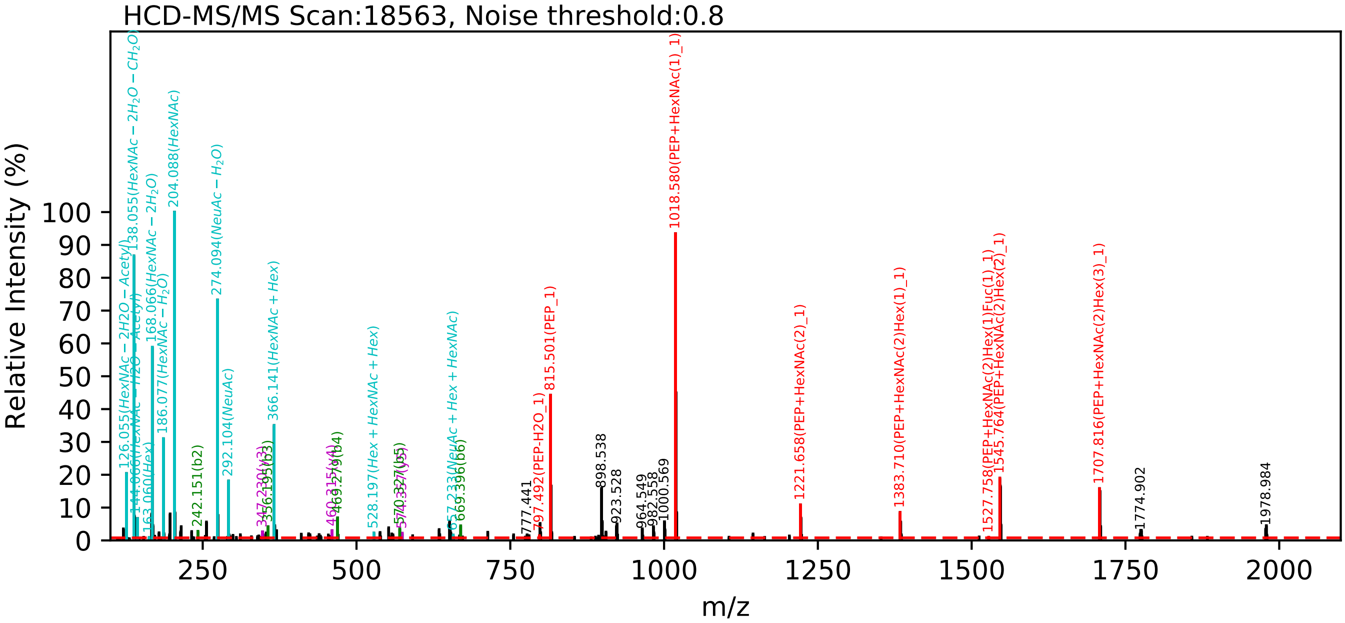

IQNLTVK(=PEP)_4_3_0_1_0, 0_None, 0_None,
m/z:1182.52(3+), RT:50.57, hcd-score:82.68

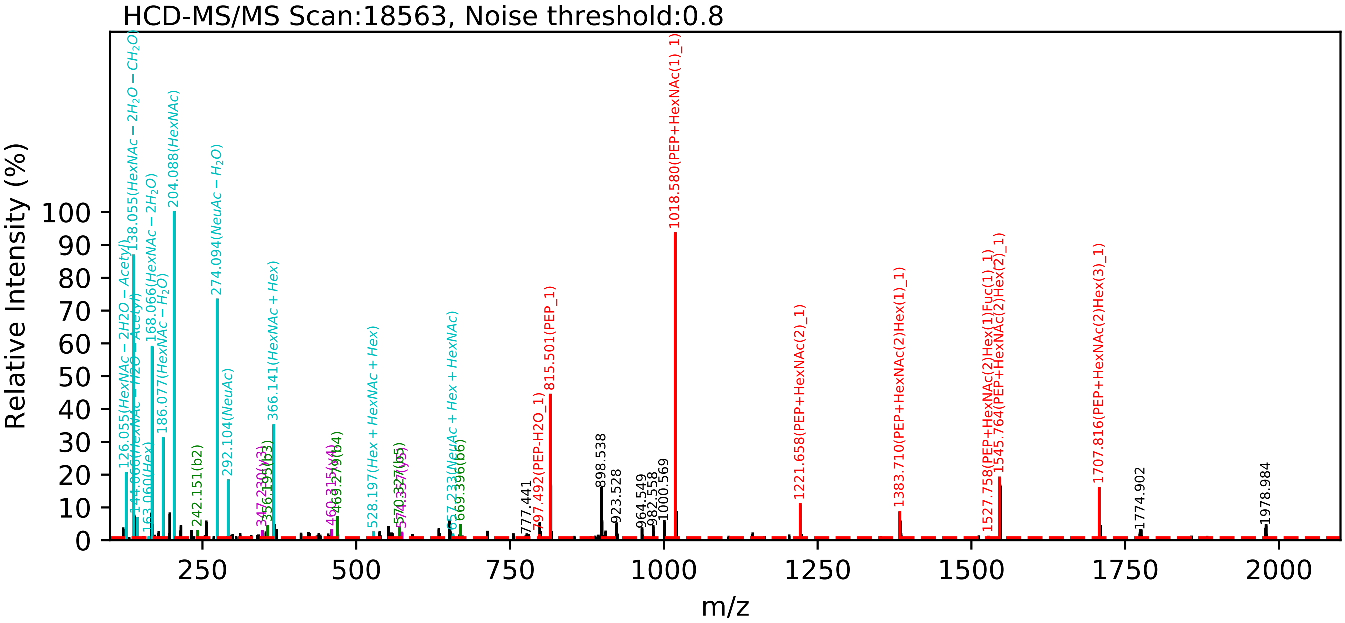

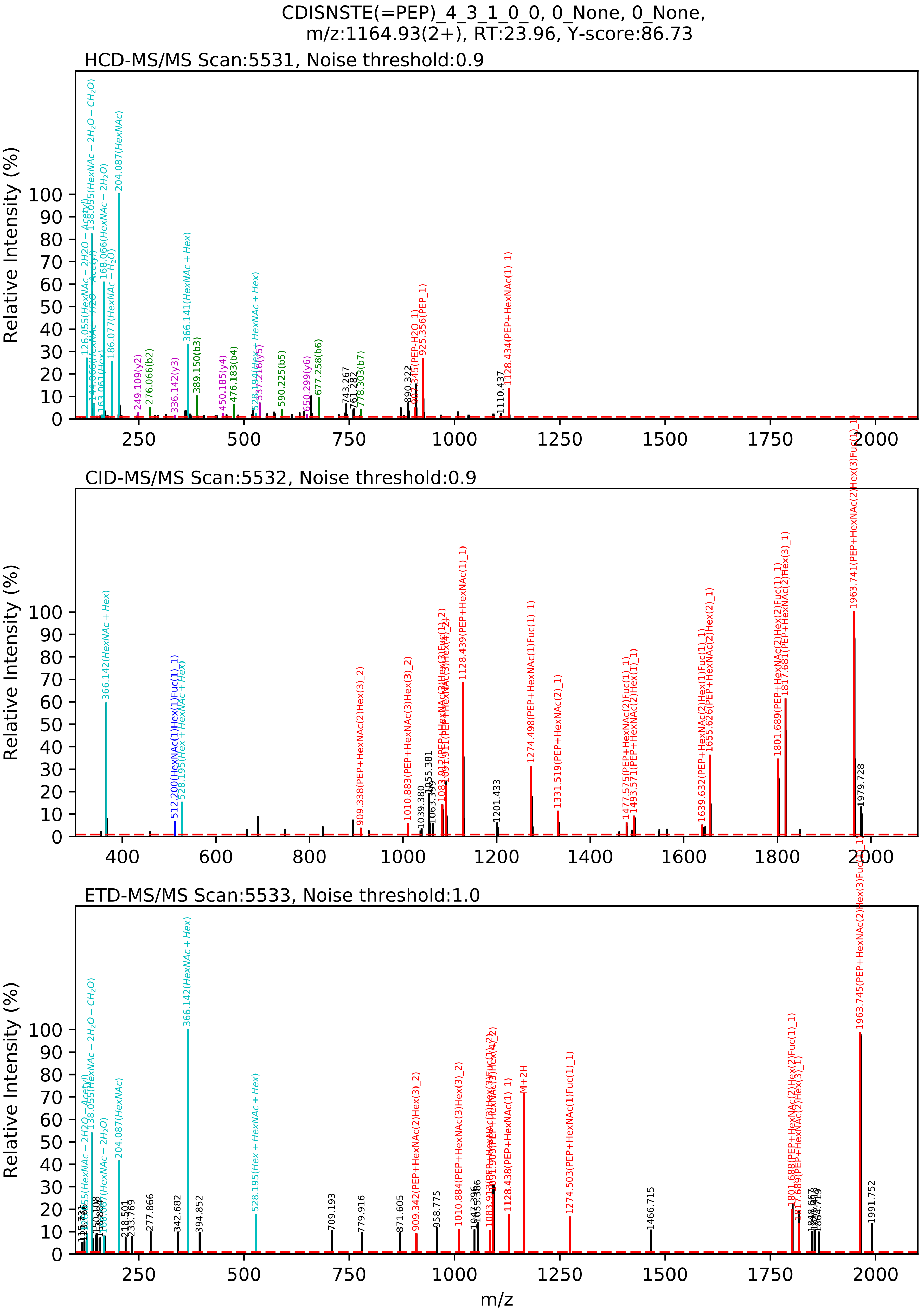

CDISNSTE(=PEP)_4_3_1_0_0, 0_None, 0_None,
m/z:1164.93(2+), RT:23.96, Y-score:86.73
HCD-MS/MS Scan:5531, Noise threshold:0.9
Relative Intensity (%)
126.055(HexNAc − 2H2O − Acetyl)
138.055(HexNAc − 2H2O − CH2O)
168.066(HexNAc − 2H2O)
186.077(HexNAc − H2O)
204.087(HexNAc)
366.141(HexNAc + Hex)
249.109(y2)
276.066(b2)
336.142(y3)
389.150(b3)
450.185(y4)
476.183(b4)
537.216(y5)
590.225(b5)
650.299(y6)
677.258(b6)
743.267
778.303(b7)
890.322
925.356(PEP_1)
1110.437
1128.434(PEP+HexNAc(1)_1)
CID-MS/MS Scan:5532, Noise threshold:0.9
366.142(HexNAc + Hex)
512.200(HexNAc(1)Hex(1)Fuc(1)_1)
528.195(Hex + HexNAc + Hex)
909.338(PEP+HexNAc(2)Hex(3)_2)
1010.883(PEP+HexNAc(3)Hex(3)_2)
1039.380
1055.381
1128.439(PEP+HexNAc(1)_1)
1201.433
1274.498(PEP+HexNAc(1)Fuc(1)_1)
1331.519(PEP+HexNAc(2)_1)
1477.575(PEP+HexNAc(2)Fuc(1)_1)
1493.571(PEP+HexNAc(2)Hex(1)_1)
1639.632(PEP+HexNAc(2)Hex(1)Fuc(1)_1)
1655.626(PEP+HexNAc(2)Hex(2)_1)
1801.689(PEP+HexNAc(2)Hex(2)Fuc(1)_1)
1817.681(PEP+HexNAc(2)Hex(3)_1)
1963.741(PEP+HexNAc(2)Hex(3)Fuc(1)_1)
1979.728
ETD-MS/MS Scan:5533, Noise threshold:1.0
138.055(HexNAc − 2H2O − CH2O)
204.087(HexNAc)
218.501
233.769
277.866
342.682
366.142(HexNAc + Hex)
394.852
528.195(Hex + HexNAc + Hex)
709.193
779.916
871.605
909.342(PEP+HexNAc(2)Hex(3)_2)
958.775
1010.884(PEP+HexNAc(3)Hex(3)_2)
1047.396
1055.386
1128.438(PEP+HexNAc(1)_1)
M+2H
1274.503(PEP+HexNAc(1)Fuc(1)_1)
1466.715
1801.688(PEP+HexNAc(2)Hex(2)Fuc(1)_1)
1817.689(PEP+HexNAc(2)Hex(3)_1)
1963.745(PEP+HexNAc(2)Hex(3)Fuc(1)_1)
1991.752
m/z

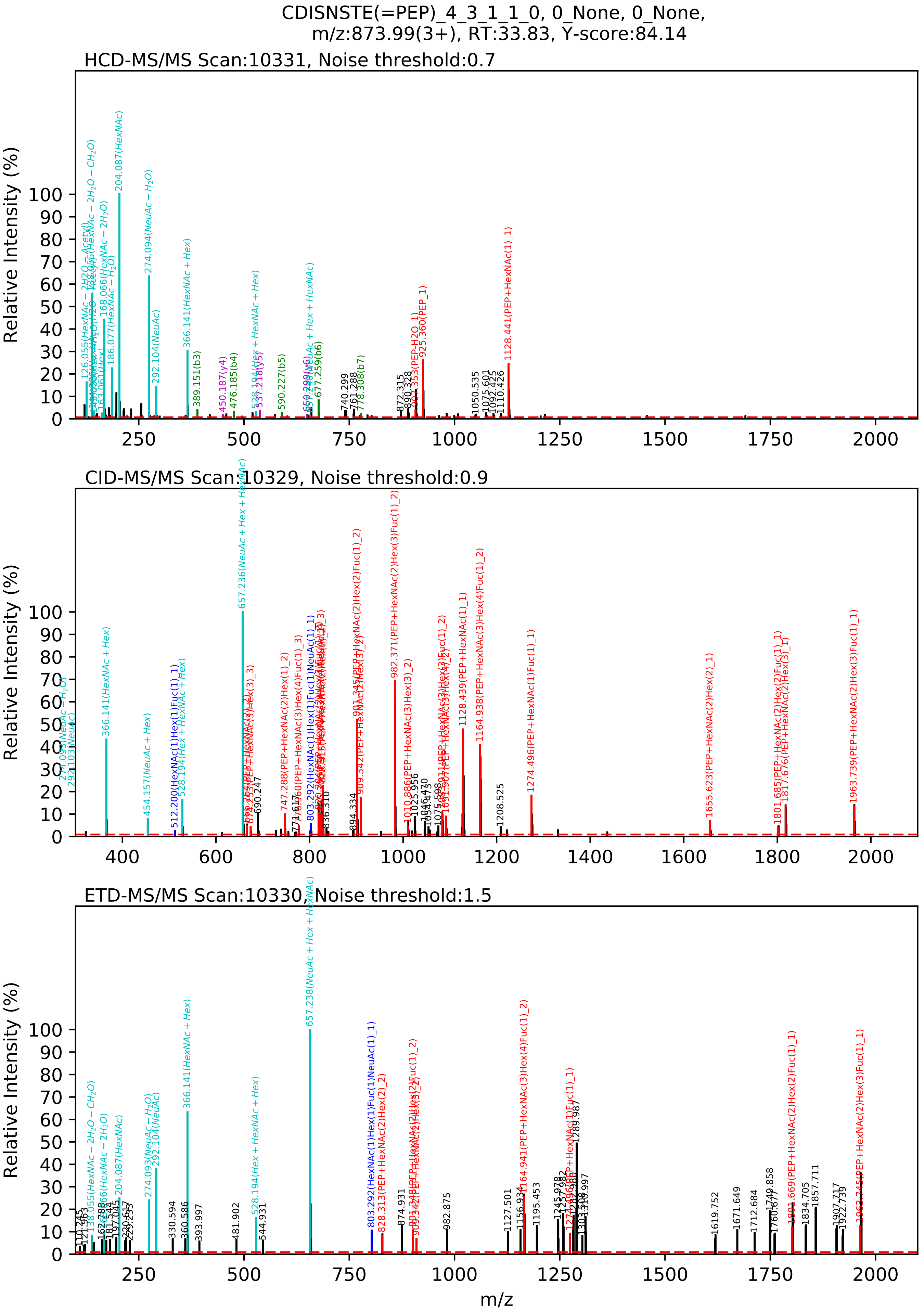
CDISNSTE(=PEP)_4_3_1_1_0, 0_None, 0_None,
m/z:873.99(3+), RT:33.83, Y-score:84.14
HCD-MS/MS Scan:10331, Noise threshold:0.7
Relative Intensity (%)
204.087(HexNAc)
274.094(NeuAc − H2O)
366.141(HexNAc + Hex)
292.104(NeuAc)
389.151(b3)
476.185(b4)
590.227(b5)
677.259(b6)
778.308(b7)
925.360(PEP_1)
1128.441(PEP+HexNAc(1)_1)
CID-MS/MS Scan:10329, Noise threshold:0.9
657.236(NeuAc + Hex + HexNAc)
366.141(HexNAc + Hex)
454.157(NeuAc + Hex)
982.371(PEP+HexNAc(2)Hex(3)Fuc(1)_2)
1128.439(PEP+HexNAc(1)_1)
1164.938(PEP+HexNAc(3)Hex(4)Fuc(1)_2)
1274.496(PEP+HexNAc(1)Fuc(1)_1)
1655.623(PEP+HexNAc(2)Hex(2)_1)
1963.739(PEP+HexNAc(2)Hex(3)Fuc(1)_1)
ETD-MS/MS Scan:10330, Noise threshold:1.5
657.238(NeuAc + Hex + HexNAc)
366.141(HexNAc + Hex)
292.104(NeuAc)
528.194(Hex + HexNAc + Hex)
803.292(HexNAc(1)Hex(1)Fuc(1)NeuAc(1)_1)
828.313(PEP+HexNAc(2)Hex(2)_2)
1164.941(PEP+HexNAc(3)Hex(4)Fuc(1)_2)
1289.987
1801.669(PEP+HexNAc(2)Hex(2)Fuc(1)_1)
1857.711
m/z

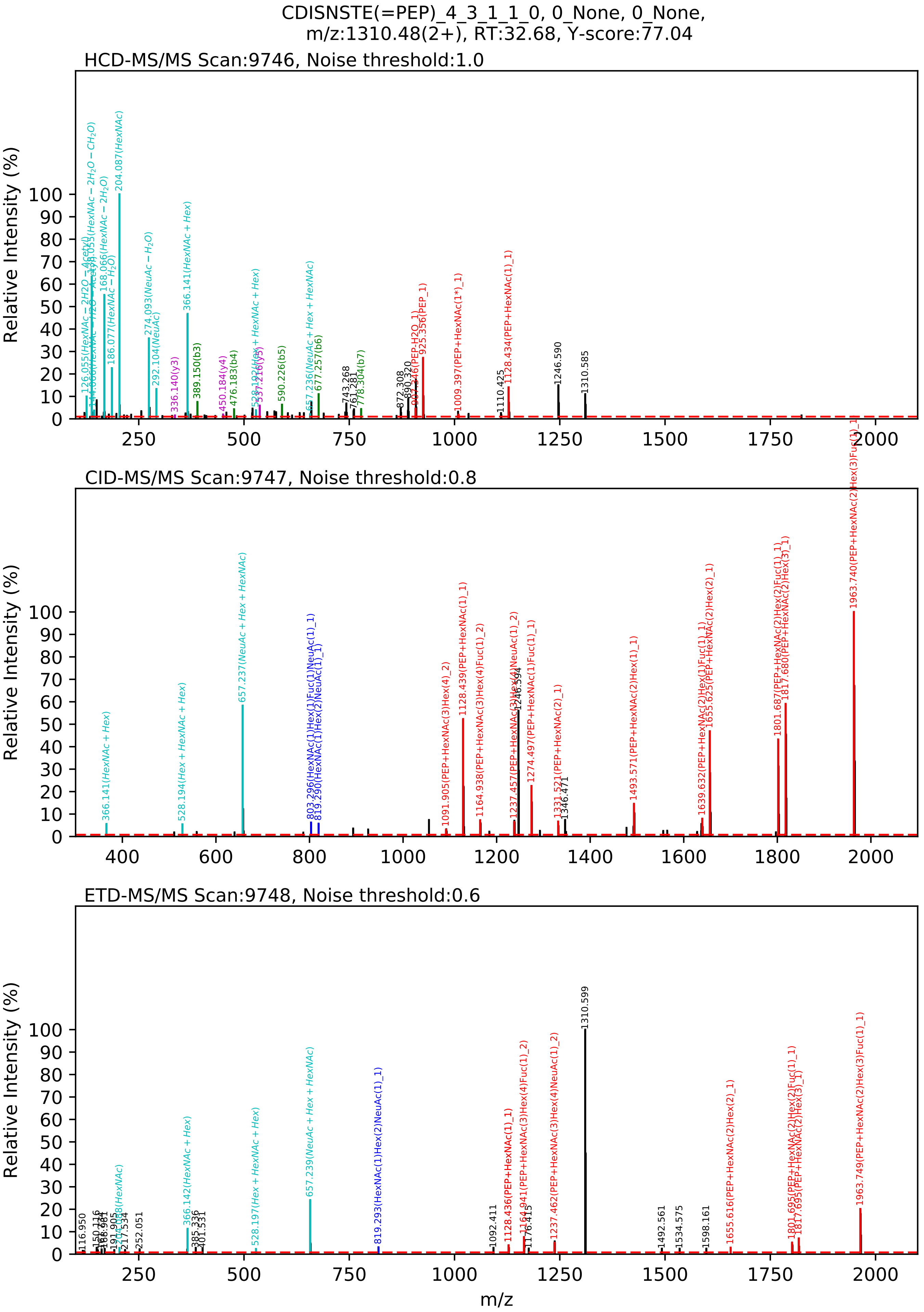

CDISNSTE(=PEP)_4_3_1_1_0, 0_None, 0_None,
m/z:1310.48(2+), RT:32.68, Y-score:77.04
HCD-MS/MS Scan:9746, Noise threshold:1.0
Relative Intensity (%)
204.087(HexNAc)
366.141(HexNAc + Hex)
274.093(NeuAc − H2O)
292.104(NeuAc)
168.066(HexNAc − 2H2O)
186.077(HexNAc − H2O)
336.140(y3)
389.150(b3)
450.184(y4)
476.183(b4)
537.216(y5)
590.226(b5)
657.236(NeuAc + Hex + HexNAc)
677.257(b6)
778.304(b7)
743.268
761.281
872.308
890.320
925.356(PEP_1)
1009.397(PEP+HexNAc(1*)_1)
1110.425
1128.434(PEP+HexNAc(1)_1)
1246.590
1310.585
CID-MS/MS Scan:9747, Noise threshold:0.8
366.141(HexNAc + Hex)
528.194(Hex + HexNAc + Hex)
657.237(NeuAc + Hex + HexNAc)
803.296(HexNAc(1)Hex(1)Fuc(1)NeuAc(1)_1)
819.290(HexNAc(1)Hex(2)NeuAc(1)_1)
1091.905(PEP+HexNAc(3)Hex(4)_2)
1128.439(PEP+HexNAc(1)_1)
1164.938(PEP+HexNAc(3)Hex(4)Fuc(1)_2)
1237.457(PEP+HexNAc(3)Hex(4)NeuAc(1)_2)
1246.594
1274.497(PEP+HexNAc(1)Fuc(1)_1)
1331.521(PEP+HexNAc(2)_1)
1346.471
1493.571(PEP+HexNAc(2)Hex(1)_1)
1639.632(PEP+HexNAc(2)Hex(1)Fuc(1)_1)
1655.625(PEP+HexNAc(2)Hex(2)_1)
1801.687(PEP+HexNAc(2)Hex(2)Fuc(1)_1)
1817.680(PEP+HexNAc(2)Hex(3)_1)
1963.740(PEP+HexNAc(2)Hex(3)Fuc(1)_1)
ETD-MS/MS Scan:9748, Noise threshold:0.6
116.950
150.116
168.981
191.905
217.534
252.051
366.142(HexNAc + Hex)
385.336
401.331
528.197(Hex + HexNAc + Hex)
657.239(NeuAc + Hex + HexNAc)
819.293(HexNAc(1)Hex(2)NeuAc(1)_1)
1092.411
1128.436(PEP+HexNAc(1)_1)
1164.941(PEP+HexNAc(3)Hex(4)Fuc(1)_2)
1176.415
1237.462(PEP+HexNAc(3)Hex(4)NeuAc(1)_2)
1310.599
1492.561
1534.575
1598.161
1655.616(PEP+HexNAc(2)Hex(2)_1)
1801.695(PEP+HexNAc(2)Hex(2)Fuc(1)_1)
1817.695(PEP+HexNAc(2)Hex(3)_1)
1963.749(PEP+HexNAc(2)Hex(3)Fuc(1)_1)
m/z

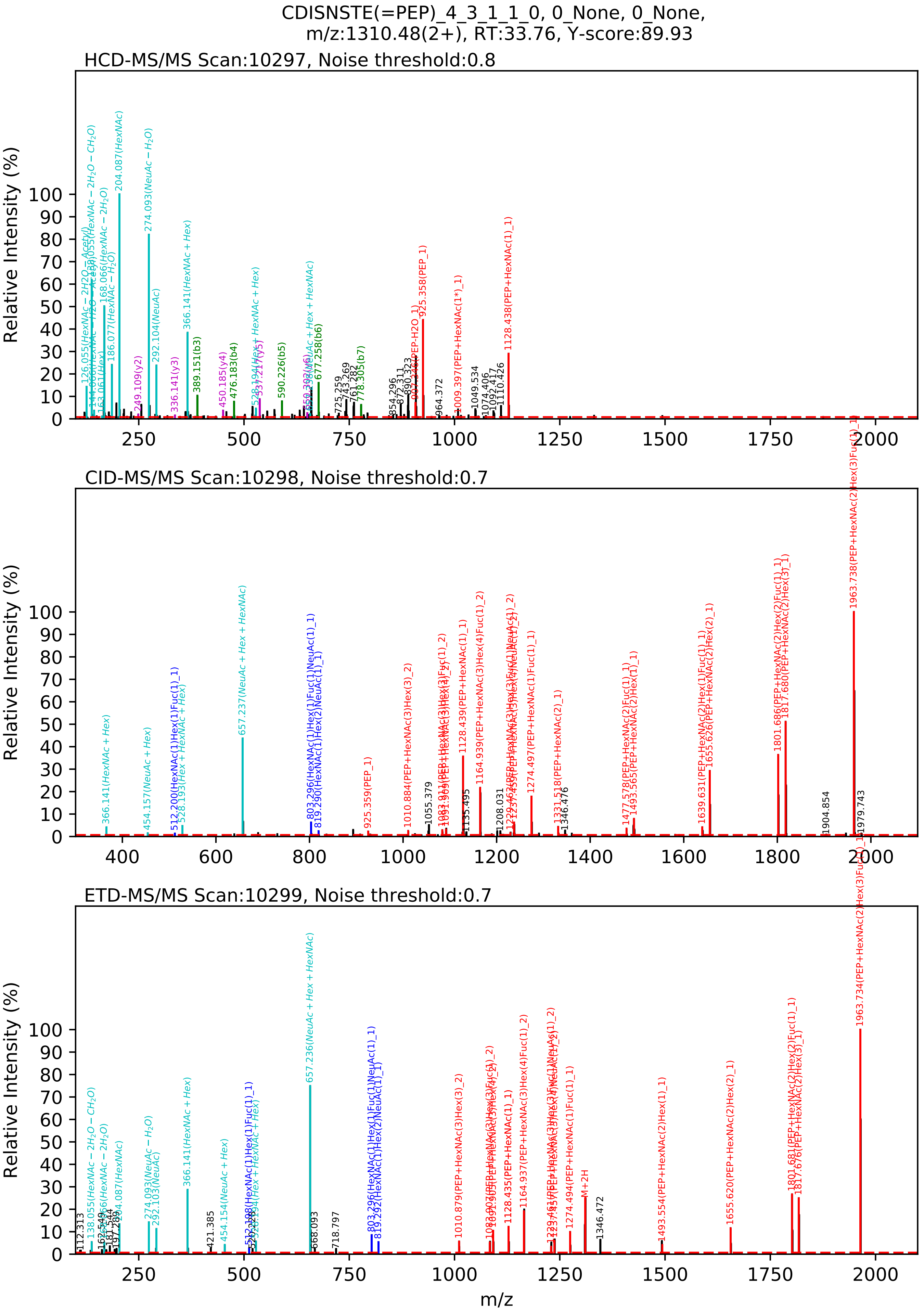
CDISNSTE(=PEP)_4_3_1_1_0, 0_None, 0_None,
m/z:1310.48(2+), RT:33.76, Y-score:89.93
HCD-MS/MS Scan:10297, Noise threshold:0.8
CID-MS/MS Scan:10298, Noise threshold:0.7
ETD-MS/MS Scan:10299, Noise threshold:0.7
Relative Intensity (%)
m/z

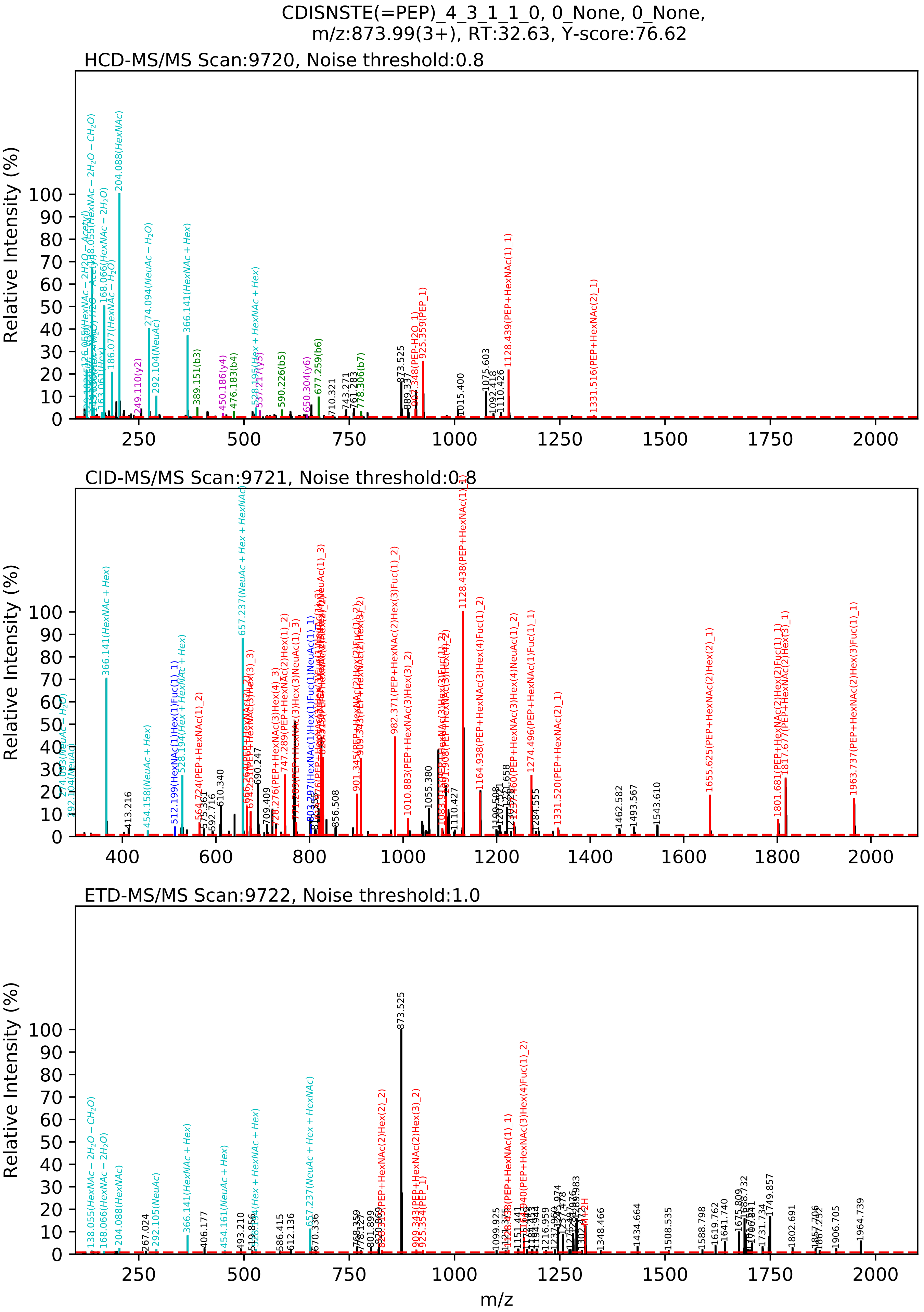
CDISNSTE(=PEP)_4_3_1_1_0, 0_None, 0_None,
m/z:873.99(3+), RT:32.63, Y-score:76.62
HCD-MS/MS Scan:9720, Noise threshold:0.8
CID-MS/MS Scan:9721, Noise threshold:0.8
ETD-MS/MS Scan:9722, Noise threshold:1.0
Relative Intensity (%)
m/z
204.088(HexNAc)
366.141(HexNAc + Hex)
274.094(NeuAc − H2O)
292.104(NeuAc)
1128.439(PEP+HexNAc(1)_1)
1331.516(PEP+HexNAc(2)_1)
925.359(PEP_1)
873.525
657.237(NeuAc + Hex + HexNAc)
1128.438(PEP+HexNAc(1)_1)
1963.737(PEP+HexNAc(2)Hex(3)Fuc(1)_1)
1655.625(PEP+HexNAc(2)Hex(2)_1)

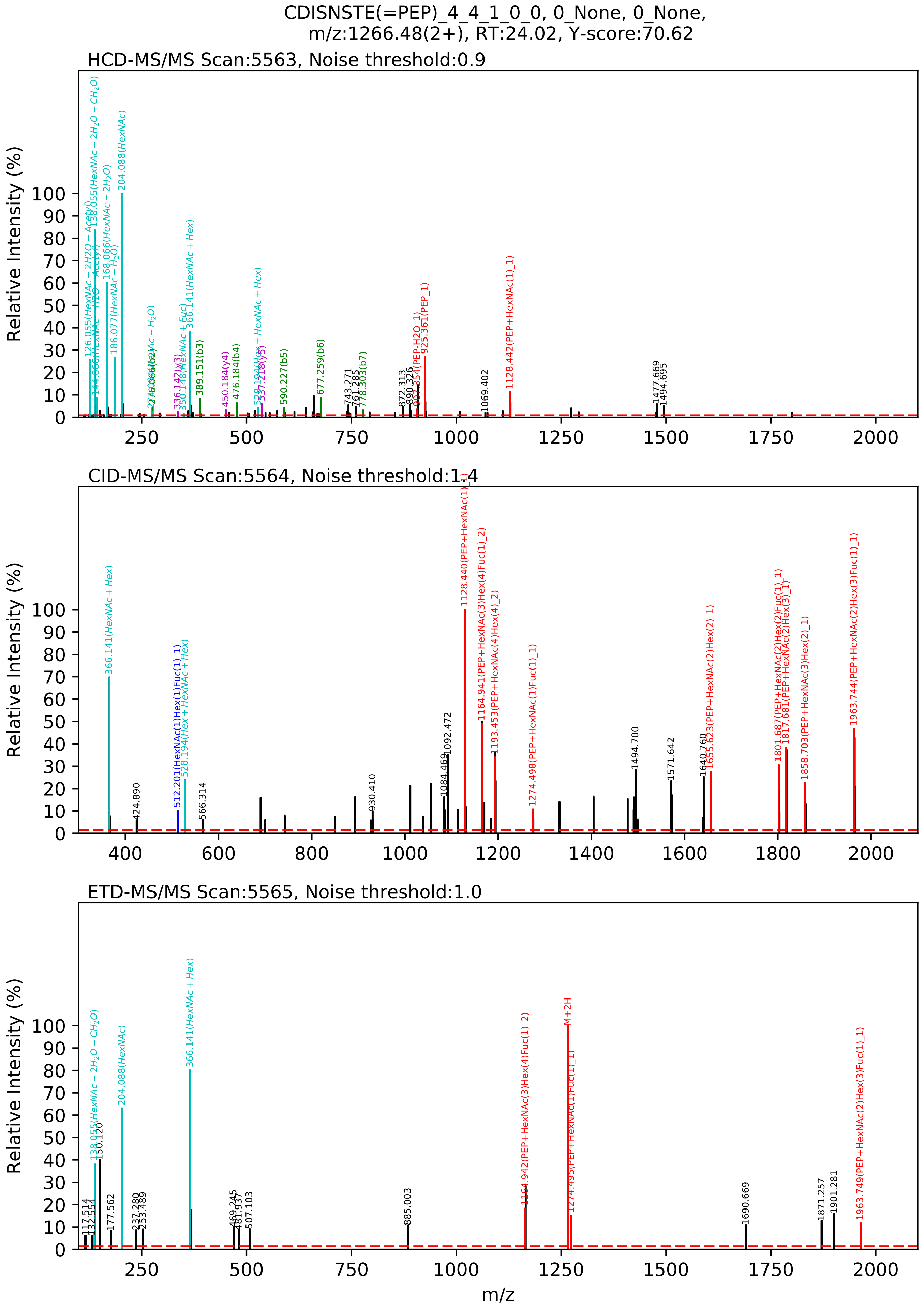

CDISNSTE(=PEP)_4_4_1_0_0, 0_None, 0_None,
m/z:1266.48(2+), RT:24.02, Y-score:70.62
HCD-MS/MS Scan:5563, Noise threshold:0.9
CID-MS/MS Scan:5564, Noise threshold:1.4
ETD-MS/MS Scan:5565, Noise threshold:1.0
Relative Intensity (%)
m/z
204.088(HexNAc)
366.141(HexNAc+Hex)
925.361(PEP_1)
1128.442(PEP+HexNAc(1)_1)
1128.440(PEP+HexNAc(1)_1)
1164.941(PEP+HexNAc(3)Hex(4)Fuc(1)_2)
1193.453(PEP+HexNAc(4)Hex(4)_2)
1274.498(PEP+HexNAc(1)Fuc(1)_1)
1655.623(PEP+HexNAc(2)Hex(2)_1)
1801.687(PEP+HexNAc(2)Hex(2)Fuc(1)_1)
1858.703(PEP+HexNAc(3)Hex(2)_1)
1963.744(PEP+HexNAc(2)Hex(3)Fuc(1)_1)
512.201(HexNAc(1)Hex(1)Fuc(1)_1)
M+2H
1164.942(PEP+HexNAc(3)Hex(4)Fuc(1)_2)
1274.495(PEP+HexNAc(1)Fuc(1)_1)
1963.749(PEP+HexNAc(2)Hex(3)Fuc(1)_1)

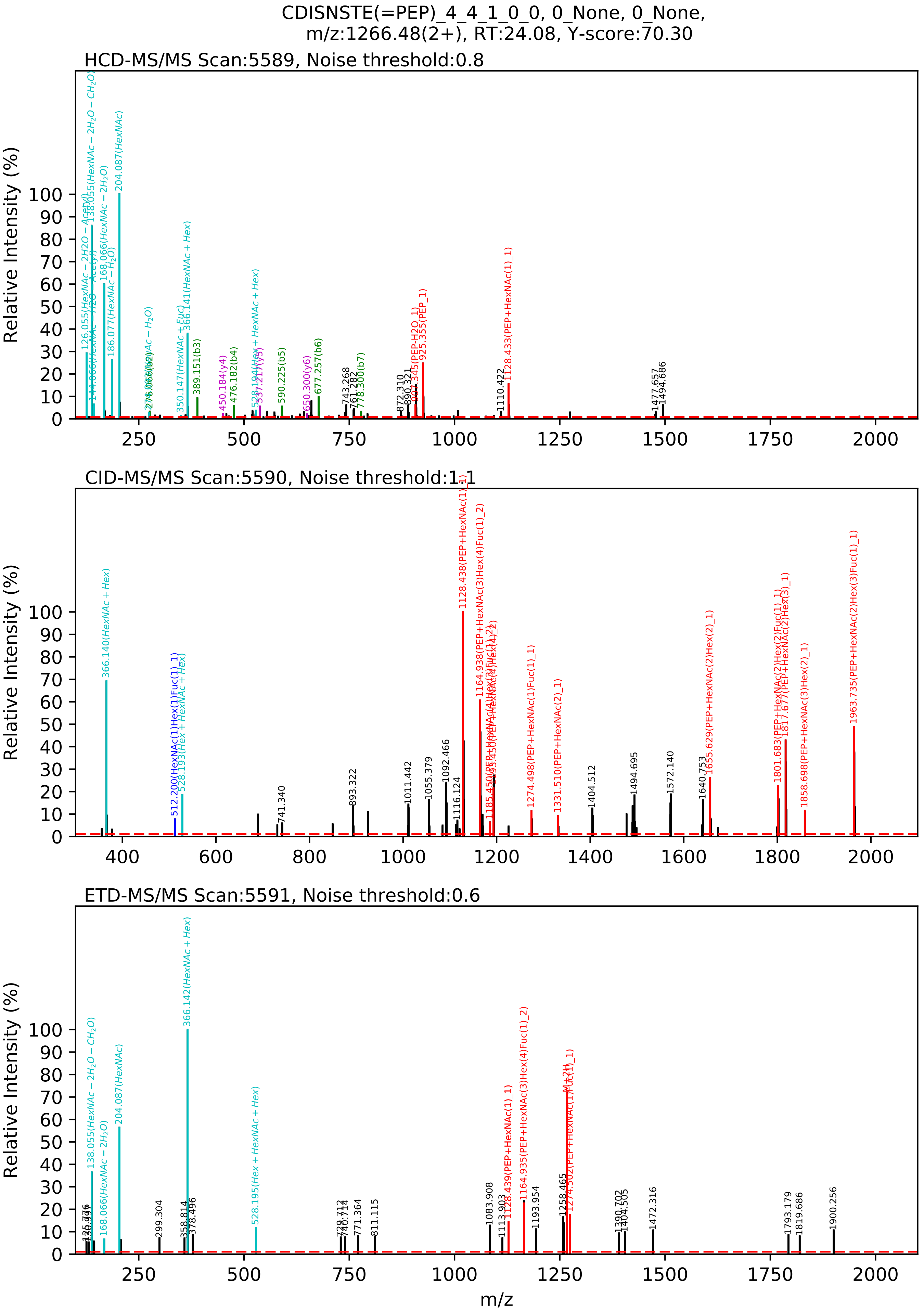

CDISNSTE(=PEP)_4_4_1_0_0, 0_None, 0_None,
m/z:1266.48(2+), RT:24.08, Y-score:70.30
HCD-MS/MS Scan:5589, Noise threshold:0.8
CID-MS/MS Scan:5590, Noise threshold:1.1
ETD-MS/MS Scan:5591, Noise threshold:0.6
Relative Intensity (%)
m/z
1128.433(PEP+HexNAc(1)_1)
925.355(PEP_1)
204.087(HexNAc)
366.141(HexNAc+Hex)
1128.438(PEP+HexNAc(1)_1)
1963.735(PEP+HexNAc(2)Hex(3)Fuc(1)_1)
366.140(HexNAc+Hex)
366.142(HexNAc+Hex)
1164.935(PEP+HexNAc(3)Hex(4)Fuc(1)_2)

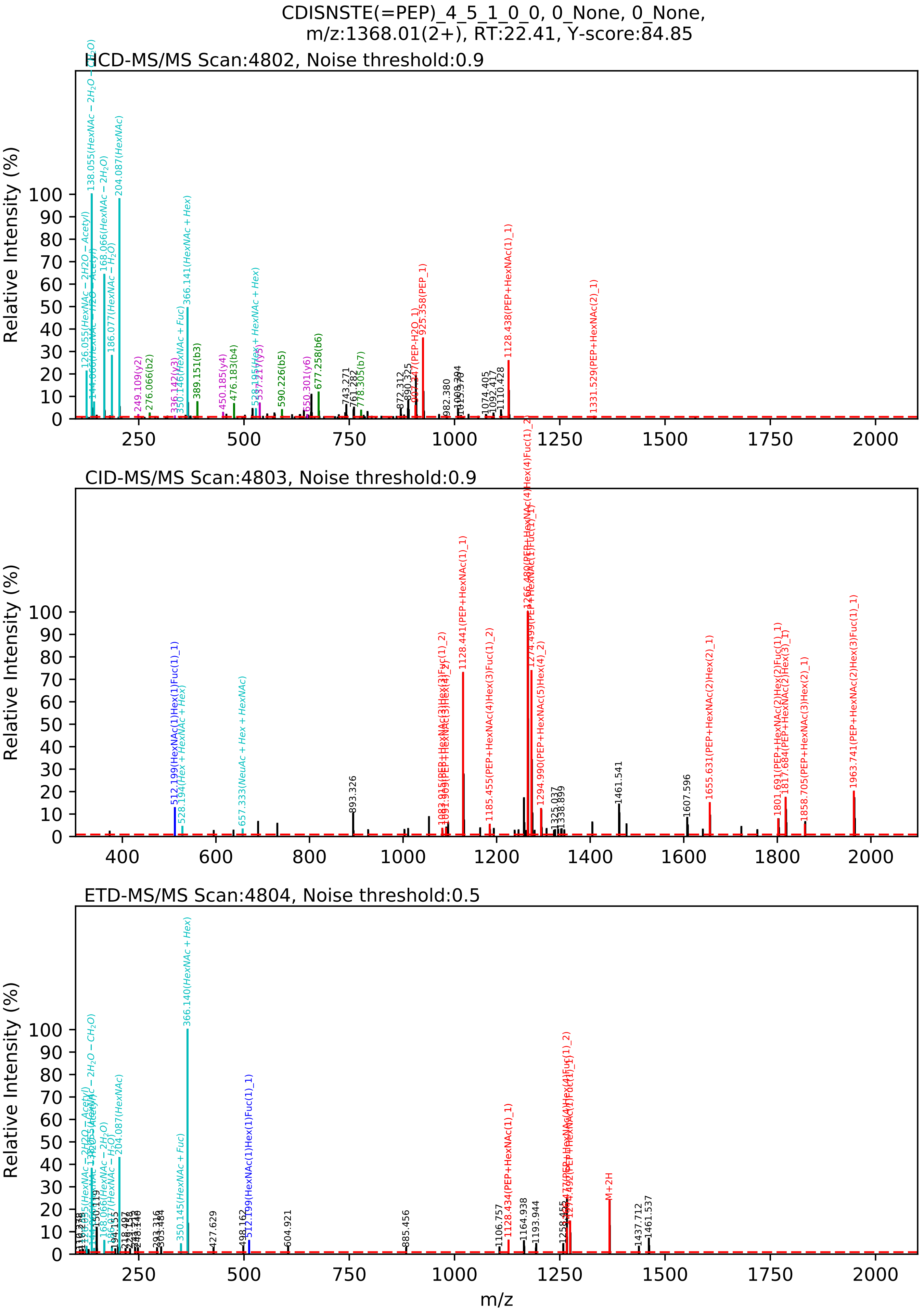

CDISNSTE(=PEP)_4_5_1_0_0, 0_None, 0_None,
m/z:1368.01(2+), RT:22.41, Y-score:84.85
HCD-MS/MS Scan:4802, Noise threshold:0.9
CID-MS/MS Scan:4803, Noise threshold:0.9
ETD-MS/MS Scan:4804, Noise threshold:0.5
Relative Intensity (%)
m/z
204.087(HexNAc)
366.141(HexNAc + Hex)
925.358(PEP_1)
1128.438(PEP+HexNAc(1)_1)
1331.529(PEP+HexNAc(2)_1)
512.199(HexNAc(1)Hex(1)Fuc(1)_1)
1128.441(PEP+HexNAc(1)_1)
1655.631(PEP+HexNAc(2)Hex(2)_1)
1963.741(PEP+HexNAc(2)Hex(3)Fuc(1)_1)
366.140(HexNAc + Hex)
M+2H

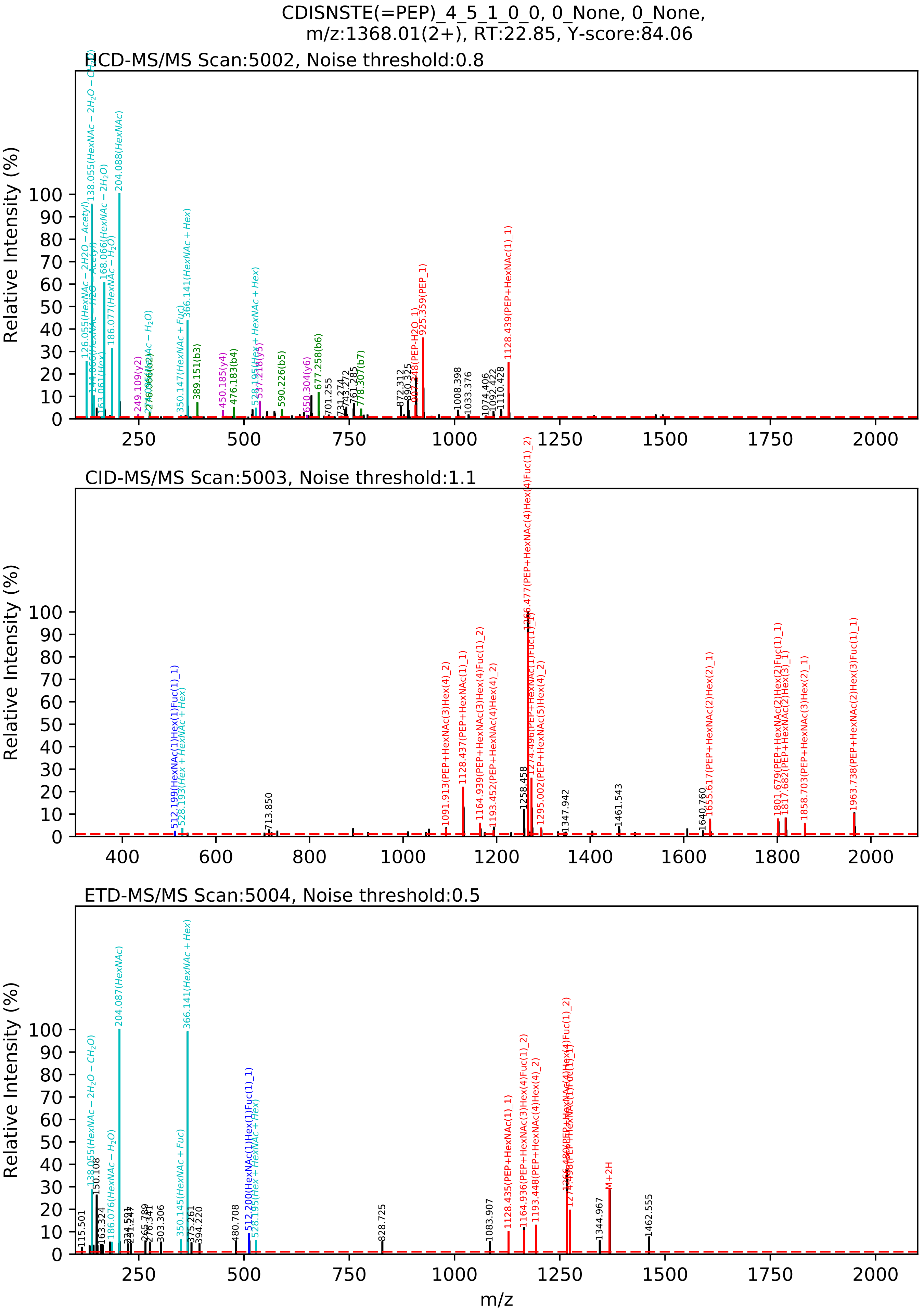
CDISNSTE(=PEP)_4_5_1_0_0, 0_None, 0_None,
m/z:1368.01(2+), RT:22.85, Y-score:84.06
HCD-MS/MS Scan:5002, Noise threshold:0.8
Relative Intensity (%)
126.055(HexNAc − 2H2O − Acetyl)
138.055(HexNAc − 2H2O − CH2O)
168.066(HexNAc − 2H2O)
186.077(HexNAc − H2O)
204.088(HexNAc)
163.061(Hex)
249.109(y2)
350.147(HexNAc + Fuc)
366.141(HexNAc + Hex)
389.151(b3)
450.185(y4)
476.183(b4)
528.195(Hex + HexNAc + Hex)
537.218(y5)
590.226(b5)
650.304(y6)
677.258(b6)
701.255
731.274
743.272
761.285
778.307(b7)
872.312
890.325
907.348(PEP-H2O_1)
925.359(PEP_1)
1008.398
1033.376
1074.406
1092.422
1110.428
1128.439(PEP+HexNAc(1)_1)
CID-MS/MS Scan:5003, Noise threshold:1.1
512.199(HexNAc(1)Hex(1)Fuc(1)_1)
528.193(Hex + HexNAc + Hex)
713.850
1091.913(PEP+HexNAc(3)Hex(4)_2)
1128.437(PEP+HexNAc(1)_1)
1164.939(PEP+HexNAc(3)Hex(4)Fuc(1)_2)
1193.452(PEP+HexNAc(4)Hex(4)_2)
1258.458
1266.477(PEP+HexNAc(4)Hex(4)Fuc(1)_2)
1274.496(PEP+HexNAc(1)Fuc(1)_1)
1295.002(PEP+HexNAc(5)Hex(4)_2)
1347.942
1461.543
1640.760
1655.617(PEP+HexNAc(2)Hex(2)_1)
1801.679(PEP+HexNAc(2)Hex(2)Fuc(1)_1)
1817.682(PEP+HexNAc(2)Hex(3)_1)
1858.703(PEP+HexNAc(3)Hex(2)_1)
1963.738(PEP+HexNAc(2)Hex(3)Fuc(1)_1)
ETD-MS/MS Scan:5004, Noise threshold:0.5
115.501
138.055(HexNAc − 2H2O − CH2O)
150.108
163.324
186.076(HexNAc − H2O)
204.087(HexNAc)
234.591
237.247
265.789
276.341
303.306
350.145(HexNAc + Fuc)
366.141(HexNAc + Hex)
375.261
394.220
480.708
512.200(HexNAc(1)Hex(1)Fuc(1)_1)
528.195(Hex + HexNAc + Hex)
828.725
1083.907
1128.435(PEP+HexNAc(1)_1)
1164.936(PEP+HexNAc(3)Hex(4)Fuc(1)_2)
1193.448(PEP+HexNAc(4)Hex(4)_2)
1266.486(PEP+HexNAc(4)Hex(4)Fuc(1)_2)
1274.498(PEP+HexNAc(1)Fuc(1)_1)
1344.967
M+2H
1462.555
m/z

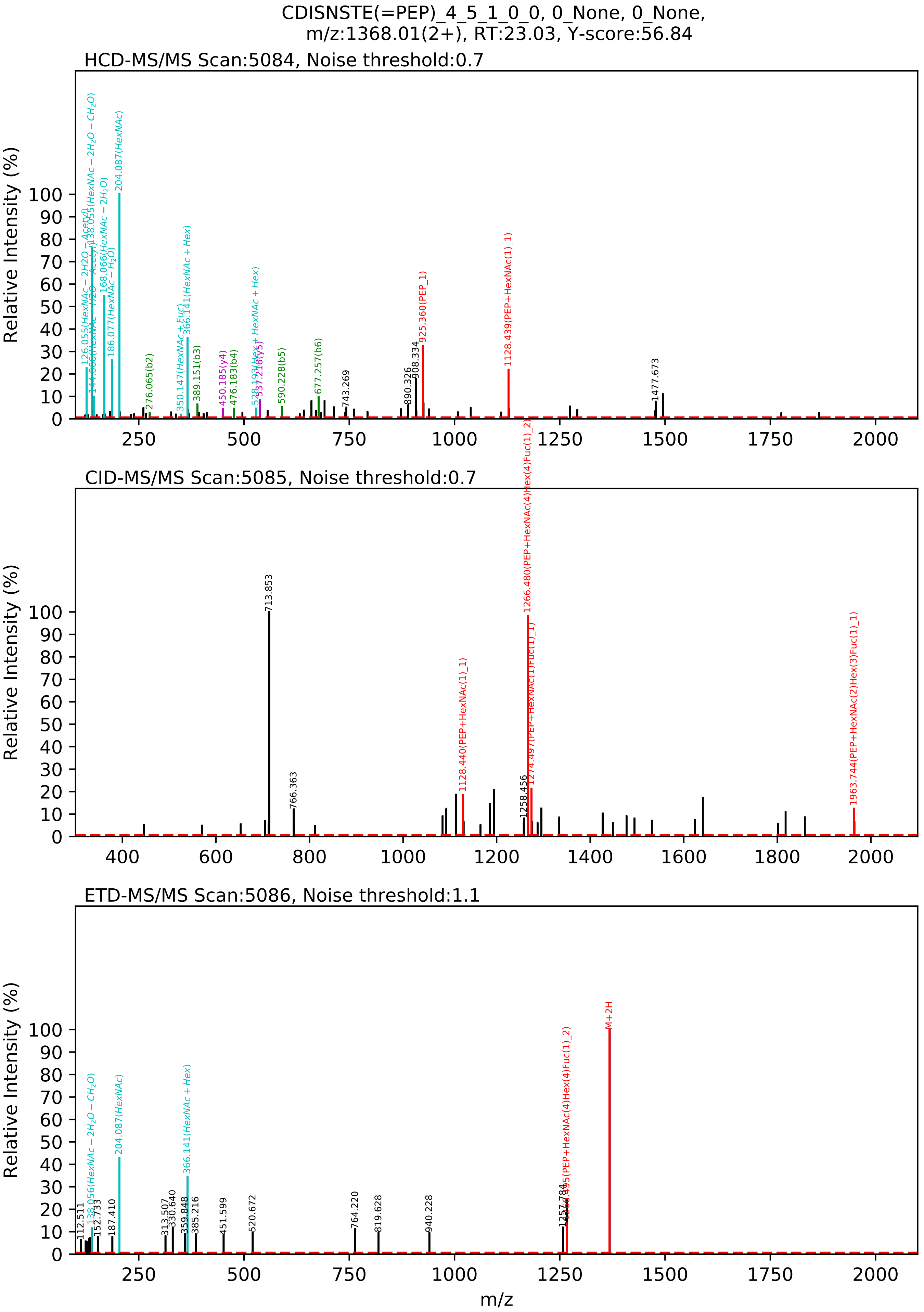

CDISNSTE(=PEP)_4_5_1_0_0, 0_None, 0_None,
m/z:1368.01(2+), RT:23.03, Y-score:56.84
HCD-MS/MS Scan:5084, Noise threshold:0.7
CID-MS/MS Scan:5085, Noise threshold:0.7
ETD-MS/MS Scan:5086, Noise threshold:1.1
Relative Intensity (%)
m/z
204.087(HexNAc)
366.141(HexNAc + Hex)
925.360(PEP_1)
1128.439(PEP+HexNAc(1)_1)
1477.673
713.853
766.363
1128.440(PEP+HexNAc(1)_1)
1266.480(PEP+HexNAc(4)Hex(4)Fuc(1)_2)
1274.497(PEP+HexNAc(1)Fuc(1)_1)
1963.744(PEP+HexNAc(2)Hex(3)Fuc(1)_1)
M+2H

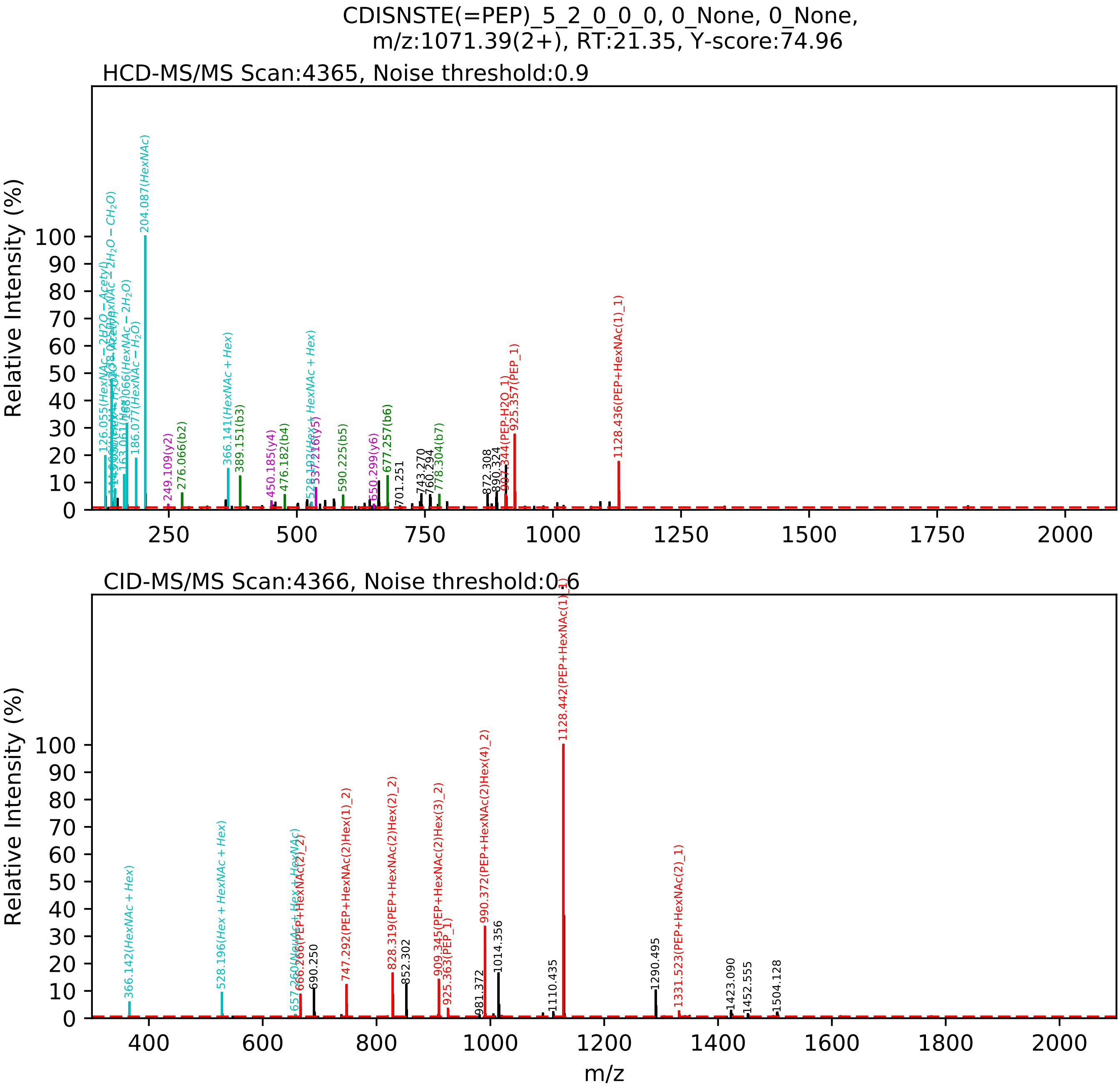

CDISNSTE(=PEP)_5_2_0_0_0, 0_None, 0_None,
m/z:1071.39(2+), RT:21.35, Y-score:74.96
HCD-MS/MS Scan:4365, Noise threshold:0.9
Relative Intensity (%)
204.087(HexNAc)
186.077(HexNAc − H2O)
126.055
249.109(y2)
276.066(b2)
366.141(HexNAc + Hex)
389.151(b3)
450.185(y4)
476.182(b4)
537.216(y5)
590.225(b5)
650.299(y6)
677.257(b6)
701.251
743.270
760.294
778.304(b7)
872.308
890.324
925.357(PEP_1)
1128.436(PEP+HexNAc(1)_1)
CID-MS/MS Scan:4366, Noise threshold:0.6
Relative Intensity (%)
366.142(HexNAc + Hex)
528.196(Hex + HexNAc + Hex)
690.250
747.292(PEP+HexNAc(2)Hex(1)_2)
828.319(PEP+HexNAc(2)Hex(2)_2)
852.302
909.345(PEP+HexNAc(2)Hex(3)_2)
925.363(PEP_1)
981.372
990.372(PEP+HexNAc(2)Hex(4)_2)
1014.356
1110.435
1128.442(PEP+HexNAc(1)_1)
1290.495
1331.523(PEP+HexNAc(2)_1)
1423.090
1452.555
1504.128
m/z

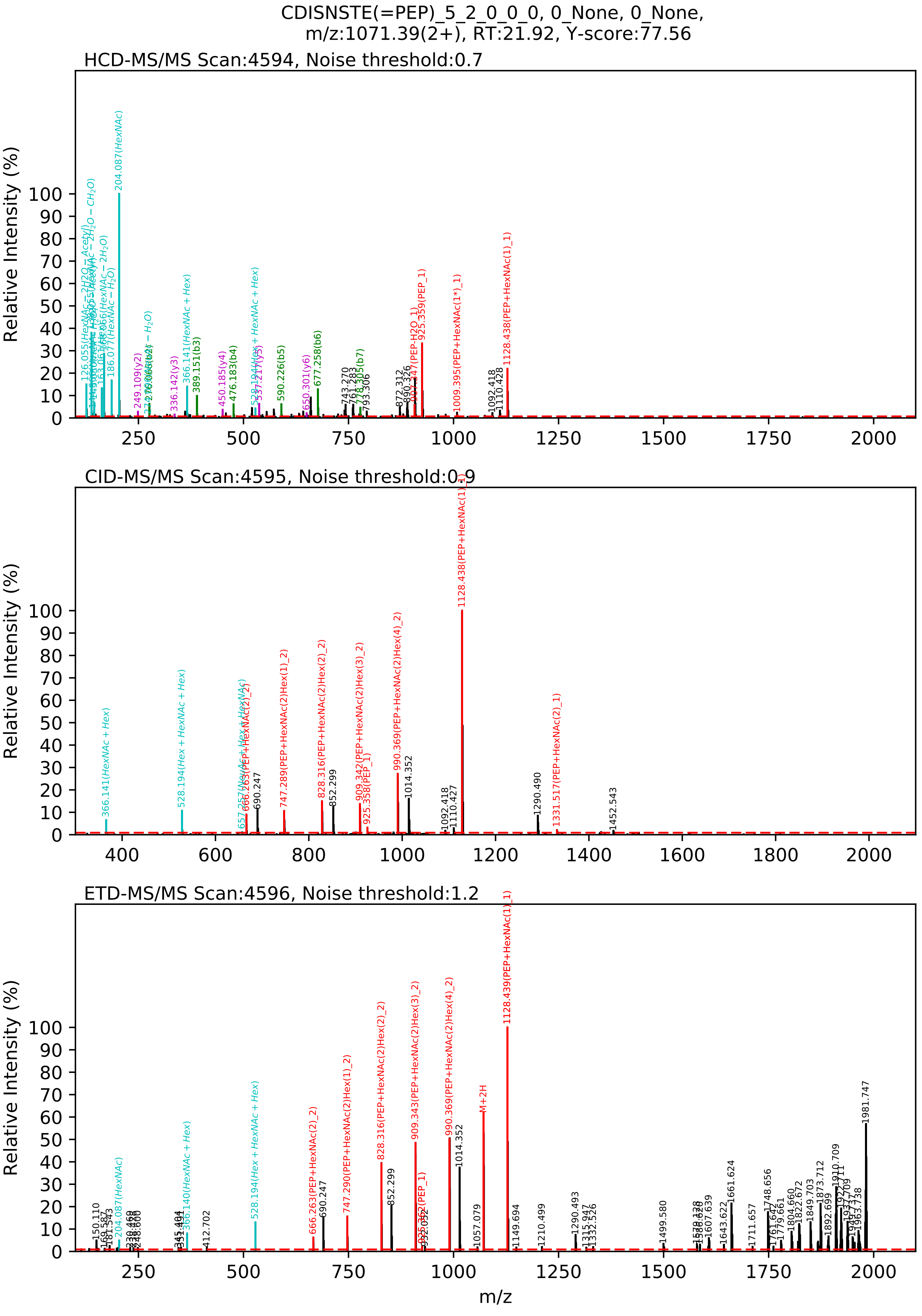
CDISNSTE(=PEP)_5_2_0_0_0, 0_None, 0_None,
m/z:1071.39(2+), RT:21.92, Y-score:77.56
HCD-MS/MS Scan:4594, Noise threshold:0.7
Relative Intensity (%)
204.087(HexNAc)
186.077(HexNAc − H2O)
366.141(HexNAc + Hex)
389.151(b3)
336.142(y3)
249.109(y2)
450.185(y4)
476.183(b4)
537.217(y5)
590.226(b5)
650.301(y6)
677.258(b6)
743.270
793.306
872.312
890.326
925.359(PEP_1)
1009.395(PEP+HexNAc(1*)_1)
1092.418
1110.428
1128.438(PEP+HexNAc(1)_1)
CID-MS/MS Scan:4595, Noise threshold:0.9
366.141(HexNAc + Hex)
528.194(Hex + HexNAc + Hex)
690.247
747.289(PEP+HexNAc(2)Hex(1)_2)
828.316(PEP+HexNAc(2)Hex(2)_2)
852.299
909.342(PEP+HexNAc(2)Hex(3)_2)
925.358(PEP_1)
990.369(PEP+HexNAc(2)Hex(4)_2)
1014.352
1092.418
1110.427
1128.438(PEP+HexNAc(1)_1)
1290.490
1331.517(PEP+HexNAc(2)_1)
1452.543
ETD-MS/MS Scan:4596, Noise threshold:1.2
150.110
204.087(HexNAc)
366.140(HexNAc + Hex)
412.702
528.194(Hex + HexNAc + Hex)
666.263(PEP+HexNAc(2)_2)
690.247
747.290(PEP+HexNAc(2)Hex(1)_2)
828.316(PEP+HexNAc(2)Hex(2)_2)
852.299
909.343(PEP+HexNAc(2)Hex(3)_2)
990.369(PEP+HexNAc(2)Hex(4)_2)
1014.352
1057.079
M+2H
1128.439(PEP+HexNAc(1)_1)
1149.694
1210.499
1290.493
1499.580
1607.639
1643.622
1661.624
1711.657
1748.656
1804.660
1822.672
1849.703
1873.712
1892.699
1910.709
1963.738
1981.747
m/z

CDISNSTE(=PEP)_5_2_0_0_0, 0_None, 0_None,
m/z:1071.39(2+), RT:22.78, Y-score:78.75

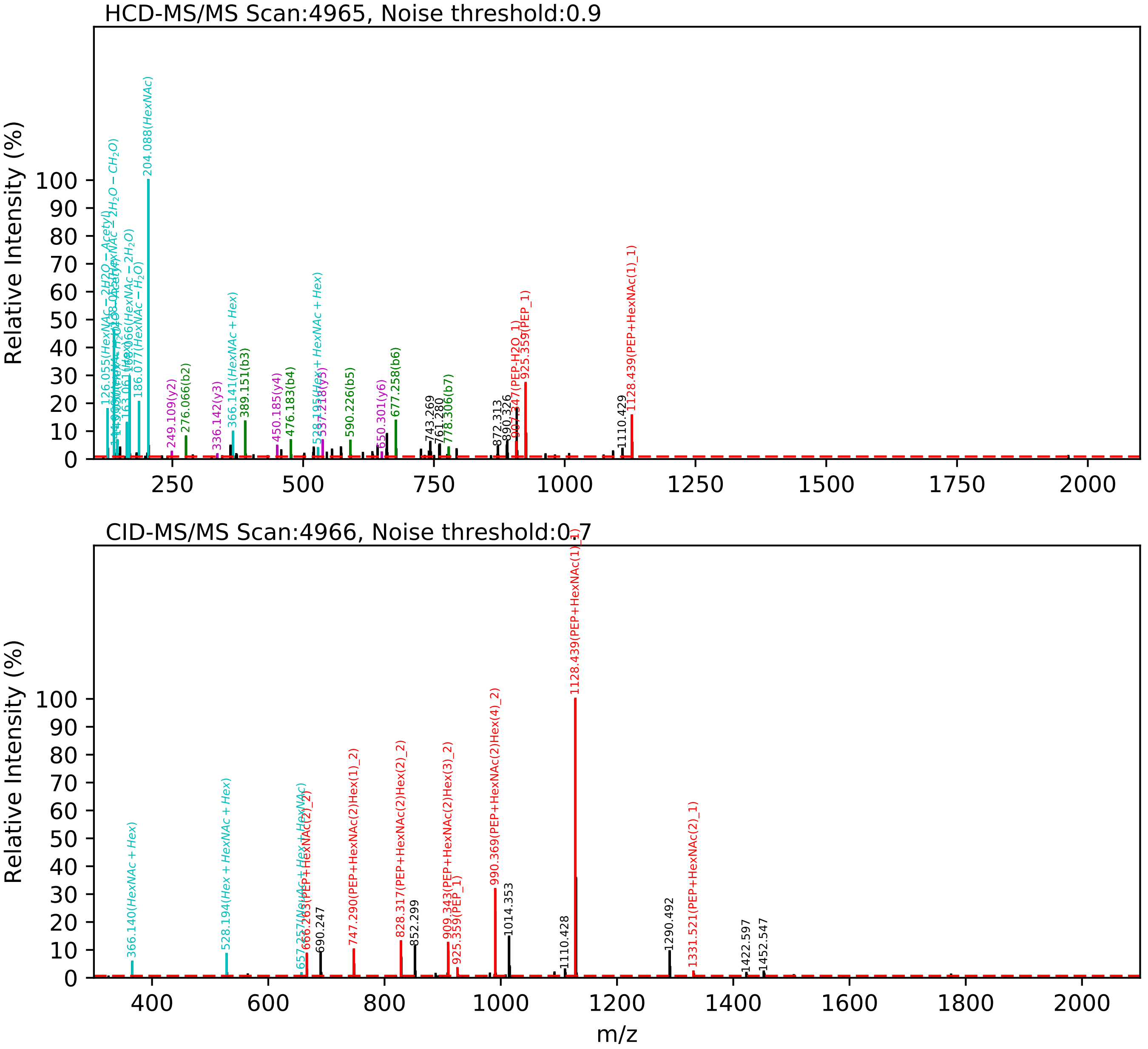

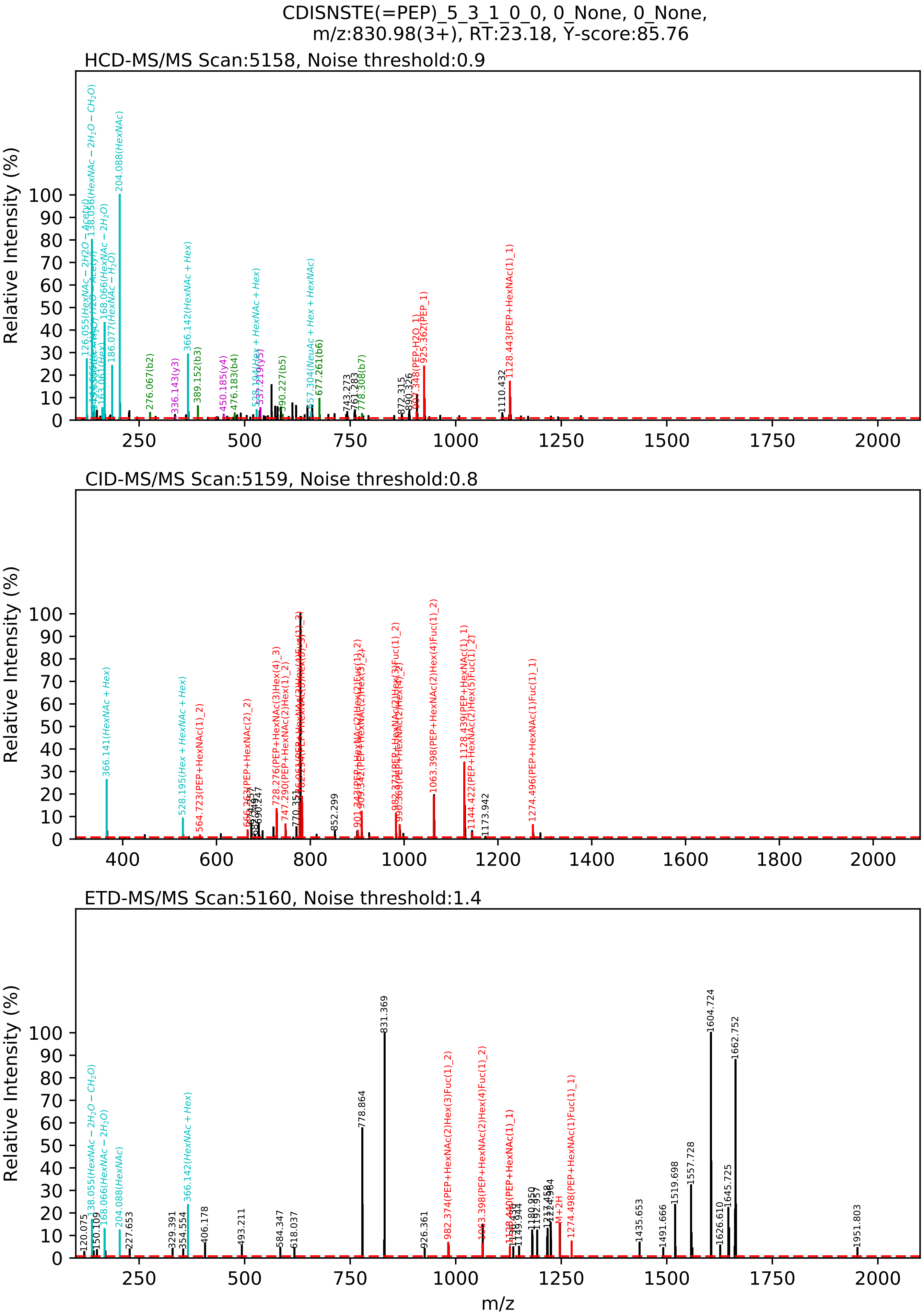

CDISNSTE(=PEP)_5_3_1_0_0, 0_None, 0_None,
m/z:830.98(3+), RT:23.18, Y-score:85.76
HCD-MS/MS Scan:5158, Noise threshold:0.9
Relative Intensity (%)
204.088(HexNAc)
1128.443(PEP+HexNAc(1)_1)
925.362(PEP_1)
366.142(HexNAc+Hex)
276.067(b2)
389.152(b3)
476.183(b4)
590.227(b5)
677.261(b6)
778.308(b7)
450.185(y4)
537.219(y5)
336.143(y3)
1110.432
CID-MS/MS Scan:5159, Noise threshold:0.8
366.141(HexNAc+Hex)
528.195(Hex+HexNAc+Hex)
564.723(PEP+HexNAc(1)_2)
728.276(PEP+HexNAc(3)Hex(4)_3)
747.290(PEP+HexNAc(2)Hex(1)_2)
770.351
852.299
1063.398(PEP+HexNAc(2)Hex(4)Fuc(1)_2)
1128.439(PEP+HexNAc(1)_1)
1144.422(PEP+HexNAc(2)Hex(5)Fuc(1)_2)
1173.942
1274.496(PEP+HexNAc(1)Fuc(1)_1)
ETD-MS/MS Scan:5160, Noise threshold:1.4
831.369
778.864
1604.724
1662.752
1557.728
1519.698
1645.725
982.374(PEP+HexNAc(2)Hex(3)Fuc(1)_2)
1063.398(PEP+HexNAc(2)Hex(4)Fuc(1)_2)
1274.498(PEP+HexNAc(1)Fuc(1)_1)
M+2H
366.142(HexNAc+Hex)
204.088(HexNAc)
168.066(HexNAc−2H2O)
120.075
227.653
329.391
354.554
406.178
493.211
584.347
618.037
926.361
1435.653
1491.666
1626.610
1951.803
m/z

CDISNSTE(=PEP)_5_3_1_0_0, 0_None, 0_None,
m/z:830.98(3+), RT:23.30, Y-score:82.35

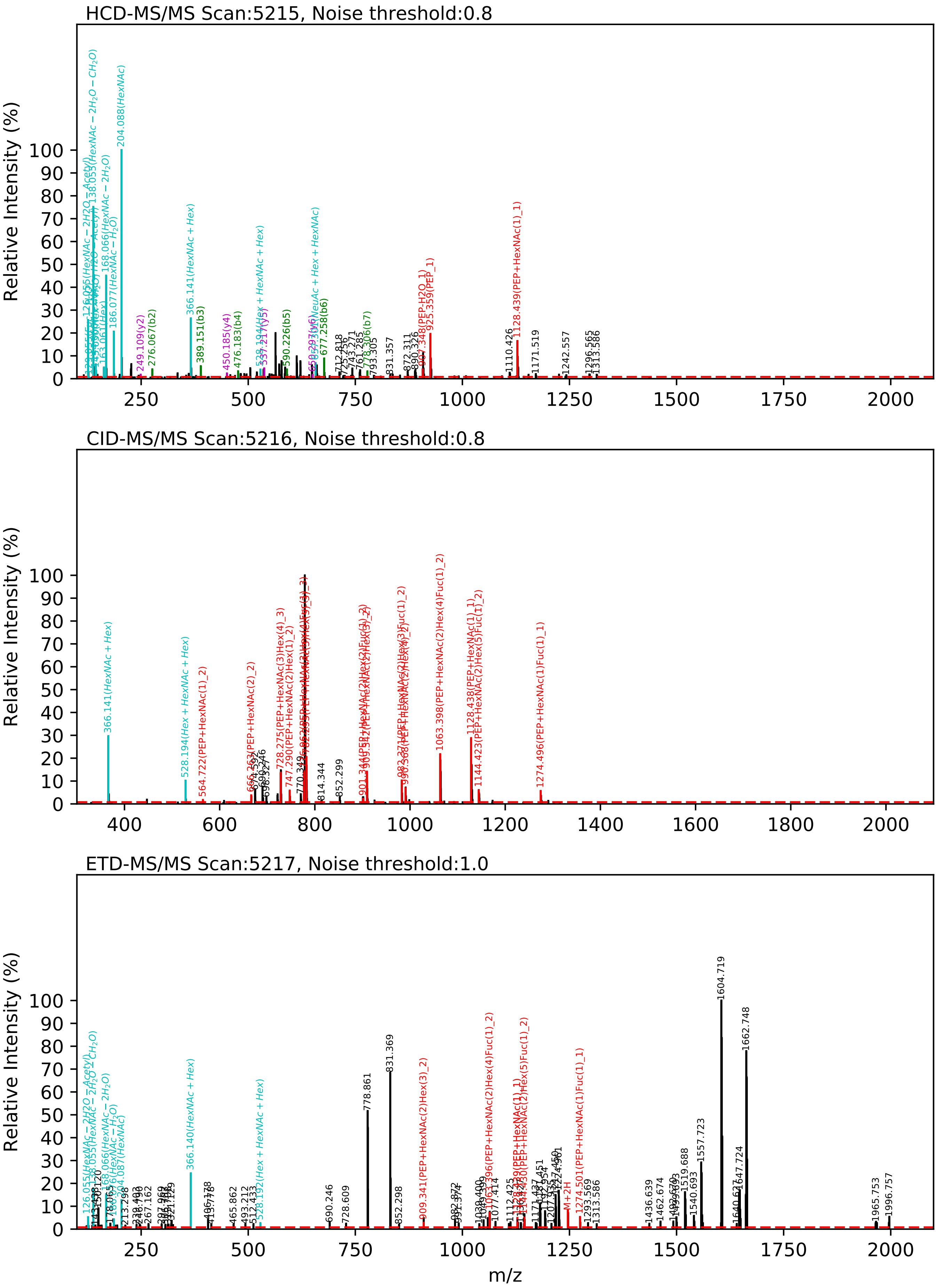

CDISNSTE(=PEP)_5_3_1_1_0, 0_None, 0_None,
m/z:1391.51(2+), RT:33.18, Y-score:80.98

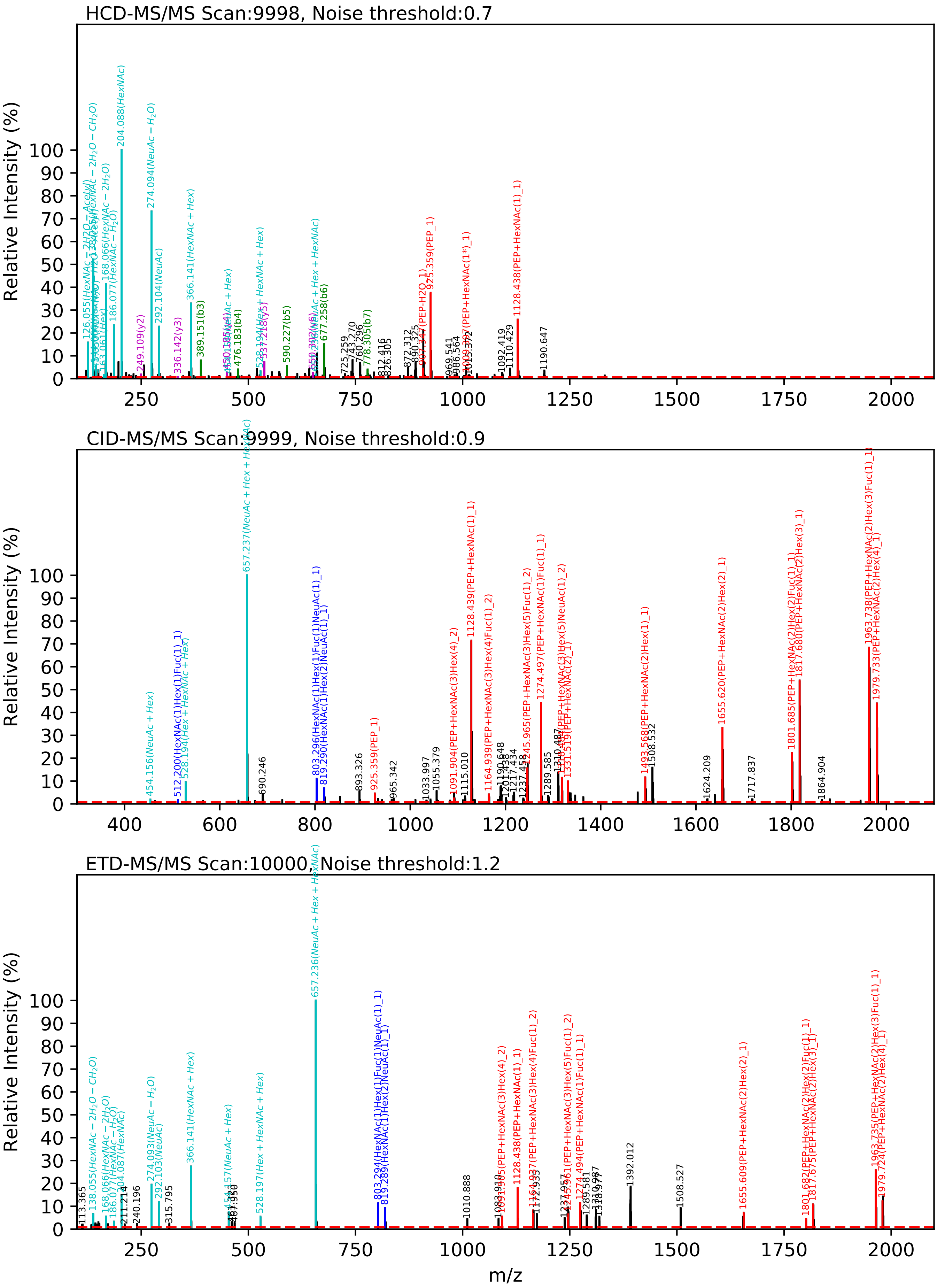

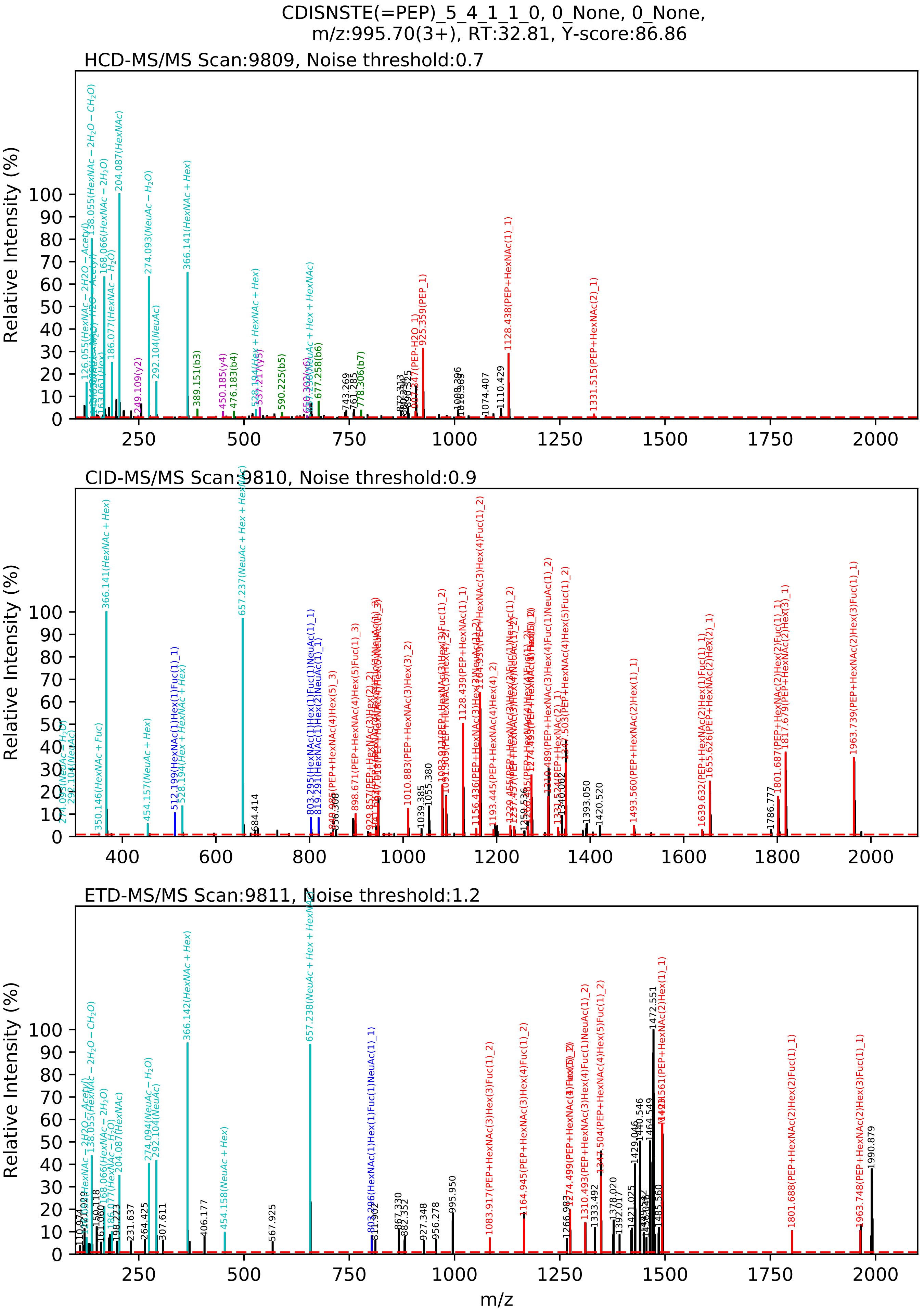

CDISNSTE(=PEP)_5_4_1_1_0, 0_None, 0_None,
m/z:995.70(3+), RT:32.81, Y-score:86.86
HCD-MS/MS Scan:9809, Noise threshold:0.7
Relative Intensity (%)
204.087(HexNAc)
138.055(HexNAc − 2H2O − CH2O)
168.066(HexNAc − 2H2O)
186.077(HexNAc − H2O)
274.093(NeuAc − H2O)
292.104(NeuAc)
366.141(HexNAc + Hex)
249.109(y2)
389.151(b3)
450.185(y4)
476.183(b4)
590.225(b5)
677.258(b6)
778.306(b7)
925.359(PEP_1)
1128.438(PEP+HexNAc(1)_1)
1331.515(PEP+HexNAc(2)_1)
CID-MS/MS Scan:9810, Noise threshold:0.9
366.141(HexNAc + Hex)
454.157(NeuAc + Hex)
512.199(HexNAc(1)Hex(1)Fuc(1)_1)
657.237(NeuAc + Hex + HexNAc)
803.295(HexNAc(1)Hex(1)Fuc(1)NeuAc(1)_1)
819.291(HexNAc(1)Hex(2)NeuAc(1)_1)
898.671(PEP+HexNAc(4)Hex(5)Fuc(1)_3)
1010.883(PEP+HexNAc(3)Hex(3)_2)
1128.439(PEP+HexNAc(1)_1)
1193.445(PEP+HexNAc(4)Hex(4)_2)
1493.560(PEP+HexNAc(2)Hex(1)_1)
1639.632(PEP+HexNAc(2)Hex(1)Fuc(1)_1)
1655.626(PEP+HexNAc(2)Hex(2)_1)
1801.687(PEP+HexNAc(2)Hex(2)Fuc(1)_1)
1817.679(PEP+HexNAc(2)Hex(3)_1)
1963.739(PEP+HexNAc(2)Hex(3)Fuc(1)_1)
ETD-MS/MS Scan:9811, Noise threshold:1.2
366.142(HexNAc + Hex)
657.238(NeuAc + Hex + HexNAc)
454.158(NeuAc + Hex)
803.296(HexNAc(1)Hex(1)Fuc(1)NeuAc(1)_1)
1083.917(PEP+HexNAc(3)Hex(3)Fuc(1)_2)
1164.945(PEP+HexNAc(3)Hex(4)Fuc(1)_2)
1310.493(PEP+HexNAc(3)Hex(4)Fuc(1)NeuAc(1)_2)
1347.504(PEP+HexNAc(4)Hex(5)Fuc(1)_2)
1472.551
1801.688(PEP+HexNAc(2)Hex(2)Fuc(1)_1)
1963.748(PEP+HexNAc(2)Hex(3)Fuc(1)_1)
1990.879
m/z

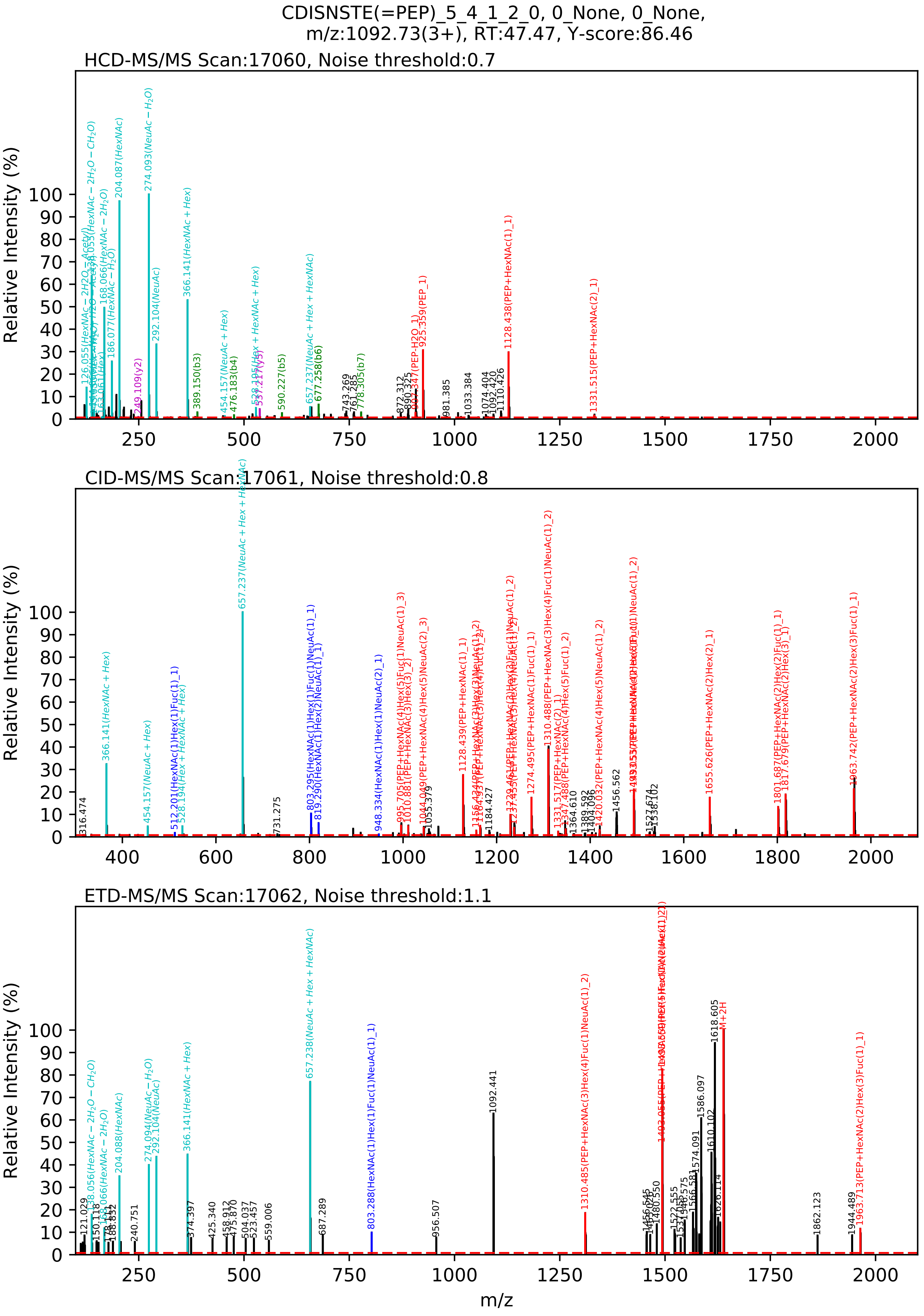
CDISNSTE(=PEP)_5_4_1_2_0, 0_None, 0_None,
m/z:1092.73(3+), RT:47.47, Y-score:86.46
HCD-MS/MS Scan:17060, Noise threshold:0.7
Relative Intensity (%)
274.093(NeuAc − H2O)
204.087(HexNAc)
366.141(HexNAc + Hex)
292.104(NeuAc)
1128.438(PEP+HexNAc(1)_1)
925.359(PEP_1)
1331.515(PEP+HexNAc(2)_1)
657.237(NeuAc + Hex + HexNAc)
CID-MS/MS Scan:17061, Noise threshold:0.8
657.237(NeuAc + Hex + HexNAc)
366.141(HexNAc + Hex)
1128.439(PEP+HexNAc(1)_1)
1274.495(PEP+HexNAc(1)Fuc(1)_1)
1655.626(PEP+HexNAc(2)Hex(2)_1)
1801.687(PEP+HexNAc(2)Hex(2)Fuc(1)_1)
1817.679(PEP+HexNAc(2)Hex(3)_1)
1963.742(PEP+HexNAc(2)Hex(3)Fuc(1)_1)
ETD-MS/MS Scan:17062, Noise threshold:1.1
657.238(NeuAc + Hex + HexNAc)
1092.441
1618.605
M+2H
1310.485(PEP+HexNAc(3)Hex(4)Fuc(1)NeuAc(1)_2)
803.288(HexNAc(1)Hex(1)Fuc(1)NeuAc(1)_1)
1963.713(PEP+HexNAc(2)Hex(3)Fuc(1)_1)
m/z

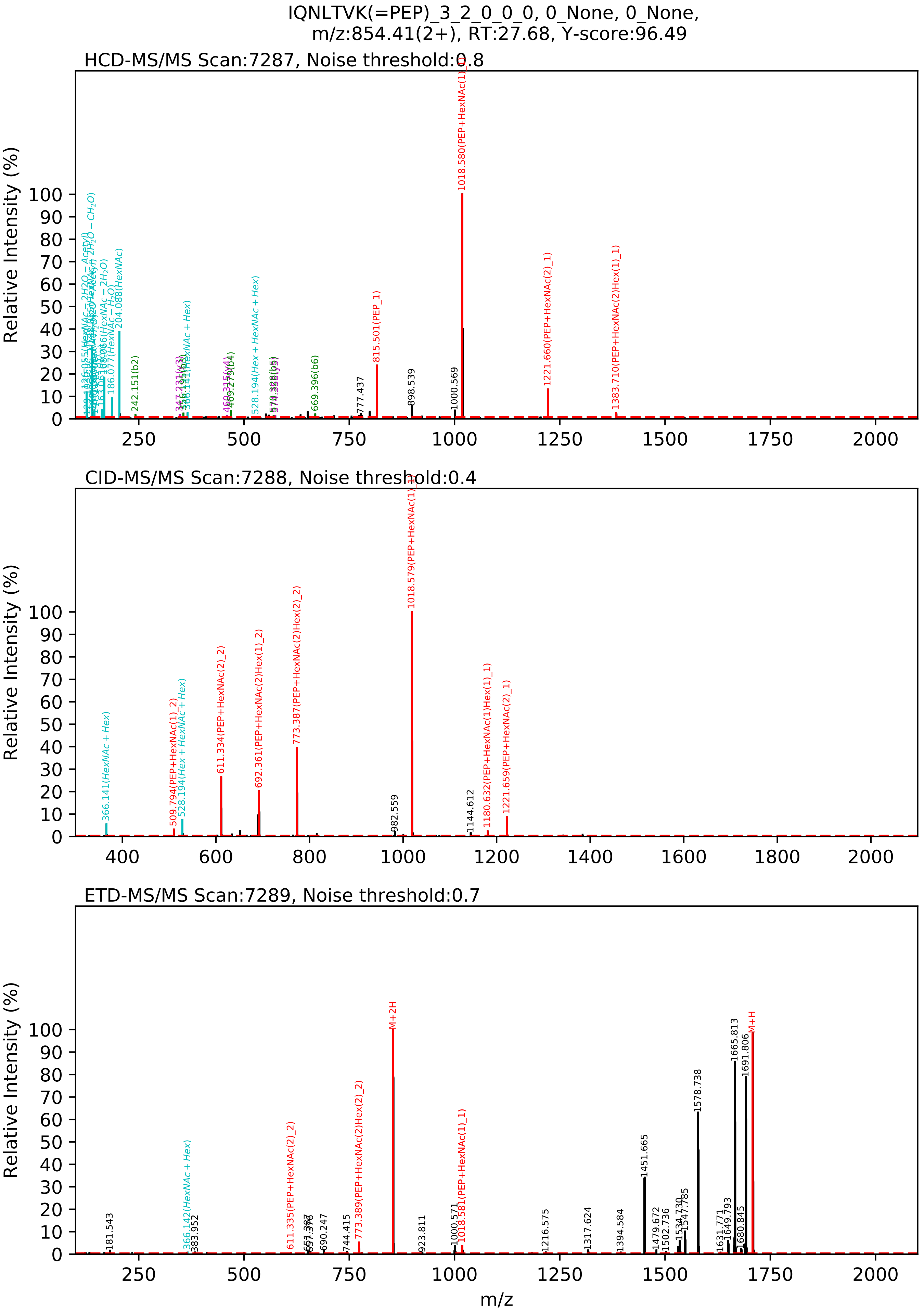

IQNLTVK(=PEP)_3_2_0_0_0, 0_None, 0_None,
m/z:854.41(2+), RT:27.68, Y-score:96.49
HCD-MS/MS Scan:7287, Noise threshold:0.8
1018.580(PEP+HexNAc(1)_1)
815.501(PEP_1)
1221.660(PEP+HexNAc(2)_1)
1383.710(PEP+HexNAc(2)Hex(1)_1)
204.088(HexNAc)
186.077(HexNAc − H2O)
242.151(b2)
669.396(b6)
528.194(Hex + HexNAc + Hex)
777.437
898.539
1000.569
CID-MS/MS Scan:7288, Noise threshold:0.4
1018.579(PEP+HexNAc(1)_1)
773.387(PEP+HexNAc(2)Hex(2)_2)
692.361(PEP+HexNAc(2)Hex(1)_2)
611.334(PEP+HexNAc(2)_2)
509.794(PEP+HexNAc(1)_2)
528.194(Hex + HexNAc + Hex)
366.141(HexNAc + Hex)
982.559
1144.612
1180.632(PEP+HexNAc(1)Hex(1)_1)
1221.659(PEP+HexNAc(2)_1)
ETD-MS/MS Scan:7289, Noise threshold:0.7
M+2H
M+H
181.543
366.142(HexNAc + Hex)
383.952
611.335(PEP+HexNAc(2)_2)
690.247
744.415
773.389(PEP+HexNAc(2)Hex(2)_2)
923.811
1000.571
1018.581(PEP+HexNAc(1)_1)
1216.575
1317.624
1394.584
1451.665
1479.672
1502.736
1534.730
1547.785
1578.738
1631.771
1649.793
1665.813
1680.845
1691.806
Relative Intensity (%)
m/z

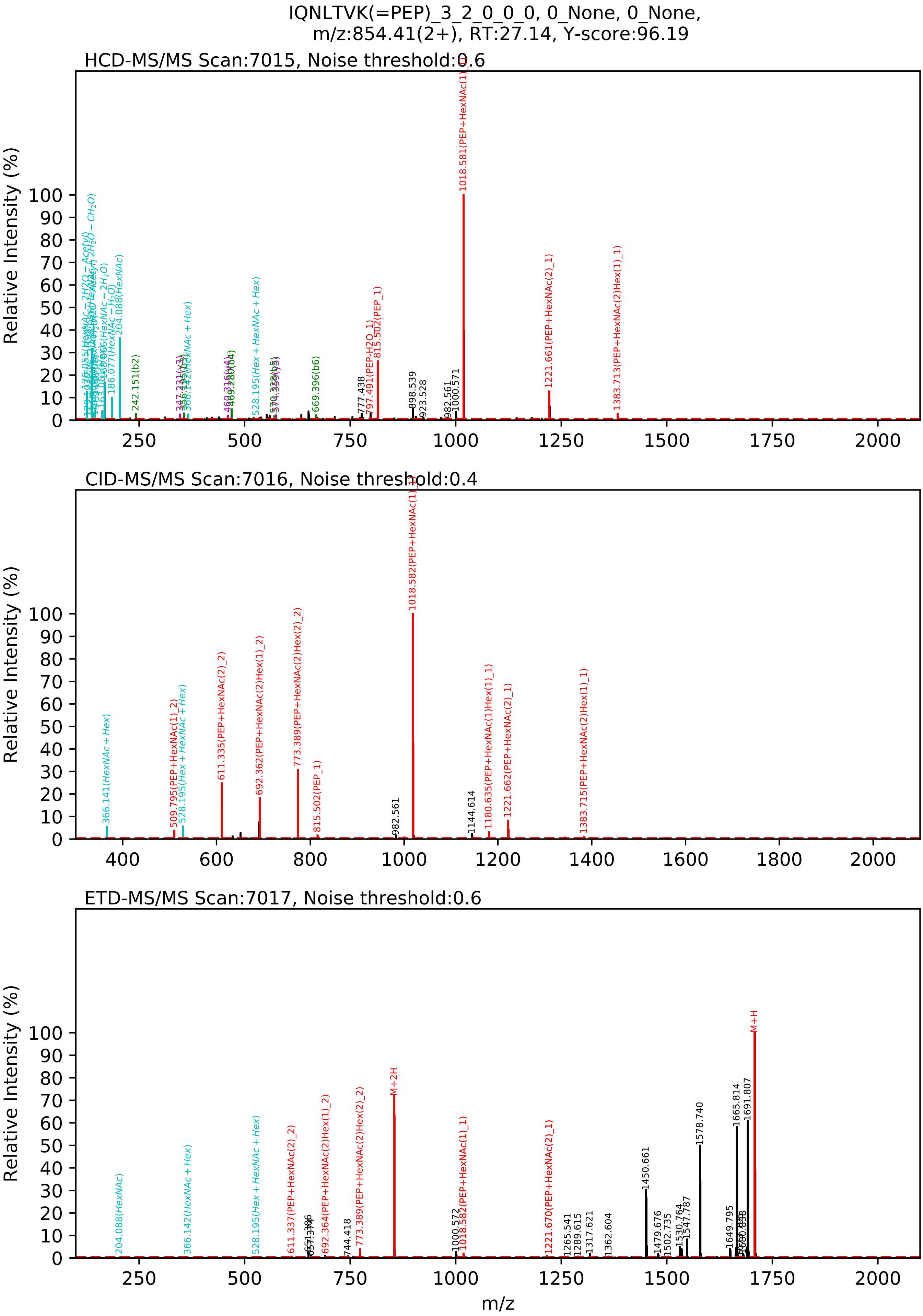

IQNLTVK(=PEP)_3_2_0_0_0, 0_None, 0_None,
m/z:854.41(2+), RT:27.14, Y-score:96.19
HCD-MS/MS Scan:7015, Noise threshold:0.6
Relative Intensity (%)
1018.581(PEP+HexNAc(1)_1)
1221.661(PEP+HexNAc(2)_1)
1383.713(PEP+HexNAc(2)Hex(1)_1)
815.502(PEP_1)
797.491(PEP-H2O_1)
204.088(HexNAc)
186.077(HexNAc − H2O)
242.151(b2)
469.280(b4)
669.396(b6)
528.195(Hex + HexNAc + Hex)
366.142(HexNAc + Hex)
898.539
923.528
982.561
1000.571
777.438
CID-MS/MS Scan:7016, Noise threshold:0.4
1018.582(PEP+HexNAc(1)_1)
773.389(PEP+HexNAc(2)Hex(2)_2)
692.362(PEP+HexNAc(2)Hex(1)_2)
611.335(PEP+HexNAc(2)_2)
509.795(PEP+HexNAc(1)_2)
528.195(Hex + HexNAc + Hex)
366.141(HexNAc + Hex)
815.502(PEP_1)
982.561
1144.614
1180.635(PEP+HexNAc(1)Hex(1)_1)
1221.662(PEP+HexNAc(2)_1)
1383.715(PEP+HexNAc(2)Hex(1)_1)
ETD-MS/MS Scan:7017, Noise threshold:0.6
M+H
M+2H
204.088(HexNAc)
366.142(HexNAc + Hex)
528.195(Hex + HexNAc + Hex)
611.337(PEP+HexNAc(2)_2)
692.364(PEP+HexNAc(2)Hex(1)_2)
773.389(PEP+HexNAc(2)Hex(2)_2)
744.418
1000.572
1018.582(PEP+HexNAc(1)_1)
1221.670(PEP+HexNAc(2)_1)
1265.541
1289.615
1317.621
1362.604
1450.661
1479.676
1502.735
1530.764
1547.787
1578.740
1649.795
1665.814
1691.807
m/z

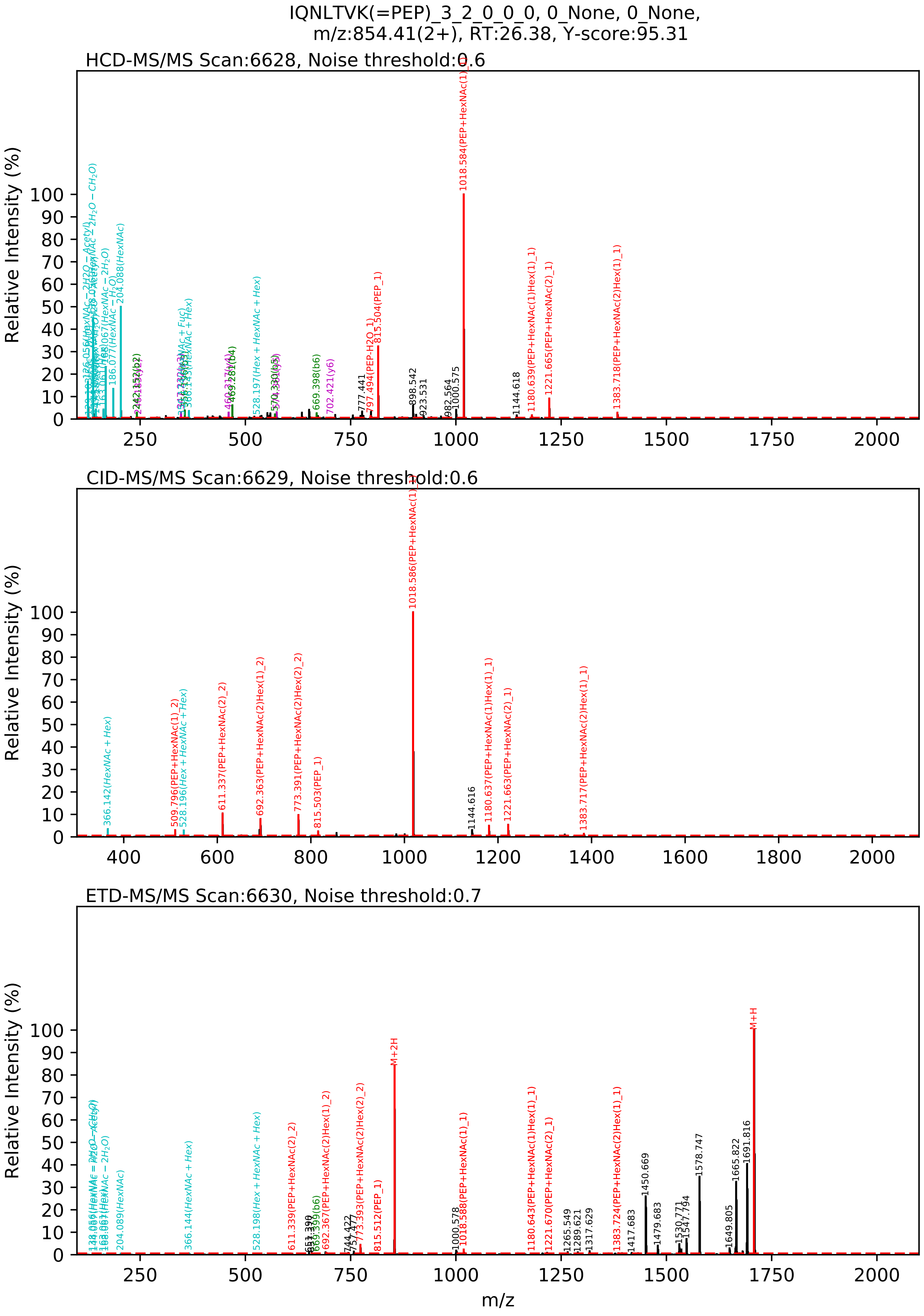
IQNLTVK(=PEP)_3_2_0_0_0, 0_None, 0_None,
m/z:854.41(2+), RT:26.38, Y-score:95.31
HCD-MS/MS Scan:6628, Noise threshold:0.6
Relative Intensity (%)
1018.584(PEP+HexNAc(1)_1)
815.504(PEP_1)
797.494(PEP-H2O_1)
1180.639(PEP+HexNAc(1)Hex(1)_1)
1221.665(PEP+HexNAc(2)_1)
1383.718(PEP+HexNAc(2)Hex(1)_1)
204.088(HexNAc)
186.077(HexNAc − H2O)
528.197(Hex + HexNAc + Hex)
669.398(b6)
702.421(y6)
777.441
898.542
923.531
982.564
1000.575
1144.618
CID-MS/MS Scan:6629, Noise threshold:0.6
Relative Intensity (%)
1018.586(PEP+HexNAc(1)_1)
366.142(HexNAc + Hex)
509.796(PEP+HexNAc(1)_2)
528.196(Hex + HexNAc + Hex)
611.337(PEP+HexNAc(2)_2)
692.363(PEP+HexNAc(2)Hex(1)_2)
773.391(PEP+HexNAc(2)Hex(2)_2)
815.503(PEP_1)
1144.616
1180.637(PEP+HexNAc(1)Hex(1)_1)
1221.663(PEP+HexNAc(2)_1)
1383.717(PEP+HexNAc(2)Hex(1)_1)
ETD-MS/MS Scan:6630, Noise threshold:0.7
Relative Intensity (%)
M+H
M+2H
204.089(HexNAc)
366.144(HexNAc + Hex)
528.198(Hex + HexNAc + Hex)
611.339(PEP+HexNAc(2)_2)
669.399(b6)
692.367(PEP+HexNAc(2)Hex(1)_2)
773.393(PEP+HexNAc(2)Hex(2)_2)
815.512(PEP_1)
1000.578
1018.588(PEP+HexNAc(1)_1)
1180.643(PEP+HexNAc(1)Hex(1)_1)
1221.670(PEP+HexNAc(2)_1)
1265.549
1289.621
1317.629
1383.724(PEP+HexNAc(2)Hex(1)_1)
1417.683
1450.669
1479.683
1530.771
1547.794
1578.747
1649.805
1665.822
1691.816
m/z

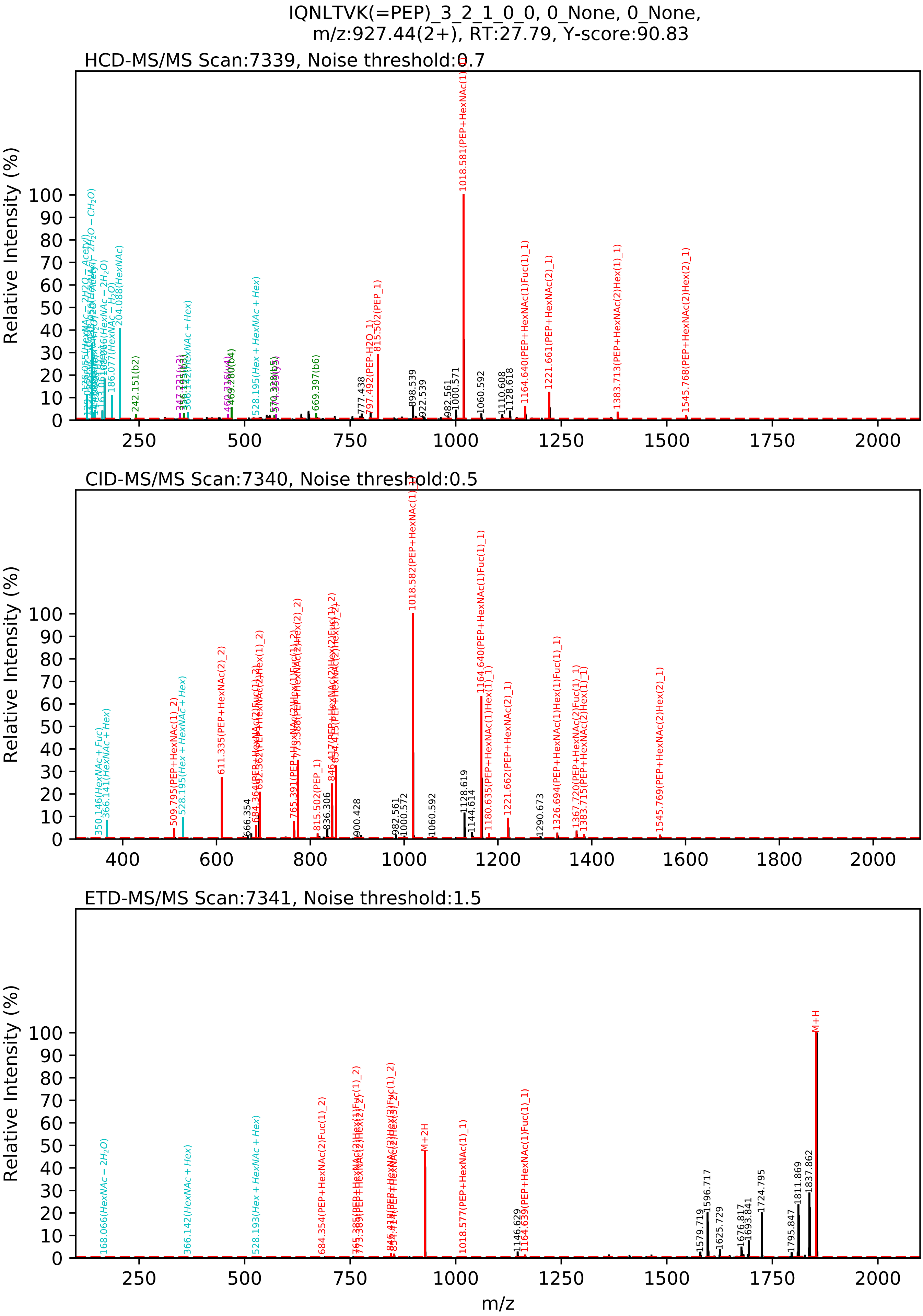

IQNLTVK(=PEP)_3_2_1_0_0, 0_None, 0_None,
m/z:927.44(2+), RT:27.79, Y-score:90.83
HCD-MS/MS Scan:7339, Noise threshold:0.7
1018.581(PEP+HexNAc(1)_1)
815.502(PEP_1)
797.492(PEP-H2O_1)
1164.640(PEP+HexNAc(1)Fuc(1)_1)
1221.661(PEP+HexNAc(2)_1)
1383.713(PEP+HexNAc(2)Hex(1)_1)
1545.768(PEP+HexNAc(2)Hex(2)_1)
204.088(HexNAc)
186.077(HexNAc − H2O)
242.151(b2)
366.142(HexNAc + Hex)
469.280(b4)
528.195(Hex + HexNAc + Hex)
669.397(b6)
777.438
898.539
922.539
982.561
1000.571
1060.592
1110.608
1128.618
CID-MS/MS Scan:7340, Noise threshold:0.5
350.146(HexNAc + Fuc)
366.141(HexNAc + Hex)
509.795(PEP+HexNAc(1)_2)
528.195(Hex + HexNAc + Hex)
611.335(PEP+HexNAc(2)_2)
666.354
765.391
773.388
815.502(PEP_1)
836.306
846.417
854.415
900.428
982.561
1000.572
1018.582(PEP+HexNAc(1)_1)
1060.592
1128.619
1144.614
1164.640(PEP+HexNAc(1)Fuc(1)_1)
1180.635(PEP+HexNAc(1)Hex(1)_1)
1221.662(PEP+HexNAc(2)_1)
1290.673
1326.694(PEP+HexNAc(1)Hex(1)Fuc(1)_1)
1367.720(PEP+HexNAc(2)Fuc(1)_1)
1383.715(PEP+HexNAc(2)Hex(1)_1)
1545.769(PEP+HexNAc(2)Hex(2)_1)
ETD-MS/MS Scan:7341, Noise threshold:1.5
168.066(HexNAc − 2H2O)
366.142(HexNAc + Hex)
528.193(Hex + HexNAc + Hex)
684.354(PEP+HexNAc(2)Fuc(1)_2)
M+2H
1018.577(PEP+HexNAc(1)_1)
1146.629
1164.639(PEP+HexNAc(1)Fuc(1)_1)
1579.719
1596.717
1625.729
1676.817
1693.841
1724.795
1795.847
1811.869
1837.862
M+H
Relative Intensity (%)
m/z

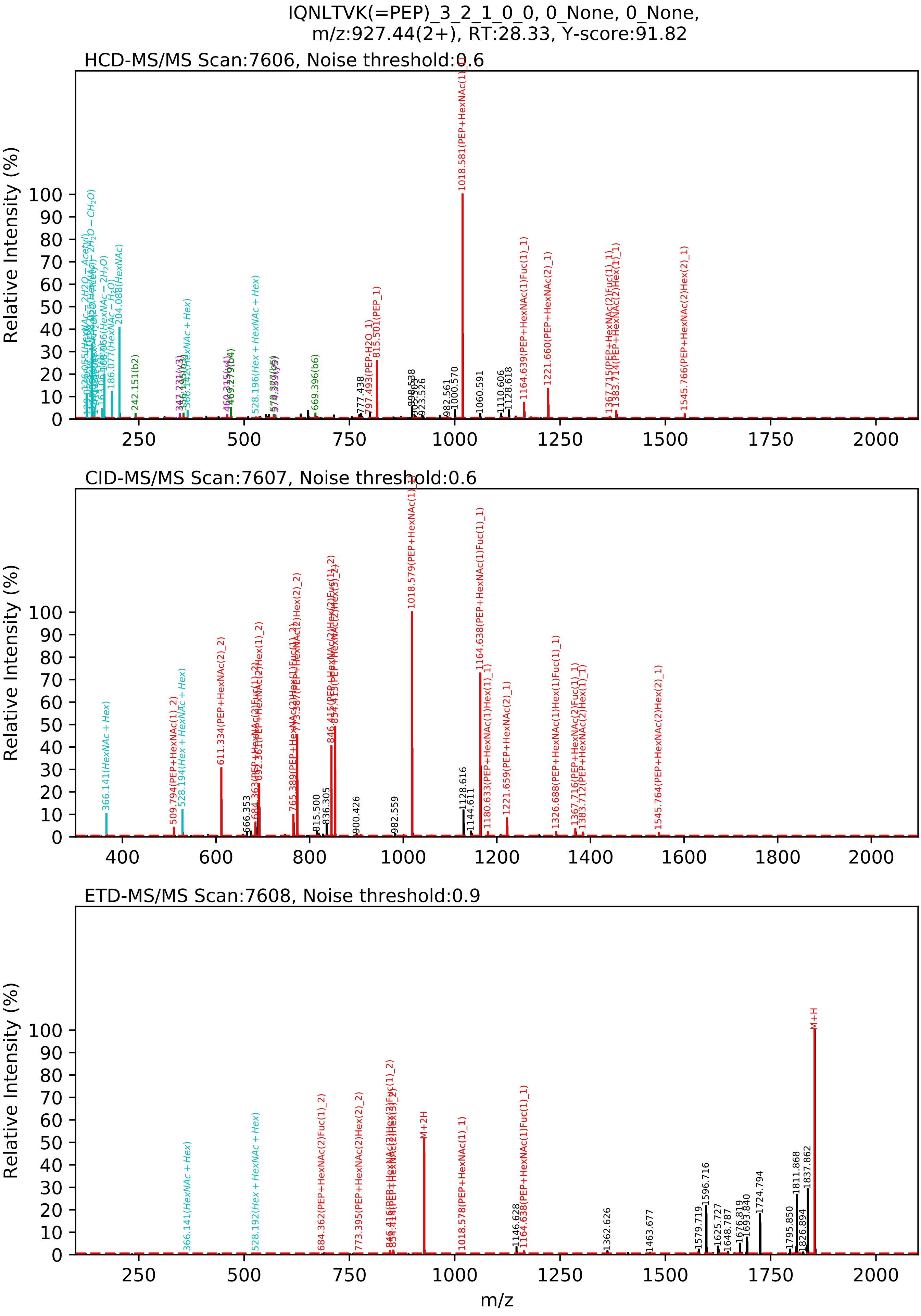
IQNLTVK(=PEP)_3_2_1_0_0, 0_None, 0_None,
m/z:927.44(2+), RT:28.33, Y-score:91.82
HCD-MS/MS Scan:7606, Noise threshold:0.6
CID-MS/MS Scan:7607, Noise threshold:0.6
ETD-MS/MS Scan:7608, Noise threshold:0.9
Relative Intensity (%)
m/z
1018.581(PEP+HexNAc(1)_1)
1164.639(PEP+HexNAc(1)Fuc(1)_1)
1221.660(PEP+HexNAc(2)_1)
1545.766(PEP+HexNAc(2)Hex(2)_1)
815.501(PEP_1)
797.493(PEP-H2O_1)
204.088(HexNAc)
669.396(b6)
242.151(b2)
1018.579(PEP+HexNAc(1)_1)
1164.638(PEP+HexNAc(1)Fuc(1)_1)
611.334(PEP+HexNAc(2)_2)
1221.659(PEP+HexNAc(2)_1)
1326.688(PEP+HexNAc(1)Hex(1)Fuc(1)_1)
1545.764(PEP+HexNAc(2)Hex(2)_1)
366.141(HexNAc + Hex)
528.194(Hex + HexNAc + Hex)
509.794(PEP+HexNAc(1)_2)
M+H
M+2H
684.362(PEP+HexNAc(2)Fuc(1)_2)
773.395(PEP+HexNAc(2)Hex(2)_2)
1018.578(PEP+HexNAc(1)_1)
1164.638(PEP+HexNAc(1)Fuc(1)_1)
1596.716
1724.794
1811.868
1837.862

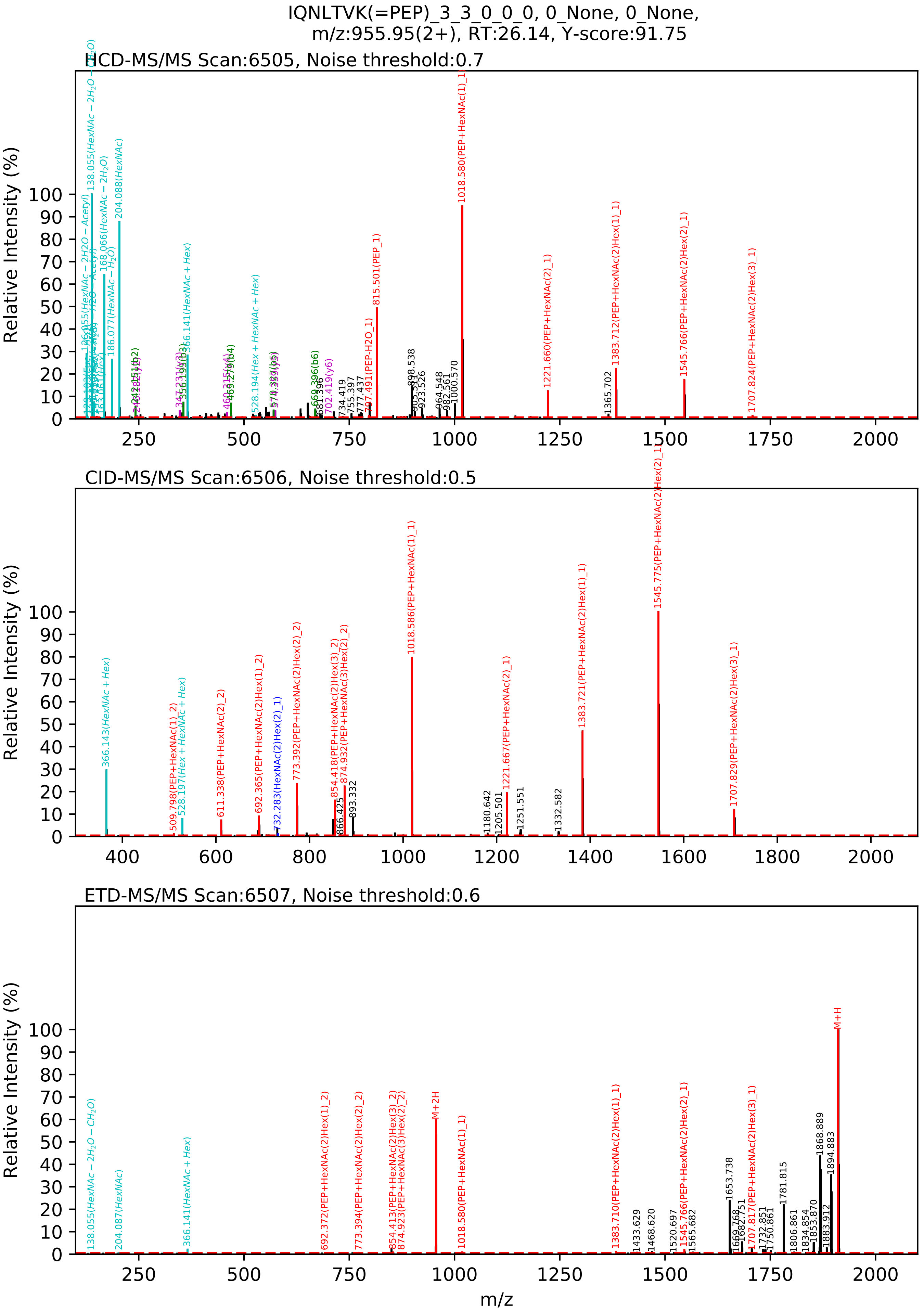
IQNLTVK(=PEP)_3_3_0_0_0, 0_None, 0_None,
m/z:955.95(2+), RT:26.14, Y-score:91.75
HCD-MS/MS Scan:6505, Noise threshold:0.7
Relative Intensity (%)
1018.580(PEP+HexNAc(1)_1)
815.501(PEP_1)
797.491(PEP-H2O_1)
1221.660(PEP+HexNAc(2)_1)
1383.712(PEP+HexNAc(2)Hex(1)_1)
1545.766(PEP+HexNAc(2)Hex(2)_1)
1707.824(PEP+HexNAc(2)Hex(3)_1)
204.088(HexNAc)
186.077(HexNAc − H2O)
168.066(HexNAc − 2H2O)
138.055(HexNAc − 2H2O − CH2O)
366.141(HexNAc + Hex)
528.194(Hex + HexNAc + Hex)
669.396(b6)
702.419(y6)
898.538
923.526
964.548
1000.570
1365.702
734.419
755.397
777.437
CID-MS/MS Scan:6506, Noise threshold:0.5
366.143(HexNAc + Hex)
509.798(PEP+HexNAc(1)_2)
528.197(Hex + HexNAc + Hex)
611.338(PEP+HexNAc(2)_2)
692.365(PEP+HexNAc(2)Hex(1)_2)
732.283(HexNAc(2)Hex(2)_1)
773.392(PEP+HexNAc(2)Hex(2)_2)
854.418(PEP+HexNAc(2)Hex(3)_2)
874.932(PEP+HexNAc(3)Hex(2)_2)
866.425
893.332
1018.586(PEP+HexNAc(1)_1)
1180.642
1205.501
1221.667(PEP+HexNAc(2)_1)
1251.551
1332.582
1383.721(PEP+HexNAc(2)Hex(1)_1)
1545.775(PEP+HexNAc(2)Hex(2)_1)
1707.829(PEP+HexNAc(2)Hex(3)_1)
ETD-MS/MS Scan:6507, Noise threshold:0.6
138.055(HexNAc − 2H2O − CH2O)
204.087(HexNAc)
366.141(HexNAc + Hex)
692.372(PEP+HexNAc(2)Hex(1)_2)
773.394(PEP+HexNAc(2)Hex(2)_2)
854.413(PEP+HexNAc(2)Hex(3)_2)
874.923(PEP+HexNAc(3)Hex(2)_2)
M+2H
1018.580(PEP+HexNAc(1)_1)
1383.710(PEP+HexNAc(2)Hex(1)_1)
1433.629
1468.620
1520.697
1545.766(PEP+HexNAc(2)Hex(2)_1)
1565.682
1653.738
1669.768
1682.751
1707.817(PEP+HexNAc(2)Hex(3)_1)
1732.851
1750.861
1781.815
1806.861
1834.854
1853.870
1868.889
1883.912
1894.883
M+H
m/z

IQNLTVK(=PEP)_3_3_0_0_0, 0_None, 0_None,
m/z:955.95(2+), RT:35.78, Y-score:89.04

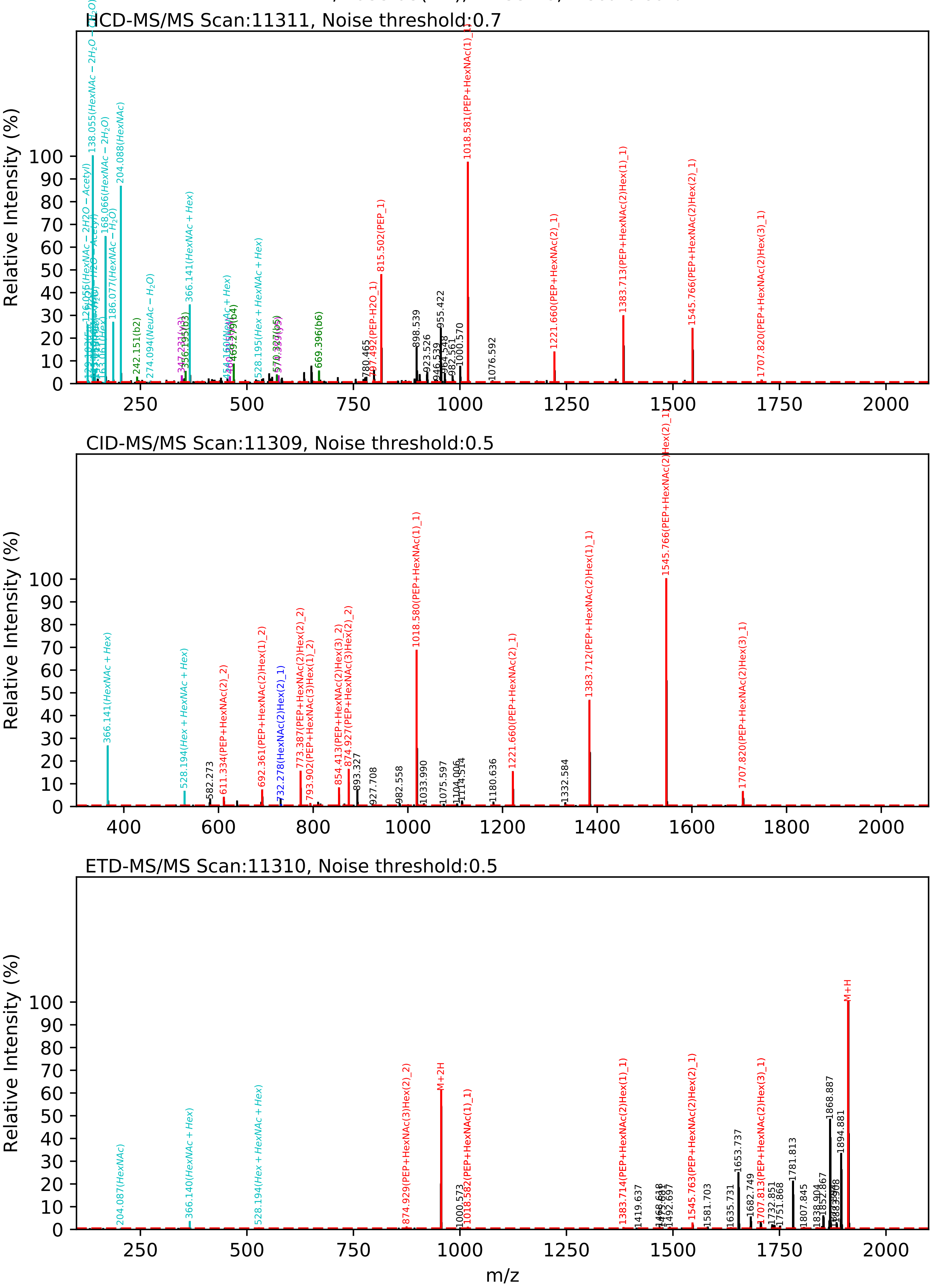

IQNLTVK(=PEP)_3_3_1_0_0, 0_None, 0_None,
m/z:1028.98(2+), RT:26.49, Y-score:92.39

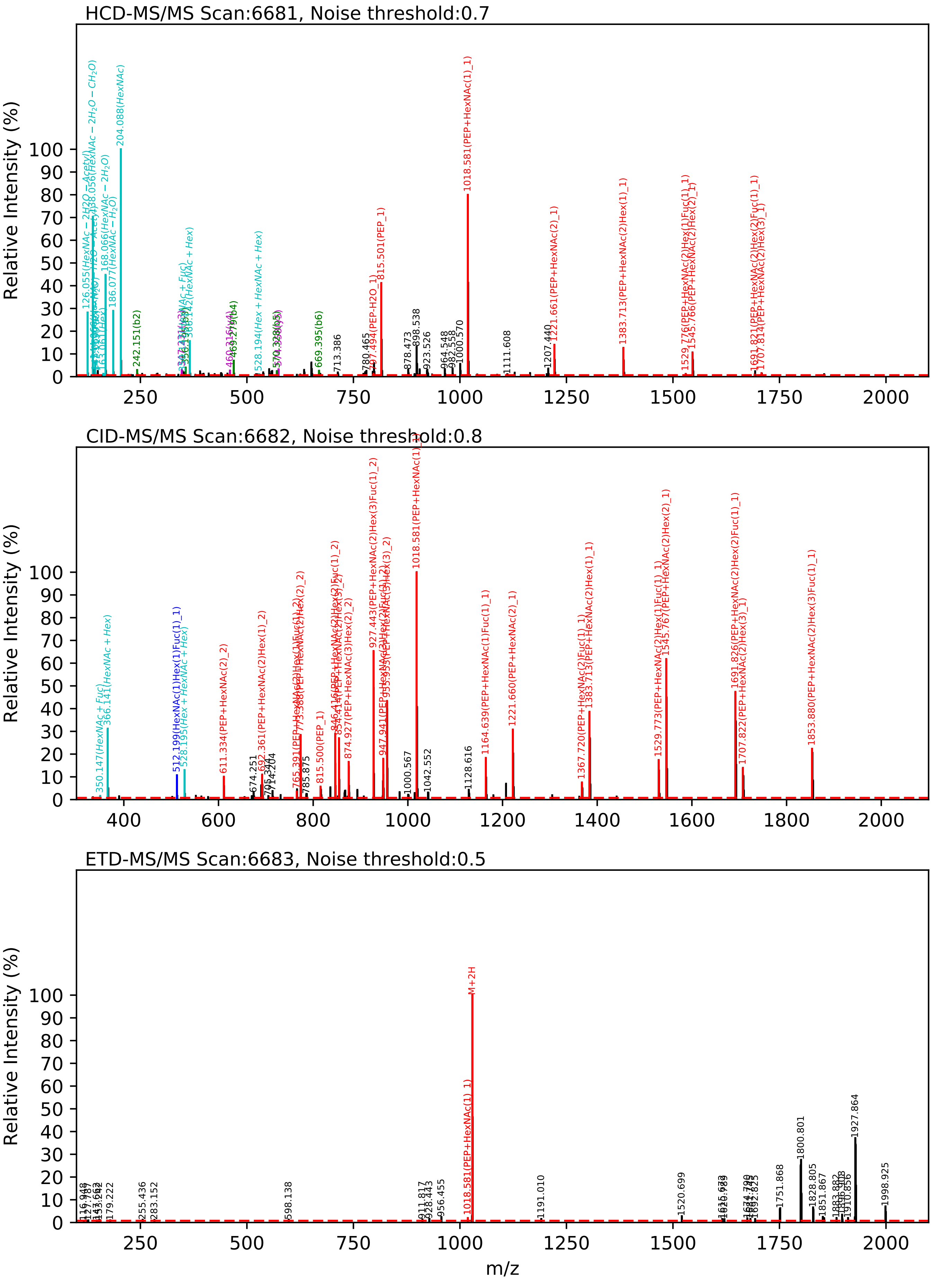

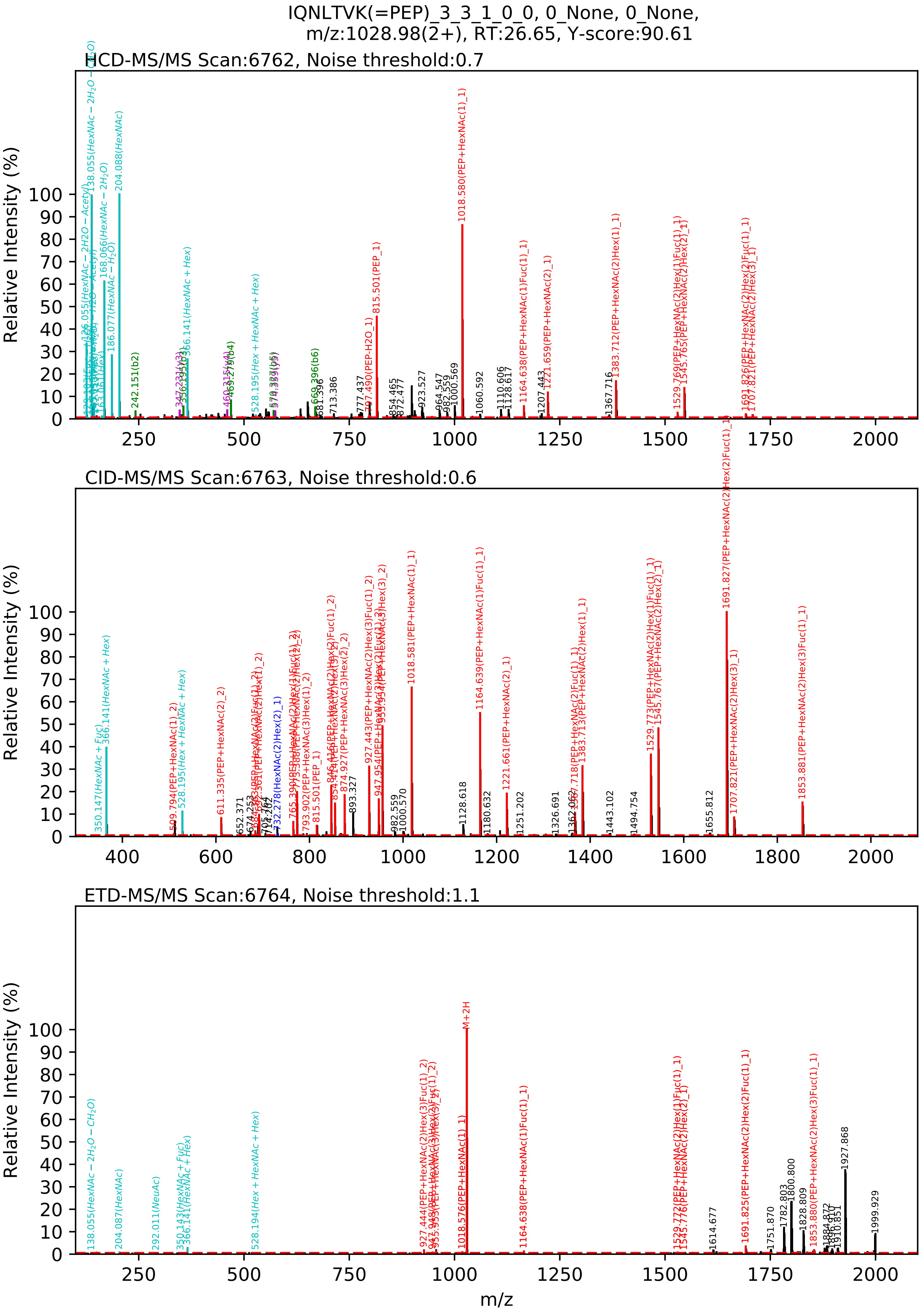

IQNLTVK(=PEP)_3_3_1_0_0, 0_None, 0_None,
m/z:1028.98(2+), RT:26.65, Y-score:90.61
HCD-MS/MS Scan:6762, Noise threshold:0.7
CID-MS/MS Scan:6763, Noise threshold:0.6
ETD-MS/MS Scan:6764, Noise threshold:1.1
Relative Intensity (%)
m/z
1018.580(PEP+HexNAc(1)_1)
815.501(PEP_1)
1691.827(PEP+HexNAc(2)Hex(2)Fuc(1)_1)
1853.881(PEP+HexNAc(2)Hex(3)Fuc(1)_1)
1164.639(PEP+HexNAc(1)Fuc(1)_1)
1018.581(PEP+HexNAc(1)_1)
M+2H
1927.868

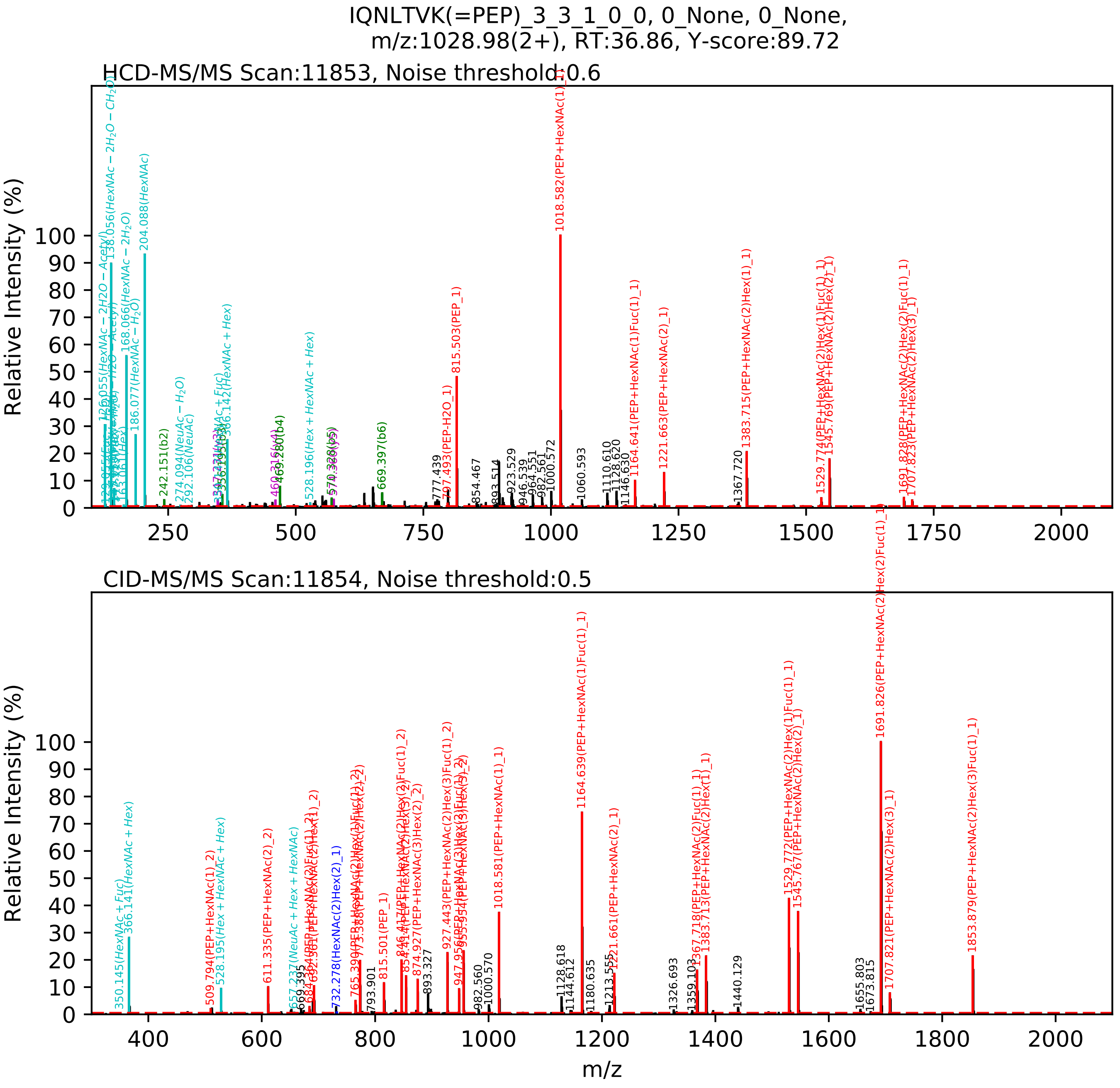
IQNLTVK(=PEP)_3_3_1_0_0, 0_None, 0_None,
m/z:1028.98(2+), RT:36.86, Y-score:89.72
HCD-MS/MS Scan:11853, Noise threshold:0.6
Relative Intensity (%)
1018.582(PEP+HexNAc(1)_1)
204.088(HexNAc)
138.056(HexNAc − 2H2O − CH2O)
168.066(HexNAc − 2H2O)
186.077(HexNAc − H2O)
163.061(Hex)
242.151(b2)
274.094(NeuAc − H2O)
292.106(NeuAc)
366.142(HexNAc + Hex)
469.280(b4)
528.196(Hex + HexNAc + Hex)
669.397(b6)
815.503(PEP_1)
797.493(PEP-H2O_1)
777.439
854.467
893.514
923.529
946.539
964.551
982.561
1000.572
1060.593
1110.610
1128.620
1146.630
1164.641(PEP+HexNAc(1)Fuc(1)_1)
1221.663(PEP+HexNAc(2)_1)
1367.720
1383.715(PEP+HexNAc(2)Hex(1)_1)
1529.774(PEP+HexNAc(2)Hex(1)Fuc(1)_1)
1545.769(PEP+HexNAc(2)Hex(2)_1)
1691.828(PEP+HexNAc(2)Hex(2)Fuc(1)_1)
1707.823(PEP+HexNAc(2)Hex(3)_1)
CID-MS/MS Scan:11854, Noise threshold:0.5
Relative Intensity (%)
1691.826(PEP+HexNAc(2)Hex(2)Fuc(1)_1)
1164.639(PEP+HexNAc(1)Fuc(1)_1)
1529.772(PEP+HexNAc(2)Hex(1)Fuc(1)_1)
1545.767(PEP+HexNAc(2)Hex(2)_1)
1018.581(PEP+HexNAc(1)_1)
1853.879(PEP+HexNAc(2)Hex(3)Fuc(1)_1)
1707.821(PEP+HexNAc(2)Hex(3)_1)
1221.661(PEP+HexNAc(2)_1)
1367.718(PEP+HexNAc(2)Fuc(1)_1)
1383.713(PEP+HexNAc(2)Hex(1)_1)
350.145(HexNAc + Fuc)
366.141(HexNAc + Hex)
509.794(PEP+HexNAc(1)_2)
528.195(Hex + HexNAc + Hex)
611.335(PEP+HexNAc(2)_2)
657.237(NeuAc + Hex + HexNAc)
669.395
692.361(PEP+HexNAc(2)Hex(1)_2)
732.278(HexNAc(2)Hex(2)_1)
765.390
793.901
815.501(PEP_1)
874.927(PEP+HexNAc(3)Hex(2)_2)
893.327
927.443(PEP+HexNAc(2)Hex(3)Fuc(1)_2)
982.560
1000.570
1128.618
1144.612
1180.635
1213.555
1326.693
1359.103
1440.129
1655.803
1673.815
m/z

IQNLTVK(=PEP)_3_3_1_0_0, 0_None, 0_None,
m/z:1028.98(2+), RT:36.88, Y-score:91.22

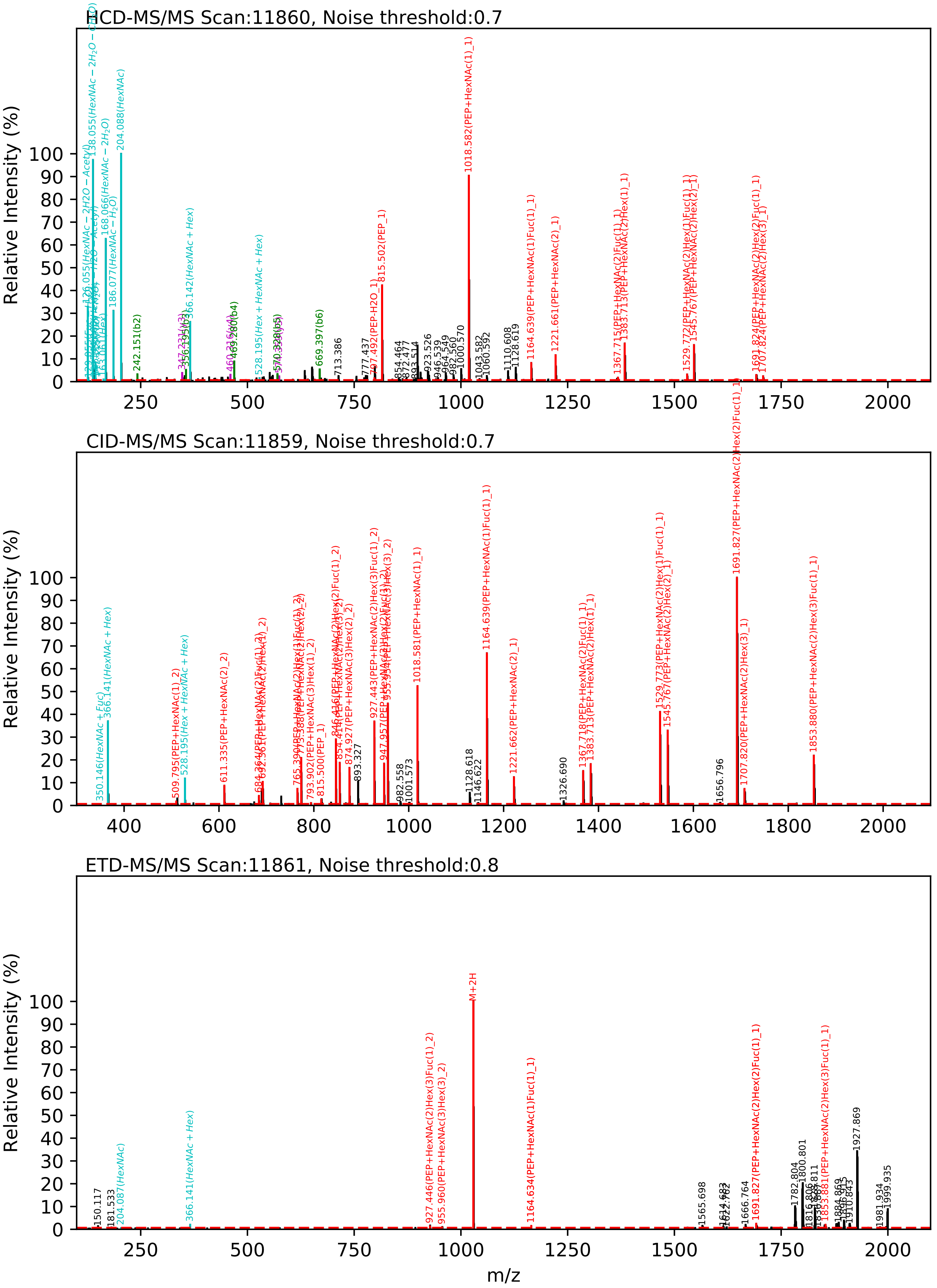

IQNLTVK(=PEP)_3_3_1_0_0, 0_None, 0_None,
m/z:1028.98(2+), RT:36.16, Y-score:88.12

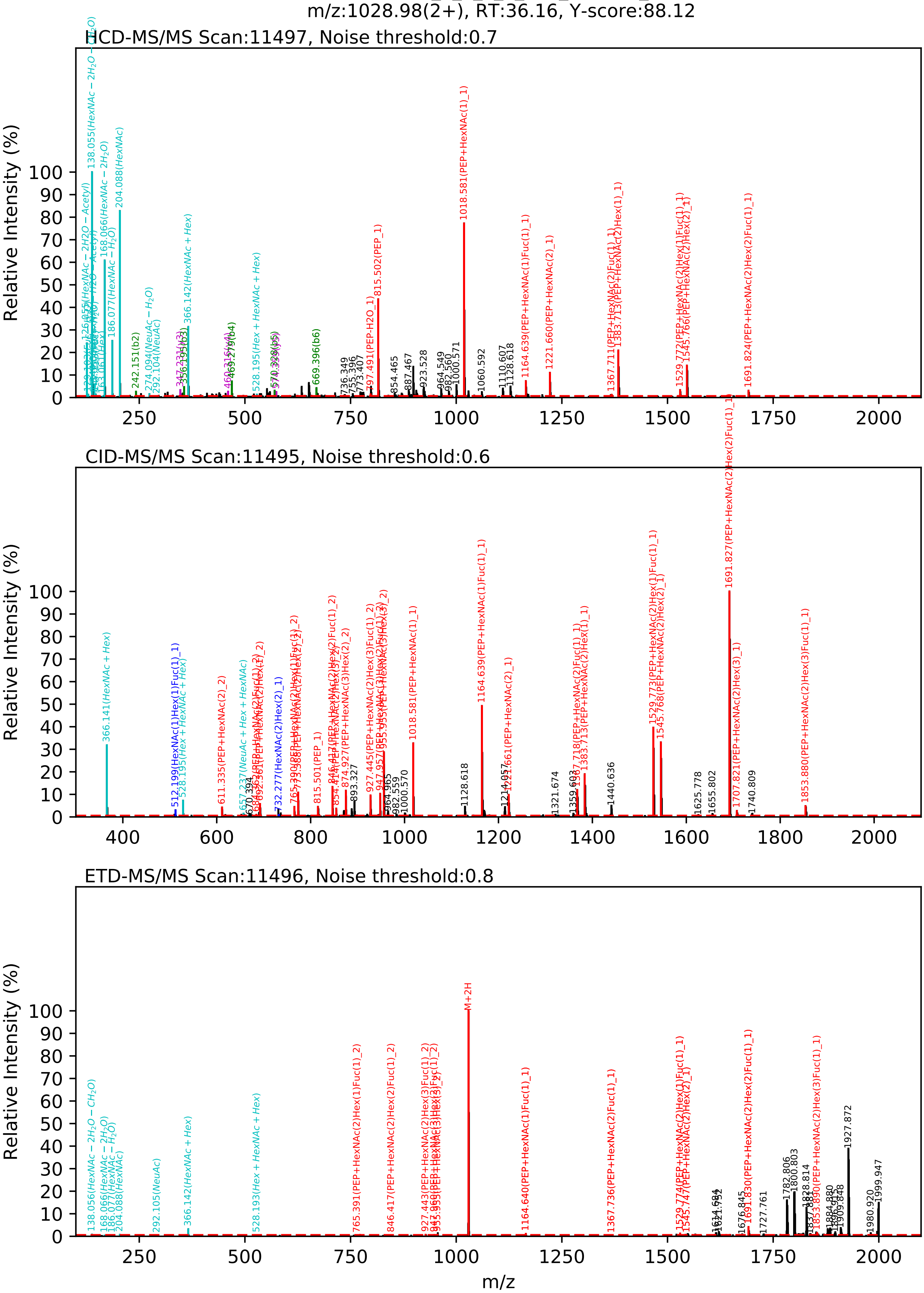

IQNLTVK(=PEP)_4_2_0_0_0, 0_None, 0_None,
m/z:935.44(2+), RT:25.50, Y-score:91.71

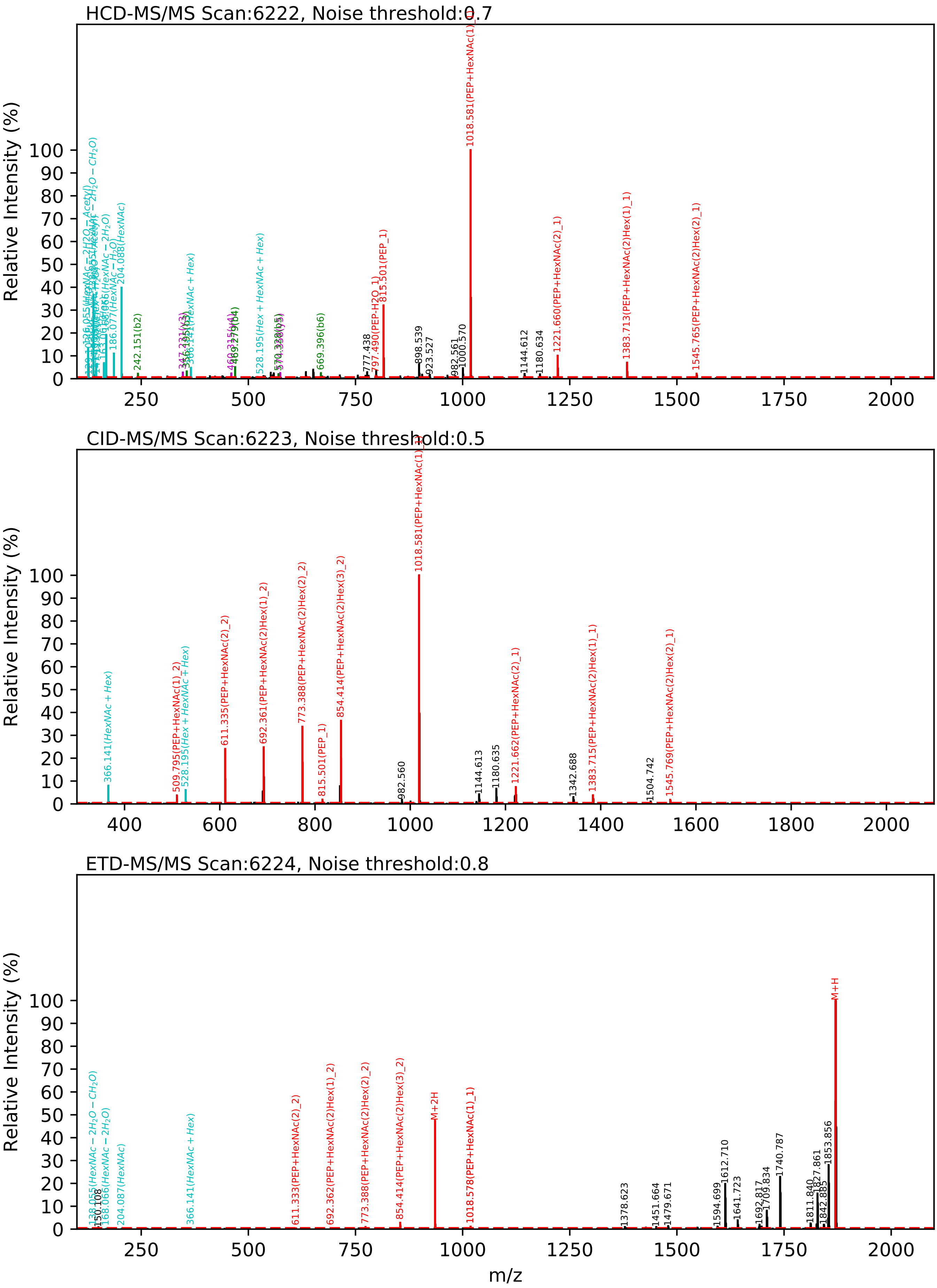

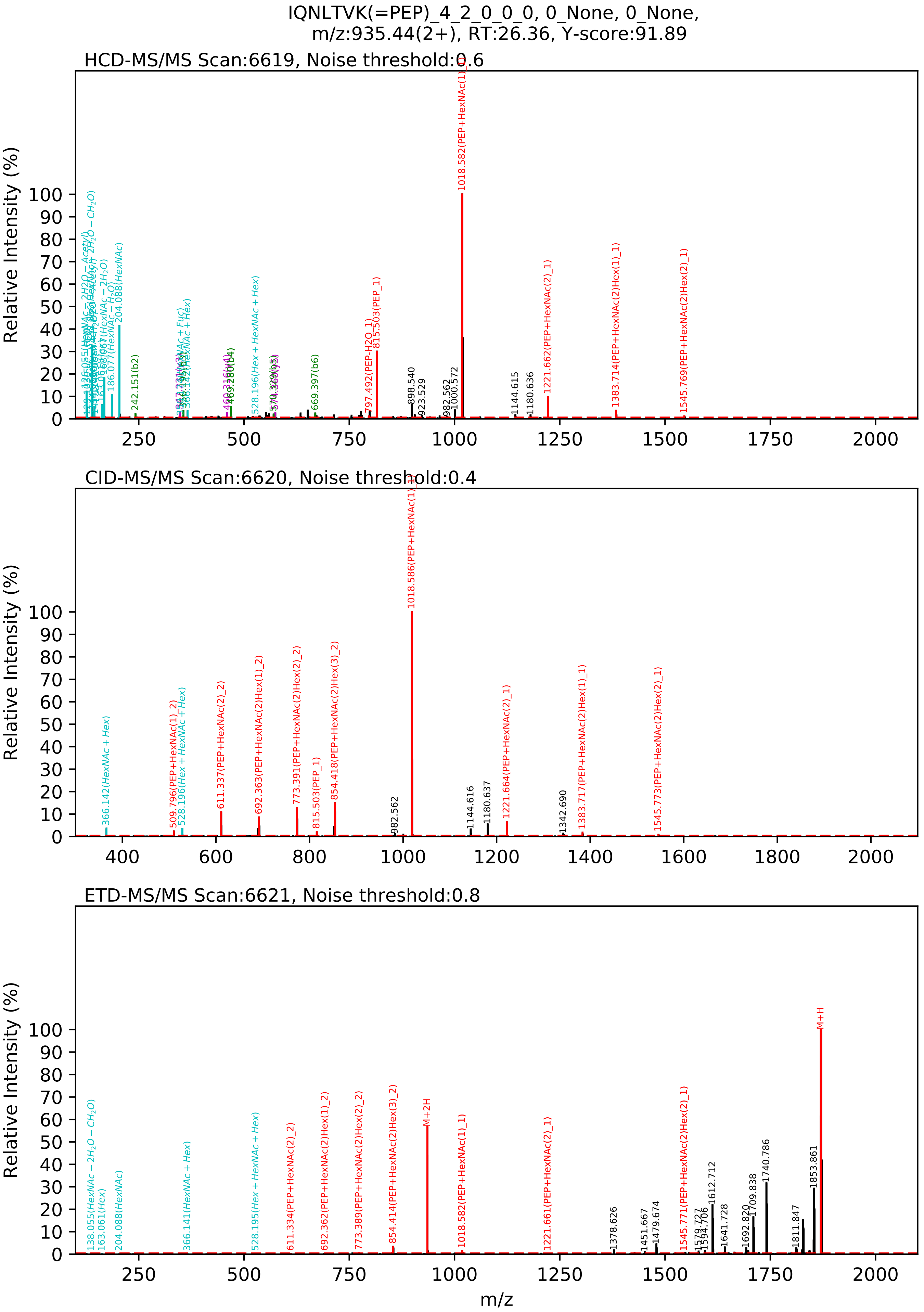
IQNLTVK(=PEP)_4_2_0_0_0, 0_None, 0_None,
m/z:935.44(2+), RT:26.36, Y-score:91.89
HCD-MS/MS Scan:6619, Noise threshold:0.6
CID-MS/MS Scan:6620, Noise threshold:0.4
ETD-MS/MS Scan:6621, Noise threshold:0.8
Relative Intensity (%)
m/z

IQNLTVK(=PEP)_4_2_0_0_0, 0_None, 0_None,
m/z:935.44(2+), RT:26.98, Y-score:94.22

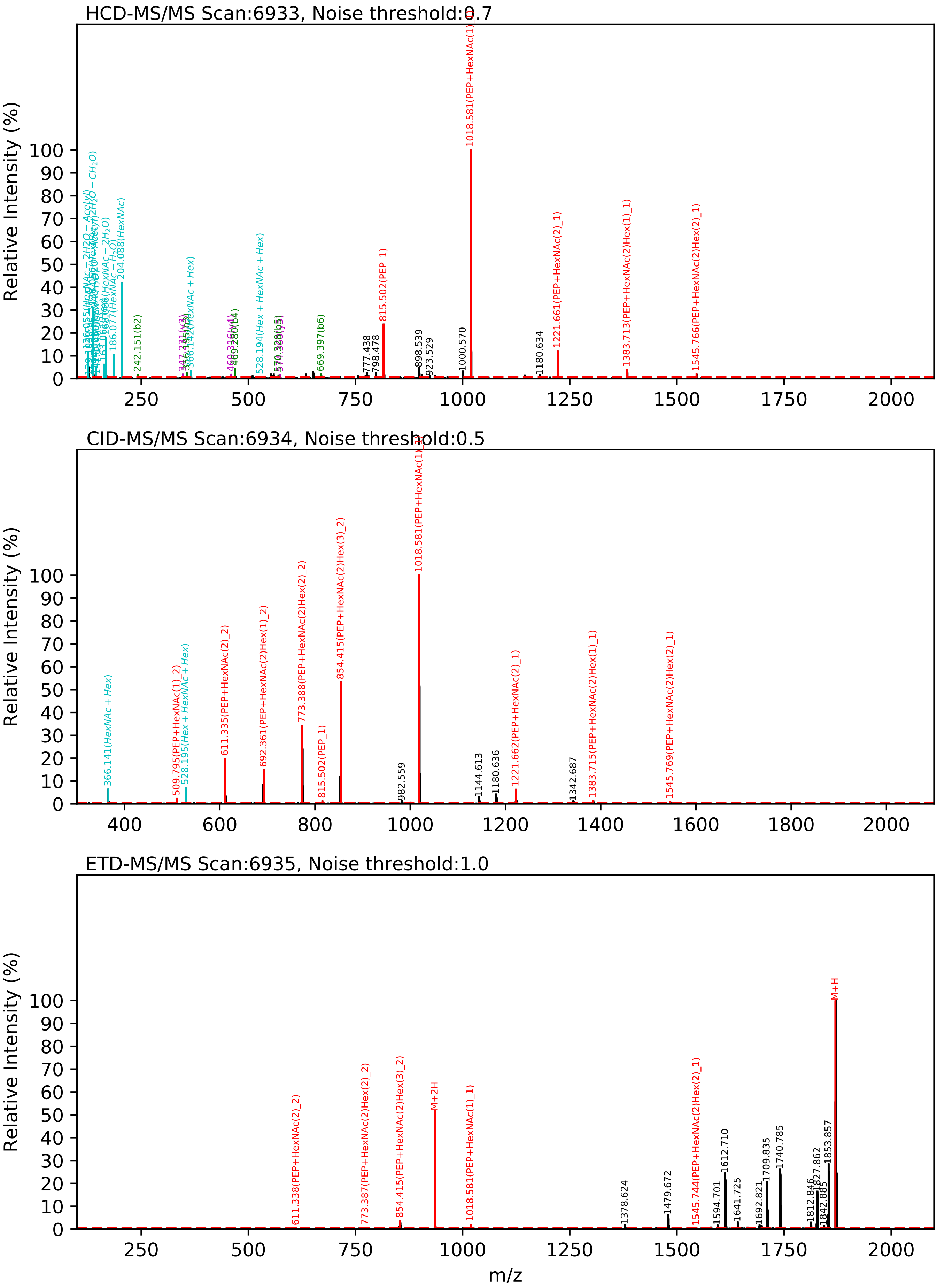

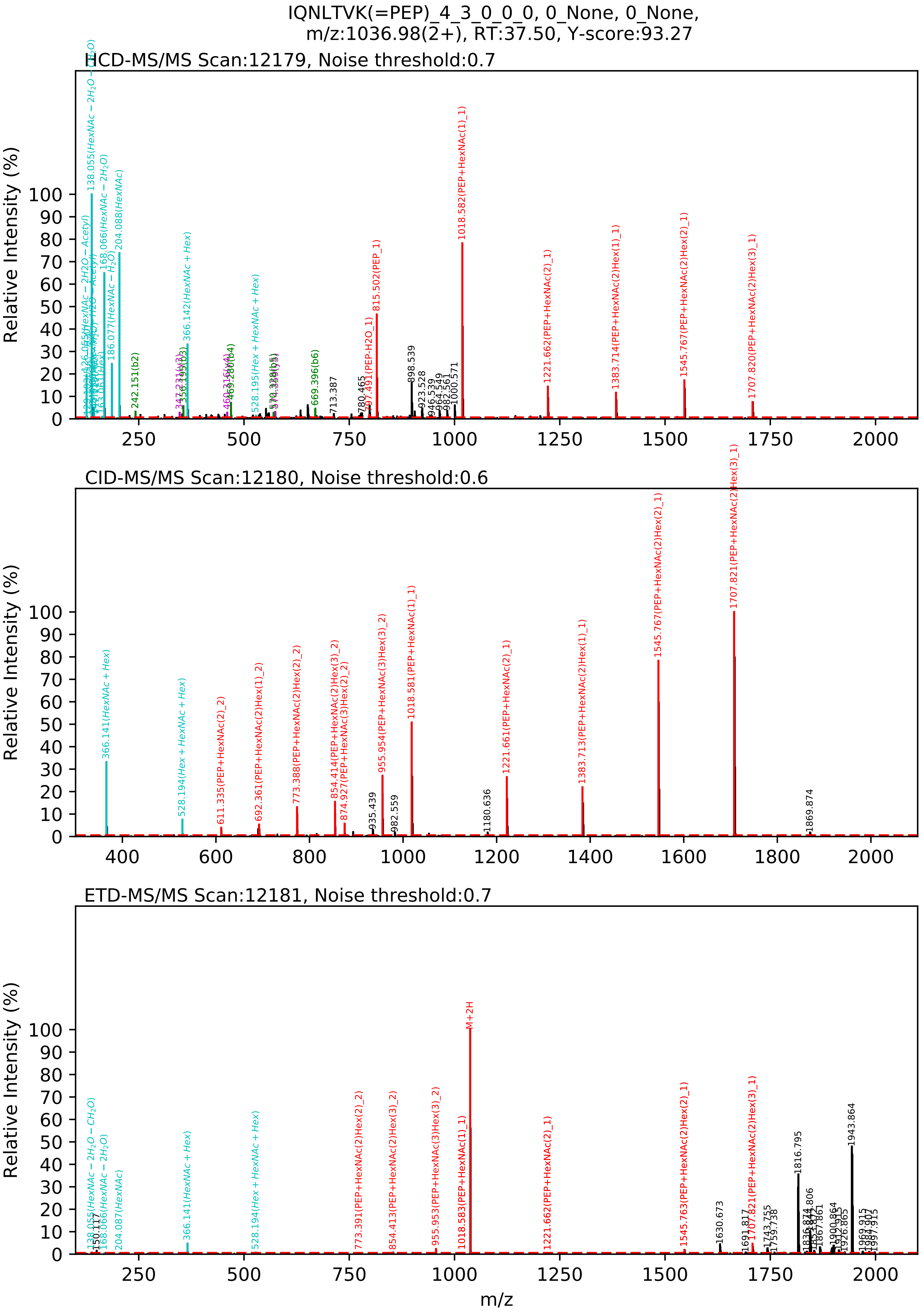
IQNLTVK(=PEP)_4_3_0_0_0, 0_None, 0_None,
m/z:1036.98(2+), RT:37.50, Y-score:93.27
HCD-MS/MS Scan:12179, Noise threshold:0.7
CID-MS/MS Scan:12180, Noise threshold:0.6
ETD-MS/MS Scan:12181, Noise threshold:0.7
Relative Intensity (%)
m/z
1018.582(PEP+HexNAc(1)_1)
1221.662(PEP+HexNAc(2)_1)
1383.714(PEP+HexNAc(2)Hex(1)_1)
1545.767(PEP+HexNAc(2)Hex(2)_1)
1707.820(PEP+HexNAc(2)Hex(3)_1)
815.502(PEP_1)
1707.821(PEP+HexNAc(2)Hex(3)_1)
1545.767(PEP+HexNAc(2)Hex(2)_1)
M+2H
1943.864
1816.795

IQNLTVK(=PEP)_4_3_0_0_0, 0_None, 0_None,
m/z:1036.98(2+), RT:37.52, Y-score:92.91

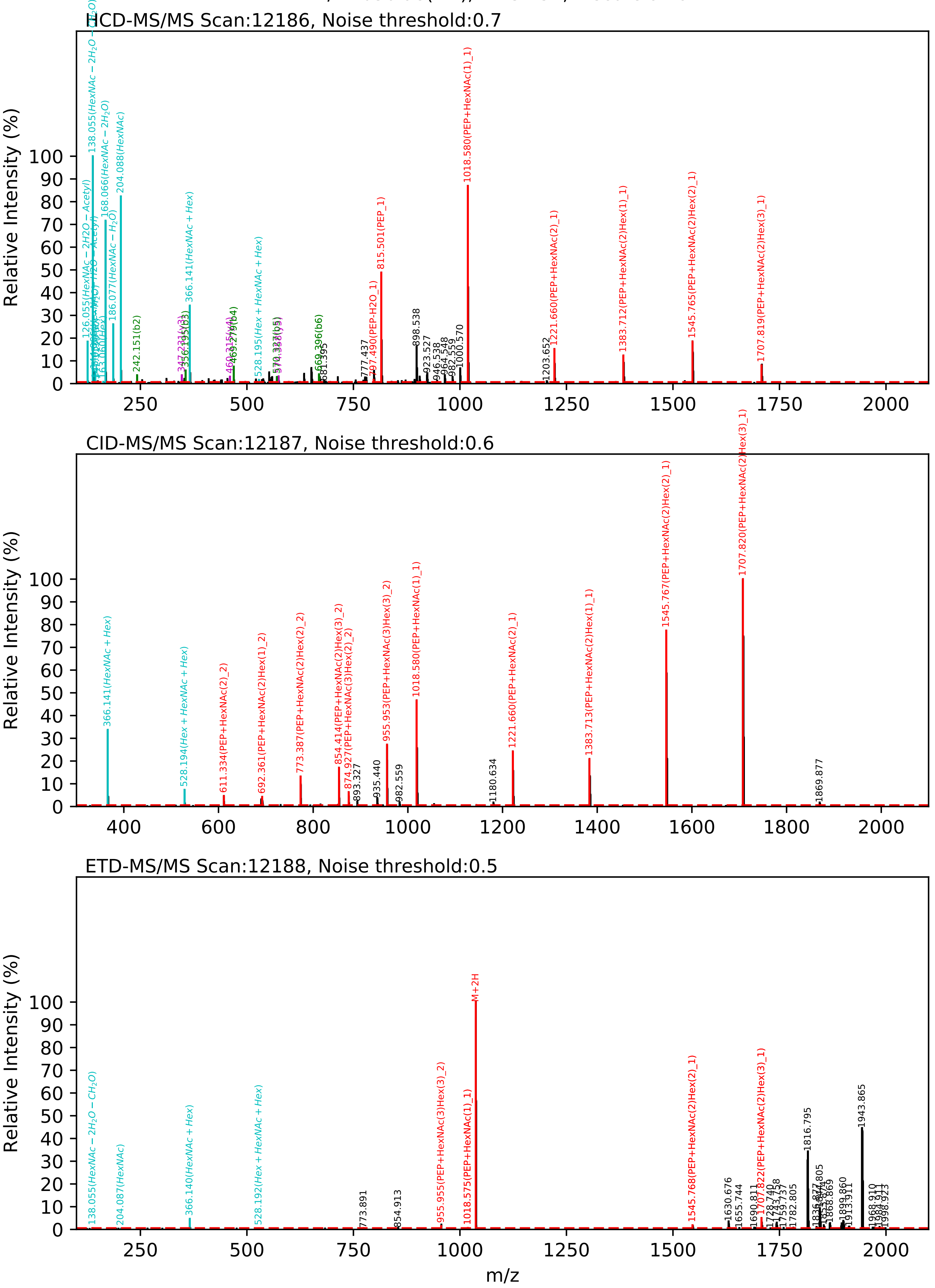

IQNLTVK(=PEP)_4_3_0_0_0, 0_None, 0_None,
m/z:1036.98(2+), RT:36.45, Y-score:95.08

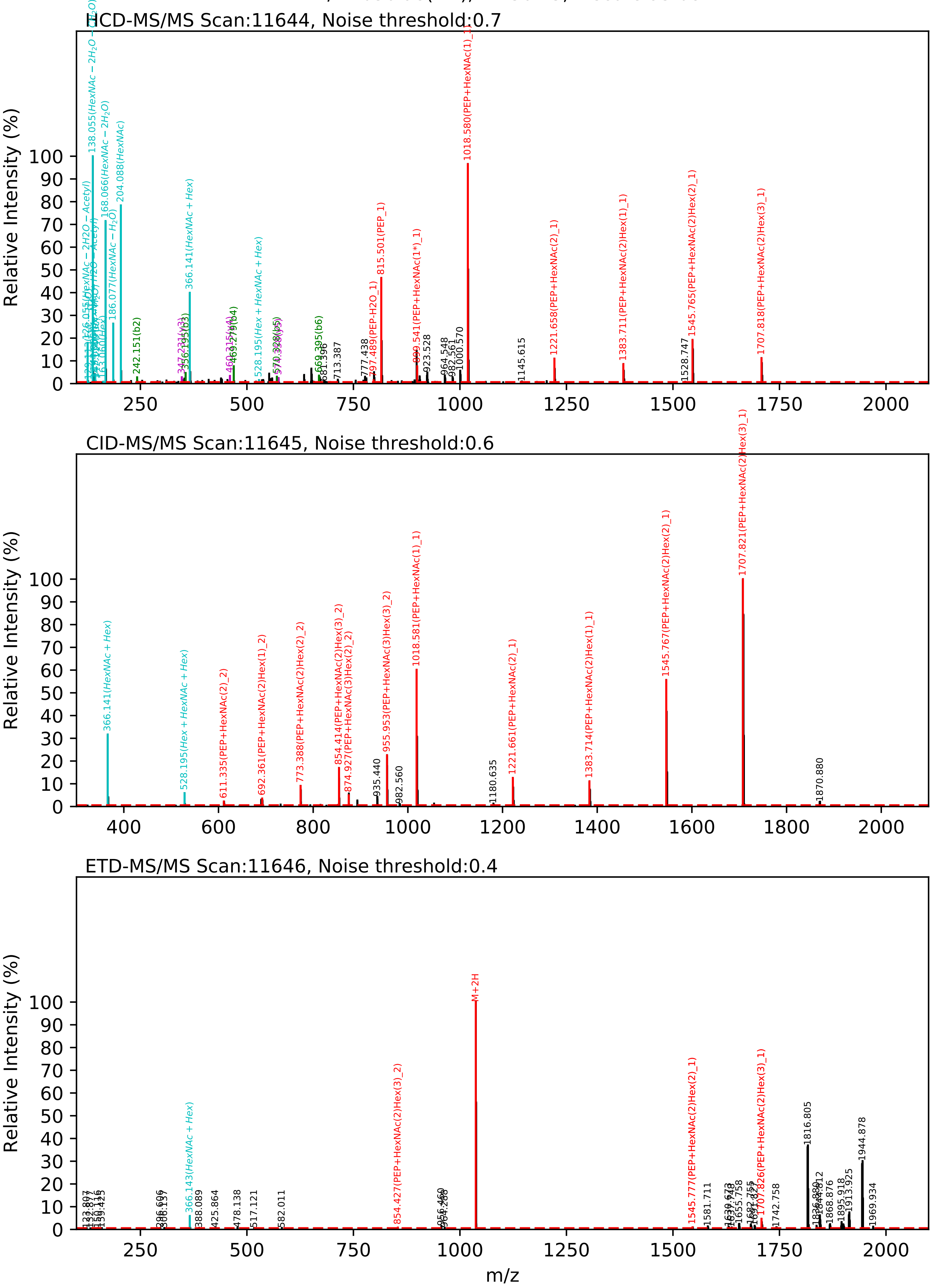

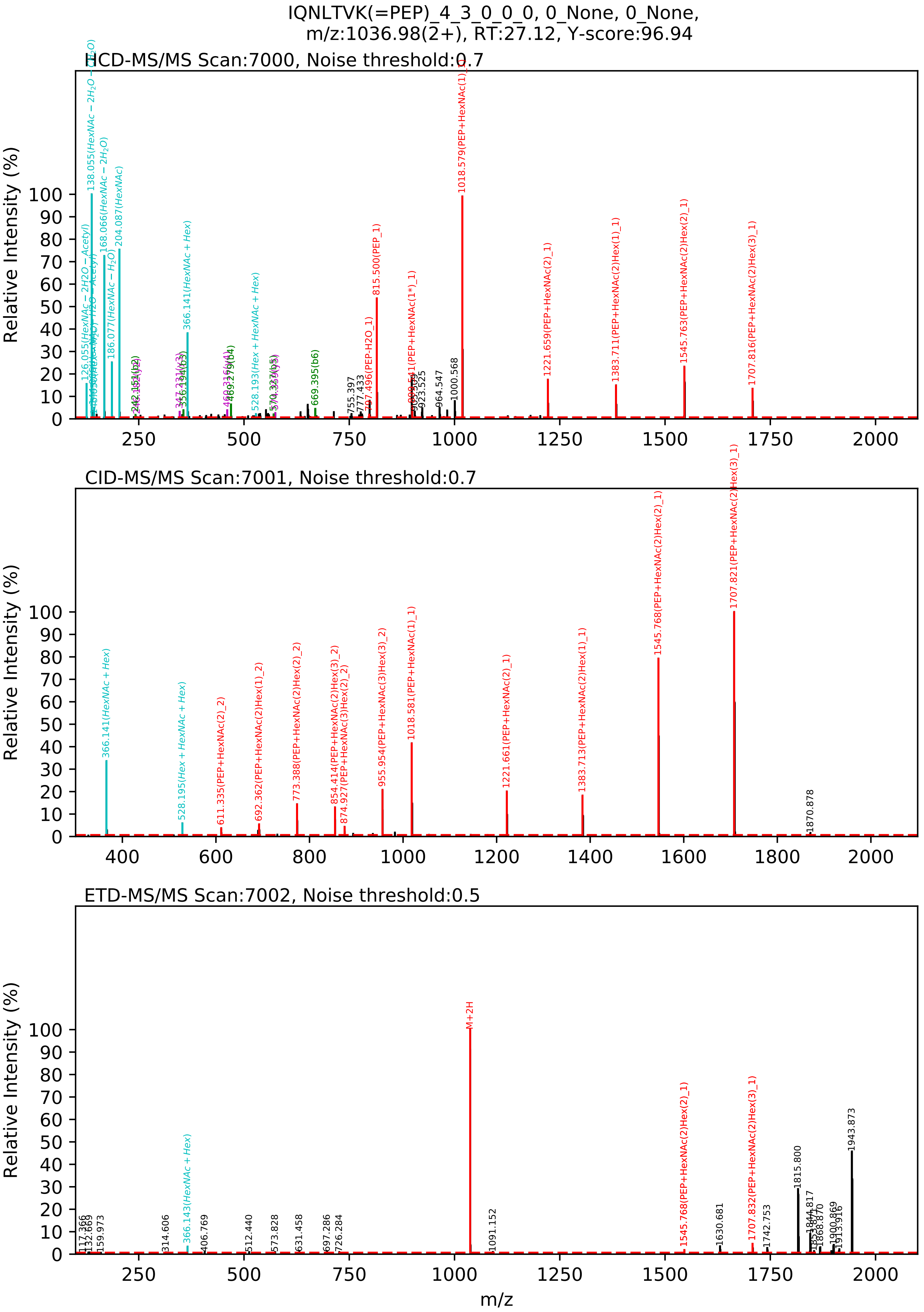

IQNLTVK(=PEP)_4_3_0_0_0, 0_None, 0_None,
m/z:1036.98(2+), RT:27.12, Y-score:96.94
HCD-MS/MS Scan:7000, Noise threshold:0.7
CID-MS/MS Scan:7001, Noise threshold:0.7
ETD-MS/MS Scan:7002, Noise threshold:0.5
Relative Intensity (%)
m/z

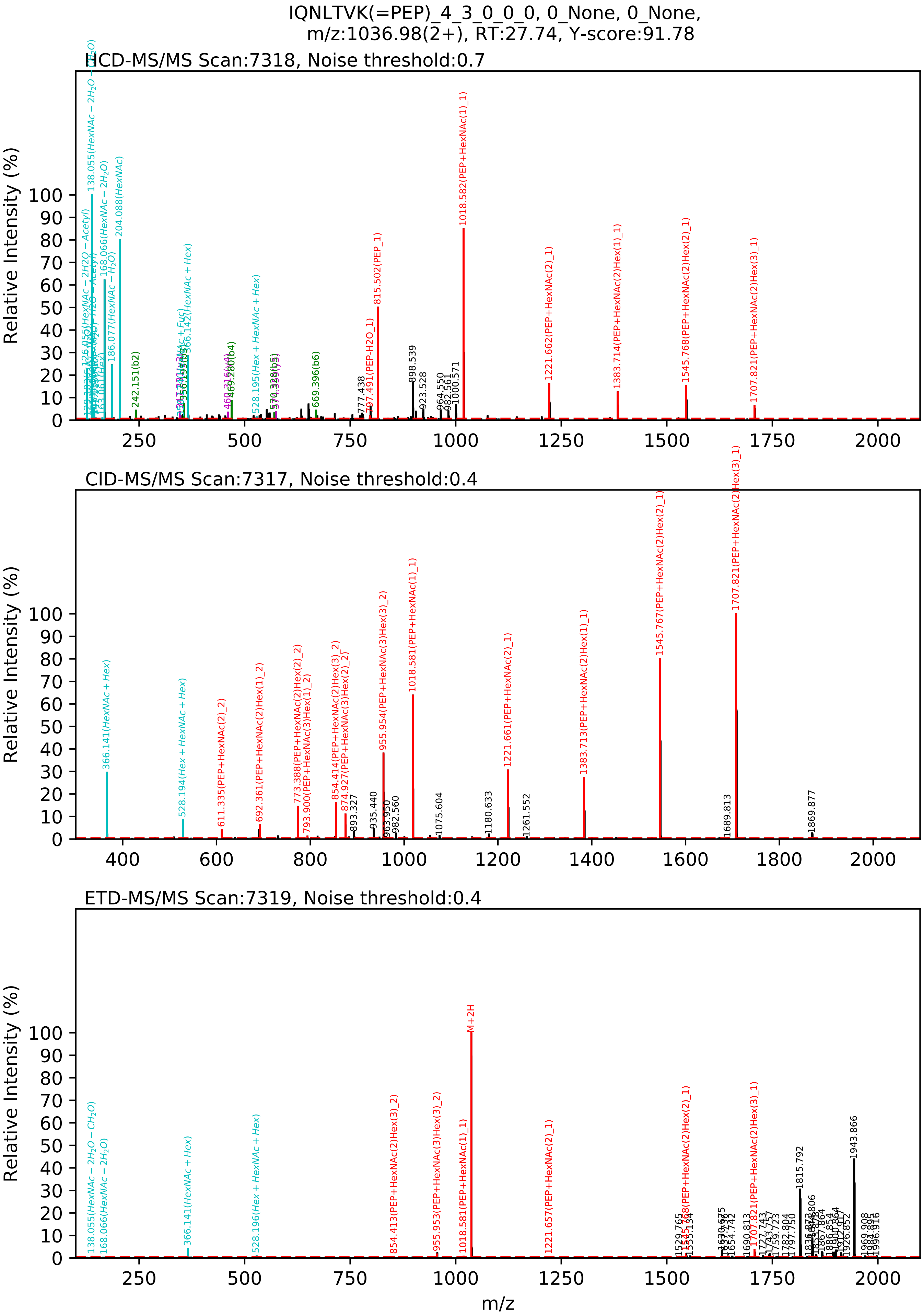

IQNLTVK(=PEP)_4_3_0_0_0, 0_None, 0_None,
m/z:1036.98(2+), RT:27.74, Y-score:91.78
HCD-MS/MS Scan:7318, Noise threshold:0.7
CID-MS/MS Scan:7317, Noise threshold:0.4
ETD-MS/MS Scan:7319, Noise threshold:0.4
Relative Intensity (%)
m/z
1018.582(PEP+HexNAc(1)_1)
815.502(PEP_1)
1221.662(PEP+HexNAc(2)_1)
1383.714(PEP+HexNAc(2)Hex(1)_1)
1545.768(PEP+HexNAc(2)Hex(2)_1)
1707.821(PEP+HexNAc(2)Hex(3)_1)
1707.821(PEP+HexNAc(2)Hex(3)_1)
1545.767(PEP+HexNAc(2)Hex(2)_1)
1383.713(PEP+HexNAc(2)Hex(1)_1)
1221.661(PEP+HexNAc(2)_1)
1018.581(PEP+HexNAc(1)_1)
955.954(PEP+HexNAc(3)Hex(3)_2)
M+2H
1943.866
1815.792

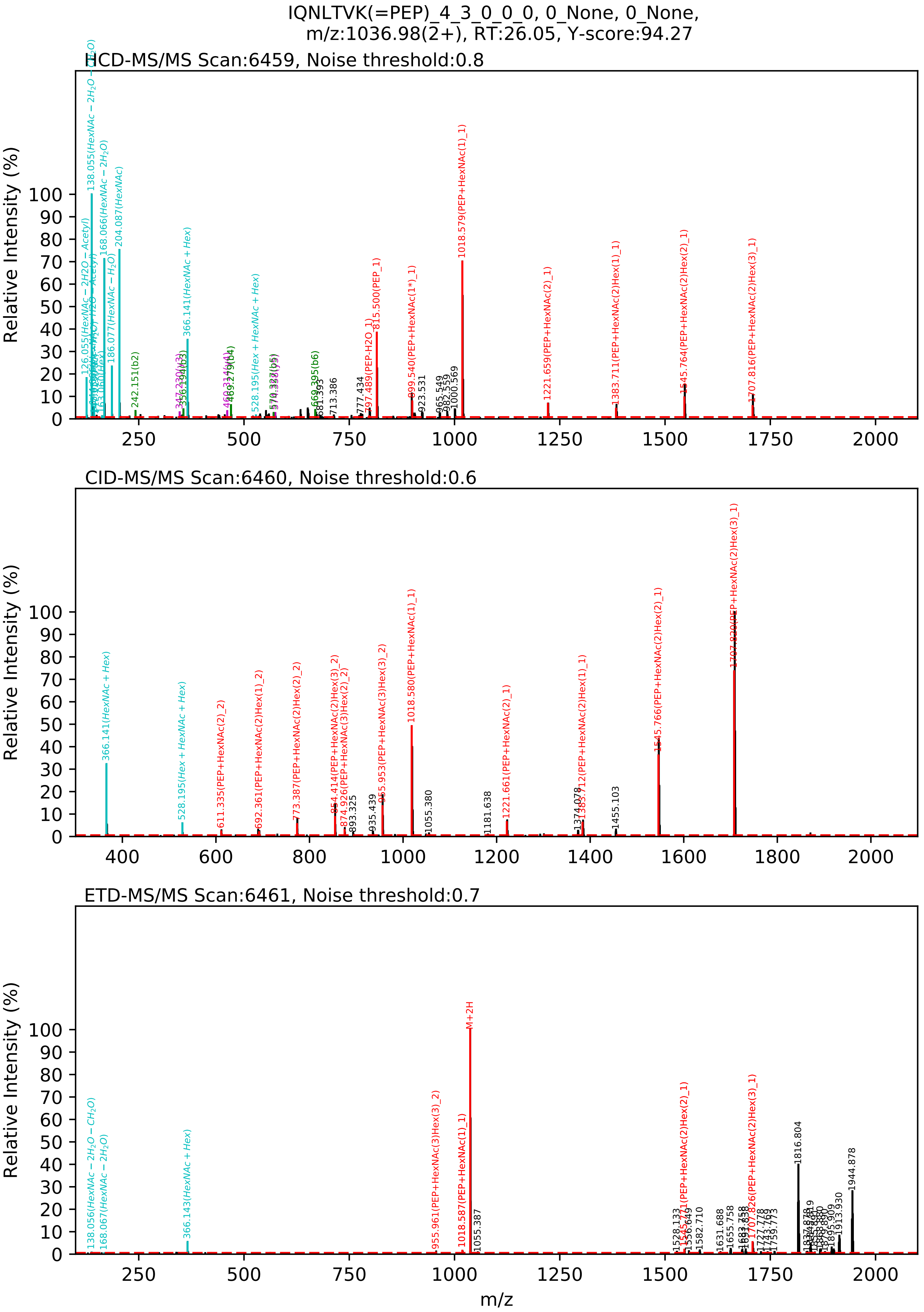

IQNLTVK(=PEP)_4_3_0_0_0, 0_None, 0_None,
m/z:1036.98(2+), RT:26.05, Y-score:94.27
HCD-MS/MS Scan:6459, Noise threshold:0.8
Relative Intensity (%)
138.055(HexNAc − 2H2O − CH2O)
168.066(HexNAc − 2H2O)
204.087(HexNAc)
186.077(HexNAc − H2O)
126.055(HexNAc − 2H2O − Acetyl)
366.141(HexNAc + Hex)
528.195(Hex + HexNAc + Hex)
242.151(b2)
469.279(b4)
669.395(b6)
815.500(PEP_1)
899.540(PEP+HexNAc(1*)_1)
1018.579(PEP+HexNAc(1)_1)
1221.659(PEP+HexNAc(2)_1)
1383.711(PEP+HexNAc(2)Hex(1)_1)
1545.764(PEP+HexNAc(2)Hex(2)_1)
1707.816(PEP+HexNAc(2)Hex(3)_1)
713.386
777.434
923.531
965.549
1000.569
CID-MS/MS Scan:6460, Noise threshold:0.6
366.141(HexNAc + Hex)
528.195(Hex + HexNAc + Hex)
611.335(PEP+HexNAc(2)_2)
692.361(PEP+HexNAc(2)Hex(1)_2)
773.387(PEP+HexNAc(2)Hex(2)_2)
854.414(PEP+HexNAc(2)Hex(3)_2)
874.926(PEP+HexNAc(3)Hex(2)_2)
893.325
935.439
955.953(PEP+HexNAc(3)Hex(3)_2)
1018.580(PEP+HexNAc(1)_1)
1055.380
1181.638
1221.661(PEP+HexNAc(2)_1)
1374.078
1455.103
1545.766(PEP+HexNAc(2)Hex(2)_1)
1707.820(PEP+HexNAc(2)Hex(3)_1)
ETD-MS/MS Scan:6461, Noise threshold:0.7
138.056(HexNAc − 2H2O − CH2O)
168.067(HexNAc − 2H2O)
366.143(HexNAc + Hex)
955.961(PEP+HexNAc(3)Hex(3)_2)
1018.587(PEP+HexNAc(1)_1)
M+2H
1055.387
1528.133
1582.710
1631.688
1655.758
1707.826(PEP+HexNAc(2)Hex(3)_1)
1727.778
1743.763
1759.773
1816.804
1913.930
1944.878
m/z

IQNLTVK(=PEP)_4_3_0_0_0, 0_None, 0_None,
m/z:1036.98(2+), RT:26.58, Y-score:90.24

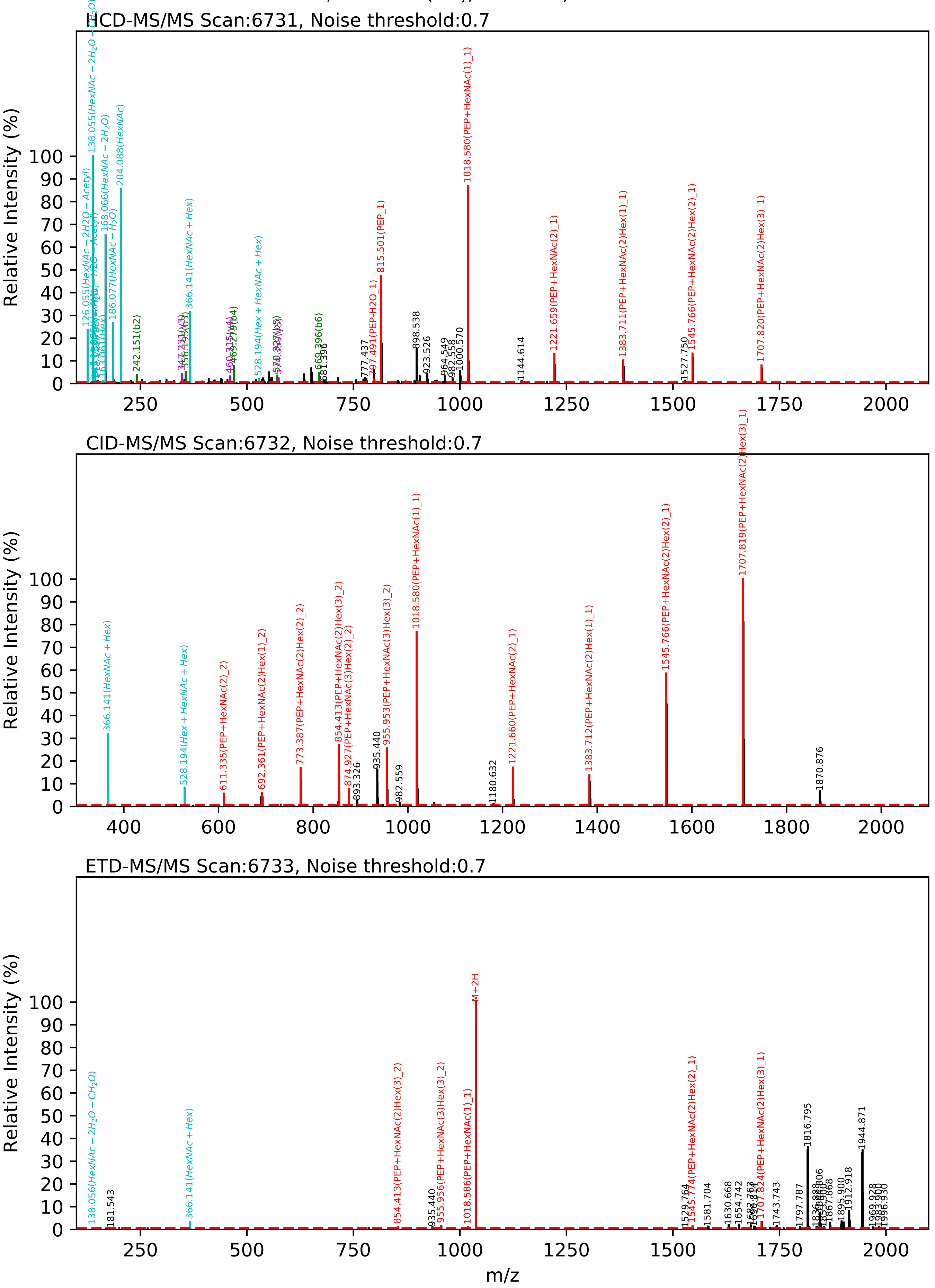

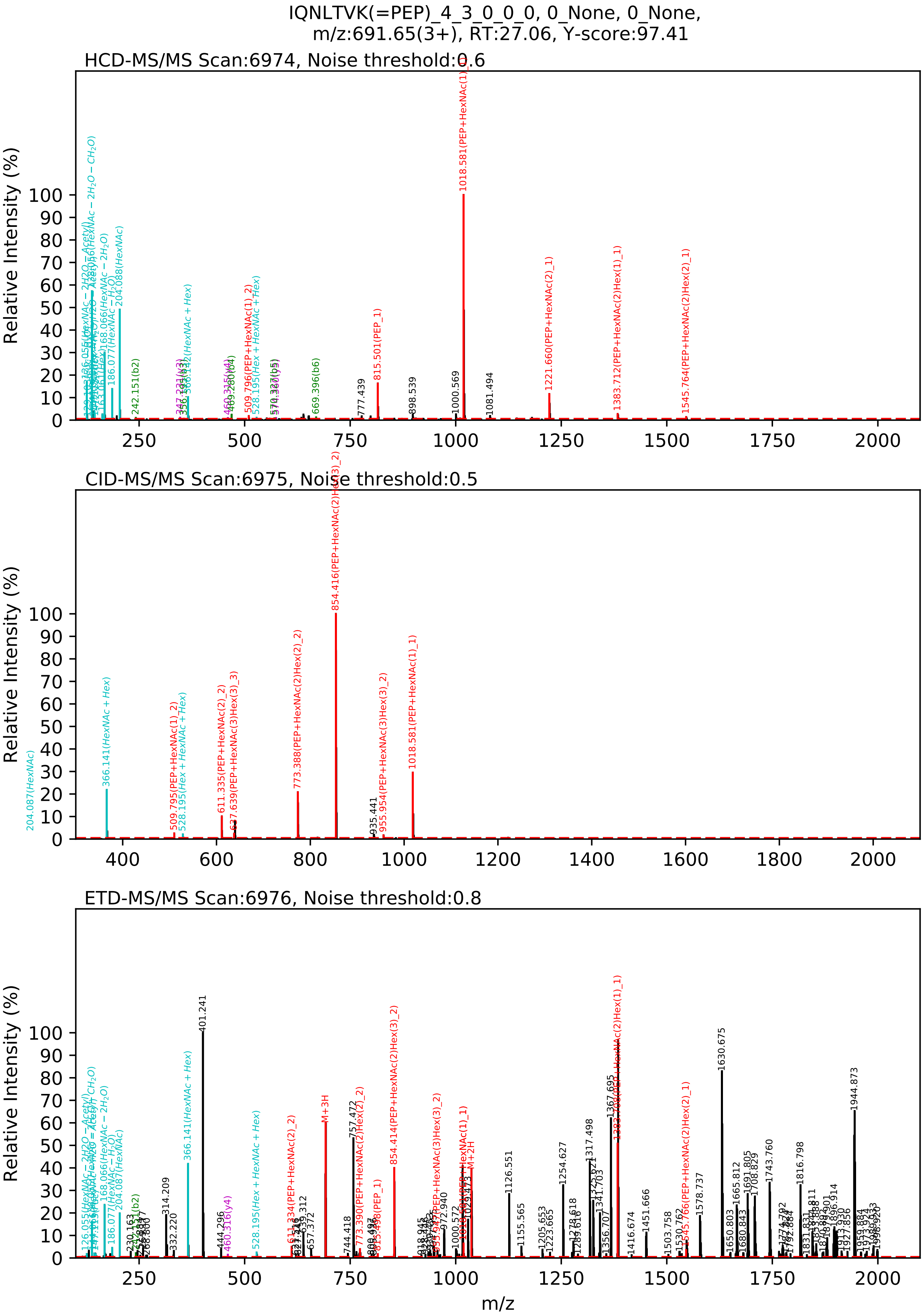

IQNLTVK(=PEP)_4_3_0_0_0, 0_None, 0_None,
m/z:691.65(3+), RT:27.06, Y-score:97.41
HCD-MS/MS Scan:6974, Noise threshold:0.6
CID-MS/MS Scan:6975, Noise threshold:0.5
ETD-MS/MS Scan:6976, Noise threshold:0.8
Relative Intensity (%)
m/z
1018.581(PEP+HexNAc(1)_1)
1221.660(PEP+HexNAc(2)_1)
1383.712(PEP+HexNAc(2)Hex(1)_1)
1545.764(PEP+HexNAc(2)Hex(2)_1)
815.501(PEP_1)
509.796(PEP+HexNAc(1)_2)
854.416(PEP+HexNAc(2)Hex(3)_2)
773.388(PEP+HexNAc(2)Hex(2)_2)
611.335(PEP+HexNAc(2)_2)
637.639(PEP+HexNAc(3)Hex(3)_3)
955.954(PEP+HexNAc(3)Hex(3)_2)
366.141(HexNAc+Hex)
204.087(HexNAc)
528.195(Hex+HexNAc+Hex)
M+3H
M+2H
401.241
1630.675
1944.873

IQNLTVK(=PEP)_4_3_0_0_0, 0_None, 0_None,
m/z:1036.98(2+), RT:34.17, Y-score:90.21

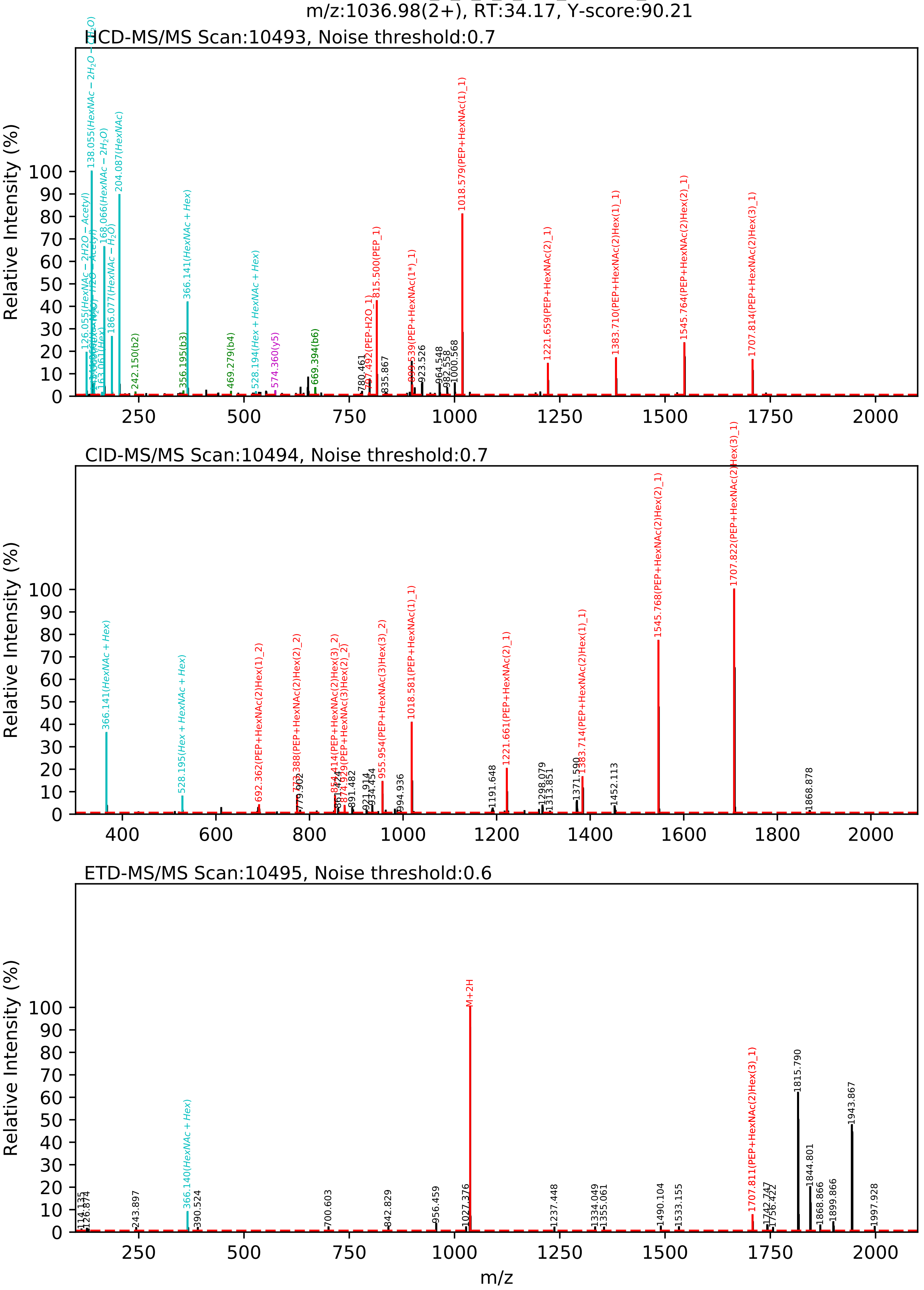

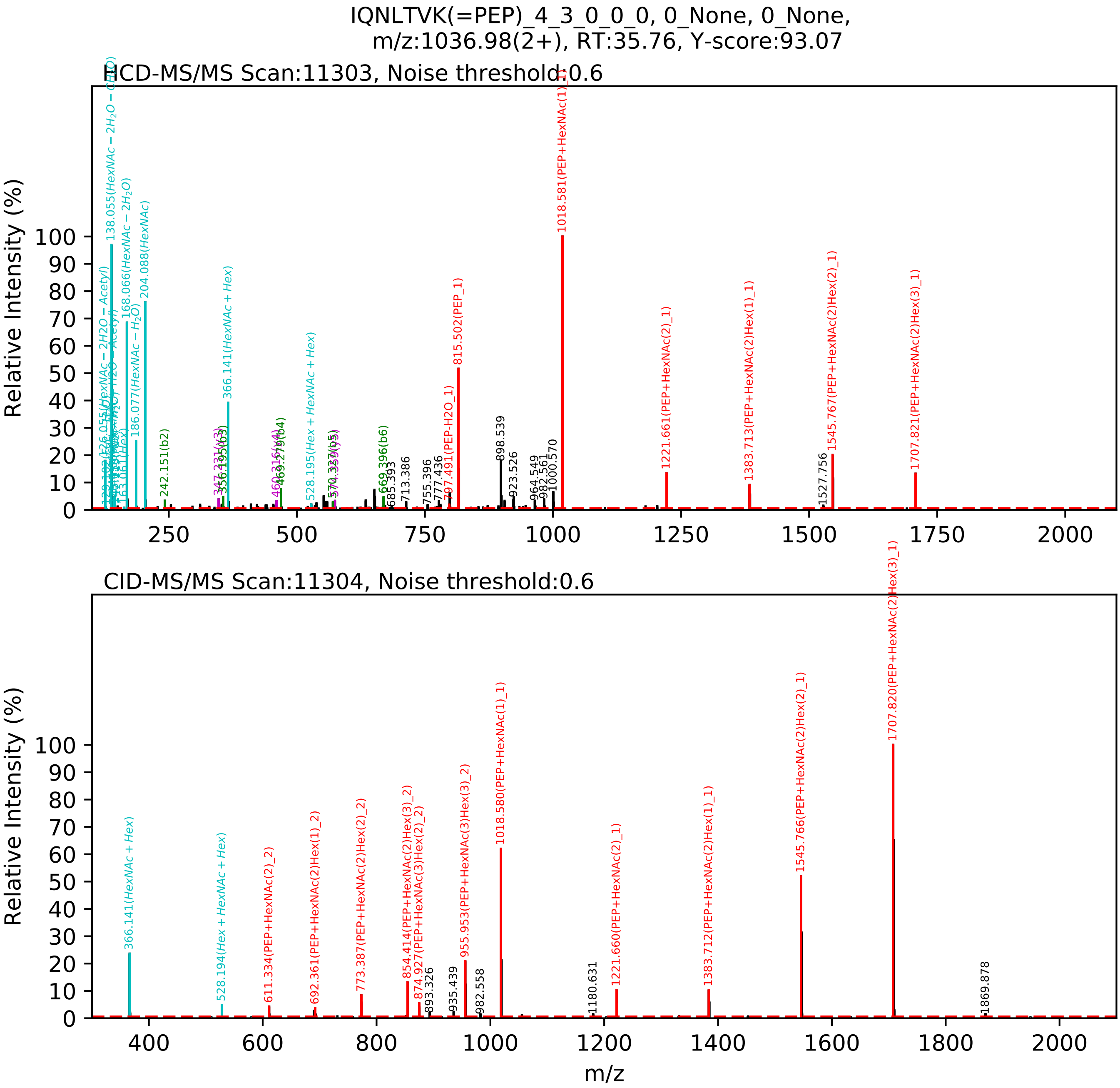

IQNLTVK(=PEP)_4_3_0_0_0, 0_None, 0_None,
m/z:1036.98(2+), RT:35.76, Y-score:93.07
HCD-MS/MS Scan:11303, Noise threshold:0.6
Relative Intensity (%)
1018.581(PEP+HexNAc(1)_1)
815.502(PEP_1)
1221.661(PEP+HexNAc(2)_1)
1383.713(PEP+HexNAc(2)Hex(1)_1)
1545.767(PEP+HexNAc(2)Hex(2)_1)
1707.821(PEP+HexNAc(2)Hex(3)_1)
204.088(HexNAc)
366.141(HexNAc + Hex)
528.195(Hex + HexNAc + Hex)
242.151(b2)
469.279(b4)
669.396(b6)
898.539
923.526
1527.756
CID-MS/MS Scan:11304, Noise threshold:0.6
Relative Intensity (%)
366.141(HexNAc + Hex)
528.194(Hex + HexNAc + Hex)
611.334(PEP+HexNAc(2)_2)
692.361(PEP+HexNAc(2)Hex(1)_2)
773.387(PEP+HexNAc(2)Hex(2)_2)
854.414(PEP+HexNAc(2)Hex(3)_2)
874.927(PEP+HexNAc(3)Hex(2)_2)
893.326
935.439
955.953(PEP+HexNAc(3)Hex(3)_2)
982.558
1018.580(PEP+HexNAc(1)_1)
1180.631
1221.660(PEP+HexNAc(2)_1)
1383.712(PEP+HexNAc(2)Hex(1)_1)
1545.766(PEP+HexNAc(2)Hex(2)_1)
1707.820(PEP+HexNAc(2)Hex(3)_1)
1869.878
m/z

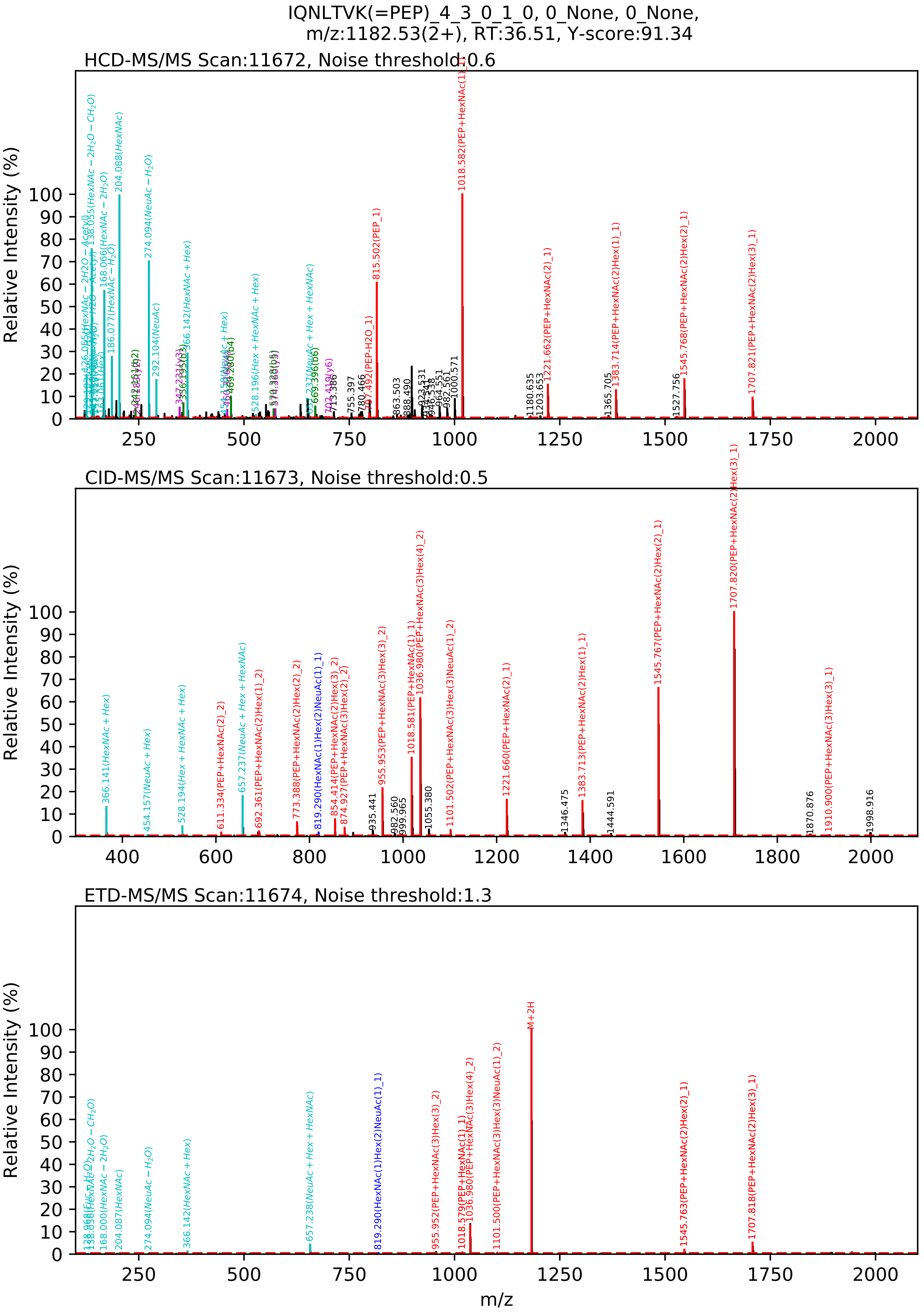
IQNLTVK(=PEP)_4_3_0_1_0, 0_None, 0_None,
m/z:1182.53(2+), RT:36.51, Y-score:91.34
HCD-MS/MS Scan:11672, Noise threshold:0.6
Relative Intensity (%)
1018.582(PEP+HexNAc(1)_1)
815.502(PEP_1)
797.492(PEP-H2O_1)
1221.662(PEP+HexNAc(2)_1)
1383.714(PEP+HexNAc(2)Hex(1)_1)
1545.768(PEP+HexNAc(2)Hex(2)_1)
1707.821(PEP+HexNAc(2)Hex(3)_1)
204.088(HexNAc)
274.094(NeuAc − H2O)
292.104(NeuAc)
366.142(HexNAc + Hex)
528.196(Hex + HexNAc + Hex)
186.077(HexNAc − H2O)
168.066(HexNAc − 2H2O)
138.055(HexNAc − 2H2O − CH2O)
469.280(b4)
669.396(b6)
702.419(y6)
755.397
780.466
863.503
888.490
923.531
946.538
964.551
982.561
1000.571
1180.635
1203.653
1365.705
1527.756
CID-MS/MS Scan:11673, Noise threshold:0.5
366.141(HexNAc + Hex)
454.157(NeuAc + Hex)
528.194(Hex + HexNAc + Hex)
611.334(PEP+HexNAc(2)_2)
657.237(NeuAc + Hex + HexNAc)
692.361(PEP+HexNAc(2)Hex(1)_2)
773.388(PEP+HexNAc(2)Hex(2)_2)
819.290(HexNAc(1)Hex(2)NeuAc(1)_1)
854.414(PEP+HexNAc(2)Hex(3)_2)
874.927(PEP+HexNAc(3)Hex(2)_2)
935.441
955.953(PEP+HexNAc(3)Hex(3)_2)
982.560
999.965
1018.581(PEP+HexNAc(1)_1)
1036.980(PEP+HexNAc(3)Hex(4)_2)
1055.380
1101.502(PEP+HexNAc(3)NeuAc(1)_2)
1221.660(PEP+HexNAc(2)_1)
1346.475
1383.713(PEP+HexNAc(2)Hex(1)_1)
1444.591
1545.767(PEP+HexNAc(2)Hex(2)_1)
1707.820(PEP+HexNAc(2)Hex(3)_1)
1870.876
1910.900(PEP+HexNAc(3)Hex(3)_1)
1998.916
ETD-MS/MS Scan:11674, Noise threshold:1.3
M+2H
138.055(HexNAc − 2H2O − CH2O)
168.000(HexNAc − 2H2O)
204.087(HexNAc)
274.094(NeuAc − H2O)
366.142(HexNAc + Hex)
657.238(NeuAc + Hex + HexNAc)
819.290(HexNAc(1)Hex(2)NeuAc(1)_1)
955.952(PEP+HexNAc(3)Hex(3)_2)
1018.579(PEP+HexNAc(1)_1)
1036.980(PEP+HexNAc(3)Hex(4)_2)
1101.500(PEP+HexNAc(3)Hex(3)NeuAc(1)_2)
1545.763(PEP+HexNAc(2)Hex(2)_1)
1707.818(PEP+HexNAc(2)Hex(3)_1)
m/z

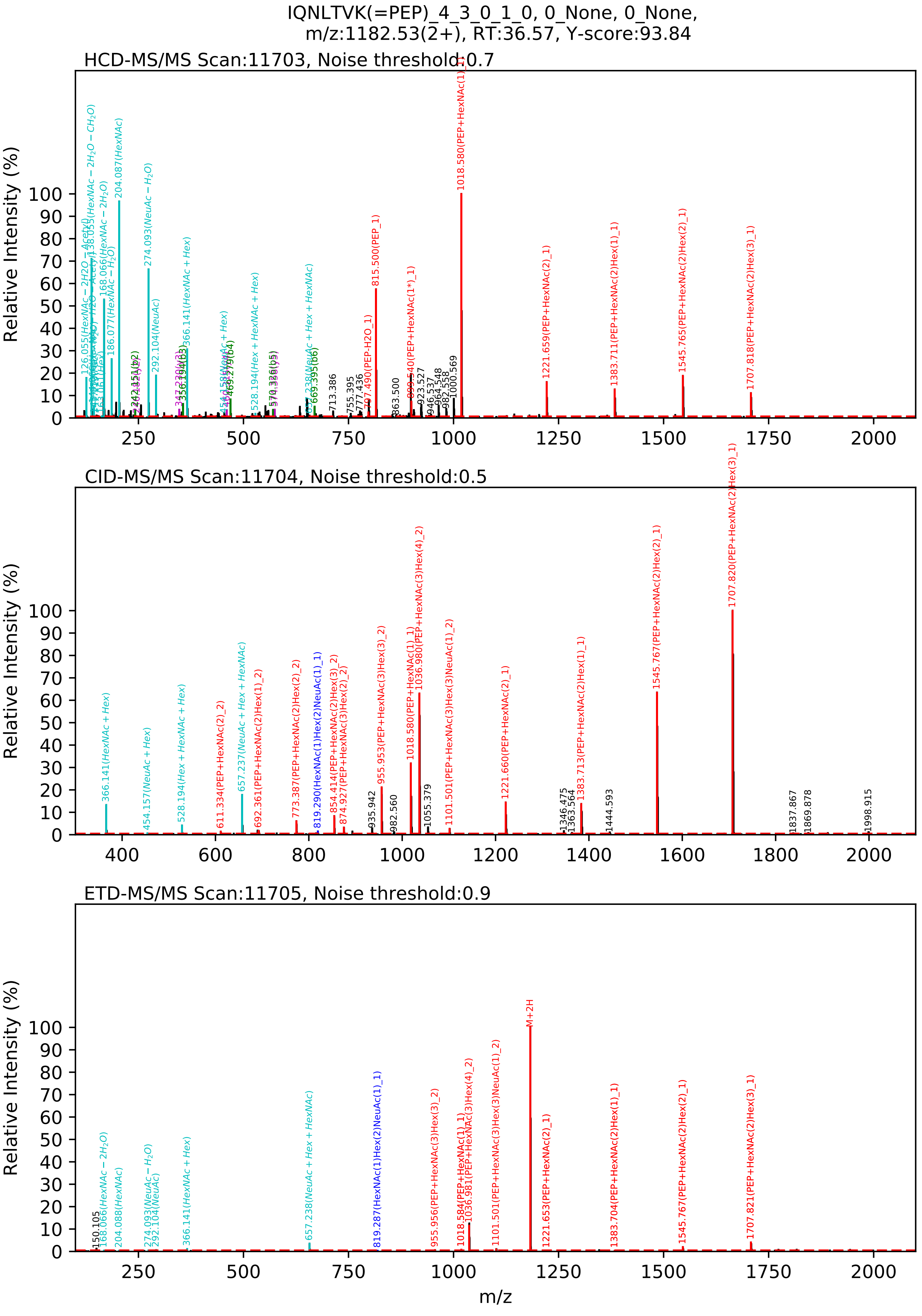
IQNLTVK(=PEP)_4_3_0_1_0, 0_None, 0_None,
m/z:1182.53(2+), RT:36.57, Y-score:93.84
HCD-MS/MS Scan:11703, Noise threshold:0.7
CID-MS/MS Scan:11704, Noise threshold:0.5
ETD-MS/MS Scan:11705, Noise threshold:0.9
Relative Intensity (%)
m/z
1018.580(PEP+HexNAc(1)_1)
815.500(PEP_1)
1221.659(PEP+HexNAc(2)_1)
1383.711(PEP+HexNAc(2)Hex(1)_1)
1545.765(PEP+HexNAc(2)Hex(2)_1)
1707.818(PEP+HexNAc(2)Hex(3)_1)
204.087(HexNAc)
274.093(NeuAc − H2O)
292.104(NeuAc)
366.141(HexNAc + Hex)
528.194(Hex + HexNAc + Hex)
899.540(PEP+HexNAc(1*)_1)
797.490(PEP-H2O_1)
1707.820(PEP+HexNAc(2)Hex(3)_1)
1545.767(PEP+HexNAc(2)Hex(2)_1)
1036.980(PEP+HexNAc(3)Hex(4)_2)
1018.580(PEP+HexNAc(1)_1)
955.953(PEP+HexNAc(3)Hex(3)_2)
1221.660(PEP+HexNAc(2)_1)
1383.713(PEP+HexNAc(2)Hex(1)_1)
1101.501(PEP+HexNAc(3)Hex(3)NeuAc(1)_2)
657.237(NeuAc + Hex + HexNAc)
819.290(HexNAc(1)Hex(2)NeuAc(1)_1)
M+2H
1036.981(PEP+HexNAc(3)Hex(4)_2)
1018.584(PEP+HexNAc(1)_1)
955.956(PEP+HexNAc(3)Hex(3)_2)
1221.653(PEP+HexNAc(2)_1)
1383.704(PEP+HexNAc(2)Hex(1)_1)
1707.821(PEP+HexNAc(2)Hex(3)_1)
819.287(HexNAc(1)Hex(2)NeuAc(1)_1)
657.238(NeuAc + Hex + HexNAc)

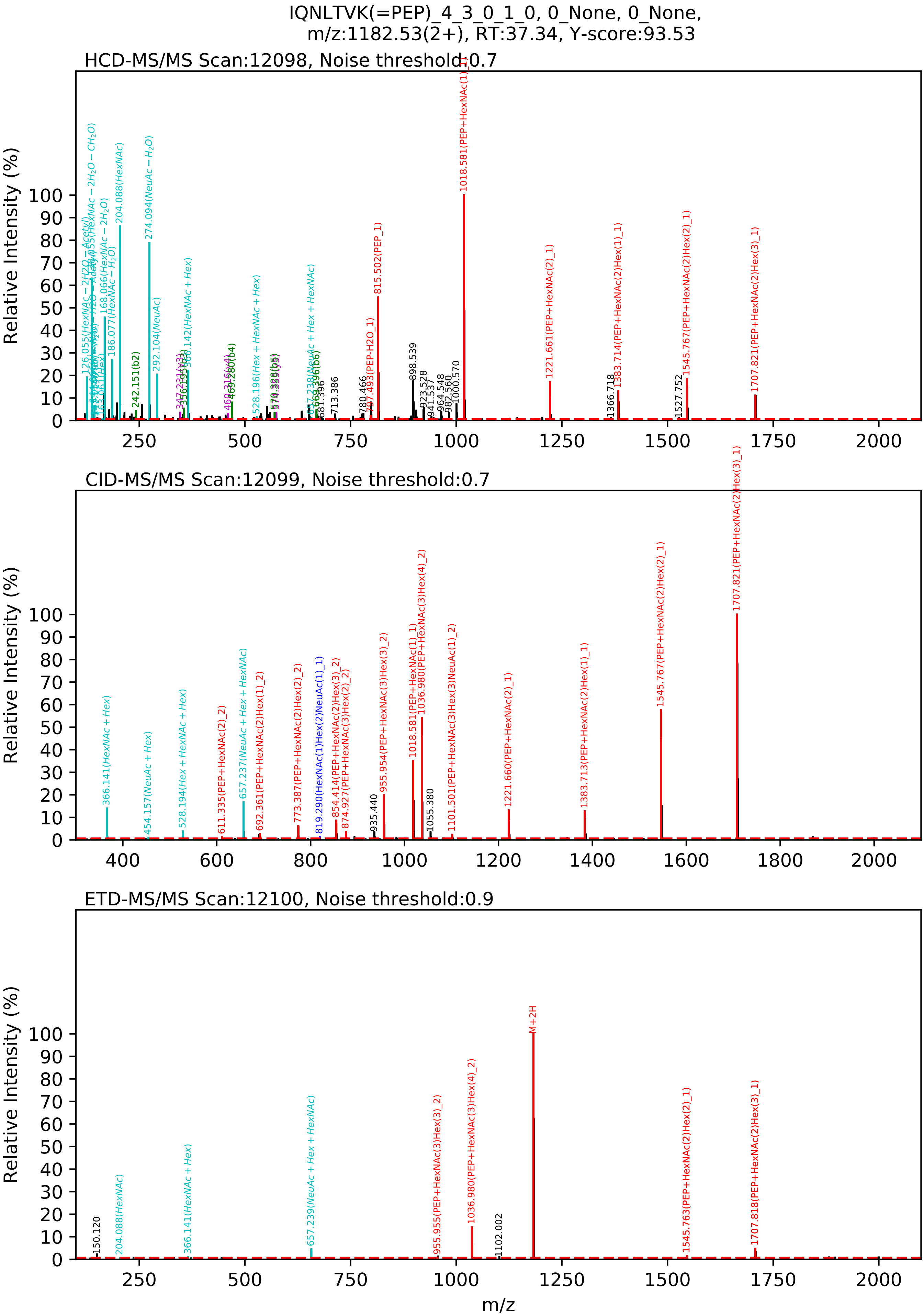

IQNLTVK(=PEP)_4_3_0_1_0, 0_None, 0_None,
m/z:1182.53(2+), RT:37.34, Y-score:93.53
HCD-MS/MS Scan:12098, Noise threshold:0.7
Relative Intensity (%)
1018.581(PEP+HexNAc(1)_1)
815.502(PEP_1)
797.493(PEP-H2O_1)
1221.661(PEP+HexNAc(2)_1)
1383.714(PEP+HexNAc(2)Hex(1)_1)
1545.767(PEP+HexNAc(2)Hex(2)_1)
1707.821(PEP+HexNAc(2)Hex(3)_1)
204.088(HexNAc)
274.094(NeuAc − H2O)
292.104(NeuAc)
186.077(HexNAc − H2O)
168.066(HexNAc − 2H2O)
126.055(HexNAc − 2H2O − CH2O)
366.142(HexNAc + Hex)
528.196(Hex + HexNAc + Hex)
657.238(NeuAc + Hex + HexNAc)
242.151(b2)
469.280(b4)
898.539
923.528
941.537
964.548
982.560
1000.570
713.386
780.466
681.396
1366.718
1527.752
CID-MS/MS Scan:12099, Noise threshold:0.7
366.141(HexNAc + Hex)
454.157(NeuAc + Hex)
528.194(Hex + HexNAc + Hex)
611.335(PEP+HexNAc(2)_2)
657.237(NeuAc + Hex + HexNAc)
692.361(PEP+HexNAc(2)Hex(1)_2)
773.387(PEP+HexNAc(2)Hex(2)_2)
819.290(HexNAc(1)Hex(2)NeuAc(1)_1)
854.414(PEP+HexNAc(2)Hex(3)_2)
874.927(PEP+HexNAc(3)Hex(2)_2)
935.440
955.954(PEP+HexNAc(3)Hex(3)_2)
1018.581(PEP+HexNAc(1)_1)
1036.980(PEP+HexNAc(3)Hex(4)_2)
1055.380
1101.501(PEP+HexNAc(3)Hex(3)NeuAc(1)_2)
1221.660(PEP+HexNAc(2)_1)
1383.713(PEP+HexNAc(2)Hex(1)_1)
1545.767(PEP+HexNAc(2)Hex(2)_1)
1707.821(PEP+HexNAc(2)Hex(3)_1)
ETD-MS/MS Scan:12100, Noise threshold:0.9
150.120
204.088(HexNAc)
366.141(HexNAc + Hex)
657.239(NeuAc + Hex + HexNAc)
955.955(PEP+HexNAc(3)Hex(3)_2)
1036.980(PEP+HexNAc(3)Hex(4)_2)
1102.002
M+2H
1545.763(PEP+HexNAc(2)Hex(2)_1)
1707.818(PEP+HexNAc(2)Hex(3)_1)
m/z

IQNLTVK(=PEP)_4_3_0_1_0, 0_None, 0_None,
m/z:1182.53(2+), RT:37.38, Y-score:94.43

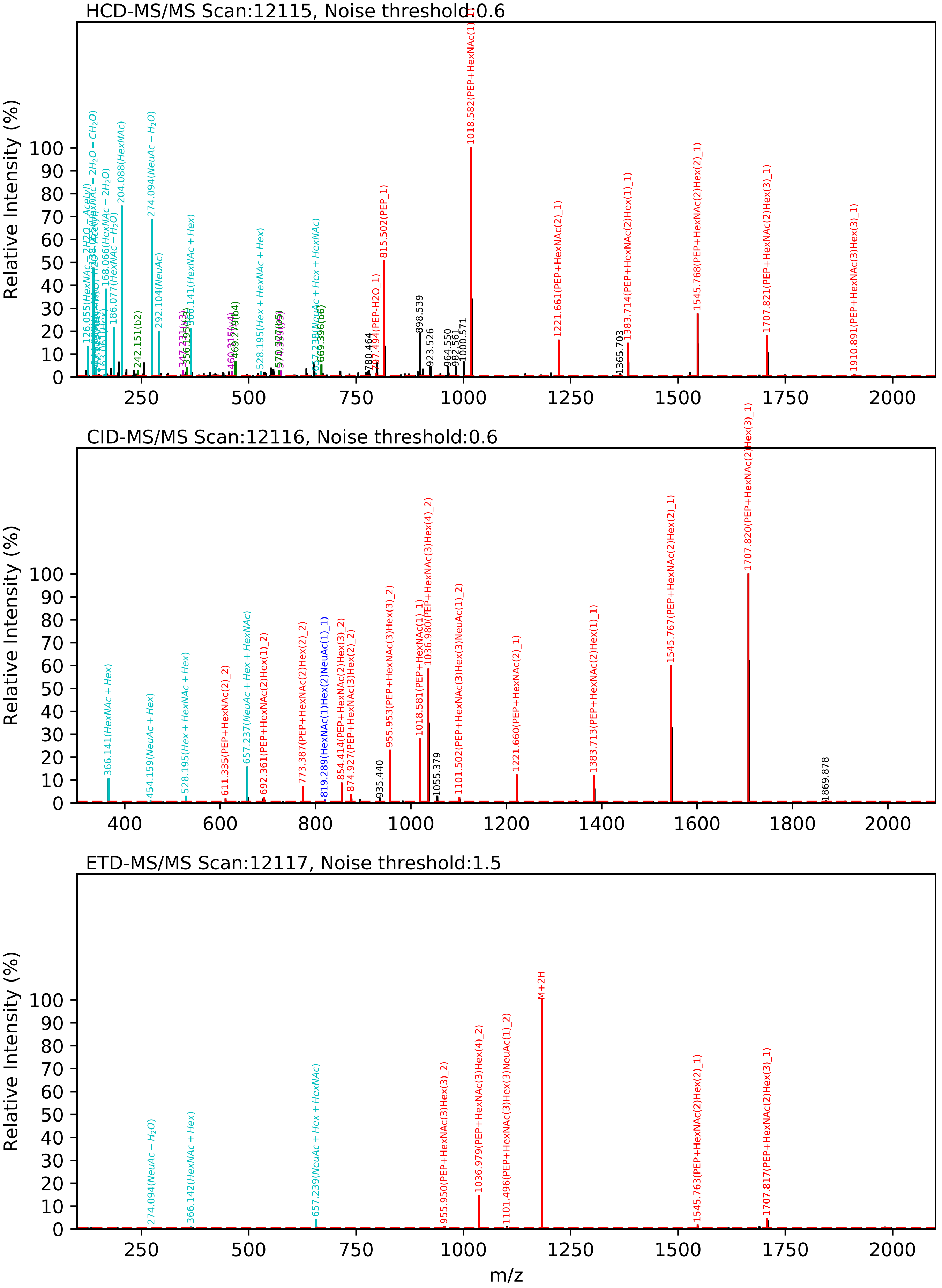

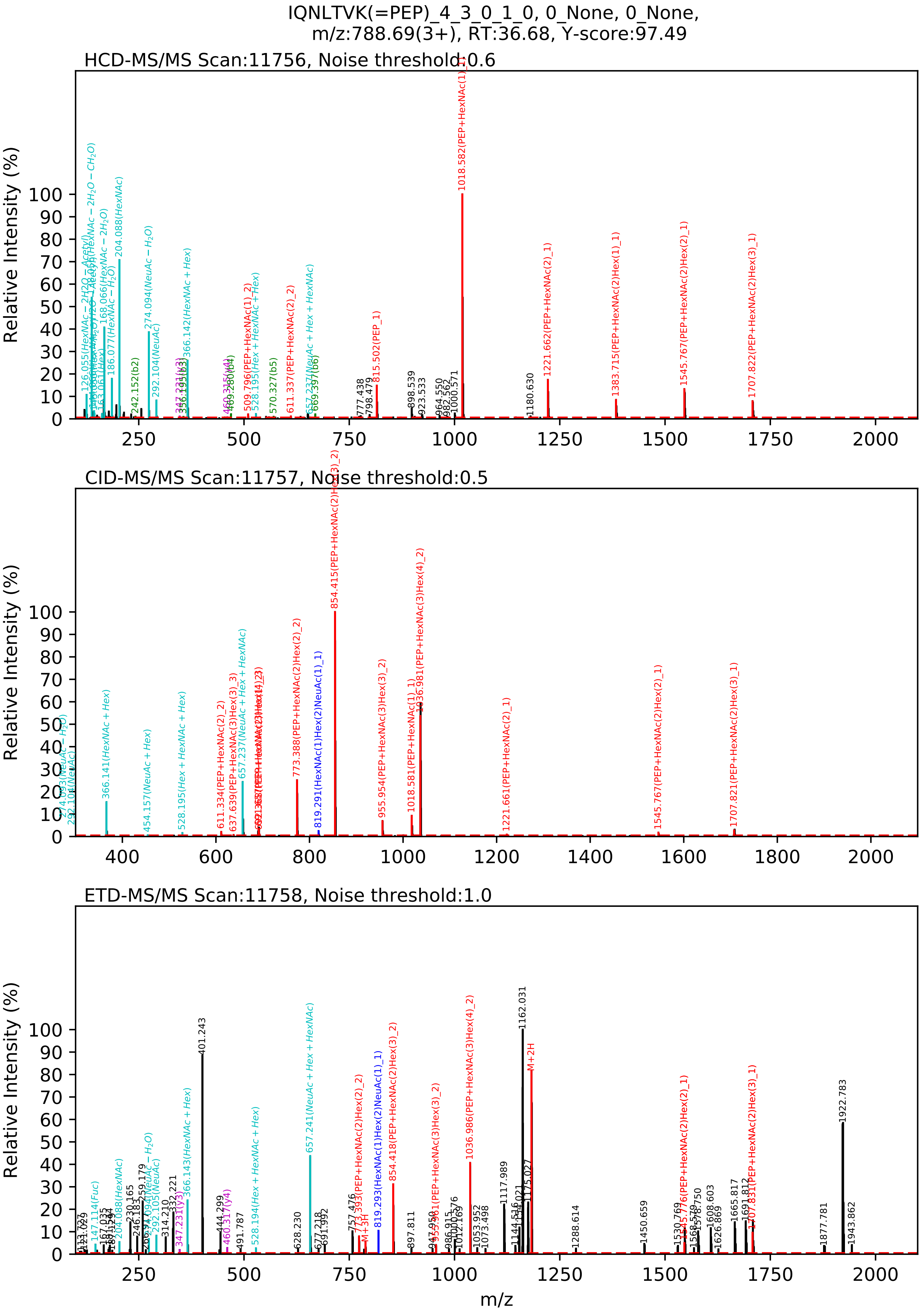

IQNLTVK(=PEP)_4_3_0_1_0, 0_None, 0_None,
m/z:788.69(3+), RT:36.68, Y-score:97.49
HCD-MS/MS Scan:11756, Noise threshold:0.6
CID-MS/MS Scan:11757, Noise threshold:0.5
ETD-MS/MS Scan:11758, Noise threshold:1.0
Relative Intensity (%)
m/z
1018.582(PEP+HexNAc(1)_1)
1221.662(PEP+HexNAc(2)_1)
1383.715(PEP+HexNAc(2)Hex(1)_1)
1545.767(PEP+HexNAc(2)Hex(2)_1)
1707.822(PEP+HexNAc(2)Hex(3)_1)
815.502(PEP_1)
854.415(PEP+HexNAc(2)Hex(3)_2)
1036.981(PEP+HexNAc(3)Hex(4)_2)
773.388(PEP+HexNAc(2)Hex(2)_2)
955.954(PEP+HexNAc(3)Hex(3)_2)
819.291(HexNAc(1)Hex(2)NeuAc(1)_1)
1162.031
M+2H
1922.783
401.243
657.241(NeuAc + Hex + HexNAc)
204.088(HexNAc)
366.142(HexNAc + Hex)
274.094(NeuAc − H2O)
292.104(NeuAc)

IQNLTVK(=PEP)_4_3_0_1_0, 0_None, 0_None,
m/z:788.69(3+), RT:36.71, Y-score:97.63

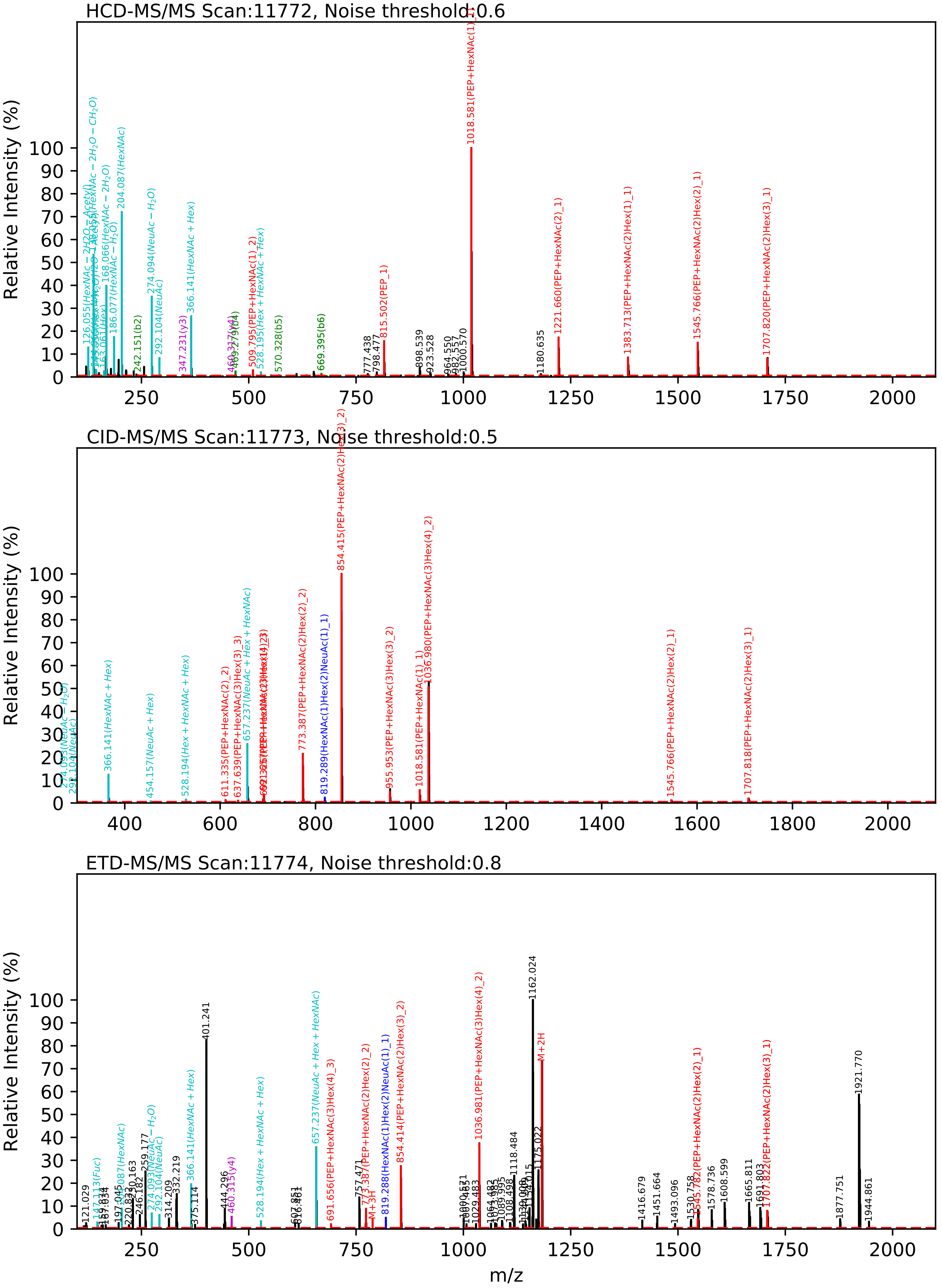

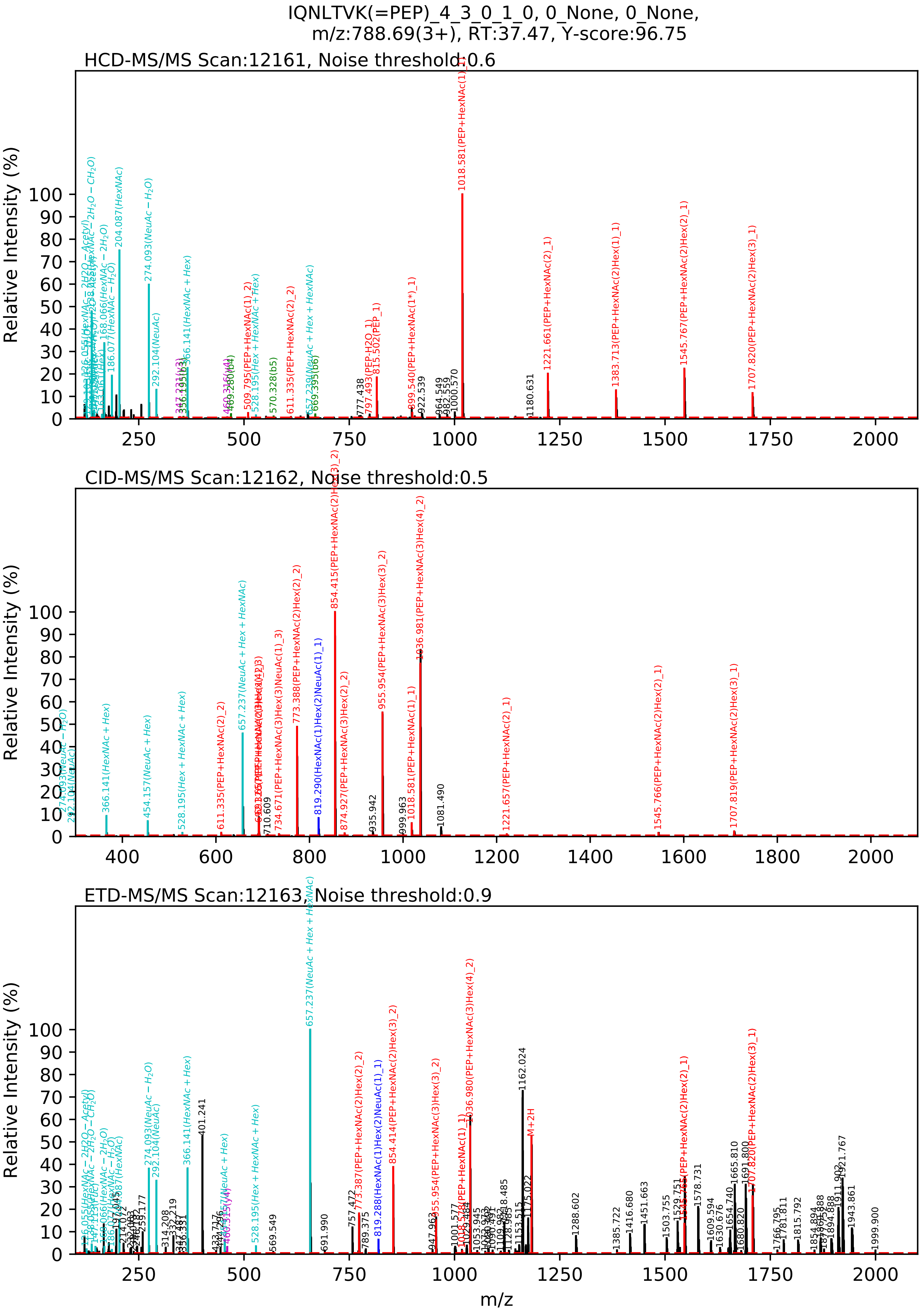
IQNLTVK(=PEP)_4_3_0_1_0, 0_None, 0_None,
m/z:788.69(3+), RT:37.47, Y-score:96.75
HCD-MS/MS Scan:12161, Noise threshold:0.6
CID-MS/MS Scan:12162, Noise threshold:0.5
ETD-MS/MS Scan:12163, Noise threshold:0.9
Relative Intensity (%)
m/z
1018.581(PEP+HexNAc(1)_1)
1221.661(PEP+HexNAc(2)_1)
1383.713(PEP+HexNAc(2)Hex(1)_1)
1545.767(PEP+HexNAc(2)Hex(2)_1)
1707.820(PEP+HexNAc(2)Hex(3)_1)
204.087(HexNAc)
274.093(NeuAc − H2O)
292.104(NeuAc)
366.141(HexNAc + Hex)
815.502(PEP_1)
899.540(PEP+HexNAc(1*)_1)
854.415(PEP+HexNAc(2)Hex(3)_2)
1036.981(PEP+HexNAc(3)Hex(4)_2)
955.954(PEP+HexNAc(3)Hex(3)_2)
773.388(PEP+HexNAc(2)Hex(2)_2)
657.237(NeuAc + Hex + HexNAc)
819.290(HexNAc(1)Hex(2)NeuAc(1)_1)
1162.024
M+2H
401.241
1921.767

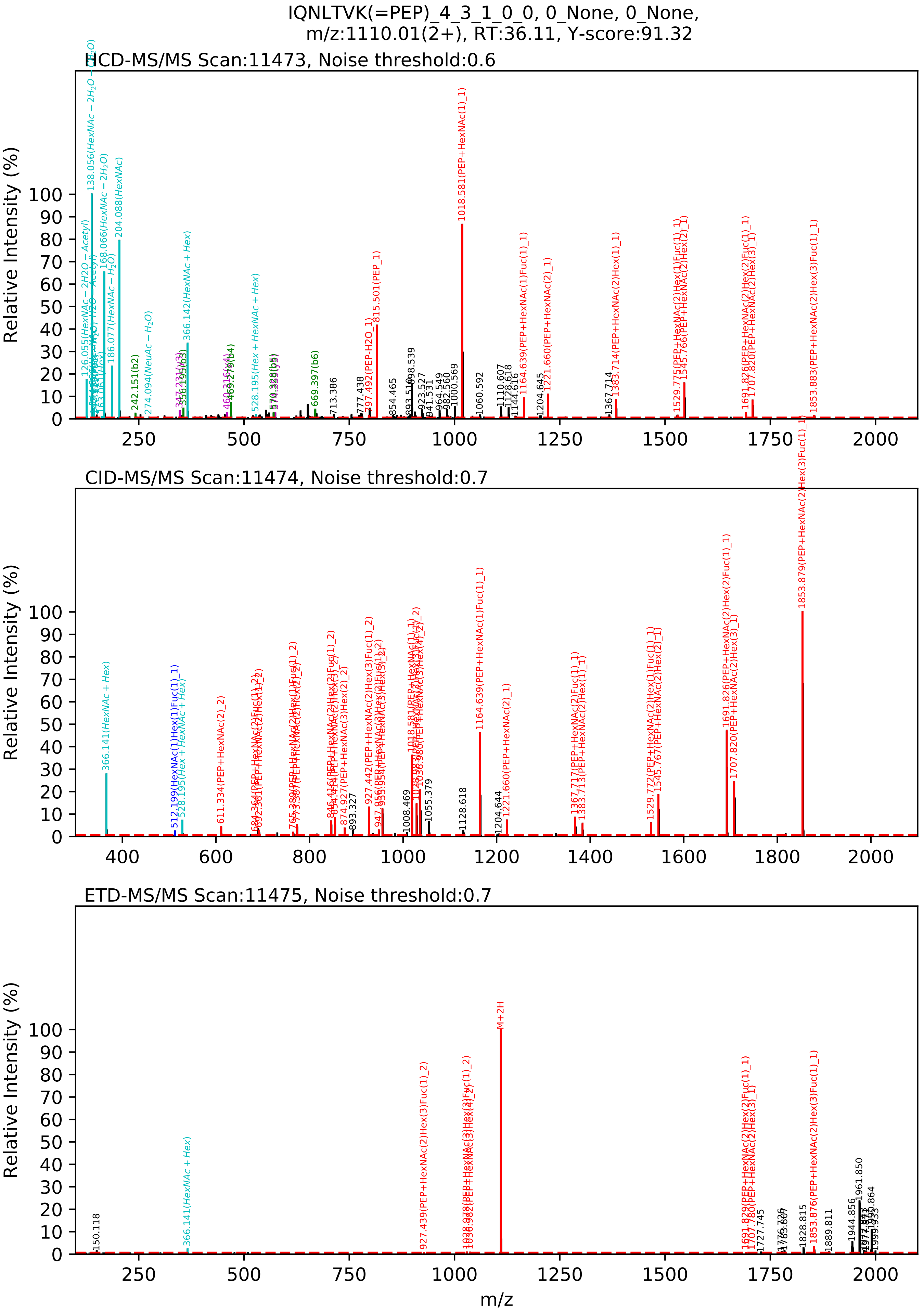
IQNLTVK(=PEP)_4_3_1_0_0, 0_None, 0_None,
m/z:1110.01(2+), RT:36.11, Y-score:91.32
HCD-MS/MS Scan:11473, Noise threshold:0.6
CID-MS/MS Scan:11474, Noise threshold:0.7
ETD-MS/MS Scan:11475, Noise threshold:0.7
Relative Intensity (%)
m/z
1018.581(PEP+HexNAc(1)_1)
815.501(PEP_1)
138.056(HexNAc − 2H2O − H2O)
204.088(HexNAc)
168.066(HexNAc − 2H2O)
186.077(HexNAc − H2O)
366.142(HexNAc + Hex)
1164.639(PEP+HexNAc(1)Fuc(1)_1)
1221.660(PEP+HexNAc(2)_1)
1383.714(PEP+HexNAc(2)Hex(1)_1)
1545.766(PEP+HexNAc(2)Hex(2)_1)
1707.820(PEP+HexNAc(2)Hex(3)_1)
1853.883(PEP+HexNAc(2)Hex(3)Fuc(1)_1)
1853.879(PEP+HexNAc(2)Hex(3)Fuc(1)_1)
1691.826(PEP+HexNAc(2)Hex(2)Fuc(1)_1)
1164.639(PEP+HexNAc(1)Fuc(1)_1)
366.141(HexNAc + Hex)
611.334(PEP+HexNAc(2)_2)
M+2H
927.439(PEP+HexNAc(2)Hex(3)Fuc(1)_2)
1853.876(PEP+HexNAc(2)Hex(3)Fuc(1)_1)
1961.850

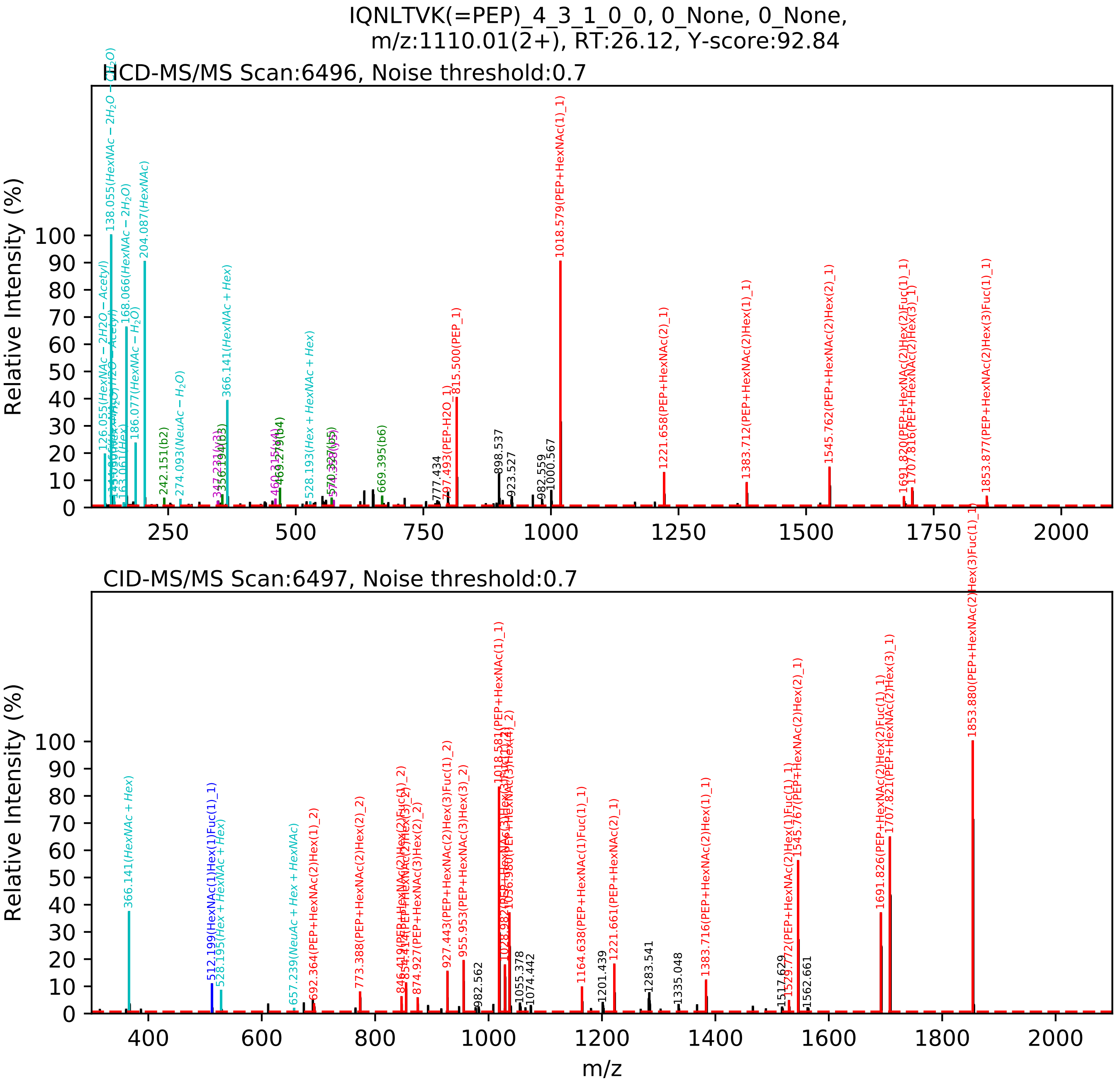
IQNLTVK(=PEP)_4_3_1_0_0, 0_None, 0_None,
m/z:1110.01(2+), RT:26.12, Y-score:92.84
HCD-MS/MS Scan:6496, Noise threshold:0.7
Relative Intensity (%)
1018.579(PEP+HexNAc(1)_1)
815.500(PEP_1)
1221.658(PEP+HexNAc(2)_1)
1383.712(PEP+HexNAc(2)Hex(1)_1)
1545.762(PEP+HexNAc(2)Hex(2)_1)
1853.877(PEP+HexNAc(2)Hex(3)Fuc(1)_1)
204.087(HexNAc)
366.141(HexNAc + Hex)
CID-MS/MS Scan:6497, Noise threshold:0.7
Relative Intensity (%)
1853.880(PEP+HexNAc(2)Hex(3)Fuc(1)_1)
1707.821(PEP+HexNAc(2)Hex(3)_1)
1691.826(PEP+HexNAc(2)Hex(2)Fuc(1)_1)
1545.767(PEP+HexNAc(2)Hex(2)_1)
1383.716(PEP+HexNAc(2)Hex(1)_1)
1221.661(PEP+HexNAc(2)_1)
1164.638(PEP+HexNAc(1)Fuc(1)_1)
366.141(HexNAc + Hex)
512.199(HexNAc(1)Hex(1)Fuc(1)_1)
m/z

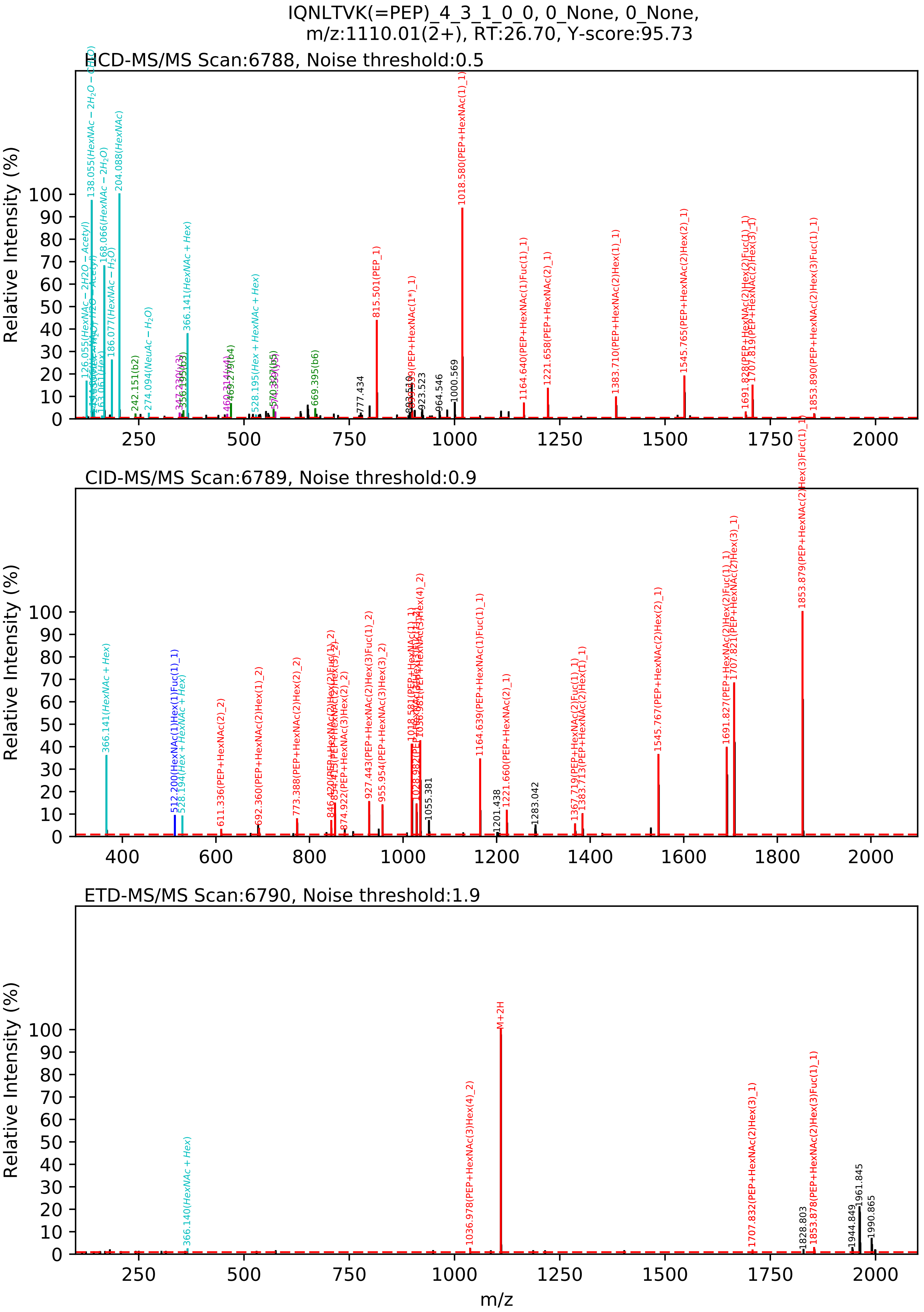
IQNLTVK(=PEP)_4_3_1_0_0, 0_None, 0_None,
m/z:1110.01(2+), RT:26.70, Y-score:95.73
HCD-MS/MS Scan:6788, Noise threshold:0.5
Relative Intensity (%)
1018.580(PEP+HexNAc(1)_1)
815.501(PEP_1)
204.088(HexNAc)
138.055(HexNAc − 2H2O − CH2O)
168.066(HexNAc − 2H2O)
186.077(HexNAc − H2O)
366.141(HexNAc + Hex)
528.195(Hex + HexNAc + Hex)
669.395(b6)
242.151(b2)
274.094(NeuAc − H2O)
1164.640(PEP+HexNAc(1)Fuc(1)_1)
1221.658(PEP+HexNAc(2)_1)
1383.710(PEP+HexNAc(2)Hex(1)_1)
1545.765(PEP+HexNAc(2)Hex(2)_1)
1853.890(PEP+HexNAc(2)Hex(3)Fuc(1)_1)
CID-MS/MS Scan:6789, Noise threshold:0.9
366.141(HexNAc + Hex)
512.200(HexNAc(1)Hex(1)Fuc(1)_1)
611.336(PEP+HexNAc(2)_2)
692.360(PEP+HexNAc(2)Hex(1)_2)
773.388(PEP+HexNAc(2)Hex(2)_2)
927.443(PEP+HexNAc(2)Hex(3)Fuc(1)_2)
955.954(PEP+HexNAc(3)Hex(3)_2)
1055.381
1164.639(PEP+HexNAc(1)Fuc(1)_1)
1221.660(PEP+HexNAc(2)_1)
1201.438
1283.042
1545.767(PEP+HexNAc(2)Hex(2)_1)
1691.827(PEP+HexNAc(2)Hex(2)Fuc(1)_1)
1707.821(PEP+HexNAc(2)Hex(3)_1)
1853.879(PEP+HexNAc(2)Hex(3)Fuc(1)_1)
ETD-MS/MS Scan:6790, Noise threshold:1.9
M+2H
366.140(HexNAc + Hex)
1036.978(PEP+HexNAc(3)Hex(4)_2)
1707.832(PEP+HexNAc(2)Hex(3)_1)
1853.878(PEP+HexNAc(2)Hex(3)Fuc(1)_1)
1828.803
1944.849
1961.845
1990.865
m/z

IQNLTVK(=PEP)_4_3_1_0_0, 0_None, 0_None,
m/z:1110.01(2+), RT:27.85, Y-score:92.28

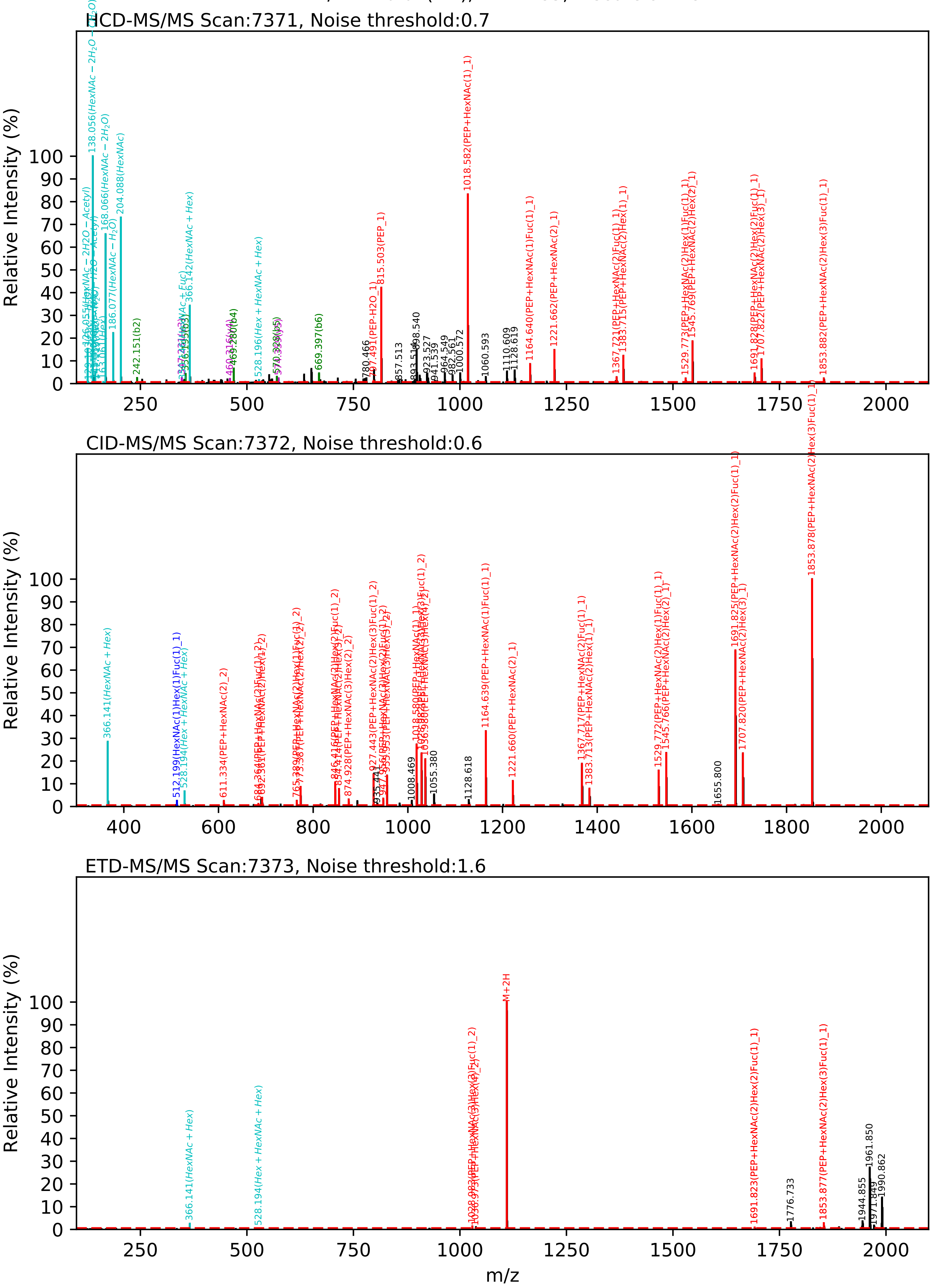

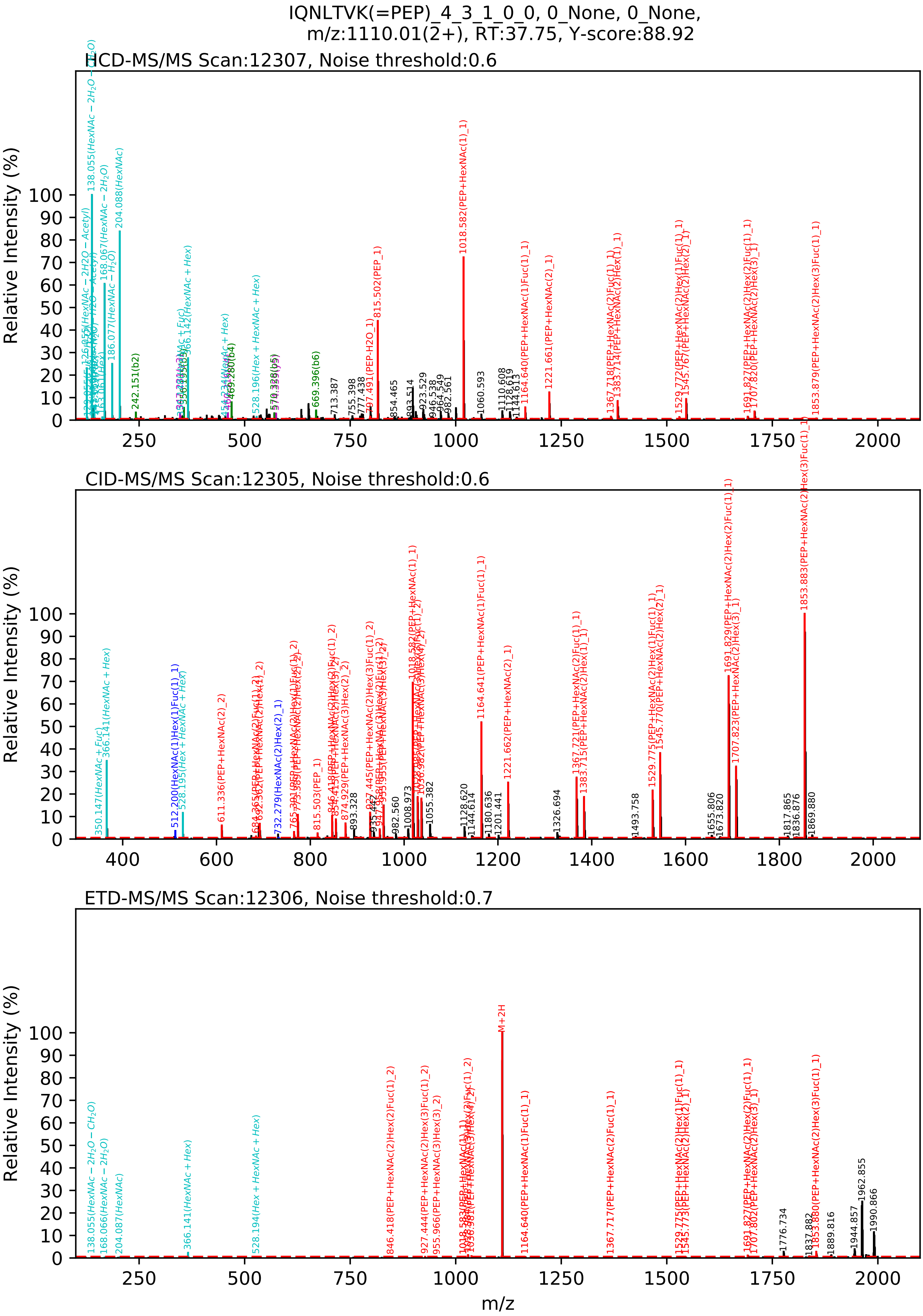
IQNLTVK(=PEP)_4_3_1_0_0, 0_None, 0_None,
m/z:1110.01(2+), RT:37.75, Y-score:88.92
HCD-MS/MS Scan:12307, Noise threshold:0.6
CID-MS/MS Scan:12305, Noise threshold:0.6
ETD-MS/MS Scan:12306, Noise threshold:0.7
Relative Intensity (%)
m/z
1018.582(PEP+HexNAc(1)_1)
815.502(PEP_1)
1853.883(PEP+HexNAc(2)Hex(3)Fuc(1)_1)
1691.829(PEP+HexNAc(2)Hex(2)Fuc(1)_1)
1164.641(PEP+HexNAc(1)Fuc(1)_1)
M+2H

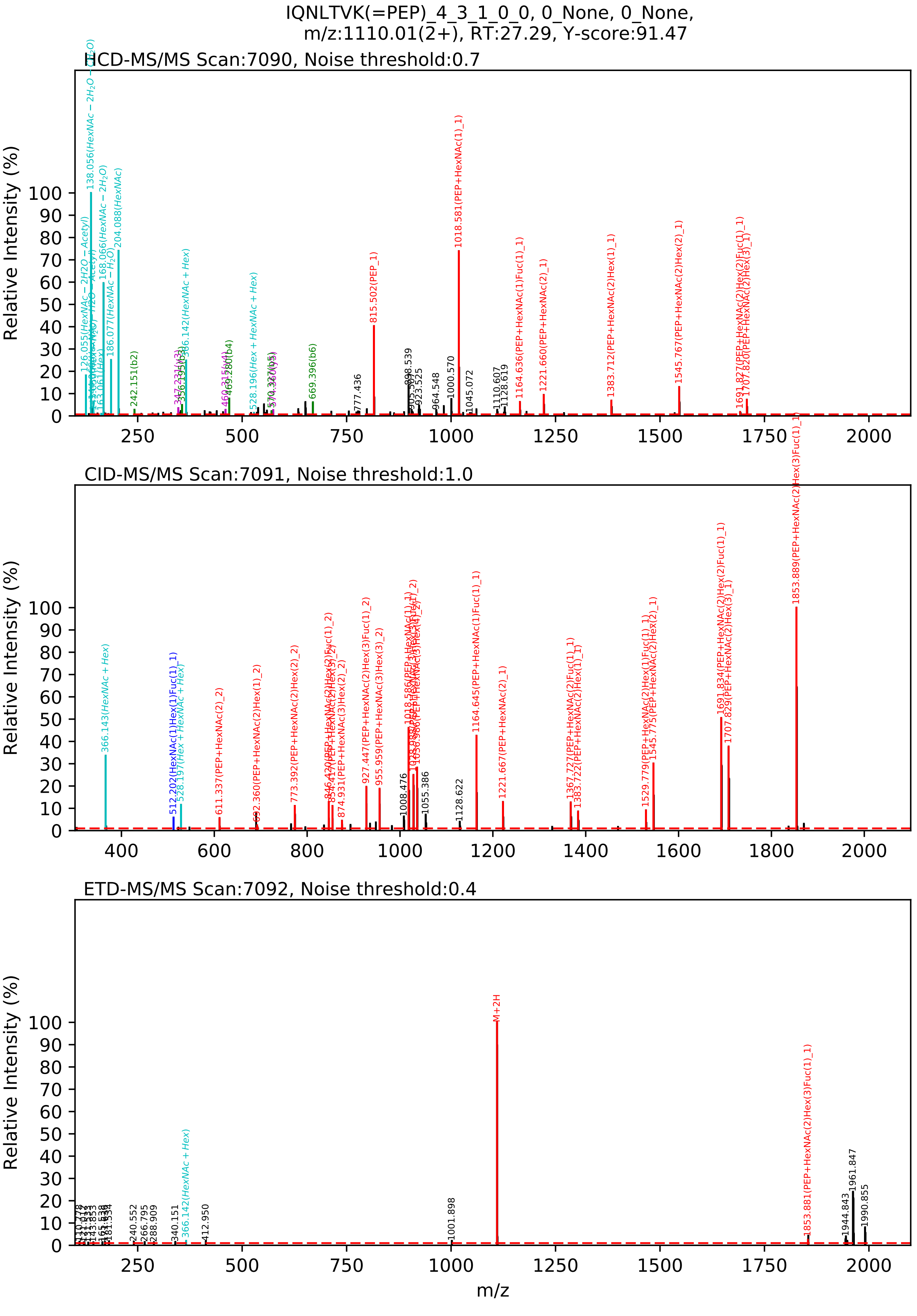
IQNLTVK(=PEP)_4_3_1_0_0, 0_None, 0_None,
m/z:1110.01(2+), RT:27.29, Y-score:91.47
HCD-MS/MS Scan:7090, Noise threshold:0.7
Relative Intensity (%)
138.056(HexNAc − 2H2O − H2O)
204.088(HexNAc)
168.066(HexNAc − 2H2O)
186.077(HexNAc − H2O)
126.055(HexNAc − 2H2O − Acetyl)
242.151(b2)
366.142(HexNAc + Hex)
469.280(b4)
528.196(Hex + HexNAc + Hex)
669.396(b6)
777.436
815.502(PEP_1)
898.539
923.525
964.548
1000.570
1018.581(PEP+HexNAc(1)_1)
1045.072
1110.607
1128.619
1164.636(PEP+HexNAc(1)Fuc(1)_1)
1221.660(PEP+HexNAc(2)_1)
1383.712(PEP+HexNAc(2)Hex(1)_1)
1545.767(PEP+HexNAc(2)Hex(2)_1)
1691.827(PEP+HexNAc(2)Hex(2)Fuc(1)_1)
1707.820(PEP+HexNAc(2)Hex(3)_1)
CID-MS/MS Scan:7091, Noise threshold:1.0
366.143(HexNAc + Hex)
512.202(HexNAc(1)Hex(1)Fuc(1)_1)
528.197(Hex + HexNAc + Hex)
611.337(PEP+HexNAc(2)_2)
692.360(PEP+HexNAc(2)Hex(1)_2)
773.392(PEP+HexNAc(2)Hex(2)_2)
874.931(PEP+HexNAc(3)Hex(2)_2)
927.447(PEP+HexNAc(2)Hex(3)Fuc(1)_2)
955.959(PEP+HexNAc(3)Hex(3)_2)
1008.476
1055.386
1128.622
1164.645(PEP+HexNAc(1)Fuc(1)_1)
1221.667(PEP+HexNAc(2)_1)
1367.727(PEP+HexNAc(2)Fuc(1)_1)
1383.722(PEP+HexNAc(2)Hex(1)_1)
1529.779(PEP+HexNAc(2)Hex(1)Fuc(1)_1)
1545.775(PEP+HexNAc(2)Hex(2)_1)
1691.834(PEP+HexNAc(2)Hex(2)Fuc(1)_1)
1707.829(PEP+HexNAc(2)Hex(3)_1)
1853.889(PEP+HexNAc(2)Hex(3)Fuc(1)_1)
ETD-MS/MS Scan:7092, Noise threshold:0.4
240.552
266.795
288.909
340.151
366.142(HexNAc + Hex)
412.950
1001.898
M+2H
1853.881(PEP+HexNAc(2)Hex(3)Fuc(1)_1)
1944.843
1961.847
1990.855
m/z

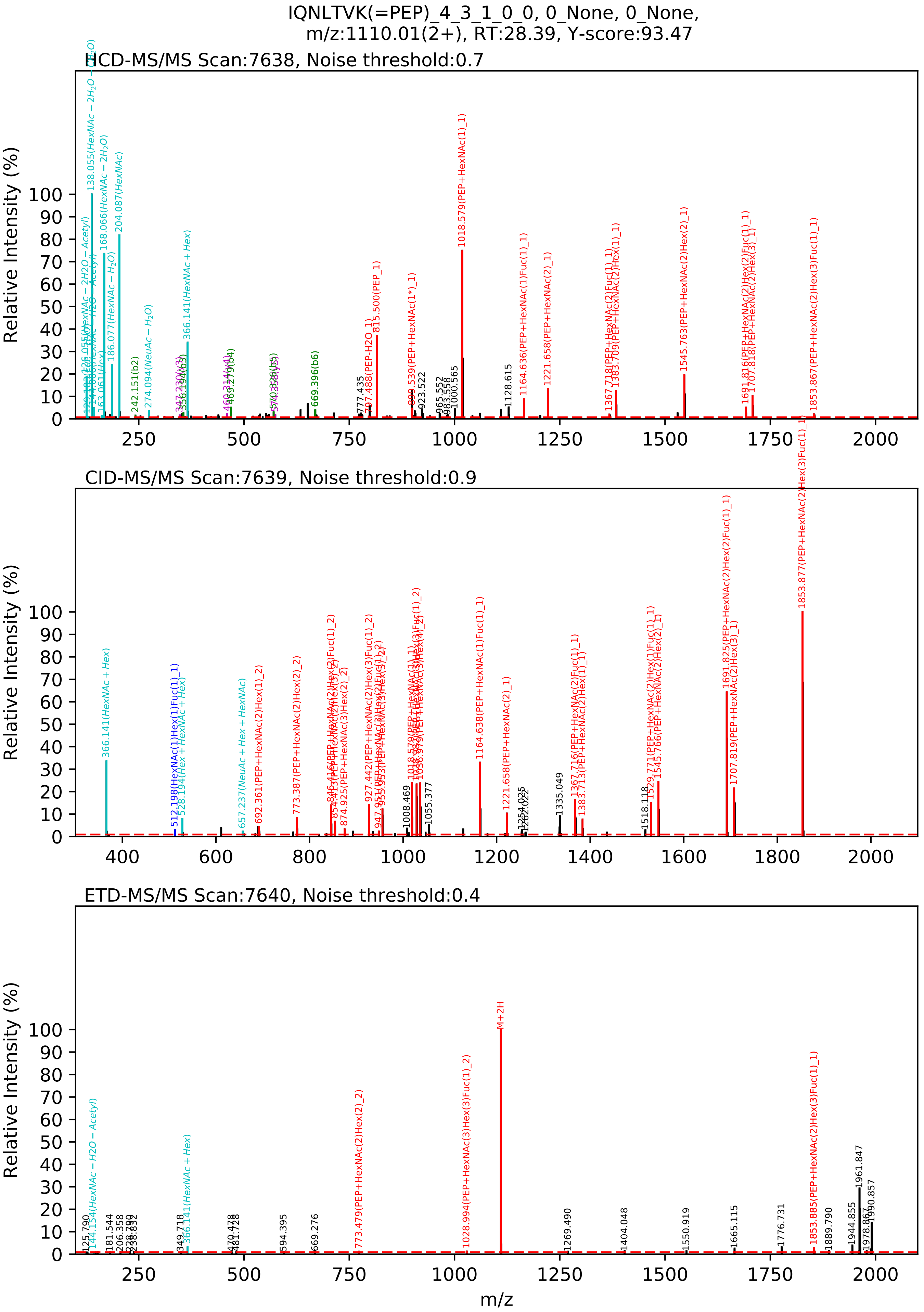
IQNLTVK(=PEP)_4_3_1_0_0, 0_None, 0_None,
m/z:1110.01(2+), RT:28.39, Y-score:93.47
HCD-MS/MS Scan:7638, Noise threshold:0.7
CID-MS/MS Scan:7639, Noise threshold:0.9
ETD-MS/MS Scan:7640, Noise threshold:0.4
Relative Intensity (%)
m/z
1018.579(PEP+HexNAc(1)_1)
815.500(PEP_1)
1853.877(PEP+HexNAc(2)Hex(3)Fuc(1)_1)
1691.825(PEP+HexNAc(2)Hex(2)Fuc(1)_1)
1164.638(PEP+HexNAc(1)Fuc(1)_1)
M+2H
1961.847

IQNLTVK(=PEP)_4_3_1_0_0, 0_None, 0_None,
m/z:1110.01(2+), RT:36.84, Y-score:92.99

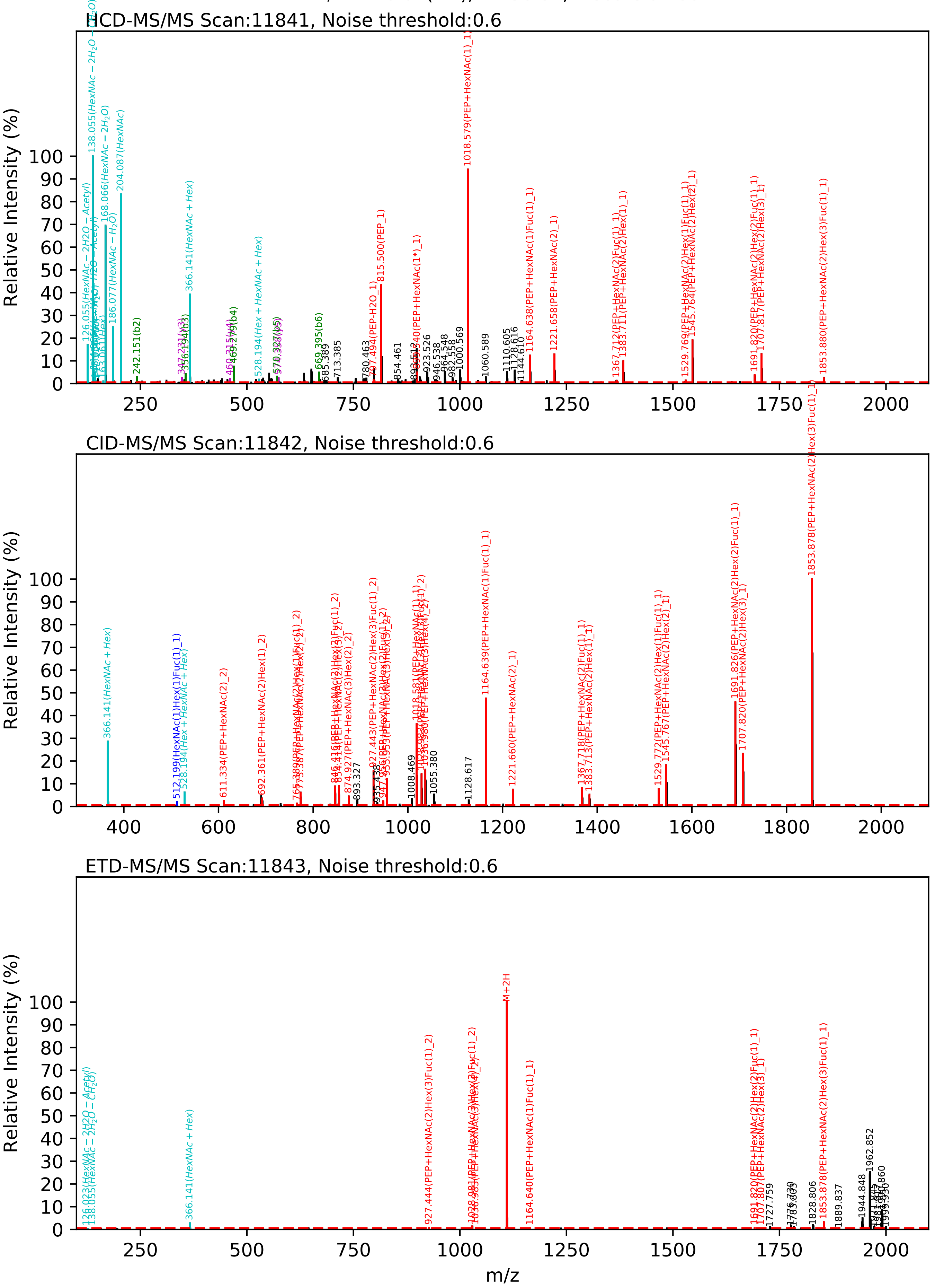

IQNLTVK(=PEP)_4_3_1_0_0, 0_None, 0_None,
m/z:1110.01(2+), RT:37.39, Y-score:90.94

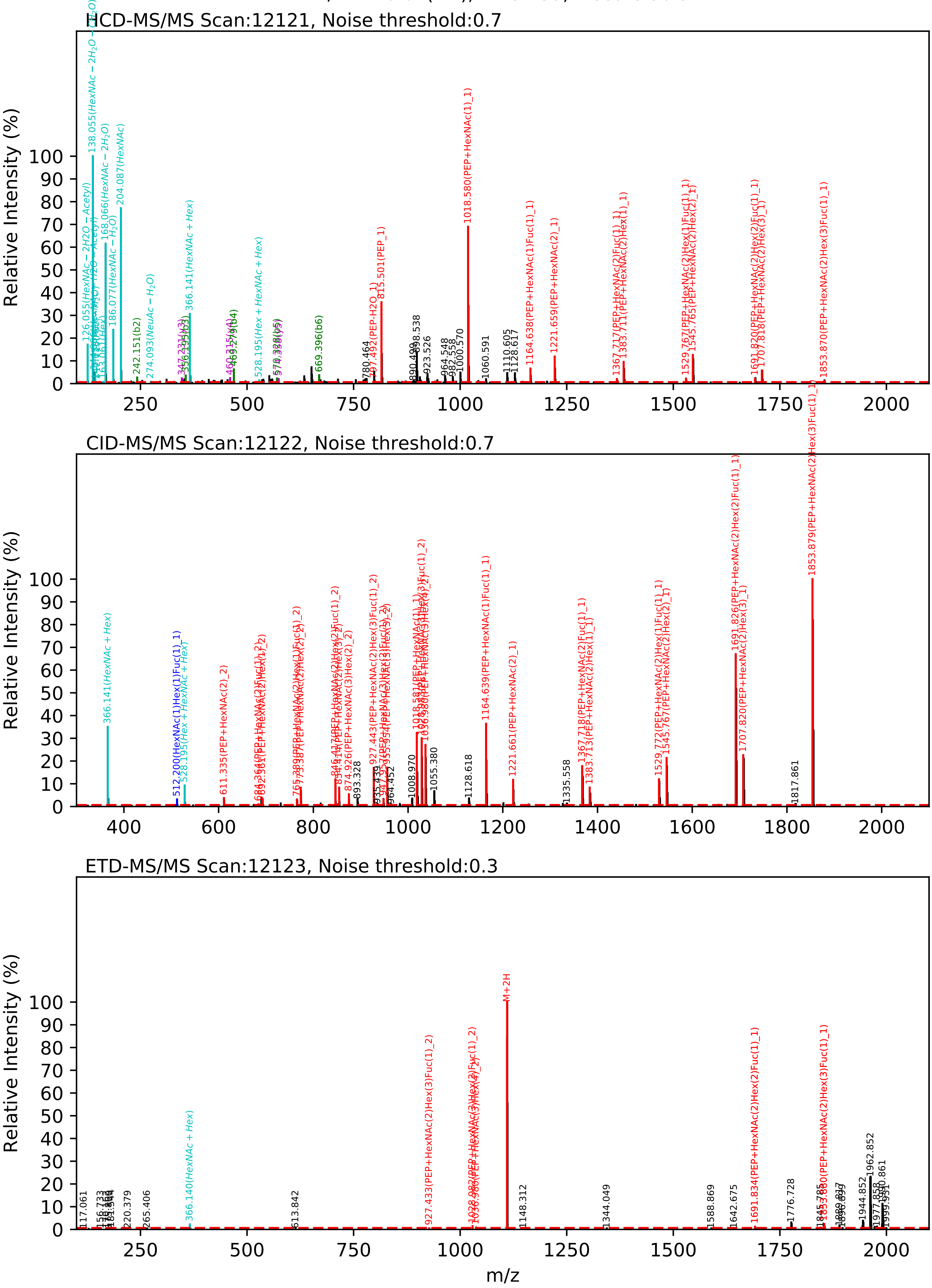

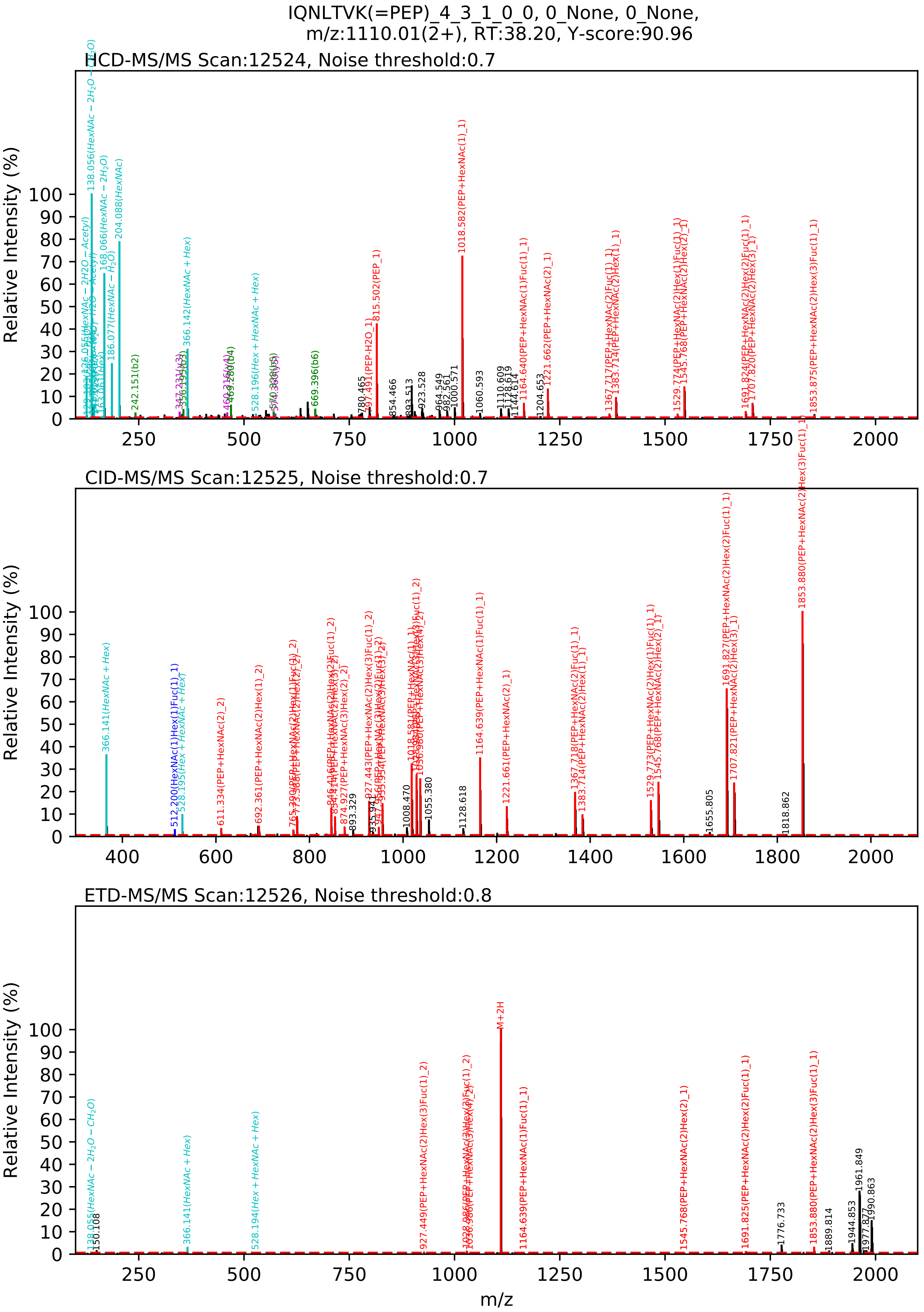

IQNLTVK(=PEP)_4_3_1_0_0, 0_None, 0_None,
m/z:1110.01(2+), RT:38.20, Y-score:90.96
HCD-MS/MS Scan:12524, Noise threshold:0.7
CID-MS/MS Scan:12525, Noise threshold:0.7
ETD-MS/MS Scan:12526, Noise threshold:0.8
Relative Intensity (%)
m/z
1018.582(PEP+HexNAc(1)_1)
815.502(PEP_1)
1853.880(PEP+HexNAc(2)Hex(3)Fuc(1)_1)
1691.827(PEP+HexNAc(2)Hex(2)Fuc(1)_1)
M+2H

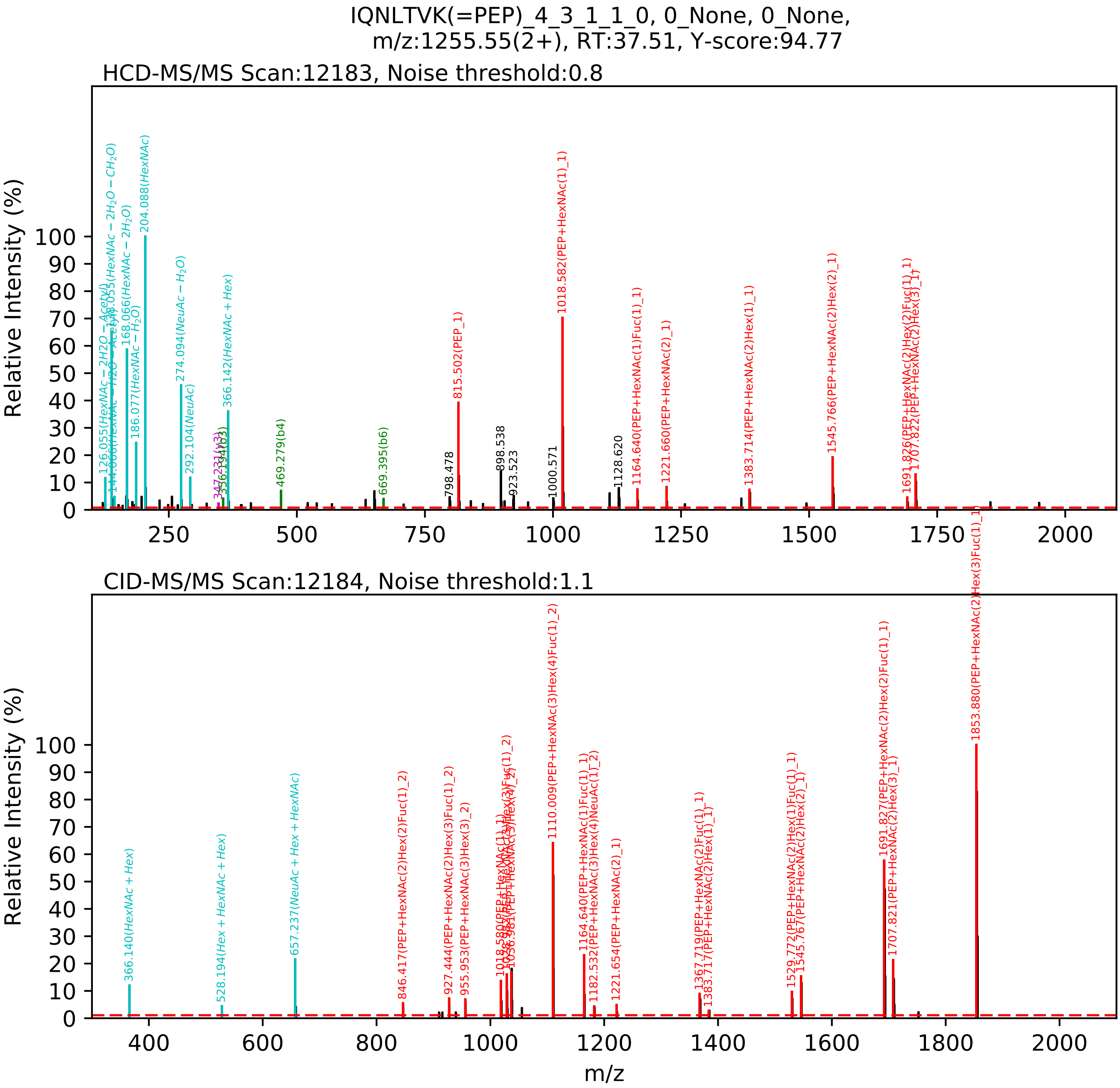

IQNLTVK(=PEP)_4_3_1_1_0, 0_None, 0_None,
m/z:1255.55(2+), RT:37.51, Y-score:94.77
HCD-MS/MS Scan:12183, Noise threshold:0.8
Relative Intensity (%)
126.055(HexNAc − 2H2O − Acetyl)
204.088(HexNAc)
186.077(HexNAc − H_2O)
168.066(HexNAc − $2H_2O$)
274.094(NeuAc − H_2O)
292.104(NeuAc)
366.142(HexNAc + Hex)
356.194(b3)
469.279(b4)
669.395(b6)
798.478
815.502(PEP_1)
898.538
923.523
1000.571
1018.582(PEP+HexNAc(1)_1)
1128.620
1164.640(PEP+HexNAc(1)Fuc(1)_1)
1221.660(PEP+HexNAc(2)_1)
1383.714(PEP+HexNAc(2)Hex(1)_1)
1545.766(PEP+HexNAc(2)Hex(2)_1)
1707.822(PEP+HexNAc(2)Hex(3)_1)
CID-MS/MS Scan:12184, Noise threshold:1.1
366.140(HexNAc + Hex)
528.194(Hex + HexNAc + Hex)
657.237(NeuAc + Hex + HexNAc)
846.417(PEP+HexNAc(2)Hex(2)Fuc(1)_2)
927.444(PEP+HexNAc(2)Hex(3)Fuc(1)_2)
955.953(PEP+HexNAc(3)Hex(3)_2)
1110.009(PEP+HexNAc(3)Hex(4)Fuc(1)_2)
1164.640(PEP+HexNAc(1)Fuc(1)_1)
1182.532(PEP+HexNAc(3)Hex(4)NeuAc(1)_2)
1221.654(PEP+HexNAc(2)_1)
1367.719(PEP+HexNAc(2)Fuc(1)_1)
1383.717(PEP+HexNAc(2)Hex(1)_1)
1529.772(PEP+HexNAc(2)Hex(1)Fuc(1)_1)
1545.767(PEP+HexNAc(2)Hex(2)_1)
1691.827(PEP+HexNAc(2)Hex(2)Fuc(1)_1)
1707.821(PEP+HexNAc(2)Hex(3)_1)
1853.880(PEP+HexNAc(2)Hex(3)Fuc(1)_1)
m/z

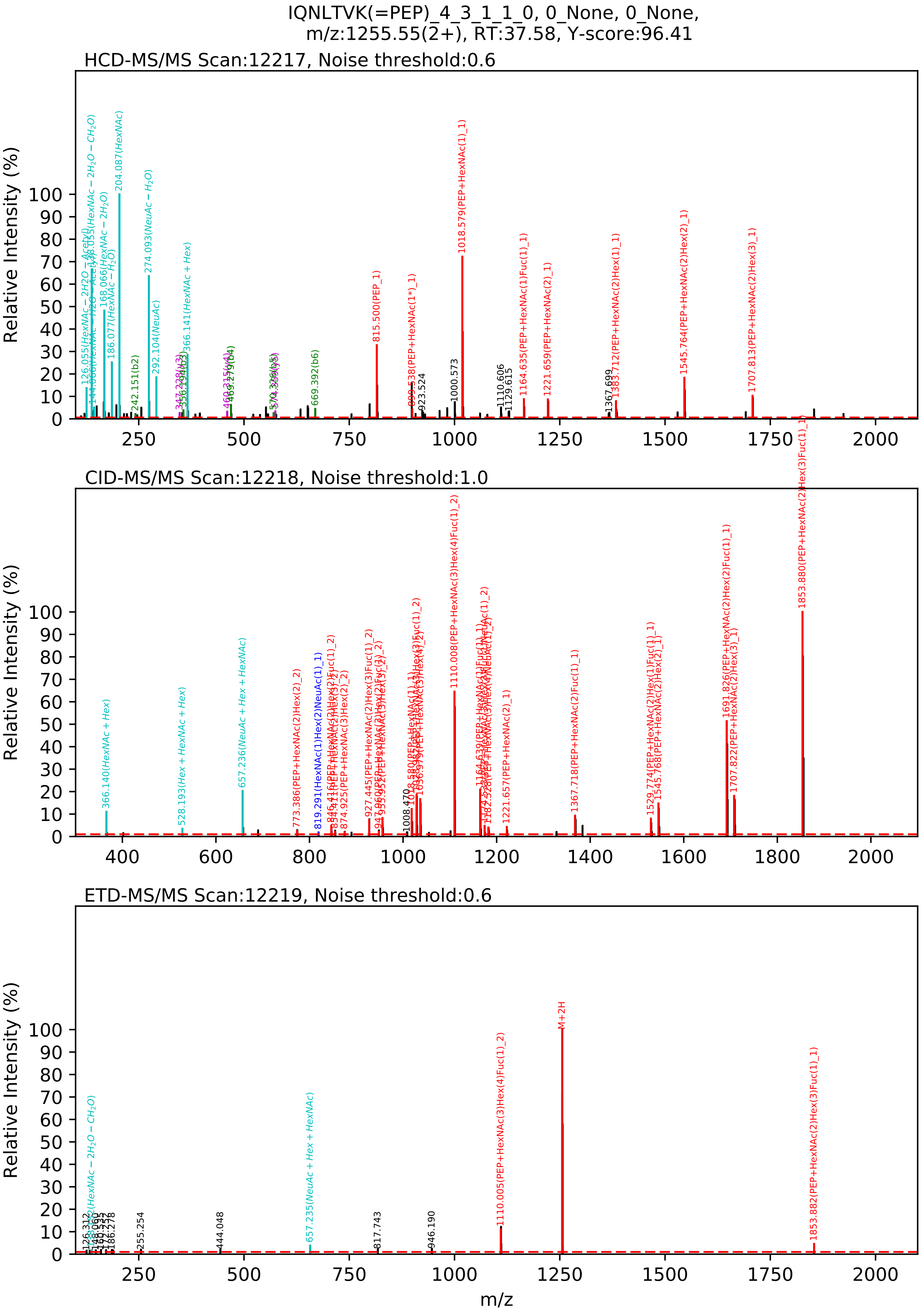
IQNLTVK(=PEP)_4_3_1_1_0, 0_None, 0_None,
m/z:1255.55(2+), RT:37.58, Y-score:96.41
HCD-MS/MS Scan:12217, Noise threshold:0.6
Relative Intensity (%)
204.087(HexNAc)
274.093(NeuAc − H2O)
292.104(NeuAc)
366.141(HexNAc + Hex)
242.151(b2)
469.279(b4)
669.392(b6)
815.500(PEP_1)
1018.579(PEP+HexNAc(1)_1)
923.524
1000.573
1110.606
1129.615
1164.635(PEP+HexNAc(1)Fuc(1)_1)
1221.659(PEP+HexNAc(2)_1)
1367.699
1383.712(PEP+HexNAc(2)Hex(1)_1)
1545.764(PEP+HexNAc(2)Hex(2)_1)
1707.813(PEP+HexNAc(2)Hex(3)_1)
CID-MS/MS Scan:12218, Noise threshold:1.0
366.140(HexNAc + Hex)
528.193(Hex + HexNAc + Hex)
657.236(NeuAc + Hex + HexNAc)
773.386(PEP+HexNAc(2)Hex(2)_2)
819.291(HexNAc(1)Hex(2)NeuAc(1)_1)
874.925(PEP+HexNAc(3)Hex(2)_2)
927.445(PEP+HexNAc(2)Hex(3)Fuc(1)_2)
1008.470
1110.008(PEP+HexNAc(3)Hex(4)Fuc(1)_2)
1221.657(PEP+HexNAc(2)_1)
1367.718(PEP+HexNAc(2)Fuc(1)_1)
1529.774(PEP+HexNAc(2)Hex(1)Fuc(1)_1)
1691.826(PEP+HexNAc(2)Hex(2)Fuc(1)_1)
1707.822(PEP+HexNAc(2)Hex(3)_1)
1853.880(PEP+HexNAc(2)Hex(3)Fuc(1)_1)
ETD-MS/MS Scan:12219, Noise threshold:0.6
126.312
255.254
444.048
657.235(NeuAc + Hex + HexNAc)
817.743
946.190
1110.005(PEP+HexNAc(3)Hex(4)Fuc(1)_2)
M+2H
1853.882(PEP+HexNAc(2)Hex(3)Fuc(1)_1)
m/z

IQNLTVK(=PEP)_4_3_1_1_0, 0_None, 0_None,
m/z:1255.55(2+), RT:36.93, Y-score:92.02

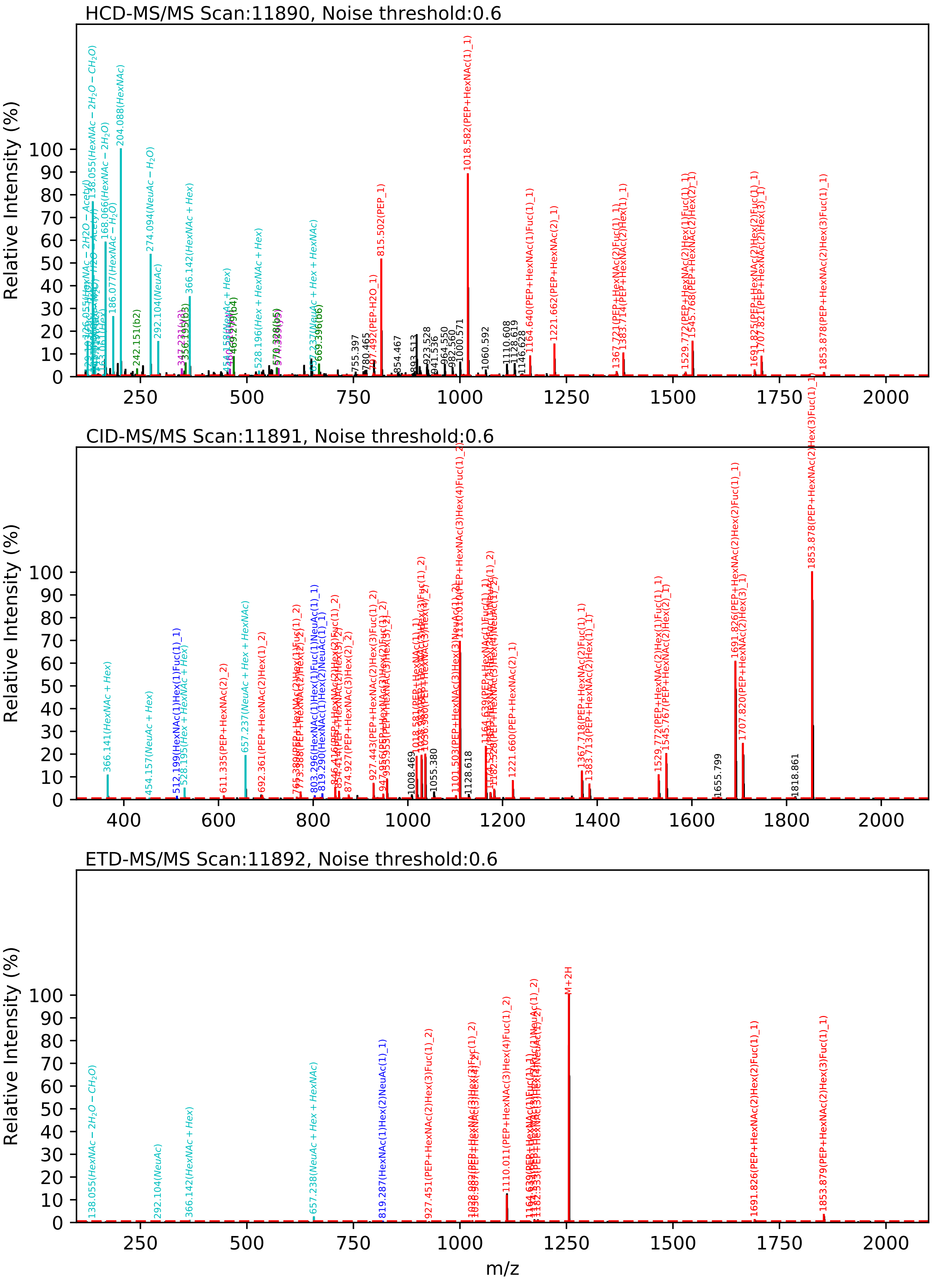

IQNLTVK(=PEP)_4_3_1_1_0, 0_None, 0_None,
m/z:1255.55(2+), RT:38.09, Y-score:92.54

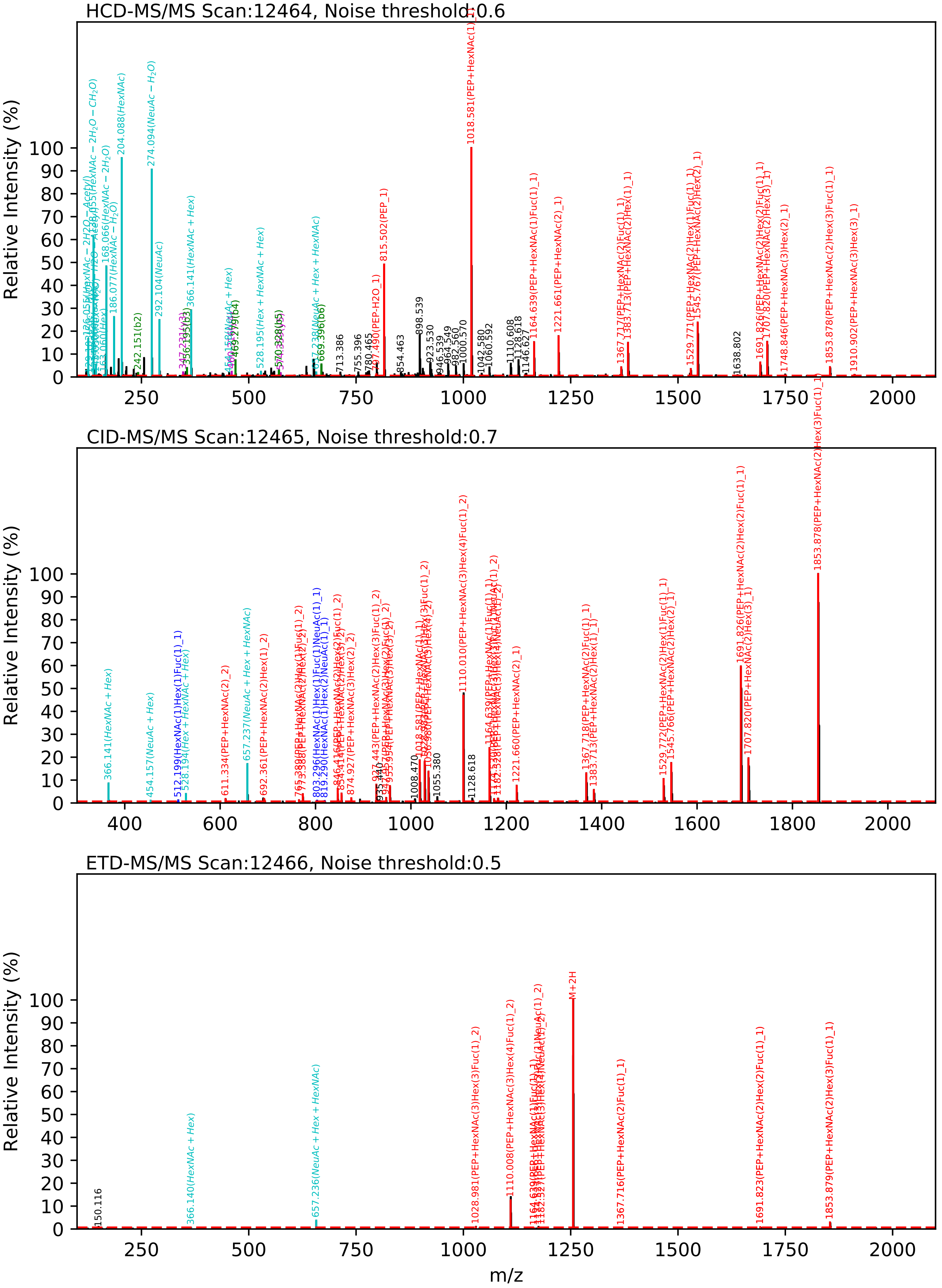

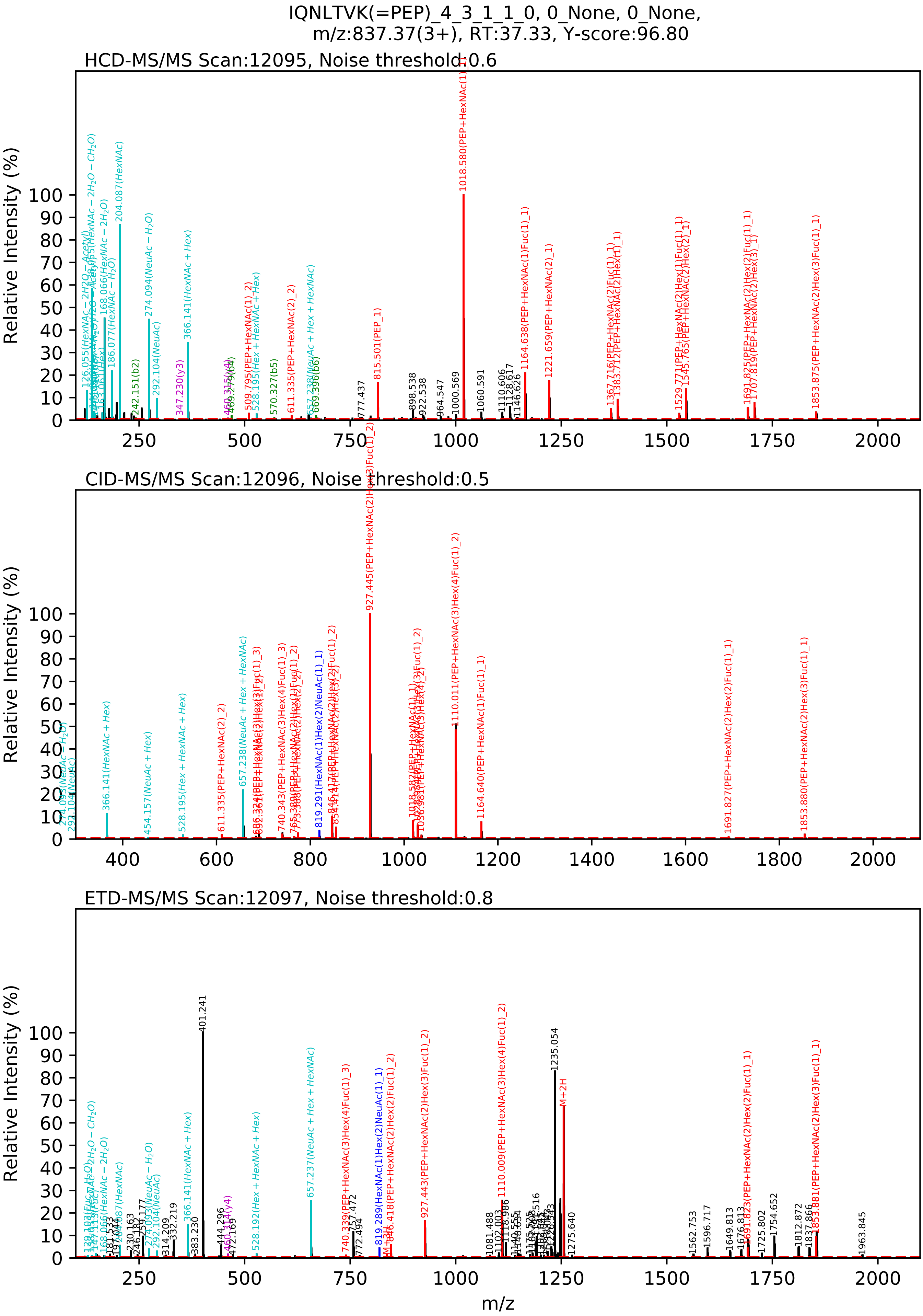

IQNLTVK(=PEP)_4_3_1_1_0, 0_None, 0_None,
m/z:837.37(3+), RT:37.33, Y-score:96.80
HCD-MS/MS Scan:12095, Noise threshold:0.6
CID-MS/MS Scan:12096, Noise threshold:0.5
ETD-MS/MS Scan:12097, Noise threshold:0.8
Relative Intensity (%)
m/z
1018.580(PEP+HexNAc(1)_1)
927.445(PEP+HexNAc(2)Hex(3)Fuc(1)_2)
1110.011(PEP+HexNAc(3)Hex(4)Fuc(1)_2)
401.241
1235.054
M+2H

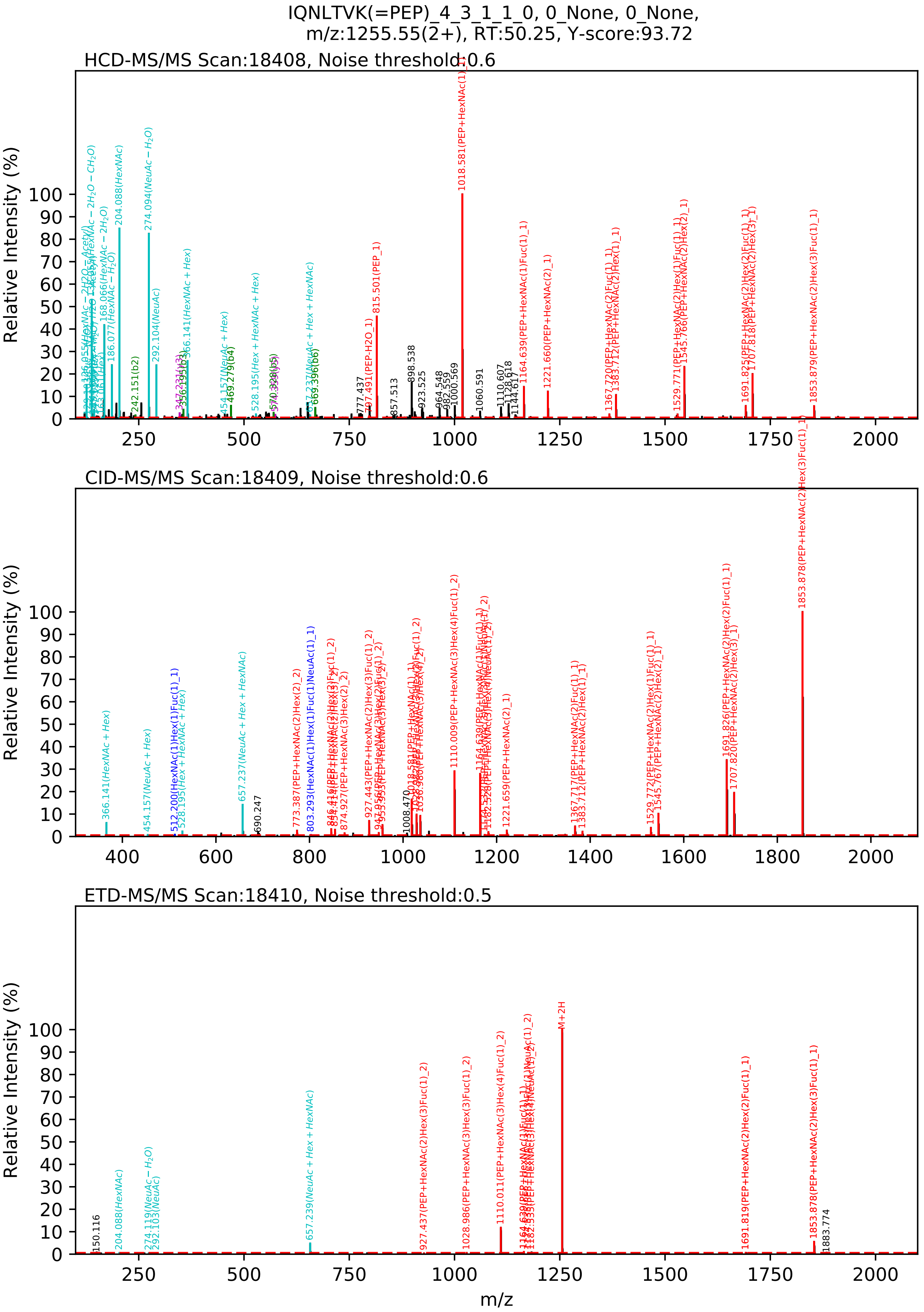

IQNLTVK(=PEP)_4_3_1_1_0, 0_None, 0_None,
m/z:1255.55(2+), RT:50.25, Y-score:93.72
HCD-MS/MS Scan:18408, Noise threshold:0.6
Relative Intensity (%)
1018.581(PEP+HexNAc(1)_1)
815.501(PEP_1)
274.094(NeuAc − H2O)
204.088(HexNAc)
292.104(NeuAc)
366.141(HexNAc + Hex)
1164.639(PEP+HexNAc(1)Fuc(1)_1)
1221.660(PEP+HexNAc(2)_1)
1853.879(PEP+HexNAc(2)Hex(3)Fuc(1)_1)
CID-MS/MS Scan:18409, Noise threshold:0.6
Relative Intensity (%)
1853.878(PEP+HexNAc(2)Hex(3)Fuc(1)_1)
1691.826(PEP+HexNAc(2)Hex(2)Fuc(1)_1)
1707.820(PEP+HexNAc(2)Hex(3)_1)
1110.009(PEP+HexNAc(3)Hex(4)Fuc(1)_2)
657.237(NeuAc + Hex + HexNAc)
366.141(HexNAc + Hex)
ETD-MS/MS Scan:18410, Noise threshold:0.5
Relative Intensity (%)
M+2H
1110.011(PEP+HexNAc(3)Hex(4)Fuc(1)_2)
1853.878(PEP+HexNAc(2)Hex(3)Fuc(1)_1)
1691.819(PEP+HexNAc(2)Hex(2)Fuc(1)_1)
927.437(PEP+HexNAc(2)Hex(3)Fuc(1)_2)
1028.986(PEP+HexNAc(3)Hex(3)Fuc(1)_2)
657.239(NeuAc + Hex + HexNAc)
204.088(HexNAc)
150.116
1883.774
m/z

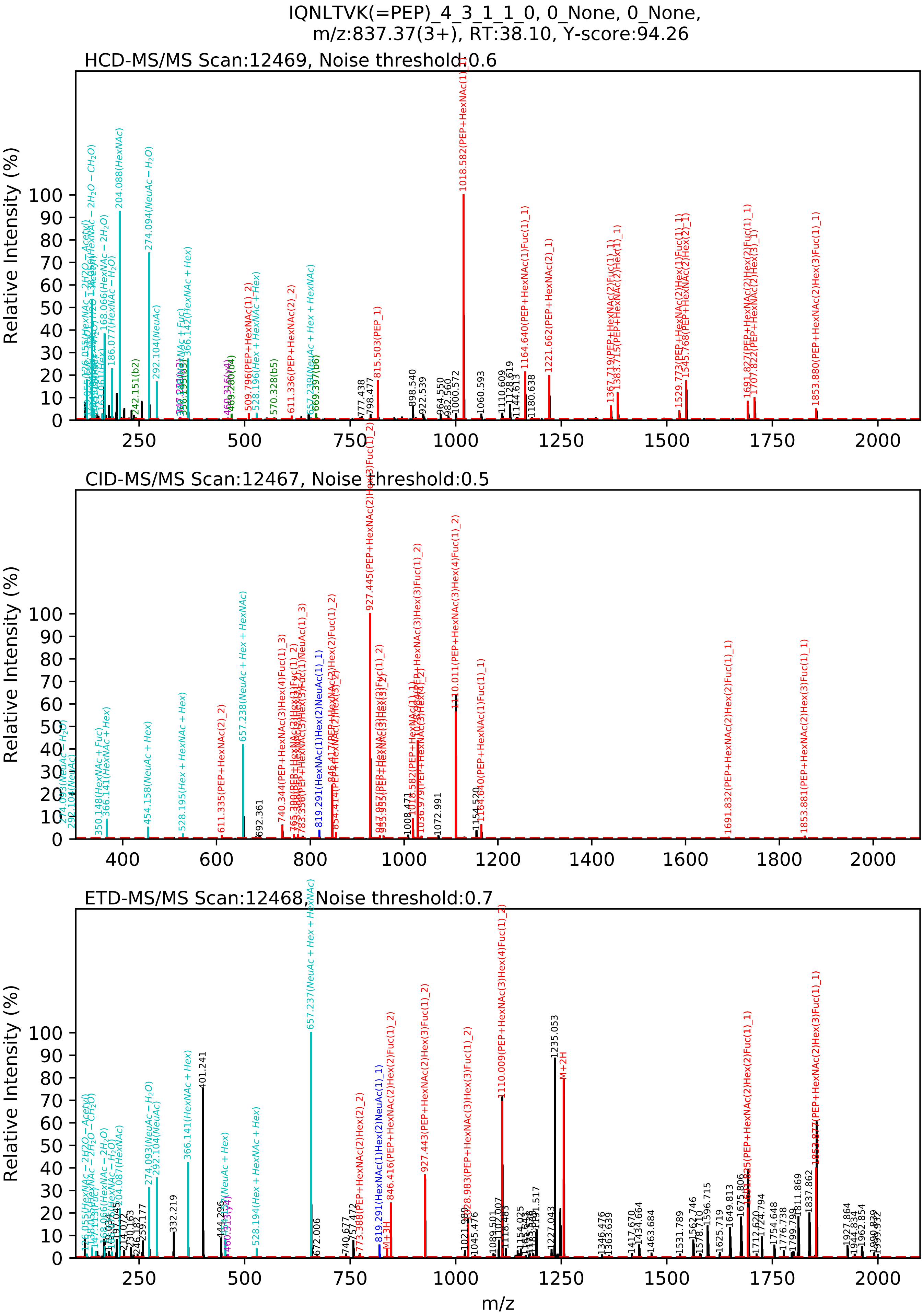

IQNLTVK(=PEP)_4_3_1_1_0, 0_None, 0_None,
m/z:837.37(3+), RT:38.10, Y-score:94.26
HCD-MS/MS Scan:12469, Noise threshold:0.6
CID-MS/MS Scan:12467, Noise threshold:0.5
ETD-MS/MS Scan:12468, Noise threshold:0.7
Relative Intensity (%)
m/z
1018.582(PEP+HexNAc(1)_1)
204.088(HexNAc)
274.094(NeuAc − H₂O)
292.104(NeuAc)
366.142(HexNAc + Hex)
815.503(PEP_1)
1164.640(PEP+HexNAc(1)Fuc(1)_1)
1221.662(PEP+HexNAc(2)_1)
1853.880(PEP+HexNAc(2)Hex(3)Fuc(1)_1)
927.445(PEP+HexNAc(2)Hex(3)Fuc(1)_2)
1110.011(PEP+HexNAc(3)Hex(4)Fuc(1)_2)
657.238(NeuAc + Hex + HexNAc)
1691.832(PEP+HexNAc(2)Hex(2)Fuc(1)_1)
1853.881(PEP+HexNAc(2)Hex(3)Fuc(1)_1)
657.237(NeuAc + Hex + HexNAc)
401.241
1235.053
M+2H
1110.009(PEP+HexNAc(3)Hex(4)Fuc(1)_2)
927.443(PEP+HexNAc(2)Hex(3)Fuc(1)_2)
846.416(PEP+HexNAc(2)Hex(2)Fuc(1)_2)
1853.877(PEP+HexNAc(2)Hex(3)Fuc(1)_1)

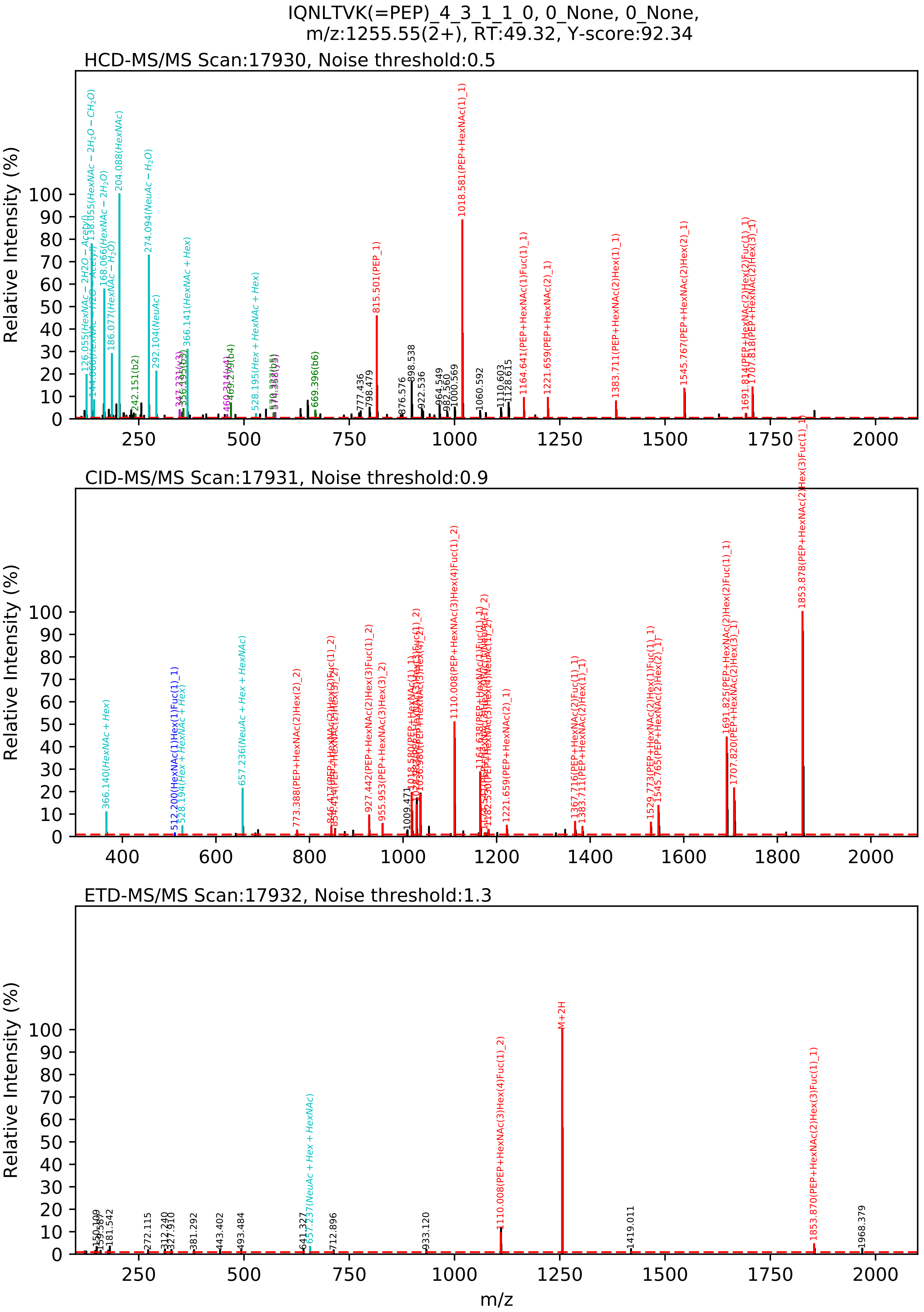

IQNLTVK(=PEP)_4_3_1_1_0, 0_None, 0_None,
m/z:1255.55(2+), RT:49.32, Y-score:92.34
HCD-MS/MS Scan:17930, Noise threshold:0.5
CID-MS/MS Scan:17931, Noise threshold:0.9
ETD-MS/MS Scan:17932, Noise threshold:1.3
Relative Intensity (%)
m/z
1018.581(PEP+HexNAc(1)_1)
815.501(PEP_1)
204.088(HexNAc)
274.094(NeuAc − H2O)
1853.878(PEP+HexNAc(2)Hex(3)Fuc(1)_1)
1110.008(PEP+HexNAc(3)Hex(4)Fuc(1)_2)
1691.825(PEP+HexNAc(2)Hex(2)Fuc(1)_1)
M+2H

IQNLTVK(=PEP)_4_3_1_1_0, 0_None, 0_None,
m/z:1255.55(2+), RT:49.42, Y-score:96.83

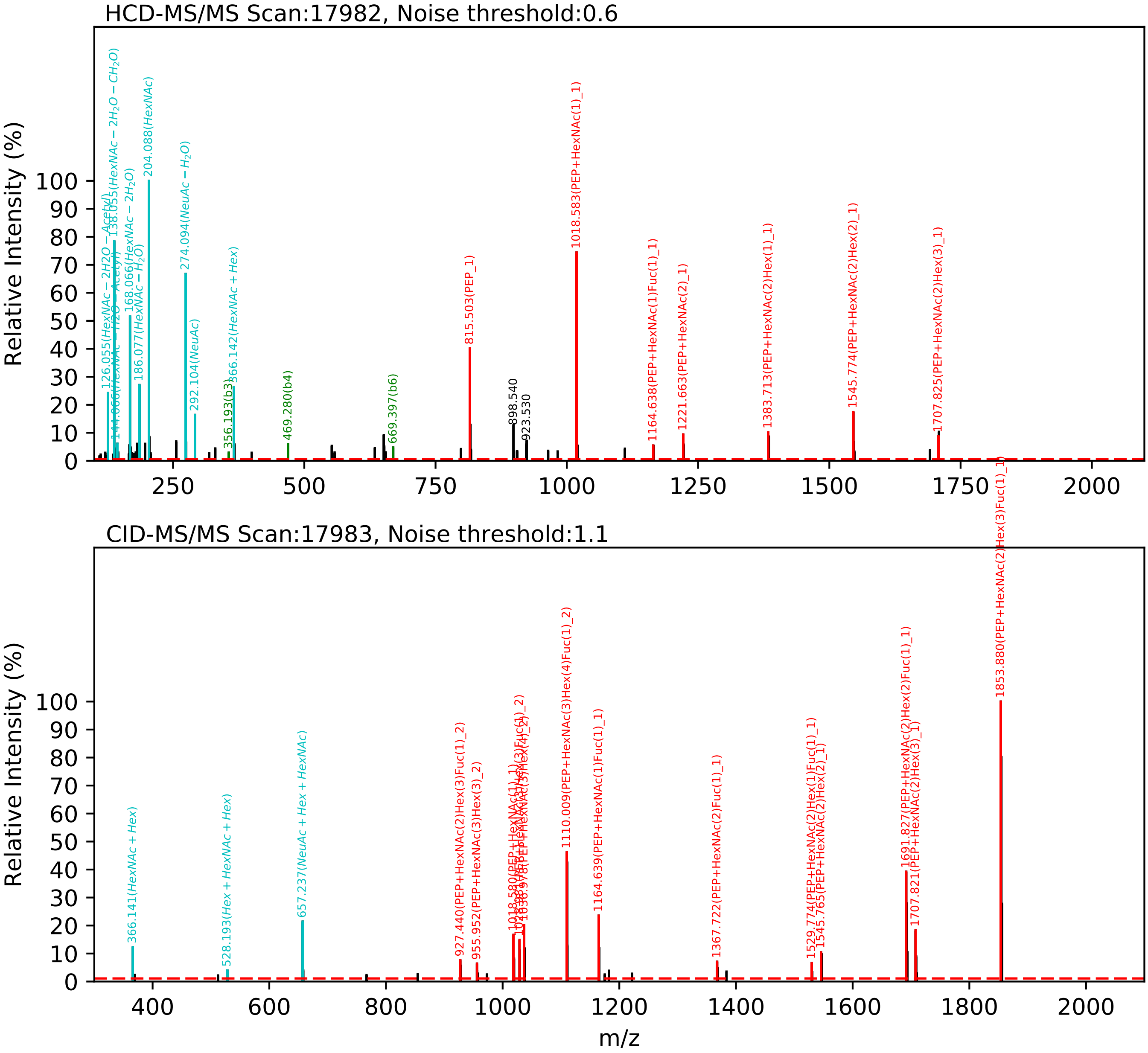

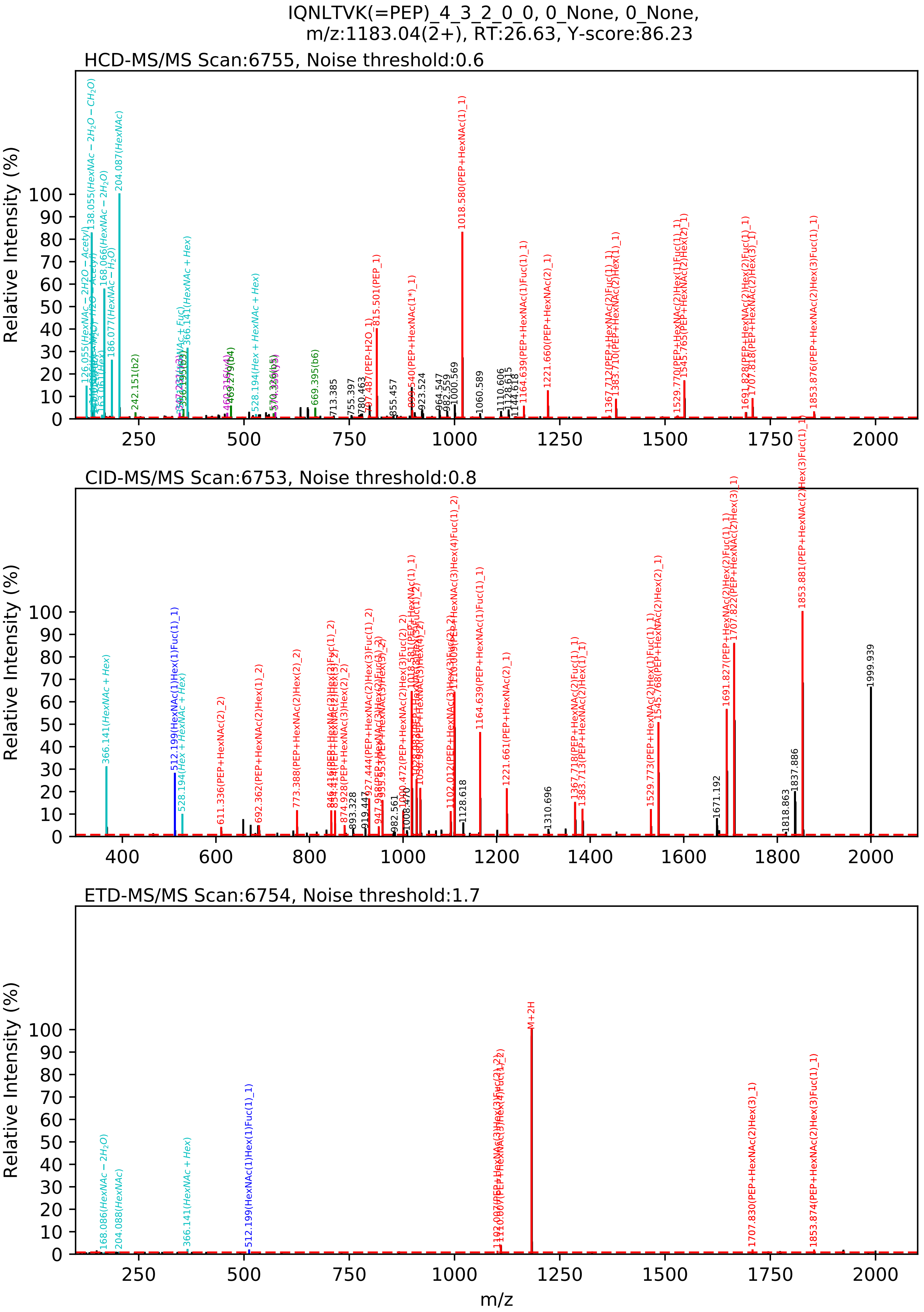

IQNLTVK(=PEP)_4_3_2_0_0, 0_None, 0_None,
m/z:1183.04(2+), RT:26.63, Y-score:86.23
HCD-MS/MS Scan:6755, Noise threshold:0.6
CID-MS/MS Scan:6753, Noise threshold:0.8
ETD-MS/MS Scan:6754, Noise threshold:1.7
Relative Intensity (%)
m/z
204.087(HexNAc)
1018.580(PEP+HexNAc(1)_1)
815.501(PEP_1)
1853.881(PEP+HexNAc(2)Hex(3)Fuc(1)_1)
1999.939
M+2H

IQNLTVK(=PEP)_4_3_2_0_0, 0_None, 0_None,
m/z:1183.04(2+), RT:27.36, Y-score:89.18

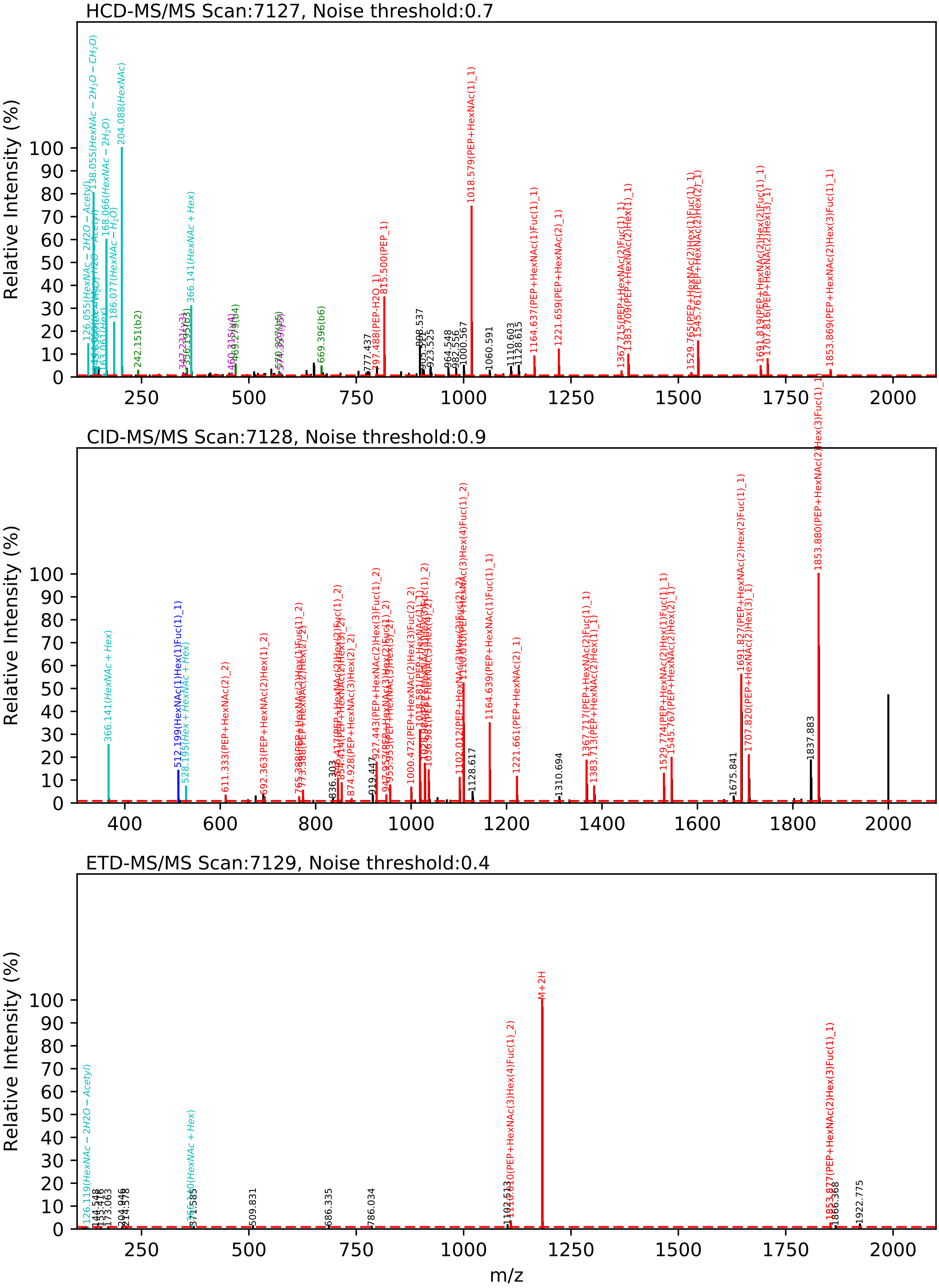

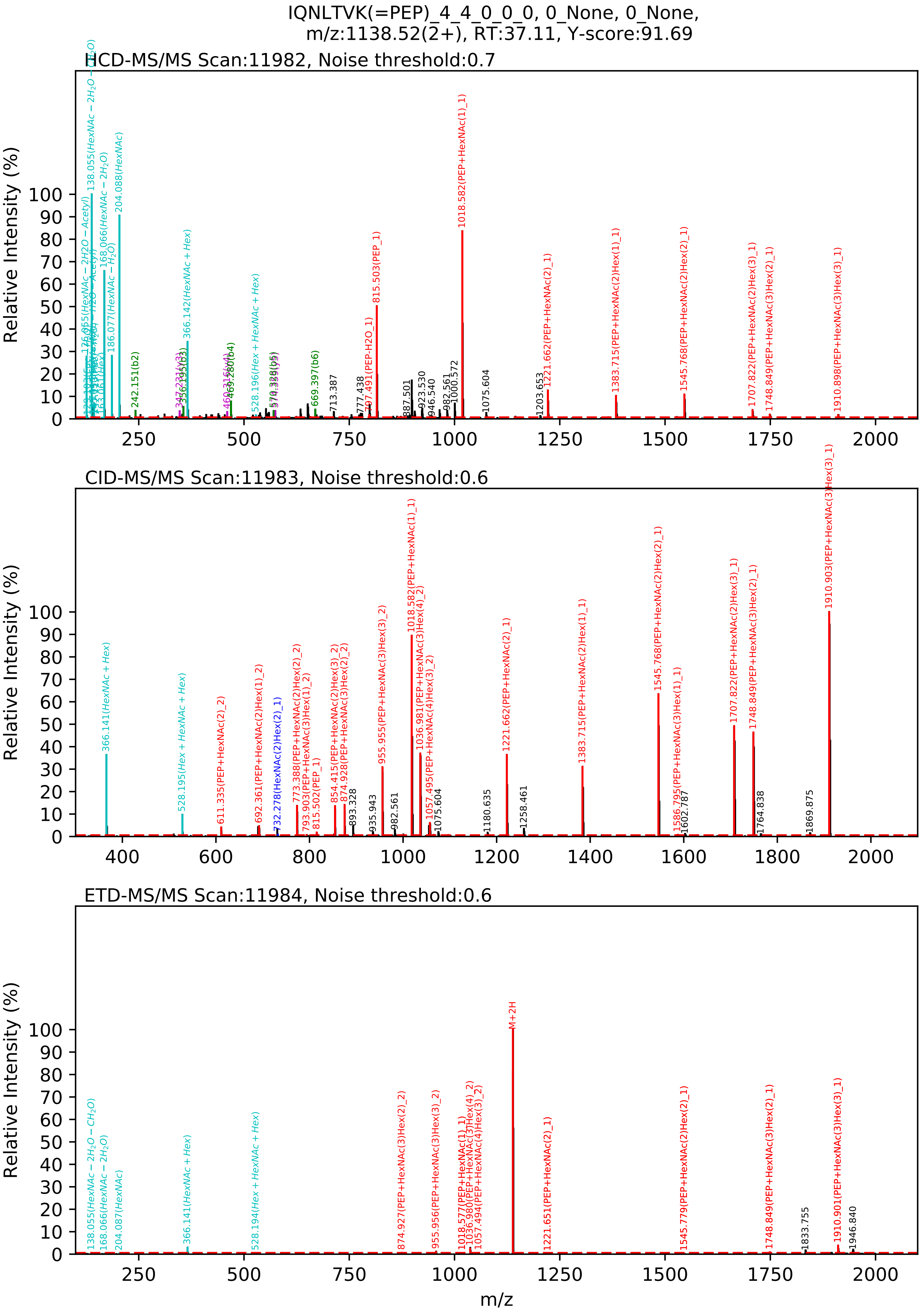
IQNLTVK(=PEP)_4_4_0_0_0, 0_None, 0_None,
m/z:1138.52(2+), RT:37.11, Y-score:91.69
HCD-MS/MS Scan:11982, Noise threshold:0.7
Relative Intensity (%)
138.055(HexNAc)
204.088(HexNAc)
168.066(HexNAc − 2H2O)
186.077(HexNAc − H2O)
366.142(HexNAc + Hex)
242.151(b2)
469.280(b4)
528.196(Hex + HexNAc + Hex)
669.397(b6)
713.387
777.438
807.491(PEP-H2O_1)
815.503(PEP_1)
887.501
923.530
946.540
982.561
1000.572
1018.582(PEP+HexNAc(1)_1)
1075.604
1203.653
1221.662(PEP+HexNAc(2)_1)
1383.715(PEP+HexNAc(2)Hex(1)_1)
1545.768(PEP+HexNAc(2)Hex(2)_1)
1707.822(PEP+HexNAc(2)Hex(3)_1)
1748.849(PEP+HexNAc(3)Hex(2)_1)
1910.898(PEP+HexNAc(3)Hex(3)_1)
CID-MS/MS Scan:11983, Noise threshold:0.6
366.141(HexNAc + Hex)
528.195(Hex + HexNAc + Hex)
611.335(PEP+HexNAc(2)_2)
692.361(PEP+HexNAc(2)Hex(1)_2)
732.278(HexNAc(2)Hex(2)_1)
773.388(PEP+HexNAc(2)Hex(2)_2)
793.903(PEP+HexNAc(3)Hex(1)_2)
815.502(PEP_1)
854.415(PEP+HexNAc(2)Hex(3)_2)
874.928(PEP+HexNAc(3)Hex(2)_2)
893.328
935.943
955.955(PEP+HexNAc(3)Hex(3)_2)
982.561
1018.582(PEP+HexNAc(1)_1)
1036.981(PEP+HexNAc(3)Hex(4)_2)
1057.495(PEP+HexNAc(4)Hex(3)_2)
1075.604
1180.635
1221.662(PEP+HexNAc(2)_1)
1258.461
1383.715(PEP+HexNAc(2)Hex(1)_1)
1545.768(PEP+HexNAc(2)Hex(2)_1)
1586.795(PEP+HexNAc(3)Hex(1)_1)
1602.787
1707.822(PEP+HexNAc(2)Hex(3)_1)
1748.849(PEP+HexNAc(3)Hex(2)_1)
1764.838
1869.875
1910.903(PEP+HexNAc(3)Hex(3)_1)
ETD-MS/MS Scan:11984, Noise threshold:0.6
138.055(HexNAc − 2H2O − CH2O)
168.066(HexNAc − 2H2O)
204.087(HexNAc)
366.141(HexNAc + Hex)
528.194(Hex + HexNAc + Hex)
874.927(PEP+HexNAc(3)Hex(2)_2)
955.956(PEP+HexNAc(3)Hex(3)_2)
1018.577(PEP+HexNAc(1)_1)
1036.980(PEP+HexNAc(3)Hex(4)_2)
1057.494(PEP+HexNAc(4)Hex(3)_2)
M+2H
1221.651(PEP+HexNAc(2)_1)
1545.779(PEP+HexNAc(2)Hex(2)_1)
1748.849(PEP+HexNAc(3)Hex(2)_1)
1833.755
1910.901(PEP+HexNAc(3)Hex(3)_1)
1946.840
m/z

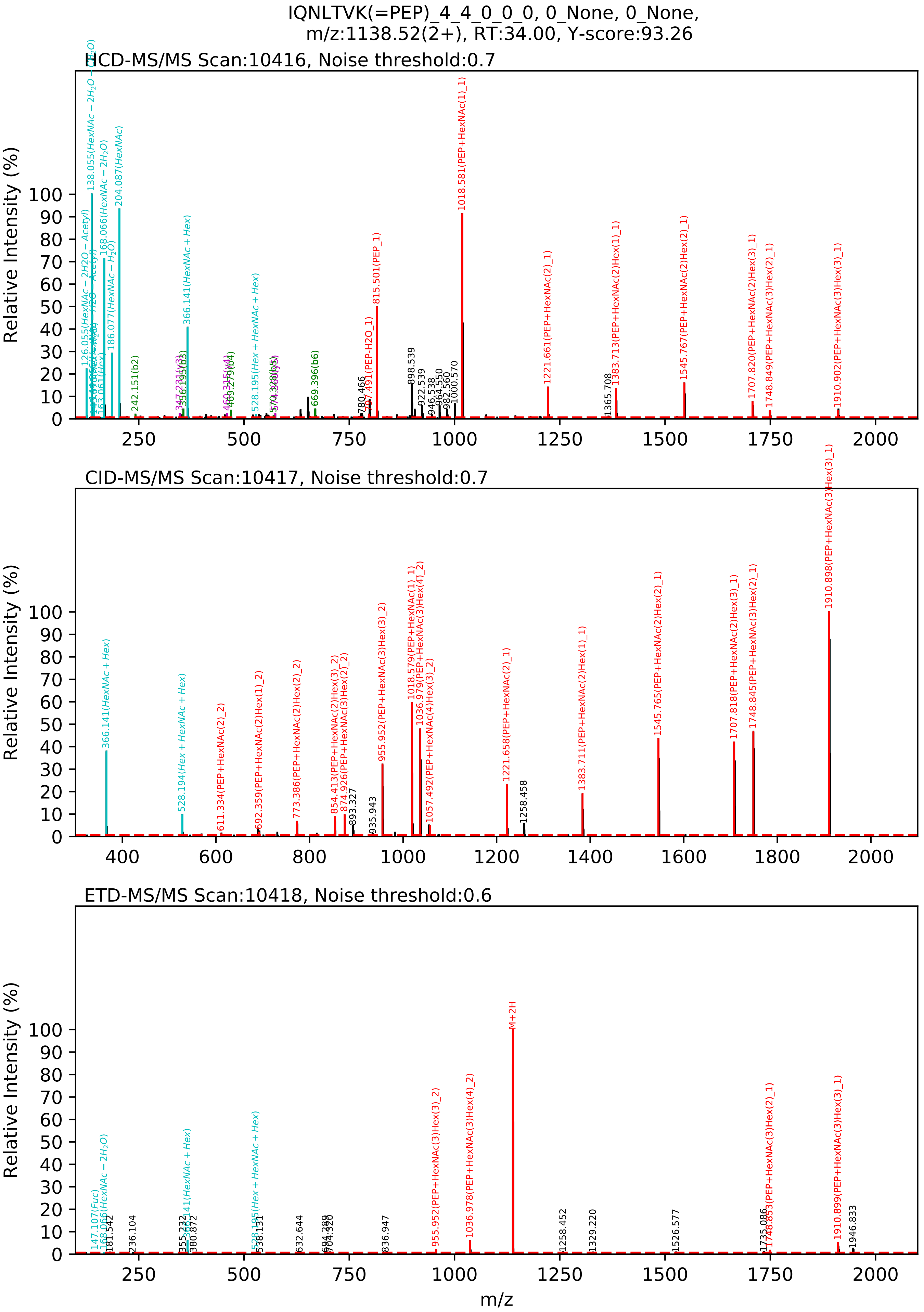

IQNLTVK(=PEP)_4_4_0_0_0, 0_None, 0_None,
m/z:1138.52(2+), RT:34.00, Y-score:93.26
HCD-MS/MS Scan:10416, Noise threshold:0.7
Relative Intensity (%)
138.055(HexNAc − 2H2O − H2O)
204.087(HexNAc)
168.066(HexNAc − 2H2O)
186.077(HexNAc − H2O)
126.055(HexNAc − 2H2O − Acetyl)
163.061(Hex)
366.141(HexNAc + Hex)
242.151(b2)
347.224(y3)
356.195(b3)
460.315(y4)
469.279(b4)
528.195(Hex + HexNAc + Hex)
570.328(b5)
574.360(y5)
669.396(b6)
780.466
797.491(PEP-H2O_1)
815.501(PEP_1)
898.539
922.539
946.538
964.550
982.560
1000.570
1018.581(PEP+HexNAc(1)_1)
1221.661(PEP+HexNAc(2)_1)
1365.708
1383.713(PEP+HexNAc(2)Hex(1)_1)
1545.767(PEP+HexNAc(2)Hex(2)_1)
1707.820(PEP+HexNAc(2)Hex(3)_1)
1748.849(PEP+HexNAc(3)Hex(2)_1)
1910.902(PEP+HexNAc(3)Hex(3)_1)
CID-MS/MS Scan:10417, Noise threshold:0.7
Relative Intensity (%)
366.141(HexNAc + Hex)
528.194(Hex + HexNAc + Hex)
611.334(PEP+HexNAc(2)_2)
692.359(PEP+HexNAc(2)Hex(1)_2)
773.386(PEP+HexNAc(2)Hex(2)_2)
854.413(PEP+HexNAc(2)Hex(3)_2)
874.926(PEP+HexNAc(3)Hex(2)_2)
893.327
935.943
955.952(PEP+HexNAc(3)Hex(3)_2)
1018.579(PEP+HexNAc(1)_1)
1036.979(PEP+HexNAc(3)Hex(4)_2)
1057.492(PEP+HexNAc(4)Hex(3)_2)
1221.658(PEP+HexNAc(2)_1)
1258.458
1383.711(PEP+HexNAc(2)Hex(1)_1)
1545.765(PEP+HexNAc(2)Hex(2)_1)
1707.818(PEP+HexNAc(2)Hex(3)_1)
1748.845(PEP+HexNAc(3)Hex(2)_1)
1910.898(PEP+HexNAc(3)Hex(3)_1)
ETD-MS/MS Scan:10418, Noise threshold:0.6
Relative Intensity (%)
147.107(Fuc)
168.066(HexNAc − 2H2O)
181.542
236.104
355.232
366.141(HexNAc + Hex)
380.872
528.195(Hex + HexNAc + Hex)
538.131
632.644
694.289
704.320
836.947
955.952(PEP+HexNAc(3)Hex(3)_2)
1036.978(PEP+HexNAc(3)Hex(4)_2)
M+2H
1258.452
1329.220
1526.577
1735.086
1748.853(PEP+HexNAc(3)Hex(2)_1)
1910.899(PEP+HexNAc(3)Hex(3)_1)
1946.833
m/z

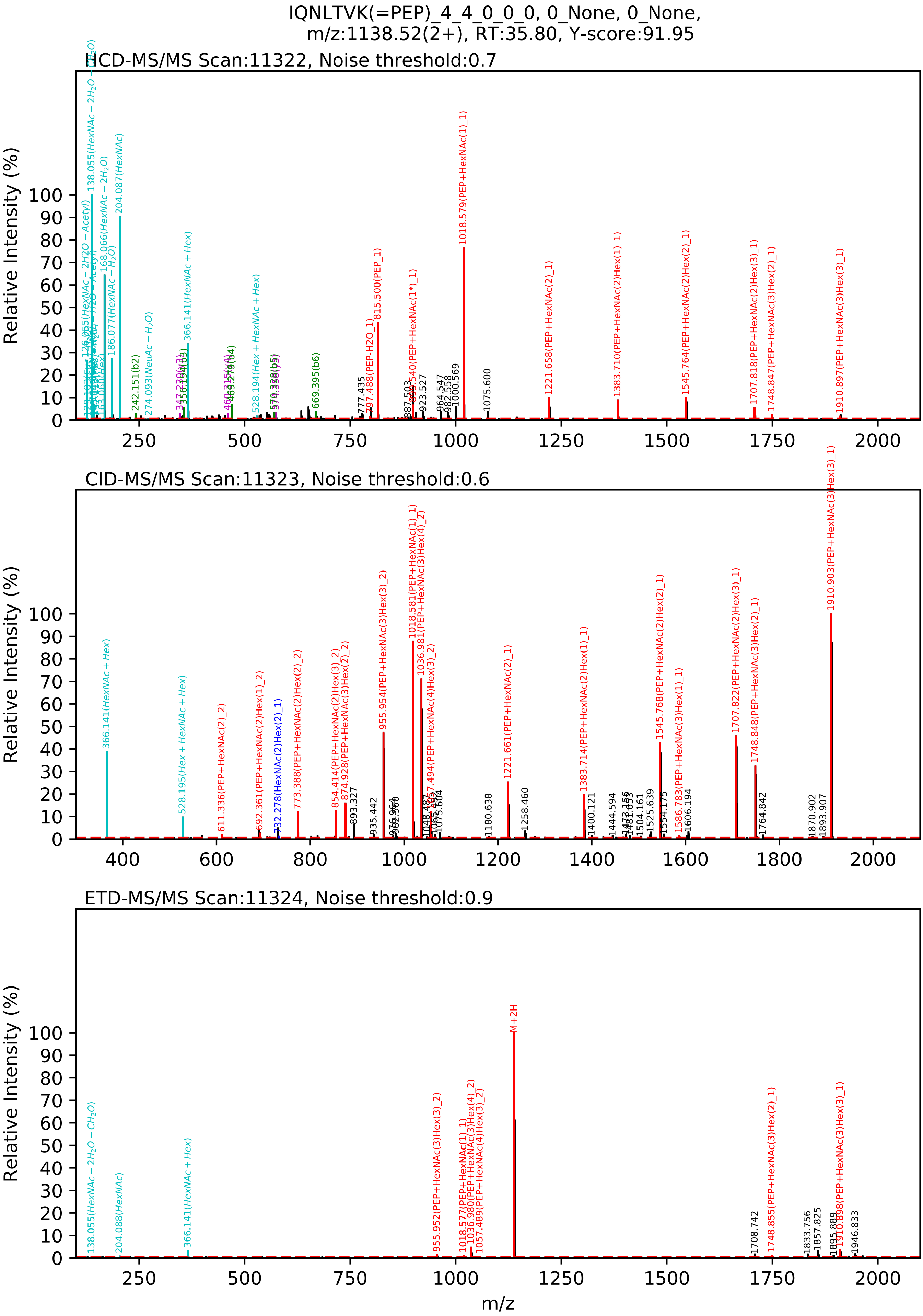
IQNLTVK(=PEP)_4_4_0_0_0, 0_None, 0_None,
m/z:1138.52(2+), RT:35.80, Y-score:91.95
HCD-MS/MS Scan:11322, Noise threshold:0.7
CID-MS/MS Scan:11323, Noise threshold:0.6
ETD-MS/MS Scan:11324, Noise threshold:0.9
Relative Intensity (%)
m/z
1018.579(PEP+HexNAc(1)_1)
815.500(PEP_1)
1221.658(PEP+HexNAc(2)_1)
1383.710(PEP+HexNAc(2)Hex(1)_1)
1545.764(PEP+HexNAc(2)Hex(2)_1)
1707.818(PEP+HexNAc(2)Hex(3)_1)
1748.847(PEP+HexNAc(3)Hex(2)_1)
1910.897(PEP+HexNAc(3)Hex(3)_1)
204.087(HexNAc)
366.141(HexNAc+Hex)
1910.903(PEP+HexNAc(3)Hex(3)_1)
1018.581(PEP+HexNAc(1)_1)
1036.981(PEP+HexNAc(3)Hex(4)_2)
955.954(PEP+HexNAc(3)Hex(3)_2)
1545.768(PEP+HexNAc(2)Hex(2)_1)
1707.822(PEP+HexNAc(2)Hex(3)_1)
1748.848(PEP+HexNAc(3)Hex(2)_1)
M+2H
1748.855(PEP+HexNAc(3)Hex(2)_1)
1910.898(PEP+HexNAc(3)Hex(3)_1)

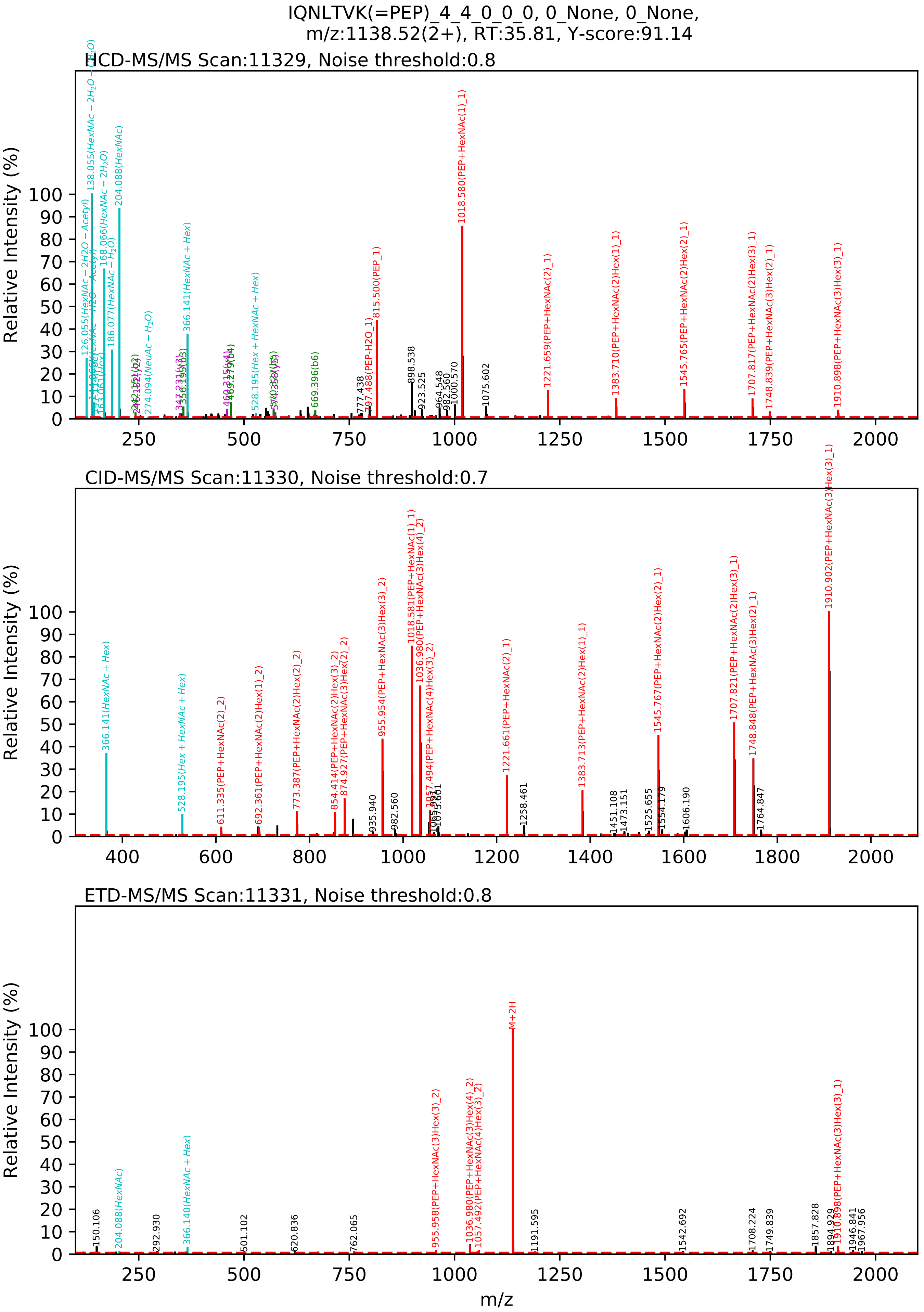

IQNLTVK(=PEP)_4_4_0_0_0, 0_None, 0_None,
m/z:1138.52(2+), RT:35.81, Y-score:91.14
HCD-MS/MS Scan:11329, Noise threshold:0.8
Relative Intensity (%)
1018.580(PEP+HexNAc(1)_1)
1221.659(PEP+HexNAc(2)_1)
1383.710(PEP+HexNAc(2)Hex(1)_1)
1545.765(PEP+HexNAc(2)Hex(2)_1)
1707.817(PEP+HexNAc(2)Hex(3)_1)
1748.839(PEP+HexNAc(3)Hex(2)_1)
1910.898(PEP+HexNAc(3)Hex(3)_1)
815.500(PEP_1)
204.088(HexNAc)
366.141(HexNAc+Hex)
528.195(Hex+HexNAc+Hex)
669.396(b6)
898.538
1075.602
CID-MS/MS Scan:11330, Noise threshold:0.7
1910.902(PEP+HexNAc(3)Hex(3)_1)
1018.581(PEP+HexNAc(1)_1)
1036.980(PEP+HexNAc(3)Hex(4)_2)
955.954(PEP+HexNAc(3)Hex(3)_2)
1707.821(PEP+HexNAc(2)Hex(3)_1)
1748.848(PEP+HexNAc(3)Hex(2)_1)
1545.767(PEP+HexNAc(2)Hex(2)_1)
1383.713(PEP+HexNAc(2)Hex(1)_1)
1221.661(PEP+HexNAc(2)_1)
773.387(PEP+HexNAc(2)Hex(2)_2)
692.361(PEP+HexNAc(2)Hex(1)_2)
611.335(PEP+HexNAc(2)_2)
854.414(PEP+HexNAc(2)Hex(3)_2)
874.927(PEP+HexNAc(3)Hex(2)_2)
1057.494(PEP+HexNAc(4)Hex(3)_2)
366.141(HexNAc+Hex)
528.195(Hex+HexNAc+Hex)
ETD-MS/MS Scan:11331, Noise threshold:0.8
M+2H
955.958(PEP+HexNAc(3)Hex(3)_2)
1036.980(PEP+HexNAc(3)Hex(4)_2)
1057.492(PEP+HexNAc(4)Hex(3)_2)
1910.898(PEP+HexNAc(3)Hex(3)_1)
204.088(HexNAc)
366.140(HexNAc+Hex)
m/z

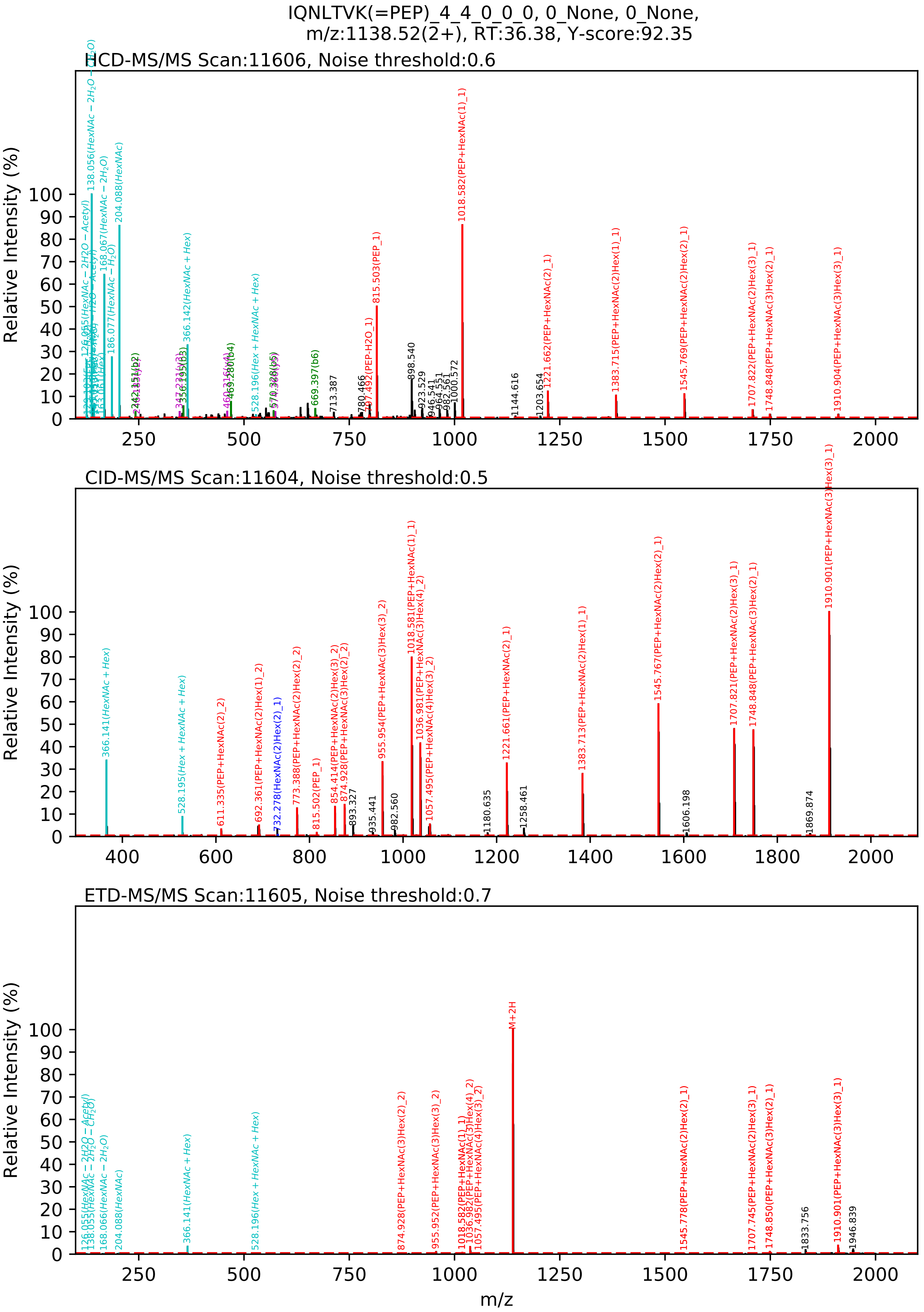

IQNLTVK(=PEP)_4_4_0_0_0, 0_None, 0_None,
m/z:1138.52(2+), RT:36.38, Y-score:92.35
HCD-MS/MS Scan:11606, Noise threshold:0.6
Relative Intensity (%)
138.056(HexNAc − 2H2O − H2O)
204.088(HexNAc)
168.067(HexNAc − 2H2O)
186.077(HexNAc − H2O)
366.142(HexNAc + Hex)
528.196(Hex + HexNAc + Hex)
669.397(b6)
815.503(PEP_1)
898.540
923.529
946.541
964.551
1000.572
1018.582(PEP+HexNAc(1)_1)
1144.616
1203.654
1221.662(PEP+HexNAc(2)_1)
1383.715(PEP+HexNAc(2)Hex(1)_1)
1545.769(PEP+HexNAc(2)Hex(2)_1)
1707.822(PEP+HexNAc(2)Hex(3)_1)
1748.848(PEP+HexNAc(3)Hex(2)_1)
1910.904(PEP+HexNAc(3)Hex(3)_1)
CID-MS/MS Scan:11604, Noise threshold:0.5
366.141(HexNAc + Hex)
528.195(Hex + HexNAc + Hex)
611.335(PEP+HexNAc(2)_2)
692.361(PEP+HexNAc(2)Hex(1)_2)
732.278(HexNAc(2)Hex(2)_1)
773.388(PEP+HexNAc(2)Hex(2)_2)
815.502(PEP_1)
854.414(PEP+HexNAc(2)Hex(3)_2)
874.928(PEP+HexNAc(3)Hex(2)_2)
893.327
935.441
955.954(PEP+HexNAc(3)Hex(3)_2)
982.560
1018.581(PEP+HexNAc(1)_1)
1036.981(PEP+HexNAc(3)Hex(4)_2)
1057.495(PEP+HexNAc(4)Hex(3)_2)
1180.635
1221.661(PEP+HexNAc(2)_1)
1258.461
1383.713(PEP+HexNAc(2)Hex(1)_1)
1545.767(PEP+HexNAc(2)Hex(2)_1)
1606.198
1707.821(PEP+HexNAc(2)Hex(3)_1)
1748.848(PEP+HexNAc(3)Hex(2)_1)
1869.874
1910.901(PEP+HexNAc(3)Hex(3)_1)
ETD-MS/MS Scan:11605, Noise threshold:0.7
204.088(HexNAc)
366.141(HexNAc + Hex)
528.196(Hex + HexNAc + Hex)
874.928(PEP+HexNAc(3)Hex(2)_2)
955.952(PEP+HexNAc(3)Hex(3)_2)
1018.582(PEP+HexNAc(1)_1)
1036.982(PEP+HexNAc(3)Hex(4)_2)
1057.495(PEP+HexNAc(4)Hex(3)_2)
M+2H
1545.778(PEP+HexNAc(2)Hex(2)_1)
1707.745(PEP+HexNAc(2)Hex(3)_1)
1748.850(PEP+HexNAc(3)Hex(2)_1)
1833.756
1910.901(PEP+HexNAc(3)Hex(3)_1)
1946.839
m/z

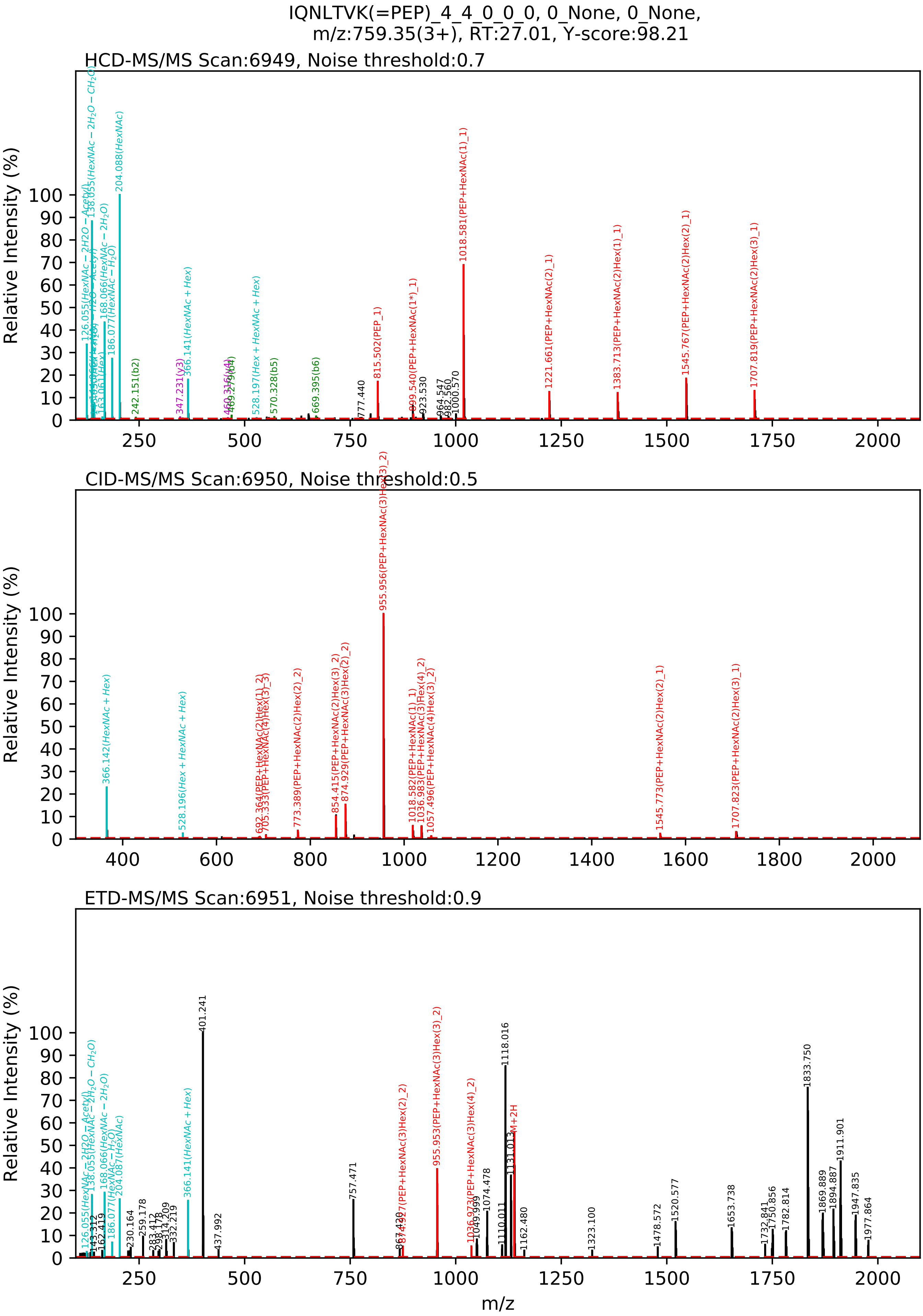

IQNLTVK(=PEP)_4_4_0_0_0, 0_None, 0_None,
m/z:759.35(3+), RT:27.01, Y-score:98.21
HCD-MS/MS Scan:6949, Noise threshold:0.7
204.088(HexNAc)
1018.581(PEP+HexNAc(1)_1)
1221.661(PEP+HexNAc(2)_1)
1383.713(PEP+HexNAc(2)Hex(1)_1)
1545.767(PEP+HexNAc(2)Hex(2)_1)
1707.819(PEP+HexNAc(2)Hex(3)_1)
815.502(PEP_1)
366.141(HexNAc+Hex)
528.197(Hex+HexNAc+Hex)
242.151(b2)
347.231(y3)
570.328(b5)
669.395(b6)
CID-MS/MS Scan:6950, Noise threshold:0.5
955.956(PEP+HexNAc(3)Hex(3)_2)
854.415(PEP+HexNAc(2)Hex(3)_2)
874.929(PEP+HexNAc(3)Hex(2)_2)
773.389(PEP+HexNAc(2)Hex(2)_2)
1545.773(PEP+HexNAc(2)Hex(2)_1)
1707.823(PEP+HexNAc(2)Hex(3)_1)
366.142(HexNAc+Hex)
528.196(Hex+HexNAc+Hex)
ETD-MS/MS Scan:6951, Noise threshold:0.9
401.241
1118.016
1833.750
955.953(PEP+HexNAc(3)Hex(3)_2)
M+2H
1911.901
757.471
Relative Intensity (%)
m/z

IQNLTVK(=PEP)_4_4_0_0_0, 0_None, 0_None,
m/z:759.35(3+), RT:27.58, Y-score:95.08

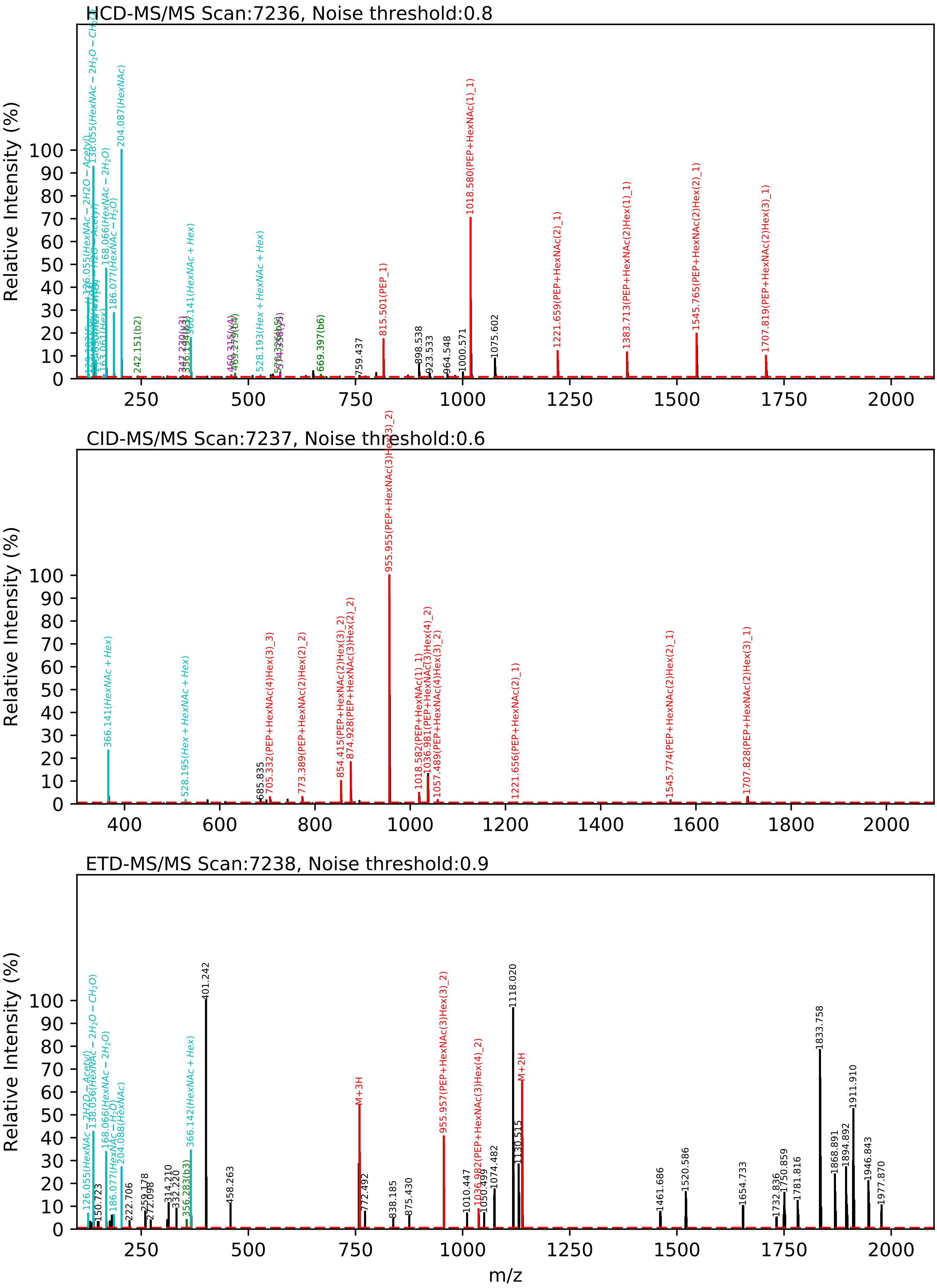

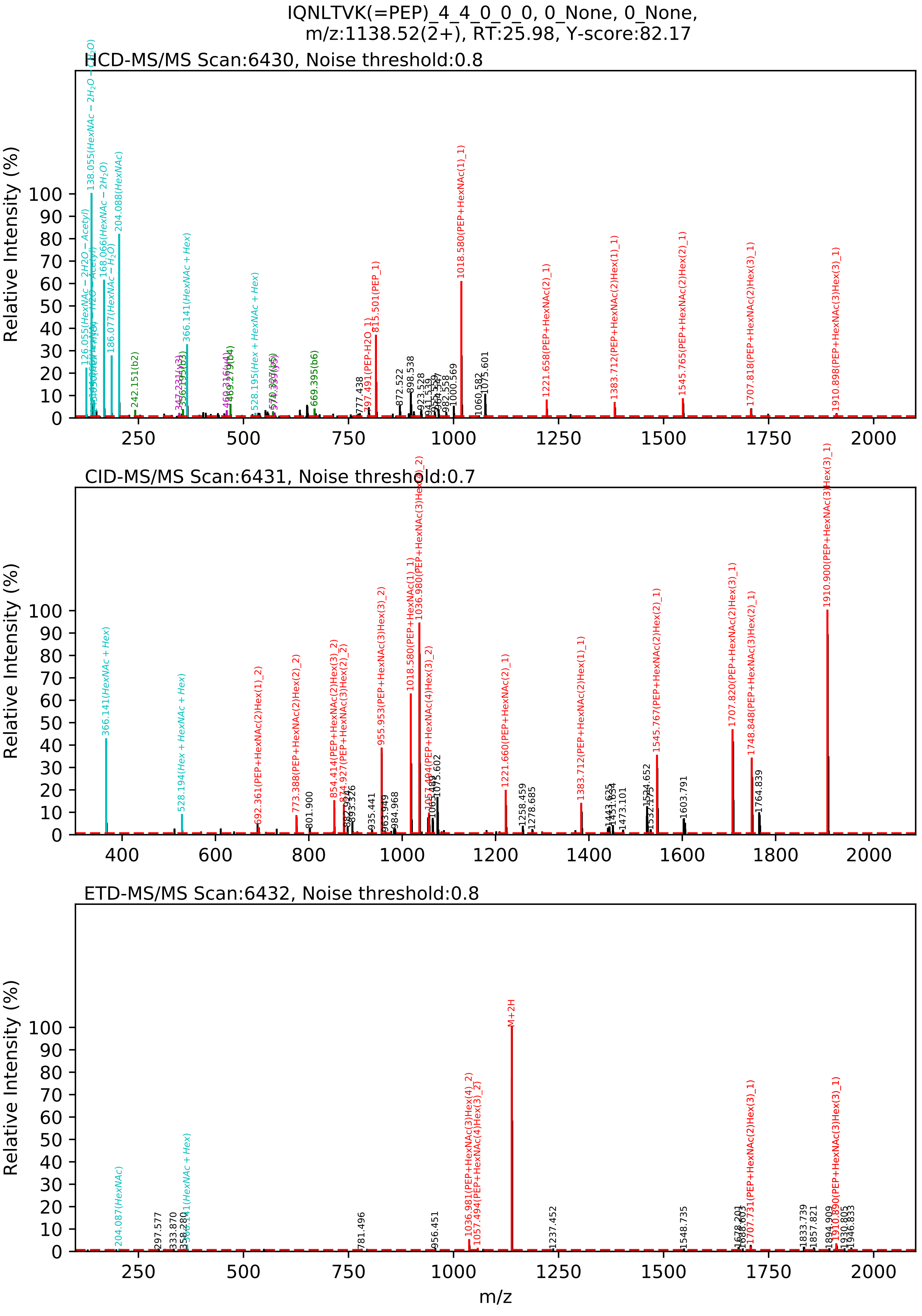
IQNLTVK(=PEP)_4_4_0_0_0, 0_None, 0_None,
m/z:1138.52(2+), RT:25.98, Y-score:82.17
HCD-MS/MS Scan:6430, Noise threshold:0.8
Relative Intensity (%)
138.055(HexNAc − 2H2O − H2O)
204.088(HexNAc)
366.141(HexNAc + Hex)
528.195(Hex + HexNAc + Hex)
242.151(b2)
669.395(b6)
815.501(PEP_1)
797.491(PEP-H2O_1)
1018.580(PEP+HexNAc(1)_1)
1221.658(PEP+HexNAc(2)_1)
1383.712(PEP+HexNAc(2)Hex(1)_1)
1545.765(PEP+HexNAc(2)Hex(2)_1)
1707.818(PEP+HexNAc(2)Hex(3)_1)
1910.898(PEP+HexNAc(3)Hex(3)_1)
CID-MS/MS Scan:6431, Noise threshold:0.7
366.141(HexNAc + Hex)
528.194(Hex + HexNAc + Hex)
692.361(PEP+HexNAc(2)Hex(1)_2)
773.388(PEP+HexNAc(2)Hex(2)_2)
854.414(PEP+HexNAc(2)Hex(3)_2)
955.953(PEP+HexNAc(3)Hex(3)_2)
1018.580(PEP+HexNAc(1)_1)
1221.660(PEP+HexNAc(2)_1)
1383.712(PEP+HexNAc(2)Hex(1)_1)
1545.767(PEP+HexNAc(2)Hex(2)_1)
1707.820(PEP+HexNAc(2)Hex(3)_1)
1748.848(PEP+HexNAc(3)Hex(2)_1)
1910.900(PEP+HexNAc(3)Hex(3)_1)
ETD-MS/MS Scan:6432, Noise threshold:0.8
204.087(HexNAc)
M+2H
1036.981(PEP+HexNAc(3)Hex(4)_2)
1057.494(PEP+HexNAc(4)Hex(3)_2)
1707.731(PEP+HexNAc(2)Hex(3)_1)
m/z

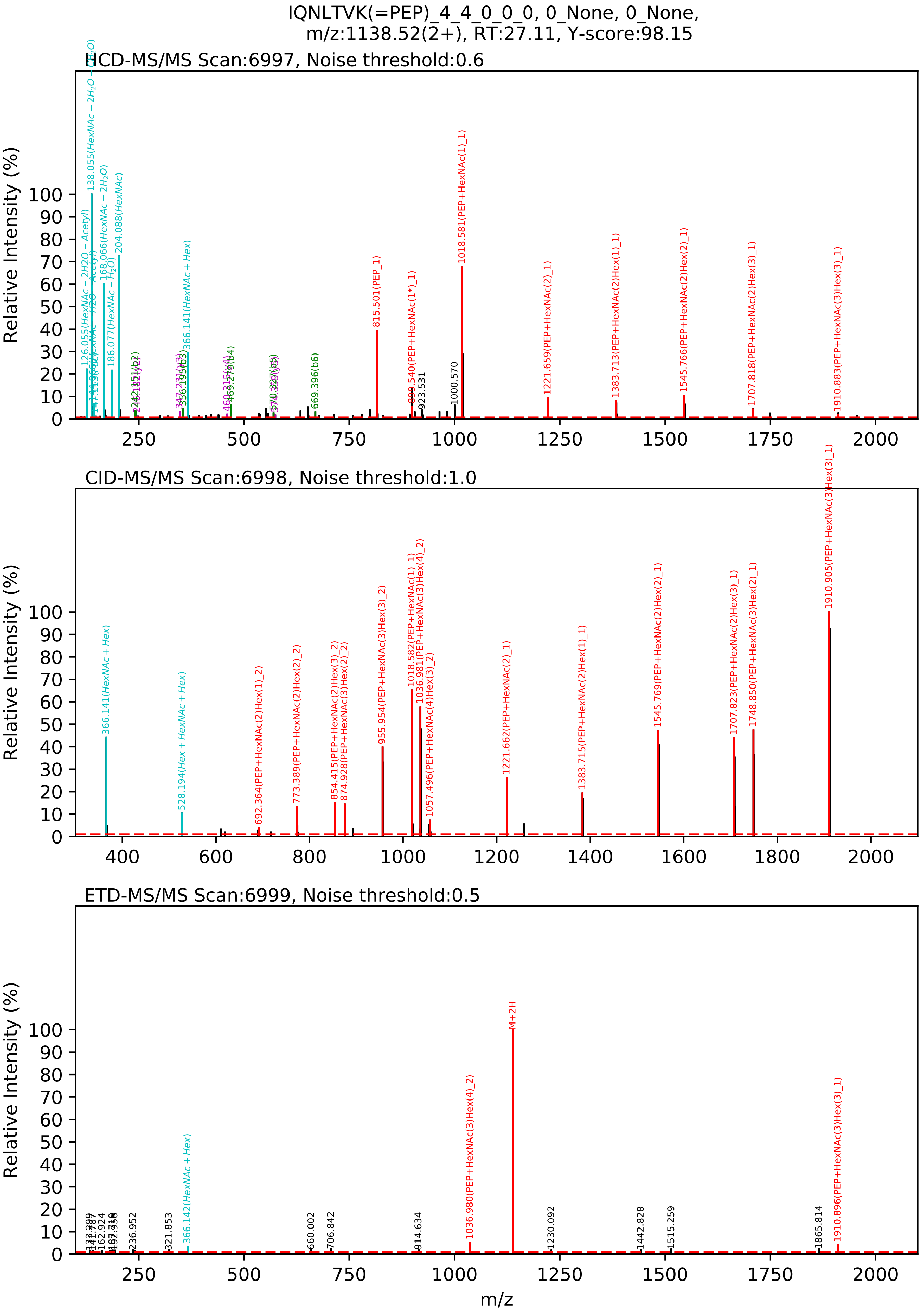
IQNLTVK(=PEP)_4_4_0_0_0, 0_None, 0_None,
m/z:1138.52(2+), RT:27.11, Y-score:98.15
HCD-MS/MS Scan:6997, Noise threshold:0.6
Relative Intensity (%)
138.055(HexNAc − 2H2O − H2O)
126.055(HexNAc − 2H2O − Acetyl)
168.066(HexNAc − 2H2O)
186.077(HexNAc − H2O)
204.088(HexNAc)
366.141(HexNAc + Hex)
669.396(b6)
815.501(PEP_1)
899.540(PEP+HexNAc(1*)_1)
923.531
1000.570
1018.581(PEP+HexNAc(1)_1)
1221.659(PEP+HexNAc(2)_1)
1383.713(PEP+HexNAc(2)Hex(1)_1)
1545.766(PEP+HexNAc(2)Hex(2)_1)
1707.818(PEP+HexNAc(2)Hex(3)_1)
1910.883(PEP+HexNAc(3)Hex(3)_1)
CID-MS/MS Scan:6998, Noise threshold:1.0
Relative Intensity (%)
366.141(HexNAc + Hex)
528.194(Hex + Hex + HexNAc + Hex)
692.364(PEP+HexNAc(2)Hex(1)_2)
773.389(PEP+HexNAc(2)Hex(2)_2)
854.415(PEP+HexNAc(2)Hex(3)_2)
874.928(PEP+HexNAc(3)Hex(2)_2)
955.954(PEP+HexNAc(3)Hex(3)_2)
1018.582(PEP+HexNAc(1)_1)
1036.981(PEP+HexNAc(3)Hex(4)_2)
1057.496(PEP+HexNAc(4)Hex(3)_2)
1221.662(PEP+HexNAc(2)_1)
1383.715(PEP+HexNAc(2)Hex(1)_1)
1545.769(PEP+HexNAc(2)Hex(2)_1)
1707.823(PEP+HexNAc(2)Hex(3)_1)
1748.850(PEP+HexNAc(3)Hex(2)_1)
1910.905(PEP+HexNAc(3)Hex(3)_1)
ETD-MS/MS Scan:6999, Noise threshold:0.5
Relative Intensity (%)
162.924
236.952
321.853
366.142(HexNAc + Hex)
660.002
706.842
914.634
1036.980(PEP+HexNAc(3)Hex(4)_2)
M+2H
1230.092
1442.828
1515.259
1865.814
1910.896(PEP+HexNAc(3)Hex(3)_1)
m/z

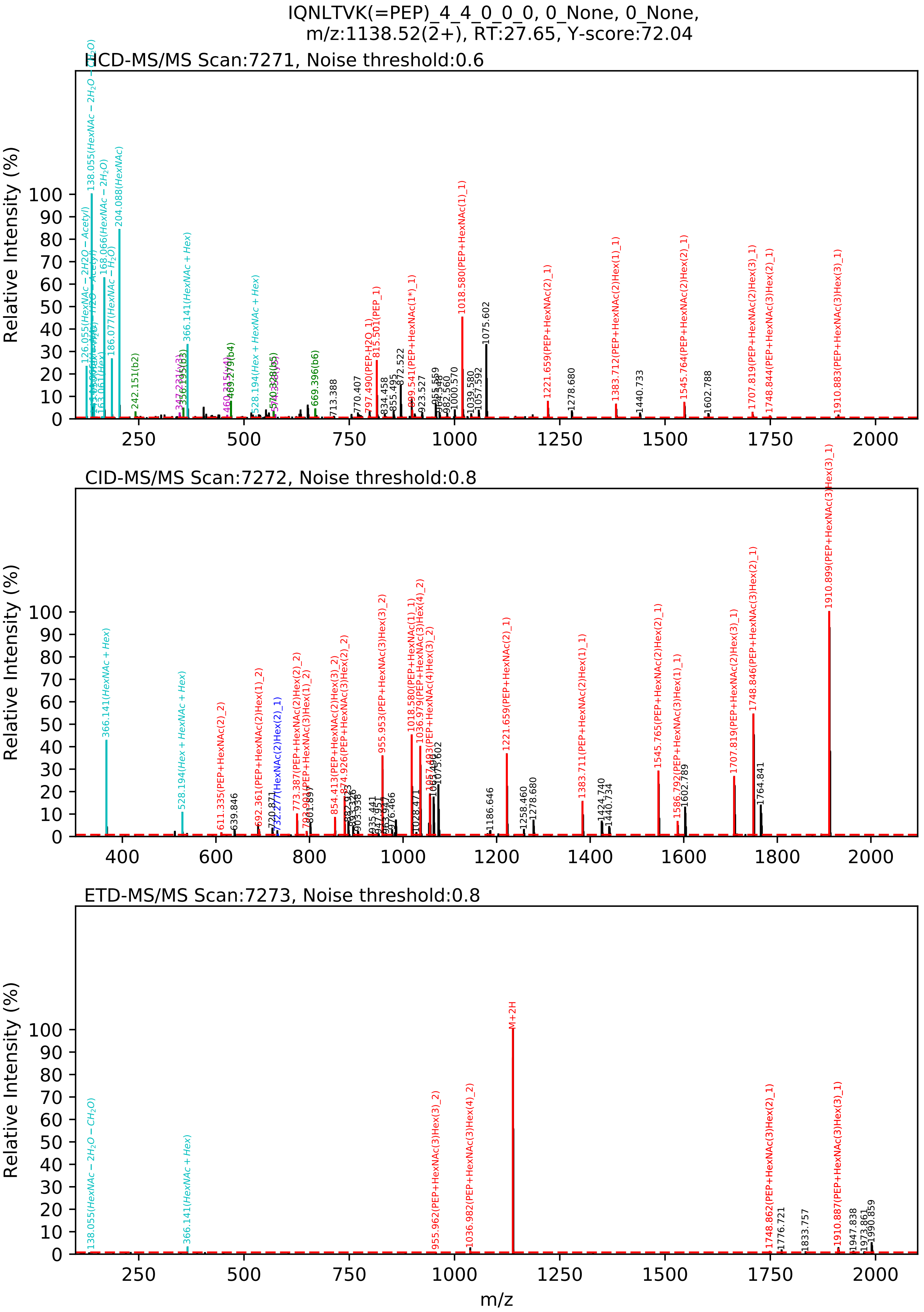
IQNLTVK(=PEP)_4_4_0_0_0, 0_None, 0_None,
m/z:1138.52(2+), RT:27.65, Y-score:72.04
HCD-MS/MS Scan:7271, Noise threshold:0.6
Relative Intensity (%)
138.055(HexNAc − 2H2O − Acetyl)
204.088(HexNAc)
168.066(HexNAc − 2H2O)
186.077(HexNAc − H2O)
126.055(HexNAc − 2H2O − Acetyl)
366.141(HexNAc + Hex)
242.151(b2)
528.194(Hex + HexNAc + Hex)
669.396(b6)
1018.580(PEP+HexNAc(1)_1)
899.541(PEP+HexNAc(1*)_1)
815.501(PEP_1)
797.490(PEP-H2O_1)
1075.602
1221.659(PEP+HexNAc(2)_1)
1278.680
1383.712(PEP+HexNAc(2)Hex(1)_1)
1440.733
1545.764(PEP+HexNAc(2)Hex(2)_1)
1602.788
1707.819(PEP+HexNAc(2)Hex(3)_1)
1748.844(PEP+HexNAc(3)Hex(2)_1)
1910.883(PEP+HexNAc(3)Hex(3)_1)
CID-MS/MS Scan:7272, Noise threshold:0.8
366.141(HexNAc + Hex)
528.194(Hex + HexNAc + Hex)
611.335(PEP+HexNAc(2)_2)
692.361(PEP+HexNAc(2)Hex(1)_2)
732.277(HexNAc(2)Hex(2)_1)
773.387(PEP+HexNAc(2)Hex(2)_2)
854.413(PEP+HexNAc(2)Hex(3)_2)
874.926(PEP+HexNAc(3)Hex(2)_2)
955.953(PEP+HexNAc(3)Hex(3)_2)
1018.580(PEP+HexNAc(1)_1)
1036.979(PEP+HexNAc(3)Hex(4)_2)
1221.659(PEP+HexNAc(2)_1)
1383.711(PEP+HexNAc(2)Hex(1)_1)
1545.765(PEP+HexNAc(2)Hex(2)_1)
1586.792(PEP+HexNAc(3)Hex(1)_1)
1707.819(PEP+HexNAc(2)Hex(3)_1)
1748.846(PEP+HexNAc(3)Hex(2)_1)
1910.899(PEP+HexNAc(3)Hex(3)_1)
1764.841
ETD-MS/MS Scan:7273, Noise threshold:0.8
138.055(HexNAc − 2H2O − CH2O)
366.141(HexNAc + Hex)
955.962(PEP+HexNAc(3)Hex(3)_2)
1036.982(PEP+HexNAc(3)Hex(4)_2)
M+2H
1748.862(PEP+HexNAc(3)Hex(2)_1)
1910.887(PEP+HexNAc(3)Hex(3)_1)
m/z

IQNLTVK(=PEP)_4_4_0_0_0, 0_None, 0_None,
m/z:1138.52(2+), RT:28.19, Y-score:84.31

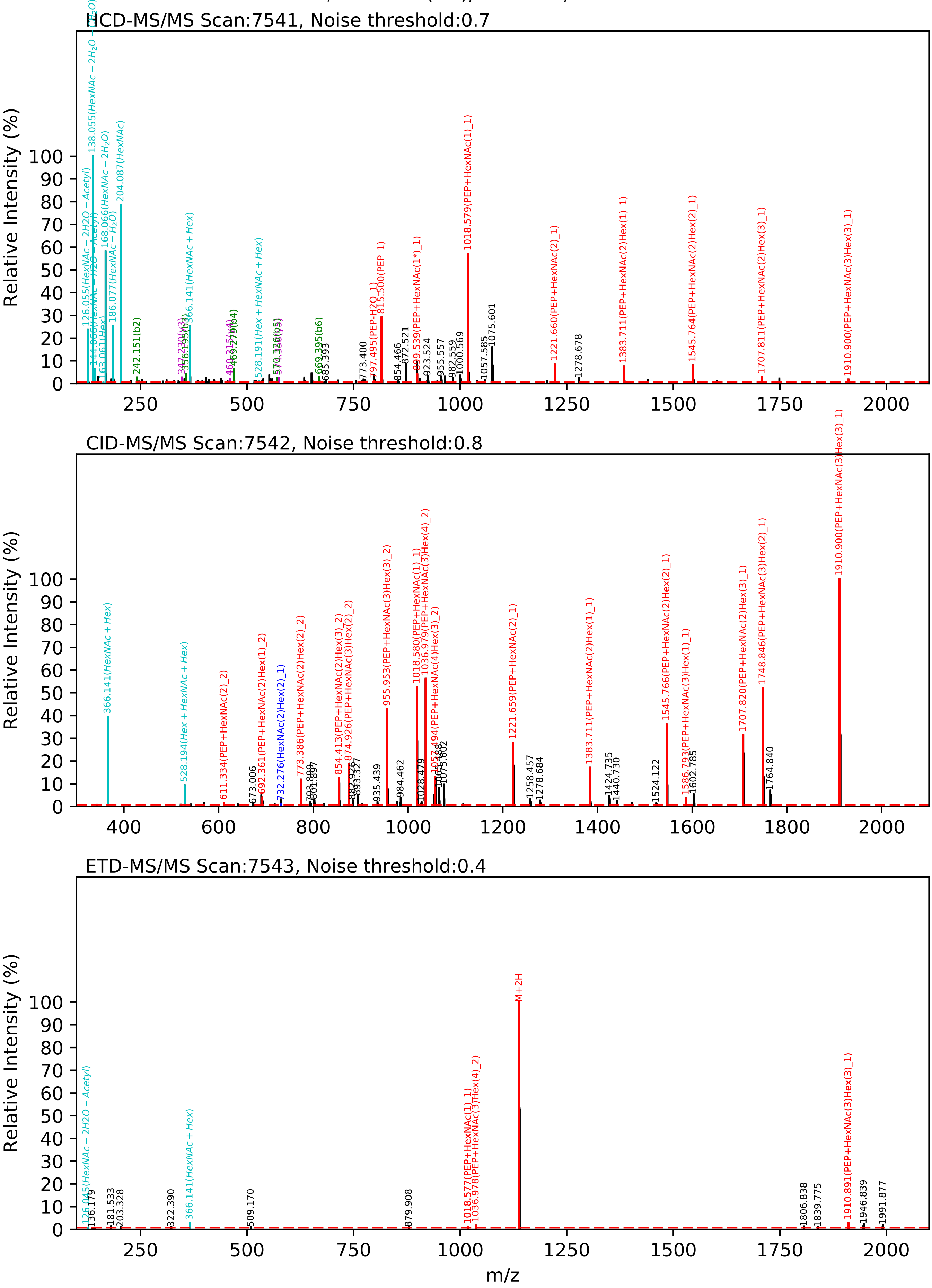

IQNLTVK(=PEP)_4_4_0_0_0, 0_None, 0_None,
m/z:1138.52(2+), RT:28.74, Y-score:79.46

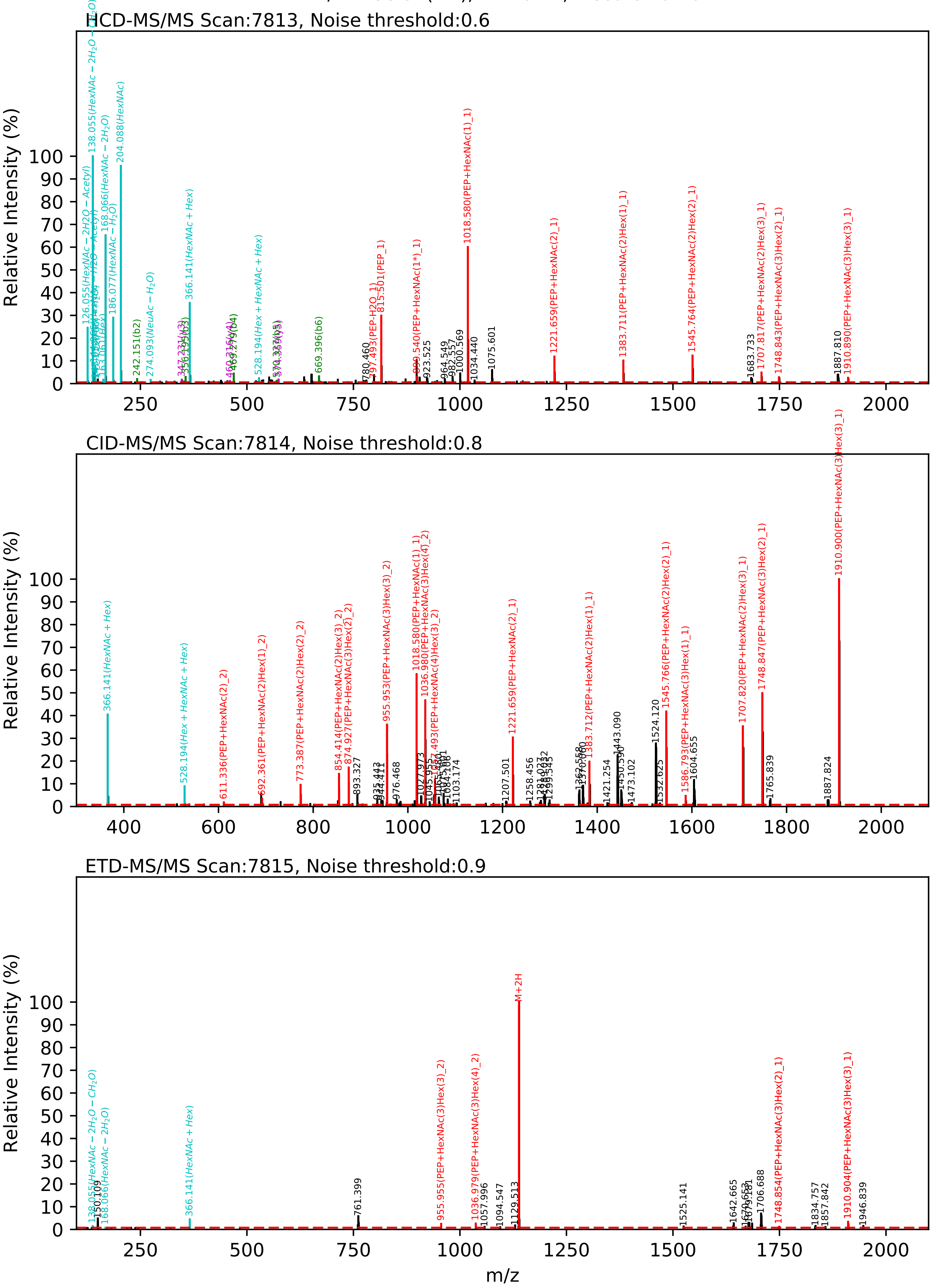

IQNLTVK(=PEP)_4_4_0_0_0, 0_None, 0_None,
m/z:1138.52(2+), RT:29.71, Y-score:69.08

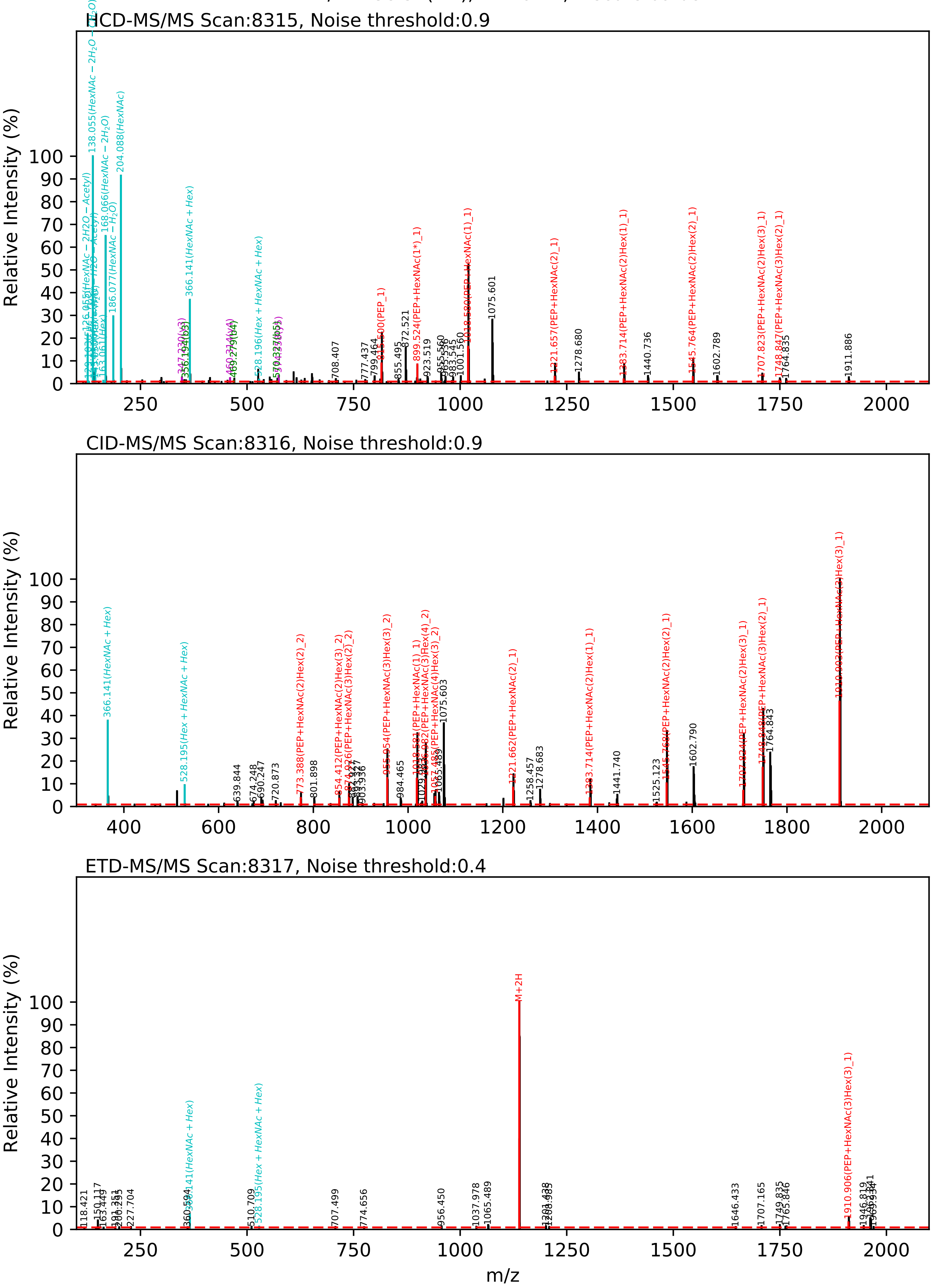

IQNLTVK(=PEP)_4_4_0_0_0, 0_None, 0_None,
m/z:1138.52(2+), RT:29.78, Y-score:75.81

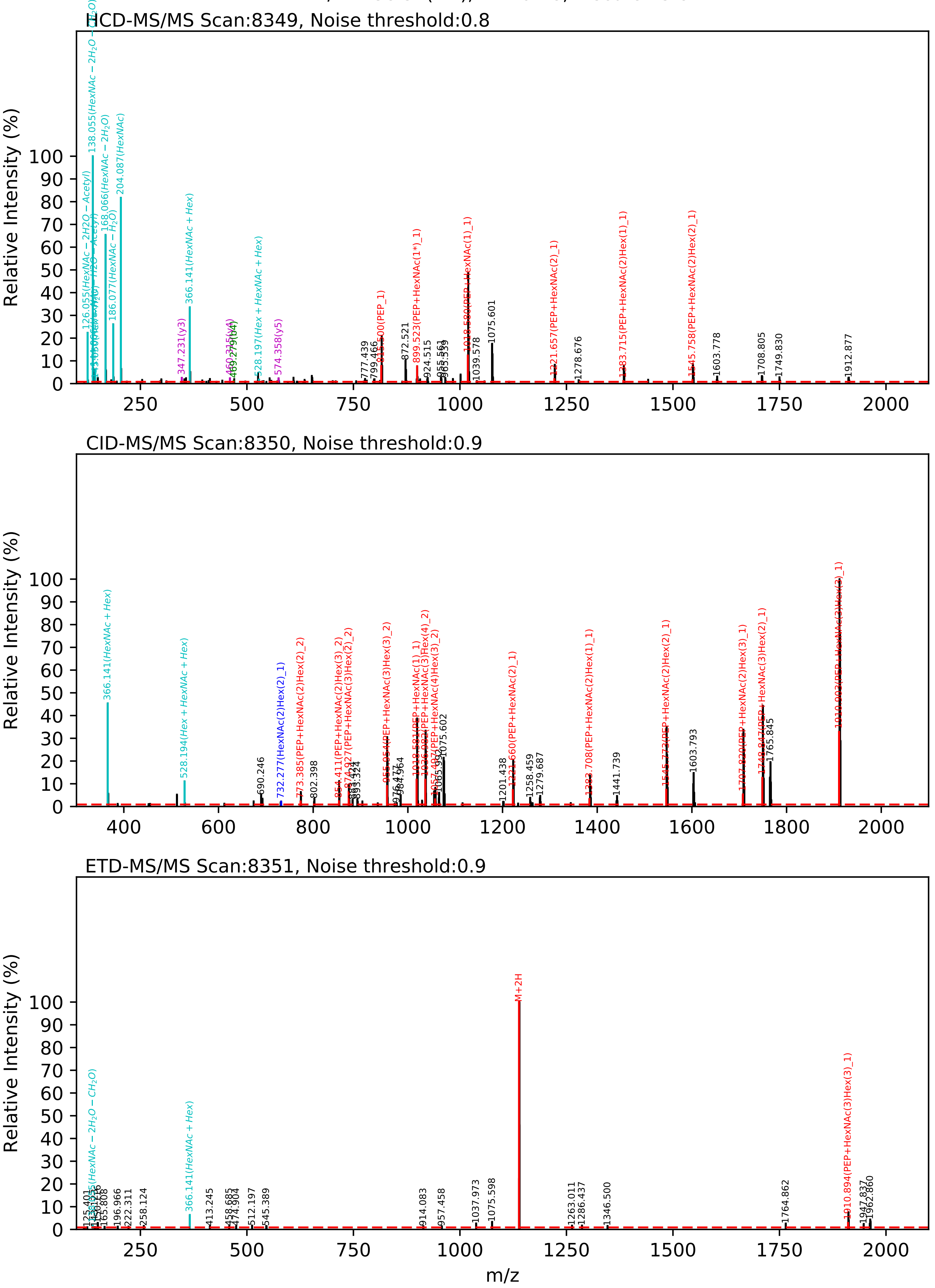

IQNLTVK(=PEP)_4_4_0_1_0, 0_None, 0_None,
m/z:1284.07(2+), RT:45.71, Y-score:93.54

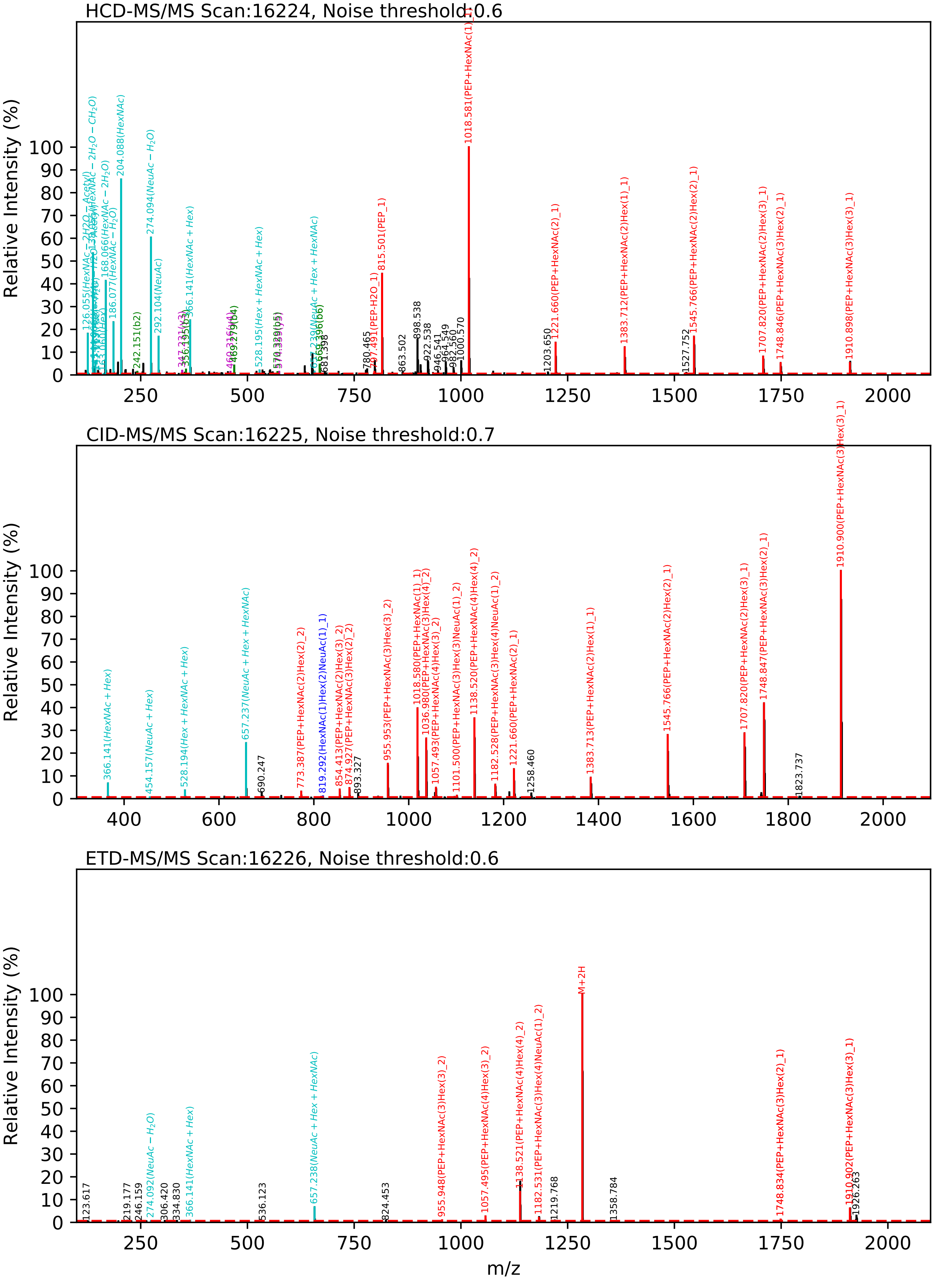

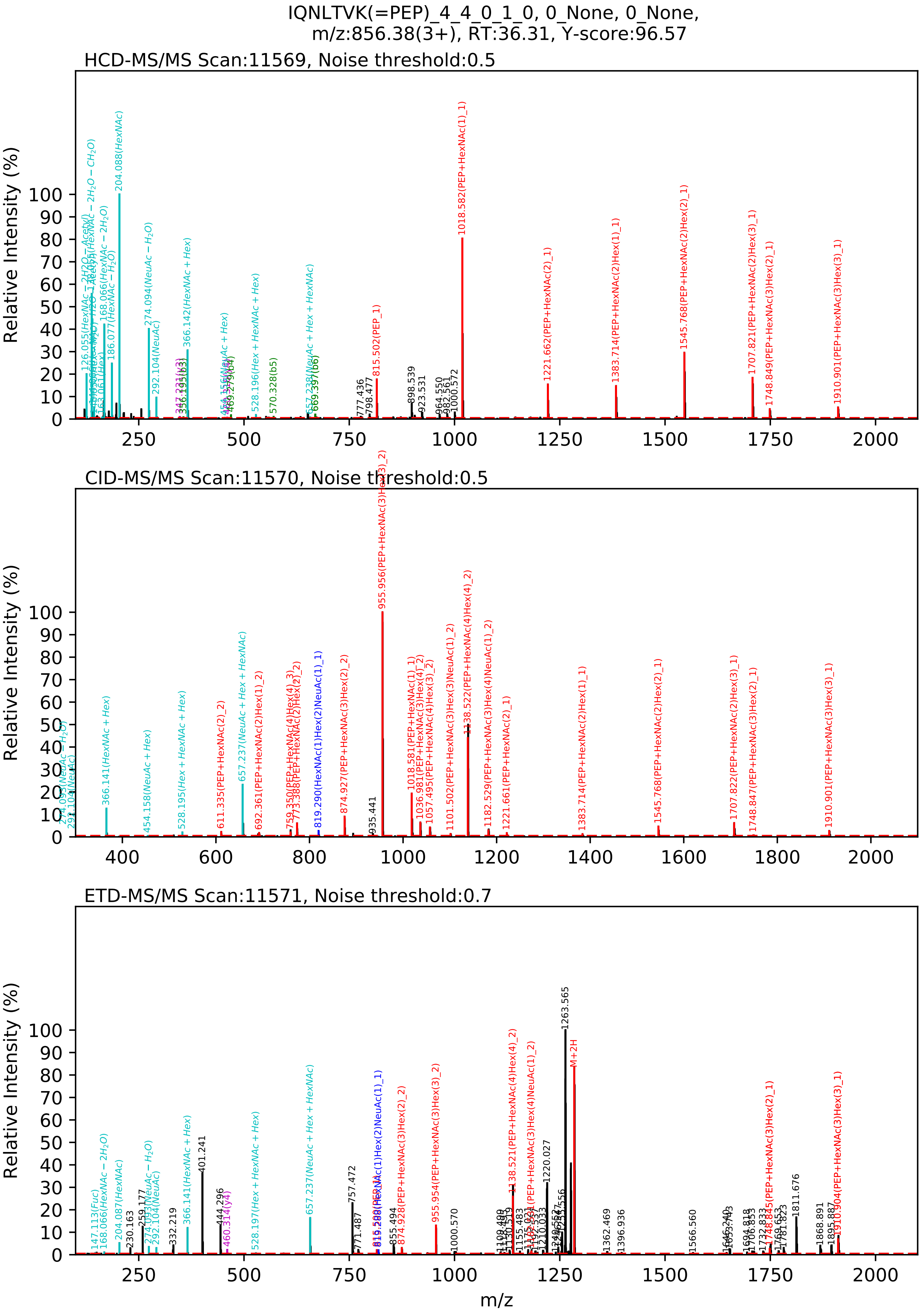

IQNLTVK(=PEP)_4_4_0_1_0, 0_None, 0_None,
m/z:856.38(3+), RT:36.31, Y-score:96.57
HCD-MS/MS Scan:11569, Noise threshold:0.5
Relative Intensity (%)
204.088(HexNAc)
274.094(NeuAc − H2O)
292.104(NeuAc)
366.142(HexNAc + Hex)
528.196(Hex + HexNAc + Hex)
570.328(b5)
815.502(PEP_1)
898.539
923.531
964.550
1000.572
1018.582(PEP+HexNAc(1)_1)
1221.662(PEP+HexNAc(2)_1)
1383.714(PEP+HexNAc(2)Hex(1)_1)
1545.768(PEP+HexNAc(2)Hex(2)_1)
1707.821(PEP+HexNAc(2)Hex(3)_1)
1748.849(PEP+HexNAc(3)Hex(2)_1)
1910.901(PEP+HexNAc(3)Hex(3)_1)
CID-MS/MS Scan:11570, Noise threshold:0.5
Relative Intensity (%)
366.141(HexNAc + Hex)
454.158(NeuAc + Hex)
528.195(Hex + HexNAc + Hex)
611.335(PEP+HexNAc(2)_2)
657.237(NeuAc + Hex + HexNAc)
692.361(PEP+HexNAc(2)Hex(1)_2)
819.290(HexNAc(1)Hex(2)NeuAc(1)_1)
874.927(PEP+HexNAc(3)Hex(2)_2)
935.441
955.956(PEP+HexNAc(3)Hex(3)_2)
1018.581(PEP+HexNAc(1)_1)
1036.981(PEP+HexNAc(3)Hex(4)_2)
1057.495(PEP+HexNAc(4)Hex(3)_2)
1101.502(PEP+HexNAc(3)Hex(3)NeuAc(1)_2)
1138.522(PEP+HexNAc(4)Hex(4)_2)
1182.529(PEP+HexNAc(3)Hex(4)NeuAc(1)_2)
1221.661(PEP+HexNAc(2)_1)
1383.714(PEP+HexNAc(2)Hex(1)_1)
1545.768(PEP+HexNAc(2)Hex(2)_1)
1707.822(PEP+HexNAc(2)Hex(3)_1)
1748.847(PEP+HexNAc(3)Hex(2)_1)
1910.901(PEP+HexNAc(3)Hex(3)_1)
ETD-MS/MS Scan:11571, Noise threshold:0.7
Relative Intensity (%)
147.113(Fuc)
168.066(HexNAc − 2H2O)
204.087(HexNAc)
230.163
259.177
292.104(NeuAc)
332.219
366.141(HexNAc + Hex)
401.241
444.296
460.314(y4)
528.197(Hex + HexNAc + Hex)
657.237(NeuAc + Hex + HexNAc)
757.472
771.487
855.494
874.928(PEP+HexNAc(3)Hex(2)_2)
955.954(PEP+HexNAc(3)Hex(3)_2)
1000.570
1155.483
1138.521(PEP+HexNAc(4)Hex(4)_2)
1220.027
1263.565
M+2H
1362.469
1396.936
1566.560
1694.818
1732.833
1748.845(PEP+HexNAc(3)Hex(2)_1)
1769.653
1781.823
1811.676
1868.891
1895.887
1910.904(PEP+HexNAc(3)Hex(3)_1)
m/z

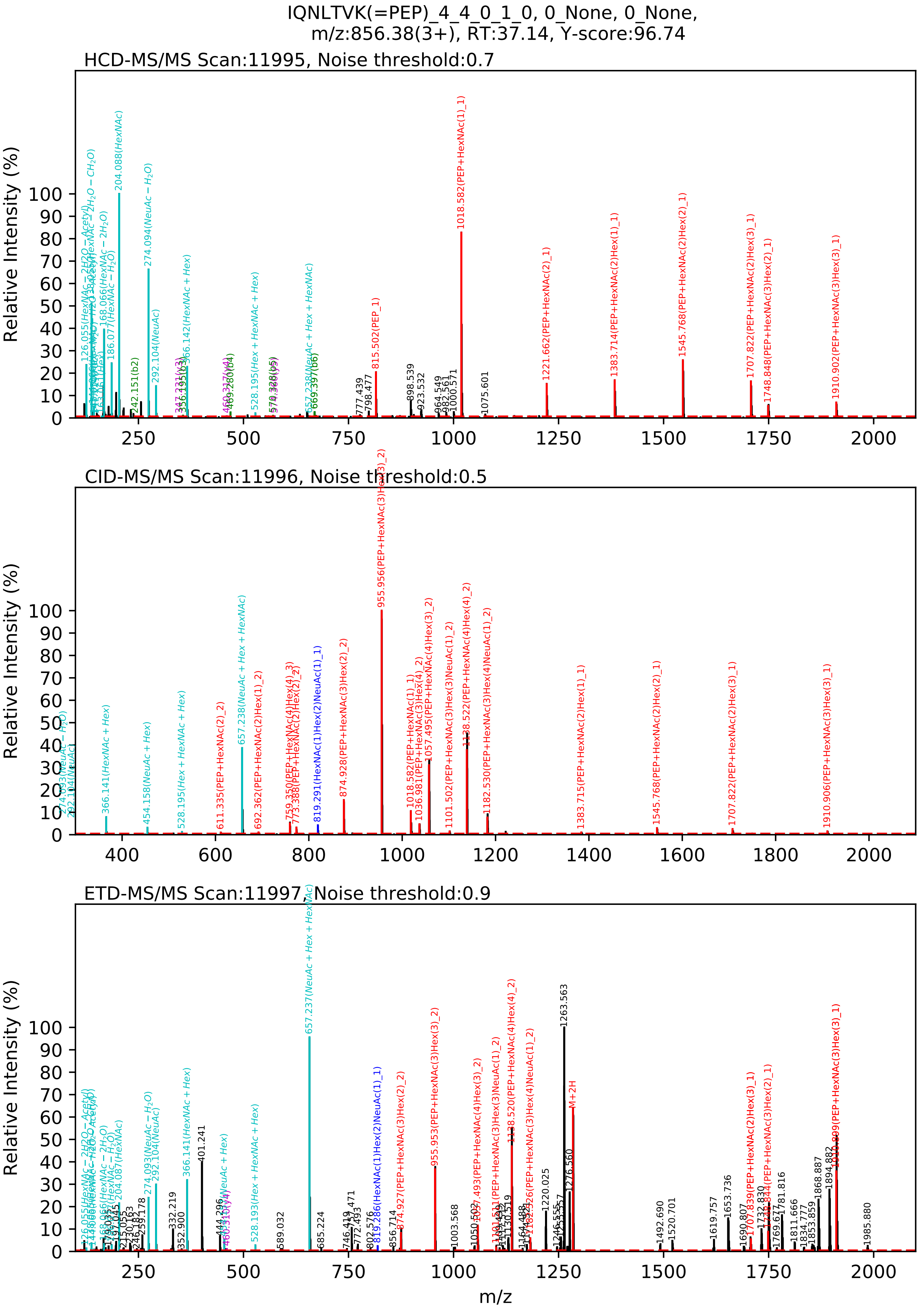
IQNLTVK(=PEP)_4_4_0_1_0, 0_None, 0_None,
m/z:856.38(3+), RT:37.14, Y-score:96.74
HCD-MS/MS Scan:11995, Noise threshold:0.7
Relative Intensity (%)
1018.582(PEP+HexNAc(1)_1)
1221.662(PEP+HexNAc(2)_1)
1383.714(PEP+HexNAc(2)Hex(1)_1)
1545.768(PEP+HexNAc(2)Hex(2)_1)
1707.822(PEP+HexNAc(2)Hex(3)_1)
1748.848(PEP+HexNAc(3)Hex(2)_1)
1910.902(PEP+HexNAc(3)Hex(3)_1)
815.502(PEP_1)
204.088(HexNAc)
274.094(NeuAc − H2O)
292.104(NeuAc)
366.142(HexNAc + Hex)
528.195(Hex + HexNAc + Hex)
657.238(NeuAc + Hex + HexNAc)
CID-MS/MS Scan:11996, Noise threshold:0.5
955.956(PEP+HexNAc(3)Hex(3)_2)
1138.522(PEP+HexNAc(4)Hex(4)_2)
1182.530(PEP+HexNAc(3)Hex(4)NeuAc(1)_2)
1057.495(PEP+HexNAc(4)Hex(3)_2)
874.928(PEP+HexNAc(3)Hex(2)_2)
819.291(HexNAc(1)Hex(2)NeuAc(1)_1)
ETD-MS/MS Scan:11997, Noise threshold:0.9
657.237(NeuAc + Hex + HexNAc)
1263.563
M+2H
401.241
m/z

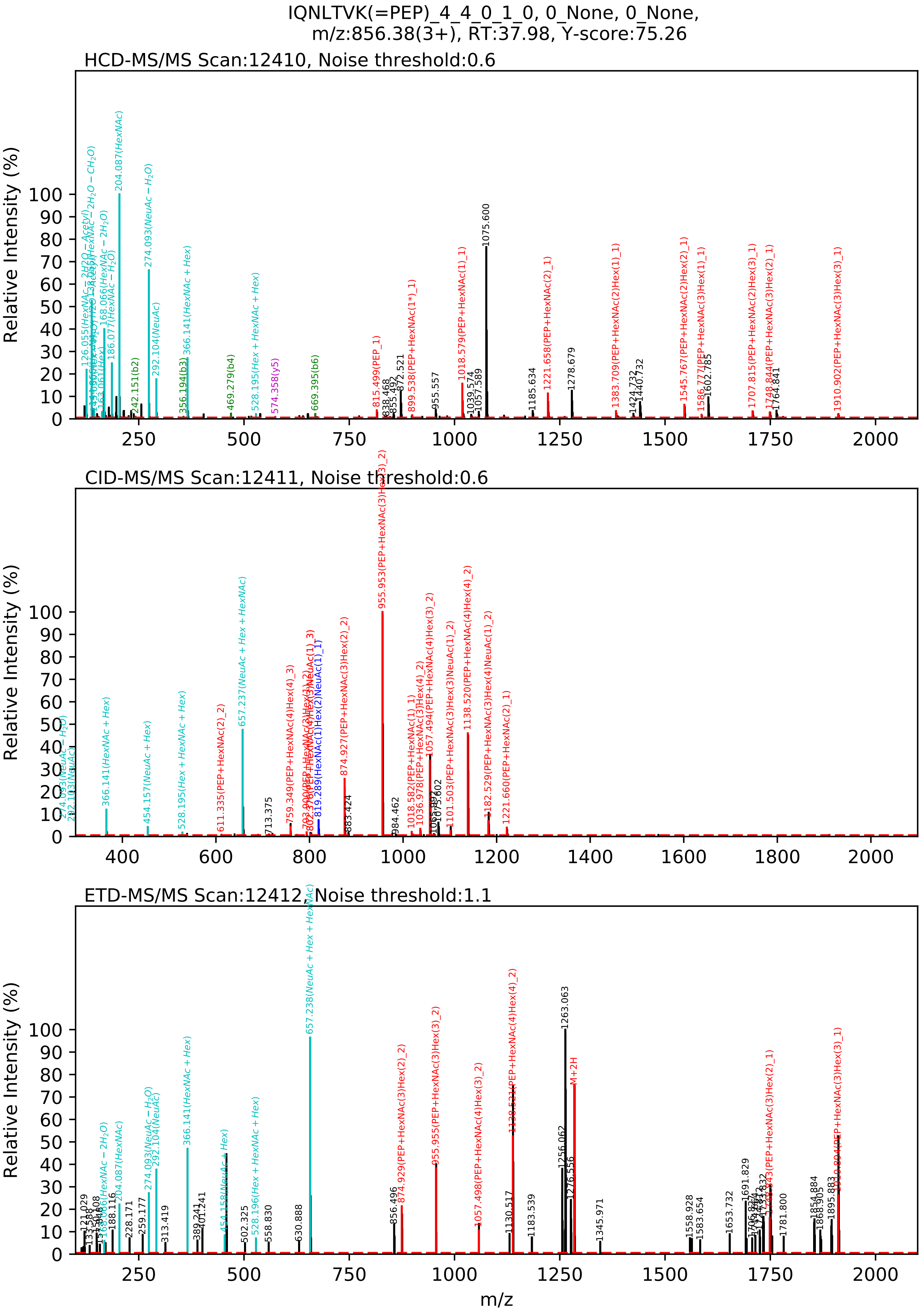
IQNLTVK(=PEP)_4_4_0_1_0, 0_None, 0_None,
m/z:856.38(3+), RT:37.98, Y-score:75.26
HCD-MS/MS Scan:12410, Noise threshold:0.6
Relative Intensity (%)
204.087(HexNAc)
274.093(NeuAc − H2O)
366.141(HexNAc + Hex)
292.104(NeuAc)
186.077(HexNAc − H2O)
168.066(HexNAc − 2H2O)
242.151(b2)
356.194(b3)
469.279(b4)
528.195(Hex + HexNAc + Hex)
574.358(y5)
669.395(b6)
815.499(PEP_1)
838.468
855.492
872.521
899.538(PEP+HexNAc(1*)_1)
955.557
1018.579(PEP+HexNAc(1)_1)
1039.574
1057.589
1075.600
1185.634
1221.658(PEP+HexNAc(2)_1)
1278.679
1383.709(PEP+HexNAc(2)Hex(1)_1)
1424.732
1440.732
1545.767(PEP+HexNAc(2)Hex(2)_1)
1586.777(PEP+HexNAc(3)Hex(1)_1)
1602.785
1707.815(PEP+HexNAc(2)Hex(3)_1)
1748.844(PEP+HexNAc(3)Hex(2)_1)
1764.841
1910.902(PEP+HexNAc(3)Hex(3)_1)
CID-MS/MS Scan:12411, Noise threshold:0.6
955.953(PEP+HexNAc(3)Hex(3)_2)
657.237(NeuAc + Hex + HexNAc)
366.141(HexNAc + Hex)
454.157(NeuAc + Hex)
528.195(Hex + HexNAc + Hex)
611.335(PEP+HexNAc(2)_2)
713.375
759.349(PEP+HexNAc(4)Hex(4)_3)
819.289(HexNAc(1)Hex(2)NeuAc(1)_1)
874.927(PEP+HexNAc(3)Hex(2)_2)
883.424
984.462
1018.582(PEP+HexNAc(1)_1)
1036.978(PEP+HexNAc(3)Hex(4)_2)
1057.494(PEP+HexNAc(4)Hex(3)_2)
1075.602
1101.503(PEP+HexNAc(3)Hex(3)NeuAc(1)_2)
1138.520(PEP+HexNAc(4)Hex(4)_2)
1182.529(PEP+HexNAc(3)Hex(4)NeuAc(1)_2)
1221.660(PEP+HexNAc(2)_1)
ETD-MS/MS Scan:12412, Noise threshold:1.1
657.238(NeuAc + Hex + HexNAc)
1263.063
M+2H
1138.523(PEP+HexNAc(4)Hex(4)_2)
955.955(PEP+HexNAc(3)Hex(3)_2)
874.929(PEP+HexNAc(3)Hex(2)_2)
1057.498(PEP+HexNAc(4)Hex(3)_2)
366.141(HexNAc + Hex)
454.158(NeuAc + Hex)
292.104(NeuAc)
274.093(NeuAc − H2O)
204.087(HexNAc)
528.196(Hex + HexNAc + Hex)
121.029
188.116
228.171
259.177
313.419
389.241
401.241
502.325
558.830
630.888
856.496
1130.517
1183.539
1256.062
1276.556
1345.971
1558.928
1583.654
1653.732
1691.829
1781.800
1854.884
1895.883
1910.904(PEP+HexNAc(3)Hex(3)_1)
m/z

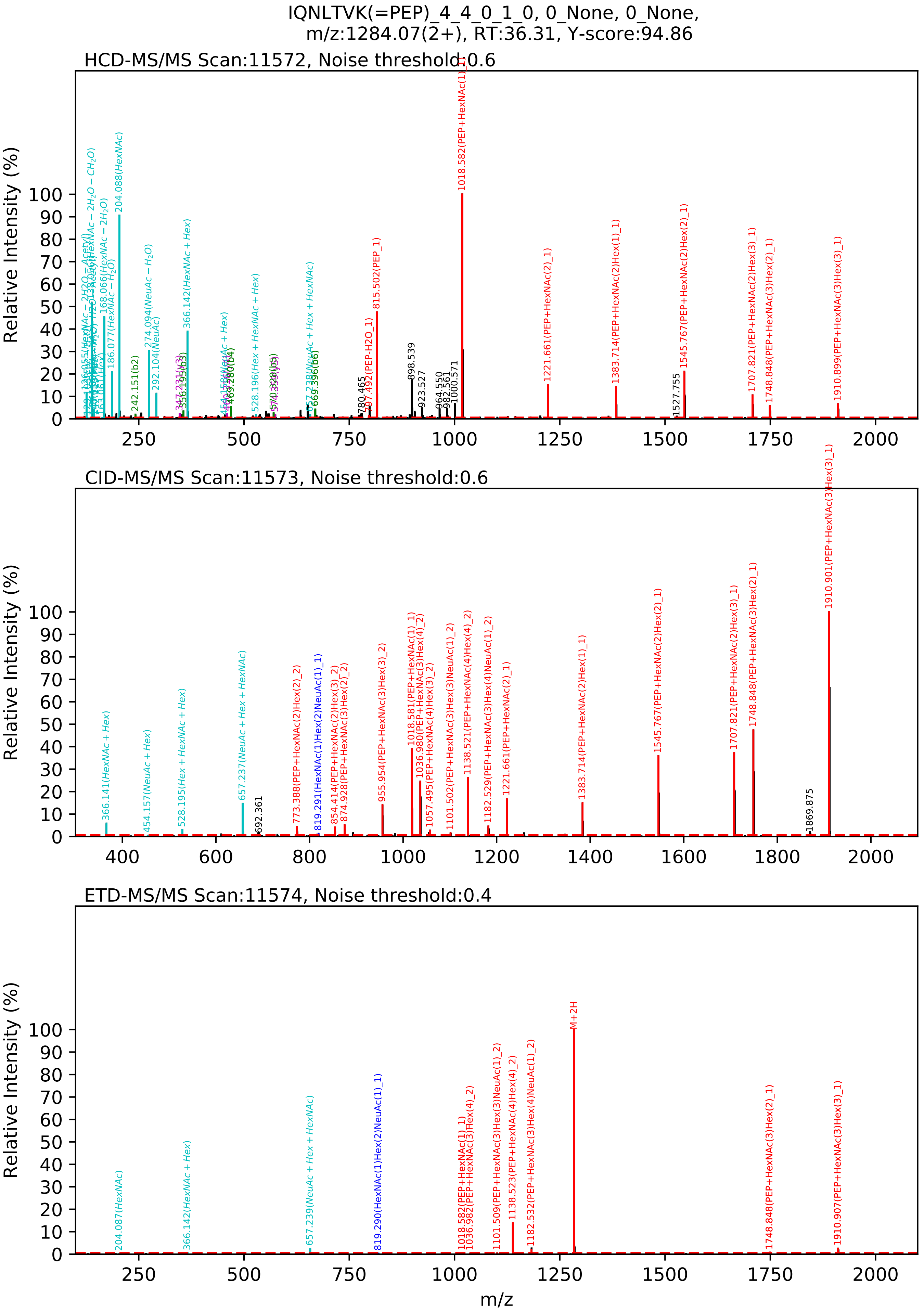

IQNLTVK(=PEP)_4_4_0_1_0, 0_None, 0_None,
m/z:1284.07(2+), RT:36.31, Y-score:94.86
HCD-MS/MS Scan:11572, Noise threshold:0.6
Relative Intensity (%)
204.088(HexNAc)
168.066(HexNAc − 2H2O)
186.077(HexNAc − H2O)
242.151(b2)
274.094(NeuAc − H2O)
292.104(NeuAc)
366.142(HexNAc + Hex)
528.196(Hex + HexNAc + Hex)
469.280(b4)
797.492(PEP-H2O_1)
815.502(PEP_1)
780.465
898.539
923.527
964.550
1000.571
1018.582(PEP+HexNAc(1)_1)
1221.661(PEP+HexNAc(2)_1)
1383.714(PEP+HexNAc(2)Hex(1)_1)
1527.755
1545.767(PEP+HexNAc(2)Hex(2)_1)
1707.821(PEP+HexNAc(2)Hex(3)_1)
1748.848(PEP+HexNAc(3)Hex(2)_1)
1910.899(PEP+HexNAc(3)Hex(3)_1)
CID-MS/MS Scan:11573, Noise threshold:0.6
Relative Intensity (%)
366.141(HexNAc + Hex)
454.157(NeuAc + Hex)
528.195(Hex + HexNAc + Hex)
657.237(NeuAc + Hex + HexNAc)
692.361
773.388(PEP+HexNAc(2)Hex(2)_2)
819.291(HexNAc(1)Hex(2)NeuAc(1)_1)
854.414(PEP+HexNAc(2)Hex(3)_2)
874.928(PEP+HexNAc(3)Hex(2)_2)
955.954(PEP+HexNAc(3)Hex(3)_2)
1018.581(PEP+HexNAc(1)_1)
1036.980(PEP+HexNAc(3)Hex(4)_2)
1057.495(PEP+HexNAc(4)Hex(3)_2)
1101.502(PEP+HexNAc(3)Hex(3)NeuAc(1)_2)
1138.521(PEP+HexNAc(4)Hex(4)_2)
1182.529(PEP+HexNAc(3)Hex(4)NeuAc(1)_2)
1221.661(PEP+HexNAc(2)_1)
1383.714(PEP+HexNAc(2)Hex(1)_1)
1545.767(PEP+HexNAc(2)Hex(2)_1)
1707.821(PEP+HexNAc(2)Hex(3)_1)
1748.848(PEP+HexNAc(3)Hex(2)_1)
1869.875
1910.901(PEP+HexNAc(3)Hex(3)_1)
ETD-MS/MS Scan:11574, Noise threshold:0.4
Relative Intensity (%)
204.087(HexNAc)
366.142(HexNAc + Hex)
657.239(NeuAc + Hex + HexNAc)
819.290(HexNAc(1)Hex(2)NeuAc(1)_1)
1018.582(PEP+HexNAc(1)_1)
1036.982(PEP+HexNAc(3)Hex(4)_2)
1101.509(PEP+HexNAc(3)Hex(3)NeuAc(1)_2)
1138.523(PEP+HexNAc(4)Hex(4)_2)
1182.532(PEP+HexNAc(3)Hex(4)NeuAc(1)_2)
M+2H
1748.848(PEP+HexNAc(3)Hex(2)_1)
1910.907(PEP+HexNAc(3)Hex(3)_1)
m/z

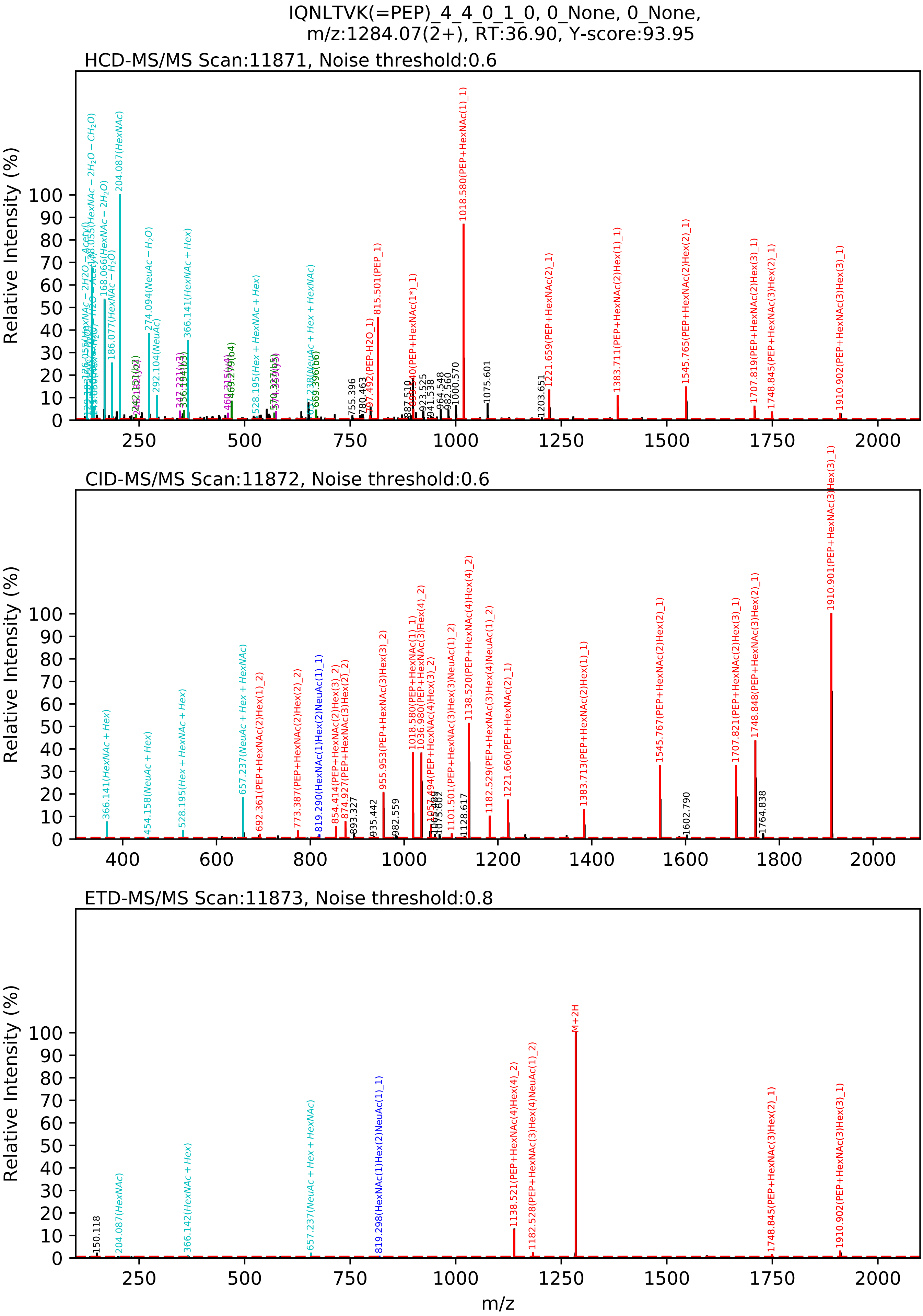

IQNLTVK(=PEP)_4_4_0_1_0, 0_None, 0_None,
m/z:1284.07(2+), RT:36.90, Y-score:93.95
HCD-MS/MS Scan:11871, Noise threshold:0.6
Relative Intensity (%)
204.087(HexNAc)
168.066(HexNAc − 2H2O)
186.077(HexNAc − H2O)
274.094(NeuAc − H2O)
292.104(NeuAc)
366.141(HexNAc + Hex)
528.195(Hex + HexNAc + Hex)
1018.580(PEP+HexNAc(1)_1)
815.501(PEP_1)
1221.659(PEP+HexNAc(2)_1)
1383.711(PEP+HexNAc(2)Hex(1)_1)
1545.765(PEP+HexNAc(2)Hex(2)_1)
1707.819(PEP+HexNAc(2)Hex(3)_1)
1748.845(PEP+HexNAc(3)Hex(2)_1)
1910.902(PEP+HexNAc(3)Hex(3)_1)
1075.601
1203.651
755.396
780.463
887.510
923.525
941.538
964.548
1000.570
CID-MS/MS Scan:11872, Noise threshold:0.6
Relative Intensity (%)
366.141(HexNAc + Hex)
454.158(NeuAc + Hex)
528.195(Hex + HexNAc + Hex)
657.237(NeuAc + Hex + HexNAc)
692.361(PEP+HexNAc(2)Hex(1)_2)
773.387(PEP+HexNAc(2)Hex(2)_2)
819.290(HexNAc(1)Hex(2)NeuAc(1)_1)
854.414(PEP+HexNAc(2)Hex(3)_2)
874.927(PEP+HexNAc(3)Hex(2)_2)
893.327
935.442
955.953(PEP+HexNAc(3)Hex(3)_2)
982.559
1018.580(PEP+HexNAc(1)_1)
1036.980(PEP+HexNAc(3)Hex(4)_2)
1101.501(PEP+HexNAc(3)Hex(3)NeuAc(1)_2)
1128.617
1138.520(PEP+HexNAc(4)Hex(4)_2)
1182.529(PEP+HexNAc(3)Hex(4)NeuAc(1)_2)
1221.660(PEP+HexNAc(2)_1)
1383.713(PEP+HexNAc(2)Hex(1)_1)
1545.767(PEP+HexNAc(2)Hex(2)_1)
1602.790
1707.821(PEP+HexNAc(2)Hex(3)_1)
1748.848(PEP+HexNAc(3)Hex(2)_1)
1764.838
1910.901(PEP+HexNAc(3)Hex(3)_1)
ETD-MS/MS Scan:11873, Noise threshold:0.8
Relative Intensity (%)
150.118
204.087(HexNAc)
366.142(HexNAc + Hex)
657.237(NeuAc + Hex + HexNAc)
819.298(HexNAc(1)Hex(2)NeuAc(1)_1)
1138.521(PEP+HexNAc(4)Hex(4)_2)
1182.528(PEP+HexNAc(3)Hex(4)NeuAc(1)_2)
M+2H
1748.845(PEP+HexNAc(3)Hex(2)_1)
1910.902(PEP+HexNAc(3)Hex(3)_1)
m/z

IQNLTVK(=PEP)_4_4_0_1_0, 0_None, 0_None,
m/z:1284.07(2+), RT:37.46, Y-score:94.03

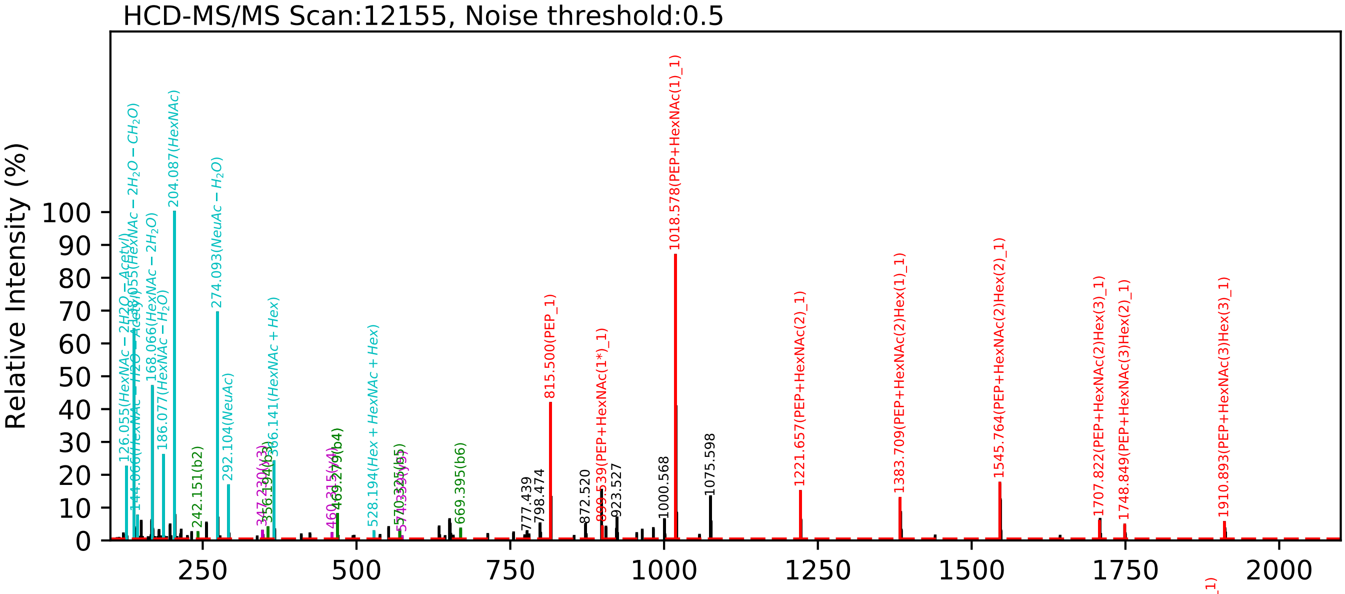

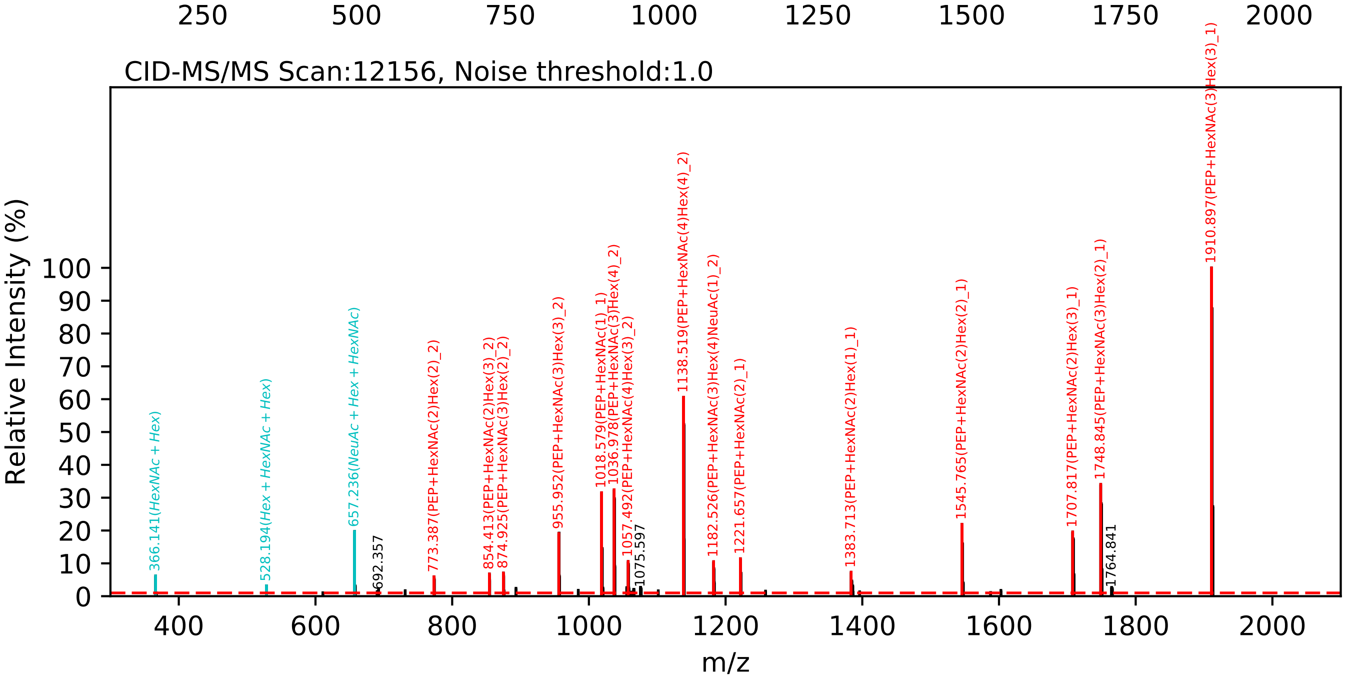

IQNLTVK(=PEP)_4_4_0_1_0, 0_None, 0_None,
m/z:1284.07(2+), RT:38.03, Y-score:62.16

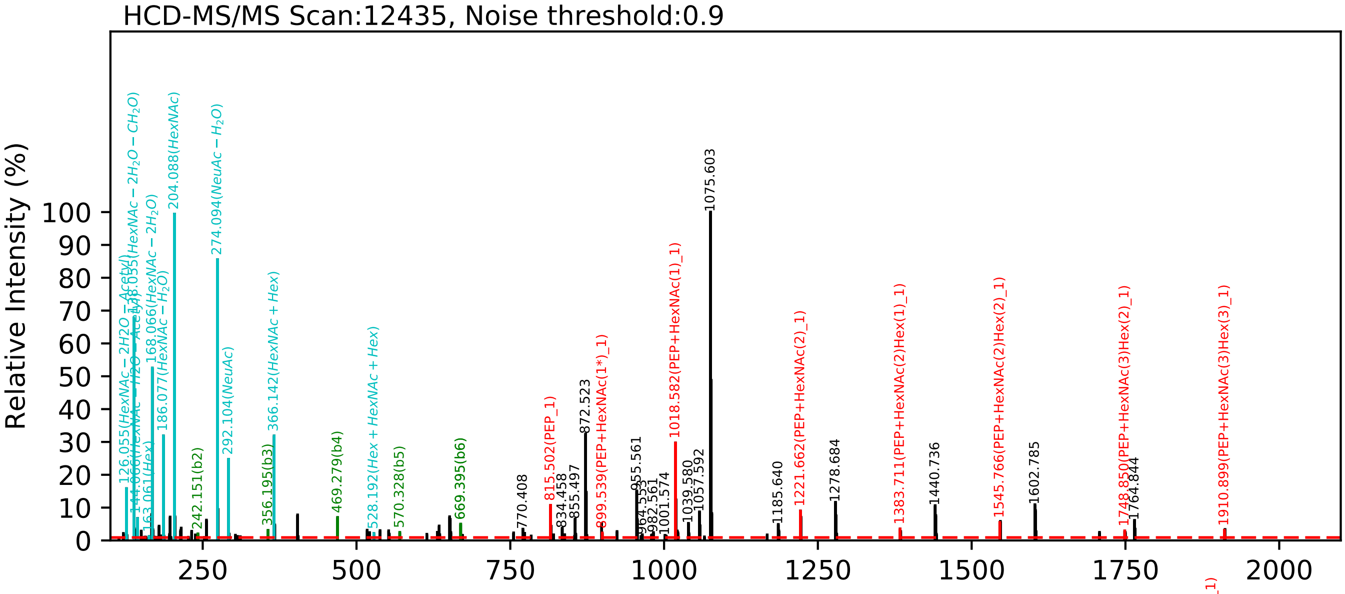

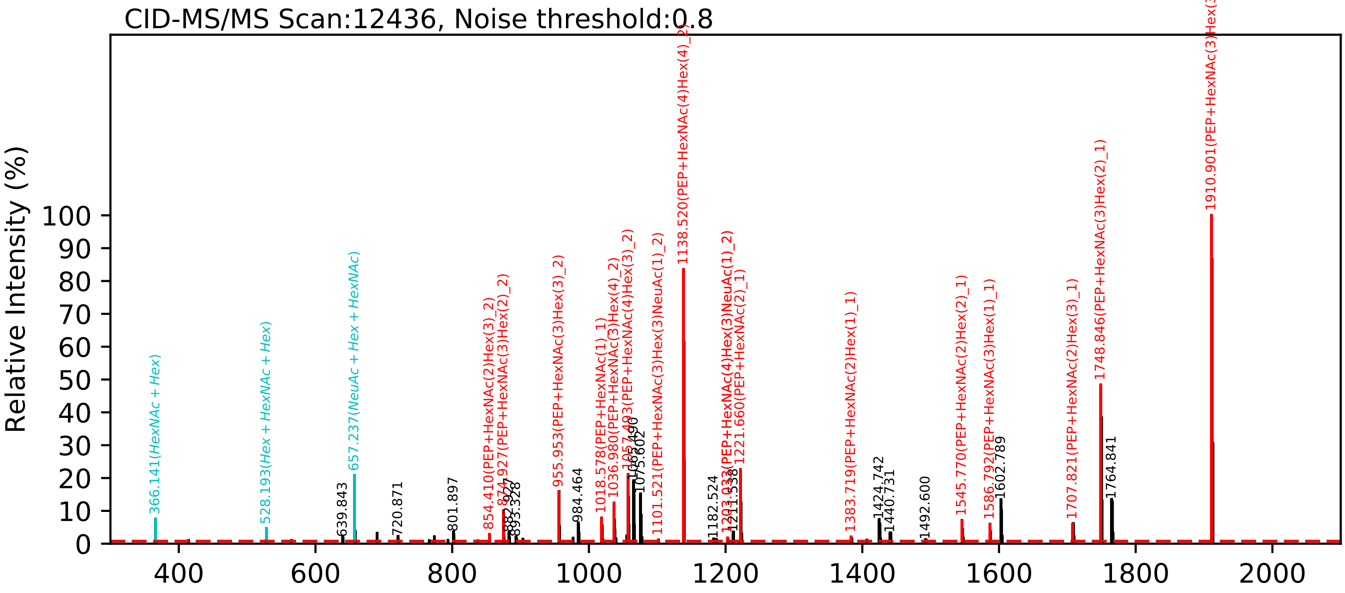

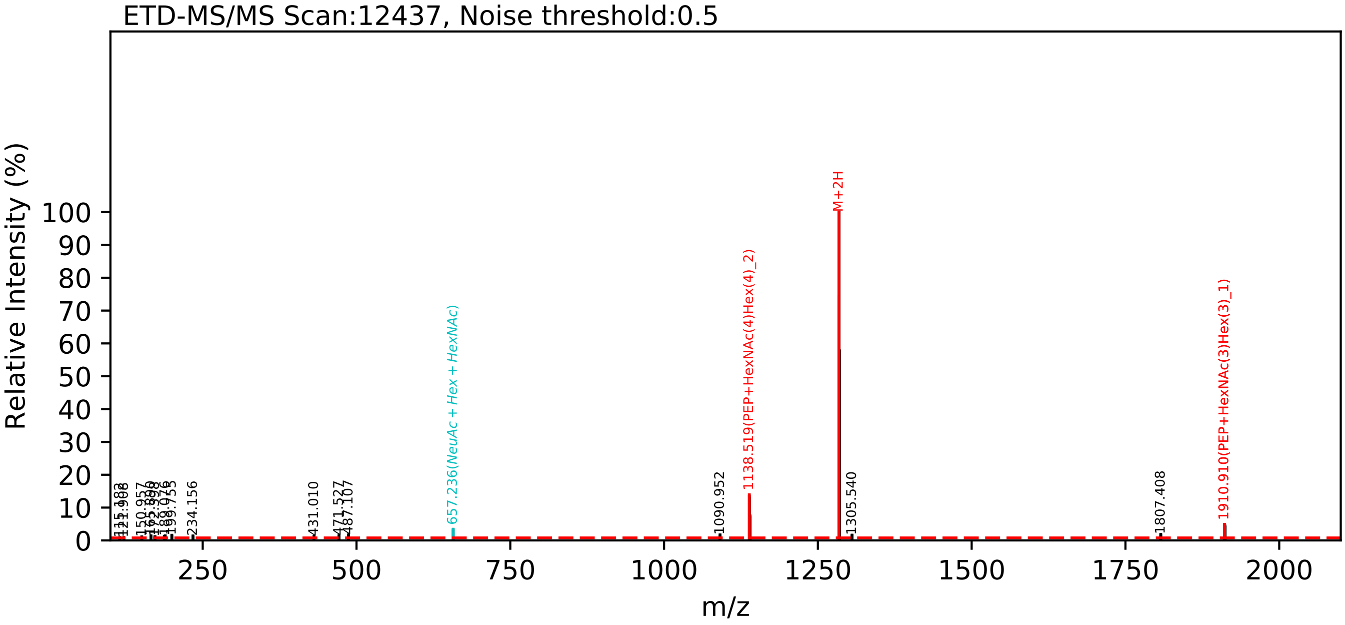

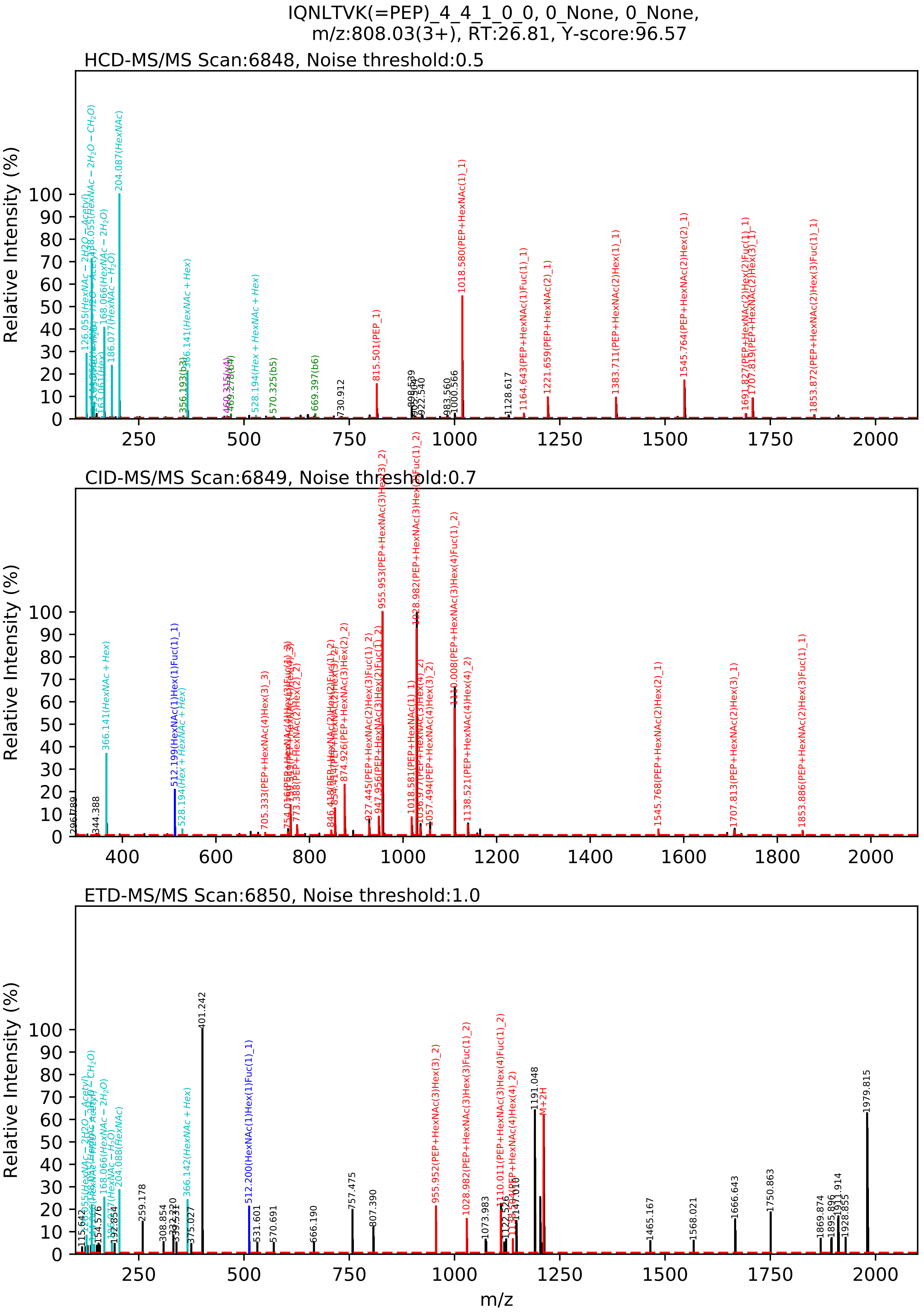
IQNLTVK(=PEP)_4_4_1_0_0, 0_None, 0_None,
m/z:808.03(3+), RT:26.81, Y-score:96.57
HCD-MS/MS Scan:6848, Noise threshold:0.5
Relative Intensity (%)
204.087(HexNAc)
168.066(HexNAc − 2H2O)
186.077(HexNAc − H2O)
366.141(HexNAc + Hex)
356.193(b3)
469.278(b4)
528.194(Hex + HexNAc + Hex)
570.325(b5)
669.397(b6)
730.912
815.501(PEP_1)
1018.580(PEP+HexNAc(1)_1)
1128.617
1164.643(PEP+HexNAc(1)Fuc(1)_1)
1221.659(PEP+HexNAc(2)_1)
1383.711(PEP+HexNAc(2)Hex(1)_1)
1545.764(PEP+HexNAc(2)Hex(2)_1)
1853.872(PEP+HexNAc(2)Hex(3)Fuc(1)_1)
CID-MS/MS Scan:6849, Noise threshold:0.7
344.388
366.141(HexNAc + Hex)
512.199(HexNAc(1)Hex(1)Fuc(1)_1)
528.194(Hex + HexNAc + Hex)
705.333(PEP+HexNAc(4)Hex(3)_3)
874.926(PEP+HexNAc(3)Hex(2)_2)
955.953(PEP+HexNAc(3)Hex(3)_2)
1028.982(PEP+HexNAc(3)Hex(3)Fuc(1)_2)
1110.008(PEP+HexNAc(3)Hex(4)Fuc(1)_2)
1138.521(PEP+HexNAc(4)Hex(4)_2)
1545.768(PEP+HexNAc(2)Hex(2)_1)
1707.813(PEP+HexNAc(2)Hex(3)_1)
1853.886(PEP+HexNAc(2)Hex(3)Fuc(1)_1)
ETD-MS/MS Scan:6850, Noise threshold:1.0
401.242
512.200(HexNAc(1)Hex(1)Fuc(1)_1)
366.142(HexNAc + Hex)
204.088(HexNAc)
259.178
308.854
375.027
531.601
570.691
666.190
757.475
807.390
955.952(PEP+HexNAc(3)Hex(3)_2)
1028.982(PEP+HexNAc(3)Hex(3)Fuc(1)_2)
1110.011(PEP+HexNAc(3)Hex(4)Fuc(1)_2)
1073.983
1191.048
M+2H
1465.167
1568.021
1666.643
1750.863
1869.874
1928.855
1979.815
m/z

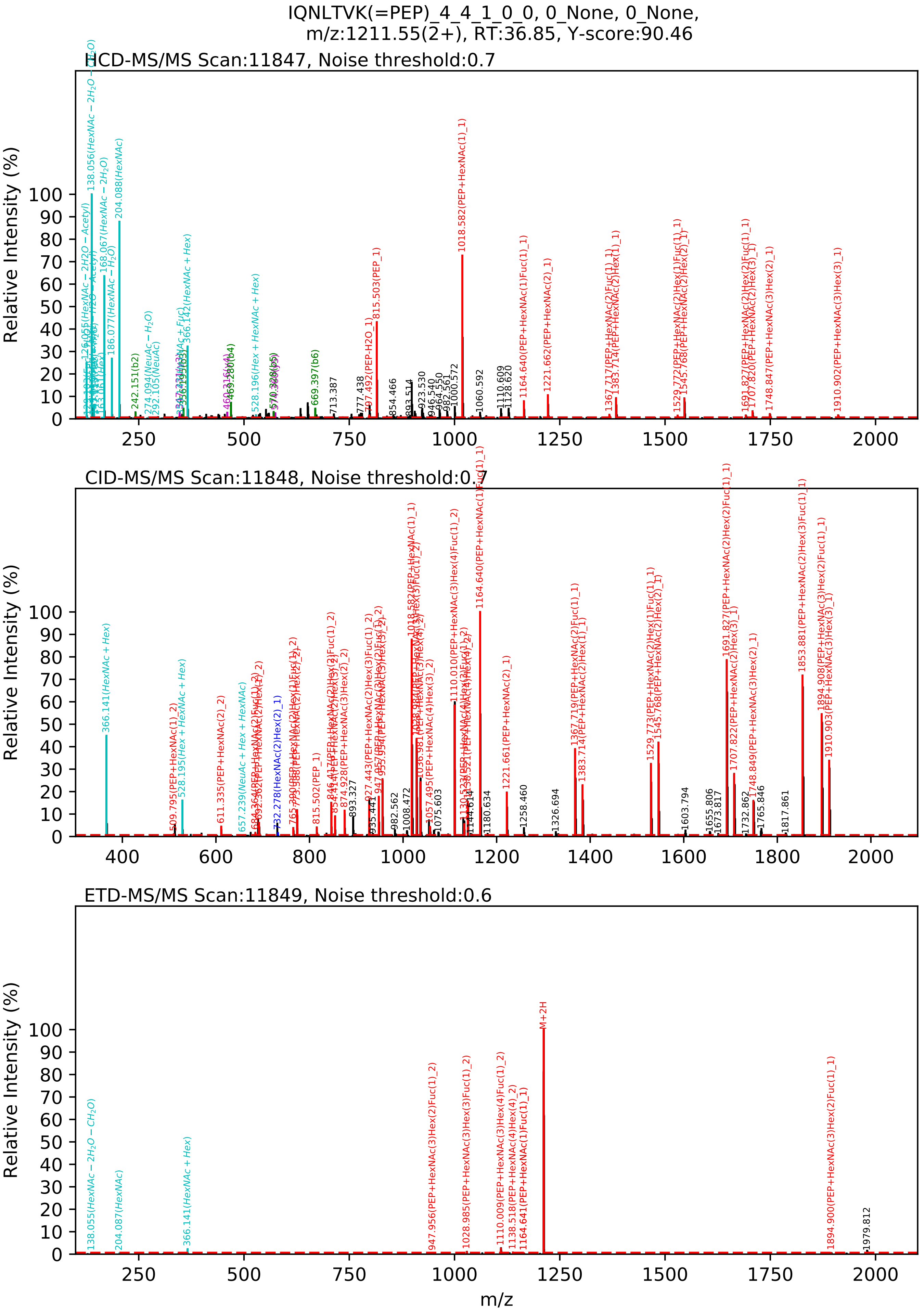
IQNLTVK(=PEP)_4_4_1_0_0, 0_None, 0_None,
m/z:1211.55(2+), RT:36.85, Y-score:90.46
HCD-MS/MS Scan:11847, Noise threshold:0.7
Relative Intensity (%)
138.056(HexNAc − 2H2O)
204.088(HexNAc)
186.077(HexNAc − H2O)
366.142(HexNAc + Hex)
528.196(Hex + HexNAc + Hex)
242.151(b2)
669.397(b6)
469.280(b4)
815.503(PEP_1)
1018.582(PEP+HexNAc(1)_1)
1164.640(PEP+HexNAc(1)Fuc(1)_1)
1221.662(PEP+HexNAc(2)_1)
1383.714(PEP+HexNAc(2)Hex(1)_1)
1545.768(PEP+HexNAc(2)Hex(2)_1)
1707.820(PEP+HexNAc(2)Hex(3)_1)
1748.847(PEP+HexNAc(3)Hex(2)_1)
1910.902(PEP+HexNAc(3)Hex(3)_1)
CID-MS/MS Scan:11848, Noise threshold:0.7
Relative Intensity (%)
366.141(HexNAc + Hex)
528.195(Hex + HexNAc + Hex)
611.335(PEP+HexNAc(2)_2)
815.502(PEP_1)
1110.010(PEP+HexNAc(3)Hex(4)Fuc(1)_2)
1164.640(PEP+HexNAc(1)Fuc(1)_1)
1221.661(PEP+HexNAc(2)_1)
1367.719(PEP+HexNAc(2)Fuc(1)_1)
1383.714(PEP+HexNAc(2)Hex(1)_1)
1529.773(PEP+HexNAc(2)Hex(1)Fuc(1)_1)
1545.768(PEP+HexNAc(2)Hex(2)_1)
1691.827(PEP+HexNAc(2)Hex(2)Fuc(1)_1)
1707.822(PEP+HexNAc(2)Hex(3)_1)
1748.849(PEP+HexNAc(3)Hex(2)_1)
1853.881(PEP+HexNAc(2)Hex(3)Fuc(1)_1)
1894.908(PEP+HexNAc(3)Hex(2)Fuc(1)_1)
1910.903(PEP+HexNAc(3)Hex(3)_1)
ETD-MS/MS Scan:11849, Noise threshold:0.6
Relative Intensity (%)
138.055(HexNAc − 2H2O − CH2O)
204.087(HexNAc)
366.141(HexNAc + Hex)
947.956(PEP+HexNAc(3)Hex(2)Fuc(1)_2)
1028.985(PEP+HexNAc(3)Hex(3)Fuc(1)_2)
1110.009(PEP+HexNAc(3)Hex(4)Fuc(1)_2)
1138.518(PEP+HexNAc(4)Hex(4)_2)
1164.641(PEP+HexNAc(1)Fuc(1)_1)
M+2H
1894.900(PEP+HexNAc(3)Hex(2)Fuc(1)_1)
1979.812
m/z

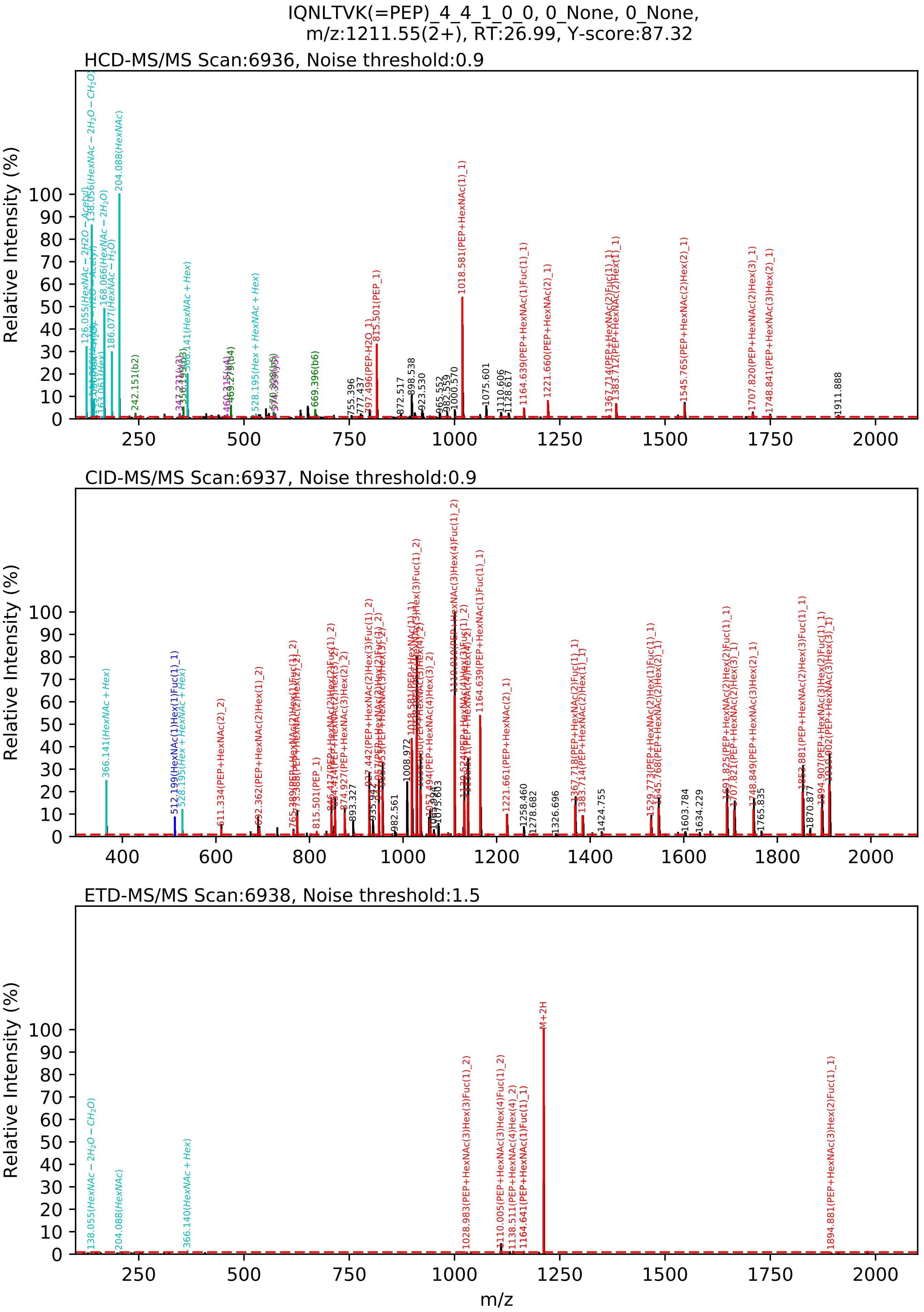
IQNLTVK(=PEP)_4_4_1_0_0, 0_None, 0_None,
m/z:1211.55(2+), RT:26.99, Y-score:87.32
HCD-MS/MS Scan:6936, Noise threshold:0.9
Relative Intensity (%)
204.088(HexNAc)
168.066(HexNAc − 2H2O)
186.077(HexNAc − H2O)
126.055(HexNAc − 2H2O − Acetyl)
242.151(b2)
366.141(HexNAc + Hex)
528.195(Hex + HexNAc + Hex)
669.396(b6)
755.396
777.437
797.496(PEP-H2O_1)
815.501(PEP_1)
872.517
898.538
923.530
965.552
1000.570
1018.581(PEP+HexNAc(1)_1)
1075.601
1110.606
1128.617
1164.639(PEP+HexNAc(1)Fuc(1)_1)
1221.660(PEP+HexNAc(2)_1)
1383.712(PEP+HexNAc(2)Hex(1)_1)
1545.765(PEP+HexNAc(2)Hex(2)_1)
1707.820(PEP+HexNAc(2)Hex(3)_1)
1748.841(PEP+HexNAc(3)Hex(2)_1)
1911.888
CID-MS/MS Scan:6937, Noise threshold:0.9
366.141(HexNAc + Hex)
512.199(HexNAc(1)Hex(1)Fuc(1)_1)
528.195(Hex + HexNAc + Hex)
611.334(PEP+HexNAc(2)_2)
692.362(PEP+HexNAc(2)Hex(1)_2)
815.501(PEP_1)
874.927(PEP+HexNAc(3)Hex(2)_2)
893.327
982.561
1008.972
1075.603
1164.639(PEP+HexNAc(1)Fuc(1)_1)
1221.661(PEP+HexNAc(2)_1)
1258.460
1278.682
1326.696
1383.714(PEP+HexNAc(2)Hex(1)_1)
1424.755
1529.773(PEP+HexNAc(2)Hex(1)Fuc(1)_1)
1545.768(PEP+HexNAc(2)Hex(2)_1)
1603.784
1634.229
1691.825(PEP+HexNAc(2)Hex(2)Fuc(1)_1)
1707.821(PEP+HexNAc(2)Hex(3)_1)
1748.849(PEP+HexNAc(3)Hex(2)_1)
1765.835
1853.881(PEP+HexNAc(2)Hex(3)Fuc(1)_1)
1870.877
1894.907(PEP+HexNAc(3)Hex(2)Fuc(1)_1)
1910.902(PEP+HexNAc(3)Hex(3)_1)
ETD-MS/MS Scan:6938, Noise threshold:1.5
138.055(HexNAc − 2H2O − CH2O)
204.088(HexNAc)
366.140(HexNAc + Hex)
1028.983(PEP+HexNAc(3)Hex(3)Fuc(1)_2)
1110.005(PEP+HexNAc(3)Hex(4)Fuc(1)_2)
1138.511(PEP+HexNAc(4)Hex(4)_2)
1164.641(PEP+HexNAc(1)Fuc(1)_1)
M+2H
1894.881(PEP+HexNAc(3)Hex(2)Fuc(1)_1)
m/z

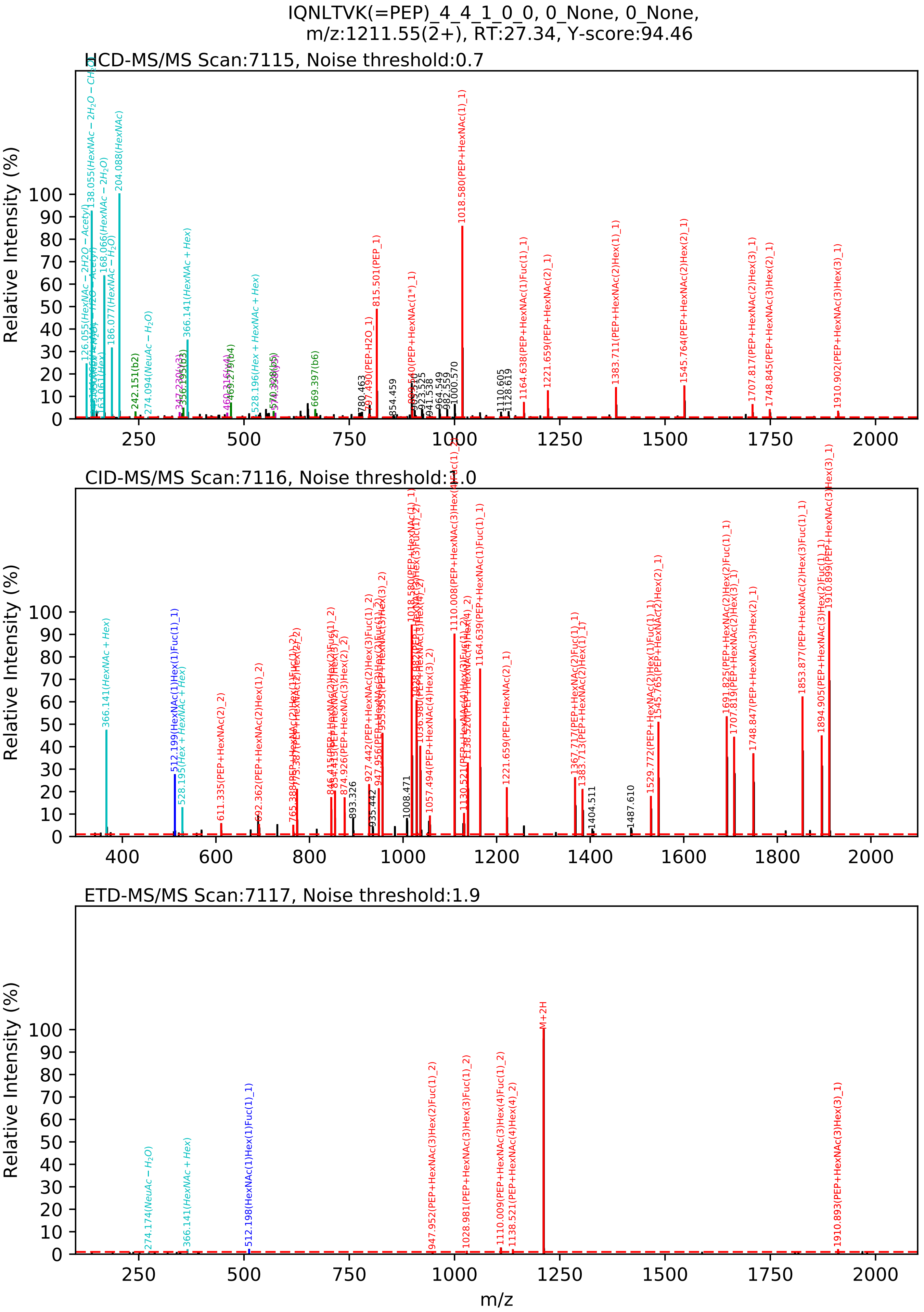

IQNLTVK(=PEP)_4_4_1_0_0, 0_None, 0_None,
m/z:1211.55(2+), RT:27.34, Y-score:94.46
HCD-MS/MS Scan:7115, Noise threshold:0.7
Relative Intensity (%)
204.088(HexNAc)
138.055(HexNAc − 2H2O − CH3)
168.066(HexNAc − 2H2O)
186.077(HexNAc − H2O)
126.055(HexNAc − 2H2O − Acetyl)
242.151(b2)
274.094(NeuAc − H2O)
366.141(HexNAc + Hex)
528.196(Hex + HexNAc + Hex)
669.397(b6)
815.501(PEP_1)
797.490(PEP-H2O_1)
780.463
854.459
1018.580(PEP+HexNAc(1)_1)
1164.638(PEP+HexNAc(1)Fuc(1)_1)
1221.659(PEP+HexNAc(2)_1)
1383.711(PEP+HexNAc(2)Hex(1)_1)
1545.764(PEP+HexNAc(2)Hex(2)_1)
1707.817(PEP+HexNAc(2)Hex(3)_1)
1748.845(PEP+HexNAc(3)Hex(2)_1)
1910.902(PEP+HexNAc(3)Hex(3)_1)
1110.605
1128.619
CID-MS/MS Scan:7116, Noise threshold:1.0
366.141(HexNAc + Hex)
512.199(HexNAc(1)Hex(1)Fuc(1)_1)
528.195(Hex + HexNAc + Hex)
611.335(PEP+HexNAc(2)_2)
692.362(PEP+HexNAc(2)Hex(1)_2)
874.926(PEP+HexNAc(3)Hex(2)_2)
893.326
927.442(PEP+HexNAc(2)Hex(3)Fuc(1)_2)
1008.471
1018.580(PEP+HexNAc(1)_1)
1057.494(PEP+HexNAc(4)Hex(3)_2)
1110.008(PEP+HexNAc(3)Hex(3)Fuc(1)_2)
1164.639(PEP+HexNAc(1)Fuc(1)_1)
1221.659(PEP+HexNAc(2)_1)
1367.717(PEP+HexNAc(2)Fuc(1)_1)
1383.713(PEP+HexNAc(2)Hex(1)_1)
1404.511
1487.610
1529.772(PEP+HexNAc(2)Hex(1)Fuc(1)_1)
1545.765(PEP+HexNAc(2)Hex(2)_1)
1691.825(PEP+HexNAc(2)Hex(2)Fuc(1)_1)
1707.819(PEP+HexNAc(2)Hex(3)_1)
1748.847(PEP+HexNAc(3)Hex(2)_1)
1853.877(PEP+HexNAc(2)Hex(3)Fuc(1)_1)
1894.905(PEP+HexNAc(3)Hex(2)Fuc(1)_1)
1910.899(PEP+HexNAc(3)Hex(3)_1)
ETD-MS/MS Scan:7117, Noise threshold:1.9
274.174(NeuAc − H2O)
366.141(HexNAc + Hex)
512.198(HexNAc(1)Hex(1)Fuc(1)_1)
947.952(PEP+HexNAc(3)Hex(2)Fuc(1)_2)
1028.981(PEP+HexNAc(3)Hex(3)Fuc(1)_2)
1110.009(PEP+HexNAc(3)Hex(4)Fuc(1)_2)
1138.521(PEP+HexNAc(4)Hex(4)_2)
M+2H
1910.893(PEP+HexNAc(3)Hex(3)_1)
m/z

IQNLTVK(=PEP)_4_4_1_0_0, 0_None, 0_None,
m/z:1211.55(2+), RT:26.18, Y-score:91.91

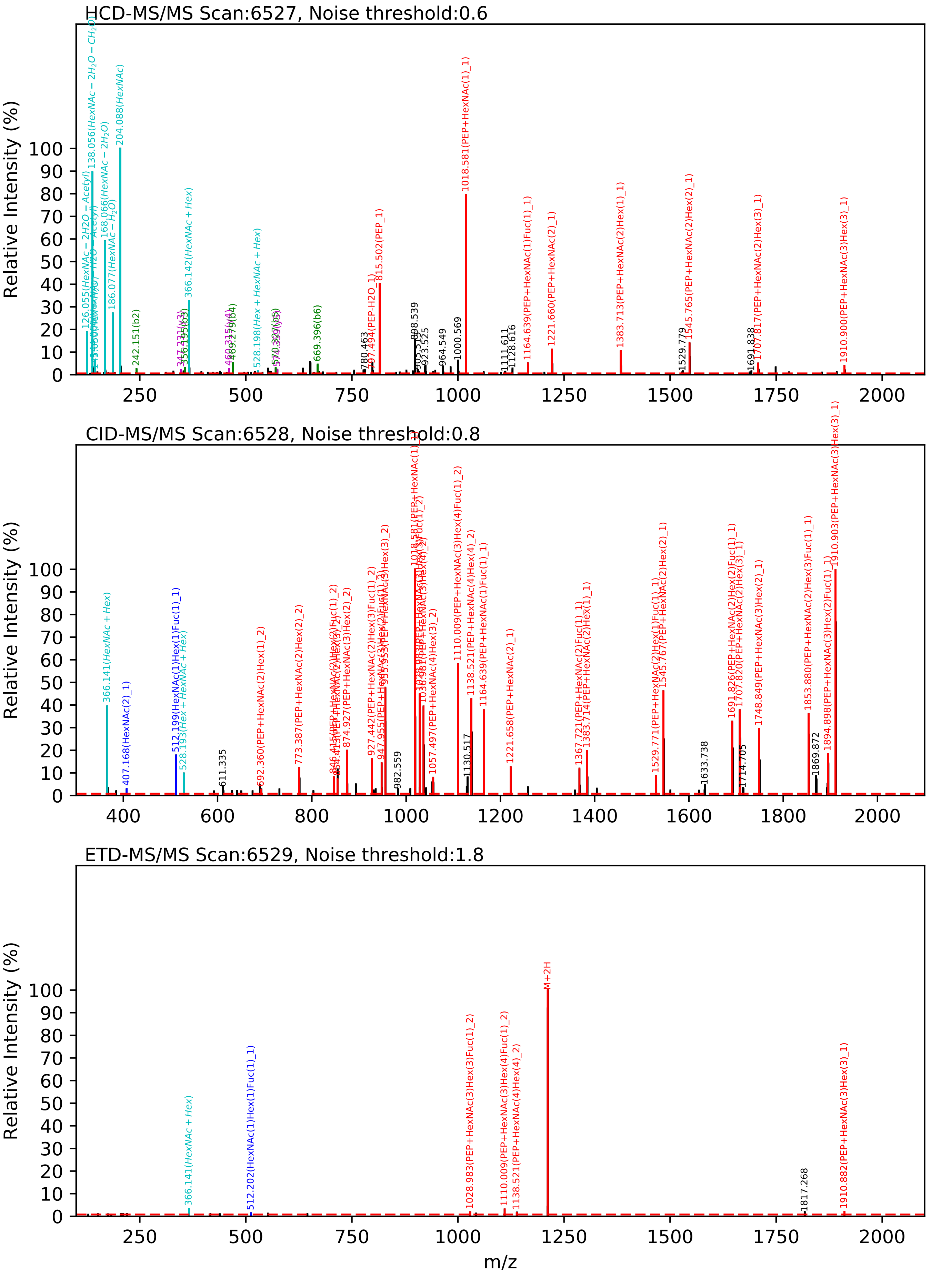

IQNLTVK(=PEP)_4_4_1_0_0, 0_None, 0_None,
m/z:1211.55(2+), RT:26.76, Y-score:95.81

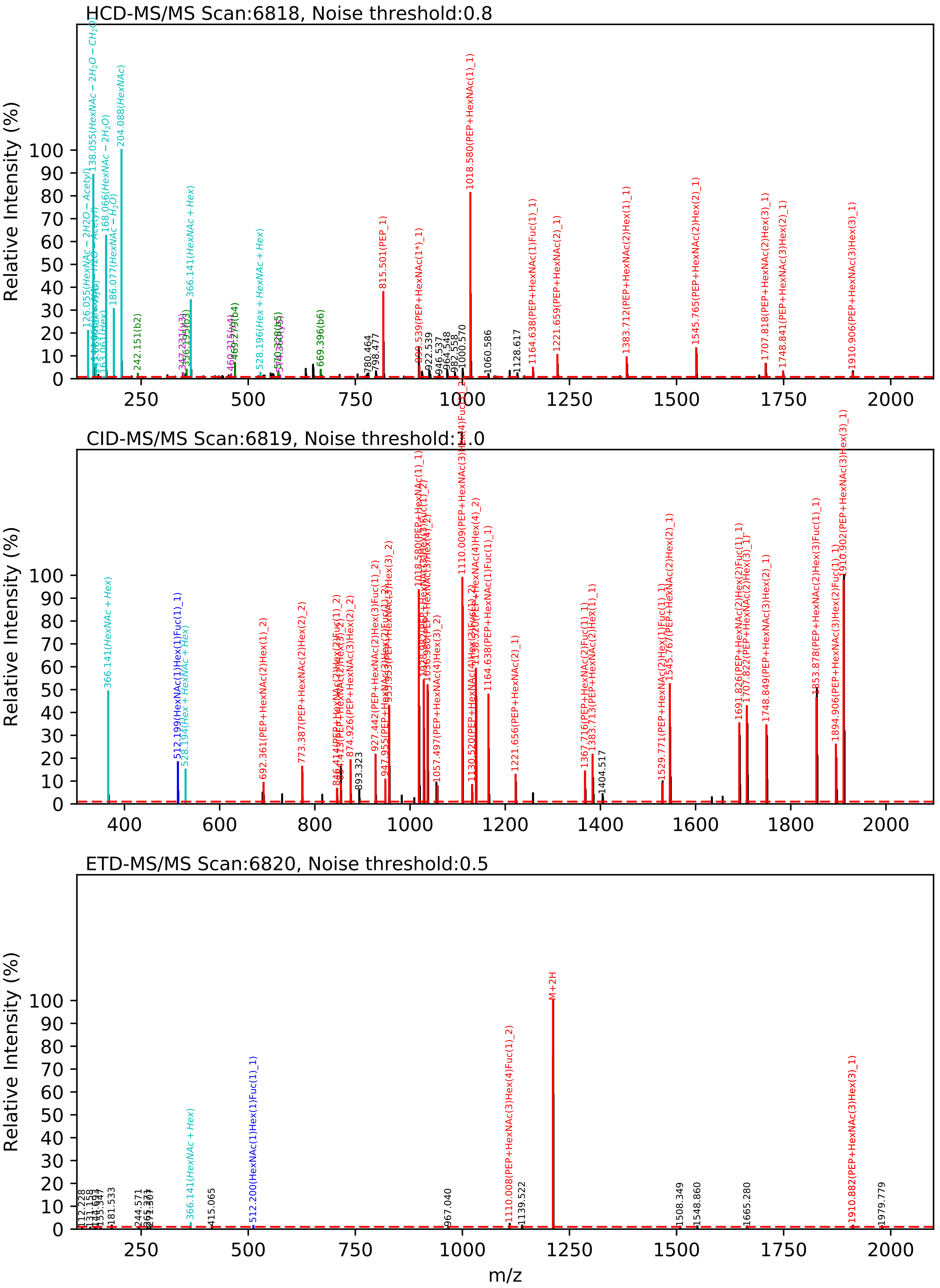

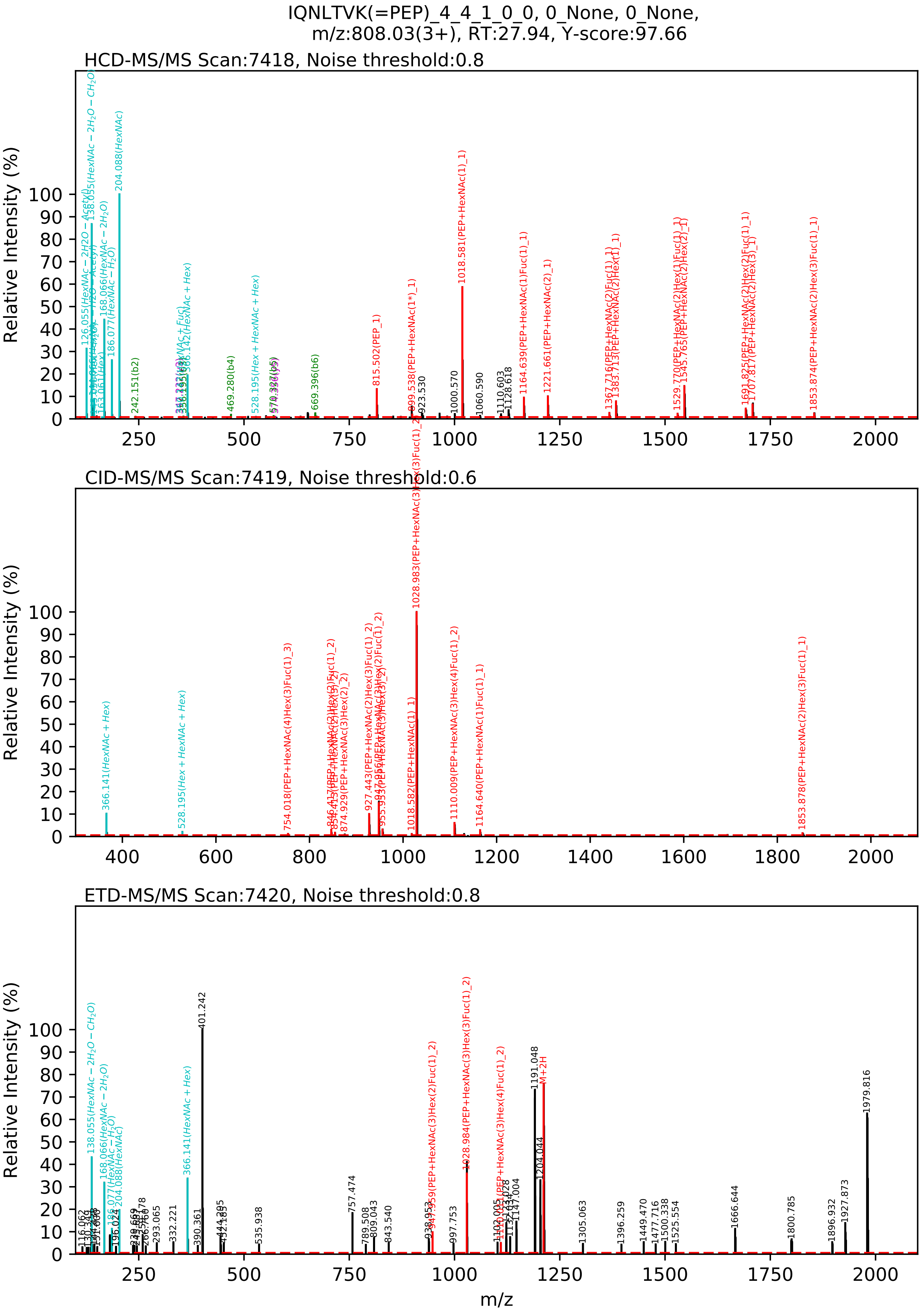
IQNLTVK(=PEP)_4_4_1_0_0, 0_None, 0_None,
m/z:808.03(3+), RT:27.94, Y-score:97.66
HCD-MS/MS Scan:7418, Noise threshold:0.8
CID-MS/MS Scan:7419, Noise threshold:0.6
ETD-MS/MS Scan:7420, Noise threshold:0.8
Relative Intensity (%)
m/z
204.088(HexNAc)
1018.581(PEP+HexNAc(1)_1)
815.502(PEP_1)
1164.639(PEP+HexNAc(1)Fuc(1)_1)
1221.661(PEP+HexNAc(2)_1)
1853.874(PEP+HexNAc(2)Hex(3)Fuc(1)_1)
1028.983(PEP+HexNAc(3)Hex(3)Fuc(1)_2)
366.141(HexNAc + Hex)
528.195(Hex + HexNAc + Hex)
754.018(PEP+HexNAc(4)Hex(3)Fuc(1)_3)
927.443(PEP+HexNAc(2)Hex(3)Fuc(1)_2)
1110.009(PEP+HexNAc(3)Hex(4)Fuc(1)_2)
1164.640(PEP+HexNAc(1)Fuc(1)_1)
1853.878(PEP+HexNAc(2)Hex(3)Fuc(1)_1)
401.242
1191.048
M+2H
1979.816
1204.044
757.474
1666.644
1800.785
1927.873

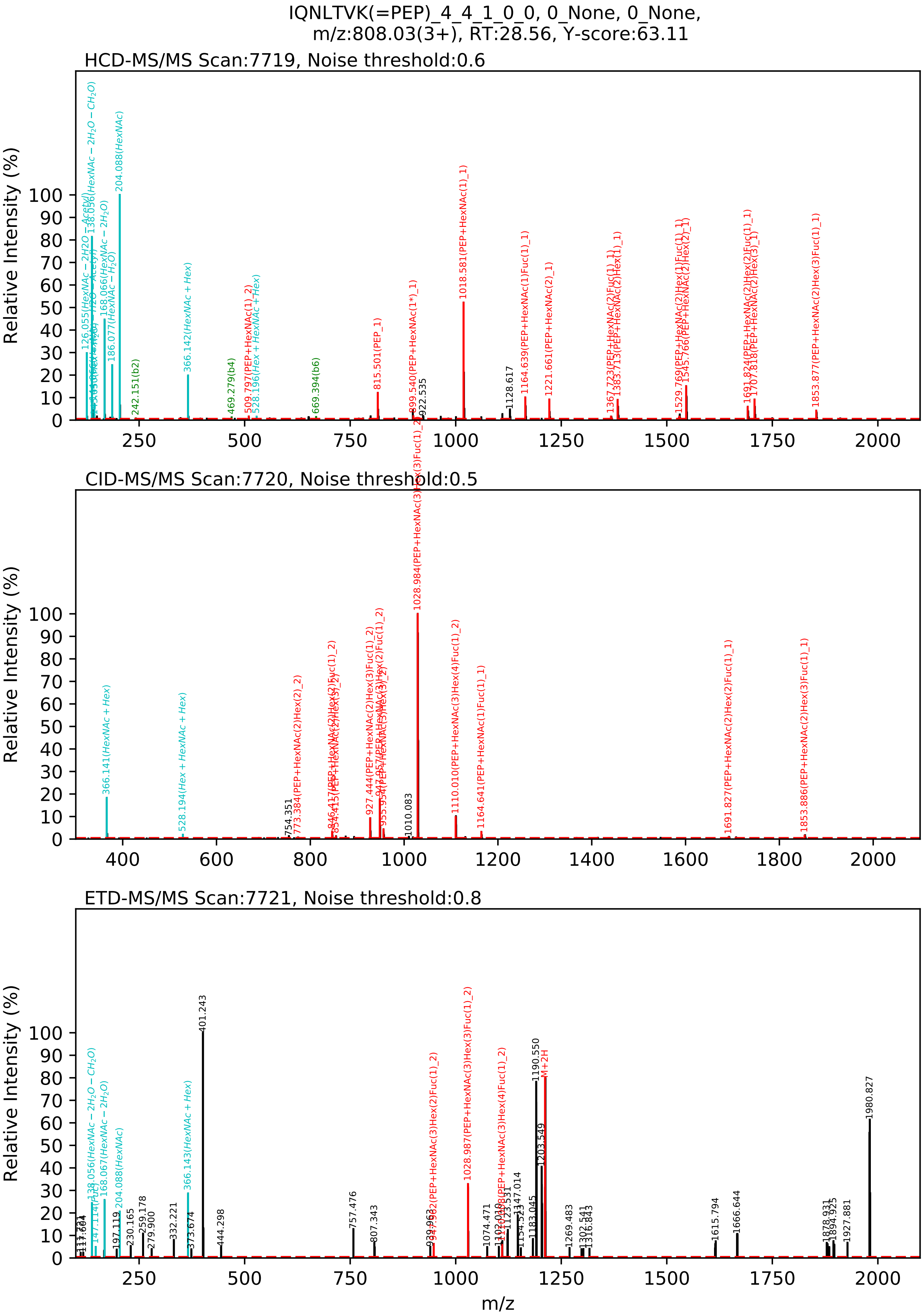

IQNLTVK(=PEP)_4_4_1_0_0, 0_None, 0_None,
m/z:808.03(3+), RT:28.56, Y-score:63.11
HCD-MS/MS Scan:7719, Noise threshold:0.6
Relative Intensity (%)
204.088(HexNAc)
168.066(HexNAc − 2H2O)
186.077(HexNAc − H2O)
126.055(HexNAc − 2H2O − Acetyl)
138.056(HexNAc − 2H2O − CH2O)
366.142(HexNAc + Hex)
242.151(b2)
469.279(b4)
509.797(PEP+HexNAc(1)_2)
528.196(Hex + HexNAc + Hex)
669.394(b6)
815.501(PEP_1)
899.540(PEP+HexNAc(1*)_1)
922.535
1018.581(PEP+HexNAc(1)_1)
1128.617
1164.639(PEP+HexNAc(1)Fuc(1)_1)
1221.661(PEP+HexNAc(2)_1)
1367.723(PEP+HexNAc(2)Fuc(1)_1)
1383.713(PEP+HexNAc(2)Hex(1)_1)
1529.769(PEP+HexNAc(2)Hex(1)Fuc(1)_1)
1545.766(PEP+HexNAc(2)Hex(2)_1)
1691.824(PEP+HexNAc(2)Hex(2)Fuc(1)_1)
1707.818(PEP+HexNAc(2)Hex(3)_1)
1853.877(PEP+HexNAc(2)Hex(3)Fuc(1)_1)
CID-MS/MS Scan:7720, Noise threshold:0.5
Relative Intensity (%)
366.141(HexNAc + Hex)
528.194(Hex + HexNAc + Hex)
754.351
773.384(PEP+HexNAc(2)Hex(2)_2)
846.417(PEP+HexNAc(2)Hex(2)Fuc(1)_2)
854.415(PEP+HexNAc(2)Hex(3)_2)
927.444(PEP+HexNAc(2)Hex(3)Fuc(1)_2)
947.957(PEP+HexNAc(3)Hex(2)Fuc(1)_2)
955.954(PEP+HexNAc(3)Hex(3)_2)
1010.083
1028.984(PEP+HexNAc(3)Hex(3)Fuc(1)_2)
1110.010(PEP+HexNAc(3)Hex(4)Fuc(1)_2)
1164.641(PEP+HexNAc(1)Fuc(1)_1)
1691.827(PEP+HexNAc(2)Hex(2)Fuc(1)_1)
1853.886(PEP+HexNAc(2)Hex(3)Fuc(1)_1)
ETD-MS/MS Scan:7721, Noise threshold:0.8
Relative Intensity (%)
138.056(HexNAc − 2H2O − CH2O)
168.067(HexNAc − 2H2O)
204.088(HexNAc)
147.114(y1)
366.143(HexNAc + Hex)
197.119
230.165
259.178
279.900
332.221
373.674
401.243
444.298
757.476
807.343
939.963
947.962(PEP+HexNAc(3)Hex(2)Fuc(1)_2)
1028.987(PEP+HexNAc(3)Hex(3)Fuc(1)_2)
1074.471
1102.010
1110.008(PEP+HexNAc(3)Hex(4)Fuc(1)_2)
1123.531
1147.014
1154.517
1183.045
1190.550
M+2H
1203.549
1269.483
1302.541
1316.843
1615.794
1666.644
1878.931
1894.925
1927.881
1980.827
m/z

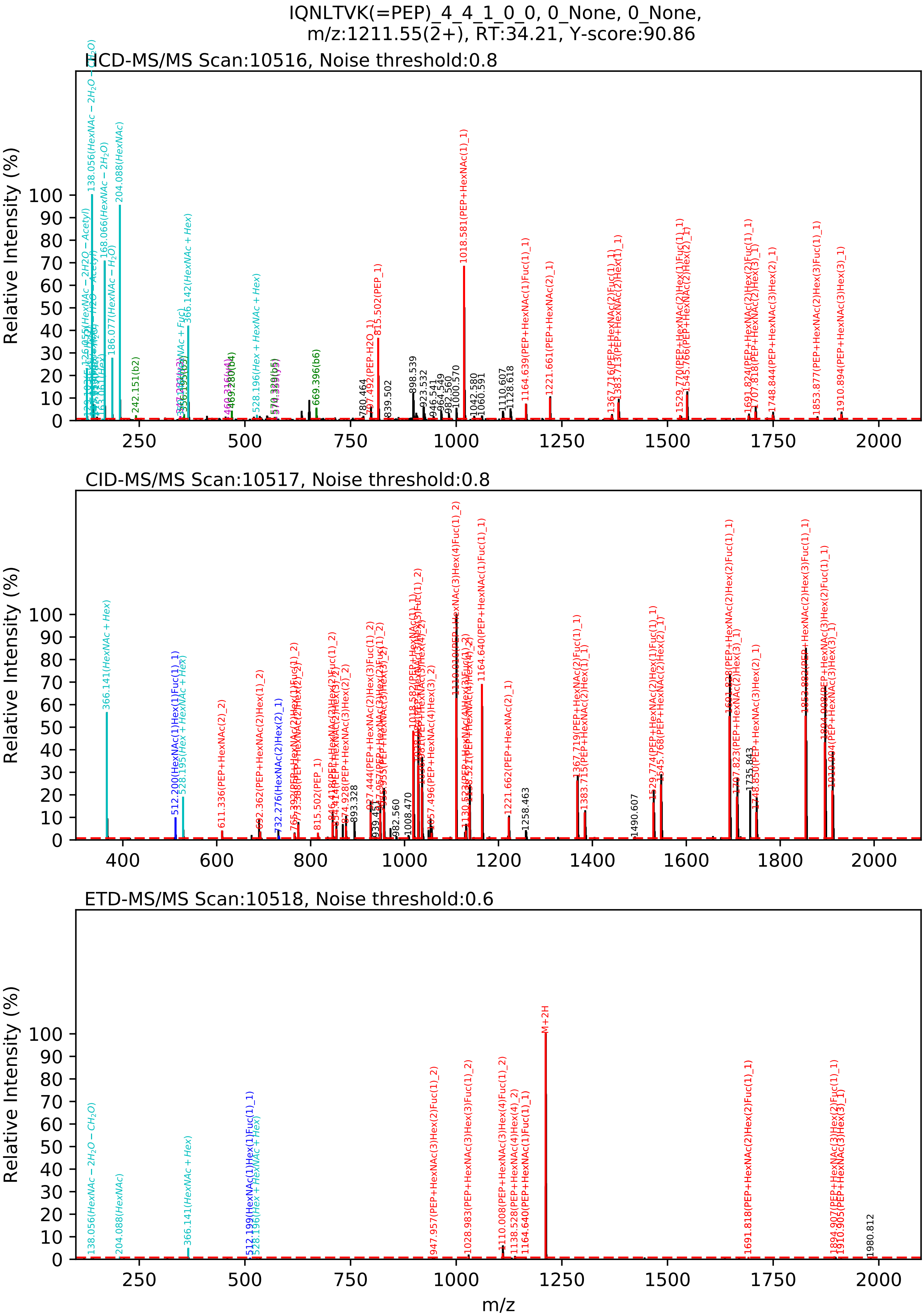

IQNLTVK(=PEP)_4_4_1_0_0, 0_None, 0_None,
m/z:1211.55(2+), RT:34.21, Y-score:90.86
HCD-MS/MS Scan:10516, Noise threshold:0.8
CID-MS/MS Scan:10517, Noise threshold:0.8
ETD-MS/MS Scan:10518, Noise threshold:0.6
Relative Intensity (%)
m/z
1018.581(PEP+HexNAc(1)_1)
815.502(PEP_1)
1164.639(PEP+HexNAc(1)Fuc(1)_1)
1221.661(PEP+HexNAc(2)_1)
138.056(HexNAc − 2H2O − H2O)
204.088(HexNAc)
366.142(HexNAc + Hex)
M+2H

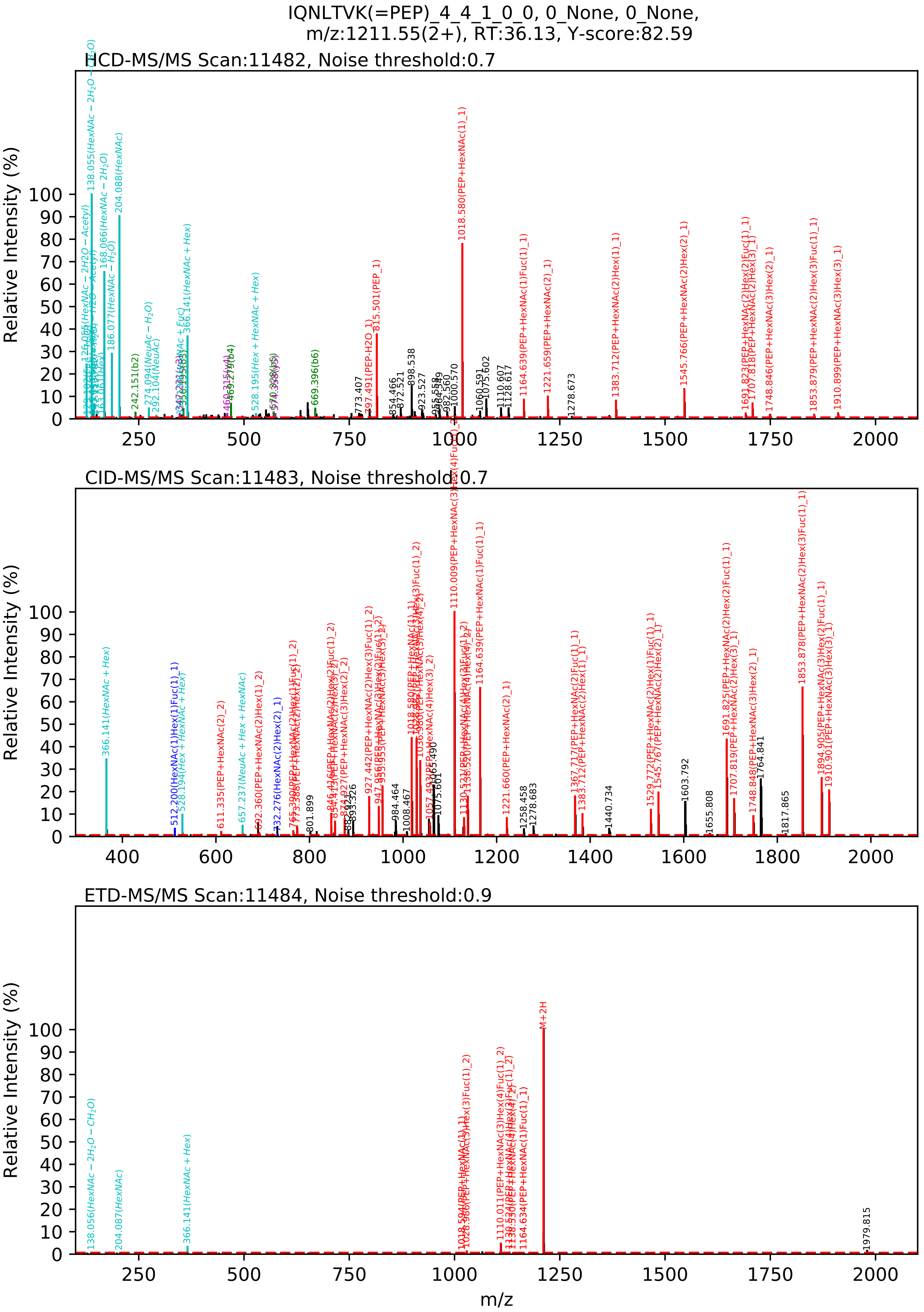

IQNLTVK(=PEP)_4_4_1_0_0, 0_None, 0_None,
m/z:1211.55(2+), RT:36.13, Y-score:82.59
HCD-MS/MS Scan:11482, Noise threshold:0.7
Relative Intensity (%)
138.055(HexNAc − 2H2O − Acetyl)
168.066(HexNAc − 2H2O)
186.077(HexNAc − H2O)
204.088(HexNAc)
242.151(b2)
274.094(NeuAc − H2O)
292.104(NeuAc)
366.141(HexNAc + Hex)
469.279(b4)
528.195(Hex + HexNAc + Hex)
669.396(b6)
773.407
797.491(PEP-H2O_1)
815.501(PEP_1)
854.466
872.521
898.538
923.527
1000.570
1018.580(PEP+HexNAc(1)_1)
1060.591
1075.602
1110.607
1128.617
1164.639(PEP+HexNAc(1)Fuc(1)_1)
1221.659(PEP+HexNAc(2)_1)
1278.673
1383.712(PEP+HexNAc(2)Hex(1)_1)
1545.766(PEP+HexNAc(2)Hex(2)_1)
1691.823(PEP+HexNAc(2)Hex(2)Fuc(1)_1)
1707.818(PEP+HexNAc(2)Hex(3)_1)
1748.846(PEP+HexNAc(3)Hex(2)_1)
1853.879(PEP+HexNAc(2)Hex(3)Fuc(1)_1)
1910.899(PEP+HexNAc(3)Hex(3)_1)
CID-MS/MS Scan:11483, Noise threshold:0.7
366.141(HexNAc + Hex)
512.200(HexNAc(1)Hex(1)Fuc(1)_1)
528.194(Hex + HexNAc + Hex)
611.335(PEP+HexNAc(2)_2)
657.237(NeuAc + Hex + HexNAc)
692.360(PEP+HexNAc(2)Hex(1)_2)
732.276(HexNAc(2)Hex(2)_1)
801.899
893.326
927.442(PEP+HexNAc(2)Hex(3)Fuc(1)_2)
984.464
1008.467
1075.601
1110.009(PEP+HexNAc(3)Hex(4)Fuc(1)_2)
1164.639(PEP+HexNAc(1)Fuc(1)_1)
1221.660(PEP+HexNAc(2)_1)
1258.458
1278.683
1367.717(PEP+HexNAc(2)Fuc(1)_1)
1383.712(PEP+HexNAc(2)Hex(1)_1)
1440.734
1529.772(PEP+HexNAc(2)Hex(1)Fuc(1)_1)
1545.767(PEP+HexNAc(2)Hex(2)_1)
1603.792
1655.808
1691.825(PEP+HexNAc(2)Hex(2)Fuc(1)_1)
1707.819(PEP+HexNAc(2)Hex(3)_1)
1748.848(PEP+HexNAc(3)Hex(2)_1)
1764.841
1817.865
1853.878(PEP+HexNAc(2)Hex(3)Fuc(1)_1)
1894.905(PEP+HexNAc(3)Hex(2)Fuc(1)_1)
1910.901(PEP+HexNAc(3)Hex(3)_1)
ETD-MS/MS Scan:11484, Noise threshold:0.9
138.056(HexNAc − 2H2O − CH2O)
204.087(HexNAc)
366.141(HexNAc + Hex)
1110.011(PEP+HexNAc(3)Hex(4)Fuc(1)_2)
1164.634(PEP+HexNAc(1)Fuc(1)_1)
M+2H
1979.815
m/z

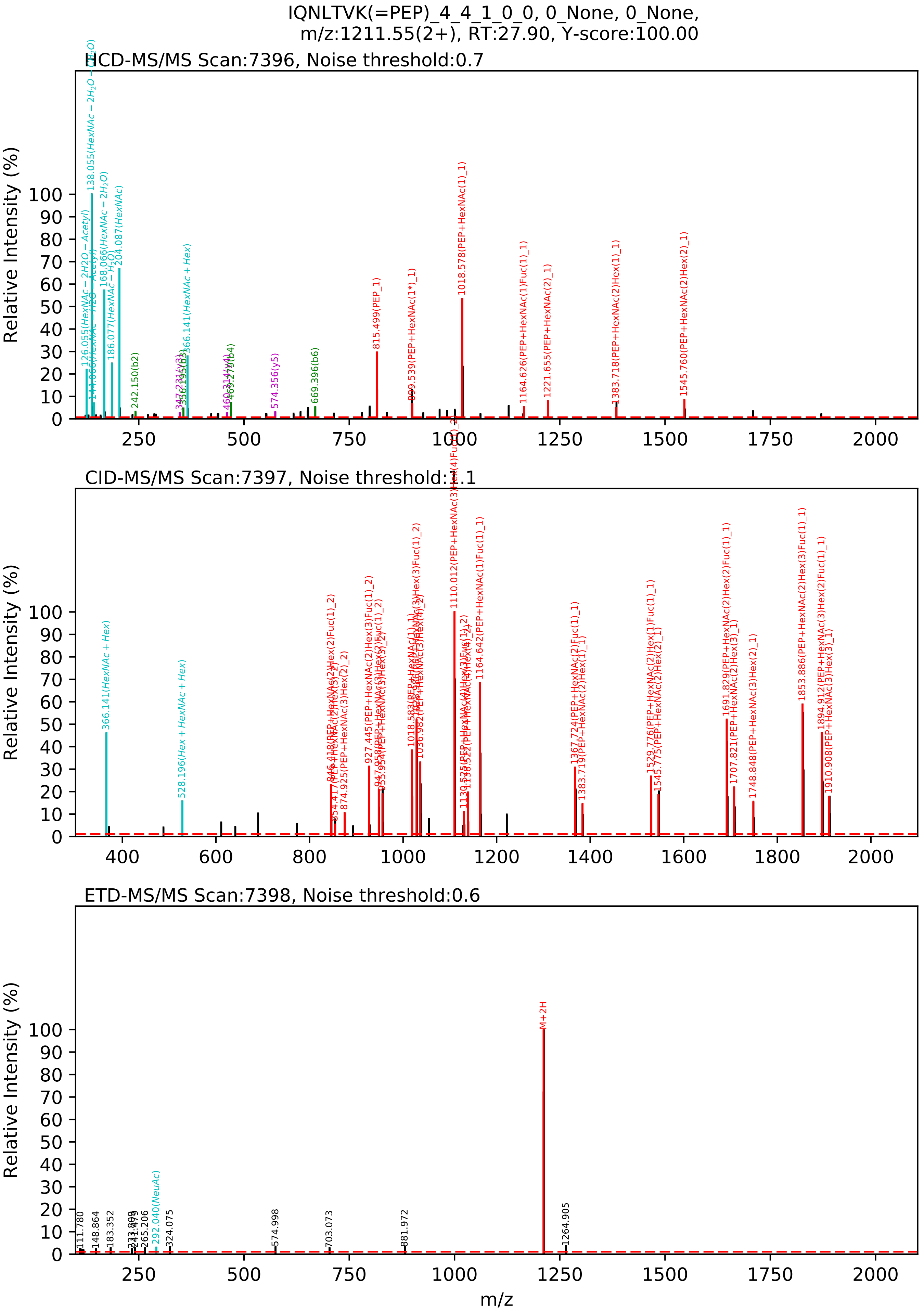
IQNLTVK(=PEP)_4_4_1_0_0, 0_None, 0_None,
m/z:1211.55(2+), RT:27.90, Y-score:100.00
HCD-MS/MS Scan:7396, Noise threshold:0.7
138.055(HexNAc − Acetyl)
126.055(HexNAc − 2H2O − Acetyl)
168.066(HexNAc − 2H2O)
186.077(HexNAc − H2O)
204.087(HexNAc)
366.141(HexNAc + Hex)
242.150(b2)
356.195(b3)
469.279(b4)
460.314(y4)
574.356(y5)
669.396(b6)
815.499(PEP_1)
899.539(PEP+HexNAc(1*)_1)
1018.578(PEP+HexNAc(1)_1)
1164.626(PEP+HexNAc(1)Fuc(1)_1)
1221.655(PEP+HexNAc(2)_1)
1383.718(PEP+HexNAc(2)Hex(1)_1)
1545.760(PEP+HexNAc(2)Hex(2)_1)
CID-MS/MS Scan:7397, Noise threshold:1.1
366.141(HexNAc + Hex)
528.196(Hex + HexNAc + Hex)
874.925(PEP+HexNAc(3)Hex(2)_2)
927.445(PEP+HexNAc(2)Hex(3)Fuc(1)_2)
1018.583
1036.982
1110.012(PEP+HexNAc(3)Hex(4)Fuc(1)_2)
1164.642(PEP+HexNAc(1)Fuc(1)_1)
1367.724(PEP+HexNAc(2)Fuc(1)_1)
1383.719(PEP+HexNAc(2)Hex(1)_1)
1529.776(PEP+HexNAc(2)Hex(1)Fuc(1)_1)
1545.775(PEP+HexNAc(2)Hex(2)_1)
1691.829(PEP+HexNAc(2)Hex(2)Fuc(1)_1)
1707.821(PEP+HexNAc(2)Hex(3)_1)
1748.848(PEP+HexNAc(3)Hex(2)_1)
1853.886(PEP+HexNAc(2)Hex(3)Fuc(1)_1)
1894.912(PEP+HexNAc(3)Hex(2)Fuc(1)_1)
1910.908(PEP+HexNAc(3)Hex(3)_1)
ETD-MS/MS Scan:7398, Noise threshold:0.6
M+2H
111.780
148.864
183.352
265.206
292.040(NeuAc)
324.075
574.998
703.073
881.972
1264.905
Relative Intensity (%)
m/z

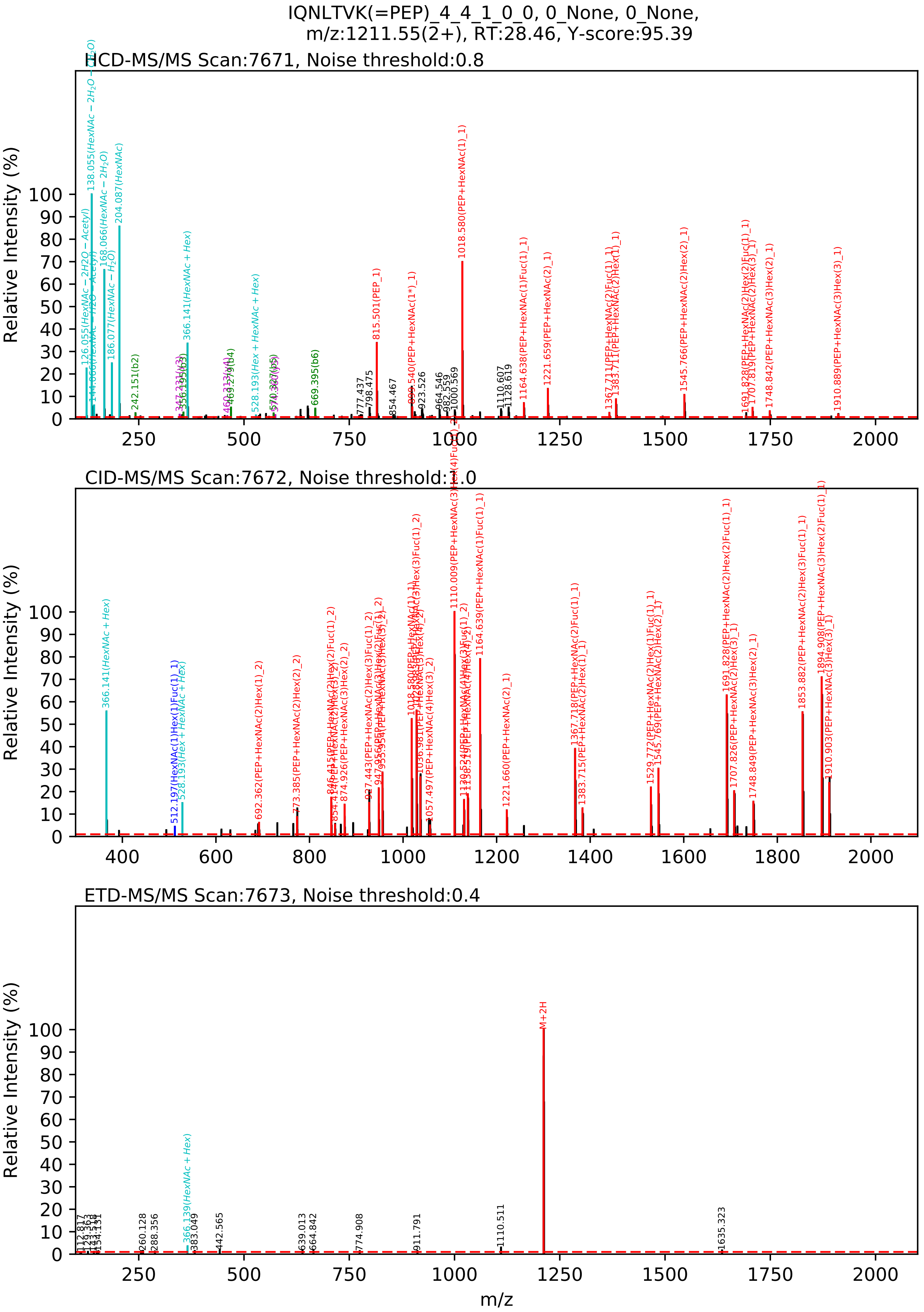
IQNLTVK(=PEP)_4_4_1_0_0, 0_None, 0_None,
m/z:1211.55(2+), RT:28.46, Y-score:95.39
HCD-MS/MS Scan:7671, Noise threshold:0.8
CID-MS/MS Scan:7672, Noise threshold:1.0
ETD-MS/MS Scan:7673, Noise threshold:0.4
Relative Intensity (%)
m/z
1018.580(PEP+HexNAc(1)_1)
815.501(PEP_1)
204.087(HexNAc)
138.055(HexNAc − 2H2O − H2O)
168.066(HexNAc − 2H2O)
366.141(HexNAc + Hex)
1110.009(PEP+HexNAc(3)Hex(4)Fuc(1)_2)
1164.639(PEP+HexNAc(1)Fuc(1)_1)
1691.828(PEP+HexNAc(2)Hex(2)Fuc(1)_1)
1853.882(PEP+HexNAc(2)Hex(3)Fuc(1)_1)
1894.908(PEP+HexNAc(3)Hex(2)Fuc(1)_1)
M+2H

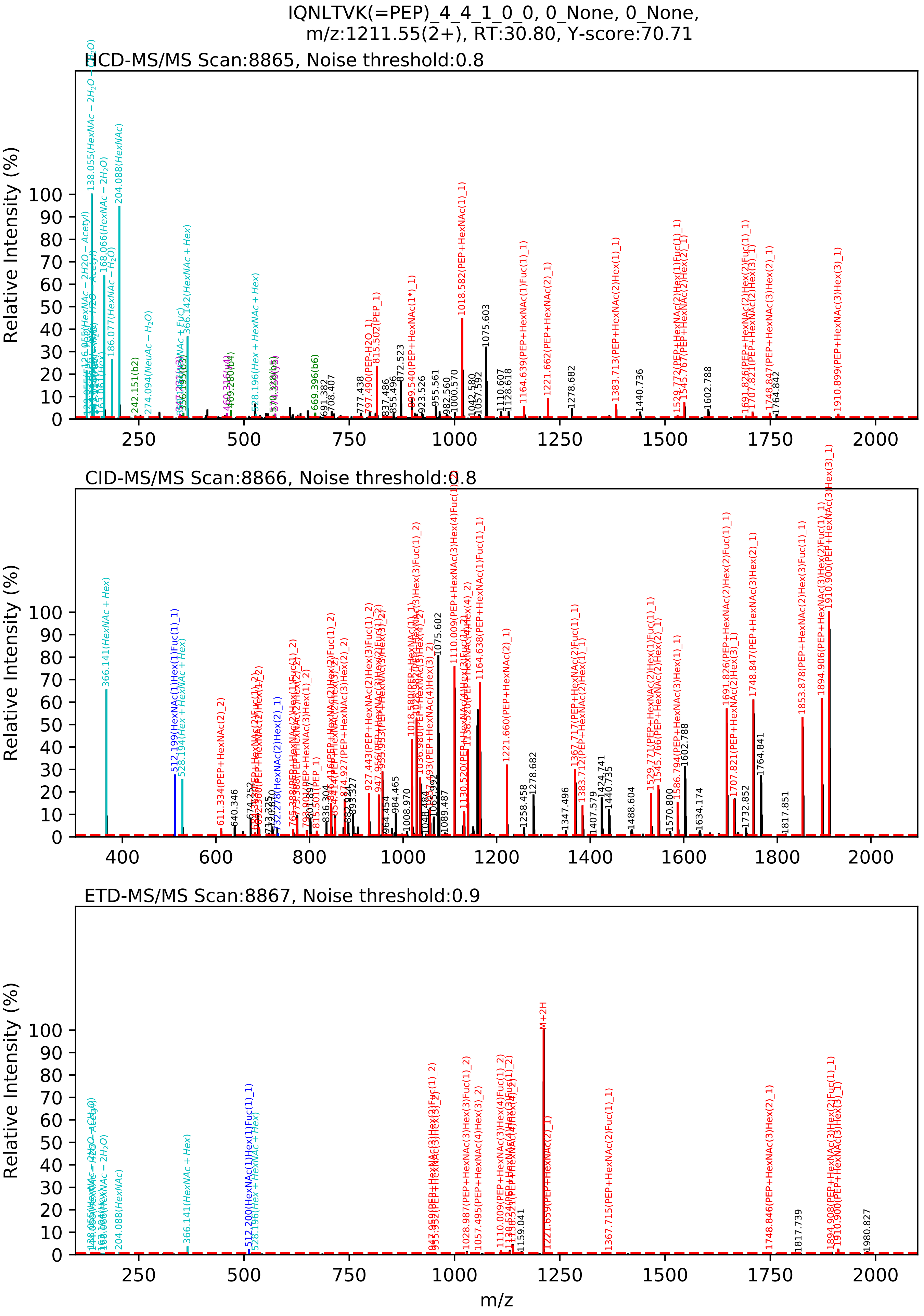

IQNLTVK(=PEP)_4_4_1_0_0, 0_None, 0_None,
m/z:1211.55(2+), RT:30.80, Y-score:70.71
HCD-MS/MS Scan:8865, Noise threshold:0.8
CID-MS/MS Scan:8866, Noise threshold:0.8
ETD-MS/MS Scan:8867, Noise threshold:0.9
Relative Intensity (%)
m/z
1018.582(PEP+HexNAc(1)_1)
1164.639(PEP+HexNAc(1)Fuc(1)_1)
1221.662(PEP+HexNAc(2)_1)
1383.713(PEP+HexNAc(2)Hex(1)_1)
1748.847(PEP+HexNAc(3)Hex(2)_1)
1910.899(PEP+HexNAc(3)Hex(3)_1)
1075.603
1110.009(PEP+HexNAc(3)Hex(4)Fuc(1)_2)
1110.009(PEP+HexNAc(3)Hex(4)Fuc(1)_2)
1164.638(PEP+HexNAc(1)Fuc(1)_1)
1221.660(PEP+HexNAc(2)_1)
1691.826(PEP+HexNAc(2)Hex(2)Fuc(1)_1)
1748.847(PEP+HexNAc(3)Hex(2)_1)
1853.878(PEP+HexNAc(2)Hex(3)Fuc(1)_1)
1894.906(PEP+HexNAc(3)Hex(2)Fuc(1)_1)
1910.900(PEP+HexNAc(3)Hex(3)_1)
366.141(HexNAc+Hex)
512.199(HexNAc(1)Hex(1)Fuc(1)_1)
M+2H
1221.659(PEP+HexNAc(2)_1)
1367.715(PEP+HexNAc(2)Fuc(1)_1)
1748.846(PEP+HexNAc(3)Hex(2)_1)

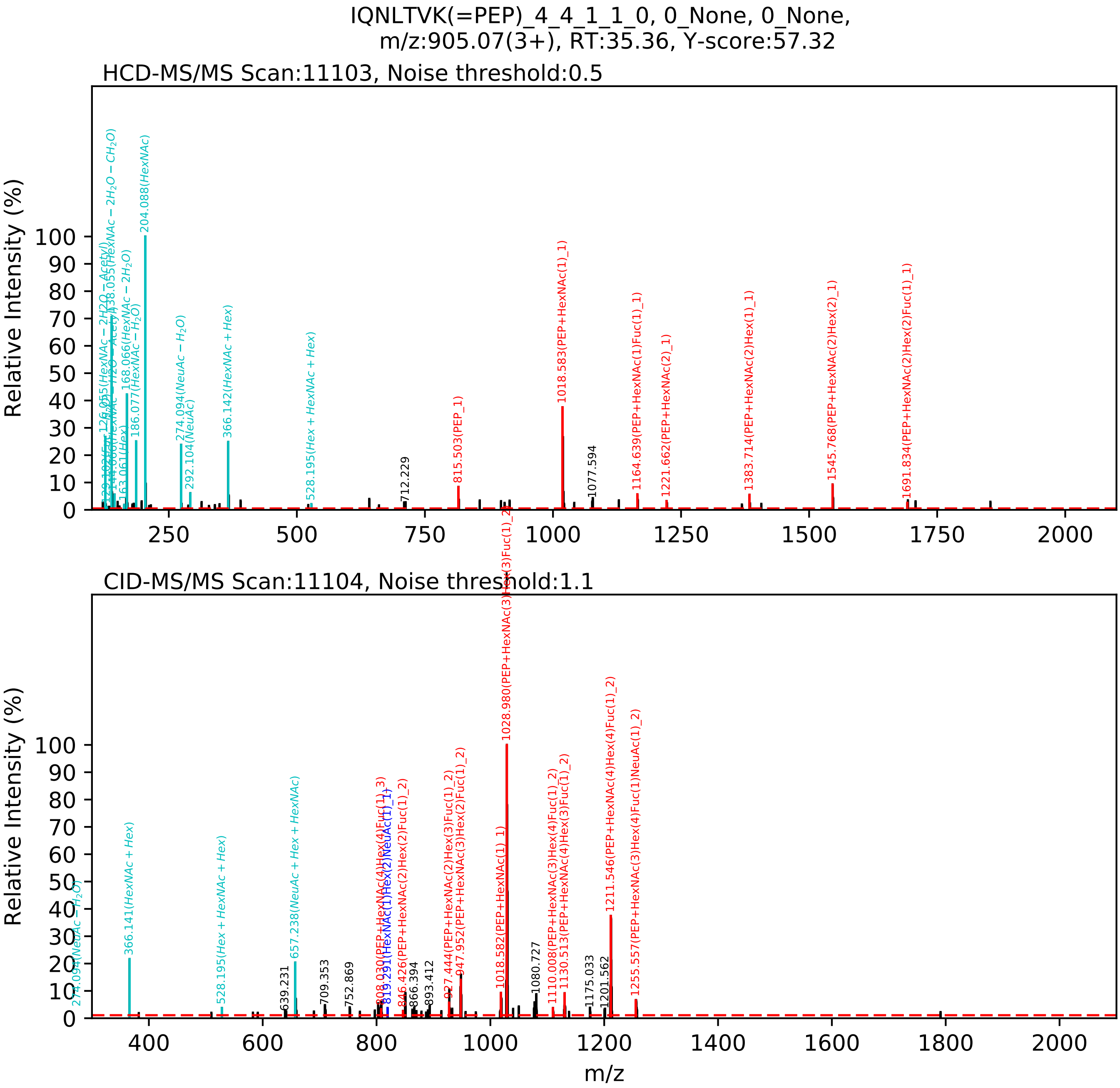

IQNLTVK(=PEP)_4_4_1_1_0, 0_None, 0_None,
m/z:905.07(3+), RT:35.36, Y-score:57.32
HCD-MS/MS Scan:11103, Noise threshold:0.5
Relative Intensity (%)
204.088(HexNAc)
186.077(HexNAc − H2O)
168.066(HexNAc − 2H2O)
274.094(NeuAc − H2O)
292.104(NeuAc)
366.142(HexNAc + Hex)
528.195(Hex + HexNAc + Hex)
712.229
815.503(PEP_1)
1018.583(PEP+HexNAc(1)_1)
1077.594
1164.639(PEP+HexNAc(1)Fuc(1)_1)
1221.662(PEP+HexNAc(2)_1)
1383.714(PEP+HexNAc(2)Hex(1)_1)
1545.768(PEP+HexNAc(2)Hex(2)_1)
1691.834(PEP+HexNAc(2)Hex(2)Fuc(1)_1)
CID-MS/MS Scan:11104, Noise threshold:1.1
1028.980(PEP+HexNAc(3)Hex(3)Fuc(1)_2)
274.094(NeuAc − H2O)
366.141(HexNAc + Hex)
528.195(Hex + HexNAc + Hex)
657.238(NeuAc + Hex + HexNAc)
639.231
709.353
752.869
808.030(PEP+HexNAc(4)Hex(4)Fuc(1)_3)
819.291(HexNAc(1)Hex(2)NeuAc(1)_1)
846.426(PEP+HexNAc(2)Hex(2)Fuc(1)_2)
866.394
893.412
927.444(PEP+HexNAc(2)Hex(3)Fuc(1)_2)
947.952(PEP+HexNAc(3)Hex(2)Fuc(1)_2)
1018.582(PEP+HexNAc(1)_1)
1080.727
1110.008(PEP+HexNAc(3)Hex(4)Fuc(1)_2)
1130.513(PEP+HexNAc(4)Hex(3)Fuc(1)_2)
1175.033
1201.562
1211.546(PEP+HexNAc(4)Hex(4)Fuc(1)_2)
1255.557(PEP+HexNAc(3)Hex(4)Fuc(1)NeuAc(1)_2)
m/z

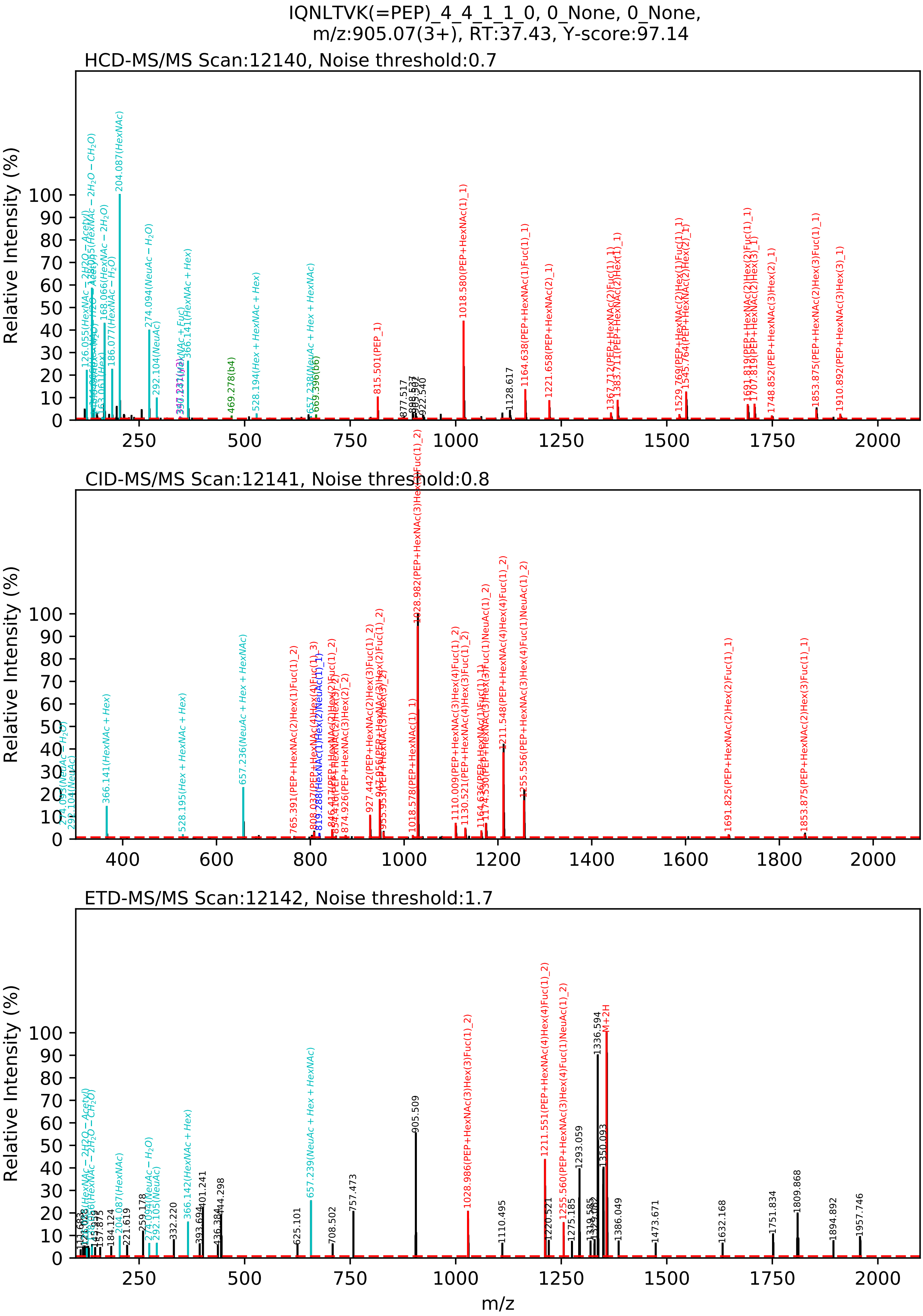
IQNLTVK(=PEP)_4_4_1_1_0, 0_None, 0_None,
m/z:905.07(3+), RT:37.43, Y-score:97.14
HCD-MS/MS Scan:12140, Noise threshold:0.7
CID-MS/MS Scan:12141, Noise threshold:0.8
ETD-MS/MS Scan:12142, Noise threshold:1.7
Relative Intensity (%)
m/z

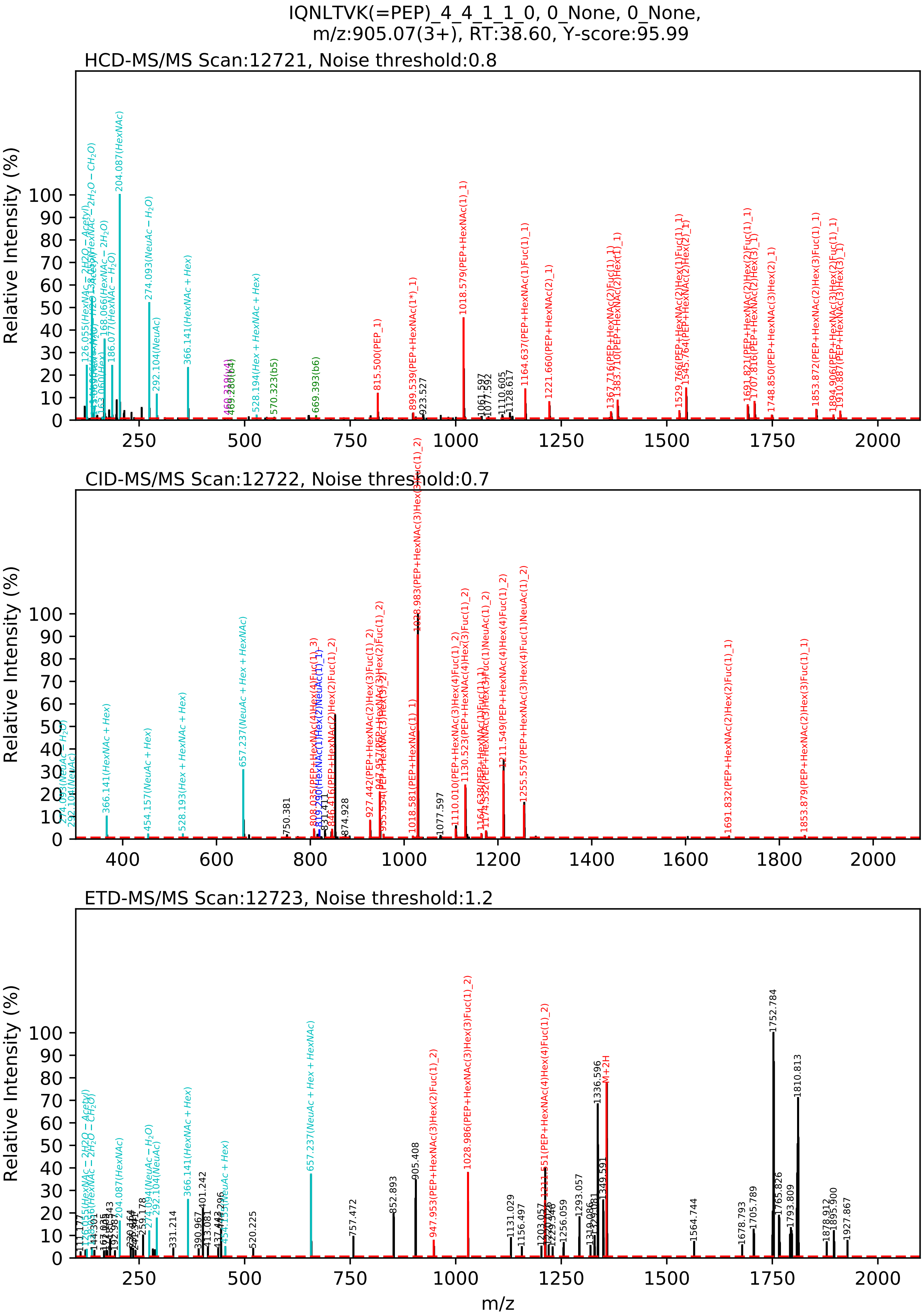
IQNLTVK(=PEP)_4_4_1_1_0, 0_None, 0_None,
m/z:905.07(3+), RT:38.60, Y-score:95.99
HCD-MS/MS Scan:12721, Noise threshold:0.8
CID-MS/MS Scan:12722, Noise threshold:0.7
ETD-MS/MS Scan:12723, Noise threshold:1.2
Relative Intensity (%)
m/z

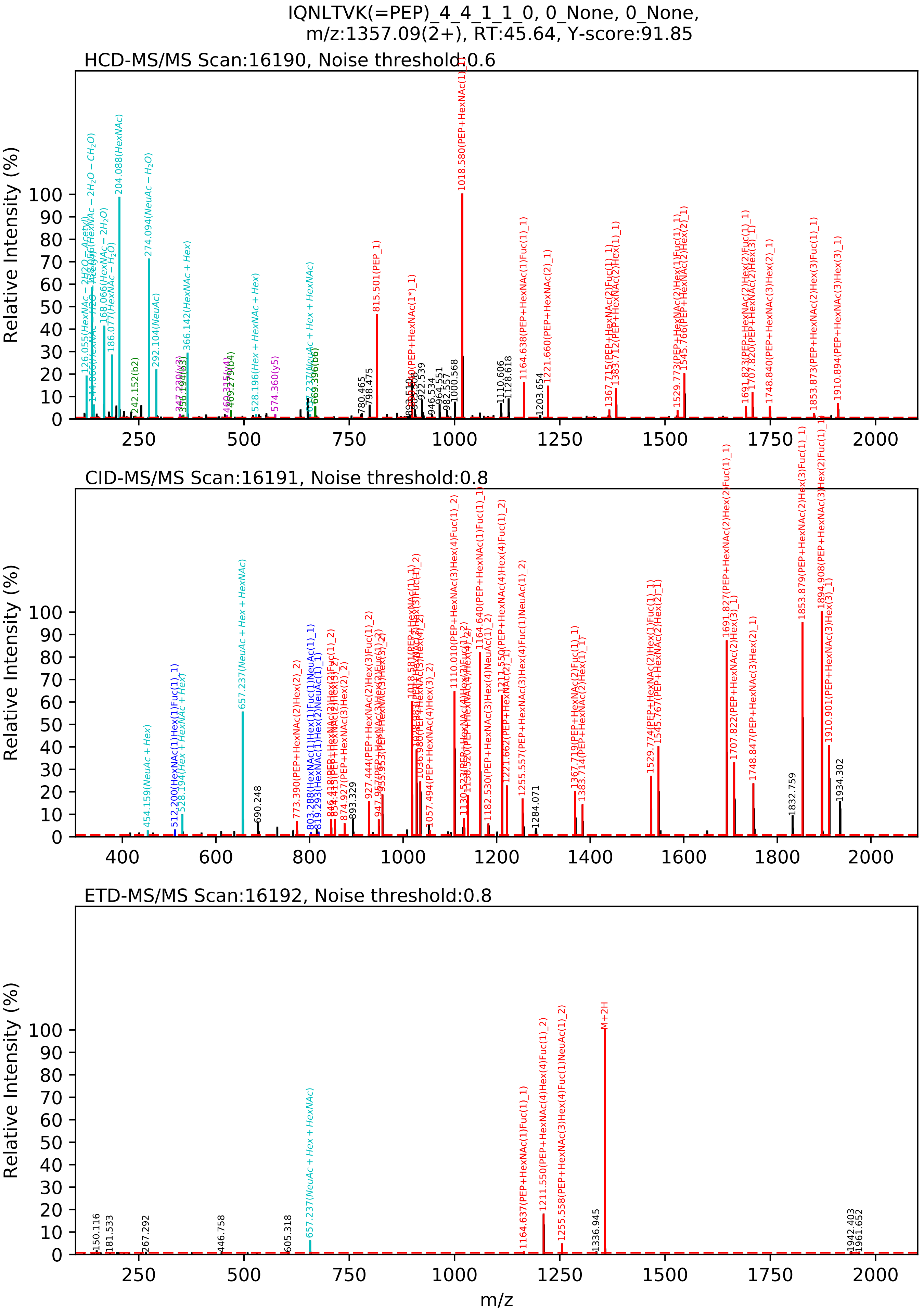

IQNLTVK(=PEP)_4_4_1_1_0, 0_None, 0_None,
m/z:1357.09(2+), RT:45.64, Y-score:91.85
HCD-MS/MS Scan:16190, Noise threshold:0.6
CID-MS/MS Scan:16191, Noise threshold:0.8
ETD-MS/MS Scan:16192, Noise threshold:0.8
Relative Intensity (%)
m/z

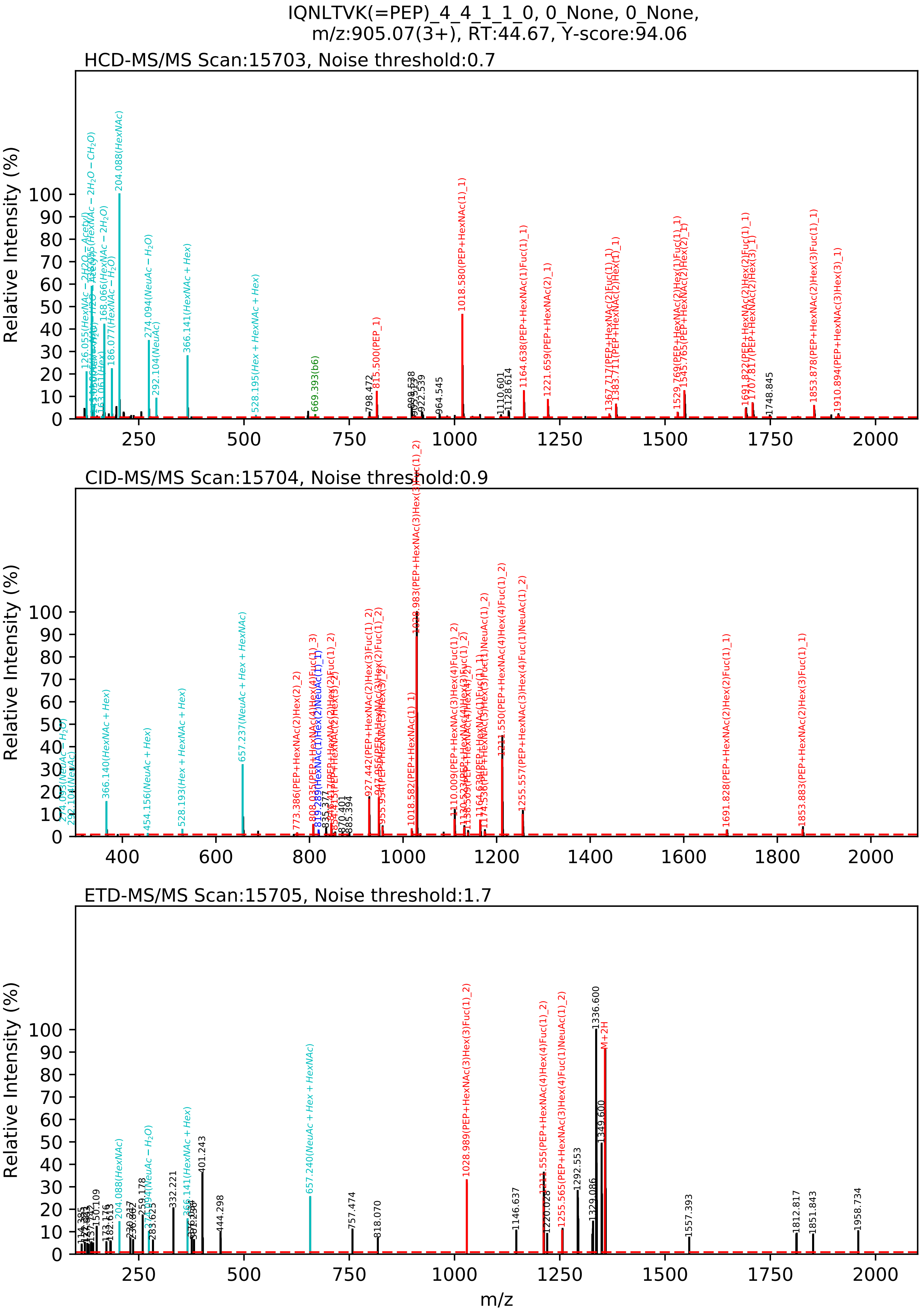

IQNLTVK(=PEP)_4_4_1_1_0, 0_None, 0_None,
m/z:905.07(3+), RT:44.67, Y-score:94.06
HCD-MS/MS Scan:15703, Noise threshold:0.7
CID-MS/MS Scan:15704, Noise threshold:0.9
ETD-MS/MS Scan:15705, Noise threshold:1.7
Relative Intensity (%)
m/z
1018.580(PEP+HexNAc(1)_1)
204.088(HexNAc)
366.141(HexNAc+Hex)
1028.983(PEP+HexNAc(3)Hex(3)Fuc(1)_2)
1211.550(PEP+HexNAc(4)Hex(4)Fuc(1)_2)
657.237(NeuAc+Hex+HexNAc)
1336.600
M+2H
1028.989(PEP+HexNAc(3)Hex(3)Fuc(1)_2)

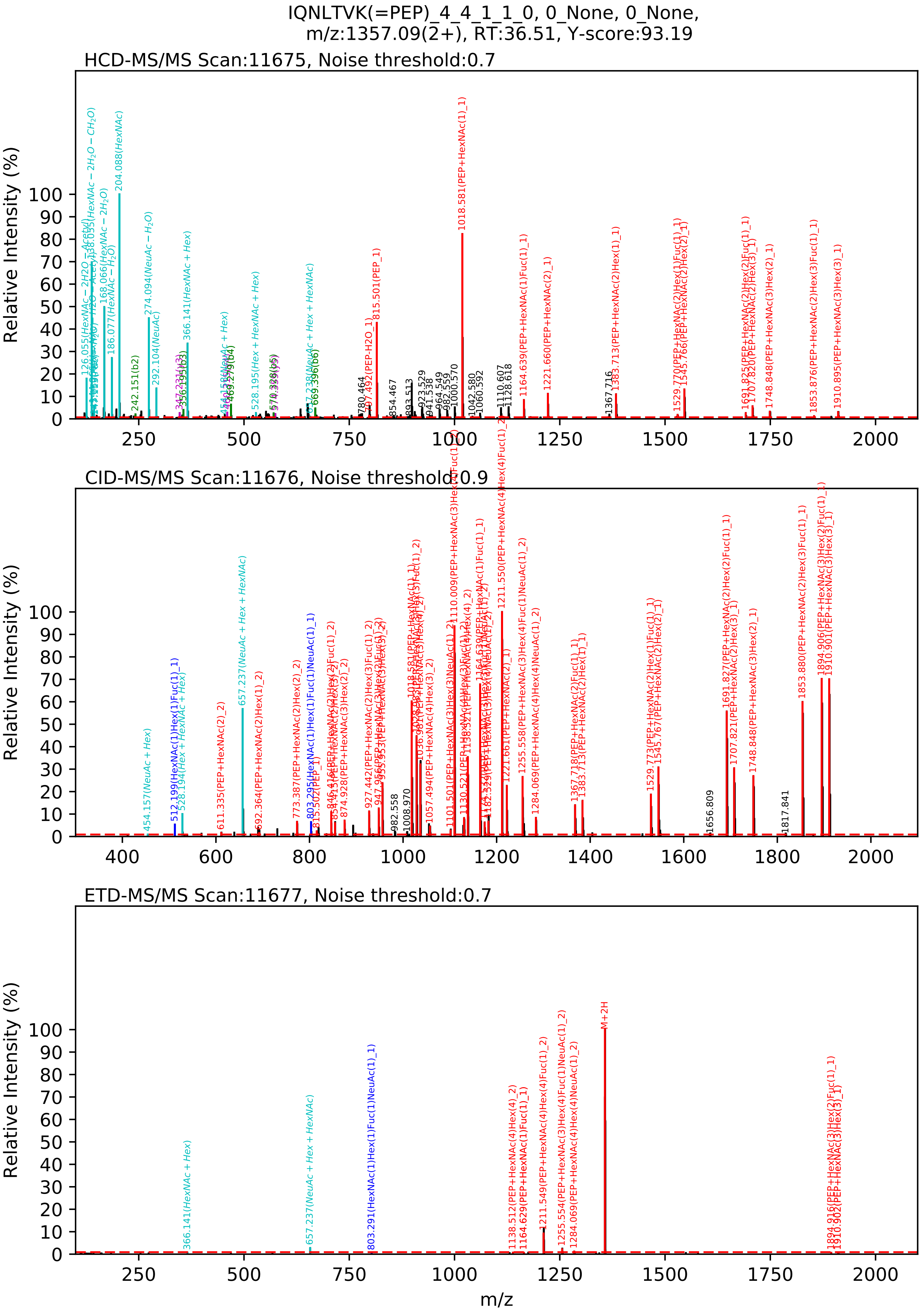

IQNLTVK(=PEP)_4_4_1_1_0, 0_None, 0_None,
m/z:1357.09(2+), RT:36.51, Y-score:93.19
HCD-MS/MS Scan:11675, Noise threshold:0.7
Relative Intensity (%)
204.088(HexNAc)
186.077(HexNAc − H2O)
168.066(HexNAc − 2H2O)
274.094(NeuAc − H2O)
292.104(NeuAc)
366.141(HexNAc + Hex)
242.151(b2)
528.195(Hex + HexNAc + Hex)
669.396(b6)
815.501(PEP_1)
797.492(PEP-H2O_1)
1018.581(PEP+HexNAc(1)_1)
1164.639(PEP+HexNAc(1)Fuc(1)_1)
1221.660(PEP+HexNAc(2)_1)
1383.713(PEP+HexNAc(2)Hex(1)_1)
1545.766(PEP+HexNAc(2)Hex(2)_1)
1707.820(PEP+HexNAc(2)Hex(3)_1)
1748.848(PEP+HexNAc(3)Hex(2)_1)
1853.876(PEP+HexNAc(2)Hex(3)Fuc(1)_1)
1910.895(PEP+HexNAc(3)Hex(3)_1)
CID-MS/MS Scan:11676, Noise threshold:0.9
454.157(NeuAc + Hex)
512.199(HexNAc(1)Hex(1)Fuc(1)_1)
528.194(Hex + HexNAc + Hex)
611.335(PEP+HexNAc(2)_2)
657.237(NeuAc + Hex + HexNAc)
692.364(PEP+HexNAc(2)Hex(1)_2)
773.387(PEP+HexNAc(2)Hex(2)_2)
803.295(HexNAc(1)Hex(1)Fuc(1)NeuAc(1)_1)
815.502(PEP_1)
874.928(PEP+HexNAc(3)Hex(2)_2)
927.442(PEP+HexNAc(2)Hex(3)Fuc(1)_2)
1036.981(PEP+HexNAc(4)Hex(3)_2)
1057.494(PEP+HexNAc(4)Hex(3)_2)
1101.501(PEP+HexNAc(3)Hex(3)NeuAc(1)_2)
1110.009(PEP+HexNAc(3)Hex(4)Fuc(1)_2)
1211.550(PEP+HexNAc(4)Hex(4)Fuc(1)_2)
1221.661(PEP+HexNAc(2)_1)
1255.558(PEP+HexNAc(3)Hex(4)Fuc(1)NeuAc(1)_2)
1284.069(PEP+HexNAc(4)Hex(4)NeuAc(1)_2)
1367.718(PEP+HexNAc(2)Fuc(1)_1)
1383.713(PEP+HexNAc(2)Hex(1)_1)
1529.773(PEP+HexNAc(2)Hex(1)Fuc(1)_1)
1545.767(PEP+HexNAc(2)Hex(2)_1)
1656.809
1691.827(PEP+HexNAc(2)Hex(2)Fuc(1)_1)
1707.821(PEP+HexNAc(2)Hex(3)_1)
1748.848(PEP+HexNAc(3)Hex(2)_1)
1817.841
1853.880(PEP+HexNAc(2)Hex(3)Fuc(1)_1)
1894.906(PEP+HexNAc(3)Hex(2)Fuc(1)_1)
1910.901(PEP+HexNAc(3)Hex(3)_1)
ETD-MS/MS Scan:11677, Noise threshold:0.7
366.141(HexNAc + Hex)
657.237(NeuAc + Hex + HexNAc)
803.291(HexNAc(1)Hex(1)Fuc(1)NeuAc(1)_1)
1138.512(PEP+HexNAc(4)Hex(4)_2)
1164.629(PEP+HexNAc(1)Fuc(1)_1)
1211.549(PEP+HexNAc(4)Hex(4)Fuc(1)_2)
1255.554(PEP+HexNAc(3)Hex(4)Fuc(1)NeuAc(1)_2)
1284.069(PEP+HexNAc(4)Hex(4)NeuAc(1)_2)
M+2H
1894.916(PEP+HexNAc(3)Hex(2)Fuc(1)_1)
1910.902(PEP+HexNAc(3)Hex(3)_1)
m/z

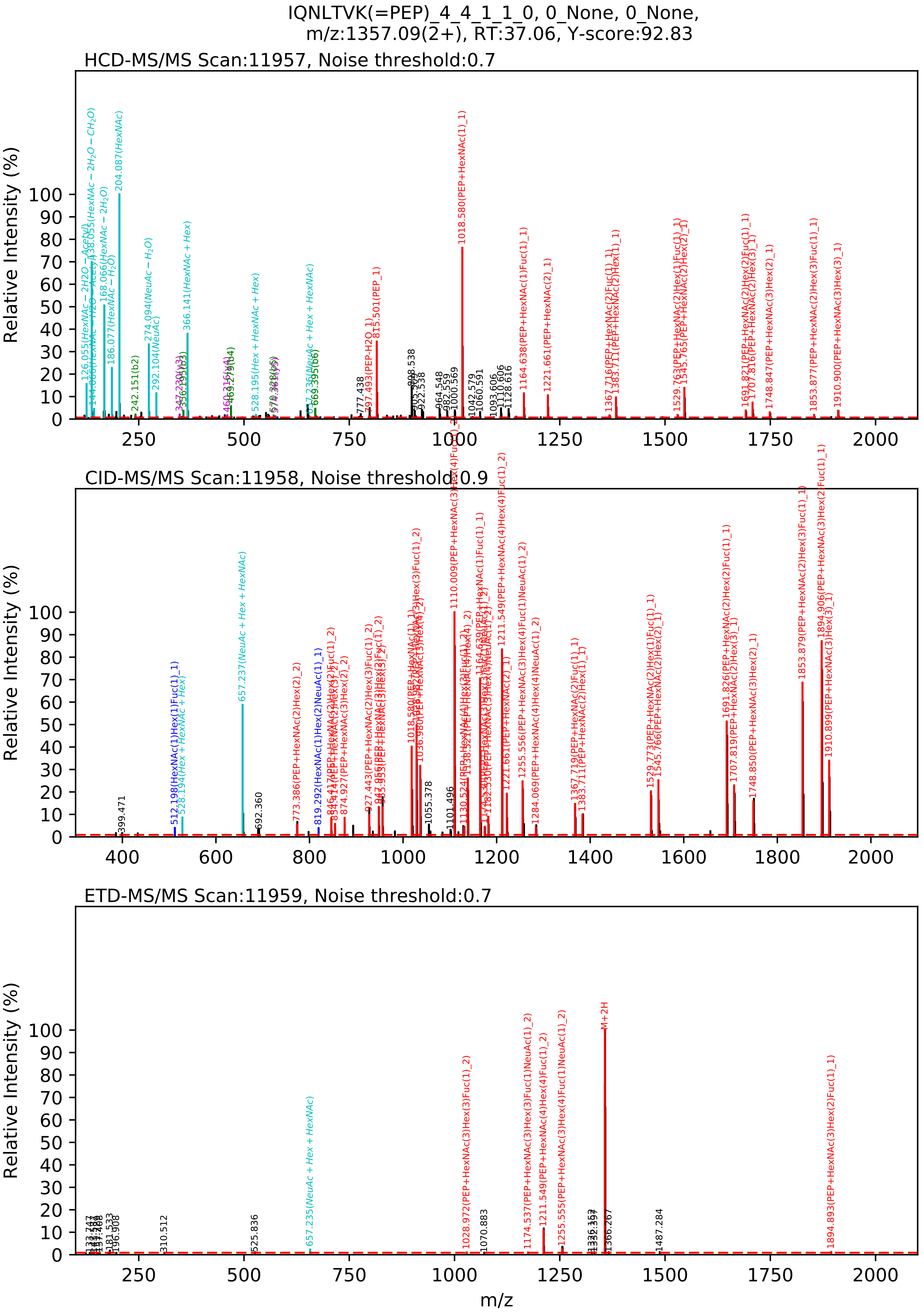
IQNLTVK(=PEP)_4_4_1_1_0, 0_None, 0_None,
m/z:1357.09(2+), RT:37.06, Y-score:92.83
HCD-MS/MS Scan:11957, Noise threshold:0.7
Relative Intensity (%)
204.087(HexNAc)
1018.580(PEP+HexNAc(1)_1)
815.501(PEP_1)
366.141(HexNAc + Hex)
274.094(NeuAc − H2O)
292.104(NeuAc)
168.066(HexNAc − 2H2O)
186.077(HexNAc − H2O)
242.151(b2)
528.195(Hex + HexNAc + Hex)
1164.638(PEP+HexNAc(1)Fuc(1)_1)
1221.661(PEP+HexNAc(2)_1)
1383.711(PEP+HexNAc(2)Hex(1)_1)
1545.765(PEP+HexNAc(2)Hex(2)_1)
1748.847(PEP+HexNAc(3)Hex(2)_1)
1853.877(PEP+HexNAc(2)Hex(3)Fuc(1)_1)
1910.900(PEP+HexNAc(3)Hex(3)_1)
CID-MS/MS Scan:11958, Noise threshold:0.9
1110.009(PEP+HexNAc(3)Hex(4)Fuc(1)_2)
657.237(NeuAc + Hex + HexNAc)
512.198(HexNAc(1)Hex(1)Fuc(1)_1)
819.292(HexNAc(1)Hex(2)NeuAc(1)_1)
773.386(PEP+HexNAc(2)Hex(2)_2)
1211.549(PEP+HexNAc(4)Hex(4)Fuc(1)_2)
1255.556(PEP+HexNAc(3)Hex(4)Fuc(1)NeuAc(1)_2)
1367.719(PEP+HexNAc(2)Fuc(1)_1)
1529.773(PEP+HexNAc(2)Hex(1)Fuc(1)_1)
1691.826(PEP+HexNAc(2)Hex(2)Fuc(1)_1)
1853.879(PEP+HexNAc(2)Hex(3)Fuc(1)_1)
1894.906(PEP+HexNAc(3)Hex(2)Fuc(1)_1)
1910.899(PEP+HexNAc(3)Hex(3)_1)
ETD-MS/MS Scan:11959, Noise threshold:0.7
M+2H
1028.972(PEP+HexNAc(3)Hex(3)Fuc(1)_2)
1174.537(PEP+HexNAc(3)Hex(3)Fuc(1)NeuAc(1)_2)
1211.549(PEP+HexNAc(4)Hex(4)Fuc(1)_2)
1255.555(PEP+HexNAc(3)Hex(4)Fuc(1)NeuAc(1)_2)
1894.893(PEP+HexNAc(3)Hex(2)Fuc(1)_1)
657.235(NeuAc + Hex + HexNAc)
m/z

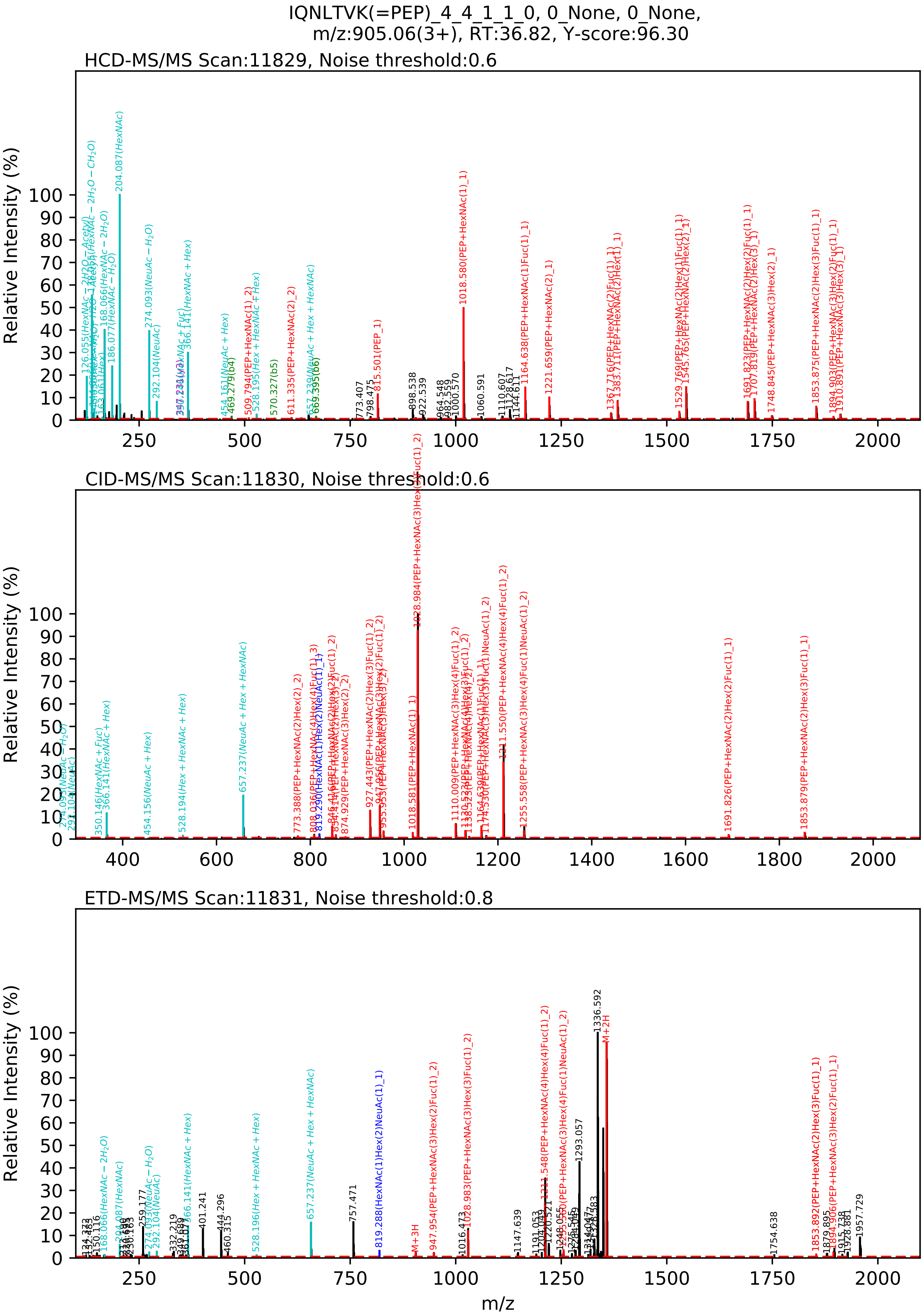
IQNLTVK(=PEP)_4_4_1_1_0, 0_None, 0_None,
m/z:905.06(3+), RT:36.82, Y-score:96.30
HCD-MS/MS Scan:11829, Noise threshold:0.6
Relative Intensity (%)
204.087(HexNAc)
274.093(NeuAc − H2O)
292.104(NeuAc)
366.141(HexNAc + Hex)
1018.580(PEP+HexNAc(1)_1)
1164.638(PEP+HexNAc(1)Fuc(1)_1)
1221.659(PEP+HexNAc(2)_1)
1545.765(PEP+HexNAc(2)Hex(2)_1)
1707.819(PEP+HexNAc(2)Hex(3)_1)
1853.875(PEP+HexNAc(2)Hex(3)Fuc(1)_1)
CID-MS/MS Scan:11830, Noise threshold:0.6
1028.984(PEP+HexNAc(3)Hex(3)Fuc(1)_2)
1211.550(PEP+HexNAc(4)Hex(4)Fuc(1)_2)
1255.558(PEP+HexNAc(3)Hex(4)Fuc(1)NeuAc(1)_2)
1691.826(PEP+HexNAc(2)Hex(2)Fuc(1)_1)
1853.879(PEP+HexNAc(2)Hex(3)Fuc(1)_1)
ETD-MS/MS Scan:11831, Noise threshold:0.8
1336.592
M+2H
1293.057
757.471
657.237(NeuAc + Hex + HexNAc)
819.288(HexNAc(1)Hex(2)NeuAc(1)_1)
1028.983(PEP+HexNAc(3)Hex(3)Fuc(1)_2)
1957.729
m/z

IQNLTVK(=PEP)_4_4_1_1_0, 0_None, 0_None,
m/z:1357.10(2+), RT:37.65, Y-score:92.60

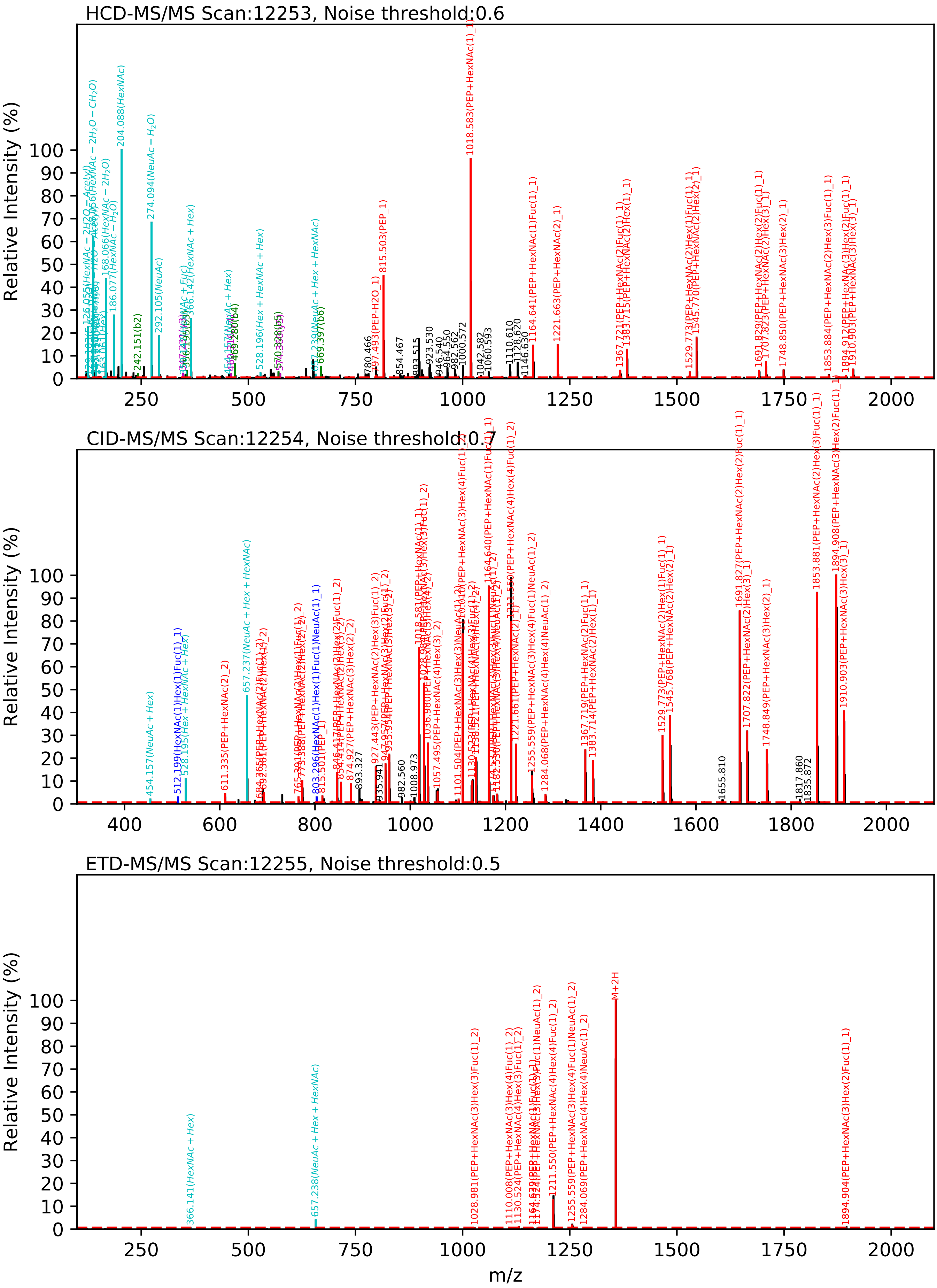

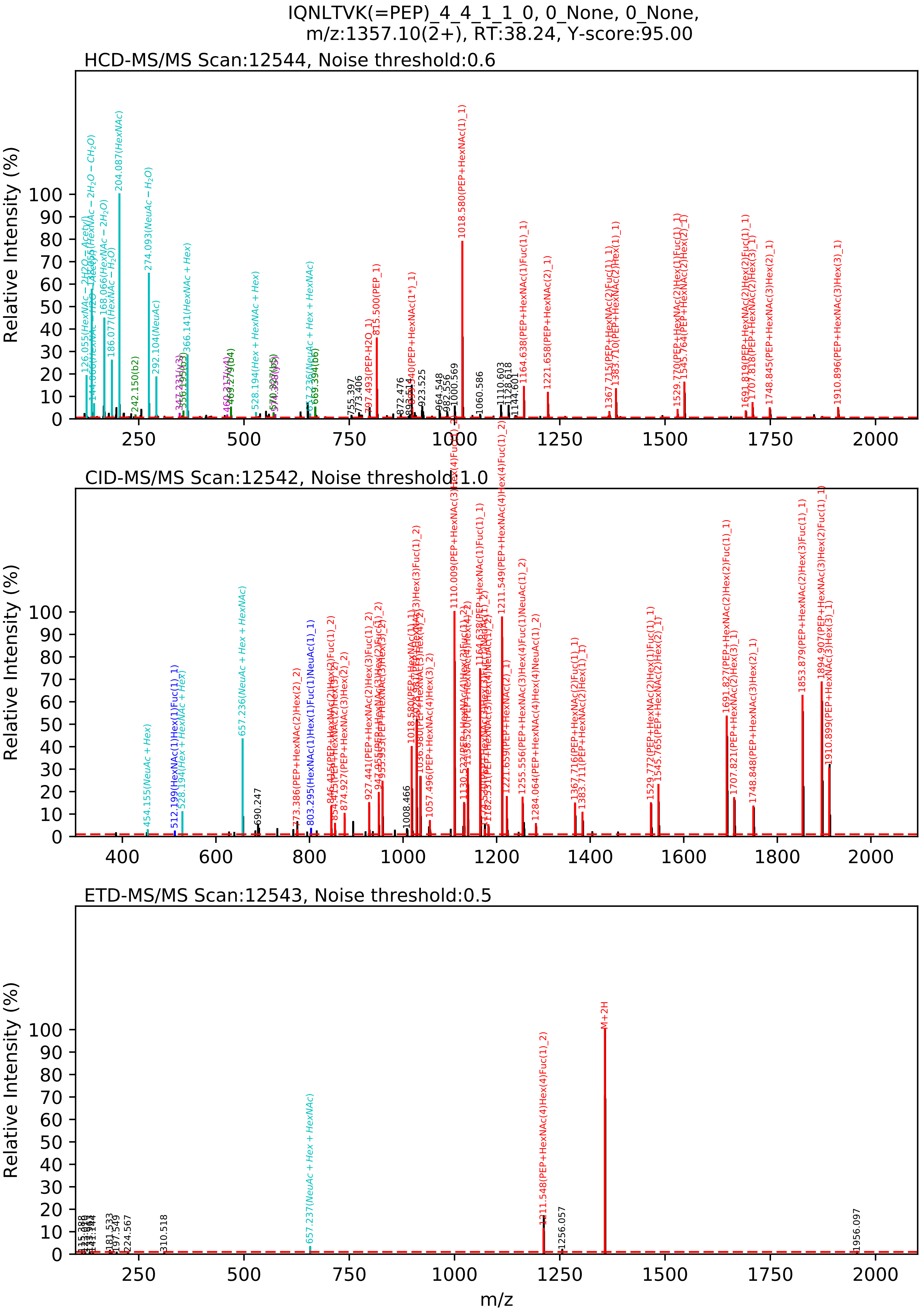
IQNLTVK(=PEP)_4_4_1_1_0, 0_None, 0_None,
m/z:1357.10(2+), RT:38.24, Y-score:95.00
HCD-MS/MS Scan:12544, Noise threshold:0.6
CID-MS/MS Scan:12542, Noise threshold:1.0
ETD-MS/MS Scan:12543, Noise threshold:0.5
Relative Intensity (%)
m/z
1018.580(PEP+HexNAc(1)_1)
815.500(PEP_1)
204.087(HexNAc)
274.093(NeuAc − H2O)
1110.009(PEP+HexNAc(3)Hex(4)Fuc(1)_2)
1211.549(PEP+HexNAc(4)Hex(4)Fuc(1)_2)
1853.879(PEP+HexNAc(2)Hex(3)Fuc(1)_1)
1894.907(PEP+HexNAc(3)Hex(2)Fuc(1)_1)
1691.827(PEP+HexNAc(2)Hex(2)Fuc(1)_1)
657.236(NeuAc + Hex + HexNAc)
M+2H
1211.548(PEP+HexNAc(4)Hex(4)Fuc(1)_2)
1956.097

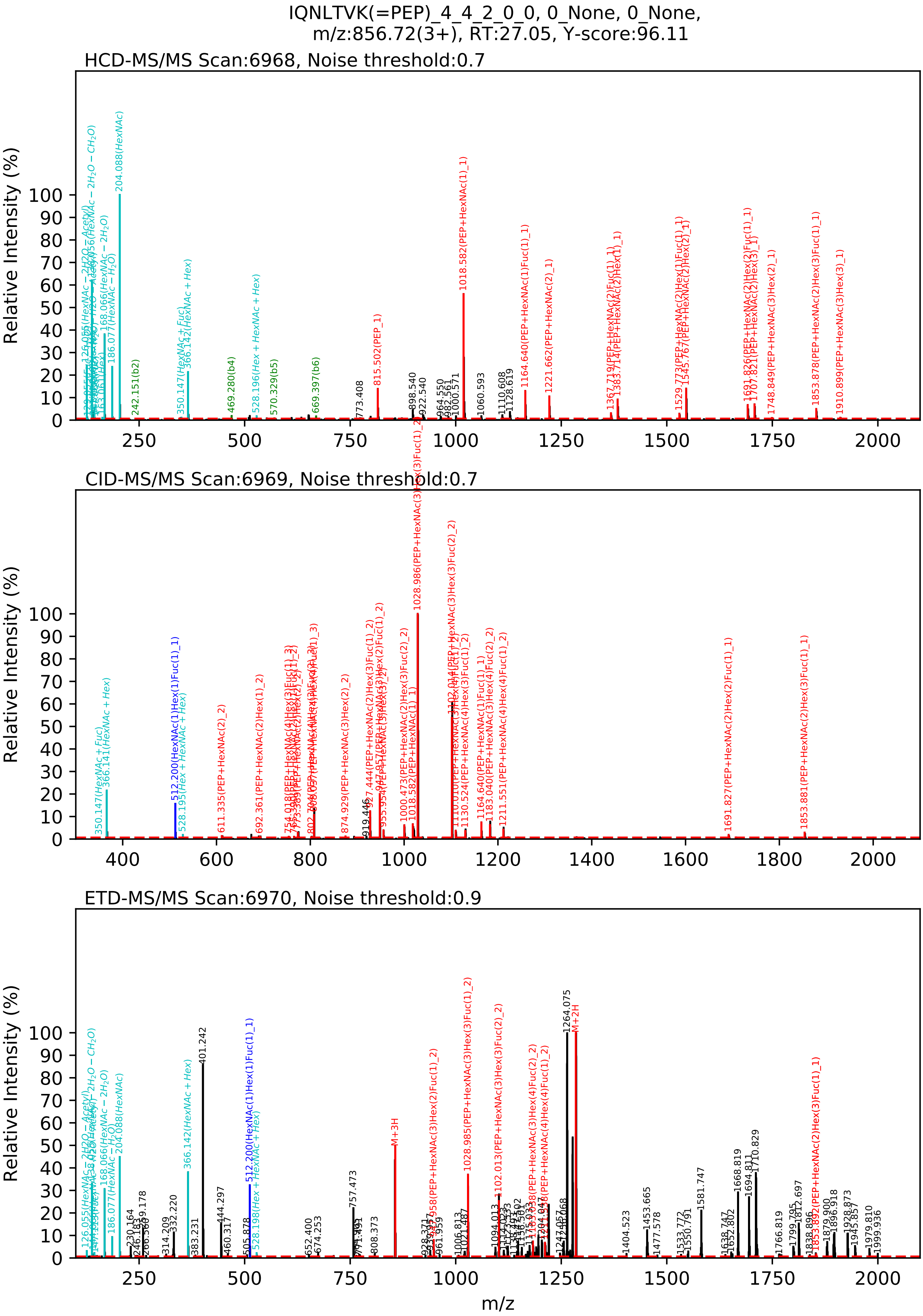
IQNLTVK(=PEP)_4_4_2_0_0, 0_None, 0_None,
m/z:856.72(3+), RT:27.05, Y-score:96.11
HCD-MS/MS Scan:6968, Noise threshold:0.7
Relative Intensity (%)
204.088(HexNAc)
168.066(HexNAc − 2H2O)
186.077(HexNAc − H2O)
366.142(HexNAc + Hex)
350.147(HexNAc + Fuc)
242.151(b2)
469.280(b4)
528.196(Hex + HexNAc + Hex)
570.329(b5)
669.397(b6)
773.408
815.502(PEP_1)
898.540
922.540
964.550
1000.571
1018.582(PEP+HexNAc(1)_1)
1060.593
1110.608
1128.619
1164.640(PEP+HexNAc(1)Fuc(1)_1)
1221.662(PEP+HexNAc(2)_1)
1367.719(PEP+HexNAc(2)Fuc(1)_1)
1383.714(PEP+HexNAc(2)Hex(1)_1)
1529.773(PEP+HexNAc(2)Hex(1)Fuc(1)_1)
1545.767(PEP+HexNAc(2)Hex(2)_1)
1691.826(PEP+HexNAc(2)Hex(2)Fuc(1)_1)
1707.821(PEP+HexNAc(2)Hex(3)_1)
1748.849(PEP+HexNAc(3)Hex(2)_1)
1853.878(PEP+HexNAc(2)Hex(3)Fuc(1)_1)
1910.899(PEP+HexNAc(3)Hex(3)_1)
CID-MS/MS Scan:6969, Noise threshold:0.7
350.147(HexNAc + Fuc)
366.141(HexNAc + Hex)
512.200(HexNAc(1)Hex(1)Fuc(1)_1)
528.195(Hex + HexNAc + Hex)
611.335(PEP+HexNAc(2)_2)
692.361(PEP+HexNAc(2)Hex(1)_2)
874.929(PEP+HexNAc(3)Hex(2)_2)
919.446
927.444(PEP+HexNAc(2)Hex(3)Fuc(1)_2)
1000.473(PEP+HexNAc(2)Hex(3)Fuc(2)_2)
1018.582(PEP+HexNAc(1)_1)
1028.986(PEP+HexNAc(3)Hex(3)Fuc(1)_2)
1102.014(PEP+HexNAc(3)Hex(3)Fuc(2)_2)
1130.524(PEP+HexNAc(4)Hex(3)Fuc(1)_2)
1164.640(PEP+HexNAc(1)Fuc(1)_1)
1183.040(PEP+HexNAc(3)Hex(4)Fuc(2)_2)
1211.551(PEP+HexNAc(4)Hex(4)Fuc(1)_2)
1691.827(PEP+HexNAc(2)Hex(2)Fuc(1)_1)
1853.881(PEP+HexNAc(2)Hex(3)Fuc(1)_1)
ETD-MS/MS Scan:6970, Noise threshold:0.9
204.088(HexNAc)
168.066(HexNAc − 2H2O)
186.077(HexNAc − H2O)
230.164
246.183
259.178
266.560
314.209
332.220
366.142(HexNAc + Hex)
383.231
401.242
444.297
460.317
505.878
512.200(HexNAc(1)Hex(1)Fuc(1)_1)
528.198(Hex + HexNAc + Hex)
652.400
674.253
757.473
808.373
M+3H
928.371
961.959
1006.813
1021.487
1028.985(PEP+HexNAc(3)Hex(3)Fuc(1)_2)
1094.013
1102.013(PEP+HexNAc(3)Hex(3)Fuc(2)_2)
1247.057
1264.075
M+2H
1404.523
1453.665
1477.578
1533.772
1550.791
1581.747
1638.747
1652.802
1668.819
1694.811
1710.829
1766.819
1799.795
1812.697
1838.896
1853.892(PEP+HexNAc(2)Hex(3)Fuc(1)_1)
1879.900
1896.918
1928.873
1945.857
1979.810
1999.936
m/z

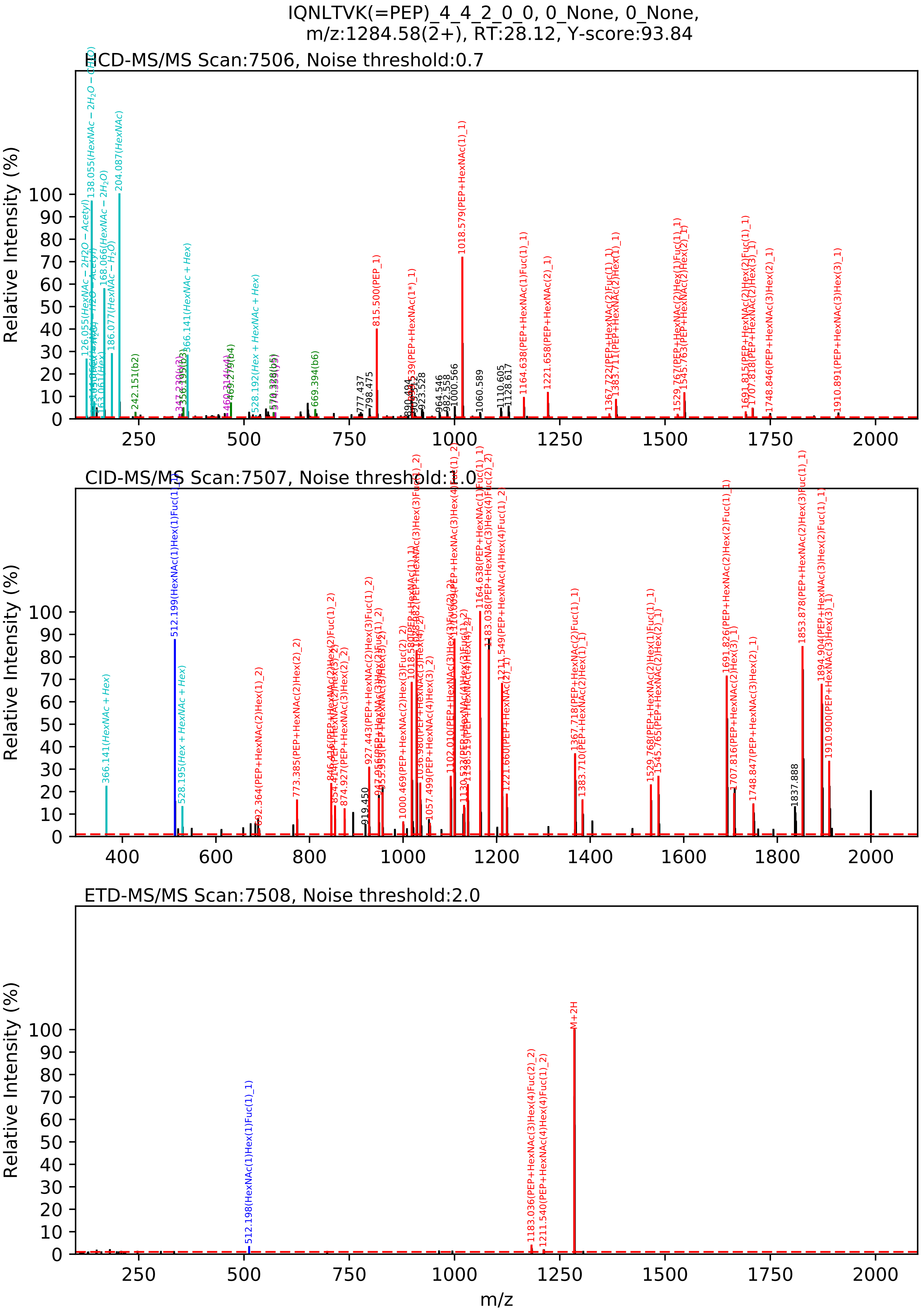
IQNLTVK(=PEP)_4_4_2_0_0, 0_None, 0_None,
m/z:1284.58(2+), RT:28.12, Y-score:93.84
HCD-MS/MS Scan:7506, Noise threshold:0.7
Relative Intensity (%)
204.087(HexNAc)
138.055(HexNAc − 2H2O − CH2O)
168.066(HexNAc − 2H2O)
186.077(HexNAc − H2O)
366.141(HexNAc + Hex)
528.192(Hex + HexNAc + Hex)
242.151(b2)
469.279(b4)
669.394(b6)
815.500(PEP_1)
1018.579(PEP+HexNAc(1)_1)
1164.638(PEP+HexNAc(1)Fuc(1)_1)
1221.658(PEP+HexNAc(2)_1)
1383.711(PEP+HexNAc(2)Hex(1)_1)
1545.763(PEP+HexNAc(2)Hex(2)_1)
1748.846(PEP+HexNAc(3)Hex(2)_1)
1910.891(PEP+HexNAc(3)Hex(3)_1)
777.437
798.475
923.528
964.546
1000.566
1060.589
1110.605
1128.617
CID-MS/MS Scan:7507, Noise threshold:1.0
512.199(HexNAc(1)Hex(1)Fuc(1)_1)
366.141(HexNAc + Hex)
528.195(Hex + HexNAc + Hex)
692.364(PEP+HexNAc(2)Hex(1)_2)
773.385(PEP+HexNAc(2)Hex(2)_2)
874.927(PEP+HexNAc(3)Hex(2)_2)
927.443(PEP+HexNAc(2)Hex(3)Fuc(1)_2)
1000.469(PEP+HexNAc(2)Hex(3)Fuc(2)_2)
1036.980(PEP+HexNAc(3)Hex(3)_2)
1057.499(PEP+HexNAc(4)Hex(3)_2)
1164.638(PEP+HexNAc(1)Fuc(1)_1)
1183.038(PEP+HexNAc(3)Hex(4)Fuc(2)_2)
1211.549(PEP+HexNAc(4)Hex(4)Fuc(1)_2)
1221.660(PEP+HexNAc(2)_1)
1367.718(PEP+HexNAc(2)Fuc(1)_1)
1383.710(PEP+HexNAc(2)Hex(1)_1)
1529.768(PEP+HexNAc(2)Hex(1)Fuc(1)_1)
1545.765(PEP+HexNAc(2)Hex(2)_1)
1691.826(PEP+HexNAc(2)Hex(2)Fuc(1)_1)
1707.816(PEP+HexNAc(2)Hex(3)_1)
1748.847(PEP+HexNAc(3)Hex(2)_1)
1853.878(PEP+HexNAc(2)Hex(3)Fuc(1)_1)
1894.904(PEP+HexNAc(3)Hex(2)Fuc(1)_1)
1910.900(PEP+HexNAc(3)Hex(3)_1)
919.450
1837.888
ETD-MS/MS Scan:7508, Noise threshold:2.0
512.198(HexNAc(1)Hex(1)Fuc(1)_1)
1183.036(PEP+HexNAc(3)Hex(4)Fuc(2)_2)
1211.540(PEP+HexNAc(4)Hex(4)Fuc(1)_2)
M+2H
m/z

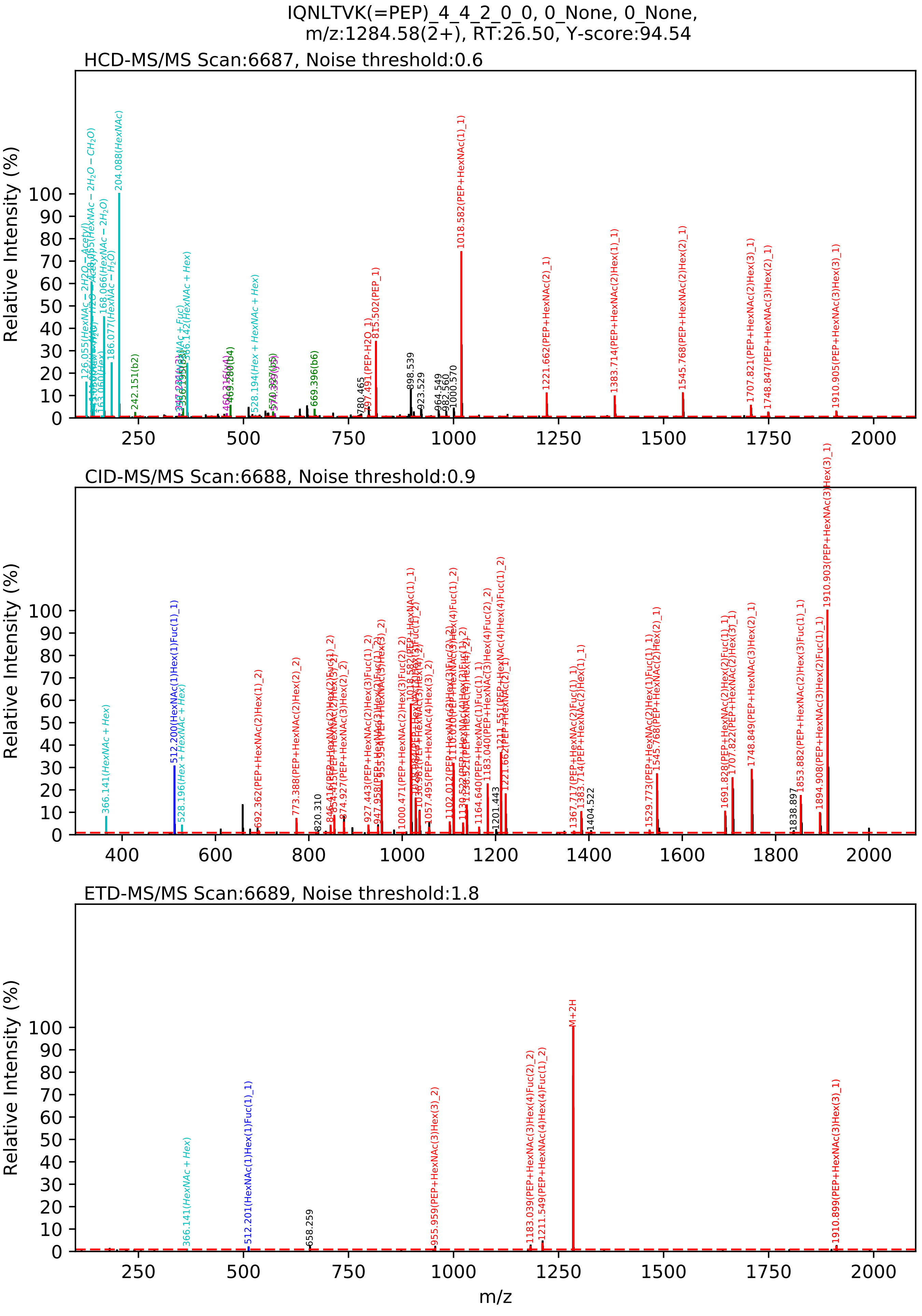

IQNLTVK(=PEP)_4_4_2_0_0, 0_None, 0_None,
m/z:1284.58(2+), RT:26.50, Y-score:94.54
HCD-MS/MS Scan:6687, Noise threshold:0.6
Relative Intensity (%)
204.088(HexNAc)
1018.582(PEP+HexNAc(1)_1)
815.502(PEP_1)
1221.662(PEP+HexNAc(2)_1)
1383.714(PEP+HexNAc(2)Hex(1)_1)
1545.768(PEP+HexNAc(2)Hex(2)_1)
1707.821(PEP+HexNAc(2)Hex(3)_1)
1748.847(PEP+HexNAc(3)Hex(2)_1)
1910.905(PEP+HexNAc(3)Hex(3)_1)
669.396(b6)
242.151(b2)
469.280(b4)
528.194(Hex + HexNAc + Hex)
898.539
923.529
964.549
1000.570
CID-MS/MS Scan:6688, Noise threshold:0.9
Relative Intensity (%)
1910.903(PEP+HexNAc(3)Hex(3)_1)
512.200(HexNAc(1)Hex(1)Fuc(1)_1)
366.141(HexNAc + Hex)
528.196(Hex + HexNAc + Hex)
692.362(PEP+HexNAc(2)Hex(1)_2)
773.388(PEP+HexNAc(2)Hex(2)_2)
874.927(PEP+HexNAc(3)Hex(2)_2)
927.443(PEP+HexNAc(2)Hex(3)Fuc(1)_2)
955.954(PEP+HexNAc(3)Hex(3)_2)
1000.471(PEP+HexNAc(2)Hex(3)Fuc(2)_2)
1057.495(PEP+HexNAc(4)Hex(3)_2)
1164.640(PEP+HexNAc(1)Fuc(1)_1)
1183.040(PEP+HexNAc(3)Hex(4)Fuc(2)_2)
1211.551(PEP+HexNAc(4)Hex(4)Fuc(1)_2)
1221.662(PEP+HexNAc(2)_1)
1367.717(PEP+HexNAc(2)Fuc(1)_1)
1383.714(PEP+HexNAc(2)Hex(1)_1)
1404.522
1529.773(PEP+HexNAc(2)Hex(1)Fuc(1)_1)
1545.768(PEP+HexNAc(2)Hex(2)_1)
1691.828(PEP+HexNAc(2)Hex(2)Fuc(1)_1)
1707.822(PEP+HexNAc(2)Hex(3)_1)
1748.849(PEP+HexNAc(3)Hex(2)_1)
1838.897
1853.882(PEP+HexNAc(2)Hex(3)Fuc(1)_1)
1894.908(PEP+HexNAc(3)Hex(2)Fuc(1)_1)
820.310
1201.443
ETD-MS/MS Scan:6689, Noise threshold:1.8
Relative Intensity (%)
M+2H
512.201(HexNAc(1)Hex(1)Fuc(1)_1)
366.141(HexNAc + Hex)
658.259
955.959(PEP+HexNAc(3)Hex(3)_2)
1183.039(PEP+HexNAc(3)Hex(4)Fuc(2)_2)
1211.549(PEP+HexNAc(4)Hex(4)Fuc(1)_2)
1910.899(PEP+HexNAc(3)Hex(3)_1)
m/z

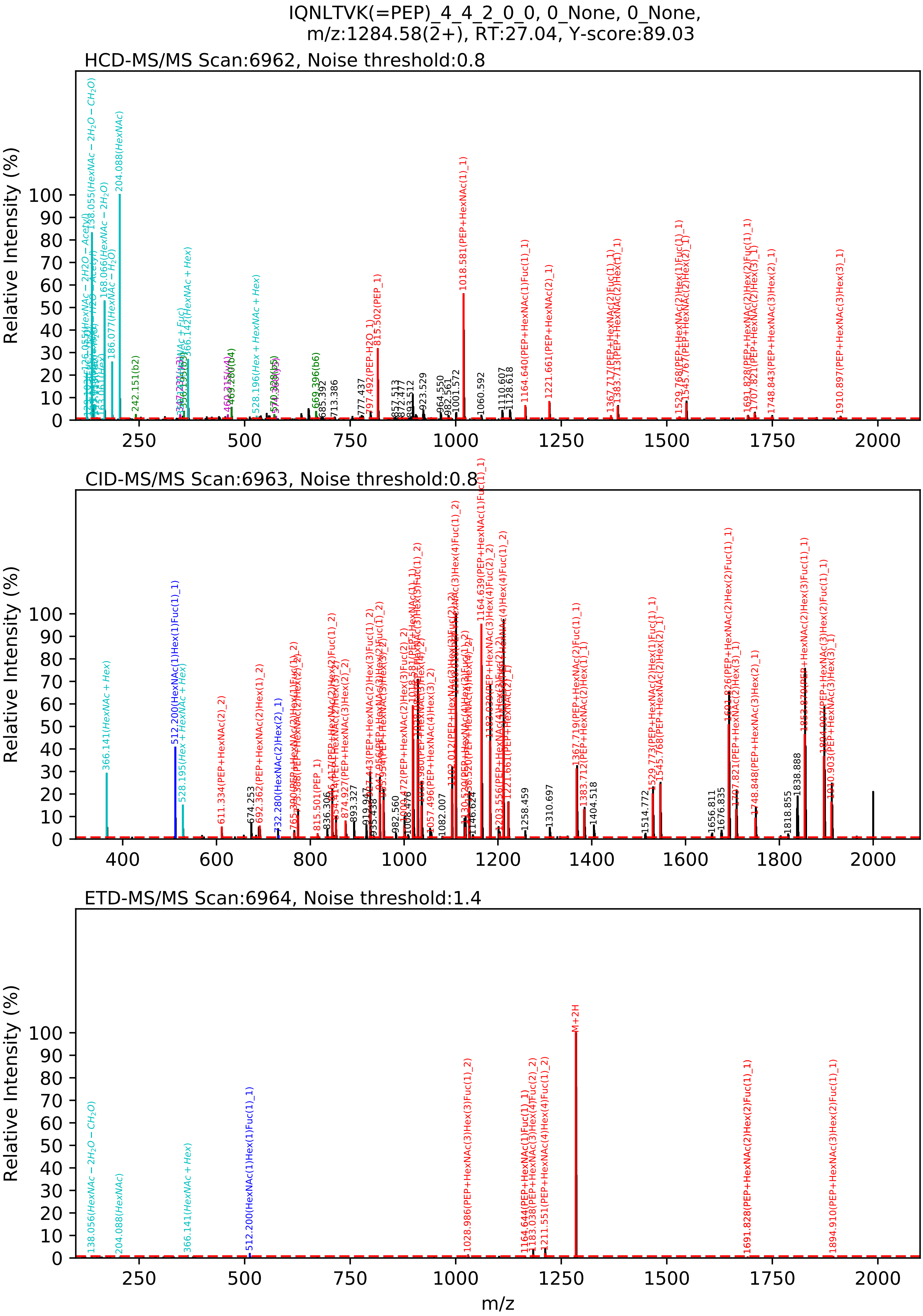

IQNLTVK(=PEP)_4_4_2_0_0, 0_None, 0_None,
m/z:1284.58(2+), RT:27.04, Y-score:89.03
HCD-MS/MS Scan:6962, Noise threshold:0.8
CID-MS/MS Scan:6963, Noise threshold:0.8
ETD-MS/MS Scan:6964, Noise threshold:1.4
Relative Intensity (%)
m/z
1018.581(PEP+HexNAc(1)_1)
815.502(PEP_1)
204.088(HexNAc)
1164.639(PEP+HexNAc(1)Fuc(1)_1)
512.200(HexNAc(1)Hex(1)Fuc(1)_1)
366.141(HexNAc+Hex)
M+2H
1691.828(PEP+HexNAc(2)Hex(2)Fuc(1)_1)
1894.910(PEP+HexNAc(3)Hex(2)Fuc(1)_1)

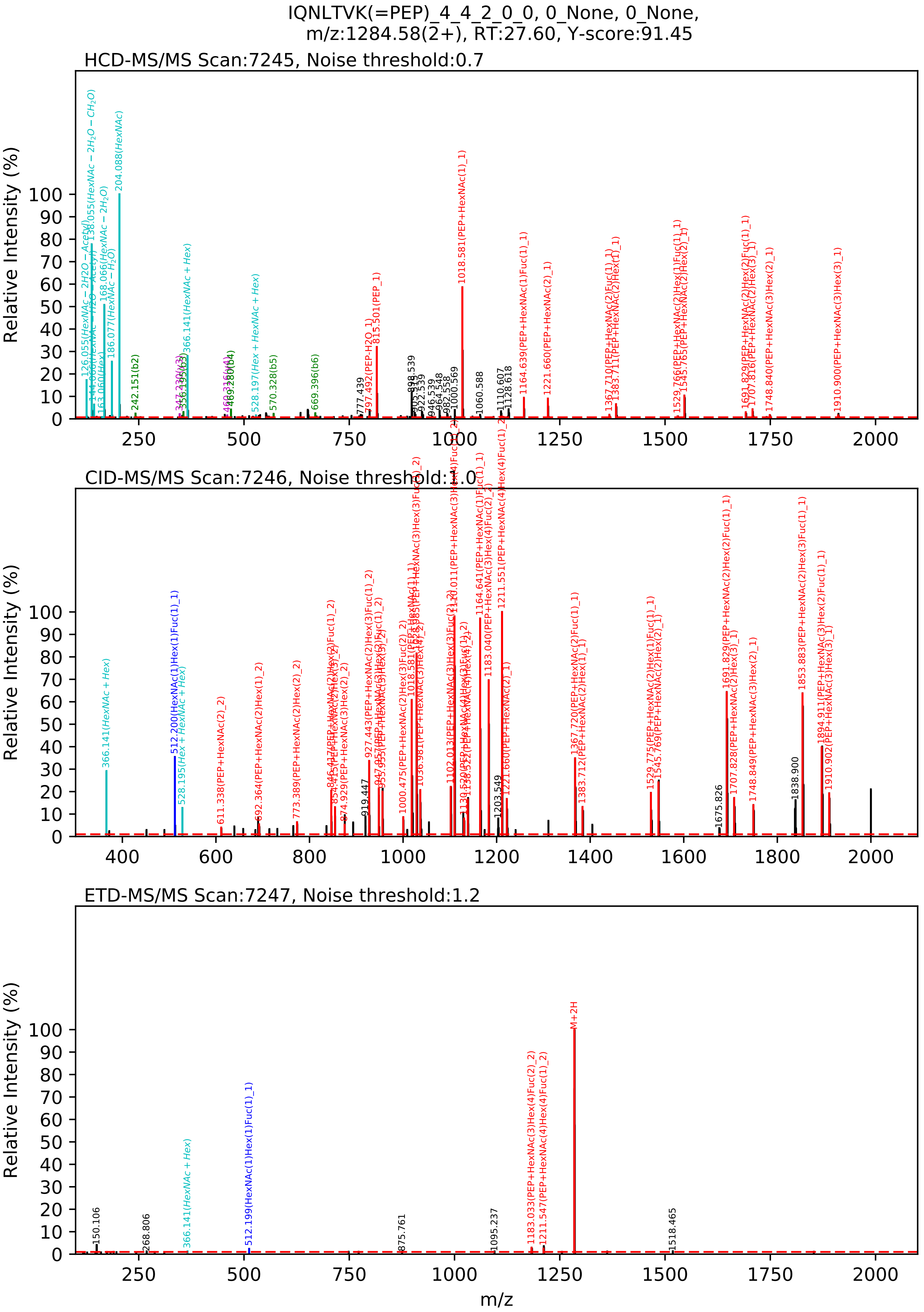
IQNLTVK(=PEP)_4_4_2_0_0, 0_None, 0_None,
m/z:1284.58(2+), RT:27.60, Y-score:91.45
HCD-MS/MS Scan:7245, Noise threshold:0.7
CID-MS/MS Scan:7246, Noise threshold:1.0
ETD-MS/MS Scan:7247, Noise threshold:1.2
Relative Intensity (%)
m/z

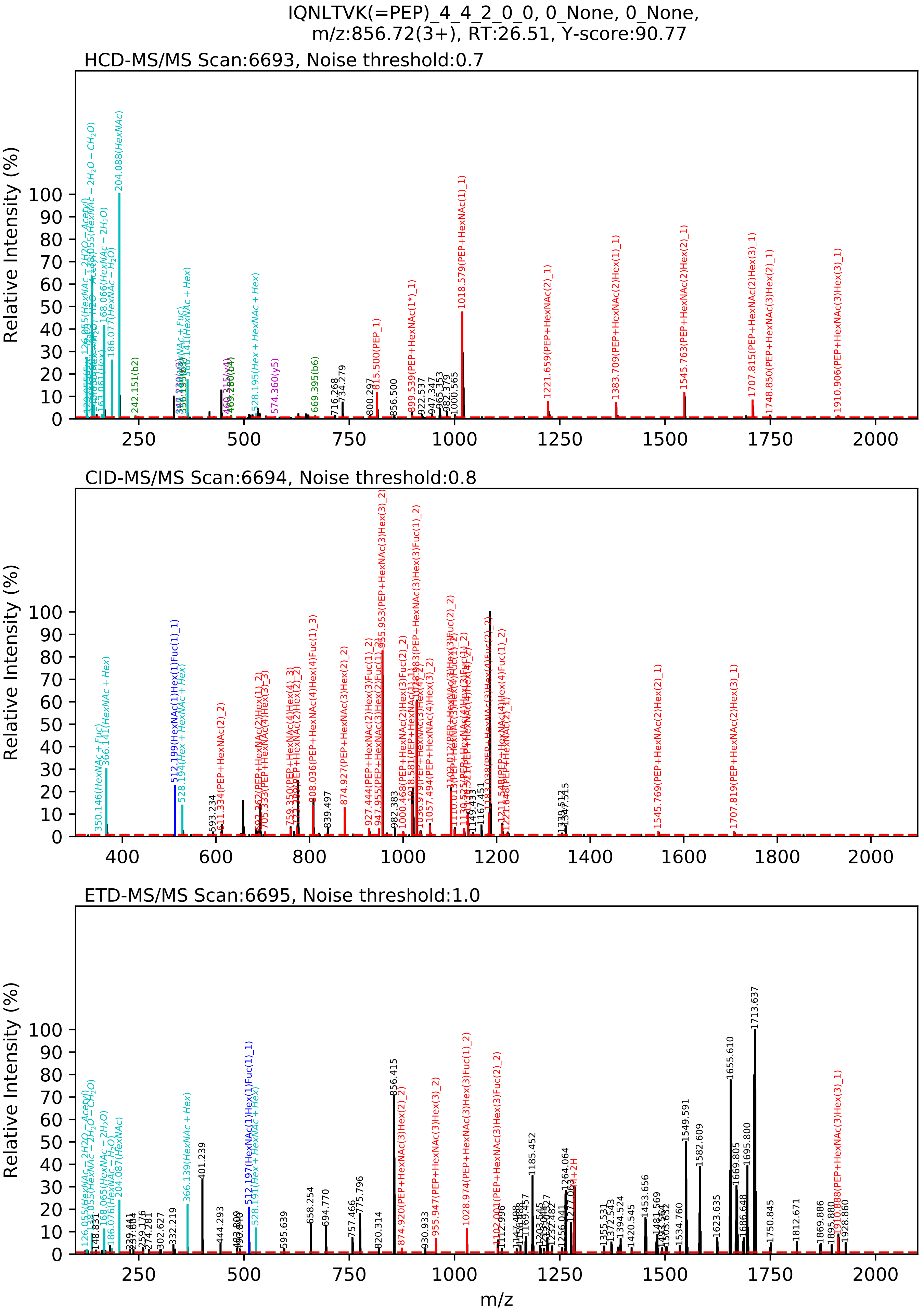
IQNLTVK(=PEP)_4_4_2_0_0, 0_None, 0_None,
m/z:856.72(3+), RT:26.51, Y-score:90.77
HCD-MS/MS Scan:6693, Noise threshold:0.7
CID-MS/MS Scan:6694, Noise threshold:0.8
ETD-MS/MS Scan:6695, Noise threshold:1.0
Relative Intensity (%)
m/z

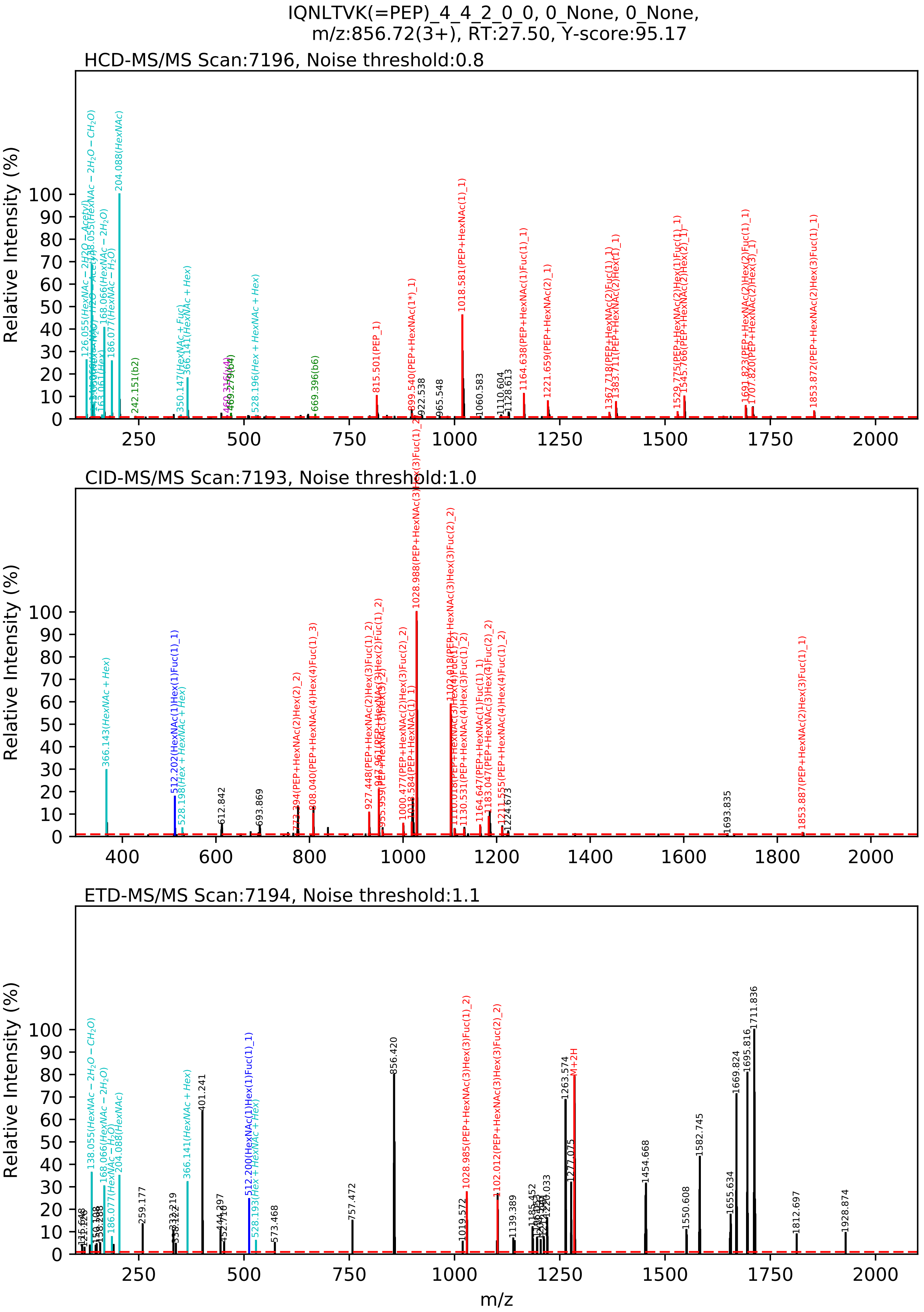

IQNLTVK(=PEP)_4_4_2_0_0, 0_None, 0_None,
m/z:856.72(3+), RT:27.50, Y-score:95.17
HCD-MS/MS Scan:7196, Noise threshold:0.8
CID-MS/MS Scan:7193, Noise threshold:1.0
ETD-MS/MS Scan:7194, Noise threshold:1.1
Relative Intensity (%)
m/z
204.088(HexNAc)
1018.581(PEP+HexNAc(1)_1)
1028.988(PEP+HexNAc(3)Hex(3)Fuc(1)_2)
1102.018(PEP+HexNAc(3)Hex(3)Fuc(2)_2)
366.143(HexNAc+Hex)
512.202(HexNAc(1)Hex(1)Fuc(1)_1)
1853.887(PEP+HexNAc(2)Hex(3)Fuc(1)_1)
1711.836
856.420
M+2H

IQNLTVK(=PEP)_4_5_0_0_0, 0_None, 0_None,
m/z:827.04(3+), RT:28.03, Y-score:81.70

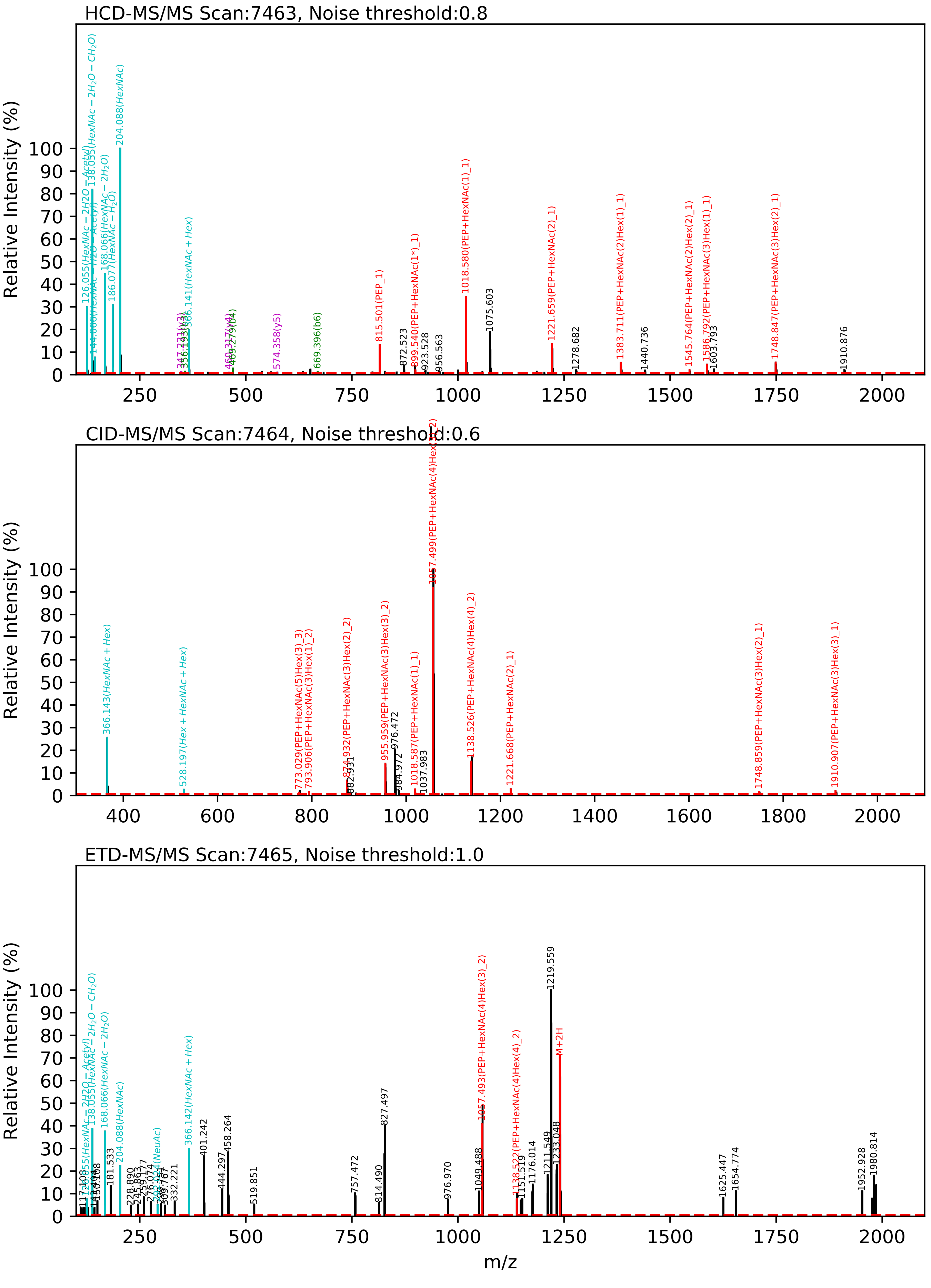

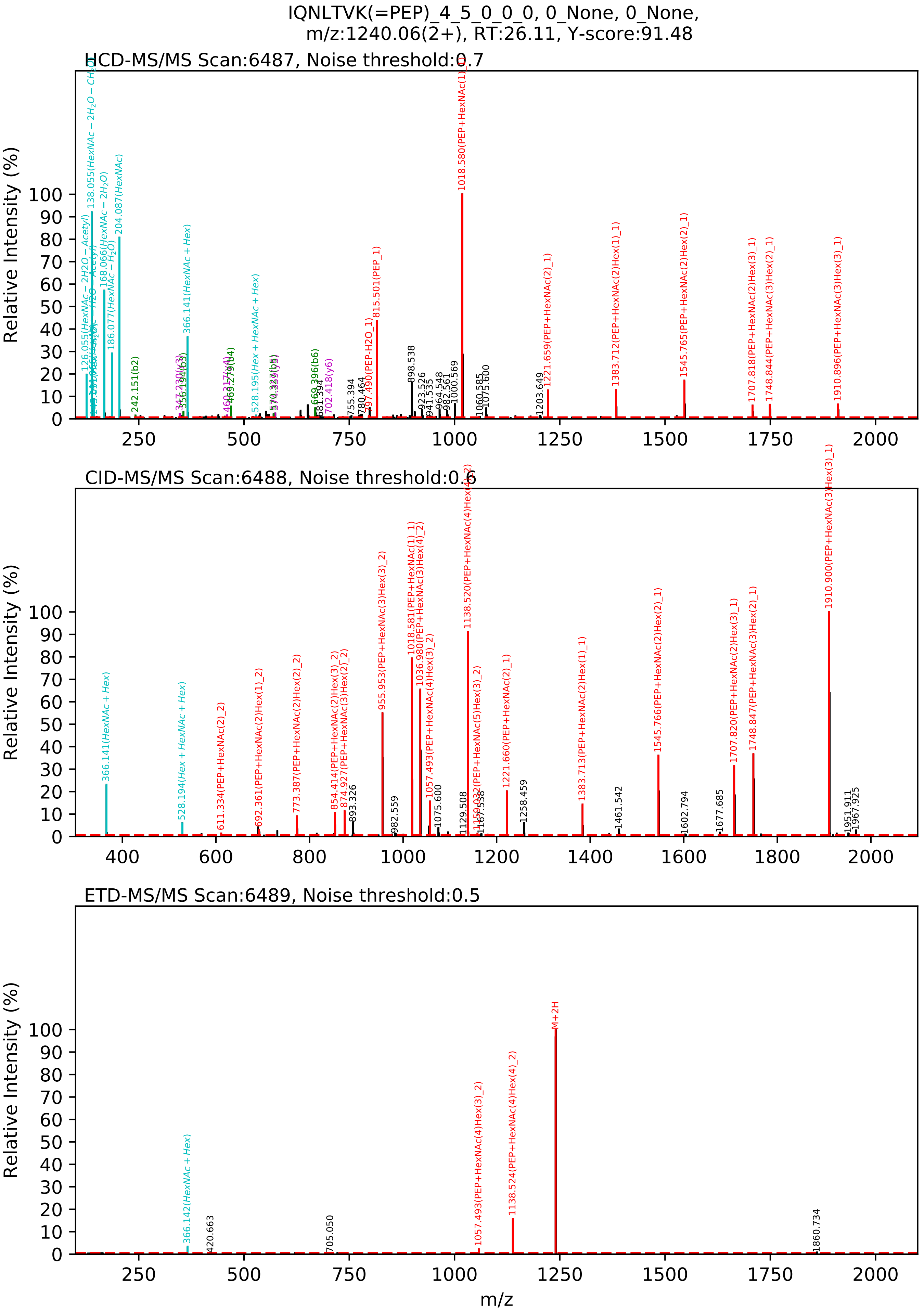

IQNLTVK(=PEP)_4_5_0_0_0, 0_None, 0_None,
m/z:1240.06(2+), RT:26.11, Y-score:91.48
HCD-MS/MS Scan:6487, Noise threshold:0.7
CID-MS/MS Scan:6488, Noise threshold:0.6
ETD-MS/MS Scan:6489, Noise threshold:0.5
Relative Intensity (%)
m/z
1018.580(PEP+HexNAc(1)_1)
815.501(PEP_1)
1221.659(PEP+HexNAc(2)_1)
1383.712(PEP+HexNAc(2)Hex(1)_1)
1545.765(PEP+HexNAc(2)Hex(2)_1)
1707.818(PEP+HexNAc(2)Hex(3)_1)
1748.844(PEP+HexNAc(3)Hex(2)_1)
1910.896(PEP+HexNAc(3)Hex(3)_1)
204.087(HexNAc)
366.141(HexNAc + Hex)
528.195(Hex + HexNAc + Hex)
1910.900(PEP+HexNAc(3)Hex(3)_1)
1138.520(PEP+HexNAc(4)Hex(4)_2)
955.953(PEP+HexNAc(3)Hex(3)_2)
1221.660(PEP+HexNAc(2)_1)
1383.713(PEP+HexNAc(2)Hex(1)_1)
1545.766(PEP+HexNAc(2)Hex(2)_1)
1707.820(PEP+HexNAc(2)Hex(3)_1)
1748.847(PEP+HexNAc(3)Hex(2)_1)
M+2H
1138.524(PEP+HexNAc(4)Hex(4)_2)
1057.493(PEP+HexNAc(4)Hex(3)_2)
366.142(HexNAc + Hex)

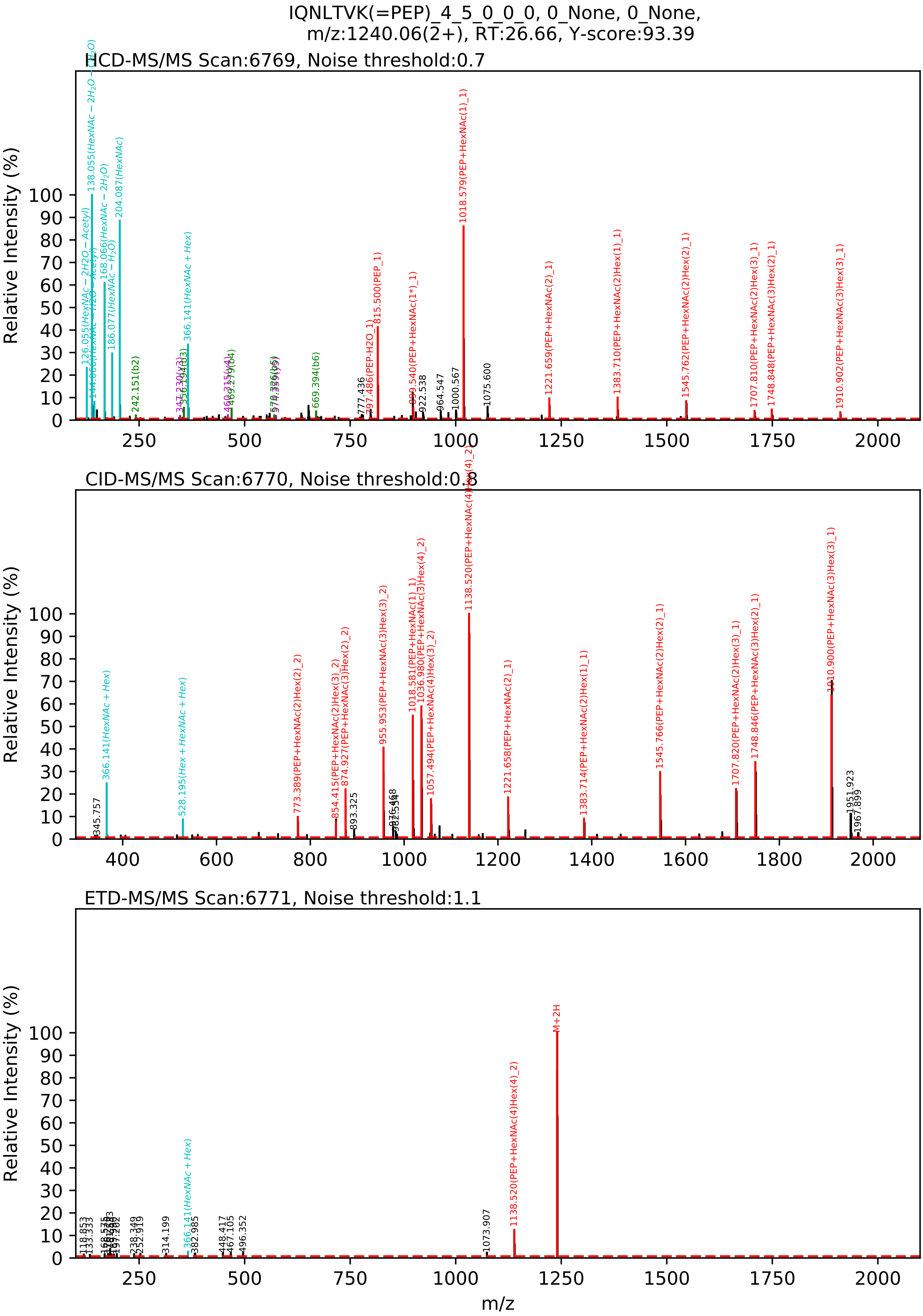
IQNLTVK(=PEP)_4_5_0_0_0, 0_None, 0_None,
m/z:1240.06(2+), RT:26.66, Y-score:93.39
HCD-MS/MS Scan:6769, Noise threshold:0.7
CID-MS/MS Scan:6770, Noise threshold:0.8
ETD-MS/MS Scan:6771, Noise threshold:1.1
Relative Intensity (%)
m/z
1018.579(PEP+HexNAc(1)_1)
1138.520(PEP+HexNAc(4)Hex(4)_2)
M+2H

IQNLTVK(=PEP)_4_5_0_0_0, 0_None, 0_None,
m/z:1240.06(2+), RT:36.05, Y-score:72.22

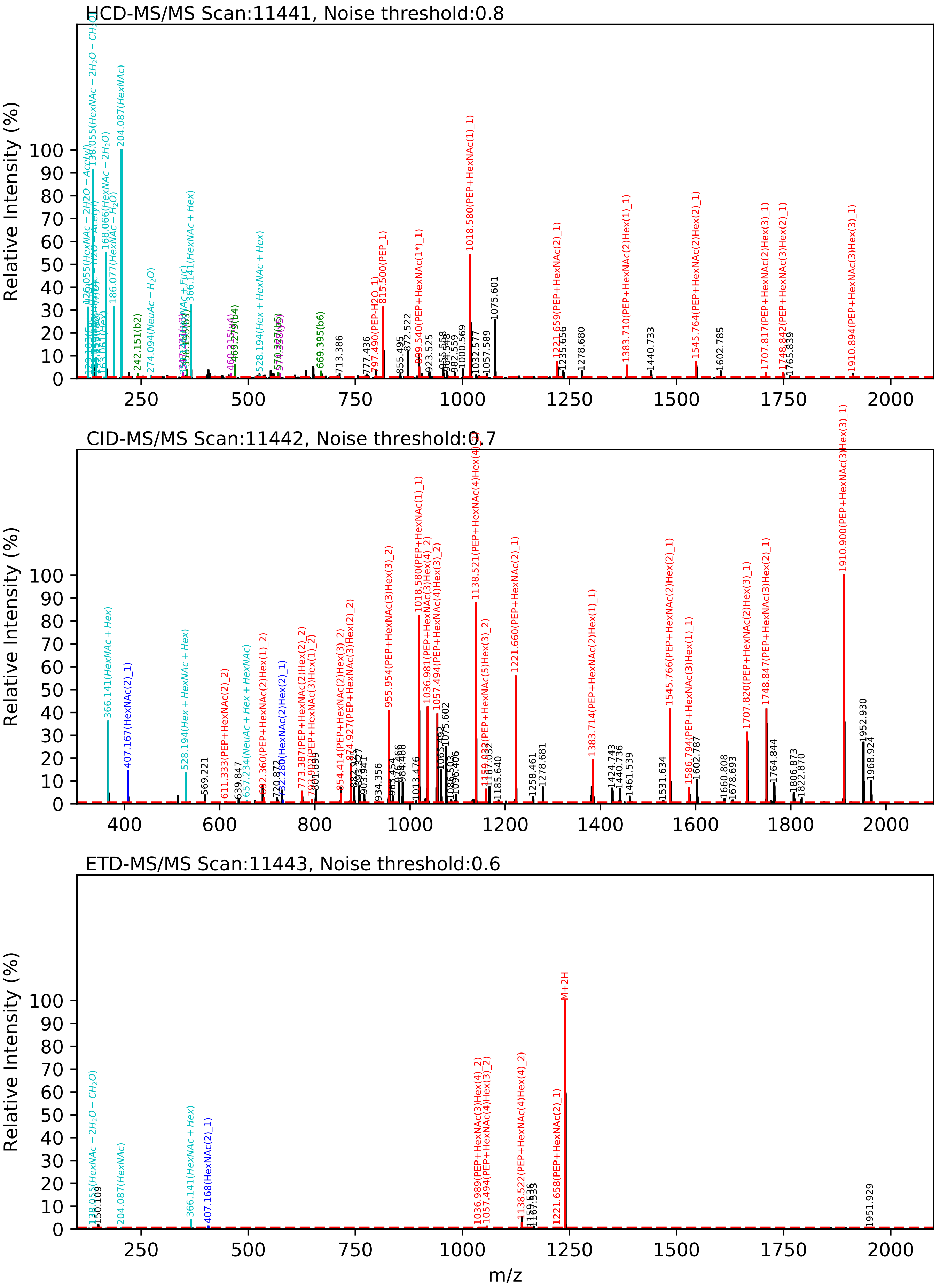

IQNLTVK(=PEP)_4_5_0_0_0, 0_None, 0_None,
m/z:1240.06(2+), RT:28.30, Y-score:57.70

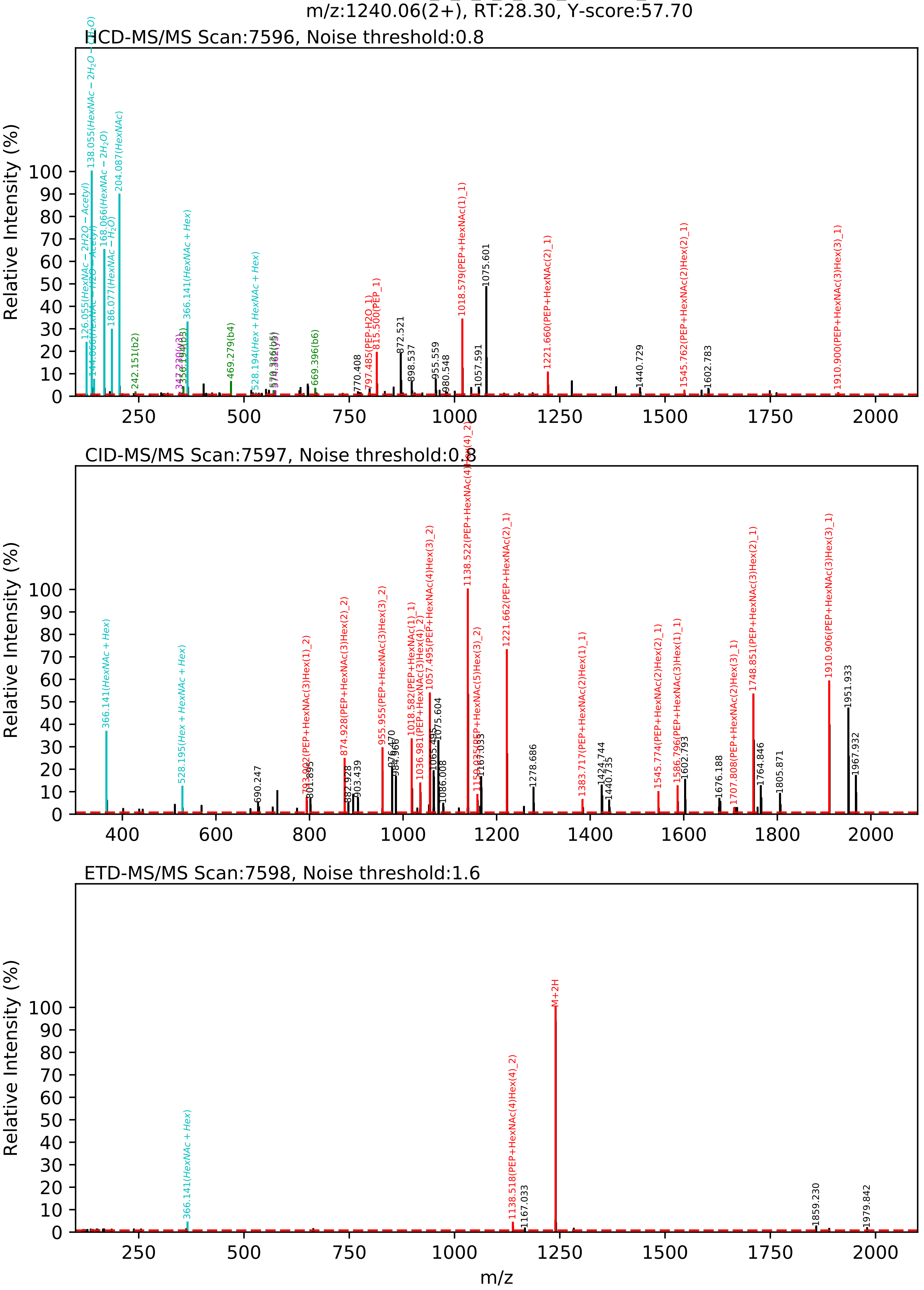

IQNLTVK(=PEP)_4_5_0_1_0, 0_None, 0_None,
m/z:1385.60(2+), RT:35.93, Y-score:93.58

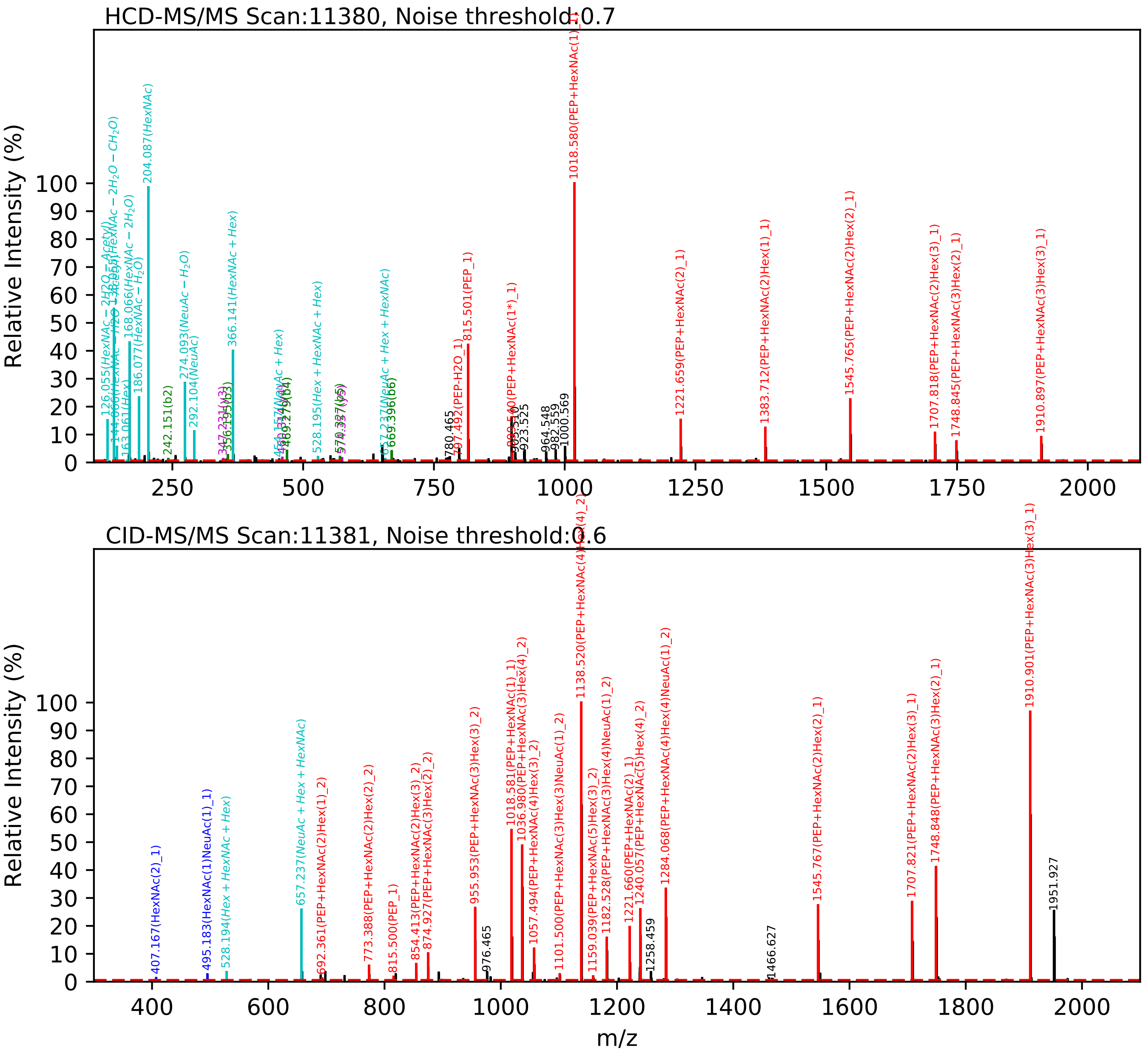

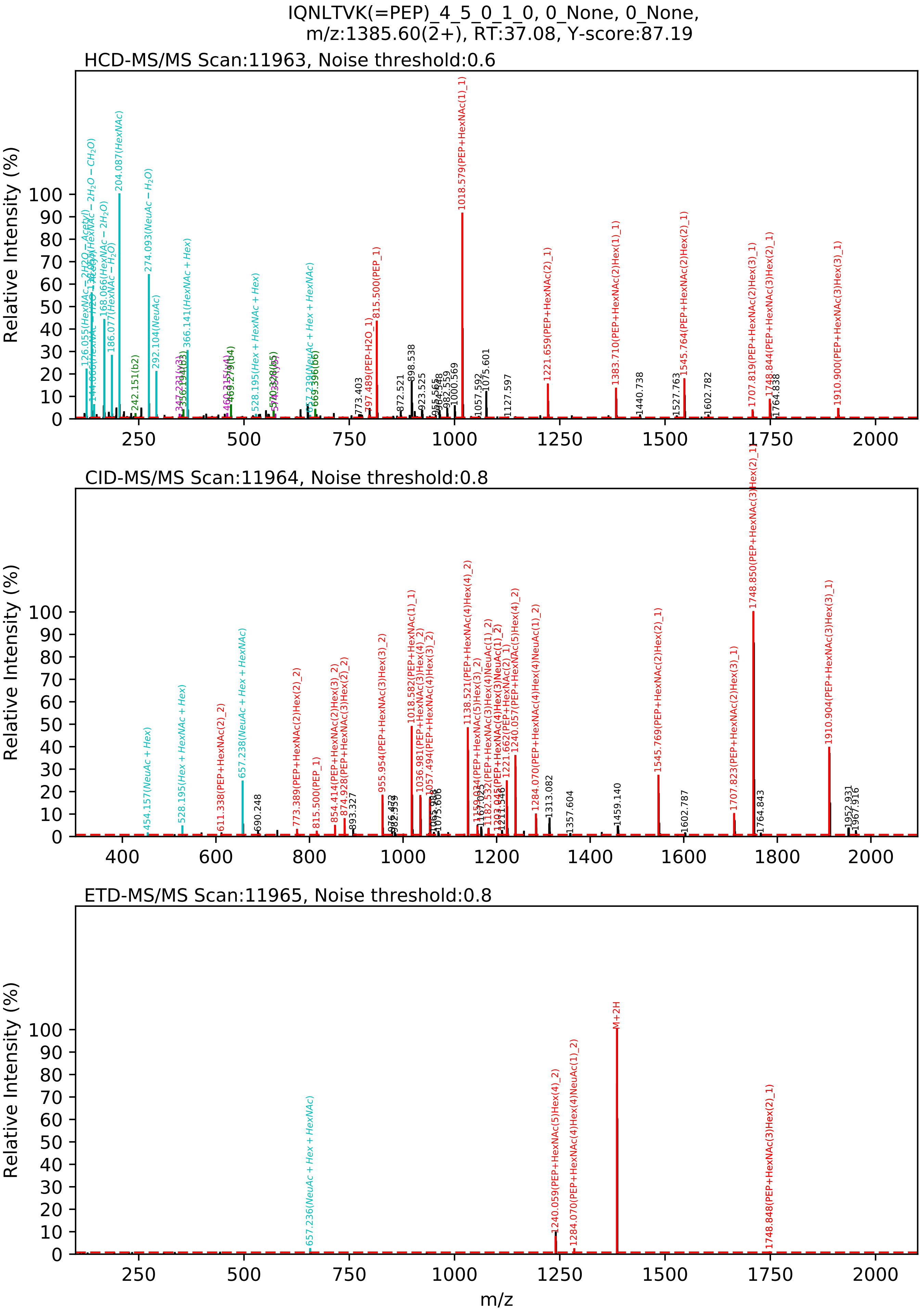

IQNLTVK(=PEP)_4_5_0_1_0, 0_None, 0_None,
m/z:1385.60(2+), RT:37.08, Y-score:87.19
HCD-MS/MS Scan:11963, Noise threshold:0.6
Relative Intensity (%)
204.087(HexNAc)
274.093(NeuAc − H2O)
292.104(NeuAc)
366.141(HexNAc + Hex)
168.066(HexNAc − 2H2O)
186.077(HexNAc − H2O)
126.055(HexNAc − 2H2O − Acetyl)
242.151(b2)
528.195(Hex + HexNAc + Hex)
657.239(NeuAc + Hex + HexNAc)
669.396(b6)
773.403
797.489(PEP-H2O_1)
815.500(PEP_1)
872.521
898.538
923.525
1000.569
1018.579(PEP+HexNAc(1)_1)
1057.592
1075.601
1127.597
1221.659(PEP+HexNAc(2)_1)
1383.710(PEP+HexNAc(2)Hex(1)_1)
1440.738
1527.763
1545.764(PEP+HexNAc(2)Hex(2)_1)
1602.782
1707.819(PEP+HexNAc(2)Hex(3)_1)
1748.844(PEP+HexNAc(3)Hex(2)_1)
1764.838
1910.900(PEP+HexNAc(3)Hex(3)_1)
CID-MS/MS Scan:11964, Noise threshold:0.8
Relative Intensity (%)
454.157(NeuAc + Hex)
528.195(Hex + HexNAc + Hex)
611.338(PEP+HexNAc(2)_2)
657.238(NeuAc + Hex + HexNAc)
690.248
773.389(PEP+HexNAc(2)Hex(2)_2)
815.500(PEP_1)
854.414(PEP+HexNAc(2)Hex(3)_2)
874.928(PEP+HexNAc(3)Hex(2)_2)
893.327
955.954(PEP+HexNAc(3)Hex(3)_2)
1018.582(PEP+HexNAc(1)_1)
1036.981(PEP+HexNAc(3)Hex(4)_2)
1057.494(PEP+HexNAc(4)Hex(3)_2)
1075.606
1138.521(PEP+HexNAc(4)Hex(4)_2)
1221.662(PEP+HexNAc(2)_1)
1240.057(PEP+HexNAc(5)Hex(4)_2)
1284.070(PEP+HexNAc(4)Hex(4)NeuAc(1)_2)
1313.082
1357.604
1459.140
1545.769(PEP+HexNAc(2)Hex(2)_1)
1602.787
1707.823(PEP+HexNAc(2)Hex(3)_1)
1748.850(PEP+HexNAc(3)Hex(2)_1)
1764.843
1910.904(PEP+HexNAc(3)Hex(3)_1)
1952.931
1967.916
ETD-MS/MS Scan:11965, Noise threshold:0.8
Relative Intensity (%)
657.236(NeuAc + Hex + HexNAc)
1240.059(PEP+HexNAc(5)Hex(4)_2)
1284.070(PEP+HexNAc(4)Hex(4)NeuAc(1)_2)
M+2H
1748.848(PEP+HexNAc(3)Hex(2)_1)
m/z

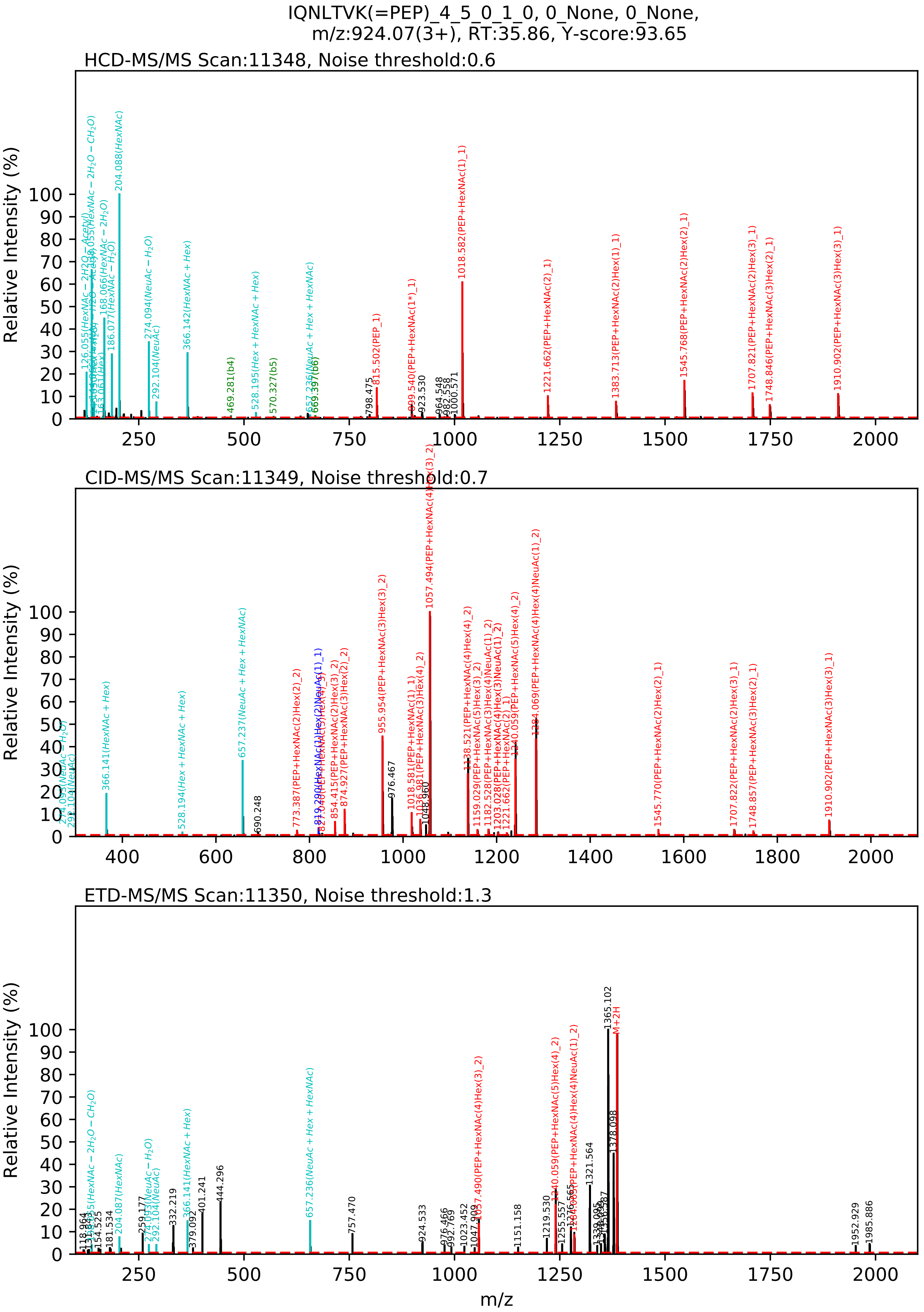

IQNLTVK(=PEP)_4_5_0_1_0, 0_None, 0_None,
m/z:924.07(3+), RT:35.86, Y-score:93.65
HCD-MS/MS Scan:11348, Noise threshold:0.6
Relative Intensity (%)
204.088(HexNAc)
1018.582(PEP+HexNAc(1)_1)
366.142(HexNAc+Hex)
274.094(NeuAc−H2O)
292.104(NeuAc)
469.281(b4)
528.195(Hex+HexNAc+Hex)
570.327(b5)
815.502(PEP_1)
909.540(PEP+HexNAc(1*)_1)
1221.662(PEP+HexNAc(2)_1)
1383.713(PEP+HexNAc(2)Hex(1)_1)
1545.768(PEP+HexNAc(2)Hex(2)_1)
1707.821(PEP+HexNAc(2)Hex(3)_1)
1748.846(PEP+HexNAc(3)Hex(2)_1)
1910.902(PEP+HexNAc(3)Hex(3)_1)
CID-MS/MS Scan:11349, Noise threshold:0.7
1057.494(PEP+HexNAc(4)Hex(3)_2)
955.954(PEP+HexNAc(3)Hex(3)_2)
1284.069(PEP+HexNAc(4)Hex(4)NeuAc(1)_2)
1138.521(PEP+HexNAc(4)Hex(4)_2)
1240.059(PEP+HexNAc(5)Hex(4)_2)
657.237(NeuAc+Hex+HexNAc)
366.141(HexNAc+Hex)
528.194(Hex+HexNAc+Hex)
773.387(PEP+HexNAc(2)Hex(2)_2)
854.415(PEP+HexNAc(2)Hex(3)_2)
874.927(PEP+HexNAc(3)Hex(2)_2)
976.467
690.248
1545.770(PEP+HexNAc(2)Hex(2)_1)
1707.822(PEP+HexNAc(2)Hex(3)_1)
1748.857(PEP+HexNAc(3)Hex(2)_1)
1910.902(PEP+HexNAc(3)Hex(3)_1)
ETD-MS/MS Scan:11350, Noise threshold:1.3
1365.102
M+2H
1378.098
1321.564
1240.059(PEP+HexNAc(5)Hex(4)_2)
1284.069(PEP+HexNAc(4)Hex(4)NeuAc(1)_2)
1057.490(PEP+HexNAc(4)Hex(3)_2)
657.236(NeuAc+Hex+HexNAc)
757.470
444.296
401.241
332.219
259.177
204.087(HexNAc)
924.533
1151.158
1219.530
1952.929
1985.886
m/z

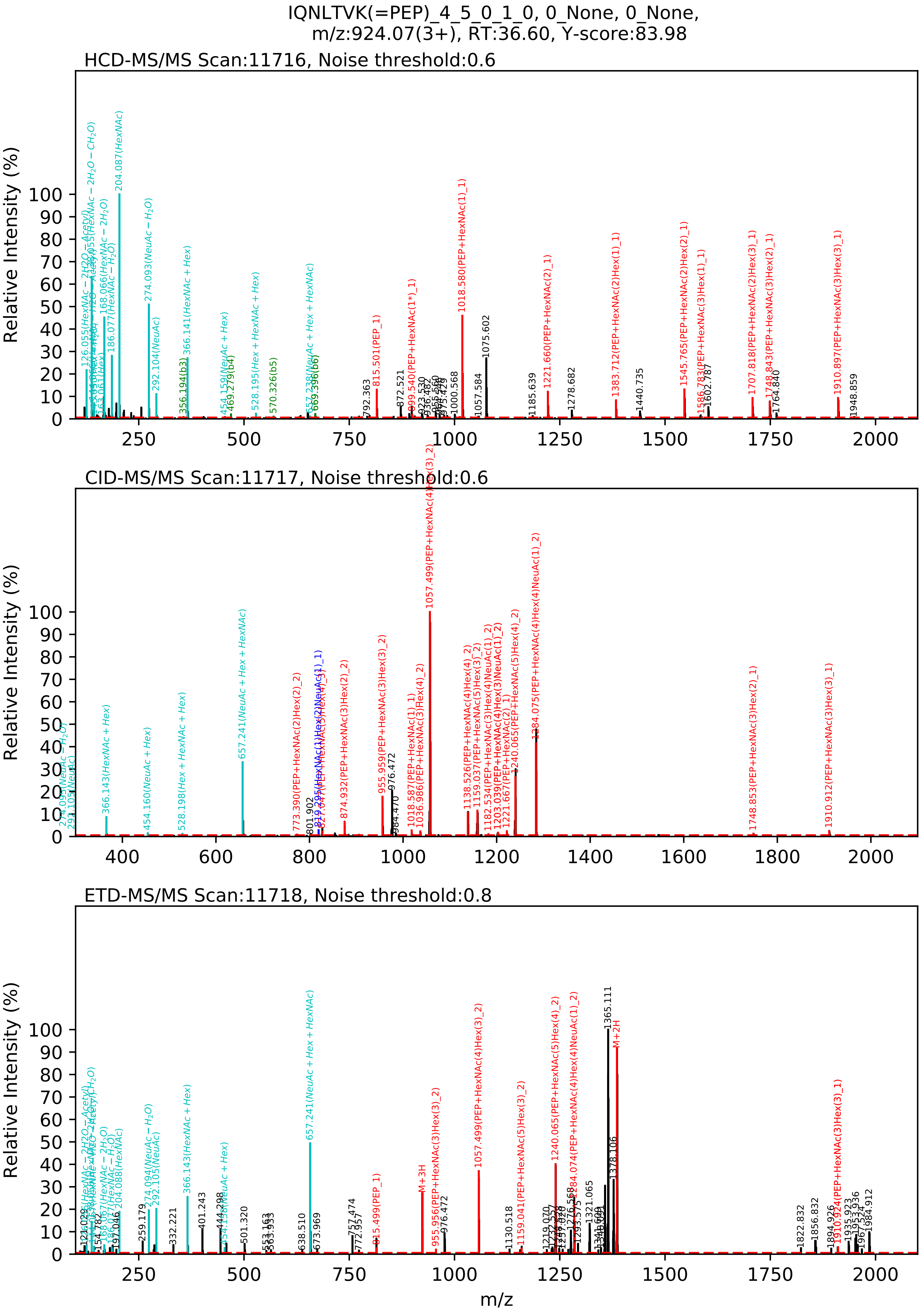
IQNLTVK(=PEP)_4_5_0_1_0, 0_None, 0_None,
m/z:924.07(3+), RT:36.60, Y-score:83.98
HCD-MS/MS Scan:11716, Noise threshold:0.6
Relative Intensity (%)
204.087(HexNAc)
274.093(NeuAc − H2O)
292.104(NeuAc)
366.141(HexNAc + Hex)
356.194(b3)
454.159(NeuAc + Hex)
469.279(b4)
528.195(Hex + HexNAc + Hex)
570.326(b5)
657.238(NeuAc + Hex + HexNAc)
669.396(b6)
186.077(HexNAc − H2O)
168.066(HexNAc − 2H2O)
126.055(HexNAc − 2H2O − CH2O)
792.363
815.501(PEP_1)
872.521
899.540(PEP+HexNAc(1*)_1)
923.530
1000.568
1018.580(PEP+HexNAc(1)_1)
1057.584
1075.602
1185.639
1221.660(PEP+HexNAc(2)_1)
1278.682
1383.712(PEP+HexNAc(2)Hex(1)_1)
1440.735
1545.765(PEP+HexNAc(2)Hex(2)_1)
1586.783(PEP+HexNAc(3)Hex(1)_1)
1602.787
1707.818(PEP+HexNAc(2)Hex(3)_1)
1748.843(PEP+HexNAc(3)Hex(2)_1)
1764.840
1910.897(PEP+HexNAc(3)Hex(3)_1)
1948.859
CID-MS/MS Scan:11717, Noise threshold:0.6
366.143(HexNAc + Hex)
454.160(NeuAc + Hex)
528.198(Hex + HexNAc + Hex)
657.241(NeuAc + Hex + HexNAc)
773.390(PEP+HexNAc(2)Hex(2)_2)
801.902
819.295(HexNAc(1)Hex(2)NeuAc(1)_1)
874.932(PEP+HexNAc(3)Hex(2)_2)
955.959(PEP+HexNAc(3)Hex(3)_2)
976.472
984.470
1018.587(PEP+HexNAc(1)_1)
1036.986(PEP+HexNAc(3)Hex(4)_2)
1057.499(PEP+HexNAc(4)Hex(3)_2)
1138.526(PEP+HexNAc(4)Hex(4)_2)
1159.037(PEP+HexNAc(5)Hex(3)_2)
1182.534(PEP+HexNAc(3)Hex(4)NeuAc(1)_2)
1203.039(PEP+HexNAc(4)Hex(3)NeuAc(1)_2)
1221.667(PEP+HexNAc(2)_1)
1240.065(PEP+HexNAc(5)Hex(4)_2)
1284.075(PEP+HexNAc(4)Hex(4)NeuAc(1)_2)
1748.853(PEP+HexNAc(3)Hex(2)_1)
1910.912(PEP+HexNAc(3)Hex(3)_1)
ETD-MS/MS Scan:11718, Noise threshold:0.8
204.088(HexNAc)
259.179
274.094(NeuAc − H2O)
292.105(NeuAc)
332.221
366.143(HexNAc + Hex)
401.243
444.298
501.320
553.163
563.933
638.510
657.241(NeuAc + Hex + HexNAc)
673.969
757.474
772.957
815.499(PEP_1)
M+3H
955.956(PEP+HexNAc(3)Hex(3)_2)
976.472
1057.499(PEP+HexNAc(4)Hex(3)_2)
1130.518
1159.041(PEP+HexNAc(5)Hex(3)_2)
1219.070
1240.065(PEP+HexNAc(5)Hex(4)_2)
1284.074(PEP+HexNAc(4)Hex(4)NeuAc(1)_2)
1276.568
1293.575
1321.065
1365.111
M+2H
1378.106
1822.832
1856.832
1894.926
1910.924(PEP+HexNAc(3)Hex(3)_1)
1935.923
1953.936
1967.524
1984.912
m/z

IQNLTVK(=PEP)_4_5_1_0_0, 0_None, 0_None,
m/z:1313.09(2+), RT:27.74, Y-score:95.45

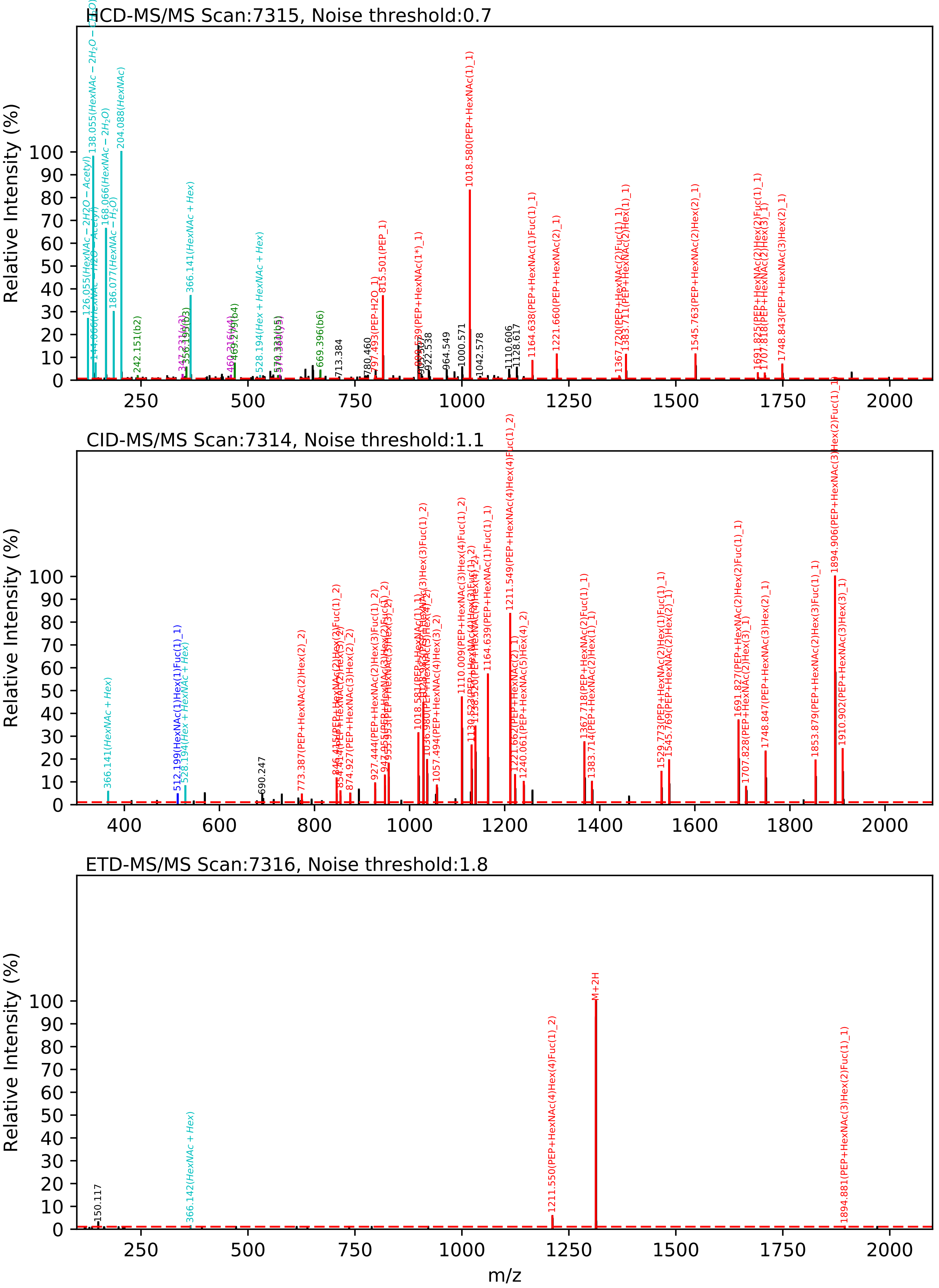

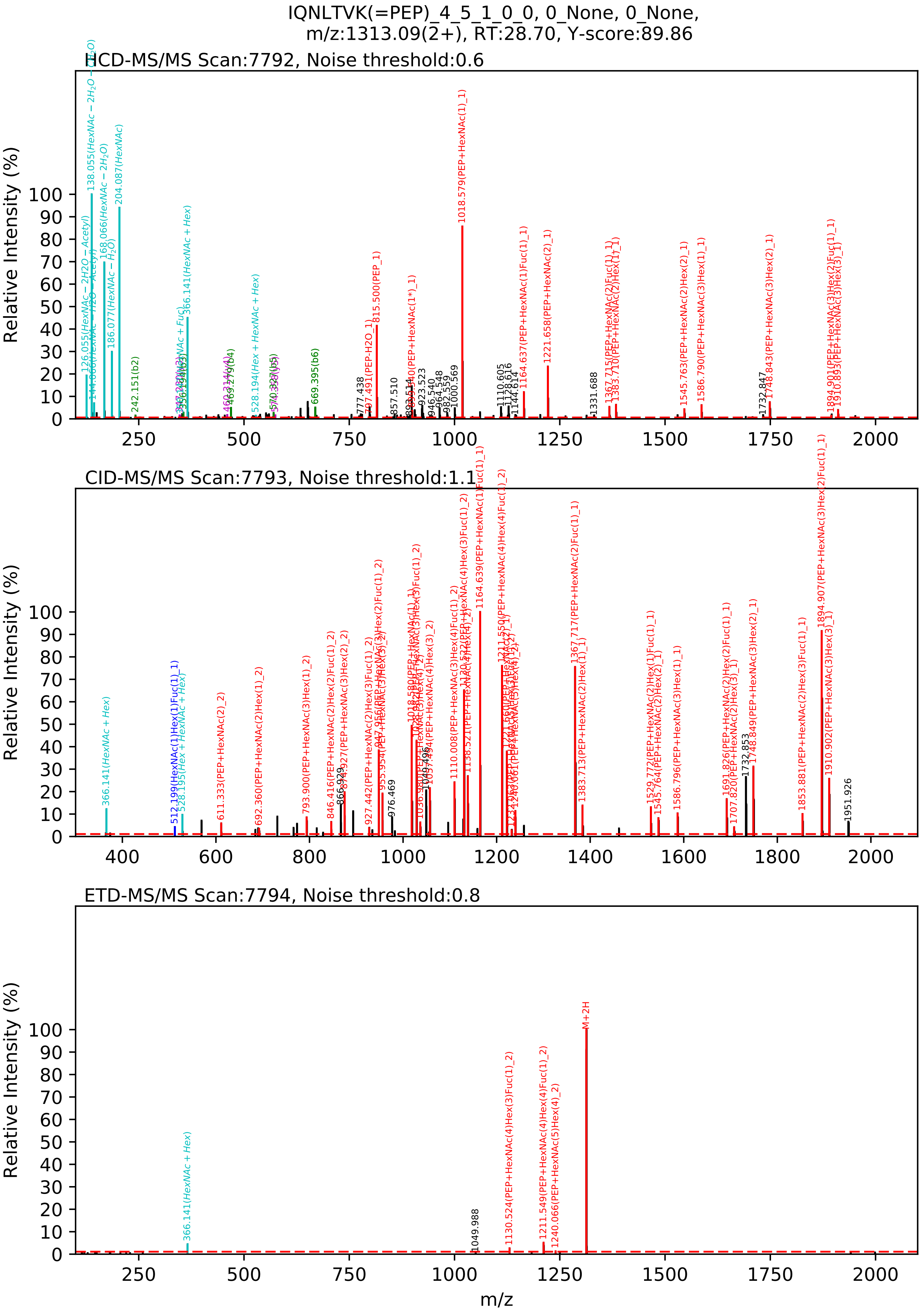

IQNLTVK(=PEP)_4_5_1_0_0, 0_None, 0_None,
m/z:1313.09(2+), RT:28.70, Y-score:89.86
HCD-MS/MS Scan:7792, Noise threshold:0.6
CID-MS/MS Scan:7793, Noise threshold:1.1
ETD-MS/MS Scan:7794, Noise threshold:0.8
Relative Intensity (%)
m/z
138.055(HexNAc − 2H2O − H2O)
204.087(HexNAc)
168.066(HexNAc − 2H2O)
186.077(HexNAc − H2O)
126.055(HexNAc − 2H2O − Acetyl)
366.141(HexNAc + Hex)
528.194(Hex + HexNAc + Hex)
242.151(b2)
469.279(b4)
669.395(b6)
815.500(PEP_1)
807.491(PEP-H2O_1)
1018.579(PEP+HexNAc(1)_1)
1164.637(PEP+HexNAc(1)Fuc(1)_1)
1221.658(PEP+HexNAc(2)_1)
1367.715(PEP+HexNAc(2)Fuc(1)_1)
1383.710(PEP+HexNAc(2)Hex(1)_1)
1545.763(PEP+HexNAc(2)Hex(2)_1)
1586.790(PEP+HexNAc(3)Hex(1)_1)
1748.843(PEP+HexNAc(3)Hex(2)_1)
1894.901(PEP+HexNAc(3)Hex(2)Fuc(1)_1)
1910.893(PEP+HexNAc(3)Hex(3)_1)
777.438
857.510
923.523
946.540
964.548
1000.569
1110.605
1128.616
1144.614
1331.688
1732.847
512.199(HexNAc(1)Hex(1)Fuc(1)_1)
611.333(PEP+HexNAc(2)_2)
692.360(PEP+HexNAc(2)Hex(1)_2)
793.900(PEP+HexNAc(3)Hex(1)_2)
846.416(PEP+HexNAc(2)Hex(2)Fuc(1)_2)
874.927(PEP+HexNAc(3)Hex(2)_2)
866.929
927.442(PEP+HexNAc(2)Hex(3)Fuc(1)_2)
955.954(PEP+HexNAc(3)Hex(3)_2)
976.469
1057.494(PEP+HexNAc(4)Hex(3)_2)
1110.008(PEP+HexNAc(3)Hex(4)Fuc(1)_2)
1130.522(PEP+HexNAc(4)Hex(3)Fuc(1)_2)
1138.521(PEP+HexNAc(4)Hex(4)_2)
1164.639(PEP+HexNAc(1)Fuc(1)_1)
1211.550(PEP+HexNAc(4)Hex(4)Fuc(1)_2)
1367.717(PEP+HexNAc(2)Fuc(1)_1)
1383.713(PEP+HexNAc(2)Hex(1)_1)
1529.772(PEP+HexNAc(2)Hex(1)Fuc(1)_1)
1545.764(PEP+HexNAc(2)Hex(2)_1)
1586.796(PEP+HexNAc(3)Hex(1)_1)
1691.826(PEP+HexNAc(2)Hex(2)Fuc(1)_1)
1707.820(PEP+HexNAc(2)Hex(3)_1)
1748.849(PEP+HexNAc(3)Hex(2)_1)
1732.853
1853.881(PEP+HexNAc(2)Hex(3)Fuc(1)_1)
1894.907(PEP+HexNAc(3)Hex(2)Fuc(1)_1)
1910.902(PEP+HexNAc(3)Hex(3)_1)
1951.926
M+2H
1049.988
1130.524(PEP+HexNAc(4)Hex(3)Fuc(1)_2)
1211.549(PEP+HexNAc(4)Hex(4)Fuc(1)_2)
1240.066(PEP+HexNAc(5)Hex(4)_2)

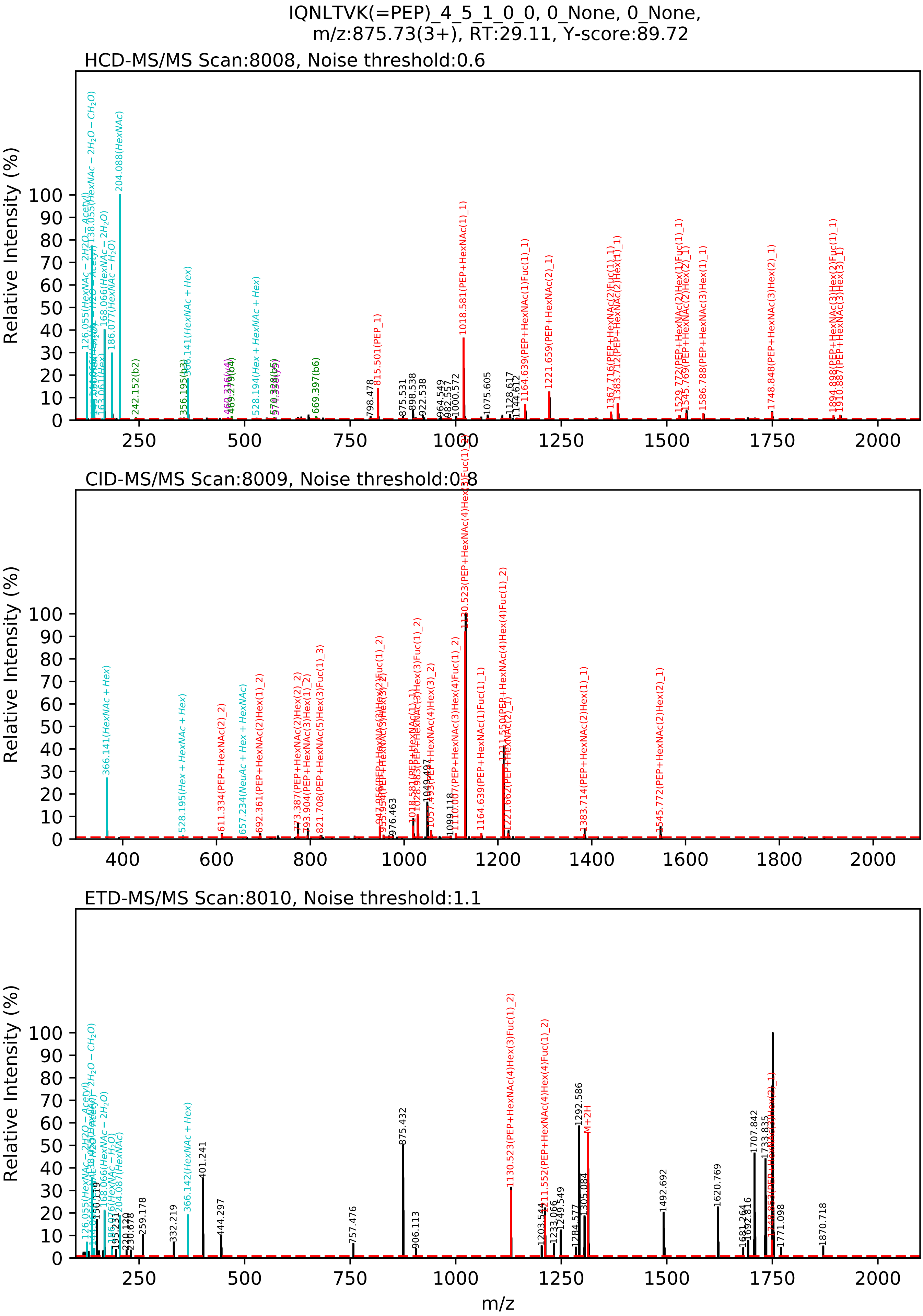

IQNLTVK(=PEP)_4_5_1_0_0, 0_None, 0_None,
m/z:875.73(3+), RT:29.11, Y-score:89.72
HCD-MS/MS Scan:8008, Noise threshold:0.6
CID-MS/MS Scan:8009, Noise threshold:0.8
ETD-MS/MS Scan:8010, Noise threshold:1.1
Relative Intensity (%)
m/z
204.088(HexNAc)
1018.581(PEP+HexNAc(1)_1)
815.501(PEP_1)
1164.639(PEP+HexNAc(1)Fuc(1)_1)
1221.659(PEP+HexNAc(2)_1)
1586.788(PEP+HexNAc(3)Hex(1)_1)
1748.848(PEP+HexNAc(3)Hex(2)_1)
366.141(HexNAc + Hex)
1130.523(PEP+HexNAc(4)Hex(3)Fuc(1)_2)
1211.550(PEP+HexNAc(4)Hex(4)Fuc(1)_2)
1383.714(PEP+HexNAc(2)Hex(1)_1)
1545.772(PEP+HexNAc(2)Hex(2)_1)
M+2H
875.432
1292.586
1707.842
1733.835

IQNLTVK(=PEP)_4_5_1_0_0, 0_None, 0_None,
m/z:875.73(3+), RT:26.04, Y-score:86.79

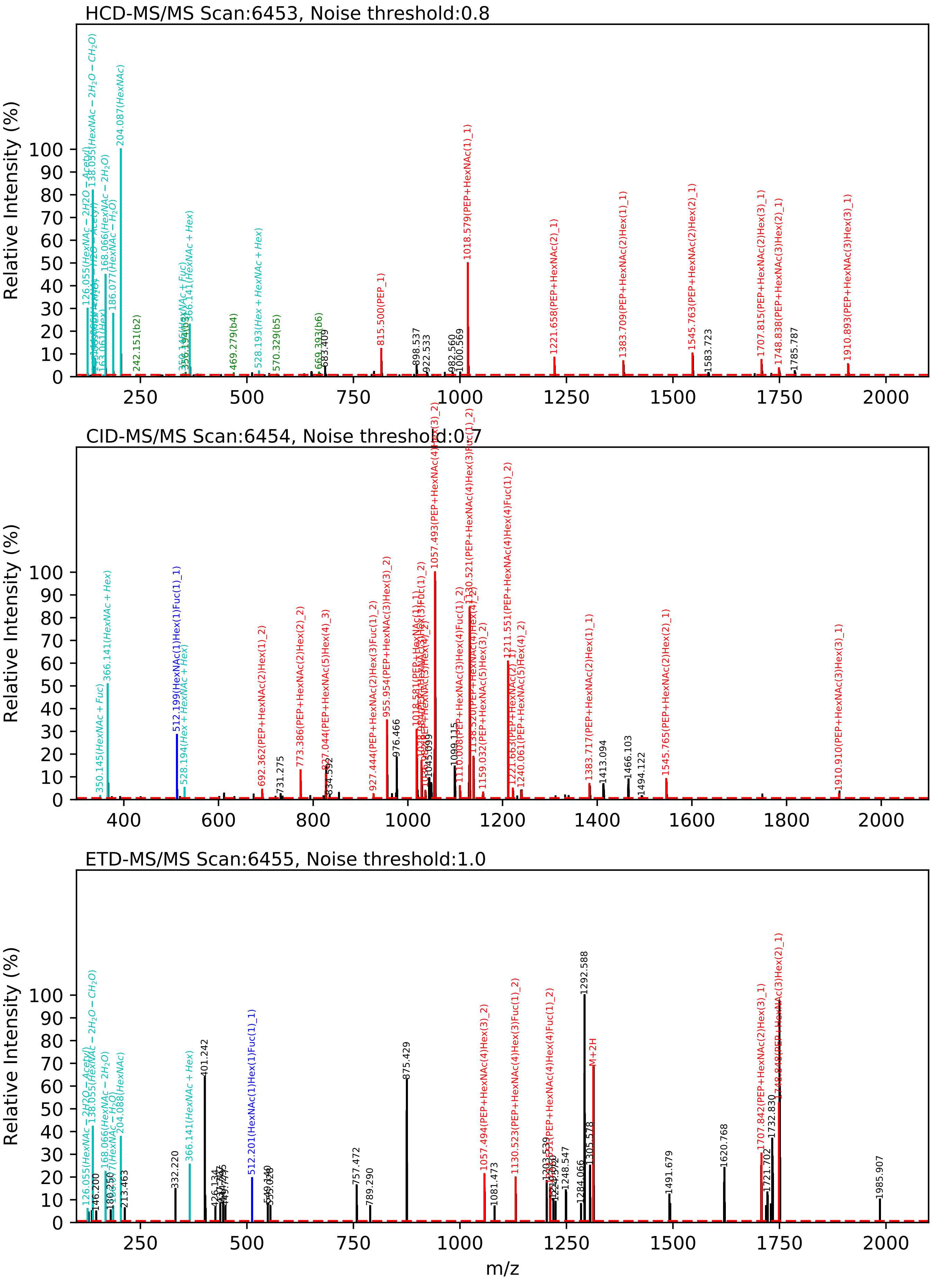

IQNLTVK(=PEP)_4_5_1_0_0, 0_None, 0_None,
m/z:1313.09(2+), RT:27.16, Y-score:90.83

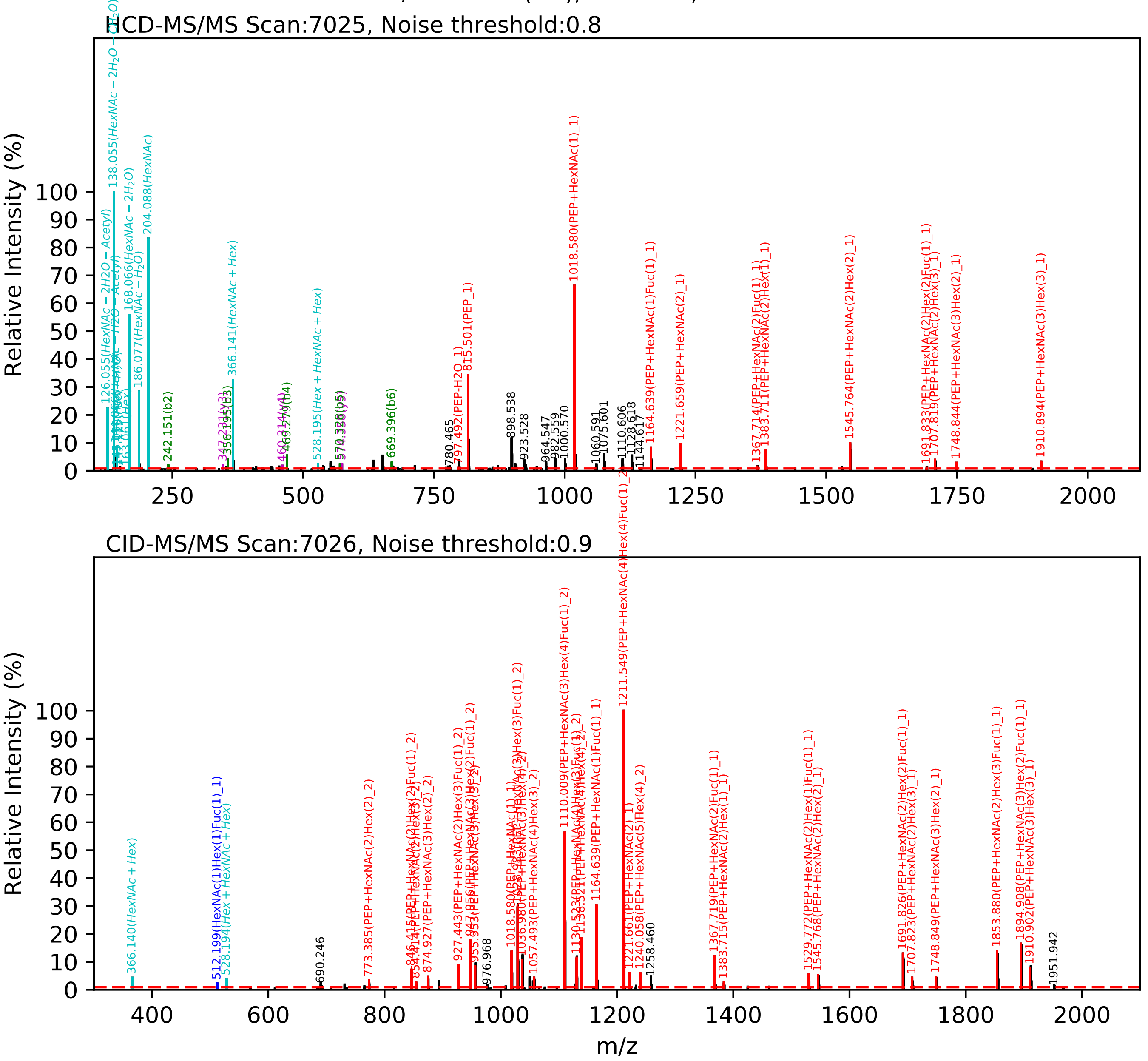

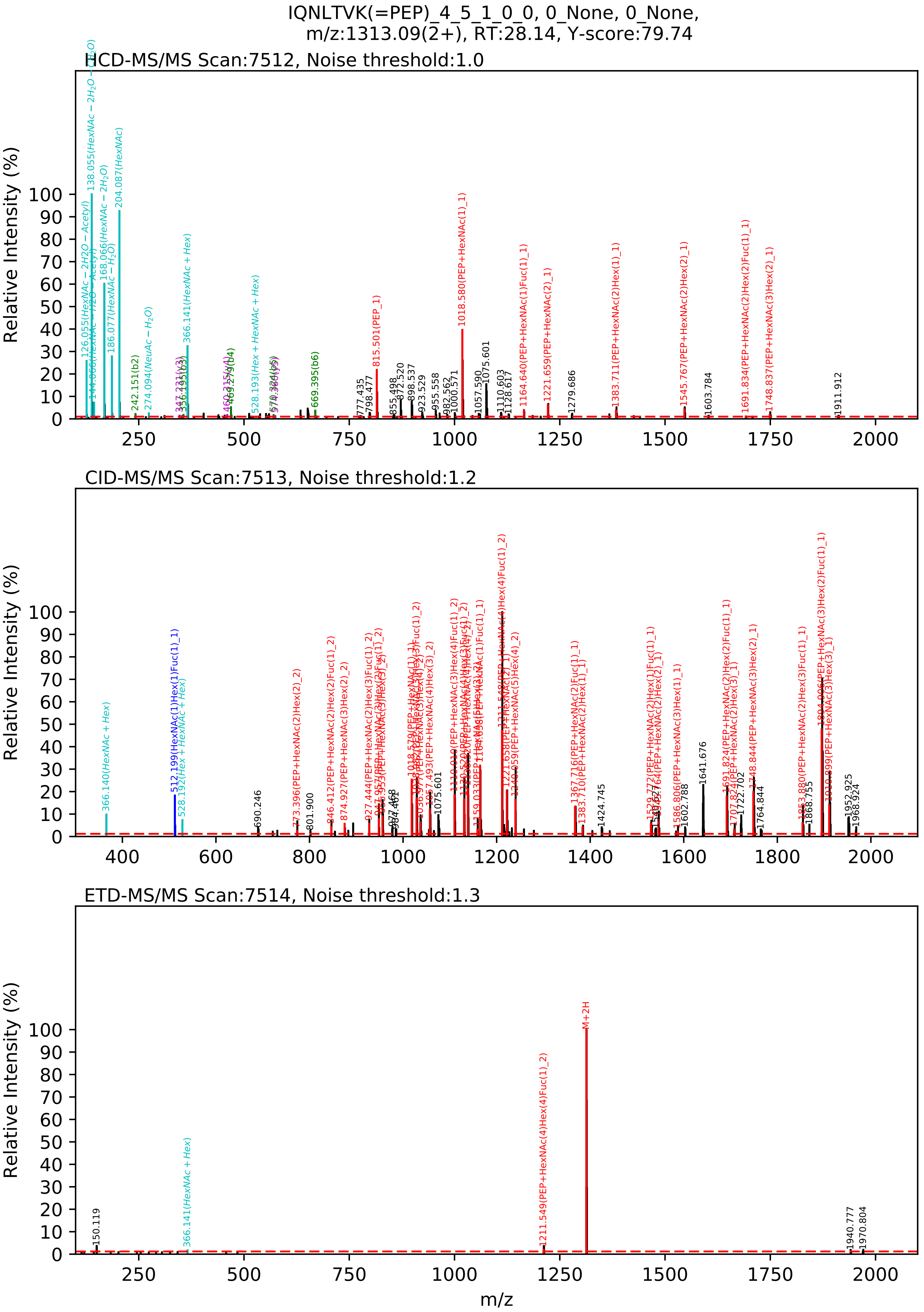

IQNLTVK(=PEP)_4_5_1_0_0, 0_None, 0_None,
m/z:1313.09(2+), RT:28.14, Y-score:79.74
HCD-MS/MS Scan:7512, Noise threshold:1.0
Relative Intensity (%)
138.055(HexNAc − 2H2O − H2O)
204.087(HexNAc)
366.141(HexNAc + Hex)
815.501(PEP_1)
1018.580(PEP+HexNAc(1)_1)
1075.601
1164.640(PEP+HexNAc(1)Fuc(1)_1)
1221.659(PEP+HexNAc(2)_1)
1279.686
1383.711(PEP+HexNAc(2)Hex(1)_1)
1545.767(PEP+HexNAc(2)Hex(2)_1)
1603.784
1691.834(PEP+HexNAc(2)Hex(2)Fuc(1)_1)
1748.837(PEP+HexNAc(3)Hex(2)_1)
1911.912
CID-MS/MS Scan:7513, Noise threshold:1.2
Relative Intensity (%)
366.140(HexNAc + Hex)
512.199(HexNAc(1)Hex(1)Fuc(1)_1)
690.246
801.900
1075.601
1211.548(PEP+HexNAc(4)Hex(4)Fuc(1)_2)
1367.716(PEP+HexNAc(2)Fuc(1)_1)
1424.745
1586.806(PEP+HexNAc(3)Hex(1)_1)
1602.788
1641.676
1691.824(PEP+HexNAc(2)Hex(2)Fuc(1)_1)
1722.702
1748.844(PEP+HexNAc(3)Hex(2)_1)
1764.844
1853.880(PEP+HexNAc(2)Hex(3)Fuc(1)_1)
1868.755
1894.906(PEP+HexNAc(3)Hex(2)Fuc(1)_1)
1952.925
1968.924
ETD-MS/MS Scan:7514, Noise threshold:1.3
Relative Intensity (%)
150.119
366.141(HexNAc + Hex)
1211.549(PEP+HexNAc(4)Hex(4)Fuc(1)_2)
M+2H
1940.777
1970.804
m/z

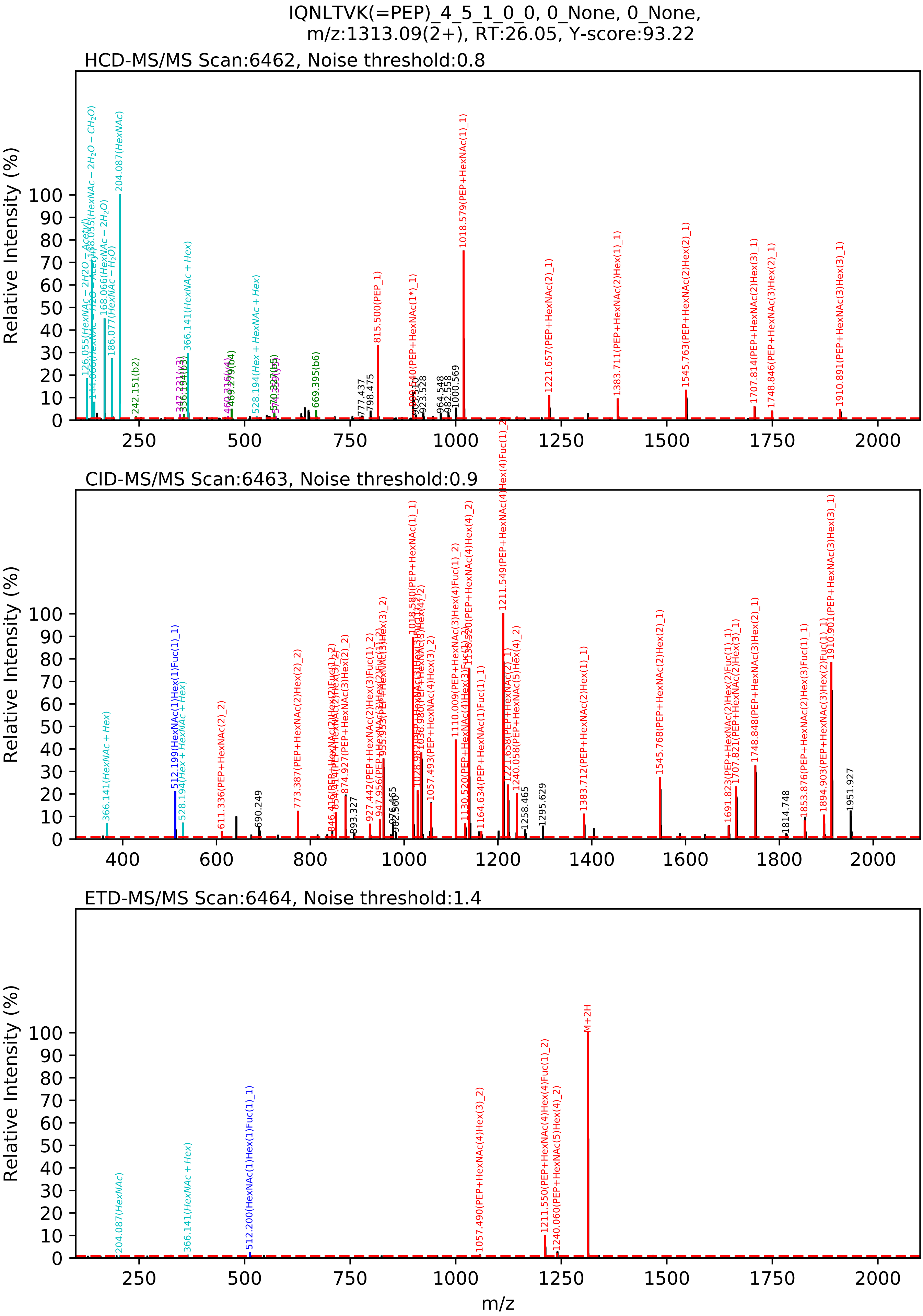

IQNLTVK(=PEP)_4_5_1_0_0, 0_None, 0_None,
m/z:1313.09(2+), RT:26.05, Y-score:93.22
HCD-MS/MS Scan:6462, Noise threshold:0.8
CID-MS/MS Scan:6463, Noise threshold:0.9
ETD-MS/MS Scan:6464, Noise threshold:1.4
Relative Intensity (%)
m/z

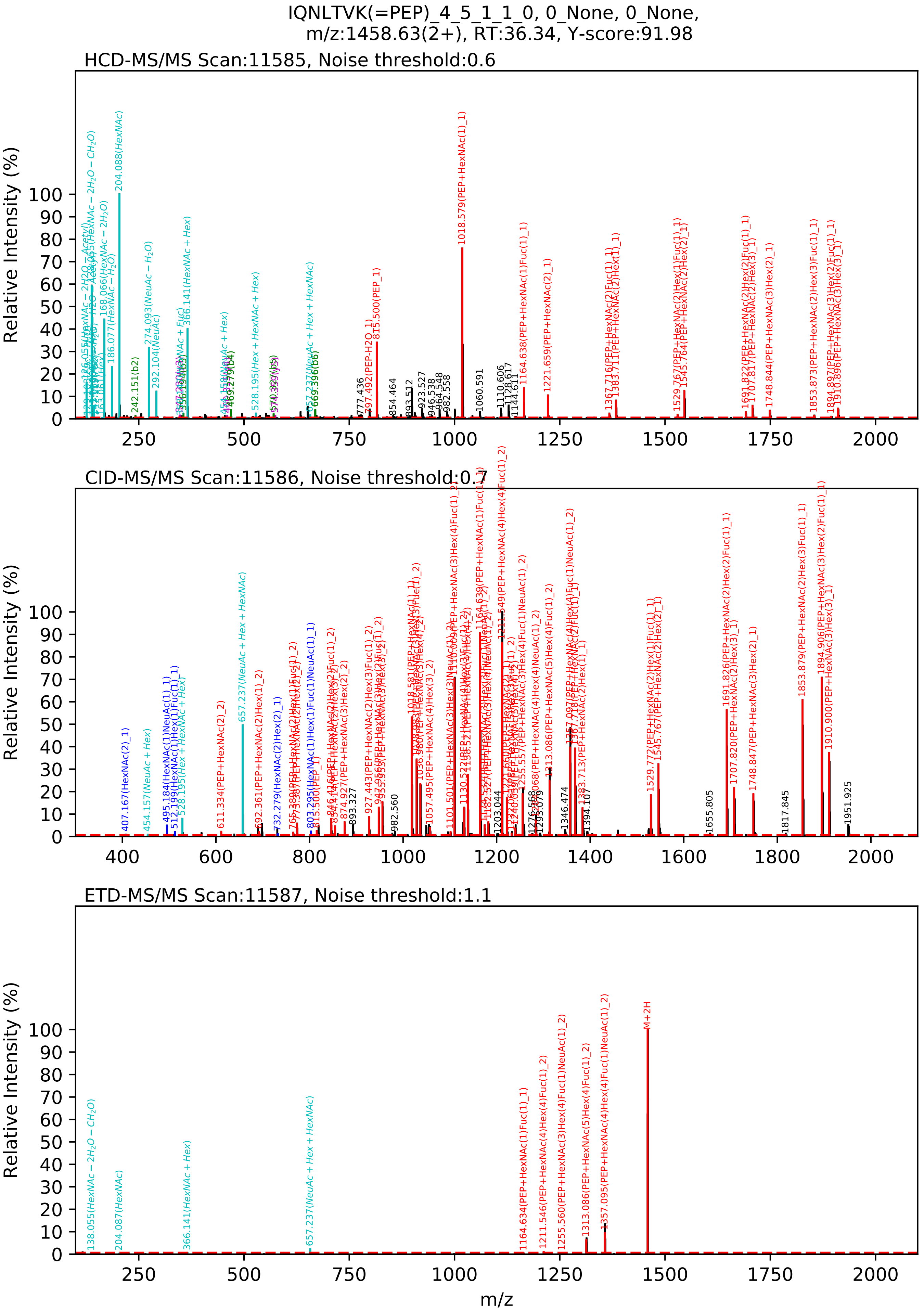

IQNLTVK(=PEP)_4_5_1_1_0, 0_None, 0_None,
m/z:1458.63(2+), RT:36.34, Y-score:91.98
HCD-MS/MS Scan:11585, Noise threshold:0.6
CID-MS/MS Scan:11586, Noise threshold:0.7
ETD-MS/MS Scan:11587, Noise threshold:1.1
Relative Intensity (%)
m/z
204.088(HexNAc)
1018.579(PEP+HexNAc(1)_1)
815.500(PEP_1)
1164.638(PEP+HexNAc(1)Fuc(1)_1)
1221.659(PEP+HexNAc(2)_1)
M+2H
1357.095(PEP+HexNAc(4)Hex(4)Fuc(1)NeuAc(1)_2)
1313.086(PEP+HexNAc(5)Hex(4)Fuc(1)_2)

IQNLTVK(=PEP)_4_5_1_1_0, 0_None, 0_None,
m/z:1458.63(2+), RT:36.42, Y-score:92.35

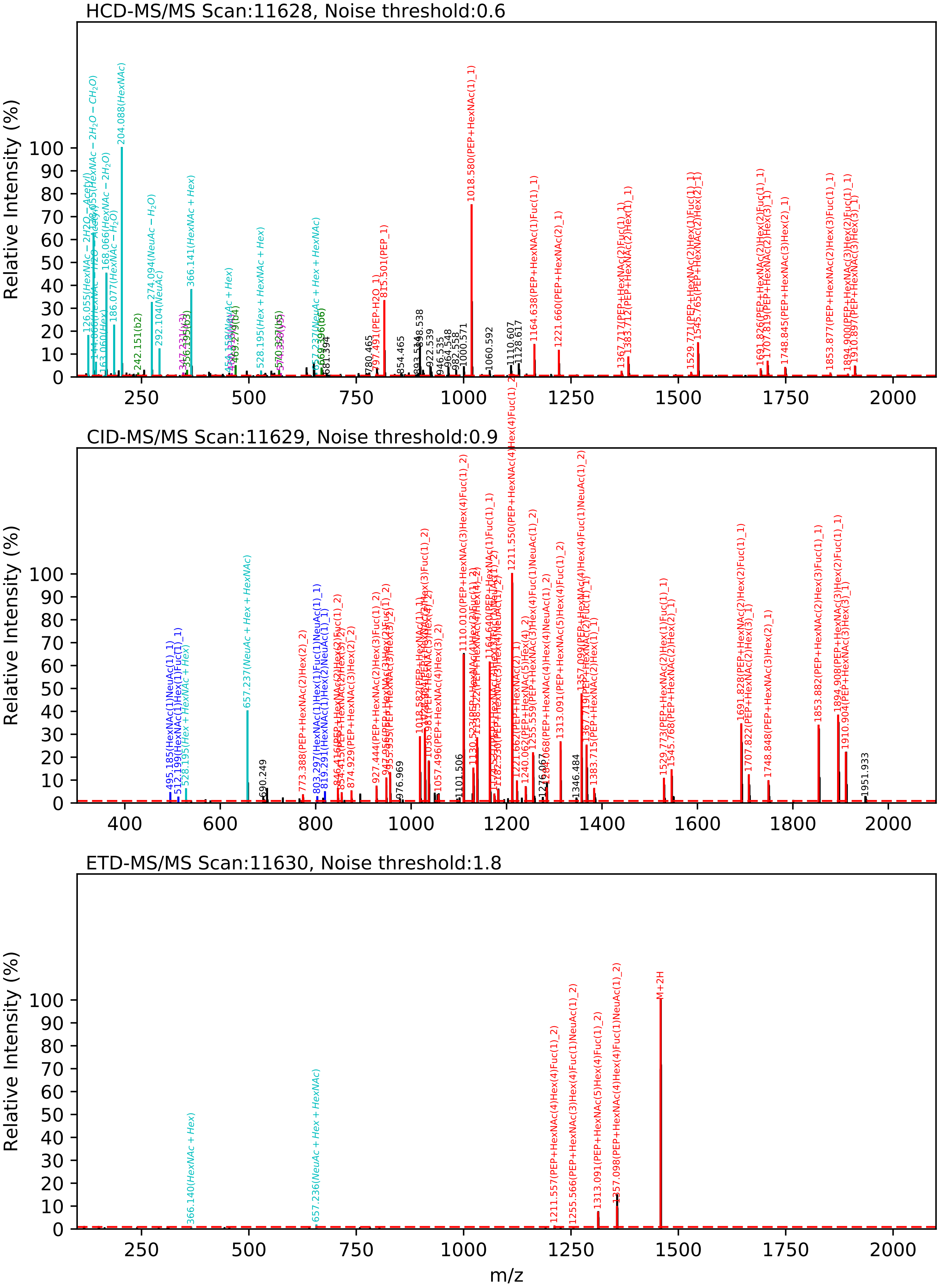

IQNLTVK(=PEP)_4_5_1_1_0, 0_None, 0_None,
m/z:972.76(3+), RT:36.37, Y-score:92.07

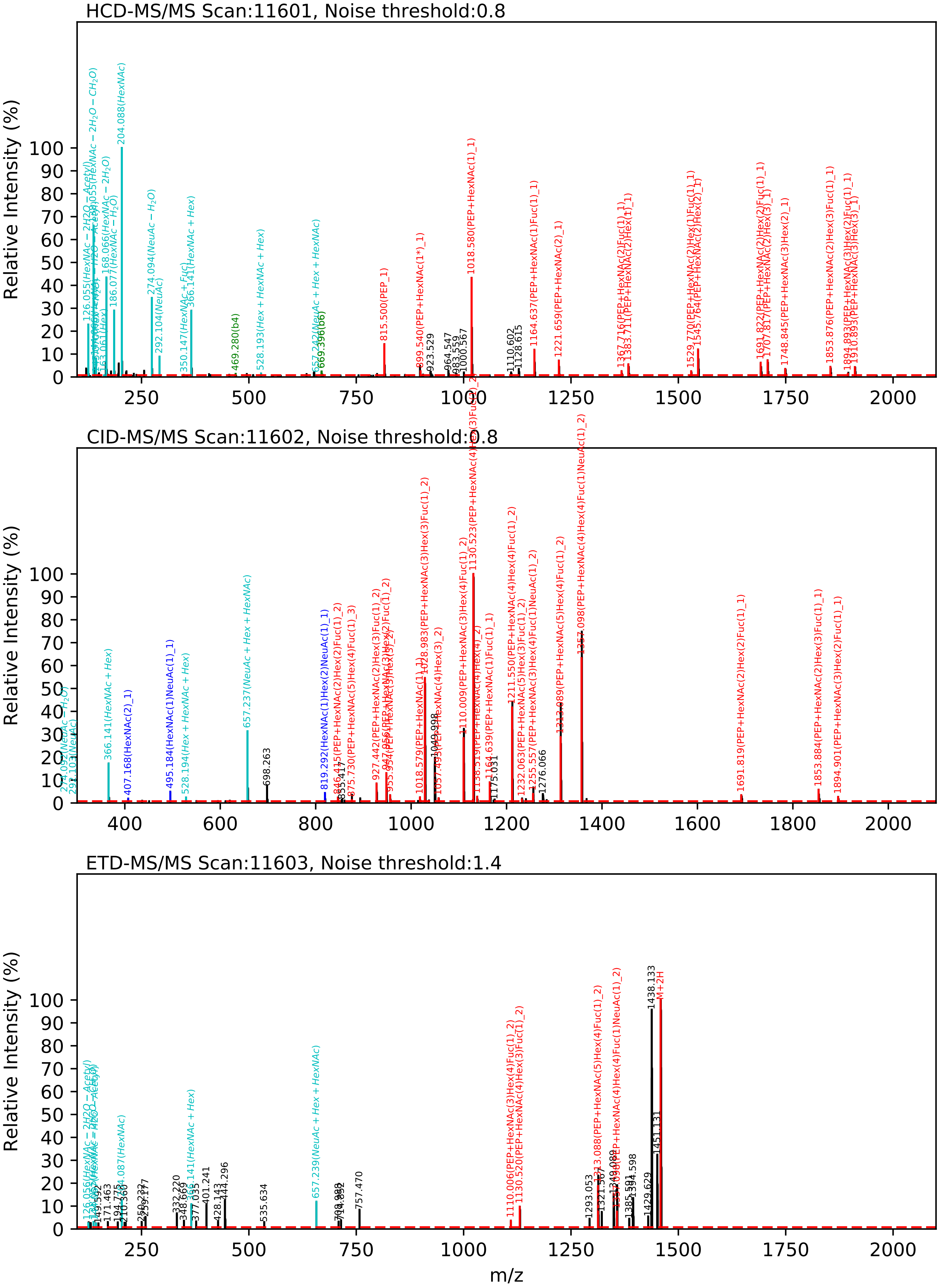

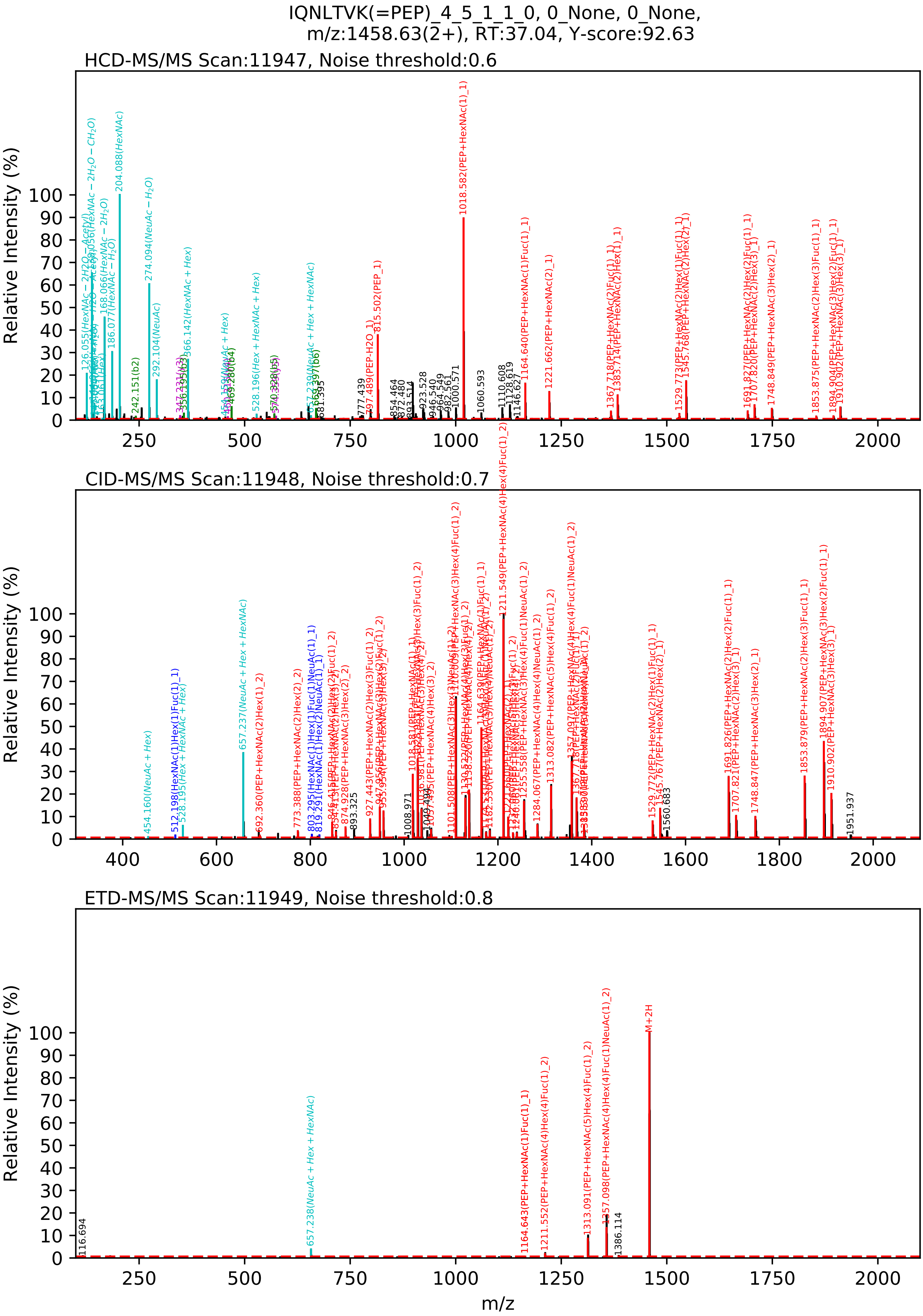
IQNLTVK(=PEP)_4_5_1_1_0, 0_None, 0_None,
m/z:1458.63(2+), RT:37.04, Y-score:92.63
HCD-MS/MS Scan:11947, Noise threshold:0.6
CID-MS/MS Scan:11948, Noise threshold:0.7
ETD-MS/MS Scan:11949, Noise threshold:0.8
Relative Intensity (%)
m/z

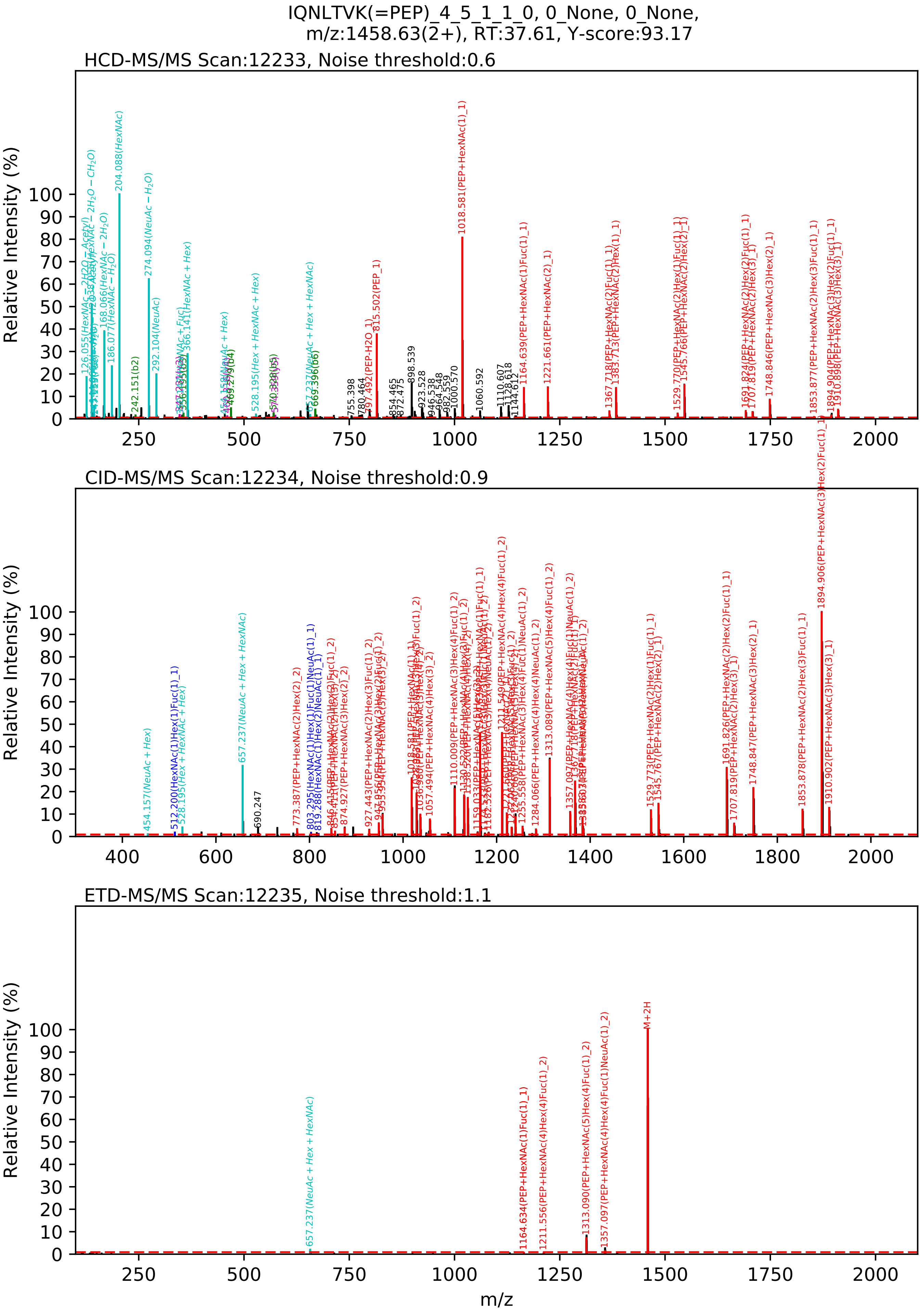
IQNLTVK(=PEP)_4_5_1_1_0, 0_None, 0_None,
m/z:1458.63(2+), RT:37.61, Y-score:93.17
HCD-MS/MS Scan:12233, Noise threshold:0.6
CID-MS/MS Scan:12234, Noise threshold:0.9
ETD-MS/MS Scan:12235, Noise threshold:1.1
Relative Intensity (%)
m/z
204.088(HexNAc)
274.094(NeuAc − H2O)
292.104(NeuAc)
168.066(HexNAc − 2H2O)
186.077(HexNAc − H2O)
366.141(HexNAc + Hex)
242.151(b2)
528.195(Hex + HexNAc + Hex)
657.237(NeuAc + Hex + HexNAc)
669.396(b6)
815.502(PEP_1)
797.492(PEP-H2O_1)
898.539
1018.581(PEP+HexNAc(1)_1)
1164.639(PEP+HexNAc(1)Fuc(1)_1)
1221.661(PEP+HexNAc(2)_1)
1383.713(PEP+HexNAc(2)Hex(1)_1)
1545.766(PEP+HexNAc(2)Hex(2)_1)
1748.846(PEP+HexNAc(3)Hex(2)_1)
1853.877(PEP+HexNAc(2)Hex(3)Fuc(1)_1)
1894.906(PEP+HexNAc(3)Hex(2)Fuc(1)_1)
454.157(NeuAc + Hex)
512.200(HexNAc(1)Hex(1)Fuc(1)_1)
803.295(HexNAc(1)Hex(1)Fuc(1)NeuAc(1)_1)
773.387(PEP+HexNAc(2)Hex(2)_2)
1313.089(PEP+HexNAc(5)Hex(4)Fuc(1)_2)
1529.773(PEP+HexNAc(2)Hex(1)Fuc(1)_1)
1691.826(PEP+HexNAc(2)Hex(2)Fuc(1)_1)
1910.902(PEP+HexNAc(3)Hex(3)_1)
1164.634(PEP+HexNAc(1)Fuc(1)_1)
1211.556(PEP+HexNAc(4)Hex(4)Fuc(1)_2)
1313.090(PEP+HexNAc(5)Hex(4)Fuc(1)_2)
1357.097(PEP+HexNAc(4)Hex(4)Fuc(1)NeuAc(1)_2)
M+2H

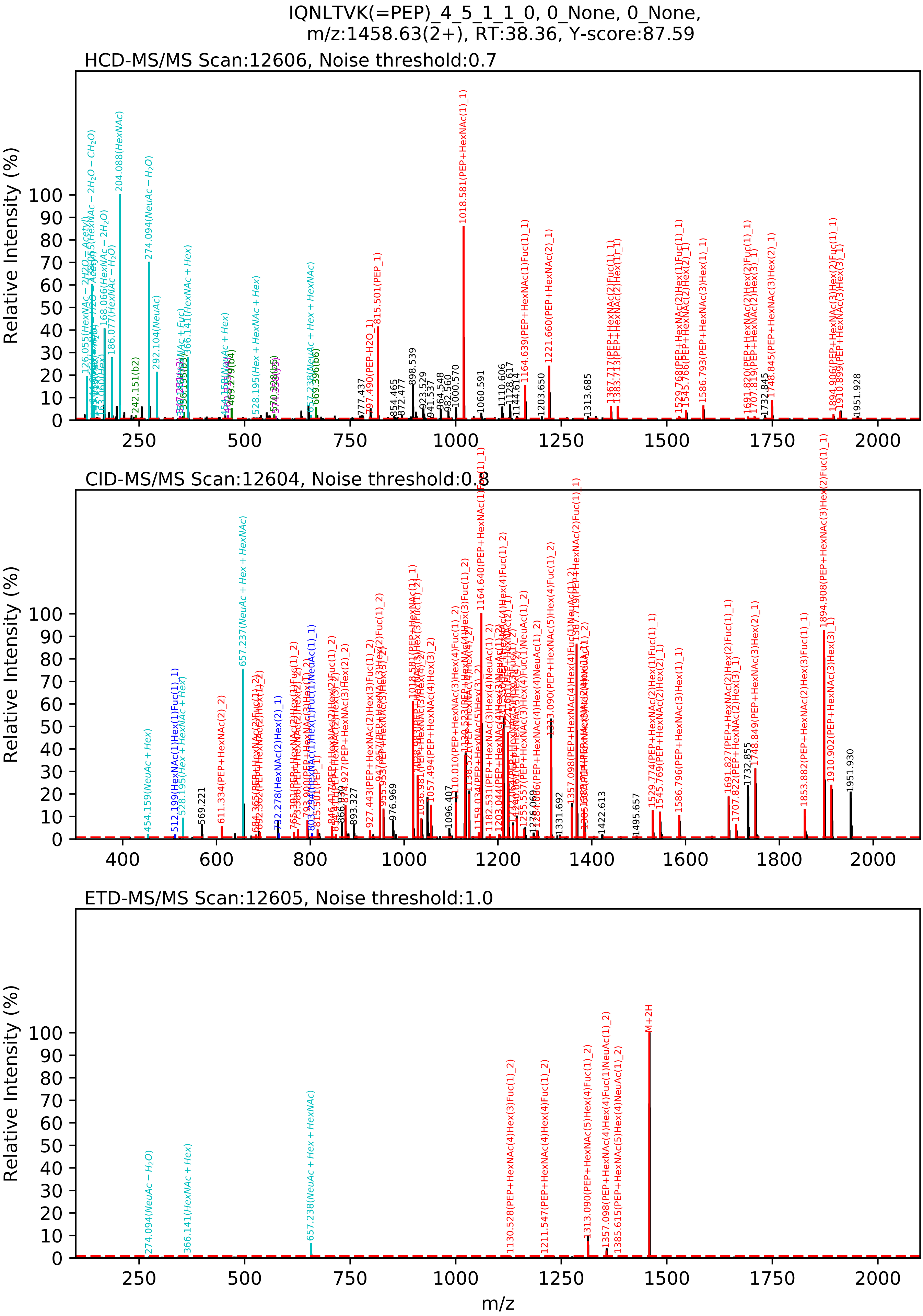
IQNLTVK(=PEP)_4_5_1_1_0, 0_None, 0_None,
m/z:1458.63(2+), RT:38.36, Y-score:87.59
HCD-MS/MS Scan:12606, Noise threshold:0.7
Relative Intensity (%)
204.088(HexNAc)
274.094(NeuAc − H2O)
292.104(NeuAc)
366.141(HexNAc + Hex)
528.195(Hex + HexNAc + Hex)
669.396(b6)
242.151(b2)
469.279(b4)
797.490(PEP-H2O_1)
815.501(PEP_1)
1018.581(PEP+HexNAc(1)_1)
1164.639(PEP+HexNAc(1)Fuc(1)_1)
1221.660(PEP+HexNAc(2)_1)
1367.717(PEP+HexNAc(2)Fuc(1)_1)
1383.713(PEP+HexNAc(2)Hex(1)_1)
1529.768(PEP+HexNAc(2)Hex(1)Fuc(1)_1)
1545.766(PEP+HexNAc(2)Hex(2)_1)
1586.793(PEP+HexNAc(3)Hex(1)_1)
1691.820(PEP+HexNAc(2)Hex(2)Fuc(1)_1)
1707.819(PEP+HexNAc(2)Hex(3)_1)
1748.845(PEP+HexNAc(3)Hex(2)_1)
1894.906(PEP+HexNAc(3)Hex(2)Fuc(1)_1)
1910.899(PEP+HexNAc(3)Hex(3)_1)
777.437
854.465
872.477
898.539
923.529
941.537
964.548
982.560
1000.570
1060.591
1110.606
1128.617
1144.614
1203.650
1313.685
1732.845
1951.928
CID-MS/MS Scan:12604, Noise threshold:0.8
Relative Intensity (%)
454.159(NeuAc + Hex)
512.199(HexNAc(1)Hex(1)Fuc(1)_1)
528.195(Hex + HexNAc + Hex)
569.221
611.334(PEP+HexNAc(2)_2)
657.237(NeuAc + Hex + HexNAc)
732.278(HexNAc(2)Hex(2)_1)
815.501(PEP_1)
866.930
893.327
927.443(PEP+HexNAc(2)Hex(3)Fuc(1)_2)
976.969
1057.494(PEP+HexNAc(4)Hex(3)_2)
1096.407
1164.640(PEP+HexNAc(1)Fuc(1)_1)
1313.090(PEP+HexNAc(5)Hex(4)Fuc(1)_2)
1331.692
1357.098(PEP+HexNAc(4)Hex(4)Fuc(1)NeuAc(1)_2)
1422.613
1495.657
1529.774(PEP+HexNAc(2)Hex(1)Fuc(1)_1)
1545.769(PEP+HexNAc(2)Hex(2)_1)
1586.796(PEP+HexNAc(3)Hex(1)_1)
1691.827(PEP+HexNAc(2)Hex(2)Fuc(1)_1)
1707.822(PEP+HexNAc(2)Hex(3)_1)
1732.855
1748.849(PEP+HexNAc(3)Hex(2)_1)
1853.882(PEP+HexNAc(2)Hex(3)Fuc(1)_1)
1894.908(PEP+HexNAc(3)Hex(2)Fuc(1)_1)
1910.902(PEP+HexNAc(3)Hex(3)_1)
1951.930
ETD-MS/MS Scan:12605, Noise threshold:1.0
Relative Intensity (%)
274.094(NeuAc − H2O)
366.141(HexNAc + Hex)
657.238(NeuAc + Hex + HexNAc)
1130.528(PEP+HexNAc(4)Hex(3)Fuc(1)_2)
1211.547(PEP+HexNAc(4)Hex(4)Fuc(1)_2)
1313.090(PEP+HexNAc(5)Hex(4)Fuc(1)_2)
1357.098(PEP+HexNAc(4)Hex(4)Fuc(1)NeuAc(1)_2)
1385.615(PEP+HexNAc(5)Hex(4)NeuAc(1)_2)
M+2H
m/z

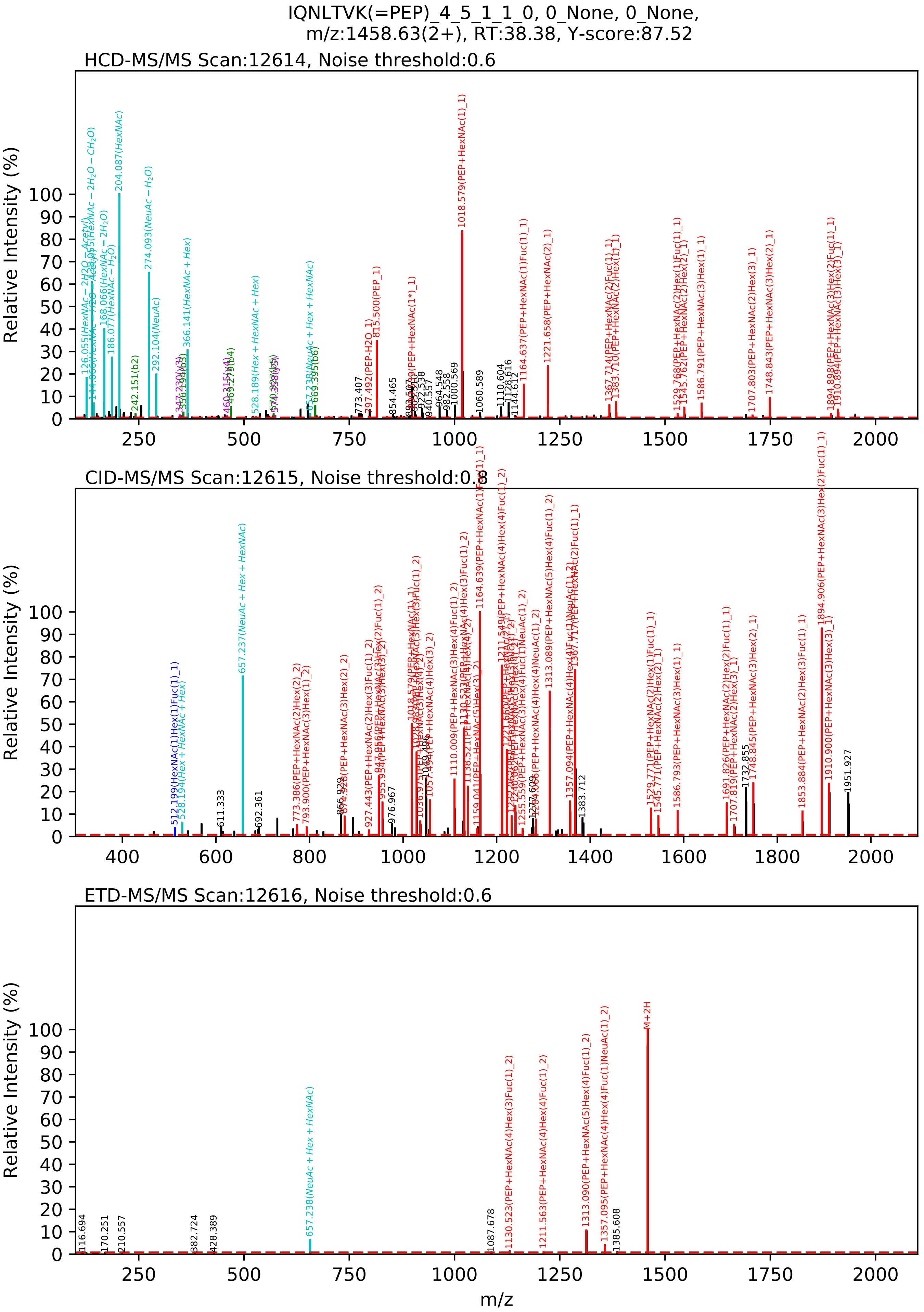

IQNLTVK(=PEP)_4_5_1_1_0, 0_None, 0_None,
m/z:1458.63(2+), RT:38.38, Y-score:87.52
HCD-MS/MS Scan:12614, Noise threshold:0.6
Relative Intensity (%)
204.087(HexNAc)
274.093(NeuAc − H2O)
366.141(HexNAc + Hex)
292.104(NeuAc)
242.151(b2)
1018.579(PEP+HexNAc(1)_1)
815.500(PEP_1)
797.492(PEP-H2O_1)
1164.637(PEP+HexNAc(1)Fuc(1)_1)
1221.658(PEP+HexNAc(2)_1)
1586.791(PEP+HexNAc(3)Hex(1)_1)
1707.803(PEP+HexNAc(2)Hex(3)_1)
1748.843(PEP+HexNAc(3)Hex(2)_1)
773.407
854.465
1060.589
1110.604
1128.616
1144.612
CID-MS/MS Scan:12615, Noise threshold:0.8
657.237(NeuAc + Hex + HexNAc)
1164.639(PEP+HexNAc(4)Hex(4)Fuc(1)_2)
1313.089(PEP+HexNAc(5)Hex(4)Fuc(1)_2)
1894.906(PEP+HexNAc(3)Hex(2)Fuc(1)_1)
1910.900(PEP+HexNAc(3)Hex(3)_1)
1951.927
1732.855
1748.845(PEP+HexNAc(3)Hex(2)_1)
1853.884(PEP+HexNAc(2)Hex(3)Fuc(1)_1)
1586.793(PEP+HexNAc(3)Hex(1)_1)
1383.712
611.333
692.361
866.929
976.967
ETD-MS/MS Scan:12616, Noise threshold:0.6
M+2H
657.238(NeuAc + Hex + HexNAc)
1130.523(PEP+HexNAc(4)Hex(3)Fuc(1)_2)
1211.563(PEP+HexNAc(4)Hex(4)Fuc(1)_2)
1313.090(PEP+HexNAc(5)Hex(4)Fuc(1)_2)
1357.095(PEP+HexNAc(4)Hex(4)Fuc(1)NeuAc(1)_2)
1385.608
1087.678
116.694
170.251
210.557
382.724
428.389
m/z

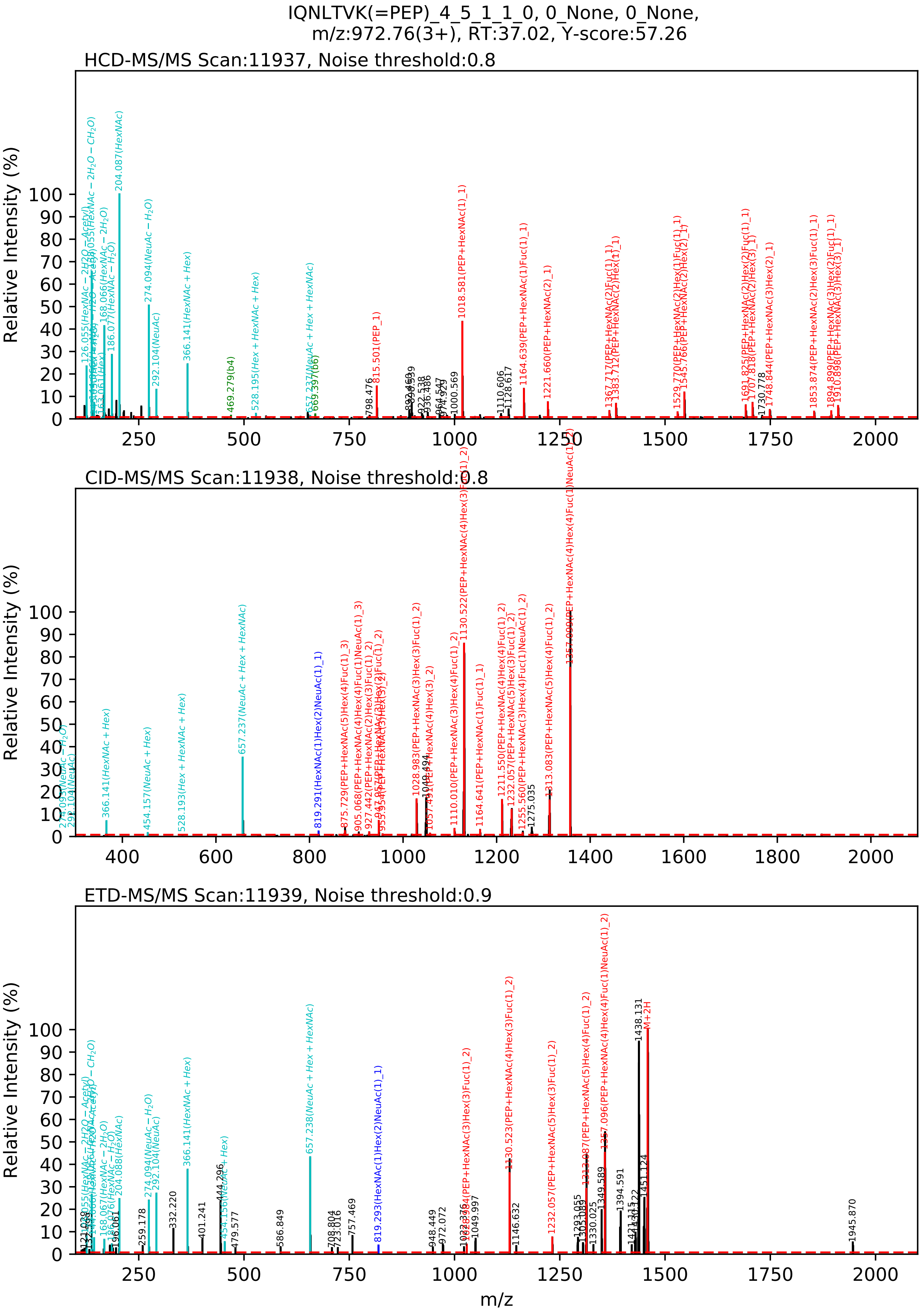
IQNLTVK(=PEP)_4_5_1_1_0, 0_None, 0_None,
m/z:972.76(3+), RT:37.02, Y-score:57.26
HCD-MS/MS Scan:11937, Noise threshold:0.8
Relative Intensity (%)
204.087(HexNAc)
274.094(NeuAc − H2O)
292.104(NeuAc)
366.141(HexNAc + Hex)
469.279(b4)
528.195(Hex + HexNAc + Hex)
657.237(NeuAc + Hex + HexNAc)
669.397(b6)
815.501(PEP_1)
1018.581(PEP+HexNAc(1)_1)
1164.639(PEP+HexNAc(1)Fuc(1)_1)
1221.660(PEP+HexNAc(2)_1)
1367.717(PEP+HexNAc(2)Fuc(1)_1)
1383.712(PEP+HexNAc(2)Hex(1)_1)
1529.770(PEP+HexNAc(2)Hex(1)Fuc(1)_1)
1545.766(PEP+HexNAc(2)Hex(2)_1)
1691.825(PEP+HexNAc(2)Hex(2)Fuc(1)_1)
1707.818(PEP+HexNAc(2)Hex(3)_1)
1748.844(PEP+HexNAc(3)Hex(2)_1)
1853.874(PEP+HexNAc(2)Hex(3)Fuc(1)_1)
1894.899(PEP+HexNAc(3)Hex(2)Fuc(1)_1)
1910.898(PEP+HexNAc(3)Hex(3)_1)
CID-MS/MS Scan:11938, Noise threshold:0.8
366.141(HexNAc + Hex)
454.157(NeuAc + Hex)
528.193(Hex + HexNAc + Hex)
657.237(NeuAc + Hex + HexNAc)
819.291(HexNAc(1)Hex(2)NeuAc(1)_1)
875.729(PEP+HexNAc(5)Hex(4)Fuc(1)_3)
905.068(PEP+HexNAc(4)Hex(4)Fuc(1)NeuAc(1)_3)
927.442(PEP+HexNAc(2)Hex(3)Fuc(1)_2)
1028.983(PEP+HexNAc(3)Hex(3)Fuc(1)_2)
1057.491(PEP+HexNAc(4)Hex(3)_2)
1110.010(PEP+HexNAc(3)Hex(4)Fuc(1)_2)
1130.522(PEP+HexNAc(4)Hex(3)Fuc(1)_2)
1164.641(PEP+HexNAc(1)Fuc(1)_1)
1211.550(PEP+HexNAc(4)Hex(4)Fuc(1)_2)
1232.057(PEP+HexNAc(5)Hex(3)Fuc(1)_2)
1255.560(PEP+HexNAc(3)Hex(4)Fuc(1)NeuAc(1)_2)
1275.035
1313.083(PEP+HexNAc(5)Hex(4)Fuc(1)_2)
1357.090(PEP+HexNAc(4)Hex(4)Fuc(1)NeuAc(1)_2)
ETD-MS/MS Scan:11939, Noise threshold:0.9
657.238(NeuAc + Hex + HexNAc)
819.293(HexNAc(1)Hex(2)NeuAc(1)_1)
1130.523(PEP+HexNAc(4)Hex(3)Fuc(1)_2)
1232.057(PEP+HexNAc(5)Hex(3)Fuc(1)_2)
1313.087(PEP+HexNAc(5)Hex(4)Fuc(1)_2)
1357.096(PEP+HexNAc(4)Hex(4)Fuc(1)NeuAc(1)_2)
1438.131
M+2H
1945.870
m/z

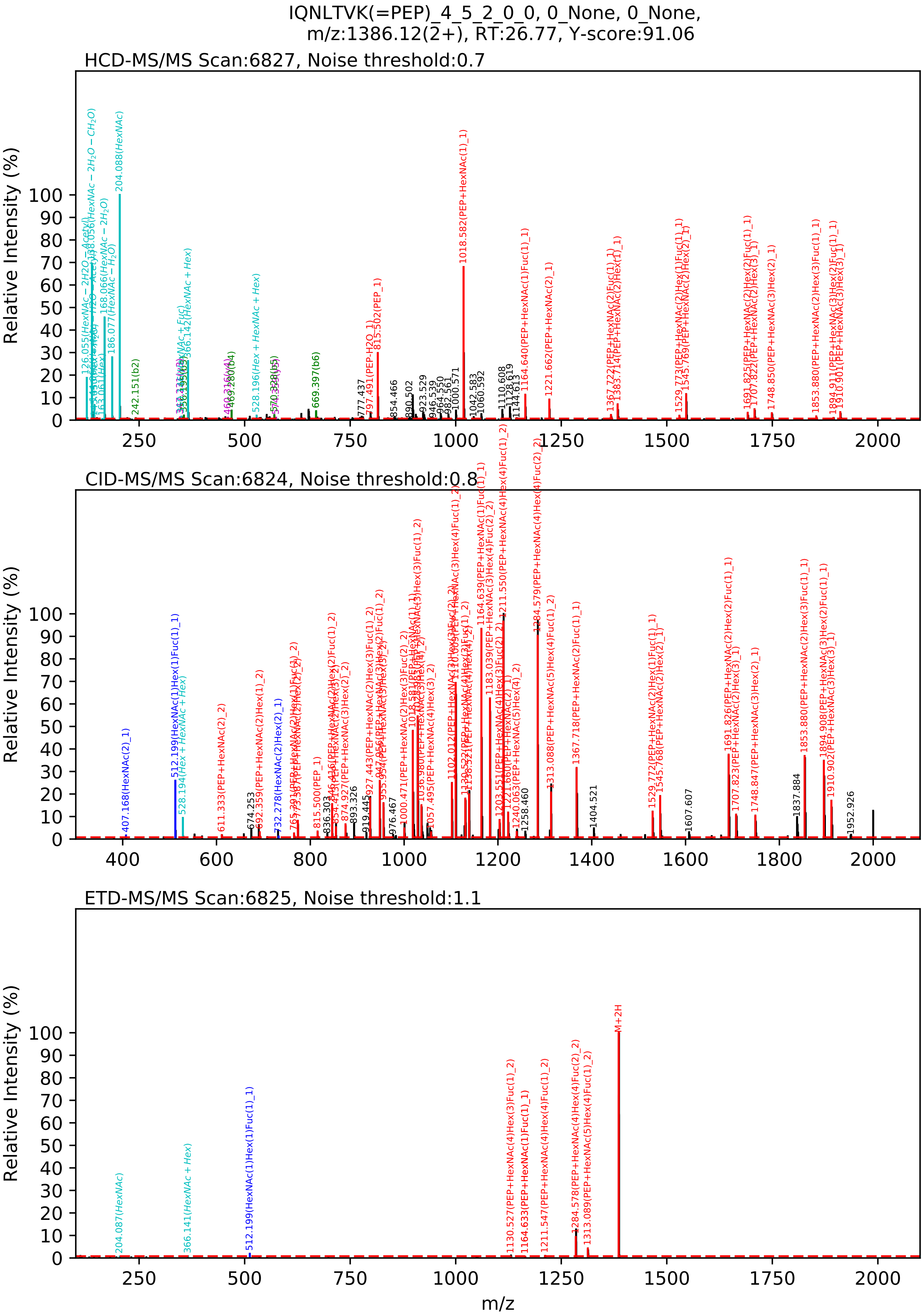

IQNLTVK(=PEP)_4_5_2_0_0, 0_None, 0_None,
m/z:1386.12(2+), RT:26.77, Y-score:91.06
HCD-MS/MS Scan:6827, Noise threshold:0.7
CID-MS/MS Scan:6824, Noise threshold:0.8
ETD-MS/MS Scan:6825, Noise threshold:1.1
Relative Intensity (%)
m/z
204.088(HexNAc)
186.077(HexNAc − H2O)
168.066(HexNAc − 2H2O)
366.142(HexNAc + Hex)
242.151(b2)
469.280(b4)
528.196(Hex + HexNAc + Hex)
669.397(b6)
815.502(PEP_1)
797.491(PEP-H2O_1)
1018.582(PEP+HexNAc(1)_1)
1164.640(PEP+HexNAc(1)Fuc(1)_1)
1221.662(PEP+HexNAc(2)_1)
1545.769(PEP+HexNAc(2)Hex(2)_1)
1748.850(PEP+HexNAc(3)Hex(2)_1)
1853.880(PEP+HexNAc(2)Hex(3)Fuc(1)_1)
512.199(HexNAc(1)Hex(1)Fuc(1)_1)
407.168(HexNAc(2)_1)
1284.579(PEP+HexNAc(4)Hex(4)Fuc(2)_2)
1164.639(PEP+HexNAc(1)Fuc(1)_1)
1367.718(PEP+HexNAc(2)Fuc(1)_1)
1691.826(PEP+HexNAc(2)Hex(2)Fuc(1)_1)
1313.088(PEP+HexNAc(5)Hex(4)Fuc(1)_2)
204.087(HexNAc)
366.141(HexNAc + Hex)
1284.578(PEP+HexNAc(4)Hex(4)Fuc(2)_2)
1313.089(PEP+HexNAc(5)Hex(4)Fuc(1)_2)
M+2H

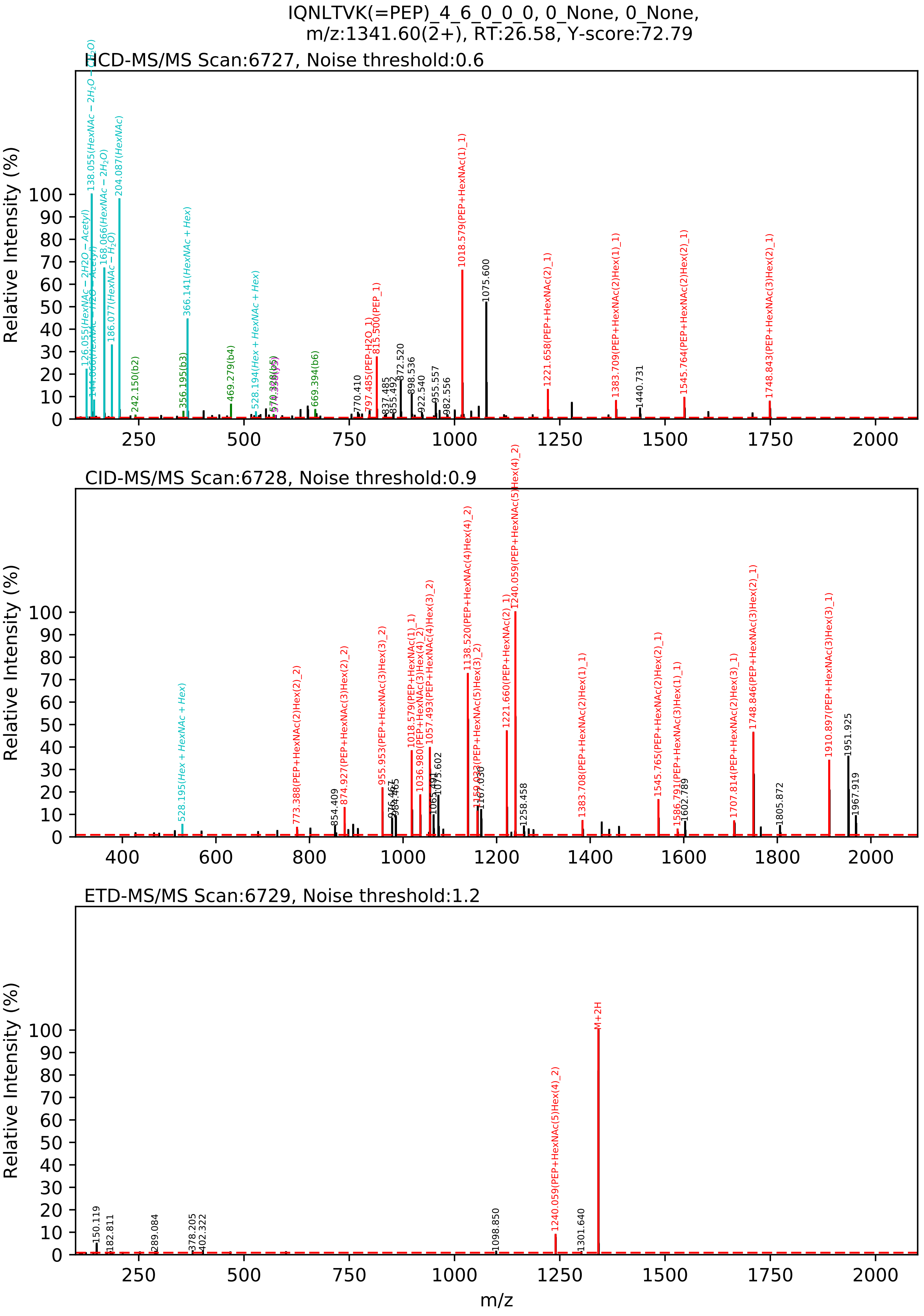

IQNLTVK(=PEP)_4_6_0_0_0, 0_None, 0_None,
m/z:1341.60(2+), RT:26.58, Y-score:72.79
HCD-MS/MS Scan:6727, Noise threshold:0.6
Relative Intensity (%)
126.055(HexNAc − 2H2O − Acetyl)
138.055(HexNAc − 2H2O − H2O)
168.066(HexNAc − 2H2O)
186.077(HexNAc − H2O)
204.087(HexNAc)
242.150(b2)
356.195(b3)
366.141(HexNAc + Hex)
469.279(b4)
528.194(Hex + HexNAc + Hex)
669.394(b6)
770.410
797.485(PEP-H2O_1)
815.500(PEP_1)
837.485
855.492
872.520
898.536
922.540
955.557
982.556
1018.579(PEP+HexNAc(1)_1)
1075.600
1221.658(PEP+HexNAc(2)_1)
1383.709(PEP+HexNAc(2)Hex(1)_1)
1440.731
1545.764(PEP+HexNAc(2)Hex(2)_1)
1748.843(PEP+HexNAc(3)Hex(2)_1)
CID-MS/MS Scan:6728, Noise threshold:0.9
528.195(Hex + HexNAc + Hex)
773.388(PEP+HexNAc(2)Hex(2)_2)
854.409
874.927(PEP+HexNAc(3)Hex(2)_2)
955.953(PEP+HexNAc(3)Hex(3)_2)
1018.579(PEP+HexNAc(1)_1)
1036.980(PEP+HexNAc(3)Hex(4)_2)
1057.493(PEP+HexNAc(4)Hex(3)_2)
1075.602
1138.520(PEP+HexNAc(4)Hex(4)_2)
1159.033(PEP+HexNAc(5)Hex(3)_2)
1167.030
1221.660(PEP+HexNAc(2)_1)
1240.059(PEP+HexNAc(5)Hex(4)_2)
1258.458
1383.708(PEP+HexNAc(2)Hex(1)_1)
1545.765(PEP+HexNAc(2)Hex(2)_1)
1586.791(PEP+HexNAc(3)Hex(1)_1)
1602.789
1707.814(PEP+HexNAc(2)Hex(3)_1)
1748.846(PEP+HexNAc(3)Hex(2)_1)
1805.872
1910.897(PEP+HexNAc(3)Hex(3)_1)
1951.925
1967.919
ETD-MS/MS Scan:6729, Noise threshold:1.2
150.119
182.811
289.084
378.205
402.322
1098.850
1240.059(PEP+HexNAc(5)Hex(4)_2)
1301.640
M+2H
m/z

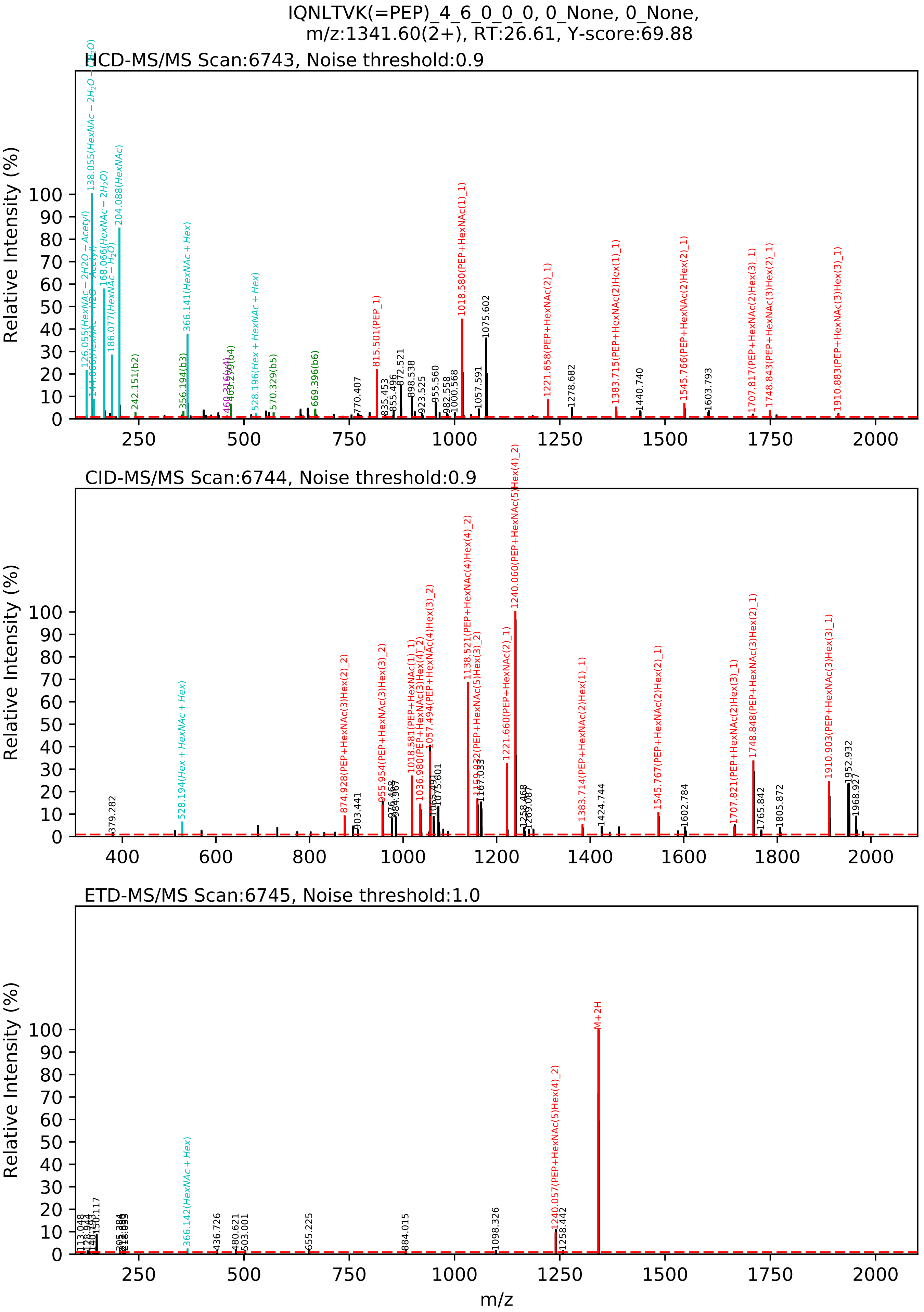

IQNLTVK(=PEP)_4_6_0_0_0, 0_None, 0_None,
m/z:1341.60(2+), RT:26.61, Y-score:69.88
HCD-MS/MS Scan:6743, Noise threshold:0.9
CID-MS/MS Scan:6744, Noise threshold:0.9
ETD-MS/MS Scan:6745, Noise threshold:1.0
Relative Intensity (%)
m/z
1018.580(PEP+HexNAc(1)_1)
1240.060(PEP+HexNAc(5)Hex(4)_2)
M+2H

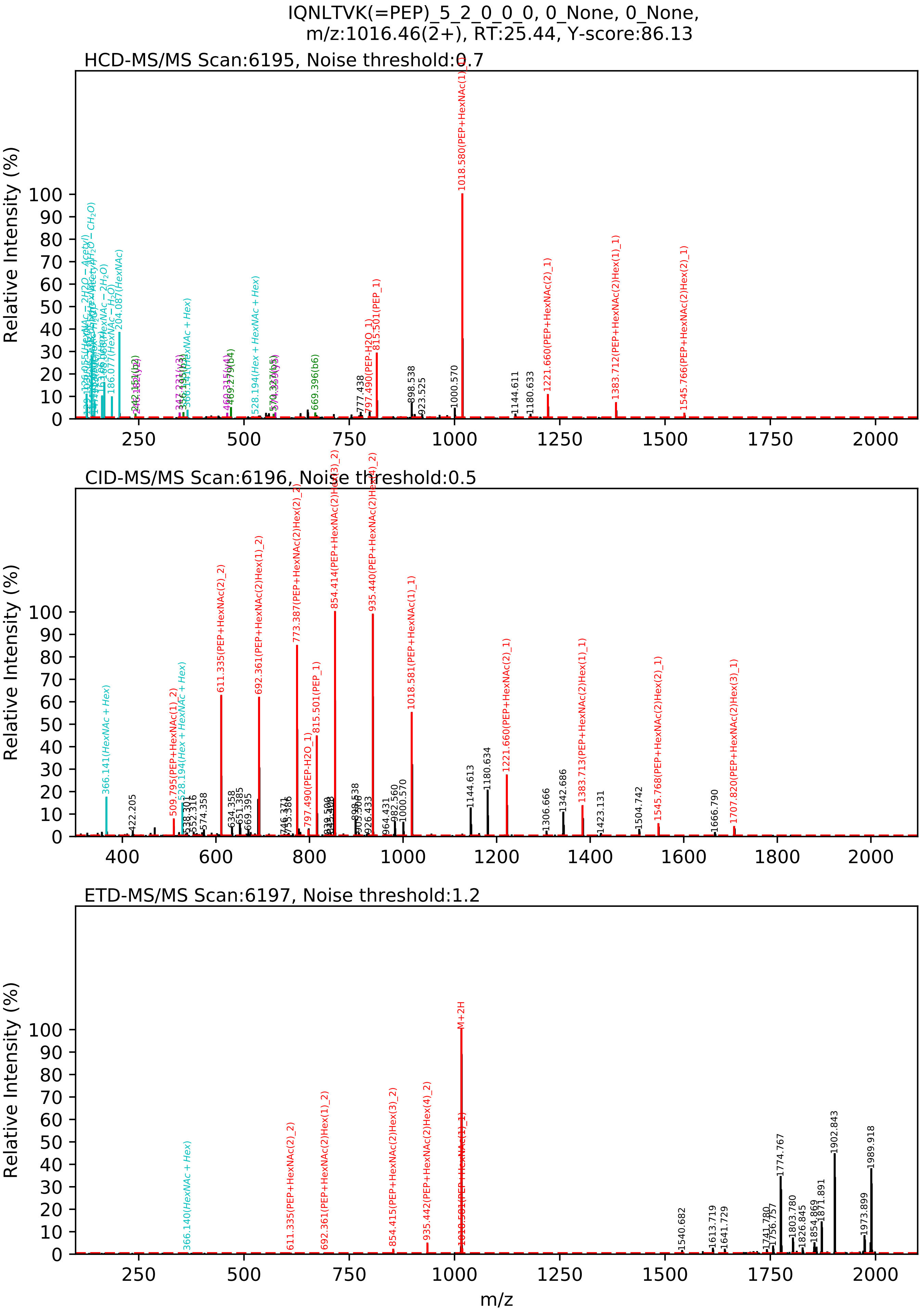

IQNLTVK(=PEP)_5_2_0_0_0, 0_None, 0_None,
m/z:1016.46(2+), RT:25.44, Y-score:86.13
HCD-MS/MS Scan:6195, Noise threshold:0.7
CID-MS/MS Scan:6196, Noise threshold:0.5
ETD-MS/MS Scan:6197, Noise threshold:1.2
Relative Intensity (%)
m/z
1018.580(PEP+HexNAc(1)_1)
815.501(PEP_1)
797.490(PEP-H2O_1)
1221.660(PEP+HexNAc(2)_1)
1383.712(PEP+HexNAc(2)Hex(1)_1)
1545.766(PEP+HexNAc(2)Hex(2)_1)
204.087(HexNAc)
528.194(Hex + HexNAc + Hex)
669.396(b6)
611.335(PEP+HexNAc(2)_2)
692.361(PEP+HexNAc(2)Hex(1)_2)
773.387(PEP+HexNAc(2)Hex(2)_2)
854.414(PEP+HexNAc(2)Hex(3)_2)
935.440(PEP+HexNAc(2)Hex(4)_2)
1018.581(PEP+HexNAc(1)_1)
1221.660(PEP+HexNAc(2)_1)
1383.713(PEP+HexNAc(2)Hex(1)_1)
1545.768(PEP+HexNAc(2)Hex(2)_1)
1707.820(PEP+HexNAc(2)Hex(3)_1)
366.141(HexNAc + Hex)
509.795(PEP+HexNAc(1)_2)
M+2H
1902.843
1989.918
1774.767

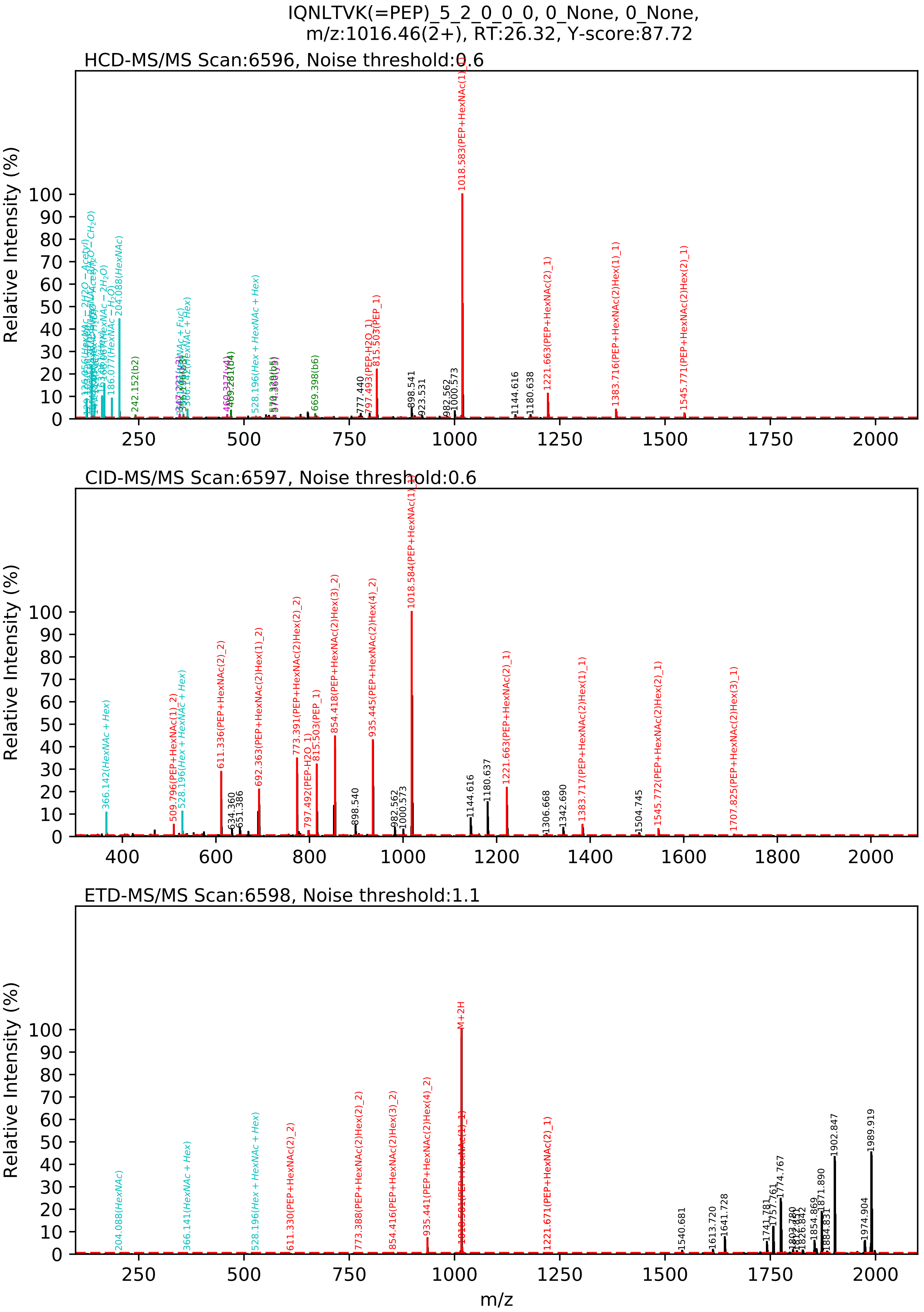
IQNLTVK(=PEP)_5_2_0_0_0, 0_None, 0_None,
m/z:1016.46(2+), RT:26.32, Y-score:87.72
HCD-MS/MS Scan:6596, Noise threshold:0.6
Relative Intensity (%)
204.088(HexNAc)
186.077(HexNAc − H2O)
242.152(b2)
366.14(HexNAc + Hex)
469.281(b4)
528.196(Hex + HexNAc + Hex)
669.398(b6)
777.440
797.493(PEP-H2O_1)
815.503(PEP_1)
898.541
923.531
982.562
1000.573
1018.583(PEP+HexNAc(1)_1)
1144.616
1180.638
1221.663(PEP+HexNAc(2)_1)
1383.716(PEP+HexNAc(2)Hex(1)_1)
1545.771(PEP+HexNAc(2)Hex(2)_1)
CID-MS/MS Scan:6597, Noise threshold:0.6
366.142(HexNAc + Hex)
509.796(PEP+HexNAc(1)_2)
528.196(Hex + HexNAc + Hex)
611.336(PEP+HexNAc(2)_2)
634.360
651.386
692.363(PEP+HexNAc(2)Hex(1)_2)
773.391(PEP+HexNAc(2)Hex(2)_2)
797.492(PEP-H2O_1)
815.503(PEP_1)
854.418(PEP+HexNAc(2)Hex(3)_2)
898.540
935.445(PEP+HexNAc(2)Hex(4)_2)
982.562
1000.573
1018.584(PEP+HexNAc(1)_1)
1144.616
1180.637
1221.663(PEP+HexNAc(2)_1)
1306.668
1342.690
1383.717(PEP+HexNAc(2)Hex(1)_1)
1504.745
1545.772(PEP+HexNAc(2)Hex(2)_1)
1707.825(PEP+HexNAc(2)Hex(3)_1)
ETD-MS/MS Scan:6598, Noise threshold:1.1
204.088(HexNAc)
366.141(HexNAc + Hex)
528.196(Hex + HexNAc + Hex)
611.330(PEP+HexNAc(2)_2)
773.388(PEP+HexNAc(2)Hex(2)_2)
854.416(PEP+HexNAc(2)Hex(3)_2)
935.441(PEP+HexNAc(2)Hex(4)_2)
M+2H
1221.671(PEP+HexNAc(2)_1)
1540.681
1613.720
1641.728
1741.781
1757.761
1774.767
1803.780
1826.842
1854.869
1871.890
1884.831
1902.847
1974.904
1989.919
m/z

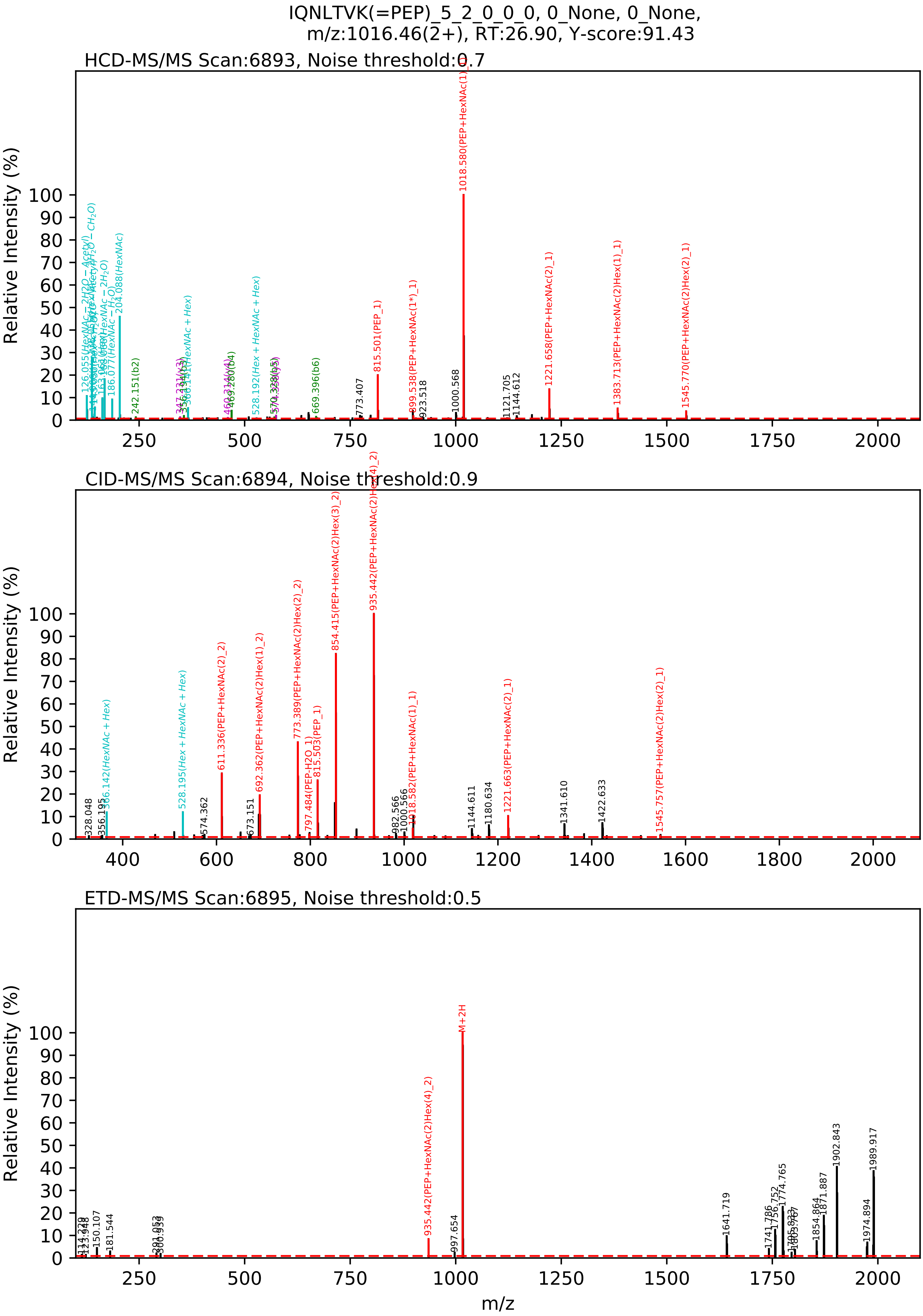
IQNLTVK(=PEP)_5_2_0_0_0, 0_None, 0_None,
m/z:1016.46(2+), RT:26.90, Y-score:91.43
HCD-MS/MS Scan:6893, Noise threshold:0.7
CID-MS/MS Scan:6894, Noise threshold:0.9
ETD-MS/MS Scan:6895, Noise threshold:0.5
Relative Intensity (%)
m/z
1018.580(PEP+HexNAc(1)_1)
815.501(PEP_1)
899.538(PEP+HexNAc(1*)_1)
1221.658(PEP+HexNAc(2)_1)
1383.713(PEP+HexNAc(2)Hex(1)_1)
1545.770(PEP+HexNAc(2)Hex(2)_1)
204.088(HexNAc)
242.151(b2)
469.280(b4)
528.192(Hex + HexNAc + Hex)
669.396(b6)
935.442(PEP+HexNAc(2)Hex(4)_2)
854.415(PEP+HexNAc(2)Hex(3)_2)
773.389(PEP+HexNAc(2)Hex(2)_2)
692.362(PEP+HexNAc(2)Hex(1)_2)
611.336(PEP+HexNAc(2)_2)
815.503(PEP_1)
797.484(PEP-H2O_1)
1018.582(PEP+HexNAc(1)_1)
1221.663(PEP+HexNAc(2)_1)
1545.757(PEP+HexNAc(2)Hex(2)_1)
528.195(Hex + HexNAc + Hex)
366.142(HexNAc + Hex)
M+2H
1902.843
1989.917
1774.765
1641.719

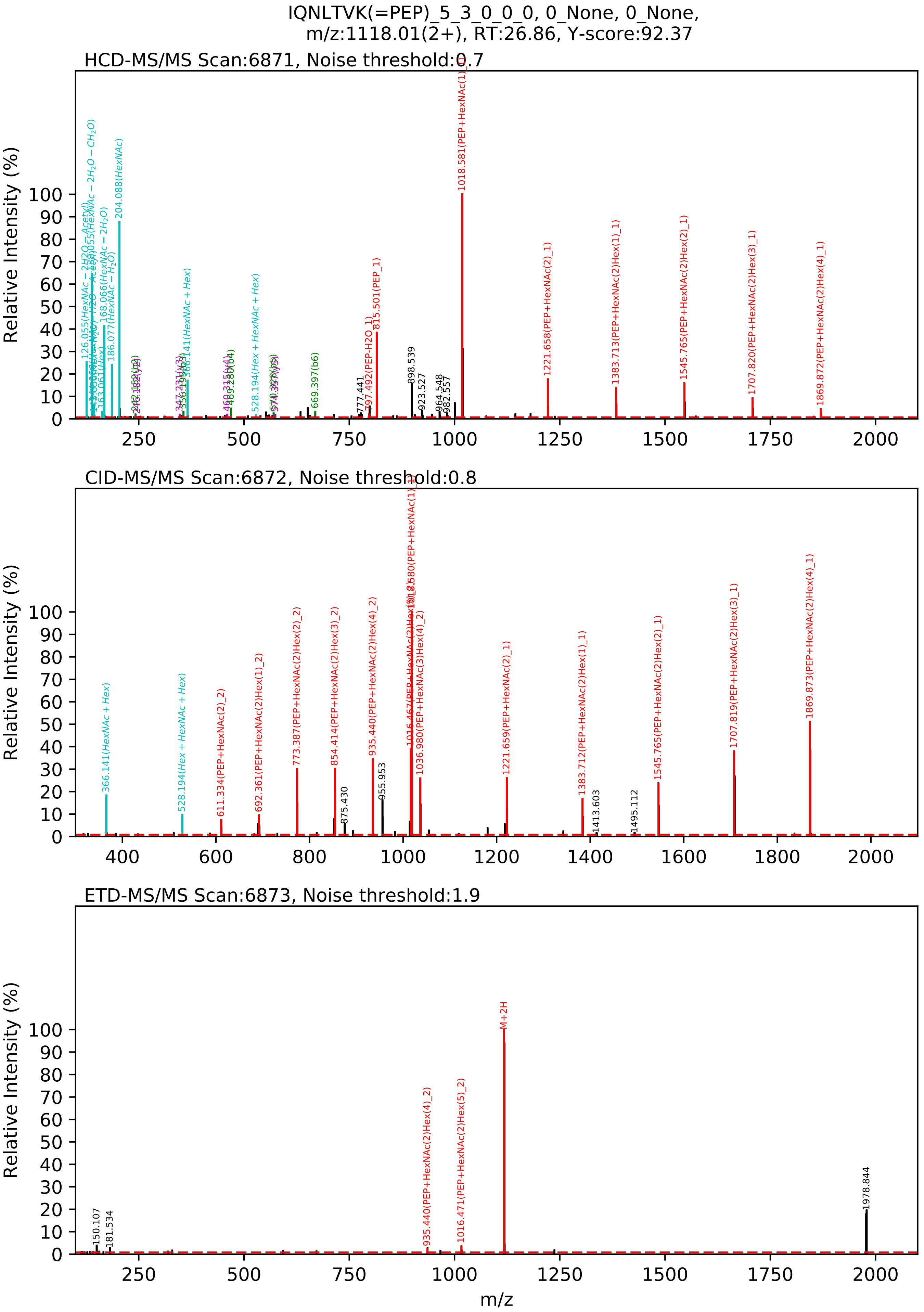
IQNLTVK(=PEP)_5_3_0_0_0, 0_None, 0_None,
m/z:1118.01(2+), RT:26.86, Y-score:92.37
HCD-MS/MS Scan:6871, Noise threshold:0.7
1018.581(PEP+HexNAc(1)_1)
1221.658(PEP+HexNAc(2)_1)
1383.713(PEP+HexNAc(2)Hex(1)_1)
1545.765(PEP+HexNAc(2)Hex(2)_1)
1707.820(PEP+HexNAc(2)Hex(3)_1)
1869.872(PEP+HexNAc(2)Hex(4)_1)
815.501(PEP_1)
797.492(PEP-H2O_1)
204.088(HexNAc)
186.077(HexNAc − H2O)
168.066(HexNAc − 2H2O)
366.141(HexNAc + Hex)
528.194(Hex + HexNAc + Hex)
669.397(b6)
469.280(b4)
898.539
923.527
964.548
982.557
777.441
CID-MS/MS Scan:6872, Noise threshold:0.8
366.141(HexNAc + Hex)
528.194(Hex + HexNAc + Hex)
611.334(PEP+HexNAc(2)_2)
692.361(PEP+HexNAc(2)Hex(1)_2)
773.387(PEP+HexNAc(2)Hex(2)_2)
854.414(PEP+HexNAc(2)Hex(3)_2)
935.440(PEP+HexNAc(2)Hex(4)_2)
1036.980(PEP+HexNAc(3)Hex(4)_2)
1221.659(PEP+HexNAc(2)_1)
1383.712(PEP+HexNAc(2)Hex(1)_1)
1545.765(PEP+HexNAc(2)Hex(2)_1)
1707.819(PEP+HexNAc(2)Hex(3)_1)
1869.873(PEP+HexNAc(2)Hex(4)_1)
875.430
955.953
1413.603
1495.112
ETD-MS/MS Scan:6873, Noise threshold:1.9
M+2H
935.440(PEP+HexNAc(2)Hex(4)_2)
1016.471(PEP+HexNAc(2)Hex(5)_2)
1978.844
150.107
181.534
Relative Intensity (%)
m/z

IQNLTVK(=PEP)_5_3_1_1_0, 0_None, 0_None,
m/z:891.39(3+), RT:37.49, Y-score:94.31

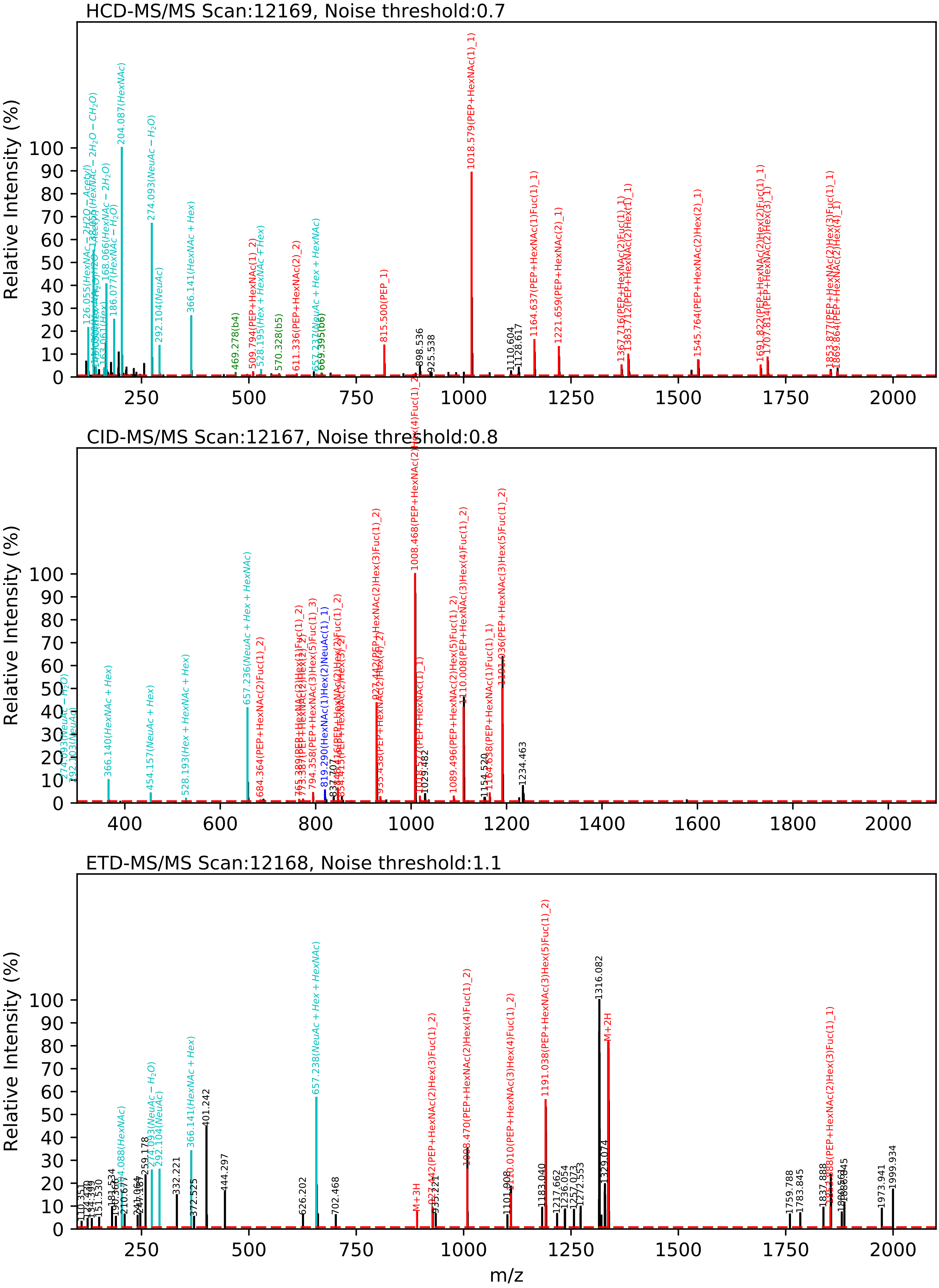

IQNLTVK(=PEP)_5_4_0_0_0, 0_None, 0_None,
m/z:1219.54(2+), RT:26.90, Y-score:91.40

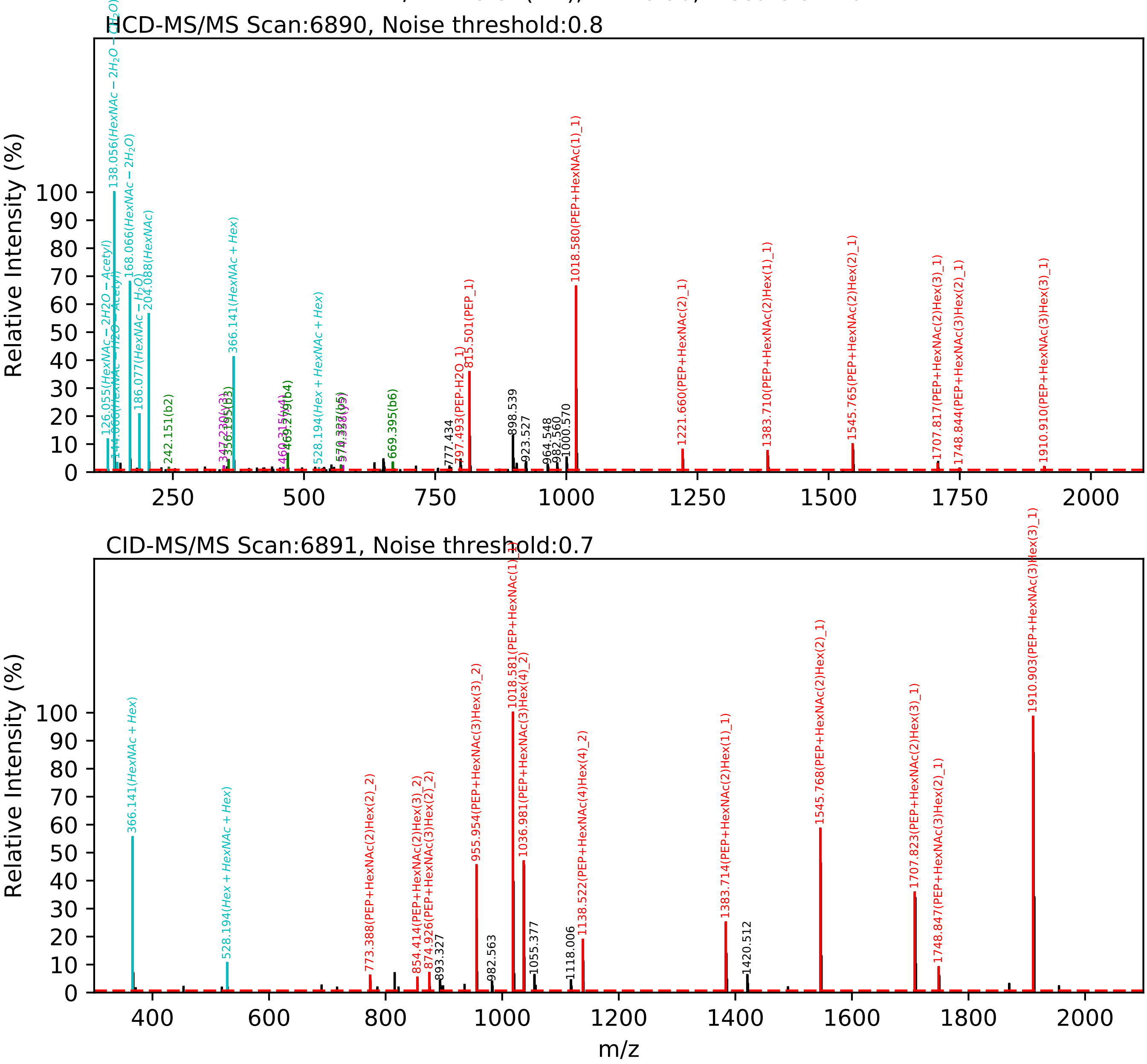

IQNLTVK(=PEP)_5_4_0_0_0, 0_None, 0_None,
m/z:1219.54(2+), RT:27.15, Y-score:89.74

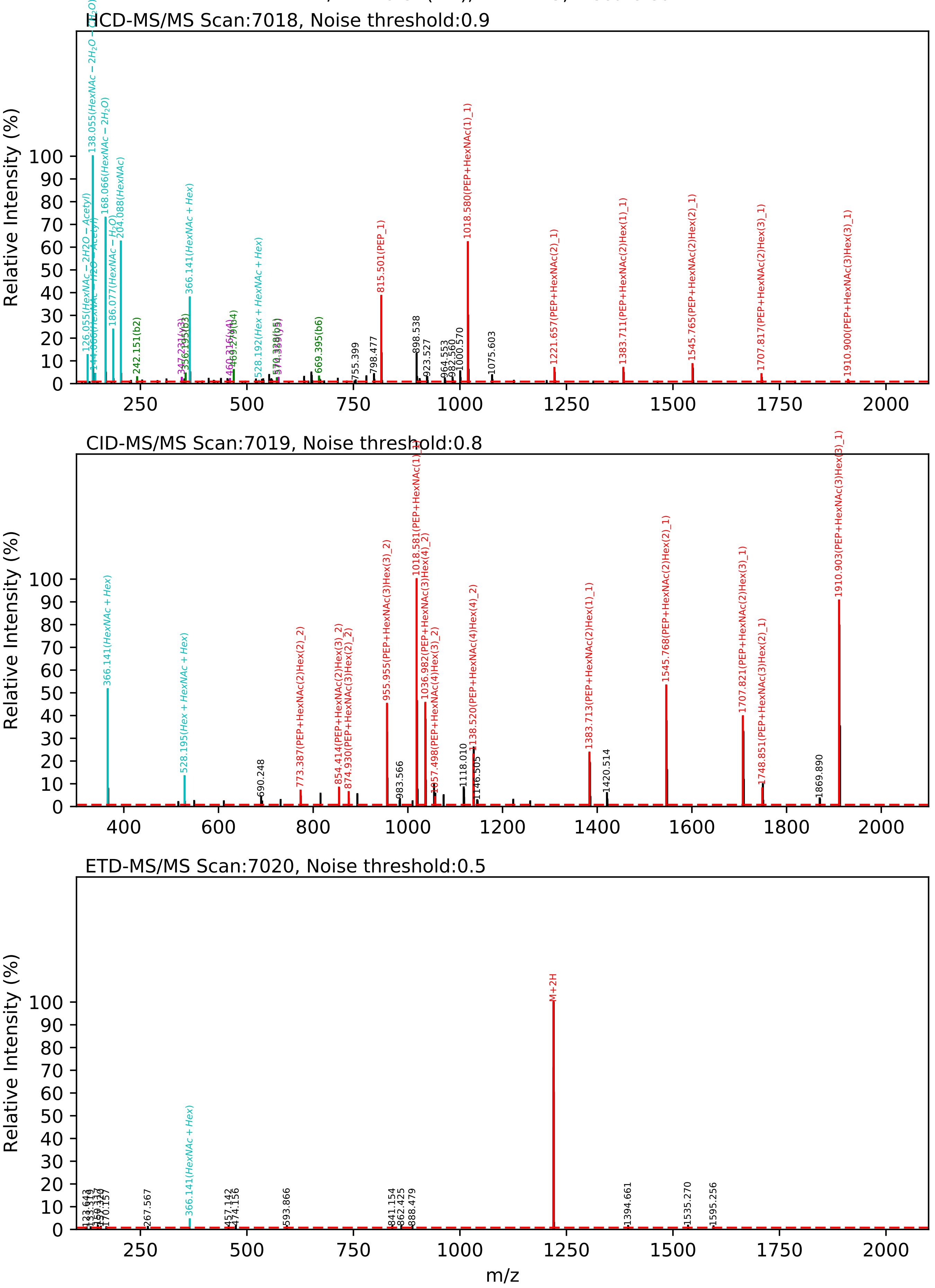

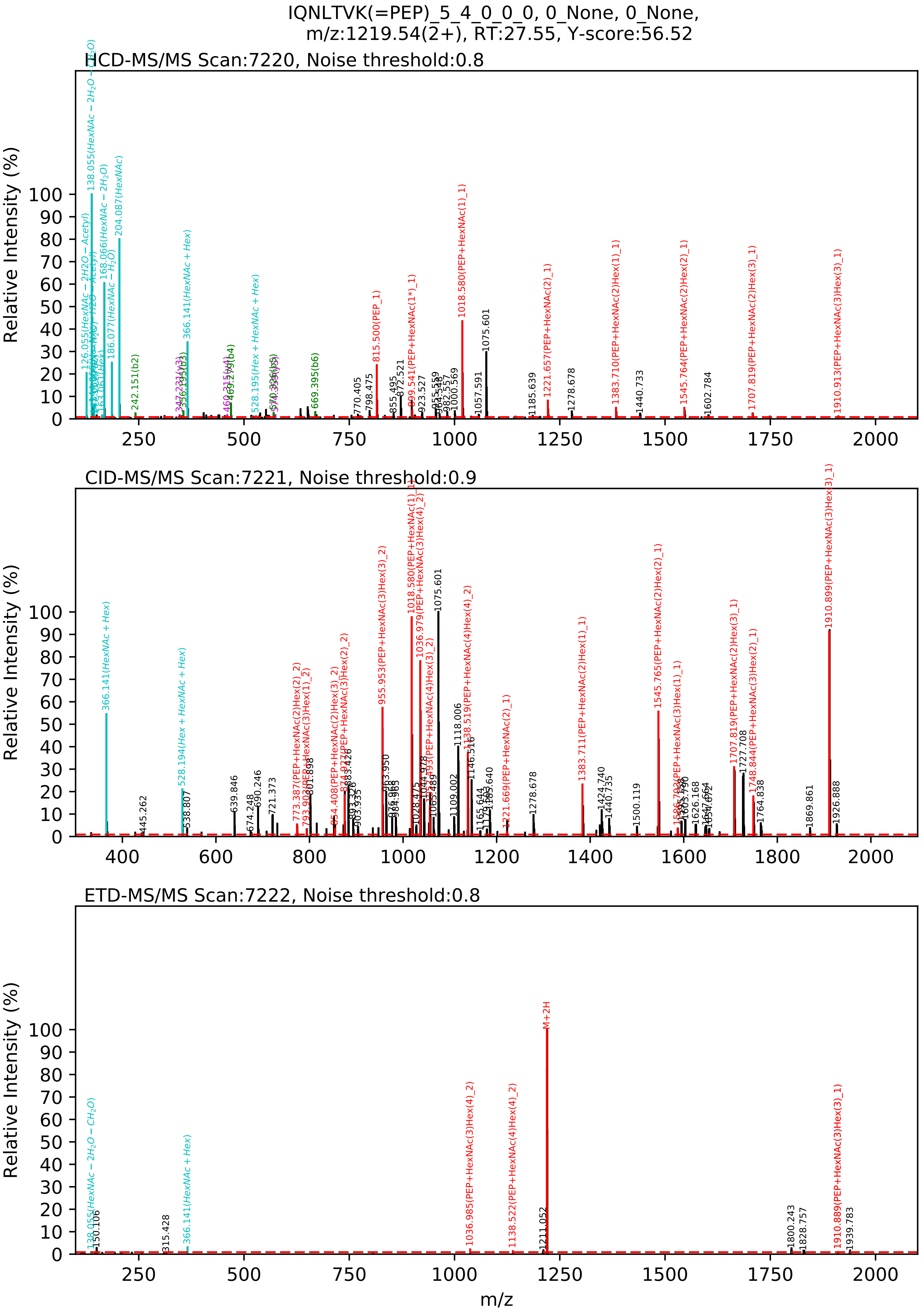

IQNLTVK(=PEP)_5_4_0_0_0, 0_None, 0_None,
m/z:1219.54(2+), RT:27.55, Y-score:56.52
HCD-MS/MS Scan:7220, Noise threshold:0.8
CID-MS/MS Scan:7221, Noise threshold:0.9
ETD-MS/MS Scan:7222, Noise threshold:0.8
Relative Intensity (%)
m/z
138.055(HexNAc − 2H2O − CH2O)
204.087(HexNAc)
366.141(HexNAc + Hex)
528.195(Hex + HexNAc + Hex)
815.500(PEP_1)
1018.580(PEP+HexNAc(1)_1)
1075.601
1221.657(PEP+HexNAc(2)_1)
1383.710(PEP+HexNAc(2)Hex(1)_1)
1545.764(PEP+HexNAc(2)Hex(2)_1)
1707.819(PEP+HexNAc(2)Hex(3)_1)
1910.913(PEP+HexNAc(3)Hex(3)_1)
955.953(PEP+HexNAc(3)Hex(3)_2)
1036.979(PEP+HexNAc(3)Hex(4)_2)
1138.519(PEP+HexNAc(4)Hex(4)_2)
1910.899(PEP+HexNAc(3)Hex(3)_1)
M+2H
1036.985(PEP+HexNAc(3)Hex(4)_2)
1138.522(PEP+HexNAc(4)Hex(4)_2)
1910.889(PEP+HexNAc(3)Hex(3)_1)

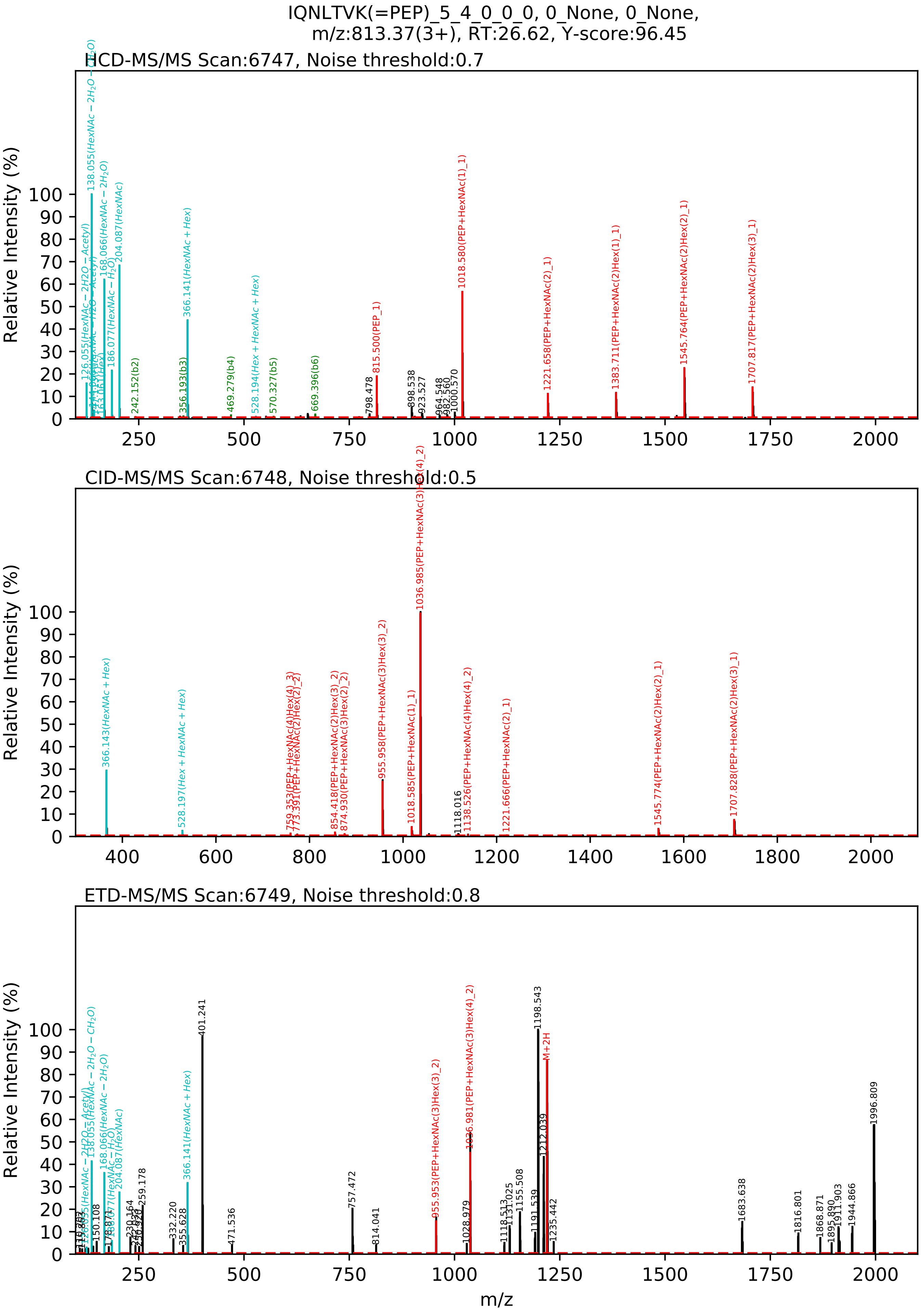

IQNLTVK(=PEP)_5_4_0_0_0, 0_None, 0_None,
m/z:813.37(3+), RT:26.62, Y-score:96.45
HCD-MS/MS Scan:6747, Noise threshold:0.7
138.055
168.066
204.087(HexNAc)
186.077(HexNAc − H2O)
126.055
366.141(HexNAc + Hex)
242.152(b2)
356.193(b3)
469.279(b4)
528.194(Hex + HexNAc + Hex)
570.327(b5)
669.396(b6)
798.478
815.500(PEP_1)
898.538
923.527
964.548
1000.570
1018.580(PEP+HexNAc(1)_1)
1221.658(PEP+HexNAc(2)_1)
1383.711(PEP+HexNAc(2)Hex(1)_1)
1545.764(PEP+HexNAc(2)Hex(2)_1)
1707.817(PEP+HexNAc(2)Hex(3)_1)
CID-MS/MS Scan:6748, Noise threshold:0.5
366.143(HexNAc + Hex)
528.197(Hex + HexNAc + Hex)
759.353(PEP+HexNAc(4)Hex(4)_3)
773.391(PEP+HexNAc(2)Hex(2)_2)
854.418(PEP+HexNAc(2)Hex(3)_2)
874.930(PEP+HexNAc(3)Hex(2)_2)
955.958(PEP+HexNAc(3)Hex(3)_2)
1018.585(PEP+HexNAc(1)_1)
1036.985(PEP+HexNAc(3)Hex(4)_2)
1118.016
1138.526(PEP+HexNAc(4)Hex(4)_2)
1221.666(PEP+HexNAc(2)_1)
1545.774(PEP+HexNAc(2)Hex(2)_1)
1707.828(PEP+HexNAc(2)Hex(3)_1)
ETD-MS/MS Scan:6749, Noise threshold:0.8
138.055
168.066
204.087(HexNAc)
150.108
259.178
332.220
355.628
366.141(HexNAc + Hex)
401.241
471.536
757.472
814.041
955.953(PEP+HexNAc(3)Hex(3)_2)
1028.979
1036.981(PEP+HexNAc(3)Hex(4)_2)
1118.513
1155.508
1191.539
1198.543
1212.039
M+2H
1235.442
1683.638
1816.801
1868.871
1944.866
1996.809
Relative Intensity (%)
m/z

IQNLTVK(=PEP)_5_4_0_0_0, 0_None, 0_None,
m/z:1219.54(2+), RT:25.64, Y-score:81.56

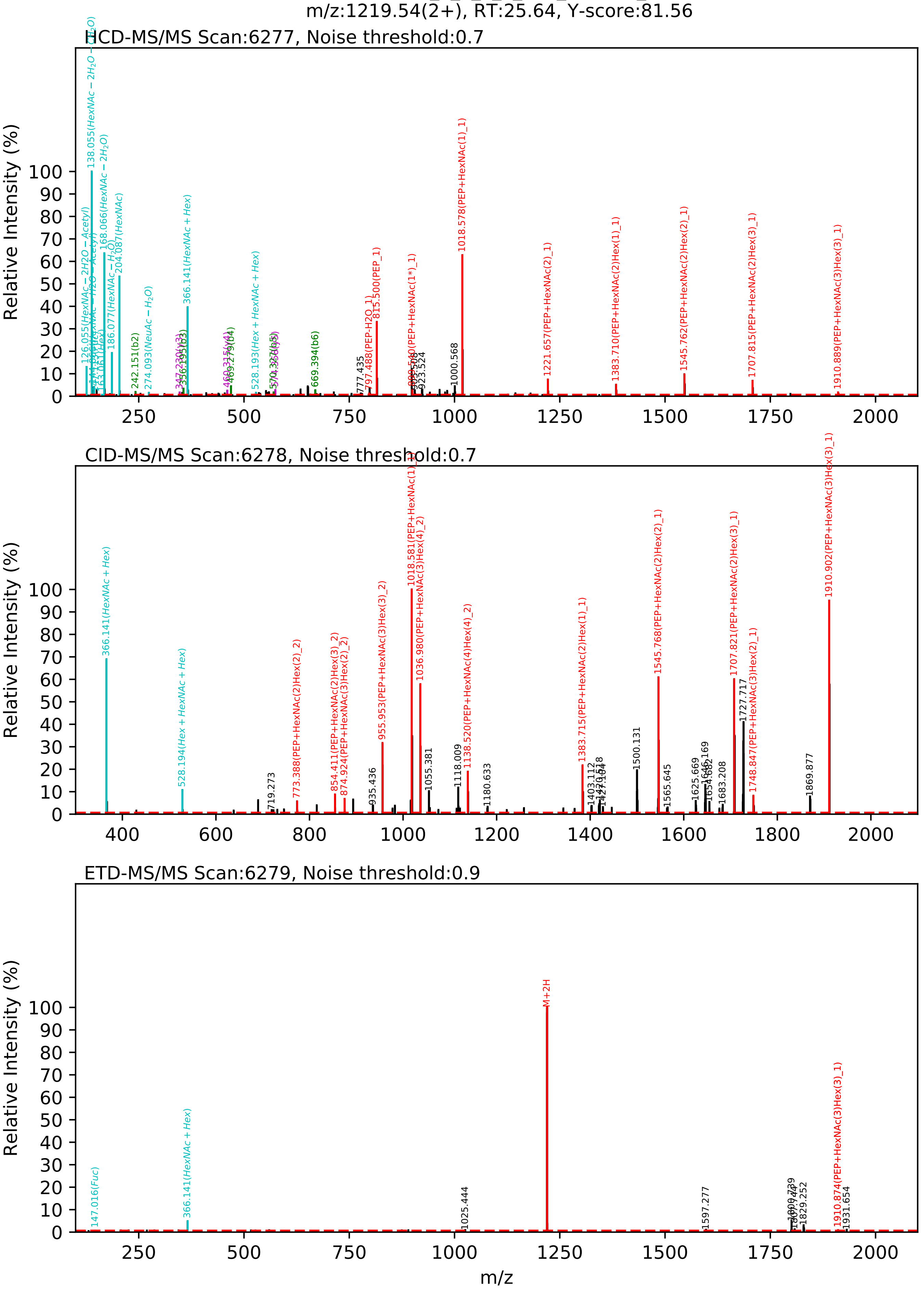

IQNLTVK(=PEP)_5_4_0_0_0, 0_None, 0_None,
m/z:1219.54(2+), RT:26.33, Y-score:81.20

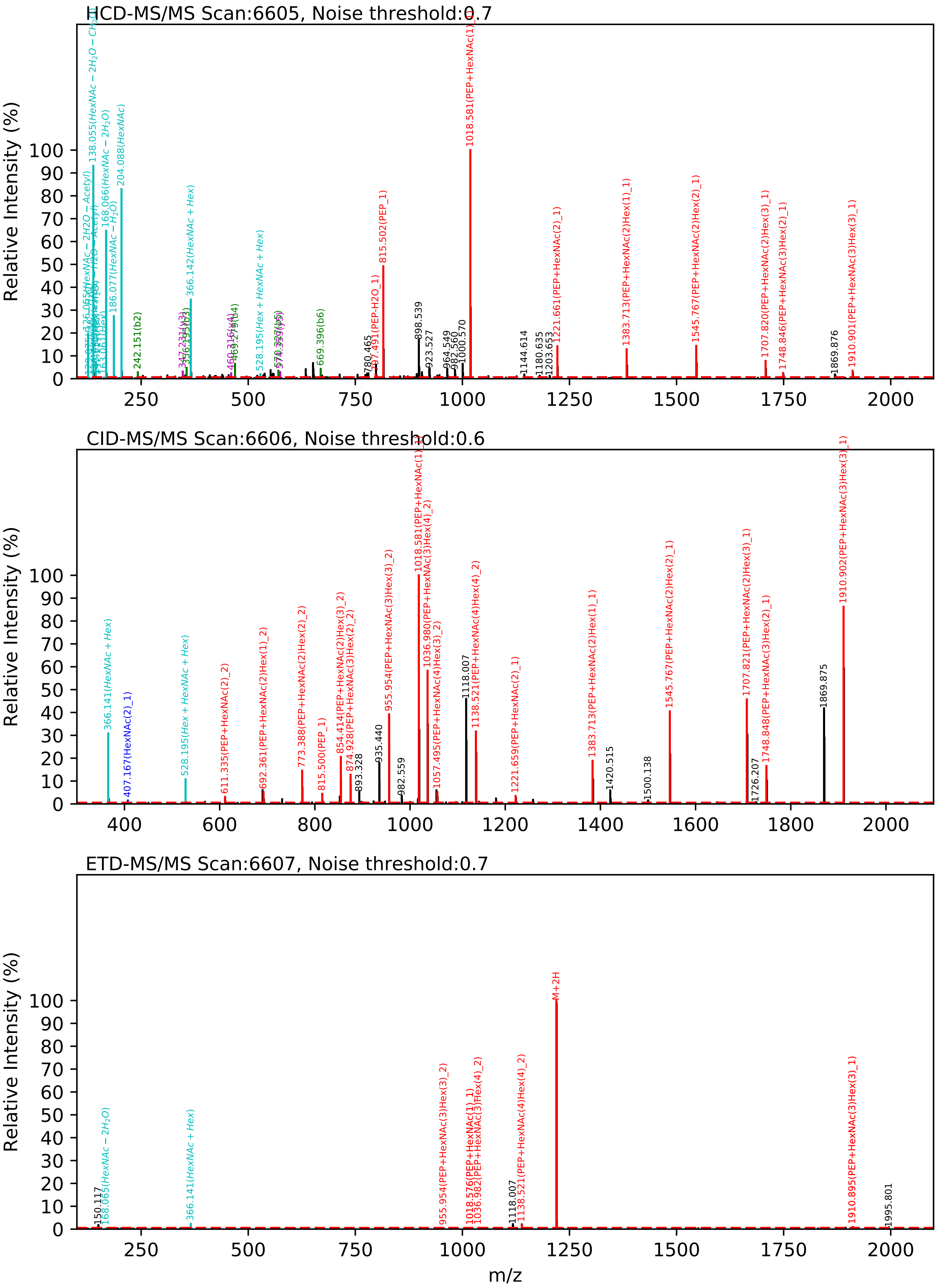

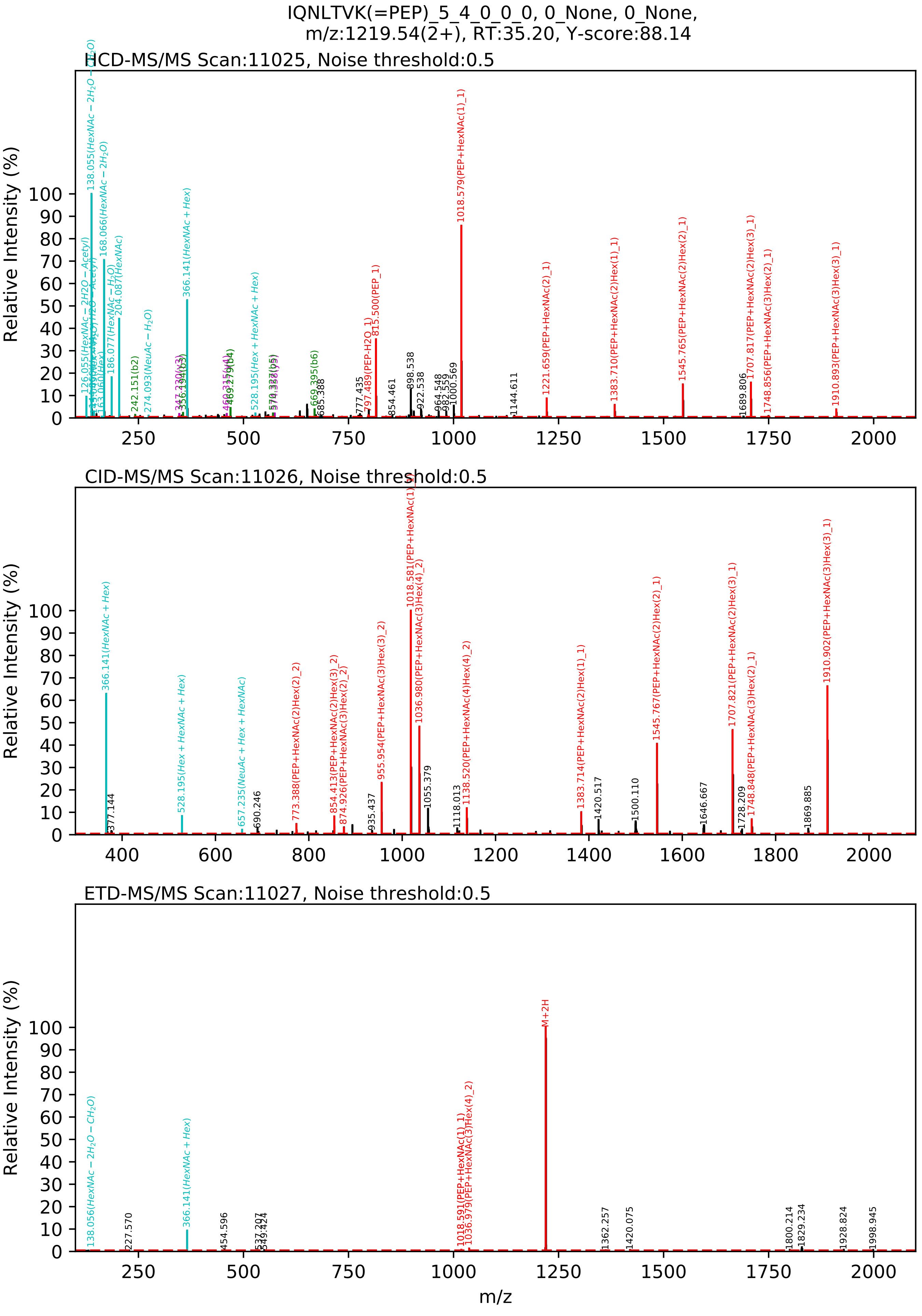

IQNLTVK(=PEP)_5_4_0_0_0, 0_None, 0_None,
m/z:1219.54(2+), RT:35.20, Y-score:88.14
HCD-MS/MS Scan:11025, Noise threshold:0.5
CID-MS/MS Scan:11026, Noise threshold:0.5
ETD-MS/MS Scan:11027, Noise threshold:0.5
Relative Intensity (%)
m/z
1018.579(PEP+HexNAc(1)_1)
1221.659(PEP+HexNAc(2)_1)
1383.710(PEP+HexNAc(2)Hex(1)_1)
1545.765(PEP+HexNAc(2)Hex(2)_1)
1707.817(PEP+HexNAc(2)Hex(3)_1)
1748.856(PEP+HexNAc(3)Hex(2)_1)
1910.893(PEP+HexNAc(3)Hex(3)_1)
815.500(PEP_1)
797.489(PEP-H2O_1)
366.141(HexNAc+Hex)
528.195(Hex+HexNAc+Hex)
204.087(HexNAc)
138.055(HexNAc−2H2O−CH2O)
168.066(HexNAc−2H2O)
186.077(HexNAc−H2O)
274.093(NeuAc−H2O)
242.151(b2)
669.395(b6)
1018.581(PEP+HexNAc(1)_1)
1036.980(PEP+HexNAc(3)Hex(4)_2)
955.954(PEP+HexNAc(3)Hex(3)_2)
773.388(PEP+HexNAc(2)Hex(2)_2)
854.413(PEP+HexNAc(2)Hex(3)_2)
874.926(PEP+HexNAc(3)Hex(2)_2)
1138.520(PEP+HexNAc(4)Hex(4)_2)
1383.714(PEP+HexNAc(2)Hex(1)_1)
1545.767(PEP+HexNAc(2)Hex(2)_1)
1707.821(PEP+HexNAc(2)Hex(3)_1)
1748.848(PEP+HexNAc(3)Hex(2)_1)
1910.902(PEP+HexNAc(3)Hex(3)_1)
657.235(NeuAc+Hex+HexNAc)
M+2H
138.056(HexNAc−2H2O−CH2O)
1018.591(PEP+HexNAc(1)_1)
1036.979(PEP+HexNAc(3)Hex(4)_2)

IQNLTVK(=PEP)_5_4_0_0_0, 0_None, 0_None,
m/z:1219.54(2+), RT:35.76, Y-score:91.41

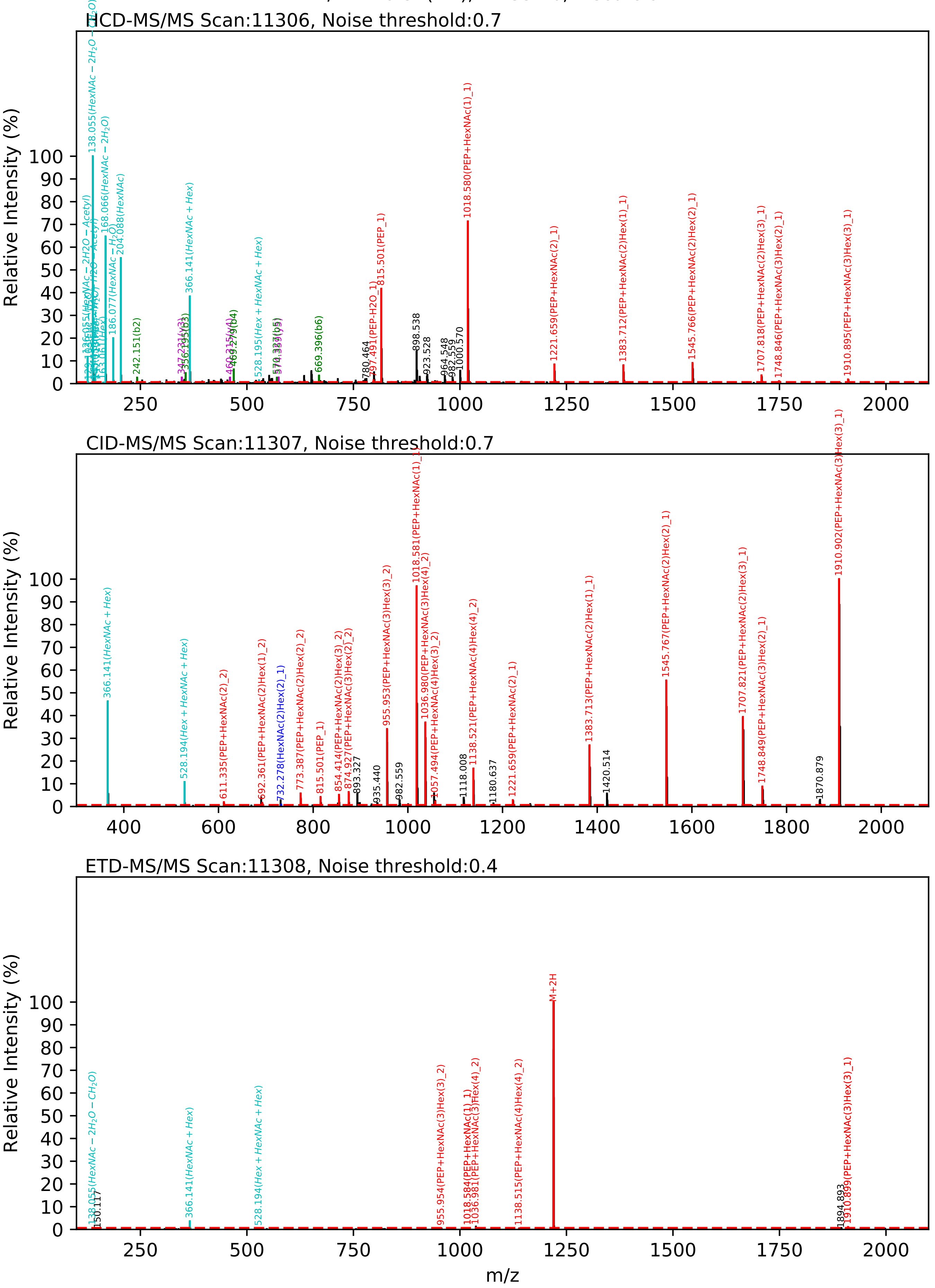

IQNLTVK(=PEP)_5_4_0_0_0, 0_None, 0_None,
m/z:1219.54(2+), RT:36.50, Y-score:91.50

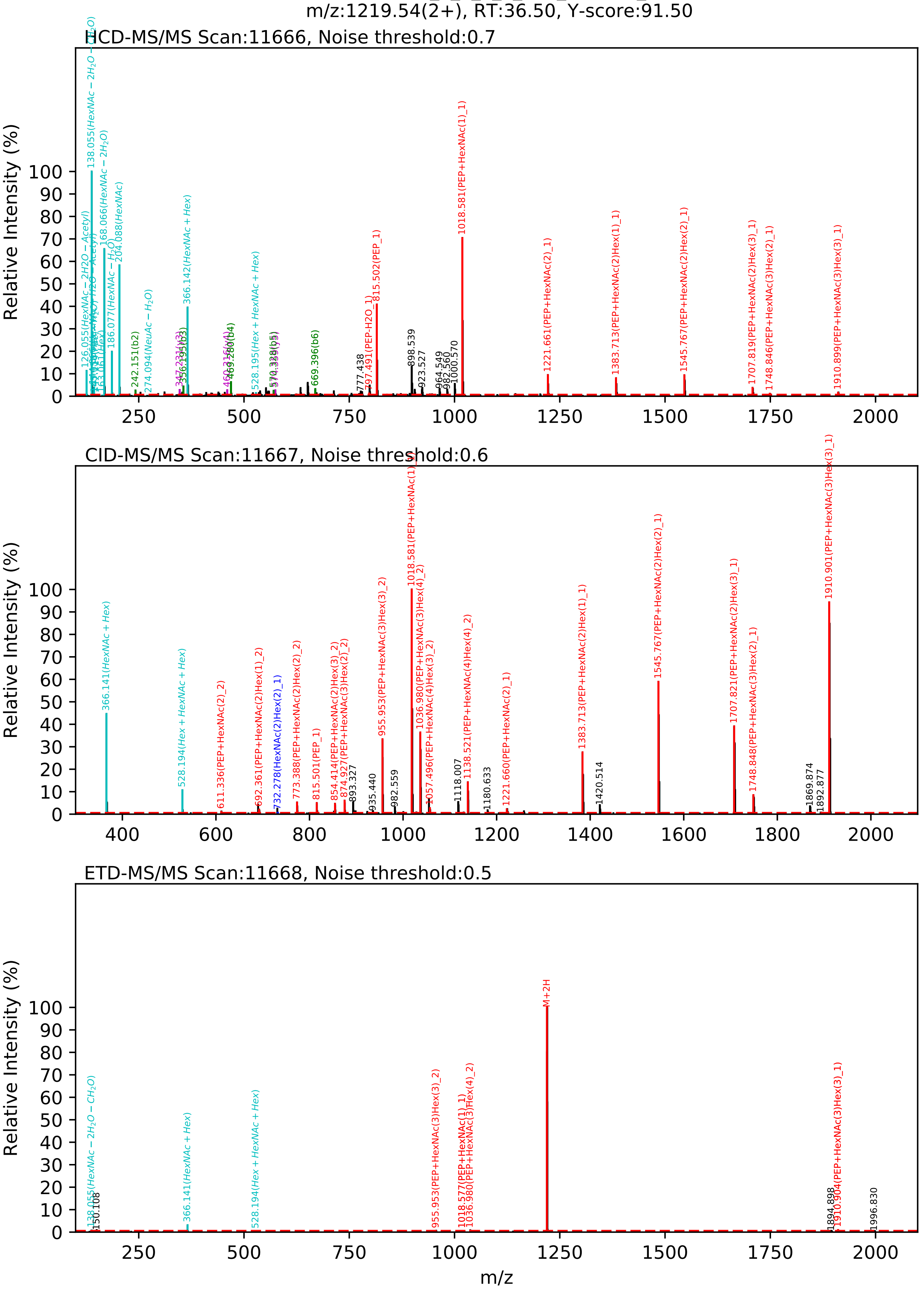

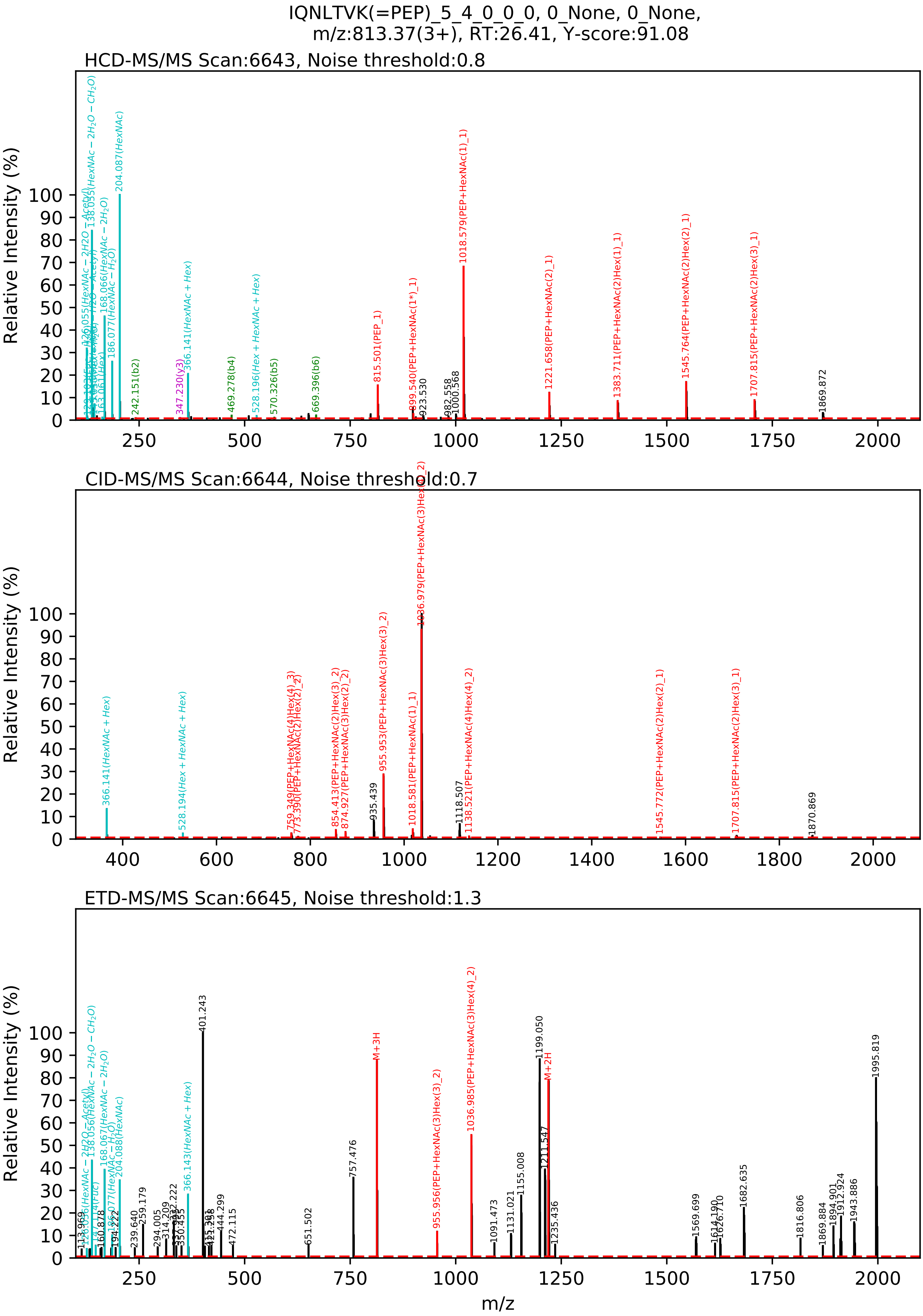

IQNLTVK(=PEP)_5_4_0_0_0, 0_None, 0_None,
m/z:813.37(3+), RT:26.41, Y-score:91.08
HCD-MS/MS Scan:6643, Noise threshold:0.8
Relative Intensity (%)
204.087(HexNAc)
168.066(HexNAc − 2H2O)
186.077(HexNAc − H2O)
366.141(HexNAc + Hex)
242.151(b2)
347.230(y3)
469.278(b4)
528.196(Hex + HexNAc + Hex)
570.326(b5)
669.396(b6)
815.501(PEP_1)
899.540(PEP+HexNAc(1*)_1)
923.530
982.558
1000.568
1018.579(PEP+HexNAc(1)_1)
1221.658(PEP+HexNAc(2)_1)
1383.711(PEP+HexNAc(2)Hex(1)_1)
1545.764(PEP+HexNAc(2)Hex(2)_1)
1707.815(PEP+HexNAc(2)Hex(3)_1)
1869.872
CID-MS/MS Scan:6644, Noise threshold:0.7
Relative Intensity (%)
366.141(HexNAc + Hex)
528.194(Hex + HexNAc + Hex)
759.349(PEP+HexNAc(4)Hex(4)_3)
773.390(PEP+HexNAc(2)Hex(2)_2)
854.413(PEP+HexNAc(2)Hex(3)_2)
874.927(PEP+HexNAc(3)Hex(2)_2)
935.439
955.953(PEP+HexNAc(3)Hex(3)_2)
1018.581(PEP+HexNAc(1)_1)
1036.979(PEP+HexNAc(3)Hex(4)_2)
1118.507
1138.521(PEP+HexNAc(4)Hex(4)_2)
1545.772(PEP+HexNAc(2)Hex(2)_1)
1707.815(PEP+HexNAc(2)Hex(3)_1)
1870.869
ETD-MS/MS Scan:6645, Noise threshold:1.3
Relative Intensity (%)
113.969
138.056(HexNAc − 2H2O − Acetyl)
168.067(HexNAc − 2H2O)
160.878
186.077(HexNAc − H2O)
204.088(HexNAc)
239.640
259.179
294.005
314.209
332.222
350.455
366.143(HexNAc + Hex)
401.243
444.299
472.115
651.502
757.476
M+3H
955.956(PEP+HexNAc(3)Hex(3)_2)
1036.985(PEP+HexNAc(3)Hex(4)_2)
1091.473
1131.021
1155.008
1199.050
1211.547
M+2H
1235.436
1569.699
1614.190
1626.710
1682.635
1816.806
1869.884
1894.901
1912.924
1943.886
1995.819
m/z

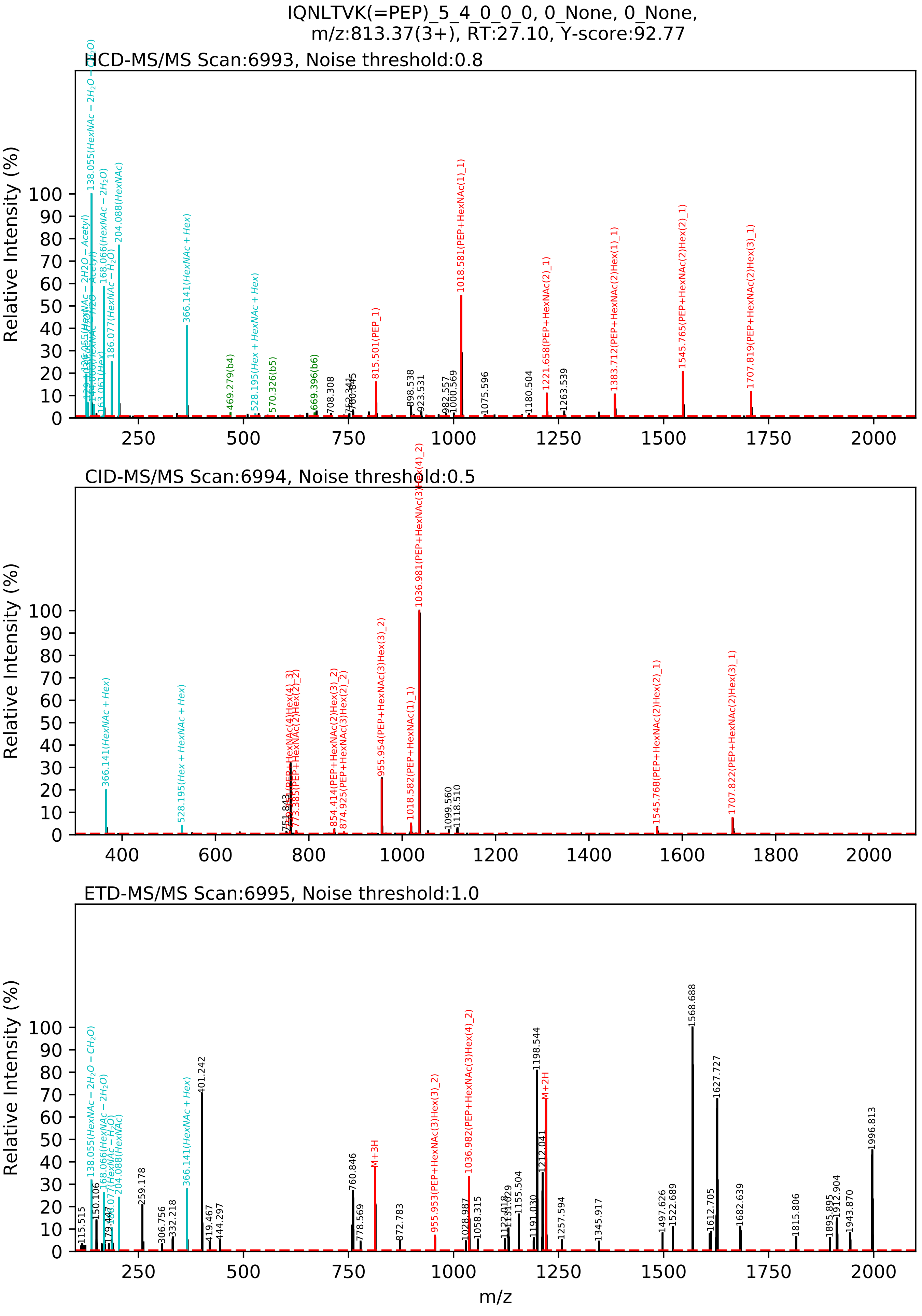
IQNLTVK(=PEP)_5_4_0_0_0, 0_None, 0_None,
m/z:813.37(3+), RT:27.10, Y-score:92.77
HCD-MS/MS Scan:6993, Noise threshold:0.8
Relative Intensity (%)
138.055(HexNAc − 2H2O − CH2O)
204.088(HexNAc)
168.066(HexNAc − 2H2O)
186.077(HexNAc − H2O)
366.141(HexNAc + Hex)
528.195(Hex + HexNAc + Hex)
469.279(b4)
570.326(b5)
669.396(b6)
708.308
815.501(PEP_1)
898.538
923.531
1018.581(PEP+HexNAc(1)_1)
1075.596
1180.504
1221.658(PEP+HexNAc(2)_1)
1263.539
1383.712(PEP+HexNAc(2)Hex(1)_1)
1545.765(PEP+HexNAc(2)Hex(2)_1)
1707.819(PEP+HexNAc(2)Hex(3)_1)
CID-MS/MS Scan:6994, Noise threshold:0.5
Relative Intensity (%)
366.141(HexNAc + Hex)
528.195(Hex + HexNAc + Hex)
751.843
773.385(PEP+HexNAc(2)Hex(2)_2)
854.414(PEP+HexNAc(2)Hex(3)_2)
874.925(PEP+HexNAc(3)Hex(2)_2)
955.954(PEP+HexNAc(3)Hex(3)_2)
1018.582(PEP+HexNAc(1)_1)
1036.981(PEP+HexNAc(3)Hex(4)_2)
1099.560
1118.510
1545.768(PEP+HexNAc(2)Hex(2)_1)
1707.822(PEP+HexNAc(2)Hex(3)_1)
ETD-MS/MS Scan:6995, Noise threshold:1.0
Relative Intensity (%)
115.515
138.055(HexNAc − 2H2O − CH2O)
150.106
168.066(HexNAc − 2H2O)
186.077(HexNAc − H2O)
204.088(HexNAc)
259.178
306.756
332.218
366.141(HexNAc + Hex)
401.242
419.467
444.297
760.846
778.569
M+3H
872.783
955.953(PEP+HexNAc(3)Hex(3)_2)
1028.987
1036.982(PEP+HexNAc(3)Hex(4)_2)
1058.315
1155.504
1191.030
1198.544
1212.041
M+2H
1257.594
1345.917
1497.626
1522.689
1568.688
1612.705
1627.727
1682.639
1815.806
1912.904
1943.870
1996.813
m/z

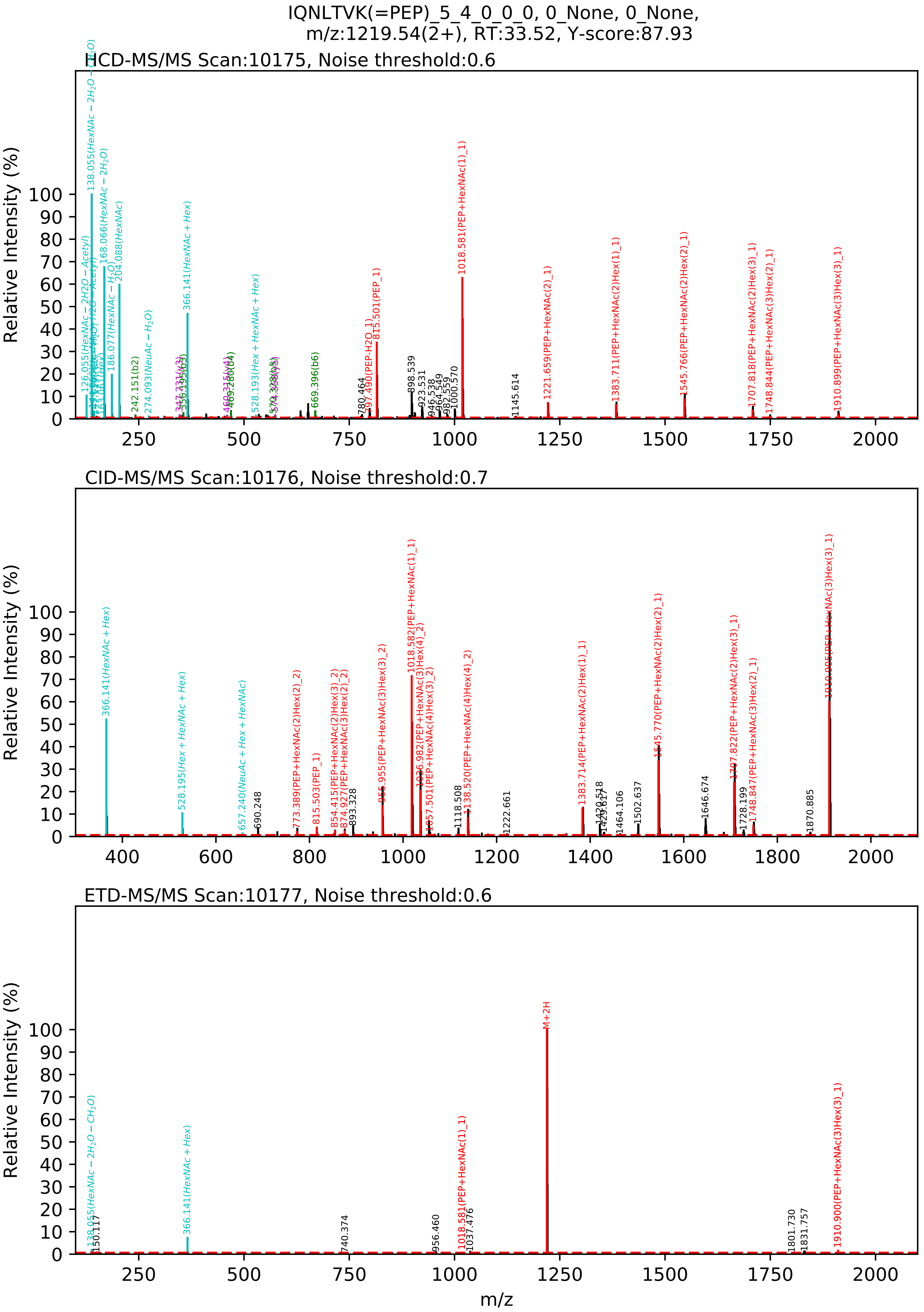
IQNLTVK(=PEP)_5_4_0_0_0, 0_None, 0_None,
m/z:1219.54(2+), RT:33.52, Y-score:87.93
HCD-MS/MS Scan:10175, Noise threshold:0.6
Relative Intensity (%)
138.055(HexNAc − 2H2O − CH2O)
168.066(HexNAc − 2H2O)
204.088(HexNAc)
186.077(HexNAc − H2O)
366.141(HexNAc + Hex)
528.193(Hex + HexNAc + Hex)
242.151(b2)
274.093(NeuAc − H2O)
469.280(b4)
669.396(b6)
815.501(PEP_1)
797.490(PEP-H2O_1)
780.464
898.539
923.531
946.538
964.549
1000.570
1018.581(PEP+HexNAc(1)_1)
1145.614
1221.659(PEP+HexNAc(2)_1)
1383.711(PEP+HexNAc(2)Hex(1)_1)
1545.766(PEP+HexNAc(2)Hex(2)_1)
1707.818(PEP+HexNAc(2)Hex(3)_1)
1748.844(PEP+HexNAc(3)Hex(2)_1)
1910.899(PEP+HexNAc(3)Hex(3)_1)
CID-MS/MS Scan:10176, Noise threshold:0.7
366.141(HexNAc + Hex)
528.195(Hex + HexNAc + Hex)
657.240(NeuAc + Hex + HexNAc)
690.248
773.389(PEP+HexNAc(2)Hex(2)_2)
815.503(PEP_1)
854.415(PEP+HexNAc(2)Hex(3)_2)
874.927(PEP+HexNAc(3)Hex(2)_2)
893.328
955.955(PEP+HexNAc(3)Hex(3)_2)
1018.582(PEP+HexNAc(1)_1)
1036.982(PEP+HexNAc(3)Hex(4)_2)
1057.501(PEP+HexNAc(4)Hex(3)_2)
1118.508
1138.520(PEP+HexNAc(4)Hex(4)_2)
1222.661
1383.714(PEP+HexNAc(2)Hex(1)_1)
1464.106
1502.637
1545.770(PEP+HexNAc(2)Hex(2)_1)
1646.674
1707.822(PEP+HexNAc(2)Hex(3)_1)
1728.199
1748.847(PEP+HexNAc(3)Hex(2)_1)
1870.885
1910.905(PEP+HexNAc(3)Hex(3)_1)
ETD-MS/MS Scan:10177, Noise threshold:0.6
138.055(HexNAc − 2H2O − CH2O)
150.117
366.141(HexNAc + Hex)
740.374
956.460
1018.581(PEP+HexNAc(1)_1)
1037.476
M+2H
1801.730
1831.757
1910.900(PEP+HexNAc(3)Hex(3)_1)
m/z

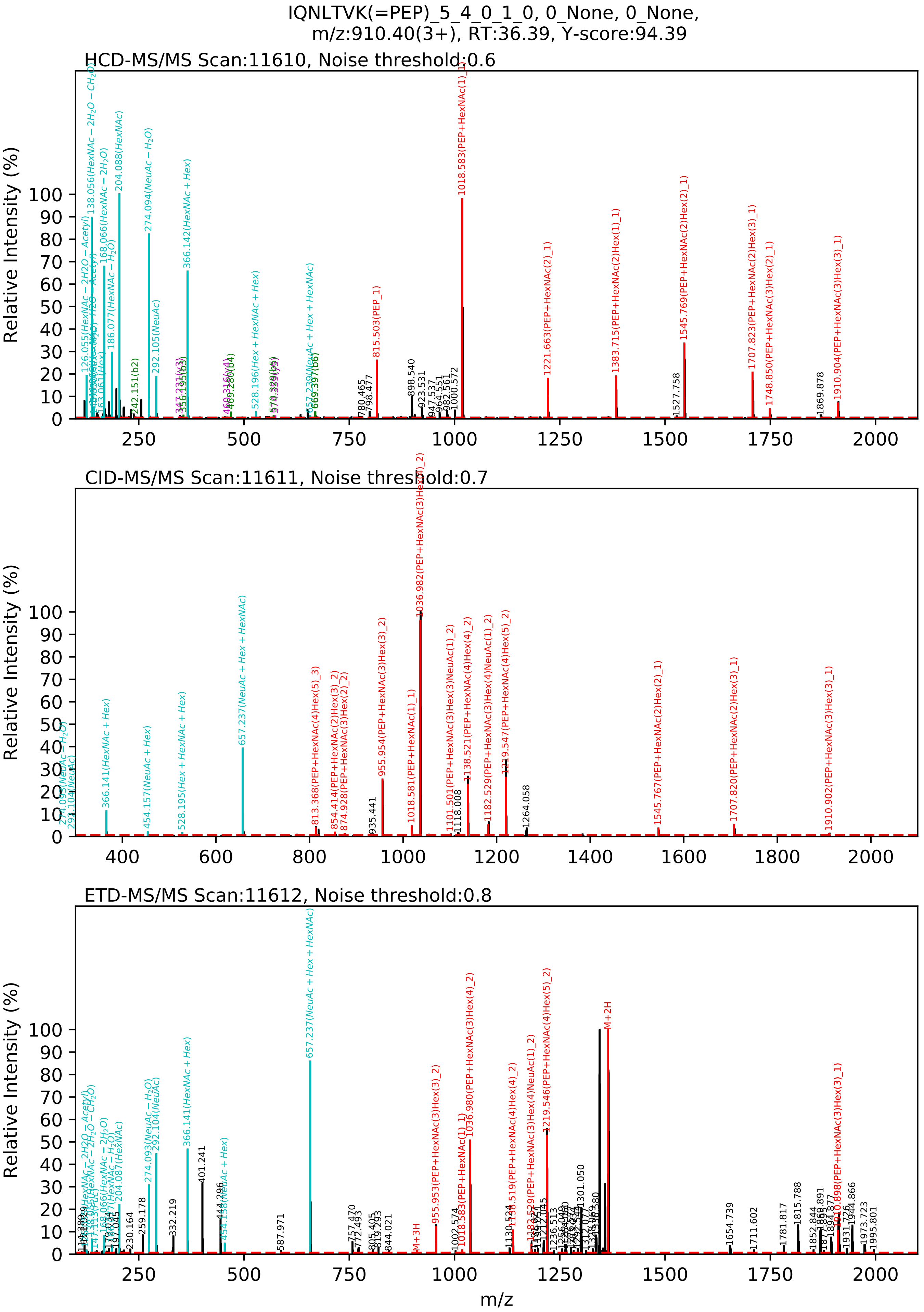
IQNLTVK(=PEP)_5_4_0_1_0, 0_None, 0_None,
m/z:910.40(3+), RT:36.39, Y-score:94.39
HCD-MS/MS Scan:11610, Noise threshold:0.6
Relative Intensity (%)
138.056(HexNAc − 2H2O − CH2O)
204.088(HexNAc)
274.094(NeuAc − H2O)
366.142(HexNAc + Hex)
168.066(HexNAc − 2H2O)
186.077(HexNAc − H2O)
292.105(NeuAc)
528.196(Hex + HexNAc + Hex)
657.239(NeuAc + Hex + HexNAc)
669.397(b6)
815.503(PEP_1)
1018.583(PEP+HexNAc(1)_1)
1221.663(PEP+HexNAc(2)_1)
1383.715(PEP+HexNAc(2)Hex(1)_1)
1545.769(PEP+HexNAc(2)Hex(2)_1)
1707.823(PEP+HexNAc(2)Hex(3)_1)
1748.850(PEP+HexNAc(3)Hex(2)_1)
1910.904(PEP+HexNAc(3)Hex(3)_1)
898.540
923.531
1527.758
1869.878
m/z
CID-MS/MS Scan:11611, Noise threshold:0.7
366.141(HexNAc + Hex)
454.157(NeuAc + Hex)
528.195(Hex + HexNAc + Hex)
657.237(NeuAc + Hex + HexNAc)
813.368(PEP+HexNAc(4)Hex(5)_3)
854.414(PEP+HexNAc(2)Hex(3)_2)
874.928(PEP+HexNAc(3)Hex(2)_2)
935.441
955.954(PEP+HexNAc(3)Hex(3)_2)
1018.581(PEP+HexNAc(1)_1)
1036.982(PEP+HexNAc(3)Hex(4)_2)
1101.501(PEP+HexNAc(3)Hex(3)NeuAc(1)_2)
1118.008
1138.521(PEP+HexNAc(4)Hex(4)_2)
1182.529(PEP+HexNAc(3)Hex(4)NeuAc(1)_2)
1219.547(PEP+HexNAc(4)Hex(5)_2)
1264.058
1545.767(PEP+HexNAc(2)Hex(2)_1)
1707.820(PEP+HexNAc(2)Hex(3)_1)
1910.902(PEP+HexNAc(3)Hex(3)_1)
ETD-MS/MS Scan:11612, Noise threshold:0.8
657.237(NeuAc + Hex + HexNAc)
366.141(HexNAc + Hex)
274.093(NeuAc − H2O)
292.104(NeuAc)
204.087(HexNAc)
259.178
332.219
401.241
444.296
454.158(NeuAc + Hex)
587.971
757.470
772.493
805.405
844.021
M+3H
955.953(PEP+HexNAc(3)Hex(3)_2)
1002.574
1018.583(PEP+HexNAc(1)_1)
1036.980(PEP+HexNAc(3)Hex(4)_2)
1130.524
1138.519(PEP+HexNAc(4)Hex(4)_2)
1182.529(PEP+HexNAc(3)Hex(4)NeuAc(1)_2)
1219.546(PEP+HexNAc(4)Hex(5)_2)
1212.045
1236.513
1301.050
M+2H
1654.739
1711.602
1781.817
1815.788
1852.844
1910.898(PEP+HexNAc(3)Hex(3)_1)
1931.726
1944.866
1973.723
1995.801
m/z

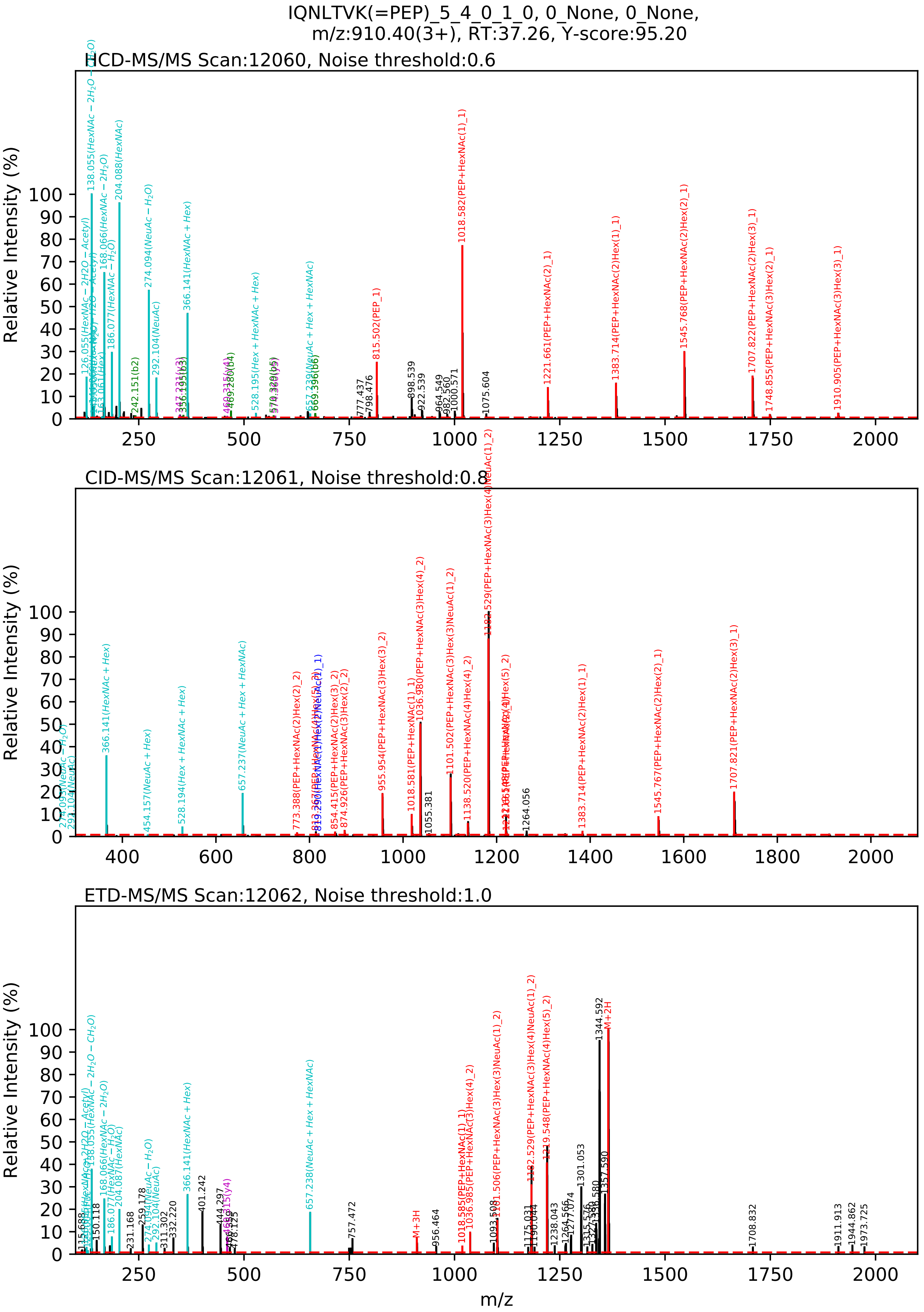

IQNLTVK(=PEP)_5_4_0_1_0, 0_None, 0_None,
m/z:910.40(3+), RT:37.26, Y-score:95.20
HCD-MS/MS Scan:12060, Noise threshold:0.6
CID-MS/MS Scan:12061, Noise threshold:0.8
ETD-MS/MS Scan:12062, Noise threshold:1.0
Relative Intensity (%)
m/z
138.055(HexNAc − 2H2O − H2O)
204.088(HexNAc)
168.066(HexNAc − 2H2O)
186.077(HexNAc − H2O)
274.094(NeuAc − H2O)
292.104(NeuAc)
366.141(HexNAc + Hex)
528.195(Hex + HexNAc + Hex)
657.238(NeuAc + Hex + HexNAc)
815.502(PEP_1)
1018.582(PEP+HexNAc(1)_1)
1221.661(PEP+HexNAc(2)_1)
1383.714(PEP+HexNAc(2)Hex(1)_1)
1545.768(PEP+HexNAc(2)Hex(2)_1)
1707.822(PEP+HexNAc(2)Hex(3)_1)
1748.855(PEP+HexNAc(3)Hex(2)_1)
1910.905(PEP+HexNAc(3)Hex(3)_1)
955.954(PEP+HexNAc(3)Hex(3)_2)
1036.980(PEP+HexNAc(3)Hex(4)_2)
1101.502(PEP+HexNAc(3)Hex(3)NeuAc(1)_2)
1138.520(PEP+HexNAc(4)Hex(4)_2)
1182.529(PEP+HexNAc(3)Hex(4)NeuAc(1)_2)
1219.548(PEP+HexNAc(4)Hex(5)_2)
1545.767(PEP+HexNAc(2)Hex(2)_1)
1707.821(PEP+HexNAc(2)Hex(3)_1)
1344.592
M+2H
M+3H

IQNLTVK(=PEP)_5_4_0_1_0, 0_None, 0_None,
m/z:910.40(3+), RT:37.31, Y-score:96.10

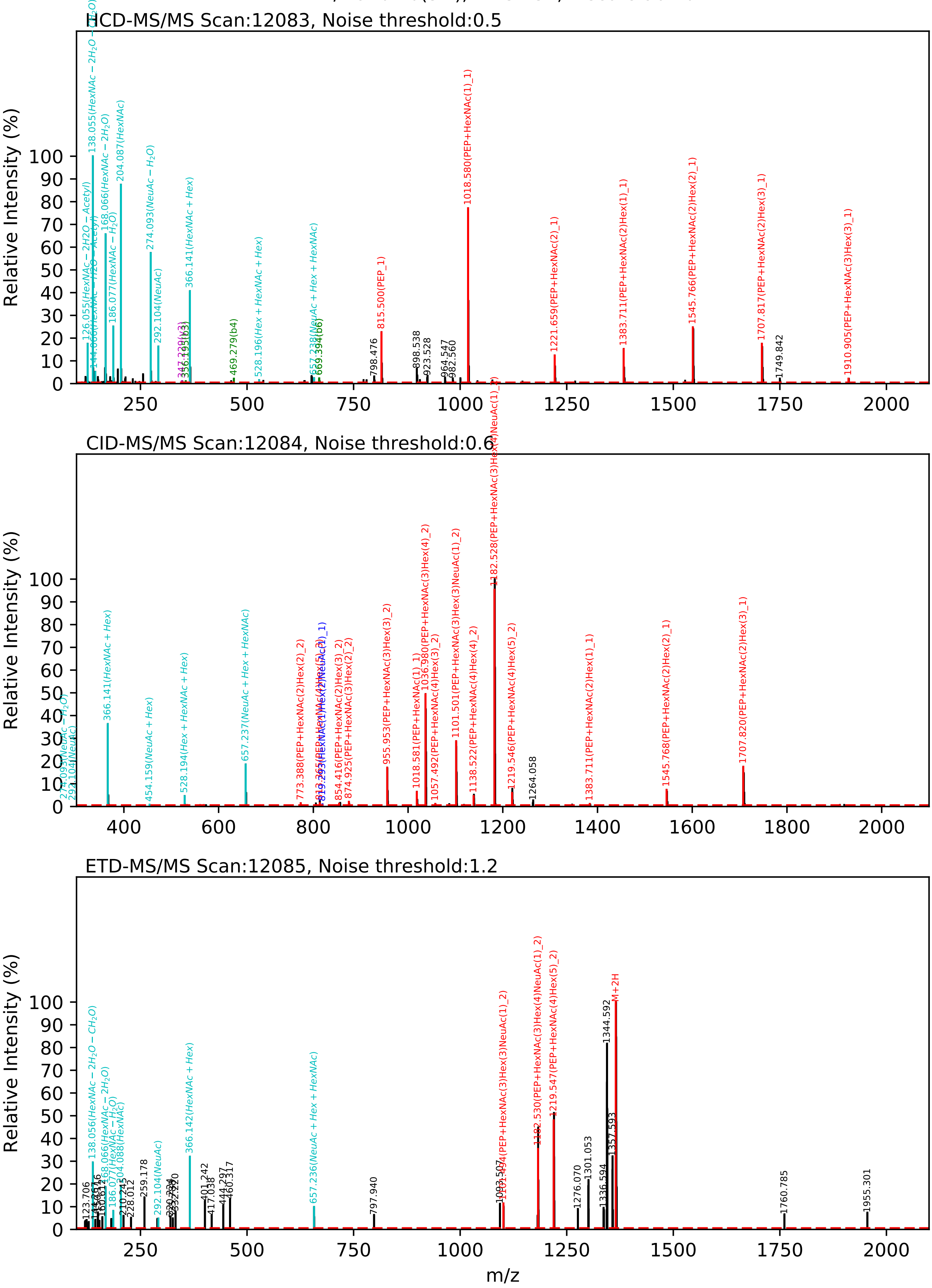

IQNLTVK(=PEP)_5_4_0_1_0, 0_None, 0_None, m/z:910.40(3+), RT:35.66, Y-score:95.74

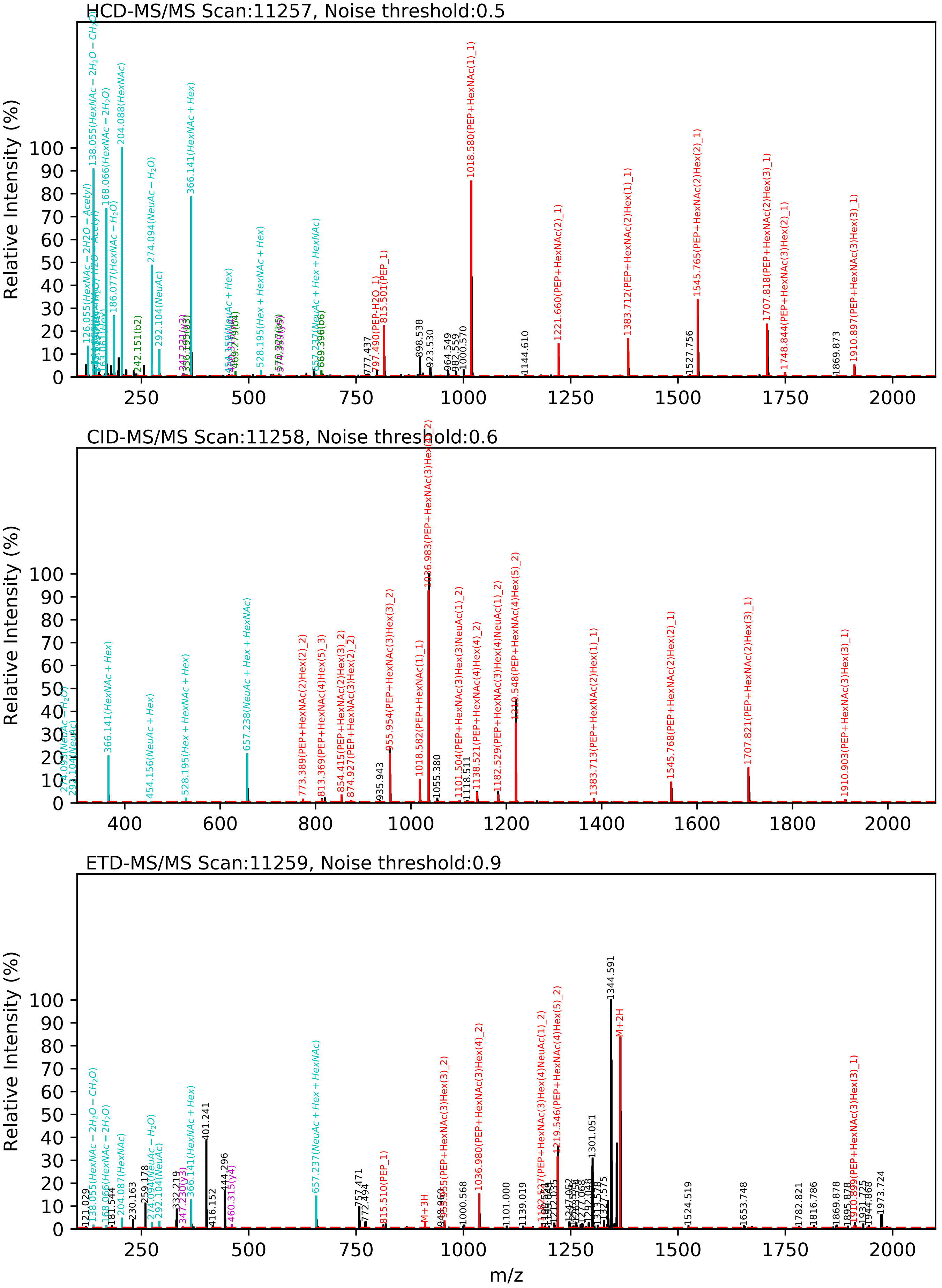

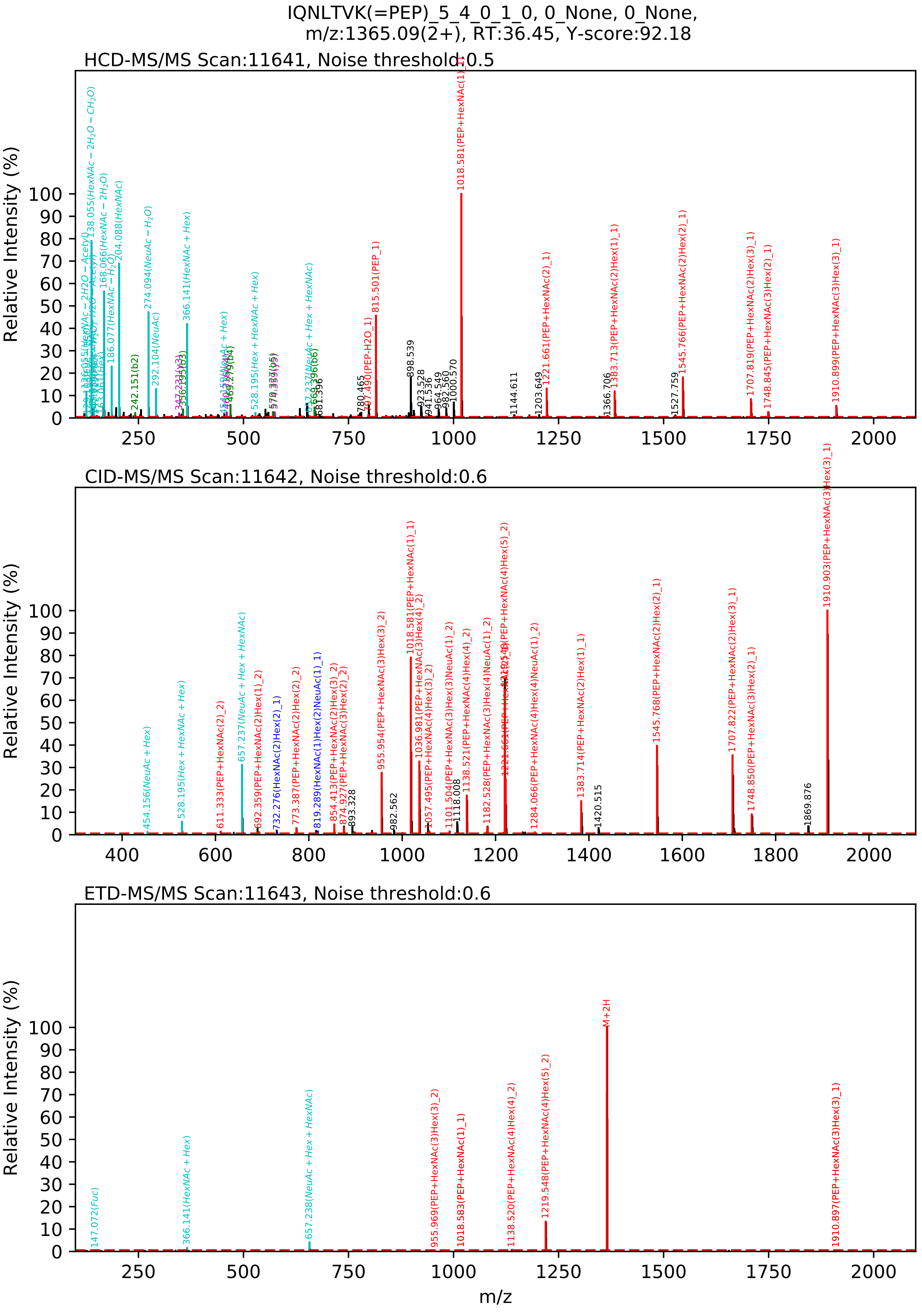
IQNLTVK(=PEP)_5_4_0_1_0, 0_None, 0_None,
m/z:1365.09(2+), RT:36.45, Y-score:92.18
HCD-MS/MS Scan:11641, Noise threshold:0.5
Relative Intensity (%)
1018.581(PEP+HexNAc(1)_1)
815.501(PEP_1)
1221.661(PEP+HexNAc(2)_1)
1383.713(PEP+HexNAc(2)Hex(1)_1)
1545.766(PEP+HexNAc(2)Hex(2)_1)
1707.819(PEP+HexNAc(2)Hex(3)_1)
1748.845(PEP+HexNAc(3)Hex(2)_1)
1910.899(PEP+HexNAc(3)Hex(3)_1)
898.539
923.528
941.536
964.549
1000.570
1144.611
1203.649
1366.706
1527.759
204.088(HexNAc)
274.094(NeuAc − H2O)
292.104(NeuAc)
366.141(HexNAc + Hex)
528.195(Hex + HexNAc + Hex)
242.151(b2)
469.279(b4)
CID-MS/MS Scan:11642, Noise threshold:0.6
1910.903(PEP+HexNAc(3)Hex(3)_1)
1018.581(PEP+HexNAc(1)_1)
955.954(PEP+HexNAc(3)Hex(3)_2)
1036.981(PEP+HexNAc(3)Hex(4)_2)
1057.495(PEP+HexNAc(4)Hex(3)_2)
1101.504(PEP+HexNAc(3)Hex(3)NeuAc(1)_2)
1138.521(PEP+HexNAc(4)Hex(4)_2)
1182.528(PEP+HexNAc(3)Hex(4)NeuAc(1)_2)
1284.066(PEP+HexNAc(4)Hex(4)NeuAc(1)_2)
1383.714(PEP+HexNAc(2)Hex(1)_1)
1545.768(PEP+HexNAc(2)Hex(2)_1)
1707.822(PEP+HexNAc(2)Hex(3)_1)
1748.850(PEP+HexNAc(3)Hex(2)_1)
1420.515
1869.876
1118.008
982.562
893.328
454.156(NeuAc + Hex)
528.195(Hex + HexNAc + Hex)
657.237(NeuAc + Hex + HexNAc)
611.333(PEP+HexNAc(2)_2)
692.359(PEP+HexNAc(2)Hex(1)_2)
732.276(HexNAc(2)Hex(2)_1)
773.387(PEP+HexNAc(2)Hex(2)_2)
819.289(HexNAc(1)Hex(2)NeuAc(1)_1)
854.413(PEP+HexNAc(2)Hex(3)_2)
874.927(PEP+HexNAc(3)Hex(2)_2)
ETD-MS/MS Scan:11643, Noise threshold:0.6
M+2H
147.072(Fuc)
366.141(HexNAc + Hex)
657.238(NeuAc + Hex + HexNAc)
955.969(PEP+HexNAc(3)Hex(3)_2)
1018.583(PEP+HexNAc(1)_1)
1138.520(PEP+HexNAc(4)Hex(4)_2)
1219.548(PEP+HexNAc(4)Hex(5)_2)
1910.897(PEP+HexNAc(3)Hex(3)_1)
m/z

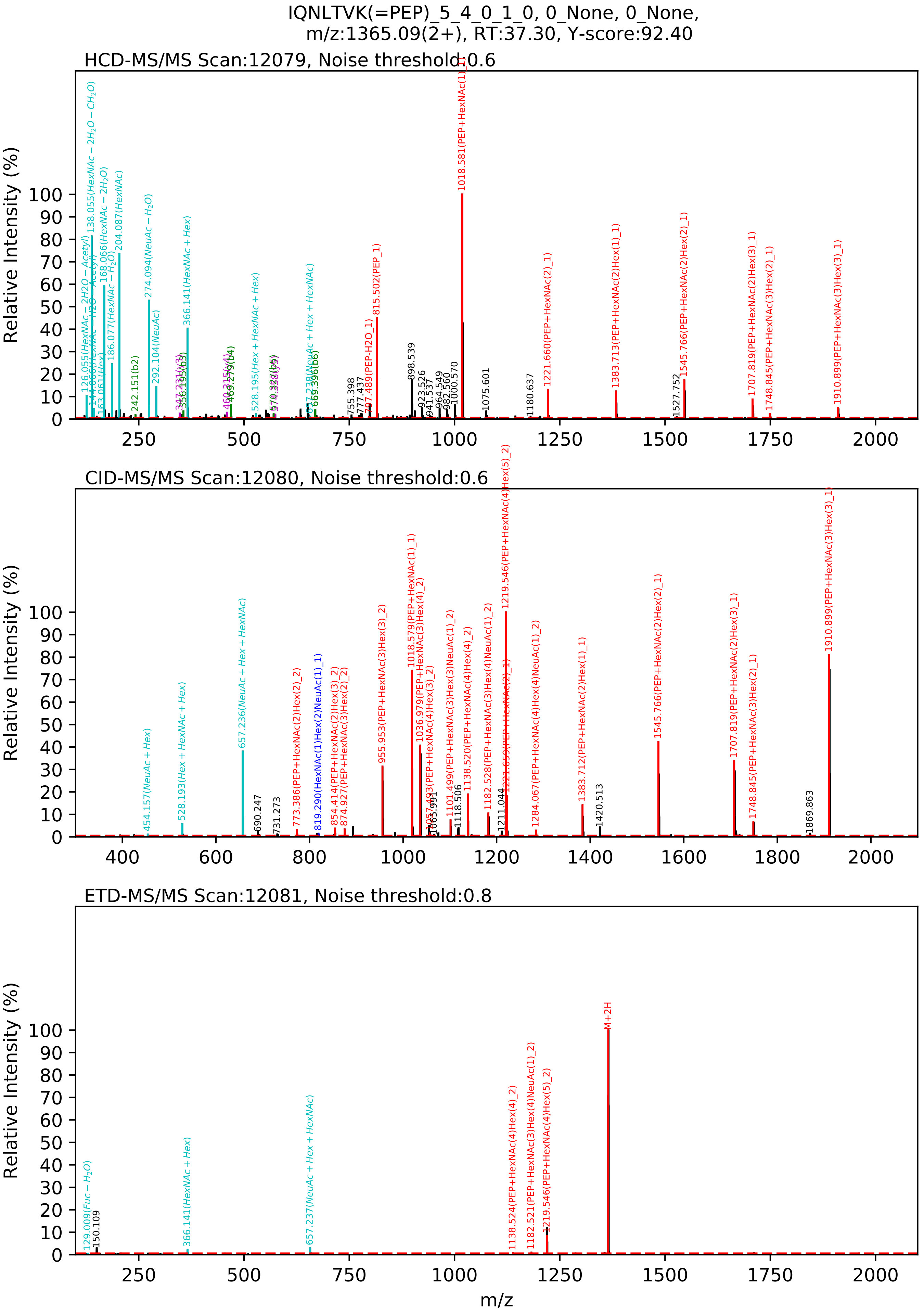

IQNLTVK(=PEP)_5_4_0_1_0, 0_None, 0_None,
m/z:1365.09(2+), RT:37.30, Y-score:92.40
HCD-MS/MS Scan:12079, Noise threshold:0.6
Relative Intensity (%)
1018.581(PEP+HexNAc(1)_1)
815.502(PEP_1)
1221.660(PEP+HexNAc(2)_1)
1383.713(PEP+HexNAc(2)Hex(1)_1)
1545.766(PEP+HexNAc(2)Hex(2)_1)
1707.819(PEP+HexNAc(2)Hex(3)_1)
1748.845(PEP+HexNAc(3)Hex(2)_1)
1910.899(PEP+HexNAc(3)Hex(3)_1)
204.087(HexNAc)
274.094(NeuAc − H2O)
292.104(NeuAc)
366.141(HexNAc + Hex)
186.077(HexNAc − H2O)
168.066(HexNAc − 2H2O)
528.195(Hex + HexNAc + Hex)
242.151(b2)
898.539
923.526
941.537
964.549
1000.570
1075.601
1180.637
1527.752
755.398
777.437
CID-MS/MS Scan:12080, Noise threshold:0.6
1219.546(PEP+HexNAc(4)Hex(5)_2)
1910.899(PEP+HexNAc(3)Hex(3)_1)
1018.579(PEP+HexNAc(1)_1)
1036.979(PEP+HexNAc(3)Hex(4)_2)
955.953(PEP+HexNAc(3)Hex(3)_2)
1101.499(PEP+HexNAc(3)Hex(3)NeuAc(1)_2)
1138.520(PEP+HexNAc(4)Hex(4)_2)
1182.528(PEP+HexNAc(3)Hex(4)NeuAc(1)_2)
1284.067(PEP+HexNAc(4)Hex(4)NeuAc(1)_2)
1383.712(PEP+HexNAc(2)Hex(1)_1)
1545.766(PEP+HexNAc(2)Hex(2)_1)
1707.819(PEP+HexNAc(2)Hex(3)_1)
1748.845(PEP+HexNAc(3)Hex(2)_1)
773.386(PEP+HexNAc(2)Hex(2)_2)
819.290(HexNAc(1)Hex(2)NeuAc(1)_1)
854.414(PEP+HexNAc(2)Hex(3)_2)
874.927(PEP+HexNAc(3)Hex(2)_2)
657.236(NeuAc + Hex + HexNAc)
528.193(Hex + HexNAc + Hex)
454.157(NeuAc + Hex)
690.247
731.273
1420.513
1869.863
ETD-MS/MS Scan:12081, Noise threshold:0.8
M+2H
1138.524(PEP+HexNAc(4)Hex(4)_2)
1182.521(PEP+HexNAc(3)Hex(4)NeuAc(1)_2)
1219.546(PEP+HexNAc(4)Hex(5)_2)
129.009(Fuc − H2O)
150.109
366.141(HexNAc + Hex)
657.237(NeuAc + Hex + HexNAc)
m/z

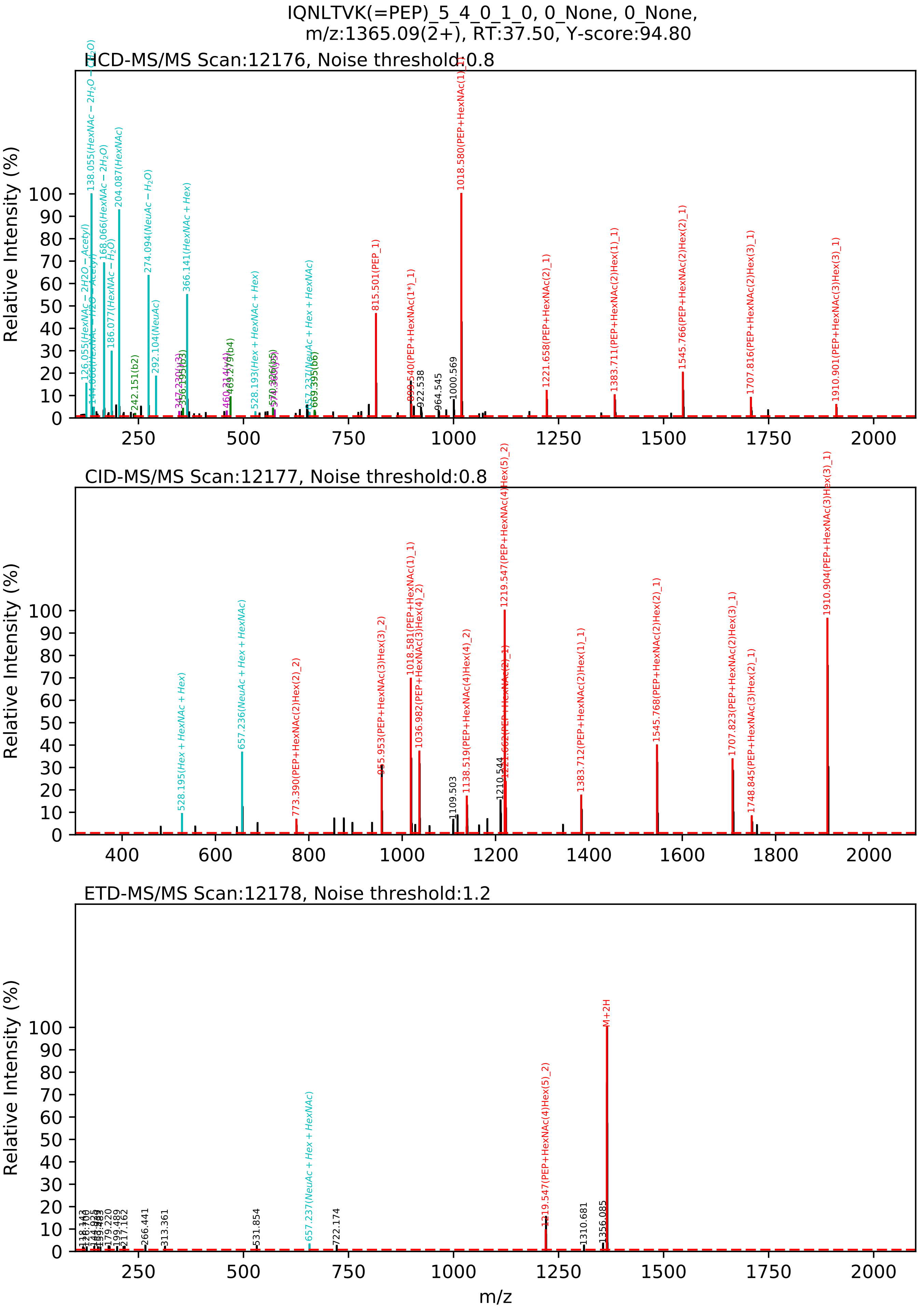
IQNLTVK(=PEP)_5_4_0_1_0, 0_None, 0_None,
m/z:1365.09(2+), RT:37.50, Y-score:94.80
HCD-MS/MS Scan:12176, Noise threshold:0.8
Relative Intensity (%)
126.055(HexNAc − 2H2O − Acetyl)
138.055(HexNAc − 2H2O)
168.066(HexNAc − 2H2O)
186.077(HexNAc − H2O)
204.087(HexNAc)
242.151(b2)
274.094(NeuAc − H2O)
292.104(NeuAc)
366.141(HexNAc + Hex)
489.279(b4)
528.193(Hex + HexNAc + Hex)
669.395(b6)
815.501(PEP_1)
922.538
964.545
1000.569
1018.580(PEP+HexNAc(1)_1)
1221.658(PEP+HexNAc(2)_1)
1383.711(PEP+HexNAc(2)Hex(1)_1)
1545.766(PEP+HexNAc(2)Hex(2)_1)
1707.816(PEP+HexNAc(2)Hex(3)_1)
1910.901(PEP+HexNAc(3)Hex(3)_1)
CID-MS/MS Scan:12177, Noise threshold:0.8
528.195(Hex + HexNAc + Hex)
657.236(NeuAc + Hex + HexNAc)
773.390(PEP+HexNAc(2)Hex(2)_2)
955.953(PEP+HexNAc(3)Hex(3)_2)
1018.581(PEP+HexNAc(1)_1)
1036.982(PEP+HexNAc(3)Hex(4)_2)
1109.503
1138.519(PEP+HexNAc(4)Hex(4)_2)
1210.544
1219.547(PEP+HexNAc(4)Hex(5)_2)
1383.712(PEP+HexNAc(2)Hex(1)_1)
1545.768(PEP+HexNAc(2)Hex(2)_1)
1707.823(PEP+HexNAc(2)Hex(3)_1)
1748.845(PEP+HexNAc(3)Hex(2)_1)
1910.904(PEP+HexNAc(3)Hex(3)_1)
ETD-MS/MS Scan:12178, Noise threshold:1.2
266.441
313.361
531.854
657.237(NeuAc + Hex + HexNAc)
722.174
1219.547(PEP+HexNAc(4)Hex(5)_2)
1310.681
1356.085
M+2H
m/z

IQNLTVK(=PEP)_5_4_0_1_0, 0_None, 0_None,
m/z:1365.09(2+), RT:35.67, Y-score:91.21

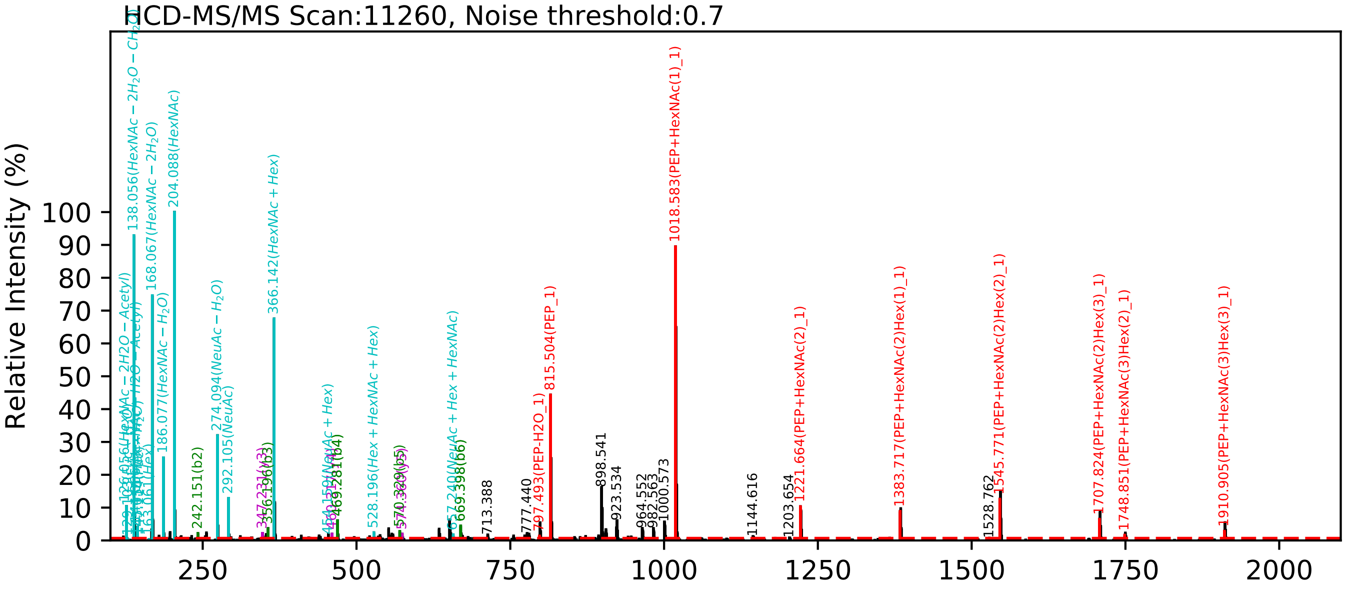

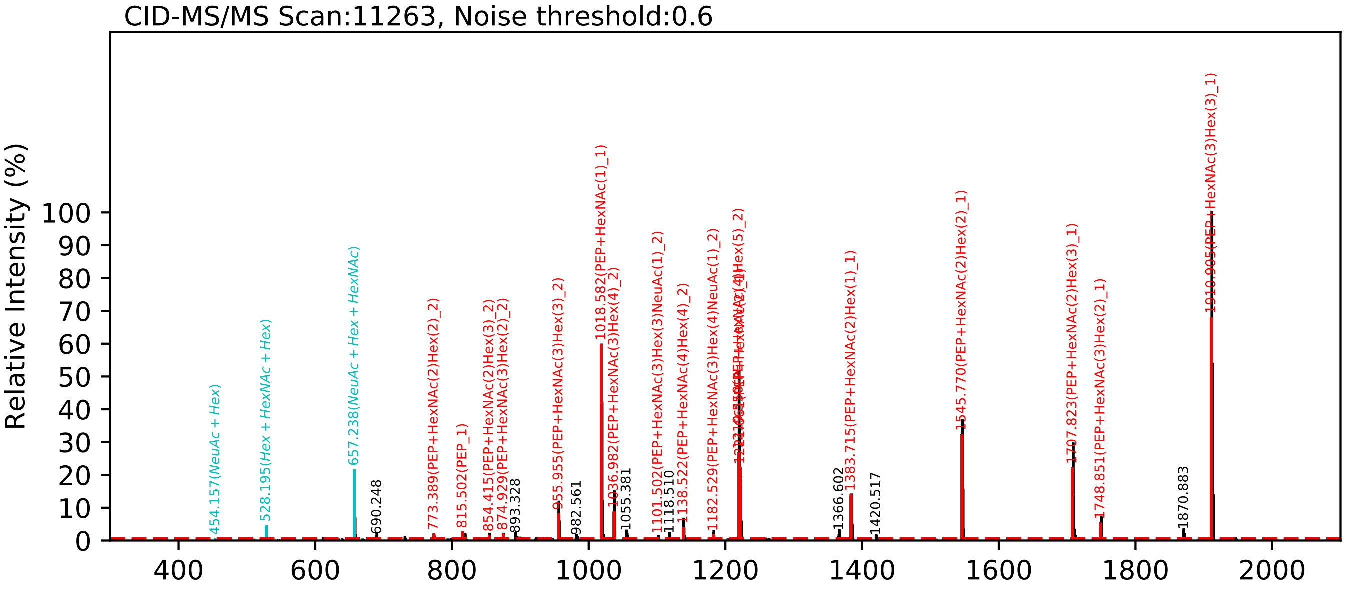

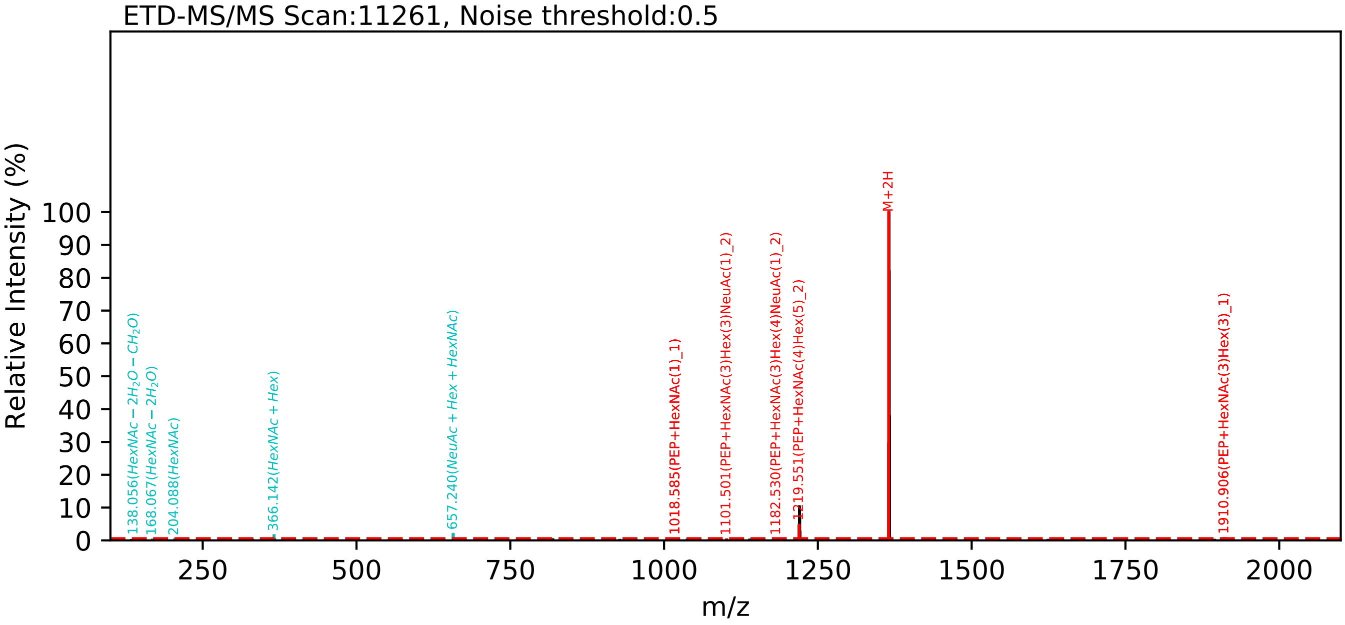

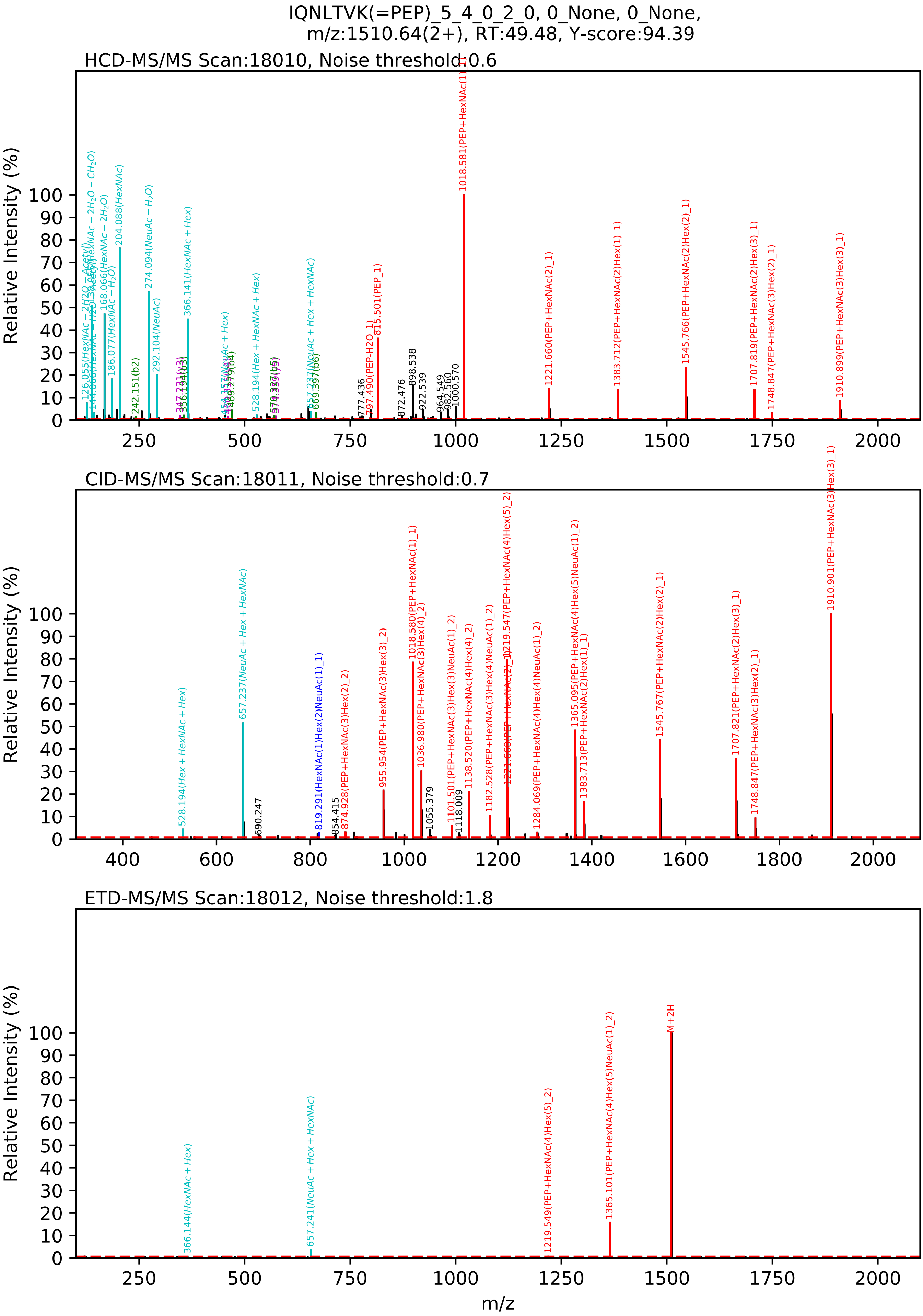

IQNLTVK(=PEP)_5_4_0_2_0, 0_None, 0_None,
m/z:1510.64(2+), RT:49.48, Y-score:94.39
HCD-MS/MS Scan:18010, Noise threshold:0.6
CID-MS/MS Scan:18011, Noise threshold:0.7
ETD-MS/MS Scan:18012, Noise threshold:1.8
Relative Intensity (%)
m/z
1018.581(PEP+HexNAc(1)_1)
204.088(HexNAc)
274.094(NeuAc − H2O)
366.141(HexNAc + Hex)
815.501(PEP_1)
1221.660(PEP+HexNAc(2)_1)
1383.712(PEP+HexNAc(2)Hex(1)_1)
1545.766(PEP+HexNAc(2)Hex(2)_1)
1707.819(PEP+HexNAc(2)Hex(3)_1)
1748.847(PEP+HexNAc(3)Hex(2)_1)
1910.899(PEP+HexNAc(3)Hex(3)_1)
1910.901(PEP+HexNAc(3)Hex(3)_1)
657.237(NeuAc + Hex + HexNAc)
1018.580(PEP+HexNAc(1)_1)
1036.980(PEP+HexNAc(3)Hex(4)_2)
1219.547(PEP+HexNAc(4)Hex(5)_2)
1365.095(PEP+HexNAc(4)Hex(5)NeuAc(1)_2)
1545.767(PEP+HexNAc(2)Hex(2)_1)
1707.821(PEP+HexNAc(2)Hex(3)_1)
1748.847(PEP+HexNAc(3)Hex(2)_1)
366.144(HexNAc + Hex)
657.241(NeuAc + Hex + HexNAc)
1219.549(PEP+HexNAc(4)Hex(5)_2)
1365.101(PEP+HexNAc(4)Hex(5)NeuAc(1)_2)
M+2H

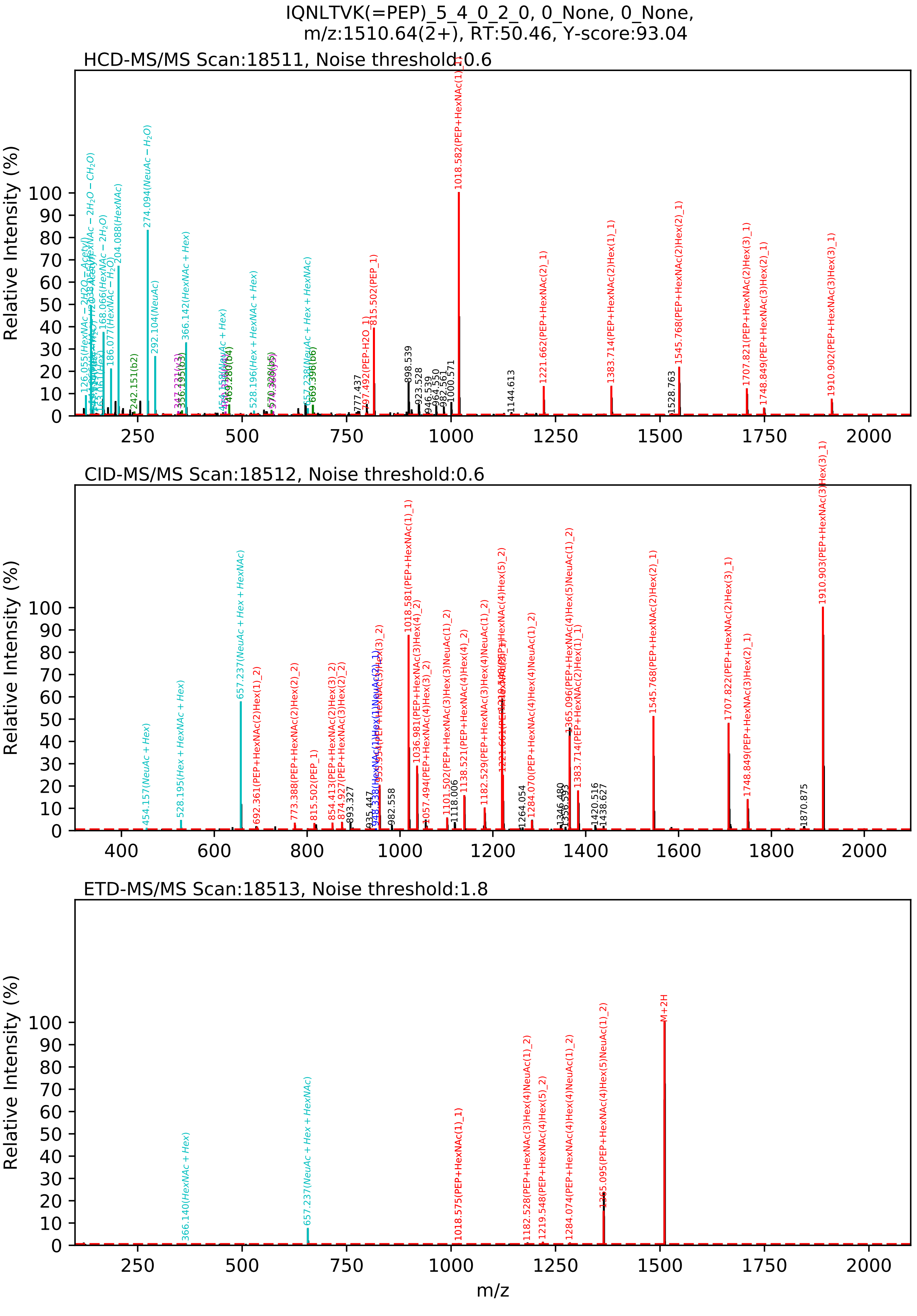

IQNLTVK(=PEP)_5_4_0_2_0, 0_None, 0_None,
m/z:1510.64(2+), RT:50.46, Y-score:93.04
HCD-MS/MS Scan:18511, Noise threshold:0.6
CID-MS/MS Scan:18512, Noise threshold:0.6
ETD-MS/MS Scan:18513, Noise threshold:1.8
Relative Intensity (%)
m/z
1018.582(PEP+HexNAc(1)_1)
274.094(NeuAc − H2O)
204.088(HexNAc)
292.104(NeuAc)
366.142(HexNAc + Hex)
815.502(PEP_1)
1221.662(PEP+HexNAc(2)_1)
1383.714(PEP+HexNAc(2)Hex(1)_1)
1545.768(PEP+HexNAc(2)Hex(2)_1)
1707.821(PEP+HexNAc(2)Hex(3)_1)
1748.849(PEP+HexNAc(3)Hex(2)_1)
1910.902(PEP+HexNAc(3)Hex(3)_1)
1910.903(PEP+HexNAc(3)Hex(3)_1)
1018.581(PEP+HexNAc(1)_1)
657.237(NeuAc + Hex + HexNAc)
1365.096(PEP+HexNAc(4)Hex(5)NeuAc(1)_2)
1545.768(PEP+HexNAc(2)Hex(2)_1)
1707.822(PEP+HexNAc(2)Hex(3)_1)
M+2H
1365.095(PEP+HexNAc(4)Hex(5)NeuAc(1)_2)
366.140(HexNAc + Hex)
1018.575(PEP+HexNAc(1)_1)

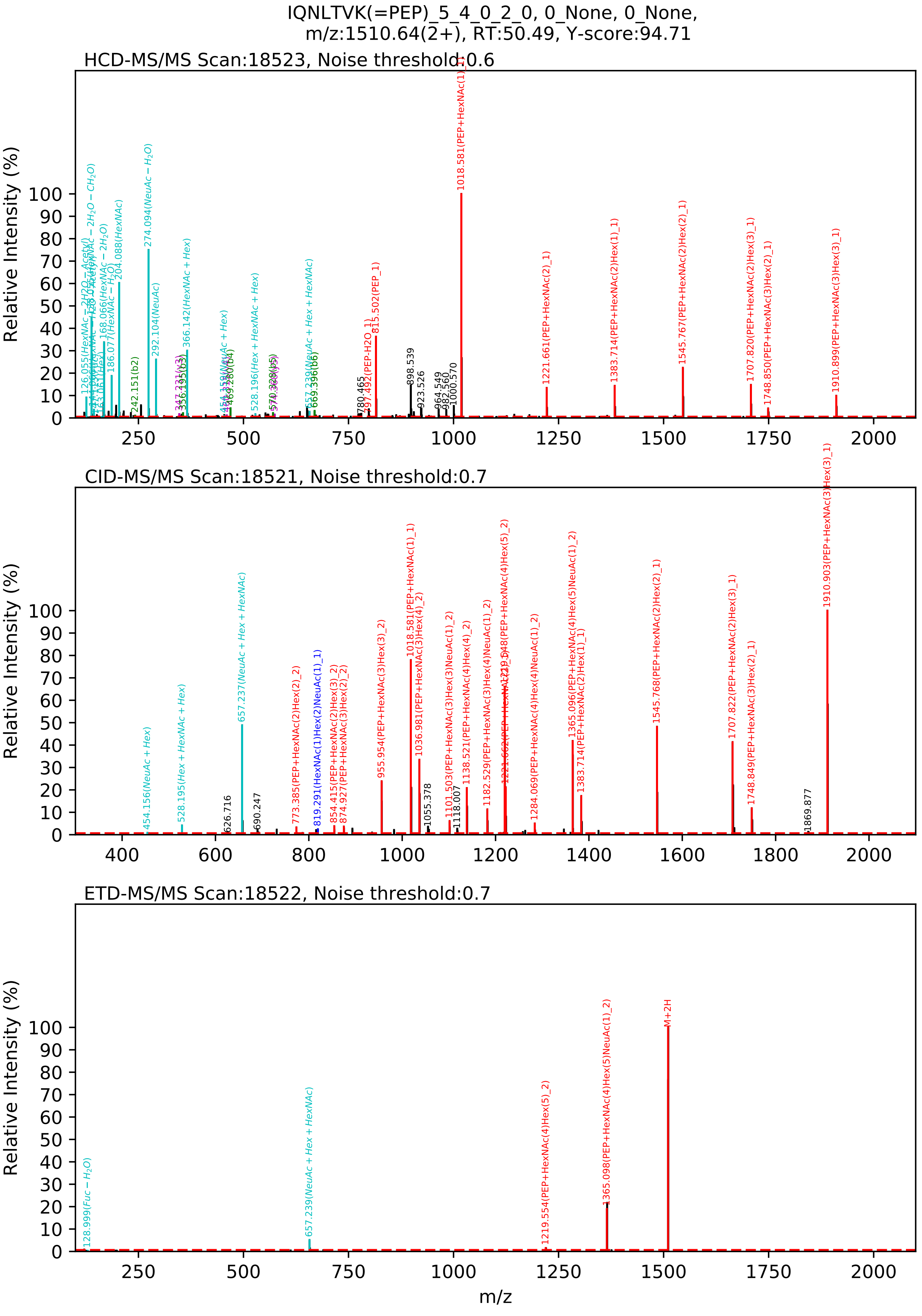

IQNLTVK(=PEP)_5_4_0_2_0, 0_None, 0_None,
m/z:1510.64(2+), RT:50.49, Y-score:94.71
HCD-MS/MS Scan:18523, Noise threshold:0.6
CID-MS/MS Scan:18521, Noise threshold:0.7
ETD-MS/MS Scan:18522, Noise threshold:0.7
Relative Intensity (%)
m/z
1018.581(PEP+HexNAc(1)_1)
815.502(PEP_1)
1221.661(PEP+HexNAc(2)_1)
1383.714(PEP+HexNAc(2)Hex(1)_1)
1545.767(PEP+HexNAc(2)Hex(2)_1)
1707.820(PEP+HexNAc(2)Hex(3)_1)
1748.850(PEP+HexNAc(3)Hex(2)_1)
1910.899(PEP+HexNAc(3)Hex(3)_1)
274.094(NeuAc − H2O)
292.104(NeuAc)
204.088(HexNAc)
366.142(HexNAc + Hex)
528.196(Hex + HexNAc + Hex)
1910.903(PEP+HexNAc(3)Hex(3)_1)
1545.768(PEP+HexNAc(2)Hex(2)_1)
1707.822(PEP+HexNAc(2)Hex(3)_1)
1365.096(PEP+HexNAc(4)Hex(5)NeuAc(1)_2)
1018.581(PEP+HexNAc(1)_1)
1036.981(PEP+HexNAc(3)Hex(4)_2)
955.954(PEP+HexNAc(3)Hex(3)_2)
657.237(NeuAc + Hex + HexNAc)
819.291(HexNAc(1)Hex(2)NeuAc(1)_1)
M+2H
1365.098(PEP+HexNAc(4)Hex(5)NeuAc(1)_2)
1219.554(PEP+HexNAc(4)Hex(5)_2)
657.239(NeuAc + Hex + HexNAc)
128.999(Fuc − H2O)

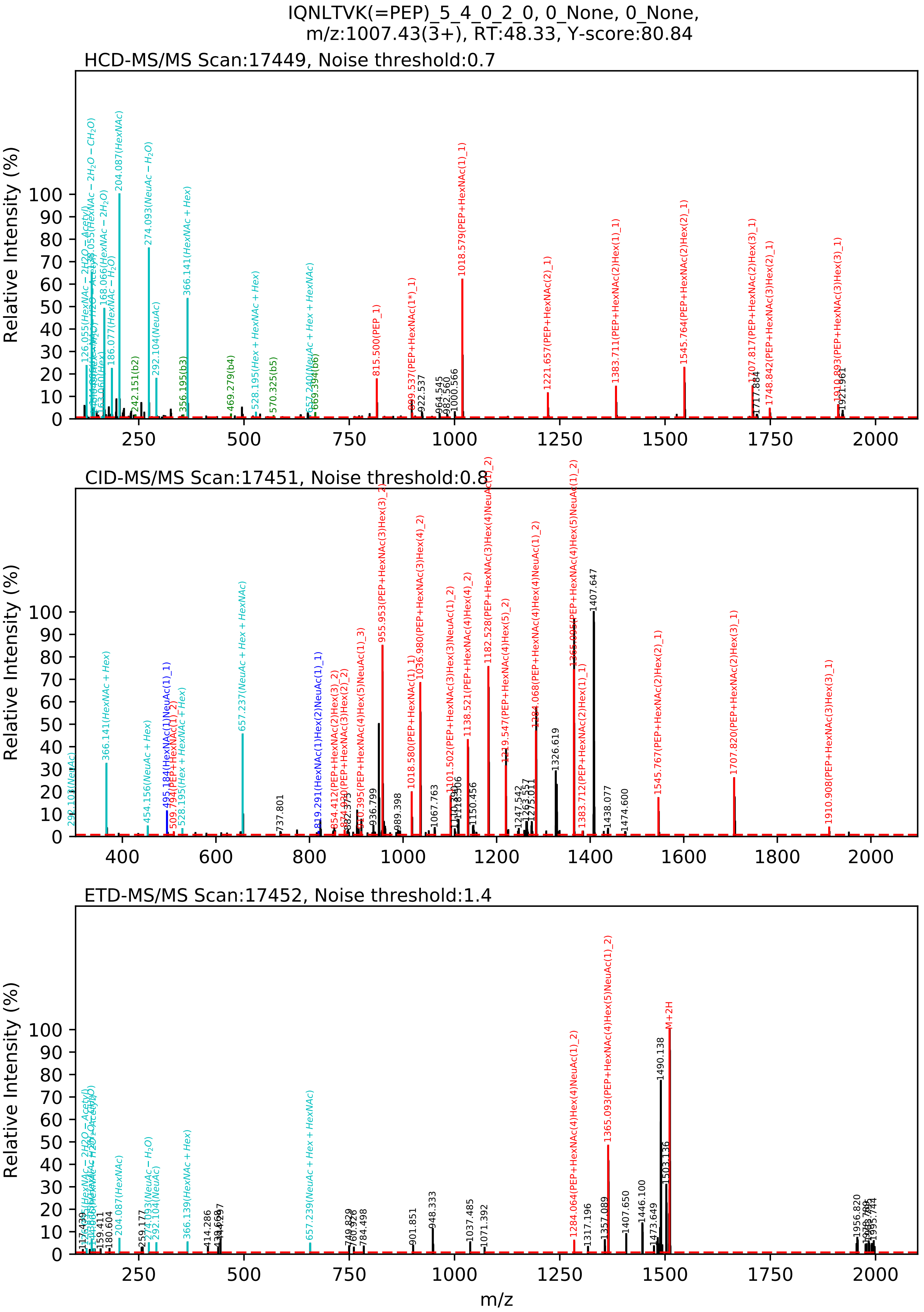
IQNLTVK(=PEP)_5_4_0_2_0, 0_None, 0_None,
m/z:1007.43(3+), RT:48.33, Y-score:80.84
HCD-MS/MS Scan:17449, Noise threshold:0.7
CID-MS/MS Scan:17451, Noise threshold:0.8
ETD-MS/MS Scan:17452, Noise threshold:1.4
Relative Intensity (%)
m/z
1018.579(PEP+HexNAc(1)_1)
815.500(PEP_1)
1221.657(PEP+HexNAc(2)_1)
1383.711(PEP+HexNAc(2)Hex(1)_1)
1545.764(PEP+HexNAc(2)Hex(2)_1)
1707.817(PEP+HexNAc(2)Hex(3)_1)
1748.842(PEP+HexNAc(3)Hex(2)_1)
1910.893(PEP+HexNAc(3)Hex(3)_1)
204.087(HexNAc)
274.093(NeuAc − H2O)
366.141(HexNAc + Hex)
292.104(NeuAc)
955.953(PEP+HexNAc(3)Hex(3)_2)
1036.980(PEP+HexNAc(3)Hex(4)_2)
1182.528(PEP+HexNAc(3)Hex(4)NeuAc(1)_2)
1365.095(PEP+HexNAc(4)Hex(5)NeuAc(1)_2)
1407.647
657.237(NeuAc + Hex + HexNAc)
1284.064(PEP+HexNAc(4)Hex(4)NeuAc(1)_2)
1365.093(PEP+HexNAc(4)Hex(5)NeuAc(1)_2)
1490.138
M+2H

IQNLTVK(=PEP)_5_4_0_2_0, 0_None, 0_None,
m/z:1007.43(3+), RT:49.14, Y-score:96.46

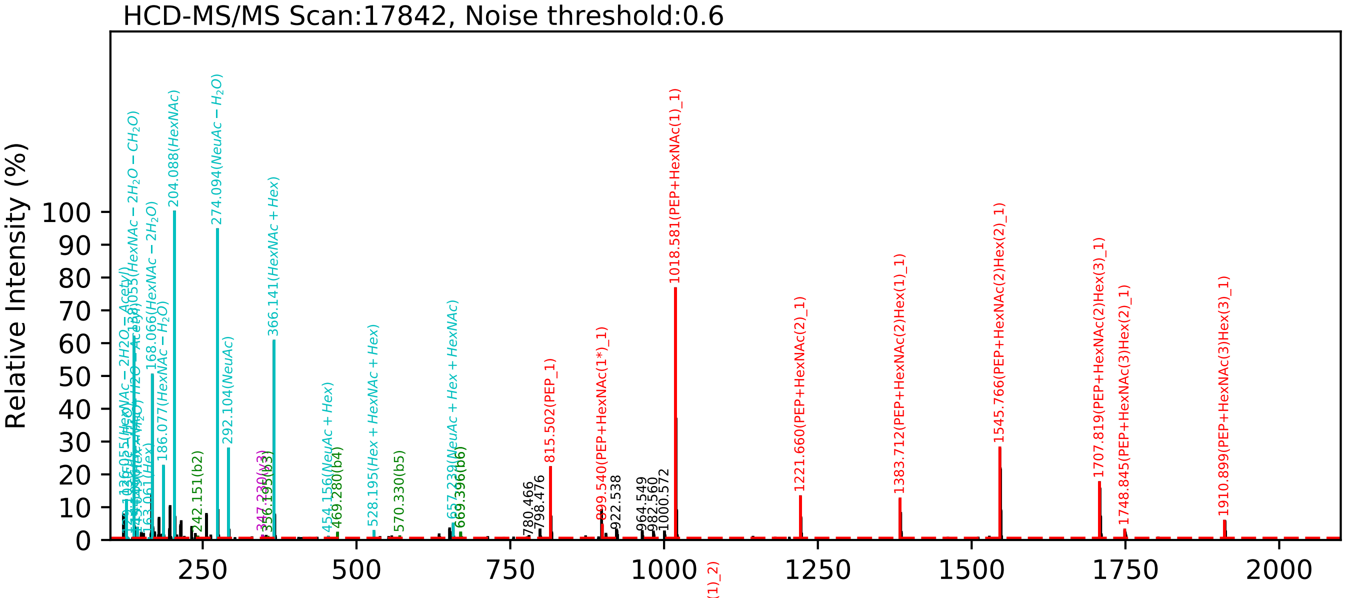

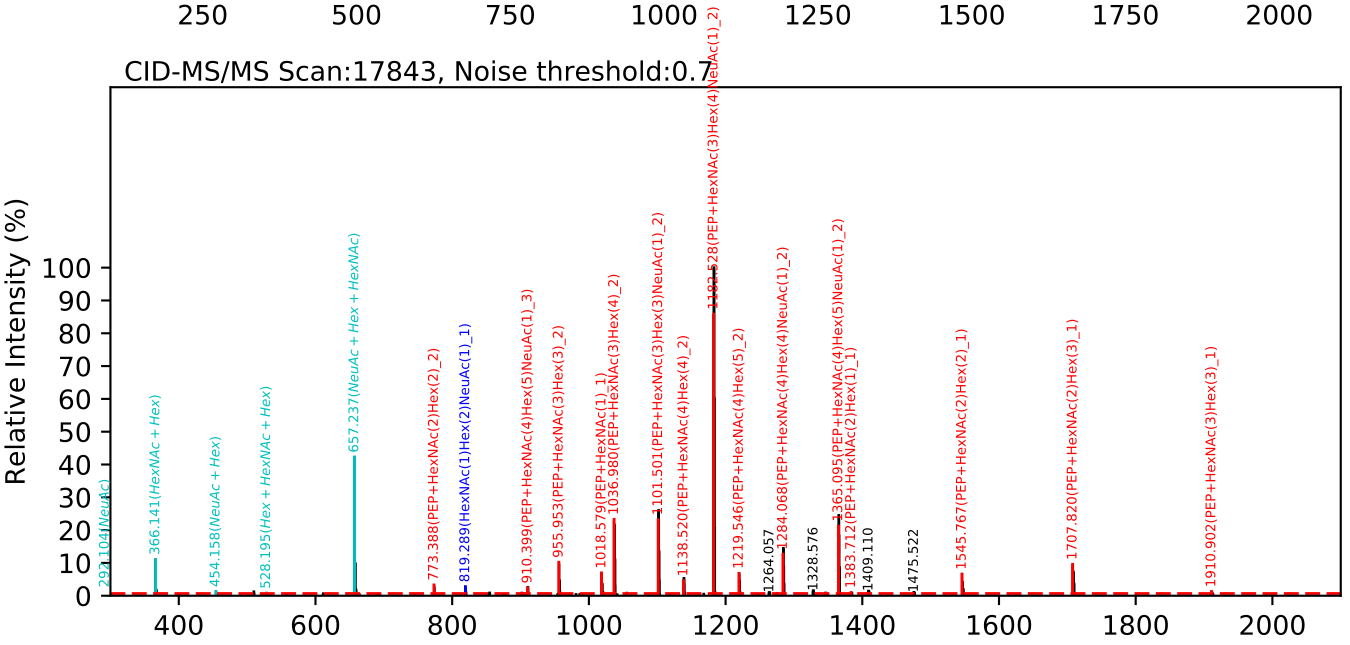

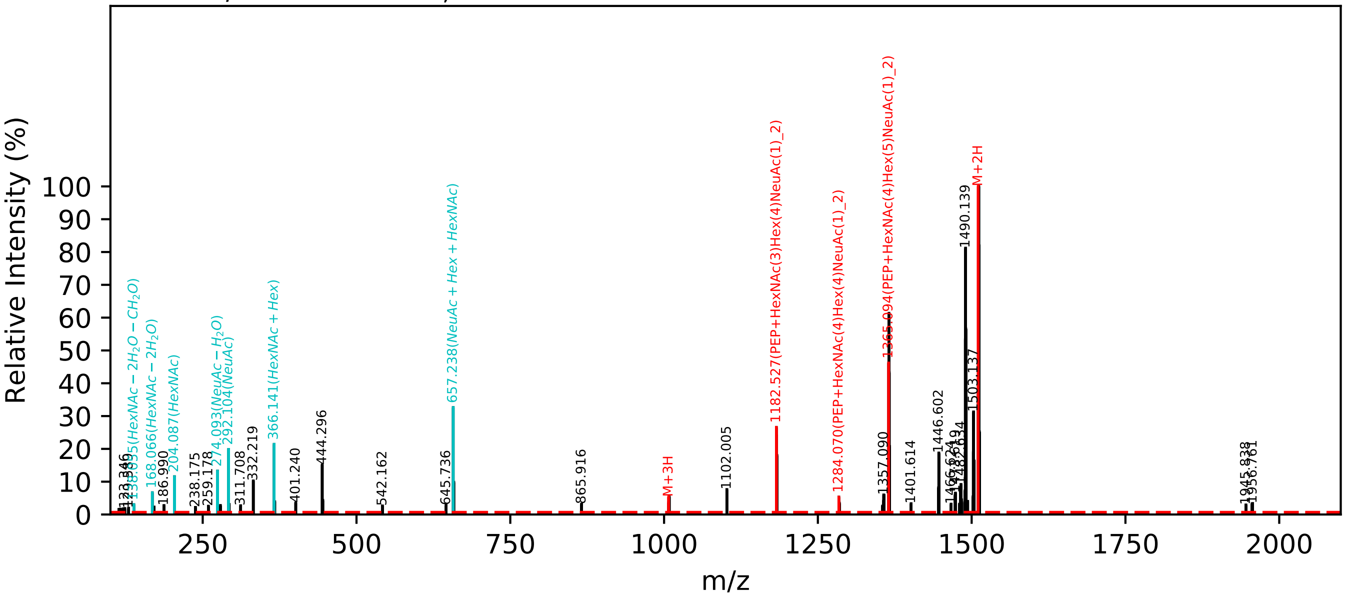

IQNLTVK(=PEP)_5_4_0_2_0, 0_None, 0_None,
m/z:1007.43(3+), RT:49.39, Y-score:96.69

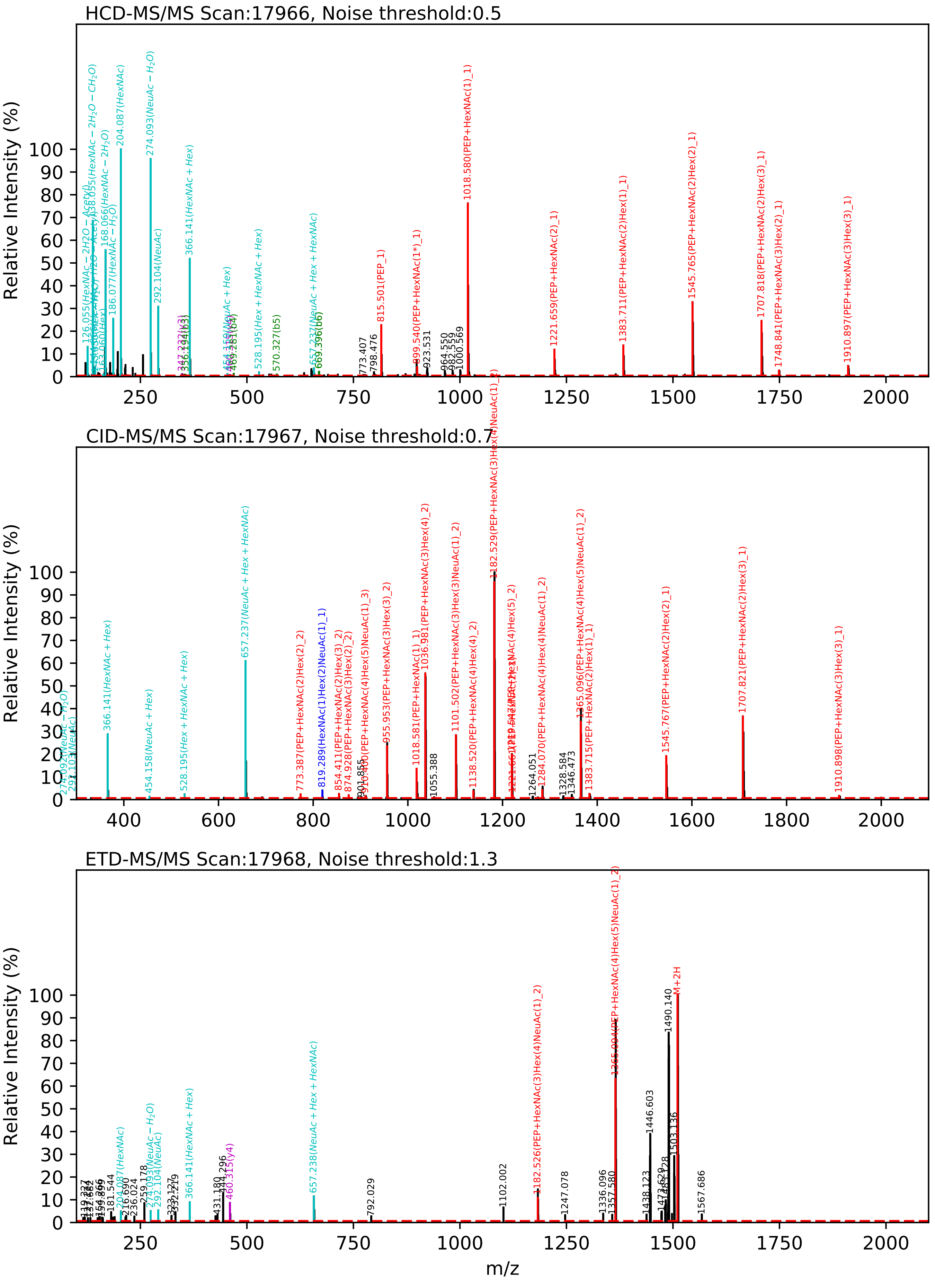

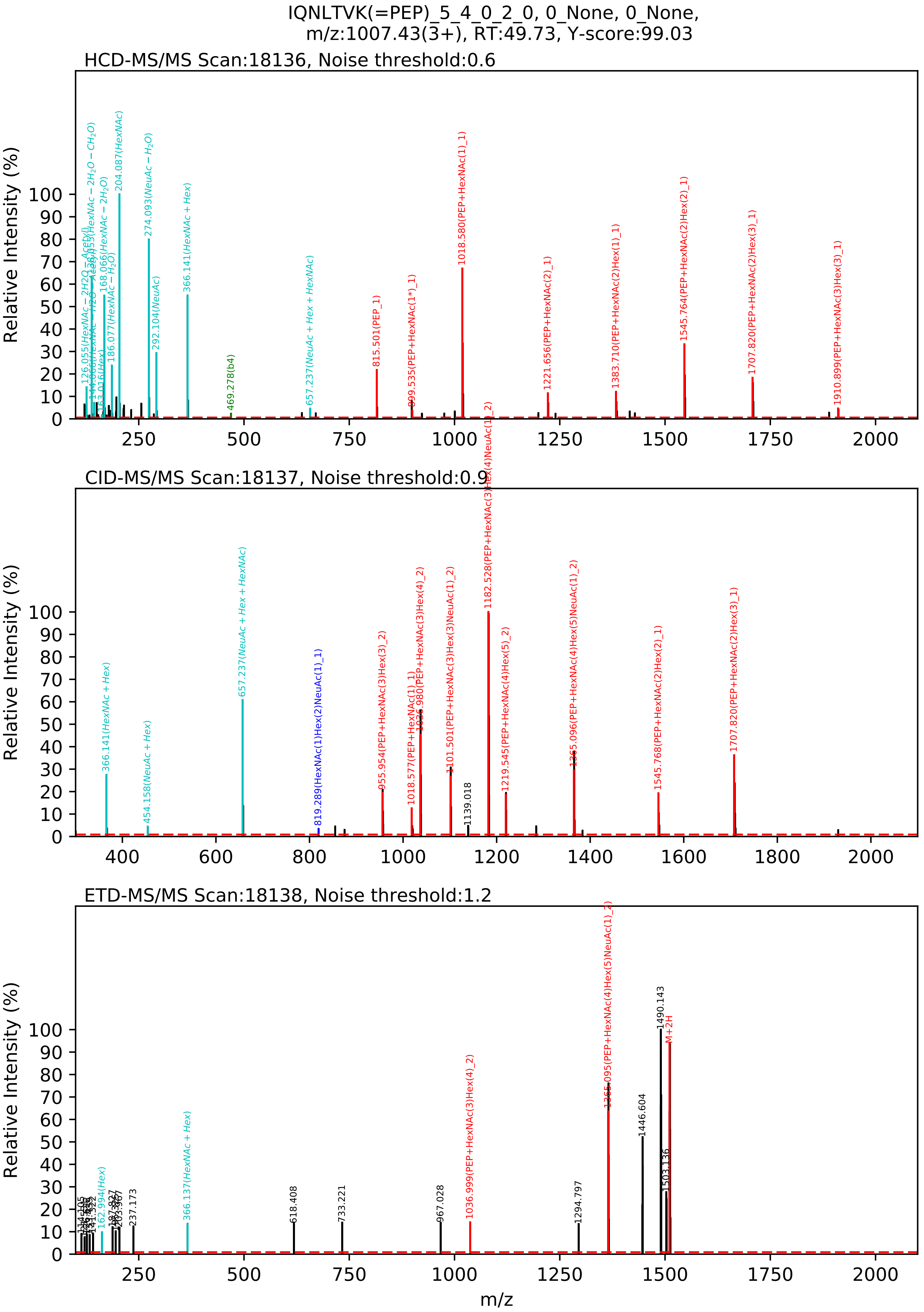
IQNLTVK(=PEP)_5_4_0_2_0, 0_None, 0_None,
m/z:1007.43(3+), RT:49.73, Y-score:99.03
HCD-MS/MS Scan:18136, Noise threshold:0.6
CID-MS/MS Scan:18137, Noise threshold:0.9
ETD-MS/MS Scan:18138, Noise threshold:1.2
Relative Intensity (%)
m/z
204.087(HexNAc)
274.093(NeuAc − H2O)
366.141(HexNAc + Hex)
292.104(NeuAc)
186.077(HexNAc − H2O)
168.066(HexNAc − 2H2O)
469.278(b4)
657.237(NeuAc + Hex + HexNAc)
815.501(PEP_1)
899.535(PEP+HexNAc(1*)_1)
1018.580(PEP+HexNAc(1)_1)
1221.656(PEP+HexNAc(2)_1)
1383.710(PEP+HexNAc(2)Hex(1)_1)
1545.764(PEP+HexNAc(2)Hex(2)_1)
1707.820(PEP+HexNAc(2)Hex(3)_1)
1910.899(PEP+HexNAc(3)Hex(3)_1)
366.141(HexNAc + Hex)
454.158(NeuAc + Hex)
657.237(NeuAc + Hex + HexNAc)
819.289(HexNAc(1)Hex(2)NeuAc(1)_1)
955.954(PEP+HexNAc(3)Hex(3)_2)
1018.577(PEP+HexNAc(1)_1)
1036.980(PEP+HexNAc(3)Hex(4)_2)
1101.501(PEP+HexNAc(3)Hex(3)NeuAc(1)_2)
1139.018
1182.528(PEP+HexNAc(3)Hex(4)NeuAc(1)_2)
1219.545(PEP+HexNAc(4)Hex(5)_2)
1365.096(PEP+HexNAc(4)Hex(5)NeuAc(1)_2)
1545.768(PEP+HexNAc(2)Hex(2)_1)
1707.820(PEP+HexNAc(2)Hex(3)_1)
162.994(Hex)
237.173
366.137(HexNAc + Hex)
618.408
733.221
967.028
1036.999(PEP+HexNAc(3)Hex(4)_2)
1294.797
1365.095(PEP+HexNAc(4)Hex(5)NeuAc(1)_2)
1446.604
1490.143
M+2H
1503.136

IQNLTVK(=PEP)_5_4_0_2_0, 0_None, 0_None,
m/z:1007.43(3+), RT:50.44, Y-score:95.72

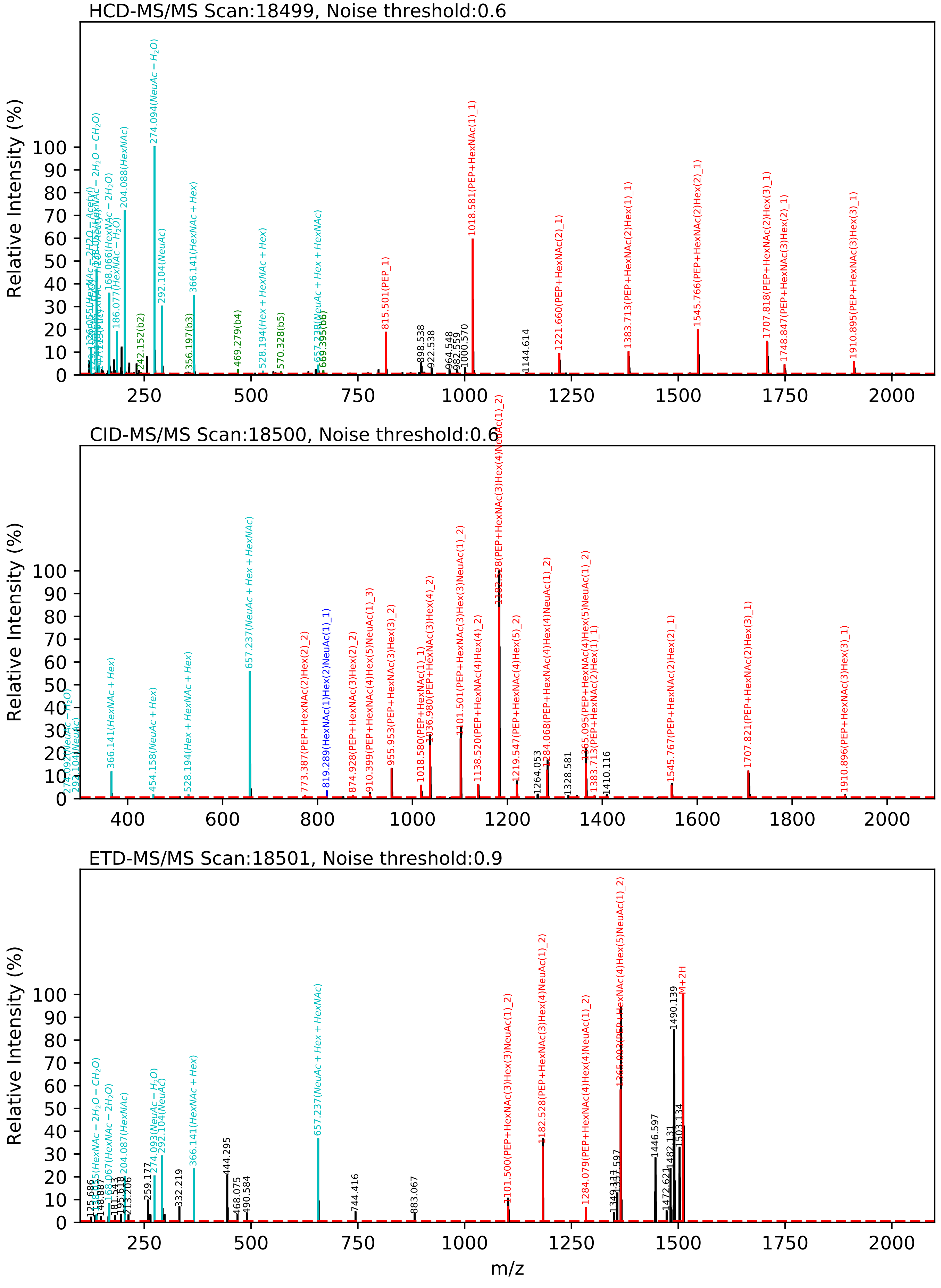

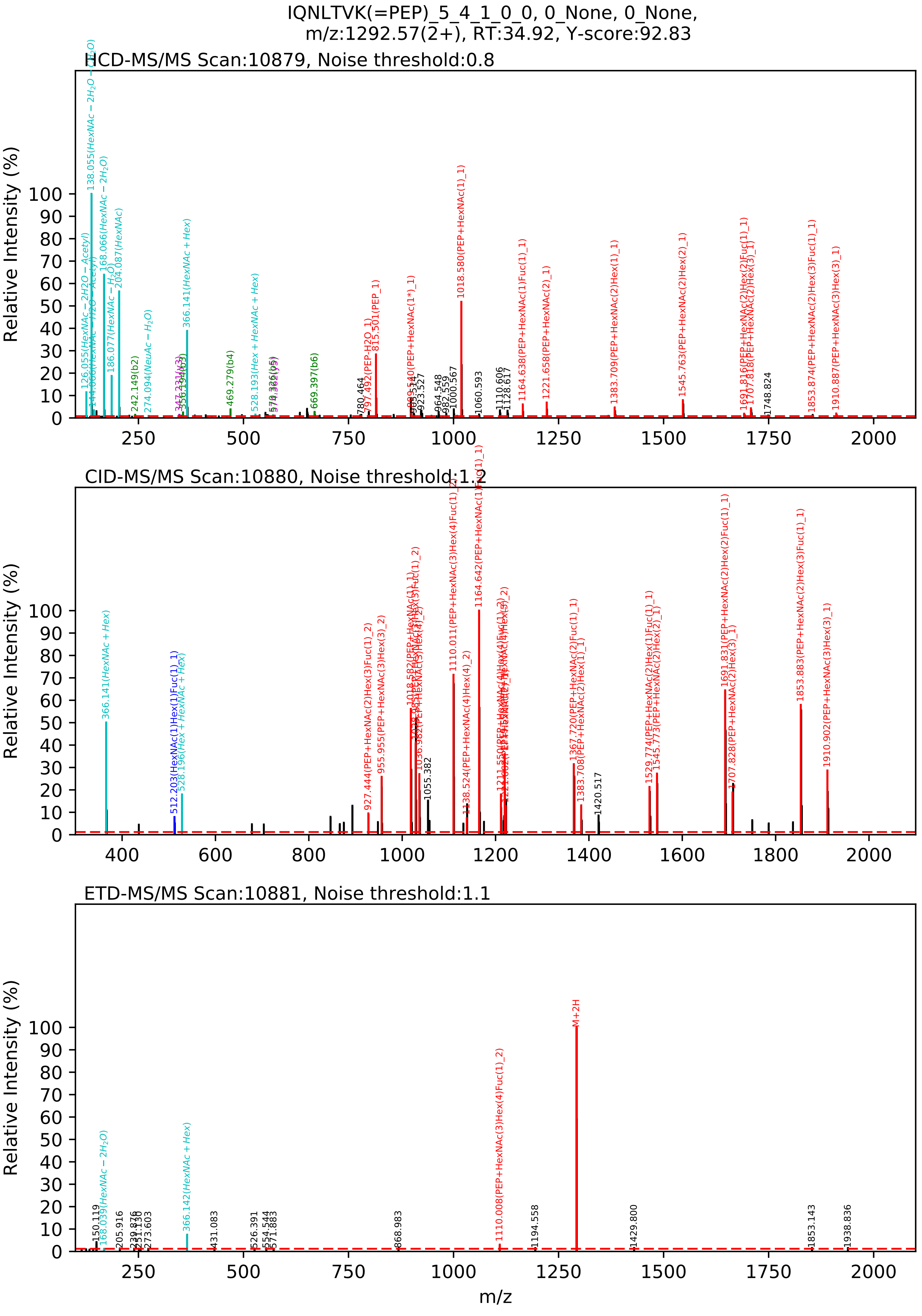

IQNLTVK(=PEP)_5_4_1_0_0, 0_None, 0_None,
m/z:1292.57(2+), RT:34.92, Y-score:92.83
HCD-MS/MS Scan:10879, Noise threshold:0.8
CID-MS/MS Scan:10880, Noise threshold:1.2
ETD-MS/MS Scan:10881, Noise threshold:1.1
Relative Intensity (%)
m/z

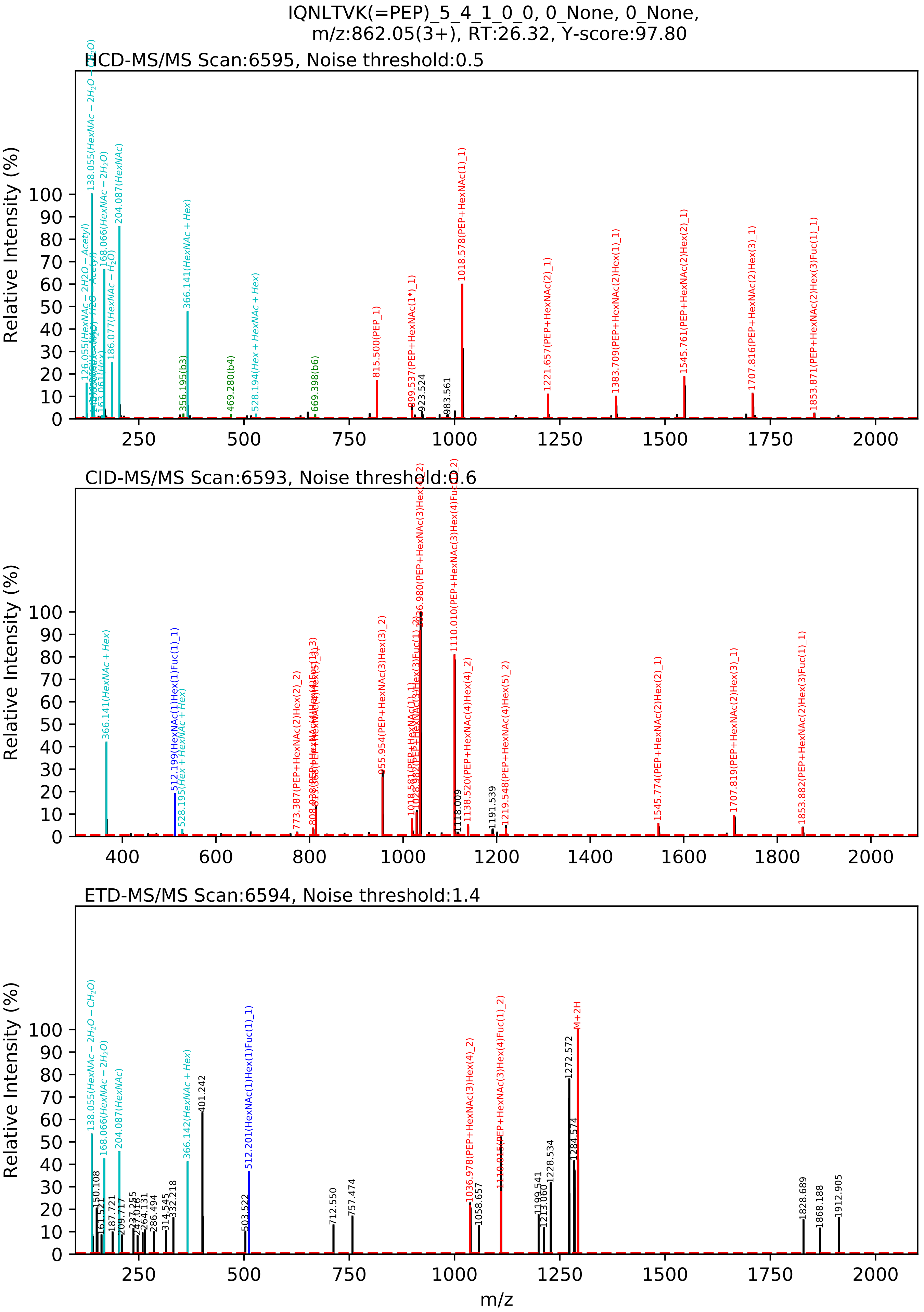
IQNLTVK(=PEP)_5_4_1_0_0, 0_None, 0_None,
m/z:862.05(3+), RT:26.32, Y-score:97.80
HCD-MS/MS Scan:6595, Noise threshold:0.5
CID-MS/MS Scan:6593, Noise threshold:0.6
ETD-MS/MS Scan:6594, Noise threshold:1.4
Relative Intensity (%)
m/z
815.500(PEP_1)
1018.578(PEP+HexNAc(1)_1)
1221.657(PEP+HexNAc(2)_1)
1383.709(PEP+HexNAc(2)Hex(1)_1)
1545.761(PEP+HexNAc(2)Hex(2)_1)
1707.816(PEP+HexNAc(2)Hex(3)_1)
1853.871(PEP+HexNAc(2)Hex(3)Fuc(1)_1)
366.141(HexNAc + Hex)
204.087(HexNAc)
1110.010(PEP+HexNAc(3)Hex(4)Fuc(1)_2)
955.954(PEP+HexNAc(3)Hex(3)_2)
512.199(HexNAc(1)Hex(1)Fuc(1)_1)
1545.774(PEP+HexNAc(2)Hex(2)_1)
1707.819(PEP+HexNAc(2)Hex(3)_1)
1853.882(PEP+HexNAc(2)Hex(3)Fuc(1)_1)
1036.978(PEP+HexNAc(3)Hex(4)_2)
512.201(HexNAc(1)Hex(1)Fuc(1)_1)
M+2H
1272.572
401.242

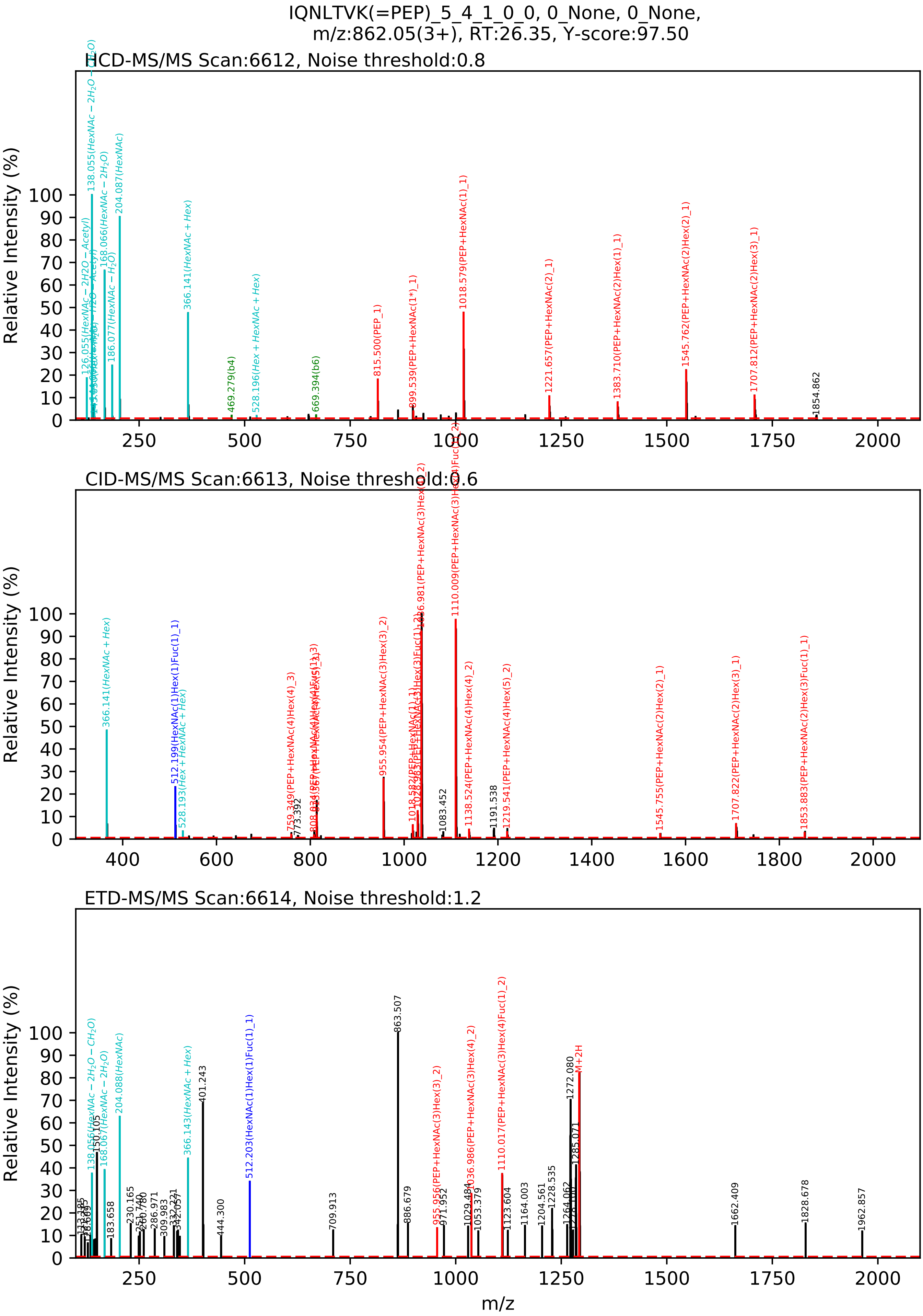

IQNLTVK(=PEP)_5_4_1_0_0, 0_None, 0_None,
m/z:862.05(3+), RT:26.35, Y-score:97.50
HCD-MS/MS Scan:6612, Noise threshold:0.8
Relative Intensity (%)
138.055(HexNAc − 2H2O − CH2O)
204.087(HexNAc)
168.066(HexNAc − 2H2O)
186.077(HexNAc − H2O)
126.055(HexNAc − 2H2O − Acetyl)
366.141(HexNAc + Hex)
469.279(b4)
528.196(Hex + HexNAc + Hex)
669.394(b6)
815.500(PEP_1)
899.539(PEP+HexNAc(1*)_1)
1018.579(PEP+HexNAc(1)_1)
1221.657(PEP+HexNAc(2)_1)
1383.710(PEP+HexNAc(2)Hex(1)_1)
1545.762(PEP+HexNAc(2)Hex(2)_1)
1707.812(PEP+HexNAc(2)Hex(3)_1)
1854.862
CID-MS/MS Scan:6613, Noise threshold:0.6
366.141(HexNAc + Hex)
512.199(HexNAc(1)Hex(1)Fuc(1)_1)
528.193(Hex + HexNAc + Hex)
759.349(PEP+HexNAc(4)Hex(4)_3)
773.392
955.954(PEP+HexNAc(3)Hex(3)_2)
1036.981(PEP+HexNAc(3)Hex(4)_2)
1110.009(PEP+HexNAc(3)Hex(4)Fuc(1)_2)
1083.452
1138.524(PEP+HexNAc(4)Hex(4)_2)
1191.538
1219.541(PEP+HexNAc(4)Hex(5)_2)
1545.755(PEP+HexNAc(2)Hex(2)_1)
1707.822(PEP+HexNAc(2)Hex(3)_1)
1853.883(PEP+HexNAc(2)Hex(3)Fuc(1)_1)
ETD-MS/MS Scan:6614, Noise threshold:1.2
138.056(HexNAc − 2H2O − CH2O)
168.067(HexNAc − 2H2O)
204.088(HexNAc)
150.105
183.658
230.165
286.971
309.983
332.221
342.057
366.143(HexNAc + Hex)
401.243
444.300
512.203(HexNAc(1)Hex(1)Fuc(1)_1)
709.913
863.507
886.679
955.956(PEP+HexNAc(3)Hex(3)_2)
971.952
1029.484
1036.986(PEP+HexNAc(3)Hex(4)_2)
1053.379
1110.017(PEP+HexNAc(3)Hex(4)Fuc(1)_2)
1123.604
1164.003
1204.561
1228.535
1264.062
1272.080
1285.071
M+2H
1662.409
1828.678
1962.857
m/z

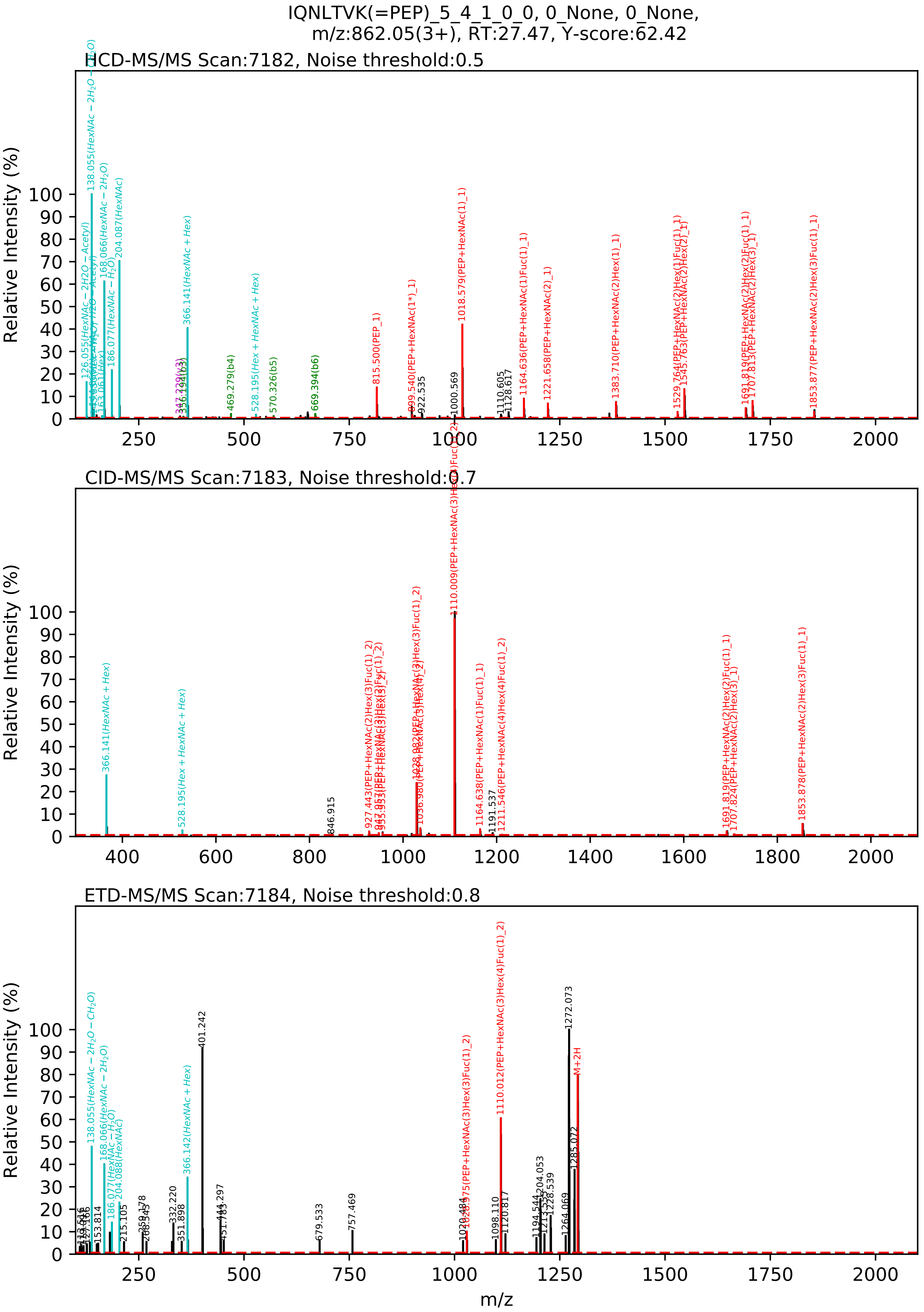

IQNLTVK(=PEP)_5_4_1_0_0, 0_None, 0_None,
m/z:862.05(3+), RT:27.47, Y-score:62.42
HCD-MS/MS Scan:7182, Noise threshold:0.5
Relative Intensity (%)
138.055(HexNAc − 2H2O − CH2O)
168.066(HexNAc − 2H2O)
204.087(HexNAc)
186.077(HexNAc − H2O)
366.141(HexNAc + Hex)
528.195(Hex + HexNAc + Hex)
469.279(b4)
570.326(b5)
669.394(b6)
815.500(PEP_1)
899.540(PEP+HexNAc(1*)_1)
922.535
1000.569
1018.579(PEP+HexNAc(1)_1)
1110.605
1128.617
1164.636(PEP+HexNAc(1)Fuc(1)_1)
1221.658(PEP+HexNAc(2)_1)
1383.710(PEP+HexNAc(2)Hex(1)_1)
1529.764(PEP+HexNAc(2)Hex(1)Fuc(1)_1)
1545.763(PEP+HexNAc(2)Hex(2)_1)
1691.819(PEP+HexNAc(2)Hex(2)Fuc(1)_1)
1707.813(PEP+HexNAc(2)Hex(3)_1)
1853.877(PEP+HexNAc(2)Hex(3)Fuc(1)_1)
CID-MS/MS Scan:7183, Noise threshold:0.7
366.141(HexNAc + Hex)
528.195(Hex + HexNAc + Hex)
846.915
927.443(PEP+HexNAc(2)Hex(3)Fuc(1)_2)
1028.982(PEP+HexNAc(3)Hex(3)Fuc(1)_2)
1110.009(PEP+HexNAc(3)Hex(4)Fuc(1)_2)
1164.638(PEP+HexNAc(1)Fuc(1)_1)
1191.537
1211.546(PEP+HexNAc(4)Hex(4)Fuc(1)_2)
1691.819(PEP+HexNAc(2)Hex(2)Fuc(1)_1)
1707.824(PEP+HexNAc(2)Hex(3)_1)
1853.878(PEP+HexNAc(2)Hex(3)Fuc(1)_1)
ETD-MS/MS Scan:7184, Noise threshold:0.8
138.055(HexNAc − 2H2O − CH2O)
168.066(HexNAc − 2H2O)
186.077(HexNAc − H2O)
204.088(HexNAc)
366.142(HexNAc + Hex)
153.814
215.105
259.178
268.345
332.220
351.898
401.242
444.297
451.783
679.533
757.469
1020.484
1028.975(PEP+HexNAc(3)Hex(3)Fuc(1)_2)
1098.110
1110.012(PEP+HexNAc(3)Hex(4)Fuc(1)_2)
1120.817
1194.544
1204.053
1228.539
1264.069
1272.073
1285.072
M+2H
m/z

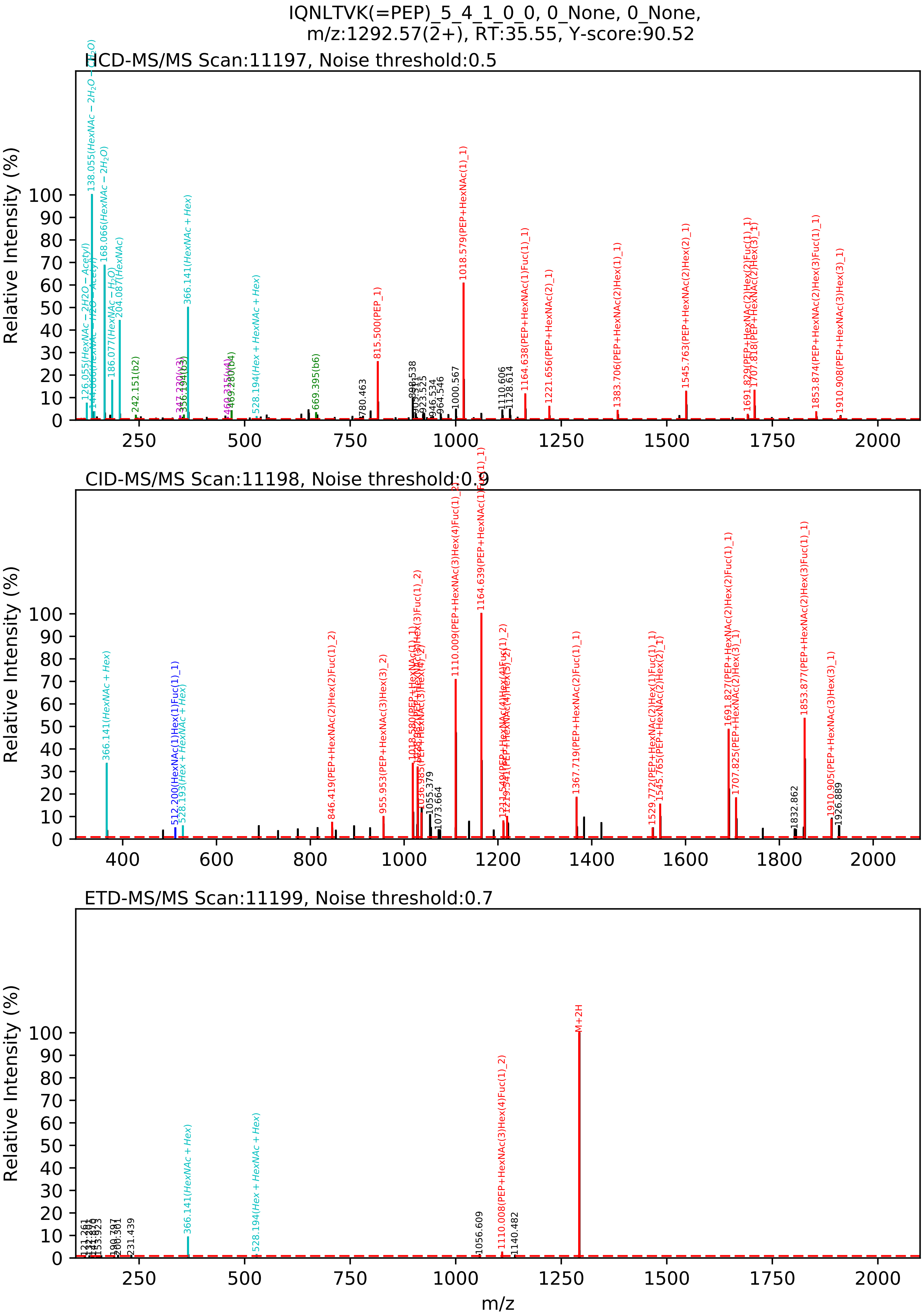
IQNLTVK(=PEP)_5_4_1_0_0, 0_None, 0_None,
m/z:1292.57(2+), RT:35.55, Y-score:90.52
HCD-MS/MS Scan:11197, Noise threshold:0.5
Relative Intensity (%)
1018.579(PEP+HexNAc(1)_1)
815.500(PEP_1)
1164.638(PEP+HexNAc(1)Fuc(1)_1)
1221.656(PEP+HexNAc(2)_1)
1383.706(PEP+HexNAc(2)Hex(1)_1)
1545.763(PEP+HexNAc(2)Hex(2)_1)
1853.874(PEP+HexNAc(2)Hex(3)Fuc(1)_1)
1910.908(PEP+HexNAc(3)Hex(3)_1)
366.141(HexNAc + Hex)
528.194(Hex + HexNAc + Hex)
204.087(HexNAc)
186.077(HexNAc − H2O)
168.066(HexNAc − 2H2O)
138.055(HexNAc − 2H2O − CH2O)
126.055(HexNAc − 2H2O − Acetyl)
242.151(b2)
669.395(b6)
780.463
1000.567
1110.606
1128.614
CID-MS/MS Scan:11198, Noise threshold:0.9
1164.639(PEP+HexNAc(1)Fuc(1)_1)
1110.009(PEP+HexNAc(3)Hex(4)Fuc(1)_2)
846.419(PEP+HexNAc(2)Hex(2)Fuc(1)_2)
955.953(PEP+HexNAc(3)Hex(3)_2)
1367.719(PEP+HexNAc(2)Fuc(1)_1)
1529.772(PEP+HexNAc(2)Hex(1)Fuc(1)_1)
1545.765(PEP+HexNAc(2)Hex(2)_1)
1691.827(PEP+HexNAc(2)Hex(2)Fuc(1)_1)
1707.825(PEP+HexNAc(2)Hex(3)_1)
1853.877(PEP+HexNAc(2)Hex(3)Fuc(1)_1)
1910.905(PEP+HexNAc(3)Hex(3)_1)
512.200(HexNAc(1)Hex(1)Fuc(1)_1)
1055.379
1073.664
1832.862
1926.889
ETD-MS/MS Scan:11199, Noise threshold:0.7
M+2H
1110.008(PEP+HexNAc(3)Hex(4)Fuc(1)_2)
1056.609
1140.482
231.439
m/z

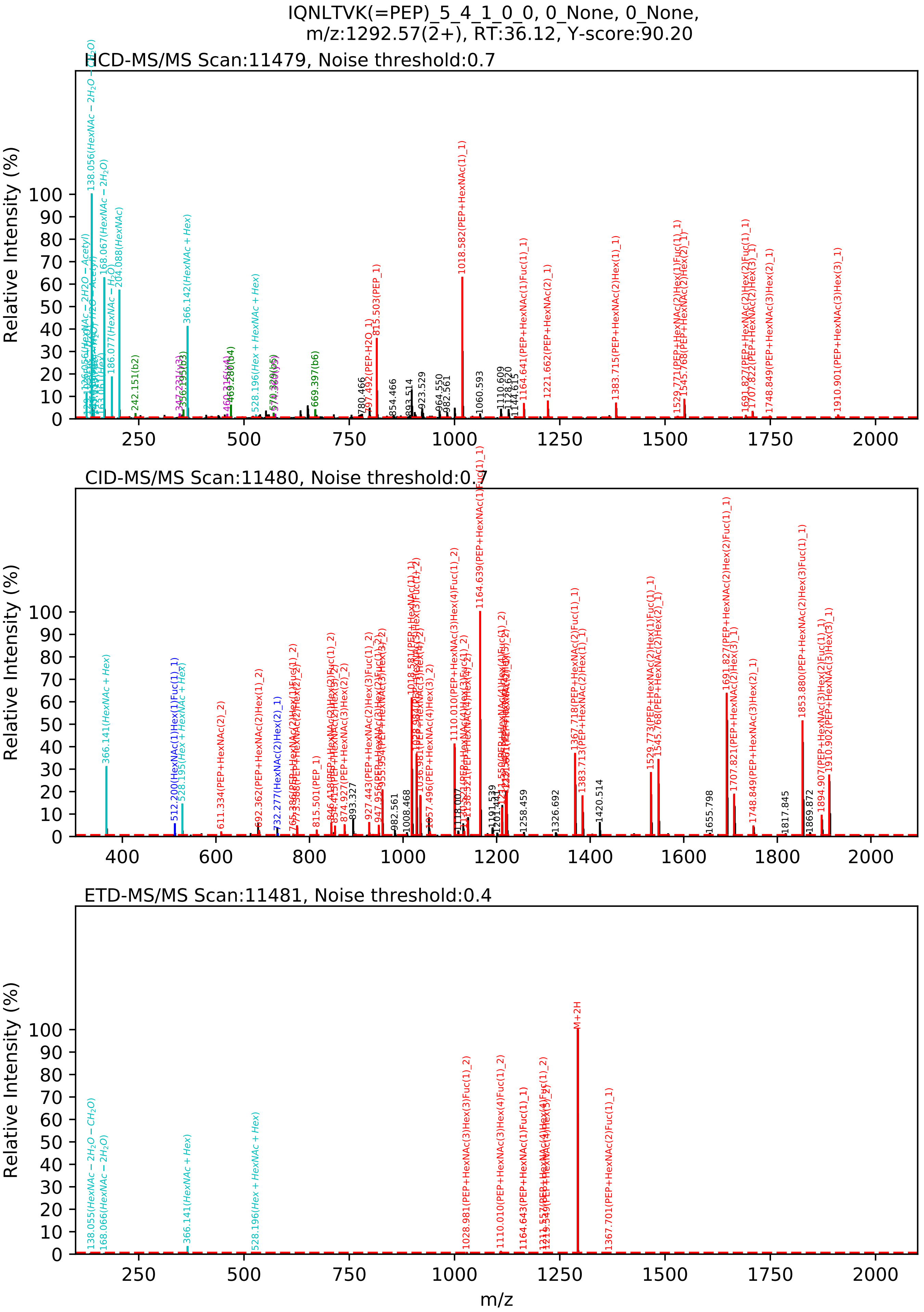

IQNLTVK(=PEP)_5_4_1_0_0, 0_None, 0_None,
m/z:1292.57(2+), RT:36.12, Y-score:90.20
HCD-MS/MS Scan:11479, Noise threshold:0.7
CID-MS/MS Scan:11480, Noise threshold:0.7
ETD-MS/MS Scan:11481, Noise threshold:0.4
Relative Intensity (%)
m/z
1018.582(PEP+HexNAc(1)_1)
1164.639(PEP+HexNAc(1)Fuc(1)_1)
M+2H

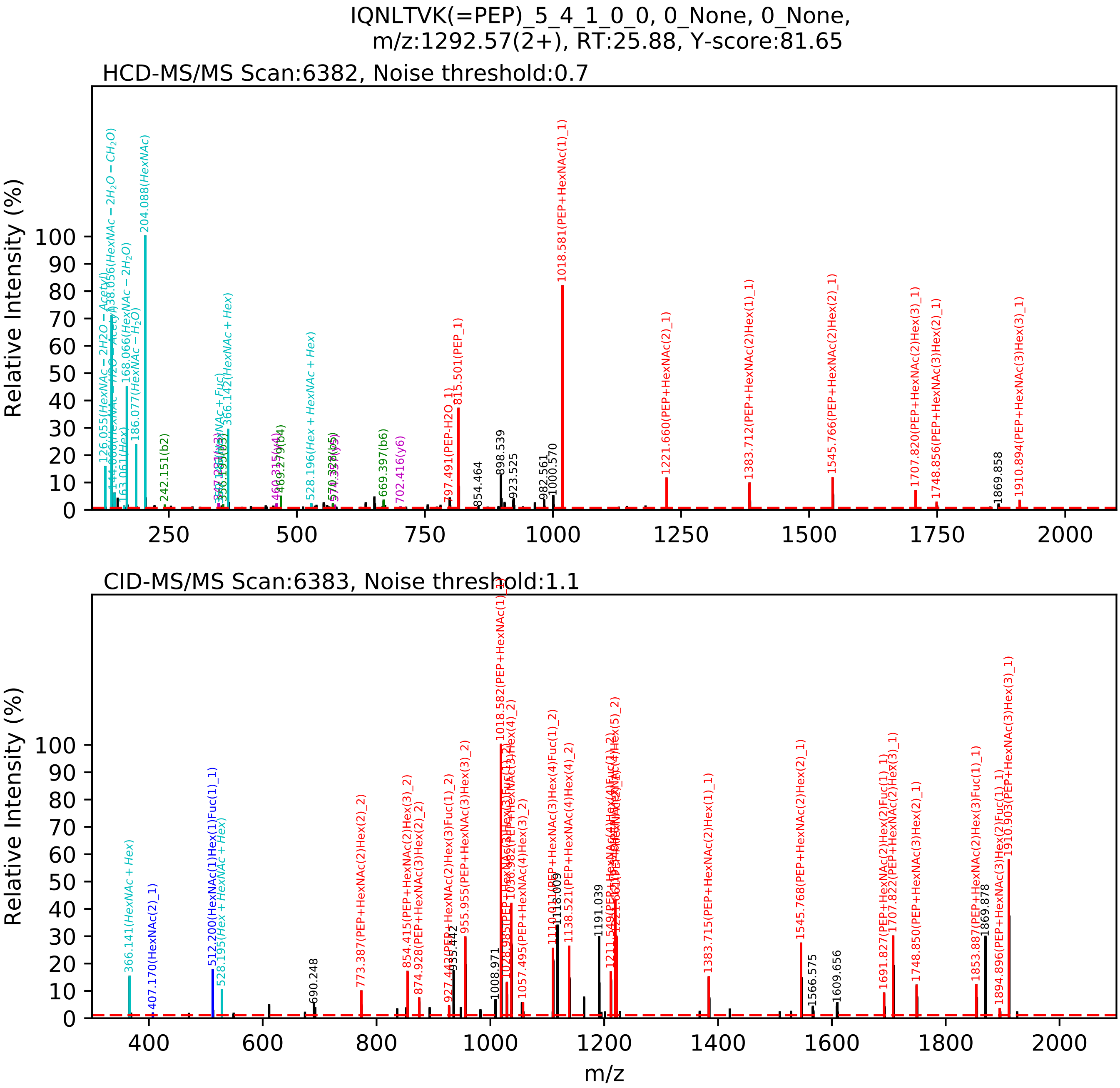
IQNLTVK(=PEP)_5_4_1_0_0, 0_None, 0_None,
m/z:1292.57(2+), RT:25.88, Y-score:81.65
HCD-MS/MS Scan:6382, Noise threshold:0.7
Relative Intensity (%)
204.088(HexNAc)
186.077(HexNAc − H2O)
168.066(HexNAc − 2H2O)
126.055(HexNAc − 2H2O − Acetyl)
163.061(Hex)
242.151(b2)
366.142(HexNAc + Hex)
469.279(b4)
528.196(Hex + HexNAc + Hex)
669.397(b6)
702.416(y6)
797.491(PEP-H2O_1)
815.501(PEP_1)
854.464
898.539
923.525
982.561
1000.570
1018.581(PEP+HexNAc(1)_1)
1221.660(PEP+HexNAc(2)_1)
1383.712(PEP+HexNAc(2)Hex(1)_1)
1545.766(PEP+HexNAc(2)Hex(2)_1)
1707.820(PEP+HexNAc(2)Hex(3)_1)
1748.856(PEP+HexNAc(3)Hex(2)_1)
1869.858
1910.894(PEP+HexNAc(3)Hex(3)_1)
CID-MS/MS Scan:6383, Noise threshold:1.1
366.141(HexNAc + Hex)
407.170(HexNAc(2)_1)
512.200(HexNAc(1)Hex(1)Fuc(1)_1)
528.195(Hex + HexNAc + Hex)
690.248
773.387(PEP+HexNAc(2)Hex(2)_2)
854.415(PEP+HexNAc(2)Hex(3)_2)
874.928(PEP+HexNAc(3)Hex(2)_2)
927.443(PEP+HexNAc(2)Hex(3)Fuc(1)_2)
935.442
955.955(PEP+HexNAc(3)Hex(3)_2)
1008.971
1018.582(PEP+HexNAc(1)_1)
1057.495(PEP+HexNAc(4)Hex(3)_2)
1110.011(PEP+HexNAc(3)Hex(4)Fuc(1)_2)
1118.009
1138.521(PEP+HexNAc(4)Hex(4)_2)
1191.039
1383.715(PEP+HexNAc(2)Hex(1)_1)
1545.768(PEP+HexNAc(2)Hex(2)_1)
1566.575
1609.656
1691.827(PEP+HexNAc(2)Hex(2)Fuc(1)_1)
1707.822(PEP+HexNAc(2)Hex(3)_1)
1748.850(PEP+HexNAc(3)Hex(2)_1)
1853.887(PEP+HexNAc(2)Hex(3)Fuc(1)_1)
1869.878
1894.896(PEP+HexNAc(3)Hex(2)Fuc(1)_1)
1910.903(PEP+HexNAc(3)Hex(3)_1)
m/z

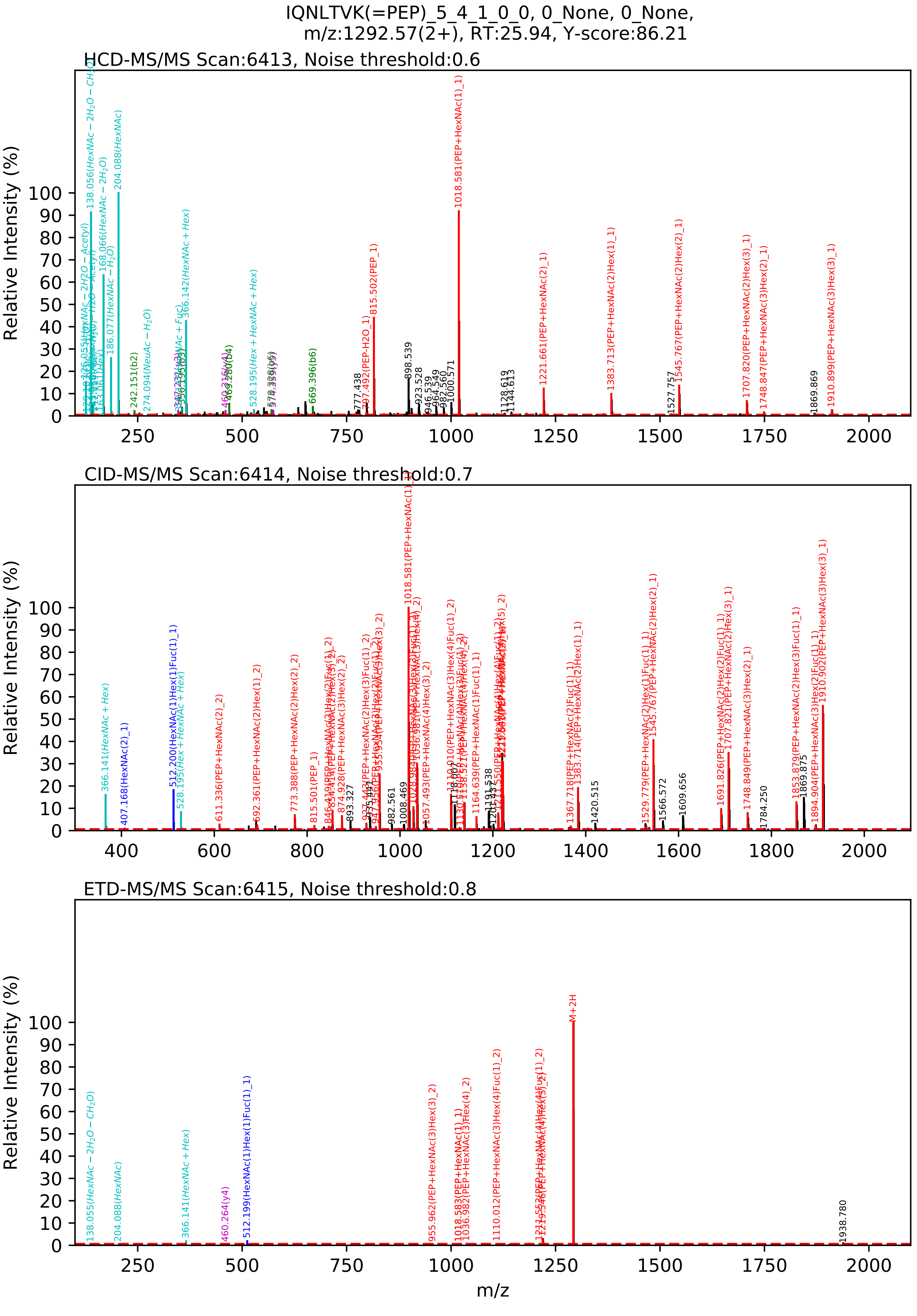
IQNLTVK(=PEP)_5_4_1_0_0, 0_None, 0_None,
m/z:1292.57(2+), RT:25.94, Y-score:86.21
HCD-MS/MS Scan:6413, Noise threshold:0.6
CID-MS/MS Scan:6414, Noise threshold:0.7
ETD-MS/MS Scan:6415, Noise threshold:0.8
Relative Intensity (%)
m/z
1018.581(PEP+HexNAc(1)_1)
815.502(PEP_1)
204.088(HexNAc)
366.142(HexNAc+Hex)
1221.661(PEP+HexNAc(2)_1)
1383.713(PEP+HexNAc(2)Hex(1)_1)
1545.767(PEP+HexNAc(2)Hex(2)_1)
1707.820(PEP+HexNAc(2)Hex(3)_1)
1748.847(PEP+HexNAc(3)Hex(2)_1)
1910.899(PEP+HexNAc(3)Hex(3)_1)
512.200(HexNAc(1)Hex(1)Fuc(1)_1)
1910.902(PEP+HexNAc(3)Hex(3)_1)
M+2H
1938.780

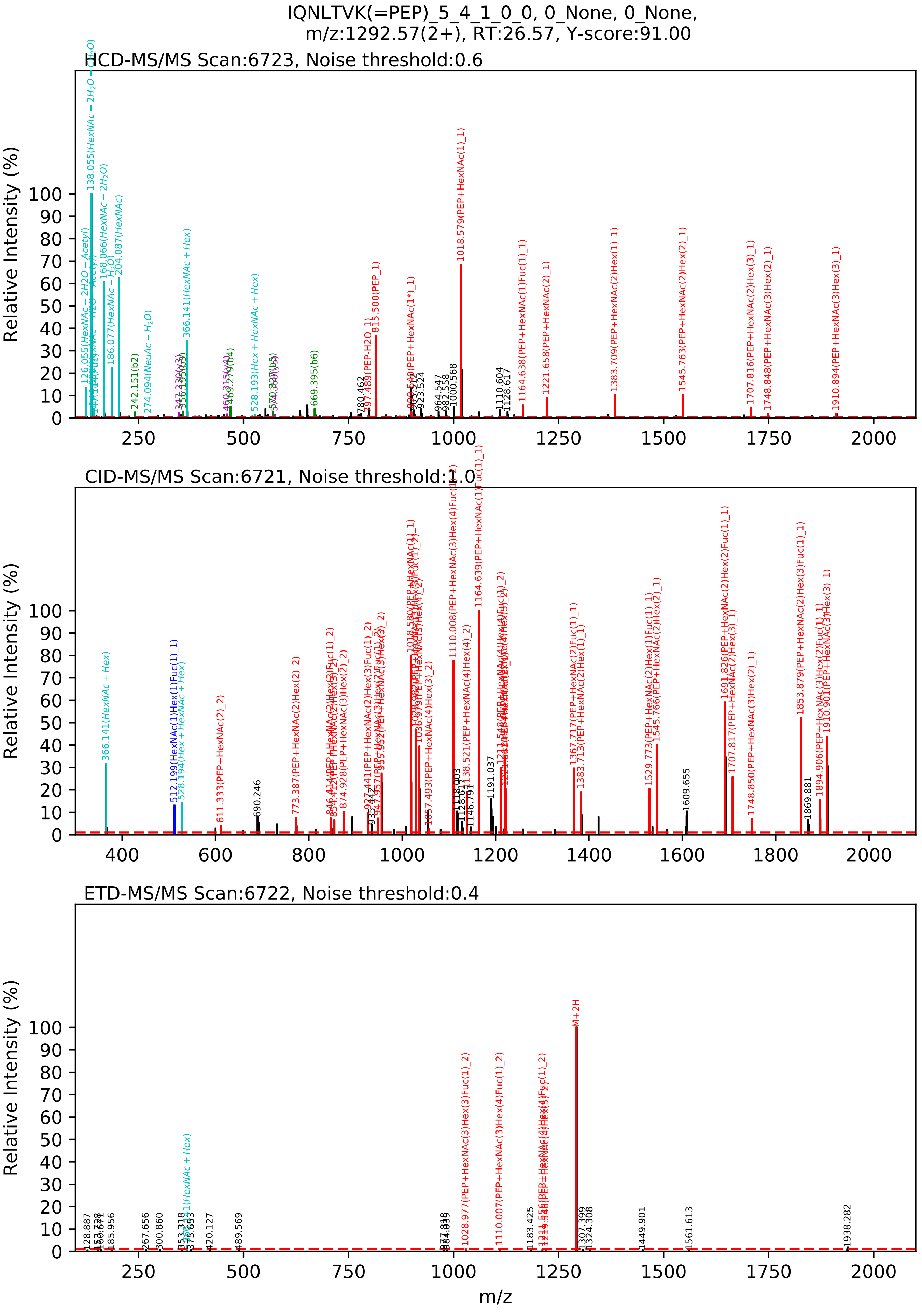
IQNLTVK(=PEP)_5_4_1_0_0, 0_None, 0_None,
m/z:1292.57(2+), RT:26.57, Y-score:91.00
HCD-MS/MS Scan:6723, Noise threshold:0.6
CID-MS/MS Scan:6721, Noise threshold:1.0
ETD-MS/MS Scan:6722, Noise threshold:0.4
Relative Intensity (%)
m/z
1018.579(PEP+HexNAc(1)_1)
815.500(PEP_1)
1164.638(PEP+HexNAc(1)Fuc(1)_1)
1221.658(PEP+HexNAc(2)_1)
1383.709(PEP+HexNAc(2)Hex(1)_1)
1545.763(PEP+HexNAc(2)Hex(2)_1)
1707.816(PEP+HexNAc(2)Hex(3)_1)
1748.848(PEP+HexNAc(3)Hex(2)_1)
1910.894(PEP+HexNAc(3)Hex(3)_1)
366.141(HexNAc + Hex)
204.087(HexNAc)
138.055(HexNAc − 2H2O − H2O)
669.395(b6)
1164.639(PEP+HexNAc(1)Fuc(1)_1)
1110.008(PEP+HexNAc(3)Hex(4)Fuc(1)_2)
1691.826(PEP+HexNAc(2)Hex(2)Fuc(1)_1)
1853.879(PEP+HexNAc(2)Hex(3)Fuc(1)_1)
512.199(HexNAc(1)Hex(1)Fuc(1)_1)
M+2H
1028.977(PEP+HexNAc(3)Hex(3)Fuc(1)_2)
1110.007(PEP+HexNAc(3)Hex(4)Fuc(1)_2)

IQNLTVK(=PEP)_5_4_1_0_0, 0_None, 0_None,
m/z:862.05(3+), RT:26.91, Y-score:92.02

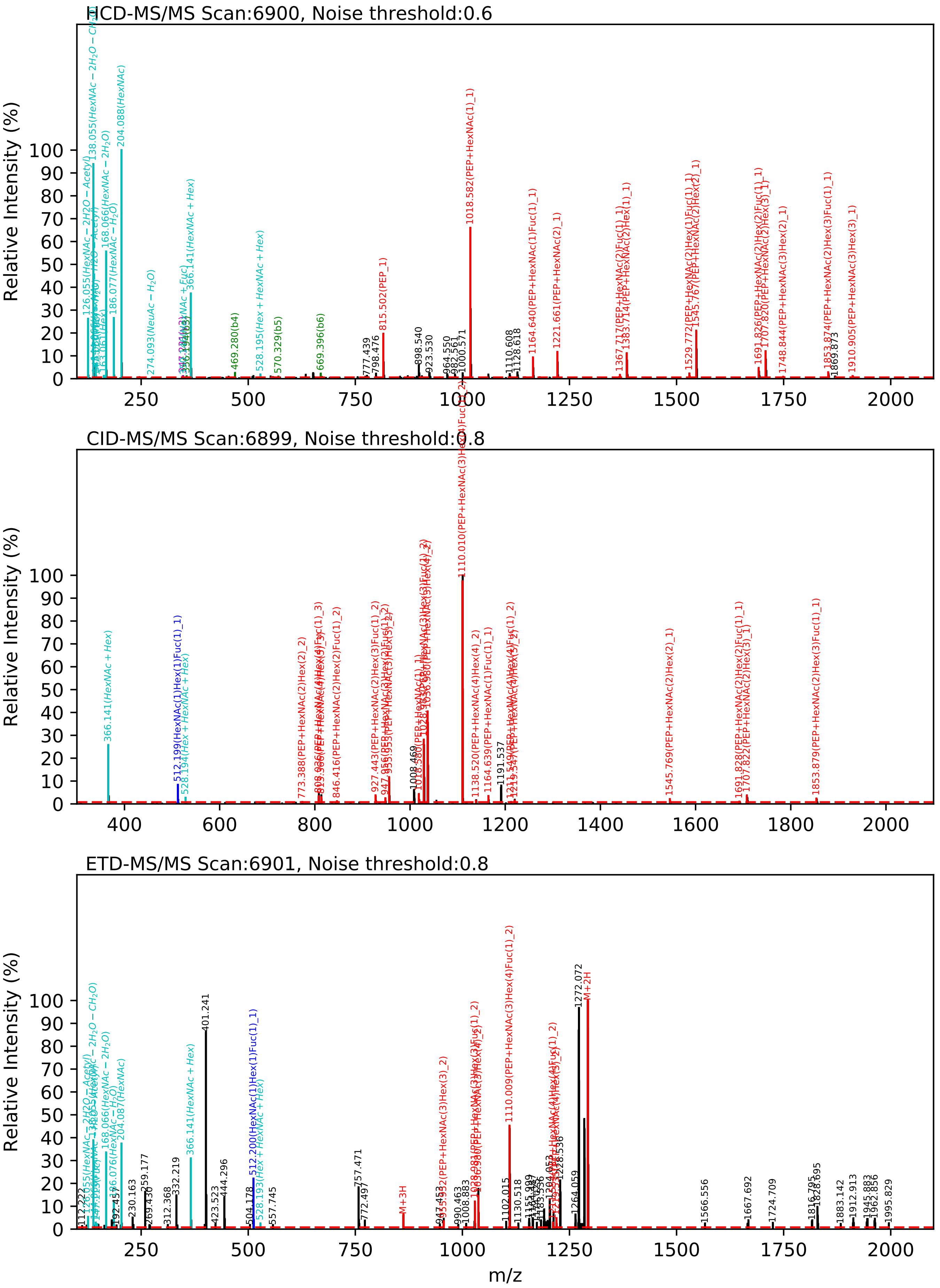

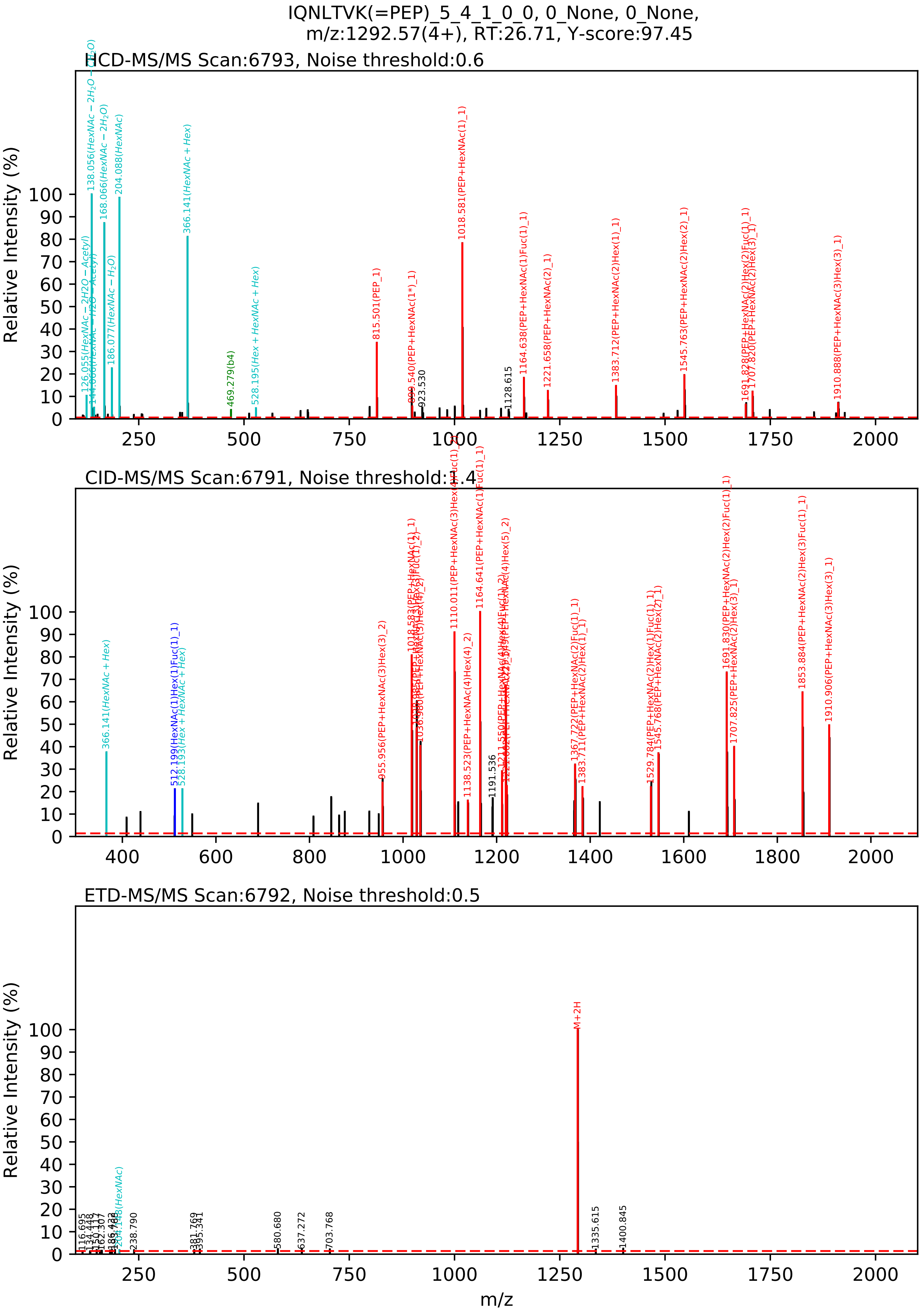

IQNLTVK(=PEP)_5_4_1_0_0, 0_None, 0_None,
m/z:1292.57(4+), RT:26.71, Y-score:97.45
HCD-MS/MS Scan:6793, Noise threshold:0.6
Relative Intensity (%)
138.056(HexNAc − 2H2O − H2O)
168.066(HexNAc − 2H2O)
204.088(HexNAc)
186.077(HexNAc − H2O)
366.141(HexNAc + Hex)
469.279(b4)
528.195(Hex + HexNAc + Hex)
815.501(PEP_1)
899.540(PEP+HexNAc(1*)_1)
923.530
1018.581(PEP+HexNAc(1)_1)
1128.615
1164.638(PEP+HexNAc(1)Fuc(1)_1)
1221.658(PEP+HexNAc(2)_1)
1383.712(PEP+HexNAc(2)Hex(1)_1)
1545.763(PEP+HexNAc(2)Hex(2)_1)
1691.828(PEP+HexNAc(2)Hex(2)Fuc(1)_1)
1707.820(PEP+HexNAc(2)Hex(3)_1)
1910.888(PEP+HexNAc(3)Hex(3)_1)
CID-MS/MS Scan:6791, Noise threshold:1.4
366.141(HexNAc + Hex)
512.199(HexNAc(1)Hex(1)Fuc(1)_1)
528.193(Hex + HexNAc + Hex)
955.956(PEP+HexNAc(3)Hex(3)_2)
1110.011(PEP+HexNAc(3)Hex
1138.523(PEP+HexNAc(4)Hex(4)_2)
1164.641(PEP+HexNAc(1)Fuc(1)_1)
1191.536
1367.722(PEP+HexNAc(2)Fuc(1)_1)
1383.711(PEP+HexNAc(2)Hex(1)_1)
1529.784(PEP+HexNAc(2)Hex(1)Fuc(1)_1)
1545.768(PEP+HexNAc(2)Hex(2)_1)
1691.830(PEP+HexNAc(2)Hex(2)Fuc(1)_1)
1707.825(PEP+HexNAc(2)Hex(3)_1)
1853.884(PEP+HexNAc(2)Hex(3)Fuc(1)_1)
1910.906(PEP+HexNAc(3)Hex(3)_1)
ETD-MS/MS Scan:6792, Noise threshold:0.5
M+2H
116.695
162.307
204.088(HexNAc)
238.790
381.769
395.341
580.680
637.272
703.768
1335.615
1400.845
m/z

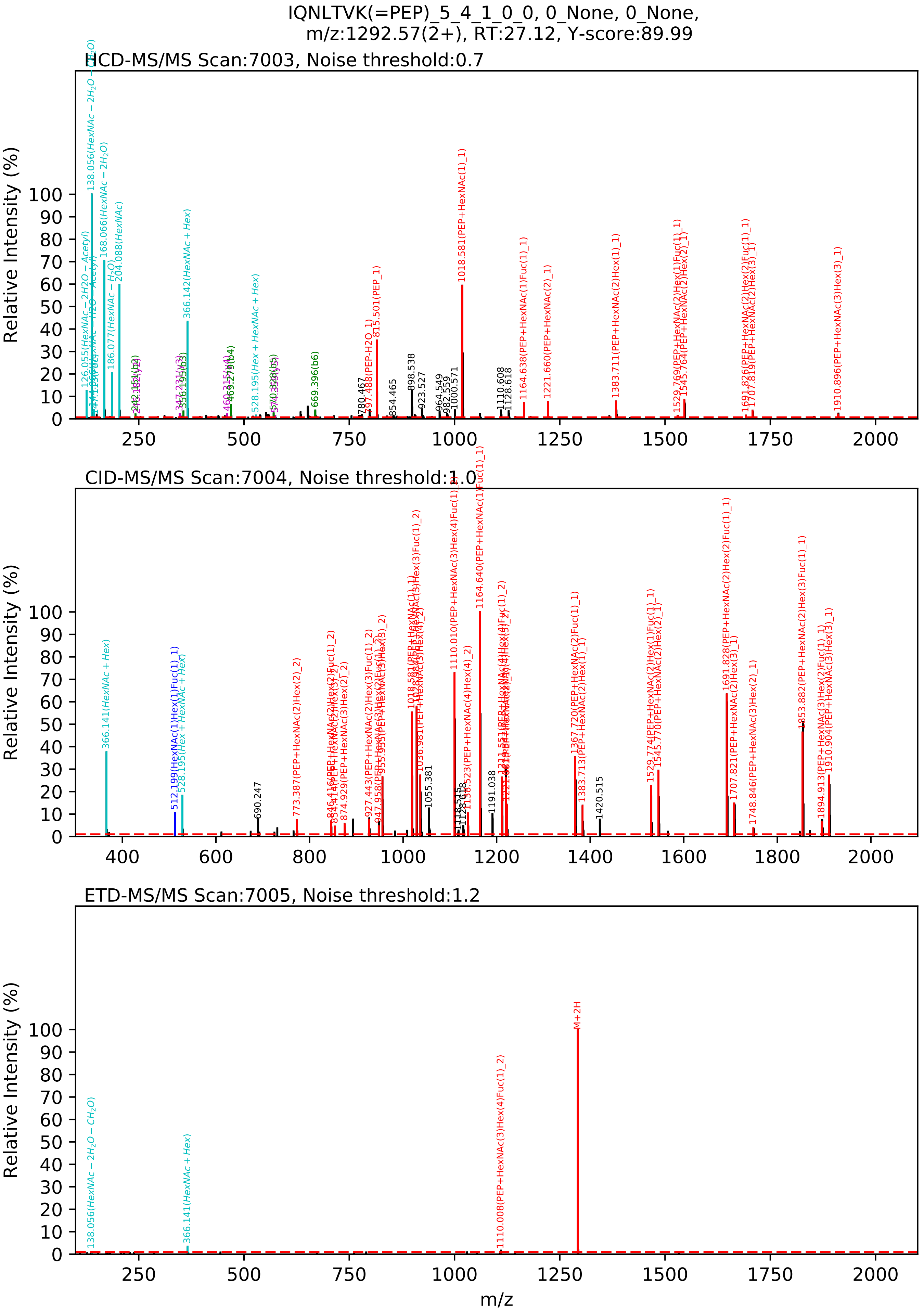

IQNLTVK(=PEP)_5_4_1_0_0, 0_None, 0_None,
m/z:1292.57(2+), RT:27.12, Y-score:89.99
HCD-MS/MS Scan:7003, Noise threshold:0.7
CID-MS/MS Scan:7004, Noise threshold:1.0
ETD-MS/MS Scan:7005, Noise threshold:1.2
Relative Intensity (%)
m/z
1018.581(PEP+HexNAc(1)_1)
815.501(PEP_1)
1164.640(PEP+HexNAc(1)Fuc(1)_1)
M+2H

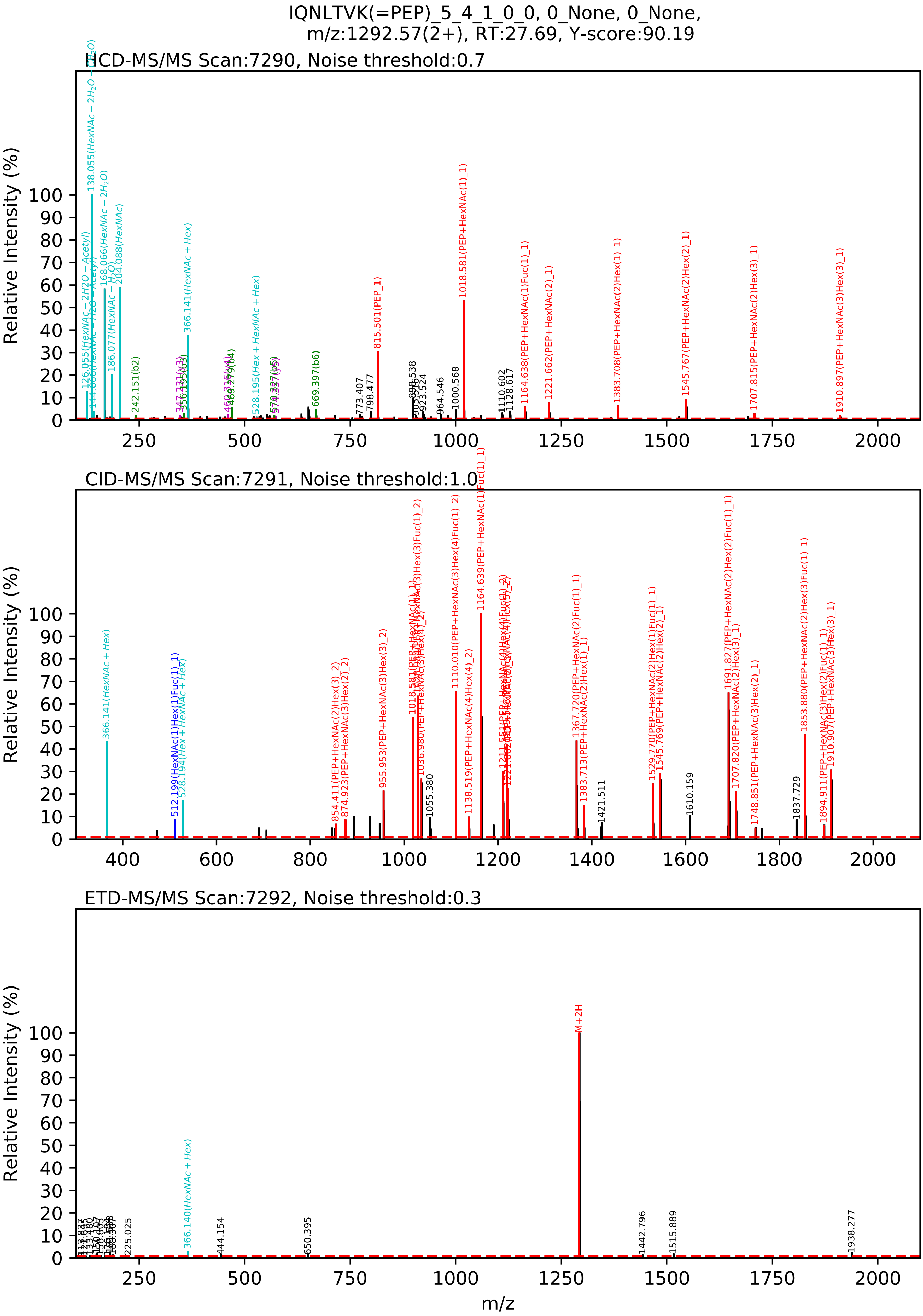

IQNLTVK(=PEP)_5_4_1_0_0, 0_None, 0_None,
m/z:1292.57(2+), RT:27.69, Y-score:90.19
HCD-MS/MS Scan:7290, Noise threshold:0.7
Relative Intensity (%)
815.501(PEP_1)
1018.581(PEP+HexNAc(1)_1)
1164.638(PEP+HexNAc(1)Fuc(1)_1)
1221.662(PEP+HexNAc(2)_1)
1383.708(PEP+HexNAc(2)Hex(1)_1)
1545.767(PEP+HexNAc(2)Hex(2)_1)
1707.815(PEP+HexNAc(2)Hex(3)_1)
1910.897(PEP+HexNAc(3)Hex(3)_1)
366.141(HexNAc + Hex)
204.088(HexNAc)
186.077(HexNAc − H2O)
168.066(HexNAc − 2H2O)
138.055(HexNAc − 2H2O − CH2O)
126.055(HexNAc − 2H2O − Acetyl)
242.151(b2)
669.397(b6)
528.195(Hex + HexNAc + Hex)
CID-MS/MS Scan:7291, Noise threshold:1.0
366.141(HexNAc + Hex)
512.199(HexNAc(1)Hex(1)Fuc(1)_1)
854.411(PEP+HexNAc(2)Hex(3)_2)
874.923(PEP+HexNAc(3)Hex(2)_2)
955.953(PEP+HexNAc(3)Hex(3)_2)
1055.380
1110.010(PEP+HexNAc(3)Hex(4)Fuc(1)_2)
1138.519(PEP+HexNAc(4)Hex(4)_2)
1164.639(PEP+HexNAc(1)Fuc(1)_1)
1367.720(PEP+HexNAc(2)Fuc(1)_1)
1383.713(PEP+HexNAc(2)Hex(1)_1)
1421.511
1529.770(PEP+HexNAc(2)Hex(1)Fuc(1)_1)
1545.769(PEP+HexNAc(2)Hex(2)_1)
1610.159
1691.827(PEP+HexNAc(2)Hex(2)Fuc(1)_1)
1707.820(PEP+HexNAc(2)Hex(3)_1)
1748.851(PEP+HexNAc(3)Hex(2)_1)
1837.729
1853.880(PEP+HexNAc(2)Hex(3)Fuc(1)_1)
1894.911(PEP+HexNAc(3)Hex(2)Fuc(1)_1)
1910.907(PEP+HexNAc(3)Hex(3)_1)
ETD-MS/MS Scan:7292, Noise threshold:0.3
M+2H
366.140(HexNAc + Hex)
225.025
444.154
650.395
1442.796
1515.889
1938.277
m/z

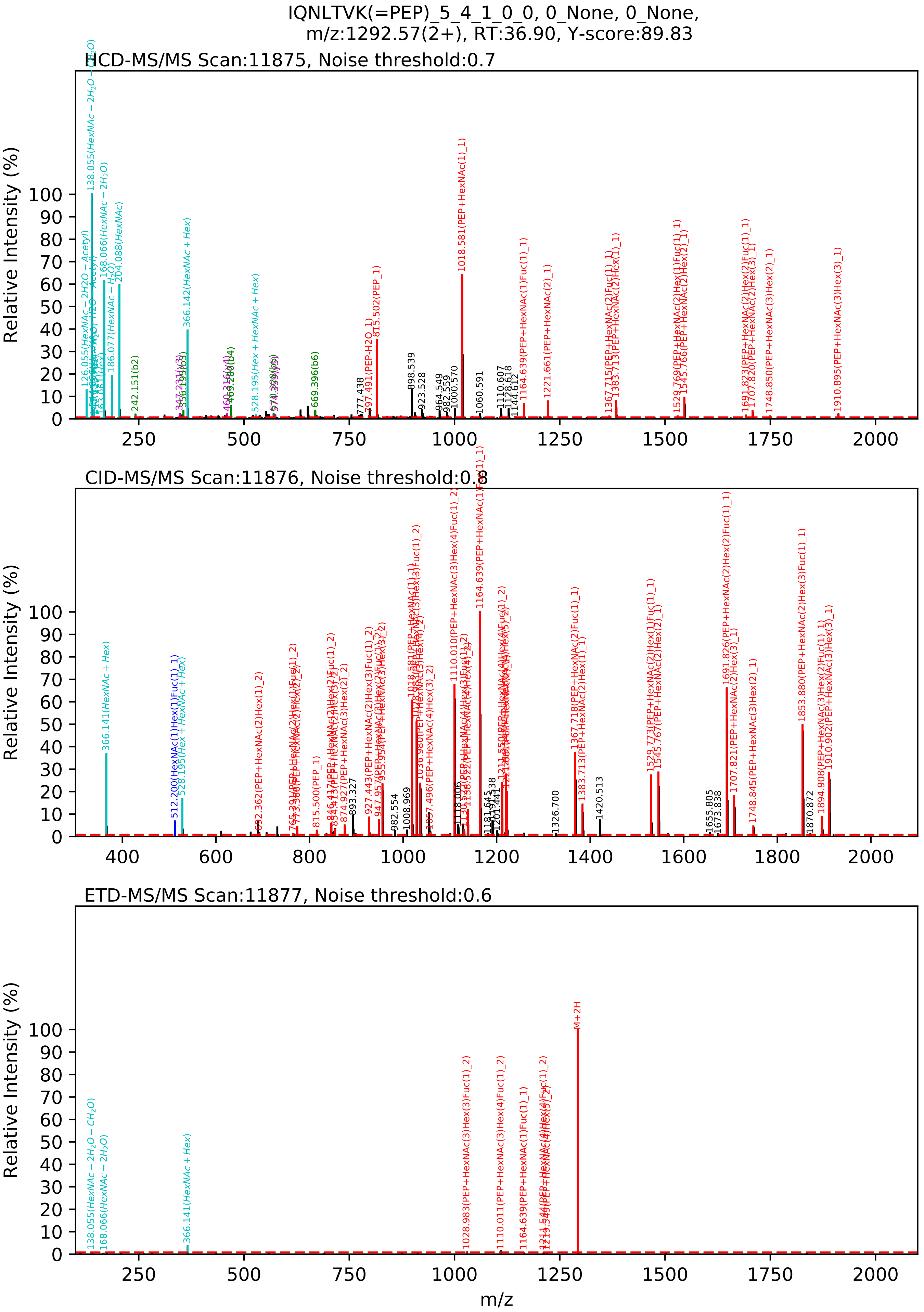

IQNLTVK(=PEP)_5_4_1_0_0, 0_None, 0_None,
m/z:1292.57(2+), RT:36.90, Y-score:89.83
HCD-MS/MS Scan:11875, Noise threshold:0.7
CID-MS/MS Scan:11876, Noise threshold:0.8
ETD-MS/MS Scan:11877, Noise threshold:0.6
Relative Intensity (%)
m/z
1018.581(PEP+HexNAc(1)_1)
815.502(PEP_1)
1164.639(PEP+HexNAc(1)Fuc(1)_1)
1221.661(PEP+HexNAc(2)_1)
1748.850(PEP+HexNAc(3)Hex(2)_1)
1910.895(PEP+HexNAc(3)Hex(3)_1)
366.142(HexNAc + Hex)
204.088(HexNAc)
669.396(b6)
242.151(b2)
1691.826(PEP+HexNAc(2)Hex(2)Fuc(1)_1)
1853.880(PEP+HexNAc(2)Hex(3)Fuc(1)_1)
1367.718(PEP+HexNAc(2)Fuc(1)_1)
1529.773(PEP+HexNAc(2)Hex(1)Fuc(1)_1)
1110.010(PEP+HexNAc(3)Hex(4)Fuc(1)_2)
512.200(HexNAc(1)Hex(1)Fuc(1)_1)
M+2H
1028.983(PEP+HexNAc(3)Hex(3)Fuc(1)_2)
1110.011(PEP+HexNAc(3)Hex(4)Fuc(1)_2)

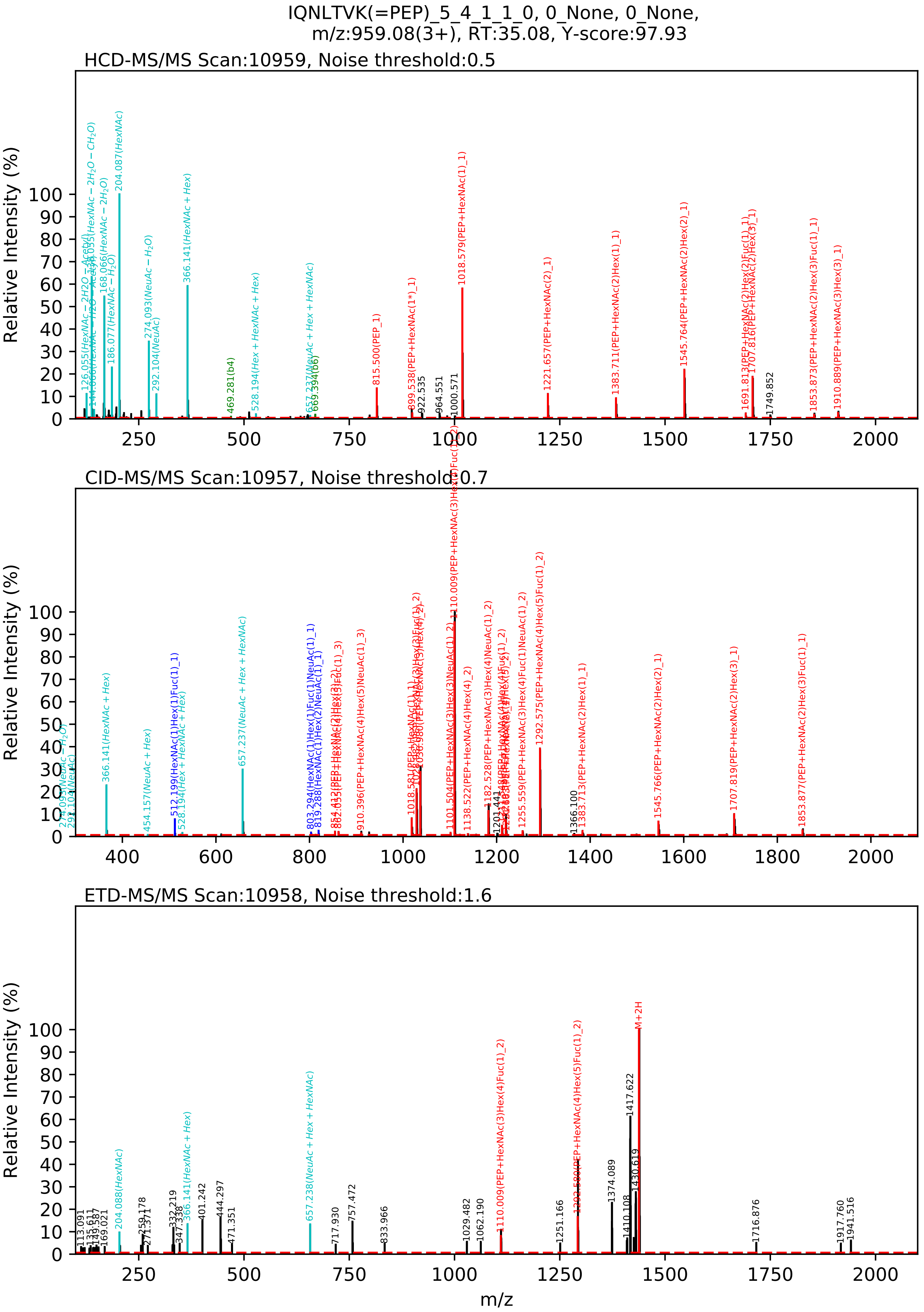
IQNLTVK(=PEP)_5_4_1_1_0, 0_None, 0_None,
m/z:959.08(3+), RT:35.08, Y-score:97.93
HCD-MS/MS Scan:10959, Noise threshold:0.5
CID-MS/MS Scan:10957, Noise threshold:0.7
ETD-MS/MS Scan:10958, Noise threshold:1.6
Relative Intensity (%)
m/z
204.087(HexNAc)
366.141(HexNAc + Hex)
1018.579(PEP+HexNAc(1)_1)
815.500(PEP_1)
1221.657(PEP+HexNAc(2)_1)
1383.711(PEP+HexNAc(2)Hex(1)_1)
1545.764(PEP+HexNAc(2)Hex(2)_1)
1707.816(PEP+HexNAc(2)Hex(3)_1)
1853.873(PEP+HexNAc(2)Hex(3)Fuc(1)_1)
1910.889(PEP+HexNAc(3)Hex(3)_1)
1110.009(PEP+HexNAc(3)Hex(4)Fuc(1)_2)
1292.575(PEP+HexNAc(4)Hex(5)Fuc(1)_2)
657.237(NeuAc + Hex + HexNAc)
M+2H
1417.622
1374.089

IQNLTVK(=PEP)_5_4_1_1_0, 0_None, 0_None,
m/z:959.08(3+), RT:36.00, Y-score:95.84

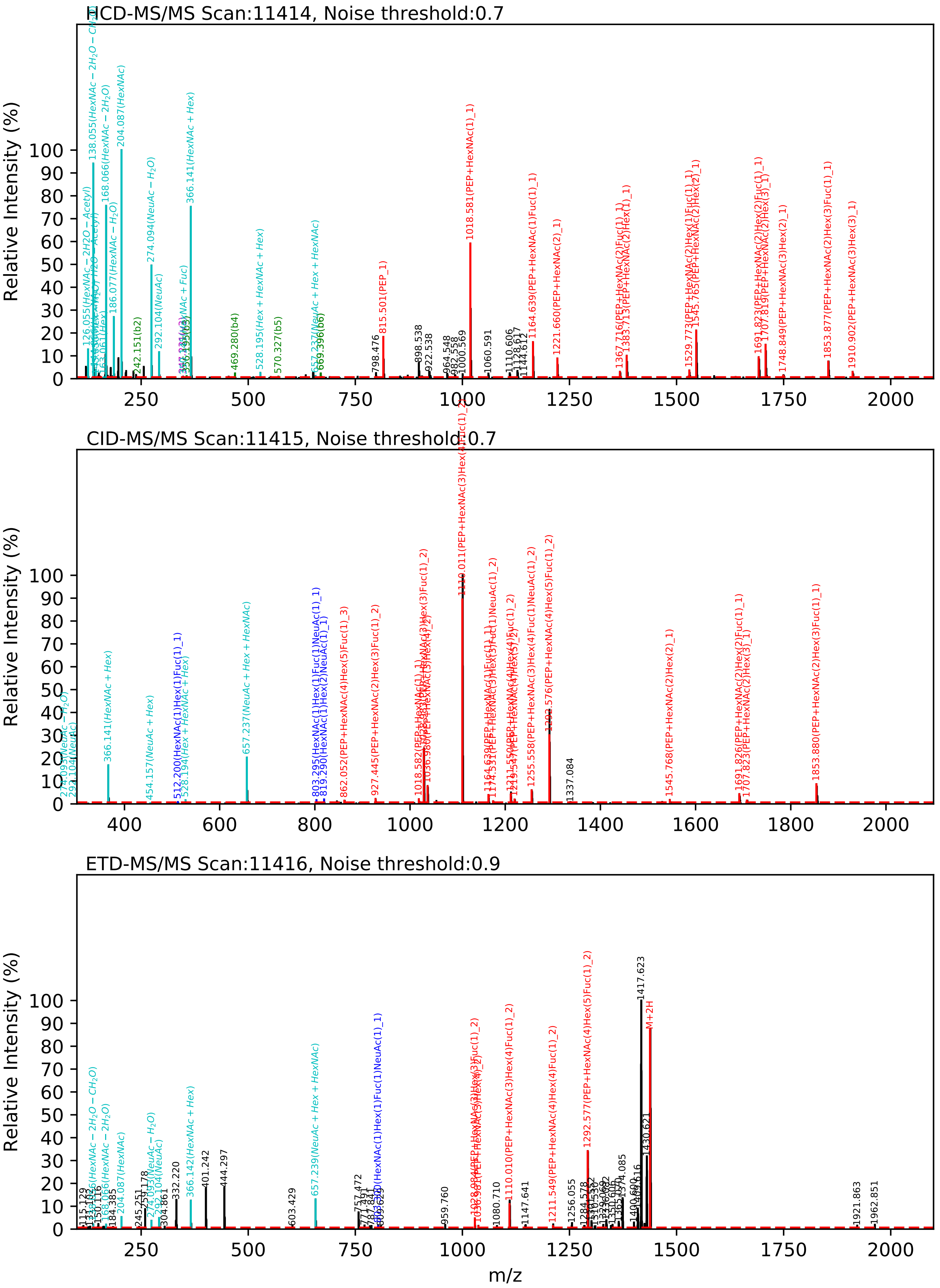

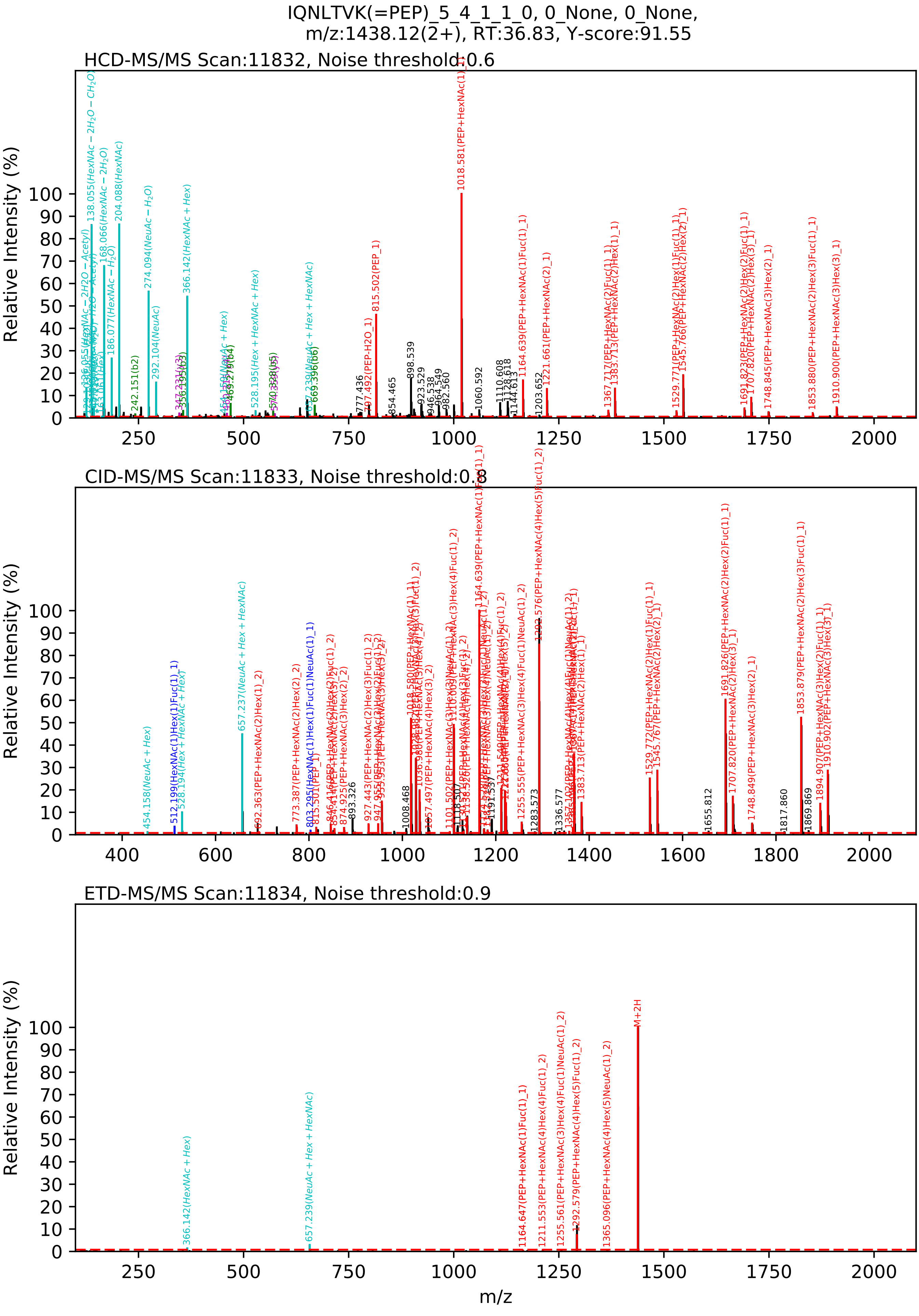
IQNLTVK(=PEP)_5_4_1_1_0, 0_None, 0_None,
m/z:1438.12(2+), RT:36.83, Y-score:91.55
HCD-MS/MS Scan:11832, Noise threshold:0.6
CID-MS/MS Scan:11833, Noise threshold:0.8
ETD-MS/MS Scan:11834, Noise threshold:0.9
Relative Intensity (%)
m/z
M+2H

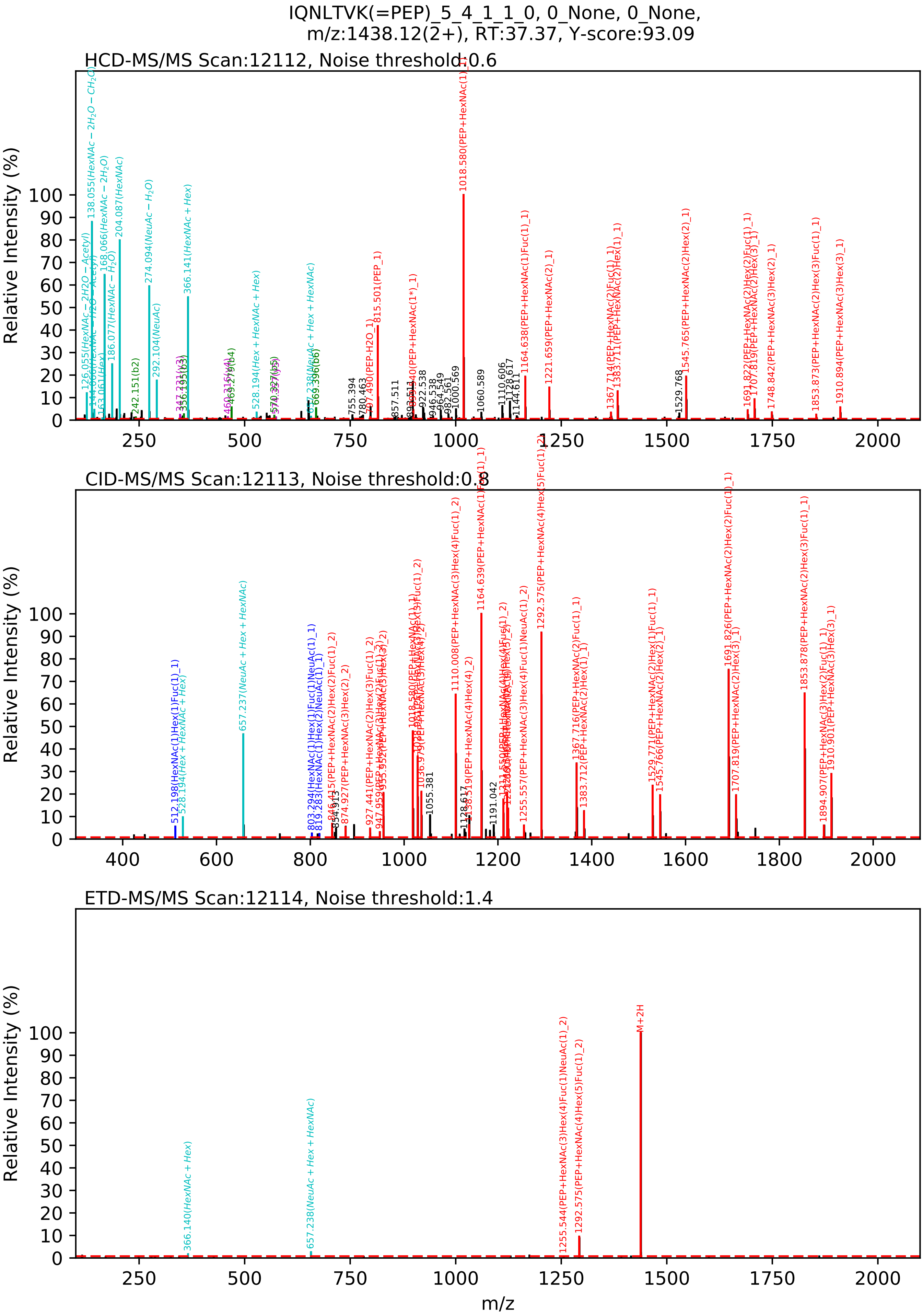

IQNLTVK(=PEP)_5_4_1_1_0, 0_None, 0_None,
m/z:1438.12(2+), RT:37.37, Y-score:93.09
HCD-MS/MS Scan:12112, Noise threshold:0.6
Relative Intensity (%)
1018.580(PEP+HexNAc(1)_1)
815.501(PEP_1)
797.490(PEP-H2O_1)
1164.638(PEP+HexNAc(1)Fuc(1)_1)
1221.659(PEP+HexNAc(2)_1)
1367.714(PEP+HexNAc(2)Fuc(1)_1)
1383.711(PEP+HexNAc(2)Hex(1)_1)
1545.765(PEP+HexNAc(2)Hex(2)_1)
1691.822(PEP+HexNAc(2)Hex(2)Fuc(1)_1)
1707.819(PEP+HexNAc(2)Hex(3)_1)
1748.842(PEP+HexNAc(3)Hex(2)_1)
1853.873(PEP+HexNAc(2)Hex(3)Fuc(1)_1)
1910.894(PEP+HexNAc(3)Hex(3)_1)
138.055(HexNAc − 2H2O − CH2O)
204.087(HexNAc)
168.066(HexNAc − 2H2O)
186.077(HexNAc − H2O)
274.094(NeuAc − H2O)
292.104(NeuAc)
366.141(HexNAc + Hex)
528.194(Hex + HexNAc + Hex)
657.238(NeuAc + Hex + HexNAc)
126.055(HexNAc − 2H2O − Acetyl)
163.061(Hex)
242.151(b2)
469.275(b4)
669.396(b6)
755.394
780.463
857.511
922.538
946.538
964.549
1000.569
1060.589
1110.606
1128.617
1144.611
1529.768
CID-MS/MS Scan:12113, Noise threshold:0.8
1164.639(PEP+HexNAc(1)Fuc(1)_1)
1292.575(PEP+HexNAc(4)Hex(5)Fuc(1)_2)
1110.008(PEP+HexNAc(3)Hex(4)Fuc(1)_2)
1691.826(PEP+HexNAc(2)Hex(2)Fuc(1)_1)
1853.878(PEP+HexNAc(2)Hex(3)Fuc(1)_1)
1707.819(PEP+HexNAc(2)Hex(3)_1)
1910.901(PEP+HexNAc(3)Hex(3)_1)
1894.907(PEP+HexNAc(3)Hex(2)Fuc(1)_1)
1529.771(PEP+HexNAc(2)Hex(1)Fuc(1)_1)
1545.766(PEP+HexNAc(2)Hex(2)_1)
1367.716(PEP+HexNAc(2)Fuc(1)_1)
1383.712(PEP+HexNAc(2)Hex(1)_1)
1255.557(PEP+HexNAc(3)Hex(4)Fuc(1)NeuAc(1)_2)
1018.580(PEP+HexNAc(1)_1)
1036.979(PEP+HexNAc(3)Hex(4)_2)
1138.519(PEP+HexNAc(4)Hex(4)_2)
927.441(PEP+HexNAc(2)Hex(3)Fuc(1)_2)
874.927(PEP+HexNAc(3)Hex(2)_2)
846.415(PEP+HexNAc(2)Hex(2)Fuc(1)_2)
854.913
1055.381
1128.617
1191.042
657.237(NeuAc + Hex + HexNAc)
512.198(HexNAc(1)Hex(1)Fuc(1)_1)
528.194(Hex + HexNAc + Hex)
803.294(HexNAc(1)Hex(1)Fuc(1)NeuAc(1)_1)
819.283(HexNAc(1)Hex(2)NeuAc(1)_1)
ETD-MS/MS Scan:12114, Noise threshold:1.4
M+2H
1255.544(PEP+HexNAc(3)Hex(4)Fuc(1)NeuAc(1)_2)
1292.575(PEP+HexNAc(4)Hex(5)Fuc(1)_2)
366.140(HexNAc + Hex)
657.238(NeuAc + Hex + HexNAc)
m/z

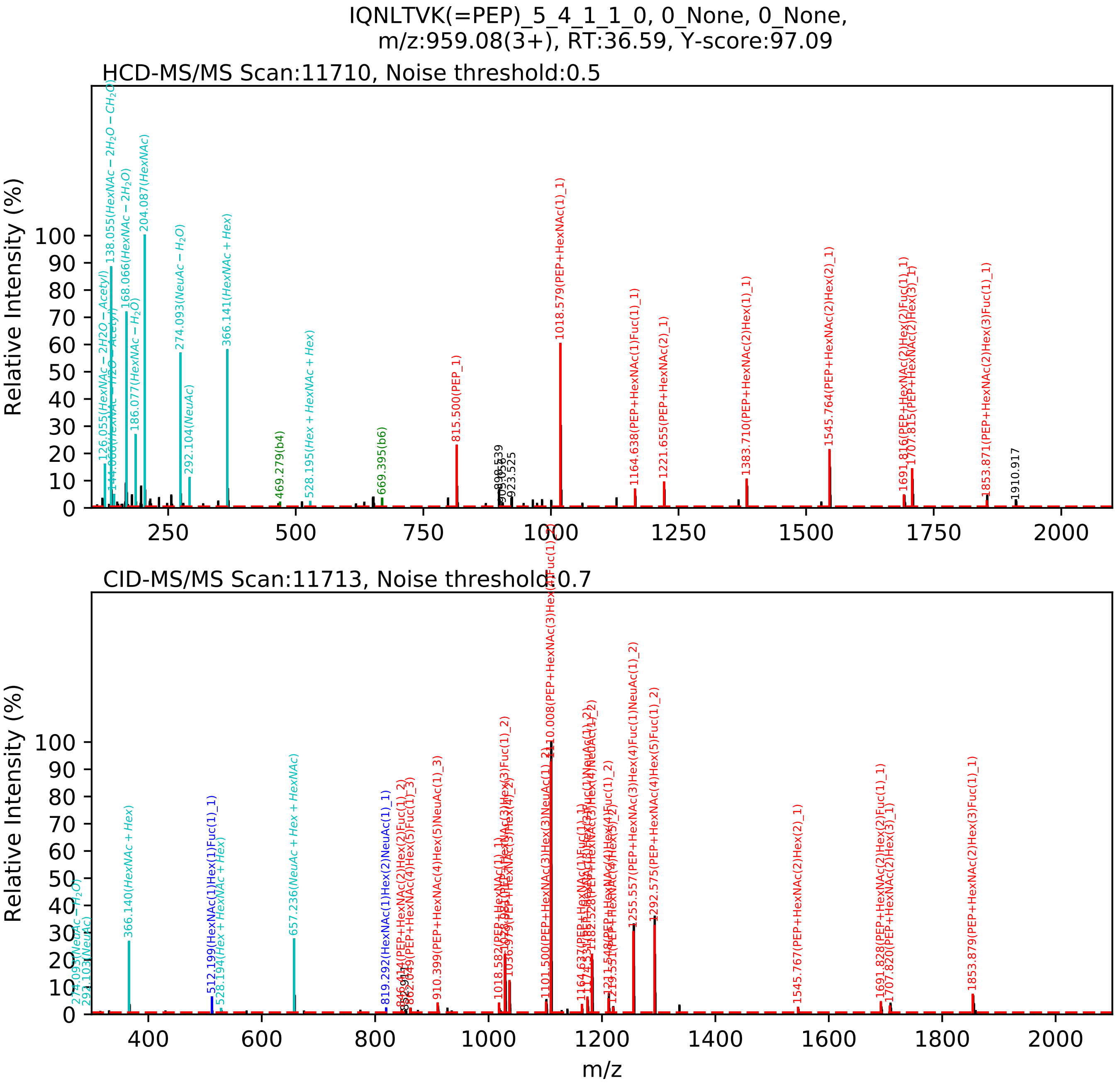

IQNLTVK(=PEP)_5_4_1_1_0, 0_None, 0_None,
m/z:959.08(3+), RT:36.59, Y-score:97.09
HCD-MS/MS Scan:11710, Noise threshold:0.5
Relative Intensity (%)
815.500(PEP_1)
1018.579(PEP+HexNAc(1)_1)
1164.638(PEP+HexNAc(1)Fuc(1)_1)
1221.655(PEP+HexNAc(2)_1)
1383.710(PEP+HexNAc(2)Hex(1)_1)
1545.764(PEP+HexNAc(2)Hex(2)_1)
1853.871(PEP+HexNAc(2)Hex(3)Fuc(1)_1)
1910.917
CID-MS/MS Scan:11713, Noise threshold:0.7
m/z

IQNLTVK(=PEP)_5_4_1_1_0, 0_None, 0_None,
m/z:959.08(3+), RT:36.64, Y-score:97.42

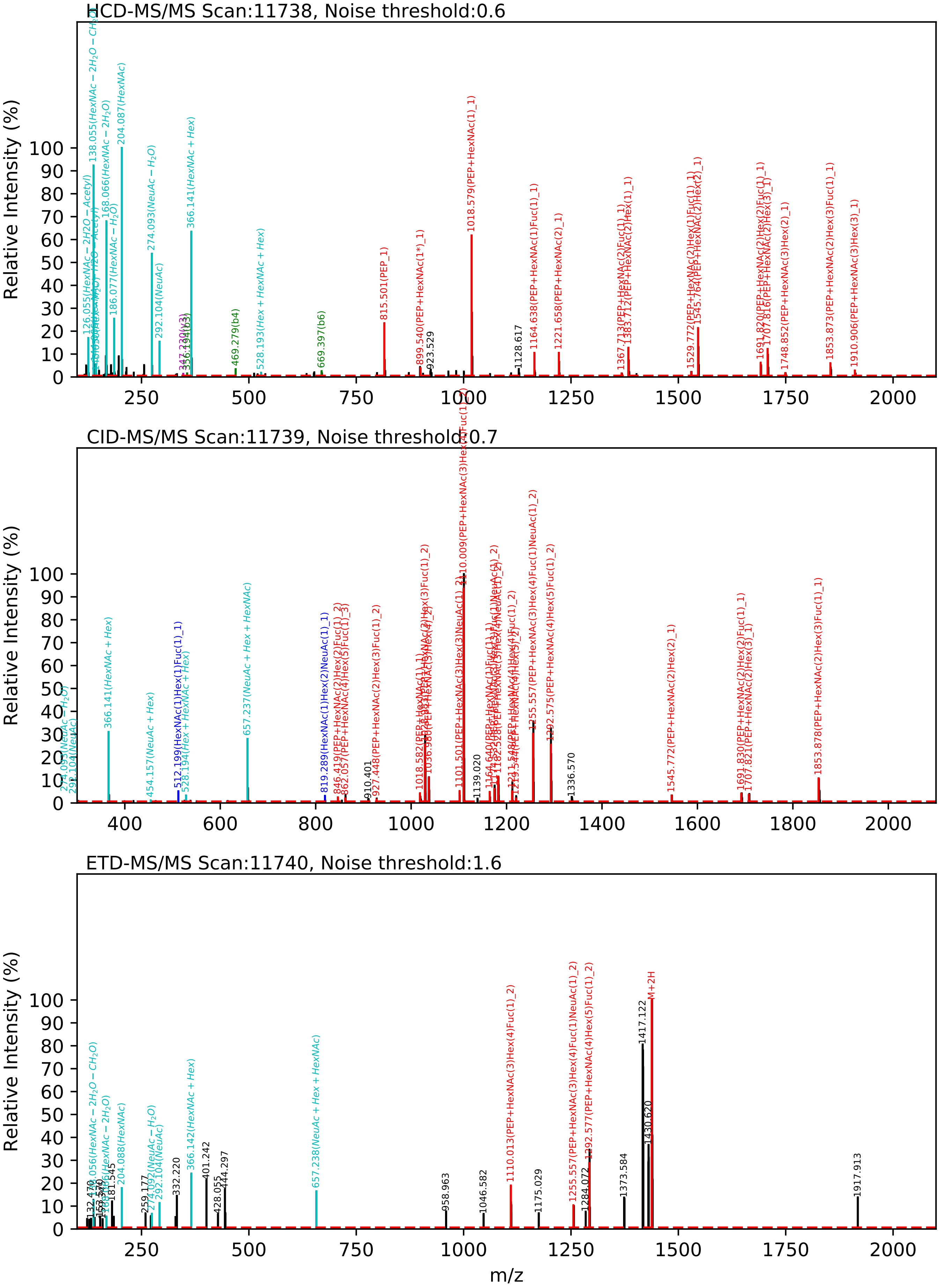

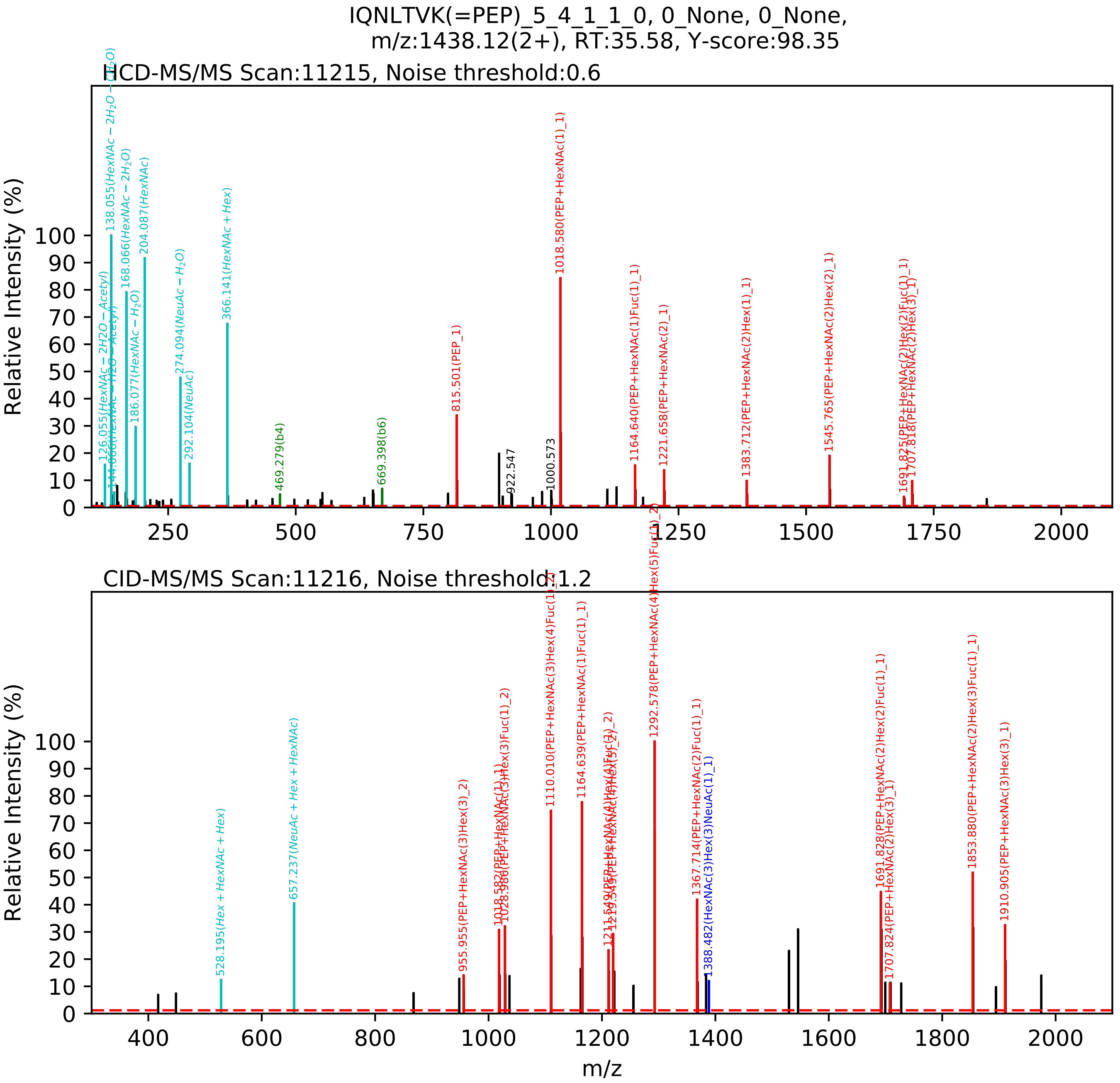
IQNLTVK(=PEP)_5_4_1_1_0, 0_None, 0_None,
m/z:1438.12(2+), RT:35.58, Y-score:98.35
HCD-MS/MS Scan:11215, Noise threshold:0.6
Relative Intensity (%)
138.055(HexNAc − 2H2O − C2H2O)
168.066(HexNAc − 2H2O)
204.087(HexNAc)
126.055(HexNAc − 2H2O − Acetyl)
186.077(HexNAc − H2O)
274.094(NeuAc − H2O)
292.104(NeuAc)
366.141(HexNAc + Hex)
469.279(b4)
669.398(b6)
815.501(PEP_1)
922.547
1000.573
1018.580(PEP+HexNAc(1)_1)
1164.640(PEP+HexNAc(1)Fuc(1)_1)
1221.658(PEP+HexNAc(2)_1)
1383.712(PEP+HexNAc(2)Hex(1)_1)
1545.765(PEP+HexNAc(2)Hex(2)_1)
1691.825(PEP+HexNAc(2)Hex(2)Fuc(1)_1)
1707.818(PEP+HexNAc(2)Hex(3)_1)
CID-MS/MS Scan:11216, Noise threshold:1.2
528.195(Hex + HexNAc + Hex)
657.237(NeuAc + Hex + HexNAc)
955.955(PEP+HexNAc(3)Hex(3)_2)
1110.010(PEP+HexNAc(3)Hex(4)Fuc(1)_2)
1164.639(PEP+HexNAc(1)Fuc(1)_1)
1292.578(PEP+HexNAc(4)Hex(5)Fuc(1)_2)
1367.714(PEP+HexNAc(2)Fuc(1)_1)
1388.482(HexNAc(3)Hex(3)NeuAc(1)_1)
1691.828(PEP+HexNAc(2)Hex(2)Fuc(1)_1)
1707.824(PEP+HexNAc(2)Hex(3)_1)
1853.880(PEP+HexNAc(2)Hex(3)Fuc(1)_1)
1910.905(PEP+HexNAc(3)Hex(3)_1)
m/z

IQNLTVK(=PEP)_5_4_1_1_0, 0_None, 0_None,
m/z:1438.12(2+), RT:36.26, Y-score:93.89

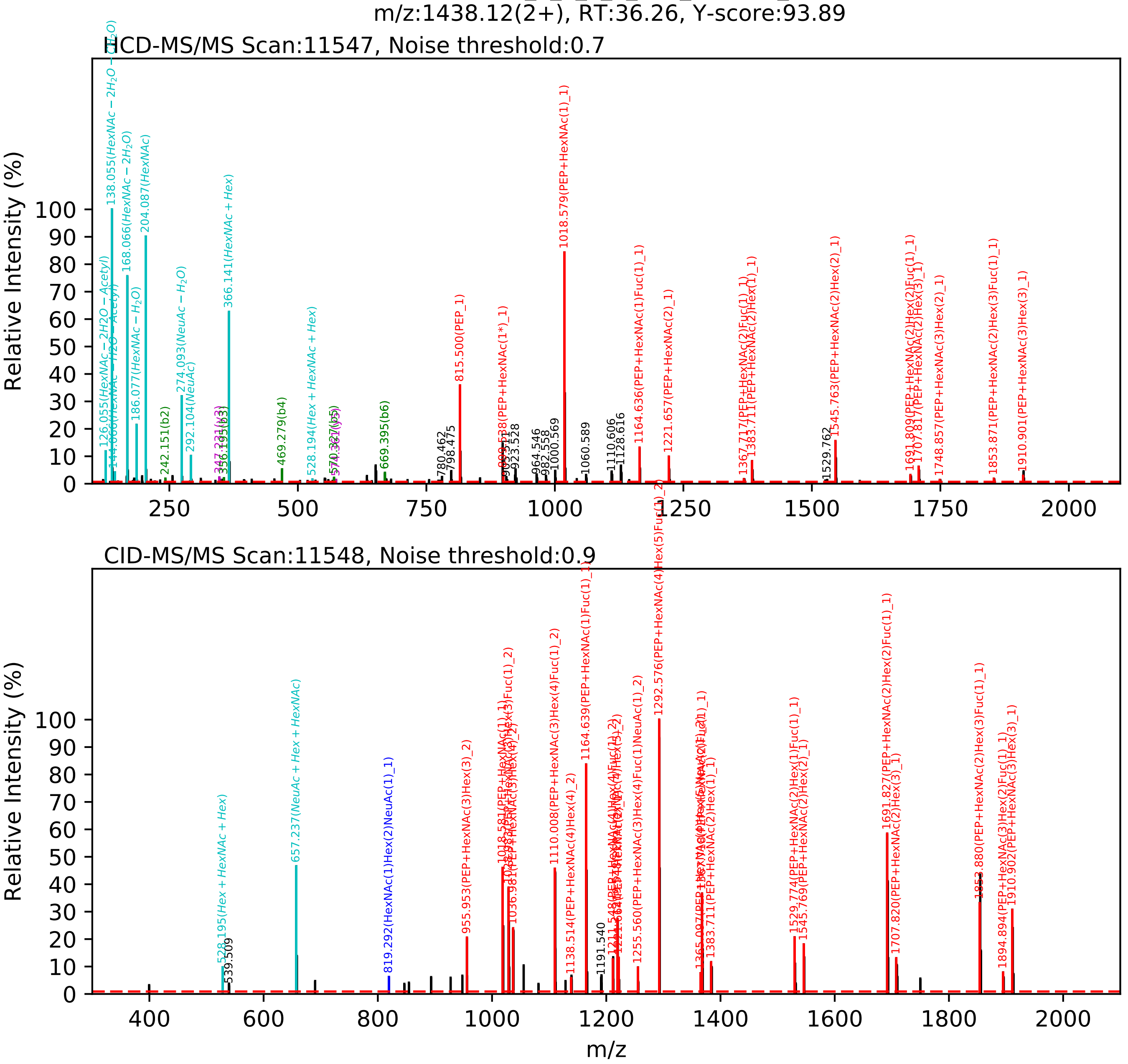

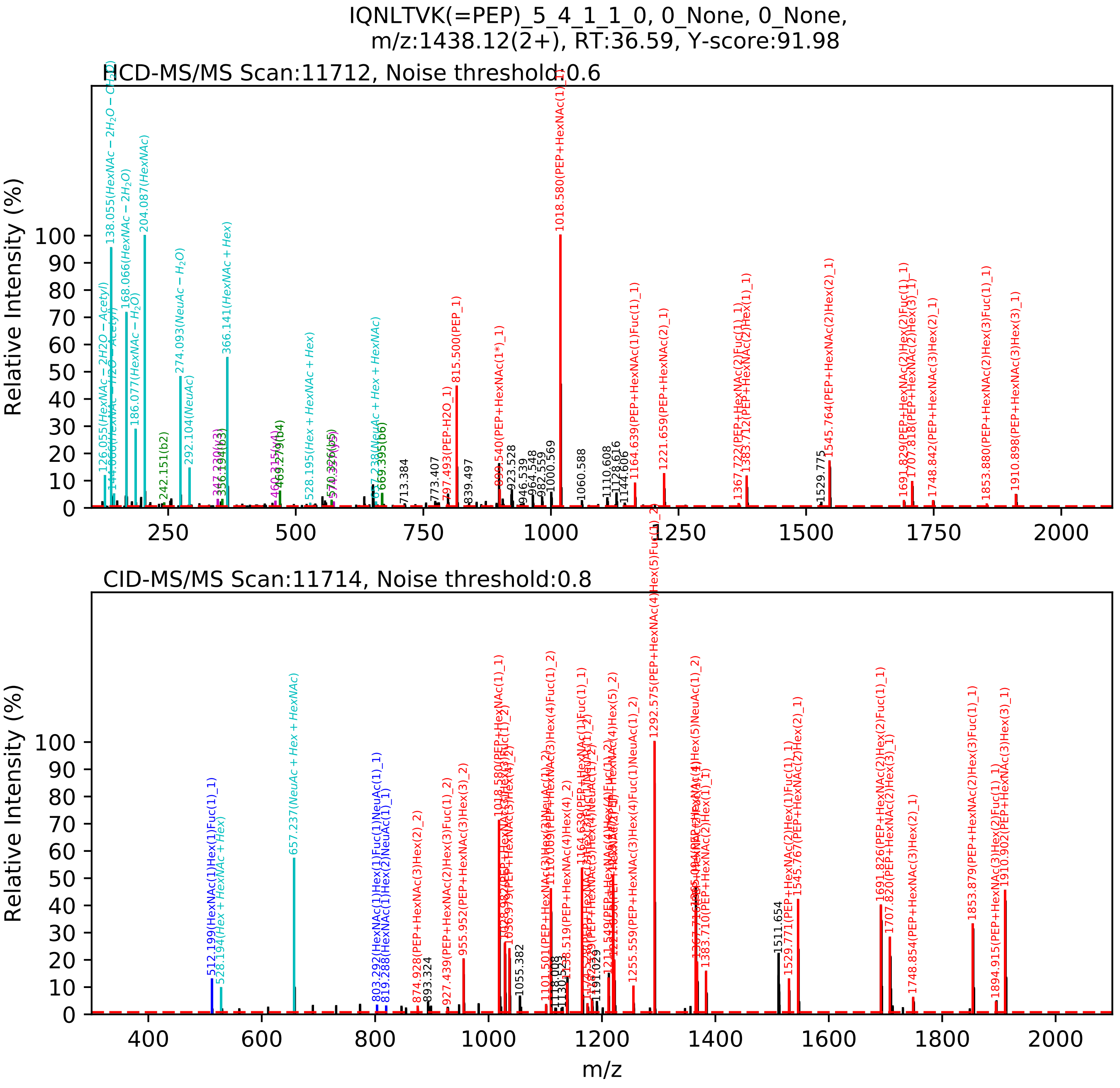
IQNLTVK(=PEP)_5_4_1_1_0, 0_None, 0_None,
m/z:1438.12(2+), RT:36.59, Y-score:91.98
HCD-MS/MS Scan:11712, Noise threshold:0.6
Relative Intensity (%)
CID-MS/MS Scan:11714, Noise threshold:0.8
Relative Intensity (%)
m/z

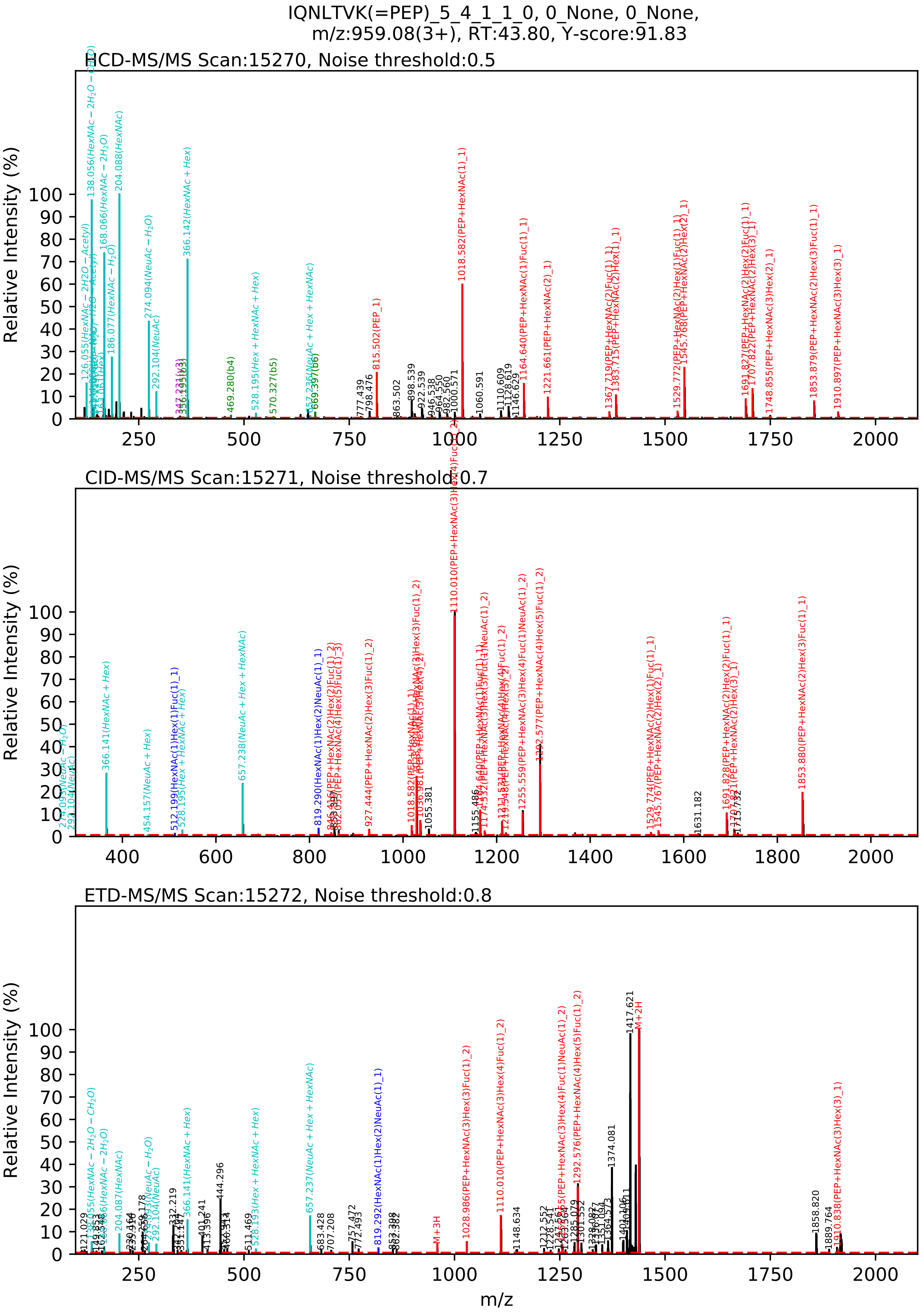
IQNLTVK(=PEP)_5_4_1_1_0, 0_None, 0_None,
m/z:959.08(3+), RT:43.80, Y-score:91.83
HCD-MS/MS Scan:15270, Noise threshold:0.5
CID-MS/MS Scan:15271, Noise threshold:0.7
ETD-MS/MS Scan:15272, Noise threshold:0.8
Relative Intensity (%)
m/z

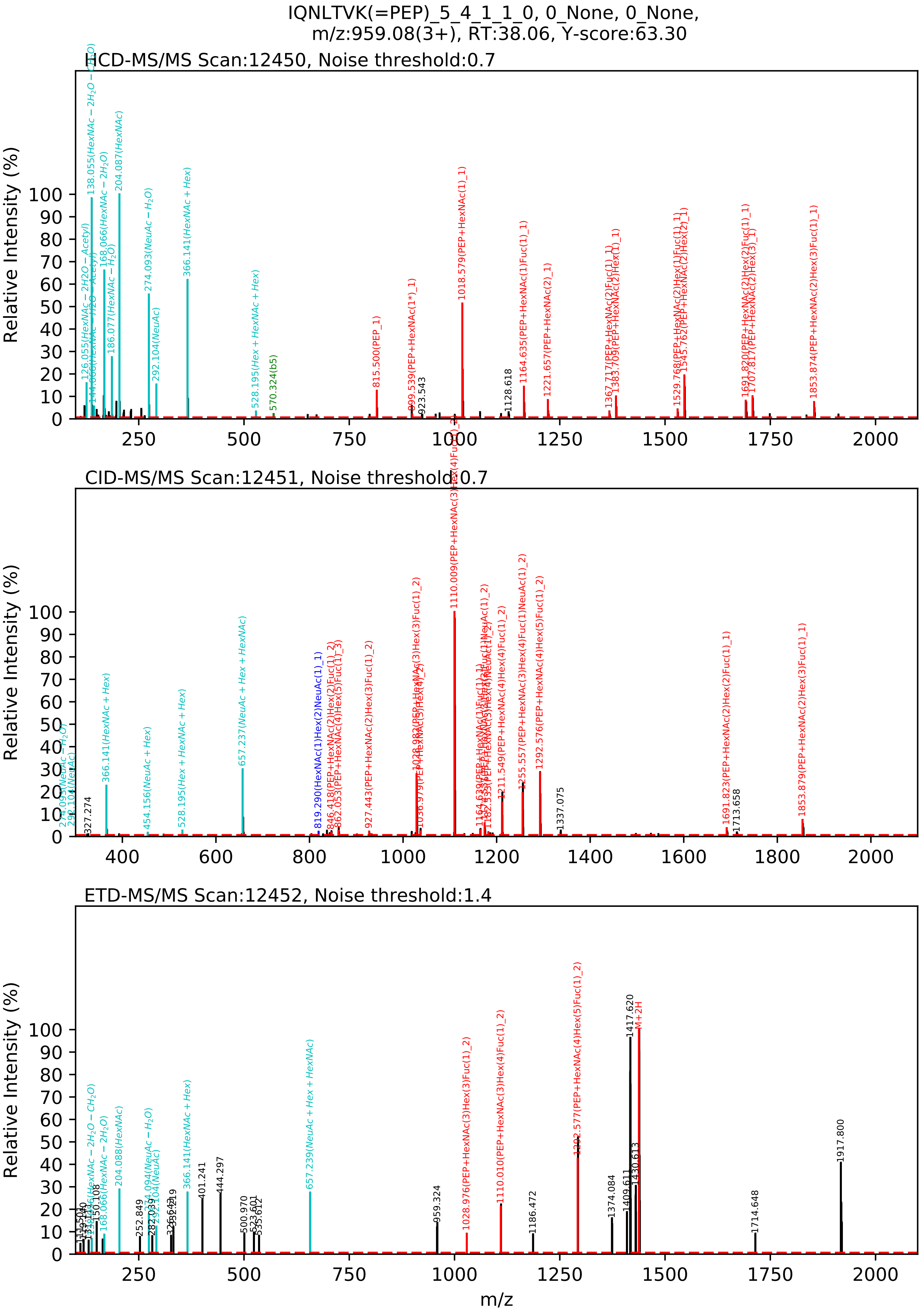
IQNLTVK(=PEP)_5_4_1_1_0, 0_None, 0_None,
m/z:959.08(3+), RT:38.06, Y-score:63.30
HCD-MS/MS Scan:12450, Noise threshold:0.7
Relative Intensity (%)
126.055(HexNAc − 2H2O − Acetyl)
138.055(HexNAc − 2H2O − CH2O)
144.066(HexNAc − H2O − Acetyl)
168.066(HexNAc − 2H2O)
186.077(HexNAc − H2O)
204.087(HexNAc)
274.093(NeuAc − H2O)
292.104(NeuAc)
366.141(HexNAc + Hex)
528.195(Hex + HexNAc + Hex)
570.324(b5)
815.500(PEP_1)
899.539(PEP+HexNAc(1*)_1)
923.543
1018.579(PEP+HexNAc(1)_1)
1128.618
1164.635(PEP+HexNAc(1)Fuc(1)_1)
1221.657(PEP+HexNAc(2)_1)
1367.717(PEP+HexNAc(2)Fuc(1)_1)
1383.709(PEP+HexNAc(2)Hex(1)_1)
1529.768(PEP+HexNAc(2)Hex(1)Fuc(1)_1)
1545.762(PEP+HexNAc(2)Hex(2)_1)
1691.820(PEP+HexNAc(2)Hex(2)Fuc(1)_1)
1707.817(PEP+HexNAc(2)Hex(3)_1)
1853.874(PEP+HexNAc(2)Hex(3)Fuc(1)_1)
CID-MS/MS Scan:12451, Noise threshold:0.7
Relative Intensity (%)
274.093(NeuAc − H2O)
292.104(NeuAc)
327.274
366.141(HexNAc + Hex)
454.156(NeuAc + Hex)
528.195(Hex + HexNAc + Hex)
657.237(NeuAc + Hex + HexNAc)
819.290(HexNAc(1)Hex(2)NeuAc(1)_1)
846.418(PEP+HexNAc(2)Hex(2)Fuc(1)_2)
862.053(PEP+HexNAc(4)Hex(5)Fuc(1)_3)
927.443(PEP+HexNAc(2)Hex(3)Fuc(1)_2)
1028.982(PEP+HexNAc(3)Hex(3)Fuc(1)_2)
1036.979(PEP+HexNAc(3)Hex(4)_2)
1110.009(PEP+HexNAc(3)Hex(4)Fuc(1)_2)
1164.639
1182.533
1211.549(PEP+HexNAc(4)Hex(4)Fuc(1)_2)
1255.557(PEP+HexNAc(3)Hex(4)Fuc(1)NeuAc(1)_2)
1292.576(PEP+HexNAc(4)Hex(5)Fuc(1)_2)
1337.075
1691.823(PEP+HexNAc(2)Hex(2)Fuc(1)_1)
1713.658
1853.879(PEP+HexNAc(2)Hex(3)Fuc(1)_1)
ETD-MS/MS Scan:12452, Noise threshold:1.4
Relative Intensity (%)
138.054(HexNAc − 2H2O − CH2O)
150.108
168.066(HexNAc − 2H2O)
204.088(HexNAc)
252.849
274.094(NeuAc − H2O)
282.039
292.104(NeuAc)
326.642
332.219
366.141(HexNAc + Hex)
401.241
444.297
500.970
523.601
535.612
657.239(NeuAc + Hex + HexNAc)
959.324
1028.976(PEP+HexNAc(3)Hex(3)Fuc(1)_2)
1110.010(PEP+HexNAc(3)Hex(4)Fuc(1)_2)
1186.472
1292.577(PEP+HexNAc(4)Hex(5)Fuc(1)_2)
1374.084
1409.611
1417.620
M+2H
1430.613
1714.648
1917.800
m/z

IQNLTVK(=PEP)_5_4_1_2_0, 0_None, 0_None,
m/z:1583.67(2+), RT:50.23, Y-score:91.73

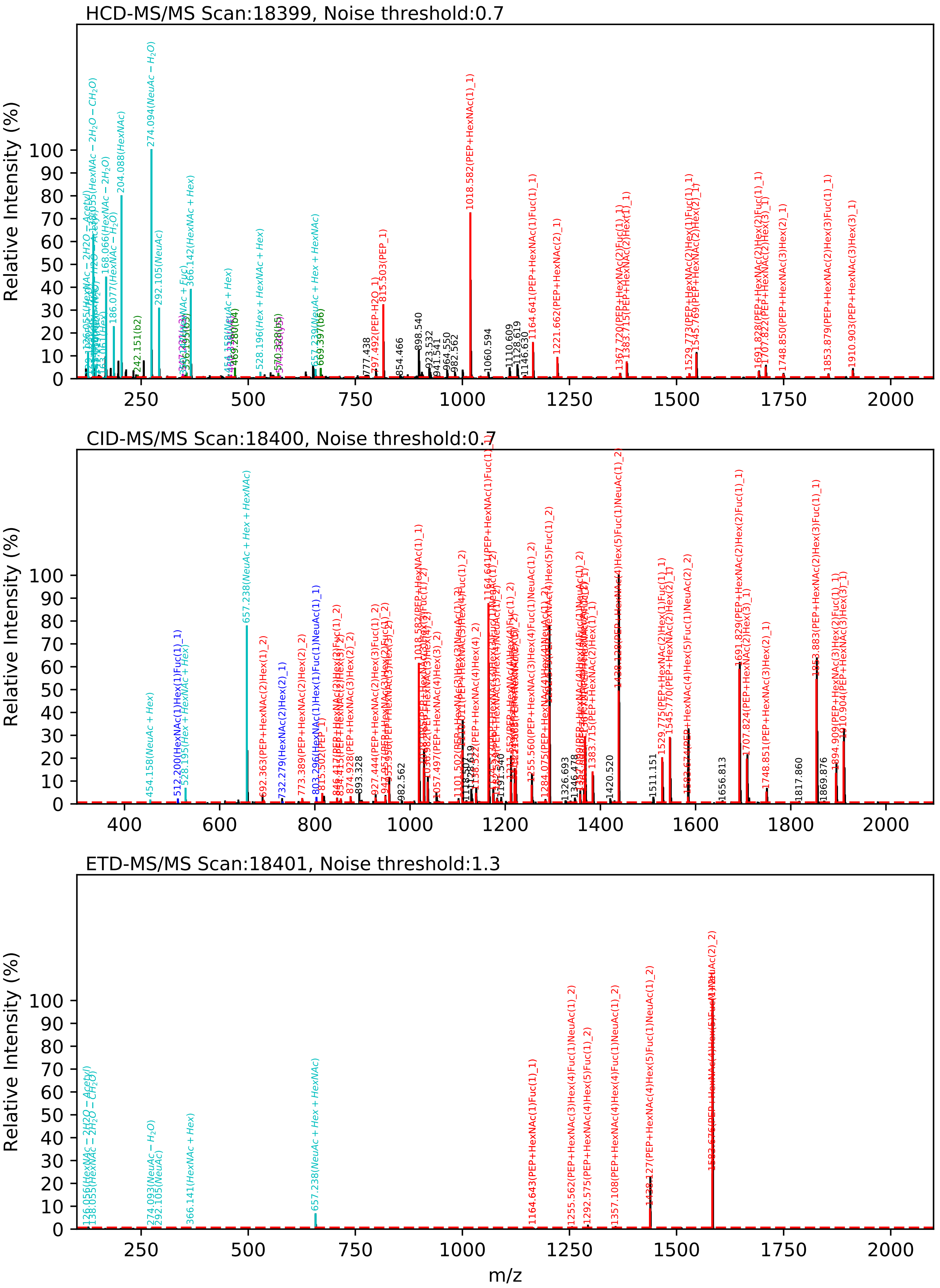

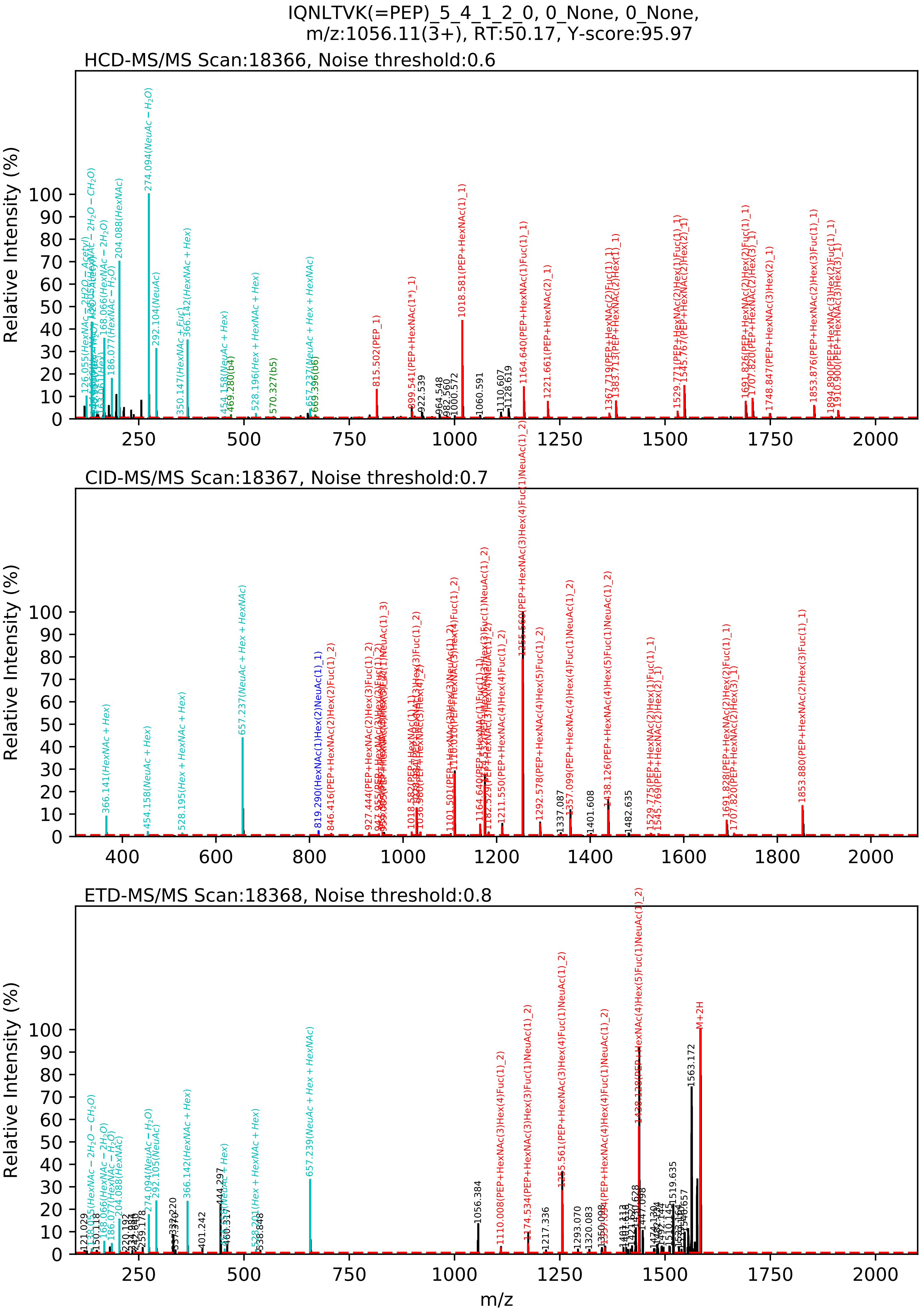

IQNLTVK(=PEP)_5_4_1_2_0, 0_None, 0_None,
m/z:1056.11(3+), RT:50.17, Y-score:95.97
HCD-MS/MS Scan:18366, Noise threshold:0.6
CID-MS/MS Scan:18367, Noise threshold:0.7
ETD-MS/MS Scan:18368, Noise threshold:0.8
Relative Intensity (%)
m/z

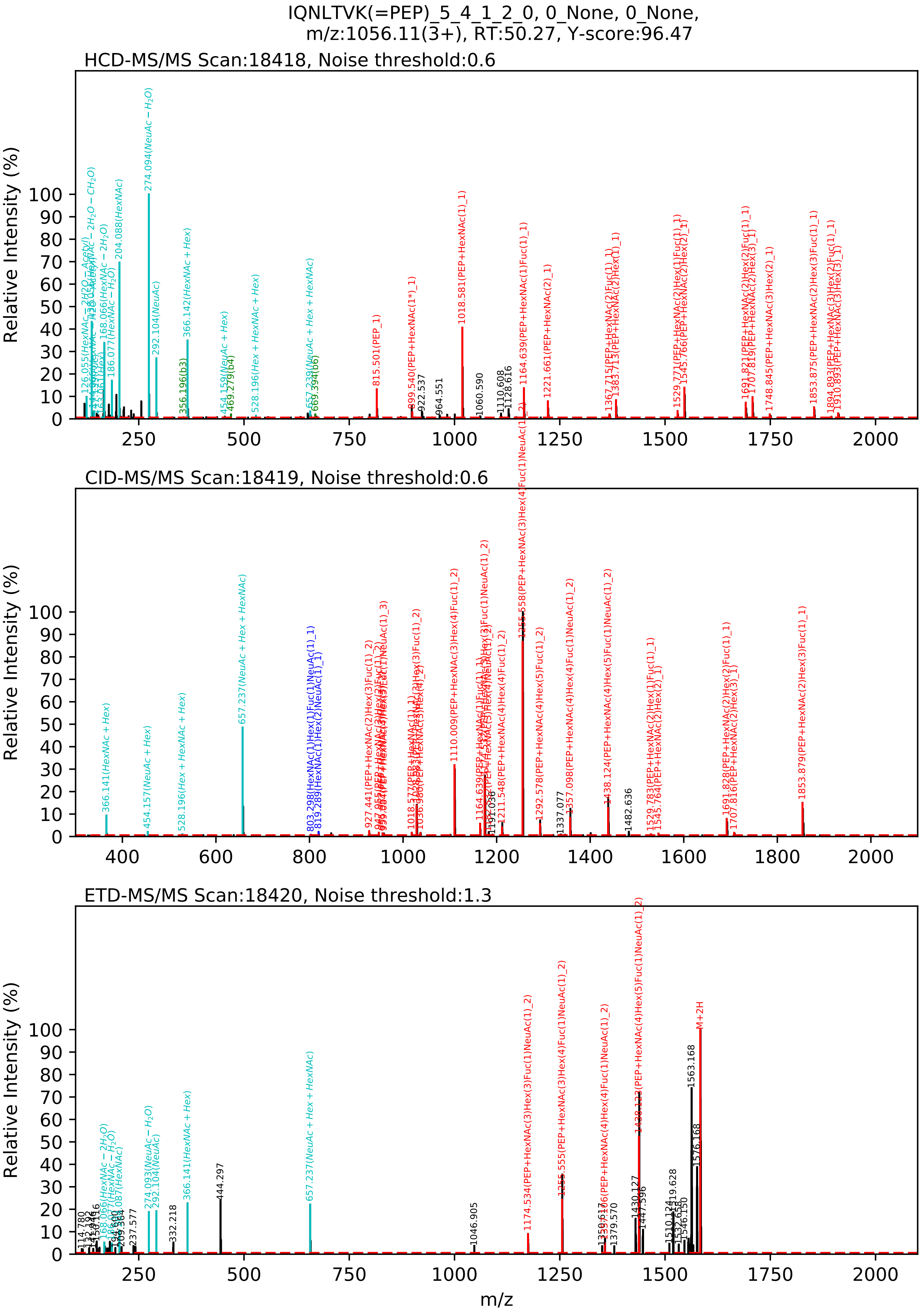

IQNLTVK(=PEP)_5_4_1_2_0, 0_None, 0_None,
m/z:1056.11(3+), RT:50.27, Y-score:96.47
HCD-MS/MS Scan:18418, Noise threshold:0.6
Relative Intensity (%)
274.094(NeuAc − H2O)
204.088(HexNAc)
292.104(NeuAc)
366.142(HexNAc + Hex)
356.196(b3)
454.159(NeuAc + Hex)
469.279(b4)
528.196(Hex + HexNAc + Hex)
657.239(NeuAc + Hex + HexNAc)
669.394(b6)
815.501(PEP_1)
899.540(PEP+HexNAc(1*)_1)
922.537
964.551
1018.581(PEP+HexNAc(1)_1)
1060.590
1110.608
1128.616
1164.639(PEP+HexNAc(1)Fuc(1)_1)
1221.661(PEP+HexNAc(2)_1)
1545.766(PEP+HexNAc(2)Hex(2)_1)
1748.845(PEP+HexNAc(3)Hex(2)_1)
1853.875(PEP+HexNAc(2)Hex(3)Fuc(1)_1)
CID-MS/MS Scan:18419, Noise threshold:0.6
366.141(HexNAc + Hex)
454.157(NeuAc + Hex)
528.196(Hex + HexNAc + Hex)
657.237(NeuAc + Hex + HexNAc)
803.298(HexNAc(1)Hex(1)Fuc(1)NeuAc(1)_1)
819.289(HexNAc(1)Hex(2)NeuAc(1)_1)
1110.009(PEP+HexNAc(3)Hex(4)Fuc(1)_2)
1255.558(PEP+HexNAc(3)Hex(4)Fuc(1)NeuAc(1)_2)
1292.578(PEP+HexNAc(4)Hex(5)Fuc(1)_2)
1337.077
1357.098(PEP+HexNAc(4)Hex(4)Fuc(1)NeuAc(1)_2)
1438.124(PEP+HexNAc(4)Hex(5)Fuc(1)NeuAc(1)_2)
1482.636
1691.828(PEP+HexNAc(2)Hex(2)Fuc(1)_1)
1853.879(PEP+HexNAc(2)Hex(3)Fuc(1)_1)
ETD-MS/MS Scan:18420, Noise threshold:1.3
274.093(NeuAc − H2O)
292.104(NeuAc)
366.141(HexNAc + Hex)
332.218
444.297
657.237(NeuAc + Hex + HexNAc)
1046.905
1174.534(PEP+HexNAc(3)Hex(3)Fuc(1)NeuAc(1)_2)
1255.555(PEP+HexNAc(3)Hex(4)Fuc(1)NeuAc(1)_2)
1379.570
1438.123(PEP+HexNAc(4)Hex(5)Fuc(1)NeuAc(1)_2)
1447.596
1563.168
1576.168
M+2H
m/z

IQNLTVK(=PEP)_5_4_1_2_0, 0_None, 0_None,
m/z:1056.11(3+), RT:50.75, Y-score:62.24

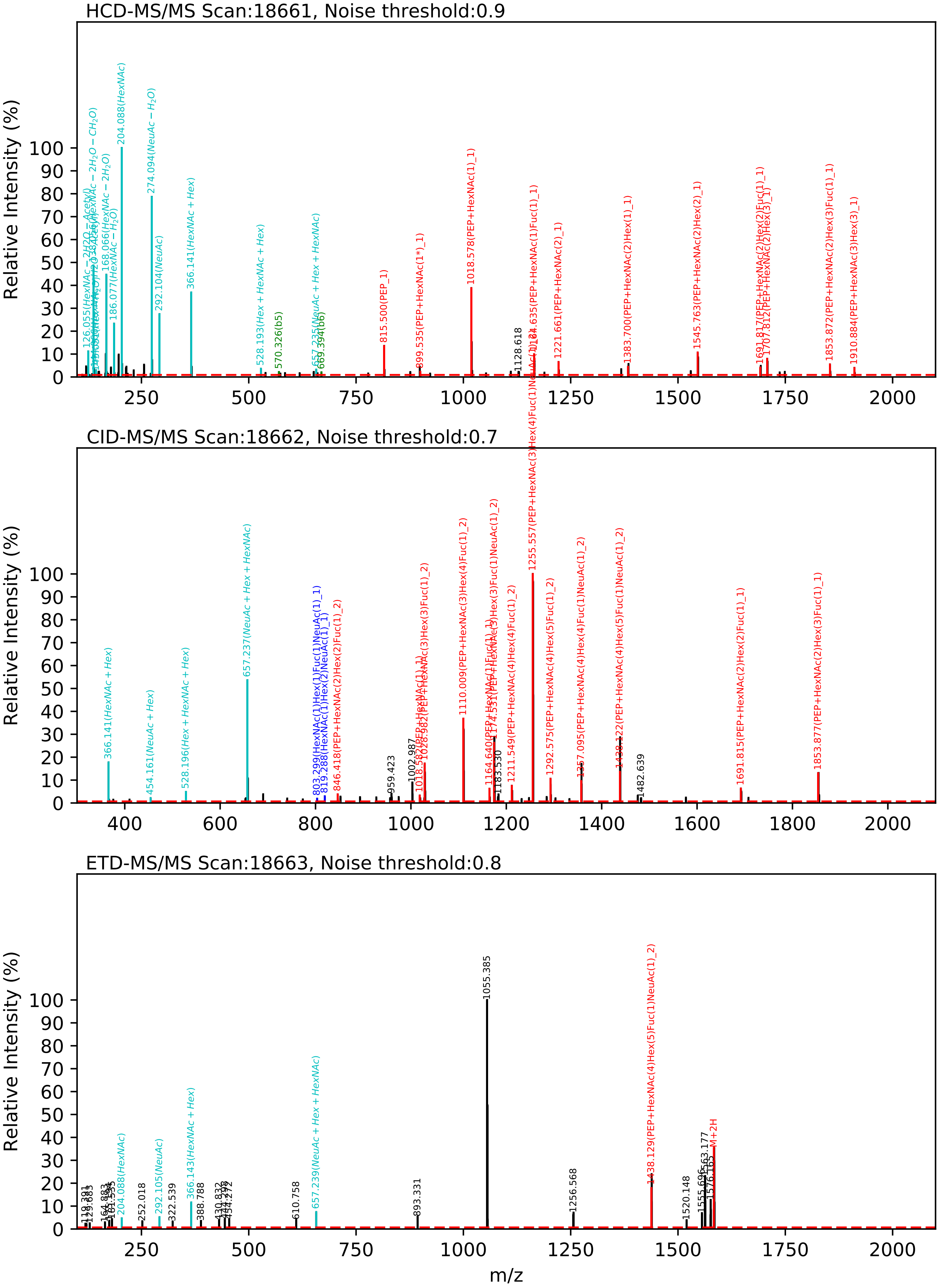

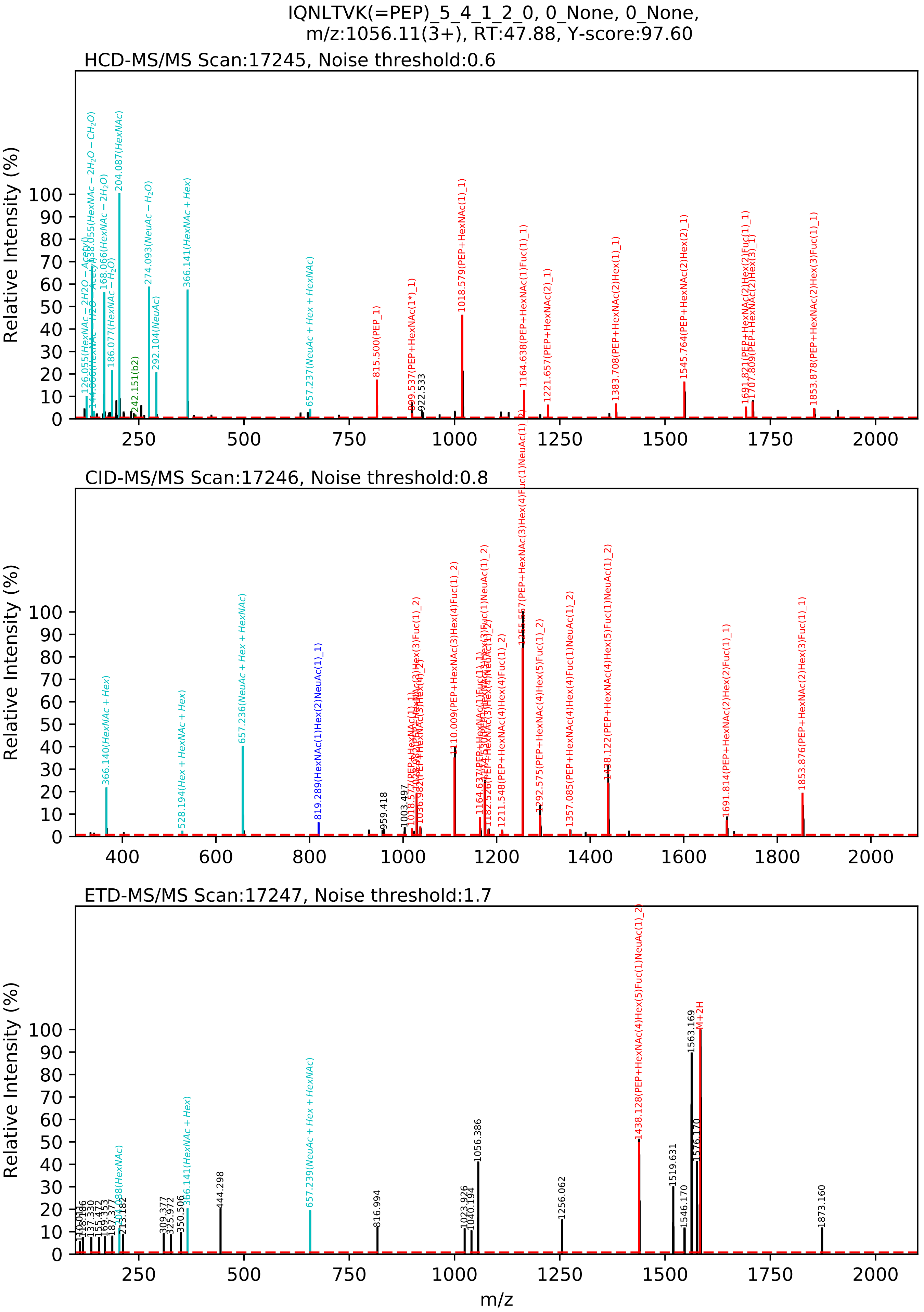

IQNLTVK(=PEP)_5_4_1_2_0, 0_None, 0_None,
m/z:1056.11(3+), RT:47.88, Y-score:97.60
HCD-MS/MS Scan:17245, Noise threshold:0.6
Relative Intensity (%)
204.087(HexNAc)
366.141(HexNAc + Hex)
274.093(NeuAc − H2O)
292.104(NeuAc)
242.151(b2)
657.237(NeuAc + Hex + HexNAc)
815.500(PEP_1)
899.537(PEP+HexNAc(1*)_1)
922.533
1018.579(PEP+HexNAc(1)_1)
1164.638(PEP+HexNAc(1)Fuc(1)_1)
1221.657(PEP+HexNAc(2)_1)
1383.708(PEP+HexNAc(2)Hex(1)_1)
1545.764(PEP+HexNAc(2)Hex(2)_1)
1691.821(PEP+HexNAc(2)Hex(2)Fuc(1)_1)
1707.809(PEP+HexNAc(2)Hex(3)_1)
1853.878(PEP+HexNAc(2)Hex(3)Fuc(1)_1)
CID-MS/MS Scan:17246, Noise threshold:0.8
366.140(HexNAc + Hex)
528.194(Hex + HexNAc + Hex)
657.236(NeuAc + Hex + HexNAc)
819.289(HexNAc(1)Hex(2)NeuAc(1)_1)
959.418
1003.497
1110.009(PEP+HexNAc(3)Hex(4)Fuc(1)_2)
1164.637
1211.548(PEP+HexNAc(4)Hex(4)Fuc(1)_2)
1255.557(PEP+HexNAc(3)Hex(4)Fuc(1)NeuAc(1)_2)
1292.575(PEP+HexNAc(4)Hex(5)Fuc(1)_2)
1357.085(PEP+HexNAc(4)Hex(4)Fuc(1)NeuAc(1)_2)
1438.122(PEP+HexNAc(4)Hex(5)Fuc(1)NeuAc(1)_2)
1691.814(PEP+HexNAc(2)Hex(2)Fuc(1)_1)
1853.876(PEP+HexNAc(2)Hex(3)Fuc(1)_1)
ETD-MS/MS Scan:17247, Noise threshold:1.7
366.141(HexNAc + Hex)
657.239(NeuAc + Hex + HexNAc)
309.377
325.972
350.506
444.298
816.994
1023.926
1040.194
1056.386
1256.062
1438.128(PEP+HexNAc(4)Hex(5)Fuc(1)NeuAc(1)_2)
1519.631
1546.170
1563.169
1576.170
M+2H
1873.160
m/z

IQNLTVK(=PEP)_5_4_1_2_0, 0_None, 0_None, m/z:1056.11(3+), RT:48.79, Y-score:57.76

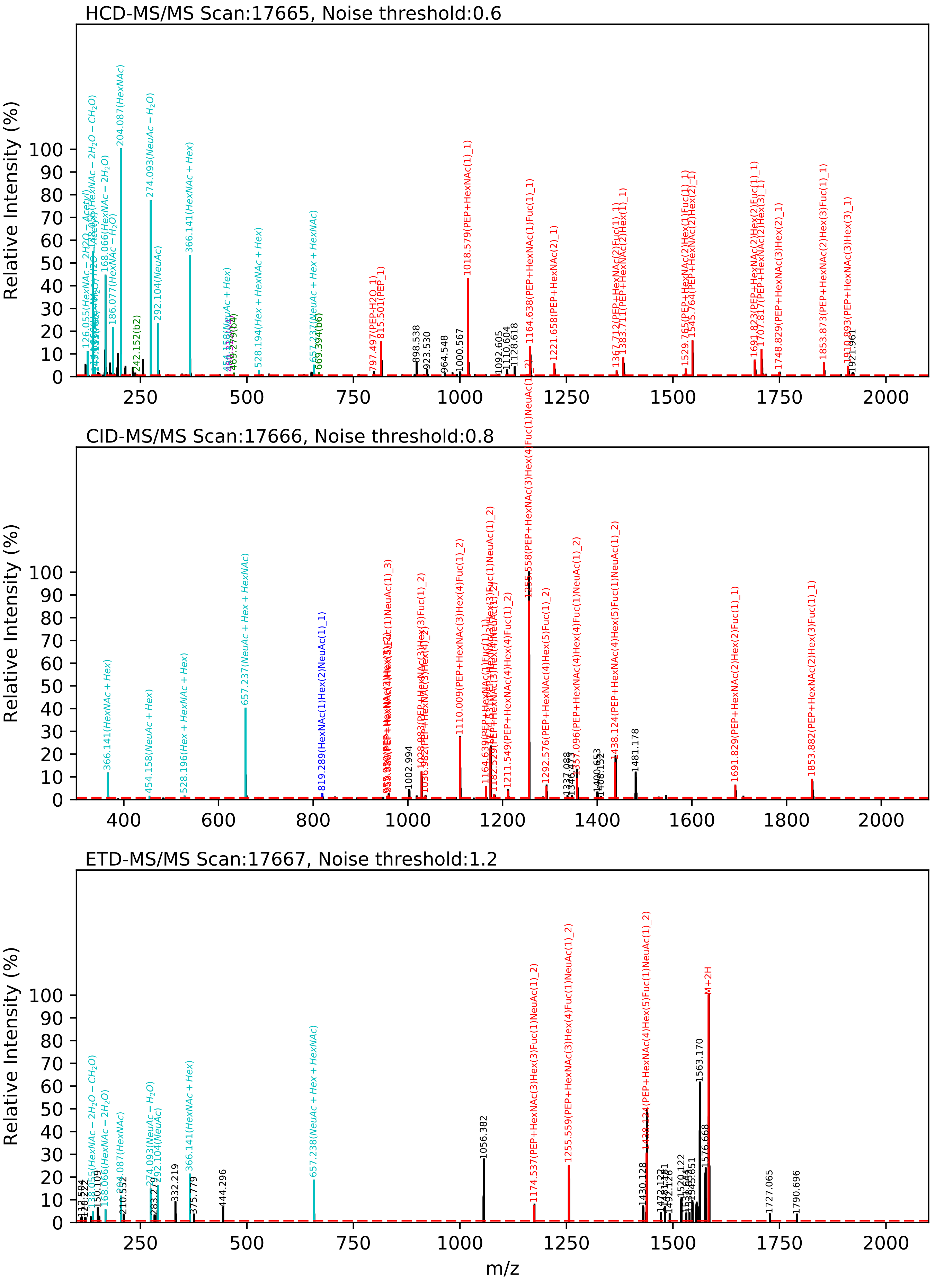

IQNLTVK(=PEP)_5_4_1_2_0, 0_None, 0_None,
m/z:1056.11(3+), RT:49.34, Y-score:96.60

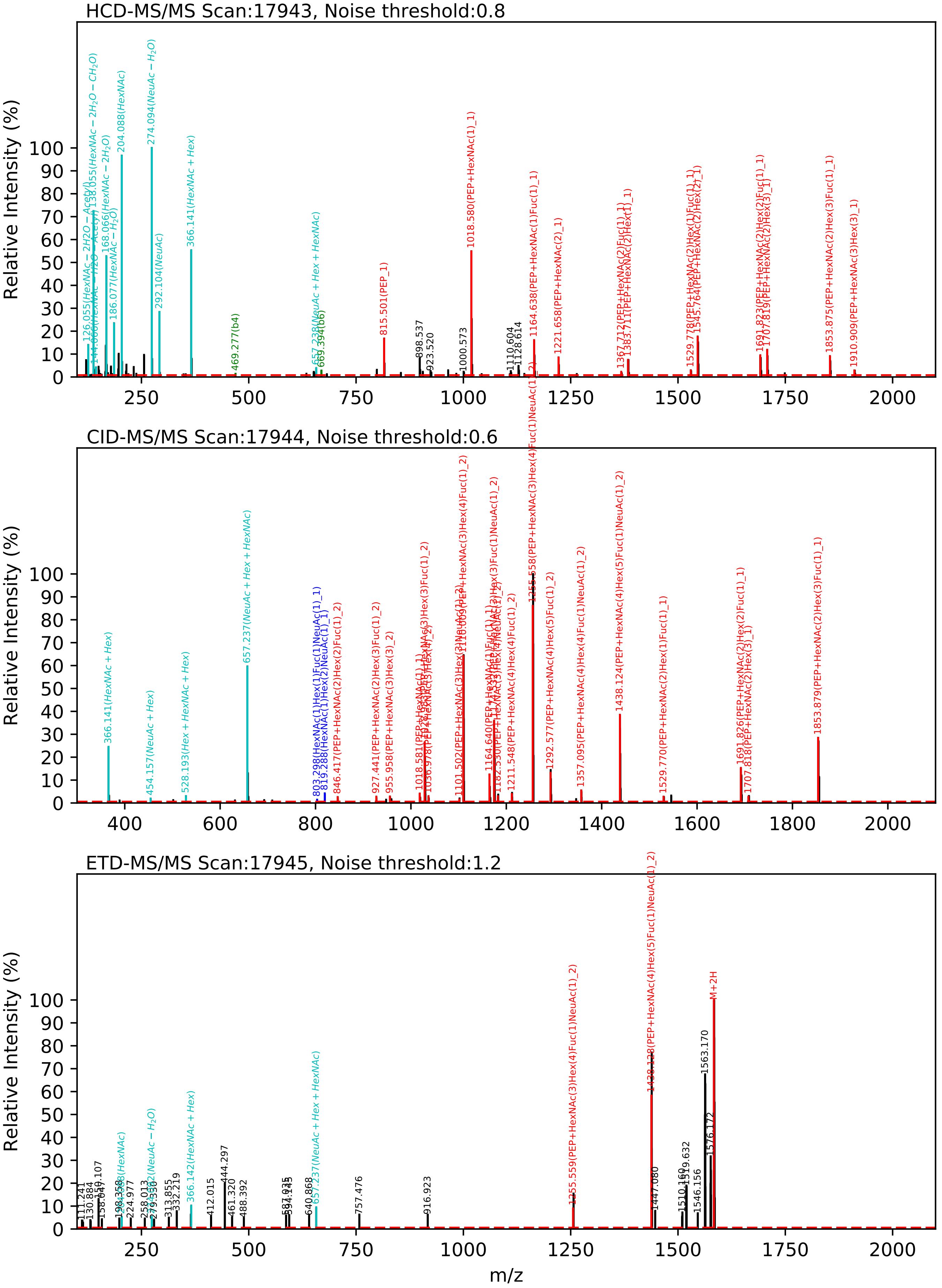

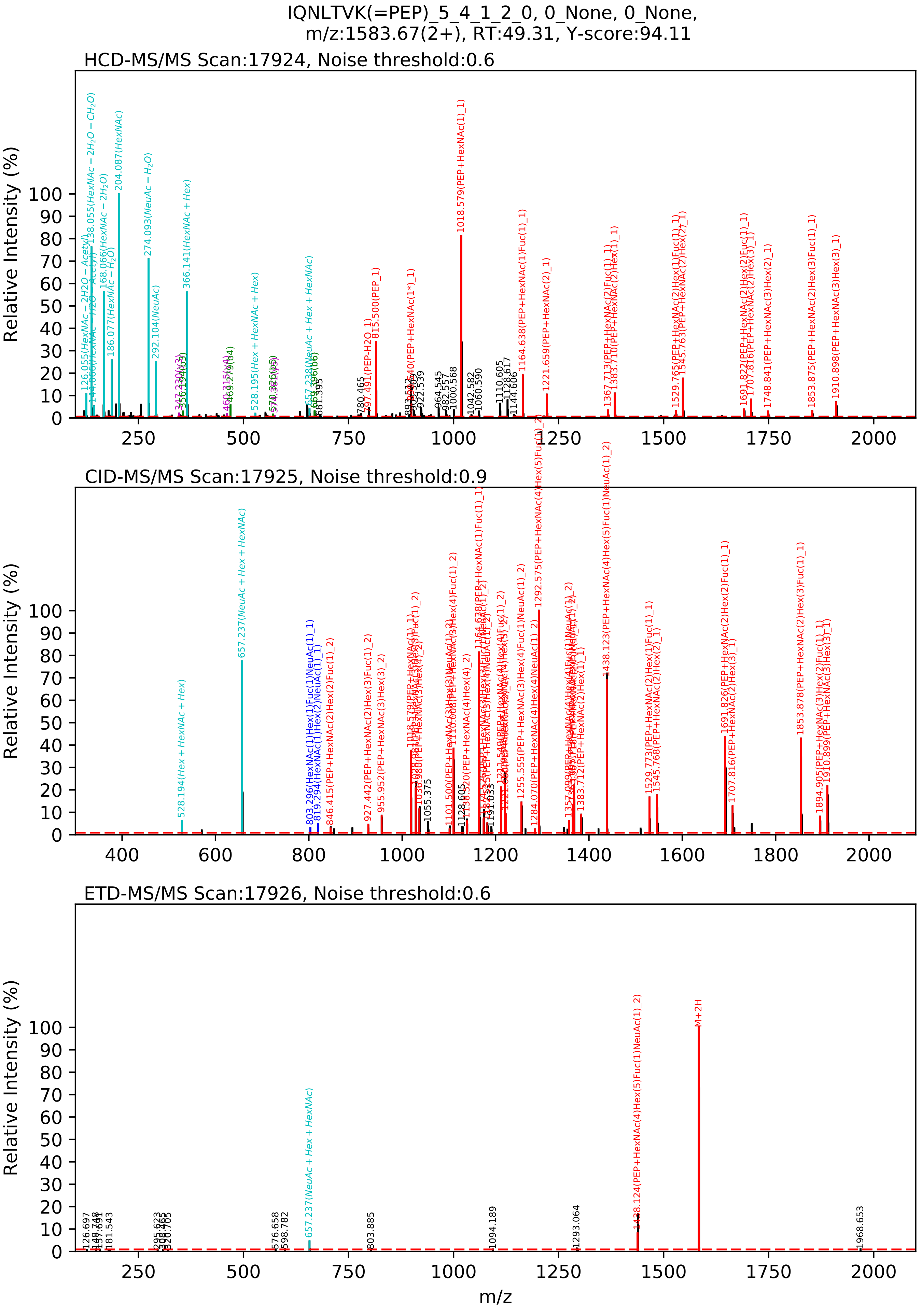

IQNLTVK(=PEP)_5_4_1_2_0, 0_None, 0_None,
m/z:1583.67(2+), RT:49.31, Y-score:94.11
HCD-MS/MS Scan:17924, Noise threshold:0.6
CID-MS/MS Scan:17925, Noise threshold:0.9
ETD-MS/MS Scan:17926, Noise threshold:0.6
Relative Intensity (%)
m/z
1018.579(PEP+HexNAc(1)_1)
204.087(HexNAc)
274.093(NeuAc − H2O)
366.141(HexNAc + Hex)
657.237(NeuAc + Hex + HexNAc)
528.194(Hex + HexNAc + Hex)
1292.575(PEP+HexNAc(4)Hex(5)Fuc(1)_2)
1438.123(PEP+HexNAc(4)Hex(5)Fuc(1)NeuAc(1)_2)
1691.826(PEP+HexNAc(2)Hex(2)Fuc(1)_1)
1853.878(PEP+HexNAc(2)Hex(3)Fuc(1)_1)
1438.124(PEP+HexNAc(4)Hex(5)Fuc(1)NeuAc(1)_2)
M+2H

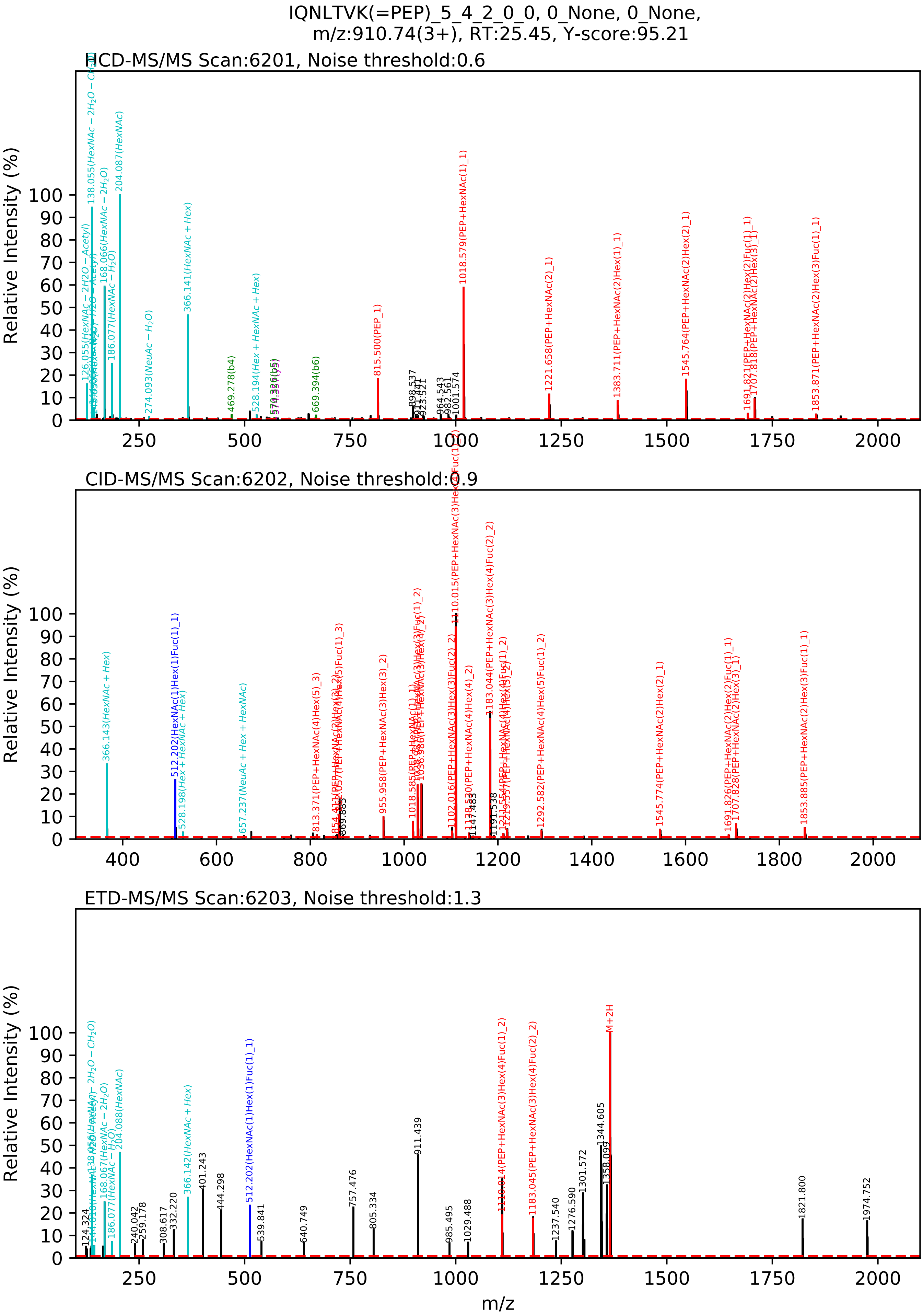
IQNLTVK(=PEP)_5_4_2_0_0, 0_None, 0_None,
m/z:910.74(3+), RT:25.45, Y-score:95.21
HCD-MS/MS Scan:6201, Noise threshold:0.6
Relative Intensity (%)
126.055(HexNAc − 2H2O − Acetyl)
138.055(HexNAc − 2H2O − CH2O)
168.066(HexNAc − 2H2O)
186.077(HexNAc − H2O)
204.087(HexNAc)
274.093(NeuAc − H2O)
366.141(HexNAc + Hex)
469.278(b4)
528.194(Hex + HexNAc + Hex)
669.394(b6)
815.500(PEP_1)
898.537
923.521
964.543
1001.574
1018.579(PEP+HexNAc(1)_1)
1221.658(PEP+HexNAc(2)_1)
1383.711(PEP+HexNAc(2)Hex(1)_1)
1545.764(PEP+HexNAc(2)Hex(2)_1)
1691.821(PEP+HexNAc(2)Hex(2)Fuc(1)_1)
1707.818(PEP+HexNAc(2)Hex(3)_1)
1853.871(PEP+HexNAc(2)Hex(3)Fuc(1)_1)
CID-MS/MS Scan:6202, Noise threshold:0.9
Relative Intensity (%)
366.143(HexNAc + Hex)
512.202(HexNAc(1)Hex(1)Fuc(1)_1)
528.198(Hex + HexNAc + Hex)
657.237(NeuAc + Hex + HexNAc)
813.371(PEP+HexNAc(4)Hex(5)_3)
869.865
955.958(PEP+HexNAc(3)Hex(3)_2)
1110.015(PEP+HexNAc(3)Hex(4)Fuc(1)_2)
1102.016(PEP+HexNAc(3)Hex(3)Fuc(2)_2)
1138.530(PEP+HexNAc(4)Hex(4)_2)
1147.483
1183.044(PEP+HexNAc(3)Hex(4)Fuc(2)_2)
1191.538
1292.582(PEP+HexNAc(4)Hex(5)Fuc(1)_2)
1545.774(PEP+HexNAc(2)Hex(2)_1)
1691.826(PEP+HexNAc(2)Hex(2)Fuc(1)_1)
1707.828(PEP+HexNAc(2)Hex(3)_1)
1853.885(PEP+HexNAc(2)Hex(3)Fuc(1)_1)
ETD-MS/MS Scan:6203, Noise threshold:1.3
Relative Intensity (%)
124.324
168.067(HexNAc − 2H2O)
186.077(HexNAc − H2O)
204.088(HexNAc)
240.042
259.178
308.617
332.220
366.142(HexNAc + Hex)
401.243
444.298
512.202(HexNAc(1)Hex(1)Fuc(1)_1)
539.841
640.749
757.476
805.334
911.439
985.495
1029.488
1110.014(PEP+HexNAc(3)Hex(4)Fuc(1)_2)
1183.045(PEP+HexNAc(3)Hex(4)Fuc(2)_2)
1237.540
1276.590
1301.572
1344.605
1358.095
M+2H
1821.800
1974.752
m/z

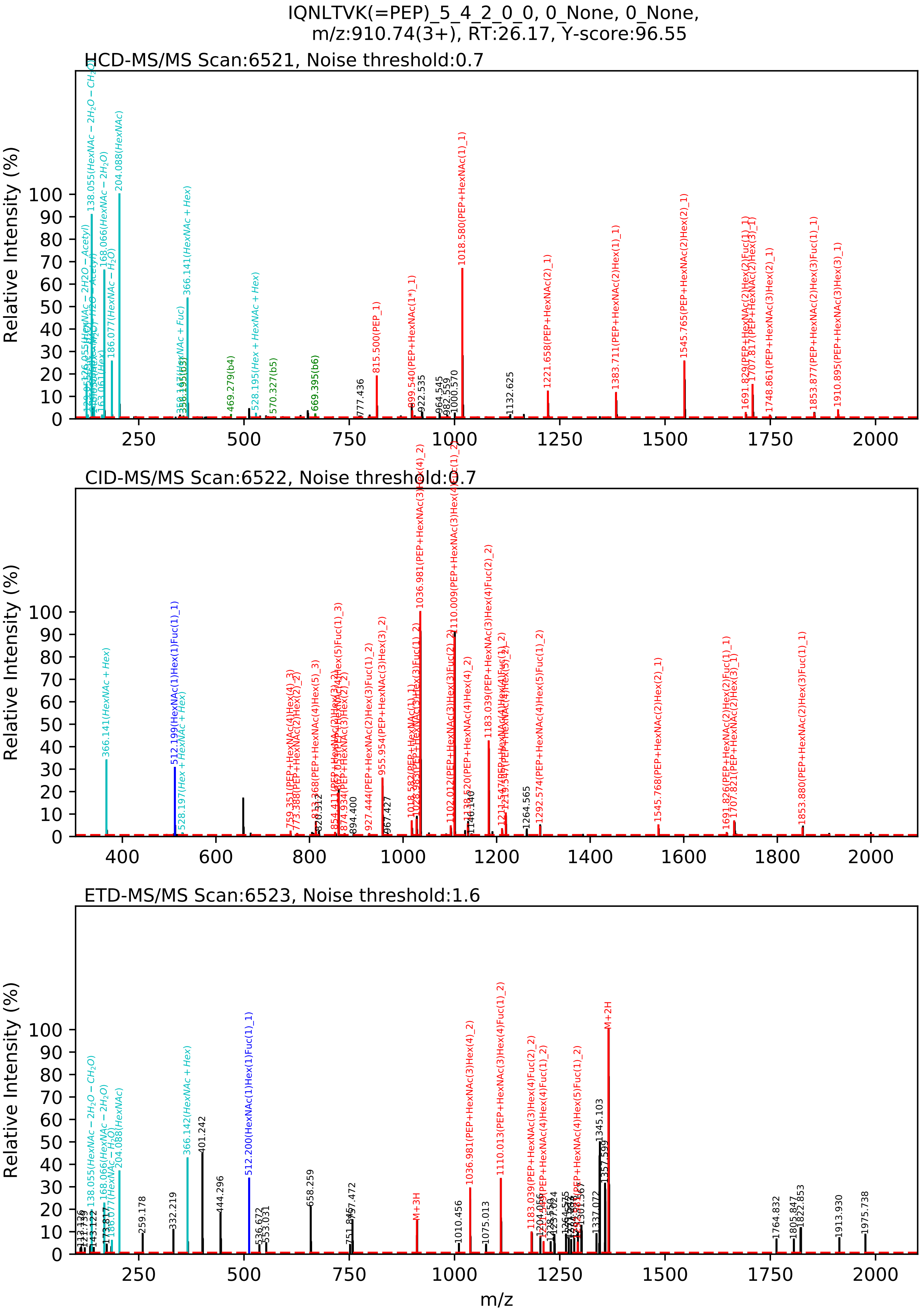
IQNLTVK(=PEP)_5_4_2_0_0, 0_None, 0_None,
m/z:910.74(3+), RT:26.17, Y-score:96.55
HCD-MS/MS Scan:6521, Noise threshold:0.7
CID-MS/MS Scan:6522, Noise threshold:0.7
ETD-MS/MS Scan:6523, Noise threshold:1.6
Relative Intensity (%)
m/z
1018.580(PEP+HexNAc(1)_1)
815.500(PEP_1)
1221.658(PEP+HexNAc(2)_1)
1383.711(PEP+HexNAc(2)Hex(1)_1)
1545.765(PEP+HexNAc(2)Hex(2)_1)
204.088(HexNAc)
366.141(HexNAc + Hex)
1036.981(PEP+HexNAc(3)Hex(4)_2)
1110.009(PEP+HexNAc(3)Hex(4)Fuc(2)_2)
512.199(HexNAc(1)Hex(1)Fuc(1)_1)
M+2H
M+3H
1110.013(PEP+HexNAc(3)Hex(4)Fuc(1)_2)
1345.103

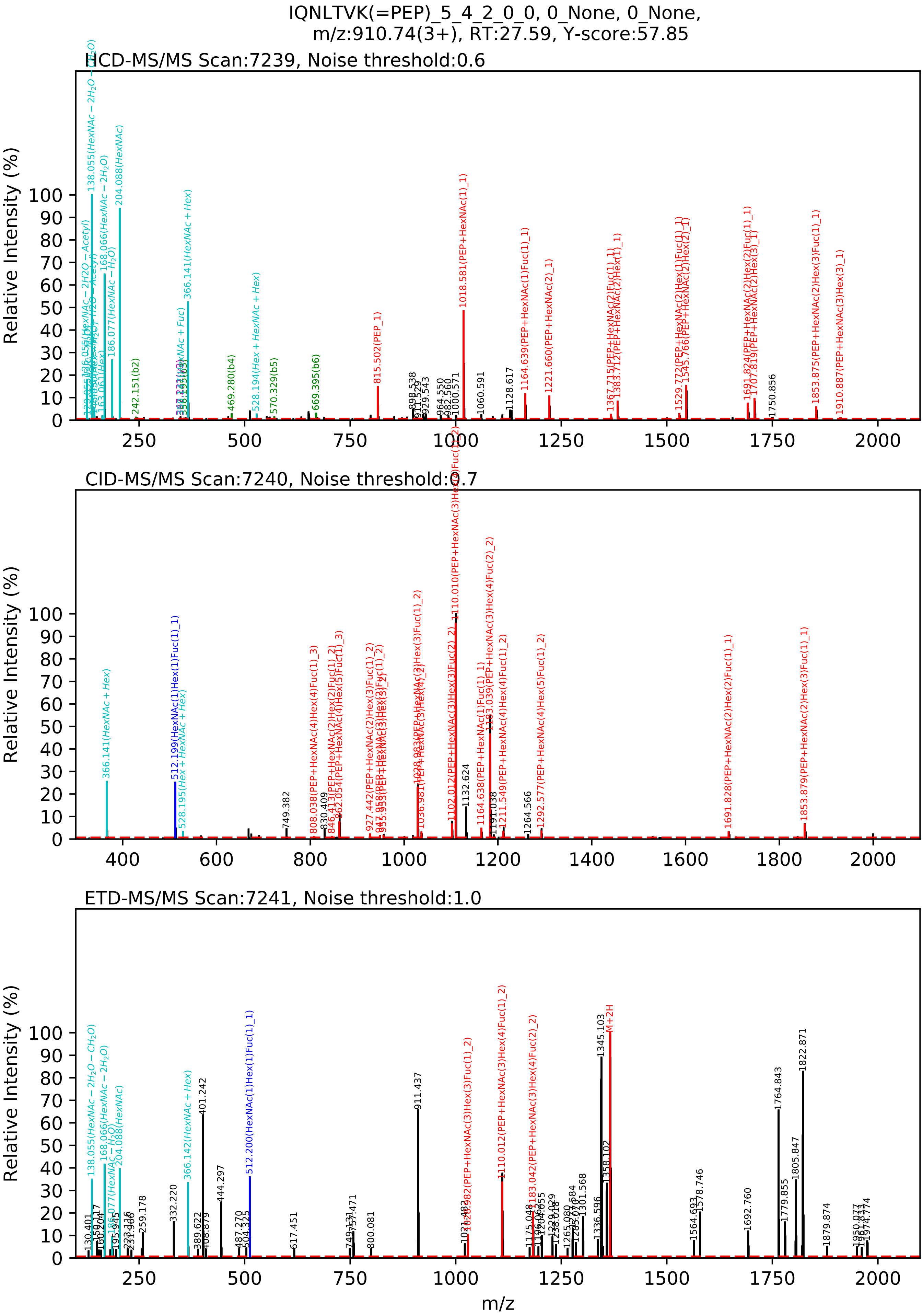
IQNLTVK(=PEP)_5_4_2_0_0, 0_None, 0_None,
m/z:910.74(3+), RT:27.59, Y-score:57.85
HCD-MS/MS Scan:7239, Noise threshold:0.6
Relative Intensity (%)
138.055(HexNAc − 2H2O − CH2O)
204.088(HexNAc)
168.066(HexNAc − 2H2O)
186.077(HexNAc − H2O)
366.141(HexNAc + Hex)
528.194(Hex + HexNAc + Hex)
242.151(b2)
469.280(b4)
570.329(b5)
669.395(b6)
815.502(PEP_1)
1018.581(PEP+HexNAc(1)_1)
1164.639(PEP+HexNAc(1)Fuc(1)_1)
1221.660(PEP+HexNAc(2)_1)
1853.875(PEP+HexNAc(2)Hex(3)Fuc(1)_1)
1910.887(PEP+HexNAc(3)Hex(3)_1)
CID-MS/MS Scan:7240, Noise threshold:0.7
366.141(HexNAc + Hex)
512.199(HexNAc(1)Hex(1)Fuc(1)_1)
528.195(Hex + HexNAc + Hex)
749.382
1132.624
1264.566
1110.010(PEP+HexNAc(3)Hex(4)Fuc(2)_2)
1183.039(PEP+HexNAc(3)Hex(4)Fuc(2)_2)
1691.828(PEP+HexNAc(2)Hex(2)Fuc(1)_1)
1853.879(PEP+HexNAc(2)Hex(3)Fuc(1)_1)
ETD-MS/MS Scan:7241, Noise threshold:1.0
401.242
444.297
512.200(HexNAc(1)Hex(1)Fuc(1)_1)
911.437
1110.012(PEP+HexNAc(3)Hex(4)Fuc(1)_2)
1183.042(PEP+HexNAc(3)Hex(4)Fuc(2)_2)
1345.103
M+2H
1358.102
1764.843
1805.847
1822.871
m/z

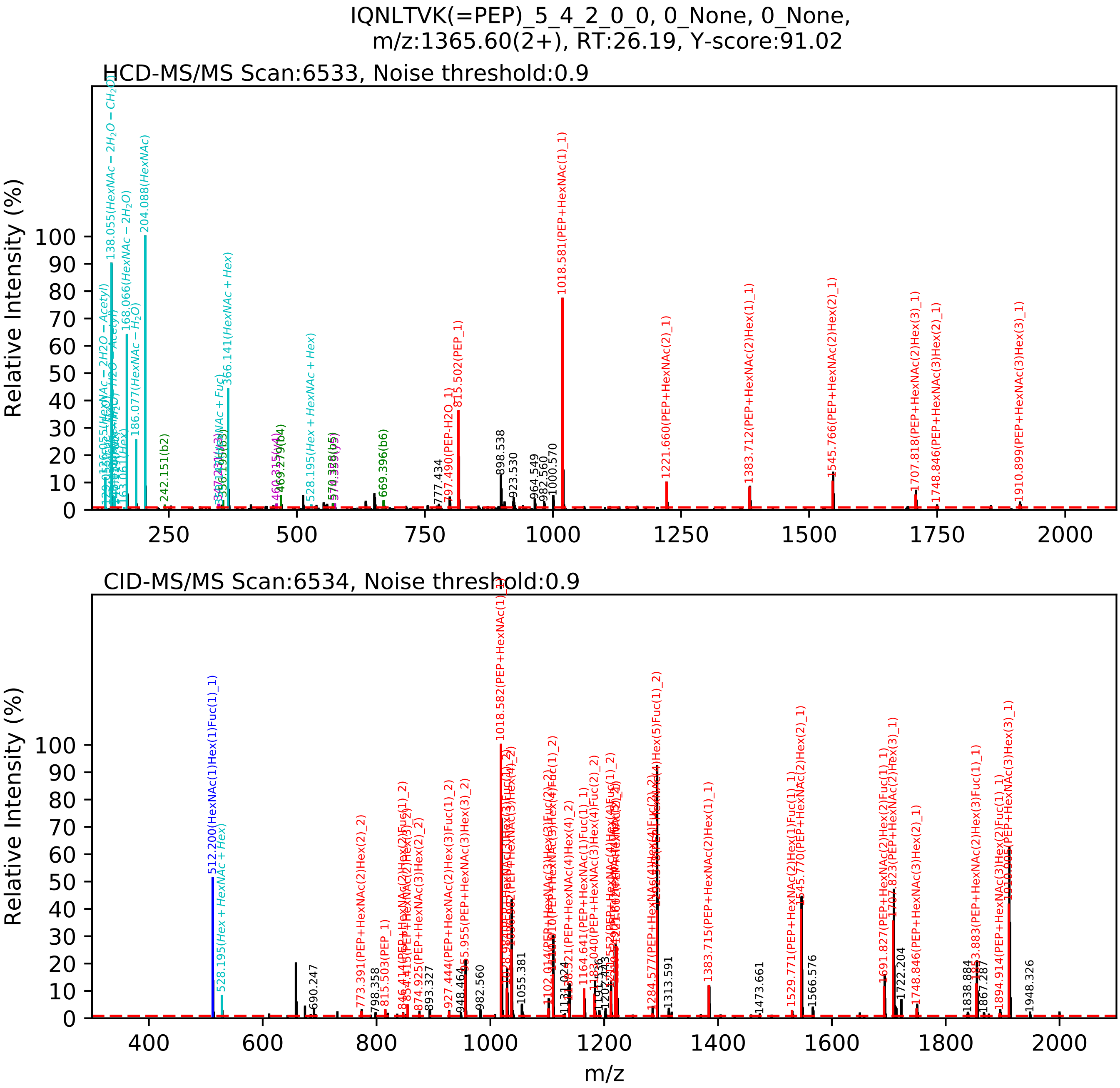
IQNLTVK(=PEP)_5_4_2_0_0, 0_None, 0_None,
m/z:1365.60(2+), RT:26.19, Y-score:91.02
HCD-MS/MS Scan:6533, Noise threshold:0.9
CID-MS/MS Scan:6534, Noise threshold:0.9
Relative Intensity (%)
m/z

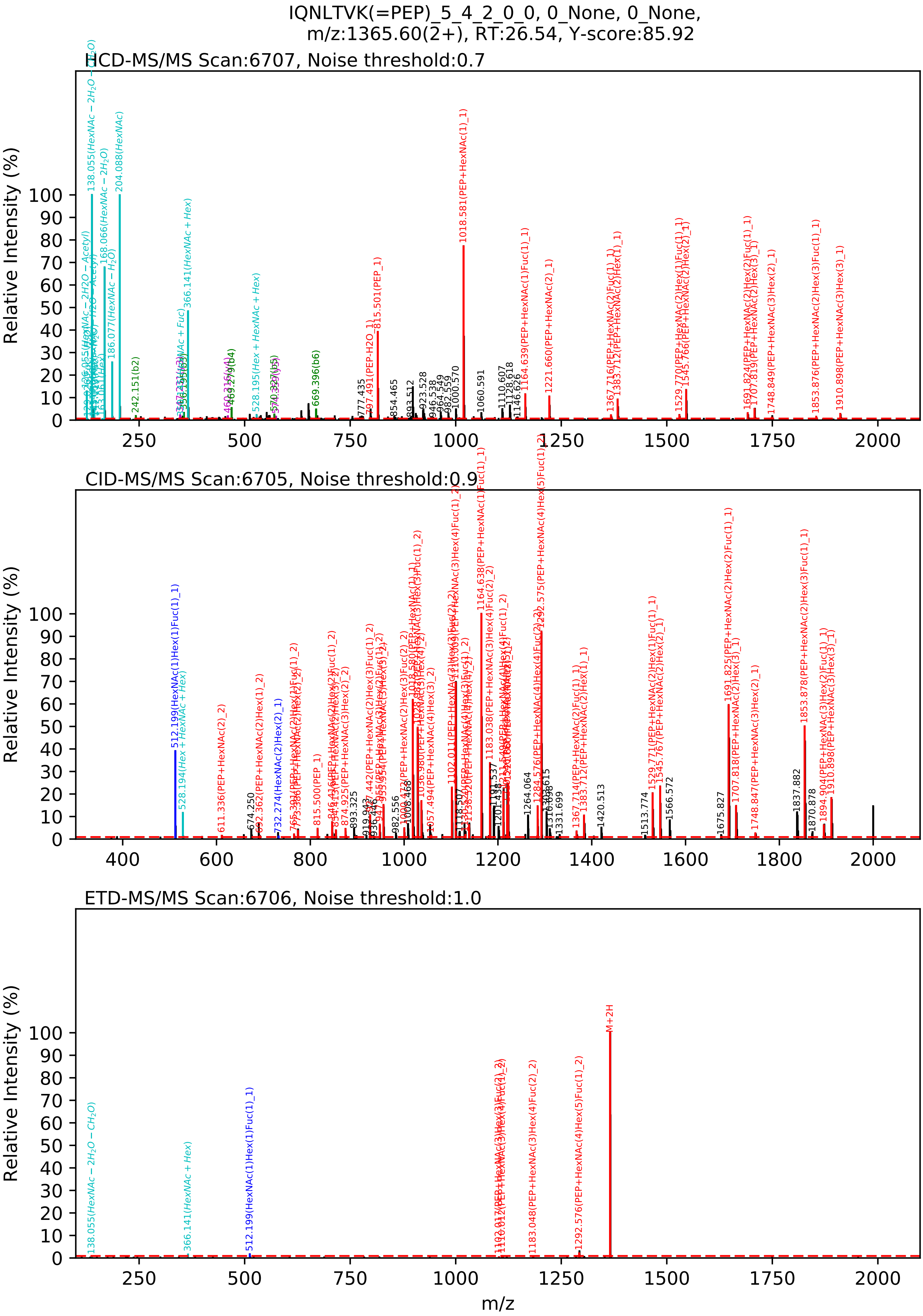
IQNLTVK(=PEP)_5_4_2_0_0, 0_None, 0_None,
m/z:1365.60(2+), RT:26.54, Y-score:85.92
HCD-MS/MS Scan:6707, Noise threshold:0.7
CID-MS/MS Scan:6705, Noise threshold:0.9
ETD-MS/MS Scan:6706, Noise threshold:1.0
Relative Intensity (%)
m/z
M+2H

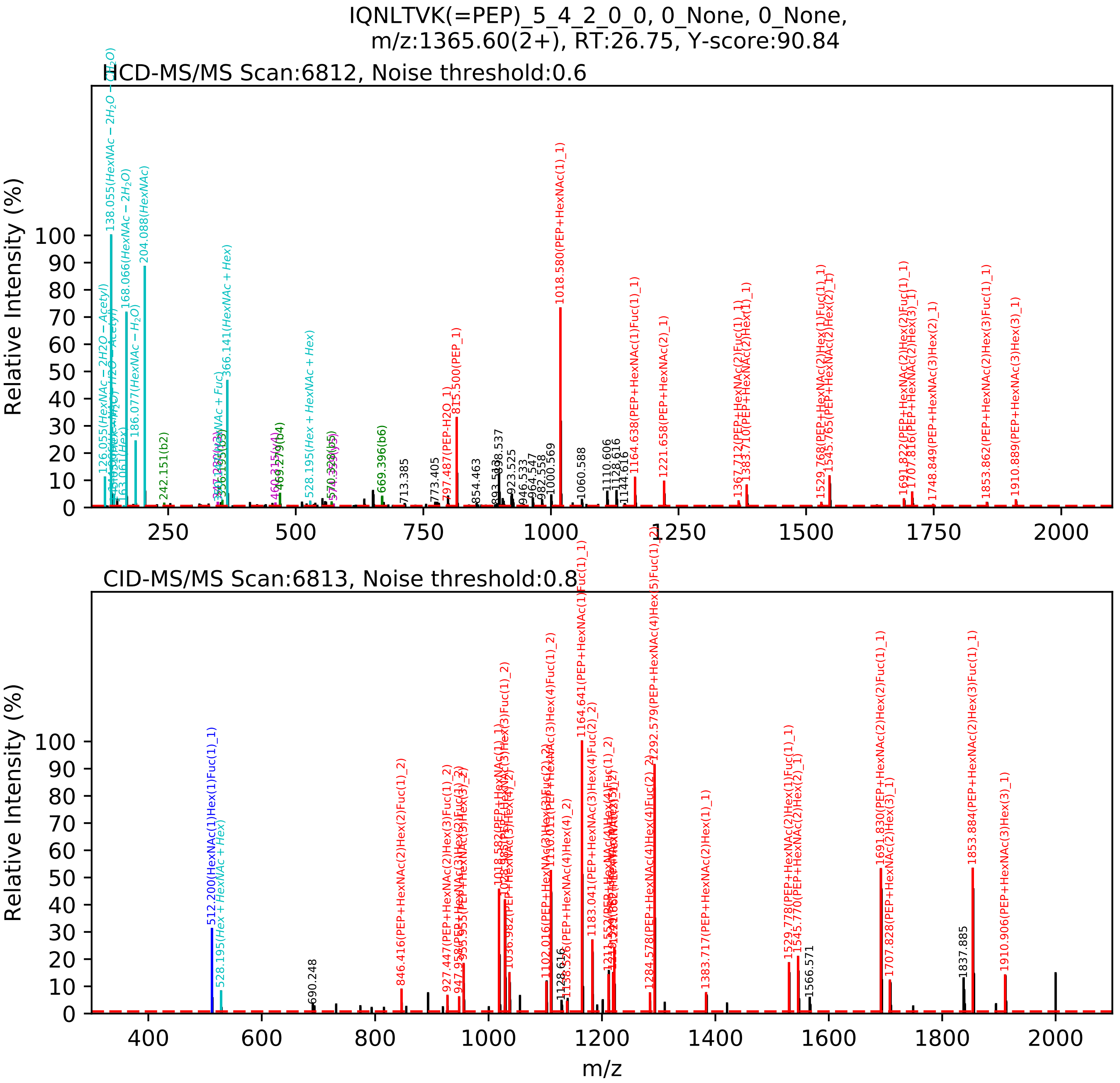

IQNLTVK(=PEP)_5_4_2_0_0, 0_None, 0_None,
m/z:1365.60(2+), RT:26.75, Y-score:90.84
HCD-MS/MS Scan:6812, Noise threshold:0.6
Relative Intensity (%)
138.055(HexNAc)
168.066(HexNAc − 2H2O)
186.077(HexNAc − H2O)
204.088(HexNAc)
242.151(b2)
366.141(HexNAc + Hex)
469.279(b4)
528.195(Hex + HexNAc + Hex)
669.396(b6)
713.385
773.405
797.487(PEP-H2O_1)
815.500(PEP_1)
854.463
898.537
923.525
946.533
964.547
1000.569
1018.580(PEP+HexNAc(1)_1)
1060.588
1110.606
1128.616
1144.616
1164.638(PEP+HexNAc(1)Fuc(1)_1)
1221.658(PEP+HexNAc(2)_1)
1383.710(PEP+HexNAc(2)Hex(1)_1)
1529.768(PEP+HexNAc(2)Hex(1)Fuc(1)_1)
1545.765(PEP+HexNAc(2)Hex(2)_1)
1691.822(PEP+HexNAc(2)Hex(2)Fuc(1)_1)
1707.816(PEP+HexNAc(2)Hex(3)_1)
1748.849(PEP+HexNAc(3)Hex(2)_1)
1853.862(PEP+HexNAc(2)Hex(3)Fuc(1)_1)
1910.889(PEP+HexNAc(3)Hex(3)_1)
CID-MS/MS Scan:6813, Noise threshold:0.8
512.200(HexNAc(1)Hex(1)Fuc(1)_1)
528.195(Hex + HexNAc + Hex)
690.248
846.416(PEP+HexNAc(2)Hex(2)Fuc(1)_2)
927.447(PEP+HexNAc(2)Hex(3)Fuc(1)_2)
1102.016(PEP+HexNAc(3)Hex(4)Fuc(1)_2)
1128.616
1164.641(PEP+HexNAc(1)Fuc(1)_1)
1183.041(PEP+HexNAc(3)Hex(4)Fuc(2)_2)
1284.578(PEP+HexNAc(4)Hex(4)Fuc(2)_2)
1292.579(PEP+HexNAc(4)Hex(5)Fuc(1)_2)
1383.717(PEP+HexNAc(2)Hex(1)_1)
1529.778(PEP+HexNAc(2)Hex(1)Fuc(1)_1)
1545.770(PEP+HexNAc(2)Hex(2)_1)
1566.571
1691.830(PEP+HexNAc(2)Hex(2)Fuc(1)_1)
1707.828(PEP+HexNAc(2)Hex(3)_1)
1837.885
1853.884(PEP+HexNAc(2)Hex(3)Fuc(1)_1)
1910.906(PEP+HexNAc(3)Hex(3)_1)
m/z

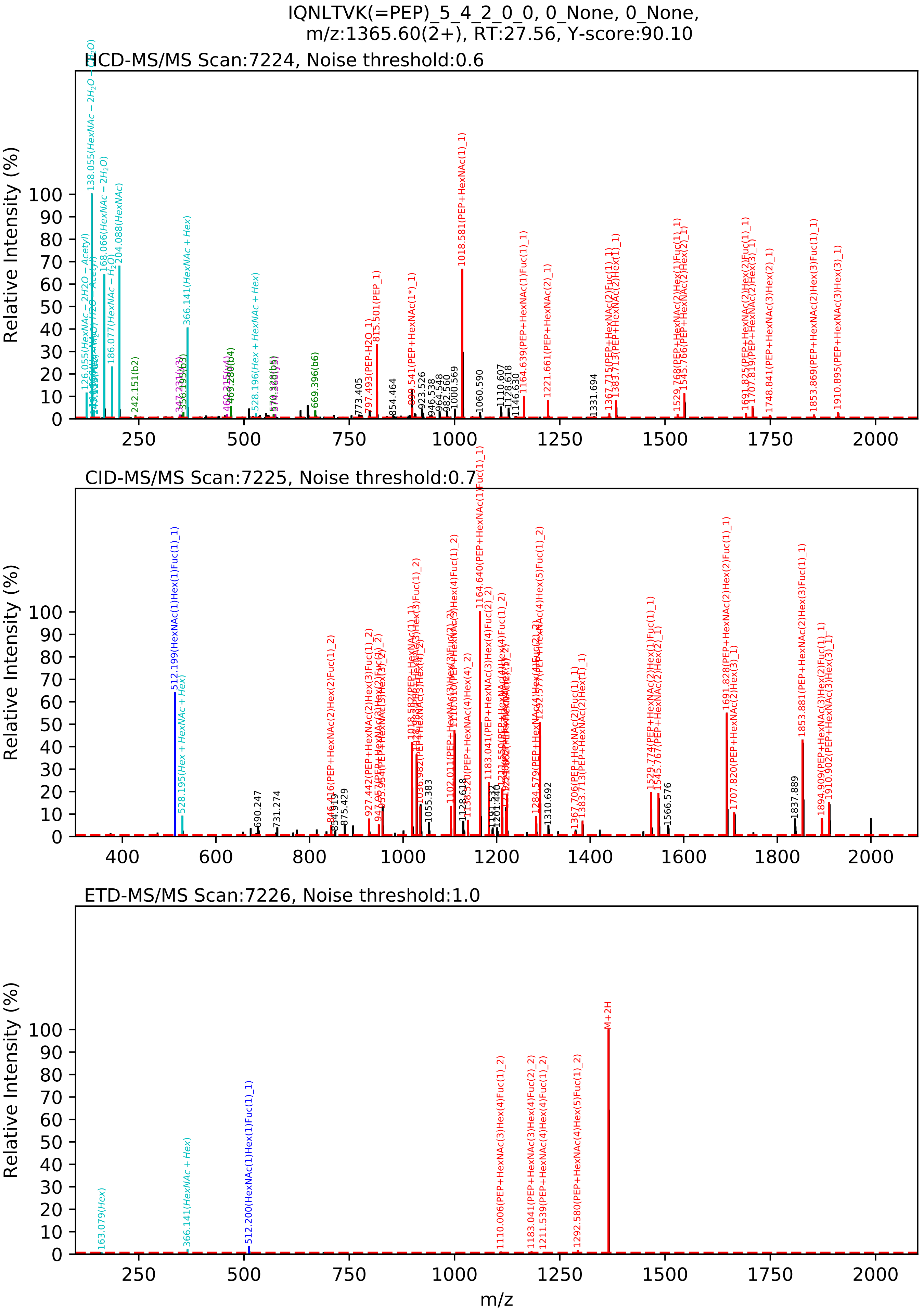
IQNLTVK(=PEP)_5_4_2_0_0, 0_None, 0_None,
m/z:1365.60(2+), RT:27.56, Y-score:90.10
HCD-MS/MS Scan:7224, Noise threshold:0.6
CID-MS/MS Scan:7225, Noise threshold:0.7
ETD-MS/MS Scan:7226, Noise threshold:1.0
Relative Intensity (%)
m/z
1018.581(PEP+HexNAc(1)_1)
1164.640(PEP+HexNAc(1)Fuc(1)_1)
512.199(HexNAc(1)Hex(1)Fuc(1)_1)
1691.828(PEP+HexNAc(2)Hex(2)Fuc(1)_1)
1853.881(PEP+HexNAc(2)Hex(3)Fuc(1)_1)
M+2H
512.200(HexNAc(1)Hex(1)Fuc(1)_1)
1110.006(PEP+HexNAc(3)Hex(4)Fuc(1)_2)
1183.041(PEP+HexNAc(3)Hex(4)Fuc(2)_2)
1211.539(PEP+HexNAc(4)Hex(4)Fuc(1)_2)
1292.580(PEP+HexNAc(4)Hex(5)Fuc(1)_2)

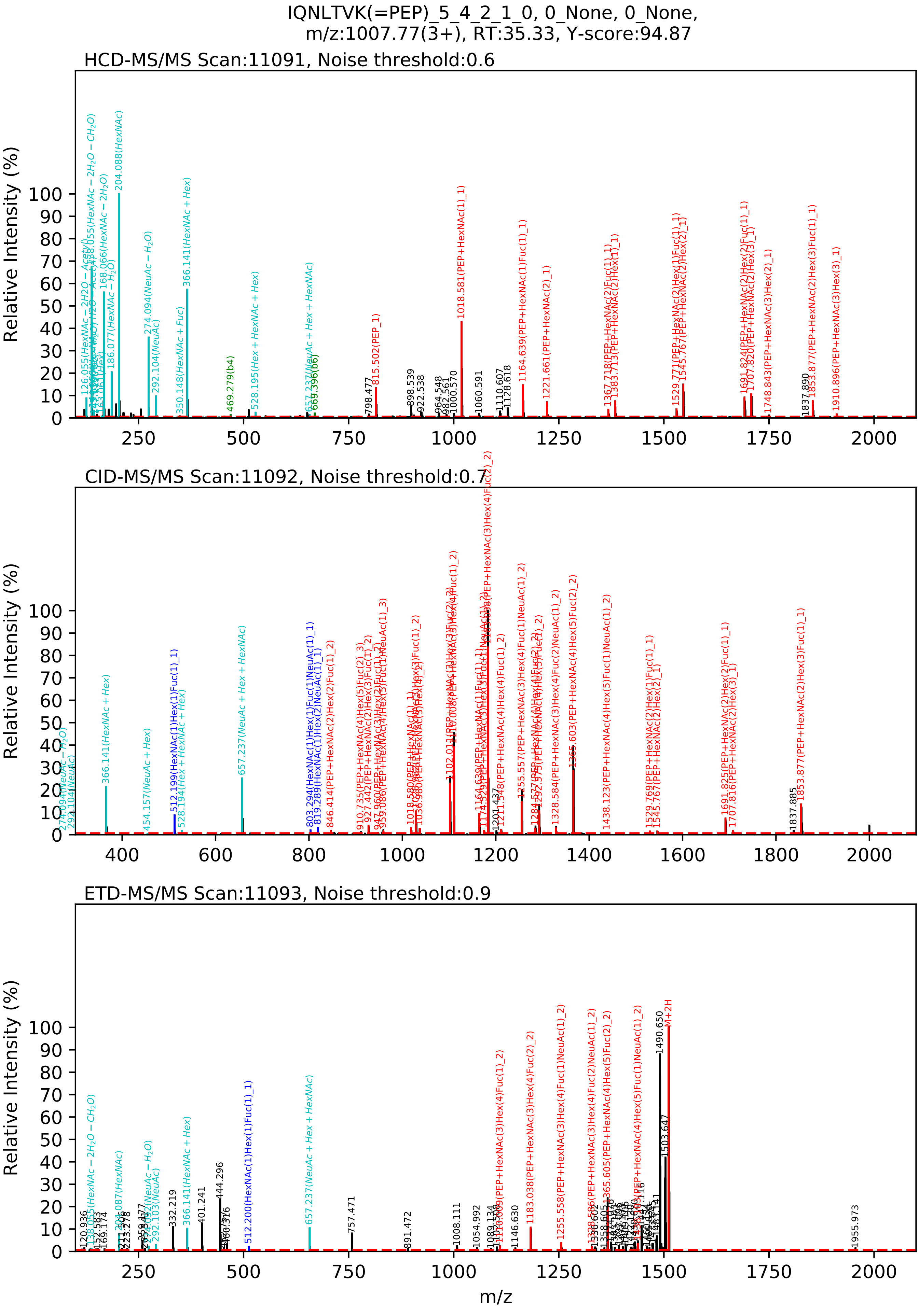

IQNLTVK(=PEP)_5_4_2_1_0, 0_None, 0_None,
m/z:1007.77(3+), RT:35.33, Y-score:94.87
HCD-MS/MS Scan:11091, Noise threshold:0.6
CID-MS/MS Scan:11092, Noise threshold:0.7
ETD-MS/MS Scan:11093, Noise threshold:0.9
Relative Intensity (%)
m/z

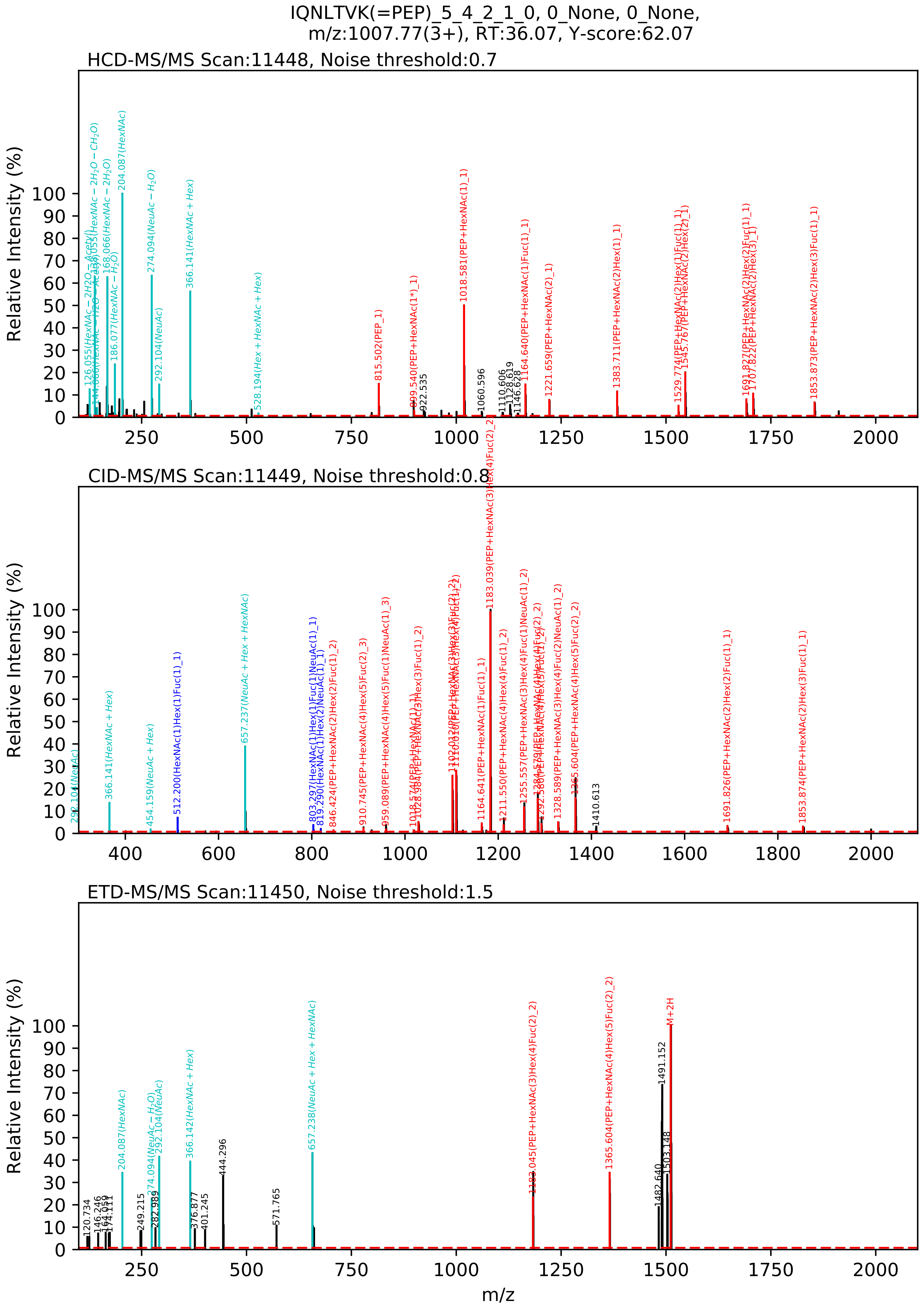
IQNLTVK(=PEP)_5_4_2_1_0, 0_None, 0_None,
m/z:1007.77(3+), RT:36.07, Y-score:62.07
HCD-MS/MS Scan:11448, Noise threshold:0.7
Relative Intensity (%)
204.087(HexNAc)
274.094(NeuAc − H2O)
366.141(HexNAc + Hex)
292.104(NeuAc)
186.077(HexNAc − H2O)
168.066(HexNAc − 2H2O)
528.194(Hex + HexNAc + Hex)
815.502(PEP_1)
899.540(PEP+HexNAc(1*)_1)
922.535
1018.581(PEP+HexNAc(1)_1)
1060.596
1110.606
1128.619
1146.628
1164.640(PEP+HexNAc(1)Fuc(1)_1)
1221.659(PEP+HexNAc(2)_1)
1383.711(PEP+HexNAc(2)Hex(1)_1)
1545.767(PEP+HexNAc(2)Hex(2)_1)
1691.827(PEP+HexNAc(2)Hex(2)Fuc(1)_1)
1707.822(PEP+HexNAc(2)Hex(3)_1)
1853.873(PEP+HexNAc(2)Hex(3)Fuc(1)_1)
CID-MS/MS Scan:11449, Noise threshold:0.8
366.141(HexNAc + Hex)
454.159(NeuAc + Hex)
512.200(HexNAc(1)Hex(1)Fuc(1)_1)
657.237(NeuAc + Hex + HexNAc)
803.297(HexNAc(1)Hex(1)Fuc(1)NeuAc(1)_1)
819.290(HexNAc(1)Hex(2)NeuAc(1)_1)
846.424(PEP+HexNAc(2)Hex(2)Fuc(1)_2)
910.745(PEP+HexNAc(4)Hex(5)Fuc(2)_3)
959.089(PEP+HexNAc(4)Hex(5)Fuc(1)NeuAc(1)_3)
1028.984(PEP+HexNAc(3)Hex(3)Fuc(1)_2)
1164.641(PEP+HexNAc(1)Fuc(1)_1)
1183.039(PEP+HexNAc(3)Hex(4)Fuc(2)_2)
1211.550(PEP+HexNAc(4)Hex(4)Fuc(1)_2)
1255.557(PEP+HexNAc(3)Hex(4)Fuc(1)NeuAc(1)_2)
1328.589(PEP+HexNAc(3)Hex(4)Fuc(2)NeuAc(1)_2)
1365.604(PEP+HexNAc(4)Hex(5)Fuc(2)_2)
1410.613
1691.826(PEP+HexNAc(2)Hex(2)Fuc(1)_1)
1853.874(PEP+HexNAc(2)Hex(3)Fuc(1)_1)
ETD-MS/MS Scan:11450, Noise threshold:1.5
120.734
146.246
164.059
174.111
204.087(HexNAc)
249.215
274.094(NeuAc − H2O)
282.989
292.104(NeuAc)
366.142(HexNAc + Hex)
376.877
401.245
444.296
571.765
657.238(NeuAc + Hex + HexNAc)
1183.045(PEP+HexNAc(3)Hex(4)Fuc(2)_2)
1365.604(PEP+HexNAc(4)Hex(5)Fuc(2)_2)
1482.640
1491.152
1503.148
M+2H
m/z

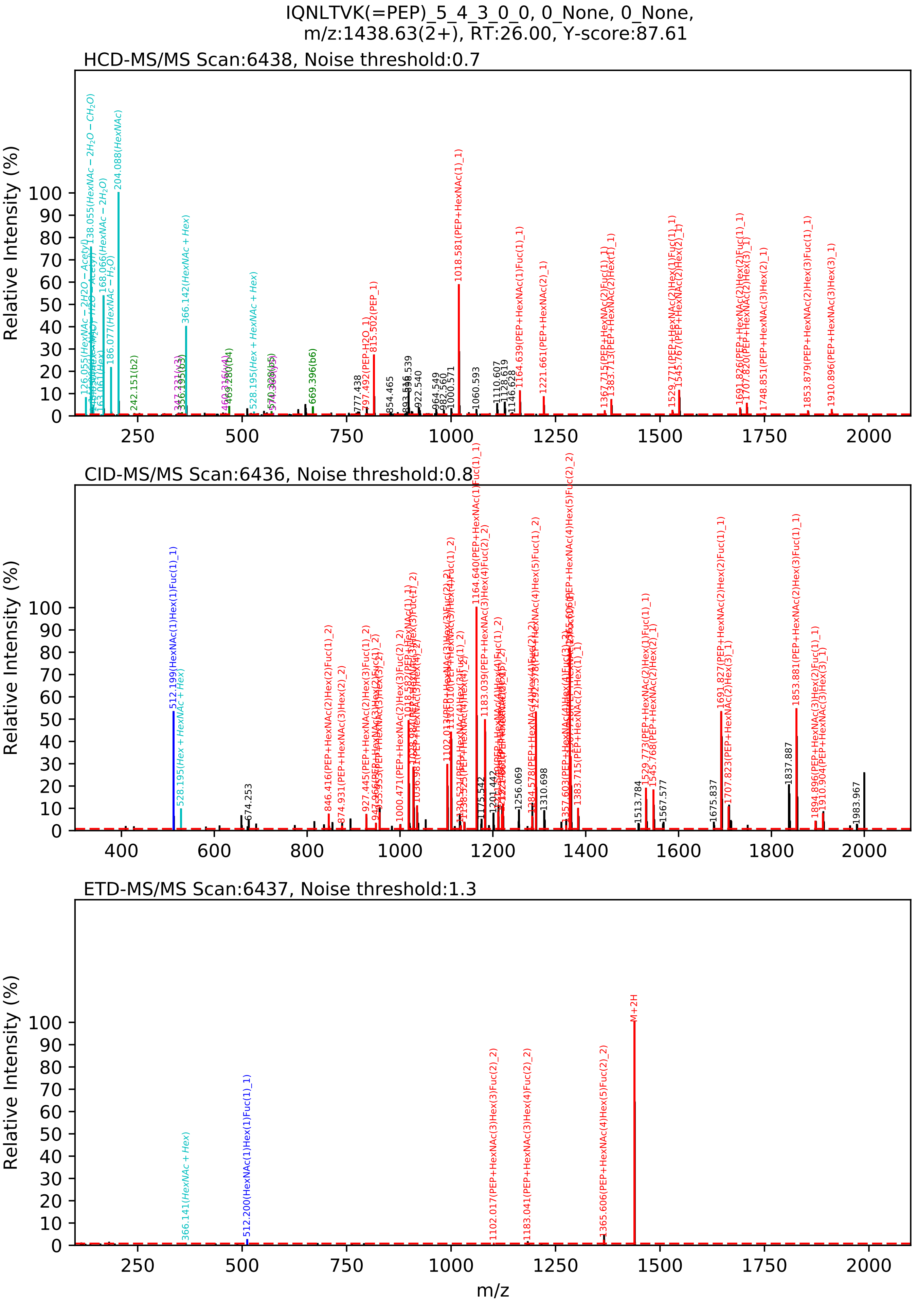
IQNLTVK(=PEP)_5_4_3_0_0, 0_None, 0_None,
m/z:1438.63(2+), RT:26.00, Y-score:87.61
HCD-MS/MS Scan:6438, Noise threshold:0.7
Relative Intensity (%)
204.088(HexNAc)
366.142(HexNAc + Hex)
528.195(Hex + HexNAc + Hex)
242.151(b2)
669.396(b6)
815.502(PEP_1)
797.492(PEP-H2O_1)
1018.581(PEP+HexNAc(1)_1)
1164.639(PEP+HexNAc(1)Fuc(1)_1)
1221.661(PEP+HexNAc(2)_1)
1748.851(PEP+HexNAc(3)Hex(2)_1)
1853.879(PEP+HexNAc(2)Hex(3)Fuc(1)_1)
1910.896(PEP+HexNAc(3)Hex(3)_1)
CID-MS/MS Scan:6436, Noise threshold:0.8
512.199(HexNAc(1)Hex(1)Fuc(1)_1)
1164.640(PEP+HexNAc(1)Fuc(1)_1)
1183.039(PEP+HexNAc(3)Hex(4)Fuc(2)_2)
1691.827(PEP+HexNAc(2)Hex(2)Fuc(1)_1)
1853.881(PEP+HexNAc(2)Hex(3)Fuc(1)_1)
1837.887
1983.967
ETD-MS/MS Scan:6437, Noise threshold:1.3
M+2H
366.141(HexNAc + Hex)
512.200(HexNAc(1)Hex(1)Fuc(1)_1)
1102.017(PEP+HexNAc(3)Hex(3)Fuc(2)_2)
1183.041(PEP+HexNAc(3)Hex(4)Fuc(2)_2)
1365.606(PEP+HexNAc(4)Hex(5)Fuc(2)_2)
m/z

IQNLTVK(=PEP)_5_4_3_0_0, 0_None, 0_None,
m/z:1438.63(2+), RT:26.06, Y-score:92.05

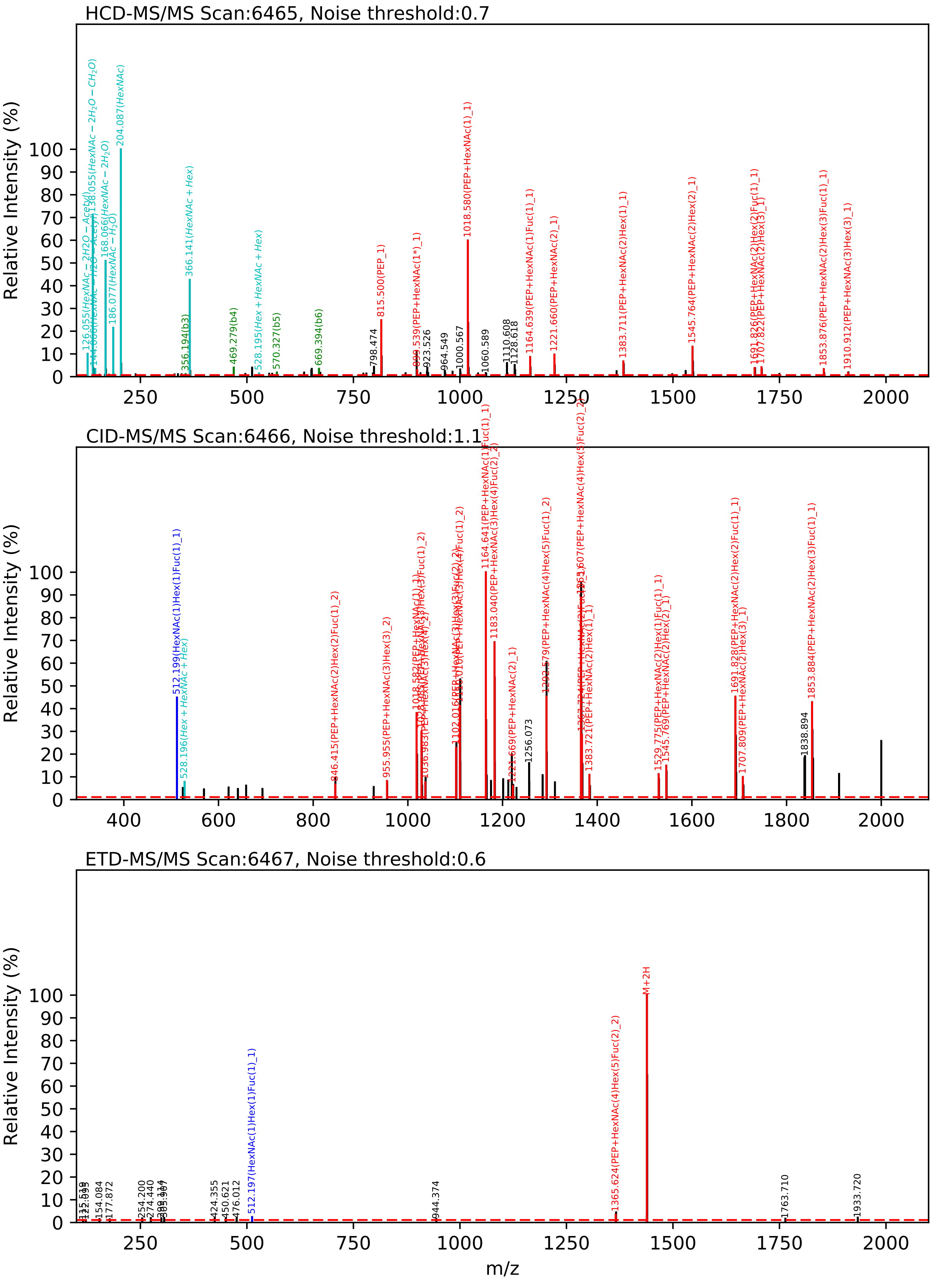

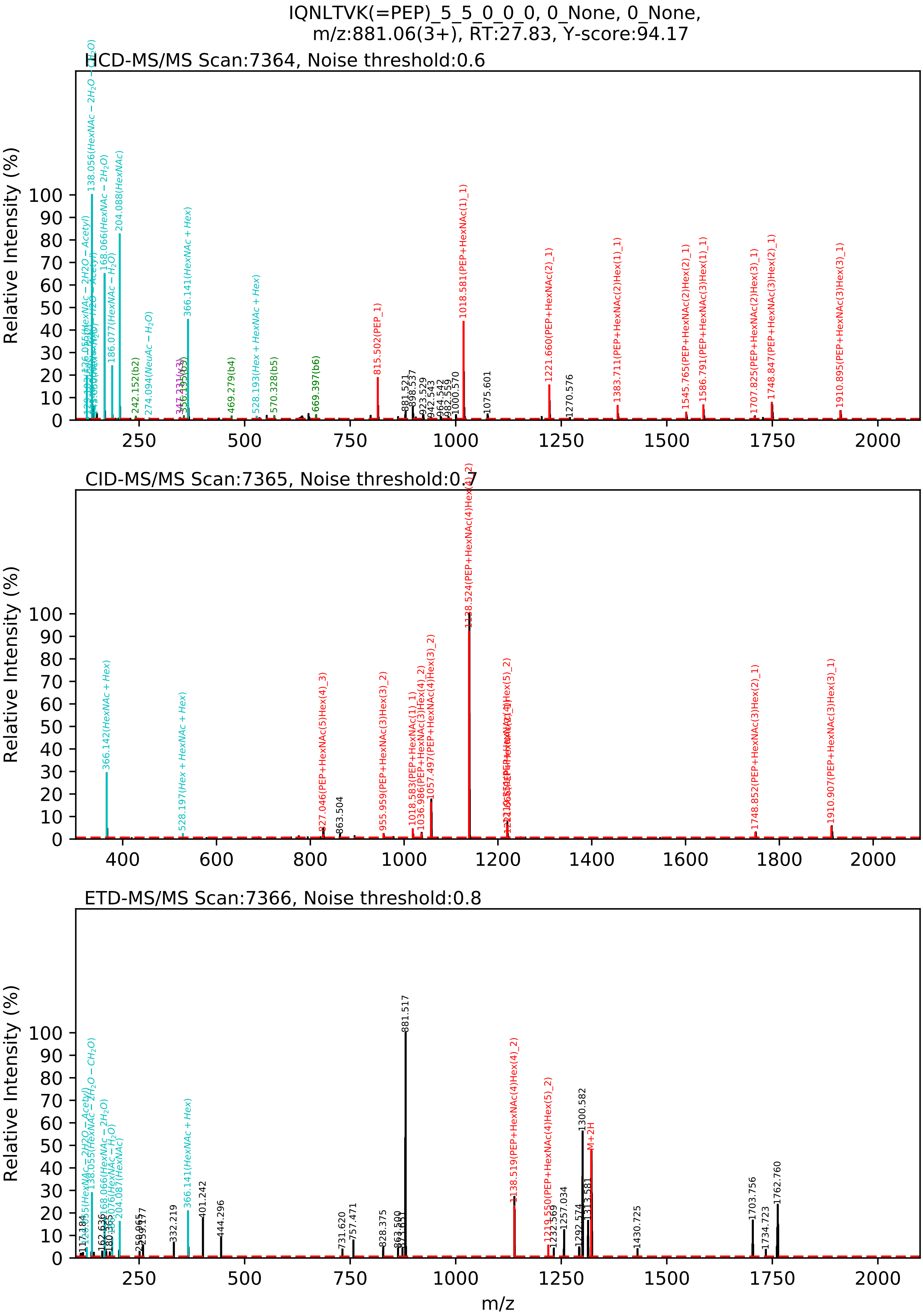

IQNLTVK(=PEP)_5_5_0_0_0, 0_None, 0_None,
m/z:881.06(3+), RT:27.83, Y-score:94.17
HCD-MS/MS Scan:7364, Noise threshold:0.6
Relative Intensity (%)
138.056(HexNAc − 2H2O − CH2O)
204.088(HexNAc)
168.066(HexNAc − 2H2O)
186.077(HexNAc − H2O)
366.141(HexNAc + Hex)
274.094(NeuAc − H2O)
242.152(b2)
469.279(b4)
528.193(Hex + HexNAc + Hex)
570.328(b5)
669.397(b6)
815.502(PEP_1)
881.521
898.537
923.529
942.543
964.542
1000.570
1018.581(PEP+HexNAc(1)_1)
1075.601
1221.660(PEP+HexNAc(2)_1)
1270.576
1383.711(PEP+HexNAc(2)Hex(1)_1)
1545.765(PEP+HexNAc(2)Hex(2)_1)
1586.791(PEP+HexNAc(3)Hex(1)_1)
1707.825(PEP+HexNAc(2)Hex(3)_1)
1748.847(PEP+HexNAc(3)Hex(2)_1)
1910.895(PEP+HexNAc(3)Hex(3)_1)
CID-MS/MS Scan:7365, Noise threshold:0.7
366.142(HexNAc + Hex)
528.197(Hex + HexNAc + Hex)
827.046(PEP+HexNAc(5)Hex(4)_3)
863.504
955.959(PEP+HexNAc(3)Hex(3)_2)
1018.583(PEP+HexNAc(1)_1)
1036.986(PEP+HexNAc(3)Hex(4)_2)
1057.497(PEP+HexNAc(4)Hex(3)_2)
1138.524(PEP+HexNAc(4)Hex(4)_2)
1748.852(PEP+HexNAc(3)Hex(2)_1)
1910.907(PEP+HexNAc(3)Hex(3)_1)
ETD-MS/MS Scan:7366, Noise threshold:0.8
117.184
138.055(HexNAc − 2H2O − CH2O)
162.636
168.066(HexNAc − 2H2O)
204.087(HexNAc)
332.219
366.141(HexNAc + Hex)
401.242
444.296
731.620
757.471
828.375
881.517
1138.519(PEP+HexNAc(4)Hex(4)_2)
1219.550(PEP+HexNAc(4)Hex(5)_2)
1232.569
1257.034
1292.574
1300.582
1313.581
M+2H
1430.725
1703.756
1734.723
1762.760
m/z

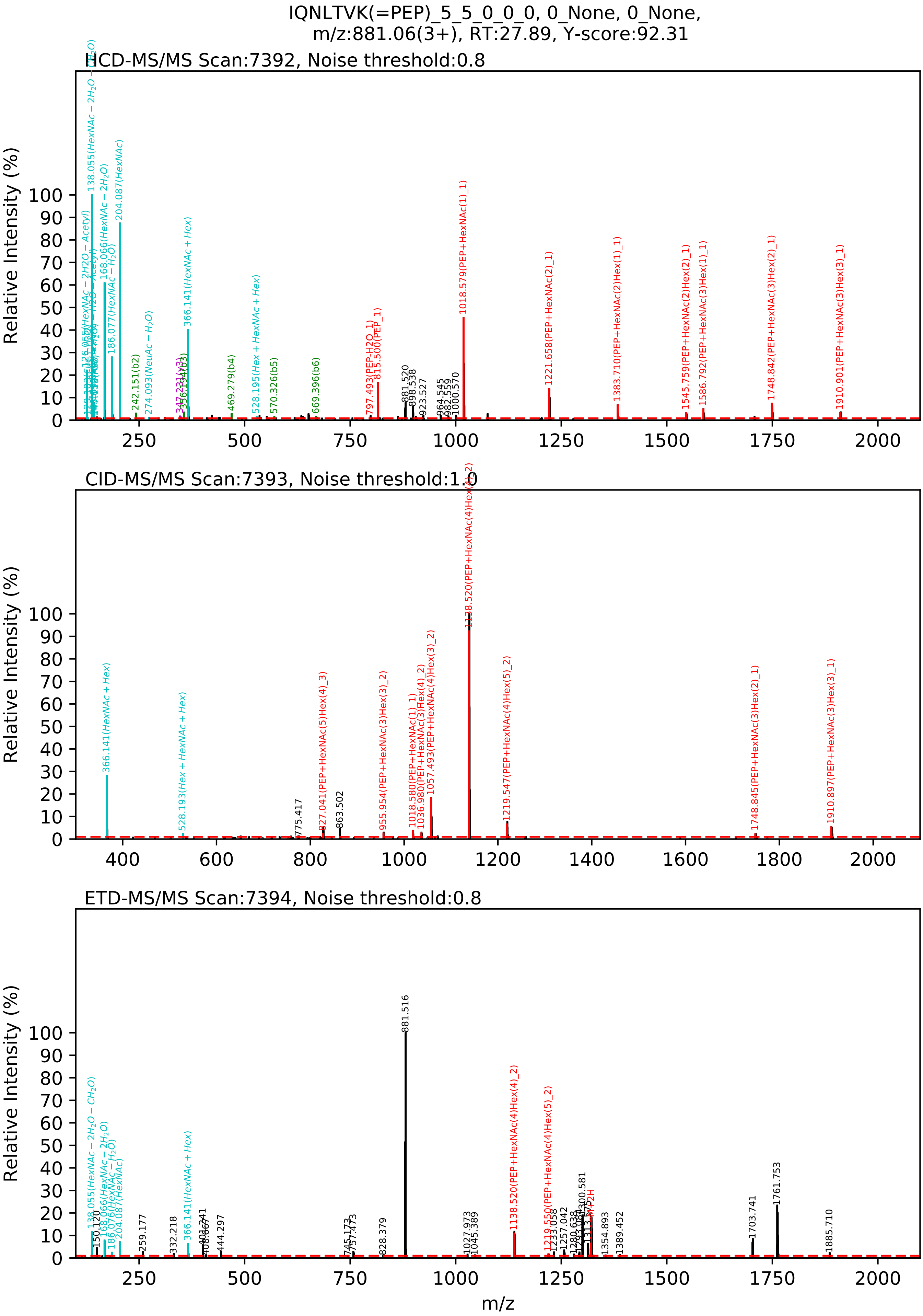

IQNLTVK(=PEP)_5_5_0_0_0, 0_None, 0_None,
m/z:881.06(3+), RT:27.89, Y-score:92.31
HCD-MS/MS Scan:7392, Noise threshold:0.8
CID-MS/MS Scan:7393, Noise threshold:1.0
ETD-MS/MS Scan:7394, Noise threshold:0.8
Relative Intensity (%)
m/z
138.055(HexNAc − 2H2O − H2O)
204.087(HexNAc)
168.066(HexNAc − 2H2O)
186.077(HexNAc − H2O)
366.141(HexNAc + Hex)
528.195(Hex + HexNAc + Hex)
242.151(b2)
274.093(NeuAc − H2O)
469.279(b4)
570.326(b5)
669.396(b6)
797.493(PEP-H2O_1)
815.500(PEP_1)
881.520
898.538
923.527
964.545
1000.570
1018.579(PEP+HexNAc(1)_1)
1221.658(PEP+HexNAc(2)_1)
1383.710(PEP+HexNAc(2)Hex(1)_1)
1545.759(PEP+HexNAc(2)Hex(2)_1)
1586.792(PEP+HexNAc(3)Hex(1)_1)
1748.842(PEP+HexNAc(3)Hex(2)_1)
1910.901(PEP+HexNAc(3)Hex(3)_1)
366.141(HexNAc + Hex)
528.193(Hex + HexNAc + Hex)
775.417
827.041(PEP+HexNAc(5)Hex(4)_3)
863.502
955.954(PEP+HexNAc(3)Hex(3)_2)
1018.580(PEP+HexNAc(1)_1)
1036.980(PEP+HexNAc(3)Hex(4)_2)
1057.493(PEP+HexNAc(4)Hex(3)_2)
1138.520(PEP+HexNAc(4)Hex(4)_2)
1219.547(PEP+HexNAc(4)Hex(5)_2)
1748.845(PEP+HexNAc(3)Hex(2)_1)
1910.897(PEP+HexNAc(3)Hex(3)_1)
138.055(HexNAc − 2H2O − CH2O)
168.066(HexNAc − 2H2O)
186.076(HexNAc − H2O)
204.087(HexNAc)
150.120
259.177
332.218
366.141(HexNAc + Hex)
401.241
444.297
745.173
757.473
828.379
881.516
1027.973
1045.389
1138.520(PEP+HexNAc(4)Hex(4)_2)
1219.550(PEP+HexNAc(4)Hex(5)_2)
1233.058
1257.042
1280.638
1300.581
M-2H
1354.893
1389.452
1703.741
1761.753
1885.710

IQNLTVK(=PEP)_5_5_0_0_0, 0_None, 0_None,
m/z:1321.08(2+), RT:26.92, Y-score:68.86

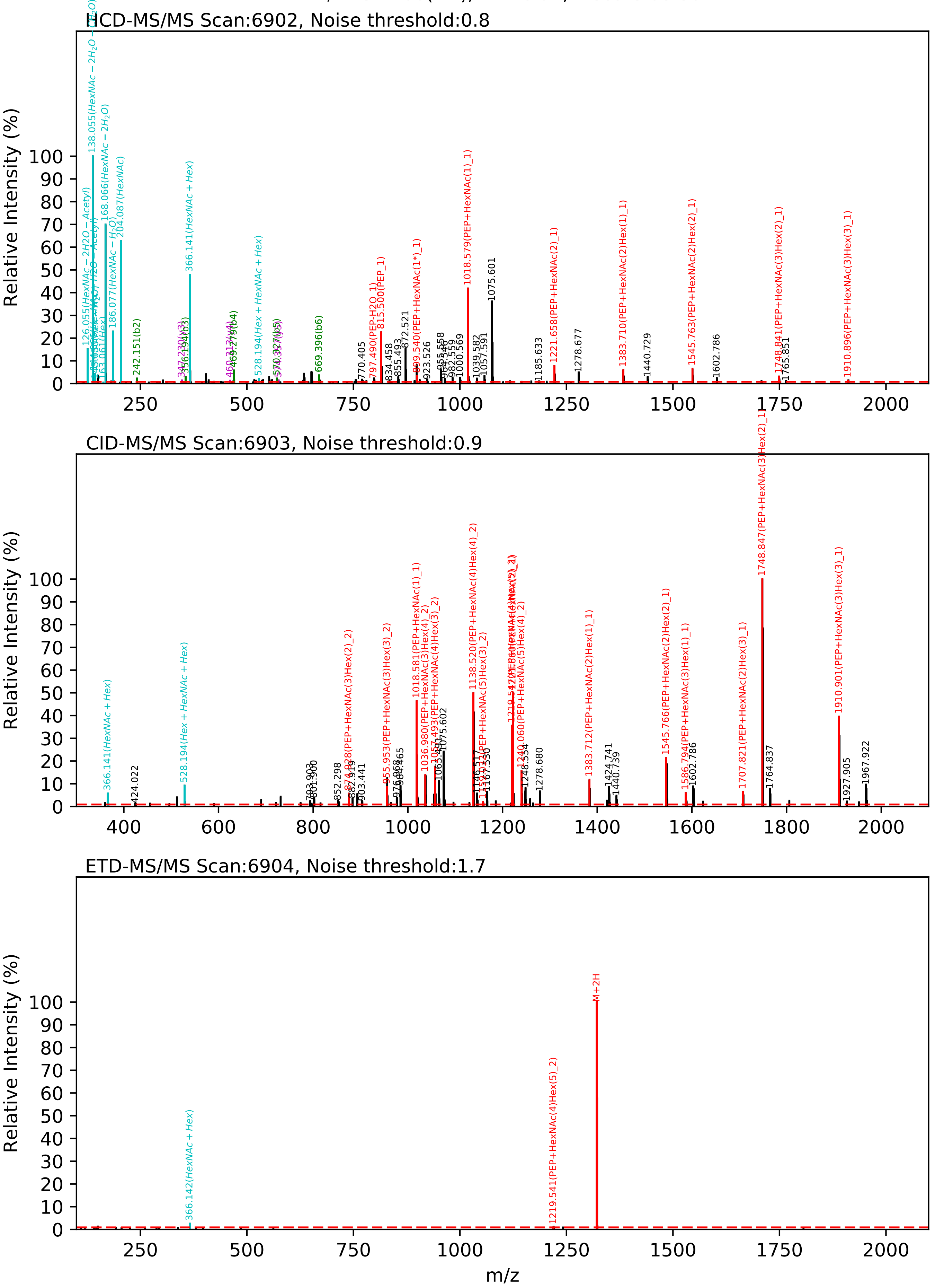

IQNLTVK(=PEP)_5_5_0_0_0, 0_None, 0_None,
m/z:1321.08(2+), RT:27.75, Y-score:94.92

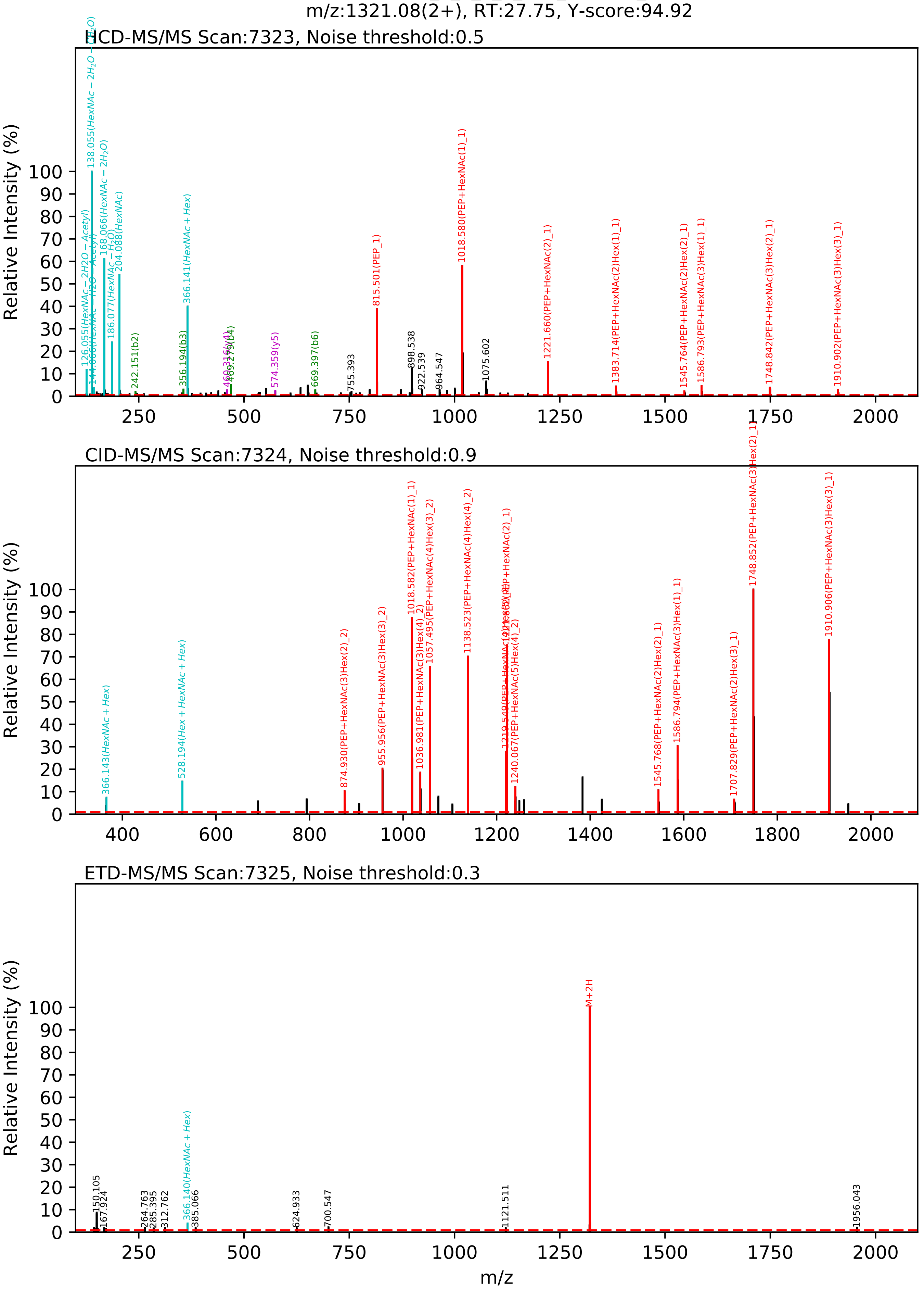

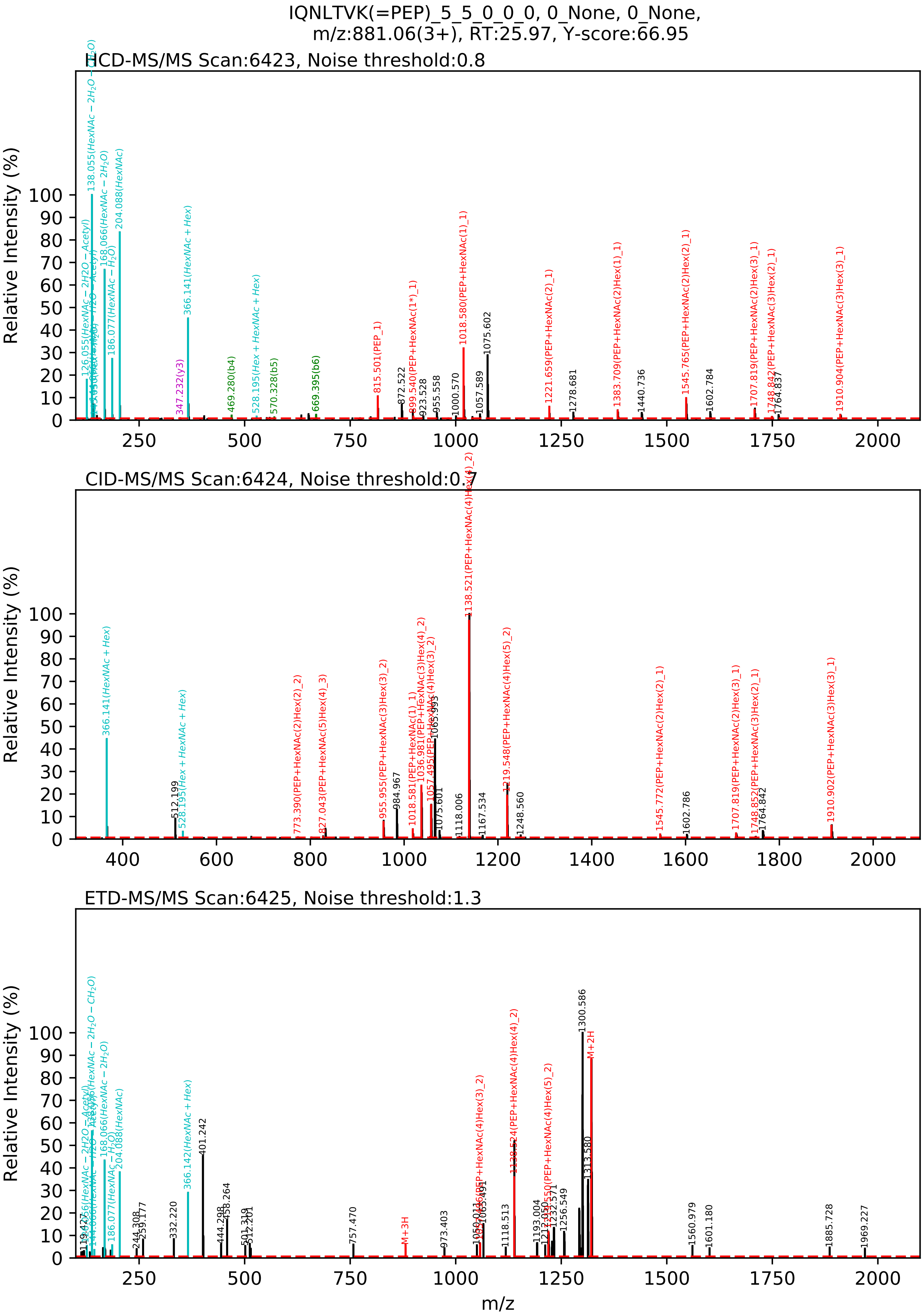
IQNLTVK(=PEP)_5_5_0_0_0, 0_None, 0_None,
m/z:881.06(3+), RT:25.97, Y-score:66.95
HCD-MS/MS Scan:6423, Noise threshold:0.8
CID-MS/MS Scan:6424, Noise threshold:0.7
ETD-MS/MS Scan:6425, Noise threshold:1.3
Relative Intensity (%)
m/z

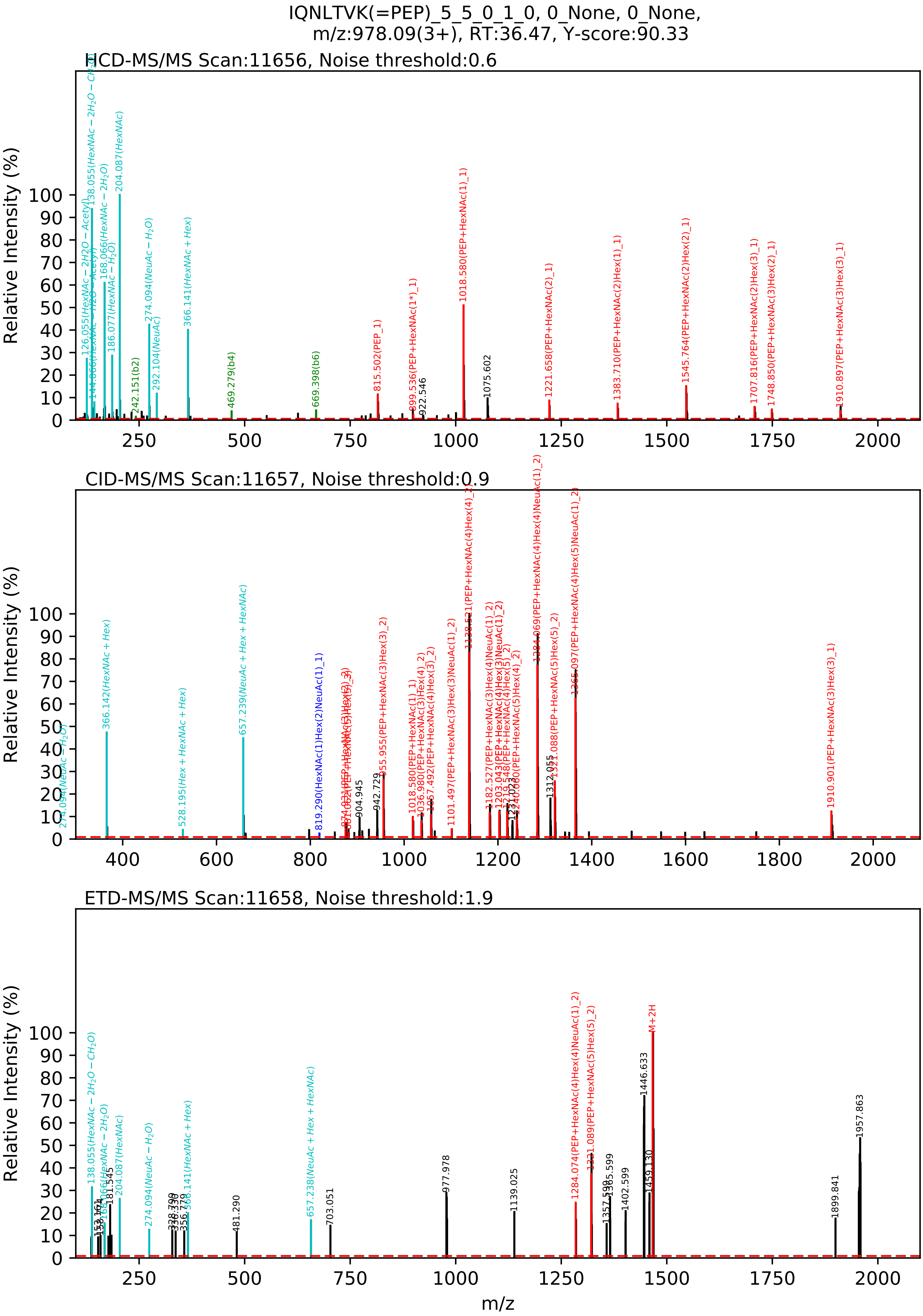
IQNLTVK(=PEP)_5_5_0_1_0, 0_None, 0_None,
m/z:978.09(3+), RT:36.47, Y-score:90.33
HCD-MS/MS Scan:11656, Noise threshold:0.6
Relative Intensity (%)
126.055(HexNAc − 2H2O − Acetyl)
138.055(HexNAc − 2H2O − CH2O)
144.066(HexNAc − H2O − Acetyl)
168.066(HexNAc − 2H2O)
186.077(HexNAc − H2O)
204.087(HexNAc)
242.151(b2)
274.094(NeuAc − H2O)
292.104(NeuAc)
366.141(HexNAc + Hex)
469.279(b4)
669.398(b6)
815.502(PEP_1)
899.536(PEP+HexNAc(1*)_1)
922.546
1018.580(PEP+HexNAc(1)_1)
1075.602
1221.658(PEP+HexNAc(2)_1)
1383.710(PEP+HexNAc(2)Hex(1)_1)
1545.764(PEP+HexNAc(2)Hex(2)_1)
1707.816(PEP+HexNAc(2)Hex(3)_1)
1748.850(PEP+HexNAc(3)Hex(2)_1)
1910.897(PEP+HexNAc(3)Hex(3)_1)
CID-MS/MS Scan:11657, Noise threshold:0.9
274.094(NeuAc − H2O)
366.142(HexNAc + Hex)
528.195(Hex + HexNAc + Hex)
657.239(NeuAc + Hex + HexNAc)
819.290(HexNAc(1)Hex(2)NeuAc(1)_1)
904.945
942.729
955.955(PEP+HexNAc(3)Hex(3)_2)
1018.580(PEP+HexNAc(1)_1)
1036.980(PEP+HexNAc(3)Hex(4)_2)
1057.492(PEP+HexNAc(4)Hex(3)_2)
1101.497(PEP+HexNAc(3)Hex(3)NeuAc(1)_2)
1138.521(PEP+HexNAc(4)Hex(4)_2)
1182.527(PEP+HexNAc(3)Hex(4)NeuAc(1)_2)
1203.043(PEP+HexNAc(4)Hex(3)NeuAc(1)_2)
1219.548(PEP+HexNAc(4)Hex(5)_2)
1240.060(PEP+HexNAc(5)Hex(4)_2)
1284.069(PEP+HexNAc(4)Hex(4)NeuAc(1)_2)
1312.055
1321.088(PEP+HexNAc(5)Hex(5)_2)
1365.097(PEP+HexNAc(4)Hex(5)NeuAc(1)_2)
1910.901(PEP+HexNAc(3)Hex(3)_1)
ETD-MS/MS Scan:11658, Noise threshold:1.9
138.055(HexNAc − 2H2O − CH2O)
168.066(HexNAc − 2H2O)
181.545
204.087(HexNAc)
274.094(NeuAc − H2O)
366.141(HexNAc + Hex)
481.290
657.238(NeuAc + Hex + HexNAc)
703.051
977.978
1139.025
1284.074(PEP+HexNAc(4)Hex(4)NeuAc(1)_2)
1321.089(PEP+HexNAc(5)Hex(5)_2)
1365.599
1402.599
1446.633
1459.130
M+2H
1899.841
1957.863
m/z

IQNLTVK(=PEP)_5_5_0_1_0, 0_None, 0_None,
m/z:978.09(3+), RT:35.13, Y-score:96.53

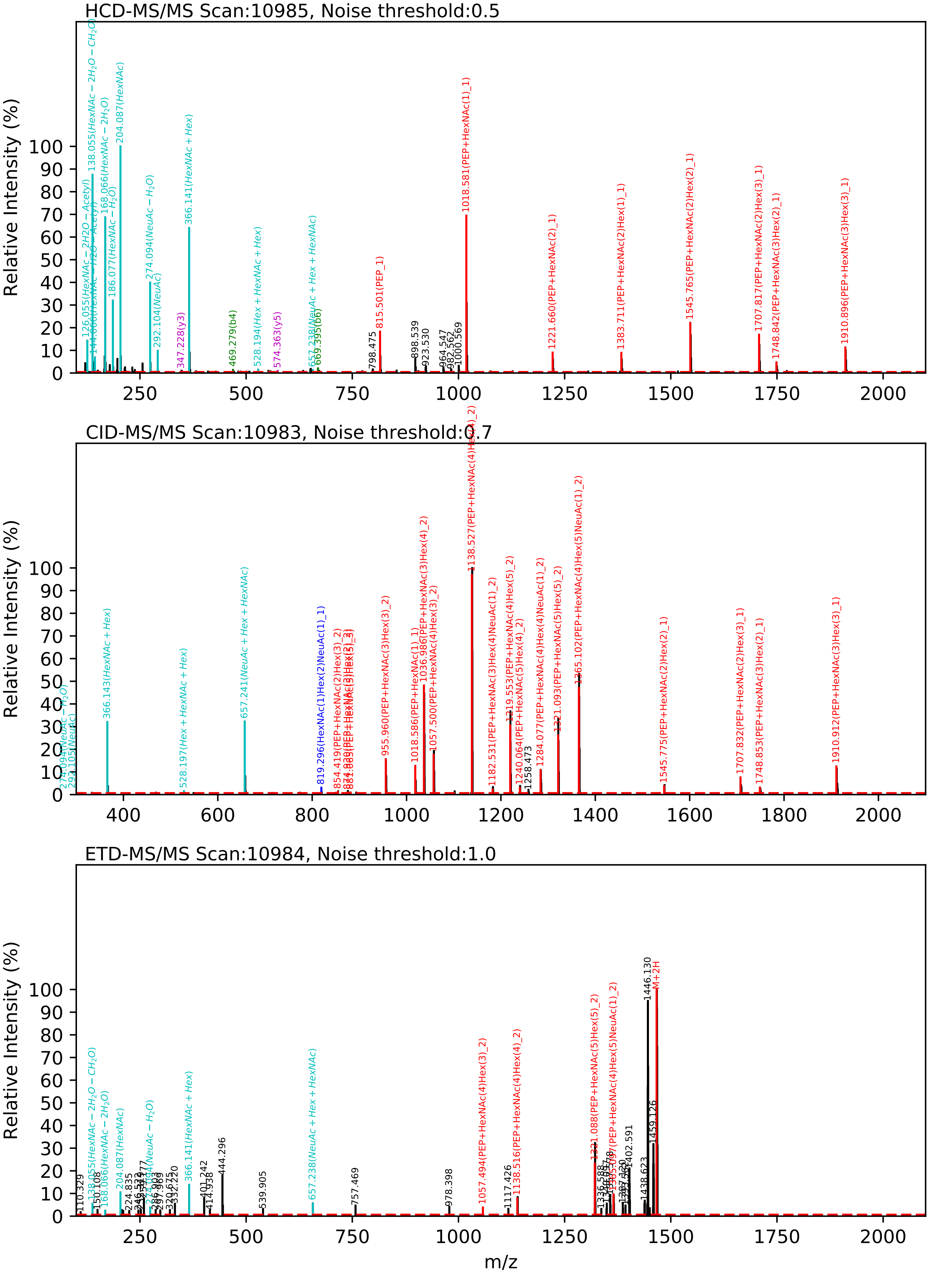

IQNLTVK(=PEP)_5_5_0_1_0, 0_None, 0_None,
m/z:978.09(3+), RT:35.73, Y-score:94.68

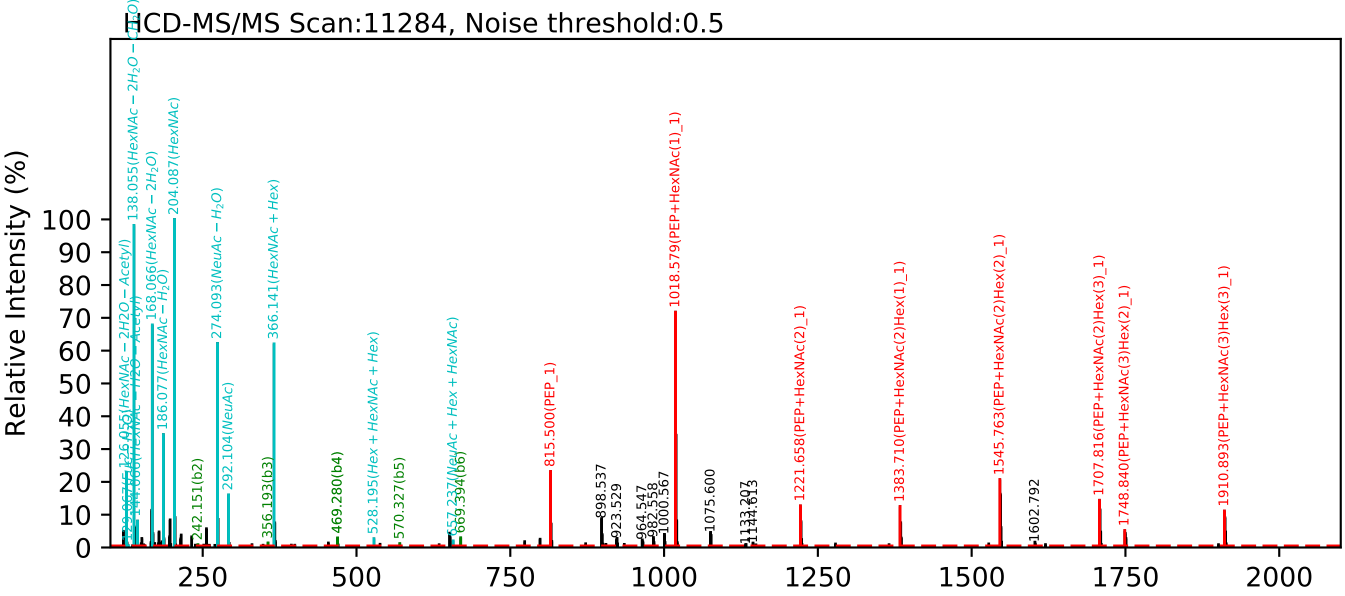

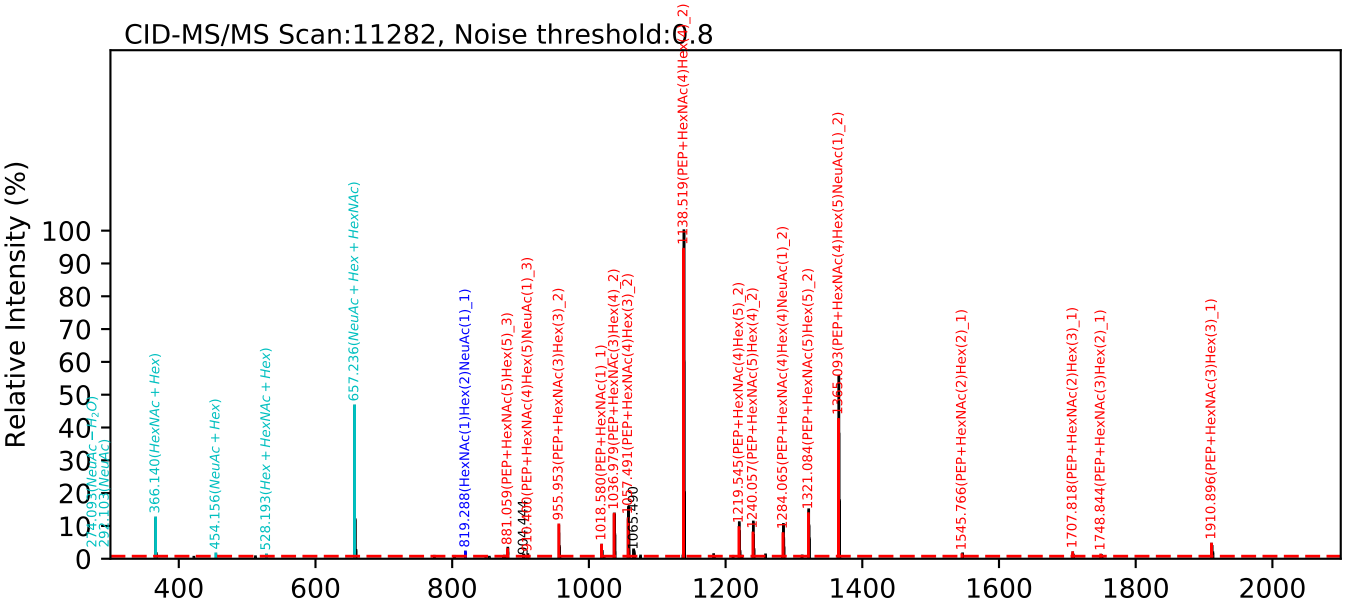

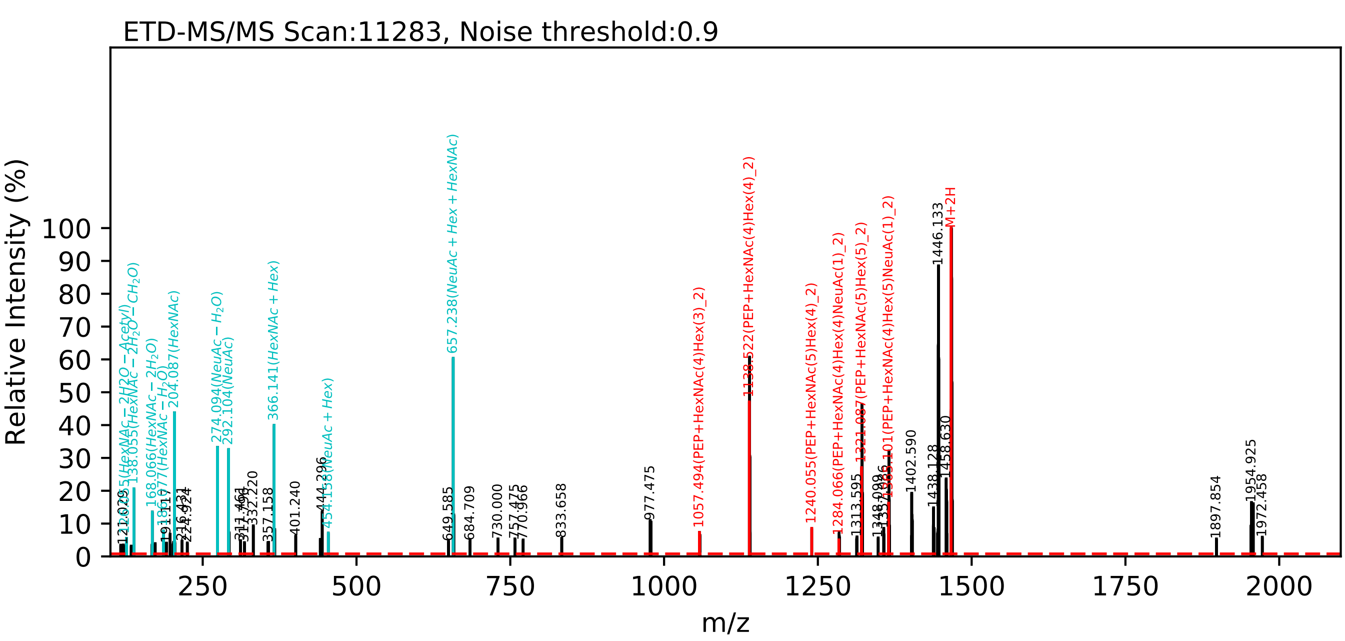

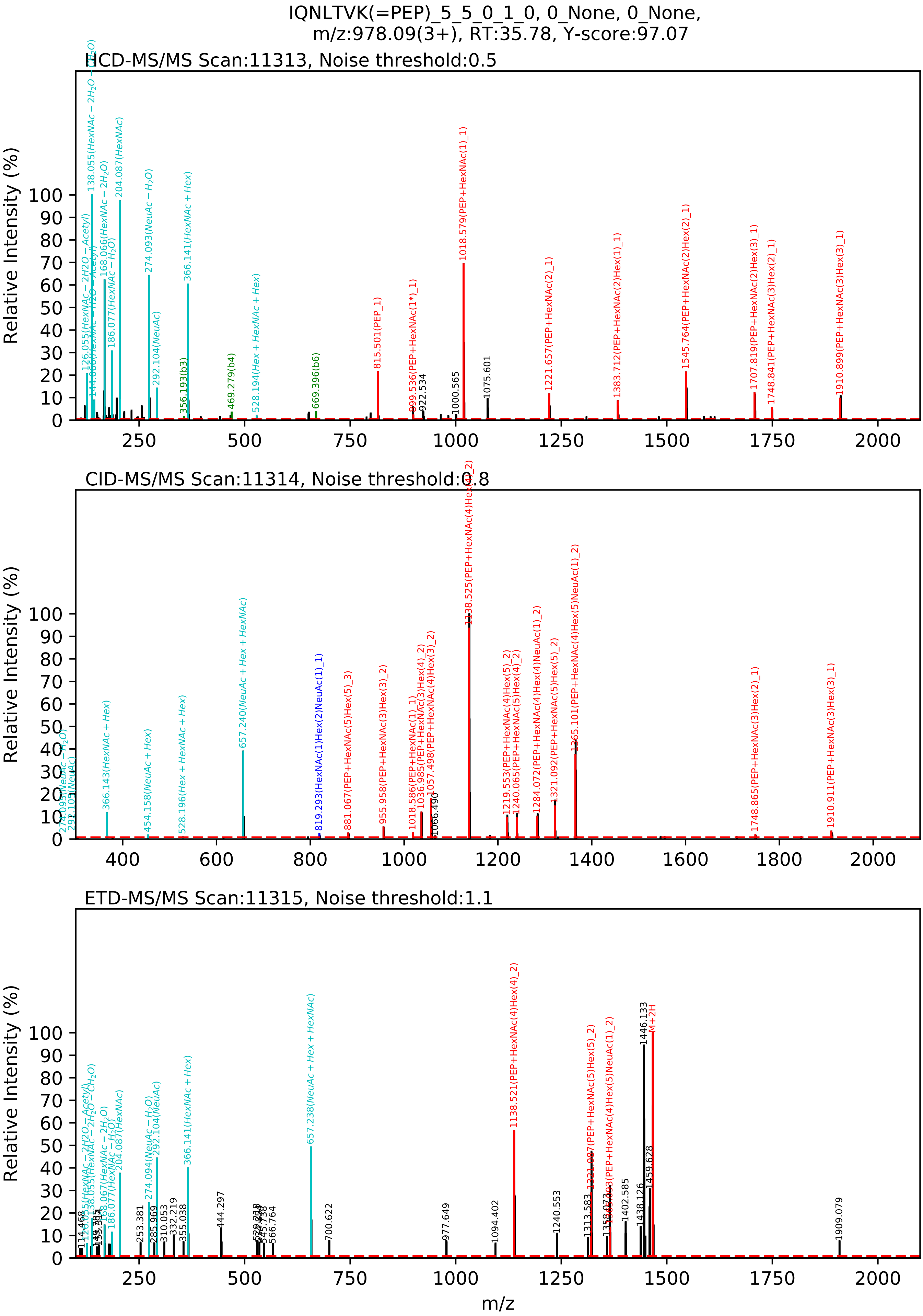
IQNLTVK(=PEP)_5_5_0_1_0, 0_None, 0_None,
m/z:978.09(3+), RT:35.78, Y-score:97.07
HCD-MS/MS Scan:11313, Noise threshold:0.5
CID-MS/MS Scan:11314, Noise threshold:0.8
ETD-MS/MS Scan:11315, Noise threshold:1.1
Relative Intensity (%)
m/z
1018.579(PEP+HexNAc(1)_1)
815.501(PEP_1)
1221.657(PEP+HexNAc(2)_1)
1383.712(PEP+HexNAc(2)Hex(1)_1)
1545.764(PEP+HexNAc(2)Hex(2)_1)
1707.819(PEP+HexNAc(2)Hex(3)_1)
1748.841(PEP+HexNAc(3)Hex(2)_1)
1910.899(PEP+HexNAc(3)Hex(3)_1)
366.141(HexNAc+Hex)
274.093(NeuAc−H2O)
292.104(NeuAc)
204.087(HexNAc)
138.055(HexNAc−2H2O−CH2O)
356.193(b3)
469.279(b4)
669.396(b6)
528.194(Hex+HexNAc+Hex)
1138.525(PEP+HexNAc(4)Hex(4)_2)
1365.101(PEP+HexNAc(4)Hex(5)NeuAc(1)_2)
1321.092(PEP+HexNAc(5)Hex(5)_2)
1284.072(PEP+HexNAc(4)Hex(4)NeuAc(1)_2)
1240.065(PEP+HexNAc(5)Hex(4)_2)
1219.553(PEP+HexNAc(4)Hex(5)_2)
657.240(NeuAc+Hex+HexNAc)
819.293(HexNAc(1)Hex(2)NeuAc(1)_1)
881.067(PEP+HexNAc(5)Hex(5)_3)
955.958(PEP+HexNAc(3)Hex(3)_2)
1748.865(PEP+HexNAc(3)Hex(2)_1)
1910.911(PEP+HexNAc(3)Hex(3)_1)
1138.521(PEP+HexNAc(4)Hex(4)_2)
1446.133
M+2H
657.238(NeuAc+Hex+HexNAc)
1909.079
1402.585
1240.553
1094.402
977.649
700.622
444.297

IQNLTVK(=PEP)_5_5_0_1_0, 0_None, 0_None,
m/z:1466.63(2+), RT:35.82, Y-score:73.96

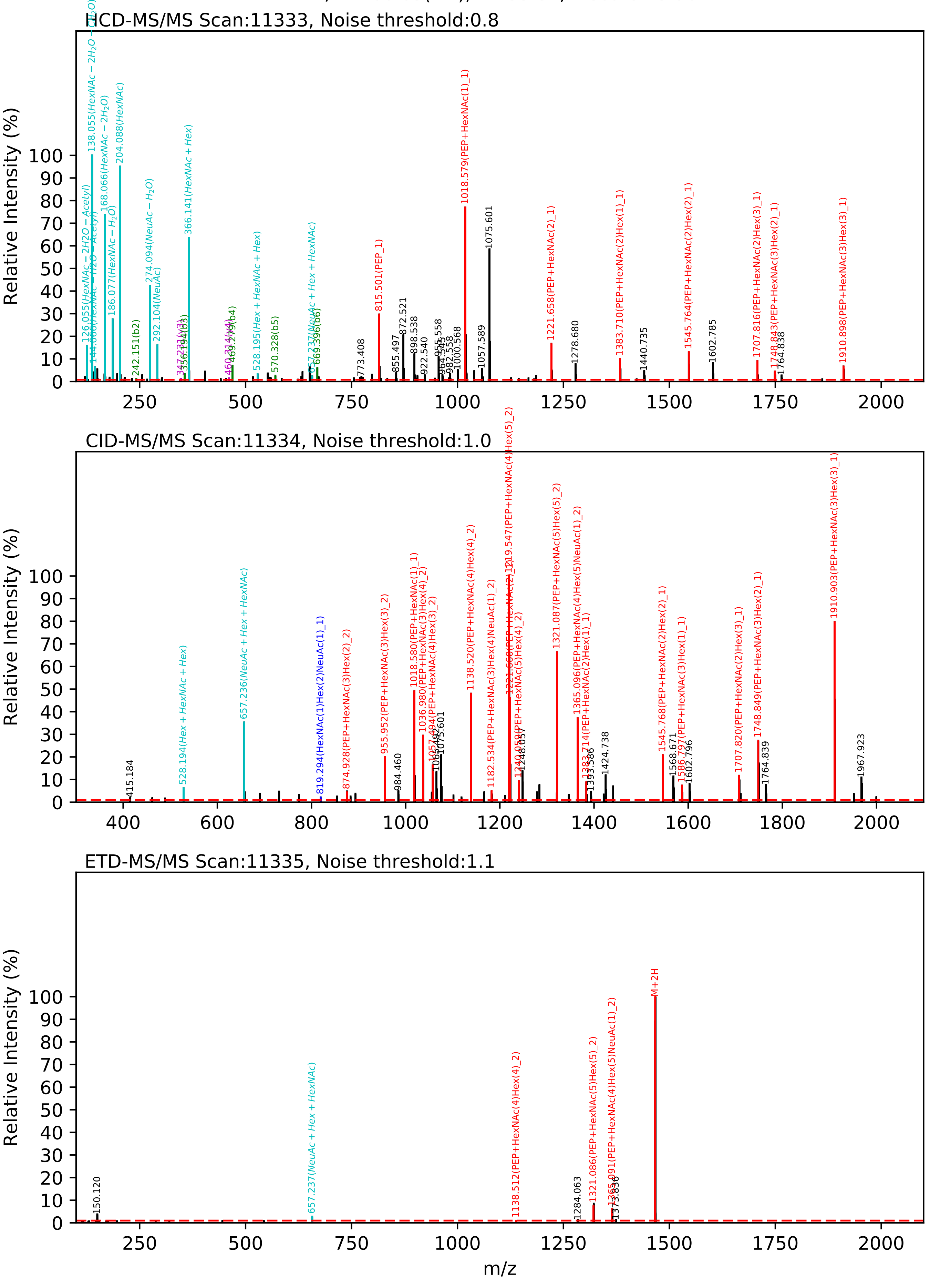

IQNLTVK(=PEP)_5_5_0_1_0, 0_None, 0_None,
m/z:978.09(3+), RT:36.32, Y-score:88.13

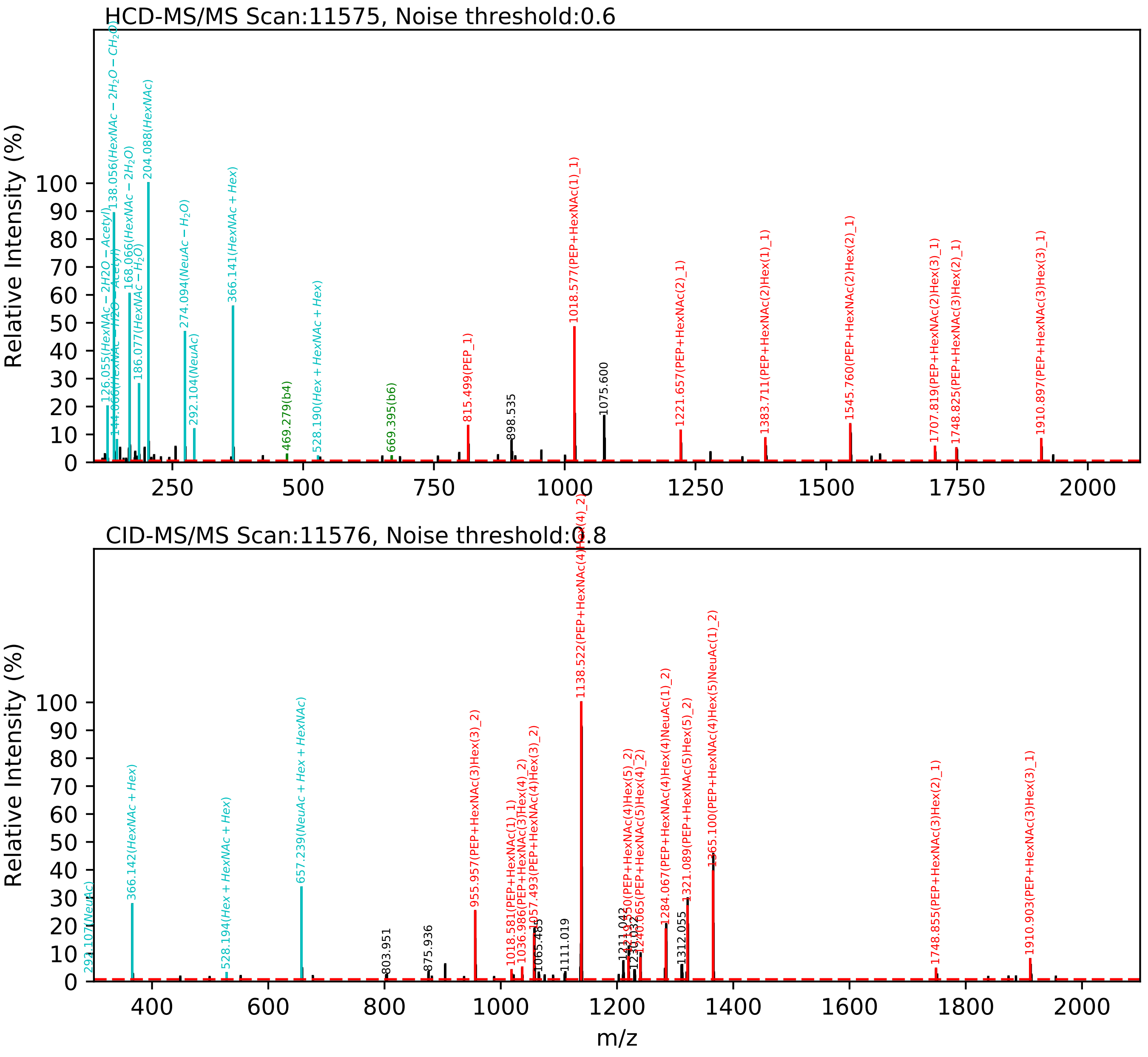

IQNLTVK(=PEP)_5_5_0_2_0, 0_None, 0_None,
m/z:1612.18(2+), RT:49.71, Y-score:94.41

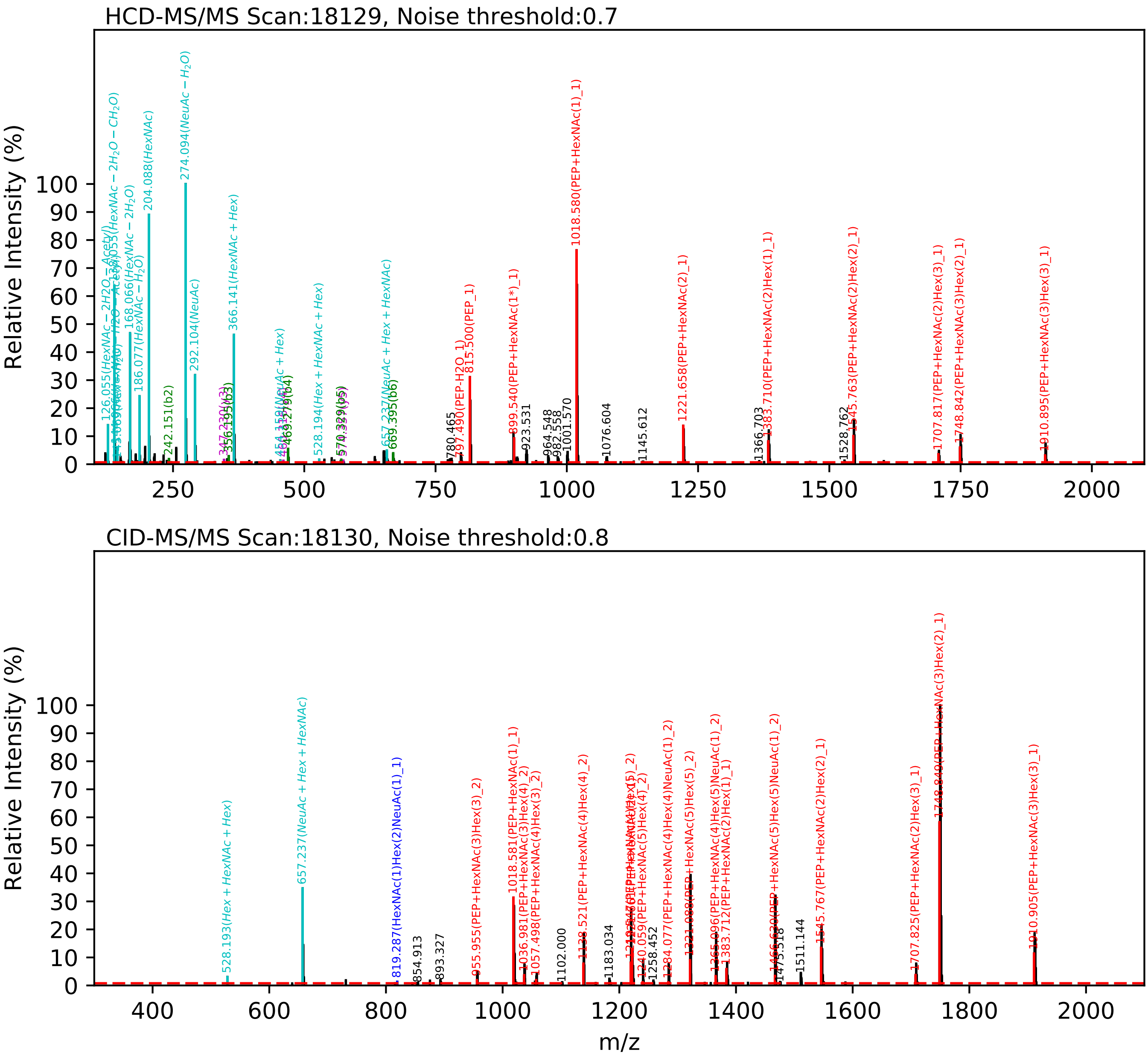

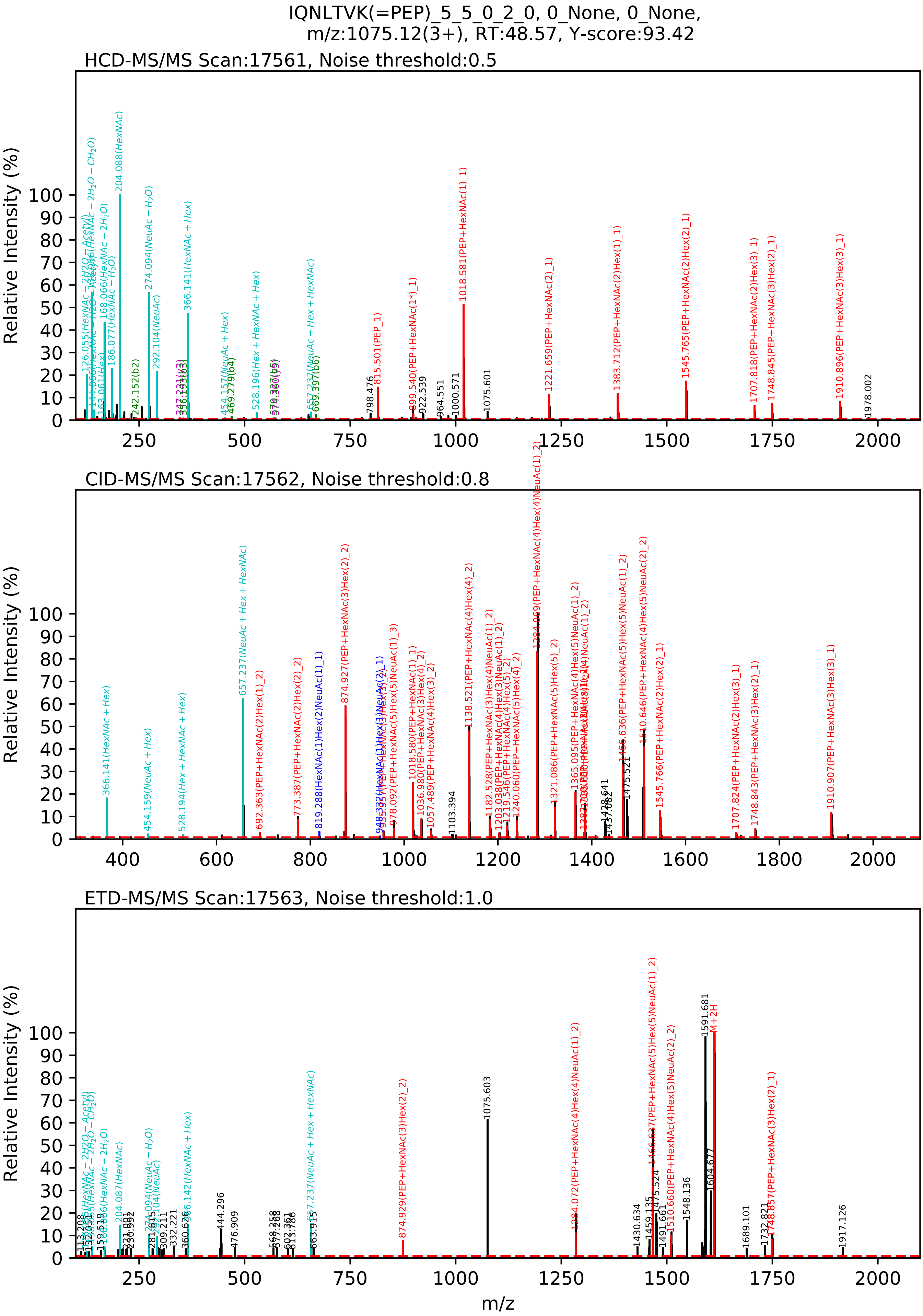
IQNLTVK(=PEP)_5_5_0_2_0, 0_None, 0_None,
m/z:1075.12(3+), RT:48.57, Y-score:93.42
HCD-MS/MS Scan:17561, Noise threshold:0.5
CID-MS/MS Scan:17562, Noise threshold:0.8
ETD-MS/MS Scan:17563, Noise threshold:1.0
Relative Intensity (%)
m/z
204.088(HexNAc)
366.141(HexNAc + Hex)
1018.581(PEP+HexNAc(1)_1)
1221.659(PEP+HexNAc(2)_1)
1383.712(PEP+HexNAc(2)Hex(1)_1)
1545.765(PEP+HexNAc(2)Hex(2)_1)
1707.818(PEP+HexNAc(2)Hex(3)_1)
1748.845(PEP+HexNAc(3)Hex(2)_1)
1910.896(PEP+HexNAc(3)Hex(3)_1)
657.237(NeuAc + Hex + HexNAc)
874.927(PEP+HexNAc(3)Hex(2)_2)
1138.521(PEP+HexNAc(4)Hex(4)_2)
1284.069(PEP+HexNAc(4)Hex(4)NeuAc(1)_2)
1466.636(PEP+HexNAc(5)Hex(5)NeuAc(1)_2)
1510.646(PEP+HexNAc(4)Hex(5)NeuAc(2)_2)
1075.603
1591.681
M+2H

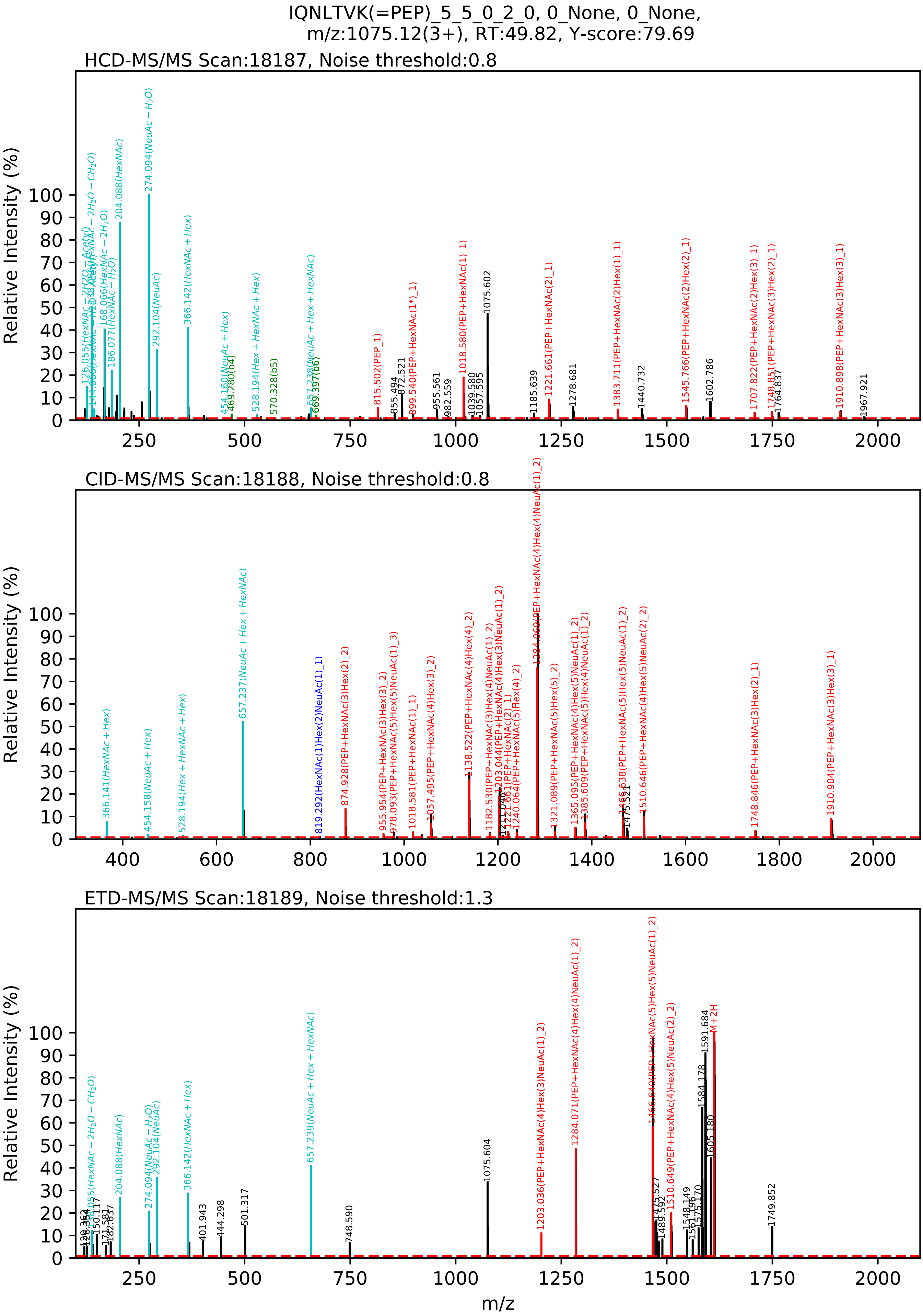

IQNLTVK(=PEP)_5_5_0_2_0, 0_None, 0_None,
m/z:1075.12(3+), RT:49.82, Y-score:79.69
HCD-MS/MS Scan:18187, Noise threshold:0.8
Relative Intensity (%)
274.094(NeuAc − H2O)
204.088(HexNAc)
168.066(HexNAc − 2H2O)
186.077(HexNAc − H2O)
126.055(HexNAc − 2H2O − CH2O)
366.142(HexNAc + Hex)
292.104(NeuAc)
454.160(NeuAc + Hex)
469.280(b4)
528.194(Hex + HexNAc + Hex)
570.328(b5)
657.238(NeuAc + Hex + HexNAc)
669.397(b6)
815.502(PEP_1)
855.494
872.521
899.540(PEP+HexNAc(1*)_1)
955.561
982.559
1018.580(PEP+HexNAc(1)_1)
1039.580
1057.595
1075.602
1185.639
1221.661(PEP+HexNAc(2)_1)
1278.681
1383.711(PEP+HexNAc(2)Hex(1)_1)
1440.732
1545.766(PEP+HexNAc(2)Hex(2)_1)
1602.786
1707.822(PEP+HexNAc(2)Hex(3)_1)
1748.851(PEP+HexNAc(3)Hex(2)_1)
1764.837
1910.898(PEP+HexNAc(3)Hex(3)_1)
1967.921
CID-MS/MS Scan:18188, Noise threshold:0.8
366.141(HexNAc + Hex)
454.158(NeuAc + Hex)
528.194(Hex + HexNAc + Hex)
657.237(NeuAc + Hex + HexNAc)
819.292(HexNAc(1)Hex(2)NeuAc(1)_1)
874.928(PEP+HexNAc(3)Hex(2)_2)
955.954(PEP+HexNAc(3)Hex(3)_2)
978.093(PEP+HexNAc(5)Hex(5)NeuAc(1)_3)
1018.581(PEP+HexNAc(1)_1)
1057.495(PEP+HexNAc(4)Hex(3)_2)
1138.522(PEP+HexNAc(4)Hex(4)_2)
1182.530(PEP+HexNAc(3)Hex(4)NeuAc(1)_2)
1203.044(PEP+HexNAc(4)Hex(3)NeuAc(1)_2)
1221.661(PEP+HexNAc(2)_1)
1240.064(PEP+HexNAc(5)Hex(4)_2)
1284.060(PEP+HexNAc(4)Hex(4)NeuAc(1)_2)
1321.089(PEP+HexNAc(5)Hex(5)_2)
1365.095(PEP+HexNAc(4)Hex(5)NeuAc(1)_2)
1385.609(PEP+HexNAc(5)Hex(4)NeuAc(1)_2)
1466.638(PEP+HexNAc(5)Hex(5)NeuAc(1)_2)
1475.521
1510.646(PEP+HexNAc(4)Hex(5)NeuAc(2)_2)
1748.846(PEP+HexNAc(3)Hex(2)_1)
1910.904(PEP+HexNAc(3)Hex(3)_1)
ETD-MS/MS Scan:18189, Noise threshold:1.3
126.055(HexNAc − 2H2O − CH2O)
204.088(HexNAc)
274.094(NeuAc − H2O)
292.104(NeuAc)
366.142(HexNAc + Hex)
401.943
444.298
501.317
657.239(NeuAc + Hex + HexNAc)
748.590
1075.604
1203.036(PEP+HexNAc(4)Hex(3)NeuAc(1)_2)
1284.071(PEP+HexNAc(4)Hex(4)NeuAc(1)_2)
1466.640(PEP+HexNAc(5)Hex(5)NeuAc(1)_2)
1510.649(PEP+HexNAc(4)Hex(5)NeuAc(2)_2)
1548.149
1584.178
1591.684
1605.180
M+2H
1749.852
m/z

IQNLTVK(=PEP)_5_5_1_0_0, 0_None, 0_None,
m/z:929.74(3+), RT:26.28, Y-score:63.13

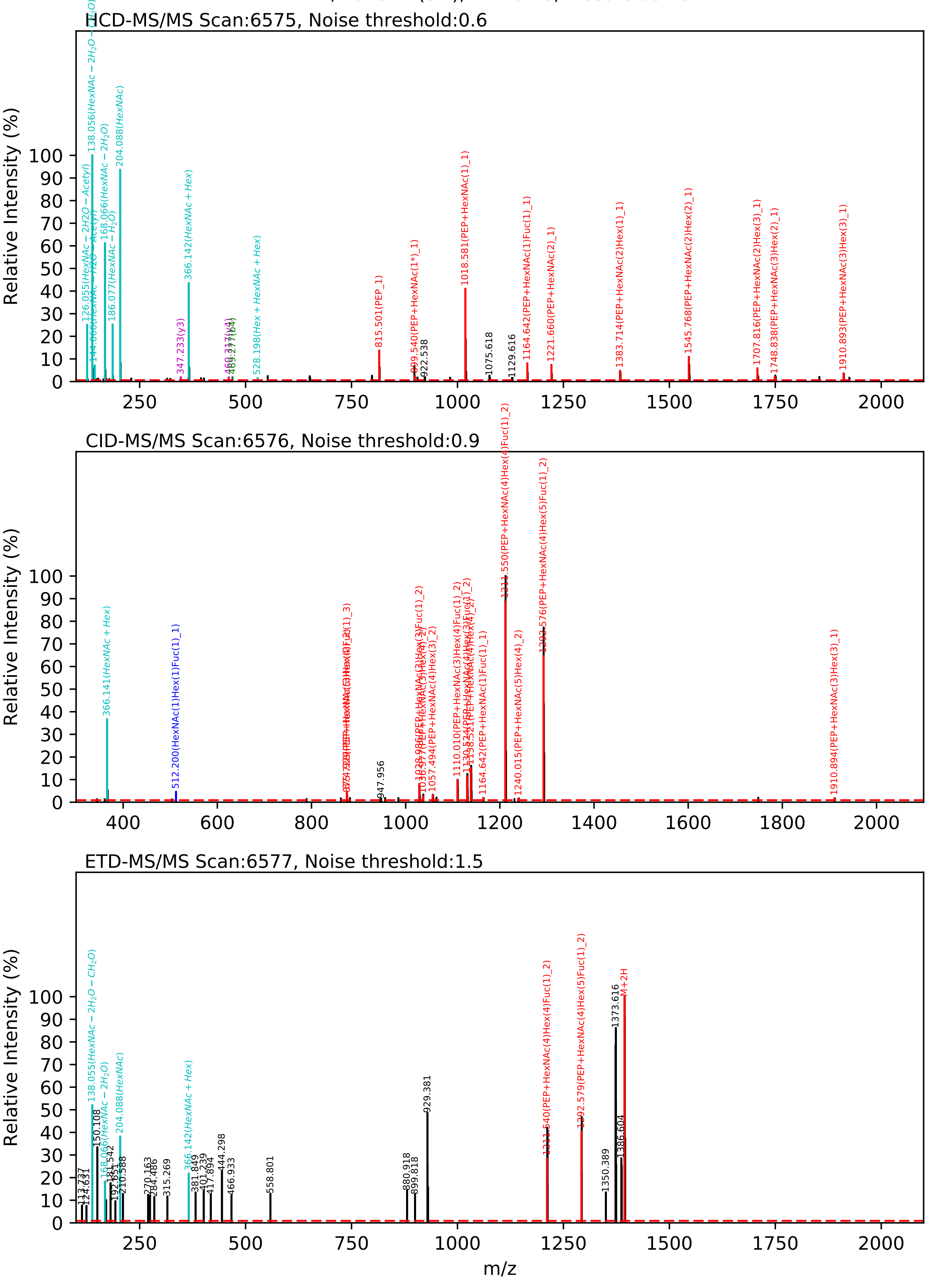

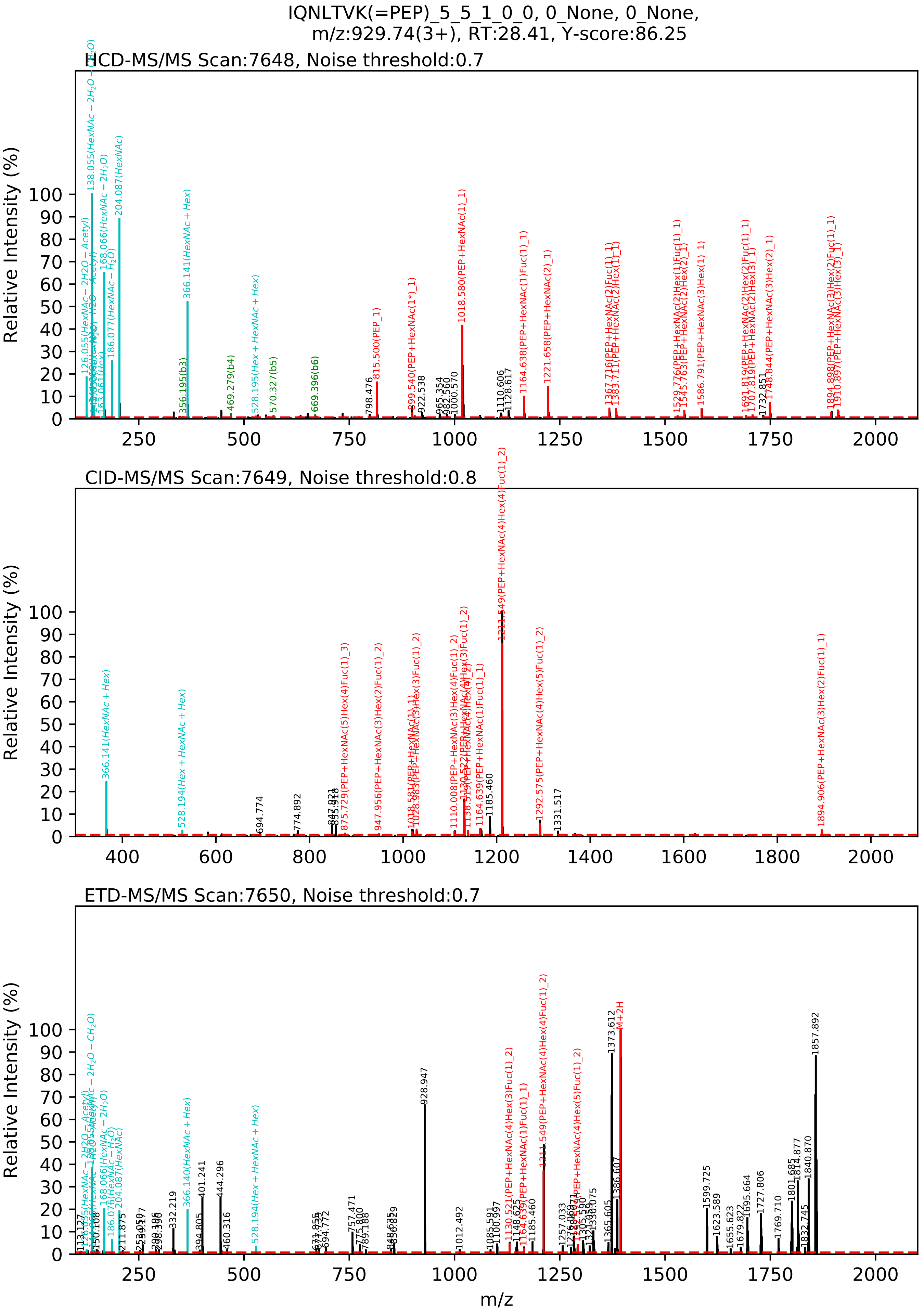
IQNLTVK(=PEP)_5_5_1_0_0, 0_None, 0_None,
m/z:929.74(3+), RT:28.41, Y-score:86.25
HCD-MS/MS Scan:7648, Noise threshold:0.7
CID-MS/MS Scan:7649, Noise threshold:0.8
ETD-MS/MS Scan:7650, Noise threshold:0.7
Relative Intensity (%)
m/z
1018.580(PEP+HexNAc(1)_1)
1221.658(PEP+HexNAc(2)_1)
1164.638(PEP+HexNAc(1)Fuc(1)_1)
815.500(PEP_1)
366.141(HexNAc+Hex)
204.087(HexNAc)
138.055(HexNAc−2H2O−CH2O)
1211.549(PEP+HexNAc(4)Hex(4)Fuc(1)_2)
1292.575(PEP+HexNAc(4)Hex(5)Fuc(1)_2)
1894.906(PEP+HexNAc(3)Hex(2)Fuc(1)_1)
M+2H
1373.612
1857.892
928.947

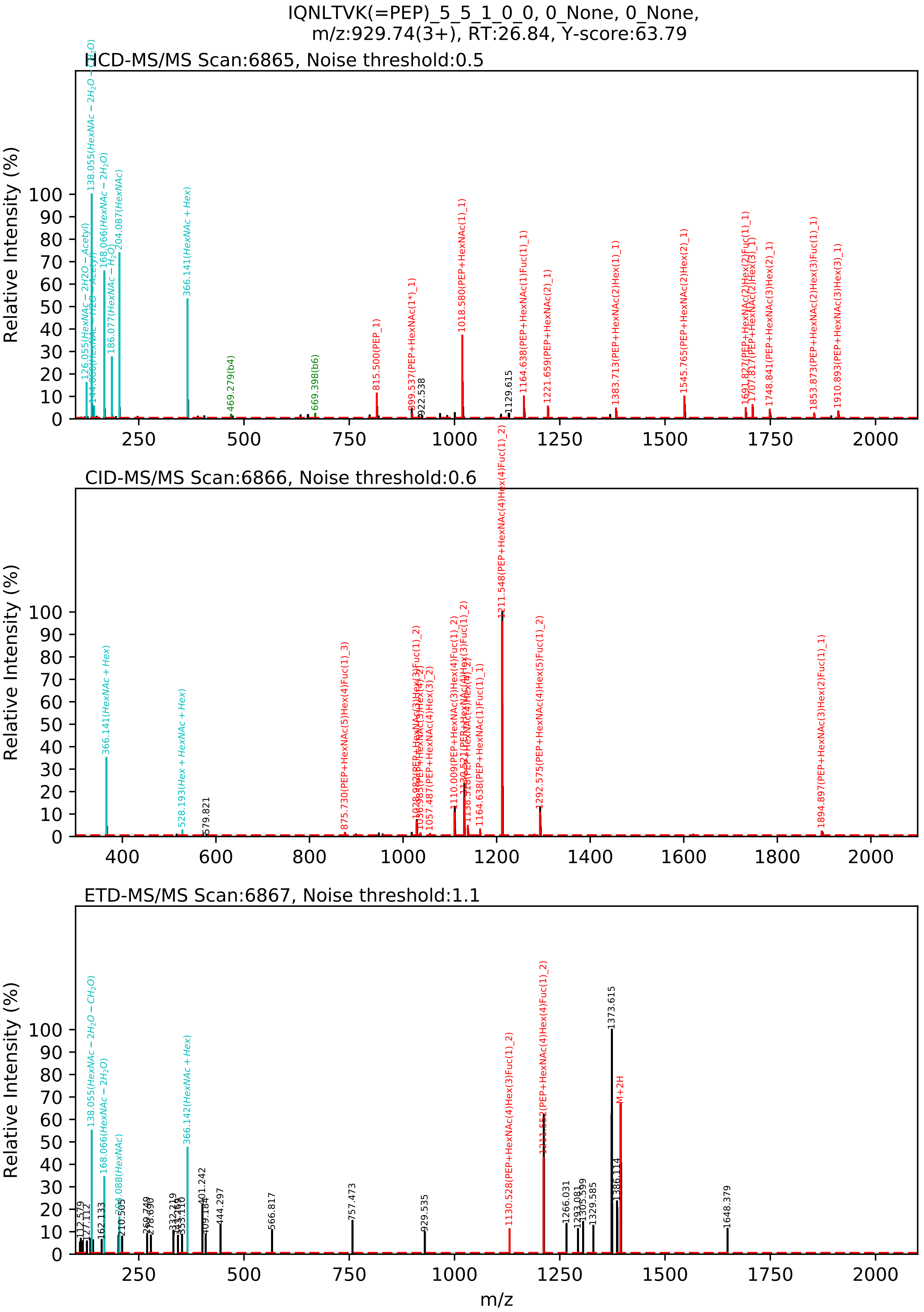
IQNLTVK(=PEP)_5_5_1_0_0, 0_None, 0_None,
m/z:929.74(3+), RT:26.84, Y-score:63.79
HCD-MS/MS Scan:6865, Noise threshold:0.5
Relative Intensity (%)
126.055(HexNAc − 2H2O − Acetyl)
138.055(HexNAc − 2H2O − CH2O)
144.066(HexNAc − H2O − Acetyl)
168.066(HexNAc − 2H2O)
186.077(HexNAc − H2O)
204.087(HexNAc)
366.141(HexNAc + Hex)
469.279(b4)
669.398(b6)
815.500(PEP_1)
899.537(PEP+HexNAc(1*)_1)
922.538
1018.580(PEP+HexNAc(1)_1)
1129.615
1164.638(PEP+HexNAc(1)Fuc(1)_1)
1221.659(PEP+HexNAc(2)_1)
1383.713(PEP+HexNAc(2)Hex(1)_1)
1545.765(PEP+HexNAc(2)Hex(2)_1)
1691.827(PEP+HexNAc(2)Hex(2)Fuc(1)_1)
1707.817(PEP+HexNAc(2)Hex(3)_1)
1748.841(PEP+HexNAc(3)Hex(2)_1)
1853.873(PEP+HexNAc(2)Hex(3)Fuc(1)_1)
1910.893(PEP+HexNAc(3)Hex(3)_1)
CID-MS/MS Scan:6866, Noise threshold:0.6
366.141(HexNAc + Hex)
528.193(Hex + HexNAc + Hex)
579.821
875.730(PEP+HexNAc(5)Hex(4)Fuc(1)_3)
1028.982(PEP+HexNAc(3)Hex(3)Fuc(1)_2)
1036.983(PEP+HexNAc(3)Hex(4)_2)
1057.487(PEP+HexNAc(4)Hex(3)_2)
1110.009(PEP+HexNAc(3)Hex(4)Fuc(1)_2)
1130.521(PEP+HexNAc(4)Hex(3)Fuc(1)_2)
1138.518(PEP+HexNAc(4)Hex(4)_2)
1164.638(PEP+HexNAc(1)Fuc(1)_1)
1211.548(PEP+HexNAc(4)Hex(4)Fuc(1)_2)
1292.575(PEP+HexNAc(4)Hex(5)Fuc(1)_2)
1894.897(PEP+HexNAc(3)Hex(2)Fuc(1)_1)
ETD-MS/MS Scan:6867, Noise threshold:1.1
112.579
127.112
138.055(HexNAc − 2H2O − CH2O)
162.133
168.066(HexNAc − 2H2O)
204.088(HexNAc)
210.505
269.749
278.690
332.219
343.265
353.110
366.142(HexNAc + Hex)
401.242
409.184
444.297
566.817
757.473
929.535
1130.528(PEP+HexNAc(4)Hex(3)Fuc(1)_2)
1211.552(PEP+HexNAc(4)Hex(4)Fuc(1)_2)
1266.031
1293.081
1305.599
1329.585
1373.615
1386.114
M+2H
1648.379
m/z

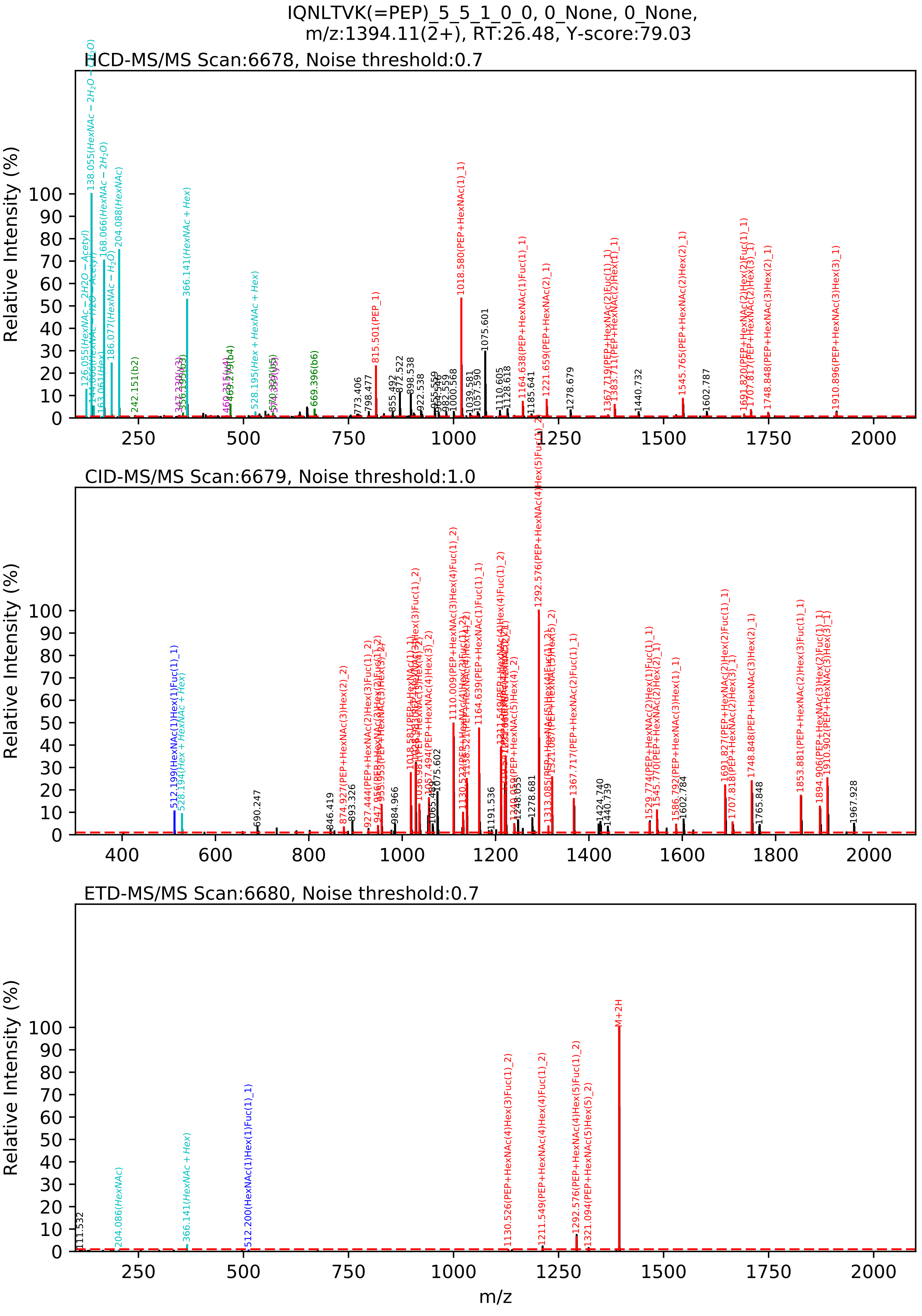
IQNLTVK(=PEP)_5_5_1_0_0, 0_None, 0_None,
m/z:1394.11(2+), RT:26.48, Y-score:79.03
HCD-MS/MS Scan:6678, Noise threshold:0.7
Relative Intensity (%)
138.055(HexNAc − 2H2O − Acetyl)
168.066(HexNAc − 2H2O)
204.088(HexNAc)
186.077(HexNAc − H2O)
366.141(HexNAc + Hex)
126.055(HexNAc − 2H2O − Acetyl)
163.061(Hex)
242.151(b2)
528.195(Hex + HexNAc + Hex)
669.396(b6)
815.501(PEP_1)
1018.580(PEP+HexNAc(1)_1)
1075.601
1164.638(PEP+HexNAc(1)Fuc(1)_1)
1221.659(PEP+HexNAc(2)_1)
1278.679
1383.711(PEP+HexNAc(2)Hex(1)_1)
1440.732
1545.765(PEP+HexNAc(2)Hex(2)_1)
1602.787
1748.848(PEP+HexNAc(3)Hex(2)_1)
1910.896(PEP+HexNAc(3)Hex(3)_1)
CID-MS/MS Scan:6679, Noise threshold:1.0
Relative Intensity (%)
512.199(HexNAc(1)Hex(1)Fuc(1)_1)
690.247
846.419
874.927(PEP+HexNAc(3)Hex(2)_2)
893.326
984.966
1075.602
1110.009(PEP+HexNAc(3)Hex(4)Fuc(1)_2)
1164.639(PEP+HexNAc(1)Fuc(1)_1)
1191.536
1278.681
1292.576(PEP+HexNAc(4)Hex(5)Fuc(1)_2)
1367.717(PEP+HexNAc(2)Fuc(1)_1)
1440.739
1545.770(PEP+HexNAc(2)Hex(2)_1)
1602.784
1748.848(PEP+HexNAc(3)Hex(2)_1)
1765.848
1853.881(PEP+HexNAc(2)Hex(3)Fuc(1)_1)
1967.928
ETD-MS/MS Scan:6680, Noise threshold:0.7
Relative Intensity (%)
111.532
204.086(HexNAc)
366.141(HexNAc + Hex)
512.200(HexNAc(1)Hex(1)Fuc(1)_1)
1130.526(PEP+HexNAc(4)Hex(3)Fuc(1)_2)
1211.549(PEP+HexNAc(4)Hex(4)Fuc(1)_2)
1292.576(PEP+HexNAc(4)Hex(5)Fuc(1)_2)
1321.094(PEP+HexNAc(5)Hex(5)_2)
M+2H
m/z

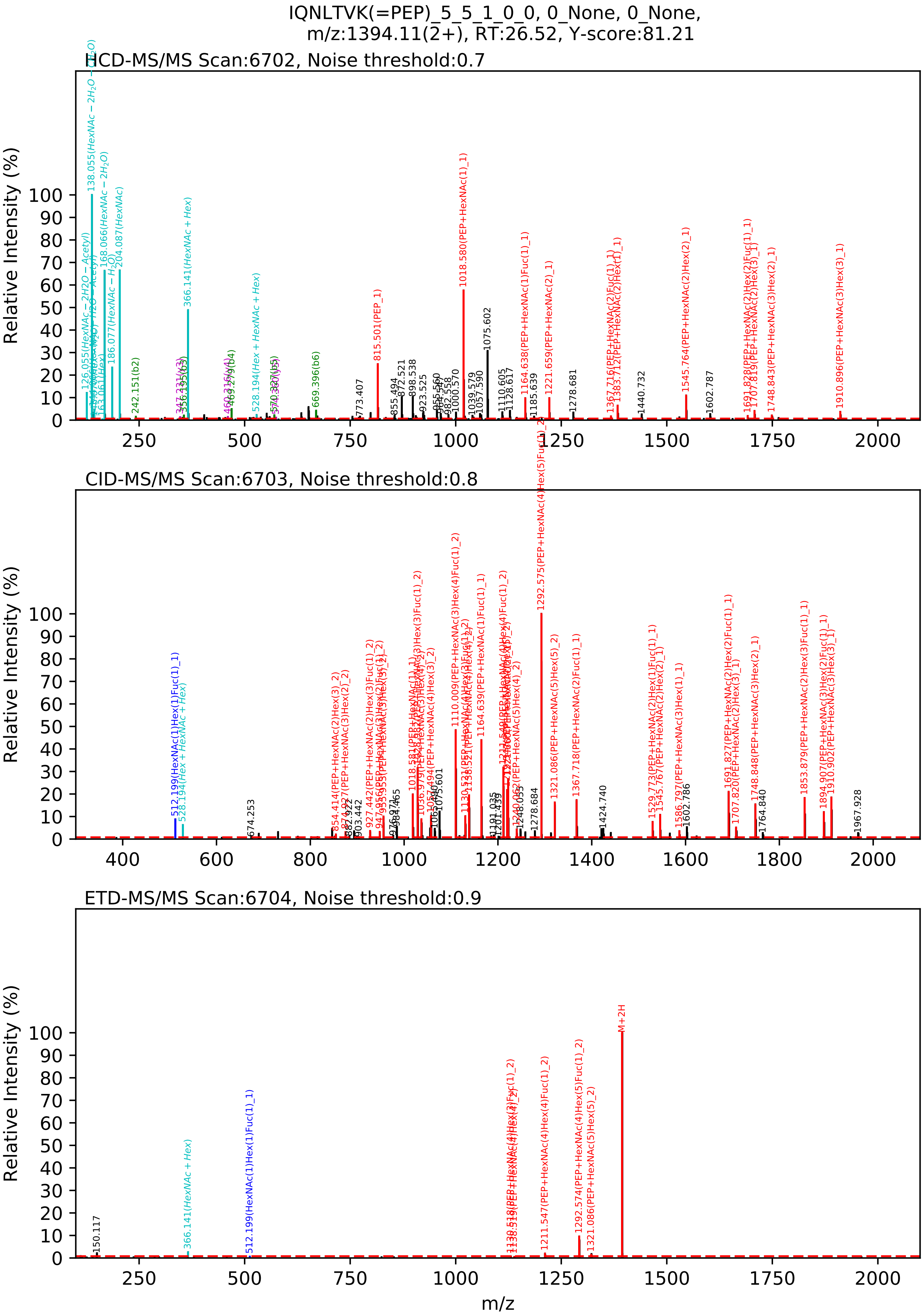

IQNLTVK(=PEP)_5_5_1_0_0, 0_None, 0_None,
m/z:1394.11(2+), RT:26.52, Y-score:81.21
HCD-MS/MS Scan:6702, Noise threshold:0.7
Relative Intensity (%)
138.055(HexNAc − 2H2O − H2O)
168.066(HexNAc − 2H2O)
204.087(HexNAc)
186.077(HexNAc − H2O)
366.141(HexNAc + Hex)
528.194(Hex + HexNAc + Hex)
242.151(b2)
469.279(b4)
669.396(b6)
815.501(PEP_1)
1018.580(PEP+HexNAc(1)_1)
1075.602
1164.638(PEP+HexNAc(1)Fuc(1)_1)
1221.659(PEP+HexNAc(2)_1)
1278.681
1383.712(PEP+HexNAc(2)Hex(1)_1)
1440.732
1545.764(PEP+HexNAc(2)Hex(2)_1)
1602.787
1748.843(PEP+HexNAc(3)Hex(2)_1)
1910.896(PEP+HexNAc(3)Hex(3)_1)
CID-MS/MS Scan:6703, Noise threshold:0.8
512.199(HexNAc(1)Hex(1)Fuc(1)_1)
674.253
1292.575(PEP+HexNAc(4)Hex(5)Fuc(1)_2)
1321.086(PEP+HexNAc(5)Hex(5)_2)
1367.718(PEP+HexNAc(2)Fuc(1)_1)
1424.740
1529.773(PEP+HexNAc(2)Hex(1)Fuc(1)_1)
1545.767(PEP+HexNAc(2)Hex(2)_1)
1586.797(PEP+HexNAc(3)Hex(1)_1)
1602.786
1691.827(PEP+HexNAc(2)Hex(2)Fuc(1)_1)
1748.848(PEP+HexNAc(3)Hex(2)_1)
1764.840
1853.879(PEP+HexNAc(2)Hex(3)Fuc(1)_1)
1967.928
ETD-MS/MS Scan:6704, Noise threshold:0.9
150.117
366.141(HexNAc + Hex)
512.199(HexNAc(1)Hex(1)Fuc(1)_1)
1211.547(PEP+HexNAc(4)Hex(4)Fuc(1)_2)
1292.574(PEP+HexNAc(4)Hex(5)Fuc(1)_2)
1321.086(PEP+HexNAc(5)Hex(5)_2)
M+2H
m/z

IQNLTVK(=PEP)_5_5_1_0_0, 0_None, 0_None,
m/z:929.74(3+), RT:27.78, Y-score:85.79

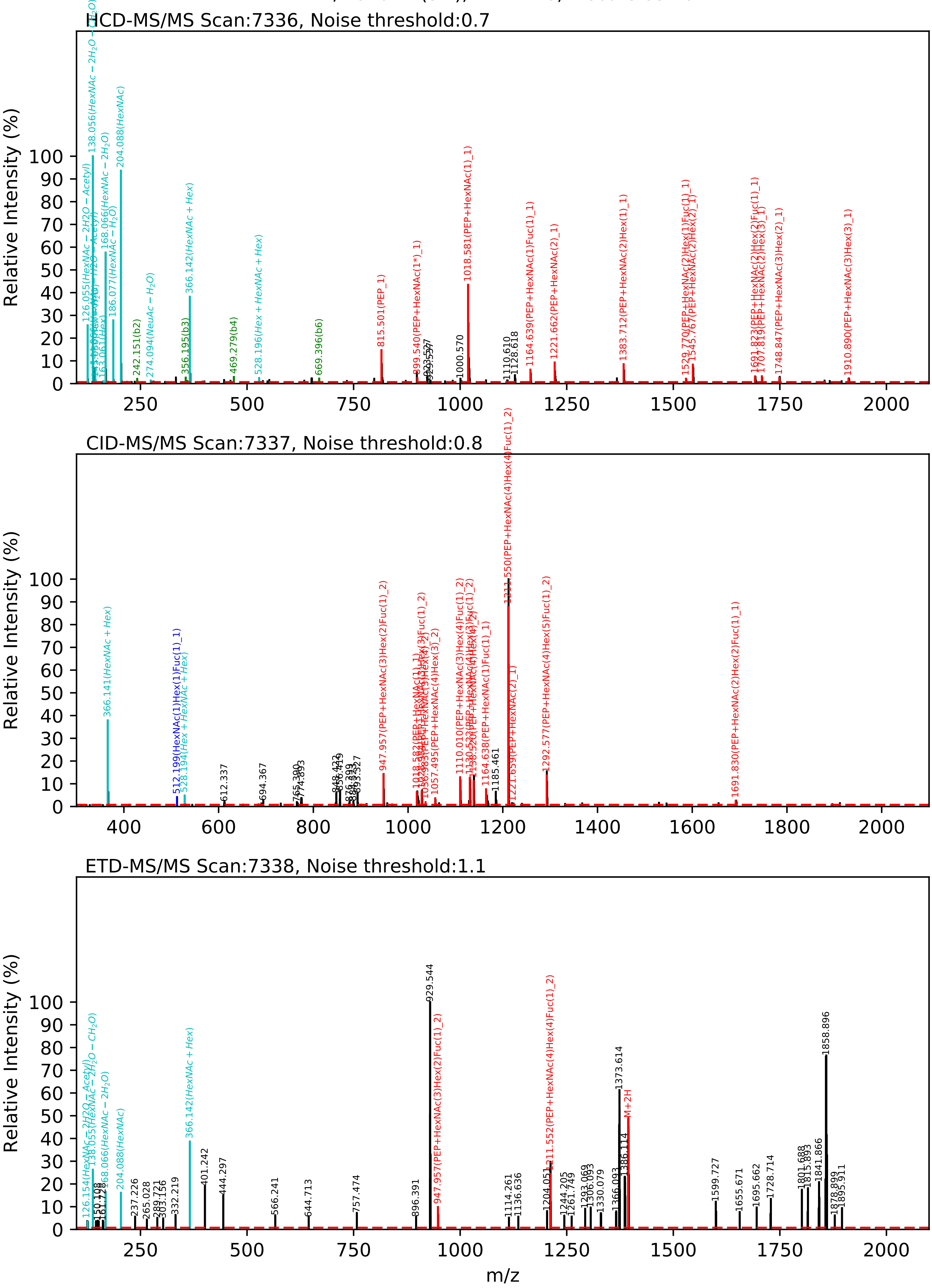

IQNLTVK(=PEP)_5_5_1_0_0, 0_None, 0_None,
m/z:1394.11(2+), RT:27.71, Y-score:92.39

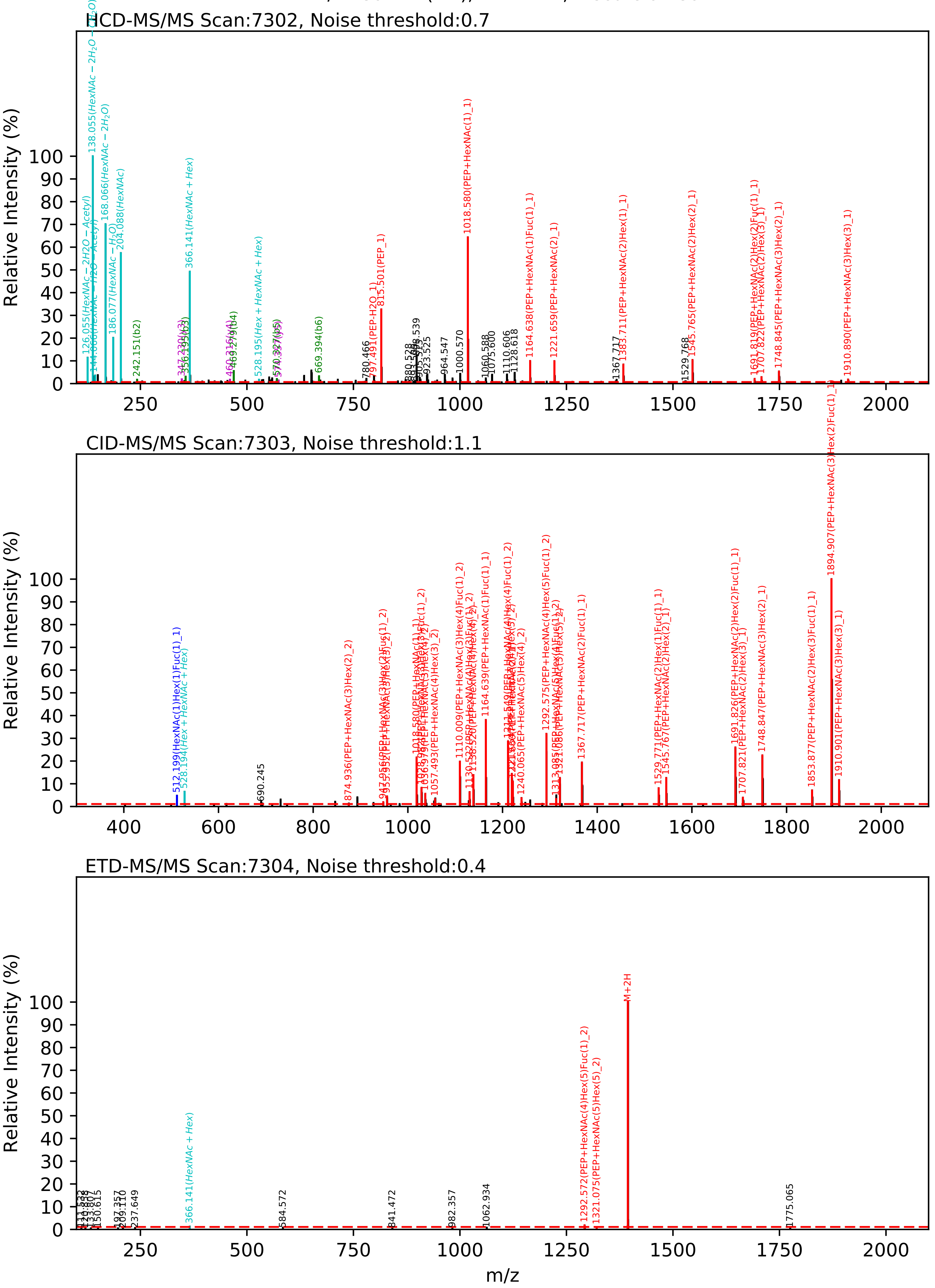

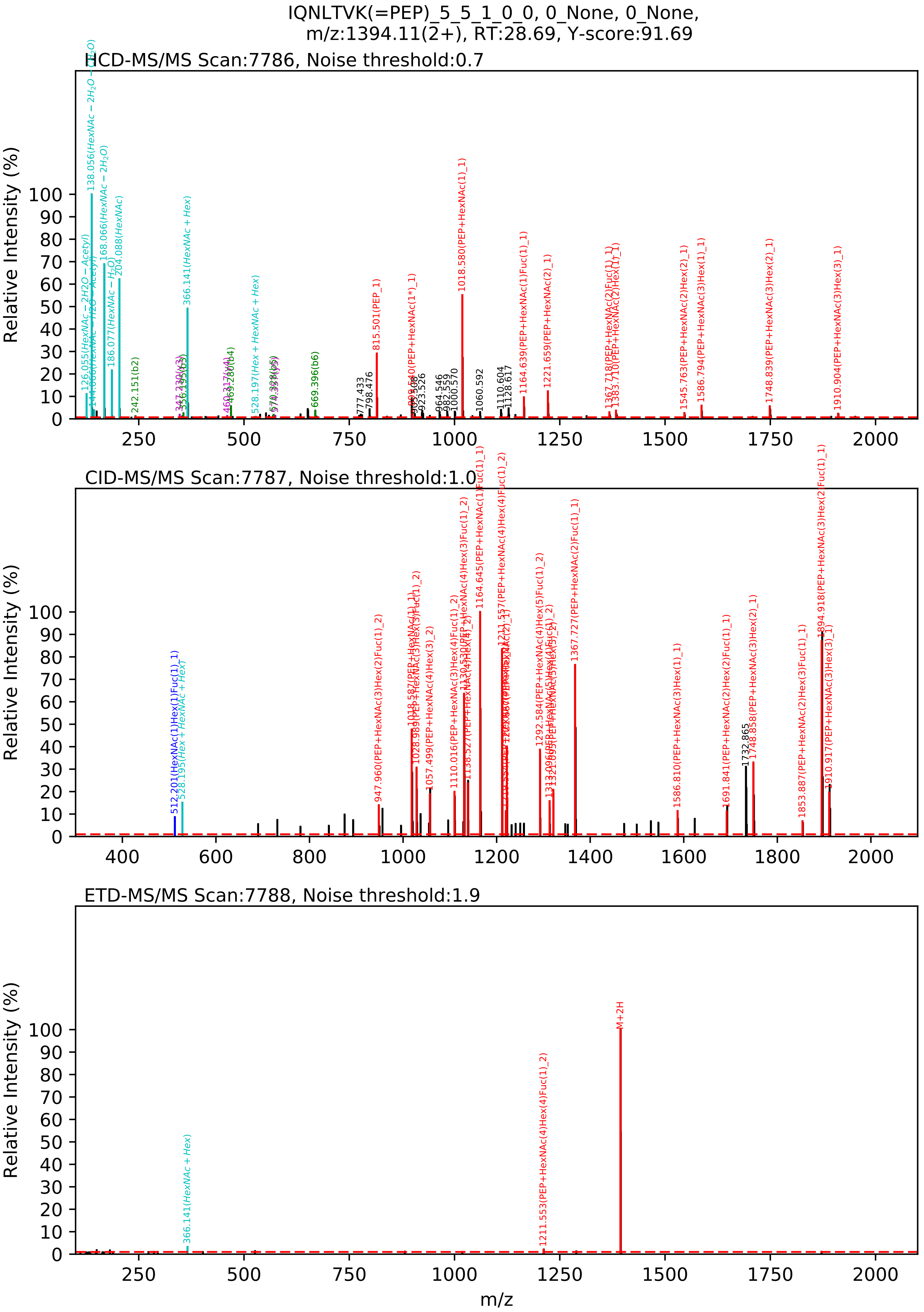

IQNLTVK(=PEP)_5_5_1_0_0, 0_None, 0_None,
m/z:1394.11(2+), RT:28.69, Y-score:91.69
HCD-MS/MS Scan:7786, Noise threshold:0.7
Relative Intensity (%)
138.056(HexNAc − 2H2O − Acetyl)
168.066(HexNAc − 2H2O)
204.088(HexNAc)
186.077(HexNAc − H2O)
366.141(HexNAc + Hex)
126.055(HexNAc − 2H2O − Acetyl)
242.151(b2)
469.280(b4)
528.197(Hex + HexNAc + Hex)
669.396(b6)
777.433
798.476
815.501(PEP_1)
1018.580(PEP+HexNAc(1)_1)
923.526
964.546
1000.570
1060.592
1110.604
1128.617
1164.639(PEP+HexNAc(1)Fuc(1)_1)
1221.659(PEP+HexNAc(2)_1)
1383.710(PEP+HexNAc(2)Hex(1)_1)
1545.763(PEP+HexNAc(2)Hex(2)_1)
1586.794(PEP+HexNAc(3)Hex(1)_1)
1748.839(PEP+HexNAc(3)Hex(2)_1)
1910.904(PEP+HexNAc(3)Hex(3)_1)
CID-MS/MS Scan:7787, Noise threshold:1.0
512.201(HexNAc(1)Hex(1)Fuc(1)_1)
528.195(Hex + HexNAc + Hex)
947.960(PEP+HexNAc(3)Hex(2)Fuc(1)_2)
1057.499(PEP+HexNAc(4)Hex(3)_2)
1110.016(PEP+HexNAc(3)Hex(4)Fuc(1)_2)
1164.645(PEP+HexNAc(1)Fuc(1)_1)
1211.557(PEP+HexNAc(4)Hex(4)Fuc(1)_2)
1292.584(PEP+HexNAc(4)Hex(5)Fuc(1)_2)
1367.727(PEP+HexNAc(2)Fuc(1)_1)
1586.810(PEP+HexNAc(3)Hex(1)_1)
1691.841(PEP+HexNAc(2)Hex(2)Fuc(1)_1)
1732.865
1748.858(PEP+HexNAc(3)Hex(2)_1)
1853.887(PEP+HexNAc(2)Hex(3)Fuc(1)_1)
1894.918(PEP+HexNAc(3)Hex(2)Fuc(1)_1)
1910.917(PEP+HexNAc(3)Hex(3)_1)
ETD-MS/MS Scan:7788, Noise threshold:1.9
366.141(HexNAc + Hex)
1211.553(PEP+HexNAc(4)Hex(4)Fuc(1)_2)
M+2H
m/z

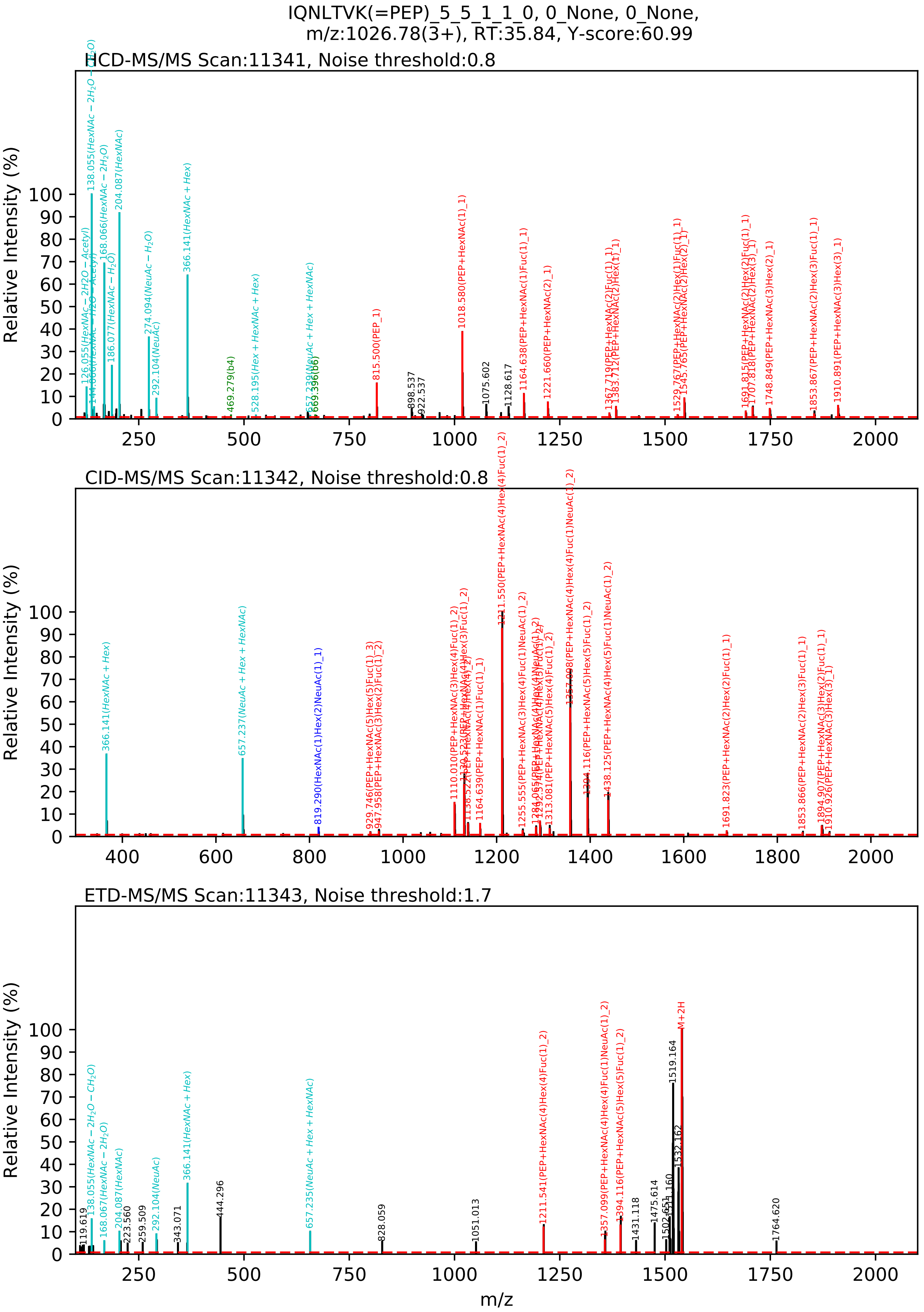

IQNLTVK(=PEP)_5_5_1_1_0, 0_None, 0_None,
m/z:1026.78(3+), RT:35.84, Y-score:60.99
HCD-MS/MS Scan:11341, Noise threshold:0.8
Relative Intensity (%)
126.055(HexNAc − 2H2O − Acetyl)
138.055(HexNAc − 2H2O − CH2O)
144.066(HexNAc − H2O − Acetyl)
168.066(HexNAc − 2H2O)
186.077(HexNAc − H2O)
204.087(HexNAc)
274.094(NeuAc − H2O)
292.104(NeuAc)
366.141(HexNAc + Hex)
469.279(b4)
528.195(Hex + HexNAc + Hex)
657.239(NeuAc + Hex + HexNAc)
669.396(b6)
815.500(PEP_1)
898.537
922.537
1018.580(PEP+HexNAc(1)_1)
1075.602
1128.617
1164.638(PEP+HexNAc(1)Fuc(1)_1)
1221.660(PEP+HexNAc(2)_1)
1367.719(PEP+HexNAc(2)Fuc(1)_1)
1383.712(PEP+HexNAc(2)Hex(1)_1)
1529.767(PEP+HexNAc(2)Hex(1)Fuc(1)_1)
1545.765(PEP+HexNAc(2)Hex(2)_1)
1691.815(PEP+HexNAc(2)Hex(2)Fuc(1)_1)
1707.818(PEP+HexNAc(2)Hex(3)_1)
1748.849(PEP+HexNAc(3)Hex(2)_1)
1853.867(PEP+HexNAc(2)Hex(3)Fuc(1)_1)
1910.891(PEP+HexNAc(3)Hex(3)_1)
CID-MS/MS Scan:11342, Noise threshold:0.8
366.141(HexNAc + Hex)
657.237(NeuAc + Hex + HexNAc)
819.290(HexNAc(1)Hex(2)NeuAc(1)_1)
929.746(PEP+HexNAc(5)Hex(5)Fuc(1)_3)
947.958(PEP+HexNAc(3)Hex(2)Fuc(1)_2)
1110.010(PEP+HexNAc(3)Hex(4)Fuc(1)_2)
1130.523(PEP+HexNAc(4)Hex(3)Fuc(1)_2)
1164.639(PEP+HexNAc(1)Fuc(1)_1)
1211.550(PEP+HexNAc(4)Hex(4)Fuc(1)_2)
1255.555(PEP+HexNAc(3)Hex(4)Fuc(1)NeuAc(1)_2)
1313.081(PEP+HexNAc(5)Hex(4)Fuc(1)_2)
1357.098(PEP+HexNAc(4)Hex(4)Fuc(1)NeuAc(1)_2)
1394.116(PEP+HexNAc(5)Hex(5)Fuc(1)_2)
1438.125(PEP+HexNAc(4)Hex(5)Fuc(1)NeuAc(1)_2)
1691.823(PEP+HexNAc(2)Hex(2)Fuc(1)_1)
1853.866(PEP+HexNAc(2)Hex(3)Fuc(1)_1)
1894.907(PEP+HexNAc(3)Hex(2)Fuc(1)_1)
1910.926(PEP+HexNAc(3)Hex(3)_1)
ETD-MS/MS Scan:11343, Noise threshold:1.7
119.619
138.055(HexNAc − 2H2O − CH2O)
168.067(HexNAc − 2H2O)
204.087(HexNAc)
223.560
259.509
292.104(NeuAc)
343.071
366.141(HexNAc + Hex)
444.296
657.235(NeuAc + Hex + HexNAc)
828.059
1051.013
1211.541(PEP+HexNAc(4)Hex(4)Fuc(1)_2)
1357.099(PEP+HexNAc(4)Hex(4)Fuc(1)NeuAc(1)_2)
1394.116(PEP+HexNAc(5)Hex(5)Fuc(1)_2)
1431.118
1475.614
1502.651
1511.160
1519.164
1532.162
M+2H
1764.620
m/z

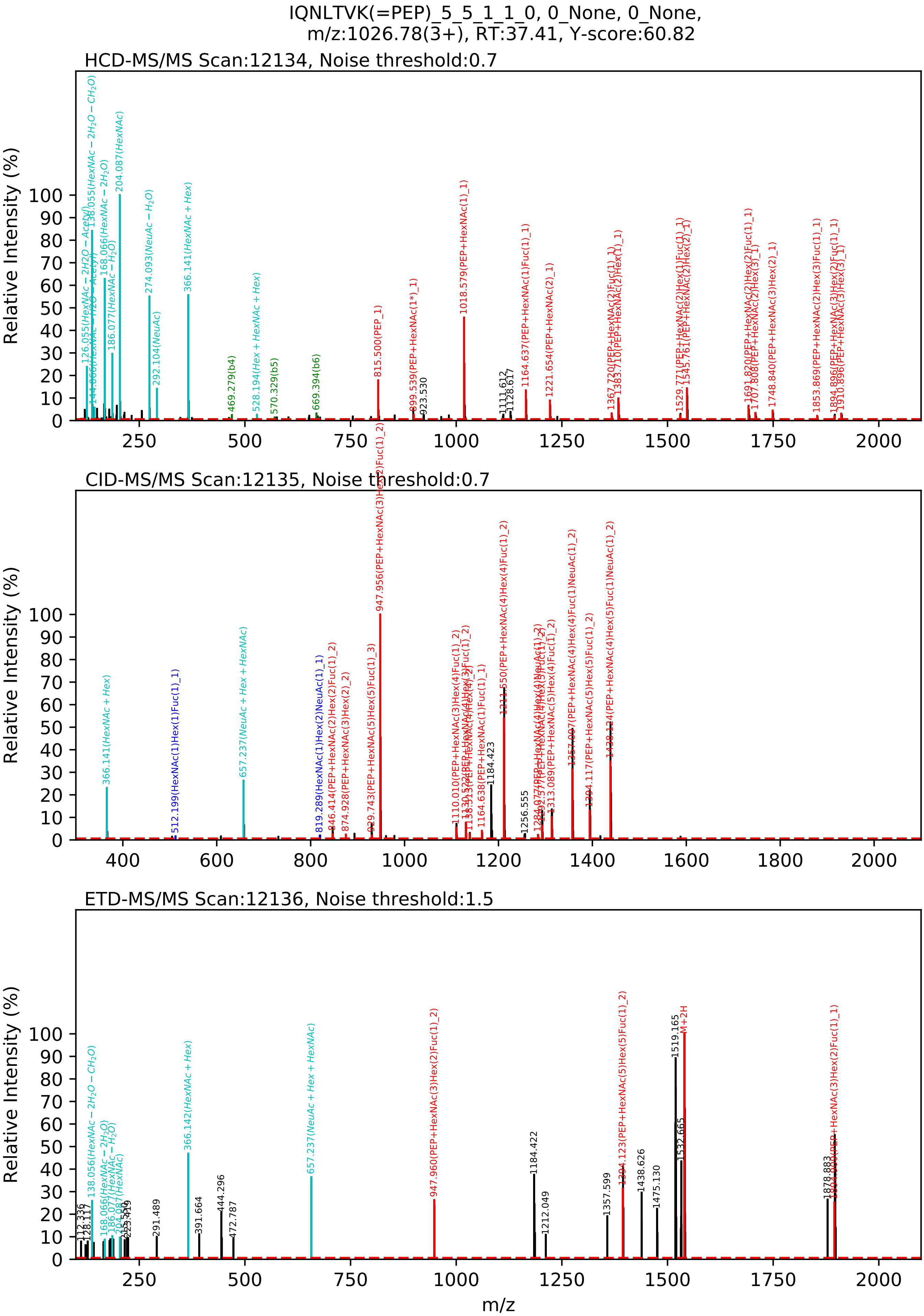

IQNLTVK(=PEP)_5_5_1_1_0, 0_None, 0_None,
m/z:1026.78(3+), RT:37.41, Y-score:60.82
HCD-MS/MS Scan:12134, Noise threshold:0.7
Relative Intensity (%)
204.087(HexNAc)
366.141(HexNAc + Hex)
274.093(NeuAc − H2O)
292.104(NeuAc)
469.279(b4)
528.194(Hex + HexNAc + Hex)
570.329(b5)
669.394(b6)
815.500(PEP_1)
899.539(PEP+HexNAc(1*)_1)
923.530
1018.579(PEP+HexNAc(1)_1)
1111.612
1128.617
1164.637(PEP+HexNAc(1)Fuc(1)_1)
1221.654(PEP+HexNAc(2)_1)
1748.840(PEP+HexNAc(3)Hex(2)_1)
1853.869(PEP+HexNAc(2)Hex(3)Fuc(1)_1)
CID-MS/MS Scan:12135, Noise threshold:0.7
366.141(HexNAc + Hex)
512.199(HexNAc(1)Hex(1)Fuc(1)_1)
657.237(NeuAc + Hex + HexNAc)
819.289(HexNAc(1)Hex(2)NeuAc(1)_1)
846.414(PEP+HexNAc(2)Hex(2)Fuc(1)_2)
874.928(PEP+HexNAc(3)Hex(2)_2)
929.743(PEP+HexNAc(5)Hex(5)Fuc(1)_3)
947.956(PEP+HexNAc(3)Hex(2)Fuc(1)_2)
1164.638(PEP+HexNAc(1)Fuc(1)_1)
1184.423
1211.550(PEP+HexNAc(4)Hex(4)Fuc(1)_2)
1256.555
1313.089(PEP+HexNAc(5)Hex(4)Fuc(1)_2)
1357.097(PEP+HexNAc(4)Hex(4)Fuc(1)NeuAc(1)_2)
1394.117(PEP+HexNAc(5)Hex(5)Fuc(1)_2)
1438.134(PEP+HexNAc(4)Hex(5)Fuc(1)NeuAc(1)_2)
ETD-MS/MS Scan:12136, Noise threshold:1.5
138.056(HexNAc − 2H2O − CH2O)
366.142(HexNAc + Hex)
657.237(NeuAc + Hex + HexNAc)
947.960(PEP+HexNAc(3)Hex(2)Fuc(1)_2)
291.489
391.664
444.296
472.787
1184.422
1212.049
1357.599
1394.123(PEP+HexNAc(5)Hex(5)Fuc(1)_2)
1438.626
1475.130
1519.165
1532.665
M+2H
1878.883
m/z

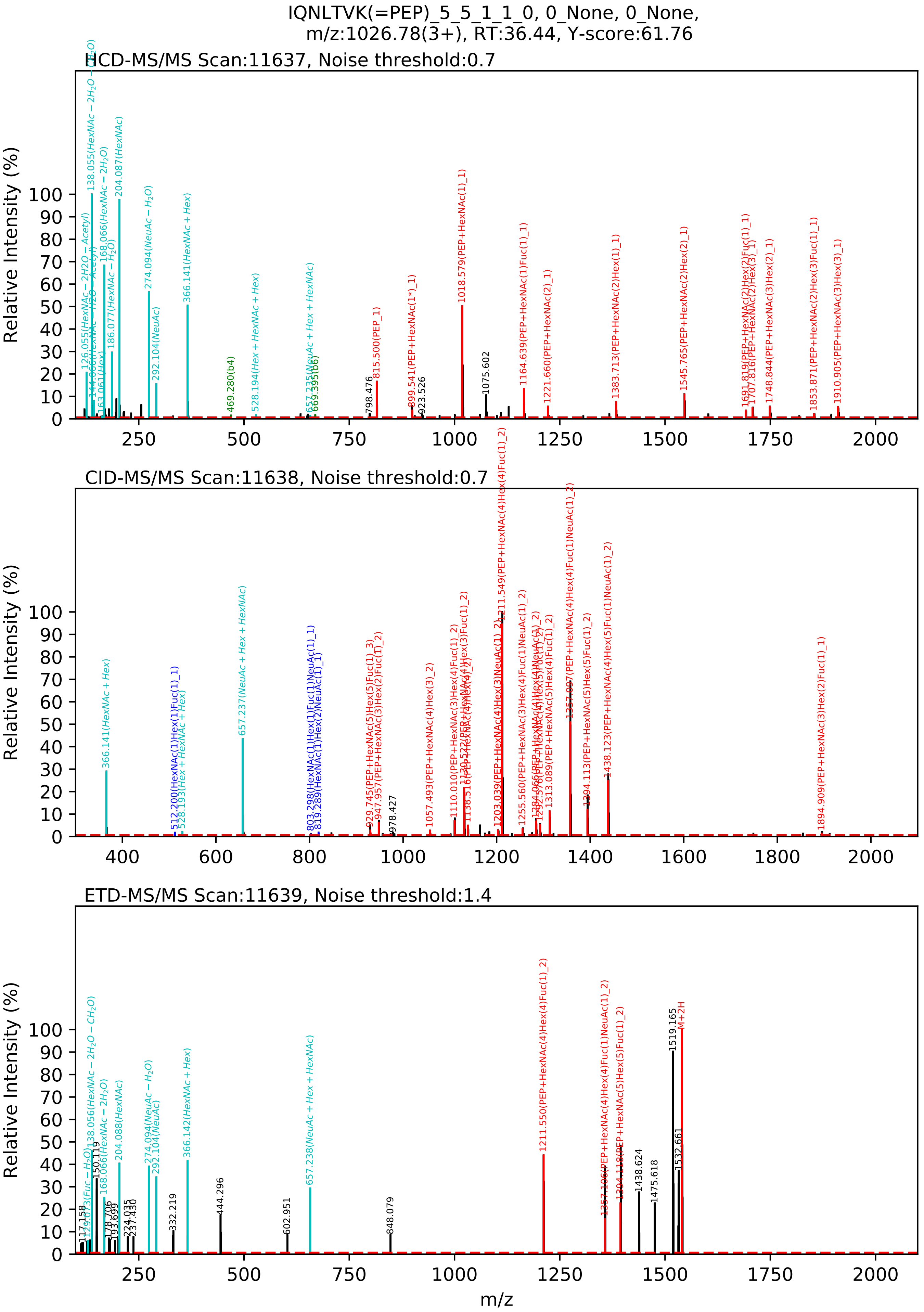

IQNLTVK(=PEP)_5_5_1_1_0, 0_None, 0_None,
m/z:1026.78(3+), RT:36.44, Y-score:61.76
HCD-MS/MS Scan:11637, Noise threshold:0.7
Relative Intensity (%)
138.055(HexNAc − 2H2O − CH2O)
204.087(HexNAc)
168.066(HexNAc − 2H2O)
186.077(HexNAc − H2O)
274.094(NeuAc − H2O)
292.104(NeuAc)
366.141(HexNAc + Hex)
469.280(b4)
528.194(Hex + HexNAc + Hex)
657.235(NeuAc + Hex + HexNAc)
669.395(b6)
798.476
815.500(PEP_1)
899.541(PEP+HexNAc(1*)_1)
923.526
1018.579(PEP+HexNAc(1)_1)
1075.602
1164.639(PEP+HexNAc(1)Fuc(1)_1)
1221.660(PEP+HexNAc(2)_1)
1383.713(PEP+HexNAc(2)Hex(1)_1)
1545.765(PEP+HexNAc(2)Hex(2)_1)
1691.819(PEP+HexNAc(2)Hex(2)Fuc(1)_1)
1707.816(PEP+HexNAc(2)Hex(3)_1)
1748.844(PEP+HexNAc(3)Hex(2)_1)
1853.871(PEP+HexNAc(2)Hex(3)Fuc(1)_1)
1910.905(PEP+HexNAc(3)Hex(3)_1)
CID-MS/MS Scan:11638, Noise threshold:0.7
366.141(HexNAc + Hex)
512.200(HexNAc(1)Hex(1)Fuc(1)_1)
528.193(Hex + HexNAc + Hex)
657.237(NeuAc + Hex + HexNAc)
803.298(HexNAc(1)Hex(1)Fuc(1)NeuAc(1)_1)
819.289(HexNAc(1)Hex(2)NeuAc(1)_1)
929.745(PEP+HexNAc(5)Hex(5)Fuc(1)_3)
947.957(PEP+HexNAc(3)Hex(2)Fuc(1)_2)
978.427
1057.493(PEP+HexNAc(4)Hex(3)_2)
1110.010(PEP+HexNAc(3)Hex(4)Fuc(1)_2)
1130.522(PEP+HexNAc(4)Hex(3)Fuc(1)_2)
1203.039(PEP+HexNAc(4)Hex(3)NeuAc(1)_2)
1211.549(PEP+HexNAc(4)Hex(4)Fuc(1)_2)
1255.560(PEP+HexNAc(3)Hex(4)Fuc(1)NeuAc(1)_2)
1313.089(PEP+HexNAc(5)Hex(4)Fuc(1)_2)
1357.097(PEP+HexNAc(4)Hex(4)Fuc(1)NeuAc(1)_2)
1394.113(PEP+HexNAc(5)Hex(5)Fuc(1)_2)
1438.123(PEP+HexNAc(4)Hex(5)Fuc(1)NeuAc(1)_2)
1894.909(PEP+HexNAc(3)Hex(2)Fuc(1)_1)
ETD-MS/MS Scan:11639, Noise threshold:1.4
117.158
150.119
138.056(HexNAc − 2H2O − CH2O)
168.066(HexNAc − 2H2O)
178.706
204.088(HexNAc)
224.035
237.430
274.094(NeuAc − H2O)
292.104(NeuAc)
332.219
366.142(HexNAc + Hex)
444.296
602.951
657.238(NeuAc + Hex + HexNAc)
848.079
1211.550(PEP+HexNAc(4)Hex(4)Fuc(1)_2)
1357.106(PEP+HexNAc(4)Hex(4)Fuc(1)NeuAc(1)_2)
1394.118(PEP+HexNAc(5)Hex(5)Fuc(1)_2)
1438.624
1475.618
1519.165
1532.661
M+2H
m/z

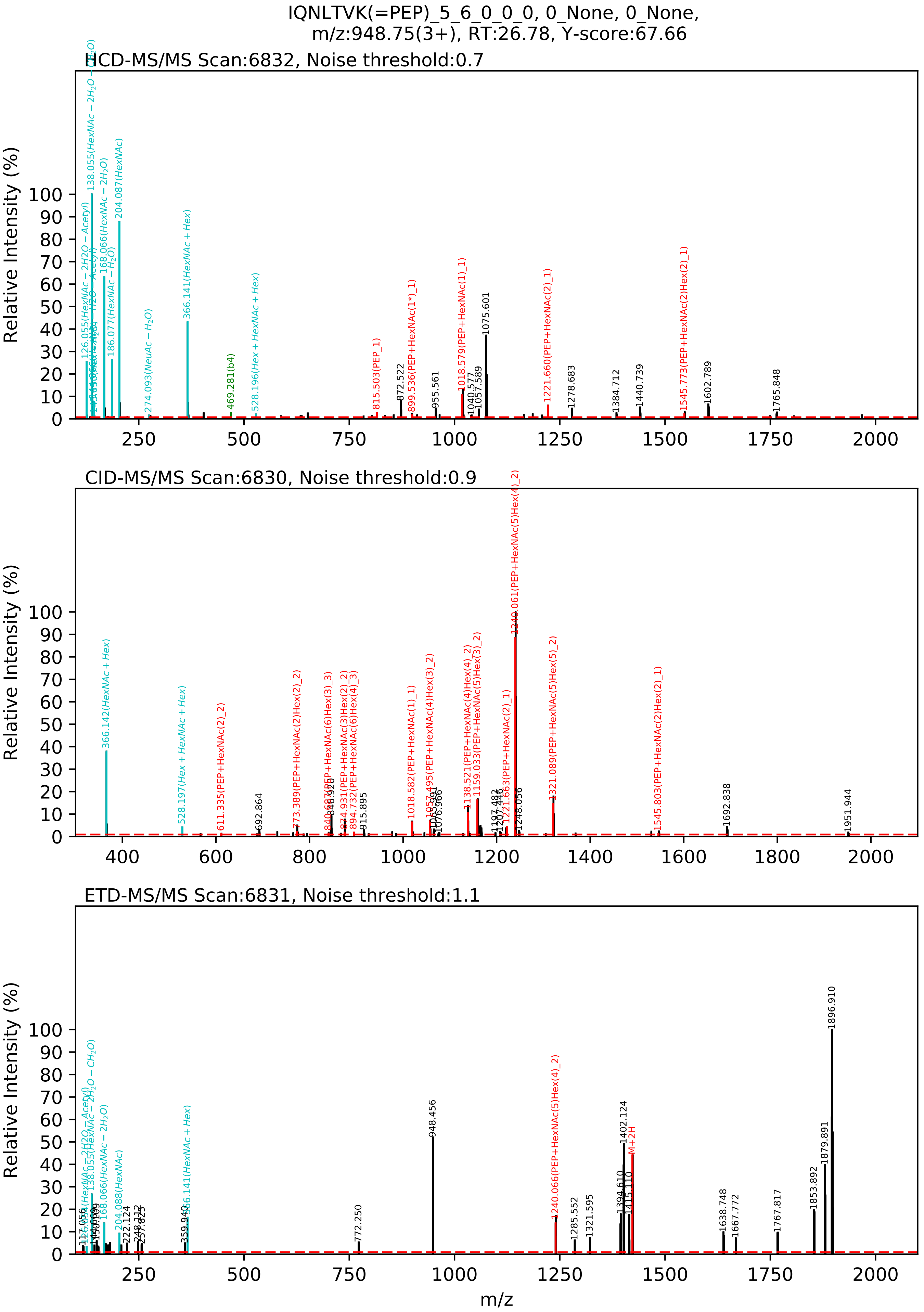

IQNLTVK(=PEP)_5_6_0_0_0, 0_None, 0_None,
m/z:948.75(3+), RT:26.78, Y-score:67.66
HCD-MS/MS Scan:6832, Noise threshold:0.7
Relative Intensity (%)
126.055(HexNAc − 2H2O − Acetyl)
138.055(HexNAc − 2H2O − CH2O)
168.066(HexNAc − 2H2O)
186.077(HexNAc − H2O)
204.087(HexNAc)
274.093(NeuAc − H2O)
366.141(HexNAc + Hex)
469.281(b4)
528.196(Hex + HexNAc + Hex)
815.503(PEP_1)
872.522
899.536(PEP+HexNAc(1*)_1)
955.561
1018.579(PEP+HexNAc(1)_1)
1040.577
1057.589
1075.601
1221.660(PEP+HexNAc(2)_1)
1278.683
1384.712
1440.739
1545.773(PEP+HexNAc(2)Hex(2)_1)
1602.789
1765.848
CID-MS/MS Scan:6830, Noise threshold:0.9
Relative Intensity (%)
366.142(HexNAc + Hex)
528.197(Hex + HexNAc + Hex)
611.335(PEP+HexNAc(2)_2)
692.864
773.389(PEP+HexNAc(2)Hex(2)_2)
874.931(PEP+HexNAc(3)Hex(2)_2)
894.732(PEP+HexNAc(6)Hex(4)_3)
915.895
1018.582(PEP+HexNAc(1)_1)
1057.495(PEP+HexNAc(4)Hex(3)_2)
1076.966
1138.521(PEP+HexNAc(4)Hex(4)_2)
1159.033(PEP+HexNAc(5)Hex(3)_2)
1221.663(PEP+HexNAc(2)_1)
1240.061(PEP+HexNAc(5)Hex(4)_2)
1248.056
1321.089(PEP+HexNAc(5)Hex(5)_2)
1545.803(PEP+HexNAc(2)Hex(2)_1)
1692.838
1951.944
ETD-MS/MS Scan:6831, Noise threshold:1.1
Relative Intensity (%)
117.056
138.055(HexNAc − 2H2O − CH2O)
168.066(HexNAc − 2H2O)
204.088(HexNAc)
222.124
248.112
257.825
359.940
366.141(HexNAc + Hex)
772.250
948.456
1240.066(PEP+HexNAc(5)Hex(4)_2)
1285.552
1321.595
1394.610
1402.124
1415.110
M+2H
1638.748
1667.772
1767.817
1853.892
1879.891
1896.910
m/z

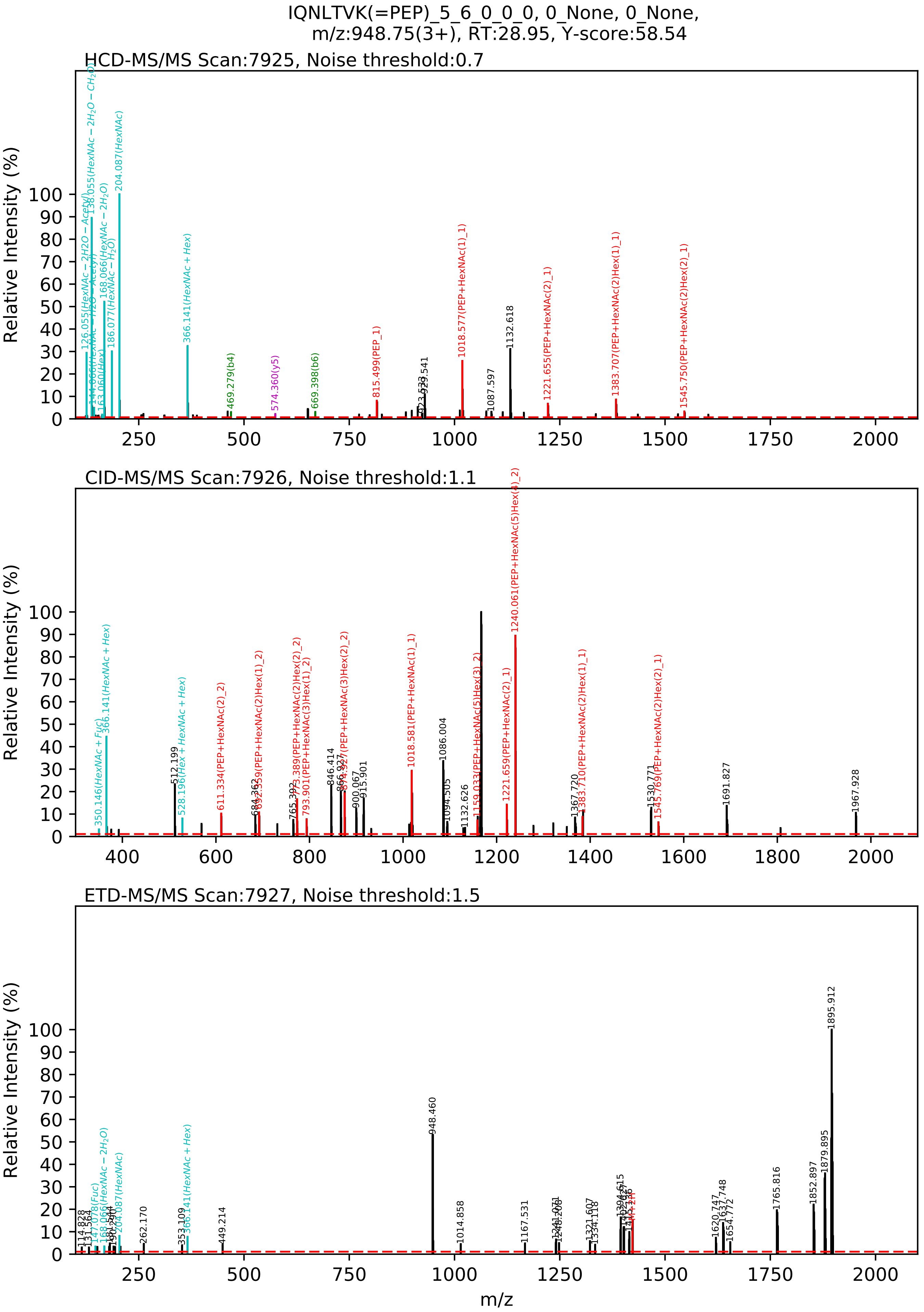
IQNLTVK(=PEP)_5_6_0_0_0, 0_None, 0_None,
m/z:948.75(3+), RT:28.95, Y-score:58.54
HCD-MS/MS Scan:7925, Noise threshold:0.7
CID-MS/MS Scan:7926, Noise threshold:1.1
ETD-MS/MS Scan:7927, Noise threshold:1.5
Relative Intensity (%)
m/z
204.087(HexNAc)
366.141(HexNAc + Hex)
469.279(b4)
574.360(y5)
669.398(b6)
815.499(PEP_1)
1018.577(PEP+HexNAc(1)_1)
1132.618
1087.597
1221.655(PEP+HexNAc(2)_1)
1383.707(PEP+HexNAc(2)Hex(1)_1)
1545.750(PEP+HexNAc(2)Hex(2)_1)
350.146(HexNAc + Fuc)
528.196(Hex + HexNAc + Hex)
512.199
611.334(PEP+HexNAc(2)_2)
692.359(PEP+HexNAc(2)Hex(1)_2)
773.389(PEP+HexNAc(2)Hex(2)_2)
793.901(PEP+HexNAc(3)Hex(1)_2)
874.927(PEP+HexNAc(3)Hex(2)_2)
765.392
846.414
866.927
900.067
915.901
1018.581(PEP+HexNAc(1)_1)
1086.004
1094.505
1132.626
1159.033(PEP+HexNAc(5)Hex(3)_2)
1221.659(PEP+HexNAc(2)_1)
1240.061(PEP+HexNAc(5)Hex(4)_2)
1367.720
1383.710(PEP+HexNAc(2)Hex(1)_1)
1530.771
1545.769(PEP+HexNAc(2)Hex(2)_1)
1691.827
1967.928
147.078(Fuc)
168.066(HexNAc − 2H2O)
204.087(HexNAc)
262.170
353.109
366.141(HexNAc + Hex)
449.214
948.460
1014.858
1167.531
1321.607
1334.118
1620.747
1637.748
1654.772
1765.816
1852.897
1879.895
1895.912

IQNLTVK(=PEP)_6_2_0_0_0, 0_None, 0_None,
m/z:1097.49(2+), RT:26.36, Y-score:94.11

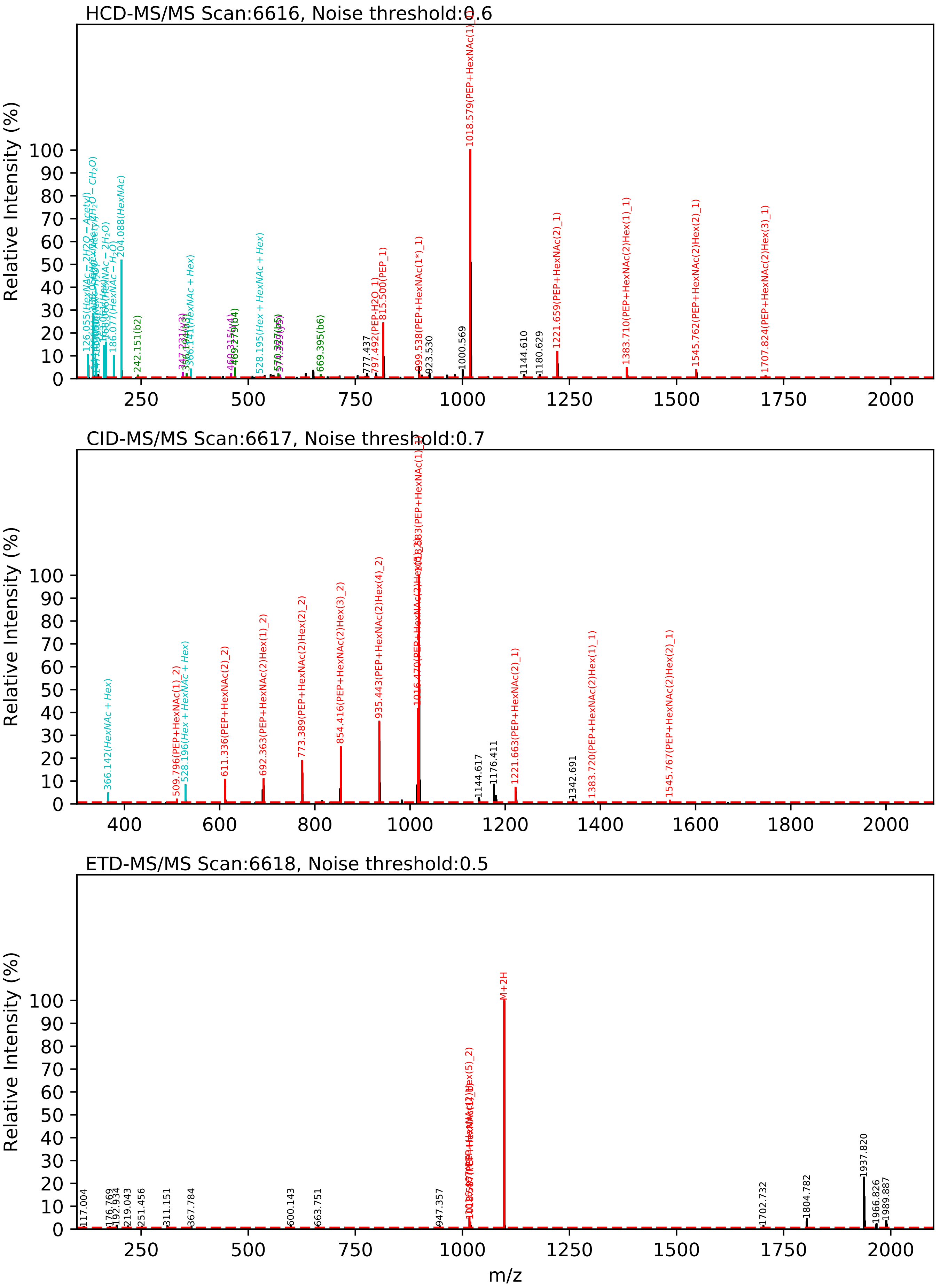

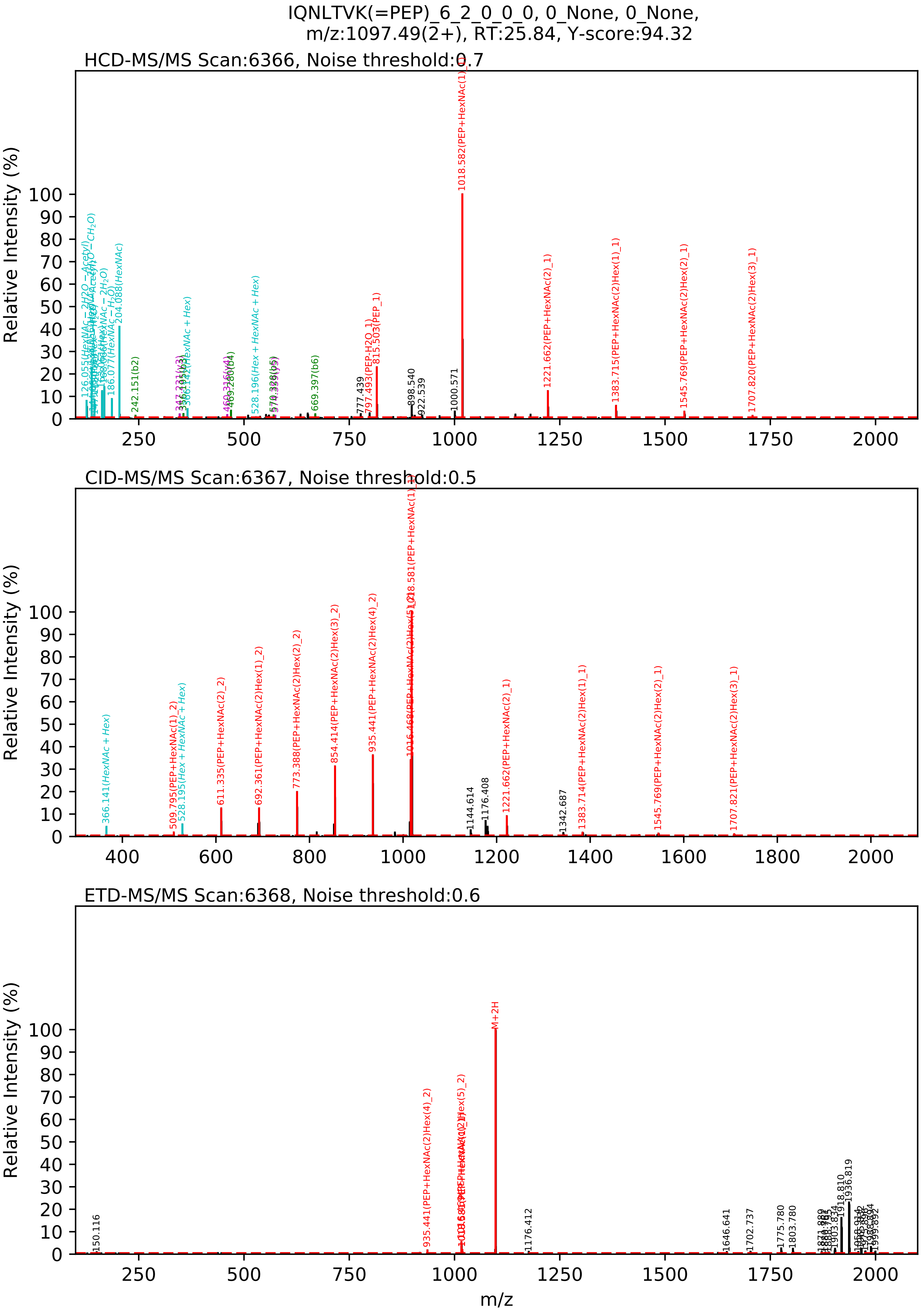
IQNLTVK(=PEP)_6_2_0_0_0, 0_None, 0_None,
m/z:1097.49(2+), RT:25.84, Y-score:94.32
HCD-MS/MS Scan:6366, Noise threshold:0.7
CID-MS/MS Scan:6367, Noise threshold:0.5
ETD-MS/MS Scan:6368, Noise threshold:0.6
Relative Intensity (%)
m/z
1018.582(PEP+HexNAc(1)_1)
1221.662(PEP+HexNAc(2)_1)
1383.715(PEP+HexNAc(2)Hex(1)_1)
1545.769(PEP+HexNAc(2)Hex(2)_1)
1707.820(PEP+HexNAc(2)Hex(3)_1)
815.503(PEP_1)
204.088(HexNAc)
242.151(b2)
669.397(b6)
1018.581(PEP+HexNAc(1)_1)
935.441(PEP+HexNAc(2)Hex(4)_2)
854.414(PEP+HexNAc(2)Hex(3)_2)
773.388(PEP+HexNAc(2)Hex(2)_2)
692.361(PEP+HexNAc(2)Hex(1)_2)
611.335(PEP+HexNAc(2)_2)
509.795(PEP+HexNAc(1)_2)
366.141(HexNAc + Hex)
1221.662(PEP+HexNAc(2)_1)
1383.714(PEP+HexNAc(2)Hex(1)_1)
1545.769(PEP+HexNAc(2)Hex(2)_1)
1707.821(PEP+HexNAc(2)Hex(3)_1)
M+2H
935.441(PEP+HexNAc(2)Hex(4)_2)
1936.819

IQNLTVK(=PEP)_6_3_0_0_0, 0_None, 0_None,
m/z:1199.03(2+), RT:26.14, Y-score:91.26

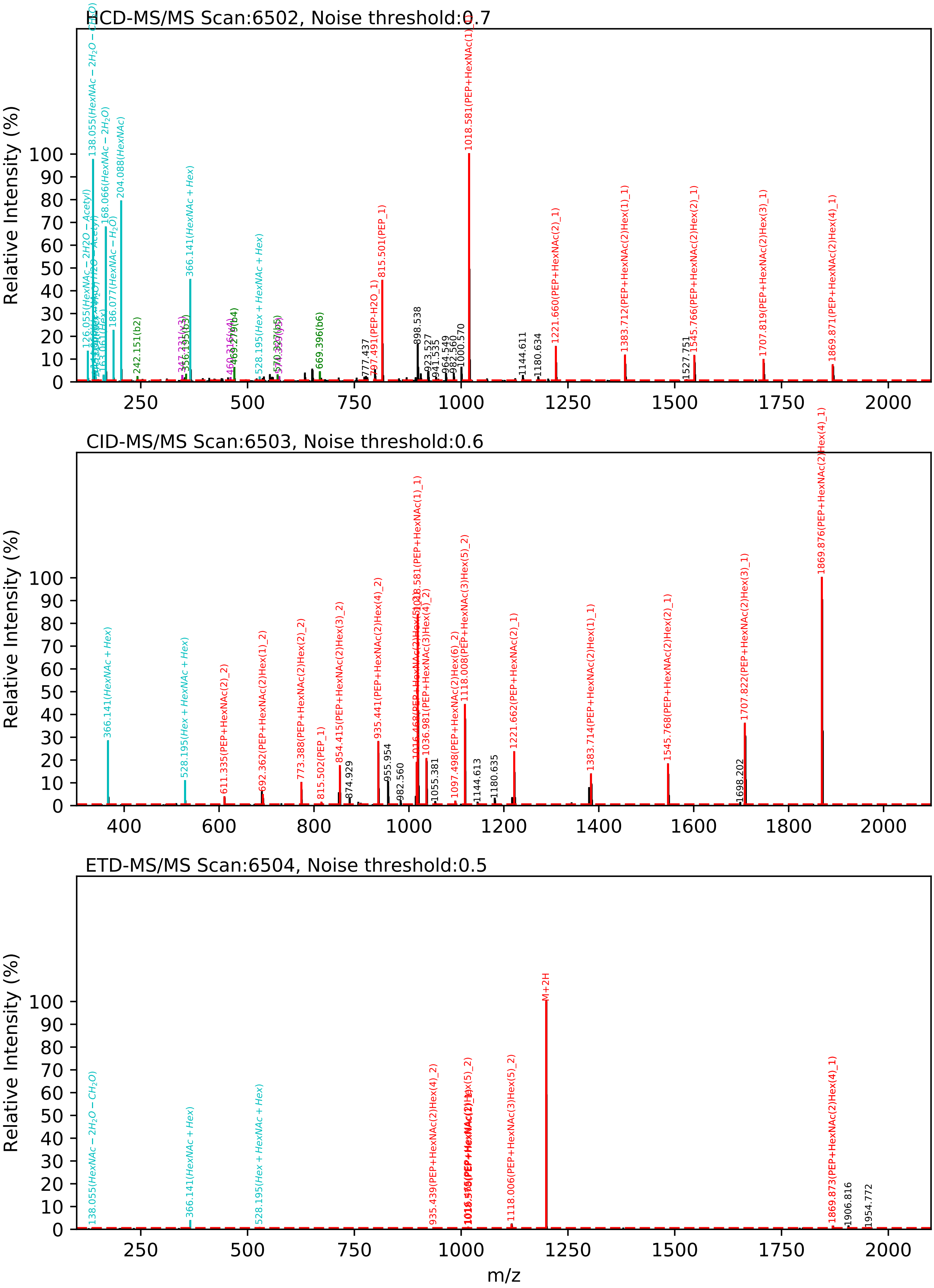

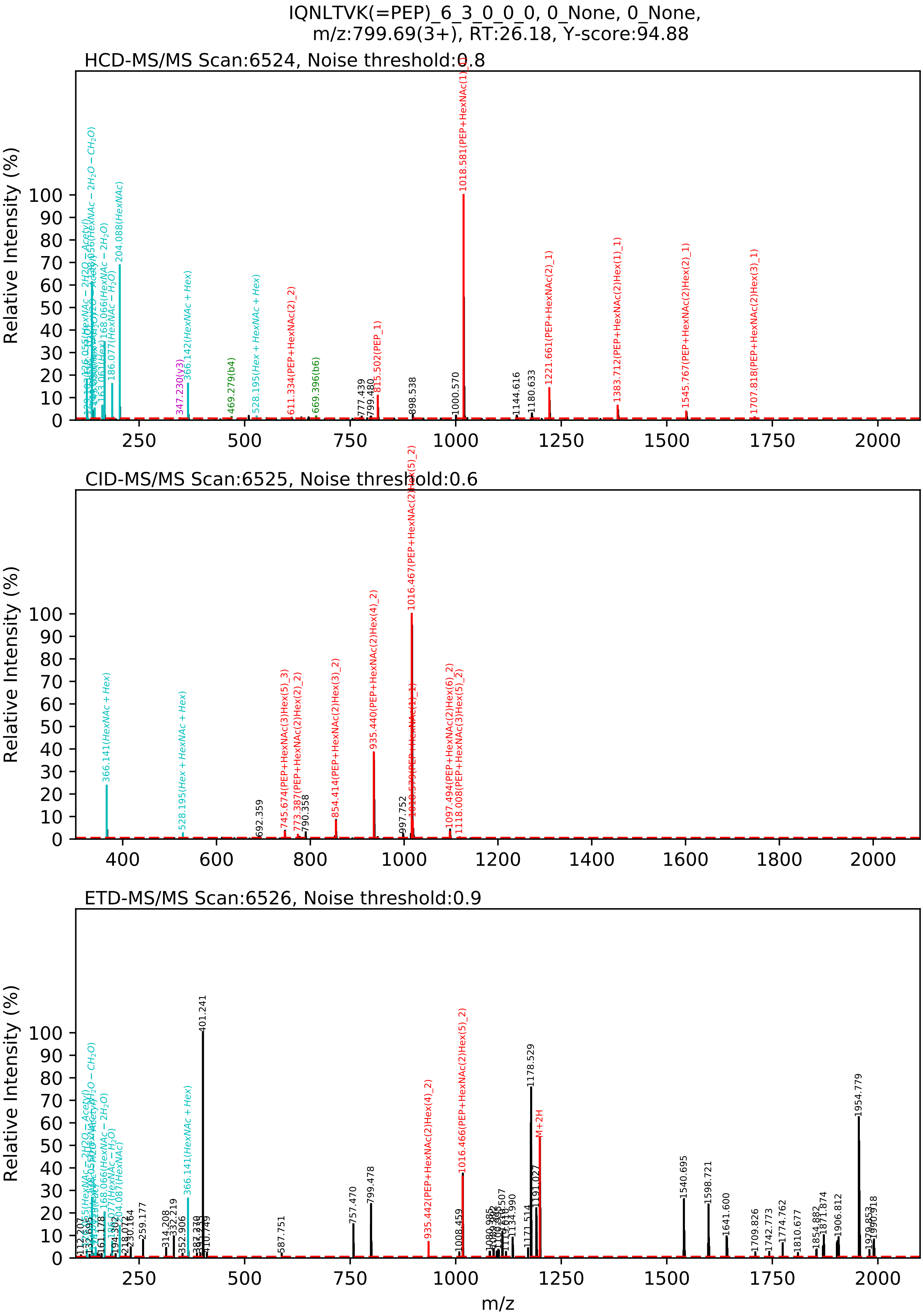

IQNLTVK(=PEP)_6_3_0_0_0, 0_None, 0_None,
m/z:799.69(3+), RT:26.18, Y-score:94.88
HCD-MS/MS Scan:6524, Noise threshold:0.8
Relative Intensity (%)
1018.581(PEP+HexNAc(1)_1)
204.088(HexNAc)
186.077(HexNAc − H2O)
168.066(HexNAc − 2H2O)
366.142(HexNAc + Hex)
347.230(y3)
469.279(b4)
528.195(Hex + HexNAc + Hex)
611.334(PEP+HexNAc(2)_2)
669.396(b6)
777.439
799.480
815.502(PEP_1)
898.538
1000.570
1144.616
1180.633
1221.661(PEP+HexNAc(2)_1)
1383.712(PEP+HexNAc(2)Hex(1)_1)
1545.767(PEP+HexNAc(2)Hex(2)_1)
1707.818(PEP+HexNAc(2)Hex(3)_1)
CID-MS/MS Scan:6525, Noise threshold:0.6
366.141(HexNAc + Hex)
528.195(Hex + HexNAc + Hex)
692.359
745.674(PEP+HexNAc(3)Hex(5)_3)
773.387(PEP+HexNAc(2)Hex(2)_2)
790.358
854.414(PEP+HexNAc(2)Hex(3)_2)
935.440(PEP+HexNAc(2)Hex(4)_2)
997.752
1016.467(PEP+HexNAc(2)Hex(5)_2)
1097.494(PEP+HexNAc(2)Hex(6)_2)
1118.008(PEP+HexNAc(3)Hex(5)_2)
ETD-MS/MS Scan:6526, Noise threshold:0.9
401.241
366.141(HexNAc + Hex)
204.087(HexNAc)
168.066(HexNAc − 2H2O)
112.207
161.178
218.072
230.164
259.177
314.208
332.219
352.906
410.749
587.751
757.470
799.478
935.442(PEP+HexNAc(2)Hex(4)_2)
1008.459
1016.466(PEP+HexNAc(2)Hex(5)_2)
1134.990
1171.514
1178.529
1191.027
M+2H
1540.695
1598.721
1641.600
1709.826
1742.773
1774.762
1810.677
1871.874
1906.812
1954.779
m/z

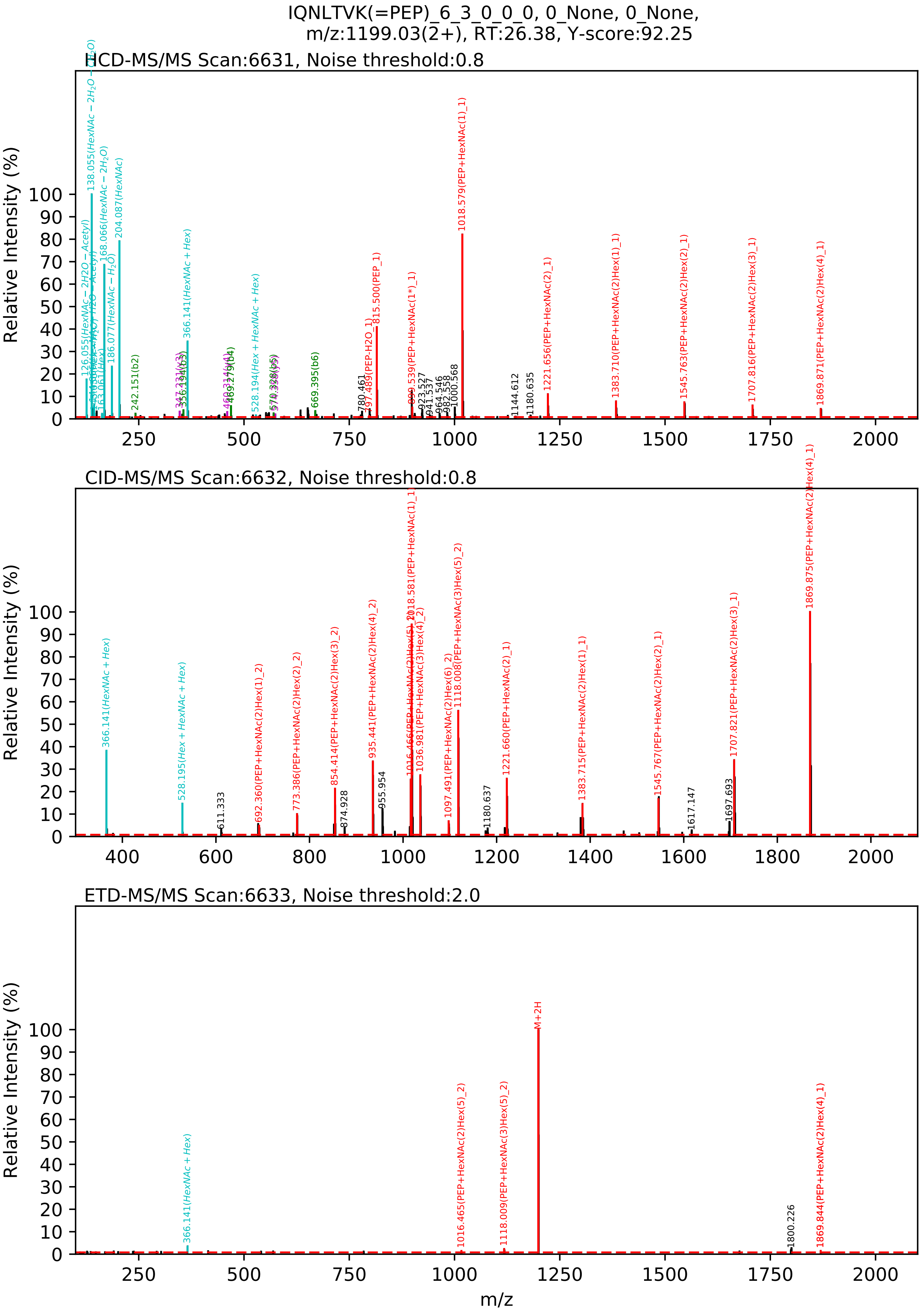

IQNLTVK(=PEP)_6_3_0_0_0, 0_None, 0_None,
m/z:1199.03(2+), RT:26.38, Y-score:92.25
HCD-MS/MS Scan:6631, Noise threshold:0.8
138.055(HexNAc − 2H2O − H2O)
168.066(HexNAc − 2H2O)
204.087(HexNAc)
186.077(HexNAc − H2O)
366.141(HexNAc + Hex)
528.194(Hex + HexNAc + Hex)
669.395(b6)
242.151(b2)
1018.579(PEP+HexNAc(1)_1)
815.500(PEP_1)
797.489(PEP-H2O_1)
899.539(PEP+HexNAc(1*)_1)
1221.656(PEP+HexNAc(2)_1)
1383.710(PEP+HexNAc(2)Hex(1)_1)
1545.763(PEP+HexNAc(2)Hex(2)_1)
1707.816(PEP+HexNAc(2)Hex(3)_1)
1869.871(PEP+HexNAc(2)Hex(4)_1)
780.461
923.527
941.537
964.546
1000.568
1144.612
1180.635
CID-MS/MS Scan:6632, Noise threshold:0.8
366.141(HexNAc + Hex)
528.195(Hex + HexNAc + Hex)
611.333
692.360(PEP+HexNAc(2)Hex(1)_2)
773.386(PEP+HexNAc(2)Hex(2)_2)
854.414(PEP+HexNAc(2)Hex(3)_2)
874.928
935.441(PEP+HexNAc(2)Hex(4)_2)
955.954
1018.581(PEP+HexNAc(1)_1)
1036.981(PEP+HexNAc(3)Hex(4)_2)
1097.491(PEP+HexNAc(2)Hex(6)_2)
1118.008(PEP+HexNAc(3)Hex(5)_2)
1180.637
1221.660(PEP+HexNAc(2)_1)
1383.715(PEP+HexNAc(2)Hex(1)_1)
1545.767(PEP+HexNAc(2)Hex(2)_1)
1617.147
1697.693
1707.821(PEP+HexNAc(2)Hex(3)_1)
1869.875(PEP+HexNAc(2)Hex(4)_1)
ETD-MS/MS Scan:6633, Noise threshold:2.0
366.141(HexNAc + Hex)
1016.465(PEP+HexNAc(2)Hex(5)_2)
1118.009(PEP+HexNAc(3)Hex(5)_2)
M+2H
1800.226
1869.844(PEP+HexNAc(2)Hex(4)_1)
Relative Intensity (%)
m/z

IQNLTVK(=PEP)_6_3_0_1_0, 0_None, 0_None,
m/z:896.72(3+), RT:35.40, Y-score:85.44

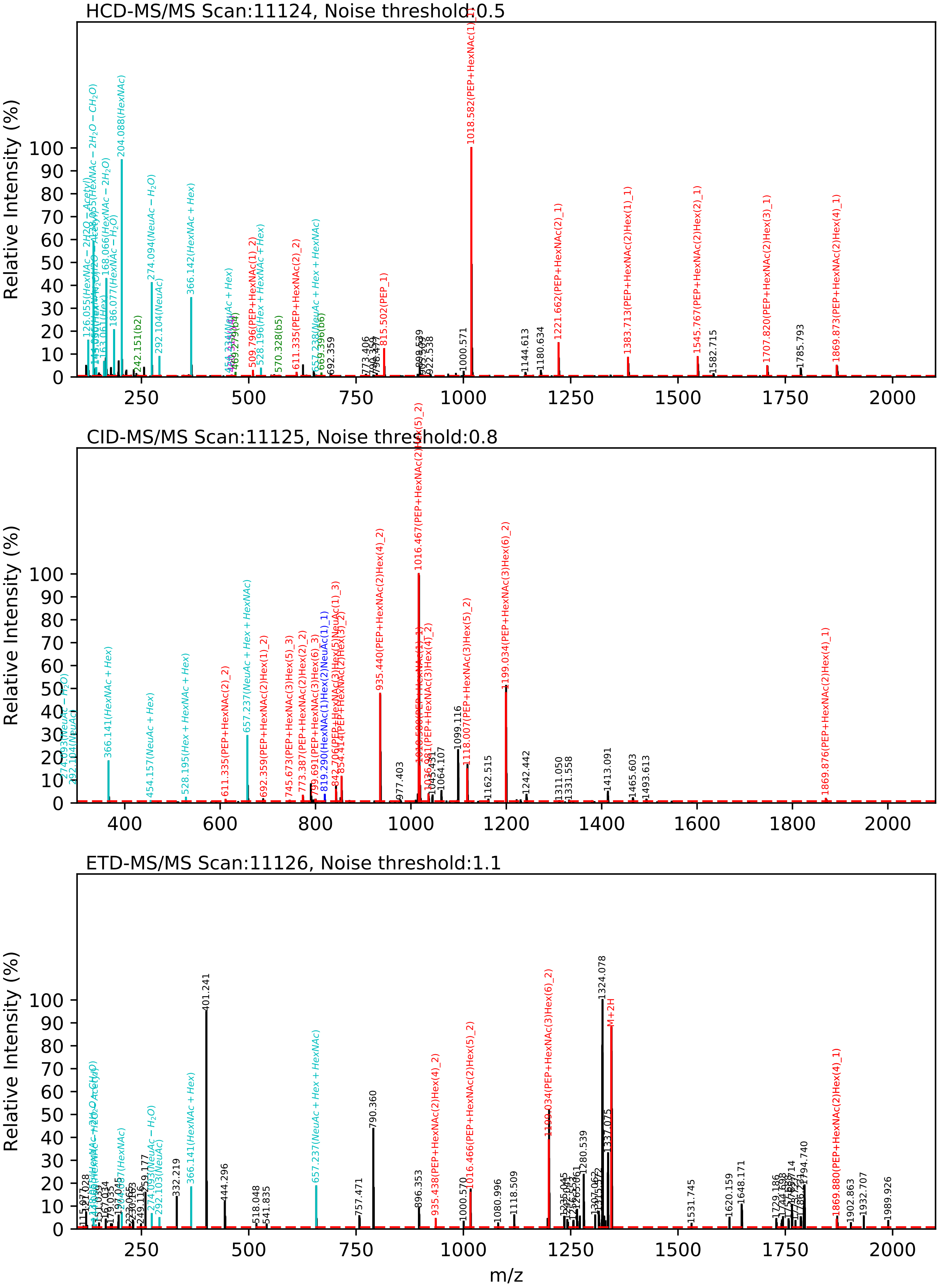

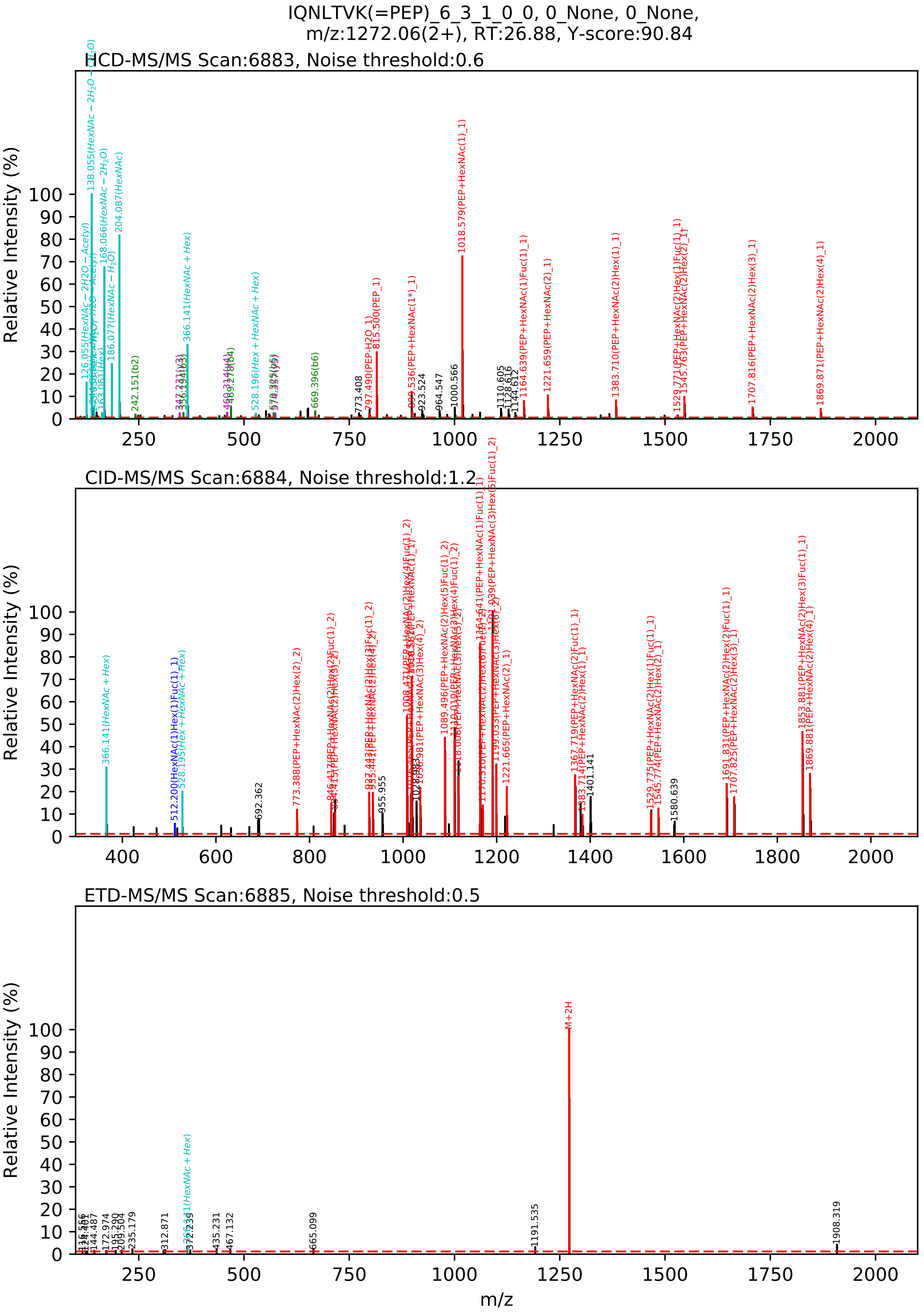
IQNLTVK(=PEP)_6_3_1_0_0, 0_None, 0_None,
m/z:1272.06(2+), RT:26.88, Y-score:90.84
HCD-MS/MS Scan:6883, Noise threshold:0.6
Relative Intensity (%)
138.055(HexNAc − 2H2O − H2O)
204.087(HexNAc)
168.066(HexNAc − 2H2O)
186.077(HexNAc − H2O)
366.141(HexNAc + Hex)
528.196(Hex + HexNAc + Hex)
242.151(b2)
669.396(b6)
773.408
797.490(PEP-H2O_1)
815.500(PEP_1)
899.536(PEP+HexNAc(1*)_1)
923.524
964.547
1000.566
1018.579(PEP+HexNAc(1)_1)
1110.605
1128.616
1144.612
1164.639(PEP+HexNAc(1)Fuc(1)_1)
1221.659(PEP+HexNAc(2)_1)
1383.710(PEP+HexNAc(2)Hex(1)_1)
1545.763(PEP+HexNAc(2)Hex(2)_1)
1707.816(PEP+HexNAc(2)Hex(3)_1)
1869.871(PEP+HexNAc(2)Hex(4)_1)
CID-MS/MS Scan:6884, Noise threshold:1.2
366.141(HexNAc + Hex)
512.200(HexNAc(1)Hex(1)Fuc(1)_1)
528.195(Hex + HexNAc + Hex)
692.362
773.388(PEP+HexNAc(2)Hex(2)_2)
955.955
1089.496(PEP+HexNAc(2)Hex(5)Fuc(1)_2)
1221.665(PEP+HexNAc(2)_1)
1367.719(PEP+HexNAc(2)Fuc(1)_1)
1383.714(PEP+HexNAc(2)Hex(1)_1)
1401.141
1529.775(PEP+HexNAc(2)Hex(1)Fuc(1)_1)
1545.774(PEP+HexNAc(2)Hex(2)_1)
1580.639
1691.831(PEP+HexNAc(2)Hex(2)Fuc(1)_1)
1707.825(PEP+HexNAc(2)Hex(3)_1)
1853.881(PEP+HexNAc(2)Hex(3)Fuc(1)_1)
1869.881(PEP+HexNAc(2)Hex(4)_1)
ETD-MS/MS Scan:6885, Noise threshold:0.5
M+2H
144.487
172.974
235.179
312.871
435.231
467.132
665.099
1191.535
1908.319
m/z

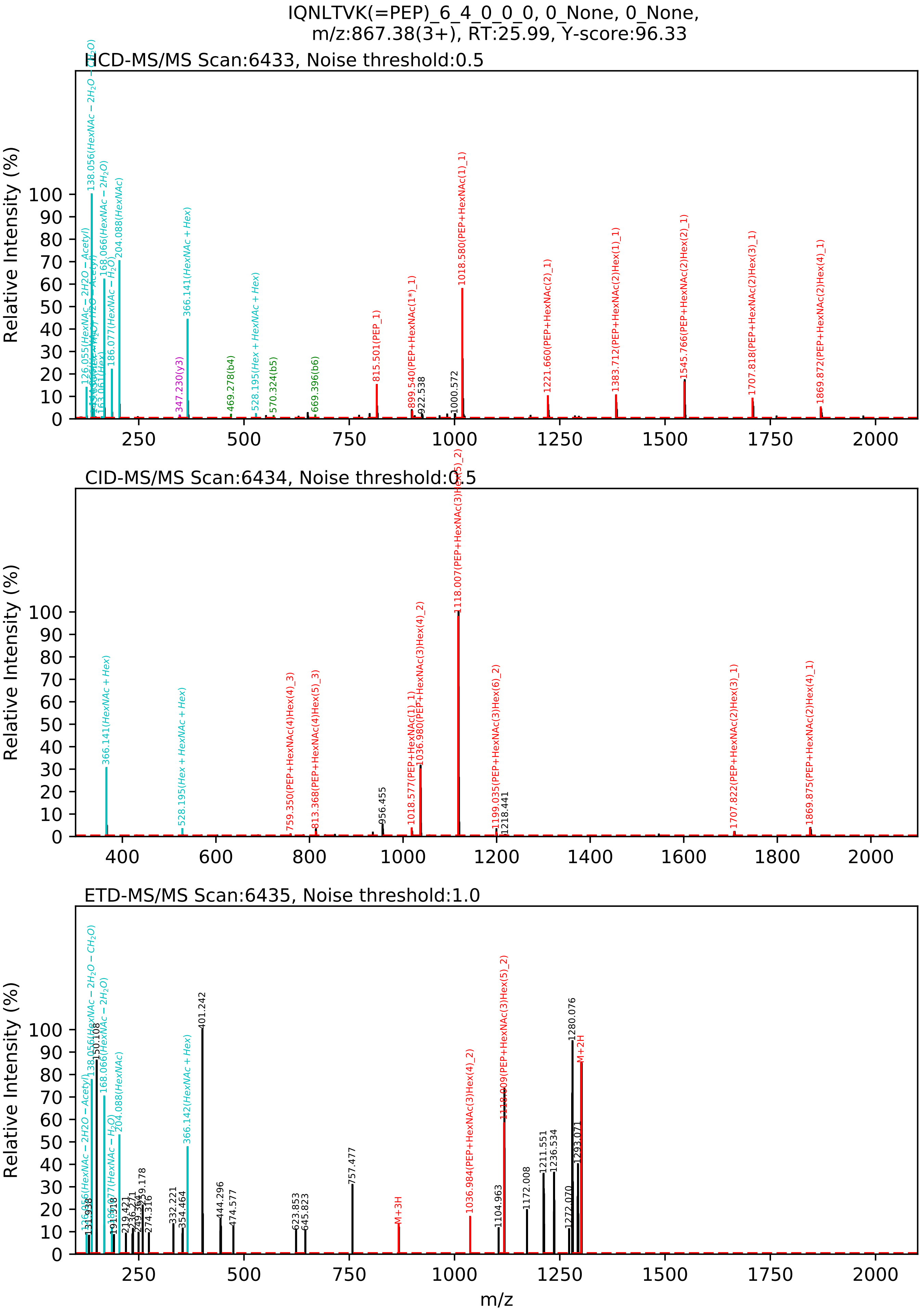

IQNLTVK(=PEP)_6_4_0_0_0, 0_None, 0_None,
m/z:867.38(3+), RT:25.99, Y-score:96.33
HCD-MS/MS Scan:6433, Noise threshold:0.5
Relative Intensity (%)
138.056(HexNAc − 2H2O − CH2O)
1018.580(PEP+HexNAc(1)_1)
1221.660(PEP+HexNAc(2)_1)
1383.712(PEP+HexNAc(2)Hex(1)_1)
1545.766(PEP+HexNAc(2)Hex(2)_1)
1707.818(PEP+HexNAc(2)Hex(3)_1)
1869.872(PEP+HexNAc(2)Hex(4)_1)
366.141(HexNAc + Hex)
204.088(HexNAc)
186.077(HexNAc − H2O)
168.066(HexNAc − 2H2O)
126.055(HexNAc − 2H2O − Acetyl)
347.230(y3)
469.278(b4)
528.195(Hex + HexNAc + Hex)
570.324(b5)
669.396(b6)
815.501(PEP_1)
899.540(PEP+HexNAc(1*)_1)
922.538
1000.572
CID-MS/MS Scan:6434, Noise threshold:0.5
1118.007(PEP+HexNAc(3)Hex(5)_2)
1036.980(PEP+HexNAc(3)Hex(4)_2)
1018.577(PEP+HexNAc(1)_1)
366.141(HexNAc + Hex)
528.195(Hex + HexNAc + Hex)
759.350(PEP+HexNAc(4)Hex(4)_3)
813.368(PEP+HexNAc(4)Hex(5)_3)
956.455
1199.035(PEP+HexNAc(3)Hex(6)_2)
1218.441
1707.822(PEP+HexNAc(2)Hex(3)_1)
1869.875(PEP+HexNAc(2)Hex(4)_1)
ETD-MS/MS Scan:6435, Noise threshold:1.0
138.056(HexNAc − 2H2O − CH2O)
168.066(HexNAc − 2H2O)
204.088(HexNAc)
366.142(HexNAc + Hex)
150.108
401.242
131.938
191.918
219.421
236.271
249.394
259.178
274.316
332.221
354.464
444.296
474.577
623.853
645.823
757.477
M+3H
1036.984(PEP+HexNAc(3)Hex(4)_2)
1118.009(PEP+HexNAc(3)Hex(5)_2)
1104.963
1172.008
1211.551
1236.534
1272.070
1280.076
1293.071
M+2H
m/z

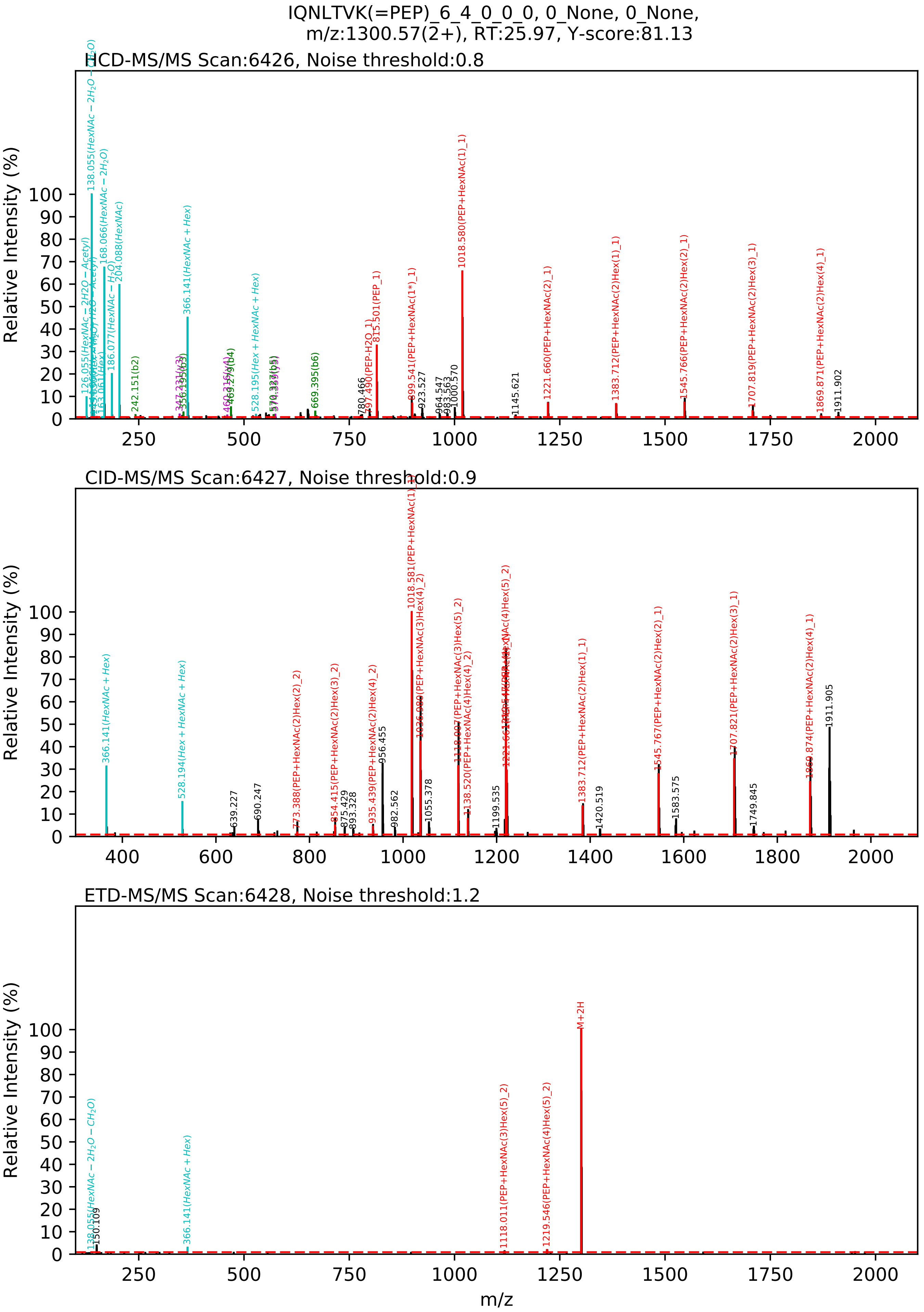

IQNLTVK(=PEP)_6_4_0_0_0, 0_None, 0_None,
m/z:1300.57(2+), RT:25.97, Y-score:81.13
HCD-MS/MS Scan:6426, Noise threshold:0.8
Relative Intensity (%)
138.055(HexNAc − 2H2O − CH2O)
168.066(HexNAc − 2H2O)
204.088(HexNAc)
186.077(HexNAc − H2O)
366.141(HexNAc + Hex)
126.055(HexNAc − 2H2O − Acetyl)
163.061(Hex)
242.151(b2)
669.395(b6)
528.195(Hex + HexNAc + Hex)
780.466
797.490(PEP-H2O_1)
815.501(PEP_1)
899.541(PEP+HexNAc(1*)_1)
923.527
964.547
1000.570
1018.580(PEP+HexNAc(1)_1)
1145.621
1221.660(PEP+HexNAc(2)_1)
1383.712(PEP+HexNAc(2)Hex(1)_1)
1545.766(PEP+HexNAc(2)Hex(2)_1)
1707.819(PEP+HexNAc(2)Hex(3)_1)
1869.871(PEP+HexNAc(2)Hex(4)_1)
1911.902
CID-MS/MS Scan:6427, Noise threshold:0.9
366.141(HexNAc + Hex)
528.194(Hex + HexNAc + Hex)
639.227
690.247
773.388(PEP+HexNAc(2)Hex(2)_2)
854.415(PEP+HexNAc(2)Hex(3)_2)
875.429
893.328
935.439(PEP+HexNAc(2)Hex(4)_2)
956.455
982.562
1018.581(PEP+HexNAc(1)_1)
1055.378
1118.007(PEP+HexNAc(3)Hex(5)_2)
1138.520(PEP+HexNAc(4)Hex(4)_2)
1199.535
1383.712(PEP+HexNAc(2)Hex(1)_1)
1420.519
1545.767(PEP+HexNAc(2)Hex(2)_1)
1583.575
1707.821(PEP+HexNAc(2)Hex(3)_1)
1749.845
1869.874(PEP+HexNAc(2)Hex(4)_1)
1911.905
ETD-MS/MS Scan:6428, Noise threshold:1.2
138.055(HexNAc − 2H2O − CH2O)
150.109
366.141(HexNAc + Hex)
1118.011(PEP+HexNAc(3)Hex(5)_2)
1219.546(PEP+HexNAc(4)Hex(5)_2)
M+2H
m/z

IQNLTVK(=PEP)_6_4_0_1_0, 0_None, 0_None,
m/z:1446.12(2+), RT:35.74, Y-score:82.62

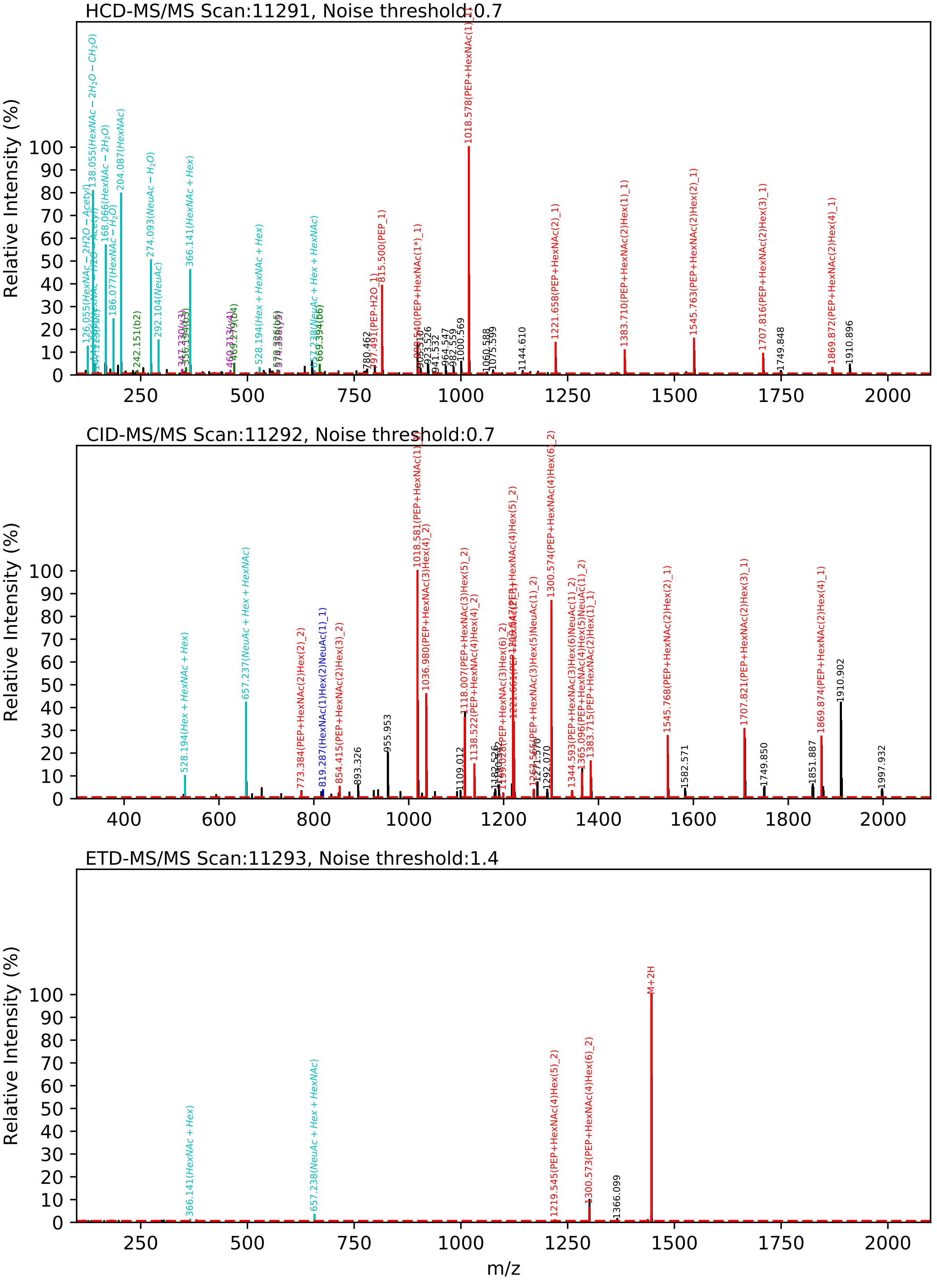

IQNLTVK(=PEP)_6_4_0_1_0, 0_None, 0_None,
m/z:1446.12(2+), RT:35.10, Y-score:83.40

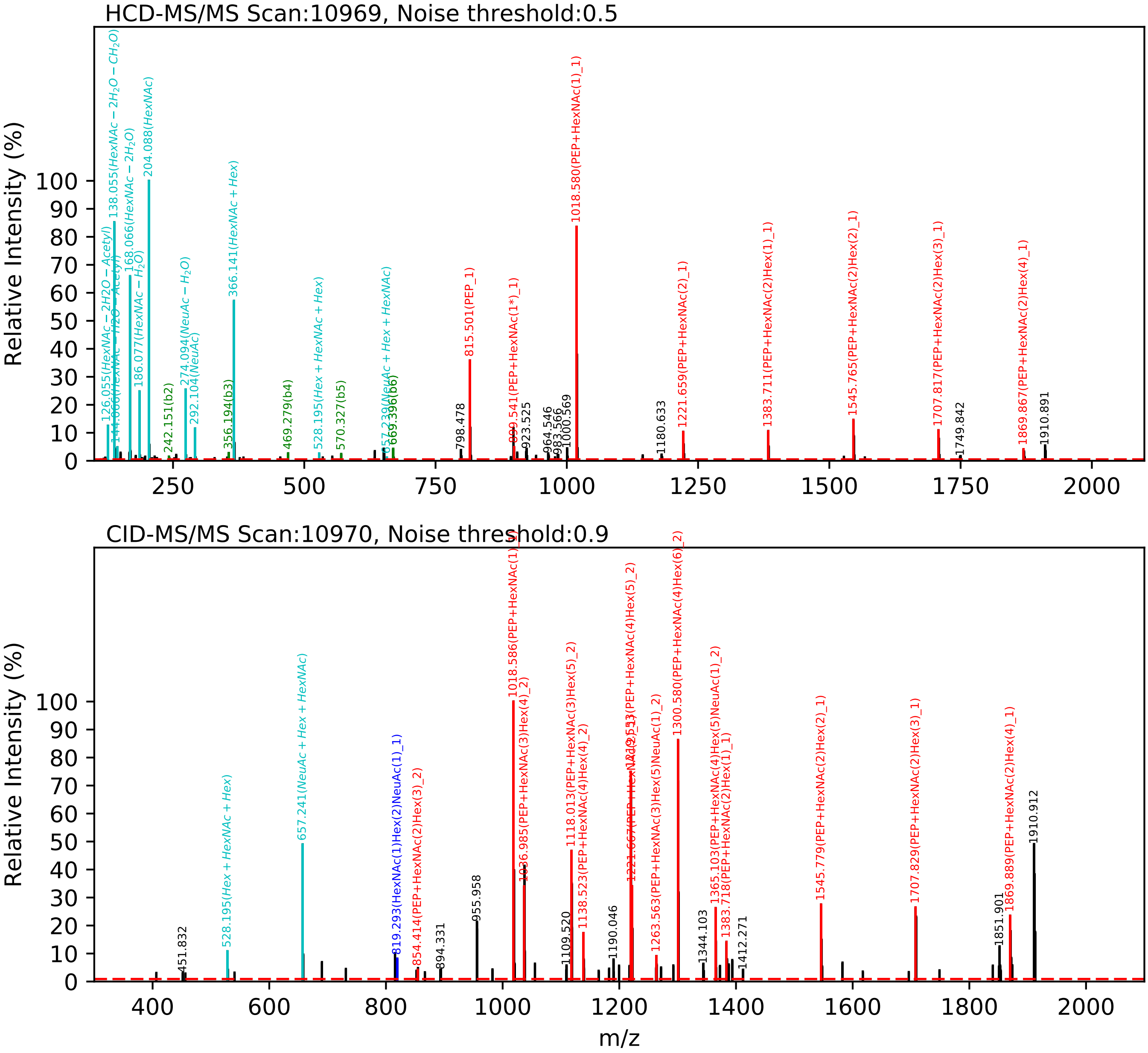

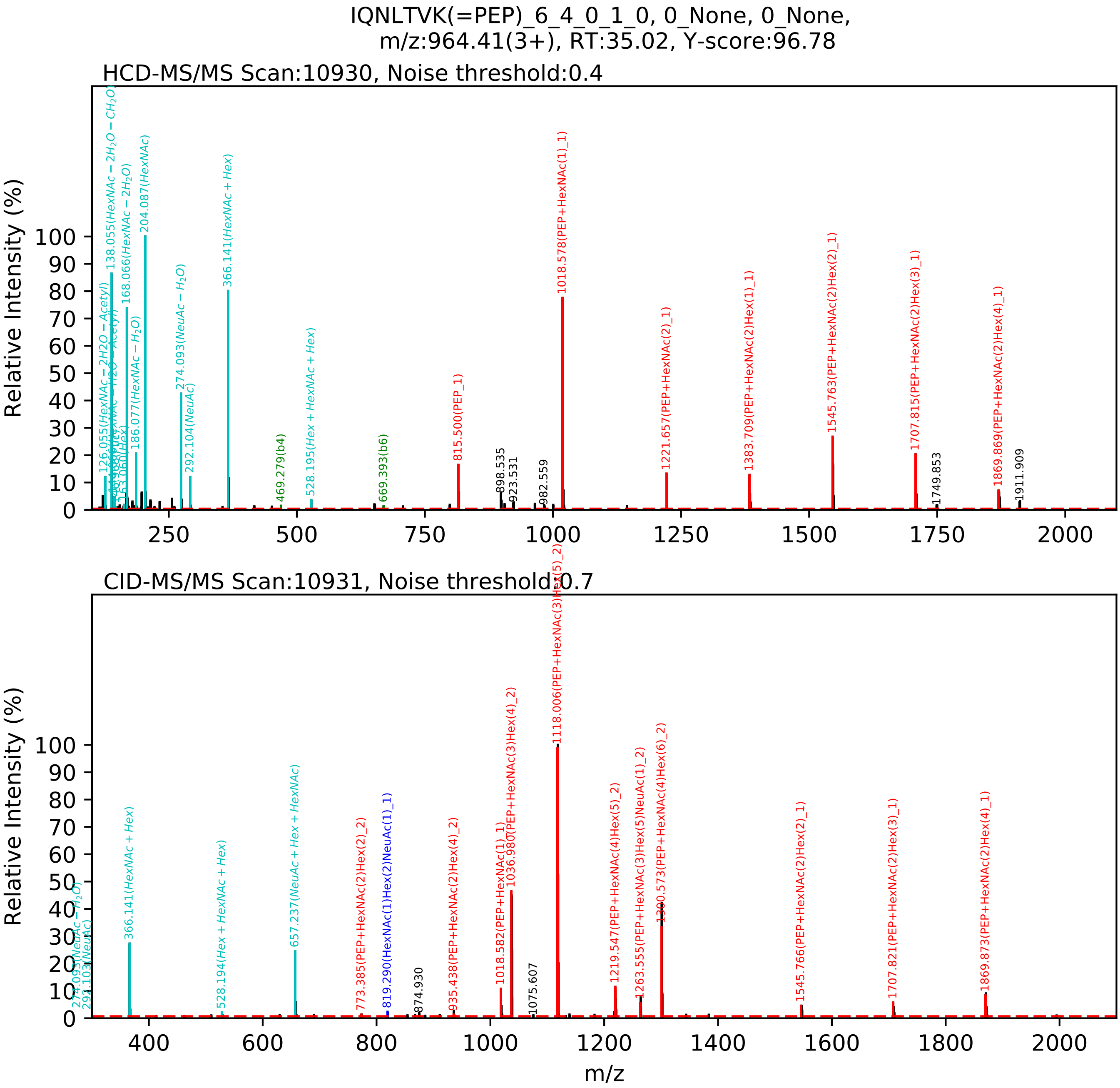

IQNLTVK(=PEP)_6_4_0_1_0, 0_None, 0_None,
m/z:964.41(3+), RT:35.02, Y-score:96.78
HCD-MS/MS Scan:10930, Noise threshold:0.4
Relative Intensity (%)
126.055(HexNAc − 2H2O − Acetyl)
138.055(HexNAc − 2H2O − CH2O)
168.066(HexNAc − 2H2O)
186.077(HexNAc − H2O)
204.087(HexNAc)
274.093(NeuAc − H2O)
292.104(NeuAc)
366.141(HexNAc + Hex)
469.279(b4)
528.195(Hex + HexNAc + Hex)
669.393(b6)
815.500(PEP_1)
898.535
923.531
982.559
1018.578(PEP+HexNAc(1)_1)
1221.657(PEP+HexNAc(2)_1)
1383.709(PEP+HexNAc(2)Hex(1)_1)
1545.763(PEP+HexNAc(2)Hex(2)_1)
1707.815(PEP+HexNAc(2)Hex(3)_1)
1749.853
1869.869(PEP+HexNAc(2)Hex(4)_1)
1911.909
CID-MS/MS Scan:10931, Noise threshold:0.7
274.093(NeuAc − H2O)
291.103(NeuAc)
366.141(HexNAc + Hex)
528.194(Hex + HexNAc + Hex)
657.237(NeuAc + Hex + HexNAc)
773.385(PEP+HexNAc(2)Hex(2)_2)
819.290(HexNAc(1)Hex(2)NeuAc(1)_1)
874.930
935.438(PEP+HexNAc(2)Hex(4)_2)
1018.582(PEP+HexNAc(1)_1)
1036.980(PEP+HexNAc(3)Hex(4)_2)
1075.607
1118.006(PEP+HexNAc(3)Hex(5)_2)
1219.547(PEP+HexNAc(4)Hex(5)_2)
1263.555(PEP+HexNAc(3)Hex(5)NeuAc(1)_2)
1300.573(PEP+HexNAc(4)Hex(6)_2)
1545.766(PEP+HexNAc(2)Hex(2)_1)
1707.821(PEP+HexNAc(2)Hex(3)_1)
1869.873(PEP+HexNAc(2)Hex(4)_1)
m/z

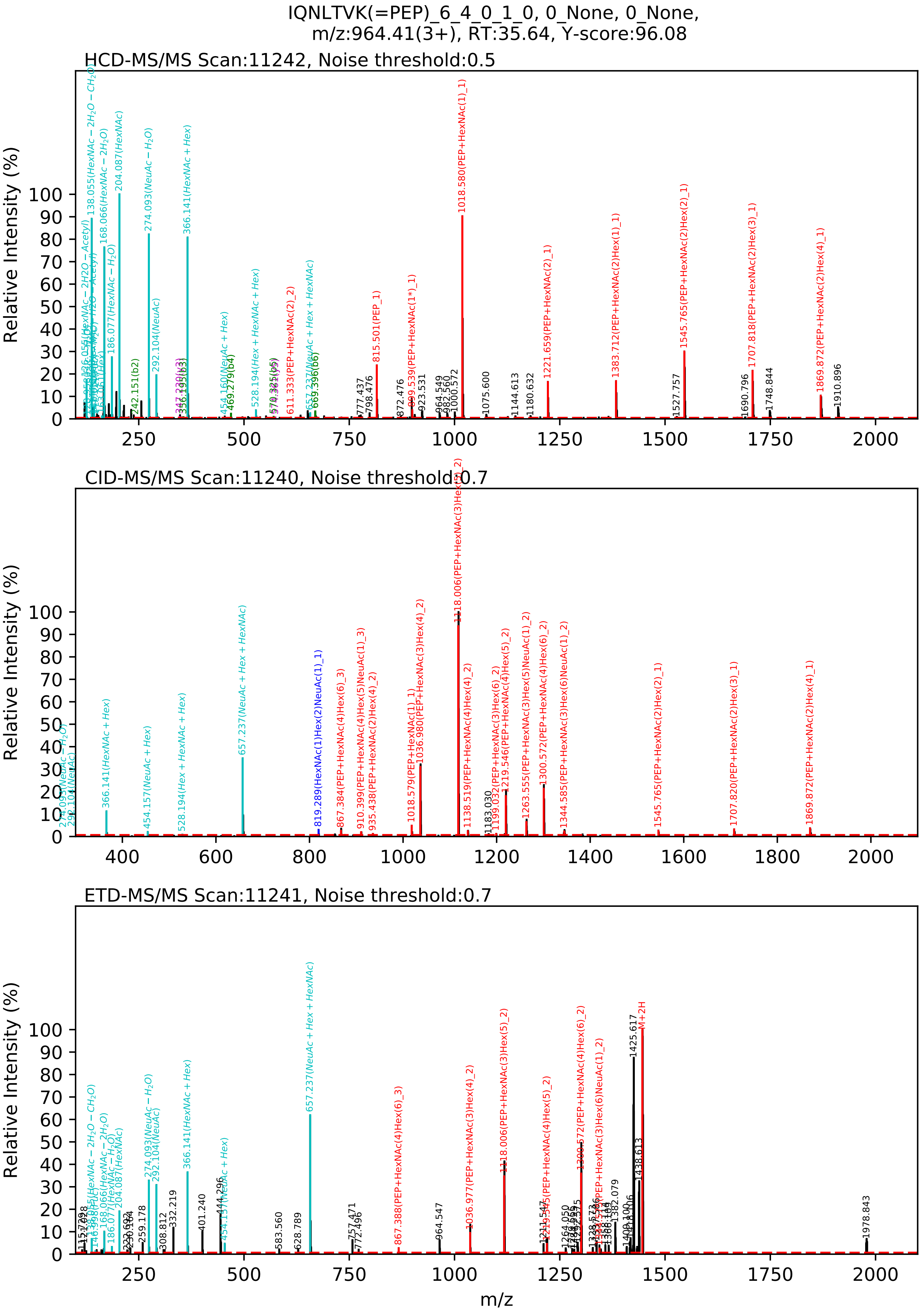
IQNLTVK(=PEP)_6_4_0_1_0, 0_None, 0_None,
m/z:964.41(3+), RT:35.64, Y-score:96.08
HCD-MS/MS Scan:11242, Noise threshold:0.5
Relative Intensity (%)
204.087(HexNAc)
274.093(NeuAc − H2O)
366.141(HexNAc + Hex)
168.066(HexNAc − 2H2O)
186.077(HexNAc − H2O)
292.104(NeuAc)
242.151(b2)
454.160(NeuAc + Hex)
469.279(b4)
528.194(Hex + HexNAc + Hex)
611.333(PEP+HexNAc(2)_2)
657.237(NeuAc + Hex + HexNAc)
669.396(b6)
815.501(PEP_1)
899.539(PEP+HexNAc(1*)_1)
1018.580(PEP+HexNAc(1)_1)
1221.659(PEP+HexNAc(2)_1)
1383.712(PEP+HexNAc(2)Hex(1)_1)
1545.765(PEP+HexNAc(2)Hex(2)_1)
1707.818(PEP+HexNAc(2)Hex(3)_1)
1869.872(PEP+HexNAc(2)Hex(4)_1)
777.437
798.476
872.476
923.531
964.549
1000.572
1075.600
1144.613
1180.632
1527.757
1690.796
1748.844
1910.896
CID-MS/MS Scan:11240, Noise threshold:0.7
366.141(HexNAc + Hex)
454.157(NeuAc + Hex)
528.194(Hex + HexNAc + Hex)
657.237(NeuAc + Hex + HexNAc)
819.289(HexNAc(1)Hex(2)NeuAc(1)_1)
867.384(PEP+HexNAc(4)Hex(6)_3)
910.399(PEP+HexNAc(4)Hex(5)NeuAc(1)_3)
935.438(PEP+HexNAc(2)Hex(4)_2)
1018.579(PEP+HexNAc(1)_1)
1036.980(PEP+HexNAc(3)Hex(4)_2)
1118.006(PEP+HexNAc(3)Hex(5)_2)
1138.519(PEP+HexNAc(4)Hex(4)_2)
1183.030
1199.032(PEP+HexNAc(3)Hex(6)_2)
1219.546(PEP+HexNAc(4)Hex(5)_2)
1263.555(PEP+HexNAc(3)Hex(5)NeuAc(1)_2)
1300.572(PEP+HexNAc(4)Hex(6)_2)
1344.585(PEP+HexNAc(3)Hex(6)NeuAc(1)_2)
1545.765(PEP+HexNAc(2)Hex(2)_1)
1707.820(PEP+HexNAc(2)Hex(3)_1)
1869.872(PEP+HexNAc(2)Hex(4)_1)
ETD-MS/MS Scan:11241, Noise threshold:0.7
274.093(NeuAc − H2O)
292.104(NeuAc)
366.141(HexNAc + Hex)
657.237(NeuAc + Hex + HexNAc)
867.388(PEP+HexNAc(4)Hex(6)_3)
1036.977(PEP+HexNAc(3)Hex(4)_2)
1118.006(PEP+HexNAc(3)Hex(5)_2)
1300.572(PEP+HexNAc(4)Hex(6)_2)
M+2H
1425.617
1438.613
1382.079
259.178
308.812
332.219
401.240
444.296
583.560
628.789
757.471
772.496
964.547
1978.843
m/z

IQNLTVK(=PEP)_6_4_0_2_0, 0_None, 0_None,
m/z:1061.45(3+), RT:48.66, Y-score:69.99

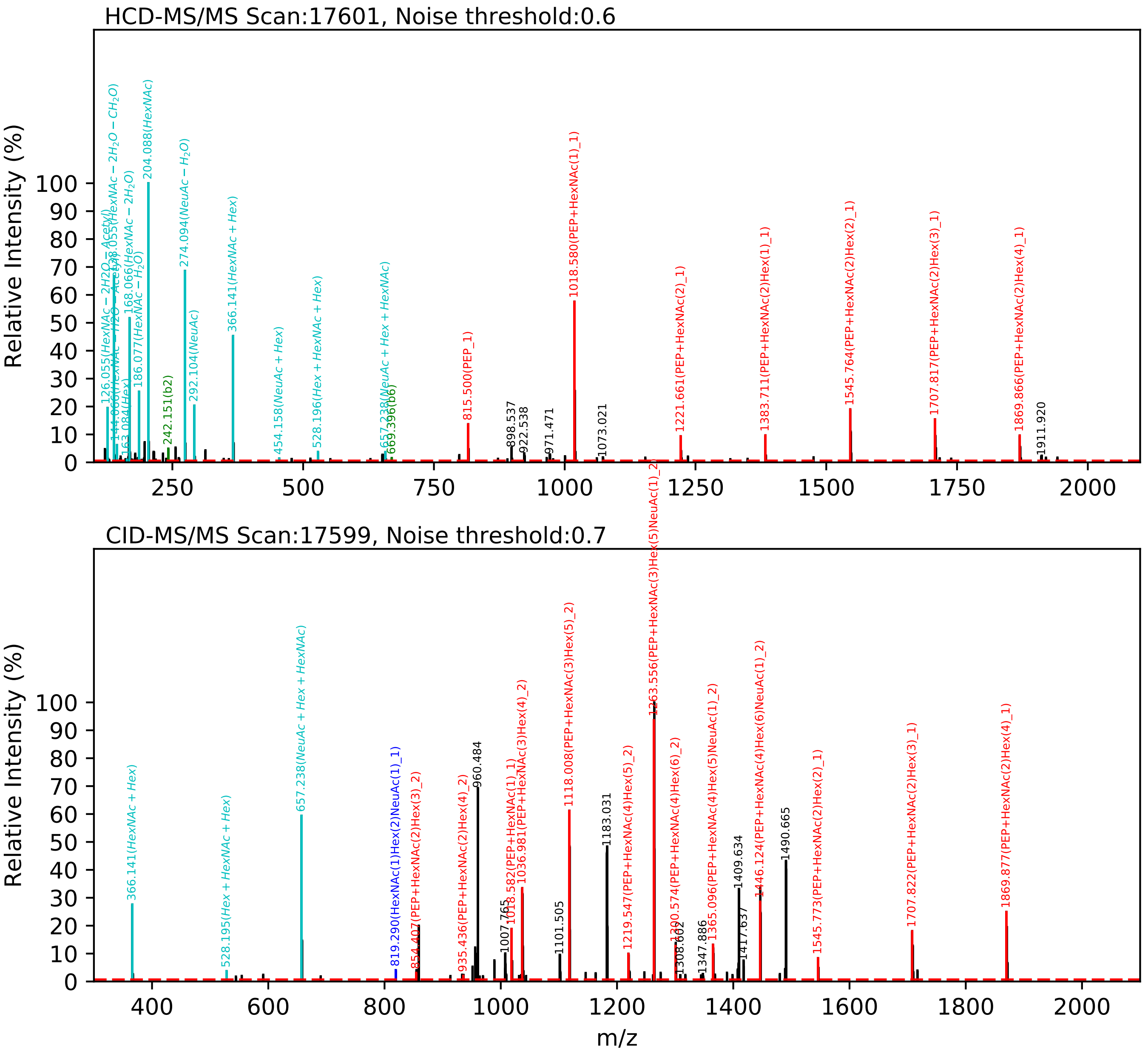

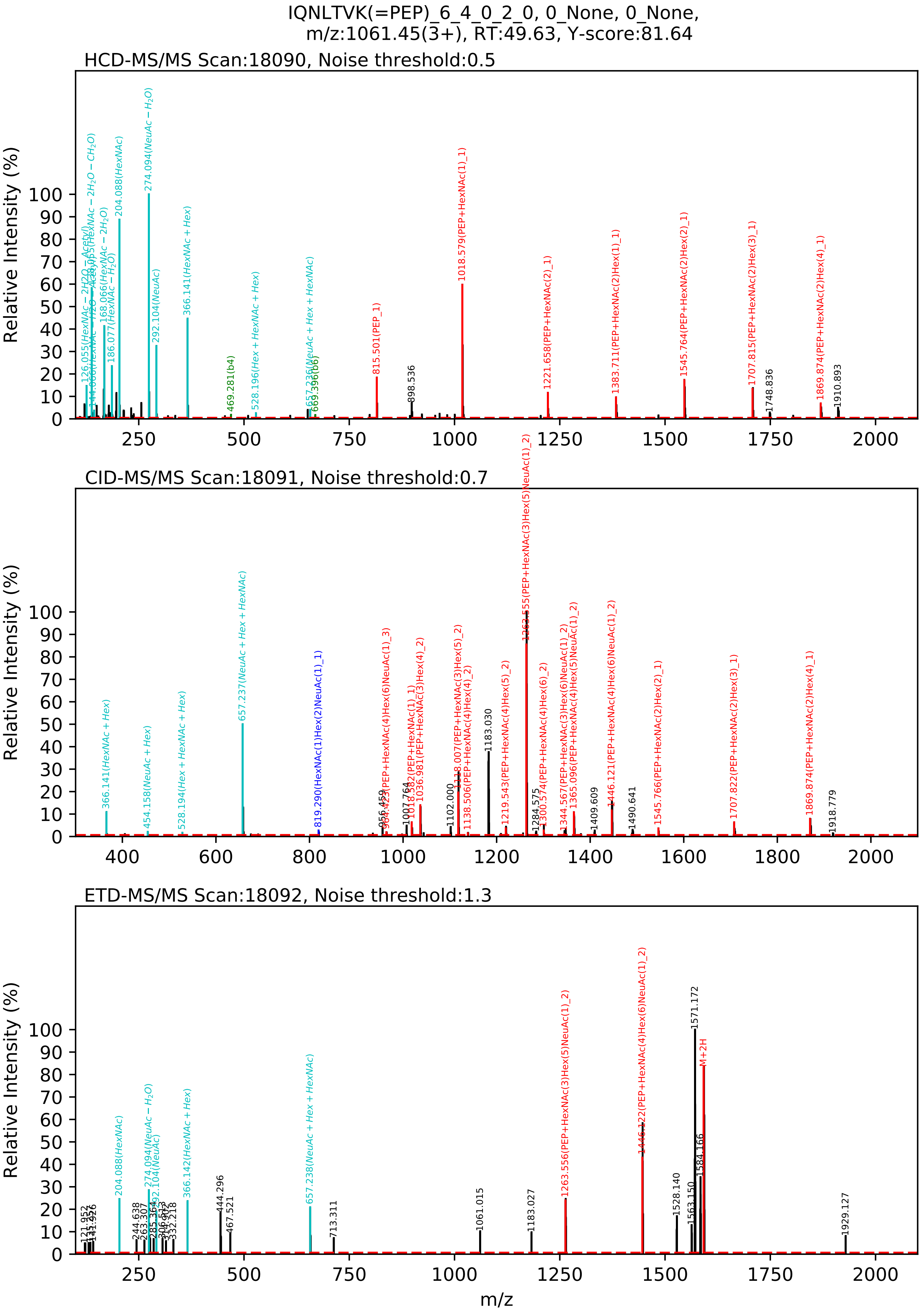

IQNLTVK(=PEP)_6_4_0_2_0, 0_None, 0_None,
m/z:1061.45(3+), RT:49.63, Y-score:81.64
HCD-MS/MS Scan:18090, Noise threshold:0.5
Relative Intensity (%)
274.094(NeuAc − H2O)
204.088(HexNAc)
366.141(HexNAc + Hex)
292.104(NeuAc)
168.066(HexNAc − 2H2O)
186.077(HexNAc − H2O)
126.055(HexNAc − 2H2O − CH2O)
469.281(b4)
528.196(Hex + HexNAc + Hex)
657.236(NeuAc + Hex + HexNAc)
669.396(b6)
815.501(PEP_1)
898.536
1018.579(PEP+HexNAc(1)_1)
1221.658(PEP+HexNAc(2)_1)
1383.711(PEP+HexNAc(2)Hex(1)_1)
1545.764(PEP+HexNAc(2)Hex(2)_1)
1707.815(PEP+HexNAc(2)Hex(3)_1)
1748.836
1869.874(PEP+HexNAc(2)Hex(4)_1)
1910.893
CID-MS/MS Scan:18091, Noise threshold:0.7
366.141(HexNAc + Hex)
454.158(NeuAc + Hex)
528.194(Hex + HexNAc + Hex)
657.237(NeuAc + Hex + HexNAc)
819.290(HexNAc(1)Hex(2)NeuAc(1)_1)
956.459
1007.764
1018.582(PEP+HexNAc(1)_1)
1036.981(PEP+HexNAc(3)Hex(4)_2)
1102.000
1118.007(PEP+HexNAc(3)Hex(5)_2)
1138.506(PEP+HexNAc(4)Hex(4)_2)
1183.030
1219.543(PEP+HexNAc(4)Hex(5)_2)
1263.555(PEP+HexNAc(3)Hex(5)NeuAc(1)_2)
1284.575
1300.574(PEP+HexNAc(4)Hex(6)_2)
1344.567(PEP+HexNAc(3)Hex(6)NeuAc(1)_2)
1365.096(PEP+HexNAc(4)Hex(5)NeuAc(1)_2)
1409.609
1446.121(PEP+HexNAc(4)Hex(6)NeuAc(1)_2)
1490.641
1545.766(PEP+HexNAc(2)Hex(2)_1)
1707.822(PEP+HexNAc(2)Hex(3)_1)
1869.874(PEP+HexNAc(2)Hex(4)_1)
1918.779
ETD-MS/MS Scan:18092, Noise threshold:1.3
121.952
141.926
204.088(HexNAc)
244.638
263.307
274.094(NeuAc − H2O)
292.104(NeuAc)
285.364
306.613
332.218
366.142(HexNAc + Hex)
444.296
467.521
657.238(NeuAc + Hex + HexNAc)
713.311
1061.015
1183.027
1263.556(PEP+HexNAc(3)Hex(5)NeuAc(1)_2)
1446.122(PEP+HexNAc(4)Hex(6)NeuAc(1)_2)
1528.140
1563.150
1571.172
1584.166
M+2H
1929.127
m/z

IQNLTVK(=PEP)_6_4_0_2_0, 0_None, 0_None,
m/z:1061.45(3+), RT:49.74, Y-score:80.38

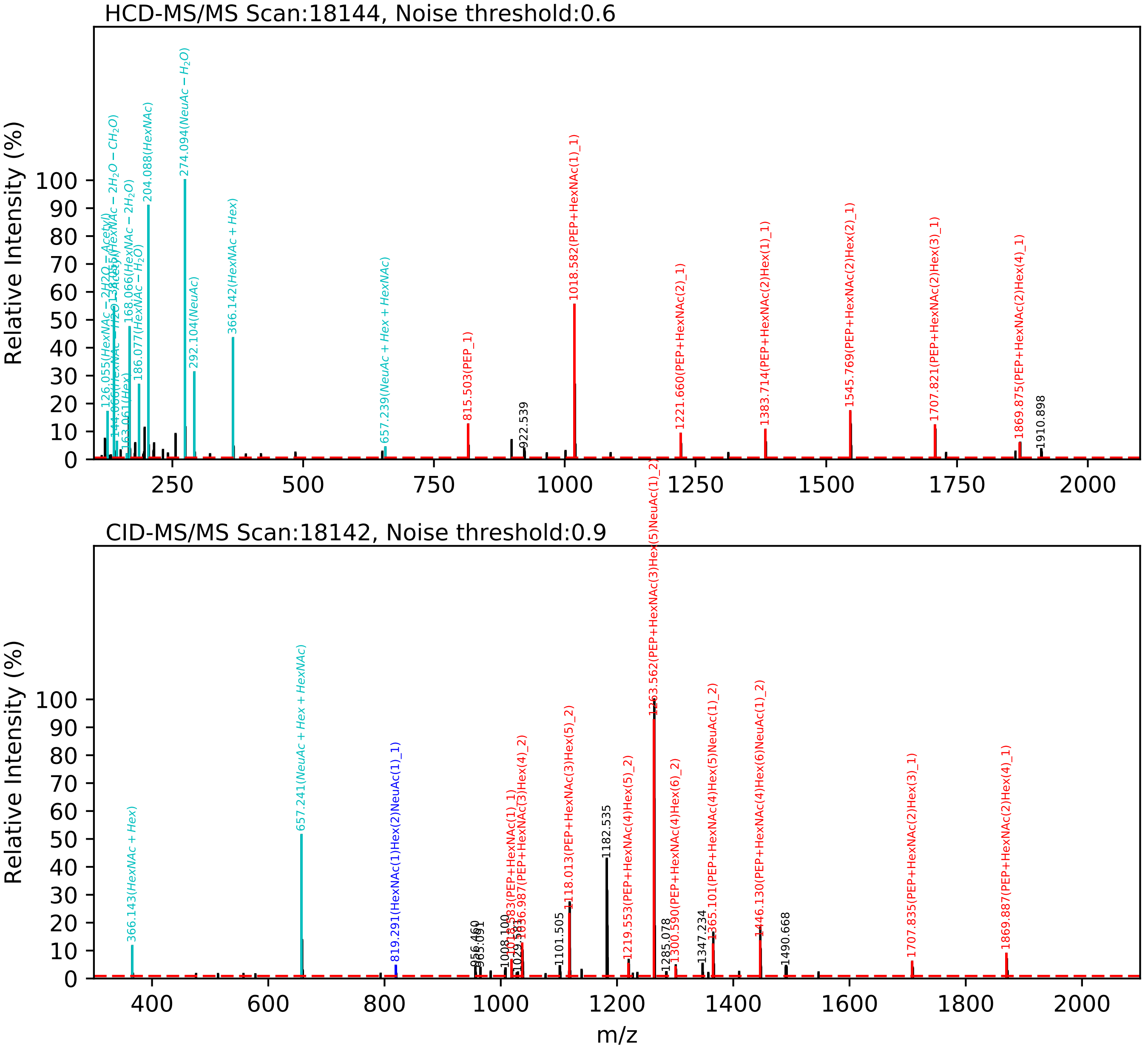

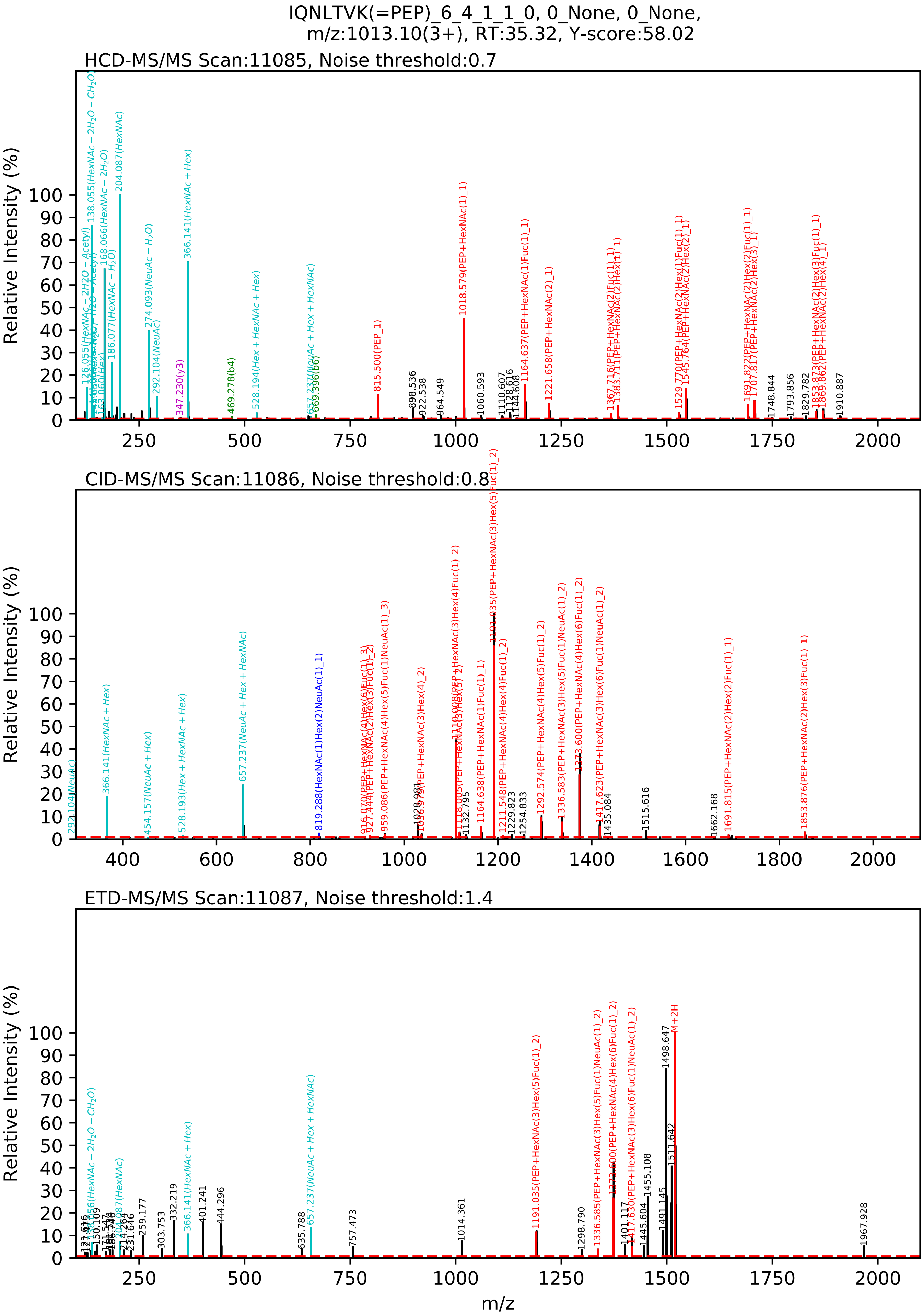

IQNLTVK(=PEP)_6_4_1_1_0, 0_None, 0_None,
m/z:1013.10(3+), RT:35.32, Y-score:58.02
HCD-MS/MS Scan:11085, Noise threshold:0.7
Relative Intensity (%)
204.087(HexNAc)
366.141(HexNAc + Hex)
274.093(NeuAc − H2O)
292.104(NeuAc)
186.077(HexNAc − H2O)
168.066(HexNAc − 2H2O)
347.230(y3)
469.278(b4)
528.194(Hex + HexNAc + Hex)
657.237(NeuAc + Hex + HexNAc)
669.396(b6)
815.500(PEP_1)
898.536
922.538
964.549
1018.579(PEP+HexNAc(1)_1)
1060.593
1110.607
1128.616
1144.608
1164.637(PEP+HexNAc(1)Fuc(1)_1)
1221.658(PEP+HexNAc(2)_1)
1545.764(PEP+HexNAc(2)Hex(2)_1)
1748.844
1793.856
1829.782
1910.887
CID-MS/MS Scan:11086, Noise threshold:0.8
366.141(HexNAc + Hex)
454.157(NeuAc + Hex)
528.193(Hex + HexNAc + Hex)
657.237(NeuAc + Hex + HexNAc)
819.288(HexNAc(1)Hex(2)NeuAc(1)_1)
959.086(PEP+HexNAc(4)Hex(5)Fuc(1)NeuAc(1)_3)
1028.981
1132.795
1164.638(PEP+HexNAc(1)Fuc(1)_1)
1191.035(PEP+HexNAc(3)Hex(5)Fuc(1)_2)
1211.548(PEP+HexNAc(4)Hex(4)Fuc(1)_2)
1229.823
1254.833
1292.574(PEP+HexNAc(4)Hex(5)Fuc(1)_2)
1336.583(PEP+HexNAc(3)Hex(5)Fuc(1)NeuAc(1)_2)
1373.600(PEP+HexNAc(4)Hex(6)Fuc(1)_2)
1417.623(PEP+HexNAc(3)Hex(6)Fuc(1)NeuAc(1)_2)
1435.084
1515.616
1662.168
1691.815(PEP+HexNAc(2)Hex(2)Fuc(1)_1)
1853.876(PEP+HexNAc(2)Hex(3)Fuc(1)_1)
ETD-MS/MS Scan:11087, Noise threshold:1.4
M+2H
1498.647
1511.642
1455.108
1491.145
1445.604
1401.117
1298.790
1191.035(PEP+HexNAc(3)Hex(5)Fuc(1)_2)
1336.585(PEP+HexNAc(3)Hex(5)Fuc(1)NeuAc(1)_2)
1373.600(PEP+HexNAc(4)Hex(6)Fuc(1)_2)
1417.630(PEP+HexNAc(3)Hex(6)Fuc(1)NeuAc(1)_2)
1014.361
757.473
657.237(NeuAc + Hex + HexNAc)
635.788
444.296
401.241
366.141(HexNAc + Hex)
332.219
303.753
259.177
231.646
1967.928
m/z

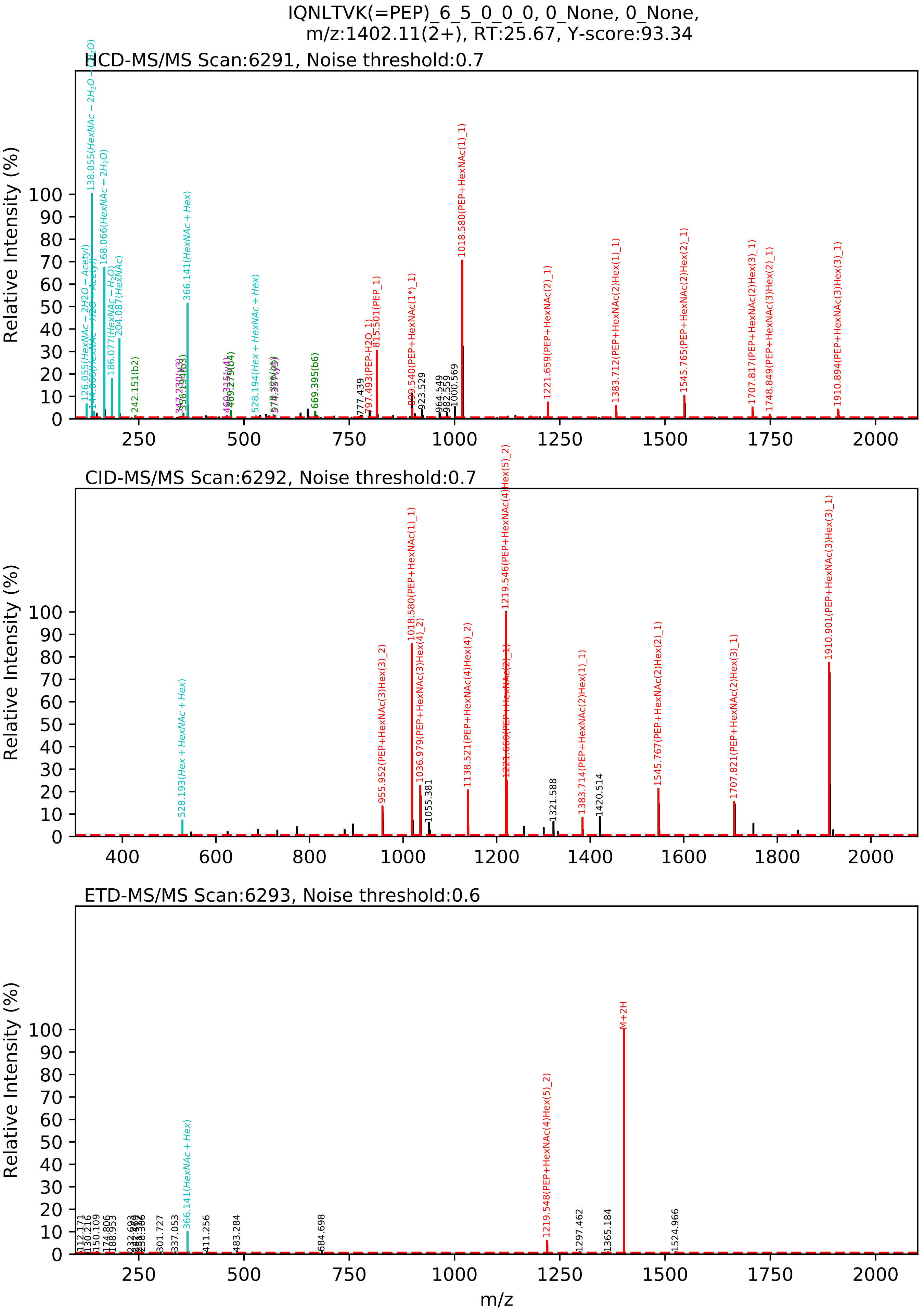
IQNLTVK(=PEP)_6_5_0_0_0, 0_None, 0_None,
m/z:1402.11(2+), RT:25.67, Y-score:93.34
HCD-MS/MS Scan:6291, Noise threshold:0.7
CID-MS/MS Scan:6292, Noise threshold:0.7
ETD-MS/MS Scan:6293, Noise threshold:0.6
Relative Intensity (%)
m/z
1018.580(PEP+HexNAc(1)_1)
1221.659(PEP+HexNAc(2)_1)
1383.712(PEP+HexNAc(2)Hex(1)_1)
1545.765(PEP+HexNAc(2)Hex(2)_1)
1707.817(PEP+HexNAc(2)Hex(3)_1)
1748.849(PEP+HexNAc(3)Hex(2)_1)
1910.894(PEP+HexNAc(3)Hex(3)_1)
815.501(PEP_1)
797.493(PEP-H2O_1)
899.540(PEP+HexNAc(1*)_1)
366.141(HexNAc+Hex)
528.194(Hex+HexNAc+Hex)
669.395(b6)
242.151(b2)
1219.546(PEP+HexNAc(4)Hex(5)_2)
1910.901(PEP+HexNAc(3)Hex(3)_1)
1036.979(PEP+HexNAc(3)Hex(4)_2)
1138.521(PEP+HexNAc(4)Hex(4)_2)
955.952(PEP+HexNAc(3)Hex(3)_2)
1383.714(PEP+HexNAc(2)Hex(1)_1)
1545.767(PEP+HexNAc(2)Hex(2)_1)
1707.821(PEP+HexNAc(2)Hex(3)_1)
528.193(Hex+HexNAc+Hex)
1219.548(PEP+HexNAc(4)Hex(5)_2)
M+2H

IQNLTVK(=PEP)_6_5_0_1_0, 0_None, 0_None,
m/z:1032.11(3+), RT:35.15, Y-score:94.84

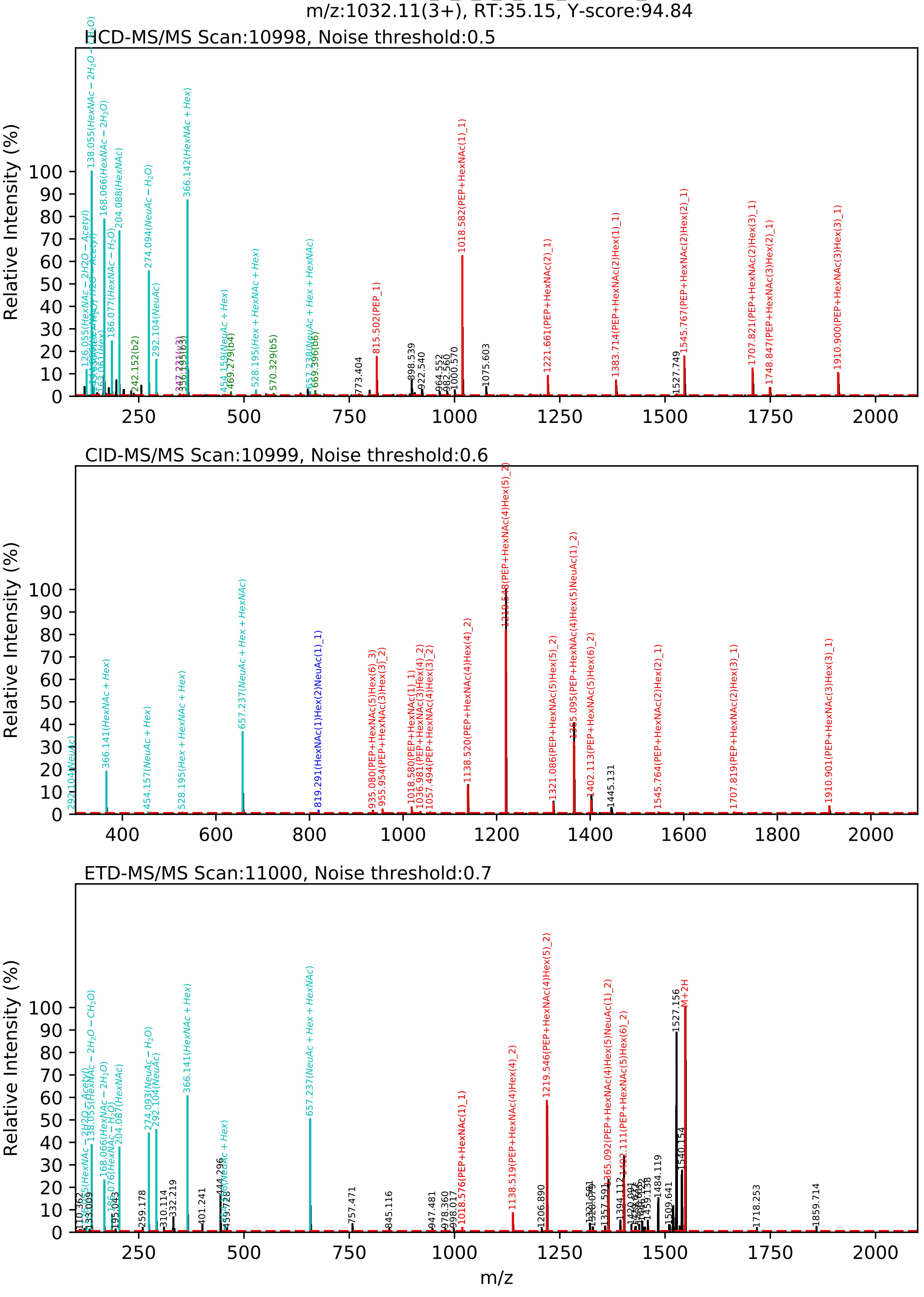

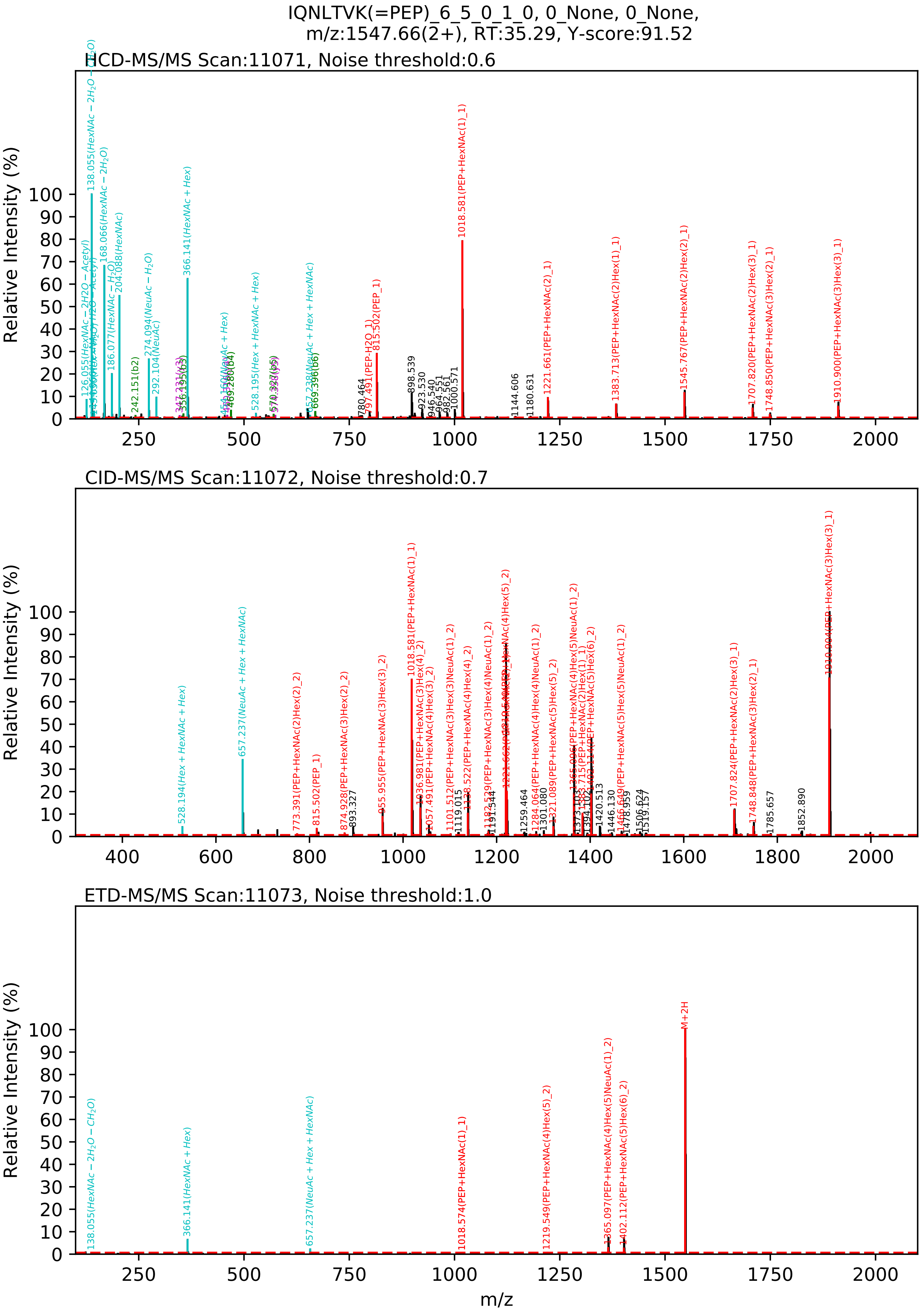
IQNLTVK(=PEP)_6_5_0_1_0, 0_None, 0_None,
m/z:1547.66(2+), RT:35.29, Y-score:91.52
HCD-MS/MS Scan:11071, Noise threshold:0.6
Relative Intensity (%)
138.055(HexNAc − 2H2O − CH2O)
168.066(HexNAc − 2H2O)
186.077(HexNAc − H2O)
204.088(HexNAc)
126.055(HexNAc − 2H2O − Acetyl)
242.151(b2)
274.094(NeuAc − H2O)
292.104(NeuAc)
366.141(HexNAc + Hex)
469.280(b4)
528.195(Hex + HexNAc + Hex)
657.238(NeuAc + Hex + HexNAc)
669.396(b6)
797.491(PEP-H2O_1)
815.502(PEP_1)
898.539
923.530
946.540
964.551
982.561
1000.571
1018.581(PEP+HexNAc(1)_1)
1144.606
1180.631
1221.661(PEP+HexNAc(2)_1)
1383.713(PEP+HexNAc(2)Hex(1)_1)
1545.767(PEP+HexNAc(2)Hex(2)_1)
1707.820(PEP+HexNAc(2)Hex(3)_1)
1748.850(PEP+HexNAc(3)Hex(2)_1)
1910.900(PEP+HexNAc(3)Hex(3)_1)
CID-MS/MS Scan:11072, Noise threshold:0.7
528.194(Hex + HexNAc + Hex)
657.237(NeuAc + Hex + HexNAc)
773.391(PEP+HexNAc(2)Hex(2)_2)
815.502(PEP_1)
874.928(PEP+HexNAc(3)Hex(2)_2)
893.327
955.955(PEP+HexNAc(3)Hex(3)_2)
1018.581(PEP+HexNAc(1)_1)
1036.981(PEP+HexNAc(3)Hex(4)_2)
1057.491(PEP+HexNAc(4)Hex(3)_2)
1101.512(PEP+HexNAc(3)Hex(3)NeuAc(1)_2)
1119.015
1138.522(PEP+HexNAc(4)Hex(4)_2)
1182.529(PEP+HexNAc(3)Hex(4)NeuAc(1)_2)
1191.544
1221.662
1259.464
1284.064(PEP+HexNAc(4)Hex(4)NeuAc(1)_2)
1301.080
1321.089(PEP+HexNAc(5)Hex(5)_2)
1365.096(PEP+HexNAc(4)Hex(5)NeuAc(1)_2)
1383.715(PEP+HexNAc(2)Hex(1)_1)
1402.114(PEP+HexNAc(5)Hex(6)_2)
1420.513
1446.130
1466.649(PEP+HexNAc(5)Hex(5)NeuAc(1)_2)
1478.959
1506.624
1519.157
1707.824(PEP+HexNAc(2)Hex(3)_1)
1748.848(PEP+HexNAc(3)Hex(2)_1)
1785.657
1852.890
1910.904(PEP+HexNAc(3)Hex(3)_1)
ETD-MS/MS Scan:11073, Noise threshold:1.0
138.055(HexNAc − 2H2O − CH2O)
366.141(HexNAc + Hex)
657.237(NeuAc + Hex + HexNAc)
1018.574(PEP+HexNAc(1)_1)
1219.549(PEP+HexNAc(4)Hex(5)_2)
1365.097(PEP+HexNAc(4)Hex(5)NeuAc(1)_2)
1402.112(PEP+HexNAc(5)Hex(6)_2)
M+2H
m/z

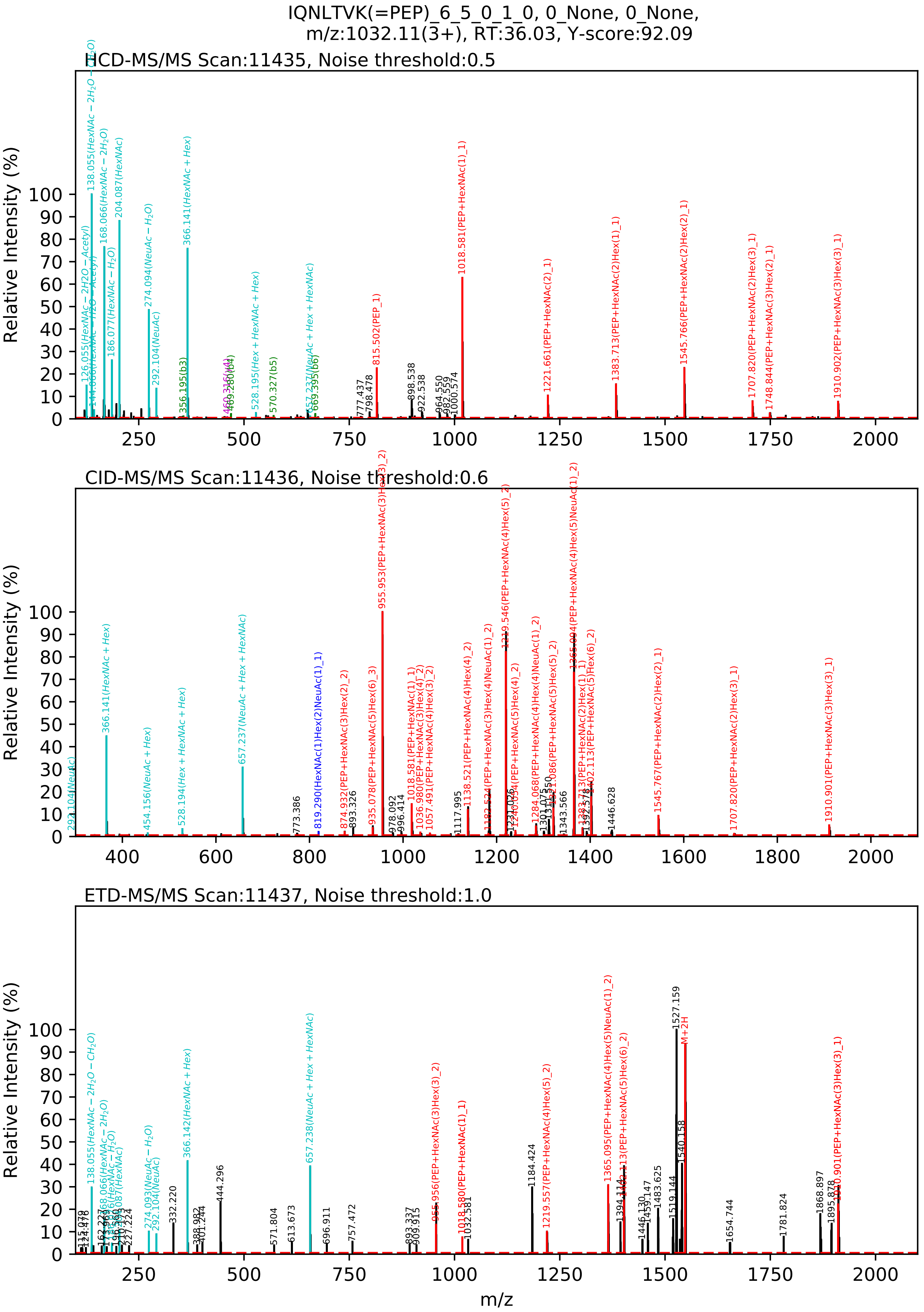
IQNLTVK(=PEP)_6_5_0_1_0, 0_None, 0_None,
m/z:1032.11(3+), RT:36.03, Y-score:92.09
HCD-MS/MS Scan:11435, Noise threshold:0.5
Relative Intensity (%)
138.055(HexNAc − 2H2O − CH2O)
168.066(HexNAc − 2H2O)
204.087(HexNAc)
186.077(HexNAc − H2O)
274.094(NeuAc − H2O)
292.104(NeuAc)
366.141(HexNAc + Hex)
356.195(b3)
469.280(b4)
528.195(Hex + HexNAc + Hex)
570.327(b5)
669.395(b6)
815.502(PEP_1)
898.538
922.538
777.437
798.478
964.550
1000.574
1018.581(PEP+HexNAc(1)_1)
1221.661(PEP+HexNAc(2)_1)
1383.713(PEP+HexNAc(2)Hex(1)_1)
1545.766(PEP+HexNAc(2)Hex(2)_1)
1707.820(PEP+HexNAc(2)Hex(3)_1)
1748.844(PEP+HexNAc(3)Hex(2)_1)
1910.902(PEP+HexNAc(3)Hex(3)_1)
CID-MS/MS Scan:11436, Noise threshold:0.6
Relative Intensity (%)
366.141(HexNAc + Hex)
454.156(NeuAc + Hex)
528.194(Hex + HexNAc + Hex)
657.237(NeuAc + Hex + HexNAc)
773.386
819.290(HexNAc(1)Hex(2)NeuAc(1)_1)
874.932(PEP+HexNAc(3)Hex(2)_2)
893.326
935.078(PEP+HexNAc(5)Hex(6)_3)
955.953(PEP+HexNAc(3)Hex(3)_2)
978.092
996.414
1117.995
1138.521(PEP+HexNAc(4)Hex(4)_2)
1219.546(PEP+HexNAc(4)Hex(5)_2)
1231.026
1343.566
1365.094(PEP+HexNAc(4)Hex(5)NeuAc(1)_2)
1446.628
1545.767(PEP+HexNAc(2)Hex(2)_1)
1707.820(PEP+HexNAc(2)Hex(3)_1)
1910.901(PEP+HexNAc(3)Hex(3)_1)
ETD-MS/MS Scan:11437, Noise threshold:1.0
Relative Intensity (%)
138.055(HexNAc − 2H2O − CH2O)
168.066(HexNAc − 2H2O)
274.093(NeuAc − H2O)
292.104(NeuAc)
366.142(HexNAc + Hex)
332.220
444.296
571.804
613.673
657.238(NeuAc + Hex + HexNAc)
696.911
757.472
955.956(PEP+HexNAc(3)Hex(3)_2)
1018.580(PEP+HexNAc(1)_1)
1032.581
1184.424
1219.557(PEP+HexNAc(4)Hex(5)_2)
1365.095(PEP+HexNAc(4)Hex(5)NeuAc(1)_2)
1483.625
1519.144
1527.159
M+2H
1540.158
1654.744
1781.824
1868.897
1910.901(PEP+HexNAc(3)Hex(3)_1)
m/z

IQNLTVK(=PEP)_6_5_0_1_0, 0_None, 0_None,
m/z:1032.11(3+), RT:34.54, Y-score:94.68

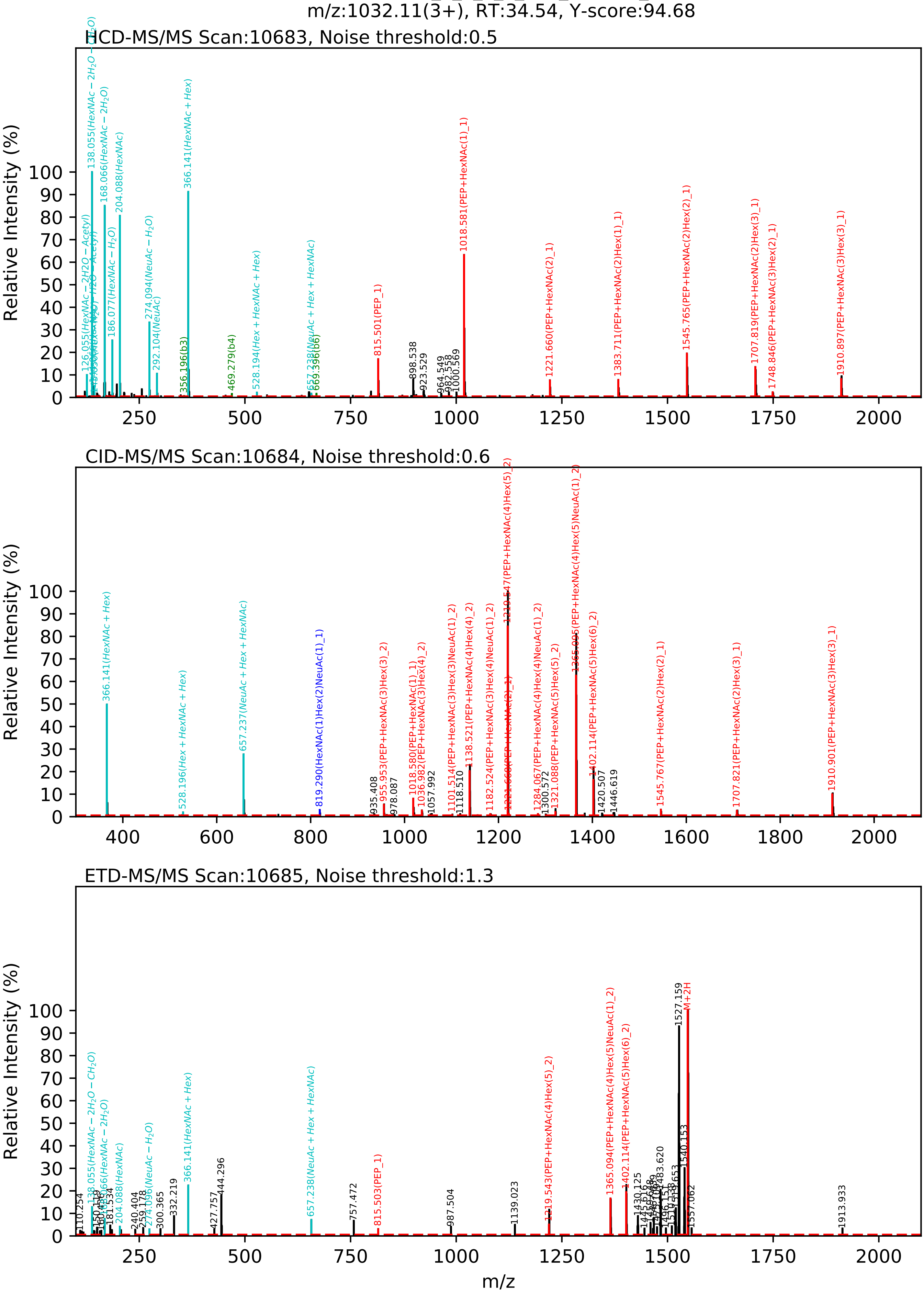

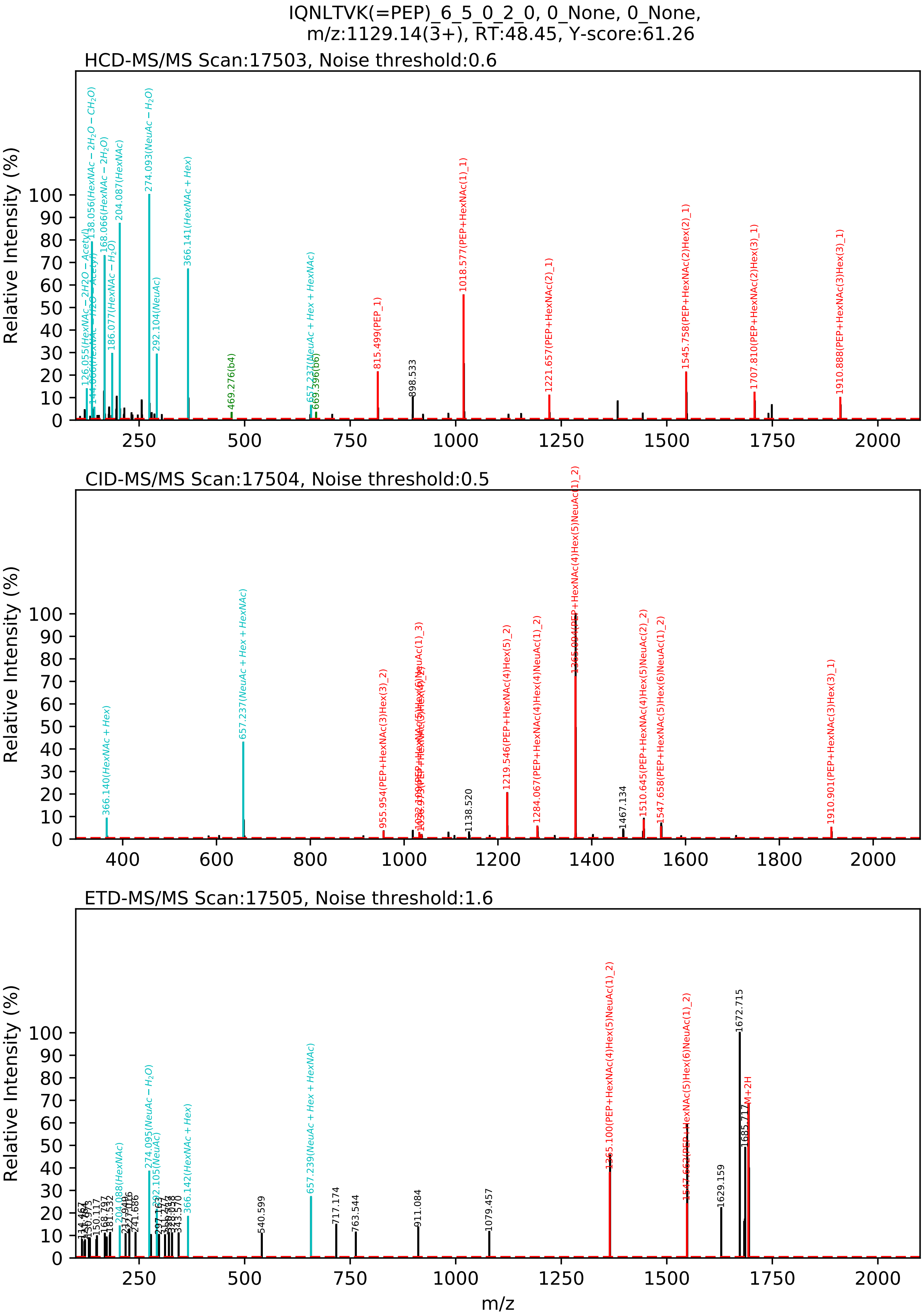

IQNLTVK(=PEP)_6_5_0_2_0, 0_None, 0_None,
m/z:1129.14(3+), RT:48.45, Y-score:61.26
HCD-MS/MS Scan:17503, Noise threshold:0.6
CID-MS/MS Scan:17504, Noise threshold:0.5
ETD-MS/MS Scan:17505, Noise threshold:1.6
Relative Intensity (%)
m/z
274.093(NeuAc − H2O)
366.141(HexNAc + Hex)
204.087(HexNAc)
292.104(NeuAc)
469.276(b4)
815.499(PEP_1)
898.533
1018.577(PEP+HexNAc(1)_1)
1221.657(PEP+HexNAc(2)_1)
1545.758(PEP+HexNAc(2)Hex(2)_1)
1707.810(PEP+HexNAc(2)Hex(3)_1)
1910.888(PEP+HexNAc(3)Hex(3)_1)
366.140(HexNAc + Hex)
657.237(NeuAc + Hex + HexNAc)
955.954(PEP+HexNAc(3)Hex(3)_2)
1138.520
1219.546(PEP+HexNAc(4)Hex(5)_2)
1284.067(PEP+HexNAc(4)Hex(4)NeuAc(1)_2)
1365.094(PEP+HexNAc(4)Hex(5)NeuAc(1)_2)
1467.134
1510.645(PEP+HexNAc(4)Hex(5)NeuAc(2)_2)
1547.658(PEP+HexNAc(5)Hex(6)NeuAc(1)_2)
1910.901(PEP+HexNAc(3)Hex(3)_1)
274.095(NeuAc − H2O)
292.105(NeuAc)
366.142(HexNAc + Hex)
540.599
657.239(NeuAc + Hex + HexNAc)
717.174
763.544
911.084
1079.457
1365.100(PEP+HexNAc(4)Hex(5)NeuAc(1)_2)
1547.662(PEP+HexNAc(5)Hex(6)NeuAc(1)_2)
1629.159
1672.715
1685.717
M+2H

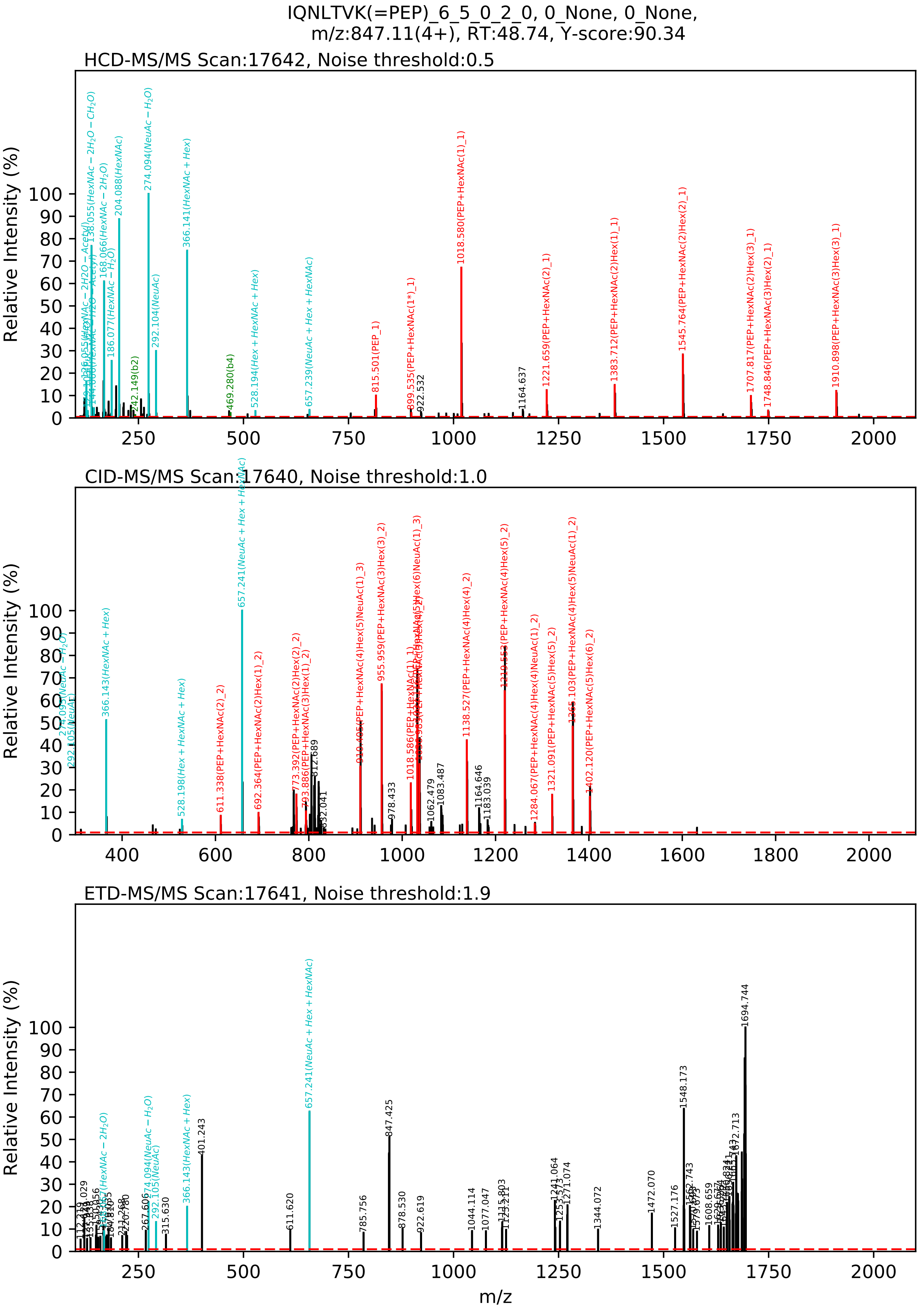

IQNLTVK(=PEP)_6_5_0_2_0, 0_None, 0_None,
m/z:847.11(4+), RT:48.74, Y-score:90.34
HCD-MS/MS Scan:17642, Noise threshold:0.5
Relative Intensity (%)
138.055(HexNAc − 2H2O − CH2O)
204.088(HexNAc)
274.094(NeuAc − H2O)
366.141(HexNAc + Hex)
292.104(NeuAc)
168.066(HexNAc − 2H2O)
186.077(HexNAc − H2O)
242.149(b2)
469.280(b4)
528.194(Hex + HexNAc + Hex)
657.239(NeuAc + Hex + HexNAc)
815.501(PEP_1)
899.535(PEP+HexNAc(1*)_1)
922.532
1018.580(PEP+HexNAc(1)_1)
1164.637
1221.659(PEP+HexNAc(2)_1)
1383.712(PEP+HexNAc(2)Hex(1)_1)
1545.764(PEP+HexNAc(2)Hex(2)_1)
1707.817(PEP+HexNAc(2)Hex(3)_1)
1748.846(PEP+HexNAc(3)Hex(2)_1)
1910.898(PEP+HexNAc(3)Hex(3)_1)
CID-MS/MS Scan:17640, Noise threshold:1.0
366.143(HexNAc + Hex)
528.198(Hex + HexNAc + Hex)
611.338(PEP+HexNAc(2)_2)
657.241(NeuAc + Hex + HexNAc)
692.364(PEP+HexNAc(2)Hex(1)_2)
773.392(PEP+HexNAc(2)Hex(2)_2)
793.886(PEP+HexNAc(3)Hex(1)_2)
812.689
832.041
910.405(PEP+HexNAc(4)Hex(5)NeuAc(1)_3)
955.959(PEP+HexNAc(3)Hex(3)_2)
978.433
1018.586(PEP+HexNAc(1)_1)
1062.479
1083.487
1138.527(PEP+HexNAc(4)Hex(4)_2)
1164.646
1183.039
1219.583(PEP+HexNAc(4)Hex(5)_2)
1284.067(PEP+HexNAc(4)Hex(4)NeuAc(1)_2)
1321.091(PEP+HexNAc(5)Hex(5)_2)
1365.103(PEP+HexNAc(4)Hex(5)NeuAc(1)_2)
1402.120(PEP+HexNAc(5)Hex(6)_2)
ETD-MS/MS Scan:17641, Noise threshold:1.9
274.094(NeuAc − H2O)
292.105(NeuAc)
366.143(HexNAc + Hex)
401.243
611.620
657.241(NeuAc + Hex + HexNAc)
785.756
847.425
878.530
922.619
1044.114
1077.047
1115.803
1241.064
1253.573
1271.074
1344.072
1472.070
1527.176
1548.173
1562.743
1608.659
1672.713
1694.744
m/z

IQNLTVK(=PEP)_6_5_1_0_0, 0_None, 0_None,
m/z:1475.14(2+), RT:26.15, Y-score:89.19

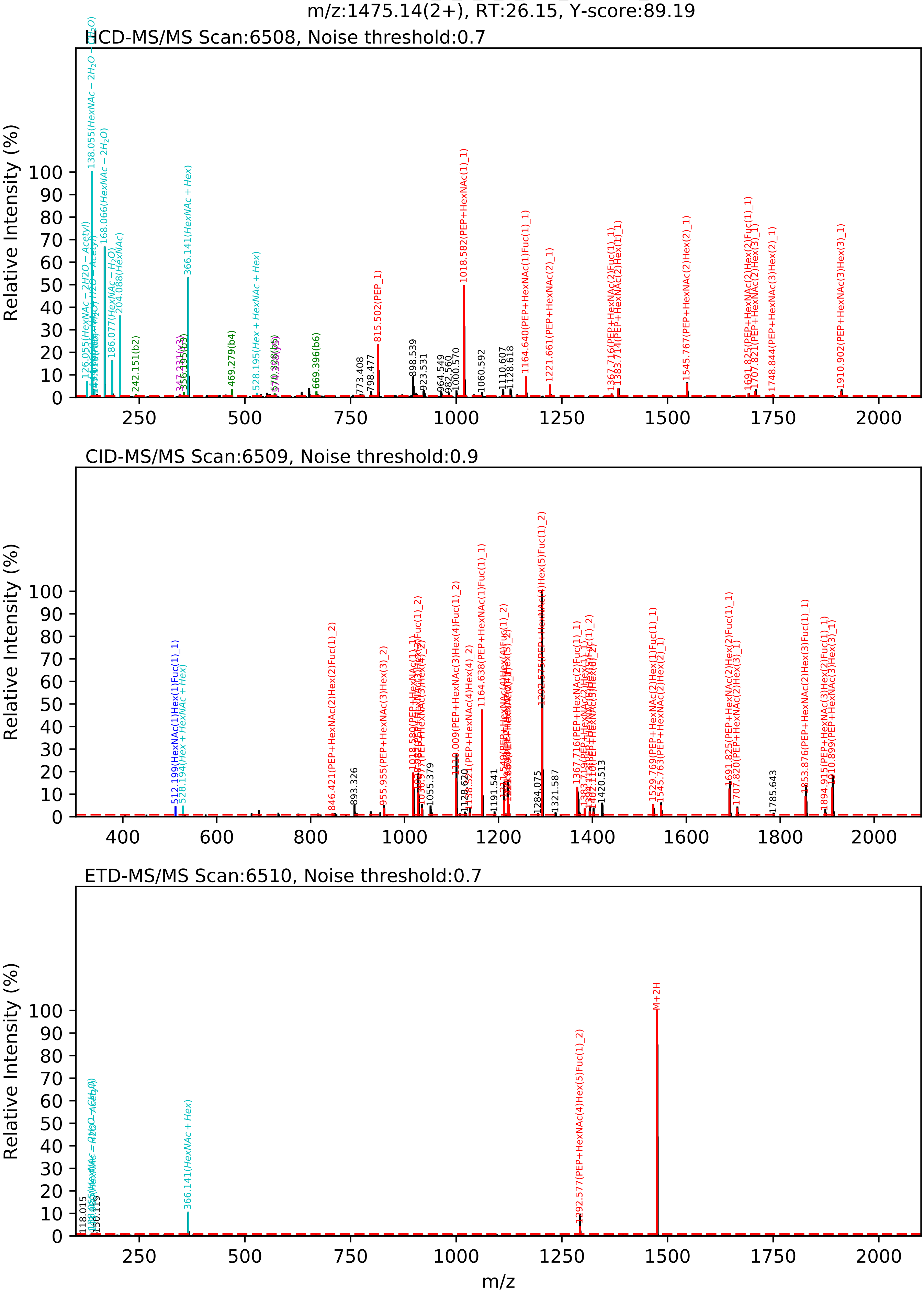

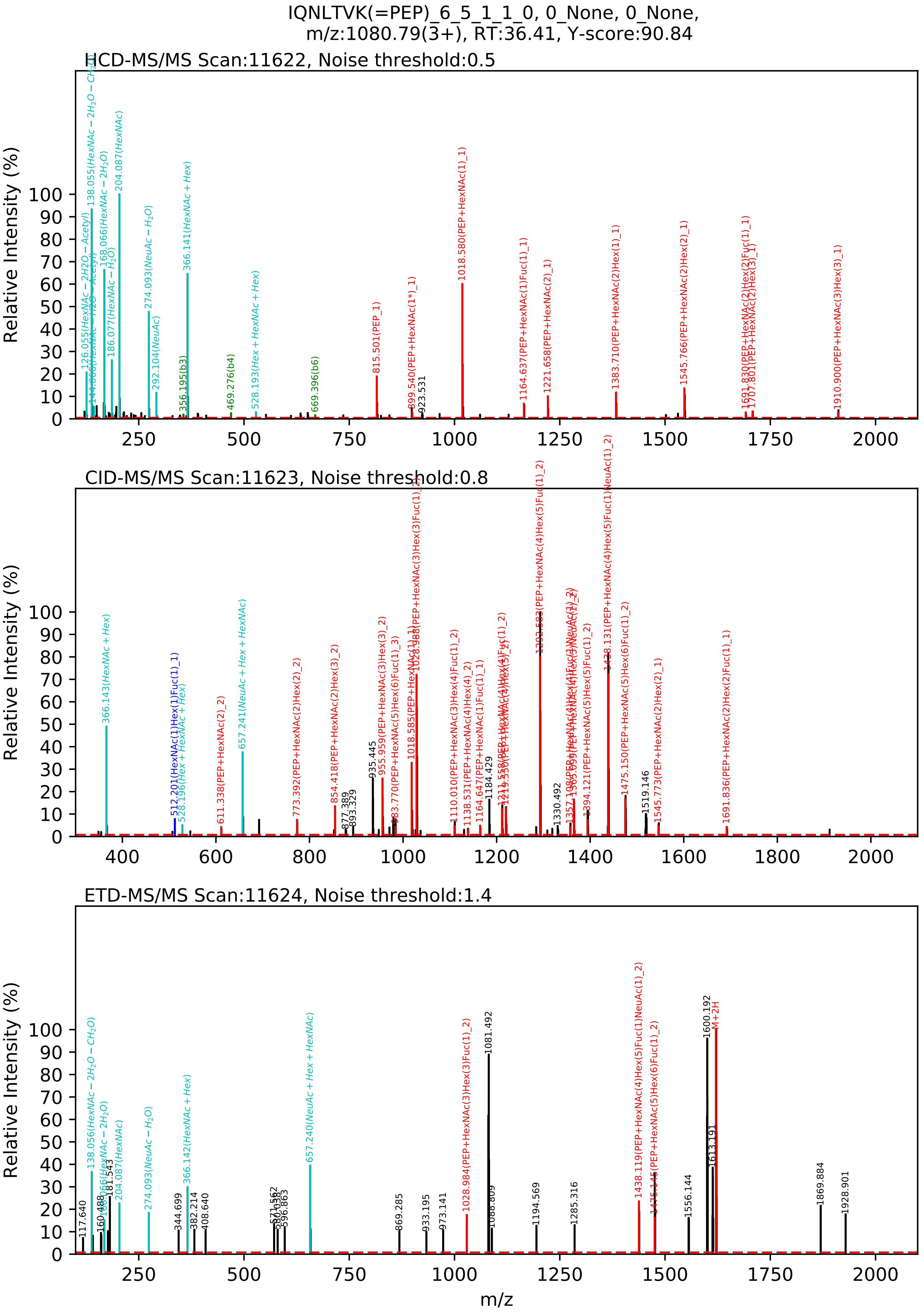
IQNLTVK(=PEP)_6_5_1_1_0, 0_None, 0_None,
m/z:1080.79(3+), RT:36.41, Y-score:90.84
HCD-MS/MS Scan:11622, Noise threshold:0.5
CID-MS/MS Scan:11623, Noise threshold:0.8
ETD-MS/MS Scan:11624, Noise threshold:1.4
Relative Intensity (%)
m/z
1018.580(PEP+HexNAc(1)_1)
1164.637(PEP+HexNAc(1)Fuc(1)_1)
1221.658(PEP+HexNAc(2)_1)
1383.710(PEP+HexNAc(2)Hex(1)_1)
1545.766(PEP+HexNAc(2)Hex(2)_1)
1910.900(PEP+HexNAc(3)Hex(3)_1)
815.501(PEP_1)
899.540(PEP+HexNAc(1*)_1)
204.087(HexNAc)
366.141(HexNAc + Hex)
274.093(NeuAc − H_2O)
292.104(NeuAc)
356.195(b3)
469.276(b4)
669.396(b6)
528.193(Hex + HexNAc + Hex)
1292.583(PEP+HexNAc(4)Hex(5)Fuc(1)_2)
1438.131(PEP+HexNAc(4)Hex(5)Fuc(1)NeuAc(1)_2)
1475.150(PEP+HexNAc(5)Hex(6)Fuc(1)_2)
1545.773(PEP+HexNAc(2)Hex(2)_1)
1691.836(PEP+HexNAc(2)Hex(2)Fuc(1)_1)
1394.121(PEP+HexNAc(5)Hex(5)Fuc(1)_2)
1164.647(PEP+HexNAc(1)Fuc(1)_1)
1138.531(PEP+HexNAc(4)Hex(4)_2)
1110.010(PEP+HexNAc(3)Hex(4)Fuc(1)_2)
955.959(PEP+HexNAc(3)Hex(3)_2)
854.418(PEP+HexNAc(2)Hex(3)_2)
773.392(PEP+HexNAc(2)Hex(2)_2)
611.338(PEP+HexNAc(2)_2)
657.241(NeuAc + Hex + HexNAc)
512.201(HexNAc(1)Hex(1)Fuc(1)_1)
366.143(HexNAc + Hex)
1600.192
M+2H
1081.492
1438.119(PEP+HexNAc(4)Hex(5)Fuc(1)NeuAc(1)_2)
1028.984(PEP+HexNAc(3)Hex(3)Fuc(1)_2)
657.240(NeuAc + Hex + HexNAc)
366.142(HexNAc + Hex)
274.093(NeuAc − H_2O)
204.087(HexNAc)
1869.884
1928.901
1556.144
1613.191
1285.316
1194.569
973.141
933.195
869.285
408.640
382.214
344.699
117.640

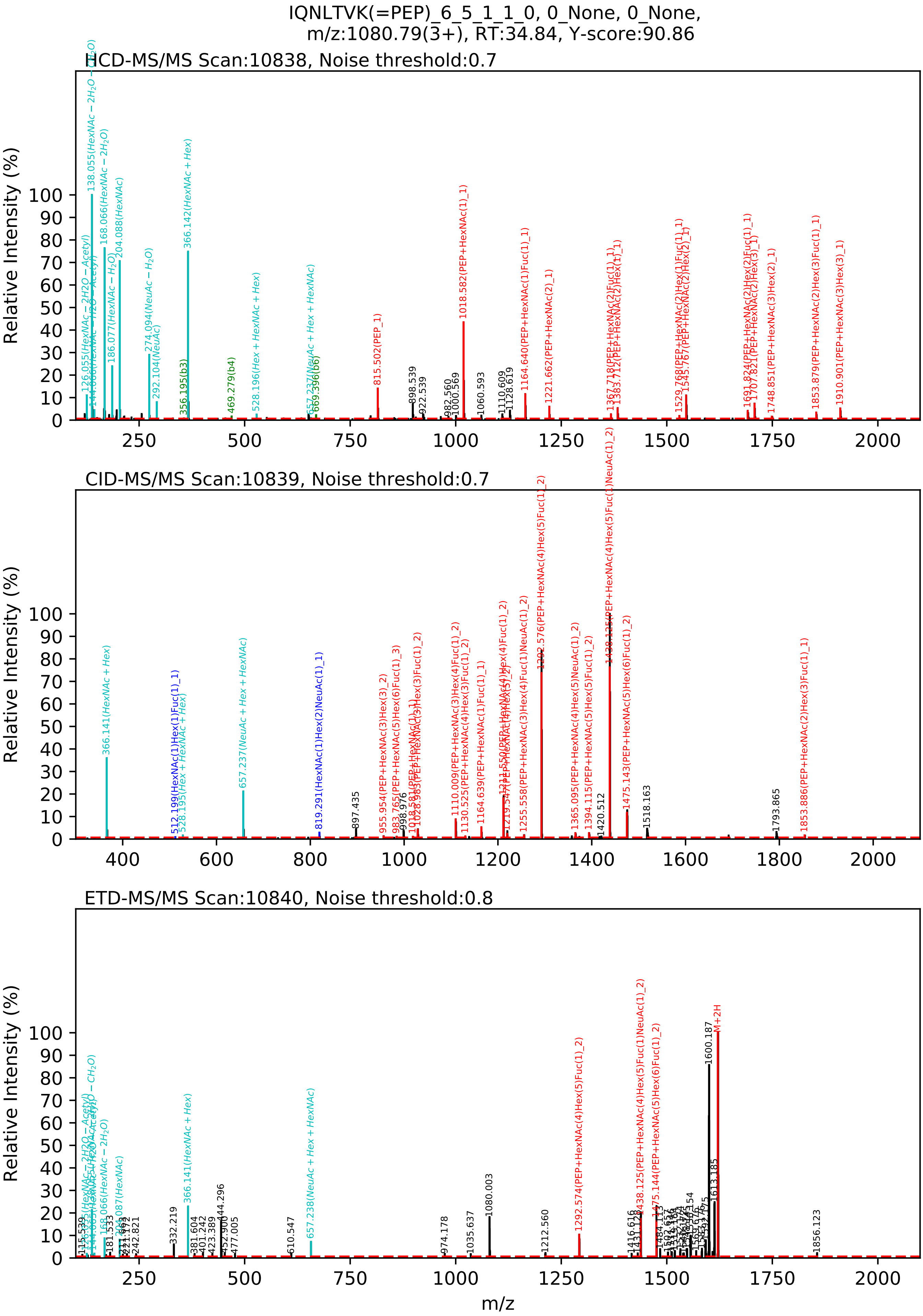
IQNLTVK(=PEP)_6_5_1_1_0, 0_None, 0_None,
m/z:1080.79(3+), RT:34.84, Y-score:90.86
HCD-MS/MS Scan:10838, Noise threshold:0.7
CID-MS/MS Scan:10839, Noise threshold:0.7
ETD-MS/MS Scan:10840, Noise threshold:0.8
Relative Intensity (%)
m/z

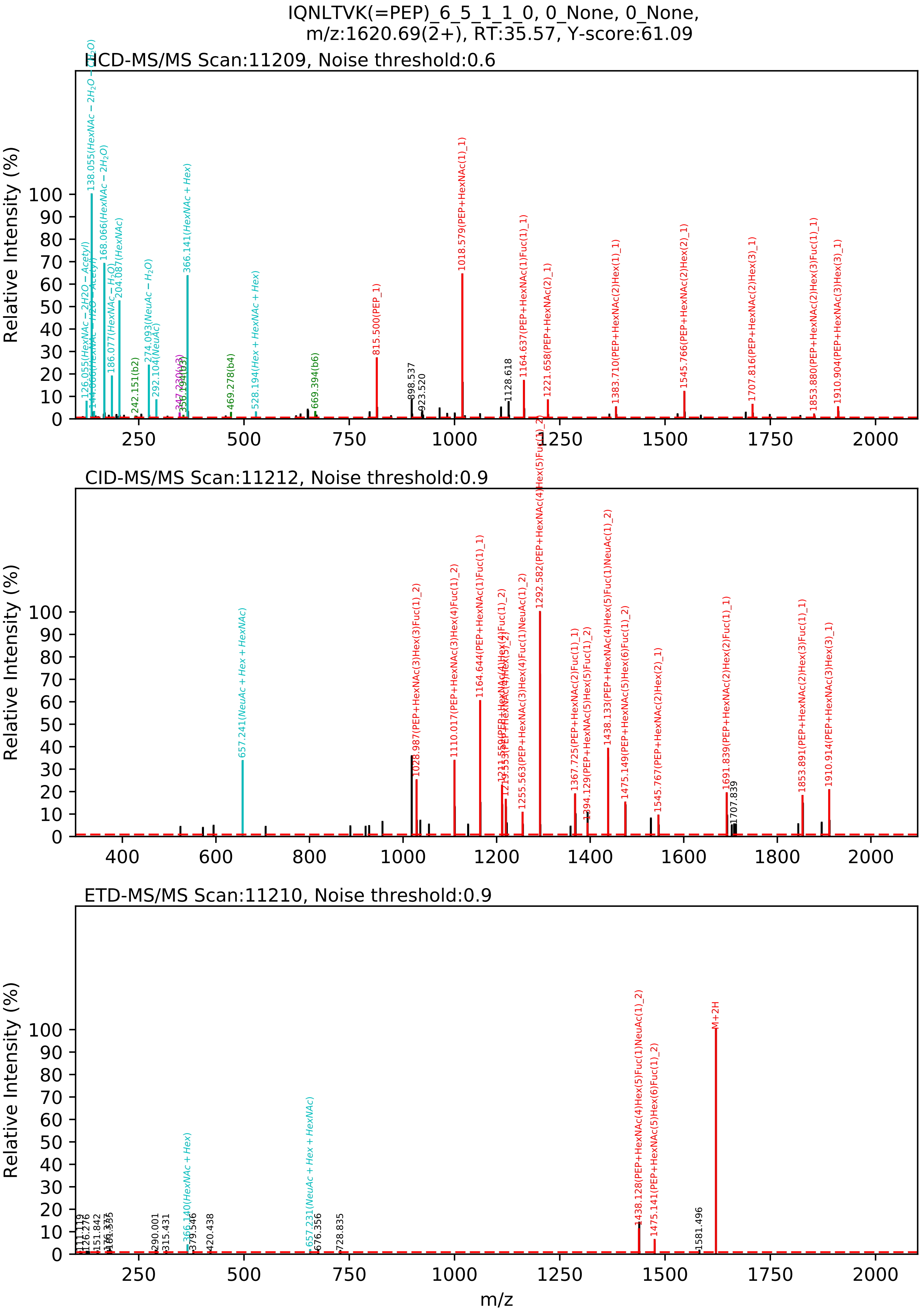

IQNLTVK(=PEP)_6_5_1_1_0, 0_None, 0_None,
m/z:1620.69(2+), RT:35.57, Y-score:61.09
HCD-MS/MS Scan:11209, Noise threshold:0.6
CID-MS/MS Scan:11212, Noise threshold:0.9
ETD-MS/MS Scan:11210, Noise threshold:0.9
Relative Intensity (%)
m/z
138.055(HexNAc − 2H2O − H2O)
168.066(HexNAc − 2H2O)
204.087(HexNAc)
366.141(HexNAc + Hex)
274.093(NeuAc − H2O)
292.104(NeuAc)
242.151(b2)
469.278(b4)
669.394(b6)
528.194(Hex + HexNAc + Hex)
815.500(PEP_1)
898.537
923.520
1018.579(PEP+HexNAc(1)_1)
1128.618
1164.637(PEP+HexNAc(1)Fuc(1)_1)
1221.658(PEP+HexNAc(2)_1)
1383.710(PEP+HexNAc(2)Hex(1)_1)
1545.766(PEP+HexNAc(2)Hex(2)_1)
1707.816(PEP+HexNAc(2)Hex(3)_1)
1853.880(PEP+HexNAc(2)Hex(3)Fuc(1)_1)
1910.904(PEP+HexNAc(3)Hex(3)_1)
657.241(NeuAc + Hex + HexNAc)
1028.987(PEP+HexNAc(3)Hex(3)Fuc(1)_2)
1110.017(PEP+HexNAc(3)Hex(4)Fuc(1)_2)
1164.644(PEP+HexNAc(1)Fuc(1)_1)
1255.563(PEP+HexNAc(3)Hex(4)Fuc(1)NeuAc(1)_2)
1292.582(PEP+HexNAc(4)Hex(5)Fuc(1)_2)
1367.725(PEP+HexNAc(2)Fuc(1)_1)
1394.129(PEP+HexNAc(5)Hex(5)Fuc(1)_2)
1438.133(PEP+HexNAc(4)Hex(5)Fuc(1)NeuAc(1)_2)
1475.149(PEP+HexNAc(5)Hex(6)Fuc(1)_2)
1545.767(PEP+HexNAc(2)Hex(2)_1)
1691.839(PEP+HexNAc(2)Hex(2)Fuc(1)_1)
1707.839
1853.891(PEP+HexNAc(2)Hex(3)Fuc(1)_1)
1910.914(PEP+HexNAc(3)Hex(3)_1)
M+2H
1438.128(PEP+HexNAc(4)Hex(5)Fuc(1)NeuAc(1)_2)
1475.141(PEP+HexNAc(5)Hex(6)Fuc(1)_2)
1581.496
366.140(HexNAc + Hex)
657.231(NeuAc + Hex + HexNAc)
676.356
728.835
290.001
315.431
379.546
420.438

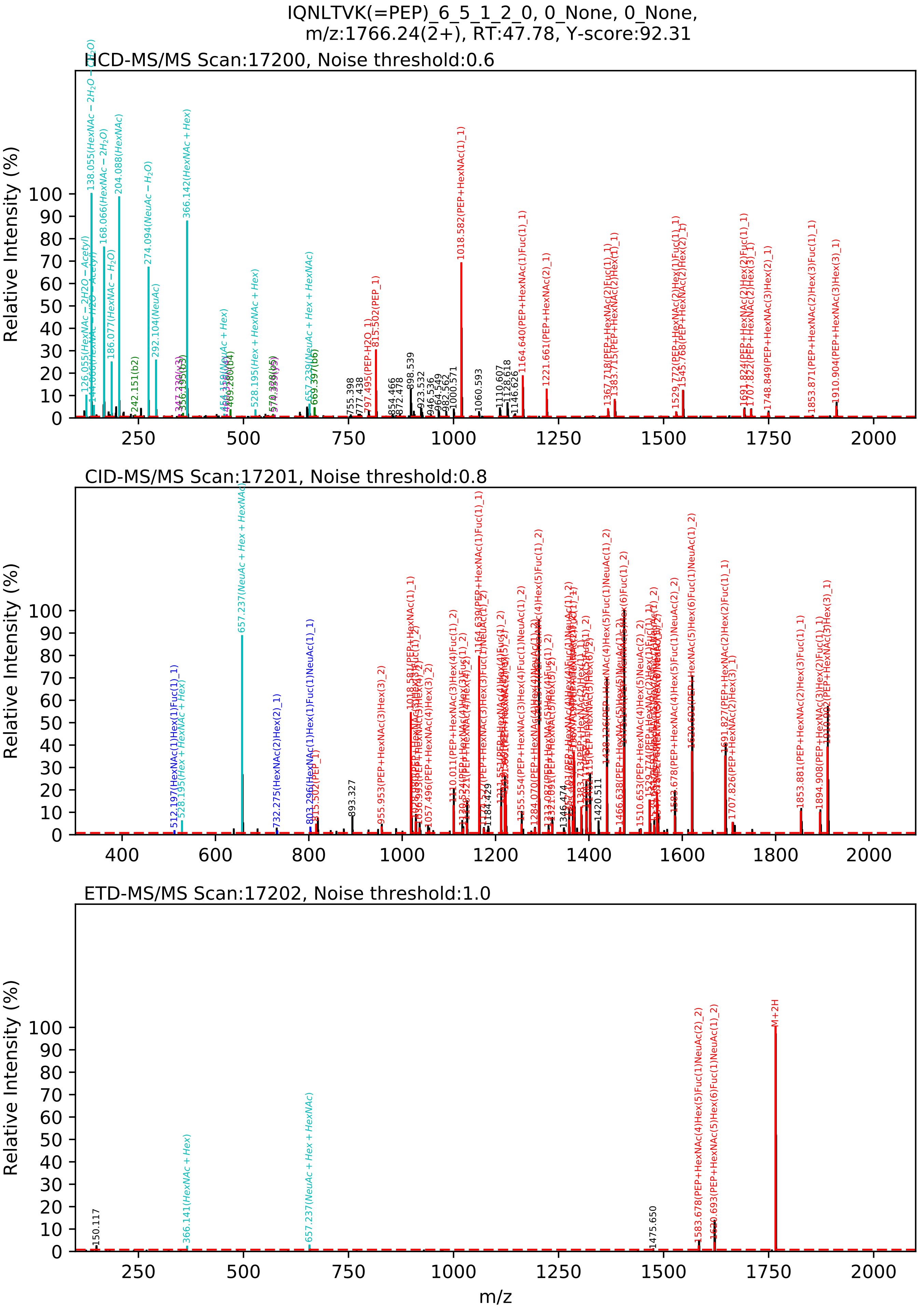
IQNLTVK(=PEP)_6_5_1_2_0, 0_None, 0_None,
m/z:1766.24(2+), RT:47.78, Y-score:92.31
HCD-MS/MS Scan:17200, Noise threshold:0.6
CID-MS/MS Scan:17201, Noise threshold:0.8
ETD-MS/MS Scan:17202, Noise threshold:1.0
Relative Intensity (%)
m/z

IQNLTVK(=PEP)_6_5_1_3_0, 0_None, 0_None,
m/z:1274.86(3+), RT:64.48, Y-score:59.31

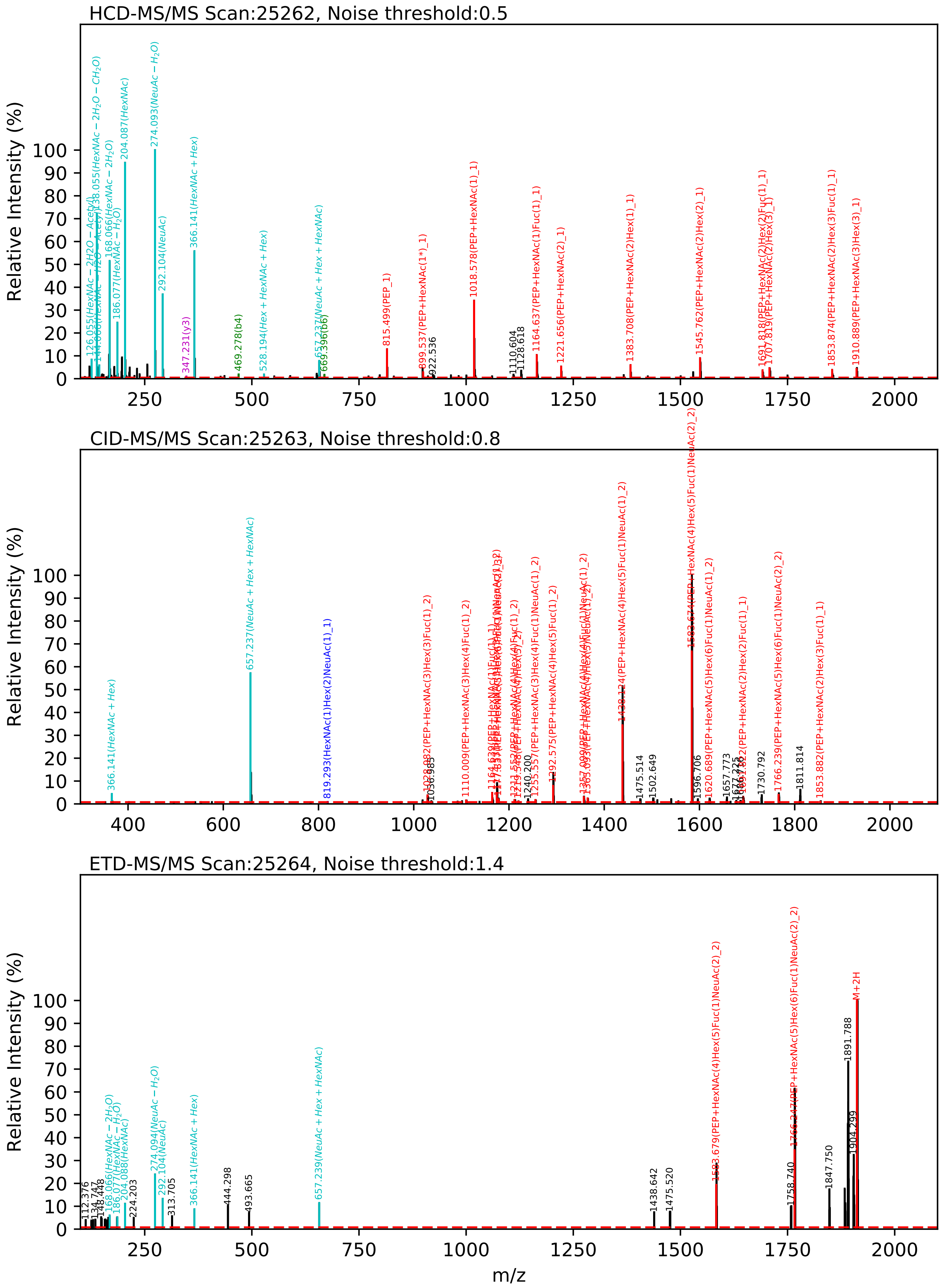

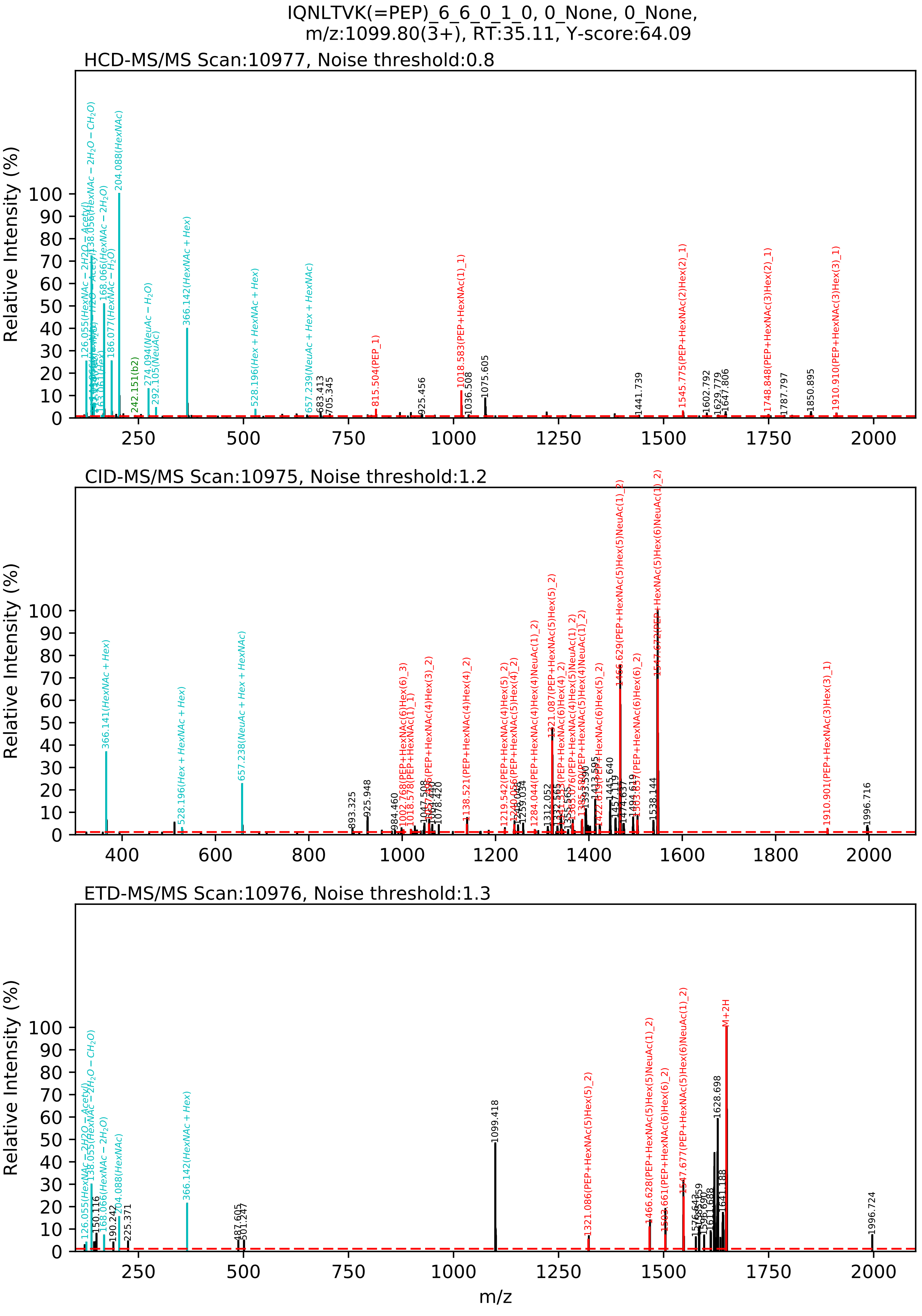
IQNLTVK(=PEP)_6_6_0_1_0, 0_None, 0_None,
m/z:1099.80(3+), RT:35.11, Y-score:64.09
HCD-MS/MS Scan:10977, Noise threshold:0.8
CID-MS/MS Scan:10975, Noise threshold:1.2
ETD-MS/MS Scan:10976, Noise threshold:1.3
Relative Intensity (%)
m/z
204.088(HexNAc)
366.142(HexNAc + Hex)
528.196(Hex + HexNAc + Hex)
657.239(NeuAc + Hex + HexNAc)
815.504(PEP_1)
1018.583(PEP+HexNAc(1)_1)
1545.775(PEP+HexNAc(2)Hex(2)_1)
1748.848(PEP+HexNAc(3)Hex(2)_1)
1910.910(PEP+HexNAc(3)Hex(3)_1)
1321.087(PEP+HexNAc(5)Hex(5)_2)
1466.629(PEP+HexNAc(5)Hex(5)NeuAc(1)_2)
1547.673(PEP+HexNAc(5)Hex(6)NeuAc(1)_2)
1910.901(PEP+HexNAc(3)Hex(3)_1)
1099.418
1628.698
1996.724
M+2H

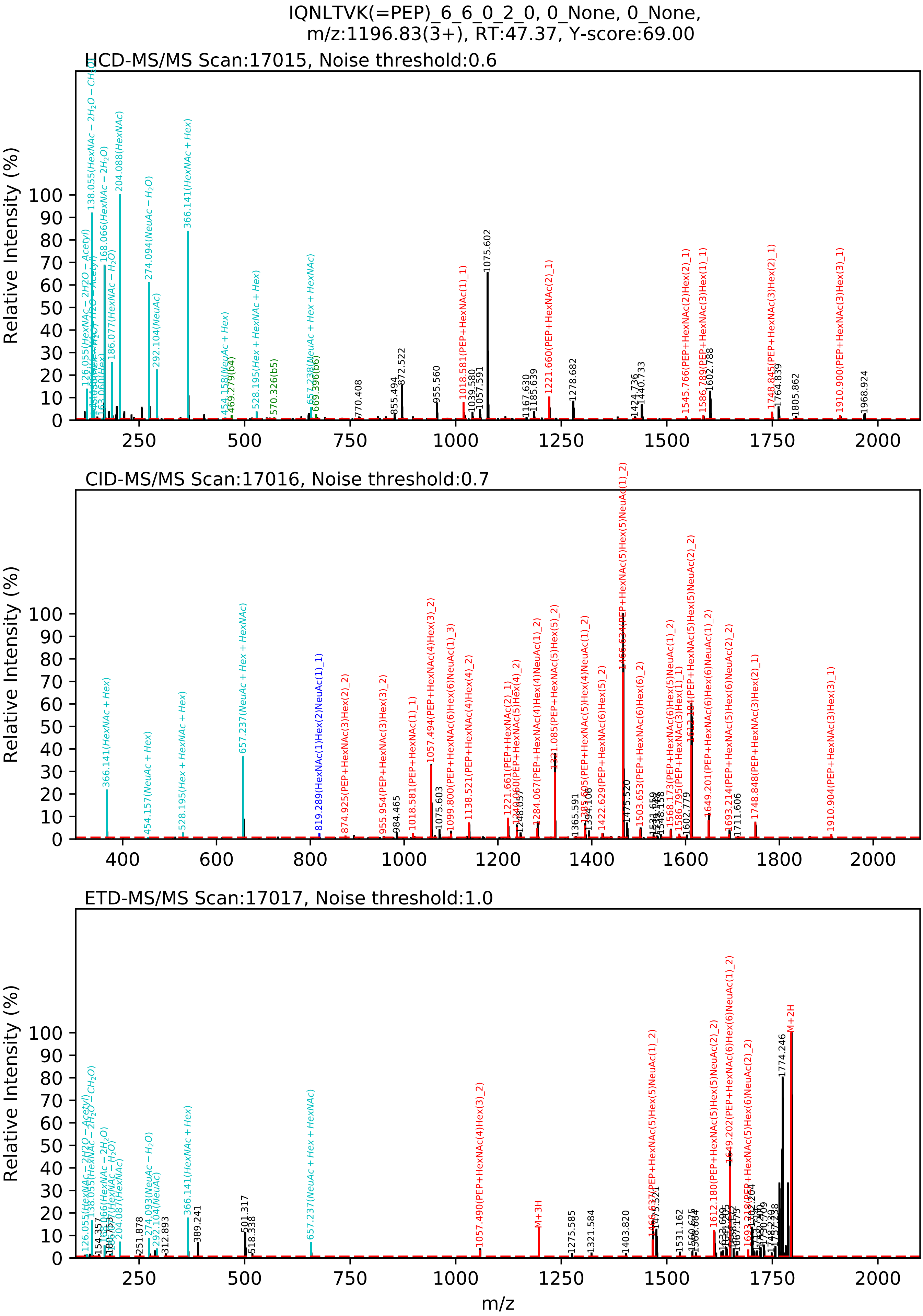

IQNLTVK(=PEP)_6_6_0_2_0, 0_None, 0_None,
m/z:1196.83(3+), RT:47.37, Y-score:69.00
HCD-MS/MS Scan:17015, Noise threshold:0.6
CID-MS/MS Scan:17016, Noise threshold:0.7
ETD-MS/MS Scan:17017, Noise threshold:1.0
Relative Intensity (%)
m/z

IQNLTVK(=PEP)_6_6_1_2_0, 0_None, 0_None,
m/z:1245.52(3+), RT:47.57, Y-score:59.69

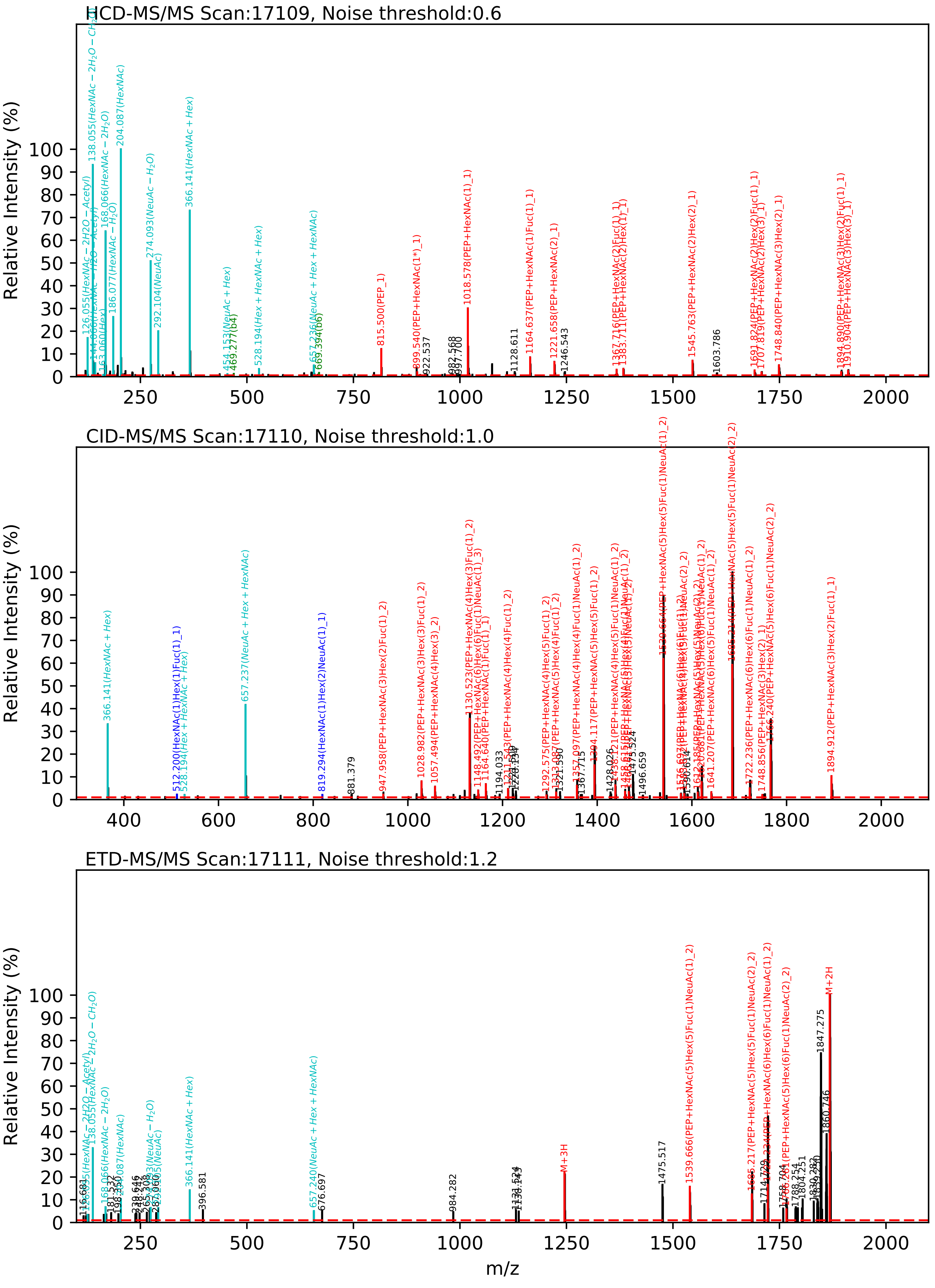

IQNLTVK(=PEP)_7_2_0_0_0, 0_None, 0_None,
m/z:1178.52(2+), RT:25.35, Y-score:94.34

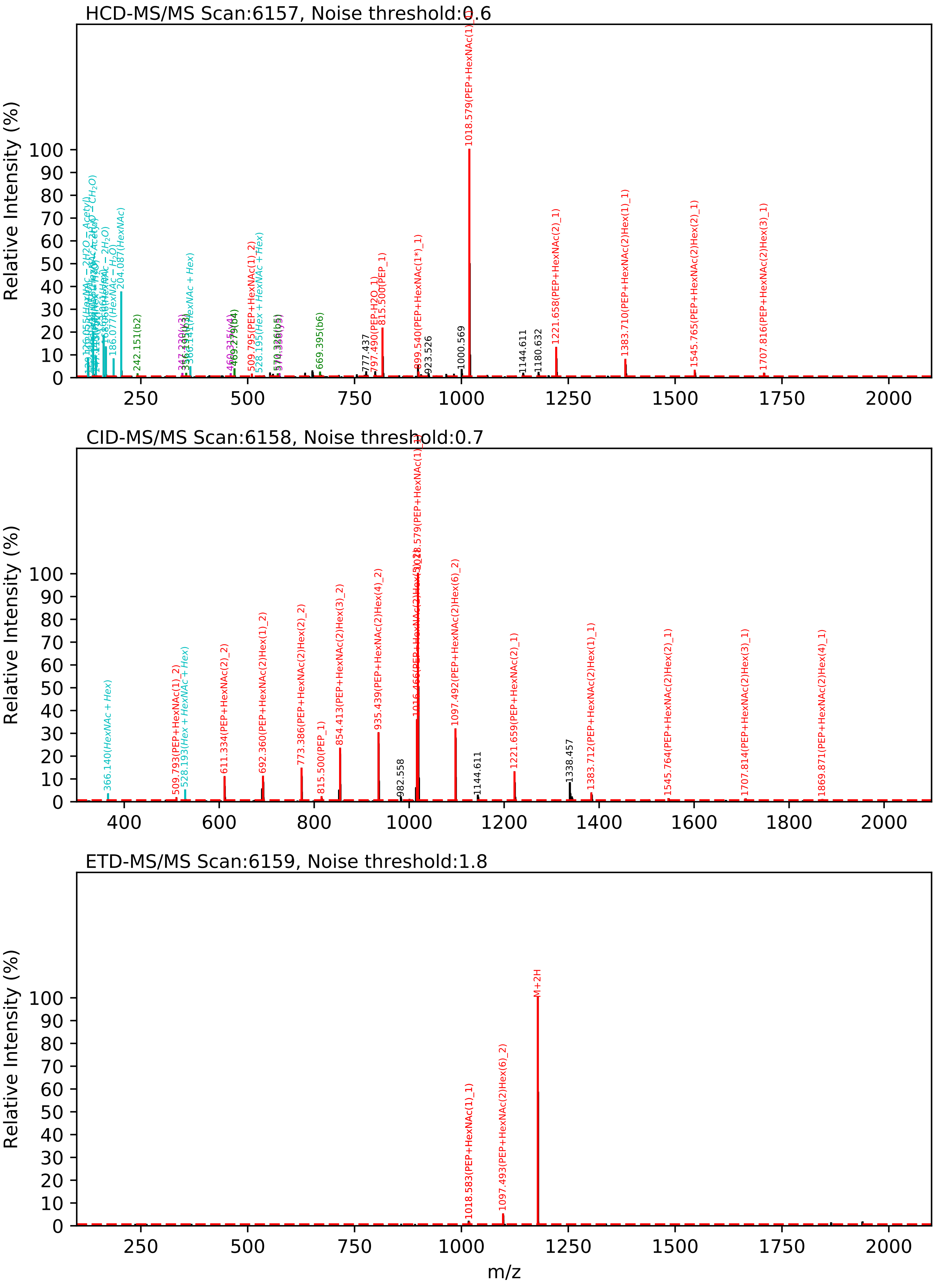

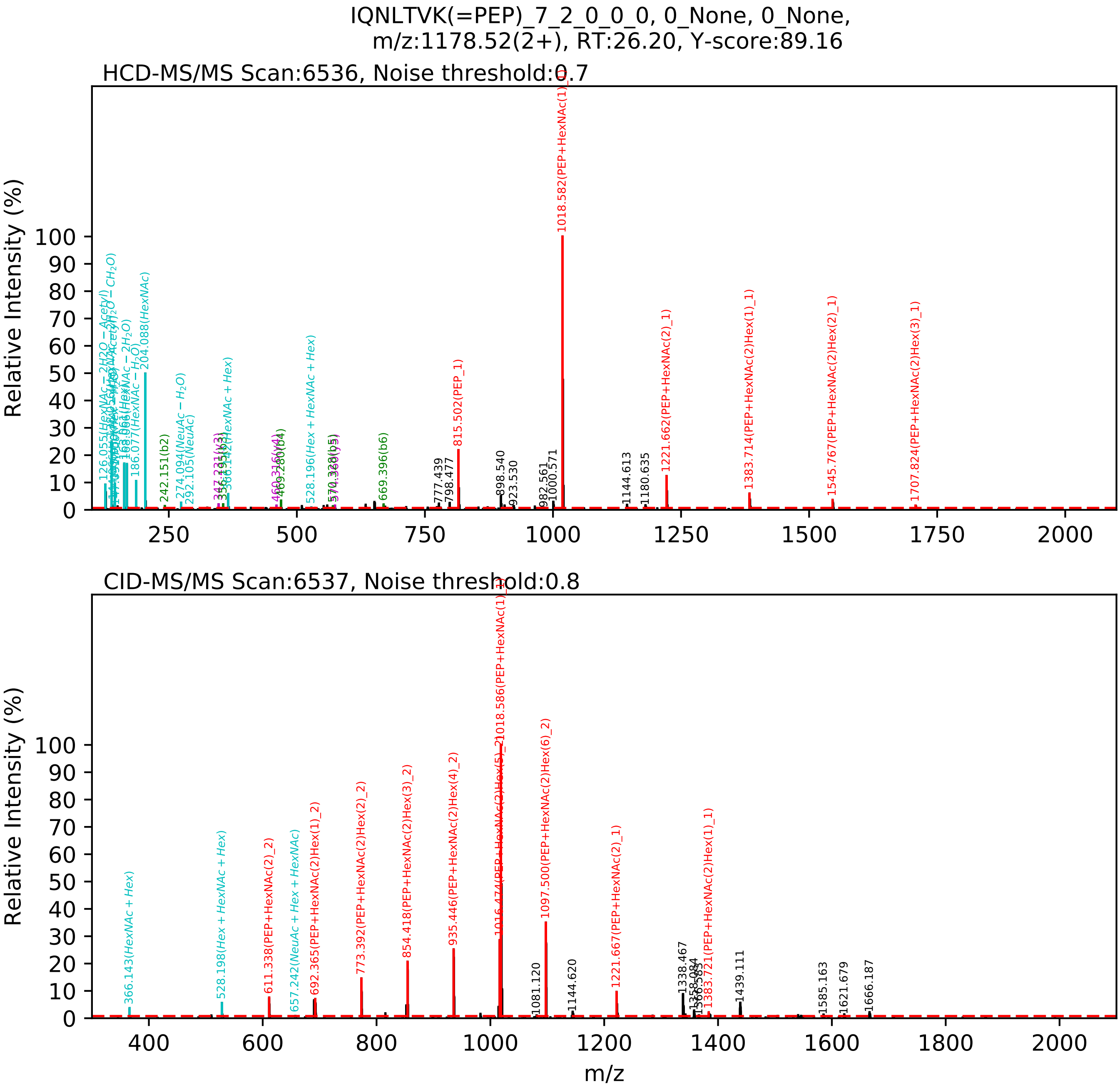

IQNLTVK(=PEP)_7_2_0_0_0, 0_None, 0_None,
m/z:1178.52(2+), RT:26.20, Y-score:89.16
HCD-MS/MS Scan:6536, Noise threshold:0.7
Relative Intensity (%)
1018.582(PEP+HexNAc(1)_1)
204.088(HexNAc)
186.077(HexNAc − H2O)
242.151(b2)
274.094(NeuAc − H2O)
292.105(NeuAc)
528.196(Hex + HexNAc + Hex)
669.396(b6)
815.502(PEP_1)
777.439
798.477
898.540
923.530
982.561
1000.571
1144.613
1180.635
1221.662(PEP+HexNAc(2)_1)
1383.714(PEP+HexNAc(2)Hex(1)_1)
1545.767(PEP+HexNAc(2)Hex(2)_1)
1707.824(PEP+HexNAc(2)Hex(3)_1)
CID-MS/MS Scan:6537, Noise threshold:0.8
366.143(HexNAc + Hex)
528.198(Hex + HexNAc + Hex)
611.338(PEP+HexNAc(2)_2)
657.242(NeuAc + Hex + HexNAc)
692.365(PEP+HexNAc(2)Hex(1)_2)
773.392(PEP+HexNAc(2)Hex(2)_2)
854.418(PEP+HexNAc(2)Hex(3)_2)
935.446(PEP+HexNAc(2)Hex(4)_2)
1018.586(PEP+HexNAc(1)_1)
1097.500(PEP+HexNAc(2)Hex(6)_2)
1081.120
1144.620
1221.667(PEP+HexNAc(2)_1)
1338.467
1383.721(PEP+HexNAc(2)Hex(1)_1)
1439.111
1585.163
1621.679
1666.187
m/z

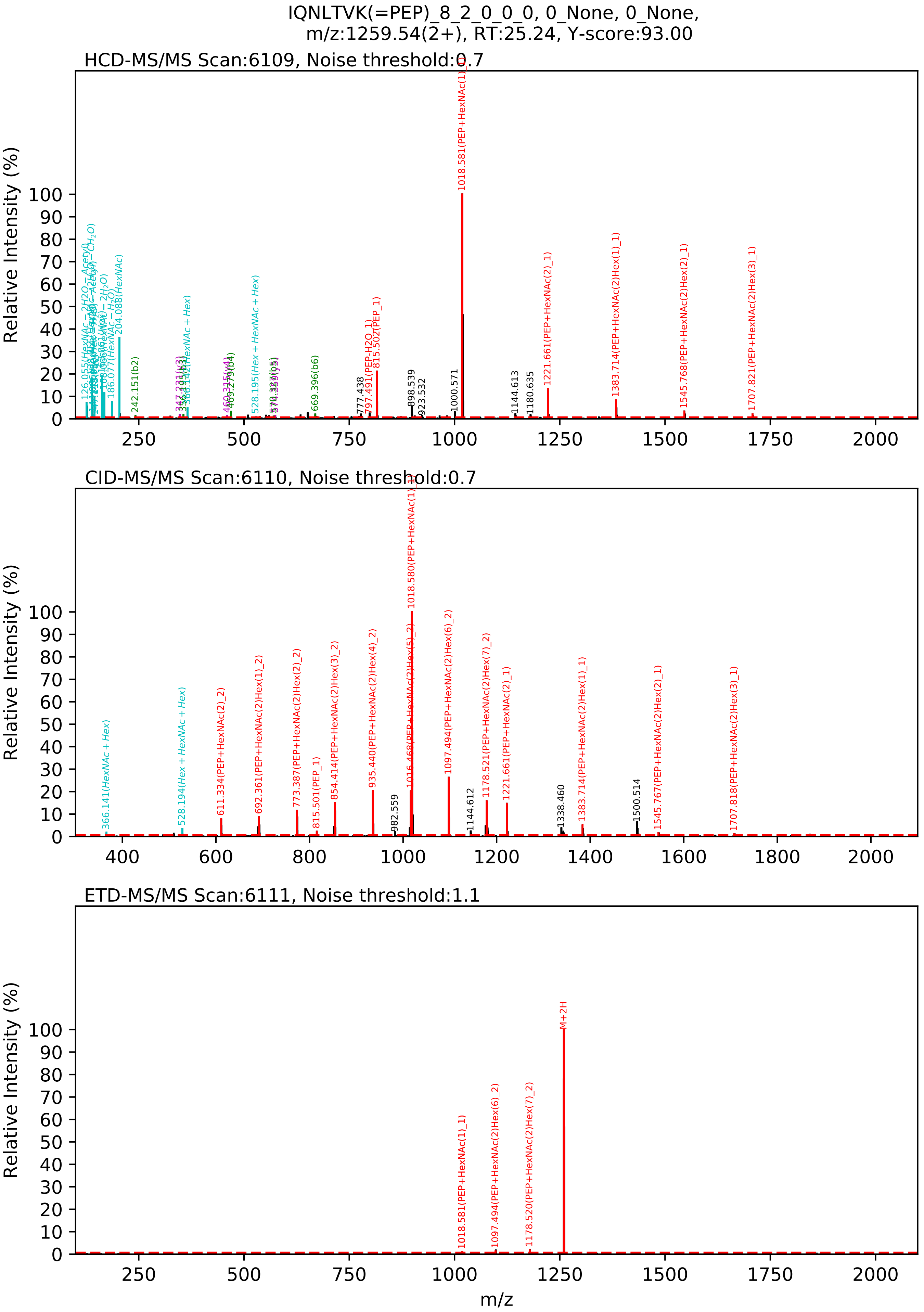

IQNLTVK(=PEP)_8_2_0_0_0, 0_None, 0_None,
m/z:1259.54(2+), RT:25.24, Y-score:93.00
HCD-MS/MS Scan:6109, Noise threshold:0.7
1018.581(PEP+HexNAc(1)_1)
1221.661(PEP+HexNAc(2)_1)
1383.714(PEP+HexNAc(2)Hex(1)_1)
1545.768(PEP+HexNAc(2)Hex(2)_1)
1707.821(PEP+HexNAc(2)Hex(3)_1)
815.502(PEP_1)
797.491(PEP-H2O_1)
204.088(HexNAc)
242.151(b2)
669.396(b6)
528.195(Hex + HexNAc + Hex)
CID-MS/MS Scan:6110, Noise threshold:0.7
1018.580(PEP+HexNAc(1)_1)
611.334(PEP+HexNAc(2)_2)
692.361(PEP+HexNAc(2)Hex(1)_2)
773.387(PEP+HexNAc(2)Hex(2)_2)
815.501(PEP_1)
854.414(PEP+HexNAc(2)Hex(3)_2)
935.440(PEP+HexNAc(2)Hex(4)_2)
1097.494(PEP+HexNAc(2)Hex(6)_2)
1178.521(PEP+HexNAc(2)Hex(7)_2)
1221.661(PEP+HexNAc(2)_1)
1383.714(PEP+HexNAc(2)Hex(1)_1)
1545.767(PEP+HexNAc(2)Hex(2)_1)
1707.818(PEP+HexNAc(2)Hex(3)_1)
366.141(HexNAc + Hex)
528.194(Hex + HexNAc + Hex)
ETD-MS/MS Scan:6111, Noise threshold:1.1
M+2H
1018.581(PEP+HexNAc(1)_1)
1097.494(PEP+HexNAc(2)Hex(6)_2)
1178.520(PEP+HexNAc(2)Hex(7)_2)
Relative Intensity (%)
m/z

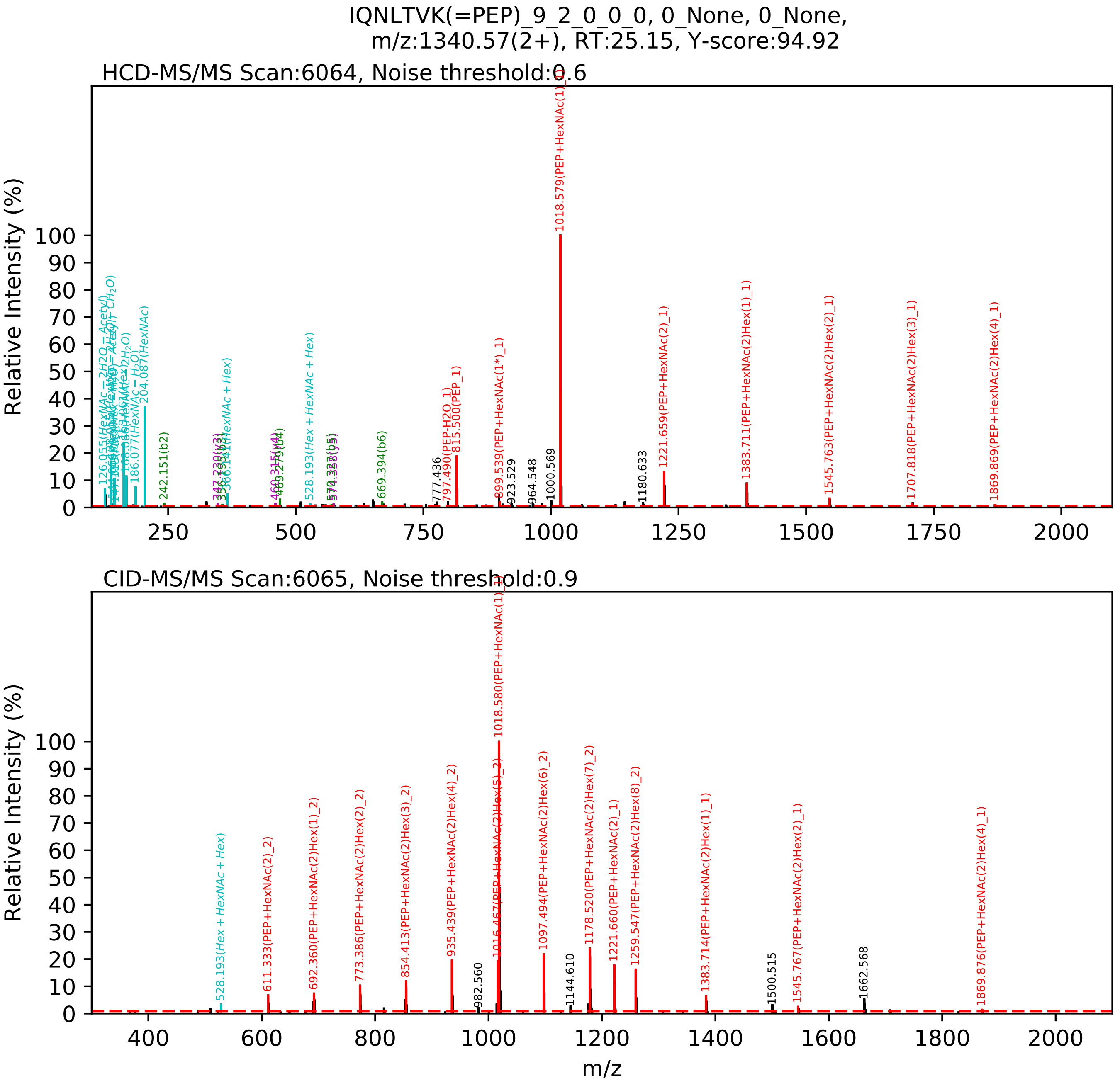

IQNLTVK(=PEP)_9_2_0_0_0, 0_None, 0_None,
m/z:1340.57(2+), RT:25.15, Y-score:94.92
HCD-MS/MS Scan:6064, Noise threshold:0.6
1018.579(PEP+HexNAc(1)_1)
1221.659(PEP+HexNAc(2)_1)
1383.711(PEP+HexNAc(2)Hex(1)_1)
1545.763(PEP+HexNAc(2)Hex(2)_1)
1707.818(PEP+HexNAc(2)Hex(3)_1)
1869.869(PEP+HexNAc(2)Hex(4)_1)
899.539(PEP+HexNAc(1*)_1)
815.500(PEP_1)
797.490(PEP-H2O_1)
204.087(HexNAc)
242.151(b2)
669.394(b6)
528.193(Hex + HexNAc + Hex)
777.436
923.529
964.548
1000.569
1180.633
Relative Intensity (%)
CID-MS/MS Scan:6065, Noise threshold:0.9
1018.580(PEP+HexNAc(1)_1)
528.193(Hex + HexNAc + Hex)
611.333(PEP+HexNAc(2)_2)
692.360(PEP+HexNAc(2)Hex(1)_2)
773.386(PEP+HexNAc(2)Hex(2)_2)
854.413(PEP+HexNAc(2)Hex(3)_2)
935.439(PEP+HexNAc(2)Hex(4)_2)
982.560
1097.494(PEP+HexNAc(2)Hex(6)_2)
1144.610
1178.520(PEP+HexNAc(2)Hex(7)_2)
1221.660(PEP+HexNAc(2)_1)
1259.547(PEP+HexNAc(2)Hex(8)_2)
1383.714(PEP+HexNAc(2)Hex(1)_1)
1500.515
1545.767(PEP+HexNAc(2)Hex(2)_1)
1662.568
1869.876(PEP+HexNAc(2)Hex(4)_1)
m/z

LQLQALQQNGSSVLSE(=PEP)_4_4_1_0_0, 0_None, 0_None,
m/z:1107.83(3+), RT:81.57, Y-score:73.74

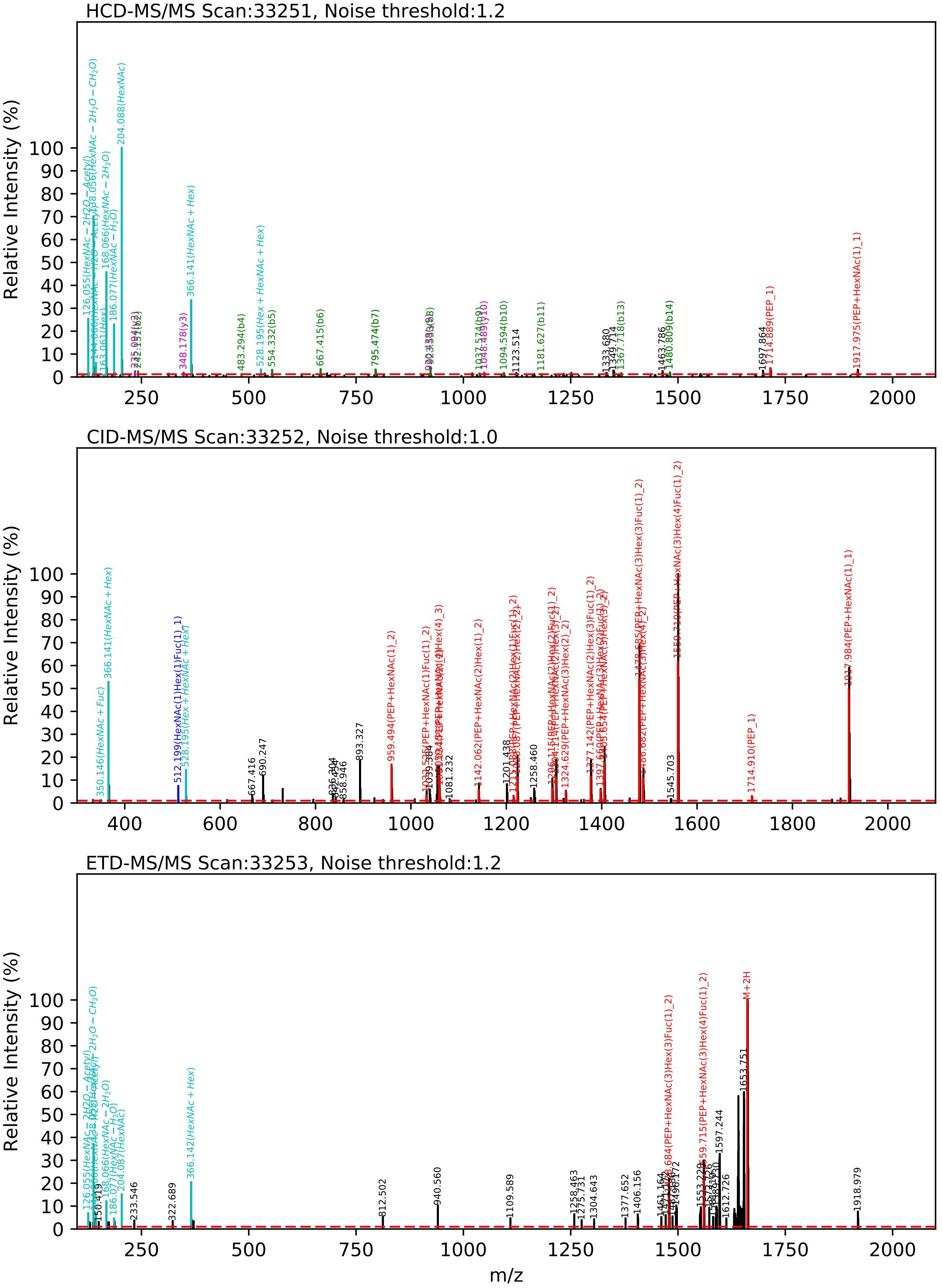

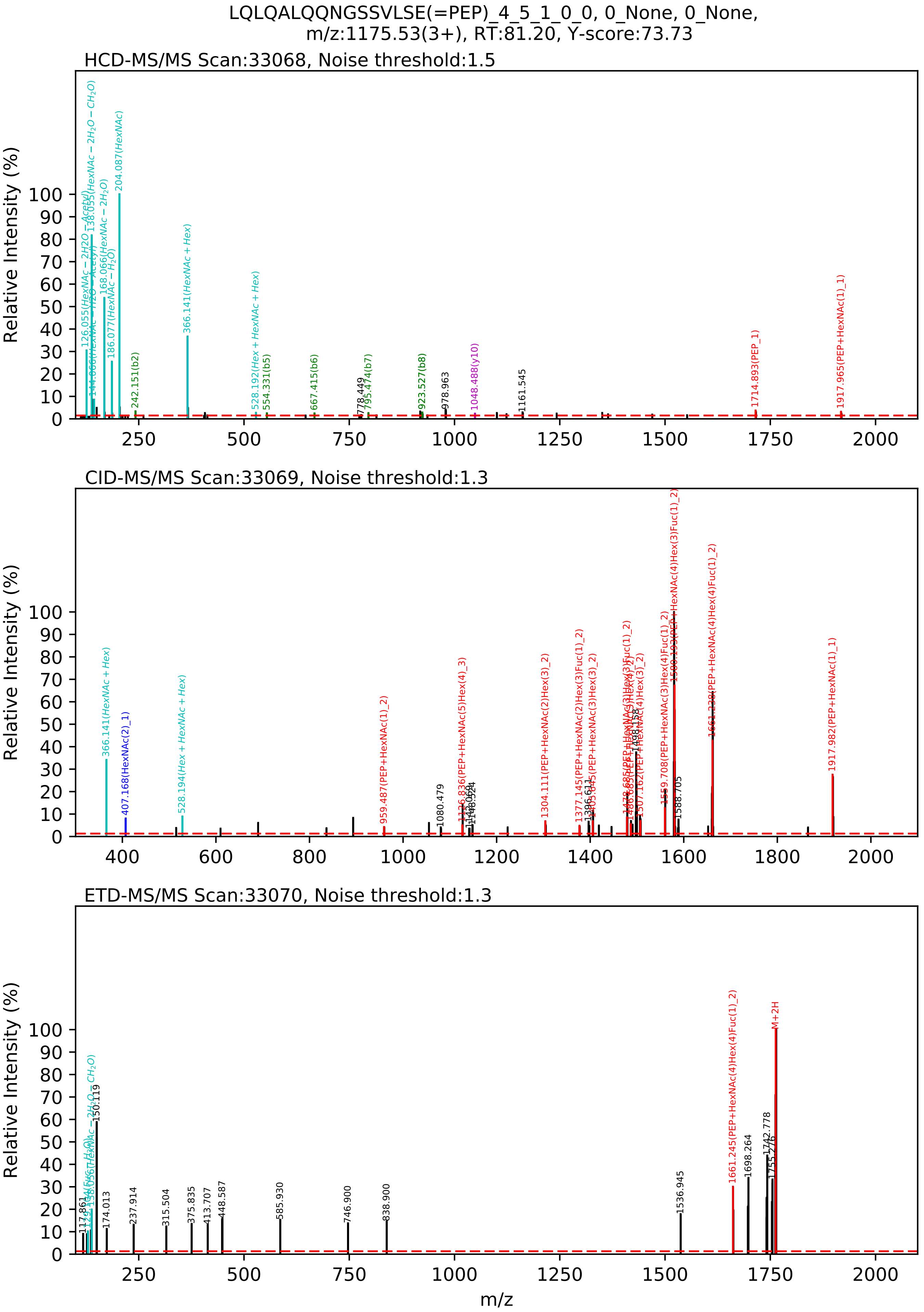
LQLQALQQNGSSVLSE(=PEP)_4_5_1_0_0, 0_None, 0_None,
m/z:1175.53(3+), RT:81.20, Y-score:73.73
HCD-MS/MS Scan:33068, Noise threshold:1.5
Relative Intensity (%)
204.087(HexNAc)
366.141(HexNAc + Hex)
528.192(Hex + HexNAc + Hex)
242.151(b2)
554.331(b5)
667.415(b6)
778.449
795.474(b7)
923.527(b8)
978.963
1048.488(y10)
1161.545
1714.893(PEP_1)
1917.965(PEP+HexNAc(1)_1)
CID-MS/MS Scan:33069, Noise threshold:1.3
366.141(HexNAc + Hex)
407.168(HexNAc(2)_1)
528.194(Hex + HexNAc + Hex)
959.487(PEP+HexNAc(1)_2)
1080.479
1126.836(PEP+HexNAc(5)Hex(4)_3)
1304.111(PEP+HexNAc(2)Hex(3)_2)
1377.145(PEP+HexNAc(2)Hex(3)Fuc(1)_2)
1559.708(PEP+HexNAc(3)Hex(4)Fuc(1)_2)
1588.705
1661.238(PEP+HexNAc(4)Hex(4)Fuc(1)_2)
1917.982(PEP+HexNAc(1)_1)
ETD-MS/MS Scan:33070, Noise threshold:1.3
117.861
150.119
174.013
237.914
315.504
375.835
413.707
448.587
585.930
746.900
838.900
1536.945
1661.245(PEP+HexNAc(4)Hex(4)Fuc(1)_2)
1698.264
1742.778
M+2H
m/z

LQLQALQQNGSSVLSE(=PEP)_4_5_1_0_0, 0_None, 0_None,
m/z:1175.53(3+), RT:81.83, Y-score:79.85

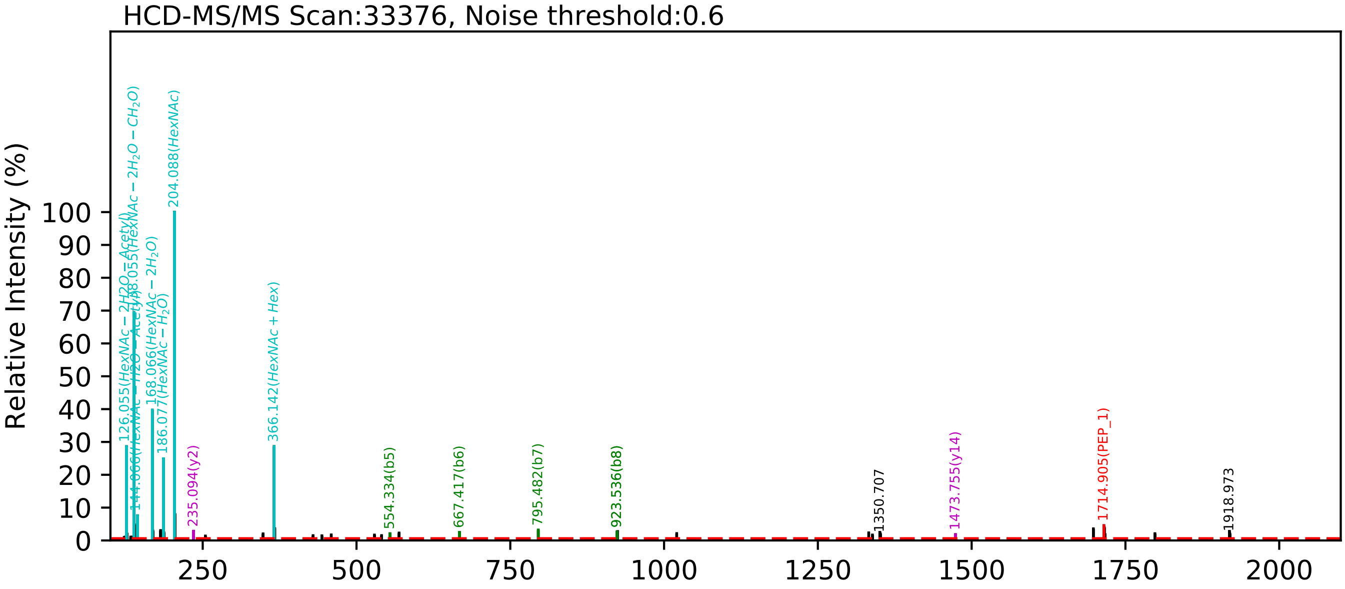

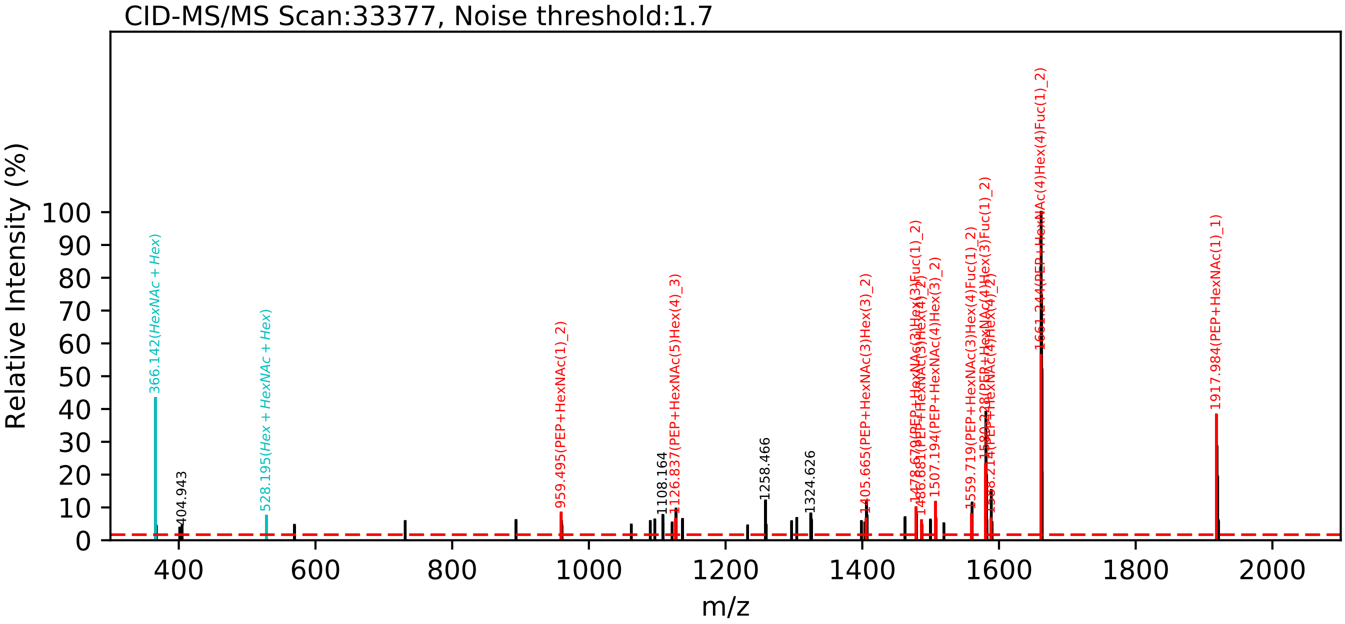

LQLQALQQNGSSVLSE(=PEP)_5_2_0_0_0, 0_None, 0_None,
m/z:1466.16(2+), RT:82.36, Y-score:74.20

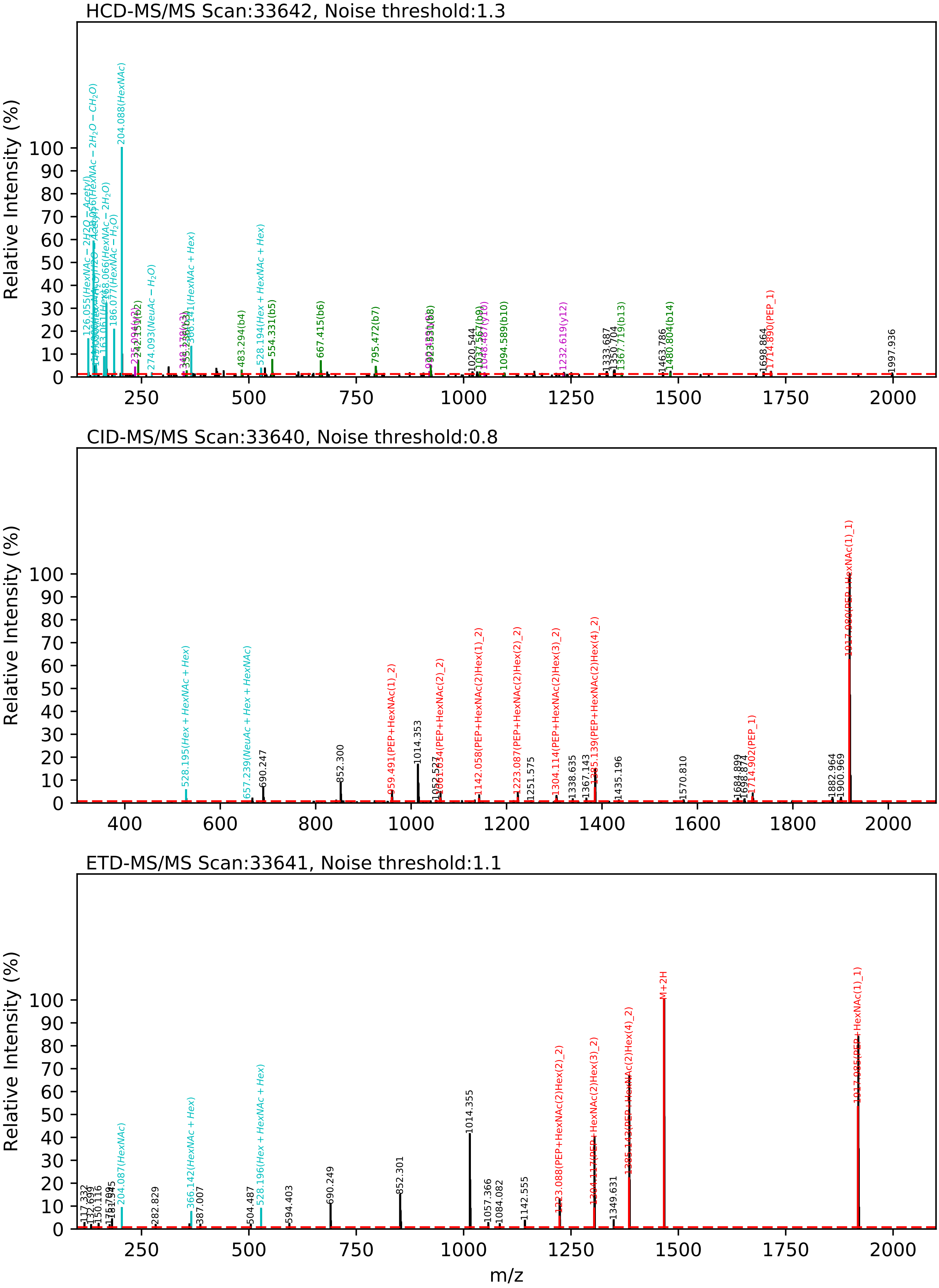

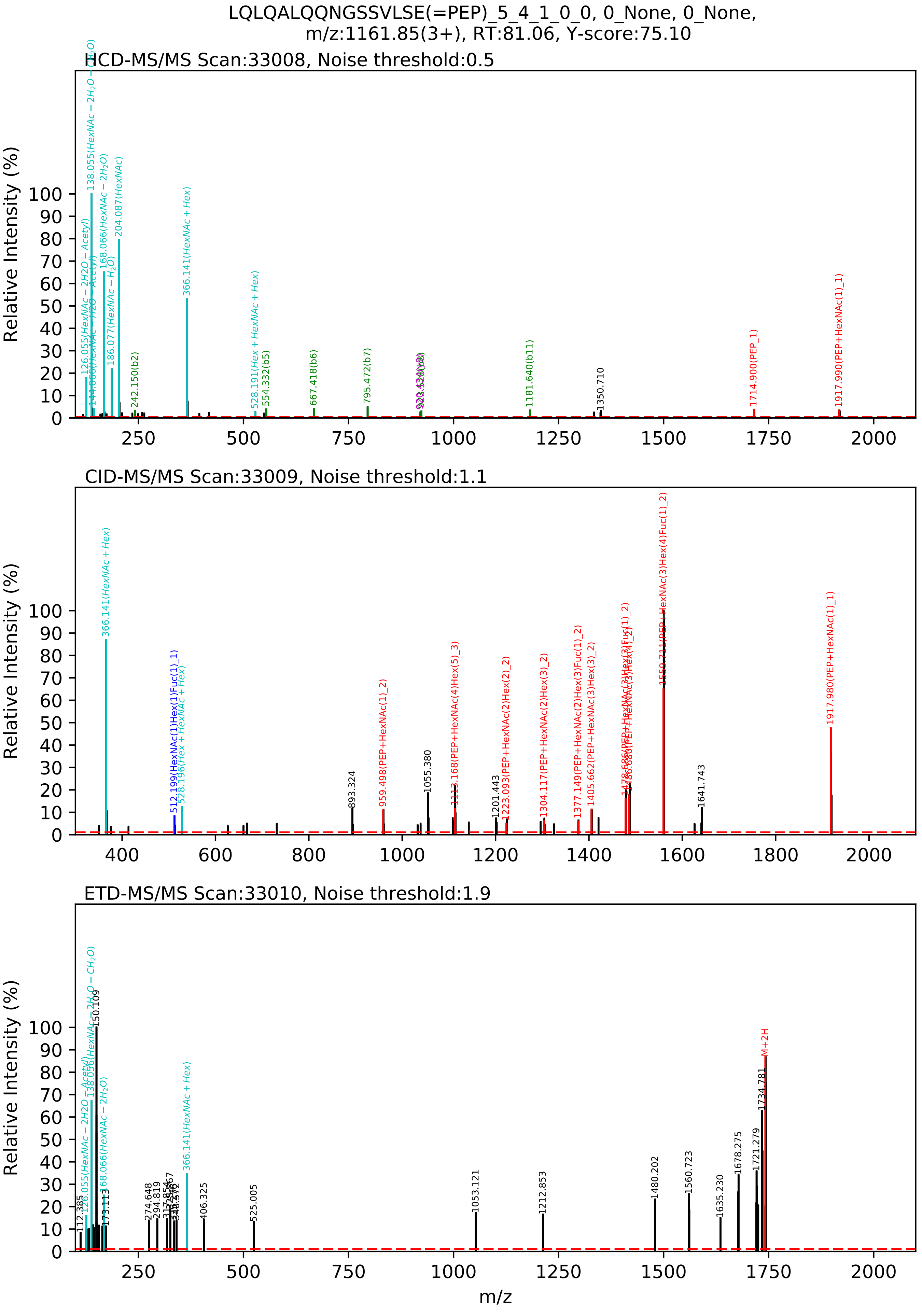
LQLQALQQNGSSVLSE(=PEP)_5_4_1_0_0, 0_None, 0_None,
m/z:1161.85(3+), RT:81.06, Y-score:75.10
HCD-MS/MS Scan:33008, Noise threshold:0.5
CID-MS/MS Scan:33009, Noise threshold:1.1
ETD-MS/MS Scan:33010, Noise threshold:1.9
Relative Intensity (%)
m/z
138.055
168.066
204.087(HexNAc)
186.077
366.141(HexNAc + Hex)
528.191(Hex + HexNAc + Hex)
242.150(b2)
554.332(b5)
667.418(b6)
795.472(b7)
1181.640(b11)
1350.710
1714.900(PEP_1)
1917.990(PEP+HexNAc(1)_1)
512.199(HexNAc(1)Hex(1)Fuc(1)_1)
893.324
959.498(PEP+HexNAc(1)_2)
1055.380
1112.168(PEP+HexNAc(4)Hex(5)_3)
1201.443
1223.093(PEP+HexNAc(2)Hex(2)_2)
1304.117(PEP+HexNAc(2)Hex(3)_2)
1377.149(PEP+HexNAc(2)Hex(3)Fuc(1)_2)
1405.662(PEP+HexNAc(3)Hex(3)_2)
1641.743
1917.980(PEP+HexNAc(1)_1)
150.109
112.385
173.113
274.648
294.819
406.325
525.005
1053.121
1212.853
1480.202
1560.723
1635.230
1678.275
1721.279
1734.781
M+2H

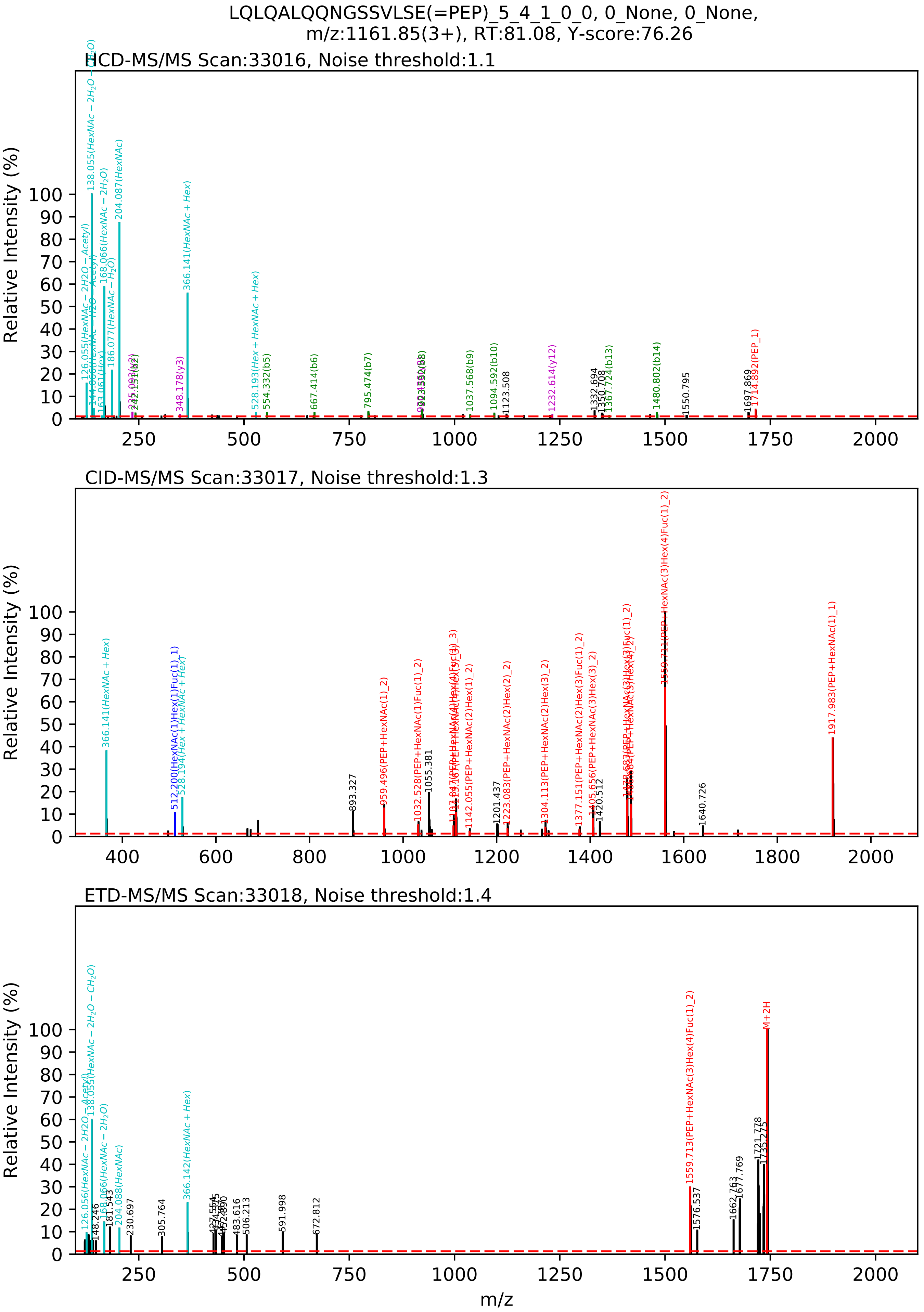

LQLQALQQNGSSVLSE(=PEP)_5_4_1_0_0, 0_None, 0_None,
m/z:1161.85(3+), RT:81.08, Y-score:76.26
HCD-MS/MS Scan:33016, Noise threshold:1.1
Relative Intensity (%)
138.055(HexNAc)
204.087(HexNAc)
168.066(HexNAc − 2H2O)
186.077(HexNAc − H2O)
366.141(HexNAc + Hex)
528.193(Hex + HexNAc + Hex)
554.332(b5)
667.414(b6)
795.474(b7)
1037.568(b9)
1094.592(b10)
1123.508
1232.614(y12)
1332.694
1350.708
1367.724(b13)
1480.802(b14)
1550.795
1697.869
1714.892(PEP_1)
CID-MS/MS Scan:33017, Noise threshold:1.3
Relative Intensity (%)
366.141(HexNAc + Hex)
512.200(HexNAc(1)Hex(1)Fuc(1)_1)
893.327
959.496(PEP+HexNAc(1)_2)
1032.528(PEP+HexNAc(1)Fuc(1)_2)
1055.381
1142.055(PEP+HexNAc(2)Hex(1)_2)
1201.437
1223.083(PEP+HexNAc(2)Hex(2)_2)
1304.113(PEP+HexNAc(2)Hex(3)_2)
1377.151(PEP+HexNAc(2)Hex(3)Fuc(1)_2)
1405.656(PEP+HexNAc(3)Hex(3)_2)
1420.512
1550.713(PEP+HexNAc(3)Hex(4)Fuc(1)_2)
1640.726
1917.983(PEP+HexNAc(1)_1)
ETD-MS/MS Scan:33018, Noise threshold:1.4
Relative Intensity (%)
126.056(HexNAc − 2H2O − Acetyl)
138.055(HexNAc − 2H2O − CH2O)
148.246
168.066(HexNAc − 2H2O)
181.543
204.088(HexNAc)
230.697
305.764
366.142(HexNAc + Hex)
483.616
506.213
591.998
672.812
1559.713(PEP+HexNAc(3)Hex(4)Fuc(1)_2)
1576.537
1662.763
1677.769
1721.778
1735.275
M+2H
m/z
