# Supplementary material for: Characterization of Site-Specific N- and O-Glycopeptides from Recombinant Spike and ACE2 Glycoproteins Using LC-MS/MS Analysis
Source: Int J Mol Sci. 2024 Dec 20;25(24):13649. doi: 10.3390/ijms252413649 (PMC11678118; doi:10.3390/ijms252413649)
Supplement: Supplementary file 1 [file ijms-25-13649-s001.zip › Supplementary Figure S2.pdf]

Supplementary Figure 2. Spectrum of 28 O-glycopeptides identified from RBD protein

1. VQP*T*ESIVR_0_1_0_0

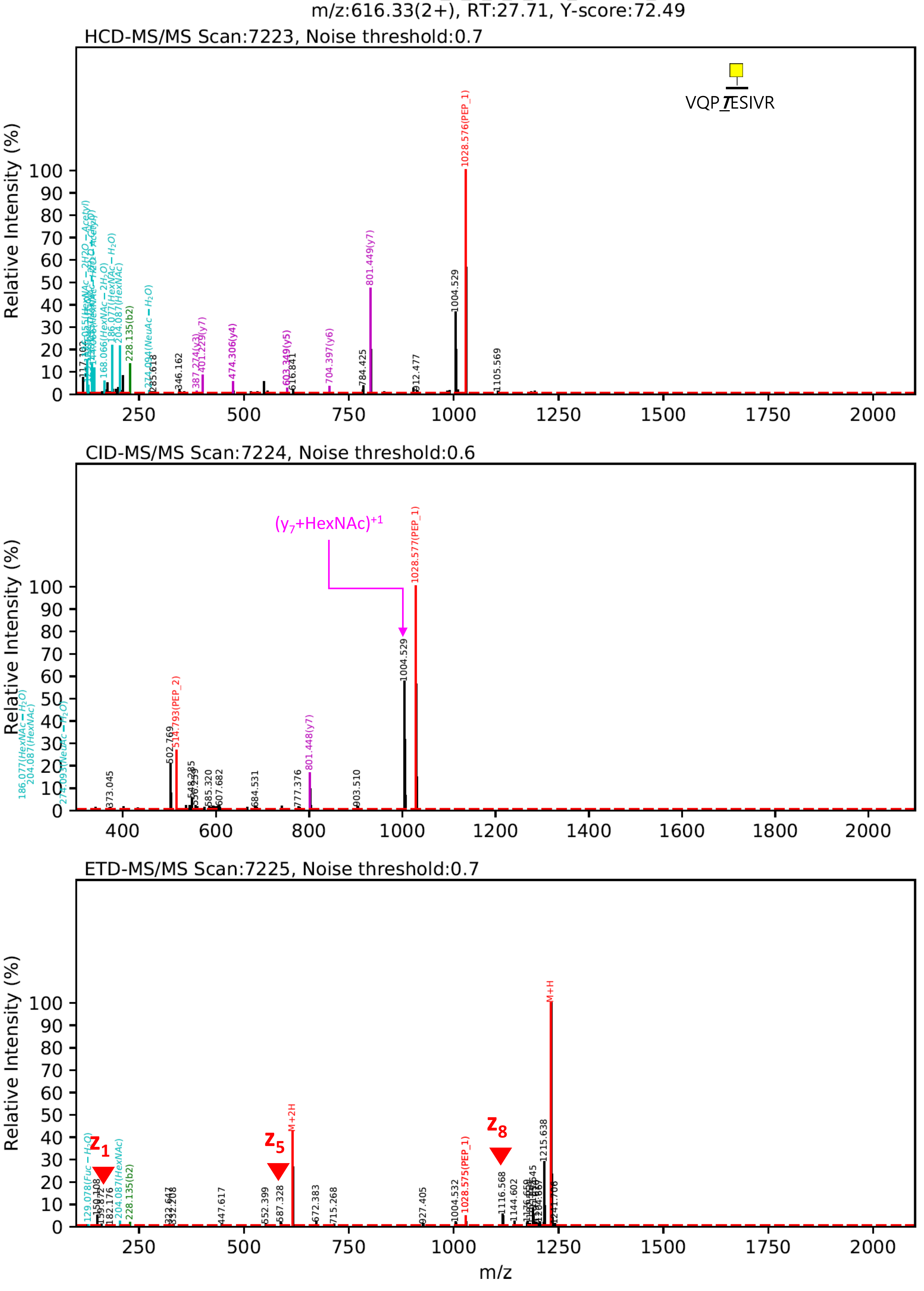

1. VQP*T*ESIVR_0_1_0_0

VQPTESIVR

MH+1(mono)	MH+2(mono)
1028.5735	514.7904

b				y	y+2
---	1	**V**	9	---	---
228.1343	2	**Q**	8	929.5051	465.2562
325.1870	3	**P**	7	801.4465	401.2269
426.2347	4	**T**	6	704.3937	352.7005
555.2773	5	**E**	5	603.3461	302.1767
642.3093	6	**S**	4	474.3035	237.6554
755.3934	7	**I**	3	387.2714	194.1394
854.4618	8	**V**	2	274.1874	137.5973
---	9	**R**	1	175.1190	88.0631

VQPT(HexNAc)ESIVR

MH+1(mono)	MH+2(mono)
1231.6529	616.3301

b	c				y	y+2	z	z+2
---	117.1022	1	V	9	---	---	---	---
228.1343	---	2	Q	8	1132.5844	566.7959	1116.5657	558.7865
325.1870	342.2136	3	P	7	1004.5259	502.7666	---	---
629.3141	646.3406	4	T(HexNAc)	6	907.4731	454.2402	891.4544	446.2308
758.3567	775.3832	5	E	5	603.3461	302.1767	587.3273	294.1673
845.3887	862.4153	6	S	4	474.3035	237.6554	458.2847	229.6460
958.4728	975.4993	7	I	3	387.2714	194.1394	371.2527	186.1300
1057.5412	1074.5677	8	V	2	274.1874	137.5973	258.1686	129.5880
---	---	9	R	1	175.1190	88.0631	159.1002	80.0538

2. VQP*T*ESIVR_0_1_0_1

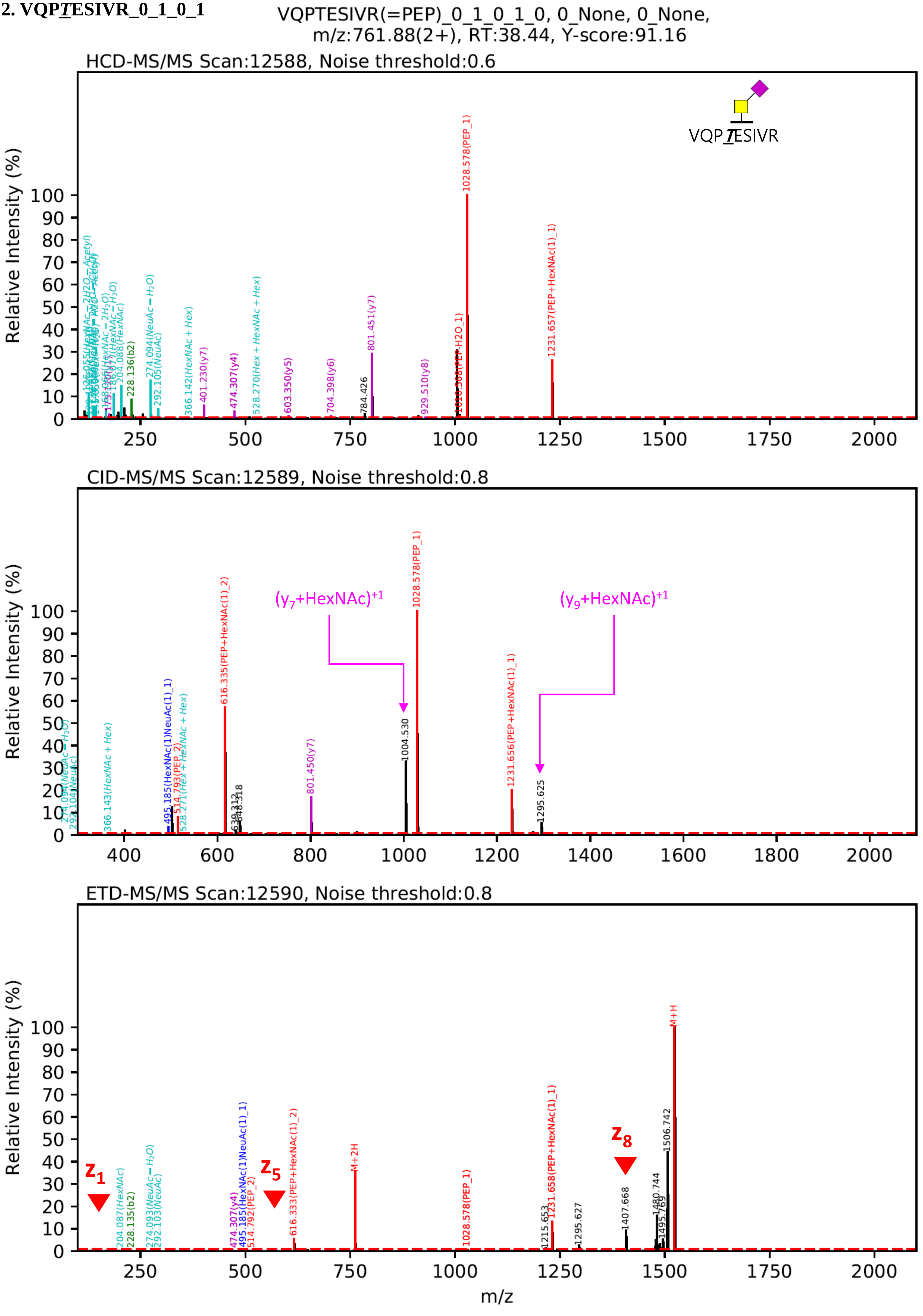

2. VQP*T*ESIVR_0_1_0_1

VQPT(HexNAcNeuAc)ESIVR

MH+1(mono)	MH+2(mono)
1522.7483	761.8778

b	c				y	y^{+2}	z	z^{+2}
---	117.1022	1	V	9	---	---	---	---
228.1343	---	2	Q	8	1423.6799	712.3436	1407.6611	704.3342
325.1870	342.2136	3	P	7	1295.6213	648.3143	---	---
920.4095	937.4360	4	T(HexNAcNeuAc)	6	1198.5685	599.7879	1182.5498	591.7785
1049.4521	1066.4786	5	E	5	603.3461	302.1767	587.3273	294.1673
1136.4841	1153.5107	6	S	4	474.3035	237.6554	458.2847	229.6460
1249.5682	1266.5947	7	I	3	387.2714	194.1394	371.2527	186.1300
1348.6366	1365.6631	8	V	2	274.1874	137.5973	258.1686	129.5880
---	---	9	R	1	175.1190	88.0631	159.1002	80.0538

3. VQP*T*ESIVR_1_1_0_0

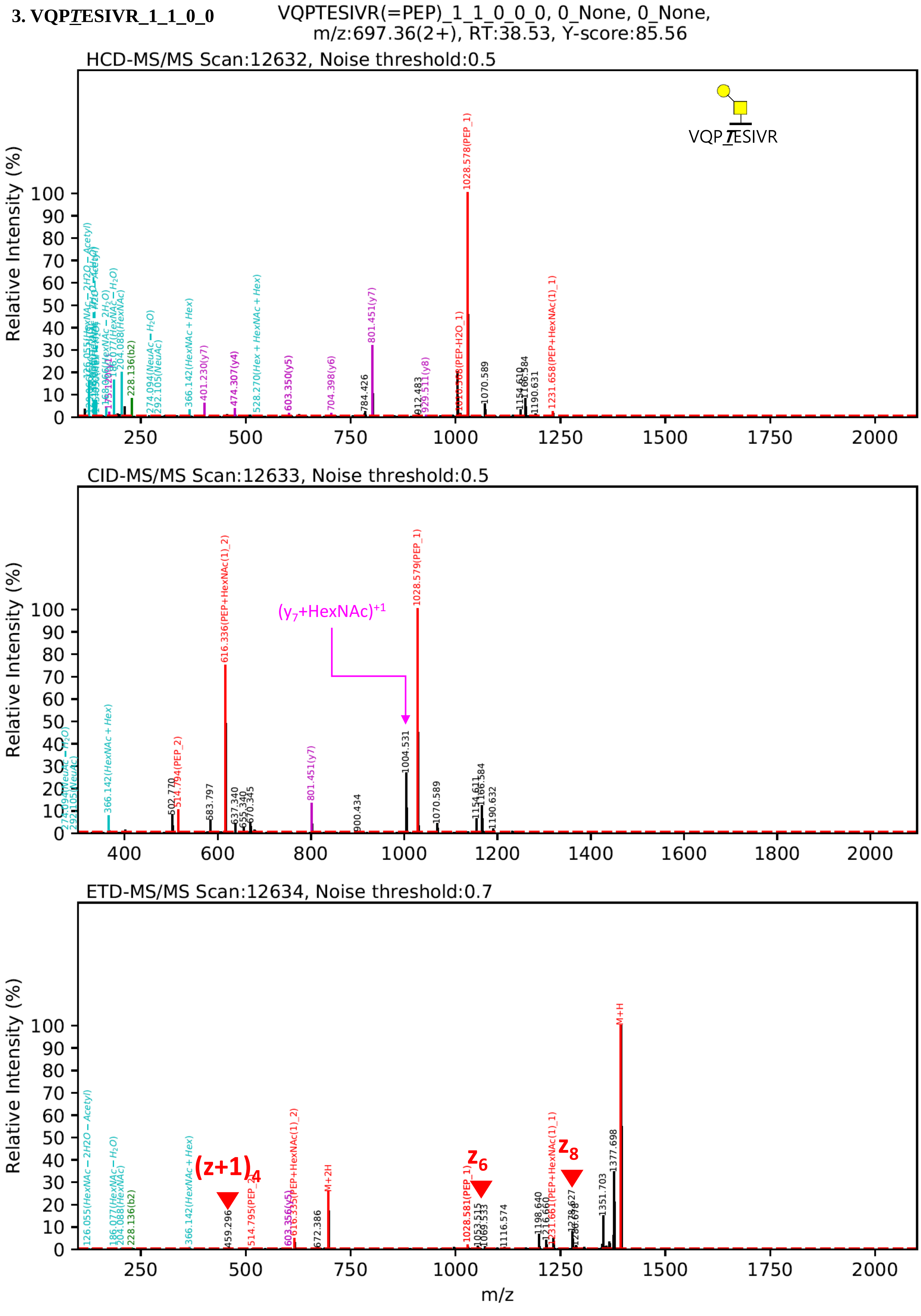

3. VQP*T*ESIVR_1_1_0_0

VQPT(HexHexNAc)ESIVR

MH+1(av)	MH+1(mono)	MH+2(av)	MH+2(mono)
1394.5277	1393.7057	697.7676	697.3565

b	c				y	y+2	z	z+2
---	117.1022	1	V	9	---	---	---	---
228.1343	---	2	Q	8	1294.6373	647.8223	1278.6185	639.8129
325.1870	342.2136	3	P	7	1166.5787	583.7930	---	---
791.3669	808.3935	4	T(HexHexNAc)	6	1069.5259	535.2666	1053.5072	527.2572
920.4095	937.4360	5	E	5	603.3461	302.1767	587.3273	294.1673
1007.4415	1024.4681	6	S	4	474.3035	237.6554	458.2847	229.6460
1120.5256	1137.5521	7	I	3	387.2714	194.1394	371.2527	186.1300
1219.5940	1236.6206	8	V	2	274.1874	137.5973	258.1686	129.5880
---	---	9	R	1	175.1190	88.0631	159.1002	80.0538

4. VQP*T*ESIVR_1_1_0_1

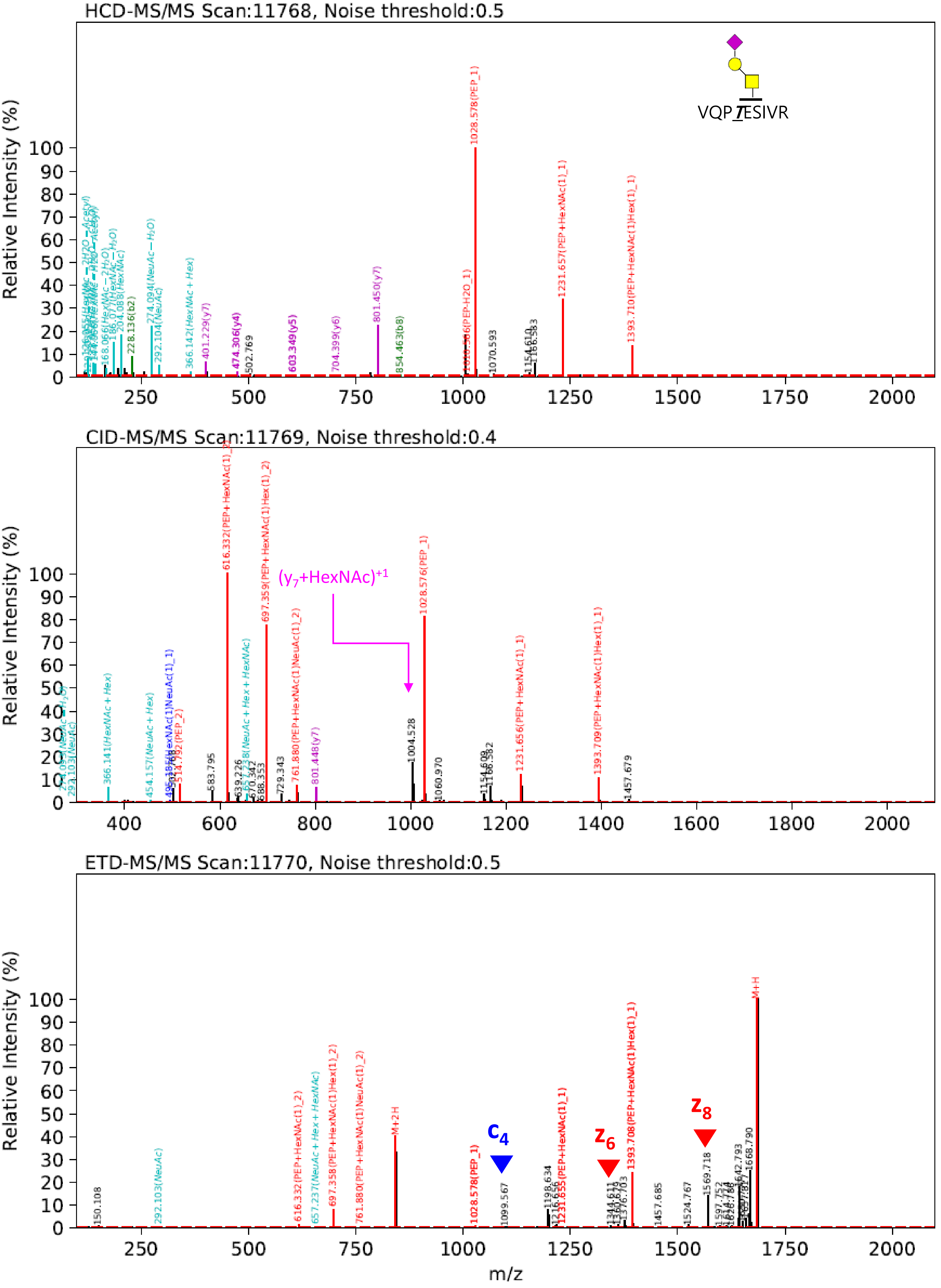

4. VQP*T*ESIVR_1_1_0_1

VQPT(<u>HexHexNAcNeuAc</u>)ESIVR

MH^{+1}(mono)	MH^{+2}(mono)
1684.8011	842.9042

b	c				y	y^{+2}	z	z^{+2}
---	117.1022	1	V	9	---	---	---	---
228.1343	---	2	Q	8	1585.7327	793.3700	1569.7140	785.3606
325.1870	342.2136	3	P	7	1457.6741	729.3407	---	---
1082.4623	1099.4889	4	T(HexHexNAcNeuAc)	6	1360.6213	680.8143	1344.6026	672.8049
1211.5049	1228.5315	5	E	5	603.3461	302.1767	587.3273	294.1673
1298.5369	1315.5635	6	S	4	474.3035	237.6554	458.2847	229.6460
1411.6210	1428.6476	7	I	3	387.2714	194.1394	371.2527	186.1300
1510.6894	1527.7160	8	V	2	274.1874	137.5973	258.1686	129.5880
---	---	9	R	1	175.1190	88.0631	159.1002	80.0538

5. VQP*T*ESIVR_1_1_0_2

VQPTESIVR(=PEP)_1_1_0_2_0, 0_None, 0_None,
m/z:659.30(3+), RT:38.40, Y-score:93.00

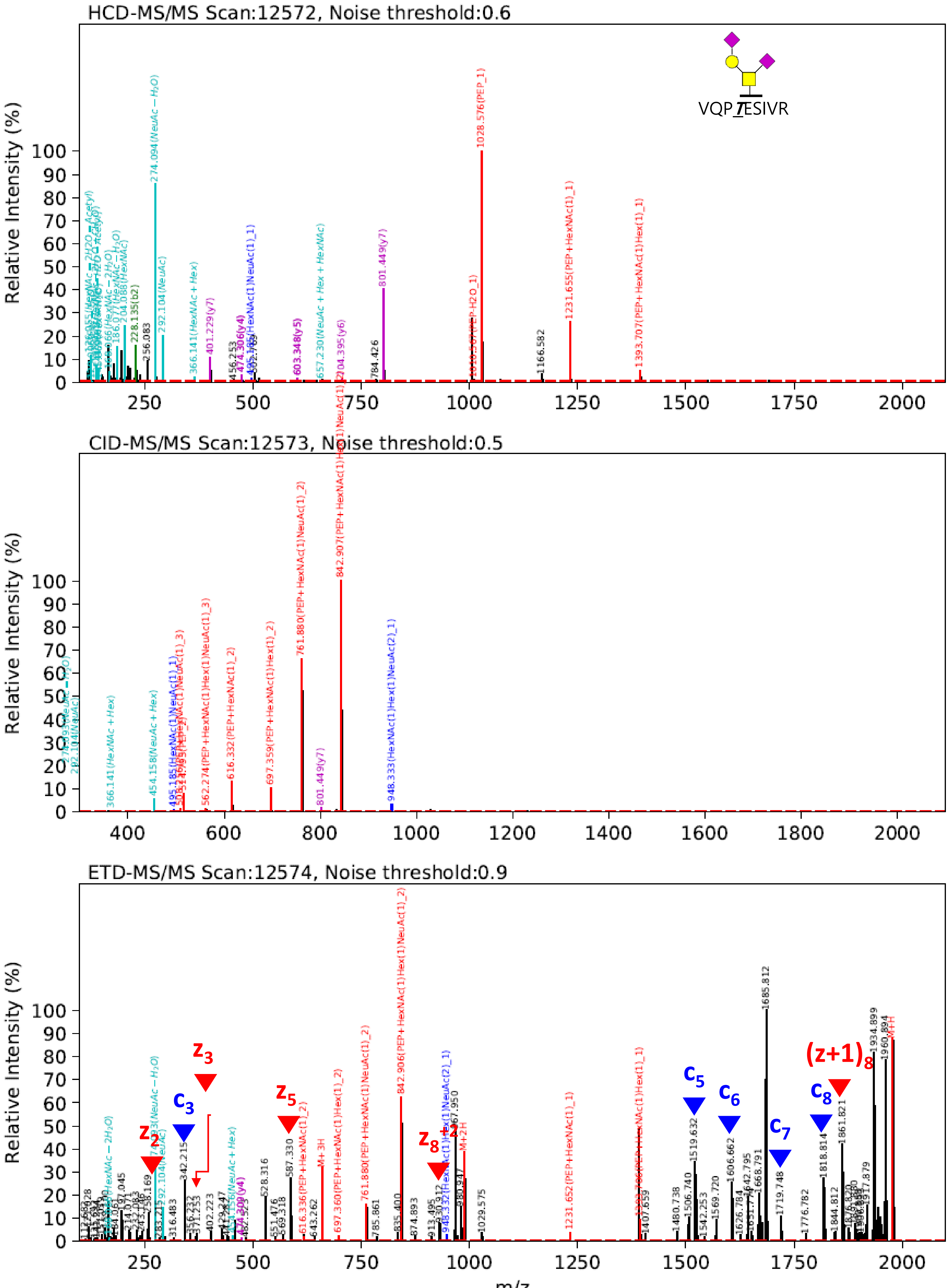

5. VQP*T*ESIVR_1_1_0_2

VQPT(HexHexNAcNeuAc2)ESIVR

MH+1(mono)	MH+2(mono)
1975.8965	988.4519

b	c				y	y+2	z	z+2
---	117.1022	1	V	9	---	---	---	---
228.1343	---	2	Q	8	1876.8281	938.9177	1860.8094	930.9083
325.1870	342.2136	3	P	7	1748.7695	874.8884	---	---
1373.5577	1390.5843	4	T(HexHexNAcNeuAc2)	6	1651.7168	826.3620	1635.6980	818.3527
1502.6003	1519.6269	5	E	5	603.3461	302.1767	587.3273	294.1673
1589.6324	1606.6589	6	S	4	474.3035	237.6554	458.2847	229.6460
1702.7164	1719.7430	7	I	3	387.2714	194.1394	371.2527	186.1300
1801.7848	1818.8114	8	V	2	274.1874	137.5973	258.1686	129.5880
---	---	9	R	1	175.1190	88.0631	159.1002	80.0538

6. VQP*T*ESIVR_1_1_0_2(Mono-Oacetylation)

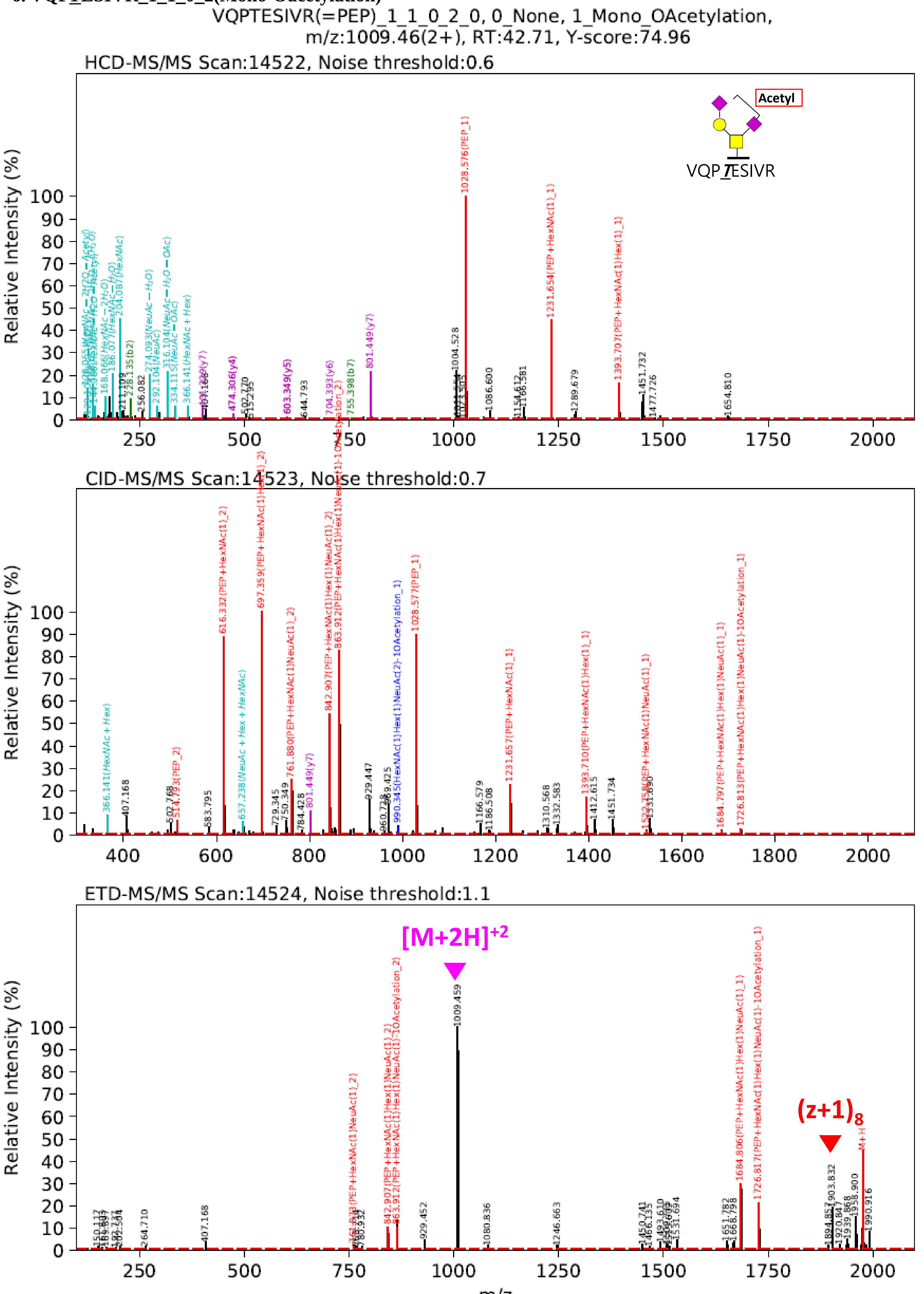

6. VQP*T*ESIVR_1_1_0_2(Mono-Oacetylation)

b	c	(c+42.0106)				y	y^{+2}	z	(z+42.0106)	z^{+2}
---	117.1022		1	V	9	---	---	---		---
228.1343	---		2	Q	8	1876.8281	938.9177	1860.8094	1902.8200	930.9083
325.187	342.2136		3	P	7	1748.7695	874.8884	---		---
1373.5577	1390.5843	1432.5949	4	T(HexNAcHexNeuAc2_Mono-OAcetyl)	6	1651.7168	826.362	1635.698	1677.7086	818.3527
1502.6003	1519.6269	1561.6375	5	E	5	603.3461	302.1767	587.3273		294.1673
1589.6324	1606.6589	1648.6695	6	S	4	474.3035	237.6554	458.2847		229.646
1702.7164	1719.743	1761.7536	7	I	3	387.2714	194.1394	371.2527		186.13
1801.7848	1818.8114	1860.822	8	V	2	274.1874	137.5973	258.1686		129.588
---	---		9	R	1	175.119	88.0631	159.1002		80.0538

7. VQP*T*E*S*IVR_1_1_1_0

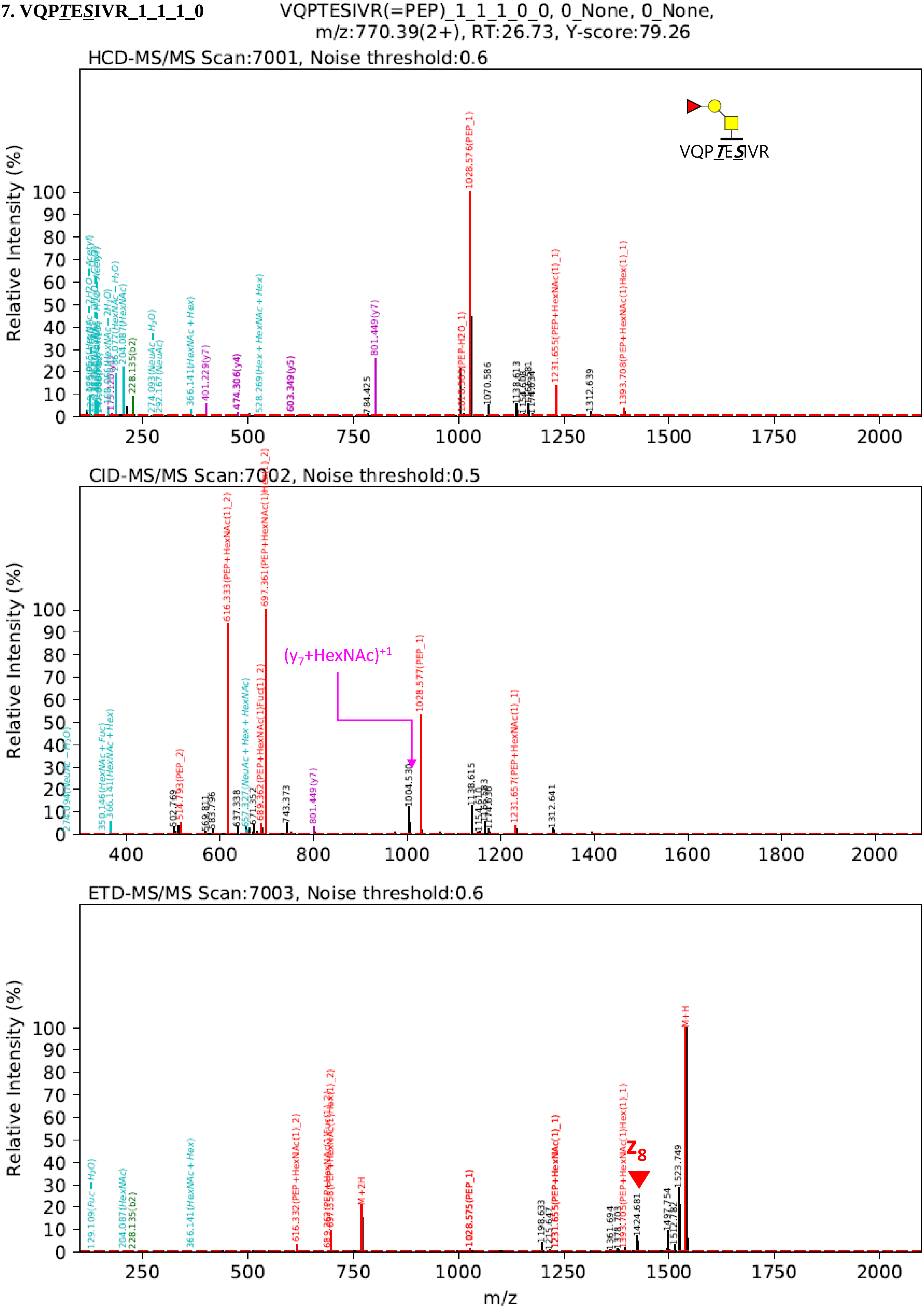

7. VQP*T*E*S*IVR_1_1_1_0

VQPT(HexHexNAcFuc)ESIVR

MH+1(mono)	MH+2(av)	MH+2(mono)
1539.7636	770.8395	770.3854

b	c				y	z	z^{+2}
---	117.1022	1	V	9	---	---	---
228.1343	---	2	Q	8	1440.6952	1424.6765	712.8419
325.1870	342.2136	3	P	7	1312.6366	---	---
937.4248	954.4514	4	T(HexHexNAcFuc)	6	1215.5838	1199.5651	600.2862
1066.4674	1083.4940	5	E	5	603.3461	587.3273	294.1673
1153.4994	1170.5260	6	S	4	474.3035	458.2847	229.6460
1266.5835	1283.6100	7	I	3	387.2714	371.2527	186.1300
1365.6519	1382.6785	8	V	2	274.1874	258.1686	129.5880
---	---	9	R	1	175.1190	159.1002	80.0538

8. VQP*T*E*S*IVR_1_1_1_1

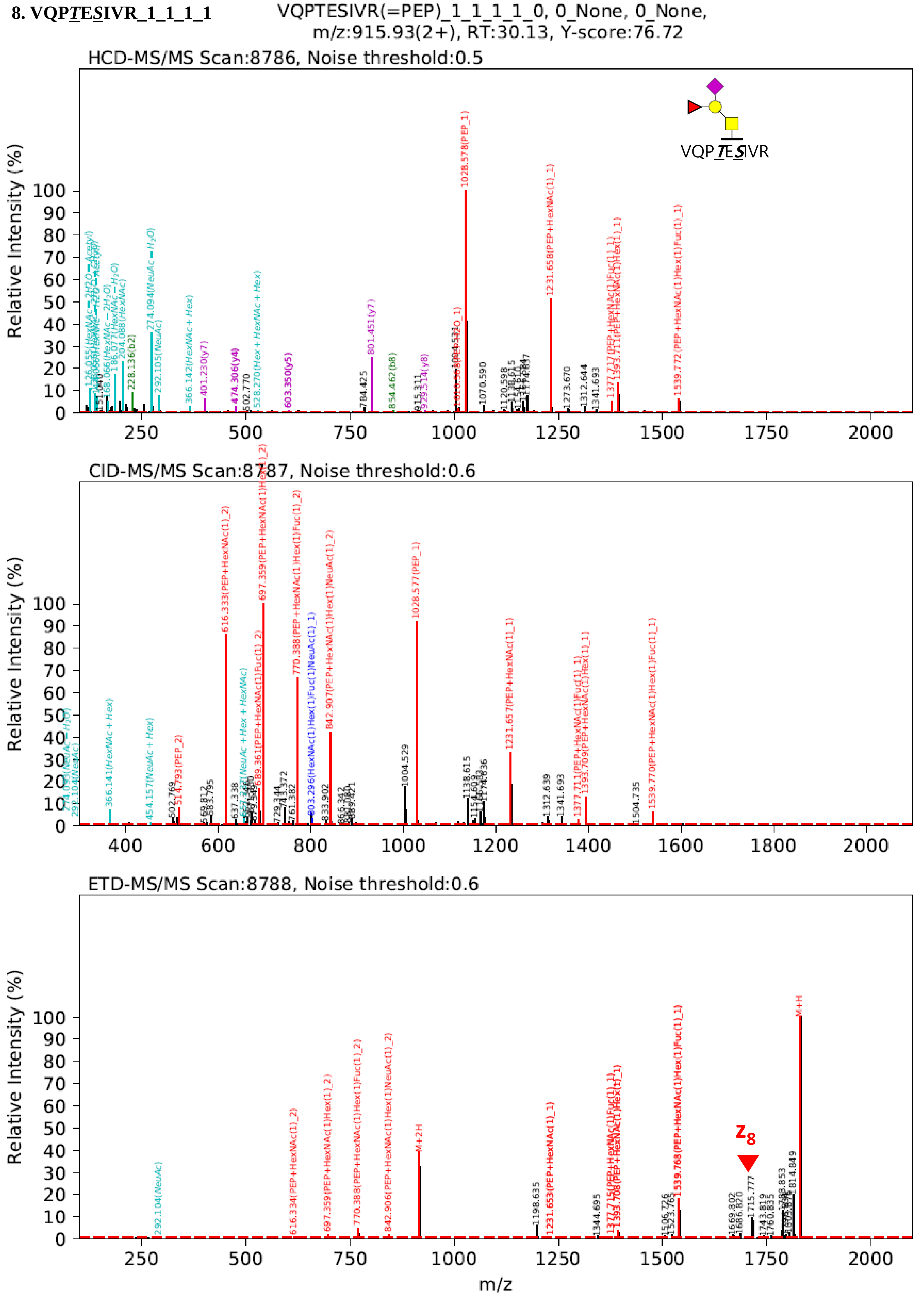

8. VQP*T*E*S*IVR_1_1_1_1

VQPT(HexHexNAcFucNeuAc)ESIVR

MH+1(mono)	MH+2(mono)
1830.8590	915.9331

b	c				y	y+2	z	z+2
---	117.1022	1	V	9	---	---	---	---
228.1343	---	2	Q	8	1731.7906	866.3989	1715.7719	858.3896
325.1870	342.2136	3	P	7	1603.7320	802.3696	---	---
1228.5202	1245.5468	4	T(HexHexNAcFucNeuAc)	6	1506.6793	753.8433	1490.6605	745.8339
1357.5628	1374.5894	5	E	5	603.3461	302.1767	587.3273	294.1673
1444.5949	1461.6214	6	S	4	474.3035	237.6554	458.2847	229.6460
1557.6789	1574.7055	7	I	3	387.2714	194.1394	371.2527	186.1300
1656.7473	1673.7739	8	V	2	274.1874	137.5973	258.1686	129.5880
---	---	9	R	1	175.1190	88.0631	159.1002	80.0538

9. VQP*T*E*S*IVR_1_2_0_0

VQPTESIVR(=PEP)_1_2_0_0_0, 0_None, 0_None,
m/z:798.90(2+), RT:39.51, Y-score:86.03

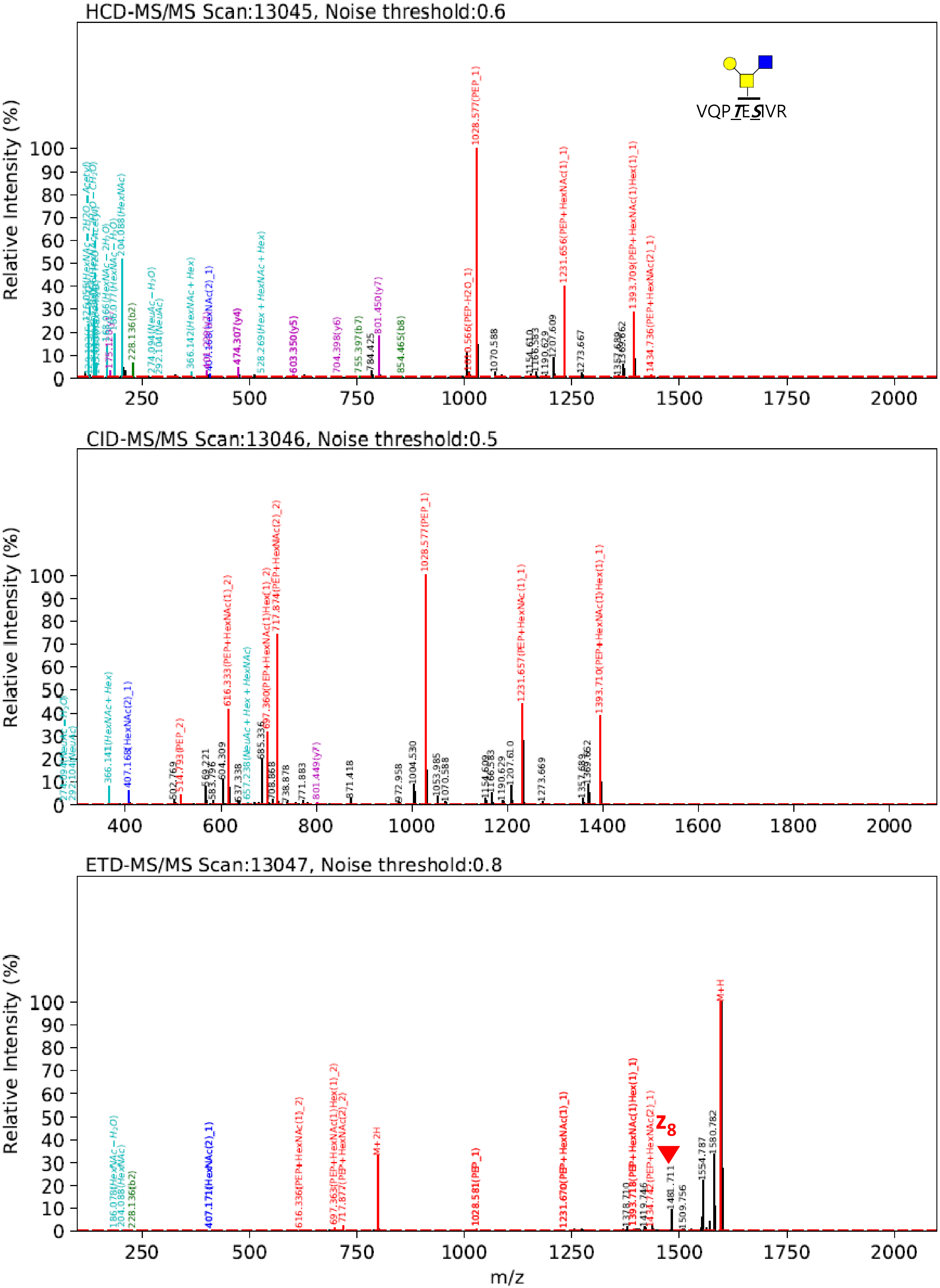

9. VQP*T*E*S*IVR_1_2_0_0

VQPT(HexHexNAc2)ESIVR

MH^{+1}(mono)	MH^{+2}(mono)
1596.7851	798.8962

b	c				y	y^{+2}	z	z^{+2}
---	117.1022	1	V	9	---	---	---	---
228.1343	---	2	Q	8	1497.7166	749.3620	1481.6979	741.3526
325.1870	342.2136	3	P	7	1369.6581	685.3327	---	---
994.4463	1011.4728	4	T(HexHexNAc2)	6	1272.6053	636.8063	1256.5866	628.7969
1123.4889	1140.5154	5	E	5	603.3461	302.1767	587.3273	294.1673
1210.5209	1227.5474	6	S	4	474.3035	237.6554	458.2847	229.6460
1323.6050	1340.6315	7	I	3	387.2714	194.1394	371.2527	186.1300
1422.6734	1439.6999	8	V	2	274.1874	137.5973	258.1686	129.5880
---	---	9	R	1	175.1190	88.0631	159.1002	80.0538

10. VQP*T*ESIVR_1_2_0_1

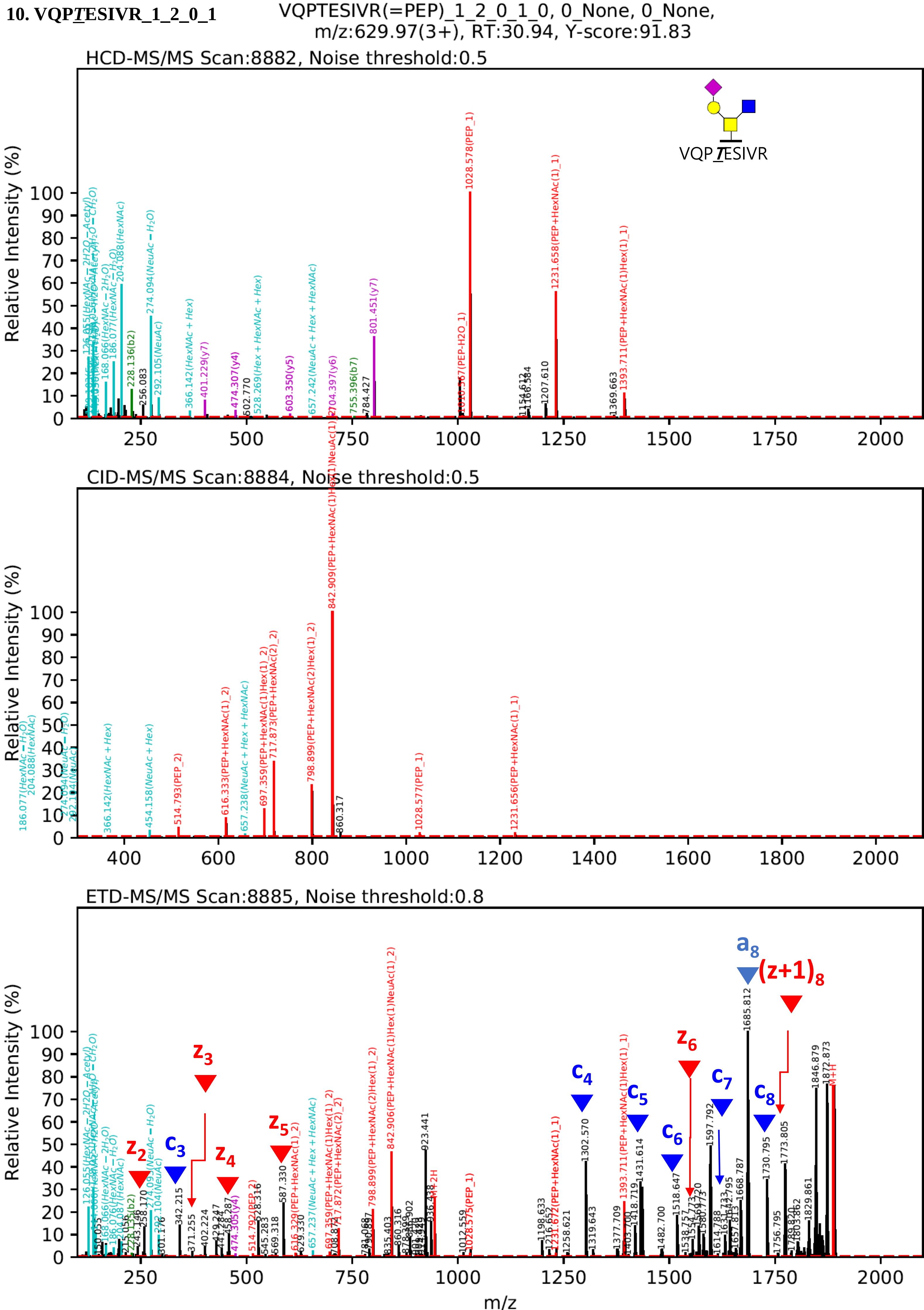

10. VQP*T*ESIVR_1_2_0_1

VQPT(HexHexNAc2NeuAc)ESIVR

MH+1(mono)	MH+2(mono)
1887.8805	944.4439

b	c				y	y+2	z	z+2
---	117.1022	1	V	9	---	---	---	---
228.1343	---	2	Q	8	1788.8121	894.9097	1772.7933	886.9003
325.1870	342.2136	3	P	7	1660.7535	830.8804	---	---
1285.5417	1302.5682	4	T(HexHexNAc2NeuAc)	6	1563.7007	782.3540	1547.6820	774.3446
1414.5843	1431.6108	5	E	5	603.3461	302.1767	587.3273	294.1673
1501.6163	1518.6429	6	S	4	474.3035	237.6554	458.2847	229.6460
1614.7004	1631.7269	7	I	3	387.2714	194.1394	371.2527	186.1300
1713.7688	1730.7953	8	V	2	274.1874	137.5973	258.1686	129.5880
---	---	9	R	1	175.1190	88.0631	159.1002	80.0538

11. VQP*T*ESIVR_1_3_0_1

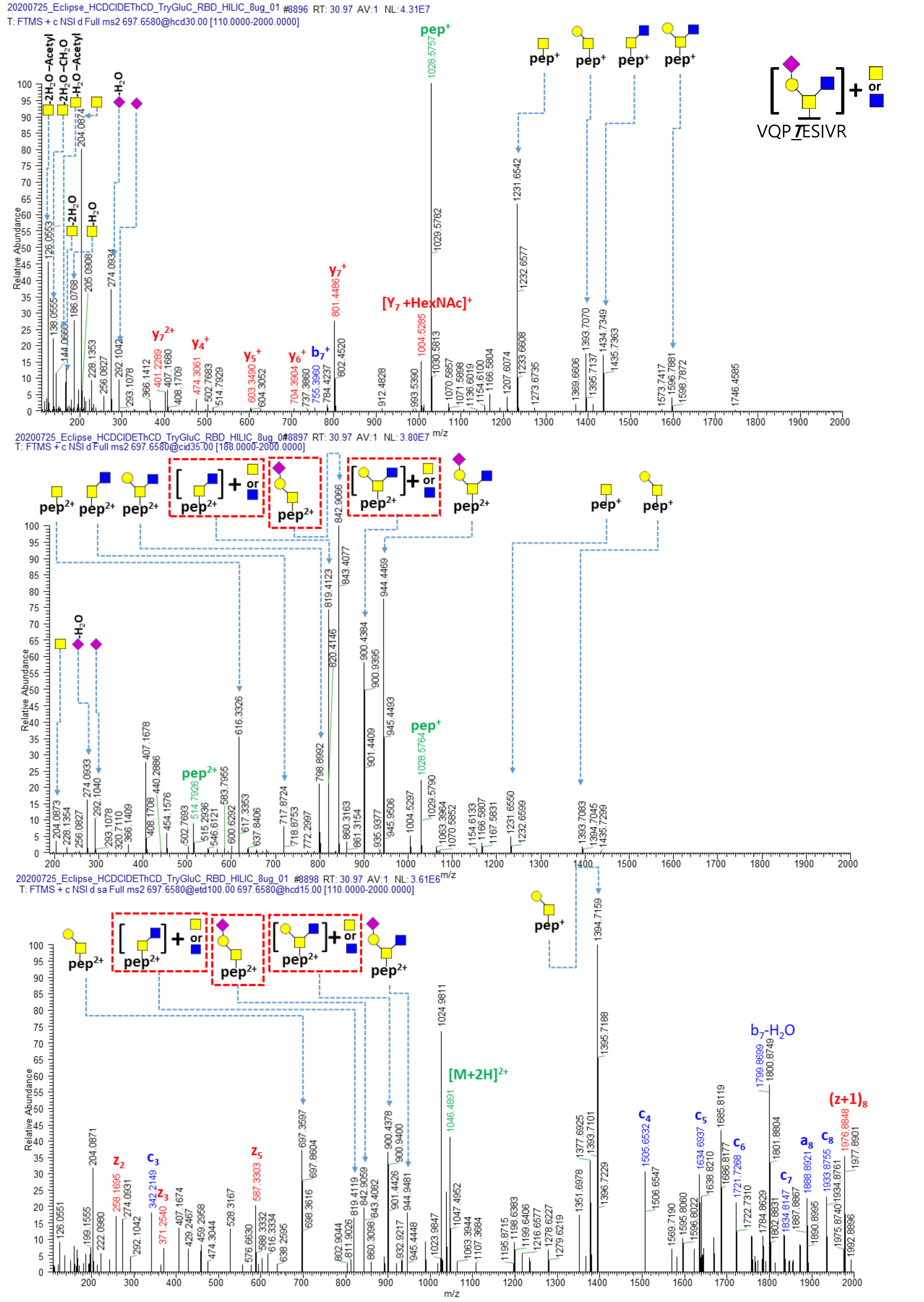

11. VQP*T*ESIVR_1_3_0_1

VQPT(HexHexNAc3NeuAc)ESIVR

MH^{+1}(mono)	MH^{+2}(mono)	MH^{+3}(mono)
2090.9598	1045.9836	697.6581

b	c				y	y^{+2}	z	z^{+2}
---	117.1022	1	V	9	---	---	---	---
228.1343	---	2	Q	8	1991.8914	996.4494	1975.8727	988.4400
325.1870	342.2136	3	P	7	1863.8329	932.4201	---	---
1488.6211	1505.6476	4	T(HexHexNAc3NeuAc)	6	1766.7801	883.8937	1750.7614	875.8843
1617.6637	1634.6902	5	E	5	603.3461	302.1767	587.3273	294.1673
1704.6957	1721.7222	6	S	4	474.3035	237.6554	458.2847	229.6460
1817.7798	1834.8063	7	I	3	387.2714	194.1394	371.2527	186.1300
1916.8482	1933.8747	8	V	2	274.1874	137.5973	258.1686	129.5880
---	---	9	R	1	175.1190	88.0631	159.1002	80.0538

12. VQP*T*E*S*IVR_2_2_0_0

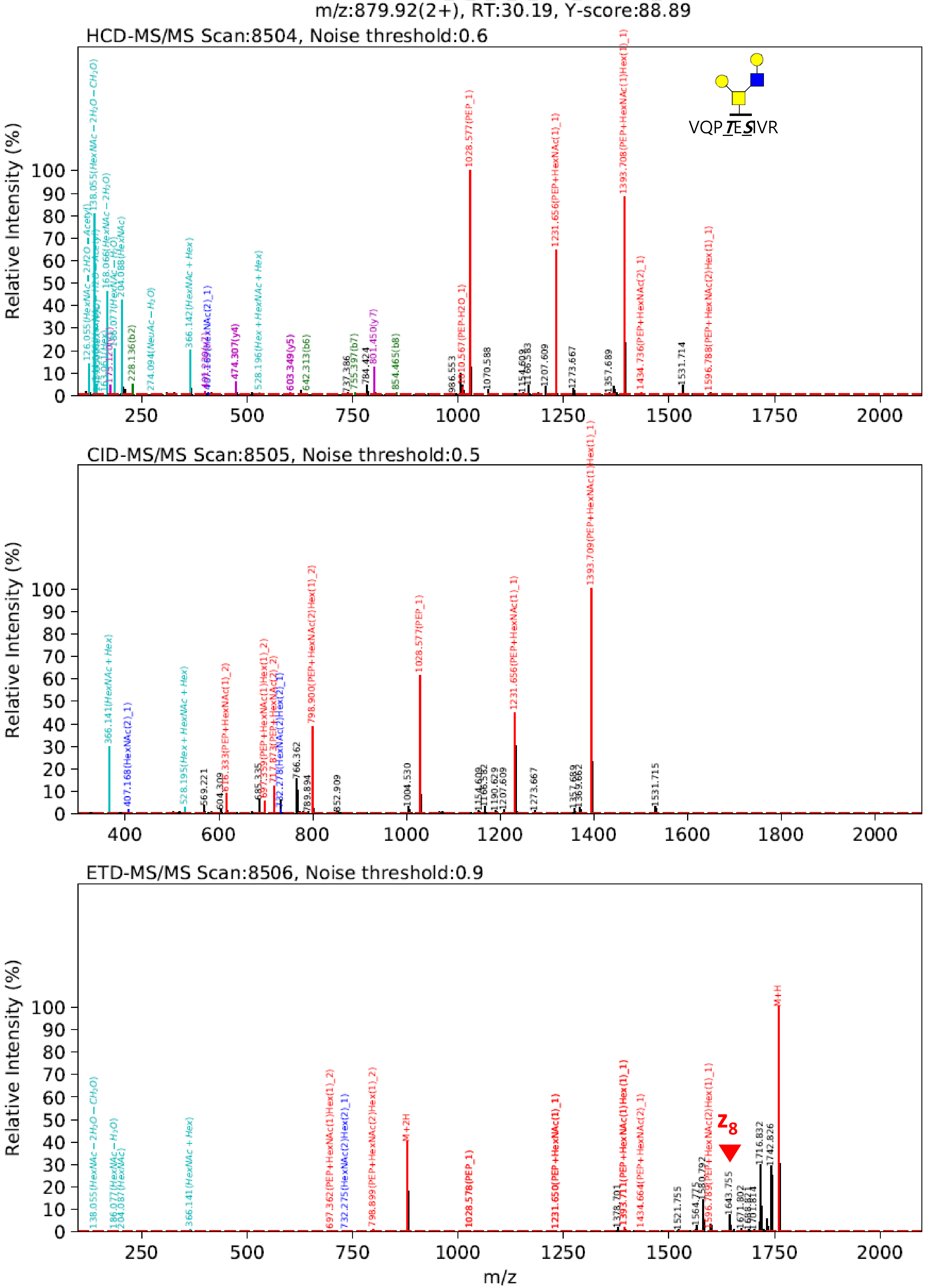

12. VQP*T*E*S*IVR_2_2_0_0

VQPT(Hex2HexNAc2)ESIVR

MH^{+1}(mono)	MH^{+2}(mono)
1758.8379	879.9226

b	c				y	z	z^{+2}
---	117.1022	1	V	9	---	---	---
228.1343	---	2	Q	8	1659.7695	1643.7507	822.3790
325.1870	342.2136	3	P	7	1531.7109	---	---
1156.4991	1173.5257	4	T(Hex2HexNAc2)	6	1434.6581	1418.6394	709.8233
1285.5417	1302.5682	5	E	5	603.3461	587.3273	294.1673
1372.5737	1389.6003	6	S	4	474.3035	458.2847	229.6460
1485.6578	1502.6843	7	I	3	387.2714	371.2527	186.1300
1584.7262	1601.7528	8	V	2	274.1874	258.1686	129.5880
---	---	9	R	1	175.1190	159.1002	80.0538

13. VQP*T*ESIVR_2_2_0_1

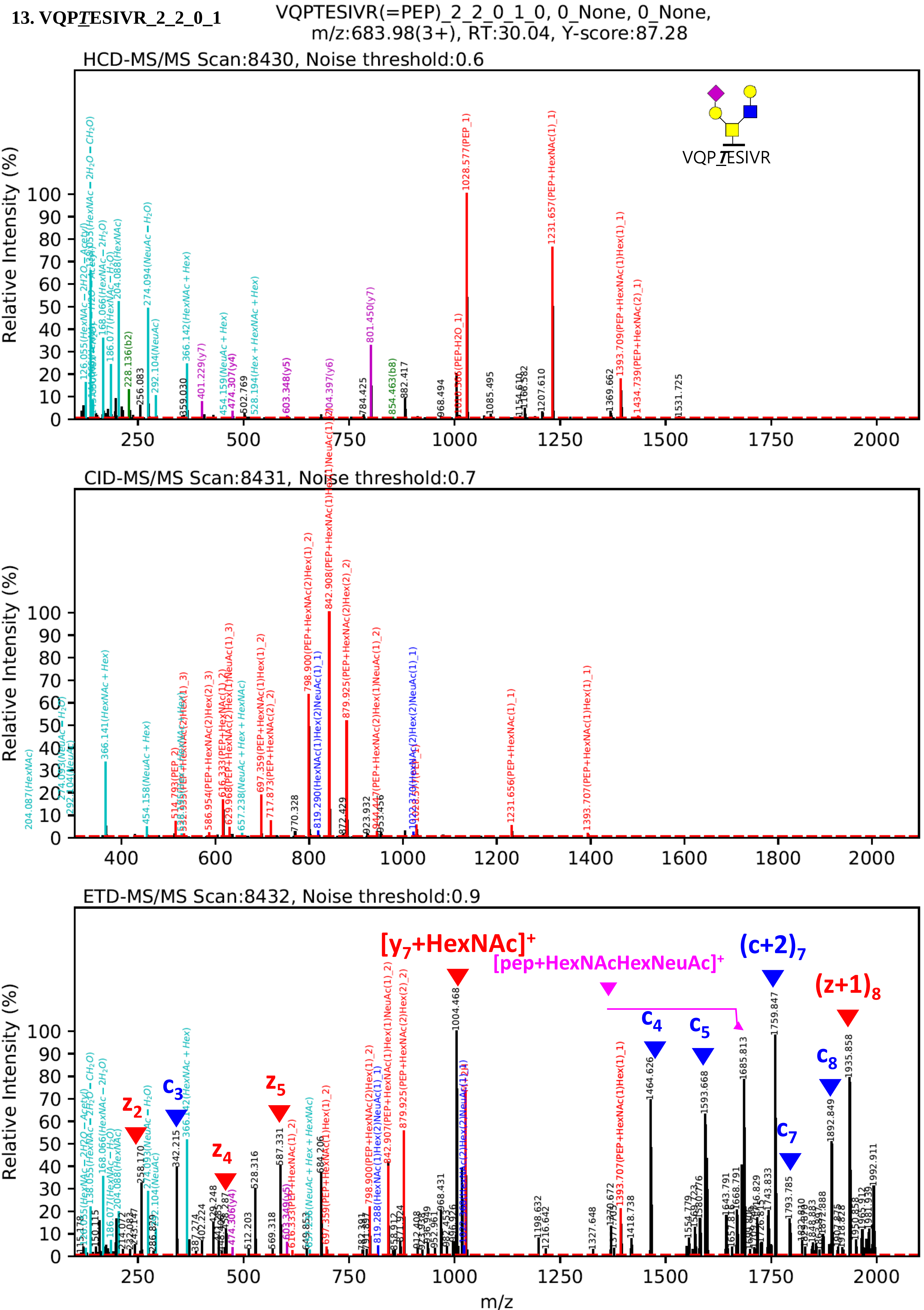

13. VQP*T*ESIVR_2_2_0_1

VQPT(Hex2HexNAc2NeuAc)ESIVR

MH^{+1}(mono)	MH^{+2}(mono)	MH^{+3}(mono)
2049.9333	1025.4703	683.9826

b	c				y	y^{+2}	z	z^{+2}
---	117.1022	1	V	9	---	---	---	---
228.1343	---	2	Q	8	1950.8649	975.9361	1934.8462	967.9267
325.1870	342.2136	3	P	7	1822.8063	911.9068	---	---
1447.5945	1464.6211	4	T(Hex2HexNAc2NeuAc)	6	1725.7535	863.3804	1709.7348	855.3710
1576.6371	1593.6637	5	E	5	603.3461	302.1767	587.3273	294.1673
1663.6691	1680.6957	6	S	4	474.3035	237.6554	458.2847	229.6460
1776.7532	1793.7798	7	I	3	387.2714	194.1394	371.2527	186.1300
1875.8216	1892.8482	8	V	2	274.1874	137.5973	258.1686	129.5880
---	---	9	R	1	175.1190	88.0631	159.1002	80.0538

14. VQP*T*ESIVR_2_2_0_2

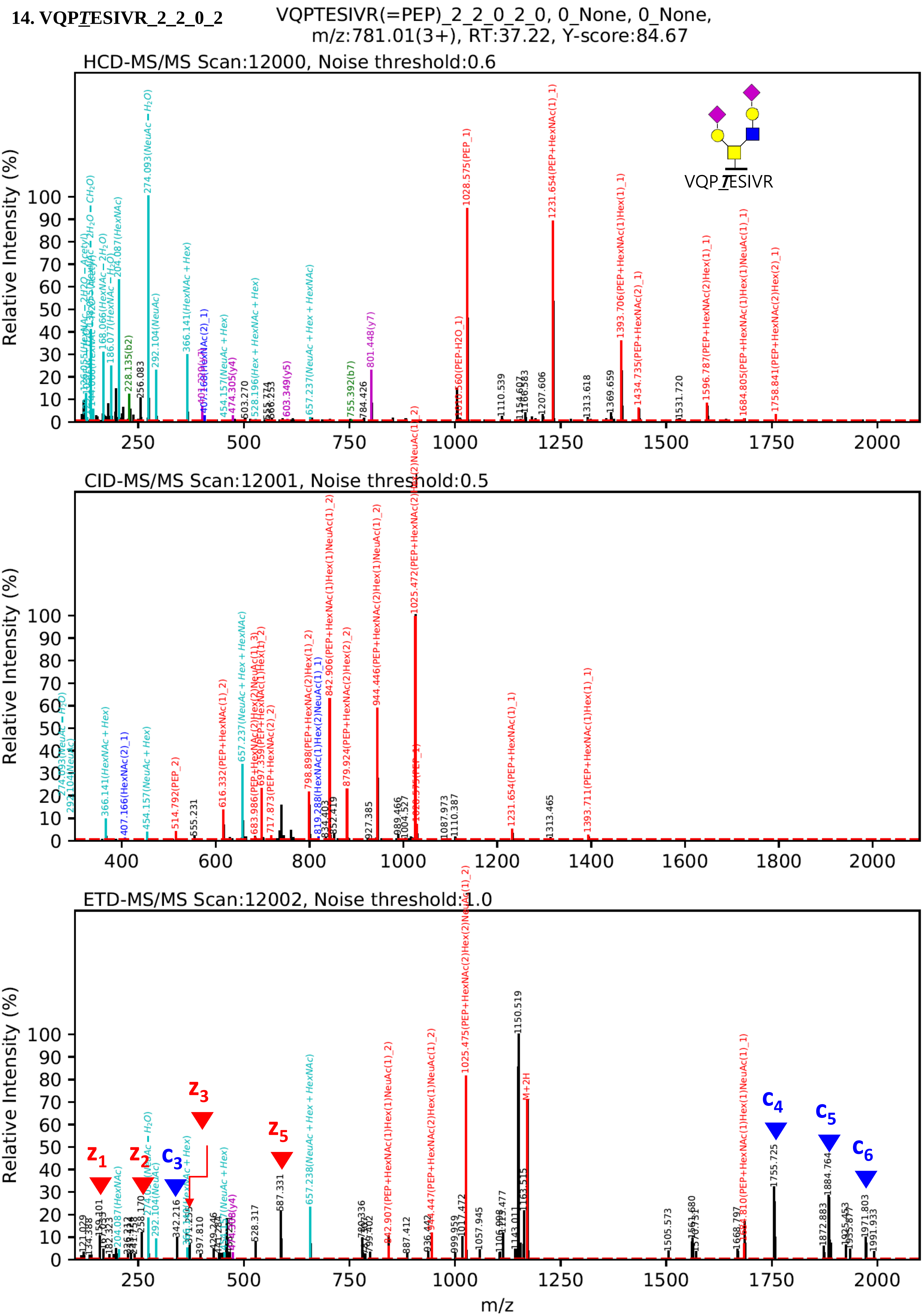

14. VQP*T*ESIVR_2_2_0_2

VQPT(Hex2HexNAc2NeuAc2)ESIVR

MH+1(mono)	MH+2(mono)	MH+3(mono)
2341.0287	1171.0180	781.0144

b	c				y	y+2	z	z+2
---	117.1022	1	V	9	---	---	---	---
228.1343	---	2	Q	8	2241.9603	1121.4838	2225.9416	1113.4744
325.1870	342.2136	3	P	7	2113.9017	1057.4545	---	---
1738.6899	1755.7165	4	T(Hex2HexNAc2NeuAc2)	6	2016.8490	1008.9281	2000.8302	1000.9188
1867.7325	1884.7591	5	E	5	603.3461	302.1767	587.3273	294.1673
1954.7646	1971.7911	6	S	4	474.3035	237.6554	458.2847	229.6460
2067.8486	2084.8752	7	I	3	387.2714	194.1394	371.2527	186.1300
2166.9170	2183.9436	8	V	2	274.1874	137.5973	258.1686	129.5880
---	---	9	R	1	175.1190	88.0631	159.1002	80.0538

15. VQP*T*ESIVR_2_2_0_3

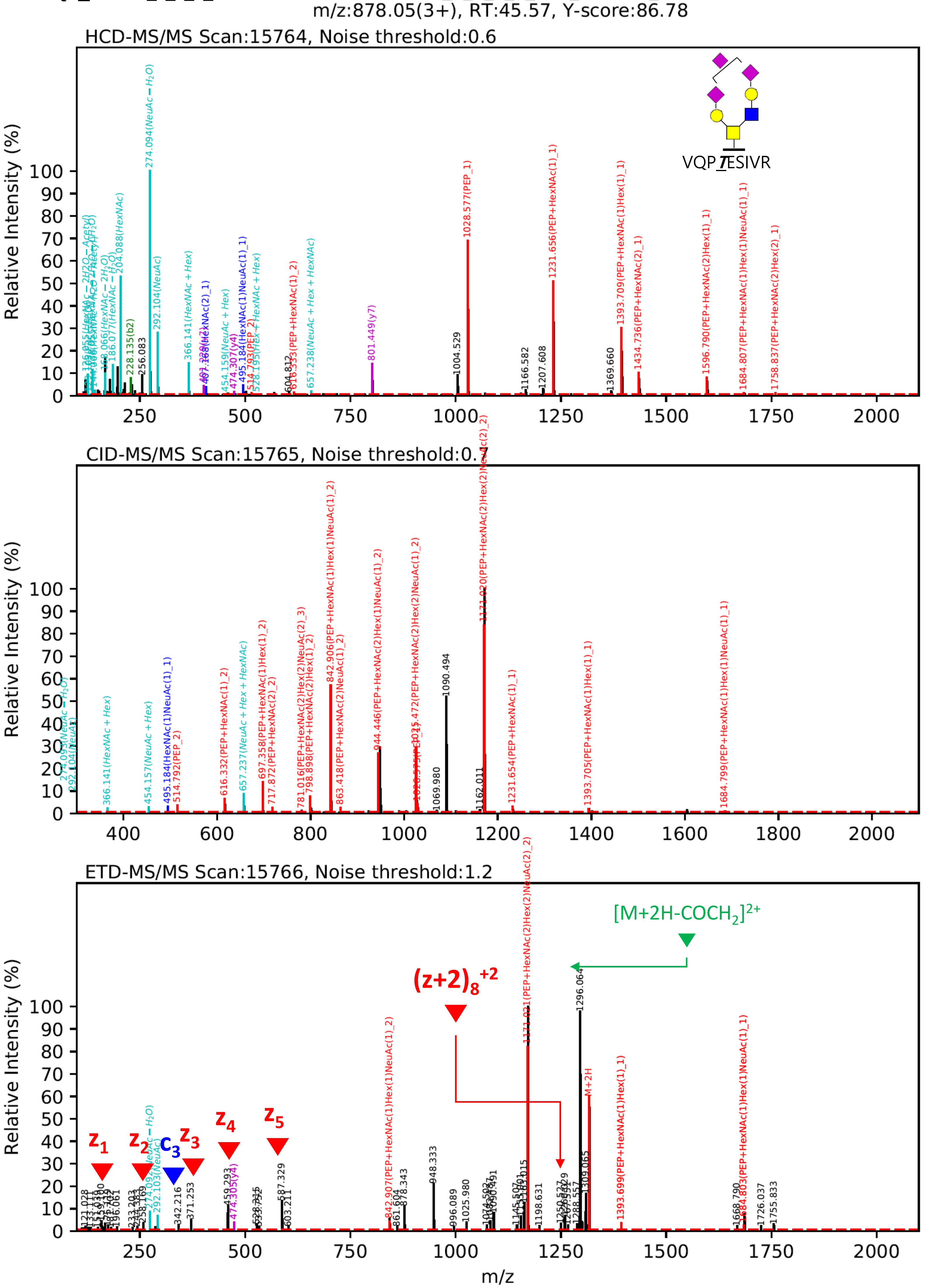

15. VQP*T*ESIVR_2_2_0_3

VQPT(Hex2HexNAc2NeuAc3)ESIVR

MH^{+1}(mono)	MH^{+2}(mono)	MH^{+3}(mono)
2632.1241	1316.5657	878.0462

b	c				y	y^{+2}	z	z^{+2}
---	117.1022	1	V	9	---	---	---	---
228.1343	---	2	Q	8	2533.0557	1267.0315	2517.0370	1259.0221
325.1870	342.2136	3	P	7	2404.9971	1203.0022	---	---
2029.7854	2046.8119	4	T(Hex2HexNAc2NeuAc3)	6	2307.9444	1154.4758	2291.9256	1146.4665
2158.8279	2175.8545	5	E	5	603.3461	302.1767	587.3273	294.1673
2245.8600	2262.8865	6	S	4	474.3035	237.6554	458.2847	229.6460
2358.9440	2375.9706	7	I	3	387.2714	194.1394	371.2527	186.1300
2458.0125	2475.0390	8	V	2	274.1874	137.5973	258.1686	129.5880
---	---	9	R	1	175.1190	88.0631	159.1002	80.0538

16. VQP*T*E*S*IVR_2_2_1_0

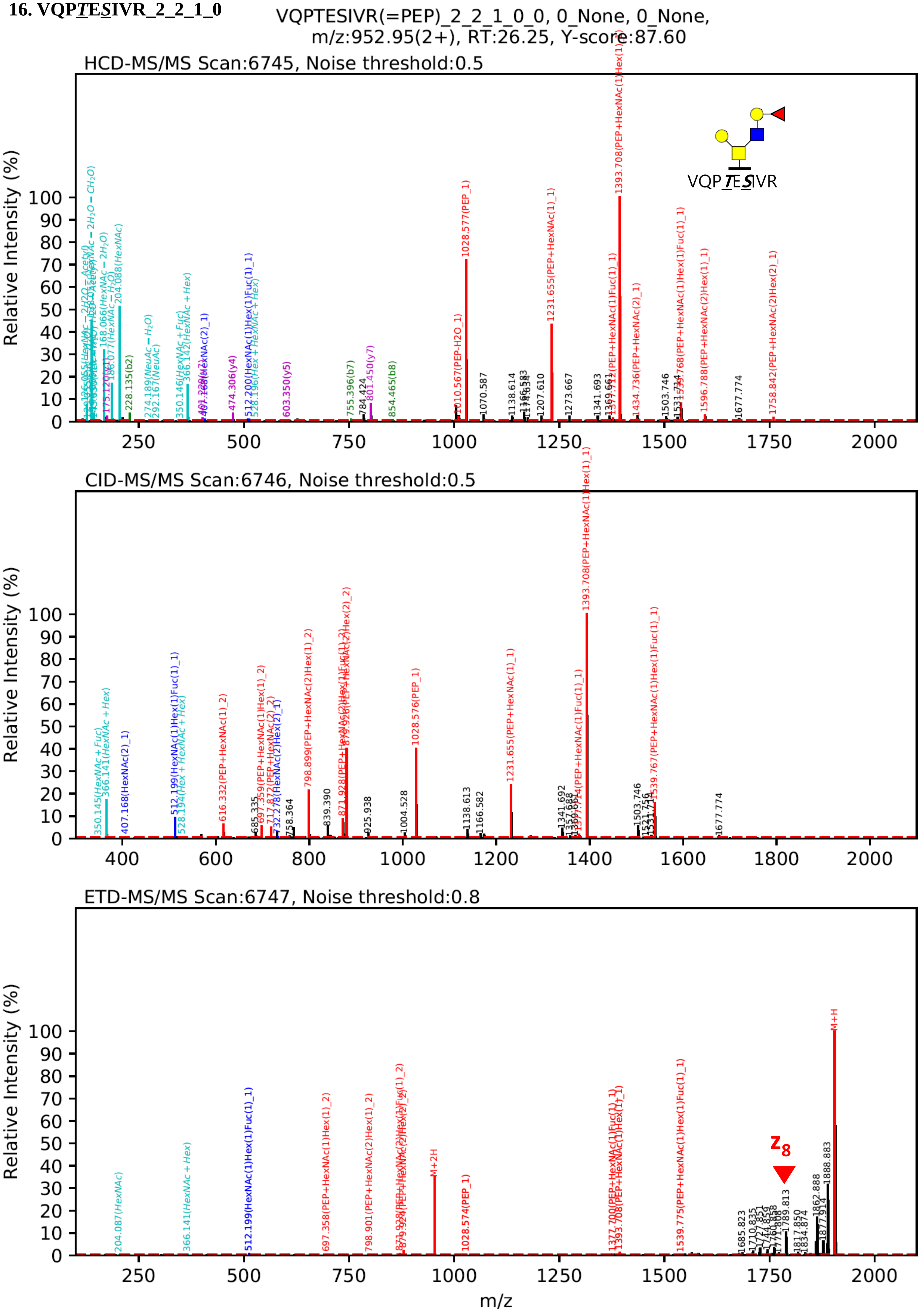

16. VQP*T*E*S*IVR_2_2_1_0

VQPT(Hex2HexNAc2Fuc)ESIVR

MH+1(mono)	MH+2(mono)	MH+3(mono)
1904.8958	952.9515	635.6368

b	c				y	y^{+2}	z	z^{+2}
---	117.1022	1	V	9	---	---	---	---
228.1343	---	2	Q	8	1805.8274	903.4173	1789.8086	895.4080
325.1870	342.2136	3	P	7	1677.7688	839.3880	---	---
1302.5570	1319.5836	4	T(Hex2HexNAc2Fuc)	6	1580.7160	790.8617	1564.6973	782.8523
1431.5996	1448.6262	5	E	5	603.3461	302.1767	587.3273	294.1673
1518.6316	1535.6582	6	S	4	474.3035	237.6554	458.2847	229.6460
1631.7157	1648.7422	7	I	3	387.2714	194.1394	371.2527	186.1300
1730.7841	1747.8107	8	V	2	274.1874	137.5973	258.1686	129.5880
---	---	9	R	1	175.1190	88.0631	159.1002	80.0538

17. VQP*T*ESIVR_2_2_1_1

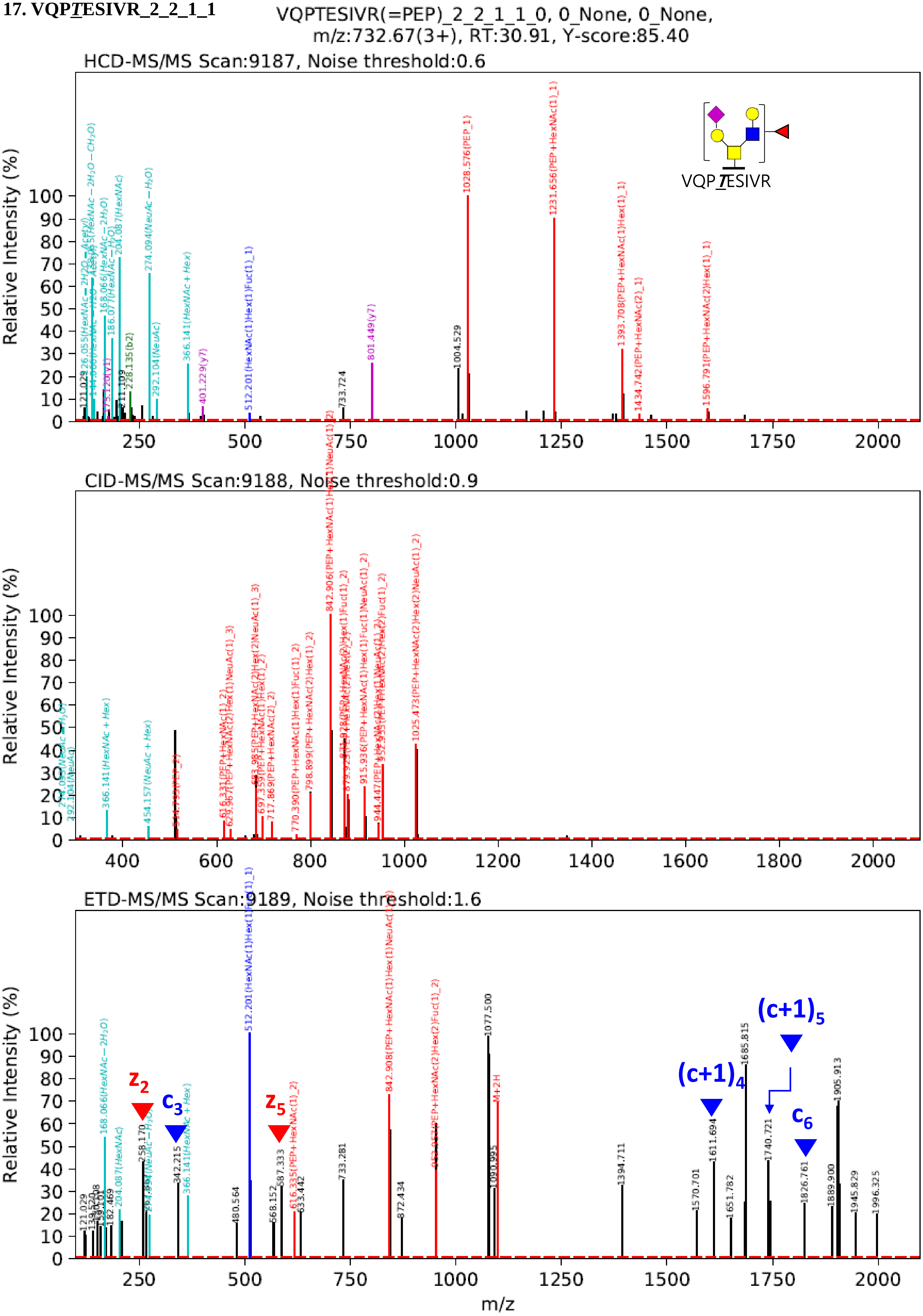

17. VQP*T*ESIVR_2_2_1_1

VQPT(Hex2HexNAc2FucNeuAc)ESIVR

MH+1(mono)	MH+2(mono)	MH+3(mono)
2195.9912	1098.4992	732.6686

b	c				y	y+2	z	z+2
---	117.1022	1	V	9	---	---	---	---
228.1343	---	2	Q	8	2096.9228	1048.9650	2080.9041	1040.9557
325.1870	342.2136	3	P	7	1968.8642	984.9357	---	---
1593.6524	1610.6790	4	T(Hex2HexNAc2FucNeuAc)	6	1871.8114	936.4094	1855.7927	928.4000
1722.6950	1739.7216	5	E	5	603.3461	302.1767	587.3273	294.1673
1809.7270	1826.7536	6	S	4	474.3035	237.6554	458.2847	229.6460
1922.8111	1939.8377	7	I	3	387.2714	194.1394	371.2527	186.1300
2021.8795	2038.9061	8	V	2	274.1874	137.5973	258.1686	129.5880
---	---	9	R	1	175.1190	88.0631	159.1002	80.0538

18. VQP*T*ESIVR_2_2_1_2

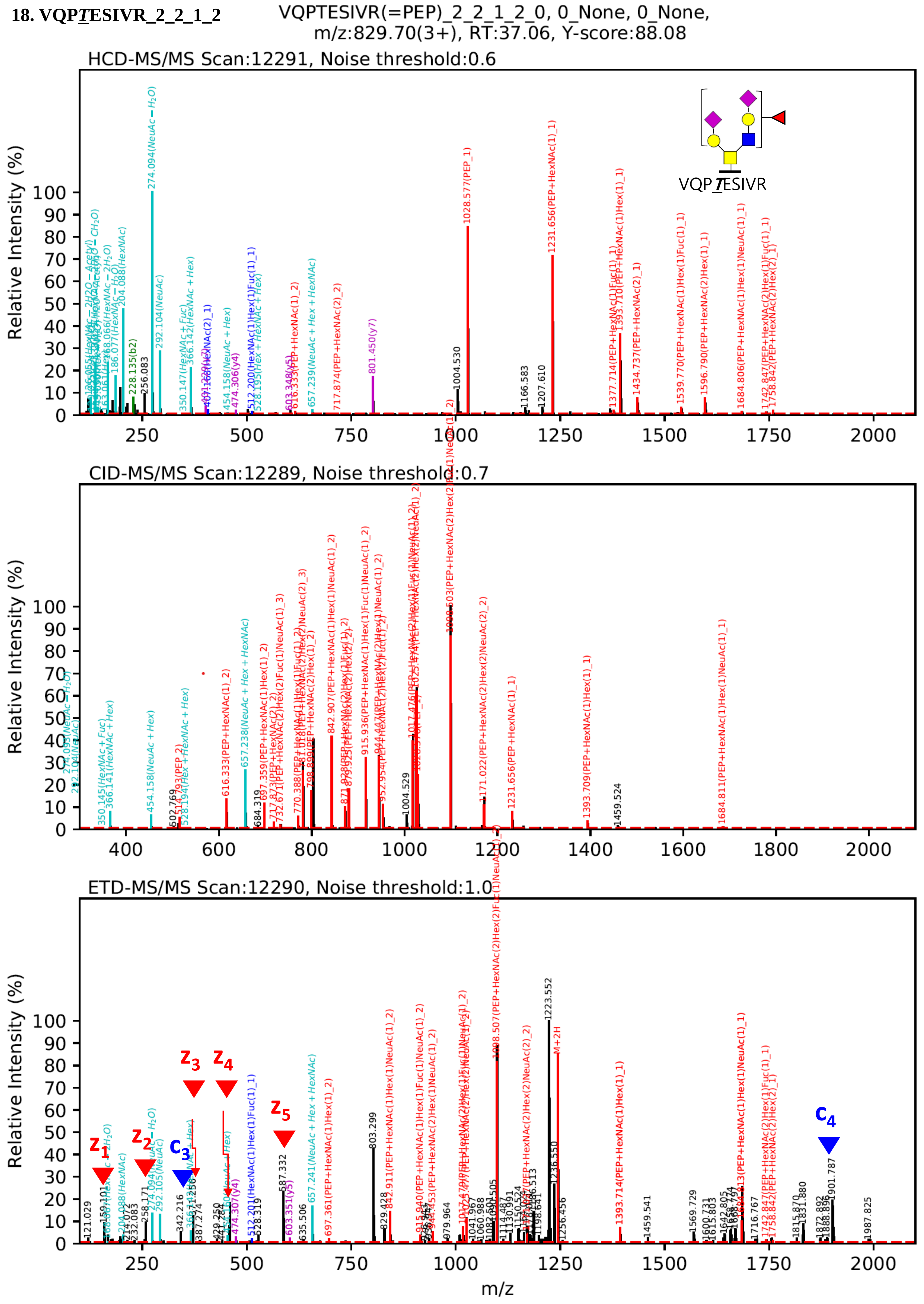

18. VQP*T*ESIVR_2_2_1_2

VQPT(Hex2HexNAc2FucNeuAc2)ESIVR

MH+1(mono)	MH+2(mono)	MH+3(mono)
2487.0866	1244.0469	829.7004

b	c				y	y+2	z	z+2
---	117.1022	1	V	9	---	---	---	---
228.1343	---	2	Q	8	2388.0182	1194.5127	2371.9995	1186.5034
325.1870	342.2136	3	P	7	2259.9596	1130.4835	---	---
1884.7478	1901.7744	4	T(Hex2HexNAc2FucNeuAc2)	6	2162.9069	1081.9571	2146.8881	1073.9477
2013.7904	2030.8170	5	E	5	603.3461	302.1767	587.3273	294.1673
2100.8225	2117.8490	6	S	4	474.3035	237.6554	458.2847	229.6460
2213.9065	2230.9331	7	I	3	387.2714	194.1394	371.2527	186.1300
2312.9749	2330.0015	8	V	2	274.1874	137.5973	258.1686	129.5880
---	---	9	R	1	175.1190	88.0631	159.1002	80.0538

19. VQP*T*ESIVR_2_2_2_1

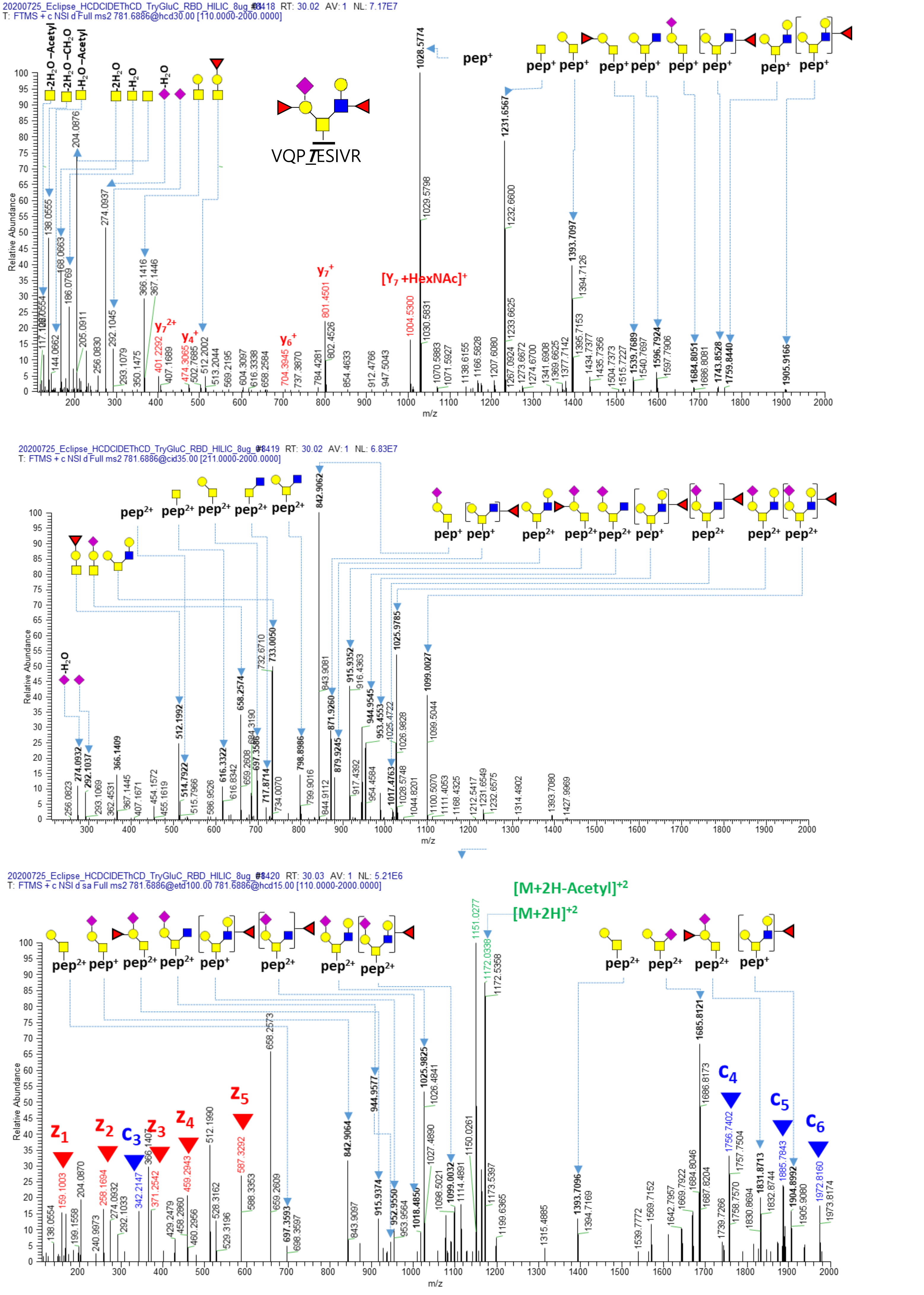

19. VQPTESIVR_2_2_2_1

VQPT(Hex2HexNAc2Fuc2NeuAc)ESIVR

MH^{+1}(mono)	MH^{+2}(mono)	MH^{+3}(mono)
2342.0491	1171.5282	781.3546

b	c				y	y^{+2}	z	z^{+2}
---	117.1022	1	V	9	---	---	---	---
228.1343	---	2	Q	8	2242.9807	1121.9940	2226.9620	1113.9846
325.1870	342.2136	3	P	7	2114.9221	1057.9647	---	---
1739.7103	1756.7369	4	T(Hex2HexNAc2Fuc2NeuAc)	6	2017.8694	1009.4383	2001.8506	1001.4290
1868.7529	1885.7795	5	E	5	603.3461	302.1767	587.3273	294.1673
1955.7850	1972.8115	6	S	4	474.3035	237.6554	458.2847	229.6460
2068.8690	2085.8956	7	I	3	387.2714	194.1394	371.2527	186.1300
2167.9374	2184.9640	8	V	2	274.1874	137.5973	258.1686	129.5880
---	---	9	R	1	175.1190	88.0631	159.1002	80.0538

20. VQP*T*ESIVR_2_3_0_2

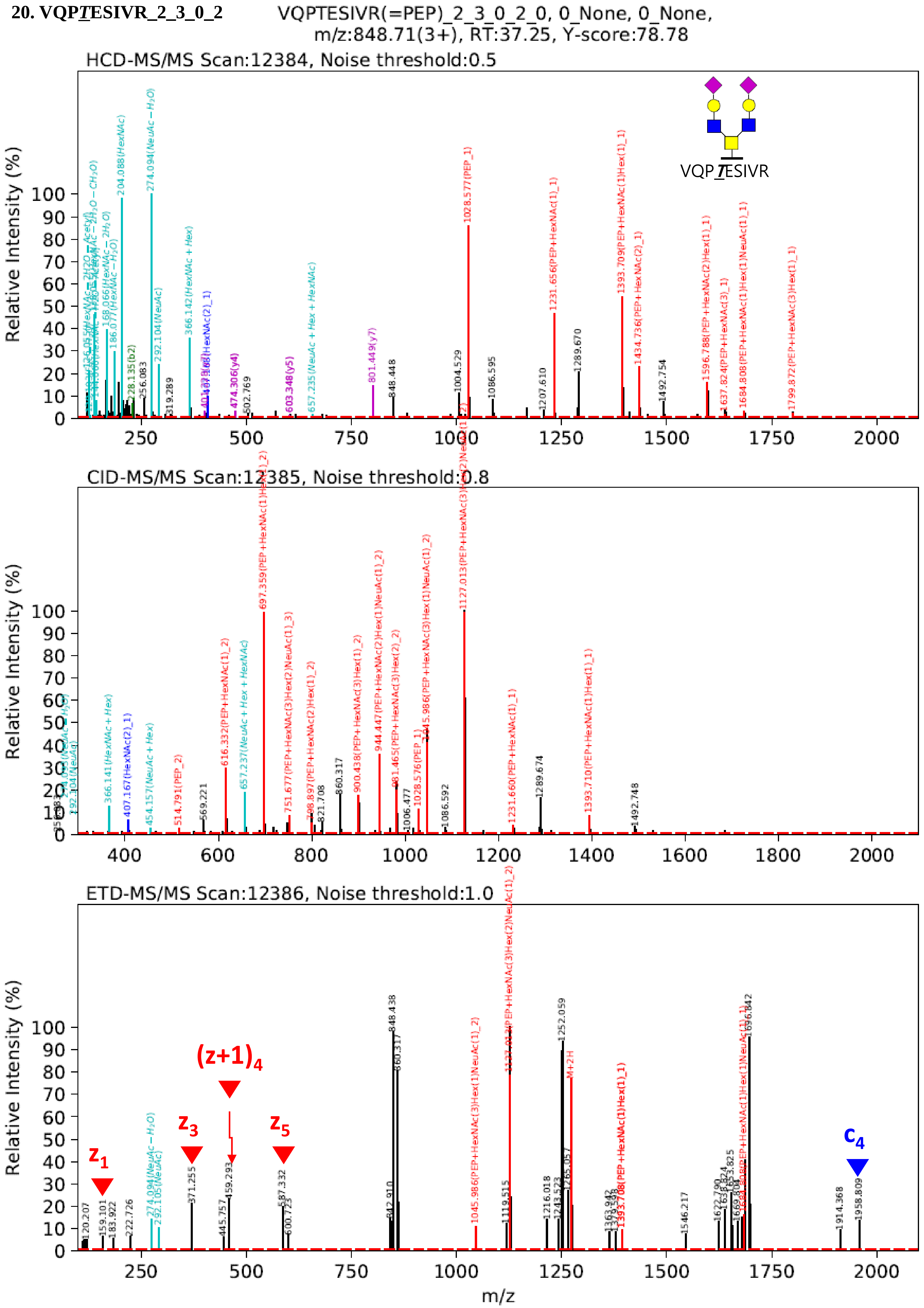

20. VQP*T*ESIVR_2_3_0_2

VQPT(Hex2HexNAc3NeuAc2)ESIVR

MH^{+1}(mono)	MH^{+2}(mono)	MH^{+3}(mono)
2544.1081	1272.5577	848.7075

b	c				y	y^{+2}	z	z^{+2}
---	117.1022	1	V	9	---	---	---	---
228.1343	---	2	Q	8	2445.0397	1223.0235	2429.0209	1215.0141
325.1870	342.2136	3	P	7	2316.9811	1158.9942	---	---
1941.7693	1958.7959	4	T(Hex2HexNAc3NeuAc2)	6	2219.9283	1110.4678	2203.9096	1102.4584
2070.8119	2087.8384	5	E	5	603.3461	302.1767	587.3273	294.1673
2157.8439	2174.8705	6	S	4	474.3035	237.6554	458.2847	229.6460
2270.9280	2287.9545	7	I	3	387.2714	194.1394	371.2527	186.1300
2369.9964	2387.0230	8	V	2	274.1874	137.5973	258.1686	129.5880
---	---	9	R	1	175.1190	88.0631	159.1002	80.0538

21. VQP*T*ESIVR_2_3_1_1

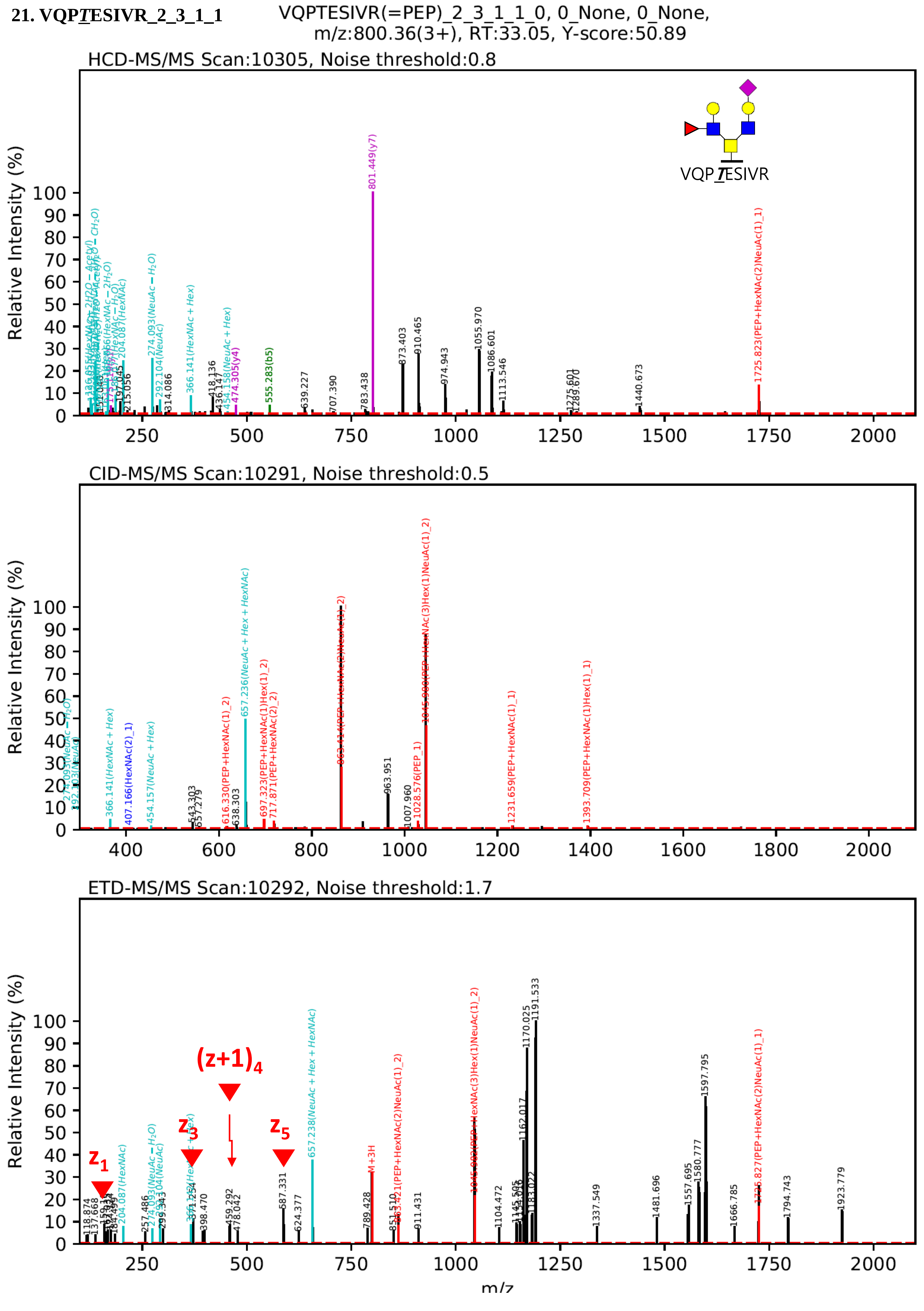

21. VQP*T*ESIVR_2_3_1_1

VQPT(Hex2HexNAc3FucNeuAc)ESIVR

MH^{+1}(mono)	MH^{+2}(mono)	MH^{+3}(mono)
2399.0706	1200.0389	800.3617

b	c				y	y^{+2}	z	z^{+2}
---	117.1022	1	V	9	---	---	---	---
228.1343	---	2	Q	8	2300.0022	1150.5047	2283.9834	1142.4954
325.1870	342.2136	3	P	7	2171.9436	1086.4754	---	---
1796.7318	1813.7583	4	T(Hex2HexNAc3FucNeuAc)	6	2074.8908	1037.9490	2058.8721	1029.9397
1925.7744	1942.8009	5	E	5	603.3461	302.1767	587.3273	294.1673
2012.8064	2029.8330	6	S	4	474.3035	237.6554	458.2847	229.6460
2125.8905	2142.9170	7	I	3	387.2714	194.1394	371.2527	186.1300
2224.9589	2241.9854	8	V	2	274.1874	137.5973	258.1686	129.5880
---	---	9	R	1	175.1190	88.0631	159.1002	80.0538

22. VQP*T*ESIVR_3_3_0_1

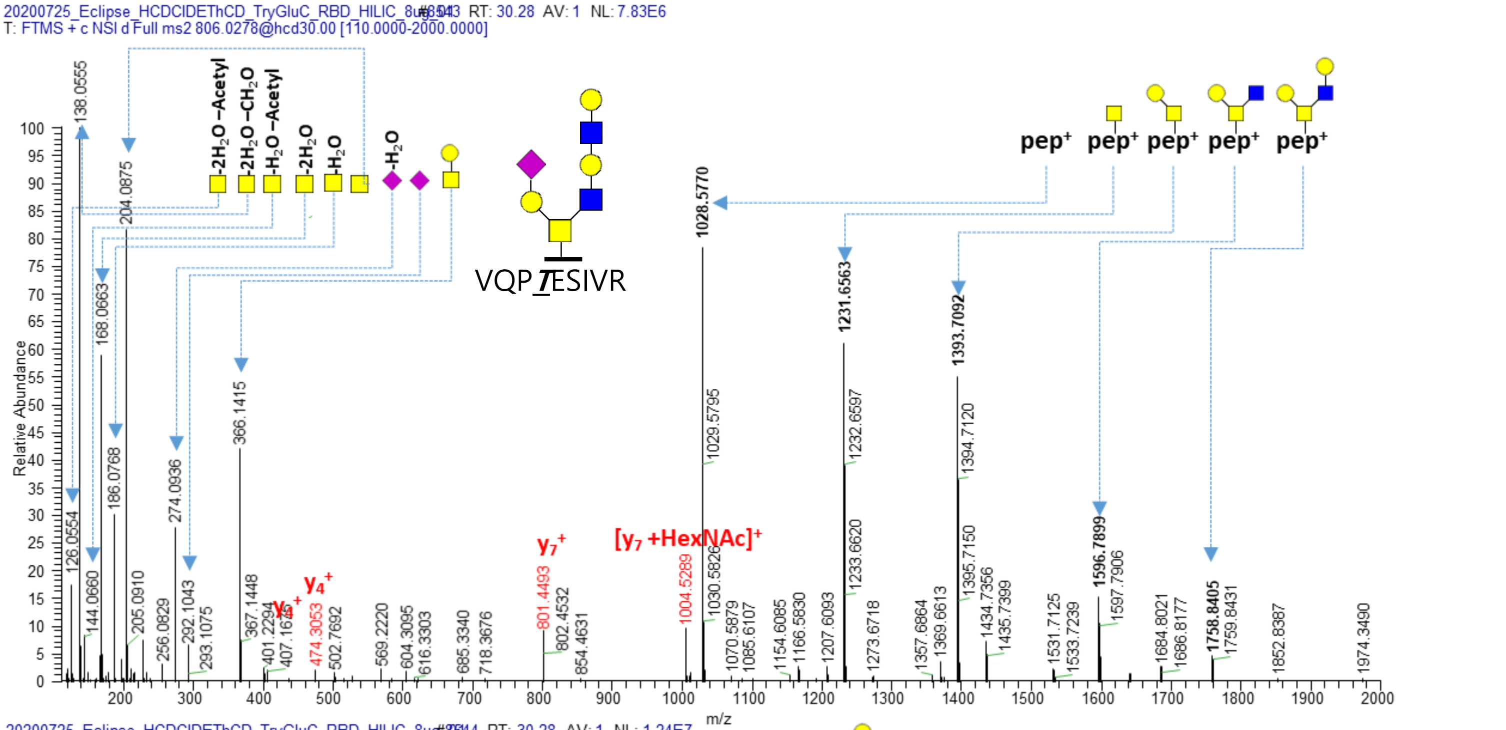

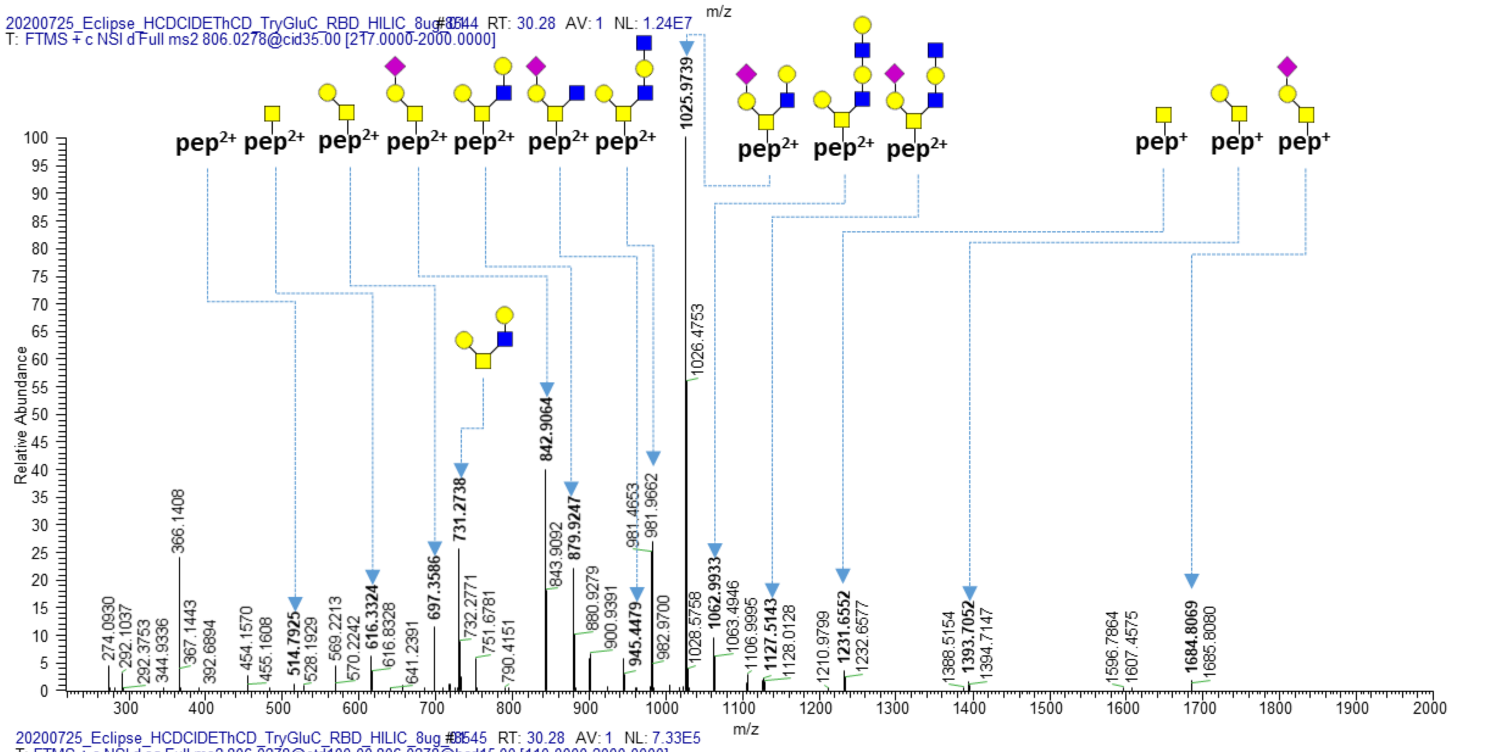

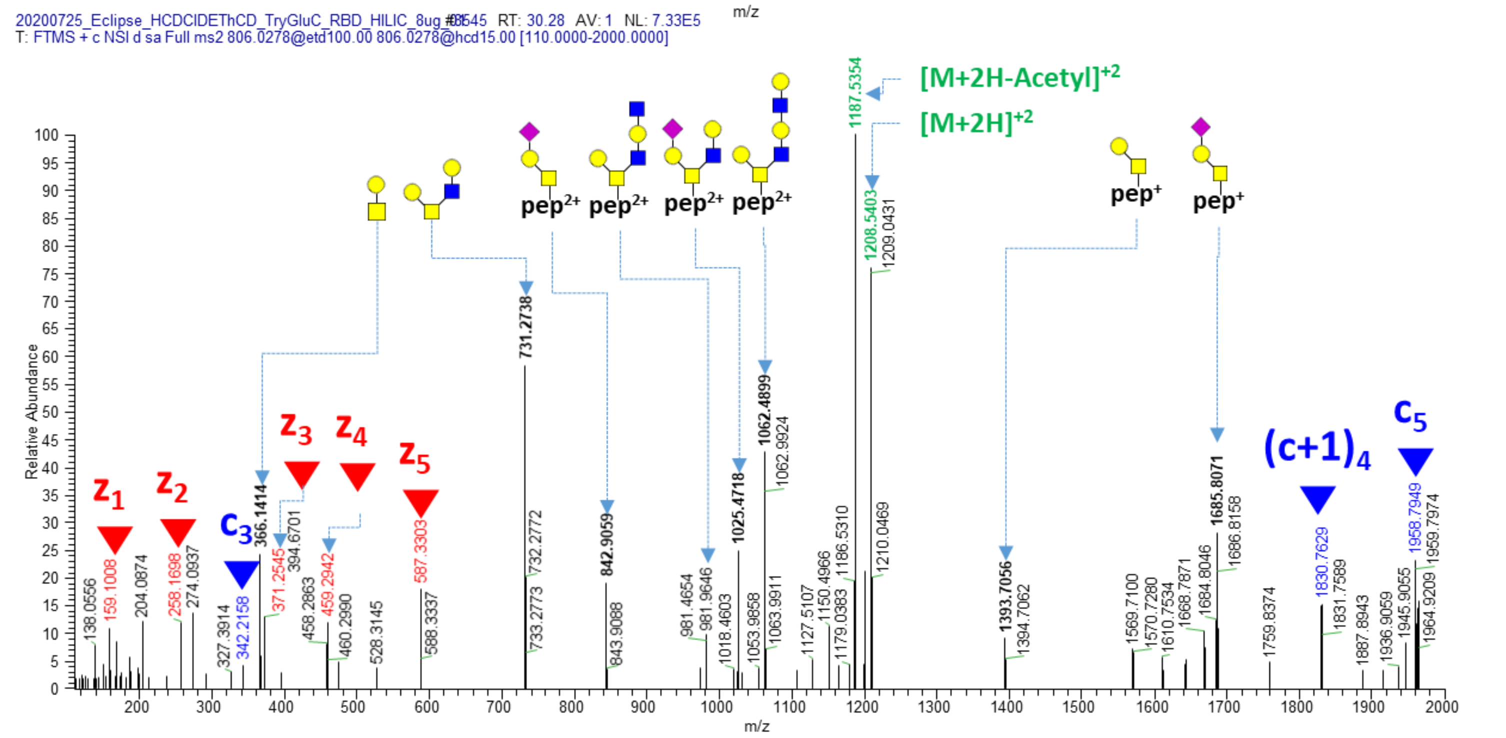

22. VQPTESIVR_3_3_0_1

VQPT(Hex3HexNAc3NeuAc)ESIVR

MH+1(mono)	MH+2(mono)	MH+3(mono)
2415.0655	1208.0364	805.6933

b	c				y	y+2	z	z+2
---	117.1022	1	V	9	---	---	---	---
228.1343	---	2	Q	8	2315.9971	1158.5022	2299.9784	1150.4928
325.1870	342.2136	3	P	7	2187.9385	1094.4729	---	---
1812.7267	1829.7533	4	T(Hex3HexNAc3NeuAc)	6	2090.8857	1045.9465	2074.8670	1037.9371
1941.7693	1958.7959	5	E	5	603.3461	302.1767	587.3273	294.1673
2028.8013	2045.8279	6	S	4	474.3035	237.6554	458.2847	229.6460
2141.8854	2158.9119	7	I	3	387.2714	194.1394	371.2527	186.1300
2240.9538	2257.9804	8	V	2	274.1874	137.5973	258.1686	129.5880
---	---	9	R	1	175.1190	88.0631	159.1002	80.0538

23. VQP*T*ESIVR_3_3_0_2

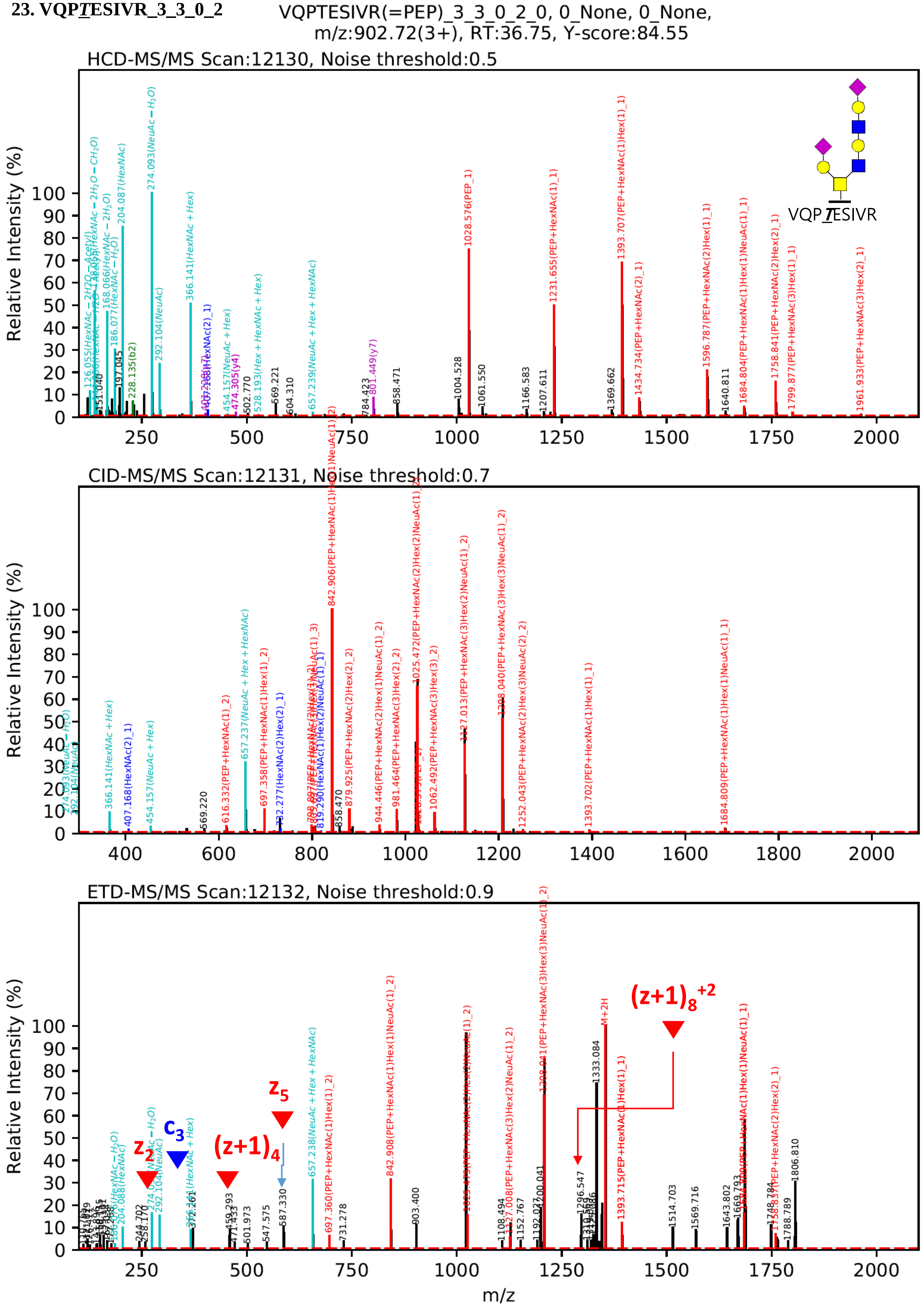

23. VQP*T*ESIVR_3_3_0_2

VQPT(Hex3HexNAc3NeuAc2)ESIVR

MH^{+1}(mono)	MH^{+2}(mono)	MH^{+3}(mono)
2706.1609	1353.5841	902.7252

b	c				y	y^{+2}	z	z^{+2}
---	117.1022	1	V	9	---	---	---	---
228.1343	---	2	Q	8	2607.0925	1304.0499	2591.0738	1296.0405
325.1870	342.2136	3	P	7	2479.0339	1240.0206	---	---
2103.8221	2120.8487	4	T(Hex3HexNAc3NeuAc2)	6	2381.9812	1191.4942	2365.9624	1183.4849
2232.8647	2249.8913	5	E	5	603.3461	302.1767	587.3273	294.1673
2319.8968	2336.9233	6	S	4	474.3035	237.6554	458.2847	229.6460
2432.9808	2450.0074	7	I	3	387.2714	194.1394	371.2527	186.1300
2532.0492	2549.0758	8	V	2	274.1874	137.5973	258.1686	129.5880
---	---	9	R	1	175.1190	88.0631	159.1002	80.0538

24. VQP*T*ESIVR_3_3_0_3

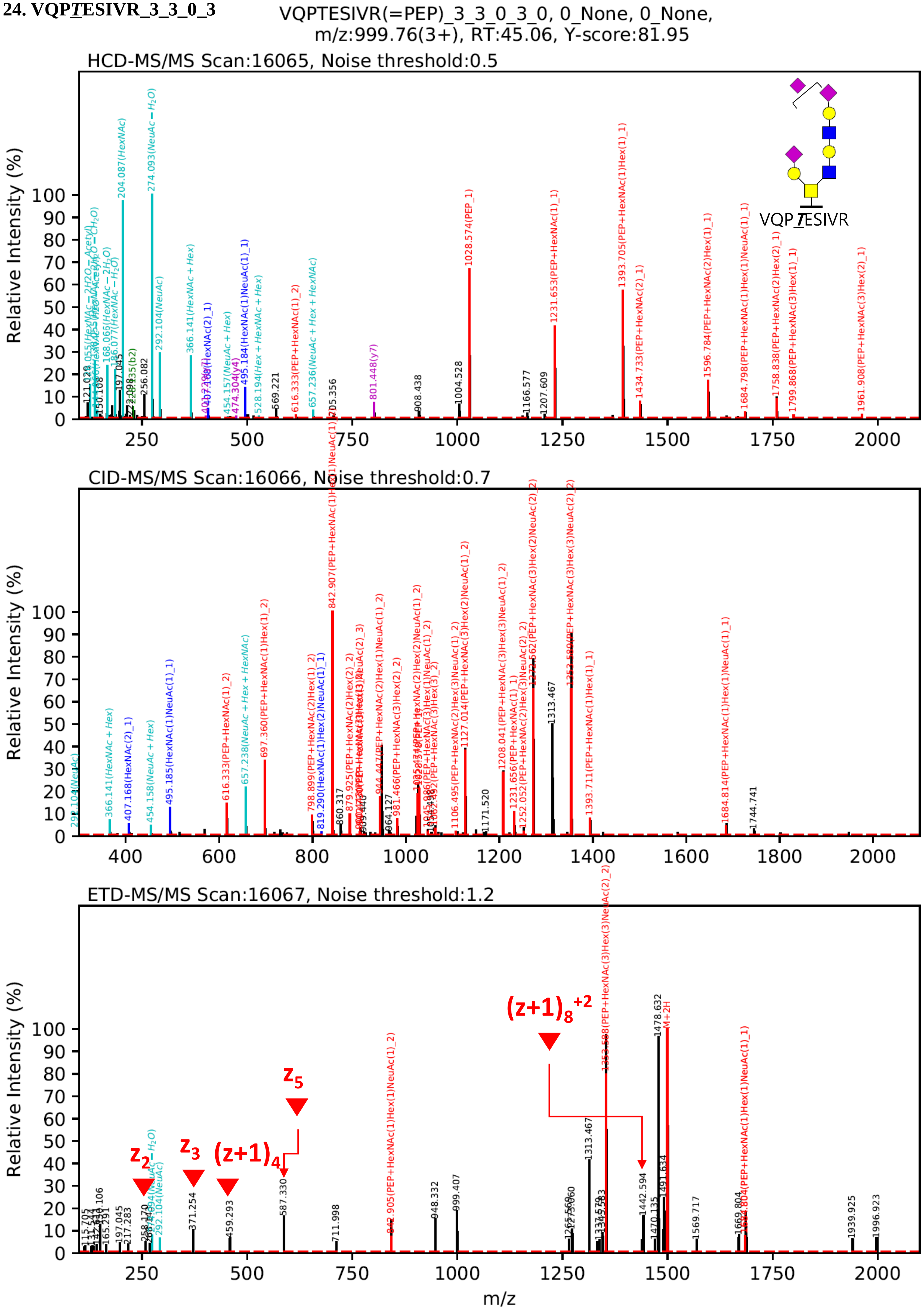

24. VQP*T*ESIVR_3_3_0_3

VQPT(Hex3HexNAc3NeuAc3)ESIVR

MH+1(mono)	MH+2(mono)	MH+3(mono)
2997.2563	1499.1318	999.7570

b	c				y	y+2	z	z+2
---	117.1022	1	V	9	---	---	---	---
228.1343	---	2	Q	8	2898.1879	1449.5976	2882.1692	1441.5882
325.1870	342.2136	3	P	7	2770.1293	1385.5683	---	---
2394.9175	2411.9441	4	T(Hex3HexNAc3NeuAc3)	6	2673.0766	1337.0419	2657.0578	1329.0326
2523.9601	2540.9867	5	E	5	603.3461	302.1767	587.3273	294.1673
2610.9922	2628.0187	6	S	4	474.3035	237.6554	458.2847	229.6460
2724.0762	2741.1028	7	I	3	387.2714	194.1394	371.2527	186.1300
2823.1446	2840.1712	8	V	2	274.1874	137.5973	258.1686	129.5880
---	---	9	R	1	175.1190	88.0631	159.1002	80.0538

25. IYQAGS*T*PCNGVE_1_1_0_2

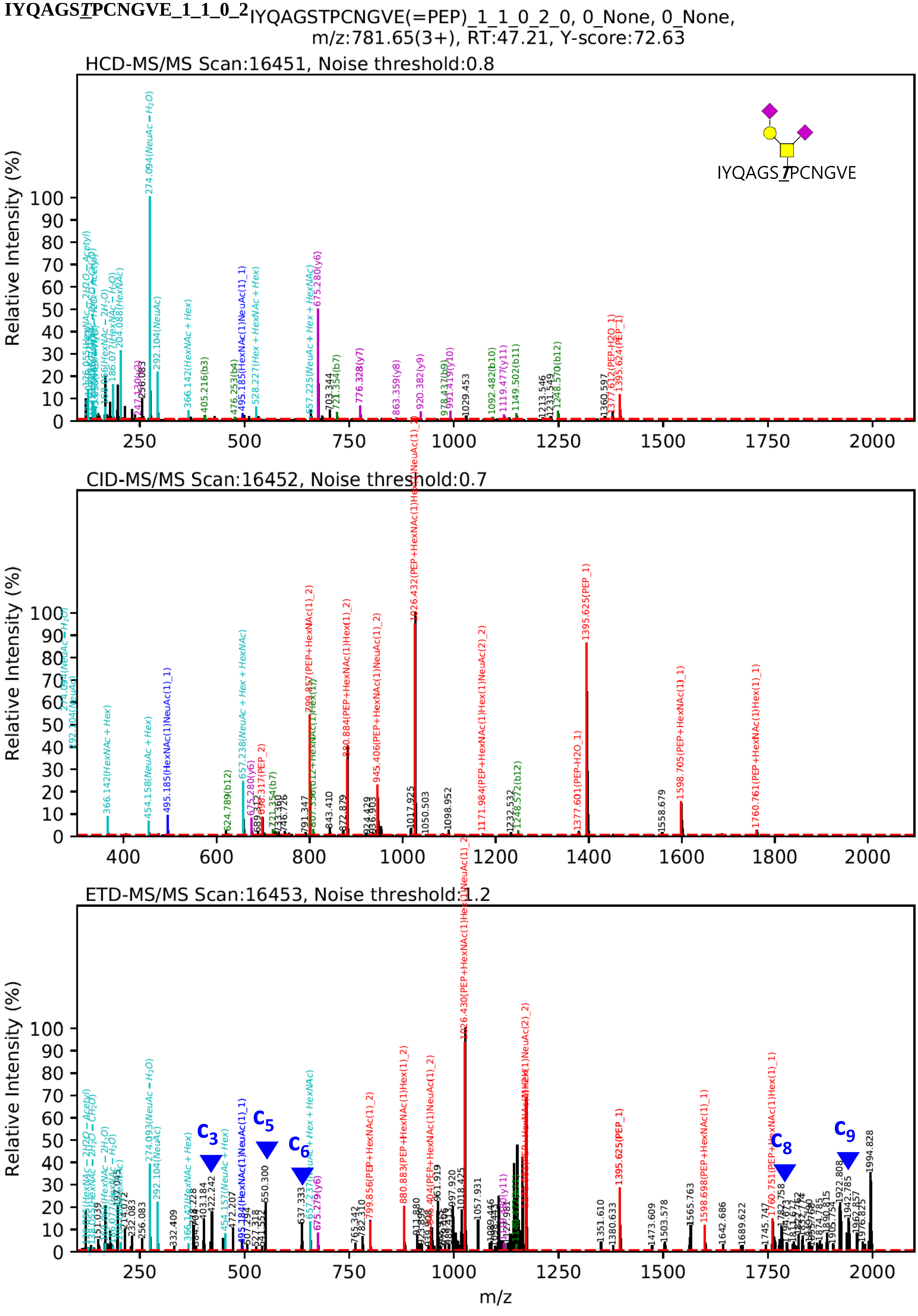

25. IYQAGS*T*PCNGVE_1_1_0_2

IYQAGSTPC(Carbamidomethyl)NGVE

MH+1(mono)	MH+2(mono)
1395.6209	698.3141

b	c				y	z
---	131.1179	1	I	13	---	---
277.1547	294.1812	2	Y	12	1282.5368	1266.5181
405.2132	422.2398	3	Q	11	1119.4735	1103.4548
476.2504	493.2769	4	A	10	991.4149	975.3962
533.2718	550.2984	5	G	9	920.3778	904.3591
620.3039	637.3304	6	S	8	863.3564	847.3376
721.3515	---	7	T	7	776.3243	760.3056
818.4043	835.4308	8	P	6	675.2767	---
978.4349	995.4615	9	C(Carbamidomethyl)	5	578.2239	562.2052
1092.4779	1109.5044	10	N	4	418.1932	402.1745
1149.4993	1166.5259	11	G	3	304.1503	288.1316
1248.5677	1265.5943	12	V	2	247.1288	231.1101
---	---	13	E	1	148.0604	132.0417

IYQAGST(HexHexNAcNeuAc2)PC(Carbamidomethyl)NGVE

MH+1(mono)	MH+2(mono)	MH+3(mono)
2342.9439	1171.9756	781.6528

b	c				y	z
---	131.1179	1	I	13	---	---
277.1547	294.1812	2	Y	12	2229.8599	2213.8411
405.2132	422.2398	3	Q	11	2066.7965	2050.7778
476.2504	493.2769	4	A	10	1938.7380	1922.7192
533.2718	550.2984	5	G	9	1867.7009	1851.6821
620.3039	637.3304	6	S	8	1810.6794	1794.6607
1668.6746	---	7	T(HexHexNAcNeuAc2)	7	1723.6474	1707.6286
1765.7273	1782.7539	8	P	6	675.2767	---
1925.7580	1942.7845	9	C(Carbamidomethyl)	5	578.2239	562.2052
2039.8009	2056.8274	10	N	4	418.1932	402.1745
2096.8224	2113.8489	11	G	3	304.1503	288.1316
2195.8908	2212.9173	12	V	2	247.1288	231.1101
---	---	13	E	1	148.0604	132.0417

26. LLHAPA*T*VCGPK_1_1_0_2

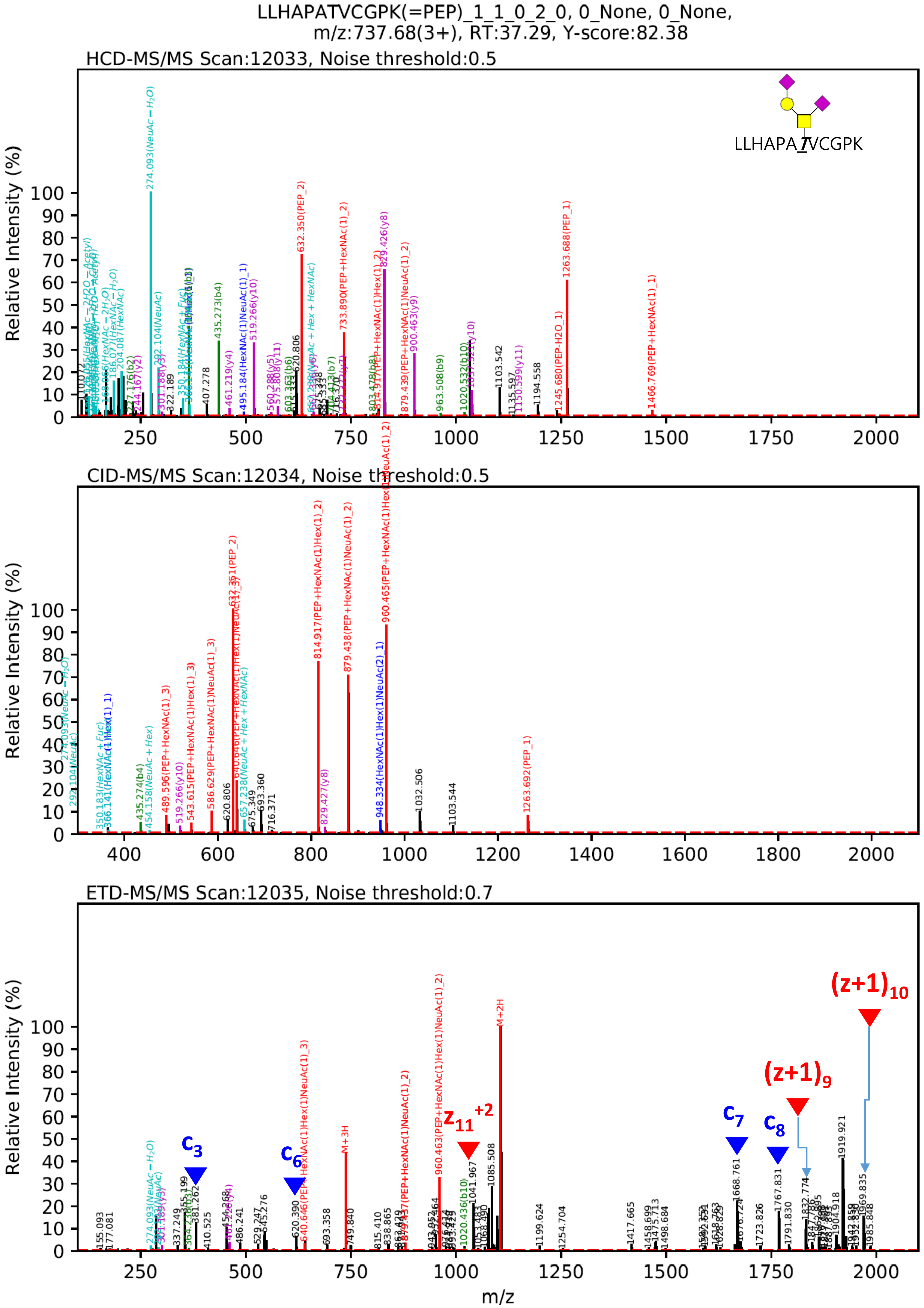

26. LLHAPA*T*VCGPK_1_1_0_2

LLHAPATVC(Carbamidomethyl)GPK

MH+1(mono)	MH+2(mono)
1263.6878	632.3475

b	c				y	z
---	131.1179	1	L	12	---	---
227.1754	244.2020	2	L	11	1150.6037	1134.5850
364.2343	381.2609	3	H	10	1037.5197	1021.5010
435.2714	---	4	A	9	900.4608	884.4420
532.3242	549.3507	5	P	8	829.4236	---
603.3613	620.3879	6	A	7	732.3709	716.3522
704.4090	721.4355	7	T	6	661.3338	645.3150
803.4774	820.5039	8	V	5	560.2861	544.2674
963.5080	980.5346	9	C(Carbamidomethyl)	4	461.2177	445.1990
1020.5295	---	10	G	3	301.1870	285.1683
1117.5823	1134.6088	11	P	2	244.1656	---
---	---	12	K	1	147.1128	131.0941

LLHAPAT(HexHexNAcNeuAc2)VC(Carbamidomethyl)GPK

MH+1(mono)	MH+2(mono)	MH+3(mono)
2211.0108	1106.0091	737.6751

b	c				y	z
---	131.1179	1	L	12	---	---
227.1754	244.2020	2	L	11	2097.9268	2081.9080
364.2343	381.2609	3	H	10	1984.8427	1968.8240
435.2714	---	4	A	9	1847.7838	1831.7651
532.3242	549.3507	5	P	8	1776.7467	---
603.3613	620.3879	6	A	7	1679.6939	1663.6752
1651.7320	1668.7586	7	T(HexHexNAcNeuAc2)	6	1608.6568	1592.6381
1750.8004	1767.8270	8	V	5	560.2861	544.2674
1910.8311	1927.8576	9	C(Carbamidomethyl)	4	461.2177	445.1990
1967.8525	---	10	G	3	301.1870	285.1683
2064.9053	2081.9319	11	P	2	244.1656	---
---	---	12	K	1	147.1128	131.0941

27. LLHAPA*T*VCGPK_2_2_0_2

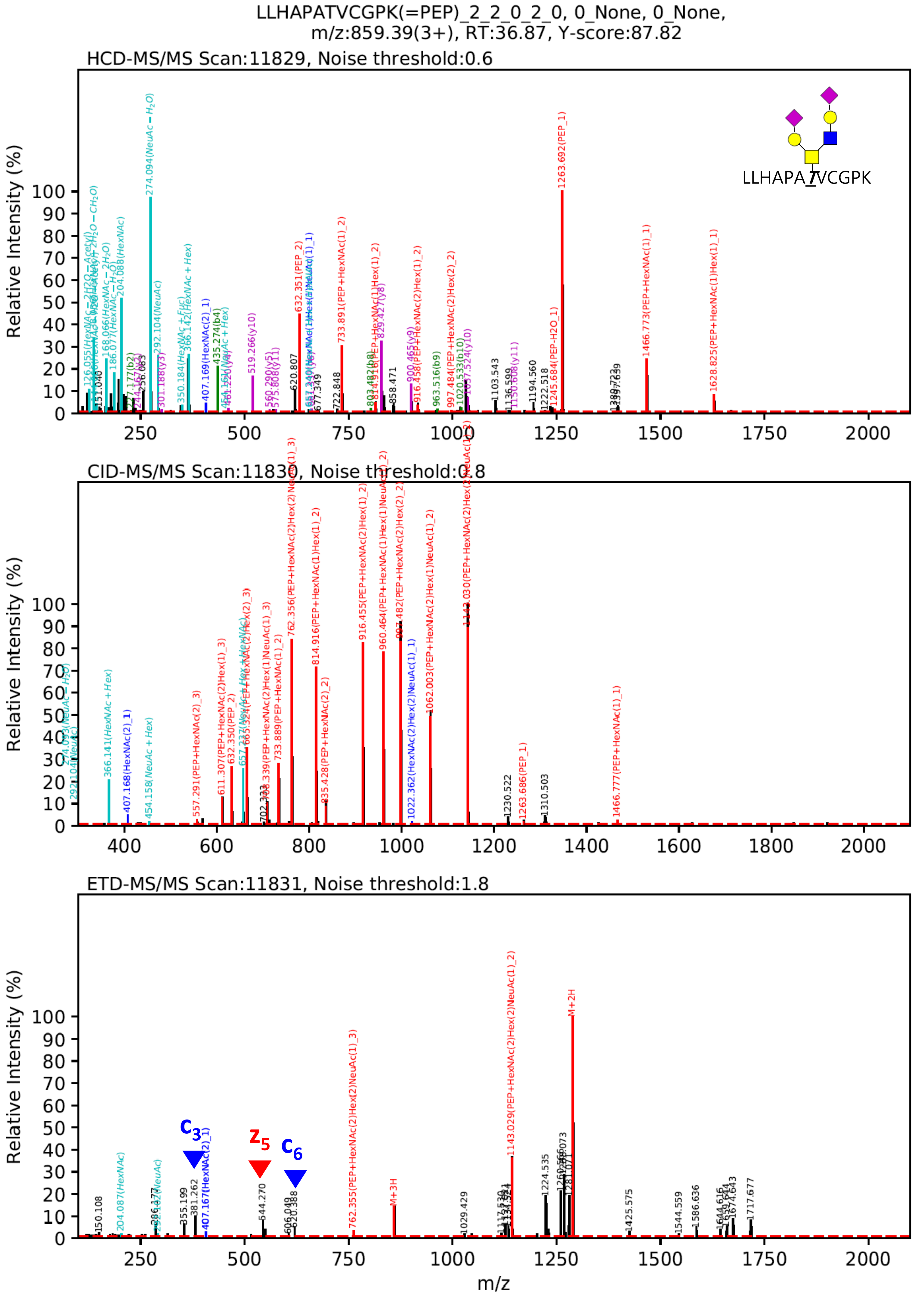

27. LLHAPA*T*VCGPK_2_2_0_2

LLHAPAT(Hex2HexNAc2NeuAc2)VC(Carbamidomethyl)GPK

MH+1(mono)	MH+2(mono)	MH+3(mono)
2576.1430	1288.5752	859.3859

b	b+2	c				y	z	z+2
---	---	131.1179	1	L	12	---	---	---
227.1754	---	244.2020	2	L	11	2463.0590	2447.0402	1224.0238
364.2343	182.6208	381.2609	3	H	10	2349.9749	2333.9562	1167.4817
435.2714	218.1394	---	4	A	9	2212.9160	2196.8973	1098.9523
532.3242	266.6657	549.3507	5	P	8	2141.8789	---	---
603.3613	302.1843	620.3879	6	A	7	2044.8261	2028.8074	1014.9073
2016.8642	1008.9357	2033.8908	7	T(Hex2HexNAc2NeuAc2)	6	1973.7890	1957.7703	979.3888
2115.9326	1058.4700	2132.9592	8	V	5	560.2861	544.2674	272.6373
2275.9633	1138.4853	2292.9898	9	C(Carbamidomethyl)	4	461.2177	445.1990	223.1031
2332.9847	1166.9960	---	10	G	3	301.1870	285.1683	143.0878
2430.0375	1215.5224	2447.0640	11	P	2	244.1656	---	---
---	---	---	12	K	1	147.1128	131.0941	66.0507
