# Supplementary material for: Characterization of Site-Specific N- and O-Glycopeptides from Recombinant Spike and ACE2 Glycoproteins Using LC-MS/MS Analysis
Source: Int J Mol Sci. 2024 Dec 20;25(24):13649. doi: 10.3390/ijms252413649 (PMC11678118; doi:10.3390/ijms252413649)
Supplement: Supplementary file 1 [file ijms-25-13649-s001.zip › Supplementary Figure S4(RBD_TG_N-glycopep_1).pdf]

FPNITNLCPFGE(=PEP)_4_3_1_0_0, 0_None, 0_None,
m/z:1406.59(3+), RT:60.03, hcd-score:74.51

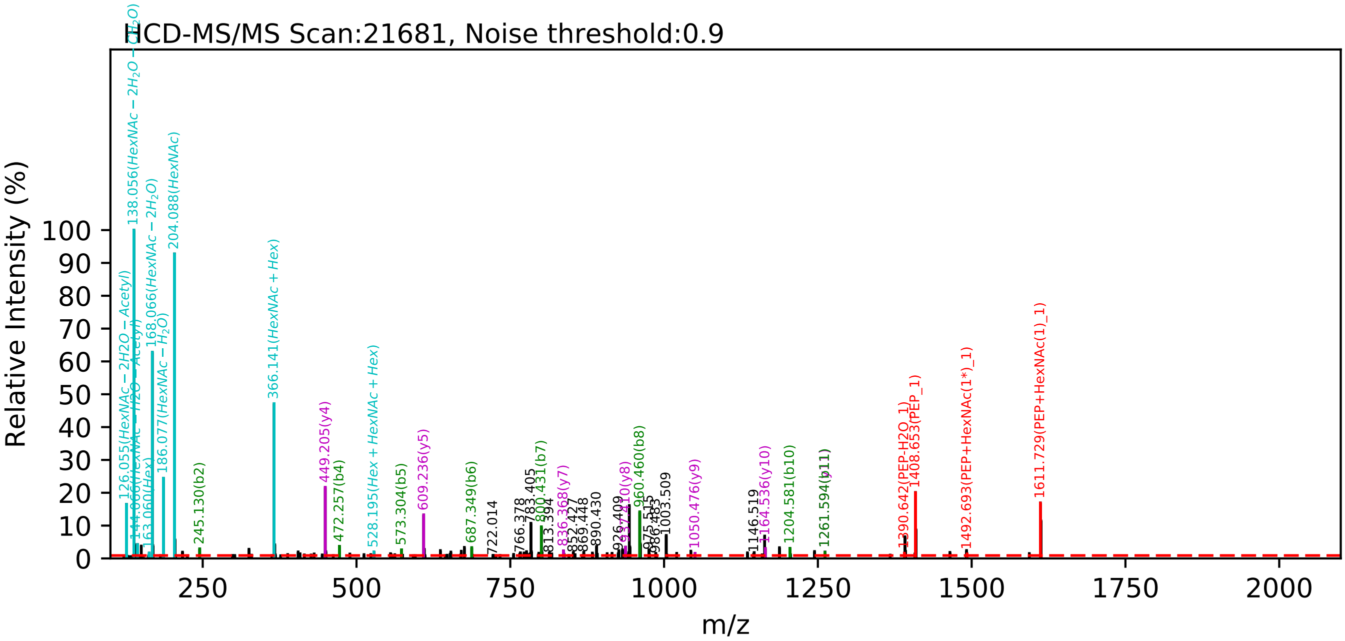

FPNITNLCPFGE(=PEP)_4_3_1_0_0, 0_None, 0_None,
m/z:1406.59(3+), RT:60.03, hcd-score:74.51

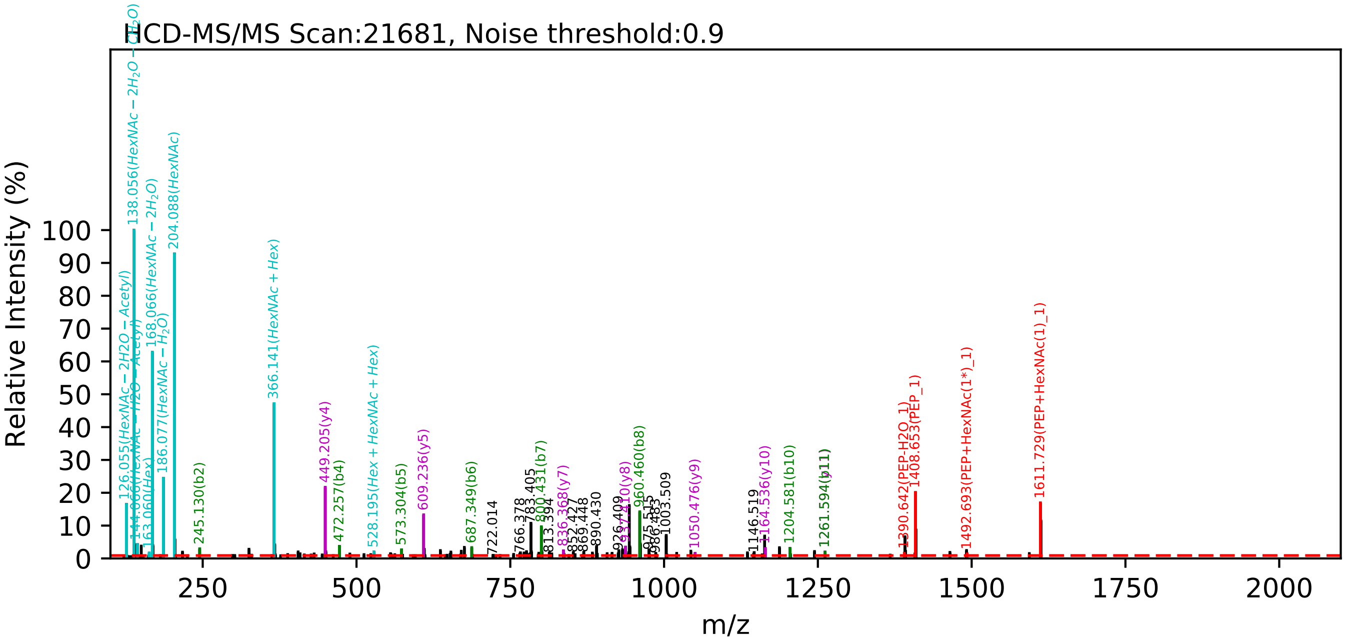

FPNITNLCPFGE(=PEP)_6_5_3_1_0, 0_None, 0_None,
m/z:1375.89(3+), RT:67.82, hcd-score:100.00

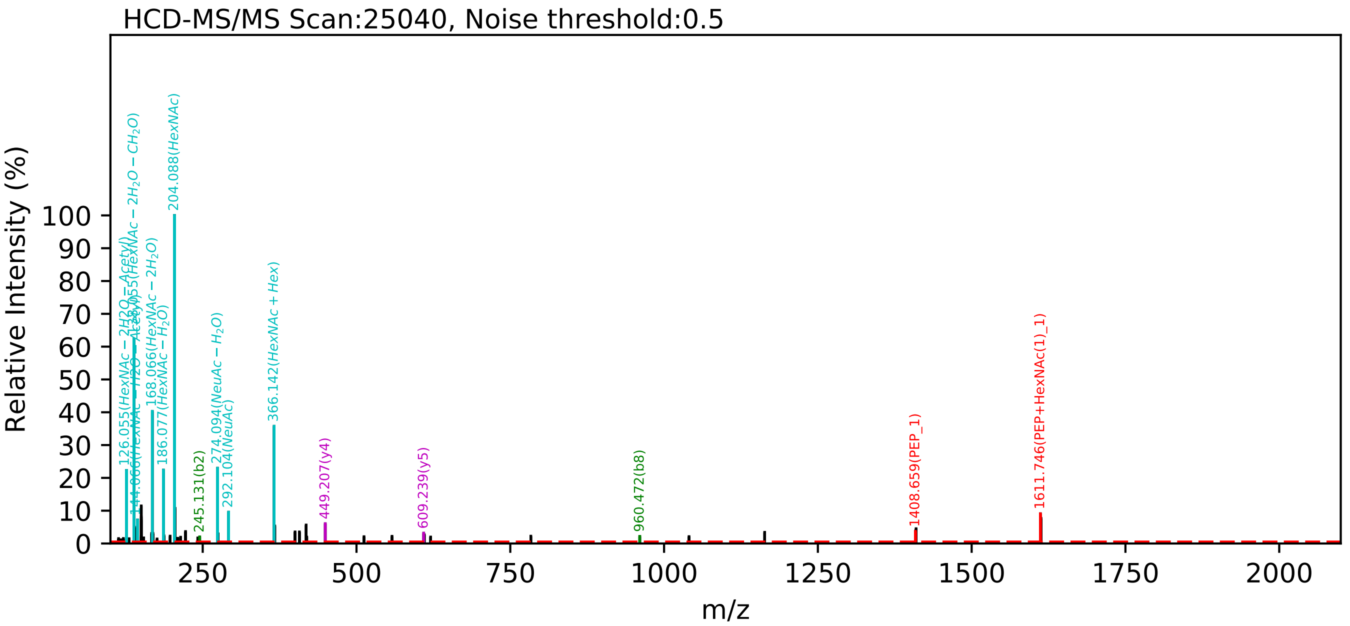

FPNITNLCPFGE(=PEP)_6_5_3_1_0, 0_None, 0_None,
m/z:1375.89(3+), RT:67.82, hcd-score:100.00

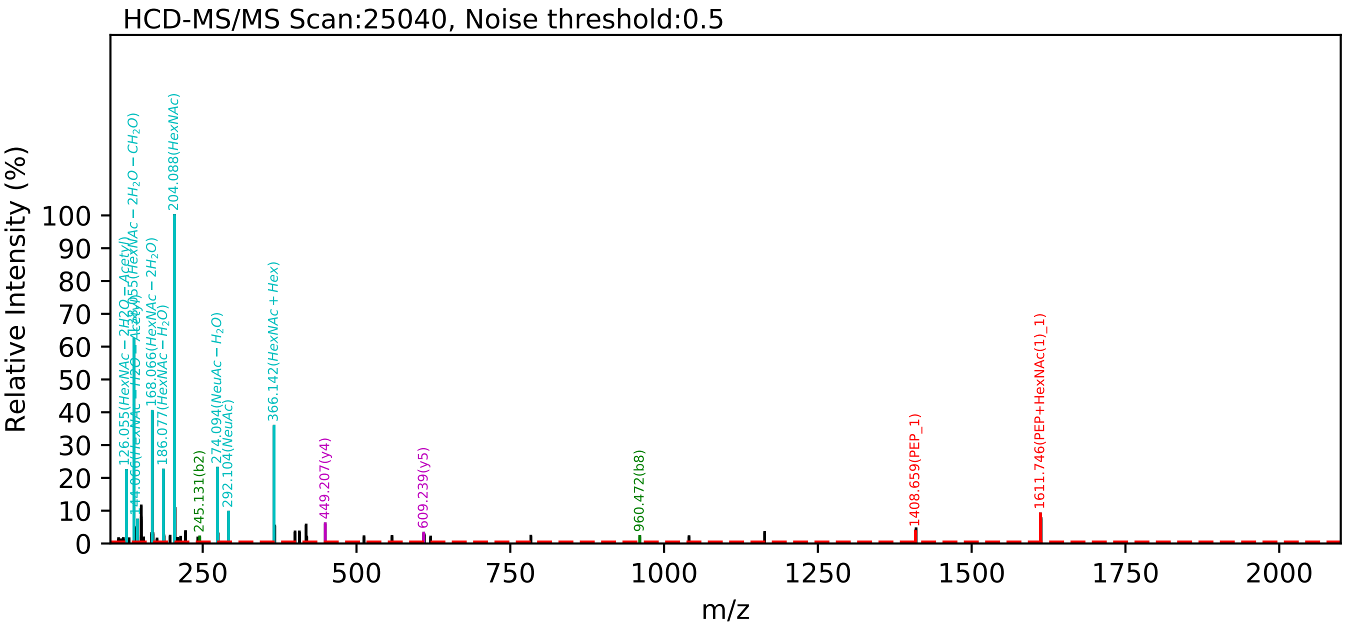

FPNITNLCPFGE(=PEP)_5_6_3_1_0, 0_None, 0_None,
m/z:1042.42(3+), RT:68.36, hcd-score:81.06

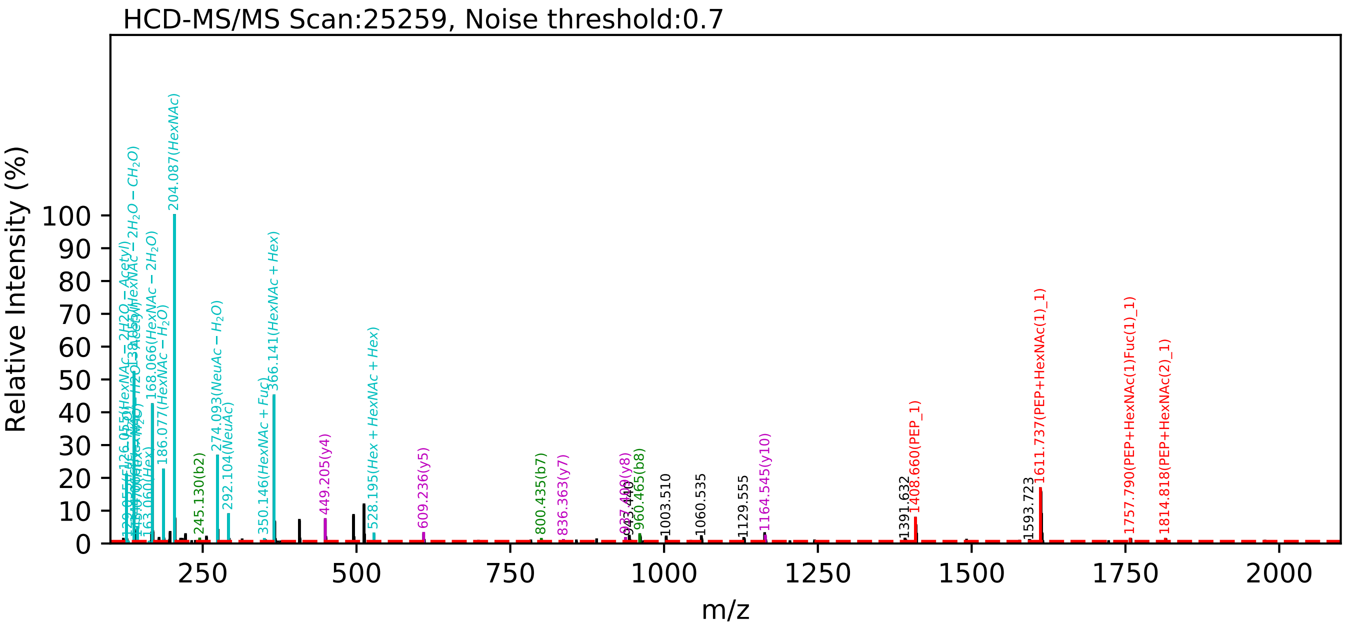

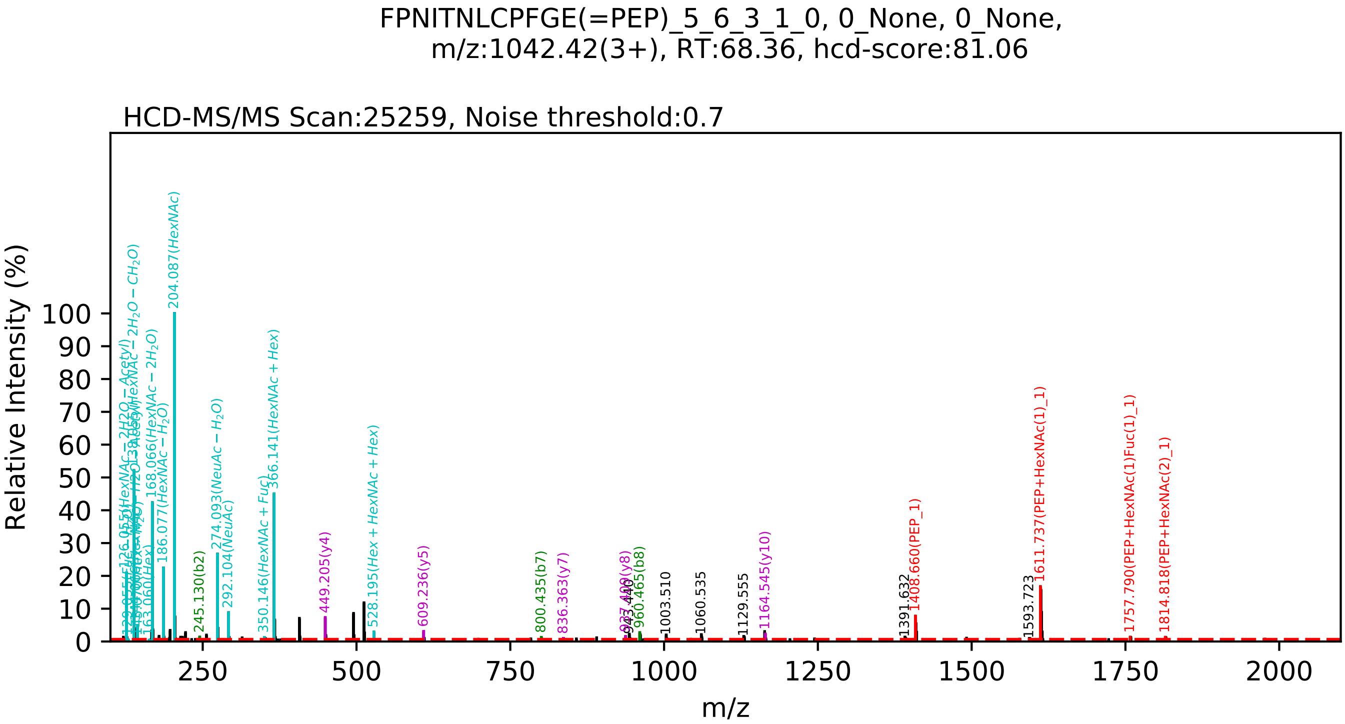
FPNITNLCPFGE(=PEP)_5_6_3_1_0, 0_None, 0_None,
m/z:1042.42(3+), RT:68.36, hcd-score:81.06
HCD-MS/MS Scan:25259, Noise threshold:0.7
Relative Intensity (%)
m/z
204.087(HexNAc)
168.066(HexNAc − 2H2O)
186.077(HexNAc − H2O)
163.060(Hex)
274.093(NeuAc − H2O)
292.104(NeuAc)
245.130(b2)
350.146(HexNAc + Fuc)
366.141(HexNAc + Hex)
449.205(y4)
528.195(Hex + HexNAc + Hex)
609.236(y5)
800.435(b7)
836.363(y7)
960.465(b8)
1003.510
1060.535
1129.555
1164.545(y10)
1391.632
1408.660(PEP_1)
1593.723
1611.737(PEP+HexNAc(1)_1)
1757.790(PEP+HexNAc(1)Fuc(1)_1)
1814.818(PEP+HexNAc(2)_1)

FPNITNLCPFGE(=PEP)_4_4_1_1_0, 0_None, 0_None,
m/z:1653.67(3+), RT:70.31, hcd-score:72.99

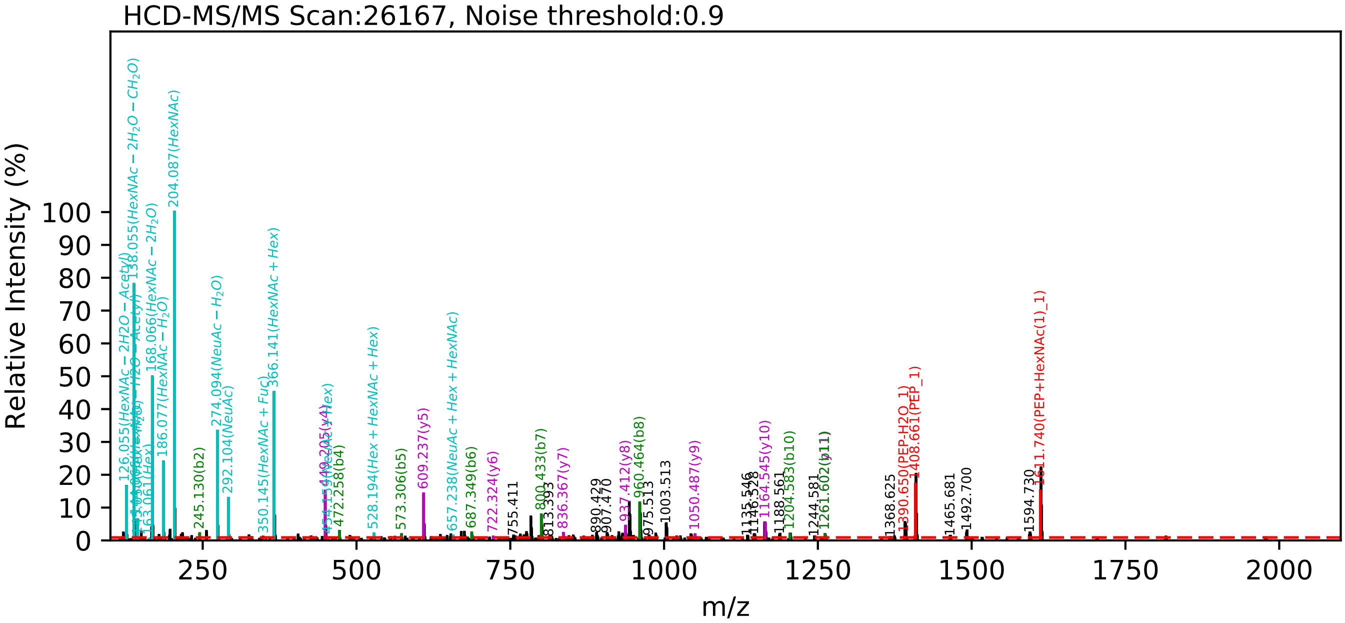

FPNITNLCPFGE(=PEP)_4_4_1_1_0, 0_None, 0_None,
m/z:1653.67(3+), RT:70.31, hcd-score:72.99

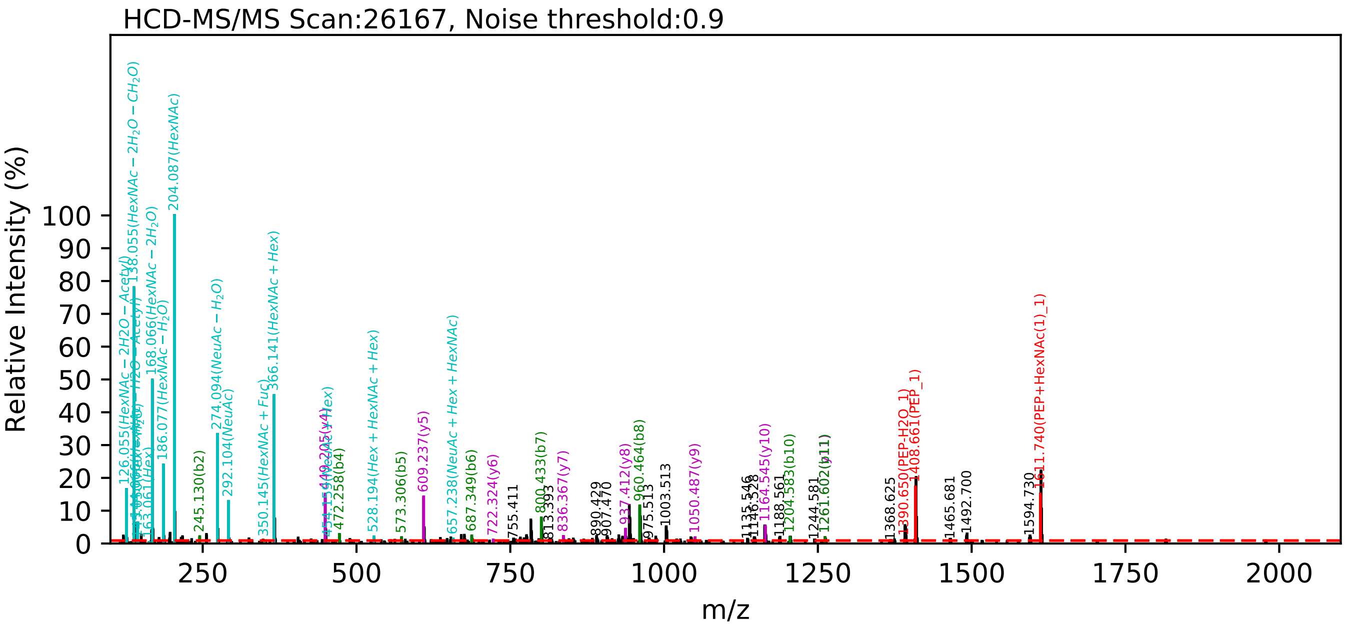

FPNITNLCPFGE(=PEP)_6_5_2_2_0, 0_None, 0_None,
m/z:1424.23(3+), RT:81.36, hcd-score:100.00

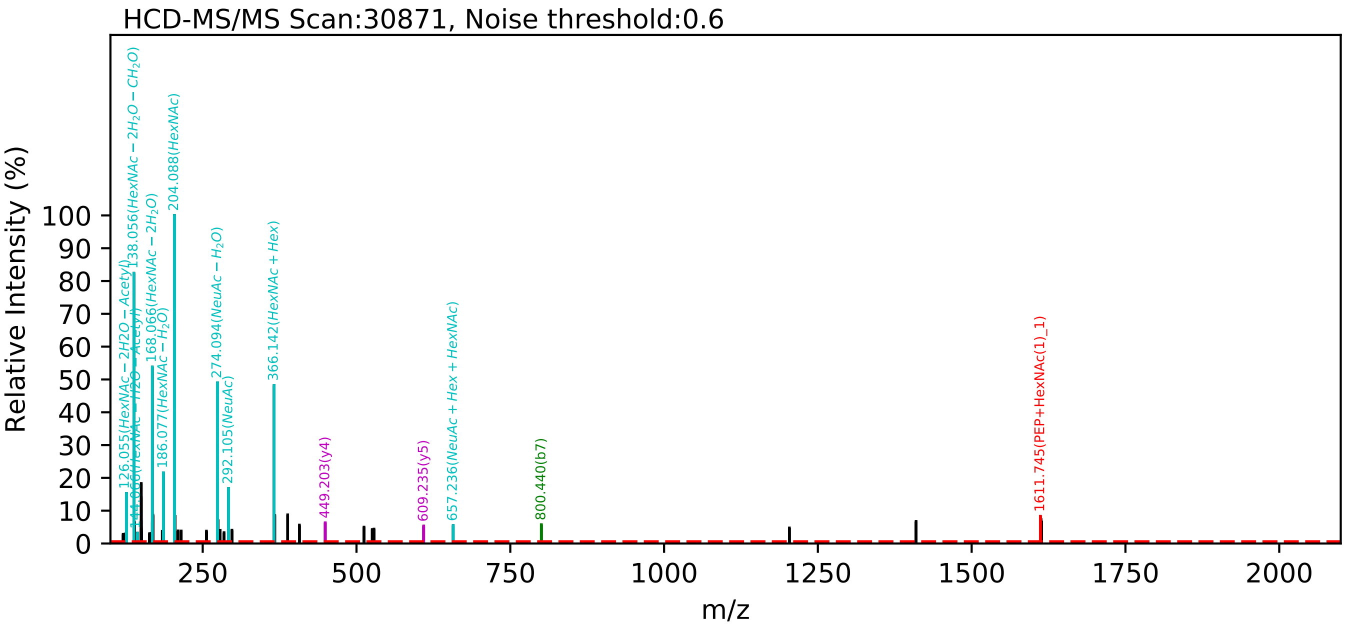

FPNITNLCPFGE(=PEP)_6_5_2_2_0, 0_None, 0_None,
m/z:1424.23(3+), RT:81.36, hcd-score:100.00

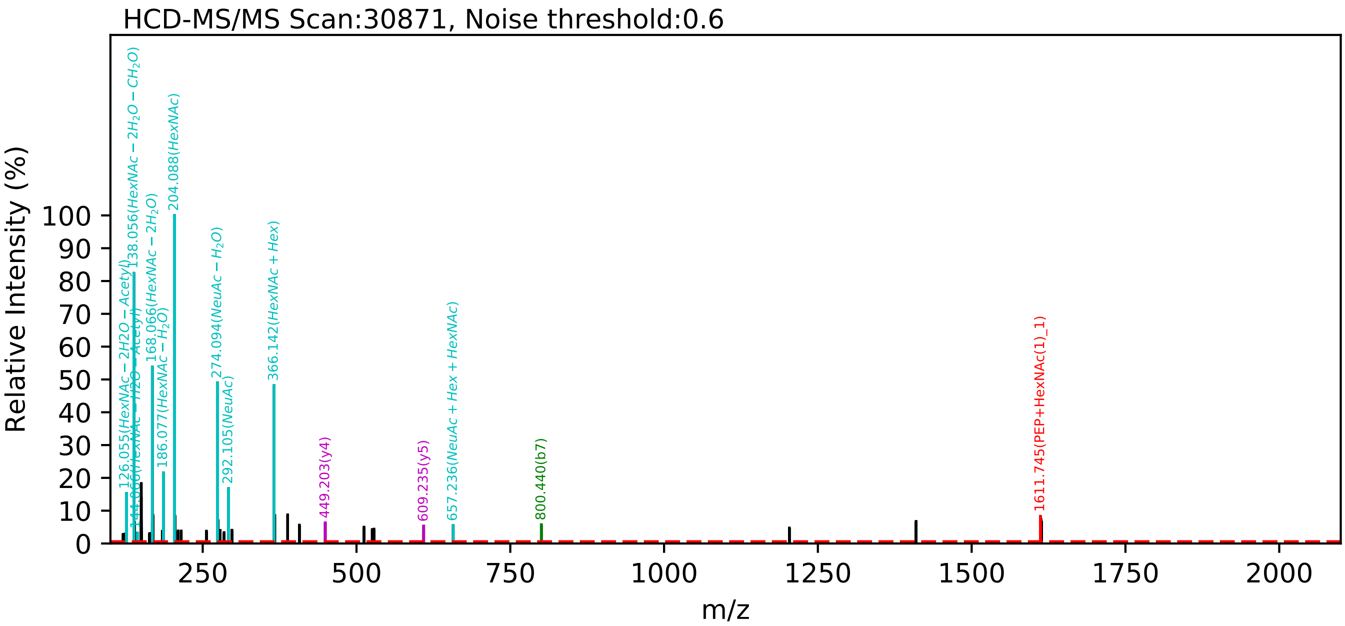

FPNITNLCPFGE(=PEP)_7_7_1_2_0, 0_None, 0_None,
m/z:1173.96(4+), RT:81.48, hcd-score:100.00

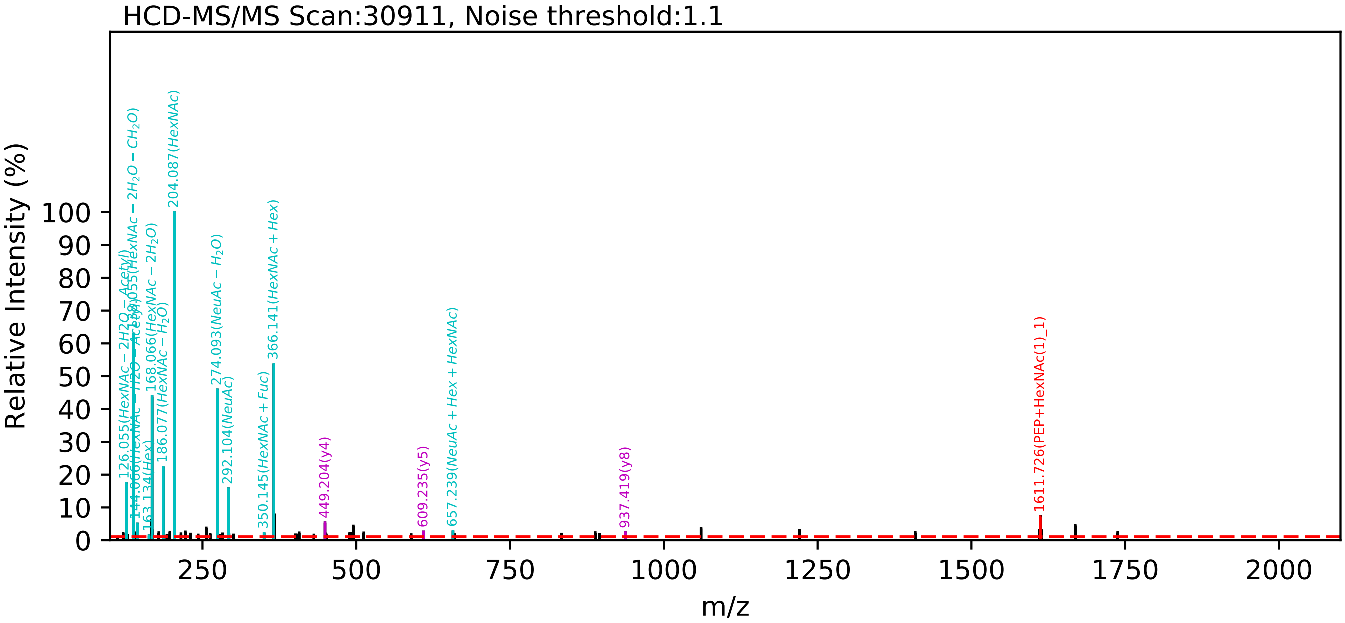

FPNITNLCPFGE(=PEP)_7_7_1_2_0, 0_None, 0_None,
m/z:1173.96(4+), RT:81.48, hcd-score:100.00

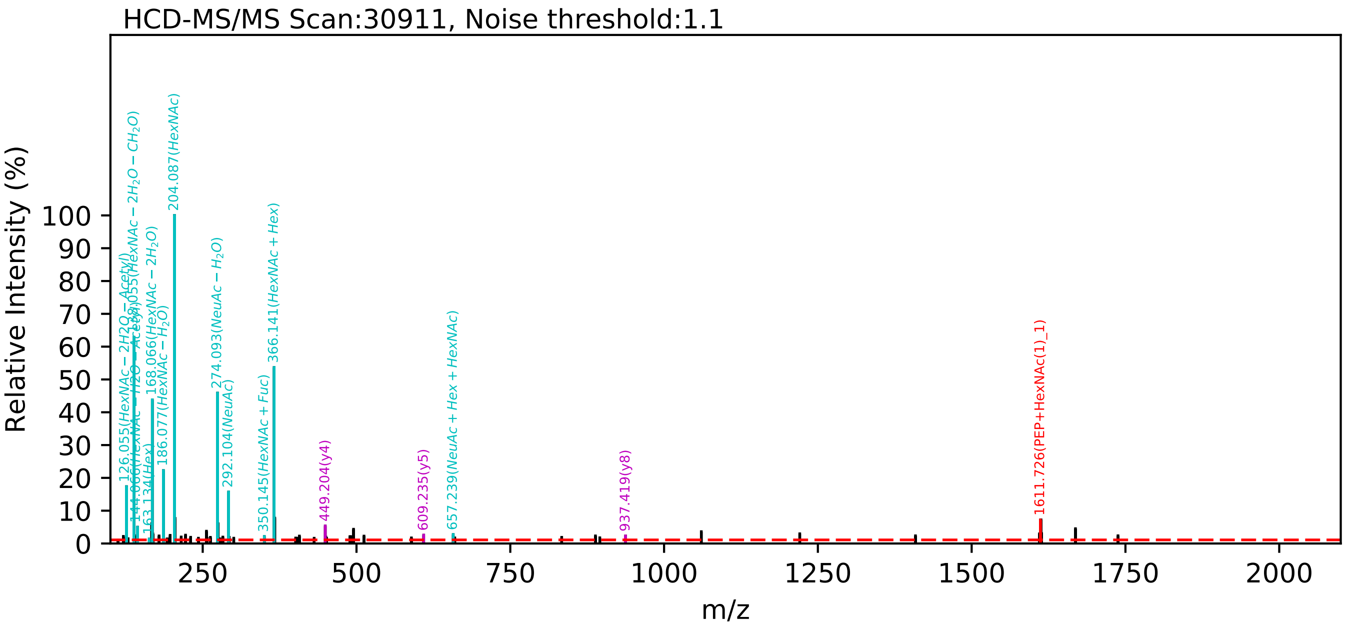

FPNITNLCPFGE(=PEP)_7_6_3_2_0, 0_None, 0_None,
m/z:1594.63(2+), RT:82.13, hcd-score:92.88

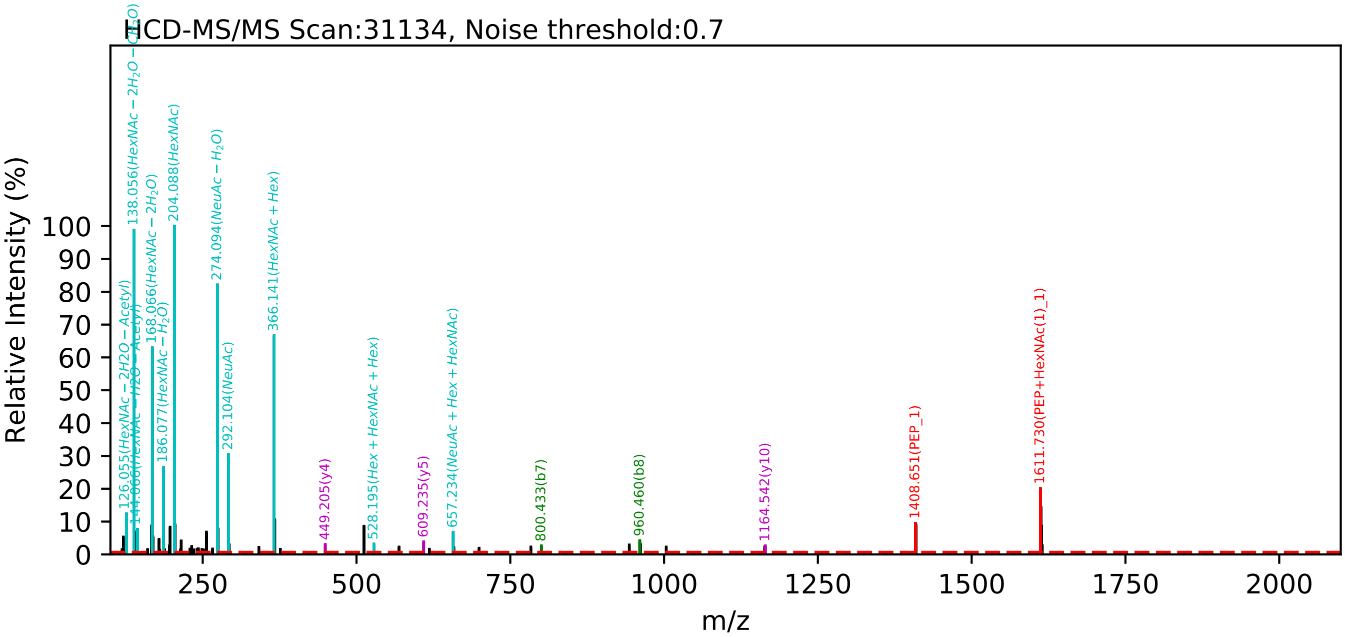

FPNITNLCPFGE(=PEP)_7_6_3_2_0, 0_None, 0_None,
m/z:1594.63(2+), RT:82.13, hcd-score:92.88

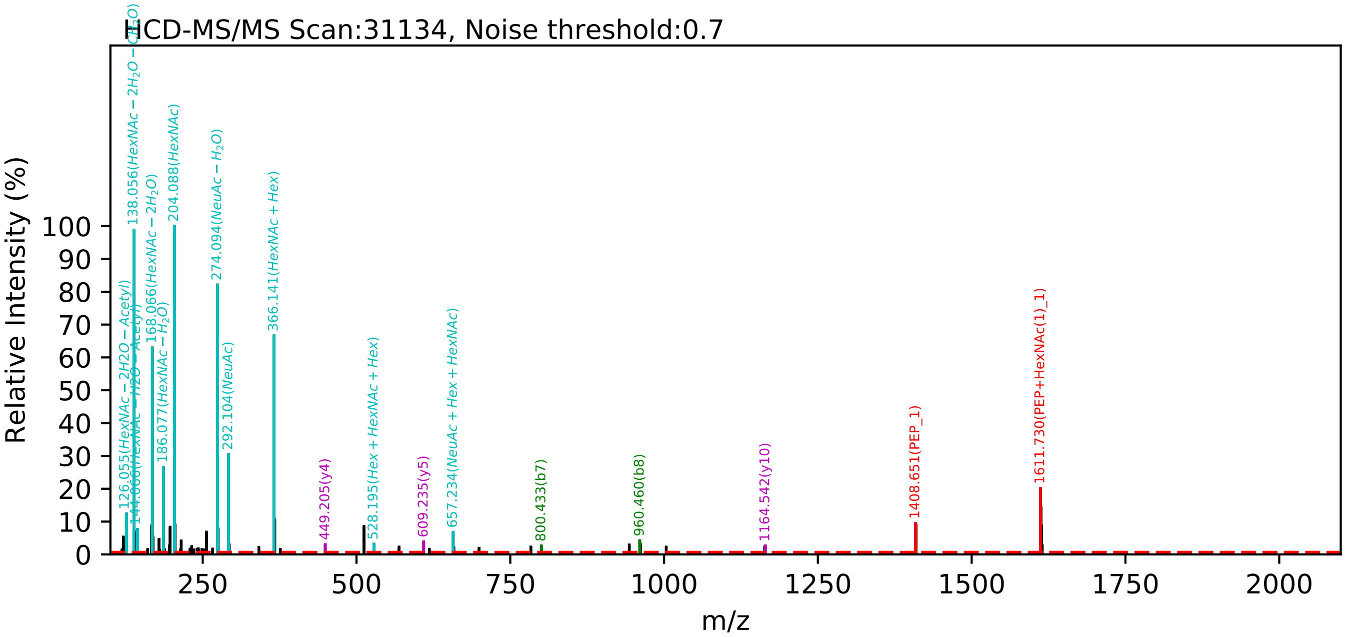

FPNITNLCPFGE(=PEP)_7_6_2_2_0, 0_None, 0_None,
m/z:1159.71(4+), RT:82.38, hcd-score:77.95

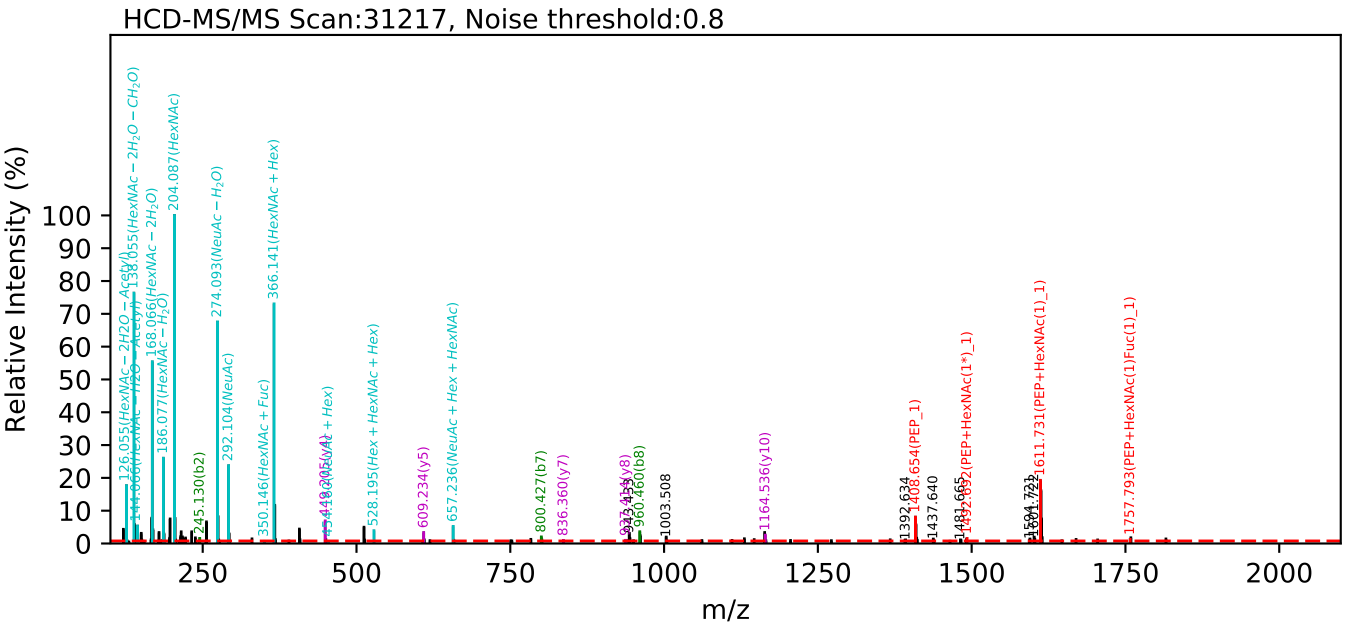

FPNITNLCPFGE(=PEP)_7_6_2_2_0, 0_None, 0_None,
m/z:1159.71(4+), RT:82.38, hcd-score:77.95

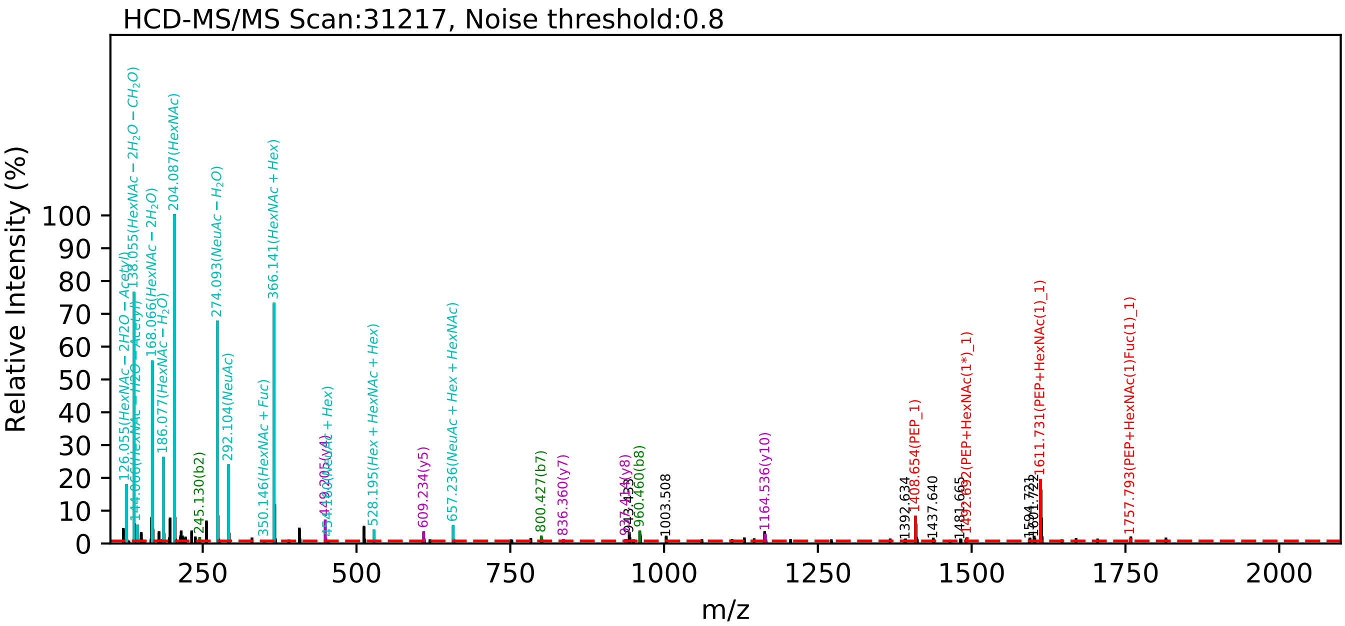

FPNITNLCPFGE(=PEP)_6_5_2_2_0, 0_None, 0_None,
m/z:1068.43(3+), RT:82.51, hcd-score:83.09

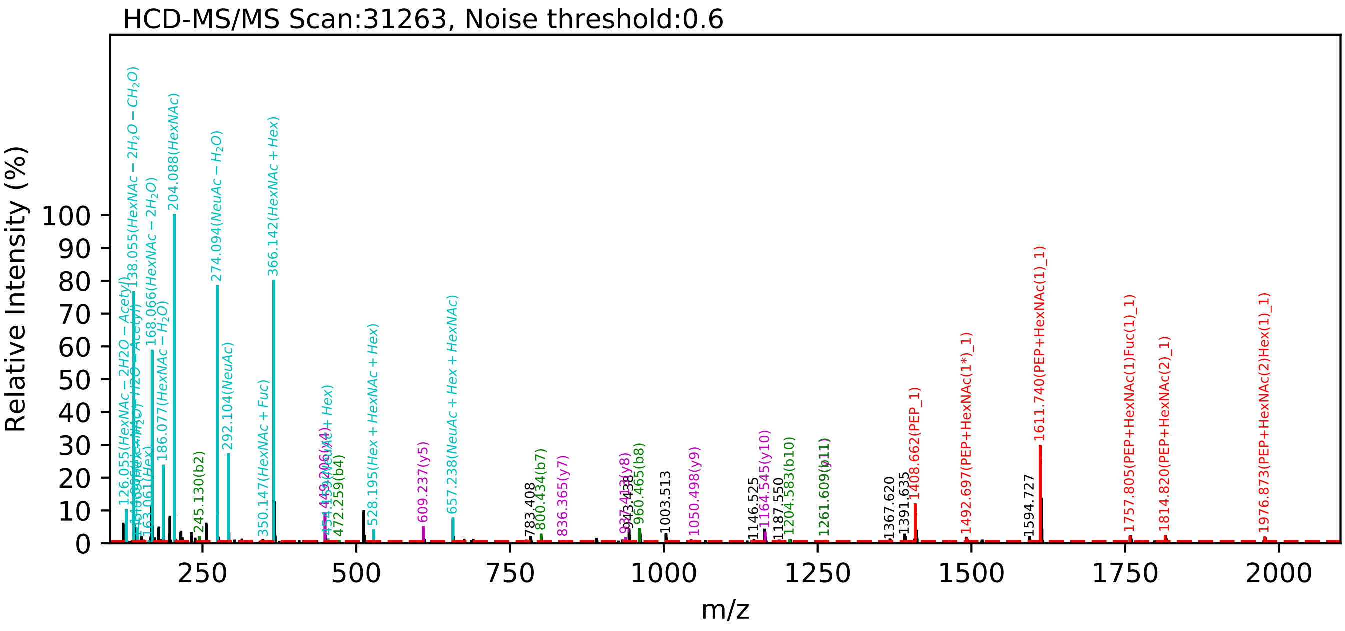

FPNITNLCPFGE(=PEP)_6_5_2_2_0, 0_None, 0_None,
m/z:1068.43(3+), RT:82.51, hcd-score:83.09

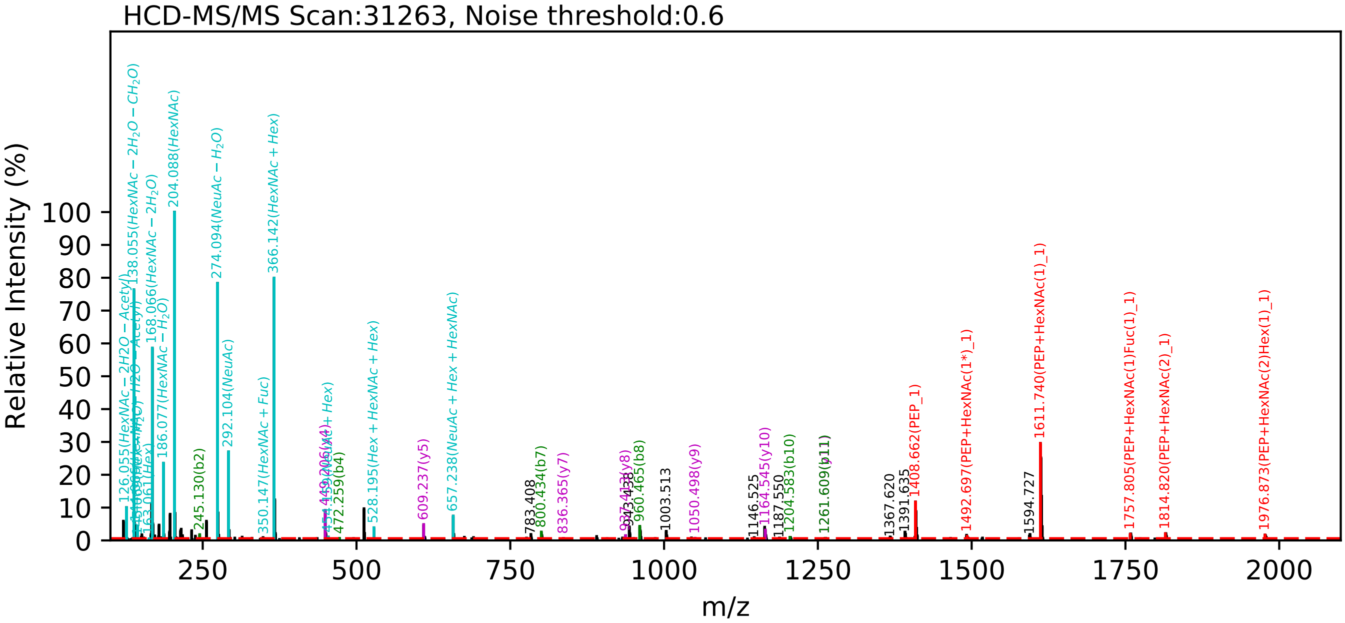

FPNITNLCPFGE(=PEP)_5_5_1_2_0, 0_None, 0_None,
m/z:1321.53(4+), RT:83.06, hcd-score:77.10

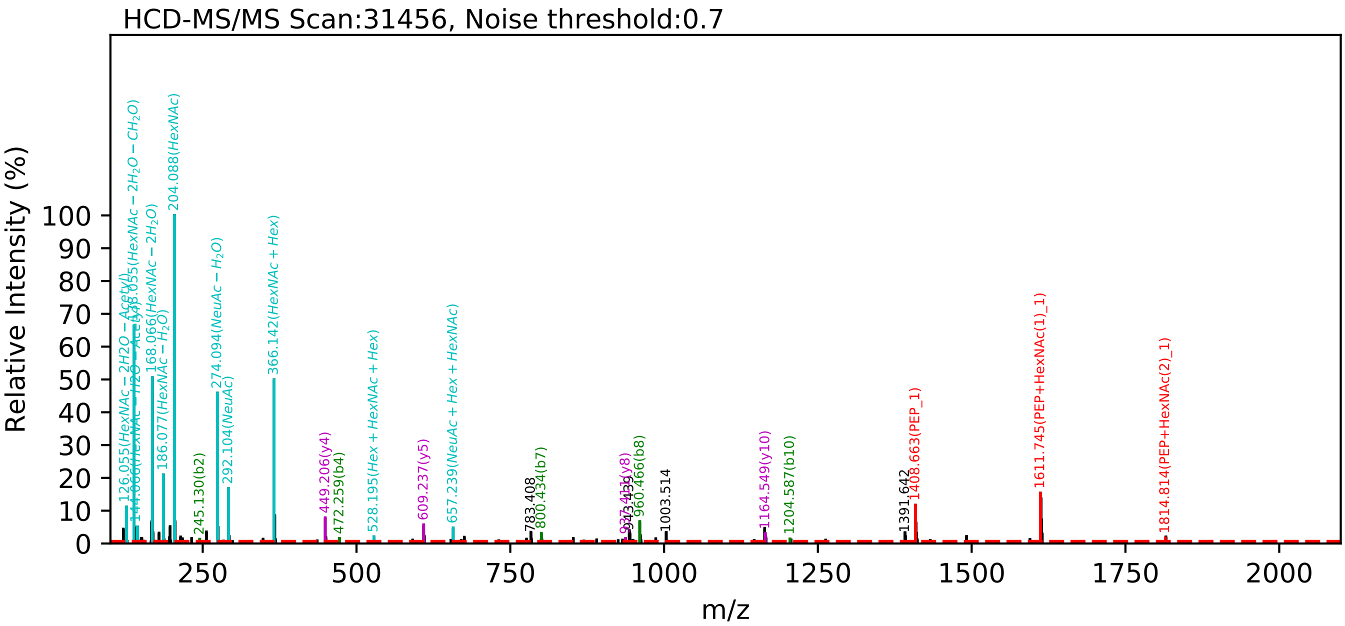

FPNITNLCPFGE(=PEP)_5_5_1_2_0, 0_None, 0_None,
m/z:1321.53(4+), RT:83.06, hcd-score:77.10

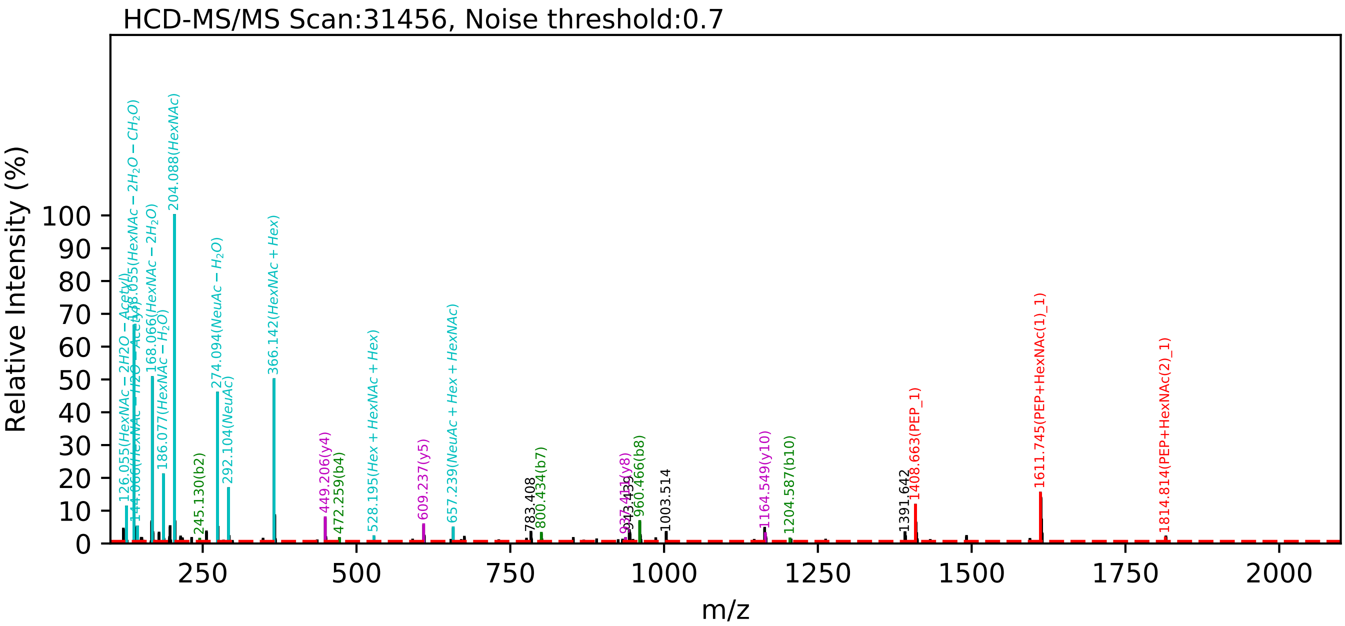

FPNITNLCPFGE(=PEP)_6_6_0_2_0, 0_None, 0_None,
m/z:1046.17(4+), RT:83.26, hcd-score:100.00

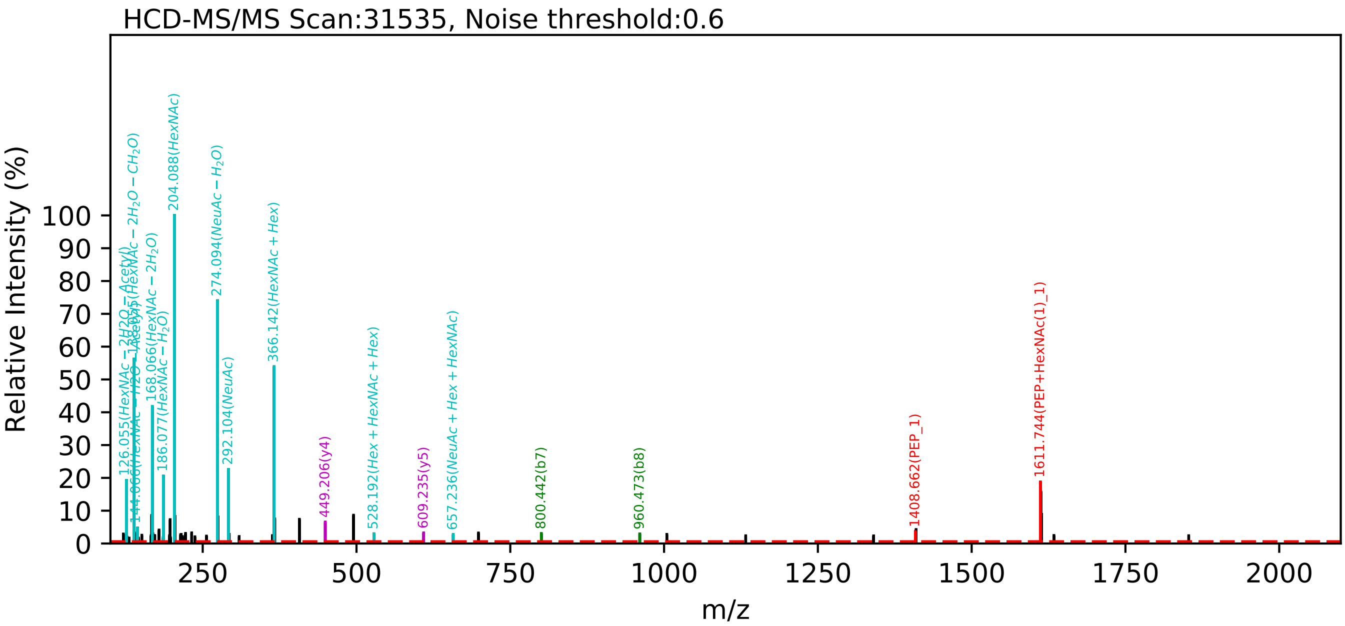

FPNITNLCPFGE(=PEP)_6_6_0_2_0, 0_None, 0_None,
m/z:1046.17(4+), RT:83.26, hcd-score:100.00

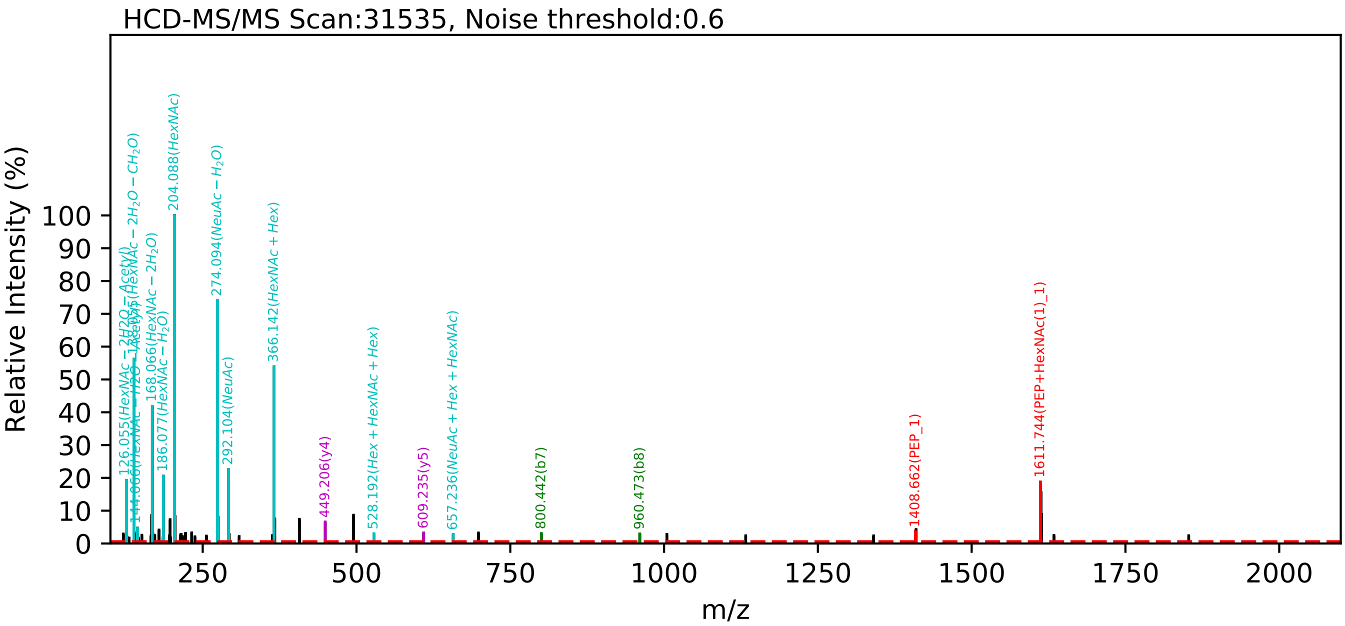

FPNITNLCPFGE(=PEP)_5_5_1_2_0, 0_None, 0_None,
m/z:1321.53(4+), RT:83.79, hcd-score:80.94

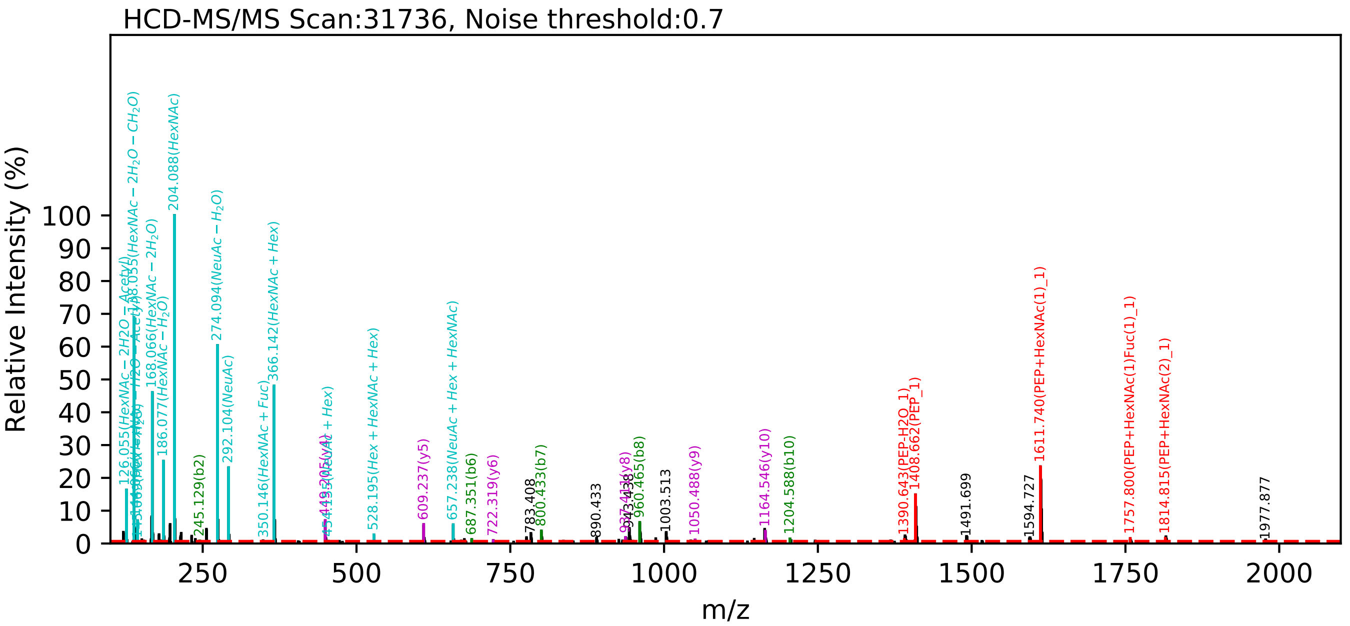

FPNITNLCPFGE(=PEP)_5_5_1_2_0, 0_None, 0_None,
m/z:1321.53(4+), RT:83.79, hcd-score:80.94

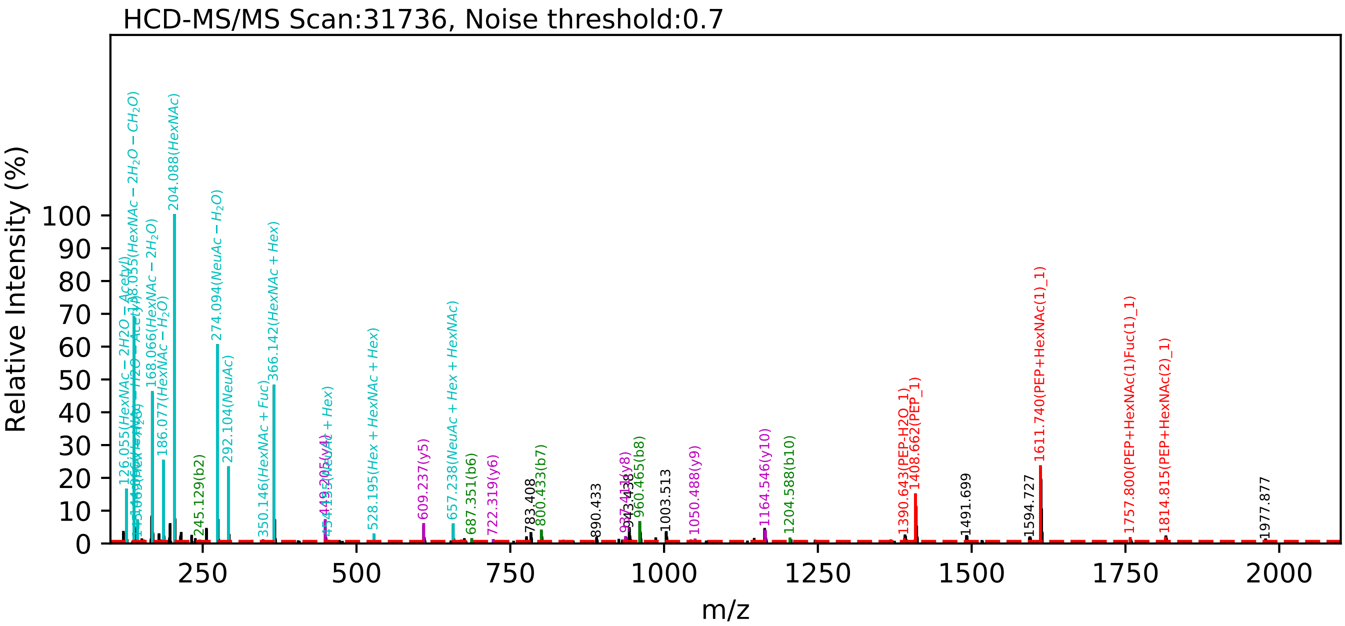

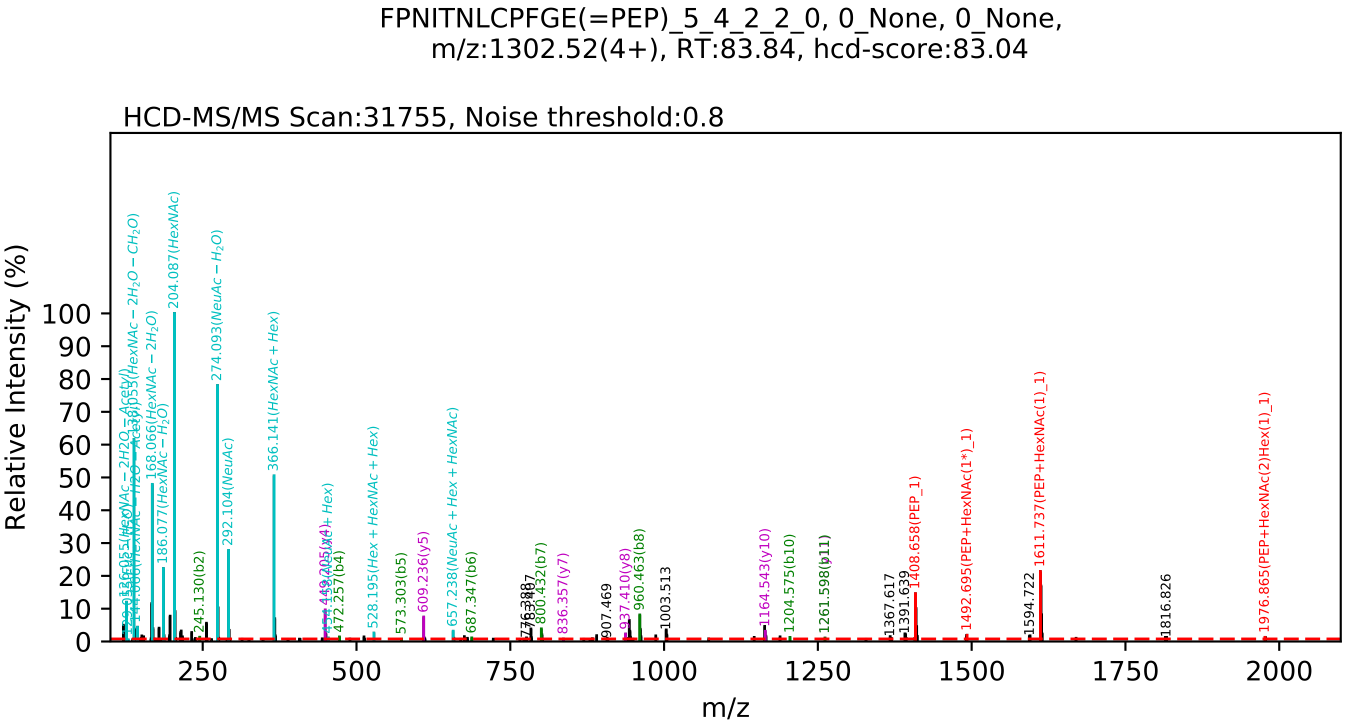

FPNITNLCPFGE(=PEP)_5_4_2_2_0, 0_None, 0_None,
m/z:1302.52(4+), RT:83.84, hcd-score:83.04
HCD-MS/MS Scan:31755, Noise threshold:0.8
Relative Intensity (%)
m/z
204.087(HexNAc)
274.093(NeuAc − H2O)
366.141(HexNAc + Hex)
292.104(NeuAc)
168.066(HexNAc − 2H2O)
186.077(HexNAc − H2O)
245.130(b2)
472.257(b4)
528.195(Hex + HexNAc + Hex)
573.303(b5)
609.236(y5)
657.238(NeuAc + Hex + HexNAc)
687.347(b6)
800.432(b7)
836.357(y7)
907.469
937.410(y8)
960.463(b8)
1003.513
1164.543(y10)
1204.575(b10)
1261.598(b11)
1367.617
1391.639
1408.658(PEP_1)
1492.695(PEP+HexNAc(1*)_1)
1594.722
1611.737(PEP+HexNAc(1)_1)
1816.826
1976.865(PEP+HexNAc(2)Hex(1)_1)

FPNITNLCPFGE(=PEP)_5_4_2_2_0, 0_None, 0_None,
m/z:1302.52(4+), RT:83.84, hcd-score:83.04

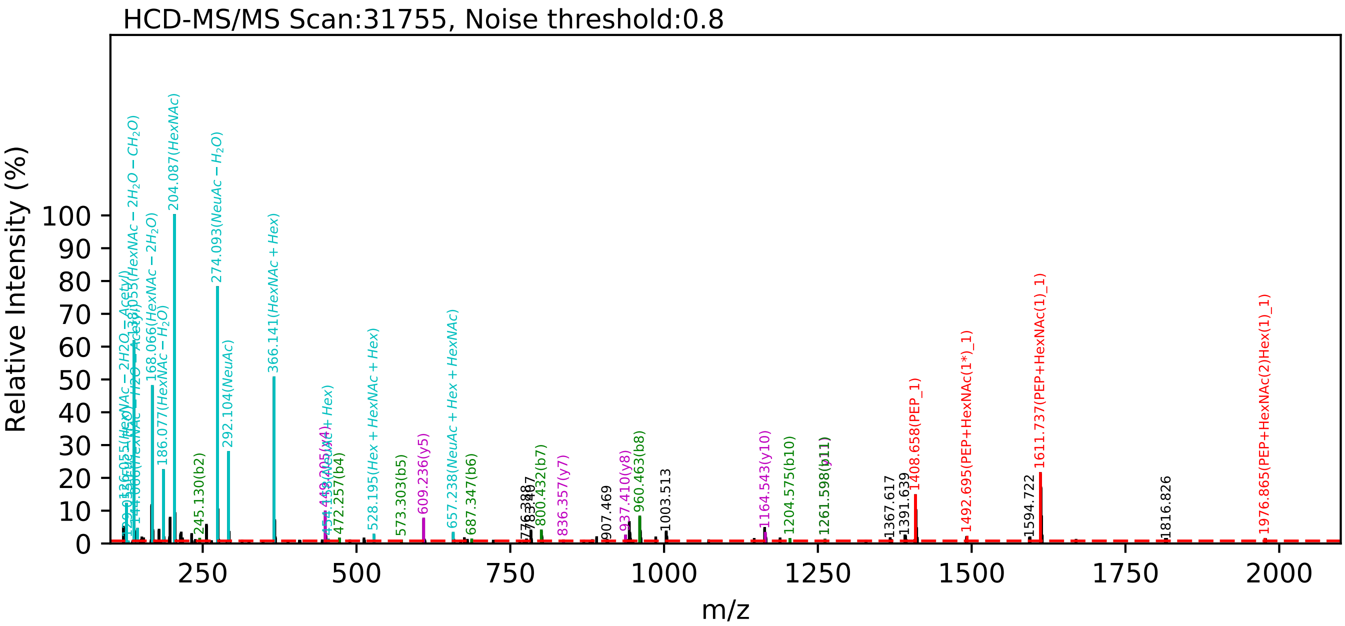

FPNITNLCPFGE(=PEP)_6_5_2_2_0, 0_None, 0_None,
m/z:1068.42(3+), RT:83.89, hcd-score:88.43

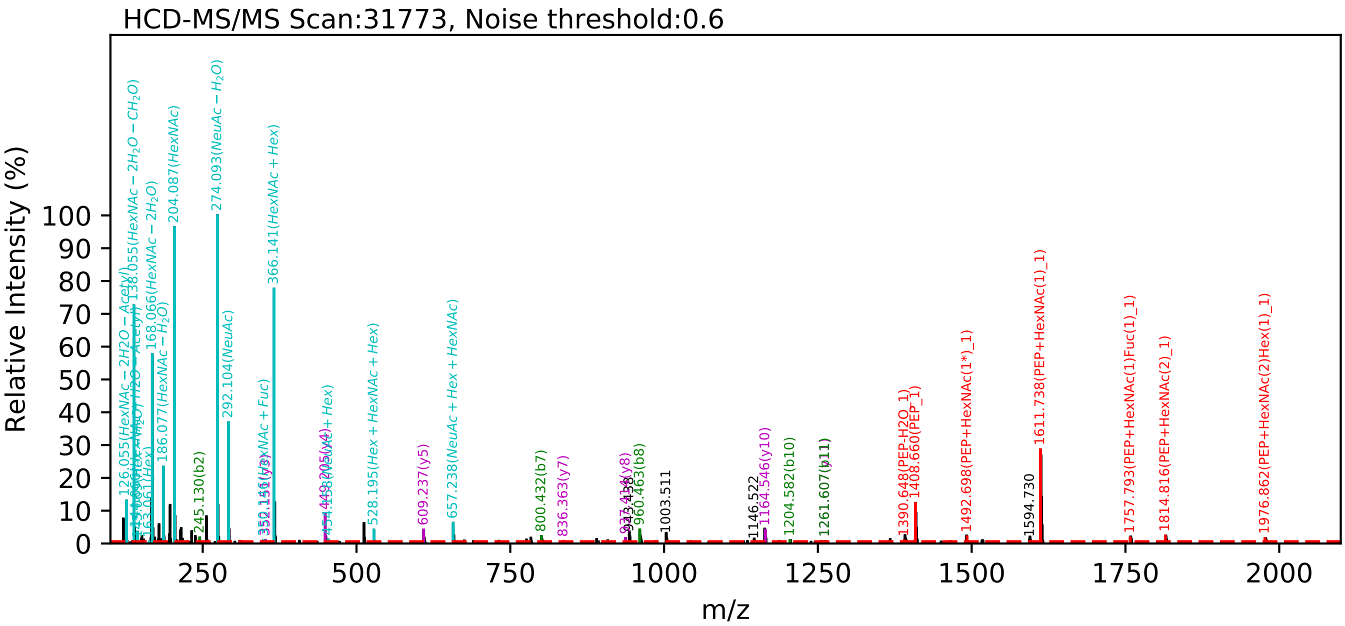

FPNITNLCPFGE(=PEP)_6_5_2_2_0, 0_None, 0_None,
m/z:1068.42(3+), RT:83.89, hcd-score:88.43

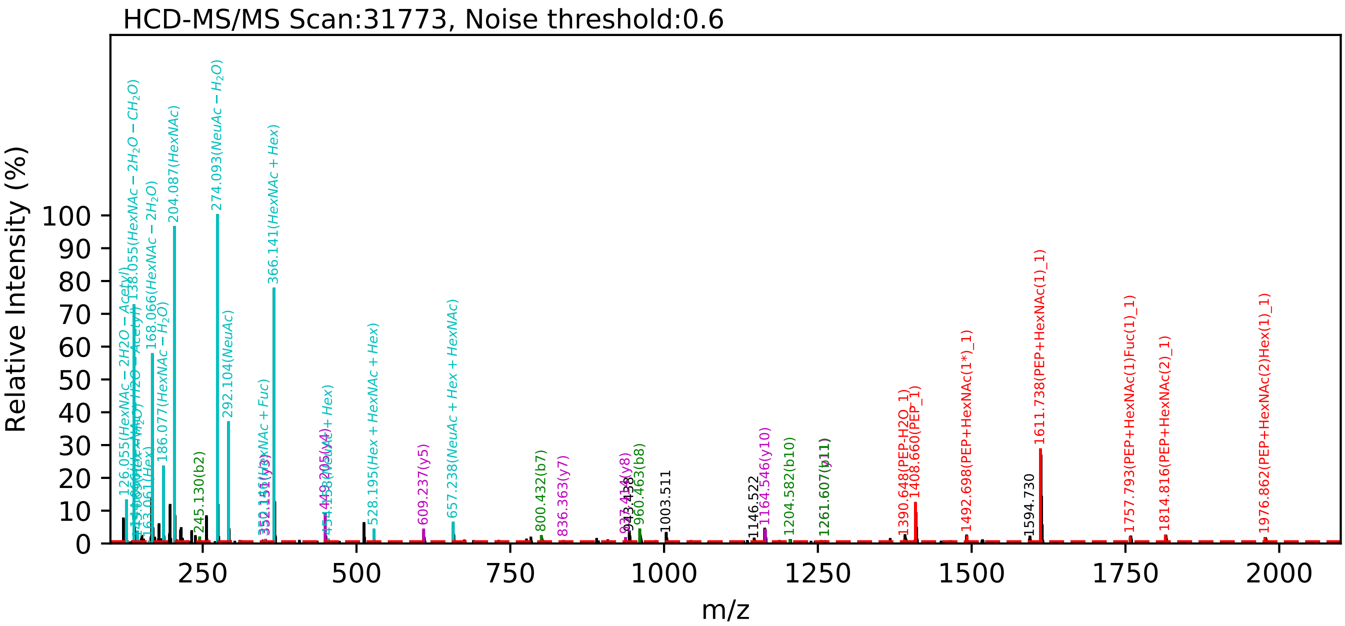

FPNITNLCPFGE(=PEP)_5_4_2_1_0, 0_None, 0_None,
m/z:1205.49(4+), RT:84.10, hcd-score:70.95

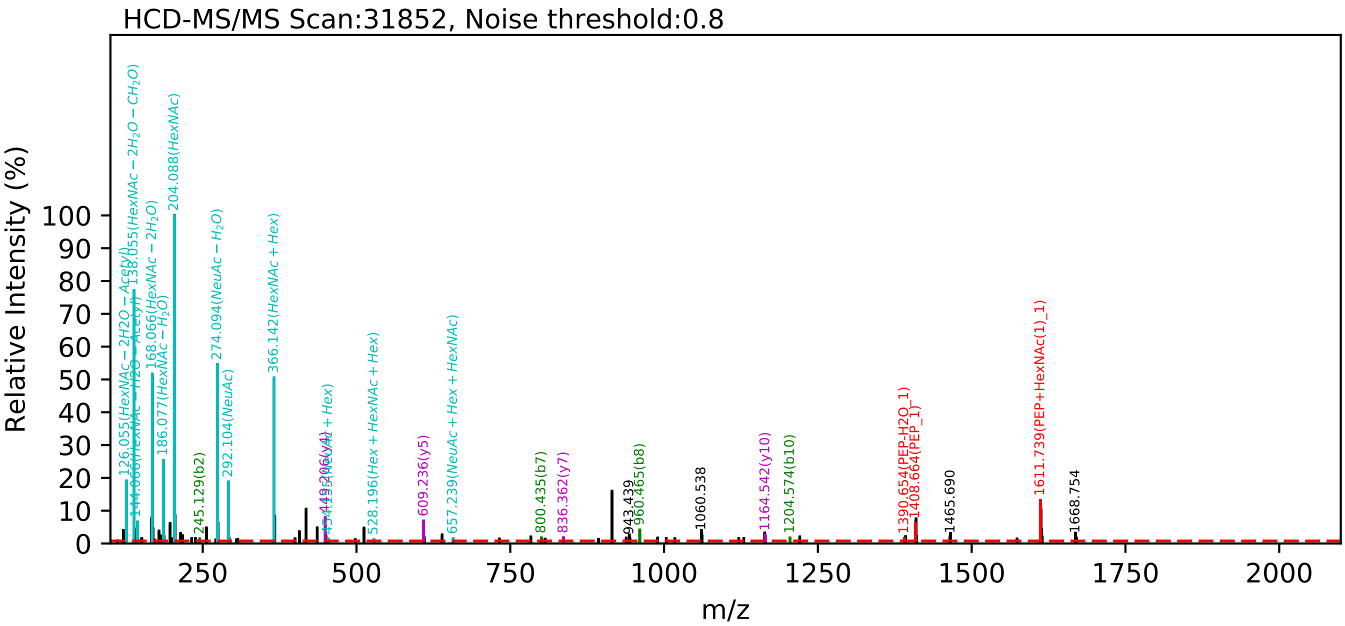

FPNITNLCPFGE(=PEP)_5_4_2_1_0, 0_None, 0_None,
m/z:1205.49(4+), RT:84.10, hcd-score:70.95

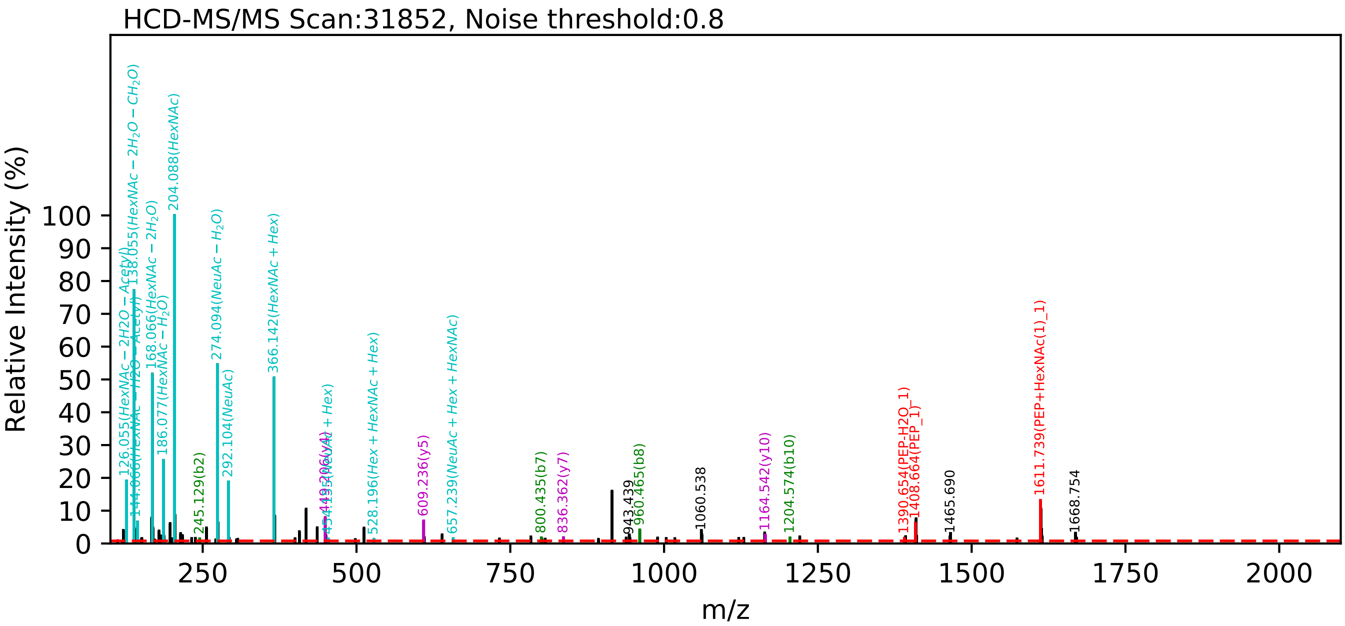

FPNITNLCPFGE(=PEP)_5_5_1_2_0, 0_None, 0_None,
m/z:1321.53(4+), RT:84.45, hcd-score:73.48

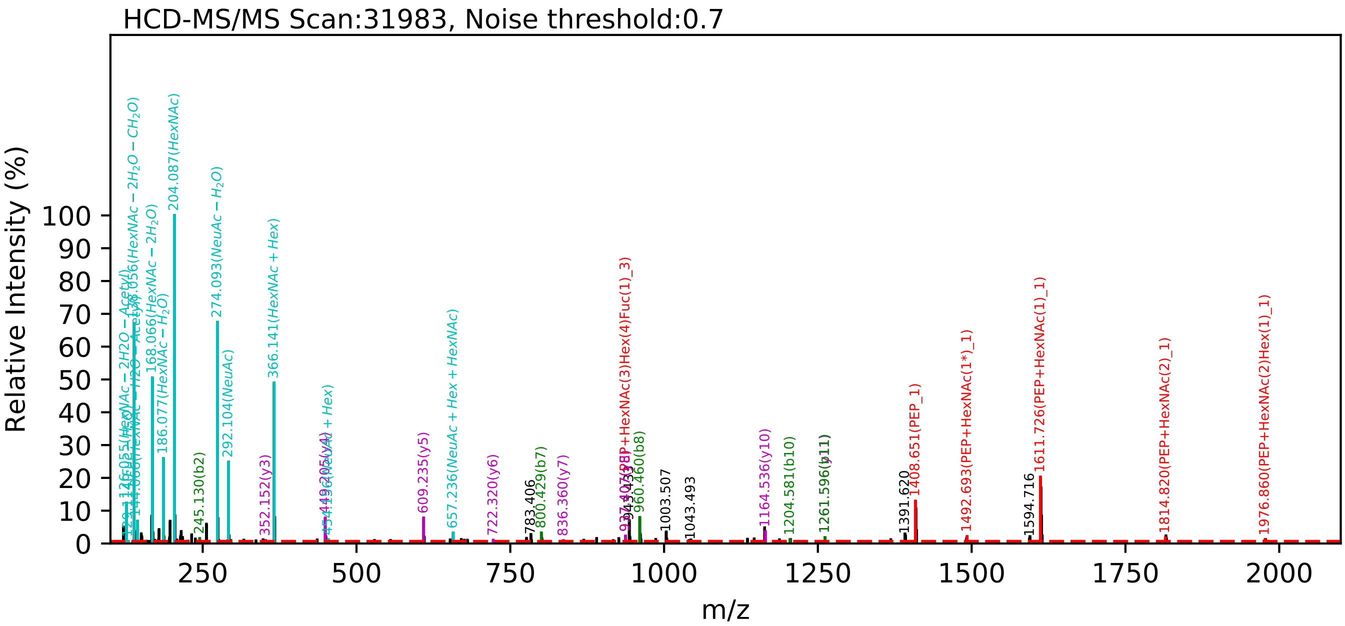

FPNITNLCPFGE(=PEP)_5_5_1_2_0, 0_None, 0_None,
m/z:1321.53(4+), RT:84.45, hcd-score:73.48

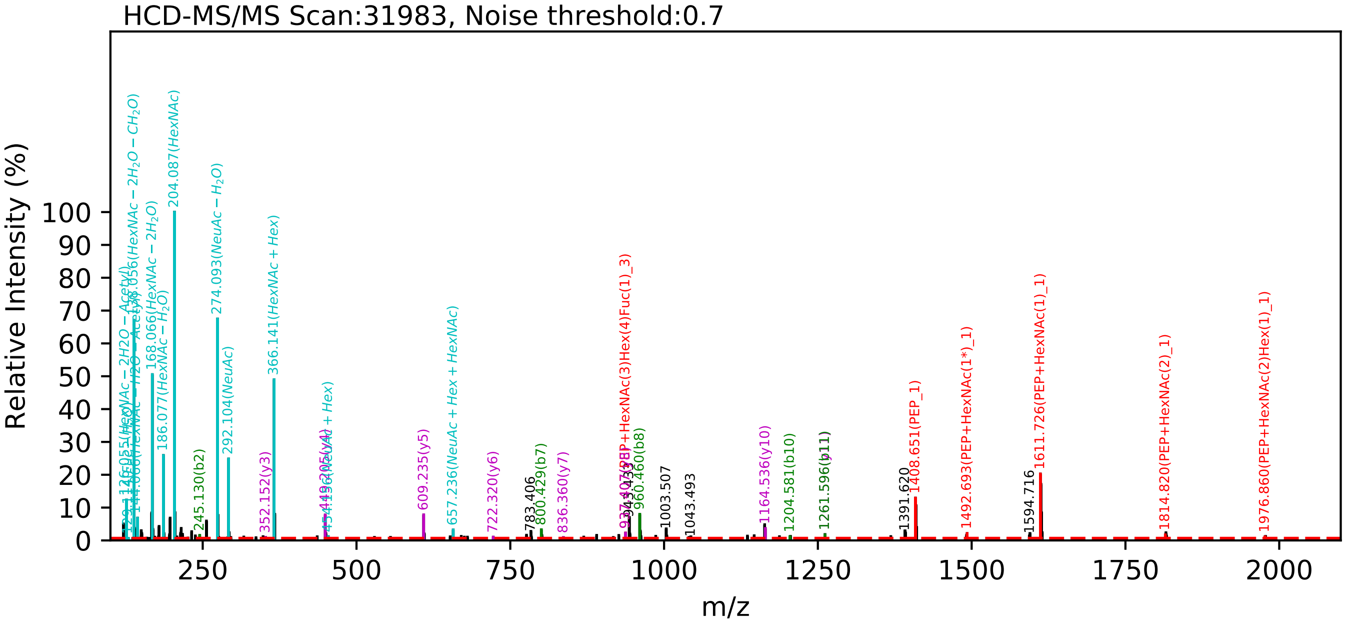

FPNITNLCPFGE(=PEP)_5_5_1_2_0, 0_None, 0_None,
m/z:1321.53(4+), RT:85.45, hcd-score:83.64

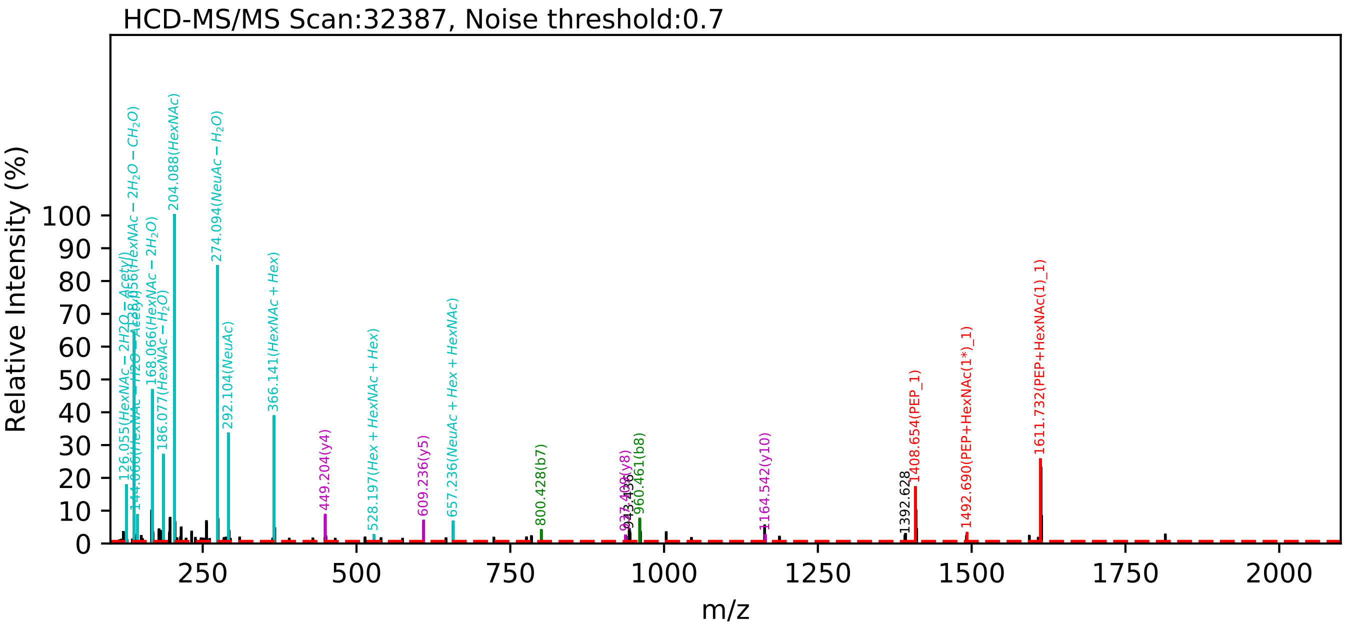

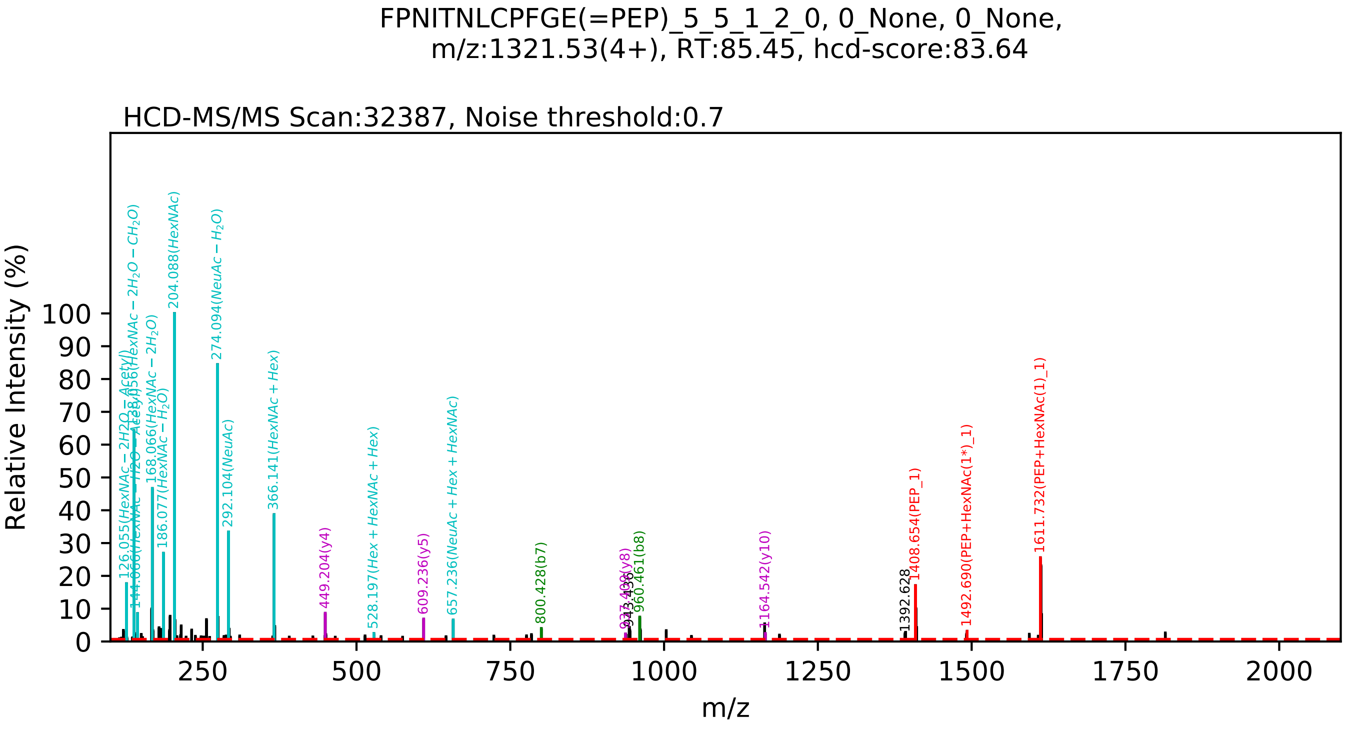
FPNITNLCPFGE(=PEP)_5_5_1_2_0, 0_None, 0_None,
m/z:1321.53(4+), RT:85.45, hcd-score:83.64
HCD-MS/MS Scan:32387, Noise threshold:0.7
Relative Intensity (%)
m/z
204.088(HexNAc)
274.094(NeuAc − H2O)
292.104(NeuAc)
366.141(HexNAc + Hex)
168.066(HexNAc − 2H2O)
186.077(HexNAc − H2O)
126.055(HexNAc − 2H2O − Acetyl)
449.204(y4)
528.197(Hex + HexNAc + Hex)
609.236(y5)
657.236(NeuAc + Hex + HexNAc)
800.428(b7)
960.461(b8)
1164.542(y10)
1392.628
1408.654(PEP_1)
1492.690(PEP+HexNAc(1*)_1)
1611.732(PEP+HexNAc(1)_1)
0
10
20
30
40
50
60
70
80
90
100
250
500
750
1000
1250
1500
1750
2000

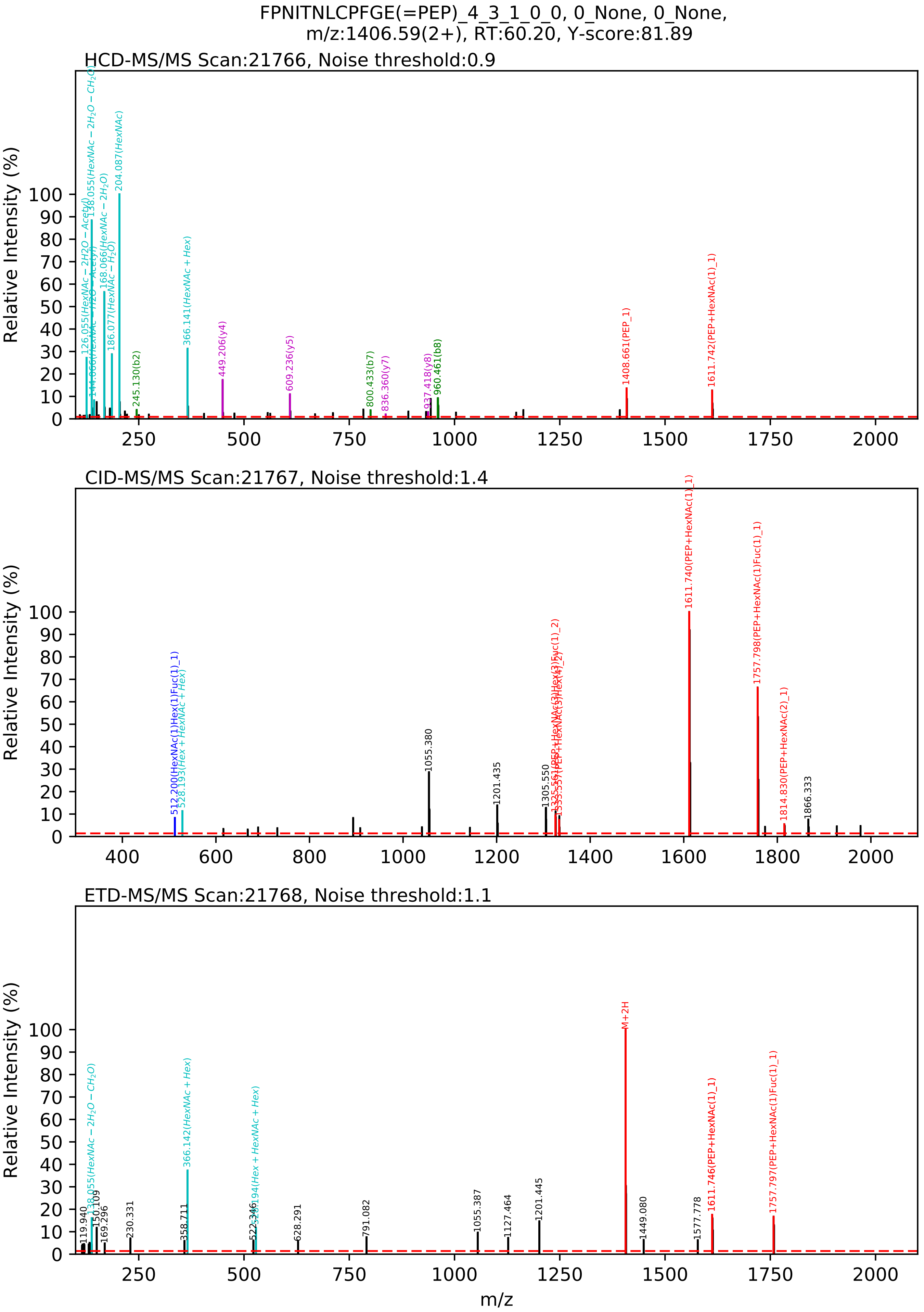

FPNITNLCPFGE(=PEP)_4_3_1_0_0, 0_None, 0_None,
m/z:1406.59(2+), RT:60.20, Y-score:81.89
HCD-MS/MS Scan:21766, Noise threshold:0.9
Relative Intensity (%)
126.055(HexNAc − 2H2O − Acetyl)
138.055(HexNAc − 2H2O − CH2O)
144.066(HexNAc − H2O − Acetyl)
168.066(HexNAc − 2H2O)
186.077(HexNAc − H2O)
204.087(HexNAc)
245.130(b2)
366.141(HexNAc + Hex)
449.206(y4)
609.236(y5)
800.433(b7)
836.360(y7)
937.418(y8)
960.461(b8)
1408.661(PEP_1)
1611.742(PEP+HexNAc(1)_1)
CID-MS/MS Scan:21767, Noise threshold:1.4
512.200(HexNAc(1)Hex(1)Fuc(1)_1)
528.193(Hex + HexNAc + Hex)
1055.380
1201.435
1305.550
1325.561(PEP+HexNAc(3)Hex(3)Fuc(1)_2)
1333.557(PEP+HexNAc(3)Hex(4)_2)
1611.740(PEP+HexNAc(1)_1)
1757.798(PEP+HexNAc(1)Fuc(1)_1)
1814.830(PEP+HexNAc(2)_1)
1866.333
ETD-MS/MS Scan:21768, Noise threshold:1.1
119.940
138.055(HexNAc − 2H2O − CH2O)
150.109
169.296
230.331
358.711
366.142(HexNAc + Hex)
522.346
528.194(Hex + HexNAc + Hex)
628.291
791.082
1055.387
1127.464
1201.445
M+2H
1449.080
1577.778
1611.746(PEP+HexNAc(1)_1)
1757.797(PEP+HexNAc(1)Fuc(1)_1)
m/z

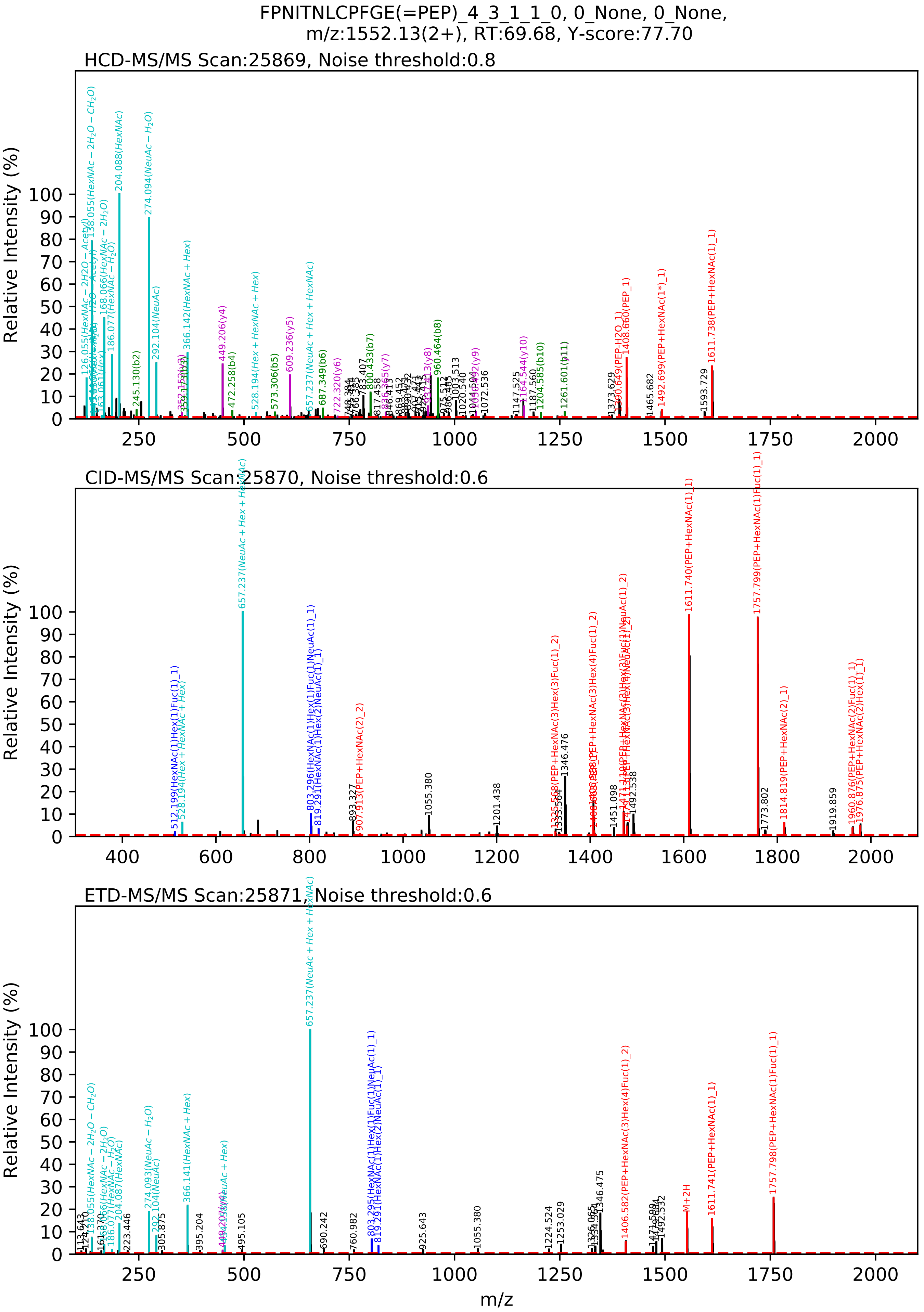
FPNITNLCPFGE(=PEP)_4_3_1_1_0, 0_None, 0_None,
m/z:1552.13(2+), RT:69.68, Y-score:77.70
HCD-MS/MS Scan:25869, Noise threshold:0.8
Relative Intensity (%)
204.088(HexNAc)
274.094(NeuAc − H2O)
292.104(NeuAc)
366.142(HexNAc + Hex)
449.206(y4)
609.236(y5)
657.237(NeuAc + Hex + HexNAc)
1408.660(PEP_1)
1492.699(PEP+HexNAc(1*)_1)
1611.738(PEP+HexNAc(1)_1)
CID-MS/MS Scan:25870, Noise threshold:0.6
512.199(HexNAc(1)Hex(1)Fuc(1)_1)
803.296(HexNAc(1)Hex(1)Fuc(1)NeuAc(1)_1)
907.913(PEP+HexNAc(2)_2)
1055.380
1201.438
1346.476
1611.740(PEP+HexNAc(1)_1)
1757.799(PEP+HexNAc(1)Fuc(1)_1)
1814.819(PEP+HexNAc(2)_1)
1919.859
ETD-MS/MS Scan:25871, Noise threshold:0.6
657.237(NeuAc + Hex + HexNAc)
803.295(HexNAc(1)Hex(1)Fuc(1)NeuAc(1)_1)
1406.582(PEP+HexNAc(3)Hex(4)Fuc(1)_2)
M+2H
1611.741(PEP+HexNAc(1)_1)
1757.798(PEP+HexNAc(1)Fuc(1)_1)
m/z

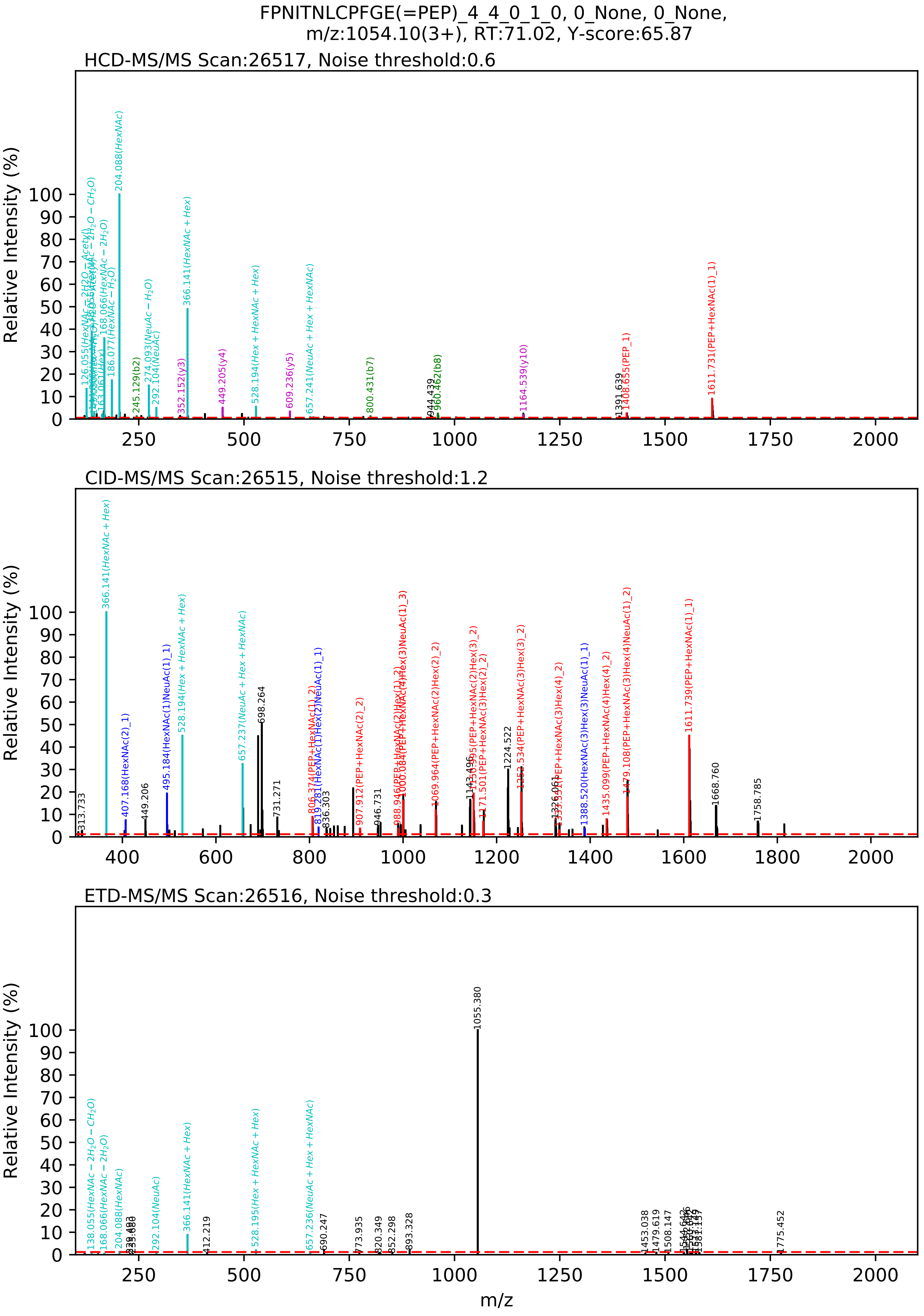

FPNITNLCPFGE(=PEP)_4_4_0_1_0, 0_None, 0_None,
m/z:1054.10(3+), RT:71.02, Y-score:65.87
HCD-MS/MS Scan:26517, Noise threshold:0.6
Relative Intensity (%)
204.088(HexNAc)
366.141(HexNAc + Hex)
168.066(HexNAc − 2H2O)
186.077(HexNAc − H2O)
163.061(Hex)
126.055
245.129(b2)
274.093(NeuAc − H2O)
292.104(NeuAc)
352.152(y3)
449.205(y4)
528.194(Hex + HexNAc + Hex)
609.236(y5)
657.241(NeuAc + Hex + HexNAc)
800.431(b7)
944.439
960.462(b8)
1164.539(y10)
1391.639
1408.655(PEP_1)
1611.731(PEP+HexNAc(1)_1)
CID-MS/MS Scan:26515, Noise threshold:1.2
313.733
366.141(HexNAc + Hex)
407.168(HexNAc(2)_1)
449.206
495.184(HexNAc(1)NeuAc(1)_1)
528.194(Hex + HexNAc + Hex)
657.237(NeuAc + Hex + HexNAc)
698.264
731.271
806.374(PEP+HexNAc(1)_2)
819.281(HexNAc(1)Hex(2)NeuAc(1)_1)
836.303
907.912(PEP+HexNAc(2)_2)
946.731
1000.084(PEP+HexNAc(4)Hex(3)NeuAc(1)_3)
1069.964(PEP+HexNAc(2)Hex(2)_2)
1143.496
1171.501(PEP+HexNAc(3)Hex(2)_2)
1224.522
1252.534(PEP+HexNAc(3)Hex(3)_2)
1326.061
1333.552(PEP+HexNAc(3)Hex(4)_2)
1388.520(HexNAc(3)Hex(3)NeuAc(1)_1)
1435.099(PEP+HexNAc(4)Hex(4)_2)
1479.108(PEP+HexNAc(3)Hex(4)NeuAc(1)_2)
1611.739(PEP+HexNAc(1)_1)
1668.760
1758.785
ETD-MS/MS Scan:26516, Noise threshold:0.3
1055.380
138.055(HexNAc − 2H2O − CH2O)
168.066(HexNAc − 2H2O)
204.088(HexNAc)
292.104(NeuAc)
366.141(HexNAc + Hex)
412.219
528.195(Hex + HexNAc + Hex)
657.236(NeuAc + Hex + HexNAc)
690.247
773.935
820.349
852.298
893.328
1453.038
1479.619
1508.147
1775.452
m/z

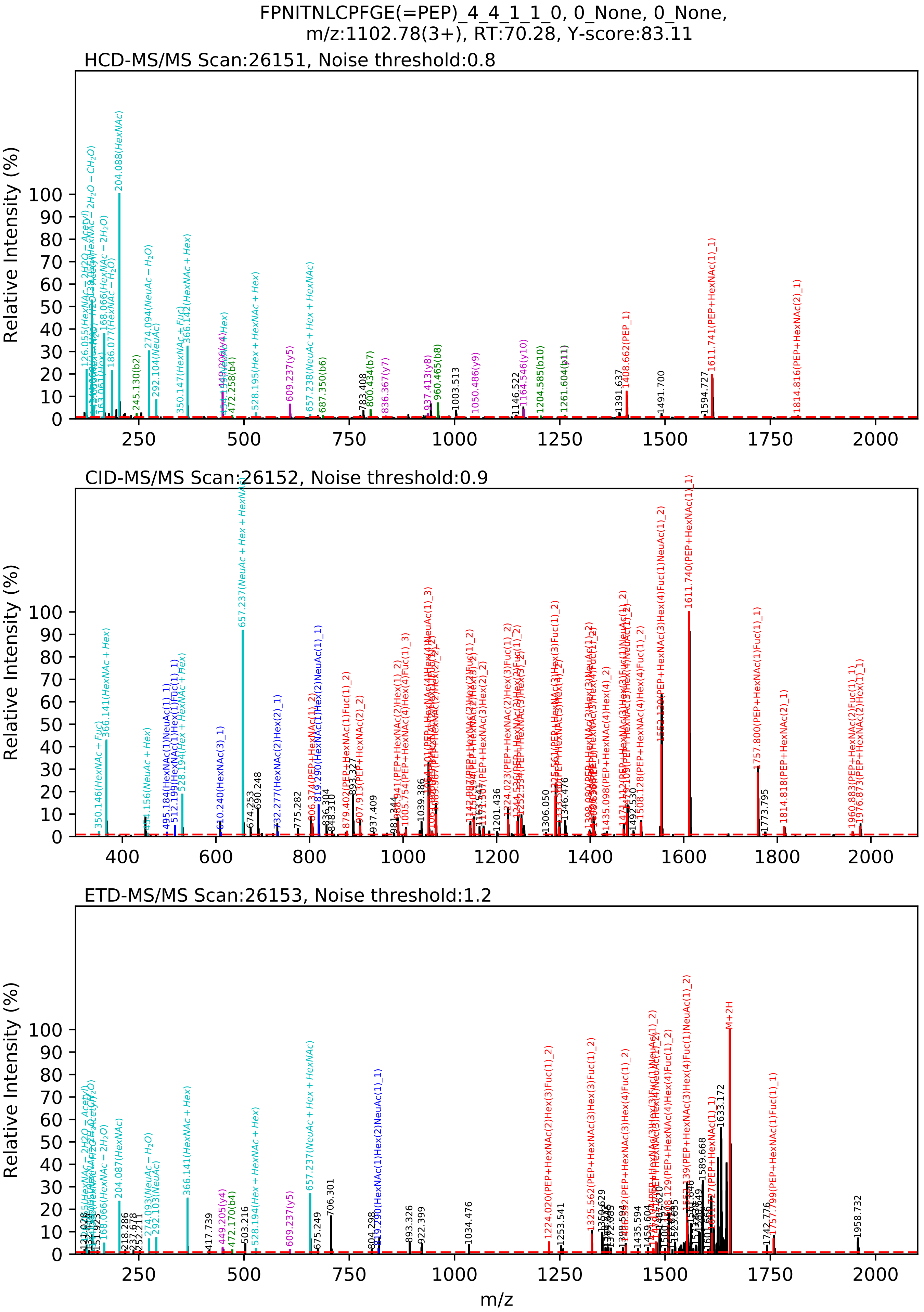
FPNITNLCPFGE(=PEP)_4_4_1_1_0, 0_None, 0_None,
m/z:1102.78(3+), RT:70.28, Y-score:83.11
HCD-MS/MS Scan:26151, Noise threshold:0.8
CID-MS/MS Scan:26152, Noise threshold:0.9
ETD-MS/MS Scan:26153, Noise threshold:1.2
Relative Intensity (%)
m/z

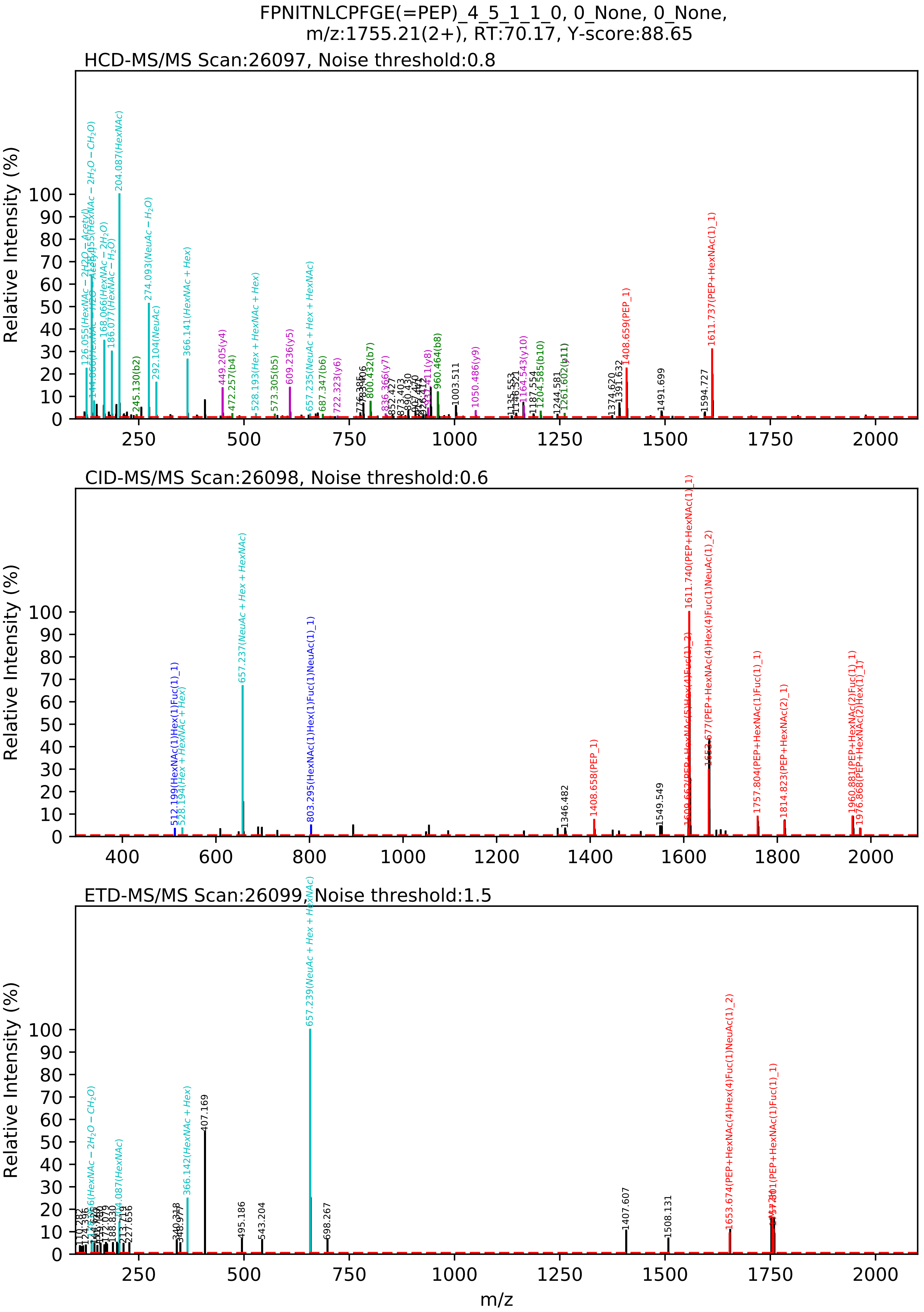

FPNITNLCPFGE(=PEP)_4_5_1_1_0, 0_None, 0_None,
m/z:1755.21(2+), RT:70.17, Y-score:88.65
HCD-MS/MS Scan:26097, Noise threshold:0.8
CID-MS/MS Scan:26098, Noise threshold:0.6
ETD-MS/MS Scan:26099, Noise threshold:1.5
Relative Intensity (%)
m/z

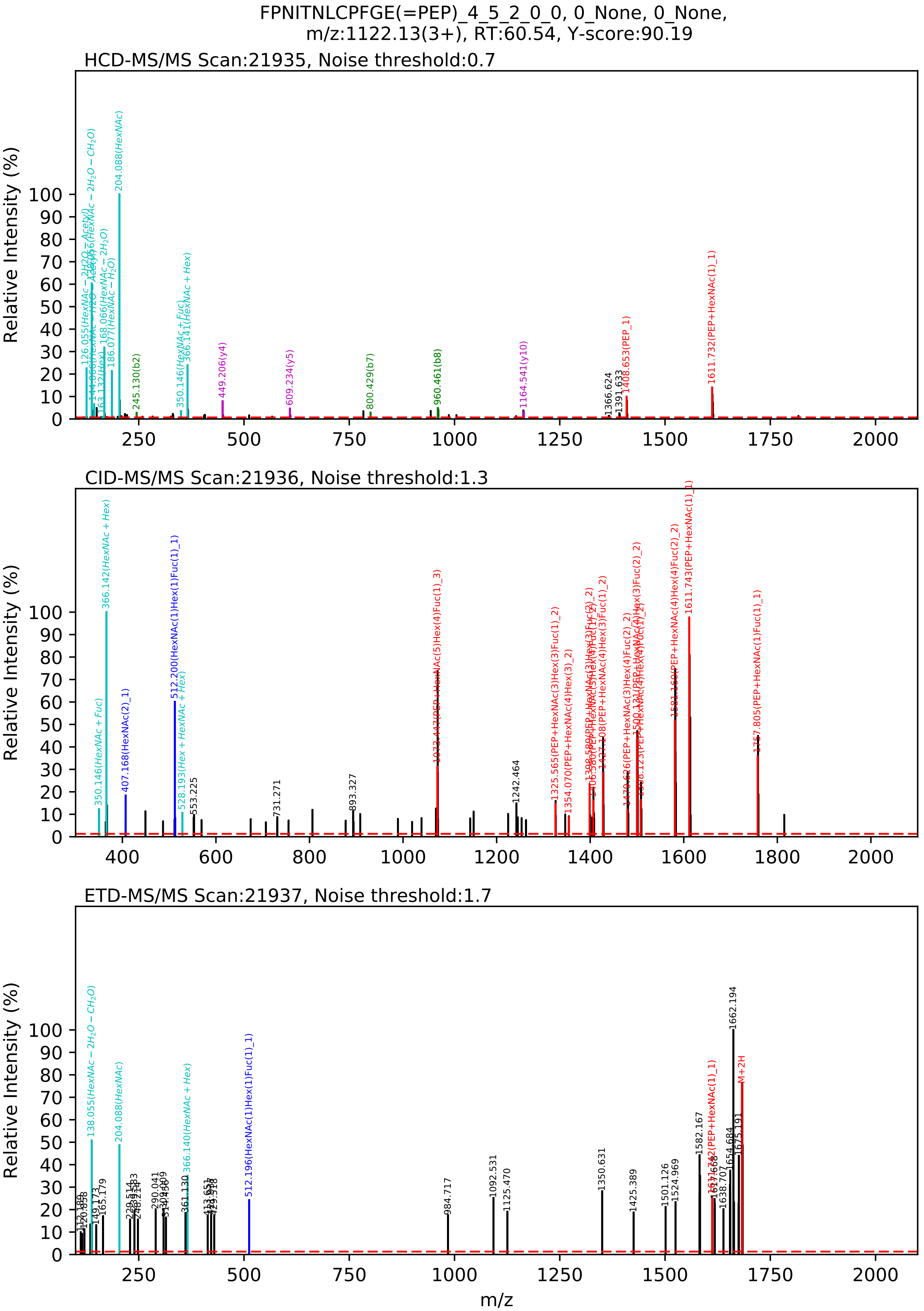

FPNITNLCPFGE(=PEP)_4_5_2_0_0, 0_None, 0_None,
m/z:1122.13(3+), RT:60.54, Y-score:90.19
HCD-MS/MS Scan:21935, Noise threshold:0.7
Relative Intensity (%)
204.088(HexNAc)
126.055(HexNAc − 2H2O − Acetyl)
168.066(HexNAc − 2H2O)
186.077(HexNAc − H2O)
245.130(b2)
350.146(HexNAc+Fuc)
366.141(HexNAc + Hex)
449.206(y4)
609.234(y5)
800.429(b7)
960.461(b8)
1164.541(y10)
1366.624
1391.633
1408.653(PEP_1)
1611.732(PEP+HexNAc(1)_1)
CID-MS/MS Scan:21936, Noise threshold:1.3
366.142(HexNAc + Hex)
350.146(HexNAc + Fuc)
407.168(HexNAc(2)_1)
512.200(HexNAc(1)Hex(1)Fuc(1)_1)
528.193(Hex + HexNAc + Hex)
553.225
731.271
893.327
1073.447(PEP+HexNAc(5)Hex(4)Fuc(1)_3)
1242.464
1325.565(PEP+HexNAc(3)Hex(3)Fuc(1)_2)
1354.070(PEP+HexNAc(4)Hex(3)_2)
1427.108(PEP+HexNAc(4)Hex(3)Fuc(1)_2)
1479.626(PEP+HexNAc(3)Hex(4)Fuc(2)_2)
1581.160(PEP+HexNAc(4)Hex(4)Fuc(2)_2)
1611.743(PEP+HexNAc(1)_1)
1757.805(PEP+HexNAc(1)Fuc(1)_1)
ETD-MS/MS Scan:21937, Noise threshold:1.7
138.055(HexNAc − 2H2O − CH2O)
204.088(HexNAc)
366.140(HexNAc + Hex)
512.196(HexNAc(1)Hex(1)Fuc(1)_1)
149.173
165.179
228.514
248.214
290.041
361.130
984.717
1092.531
1125.470
1350.631
1425.389
1501.126
1524.969
1582.167
1638.707
1654.684
1662.194
1675.191
M+2H
m/z

FPNITNLCPFGE(=PEP)_5_2_0_0_0, 0_None, 1_Hex_Phosphorylation,
m/z:1353.03(2+), RT:74.83, Y-score:71.56

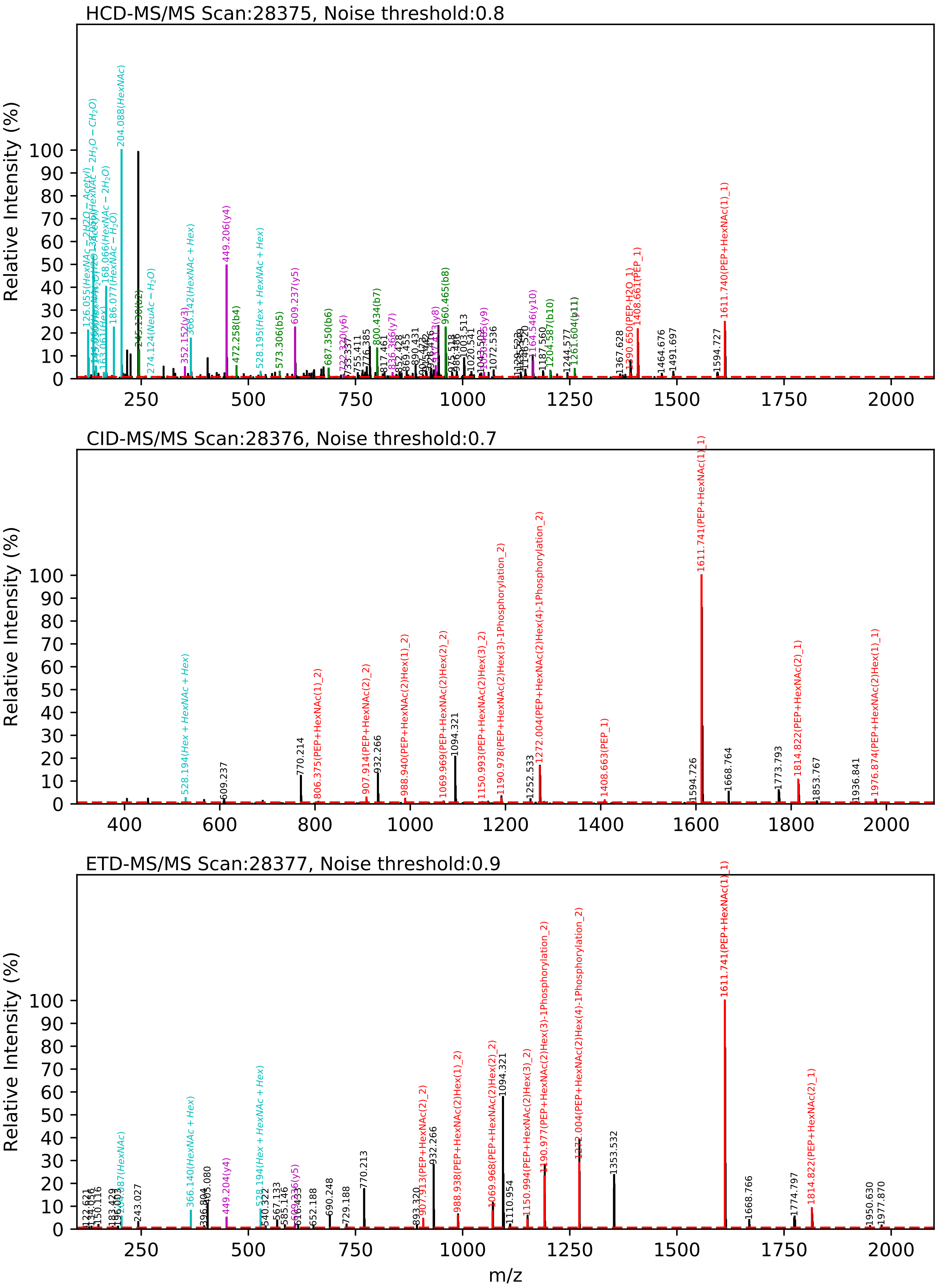

FPNITNLCPFGE(=PEP)_5_3_1_1_0, 0_None, 0_None,
m/z:1089.11(3+), RT:70.38, Y-score:83.19

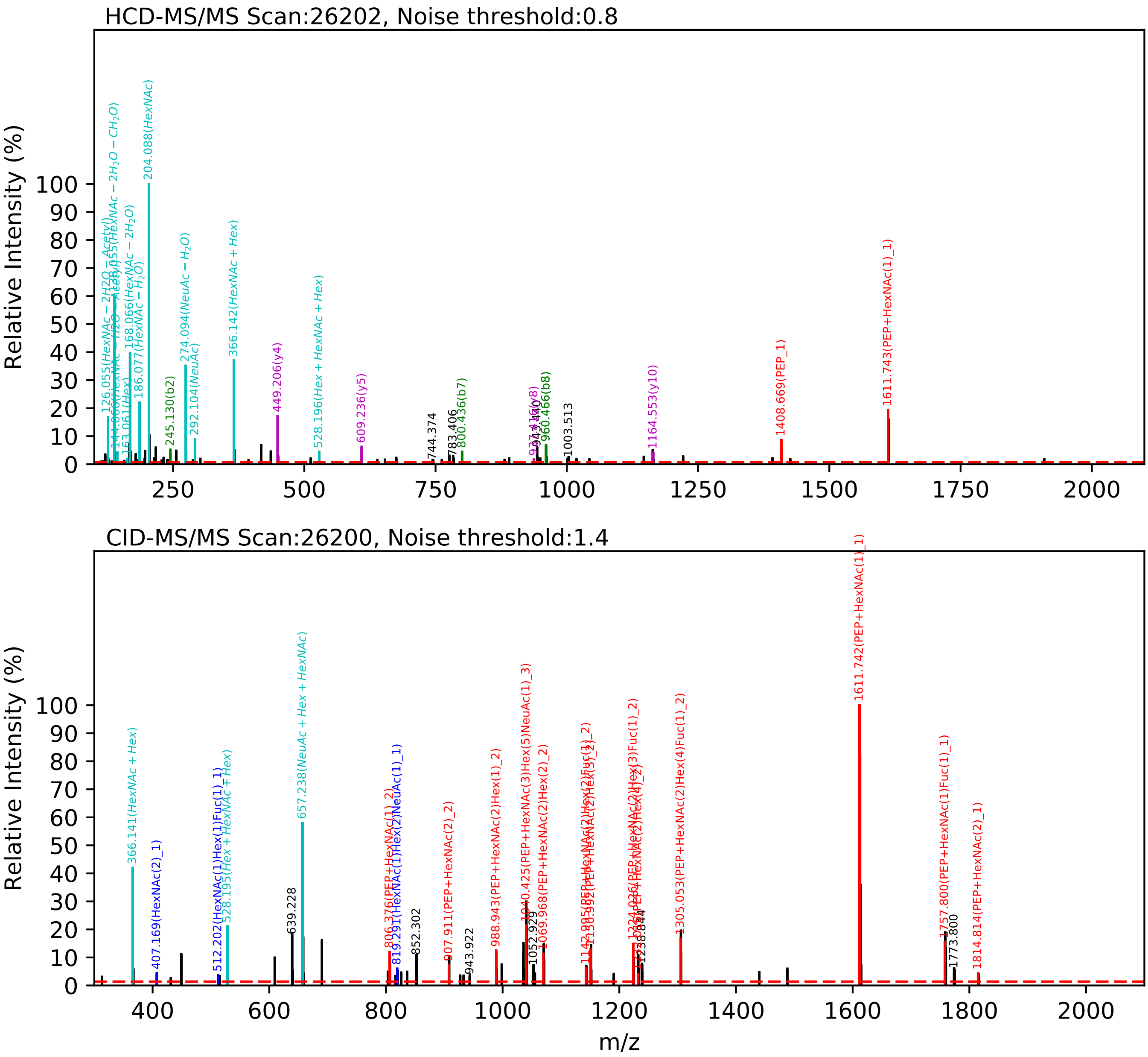

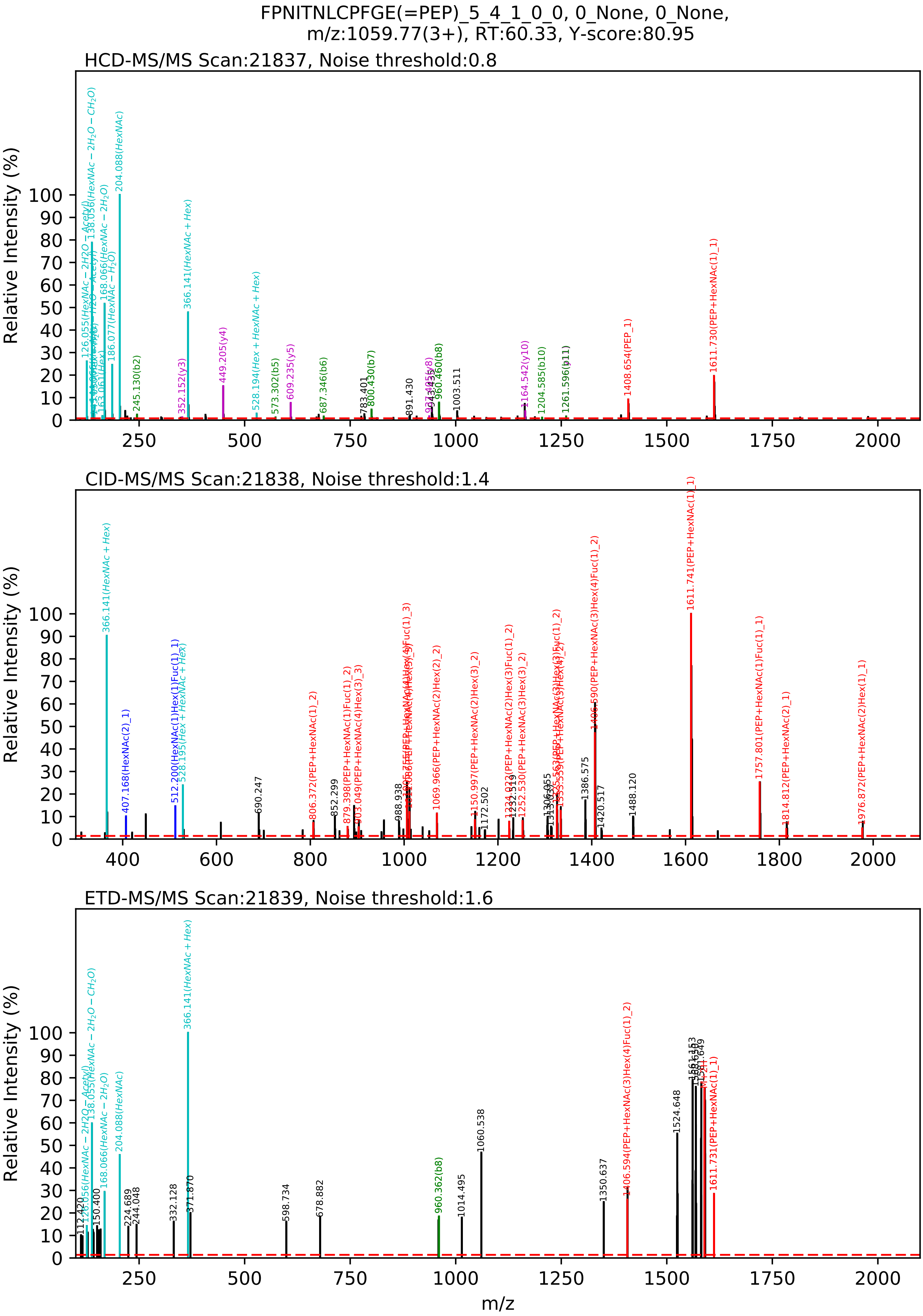

FPNITNLCPFGE(=PEP)_5_4_1_0_0, 0_None, 0_None,
m/z:1059.77(3+), RT:60.33, Y-score:80.95
HCD-MS/MS Scan:21837, Noise threshold:0.8
Relative Intensity (%)
204.088(HexNAc)
366.141(HexNAc + Hex)
168.066(HexNAc − 2H2O)
186.077(HexNAc − H2O)
126.055(HexNAc − 2H2O − Acetyl)
138.056(HexNAc − 2H2O − CH2O)
163.061(Hex)
245.130(b2)
352.152(y3)
449.205(y4)
528.194(Hex + HexNAc + Hex)
573.302(b5)
609.235(y5)
687.346(b6)
783.401
800.430(b7)
891.430
960.460(b8)
1003.511
1164.542(y10)
1204.585(b10)
1261.596(b11)
1408.654(PEP_1)
1611.730(PEP+HexNAc(1)_1)
CID-MS/MS Scan:21838, Noise threshold:1.4
366.141(HexNAc + Hex)
407.168(HexNAc(2)_1)
512.200(HexNAc(1)Hex(1)Fuc(1)_1)
528.195(Hex + HexNAc + Hex)
690.247
806.372(PEP+HexNAc(1)_2)
852.299
879.398(PEP+HexNAc(1)Fuc(1)_2)
903.049(PEP+HexNAc(4)Hex(3)_3)
988.938
1069.966(PEP+HexNAc(2)Hex(2)_2)
1150.997(PEP+HexNAc(2)Hex(3)_2)
1172.502
1223.923(PEP+HexNAc(2)Hex(3)Fuc(1)_2)
1252.530(PEP+HexNAc(3)Hex(3)_2)
1386.575
1406.590(PEP+HexNAc(3)Hex(4)Fuc(1)_2)
1420.517
1488.120
1611.741(PEP+HexNAc(1)_1)
1757.801(PEP+HexNAc(1)Fuc(1)_1)
1814.812(PEP+HexNAc(2)_1)
1976.872(PEP+HexNAc(2)Hex(1)_1)
ETD-MS/MS Scan:21839, Noise threshold:1.6
112.420
126.056(HexNAc − 2H2O − Acetyl)
138.055(HexNAc − 2H2O − CH2O)
150.400
168.066(HexNAc − 2H2O)
204.088(HexNAc)
224.689
244.048
332.128
366.141(HexNAc + Hex)
371.870
598.734
678.882
960.362(b8)
1014.495
1060.538
1350.637
1406.594(PEP+HexNAc(3)Hex(4)Fuc(1)_2)
1524.648
1561.153
1581.649
1611.731(PEP+HexNAc(1)_1)
m/z

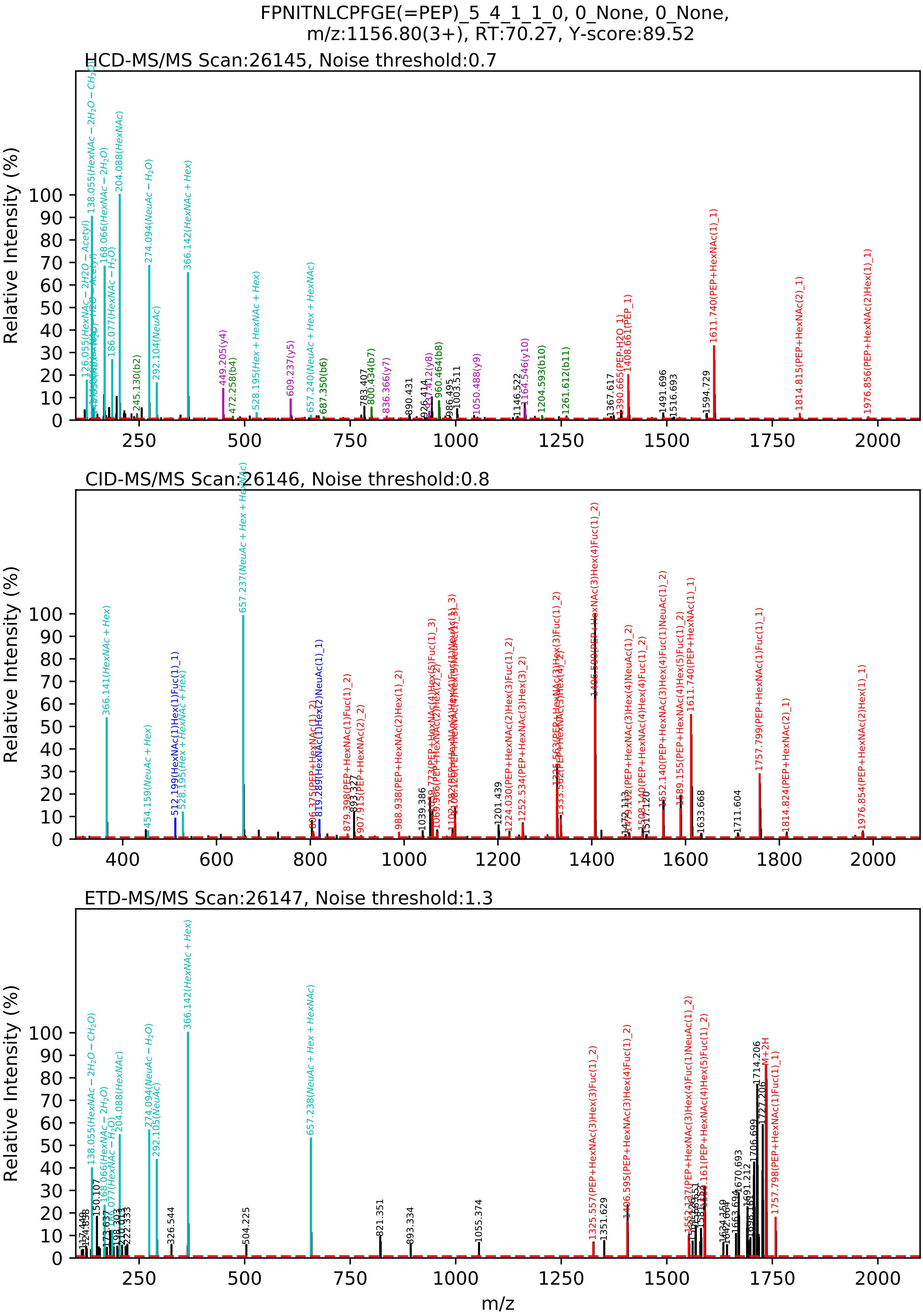
FPNITNLCPFGE(=PEP)_5_4_1_1_0, 0_None, 0_None,
m/z:1156.80(3+), RT:70.27, Y-score:89.52
HCD-MS/MS Scan:26145, Noise threshold:0.7
Relative Intensity (%)
138.055(HexNAc − 2H2O − CH2O)
204.088(HexNAc)
168.066(HexNAc − 2H2O)
186.077(HexNAc − H2O)
274.094(NeuAc − H2O)
292.104(NeuAc)
366.142(HexNAc + Hex)
245.130(b2)
449.205(y4)
472.258(b4)
528.195(Hex + HexNAc + Hex)
609.237(y5)
657.240(NeuAc + Hex + HexNAc)
687.350(b6)
783.407
800.434(b7)
836.366(y7)
890.431
960.464(b8)
1003.511
1050.488(y9)
1146.522
1164.546(y10)
1204.593(b10)
1261.612(b11)
1367.617
1408.661(PEP_1)
1491.696
1516.693
1594.729
1611.740(PEP+HexNAc(1)_1)
1814.815(PEP+HexNAc(2)_1)
1976.856(PEP+HexNAc(2)Hex(1)_1)
CID-MS/MS Scan:26146, Noise threshold:0.8
366.141(HexNAc + Hex)
454.159(NeuAc + Hex)
512.199(HexNAc(1)Hex(1)Fuc(1)_1)
528.195(Hex + HexNAc + Hex)
657.237(NeuAc + Hex + HexNAc)
819.289(HexNAc(1)Hex(2)NeuAc(1)_1)
879.398(PEP+HexNAc(1)Fuc(1)_2)
907.915(PEP+HexNAc(2)_2)
988.938(PEP+HexNAc(2)Hex(1)_2)
1039.386
1201.439
1224.030(PEP+HexNAc(2)Hex(3)Fuc(1)_2)
1252.534(PEP+HexNAc(3)Hex(3)_2)
1406.590(PEP+HexNAc(3)Hex(4)Fuc(1)_2)
1552.140(PEP+HexNAc(3)Hex(4)Fuc(1)NeuAc(1)_2)
1589.155(PEP+HexNAc(4)Hex(5)Fuc(1)_2)
1611.740(PEP+HexNAc(1)_1)
1633.668
1711.604
1757.799(PEP+HexNAc(1)Fuc(1)_1)
1814.824(PEP+HexNAc(2)_1)
1976.854(PEP+HexNAc(2)Hex(1)_1)
ETD-MS/MS Scan:26147, Noise threshold:1.3
138.055(HexNAc − 2H2O − CH2O)
168.066(HexNAc − 2H2O)
204.088(HexNAc)
274.094(NeuAc − H2O)
292.105(NeuAc)
366.142(HexNAc + Hex)
150.107
326.544
504.225
657.238(NeuAc + Hex + HexNAc)
821.351
893.334
1055.374
1325.557(PEP+HexNAc(3)Hex(3)Fuc(1)_2)
1351.629
1406.595(PEP+HexNAc(3)Hex(4)Fuc(1)_2)
1552.137(PEP+HexNAc(3)Hex(4)Fuc(1)NeuAc(1)_2)
1589.161(PEP+HexNAc(4)Hex(5)Fuc(1)_2)
1670.693
1691.212
1706.699
1714.206
1727.206
M+2H
1757.798(PEP+HexNAc(1)Fuc(1)_1)
m/z

FPNITNLCPFGE(=PEP)_5_4_1_1_0, 0_None, 0_None,
m/z:1734.70(2+), RT:68.75, Y-score:83.51

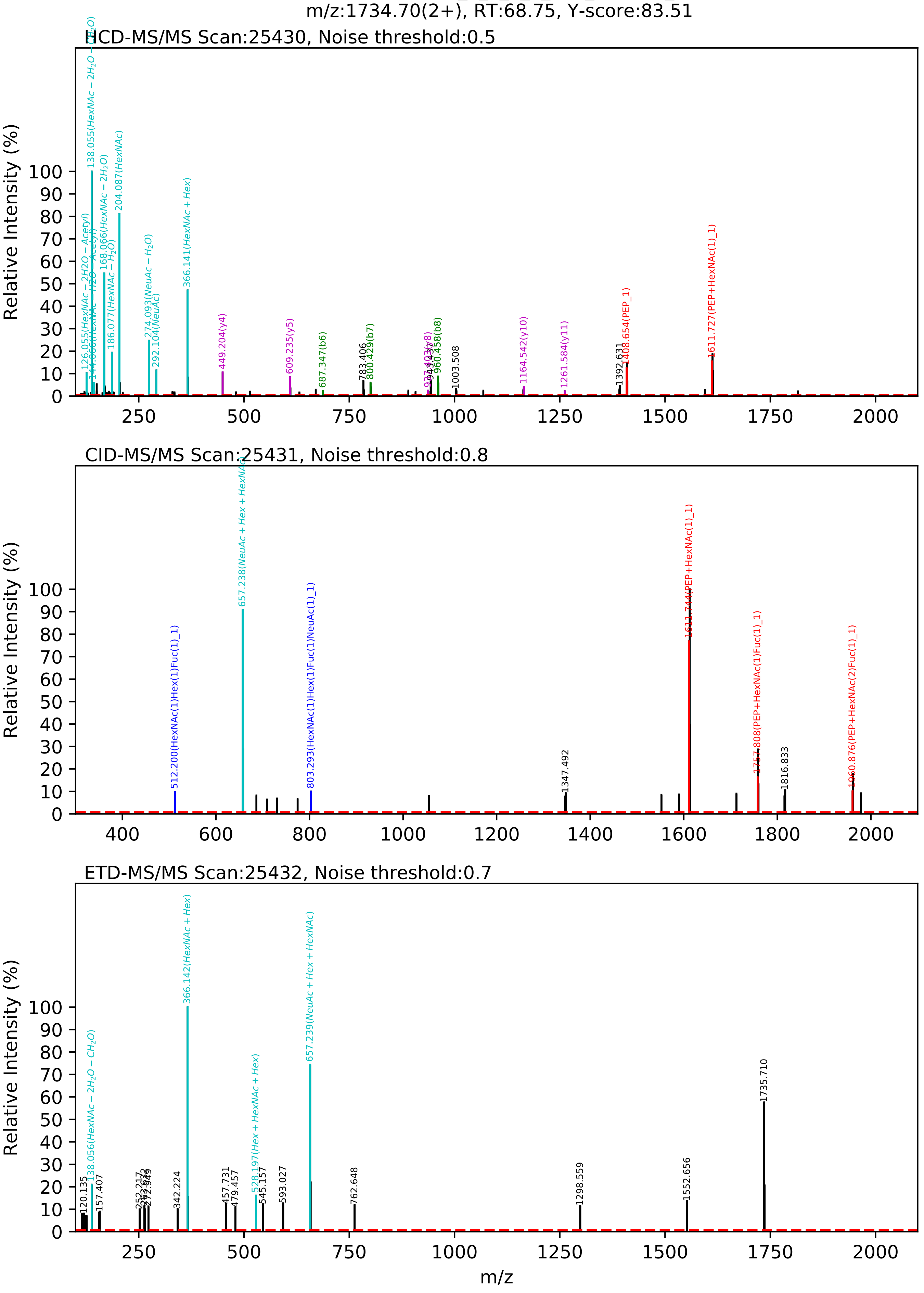

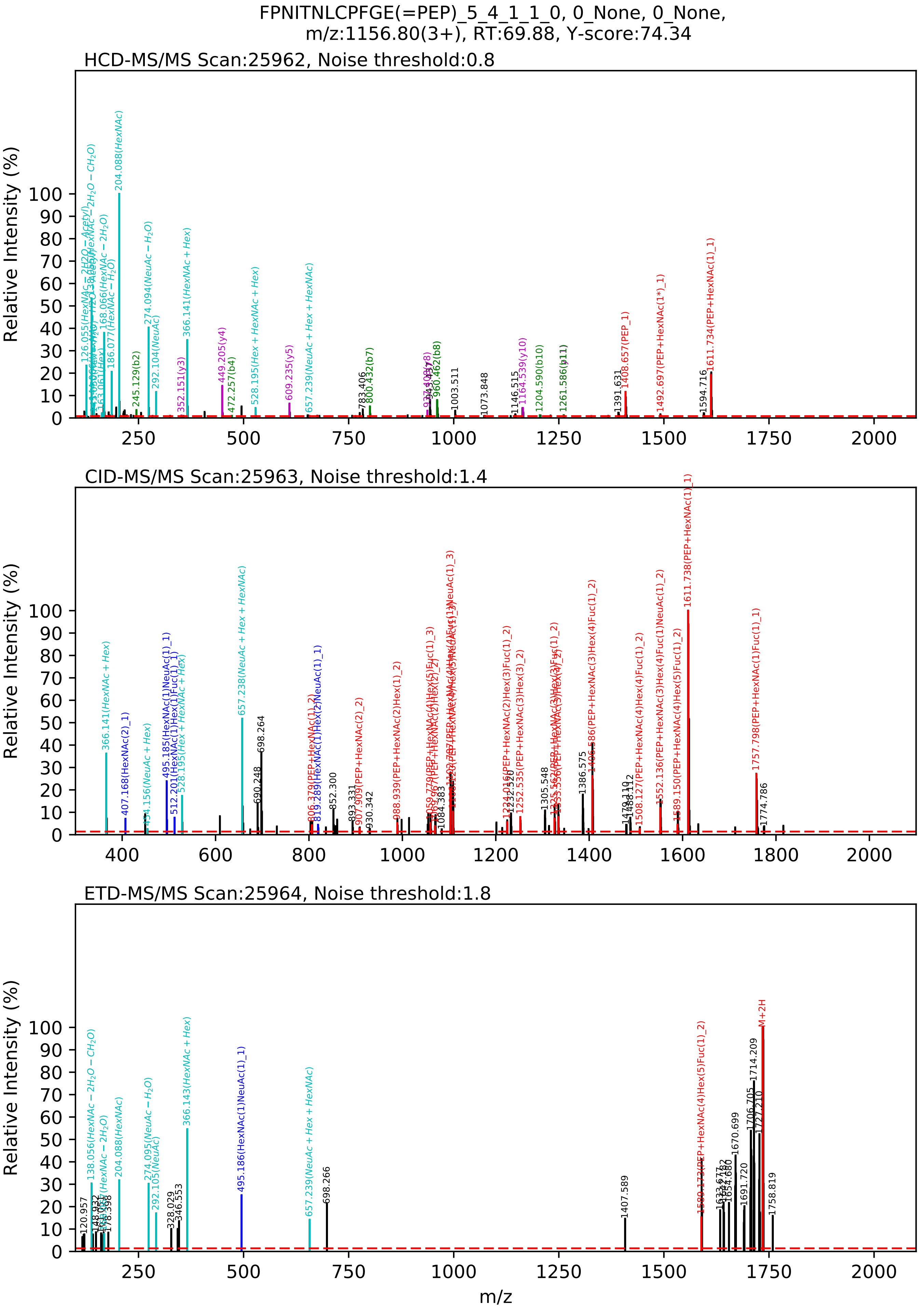

FPNITNLCPFGE(=PEP)_5_4_1_1_0, 0_None, 0_None,
m/z:1156.80(3+), RT:69.88, Y-score:74.34
HCD-MS/MS Scan:25962, Noise threshold:0.8
Relative Intensity (%)
204.088(HexNAc)
274.094(NeuAc − H2O)
292.104(NeuAc)
366.141(HexNAc + Hex)
245.129(b2)
352.151(y3)
449.205(y4)
472.257(b4)
528.195(Hex + HexNAc + Hex)
609.235(y5)
657.239(NeuAc + Hex + HexNAc)
783.406
800.432(b7)
960.462(b8)
1003.511
1073.848
1146.515
1164.539(y10)
1204.590(b10)
1261.586(b11)
1391.631
1408.657(PEP_1)
1492.697(PEP+HexNAc(1*)_1)
1594.716
1611.734(PEP+HexNAc(1)_1)
CID-MS/MS Scan:25963, Noise threshold:1.4
366.141(HexNAc + Hex)
407.168(HexNAc(2)_1)
454.156(NeuAc + Hex)
495.185(HexNAc(1)NeuAc(1)_1)
512.201(HexNAc(1)Hex(1)Fuc(1)_1)
528.195(Hex + HexNAc + Hex)
657.238(NeuAc + Hex + HexNAc)
690.248
698.264
819.289(HexNAc(1)Hex(2)NeuAc(1)_1)
852.300
893.331
907.909(PEP+HexNAc(2)_2)
930.342
988.939(PEP+HexNAc(2)Hex(1)_2)
1084.383
1224.016(PEP+HexNAc(2)Hex(3)Fuc(1)_2)
1252.535(PEP+HexNAc(3)Hex(3)_2)
1305.548
1386.575
1406.586(PEP+HexNAc(3)Hex(4)Fuc(1)_2)
1479.110
1508.127(PEP+HexNAc(4)Hex(4)Fuc(1)_2)
1552.136(PEP+HexNAc(3)Hex(4)Fuc(1)NeuAc(1)_2)
1589.150(PEP+HexNAc(4)Hex(5)Fuc(1)_2)
1611.738(PEP+HexNAc(1)_1)
1757.798(PEP+HexNAc(1)Fuc(1)_1)
1774.786
ETD-MS/MS Scan:25964, Noise threshold:1.8
120.957
138.056(HexNAc − 2H2O − CH2O)
148.932
178.398
204.088(HexNAc)
274.095(NeuAc − H2O)
292.105(NeuAc)
328.029
346.553
366.143(HexNAc + Hex)
495.186(HexNAc(1)NeuAc(1)_1)
657.239(NeuAc + Hex + HexNAc)
698.266
1407.589
1589.173(PEP+HexNAc(4)Hex(5)Fuc(1)_2)
1670.699
1691.720
1706.705
1714.209
1727.210
1758.819
M+2H
m/z

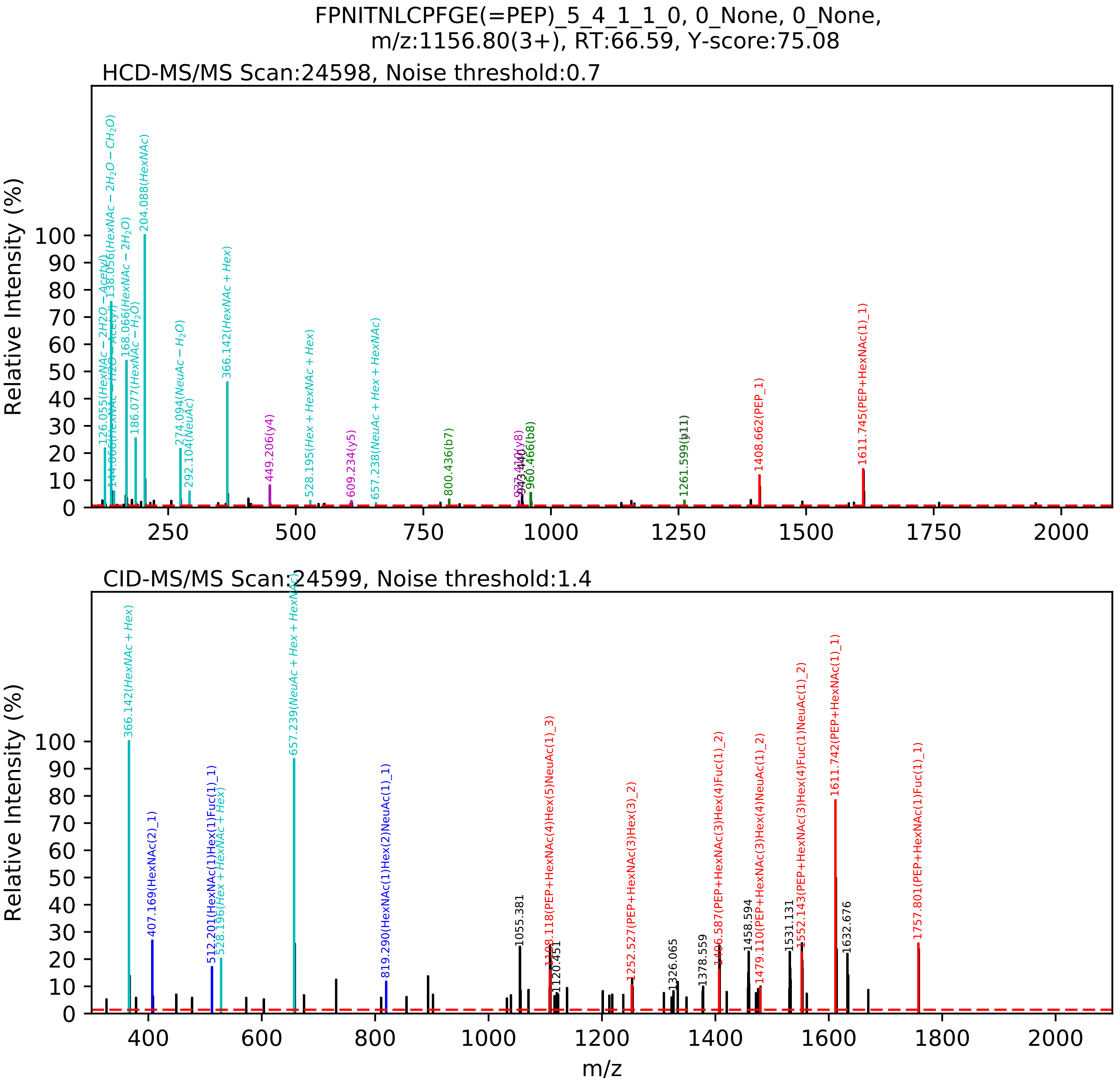
FPNITNLCPFGE(=PEP)_5_4_1_1_0, 0_None, 0_None,
m/z:1156.80(3+), RT:66.59, Y-score:75.08
HCD-MS/MS Scan:24598, Noise threshold:0.7
CID-MS/MS Scan:24599, Noise threshold:1.4
Relative Intensity (%)
m/z
204.088(HexNAc)
366.142(HexNAc + Hex)
168.066(HexNAc − 2H2O)
186.077(HexNAc − H2O)
274.094(NeuAc − H2O)
292.104(NeuAc)
449.206(y4)
528.195(Hex + HexNAc + Hex)
609.234(y5)
657.238(NeuAc + Hex + HexNAc)
800.436(b7)
960.466(b8)
1261.599(b11)
1408.662(PEP_1)
1611.745(PEP+HexNAc(1)_1)
657.239(NeuAc + Hex + HexNAc)
407.169(HexNAc(2)_1)
512.201(HexNAc(1)Hex(1)Fuc(1)_1)
528.196(Hex + HexNAc + Hex)
819.290(HexNAc(1)Hex(2)NeuAc(1)_1)
1055.381
1108.118(PEP+HexNAc(4)Hex(5)NeuAc(1)_3)
1120.451
1252.527(PEP+HexNAc(3)Hex(3)_2)
1326.065
1378.559
1406.587(PEP+HexNAc(3)Hex(4)Fuc(1)_2)
1458.594
1479.110(PEP+HexNAc(3)Hex(4)NeuAc(1)_2)
1531.131
1552.143(PEP+HexNAc(3)Hex(4)Fuc(1)NeuAc(1)_2)
1611.742(PEP+HexNAc(1)_1)
1632.676
1757.801(PEP+HexNAc(1)Fuc(1)_1)

FPNITNLCPFGE(=PEP)_5_4_1_1_0, 0_None, 0_None,
m/z:1156.80(3+), RT:68.74, Y-score:80.90

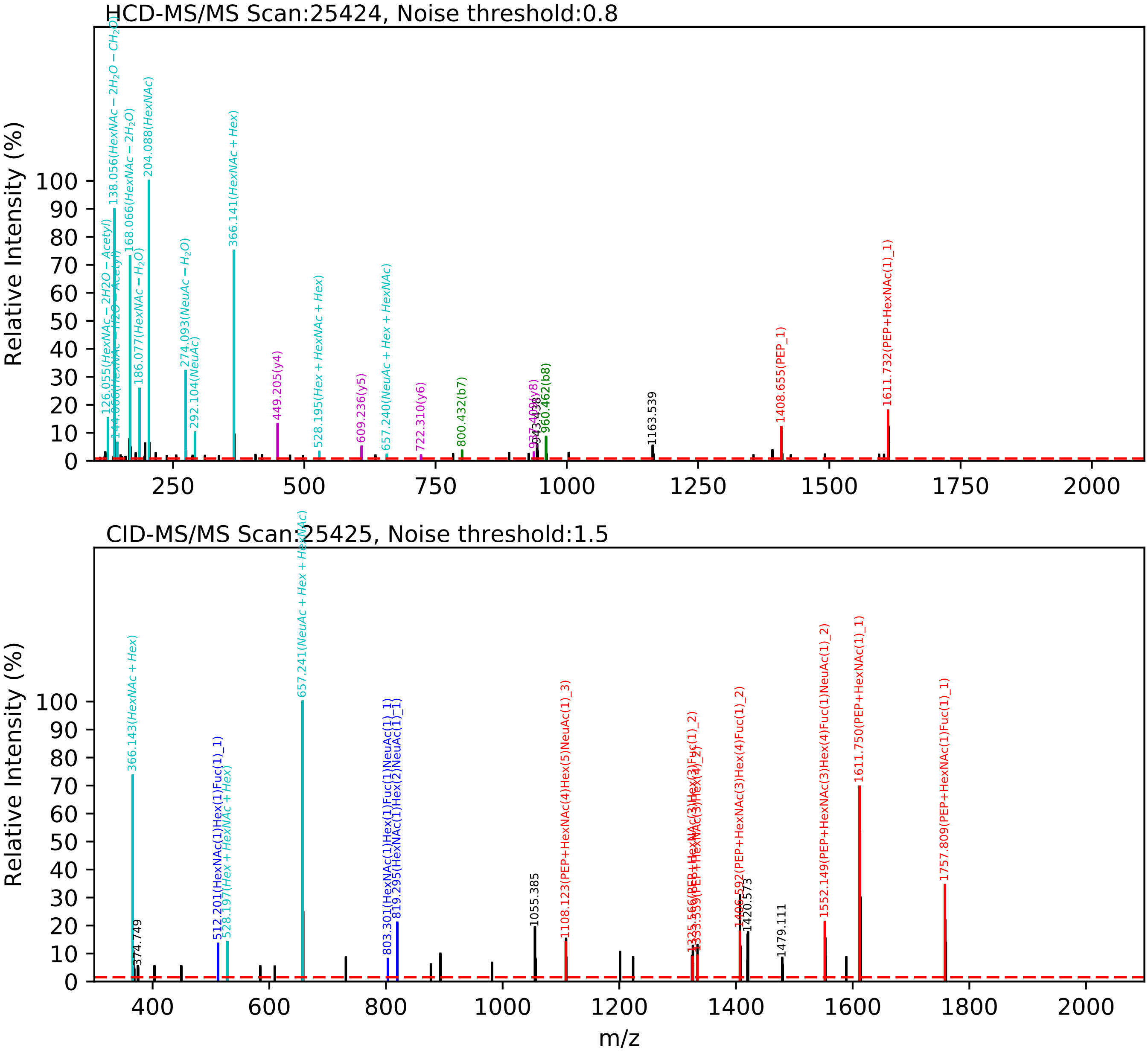

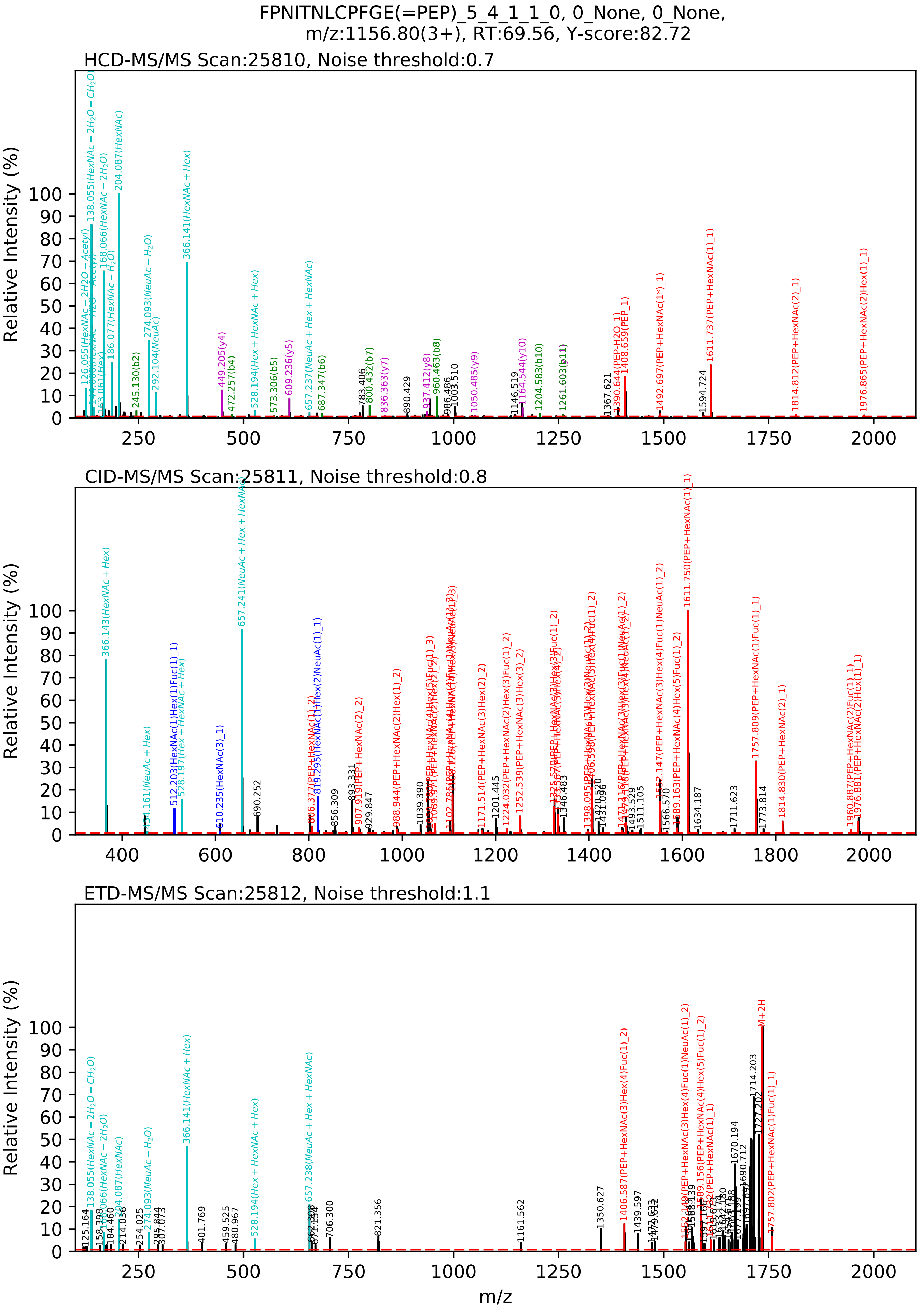
FPNITNLCPFGE(=PEP)_5_4_1_1_0, 0_None, 0_None,
m/z:1156.80(3+), RT:69.56, Y-score:82.72
HCD-MS/MS Scan:25810, Noise threshold:0.7
Relative Intensity (%)
CID-MS/MS Scan:25811, Noise threshold:0.8
Relative Intensity (%)
ETD-MS/MS Scan:25812, Noise threshold:1.1
Relative Intensity (%)
m/z

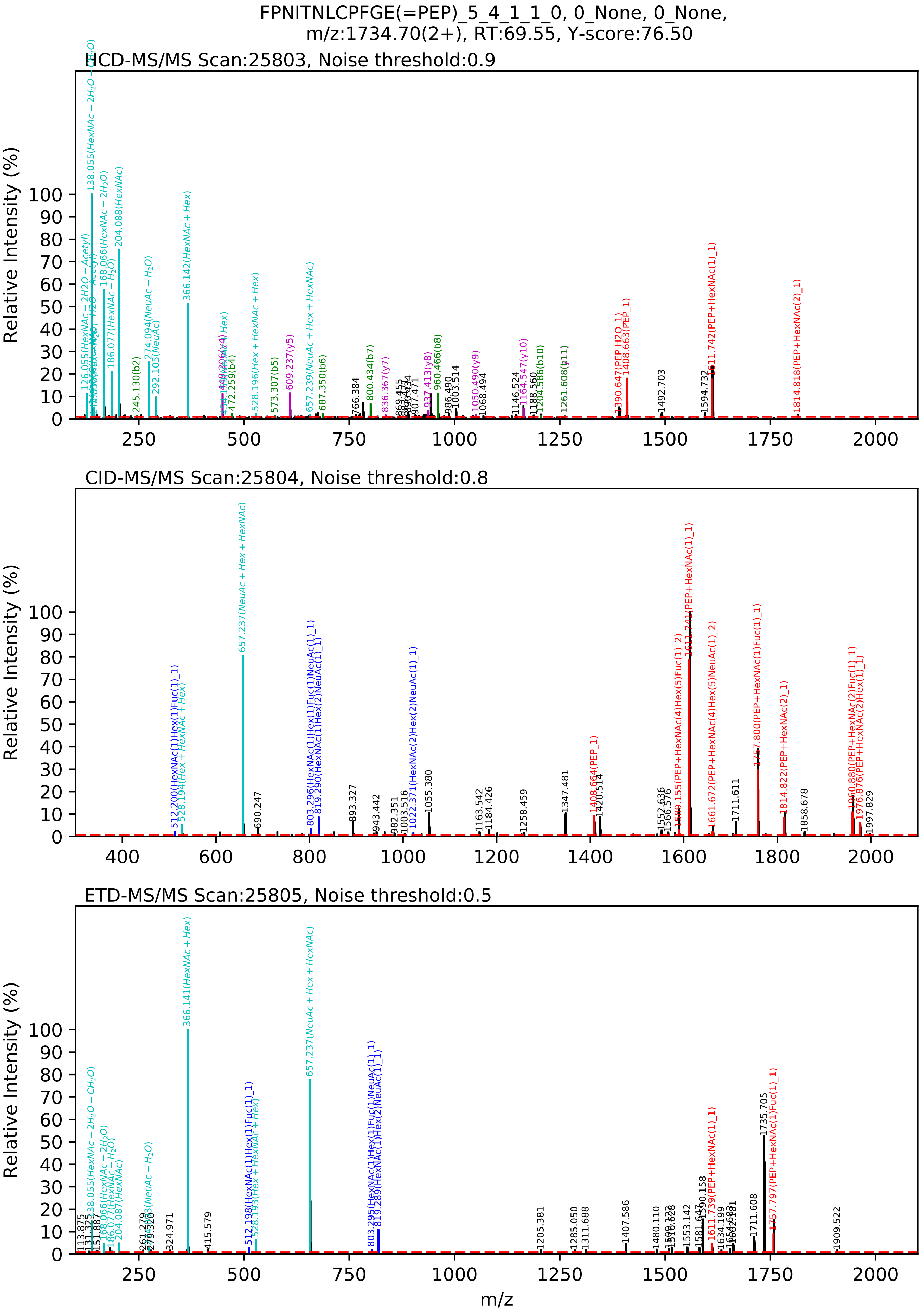

FPNITNLCPFGE(=PEP)_5_4_1_1_0, 0_None, 0_None,
m/z:1734.70(2+), RT:69.55, Y-score:76.50
HCD-MS/MS Scan:25803, Noise threshold:0.9
CID-MS/MS Scan:25804, Noise threshold:0.8
ETD-MS/MS Scan:25805, Noise threshold:0.5
Relative Intensity (%)
m/z

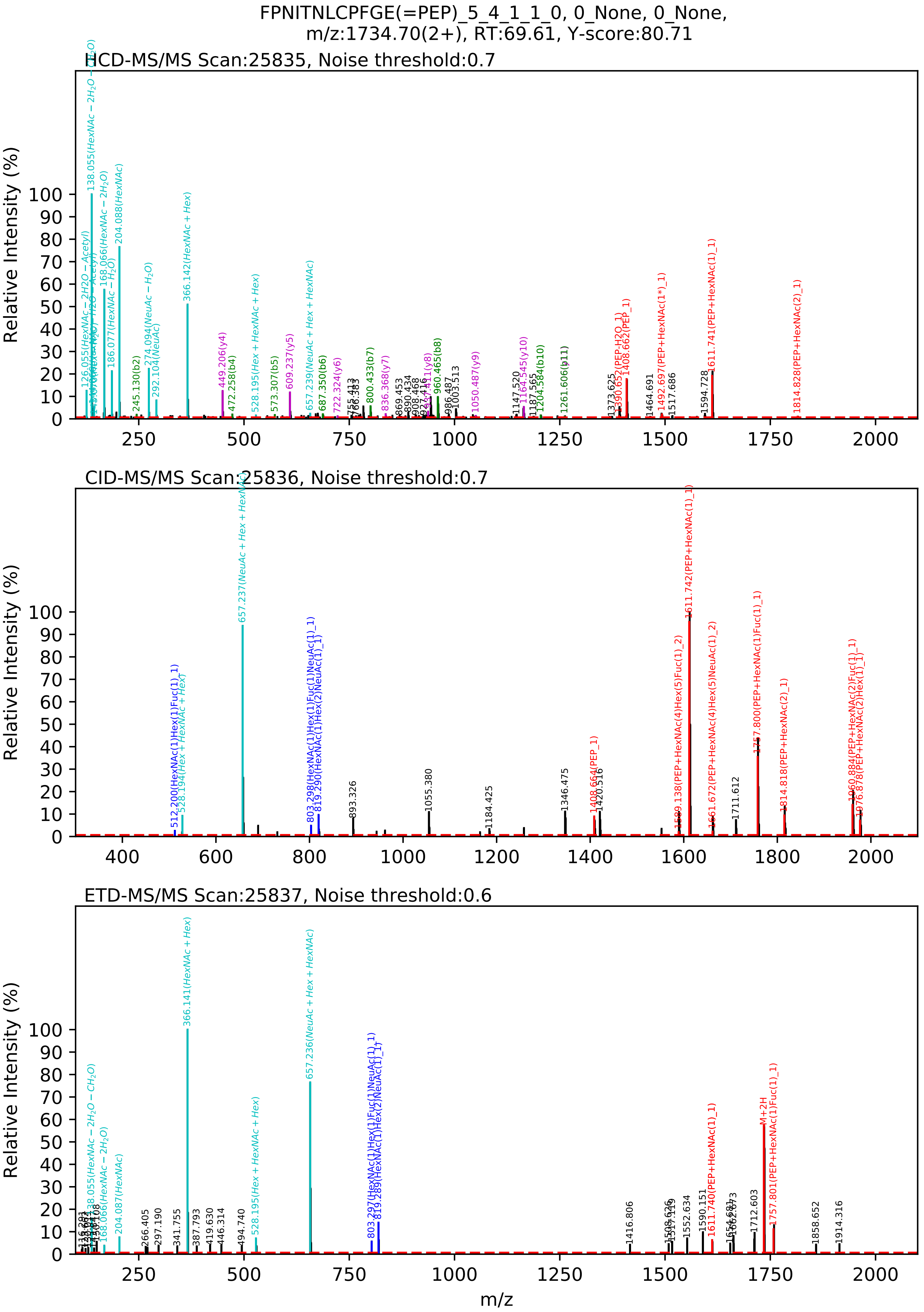

FPNITNLCPFGE(=PEP)_5_4_1_1_0, 0_None, 0_None,
m/z:1734.70(2+), RT:69.61, Y-score:80.71
HCD-MS/MS Scan:25835, Noise threshold:0.7
Relative Intensity (%)
138.055(HexNAc − 2H2O − CH2O)
204.088(HexNAc)
168.066(HexNAc − 2H2O)
186.077(HexNAc − H2O)
126.055(HexNAc − 2H2O − Acetyl)
274.094(NeuAc − H2O)
292.104(NeuAc)
245.130(b2)
366.142(HexNAc + Hex)
449.206(y4)
472.258(b4)
528.195(Hex + HexNAc + Hex)
573.307(b5)
609.237(y5)
657.239(NeuAc + Hex + HexNAc)
687.350(b6)
722.324(y6)
800.433(b7)
836.368(y7)
960.465(b8)
1003.513
1050.487(y9)
1164.545(y10)
1204.584(b10)
1261.606(b11)
1373.625
1408.662(PEP_1)
1464.691
1492.697(PEP+HexNAc(1*)_1)
1517.686
1594.728
1611.741(PEP+HexNAc(1)_1)
1814.828(PEP+HexNAc(2)_1)
CID-MS/MS Scan:25836, Noise threshold:0.7
512.200(HexNAc(1)Hex(1)Fuc(1)_1)
528.194(Hex + HexNAc + Hex)
657.237(NeuAc + Hex + HexNAc)
803.298(HexNAc(1)Hex(1)Fuc(1)NeuAc(1)_1)
819.290(HexNAc(1)Hex(2)NeuAc(1)_1)
893.326
1055.380
1184.425
1346.475
1408.664(PEP_1)
1420.516
1599.138(PEP+HexNAc(4)Hex(5)Fuc(1)_2)
1611.742(PEP+HexNAc(1)_1)
1661.672(PEP+HexNAc(4)Hex(5)NeuAc(1)_2)
1711.612
1757.800(PEP+HexNAc(1)Fuc(1)_1)
1814.818(PEP+HexNAc(2)_1)
1960.884(PEP+HexNAc(2)Fuc(1)_1)
1976.878(PEP+HexNAc(2)Hex(1)_1)
ETD-MS/MS Scan:25837, Noise threshold:0.6
138.055(HexNAc − 2H2O − CH2O)
168.066(HexNAc − 2H2O)
204.087(HexNAc)
266.405
297.190
341.755
366.141(HexNAc + Hex)
387.793
419.630
446.314
494.740
528.195(Hex + HexNAc + Hex)
657.236(NeuAc + Hex + HexNAc)
803.297(HexNAc(1)Hex(1)Fuc(1)NeuAc(1)_1)
819.289(HexNAc(1)Hex(2)NeuAc(1)_1)
1416.806
1517.119
1552.634
1590.151
1611.740(PEP+HexNAc(1)_1)
1712.603
M+2H
1757.801(PEP+HexNAc(1)Fuc(1)_1)
1858.652
1914.316
m/z

FPNITNLCPFGE(=PEP)_5_4_1_1_0, 0_None, 0_None,
m/z:1156.81(3+), RT:81.04, Y-score:79.99

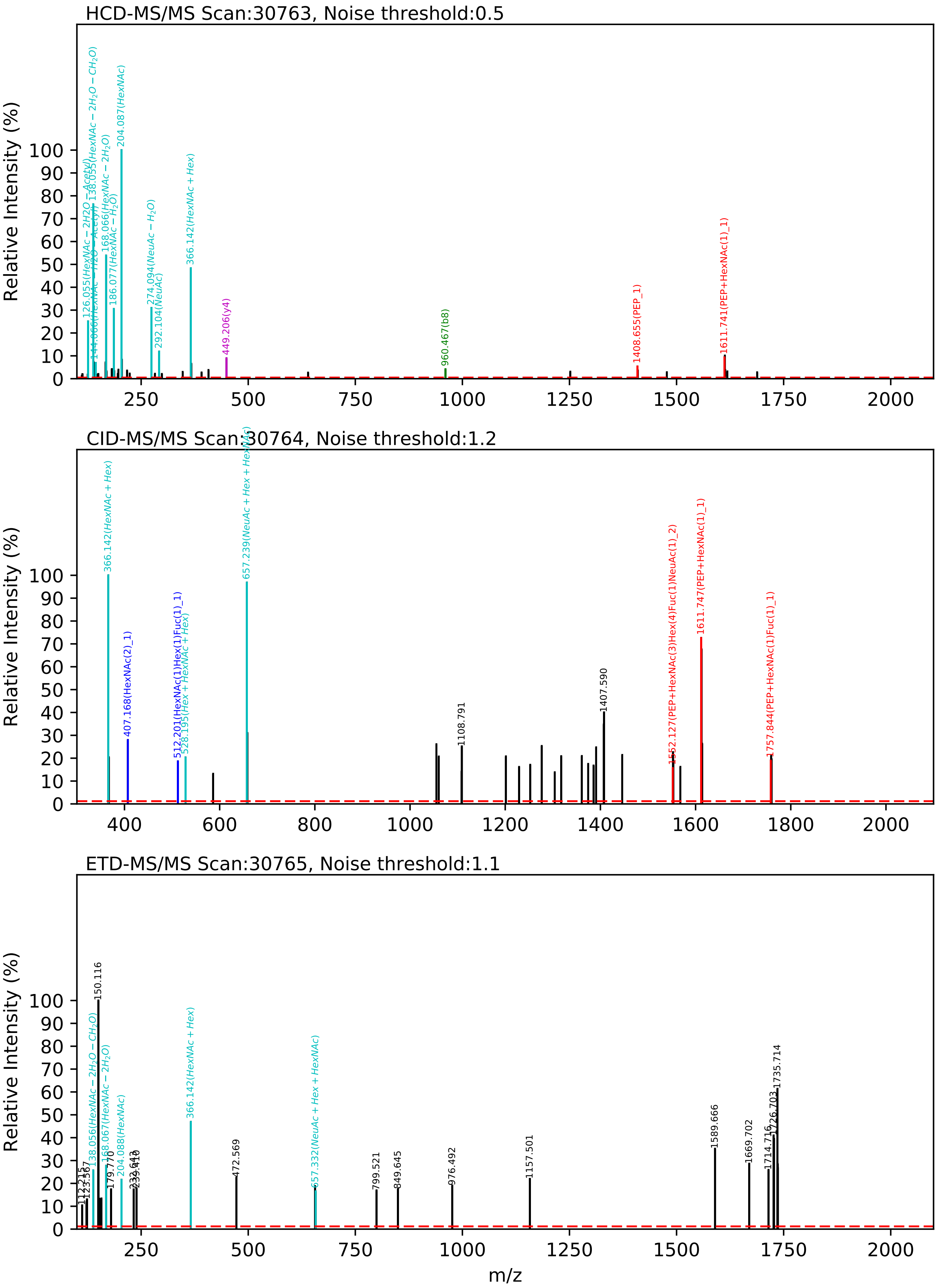

FPNITNLCPFGE(=PEP)_5_4_1_2_0, 0_None, 0_None,
m/z:1253.83(3+), RT:81.54, Y-score:94.91

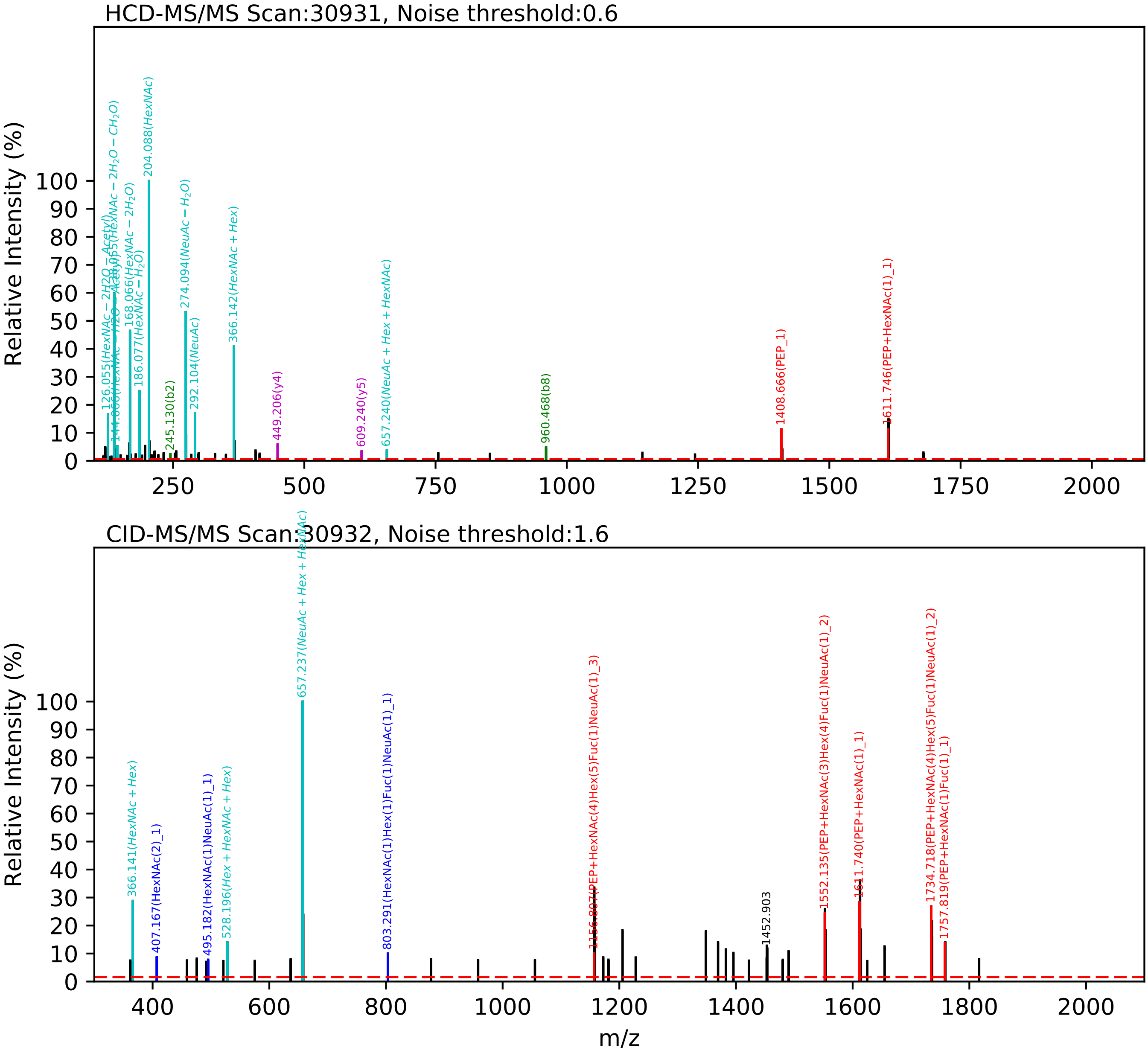

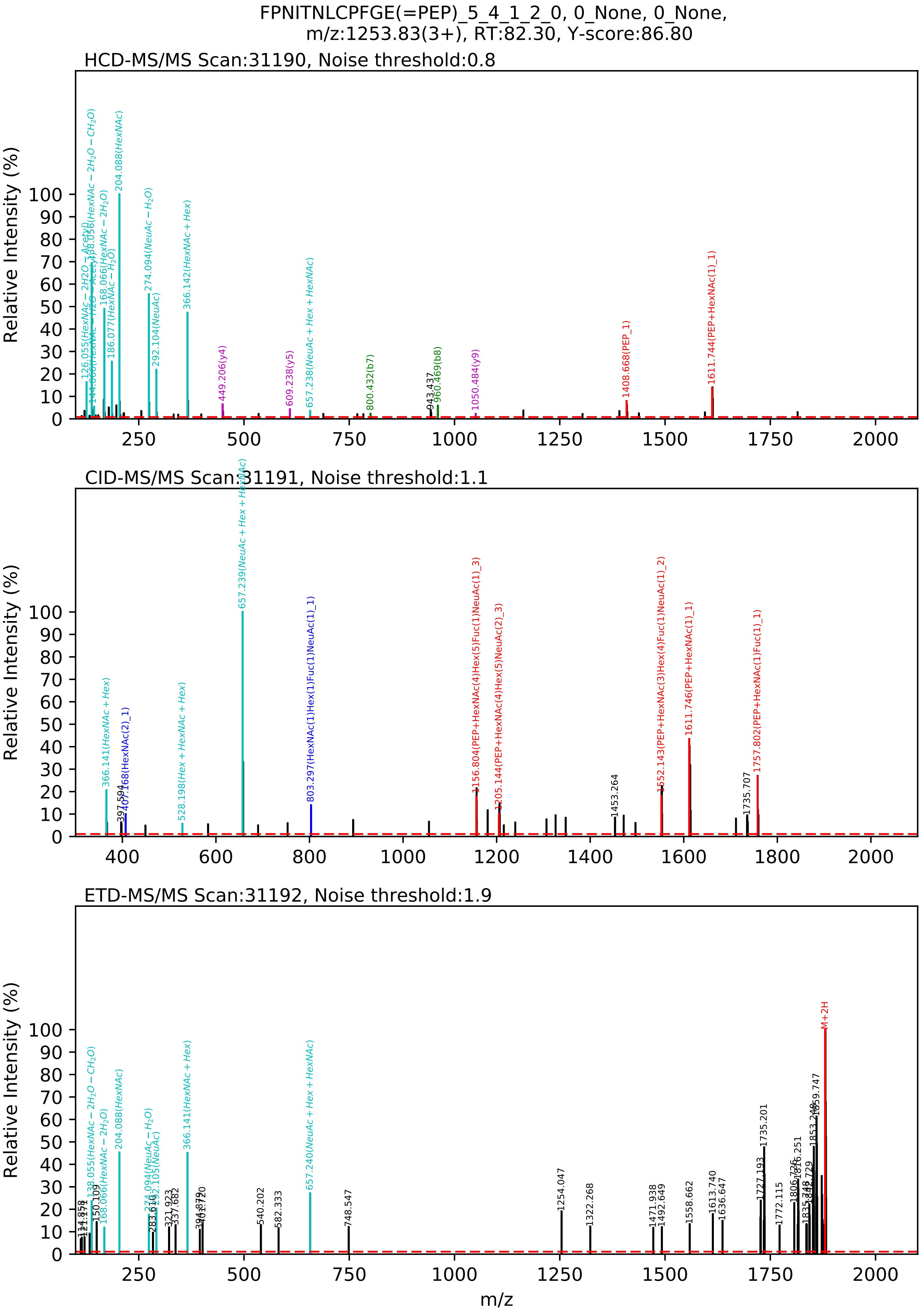
FPNITNLCPFGE(=PEP)_5_4_1_2_0, 0_None, 0_None,
m/z:1253.83(3+), RT:82.30, Y-score:86.80
HCD-MS/MS Scan:31190, Noise threshold:0.8
Relative Intensity (%)
204.088(HexNAc)
274.094(NeuAc − H2O)
366.142(HexNAc + Hex)
292.104(NeuAc)
168.066(HexNAc − 2H2O)
186.077(HexNAc − H2O)
126.055(HexNAc − 2H2O − Acetyl)
449.206(y4)
609.238(y5)
657.238(NeuAc + Hex + HexNAc)
800.432(b7)
943.437
960.469(b8)
1050.484(y9)
1408.668(PEP_1)
1611.744(PEP+HexNAc(1)_1)
CID-MS/MS Scan:31191, Noise threshold:1.1
657.239(NeuAc + Hex + HexNAc)
366.141(HexNAc + Hex)
397.591
407.168(HexNAc(2)_1)
528.198(Hex + HexNAc + Hex)
803.297(HexNAc(1)Hex(1)Fuc(1)NeuAc(1)_1)
1156.804(PEP+HexNAc(4)Hex(5)Fuc(1)NeuAc(1)_3)
1205.144(PEP+HexNAc(4)Hex(5)NeuAc(2)_3)
1453.264
1552.143(PEP+HexNAc(3)Hex(4)Fuc(1)NeuAc(1)_2)
1611.746(PEP+HexNAc(1)_1)
1735.707
1757.802(PEP+HexNAc(1)Fuc(1)_1)
ETD-MS/MS Scan:31192, Noise threshold:1.9
M+2H
204.088(HexNAc)
366.141(HexNAc + Hex)
657.240(NeuAc + Hex + HexNAc)
138.055(HexNAc − 2H2O − CH2O)
168.066(HexNAc − 2H2O)
274.094(NeuAc − H2O)
292.105(NeuAc)
150.109
114.858
283.610
321.923
337.682
394.879
401.720
540.202
582.333
748.547
1254.047
1322.268
1471.938
1492.649
1558.662
1613.740
1636.647
1727.193
1735.201
1772.115
1806.726
1816.251
1835.348
1853.248
1859.747
m/z

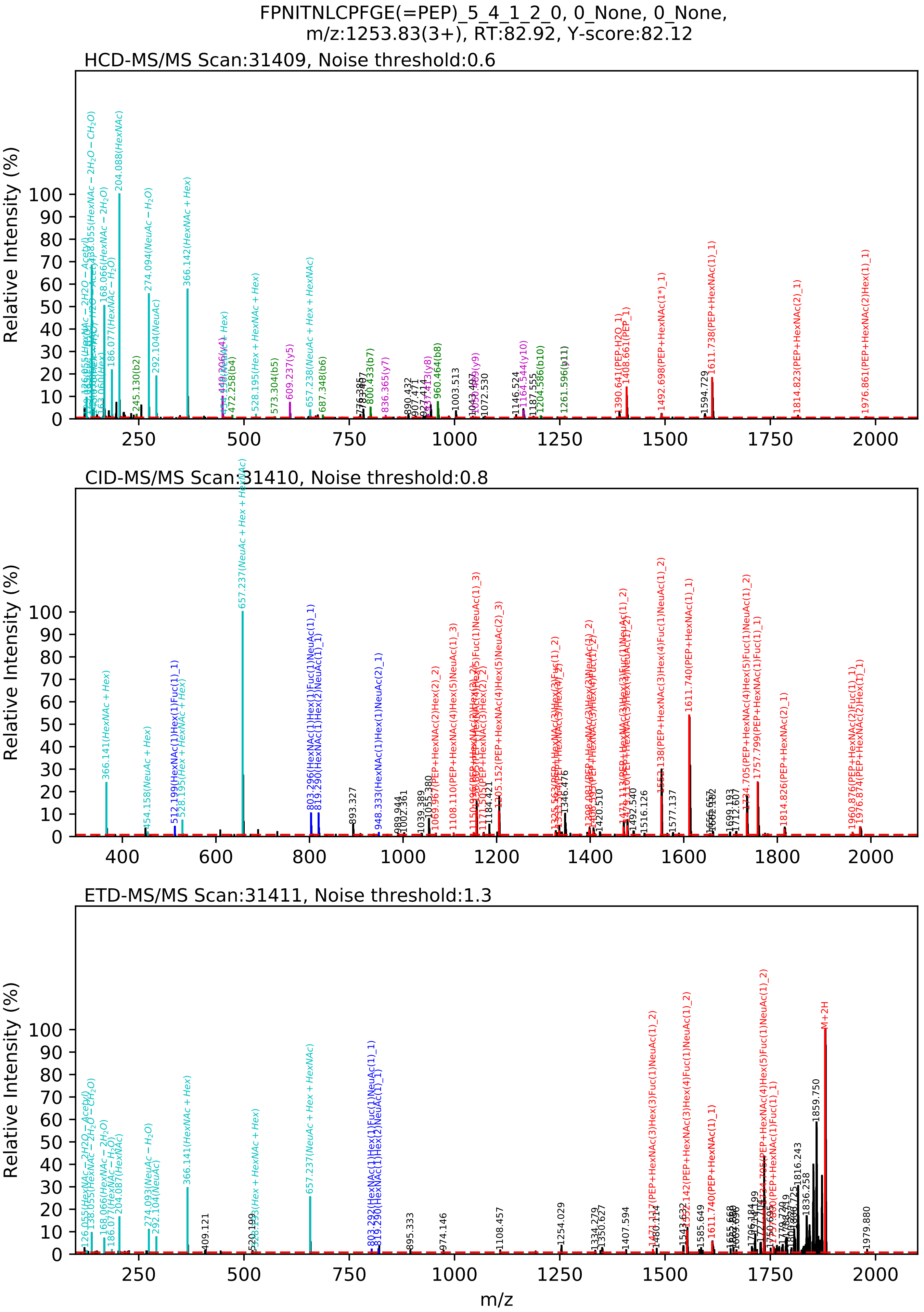

FPNITNLCPFGE(=PEP)_5_4_1_2_0, 0_None, 0_None,
m/z:1253.83(3+), RT:82.92, Y-score:82.12
HCD-MS/MS Scan:31409, Noise threshold:0.6
Relative Intensity (%)
204.088(HexNAc)
274.094(NeuAc − H2O)
292.104(NeuAc)
366.142(HexNAc + Hex)
168.066(HexNAc − 2H2O)
186.077(HexNAc − H2O)
245.130(b2)
472.258(b4)
528.195(Hex + HexNAc + Hex)
573.304(b5)
609.237(y5)
657.238(NeuAc + Hex + HexNAc)
687.348(b6)
800.433(b7)
836.365(y7)
960.464(b8)
1003.513
1072.530
1146.524
1164.544(y10)
1204.586(b10)
1261.596(b11)
1390.641(PEP-H2O_1)
1408.661(PEP_1)
1492.698(PEP+HexNAc(1*)_1)
1594.729
1611.738(PEP+HexNAc(1)_1)
1814.823(PEP+HexNAc(2)_1)
1976.861(PEP+HexNAc(2)Hex(1)_1)
CID-MS/MS Scan:31410, Noise threshold:0.8
366.141(HexNAc + Hex)
454.158(NeuAc + Hex)
512.199(HexNAc(1)Hex(1)Fuc(1)_1)
657.237(NeuAc + Hex + HexNAc)
803.296(HexNAc(1)Hex(1)Fuc(1)NeuAc(1)_1)
819.290(HexNAc(1)Hex(2)NeuAc(1)_1)
893.327
948.333(HexNAc(1)Hex(1)NeuAc(2)_1)
1108.110(PEP+HexNAc(4)Hex(5)NeuAc(1)_3)
1205.152(PEP+HexNAc(4)Hex(5)NeuAc(2)_3)
1346.476
1420.510
1516.126
1552.138(PEP+HexNAc(3)Hex(4)Fuc(1)NeuAc(1)_2)
1577.137
1611.740(PEP+HexNAc(1)_1)
1734.705(PEP+HexNAc(4)Hex(5)Fuc(1)NeuAc(1)_2)
1757.799(PEP+HexNAc(1)Fuc(1)_1)
1814.826(PEP+HexNAc(2)_1)
1960.876(PEP+HexNAc(2)Fuc(1)_1)
1976.874(PEP+HexNAc(2)Hex(1)_1)
ETD-MS/MS Scan:31411, Noise threshold:1.3
366.141(HexNAc + Hex)
409.121
657.237(NeuAc + Hex + HexNAc)
895.333
974.146
1108.457
1254.029
1407.594
1585.649
1611.740(PEP+HexNAc(1)_1)
1816.243
1836.258
1859.750
M+2H
1979.880
m/z

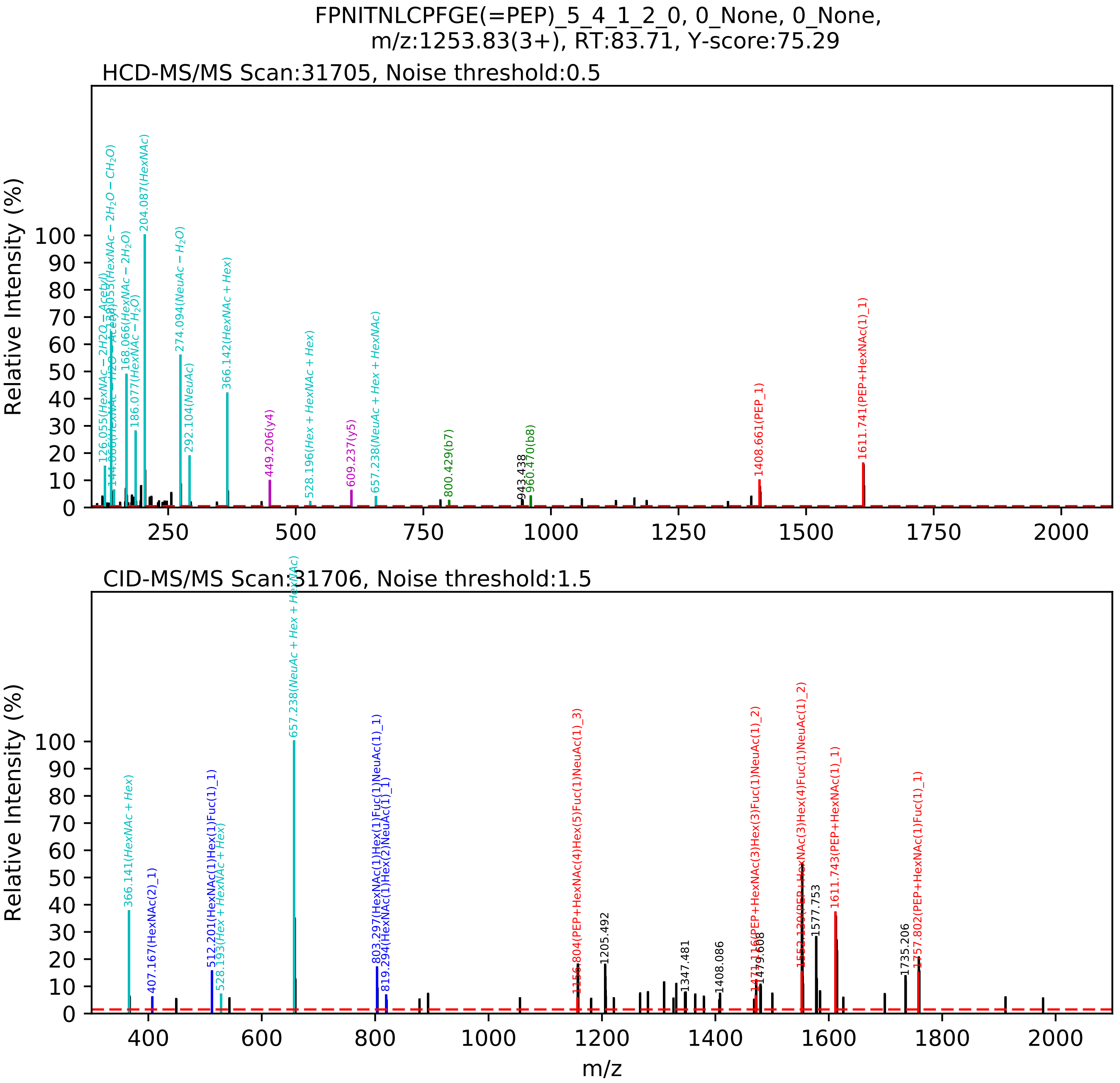

FPNITNLCPFGE(=PEP)_5_4_1_2_0, 0_None, 0_None,
m/z:1253.83(3+), RT:83.71, Y-score:75.29
HCD-MS/MS Scan:31705, Noise threshold:0.5
Relative Intensity (%)
204.087(HexNAc)
274.094(NeuAc − H2O)
366.142(HexNAc + Hex)
292.104(NeuAc)
186.077(HexNAc − H2O)
168.066(HexNAc − 2H2O)
126.055(HexNAc − 2H2O − Acetyl)
449.206(y4)
528.196(Hex + HexNAc + Hex)
609.237(y5)
657.238(NeuAc + Hex + HexNAc)
800.429(b7)
943.438
960.470(b8)
1408.661(PEP_1)
1611.741(PEP+HexNAc(1)_1)
CID-MS/MS Scan:31706, Noise threshold:1.5
657.238(NeuAc + Hex + HexNAc)
366.141(HexNAc + Hex)
407.167(HexNAc(2)_1)
512.201(HexNAc(1)Hex(1)Fuc(1)_1)
528.193(Hex + HexNAc + Hex)
803.297(HexNAc(1)Hex(1)Fuc(1)NeuAc(1)_1)
819.294(HexNAc(1)Hex(2)NeuAc(1)_1)
1156.804(PEP+HexNAc(4)Hex(5)Fuc(1)NeuAc(1)_3)
1205.492
1347.481
1408.086
1471.116(PEP+HexNAc(3)Hex(3)Fuc(1)NeuAc(1)_2)
1479.608
1552.129(PEP+HexNAc(3)Hex(4)Fuc(1)NeuAc(1)_2)
1577.753
1611.743(PEP+HexNAc(1)_1)
1735.206
1757.802(PEP+HexNAc(1)Fuc(1)_1)
m/z

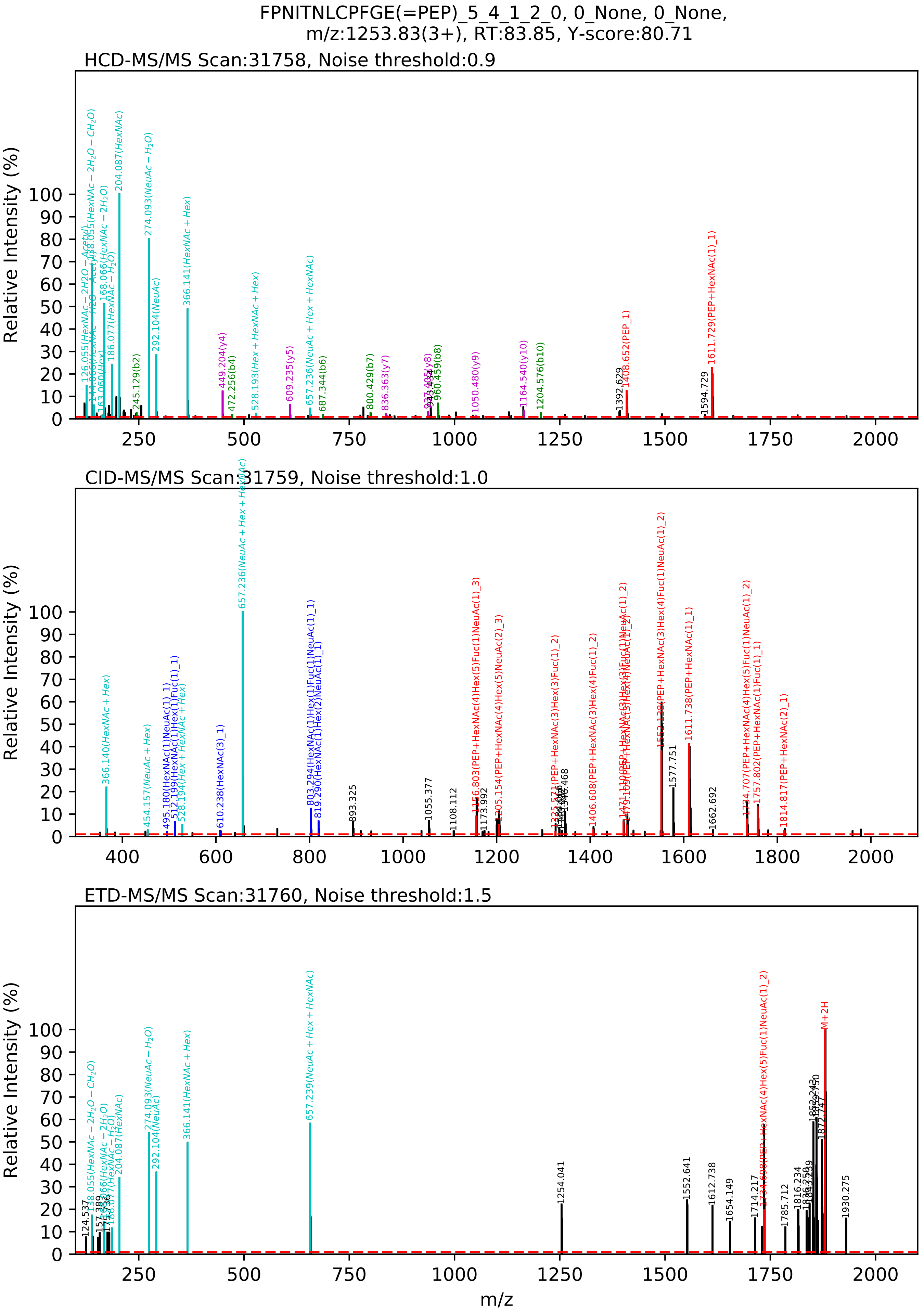
FPNITNLCPFGE(=PEP)_5_4_1_2_0, 0_None, 0_None,
m/z:1253.83(3+), RT:83.85, Y-score:80.71
HCD-MS/MS Scan:31758, Noise threshold:0.9
Relative Intensity (%)
204.087(HexNAc)
274.093(NeuAc − H2O)
366.141(HexNAc + Hex)
292.104(NeuAc)
168.066(HexNAc − 2H2O)
186.077(HexNAc − H2O)
245.129(b2)
449.204(y4)
472.256(b4)
528.193(Hex + HexNAc + Hex)
609.235(y5)
657.236(NeuAc + Hex + HexNAc)
687.344(b6)
800.429(b7)
836.363(y7)
960.459(b8)
1050.480(y9)
1164.540(y10)
1204.576(b10)
1392.629
1408.652(PEP_1)
1594.729
1611.729(PEP+HexNAc(1)_1)
CID-MS/MS Scan:31759, Noise threshold:1.0
Relative Intensity (%)
657.236(NeuAc + Hex + HexNAc)
366.140(HexNAc + Hex)
454.157(NeuAc + Hex)
610.238(HexNAc(3)_1)
803.294(HexNAc(1)Hex(1)Fuc(1)NeuAc(1)_1)
819.290(HexNAc(1)Hex(2)NeuAc(1)_1)
893.325
1055.377
1108.112
1156.803(PEP+HexNAc(4)Hex(5)Fuc(1)NeuAc(1)_3)
1173.992
1205.154(PEP+HexNAc(4)Hex(5)NeuAc(2)_3)
1346.468
1406.608(PEP+HexNAc(3)Hex(4)Fuc(1)_2)
1552.138(PEP+HexNAc(3)Hex(4)Fuc(1)NeuAc(1)_2)
1577.751
1611.738(PEP+HexNAc(1)_1)
1662.692
1734.707(PEP+HexNAc(4)Hex(5)Fuc(1)NeuAc(1)_2)
1757.802(PEP+HexNAc(1)Fuc(1)_1)
1814.817(PEP+HexNAc(2)_1)
ETD-MS/MS Scan:31760, Noise threshold:1.5
Relative Intensity (%)
124.537
274.093(NeuAc − H2O)
292.104(NeuAc)
366.141(HexNAc + Hex)
657.239(NeuAc + Hex + HexNAc)
1254.041
1552.641
1612.738
1654.149
1714.217
1785.712
1816.234
1930.275
M+2H
m/z

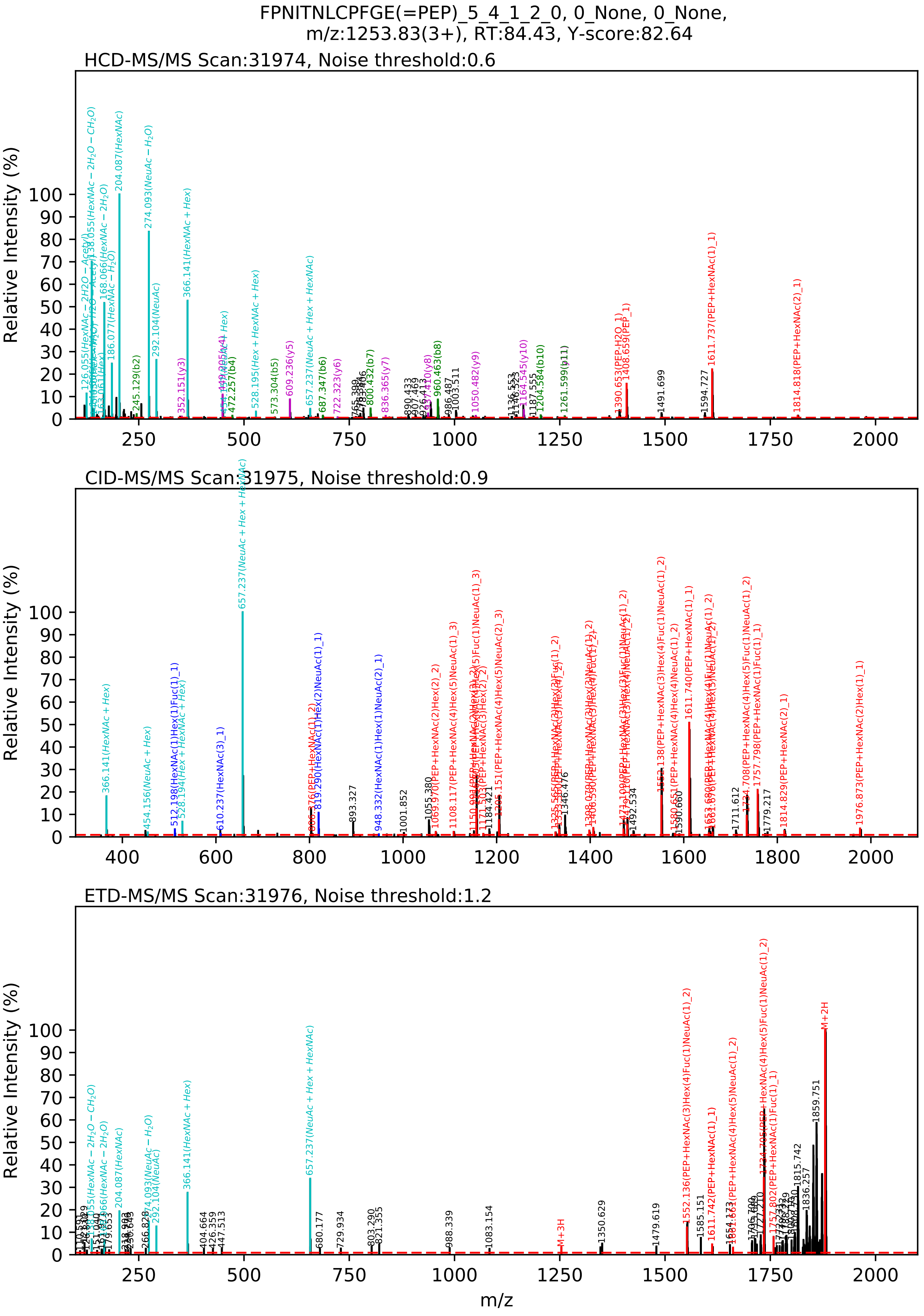

FPNITNLCPFGE(=PEP)_5_4_1_2_0, 0_None, 0_None,
m/z:1253.83(3+), RT:84.43, Y-score:82.64
HCD-MS/MS Scan:31974, Noise threshold:0.6
Relative Intensity (%)
204.087(HexNAc)
274.093(NeuAc − H2O)
366.141(HexNAc + Hex)
292.104(NeuAc)
186.077(HexNAc − H2O)
168.066(HexNAc − 2H2O)
245.129(b2)
352.151(y3)
472.257(b4)
528.195(Hex + HexNAc + Hex)
573.304(b5)
609.236(y5)
657.237(NeuAc + Hex + HexNAc)
687.347(b6)
722.323(y6)
800.432(b7)
836.365(y7)
960.463(b8)
1003.511
1050.482(y9)
1164.545(y10)
1204.584(b10)
1261.599(b11)
1390.653(PEP-H2O_1)
1408.659(PEP_1)
1491.699
1594.727
1611.737(PEP+HexNAc(1)_1)
1814.818(PEP+HexNAc(2)_1)
CID-MS/MS Scan:31975, Noise threshold:0.9
366.141(HexNAc + Hex)
454.156(NeuAc + Hex)
512.198(HexNAc(1)Hex(1)Fuc(1)_1)
610.237(HexNAc(3)_1)
657.237(NeuAc + Hex + HexNAc)
819.290(HexNAc(1)Hex(2)NeuAc(1)_1)
893.327
948.332(HexNAc(1)Hex(1)NeuAc(2)_1)
1001.852
1055.380
1108.117(PEP+HexNAc(4)Hex(5)NeuAc(1)_3)
1205.151(PEP+HexNAc(4)Hex(5)NeuAc(2)_3)
1346.476
1492.534
1552.138(PEP+HexNAc(3)Hex(4)Fuc(1)NeuAc(1)_2)
1611.740(PEP+HexNAc(1)_1)
1711.612
1734.708(PEP+HexNAc(4)Hex(5)Fuc(1)NeuAc(1)_2)
1757.798(PEP+HexNAc(1)Fuc(1)_1)
1779.217
1814.829(PEP+HexNAc(2)_1)
1976.873(PEP+HexNAc(2)Hex(1)_1)
ETD-MS/MS Scan:31976, Noise threshold:1.2
M+3H
M+2H
1350.629
1479.619
1552.136(PEP+HexNAc(3)Hex(4)Fuc(1)NeuAc(1)_2)
1585.151
1611.742(PEP+HexNAc(1)_1)
1654.173
1757.802(PEP+HexNAc(1)Fuc(1)_1)
1815.742
1836.257
1859.751
m/z

FPNITNLCPFGE(=PEP)_5_4_1_2_0, 0_None, 0_None,
m/z:1253.83(3+), RT:85.00, Y-score:79.72

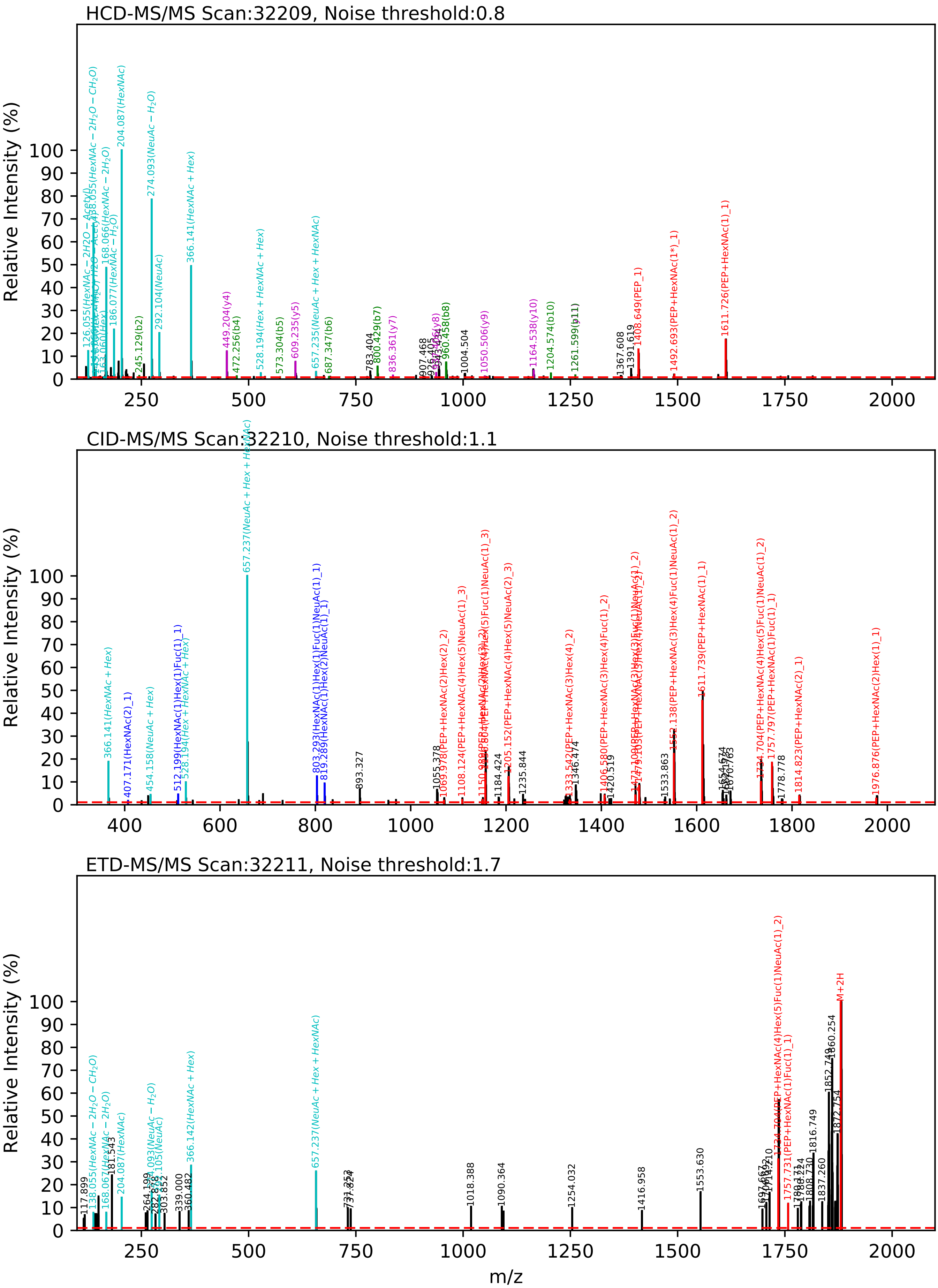

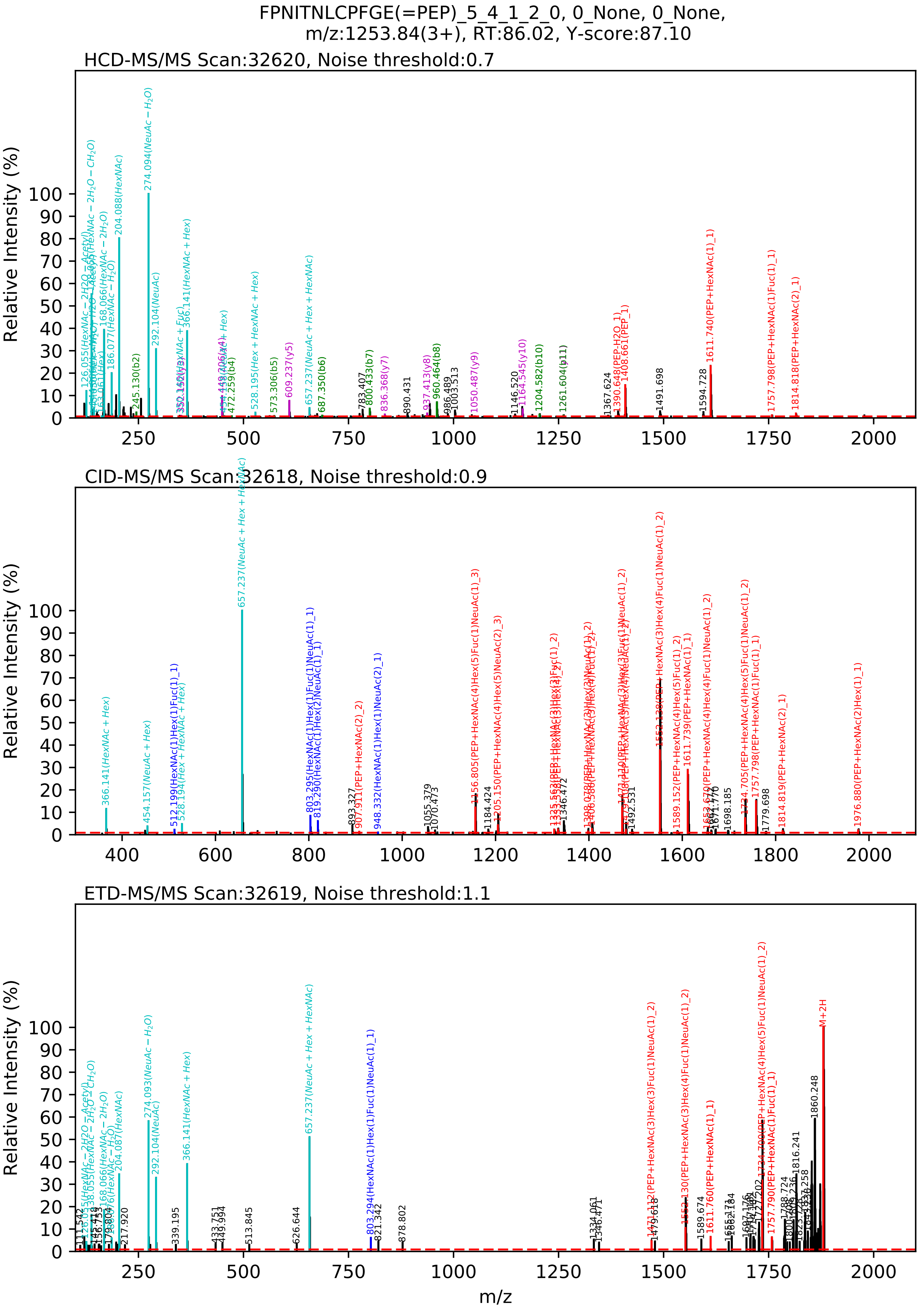

FPNITNLCPFGE(=PEP)_5_4_1_2_0, 0_None, 0_None,
m/z:1253.84(3+), RT:86.02, Y-score:87.10
HCD-MS/MS Scan:32620, Noise threshold:0.7
CID-MS/MS Scan:32618, Noise threshold:0.9
ETD-MS/MS Scan:32619, Noise threshold:1.1
Relative Intensity (%)
m/z
274.094(NeuAc − H2O)
204.088(HexNAc)
366.141(HexNAc + Hex)
292.104(NeuAc)
657.237(NeuAc + Hex + HexNAc)
1611.740(PEP+HexNAc(1)_1)
1757.798(PEP+HexNAc(1)Fuc(1)_1)
1814.818(PEP+HexNAc(2)_1)
1408.661(PEP_1)
1552.129(PEP+HexNAc(3)Hex(4)Fuc(1)NeuAc(1)_2)
1156.805(PEP+HexNAc(4)Hex(5)Fuc(1)NeuAc(1)_3)
1205.150(PEP+HexNAc(4)Hex(5)NeuAc(2)_3)
1976.880(PEP+HexNAc(2)Hex(1)_1)
803.295(HexNAc(1)Hex(1)Fuc(1)NeuAc(1)_1)
948.332(HexNAc(1)Hex(1)NeuAc(2)_1)
1860.248
M+2H

FPNITNLCPFGE(=PEP)_5_4_1_2_0, 0_None, 0_None,
m/z:1253.84(3+), RT:86.79, Y-score:80.25

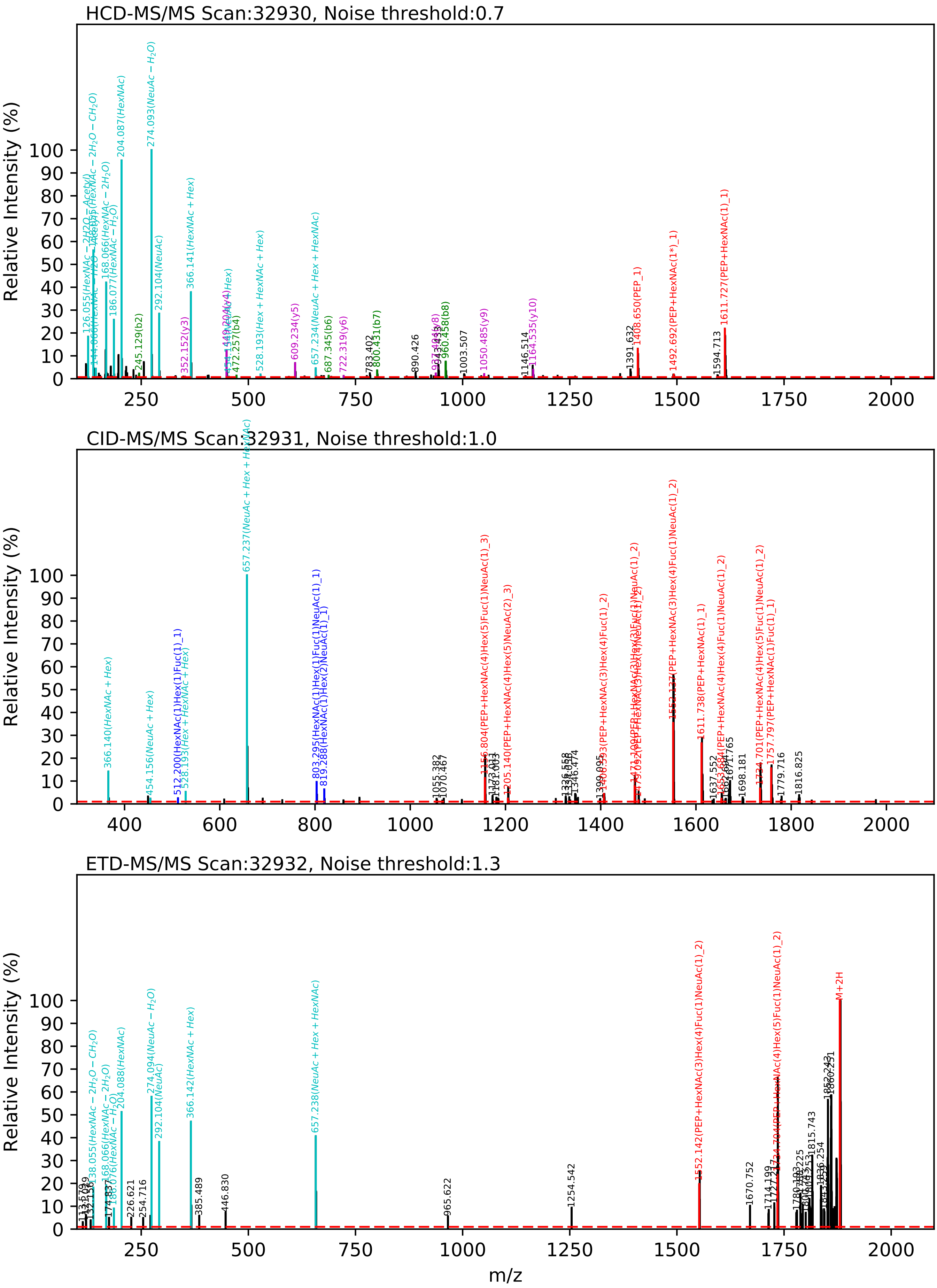

FPNITNLCPFGE(=PEP)_5_4_1_2_0, 0_None, 0_None,
m/z:940.63(4+), RT:86.32, Y-score:84.76

HCD-MS/MS Scan:32745, Noise threshold:0.8

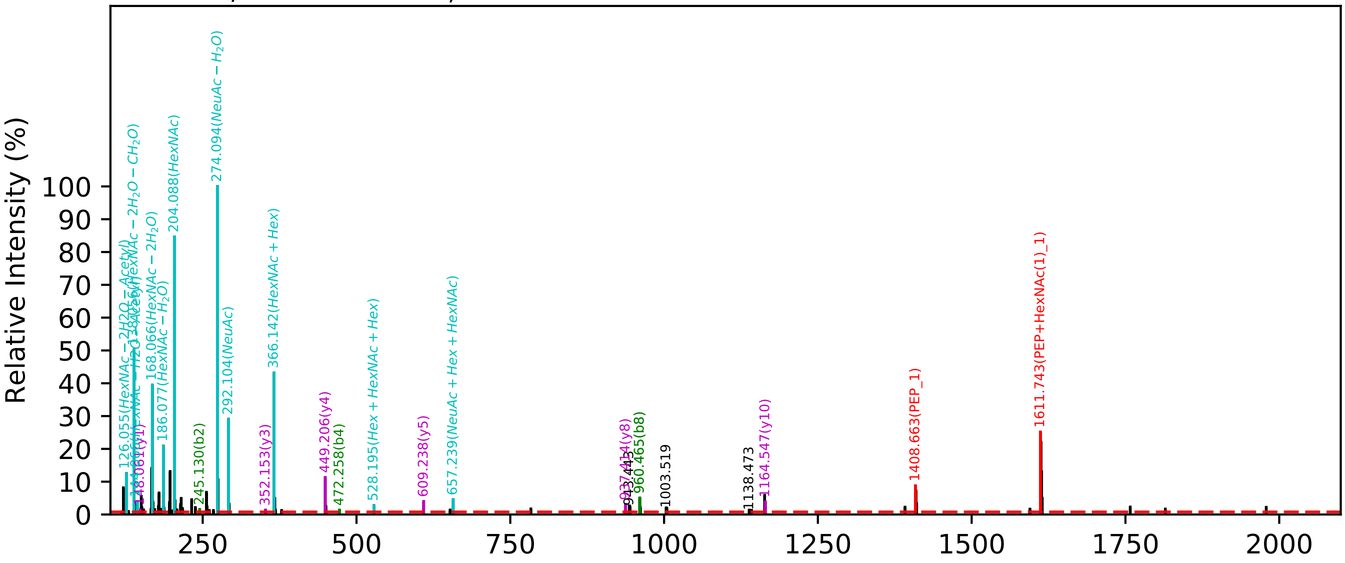

CID-MS/MS Scan:32746, Noise threshold:1.0

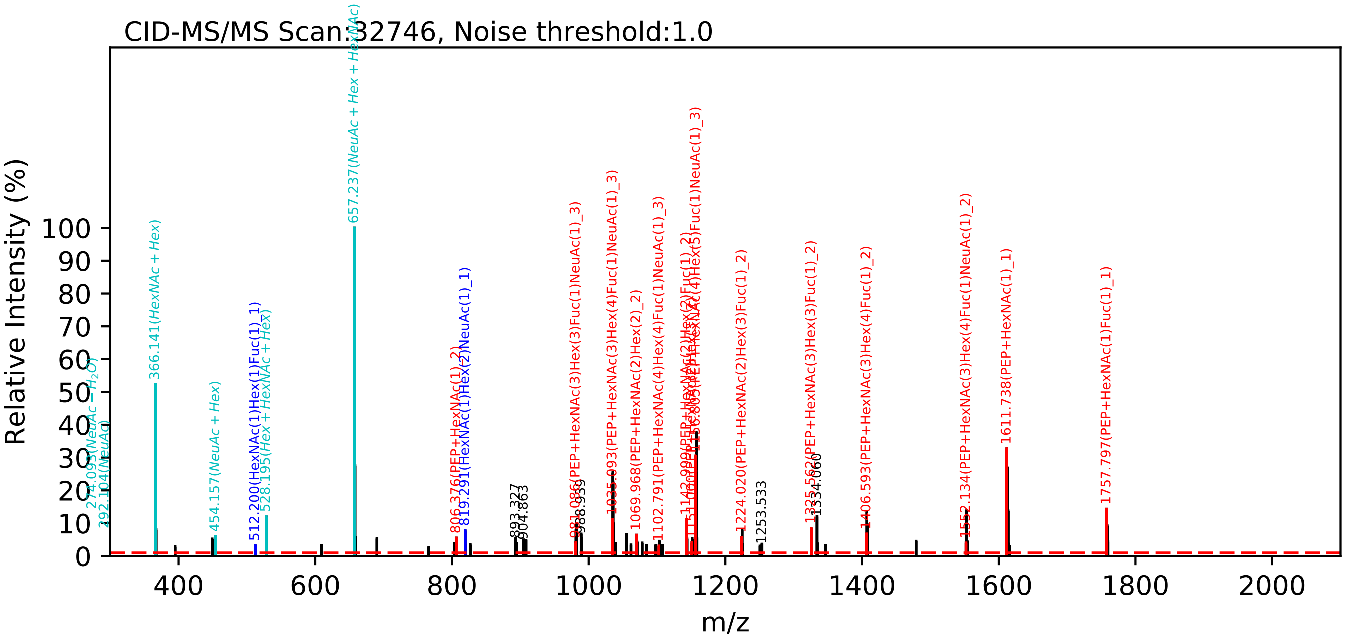

FPNITNLCPFGE(=PEP)_5_4_2_0_0, 0_None, 0_None,
m/z:1108.46(3+), RT:59.98, Y-score:82.52

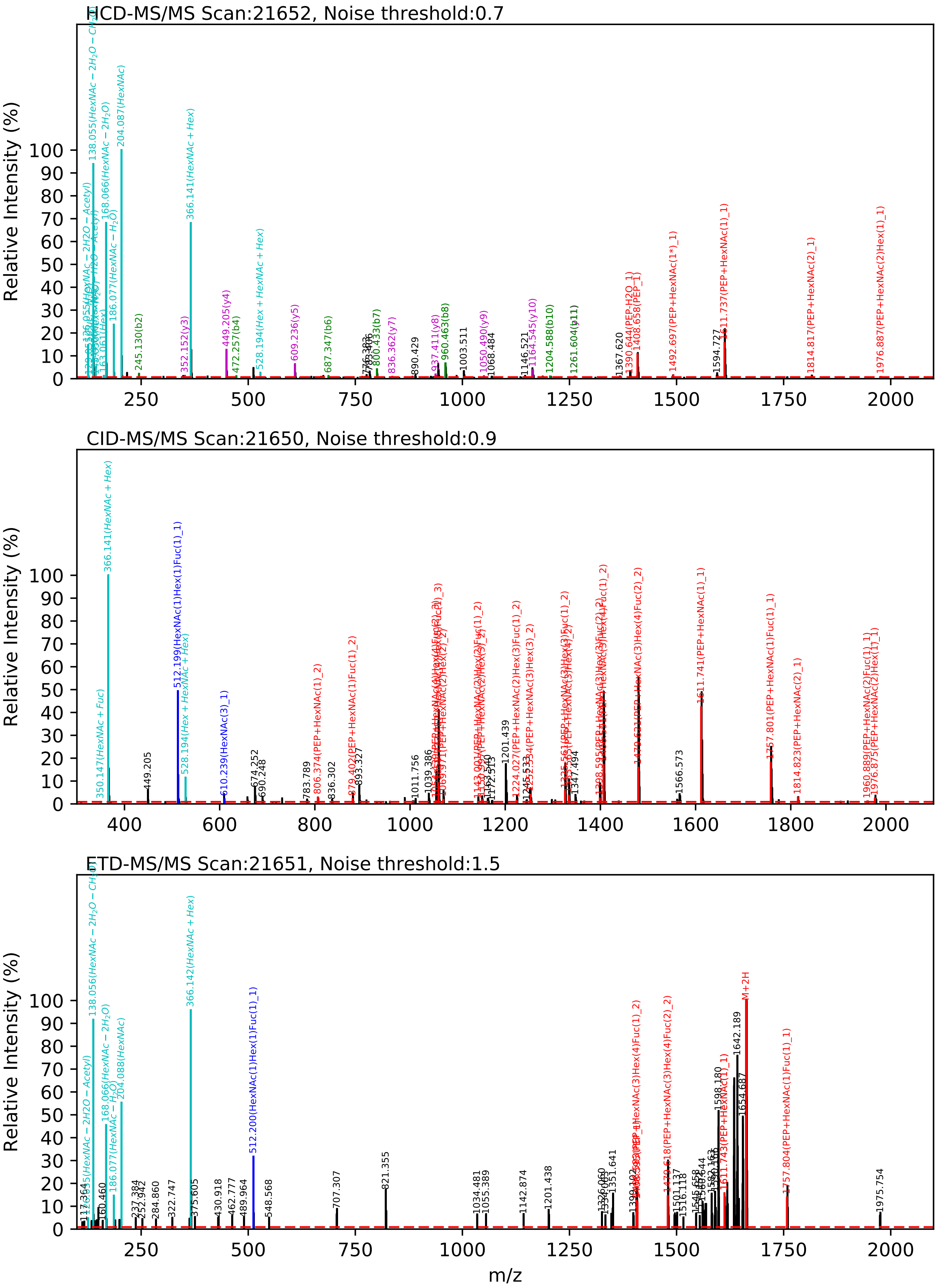

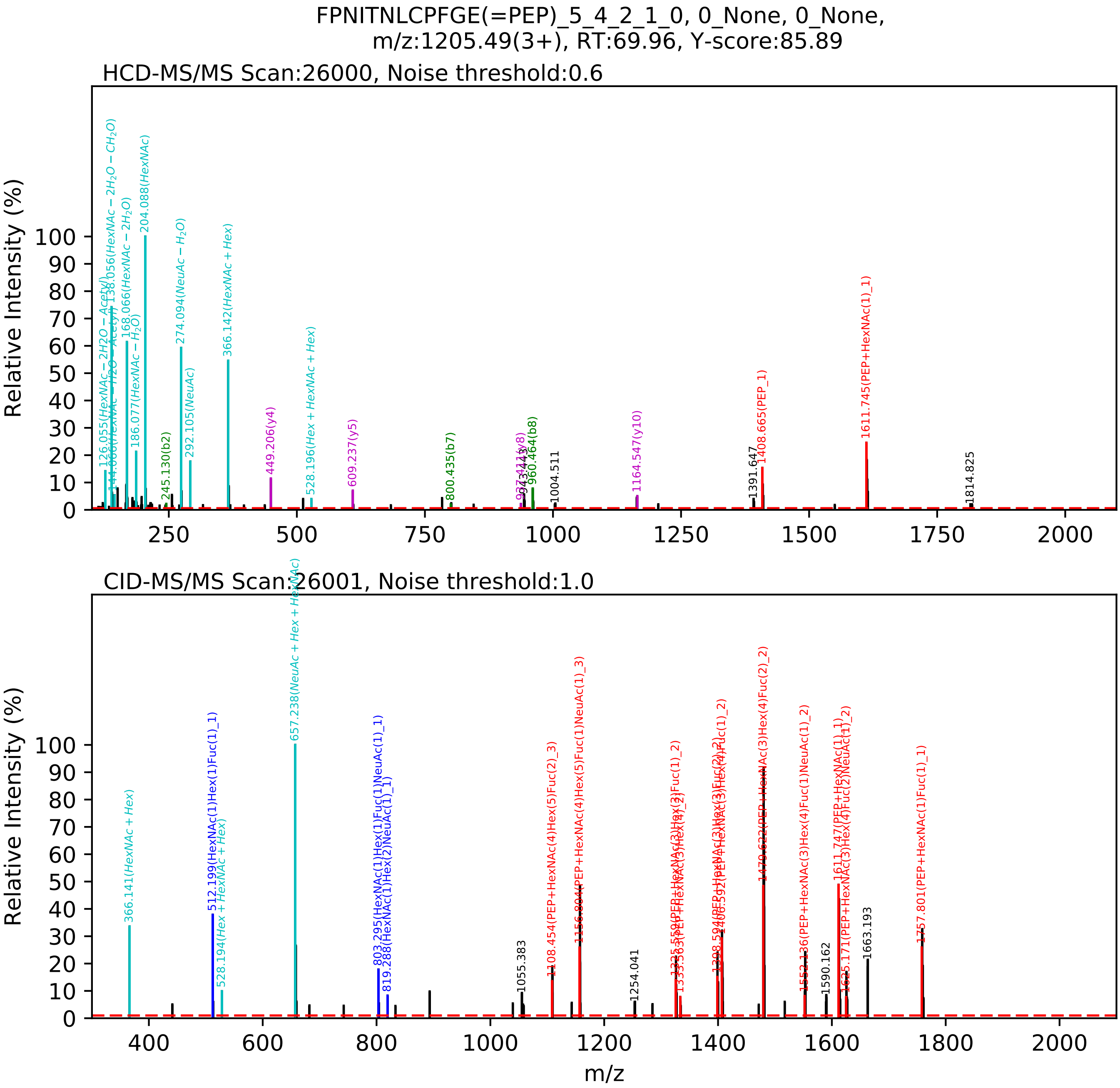
FPNITNLCPFGE(=PEP)_5_4_2_1_0, 0_None, 0_None,
m/z:1205.49(3+), RT:69.96, Y-score:85.89
HCD-MS/MS Scan:26000, Noise threshold:0.6
Relative Intensity (%)
204.088(HexNAc)
366.142(HexNAc+Hex)
274.094(NeuAc−H2O)
292.105(NeuAc)
245.130(b2)
449.206(y4)
528.196(Hex+HexNAc+Hex)
609.237(y5)
800.435(b7)
960.464(b8)
1004.511
1164.547(y10)
1391.647
1408.665(PEP_1)
1611.745(PEP+HexNAc(1)_1)
1814.825
CID-MS/MS Scan:26001, Noise threshold:1.0
366.141(HexNAc+Hex)
512.199(HexNAc(1)Hex(1)Fuc(1)_1)
528.194(Hex+HexNAc+Hex)
657.238(NeuAc+Hex+HexNAc)
803.295(HexNAc(1)Hex(1)Fuc(1)NeuAc(1)_1)
819.288(HexNAc(1)Hex(2)NeuAc(1)_1)
1055.383
1108.454(PEP+HexNAc(4)Hex(5)Fuc(2)_3)
1156.804(PEP+HexNAc(4)Hex(5)Fuc(1)NeuAc(1)_3)
1254.041
1479.632(PEP+HexNAc(3)Hex(4)Fuc(2)_2)
1552.136(PEP+HexNAc(3)Hex(4)Fuc(1)NeuAc(1)_2)
1590.162
1611.747(PEP+HexNAc(1)_1)
1663.193
1757.801(PEP+HexNAc(1)Fuc(1)_1)
m/z

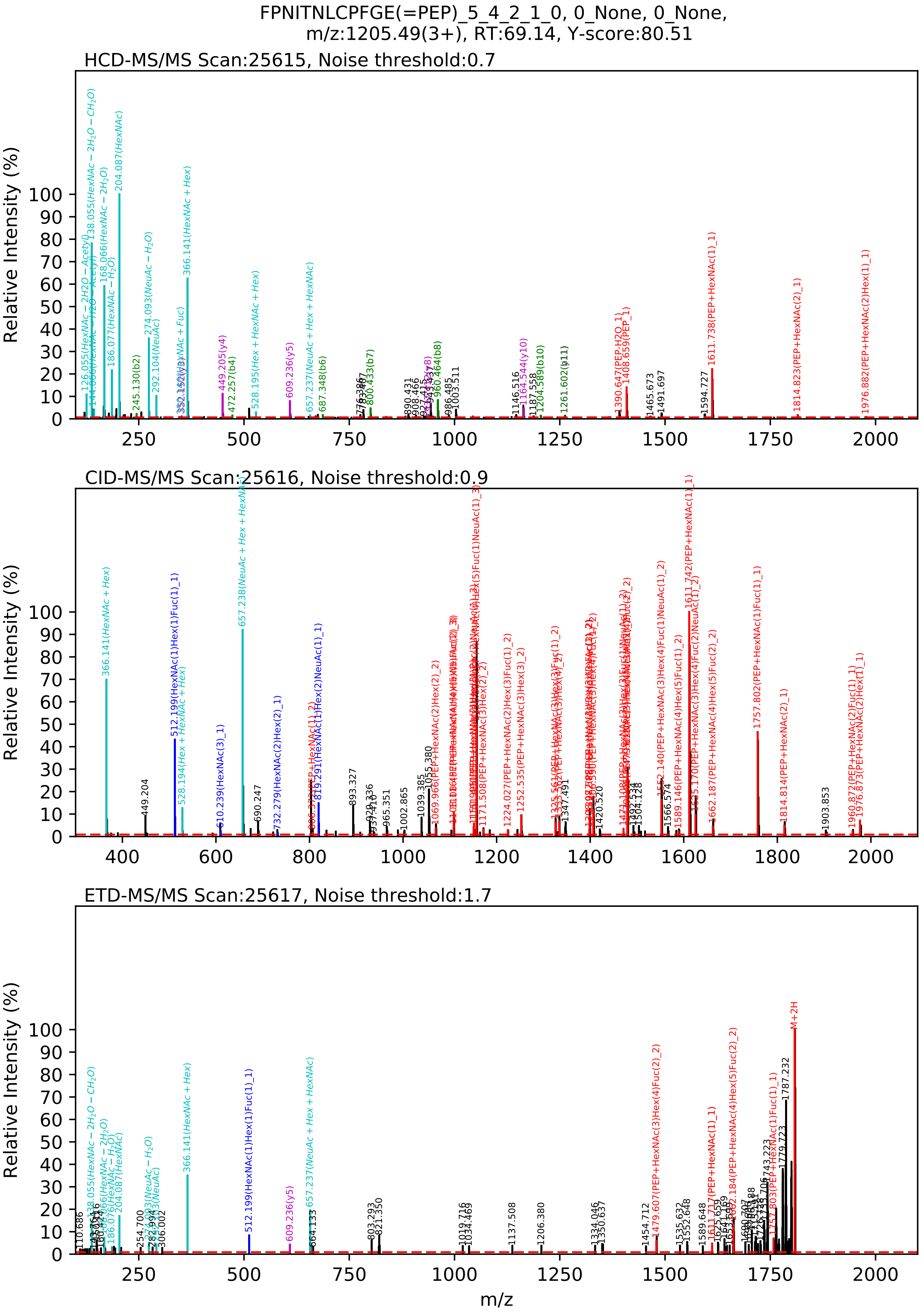
FPNITNLCPFGE(=PEP)_5_4_2_1_0, 0_None, 0_None,
m/z:1205.49(3+), RT:69.14, Y-score:80.51
HCD-MS/MS Scan:25615, Noise threshold:0.7
CID-MS/MS Scan:25616, Noise threshold:0.9
ETD-MS/MS Scan:25617, Noise threshold:1.7
Relative Intensity (%)
m/z

FPNITNLCPFGE(=PEP)_5_4_2_2_0, 0_None, 0_None,
m/z:1302.52(3+), RT:85.35, Y-score:88.78

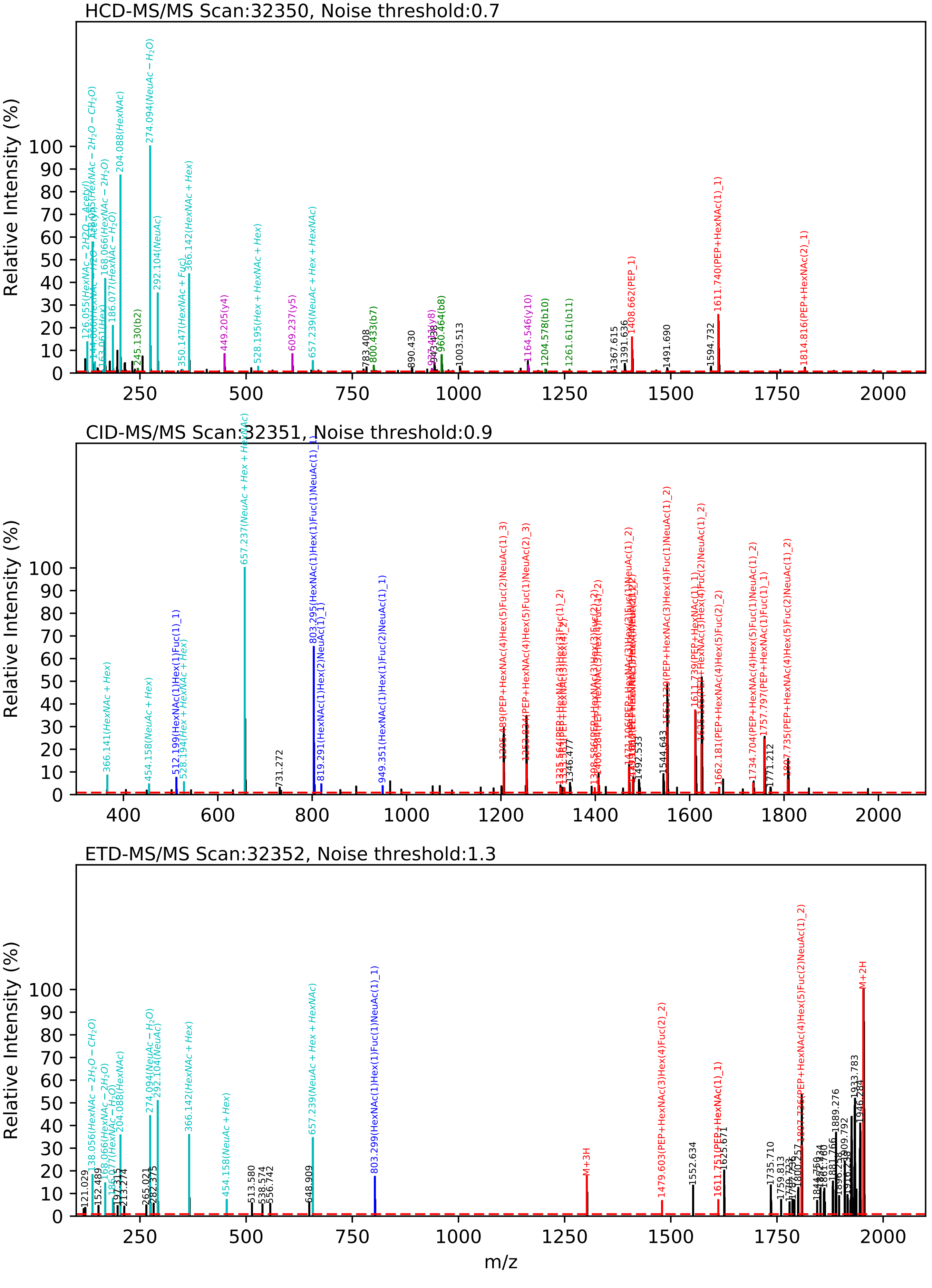

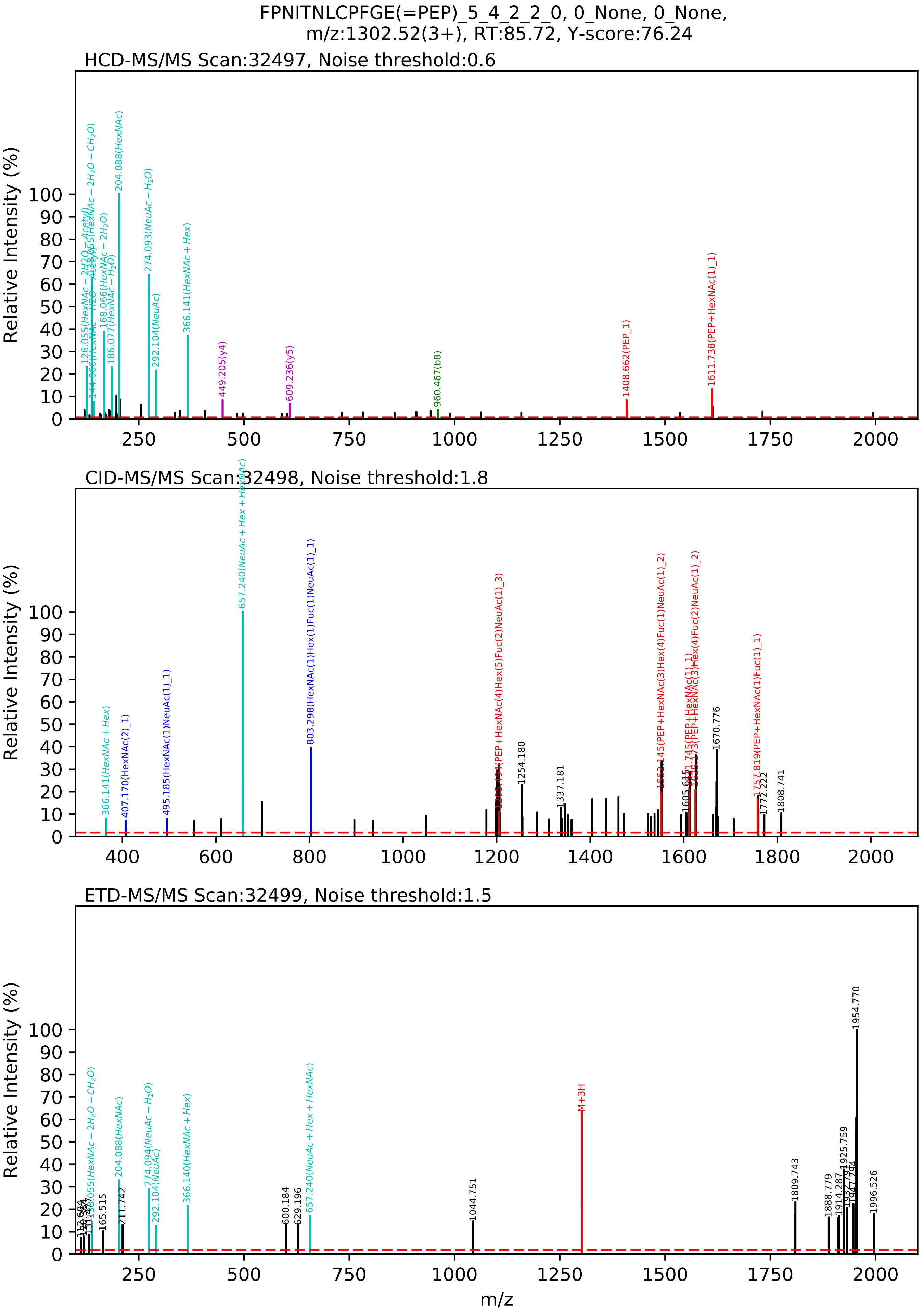
FPNITNLCPFGE(=PEP)_5_4_2_2_0, 0_None, 0_None,
m/z:1302.52(3+), RT:85.72, Y-score:76.24
HCD-MS/MS Scan:32497, Noise threshold:0.6
Relative Intensity (%)
204.088(HexNAc)
274.093(NeuAc − H2O)
366.141(HexNAc + Hex)
292.104(NeuAc)
168.066(HexNAc − 2H2O)
186.077(HexNAc − H2O)
126.055(HexNAc − 2H2O − CH2O)
449.205(y4)
609.236(y5)
960.467(b8)
1408.662(PEP_1)
1611.738(PEP+HexNAc(1)_1)
CID-MS/MS Scan:32498, Noise threshold:1.8
657.240(NeuAc + Hex + HexNAc)
366.141(HexNAc + Hex)
407.170(HexNAc(2)_1)
495.185(HexNAc(1)NeuAc(1)_1)
803.298(HexNAc(1)Hex(1)Fuc(1)NeuAc(1)_1)
(PEP+HexNAc(4)Hex(5)Fuc(2)NeuAc(1)_3)
1254.180
1337.181
1552.145(PEP+HexNAc(3)Hex(4)Fuc(1)NeuAc(1)_2)
1605.615
1670.776
1757.819(PEP+HexNAc(1)Fuc(1)_1)
1772.222
1808.741
ETD-MS/MS Scan:32499, Noise threshold:1.5
204.088(HexNAc)
211.742
165.515
292.104(NeuAc)
366.140(HexNAc + Hex)
600.184
629.196
657.240(NeuAc + Hex + HexNAc)
1044.751
M+3H
1809.743
1888.779
1914.287
1925.759
1954.770
1996.526
m/z

FPNITNLCPFGE(=PEP)_5_4_3_0_0, 0_None, 0_None,
m/z:1157.14(3+), RT:59.76, Y-score:88.82

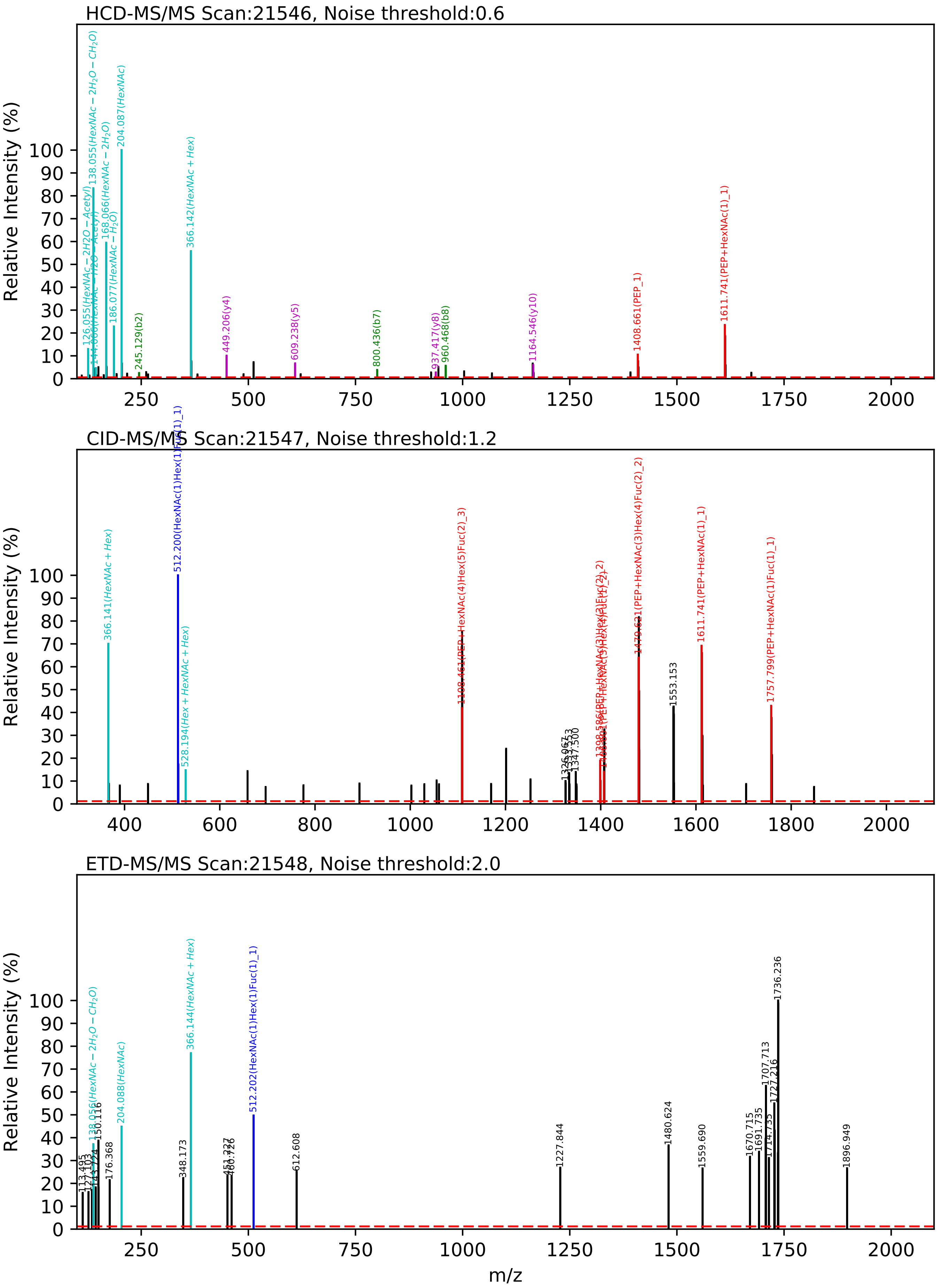

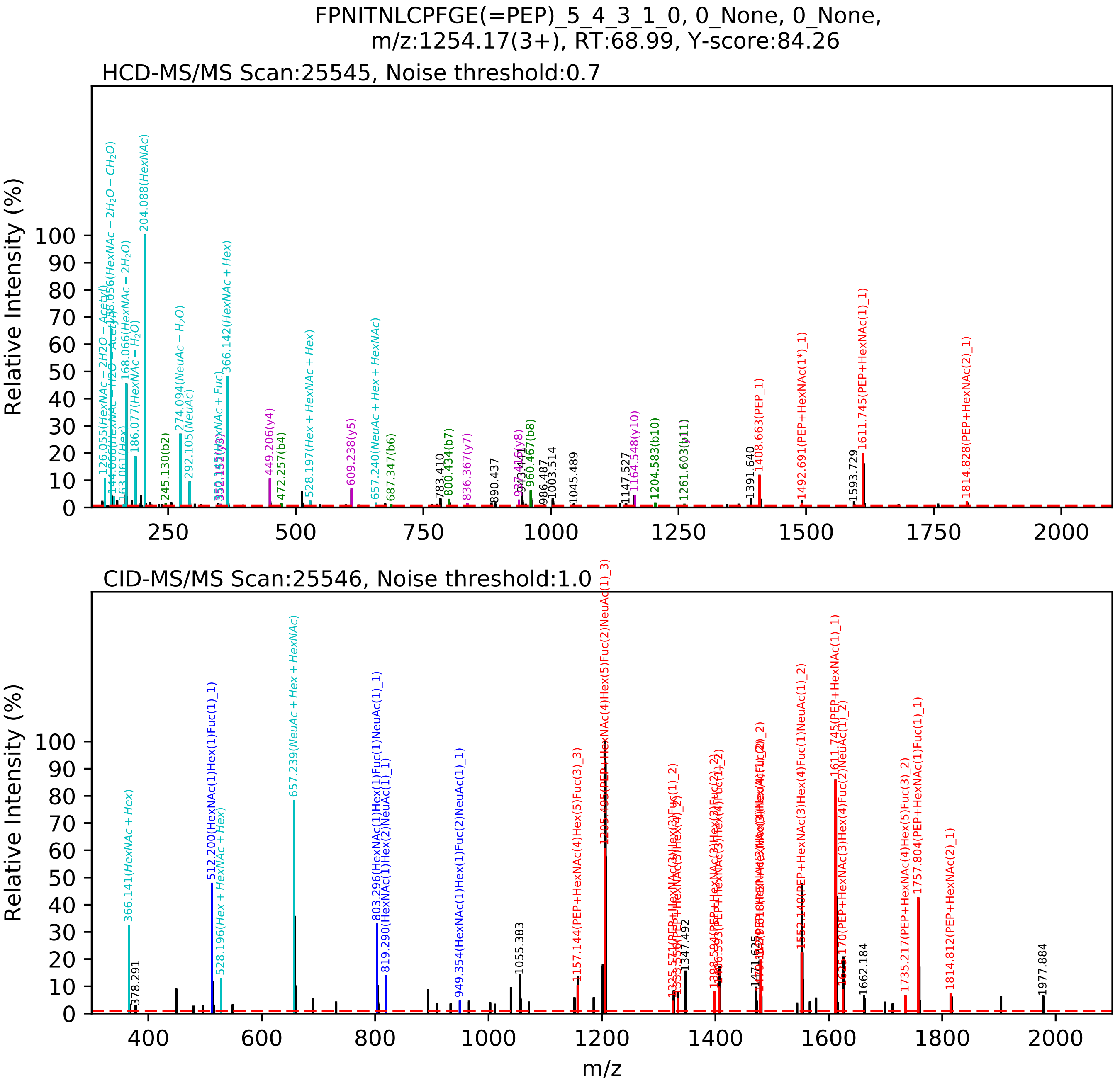
FPNITNLCPFGE(=PEP)_5_4_3_1_0, 0_None, 0_None,
m/z:1254.17(3+), RT:68.99, Y-score:84.26
HCD-MS/MS Scan:25545, Noise threshold:0.7
Relative Intensity (%)
126.055(HexNAc − 2H2O − Acetyl)
204.088(HexNAc)
168.066(HexNAc − 2H2O)
186.077(HexNAc − H2O)
163.061(Hex)
245.130(b2)
274.094(NeuAc − H2O)
292.105(NeuAc)
366.142(HexNAc + Hex)
449.206(y4)
472.257(b4)
528.197(Hex + HexNAc + Hex)
609.238(y5)
657.240(NeuAc + Hex + HexNAc)
687.347(b6)
783.410
800.434(b7)
836.367(y7)
890.437
960.467(b8)
986.487
1003.514
1045.489
1147.527
1164.548(y10)
1204.583(b10)
1261.603(b11)
1391.640
1408.663(PEP_1)
1492.691(PEP+HexNAc(1*)_1)
1593.729
1611.745(PEP+HexNAc(1)_1)
1814.828(PEP+HexNAc(2)_1)
CID-MS/MS Scan:25546, Noise threshold:1.0
366.141(HexNAc + Hex)
378.291
512.200(HexNAc(1)Hex(1)Fuc(1)_1)
528.196(Hex + HexNAc + Hex)
657.239(NeuAc + Hex + HexNAc)
803.296(HexNAc(1)Hex(1)Fuc(1)NeuAc(1)_1)
819.290(HexNAc(1)Hex(2)NeuAc(1)_1)
949.354(HexNAc(1)Hex(1)Fuc(2)NeuAc(1)_1)
1055.383
1157.144(PEP+HexNAc(4)Hex(5)Fuc(3)_3)
1205.405(PEP+HexNAc(4)Hex(5)Fuc(2)NeuAc(1)_3)
1347.492
1471.625
1552.140(PEP+HexNAc(3)Hex(4)Fuc(1)NeuAc(1)_2)
1611.745(PEP+HexNAc(1)_1)
1662.184
1735.217(PEP+HexNAc(4)Hex(5)Fuc(3)_2)
1757.804(PEP+HexNAc(1)Fuc(1)_1)
1814.812(PEP+HexNAc(2)_1)
1977.884
m/z

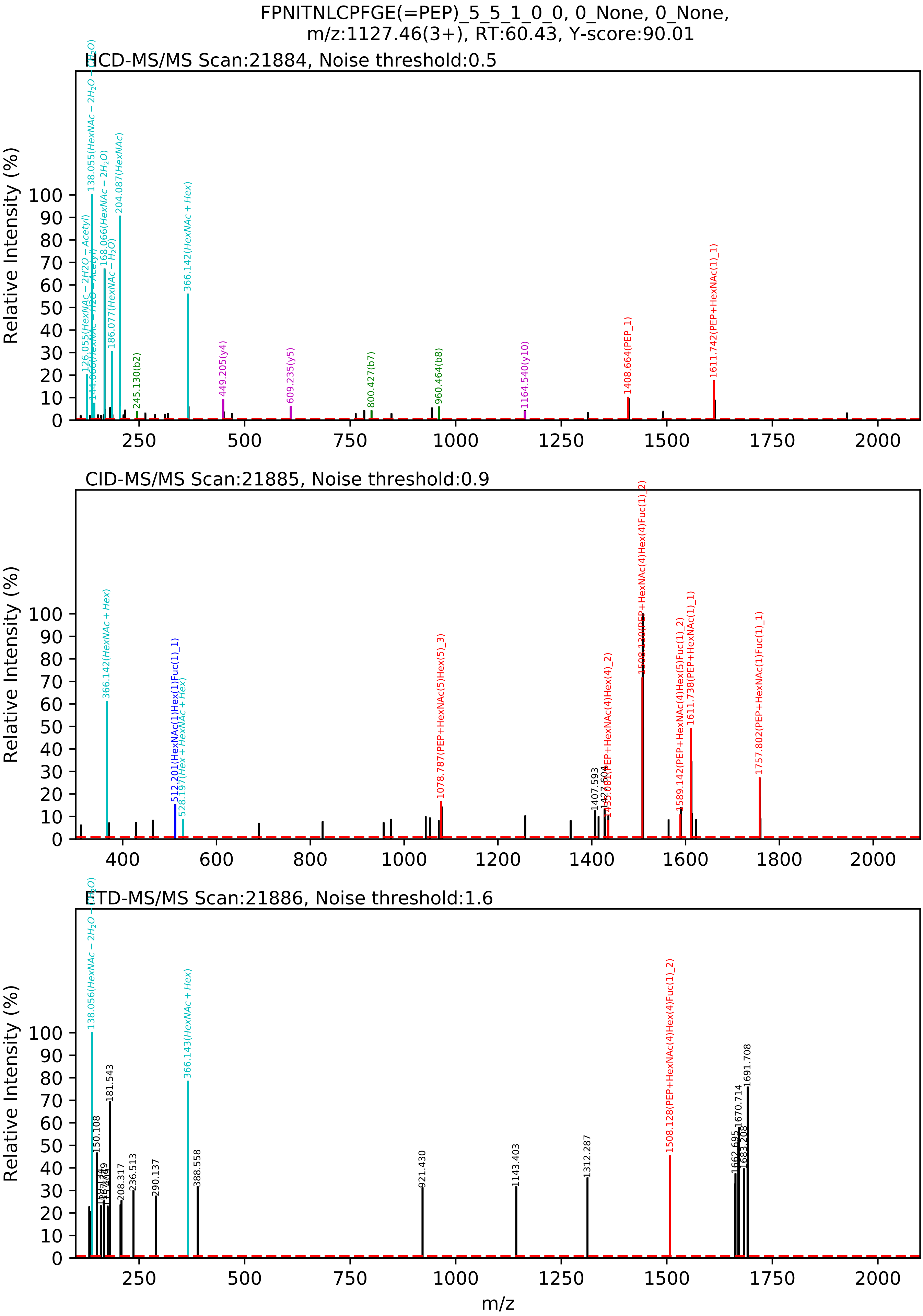
FPNITNLCPFGE(=PEP)_5_5_1_0_0, 0_None, 0_None,
m/z:1127.46(3+), RT:60.43, Y-score:90.01
HCD-MS/MS Scan:21884, Noise threshold:0.5
Relative Intensity (%)
126.055(HexNAc − 2H2O − Acetyl)
144.066(HexNAc − H2O − Acetyl)
138.055(HexNAc − 2H2O − H2O)
168.066(HexNAc − 2H2O)
186.077(HexNAc − H2O)
204.087(HexNAc)
245.130(b2)
366.142(HexNAc + Hex)
449.205(y4)
609.235(y5)
800.427(b7)
960.464(b8)
1164.540(y10)
1408.664(PEP_1)
1611.742(PEP+HexNAc(1)_1)
CID-MS/MS Scan:21885, Noise threshold:0.9
366.142(HexNAc + Hex)
512.201(HexNAc(1)Hex(1)Fuc(1)_1)
528.197(Hex + HexNAc + Hex)
1078.787(PEP+HexNAc(5)Hex(5)_3)
1407.593
1427.604
1433.081(PEP+HexNAc(4)Hex(4)_2)
1508.130(PEP+HexNAc(4)Hex(4)Fuc(1)_2)
1589.142(PEP+HexNAc(4)Hex(5)Fuc(1)_2)
1611.738(PEP+HexNAc(1)_1)
1757.802(PEP+HexNAc(1)Fuc(1)_1)
ETD-MS/MS Scan:21886, Noise threshold:1.6
138.056(HexNAc − 2H2O − H2O)
150.108
181.543
208.317
236.513
290.137
366.143(HexNAc + Hex)
388.558
921.430
1143.403
1312.287
1508.128(PEP+HexNAc(4)Hex(4)Fuc(1)_2)
1662.695
1670.714
1683.208
1691.708
m/z

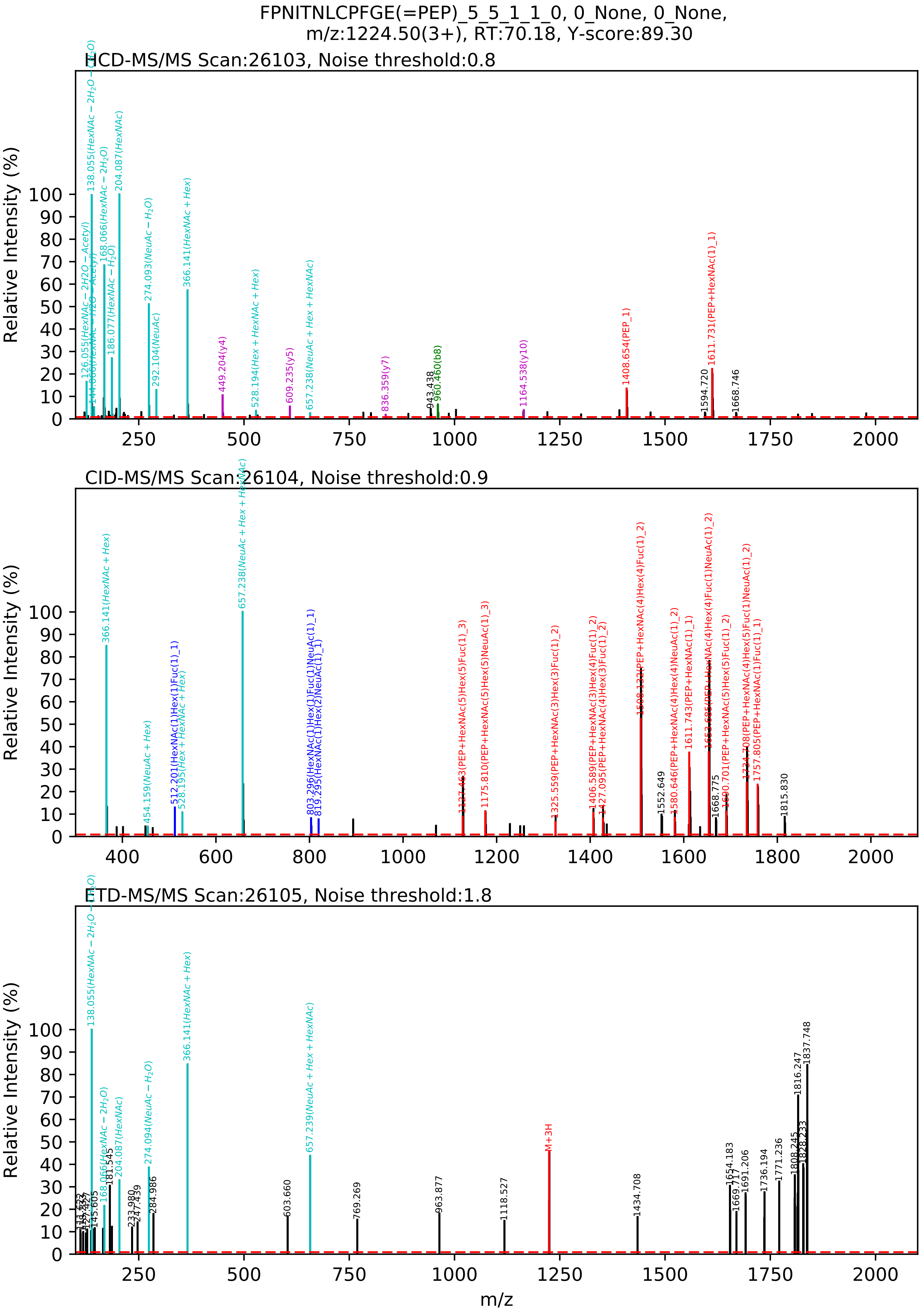

FPNITNLCPFGE(=PEP)_5_5_1_1_0, 0_None, 0_None,
m/z:1224.50(3+), RT:70.18, Y-score:89.30
HCD-MS/MS Scan:26103, Noise threshold:0.8
CID-MS/MS Scan:26104, Noise threshold:0.9
ETD-MS/MS Scan:26105, Noise threshold:1.8
Relative Intensity (%)
m/z
138.055(HexNAc − 2H2O − H2O)
204.087(HexNAc)
168.066(HexNAc − 2H2O)
186.077(HexNAc − H2O)
274.093(NeuAc − H2O)
292.104(NeuAc)
366.141(HexNAc + Hex)
449.204(y4)
528.194(Hex + HexNAc + Hex)
609.235(y5)
657.238(NeuAc + Hex + HexNAc)
836.359(y7)
943.438
960.460(b8)
1164.538(y10)
1408.654(PEP_1)
1594.720
1611.731(PEP+HexNAc(1)_1)
1668.746
454.159(NeuAc + Hex)
512.201(HexNAc(1)Hex(1)Fuc(1)_1)
528.195(Hex + HexNAc + Hex)
803.296(HexNAc(1)Hex(1)Fuc(1)NeuAc(1)_1)
819.295(HexNAc(1)Hex(2)NeuAc(1)_1)
1127.463(PEP+HexNAc(5)Hex(5)Fuc(1)_3)
1175.810(PEP+HexNAc(5)Hex(5)NeuAc(1)_3)
1325.559(PEP+HexNAc(3)Hex(3)Fuc(1)_2)
1406.589(PEP+HexNAc(3)Hex(4)Fuc(1)_2)
1427.095(PEP+HexNAc(4)Hex(3)Fuc(1)_2)
1508.122(PEP+HexNAc(4)Hex(4)Fuc(1)_2)
1552.649
1580.646(PEP+HexNAc(4)Hex(4)NeuAc(1)_2)
1611.743(PEP+HexNAc(1)_1)
1653.685(PEP+HexNAc(4)Hex(4)Fuc(1)NeuAc(1)_2)
1668.775
1690.701(PEP+HexNAc(5)Hex(5)Fuc(1)_2)
1734.708(PEP+HexNAc(4)Hex(5)Fuc(1)NeuAc(1)_2)
1757.805(PEP+HexNAc(1)Fuc(1)_1)
1815.830
168.066(HexNAc − 2H2O)
204.087(HexNAc)
181.545
233.980
247.439
284.986
274.094(NeuAc − H2O)
366.141(HexNAc + Hex)
603.660
657.239(NeuAc + Hex + HexNAc)
769.269
963.877
1118.527
M+3H
1434.708
1654.183
1669.717
1691.206
1736.194
1771.236
1808.245
1816.247
1828.233
1837.748

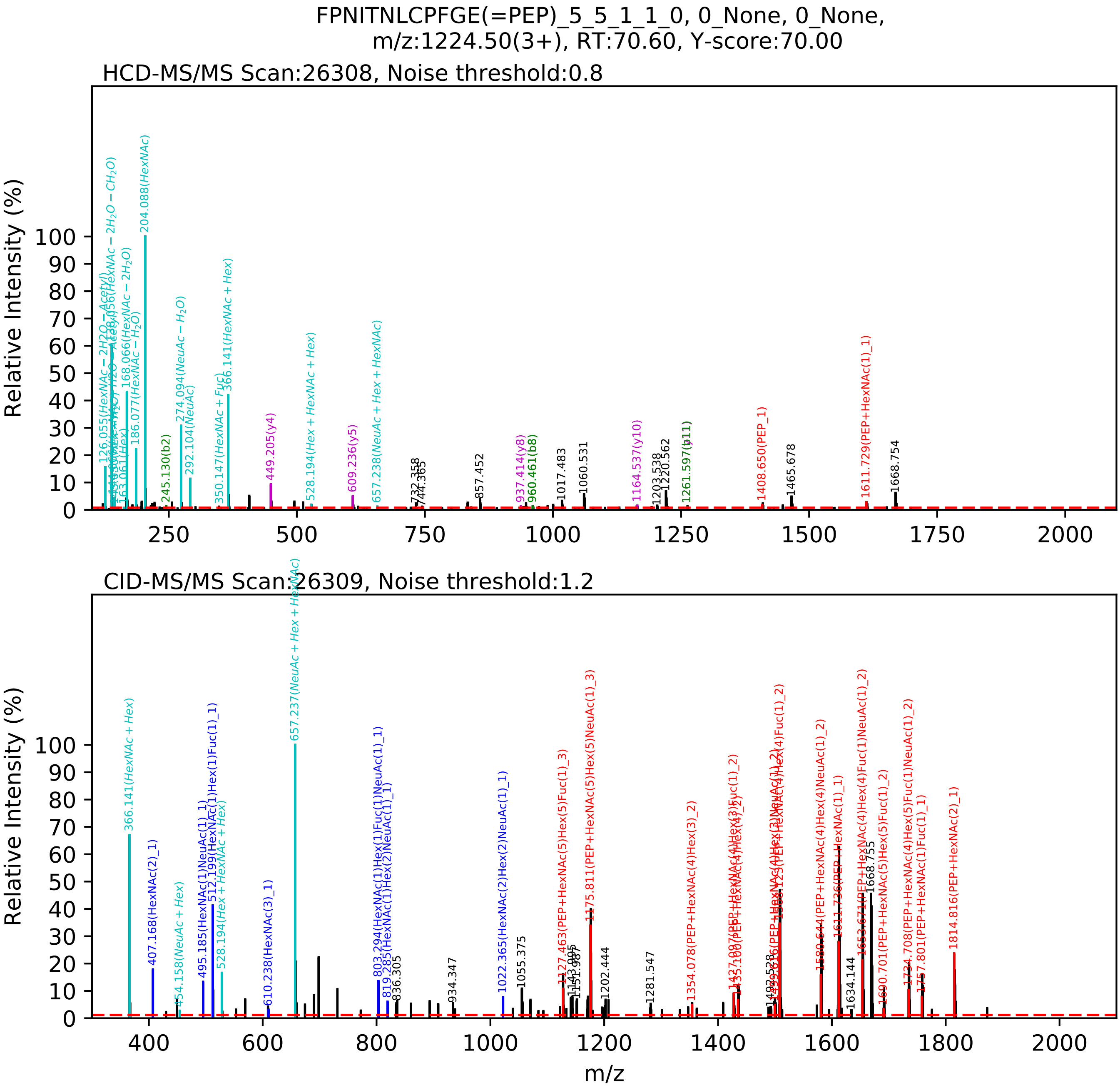
FPNITNLCPFGE(=PEP)_5_5_1_1_0, 0_None, 0_None,
m/z:1224.50(3+), RT:70.60, Y-score:70.00
HCD-MS/MS Scan:26308, Noise threshold:0.8
Relative Intensity (%)
204.088(HexNAc)
366.141(HexNAc+Hex)
274.094(NeuAc−H2O)
292.104(NeuAc)
245.130(b2)
449.205(y4)
528.194(Hex+HexNAc+Hex)
609.236(y5)
657.238(NeuAc+Hex+HexNAc)
857.452
937.414(y8)
960.461(b8)
1017.483
1060.531
1164.537(y10)
1203.538
1220.562
1261.597(b11)
1408.650(PEP_1)
1465.678
1611.729(PEP+HexNAc(1)_1)
1668.754
CID-MS/MS Scan:26309, Noise threshold:1.2
Relative Intensity (%)
366.141(HexNAc+Hex)
407.168(HexNAc(2)_1)
454.158(NeuAc+Hex)
495.185(HexNAc(1)NeuAc(1)_1)
528.194(Hex+HexNAc+Hex)
610.238(HexNAc(3)_1)
657.237(NeuAc+Hex+HexNAc)
803.294(HexNAc(1)Hex(1)Fuc(1)NeuAc(1)_1)
819.285(HexNAc(1)Hex(2)NeuAc(1)_1)
836.305
934.347
1022.365(HexNAc(2)Hex(2)NeuAc(1)_1)
1055.375
1127.463(PEP+HexNAc(5)Hex(5)Fuc(1)_3)
1175.811(PEP+HexNAc(5)Hex(5)NeuAc(1)_3)
1202.444
1281.547
1354.078(PEP+HexNAc(4)Hex(3)_2)
1492.528
1580.644(PEP+HexNAc(4)Hex(4)NeuAc(1)_2)
1611.726(PEP+HexNAc(1)_1)
1634.144
1652.673(PEP+HexNAc(4)Hex(4)Fuc(1)NeuAc(1)_2)
1668.755
1690.701(PEP+HexNAc(5)Hex(5)Fuc(1)_2)
1734.708(PEP+HexNAc(4)Hex(5)Fuc(1)NeuAc(1)_2)
1757.801(PEP+HexNAc(1)Fuc(1)_1)
1814.816(PEP+HexNAc(2)_1)
m/z

FPNITNLCPFGE(=PEP)_5_5_1_1_0, 0_None, 0_None,
m/z:1224.50(3+), RT:68.97, Y-score:74.55

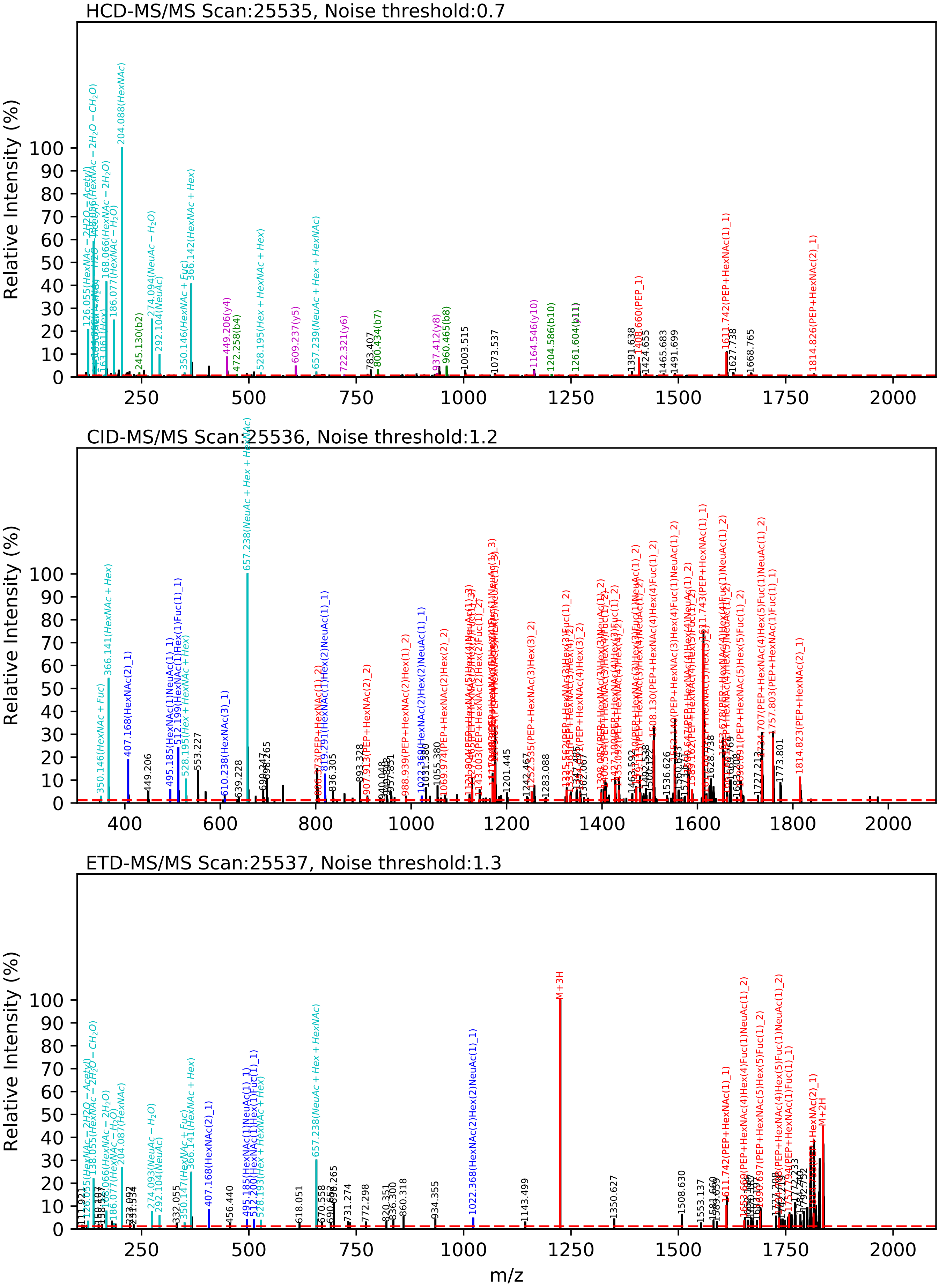

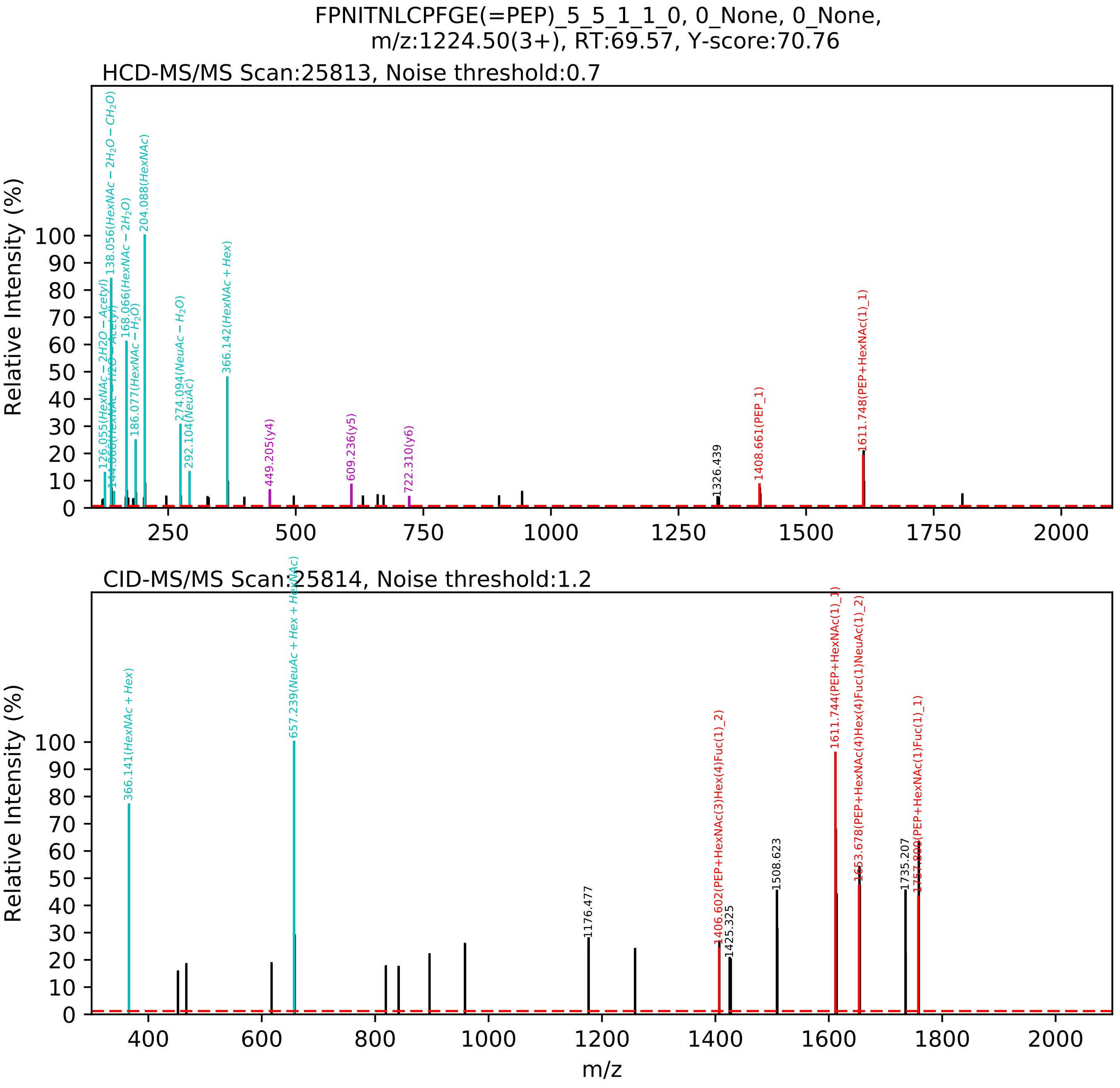

FPNITNLCPFGE(=PEP)_5_5_1_1_0, 0_None, 0_None,
m/z:1224.50(3+), RT:69.57, Y-score:70.76
HCD-MS/MS Scan:25813, Noise threshold:0.7
Relative Intensity (%)
204.088(HexNAc)
366.142(HexNAc + Hex)
274.094(NeuAc − H2O)
292.104(NeuAc)
449.205(y4)
609.236(y5)
722.310(y6)
1326.439
1408.661(PEP_1)
1611.748(PEP+HexNAc(1)_1)
CID-MS/MS Scan:25814, Noise threshold:1.2
366.141(HexNAc + Hex)
657.239(NeuAc + Hex + HexNAc)
1176.477
1406.602(PEP+HexNAc(3)Hex(4)Fuc(1)_2)
1425.325
1508.623
1611.744(PEP+HexNAc(1)_1)
1653.678(PEP+HexNAc(4)Hex(4)Fuc(1)NeuAc(1)_2)
1735.207
1757.800(PEP+HexNAc(1)Fuc(1)_1)
m/z

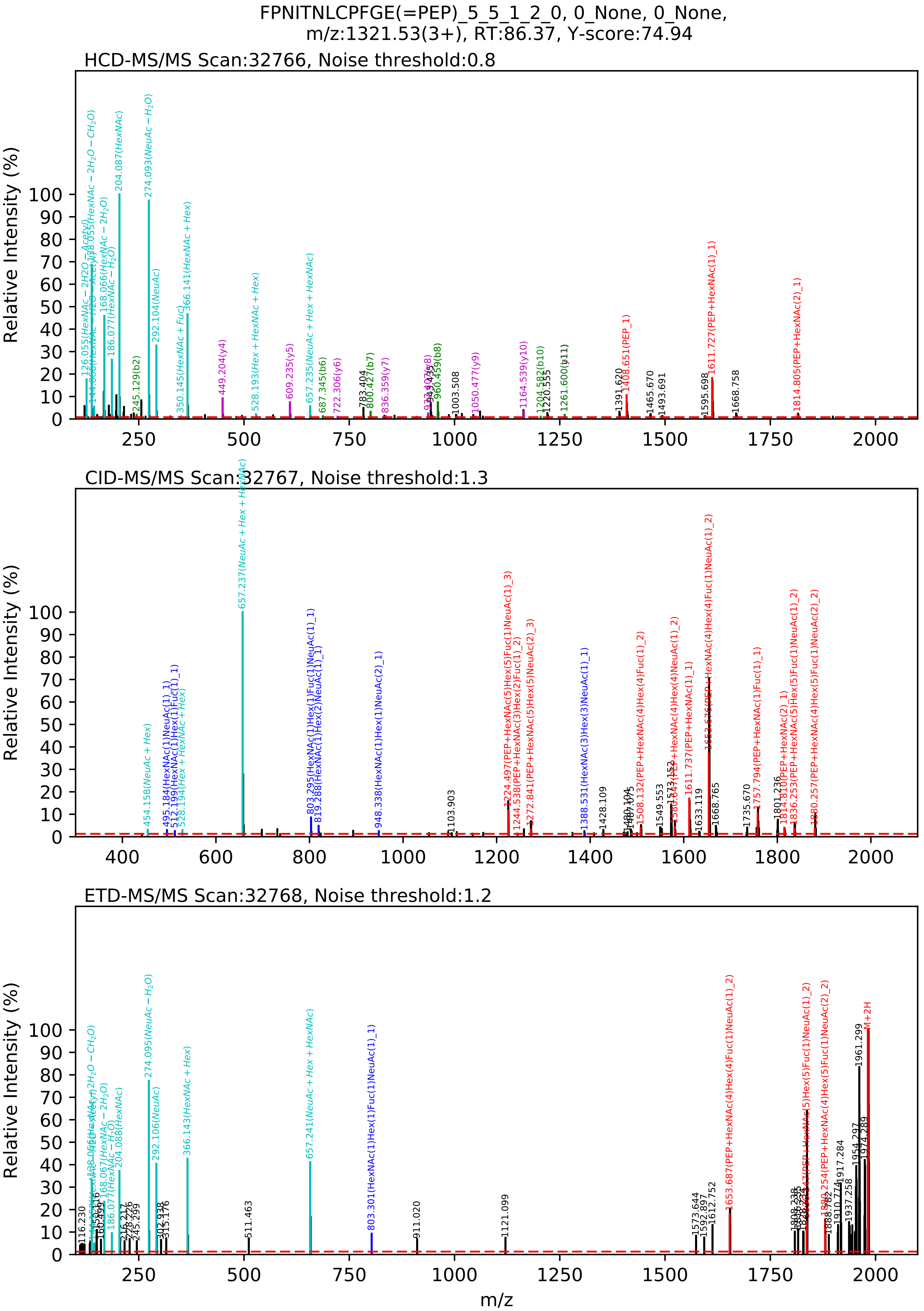

FPNITNLCPFGE(=PEP)_5_5_1_2_0, 0_None, 0_None,
m/z:1321.53(3+), RT:86.37, Y-score:74.94
HCD-MS/MS Scan:32766, Noise threshold:0.8
CID-MS/MS Scan:32767, Noise threshold:1.3
ETD-MS/MS Scan:32768, Noise threshold:1.2
Relative Intensity (%)
m/z
204.087(HexNAc)
274.093(NeuAc − H2O)
292.104(NeuAc)
366.141(HexNAc + Hex)
657.235(NeuAc + Hex + HexNAc)
1408.651(PEP_1)
1611.727(PEP+HexNAc(1)_1)
1814.805(PEP+HexNAc(2)_1)
657.237(NeuAc + Hex + HexNAc)
1653.676(PEP+HexNAc(4)Hex(4)Fuc(1)NeuAc(1)_2)
1224.497(PEP+HexNAc(5)Hex(5)Fuc(1)NeuAc(1)_3)
1880.257(PEP+HexNAc(4)Hex(5)Fuc(1)NeuAc(2)_2)
1653.687(PEP+HexNAc(4)Hex(4)Fuc(1)NeuAc(1)_2)
803.301(HexNAc(1)Hex(1)Fuc(1)NeuAc(1)_1)
1961.299
M+2H

FPNITNLCPFGE(=PEP)_5_5_1_2_0, 0_None, 0_None,
m/z:1321.53(3+), RT:86.47, Y-score:79.80

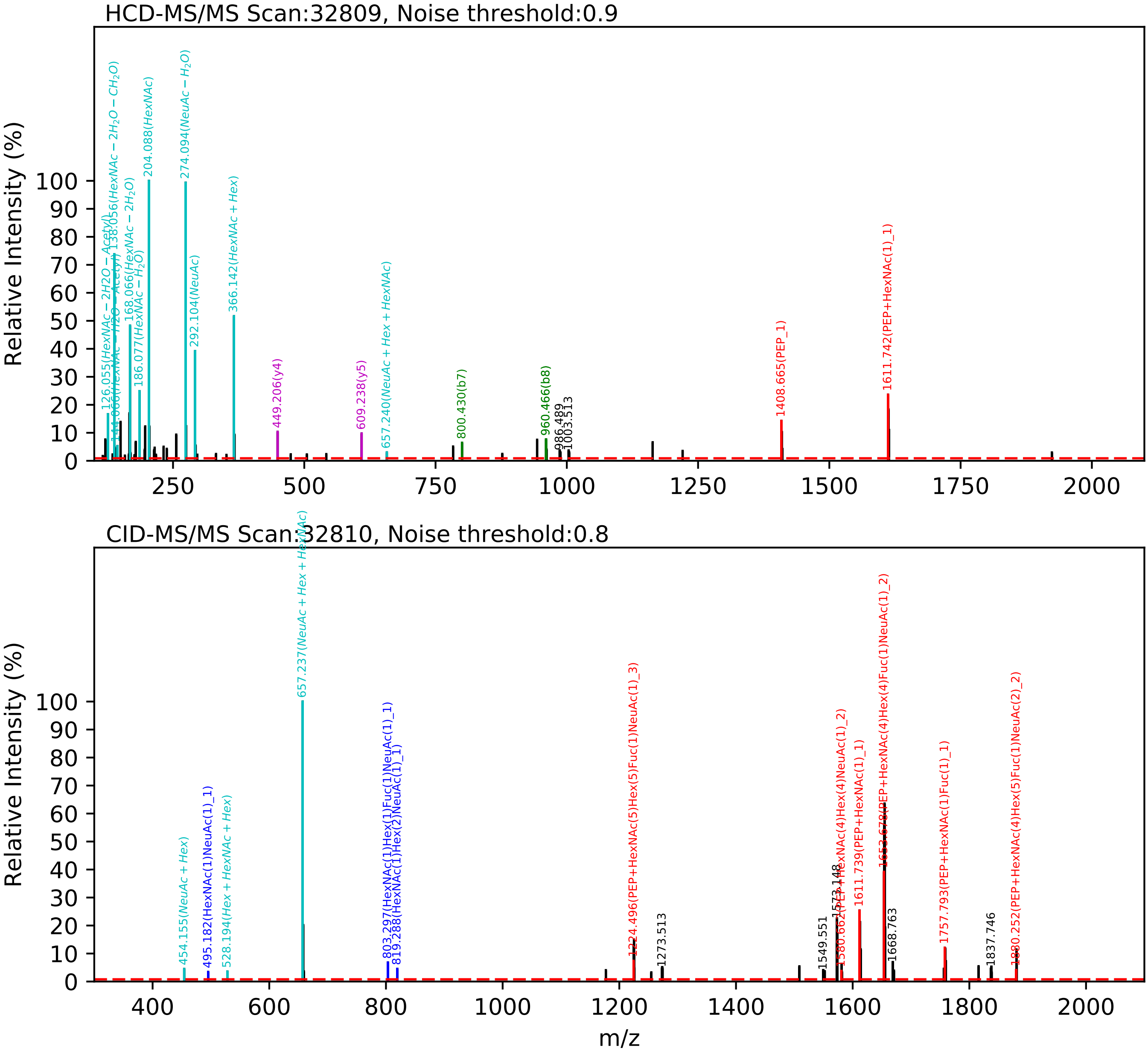

FPNITNLCPFGE(=PEP)_5_5_1_2_0, 0_None, 0_None,
m/z:991.40(4+), RT:85.51, Y-score:86.31

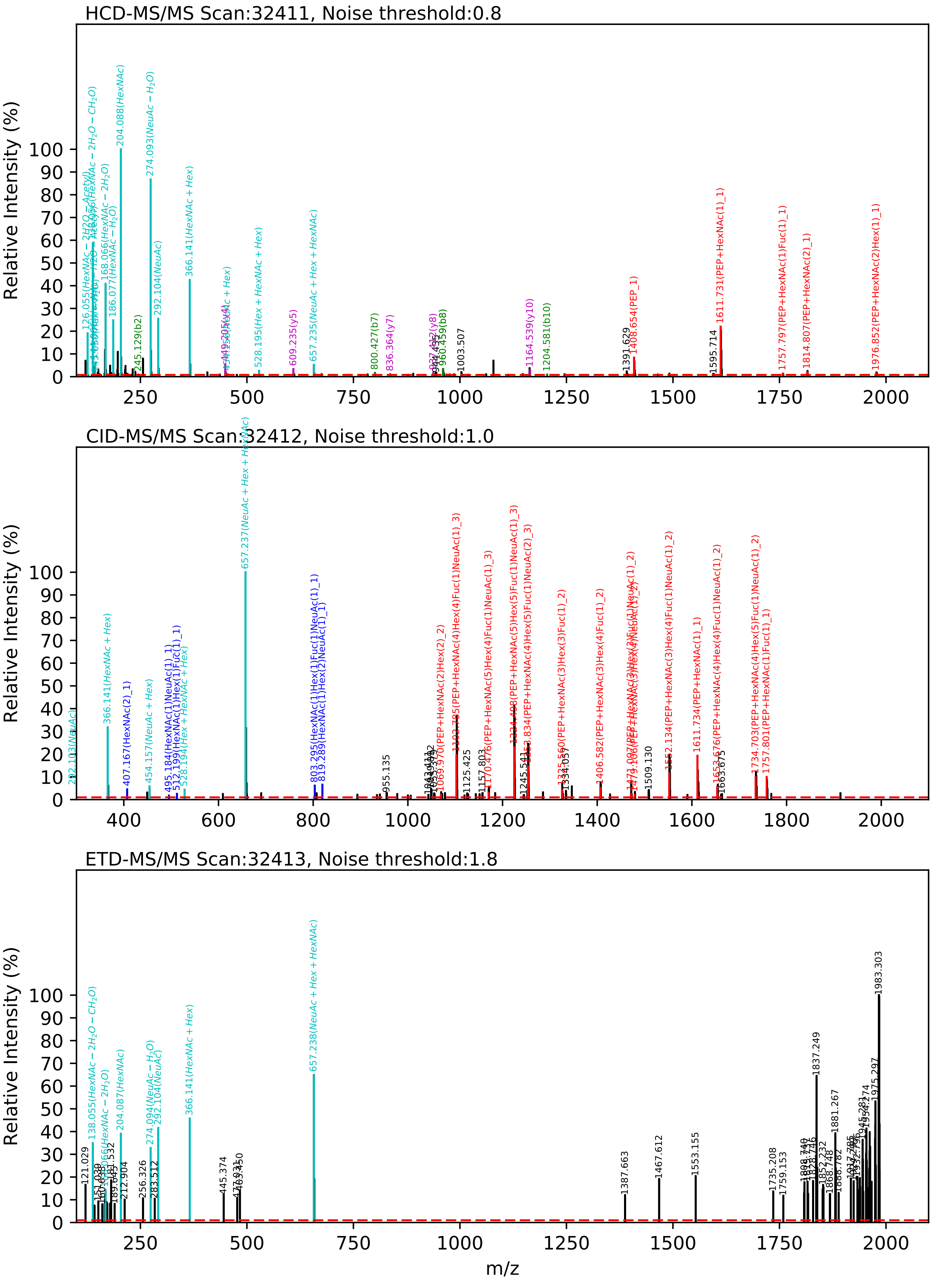

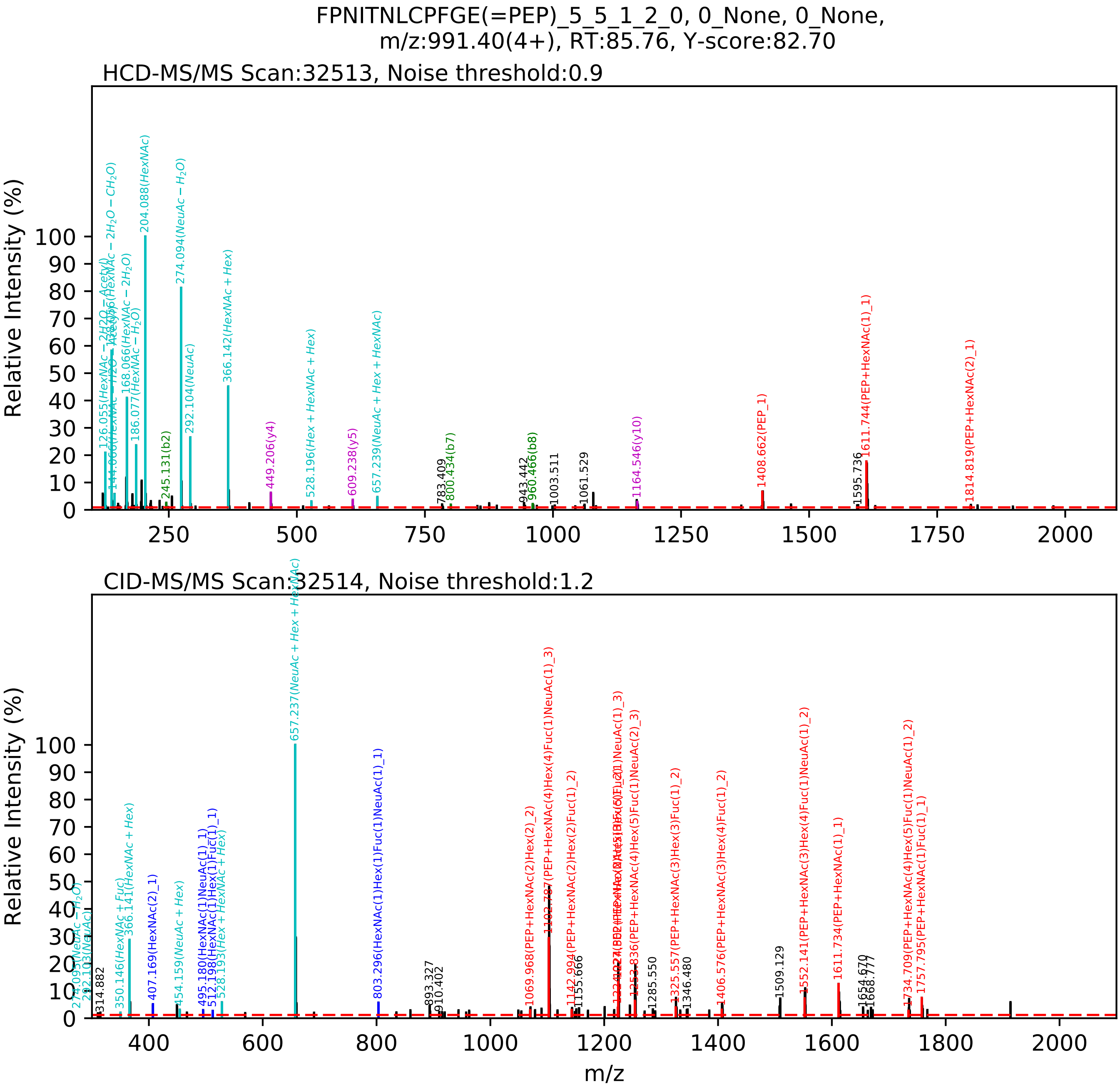
FPNITNLCPFGE(=PEP)_5_5_1_2_0, 0_None, 0_None,
m/z:991.40(4+), RT:85.76, Y-score:82.70
HCD-MS/MS Scan:32513, Noise threshold:0.9
CID-MS/MS Scan:32514, Noise threshold:1.2
Relative Intensity (%)
m/z
204.088(HexNAc)
274.094(NeuAc − H2O)
366.142(HexNAc + Hex)
292.104(NeuAc)
245.131(b2)
449.206(y4)
528.196(Hex + HexNAc + Hex)
609.238(y5)
657.239(NeuAc + Hex + HexNAc)
800.434(b7)
960.466(b8)
1164.546(y10)
1408.662(PEP_1)
1611.744(PEP+HexNAc(1)_1)
1814.819(PEP+HexNAc(2)_1)
657.237(NeuAc + Hex + HexNAc)
803.296(HexNAc(1)Hex(1)Fuc(1)NeuAc(1)_1)
1102.787(PEP+HexNAc(4)Hex(4)Fuc(1)NeuAc(1)_3)
1552.141(PEP+HexNAc(3)Hex(4)Fuc(1)NeuAc(1)_2)
1611.734(PEP+HexNAc(1)_1)
1734.709(PEP+HexNAc(4)Hex(5)Fuc(1)NeuAc(1)_2)
1757.795(PEP+HexNAc(1)Fuc(1)_1)

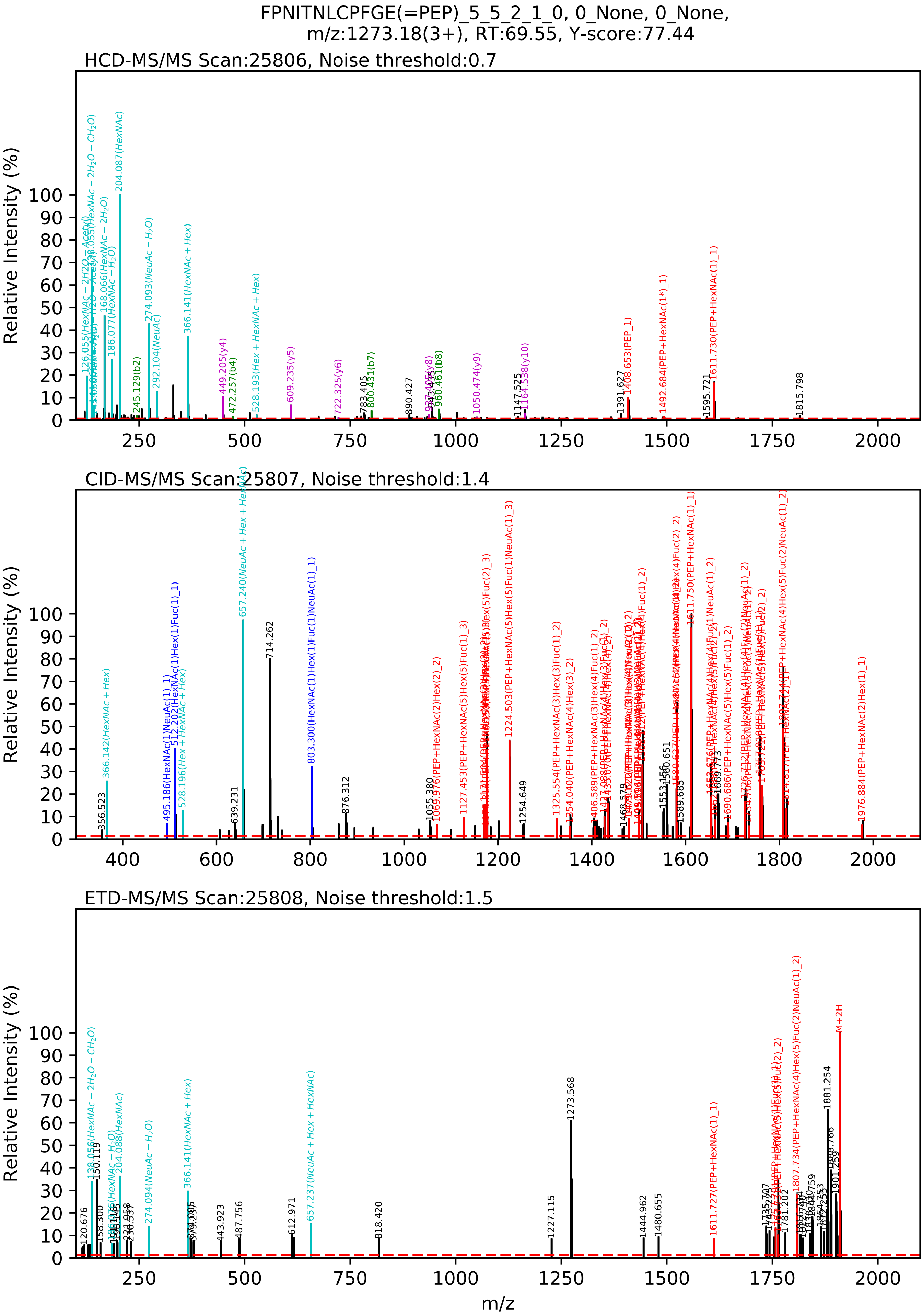
FPNITNLCPFGE(=PEP)_5_5_2_1_0, 0_None, 0_None,
m/z:1273.18(3+), RT:69.55, Y-score:77.44
HCD-MS/MS Scan:25806, Noise threshold:0.7
CID-MS/MS Scan:25807, Noise threshold:1.4
ETD-MS/MS Scan:25808, Noise threshold:1.5
Relative Intensity (%)
m/z
204.087(HexNAc)
366.141(HexNAc + Hex)
274.093(NeuAc − H2O)
292.104(NeuAc)
1611.730(PEP+HexNAc(1)_1)
1408.653(PEP_1)
1492.684(PEP+HexNAc(1*)_1)
657.240(NeuAc + Hex + HexNAc)
714.262
803.300(HexNAc(1)Hex(1)Fuc(1)NeuAc(1)_1)
1224.503(PEP+HexNAc(5)Hex(5)Fuc(1)NeuAc(1)_3)
1611.750(PEP+HexNAc(1)_1)
1976.884(PEP+HexNAc(2)Hex(1)_1)
1273.568
1881.254
1807.734(PEP+HexNAc(4)Hex(5)Fuc(2)NeuAc(1)_2)
M+2H

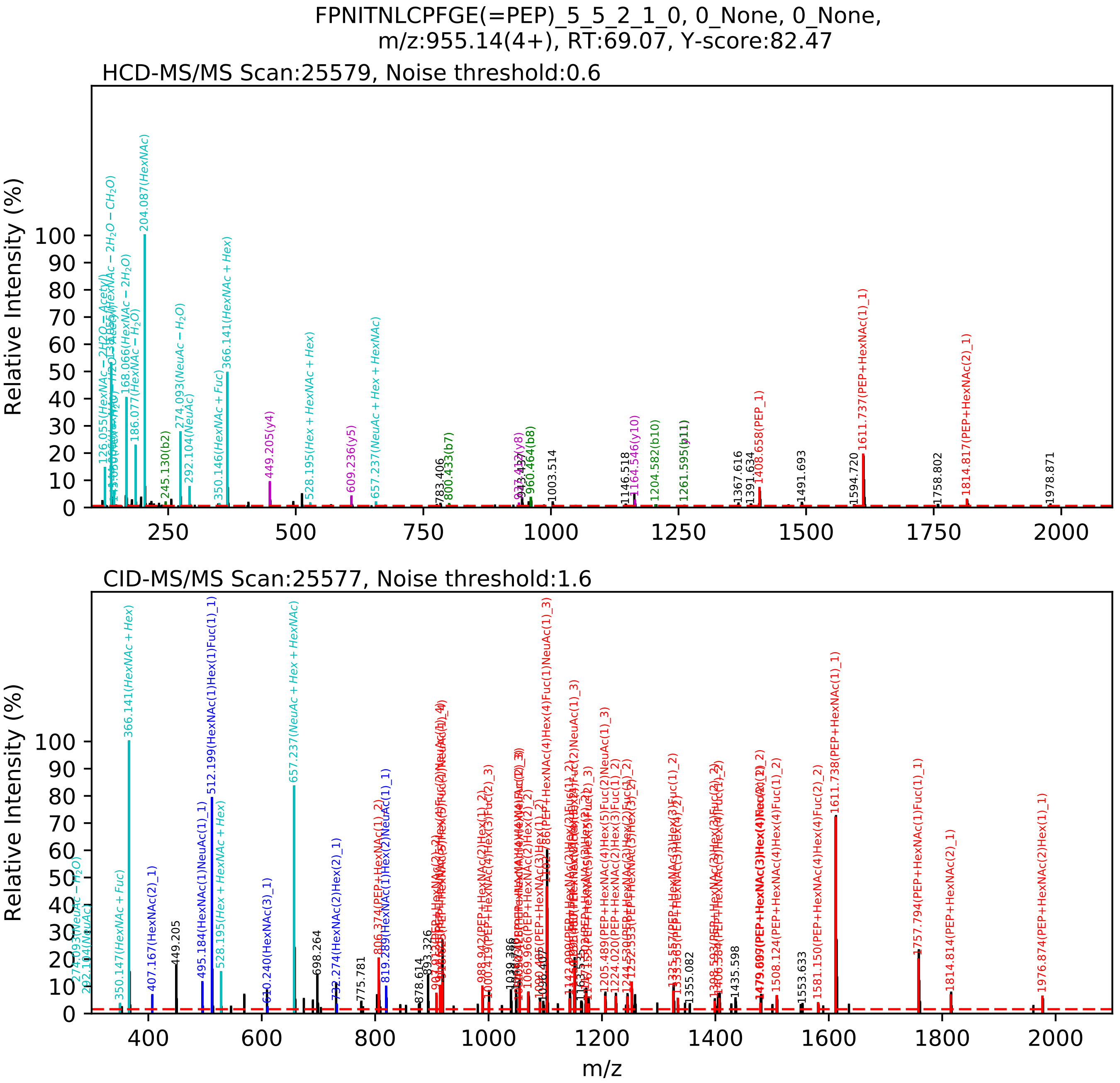
FPNITNLCPFGE(=PEP)_5_5_2_1_0, 0_None, 0_None,
m/z:955.14(4+), RT:69.07, Y-score:82.47
HCD-MS/MS Scan:25579, Noise threshold:0.6
Relative Intensity (%)
204.087(HexNAc)
366.141(HexNAc + Hex)
449.205(y4)
528.195(Hex + HexNAc + Hex)
609.236(y5)
657.237(NeuAc + Hex + HexNAc)
1003.514
1408.658(PEP_1)
1611.737(PEP+HexNAc(1)_1)
1814.817(PEP+HexNAc(2)_1)
1978.871
CID-MS/MS Scan:25577, Noise threshold:1.6
Relative Intensity (%)
366.141(HexNAc + Hex)
512.199(HexNAc(1)Hex(1)Fuc(1)_1)
657.237(NeuAc + Hex + HexNAc)
1611.738(PEP+HexNAc(1)_1)
1757.794(PEP+HexNAc(1)Fuc(1)_1)
1814.814(PEP+HexNAc(2)_1)
1976.874(PEP+HexNAc(2)Hex(1)_1)
m/z

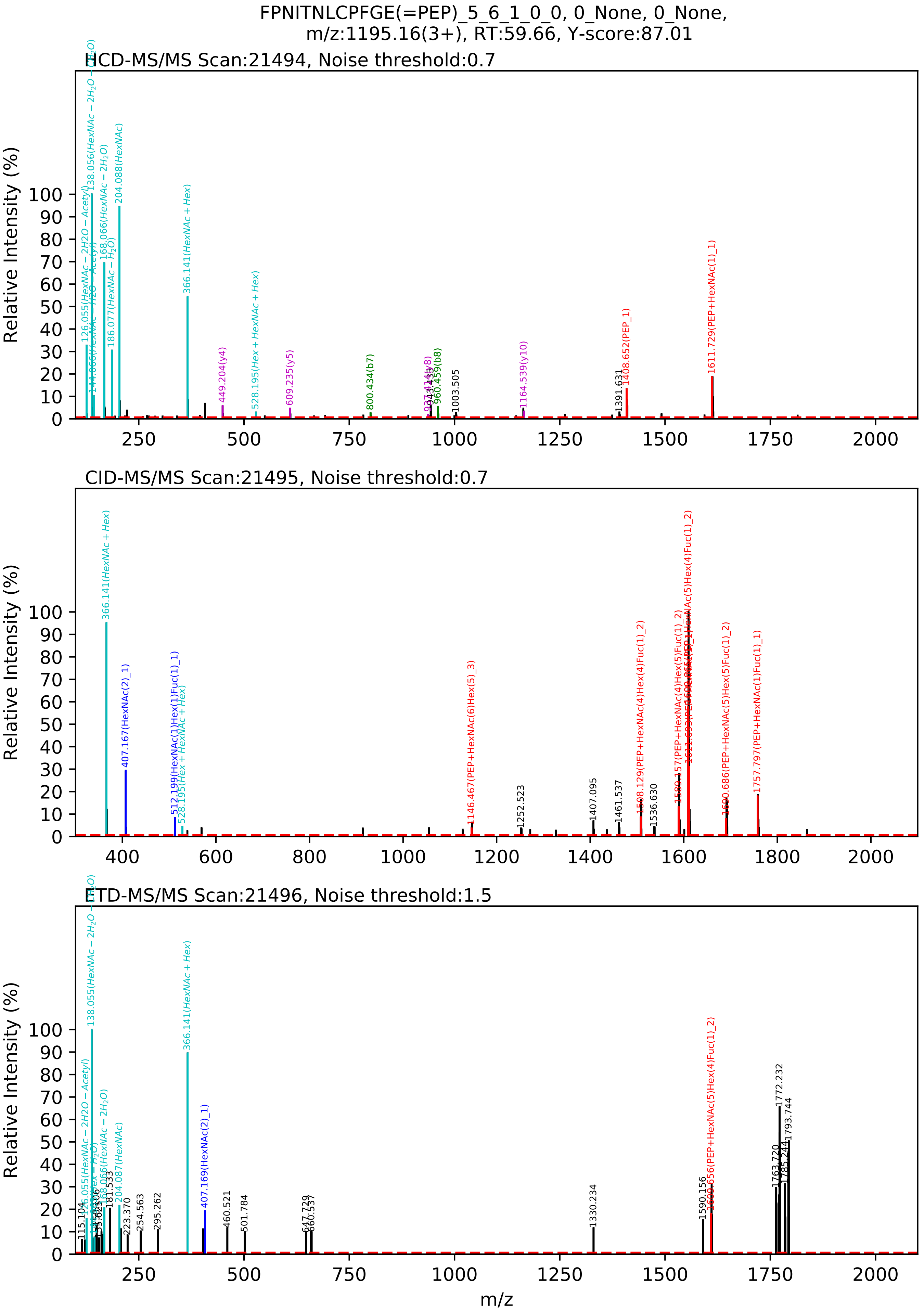
FPNITNLCPFGE(=PEP)_5_6_1_0_0, 0_None, 0_None,
m/z:1195.16(3+), RT:59.66, Y-score:87.01
HCD-MS/MS Scan:21494, Noise threshold:0.7
CID-MS/MS Scan:21495, Noise threshold:0.7
ETD-MS/MS Scan:21496, Noise threshold:1.5
Relative Intensity (%)
m/z
1611.729(PEP+HexNAc(1)_1)
1408.652(PEP_1)
366.141(HexNAc+Hex)
204.088(HexNAc)
138.056(HexNAc − 2H2O − H2O)
1164.539(y10)
960.459(b8)
800.434(b7)
609.235(y5)
449.204(y4)
1757.797(PEP+HexNAc(1)Fuc(1)_1)
1690.686(PEP+HexNAc(5)Hex(5)Fuc(1)_2)
1508.129(PEP+HexNAc(4)Hex(4)Fuc(1)_2)
1146.467(PEP+HexNAc(6)Hex(5)_3)
512.199(HexNAc(1)Hex(1)Fuc(1)_1)
407.167(HexNAc(2)_1)
1772.232
1793.744
1763.720
1330.234
1590.156
407.169(HexNAc(2)_1)

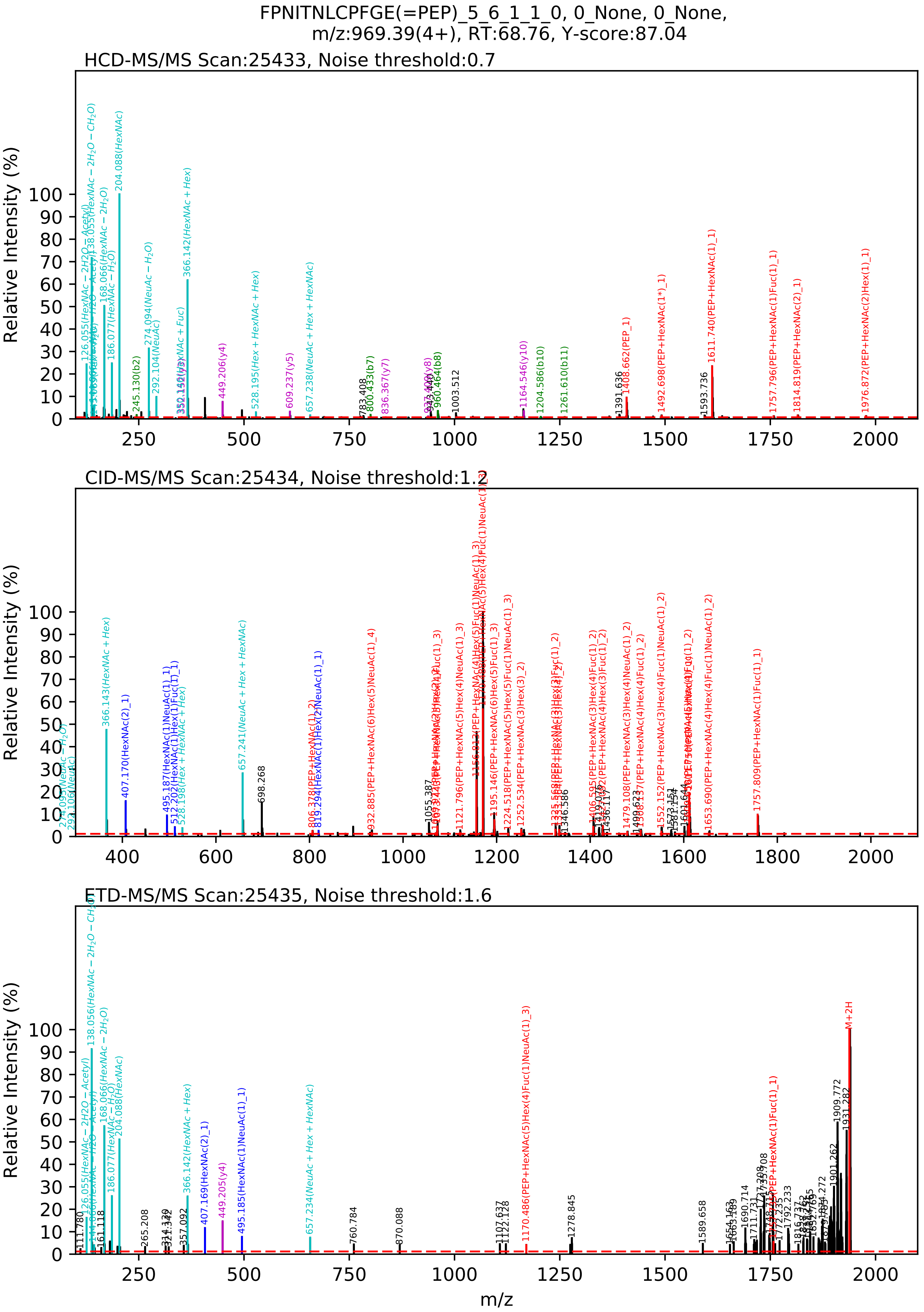
FPNITNLCPFGE(=PEP)_5_6_1_1_0, 0_None, 0_None,
m/z:969.39(4+), RT:68.76, Y-score:87.04
HCD-MS/MS Scan:25433, Noise threshold:0.7
Relative Intensity (%)
204.088(HexNAc)
366.142(HexNAc + Hex)
274.094(NeuAc − H2O)
292.104(NeuAc)
245.130(b2)
449.206(y4)
528.195(Hex + HexNAc + Hex)
609.237(y5)
657.238(NeuAc + Hex + HexNAc)
783.408
800.433(b7)
836.367(y7)
960.464(b8)
1003.512
1164.546(y10)
1204.586(b10)
1261.610(b11)
1391.636
1408.662(PEP_1)
1492.698(PEP+HexNAc(1*)_1)
1593.736
1611.740(PEP+HexNAc(1)_1)
1757.796(PEP+HexNAc(1)Fuc(1)_1)
1814.819(PEP+HexNAc(2)_1)
1976.872(PEP+HexNAc(2)Hex(1)_1)
CID-MS/MS Scan:25434, Noise threshold:1.2
366.143(HexNAc + Hex)
407.170(HexNAc(2)_1)
495.187(HexNAc(1)NeuAc(1)_1)
657.241(NeuAc + Hex + HexNAc)
698.268
806.378(PEP+HexNAc(1)_2)
819.294(HexNAc(1)Hex(2)NeuAc(1)_1)
932.885(PEP+HexNAc(6)Hex(5)NeuAc(1)_4)
1055.387
1121.796(PEP+HexNAc(5)Hex(4)NeuAc(1)_3)
1195.146(PEP+HexNAc(6)Hex(5)Fuc(1)NeuAc(1)_3)
1224.518(PEP+HexNAc(5)Hex(5)Fuc(1)NeuAc(1)_3)
1252.534(PEP+HexNAc(3)Hex(3)_2)
1346.586
1406.595(PEP+HexNAc(3)Hex(4)Fuc(1)_2)
1436.117
1479.108(PEP+HexNAc(3)Hex(4)NeuAc(1)_2)
1552.152(PEP+HexNAc(3)Hex(4)Fuc(1)NeuAc(1)_2)
1573.151
1653.690(PEP+HexNAc(4)Hex(4)Fuc(1)NeuAc(1)_2)
1757.809(PEP+HexNAc(1)Fuc(1)_1)
ETD-MS/MS Scan:25435, Noise threshold:1.6
111.780
138.056(HexNAc − 2H2O)
168.066(HexNAc − 2H2O)
186.077(HexNAc − H2O)
204.088(HexNAc)
161.118
265.208
314.139
357.092
366.142(HexNAc + Hex)
407.169(HexNAc(2)_1)
449.205(y4)
495.185(HexNAc(1)NeuAc(1)_1)
657.234(NeuAc + Hex + HexNAc)
760.784
870.088
1107.637
1122.128
1170.486(PEP+HexNAc(5)Hex(4)Fuc(1)NeuAc(1)_3)
1278.845
1589.658
1690.714
1711.731
1772.235
1792.233
1901.262
1909.772
1931.282
M+2H
m/z

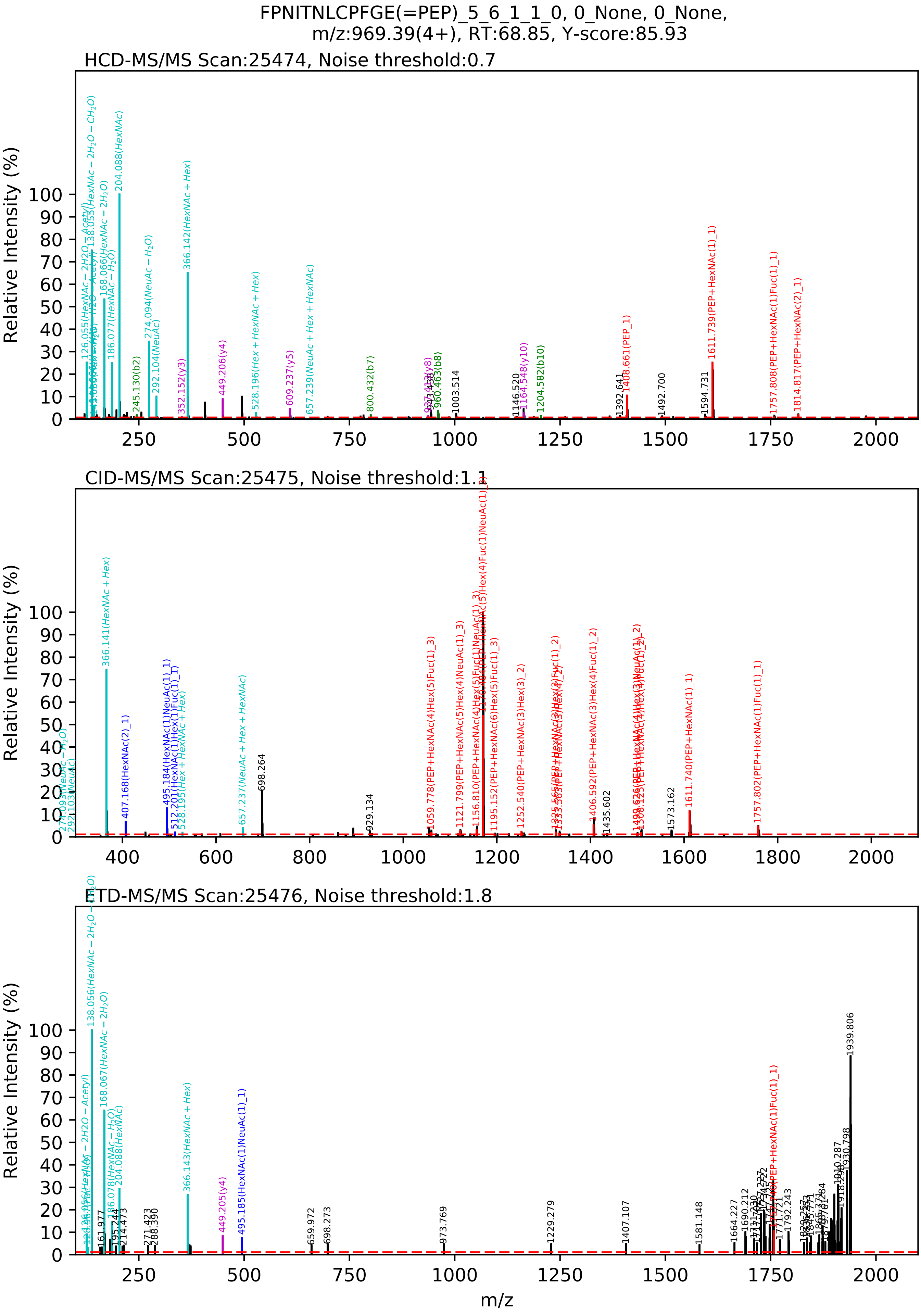
FPNITNLCPFGE(=PEP)_5_6_1_1_0, 0_None, 0_None,
m/z:969.39(4+), RT:68.85, Y-score:85.93
HCD-MS/MS Scan:25474, Noise threshold:0.7
CID-MS/MS Scan:25475, Noise threshold:1.1
ETD-MS/MS Scan:25476, Noise threshold:1.8
Relative Intensity (%)
m/z

FPNITNLCPFGE(=PEP)_5_6_1_1_0, 0_None, 0_None,
m/z:1292.19(3+), RT:89.42, Y-score:95.82

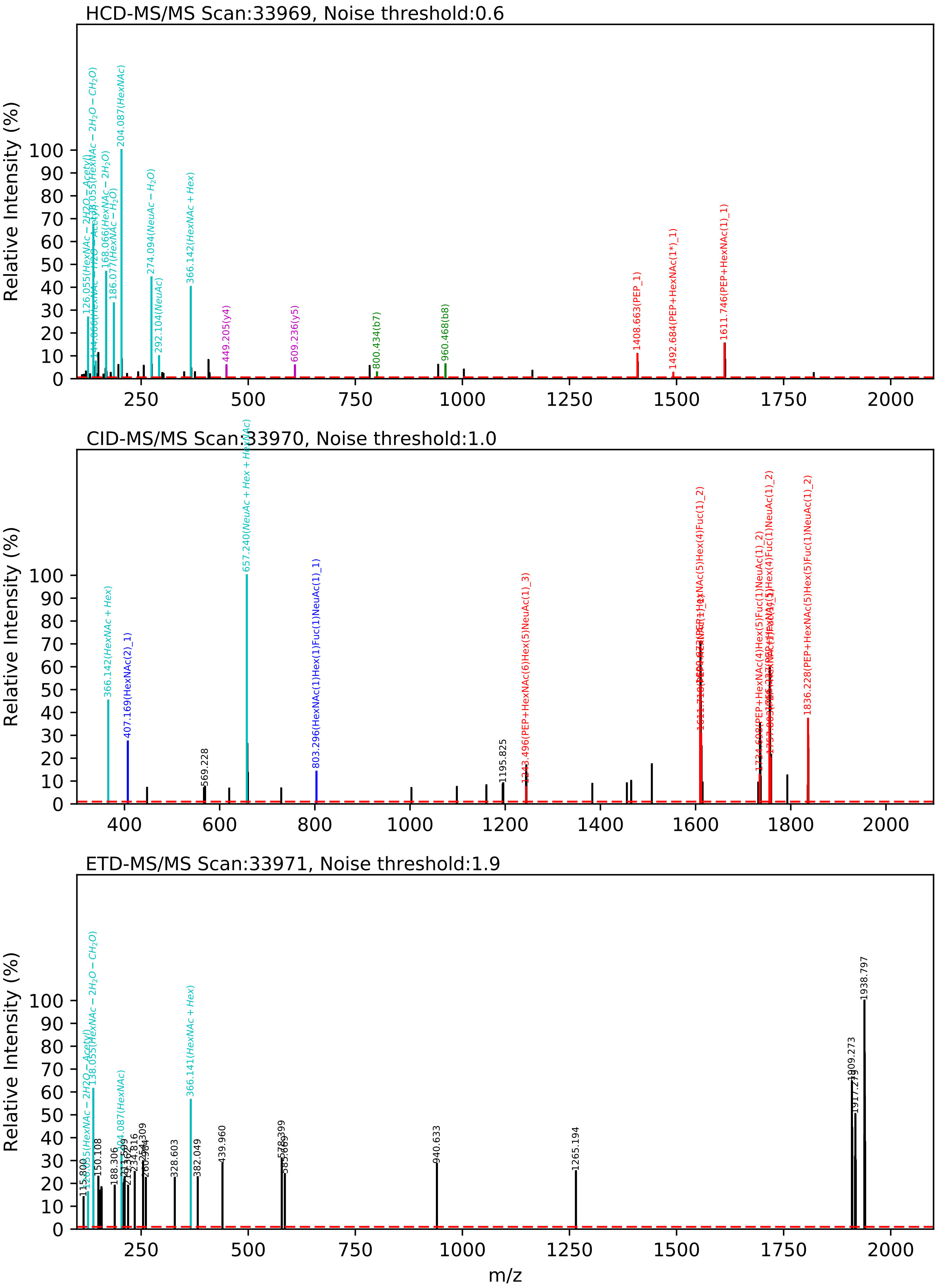

FPNITNLCPFGE(=PEP)_5_6_1_2_0, 0_None, 0_None,
m/z:1042.17(4+), RT:83.16, Y-score:82.01

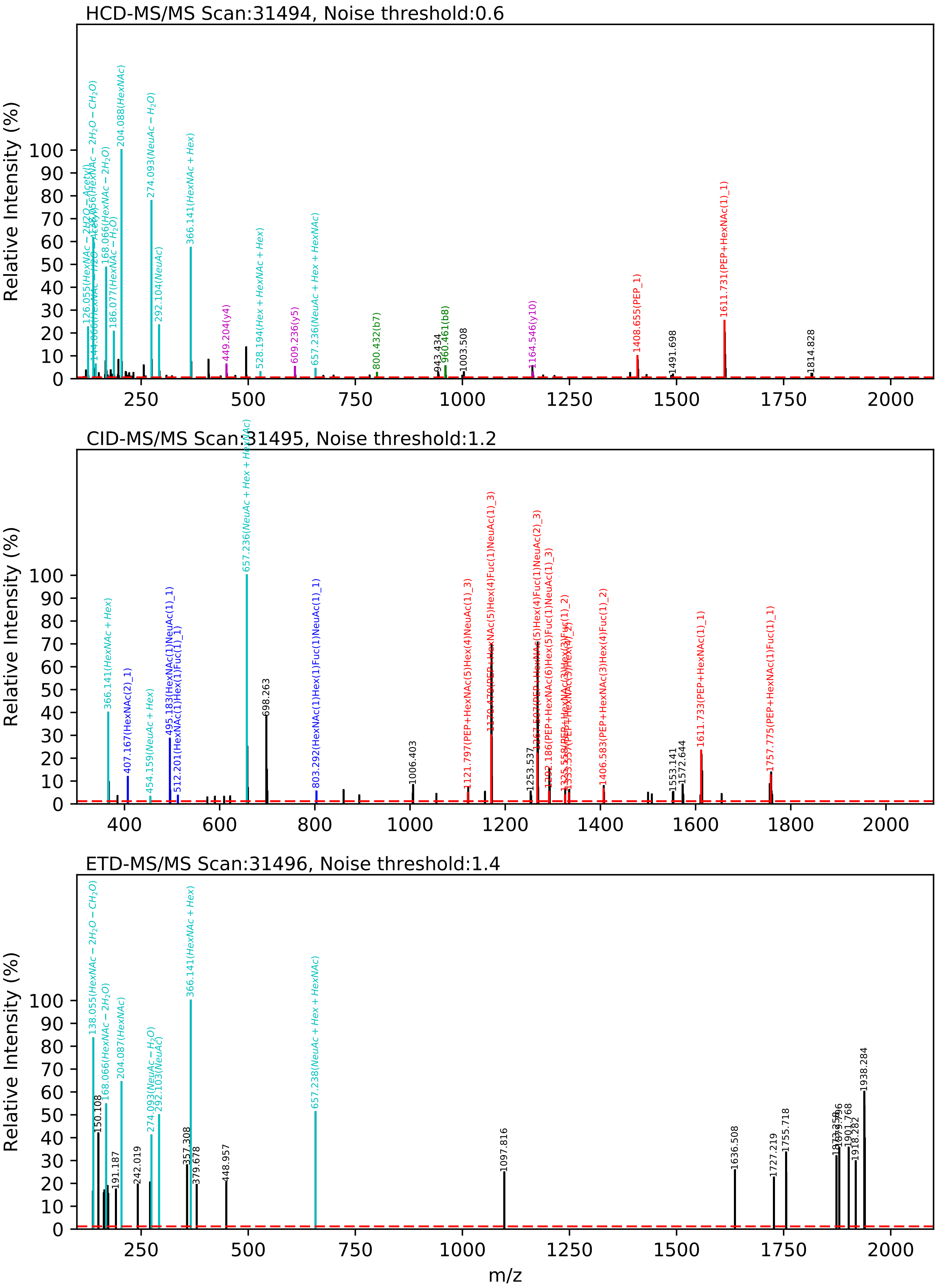

FPNITNLCPFGE(=PEP)_5_6_1_2_0, 0_None, 0_None,
m/z:1389.22(3+), RT:82.84, Y-score:77.72

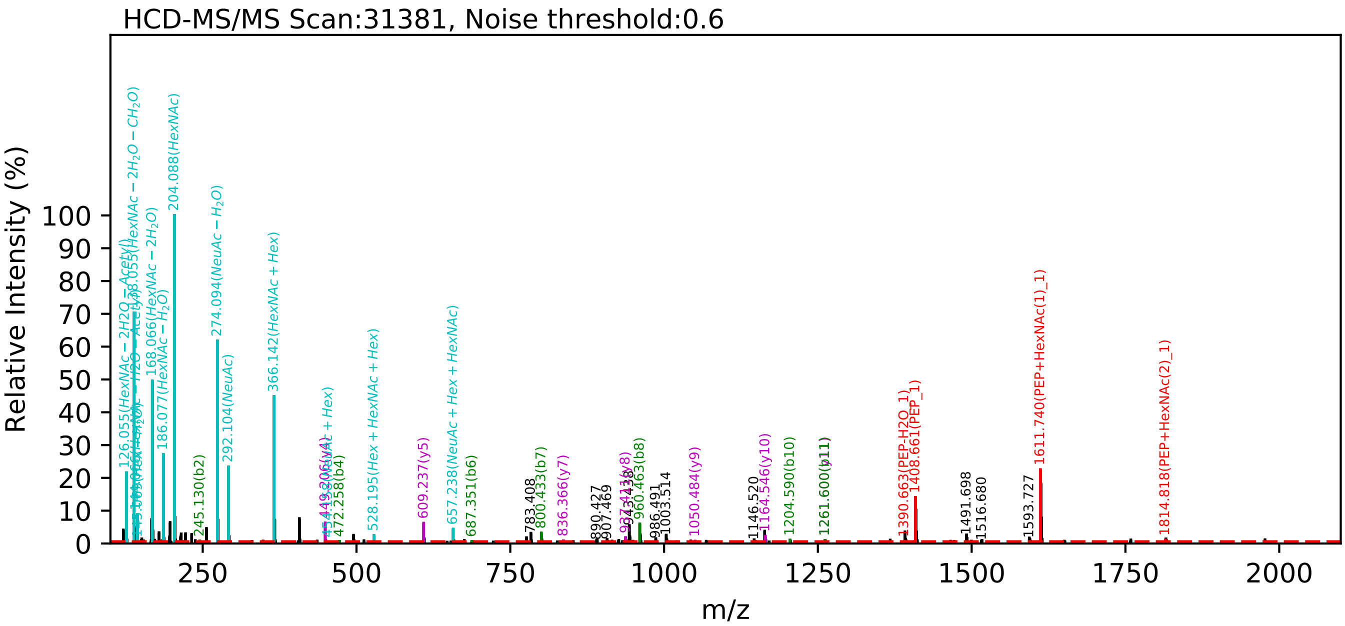

FPNITNLCPFGE(=PEP)_5_6_1_2_0, 0_None, 0_None,
m/z:1389.22(3+), RT:82.84, Y-score:77.72

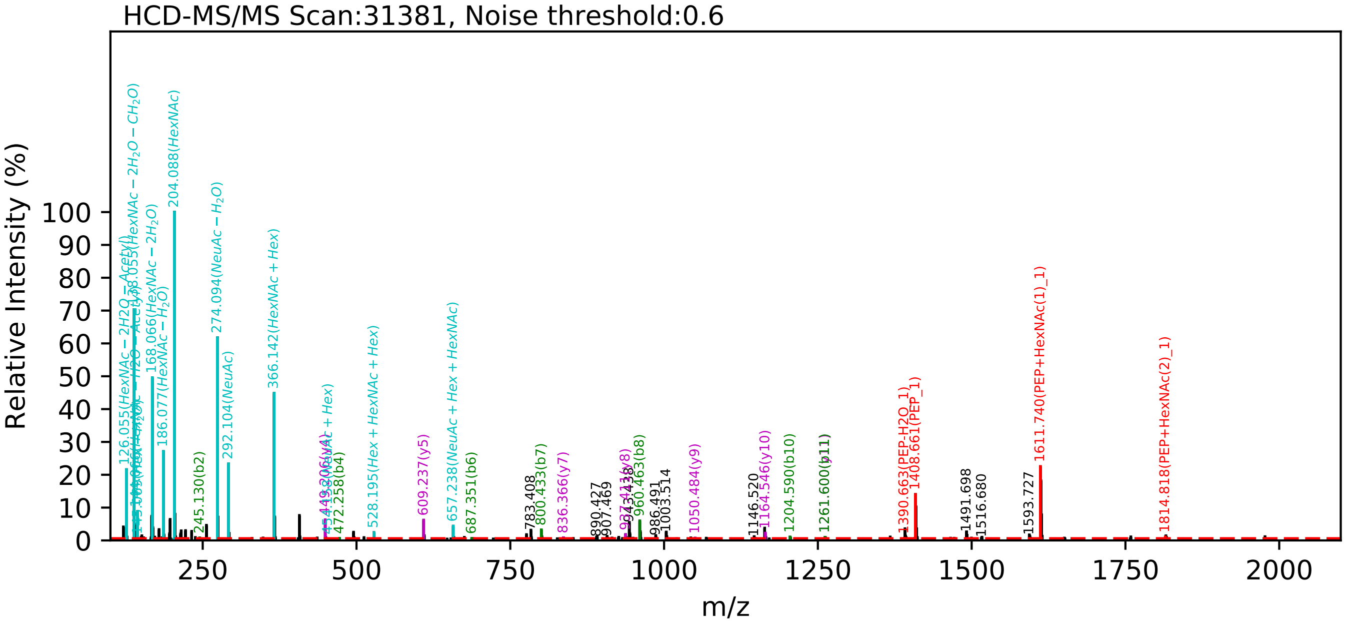

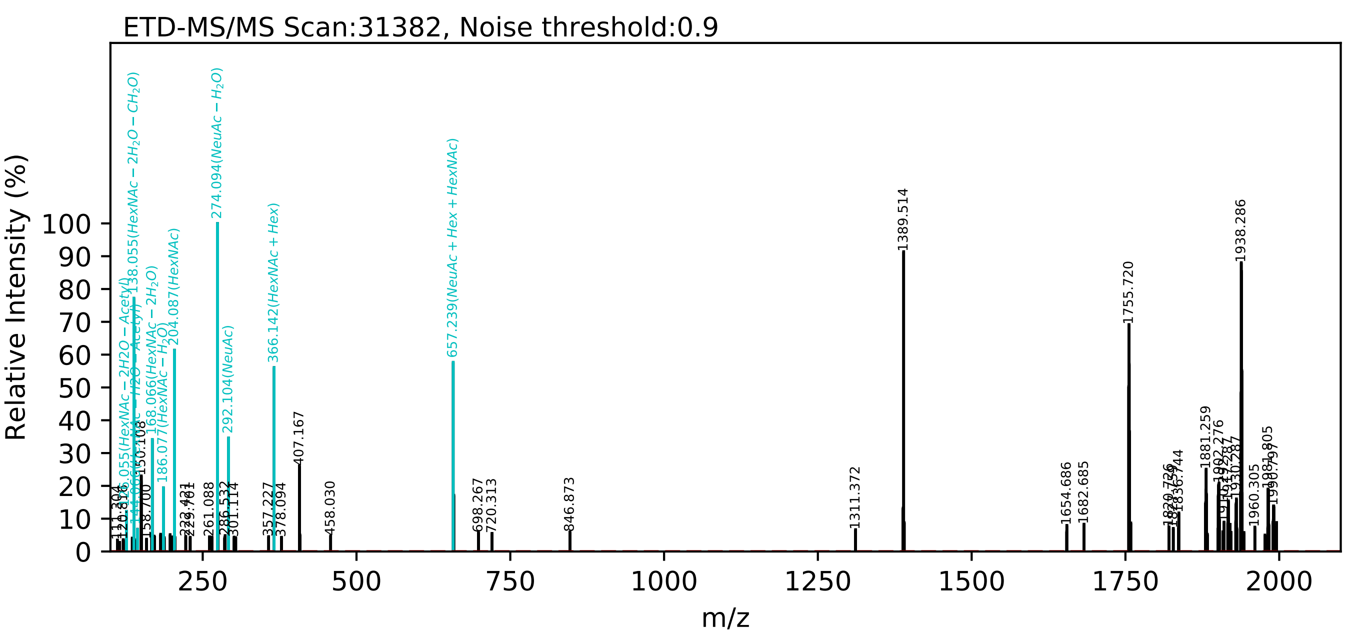

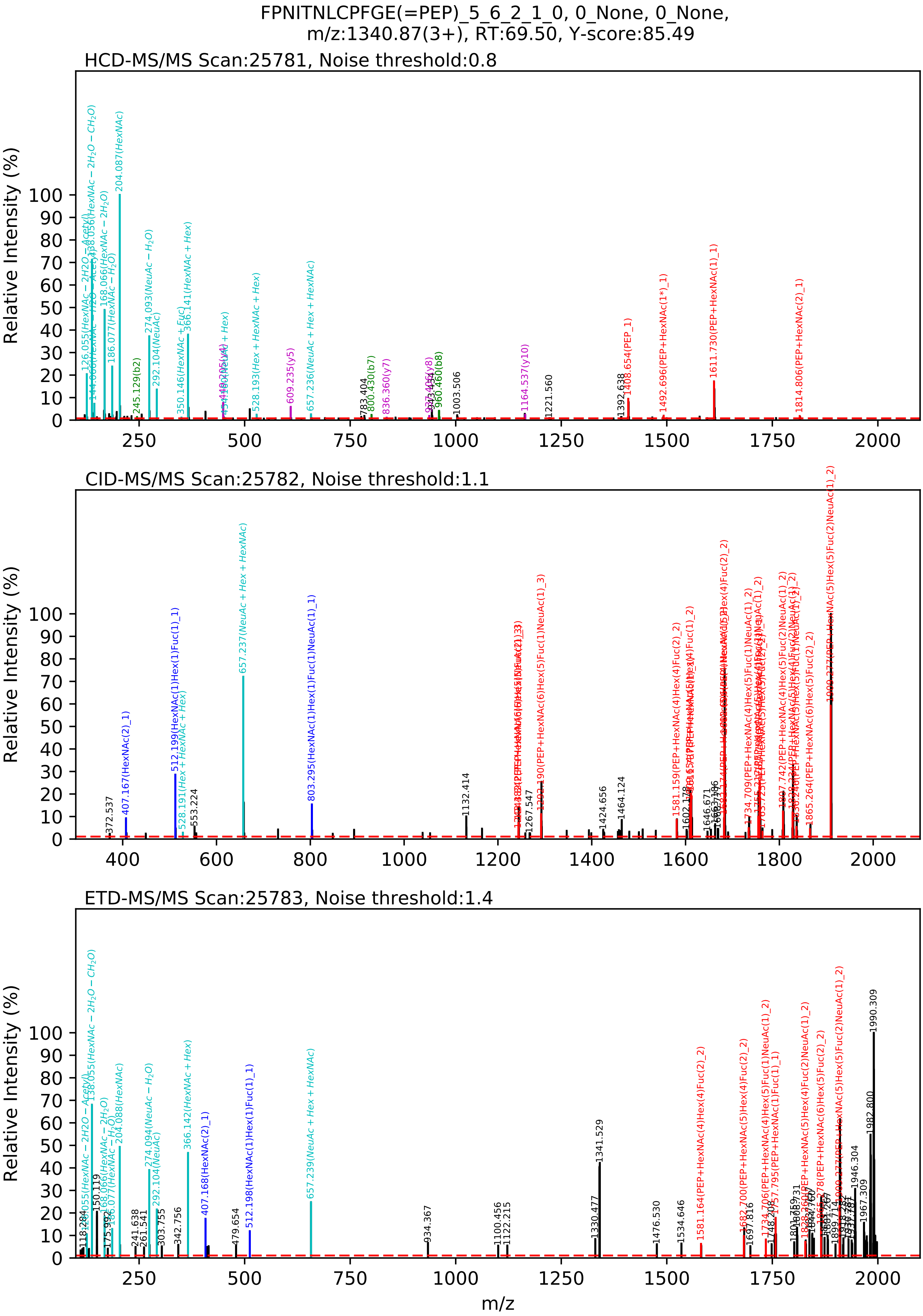
FPNITNLCPFGE(=PEP)_5_6_2_1_0, 0_None, 0_None,
m/z:1340.87(3+), RT:69.50, Y-score:85.49
HCD-MS/MS Scan:25781, Noise threshold:0.8
Relative Intensity (%)
204.087(HexNAc)
168.066(HexNAc − 2H2O)
186.077(HexNAc − H2O)
274.093(NeuAc − H2O)
292.104(NeuAc)
366.141(HexNAc + Hex)
245.129(b2)
528.193(Hex + HexNAc + Hex)
609.235(y5)
657.236(NeuAc + Hex + HexNAc)
800.430(b7)
836.360(y7)
960.460(b8)
1003.506
1164.537(y10)
1221.560
1392.638
1408.654(PEP_1)
1492.696(PEP+HexNAc(1*)_1)
1611.730(PEP+HexNAc(1)_1)
1814.806(PEP+HexNAc(2)_1)
CID-MS/MS Scan:25782, Noise threshold:1.1
Relative Intensity (%)
372.537
407.167(HexNAc(2)_1)
512.199(HexNAc(1)Hex(1)Fuc(1)_1)
528.191(Hex + HexNAc + Hex)
553.224
657.237(NeuAc + Hex + HexNAc)
803.295(HexNAc(1)Hex(1)Fuc(1)NeuAc(1)_1)
1132.414
1267.547
1293.190(PEP+HexNAc(6)Hex(5)Fuc(1)NeuAc(1)_3)
1424.656
1464.124
1581.159(PEP+HexNAc(4)Hex(4)Fuc(2)_2)
1602.178
1646.671
1734.709(PEP+HexNAc(4)Hex(5)Fuc(1)NeuAc(1)_2)
1807.742(PEP+HexNAc(4)Hex(5)Fuc(2)NeuAc(1)_2)
1865.264(PEP+HexNAc(6)Hex(5)Fuc(2)_2)
ETD-MS/MS Scan:25783, Noise threshold:1.4
Relative Intensity (%)
138.055(HexNAc − 2H2O − CH2O)
150.119
168.066(HexNAc − 2H2O)
186.077(HexNAc − H2O)
204.088(HexNAc)
241.638
261.541
274.094(NeuAc − H2O)
292.104(NeuAc)
303.755
342.756
366.142(HexNAc + Hex)
407.168(HexNAc(2)_1)
479.654
512.198(HexNAc(1)Hex(1)Fuc(1)_1)
657.239(NeuAc + Hex + HexNAc)
934.367
1100.456
1122.215
1330.477
1341.529
1476.530
1534.646
1581.164(PEP+HexNAc(4)Hex(4)Fuc(2)_2)
1682.700(PEP+HexNAc(5)Hex(4)Fuc(2)_2)
1697.816
1734.706(PEP+HexNAc(4)Hex(5)Fuc(1)NeuAc(1)_2)
1748.209
1757.795(PEP+HexNAc(1)Fuc(1)_1)
1865.278(PEP+HexNAc(6)Hex(5)Fuc(2)_2)
1946.304
1967.309
1982.800
1990.309
m/z

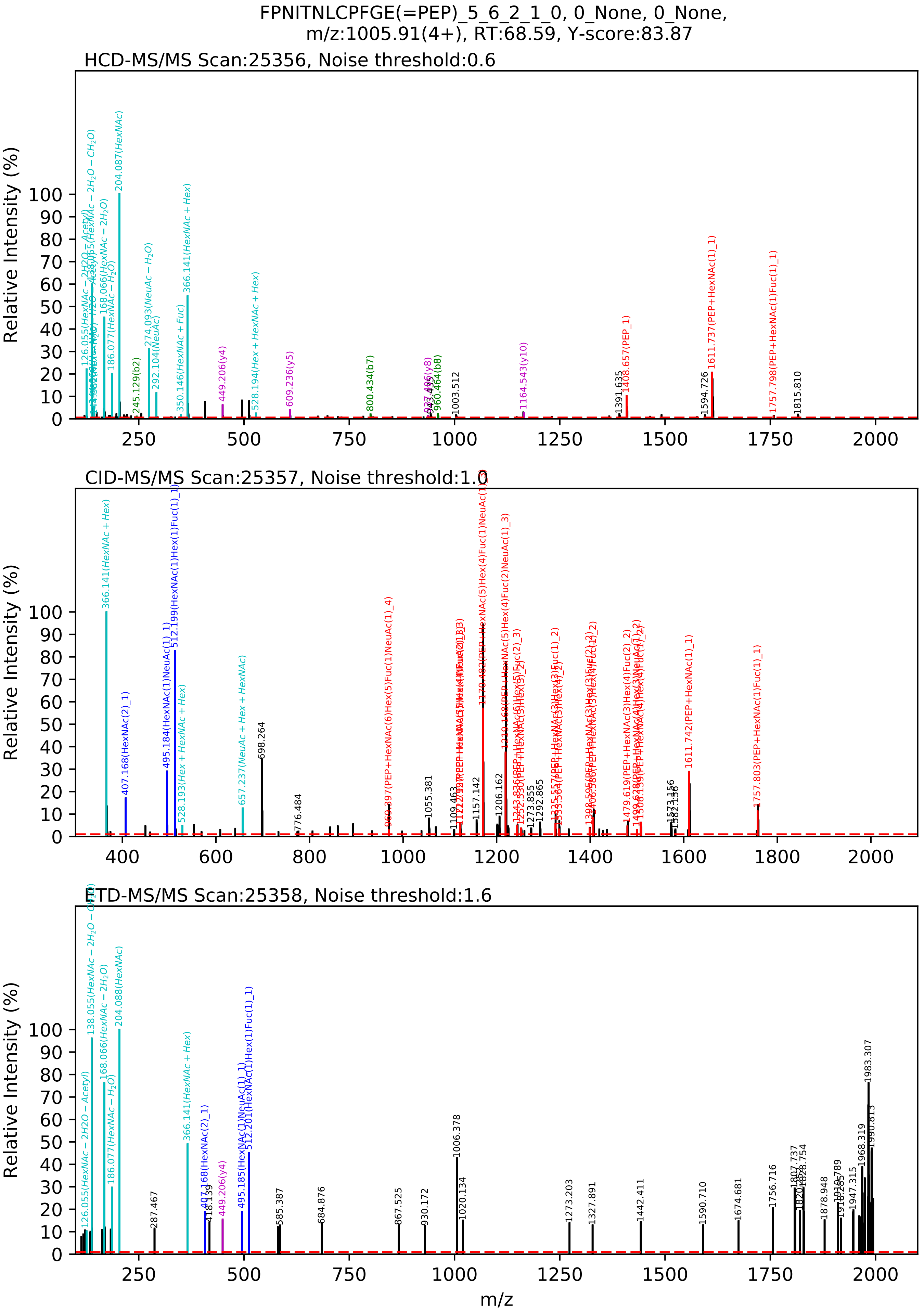

FPNITNLCPFGE(=PEP)_5_6_2_1_0, 0_None, 0_None,
m/z:1005.91(4+), RT:68.59, Y-score:83.87
HCD-MS/MS Scan:25356, Noise threshold:0.6
CID-MS/MS Scan:25357, Noise threshold:1.0
ETD-MS/MS Scan:25358, Noise threshold:1.6
Relative Intensity (%)
m/z

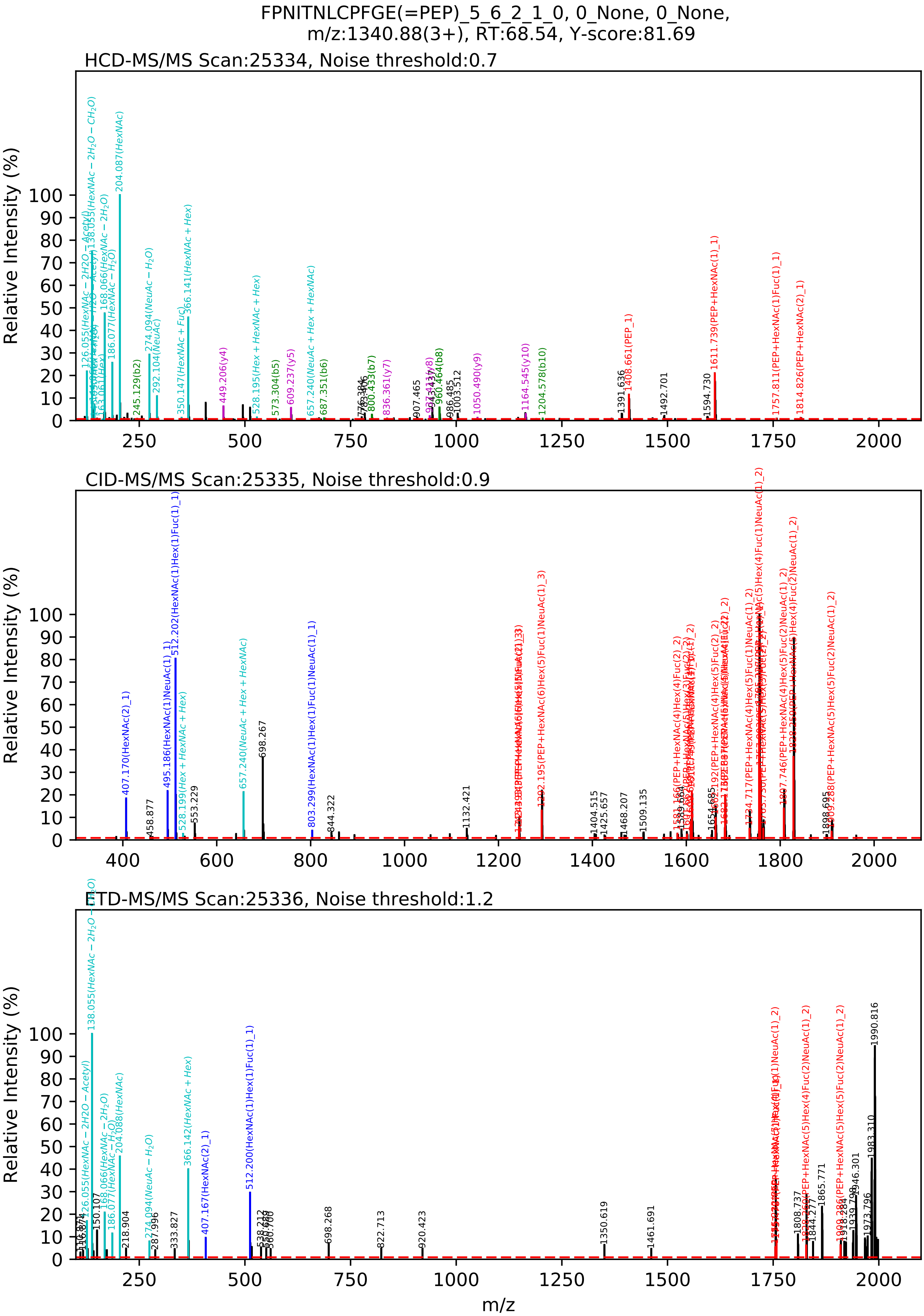

FPNITNLCPFGE(=PEP)_5_6_2_1_0, 0_None, 0_None,
m/z:1340.88(3+), RT:68.54, Y-score:81.69
HCD-MS/MS Scan:25334, Noise threshold:0.7
CID-MS/MS Scan:25335, Noise threshold:0.9
ETD-MS/MS Scan:25336, Noise threshold:1.2
Relative Intensity (%)
m/z
204.087(HexNAc)
366.141(HexNAc + Hex)
1611.739(PEP+HexNAc(1)_1)
1408.661(PEP_1)
512.202(HexNAc(1)Hex(1)Fuc(1)_1)
698.267
1990.816

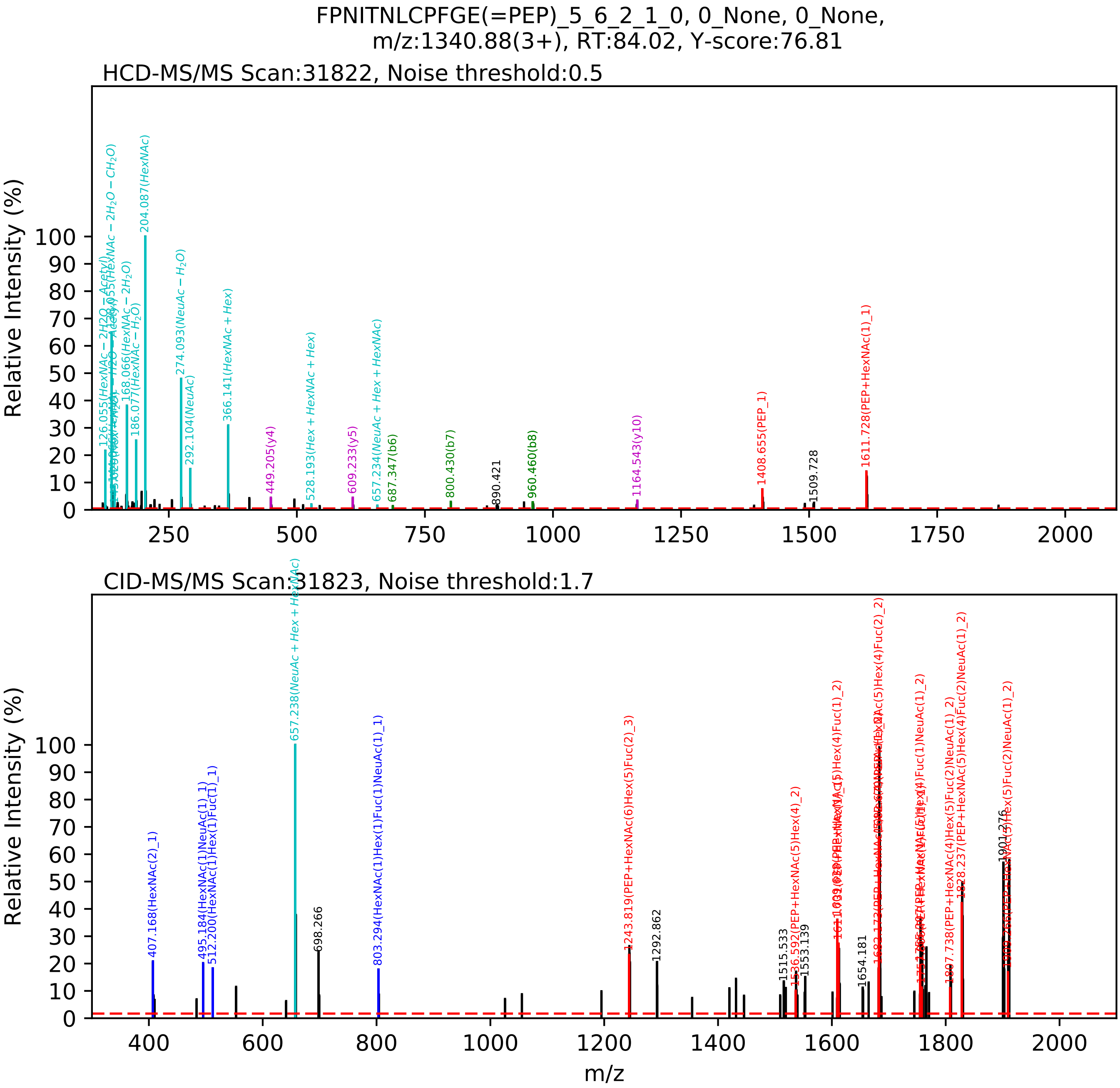
FPNITNLCPFGE(=PEP)_5_6_2_1_0, 0_None, 0_None,
m/z:1340.88(3+), RT:84.02, Y-score:76.81
HCD-MS/MS Scan:31822, Noise threshold:0.5
Relative Intensity (%)
204.087(HexNAc)
274.093(NeuAc − H2O)
292.104(NeuAc)
366.141(HexNAc + Hex)
449.205(y4)
528.193(Hex + HexNAc + Hex)
609.233(y5)
657.234(NeuAc + Hex + HexNAc)
687.347(b6)
800.430(b7)
890.421
960.460(b8)
1164.543(y10)
1408.655(PEP_1)
1509.728
1611.728(PEP+HexNAc(1)_1)
CID-MS/MS Scan:31823, Noise threshold:1.7
Relative Intensity (%)
407.168(HexNAc(2)_1)
495.184(HexNAc(1)NeuAc(1)_1)
512.200(HexNAc(1)Hex(1)Fuc(1)_1)
657.238(NeuAc + Hex + HexNAc)
698.266
803.294(HexNAc(1)Hex(1)Fuc(1)NeuAc(1)_1)
1243.819(PEP+HexNAc(6)Hex(5)Fuc(2)_3)
1292.862
1515.533
1553.139
1654.181
1807.738(PEP+HexNAc(4)Hex(5)Fuc(2)NeuAc(1)_2)
1828.237(PEP+HexNAc(5)Hex(4)Fuc(2)NeuAc(1)_2)
1901.276
m/z

FPNITNLCPFGE(=PEP)_5_6_2_2_0, 0_None, 0_None,
m/z:1437.91(3+), RT:82.80, Y-score:78.80

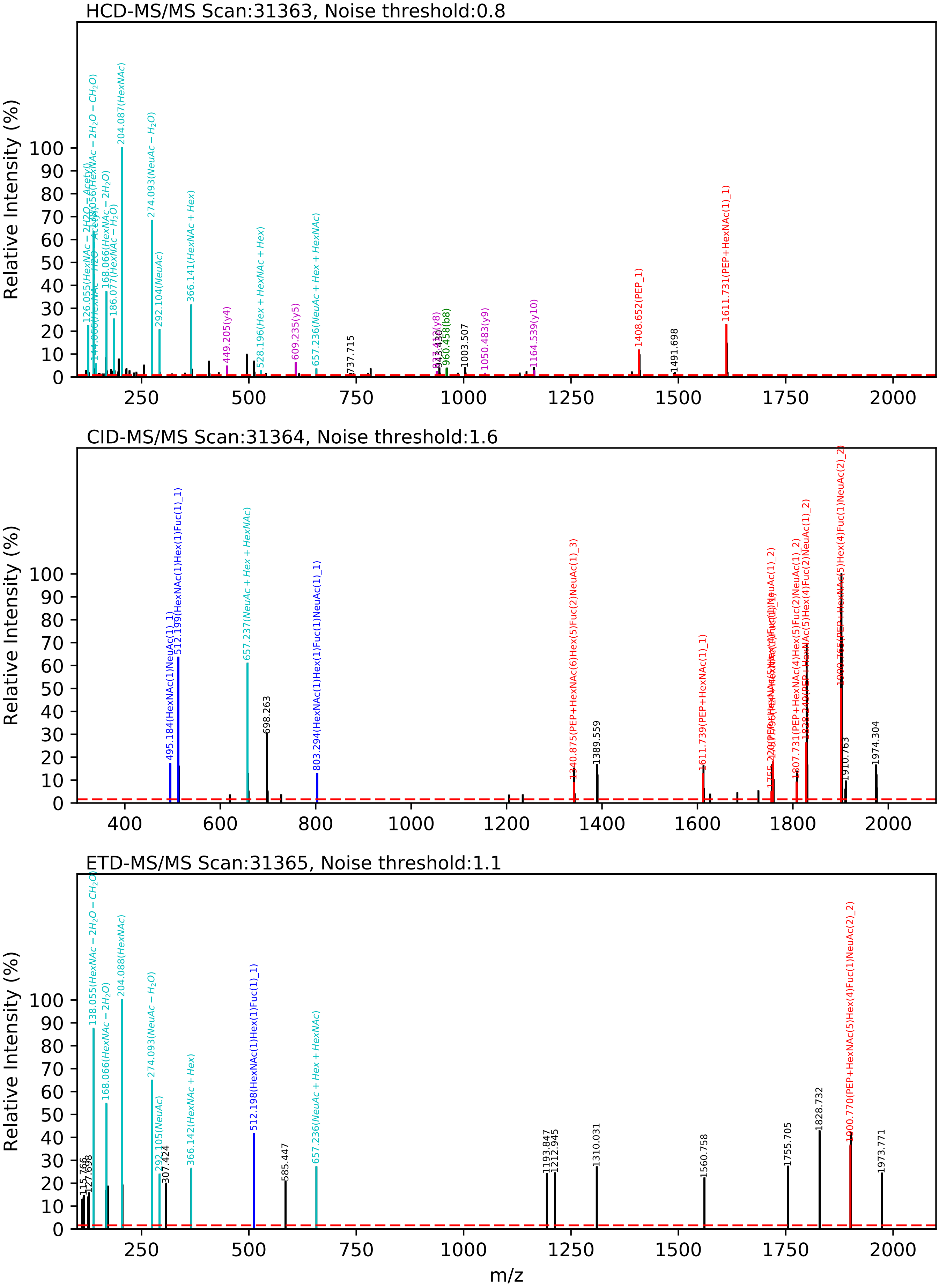

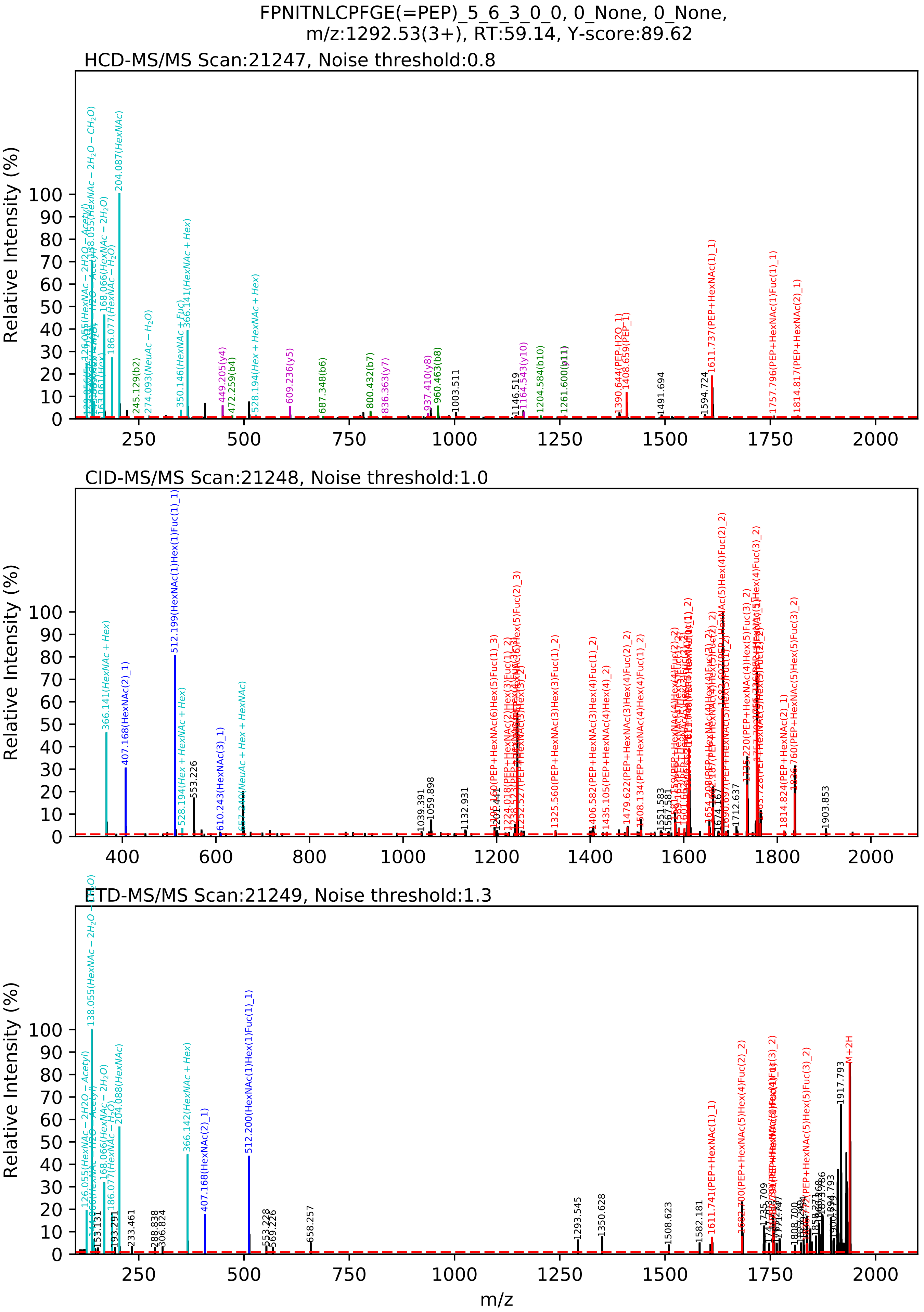

FPNITNLCPFGE(=PEP)_5_6_3_0_0, 0_None, 0_None,
m/z:1292.53(3+), RT:59.14, Y-score:89.62
HCD-MS/MS Scan:21247, Noise threshold:0.8
Relative Intensity (%)
204.087(HexNAc)
168.066(HexNAc − 2H2O)
186.077(HexNAc − H2O)
245.129(b2)
274.093(NeuAc − H2O)
350.146(HexNAc + Fuc)
366.141(HexNAc + Hex)
449.205(y4)
472.259(b4)
528.194(Hex + HexNAc + Hex)
609.236(y5)
687.348(b6)
800.432(b7)
836.363(y7)
937.410(y8)
960.463(b8)
1003.511
1146.519
1164.543(y10)
1204.584(b10)
1261.600(b11)
1390.644(PEP-H2O_1)
1408.659(PEP_1)
1491.694
1594.724
1611.737(PEP+HexNAc(1)_1)
1757.796(PEP+HexNAc(1)Fuc(1)_1)
1814.817(PEP+HexNAc(2)_1)
CID-MS/MS Scan:21248, Noise threshold:1.0
Relative Intensity (%)
366.141(HexNAc + Hex)
407.168(HexNAc(2)_1)
512.199(HexNAc(1)Hex(1)Fuc(1)_1)
528.194(Hex + HexNAc + Hex)
553.226
610.243(HexNAc(3)_1)
657.240(NeuAc + Hex + HexNAc)
1039.391
1059.898
1132.931
1201.442
1325.560(PEP+HexNAc(3)Hex(3)Fuc(1)_2)
1406.582(PEP+HexNAc(3)Hex(4)Fuc(1)_2)
1435.105(PEP+HexNAc(4)Hex(4)_2)
1479.622(PEP+HexNAc(3)Hex(4)Fuc(2)_2)
1508.134(PEP+HexNAc(4)Hex(4)Fuc(1)_2)
1551.583
1567.581
1712.637
1725.220(PEP+HexNAc(4)Hex(5)Fuc(3)_2)
1814.824(PEP+HexNAc(2)_1)
1836.760(PEP+HexNAc(5)Hex(5)Fuc(3)_2)
1903.853
ETD-MS/MS Scan:21249, Noise threshold:1.3
Relative Intensity (%)
138.055(HexNAc − 2H2O − CH2O)
126.055(HexNAc − 2H2O − Acetyl)
168.066(HexNAc − 2H2O)
186.077(HexNAc − H2O)
204.088(HexNAc)
153.131
193.291
233.461
288.838
306.824
366.142(HexNAc + Hex)
407.168(HexNAc(2)_1)
512.200(HexNAc(1)Hex(1)Fuc(1)_1)
553.228
569.226
658.257
1293.545
1350.628
1508.623
1582.181
1611.741(PEP+HexNAc(1)_1)
1682.700(PEP+HexNAc(5)Hex(4)Fuc(2)_2)
1735.709
1917.793
M+2H
m/z

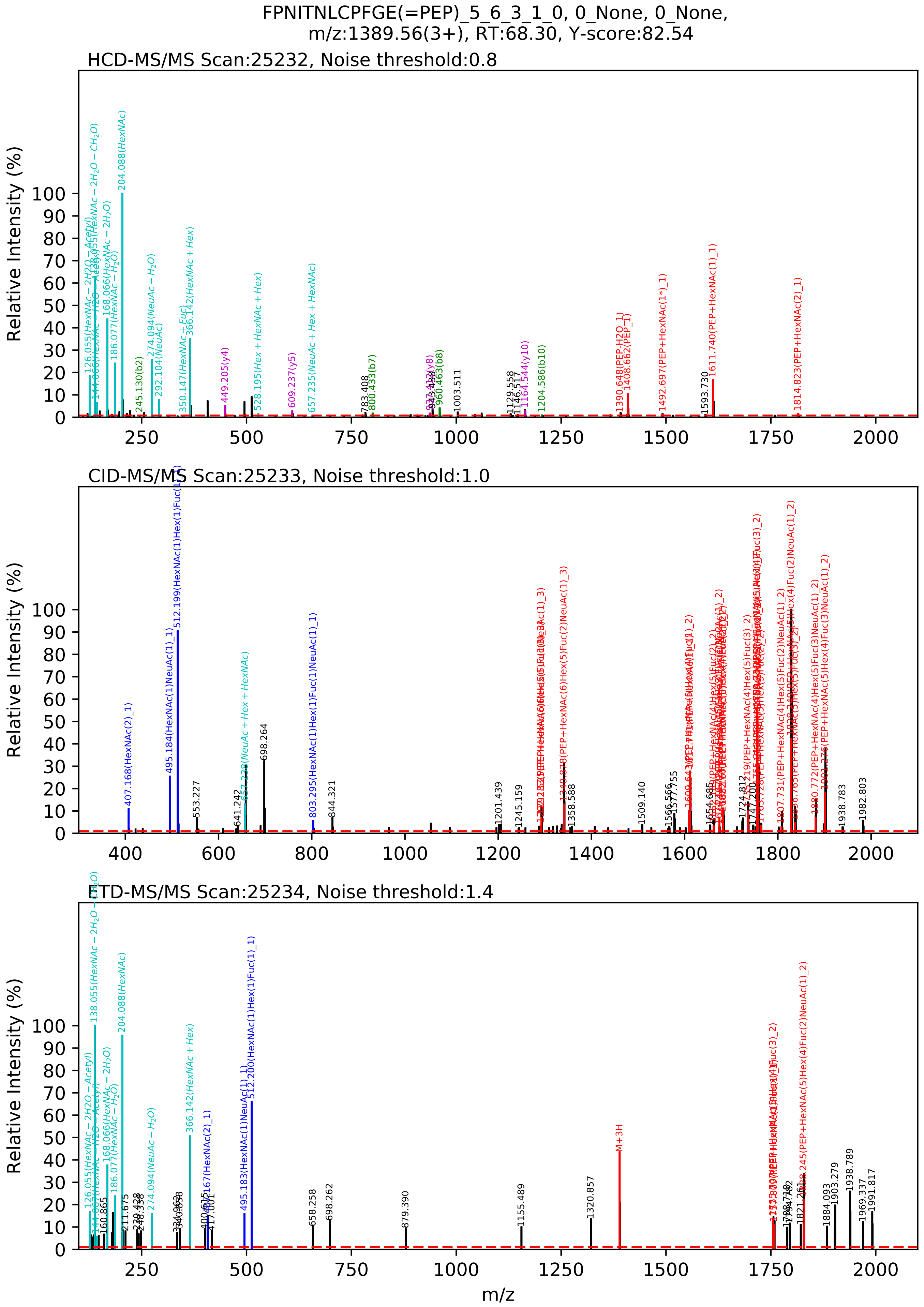
FPNITNLCPFGE(=PEP)_5_6_3_1_0, 0_None, 0_None,
m/z:1389.56(3+), RT:68.30, Y-score:82.54
HCD-MS/MS Scan:25232, Noise threshold:0.8
Relative Intensity (%)
126.055
144.066
168.066
186.077
204.088(HexNAc)
245.130(b2)
274.094
292.104(NeuAc)
350.147
366.142(HexNAc + Hex)
449.205(y4)
528.195(Hex + HexNAc + Hex)
609.237(y5)
657.235(NeuAc + Hex + HexNAc)
783.408
800.433(b7)
943.438
960.463(b8)
1003.511
1129.558
1146.517
1164.544(y10)
1204.586(b10)
1390.648(PEP-H2O_1)
1408.662(PEP_1)
1492.697(PEP+HexNAc(1*)_1)
1593.730
1611.740(PEP+HexNAc(1)_1)
1814.823(PEP+HexNAc(2)_1)
CID-MS/MS Scan:25233, Noise threshold:1.0
407.168(HexNAc(2)_1)
495.184(HexNAc(1)NeuAc(1)_1)
512.199(HexNAc(1)Hex(1)Fuc(1)_1)
553.227
641.242
698.264
803.295(HexNAc(1)Hex(1)Fuc(1)NeuAc(1)_1)
844.321
1201.439
1245.159
1340.678(PEP+HexNAc(6)Hex(5)Fuc(2)NeuAc(1)_3)
1358.588
1509.140
1566.566
1577.755
1654.685
1724.812
1747.200
1807.731(PEP+HexNAc(4)Hex(5)Fuc(2)NeuAc(1)_2)
1828.249
1836.765
1880.772(PEP+HexNAc(4)Hex(5)Fuc(3)NeuAc(1)_2)
1901.776(PEP+HexNAc(5)Hex(4)Fuc(3)NeuAc(1)_2)
1938.783
1982.803
ETD-MS/MS Scan:25234, Noise threshold:1.4
138.055
160.865
168.066
186.077
204.088(HexNAc)
211.675
239.428
248.338
274.094
334.962
340.838
366.142(HexNAc + Hex)
400.615
407.167(HexNAc(2)_1)
417.001
495.183(HexNAc(1)NeuAc(1)_1)
512.200(HexNAc(1)Hex(1)Fuc(1)_1)
658.258
698.262
879.390
1155.489
1320.857
M+3H
1794.742
1821.266
1828.245(PEP+HexNAc(5)Hex(4)Fuc(2)NeuAc(1)_2)
1884.093
1903.279
1938.789
1969.337
1991.817
m/z

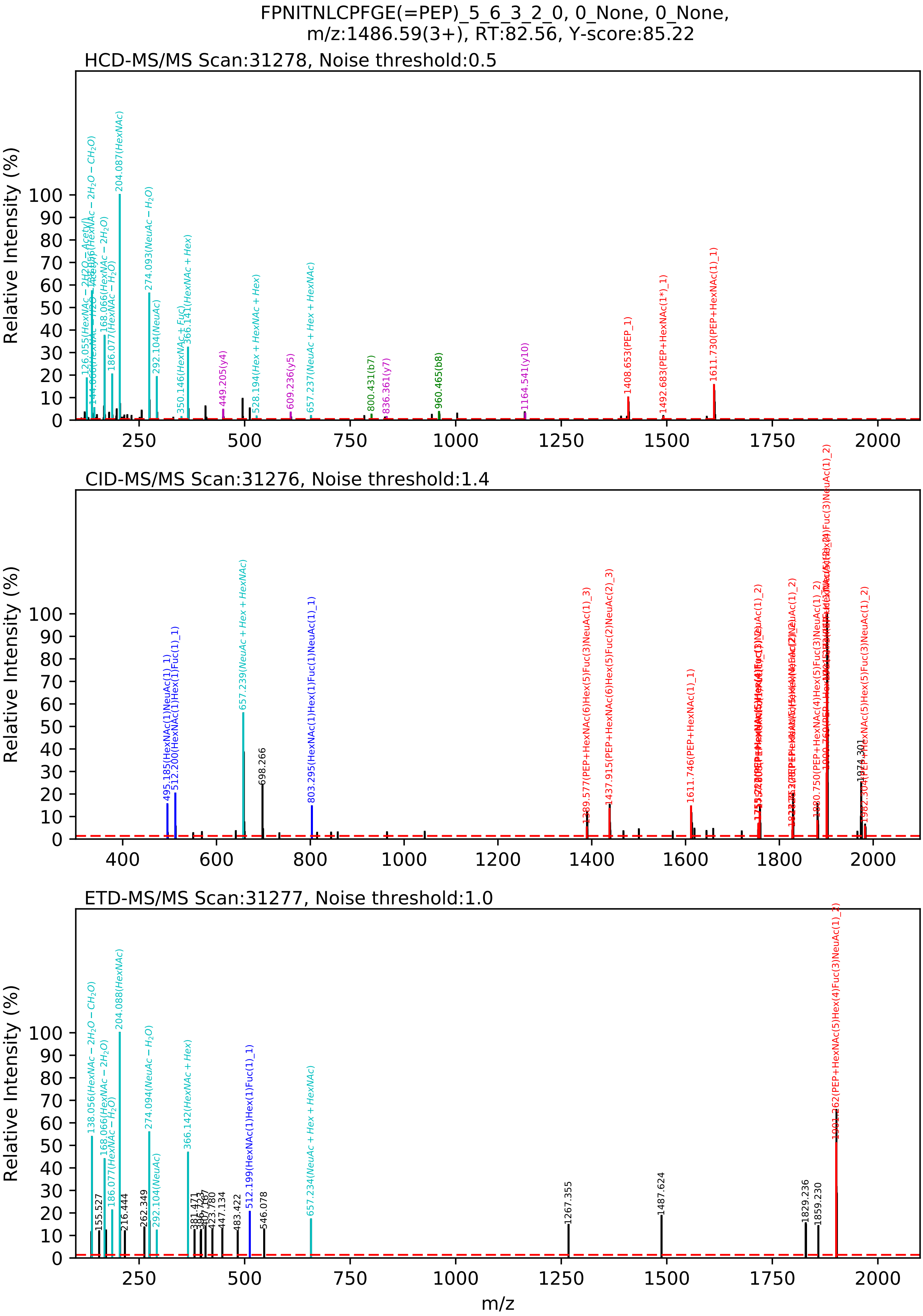

FPNITNLCPFGE(=PEP)_5_6_3_2_0, 0_None, 0_None,
m/z:1486.59(3+), RT:82.56, Y-score:85.22
HCD-MS/MS Scan:31278, Noise threshold:0.5
CID-MS/MS Scan:31276, Noise threshold:1.4
ETD-MS/MS Scan:31277, Noise threshold:1.0
Relative Intensity (%)
m/z
204.087(HexNAc)
274.093(NeuAc − H2O)
292.104(NeuAc)
366.141(HexNAc + Hex)
168.066(HexNAc − 2H2O)
186.077(HexNAc − H2O)
126.055(HexNAc − 2H2O − CH2O)
449.205(y4)
528.194(Hex + HexNAc + Hex)
609.236(y5)
657.237(NeuAc + Hex + HexNAc)
800.431(b7)
836.361(y7)
960.465(b8)
1164.541(y10)
1408.653(PEP_1)
1492.683(PEP+HexNAc(1*)_1)
1611.730(PEP+HexNAc(1)_1)
495.185(HexNAc(1)NeuAc(1)_1)
512.200(HexNAc(1)Hex(1)Fuc(1)_1)
657.239(NeuAc + Hex + HexNAc)
698.266
803.295(HexNAc(1)Hex(1)Fuc(1)NeuAc(1)_1)
1389.577(PEP+HexNAc(6)Hex(5)Fuc(3)NeuAc(1)_3)
1437.915(PEP+HexNAc(6)Hex(5)Fuc(2)NeuAc(2)_3)
1611.746(PEP+HexNAc(1)_1)
1880.750(PEP+HexNAc(4)Hex(5)Fuc(3)NeuAc(1)_2)
1974.301
1982.304(PEP+HexNAc(5)Hex(5)Fuc(3)NeuAc(1)_2)
138.056(HexNAc − 2H2O − CH2O)
168.066(HexNAc − 2H2O)
186.077(HexNAc − H2O)
204.088(HexNAc)
274.094(NeuAc − H2O)
292.104(NeuAc)
366.142(HexNAc + Hex)
512.199(HexNAc(1)Hex(1)Fuc(1)_1)
657.234(NeuAc + Hex + HexNAc)
155.527
216.444
262.349
381.471
447.134
483.422
546.078
1267.355
1487.624
1829.236
1859.230
1901.262(PEP+HexNAc(5)Hex(4)Fuc(3)NeuAc(1)_2)

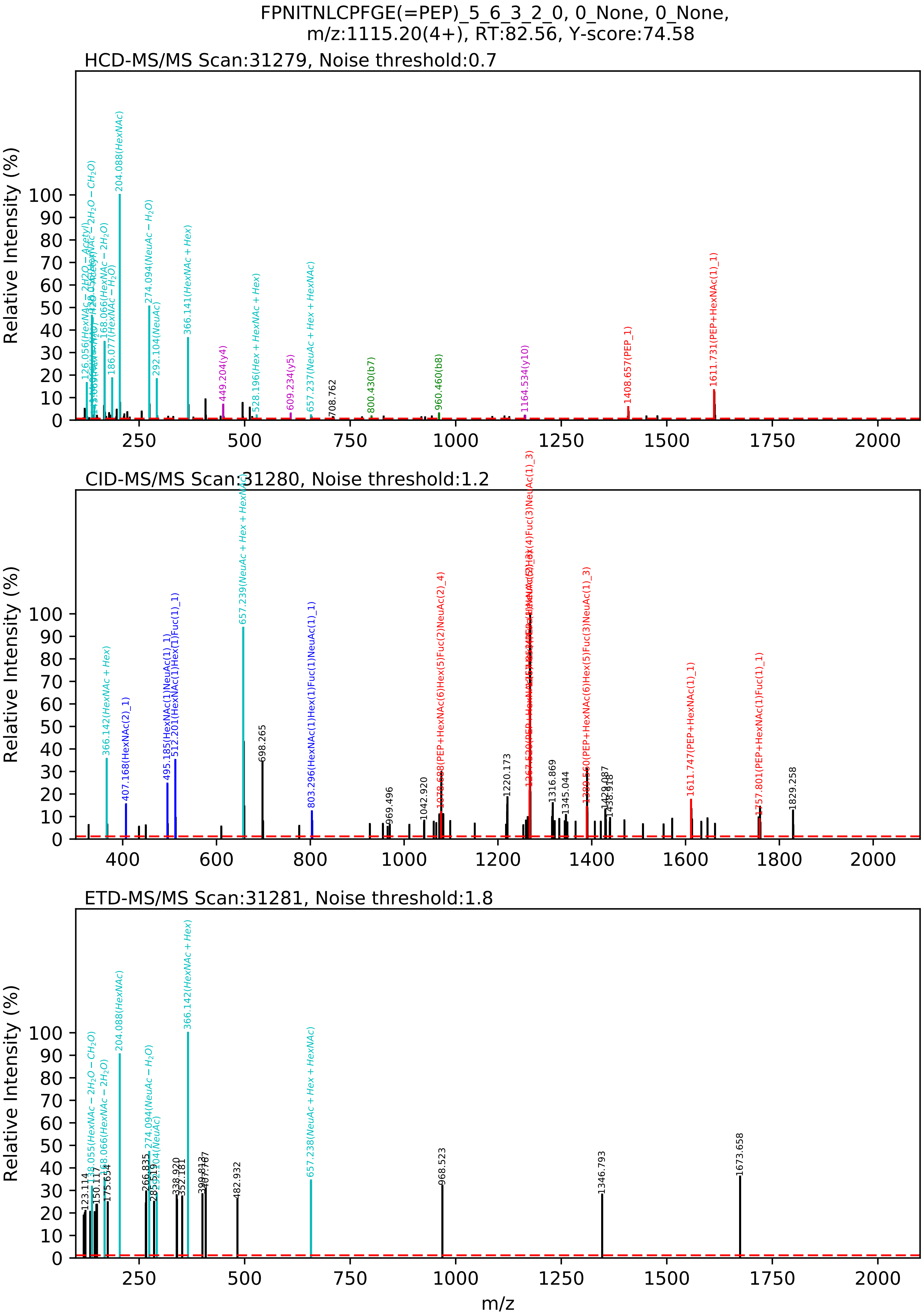

FPNITNLCPFGE(=PEP)_5_6_3_2_0, 0_None, 0_None,
m/z:1115.20(4+), RT:82.56, Y-score:74.58
HCD-MS/MS Scan:31279, Noise threshold:0.7
CID-MS/MS Scan:31280, Noise threshold:1.2
ETD-MS/MS Scan:31281, Noise threshold:1.8
Relative Intensity (%)
m/z
204.088(HexNAc)
366.141(HexNAc + Hex)
274.094(NeuAc − H2O)
292.104(NeuAc)
1611.731(PEP+HexNAc(1)_1)
1408.657(PEP_1)
1164.534(y10)
960.460(b8)
800.430(b7)
657.237(NeuAc + Hex + HexNAc)
609.234(y5)
528.196(Hex + HexNAc + Hex)
449.204(y4)
708.762
657.239(NeuAc + Hex + HexNAc)
366.142(HexNAc + Hex)
407.168(HexNAc(2)_1)
495.185(HexNAc(1)NeuAc(1)_1)
512.201(HexNAc(1)Hex(1)Fuc(1)_1)
803.296(HexNAc(1)Hex(1)Fuc(1)NeuAc(1)_1)
698.265
969.496
1042.920
1078.688(PEP+HexNAc(6)Hex(5)Fuc(2)NeuAc(2)_4)
1220.173
1316.869
1345.044
1389.560(PEP+HexNAc(6)Hex(5)Fuc(3)NeuAc(1)_3)
1429.087
1438.918
1611.747(PEP+HexNAc(1)_1)
1757.801(PEP+HexNAc(1)Fuc(1)_1)
1829.258
366.142(HexNAc + Hex)
204.088(HexNAc)
168.066(HexNAc − 2H2O)
274.094(NeuAc − H2O)
292.104(NeuAc)
657.238(NeuAc + Hex + HexNAc)
123.114
150.117
175.654
266.835
285.519
338.920
352.181
399.812
407.767
482.932
968.523
1346.793
1673.658

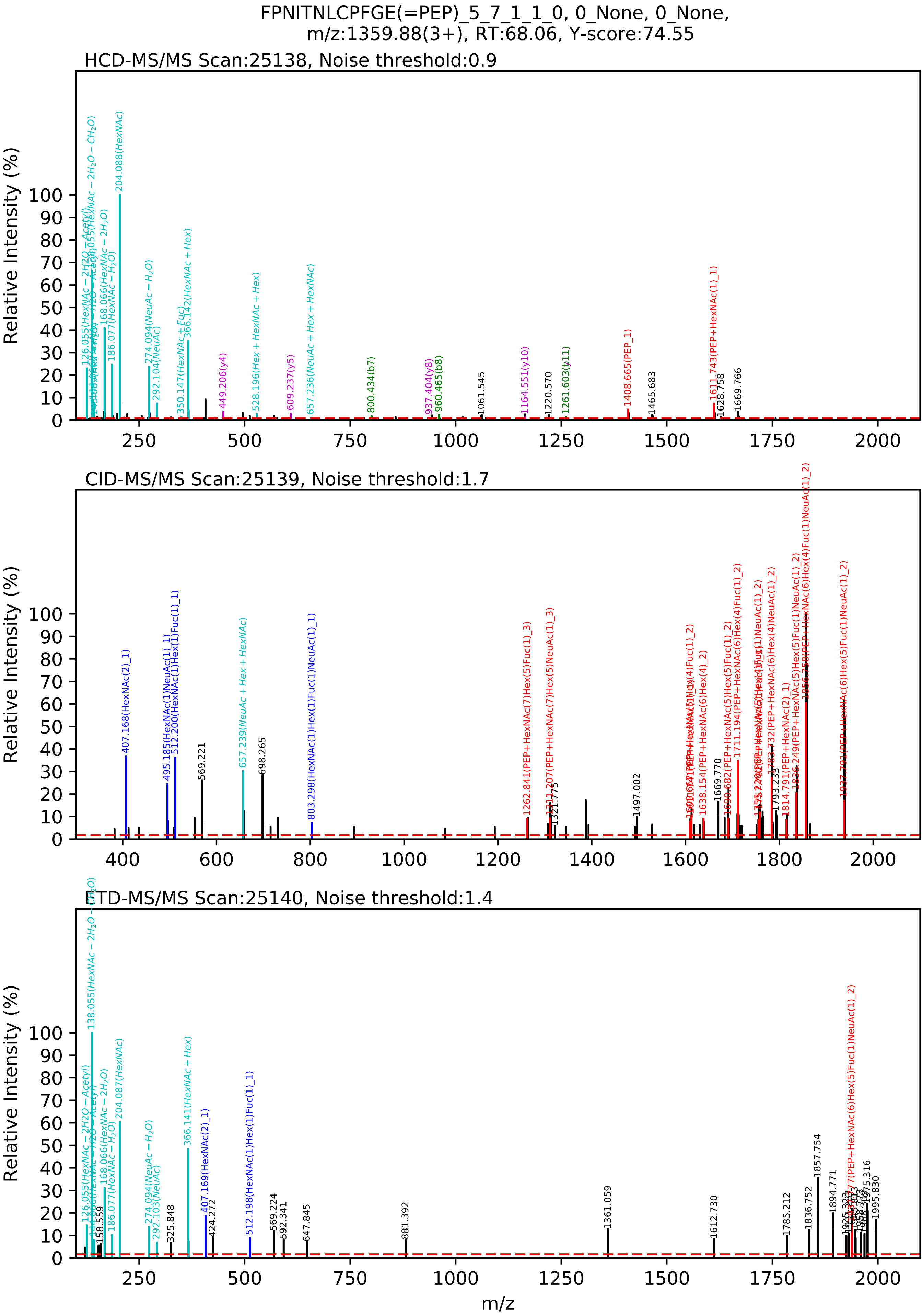
FPNITNLCPFGE(=PEP)_5_7_1_1_0, 0_None, 0_None,
m/z:1359.88(3+), RT:68.06, Y-score:74.55
HCD-MS/MS Scan:25138, Noise threshold:0.9
CID-MS/MS Scan:25139, Noise threshold:1.7
ETD-MS/MS Scan:25140, Noise threshold:1.4
Relative Intensity (%)
m/z

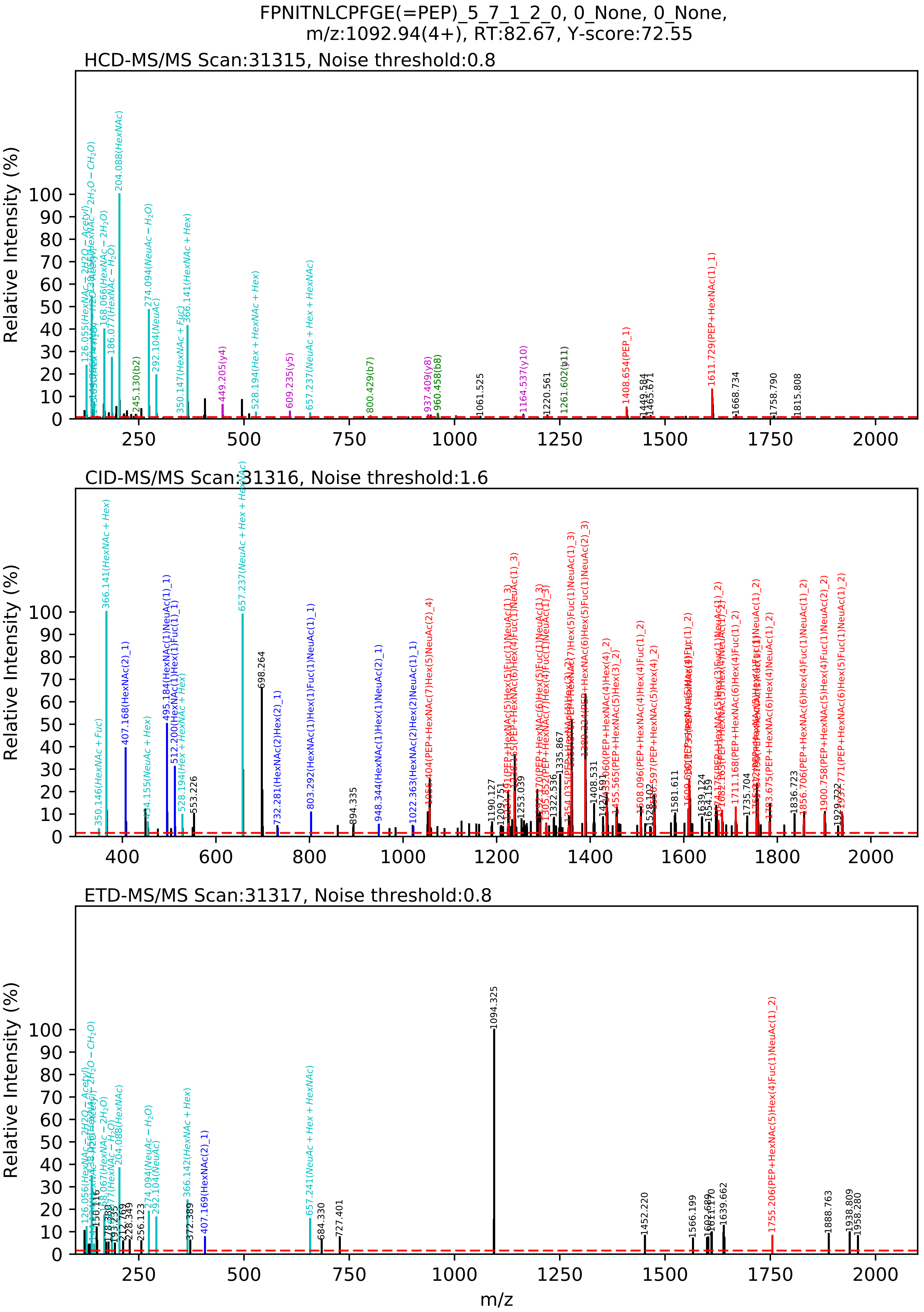
FPNITNLCPFGE(=PEP)_5_7_1_2_0, 0_None, 0_None,
m/z:1092.94(4+), RT:82.67, Y-score:72.55
HCD-MS/MS Scan:31315, Noise threshold:0.8
Relative Intensity (%)
204.088(HexNAc)
168.066(HexNAc − 2H2O)
186.077(HexNAc − H2O)
126.055(HexNAc − 2H2O − CH2O)
274.094(NeuAc − H2O)
292.104(NeuAc)
245.130(b2)
350.147(HexNAc + Fuc)
366.141(HexNAc + Hex)
449.205(y4)
528.194(Hex + HexNAc + Hex)
609.235(y5)
657.237(NeuAc + Hex + HexNAc)
800.429(b7)
937.409(y8)
960.458(b8)
1061.525
1164.537(y10)
1220.561
1261.602(b11)
1408.654(PEP_1)
1449.584
1465.671
1611.729(PEP+HexNAc(1)_1)
1668.734
1758.790
1815.808
CID-MS/MS Scan:31316, Noise threshold:1.6
Relative Intensity (%)
350.146(HexNAc + Fuc)
366.141(HexNAc + Hex)
407.168(HexNAc(2)_1)
454.155(NeuAc + Hex)
495.184(HexNAc(1)NeuAc(1)_1)
512.200(HexNAc(1)Hex(1)Fuc(1)_1)
528.194(Hex + HexNAc + Hex)
553.226
657.237(NeuAc + Hex + HexNAc)
698.264
732.281(HexNAc(2)Hex(2)_1)
803.292(HexNAc(1)Hex(1)Fuc(1)NeuAc(1)_1)
894.335
948.344(HexNAc(1)Hex(1)NeuAc(2)_1)
1022.363(HexNAc(2)Hex(2)NeuAc(1)_1)
1056.404(PEP+HexNAc(7)Hex(5)NeuAc(2)_4)
1190.127
1209.751
1253.039
1322.536
1335.867
1389.224(PEP+HexNAc(6)Hex(5)Fuc(1)NeuAc(2)_3)
1408.531
1427.591
1443.060(PEP+HexNAc(4)Hex(4)_2)
1455.565(PEP+HexNAc(5)Hex(3)_2)
1508.096(PEP+HexNAc(4)Hex(4)Fuc(1)_2)
1528.102
1536.597(PEP+HexNAc(5)Hex(4)_2)
1581.611
1639.124
1654.159
1711.168(PEP+HexNAc(6)Hex(4)Fuc(1)_2)
1735.704
1783.675(PEP+HexNAc(6)Hex(4)NeuAc(1)_2)
1836.723
1856.706(PEP+HexNAc(6)Hex(4)Fuc(1)NeuAc(1)_2)
1900.758(PEP+HexNAc(5)Hex(4)Fuc(1)NeuAc(2)_2)
1929.722
1937.771(PEP+HexNAc(6)Hex(5)Fuc(1)NeuAc(1)_2)
ETD-MS/MS Scan:31317, Noise threshold:0.8
Relative Intensity (%)
126.056(HexNAc − 2H2O − CH2O)
150.116
178.380
193.755
204.088(HexNAc)
212.769
228.349
256.123
274.094(NeuAc − H2O)
292.104(NeuAc)
366.142(HexNAc + Hex)
372.389
407.169(HexNAc(2)_1)
657.241(NeuAc + Hex + HexNAc)
684.330
727.401
1094.325
1452.220
1566.199
1602.689
1611.170
1639.662
1755.206(PEP+HexNAc(5)Hex(4)Fuc(1)NeuAc(1)_2)
1888.763
1938.809
1958.280
m/z

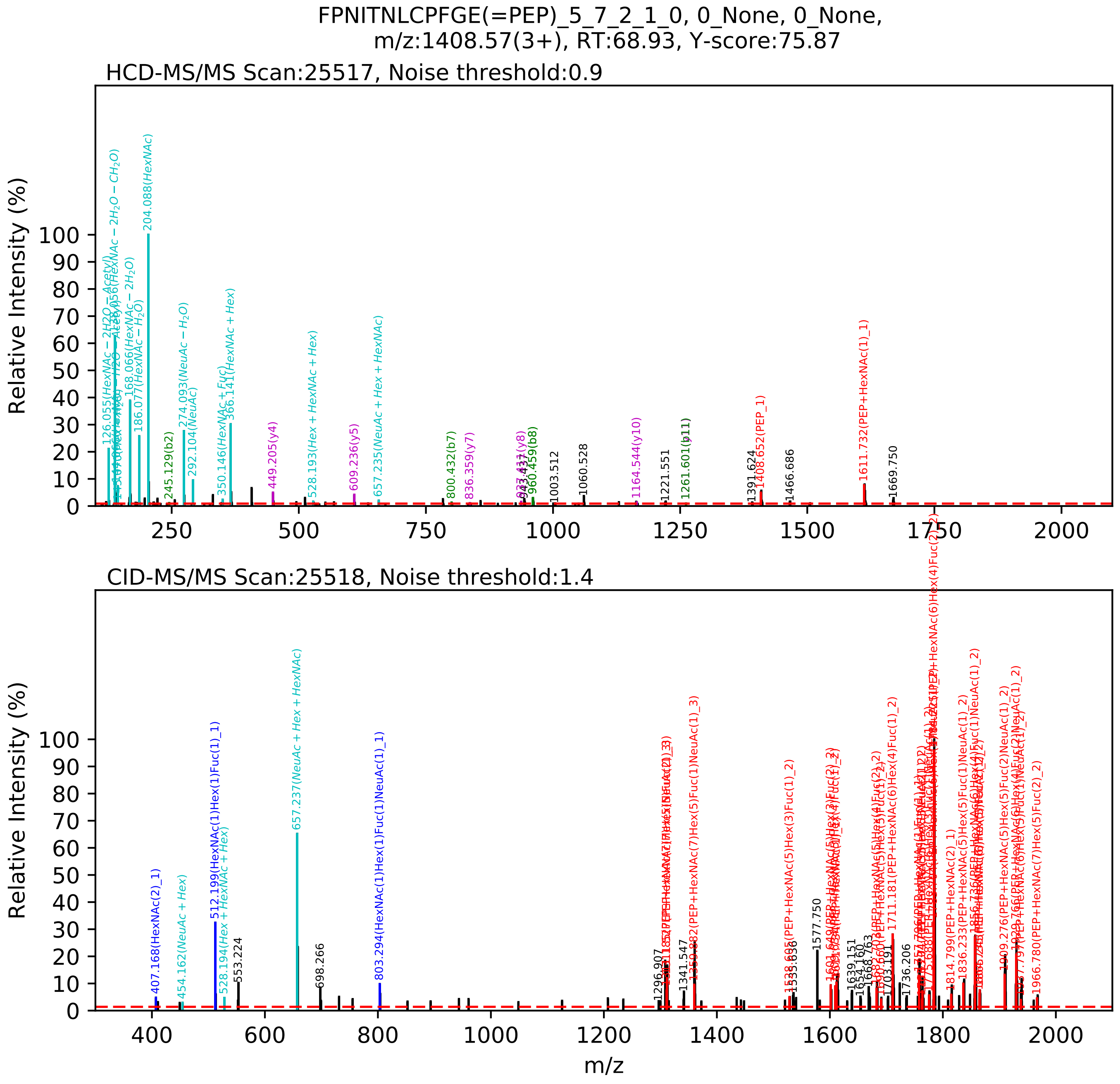
FPNITNLCPFGE(=PEP)_5_7_2_1_0, 0_None, 0_None,
m/z:1408.57(3+), RT:68.93, Y-score:75.87
HCD-MS/MS Scan:25517, Noise threshold:0.9
CID-MS/MS Scan:25518, Noise threshold:1.4
Relative Intensity (%)
m/z
204.088(HexNAc)
168.066(HexNAc − 2H2O)
186.077(HexNAc − H2O)
274.093(NeuAc − H2O)
292.104(NeuAc)
366.141(HexNAc + Hex)
350.146(HexNAc + Fuc)
245.129(b2)
449.205(y4)
528.193(Hex + HexNAc + Hex)
609.236(y5)
657.235(NeuAc + Hex + HexNAc)
800.432(b7)
836.359(y7)
960.459(b8)
1003.512
1060.528
1164.544(y10)
1221.551
1261.601(b11)
1391.624
1408.652(PEP_1)
1466.686
1611.732(PEP+HexNAc(1)_1)
1669.750
407.168(HexNAc(2)_1)
454.162(NeuAc + Hex)
512.199(HexNAc(1)Hex(1)Fuc(1)_1)
528.194(Hex + HexNAc + Hex)
553.224
657.237(NeuAc + Hex + HexNAc)
698.266
803.294(HexNAc(1)Hex(1)Fuc(1)NeuAc(1)_1)
1296.907
1341.547
1360.882(PEP+HexNAc(7)Hex(5)Fuc(1)NeuAc(1)_3)
1535.656
1577.750
1639.151
1711.181(PEP+HexNAc(6)Hex(4)Fuc(1)_2)
1736.206
1814.799(PEP+HexNAc(2)_1)
1836.233(PEP+HexNAc(5)Hex(5)Fuc(1)NeuAc(1)_2)
1909.276(PEP+HexNAc(5)Hex(5)Fuc(2)NeuAc(1)_2)
1966.780(PEP+HexNAc(7)Hex(5)Fuc(2)_2)

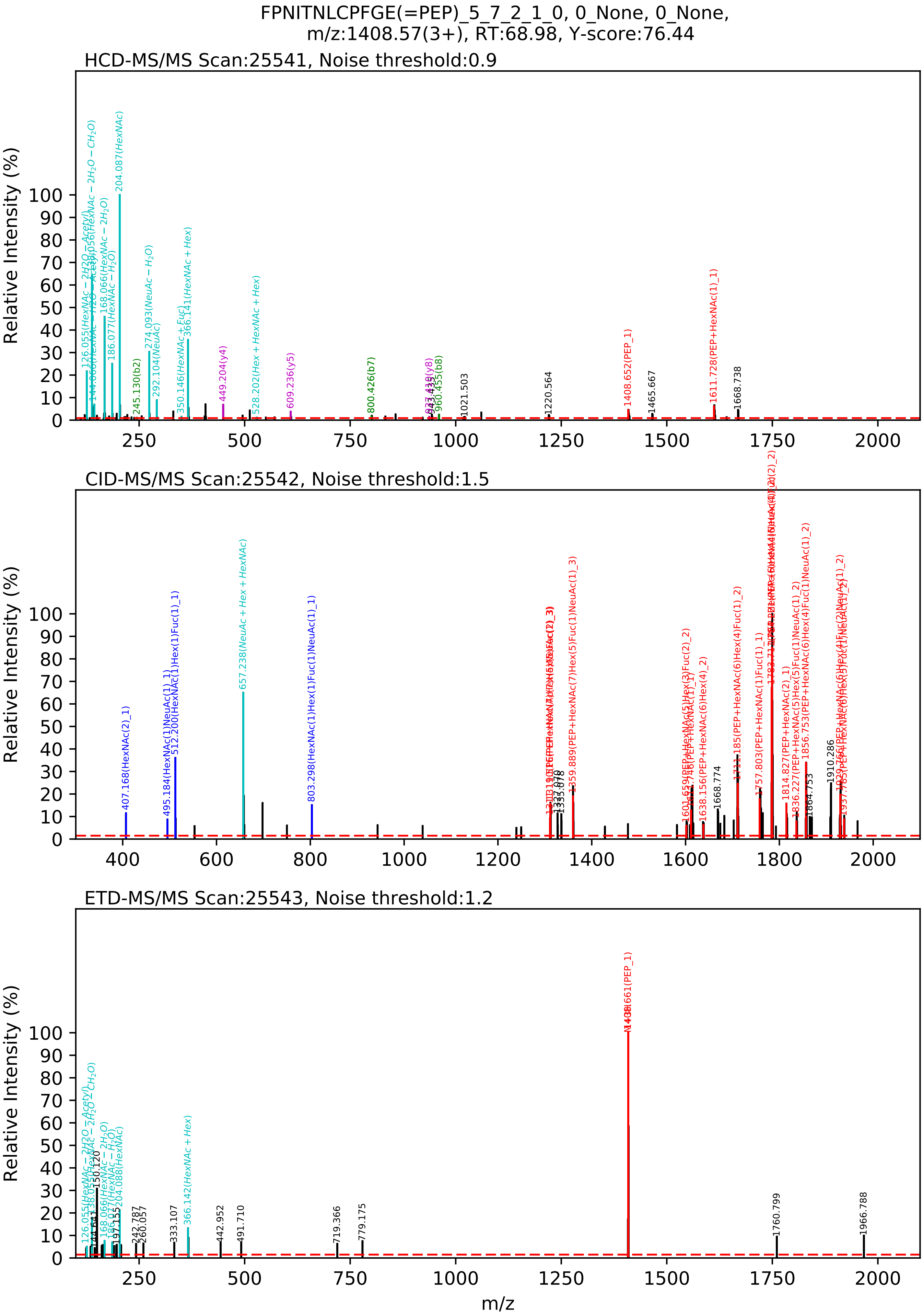

FPNITNLCPFGE(=PEP)_5_7_2_1_0, 0_None, 0_None,
m/z:1408.57(3+), RT:68.98, Y-score:76.44
HCD-MS/MS Scan:25541, Noise threshold:0.9
Relative Intensity (%)
204.087(HexNAc)
168.066(HexNAc − 2H2O)
186.077(HexNAc − H2O)
274.093(NeuAc − H2O)
292.104(NeuAc)
366.141(HexNAc + Hex)
350.146(HexNAc + Fuc)
245.130(b2)
449.204(y4)
528.202(Hex + HexNAc + Hex)
609.236(y5)
800.426(b7)
960.455(b8)
1021.503
1220.564
1408.652(PEP_1)
1465.667
1611.728(PEP+HexNAc(1)_1)
1668.738
CID-MS/MS Scan:25542, Noise threshold:1.5
407.168(HexNAc(2)_1)
495.184(HexNAc(1)NeuAc(1)_1)
512.200(HexNAc(1)Hex(1)Fuc(1)_1)
657.238(NeuAc + Hex + HexNAc)
803.298(HexNAc(1)Hex(1)Fuc(1)NeuAc(1)_1)
1327.070
1335.078
1359.889(PEP+HexNAc(7)Hex(5)Fuc(1)NeuAc(1)_3)
1638.156(PEP+HexNAc(6)Hex(4)_2)
1668.774
1711.185(PEP+HexNAc(6)Hex(4)Fuc(1)_2)
1757.803(PEP+HexNAc(1)Fuc(1)_1)
1814.827(PEP+HexNAc(2)_1)
1836.227(PEP+HexNAc(5)Hex(5)Fuc(1)NeuAc(1)_2)
1856.753(PEP+HexNAc(6)Hex(4)Fuc(1)NeuAc(1)_2)
1864.753
1910.286
ETD-MS/MS Scan:25543, Noise threshold:1.2
1408.661(PEP_1)
150.120
242.787
260.057
333.107
366.142(HexNAc + Hex)
442.952
491.710
719.366
779.175
1760.799
1966.788
m/z

FPNITNLCPFGE(=PEP)_6_2_0_0_0, 0_None, 1_Hex_Phosphorylation,
m/z:956.37(3+), RT:74.42, Y-score:86.03

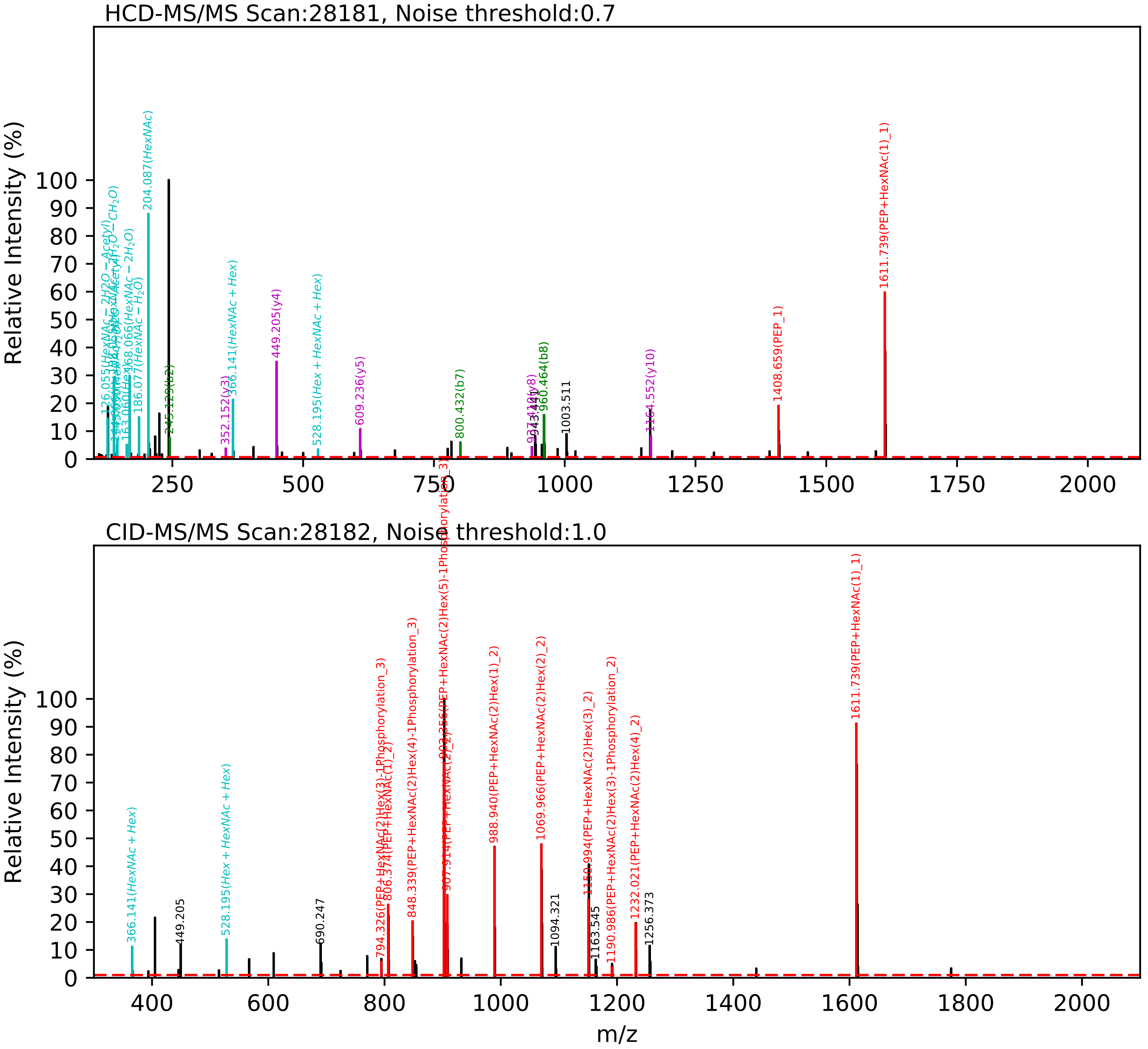

FPNITNLCPFGE(=PEP)_6_2_0_0_0, 0_None, 1_Hex_Phosphorylation,
m/z:956.37(3+), RT:74.47, Y-score:84.78

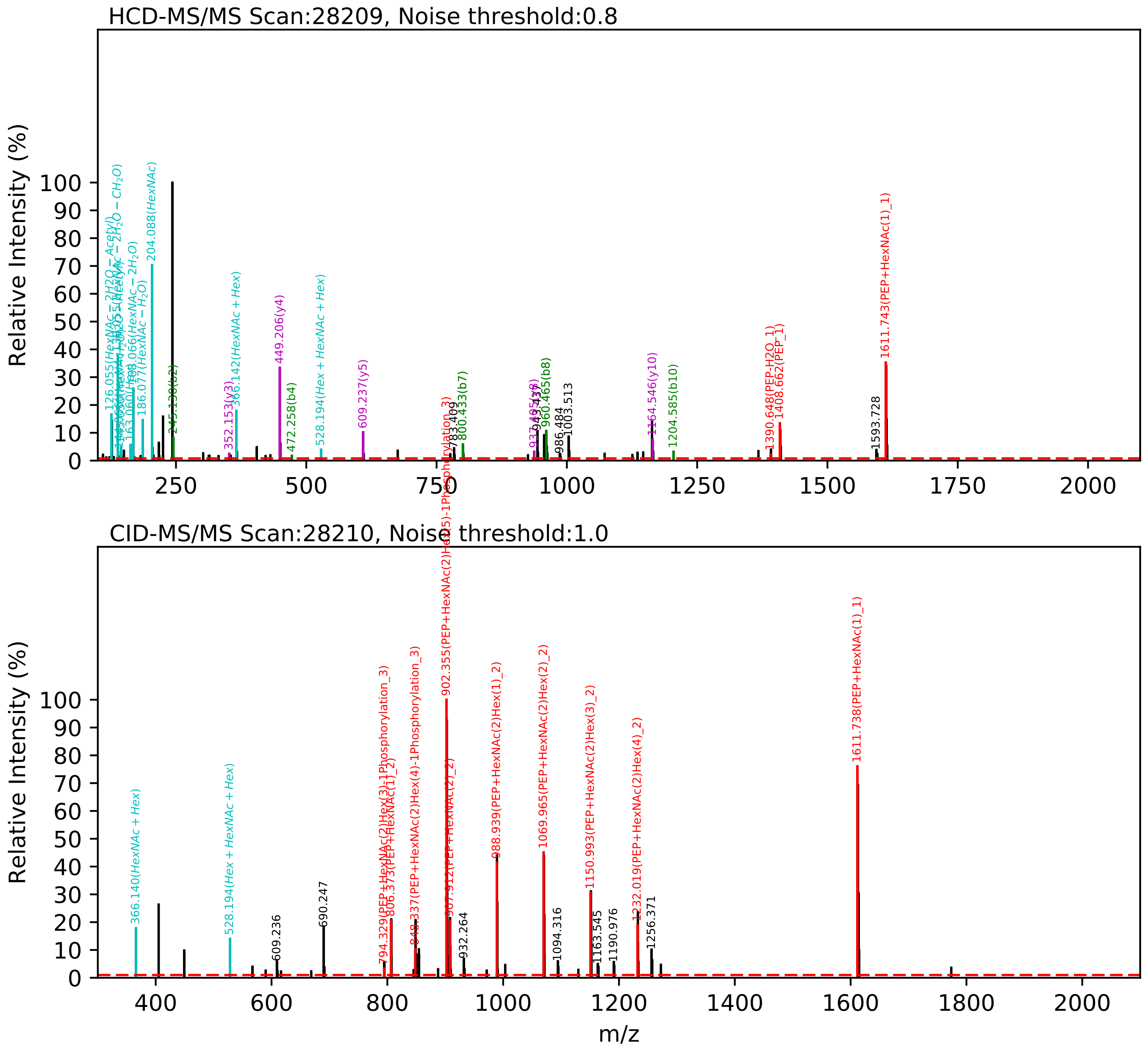

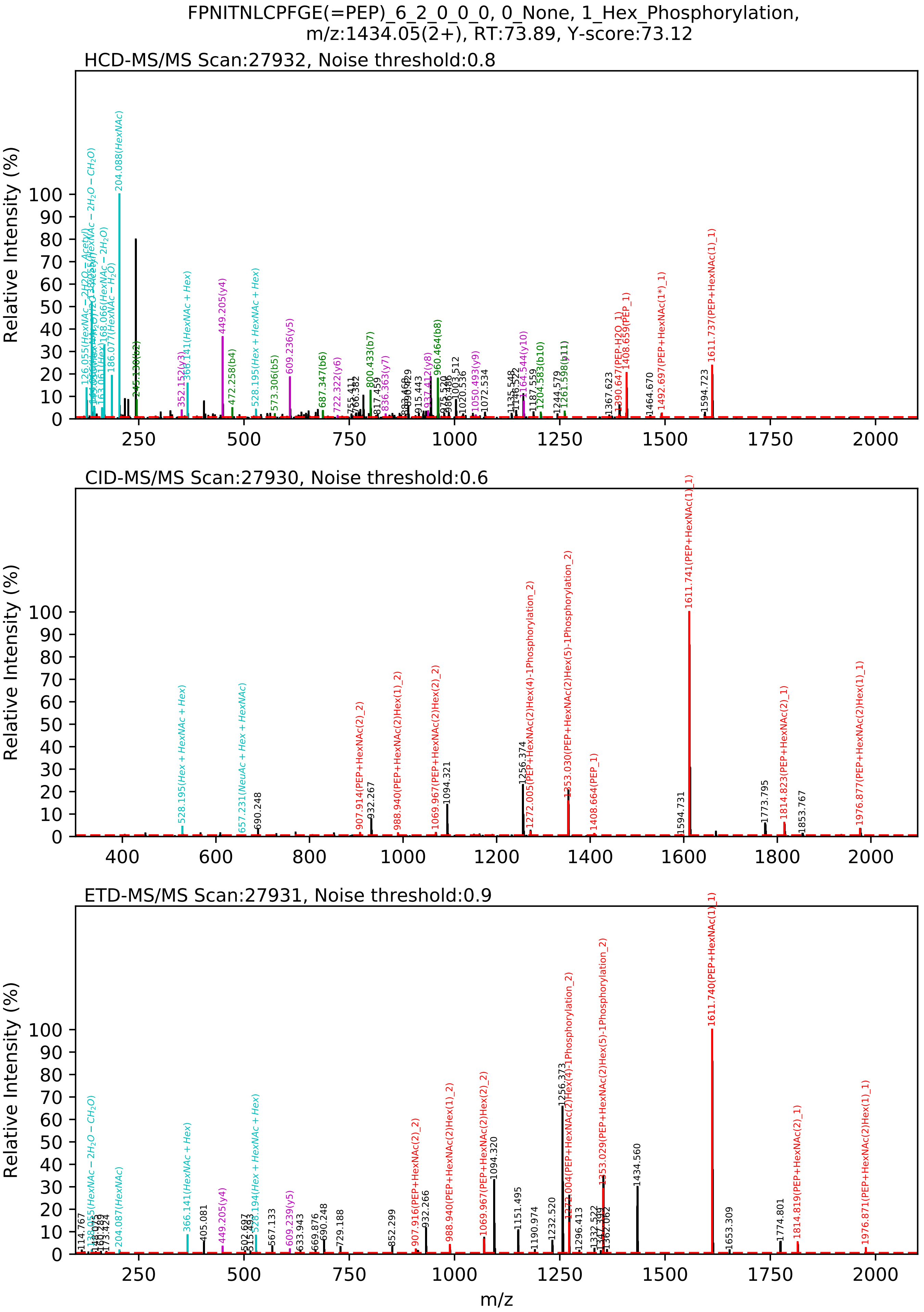

FPNITNLCPFGE(=PEP)_6_2_0_0_0, 0_None, 1_Hex_Phosphorylation,
m/z:1434.05(2+), RT:73.89, Y-score:73.12
HCD-MS/MS Scan:27932, Noise threshold:0.8
Relative Intensity (%)
204.088(HexNAc)
186.077(HexNAc − H2O)
168.066(HexNAc − 2H2O)
366.141(HexNAc + Hex)
449.205(y4)
352.152(y3)
472.258(b4)
528.195(Hex + HexNAc + Hex)
573.306(b5)
609.236(y5)
687.347(b6)
722.322(y6)
800.433(b7)
836.363(y7)
960.464(b8)
1050.493(y9)
1164.544(y10)
1204.583(b10)
1261.598(b11)
1408.659(PEP_1)
1492.697(PEP+HexNAc(1*)_1)
1611.737(PEP+HexNAc(1)_1)
1594.723
1464.670
1367.623
CID-MS/MS Scan:27930, Noise threshold:0.6
528.195(Hex + HexNAc + Hex)
657.231(NeuAc + Hex + HexNAc)
690.248
907.914(PEP+HexNAc(2)_2)
932.267
988.940(PEP+HexNAc(2)Hex(1)_2)
1069.967(PEP+HexNAc(2)Hex(2)_2)
1094.321
1256.374
1272.005(PEP+HexNAc(2)Hex(4)-1Phosphorylation_2)
1353.030(PEP+HexNAc(2)Hex(5)-1Phosphorylation_2)
1408.664(PEP_1)
1594.731
1611.741(PEP+HexNAc(1)_1)
1773.795
1814.823(PEP+HexNAc(2)_1)
1853.767
1976.877(PEP+HexNAc(2)Hex(1)_1)
ETD-MS/MS Scan:27931, Noise threshold:0.9
204.087(HexNAc)
366.141(HexNAc + Hex)
405.081
449.205(y4)
528.194(Hex + HexNAc + Hex)
567.133
609.239(y5)
633.943
669.876
690.248
729.188
852.299
907.916(PEP+HexNAc(2)_2)
932.266
988.940(PEP+HexNAc(2)Hex(1)_2)
1069.967(PEP+HexNAc(2)Hex(2)_2)
1094.320
1151.495
1190.974
1232.520
1256.373
1272.004(PEP+HexNAc(2)Hex(4)-1Phosphorylation_2)
1296.413
1332.522
1353.029(PEP+HexNAc(2)Hex(5)-1Phosphorylation_2)
1362.062
1434.560
1611.740(PEP+HexNAc(1)_1)
1653.309
1774.801
1814.819(PEP+HexNAc(2)_1)
1976.871(PEP+HexNAc(2)Hex(1)_1)
m/z

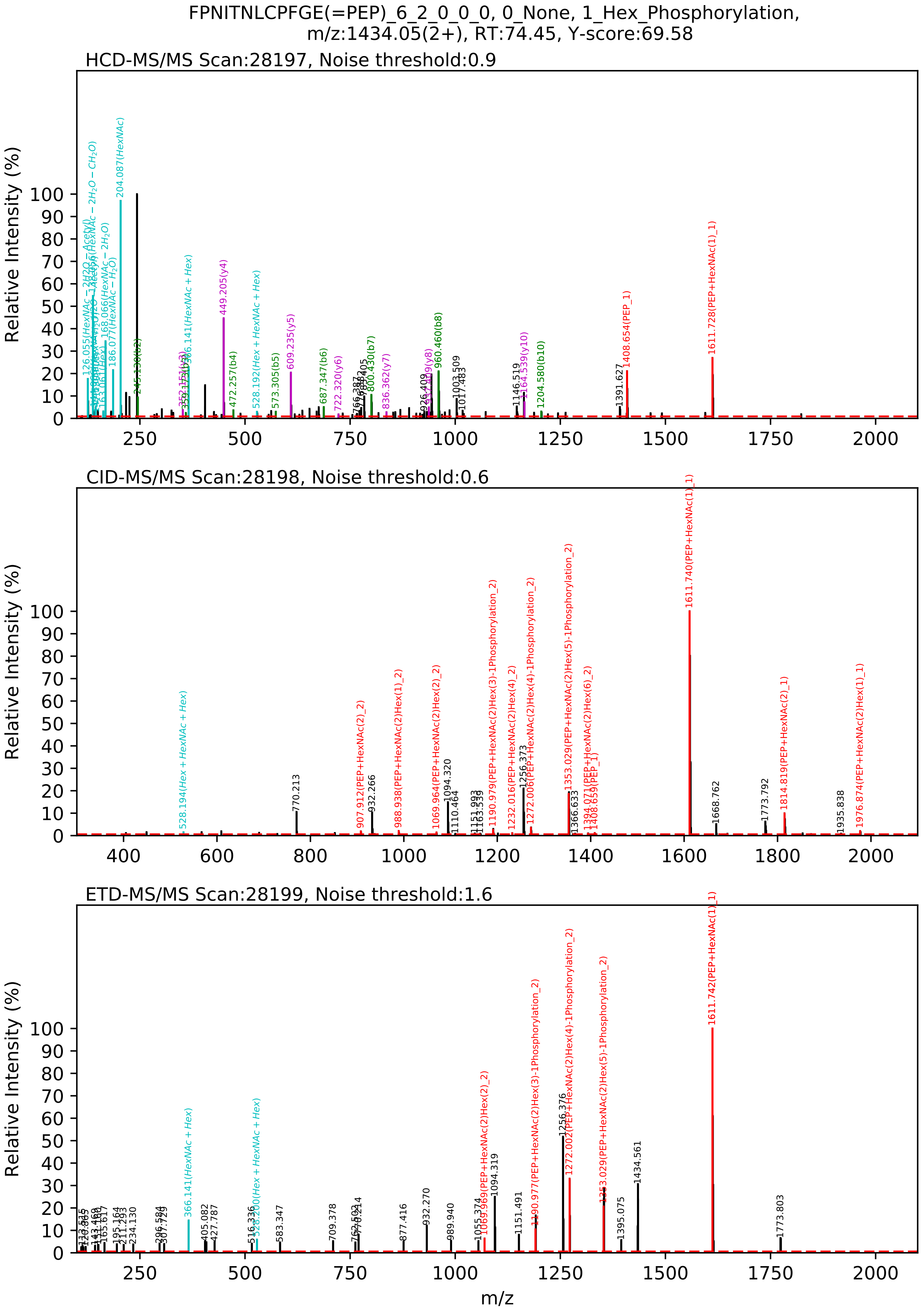
FPNITNLCPFGE(=PEP)_6_2_0_0_0, 0_None, 1_Hex_Phosphorylation,
m/z:1434.05(2+), RT:74.45, Y-score:69.58
HCD-MS/MS Scan:28197, Noise threshold:0.9
Relative Intensity (%)
204.087(HexNAc)
186.077(HexNAc − H2O)
168.066(HexNAc − 2H2O)
126.055(HexNAc − 2H2O − Acetyl)
245.130(b2)
366.141(HexNAc + Hex)
449.205(y4)
472.257(b4)
528.192(Hex + HexNAc + Hex)
573.305(b5)
609.235(y5)
687.347(b6)
722.320(y6)
800.430(b7)
836.362(y7)
960.460(b8)
1003.509
1017.483
1146.519
1164.539(y10)
1204.580(b10)
1391.627
1408.654(PEP_1)
1611.728(PEP+HexNAc(1)_1)
CID-MS/MS Scan:28198, Noise threshold:0.6
528.194(Hex + HexNAc + Hex)
770.213
907.912(PEP+HexNAc(2)_2)
932.266
988.938(PEP+HexNAc(2)Hex(1)_2)
1069.964(PEP+HexNAc(2)Hex(2)_2)
1094.320
1110.464
1151.993
1163.539
1190.979(PEP+HexNAc(2)Hex(3)-1Phosphorylation_2)
1232.016(PEP+HexNAc(2)Hex(4)_2)
1256.373
1272.006(PEP+HexNAc(2)Hex(4)-1Phosphorylation_2)
1353.029(PEP+HexNAc(2)Hex(5)-1Phosphorylation_2)
1366.633
1394.071(PEP+HexNAc(2)Hex(6)_2)
1611.740(PEP+HexNAc(1)_1)
1668.762
1773.792
1814.819(PEP+HexNAc(2)_1)
1935.838
1976.874(PEP+HexNAc(2)Hex(1)_1)
ETD-MS/MS Scan:28199, Noise threshold:1.6
366.141(HexNAc + Hex)
528.200(Hex + HexNAc + Hex)
405.082
427.787
516.336
583.347
709.378
877.416
932.270
989.940
1055.374
1069.969(PEP+HexNAc(2)Hex(2)_2)
1094.319
1151.491
1190.977(PEP+HexNAc(2)Hex(3)-1Phosphorylation_2)
1256.376
1272.002(PEP+HexNAc(2)Hex(4)-1Phosphorylation_2)
1353.029(PEP+HexNAc(2)Hex(5)-1Phosphorylation_2)
1395.075
1434.561
1611.742(PEP+HexNAc(1)_1)
1773.803
m/z

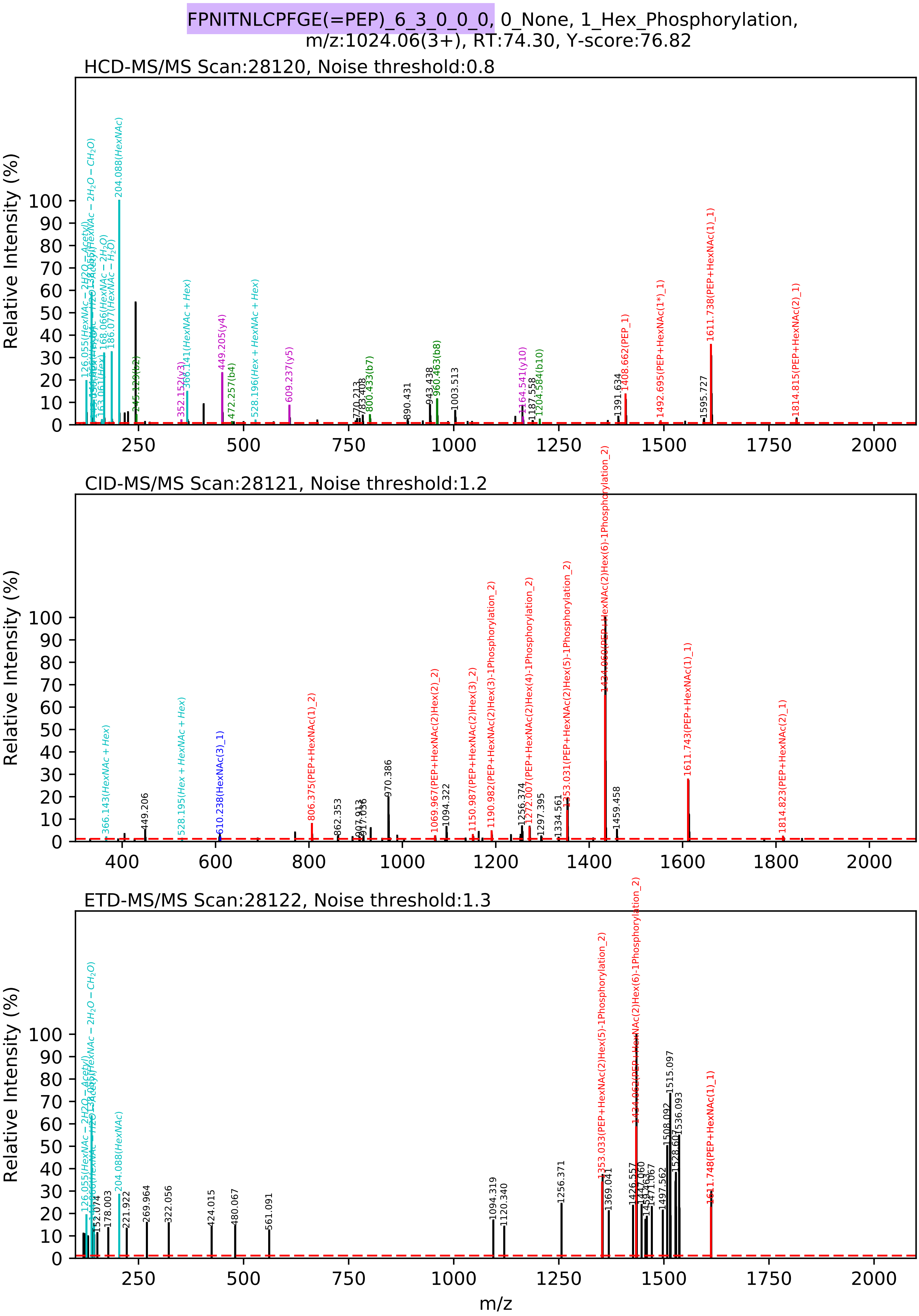
FPNITNLCPFGE(=PEP)_6_3_0_0_0, 0_None, 1_Hex_Phosphorylation,
m/z:1024.06(3+), RT:74.30, Y-score:76.82
HCD-MS/MS Scan:28120, Noise threshold:0.8
CID-MS/MS Scan:28121, Noise threshold:1.2
ETD-MS/MS Scan:28122, Noise threshold:1.3
Relative Intensity (%)
m/z
204.088(HexNAc)
366.141(HexNAc + Hex)
449.205(y4)
609.237(y5)
960.463(b8)
1408.662(PEP_1)
1492.695(PEP+HexNAc(1*)_1)
1611.738(PEP+HexNAc(1)_1)
1814.815(PEP+HexNAc(2)_1)
806.375(PEP+HexNAc(1)_2)
1069.967(PEP+HexNAc(2)Hex(2)_2)
1150.987(PEP+HexNAc(2)Hex(3)_2)
1190.982(PEP+HexNAc(2)Hex(3)-1Phosphorylation_2)
1272.007(PEP+HexNAc(2)Hex(4)-1Phosphorylation_2)
1353.031(PEP+HexNAc(2)Hex(5)-1Phosphorylation_2)
1434.060(PEP+HexNAc(2)Hex(6)-1Phosphorylation_2)
1611.743(PEP+HexNAc(1)_1)
1814.823(PEP+HexNAc(2)_1)
1353.033(PEP+HexNAc(2)Hex(5)-1Phosphorylation_2)
1434.062(PEP+HexNAc(2)Hex(6)-1Phosphorylation_2)
1611.748(PEP+HexNAc(1)_1)

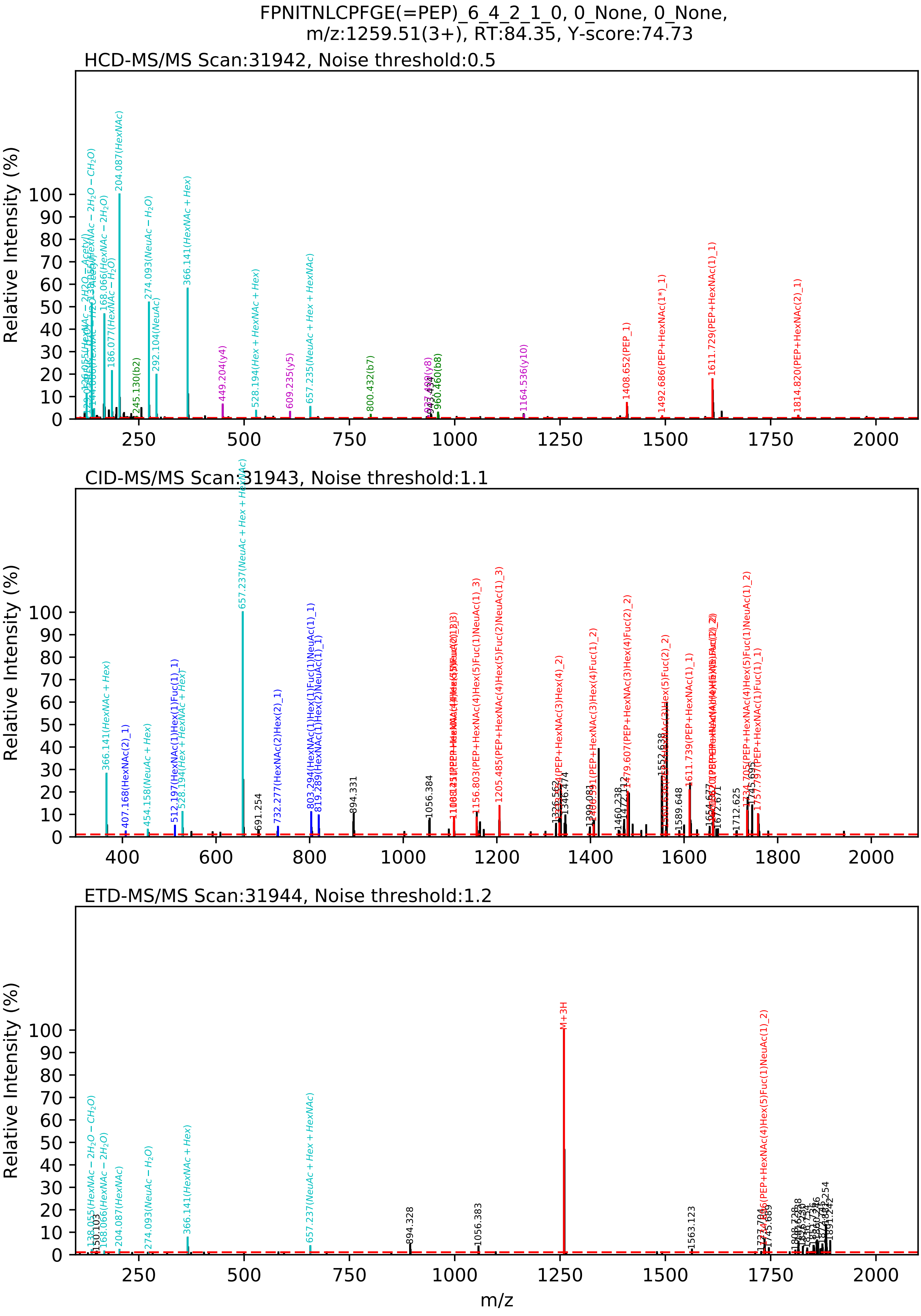
FPNITNLCPFGE(=PEP)_6_4_2_1_0, 0_None, 0_None,
m/z:1259.51(3+), RT:84.35, Y-score:74.73
HCD-MS/MS Scan:31942, Noise threshold:0.5
Relative Intensity (%)
204.087(HexNAc)
168.066(HexNAc − 2H2O)
186.077(HexNAc − H2O)
274.093(NeuAc − H2O)
292.104(NeuAc)
366.141(HexNAc + Hex)
245.130(b2)
449.204(y4)
528.194(Hex + HexNAc + Hex)
609.235(y5)
657.235(NeuAc + Hex + HexNAc)
800.432(b7)
960.460(b8)
1164.536(y10)
1408.652(PEP_1)
1492.686(PEP+HexNAc(1*)_1)
1611.729(PEP+HexNAc(1)_1)
1814.820(PEP+HexNAc(2)_1)
CID-MS/MS Scan:31943, Noise threshold:1.1
657.237(NeuAc + Hex + HexNAc)
366.141(HexNAc + Hex)
407.168(HexNAc(2)_1)
454.158(NeuAc + Hex)
512.197(HexNAc(1)Hex(1)Fuc(1)_1)
528.194(Hex + HexNAc + Hex)
691.254
732.277(HexNAc(2)Hex(2)_1)
803.294(HexNAc(1)Hex(1)Fuc(1)NeuAc(1)_1)
819.289(HexNAc(1)Hex(2)NeuAc(1)_1)
894.331
1056.384
1156.803(PEP+HexNAc(4)Hex(5)Fuc(1)NeuAc(1)_3)
1205.485(PEP+HexNAc(4)Hex(5)Fuc(2)NeuAc(1)_3)
1326.562
1346.474
1399.091
1460.238
1479.607(PEP+HexNAc(3)Hex(4)Fuc(2)_2)
1552.638
1589.648
1611.739(PEP+HexNAc(1)_1)
1654.677
1672.671
1712.625
1734.705(PEP+HexNAc(4)Hex(5)Fuc(1)NeuAc(1)_2)
1745.695
1757.797(PEP+HexNAc(1)Fuc(1)_1)
ETD-MS/MS Scan:31944, Noise threshold:1.2
138.055(HexNAc − 2H2O − CH2O)
150.103
168.066(HexNAc − 2H2O)
204.087(HexNAc)
274.093(NeuAc − H2O)
366.141(HexNAc + Hex)
657.237(NeuAc + Hex + HexNAc)
894.328
1056.383
M+3H
1563.123
1745.689
1891.242
m/z

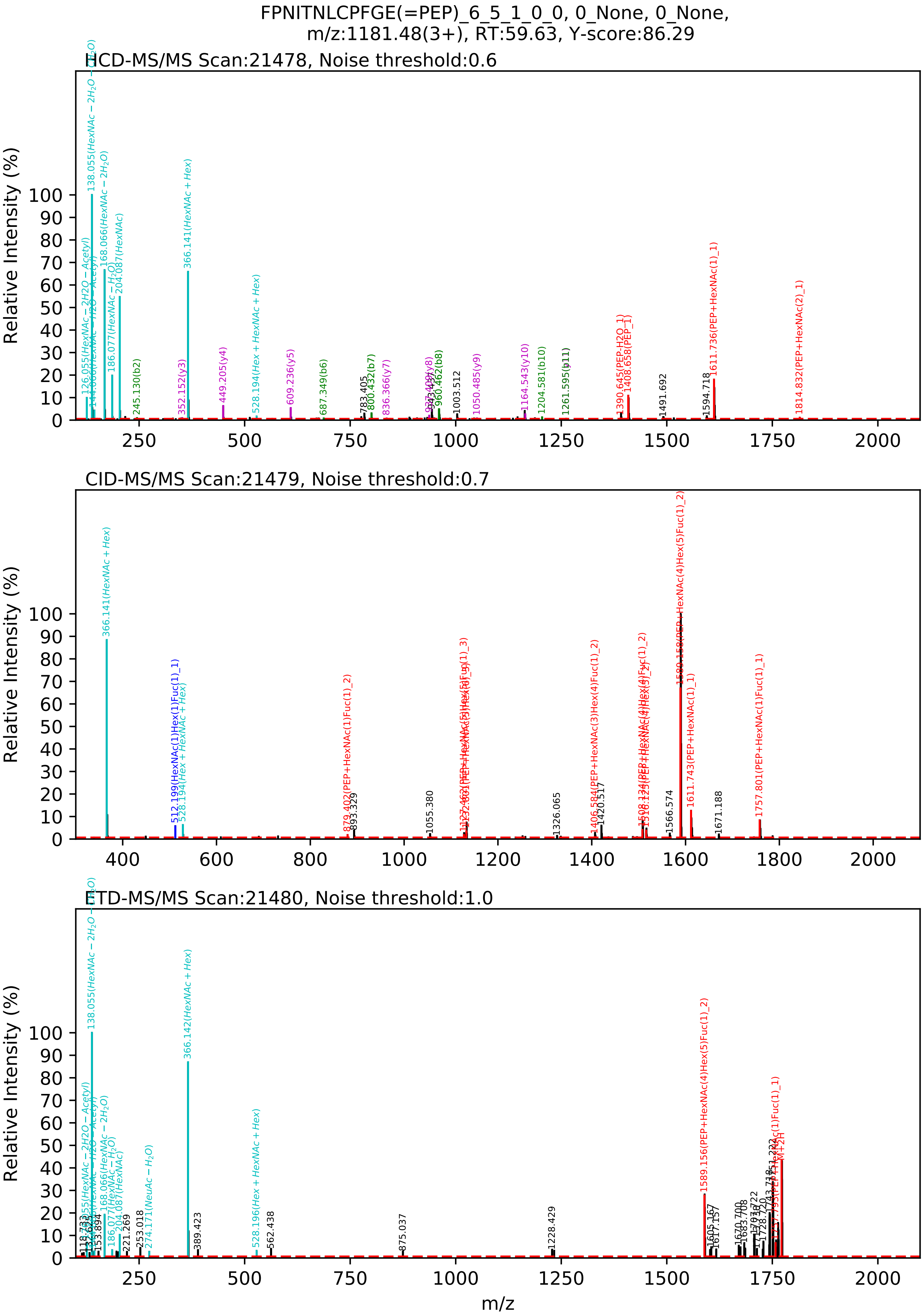

FPNITNLCPFGE(=PEP)_6_5_1_0_0, 0_None, 0_None,
m/z:1181.48(3+), RT:59.63, Y-score:86.29
HCD-MS/MS Scan:21478, Noise threshold:0.6
CID-MS/MS Scan:21479, Noise threshold:0.7
ETD-MS/MS Scan:21480, Noise threshold:1.0
Relative Intensity (%)
m/z

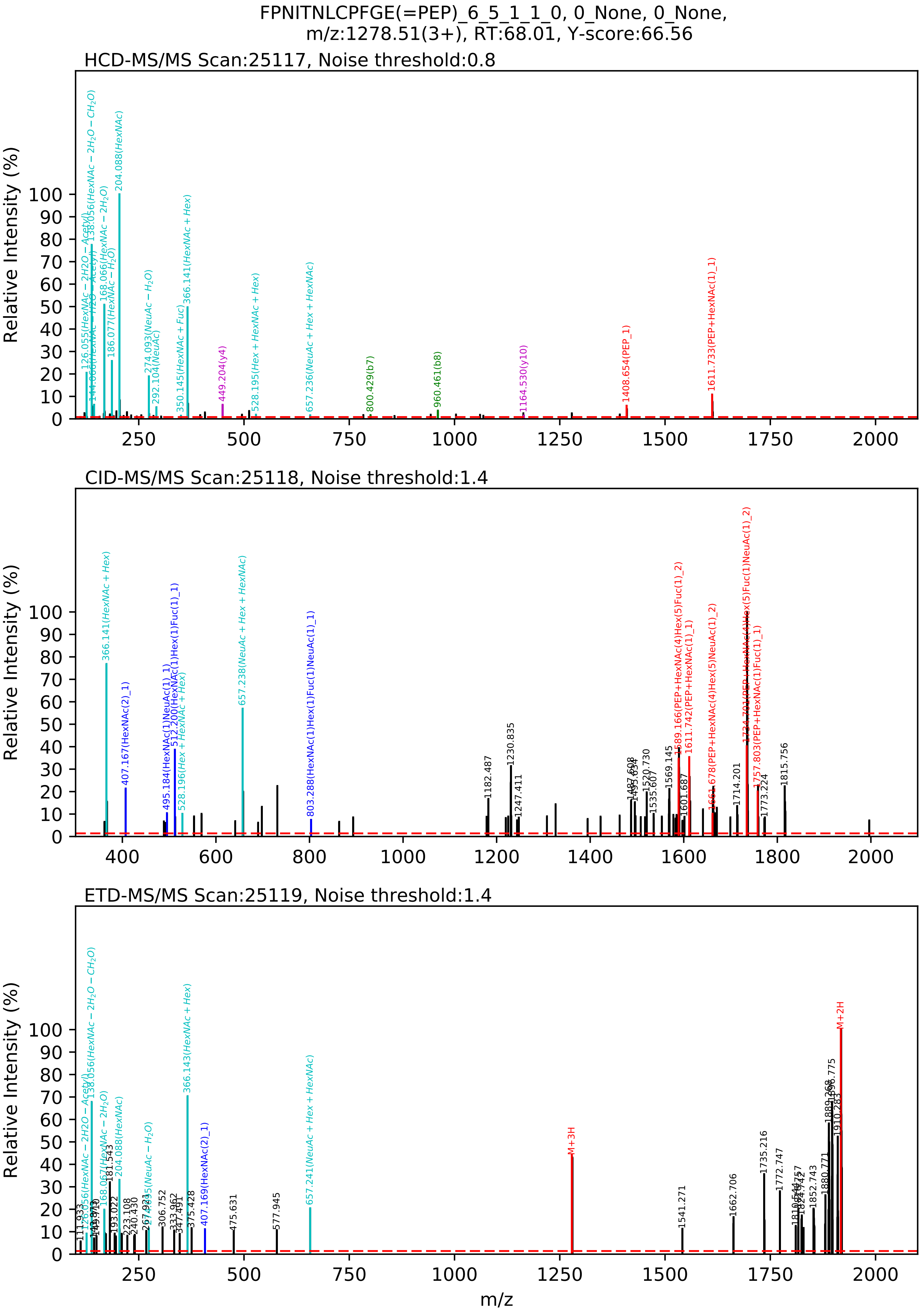
FPNITNLCPFGE(=PEP)_6_5_1_1_0, 0_None, 0_None,
m/z:1278.51(3+), RT:68.01, Y-score:66.56
HCD-MS/MS Scan:25117, Noise threshold:0.8
Relative Intensity (%)
204.088(HexNAc)
366.141(HexNAc + Hex)
1408.654(PEP_1)
1611.733(PEP+HexNAc(1)_1)
449.204(y4)
800.429(b7)
960.461(b8)
1164.530(y10)
528.195(Hex + HexNAc + Hex)
657.236(NeuAc + Hex + HexNAc)
274.093(NeuAc − H2O)
292.104(NeuAc)
350.145(HexNAc + Fuc)
CID-MS/MS Scan:25118, Noise threshold:1.4
366.141(HexNAc + Hex)
407.167(HexNAc(2)_1)
657.238(NeuAc + Hex + HexNAc)
803.288(HexNAc(1)Hex(1)Fuc(1)NeuAc(1)_1)
1182.487
1230.835
1247.411
1569.145
1601.687
1714.201
1773.224
1815.756
ETD-MS/MS Scan:25119, Noise threshold:1.4
M+3H
M+2H
366.143(HexNAc + Hex)
407.169(HexNAc(2)_1)
657.241(NeuAc + Hex + HexNAc)
181.543
223.108
240.430
306.752
333.962
347.491
375.428
475.631
577.945
1541.271
1662.706
1735.216
1772.747
1852.743
1880.771
m/z

FPNITNLCPFGE(=PEP)_6_5_1_1_0, 0_None, 0_None,
m/z:1278.51(3+), RT:68.79, Y-score:89.73

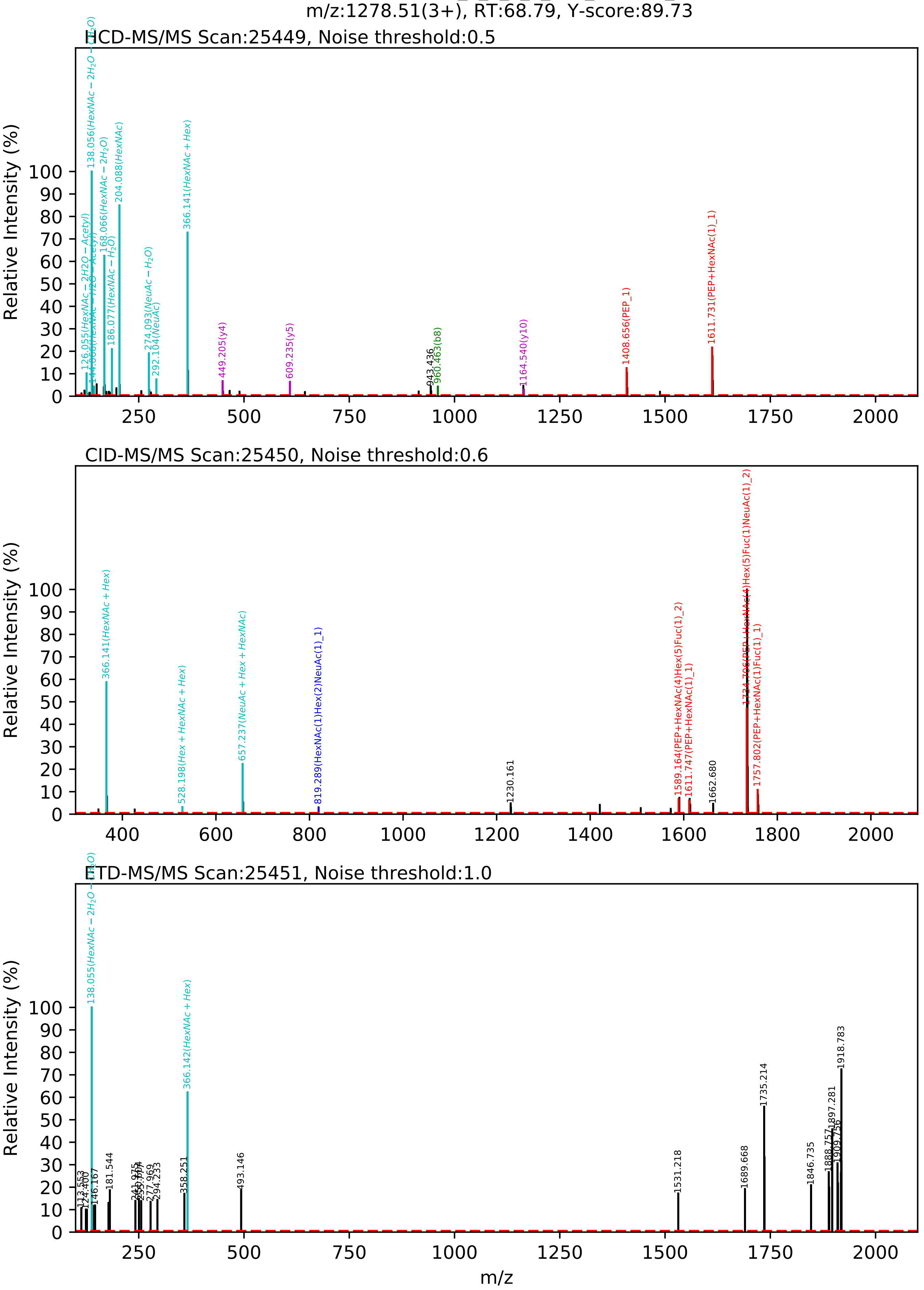

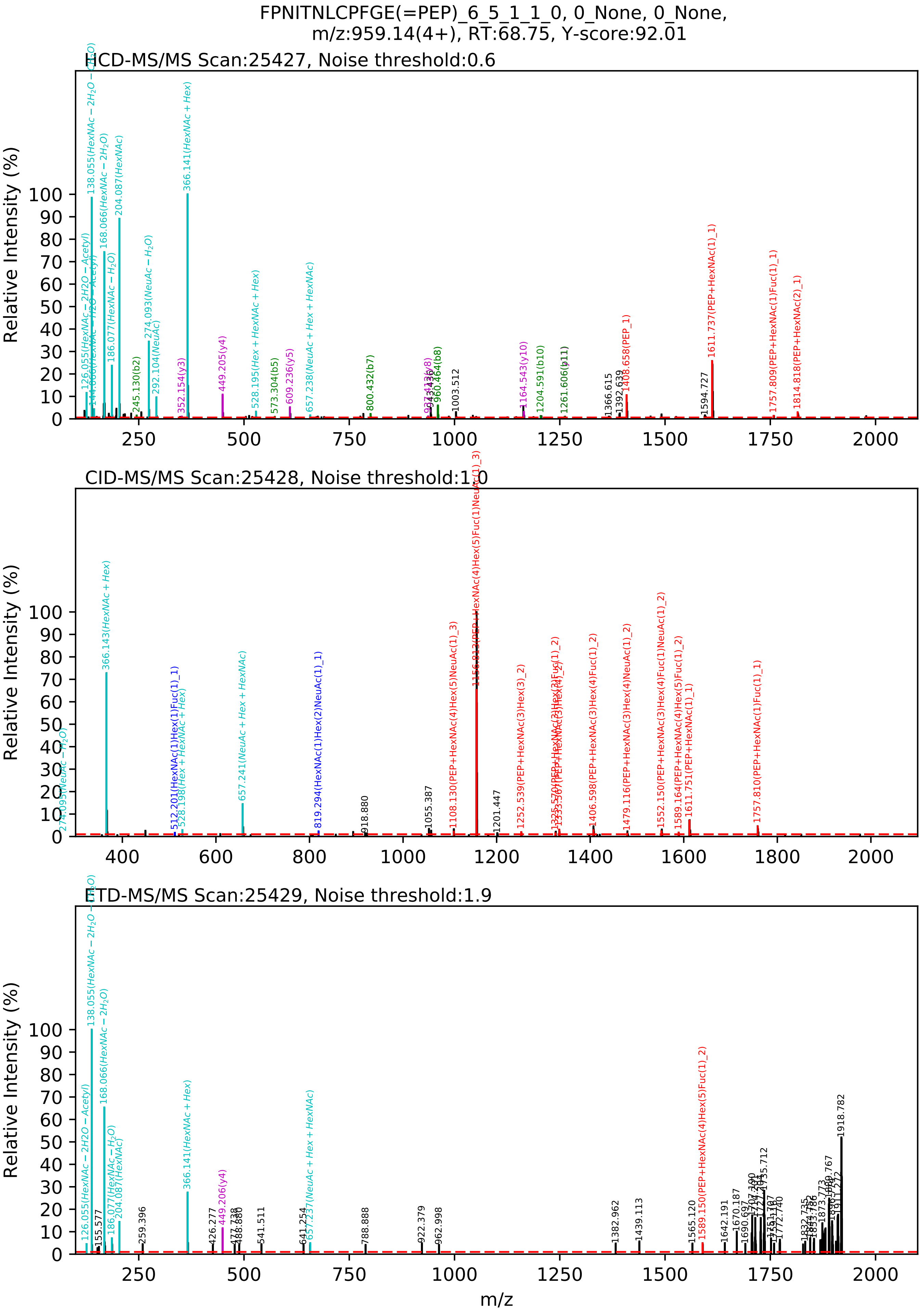

FPNITNLCPFGE(=PEP)_6_5_1_1_0, 0_None, 0_None,
m/z:959.14(4+), RT:68.75, Y-score:92.01
HCD-MS/MS Scan:25427, Noise threshold:0.6
CID-MS/MS Scan:25428, Noise threshold:1.0
ETD-MS/MS Scan:25429, Noise threshold:1.9
Relative Intensity (%)
m/z

FPNITNLCPFGE(=PEP)_6_5_1_1_0, 0_None, 0_None,
m/z:959.14(4+), RT:69.47, Y-score:89.17

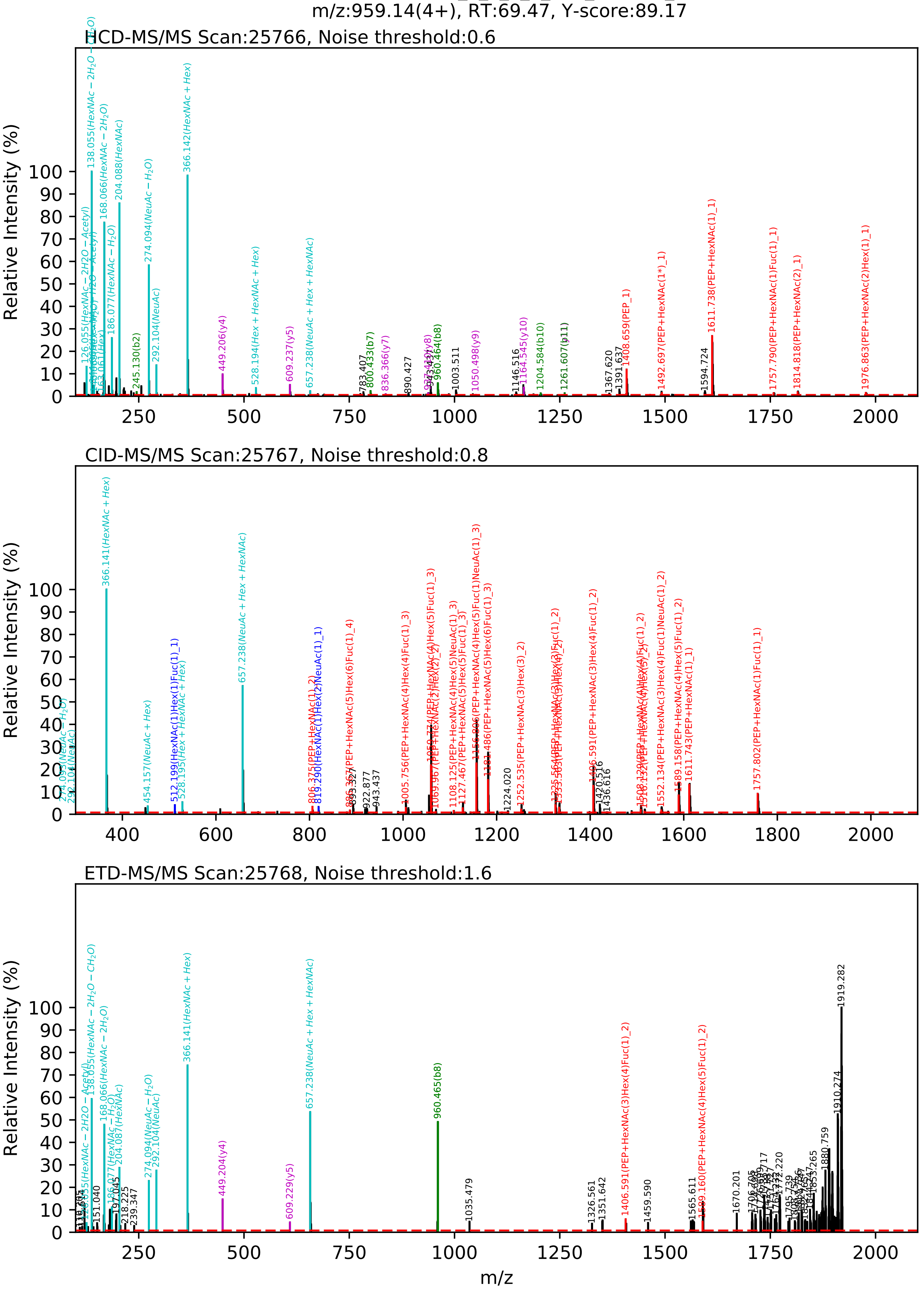

FPNITNLCPFGE(=PEP)_6_5_1_1_0, 0_None, 0_None,
m/z:1278.51(3+), RT:69.40, Y-score:89.64

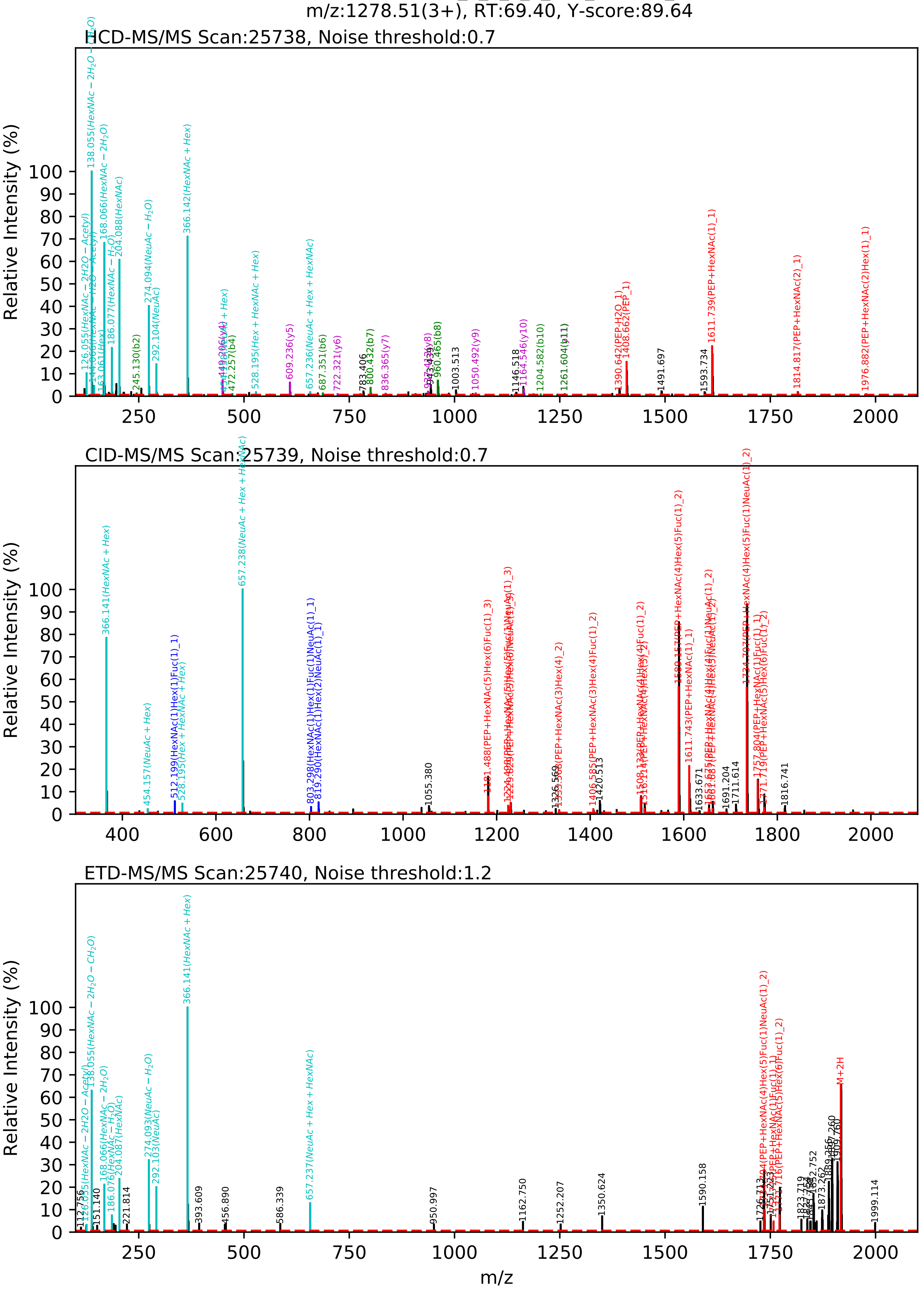

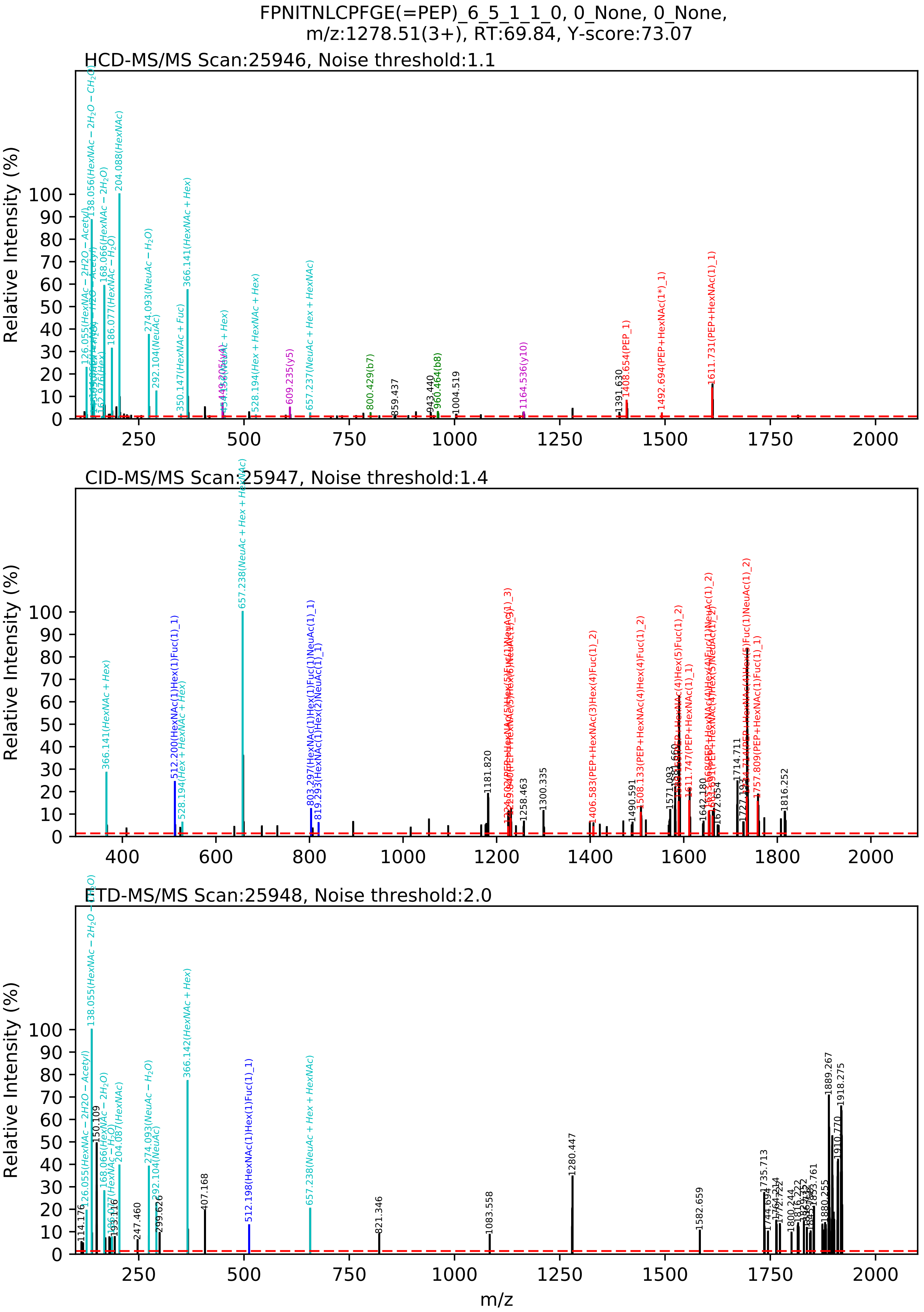
FPNITNLCPFGE(=PEP)_6_5_1_1_0, 0_None, 0_None,
m/z:1278.51(3+), RT:69.84, Y-score:73.07
HCD-MS/MS Scan:25946, Noise threshold:1.1
Relative Intensity (%)
204.088(HexNAc)
138.056(HexNAc − 2H2O − CH2O)
168.066(HexNAc − 2H2O)
186.077(HexNAc − H2O)
274.093(NeuAc − H2O)
292.104(NeuAc)
366.141(HexNAc + Hex)
350.147(HexNAc + Fuc)
528.194(Hex + HexNAc + Hex)
609.235(y5)
657.237(NeuAc + Hex + HexNAc)
800.429(b7)
859.437
943.440
960.464(b8)
1004.519
1164.536(y10)
1391.630
1408.654(PEP_1)
1492.694(PEP+HexNAc(1*)_1)
1611.731(PEP+HexNAc(1)_1)
CID-MS/MS Scan:25947, Noise threshold:1.4
366.141(HexNAc + Hex)
512.200(HexNAc(1)Hex(1)Fuc(1)_1)
528.194(Hex + HexNAc + Hex)
657.238(NeuAc + Hex + HexNAc)
803.297(HexNAc(1)Hex(1)Fuc(1)NeuAc(1)_1)
819.293(HexNAc(1)Hex(2)NeuAc(1)_1)
1181.820
1258.463
1300.335
1406.583(PEP+HexNAc(3)Hex(4)Fuc(1)_2)
1490.591
1508.133(PEP+HexNAc(4)Hex(4)Fuc(1)_2)
1571.093
1611.747(PEP+HexNAc(1)_1)
1642.180
1672.654
1714.711
1727.193
1757.809(PEP+HexNAc(1)Fuc(1)_1)
1816.252
ETD-MS/MS Scan:25948, Noise threshold:2.0
114.176
126.055(HexNAc − 2H2O − Acetyl)
138.055(HexNAc − 2H2O − CH2O)
150.109
168.066(HexNAc − 2H2O)
186.077(HexNAc − H2O)
193.116
204.087(HexNAc)
247.460
274.093(NeuAc − H2O)
292.104(NeuAc)
299.626
366.142(HexNAc + Hex)
407.168
512.198(HexNAc(1)Hex(1)Fuc(1)_1)
657.238(NeuAc + Hex + HexNAc)
821.346
1083.558
1280.447
1582.659
1735.713
1744.694
1800.244
1816.222
1853.761
1880.255
1889.267
1910.770
1918.275
m/z

FPNITNLCPFGE(=PEP)_6_5_1_2_0, 0_None, 0_None,
m/z:1031.91(4+), RT:84.34, Y-score:90.31

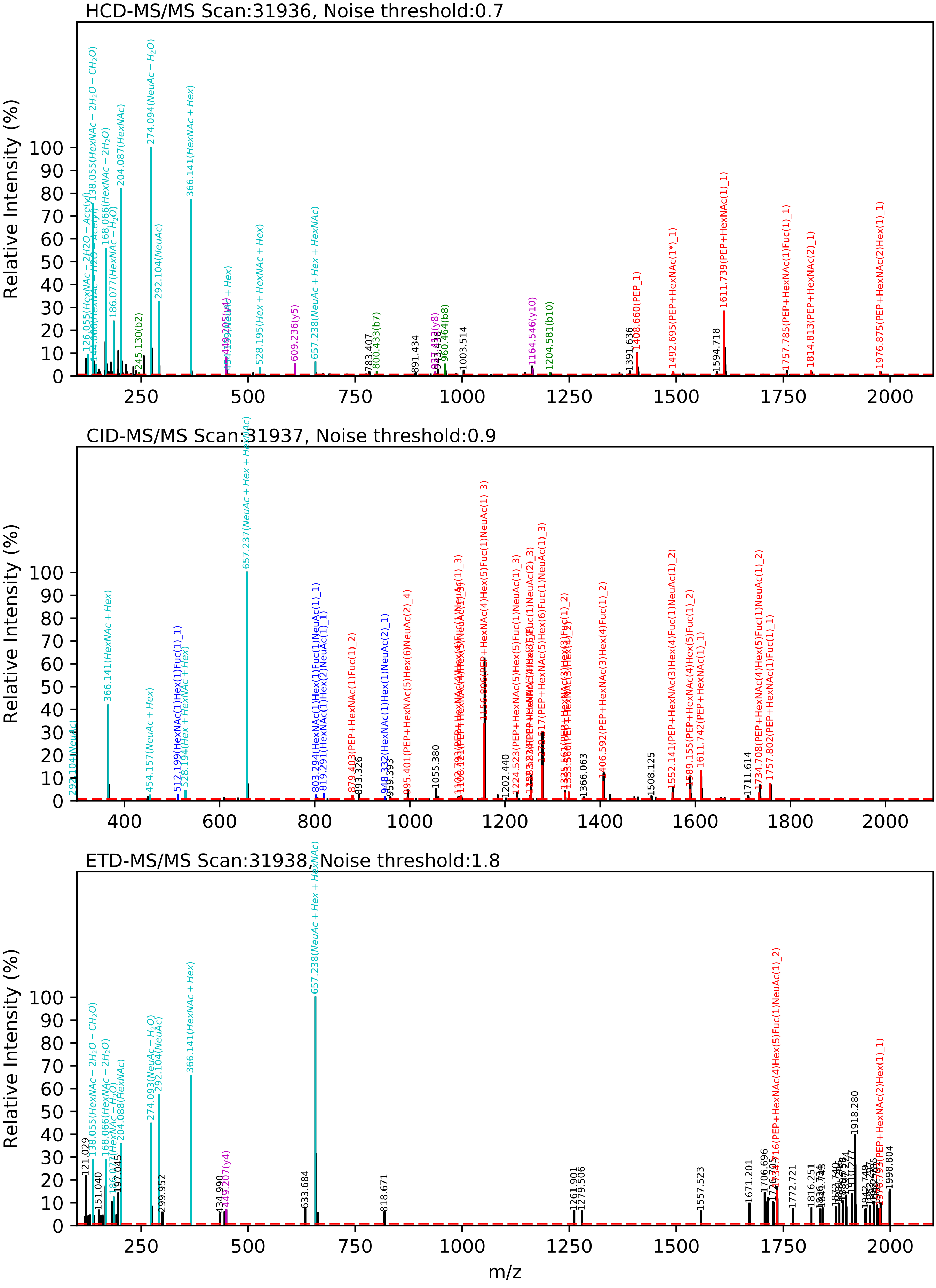

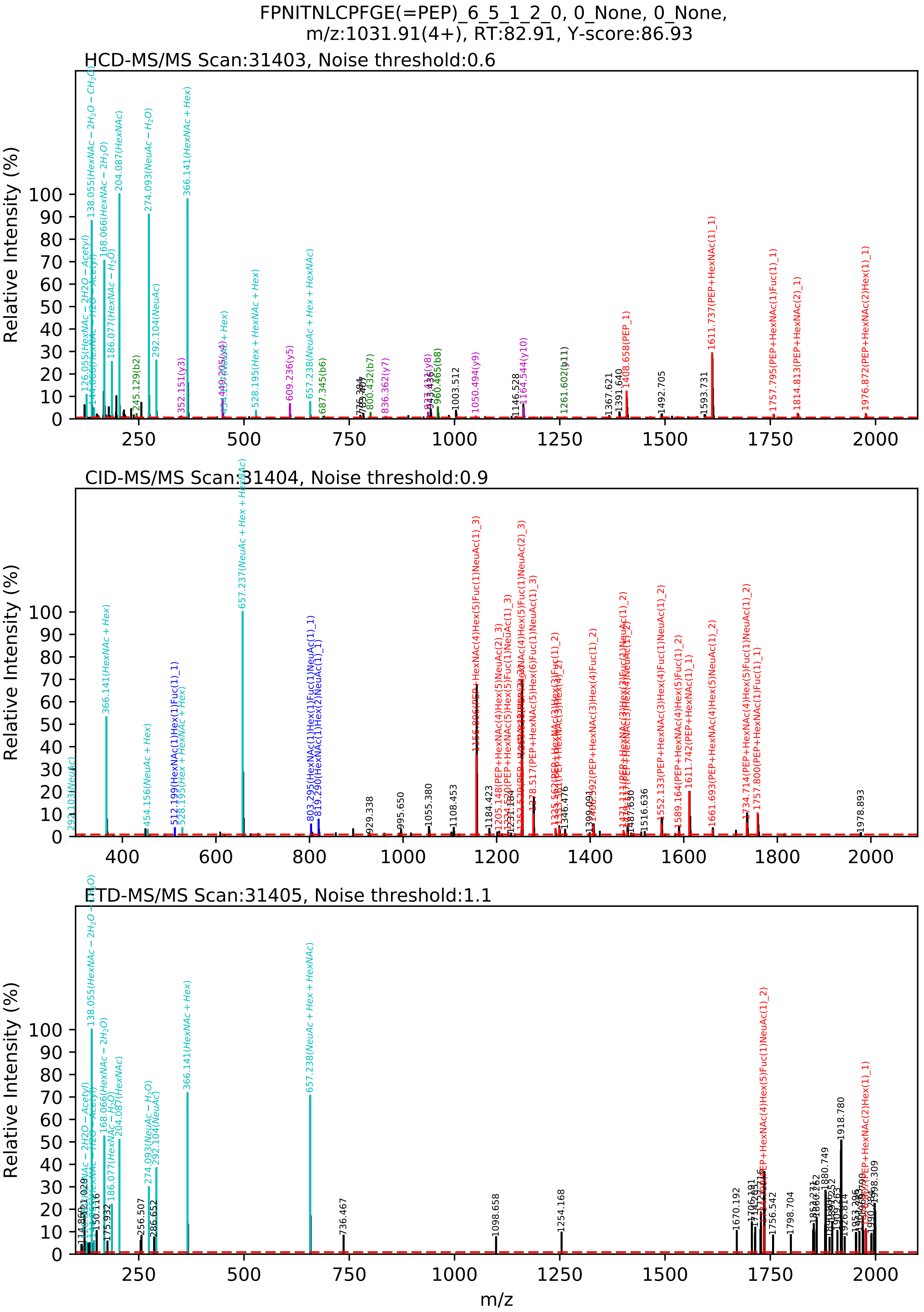
FPNITNLCPFGE(=PEP)_6_5_1_2_0, 0_None, 0_None,
m/z:1031.91(4+), RT:82.91, Y-score:86.93
HCD-MS/MS Scan:31403, Noise threshold:0.6
Relative Intensity (%)
138.055(HexNAc − 2H2O − CH2O)
204.087(HexNAc)
274.093(NeuAc − H2O)
366.141(HexNAc + Hex)
168.066(HexNAc − 2H2O)
186.077(HexNAc − H2O)
292.104(NeuAc)
245.129(b2)
352.151(y3)
528.195(Hex + HexNAc + Hex)
609.236(y5)
657.238(NeuAc + Hex + HexNAc)
687.345(b6)
800.432(b7)
836.362(y7)
960.465(b8)
1003.512
1050.494(y9)
1146.528
1164.544(y10)
1261.602(b11)
1367.621
1391.640
1408.658(PEP_1)
1492.705
1593.731
1611.737(PEP+HexNAc(1)_1)
1757.795(PEP+HexNAc(1)Fuc(1)_1)
1814.813(PEP+HexNAc(2)_1)
1976.872(PEP+HexNAc(2)Hex(1)_1)
CID-MS/MS Scan:31404, Noise threshold:0.9
366.141(HexNAc + Hex)
454.156(NeuAc + Hex)
512.199(HexNAc(1)Hex(1)Fuc(1)_1)
528.195(Hex + HexNAc + Hex)
657.237(NeuAc + Hex + HexNAc)
803.295(HexNAc(1)Hex(1)Fuc(1)NeuAc(1)_1)
819.290(HexNAc(1)Hex(2)NeuAc(1)_1)
929.338
995.650
1055.380
1108.453
1156.806(PEP+HexNAc(4)Hex(5)Fuc(1)NeuAc(1)_3)
1184.423
1205.148(PEP+HexNAc(4)Hex(5)NeuAc(2)_3)
1278.517(PEP+HexNAc(5)Hex(6)Fuc(1)NeuAc(1)_3)
1346.476
1399.091
1516.636
1552.133(PEP+HexNAc(3)Hex(4)Fuc(1)NeuAc(1)_2)
1589.164(PEP+HexNAc(4)Hex(5)Fuc(1)_2)
1611.742(PEP+HexNAc(1)_1)
1661.693(PEP+HexNAc(4)Hex(5)NeuAc(1)_2)
1734.714(PEP+HexNAc(4)Hex(5)Fuc(1)NeuAc(1)_2)
1757.800(PEP+HexNAc(1)Fuc(1)_1)
1978.893
ETD-MS/MS Scan:31405, Noise threshold:1.1
138.055(HexNAc − 2H2O − CH2O)
168.066(HexNAc − 2H2O)
186.077(HexNAc − H2O)
204.087(HexNAc)
274.093(NeuAc − H2O)
292.104(NeuAc)
366.141(HexNAc + Hex)
657.238(NeuAc + Hex + HexNAc)
150.116
175.932
256.507
286.652
736.467
1098.658
1254.168
1670.192
1756.542
1798.704
1880.749
1918.780
1998.309
m/z

FPNITNLCPFGE(=PEP)_6_5_1_2_0, 0_None, 0_None,
m/z:1375.55(3+), RT:82.86, Y-score:88.62

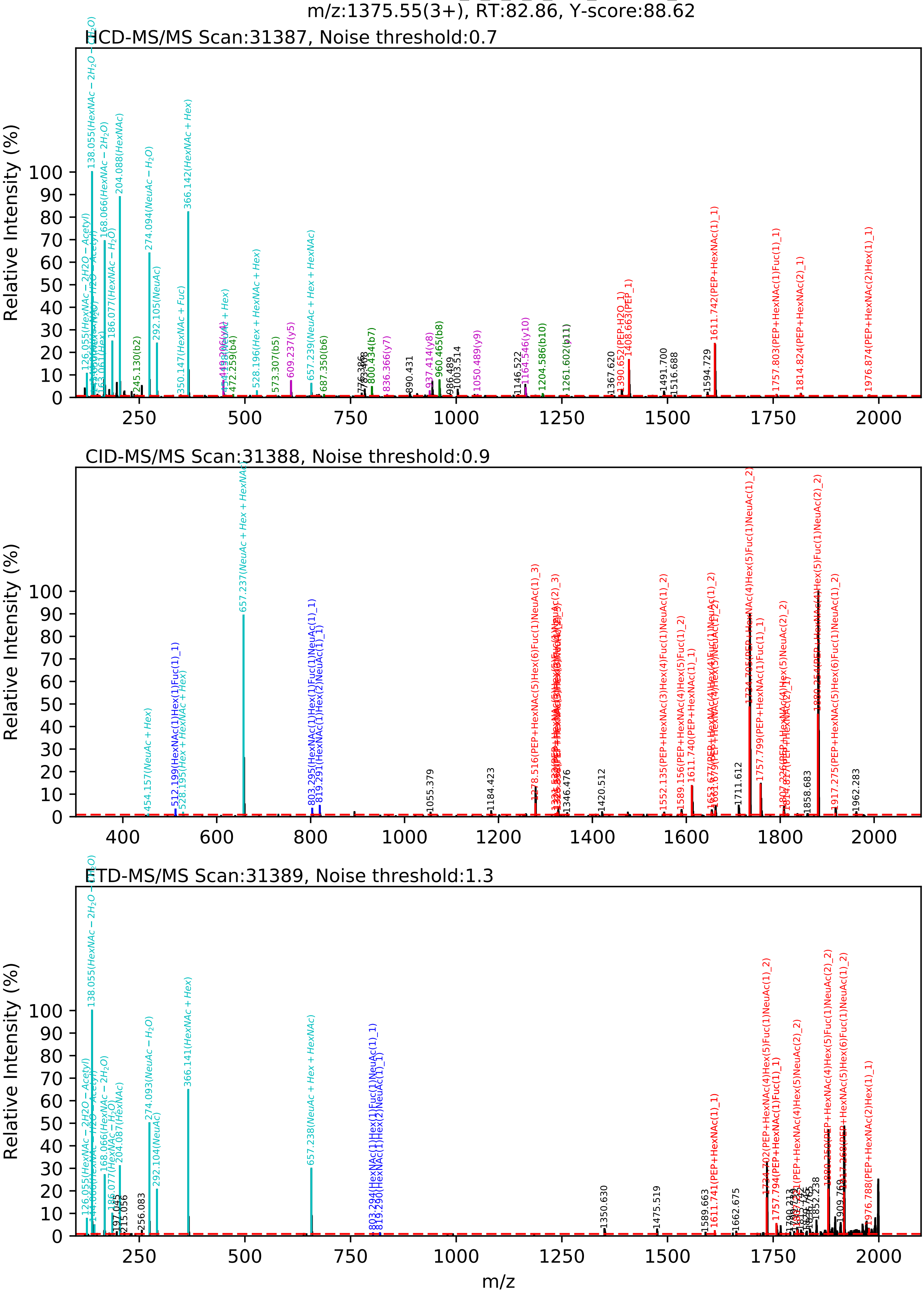

FPNITNLCPFGE(=PEP)_6_5_2_0_0, 0_None, 0_None,
m/z:1230.17(3+), RT:59.41, Y-score:90.99

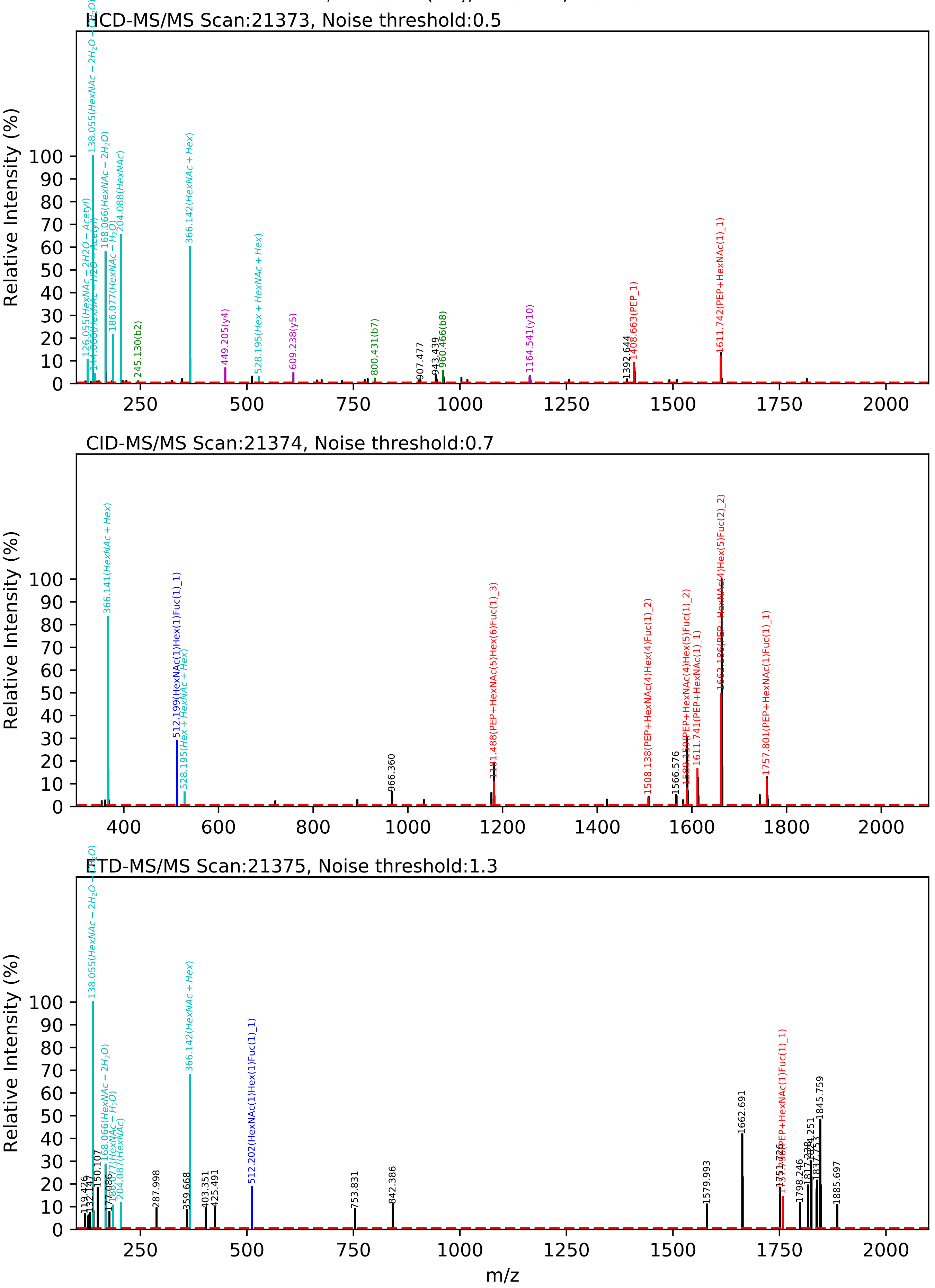

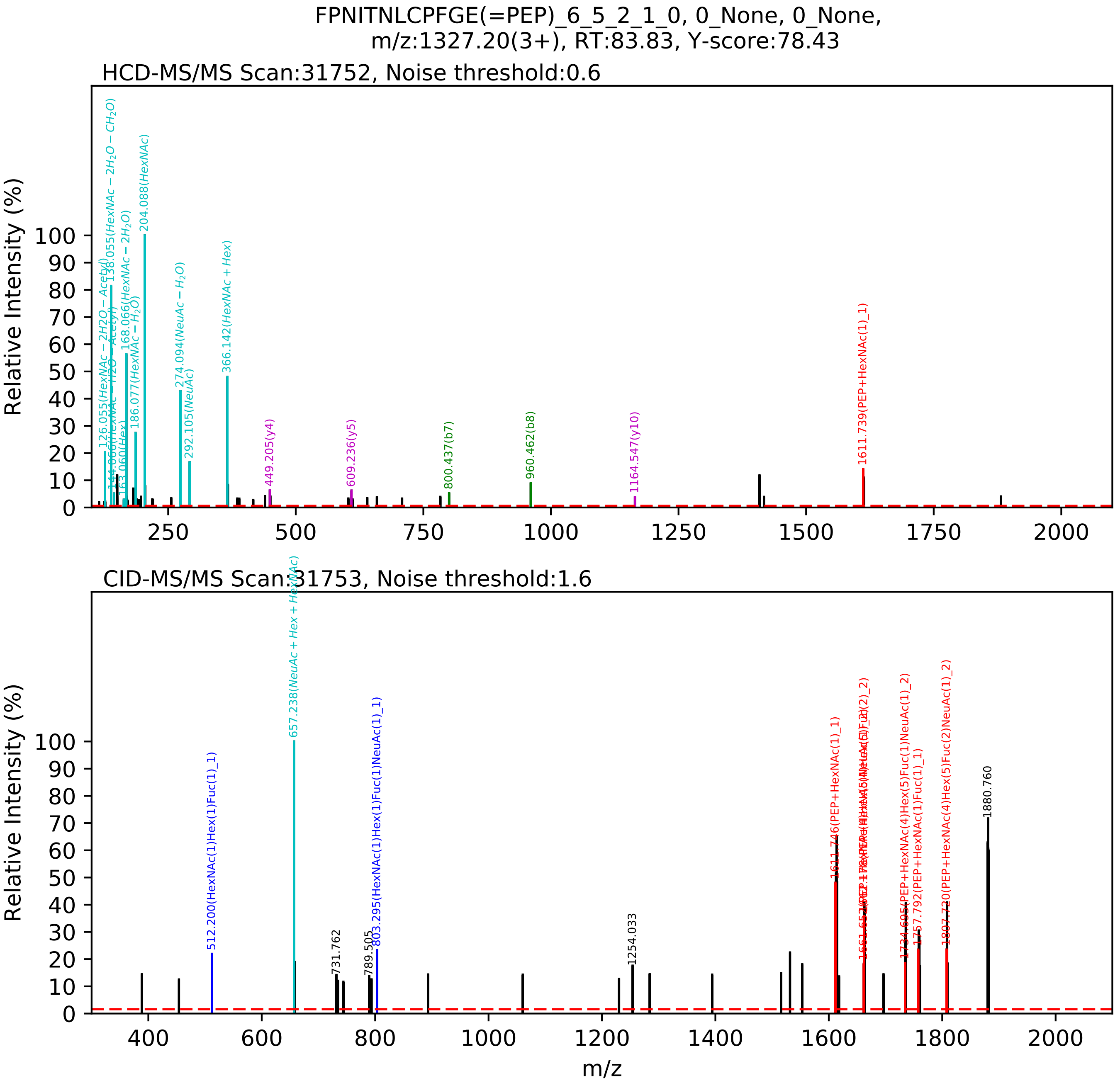
FPNITNLCPFGE(=PEP)_6_5_2_1_0, 0_None, 0_None,
m/z:1327.20(3+), RT:83.83, Y-score:78.43
HCD-MS/MS Scan:31752, Noise threshold:0.6
Relative Intensity (%)
126.055(HexNAc − 2H2O − Acetyl)
138.055(HexNAc − 2H2O − CH2O)
144.066(HexNAc − H2O − Acetyl)
163.060(Hex)
168.066(HexNAc − 2H2O)
186.077(HexNAc − H2O)
204.088(HexNAc)
274.094(NeuAc − H2O)
292.105(NeuAc)
366.142(HexNAc + Hex)
449.205(y4)
609.236(y5)
800.437(b7)
960.462(b8)
1164.547(y10)
1611.739(PEP+HexNAc(1)_1)
CID-MS/MS Scan:31753, Noise threshold:1.6
657.238(NeuAc + Hex + HexNAc)
512.200(HexNAc(1)Hex(1)Fuc(1)_1)
803.295(HexNAc(1)Hex(1)Fuc(1)NeuAc(1)_1)
731.762
789.505
1254.033
1611.746(PEP+HexNAc(1)_1)
1724.605(PEP+HexNAc(4)Hex(5)Fuc(1)NeuAc(1)_2)
1757.792(PEP+HexNAc(1)Fuc(1)_1)
1807.720(PEP+HexNAc(4)Hex(5)Fuc(2)NeuAc(1)_2)
1880.760
m/z

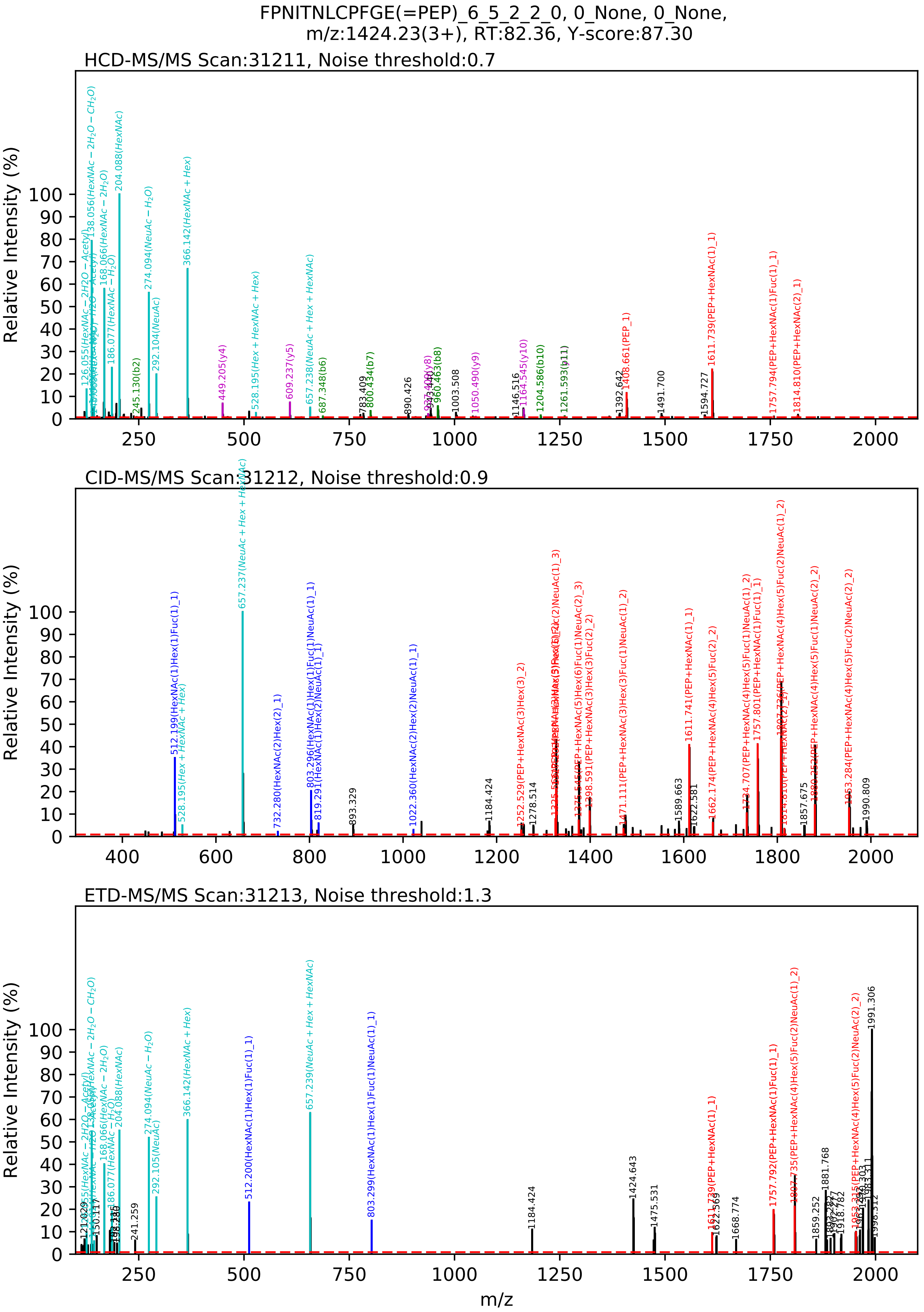

FPNITNLCPFGE(=PEP)_6_5_2_2_0, 0_None, 0_None,
m/z:1424.23(3+), RT:82.36, Y-score:87.30
HCD-MS/MS Scan:31211, Noise threshold:0.7
CID-MS/MS Scan:31212, Noise threshold:0.9
ETD-MS/MS Scan:31213, Noise threshold:1.3
Relative Intensity (%)
m/z
204.088(HexNAc)
366.142(HexNAc + Hex)
274.094(NeuAc − H2O)
292.104(NeuAc)
1611.739(PEP+HexNAc(1)_1)
1408.661(PEP_1)
1757.794(PEP+HexNAc(1)Fuc(1)_1)
1814.810(PEP+HexNAc(2)_1)
657.237(NeuAc + Hex + HexNAc)
512.199(HexNAc(1)Hex(1)Fuc(1)_1)
1991.306

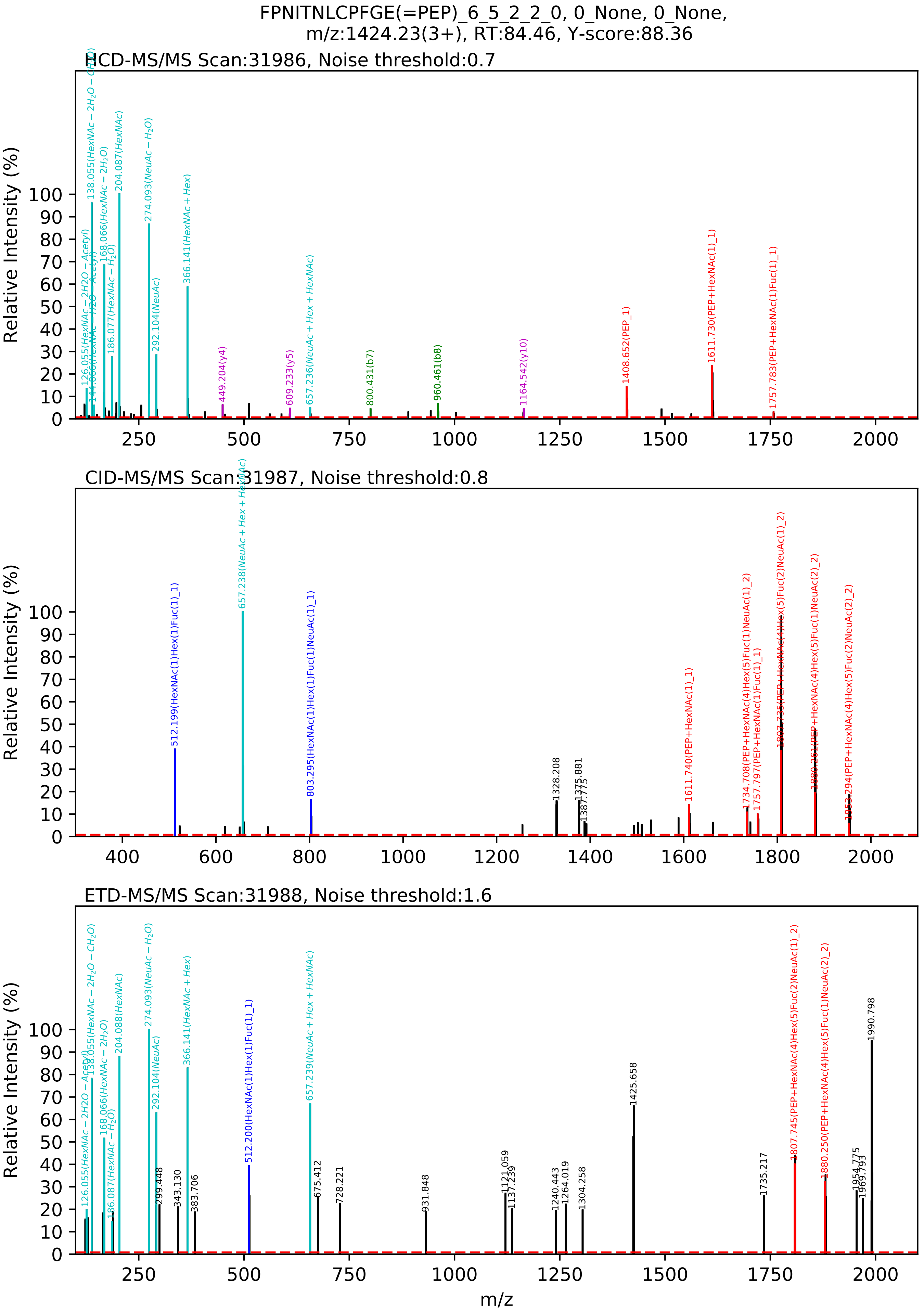
FPNITNLCPFGE(=PEP)_6_5_2_2_0, 0_None, 0_None,
m/z:1424.23(3+), RT:84.46, Y-score:88.36
HCD-MS/MS Scan:31986, Noise threshold:0.7
Relative Intensity (%)
126.055(HexNAc − 2H2O − Acetyl)
138.055(HexNAc − 2H2O − CH2O)
168.066(HexNAc − 2H2O)
186.077(HexNAc − H2O)
204.087(HexNAc)
274.093(NeuAc − H2O)
292.104(NeuAc)
366.141(HexNAc + Hex)
449.204(y4)
609.233(y5)
657.236(NeuAc + Hex + HexNAc)
800.431(b7)
960.461(b8)
1164.542(y10)
1408.652(PEP_1)
1611.730(PEP+HexNAc(1)_1)
1757.783(PEP+HexNAc(1)Fuc(1)_1)
CID-MS/MS Scan:31987, Noise threshold:0.8
512.199(HexNAc(1)Hex(1)Fuc(1)_1)
657.238(NeuAc + Hex + HexNAc)
803.295(HexNAc(1)Hex(1)Fuc(1)NeuAc(1)_1)
1328.208
1375.881
1387.775
1611.740(PEP+HexNAc(1)_1)
1734.708(PEP+HexNAc(4)Hex(5)Fuc(1)NeuAc(1)_2)
1757.797(PEP+HexNAc(1)Fuc(1)_1)
1807.725(PEP+HexNAc(4)Hex(5)Fuc(2)NeuAc(1)_2)
1880.261(PEP+HexNAc(4)Hex(5)Fuc(1)NeuAc(2)_2)
1953.294(PEP+HexNAc(4)Hex(5)Fuc(2)NeuAc(2)_2)
ETD-MS/MS Scan:31988, Noise threshold:1.6
126.055(HexNAc − 2H2O − Acetyl)
138.055(HexNAc − 2H2O − CH2O)
168.066(HexNAc − 2H2O)
186.087(HexNAc − H2O)
204.088(HexNAc)
274.093(NeuAc − H2O)
292.104(NeuAc)
299.448
343.130
366.141(HexNAc + Hex)
383.706
512.200(HexNAc(1)Hex(1)Fuc(1)_1)
657.239(NeuAc + Hex + HexNAc)
675.412
728.221
931.848
1121.059
1137.239
1240.443
1264.019
1304.258
1425.658
1735.217
1807.745(PEP+HexNAc(4)Hex(5)Fuc(2)NeuAc(1)_2)
1880.250(PEP+HexNAc(4)Hex(5)Fuc(1)NeuAc(2)_2)
1954.775
1969.793
1990.798
m/z

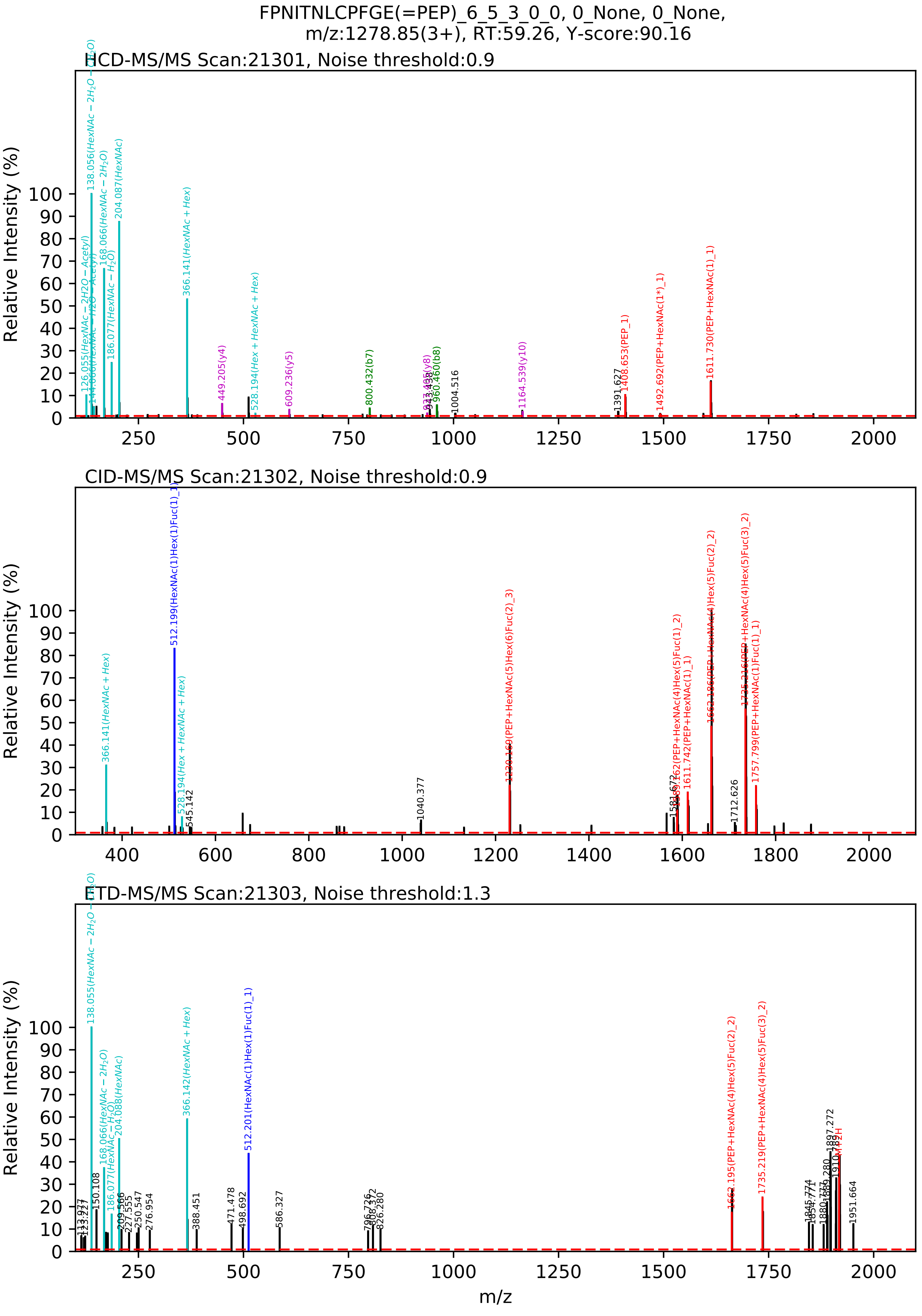
FPNITNLCPFGE(=PEP)_6_5_3_0_0, 0_None, 0_None,
m/z:1278.85(3+), RT:59.26, Y-score:90.16
HCD-MS/MS Scan:21301, Noise threshold:0.9
CID-MS/MS Scan:21302, Noise threshold:0.9
ETD-MS/MS Scan:21303, Noise threshold:1.3
Relative Intensity (%)
m/z
138.056(HexNAc − 2H2O − H2O)
204.087(HexNAc)
366.141(HexNAc + Hex)
528.194(Hex + HexNAc + Hex)
449.205(y4)
609.236(y5)
800.432(b7)
960.460(b8)
1004.516
1164.539(y10)
1391.627
1408.653(PEP_1)
1492.692(PEP+HexNAc(1*)_1)
1611.730(PEP+HexNAc(1)_1)
512.199(HexNAc(1)Hex(1)Fuc(1)_1)
545.142
1040.377
1712.626
1757.799(PEP+HexNAc(1)Fuc(1)_1)
1611.742(PEP+HexNAc(1)_1)
512.201(HexNAc(1)Hex(1)Fuc(1)_1)
1662.195(PEP+HexNAc(4)Hex(5)Fuc(2)_2)
1735.219(PEP+HexNAc(4)Hex(5)Fuc(3)_2)
150.108
388.451
471.478
498.692
586.327
826.280
1951.664
1897.272
M+2H

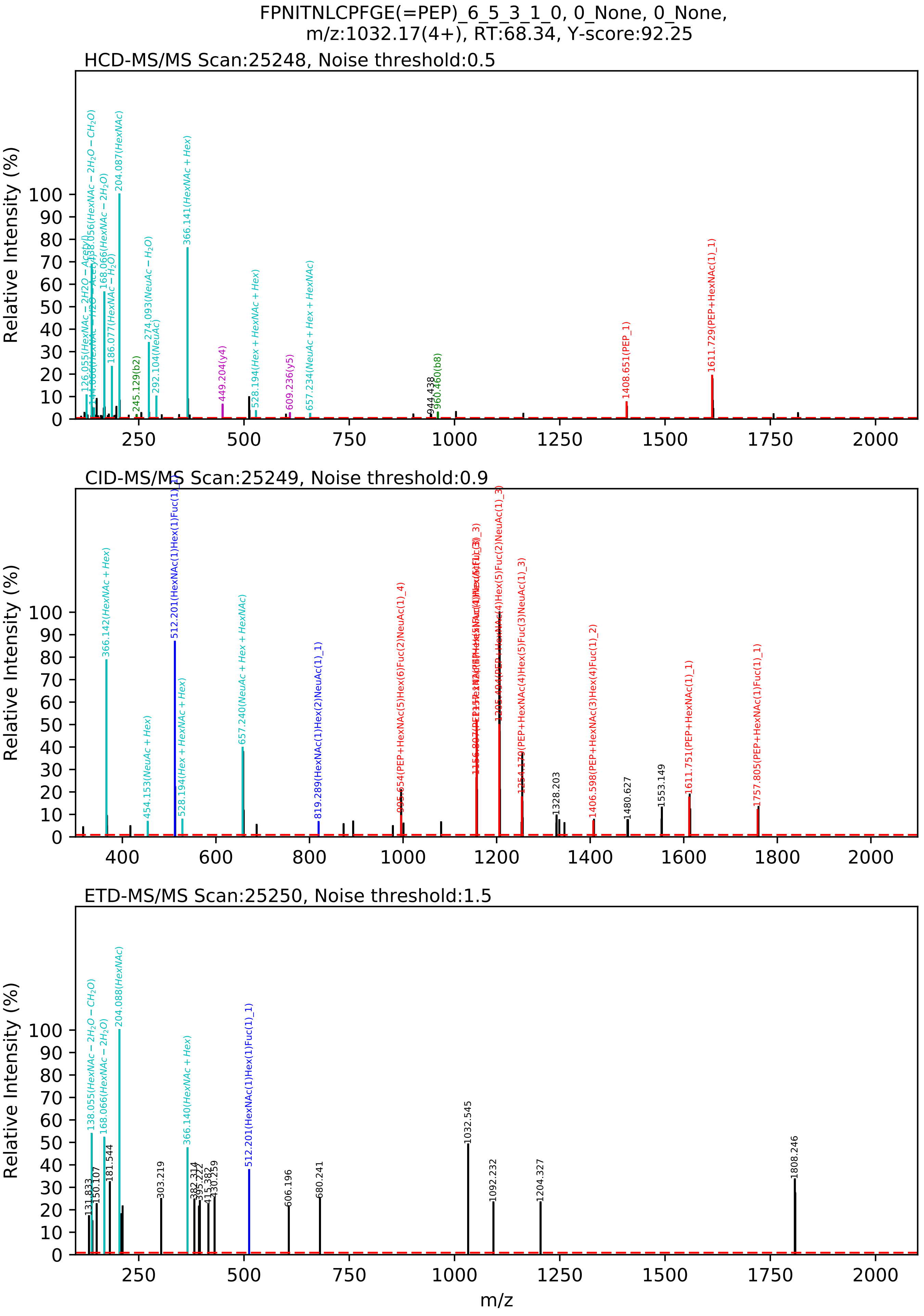
FPNITNLCPFGE(=PEP)_6_5_3_1_0, 0_None, 0_None,
m/z:1032.17(4+), RT:68.34, Y-score:92.25
HCD-MS/MS Scan:25248, Noise threshold:0.5
Relative Intensity (%)
126.055(HexNAc − 2H2O − Acetyl)
144.066(HexNAc − H2O − Acetyl)
138.056(HexNAc − 2H2O − CH2O)
168.066(HexNAc − 2H2O)
186.077(HexNAc − H2O)
204.087(HexNAc)
245.129(b2)
274.093(NeuAc − H2O)
292.104(NeuAc)
366.141(HexNAc + Hex)
449.204(y4)
528.194(Hex + HexNAc + Hex)
609.236(y5)
657.234(NeuAc + Hex + HexNAc)
944.438
960.460(b8)
1408.651(PEP_1)
1611.729(PEP+HexNAc(1)_1)
CID-MS/MS Scan:25249, Noise threshold:0.9
Relative Intensity (%)
366.142(HexNAc + Hex)
454.153(NeuAc + Hex)
512.201(HexNAc(1)Hex(1)Fuc(1)_1)
528.194(Hex + HexNAc + Hex)
657.240(NeuAc + Hex + HexNAc)
819.289(HexNAc(1)Hex(2)NeuAc(1)_1)
995.654(PEP+HexNAc(5)Hex(6)Fuc(2)NeuAc(1)_4)
1205.494(PEP+HexNAc(4)Hex(5)Fuc(2)NeuAc(1)_3)
1254.170(PEP+HexNAc(4)Hex(5)Fuc(3)NeuAc(1)_3)
1328.203
1406.598(PEP+HexNAc(3)Hex(4)Fuc(1)_2)
1480.627
1553.149
1611.751(PEP+HexNAc(1)_1)
1757.805(PEP+HexNAc(1)Fuc(1)_1)
ETD-MS/MS Scan:25250, Noise threshold:1.5
Relative Intensity (%)
131.833
138.055(HexNAc − 2H2O − CH2O)
150.107
168.066(HexNAc − 2H2O)
181.544
204.088(HexNAc)
303.219
366.140(HexNAc + Hex)
382.314
395.222
415.382
430.259
512.201(HexNAc(1)Hex(1)Fuc(1)_1)
606.196
680.241
1032.545
1092.232
1204.327
1808.246
m/z

FPNITNLCPFGE(=PEP)_6_5_3_1_0, 0_None, 0_None, m/z:1375.89(3+), RT:68.19, Y-score:88.23

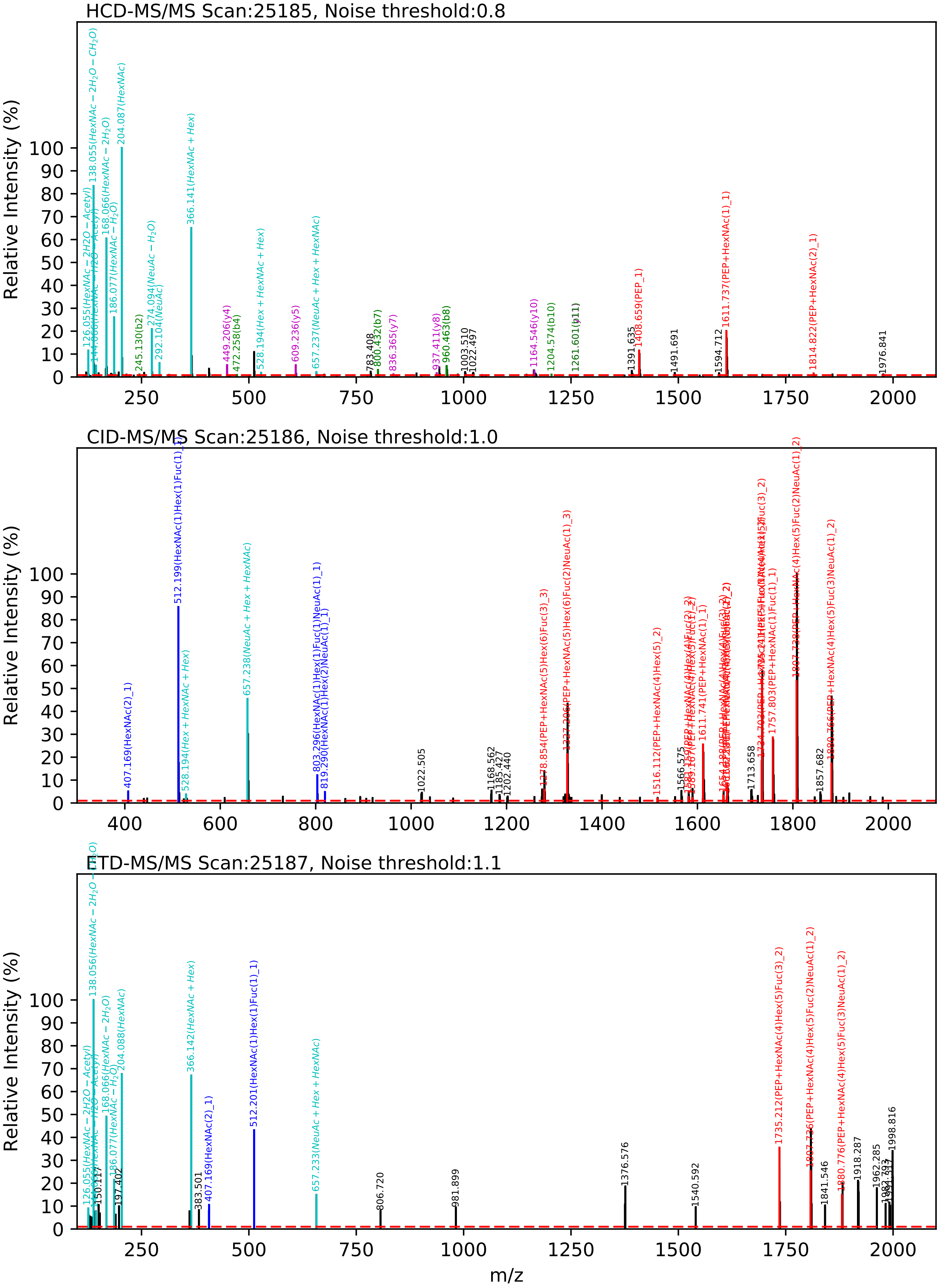

FPNITNLCPFGE(=PEP)_6_6_1_1_0, 0_None, 0_None,
m/z:1009.91(4+), RT:68.62, Y-score:82.48

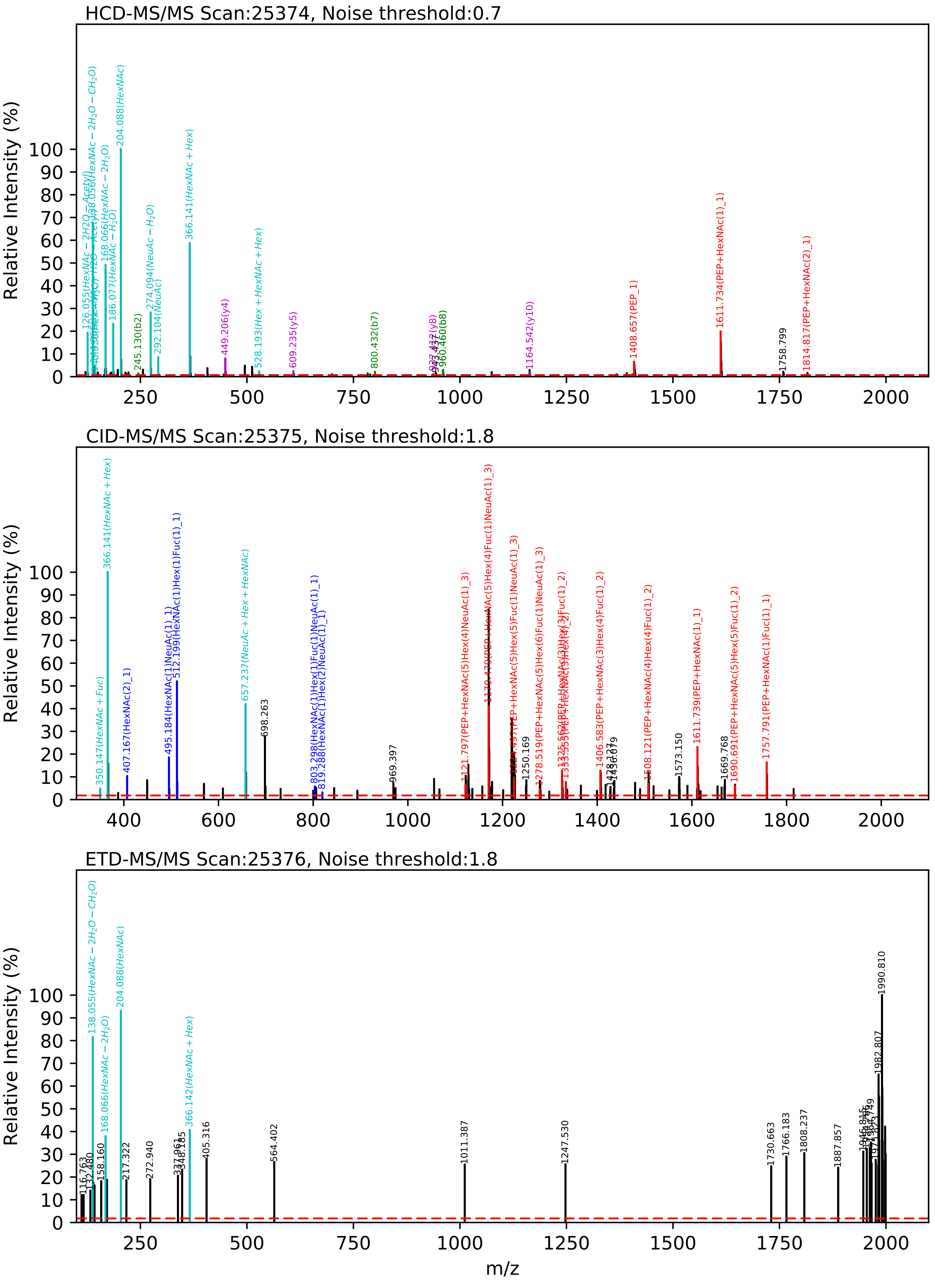

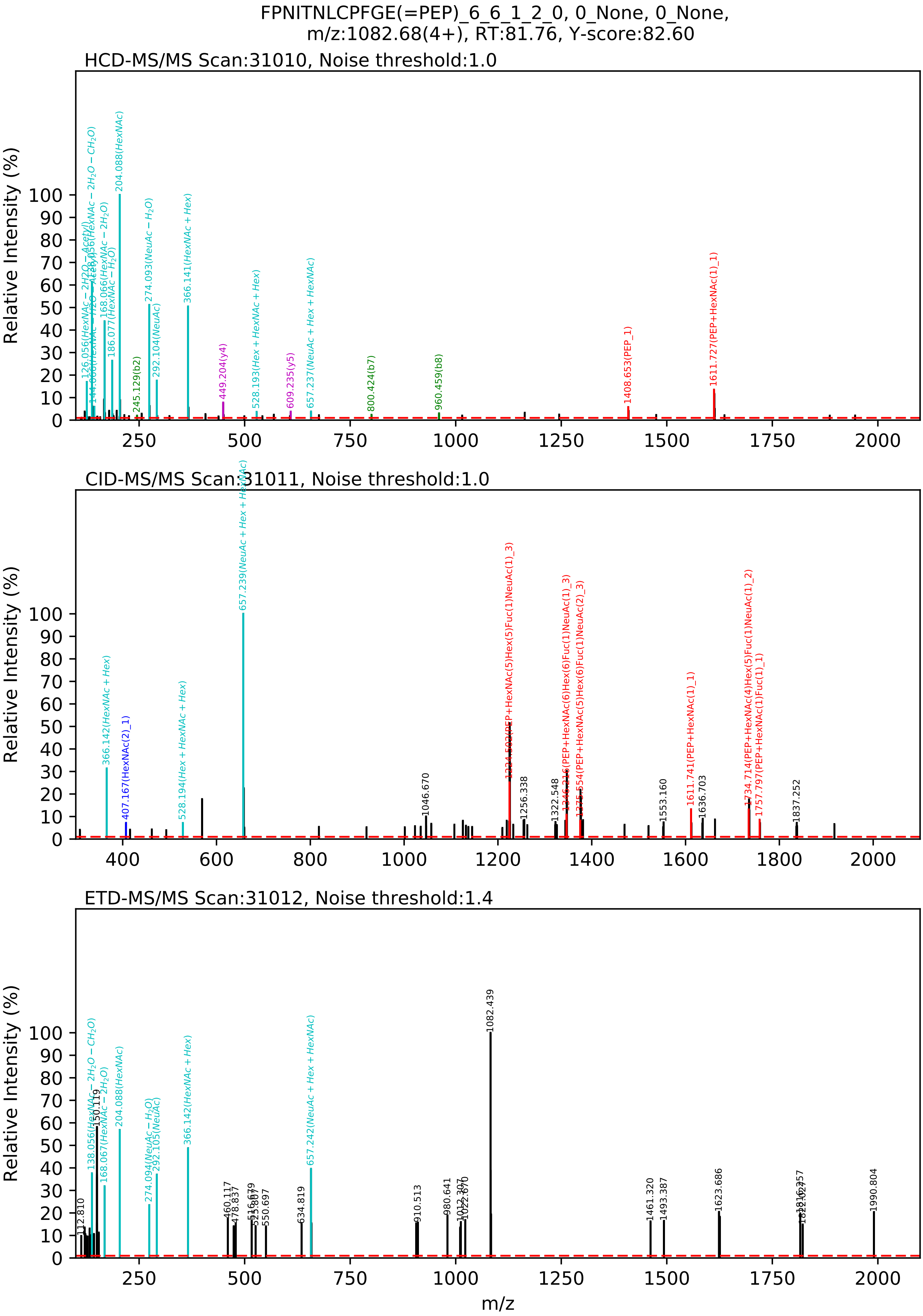

FPNITNLCPFGE(=PEP)_6_6_1_2_0, 0_None, 0_None,
m/z:1082.68(4+), RT:81.76, Y-score:82.60
HCD-MS/MS Scan:31010, Noise threshold:1.0
Relative Intensity (%)
204.088(HexNAc)
366.141(HexNAc + Hex)
274.093(NeuAc − H2O)
292.104(NeuAc)
168.066(HexNAc − 2H2O)
186.077(HexNAc − H2O)
245.129(b2)
449.204(y4)
528.193(Hex + HexNAc + Hex)
609.235(y5)
657.237(NeuAc + Hex + HexNAc)
800.424(b7)
960.459(b8)
1408.653(PEP_1)
1611.727(PEP+HexNAc(1)_1)
CID-MS/MS Scan:31011, Noise threshold:1.0
366.142(HexNAc + Hex)
407.167(HexNAc(2)_1)
528.194(Hex + HexNAc + Hex)
657.239(NeuAc + Hex + HexNAc)
1046.670
1224.502(PEP+HexNAc(5)Hex(5)Fuc(1)NeuAc(1)_3)
1256.338
1322.548
1346.216(PEP+HexNAc(6)Hex(6)Fuc(1)NeuAc(1)_3)
1375.554(PEP+HexNAc(5)Hex(6)Fuc(1)NeuAc(2)_3)
1553.160
1611.741(PEP+HexNAc(1)_1)
1636.703
1734.714(PEP+HexNAc(4)Hex(5)Fuc(1)NeuAc(1)_2)
1757.797(PEP+HexNAc(1)Fuc(1)_1)
1837.252
ETD-MS/MS Scan:31012, Noise threshold:1.4
112.810
138.056(HexNAc − 2H2O − CH2O)
150.119
168.067(HexNAc − 2H2O)
204.088(HexNAc)
274.094(NeuAc − H2O)
292.105(NeuAc)
366.142(HexNAc + Hex)
460.117
478.837
516.679
550.697
634.819
657.242(NeuAc + Hex + HexNAc)
910.513
980.641
1022.670
1082.439
1461.320
1493.387
1623.686
1990.804
m/z

FPNITNLCPFGE(=PEP)_6_6_1_2_0, 0_None, 0_None,
m/z:1082.68(4+), RT:82.35, Y-score:86.08

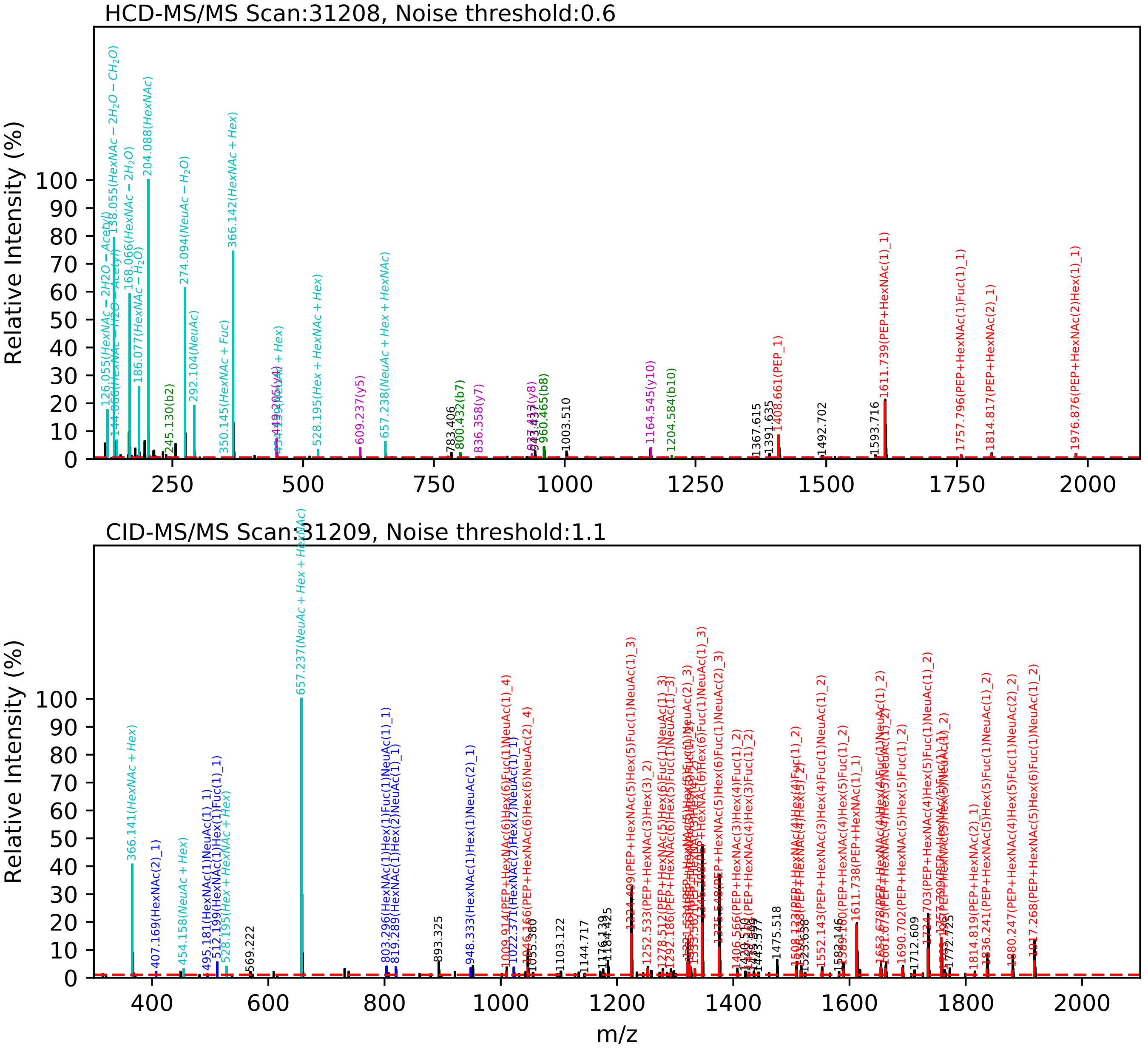

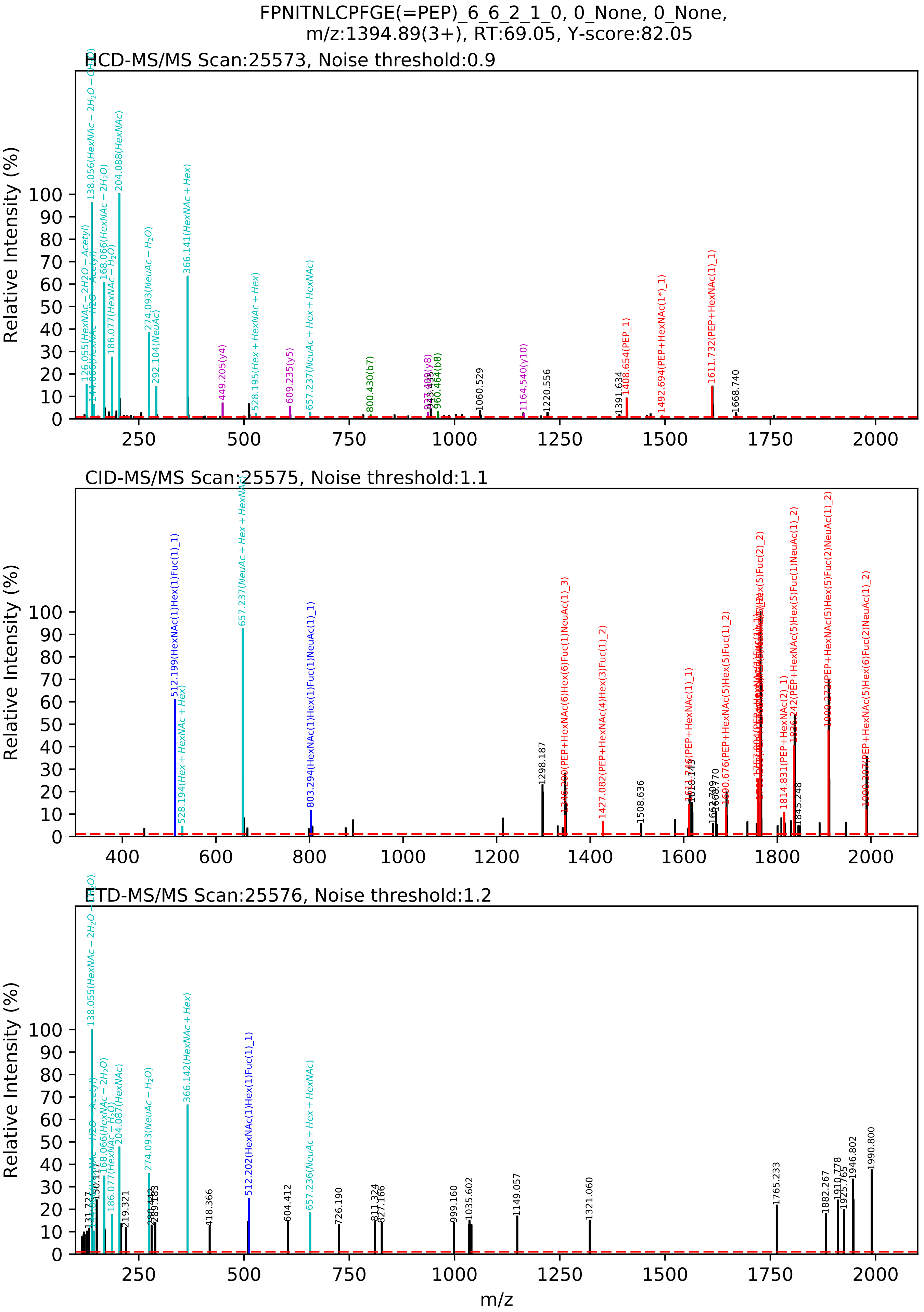
FPNITNLCPFGE(=PEP)_6_6_2_1_0, 0_None, 0_None,
m/z:1394.89(3+), RT:69.05, Y-score:82.05
HCD-MS/MS Scan:25573, Noise threshold:0.9
Relative Intensity (%)
204.088(HexNAc)
138.056(HexNAc − 2H2O − CH4O)
168.066(HexNAc − 2H2O)
186.077(HexNAc − H2O)
366.141(HexNAc + Hex)
274.093(NeuAc − H2O)
292.104(NeuAc)
126.055(HexNAc − 2H2O − Acetyl)
449.205(y4)
528.195(Hex + HexNAc + Hex)
609.235(y5)
657.237(NeuAc + Hex + HexNAc)
800.430(b7)
960.464(b8)
1060.529
1164.540(y10)
1220.556
1391.634
1408.654(PEP_1)
1492.694(PEP+HexNAc(1*)_1)
1611.732(PEP+HexNAc(1)_1)
1668.740
CID-MS/MS Scan:25575, Noise threshold:1.1
512.199(HexNAc(1)Hex(1)Fuc(1)_1)
528.194(Hex + HexNAc + Hex)
657.237(NeuAc + Hex + HexNAc)
803.294(HexNAc(1)Hex(1)Fuc(1)NeuAc(1)_1)
1298.187
1346.200(PEP+HexNAc(6)Hex(6)Fuc(1)NeuAc(1)_3)
1427.082(PEP+HexNAc(4)Hex(3)Fuc(1)_2)
1508.636
1611.746(PEP+HexNAc(1)_1)
1618.143
1690.676(PEP+HexNAc(5)Hex(5)Fuc(1)_2)
1814.831(PEP+HexNAc(2)_1)
1836.242(PEP+HexNAc(5)Hex(5)Fuc(1)NeuAc(1)_2)
1845.248
1909.272(PEP+HexNAc(5)Hex(5)Fuc(2)NeuAc(1)_2)
1990.297(PEP+HexNAc(5)Hex(6)Fuc(2)NeuAc(1)_2)
ETD-MS/MS Scan:25576, Noise threshold:1.2
138.055(HexNAc − 2H2O − CH4O)
168.066(HexNAc − 2H2O)
186.077(HexNAc − H2O)
204.087(HexNAc)
274.093(NeuAc − H2O)
366.142(HexNAc + Hex)
512.202(HexNAc(1)Hex(1)Fuc(1)_1)
657.236(NeuAc + Hex + HexNAc)
131.727
150.117
219.321
280.442
289.163
418.366
604.412
726.190
811.324
827.166
999.160
1035.602
1149.057
1321.060
1765.233
1882.267
1910.778
1925.765
1946.802
1990.800
m/z

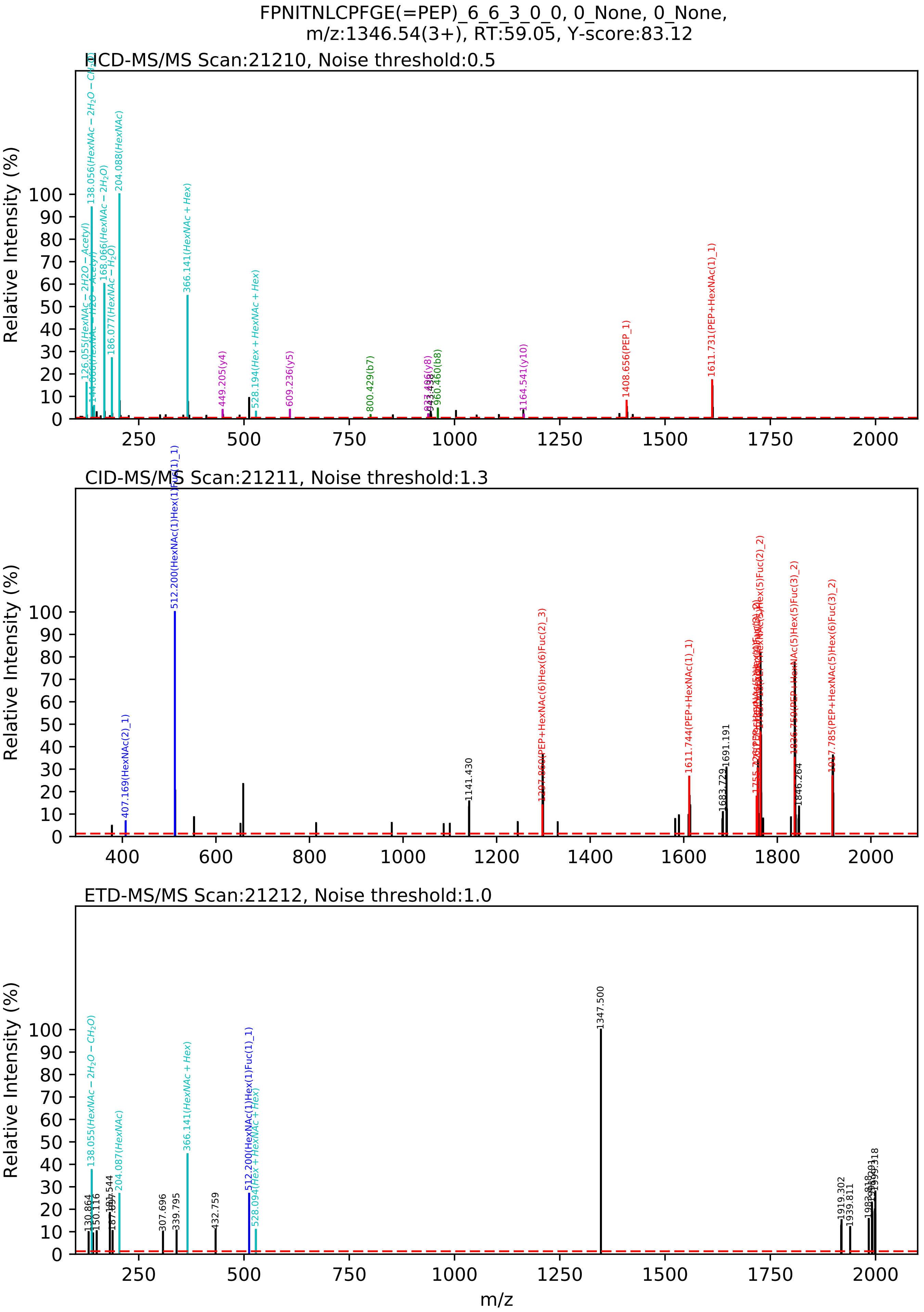

FPNITNLCPFGE(=PEP)_6_6_3_0_0, 0_None, 0_None,
m/z:1346.54(3+), RT:59.05, Y-score:83.12
HCD-MS/MS Scan:21210, Noise threshold:0.5
Relative Intensity (%)
126.055(HexNAc − 2H2O − Acetyl)
138.056(HexNAc − 2H2O − CH2O)
168.066(HexNAc − 2H2O)
186.077(HexNAc − H2O)
204.088(HexNAc)
366.141(HexNAc + Hex)
449.205(y4)
528.194(Hex + HexNAc + Hex)
609.236(y5)
800.429(b7)
960.460(b8)
1164.541(y10)
1408.656(PEP_1)
1611.731(PEP+HexNAc(1)_1)
CID-MS/MS Scan:21211, Noise threshold:1.3
407.169(HexNAc(2)_1)
512.200(HexNAc(1)Hex(1)Fuc(1)_1)
1141.430
1297.860(PEP+HexNAc(6)Hex(6)Fuc(2)_3)
1611.744(PEP+HexNAc(1)_1)
1683.729
1691.191
1836.750(PEP+HexNAc(5)Hex(5)Fuc(3)_2)
1846.264
1917.785(PEP+HexNAc(5)Hex(6)Fuc(3)_2)
ETD-MS/MS Scan:21212, Noise threshold:1.0
138.055(HexNAc − 2H2O − CH2O)
204.087(HexNAc)
130.864
150.116
181.544
187.097
307.696
339.795
366.141(HexNAc + Hex)
432.759
512.200(HexNAc(1)Hex(1)Fuc(1)_1)
528.094(Hex + HexNAc + Hex)
1347.500
1919.302
1939.811
1983.818
1999.318
m/z

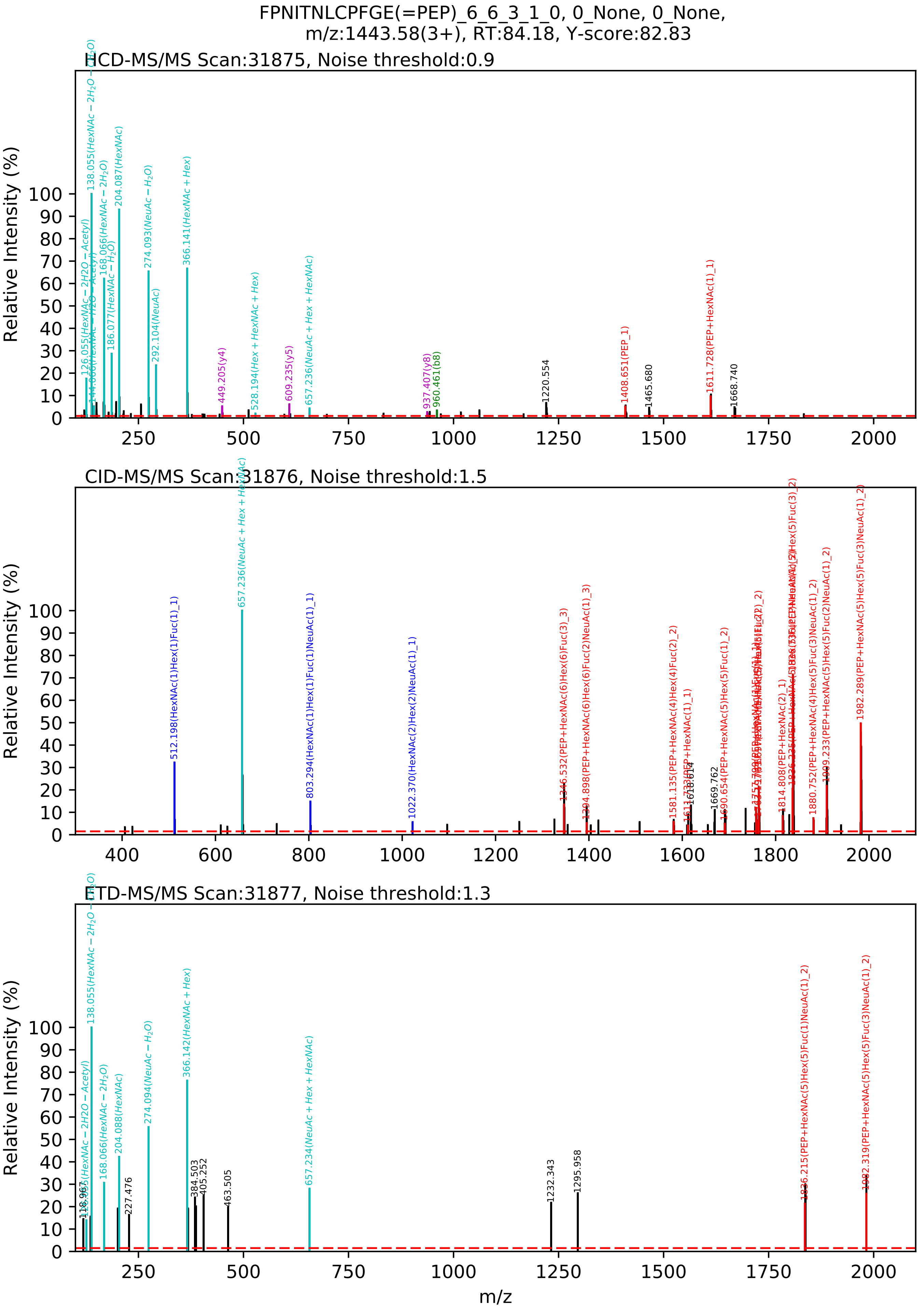

FPNITNLCPFGE(=PEP)_6_6_3_1_0, 0_None, 0_None,
m/z:1443.58(3+), RT:84.18, Y-score:82.83
HCD-MS/MS Scan:31875, Noise threshold:0.9
Relative Intensity (%)
138.055(HexNAc − 2H₂O − H₂O)
204.087(HexNAc)
168.066(HexNAc − 2H₂O)
186.077(HexNAc − H₂O)
274.093(NeuAc − H₂O)
292.104(NeuAc)
366.141(HexNAc + Hex)
449.205(y4)
528.194(Hex + HexNAc + Hex)
609.235(y5)
657.236(NeuAc + Hex + HexNAc)
937.407(y8)
960.461(b8)
1220.554
1408.651(PEP_1)
1465.680
1611.728(PEP+HexNAc(1)_1)
1668.740
CID-MS/MS Scan:31876, Noise threshold:1.5
512.198(HexNAc(1)Hex(1)Fuc(1)_1)
657.236(NeuAc + Hex + HexNAc)
803.294(HexNAc(1)Hex(1)Fuc(1)NeuAc(1)_1)
1022.370(HexNAc(2)Hex(2)NeuAc(1)_1)
1346.532(PEP+HexNAc(6)Hex(6)Fuc(3)_3)
1394.898(PEP+HexNAc(6)Hex(6)Fuc(2)NeuAc(1)_3)
1581.135(PEP+HexNAc(4)Hex(4)Fuc(2)_2)
1611.733(PEP+HexNAc(1)_1)
1618.614
1669.762
1690.654(PEP+HexNAc(5)Hex(5)Fuc(1)_2)
1814.808(PEP+HexNAc(2)_1)
1880.752(PEP+HexNAc(4)Hex(5)Fuc(3)NeuAc(1)_2)
1909.233(PEP+HexNAc(5)Hex(5)Fuc(2)NeuAc(1)_2)
1982.289(PEP+HexNAc(5)Hex(5)Fuc(3)NeuAc(1)_2)
ETD-MS/MS Scan:31877, Noise threshold:1.3
118.967
138.055(HexNAc − 2H₂O − H₂O)
168.066(HexNAc − 2H₂O)
204.088(HexNAc)
227.476
274.094(NeuAc − H₂O)
366.142(HexNAc + Hex)
384.503
405.252
463.505
657.234(NeuAc + Hex + HexNAc)
1232.343
1295.958
1836.215(PEP+HexNAc(5)Hex(5)Fuc(1)NeuAc(1)_2)
1982.319(PEP+HexNAc(5)Hex(5)Fuc(3)NeuAc(1)_2)
m/z

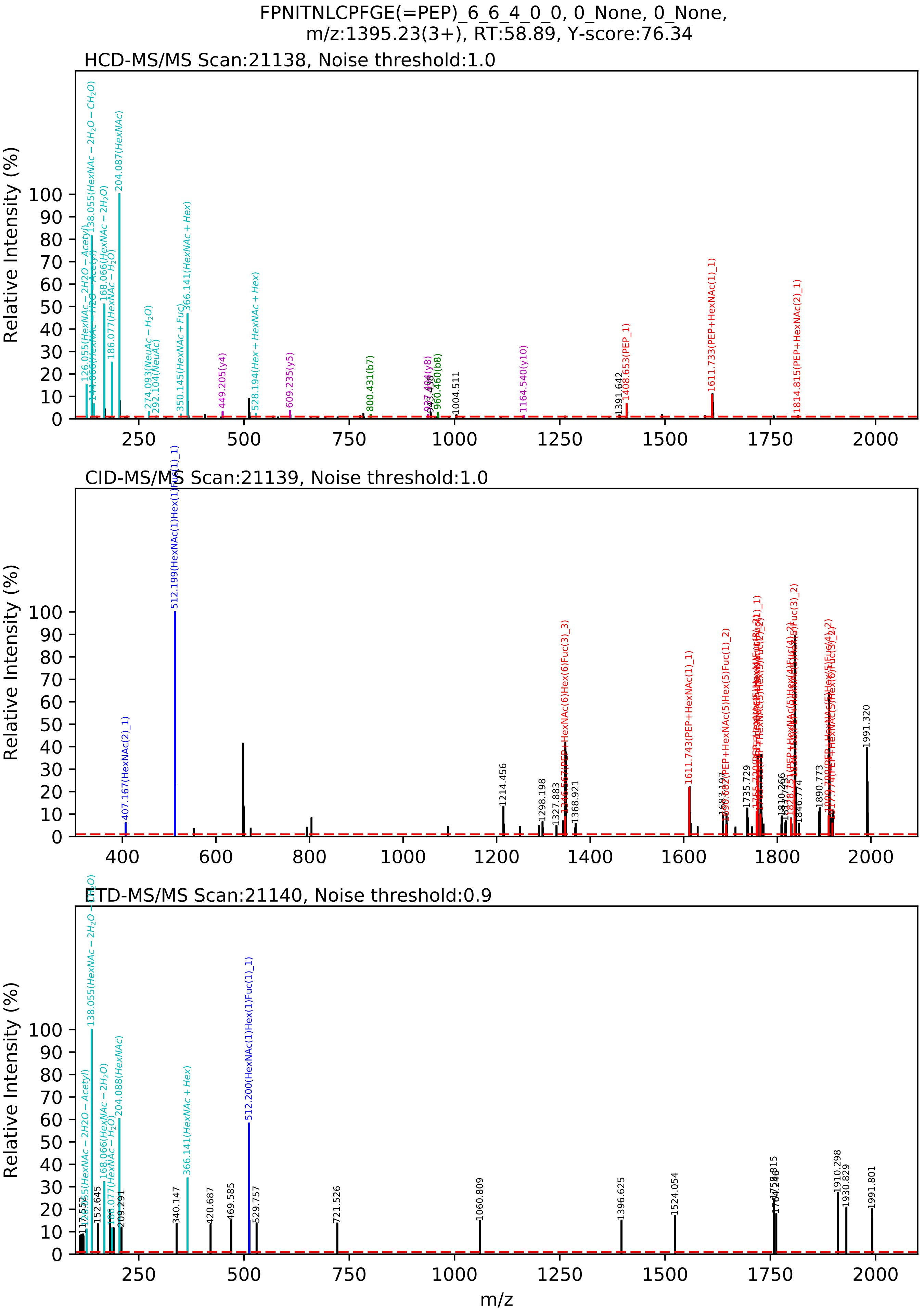
FPNITNLCPFGE(=PEP)_6_6_4_0_0, 0_None, 0_None,
m/z:1395.23(3+), RT:58.89, Y-score:76.34
HCD-MS/MS Scan:21138, Noise threshold:1.0
Relative Intensity (%)
204.087(HexNAc)
366.141(HexNAc + Hex)
528.194(Hex + HexNAc + Hex)
449.205(y4)
609.235(y5)
800.431(b7)
960.460(b8)
1004.511
1164.540(y10)
1391.642
1408.653(PEP_1)
1611.733(PEP+HexNAc(1)_1)
1814.815(PEP+HexNAc(2)_1)
CID-MS/MS Scan:21139, Noise threshold:1.0
407.167(HexNAc(2)_1)
512.199(HexNAc(1)Hex(1)Fuc(1)_1)
1214.456
1298.198
1327.883
1368.921
1611.743(PEP+HexNAc(1)_1)
1683.197
1735.729
1890.773
1991.320
ETD-MS/MS Scan:21140, Noise threshold:0.9
138.055(HexNAc − 2H2O − CH2O)
204.088(HexNAc)
366.141(HexNAc + Hex)
512.200(HexNAc(1)Hex(1)Fuc(1)_1)
152.645
209.291
340.147
420.687
469.585
529.757
721.526
1060.809
1396.625
1524.054
1910.298
1930.829
1991.801
m/z

FPNITNLCPFGE(=PEP)_6_7_1_0_0, 0_None, 0_None,
m/z:1316.87(3+), RT:59.28, Y-score:70.87

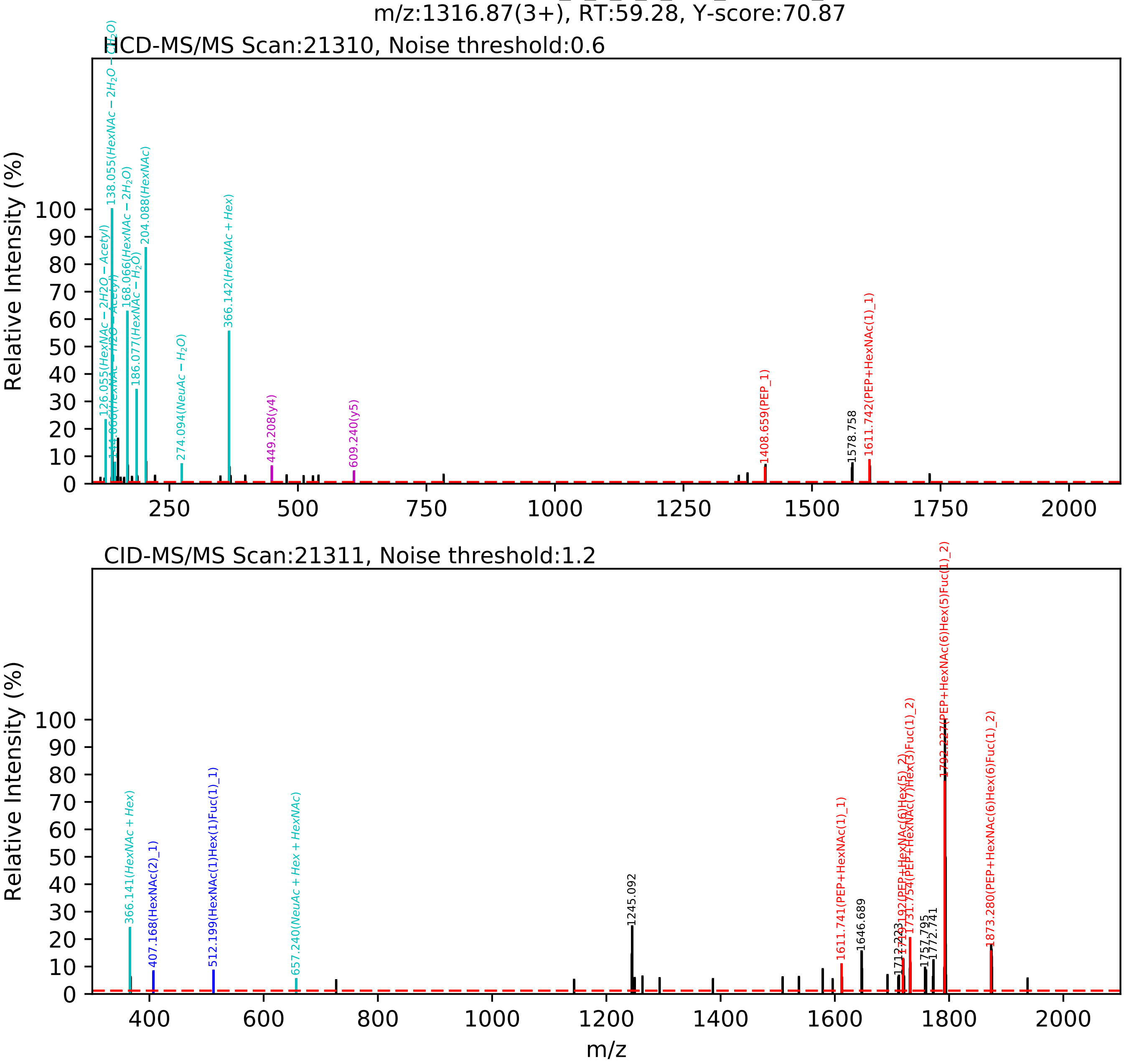

FPNITNLCPFGE(=PEP)_6_7_1_1_0, 0_None, 0_None,
m/z:1413.90(3+), RT:67.27, Y-score:86.08

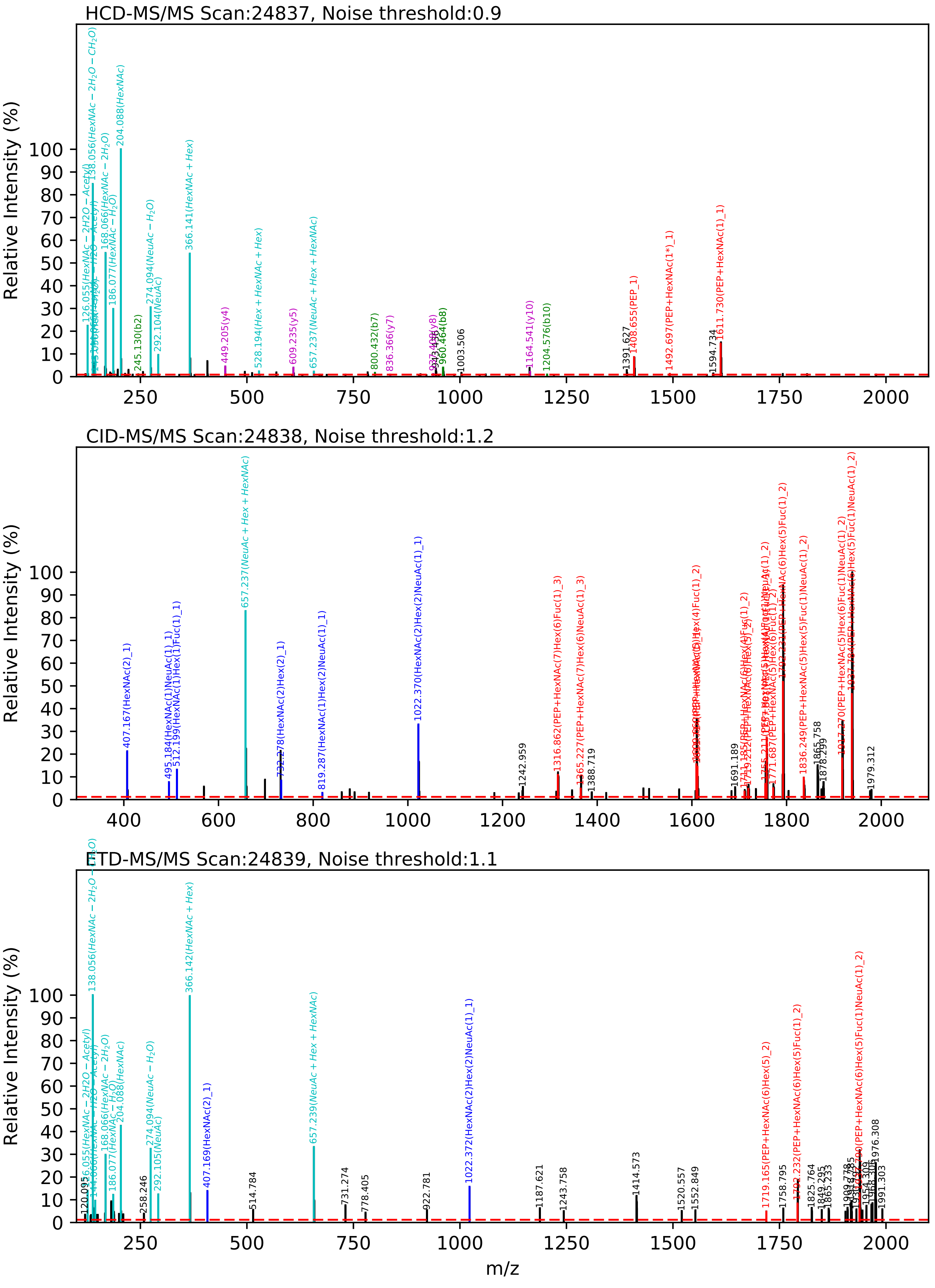

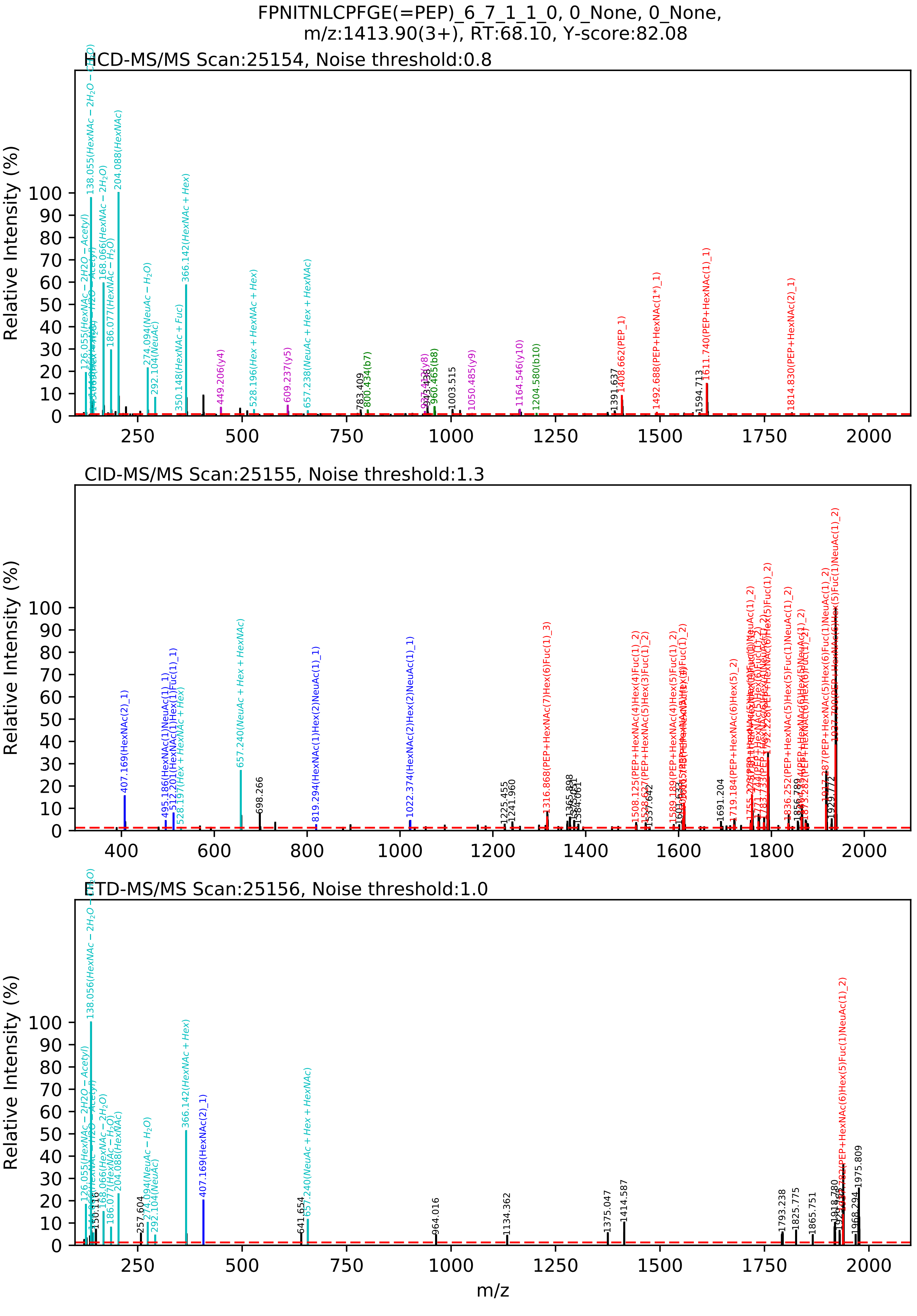

FPNITNLCPFGE(=PEP)_6_7_1_1_0, 0_None, 0_None,
m/z:1413.90(3+), RT:68.10, Y-score:82.08
HCD-MS/MS Scan:25154, Noise threshold:0.8
CID-MS/MS Scan:25155, Noise threshold:1.3
ETD-MS/MS Scan:25156, Noise threshold:1.0
Relative Intensity (%)
m/z

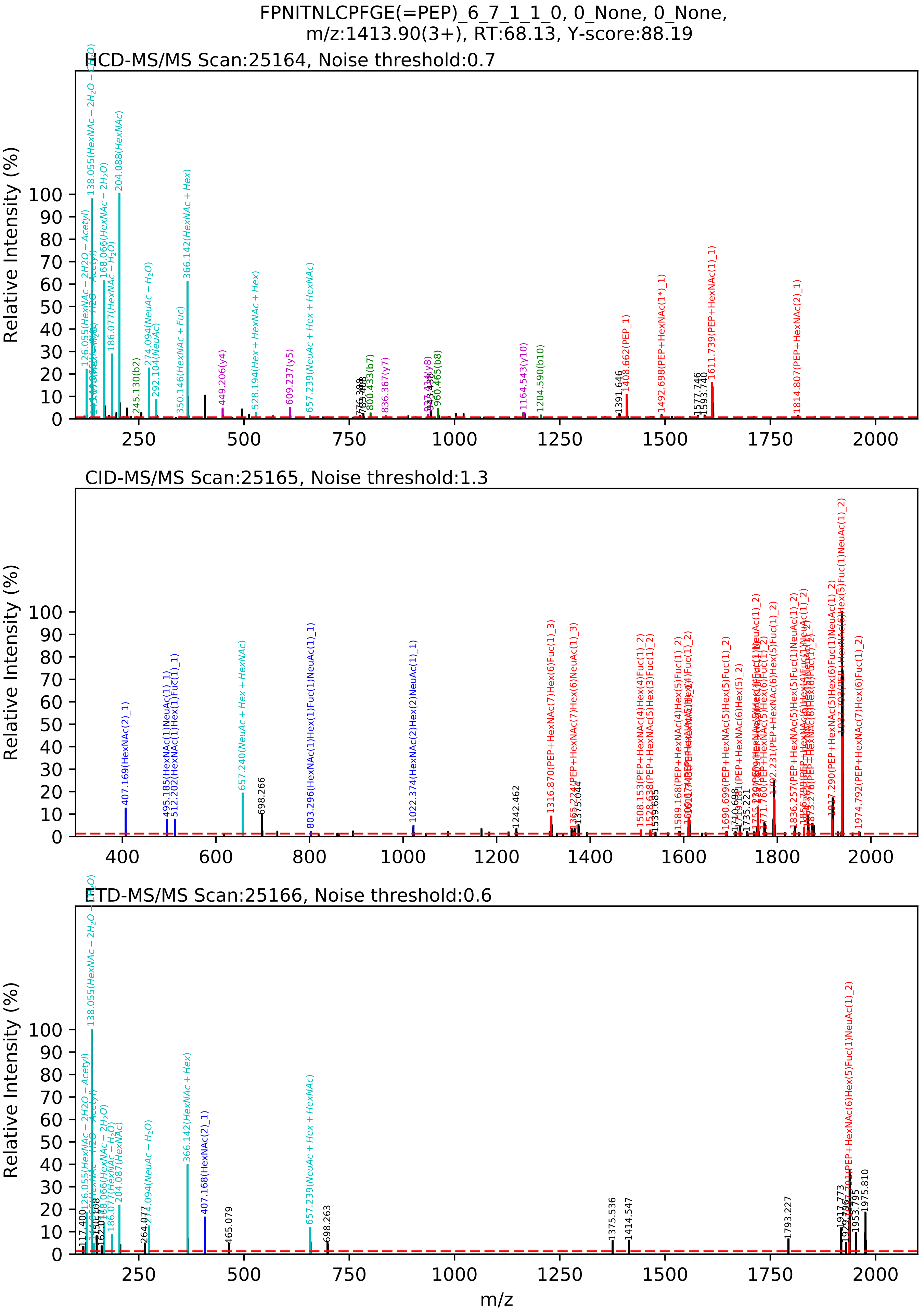

FPNITNLCPFGE(=PEP)_6_7_1_1_0, 0_None, 0_None,
m/z:1413.90(3+), RT:68.13, Y-score:88.19
HCD-MS/MS Scan:25164, Noise threshold:0.7
CID-MS/MS Scan:25165, Noise threshold:1.3
ETD-MS/MS Scan:25166, Noise threshold:0.6
Relative Intensity (%)
m/z

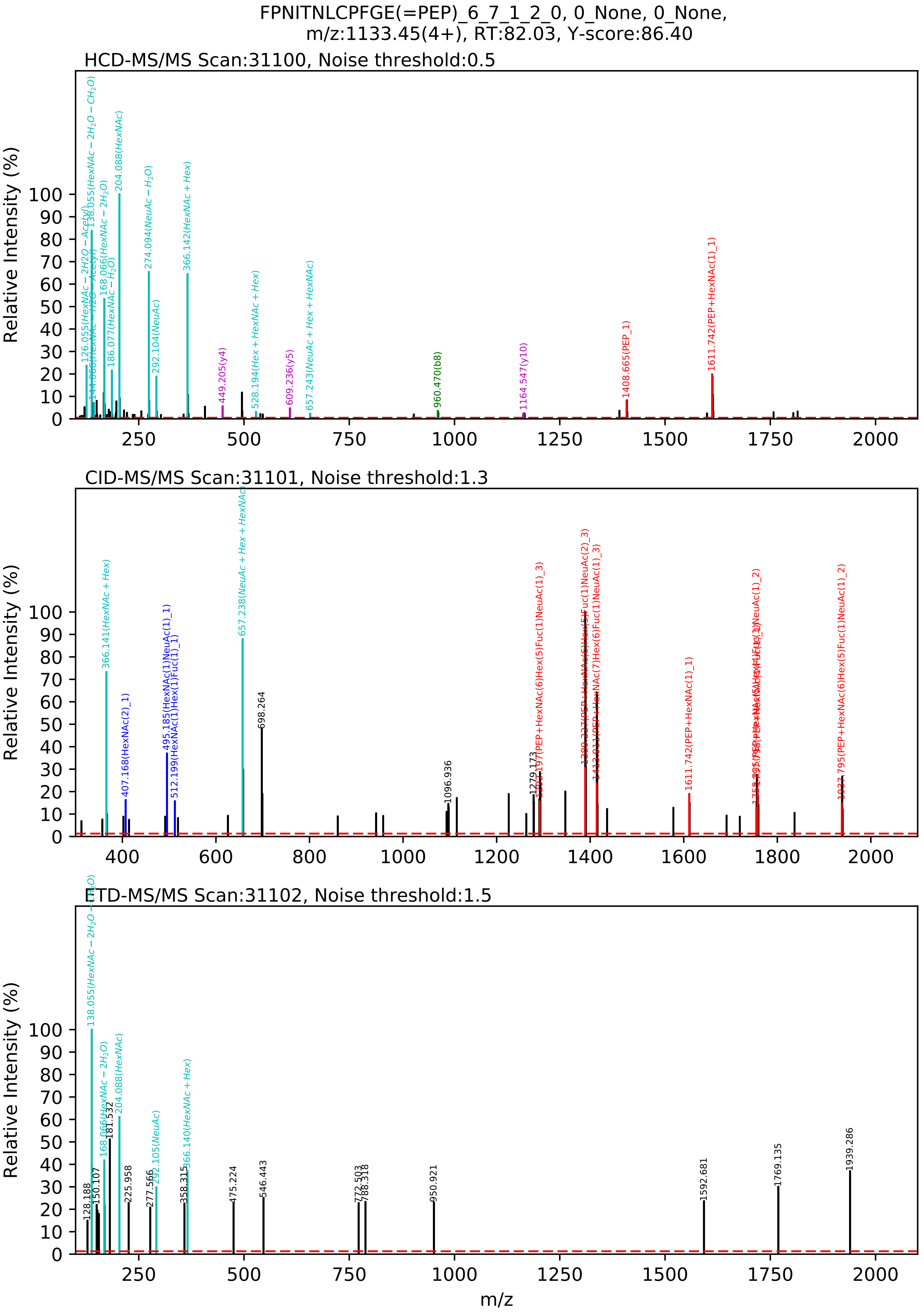

FPNITNLCPFGE(=PEP)_6_7_1_2_0, 0_None, 0_None,
m/z:1133.45(4+), RT:82.03, Y-score:86.40
HCD-MS/MS Scan:31100, Noise threshold:0.5
Relative Intensity (%)
126.055(HexNAc − 2H2O − Acetyl)
138.055(HexNAc − 2H2O − CH2O)
168.066(HexNAc − 2H2O)
186.077(HexNAc − H2O)
204.088(HexNAc)
274.094(NeuAc − H2O)
292.104(NeuAc)
366.142(HexNAc + Hex)
449.205(y4)
528.194(Hex + HexNAc + Hex)
609.236(y5)
657.243(NeuAc + Hex + HexNAc)
960.470(b8)
1164.547(y10)
1408.665(PEP_1)
1611.742(PEP+HexNAc(1)_1)
CID-MS/MS Scan:31101, Noise threshold:1.3
366.141(HexNAc + Hex)
407.168(HexNAc(2)_1)
495.185(HexNAc(1)NeuAc(1)_1)
512.199(HexNAc(1)Hex(1)Fuc(1)_1)
657.238(NeuAc + Hex + HexNAc)
698.264
1096.936
1279.173
1611.742(PEP+HexNAc(1)_1)
1937.795(PEP+HexNAc(6)Hex(5)Fuc(1)NeuAc(1)_2)
ETD-MS/MS Scan:31102, Noise threshold:1.5
128.188
138.055(HexNAc − 2H2O − CH2O)
150.107
168.066(HexNAc − 2H2O)
181.532
204.088(HexNAc)
225.958
277.566
292.105(NeuAc)
358.315
366.140(HexNAc + Hex)
475.224
546.443
772.503
788.318
950.921
1592.681
1769.135
1939.286
m/z

FPNITNLCPFGE(=PEP)_6_7_1_2_0, 0_None, 0_None,
m/z:1510.93(3+), RT:83.02, Y-score:87.06

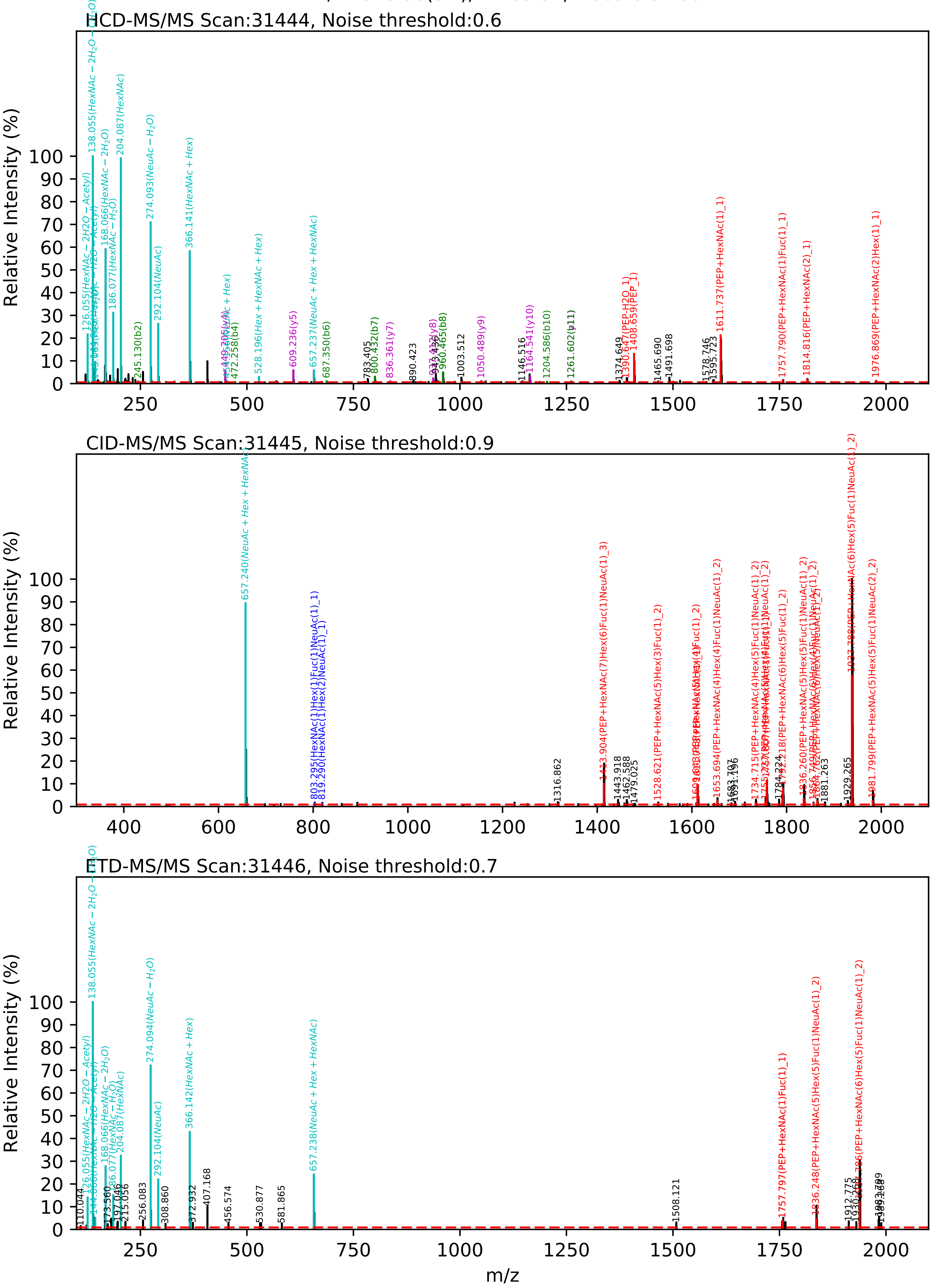

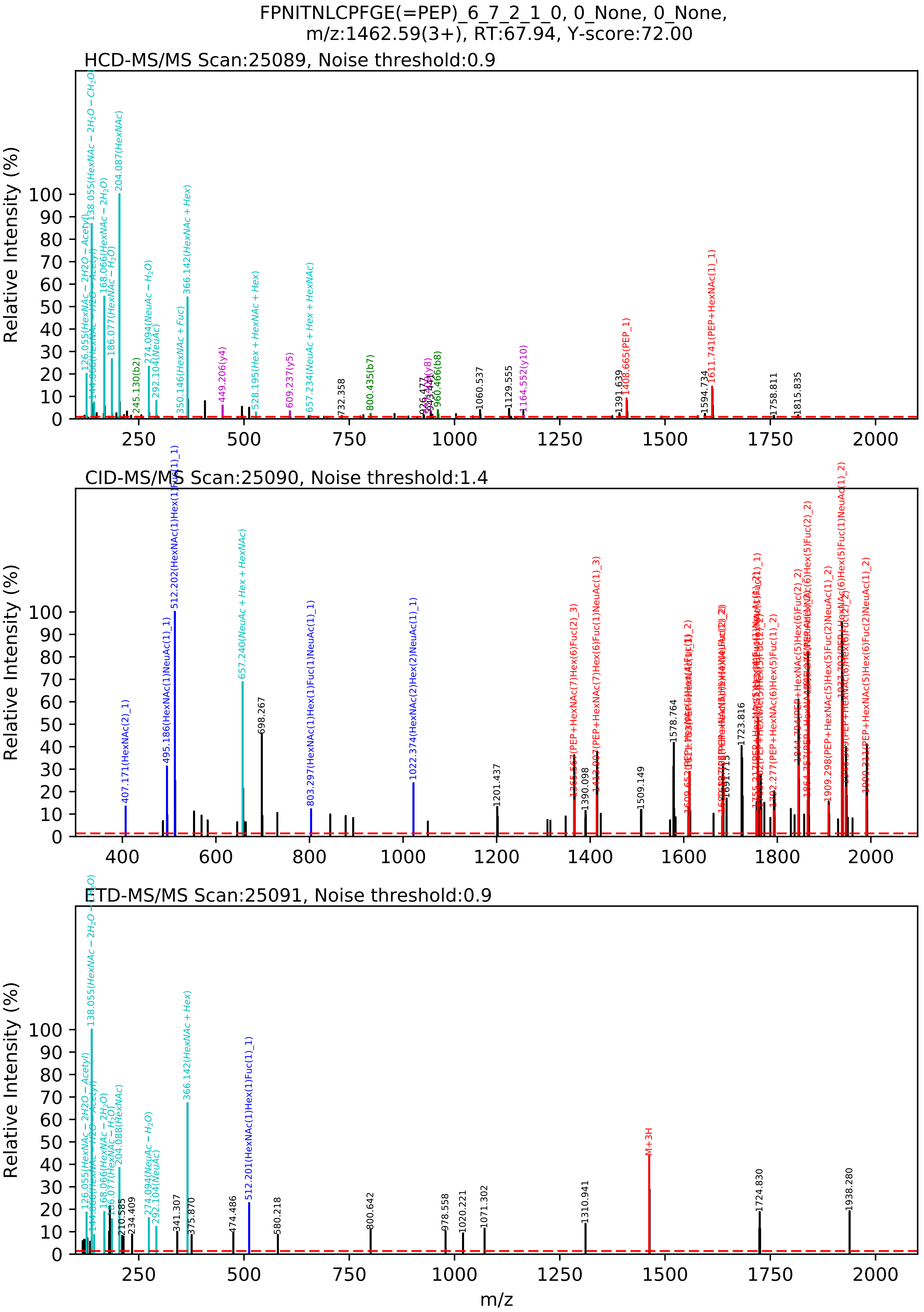

FPNITNLCPFGE(=PEP)_6_7_2_1_0, 0_None, 0_None,
m/z:1462.59(3+), RT:67.94, Y-score:72.00
HCD-MS/MS Scan:25089, Noise threshold:0.9
CID-MS/MS Scan:25090, Noise threshold:1.4
ETD-MS/MS Scan:25091, Noise threshold:0.9
Relative Intensity (%)
m/z
204.087(HexNAc)
366.142(HexNAc + Hex)
274.094(NeuAc − H2O)
292.104(NeuAc)
245.130(b2)
449.206(y4)
528.195(Hex + HexNAc + Hex)
609.237(y5)
657.234(NeuAc + Hex + HexNAc)
732.358
800.435(b7)
960.466(b8)
1060.537
1129.555
1164.552(y10)
1391.639
1408.665(PEP_1)
1594.734
1611.741(PEP+HexNAc(1)_1)
1758.811
1815.835
407.171(HexNAc(2)_1)
495.186(HexNAc(1)NeuAc(1)_1)
657.240(NeuAc + Hex + HexNAc)
698.267
803.297(HexNAc(1)Hex(1)Fuc(1)NeuAc(1)_1)
1022.374(HexNAc(2)Hex(2)NeuAc(1)_1)
1201.437
1390.098
1509.149
1578.764
1691.715
1723.816
1792.277(PEP+HexNAc(6)Hex(5)Fuc(1)_2)
1909.298(PEP+HexNAc(5)Hex(5)Fuc(2)NeuAc(1)_2)
1990.211(PEP+HexNAc(5)Hex(6)Fuc(2)NeuAc(1)_2)
138.055(HexNAc − 2H2O − CH2O)
512.201(HexNAc(1)Hex(1)Fuc(1)_1)
M+3H
210.585
234.409
341.307
375.870
474.486
580.218
800.642
978.558
1020.221
1071.302
1310.941
1724.830
1938.280

FPNITNLCPFGE(=PEP)_6_7_2_1_0, 0_None, 0_None,
m/z:1462.59(3+), RT:67.28, Y-score:76.93

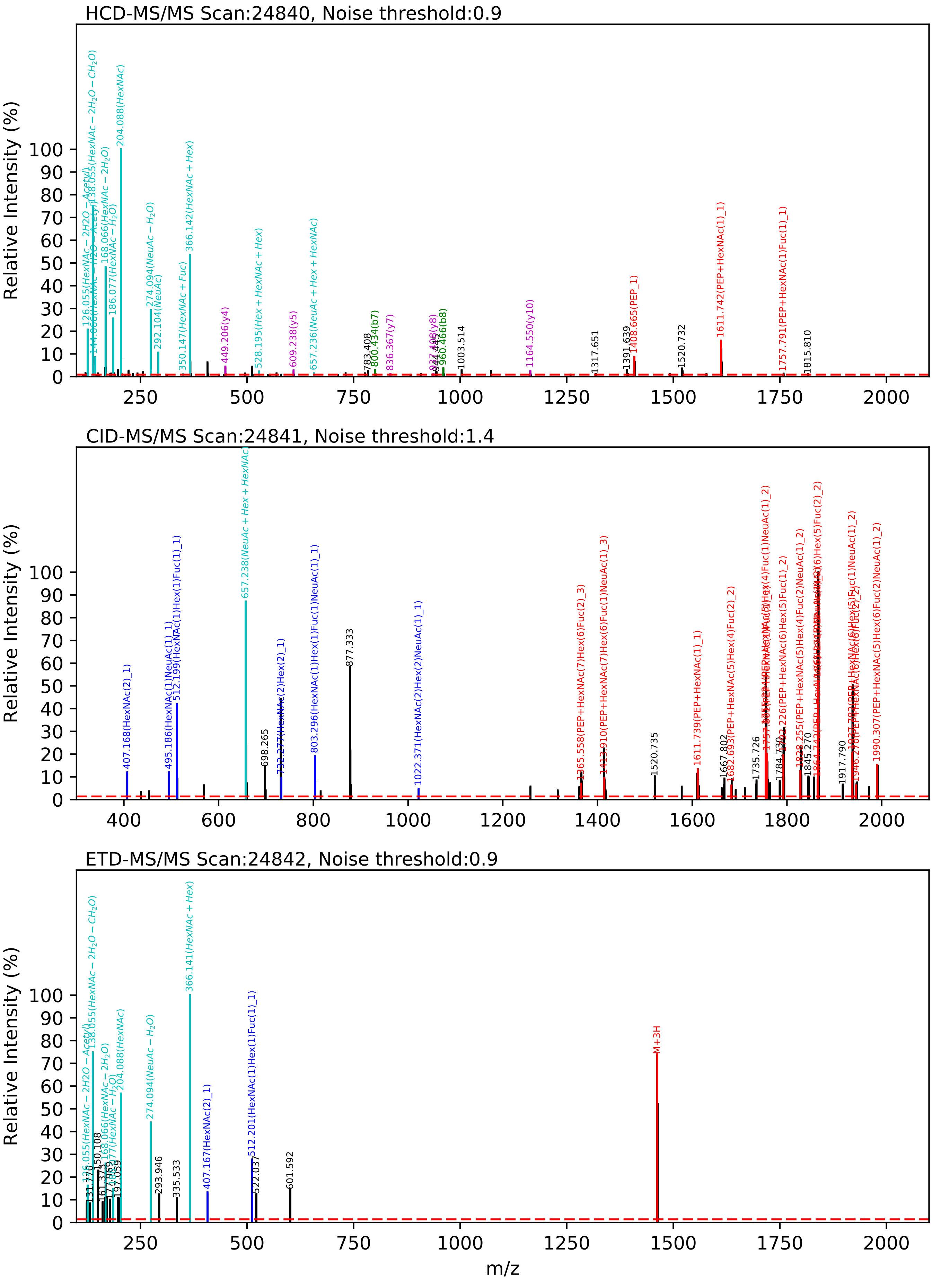

FPNITNLCPFGE(=PEP)_6_7_2_2_0, 0_None, 0_None, m/z:1559.62(3+), RT:82.65, Y-score:84.23

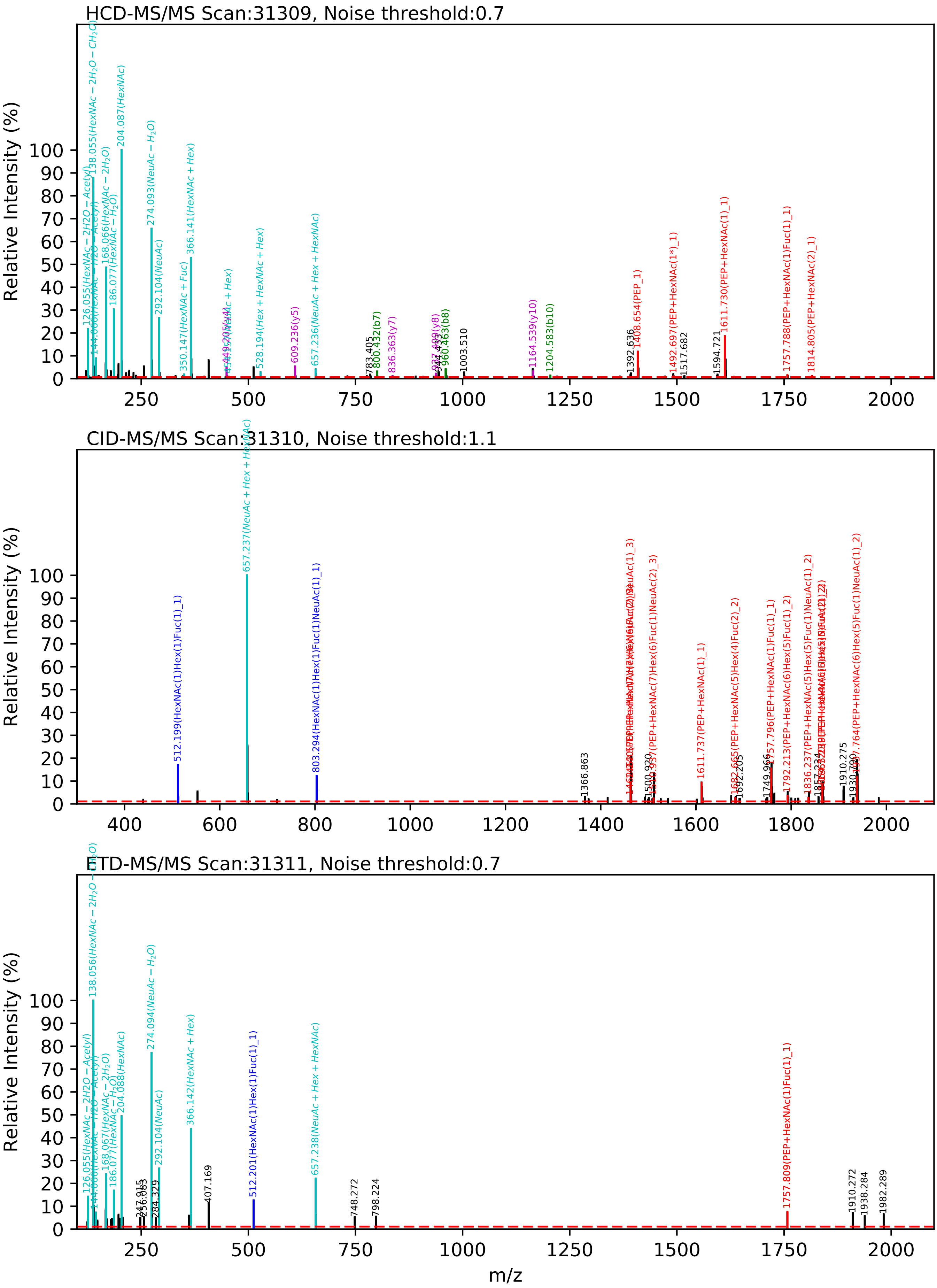

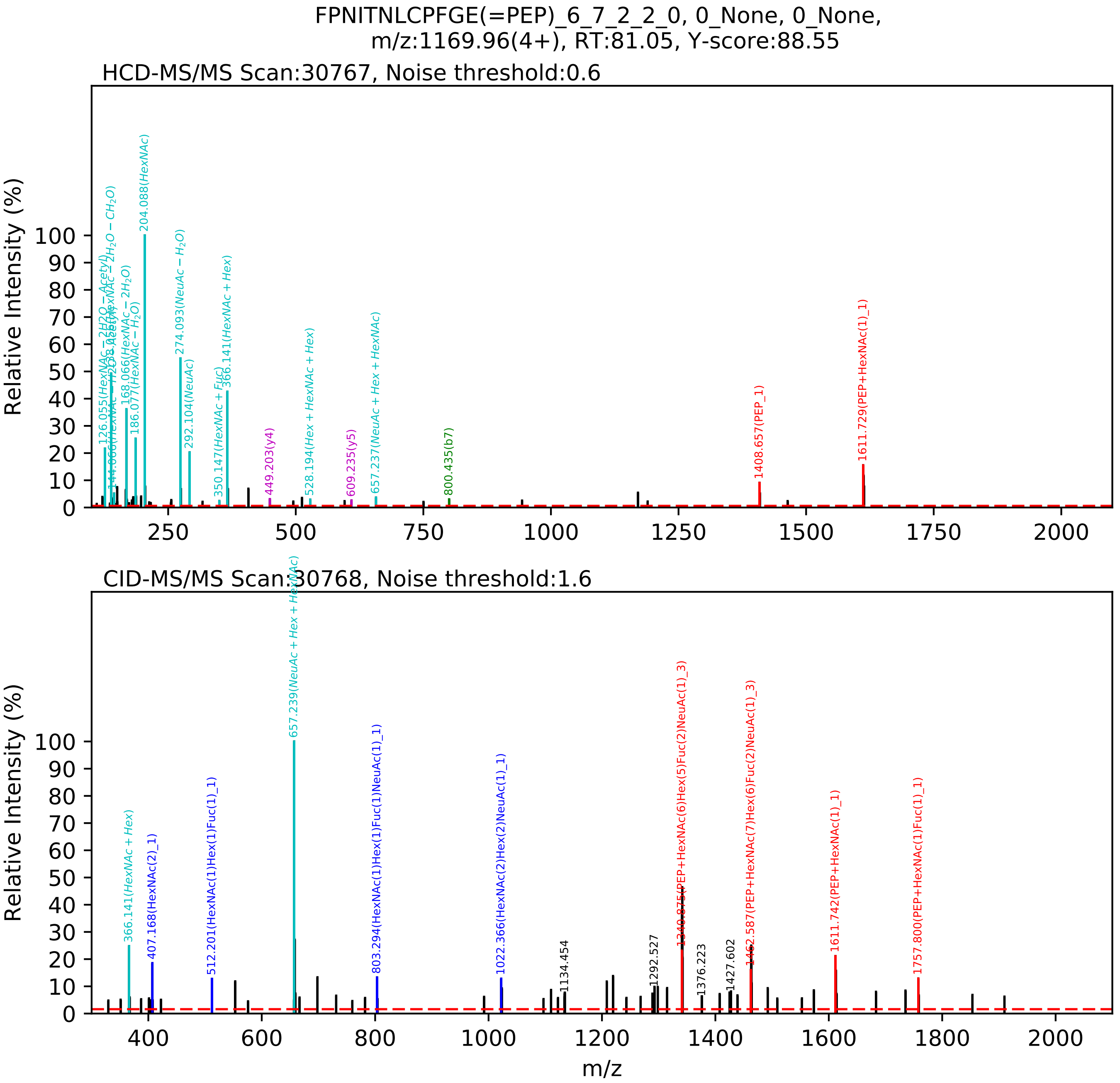
FPNITNLCPFGE(=PEP)_6_7_2_2_0, 0_None, 0_None,
m/z:1169.96(4+), RT:81.05, Y-score:88.55
HCD-MS/MS Scan:30767, Noise threshold:0.6
Relative Intensity (%)
204.088(HexNAc)
274.093(NeuAc − H2O)
292.104(NeuAc)
366.141(HexNAc + Hex)
350.147(HexNAc + Fuc)
449.203(y4)
528.194(Hex + HexNAc + Hex)
609.235(y5)
657.237(NeuAc + Hex + HexNAc)
800.435(b7)
1408.657(PEP_1)
1611.729(PEP+HexNAc(1)_1)
CID-MS/MS Scan:30768, Noise threshold:1.6
657.239(NeuAc + Hex + HexNAc)
366.141(HexNAc + Hex)
407.168(HexNAc(2)_1)
512.201(HexNAc(1)Hex(1)Fuc(1)_1)
803.294(HexNAc(1)Hex(1)Fuc(1)NeuAc(1)_1)
1022.366(HexNAc(2)Hex(2)NeuAc(1)_1)
1134.454
1292.527
1340.875(PEP+HexNAc(6)Hex(5)Fuc(2)NeuAc(1)_3)
1376.223
1427.602
1462.587(PEP+HexNAc(7)Hex(6)Fuc(2)NeuAc(1)_3)
1611.742(PEP+HexNAc(1)_1)
1757.800(PEP+HexNAc(1)Fuc(1)_1)
m/z

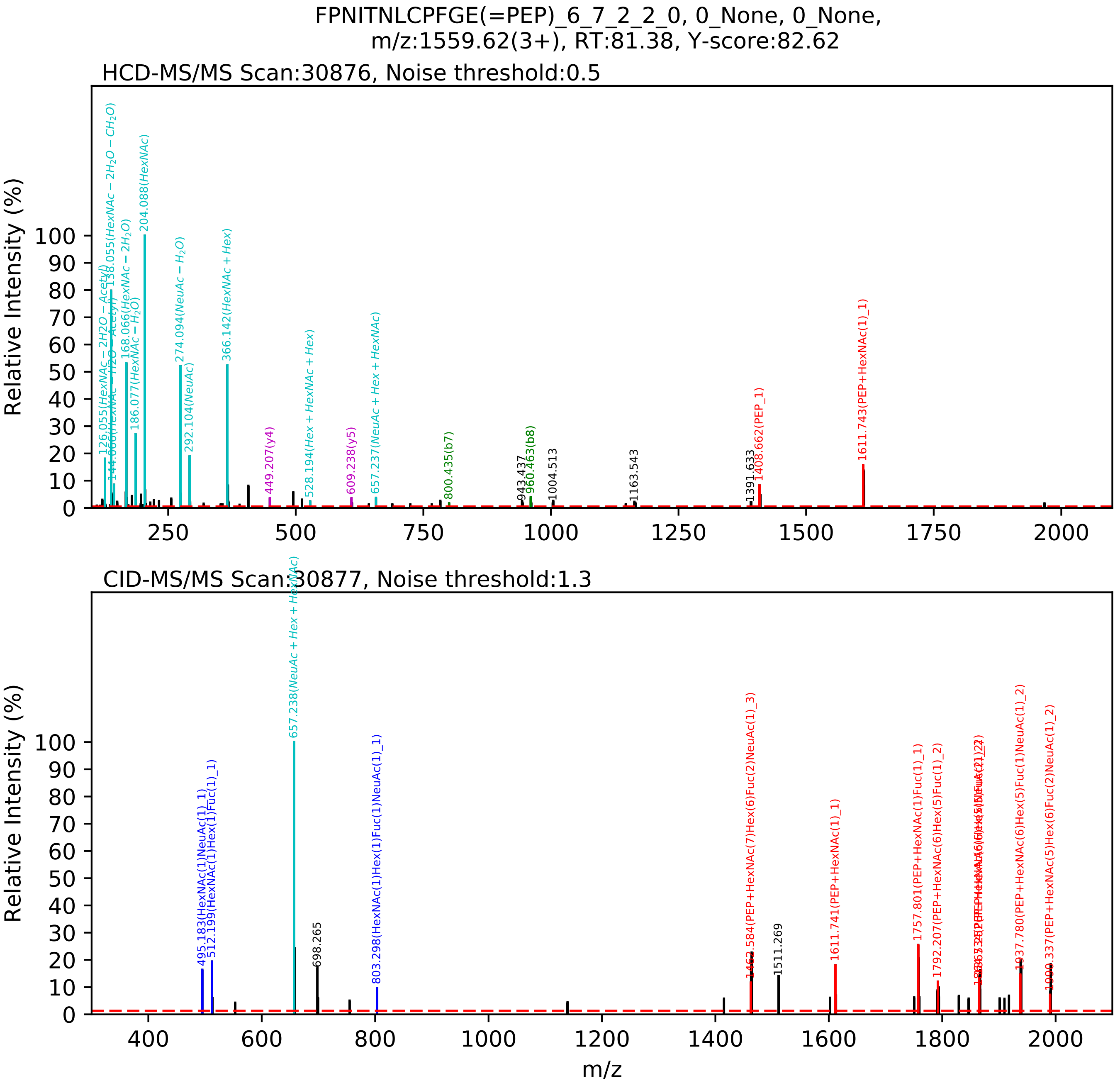

FPNITNLCPFGE(=PEP)_6_7_2_2_0, 0_None, 0_None,
m/z:1559.62(3+), RT:81.38, Y-score:82.62
HCD-MS/MS Scan:30876, Noise threshold:0.5
CID-MS/MS Scan:30877, Noise threshold:1.3
Relative Intensity (%)
m/z
204.088(HexNAc)
366.142(HexNAc + Hex)
274.094(NeuAc − H2O)
292.104(NeuAc)
168.066(HexNAc − 2H2O)
186.077(HexNAc − H2O)
126.055(HexNAc − 2H2O − Acetyl)
449.207(y4)
528.194(Hex + HexNAc + Hex)
609.238(y5)
657.237(NeuAc + Hex + HexNAc)
800.435(b7)
943.437
960.463(b8)
1004.513
1163.543
1391.633
1408.662(PEP_1)
1611.743(PEP+HexNAc(1)_1)
657.238(NeuAc + Hex + HexNAc)
495.183(HexNAc(1)NeuAc(1)_1)
512.199(HexNAc(1)Hex(1)Fuc(1)_1)
698.265
803.298(HexNAc(1)Hex(1)Fuc(1)NeuAc(1)_1)
1462.584(PEP+HexNAc(7)Hex(6)Fuc(2)NeuAc(1)_3)
1511.269
1611.741(PEP+HexNAc(1)_1)
1757.801(PEP+HexNAc(1)Fuc(1)_1)
1792.207(PEP+HexNAc(6)Hex(5)Fuc(1)_2)
1937.780(PEP+HexNAc(6)Hex(5)Fuc(1)NeuAc(1)_2)
1990.337(PEP+HexNAc(5)Hex(6)Fuc(2)NeuAc(1)_2)

FPNITNLCPFGE(=PEP)_6_7_4_1_0, 0_None, 0_None,
m/z:1559.96(3+), RT:67.64, Y-score:82.68

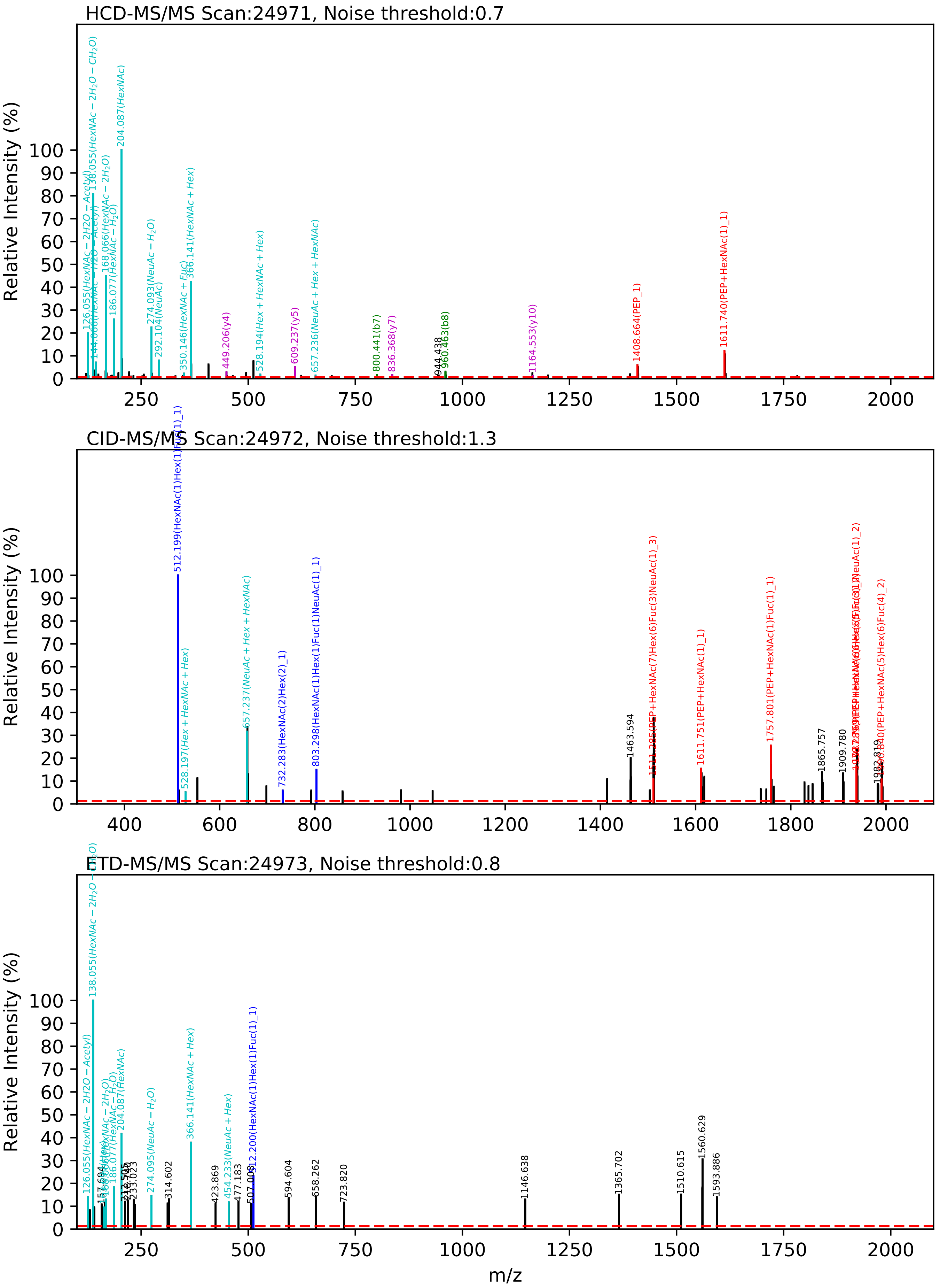

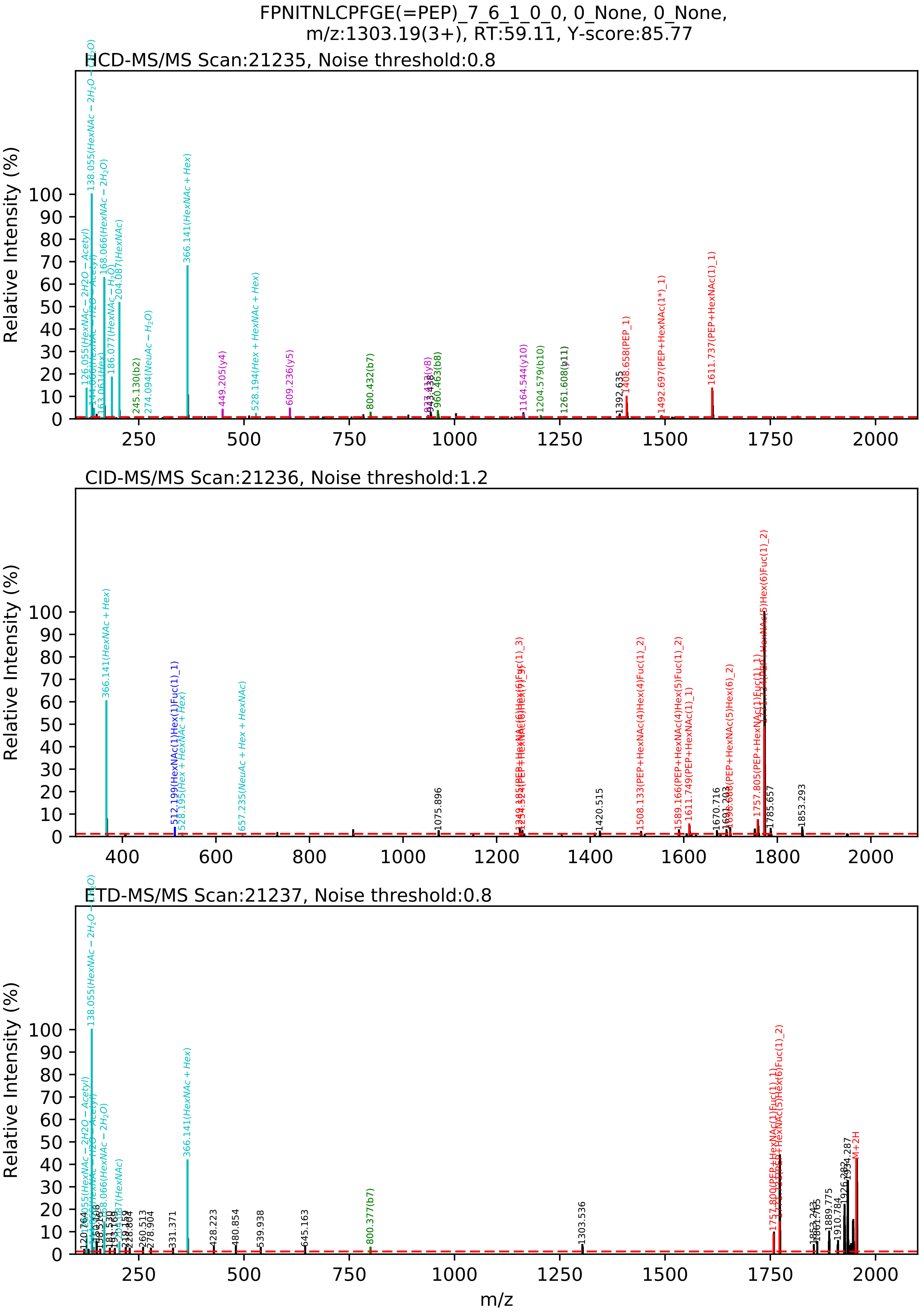
FPNITNLCPFGE(=PEP)_7_6_1_0_0, 0_None, 0_None,
m/z:1303.19(3+), RT:59.11, Y-score:85.77
HCD-MS/MS Scan:21235, Noise threshold:0.8
Relative Intensity (%)
138.055(HexNAc − 2H2O − H2O)
168.066(HexNAc − 2H2O)
204.087(HexNAc)
186.077(HexNAc − H2O)
366.141(HexNAc + Hex)
126.055(HexNAc − 2H2O − Acetyl)
163.061(Hex)
245.130(b2)
274.094(NeuAc − H2O)
449.205(y4)
528.194(Hex + HexNAc + Hex)
609.236(y5)
800.432(b7)
960.463(b8)
1164.544(y10)
1204.579(b10)
1261.608(b11)
1392.635
1408.658(PEP_1)
1492.697(PEP+HexNAc(1*)_1)
1611.737(PEP+HexNAc(1)_1)
CID-MS/MS Scan:21236, Noise threshold:1.2
366.141(HexNAc + Hex)
512.199(HexNAc(1)Hex(1)Fuc(1)_1)
528.195(Hex + HexNAc + Hex)
657.235(NeuAc + Hex + HexNAc)
1075.896
1420.515
1508.133(PEP+HexNAc(4)Hex(4)Fuc(1)_2)
1589.166(PEP+HexNAc(4)Hex(5)Fuc(1)_2)
1611.749(PEP+HexNAc(1)_1)
1670.716
1757.805(PEP+HexNAc(1)Fuc(1)_1)
1785.657
1853.293
ETD-MS/MS Scan:21237, Noise threshold:0.8
138.055(HexNAc − 2H2O − H2O)
366.141(HexNAc + Hex)
260.513
278.904
331.371
428.223
480.854
539.938
645.163
800.377(b7)
1303.536
1757.800(PEP+HexNAc(1)Fuc(1)_1)
1889.775
1910.784
M+2H
m/z

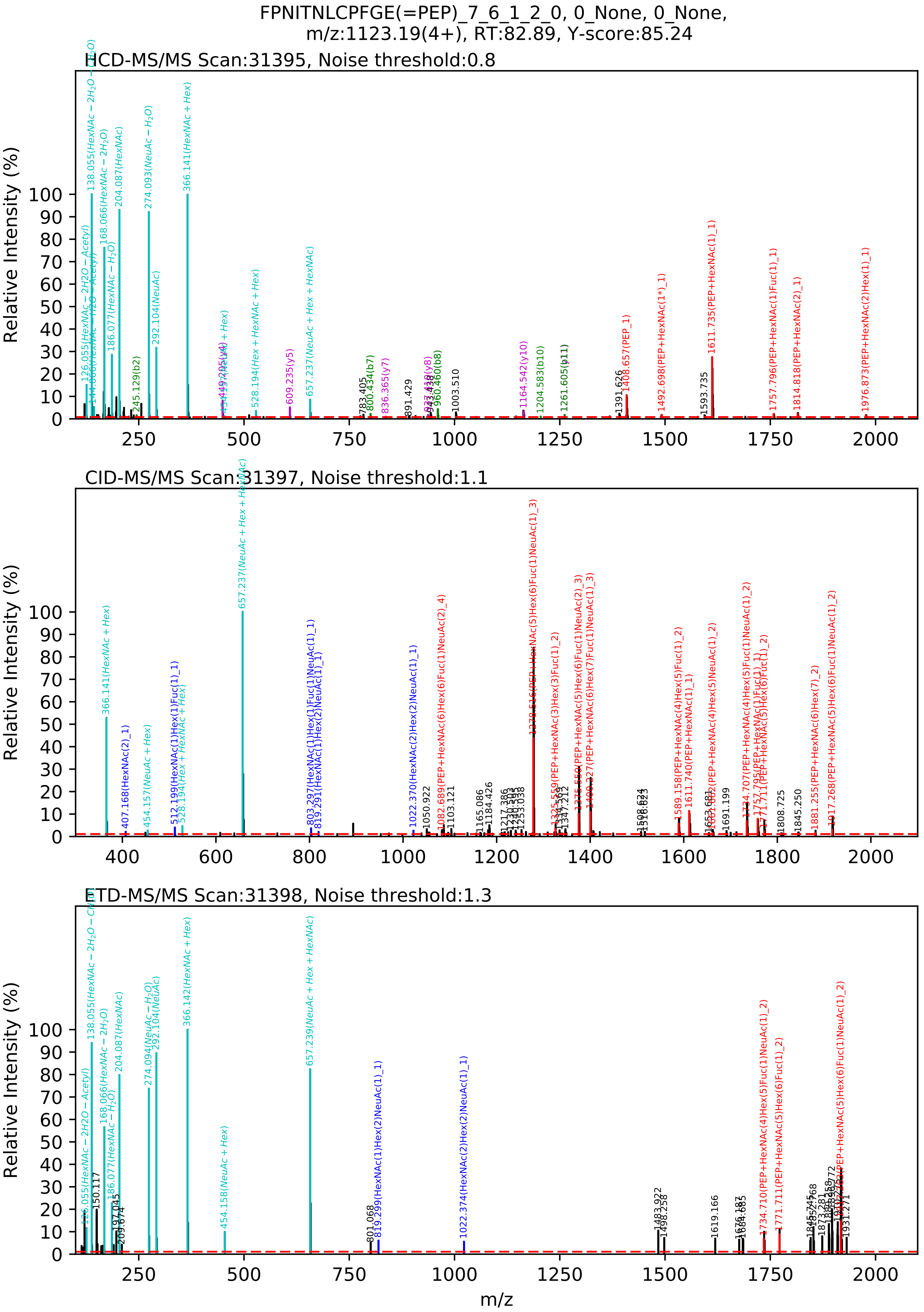
FPNITNLCPFGE(=PEP)_7_6_1_2_0, 0_None, 0_None,
m/z:1123.19(4+), RT:82.89, Y-score:85.24
HCD-MS/MS Scan:31395, Noise threshold:0.8
CID-MS/MS Scan:31397, Noise threshold:1.1
ETD-MS/MS Scan:31398, Noise threshold:1.3
Relative Intensity (%)
m/z
366.141(HexNAc + Hex)
657.237(NeuAc + Hex + HexNAc)
204.087(HexNAc)
274.093(NeuAc − H2O)
292.104(NeuAc)
168.066(HexNAc − 2H2O)
186.077(HexNAc − H2O)
528.194(Hex + HexNAc + Hex)
1611.735(PEP+HexNAc(1)_1)
1408.657(PEP_1)
1492.698(PEP+HexNAc(1*)_1)
1757.796(PEP+HexNAc(1)Fuc(1)_1)
1814.818(PEP+HexNAc(2)_1)
1976.873(PEP+HexNAc(2)Hex(1)_1)
1278.516(PEP+HexNAc(5)Hex(6)Fuc(1)NeuAc(1)_3)
1375.550(PEP+HexNAc(5)Hex(6)Fuc(1)NeuAc(2)_3)
1400.227(PEP+HexNAc(6)Hex(7)Fuc(1)NeuAc(1)_3)
1082.689(PEP+HexNAc(6)Hex(6)Fuc(1)NeuAc(2)_4)
1022.370(HexNAc(2)Hex(2)NeuAc(1)_1)
803.297(HexNAc(1)Hex(1)Fuc(1)NeuAc(1)_1)
819.291(HexNAc(1)Hex(2)NeuAc(1)_1)
512.199(HexNAc(1)Hex(1)Fuc(1)_1)
407.168(HexNAc(2)_1)
454.157(NeuAc + Hex)
1589.158(PEP+HexNAc(4)Hex(5)Fuc(1)_2)
1611.740(PEP+HexNAc(1)_1)
1734.707(PEP+HexNAc(4)Hex(5)Fuc(1)NeuAc(1)_2)
1881.255(PEP+HexNAc(6)Hex(7)_2)
1917.268(PEP+HexNAc(5)Hex(6)Fuc(1)NeuAc(1)_2)
1734.710(PEP+HexNAc(4)Hex(5)Fuc(1)NeuAc(1)_2)
1771.711(PEP+HexNAc(5)Hex(6)Fuc(1)_2)
819.299(HexNAc(1)Hex(2)NeuAc(1)_1)
1022.374(HexNAc(2)Hex(2)NeuAc(1)_1)

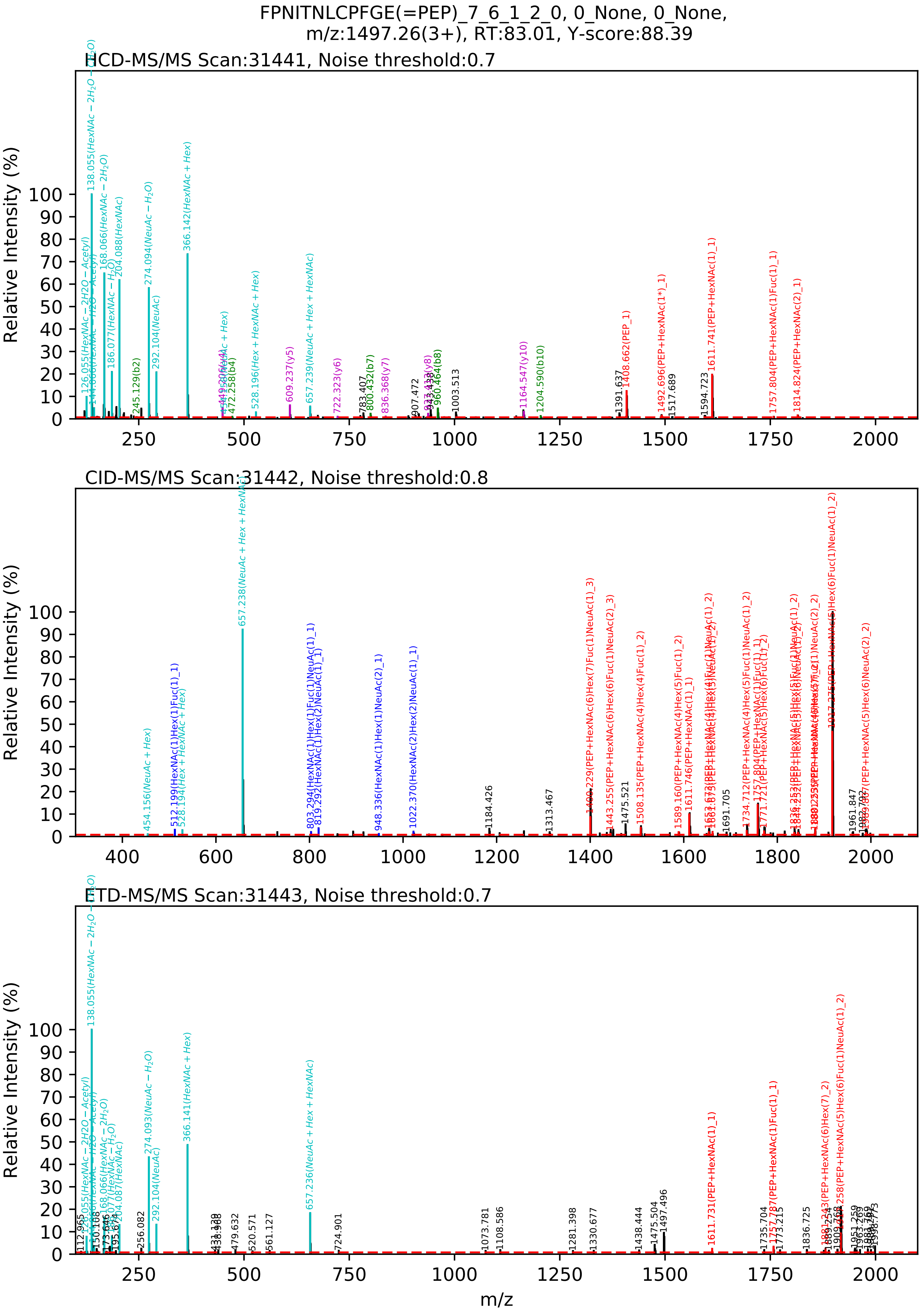
FPNITNLCPFGE(=PEP)_7_6_1_2_0, 0_None, 0_None,
m/z:1497.26(3+), RT:83.01, Y-score:88.39
HCD-MS/MS Scan:31441, Noise threshold:0.7
CID-MS/MS Scan:31442, Noise threshold:0.8
ETD-MS/MS Scan:31443, Noise threshold:0.7
Relative Intensity (%)
m/z

FPNITNLCPFGE(=PEP)_7_6_1_2_0, 0_None, 0_None,
m/z:1123.19(4+), RT:81.57, Y-score:88.28

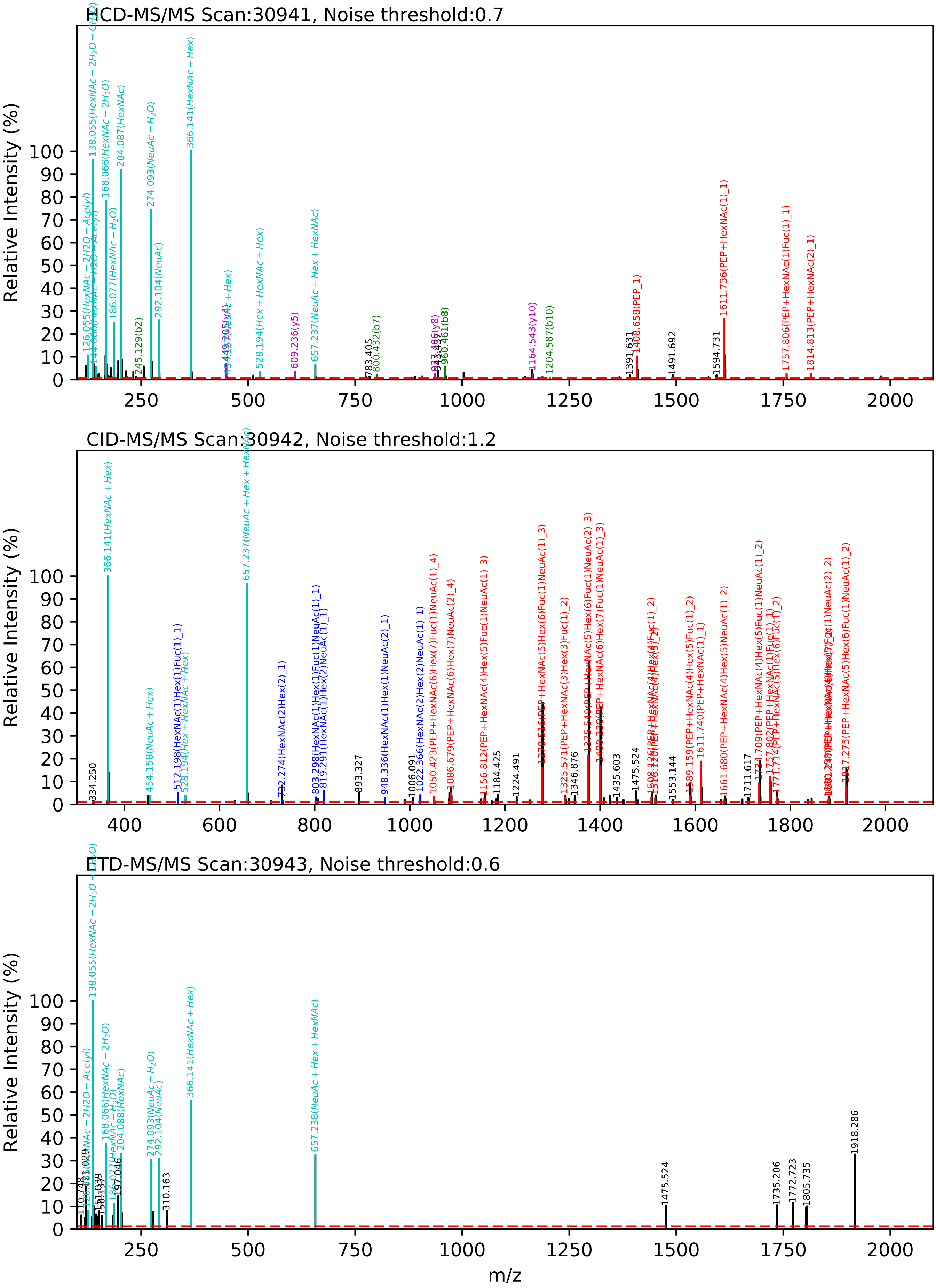

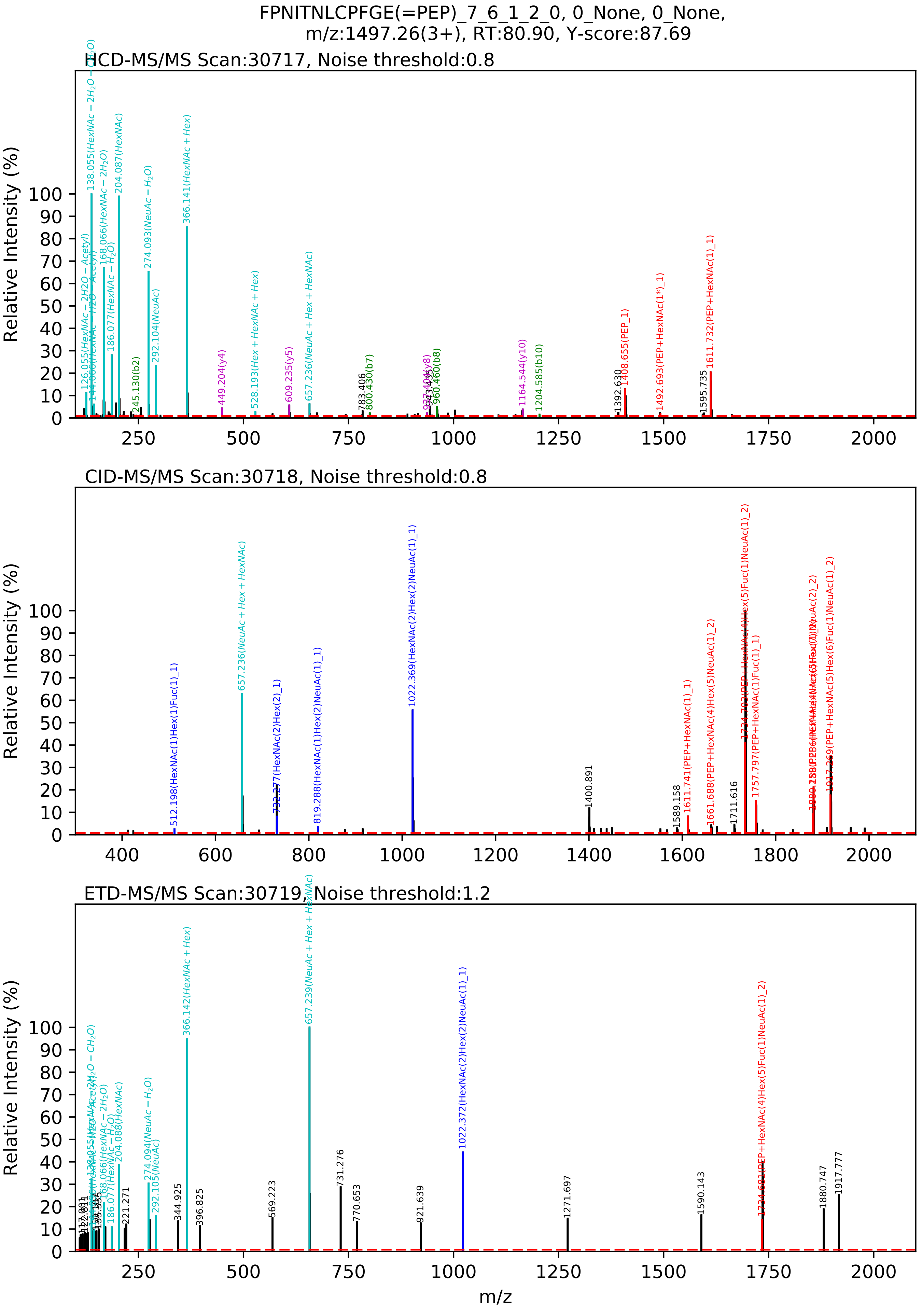

FPNITNLCPFGE(=PEP)_7_6_1_2_0, 0_None, 0_None,
m/z:1497.26(3+), RT:80.90, Y-score:87.69
HCD-MS/MS Scan:30717, Noise threshold:0.8
CID-MS/MS Scan:30718, Noise threshold:0.8
ETD-MS/MS Scan:30719, Noise threshold:1.2
Relative Intensity (%)
m/z

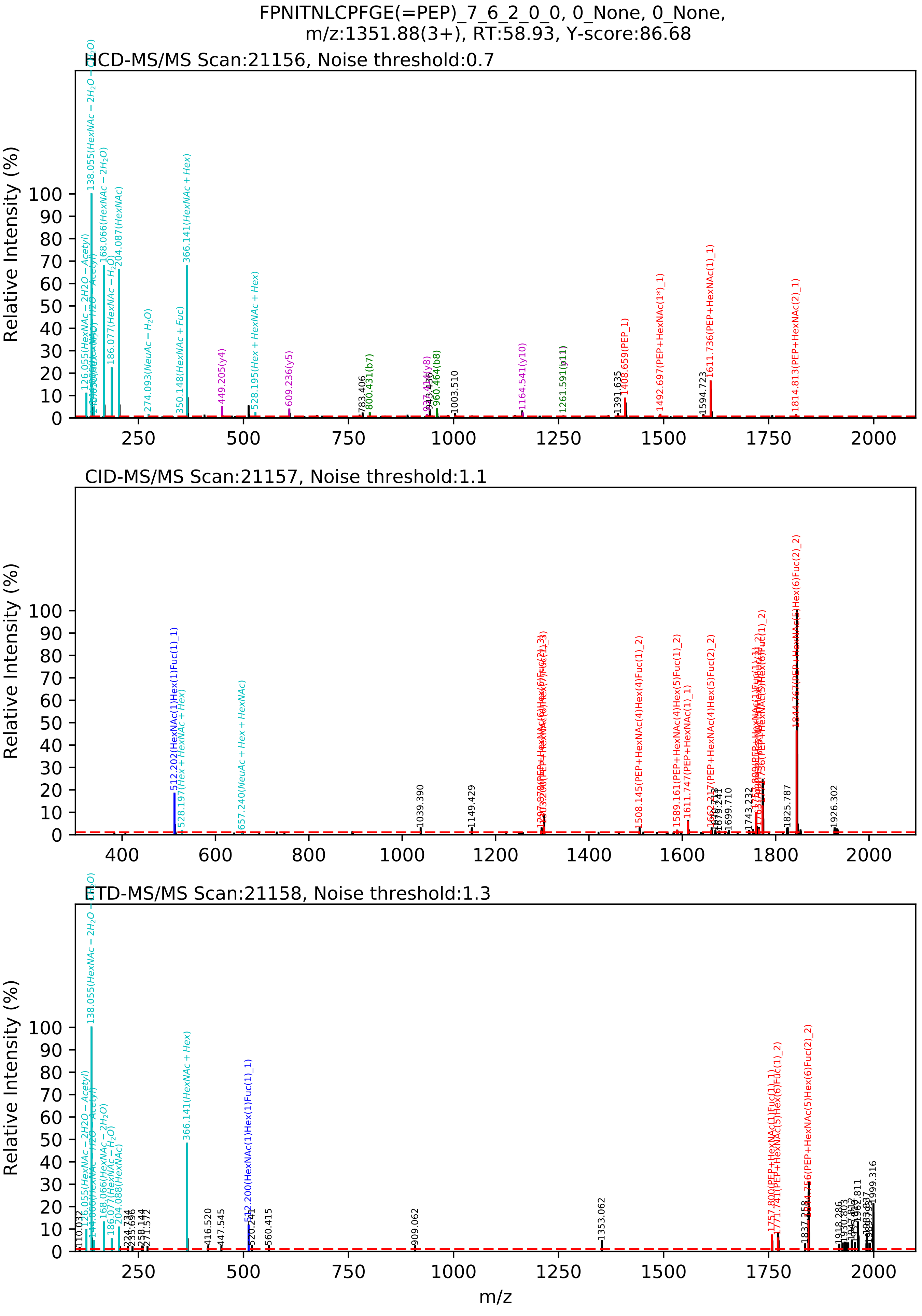
FPNITNLCPFGE(=PEP)_7_6_2_0_0, 0_None, 0_None,
m/z:1351.88(3+), RT:58.93, Y-score:86.68
HCD-MS/MS Scan:21156, Noise threshold:0.7
CID-MS/MS Scan:21157, Noise threshold:1.1
ETD-MS/MS Scan:21158, Noise threshold:1.3
Relative Intensity (%)
m/z
138.055(HexNAc − 2H2O − H2O)
168.066(HexNAc − 2H2O)
204.087(HexNAc)
186.077(HexNAc − H2O)
366.141(HexNAc + Hex)
350.148(HexNAc + Fuc)
274.093(NeuAc − H2O)
449.205(y4)
528.195(Hex + HexNAc + Hex)
609.236(y5)
800.431(b7)
960.464(b8)
1003.510
1164.541(y10)
1261.591(b11)
1391.635
1408.659(PEP_1)
1492.697(PEP+HexNAc(1*)_1)
1594.723
1611.736(PEP+HexNAc(1)_1)
1814.813(PEP+HexNAc(2)_1)
512.202(HexNAc(1)Hex(1)Fuc(1)_1)
528.197(Hex + HexNAc + Hex)
657.240(NeuAc + Hex + HexNAc)
1039.390
1149.429
1508.145(PEP+HexNAc(4)Hex(4)Fuc(1)_2)
1589.161(PEP+HexNAc(4)Hex(5)Fuc(1)_2)
1611.747(PEP+HexNAc(1)_1)
1699.710
1743.232
1825.787
1926.302
512.200(HexNAc(1)Hex(1)Fuc(1)_1)
416.520
447.545
560.415
909.062
1353.062
1757.800(PEP+HexNAc(1)Fuc(1)_1)
1837.258
1918.286
1962.811
1999.316

FPNITNLCPFGE(=PEP)_7_6_2_1_0, 0_None, 0_None,
m/z:1448.91(3+), RT:68.48, Y-score:86.98

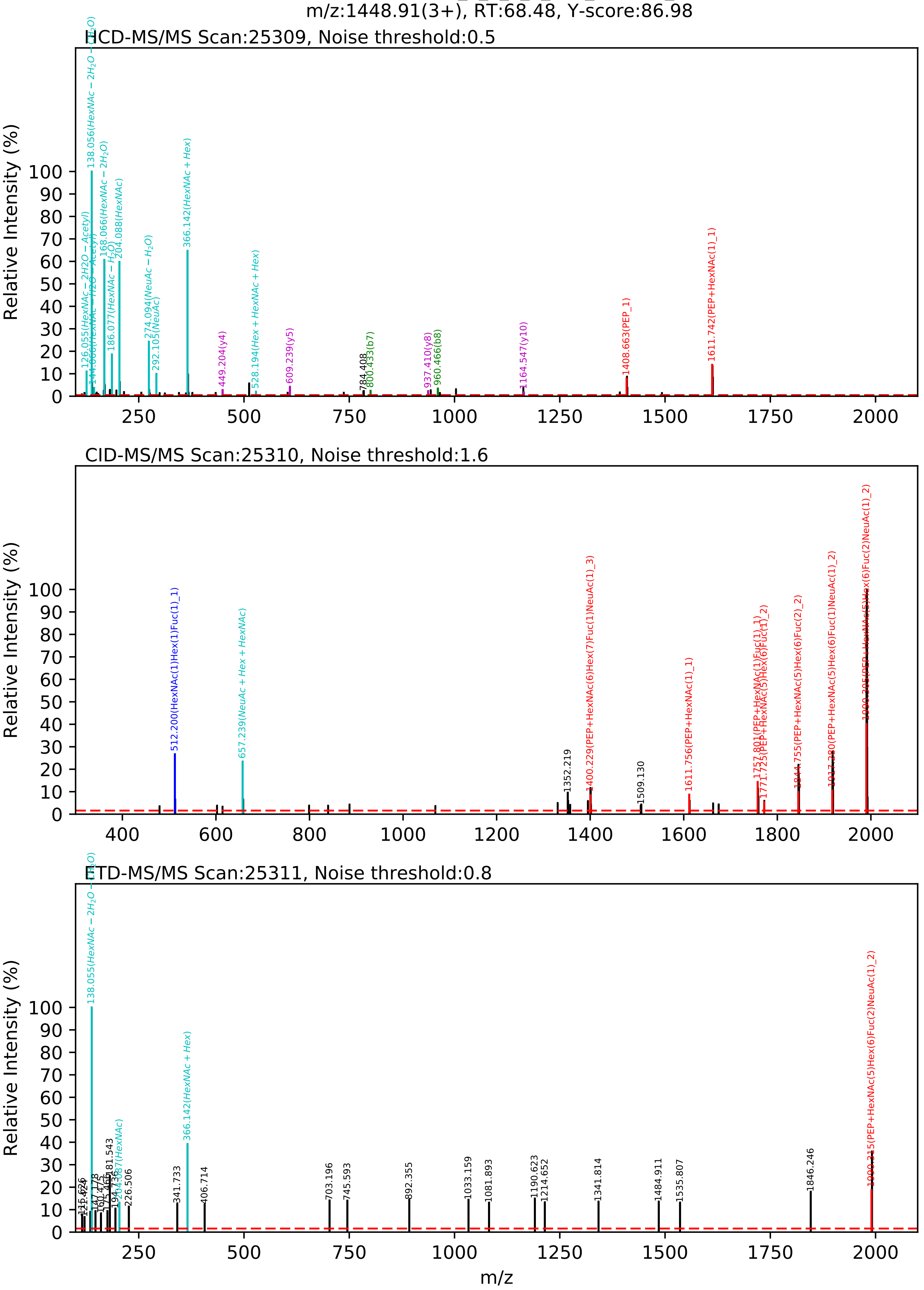

FPNITNLCPFGE(=PEP)_7_6_2_1_0, 0_None, 0_None,
m/z:1448.91(3+), RT:82.44, Y-score:77.96

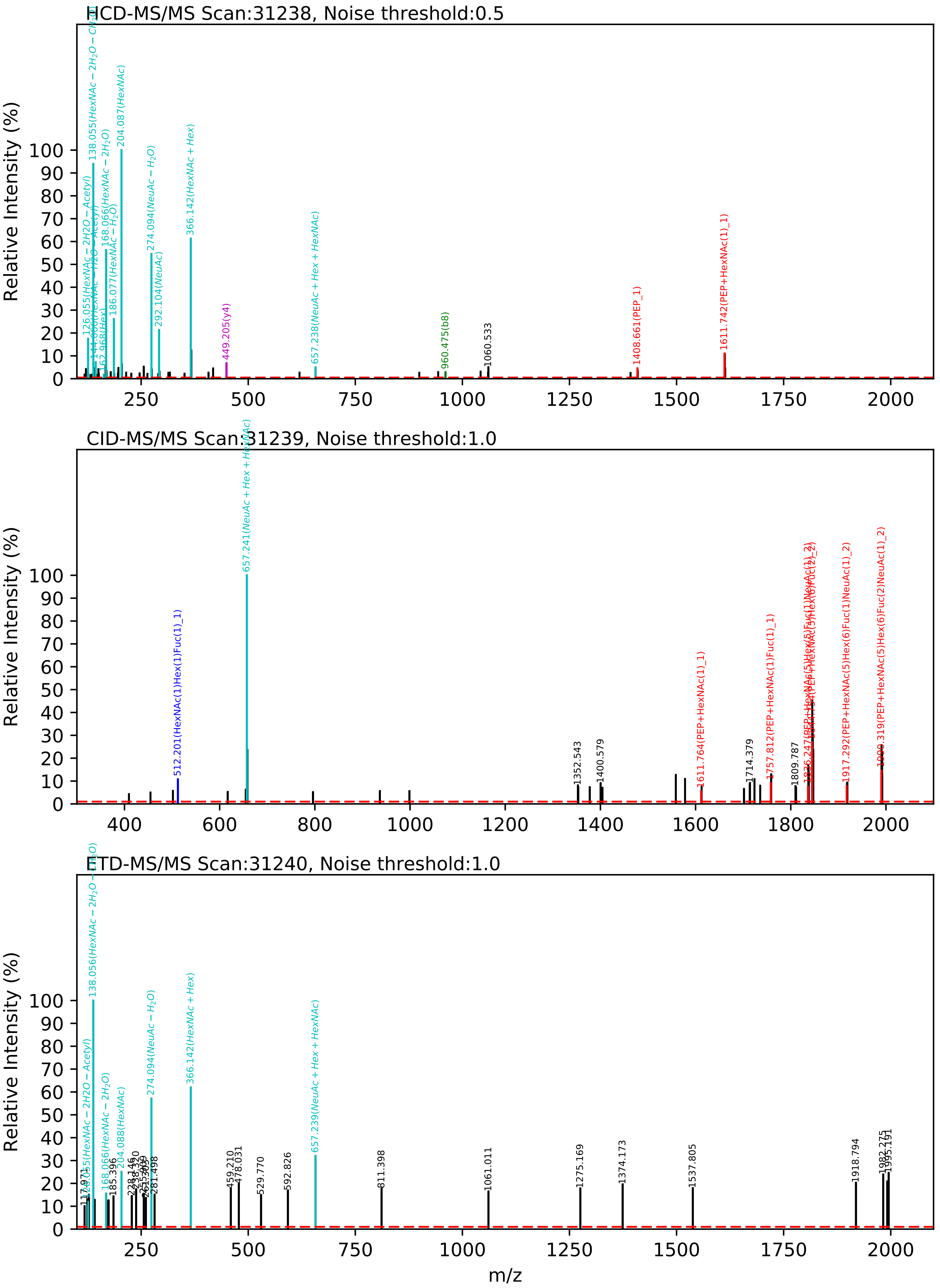

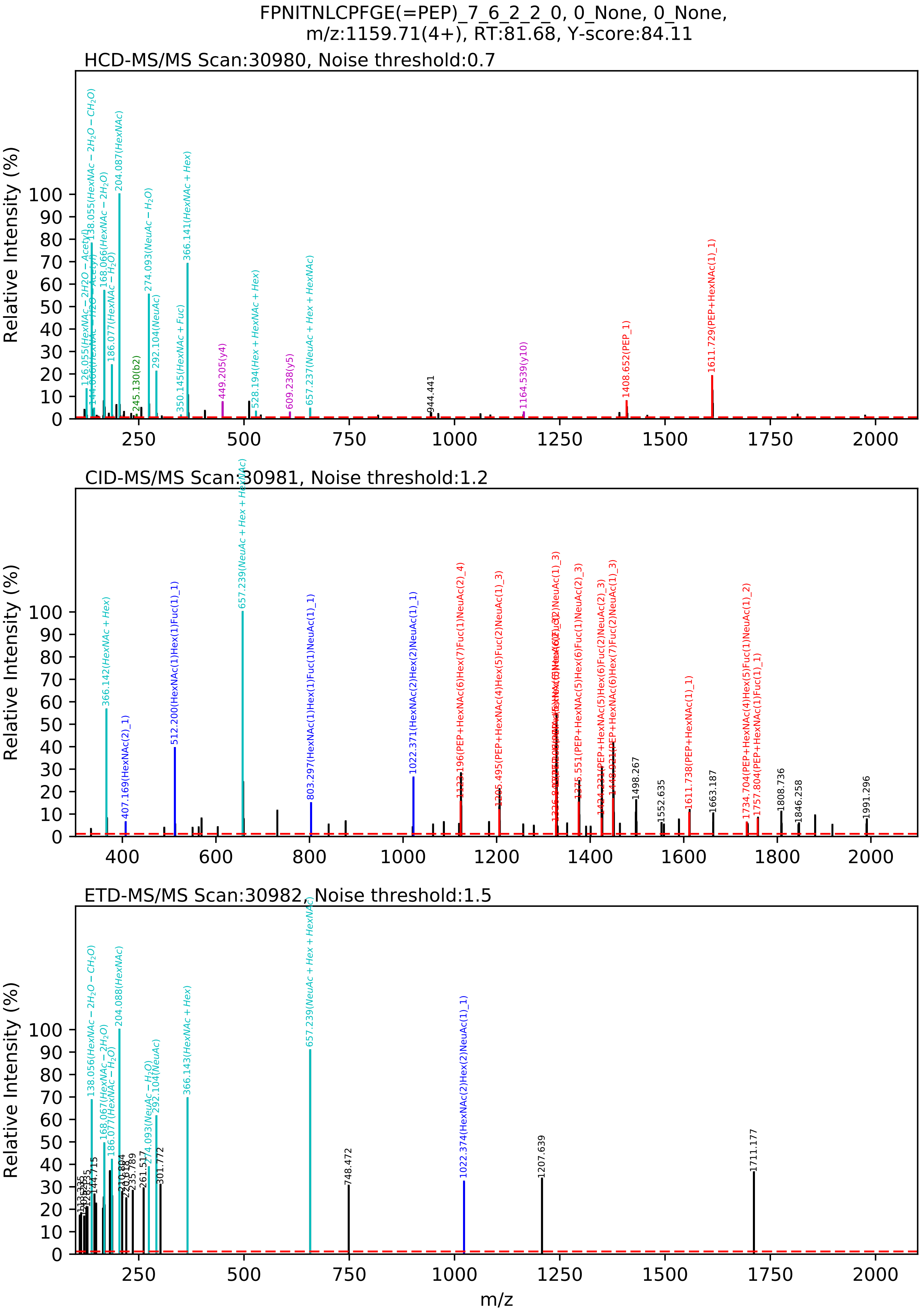
FPNITNLCPFGE(=PEP)_7_6_2_2_0, 0_None, 0_None,
m/z:1159.71(4+), RT:81.68, Y-score:84.11
HCD-MS/MS Scan:30980, Noise threshold:0.7
CID-MS/MS Scan:30981, Noise threshold:1.2
ETD-MS/MS Scan:30982, Noise threshold:1.5
Relative Intensity (%)
m/z
204.087(HexNAc)
366.141(HexNAc + Hex)
274.093(NeuAc − H2O)
292.104(NeuAc)
168.066(HexNAc − 2H2O)
186.077(HexNAc − H2O)
245.130(b2)
449.205(y4)
528.194(Hex + HexNAc + Hex)
609.238(y5)
657.237(NeuAc + Hex + HexNAc)
944.441
1164.539(y10)
1408.652(PEP_1)
1611.729(PEP+HexNAc(1)_1)
366.142(HexNAc + Hex)
407.169(HexNAc(2)_1)
512.200(HexNAc(1)Hex(1)Fuc(1)_1)
657.239(NeuAc + Hex + HexNAc)
803.297(HexNAc(1)Hex(1)Fuc(1)NeuAc(1)_1)
1022.371(HexNAc(2)Hex(2)NeuAc(1)_1)
1498.267
1552.635
1611.738(PEP+HexNAc(1)_1)
1663.187
1734.704(PEP+HexNAc(4)Hex(5)Fuc(1)NeuAc(1)_2)
1757.804(PEP+HexNAc(1)Fuc(1)_1)
1808.736
1846.258
1991.296
138.056(HexNAc − 2H2O − CH2O)
204.088(HexNAc)
168.067(HexNAc − 2H2O)
186.077(HexNAc − H2O)
274.093(NeuAc − H2O)
292.104(NeuAc)
366.143(HexNAc + Hex)
657.239(NeuAc + Hex + HexNAc)
144.715
261.517
301.772
748.472
1022.374(HexNAc(2)Hex(2)NeuAc(1)_1)
1207.639
1711.177

FPNITNLCPFGE(=PEP)_7_6_2_2_0, 0_None, 0_None,
m/z:1159.71(4+), RT:81.06, Y-score:77.59

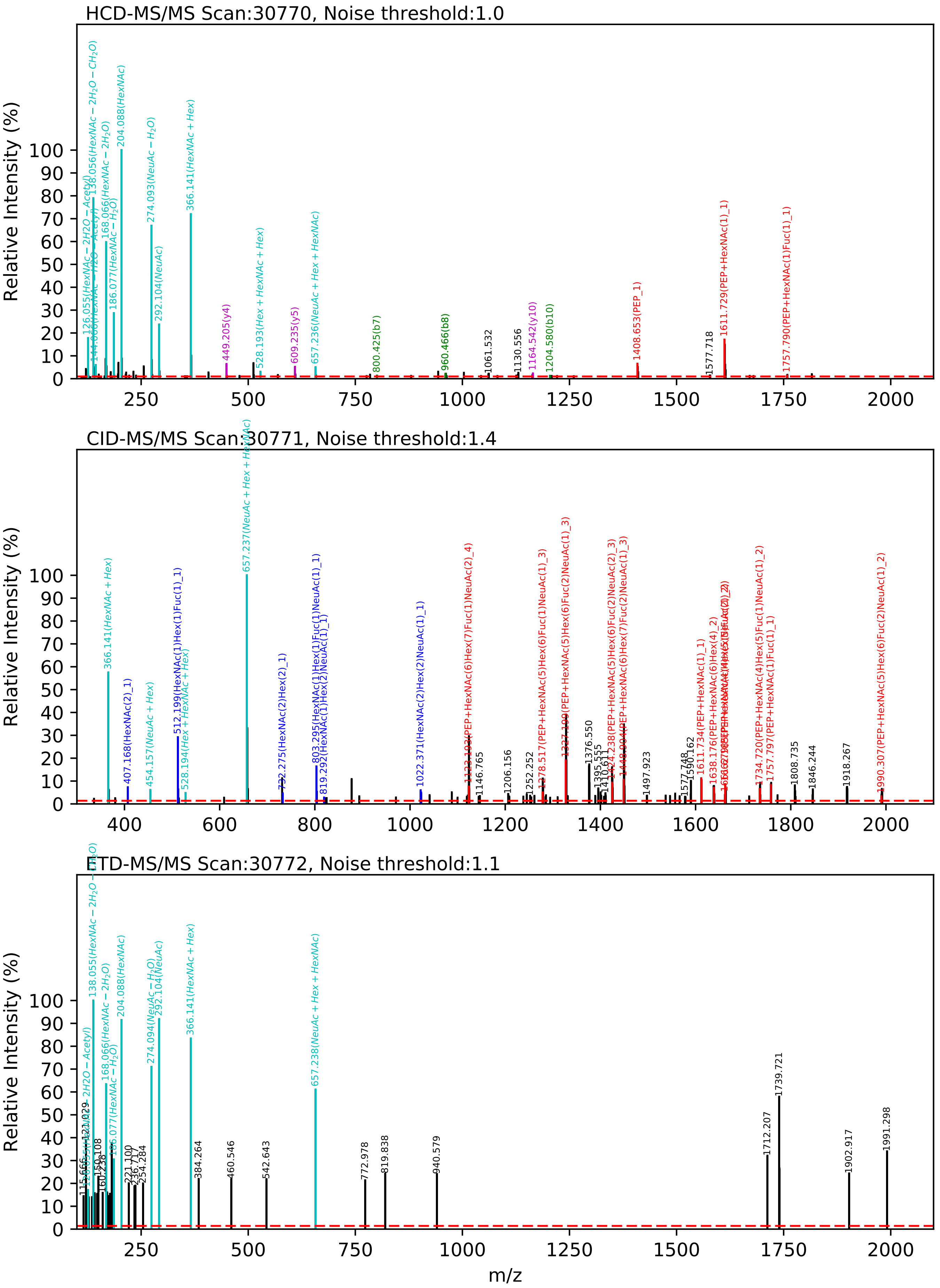

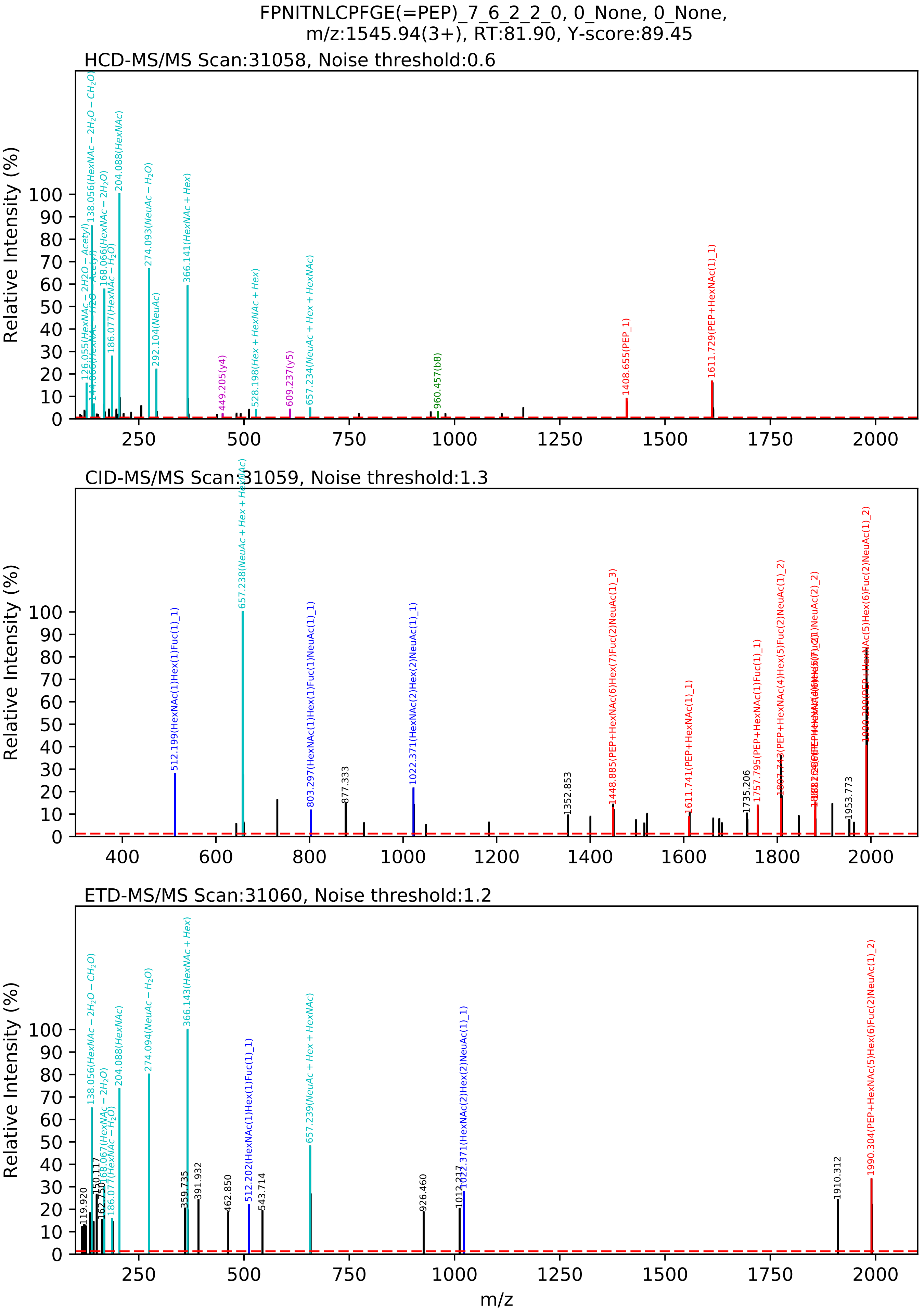
FPNITNLCPFGE(=PEP)_7_6_2_2_0, 0_None, 0_None,
m/z:1545.94(3+), RT:81.90, Y-score:89.45
HCD-MS/MS Scan:31058, Noise threshold:0.6
Relative Intensity (%)
126.055(HexNAc − 2H2O − Acetyl)
138.056(HexNAc − 2H2O − CH2O)
144.066(HexNAc − H2O − Acetyl)
168.066(HexNAc − 2H2O)
186.077(HexNAc − H2O)
204.088(HexNAc)
274.093(NeuAc − H2O)
292.104(NeuAc)
366.141(HexNAc + Hex)
449.205(y4)
528.198(Hex + HexNAc + Hex)
609.237(y5)
657.234(NeuAc + Hex + HexNAc)
960.457(b8)
1408.655(PEP_1)
1611.729(PEP+HexNAc(1)_1)
CID-MS/MS Scan:31059, Noise threshold:1.3
512.199(HexNAc(1)Hex(1)Fuc(1)_1)
657.238(NeuAc + Hex + HexNAc)
803.297(HexNAc(1)Hex(1)Fuc(1)NeuAc(1)_1)
877.333
1022.371(HexNAc(2)Hex(2)NeuAc(1)_1)
1352.853
1448.885(PEP+HexNAc(6)Hex(7)Fuc(2)NeuAc(1)_3)
1611.741(PEP+HexNAc(1)_1)
1735.206
1757.795(PEP+HexNAc(1)Fuc(1)_1)
1807.743(PEP+HexNAc(4)Hex(5)Fuc(2)NeuAc(1)_2)
1953.773
1990.299(PEP+HexNAc(5)Hex(6)Fuc(2)NeuAc(1)_2)
ETD-MS/MS Scan:31060, Noise threshold:1.2
119.920
138.056(HexNAc − 2H2O − CH2O)
150.117
162.750
168.067(HexNAc − 2H2O)
186.077(HexNAc − H2O)
204.088(HexNAc)
274.094(NeuAc − H2O)
359.735
366.143(HexNAc + Hex)
391.932
462.850
512.202(HexNAc(1)Hex(1)Fuc(1)_1)
543.714
657.239(NeuAc + Hex + HexNAc)
926.460
1012.317
1022.371(HexNAc(2)Hex(2)NeuAc(1)_1)
1910.312
1990.304(PEP+HexNAc(5)Hex(6)Fuc(2)NeuAc(1)_2)
m/z

FPNITNLCPFGE(=PEP)_7_7_0_2_0, 0_None, 0_None,
m/z:1516.26(3+), RT:82.85, Y-score:64.78

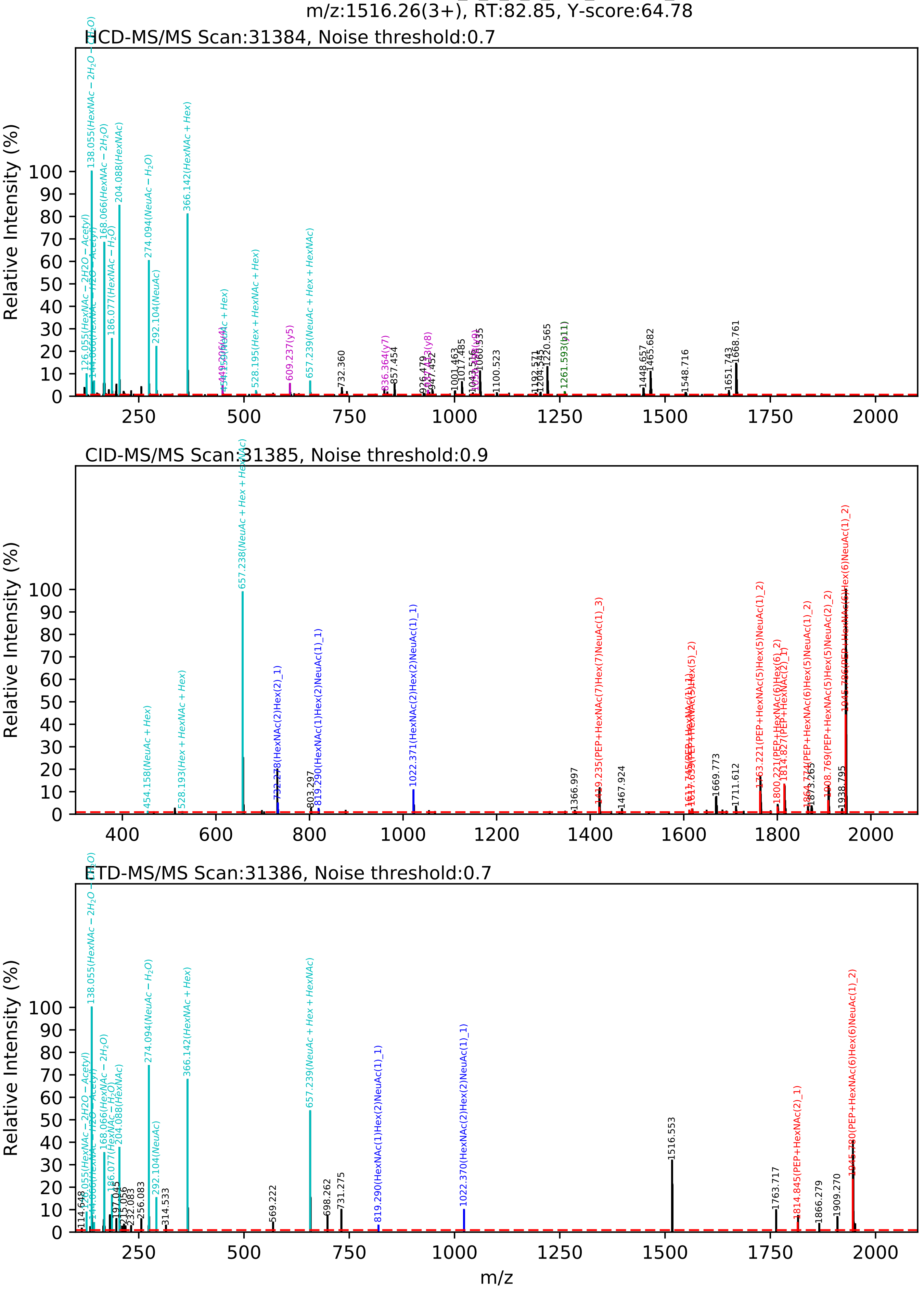

FPNITNLCPFGE(=PEP)_7_7_1_1_0, 0_None, 0_None,
m/z:1467.92(3+), RT:68.37, Y-score:70.29

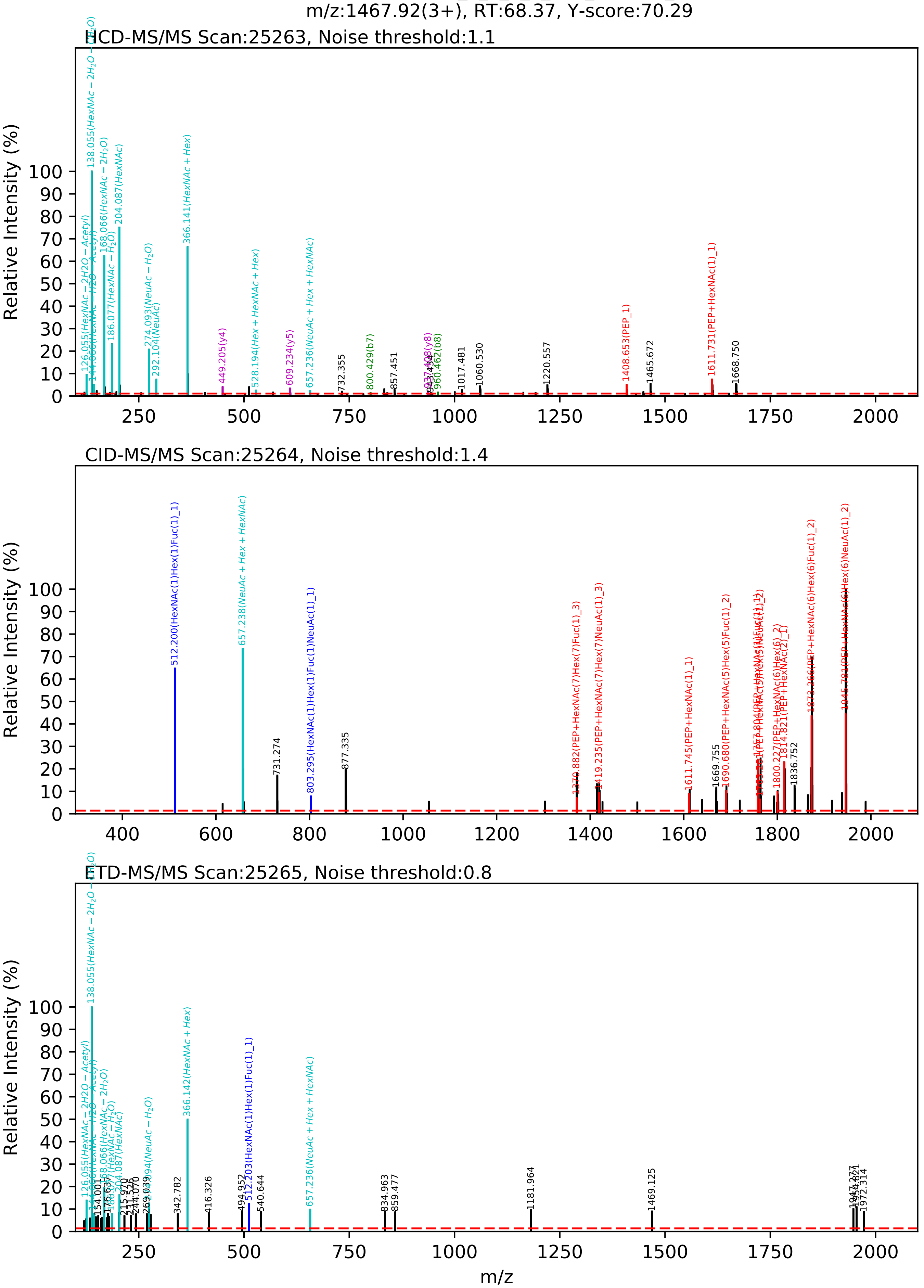

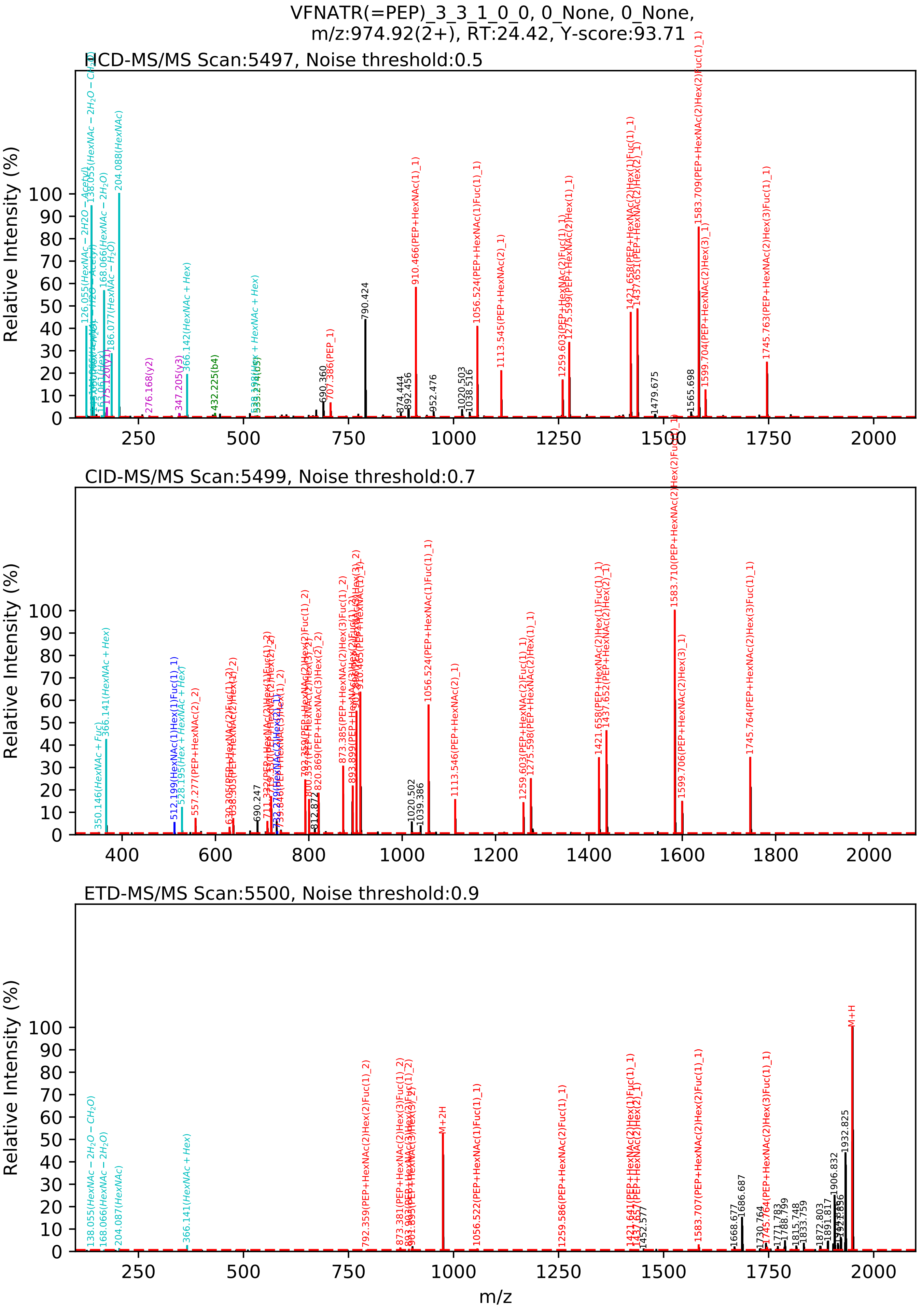
VFNATR(=PEP)_3_3_1_0_0, 0_None, 0_None,
m/z:974.92(2+), RT:24.42, Y-score:93.71
HCD-MS/MS Scan:5497, Noise threshold:0.5
Relative Intensity (%)
204.088(HexNAc)
138.055(HexNAc − 2H2O − CH2O)
168.066(HexNAc − 2H2O)
186.077(HexNAc − H2O)
126.055(HexNAc − 2H2O − Acetyl)
366.142(HexNAc + Hex)
276.168(y2)
347.205(y3)
432.225(b4)
790.424
707.386(PEP_1)
690.360
874.444
892.456
910.466(PEP+HexNAc(1)_1)
952.476
1020.503
1038.516
1056.524(PEP+HexNAc(1)Fuc(1)_1)
1113.545(PEP+HexNAc(2)_1)
1259.603(PEP+HexNAc(2)Fuc(1)_1)
1275.599(PEP+HexNAc(2)Hex(1)_1)
1421.658(PEP+HexNAc(2)Hex(1)Fuc(1)_1)
1437.651(PEP+HexNAc(2)Hex(2)_1)
1479.675
1565.698
1583.709(PEP+HexNAc(2)Hex(2)Fuc(1)_1)
1599.704(PEP+HexNAc(2)Hex(3)_1)
1745.763(PEP+HexNAc(2)Hex(3)Fuc(1)_1)
CID-MS/MS Scan:5499, Noise threshold:0.7
350.146(HexNAc + Fuc)
366.141(HexNAc + Hex)
512.199(HexNAc(1)Hex(1)Fuc(1)_1)
528.195(Hex + HexNAc + Hex)
557.277(PEP+HexNAc(2)_2)
690.247
792.359(PEP+HexNAc(2)Hex(2)Fuc(1)_2)
820.869(PEP+HexNAc(3)Hex(2)_2)
812.872
873.385(PEP+HexNAc(2)Hex(3)Fuc(1)_2)
893.899
910.465(PEP+HexNAc(1)_1)
1020.502
1039.386
1056.524(PEP+HexNAc(1)Fuc(1)_1)
1113.546(PEP+HexNAc(2)_1)
1259.603(PEP+HexNAc(2)Fuc(1)_1)
1275.598(PEP+HexNAc(2)Hex(1)_1)
1421.658(PEP+HexNAc(2)Hex(1)Fuc(1)_1)
1437.652(PEP+HexNAc(2)Hex(2)_1)
1583.710(PEP+HexNAc(2)Hex(2)Fuc(1)_1)
1599.706(PEP+HexNAc(2)Hex(3)_1)
1745.764(PEP+HexNAc(2)Hex(3)Fuc(1)_1)
ETD-MS/MS Scan:5500, Noise threshold:0.9
138.055(HexNAc − 2H2O − CH2O)
168.066(HexNAc − 2H2O)
204.087(HexNAc)
366.141(HexNAc + Hex)
792.359(PEP+HexNAc(2)Hex(2)Fuc(1)_2)
873.381(PEP+HexNAc(2)Hex(3)Fuc(1)_2)
M+2H
1056.522(PEP+HexNAc(1)Fuc(1)_1)
1259.586(PEP+HexNAc(2)Fuc(1)_1)
1421.641(PEP+HexNAc(2)Hex(1)Fuc(1)_1)
1452.577
1583.707(PEP+HexNAc(2)Hex(2)Fuc(1)_1)
1668.677
1686.687
1730.764
1745.764(PEP+HexNAc(2)Hex(3)Fuc(1)_1)
1771.783
1788.799
1815.748
1833.759
1872.803
1891.817
1906.832
1932.825
M+H
m/z

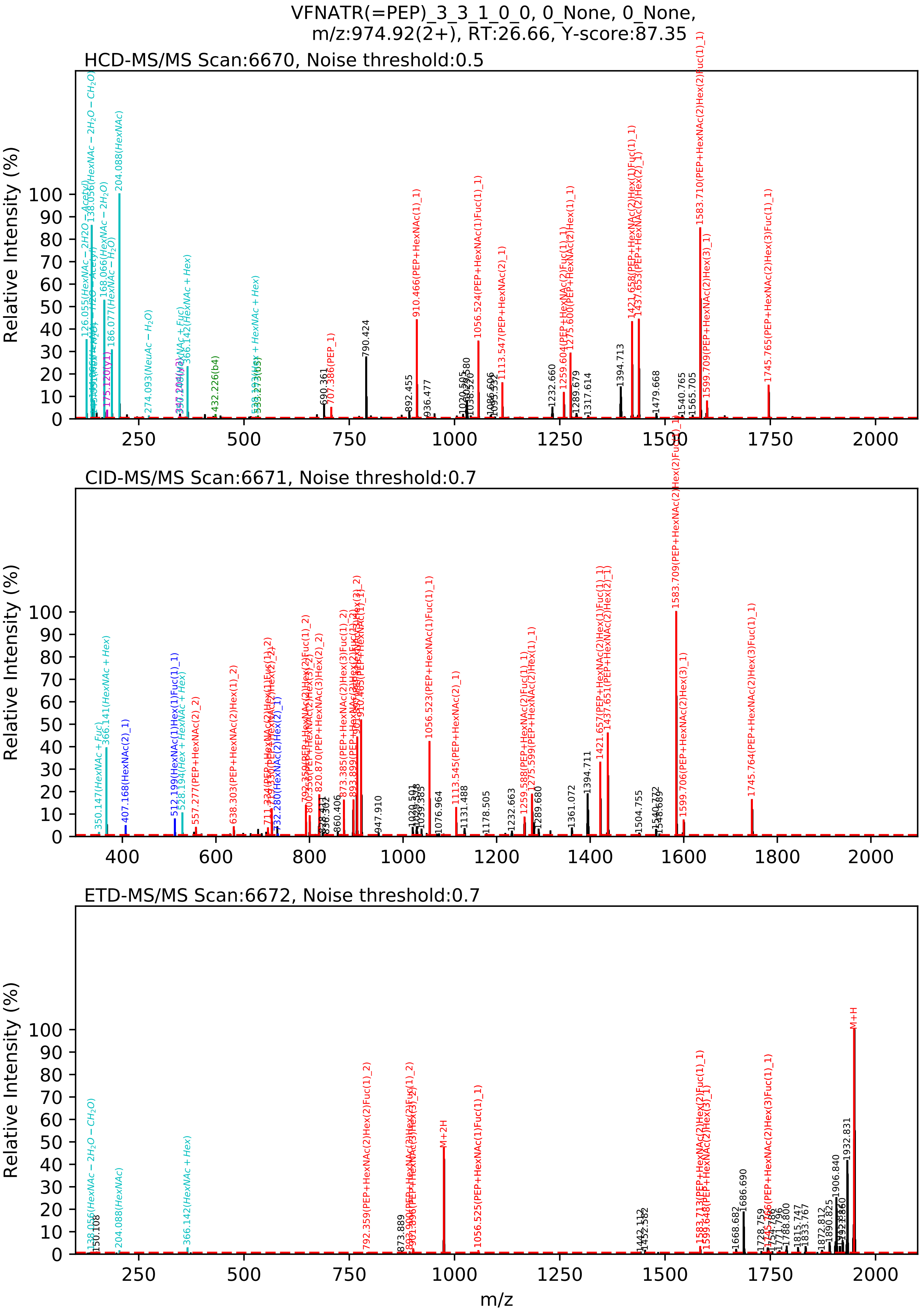
VFNATR(=PEP)_3_3_1_0_0, 0_None, 0_None,
m/z:974.92(2+), RT:26.66, Y-score:87.35
HCD-MS/MS Scan:6670, Noise threshold:0.5
CID-MS/MS Scan:6671, Noise threshold:0.7
ETD-MS/MS Scan:6672, Noise threshold:0.7
Relative Intensity (%)
m/z

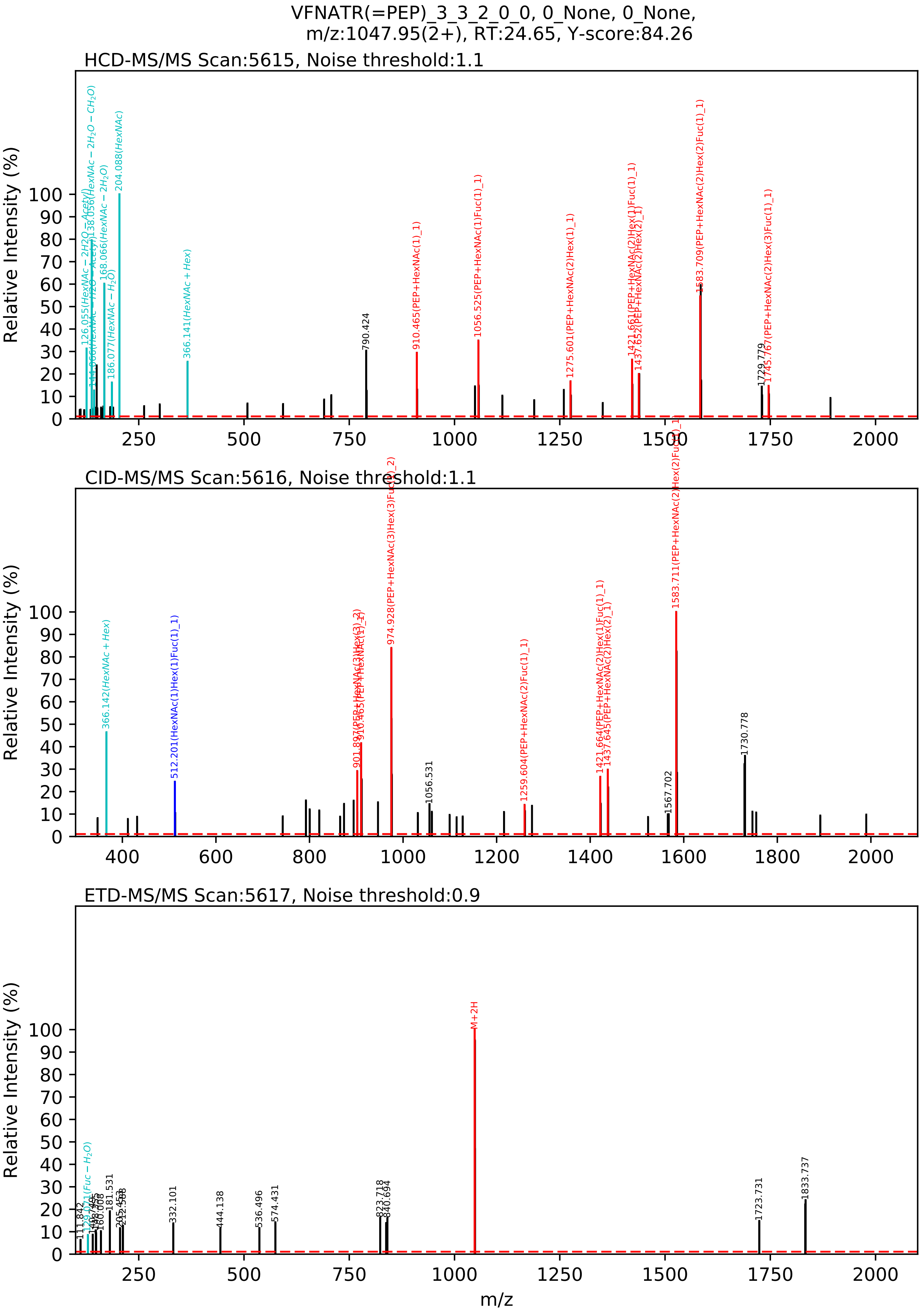
VFNATR(=PEP)_3_3_2_0_0, 0_None, 0_None,
m/z:1047.95(2+), RT:24.65, Y-score:84.26
HCD-MS/MS Scan:5615, Noise threshold:1.1
CID-MS/MS Scan:5616, Noise threshold:1.1
ETD-MS/MS Scan:5617, Noise threshold:0.9
Relative Intensity (%)
m/z
204.088(HexNAc)
366.141(HexNAc+Hex)
790.424
910.465(PEP+HexNAc(1)_1)
1056.525(PEP+HexNAc(1)Fuc(1)_1)
1275.601(PEP+HexNAc(2)Hex(1)_1)
1583.709(PEP+HexNAc(2)Hex(2)Fuc(1)_1)
1745.767(PEP+HexNAc(2)Hex(3)Fuc(1)_1)
366.142(HexNAc+Hex)
512.201(HexNAc(1)Hex(1)Fuc(1)_1)
974.928(PEP+HexNAc(3)Hex(3)Fuc(1)_2)
1056.531
1259.604(PEP+HexNAc(2)Fuc(1)_1)
1567.702
1583.711(PEP+HexNAc(2)Hex(2)Fuc(1)_1)
1730.778
M+2H
332.101
444.138
536.496
574.431
823.718
840.694
1723.731
1833.737

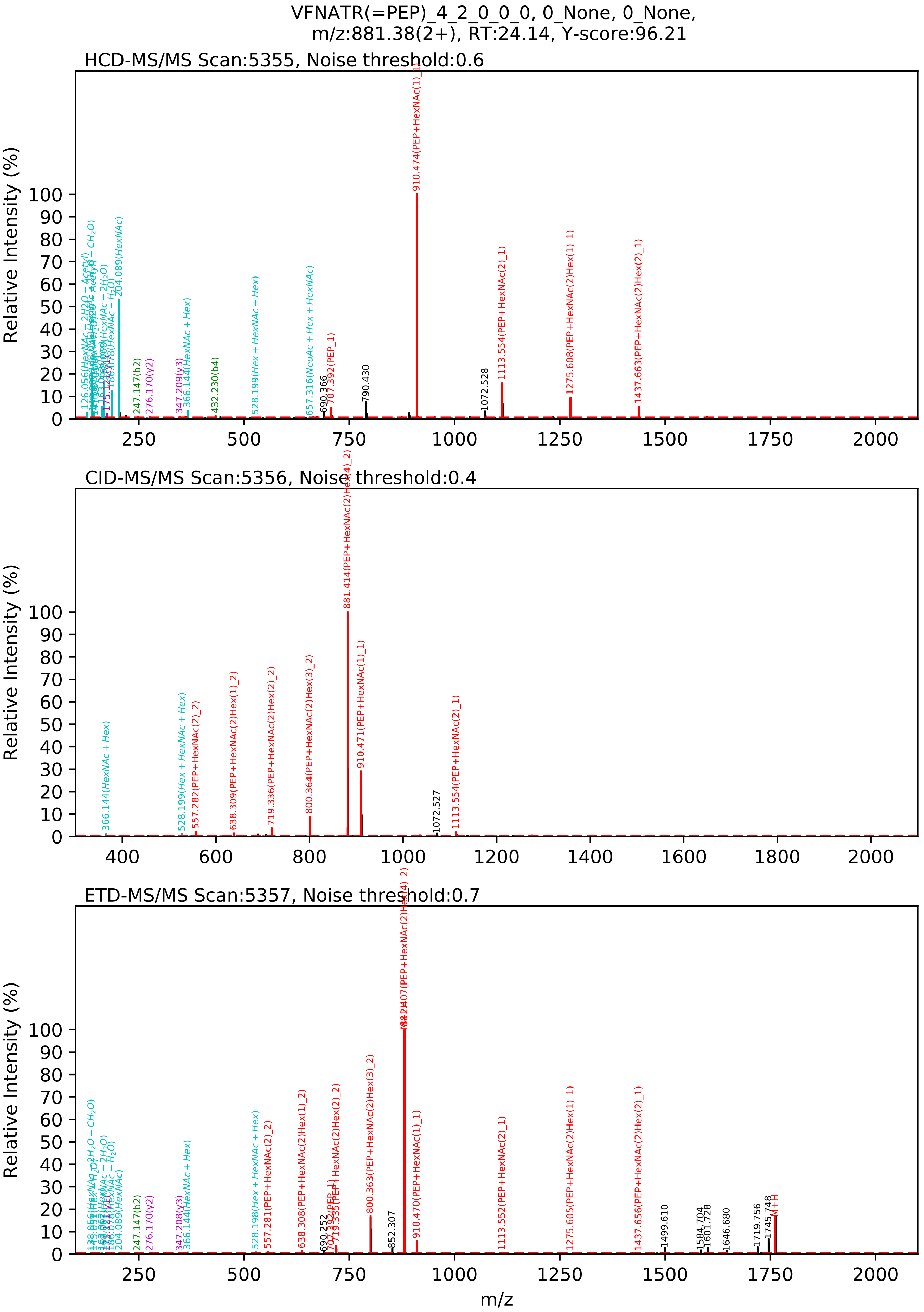
VFNATR(=PEP)_4_2_0_0_0, 0_None, 0_None,
m/z:881.38(2+), RT:24.14, Y-score:96.21
HCD-MS/MS Scan:5355, Noise threshold:0.6
CID-MS/MS Scan:5356, Noise threshold:0.4
ETD-MS/MS Scan:5357, Noise threshold:0.7
Relative Intensity (%)
m/z
910.474(PEP+HexNAc(1)_1)
1113.554(PEP+HexNAc(2)_1)
1275.608(PEP+HexNAc(2)Hex(1)_1)
1437.663(PEP+HexNAc(2)Hex(2)_1)
204.089(HexNAc)
247.147(b2)
276.170(y2)
347.209(y3)
366.144(HexNAc+Hex)
432.230(b4)
528.199(Hex+HexNAc+Hex)
657.316(NeuAc+Hex+HexNAc)
690.366
707.392(PEP_1)
790.430
1072.528
881.414(PEP+HexNAc(2)Hex(4)_2)
910.471(PEP+HexNAc(1)_1)
557.282(PEP+HexNAc(2)_2)
638.309(PEP+HexNAc(2)Hex(1)_2)
719.336(PEP+HexNAc(2)Hex(2)_2)
800.364(PEP+HexNAc(2)Hex(3)_2)
1072.527
881.407(PEP+HexNAc(2)Hex(4)_2)
800.363(PEP+HexNAc(2)Hex(3)_2)
910.470(PEP+HexNAc(1)_1)
1113.552(PEP+HexNAc(2)_1)
1275.605(PEP+HexNAc(2)Hex(1)_1)
1437.656(PEP+HexNAc(2)Hex(2)_1)
1499.610
1584.704
1601.728
1646.680
1719.756
1745.748
M+H

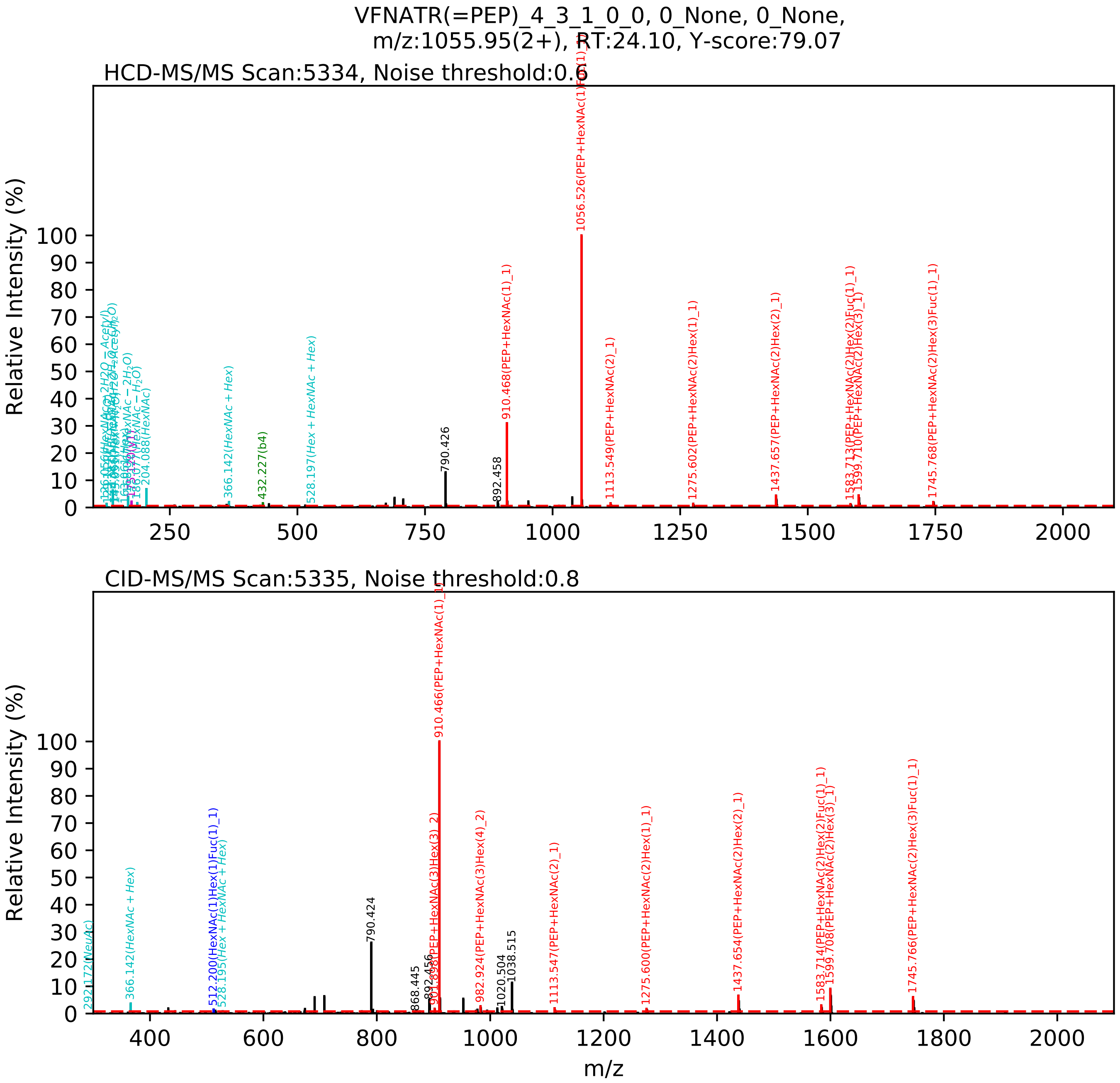
VFNATR(=PEP)_4_3_1_0_0, 0_None, 0_None,
m/z:1055.95(2+), RT:24.10, Y-score:79.07
HCD-MS/MS Scan:5334, Noise threshold:0.6
Relative Intensity (%)
1056.526(PEP+HexNAc(1)Fuc(1)_1)
910.468(PEP+HexNAc(1)_1)
790.426
892.458
1113.549(PEP+HexNAc(2)_1)
1275.602(PEP+HexNAc(2)Hex(1)_1)
1437.657(PEP+HexNAc(2)Hex(2)_1)
1583.713(PEP+HexNAc(2)Hex(2)Fuc(1)_1)
1599.710(PEP+HexNAc(2)Hex(3)_1)
1745.768(PEP+HexNAc(2)Hex(3)Fuc(1)_1)
366.142(HexNAc + Hex)
432.227(b4)
528.197(Hex + HexNAc + Hex)
204.088(HexNAc)
CID-MS/MS Scan:5335, Noise threshold:0.8
Relative Intensity (%)
910.466(PEP+HexNAc(1)_1)
790.424
868.445
892.456
982.924(PEP+HexNAc(3)Hex(4)_2)
1020.504
1038.515
1113.547(PEP+HexNAc(2)_1)
1275.600(PEP+HexNAc(2)Hex(1)_1)
1437.654(PEP+HexNAc(2)Hex(2)_1)
1583.714(PEP+HexNAc(2)Hex(2)Fuc(1)_1)
1599.708(PEP+HexNAc(2)Hex(3)_1)
1745.766(PEP+HexNAc(2)Hex(3)Fuc(1)_1)
366.142(HexNAc + Hex)
512.200(HexNAc(1)Hex(1)Fuc(1)_1)
528.195(Hex + HexNAc + Hex)
m/z

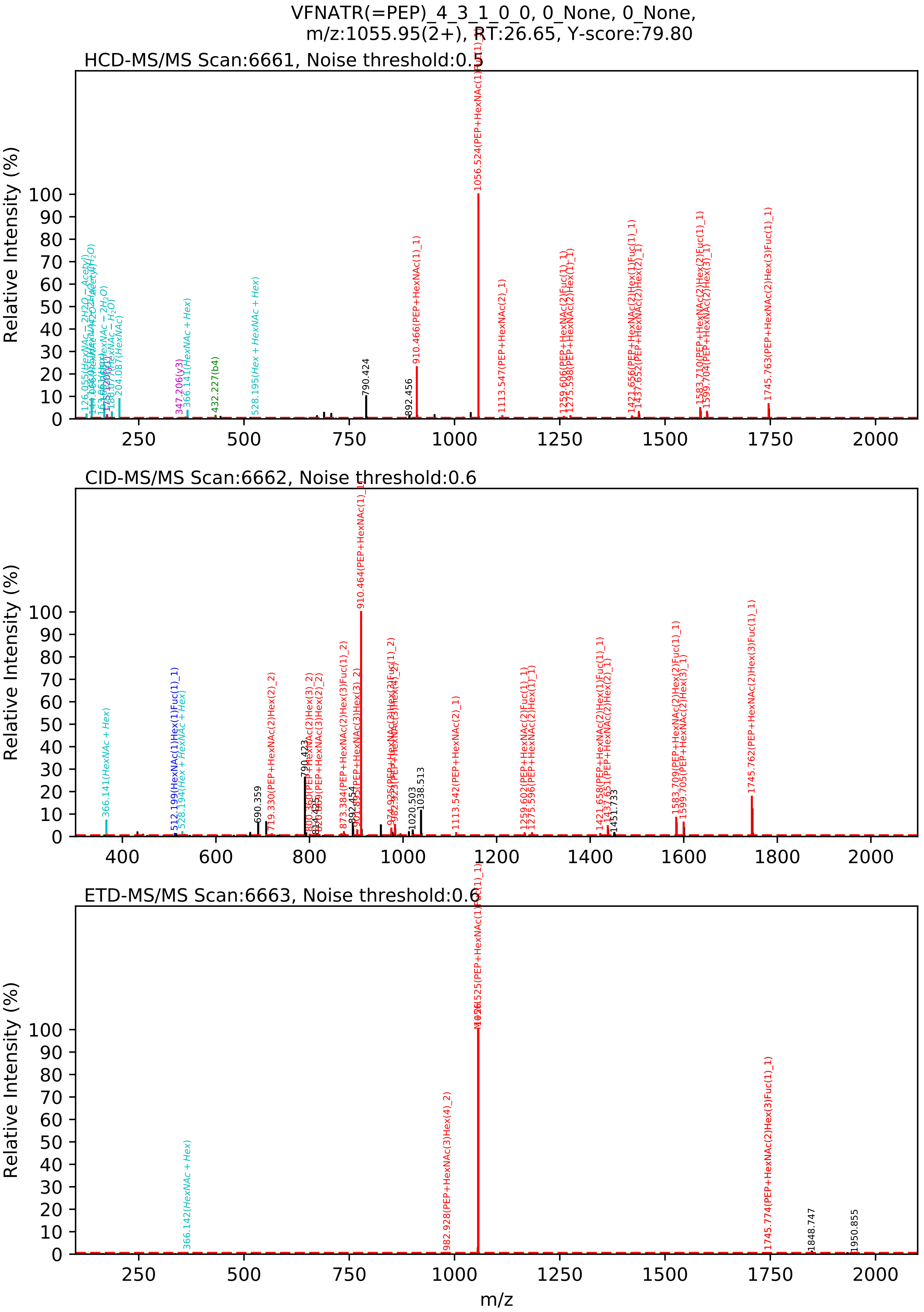
VFNATR(=PEP)_4_3_1_0_0, 0_None, 0_None,
m/z:1055.95(2+), RT:26.65, Y-score:79.80
HCD-MS/MS Scan:6661, Noise threshold:0.5
Relative Intensity (%)
1056.524(PEP+HexNAc(1)Fuc(1)_1)
910.466(PEP+HexNAc(1)_1)
1113.547(PEP+HexNAc(2)_1)
1259.606(PEP+HexNAc(2)Fuc(1)_1)
1275.598(PEP+HexNAc(2)Hex(1)_1)
1421.656(PEP+HexNAc(2)Hex(1)Fuc(1)_1)
1437.652(PEP+HexNAc(2)Hex(2)_1)
1583.710(PEP+HexNAc(2)Hex(2)Fuc(1)_1)
1599.704(PEP+HexNAc(2)Hex(3)_1)
1745.763(PEP+HexNAc(2)Hex(3)Fuc(1)_1)
790.424
892.456
432.227(b4)
347.206(y3)
366.141(HexNAc + Hex)
528.195(Hex + HexNAc + Hex)
204.087(HexNAc)
CID-MS/MS Scan:6662, Noise threshold:0.6
910.464(PEP+HexNAc(1)_1)
366.141(HexNAc + Hex)
512.199(HexNAc(1)Hex(1)Fuc(1)_1)
528.194(Hex + HexNAc + Hex)
690.359
719.330(PEP+HexNAc(2)Hex(2)_2)
790.423
873.384(PEP+HexNAc(2)Hex(3)Fuc(1)_2)
892.454
1020.503
1038.513
1113.542(PEP+HexNAc(2)_1)
1259.602(PEP+HexNAc(2)Fuc(1)_1)
1275.596(PEP+HexNAc(2)Hex(1)_1)
1421.658(PEP+HexNAc(2)Hex(1)Fuc(1)_1)
1451.733
1583.709(PEP+HexNAc(2)Hex(2)Fuc(1)_1)
1599.705(PEP+HexNAc(2)Hex(3)_1)
1745.762(PEP+HexNAc(2)Hex(3)Fuc(1)_1)
ETD-MS/MS Scan:6663, Noise threshold:0.6
366.142(HexNAc + Hex)
982.928(PEP+HexNAc(3)Hex(4)_2)
1745.774(PEP+HexNAc(2)Hex(3)Fuc(1)_1)
1848.747
1950.855
m/z

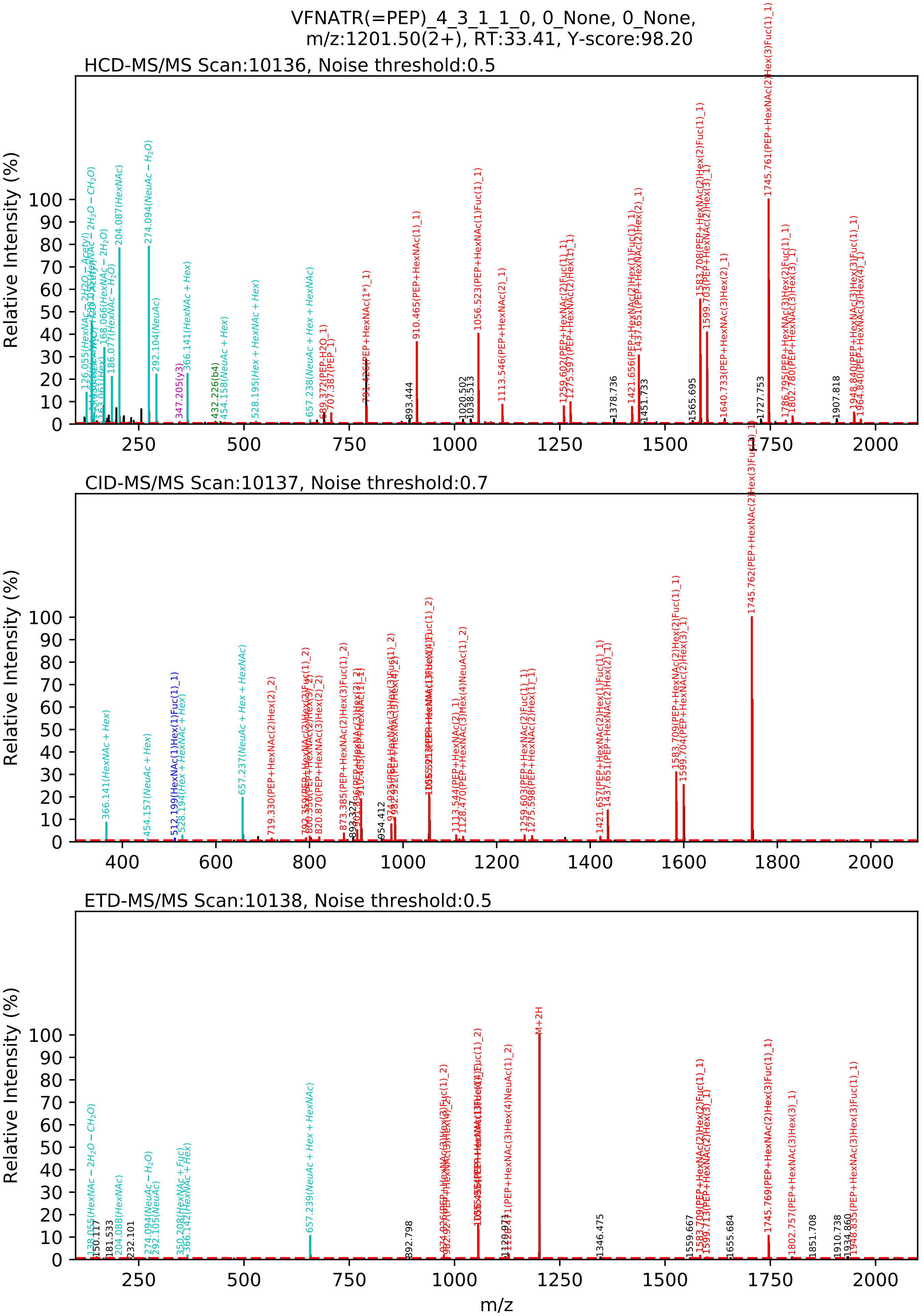
VFNATR(=PEP)_4_3_1_1_0, 0_None, 0_None,
m/z:1201.50(2+), RT:33.41, Y-score:98.20
HCD-MS/MS Scan:10136, Noise threshold:0.5
Relative Intensity (%)
204.087(HexNAc)
274.094(NeuAc − H2O)
292.104(NeuAc)
366.141(HexNAc + Hex)
347.205(y3)
432.226(b4)
454.158(NeuAc + Hex)
528.195(Hex + HexNAc + Hex)
657.238(NeuAc + Hex + HexNAc)
689.372(PEP-H2O_1)
707.387(PEP_1)
893.444
910.465(PEP+HexNAc(1)_1)
1020.502
1038.513
1056.523(PEP+HexNAc(1)Fuc(1)_1)
1113.546(PEP+HexNAc(2)_1)
1259.602(PEP+HexNAc(2)Fuc(1)_1)
1275.597(PEP+HexNAc(2)Hex(1)_1)
1378.736
1421.656(PEP+HexNAc(2)Hex(1)Fuc(1)_1)
1437.651(PEP+HexNAc(2)Hex(2)_1)
1451.733
1565.695
1583.708(PEP+HexNAc(2)Hex(2)Fuc(1)_1)
1599.703(PEP+HexNAc(2)Hex(3)_1)
1640.733(PEP+HexNAc(3)Hex(2)_1)
1727.753
1745.761(PEP+HexNAc(2)Hex(3)Fuc(1)_1)
1786.795(PEP+HexNAc(3)Hex(2)Fuc(1)_1)
1802.780(PEP+HexNAc(3)Hex(3)_1)
1907.818
1948.840(PEP+HexNAc(3)Hex(3)Fuc(1)_1)
1964.840(PEP+HexNAc(3)Hex(4)_1)
CID-MS/MS Scan:10137, Noise threshold:0.7
366.141(HexNAc + Hex)
454.157(NeuAc + Hex)
512.199(HexNAc(1)Hex(1)Fuc(1)_1)
657.237(NeuAc + Hex + HexNAc)
719.330(PEP+HexNAc(2)Hex(2)_2)
820.870(PEP+HexNAc(3)Hex(2)_2)
873.385(PEP+HexNAc(2)Hex(3)Fuc(1)_2)
893.327
954.412
1113.544(PEP+HexNAc(2)_1)
1128.470(PEP+HexNAc(3)Hex(4)NeuAc(1)_2)
1259.603(PEP+HexNAc(2)Fuc(1)_1)
1275.598(PEP+HexNAc(2)Hex(1)_1)
1421.657(PEP+HexNAc(2)Hex(1)Fuc(1)_1)
1437.651(PEP+HexNAc(2)Hex(2)_1)
1583.709(PEP+HexNAc(2)Hex(2)Fuc(1)_1)
1599.704(PEP+HexNAc(2)Hex(3)_1)
1745.762(PEP+HexNAc(2)Hex(3)Fuc(1)_1)
ETD-MS/MS Scan:10138, Noise threshold:0.5
150.117
181.533
204.088(HexNAc)
232.101
274.094(NeuAc − H2O)
292.105(NeuAc)
350.208(HexNAc + Fuc)
366.142(HexNAc + Hex)
657.239(NeuAc + Hex + HexNAc)
892.798
M+2H
1346.475
1559.667
1655.684
1745.769(PEP+HexNAc(2)Hex(3)Fuc(1)_1)
1802.757(PEP+HexNAc(3)Hex(3)_1)
1851.708
1910.738
1934.860
1948.835(PEP+HexNAc(3)Hex(3)Fuc(1)_1)
m/z

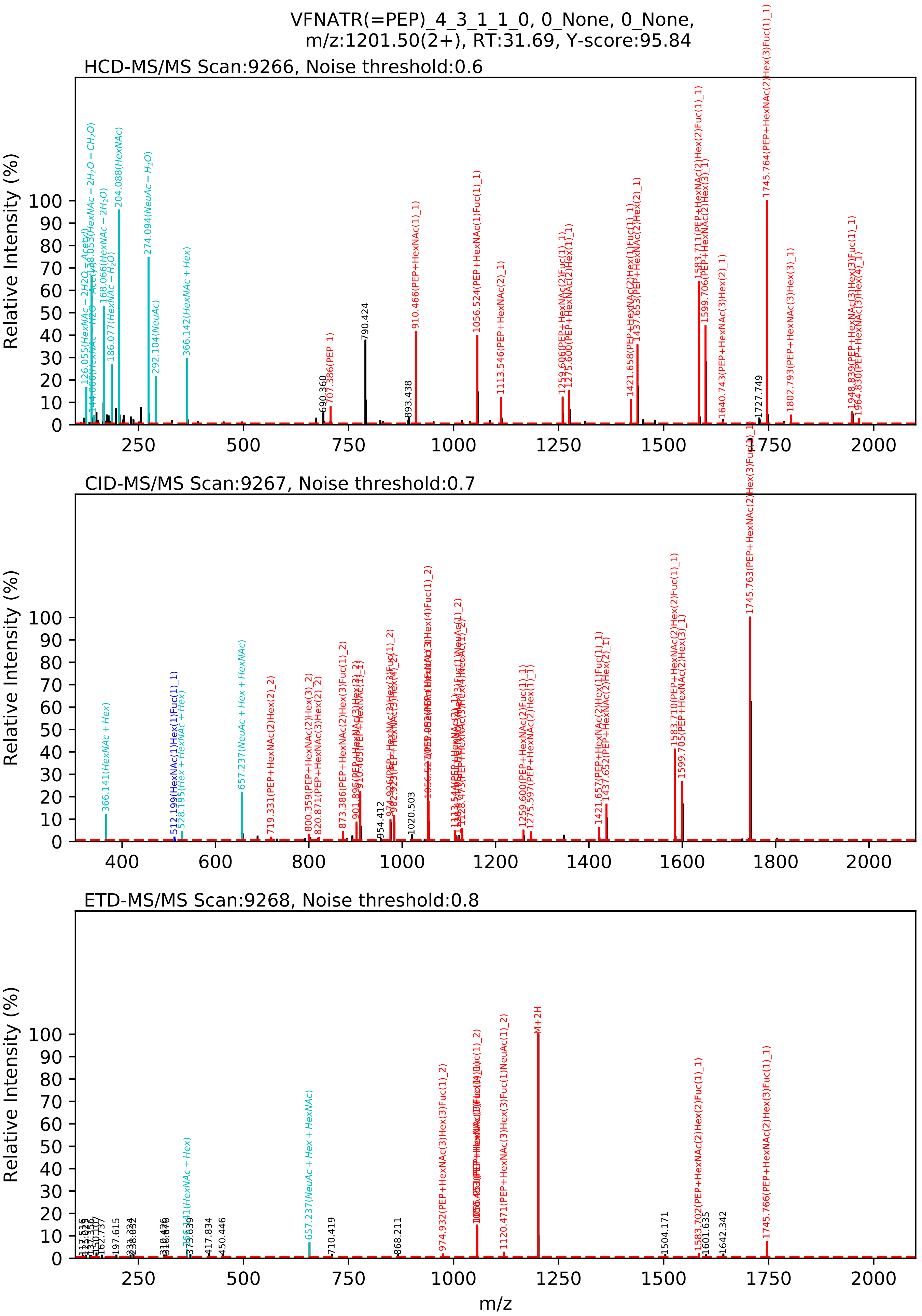

VFNATR(=PEP)_4_3_1_1_0, 0_None, 0_None,
m/z:1201.50(2+), RT:31.69, Y-score:95.84
HCD-MS/MS Scan:9266, Noise threshold:0.6
CID-MS/MS Scan:9267, Noise threshold:0.7
ETD-MS/MS Scan:9268, Noise threshold:0.8
Relative Intensity (%)
m/z
1745.764(PEP+HexNAc(2)Hex(3)Fuc(1)_1)
1583.711(PEP+HexNAc(2)Hex(2)Fuc(1)_1)
1599.706(PEP+HexNAc(2)Hex(3)_1)
910.466(PEP+HexNAc(1)_1)
1056.524(PEP+HexNAc(1)Fuc(1)_1)
790.424
204.088(HexNAc)
274.094(NeuAc − H2O)
366.142(HexNAc + Hex)
1745.763(PEP+HexNAc(2)Hex(3)Fuc(1)_1)
1583.710(PEP+HexNAc(2)Hex(2)Fuc(1)_1)
1599.705(PEP+HexNAc(2)Hex(3)_1)
657.237(NeuAc + Hex + HexNAc)
M+2H
1120.471(PEP+HexNAc(3)Hex(3)Fuc(1)NeuAc(1)_2)

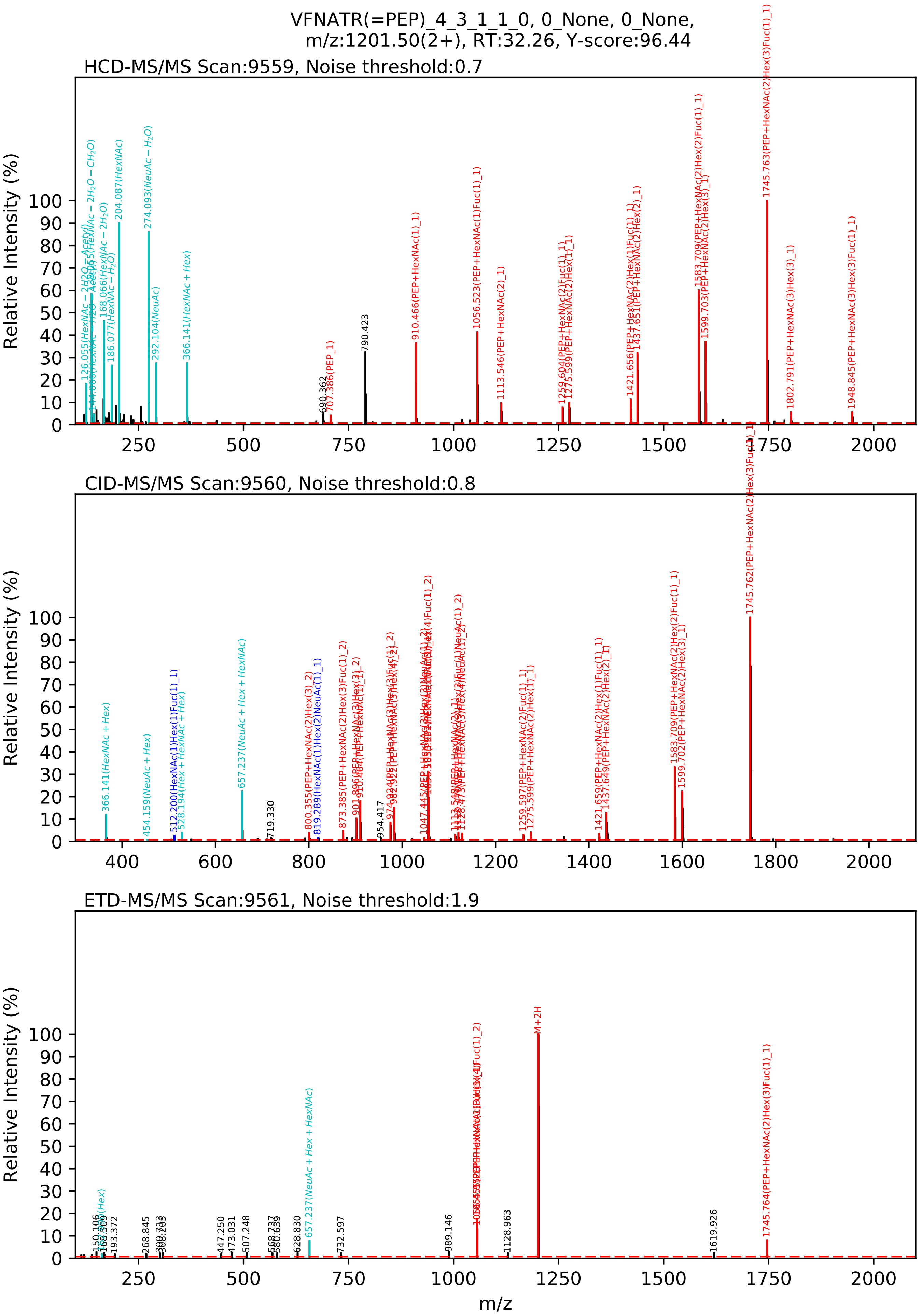
VFNATR(=PEP)_4_3_1_1_0, 0_None, 0_None,
m/z:1201.50(2+), RT:32.26, Y-score:96.44
HCD-MS/MS Scan:9559, Noise threshold:0.7
CID-MS/MS Scan:9560, Noise threshold:0.8
ETD-MS/MS Scan:9561, Noise threshold:1.9
Relative Intensity (%)
m/z
910.466(PEP+HexNAc(1)_1)
1056.523(PEP+HexNAc(1)Fuc(1)_1)
1113.546(PEP+HexNAc(2)_1)
1745.763(PEP+HexNAc(2)Hex(3)Fuc(1)_1)
1802.791(PEP+HexNAc(3)Hex(3)_1)
1948.845(PEP+HexNAc(3)Hex(3)Fuc(1)_1)
1745.762(PEP+HexNAc(2)Hex(3)Fuc(1)_1)
1583.709(PEP+HexNAc(2)Hex(2)Fuc(1)_1)
M+2H
1745.764(PEP+HexNAc(2)Hex(3)Fuc(1)_1)

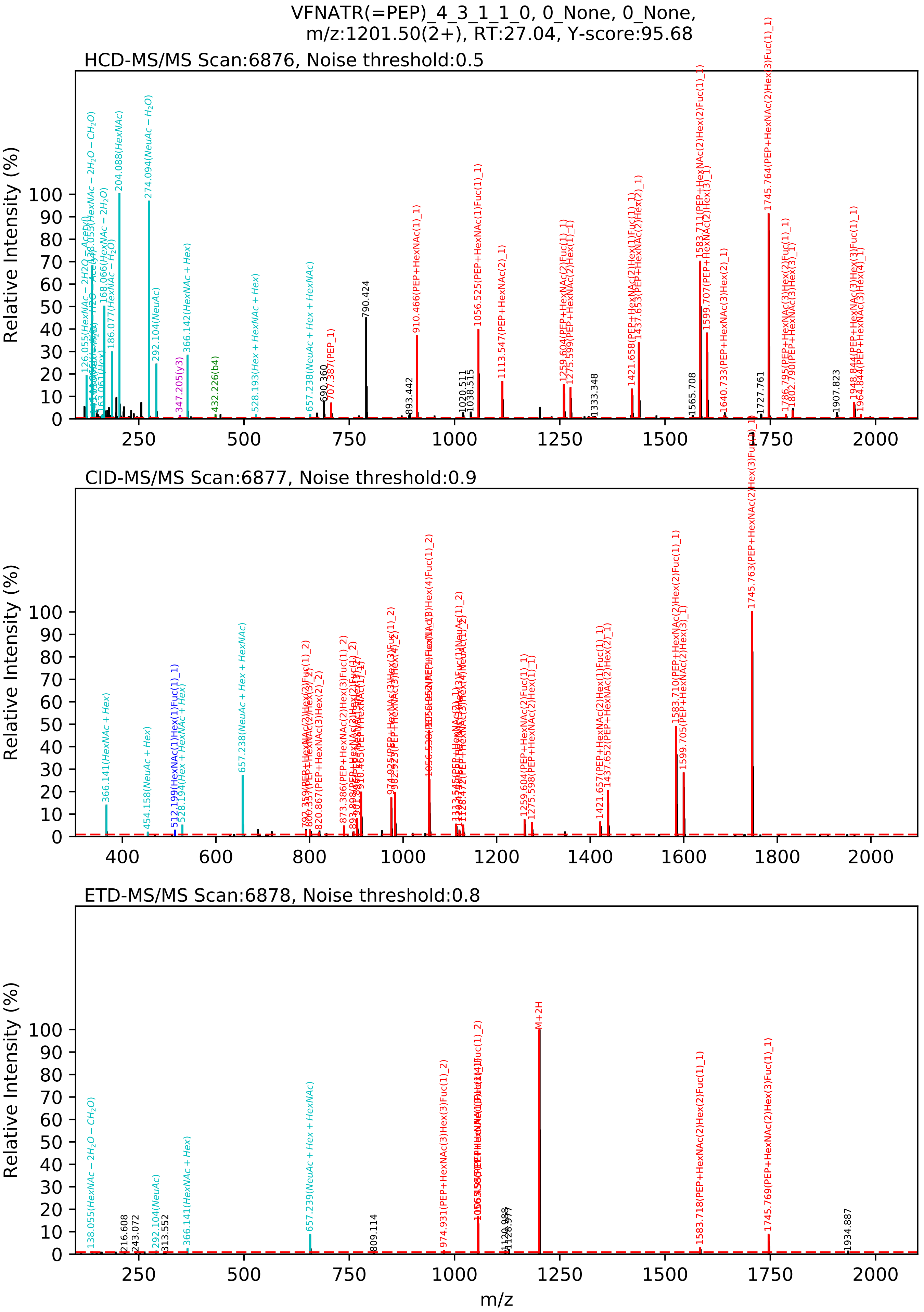
VFNATR(=PEP)_4_3_1_1_0, 0_None, 0_None,
m/z:1201.50(2+), RT:27.04, Y-score:95.68
HCD-MS/MS Scan:6876, Noise threshold:0.5
CID-MS/MS Scan:6877, Noise threshold:0.9
ETD-MS/MS Scan:6878, Noise threshold:0.8
Relative Intensity (%)
m/z
204.088(HexNAc)
274.094(NeuAc − H2O)
292.104(NeuAc)
366.142(HexNAc + Hex)
347.205(y3)
432.226(b4)
528.193(Hex + HexNAc + Hex)
657.238(NeuAc + Hex + HexNAc)
690.360
707.387(PEP_1)
790.424
893.442
910.466(PEP+HexNAc(1)_1)
1020.511
1038.515
1056.525(PEP+HexNAc(1)Fuc(1)_1)
1113.547(PEP+HexNAc(2)_1)
1259.604(PEP+HexNAc(2)Fuc(1)_1)
1275.599(PEP+HexNAc(2)Hex(1)_1)
1333.348
1421.658(PEP+HexNAc(2)Hex(1)Fuc(1)_1)
1437.653(PEP+HexNAc(2)Hex(2)_1)
1565.708
1583.711(PEP+HexNAc(2)Hex(2)Fuc(1)_1)
1599.707(PEP+HexNAc(2)Hex(3)_1)
1640.733(PEP+HexNAc(3)Hex(2)_1)
1727.761
1745.764(PEP+HexNAc(2)Hex(3)Fuc(1)_1)
1786.795(PEP+HexNAc(3)Hex(2)Fuc(1)_1)
1802.790(PEP+HexNAc(3)Hex(3)_1)
1907.823
1948.844(PEP+HexNAc(3)Hex(3)Fuc(1)_1)
1964.844(PEP+HexNAc(3)Hex(4)_1)
366.141(HexNAc + Hex)
454.158(NeuAc + Hex)
512.199(HexNAc(1)Hex(1)Fuc(1)_1)
528.194(Hex + HexNAc + Hex)
657.238(NeuAc + Hex + HexNAc)
820.867(PEP+HexNAc(3)Hex(2)_2)
873.386(PEP+HexNAc(2)Hex(3)Fuc(1)_2)
910.465(PEP+HexNAc(1)_1)
1056.530(PEP+HexNAc(1)Fuc(1)_1)
1259.604(PEP+HexNAc(2)Fuc(1)_1)
1275.598(PEP+HexNAc(2)Hex(1)_1)
1421.657(PEP+HexNAc(2)Hex(1)Fuc(1)_1)
1437.652(PEP+HexNAc(2)Hex(2)_1)
1583.710(PEP+HexNAc(2)Hex(2)Fuc(1)_1)
1599.705(PEP+HexNAc(2)Hex(3)_1)
1745.763(PEP+HexNAc(2)Hex(3)Fuc(1)_1)
138.055(HexNAc − 2H2O − CH2O)
216.608
243.072
292.104(NeuAc)
313.552
366.141(HexNAc + Hex)
657.239(NeuAc + Hex + HexNAc)
809.114
974.931(PEP+HexNAc(3)Hex(3)Fuc(1)_2)
M+2H
1583.718(PEP+HexNAc(2)Hex(2)Fuc(1)_1)
1745.769(PEP+HexNAc(2)Hex(3)Fuc(1)_1)
1934.887

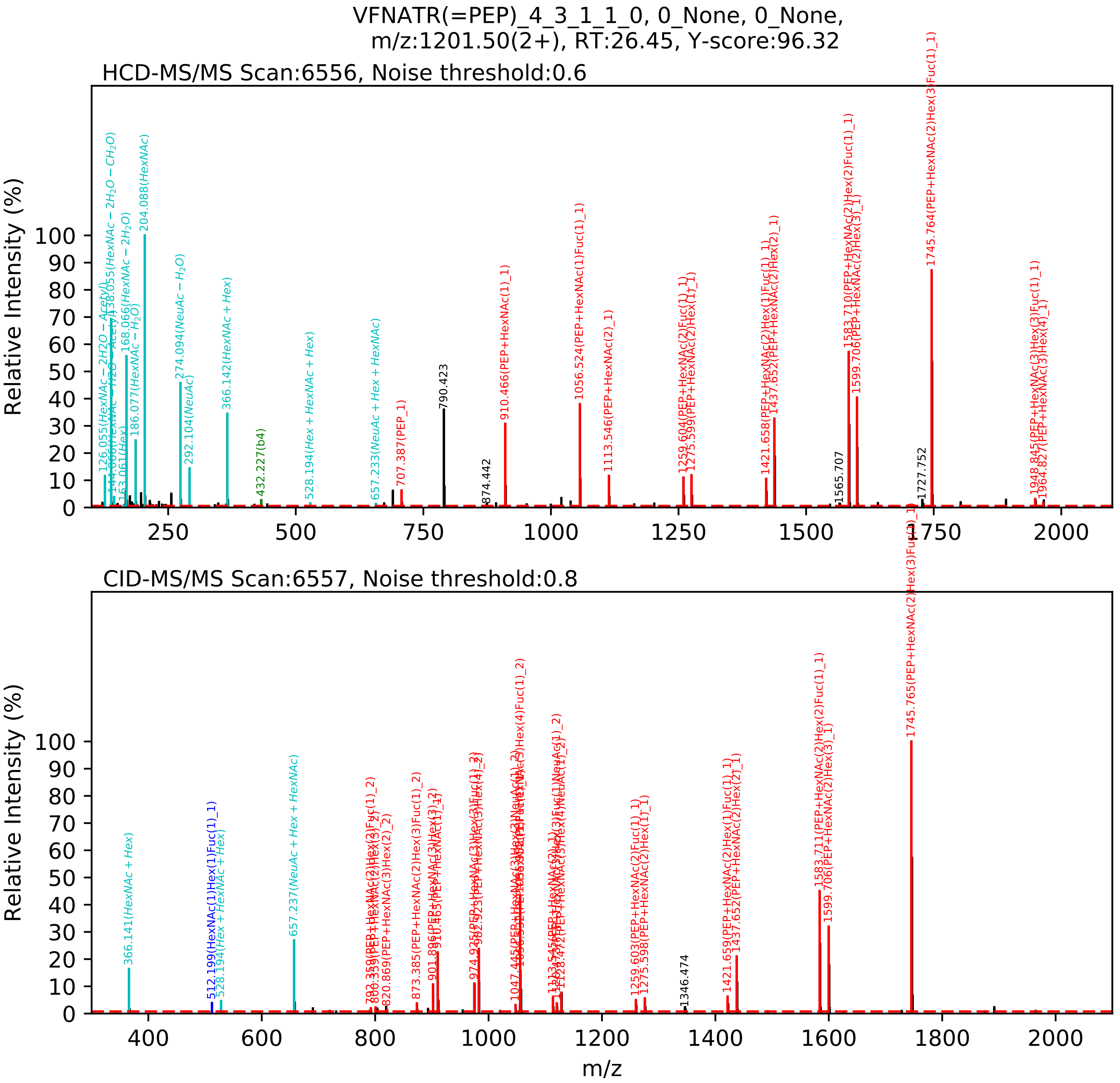

VFNATR(=PEP)_4_3_1_1_0, 0_None, 0_None,
m/z:1201.50(2+), RT:26.45, Y-score:96.32
HCD-MS/MS Scan:6556, Noise threshold:0.6
Relative Intensity (%)
204.088(HexNAc)
274.094(NeuAc − H2O)
292.104(NeuAc)
366.142(HexNAc + Hex)
432.227(b4)
528.194(Hex + HexNAc + Hex)
657.233(NeuAc + Hex + HexNAc)
707.387(PEP_1)
790.423
874.442
910.466(PEP+HexNAc(1)_1)
1056.524(PEP+HexNAc(1)Fuc(1)_1)
1113.546(PEP+HexNAc(2)_1)
1259.604(PEP+HexNAc(2)Fuc(1)_1)
1275.599(PEP+HexNAc(2)Hex(1)_1)
1421.658(PEP+HexNAc(2)Hex(1)Fuc(1)_1)
1437.652(PEP+HexNAc(2)Hex(2)_1)
1565.707
1583.710(PEP+HexNAc(2)Hex(2)Fuc(1)_1)
1599.706(PEP+HexNAc(2)Hex(3)_1)
1727.752
1745.764(PEP+HexNAc(2)Hex(3)Fuc(1)_1)
1948.845(PEP+HexNAc(3)Hex(3)Fuc(1)_1)
1964.827(PEP+HexNAc(3)Hex(4)_1)
CID-MS/MS Scan:6557, Noise threshold:0.8
366.141(HexNAc + Hex)
512.199(HexNAc(1)Hex(1)Fuc(1)_1)
528.194(Hex + HexNAc + Hex)
657.237(NeuAc + Hex + HexNAc)
820.869(PEP+HexNAc(3)Hex(2)_2)
873.385(PEP+HexNAc(2)Hex(3)Fuc(1)_2)
910.465(PEP+HexNAc(1)_1)
1346.474
1259.603(PEP+HexNAc(2)Fuc(1)_1)
1275.598(PEP+HexNAc(2)Hex(1)_1)
1421.659(PEP+HexNAc(2)Hex(1)Fuc(1)_1)
1437.652(PEP+HexNAc(2)Hex(2)_1)
1583.711(PEP+HexNAc(2)Hex(2)Fuc(1)_1)
1599.706(PEP+HexNAc(2)Hex(3)_1)
1745.765(PEP+HexNAc(2)Hex(3)Fuc(1)_1)
m/z

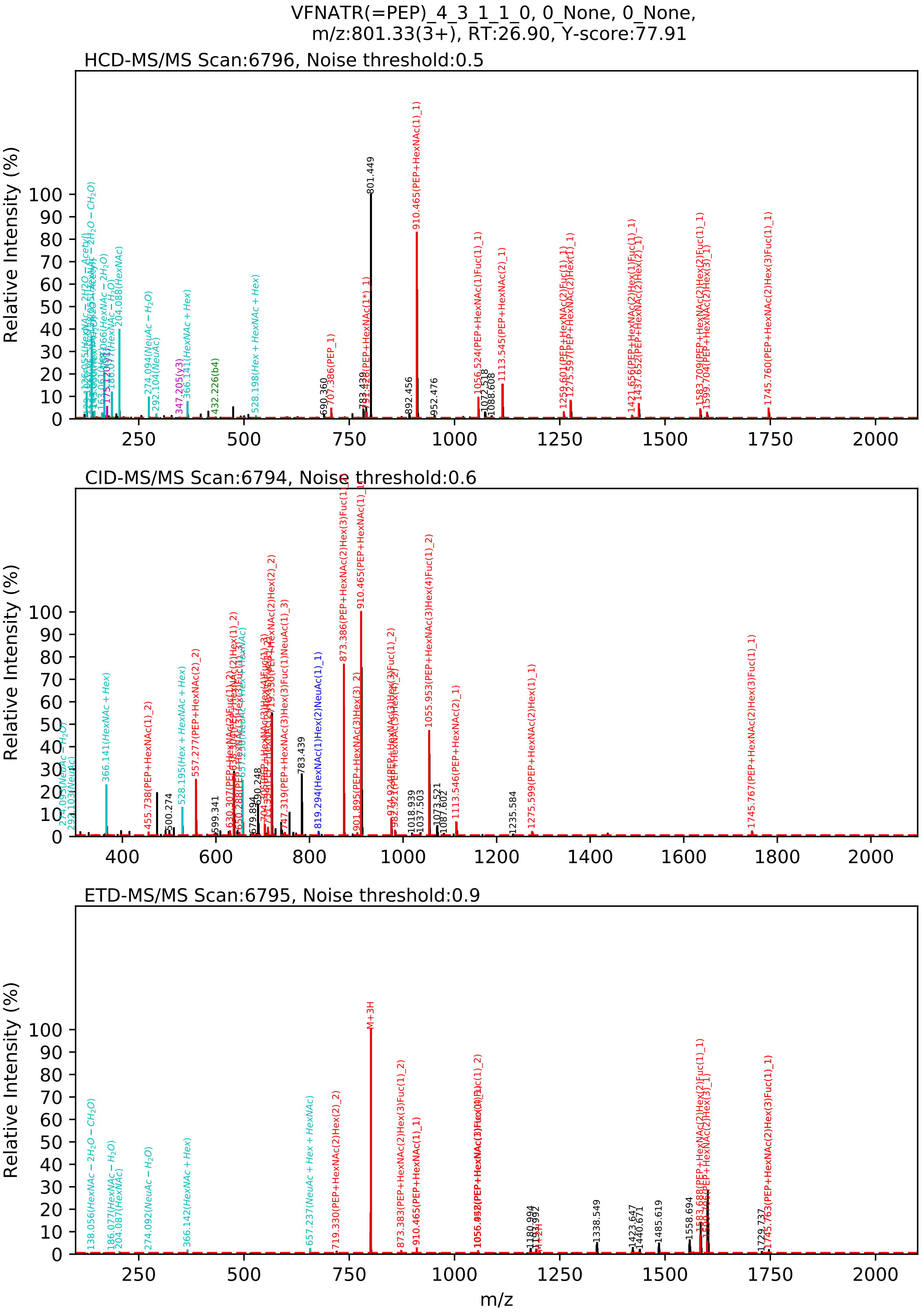

VFNATR(=PEP)_4_3_1_1_0, 0_None, 0_None,
m/z:801.33(3+), RT:26.90, Y-score:77.91
HCD-MS/MS Scan:6796, Noise threshold:0.5
Relative Intensity (%)
801.449
910.465(PEP+HexNAc(1)_1)
204.088(HexNAc)
274.094(NeuAc − H2O)
292.104(NeuAc)
347.205(y3)
366.141(HexNAc + Hex)
432.226(b4)
528.198(Hex + HexNAc + Hex)
690.360
707.386(PEP_1)
783.439
892.456
952.476
1056.524(PEP+HexNAc(1)Fuc(1)_1)
1113.545(PEP+HexNAc(2)_1)
1259.601(PEP+HexNAc(2)Fuc(1)_1)
1275.597(PEP+HexNAc(2)Hex(1)_1)
1421.656(PEP+HexNAc(2)Hex(1)Fuc(1)_1)
1437.652(PEP+HexNAc(2)Hex(2)_1)
1583.709(PEP+HexNAc(2)Hex(2)Fuc(1)_1)
1599.704(PEP+HexNAc(2)Hex(3)_1)
1745.760(PEP+HexNAc(2)Hex(3)Fuc(1)_1)
CID-MS/MS Scan:6794, Noise threshold:0.6
366.141(HexNAc + Hex)
455.738(PEP+HexNAc(1)_2)
500.274
528.195(Hex + HexNAc + Hex)
557.277(PEP+HexNAc(2)_2)
599.341
783.439
819.294(HexNAc(1)Hex(2)NeuAc(1)_1)
873.386(PEP+HexNAc(2)Hex(3)Fuc(1)_2)
910.465(PEP+HexNAc(1)_1)
901.895(PEP+HexNAc(3)Hex(3)_2)
1018.939
1037.503
1055.953(PEP+HexNAc(3)Hex(4)Fuc(1)_2)
1073.521
1087.602
1113.546(PEP+HexNAc(2)_1)
1235.584
1275.599(PEP+HexNAc(2)Hex(1)_1)
1745.767(PEP+HexNAc(2)Hex(3)Fuc(1)_1)
ETD-MS/MS Scan:6795, Noise threshold:0.9
M+3H
138.056(HexNAc − 2H2O − CH2O)
186.077(HexNAc − H2O)
204.087(HexNAc)
274.092(NeuAc − H2O)
366.142(HexNAc + Hex)
657.237(NeuAc + Hex + HexNAc)
719.330(PEP+HexNAc(2)Hex(2)_2)
873.383(PEP+HexNAc(2)Hex(3)Fuc(1)_2)
910.465(PEP+HexNAc(1)_1)
1180.994
1338.549
1423.647
1440.671
1485.619
1558.694
1729.737
1745.763(PEP+HexNAc(2)Hex(3)Fuc(1)_1)
m/z

VFNATR(=PEP)_4_3_2_0_0, 0_None, 0_None,
m/z:1128.98(2+), RT:23.96, Y-score:92.40

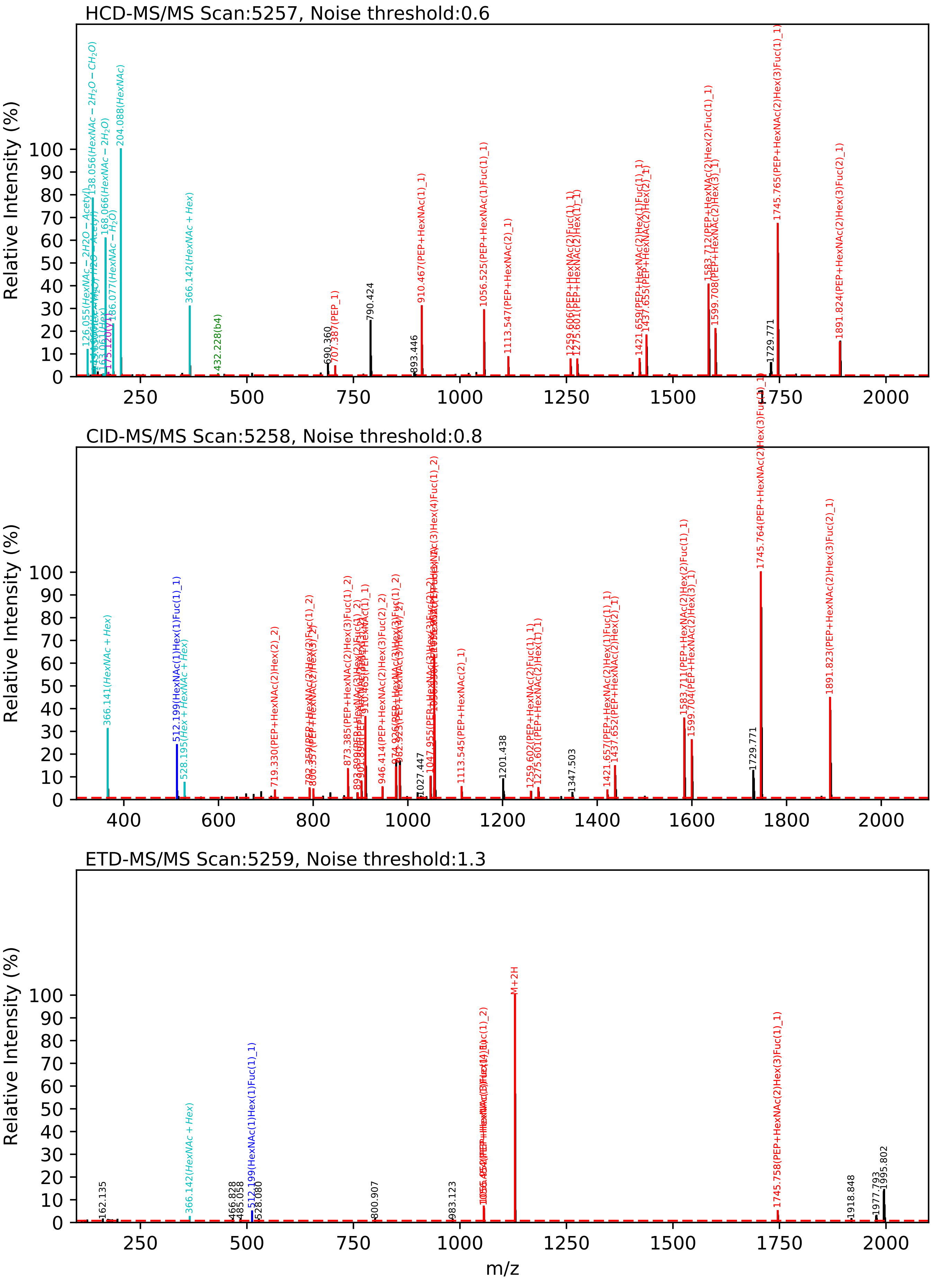

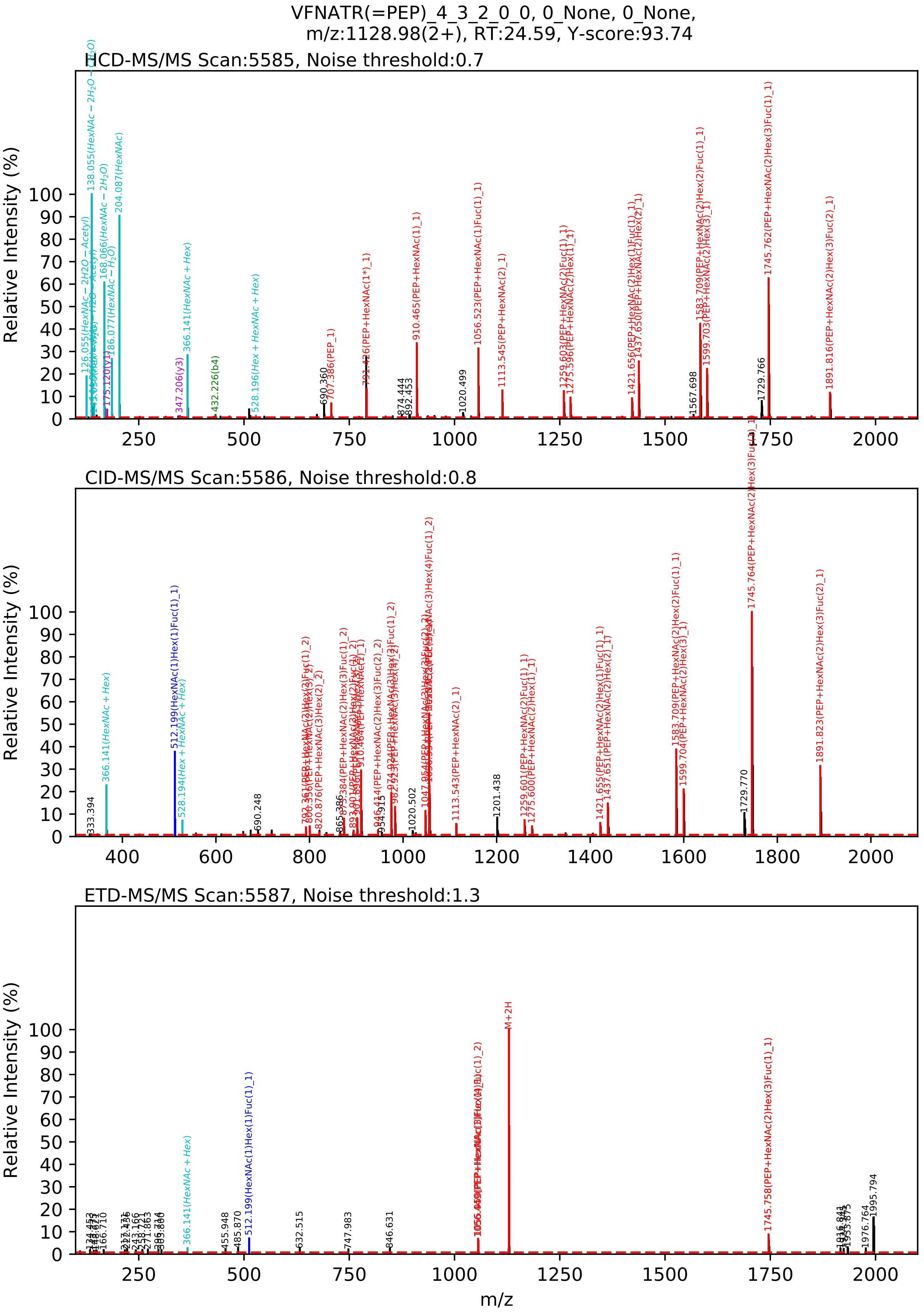
VFNATR(=PEP)_4_3_2_0_0, 0_None, 0_None,
m/z:1128.98(2+), RT:24.59, Y-score:93.74
HCD-MS/MS Scan:5585, Noise threshold:0.7
CID-MS/MS Scan:5586, Noise threshold:0.8
ETD-MS/MS Scan:5587, Noise threshold:1.3
Relative Intensity (%)
m/z
138.055(HexNAc − 2H2O − H2O)
204.087(HexNAc)
168.066(HexNAc − 2H2O)
186.077(HexNAc − H2O)
366.141(HexNAc + Hex)
528.196(Hex + HexNAc + Hex)
347.206(y3)
432.226(b4)
175.120(y1)
707.386(PEP_1)
791.426(PEP+HexNAc(1*)_1)
910.465(PEP+HexNAc(1)_1)
1056.523(PEP+HexNAc(1)Fuc(1)_1)
1113.545(PEP+HexNAc(2)_1)
1259.603(PEP+HexNAc(2)Fuc(1)_1)
1275.596(PEP+HexNAc(2)Hex(1)_1)
1421.656(PEP+HexNAc(2)Hex(1)Fuc(1)_1)
1437.650(PEP+HexNAc(2)Hex(2)_1)
1583.709(PEP+HexNAc(2)Hex(2)Fuc(1)_1)
1599.703(PEP+HexNAc(2)Hex(3)_1)
1745.762(PEP+HexNAc(2)Hex(3)Fuc(1)_1)
1891.816(PEP+HexNAc(2)Hex(3)Fuc(2)_1)
690.360
874.444
892.453
1020.499
1567.698
1729.766
512.199(HexNAc(1)Hex(1)Fuc(1)_1)
528.194(Hex + HexNAc + Hex)
333.394
690.248
865.386
934.915
1020.502
1201.438
1113.543(PEP+HexNAc(2)_1)
1259.601(PEP+HexNAc(2)Fuc(1)_1)
1275.600(PEP+HexNAc(2)Hex(1)_1)
1421.655(PEP+HexNAc(2)Hex(1)Fuc(1)_1)
1437.651(PEP+HexNAc(2)Hex(2)_1)
1583.709(PEP+HexNAc(2)Hex(2)Fuc(1)_1)
1599.704(PEP+HexNAc(2)Hex(3)_1)
1745.764(PEP+HexNAc(2)Hex(3)Fuc(1)_1)
1891.823(PEP+HexNAc(2)Hex(3)Fuc(2)_1)
1729.770
M+2H
1745.758(PEP+HexNAc(2)Hex(3)Fuc(1)_1)
512.199(HexNAc(1)Hex(1)Fuc(1)_1)
455.948
485.870
632.515
747.983
846.631
1976.764
1995.794

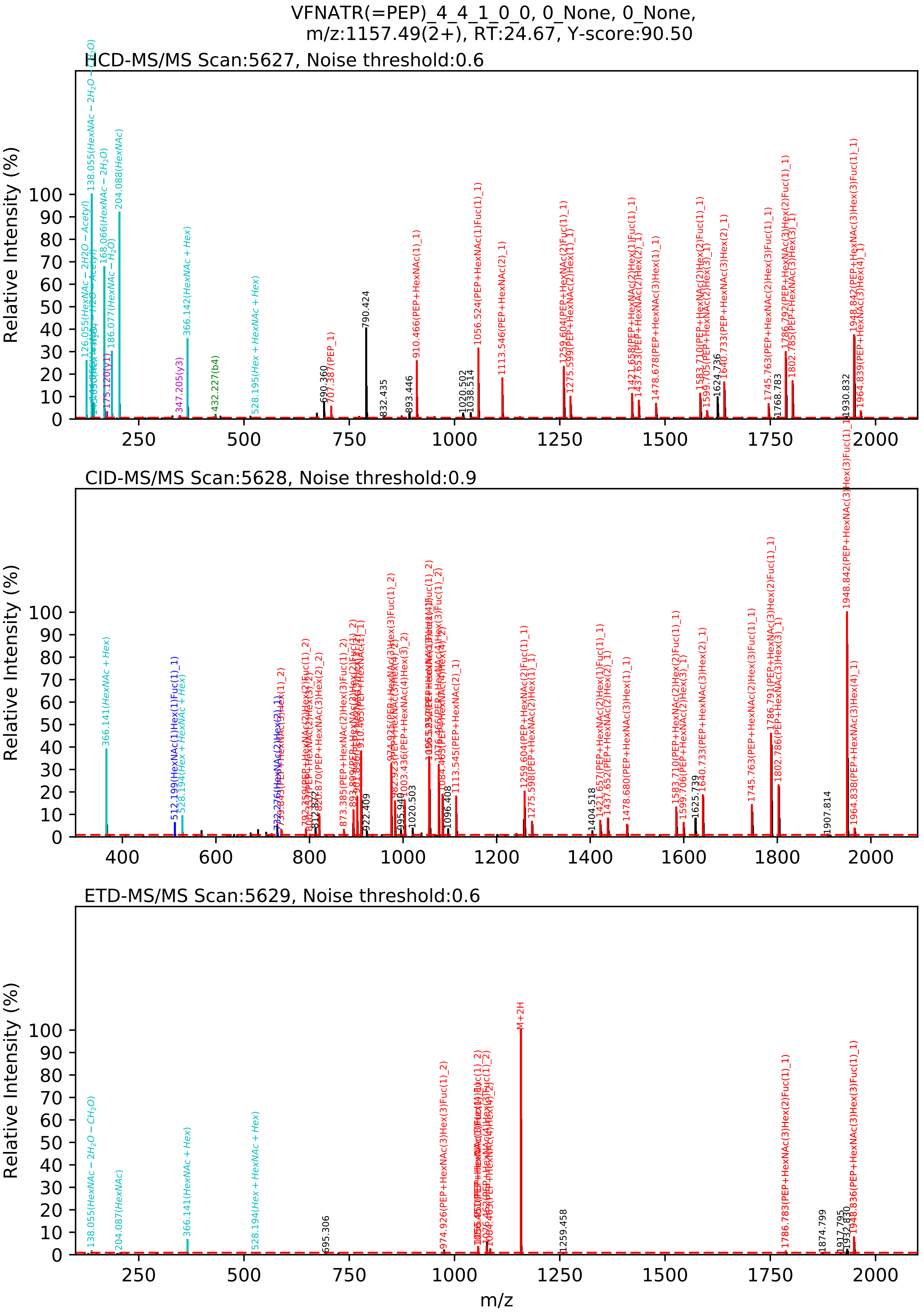
VFNATR(=PEP)_4_4_1_0_0, 0_None, 0_None,
m/z:1157.49(2+), RT:24.67, Y-score:90.50
HCD-MS/MS Scan:5627, Noise threshold:0.6
Relative Intensity (%)
138.055(HexNAc − 2H2O − H2O)
204.088(HexNAc)
168.066(HexNAc − 2H2O)
186.077(HexNAc − H2O)
126.055(HexNAc − 2H2O − Acetyl)
175.120(y1)
366.142(HexNAc + Hex)
347.205(y3)
432.227(b4)
528.195(Hex + HexNAc + Hex)
690.360
707.387(PEP_1)
790.424
832.435
893.446
910.466(PEP+HexNAc(1)_1)
1020.502
1038.514
1056.524(PEP+HexNAc(1)Fuc(1)_1)
1113.546(PEP+HexNAc(2)_1)
1259.604(PEP+HexNAc(2)Fuc(1)_1)
1275.599(PEP+HexNAc(2)Hex(1)_1)
1421.658(PEP+HexNAc(2)Hex(1)Fuc(1)_1)
1437.653(PEP+HexNAc(2)Hex(2)_1)
1478.678(PEP+HexNAc(3)Hex(1)_1)
1583.710(PEP+HexNAc(2)Hex(2)Fuc(1)_1)
1599.705(PEP+HexNAc(2)Hex(3)_1)
1624.736
1640.733(PEP+HexNAc(3)Hex(2)_1)
1745.763(PEP+HexNAc(2)Hex(3)Fuc(1)_1)
1768.783
1786.792(PEP+HexNAc(3)Hex(2)Fuc(1)_1)
1802.785(PEP+HexNAc(3)Hex(3)_1)
1930.832
1948.842(PEP+HexNAc(3)Hex(3)Fuc(1)_1)
1964.839(PEP+HexNAc(3)Hex(4)_1)
CID-MS/MS Scan:5628, Noise threshold:0.9
Relative Intensity (%)
366.141(HexNAc + Hex)
512.199(HexNAc(1)Hex(1)Fuc(1)_1)
528.194(Hex + HexNAc + Hex)
732.276(HexNAc(2)Hex(2)_1)
792.359(PEP+HexNAc(2)Hex(2)Fuc(1)_2)
827.870(PEP+HexNAc(3)Hex(2)_2)
873.385(PEP+HexNAc(2)Hex(3)Fuc(1)_2)
910.465(PEP+HexNAc(1)_1)
922.409
974.925(PEP+HexNAc(3)Hex(3)Fuc(1)_2)
982.923(PEP+HexNAc(3)Hex(4)_2)
995.940
1003.436(PEP+HexNAc(4)Hex(3)_2)
1020.503
1084.463(PEP+HexNAc(4)Hex(3)Fuc(1)_2)
1096.408
1113.545(PEP+HexNAc(2)_1)
1259.604(PEP+HexNAc(2)Fuc(1)_1)
1275.598(PEP+HexNAc(2)Hex(1)_1)
1404.518
1421.657(PEP+HexNAc(2)Hex(1)Fuc(1)_1)
1437.652(PEP+HexNAc(2)Hex(2)_1)
1478.680(PEP+HexNAc(3)Hex(1)_1)
1583.710(PEP+HexNAc(2)Hex(2)Fuc(1)_1)
1599.706(PEP+HexNAc(2)Hex(3)_1)
1625.739
1640.733(PEP+HexNAc(3)Hex(2)_1)
1745.763(PEP+HexNAc(2)Hex(3)Fuc(1)_1)
1786.791(PEP+HexNAc(3)Hex(2)Fuc(1)_1)
1802.786(PEP+HexNAc(3)Hex(3)_1)
1907.814
1948.842(PEP+HexNAc(3)Hex(3)Fuc(1)_1)
1964.838(PEP+HexNAc(3)Hex(4)_1)
ETD-MS/MS Scan:5629, Noise threshold:0.6
Relative Intensity (%)
138.055(HexNAc − 2H2O − CH2O)
204.087(HexNAc)
366.141(HexNAc + Hex)
528.194(Hex + HexNAc + Hex)
695.306
974.926(PEP+HexNAc(3)Hex(3)Fuc(1)_2)
M+2H
1259.458
1786.783(PEP+HexNAc(3)Hex(2)Fuc(1)_1)
1874.799
1917.795
1932.830
1948.836(PEP+HexNAc(3)Hex(3)Fuc(1)_1)
m/z

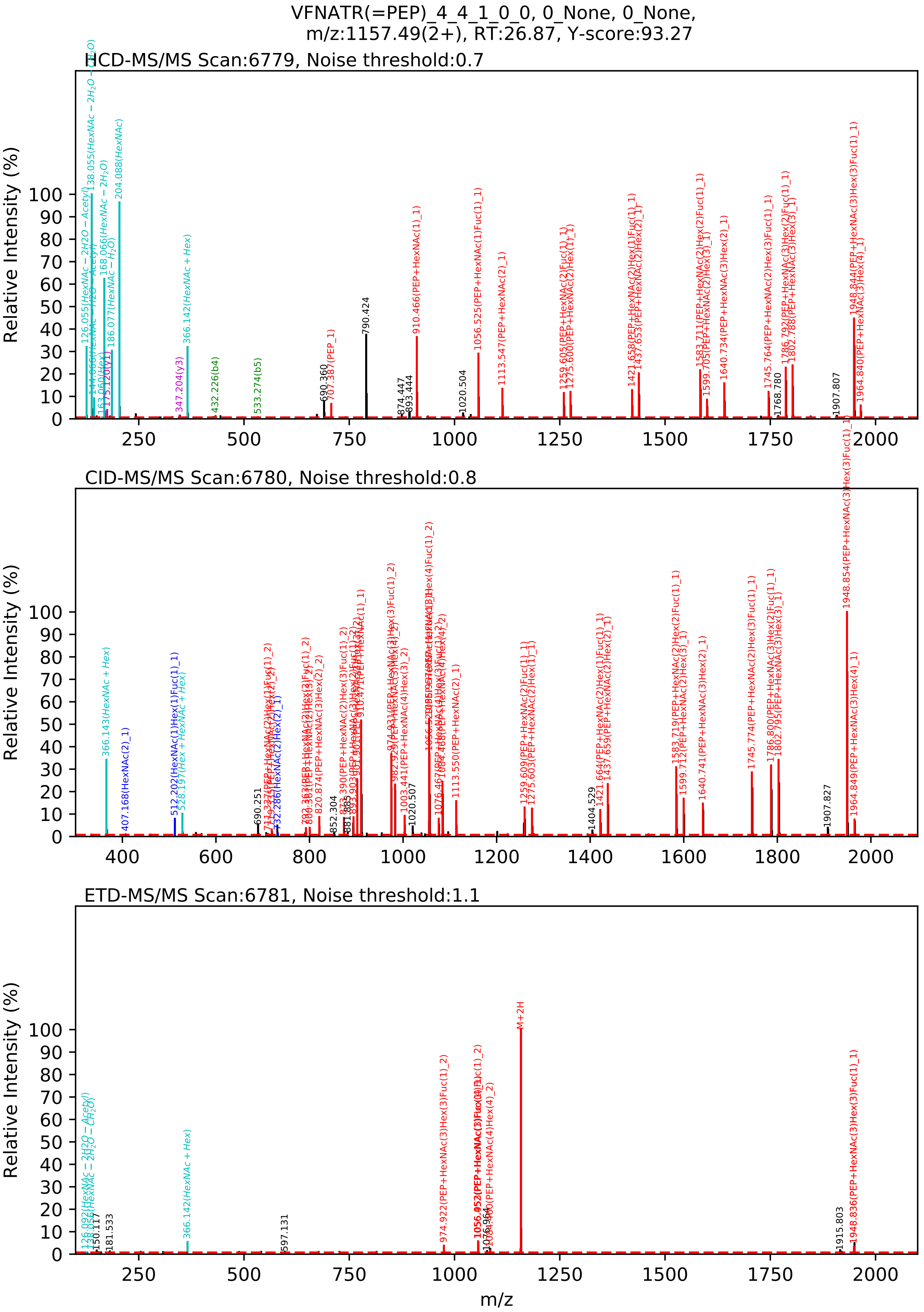

VFNATR(=PEP)_4_4_1_0_0, 0_None, 0_None,
m/z:1157.49(2+), RT:26.87, Y-score:93.27
HCD-MS/MS Scan:6779, Noise threshold:0.7
CID-MS/MS Scan:6780, Noise threshold:0.8
ETD-MS/MS Scan:6781, Noise threshold:1.1
Relative Intensity (%)
m/z
910.466(PEP+HexNAc(1)_1)
1056.525(PEP+HexNAc(1)Fuc(1)_1)
1113.547(PEP+HexNAc(2)_1)
1948.844(PEP+HexNAc(3)Hex(3)Fuc(1)_1)
1948.854(PEP+HexNAc(3)Hex(3)Fuc(1)_1)
M+2H

VFNATR(=PEP)_4_4_1_0_0, 0_None, 0_None,
m/z:1157.49(2+), RT:26.90, Y-score:93.37

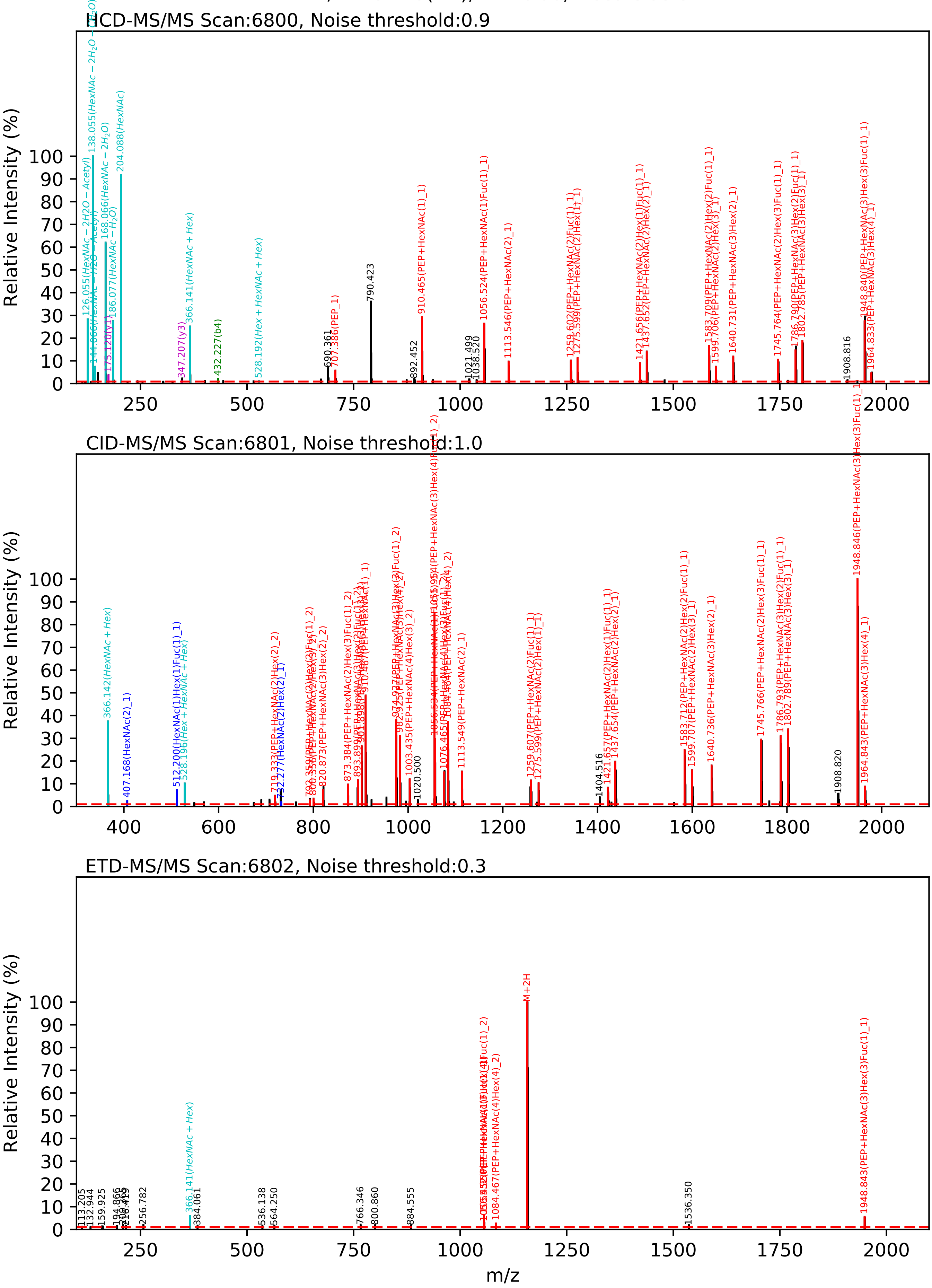

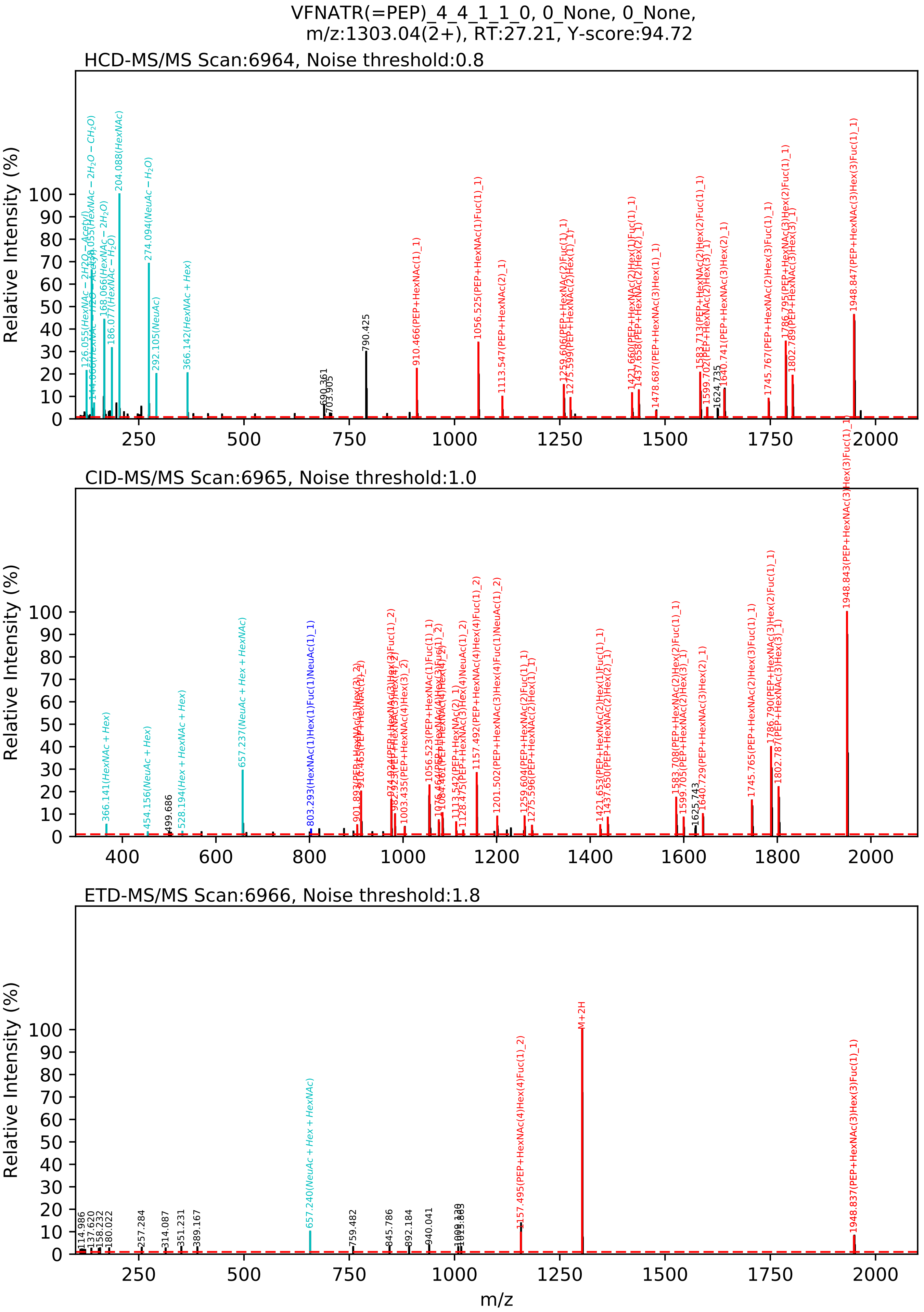
VFNATR(=PEP)_4_4_1_1_0, 0_None, 0_None,
m/z:1303.04(2+), RT:27.21, Y-score:94.72
HCD-MS/MS Scan:6964, Noise threshold:0.8
Relative Intensity (%)
126.055(HexNAc − 2H2O − Acetyl)
144.066(HexNAc − H2O − Acetyl)
168.066(HexNAc − 2H2O)
186.077(HexNAc − H2O)
204.088(HexNAc)
274.094(NeuAc − H2O)
292.105(NeuAc)
366.142(HexNAc + Hex)
690.361
703.905
790.425
910.466(PEP+HexNAc(1)_1)
1056.525(PEP+HexNAc(1)Fuc(1)_1)
1113.547(PEP+HexNAc(2)_1)
1259.606(PEP+HexNAc(2)Fuc(1)_1)
1275.599(PEP+HexNAc(2)Hex(1)_1)
1421.660(PEP+HexNAc(2)Hex(1)Fuc(1)_1)
1437.658(PEP+HexNAc(2)Hex(2)_1)
1478.687(PEP+HexNAc(3)Hex(1)_1)
1583.713(PEP+HexNAc(2)Hex(2)Fuc(1)_1)
1599.702(PEP+HexNAc(2)Hex(3)_1)
1624.735
1640.741(PEP+HexNAc(3)Hex(2)_1)
1745.767(PEP+HexNAc(2)Hex(3)Fuc(1)_1)
1786.795(PEP+HexNAc(3)Hex(2)Fuc(1)_1)
1802.789(PEP+HexNAc(3)Hex(3)_1)
1948.847(PEP+HexNAc(3)Hex(3)Fuc(1)_1)
CID-MS/MS Scan:6965, Noise threshold:1.0
Relative Intensity (%)
366.141(HexNAc + Hex)
454.156(NeuAc + Hex)
499.686
528.194(Hex + HexNAc + Hex)
657.237(NeuAc + Hex + HexNAc)
803.293(HexNAc(1)Hex(1)Fuc(1)NeuAc(1)_1)
910.465(PEP+HexNAc(1)_1)
1003.435(PEP+HexNAc(4)Hex(3)_2)
1056.523(PEP+HexNAc(1)Fuc(1)_1)
1113.542(PEP+HexNAc(2)_1)
1157.492(PEP+HexNAc(4)Hex(4)Fuc(1)_2)
1201.502(PEP+HexNAc(3)Hex(4)Fuc(1)NeuAc(1)_2)
1259.604(PEP+HexNAc(2)Fuc(1)_1)
1275.596(PEP+HexNAc(2)Hex(1)_1)
1421.653(PEP+HexNAc(2)Hex(1)Fuc(1)_1)
1437.650(PEP+HexNAc(2)Hex(2)_1)
1583.708(PEP+HexNAc(2)Hex(2)Fuc(1)_1)
1599.705(PEP+HexNAc(2)Hex(3)_1)
1625.743
1640.729(PEP+HexNAc(3)Hex(2)_1)
1745.765(PEP+HexNAc(2)Hex(3)Fuc(1)_1)
1786.790(PEP+HexNAc(3)Hex(2)Fuc(1)_1)
1802.787(PEP+HexNAc(3)Hex(3)_1)
1948.843(PEP+HexNAc(3)Hex(3)Fuc(1)_1)
ETD-MS/MS Scan:6966, Noise threshold:1.8
Relative Intensity (%)
114.986
137.620
158.232
180.022
257.284
314.087
351.231
389.167
657.240(NeuAc + Hex + HexNAc)
759.482
845.786
892.184
940.041
1157.495(PEP+HexNAc(4)Hex(4)Fuc(1)_2)
M+2H
1948.837(PEP+HexNAc(3)Hex(3)Fuc(1)_1)
m/z

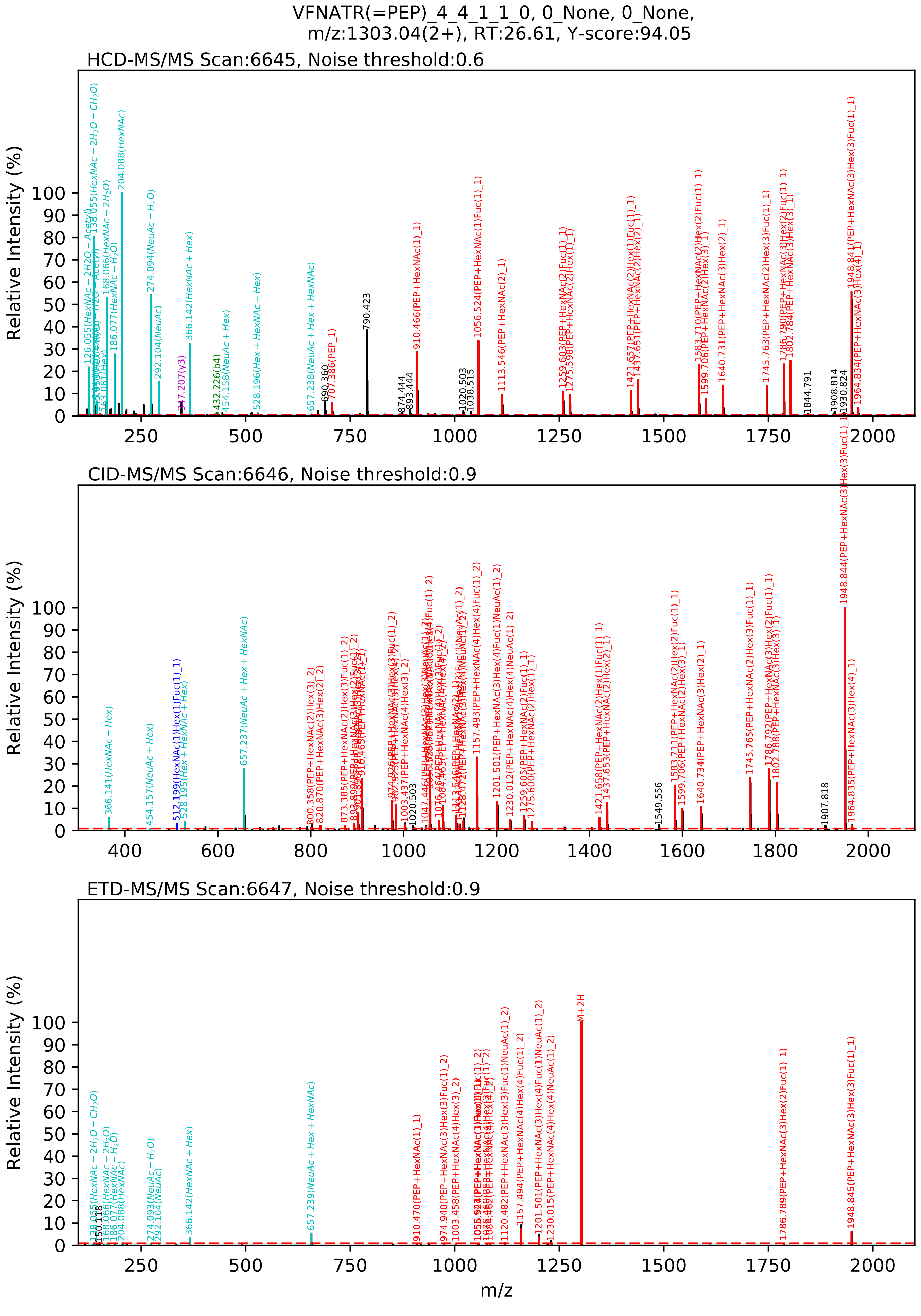
VFNATR(=PEP)_4_4_1_1_0, 0_None, 0_None,
m/z:1303.04(2+), RT:26.61, Y-score:94.05
HCD-MS/MS Scan:6645, Noise threshold:0.6
CID-MS/MS Scan:6646, Noise threshold:0.9
ETD-MS/MS Scan:6647, Noise threshold:0.9
Relative Intensity (%)
m/z
M+2H

VFNATR(=PEP)_4_4_1_1_0, 0_None, 0_None,
m/z:869.03(3+), RT:26.78, Y-score:93.13

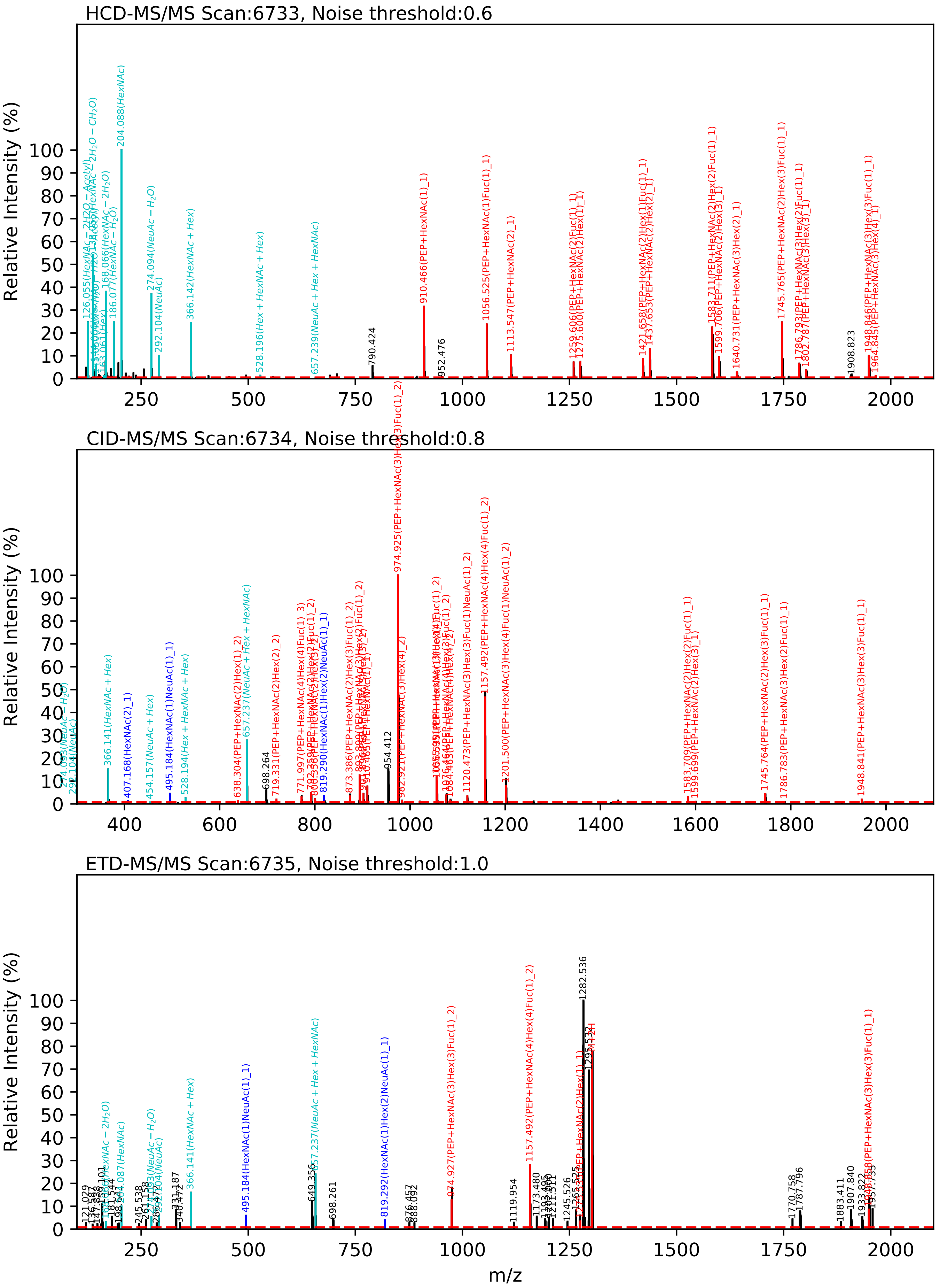

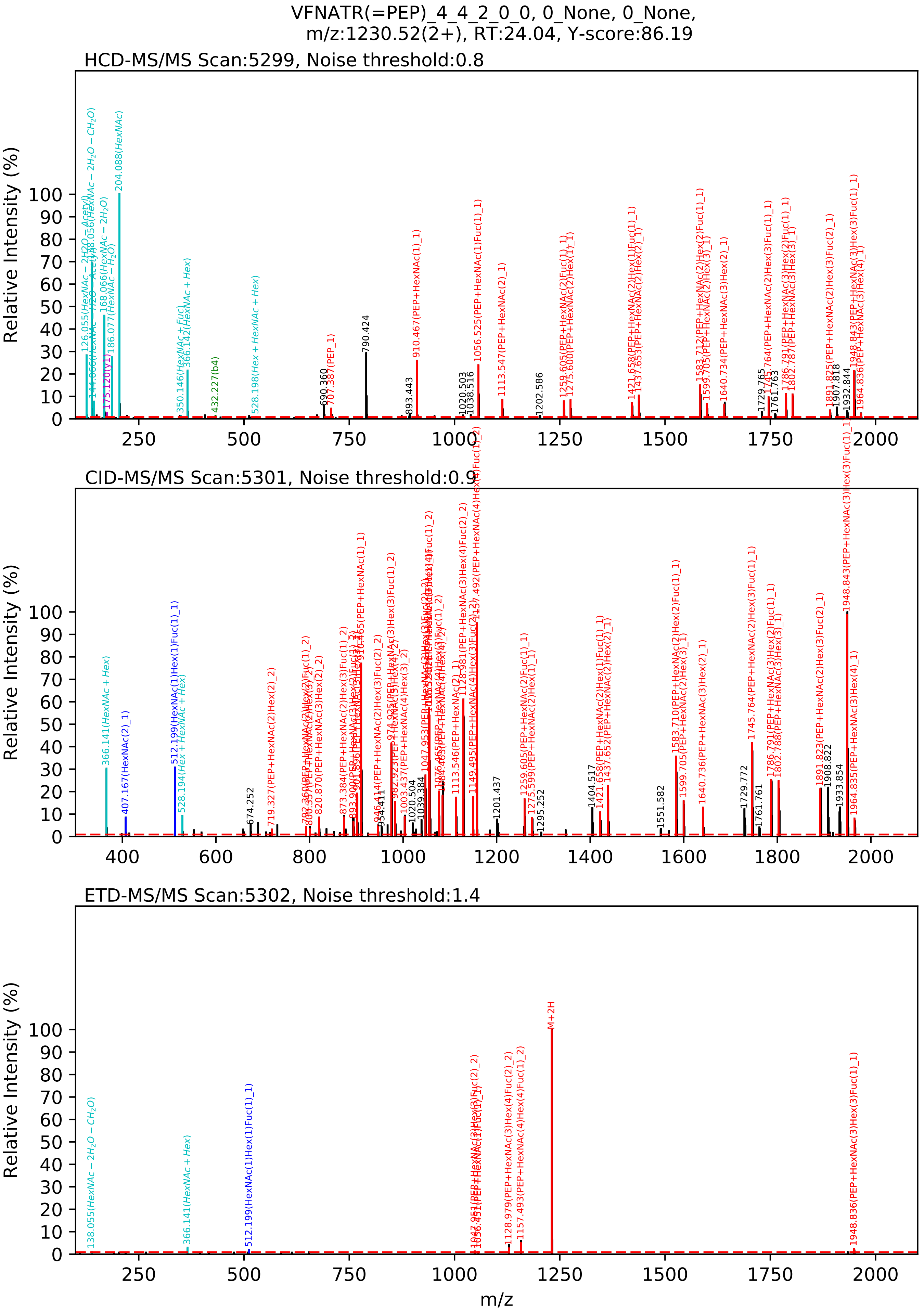

VFNATR(=PEP)_4_4_2_0_0, 0_None, 0_None,
m/z:1230.52(2+), RT:24.04, Y-score:86.19
HCD-MS/MS Scan:5299, Noise threshold:0.8
Relative Intensity (%)
CID-MS/MS Scan:5301, Noise threshold:0.9
Relative Intensity (%)
ETD-MS/MS Scan:5302, Noise threshold:1.4
Relative Intensity (%)
M+2H
m/z

VFNATR(=PEP)_4_4_3_0_0, 0_None, 0_None,
m/z:1303.55(2+), RT:24.08, Y-score:88.61

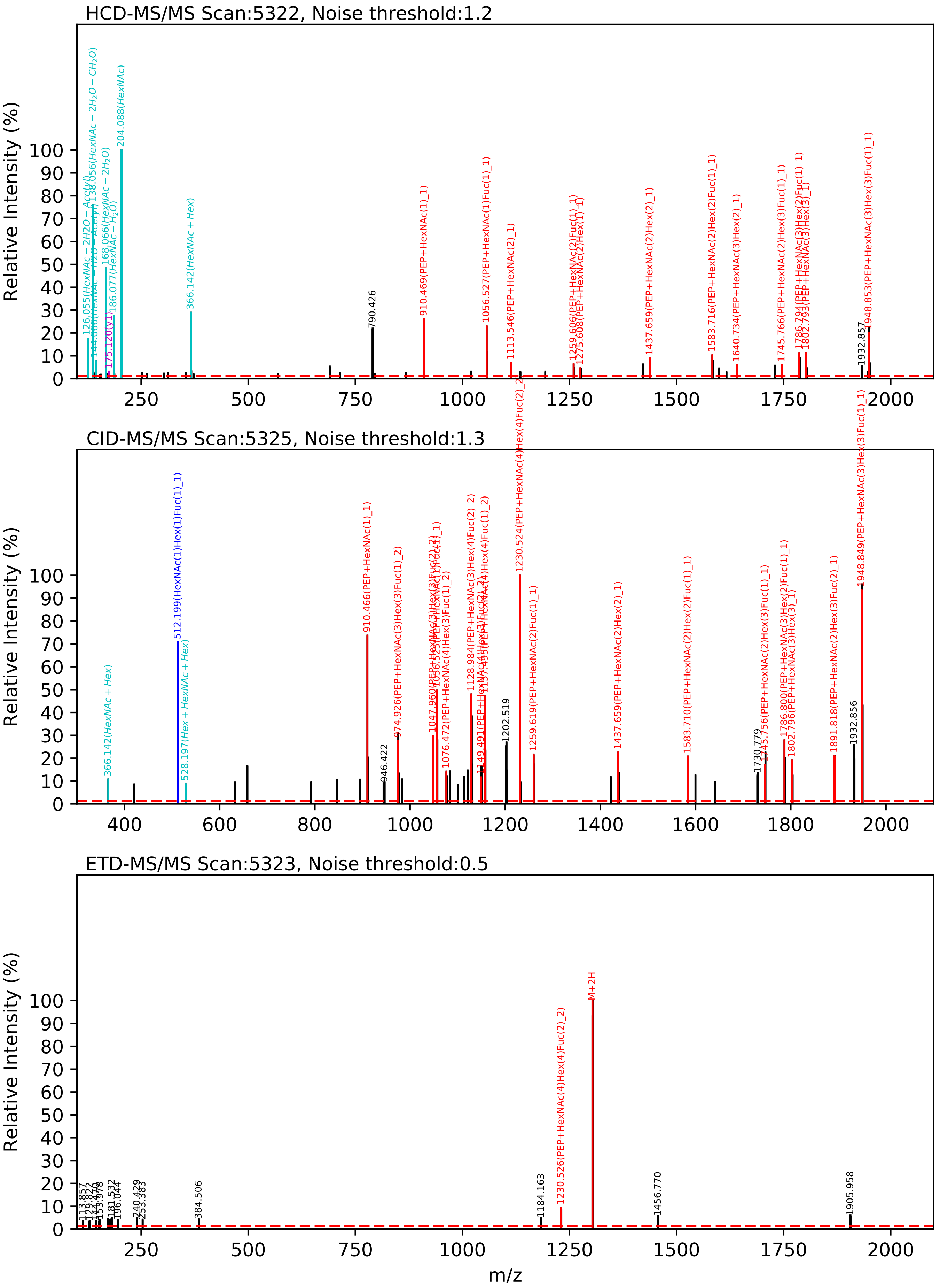

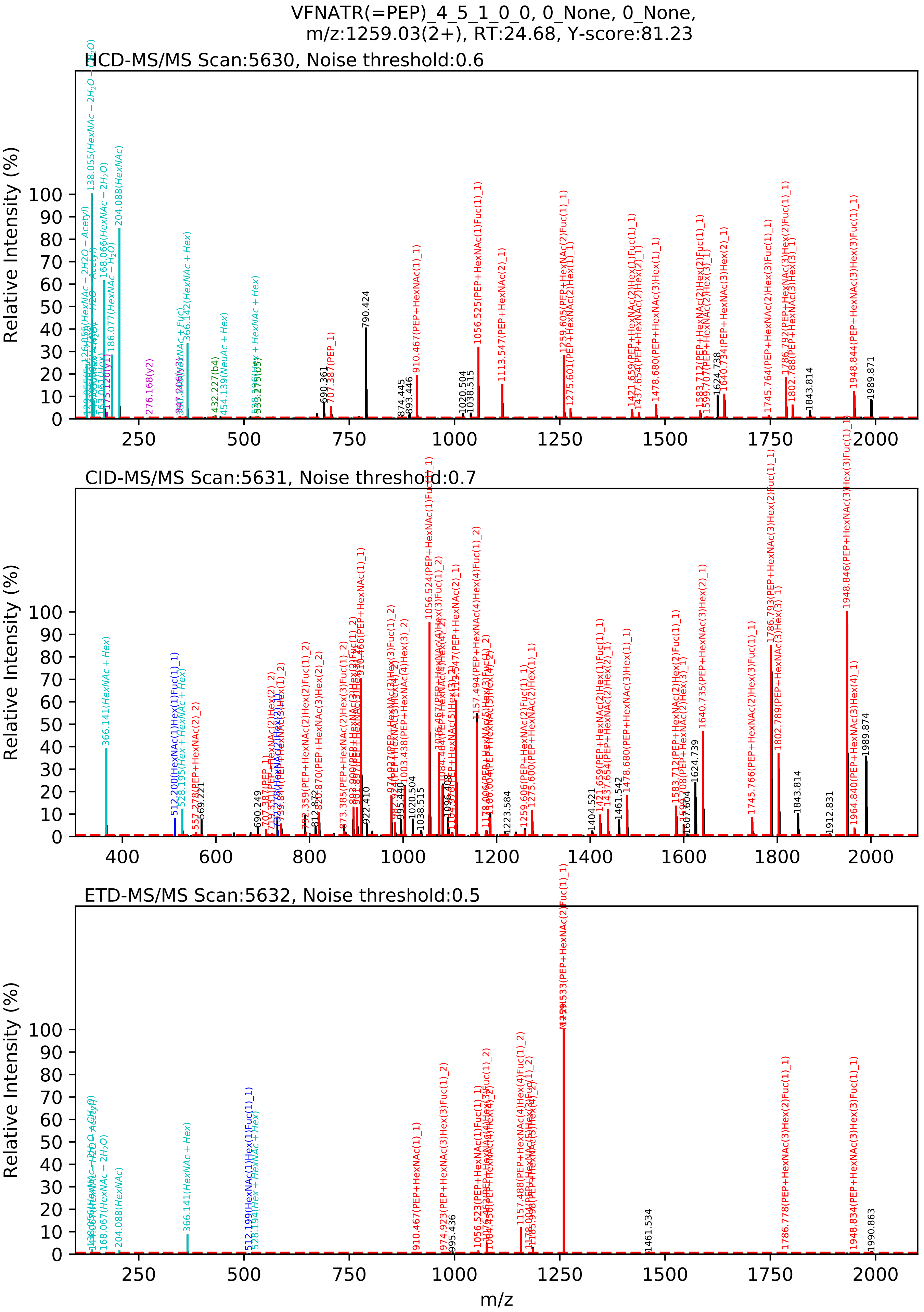
VFNATR(=PEP)_4_5_1_0_0, 0_None, 0_None,
m/z:1259.03(2+), RT:24.68, Y-score:81.23
HCD-MS/MS Scan:5630, Noise threshold:0.6
Relative Intensity (%)
138.055(HexNAc)
204.088(HexNAc)
168.066(HexNAc − 2H2O)
186.077(HexNAc − H2O)
276.168(y2)
366.142(HexNAc + Hex)
432.227(b4)
454.139(NeuAc + Hex)
690.361
707.387(PEP_1)
790.424
874.445
893.446
910.467(PEP+HexNAc(1)_1)
1020.504
1038.515
1056.525(PEP+HexNAc(1)Fuc(1)_1)
1113.547(PEP+HexNAc(2)_1)
1259.605(PEP+HexNAc(2)Fuc(1)_1)
1275.601(PEP+HexNAc(2)Hex(1)_1)
1421.659(PEP+HexNAc(2)Hex(1)Fuc(1)_1)
1437.654(PEP+HexNAc(2)Hex(2)_1)
1478.680(PEP+HexNAc(3)Hex(1)_1)
1583.712(PEP+HexNAc(2)Hex(2)Fuc(1)_1)
1624.738
1640.734(PEP+HexNAc(3)Hex(2)_1)
1745.764(PEP+HexNAc(2)Hex(3)Fuc(1)_1)
1786.792(PEP+HexNAc(3)Hex(2)Fuc(1)_1)
1802.788(PEP+HexNAc(3)Hex(3)_1)
1843.814
1948.844(PEP+HexNAc(3)Hex(3)Fuc(1)_1)
1989.871
CID-MS/MS Scan:5631, Noise threshold:0.7
366.141(HexNAc + Hex)
512.200(HexNAc(1)Hex(1)Fuc(1)_1)
528.195(Hex + HexNAc + Hex)
557.778(PEP+HexNAc(2)_2)
569.221
690.249
707.387(PEP_1)
792.359(PEP+HexNAc(2)Hex(2)Fuc(1)_2)
812.872
817.870(PEP+HexNAc(3)Hex(2)_2)
873.385(PEP+HexNAc(2)Hex(3)Fuc(1)_2)
910.466(PEP+HexNAc(1)_1)
922.410
974.927(PEP+HexNAc(3)Hex(3)Fuc(1)_2)
995.440
1003.438(PEP+HexNAc(4)Hex(3)_2)
1020.504
1038.515
1056.524(PEP+HexNAc(1)Fuc(1)_1)
1113.547(PEP+HexNAc(2)_1)
1157.494(PEP+HexNAc(4)Hex(4)Fuc(1)_2)
1223.584
1259.606(PEP+HexNAc(2)Fuc(1)_1)
1275.600(PEP+HexNAc(2)Hex(1)_1)
1404.521
1421.659(PEP+HexNAc(2)Hex(1)Fuc(1)_1)
1437.654(PEP+HexNAc(2)Hex(2)_1)
1461.542
1478.680(PEP+HexNAc(3)Hex(1)_1)
1583.712(PEP+HexNAc(2)Hex(2)Fuc(1)_1)
1624.739
1640.735(PEP+HexNAc(3)Hex(2)_1)
1745.766(PEP+HexNAc(2)Hex(3)Fuc(1)_1)
1786.793(PEP+HexNAc(3)Hex(2)Fuc(1)_1)
1802.789(PEP+HexNAc(3)Hex(3)_1)
1843.814
1912.831
1948.846(PEP+HexNAc(3)Hex(3)Fuc(1)_1)
1964.840(PEP+HexNAc(3)Hex(4)_1)
1989.874
ETD-MS/MS Scan:5632, Noise threshold:0.5
168.067(HexNAc − 2H2O)
204.088(HexNAc)
366.141(HexNAc + Hex)
512.199(HexNAc(1)Hex(1)Fuc(1)_1)
528.194(Hex + HexNAc + Hex)
910.467(PEP+HexNAc(1)_1)
974.923(PEP+HexNAc(3)Hex(3)Fuc(1)_2)
995.436
1056.523(PEP+HexNAc(1)Fuc(1)_1)
1157.488(PEP+HexNAc(4)Hex(4)Fuc(1)_2)
1259.533(PEP+HexNAc(2)Fuc(1)_1)
1461.534
1786.778(PEP+HexNAc(3)Hex(2)Fuc(1)_1)
1948.834(PEP+HexNAc(3)Hex(3)Fuc(1)_1)
1990.863
m/z

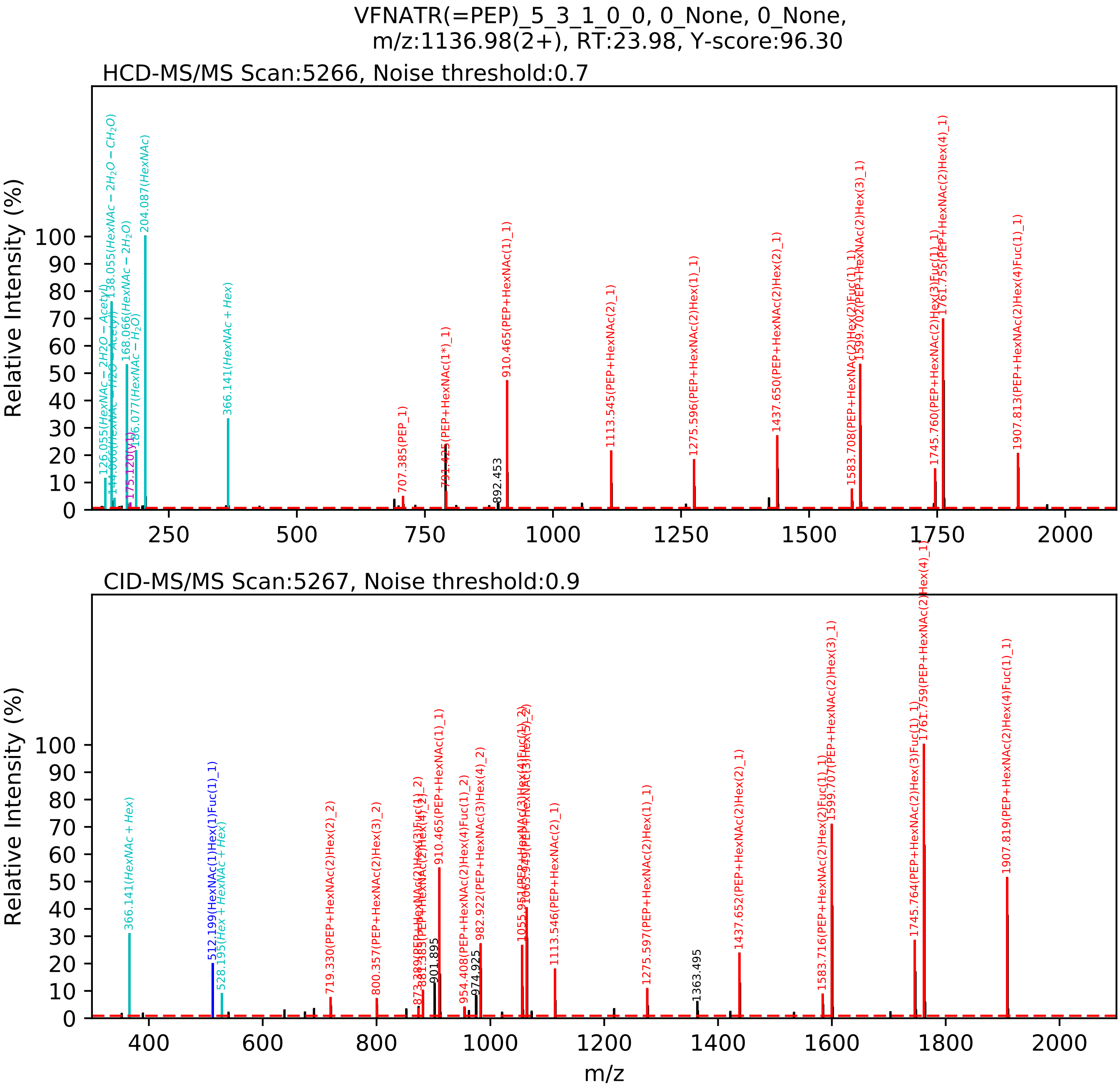
VFNATR(=PEP)_5_3_1_0_0, 0_None, 0_None,
m/z:1136.98(2+), RT:23.98, Y-score:96.30
HCD-MS/MS Scan:5266, Noise threshold:0.7
Relative Intensity (%)
204.087(HexNAc)
366.141(HexNAc + Hex)
707.385(PEP_1)
791.425(PEP+HexNAc(1*)_1)
892.453
910.465(PEP+HexNAc(1)_1)
1113.545(PEP+HexNAc(2)_1)
1275.596(PEP+HexNAc(2)Hex(1)_1)
1437.650(PEP+HexNAc(2)Hex(2)_1)
1583.708(PEP+HexNAc(2)Hex(2)Fuc(1)_1)
1599.702(PEP+HexNAc(2)Hex(3)_1)
1745.760(PEP+HexNAc(2)Hex(3)Fuc(1)_1)
1761.755(PEP+HexNAc(2)Hex(4)_1)
1907.813(PEP+HexNAc(2)Hex(4)Fuc(1)_1)
CID-MS/MS Scan:5267, Noise threshold:0.9
Relative Intensity (%)
366.141(HexNAc + Hex)
512.199(HexNAc(1)Hex(1)Fuc(1)_1)
528.195(Hex + HexNAc + Hex)
719.330(PEP+HexNAc(2)Hex(2)_2)
800.357(PEP+HexNAc(2)Hex(3)_2)
901.895
910.465(PEP+HexNAc(1)_1)
954.408(PEP+HexNAc(2)Hex(4)Fuc(1)_2)
974.925
982.922(PEP+HexNAc(3)Hex(4)_2)
1113.546(PEP+HexNAc(2)_1)
1275.597(PEP+HexNAc(2)Hex(1)_1)
1363.495
1437.652(PEP+HexNAc(2)Hex(2)_1)
1583.716(PEP+HexNAc(2)Hex(2)Fuc(1)_1)
1599.707(PEP+HexNAc(2)Hex(3)_1)
1745.764(PEP+HexNAc(2)Hex(3)Fuc(1)_1)
1761.759(PEP+HexNAc(2)Hex(4)_1)
1907.819(PEP+HexNAc(2)Hex(4)Fuc(1)_1)
m/z

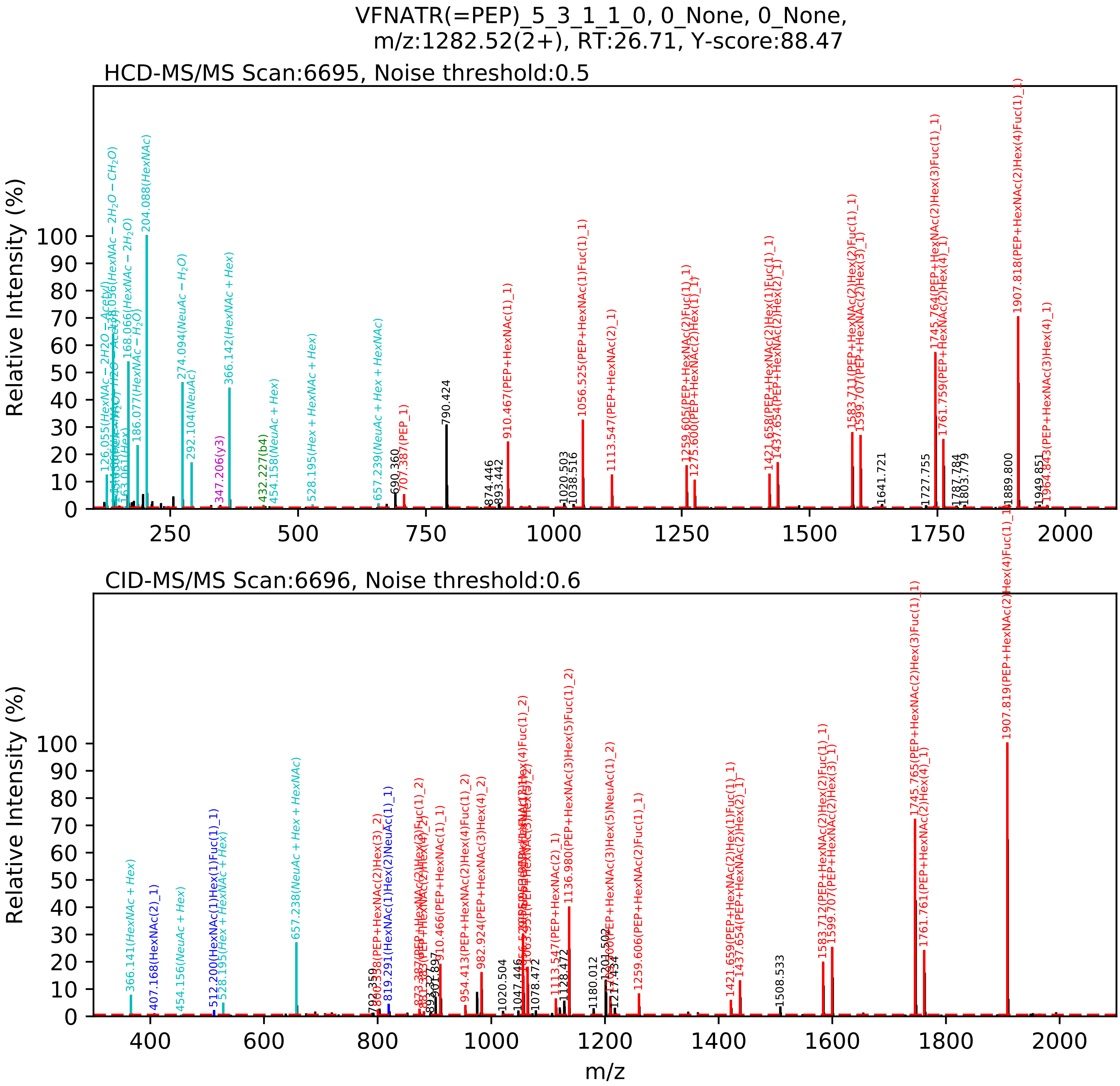

VFNATR(=PEP)_5_3_1_1_0, 0_None, 0_None,
m/z:1282.52(2+), RT:26.71, Y-score:88.47
HCD-MS/MS Scan:6695, Noise threshold:0.5
Relative Intensity (%)
204.088(HexNAc)
366.142(HexNAc + Hex)
274.094(NeuAc − H2O)
292.104(NeuAc)
347.206(y3)
432.227(b4)
454.158(NeuAc + Hex)
528.195(Hex + HexNAc + Hex)
657.239(NeuAc + Hex + HexNAc)
707.387(PEP_1)
790.424
910.467(PEP+HexNAc(1)_1)
1056.525(PEP+HexNAc(1)Fuc(1)_1)
1113.547(PEP+HexNAc(2)_1)
1641.721
1727.755
1889.800
1907.818(PEP+HexNAc(2)Hex(4)Fuc(1)_1)
1964.843(PEP+HexNAc(3)Hex(4)_1)
CID-MS/MS Scan:6696, Noise threshold:0.6
366.141(HexNAc + Hex)
407.168(HexNAc(2)_1)
454.156(NeuAc + Hex)
657.238(NeuAc + Hex + HexNAc)
819.291(HexNAc(1)Hex(2)NeuAc(1)_1)
910.466(PEP+HexNAc(1)_1)
954.413(PEP+HexNAc(2)Hex(4)Fuc(1)_2)
982.924(PEP+HexNAc(3)Hex(4)_2)
1136.980(PEP+HexNAc(3)Hex(5)Fuc(1)_2)
1259.606(PEP+HexNAc(2)Fuc(1)_1)
1508.533
1583.712(PEP+HexNAc(2)Hex(2)Fuc(1)_1)
1599.707(PEP+HexNAc(2)Hex(3)_1)
1745.765(PEP+HexNAc(2)Hex(3)Fuc(1)_1)
1761.761(PEP+HexNAc(2)Hex(4)_1)
1907.819(PEP+HexNAc(2)Hex(4)Fuc(1)_1)
m/z

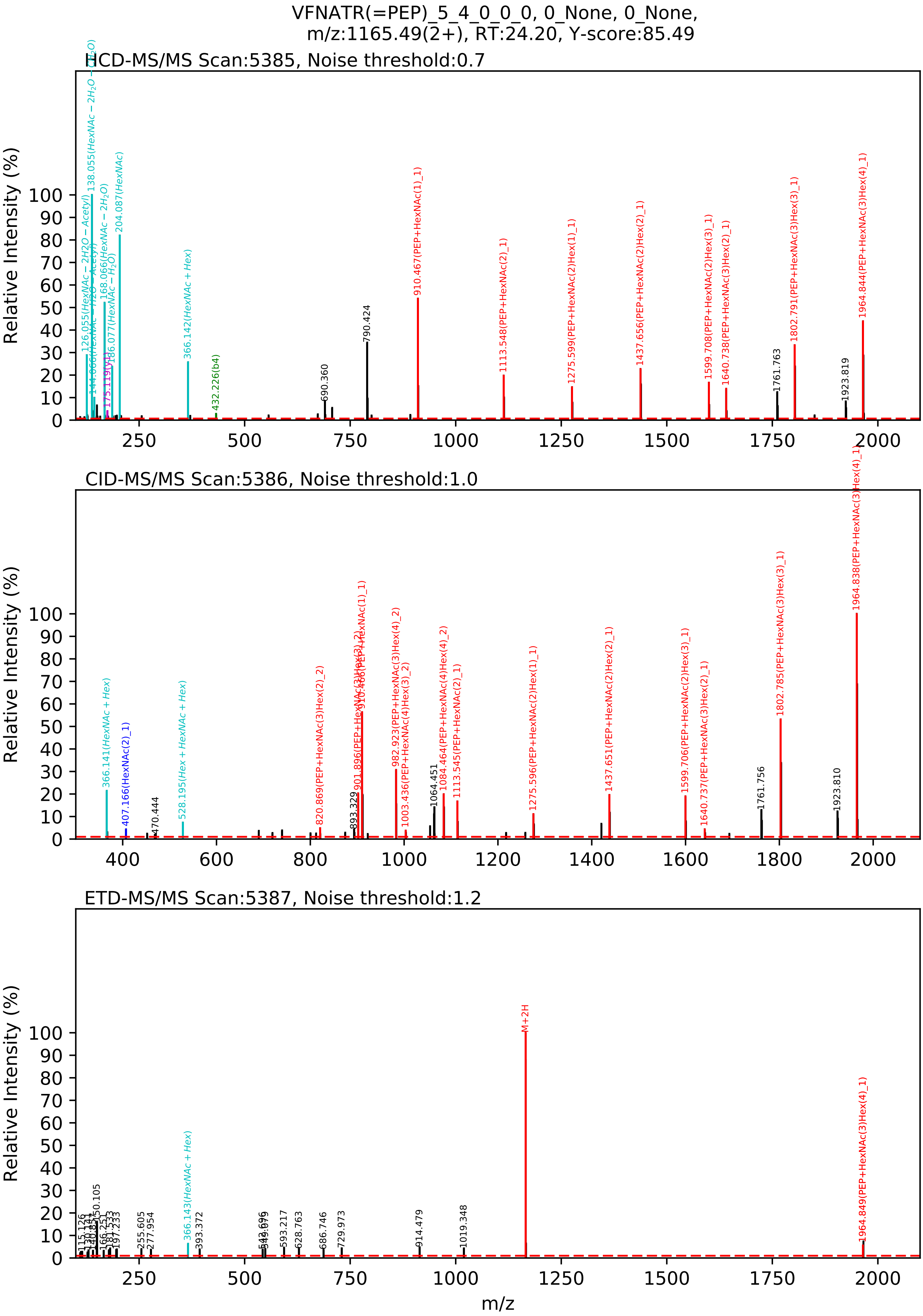

VFNATR(=PEP)_5_4_0_0_0, 0_None, 0_None,
m/z:1165.49(2+), RT:24.20, Y-score:85.49
HCD-MS/MS Scan:5385, Noise threshold:0.7
Relative Intensity (%)
126.055(HexNAc − 2H2O − Acetyl)
138.055(HexNAc − 2H2O − H2O)
144.066(HexNAc − H2O − Acetyl)
168.066(HexNAc − 2H2O)
186.077(HexNAc − H2O)
175.119(y1)
204.087(HexNAc)
366.142(HexNAc + Hex)
432.226(b4)
690.360
790.424
910.467(PEP+HexNAc(1)_1)
1113.548(PEP+HexNAc(2)_1)
1275.599(PEP+HexNAc(2)Hex(1)_1)
1437.656(PEP+HexNAc(2)Hex(2)_1)
1599.708(PEP+HexNAc(2)Hex(3)_1)
1640.738(PEP+HexNAc(3)Hex(2)_1)
1761.763
1802.791(PEP+HexNAc(3)Hex(3)_1)
1923.819
1964.844(PEP+HexNAc(3)Hex(4)_1)
CID-MS/MS Scan:5386, Noise threshold:1.0
366.141(HexNAc + Hex)
407.166(HexNAc(2)_1)
470.444
528.195(Hex + HexNAc + Hex)
820.869(PEP+HexNAc(3)Hex(2)_2)
893.329
901.896(PEP+HexNAc(3)Hex(3)_2)
910.466(PEP+HexNAc(1)_1)
982.923(PEP+HexNAc(3)Hex(4)_2)
1003.436(PEP+HexNAc(4)Hex(3)_2)
1064.451
1084.464(PEP+HexNAc(4)Hex(4)_2)
1113.545(PEP+HexNAc(2)_1)
1275.596(PEP+HexNAc(2)Hex(1)_1)
1437.651(PEP+HexNAc(2)Hex(2)_1)
1599.706(PEP+HexNAc(2)Hex(3)_1)
1640.737(PEP+HexNAc(3)Hex(2)_1)
1761.756
1802.785(PEP+HexNAc(3)Hex(3)_1)
1923.810
1964.838(PEP+HexNAc(3)Hex(4)_1)
ETD-MS/MS Scan:5387, Noise threshold:1.2
M+2H
115.126
130.141
140.851
150.105
166.251
181.533
197.233
255.605
277.954
366.143(HexNAc + Hex)
393.372
543.696
549.676
593.217
628.763
686.746
729.973
914.479
1019.348
1964.849(PEP+HexNAc(3)Hex(4)_1)
m/z

VFNATR(=PEP)_5_4_0_0_0, 0_None, 0_None,
m/z:1165.49(2+), RT:24.24, Y-score:83.06

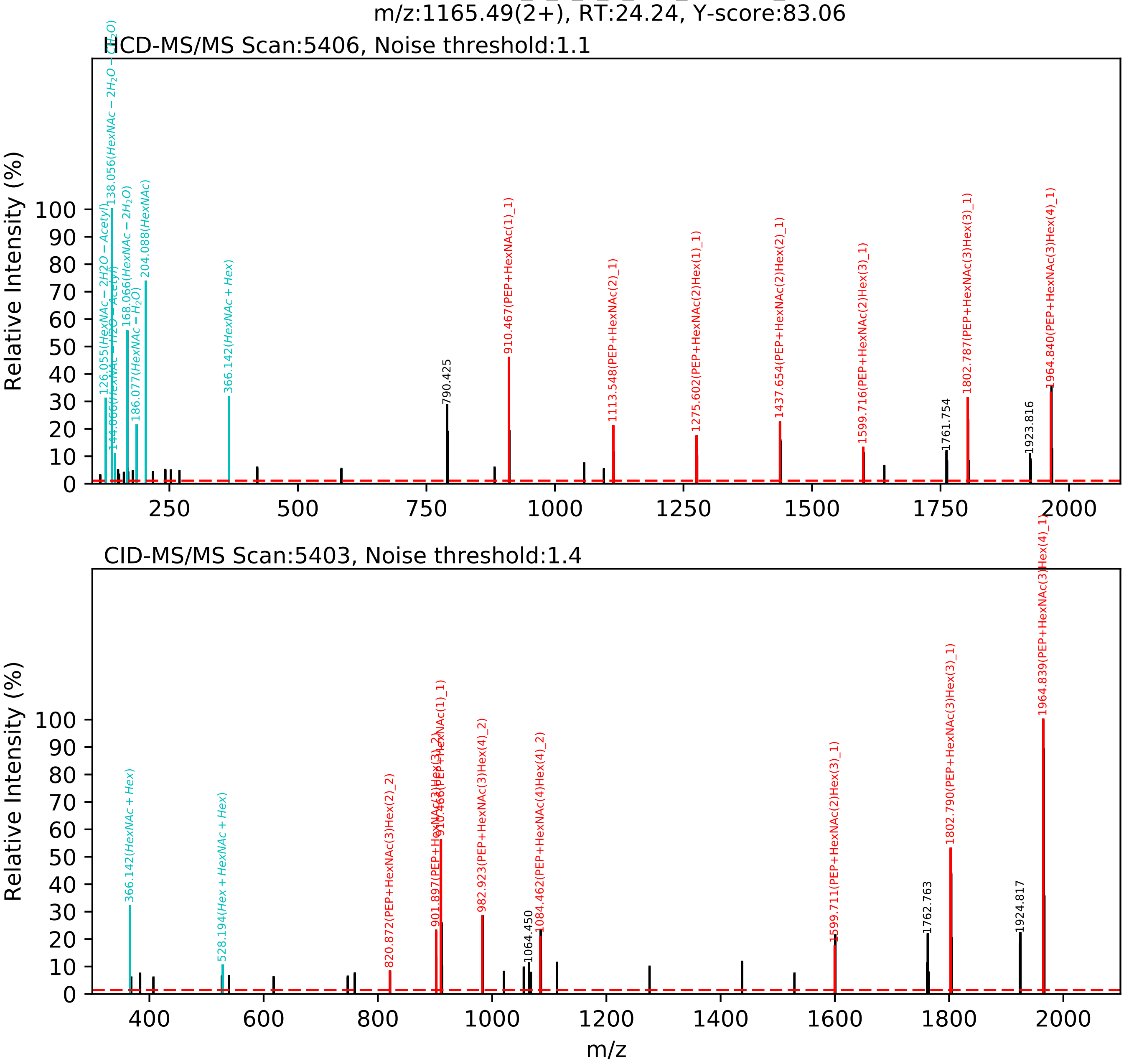

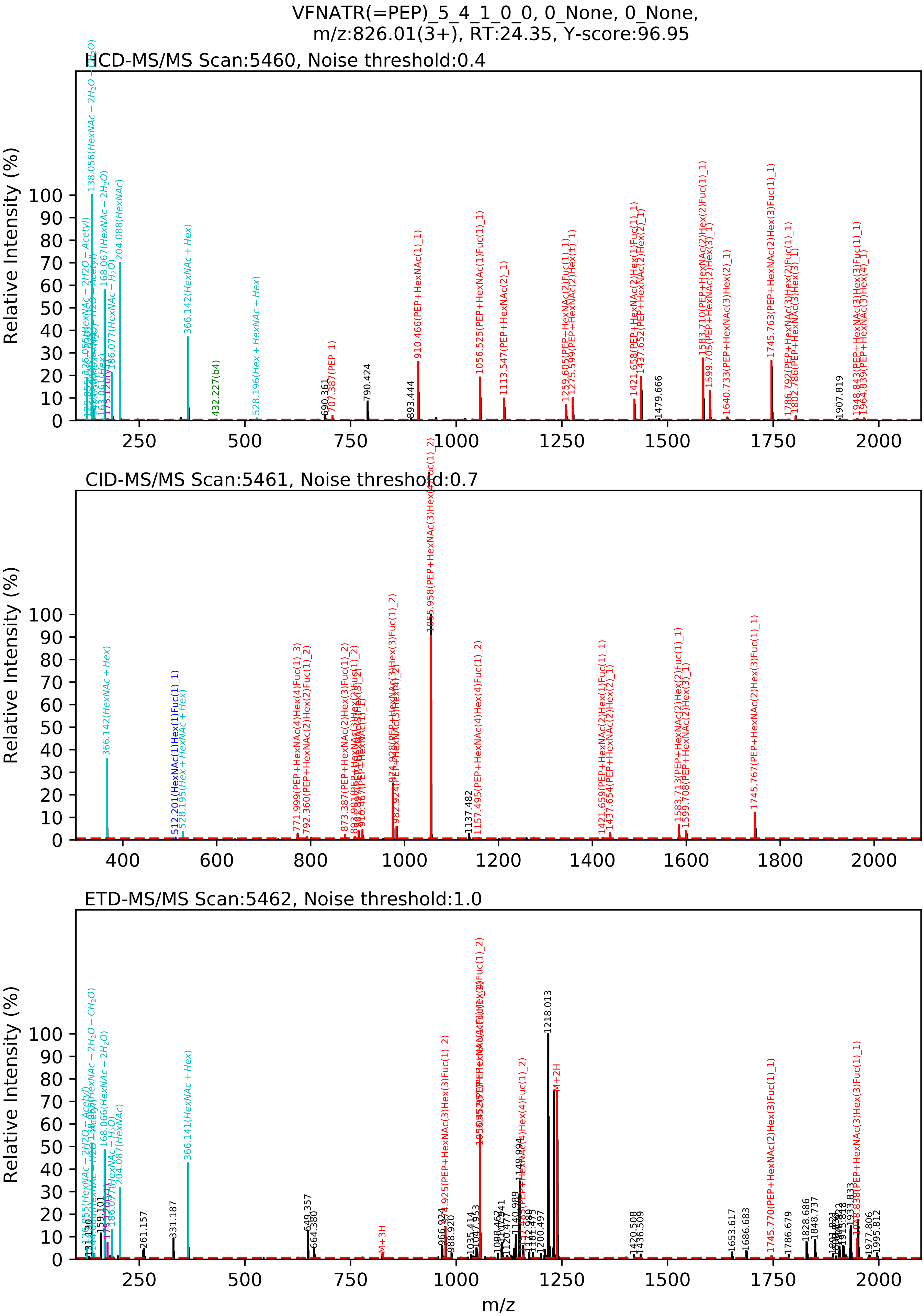
VFNATR(=PEP)_5_4_1_0_0, 0_None, 0_None,
m/z:826.01(3+), RT:24.35, Y-score:96.95
HCD-MS/MS Scan:5460, Noise threshold:0.4
CID-MS/MS Scan:5461, Noise threshold:0.7
ETD-MS/MS Scan:5462, Noise threshold:1.0
Relative Intensity (%)
m/z

VFNATR(=PEP)_5_4_1_0_0, 0_None, 0_None,
m/z:826.01(3+), RT:24.47, Y-score:98.53

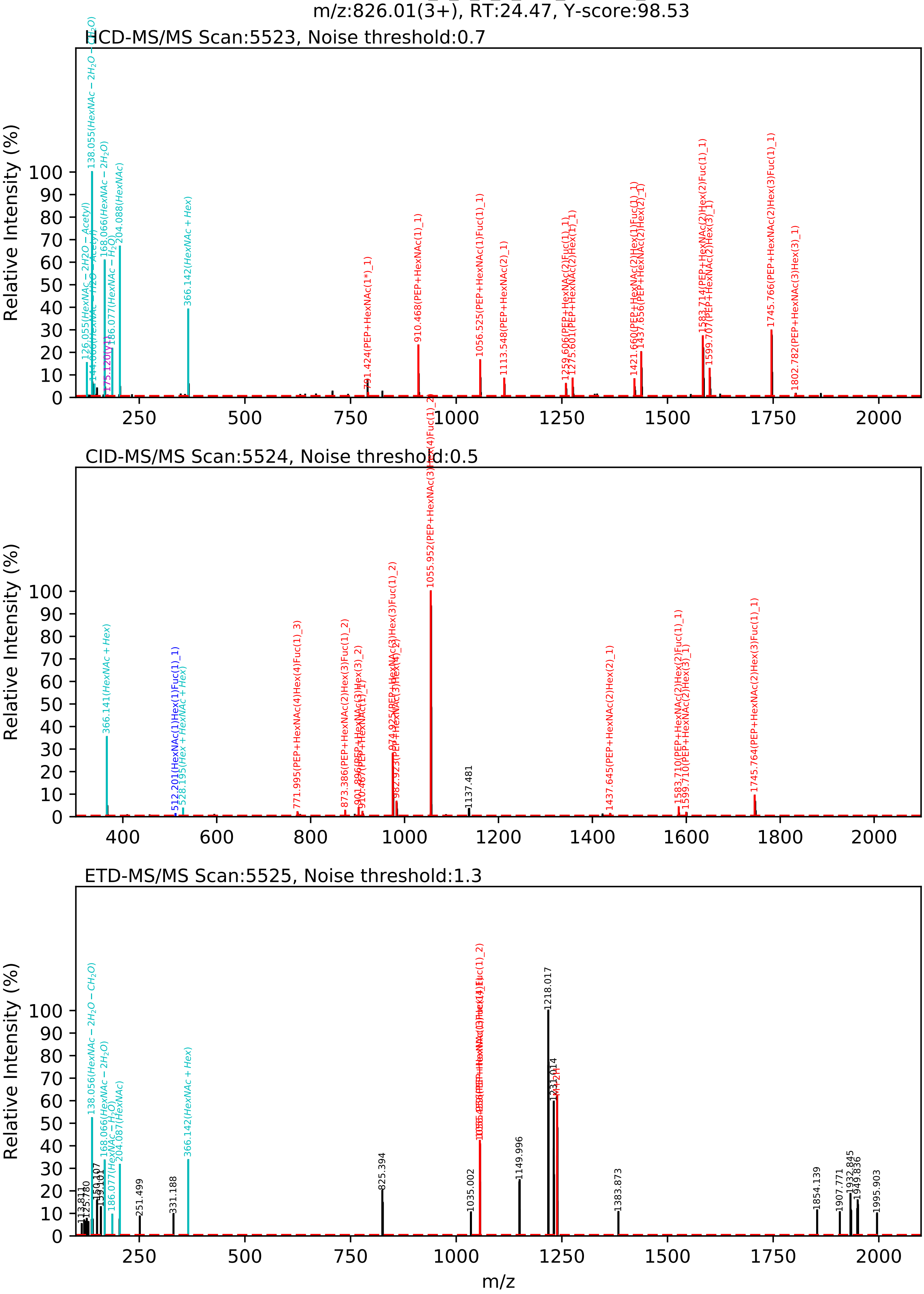

VFNATR(=PEP)_5_4_1_0_0, 0_None, 0_None,
m/z:1238.52(2+), RT:23.89, Y-score:70.31

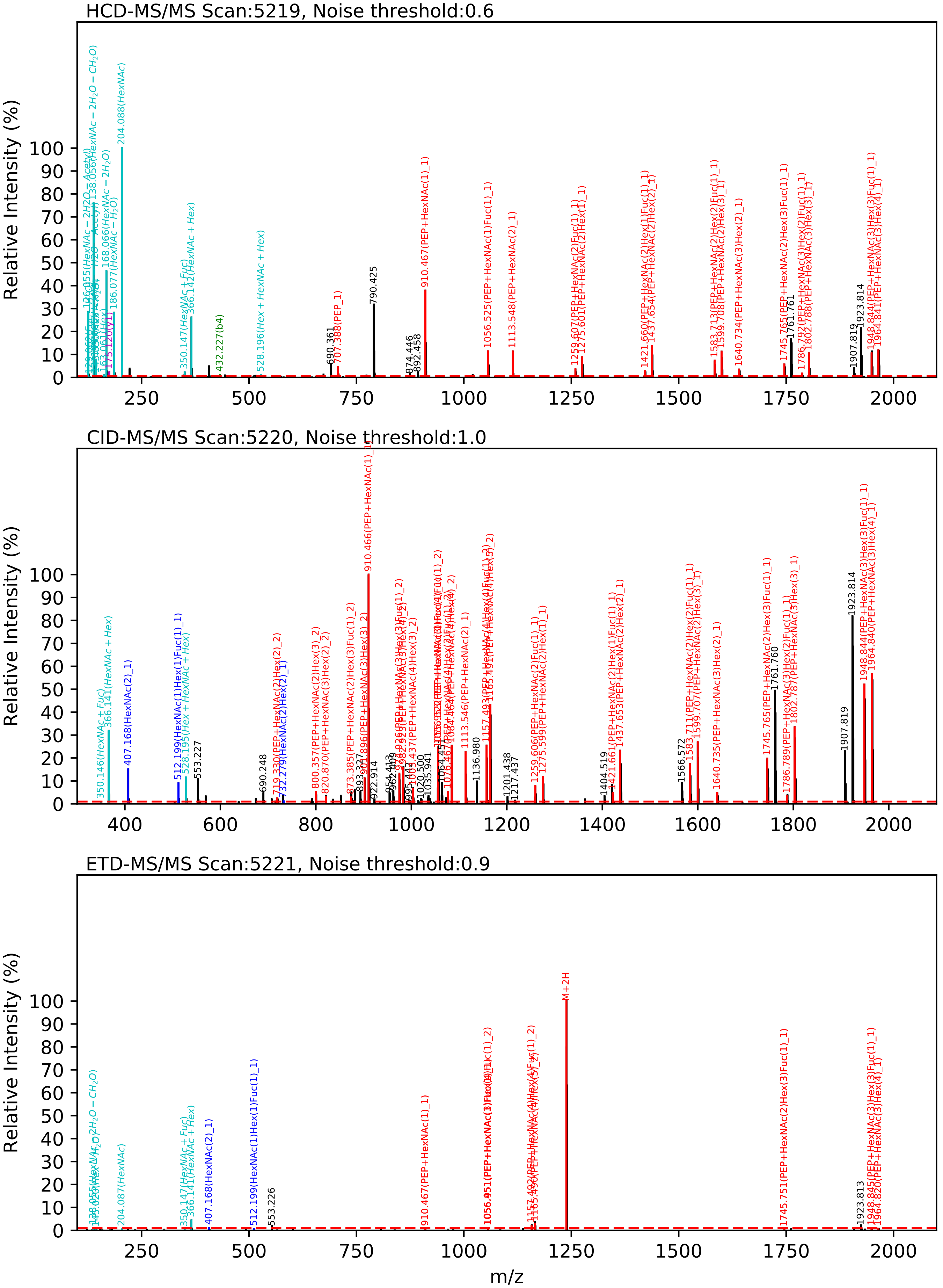

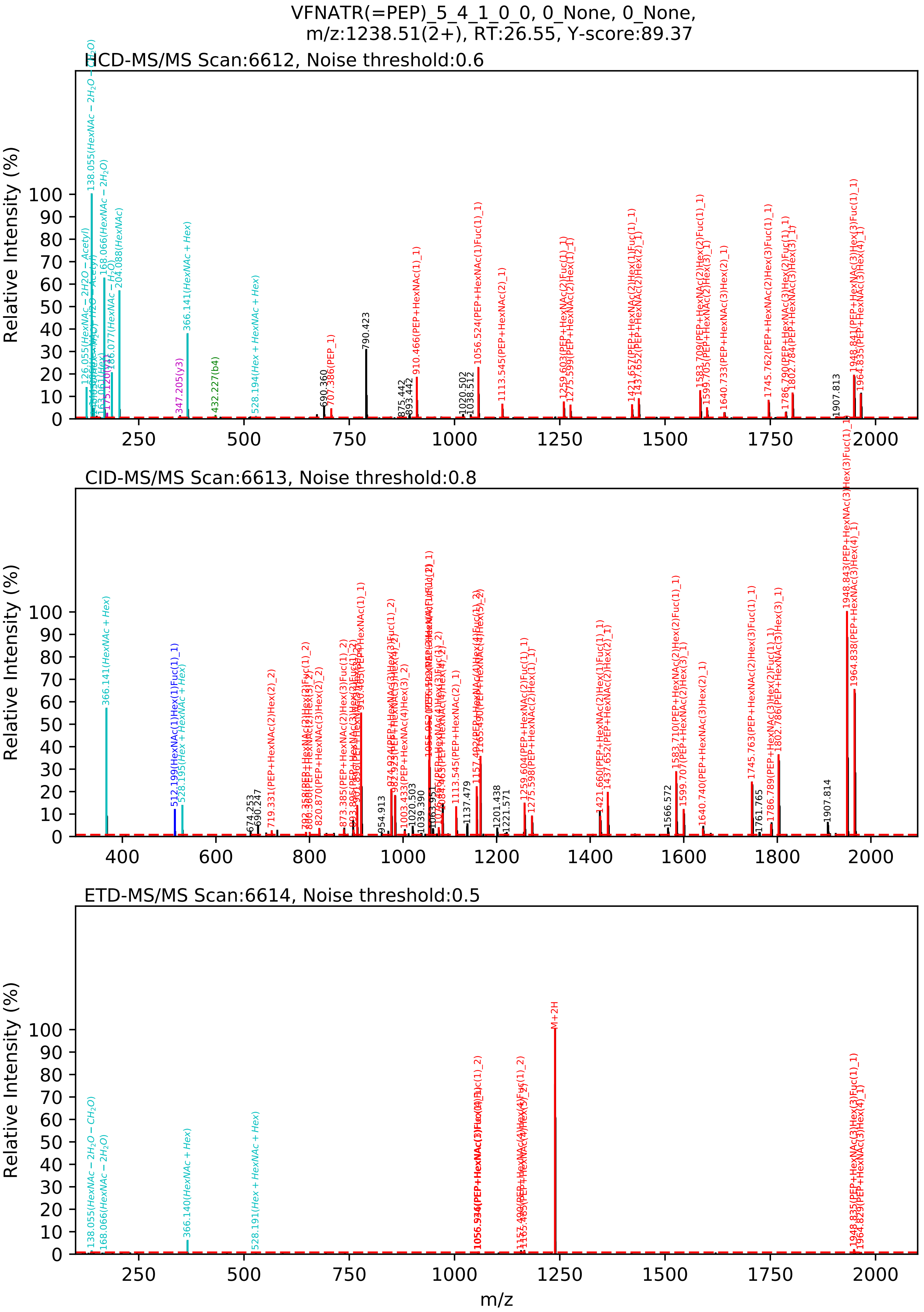

VFNATR(=PEP)_5_4_1_0_0, 0_None, 0_None,
m/z:1238.51(2+), RT:26.55, Y-score:89.37
HCD-MS/MS Scan:6612, Noise threshold:0.6
Relative Intensity (%)
138.055(HexNAc − 2H2O − CH2O)
168.066(HexNAc − 2H2O)
204.088(HexNAc)
366.141(HexNAc + Hex)
347.205(y3)
432.227(b4)
528.194(Hex + HexNAc + Hex)
690.360
707.386(PEP_1)
790.423
875.442
893.442
910.466(PEP+HexNAc(1)_1)
1020.502
1038.512
1056.524(PEP+HexNAc(1)Fuc(1)_1)
1113.545(PEP+HexNAc(2)_1)
1259.603(PEP+HexNAc(2)Fuc(1)_1)
1275.599(PEP+HexNAc(2)Hex(1)_1)
1421.657(PEP+HexNAc(2)Hex(1)Fuc(1)_1)
1437.652(PEP+HexNAc(2)Hex(2)_1)
1583.709(PEP+HexNAc(2)Hex(2)Fuc(1)_1)
1599.705(PEP+HexNAc(2)Hex(3)_1)
1640.733(PEP+HexNAc(3)Hex(2)_1)
1745.762(PEP+HexNAc(2)Hex(3)Fuc(1)_1)
1786.790(PEP+HexNAc(3)Hex(2)Fuc(1)_1)
1802.784(PEP+HexNAc(3)Hex(3)_1)
1907.813
1948.841(PEP+HexNAc(3)Hex(3)Fuc(1)_1)
1964.835(PEP+HexNAc(3)Hex(4)_1)
CID-MS/MS Scan:6613, Noise threshold:0.8
366.141(HexNAc + Hex)
512.199(HexNAc(1)Hex(1)Fuc(1)_1)
528.195(Hex + HexNAc + Hex)
674.253
690.247
719.331(PEP+HexNAc(2)Hex(2)_2)
820.870(PEP+HexNAc(3)Hex(2)_2)
873.385(PEP+HexNAc(2)Hex(3)Fuc(1)_2)
910.465(PEP+HexNAc(1)_1)
954.913
1003.433(PEP+HexNAc(4)Hex(3)_2)
1020.503
1039.390
1113.545(PEP+HexNAc(2)_1)
1137.479
1201.438
1221.571
1259.604(PEP+HexNAc(2)Fuc(1)_1)
1275.598(PEP+HexNAc(2)Hex(1)_1)
1421.660(PEP+HexNAc(2)Hex(1)Fuc(1)_1)
1437.652(PEP+HexNAc(2)Hex(2)_1)
1566.572
1583.710(PEP+HexNAc(2)Hex(2)Fuc(1)_1)
1599.707(PEP+HexNAc(2)Hex(3)_1)
1640.740(PEP+HexNAc(3)Hex(2)_1)
1745.763(PEP+HexNAc(2)Hex(3)Fuc(1)_1)
1761.765
1786.789(PEP+HexNAc(3)Hex(2)Fuc(1)_1)
1802.786(PEP+HexNAc(3)Hex(3)_1)
1907.814
1948.843(PEP+HexNAc(3)Hex(3)Fuc(1)_1)
1964.838(PEP+HexNAc(3)Hex(4)_1)
ETD-MS/MS Scan:6614, Noise threshold:0.5
138.055(HexNAc − 2H2O − CH2O)
168.066(HexNAc − 2H2O)
366.140(HexNAc + Hex)
528.191(Hex + HexNAc + Hex)
M+2H
1948.835(PEP+HexNAc(3)Hex(3)Fuc(1)_1)
1964.829(PEP+HexNAc(3)Hex(4)_1)
m/z

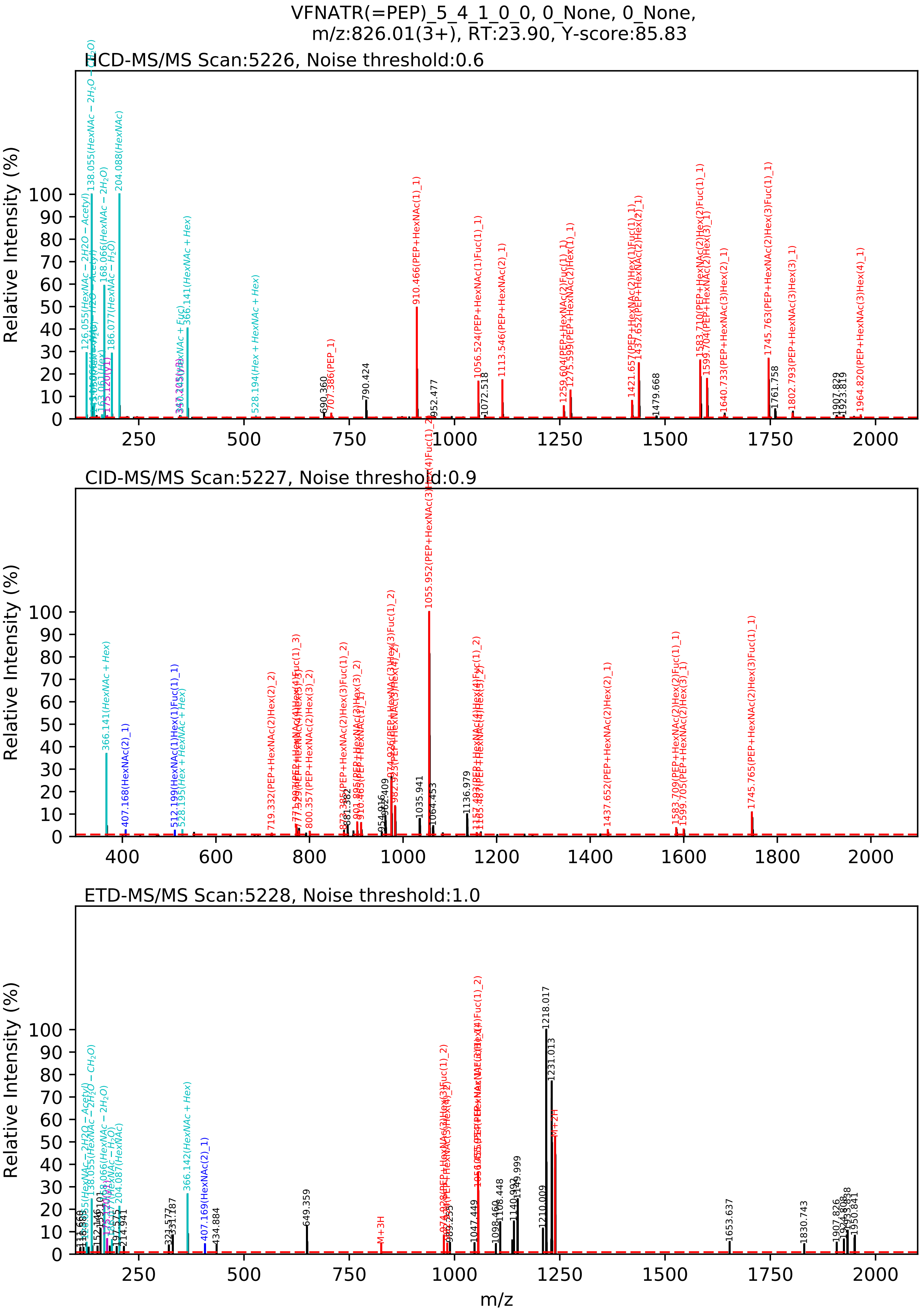

VFNATR(=PEP)_5_4_1_0_0, 0_None, 0_None,
m/z:826.01(3+), RT:23.90, Y-score:85.83
HCD-MS/MS Scan:5226, Noise threshold:0.6
CID-MS/MS Scan:5227, Noise threshold:0.9
ETD-MS/MS Scan:5228, Noise threshold:1.0
Relative Intensity (%)
m/z
910.466(PEP+HexNAc(1)_1)
1056.524(PEP+HexNAc(1)Fuc(1)_1)
1113.546(PEP+HexNAc(2)_1)
1583.710(PEP+HexNAc(2)Hex(2)Fuc(1)_1)
1745.763(PEP+HexNAc(2)Hex(3)Fuc(1)_1)
1964.820(PEP+HexNAc(3)Hex(4)_1)
1055.952(PEP+HexNAc(3)Hex(4)Fuc(1)_2)
1437.652(PEP+HexNAc(2)Hex(2)_1)
1745.765(PEP+HexNAc(2)Hex(3)Fuc(1)_1)
366.141(HexNAc+Hex)
407.168(HexNAc(2)_1)
1218.017
1231.013
M+2H
M+3H

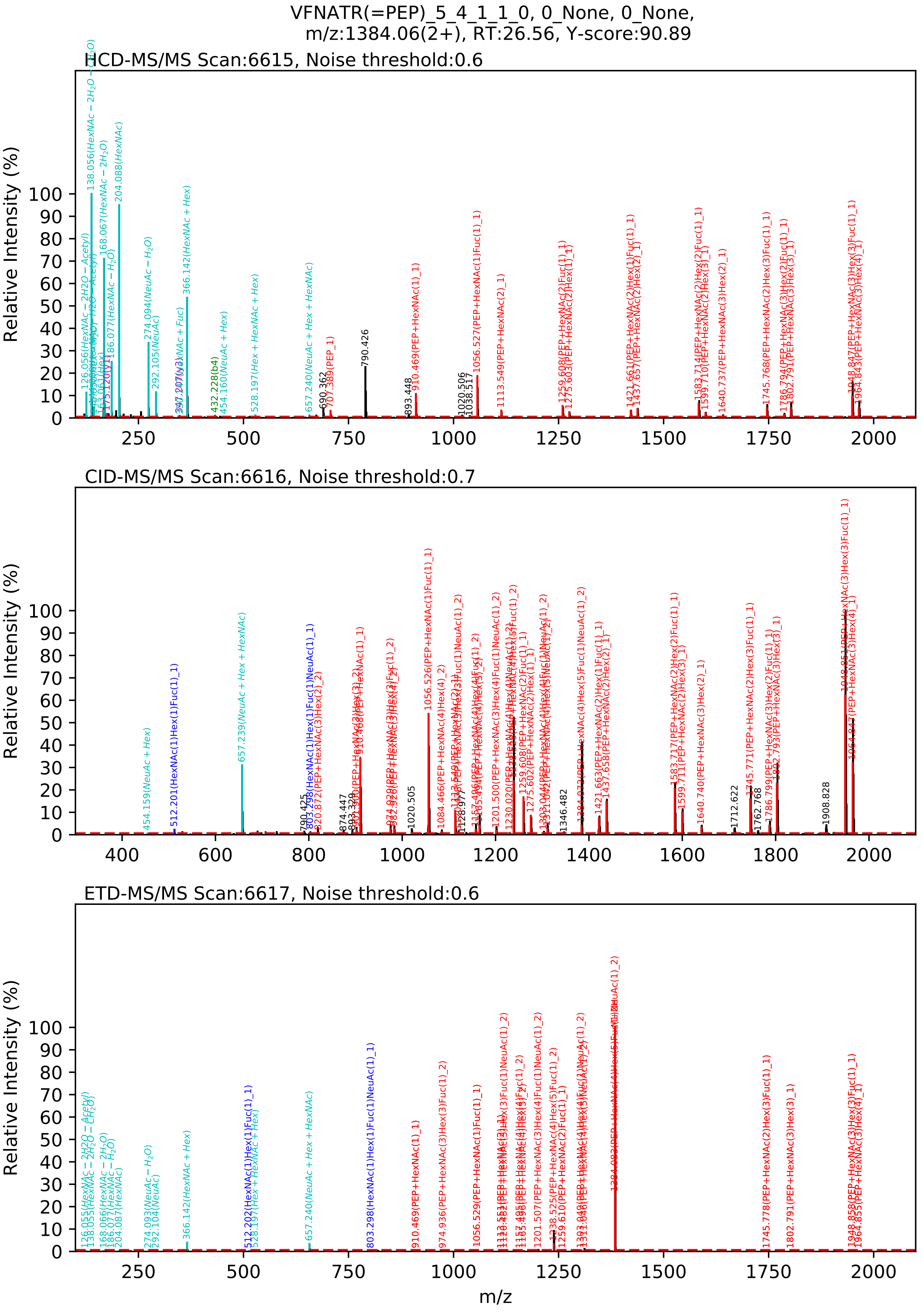

VFNATR(=PEP)_5_4_1_1_0, 0_None, 0_None,
m/z:1384.06(2+), RT:26.56, Y-score:90.89
HCD-MS/MS Scan:6615, Noise threshold:0.6
CID-MS/MS Scan:6616, Noise threshold:0.7
ETD-MS/MS Scan:6617, Noise threshold:0.6
Relative Intensity (%)
m/z

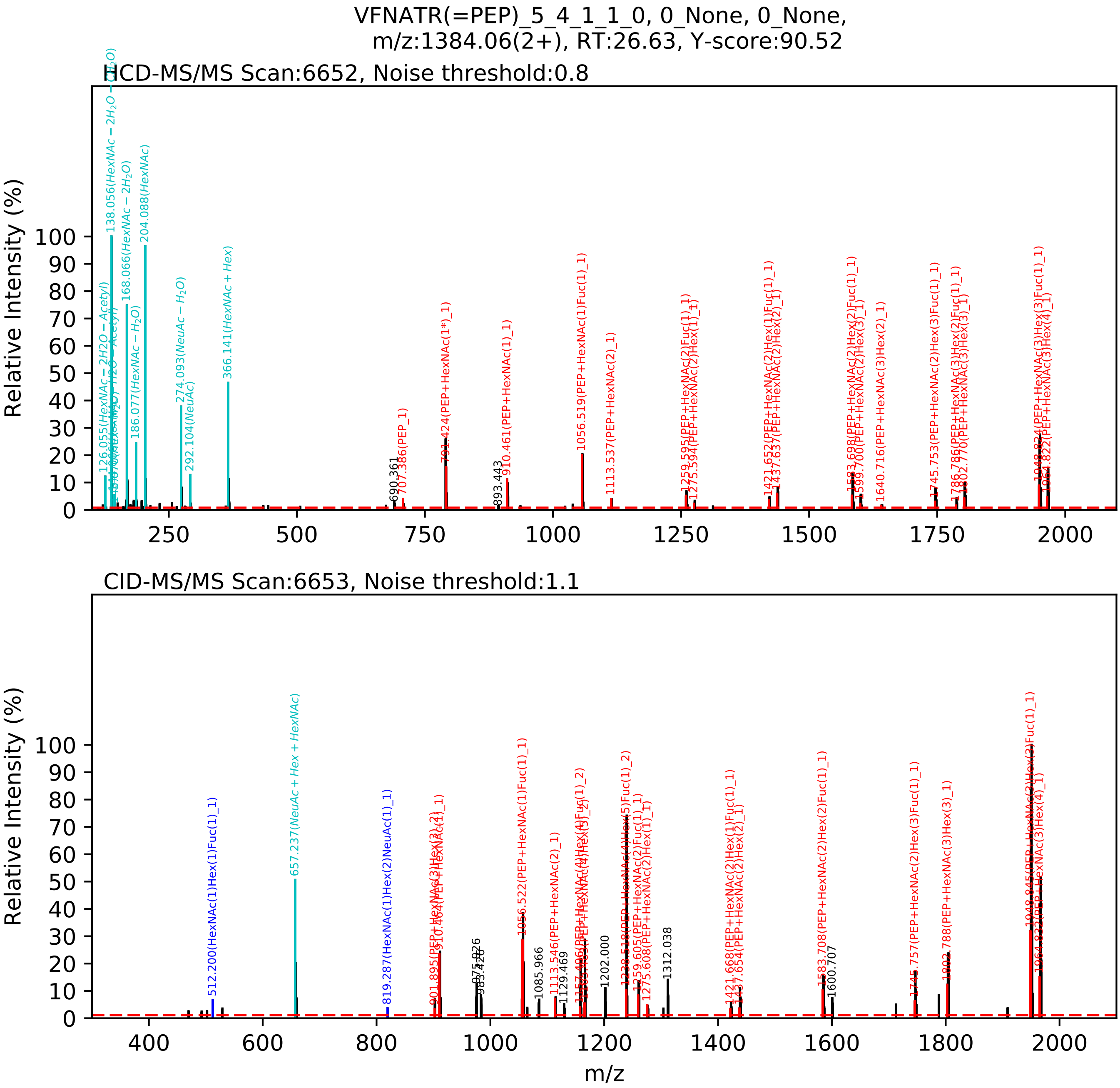
VFNATR(=PEP)_5_4_1_1_0, 0_None, 0_None,
m/z:1384.06(2+), RT:26.63, Y-score:90.52
HCD-MS/MS Scan:6652, Noise threshold:0.8
CID-MS/MS Scan:6653, Noise threshold:1.1
Relative Intensity (%)
m/z

VFNATR(=PEP)_5_4_1_1_0, 0_None, 0_None,
m/z:1384.06(2+), RT:27.17, Y-score:91.12

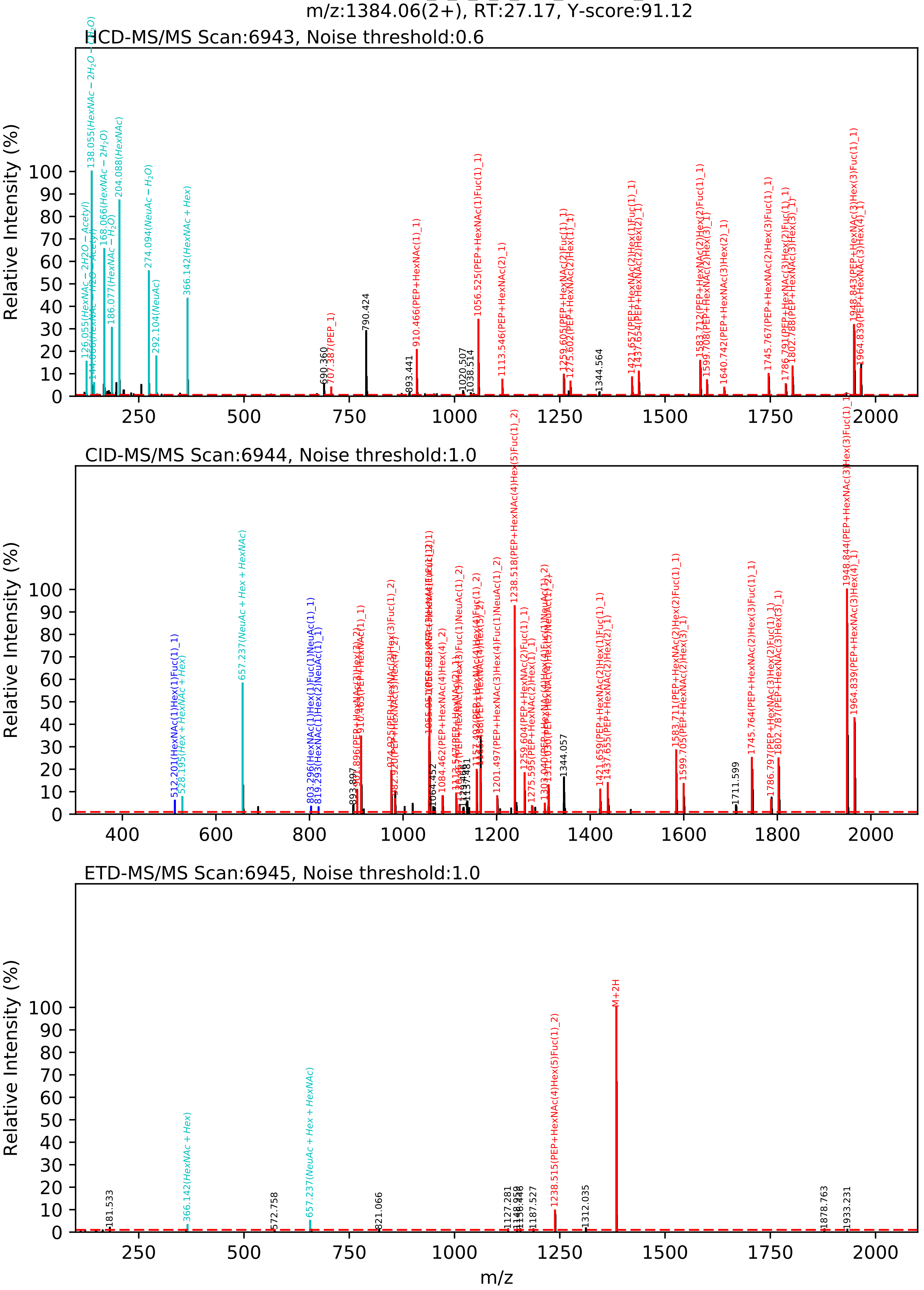

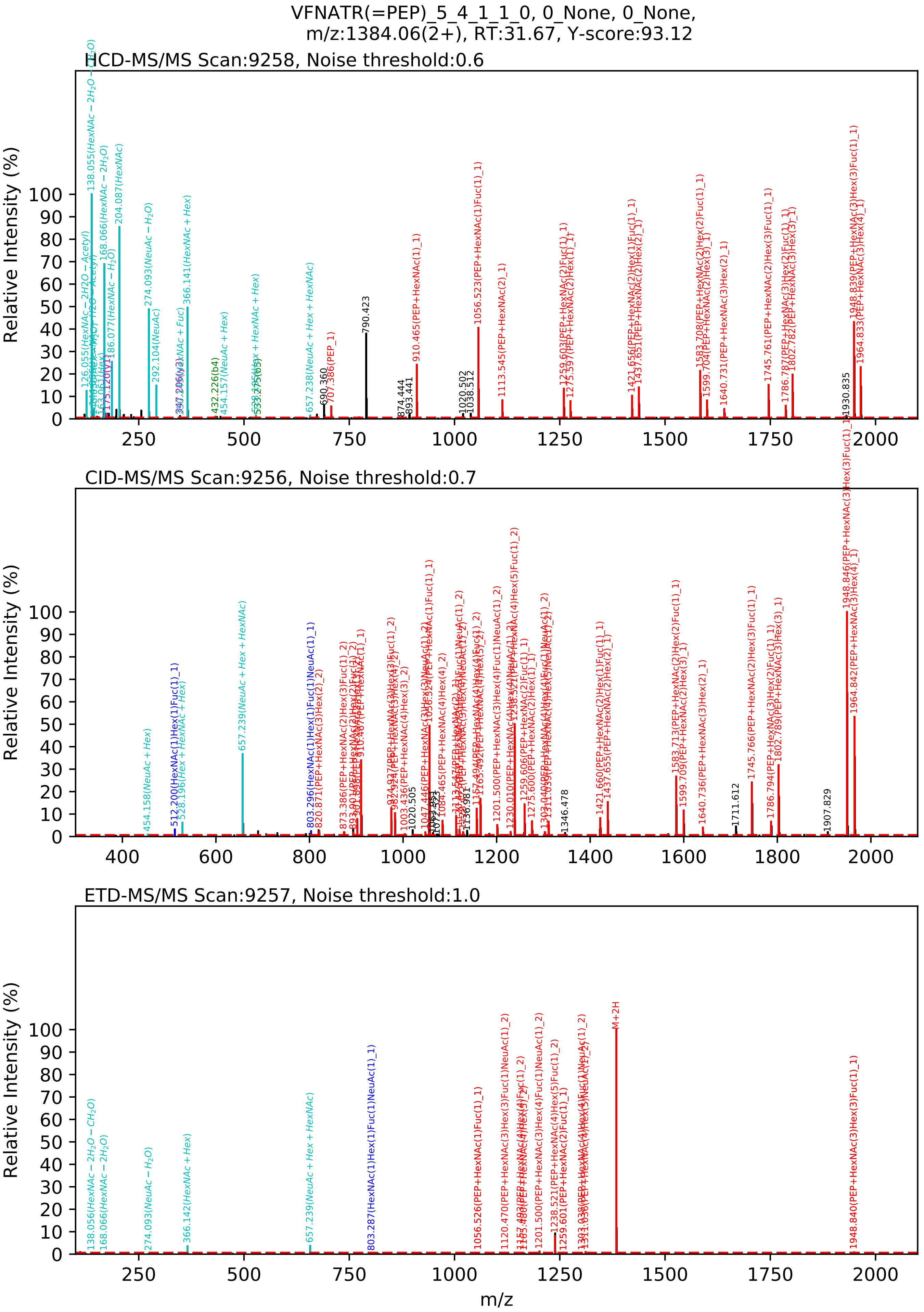

VFNATR(=PEP)_5_4_1_1_0, 0_None, 0_None,
m/z:1384.06(2+), RT:31.67, Y-score:93.12
HCD-MS/MS Scan:9258, Noise threshold:0.6
CID-MS/MS Scan:9256, Noise threshold:0.7
ETD-MS/MS Scan:9257, Noise threshold:1.0
Relative Intensity (%)
m/z

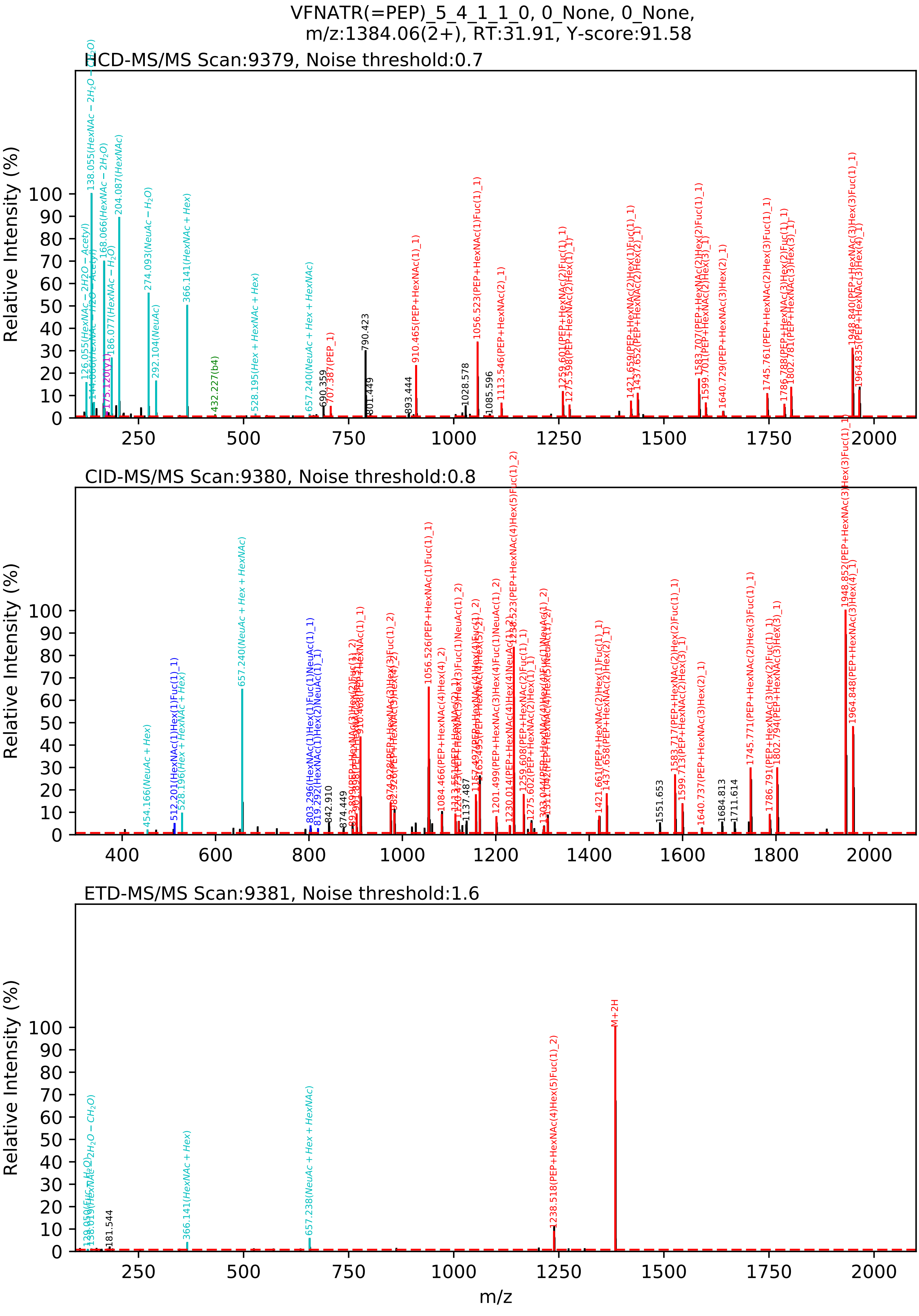
VFNATR(=PEP)_5_4_1_1_0, 0_None, 0_None,
m/z:1384.06(2+), RT:31.91, Y-score:91.58
HCD-MS/MS Scan:9379, Noise threshold:0.7
Relative Intensity (%)
138.055(HexNAc)
204.087(HexNAc)
168.066(HexNAc − 2H2O)
186.077(HexNAc − H2O)
274.093(NeuAc − H2O)
292.104(NeuAc)
366.141(HexNAc + Hex)
432.227(b4)
528.195(Hex + HexNAc + Hex)
657.240(NeuAc + Hex + HexNAc)
690.359
707.387(PEP_1)
790.423
801.449
893.444
910.465(PEP+HexNAc(1)_1)
1028.578
1056.523(PEP+HexNAc(1)Fuc(1)_1)
1085.596
1113.546(PEP+HexNAc(2)_1)
1259.601(PEP+HexNAc(2)Fuc(1)_1)
1275.598(PEP+HexNAc(2)Hex(1)_1)
1421.659(PEP+HexNAc(2)Hex(1)Fuc(1)_1)
1437.652(PEP+HexNAc(2)Hex(2)_1)
1583.707(PEP+HexNAc(2)Hex(2)Fuc(1)_1)
1599.701(PEP+HexNAc(2)Hex(3)_1)
1640.729(PEP+HexNAc(3)Hex(2)_1)
1745.761(PEP+HexNAc(2)Hex(3)Fuc(1)_1)
1786.788(PEP+HexNAc(3)Hex(2)Fuc(1)_1)
1802.781(PEP+HexNAc(3)Hex(3)_1)
1948.840(PEP+HexNAc(3)Hex(3)Fuc(1)_1)
1964.835(PEP+HexNAc(3)Hex(4)_1)
CID-MS/MS Scan:9380, Noise threshold:0.8
454.166(NeuAc + Hex)
512.201(HexNAc(1)Hex(1)Fuc(1)_1)
528.196(Hex + HexNAc + Hex)
657.240(NeuAc + Hex + HexNAc)
803.296(HexNAc(1)Hex(1)Fuc(1)NeuAc(1)_1)
819.292(HexNAc(1)Hex(2)NeuAc(1)_1)
842.910
874.449
910.468(PEP+HexNAc(1)_1)
1056.526(PEP+HexNAc(1)Fuc(1)_1)
1084.466(PEP+HexNAc(4)Hex(4)_2)
1137.487
1201.499(PEP+HexNAc(3)Hex(4)Fuc(1)NeuAc(1)_2)
1238.523(PEP+HexNAc(4)Hex(5)Fuc(1)_2)
1259.608(PEP+HexNAc(2)Fuc(1)_1)
1275.602(PEP+HexNAc(2)Hex(1)_1)
1421.661(PEP+HexNAc(2)Hex(1)Fuc(1)_1)
1437.658(PEP+HexNAc(2)Hex(2)_1)
1551.653
1583.717(PEP+HexNAc(2)Hex(2)Fuc(1)_1)
1599.713(PEP+HexNAc(2)Hex(3)_1)
1640.737(PEP+HexNAc(3)Hex(2)_1)
1684.813
1711.614
1745.771(PEP+HexNAc(2)Hex(3)Fuc(1)_1)
1786.791(PEP+HexNAc(3)Hex(2)Fuc(1)_1)
1802.794(PEP+HexNAc(3)Hex(3)_1)
1948.852(PEP+HexNAc(3)Hex(3)Fuc(1)_1)
1964.848(PEP+HexNAc(3)Hex(4)_1)
ETD-MS/MS Scan:9381, Noise threshold:1.6
181.544
366.141(HexNAc + Hex)
657.238(NeuAc + Hex + HexNAc)
1238.518(PEP+HexNAc(4)Hex(5)Fuc(1)_2)
M+2H
m/z

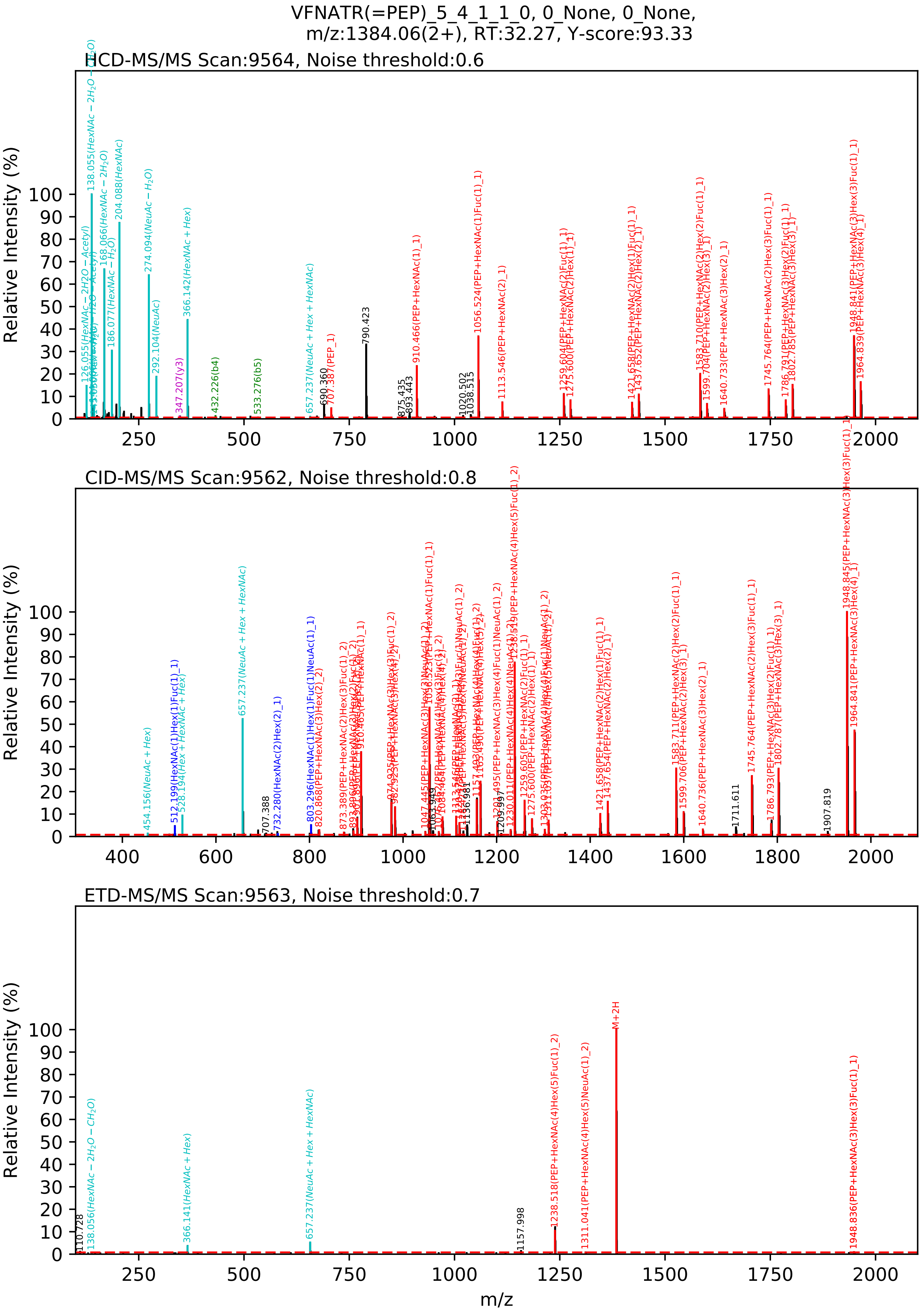
VFNATR(=PEP)_5_4_1_1_0, 0_None, 0_None,
m/z:1384.06(2+), RT:32.27, Y-score:93.33
HCD-MS/MS Scan:9564, Noise threshold:0.6
Relative Intensity (%)
138.055(HexNAc − 2H2O − Acetyl)
168.066(HexNAc − 2H2O)
186.077(HexNAc − H2O)
204.088(HexNAc)
274.094(NeuAc − H2O)
292.104(NeuAc)
366.142(HexNAc + Hex)
347.207(y3)
432.226(b4)
533.276(b5)
657.237(NeuAc + Hex + HexNAc)
690.360
707.387(PEP_1)
790.423
875.435
893.443
910.466(PEP+HexNAc(1)_1)
1020.502
1038.515
1056.524(PEP+HexNAc(1)Fuc(1)_1)
1113.546(PEP+HexNAc(2)_1)
1259.604(PEP+HexNAc(2)Fuc(1)_1)
1275.600(PEP+HexNAc(2)Hex(1)_1)
1421.658(PEP+HexNAc(2)Hex(1)Fuc(1)_1)
1437.652(PEP+HexNAc(2)Hex(2)_1)
1583.710(PEP+HexNAc(2)Hex(2)Fuc(1)_1)
1599.704(PEP+HexNAc(2)Hex(3)_1)
1640.733(PEP+HexNAc(3)Hex(2)_1)
1745.764(PEP+HexNAc(2)Hex(3)Fuc(1)_1)
1786.791(PEP+HexNAc(3)Hex(2)Fuc(1)_1)
1802.785(PEP+HexNAc(3)Hex(3)_1)
1948.841(PEP+HexNAc(3)Hex(3)Fuc(1)_1)
1964.839(PEP+HexNAc(3)Hex(4)_1)
CID-MS/MS Scan:9562, Noise threshold:0.8
Relative Intensity (%)
454.156(NeuAc + Hex)
512.199(HexNAc(1)Hex(1)Fuc(1)_1)
528.194(Hex + HexNAc + Hex)
657.237(NeuAc + Hex + HexNAc)
707.388
732.280(HexNAc(2)Hex(2)_1)
803.296(HexNAc(1)Hex(1)Fuc(1)NeuAc(1)_1)
820.868(PEP+HexNAc(3)Hex(2)_2)
1421.658(PEP+HexNAc(2)Hex(1)Fuc(1)_1)
1437.654(PEP+HexNAc(2)Hex(2)_1)
1583.711(PEP+HexNAc(2)Hex(2)Fuc(1)_1)
1599.706(PEP+HexNAc(2)Hex(3)_1)
1640.736(PEP+HexNAc(3)Hex(2)_1)
1711.611
1745.764(PEP+HexNAc(2)Hex(3)Fuc(1)_1)
1786.793(PEP+HexNAc(3)Hex(2)Fuc(1)_1)
1802.787(PEP+HexNAc(3)Hex(3)_1)
1907.819
1948.845(PEP+HexNAc(3)Hex(3)Fuc(1)_1)
1964.841(PEP+HexNAc(3)Hex(4)_1)
ETD-MS/MS Scan:9563, Noise threshold:0.7
Relative Intensity (%)
110.728
138.056(HexNAc − 2H2O − CH2O)
366.141(HexNAc + Hex)
657.237(NeuAc + Hex + HexNAc)
1157.998
1238.518(PEP+HexNAc(4)Hex(5)Fuc(1)_2)
1311.041(PEP+HexNAc(4)Hex(5)NeuAc(1)_2)
M+2H
1948.836(PEP+HexNAc(3)Hex(3)Fuc(1)_1)
m/z

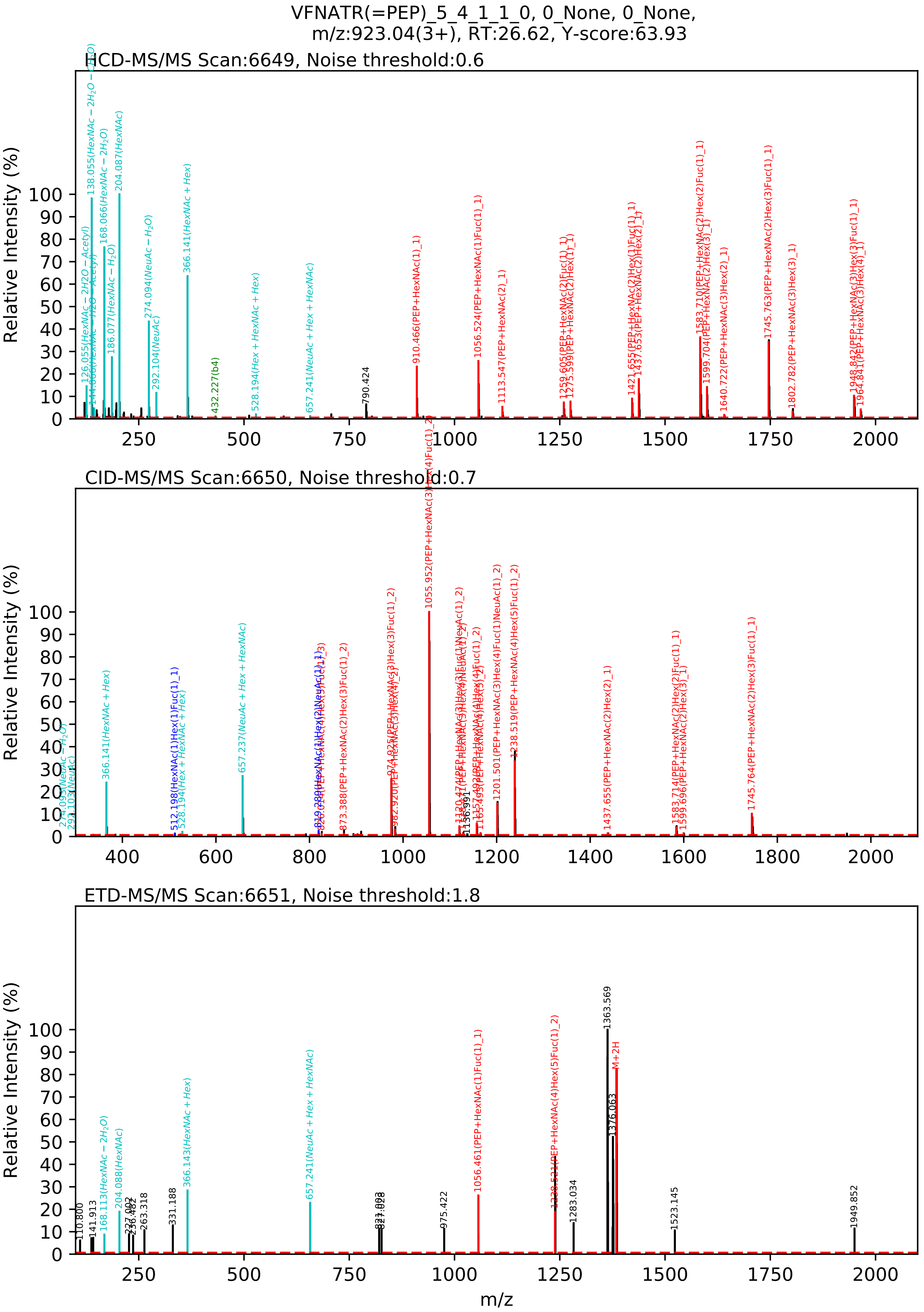
VFNATR(=PEP)_5_4_1_1_0, 0_None, 0_None,
m/z:923.04(3+), RT:26.62, Y-score:63.93
HCD-MS/MS Scan:6649, Noise threshold:0.6
Relative Intensity (%)
138.055(HexNAc − 2H2O − C2H4O)
204.087(HexNAc)
168.066(HexNAc − 2H2O)
186.077(HexNAc − H2O)
274.094(NeuAc − H2O)
292.104(NeuAc)
366.141(HexNAc + Hex)
432.227(b4)
528.194(Hex + HexNAc + Hex)
657.241(NeuAc + Hex + HexNAc)
790.424
910.466(PEP+HexNAc(1)_1)
1056.524(PEP+HexNAc(1)Fuc(1)_1)
1113.547(PEP+HexNAc(2)_1)
1745.763(PEP+HexNAc(2)Hex(3)Fuc(1)_1)
1802.782(PEP+HexNAc(3)Hex(3)_1)
1640.722(PEP+HexNAc(3)Hex(2)_1)
CID-MS/MS Scan:6650, Noise threshold:0.7
1055.952(PEP+HexNAc(3)Hex(4)Fuc(1)_2)
1238.519(PEP+HexNAc(4)Hex(5)Fuc(1)_2)
1201.501(PEP+HexNAc(3)Hex(4)Fuc(1)NeuAc(1)_2)
1437.655(PEP+HexNAc(2)Hex(2)_1)
1745.764(PEP+HexNAc(2)Hex(3)Fuc(1)_1)
873.388(PEP+HexNAc(2)Hex(3)Fuc(1)_2)
657.237(NeuAc + Hex + HexNAc)
366.141(HexNAc + Hex)
512.198(HexNAc(1)Hex(1)Fuc(1)_1)
1136.991
ETD-MS/MS Scan:6651, Noise threshold:1.8
1363.569
M+2H
1376.063
1056.461(PEP+HexNAc(1)Fuc(1)_1)
1283.034
1523.145
1949.852
975.422
331.188
263.318
141.913
110.800
366.143(HexNAc + Hex)
657.241(NeuAc + Hex + HexNAc)
204.088(HexNAc)
168.113(HexNAc − 2H2O)
m/z

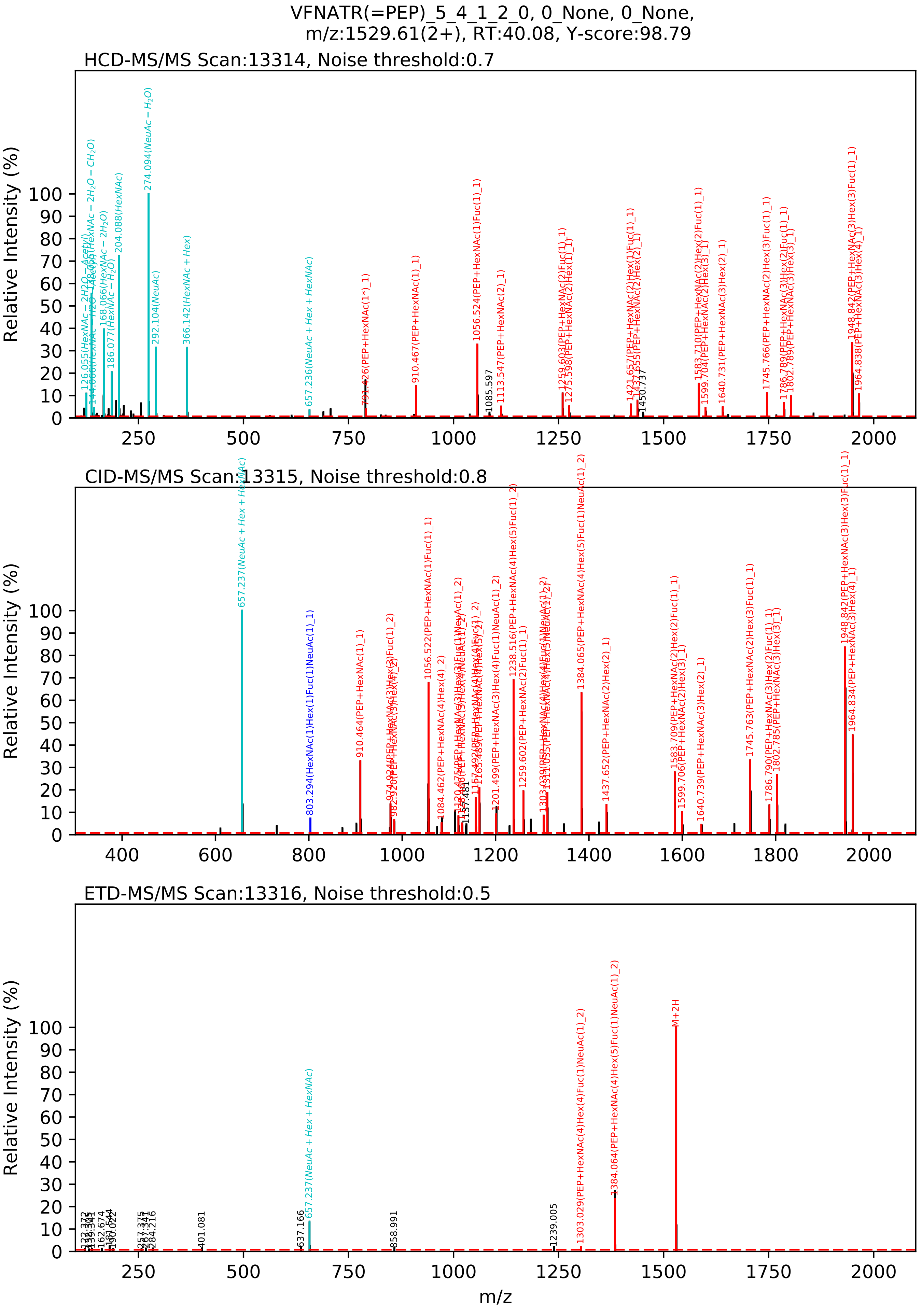

VFNATR(=PEP)_5_4_1_2_0, 0_None, 0_None,
m/z:1529.61(2+), RT:40.08, Y-score:98.79
HCD-MS/MS Scan:13314, Noise threshold:0.7
CID-MS/MS Scan:13315, Noise threshold:0.8
ETD-MS/MS Scan:13316, Noise threshold:0.5
Relative Intensity (%)
m/z

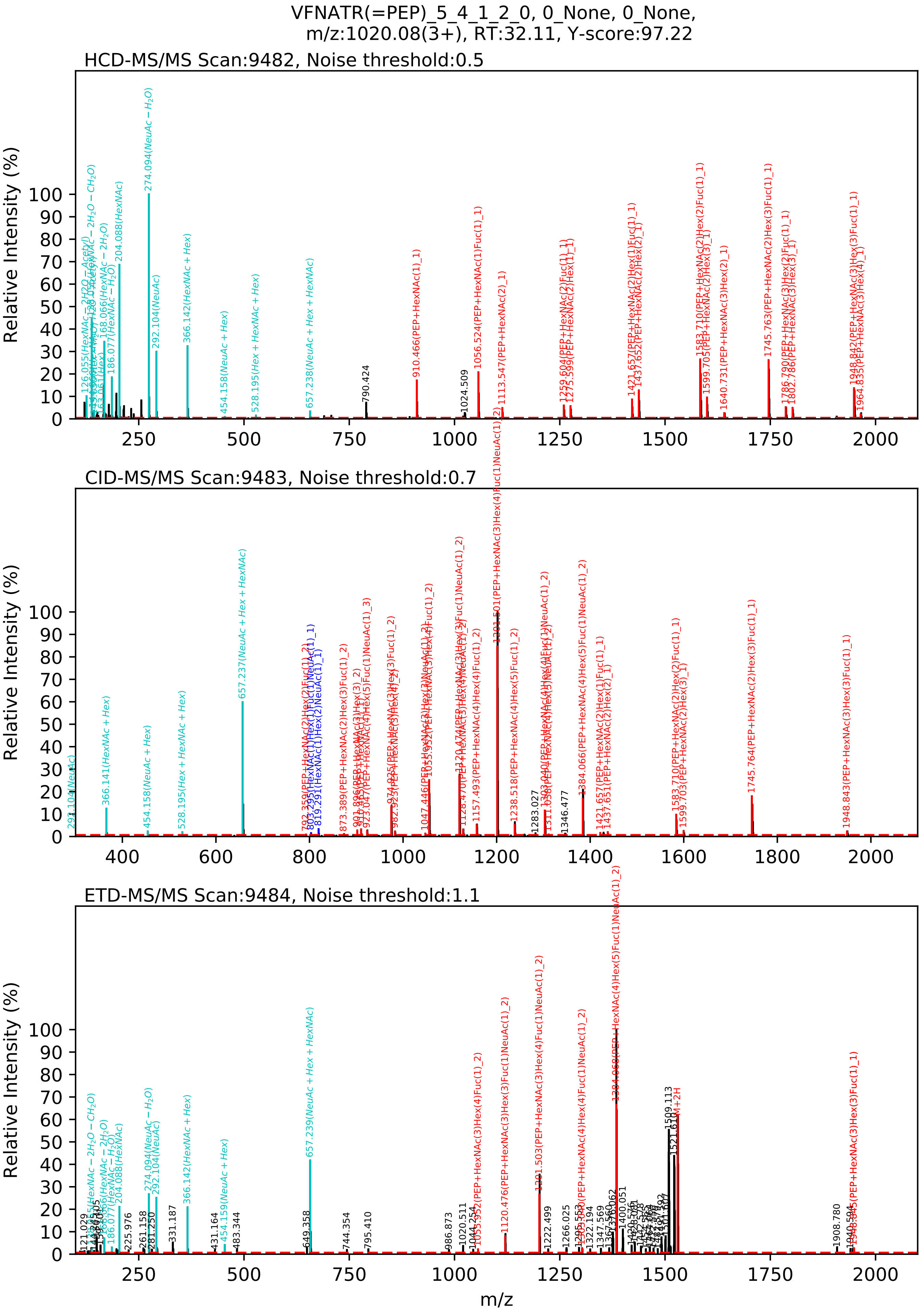

VFNATR(=PEP)_5_4_1_2_0, 0_None, 0_None,
m/z:1020.08(3+), RT:32.11, Y-score:97.22
HCD-MS/MS Scan:9482, Noise threshold:0.5
CID-MS/MS Scan:9483, Noise threshold:0.7
ETD-MS/MS Scan:9484, Noise threshold:1.1
Relative Intensity (%)
m/z
274.094(NeuAc − H2O)
204.088(HexNAc)
366.142(HexNAc + Hex)
292.104(NeuAc)
657.238(NeuAc + Hex + HexNAc)
910.466(PEP+HexNAc(1)_1)
1056.524(PEP+HexNAc(1)Fuc(1)_1)
1583.710(PEP+HexNAc(2)Hex(2)Fuc(1)_1)
1745.763(PEP+HexNAc(2)Hex(3)Fuc(1)_1)
1948.842(PEP+HexNAc(3)Hex(3)Fuc(1)_1)
657.237(NeuAc + Hex + HexNAc)
1201.501(PEP+HexNAc(3)Hex(4)Fuc(1)NeuAc(1)_2)
1384.066(PEP+HexNAc(4)Hex(5)Fuc(1)NeuAc(1)_2)
1745.764(PEP+HexNAc(2)Hex(3)Fuc(1)_1)
657.239(NeuAc + Hex + HexNAc)
1201.503(PEP+HexNAc(3)Hex(4)Fuc(1)NeuAc(1)_2)
1384.068(PEP+HexNAc(4)Hex(5)Fuc(1)NeuAc(1)_2)
1509.113
1521.610
M+2H

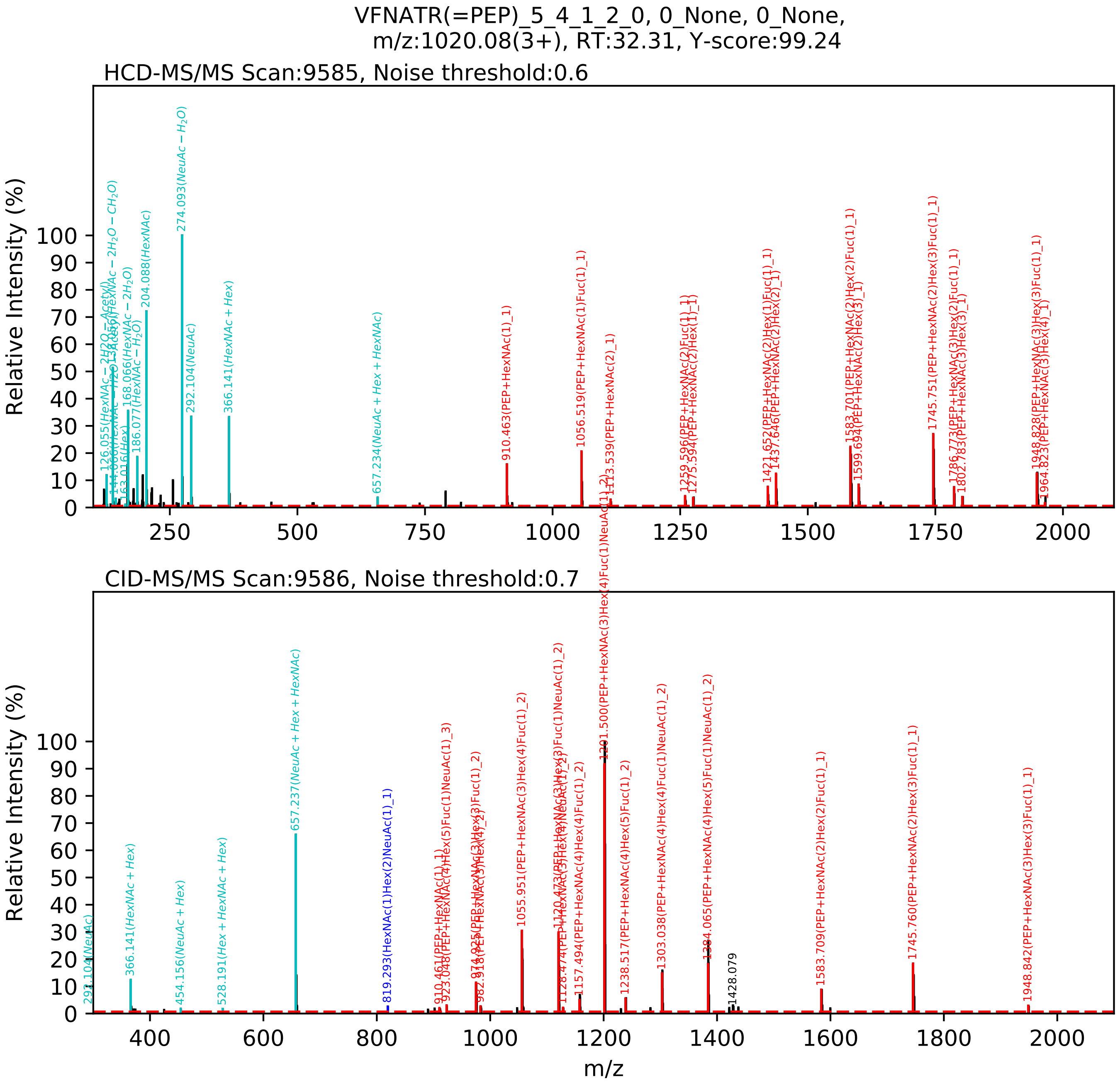
VFNATR(=PEP)_5_4_1_2_0, 0_None, 0_None,
m/z:1020.08(3+), RT:32.31, Y-score:99.24
HCD-MS/MS Scan:9585, Noise threshold:0.6
Relative Intensity (%)
274.093(NeuAc − H2O)
204.088(HexNAc)
292.104(NeuAc)
366.141(HexNAc + Hex)
657.234(NeuAc + Hex + HexNAc)
910.463(PEP+HexNAc(1)_1)
1056.519(PEP+HexNAc(1)Fuc(1)_1)
1113.539(PEP+HexNAc(2)_1)
1745.751(PEP+HexNAc(2)Hex(3)Fuc(1)_1)
CID-MS/MS Scan:9586, Noise threshold:0.7
Relative Intensity (%)
366.141(HexNAc + Hex)
454.156(NeuAc + Hex)
528.191(Hex + HexNAc + Hex)
657.237(NeuAc + Hex + HexNAc)
819.293(HexNAc(1)Hex(2)NeuAc(1)_1)
1055.951(PEP+HexNAc(3)Hex(4)Fuc(1)_2)
1157.494(PEP+HexNAc(4)Hex(4)Fuc(1)_2)
1201.500(PEP+HexNAc(3)Hex(4)Fuc(1)NeuAc(1)_2)
1238.517(PEP+HexNAc(4)Hex(5)Fuc(1)_2)
1303.038(PEP+HexNAc(4)Hex(4)Fuc(1)NeuAc(1)_2)
1384.065(PEP+HexNAc(4)Hex(5)Fuc(1)NeuAc(1)_2)
1428.079
1583.709(PEP+HexNAc(2)Hex(2)Fuc(1)_1)
1745.760(PEP+HexNAc(2)Hex(3)Fuc(1)_1)
1948.842(PEP+HexNAc(3)Hex(3)Fuc(1)_1)
m/z

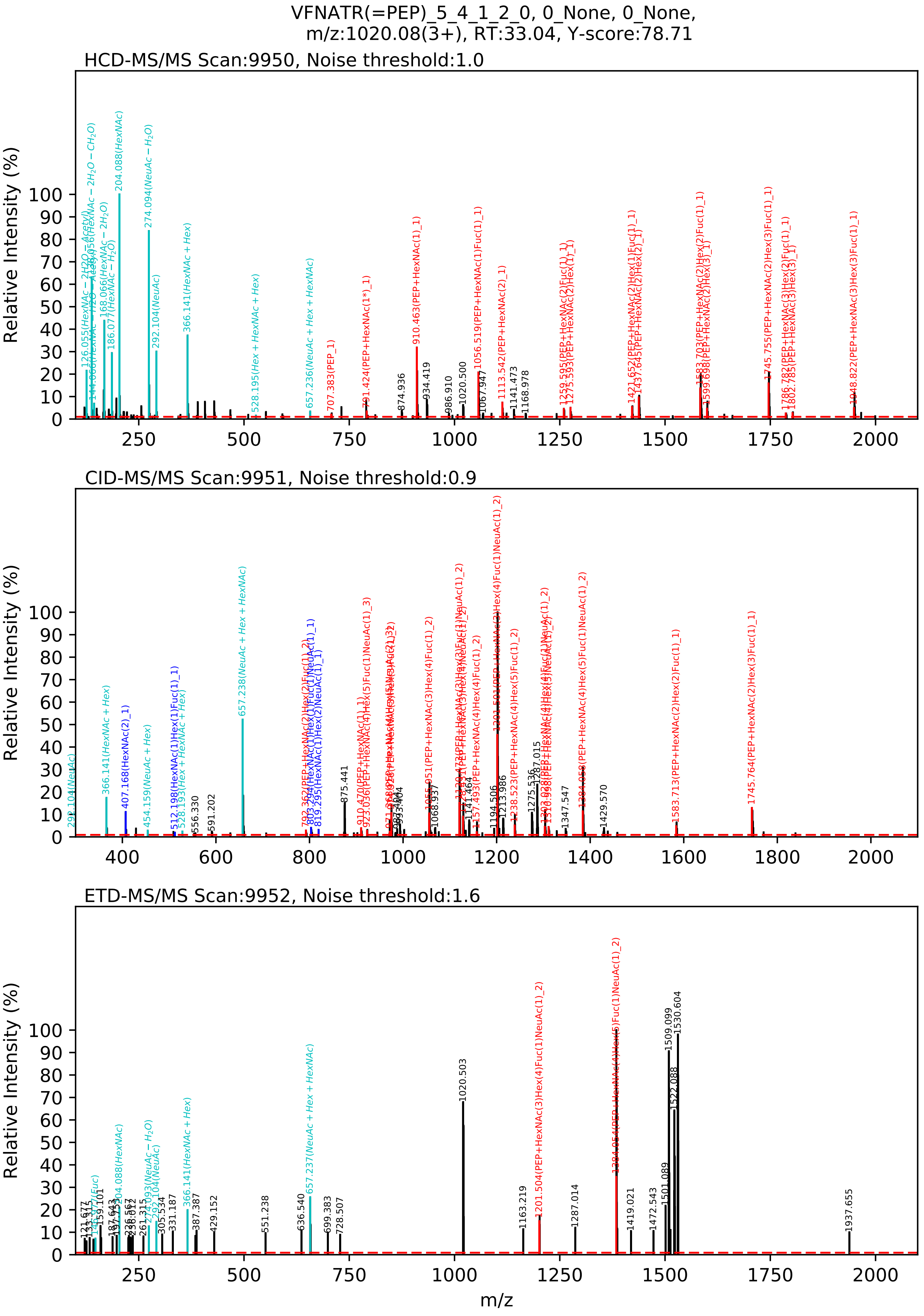
VFNATR(=PEP)_5_4_1_2_0, 0_None, 0_None,
m/z:1020.08(3+), RT:33.04, Y-score:78.71
HCD-MS/MS Scan:9950, Noise threshold:1.0
Relative Intensity (%)
126.055(HexNAc − 2H2O − Acetyl)
168.066(HexNAc − 2H2O)
186.077(HexNAc − H2O)
204.088(HexNAc)
274.094(NeuAc − H2O)
292.104(NeuAc)
366.141(HexNAc + Hex)
528.195(Hex + HexNAc + Hex)
657.236(NeuAc + Hex + HexNAc)
707.383(PEP_1)
791.424(PEP+HexNAc(1*)_1)
910.463(PEP+HexNAc(1)_1)
1056.519(PEP+HexNAc(1)Fuc(1)_1)
1113.542(PEP+HexNAc(2)_1)
1259.595(PEP+HexNAc(2)Fuc(1)_1)
1275.593(PEP+HexNAc(2)Hex(1)_1)
1421.652(PEP+HexNAc(2)Hex(1)Fuc(1)_1)
1437.645(PEP+HexNAc(2)Hex(2)_1)
1583.703(PEP+HexNAc(2)Hex(2)Fuc(1)_1)
1599.698(PEP+HexNAc(2)Hex(3)_1)
1745.755(PEP+HexNAc(2)Hex(3)Fuc(1)_1)
1786.782(PEP+HexNAc(3)Hex(2)Fuc(1)_1)
1802.785(PEP+HexNAc(3)Hex(3)_1)
1948.822(PEP+HexNAc(3)Hex(3)Fuc(1)_1)
874.936
934.419
986.910
1020.500
1067.947
1141.473
1168.978
CID-MS/MS Scan:9951, Noise threshold:0.9
292.104(NeuAc)
366.141(HexNAc + Hex)
407.168(HexNAc(2)_1)
454.159(NeuAc + Hex)
512.198(HexNAc(1)Hex(1)Fuc(1)_1)
528.193(Hex + HexNAc + Hex)
556.330
591.202
657.238(NeuAc + Hex + HexNAc)
875.441
1068.937
1141.464
1157.493(PEP+HexNAc(4)Hex(4)Fuc(1)_2)
1194.506
1213.986
1238.523(PEP+HexNAc(4)Hex(5)Fuc(1)_2)
1275.536
1287.015
1347.547
1384.058(PEP+HexNAc(4)Hex(5)Fuc(1)NeuAc(1)_2)
1429.570
1583.713(PEP+HexNAc(2)Hex(2)Fuc(1)_1)
1745.764(PEP+HexNAc(2)Hex(3)Fuc(1)_1)
ETD-MS/MS Scan:9952, Noise threshold:1.6
204.088(HexNAc)
274.093(NeuAc − H2O)
292.104(NeuAc)
366.141(HexNAc + Hex)
657.237(NeuAc + Hex + HexNAc)
139.101
261.315
305.534
331.187
387.387
429.152
551.238
636.540
699.383
728.507
1020.503
1163.219
1201.504(PEP+HexNAc(3)Hex(4)Fuc(1)NeuAc(1)_2)
1287.014
1419.021
1472.543
1501.089
1509.099
1522.088
1530.604
1937.655
m/z

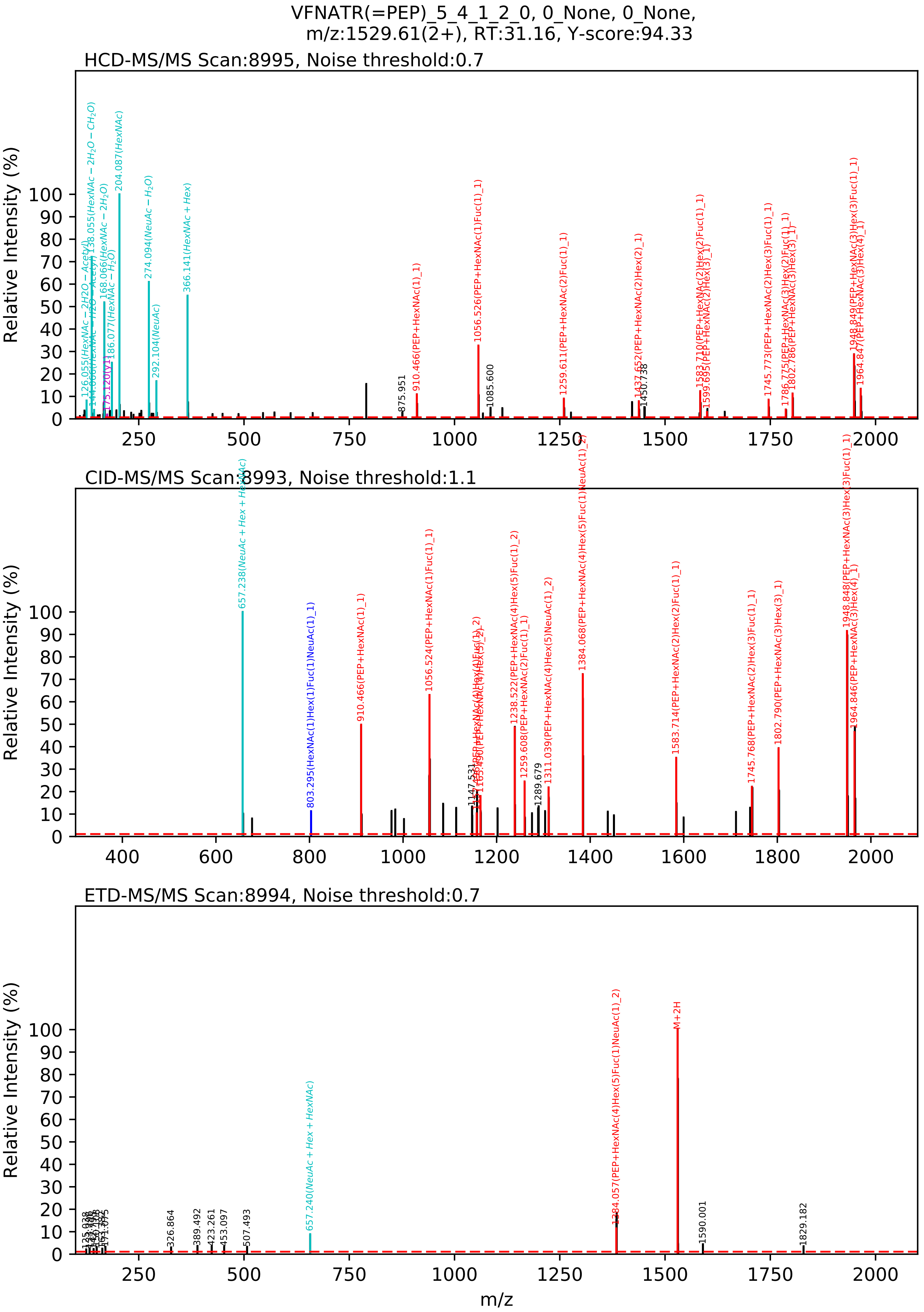

VFNATR(=PEP)_5_4_1_2_0, 0_None, 0_None,
m/z:1529.61(2+), RT:31.16, Y-score:94.33
HCD-MS/MS Scan:8995, Noise threshold:0.7
CID-MS/MS Scan:8993, Noise threshold:1.1
ETD-MS/MS Scan:8994, Noise threshold:0.7
Relative Intensity (%)
m/z
1056.526(PEP+HexNAc(1)Fuc(1)_1)
1948.849(PEP+HexNAc(3)Hex(3)Fuc(1)_1)
1384.068(PEP+HexNAc(4)Hex(5)Fuc(1)NeuAc(1)_2)
657.238(NeuAc + Hex + HexNAc)
M+2H

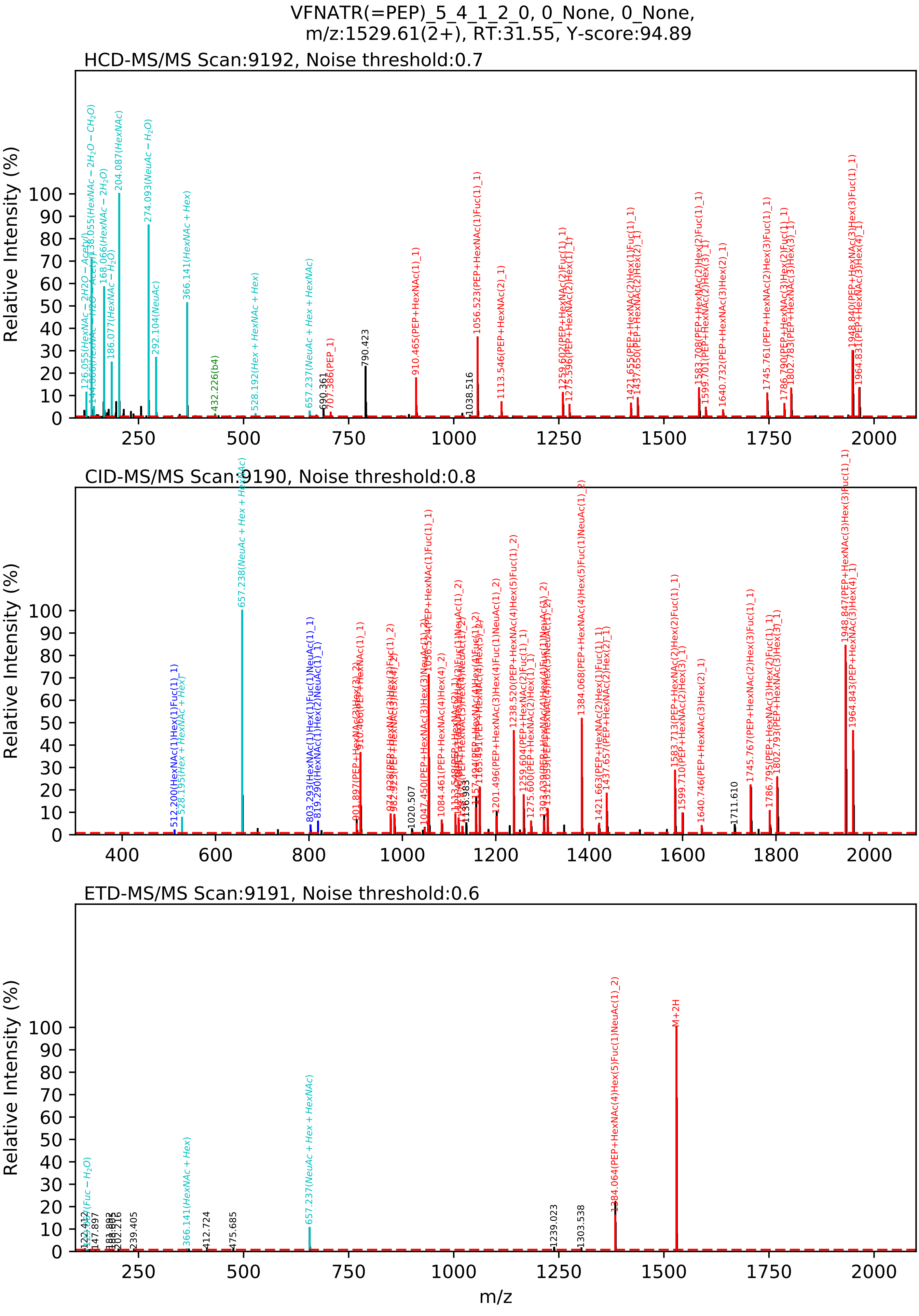

VFNATR(=PEP)_5_4_1_2_0, 0_None, 0_None,
m/z:1529.61(2+), RT:31.55, Y-score:94.89
HCD-MS/MS Scan:9192, Noise threshold:0.7
Relative Intensity (%)
204.087(HexNAc)
274.093(NeuAc − H2O)
366.141(HexNAc + Hex)
292.104(NeuAc)
186.077(HexNAc − H2O)
168.066(HexNAc − 2H2O)
432.226(b4)
528.192(Hex + HexNAc + Hex)
657.237(NeuAc + Hex + HexNAc)
690.361
707.386(PEP_1)
790.423
910.465(PEP+HexNAc(1)_1)
1038.516
1056.523(PEP+HexNAc(1)Fuc(1)_1)
1113.546(PEP+HexNAc(2)_1)
1259.602(PEP+HexNAc(2)Fuc(1)_1)
1275.596(PEP+HexNAc(2)Hex(1)_1)
1421.655(PEP+HexNAc(2)Hex(1)Fuc(1)_1)
1437.650(PEP+HexNAc(2)Hex(2)_1)
1583.708(PEP+HexNAc(2)Hex(2)Fuc(1)_1)
1599.701(PEP+HexNAc(2)Hex(3)_1)
1640.732(PEP+HexNAc(3)Hex(2)_1)
1745.761(PEP+HexNAc(2)Hex(3)Fuc(1)_1)
1786.790(PEP+HexNAc(3)Hex(2)Fuc(1)_1)
1802.783(PEP+HexNAc(3)Hex(3)_1)
1948.840(PEP+HexNAc(3)Hex(3)Fuc(1)_1)
1964.831(PEP+HexNAc(3)Hex(4)_1)
CID-MS/MS Scan:9190, Noise threshold:0.8
657.238(NeuAc + Hex + HexNAc)
512.200(HexNAc(1)Hex(1)Fuc(1)_1)
528.195(Hex + HexNAc + Hex)
803.293(HexNAc(1)Hex(1)Fuc(1)NeuAc(1)_1)
819.290(HexNAc(1)Hex(2)NeuAc(1)_1)
1020.507
1047.450(PEP+HexNAc(3)Hex(3)NeuAc(1)_2)
1056.524(PEP+HexNAc(1)Fuc(1)_1)
1084.461(PEP+HexNAc(4)Hex(4)_2)
1136.983
1201.496(PEP+HexNAc(3)Hex(4)Fuc(1)NeuAc(1)_2)
1238.520(PEP+HexNAc(4)Hex(5)Fuc(1)_2)
1259.604(PEP+HexNAc(2)Fuc(1)_1)
1275.600(PEP+HexNAc(2)Hex(1)_1)
1384.068(PEP+HexNAc(4)Hex(5)Fuc(1)NeuAc(1)_2)
1421.663(PEP+HexNAc(2)Hex(1)Fuc(1)_1)
1437.657(PEP+HexNAc(2)Hex(2)_1)
1583.713(PEP+HexNAc(2)Hex(2)Fuc(1)_1)
1599.710(PEP+HexNAc(2)Hex(3)_1)
1640.746(PEP+HexNAc(3)Hex(2)_1)
1711.610
1745.767(PEP+HexNAc(2)Hex(3)Fuc(1)_1)
1786.795(PEP+HexNAc(3)Hex(2)Fuc(1)_1)
1802.793(PEP+HexNAc(3)Hex(3)_1)
1948.847(PEP+HexNAc(3)Hex(3)Fuc(1)_1)
1964.843(PEP+HexNAc(3)Hex(4)_1)
ETD-MS/MS Scan:9191, Noise threshold:0.6
147.897
202.216
239.405
366.141(HexNAc + Hex)
412.724
475.685
657.237(NeuAc + Hex + HexNAc)
1239.023
1303.538
1384.064(PEP+HexNAc(4)Hex(5)Fuc(1)NeuAc(1)_2)
M+2H
m/z

VFNATR(=PEP)_5_4_1_2_0, 0_None, 0_None,
m/z:1529.61(2+), RT:32.17, Y-score:94.34

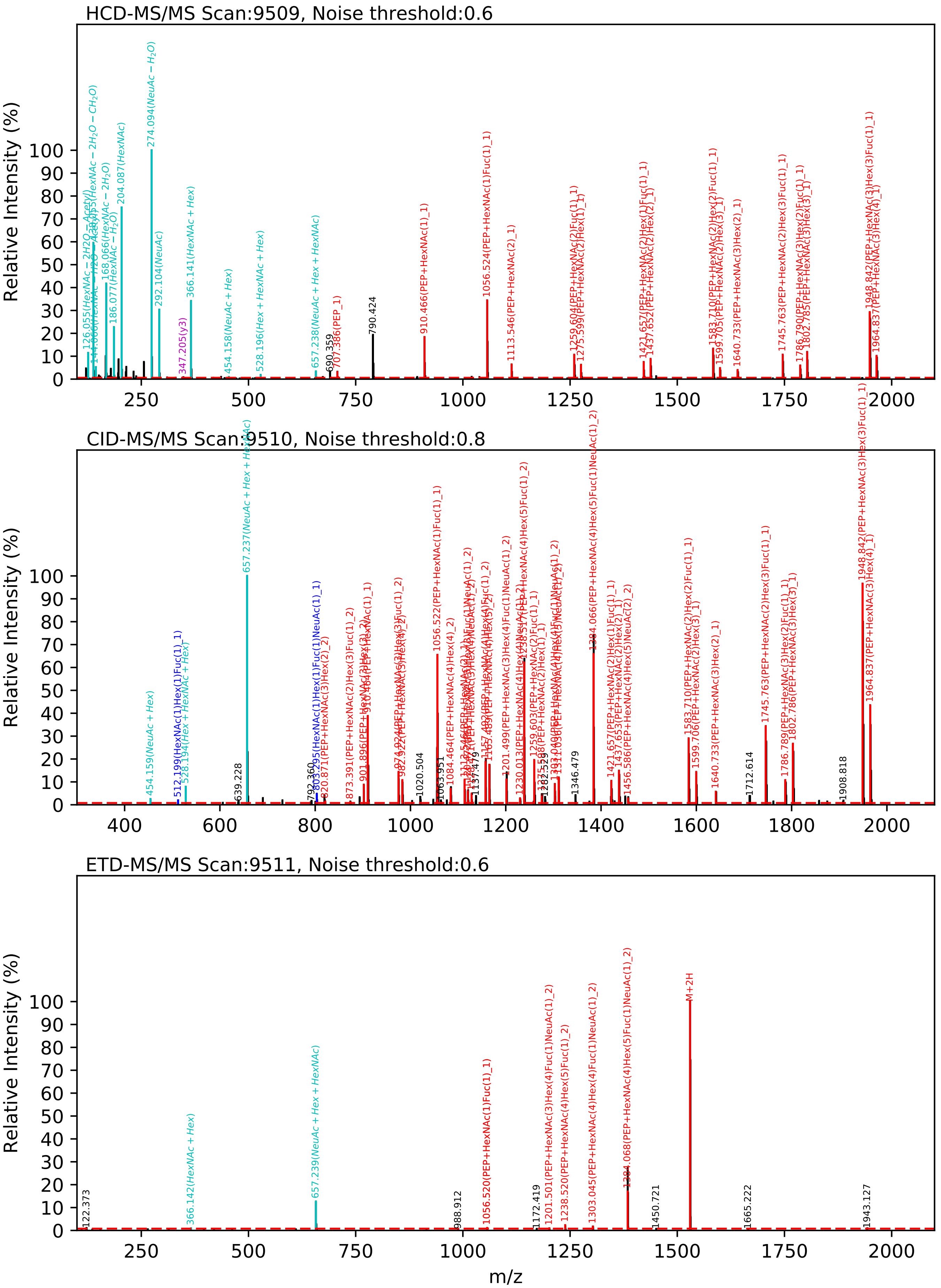

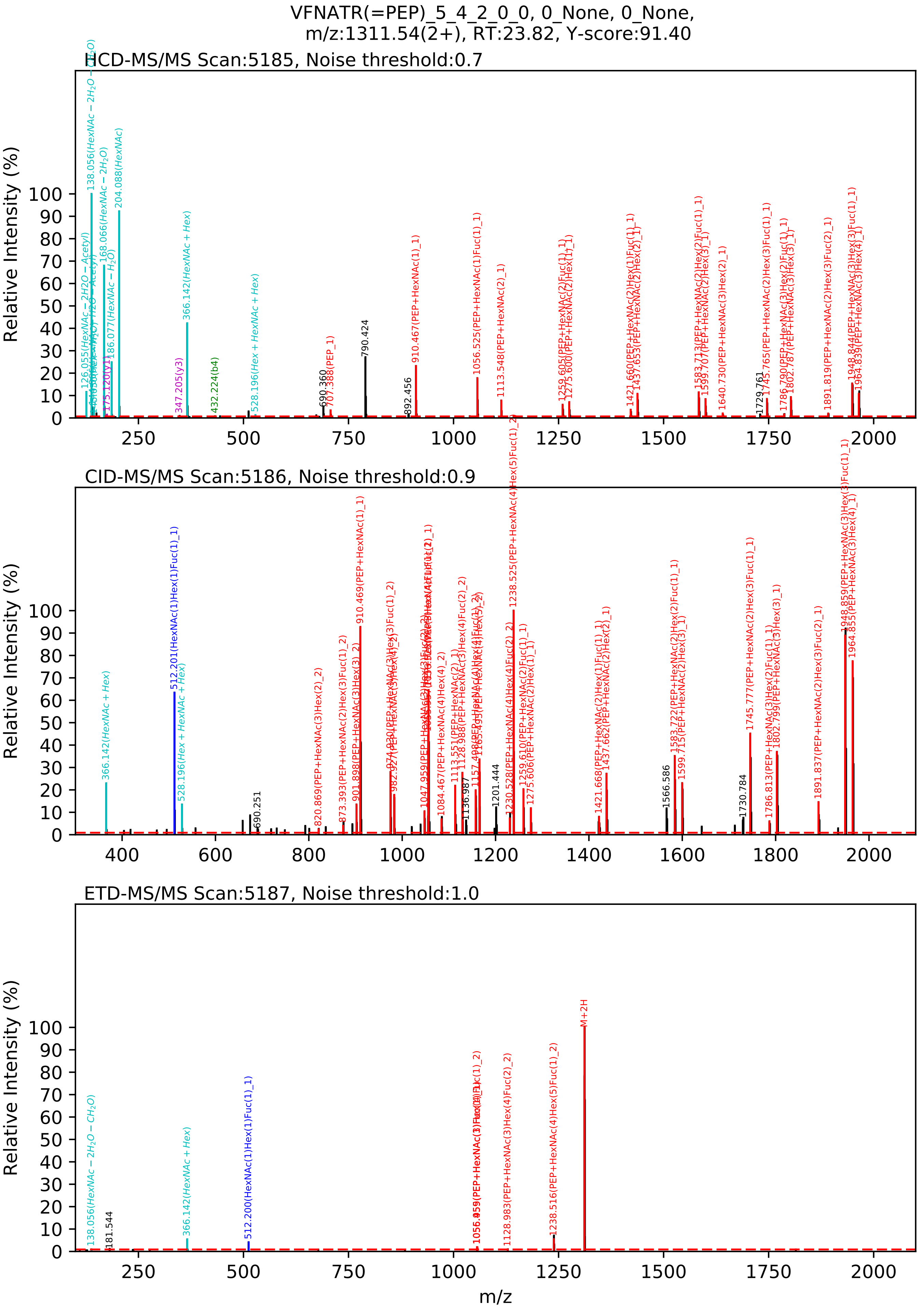

VFNATR(=PEP)_5_4_2_0_0, 0_None, 0_None,
m/z:1311.54(2+), RT:23.82, Y-score:91.40
HCD-MS/MS Scan:5185, Noise threshold:0.7
CID-MS/MS Scan:5186, Noise threshold:0.9
ETD-MS/MS Scan:5187, Noise threshold:1.0
Relative Intensity (%)
m/z
910.467(PEP+HexNAc(1)_1)
1056.525(PEP+HexNAc(1)Fuc(1)_1)
1113.548(PEP+HexNAc(2)_1)
790.424
1238.525(PEP+HexNAc(4)Hex(5)Fuc(1)_2)
512.201(HexNAc(1)Hex(1)Fuc(1)_1)
1948.859(PEP+HexNAc(3)Hex(3)Fuc(1)_1)
1964.855(PEP+HexNAc(3)Hex(4)_1)
M+2H
1238.516(PEP+HexNAc(4)Hex(5)Fuc(1)_2)
1128.983(PEP+HexNAc(3)Hex(4)Fuc(2)_2)
512.200(HexNAc(1)Hex(1)Fuc(1)_1)
366.142(HexNAc+Hex)

VFNATR(=PEP)_5_4_2_0_0, 0_None, 0_None,
m/z:1311.54(2+), RT:24.43, Y-score:98.56

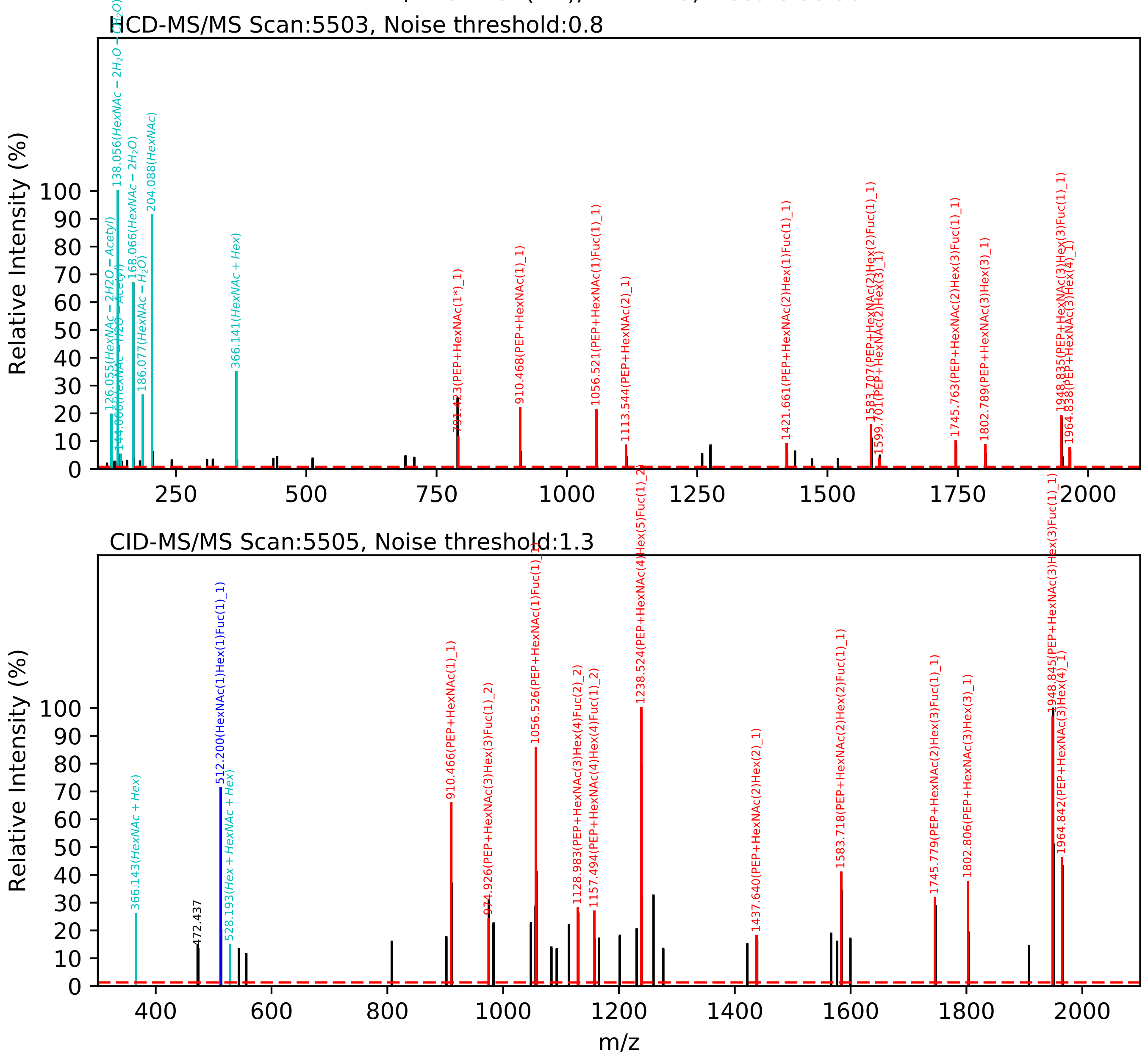

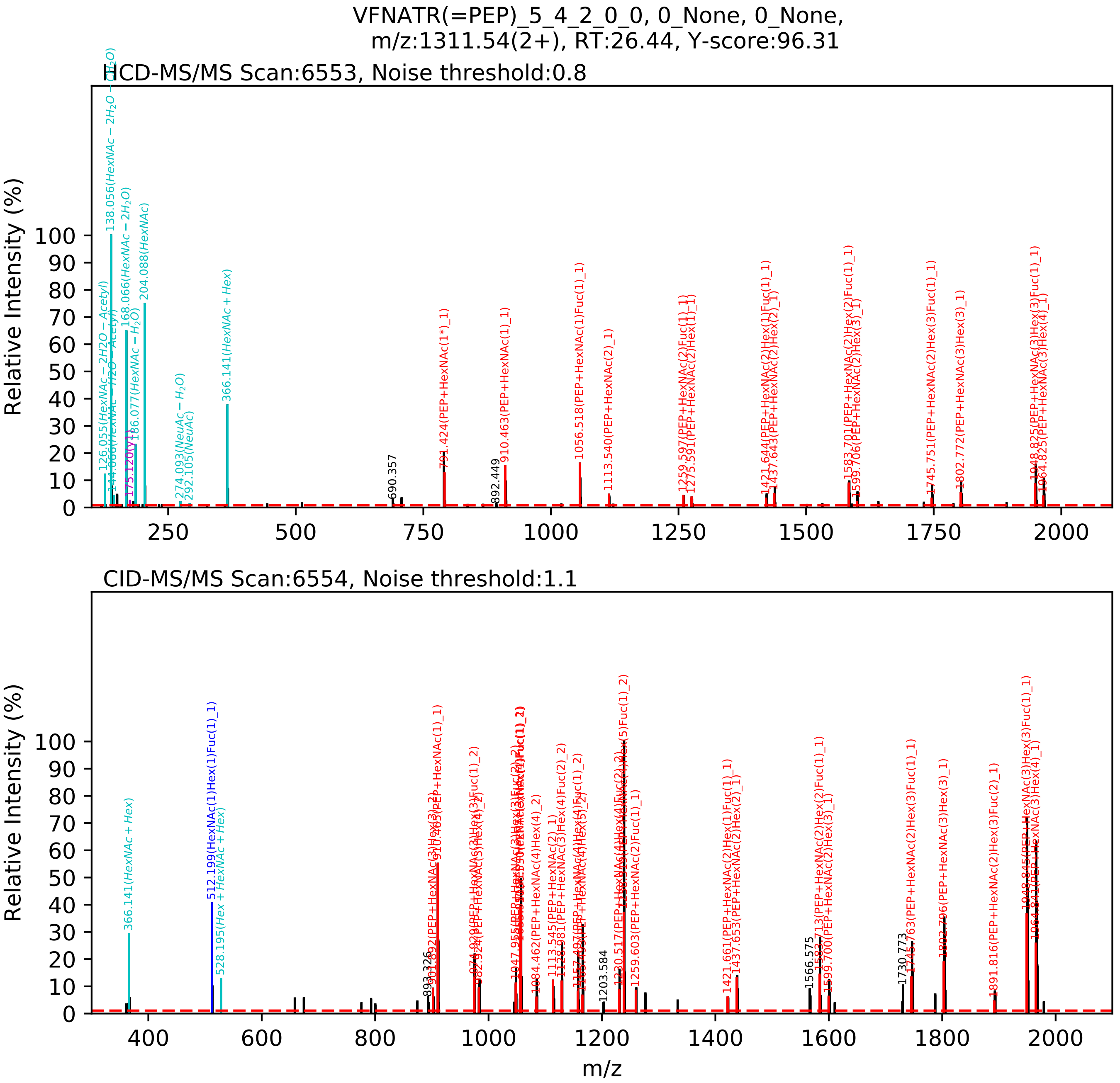
VFNATR(=PEP)_5_4_2_0_0, 0_None, 0_None,
m/z:1311.54(2+), RT:26.44, Y-score:96.31
HCD-MS/MS Scan:6553, Noise threshold:0.8
Relative Intensity (%)
138.056(HexNAc − 2H2O − Acetyl)
168.066(HexNAc − 2H2O)
204.088(HexNAc)
186.077(HexNAc − H2O)
175.120(y1)
274.093(NeuAc − H2O)
292.105(NeuAc)
366.141(HexNAc + Hex)
690.357
791.424(PEP+HexNAc(1*)_1)
892.449
910.463(PEP+HexNAc(1)_1)
1056.518(PEP+HexNAc(1)Fuc(1)_1)
1113.540(PEP+HexNAc(2)_1)
1259.597(PEP+HexNAc(2)Fuc(1)_1)
1275.591(PEP+HexNAc(2)Hex(1)_1)
1421.644(PEP+HexNAc(2)Hex(1)Fuc(1)_1)
1437.643(PEP+HexNAc(2)Hex(2)_1)
1583.701(PEP+HexNAc(2)Hex(2)Fuc(1)_1)
1745.751(PEP+HexNAc(2)Hex(3)Fuc(1)_1)
1802.772(PEP+HexNAc(3)Hex(3)_1)
1948.825(PEP+HexNAc(3)Hex(3)Fuc(1)_1)
1964.825(PEP+HexNAc(3)Hex(4)_1)
CID-MS/MS Scan:6554, Noise threshold:1.1
366.141(HexNAc + Hex)
512.199(HexNAc(1)Hex(1)Fuc(1)_1)
528.195(Hex + HexNAc + Hex)
893.326
910.463(PEP+HexNAc(1)_1)
1084.462(PEP+HexNAc(4)Hex(4)_2)
1203.584
1259.603(PEP+HexNAc(2)Fuc(1)_1)
1421.661(PEP+HexNAc(2)Hex(1)Fuc(1)_1)
1437.653(PEP+HexNAc(2)Hex(2)_1)
1566.575
1599.700(PEP+HexNAc(2)Hex(3)_1)
1730.773
1802.796(PEP+HexNAc(3)Hex(3)_1)
1891.816(PEP+HexNAc(2)Hex(3)Fuc(2)_1)
m/z
0
10
20
30
40
50
60
70
80
90
100
250
500
750
1000
1250
1500
1750
2000
400
600
800
1200
1400
1600
1800

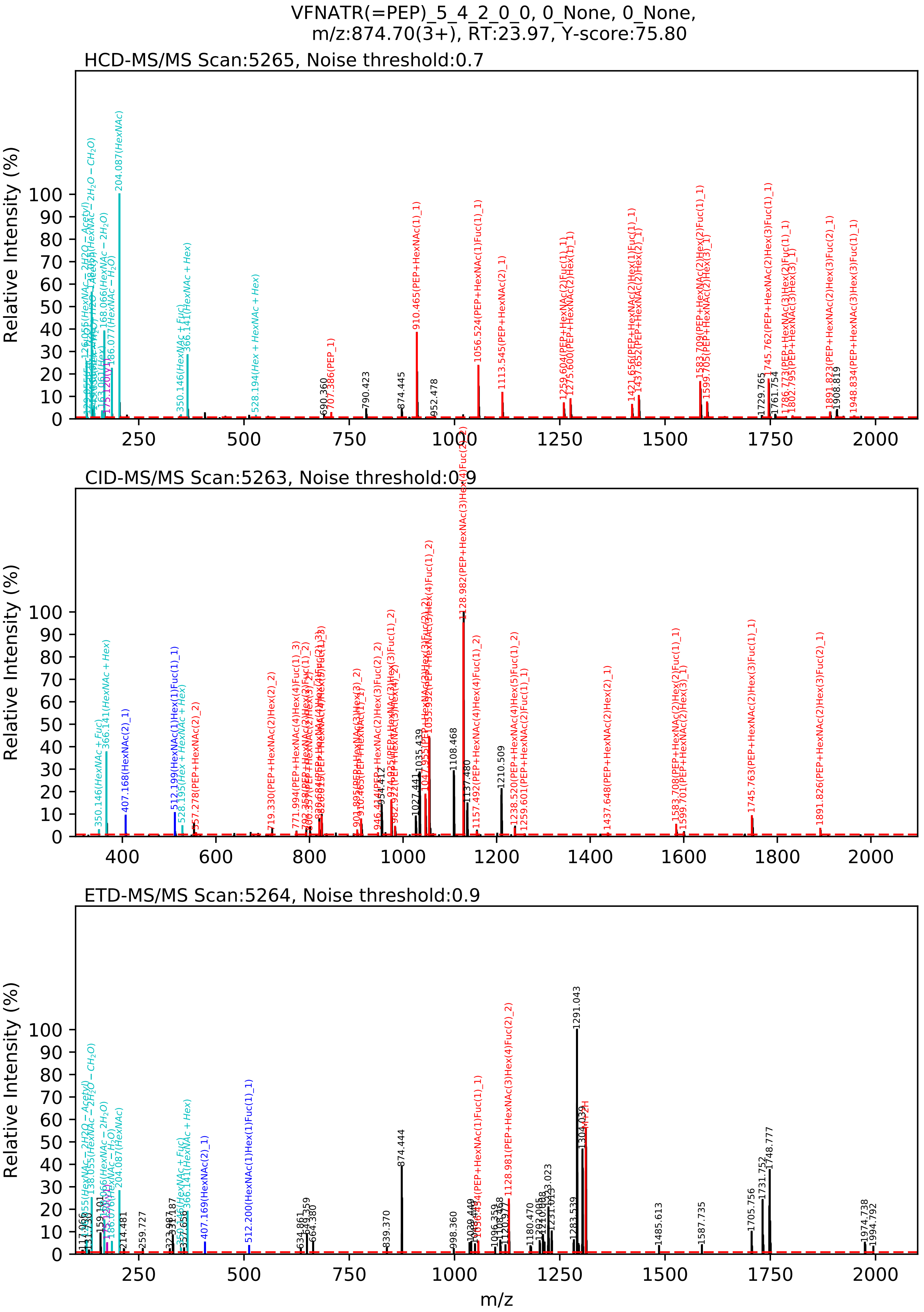
VFNATR(=PEP)_5_4_2_0_0, 0_None, 0_None,
m/z:874.70(3+), RT:23.97, Y-score:75.80
HCD-MS/MS Scan:5265, Noise threshold:0.7
CID-MS/MS Scan:5263, Noise threshold:0.9
ETD-MS/MS Scan:5264, Noise threshold:0.9
Relative Intensity (%)
m/z

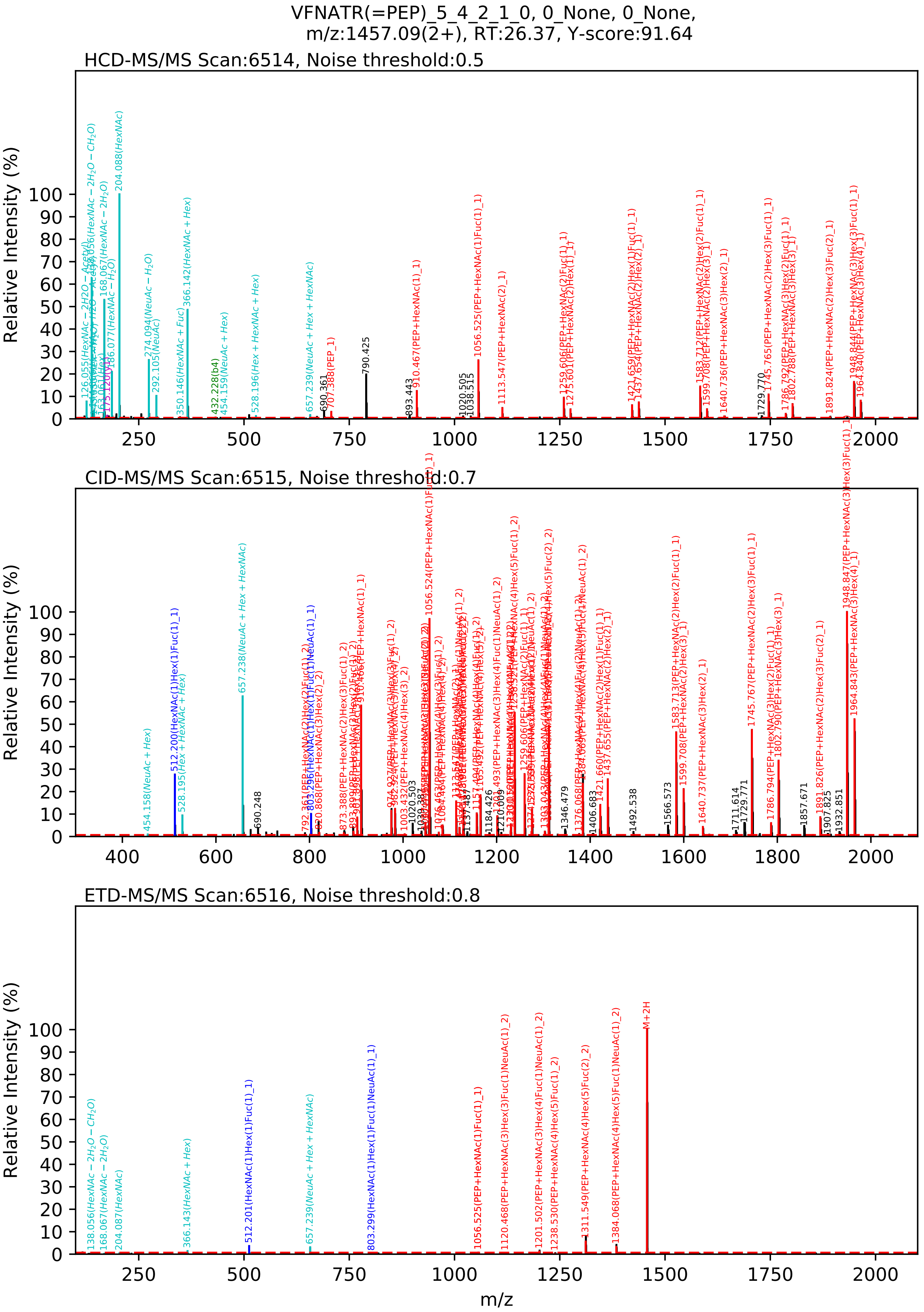
VFNATR(=PEP)_5_4_2_1_0, 0_None, 0_None,
m/z:1457.09(2+), RT:26.37, Y-score:91.64
HCD-MS/MS Scan:6514, Noise threshold:0.5
Relative Intensity (%)
204.088(HexNAc)
168.067(HexNAc − 2H2O)
186.077(HexNAc − H2O)
274.094(NeuAc − H2O)
292.105(NeuAc)
366.142(HexNAc + Hex)
350.146(HexNAc + Fuc)
432.228(b4)
454.159(NeuAc + Hex)
528.196(Hex + HexNAc + Hex)
657.239(NeuAc + Hex + HexNAc)
690.361
707.388(PEP_1)
790.425
893.443
910.467(PEP+HexNAc(1)_1)
1020.505
1038.515
1056.525(PEP+HexNAc(1)Fuc(1)_1)
1113.547(PEP+HexNAc(2)_1)
1259.606(PEP+HexNAc(2)Fuc(1)_1)
1275.601(PEP+HexNAc(2)Hex(1)_1)
1421.659(PEP+HexNAc(2)Hex(1)Fuc(1)_1)
1437.654(PEP+HexNAc(2)Hex(2)_1)
1583.712(PEP+HexNAc(2)Hex(2)Fuc(1)_1)
1599.708(PEP+HexNAc(2)Hex(3)_1)
1640.736(PEP+HexNAc(3)Hex(2)_1)
1729.770
1745.765(PEP+HexNAc(2)Hex(3)Fuc(1)_1)
1786.792(PEP+HexNAc(3)Hex(2)Fuc(1)_1)
1802.788(PEP+HexNAc(3)Hex(3)_1)
1891.824(PEP+HexNAc(2)Hex(3)Fuc(2)_1)
1948.844(PEP+HexNAc(3)Hex(3)Fuc(1)_1)
1964.840(PEP+HexNAc(3)Hex(4)_1)
CID-MS/MS Scan:6515, Noise threshold:0.7
454.158(NeuAc + Hex)
512.200(HexNAc(1)Hex(1)Fuc(1)_1)
528.195(Hex + HexNAc + Hex)
657.238(NeuAc + Hex + HexNAc)
690.248
803.296(HexNAc(1)Hex(1)Fuc(1)NeuAc(1)_1)
820.868(PEP+HexNAc(3)Hex(2)_2)
1056.524(PEP+HexNAc(1)Fuc(1)_1)
1583.713(PEP+HexNAc(2)Hex(2)Fuc(1)_1)
1599.708(PEP+HexNAc(2)Hex(3)_1)
1640.737(PEP+HexNAc(3)Hex(2)_1)
1745.767(PEP+HexNAc(2)Hex(3)Fuc(1)_1)
1786.794(PEP+HexNAc(3)Hex(2)Fuc(1)_1)
1802.790(PEP+HexNAc(3)Hex(3)_1)
1891.826(PEP+HexNAc(2)Hex(3)Fuc(2)_1)
1948.847(PEP+HexNAc(3)Hex(3)Fuc(1)_1)
1964.843(PEP+HexNAc(3)Hex(4)_1)
ETD-MS/MS Scan:6516, Noise threshold:0.8
138.056(HexNAc − 2H2O − CH2O)
168.067(HexNAc − 2H2O)
204.087(HexNAc)
366.143(HexNAc + Hex)
512.201(HexNAc(1)Hex(1)Fuc(1)_1)
657.239(NeuAc + Hex + HexNAc)
803.299(HexNAc(1)Hex(1)Fuc(1)NeuAc(1)_1)
1056.525(PEP+HexNAc(1)Fuc(1)_1)
1120.468(PEP+HexNAc(3)Hex(3)Fuc(1)NeuAc(1)_2)
1201.502(PEP+HexNAc(3)Hex(4)Fuc(1)NeuAc(1)_2)
1238.530(PEP+HexNAc(4)Hex(5)Fuc(1)_2)
1311.549(PEP+HexNAc(4)Hex(5)Fuc(2)_2)
1384.068(PEP+HexNAc(4)Hex(5)Fuc(1)NeuAc(1)_2)
M+2H
m/z

VFNATR(=PEP)_5_4_2_1_0, 0_None, 0_None,
m/z:1457.09(2+), RT:26.94, Y-score:95.07

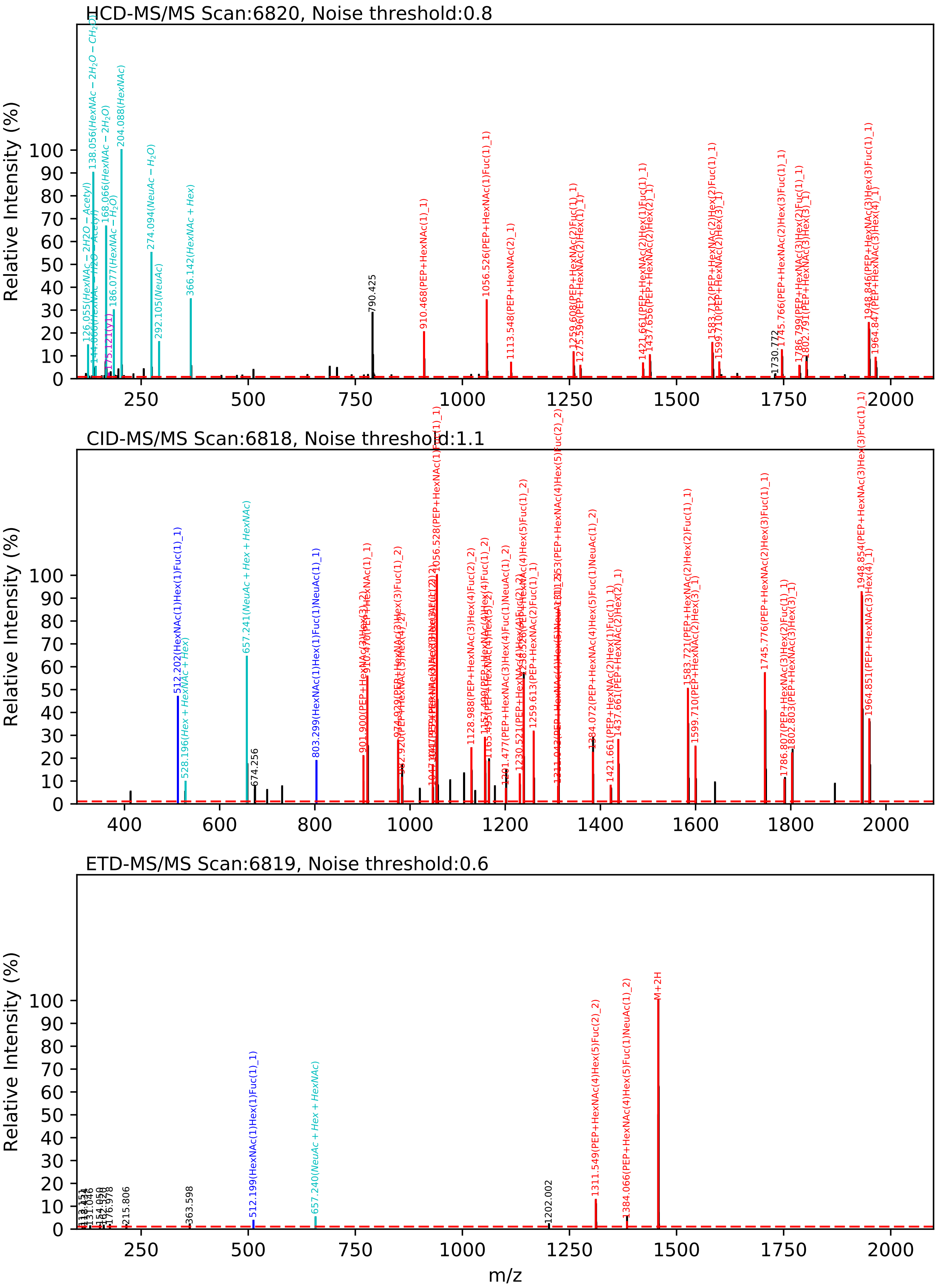

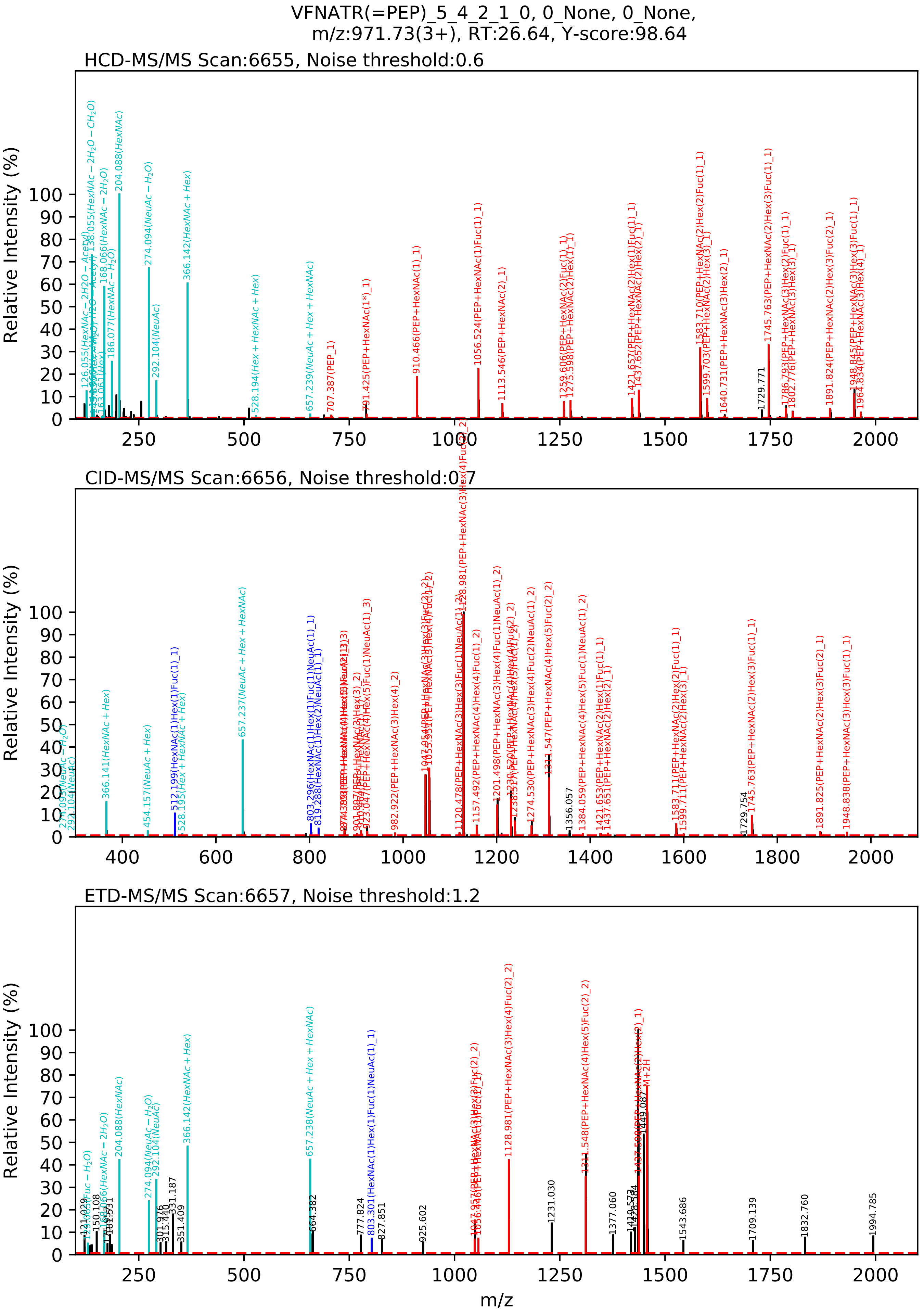
VFNATR(=PEP)_5_4_2_1_0, 0_None, 0_None,
m/z:971.73(3+), RT:26.64, Y-score:98.64
HCD-MS/MS Scan:6655, Noise threshold:0.6
Relative Intensity (%)
204.088(HexNAc)
274.094(NeuAc − H2O)
292.104(NeuAc)
366.142(HexNAc + Hex)
528.194(Hex + HexNAc + Hex)
657.239(NeuAc + Hex + HexNAc)
707.387(PEP_1)
791.425(PEP+HexNAc(1*)_1)
910.466(PEP+HexNAc(1)_1)
1056.524(PEP+HexNAc(1)Fuc(1)_1)
1113.546(PEP+HexNAc(2)_1)
1259.606(PEP+HexNAc(2)Fuc(1)_1)
1275.598(PEP+HexNAc(2)Hex(1)_1)
1421.657(PEP+HexNAc(2)Hex(1)Fuc(1)_1)
1437.652(PEP+HexNAc(2)Hex(2)_1)
1583.710(PEP+HexNAc(2)Hex(2)Fuc(1)_1)
1599.703(PEP+HexNAc(2)Hex(3)_1)
1640.731(PEP+HexNAc(3)Hex(2)_1)
1729.771
1745.763(PEP+HexNAc(2)Hex(3)Fuc(1)_1)
1891.824(PEP+HexNAc(2)Hex(3)Fuc(2)_1)
1948.845(PEP+HexNAc(3)Hex(3)Fuc(1)_1)
1964.834(PEP+HexNAc(3)Hex(4)_1)
CID-MS/MS Scan:6656, Noise threshold:0.7
Relative Intensity (%)
366.141(HexNAc + Hex)
454.157(NeuAc + Hex)
512.199(HexNAc(1)Hex(1)Fuc(1)_1)
528.195(Hex + HexNAc + Hex)
657.237(NeuAc + Hex + HexNAc)
803.296(HexNAc(1)Hex(1)Fuc(1)NeuAc(1)_1)
819.288(HexNAc(1)Hex(2)NeuAc(1)_1)
982.922(PEP+HexNAc(3)Hex(4)_2)
1047.954(PEP+HexNAc(3)Hex(3)Fuc(2)_2)
1120.478(PEP+HexNAc(3)Hex(3)Fuc(1)NeuAc(1)_3)
1128.981(PEP+HexNAc(3)Hex(4)Fuc(1)_2)
1157.492(PEP+HexNAc(4)Hex(4)Fuc(1)_2)
1201.498(PEP+HexNAc(3)Hex(4)Fuc(1)NeuAc(1)_2)
1274.530(PEP+HexNAc(3)Hex(4)Fuc(2)NeuAc(1)_2)
1311.547(PEP+HexNAc(4)Hex(5)Fuc(2)_2)
1356.057
1384.059(PEP+HexNAc(4)Hex(5)Fuc(1)NeuAc(1)_2)
1421.653(PEP+HexNAc(2)Hex(1)Fuc(1)_1)
1437.651(PEP+HexNAc(2)Hex(2)_1)
1583.711(PEP+HexNAc(2)Hex(2)Fuc(1)_1)
1599.711(PEP+HexNAc(2)Hex(3)_1)
1729.754
1745.763(PEP+HexNAc(2)Hex(3)Fuc(1)_1)
1891.825(PEP+HexNAc(2)Hex(3)Fuc(2)_1)
1948.838(PEP+HexNAc(3)Hex(3)Fuc(1)_1)
ETD-MS/MS Scan:6657, Noise threshold:1.2
Relative Intensity (%)
121.029
150.108
204.088(HexNAc)
274.094(NeuAc − H2O)
292.104(NeuAc)
301.976
315.440
331.187
351.409
366.142(HexNAc + Hex)
657.238(NeuAc + Hex + HexNAc)
664.382
777.824
803.301(HexNAc(1)Hex(1)Fuc(1)NeuAc(1)_1)
827.851
925.602
1128.981(PEP+HexNAc(3)Hex(4)Fuc(2)_2)
1231.030
1311.548(PEP+HexNAc(4)Hex(5)Fuc(2)_2)
1377.060
1449.087
M+2H
1543.686
1709.139
1832.760
1994.785
m/z

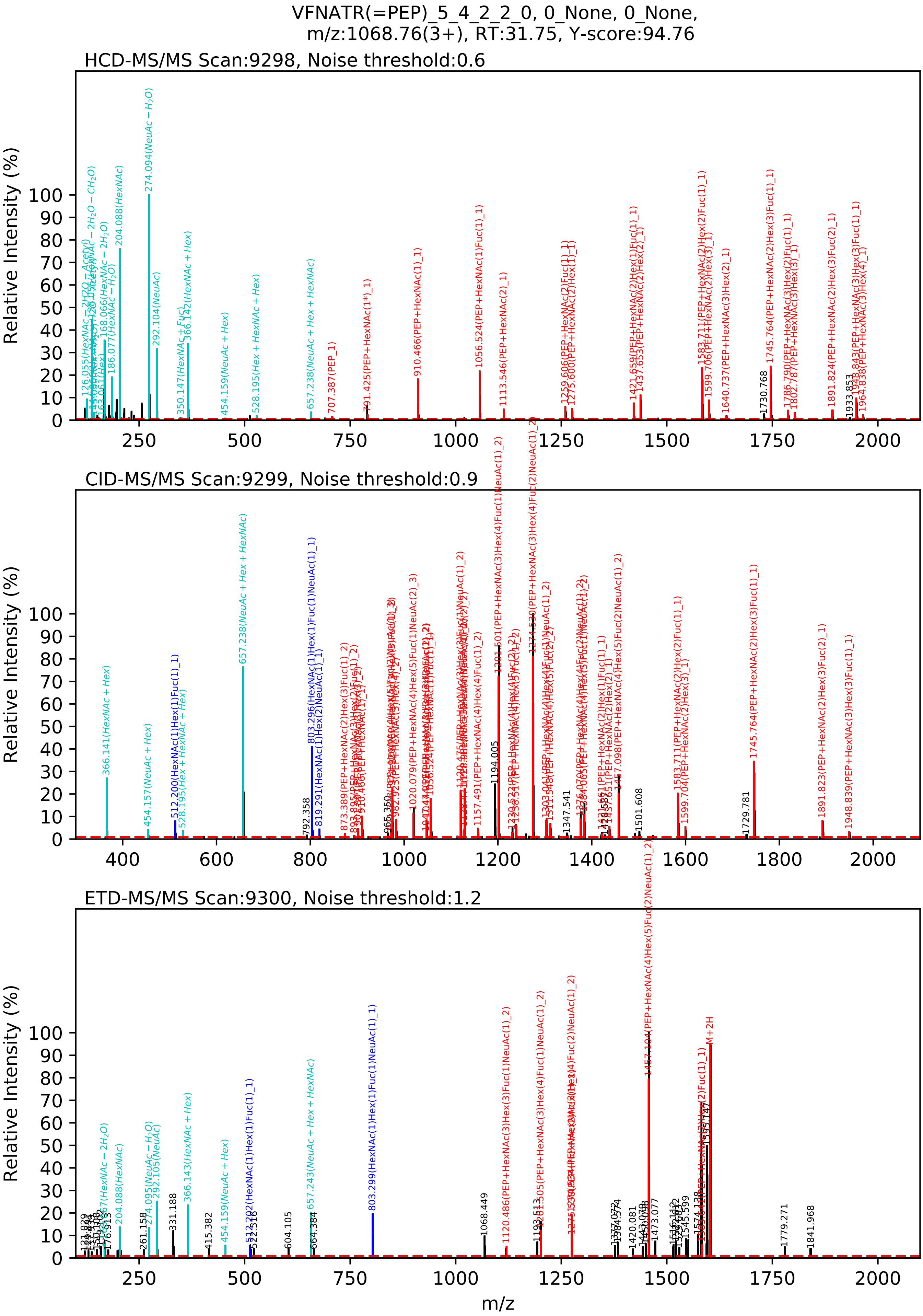

VFNATR(=PEP)_5_4_2_2_0, 0_None, 0_None,
m/z:1068.76(3+), RT:31.75, Y-score:94.76
HCD-MS/MS Scan:9298, Noise threshold:0.6
CID-MS/MS Scan:9299, Noise threshold:0.9
ETD-MS/MS Scan:9300, Noise threshold:1.2
Relative Intensity (%)
m/z
274.094(NeuAc − H2O)
204.088(HexNAc)
292.104(NeuAc)
366.142(HexNAc + Hex)
454.159(NeuAc + Hex)
528.195(Hex + HexNAc + Hex)
657.238(NeuAc + Hex + HexNAc)
707.387(PEP_1)
791.425(PEP+HexNAc(1*)_1)
910.466(PEP+HexNAc(1)_1)
1056.524(PEP+HexNAc(1)Fuc(1)_1)
1113.546(PEP+HexNAc(2)_1)
1599.704(PEP+HexNAc(2)Hex(3)_1)
1640.737(PEP+HexNAc(3)Hex(2)_1)
1730.768
1745.764(PEP+HexNAc(2)Hex(3)Fuc(1)_1)
1891.824(PEP+HexNAc(2)Hex(3)Fuc(2)_1)
1933.853
366.141(HexNAc + Hex)
454.157(NeuAc + Hex)
512.200(HexNAc(1)Hex(1)Fuc(1)_1)
657.238(NeuAc + Hex + HexNAc)
792.358
803.296(HexNAc(1)Hex(1)Fuc(1)NeuAc(1)_1)
819.291(HexNAc(1)Hex(2)NeuAc(1)_1)
1020.079(PEP+HexNAc(4)Hex(5)Fuc(1)NeuAc(2)_3)
1157.491(PEP+HexNAc(4)Hex(4)Fuc(1)_2)
1194.005
1347.541
1501.608
1583.711(PEP+HexNAc(2)Hex(2)Fuc(1)_1)
1599.704(PEP+HexNAc(2)Hex(3)_1)
1729.781
1745.764(PEP+HexNAc(2)Hex(3)Fuc(1)_1)
1891.823(PEP+HexNAc(2)Hex(3)Fuc(2)_1)
1948.839(PEP+HexNAc(3)Hex(3)Fuc(1)_1)
261.158
331.188
415.382
604.105
664.384
803.299(HexNAc(1)Hex(1)Fuc(1)NeuAc(1)_1)
1068.449
1120.486(PEP+HexNAc(3)Hex(3)Fuc(1)NeuAc(1)_2)
1473.077
1779.271
1841.968
M+2H

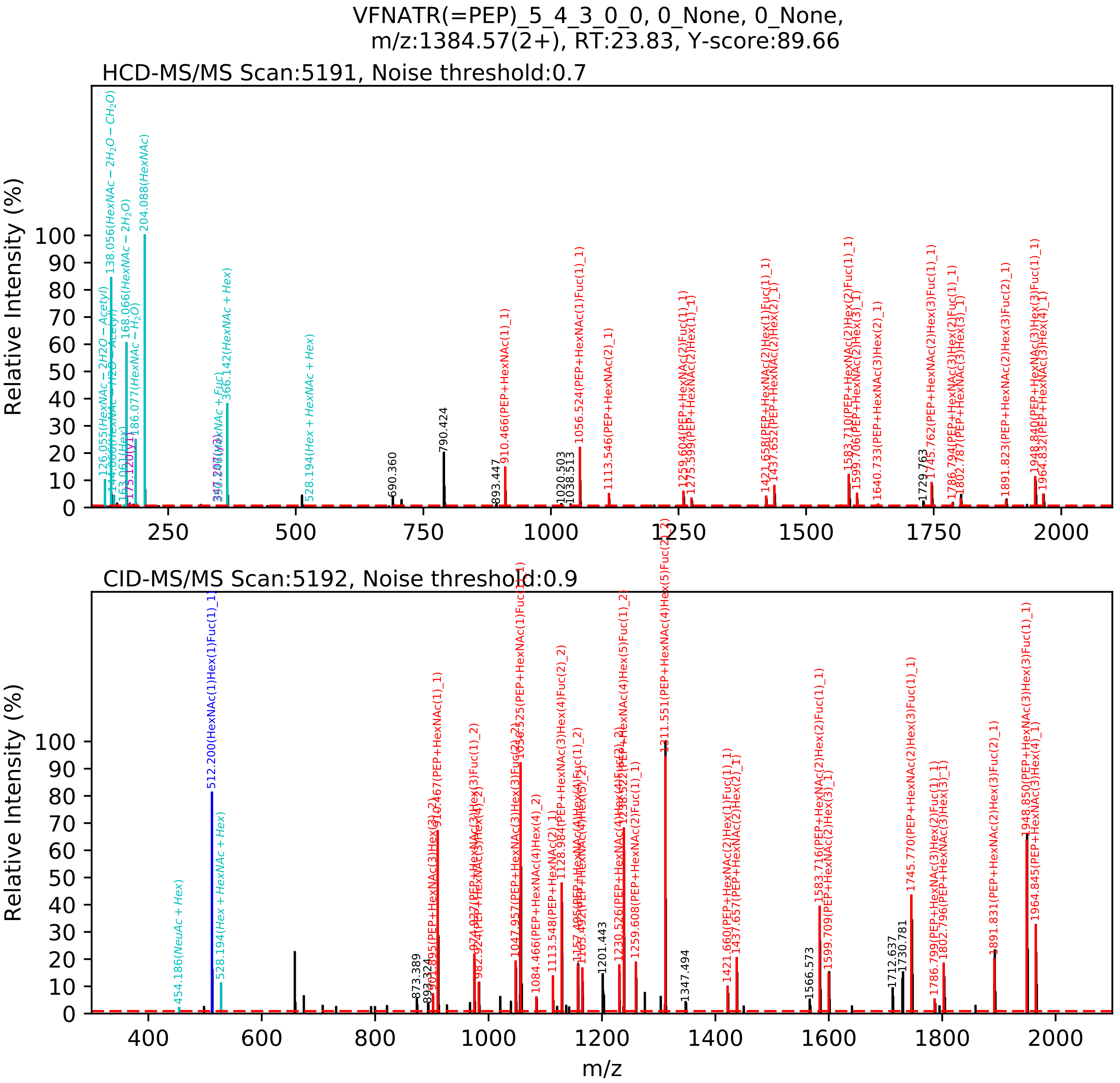
VFNATR(=PEP)_5_4_3_0_0, 0_None, 0_None,
m/z:1384.57(2+), RT:23.83, Y-score:89.66
HCD-MS/MS Scan:5191, Noise threshold:0.7
Relative Intensity (%)
CID-MS/MS Scan:5192, Noise threshold:0.9
Relative Intensity (%)
m/z

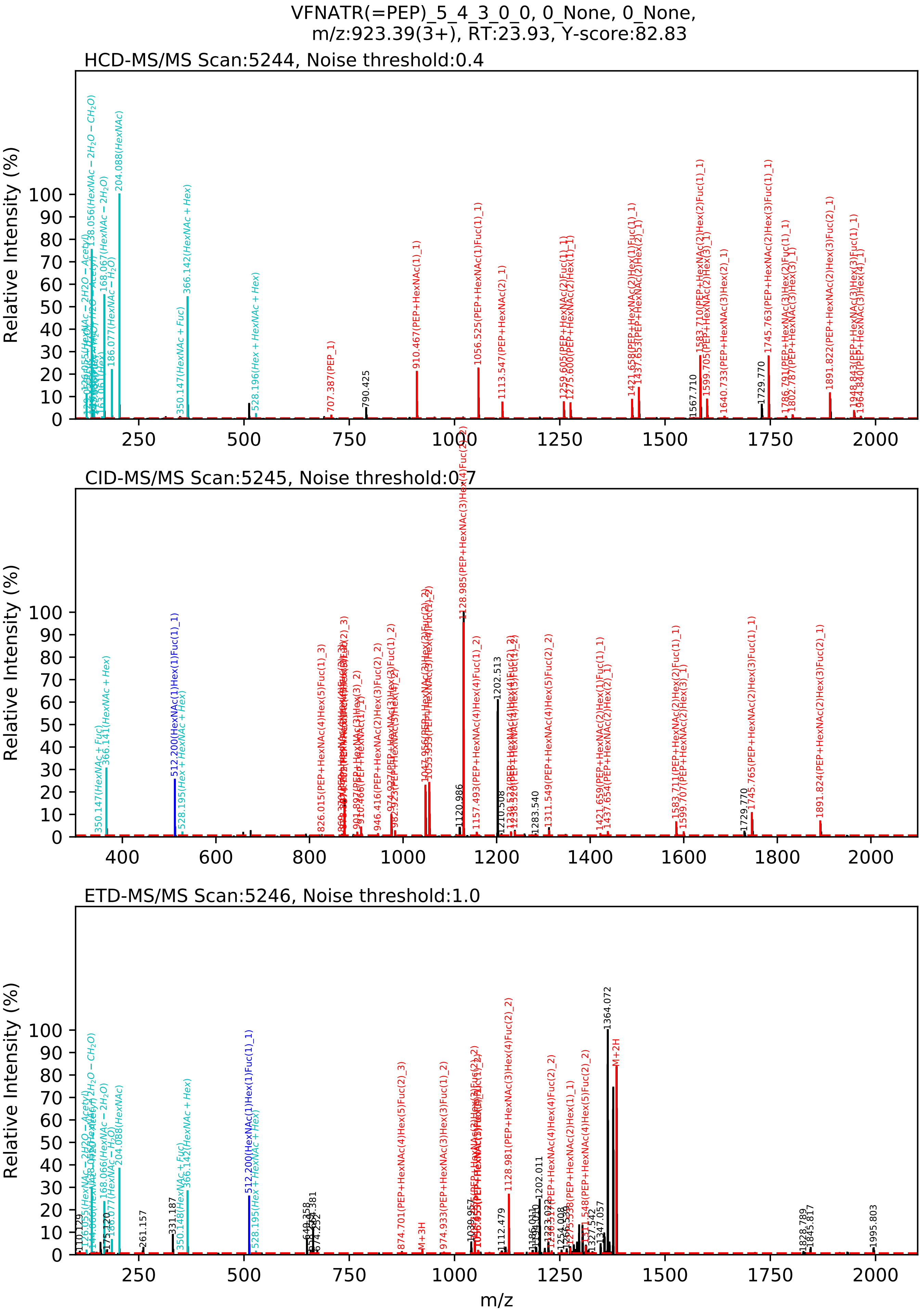

VFNATR(=PEP)_5_4_3_0_0, 0_None, 0_None,
m/z:923.39(3+), RT:23.93, Y-score:82.83
HCD-MS/MS Scan:5244, Noise threshold:0.4
Relative Intensity (%)
204.088(HexNAc)
366.142(HexNAc + Hex)
350.147(HexNAc + Fuc)
528.196(Hex + HexNAc + Hex)
707.387(PEP_1)
790.425
910.467(PEP+HexNAc(1)_1)
1056.525(PEP+HexNAc(1)Fuc(1)_1)
1113.547(PEP+HexNAc(2)_1)
1421.658(PEP+HexNAc(2)Hex(1)Fuc(1)_1)
1567.710
1640.733(PEP+HexNAc(3)Hex(2)_1)
1729.770
1745.763(PEP+HexNAc(2)Hex(3)Fuc(1)_1)
1891.822(PEP+HexNAc(2)Hex(3)Fuc(2)_1)
CID-MS/MS Scan:5245, Noise threshold:0.7
512.200(HexNAc(1)Hex(1)Fuc(1)_1)
826.015(PEP+HexNAc(4)Hex(5)Fuc(1)_3)
1128.985(PEP+HexNAc(3)Hex(4)Fuc(2)_2)
1120.986
1157.493(PEP+HexNAc(4)Hex(4)Fuc(1)_2)
1202.513
1283.540
1311.549(PEP+HexNAc(4)Hex(5)Fuc(2)_2)
1583.711(PEP+HexNAc(2)Hex(2)Fuc(1)_1)
1729.770
1745.765(PEP+HexNAc(2)Hex(3)Fuc(1)_1)
1891.824(PEP+HexNAc(2)Hex(3)Fuc(2)_1)
ETD-MS/MS Scan:5246, Noise threshold:1.0
1364.072
M+2H
M+3H
261.157
331.187
874.701(PEP+HexNAc(4)Hex(5)Fuc(2)_3)
974.933(PEP+HexNAc(3)Hex(3)Fuc(1)_2)
1128.981(PEP+HexNAc(3)Hex(4)Fuc(2)_2)
1202.011
1347.057
1828.789
1845.817
1995.803
m/z

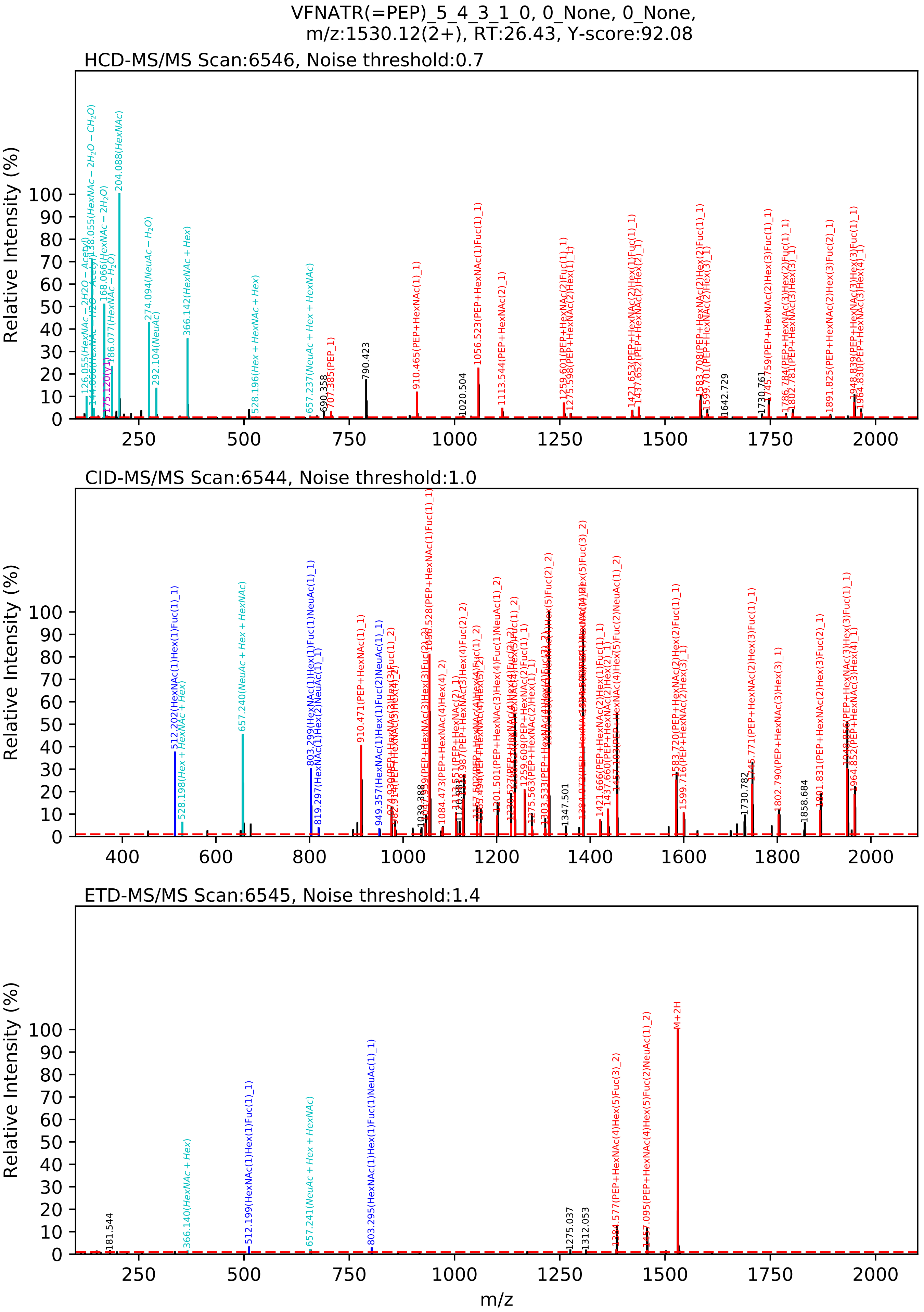
VFNATR(=PEP)_5_4_3_1_0, 0_None, 0_None,
m/z:1530.12(2+), RT:26.43, Y-score:92.08
HCD-MS/MS Scan:6546, Noise threshold:0.7
CID-MS/MS Scan:6544, Noise threshold:1.0
ETD-MS/MS Scan:6545, Noise threshold:1.4
Relative Intensity (%)
m/z
204.088(HexNAc)
274.094(NeuAc − H2O)
292.104(NeuAc)
366.142(HexNAc + Hex)
528.196(Hex + HexNAc + Hex)
657.237(NeuAc + Hex + HexNAc)
790.423
910.465(PEP+HexNAc(1)_1)
1056.523(PEP+HexNAc(1)Fuc(1)_1)
1113.544(PEP+HexNAc(2)_1)
512.202(HexNAc(1)Hex(1)Fuc(1)_1)
657.240(NeuAc + Hex + HexNAc)
803.299(HexNAc(1)Hex(1)Fuc(1)NeuAc(1)_1)
910.471(PEP+HexNAc(1)_1)
1347.501
1730.782
1858.684
181.544
366.140(HexNAc + Hex)
512.199(HexNAc(1)Hex(1)Fuc(1)_1)
657.241(NeuAc + Hex + HexNAc)
803.295(HexNAc(1)Hex(1)Fuc(1)NeuAc(1)_1)
1275.037
1312.053
1384.577(PEP+HexNAc(4)Hex(5)Fuc(3)_2)
1457.095(PEP+HexNAc(4)Hex(5)Fuc(2)NeuAc(1)_2)
M+2H

VFNATR(=PEP)_5_5_1_0_0, 0_None, 0_None,
m/z:893.71(3+), RT:24.15, Y-score:92.85

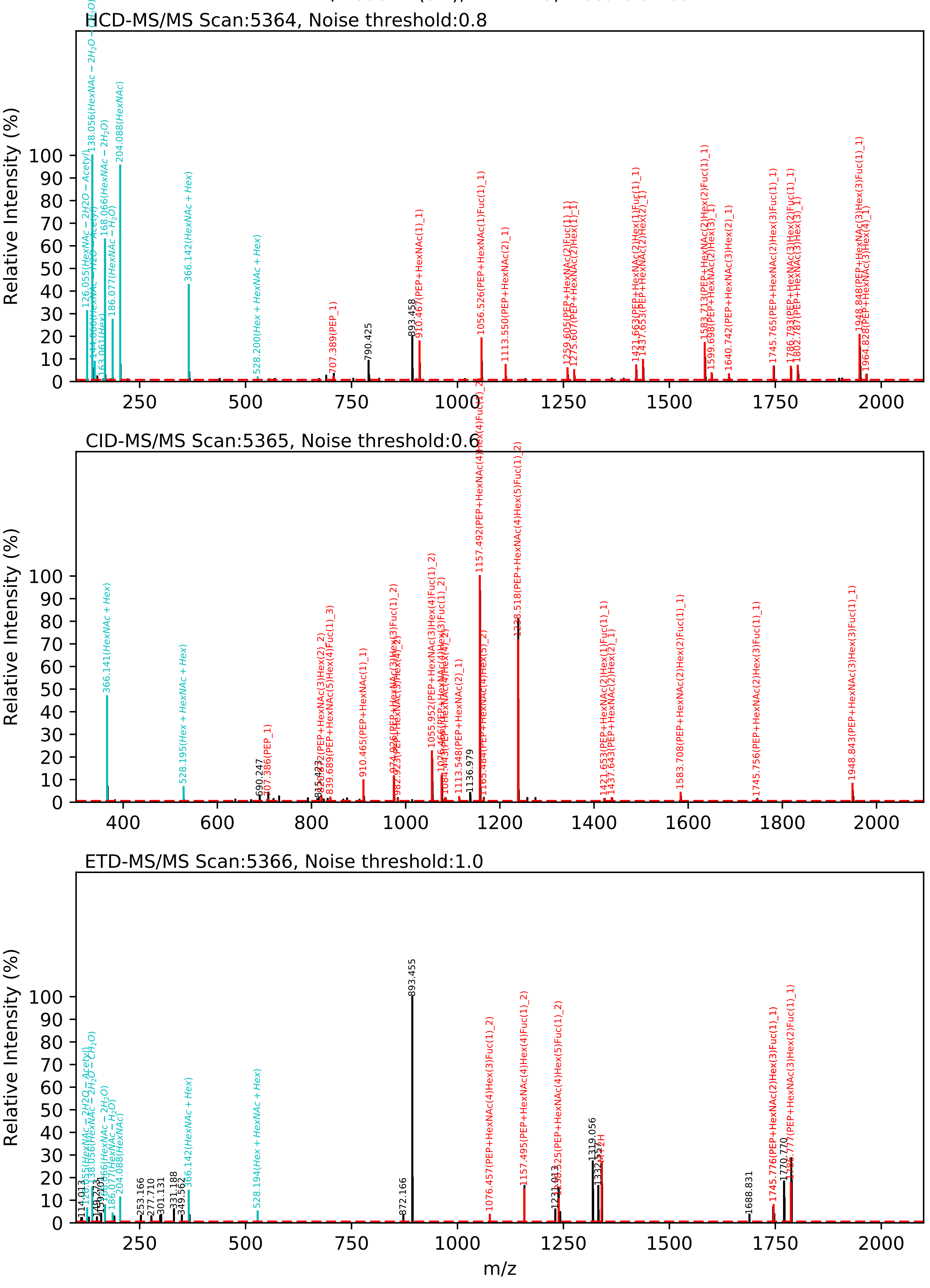

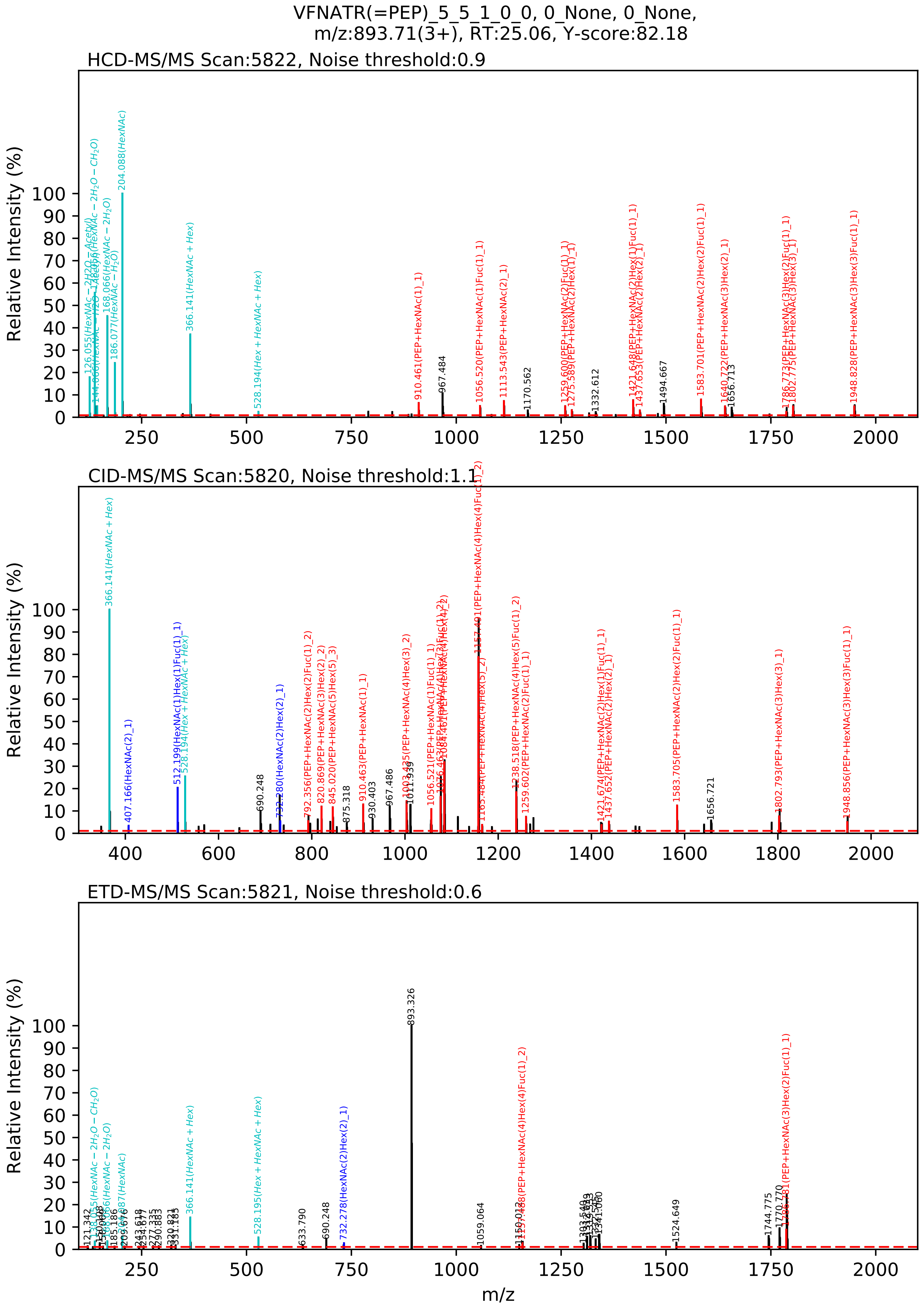

VFNATR(=PEP)_5_5_1_0_0, 0_None, 0_None,
m/z:893.71(3+), RT:25.06, Y-score:82.18
HCD-MS/MS Scan:5822, Noise threshold:0.9
CID-MS/MS Scan:5820, Noise threshold:1.1
ETD-MS/MS Scan:5821, Noise threshold:0.6
Relative Intensity (%)
m/z
204.088(HexNAc)
366.141(HexNAc + Hex)
528.194(Hex + HexNAc + Hex)
910.461(PEP+HexNAc(1)_1)
967.484
1056.520(PEP+HexNAc(1)Fuc(1)_1)
1113.543(PEP+HexNAc(2)_1)
1170.562
1332.612
1494.667
1583.701(PEP+HexNAc(2)Hex(2)Fuc(1)_1)
1640.722(PEP+HexNAc(3)Hex(2)_1)
1656.713
1948.828(PEP+HexNAc(3)Hex(3)Fuc(1)_1)
407.166(HexNAc(2)_1)
512.199(HexNAc(1)Hex(1)Fuc(1)_1)
732.280(HexNAc(2)Hex(2)_1)
690.248
792.356(PEP+HexNAc(2)Hex(2)Fuc(1)_2)
820.869(PEP+HexNAc(3)Hex(2)_2)
845.020(PEP+HexNAc(5)Hex(5)_3)
875.318
910.463(PEP+HexNAc(1)_1)
930.403
967.486
1011.939
1056.521(PEP+HexNAc(1)Fuc(1)_1)
1157.491(PEP+HexNAc(4)Hex(4)Fuc(1)_2)
1238.518(PEP+HexNAc(4)Hex(5)Fuc(1)_2)
1259.602(PEP+HexNAc(2)Fuc(1)_1)
1421.674(PEP+HexNAc(2)Hex(1)Fuc(1)_1)
1437.652(PEP+HexNAc(2)Hex(2)_1)
1583.705(PEP+HexNAc(2)Hex(2)Fuc(1)_1)
1656.721
1802.793(PEP+HexNAc(3)Hex(3)_1)
1948.856(PEP+HexNAc(3)Hex(3)Fuc(1)_1)
893.326
121.342
185.186
209.676
243.618
254.677
277.335
290.883
331.185
366.141(HexNAc + Hex)
528.195(Hex + HexNAc + Hex)
633.790
690.248
732.278(HexNAc(2)Hex(2)_1)
1059.064
1157.488(PEP+HexNAc(4)Hex(4)Fuc(1)_2)
1341.060
1524.649
1744.775
1770.770

VFNATR(=PEP)_5_5_1_0_0, 0_None, 0_None,
m/z:1340.06(2+), RT:24.11, Y-score:96.07

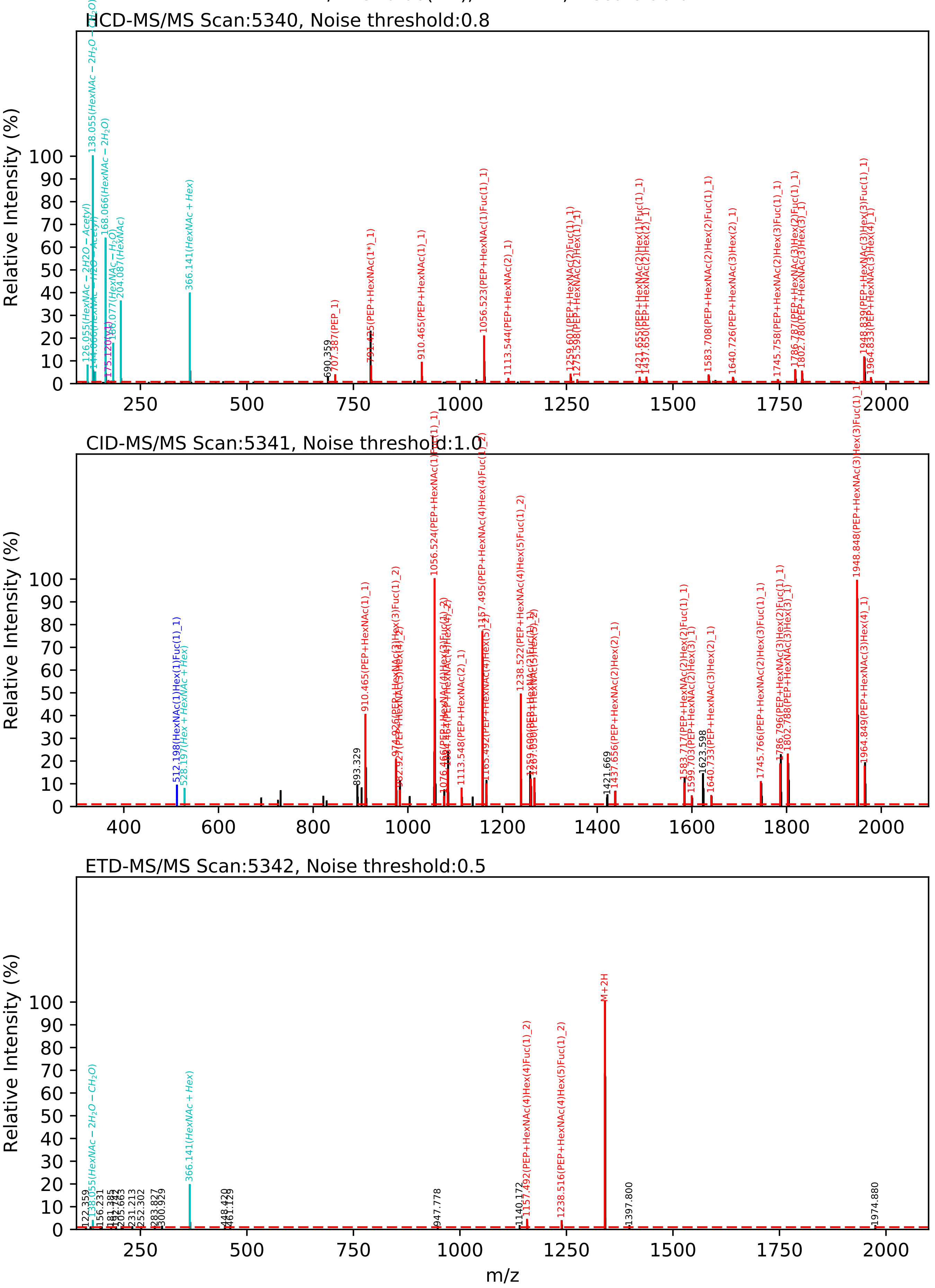

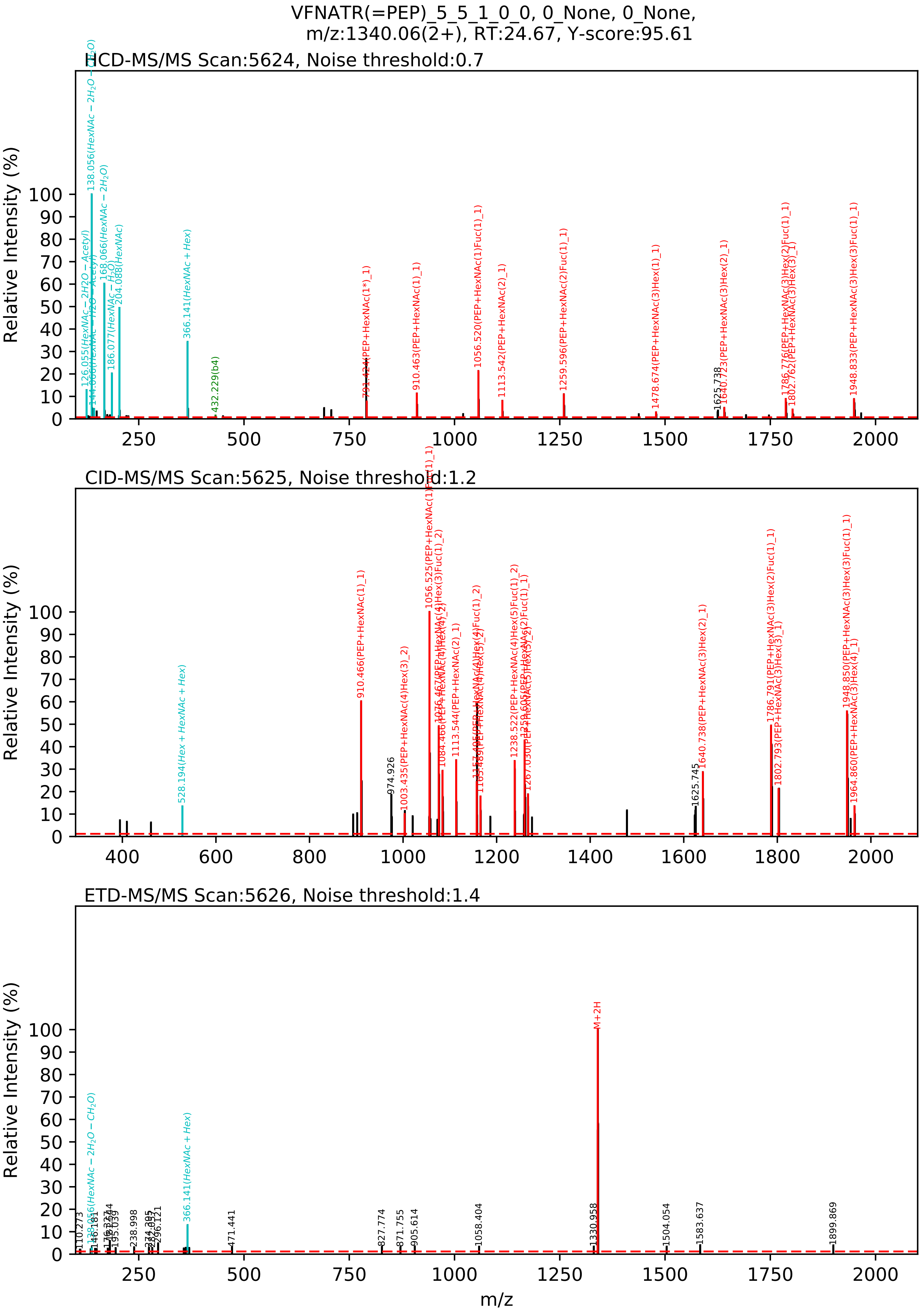
VFNATR(=PEP)_5_5_1_0_0, 0_None, 0_None,
m/z:1340.06(2+), RT:24.67, Y-score:95.61
HCD-MS/MS Scan:5624, Noise threshold:0.7
CID-MS/MS Scan:5625, Noise threshold:1.2
ETD-MS/MS Scan:5626, Noise threshold:1.4
Relative Intensity (%)
m/z
138.056
366.141(HexNAc + Hex)
432.229(b4)
910.463(PEP+HexNAc(1)_1)
1056.520(PEP+HexNAc(1)Fuc(1)_1)
1113.542(PEP+HexNAc(2)_1)
1259.596(PEP+HexNAc(2)Fuc(1)_1)
1478.674(PEP+HexNAc(3)Hex(1)_1)
1625.738
1948.833(PEP+HexNAc(3)Hex(3)Fuc(1)_1)
528.194(Hex + HexNAc + Hex)
910.466(PEP+HexNAc(1)_1)
974.926
1003.435(PEP+HexNAc(4)Hex(3)_2)
1113.544(PEP+HexNAc(2)_1)
1238.522(PEP+HexNAc(4)Hex(5)Fuc(1)_2)
1625.745
1640.738(PEP+HexNAc(3)Hex(2)_1)
1786.791(PEP+HexNAc(3)Hex(2)Fuc(1)_1)
1802.793(PEP+HexNAc(3)Hex(3)_1)
1948.850(PEP+HexNAc(3)Hex(3)Fuc(1)_1)
1964.860(PEP+HexNAc(3)Hex(4)_1)
M+2H
471.441
827.774
871.755
905.614
1058.404
1330.958
1504.054
1583.637
1899.869

VFNATR(=PEP)_5_5_1_1_0, 0_None, 0_None,
m/z:1485.60(2+), RT:26.74, Y-score:98.86

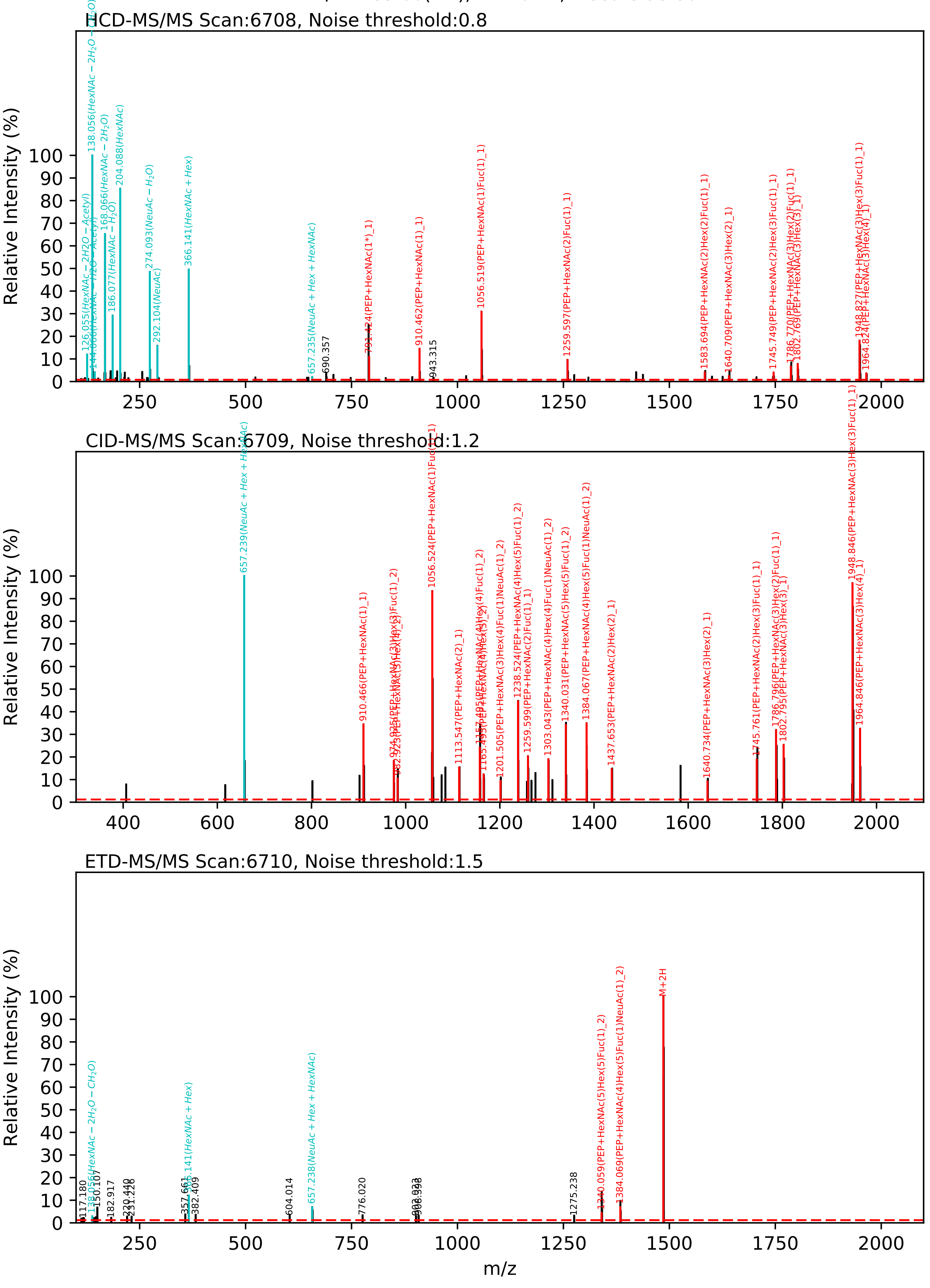

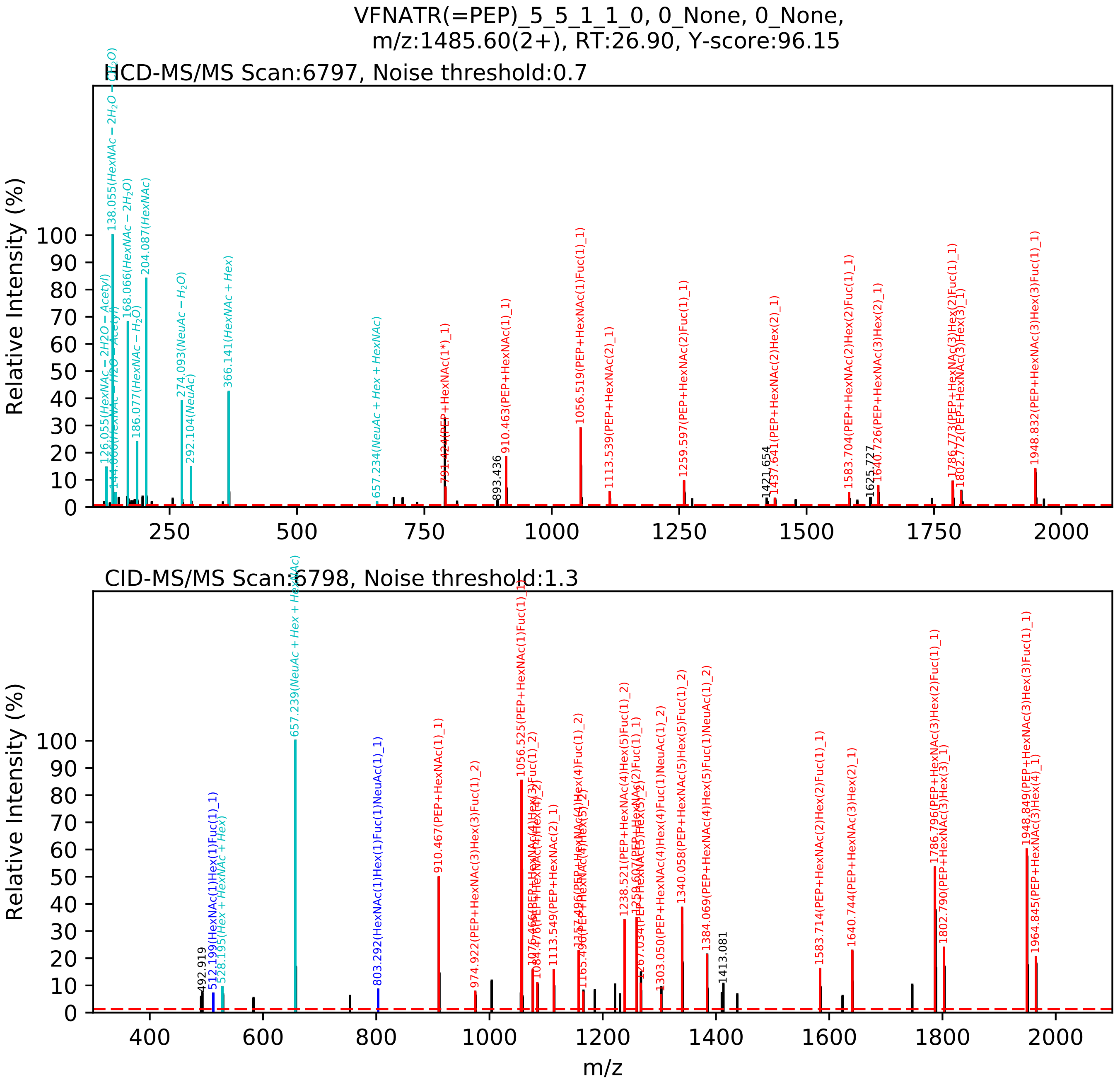
VFNATR(=PEP)_5_5_1_1_0, 0_None, 0_None,
m/z:1485.60(2+), RT:26.90, Y-score:96.15
HCD-MS/MS Scan:6797, Noise threshold:0.7
Relative Intensity (%)
126.055(HexNAc − 2H2O − Acetyl)
138.055(HexNAc − 2H2O − H2O)
168.066(HexNAc − 2H2O)
186.077(HexNAc − H2O)
204.087(HexNAc)
274.093(NeuAc − H2O)
292.104(NeuAc)
366.141(HexNAc + Hex)
657.234(NeuAc + Hex + HexNAc)
910.463(PEP+HexNAc(1)_1)
893.436
1056.519(PEP+HexNAc(1)Fuc(1)_1)
1113.539(PEP+HexNAc(2)_1)
1259.597(PEP+HexNAc(2)Fuc(1)_1)
1421.654
1583.704(PEP+HexNAc(2)Hex(2)Fuc(1)_1)
1625.727
1948.832(PEP+HexNAc(3)Hex(3)Fuc(1)_1)
CID-MS/MS Scan:6798, Noise threshold:1.3
492.919
512.199(HexNAc(1)Hex(1)Fuc(1)_1)
657.239(NeuAc + Hex + HexNAc)
803.292(HexNAc(1)Hex(1)Fuc(1)NeuAc(1)_1)
910.467(PEP+HexNAc(1)_1)
974.922(PEP+HexNAc(3)Hex(3)Fuc(1)_2)
1056.525(PEP+HexNAc(1)Fuc(1)_1)
1113.549(PEP+HexNAc(2)_1)
1238.521(PEP+HexNAc(4)Hex(5)Fuc(1)_2)
1303.050(PEP+HexNAc(4)Hex(4)Fuc(1)NeuAc(1)_2)
1340.058(PEP+HexNAc(5)Hex(5)Fuc(1)_2)
1384.069(PEP+HexNAc(4)Hex(5)Fuc(1)NeuAc(1)_2)
1413.081
1583.714(PEP+HexNAc(2)Hex(2)Fuc(1)_1)
1640.744(PEP+HexNAc(3)Hex(2)_1)
1786.796(PEP+HexNAc(3)Hex(2)Fuc(1)_1)
1802.790(PEP+HexNAc(3)Hex(3)_1)
1948.849(PEP+HexNAc(3)Hex(3)Fuc(1)_1)
1964.845(PEP+HexNAc(3)Hex(4)_1)
m/z

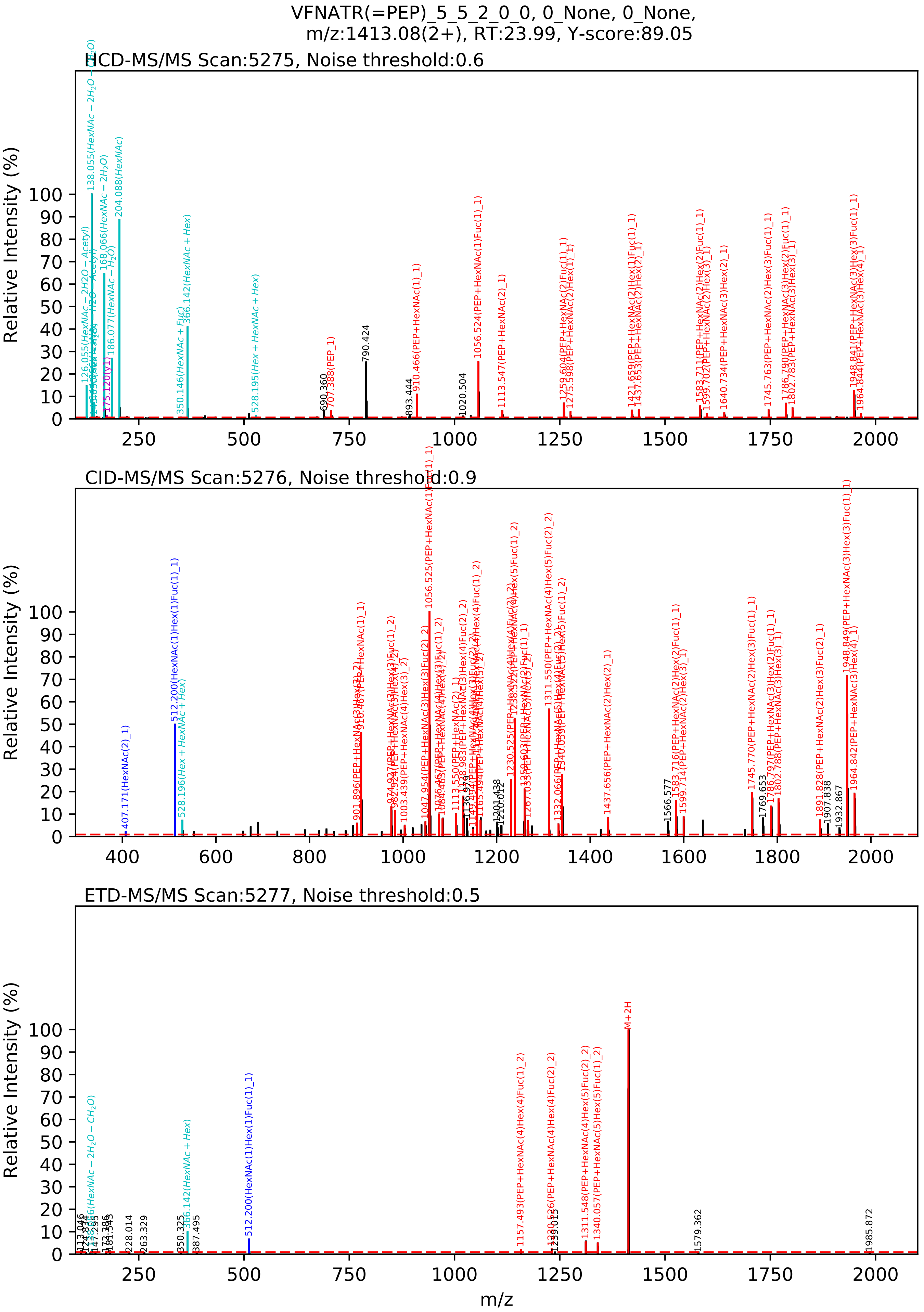

VFNATR(=PEP)_5_5_2_0_0, 0_None, 0_None,
m/z:1413.08(2+), RT:23.99, Y-score:89.05
HCD-MS/MS Scan:5275, Noise threshold:0.6
CID-MS/MS Scan:5276, Noise threshold:0.9
ETD-MS/MS Scan:5277, Noise threshold:0.5
Relative Intensity (%)
m/z

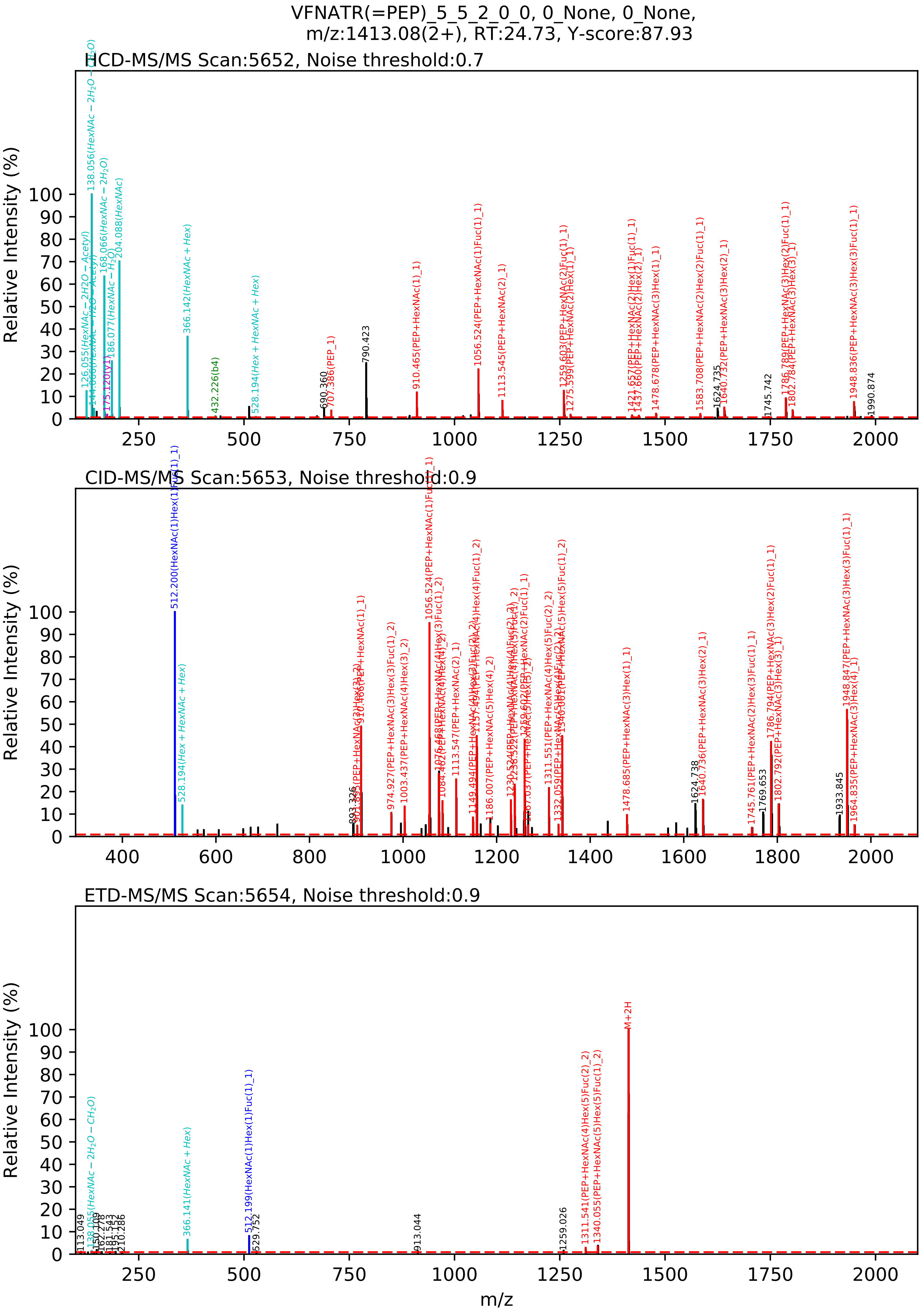
VFNATR(=PEP)_5_5_2_0_0, 0_None, 0_None,
m/z:1413.08(2+), RT:24.73, Y-score:87.93
HCD-MS/MS Scan:5652, Noise threshold:0.7
CID-MS/MS Scan:5653, Noise threshold:0.9
ETD-MS/MS Scan:5654, Noise threshold:0.9
Relative Intensity (%)
m/z
M+2H

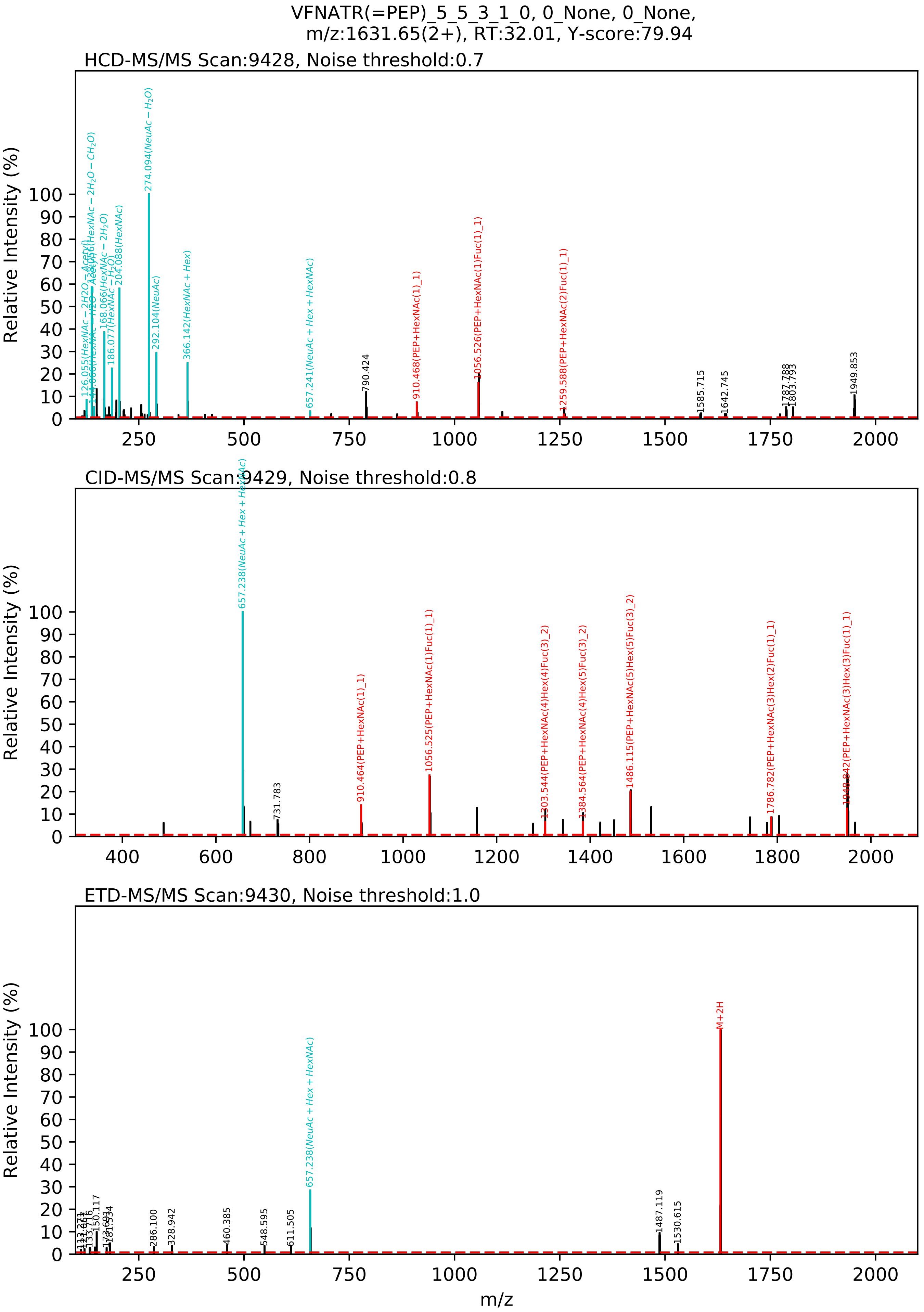
VFNATR(=PEP)_5_5_3_1_0, 0_None, 0_None,
m/z:1631.65(2+), RT:32.01, Y-score:79.94
HCD-MS/MS Scan:9428, Noise threshold:0.7
Relative Intensity (%)
274.094(NeuAc − H2O)
204.088(HexNAc)
168.066(HexNAc − 2H2O)
186.077(HexNAc − H2O)
126.055(HexNAc − 2H2O − CH2O)
292.104(NeuAc)
366.142(HexNAc + Hex)
657.241(NeuAc + Hex + HexNAc)
790.424
910.468(PEP+HexNAc(1)_1)
1056.526(PEP+HexNAc(1)Fuc(1)_1)
1259.588(PEP+HexNAc(2)Fuc(1)_1)
1585.715
1642.745
1787.788
1803.793
1949.853
CID-MS/MS Scan:9429, Noise threshold:0.8
Relative Intensity (%)
657.238(NeuAc + Hex + HexNAc)
731.783
910.464(PEP+HexNAc(1)_1)
1056.525(PEP+HexNAc(1)Fuc(1)_1)
1303.544(PEP+HexNAc(4)Hex(4)Fuc(3)_2)
1384.564(PEP+HexNAc(4)Hex(5)Fuc(3)_2)
1486.115(PEP+HexNAc(5)Hex(5)Fuc(3)_2)
1786.782(PEP+HexNAc(3)Hex(2)Fuc(1)_1)
1948.842(PEP+HexNAc(3)Hex(3)Fuc(1)_1)
ETD-MS/MS Scan:9430, Noise threshold:1.0
Relative Intensity (%)
M+2H
657.238(NeuAc + Hex + HexNAc)
150.117
181.934
286.100
328.942
460.385
548.595
611.505
1487.119
1530.615
m/z

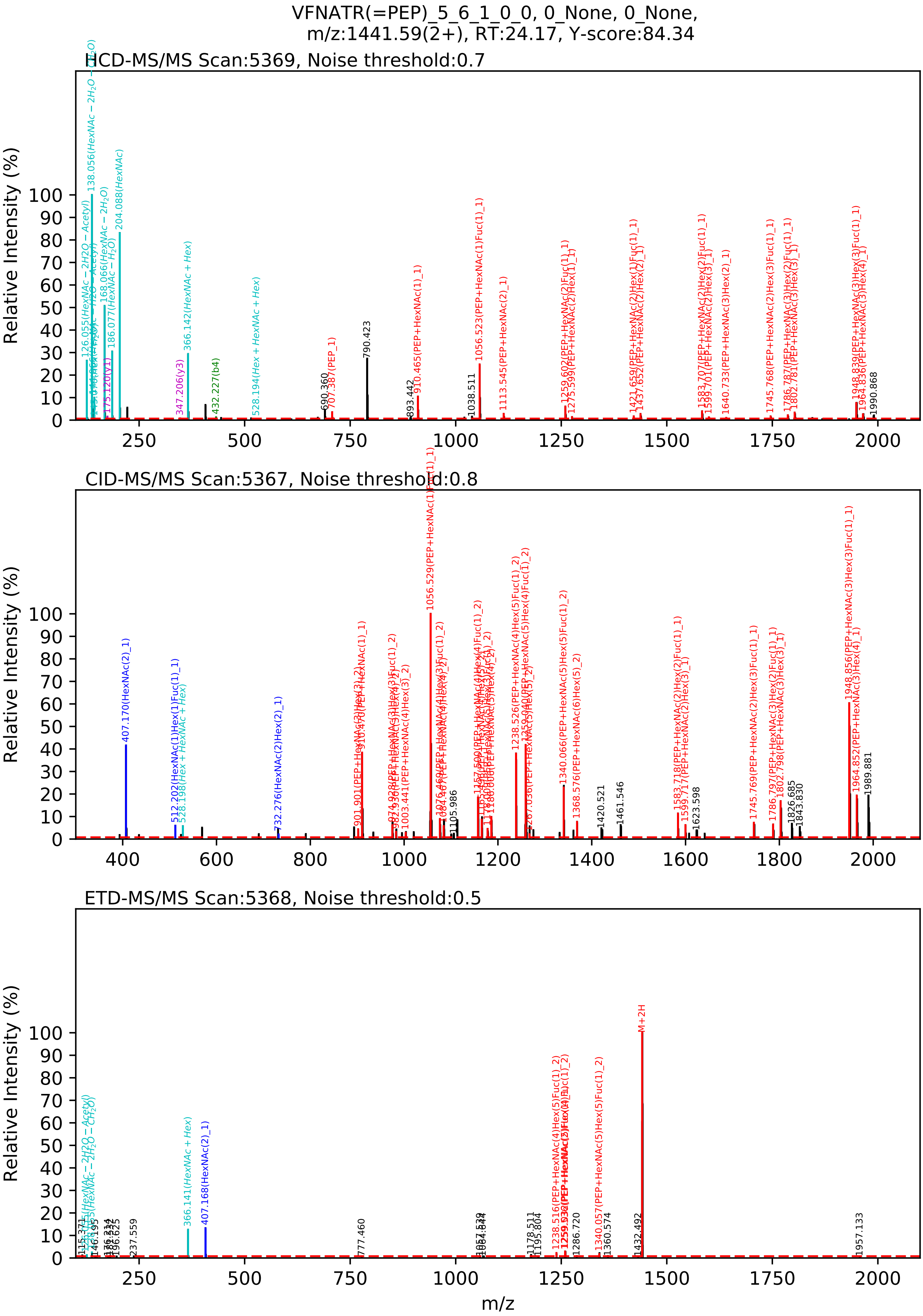

VFNATR(=PEP)_5_6_1_0_0, 0_None, 0_None,
m/z:1441.59(2+), RT:24.17, Y-score:84.34
HCD-MS/MS Scan:5369, Noise threshold:0.7
Relative Intensity (%)
138.056(HexNAc − 2H2O − H2O)
204.088(HexNAc)
366.142(HexNAc + Hex)
347.206(y3)
175.120(y1)
432.227(b4)
528.194(Hex + HexNAc + Hex)
690.360
707.387(PEP_1)
790.423
893.442
910.465(PEP+HexNAc(1)_1)
1038.511
1056.523(PEP+HexNAc(1)Fuc(1)_1)
1113.545(PEP+HexNAc(2)_1)
1259.602(PEP+HexNAc(2)Fuc(1)_1)
1275.599(PEP+HexNAc(2)Hex(1)_1)
1421.659(PEP+HexNAc(2)Hex(1)Fuc(1)_1)
1437.652(PEP+HexNAc(2)Hex(2)_1)
1583.707(PEP+HexNAc(2)Hex(2)Fuc(1)_1)
1640.733(PEP+HexNAc(3)Hex(2)_1)
1745.768(PEP+HexNAc(2)Hex(3)Fuc(1)_1)
1786.787(PEP+HexNAc(3)Hex(2)Fuc(1)_1)
1802.781(PEP+HexNAc(3)Hex(3)_1)
1948.839(PEP+HexNAc(3)Hex(3)Fuc(1)_1)
1964.836(PEP+HexNAc(3)Hex(4)_1)
1990.868
CID-MS/MS Scan:5367, Noise threshold:0.8
Relative Intensity (%)
407.170(HexNAc(2)_1)
512.202(HexNAc(1)Hex(1)Fuc(1)_1)
528.198(Hex + HexNAc + Hex)
732.276(HexNAc(2)Hex(2)_1)
910.470(PEP+HexNAc(1)_1)
1003.441(PEP+HexNAc(4)Hex(3)_2)
1056.529(PEP+HexNAc(1)Fuc(1)_1)
1105.986
1238.526(PEP+HexNAc(4)Hex(5)Fuc(1)_2)
1340.066(PEP+HexNAc(5)Hex(5)Fuc(1)_2)
1368.576(PEP+HexNAc(6)Hex(5)_2)
1420.521
1461.546
1583.718(PEP+HexNAc(2)Hex(2)Fuc(1)_1)
1599.717(PEP+HexNAc(2)Hex(3)_1)
1623.598
1745.769(PEP+HexNAc(2)Hex(3)Fuc(1)_1)
1786.797(PEP+HexNAc(3)Hex(2)Fuc(1)_1)
1802.798(PEP+HexNAc(3)Hex(3)_1)
1826.685
1843.830
1948.856(PEP+HexNAc(3)Hex(3)Fuc(1)_1)
1964.852(PEP+HexNAc(3)Hex(4)_1)
1989.881
ETD-MS/MS Scan:5368, Noise threshold:0.5
Relative Intensity (%)
366.141(HexNAc + Hex)
407.168(HexNAc(2)_1)
777.460
1178.511
1195.804
1238.516(PEP+HexNAc(4)Hex(5)Fuc(1)_2)
1286.720
1340.057(PEP+HexNAc(5)Hex(5)Fuc(1)_2)
1360.574
1432.492
M+2H
1957.133
m/z

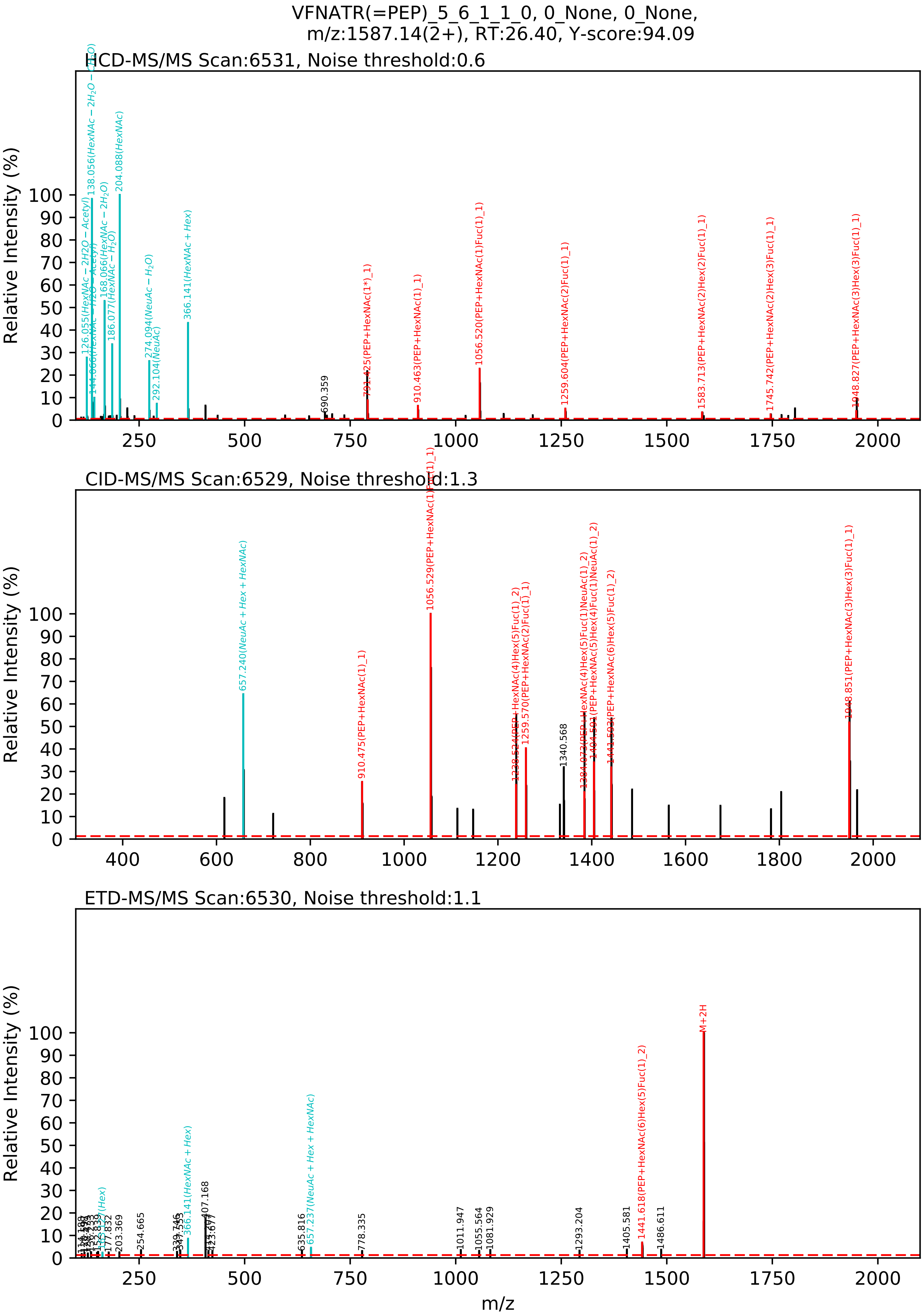

VFNATR(=PEP)_5_6_1_1_0, 0_None, 0_None,
m/z:1587.14(2+), RT:26.40, Y-score:94.09
HCD-MS/MS Scan:6531, Noise threshold:0.6
204.088(HexNAc)
366.141(HexNAc + Hex)
274.094(NeuAc − H2O)
292.104(NeuAc)
186.077(HexNAc − H2O)
168.066(HexNAc − 2H2O)
126.055(HexNAc − 2H2O − Acetyl)
690.359
910.463(PEP+HexNAc(1)_1)
1056.520(PEP+HexNAc(1)Fuc(1)_1)
1259.604(PEP+HexNAc(2)Fuc(1)_1)
1583.713(PEP+HexNAc(2)Hex(2)Fuc(1)_1)
1745.742(PEP+HexNAc(2)Hex(3)Fuc(1)_1)
1948.827(PEP+HexNAc(3)Hex(3)Fuc(1)_1)
Relative Intensity (%)
CID-MS/MS Scan:6529, Noise threshold:1.3
657.240(NeuAc + Hex + HexNAc)
910.475(PEP+HexNAc(1)_1)
1056.529(PEP+HexNAc(1)Fuc(1)_1)
1259.570(PEP+HexNAc(2)Fuc(1)_1)
1340.568
1948.851(PEP+HexNAc(3)Hex(3)Fuc(1)_1)
ETD-MS/MS Scan:6530, Noise threshold:1.1
366.141(HexNAc + Hex)
657.237(NeuAc + Hex + HexNAc)
407.168
635.816
778.335
1011.947
1055.564
1081.929
1293.204
1405.581
1441.618(PEP+HexNAc(6)Hex(5)Fuc(1)_2)
1486.611
M+2H
m/z

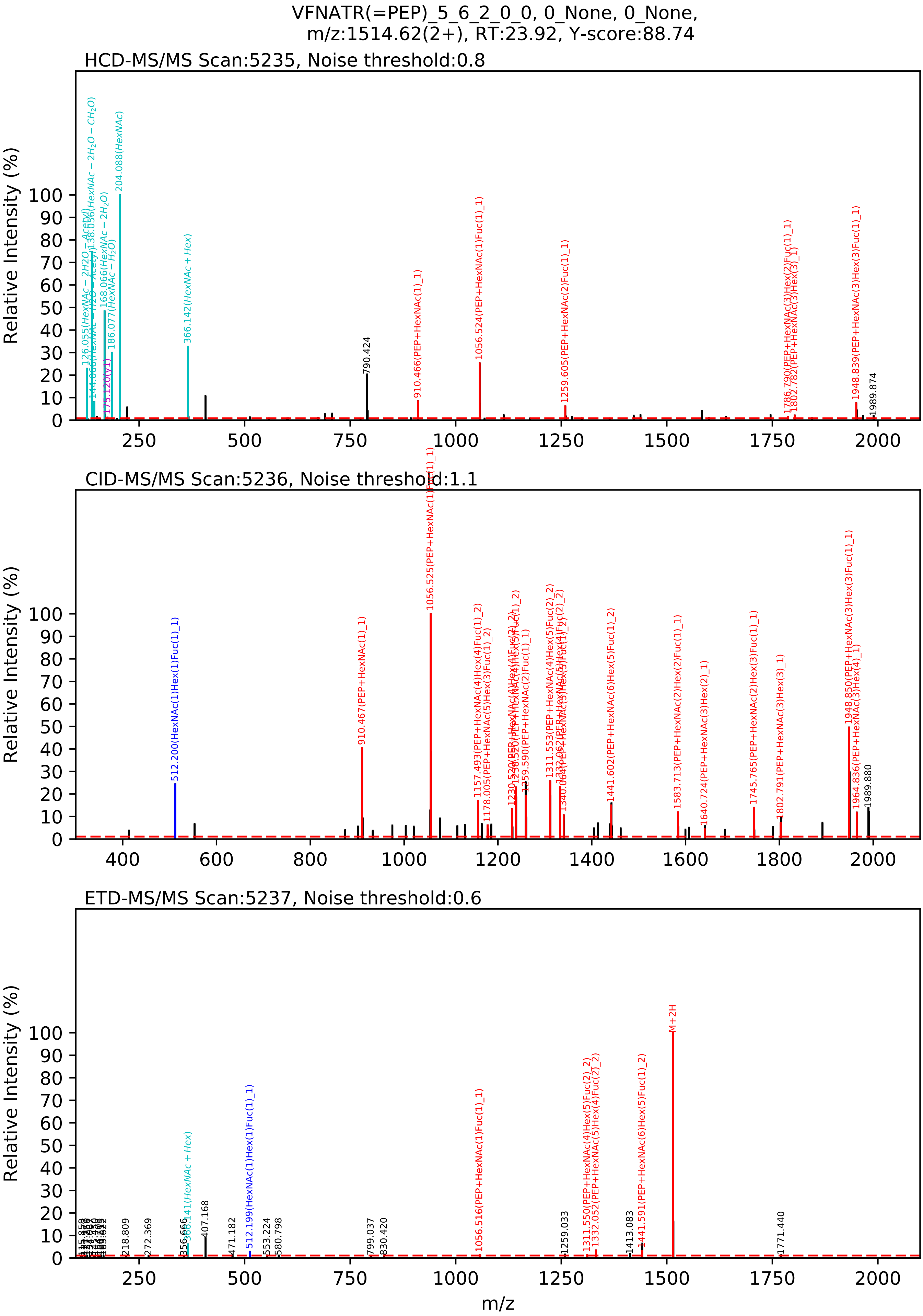

VFNATR(=PEP)_5_6_2_0_0, 0_None, 0_None,
m/z:1514.62(2+), RT:23.92, Y-score:88.74
HCD-MS/MS Scan:5235, Noise threshold:0.8
Relative Intensity (%)
126.055(HexNAc − 2H2O − Acetyl)
138.056(HexNAc − 2H2O − CH2O)
144.066(HexNAc − H2O − Acetyl)
168.066(HexNAc − 2H2O)
186.077(HexNAc − H2O)
175.120(y1)
204.088(HexNAc)
366.142(HexNAc + Hex)
790.424
910.466(PEP+HexNAc(1)_1)
1056.524(PEP+HexNAc(1)Fuc(1)_1)
1259.605(PEP+HexNAc(2)Fuc(1)_1)
1786.790(PEP+HexNAc(3)Hex(2)Fuc(1)_1)
1802.782(PEP+HexNAc(3)Hex(3)_1)
1948.839(PEP+HexNAc(3)Hex(3)Fuc(1)_1)
1989.874
CID-MS/MS Scan:5236, Noise threshold:1.1
512.200(HexNAc(1)Hex(1)Fuc(1)_1)
910.467(PEP+HexNAc(1)_1)
1056.525(PEP+HexNAc(1)Fuc(1)_1)
1157.493(PEP+HexNAc(4)Hex(4)Fuc(1)_2)
1178.005(PEP+HexNAc(5)Hex(3)Fuc(1)_2)
1230.520(PEP+HexNAc(4)Hex(4)Fuc(2)_2)
1238.520(PEP+HexNAc(4)Hex(5)Fuc(1)_2)
1259.590(PEP+HexNAc(2)Fuc(1)_1)
1311.553(PEP+HexNAc(4)Hex(5)Fuc(2)_2)
1332.062(PEP+HexNAc(5)Hex(4)Fuc(2)_2)
1340.064(PEP+HexNAc(5)Hex(5)Fuc(1)_2)
1441.602(PEP+HexNAc(6)Hex(5)Fuc(1)_2)
1583.713(PEP+HexNAc(2)Hex(2)Fuc(1)_1)
1640.724(PEP+HexNAc(3)Hex(2)_1)
1745.765(PEP+HexNAc(2)Hex(3)Fuc(1)_1)
1802.791(PEP+HexNAc(3)Hex(3)_1)
1948.850(PEP+HexNAc(3)Hex(3)Fuc(1)_1)
1964.836(PEP+HexNAc(3)Hex(4)_1)
1989.880
ETD-MS/MS Scan:5237, Noise threshold:0.6
218.809
272.369
356.666
366.141(HexNAc + Hex)
407.168
471.182
512.199(HexNAc(1)Hex(1)Fuc(1)_1)
553.224
580.798
799.037
830.420
1056.516(PEP+HexNAc(1)Fuc(1)_1)
1259.033
1311.550(PEP+HexNAc(4)Hex(5)Fuc(2)_2)
1332.052(PEP+HexNAc(5)Hex(4)Fuc(2)_2)
1413.083
1441.591(PEP+HexNAc(6)Hex(5)Fuc(1)_2)
M+2H
1771.440
m/z

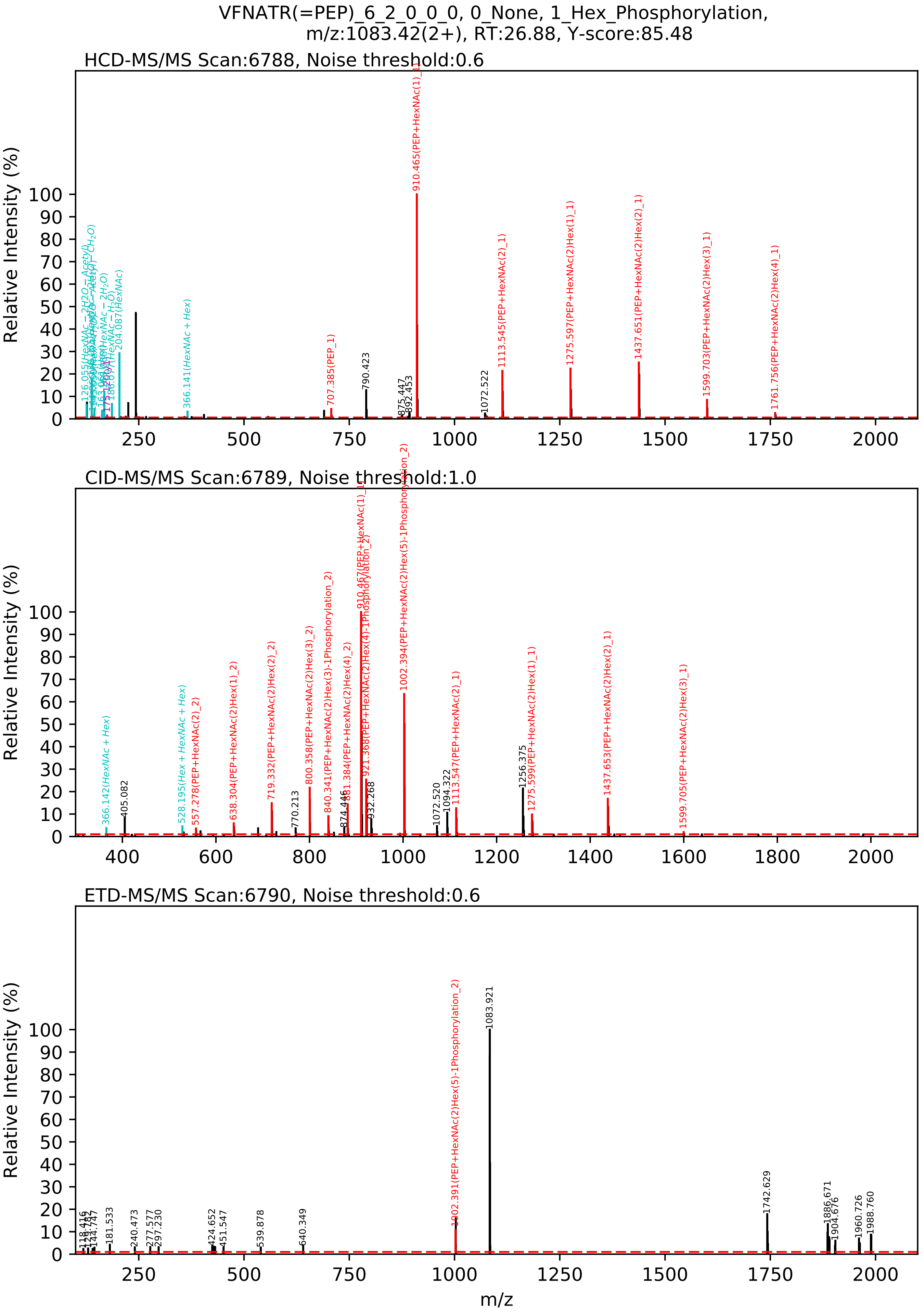
VFNATR(=PEP)_6_2_0_0_0, 0_None, 1_Hex_Phosphorylation,
m/z:1083.42(2+), RT:26.88, Y-score:85.48
HCD-MS/MS Scan:6788, Noise threshold:0.6
910.465(PEP+HexNAc(1)_1)
1113.545(PEP+HexNAc(2)_1)
1275.597(PEP+HexNAc(2)Hex(1)_1)
1437.651(PEP+HexNAc(2)Hex(2)_1)
1599.703(PEP+HexNAc(2)Hex(3)_1)
1761.756(PEP+HexNAc(2)Hex(4)_1)
707.385(PEP_1)
790.423
875.447
892.453
1072.522
366.141(HexNAc + Hex)
204.087(HexNAc)
CID-MS/MS Scan:6789, Noise threshold:1.0
366.142(HexNAc + Hex)
405.082
528.195(Hex + HexNAc + Hex)
557.278(PEP+HexNAc(2)_2)
638.304(PEP+HexNAc(2)Hex(1)_2)
719.332(PEP+HexNAc(2)Hex(2)_2)
770.213
800.358(PEP+HexNAc(2)Hex(3)_2)
840.341(PEP+HexNAc(2)Hex(3)-1Phosphorylation_2)
874.446
881.384(PEP+HexNAc(2)Hex(4)_2)
910.467(PEP+HexNAc(1)_1)
921.368(PEP+HexNAc(2)Hex(4)-1Phosphorylation_2)
932.268
1002.394(PEP+HexNAc(2)Hex(5)-1Phosphorylation_2)
1072.520
1094.322
1113.547(PEP+HexNAc(2)_1)
1256.375
1275.599(PEP+HexNAc(2)Hex(1)_1)
1437.653(PEP+HexNAc(2)Hex(2)_1)
1599.705(PEP+HexNAc(2)Hex(3)_1)
ETD-MS/MS Scan:6790, Noise threshold:0.6
128.416
144.747
181.533
240.473
277.577
297.230
424.652
451.547
539.878
640.349
1002.391(PEP+HexNAc(2)Hex(5)-1Phosphorylation_2)
1083.921
1742.629
1886.671
1904.676
1960.726
1988.760
Relative Intensity (%)
m/z

VFNATR(=PEP)_6_3_0_0_0, 0_None, 1_Hex_Phosphorylation,
m/z:1184.96(2+), RT:26.97, Y-score:85.69

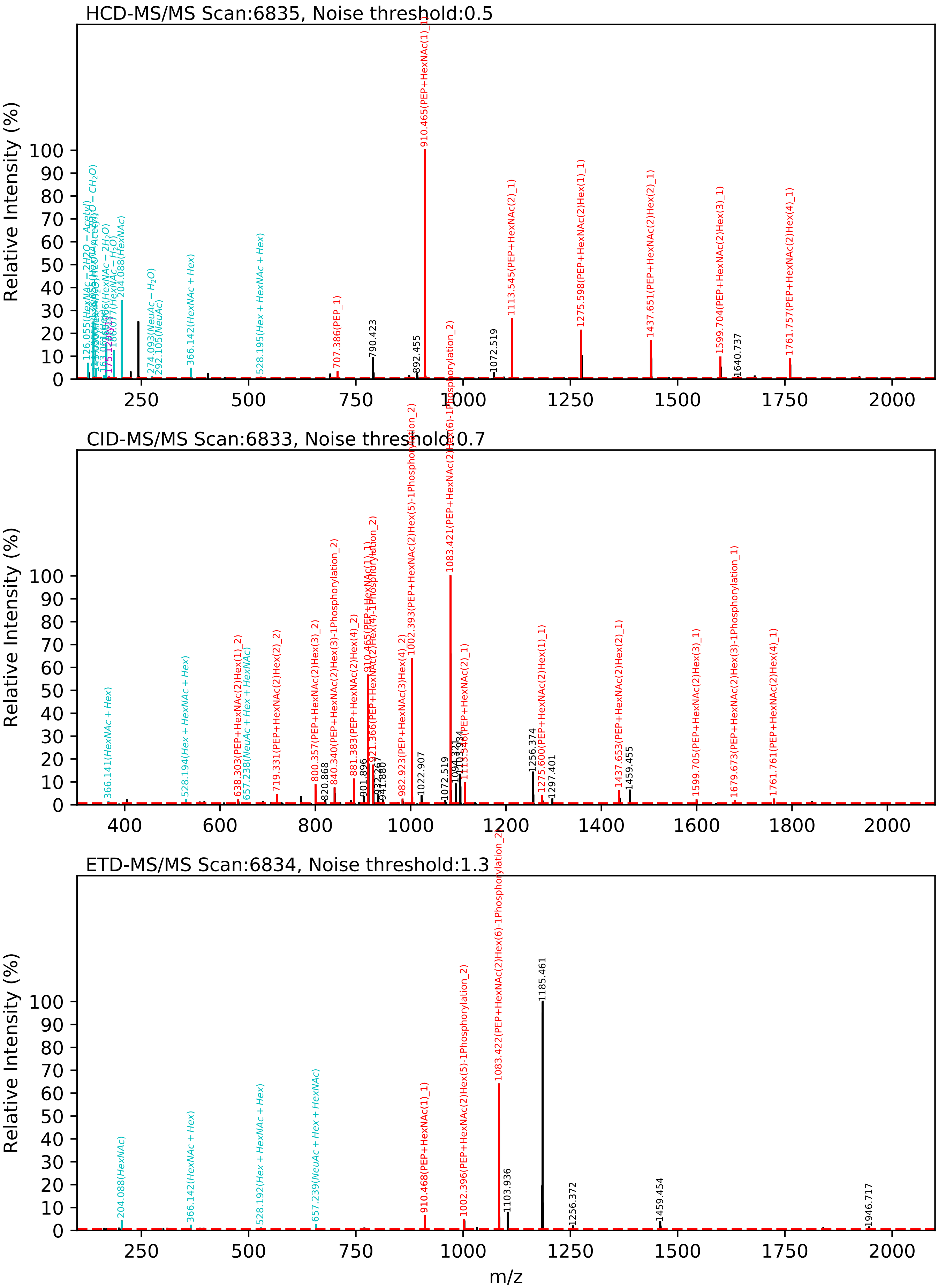

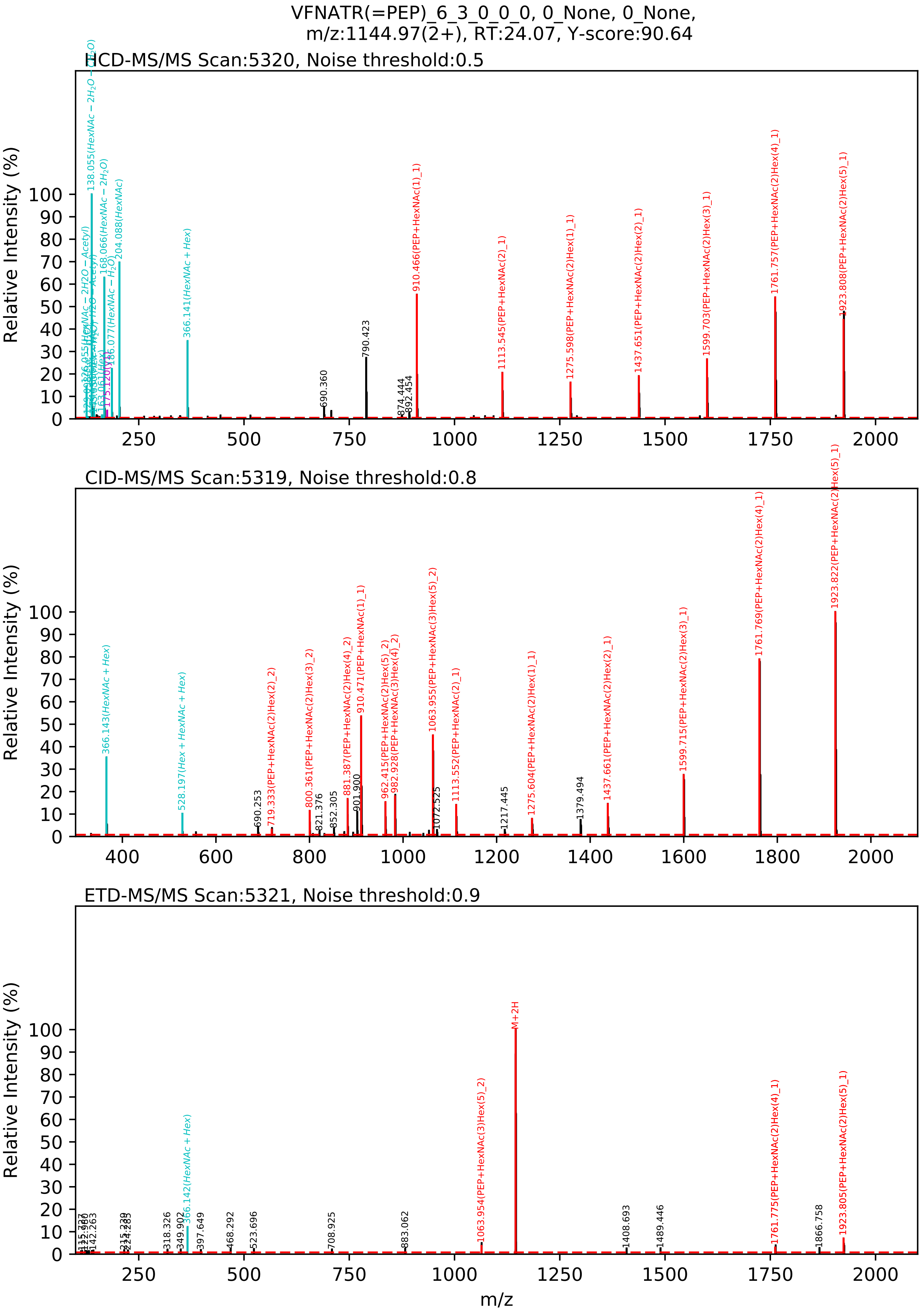
VFNATR(=PEP)_6_3_0_0_0, 0_None, 0_None,
m/z:1144.97(2+), RT:24.07, Y-score:90.64
HCD-MS/MS Scan:5320, Noise threshold:0.5
Relative Intensity (%)
138.055(HexNAc − 2H2O − H2O)
168.066(HexNAc − 2H2O)
204.088(HexNAc)
186.077(HexNAc − H2O)
366.141(HexNAc + Hex)
690.360
790.423
874.444
892.454
910.466(PEP+HexNAc(1)_1)
1113.545(PEP+HexNAc(2)_1)
1275.598(PEP+HexNAc(2)Hex(1)_1)
1437.651(PEP+HexNAc(2)Hex(2)_1)
1599.703(PEP+HexNAc(2)Hex(3)_1)
1761.757(PEP+HexNAc(2)Hex(4)_1)
1923.808(PEP+HexNAc(2)Hex(5)_1)
CID-MS/MS Scan:5319, Noise threshold:0.8
Relative Intensity (%)
366.143(HexNAc + Hex)
528.197(Hex + HexNAc + Hex)
690.253
719.333(PEP+HexNAc(2)Hex(2)_2)
800.361(PEP+HexNAc(2)Hex(3)_2)
821.376
852.305
881.387(PEP+HexNAc(2)Hex(4)_2)
901.900
910.471(PEP+HexNAc(1)_1)
962.415(PEP+HexNAc(2)Hex(5)_2)
982.928(PEP+HexNAc(3)Hex(4)_2)
1063.955(PEP+HexNAc(3)Hex(5)_2)
1072.525
1113.552(PEP+HexNAc(2)_1)
1217.445
1275.604(PEP+HexNAc(2)Hex(1)_1)
1379.494
1437.661(PEP+HexNAc(2)Hex(2)_1)
1599.715(PEP+HexNAc(2)Hex(3)_1)
1761.769(PEP+HexNAc(2)Hex(4)_1)
1923.822(PEP+HexNAc(2)Hex(5)_1)
ETD-MS/MS Scan:5321, Noise threshold:0.9
Relative Intensity (%)
142.263
224.285
318.326
349.902
366.142(HexNAc + Hex)
397.649
468.292
523.696
708.925
883.062
1063.954(PEP+HexNAc(3)Hex(5)_2)
M+2H
1408.693
1489.446
1761.775(PEP+HexNAc(2)Hex(4)_1)
1866.758
1923.805(PEP+HexNAc(2)Hex(5)_1)
m/z

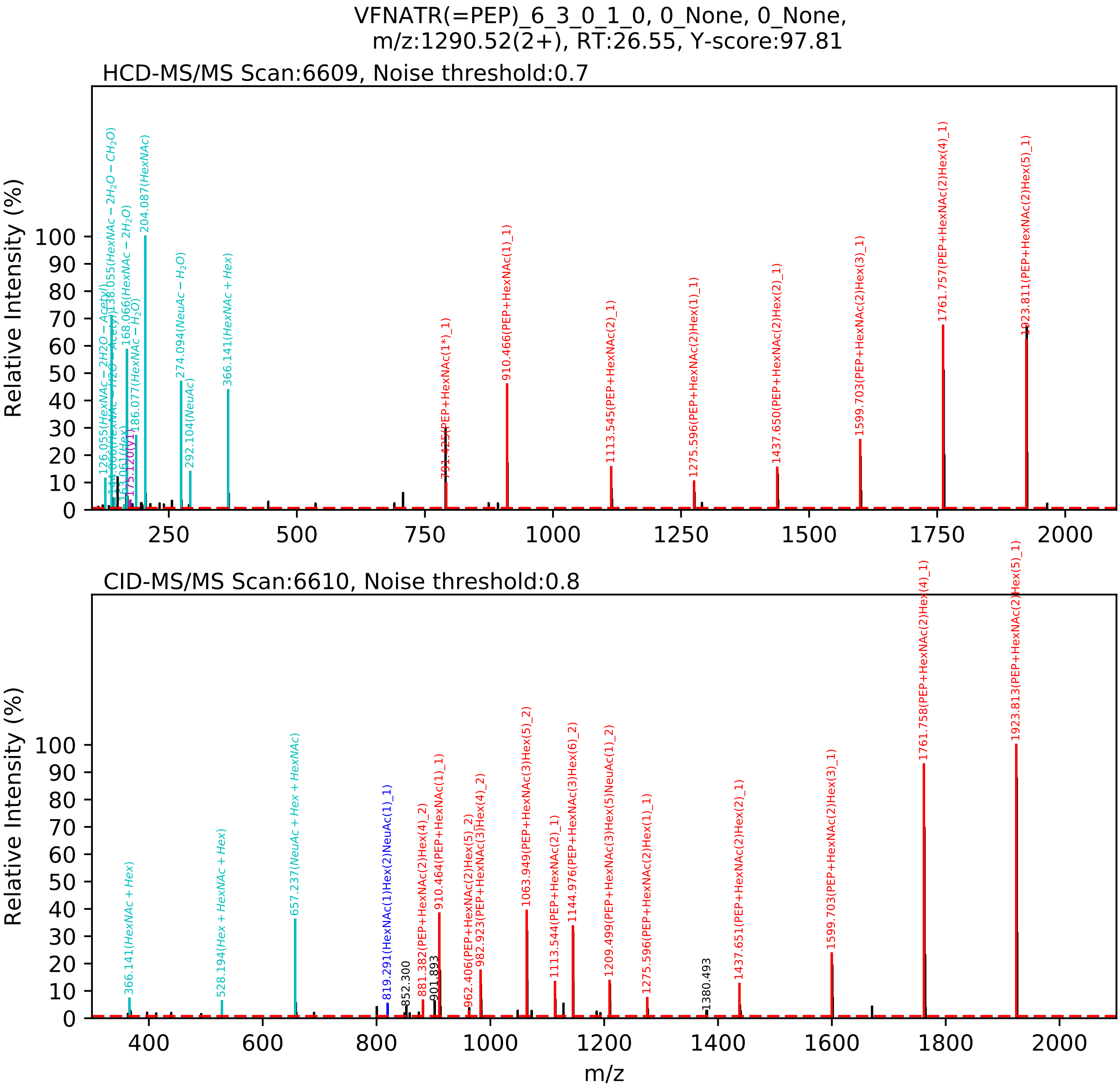

VFNATR(=PEP)_6_3_0_1_0, 0_None, 0_None,
m/z:1290.52(2+), RT:26.55, Y-score:97.81
HCD-MS/MS Scan:6609, Noise threshold:0.7
Relative Intensity (%)
204.087(HexNAc)
168.066(HexNAc − 2H2O)
186.077(HexNAc − H2O)
274.094(NeuAc − H2O)
292.104(NeuAc)
366.141(HexNAc + Hex)
910.466(PEP+HexNAc(1)_1)
1113.545(PEP+HexNAc(2)_1)
1275.596(PEP+HexNAc(2)Hex(1)_1)
1437.650(PEP+HexNAc(2)Hex(2)_1)
1599.703(PEP+HexNAc(2)Hex(3)_1)
1761.757(PEP+HexNAc(2)Hex(4)_1)
1923.811(PEP+HexNAc(2)Hex(5)_1)
CID-MS/MS Scan:6610, Noise threshold:0.8
Relative Intensity (%)
366.141(HexNAc + Hex)
528.194(Hex + HexNAc + Hex)
657.237(NeuAc + Hex + HexNAc)
819.291(HexNAc(1)Hex(2)NeuAc(1)_1)
852.300
881.382(PEP+HexNAc(2)Hex(4)_2)
901.893
910.464(PEP+HexNAc(1)_1)
962.406(PEP+HexNAc(2)Hex(5)_2)
982.923(PEP+HexNAc(3)Hex(4)_2)
1063.949(PEP+HexNAc(3)Hex(5)_2)
1113.544(PEP+HexNAc(2)_1)
1144.976(PEP+HexNAc(3)Hex(6)_2)
1209.499(PEP+HexNAc(3)Hex(5)NeuAc(1)_2)
1275.596(PEP+HexNAc(2)Hex(1)_1)
1380.493
1437.651(PEP+HexNAc(2)Hex(2)_1)
1599.703(PEP+HexNAc(2)Hex(3)_1)
1761.758(PEP+HexNAc(2)Hex(4)_1)
1923.813(PEP+HexNAc(2)Hex(5)_1)
m/z

VFNATR(=PEP)_6_3_1_0_0, 0_None, 0_None,
m/z:1218.00(2+), RT:23.83, Y-score:91.87

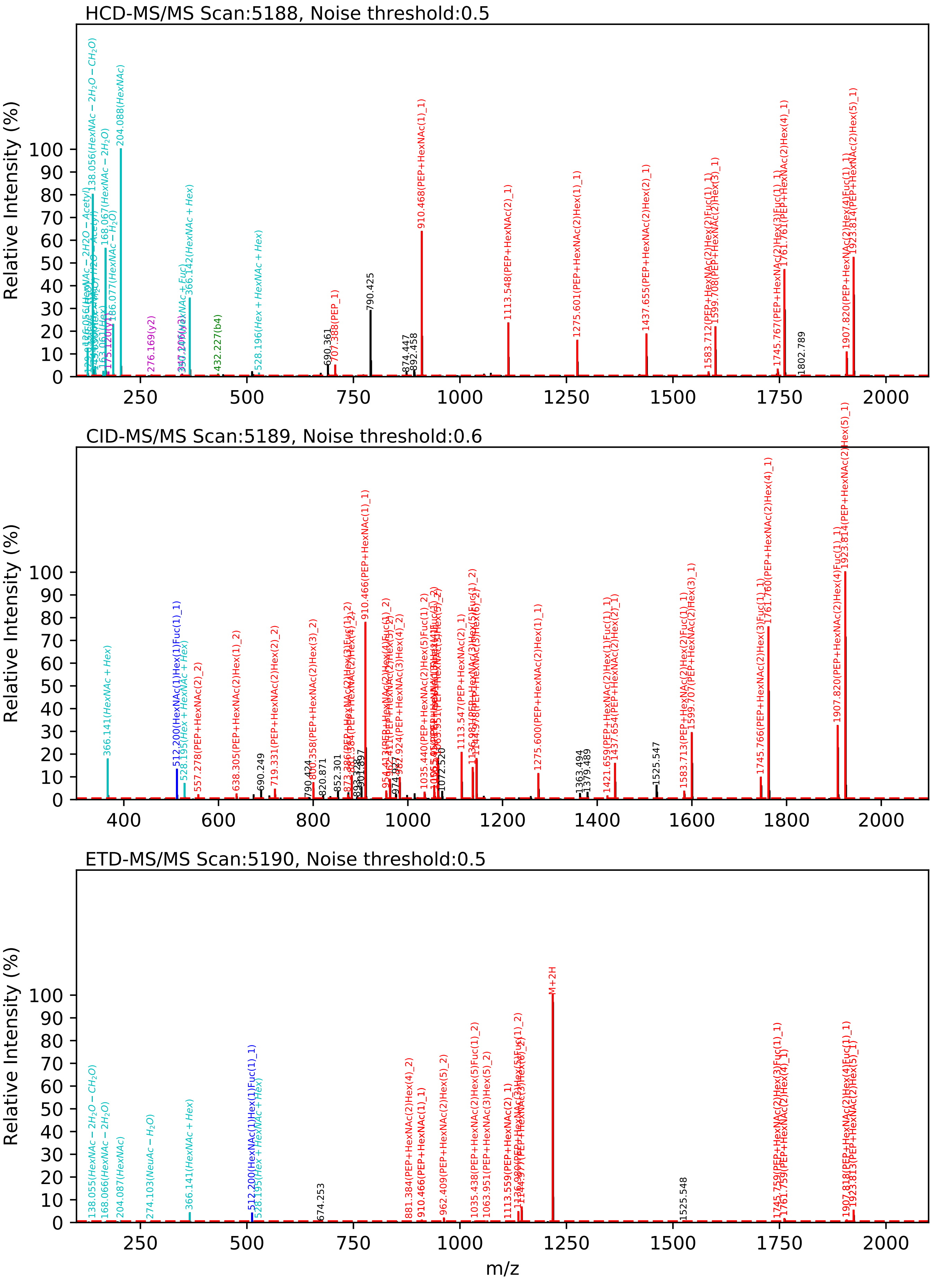

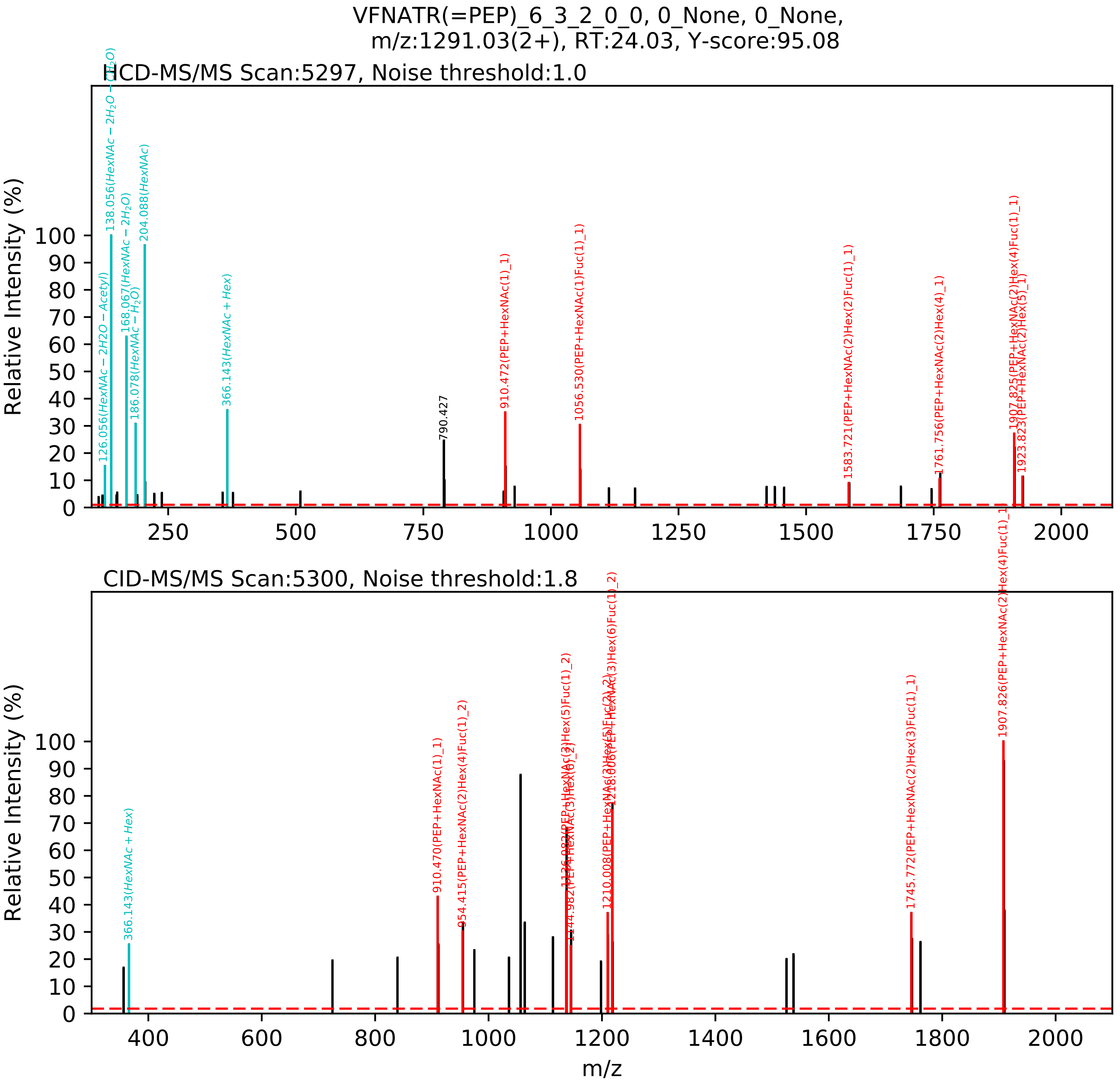

VFNATR(=PEP)_6_3_2_0_0, 0_None, 0_None,
m/z:1291.03(2+), RT:24.03, Y-score:95.08
HCD-MS/MS Scan:5297, Noise threshold:1.0
Relative Intensity (%)
126.056(HexNAc − 2H2O − Acetyl)
138.056(HexNAc − 2H2O − H2O)
168.067(HexNAc − 2H2O)
186.078(HexNAc − H2O)
204.088(HexNAc)
366.143(HexNAc + Hex)
790.427
910.472(PEP+HexNAc(1)_1)
1056.530(PEP+HexNAc(1)Fuc(1)_1)
1583.721(PEP+HexNAc(2)Hex(2)Fuc(1)_1)
1761.756(PEP+HexNAc(2)Hex(4)_1)
1907.825(PEP+HexNAc(2)Hex(4)Fuc(1)_1)
1923.823(PEP+HexNAc(2)Hex(5)_1)
CID-MS/MS Scan:5300, Noise threshold:1.8
366.143(HexNAc + Hex)
910.470(PEP+HexNAc(1)_1)
954.415(PEP+HexNAc(2)Hex(4)Fuc(1)_2)
1745.772(PEP+HexNAc(2)Hex(3)Fuc(1)_1)
1907.826(PEP+HexNAc(2)Hex(4)Fuc(1)_1)
m/z

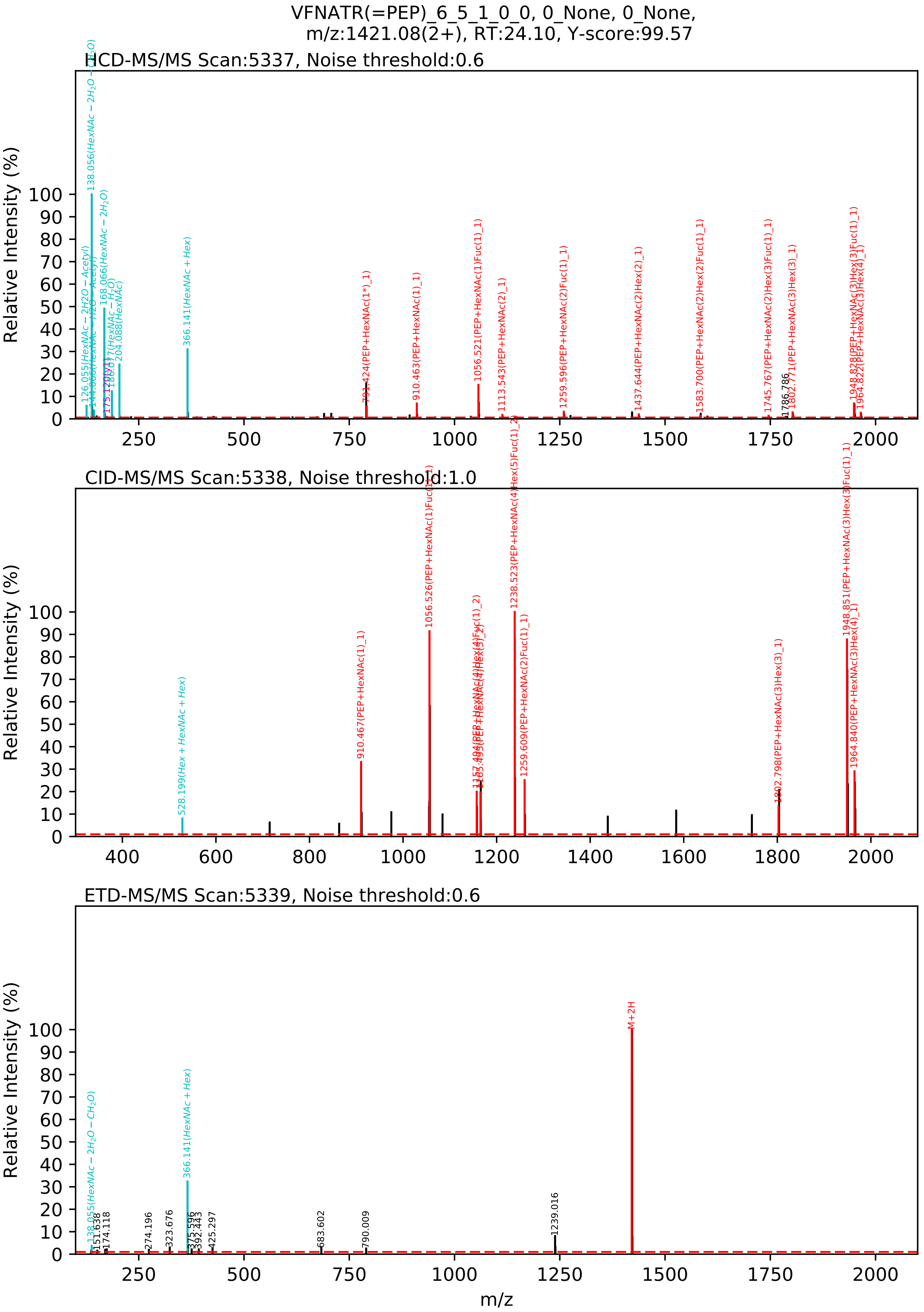

VFNATR(=PEP)_6_5_1_0_0, 0_None, 0_None,
m/z:1421.08(2+), RT:24.10, Y-score:99.57
HCD-MS/MS Scan:5337, Noise threshold:0.6
Relative Intensity (%)
138.056(HexNAc − 2H2O − CH2O)
168.066(HexNAc − 2H2O)
366.141(HexNAc + Hex)
204.088(HexNAc)
791.424(PEP+HexNAc(1*)_1)
910.463(PEP+HexNAc(1)_1)
1056.521(PEP+HexNAc(1)Fuc(1)_1)
1113.543(PEP+HexNAc(2)_1)
1259.596(PEP+HexNAc(2)Fuc(1)_1)
1437.644(PEP+HexNAc(2)Hex(2)_1)
1583.700(PEP+HexNAc(2)Hex(2)Fuc(1)_1)
1745.767(PEP+HexNAc(2)Hex(3)Fuc(1)_1)
1786.786
1802.771(PEP+HexNAc(3)Hex(3)_1)
1948.828(PEP+HexNAc(3)Hex(3)Fuc(1)_1)
1964.822(PEP+HexNAc(3)Hex(4)_1)
CID-MS/MS Scan:5338, Noise threshold:1.0
528.199(Hex + HexNAc + Hex)
910.467(PEP+HexNAc(1)_1)
1056.526(PEP+HexNAc(1)Fuc(1)_1)
1238.523(PEP+HexNAc(4)Hex(5)Fuc(1)_2)
1259.609(PEP+HexNAc(2)Fuc(1)_1)
1802.798(PEP+HexNAc(3)Hex(3)_1)
1948.851(PEP+HexNAc(3)Hex(3)Fuc(1)_1)
1964.840(PEP+HexNAc(3)Hex(4)_1)
ETD-MS/MS Scan:5339, Noise threshold:0.6
M+2H
138.055(HexNAc − 2H2O − CH2O)
151.638
174.118
274.196
323.676
366.141(HexNAc + Hex)
375.596
392.443
425.297
683.602
790.009
1239.016
m/z

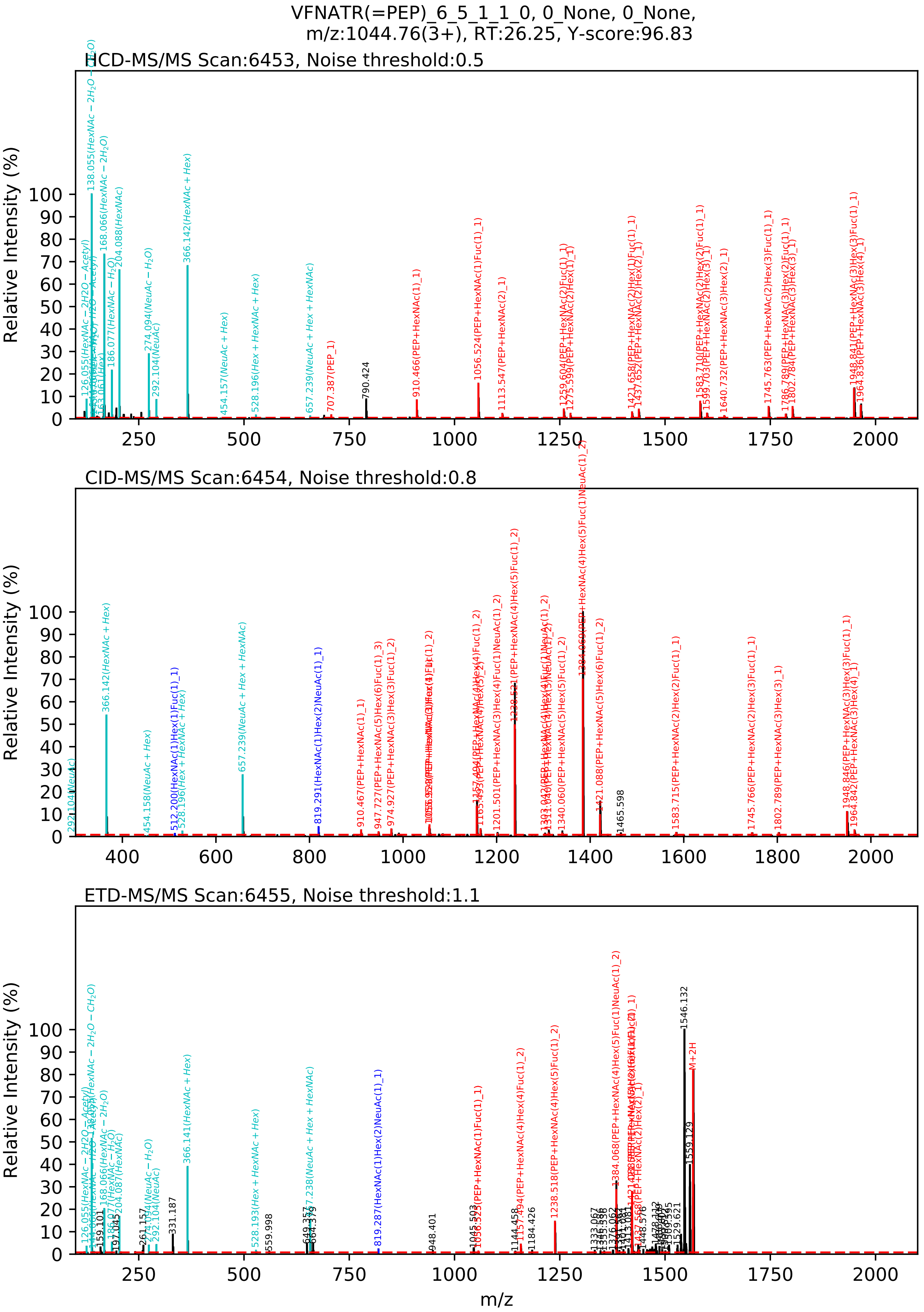
VFNATR(=PEP)_6_5_1_1_0, 0_None, 0_None,
m/z:1044.76(3+), RT:26.25, Y-score:96.83
HCD-MS/MS Scan:6453, Noise threshold:0.5
CID-MS/MS Scan:6454, Noise threshold:0.8
ETD-MS/MS Scan:6455, Noise threshold:1.1
Relative Intensity (%)
m/z
138.055(HexNAc − 2H2O − H2O)
168.066(HexNAc − 2H2O)
204.088(HexNAc)
366.142(HexNAc + Hex)
454.157(NeuAc + Hex)
528.196(Hex + HexNAc + Hex)
657.239(NeuAc + Hex + HexNAc)
707.387(PEP_1)
790.424
910.466(PEP+HexNAc(1)_1)
1056.524(PEP+HexNAc(1)Fuc(1)_1)
1113.547(PEP+HexNAc(2)_1)
1745.763(PEP+HexNAc(2)Hex(3)Fuc(1)_1)
819.291(HexNAc(1)Hex(2)NeuAc(1)_1)
1421.088(PEP+HexNAc(5)Hex(6)Fuc(1)_2)
1465.598
1583.715(PEP+HexNAc(2)Hex(2)Fuc(1)_1)
1745.766(PEP+HexNAc(2)Hex(3)Fuc(1)_1)
1802.789(PEP+HexNAc(3)Hex(3)_1)
1546.132
1559.129
M+2H
1238.518(PEP+HexNAc(4)Hex(5)Fuc(1)_2)
819.287(HexNAc(1)Hex(2)NeuAc(1)_1)
331.187
559.998
948.401

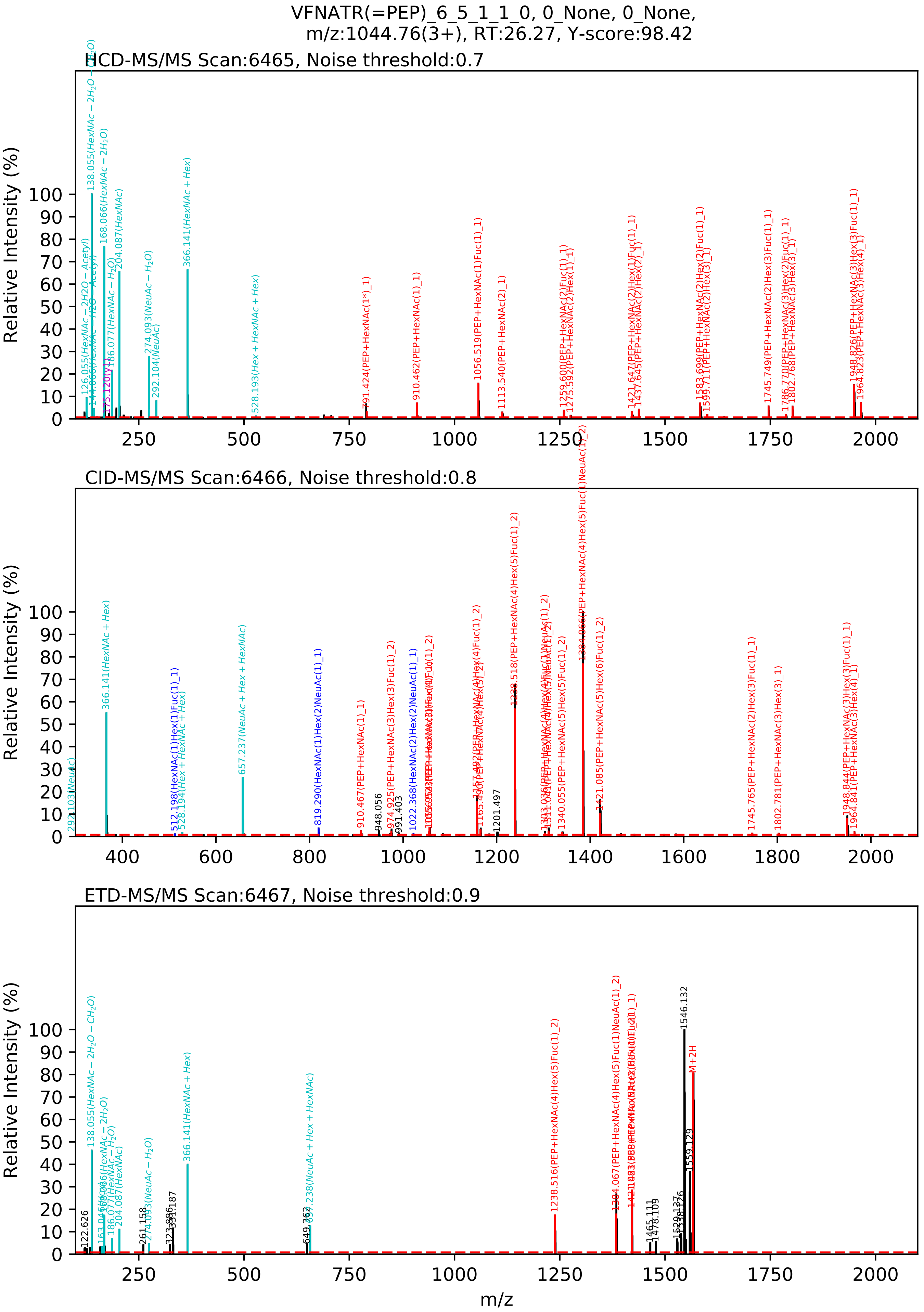
VFNATR(=PEP)_6_5_1_1_0, 0_None, 0_None,
m/z:1044.76(3+), RT:26.27, Y-score:98.42
HCD-MS/MS Scan:6465, Noise threshold:0.7
Relative Intensity (%)
138.055(HexNAc − 2H2O − CH2O)
168.066(HexNAc − 2H2O)
204.087(HexNAc)
186.077(HexNAc − H2O)
274.093(NeuAc − H2O)
292.104(NeuAc)
366.141(HexNAc + Hex)
528.193(Hex + HexNAc + Hex)
175.120(y1)
791.424(PEP+HexNAc(1*)_1)
910.462(PEP+HexNAc(1)_1)
1056.519(PEP+HexNAc(1)Fuc(1)_1)
1113.540(PEP+HexNAc(2)_1)
1745.749(PEP+HexNAc(2)Hex(3)Fuc(1)_1)
1948.826(PEP+HexNAc(3)Hex(3)Fuc(1)_1)
1964.823(PEP+HexNAc(3)Hex(4)_1)
CID-MS/MS Scan:6466, Noise threshold:0.8
366.141(HexNAc + Hex)
512.198(HexNAc(1)Hex(1)Fuc(1)_1)
657.237(NeuAc + Hex + HexNAc)
819.290(HexNAc(1)Hex(2)NeuAc(1)_1)
910.467(PEP+HexNAc(1)_1)
948.056
991.403
974.925(PEP+HexNAc(3)Hex(3)Fuc(1)_2)
1022.368(HexNAc(2)Hex(2)NeuAc(1)_1)
1201.497
1238.518(PEP+HexNAc(4)Hex(5)Fuc(1)_2)
1340.055(PEP+HexNAc(5)Hex(5)Fuc(1)_2)
1384.066(PEP+HexNAc(4)Hex(5)Fuc(1)NeuAc(1)_2)
1421.085(PEP+HexNAc(5)Hex(6)Fuc(1)_2)
1745.765(PEP+HexNAc(2)Hex(3)Fuc(1)_1)
1802.781(PEP+HexNAc(3)Hex(3)_1)
1948.844(PEP+HexNAc(3)Hex(3)Fuc(1)_1)
1964.841(PEP+HexNAc(3)Hex(4)_1)
ETD-MS/MS Scan:6467, Noise threshold:0.9
138.055(HexNAc − 2H2O − CH2O)
366.141(HexNAc + Hex)
657.238(NeuAc + Hex + HexNAc)
122.626
261.158
649.362
1238.516(PEP+HexNAc(4)Hex(5)Fuc(1)_2)
1384.067(PEP+HexNAc(4)Hex(5)Fuc(1)NeuAc(1)_2)
1465.111
1478.109
1546.132
1559.129
M+2H
m/z

VFNATR(=PEP)_6_5_1_1_0, 0_None, 0_None,
m/z:1566.63(2+), RT:26.54, Y-score:86.97

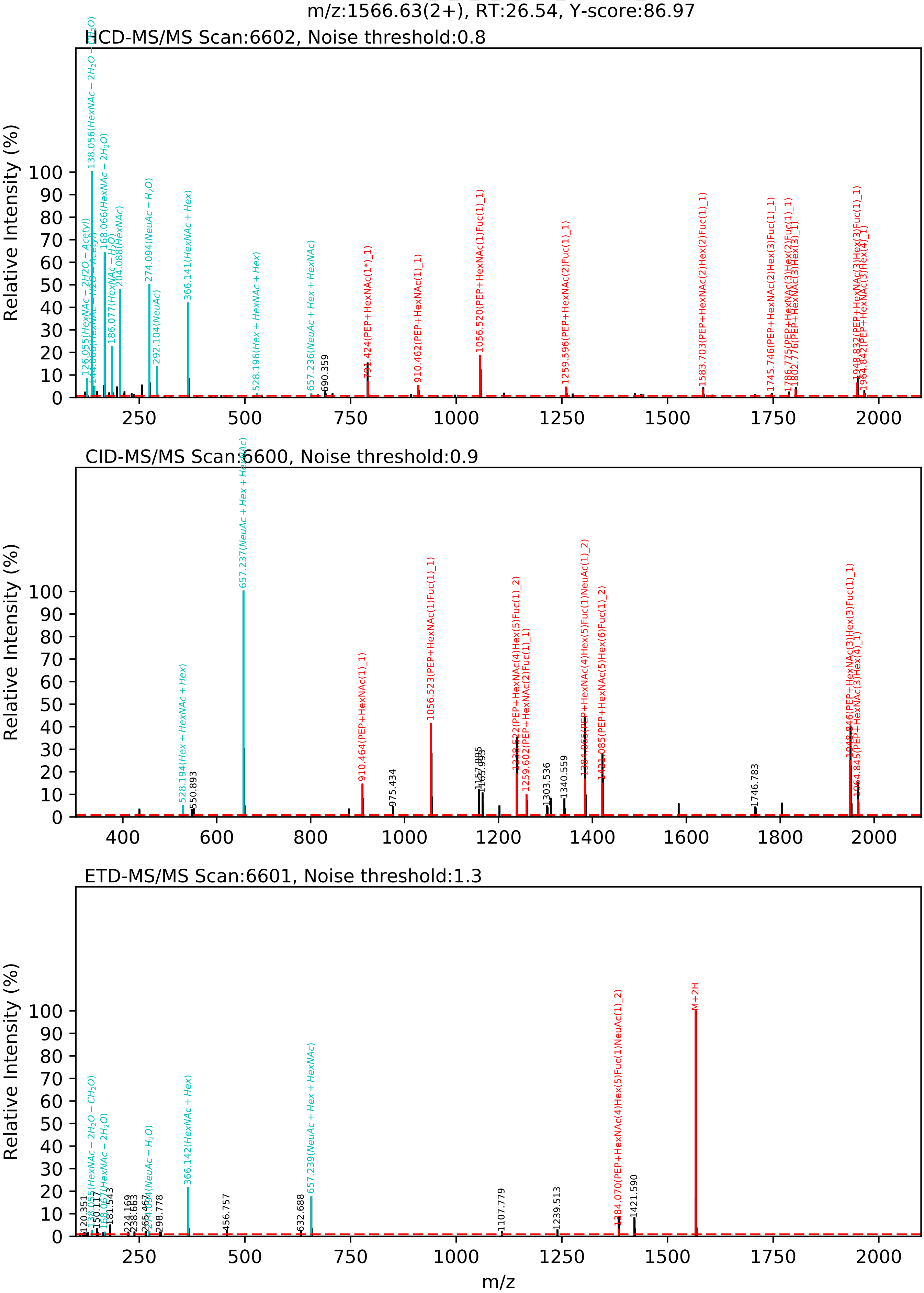

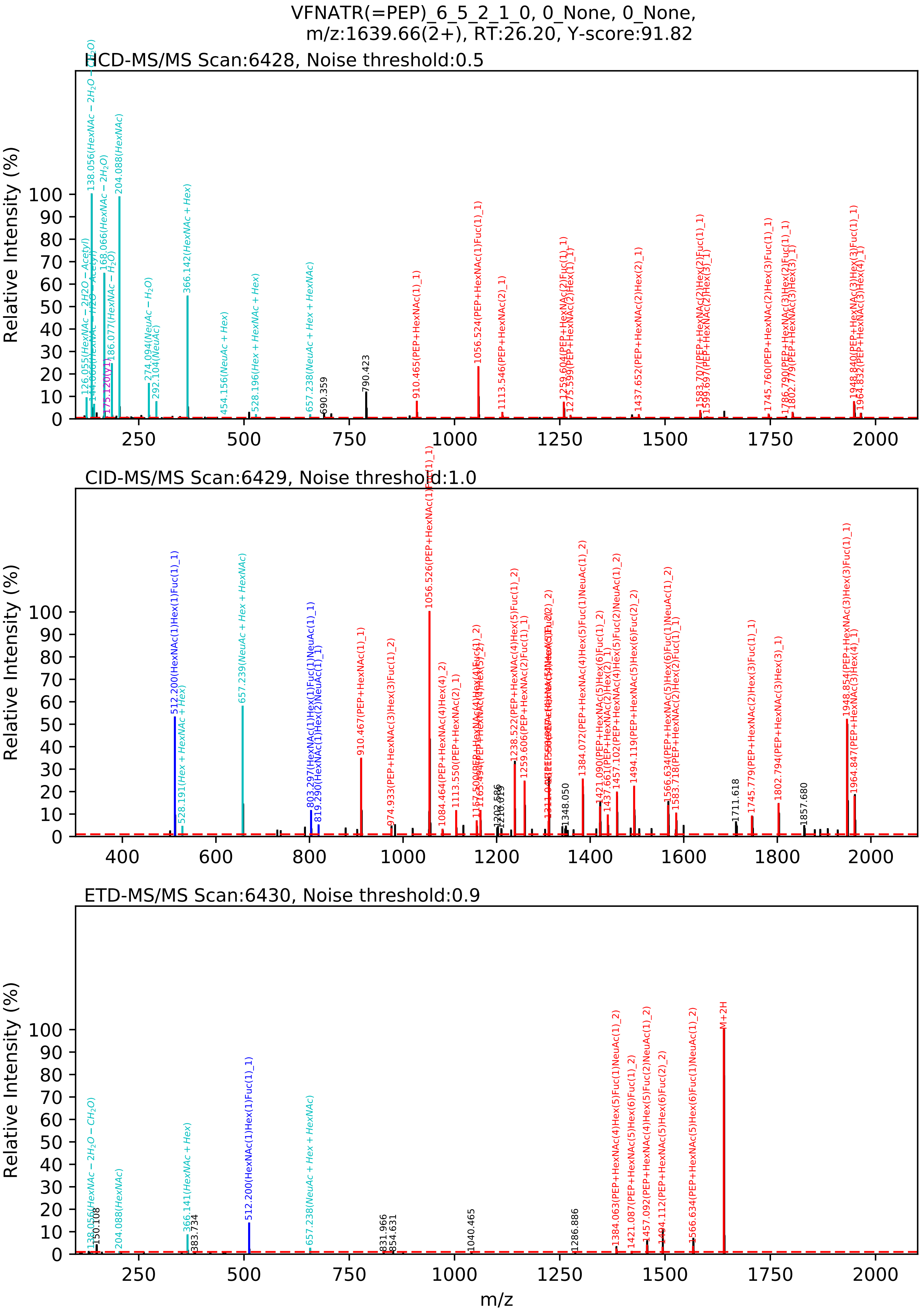
VFNATR(=PEP)_6_5_2_1_0, 0_None, 0_None,
m/z:1639.66(2+), RT:26.20, Y-score:91.82
HCD-MS/MS Scan:6428, Noise threshold:0.5
CID-MS/MS Scan:6429, Noise threshold:1.0
ETD-MS/MS Scan:6430, Noise threshold:0.9
Relative Intensity (%)
m/z
1056.524(PEP+HexNAc(1)Fuc(1)_1)
910.465(PEP+HexNAc(1)_1)
1056.526(PEP+HexNAc(1)Fuc(1)_1)
512.200(HexNAc(1)Hex(1)Fuc(1)_1)
657.239(NeuAc + Hex + HexNAc)
1948.854(PEP+HexNAc(3)Hex(3)Fuc(1)_1)
M+2H

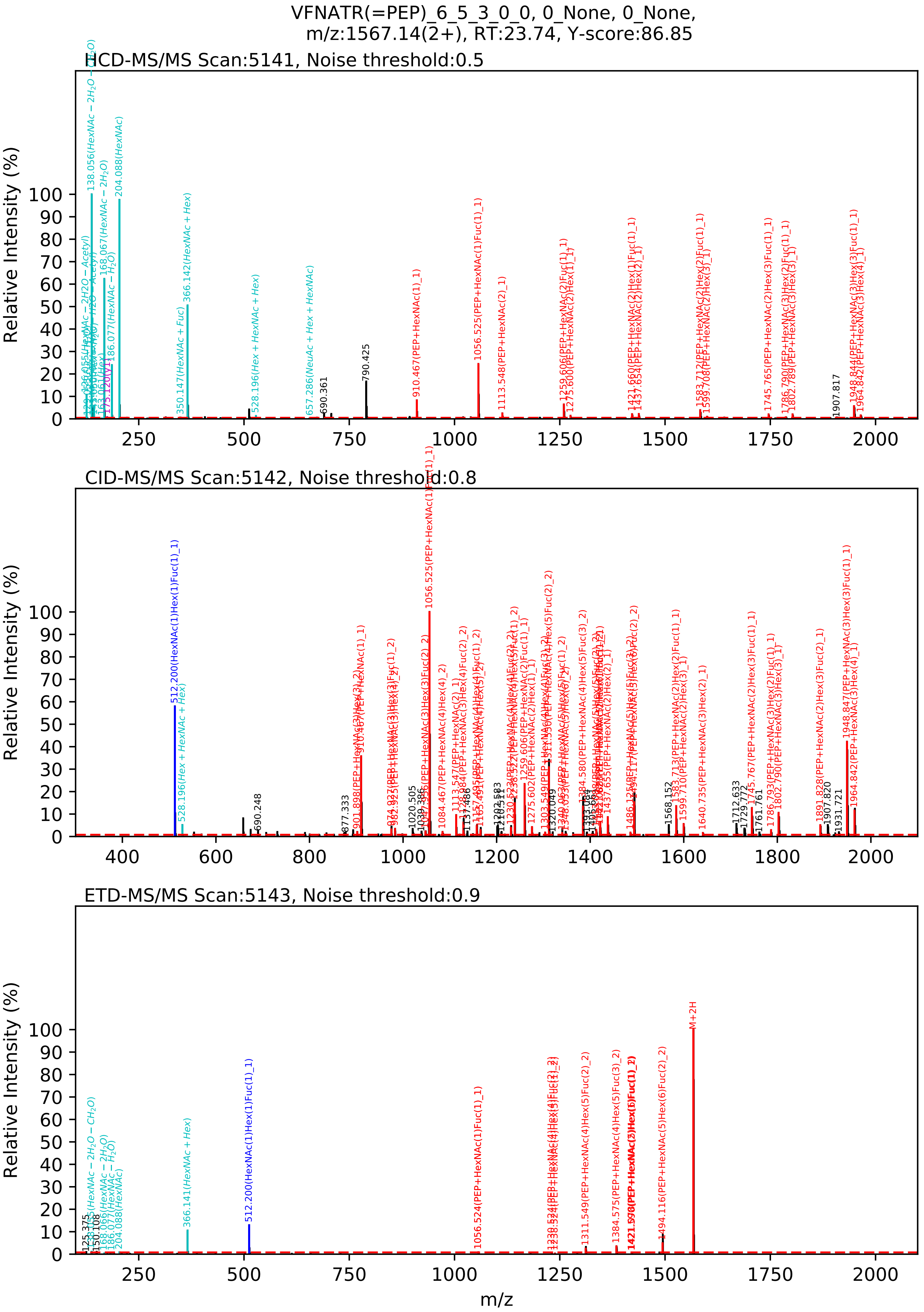
VFNATR(=PEP)_6_5_3_0_0, 0_None, 0_None,
m/z:1567.14(2+), RT:23.74, Y-score:86.85
HCD-MS/MS Scan:5141, Noise threshold:0.5
CID-MS/MS Scan:5142, Noise threshold:0.8
ETD-MS/MS Scan:5143, Noise threshold:0.9
Relative Intensity (%)
m/z

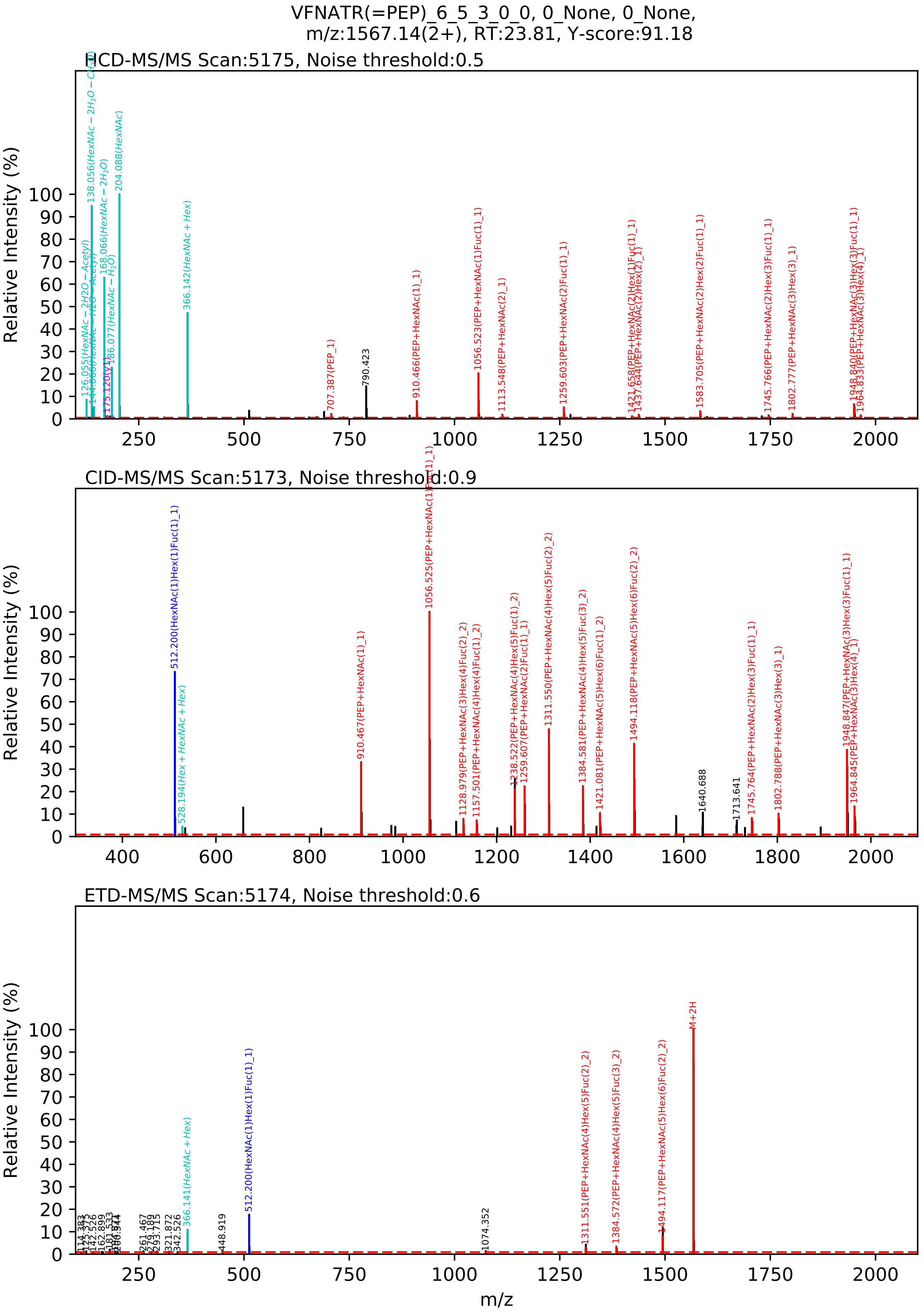
VFNATR(=PEP)_6_5_3_0_0, 0_None, 0_None,
m/z:1567.14(2+), RT:23.81, Y-score:91.18
HCD-MS/MS Scan:5175, Noise threshold:0.5
CID-MS/MS Scan:5173, Noise threshold:0.9
ETD-MS/MS Scan:5174, Noise threshold:0.6
Relative Intensity (%)
m/z

VFNATR(=PEP)_6_5_3_0_0, 0_None, 0_None,
m/z:1045.10(3+), RT:23.76, Y-score:95.61

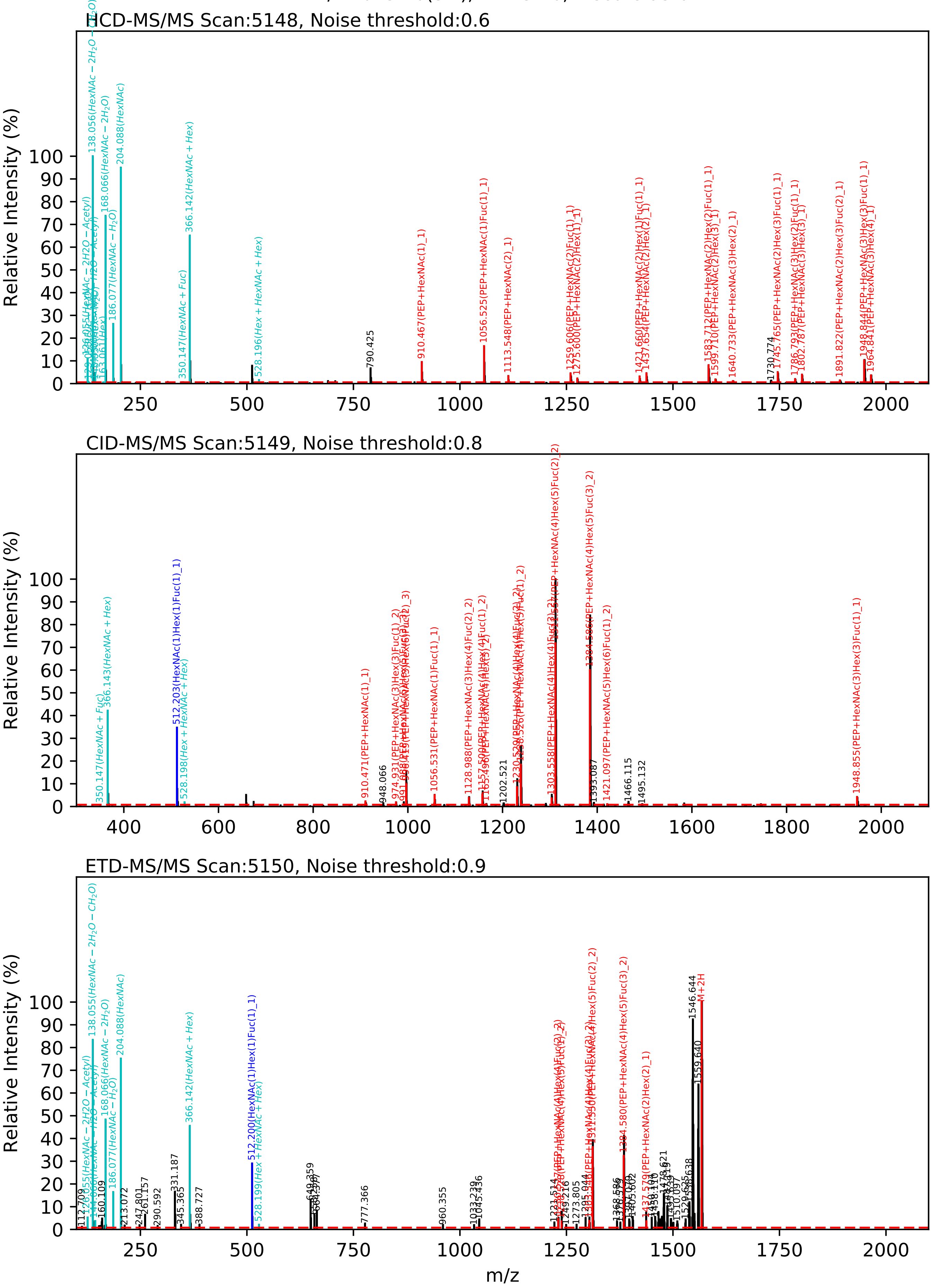

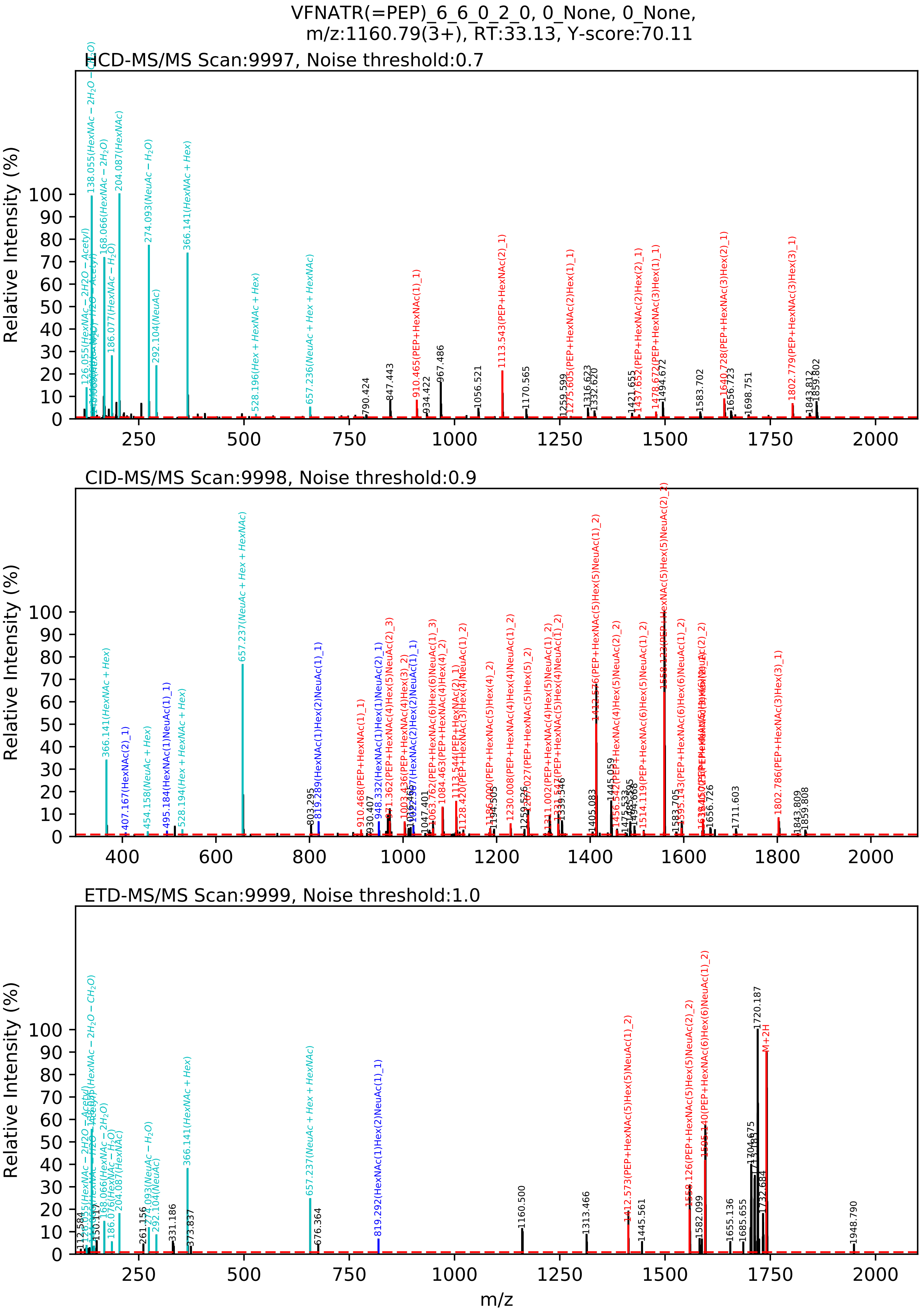

VFNATR(=PEP)_6_6_0_2_0, 0_None, 0_None,
m/z:1160.79(3+), RT:33.13, Y-score:70.11
HCD-MS/MS Scan:9997, Noise threshold:0.7
CID-MS/MS Scan:9998, Noise threshold:0.9
ETD-MS/MS Scan:9999, Noise threshold:1.0
Relative Intensity (%)
m/z

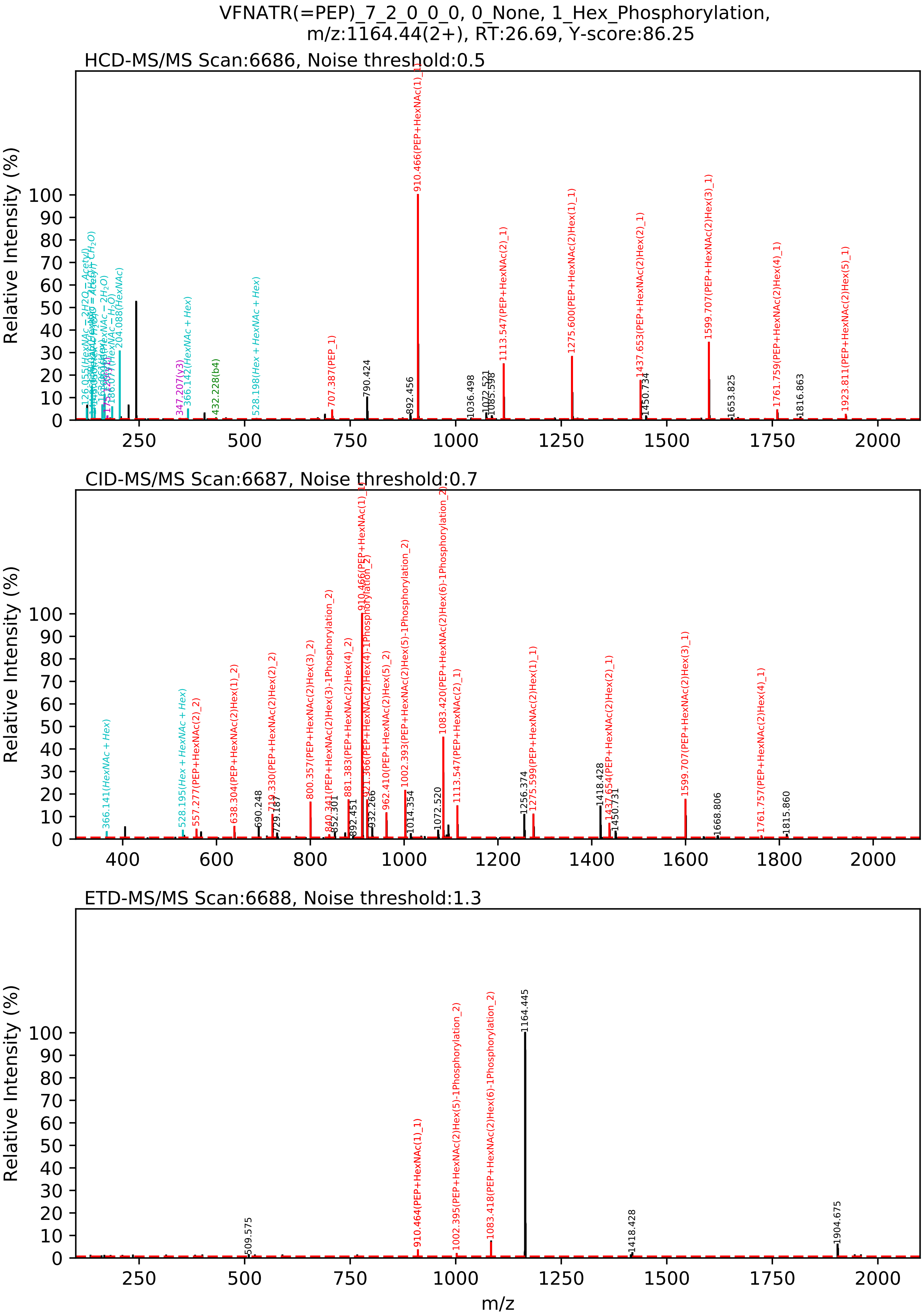

VFNATR(=PEP)_7_2_0_0_0, 0_None, 1_Hex_Phosphorylation,
m/z:1164.44(2+), RT:26.69, Y-score:86.25
HCD-MS/MS Scan:6686, Noise threshold:0.5
Relative Intensity (%)
910.466(PEP+HexNAc(1)_1)
1113.547(PEP+HexNAc(2)_1)
1275.600(PEP+HexNAc(2)Hex(1)_1)
1437.653(PEP+HexNAc(2)Hex(2)_1)
1599.707(PEP+HexNAc(2)Hex(3)_1)
1761.759(PEP+HexNAc(2)Hex(4)_1)
1923.811(PEP+HexNAc(2)Hex(5)_1)
707.387(PEP_1)
790.424
892.456
1036.498
1072.521
1085.598
1450.734
1653.825
1816.863
204.088(HexNAc)
347.207(y3)
366.142(HexNAc + Hex)
432.228(b4)
528.198(Hex + HexNAc + Hex)
CID-MS/MS Scan:6687, Noise threshold:0.7
366.141(HexNAc + Hex)
528.195(Hex + HexNAc + Hex)
557.277(PEP+HexNAc(2)_2)
638.304(PEP+HexNAc(2)Hex(1)_2)
690.248
719.330(PEP+HexNAc(2)Hex(2)_2)
729.187
800.357(PEP+HexNAc(2)Hex(3)_2)
840.341(PEP+HexNAc(2)Hex(3)-1Phosphorylation_2)
852.301
881.383(PEP+HexNAc(2)Hex(4)_2)
892.451
910.466(PEP+HexNAc(1)_1)
921.366(PEP+HexNAc(2)Hex(4)-1Phosphorylation_2)
932.266
962.410(PEP+HexNAc(2)Hex(5)_2)
1002.393(PEP+HexNAc(2)Hex(5)-1Phosphorylation_2)
1014.354
1072.520
1083.420(PEP+HexNAc(2)Hex(6)-1Phosphorylation_2)
1113.547(PEP+HexNAc(2)_1)
1256.374
1275.599(PEP+HexNAc(2)Hex(1)_1)
1418.428
1437.654(PEP+HexNAc(2)Hex(2)_1)
1450.731
1599.707(PEP+HexNAc(2)Hex(3)_1)
1668.806
1761.757(PEP+HexNAc(2)Hex(4)_1)
1815.860
ETD-MS/MS Scan:6688, Noise threshold:1.3
509.575
910.464(PEP+HexNAc(1)_1)
1002.395(PEP+HexNAc(2)Hex(5)-1Phosphorylation_2)
1083.418(PEP+HexNAc(2)Hex(6)-1Phosphorylation_2)
1164.445
1418.428
1904.675
m/z

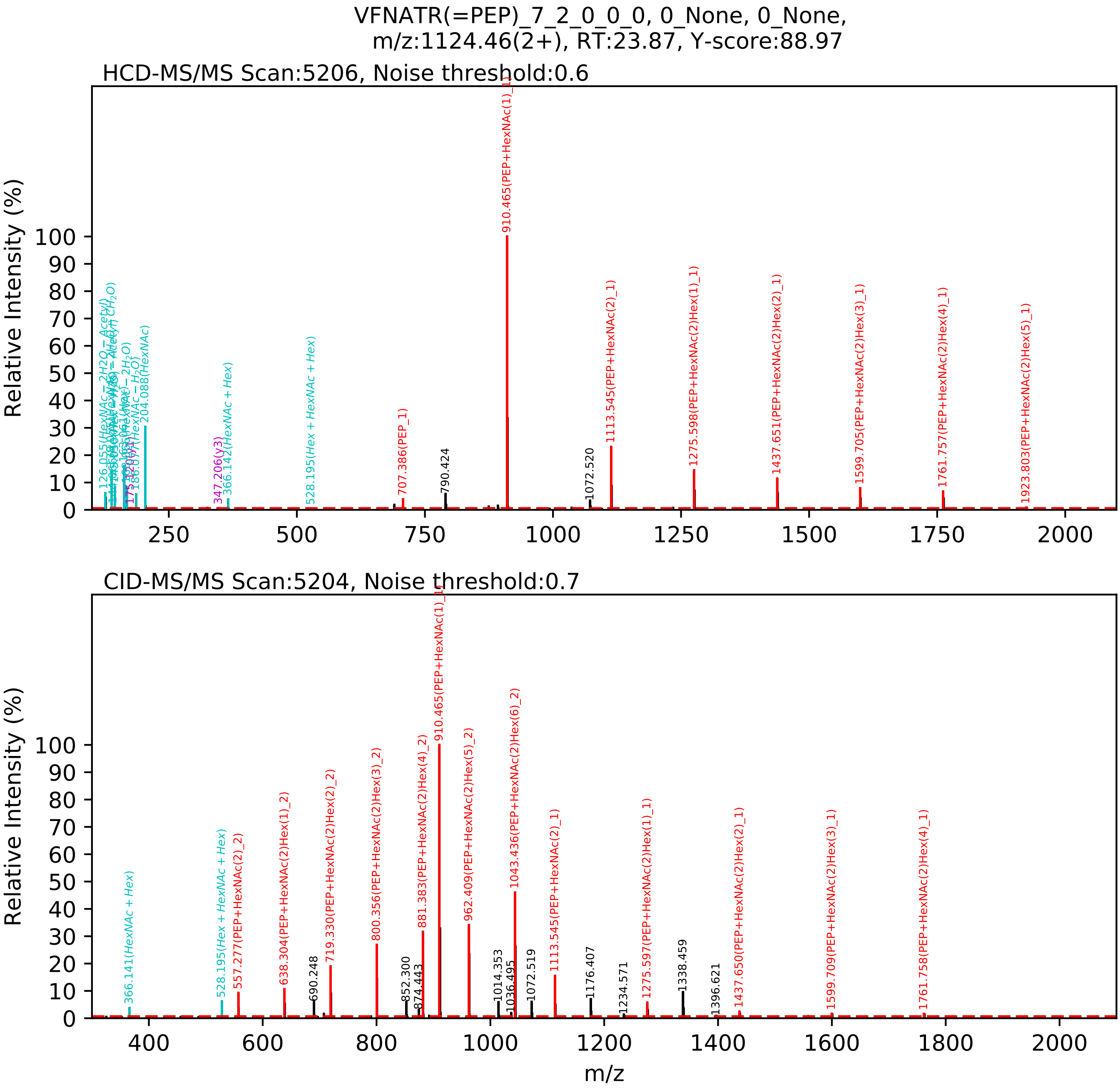
VFNATR(=PEP)_7_2_0_0_0, 0_None, 0_None,
m/z:1124.46(2+), RT:23.87, Y-score:88.97
HCD-MS/MS Scan:5206, Noise threshold:0.6
Relative Intensity (%)
910.465(PEP+HexNAc(1)_1)
1113.545(PEP+HexNAc(2)_1)
1275.598(PEP+HexNAc(2)Hex(1)_1)
1437.651(PEP+HexNAc(2)Hex(2)_1)
1599.705(PEP+HexNAc(2)Hex(3)_1)
1761.757(PEP+HexNAc(2)Hex(4)_1)
1923.803(PEP+HexNAc(2)Hex(5)_1)
707.386(PEP_1)
790.424
1072.520
528.195(Hex + HexNAc + Hex)
366.142(HexNAc + Hex)
347.206(y3)
204.088(HexNAc)
126.055(HexNAc − 2H2O − Acetyl)
CID-MS/MS Scan:5204, Noise threshold:0.7
Relative Intensity (%)
366.141(HexNAc + Hex)
528.195(Hex + HexNAc + Hex)
557.277(PEP+HexNAc(2)_2)
638.304(PEP+HexNAc(2)Hex(1)_2)
690.248
719.330(PEP+HexNAc(2)Hex(2)_2)
800.356(PEP+HexNAc(2)Hex(3)_2)
852.300
874.443
881.383(PEP+HexNAc(2)Hex(4)_2)
910.465(PEP+HexNAc(1)_1)
962.409(PEP+HexNAc(2)Hex(5)_2)
1014.353
1036.495
1043.436(PEP+HexNAc(2)Hex(6)_2)
1072.519
1113.545(PEP+HexNAc(2)_1)
1176.407
1234.571
1275.597(PEP+HexNAc(2)Hex(1)_1)
1338.459
1396.621
1437.650(PEP+HexNAc(2)Hex(2)_1)
1599.709(PEP+HexNAc(2)Hex(3)_1)
1761.758(PEP+HexNAc(2)Hex(4)_1)
m/z

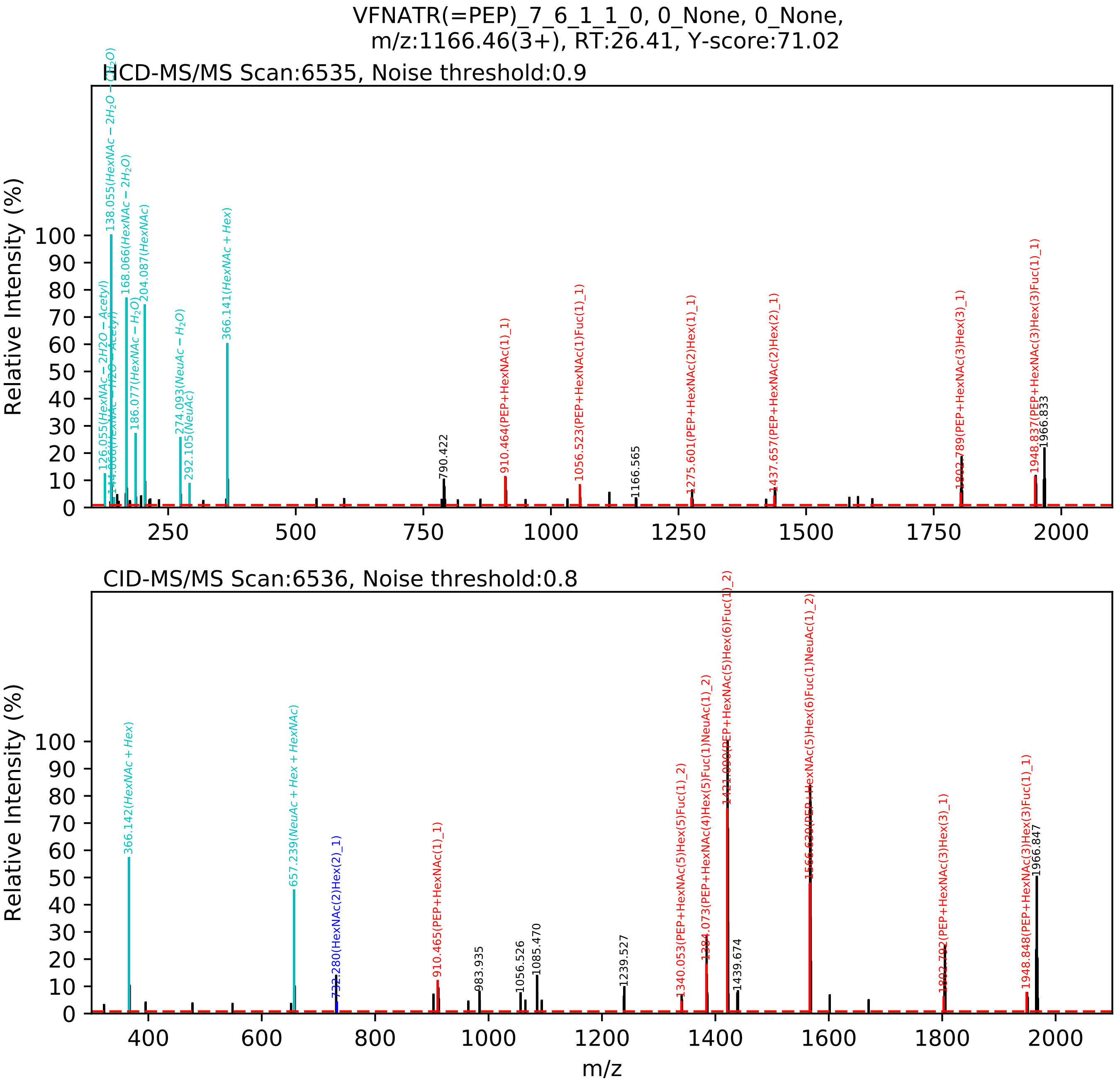

VFNATR(=PEP)_7_6_1_1_0, 0_None, 0_None,
m/z:1166.46(3+), RT:26.41, Y-score:71.02
HCD-MS/MS Scan:6535, Noise threshold:0.9
Relative Intensity (%)
138.055(HexNAc − 2H2O − H2O)
168.066(HexNAc − 2H2O)
204.087(HexNAc)
186.077(HexNAc − H2O)
126.055(HexNAc − 2H2O − Acetyl)
366.141(HexNAc + Hex)
274.093(NeuAc − H2O)
292.105(NeuAc)
790.422
910.464(PEP+HexNAc(1)_1)
1056.523(PEP+HexNAc(1)Fuc(1)_1)
1166.565
1275.601(PEP+HexNAc(2)Hex(1)_1)
1437.657(PEP+HexNAc(2)Hex(2)_1)
1802.789(PEP+HexNAc(3)Hex(3)_1)
1948.837(PEP+HexNAc(3)Hex(3)Fuc(1)_1)
1966.833
CID-MS/MS Scan:6536, Noise threshold:0.8
Relative Intensity (%)
366.142(HexNAc + Hex)
657.239(NeuAc + Hex + HexNAc)
732.280(HexNAc(2)Hex(2)_1)
910.465(PEP+HexNAc(1)_1)
983.935
1056.526
1085.470
1239.527
1340.053(PEP+HexNAc(5)Hex(5)Fuc(1)_2)
1384.073(PEP+HexNAc(4)Hex(5)Fuc(1)NeuAc(1)_2)
1421.090(PEP+HexNAc(5)Hex(6)Fuc(1)_2)
1439.674
1566.630(PEP+HexNAc(5)Hex(6)Fuc(1)NeuAc(1)_2)
1802.792(PEP+HexNAc(3)Hex(3)_1)
1948.848(PEP+HexNAc(3)Hex(3)Fuc(1)_1)
1966.847
m/z

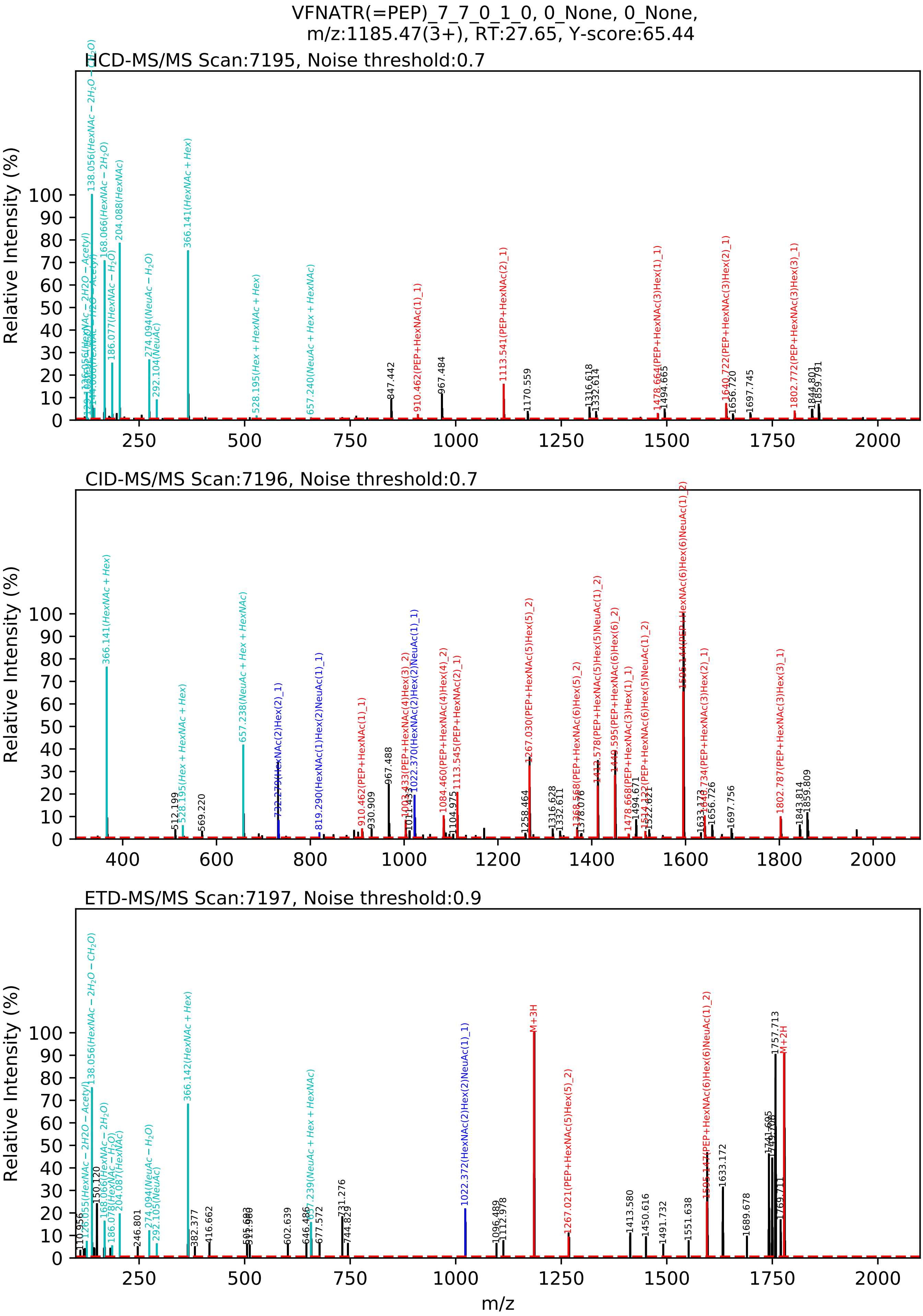

VFNATR(=PEP)_7_7_0_1_0, 0_None, 0_None,
m/z:1185.47(3+), RT:27.65, Y-score:65.44
HCD-MS/MS Scan:7195, Noise threshold:0.7
CID-MS/MS Scan:7196, Noise threshold:0.7
ETD-MS/MS Scan:7197, Noise threshold:0.9
Relative Intensity (%)
m/z
366.141(HexNAc + Hex)
657.240(NeuAc + Hex + HexNAc)
1113.541(PEP+HexNAc(2)_1)
1802.772(PEP+HexNAc(3)Hex(3)_1)
1595.144(PEP+HexNAc(6)Hex(6)NeuAc(1)_2)
1022.370(HexNAc(2)Hex(2)NeuAc(1)_1)
1267.030(PEP+HexNAc(5)Hex(5)_2)
M+3H
M+2H
1757.713
