# Supplementary material for: Characterization of Site-Specific N- and O-Glycopeptides from Recombinant Spike and ACE2 Glycoproteins Using LC-MS/MS Analysis
Source: Int J Mol Sci. 2024 Dec 20;25(24):13649. doi: 10.3390/ijms252413649 (PMC11678118; doi:10.3390/ijms252413649)
Supplement: Supplementary file 1 [file ijms-25-13649-s001.zip › Supplementary Figure S5(RBD_TG_N-glycopep_2).pdf]

VFNATR(=PEP)_8_2_0_0_0, 0_None, 0_None,
m/z:1205.49(3+), RT:23.74, hcd-score:88.05

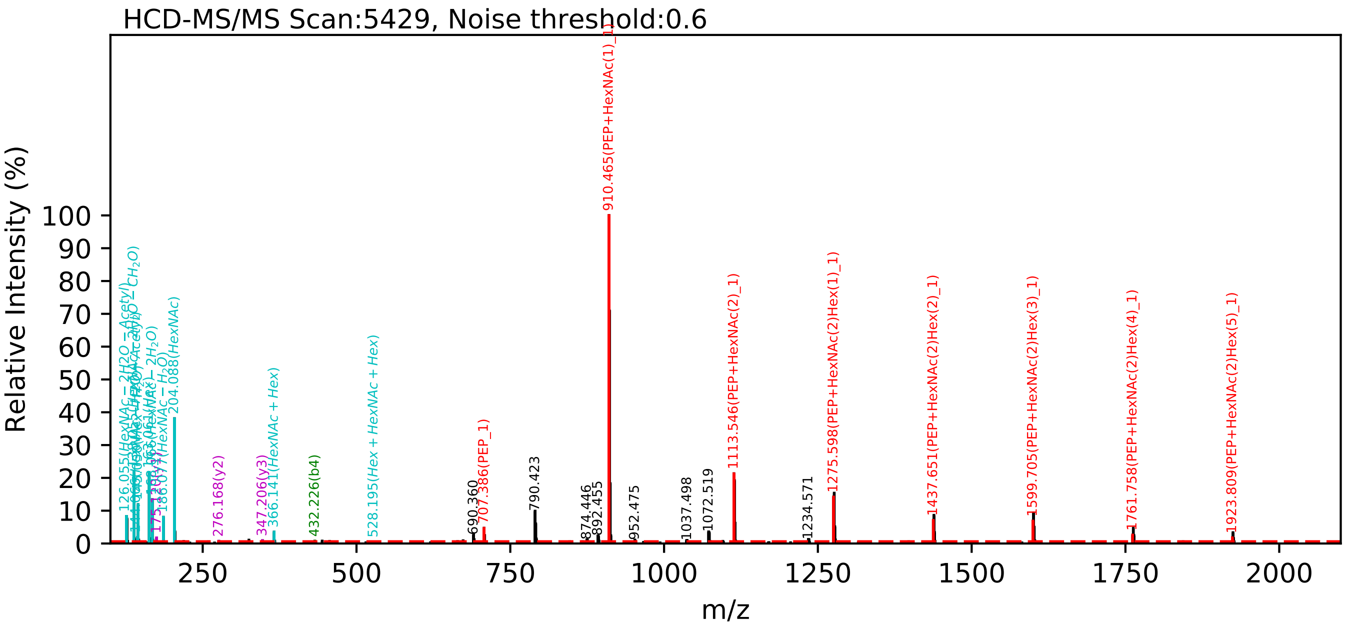

VFNATR(=PEP)_8_2_0_0_0, 0_None, 0_None,
m/z:1205.49(3+), RT:23.74, hcd-score:88.05

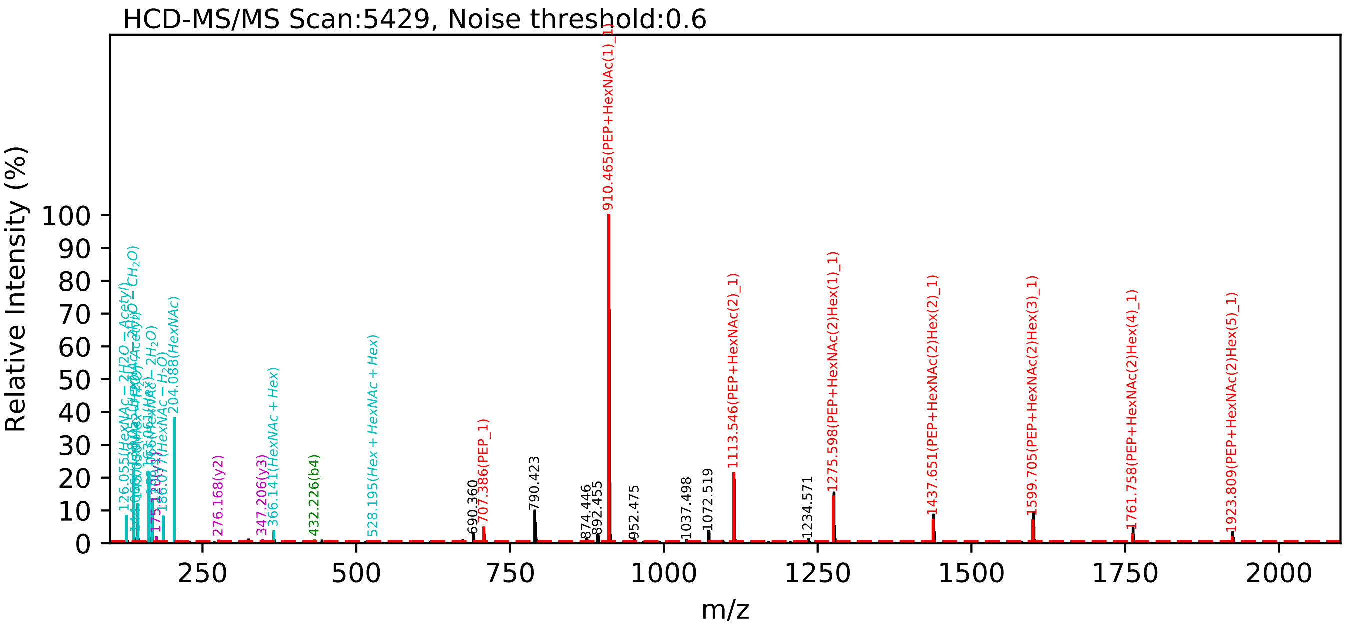

VFNATR(=PEP)_5_2_0_0_0, 0_None, 0_None,
m/z:962.41(3+), RT:23.92, hcd-score:94.59

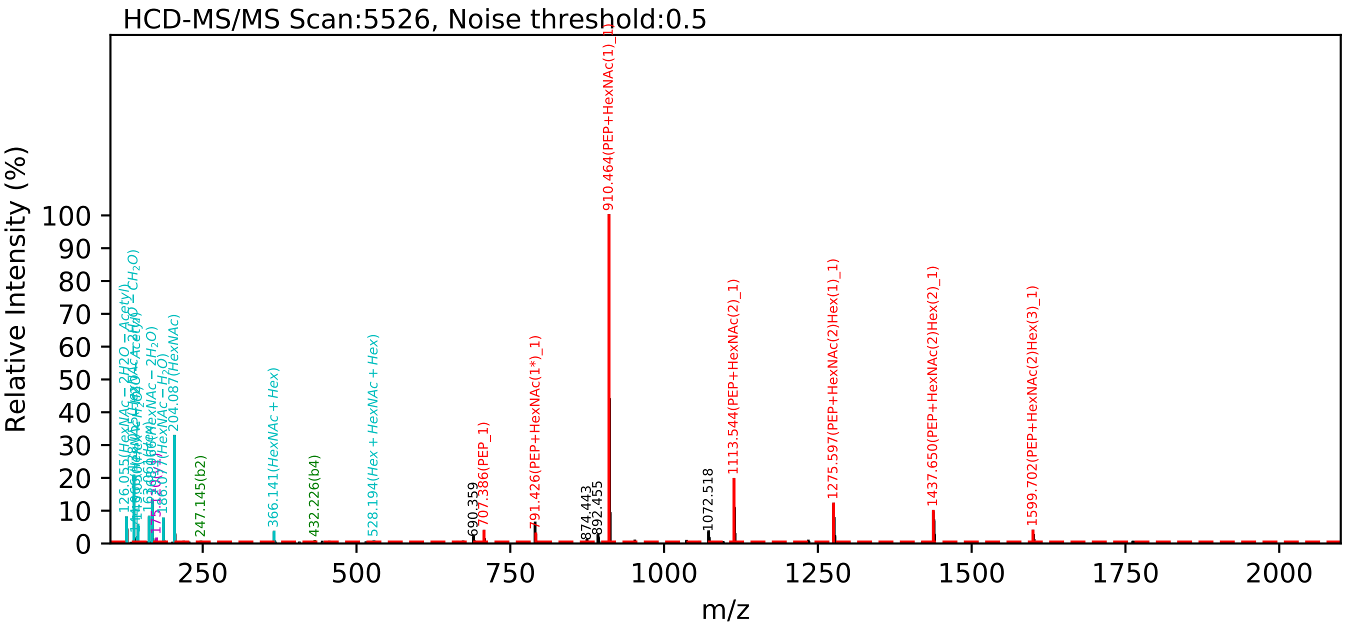

VFNATR(=PEP)_5_2_0_0_0, 0_None, 0_None,
m/z:962.41(3+), RT:23.92, hcd-score:94.59

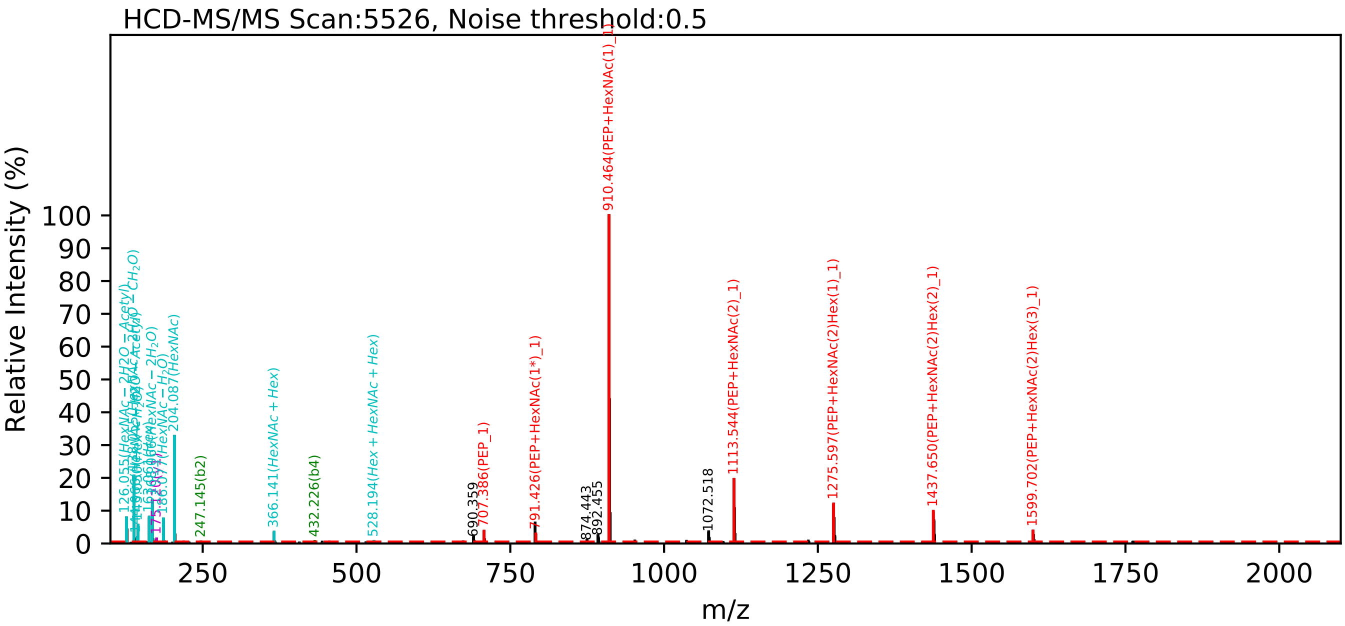

VFNATR(=PEP)_4_2_0_0_0, 0_None, 0_None,
m/z:881.38(3+), RT:24.19, hcd-score:89.78

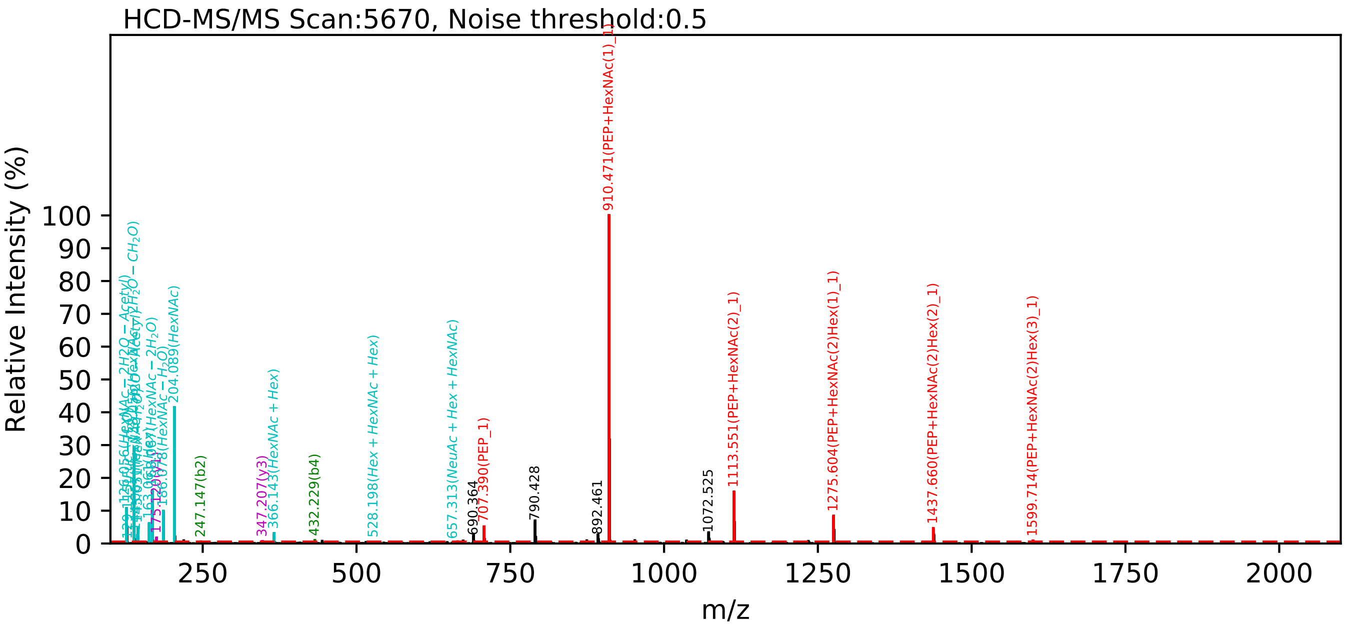

VFNATR(=PEP)_4_2_0_0_0, 0_None, 0_None,
m/z:881.38(3+), RT:24.19, hcd-score:89.78

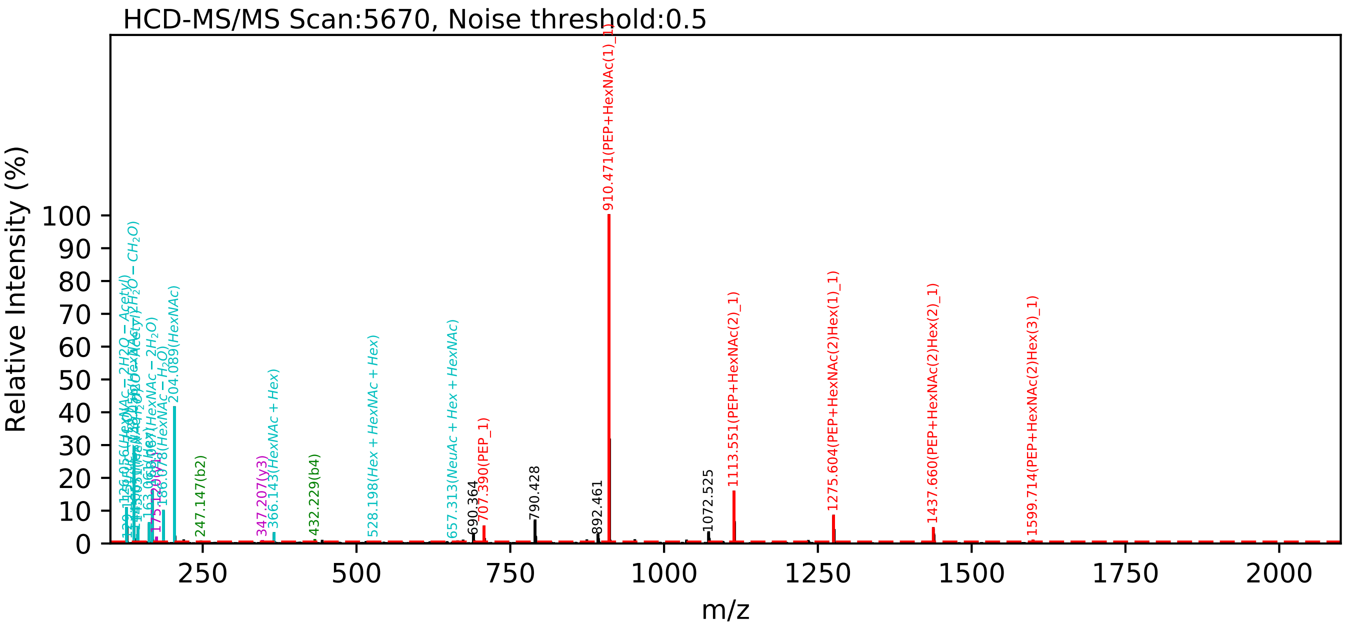

FPNITNLCPFGE(=PEP)_4_3_2_0_0, 0_None, 0_None,
m/z:1479.61(3+), RT:59.79, hcd-score:67.49

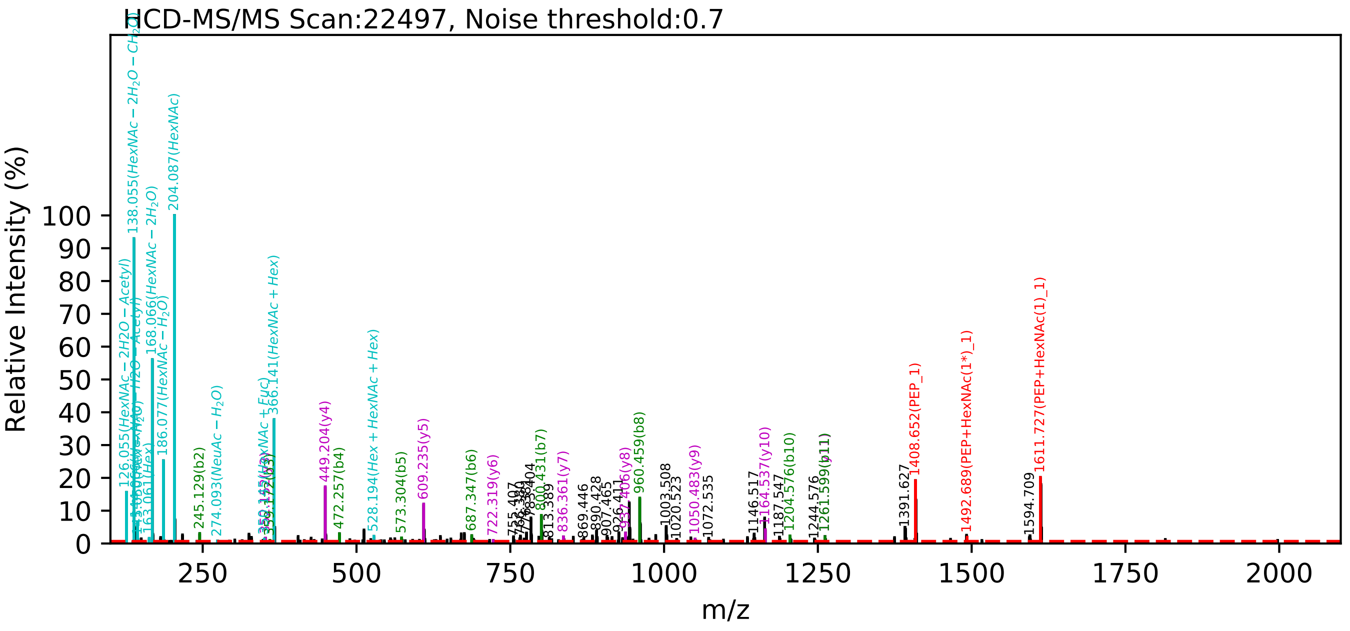

FPNITNLCPFGE(=PEP)_4_3_2_0_0, 0_None, 0_None,
m/z:1479.61(3+), RT:59.79, hcd-score:67.49

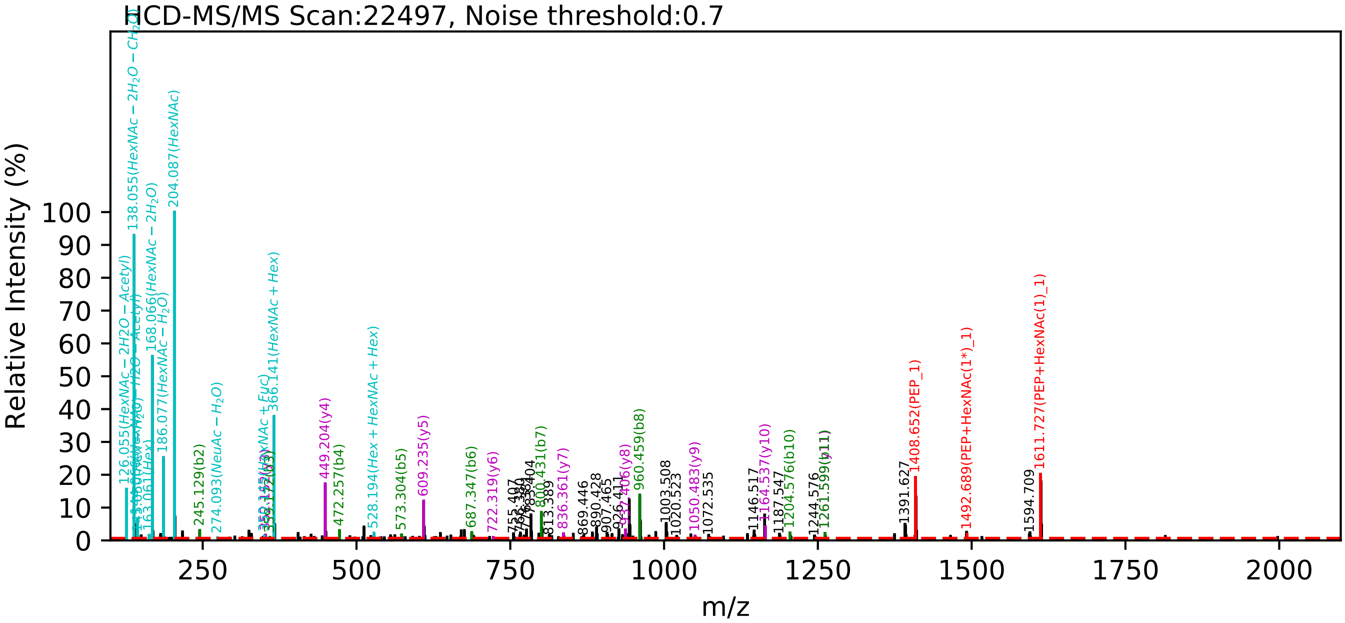

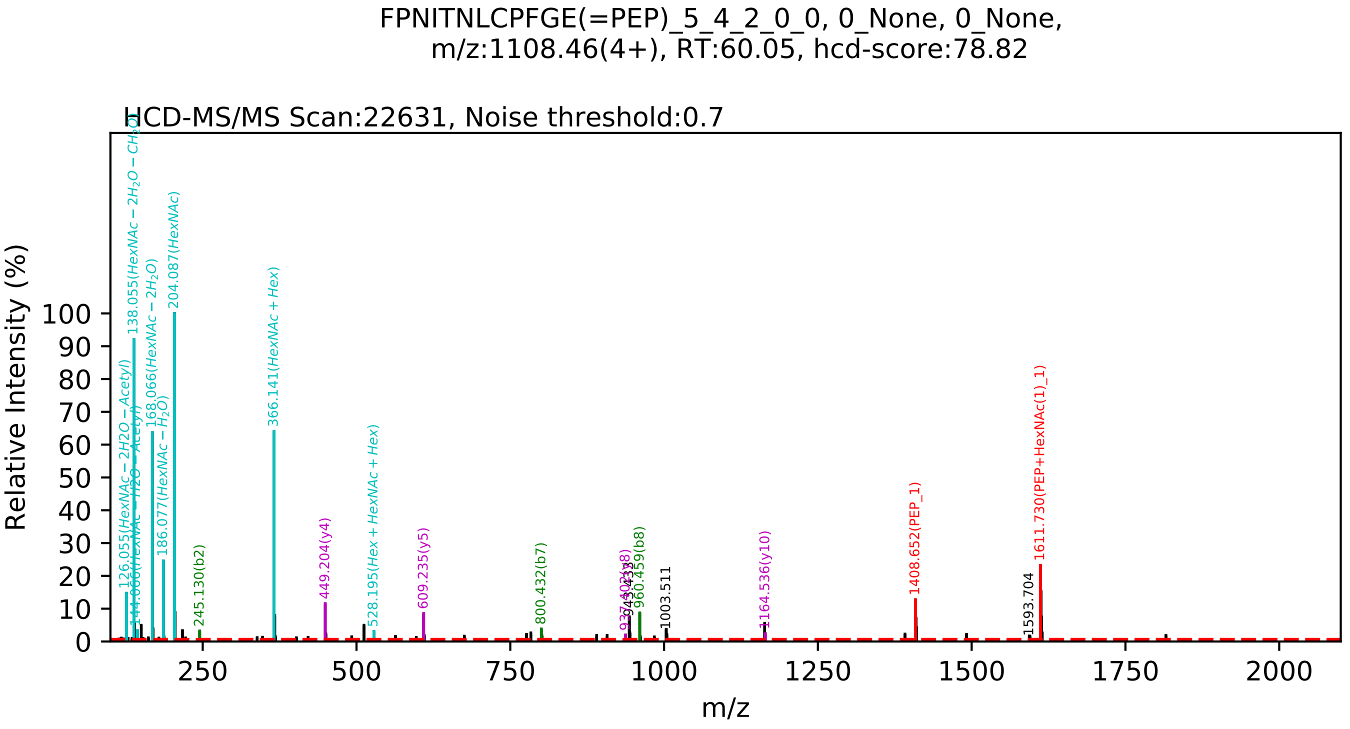

FPNITNLCPFGE(=PEP)_5_4_2_0_0, 0_None, 0_None,
m/z:1108.46(4+), RT:60.05, hcd-score:78.82
HCD-MS/MS Scan:22631, Noise threshold:0.7
Relative Intensity (%)
m/z
126.055(HexNAc − 2H2O − Acetyl)
138.055(HexNAc − 2H2O − CH3O)
168.066(HexNAc − 2H2O)
186.077(HexNAc − H2O)
204.087(HexNAc)
245.130(b2)
366.141(HexNAc + Hex)
449.204(y4)
528.195(Hex + HexNAc + Hex)
609.235(y5)
800.432(b7)
960.459(b8)
1003.511
1164.536(y10)
1408.652(PEP_1)
1593.704
1611.730(PEP+HexNAc(1)_1)

FPNITNLCPFGE(=PEP)_5_4_2_0_0, 0_None, 0_None,
m/z:1108.46(4+), RT:60.05, hcd-score:78.82

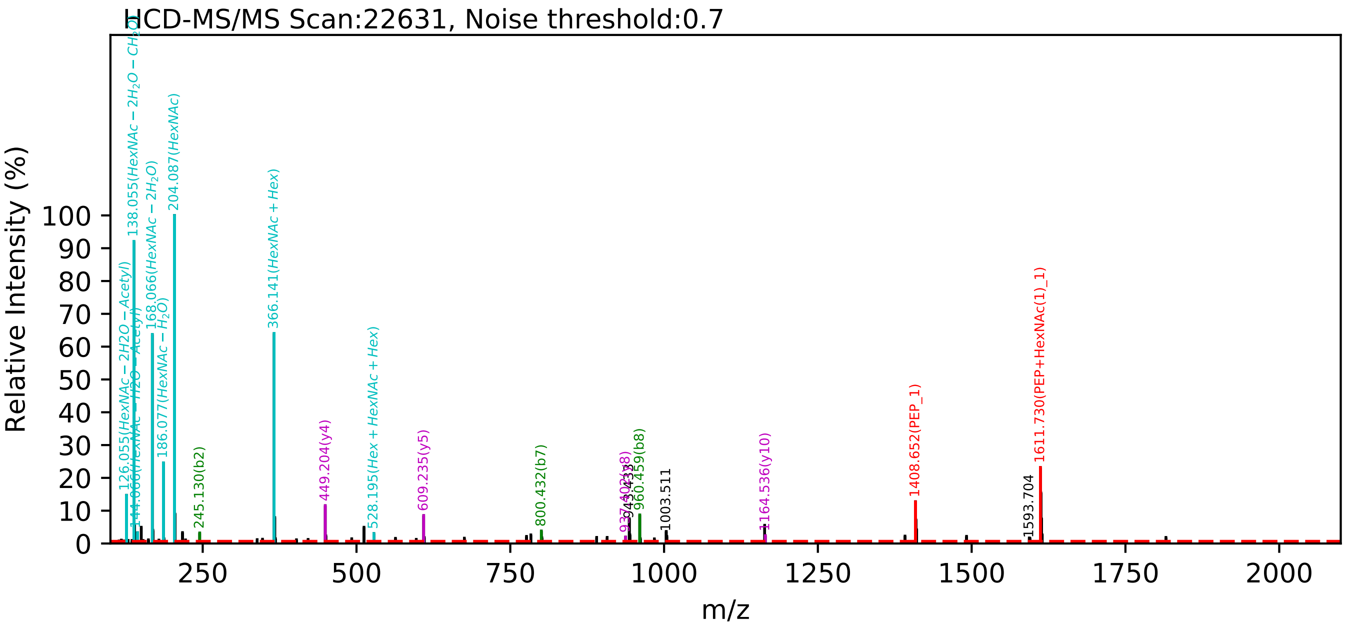

FPNITNLCPFGE(=PEP)_4_5_1_1_0, 0_None, 0_None,
m/z:1170.48(3+), RT:66.44, hcd-score:77.72

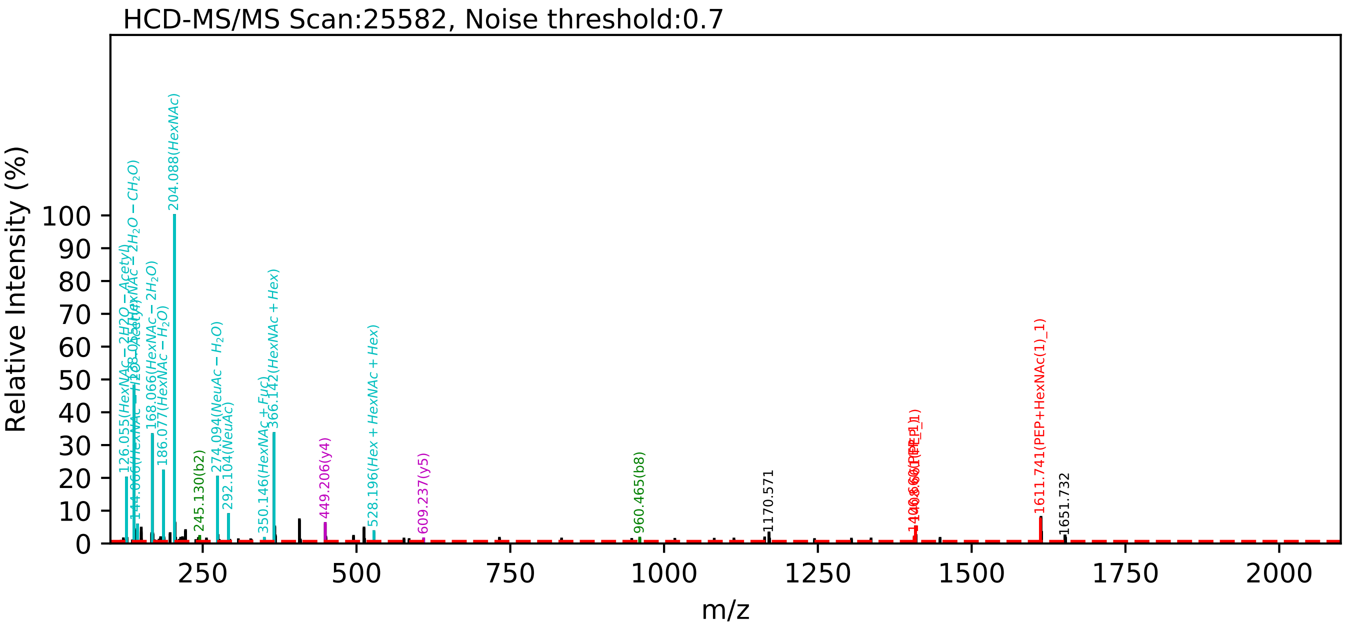

FPNITNLCPFGE(=PEP)_4_5_1_1_0, 0_None, 0_None,
m/z:1170.48(3+), RT:66.44, hcd-score:77.72

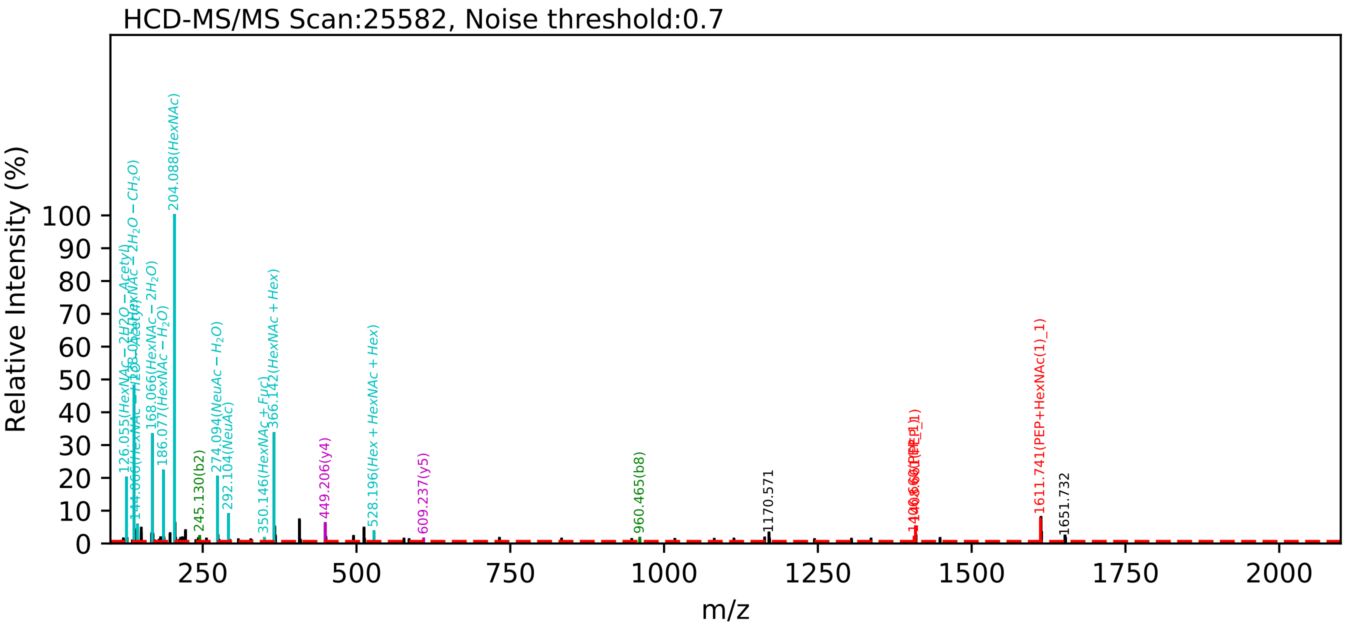

FPNITNLCPFGE(=PEP)_7_6_3_1_0, 0_None, 0_None,
m/z:1497.60(4+), RT:66.54, hcd-score:93.51

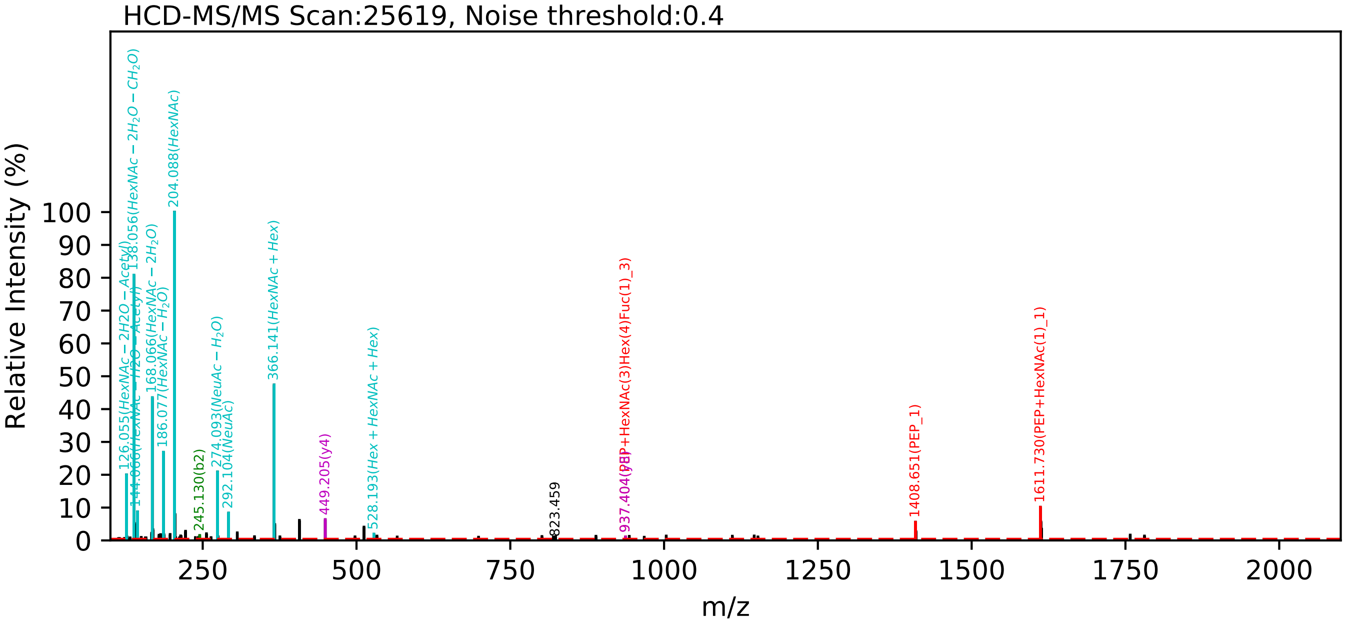

FPNITNLCPFGE(=PEP)_7_6_3_1_0, 0_None, 0_None,
m/z:1497.60(4+), RT:66.54, hcd-score:93.51

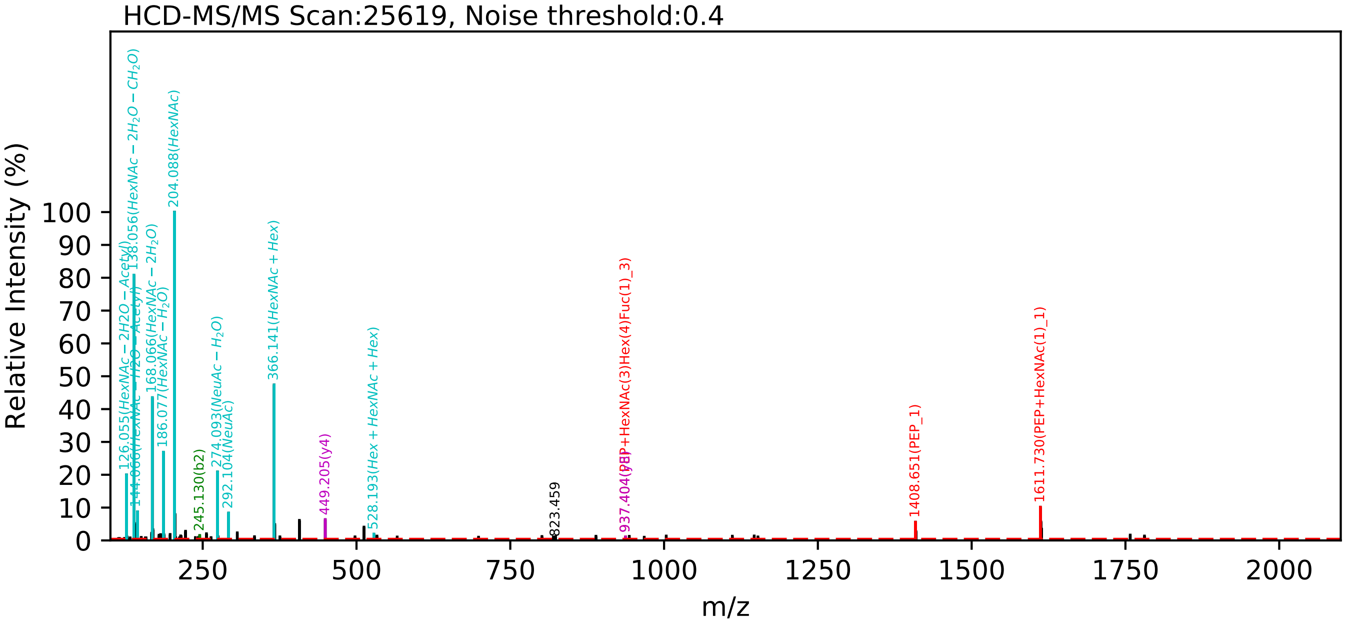

FPNITNLCPFGE(=PEP)_4_5_1_1_0, 0_None, 0_None,
m/z:1170.48(4+), RT:66.74, hcd-score:88.55

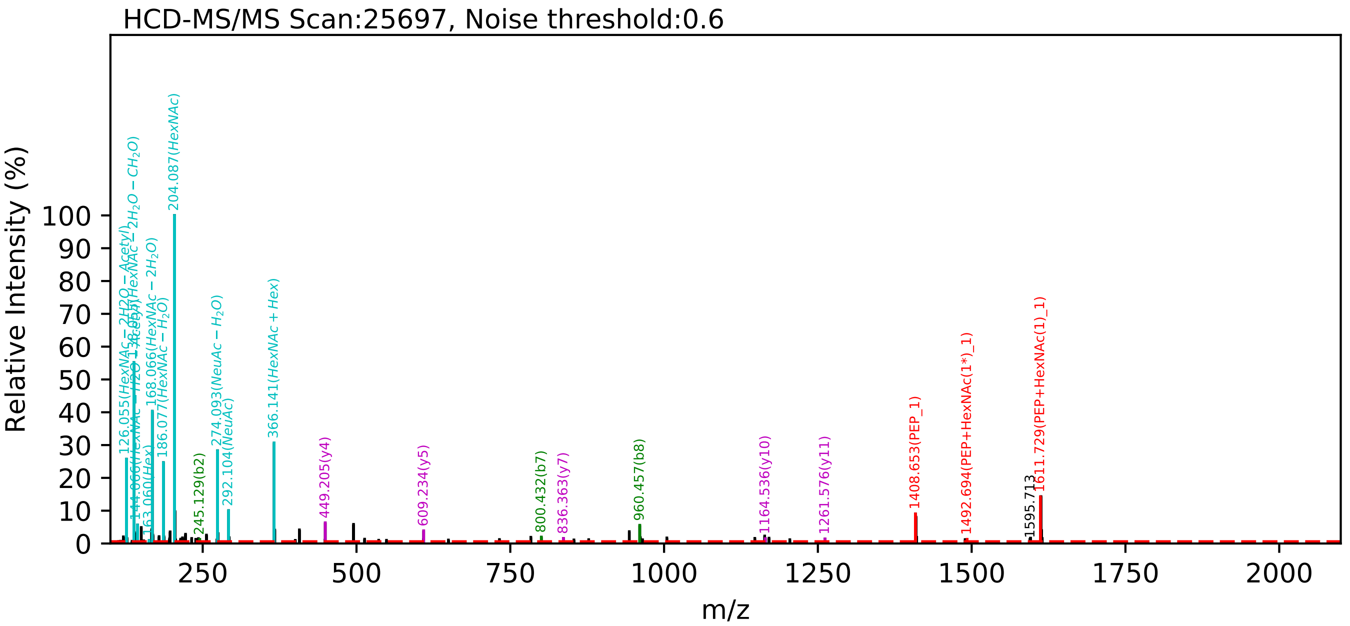

FPNITNLCPFGE(=PEP)_4_5_1_1_0, 0_None, 0_None,
m/z:1170.48(4+), RT:66.74, hcd-score:88.55

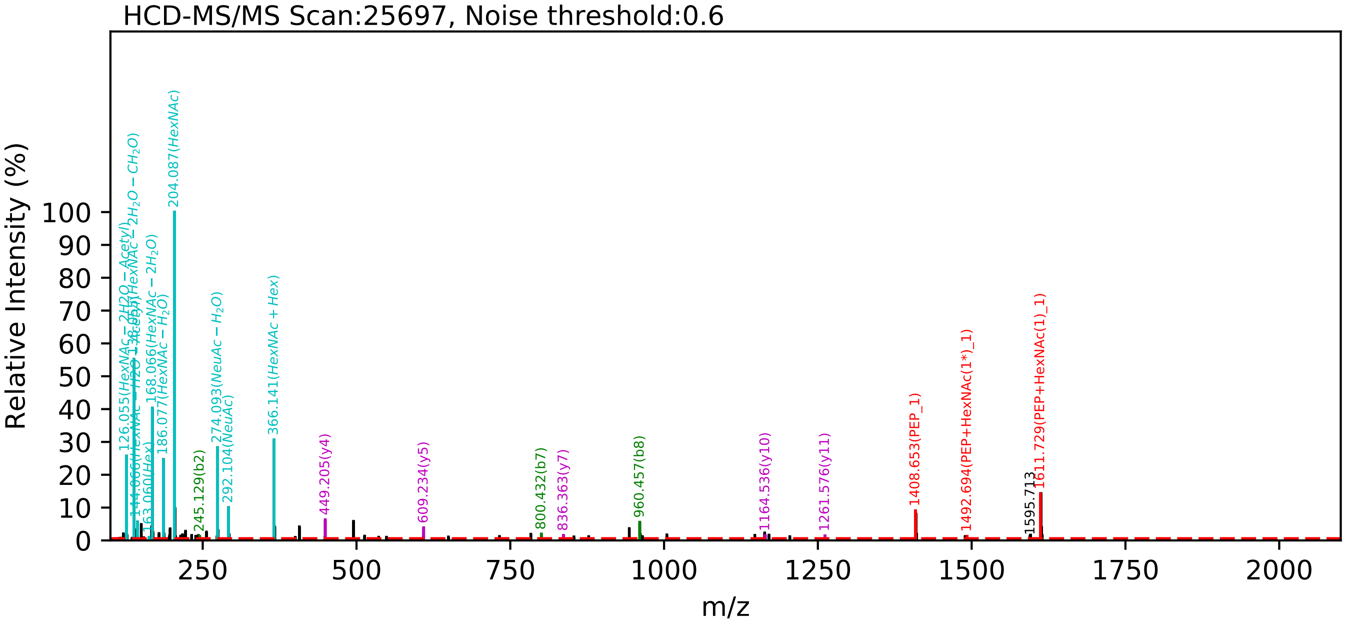

FPNITNLCPFGE(=PEP)_7_6_3_1_0, 0_None, 0_None,
m/z:1497.60(4+), RT:67.05, hcd-score:68.23

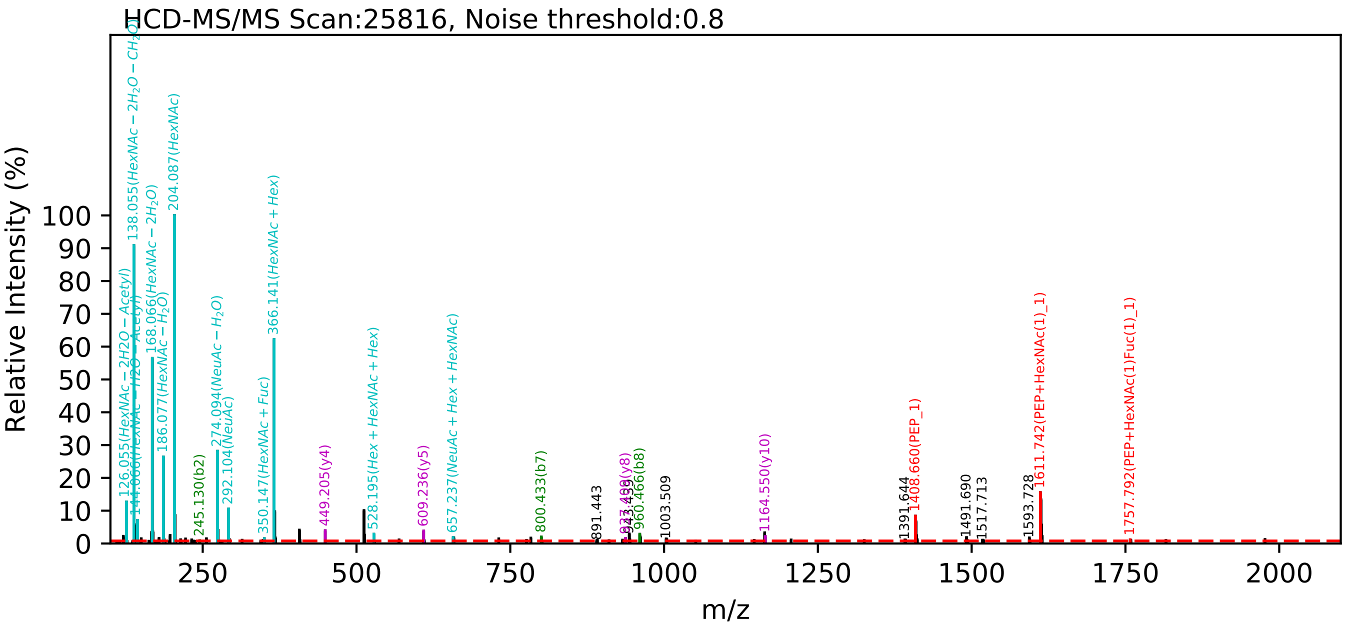

FPNITNLCPFGE(=PEP)_7_6_3_1_0, 0_None, 0_None,
m/z:1497.60(4+), RT:67.05, hcd-score:68.23

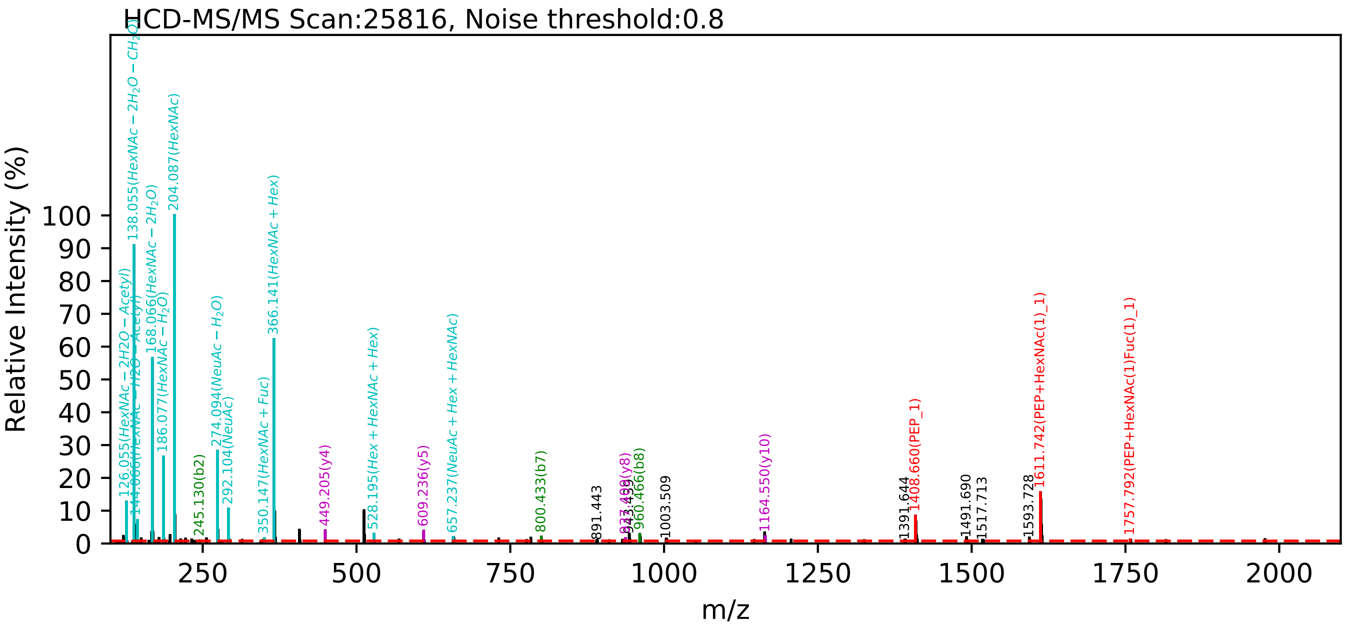

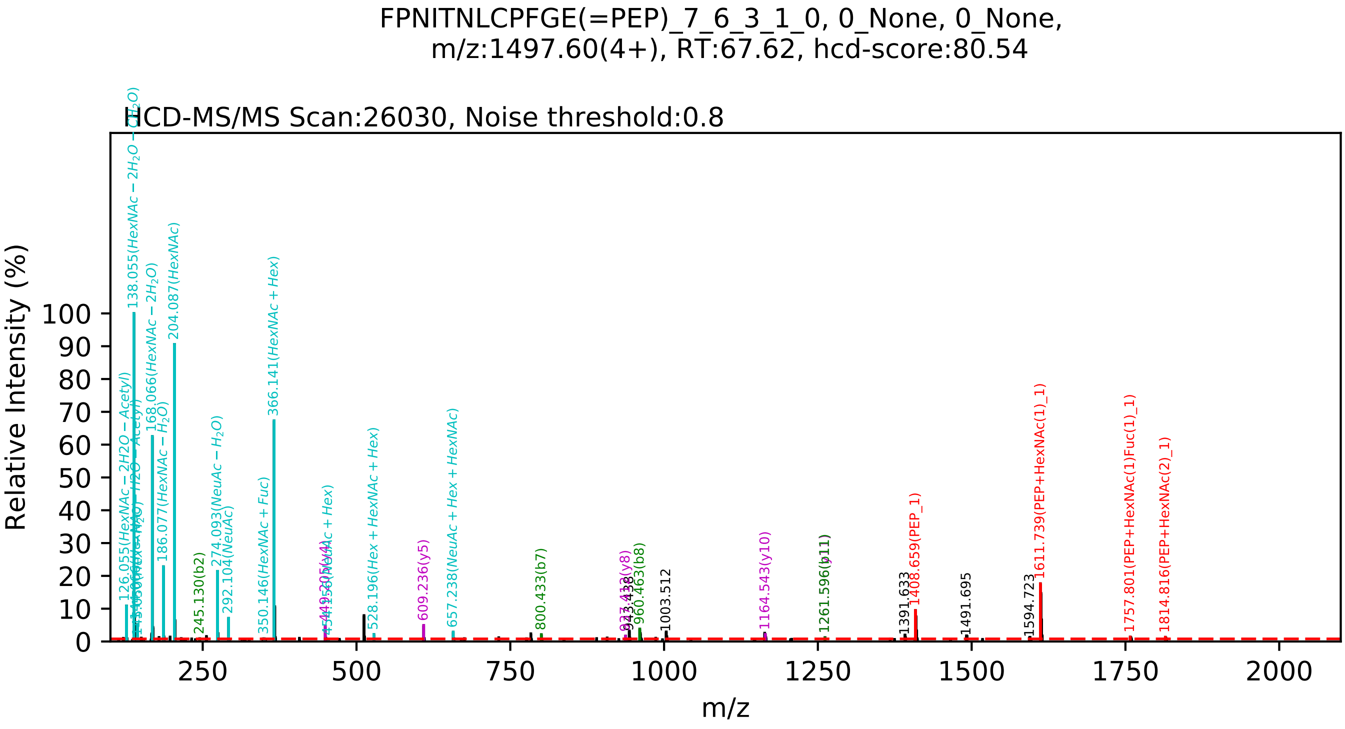

FPNITNLCPFGE(=PEP)_7_6_3_1_0, 0_None, 0_None,
m/z:1497.60(4+), RT:67.62, hcd-score:80.54
HCD-MS/MS Scan:26030, Noise threshold:0.8
Relative Intensity (%)
m/z
138.055(HexNAc − 2H2O − H2O)
204.087(HexNAc)
168.066(HexNAc − 2H2O)
186.077(HexNAc − H2O)
126.055(HexNAc − 2H2O − Acetyl)
366.141(HexNAc + Hex)
274.093(NeuAc − H2O)
292.104(NeuAc)
245.130(b2)
350.146(HexNAc + Fuc)
528.196(Hex + HexNAc + Hex)
657.238(NeuAc + Hex + HexNAc)
609.236(y5)
800.433(b7)
960.463(b8)
1003.512
1164.543(y10)
1261.596(b11)
1391.633
1408.659(PEP_1)
1491.695
1594.723
1611.739(PEP+HexNAc(1)_1)
1757.801(PEP+HexNAc(1)Fuc(1)_1)
1814.816(PEP+HexNAc(2)_1)

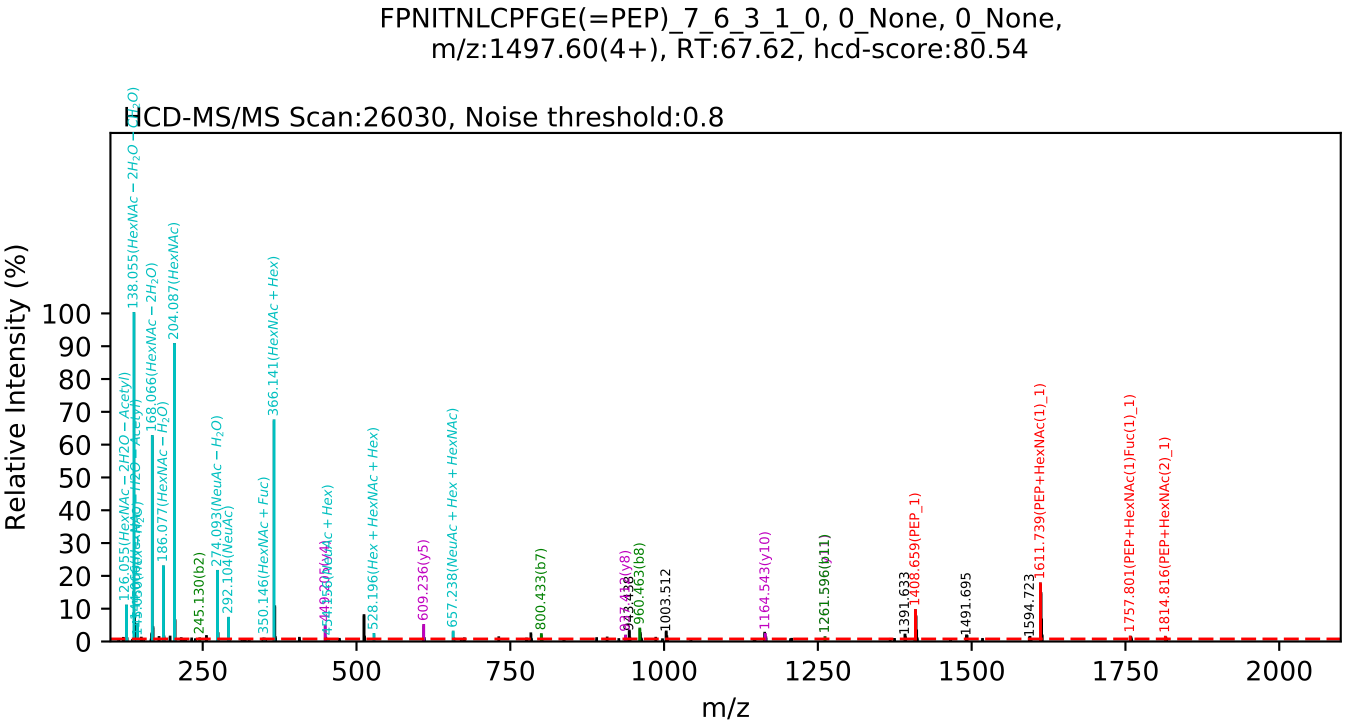
FPNITNLCPFGE(=PEP)_7_6_3_1_0, 0_None, 0_None,
m/z:1497.60(4+), RT:67.62, hcd-score:80.54
HCD-MS/MS Scan:26030, Noise threshold:0.8
Relative Intensity (%)
m/z
138.055(HexNAc − 2H2O − H2O)
204.087(HexNAc)
168.066(HexNAc − 2H2O)
186.077(HexNAc − H2O)
366.141(HexNAc + Hex)
126.055(HexNAc − 2H2O − Acetyl)
245.130(b2)
274.093(NeuAc − H2O)
292.104(NeuAc)
350.146(HexNAc + Fuc)
528.196(Hex + HexNAc + Hex)
609.236(y5)
657.238(NeuAc + Hex + HexNAc)
800.433(b7)
960.463(b8)
1003.512
1164.543(y10)
1261.596(b11)
1391.633
1408.659(PEP_1)
1491.695
1594.723
1611.739(PEP+HexNAc(1)_1)
1757.801(PEP+HexNAc(1)Fuc(1)_1)
1814.816(PEP+HexNAc(2)_1)

FPNITNLCPFGE(=PEP)_5_6_1_1_0, 0_None, 0_None,
m/z:1292.19(2+), RT:68.02, hcd-score:76.39

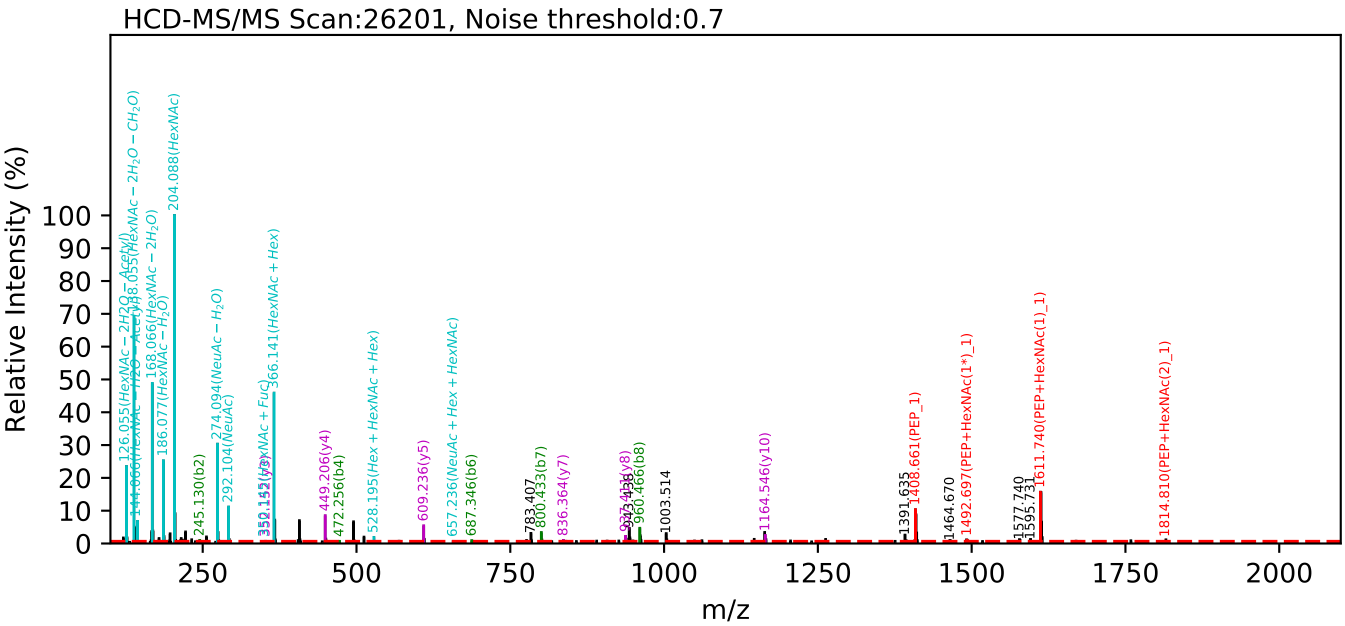

FPNITNLCPFGE(=PEP)_5_6_1_1_0, 0_None, 0_None,
m/z:1292.19(2+), RT:68.02, hcd-score:76.39

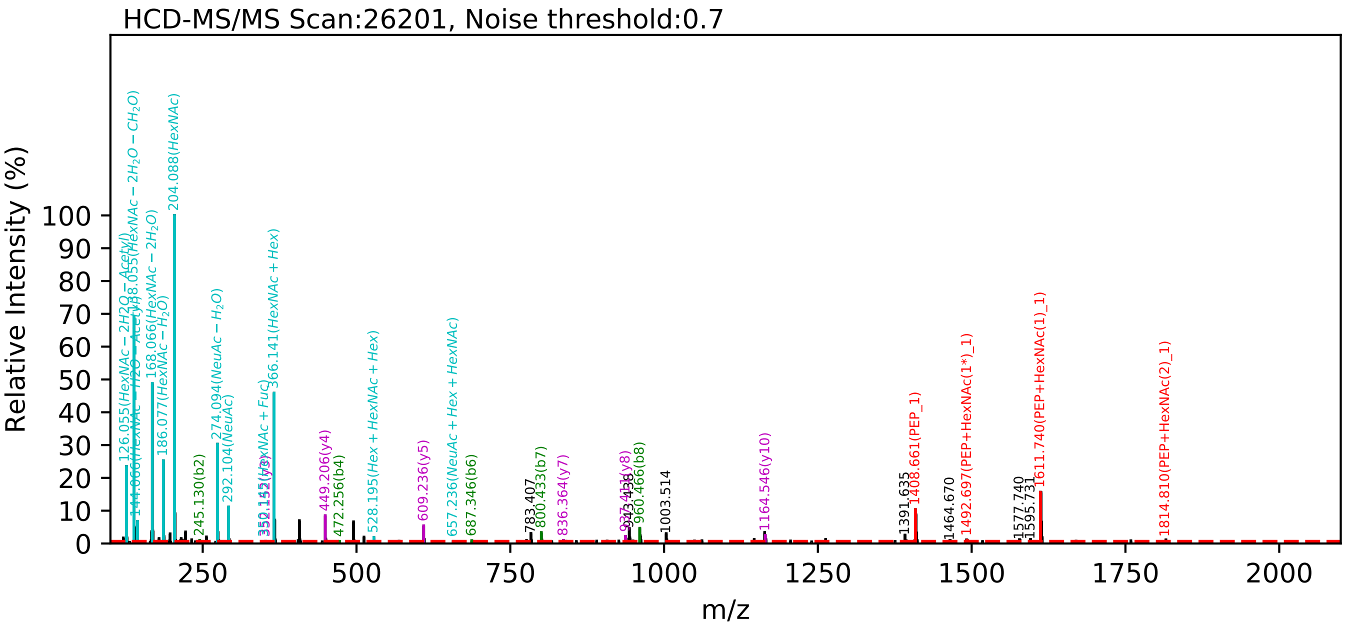

FPNITNLCPFGE(=PEP)_6_6_2_1_0, 0_None, 0_None,
m/z:1046.42(3+), RT:68.17, hcd-score:84.24

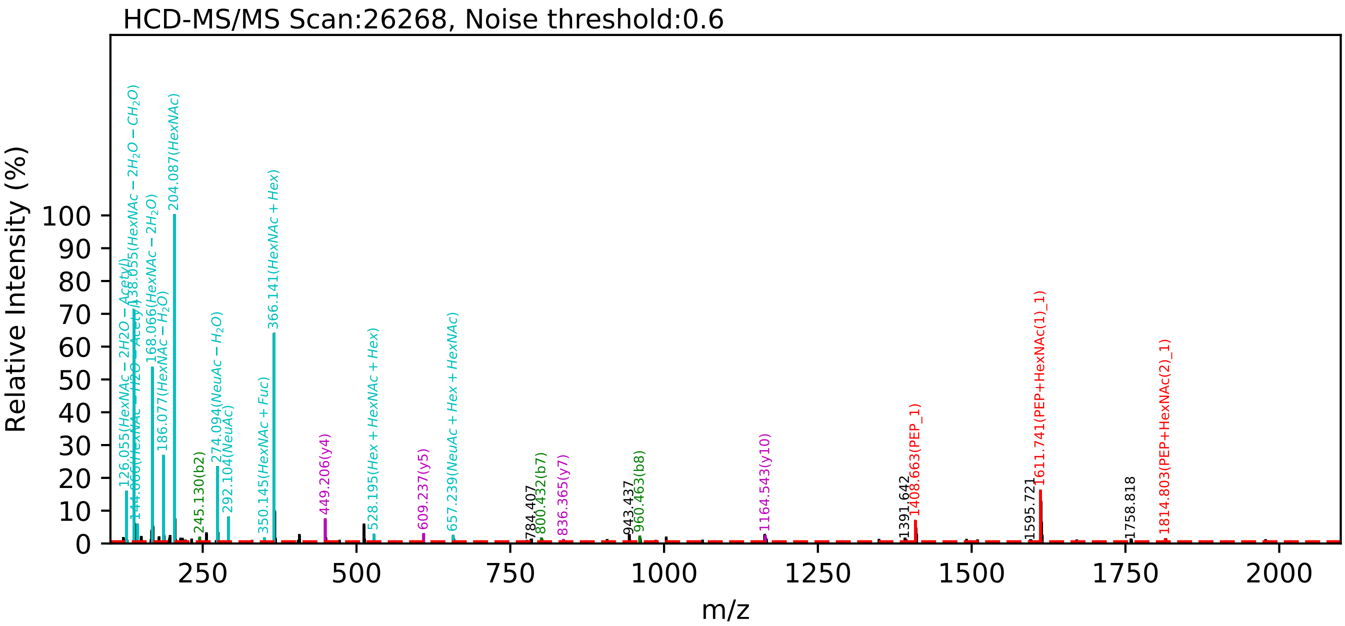

FPNITNLCPFGE(=PEP)_6_6_2_1_0, 0_None, 0_None,
m/z:1046.42(3+), RT:68.17, hcd-score:84.24

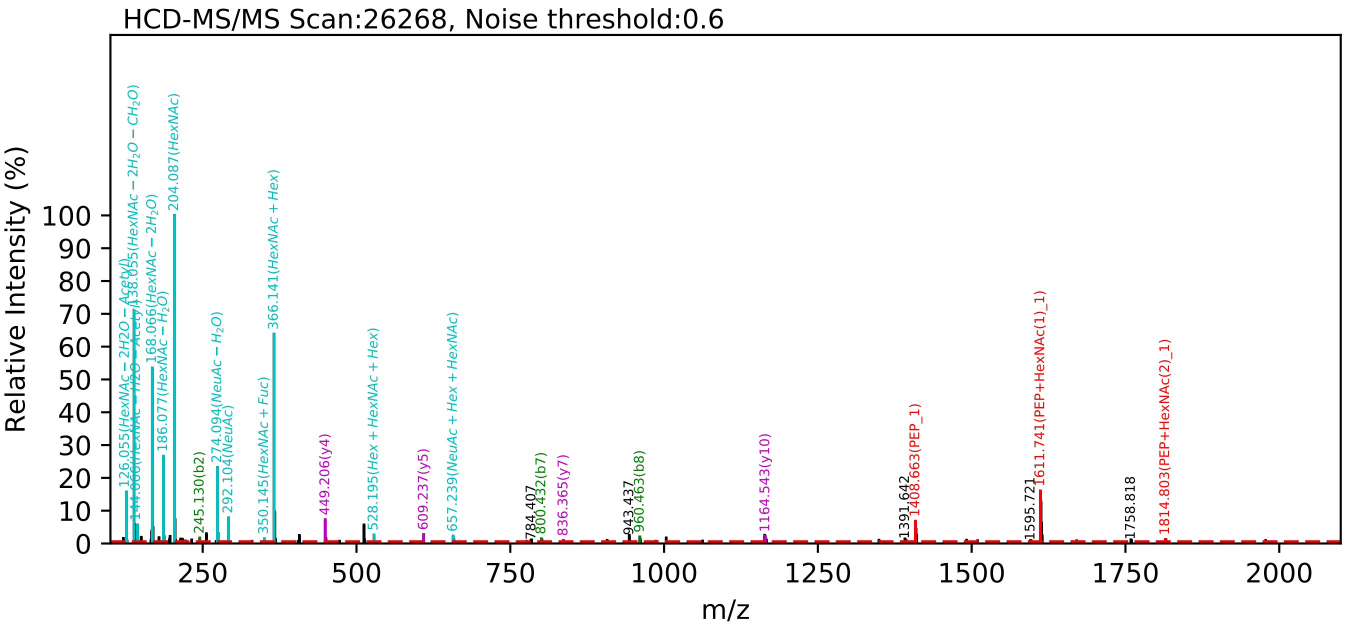

FPNITNLCPFGE(=PEP)_4_3_1_1_0, 0_None, 0_None,
m/z:1552.13(3+), RT:69.67, hcd-score:65.51

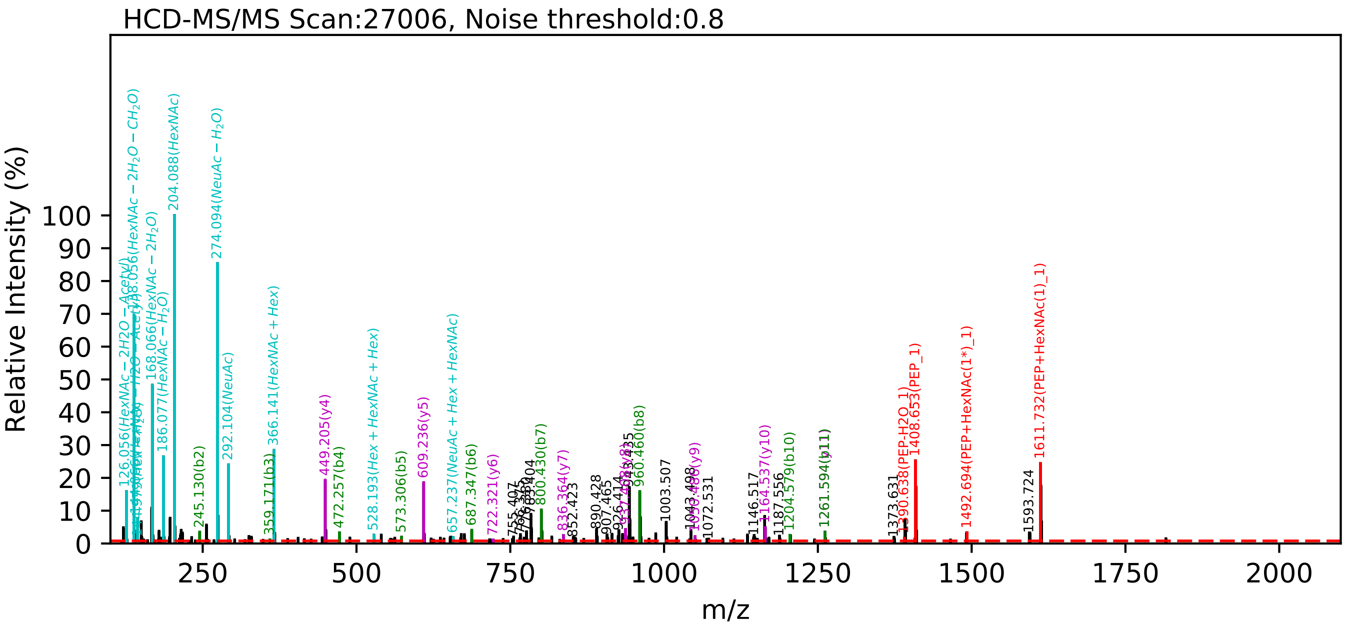

FPNITNLCPFGE(=PEP)_4_3_1_1_0, 0_None, 0_None,
m/z:1552.13(3+), RT:69.67, hcd-score:65.51

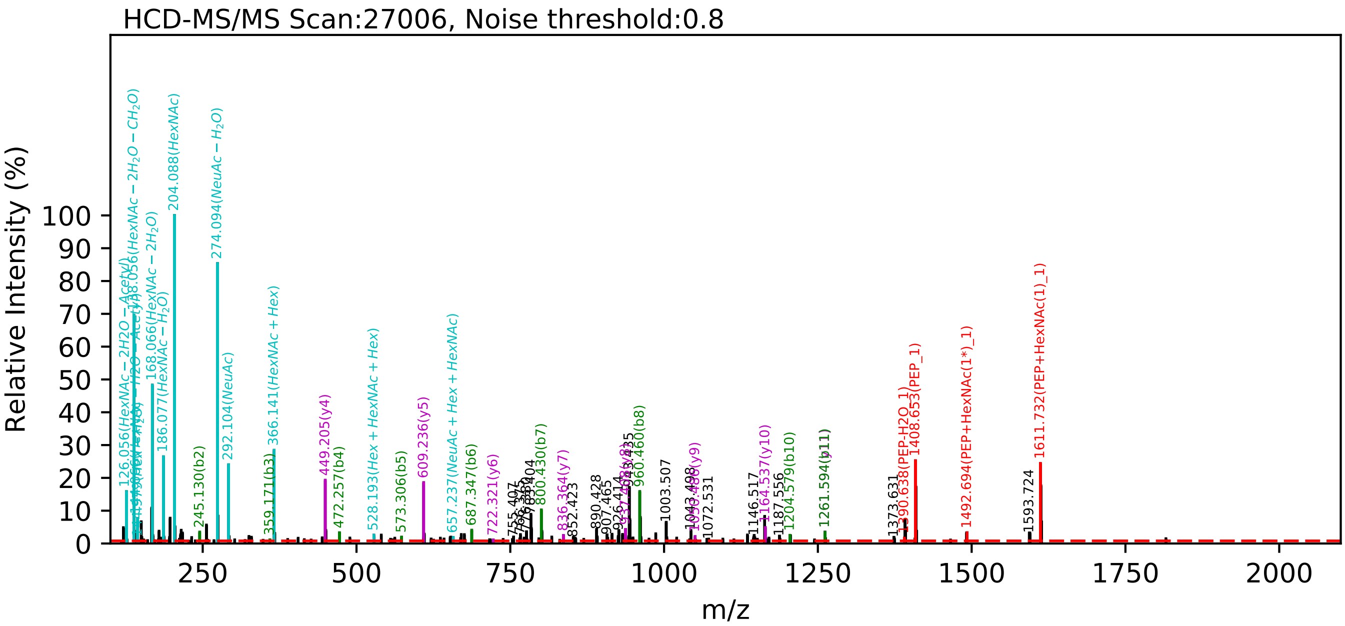

FPNITNLCPFGE(=PEP)_4_3_1_1_0, 0_None, 0_None,
m/z:1552.14(4+), RT:69.78, hcd-score:71.33

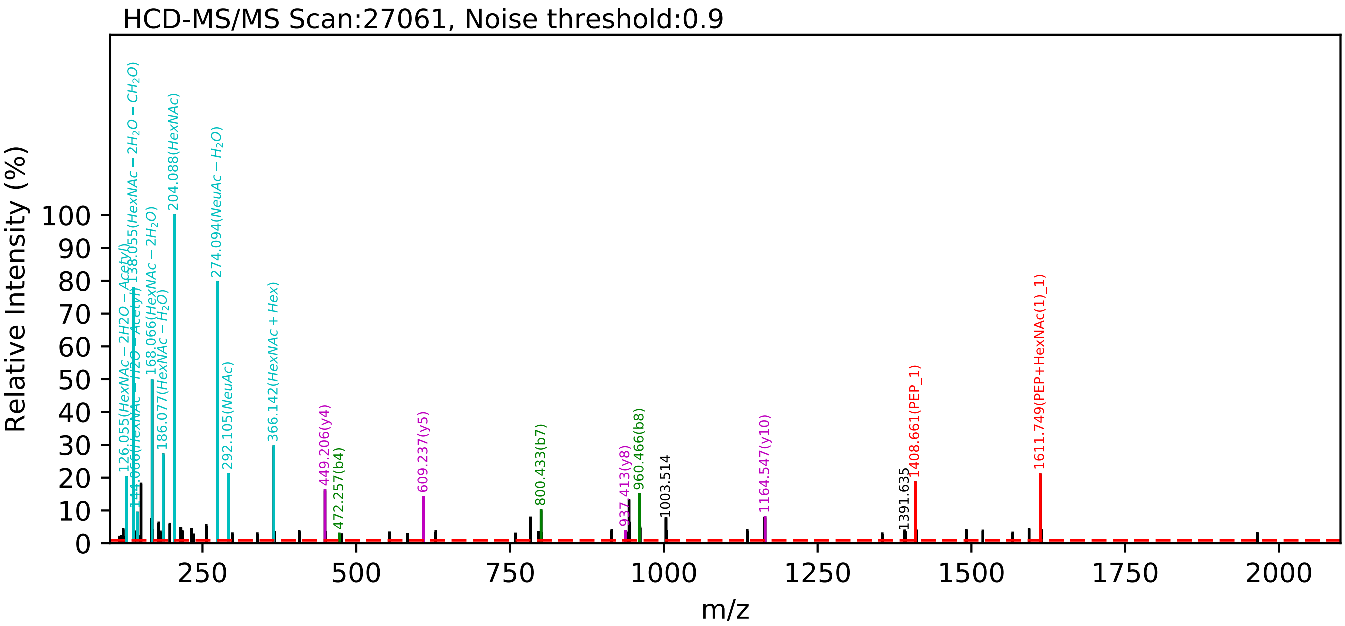

FPNITNLCPFGE(=PEP)_4_3_1_1_0, 0_None, 0_None,
m/z:1552.14(4+), RT:69.78, hcd-score:71.33

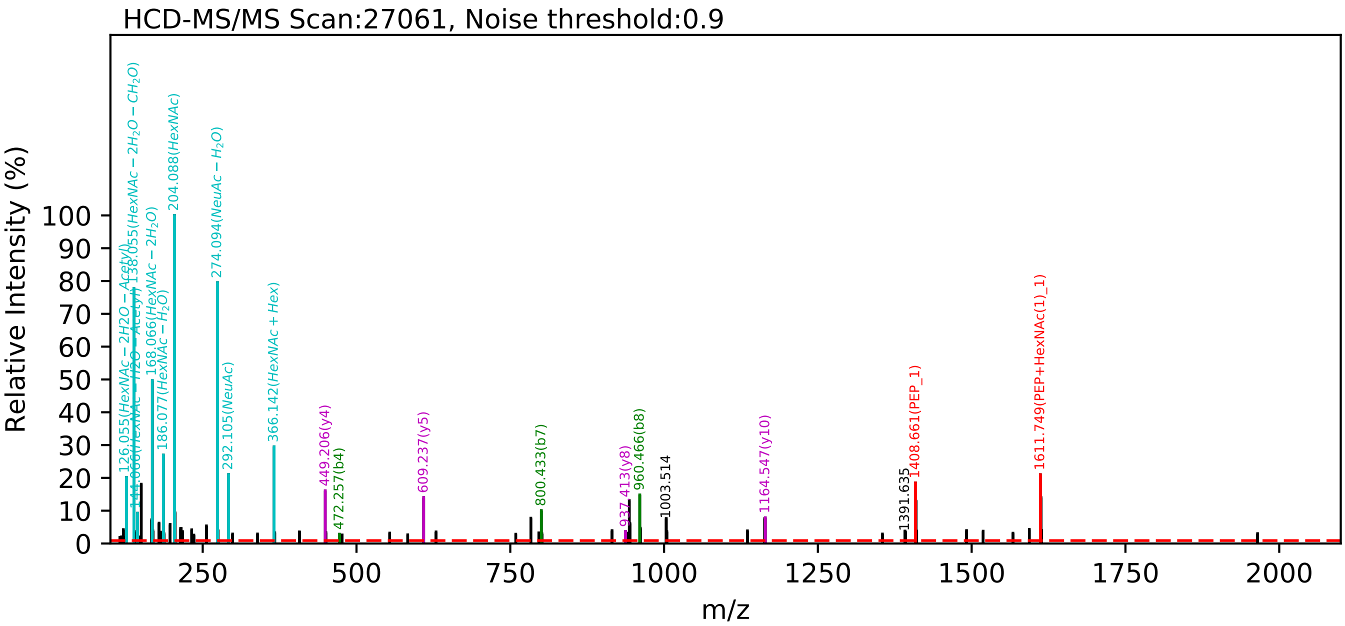

FPNITNLCPFGE(=PEP)_6_7_1_2_0, 0_None, 0_None,
m/z:1133.45(3+), RT:80.77, hcd-score:90.57

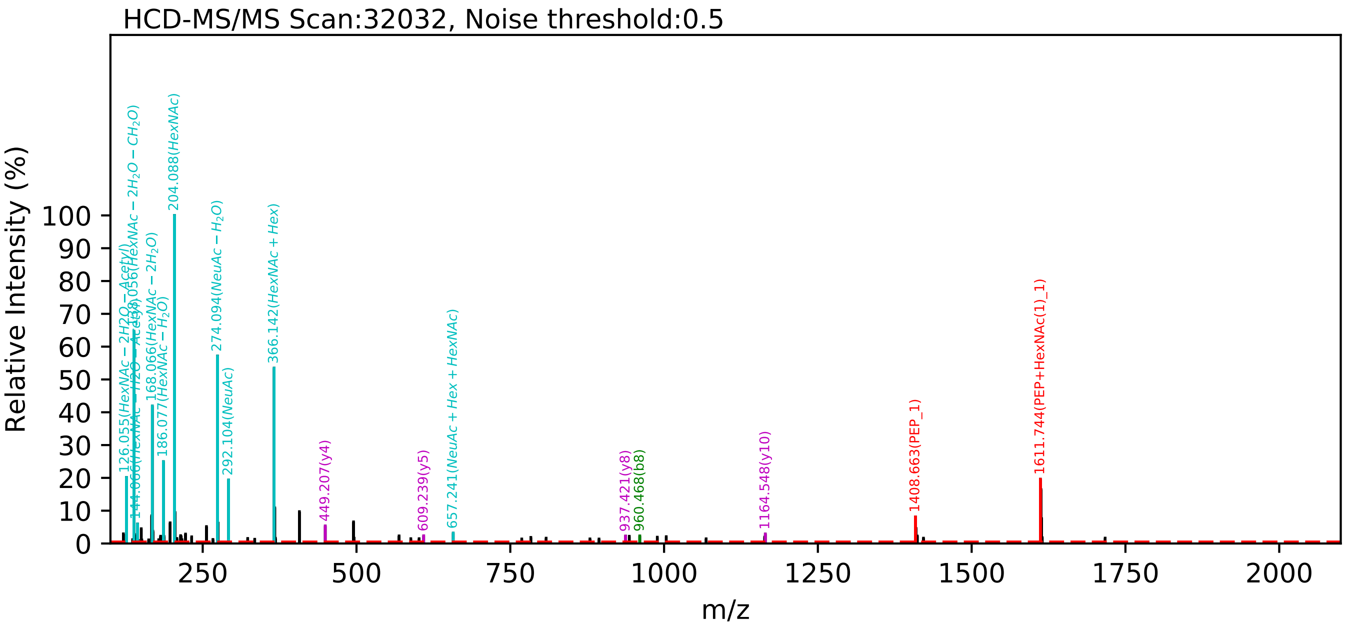

FPNITNLCPFGE(=PEP)_6_7_1_2_0, 0_None, 0_None,
m/z:1133.45(3+), RT:80.77, hcd-score:90.57

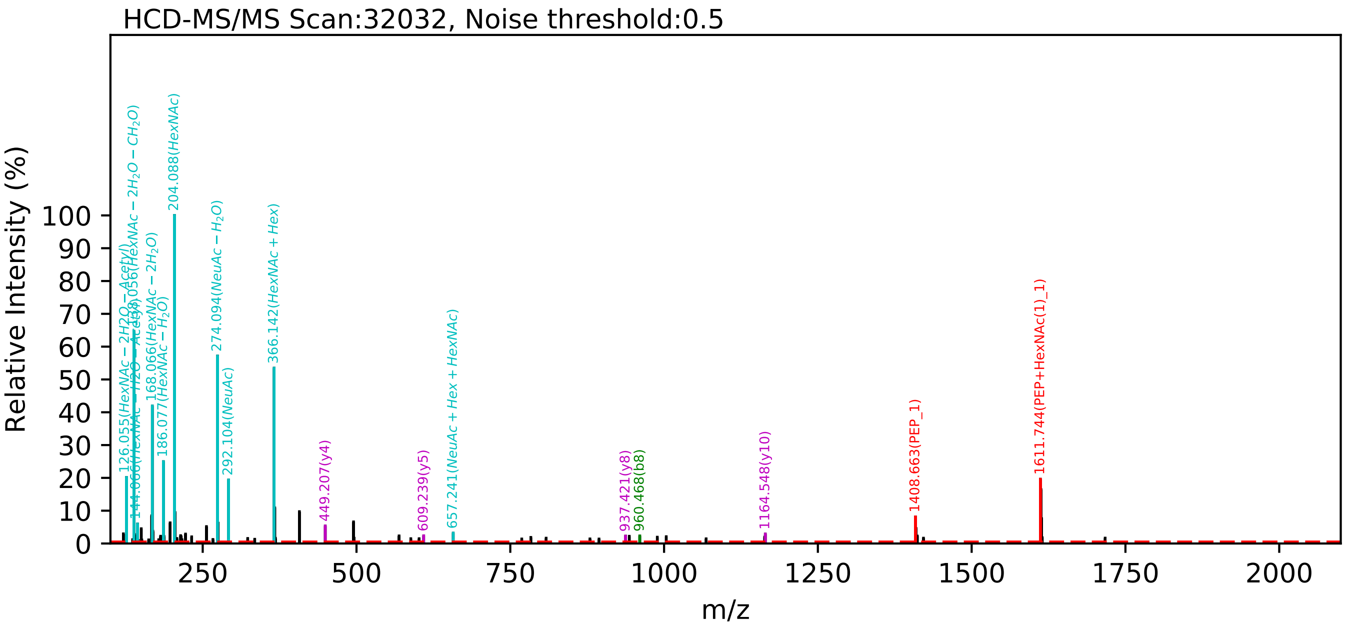

FPNITNLCPFGE(=PEP)_5_7_1_2_0, 0_None, 0_None,
m/z:1456.91(3+), RT:81.02, hcd-score:80.19

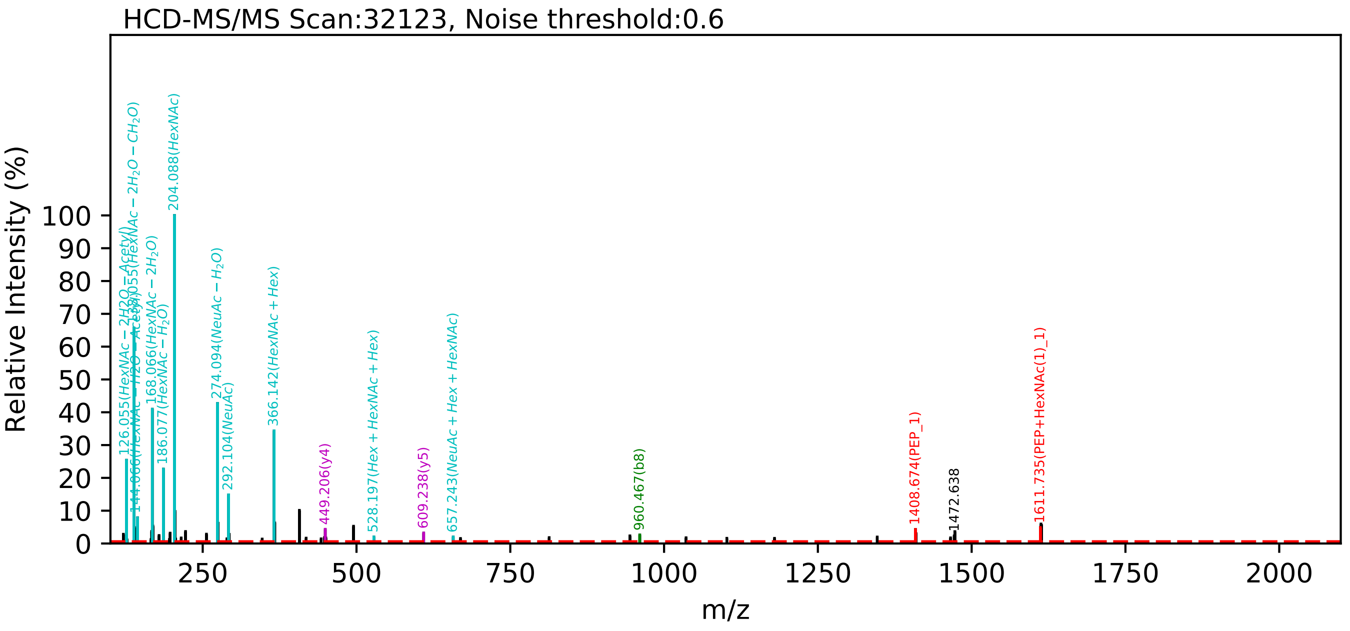

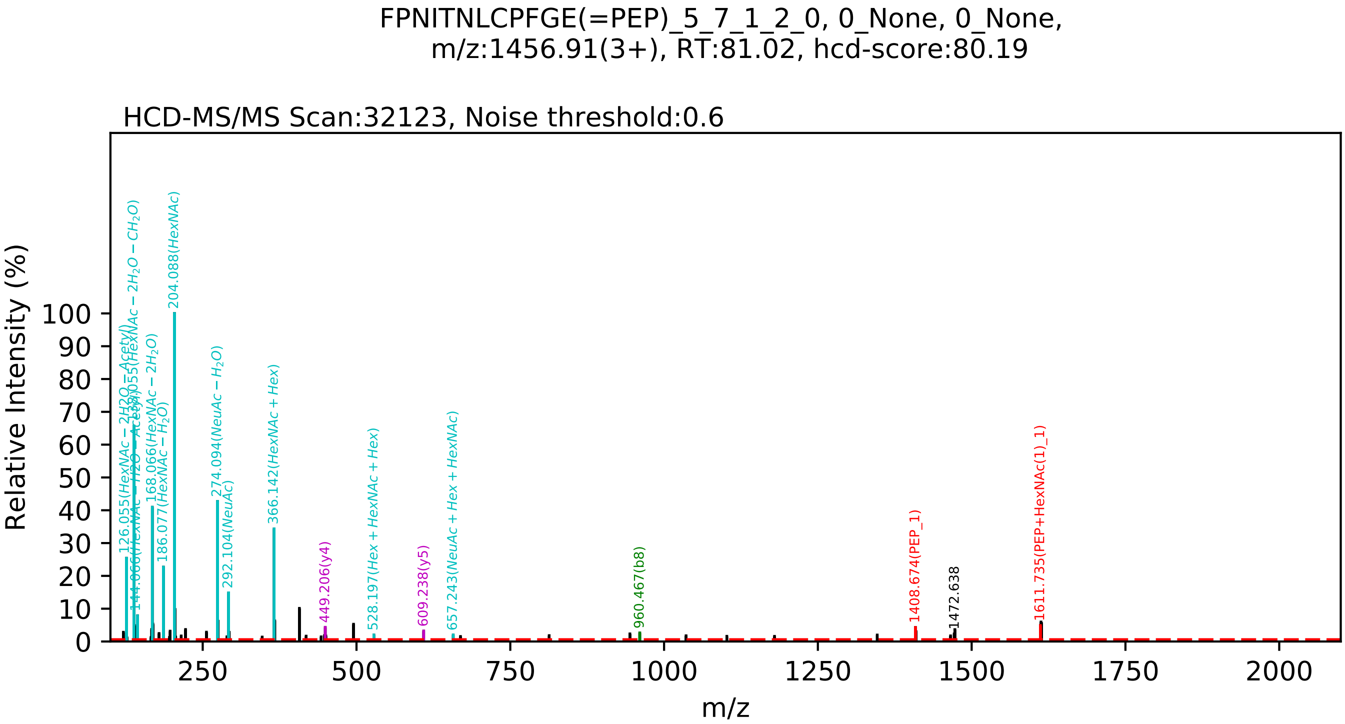
FPNITNLCPFGE(=PEP)_5_7_1_2_0, 0_None, 0_None,
m/z:1456.91(3+), RT:81.02, hcd-score:80.19
HCD-MS/MS Scan:32123, Noise threshold:0.6
Relative Intensity (%)
m/z
204.088(HexNAc)
274.094(NeuAc − H2O)
292.104(NeuAc)
366.142(HexNAc + Hex)
449.206(y4)
528.197(Hex + HexNAc + Hex)
609.238(y5)
657.243(NeuAc + Hex + HexNAc)
960.467(b8)
1408.674(PEP_1)
1472.638
1611.735(PEP+HexNAc(1)_1)

FPNITNLCPFGE(=PEP)_7_6_1_2_0, 0_None, 0_None,
m/z:1123.20(4+), RT:81.17, hcd-score:80.69

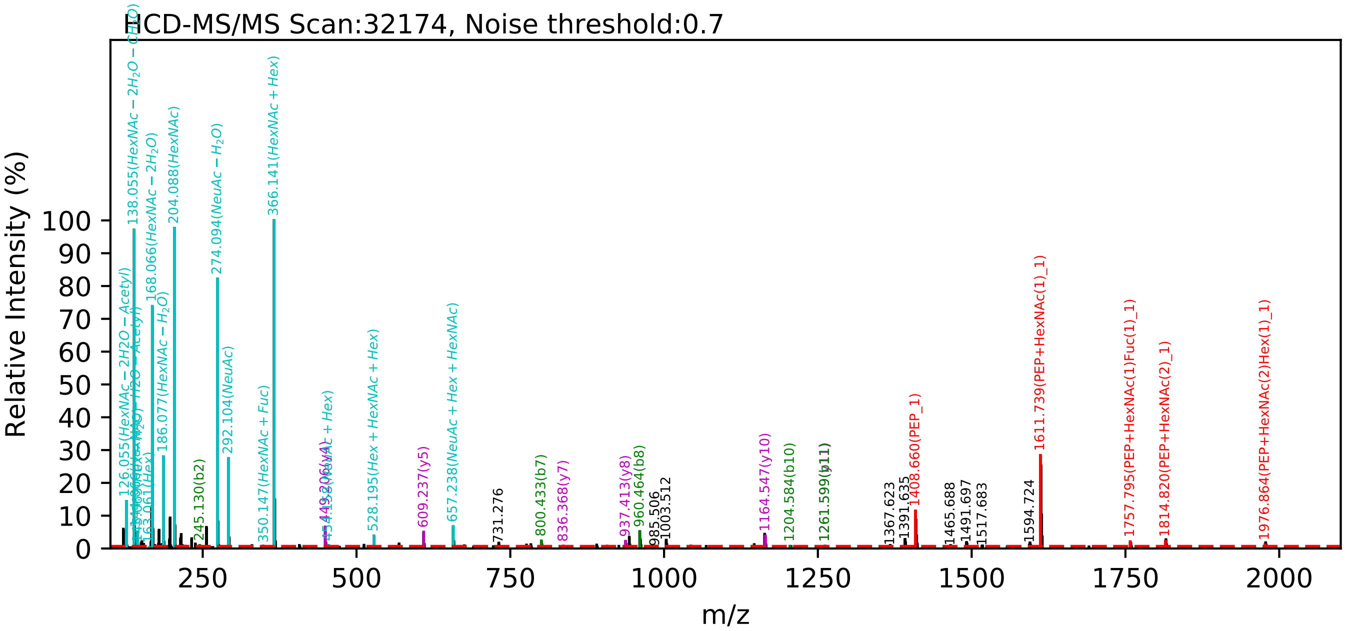

FPNITNLCPFGE(=PEP)_7_6_1_2_0, 0_None, 0_None,
m/z:1123.20(4+), RT:81.17, hcd-score:80.69

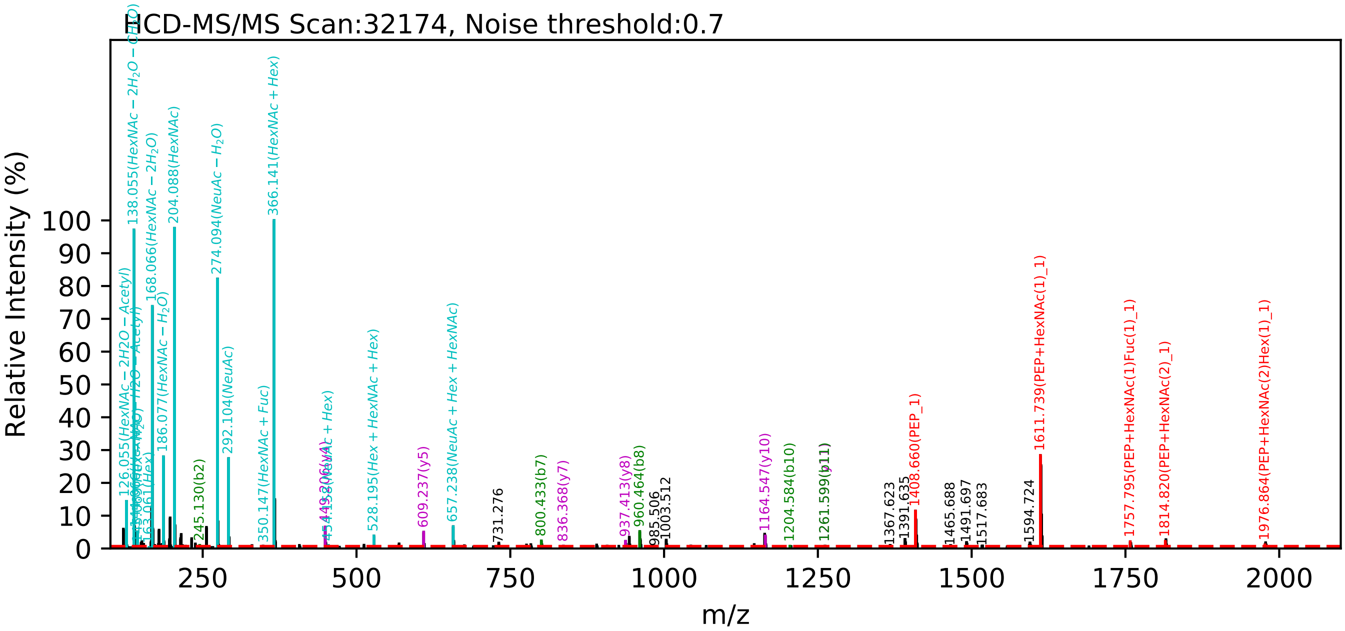

FPNITNLCPFGE(=PEP)_6_5_2_2_0, 0_None, 0_None,
m/z:1068.43(3+), RT:82.17, hcd-score:82.13

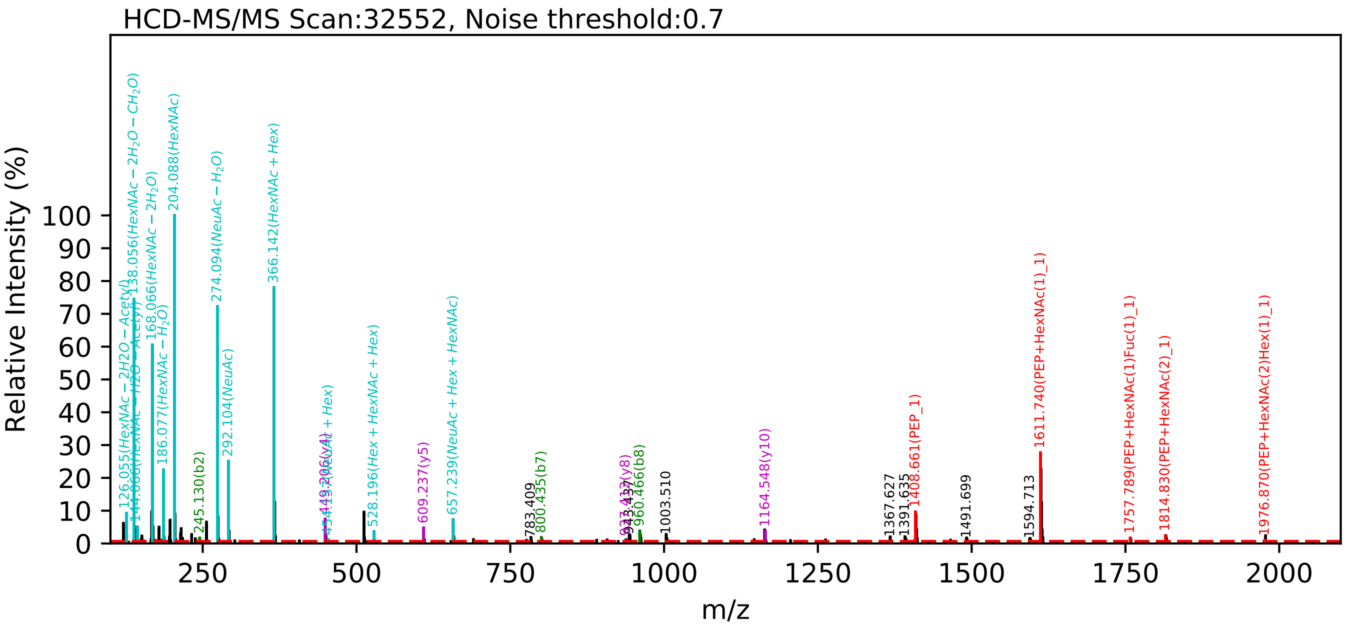

FPNITNLCPFGE(=PEP)_6_5_2_2_0, 0_None, 0_None,
m/z:1068.43(3+), RT:82.17, hcd-score:82.13

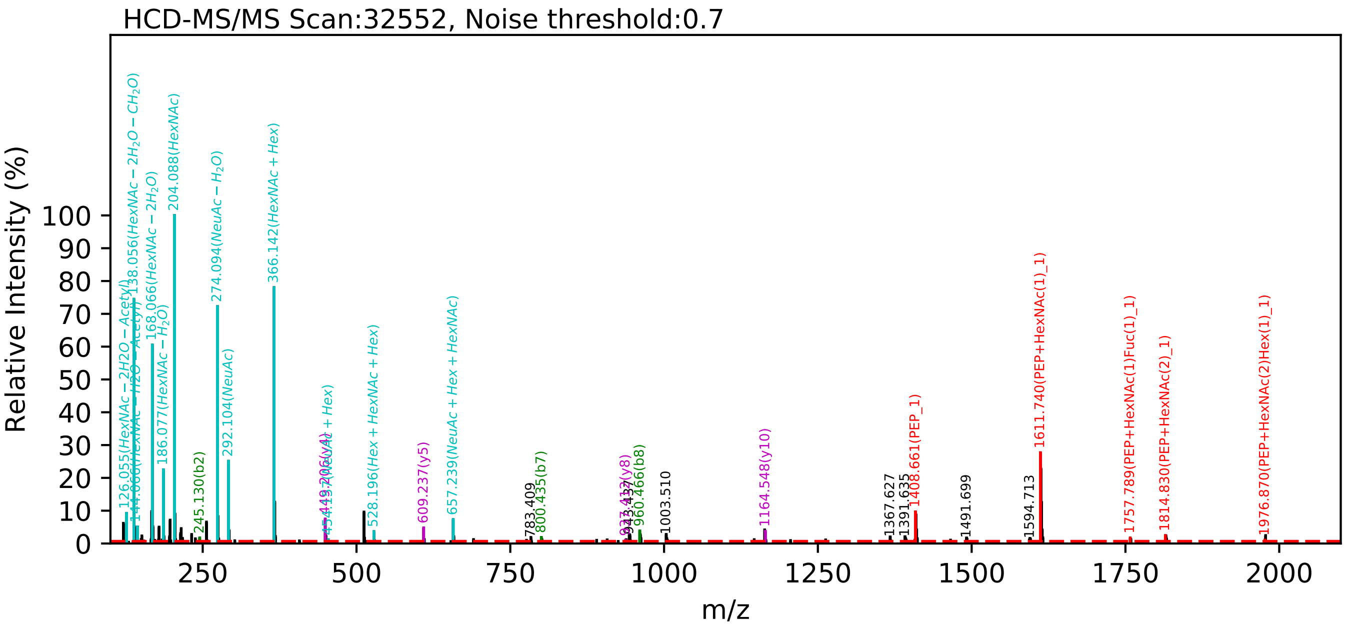

FPNITNLCPFGE(=PEP)_5_6_0_2_0, 0_None, 0_None,
m/z:1340.53(4+), RT:82.44, hcd-score:77.34

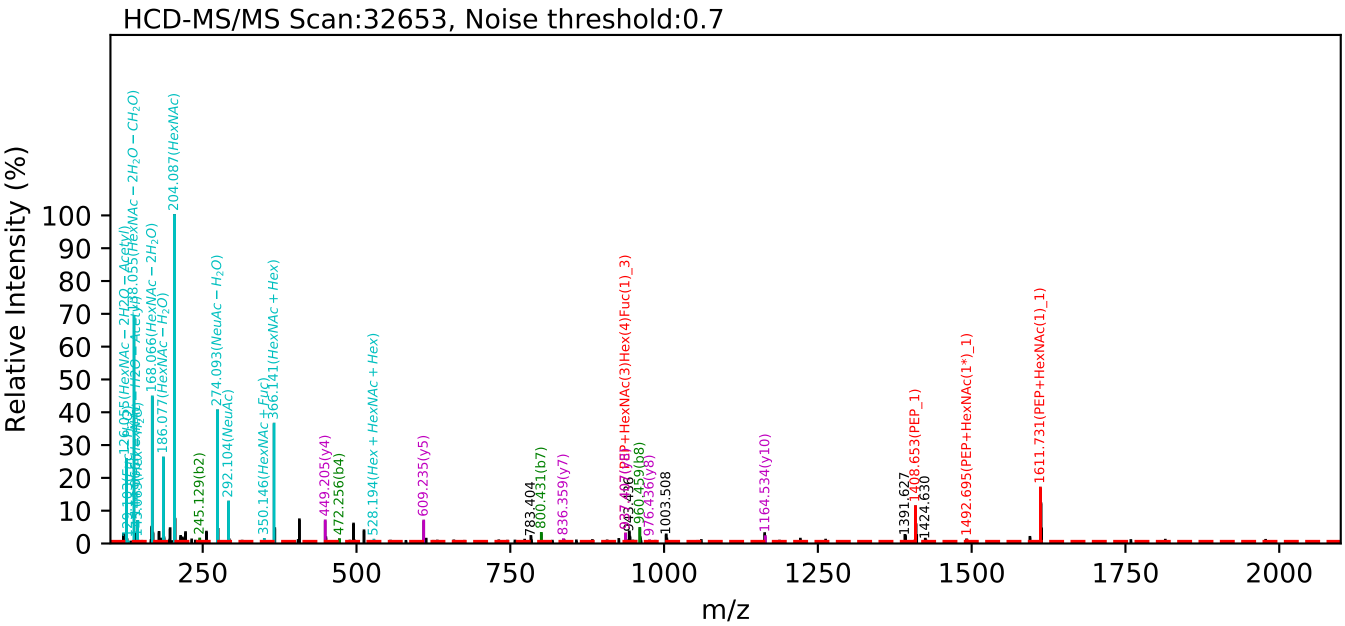

FPNITNLCPFGE(=PEP)_5_6_0_2_0, 0_None, 0_None,
m/z:1340.53(4+), RT:82.44, hcd-score:77.34

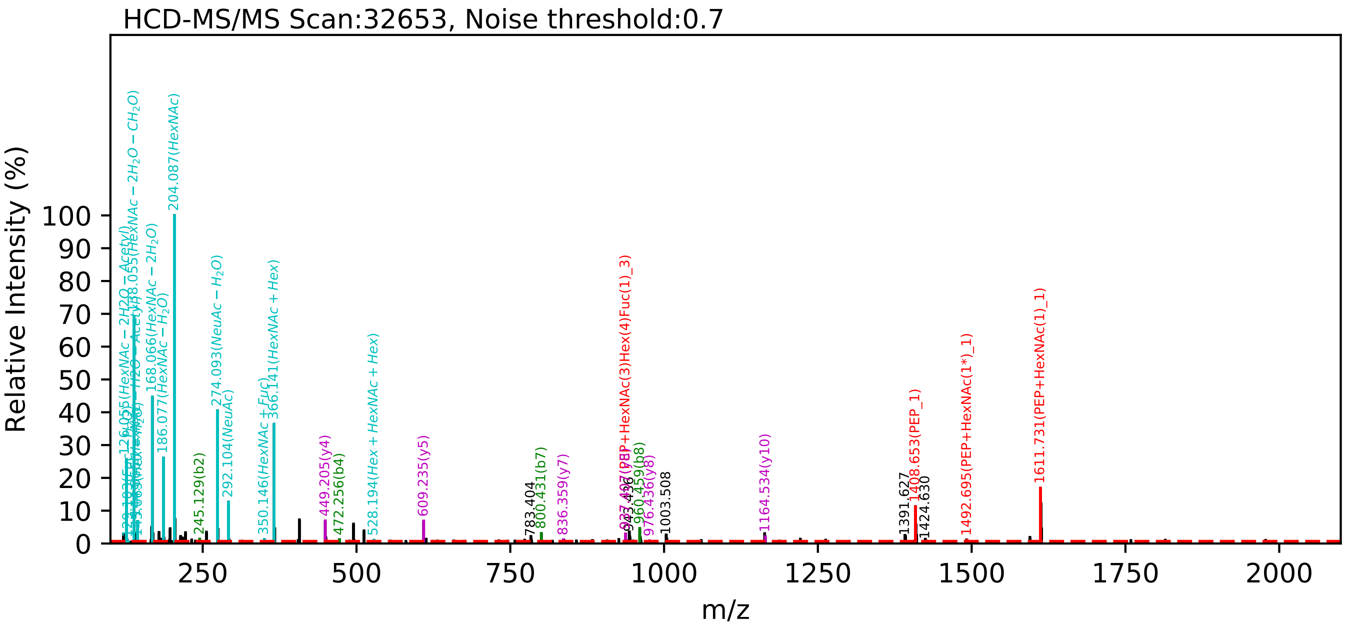

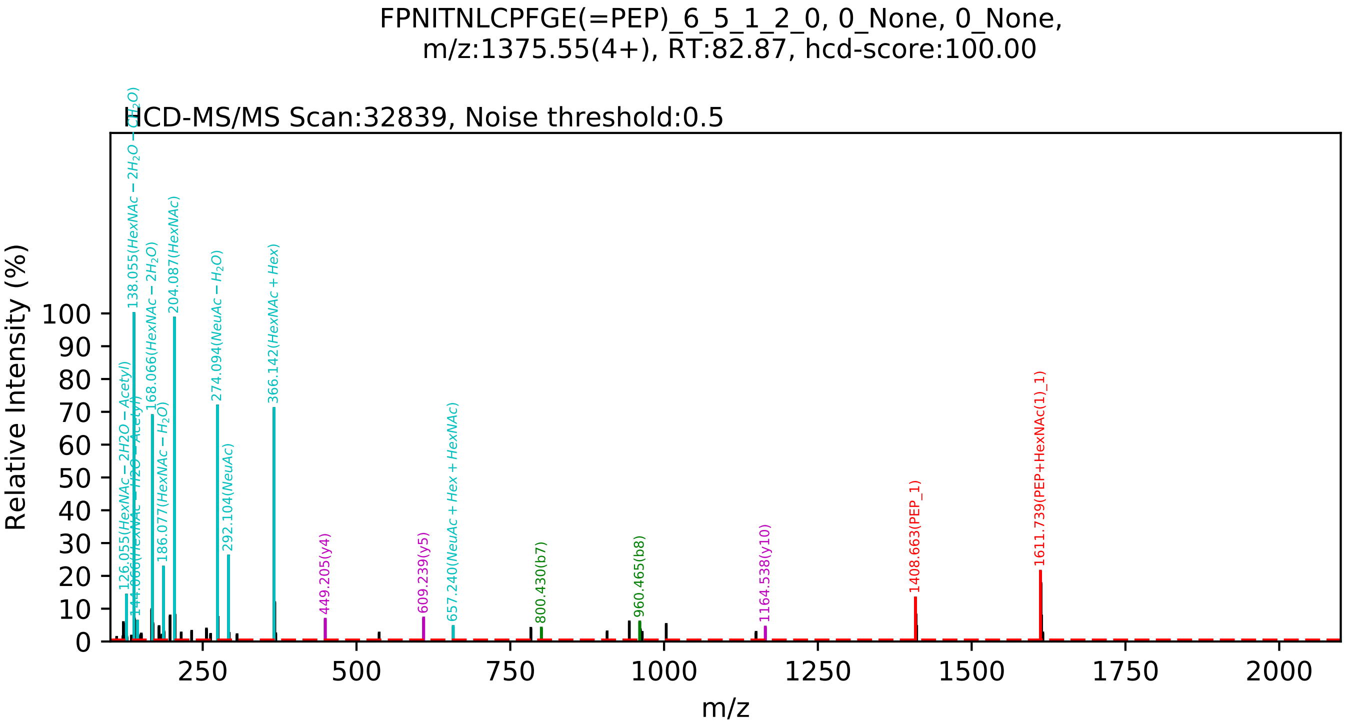
FPNITNLCPFGE(=PEP)_6_5_1_2_0, 0_None, 0_None,
m/z:1375.55(4+), RT:82.87, hcd-score:100.00
HCD-MS/MS Scan:32839, Noise threshold:0.5
Relative Intensity (%)
m/z
138.055(HexNAc − 2H2O − H2O)
168.066(HexNAc − 2H2O)
204.087(HexNAc)
126.055(HexNAc − 2H2O − Acetyl)
144.066(HexNAc − H2O − Acetyl)
186.077(HexNAc − H2O)
274.094(NeuAc − H2O)
292.104(NeuAc)
366.142(HexNAc + Hex)
449.205(y4)
609.239(y5)
657.240(NeuAc + Hex + HexNAc)
800.430(b7)
960.465(b8)
1164.538(y10)
1408.663(PEP_1)
1611.739(PEP+HexNAc(1)_1)

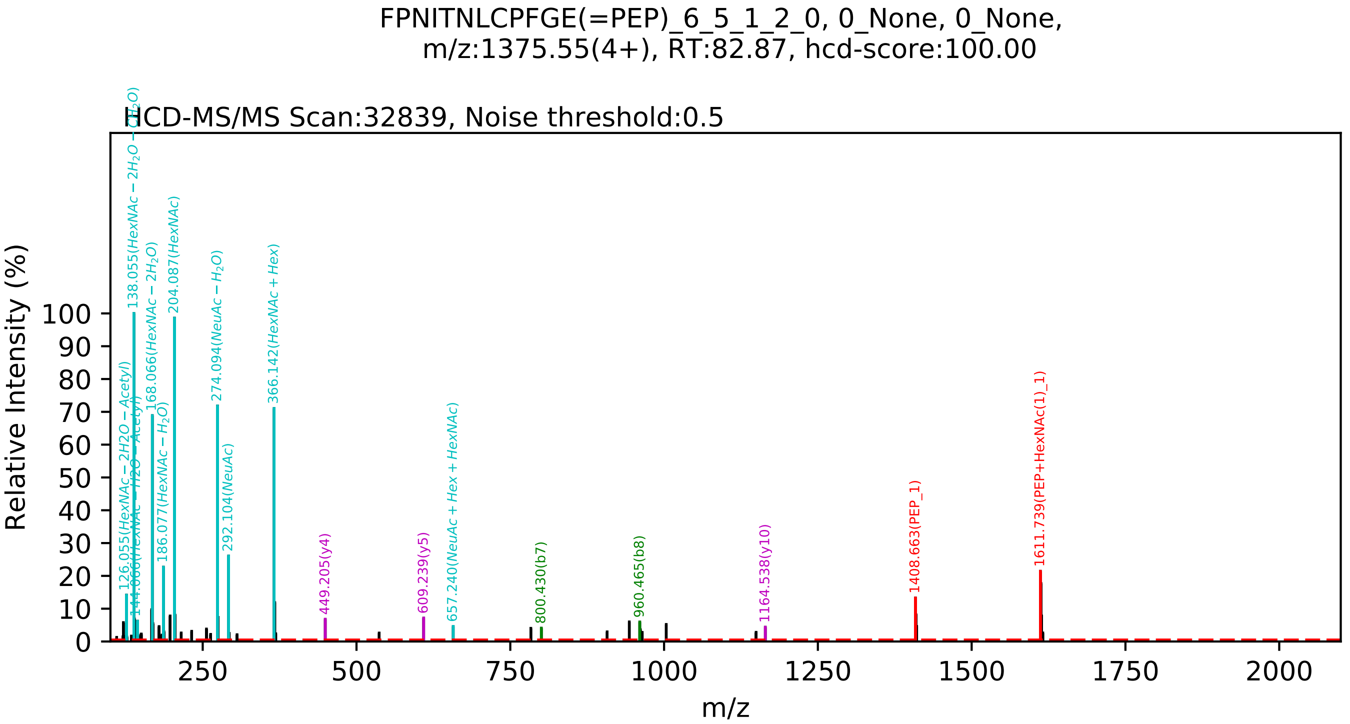
FPNITNLCPFGE(=PEP)_6_5_1_2_0, 0_None, 0_None,
m/z:1375.55(4+), RT:82.87, hcd-score:100.00
HCD-MS/MS Scan:32839, Noise threshold:0.5
Relative Intensity (%)
m/z
126.055(HexNAc − 2H2O − Acetyl)
138.055(HexNAc − 2H2O − CH2O)
168.066(HexNAc − 2H2O)
186.077(HexNAc − H2O)
204.087(HexNAc)
274.094(NeuAc − H2O)
292.104(NeuAc)
366.142(HexNAc + Hex)
449.205(y4)
609.239(y5)
657.240(NeuAc + Hex + HexNAc)
800.430(b7)
960.465(b8)
1164.538(y10)
1408.663(PEP_1)
1611.739(PEP+HexNAc(1)_1)

FPNITNLCPFGE(=PEP)_6_5_1_2_0, 0_None, 0_None,
m/z:1375.55(4+), RT:82.94, hcd-score:82.93

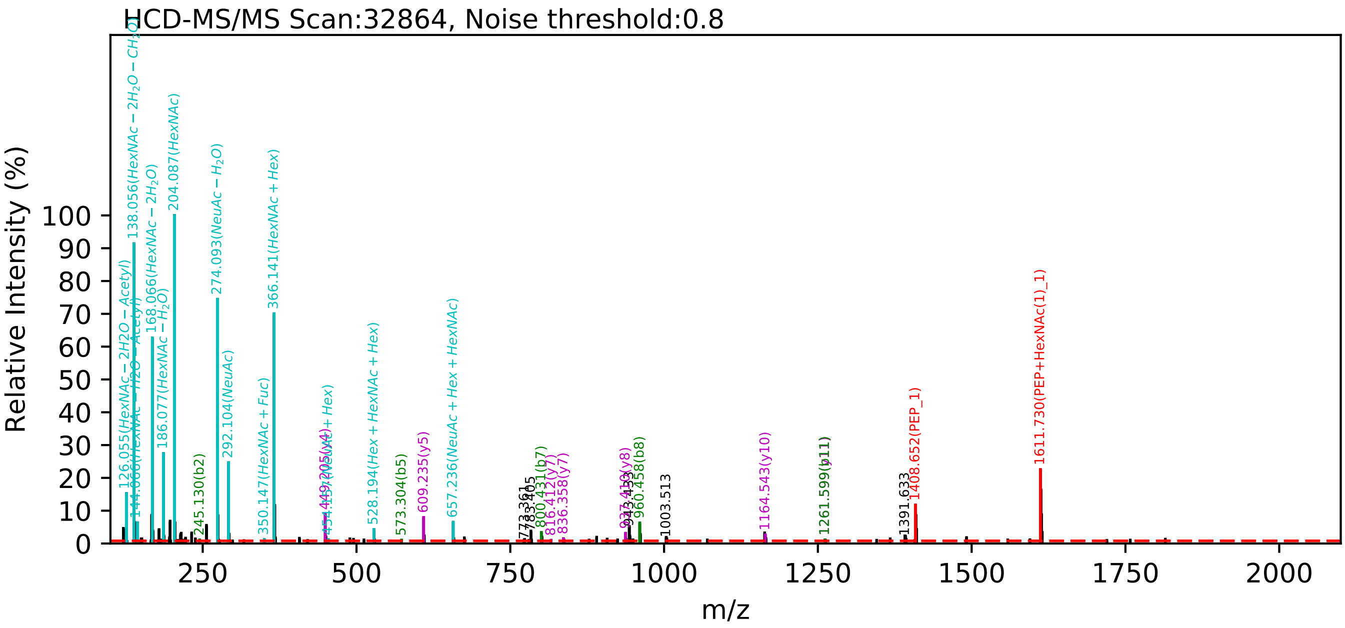

FPNITNLCPFGE(=PEP)_6_5_1_2_0, 0_None, 0_None,
m/z:1375.55(4+), RT:82.94, hcd-score:82.93

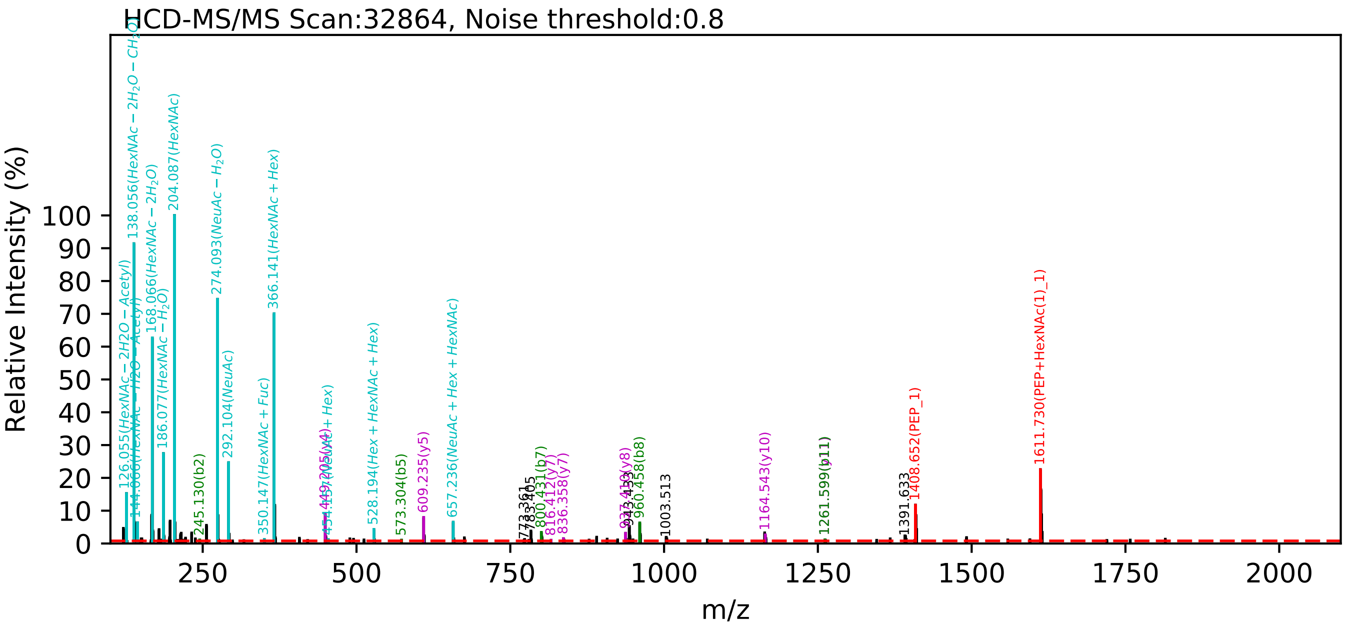

FPNITNLCPFGE(=PEP)_5_5_1_2_0, 0_None, 0_None,
m/z:1321.53(4+), RT:83.38, hcd-score:78.71

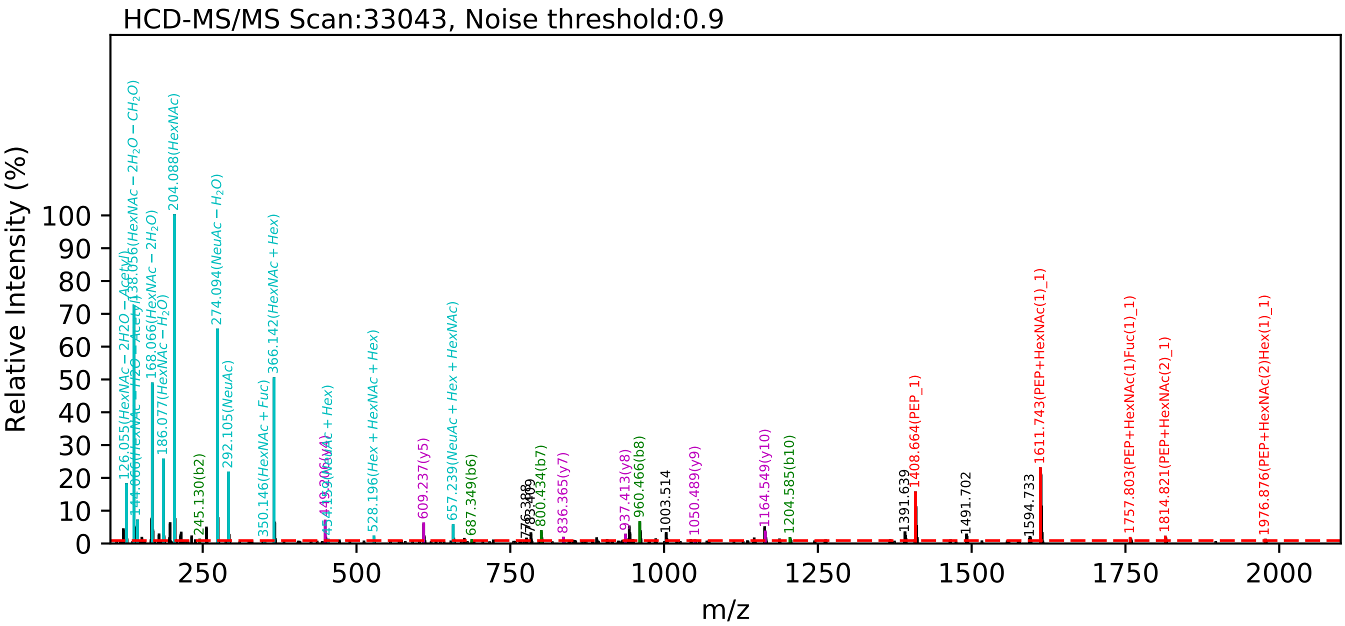

FPNITNLCPFGE(=PEP)_5_5_1_2_0, 0_None, 0_None,
m/z:1321.53(4+), RT:83.38, hcd-score:78.71

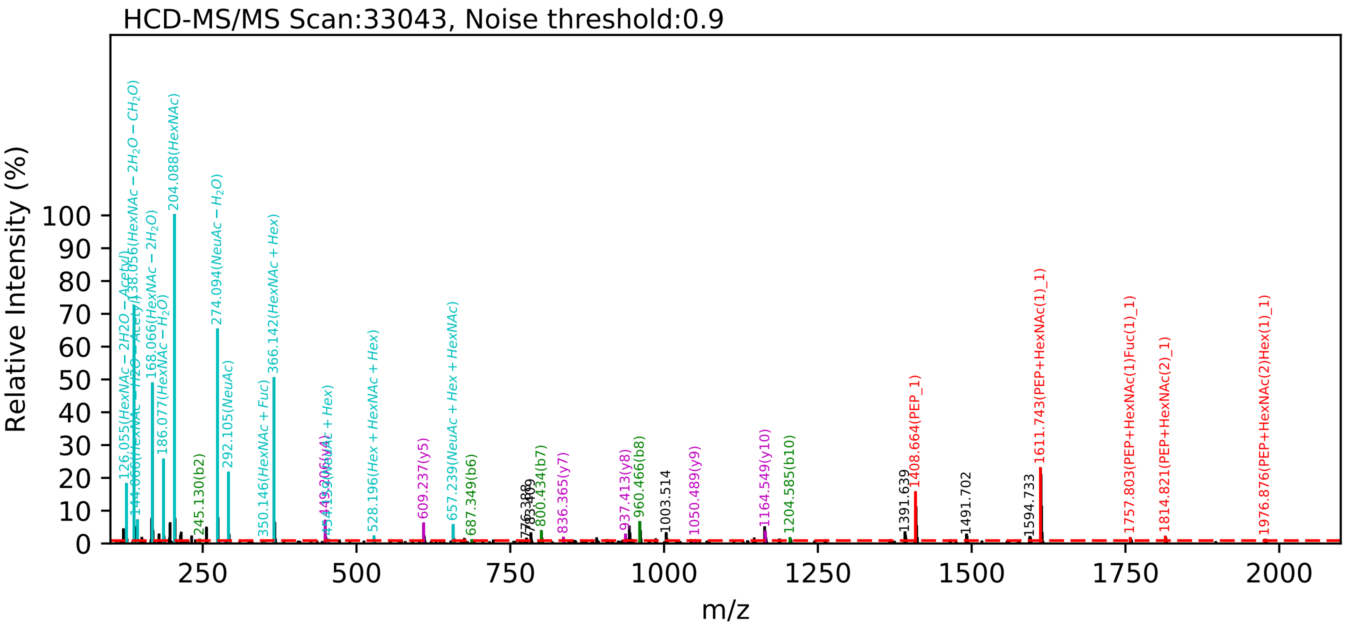

FPNITNLCPFGE(=PEP)_6_5_1_2_0, 0_None, 0_None,
m/z:1375.54(3+), RT:83.43, hcd-score:78.18

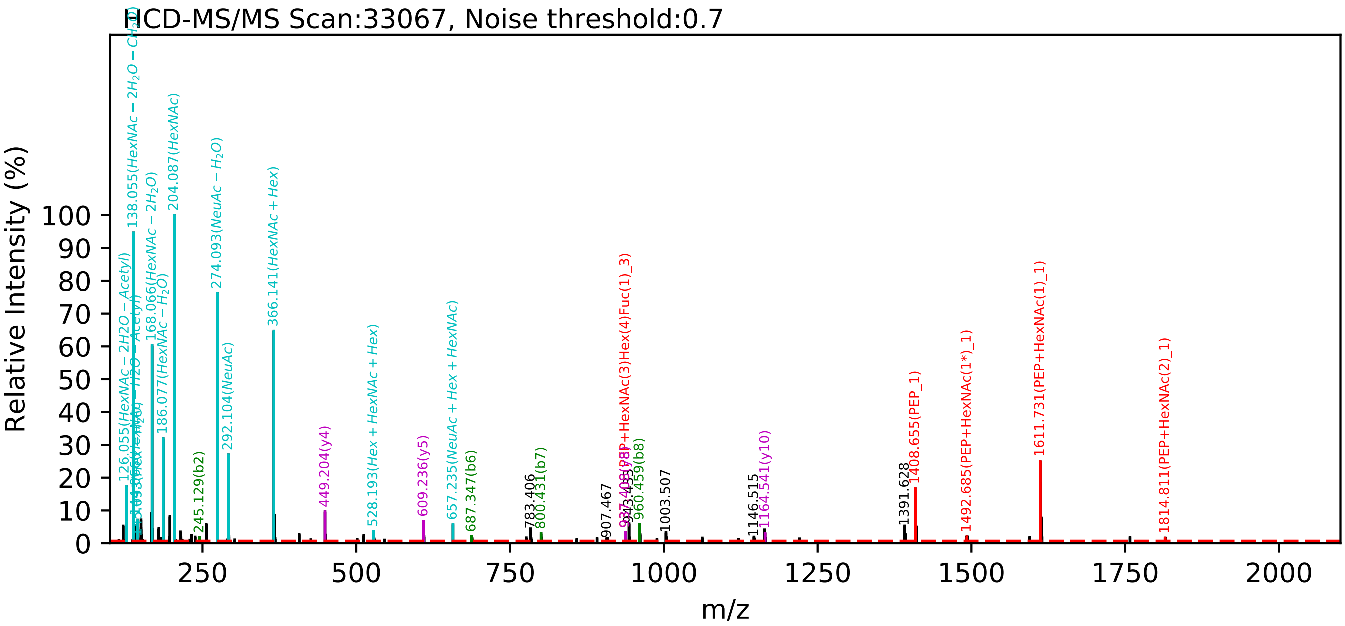

FPNITNLCPFGE(=PEP)_6_5_1_2_0, 0_None, 0_None,
m/z:1375.54(3+), RT:83.43, hcd-score:78.18

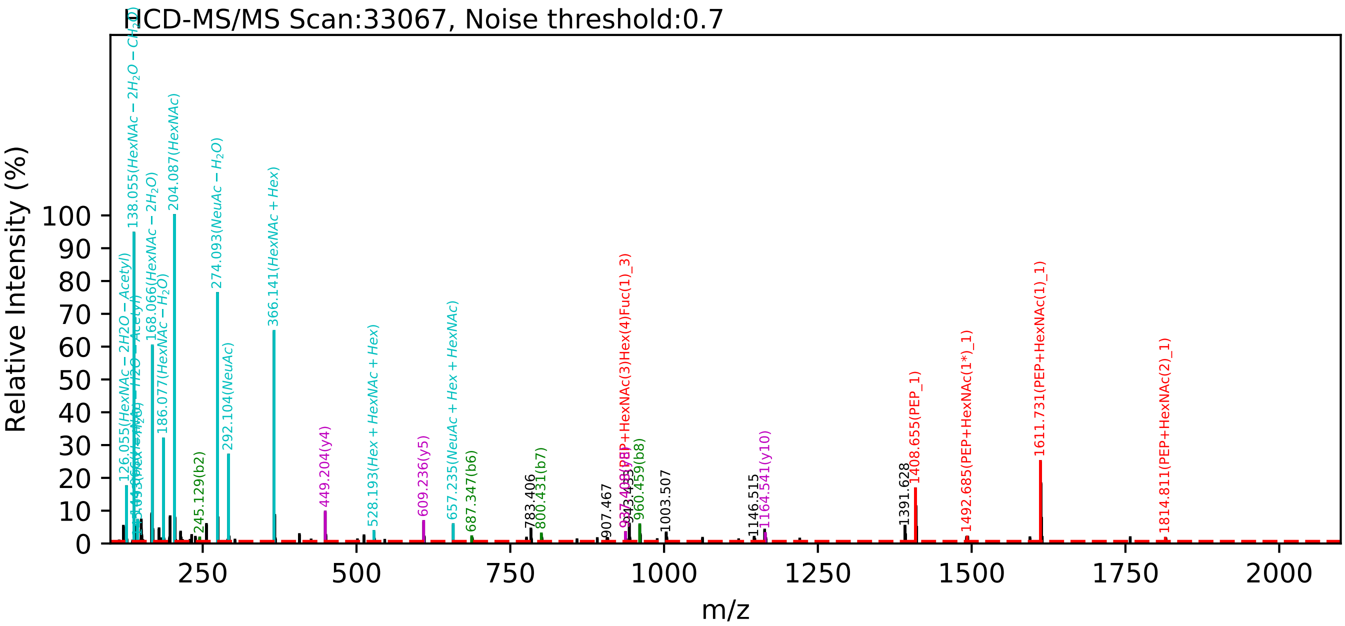

FPNITNLCPFGE(=PEP)_6_5_2_2_0, 0_None, 0_None,
m/z:1068.43(3+), RT:83.54, hcd-score:82.07

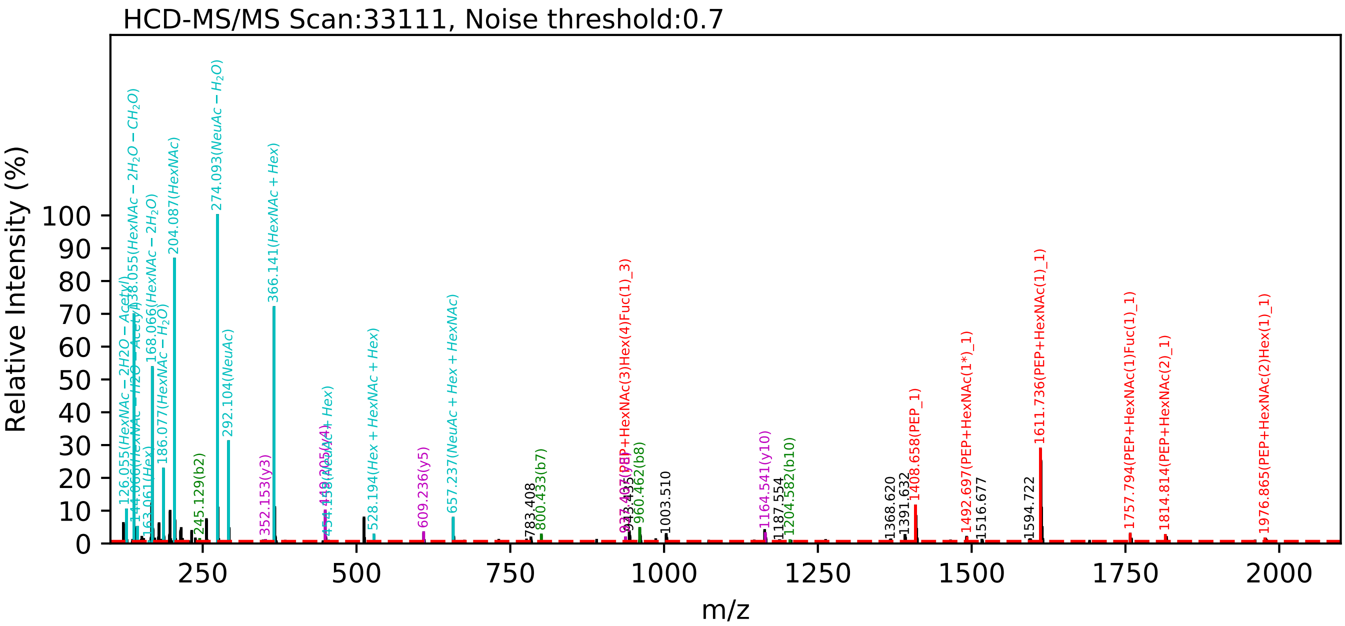

FPNITNLCPFGE(=PEP)_6_5_2_2_0, 0_None, 0_None,
m/z:1068.43(3+), RT:83.54, hcd-score:82.07

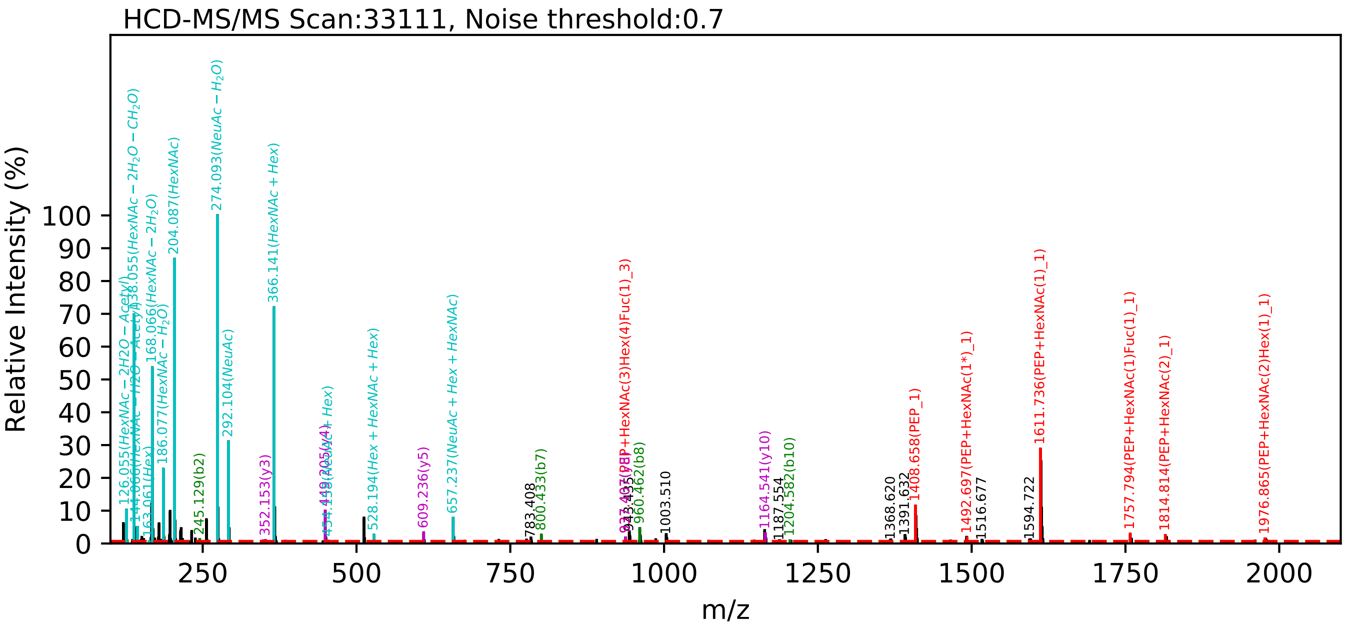

FPNITNLCPFGE(=PEP)_5_4_2_1_0, 0_None, 0_None,
m/z:1205.49(4+), RT:83.58, hcd-score:71.03

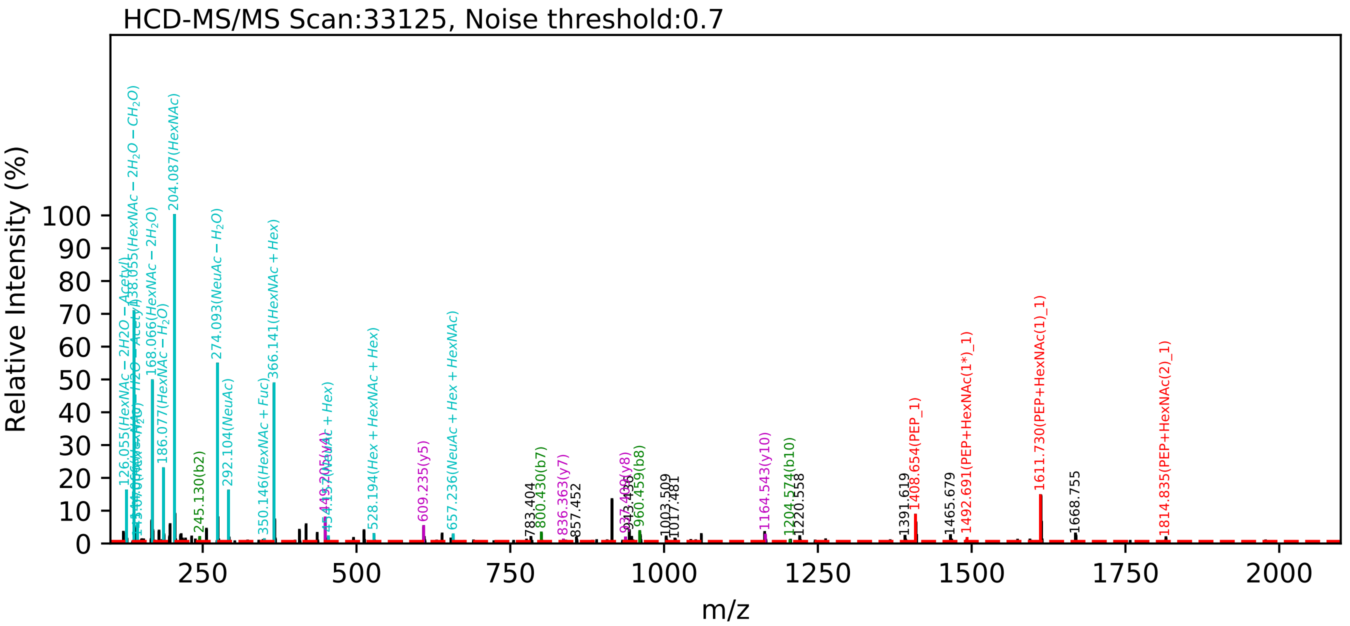

FPNITNLCPFGE(=PEP)_5_4_2_1_0, 0_None, 0_None,
m/z:1205.49(4+), RT:83.58, hcd-score:71.03

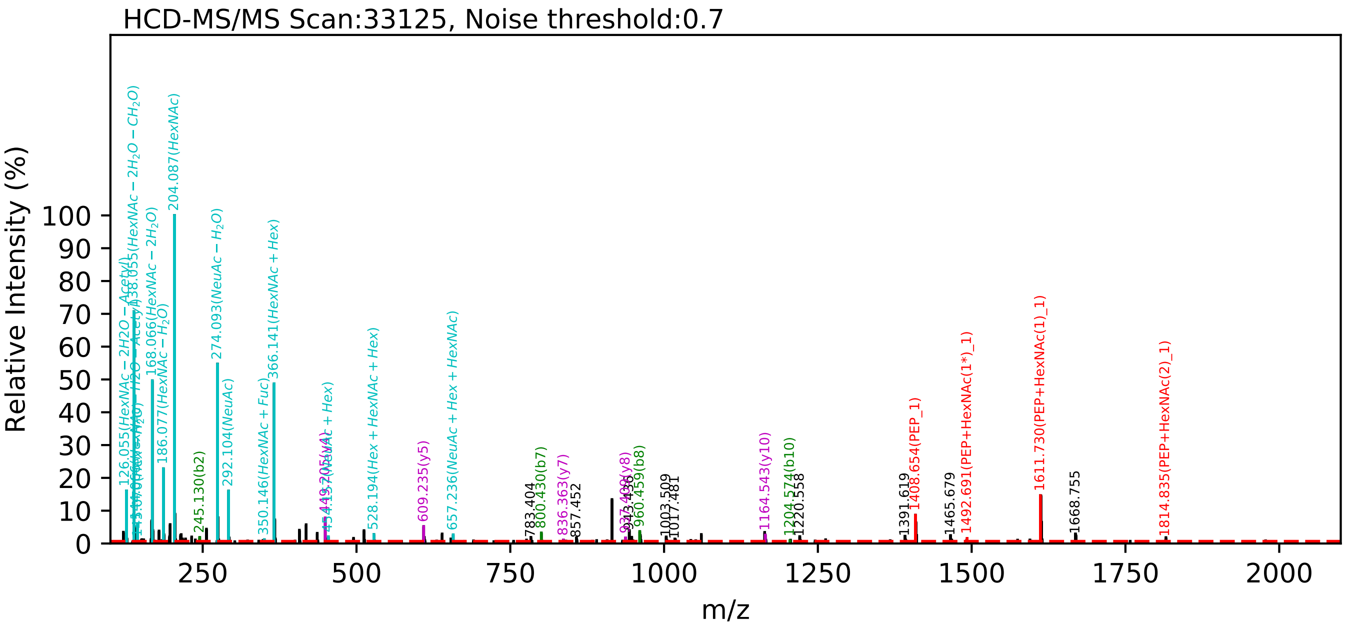

FPNITNLCPFGE(=PEP)_6_5_1_2_0, 0_None, 0_None,
m/z:1375.55(4+), RT:83.85, hcd-score:81.66

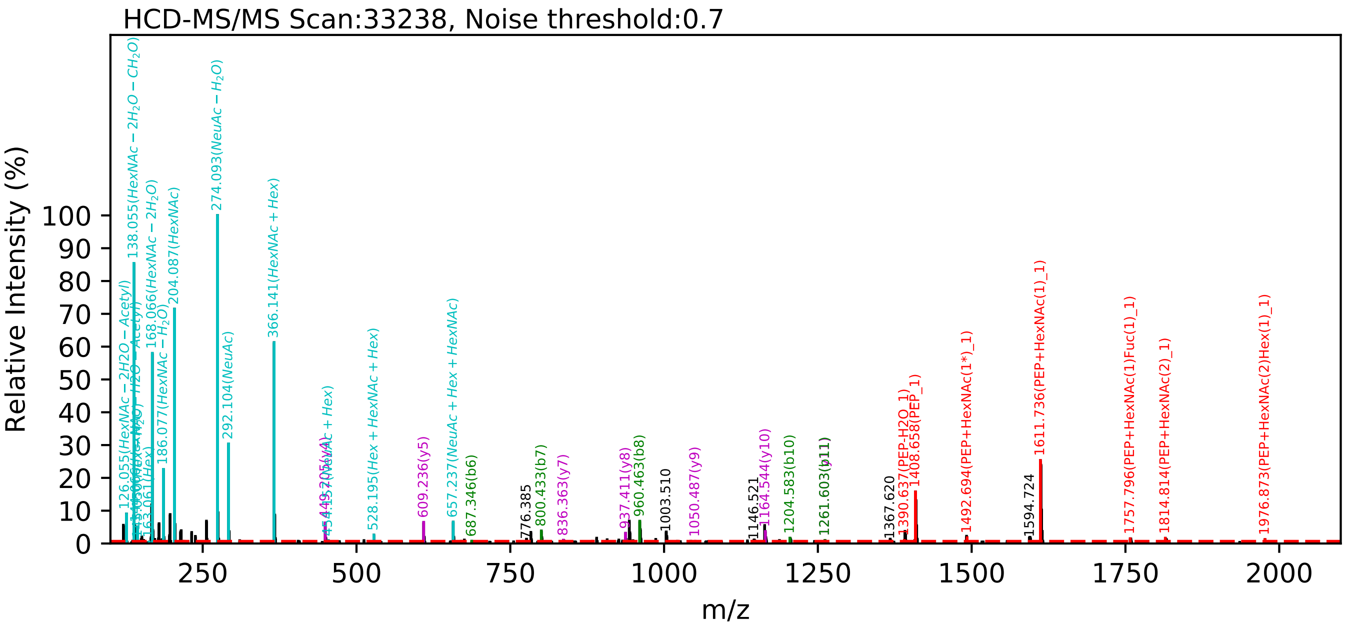

FPNITNLCPFGE(=PEP)_6_5_1_2_0, 0_None, 0_None,
m/z:1375.55(4+), RT:83.85, hcd-score:81.66

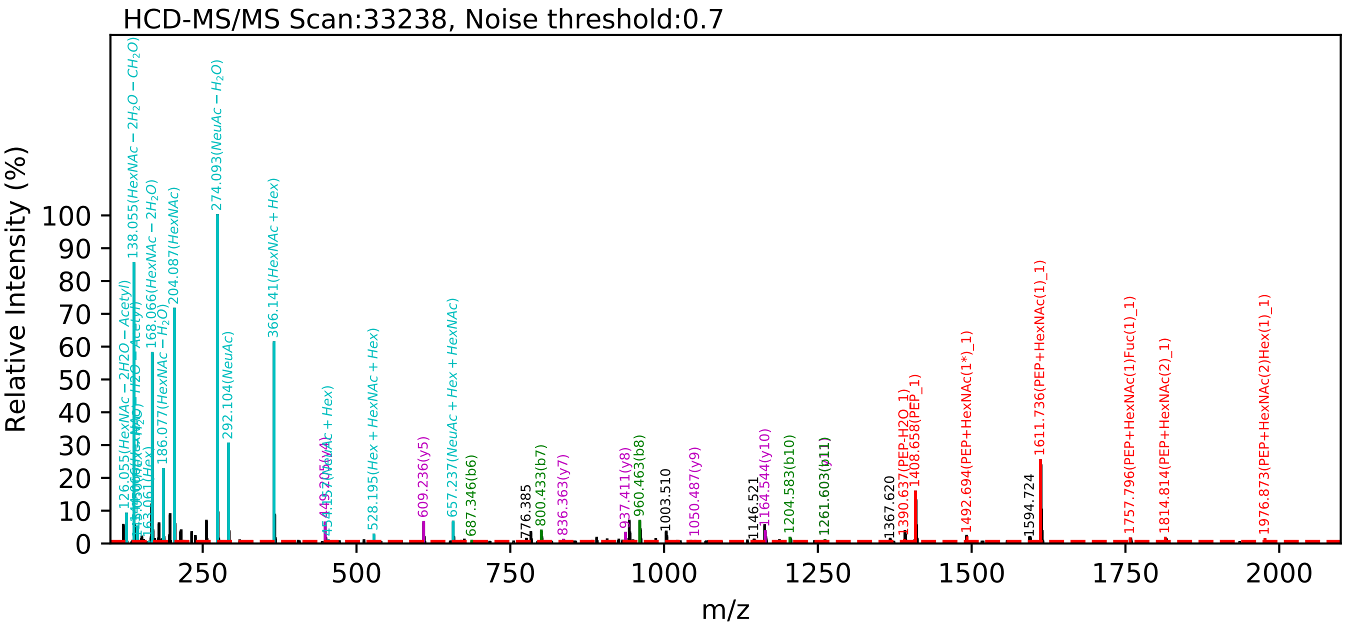

FPNITNLCPFGE(=PEP)_5_5_1_2_0, 0_None, 0_None,
m/z:1321.53(4+), RT:84.98, hcd-score:79.37

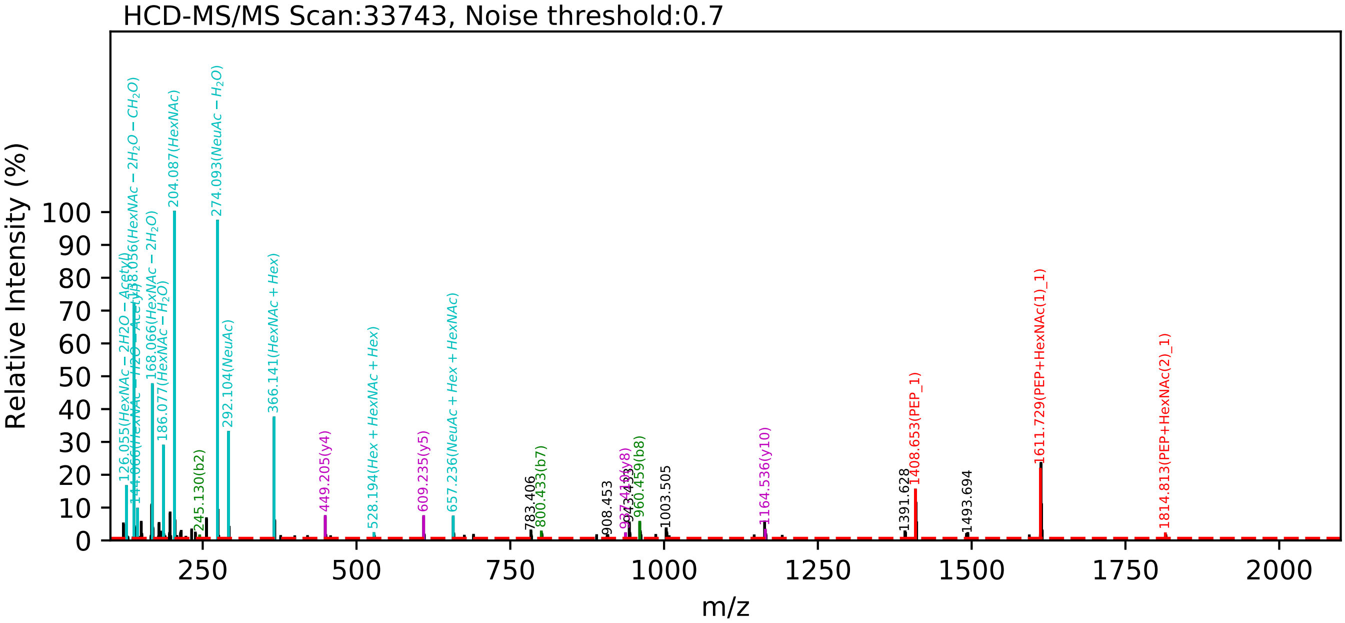

FPNITNLCPFGE(=PEP)_5_5_1_2_0, 0_None, 0_None,
m/z:1321.53(4+), RT:84.98, hcd-score:79.37

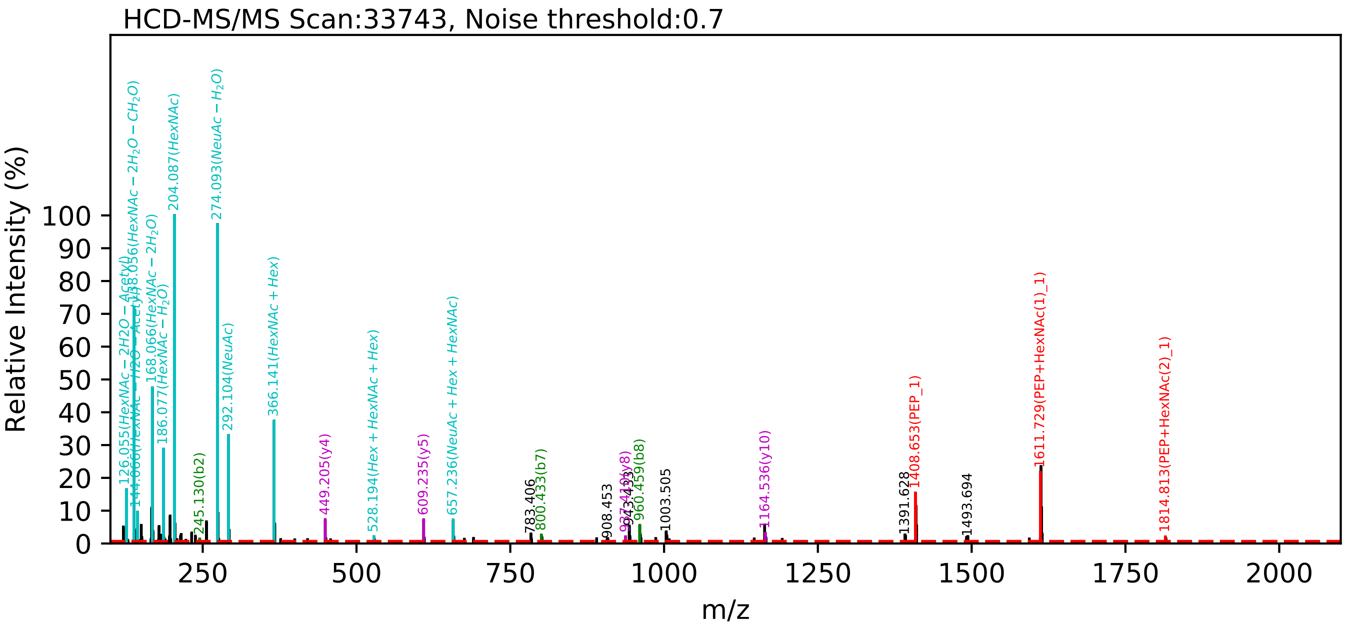

FPNITNLCPFGE(=PEP)_4_4_1_1_0, 0_None, 0_None,
m/z:1102.78(3+), RT:70.25, Y-score:86.13

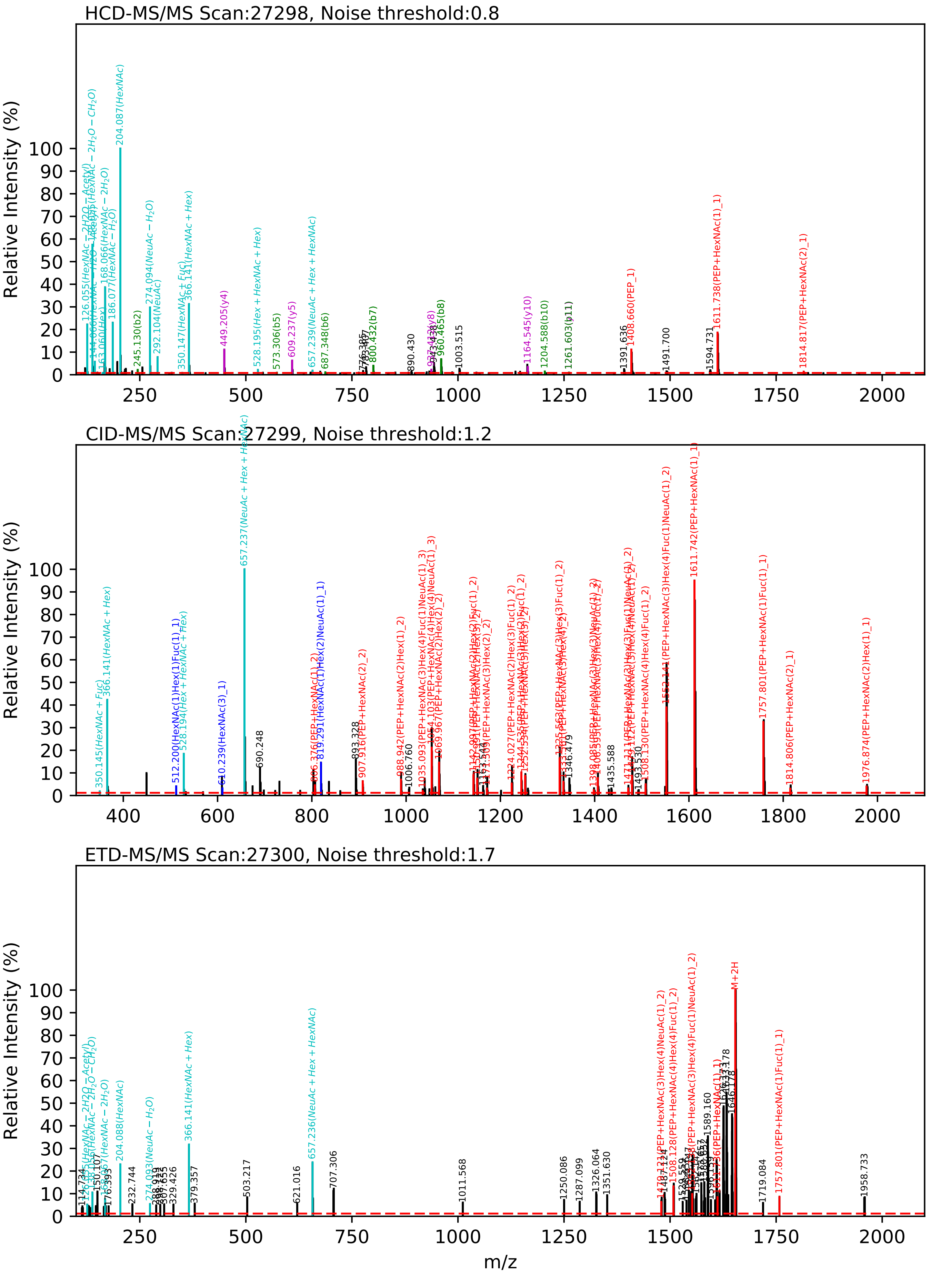

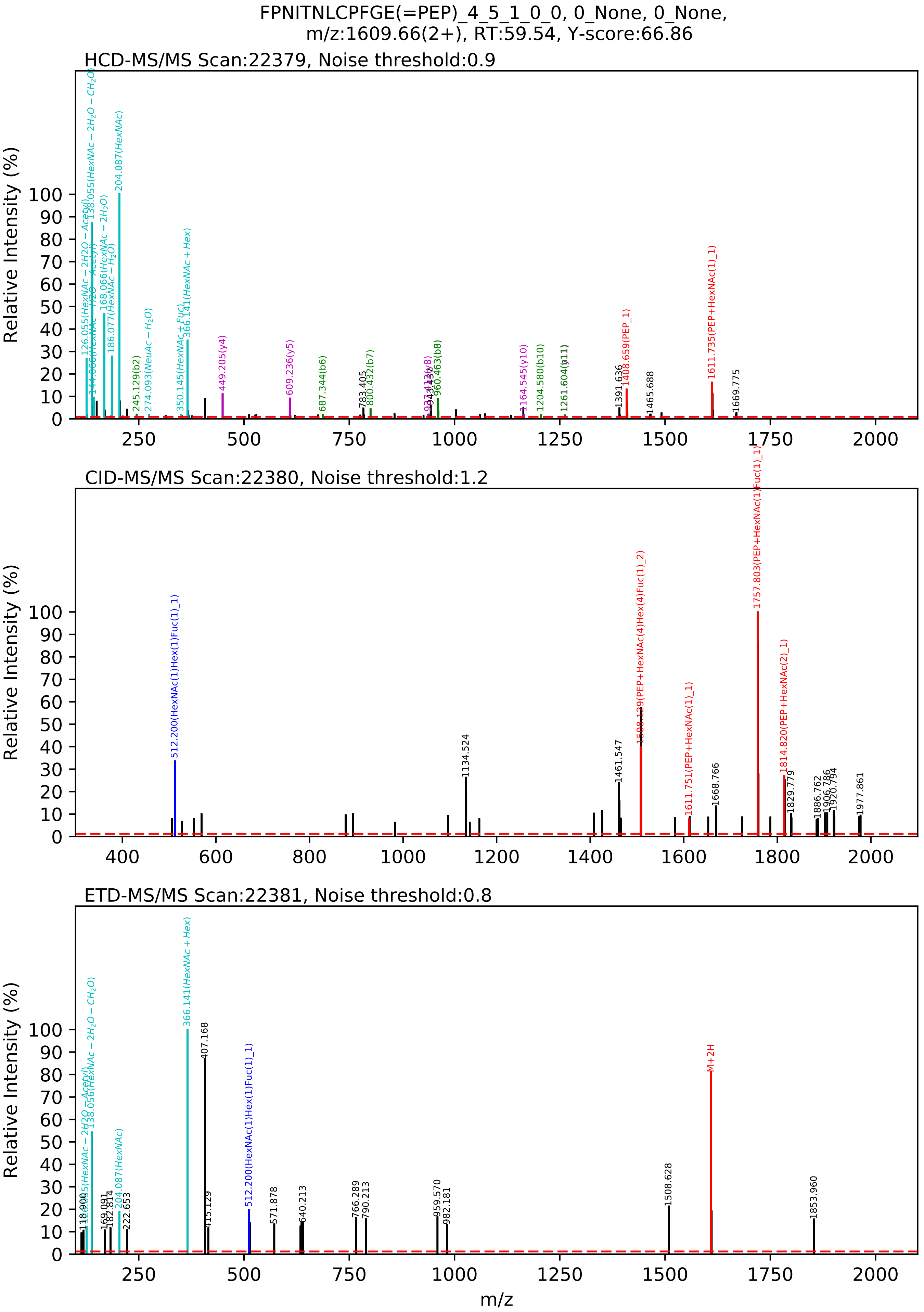
FPNITNLCPFGE(=PEP)_4_5_1_0_0, 0_None, 0_None,
m/z:1609.66(2+), RT:59.54, Y-score:66.86
HCD-MS/MS Scan:22379, Noise threshold:0.9
CID-MS/MS Scan:22380, Noise threshold:1.2
ETD-MS/MS Scan:22381, Noise threshold:0.8
Relative Intensity (%)
m/z
204.087(HexNAc)
186.077(HexNAc − H2O)
168.066(HexNAc − 2H2O)
366.141(HexNAc + Hex)
350.145(HexNAc + Fuc)
274.093(NeuAc − H2O)
245.129(b2)
449.205(y4)
609.236(y5)
687.344(b6)
800.432(b7)
960.463(b8)
1164.545(y10)
1204.580(b10)
1261.604(b11)
1408.659(PEP_1)
1611.735(PEP+HexNAc(1)_1)
1391.636
1465.688
1669.775
783.405
512.200(HexNAc(1)Hex(1)Fuc(1)_1)
1134.524
1461.547
1508.139(PEP+HexNAc(4)Hex(4)Fuc(1)_2)
1611.751(PEP+HexNAc(1)_1)
1668.766
1757.803(PEP+HexNAc(1)Fuc(1)_1)
1814.820(PEP+HexNAc(2)_1)
1829.779
1886.762
1906.786
1920.794
1977.861
407.168
415.129
571.878
640.213
766.289
790.213
959.570
982.181
1508.628
M+2H
1853.960
169.091
182.814
222.653
118.900

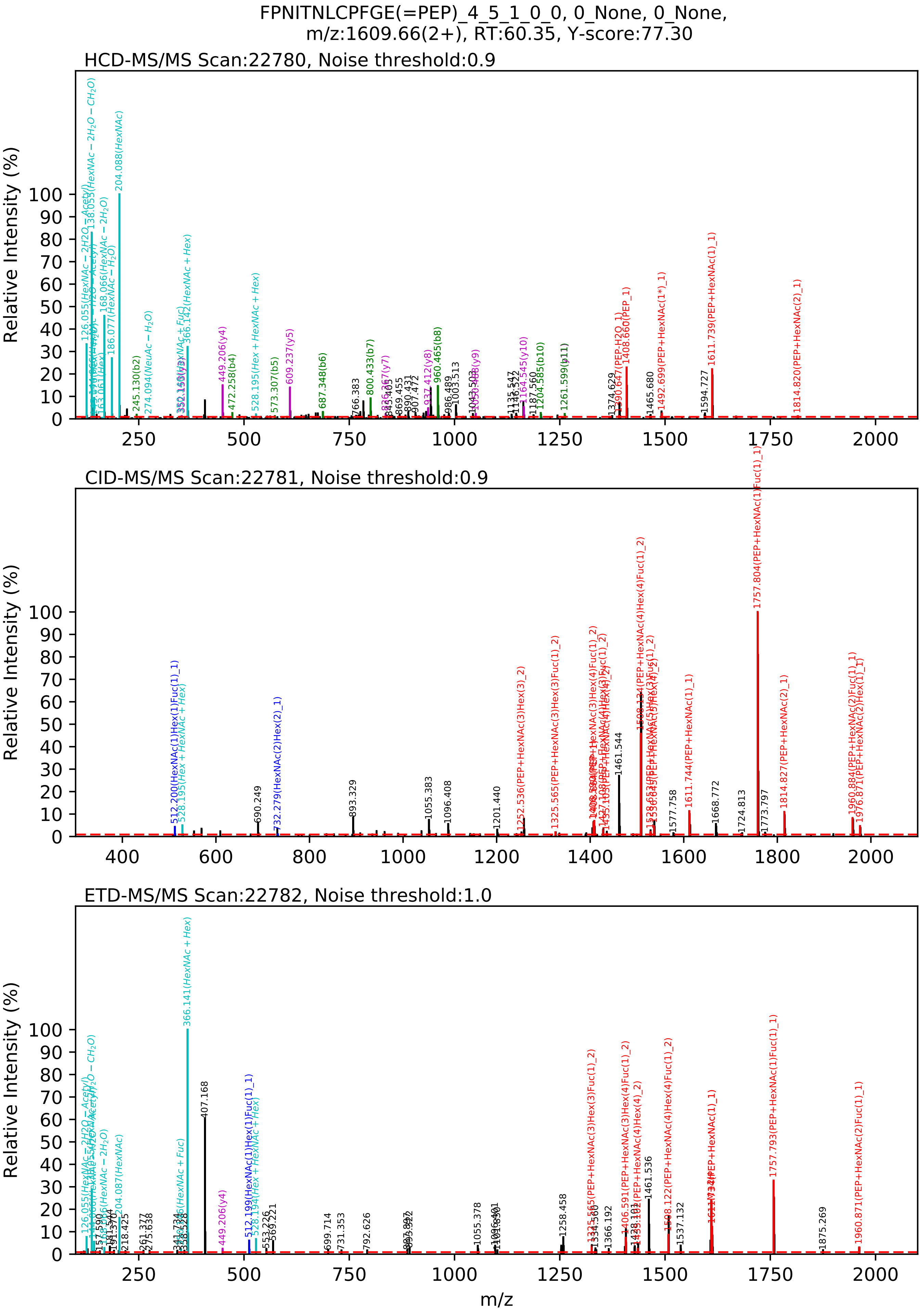
FPNITNLCPFGE(=PEP)_4_5_1_0_0, 0_None, 0_None,
m/z:1609.66(2+), RT:60.35, Y-score:77.30
HCD-MS/MS Scan:22780, Noise threshold:0.9
CID-MS/MS Scan:22781, Noise threshold:0.9
ETD-MS/MS Scan:22782, Noise threshold:1.0
Relative Intensity (%)
m/z

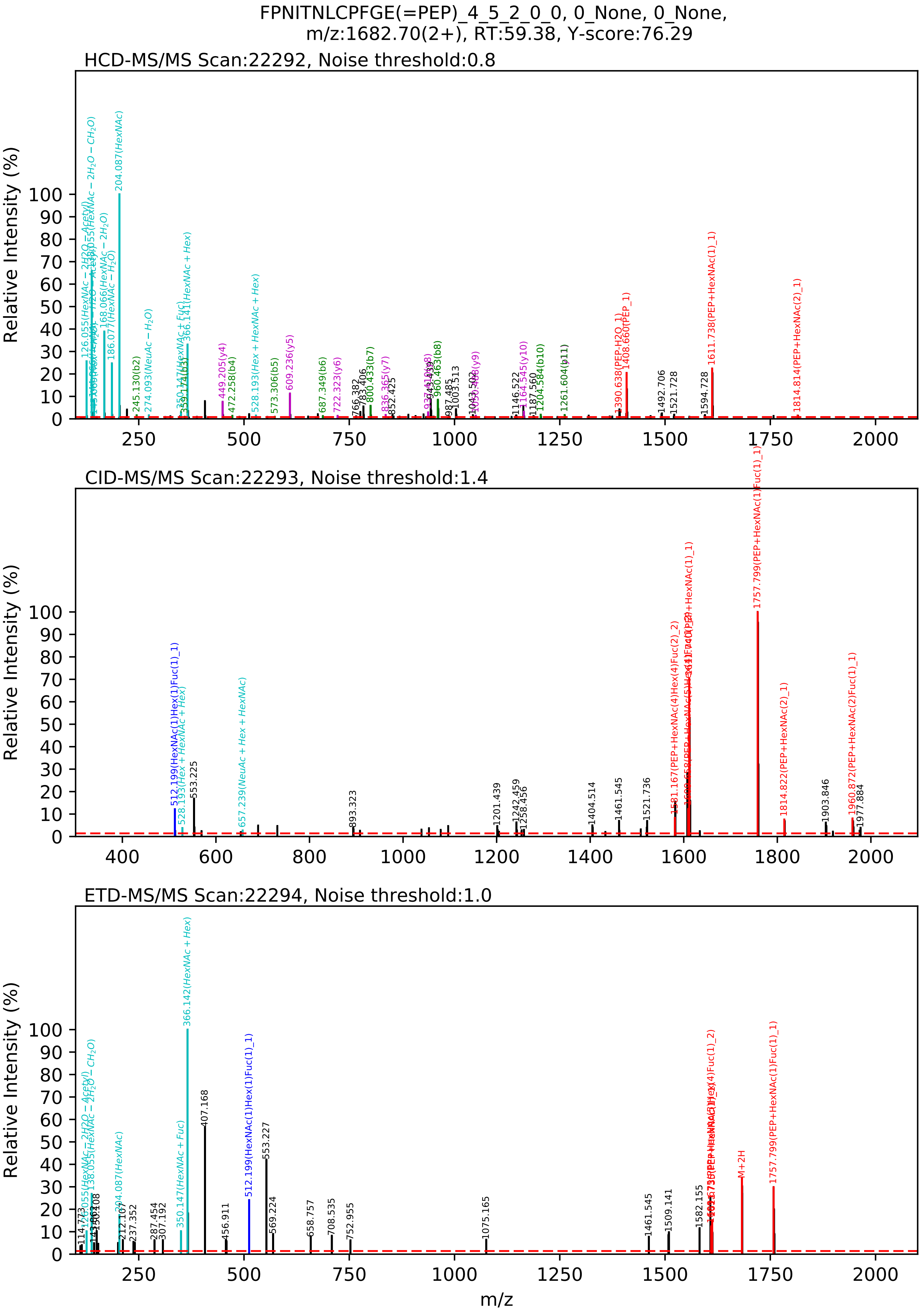
FPNITNLCPFGE(=PEP)_4_5_2_0_0, 0_None, 0_None,
m/z:1682.70(2+), RT:59.38, Y-score:76.29
HCD-MS/MS Scan:22292, Noise threshold:0.8
Relative Intensity (%)
204.087(HexNAc)
186.077(HexNAc − H2O)
168.066(HexNAc − 2H2O)
245.130(b2)
274.093(NeuAc − H2O)
366.141(HexNAc + Hex)
449.205(y4)
472.258(b4)
528.193(Hex + HexNAc + Hex)
573.306(b5)
609.236(y5)
687.349(b6)
722.323(y6)
766.382
783.406
800.433(b7)
836.365(y7)
852.425
960.463(b8)
987.485
1003.513
1043.502
1146.522
1164.545(y10)
1187.560
1204.584(b10)
1261.604(b11)
1390.638(PEP-H2O_1)
1408.660(PEP_1)
1492.706
1521.728
1594.728
1611.738(PEP+HexNAc(1)_1)
1814.814(PEP+HexNAc(2)_1)
CID-MS/MS Scan:22293, Noise threshold:1.4
512.199(HexNAc(1)Hex(1)Fuc(1)_1)
528.193(Hex + HexNAc + Hex)
553.225
657.239(NeuAc + Hex + HexNAc)
893.323
1201.439
1242.459
1258.456
1404.514
1461.545
1521.736
1581.167(PEP+HexNAc(4)Hex(4)Fuc(2)_2)
1757.799(PEP+HexNAc(1)Fuc(1)_1)
1814.822(PEP+HexNAc(2)_1)
1903.846
1960.872(PEP+HexNAc(2)Fuc(1)_1)
1977.884
ETD-MS/MS Scan:22294, Noise threshold:1.0
114.773
138.055(HexNAc − 2H2O − CH2O)
150.108
204.087(HexNAc)
212.107
237.352
287.454
307.192
350.147(HexNAc + Fuc)
366.142(HexNAc + Hex)
407.168
456.911
512.199(HexNAc(1)Hex(1)Fuc(1)_1)
553.227
569.224
658.757
708.535
752.955
1075.165
1461.545
1509.141
1582.155
M+2H
1757.799(PEP+HexNAc(1)Fuc(1)_1)
m/z

FPNITNLCPFGE(=PEP)_4_5_2_1_0, 0_None, 0_None,
m/z:1219.16(3+), RT:66.34, Y-score:69.26

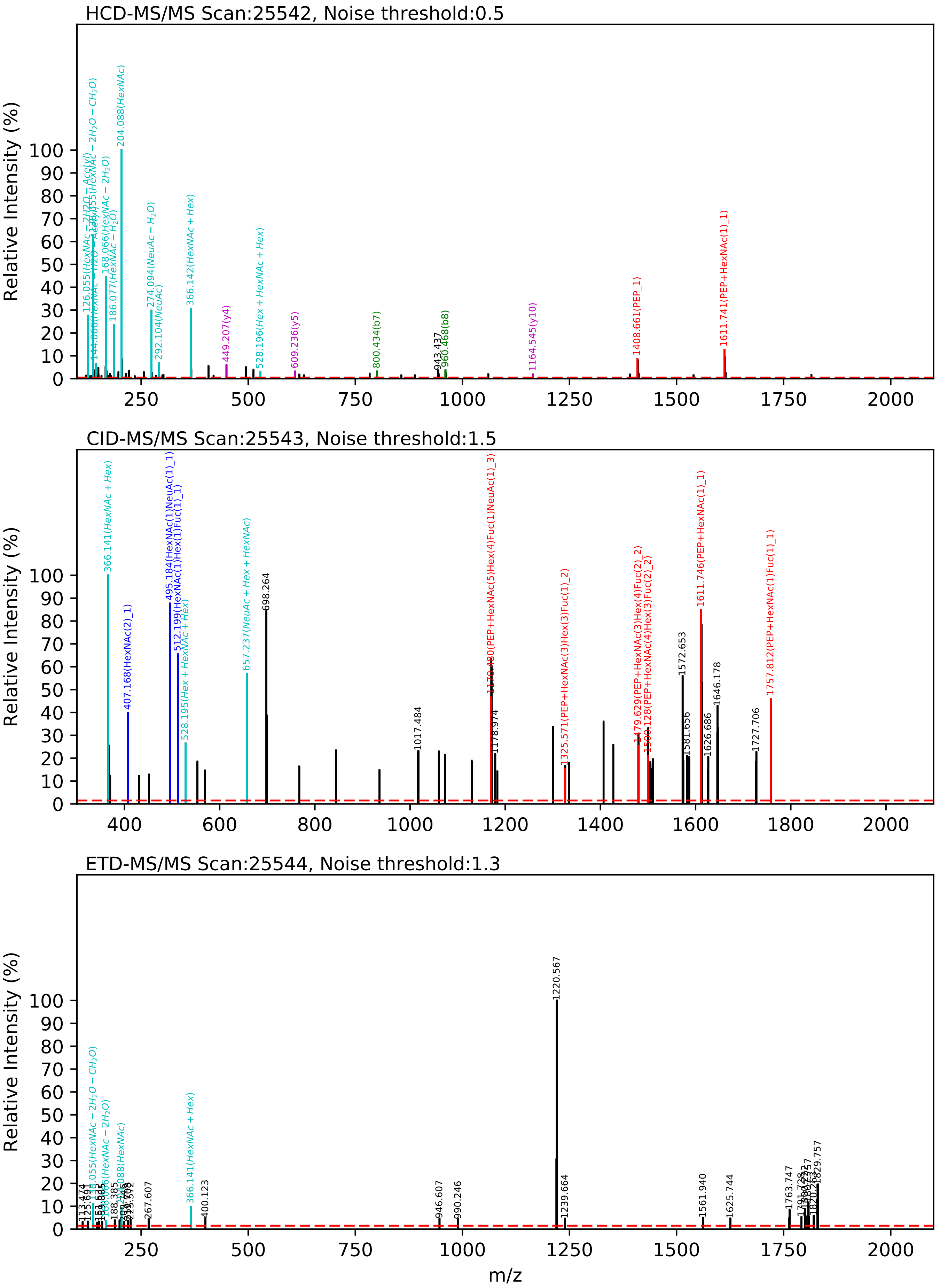

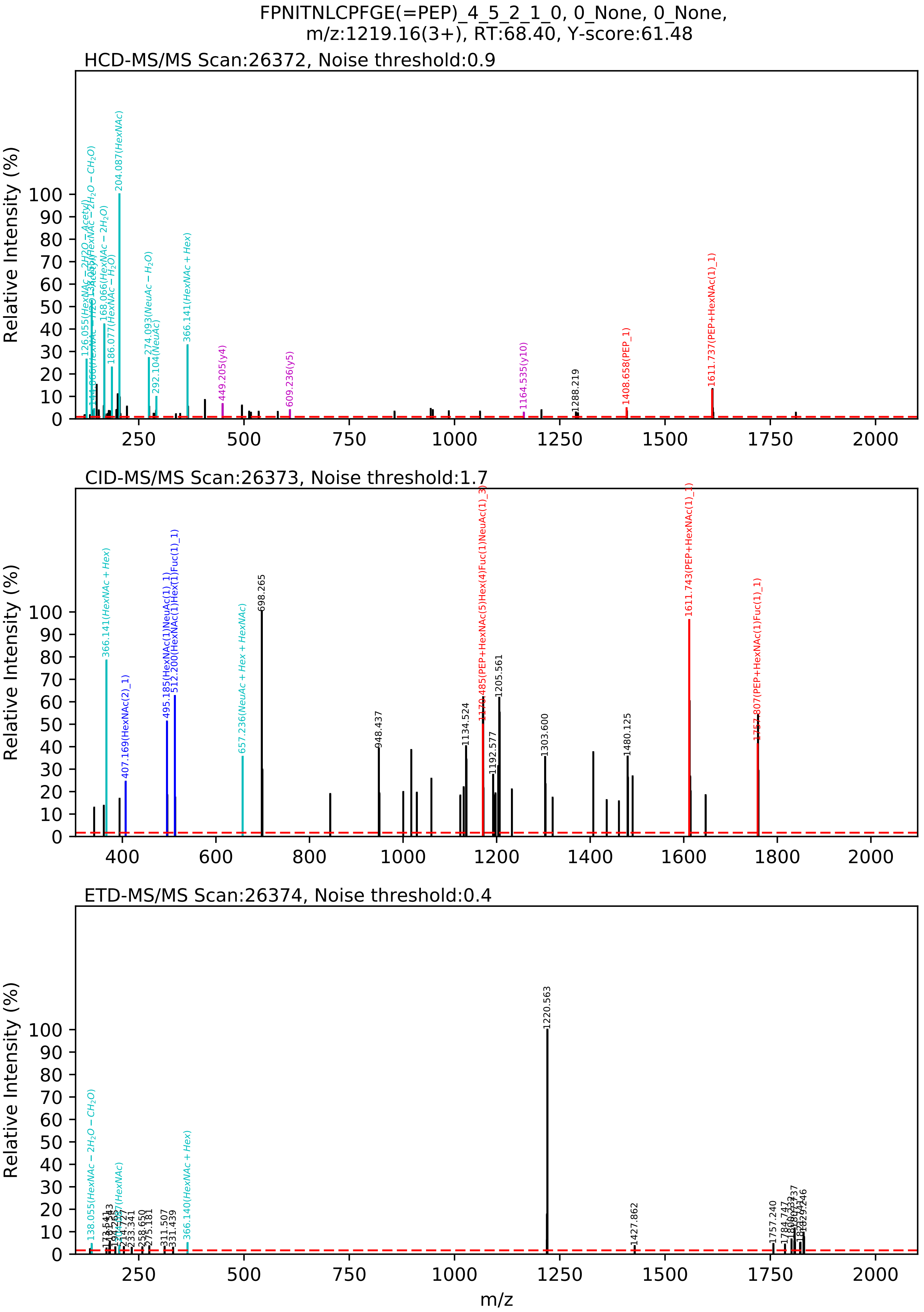
FPNITNLCPFGE(=PEP)_4_5_2_1_0, 0_None, 0_None,
m/z:1219.16(3+), RT:68.40, Y-score:61.48
HCD-MS/MS Scan:26372, Noise threshold:0.9
CID-MS/MS Scan:26373, Noise threshold:1.7
ETD-MS/MS Scan:26374, Noise threshold:0.4
Relative Intensity (%)
m/z

FPNITNLCPFGE(=PEP)_4_6_1_1_0, 0_None, 0_None,
m/z:1238.17(3+), RT:69.46, Y-score:96.78

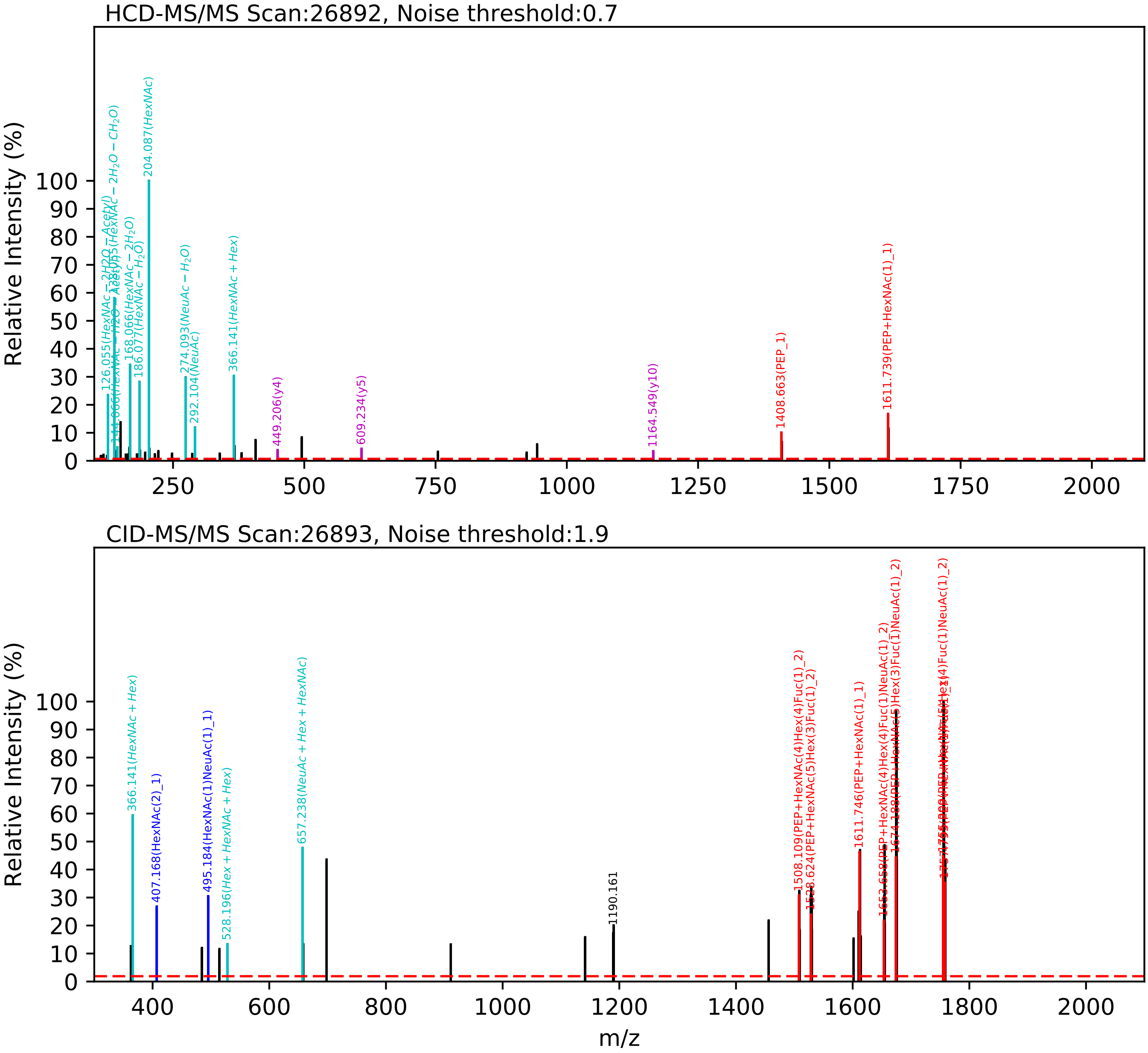

FPNITNLCPFGE(=PEP)_5_2_0_0_0, 0_None, 0_None,
m/z:1313.04(2+), RT:62.49, Y-score:73.85

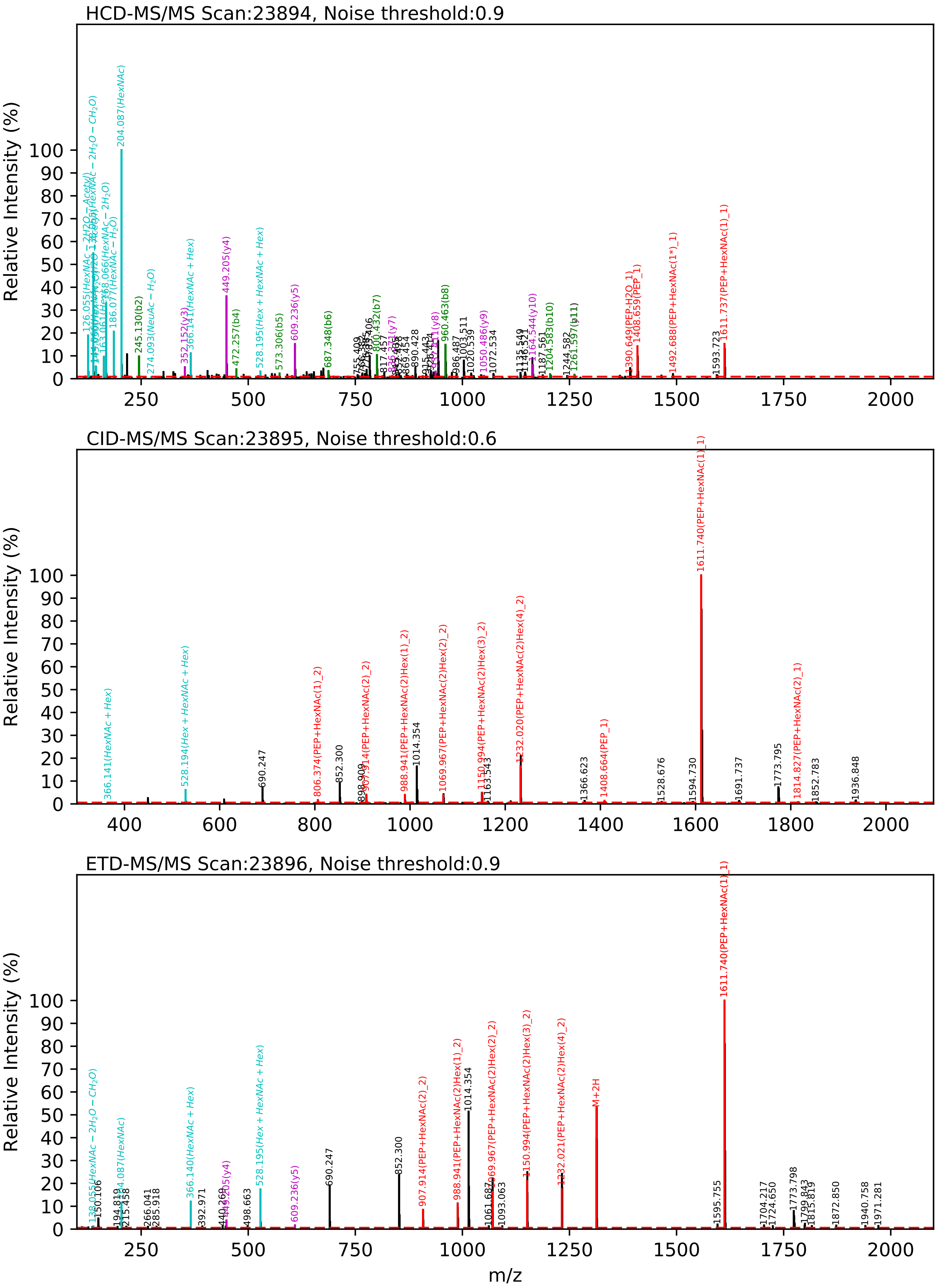

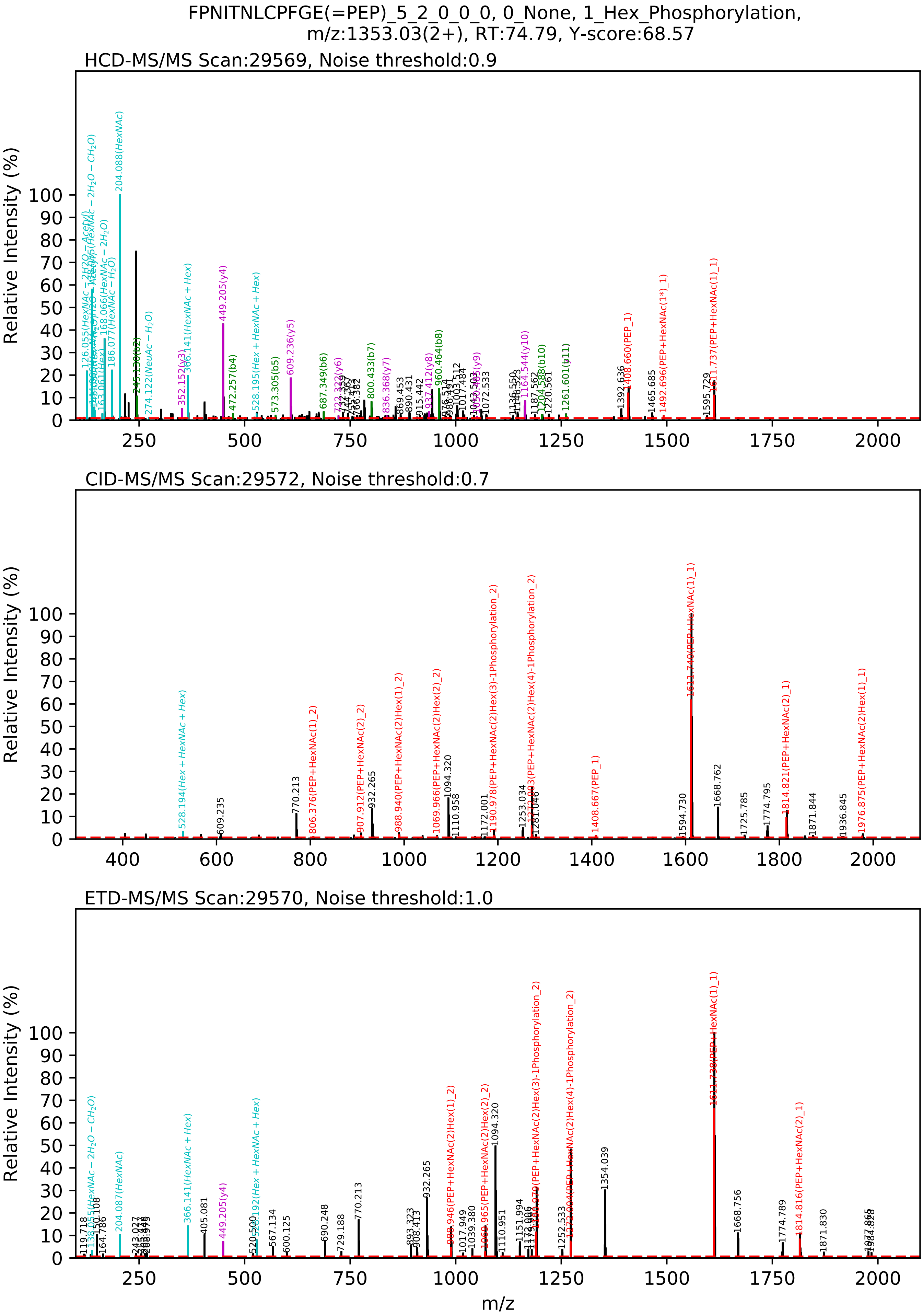
FPNITNLCPFGE(=PEP)_5_2_0_0_0, 0_None, 1_Hex_Phosphorylation,
m/z:1353.03(2+), RT:74.79, Y-score:68.57
HCD-MS/MS Scan:29569, Noise threshold:0.9
CID-MS/MS Scan:29572, Noise threshold:0.7
ETD-MS/MS Scan:29570, Noise threshold:1.0
Relative Intensity (%)
m/z

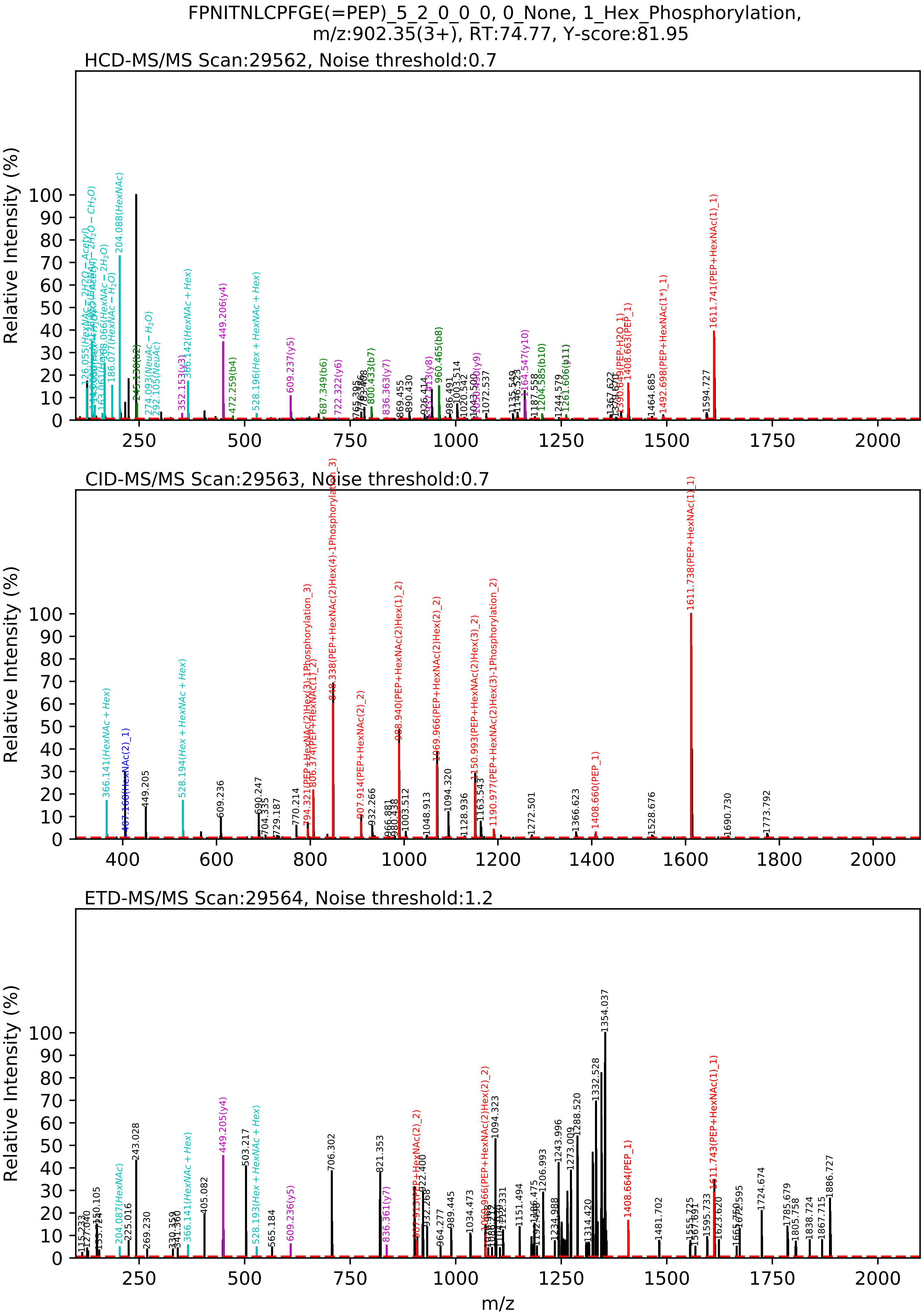

FPNITNLCPFGE(=PEP)_5_2_0_0_0, 0_None, 1_Hex_Phosphorylation,
m/z:902.35(3+), RT:74.77, Y-score:81.95
HCD-MS/MS Scan:29562, Noise threshold:0.7
CID-MS/MS Scan:29563, Noise threshold:0.7
ETD-MS/MS Scan:29564, Noise threshold:1.2
Relative Intensity (%)
m/z
204.088(HexNAc)
449.206(y4)
609.237(y5)
960.465(b8)
1408.663(PEP_1)
1611.741(PEP+HexNAc(1)_1)
848.338(PEP+HexNAc(2)Hex(4)-1Phosphorylation_3)
988.940(PEP+HexNAc(2)Hex(1)_2)
1069.966(PEP+HexNAc(2)Hex(2)_2)
1150.993(PEP+HexNAc(2)Hex(3)_2)
1611.738(PEP+HexNAc(1)_1)
1354.037
1332.528
1094.323
1611.743(PEP+HexNAc(1)_1)

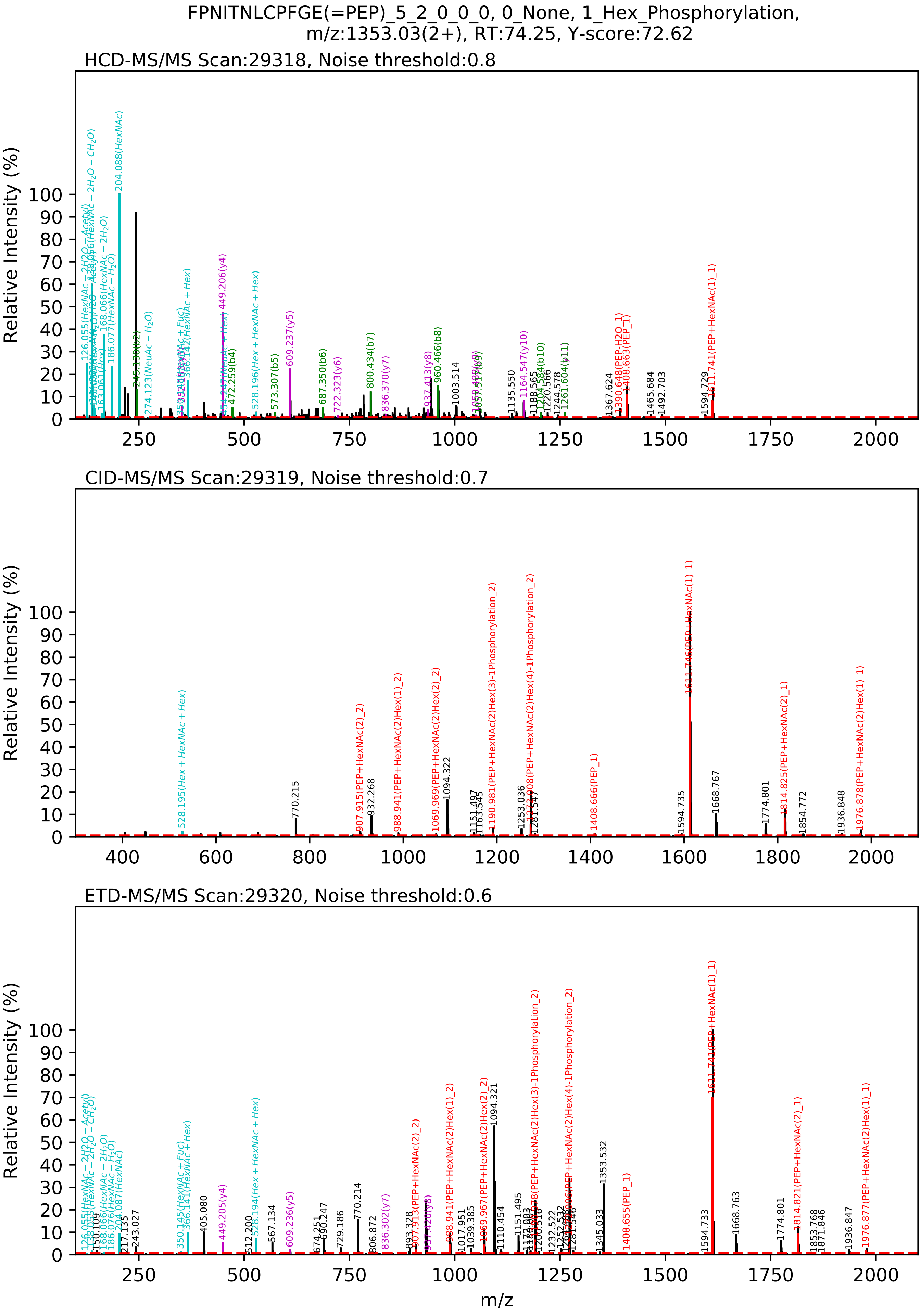
FPNITNLCPFGE(=PEP)_5_2_0_0_0, 0_None, 1_Hex_Phosphorylation,
m/z:1353.03(2+), RT:74.25, Y-score:72.62
HCD-MS/MS Scan:29318, Noise threshold:0.8
CID-MS/MS Scan:29319, Noise threshold:0.7
ETD-MS/MS Scan:29320, Noise threshold:0.6
Relative Intensity (%)
m/z
204.088(HexNAc)
609.237(y5)
449.206(y4)
800.434(b7)
960.466(b8)
1611.741(PEP+HexNAc(1)_1)
1611.746(PEP+HexNAc(1)_1)
1094.322
1668.767
1814.825(PEP+HexNAc(2)_1)
1976.878(PEP+HexNAc(2)Hex(1)_1)
1408.666(PEP_1)
1094.321
1353.532
1668.763
1814.821(PEP+HexNAc(2)_1)
1976.877(PEP+HexNAc(2)Hex(1)_1)
1408.655(PEP_1)

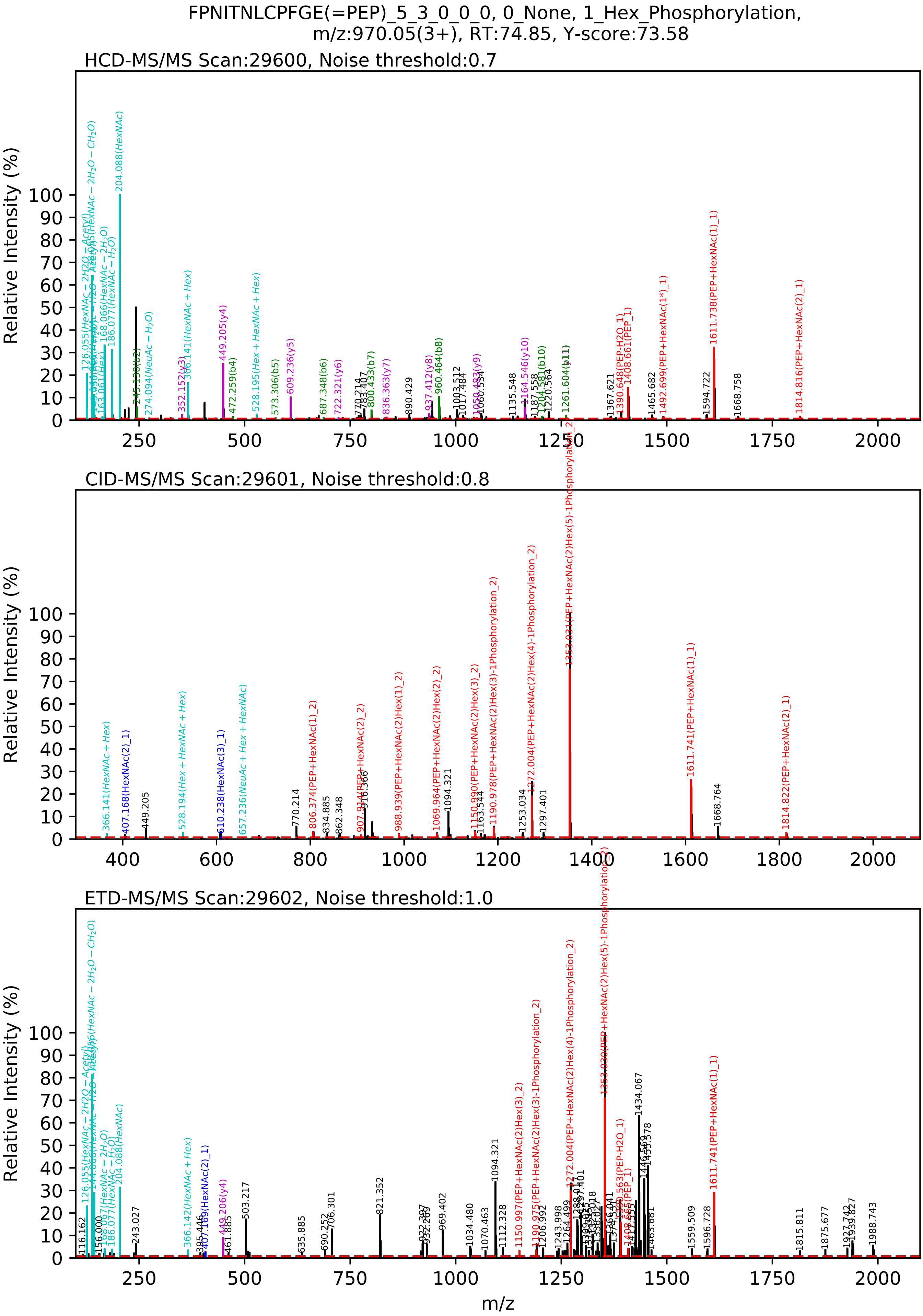

FPNITNLCPFGE(=PEP)_5_3_0_0_0, 0_None, 1_Hex_Phosphorylation,
m/z:970.05(3+), RT:74.85, Y-score:73.58
HCD-MS/MS Scan:29600, Noise threshold:0.7
CID-MS/MS Scan:29601, Noise threshold:0.8
ETD-MS/MS Scan:29602, Noise threshold:1.0
Relative Intensity (%)
m/z
204.088(HexNAc)
1611.738(PEP+HexNAc(1)_1)
1814.816(PEP+HexNAc(2)_1)
1492.699(PEP+HexNAc(1*)_1)
1408.661(PEP_1)
1353.021(PEP+HexNAc(2)Hex(5)-1Phosphorylation_2)
1611.741(PEP+HexNAc(1)_1)
1814.822(PEP+HexNAc(2)_1)
1272.004(PEP+HexNAc(2)Hex(4)-1Phosphorylation_2)
1190.978(PEP+HexNAc(2)Hex(3)-1Phosphorylation_2)
1150.990(PEP+HexNAc(2)Hex(3)_2)
1069.964(PEP+HexNAc(2)Hex(2)_2)
988.939(PEP+HexNAc(2)Hex(1)_2)
806.374(PEP+HexNAc(1)_2)
1434.067

FPNITNLCPFGE(=PEP)_5_4_1_1_0, 0_None, 0_None,
m/z:1156.80(3+), RT:69.49, Y-score:83.97

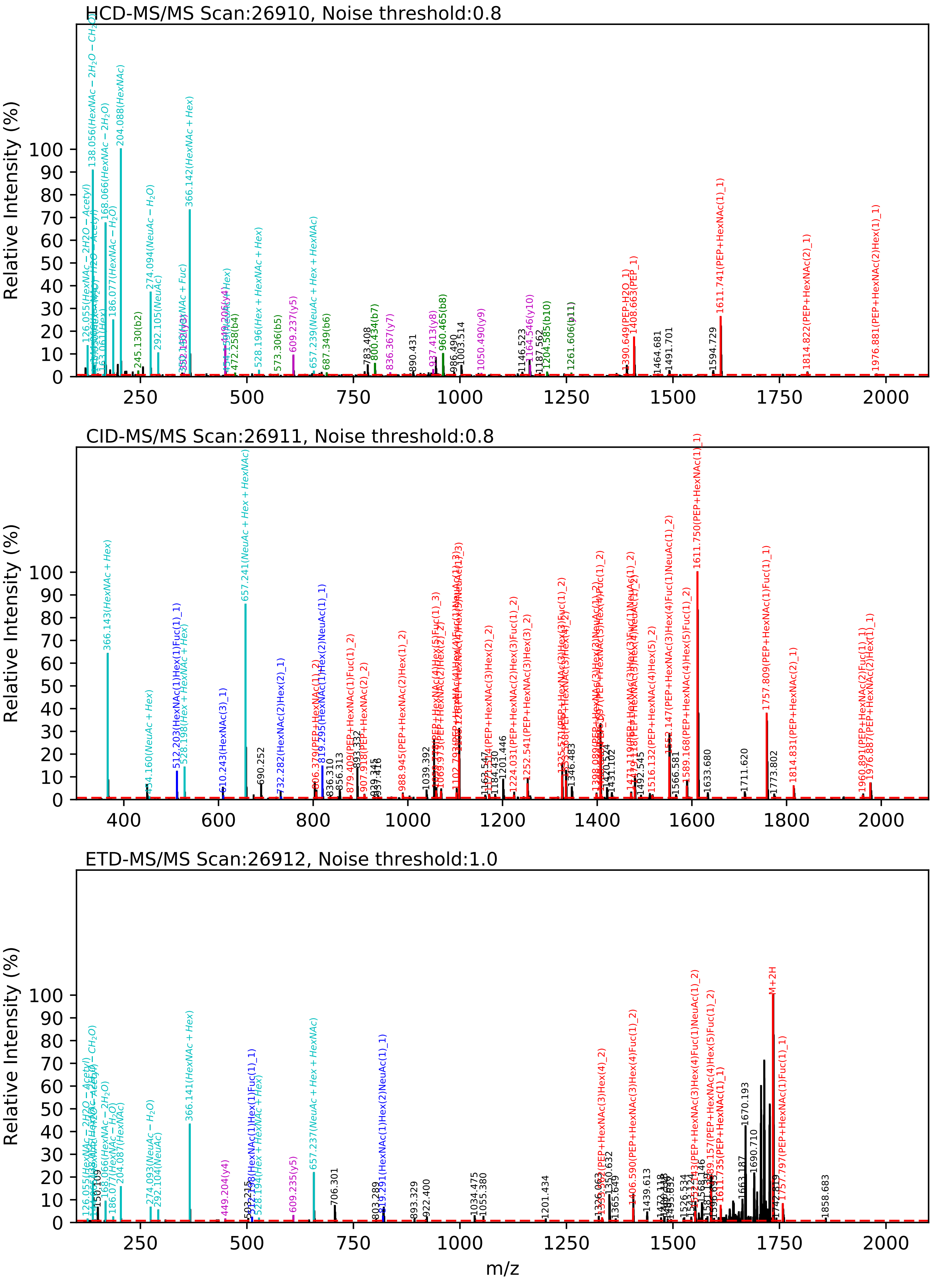

FPNITNLCPFGE(=PEP)_5_4_1_1_0, 0_None, 0_None,
m/z:1734.70(2+), RT:68.51, Y-score:83.64

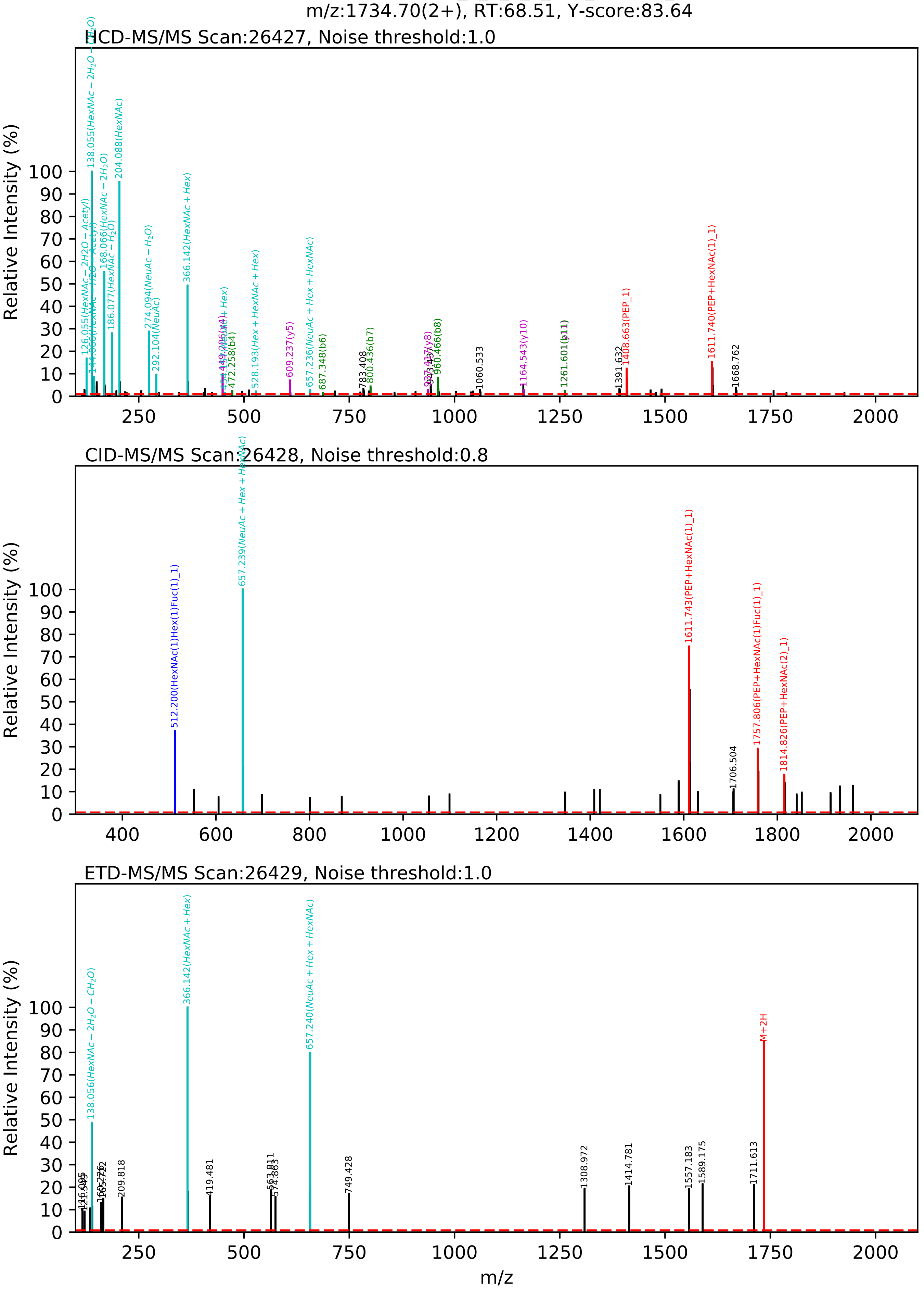

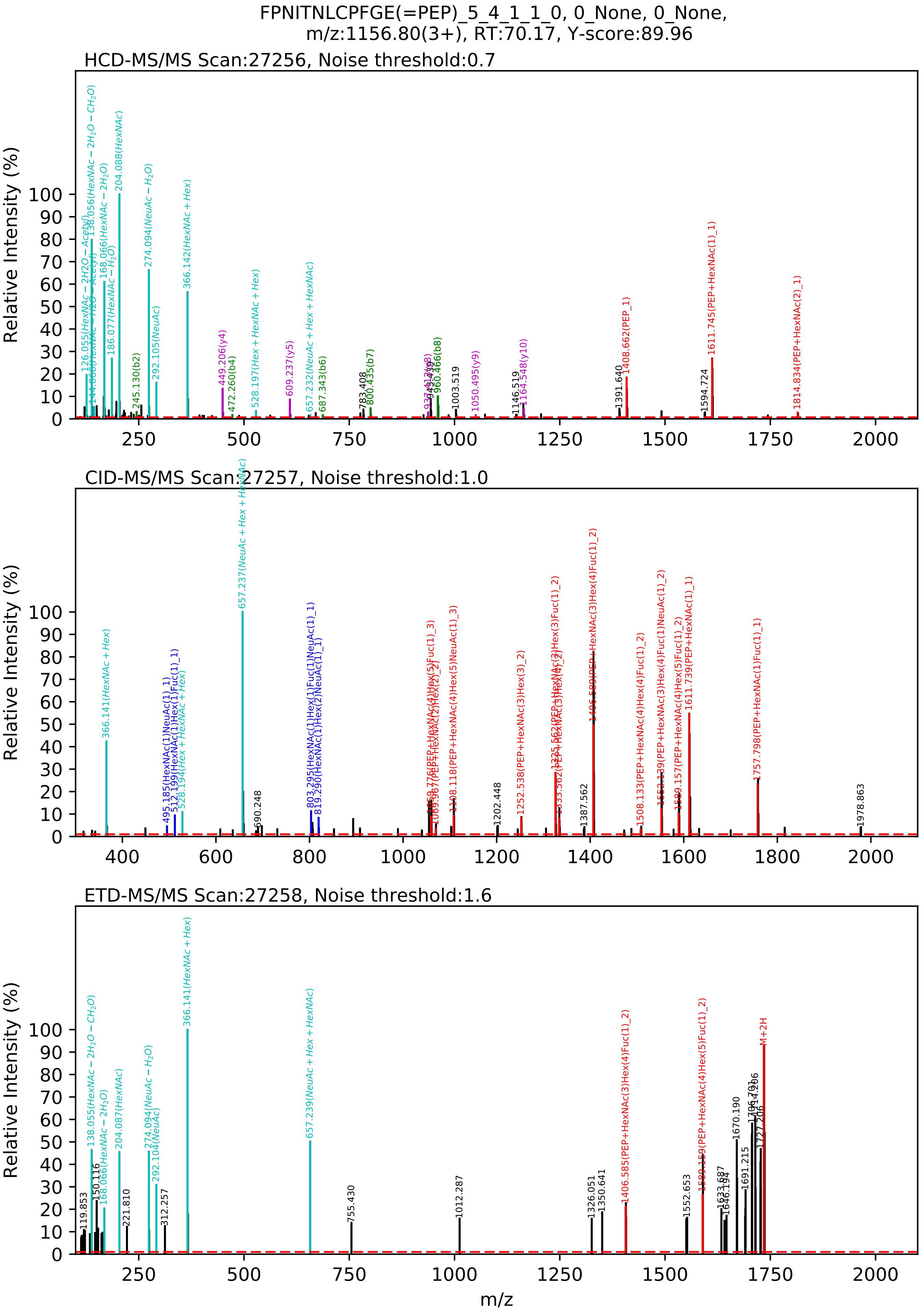
FPNITNLCPFGE(=PEP)_5_4_1_1_0, 0_None, 0_None,
m/z:1156.80(3+), RT:70.17, Y-score:89.96
HCD-MS/MS Scan:27256, Noise threshold:0.7
CID-MS/MS Scan:27257, Noise threshold:1.0
ETD-MS/MS Scan:27258, Noise threshold:1.6
Relative Intensity (%)
m/z
204.088(HexNAc)
274.094(NeuAc − H2O)
366.142(HexNAc + Hex)
292.105(NeuAc)
1408.662(PEP_1)
1611.745(PEP+HexNAc(1)_1)
1814.834(PEP+HexNAc(2)_1)
657.237(NeuAc + Hex + HexNAc)
366.141(HexNAc + Hex)
1406.580(PEP+HexNAc(3)Hex(4)Fuc(1)_2)
1611.739(PEP+HexNAc(1)_1)
1757.798(PEP+HexNAc(1)Fuc(1)_1)
1978.863
366.141(HexNAc + Hex)
657.239(NeuAc + Hex + HexNAc)
1406.585(PEP+HexNAc(3)Hex(4)Fuc(1)_2)
1589.159(PEP+HexNAc(4)Hex(5)Fuc(1)_2)
M+2H

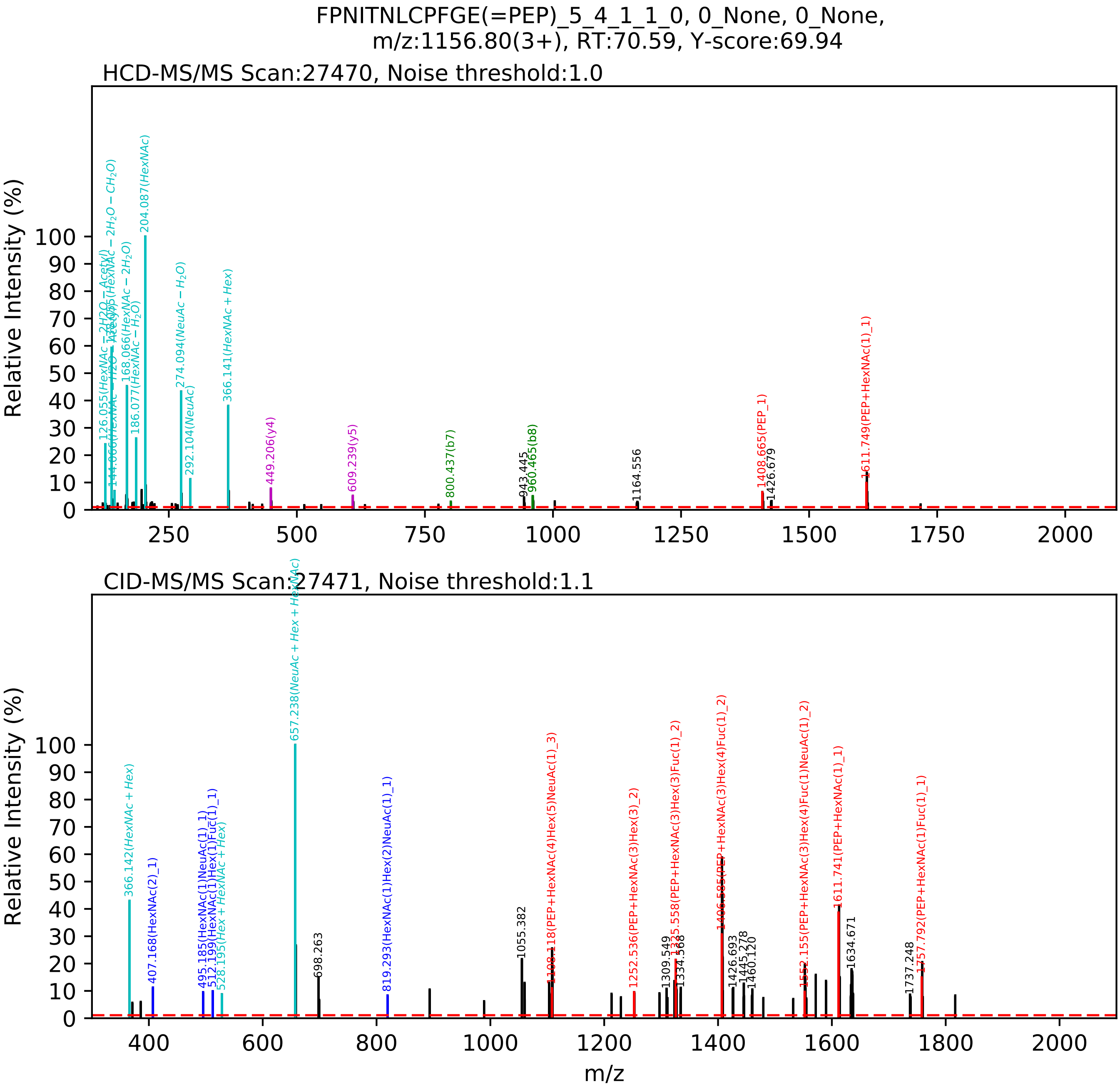
FPNITNLCPFGE(=PEP)_5_4_1_1_0, 0_None, 0_None,
m/z:1156.80(3+), RT:70.59, Y-score:69.94
HCD-MS/MS Scan:27470, Noise threshold:1.0
Relative Intensity (%)
126.055(HexNAc − 2H2O − Acetyl)
144.066(HexNAc − 2H2O)
168.066(HexNAc − 2H2O)
186.077(HexNAc − H2O)
204.087(HexNAc)
274.094(NeuAc − H2O)
292.104(NeuAc)
366.141(HexNAc + Hex)
449.206(y4)
609.239(y5)
800.437(b7)
943.445
960.465(b8)
1164.556
1408.665(PEP_1)
1426.679
1611.749(PEP+HexNAc(1)_1)
CID-MS/MS Scan:27471, Noise threshold:1.1
366.142(HexNAc + Hex)
407.168(HexNAc(2)_1)
495.185(HexNAc(1)NeuAc(1)_1)
512.199(HexNAc(1)Hex(1)Fuc(1)_1)
528.195(Hex + HexNAc + Hex)
657.238(NeuAc + Hex + HexNAc)
698.263
819.293(HexNAc(1)Hex(2)NeuAc(1)_1)
1055.382
1108.118(PEP+HexNAc(4)Hex(5)NeuAc(1)_3)
1252.536(PEP+HexNAc(3)Hex(3)_2)
1309.549
1325.558(PEP+HexNAc(3)Hex(3)Fuc(1)_2)
1334.568
1406.585(PEP+HexNAc(3)Hex(4)Fuc(1)_2)
1426.693
1445.278
1460.120
1552.155(PEP+HexNAc(3)Hex(4)Fuc(1)NeuAc(1)_2)
1611.741(PEP+HexNAc(1)_1)
1634.671
1737.248
1757.792(PEP+HexNAc(1)Fuc(1)_1)
m/z

FPNITNLCPFGE(=PEP)_5_4_1_1_0, 0_None, 0_None,
m/z:1734.70(2+), RT:70.27, Y-score:78.57

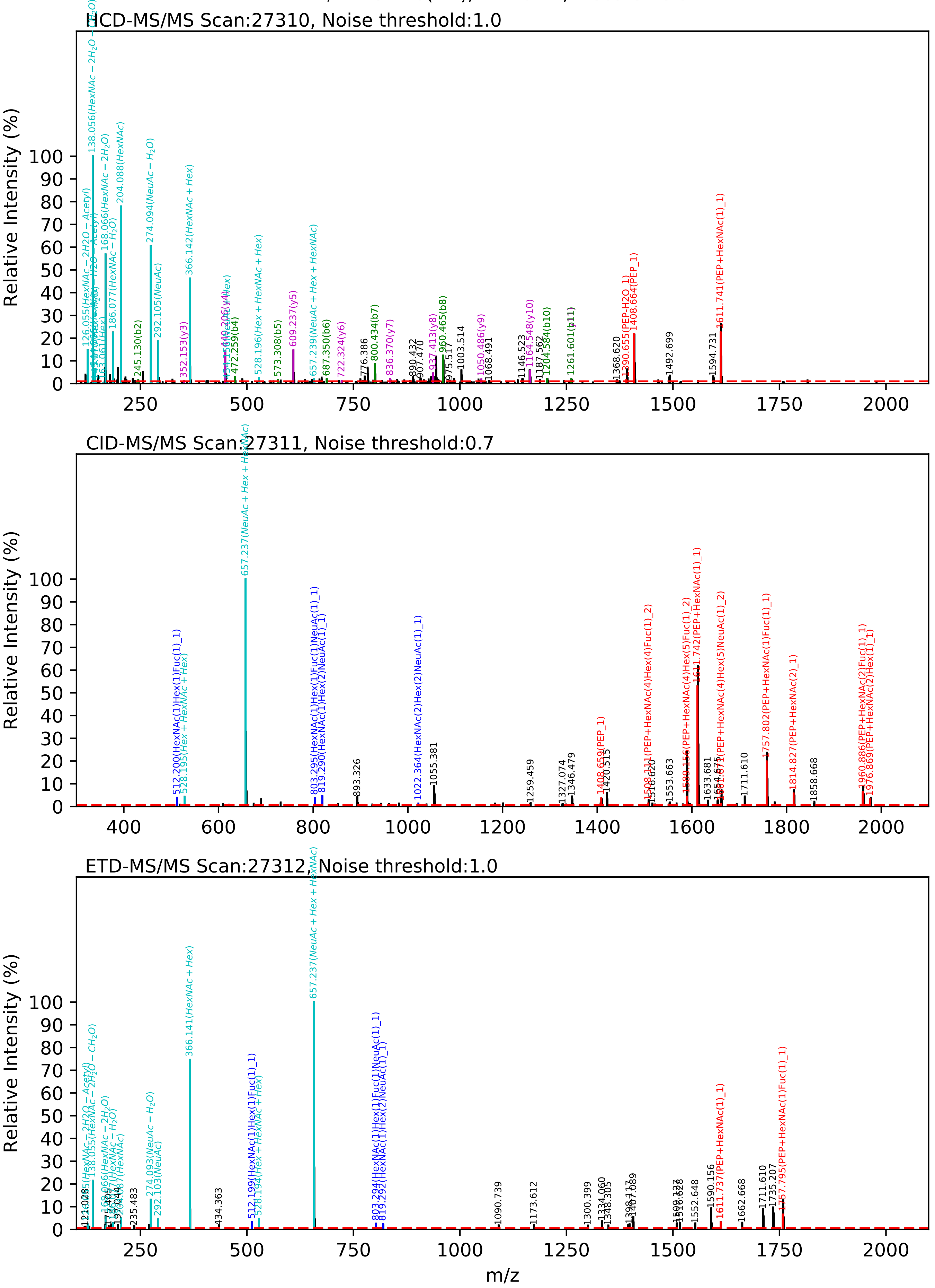

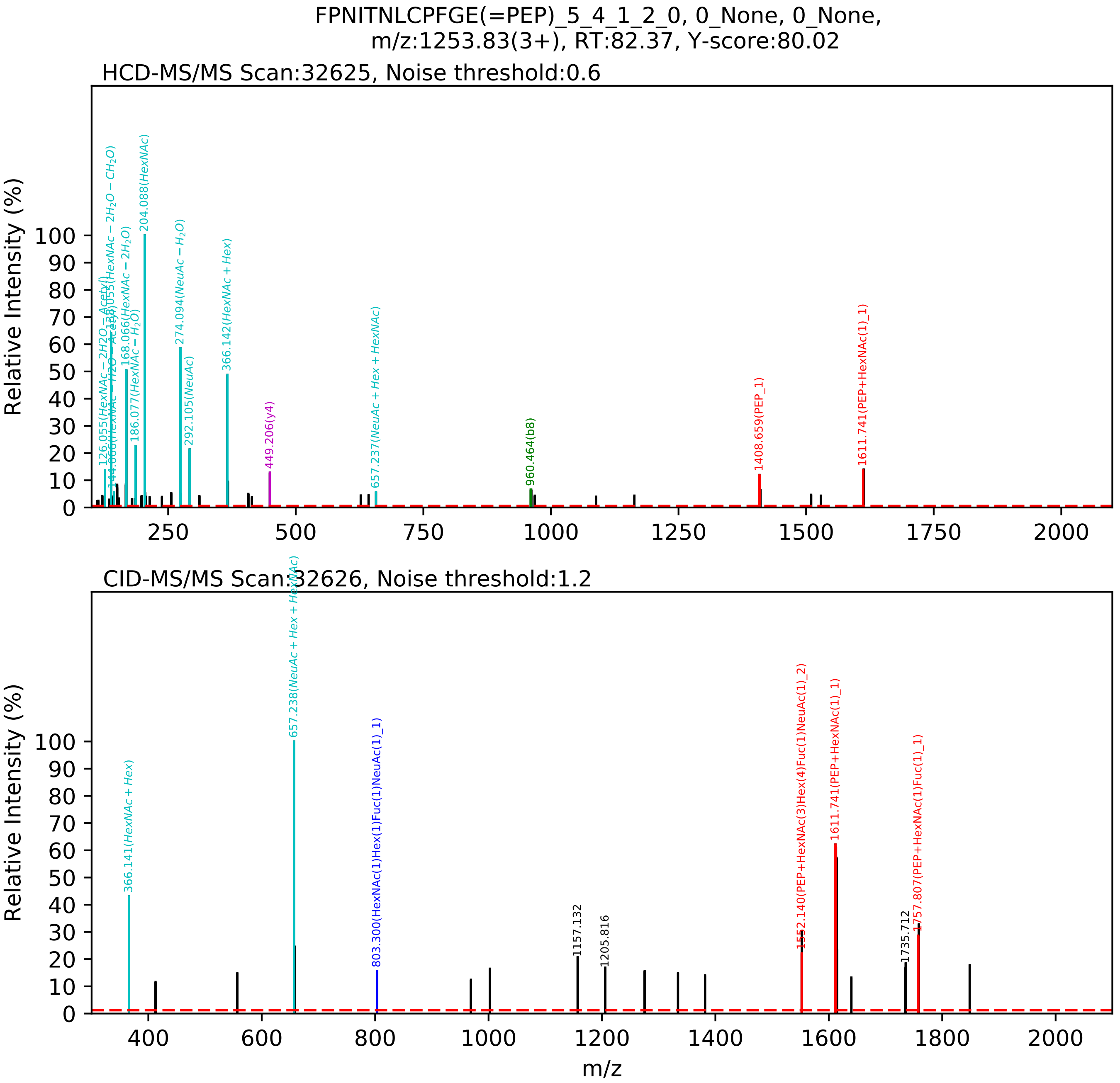
FPNITNLCPFGE(=PEP)_5_4_1_2_0, 0_None, 0_None,
m/z:1253.83(3+), RT:82.37, Y-score:80.02
HCD-MS/MS Scan:32625, Noise threshold:0.6
Relative Intensity (%)
204.088(HexNAc)
168.066(HexNAc − 2H2O)
186.077(HexNAc − H2O)
126.055(HexNAc − 2H2O − Acetyl)
274.094(NeuAc − H2O)
292.105(NeuAc)
366.142(HexNAc + Hex)
449.206(y4)
657.237(NeuAc + Hex + HexNAc)
960.464(b8)
1408.659(PEP_1)
1611.741(PEP+HexNAc(1)_1)
CID-MS/MS Scan:32626, Noise threshold:1.2
366.141(HexNAc + Hex)
657.238(NeuAc + Hex + HexNAc)
803.300(HexNAc(1)Hex(1)Fuc(1)NeuAc(1)_1)
1157.132
1205.816
1552.140(PEP+HexNAc(3)Hex(4)Fuc(1)NeuAc(1)_2)
1611.741(PEP+HexNAc(1)_1)
1735.712
1757.807(PEP+HexNAc(1)Fuc(1)_1)
m/z

FPNITNLCPFGE(=PEP)_5_4_1_2_0, 0_None, 0_None,
m/z:1253.83(3+), RT:85.56, Y-score:82.41

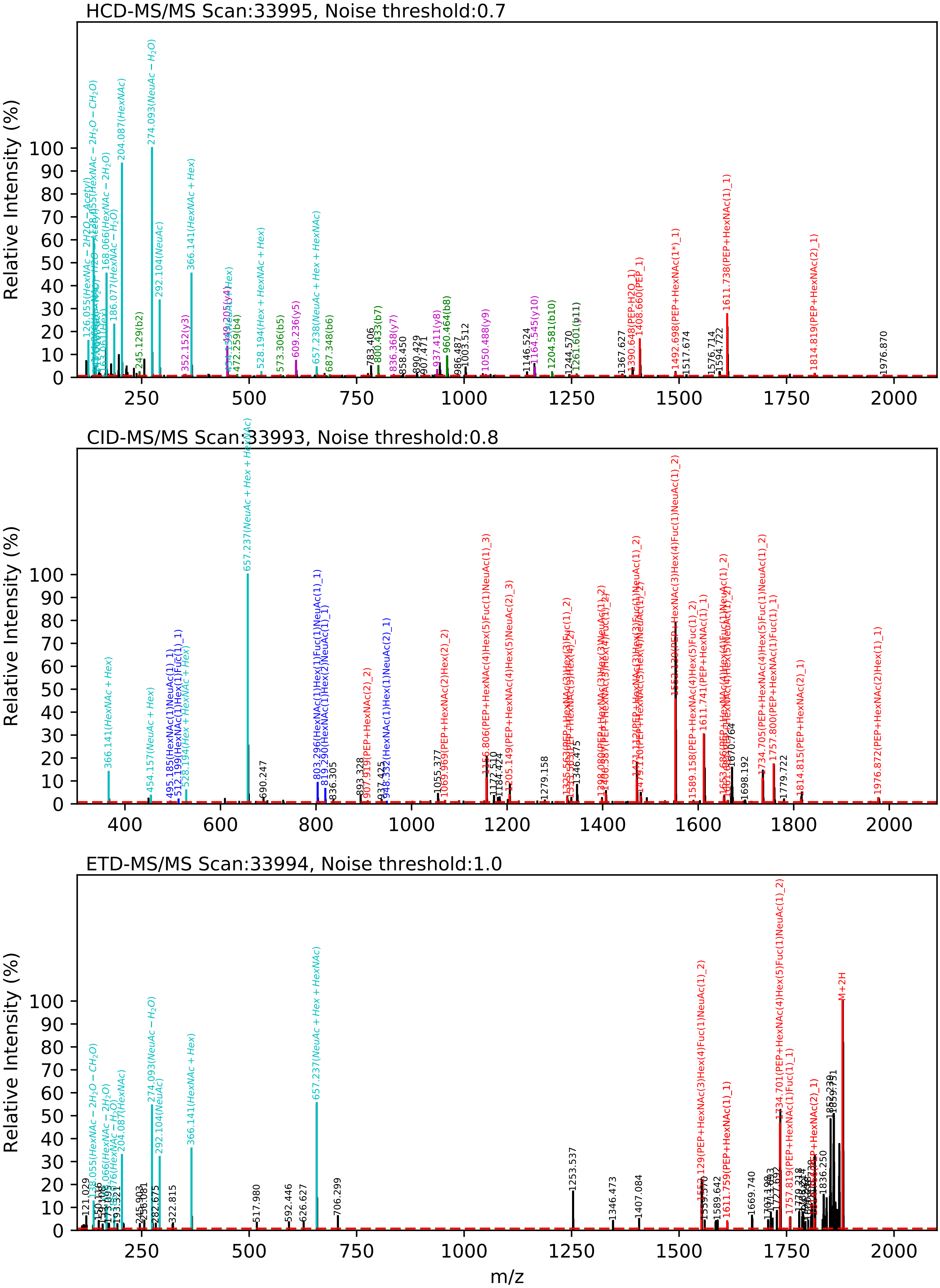

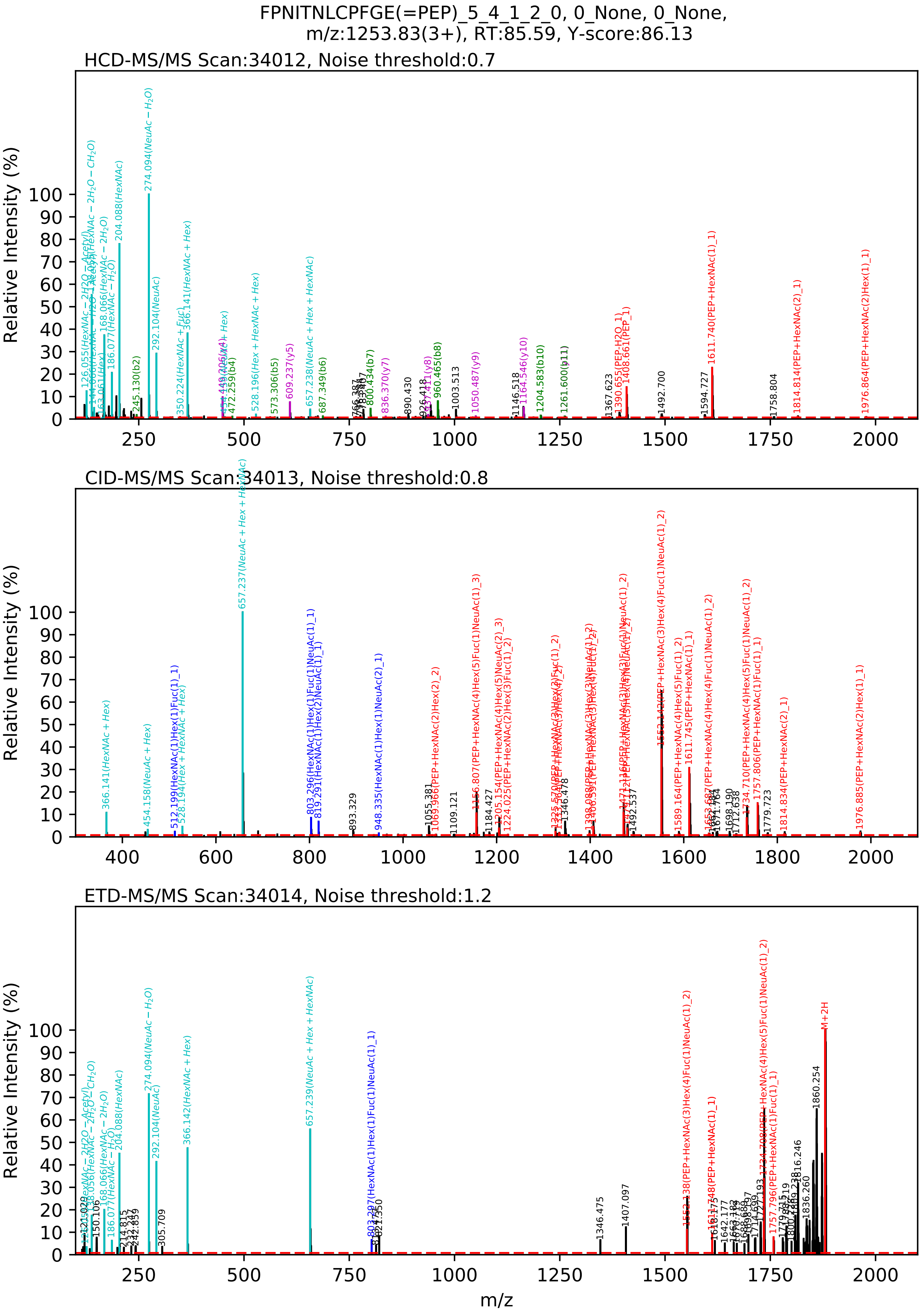
FPNITNLCPFGE(=PEP)_5_4_1_2_0, 0_None, 0_None,
m/z:1253.83(3+), RT:85.59, Y-score:86.13
HCD-MS/MS Scan:34012, Noise threshold:0.7
CID-MS/MS Scan:34013, Noise threshold:0.8
ETD-MS/MS Scan:34014, Noise threshold:1.2
Relative Intensity (%)
m/z

FPNITNLCPFGE(=PEP)_5_4_1_2_0, 0_None, 0_None,
m/z:1253.83(3+), RT:86.14, Y-score:75.80

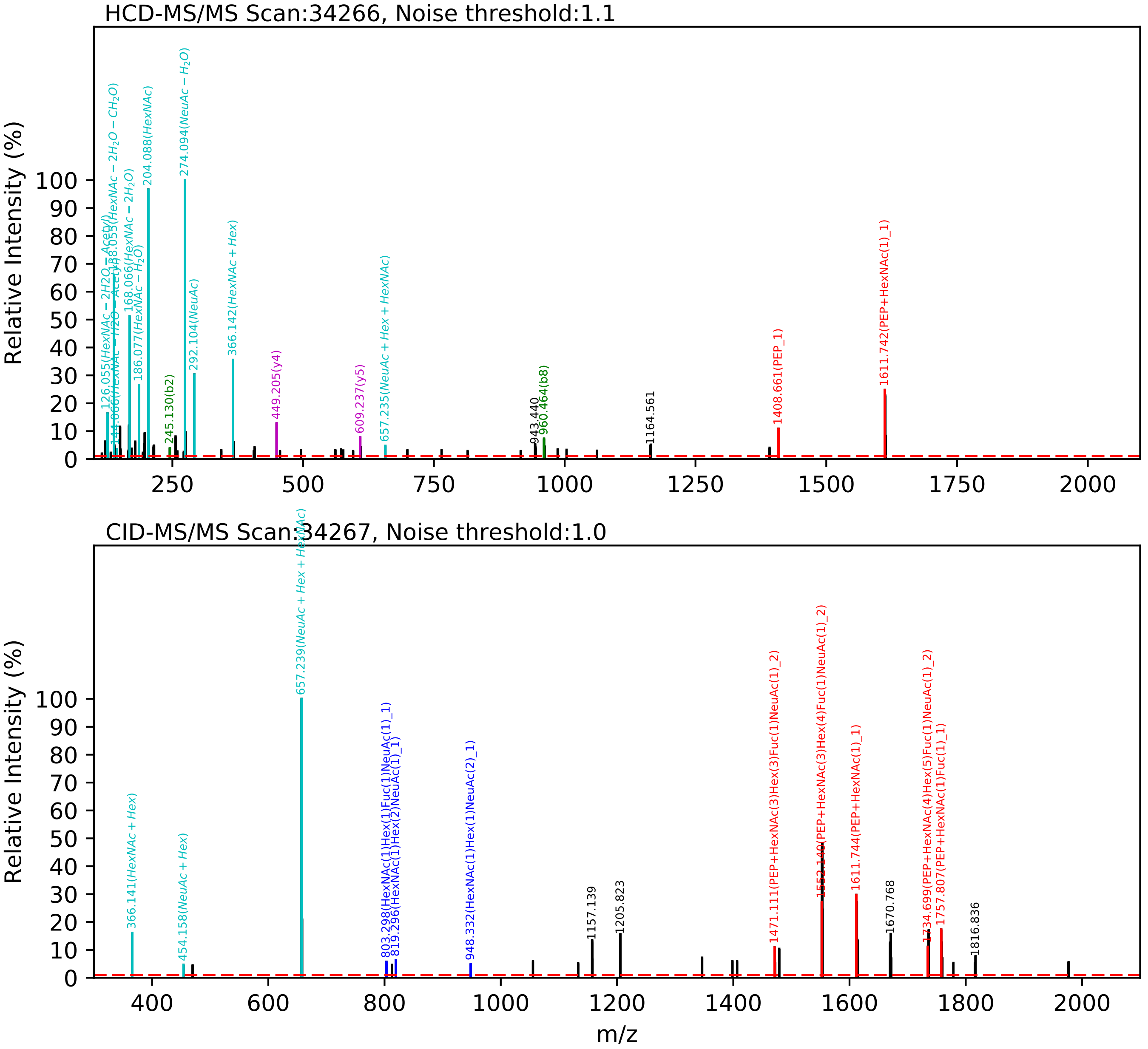

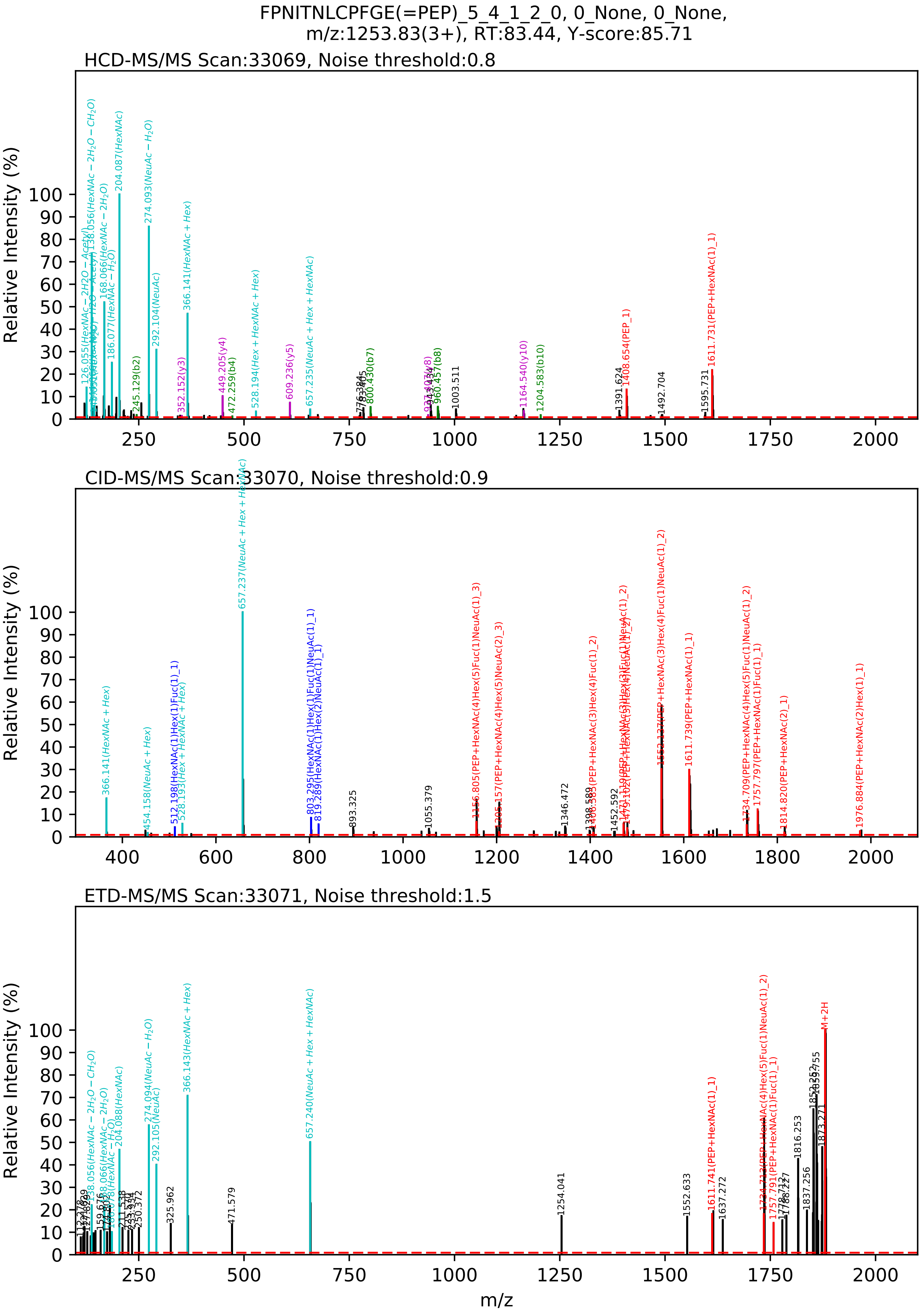

FPNITNLCPFGE(=PEP)_5_4_1_2_0, 0_None, 0_None,
m/z:1253.83(3+), RT:83.44, Y-score:85.71
HCD-MS/MS Scan:33069, Noise threshold:0.8
CID-MS/MS Scan:33070, Noise threshold:0.9
ETD-MS/MS Scan:33071, Noise threshold:1.5
Relative Intensity (%)
m/z

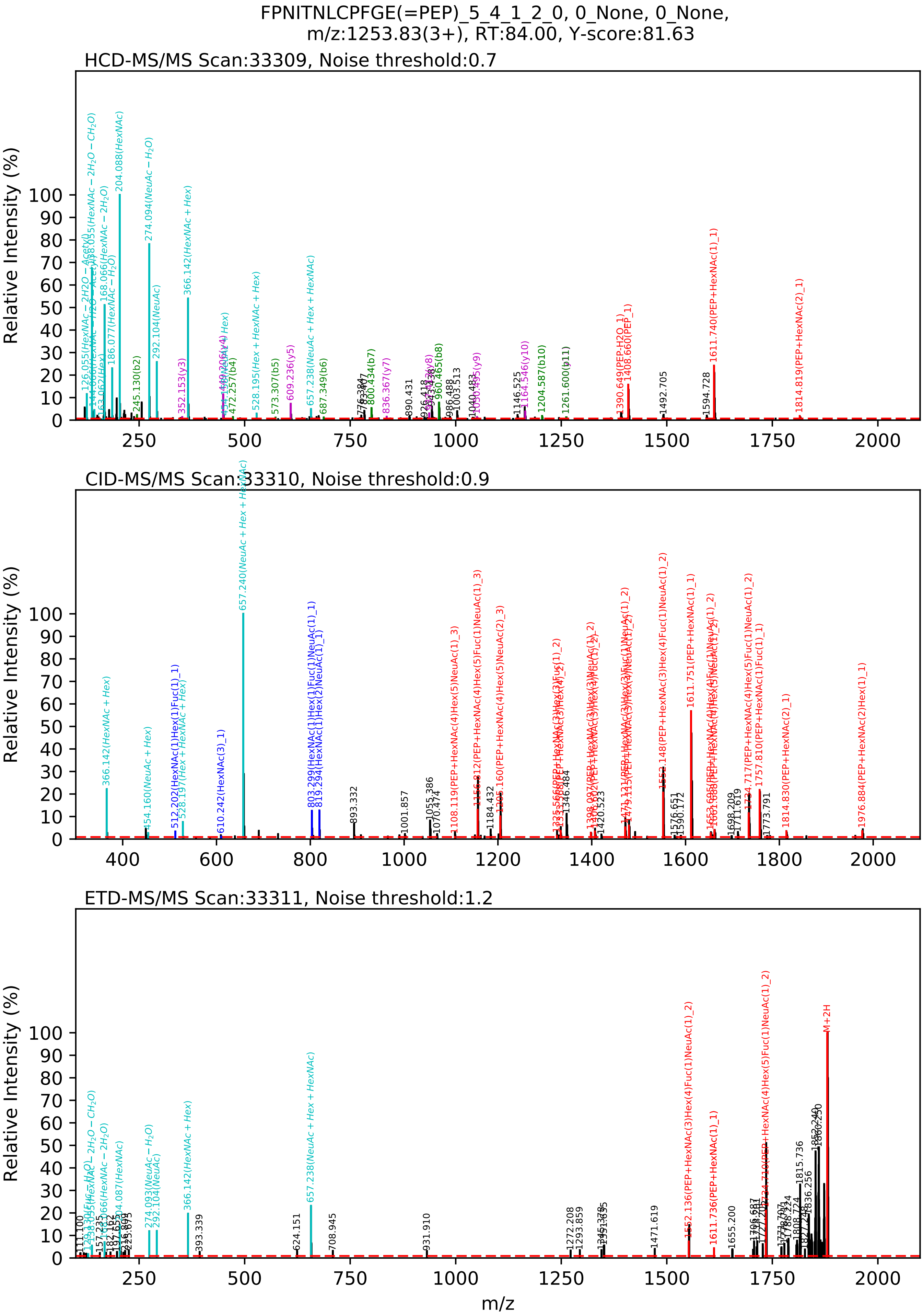

FPNITNLCPFGE(=PEP)_5_4_1_2_0, 0_None, 0_None,
m/z:1253.83(3+), RT:84.00, Y-score:81.63
HCD-MS/MS Scan:33309, Noise threshold:0.7
CID-MS/MS Scan:33310, Noise threshold:0.9
ETD-MS/MS Scan:33311, Noise threshold:1.2
Relative Intensity (%)
m/z

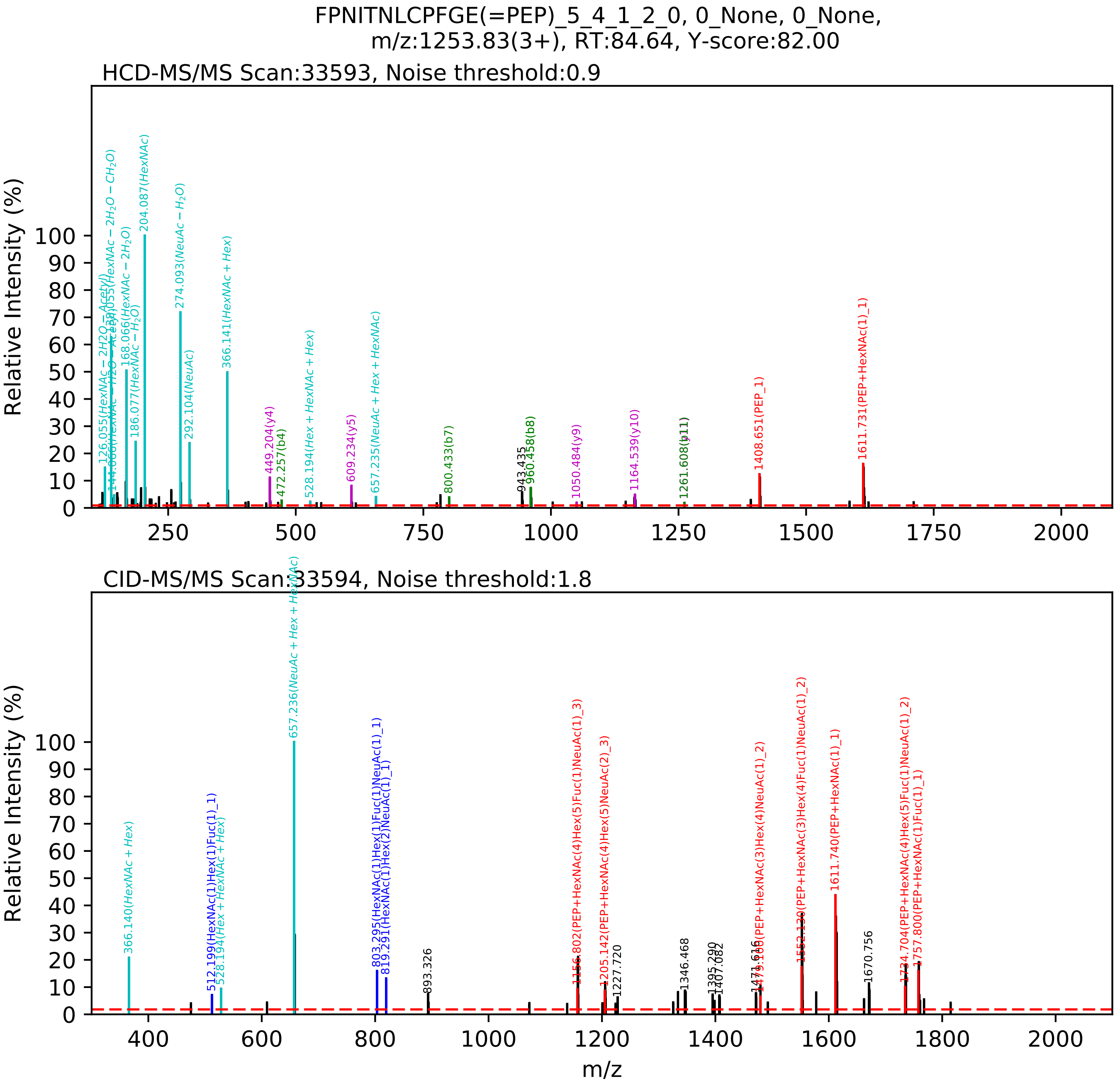
FPNITNLCPFGE(=PEP)_5_4_1_2_0, 0_None, 0_None,
m/z:1253.83(3+), RT:84.64, Y-score:82.00
HCD-MS/MS Scan:33593, Noise threshold:0.9
Relative Intensity (%)
204.087(HexNAc)
274.093(NeuAc − H2O)
366.141(HexNAc + Hex)
292.104(NeuAc)
186.077(HexNAc − H2O)
168.066(HexNAc − 2H2O)
126.055(HexNAc − 2H2O − Acetyl)
449.204(y4)
472.257(b4)
528.194(Hex + HexNAc + Hex)
609.234(y5)
657.235(NeuAc + Hex + HexNAc)
800.433(b7)
943.435
960.458(b8)
1050.484(y9)
1164.539(y10)
1261.608(b11)
1408.651(PEP_1)
1611.731(PEP+HexNAc(1)_1)
CID-MS/MS Scan:33594, Noise threshold:1.8
Relative Intensity (%)
366.140(HexNAc + Hex)
512.199(HexNAc(1)Hex(1)Fuc(1)_1)
528.194(Hex + HexNAc + Hex)
657.236(NeuAc + Hex + HexNAc)
803.295(HexNAc(1)Hex(1)Fuc(1)NeuAc(1)_1)
819.291(HexNAc(1)Hex(2)NeuAc(1)_1)
893.326
1156.802(PEP+HexNAc(4)Hex(5)Fuc(1)NeuAc(1)_3)
1205.142(PEP+HexNAc(4)Hex(5)NeuAc(2)_3)
1227.720
1346.468
1395.280
1407.082
1471.616
1479.100(PEP+HexNAc(3)Hex(4)NeuAc(1)_2)
1552.120(PEP+HexNAc(3)Hex(4)Fuc(1)NeuAc(1)_2)
1611.740(PEP+HexNAc(1)_1)
1670.756
1734.704(PEP+HexNAc(4)Hex(5)Fuc(1)NeuAc(1)_2)
1757.800(PEP+HexNAc(1)Fuc(1)_1)
m/z

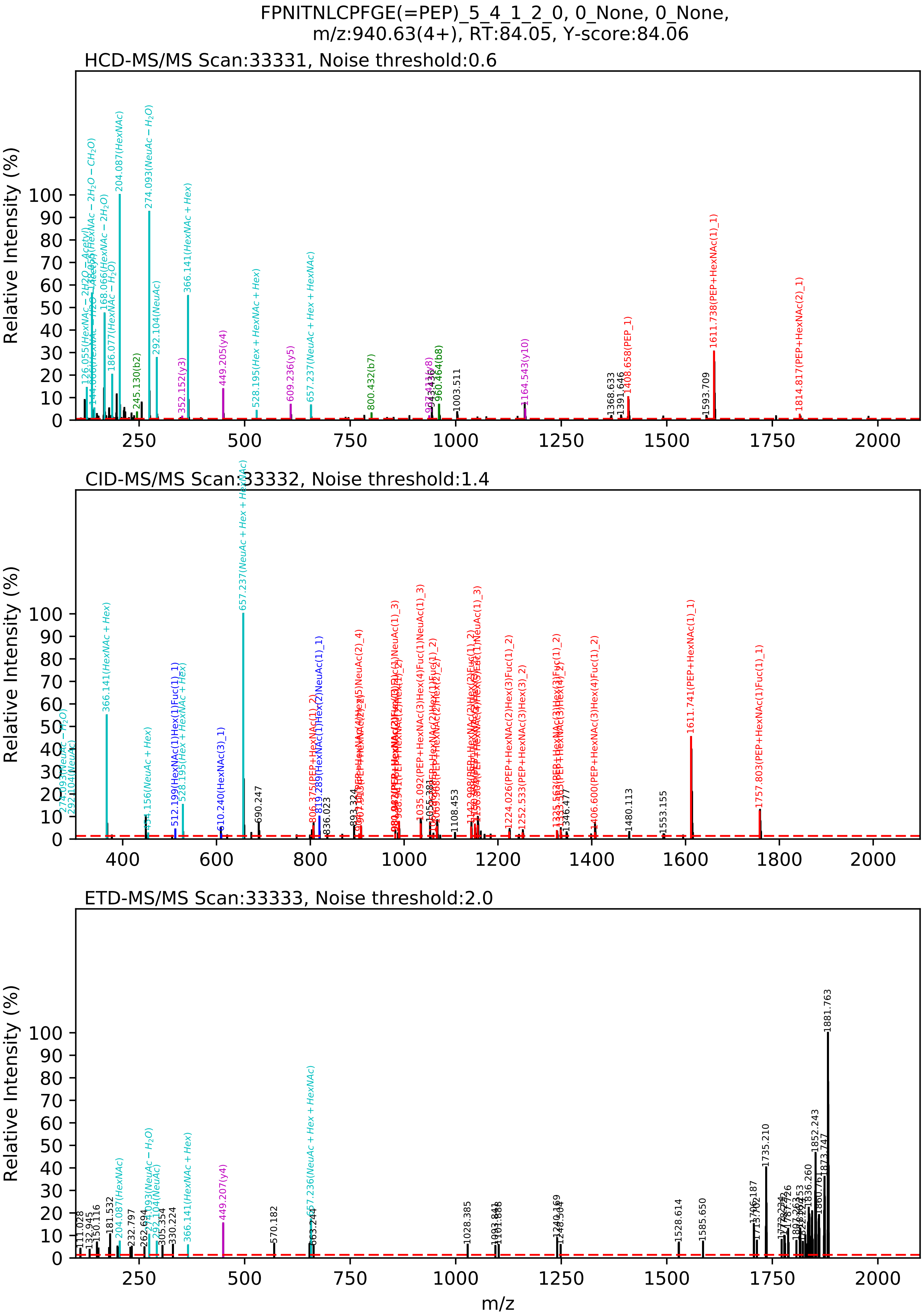
FPNITNLCPFGE(=PEP)_5_4_1_2_0, 0_None, 0_None,
m/z:940.63(4+), RT:84.05, Y-score:84.06
HCD-MS/MS Scan:33331, Noise threshold:0.6
Relative Intensity (%)
204.087(HexNAc)
274.093(NeuAc − H2O)
366.141(HexNAc + Hex)
292.104(NeuAc)
168.066(HexNAc − 2H2O)
186.077(HexNAc − H2O)
245.130(b2)
352.152(y3)
449.205(y4)
528.195(Hex + HexNAc + Hex)
609.236(y5)
657.237(NeuAc + Hex + HexNAc)
800.432(b7)
960.464(b8)
1003.511
1164.543(y10)
1368.633
1391.646
1408.658(PEP_1)
1593.709
1611.738(PEP+HexNAc(1)_1)
1814.817(PEP+HexNAc(2)_1)
CID-MS/MS Scan:33332, Noise threshold:1.4
Relative Intensity (%)
657.237(NeuAc + Hex + HexNAc)
366.141(HexNAc + Hex)
454.156(NeuAc + Hex)
512.199(HexNAc(1)Hex(1)Fuc(1)_1)
528.195(Hex + HexNAc + Hex)
610.240(HexNAc(3)_1)
690.247
819.289(HexNAc(1)Hex(2)NeuAc(1)_1)
836.023
893.324
1035.092(PEP+HexNAc(3)Hex(4)Fuc(1)NeuAc(1)_3)
1108.453
1224.026(PEP+HexNAc(2)Hex(3)Fuc(1)_2)
1252.533(PEP+HexNAc(3)Hex(3)_2)
1346.477
1406.600(PEP+HexNAc(3)Hex(4)Fuc(1)_2)
1480.113
1553.155
1611.741(PEP+HexNAc(1)_1)
1757.803(PEP+HexNAc(1)Fuc(1)_1)
ETD-MS/MS Scan:33333, Noise threshold:2.0
Relative Intensity (%)
1881.763
1735.210
1852.243
1873.747
1836.260
204.087(HexNAc)
274.093(NeuAc − H2O)
366.141(HexNAc + Hex)
449.207(y4)
570.182
657.236(NeuAc + Hex + HexNAc)
663.244
1028.385
1240.169
1248.504
1528.614
1585.650
1715.702
181.532
232.797
262.694
305.354
330.224
m/z

FPNITNLCPFGE(=PEP)_5_4_1_2_0, 0_None, 0_None,
m/z:940.63(4+), RT:85.76, Y-score:89.60

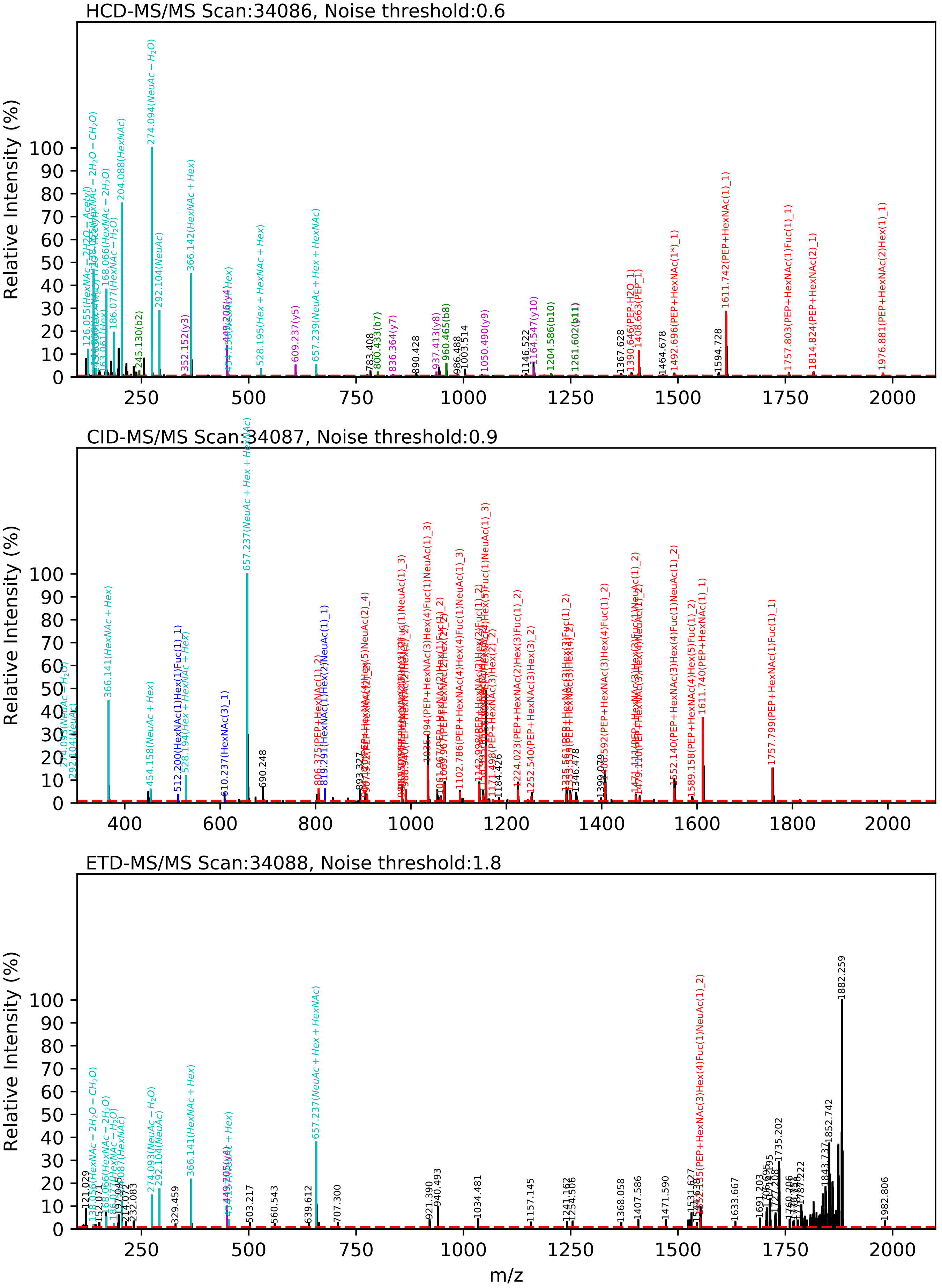

FPNITNLCPFGE(=PEP)_5_4_2_1_0, 0_None, 0_None,
m/z:1205.49(3+), RT:69.75, Y-score:90.29

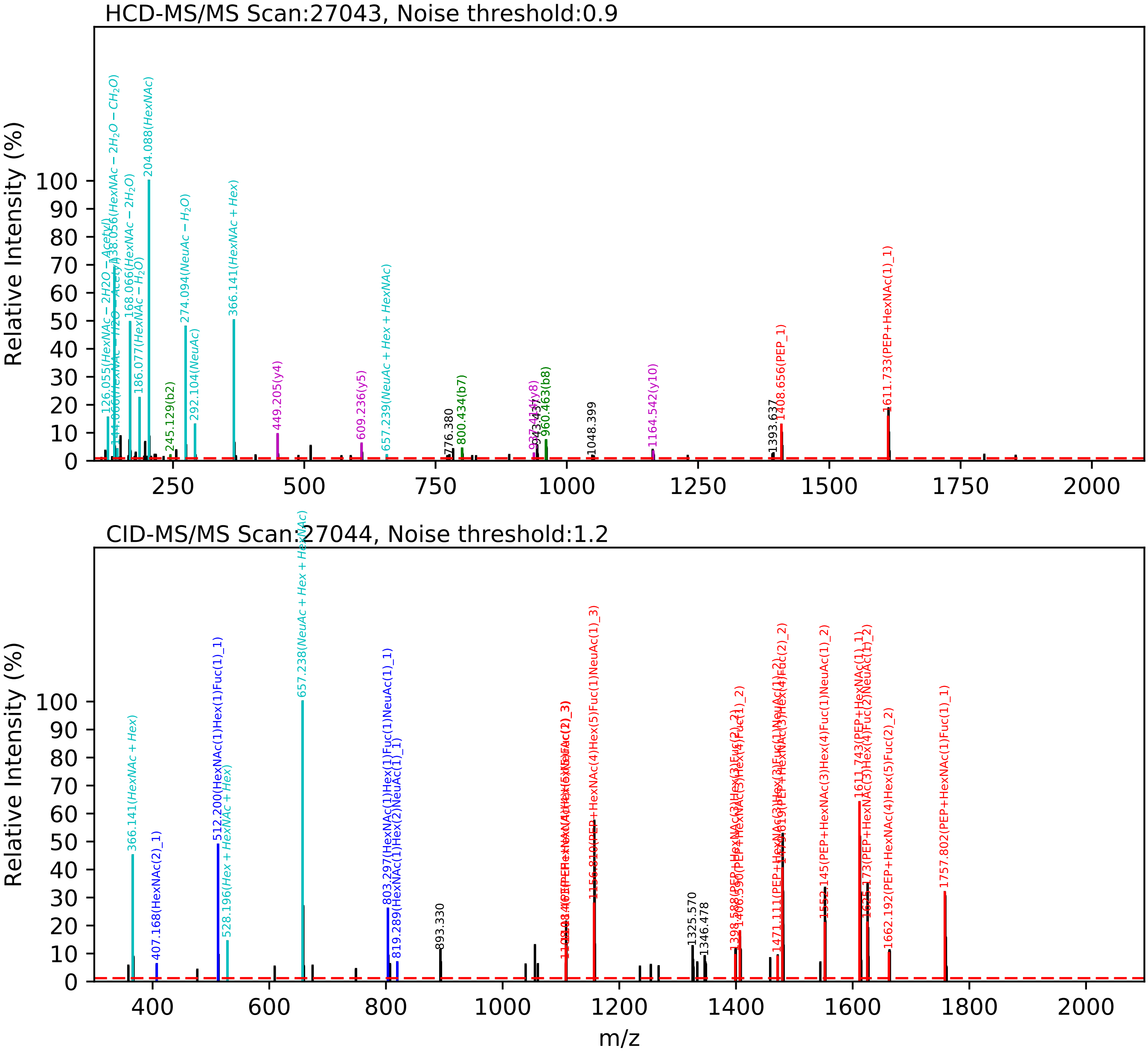

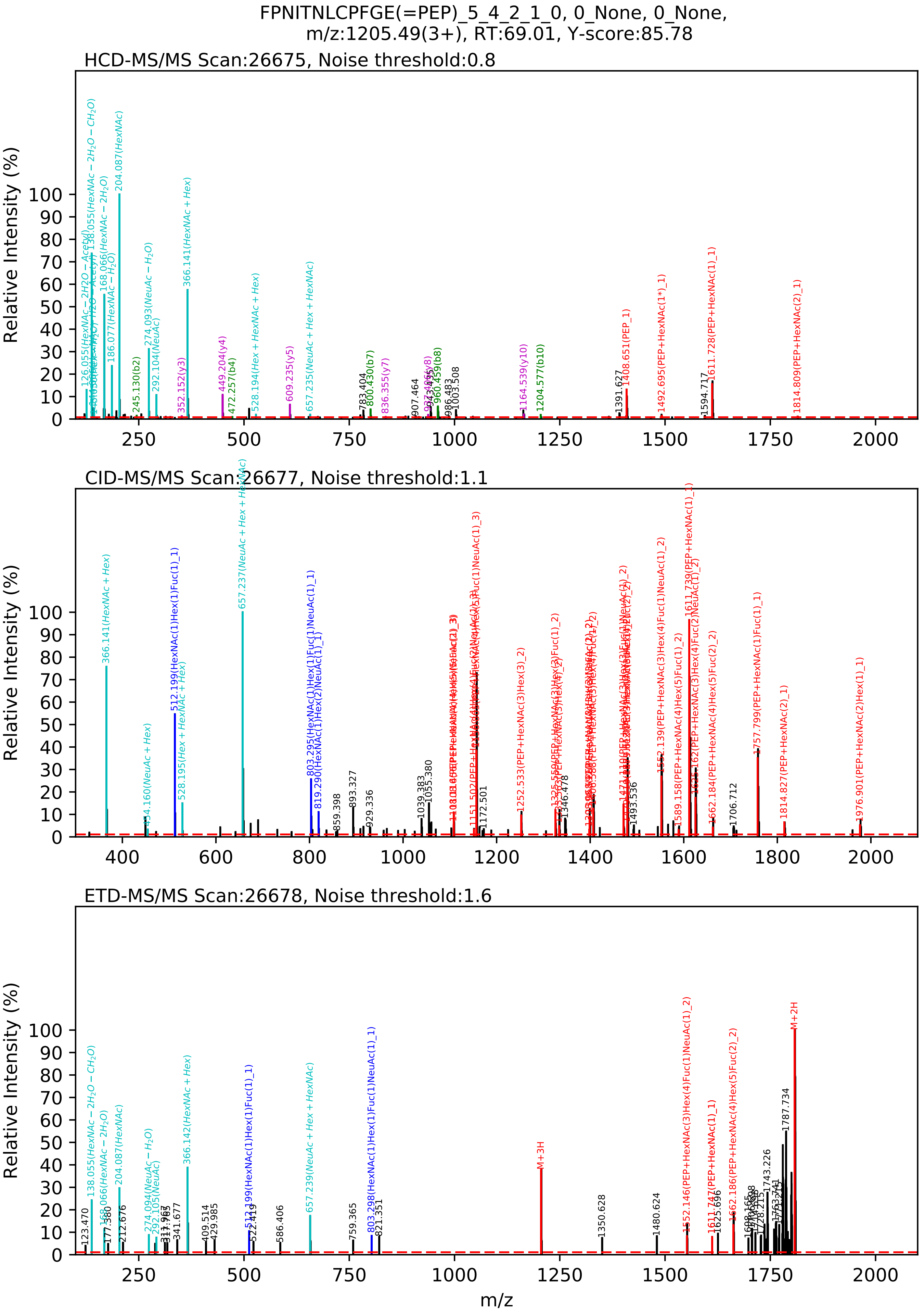
FPNITNLCPFGE(=PEP)_5_4_2_1_0, 0_None, 0_None,
m/z:1205.49(3+), RT:69.01, Y-score:85.78
HCD-MS/MS Scan:26675, Noise threshold:0.8
CID-MS/MS Scan:26677, Noise threshold:1.1
ETD-MS/MS Scan:26678, Noise threshold:1.6
Relative Intensity (%)
m/z

FPNITNLCPFGE(=PEP)_5_4_2_2_0, 0_None, 0_None,
m/z:1302.52(3+), RT:85.00, Y-score:85.67

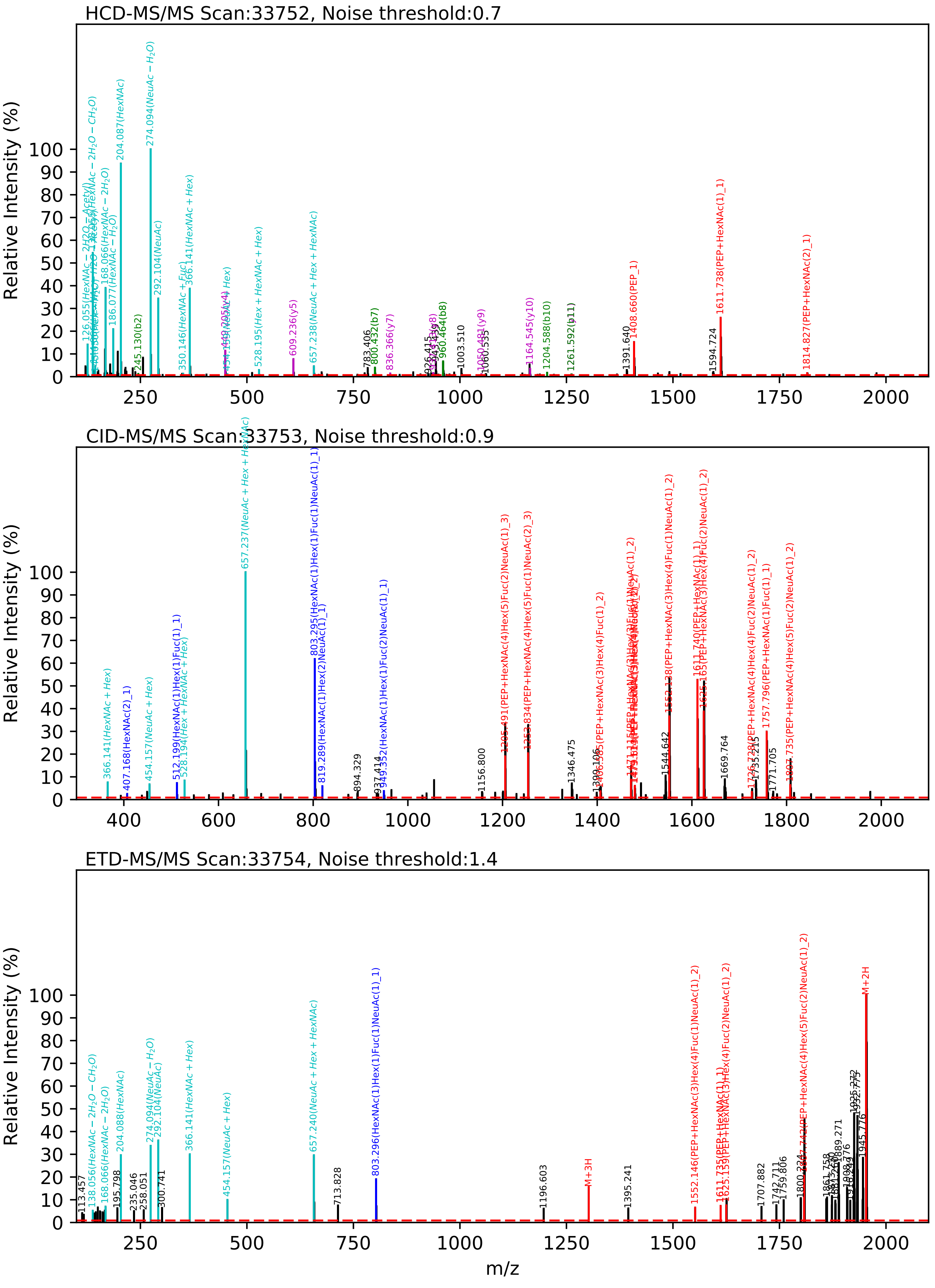

FPNITNLCPFGE(=PEP)_5_4_3_1_0, 0_None, 0_None,
m/z:1254.17(3+), RT:68.96, Y-score:87.12

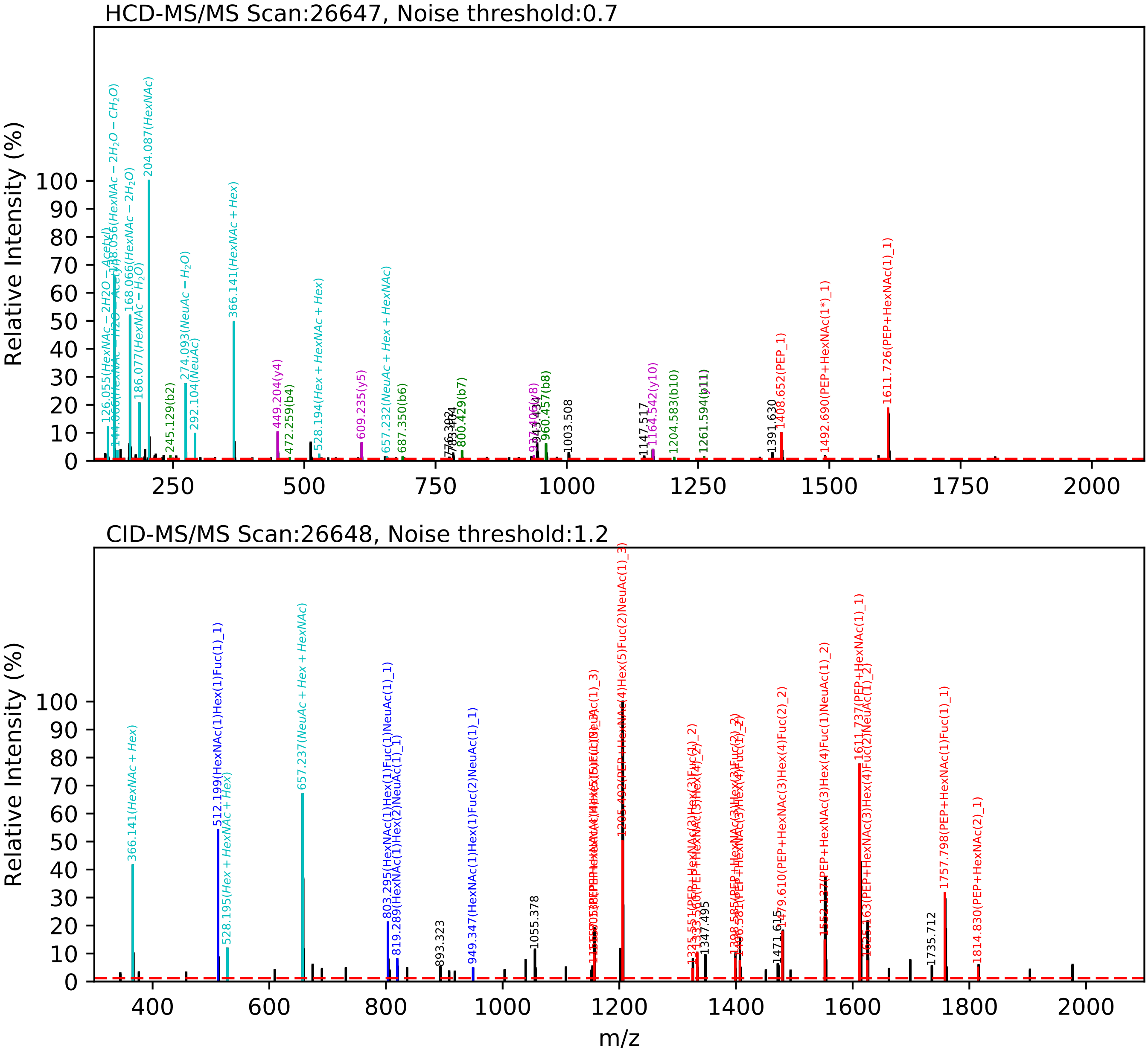

FPNITNLCPFGE(=PEP)_5_5_1_0_0, 0_None, 0_None,
m/z:1127.46(3+), RT:60.44, Y-score:81.92

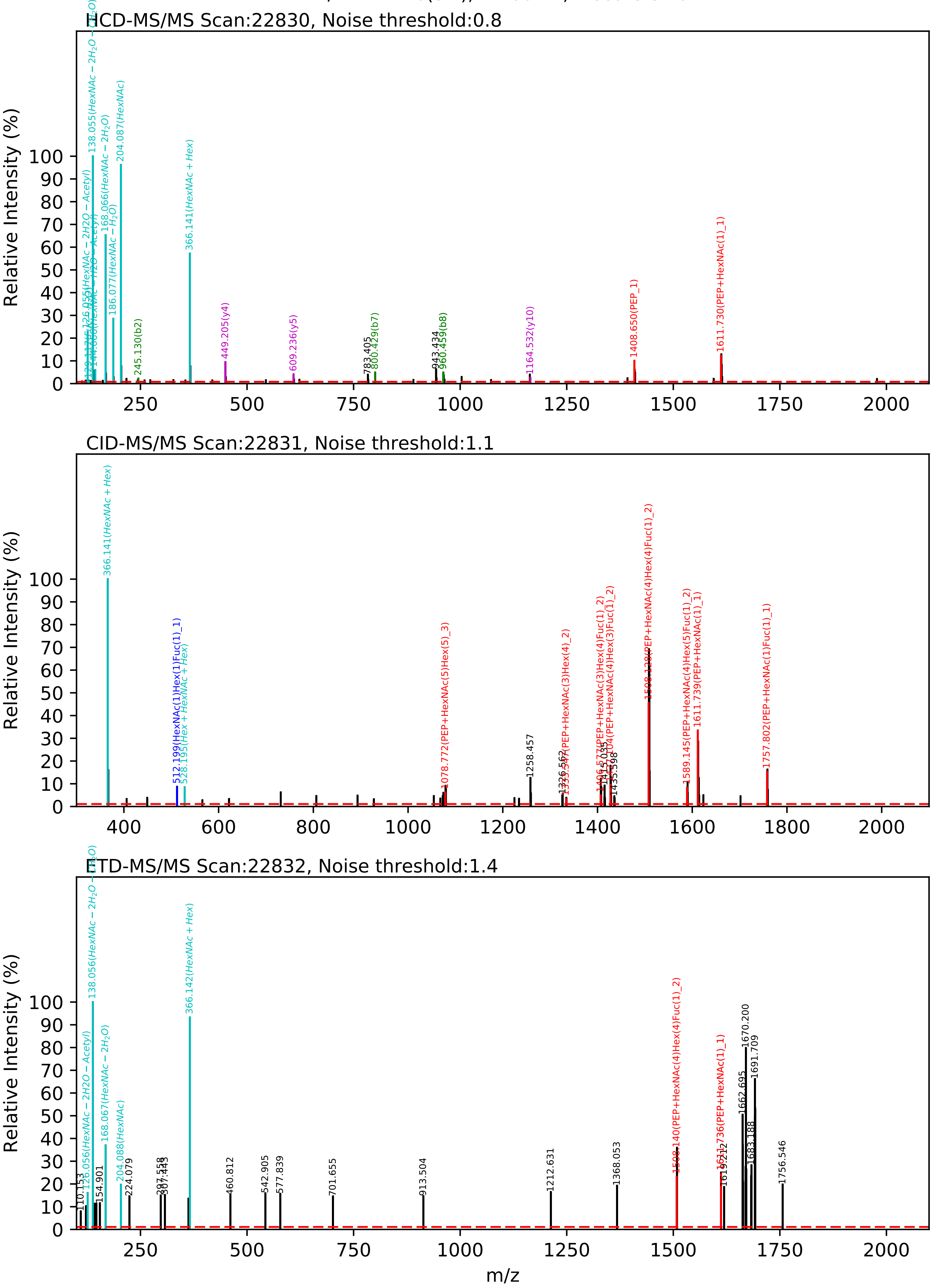

FPNITNLCPFGE(=PEP)_5_5_1_1_0, 0_None, 0_None,
m/z:1224.50(3+), RT:69.90, Y-score:93.04

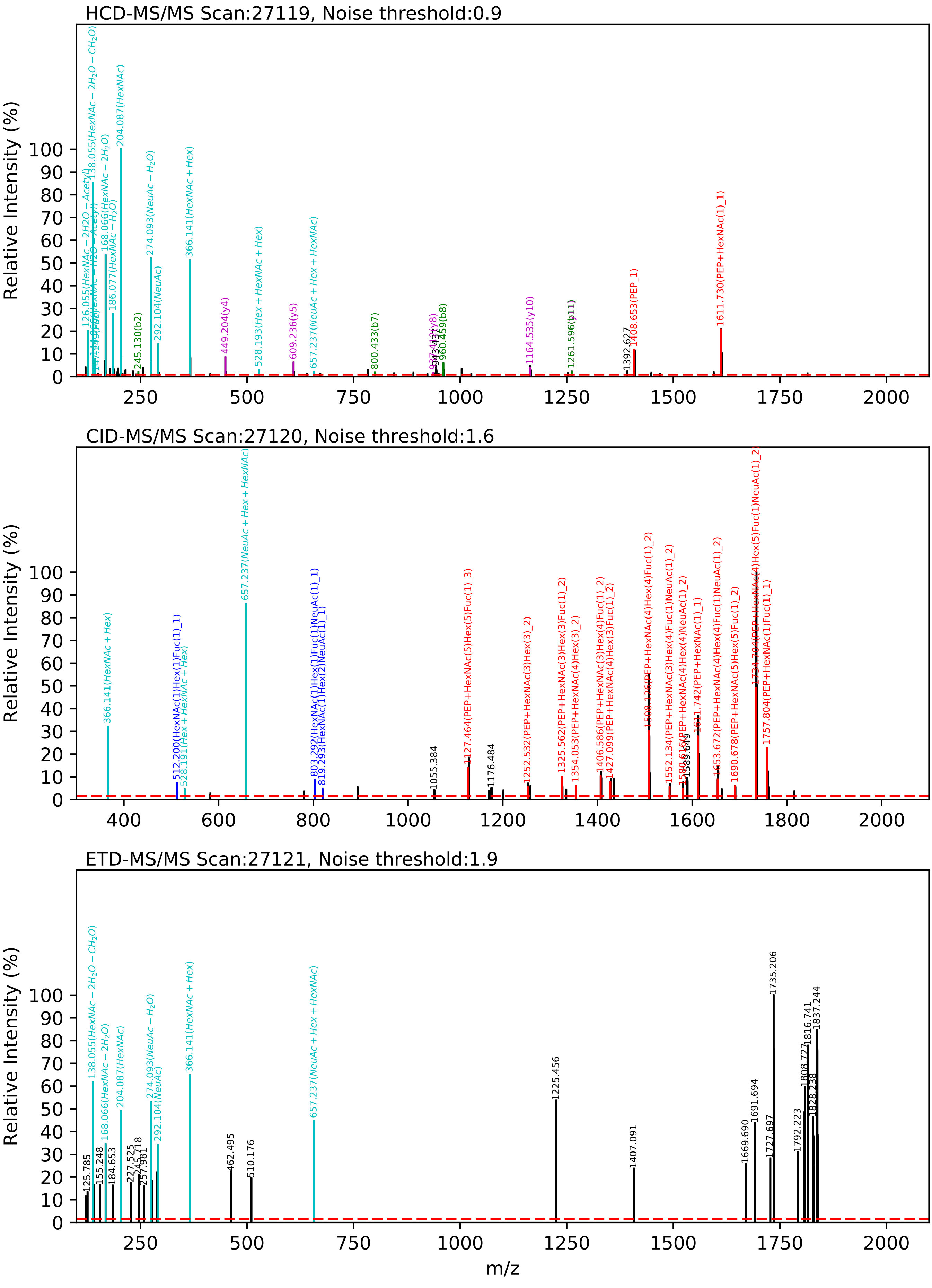

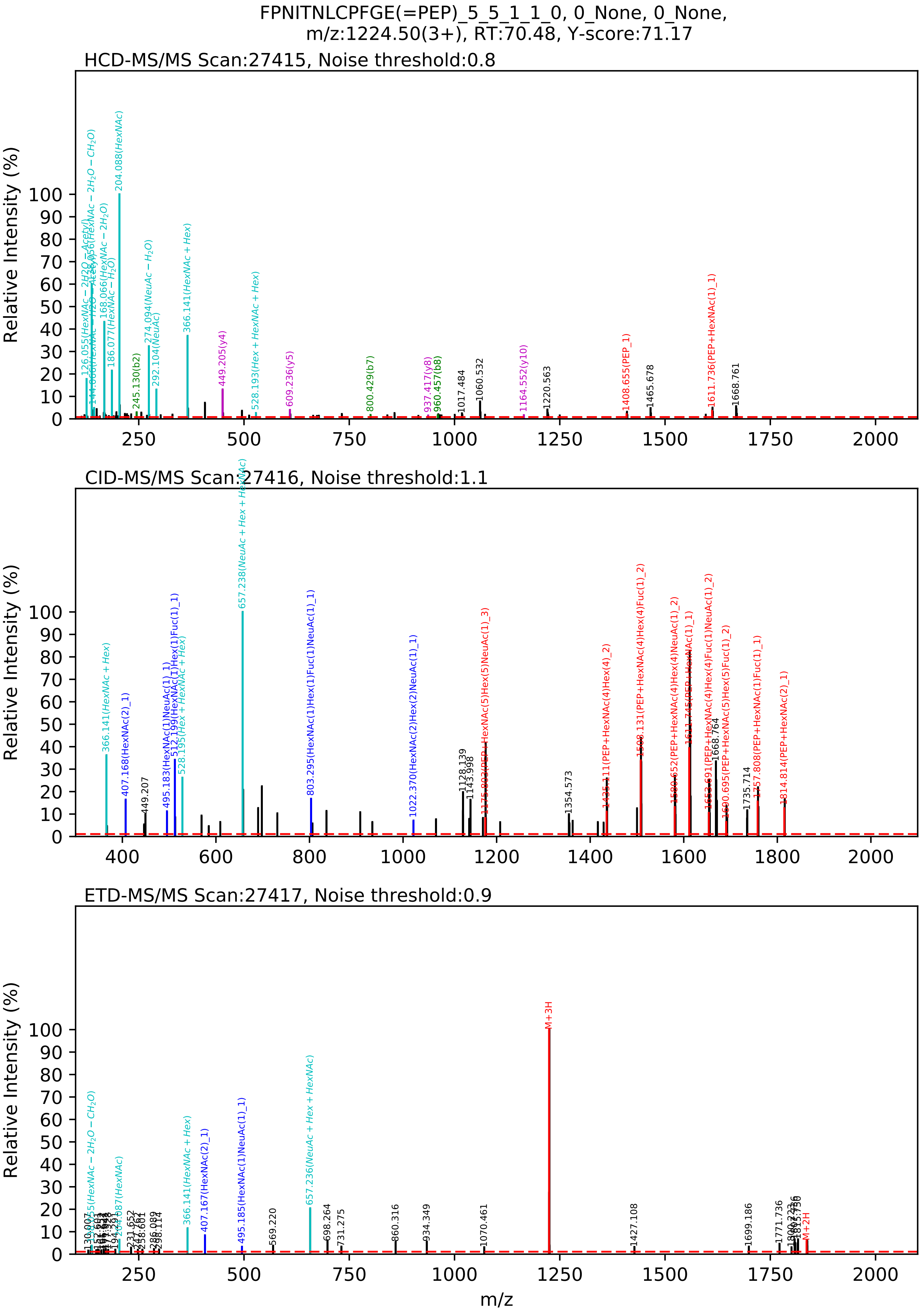

FPNITNLCPFGE(=PEP)_5_5_1_1_0, 0_None, 0_None,
m/z:1224.50(3+), RT:70.48, Y-score:71.17
HCD-MS/MS Scan:27415, Noise threshold:0.8
CID-MS/MS Scan:27416, Noise threshold:1.1
ETD-MS/MS Scan:27417, Noise threshold:0.9
Relative Intensity (%)
m/z

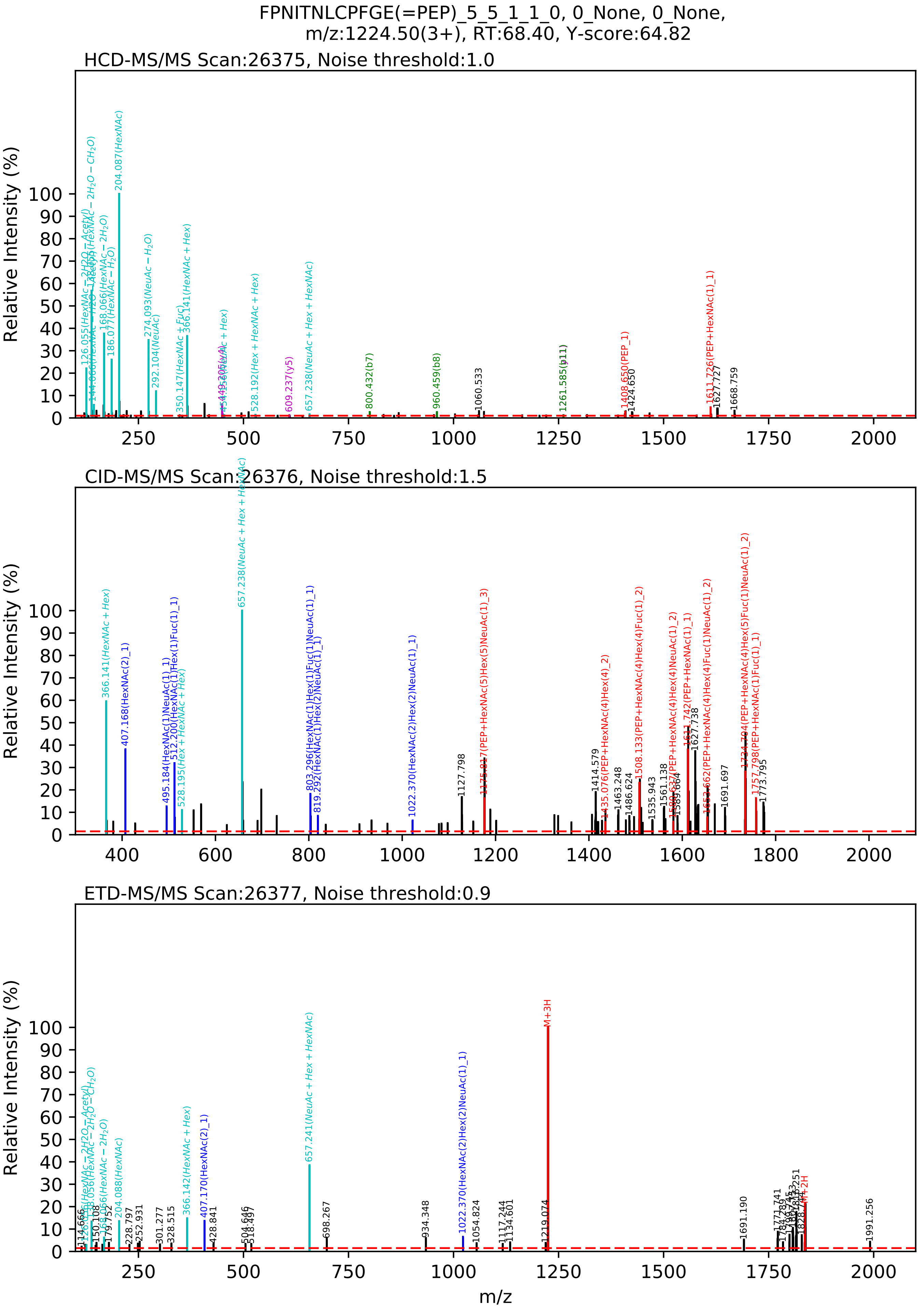

FPNITNLCPFGE(=PEP)_5_5_1_1_0, 0_None, 0_None,
m/z:1224.50(3+), RT:68.40, Y-score:64.82
HCD-MS/MS Scan:26375, Noise threshold:1.0
CID-MS/MS Scan:26376, Noise threshold:1.5
ETD-MS/MS Scan:26377, Noise threshold:0.9
Relative Intensity (%)
m/z
204.087(HexNAc)
274.093(NeuAc − H2O)
366.141(HexNAc + Hex)
657.238(NeuAc + Hex + HexNAc)
800.432(b7)
960.459(b8)
1060.533
1261.585(b11)
1408.650(PEP_1)
1611.726(PEP+HexNAc(1)_1)
407.168(HexNAc(2)_1)
495.184(HexNAc(1)NeuAc(1)_1)
512.200(HexNAc(1)Hex(1)Fuc(1)_1)
803.296(HexNAc(1)Hex(1)Fuc(1)NeuAc(1)_1)
819.292(HexNAc(1)Hex(2)NeuAc(1)_1)
1022.370(HexNAc(2)Hex(2)NeuAc(1)_1)
1175.817(PEP+HexNAc(5)Hex(5)NeuAc(1)_3)
1435.076(PEP+HexNAc(4)Hex(4)_2)
1508.133(PEP+HexNAc(4)Hex(4)Fuc(1)_2)
1611.742(PEP+HexNAc(1)_1)
1652.662(PEP+HexNAc(4)Hex(4)Fuc(1)NeuAc(1)_2)
1734.704(PEP+HexNAc(4)Hex(5)Fuc(1)NeuAc(1)_2)
1757.798(PEP+HexNAc(1)Fuc(1)_1)
M+3H
M+2H

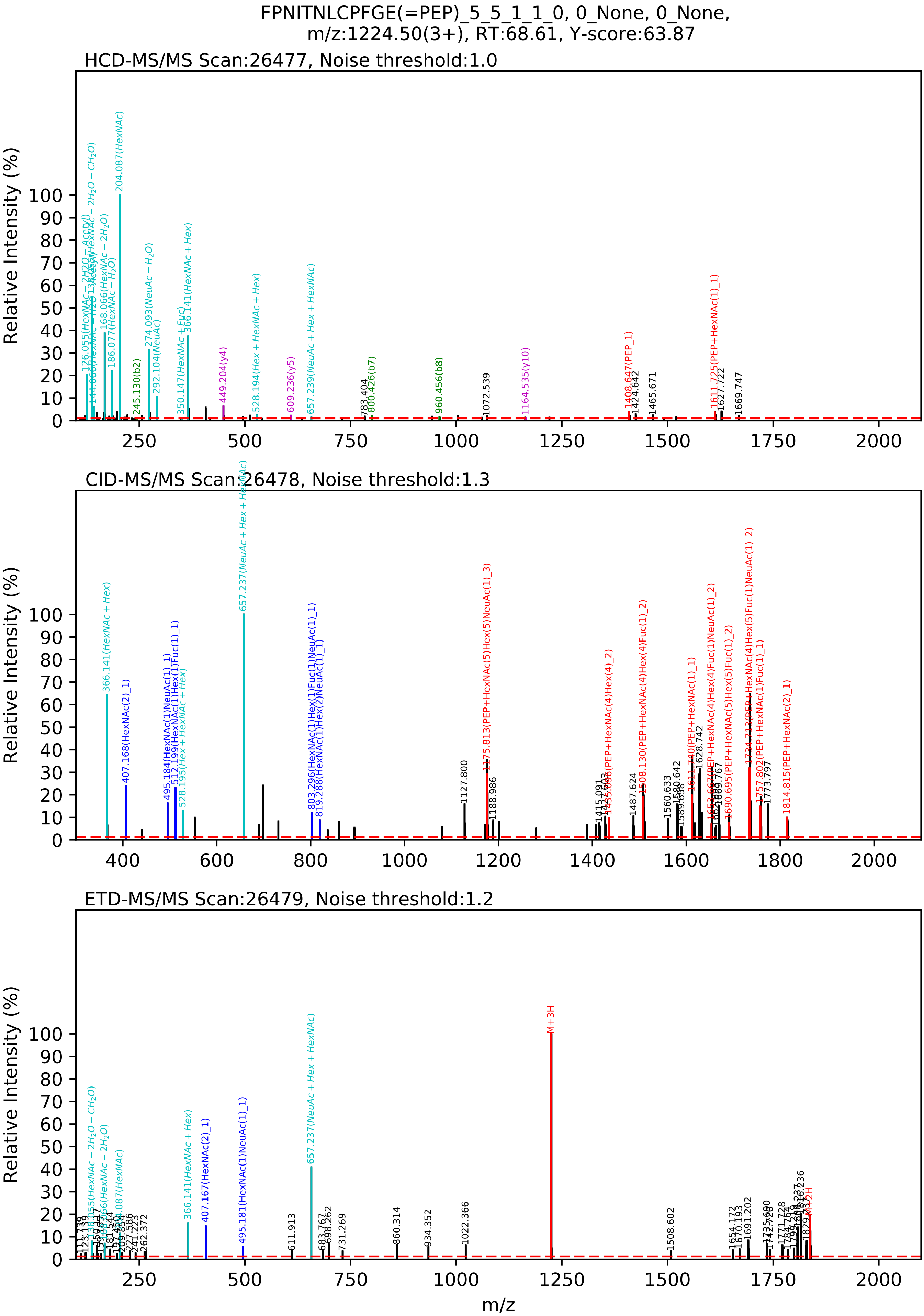

FPNITNLCPFGE(=PEP)_5_5_1_1_0, 0_None, 0_None,
m/z:1224.50(3+), RT:68.61, Y-score:63.87
HCD-MS/MS Scan:26477, Noise threshold:1.0
Relative Intensity (%)
204.087(HexNAc)
168.066(HexNAc − 2H2O)
186.077(HexNAc − H2O)
274.093(NeuAc − H2O)
292.104(NeuAc)
366.141(HexNAc + Hex)
350.147(HexNAc + Fuc)
245.130(b2)
449.204(y4)
528.194(Hex + HexNAc + Hex)
609.236(y5)
657.239(NeuAc + Hex + HexNAc)
783.404
800.426(b7)
960.456(b8)
1072.539
1164.535(y10)
1408.647(PEP_1)
1424.642
1465.671
1611.725(PEP+HexNAc(1)_1)
1627.722
1669.747
CID-MS/MS Scan:26478, Noise threshold:1.3
366.141(HexNAc + Hex)
407.168(HexNAc(2)_1)
495.184(HexNAc(1)NeuAc(1)_1)
512.199(HexNAc(1)Hex(1)Fuc(1)_1)
528.195(Hex + HexNAc + Hex)
657.237(NeuAc + Hex + HexNAc)
803.296(HexNAc(1)Hex(1)Fuc(1)NeuAc(1)_1)
819.288(HexNAc(1)Hex(2)NeuAc(1)_1)
1127.800
1175.813(PEP+HexNAc(5)Hex(5)NeuAc(1)_3)
1188.986
1435.098(PEP+HexNAc(4)Hex(4)_2)
1487.624
1508.130(PEP+HexNAc(4)Hex(4)Fuc(1)_2)
1560.633
1611.740(PEP+HexNAc(1)_1)
1628.742
1690.695(PEP+HexNAc(5)Hex(5)Fuc(1)_2)
1724.712(PEP+HexNAc(4)Hex(5)Fuc(1)NeuAc(1)_2)
1757.802(PEP+HexNAc(1)Fuc(1)_1)
1773.797
1814.815(PEP+HexNAc(2)_1)
ETD-MS/MS Scan:26479, Noise threshold:1.2
366.141(HexNAc + Hex)
407.167(HexNAc(2)_1)
495.181(HexNAc(1)NeuAc(1)_1)
657.237(NeuAc + Hex + HexNAc)
611.913
683.767
698.262
731.269
860.314
934.352
1022.366
M+3H
1508.602
1654.172
1691.202
1735.690
1771.728
M+2H
m/z

FPNITNLCPFGE(=PEP)_5_5_1_1_0, 0_None, 0_None,
m/z:1224.50(3+), RT:69.14, Y-score:90.78

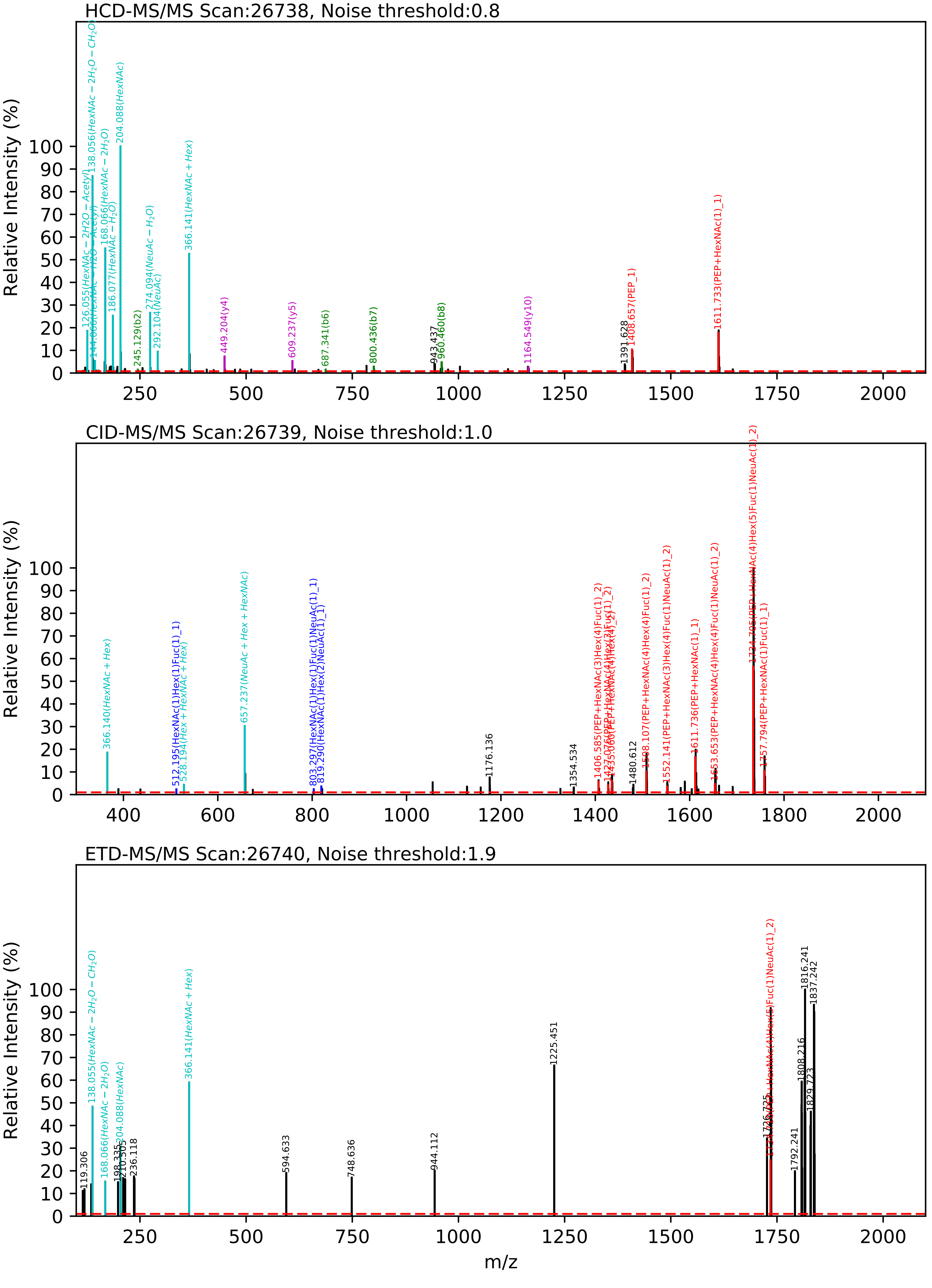

FPNITNLCPFGE(=PEP)_5_5_1_2_0, 0_None, 0_None,
m/z:1321.53(3+), RT:86.06, Y-score:81.12

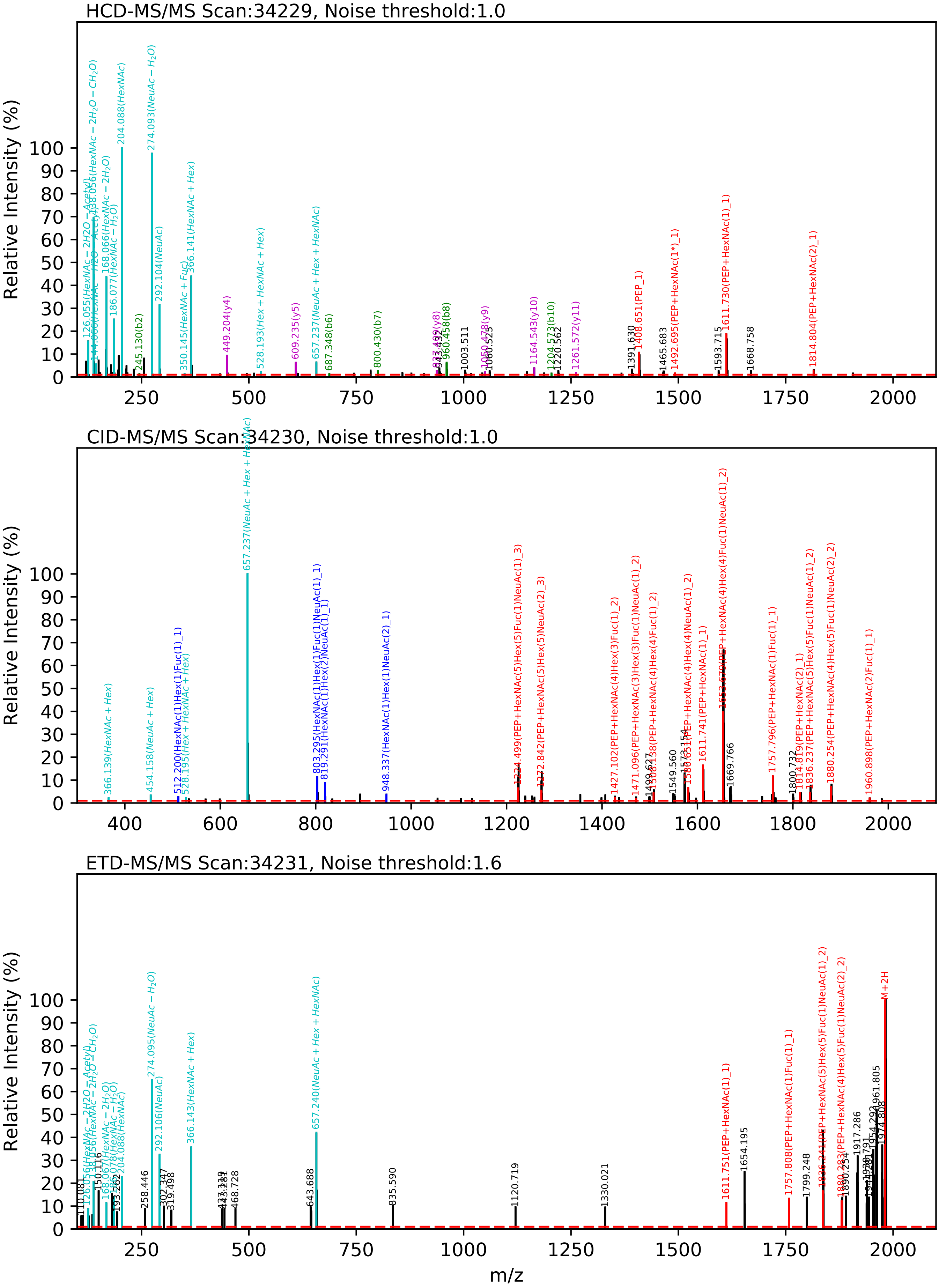

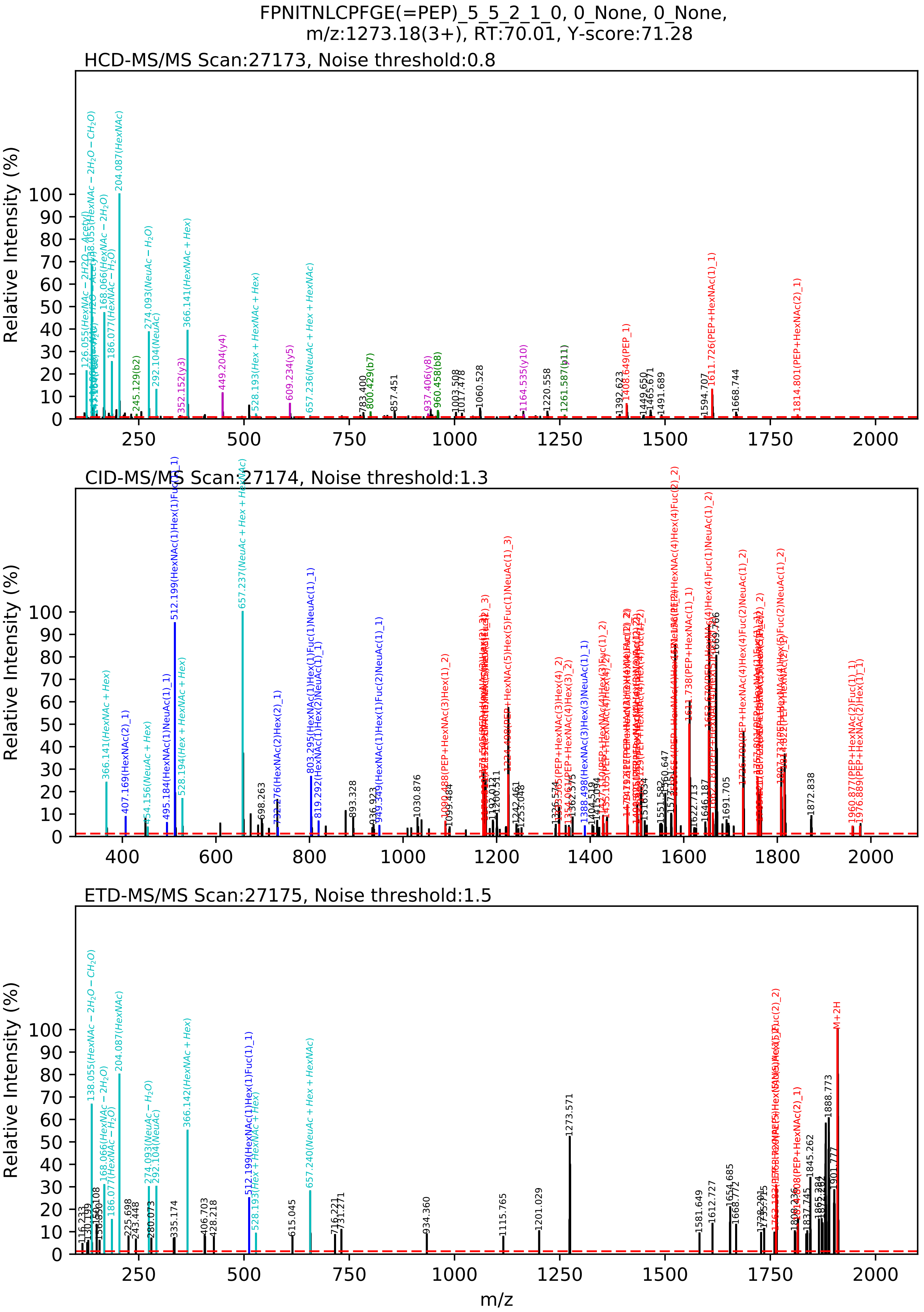
FPNITNLCPFGE(=PEP)_5_5_2_1_0, 0_None, 0_None,
m/z:1273.18(3+), RT:70.01, Y-score:71.28
HCD-MS/MS Scan:27173, Noise threshold:0.8
CID-MS/MS Scan:27174, Noise threshold:1.3
ETD-MS/MS Scan:27175, Noise threshold:1.5
Relative Intensity (%)
m/z

FPNITNLCPFGE(=PEP)_5_5_2_1_0, 0_None, 0_None,
m/z:1273.18(3+), RT:84.21, Y-score:83.64

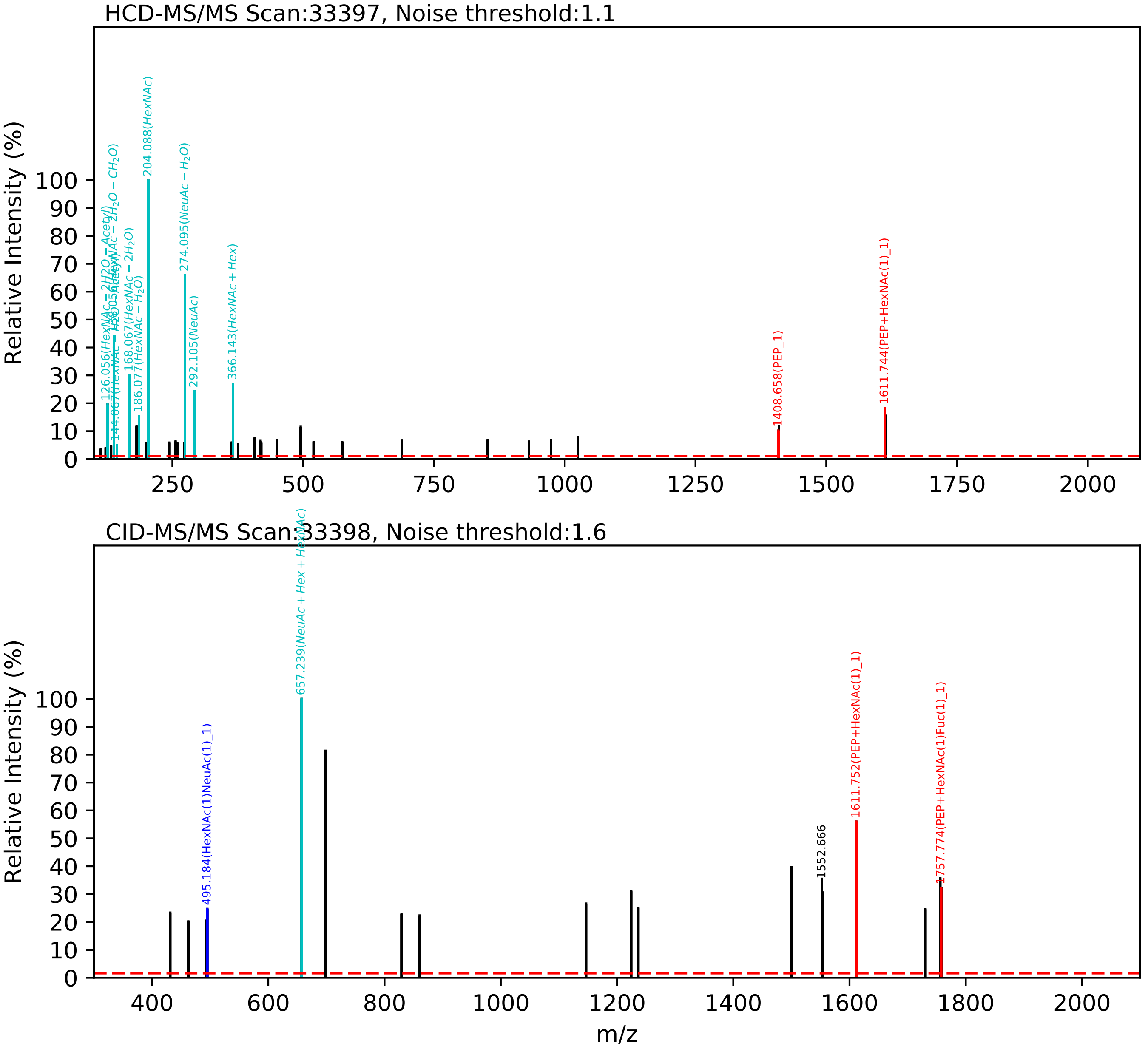

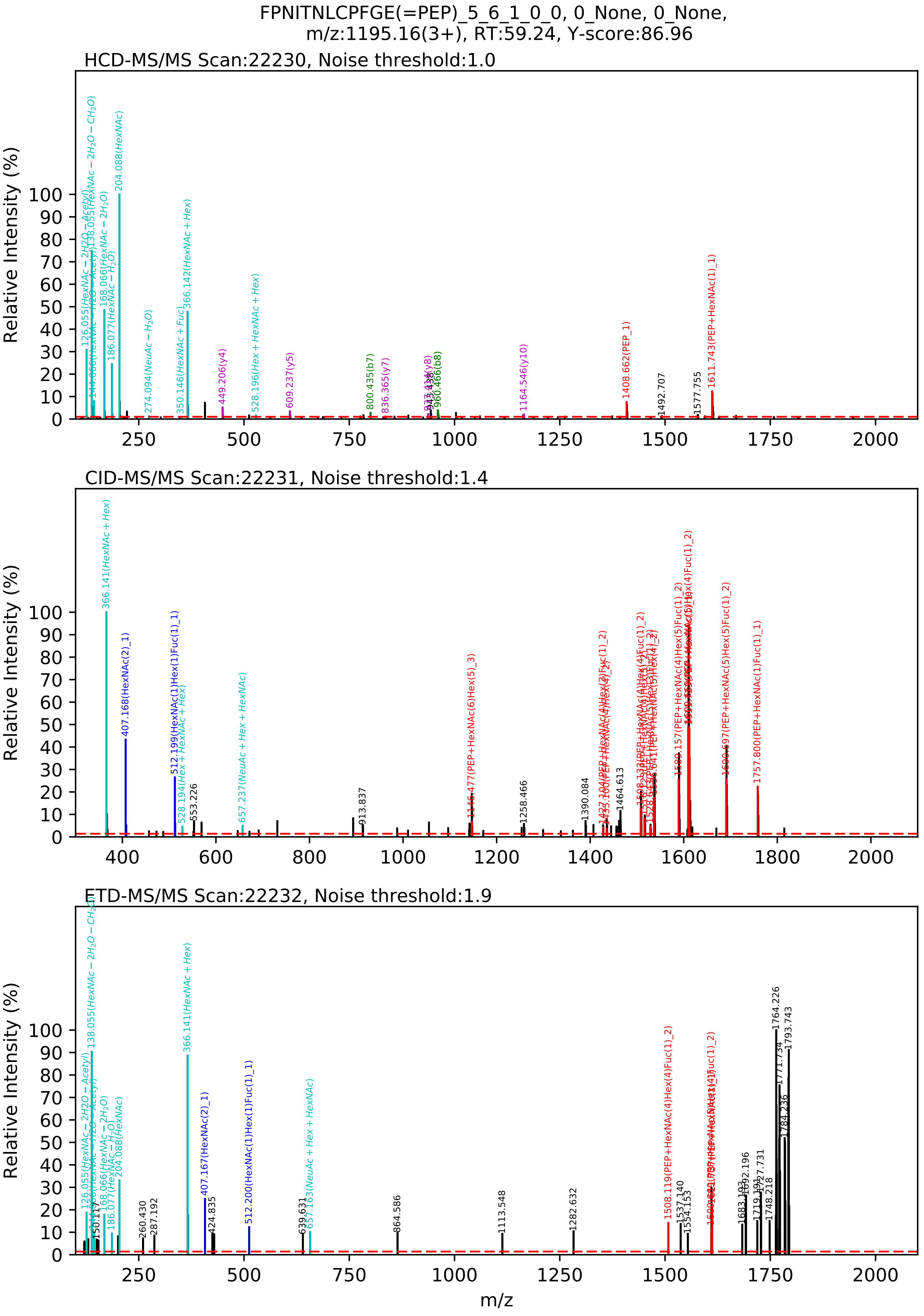

FPNITNLCPFGE(=PEP)_5_6_1_0_0, 0_None, 0_None,
m/z:1195.16(3+), RT:59.24, Y-score:86.96
HCD-MS/MS Scan:22230, Noise threshold:1.0
Relative Intensity (%)
CID-MS/MS Scan:22231, Noise threshold:1.4
Relative Intensity (%)
ETD-MS/MS Scan:22232, Noise threshold:1.9
Relative Intensity (%)
m/z

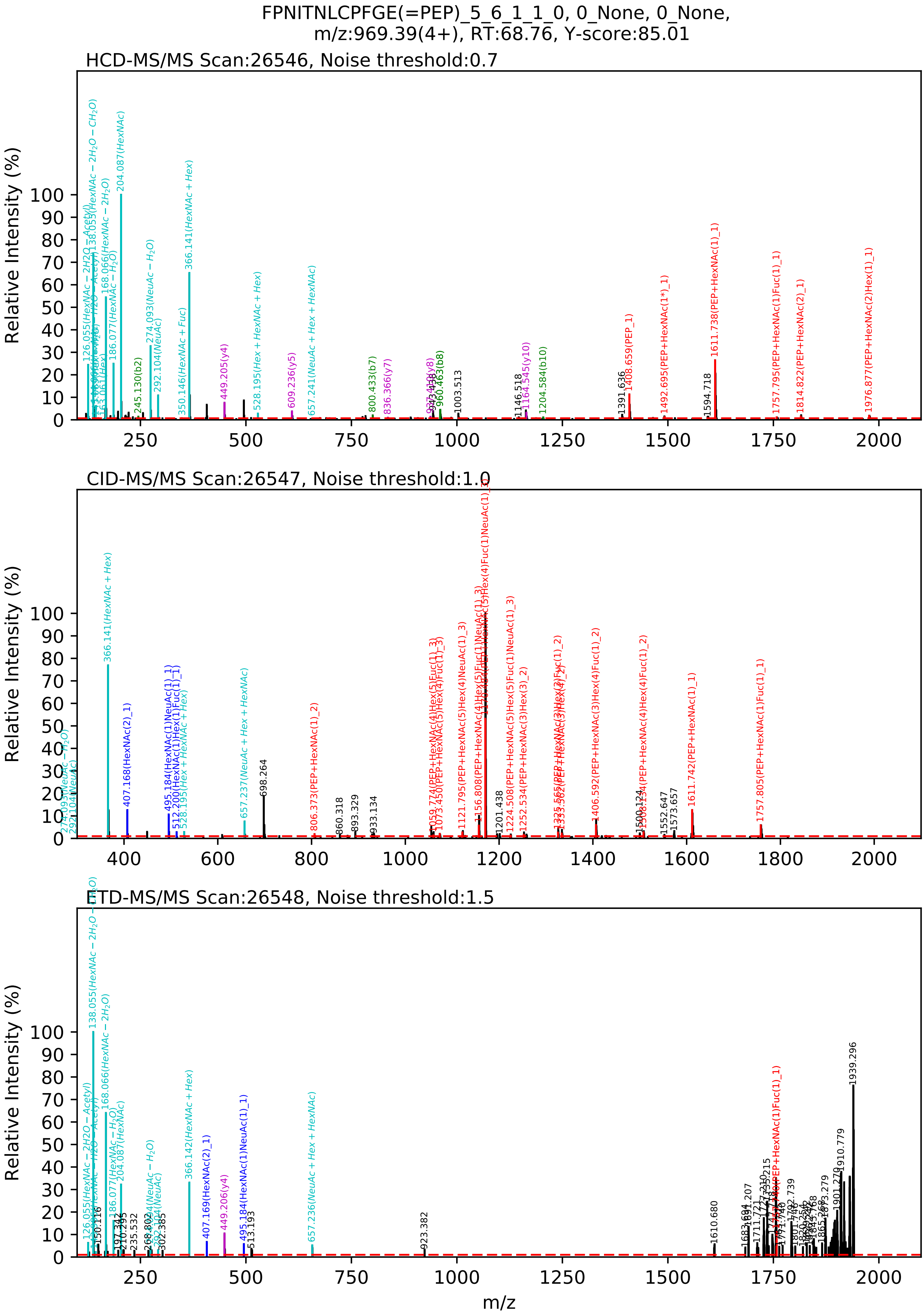

FPNITNLCPFGE(=PEP)_5_6_1_1_0, 0_None, 0_None,
m/z:969.39(4+), RT:68.76, Y-score:85.01
HCD-MS/MS Scan:26546, Noise threshold:0.7
Relative Intensity (%)
204.087(HexNAc)
366.141(HexNAc + Hex)
274.093(NeuAc − H2O)
292.104(NeuAc)
245.130(b2)
350.146(HexNAc + Fuc)
449.205(y4)
528.195(Hex + HexNAc + Hex)
609.236(y5)
657.241(NeuAc + Hex + HexNAc)
800.433(b7)
836.366(y7)
960.463(b8)
1003.513
1146.518
1164.545(y10)
1204.584(b10)
1391.636
1408.659(PEP_1)
1492.695(PEP+HexNAc(1*)_1)
1594.718
1611.738(PEP+HexNAc(1)_1)
1757.795(PEP+HexNAc(1)Fuc(1)_1)
1814.822(PEP+HexNAc(2)_1)
1976.877(PEP+HexNAc(2)Hex(1)_1)
CID-MS/MS Scan:26547, Noise threshold:1.0
366.141(HexNAc + Hex)
407.168(HexNAc(2)_1)
495.184(HexNAc(1)NeuAc(1)_1)
528.195(Hex + HexNAc + Hex)
657.237(NeuAc + Hex + HexNAc)
698.264
806.373(PEP+HexNAc(1)_2)
860.318
893.329
933.134
1121.795(PEP+HexNAc(5)Hex(4)NeuAc(1)_3)
1156.808(PEP+HexNAc(4)Hex(5)Fuc(1)NeuAc(1)_3)
1201.438
1224.508(PEP+HexNAc(5)Hex(5)Fuc(1)NeuAc(1)_3)
1252.534(PEP+HexNAc(3)Hex(3)_2)
1406.592(PEP+HexNAc(3)Hex(4)Fuc(1)_2)
1508.134(PEP+HexNAc(4)Hex(4)Fuc(1)_2)
1552.647
1573.657
1611.742(PEP+HexNAc(1)_1)
1757.805(PEP+HexNAc(1)Fuc(1)_1)
ETD-MS/MS Scan:26548, Noise threshold:1.5
138.055(HexNAc − 2H2O − CH2O)
168.066(HexNAc − 2H2O)
204.087(HexNAc)
366.142(HexNAc + Hex)
407.169(HexNAc(2)_1)
449.206(y4)
495.184(HexNAc(1)NeuAc(1)_1)
513.193
657.236(NeuAc + Hex + HexNAc)
923.382
1610.680
1939.296
1910.779
1873.279
m/z

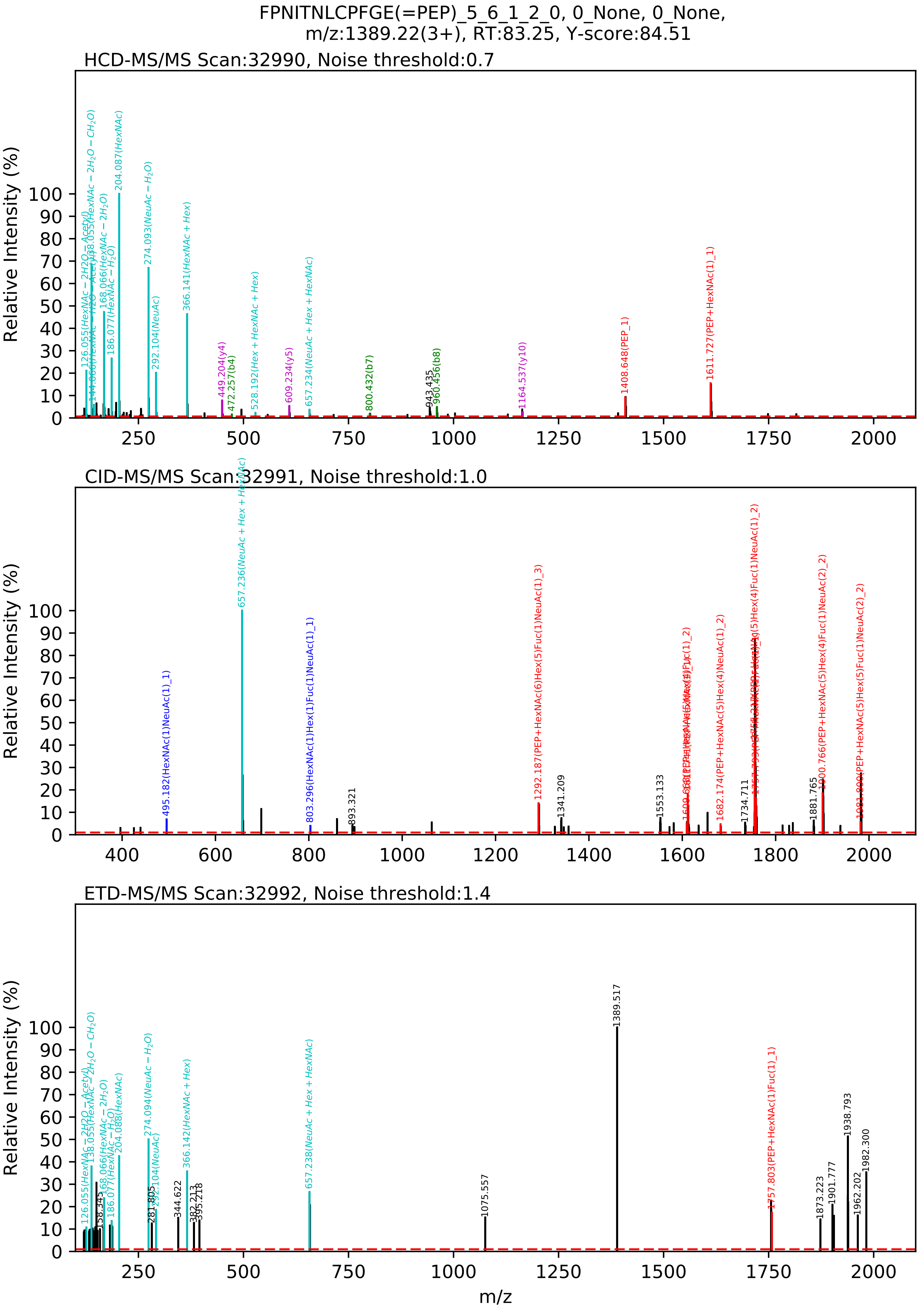

FPNITNLCPFGE(=PEP)_5_6_1_2_0, 0_None, 0_None,
m/z:1389.22(3+), RT:83.25, Y-score:84.51
HCD-MS/MS Scan:32990, Noise threshold:0.7
CID-MS/MS Scan:32991, Noise threshold:1.0
ETD-MS/MS Scan:32992, Noise threshold:1.4
Relative Intensity (%)
m/z
204.087(HexNAc)
274.093(NeuAc − H2O)
366.141(HexNAc + Hex)
292.104(NeuAc)
449.204(y4)
472.257(b4)
528.192(Hex + HexNAc + Hex)
609.234(y5)
657.234(NeuAc + Hex + HexNAc)
800.432(b7)
943.435
960.456(b8)
1164.537(y10)
1408.648(PEP_1)
1611.727(PEP+HexNAc(1)_1)
657.236(NeuAc + Hex + HexNAc)
495.182(HexNAc(1)NeuAc(1)_1)
803.296(HexNAc(1)Hex(1)Fuc(1)NeuAc(1)_1)
893.321
1292.187(PEP+HexNAc(6)Hex(5)Fuc(1)NeuAc(1)_3)
1341.209
1553.133
1682.174(PEP+HexNAc(5)Hex(4)NeuAc(1)_2)
1734.711
1881.765
1900.766(PEP+HexNAc(5)Hex(4)Fuc(1)NeuAc(2)_2)
1389.517
1075.557
1757.803(PEP+HexNAc(1)Fuc(1)_1)
1873.223
1901.777
1938.793
1962.202
1982.300
281.805
344.622
382.213
395.218

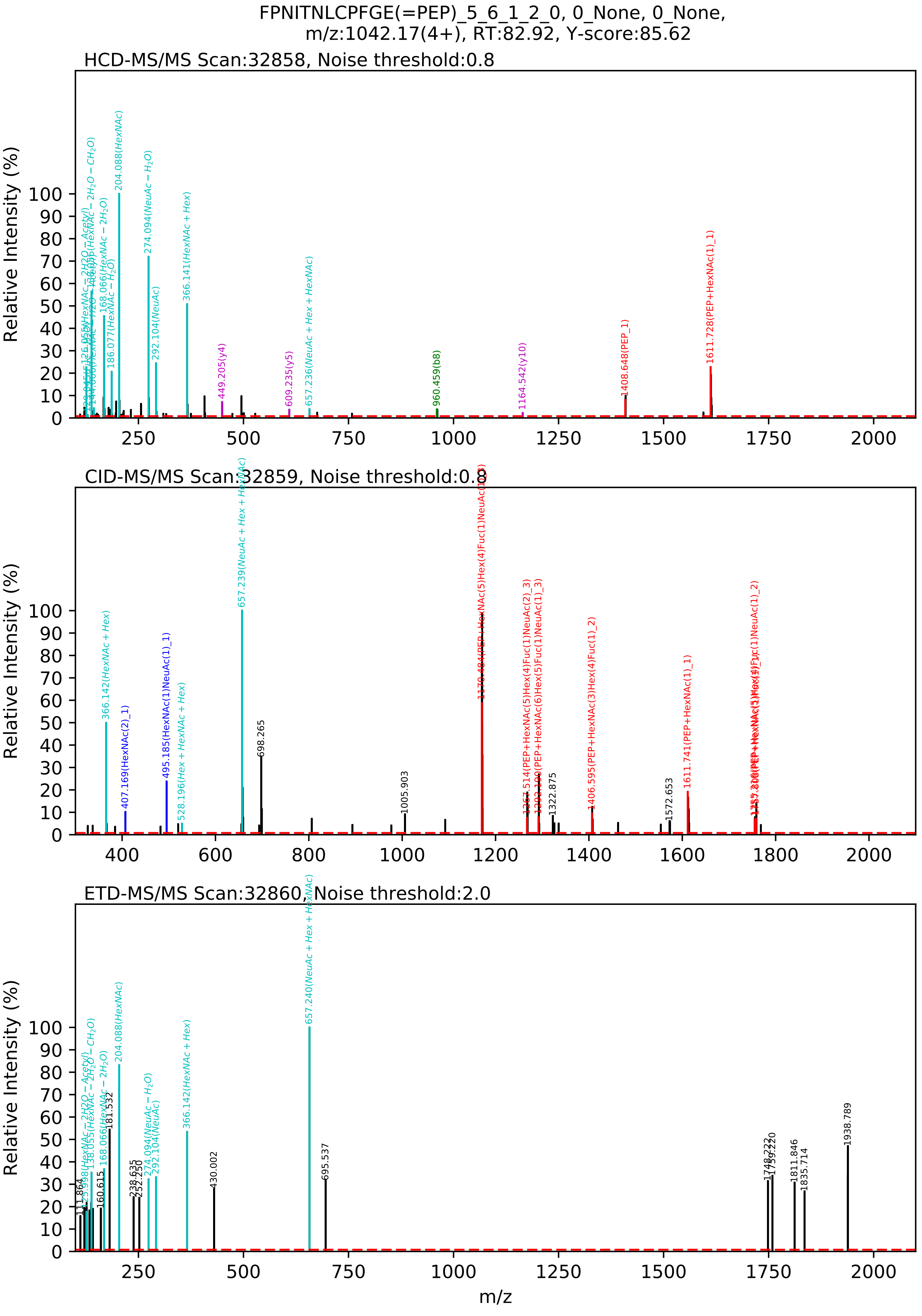

FPNITNLCPFGE(=PEP)_5_6_1_2_0, 0_None, 0_None,
m/z:1042.17(4+), RT:82.92, Y-score:85.62
HCD-MS/MS Scan:32858, Noise threshold:0.8
Relative Intensity (%)
204.088(HexNAc)
274.094(NeuAc − H2O)
366.141(HexNAc + Hex)
292.104(NeuAc)
168.066(HexNAc − 2H2O)
186.077(HexNAc − H2O)
449.205(y4)
609.235(y5)
657.236(NeuAc + Hex + HexNAc)
960.459(b8)
1164.542(y10)
1408.648(PEP_1)
1611.728(PEP+HexNAc(1)_1)
CID-MS/MS Scan:32859, Noise threshold:0.8
657.239(NeuAc + Hex + HexNAc)
366.142(HexNAc + Hex)
407.169(HexNAc(2)_1)
495.185(HexNAc(1)NeuAc(1)_1)
528.196(Hex + HexNAc + Hex)
698.265
1005.903
1322.875
1406.595(PEP+HexNAc(3)Hex(4)Fuc(1)_2)
1611.741(PEP+HexNAc(1)_1)
1572.653
ETD-MS/MS Scan:32860, Noise threshold:2.0
657.240(NeuAc + Hex + HexNAc)
204.088(HexNAc)
366.142(HexNAc + Hex)
181.532
168.066(HexNAc − 2H2O)
160.615
111.864
238.635
252.250
274.094(NeuAc − H2O)
292.104(NeuAc)
430.002
695.537
1748.222
1759.220
1811.846
1835.714
1938.789
m/z

FPNITNLCPFGE(=PEP)_5_6_2_0_0, 0_None, 0_None,
m/z:1243.84(3+), RT:59.28, Y-score:83.67

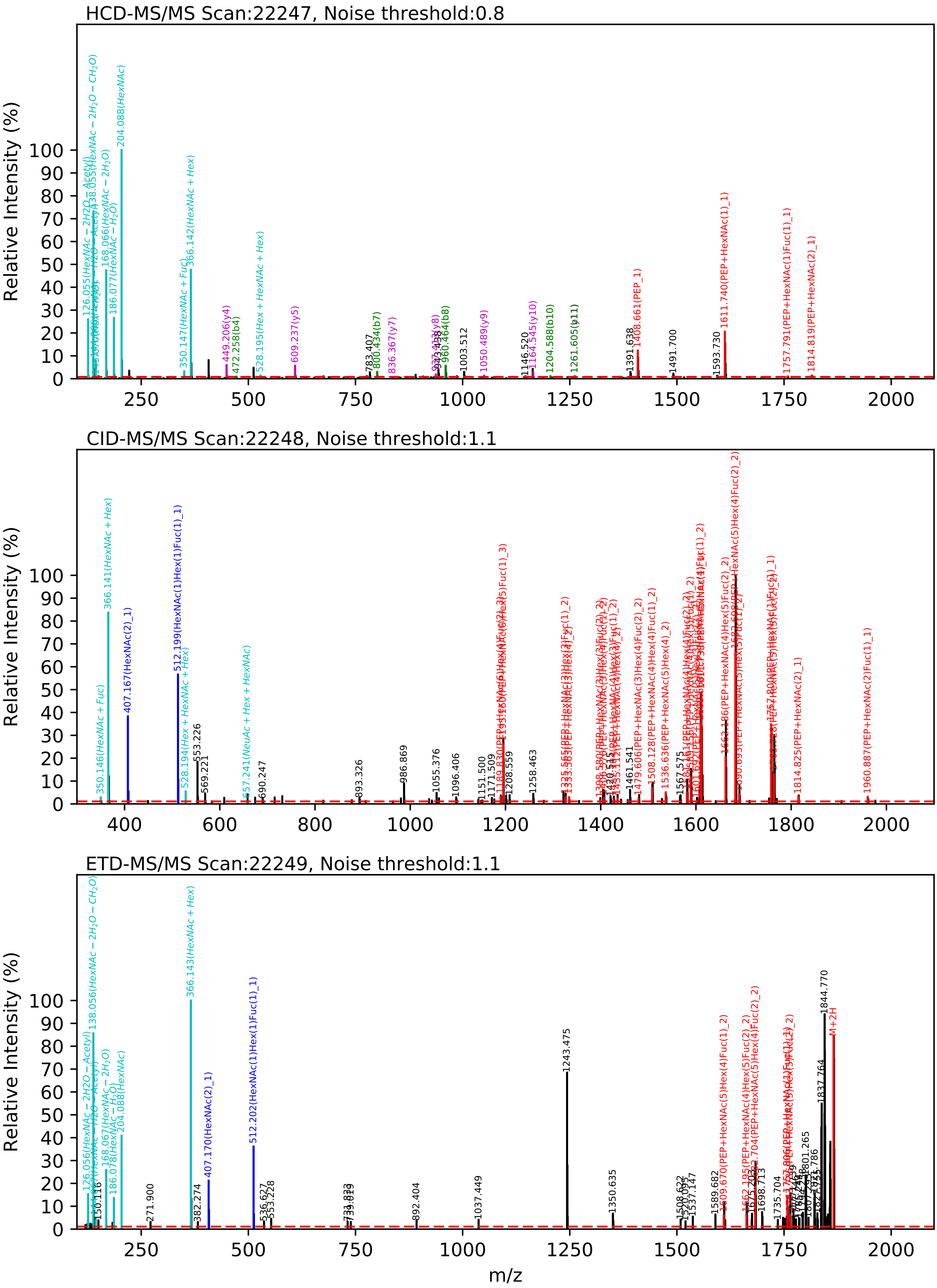

FPNITNLCPFGE(=PEP)_5_6_2_1_0, 0_None, 0_None,
m/z:1340.87(3+), RT:67.69, Y-score:78.88

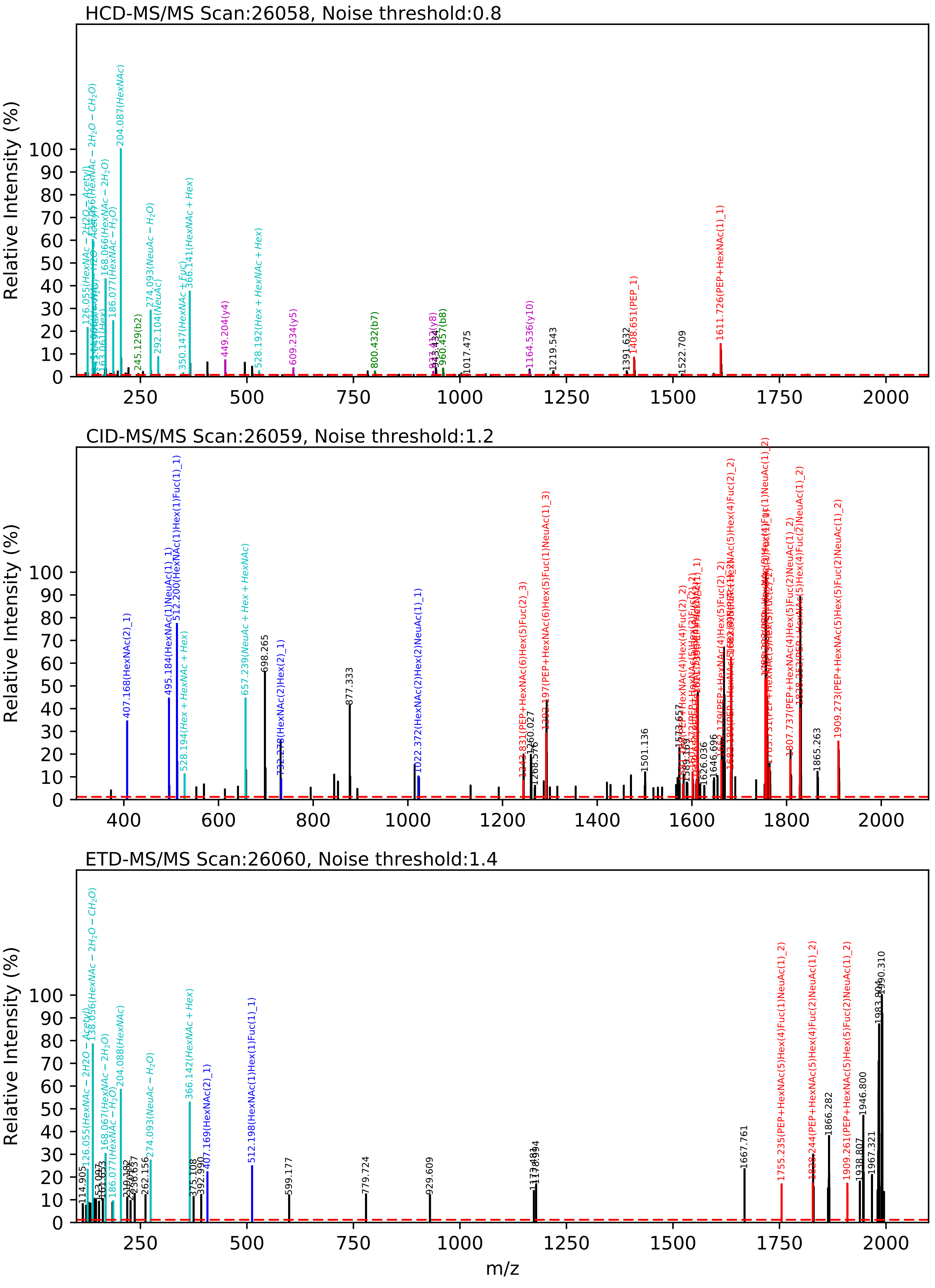

FPNITNLCPFGE(=PEP)_5_6_2_1_0, 0_None, 0_None,
m/z:1340.87(3+), RT:68.45, Y-score:83.81

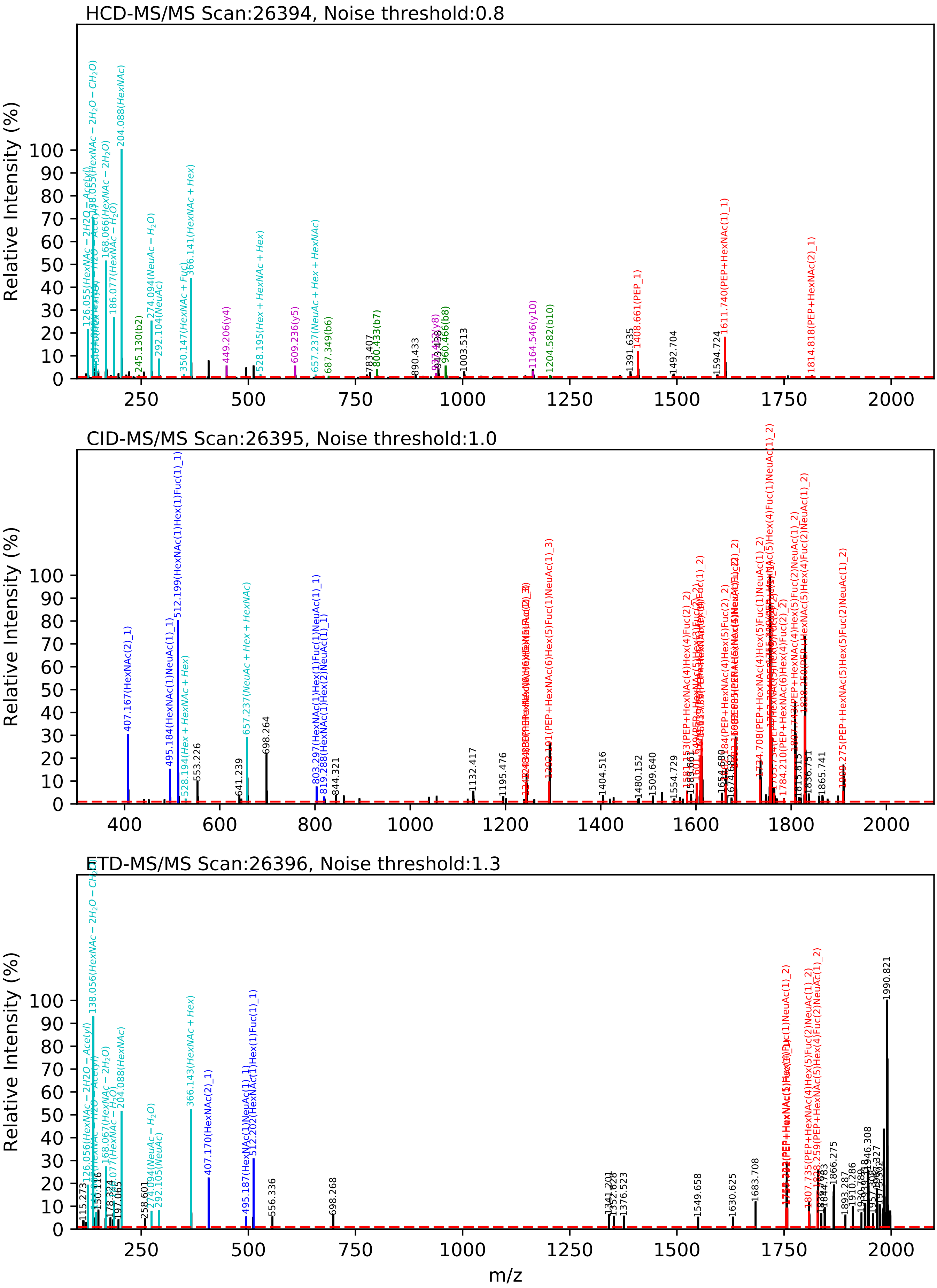

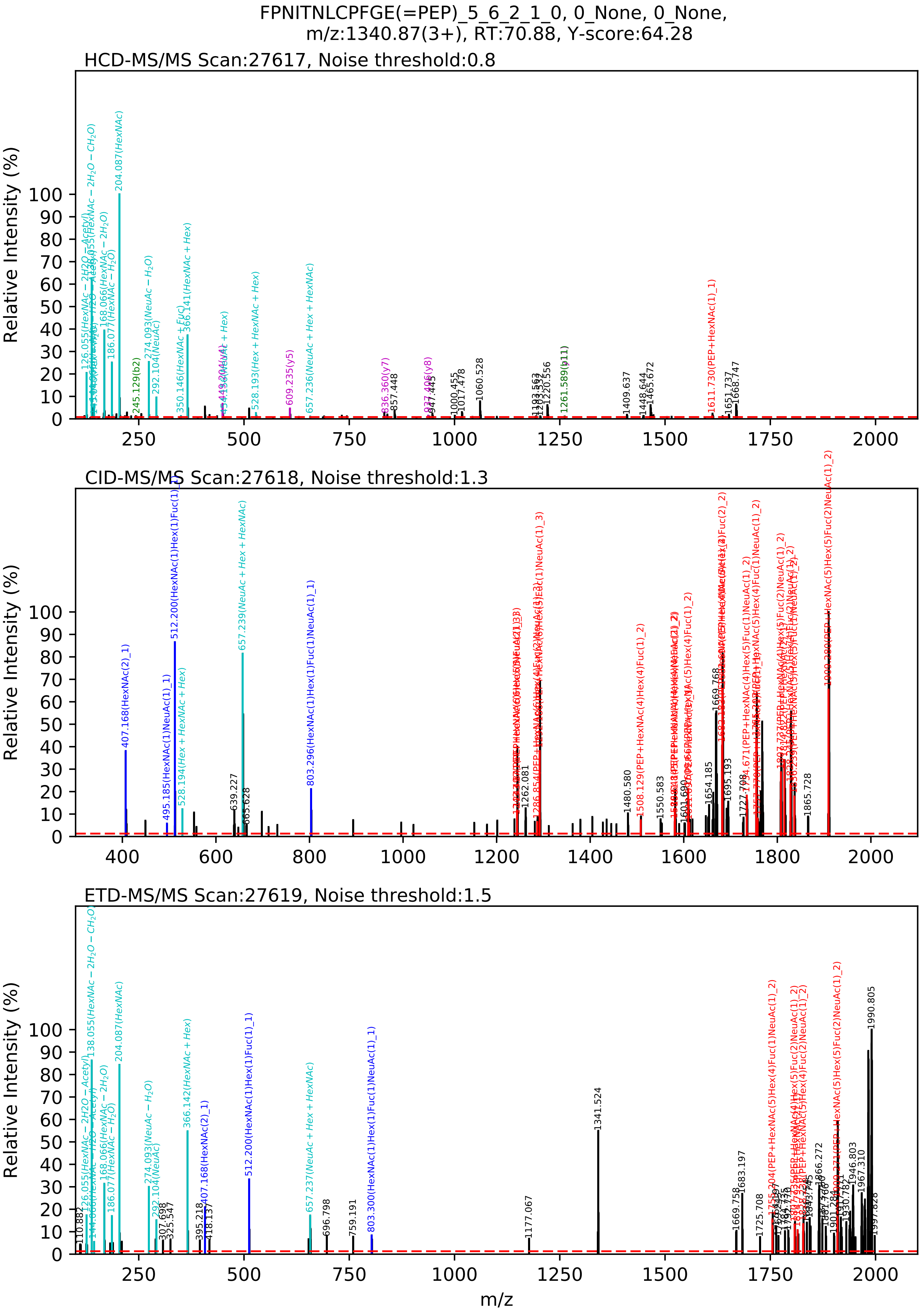
FPNITNLCPFGE(=PEP)_5_6_2_1_0, 0_None, 0_None,
m/z:1340.87(3+), RT:70.88, Y-score:64.28
HCD-MS/MS Scan:27617, Noise threshold:0.8
CID-MS/MS Scan:27618, Noise threshold:1.3
ETD-MS/MS Scan:27619, Noise threshold:1.5
Relative Intensity (%)
m/z
204.087(HexNAc)
366.141(HexNAc + Hex)
274.093(NeuAc − H2O)
292.104(NeuAc)
1611.730(PEP+HexNAc(1)_1)
512.200(HexNAc(1)Hex(1)Fuc(1)_1)
657.239(NeuAc + Hex + HexNAc)
407.168(HexNAc(2)_1)
803.296(HexNAc(1)Hex(1)Fuc(1)NeuAc(1)_1)
1341.524
1990.805

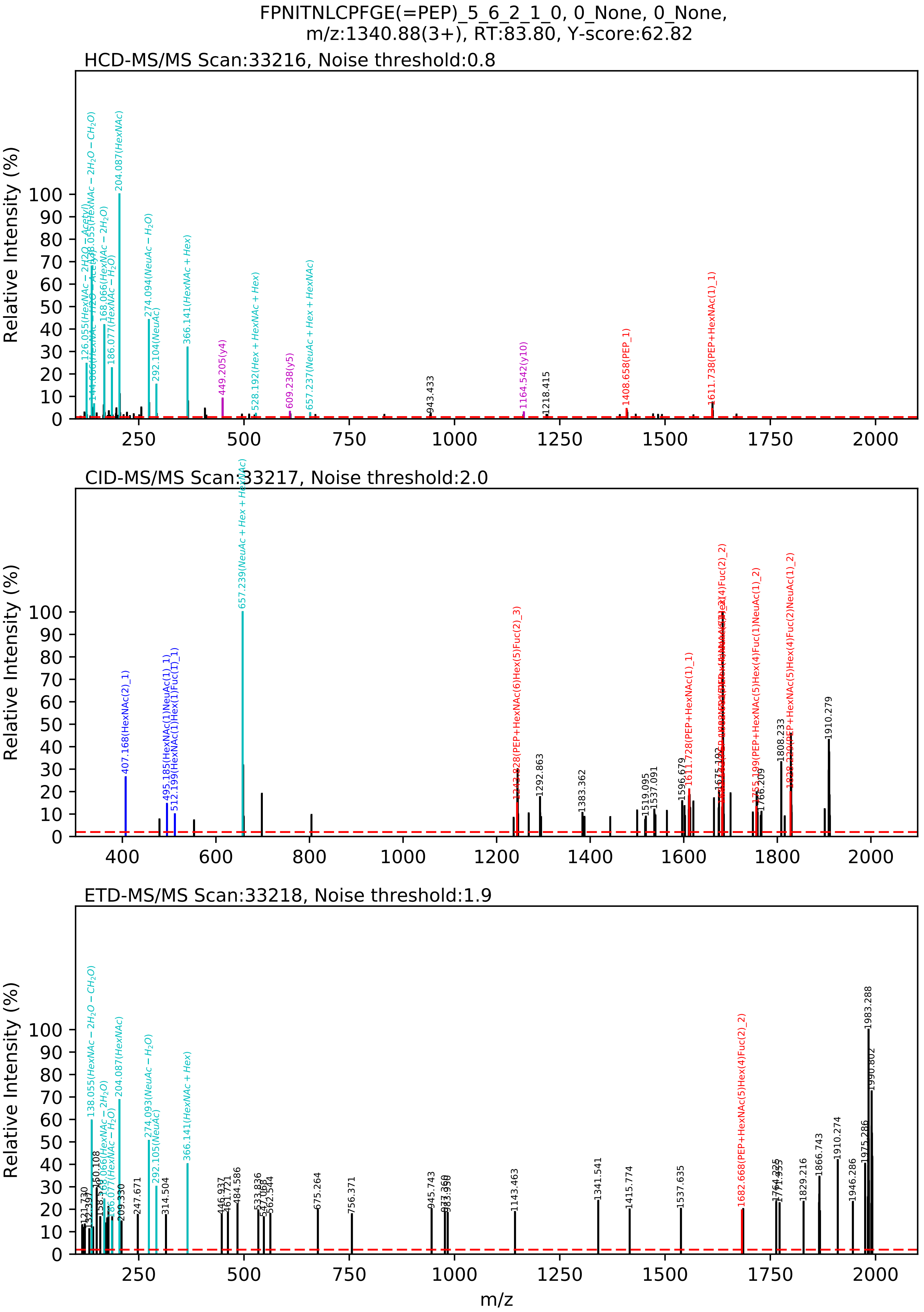
FPNITNLCPFGE(=PEP)_5_6_2_1_0, 0_None, 0_None,
m/z:1340.88(3+), RT:83.80, Y-score:62.82
HCD-MS/MS Scan:33216, Noise threshold:0.8
CID-MS/MS Scan:33217, Noise threshold:2.0
ETD-MS/MS Scan:33218, Noise threshold:1.9
Relative Intensity (%)
m/z
204.087(HexNAc)
366.141(HexNAc + Hex)
274.094(NeuAc − H2O)
292.104(NeuAc)
449.205(y4)
528.192(Hex + HexNAc + Hex)
609.238(y5)
657.237(NeuAc + Hex + HexNAc)
943.433
1164.542(y10)
1218.415
1408.658(PEP_1)
1611.738(PEP+HexNAc(1)_1)
407.168(HexNAc(2)_1)
495.185(HexNAc(1)NeuAc(1)_1)
512.199(HexNAc(1)Hex(1)Fuc(1)_1)
657.239(NeuAc + Hex + HexNAc)
1243.828(PEP+HexNAc(6)Hex(5)Fuc(2)_3)
1292.863
1383.362
1519.095
1537.091
1596.679
1611.728(PEP+HexNAc(1)_1)
1675.192
1755.199(PEP+HexNAc(5)Hex(4)Fuc(1)NeuAc(1)_2)
1766.209
1808.233
1828.220(PEP+HexNAc(5)Hex(4)Fuc(2)NeuAc(1)_2)
1910.279
138.055(HexNAc − 2H2O − CH2O)
204.087(HexNAc)
274.093(NeuAc − H2O)
292.105(NeuAc)
366.141(HexNAc + Hex)
160.108
209.330
247.671
314.504
446.937
461.721
484.586
533.836
547.098
562.544
675.264
756.371
945.743
1143.463
1341.541
1415.774
1537.635
1682.668(PEP+HexNAc(5)Hex(4)Fuc(2)_2)
1764.335
1829.216
1866.743
1910.274
1946.286
1975.286
1983.288
1990.802

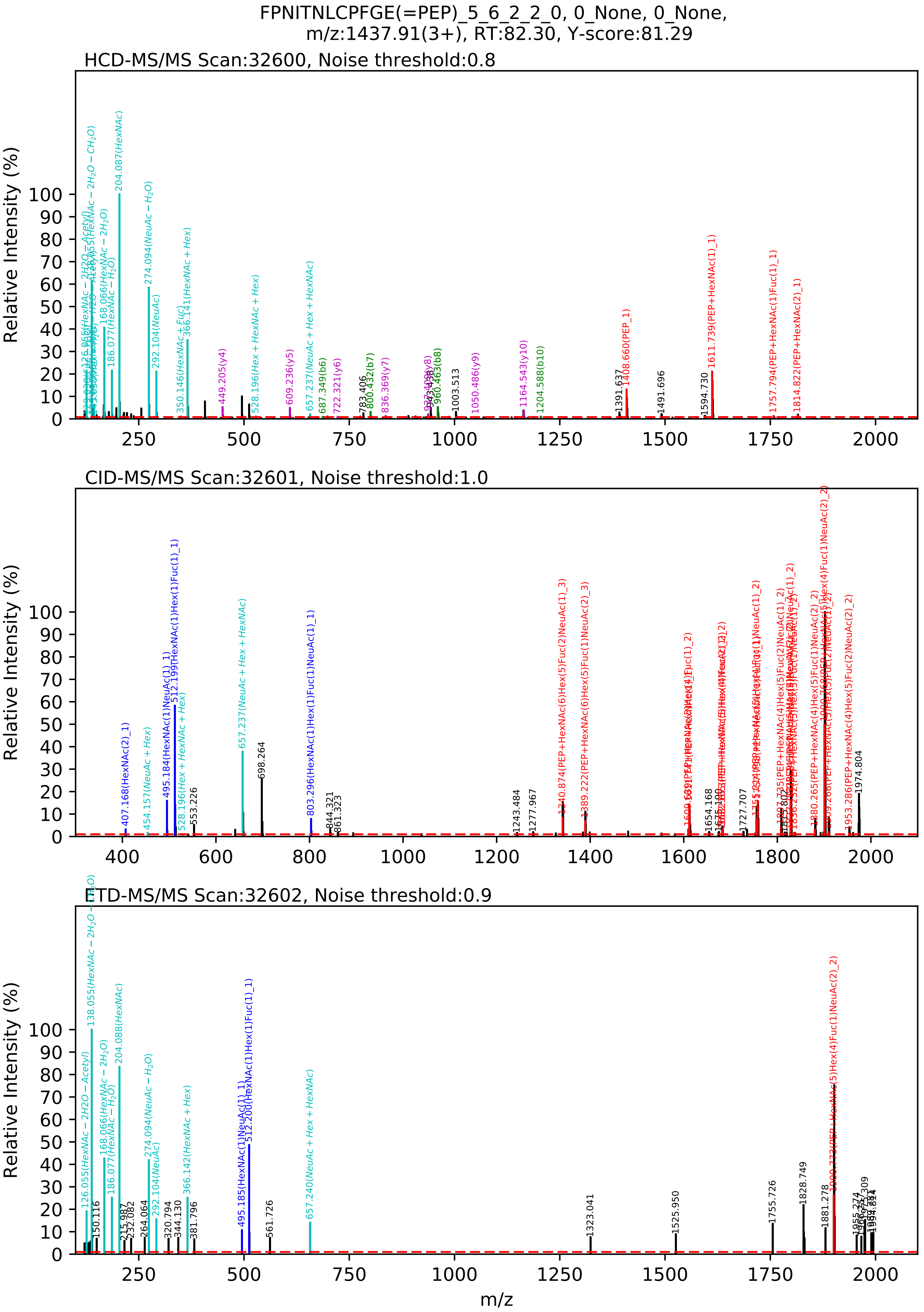
FPNITNLCPFGE(=PEP)_5_6_2_2_0, 0_None, 0_None,
m/z:1437.91(3+), RT:82.30, Y-score:81.29
HCD-MS/MS Scan:32600, Noise threshold:0.8
CID-MS/MS Scan:32601, Noise threshold:1.0
ETD-MS/MS Scan:32602, Noise threshold:0.9
Relative Intensity (%)
m/z

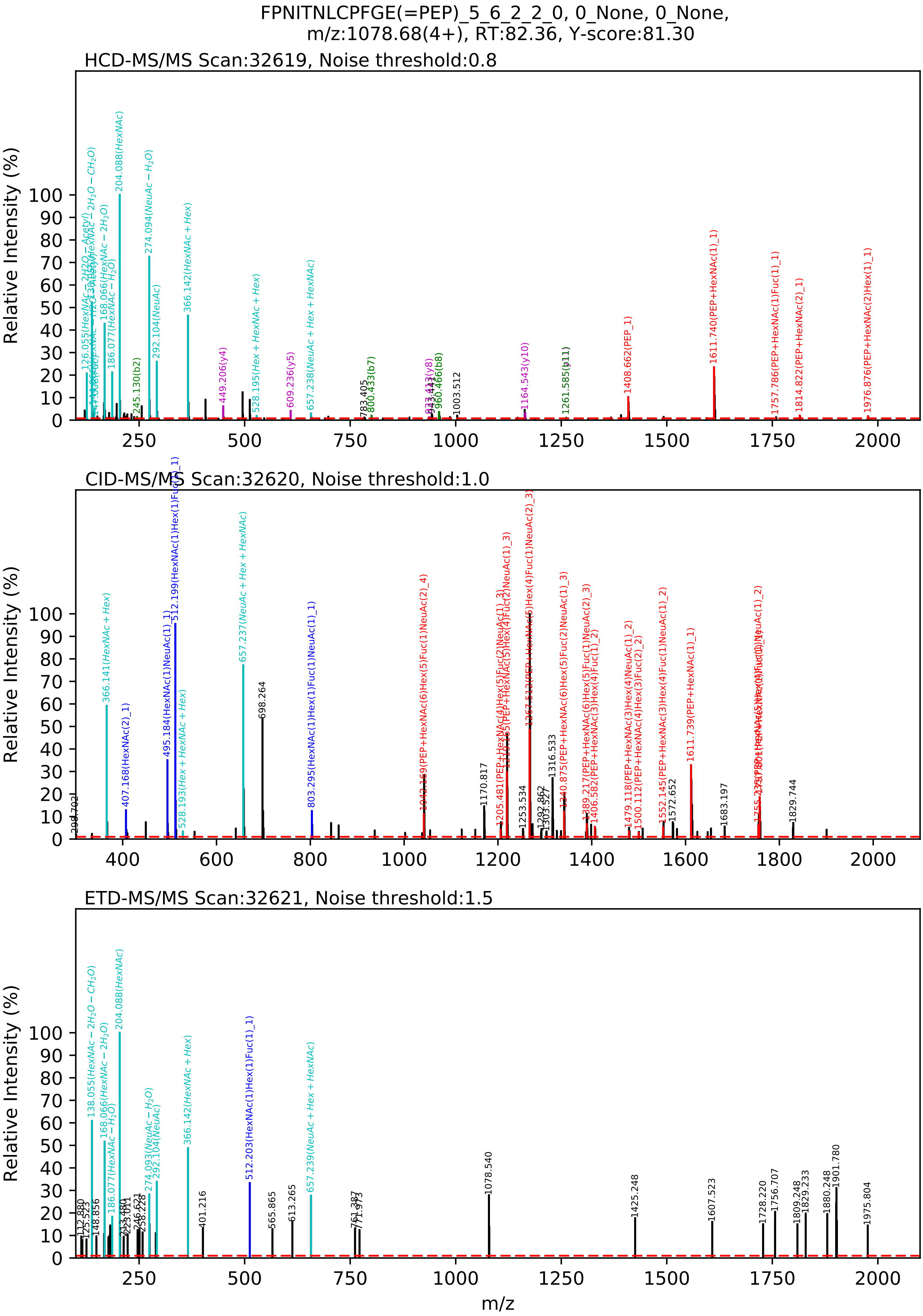

FPNITNLCPFGE(=PEP)_5_6_2_2_0, 0_None, 0_None,
m/z:1078.68(4+), RT:82.36, Y-score:81.30
HCD-MS/MS Scan:32619, Noise threshold:0.8
Relative Intensity (%)
204.088(HexNAc)
274.094(NeuAc − H2O)
366.142(HexNAc + Hex)
168.066(HexNAc − 2H2O)
186.077(HexNAc − H2O)
126.055(HexNAc − 2H2O − CH2O)
292.104(NeuAc)
245.130(b2)
449.206(y4)
528.195(Hex + HexNAc + Hex)
609.236(y5)
657.238(NeuAc + Hex + HexNAc)
783.405
800.433(b7)
960.466(b8)
1003.512
1164.543(y10)
1261.585(b11)
1408.662(PEP_1)
1611.740(PEP+HexNAc(1)_1)
1757.786(PEP+HexNAc(1)Fuc(1)_1)
1814.822(PEP+HexNAc(2)_1)
1976.876(PEP+HexNAc(2)Hex(1)_1)
CID-MS/MS Scan:32620, Noise threshold:1.0
Relative Intensity (%)
366.141(HexNAc + Hex)
407.168(HexNAc(2)_1)
495.184(HexNAc(1)NeuAc(1)_1)
512.199(HexNAc(1)Hex(1)Fuc(1)_1)
528.193(Hex + HexNAc + Hex)
657.237(NeuAc + Hex + HexNAc)
698.264
803.295(HexNAc(1)Hex(1)Fuc(1)NeuAc(1)_1)
1170.817
1253.534
1316.533
1240.875(PEP+HexNAc(6)Hex(5)Fuc(2)NeuAc(1)_3)
1389.217(PEP+HexNAc(6)Hex(5)Fuc(1)NeuAc(2)_3)
1406.582(PEP+HexNAc(3)Hex(4)Fuc(1)_2)
1479.118(PEP+HexNAc(3)Hex(4)NeuAc(1)_2)
1500.112(PEP+HexNAc(4)Hex(3)Fuc(2)_2)
1552.145(PEP+HexNAc(3)Hex(4)Fuc(1)NeuAc(1)_2)
1572.652
1611.739(PEP+HexNAc(1)_1)
1683.197
1829.744
ETD-MS/MS Scan:32621, Noise threshold:1.5
Relative Intensity (%)
138.055(HexNAc − 2H2O − CH2O)
168.066(HexNAc − 2H2O)
186.077(HexNAc − H2O)
204.088(HexNAc)
274.093(NeuAc − H2O)
292.104(NeuAc)
366.142(HexNAc + Hex)
401.216
512.203(HexNAc(1)Hex(1)Fuc(1)_1)
565.865
613.265
657.239(NeuAc + Hex + HexNAc)
1078.540
1425.248
1607.523
1728.220
1756.707
1809.248
1829.233
1880.248
1901.780
1975.804
m/z

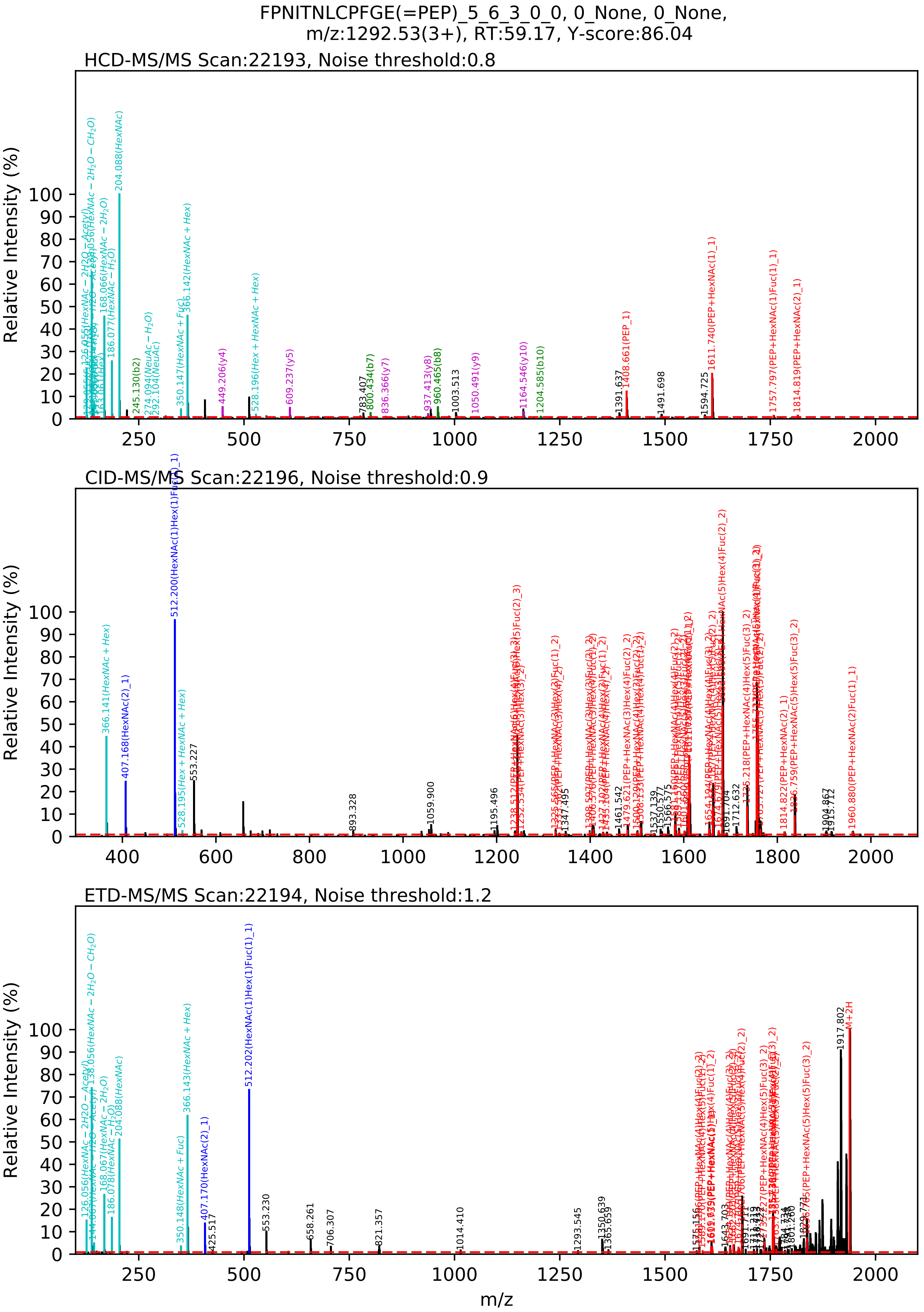
FPNITNLCPFGE(=PEP)_5_6_3_0_0, 0_None, 0_None,
m/z:1292.53(3+), RT:59.17, Y-score:86.04
HCD-MS/MS Scan:22193, Noise threshold:0.8
CID-MS/MS Scan:22196, Noise threshold:0.9
ETD-MS/MS Scan:22194, Noise threshold:1.2
Relative Intensity (%)
m/z
204.088(HexNAc)
366.142(HexNAc+Hex)
1611.740(PEP+HexNAc(1)_1)
1408.661(PEP_1)
512.200(HexNAc(1)Hex(1)Fuc(1)_1)
407.168(HexNAc(2)_1)
512.202(HexNAc(1)Hex(1)Fuc(1)_1)
1917.802
M+2H

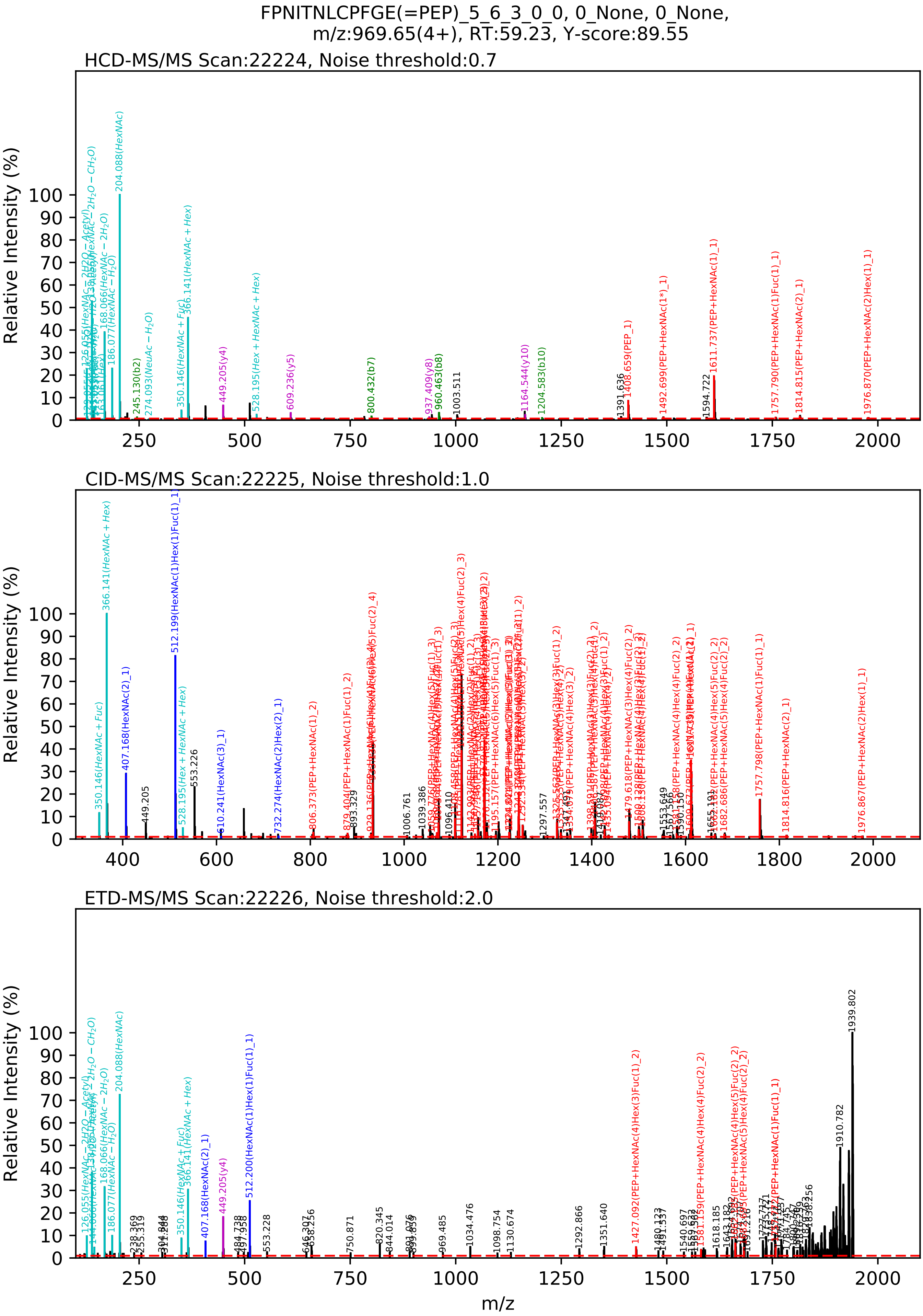

FPNITNLCPFGE(=PEP)_5_6_3_0_0, 0_None, 0_None,
m/z:969.65(4+), RT:59.23, Y-score:89.55
HCD-MS/MS Scan:22224, Noise threshold:0.7
204.088(HexNAc)
366.141(HexNAc + Hex)
350.146(HexNAc + Fuc)
168.066(HexNAc − 2H2O)
186.077(HexNAc − H2O)
245.130(b2)
274.093(NeuAc − H2O)
449.205(y4)
528.195(Hex + HexNAc + Hex)
609.236(y5)
800.432(b7)
937.409(y8)
960.463(b8)
1003.511
1164.544(y10)
1204.583(b10)
1391.636
1408.659(PEP_1)
1492.699(PEP+HexNAc(1*)_1)
1594.722
1611.737(PEP+HexNAc(1)_1)
1757.790(PEP+HexNAc(1)Fuc(1)_1)
1814.815(PEP+HexNAc(2)_1)
1976.870(PEP+HexNAc(2)Hex(1)_1)
CID-MS/MS Scan:22225, Noise threshold:1.0
366.141(HexNAc + Hex)
350.146(HexNAc + Fuc)
407.168(HexNAc(2)_1)
449.205
512.199(HexNAc(1)Hex(1)Fuc(1)_1)
528.195(Hex + HexNAc + Hex)
553.226
610.241(HexNAc(3)_1)
732.274(HexNAc(2)Hex(2)_1)
806.373(PEP+HexNAc(1)_2)
879.404(PEP+HexNAc(1)Fuc(1)_2)
893.329
1006.761
1039.386
1096.410
1297.557
1553.649
1655.191
1682.686(PEP+HexNAc(5)Hex(4)Fuc(2)_2)
1757.798(PEP+HexNAc(1)Fuc(1)_1)
1814.816(PEP+HexNAc(2)_1)
1976.867(PEP+HexNAc(2)Hex(1)_1)
ETD-MS/MS Scan:22226, Noise threshold:2.0
204.088(HexNAc)
168.066(HexNAc − 2H2O)
186.077(HexNAc − H2O)
238.369
255.319
350.146(HexNAc + Fuc)
366.141(HexNAc + Hex)
407.168(HexNAc(2)_1)
449.205(y4)
484.738
497.958
512.200(HexNAc(1)Hex(1)Fuc(1)_1)
553.228
646.307
658.256
750.871
820.345
844.014
969.485
1034.476
1098.754
1130.674
1292.866
1351.640
1427.092(PEP+HexNAc(4)Hex(3)Fuc(1)_2)
1480.123
1491.537
1540.697
1618.185
1643.182
1691.216
1910.782
1939.802
Relative Intensity (%)
m/z

FPNITNLCPFGE(=PEP)_5_6_3_1_0, 0_None, 0_None, m/z:1042.42(4+), RT:68.30, Y-score:86.70

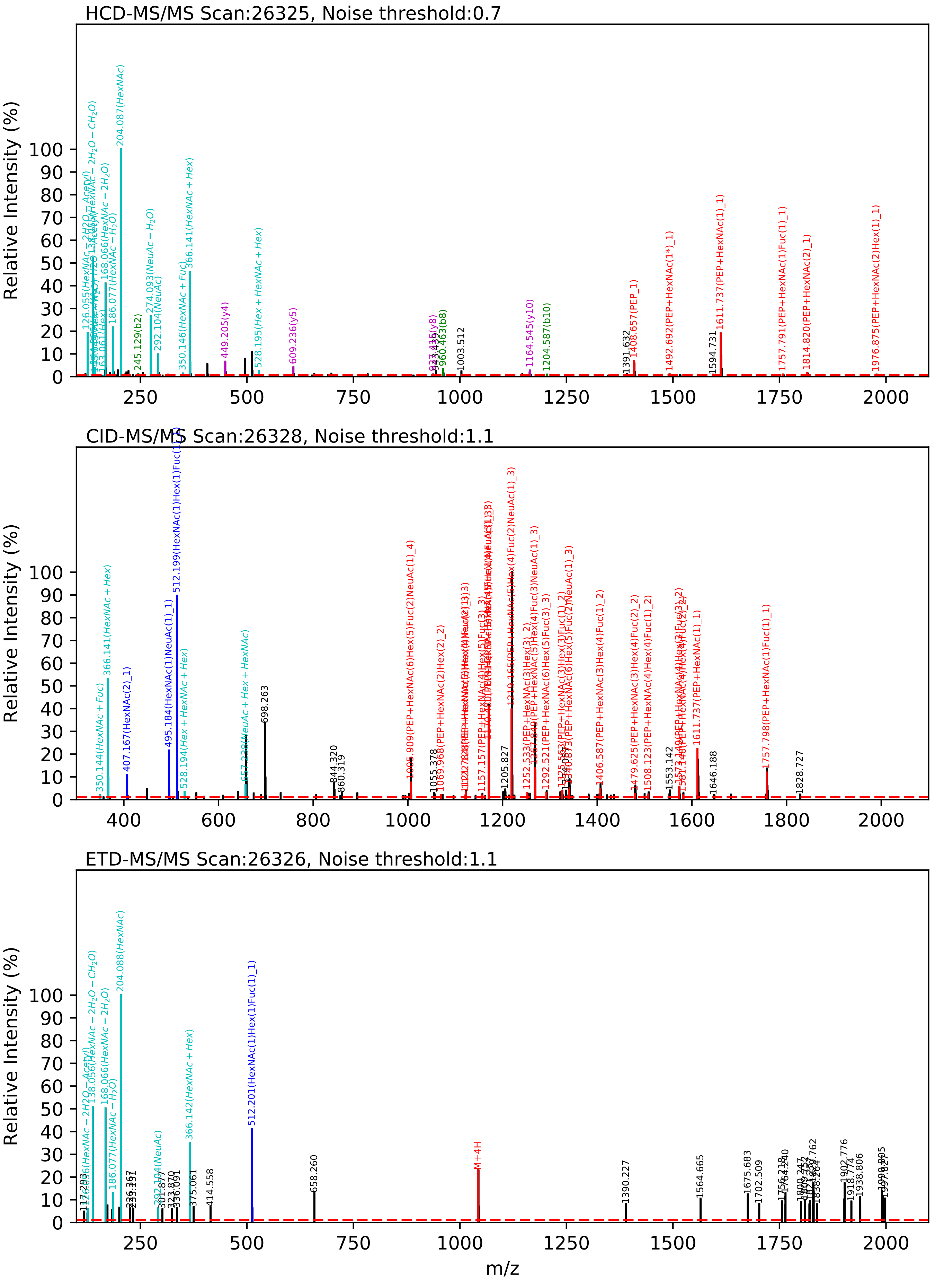

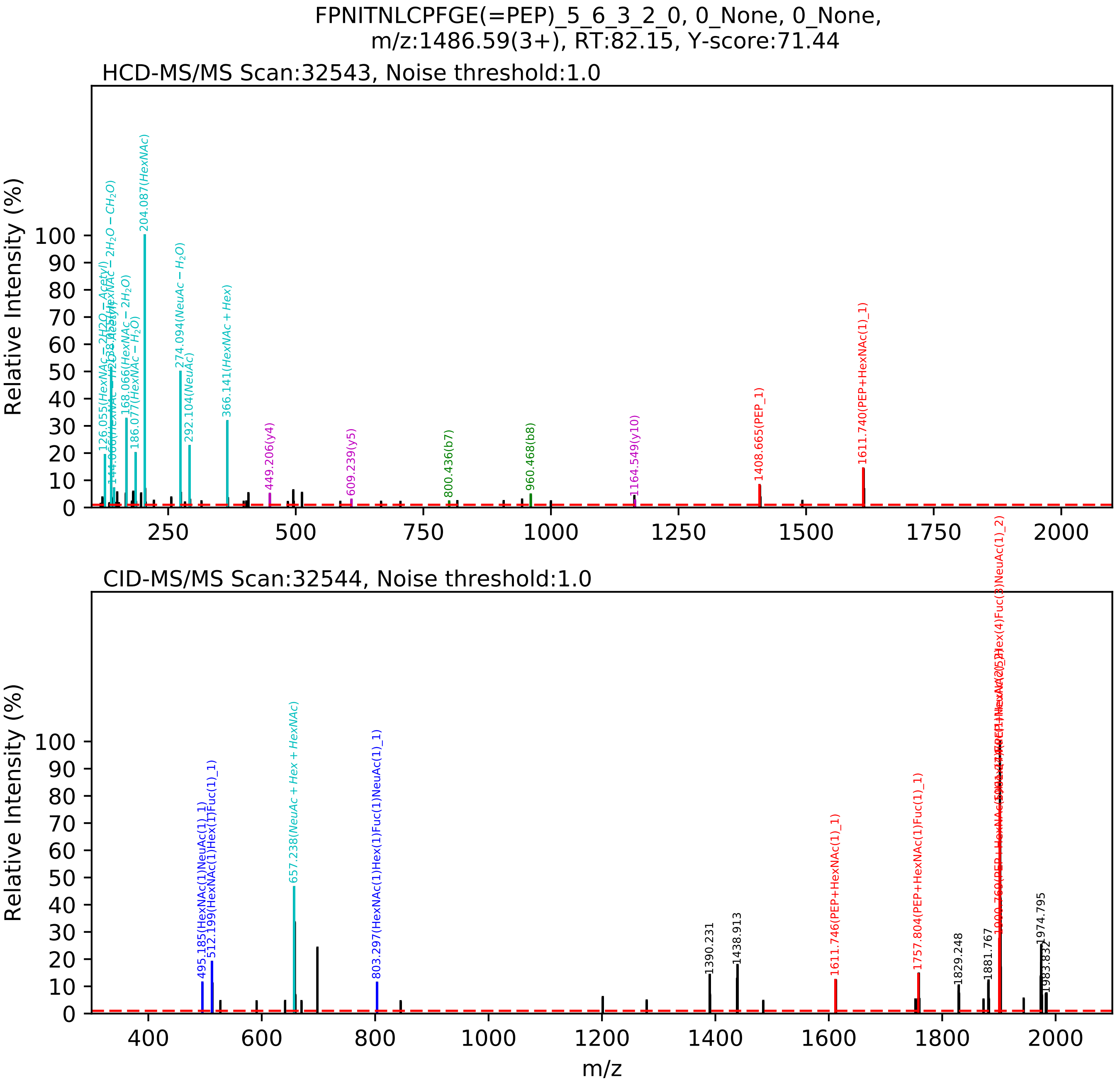
FPNITNLCPFGE(=PEP)_5_6_3_2_0, 0_None, 0_None,
m/z:1486.59(3+), RT:82.15, Y-score:71.44
HCD-MS/MS Scan:32543, Noise threshold:1.0
Relative Intensity (%)
126.055(HexNAc − 2H2O − Acetyl)
144.066(HexNAc − H2O − Acetyl)
168.066(HexNAc − 2H2O)
186.077(HexNAc − H2O)
204.087(HexNAc)
274.094(NeuAc − H2O)
292.104(NeuAc)
366.141(HexNAc + Hex)
449.206(y4)
609.239(y5)
800.436(b7)
960.468(b8)
1164.549(y10)
1408.665(PEP_1)
1611.740(PEP+HexNAc(1)_1)
CID-MS/MS Scan:32544, Noise threshold:1.0
495.185(HexNAc(1)NeuAc(1)_1)
512.199(HexNAc(1)Hex(1)Fuc(1)_1)
657.238(NeuAc + Hex + HexNAc)
803.297(HexNAc(1)Hex(1)Fuc(1)NeuAc(1)_1)
1390.231
1438.913
1611.746(PEP+HexNAc(1)_1)
1757.804(PEP+HexNAc(1)Fuc(1)_1)
1829.248
1881.767
1974.795
1983.832
m/z

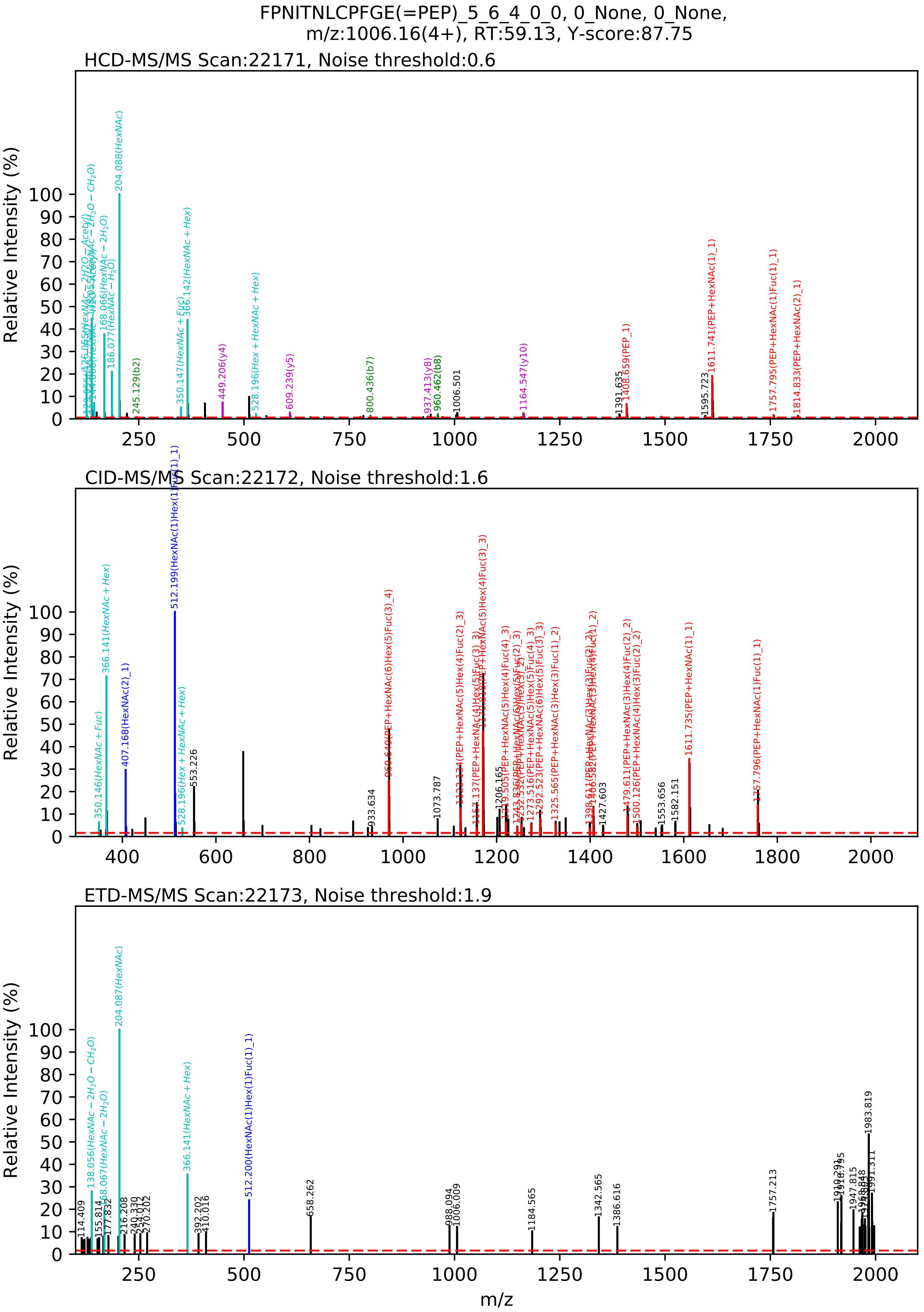
FPNITNLCPFGE(=PEP)_5_6_4_0_0, 0_None, 0_None,
m/z:1006.16(4+), RT:59.13, Y-score:87.75
HCD-MS/MS Scan:22171, Noise threshold:0.6
Relative Intensity (%)
204.088(HexNAc)
366.142(HexNAc + Hex)
350.147(HexNAc + Fuc)
168.066(HexNAc − 2H2O)
186.077(HexNAc − H2O)
245.129(b2)
449.206(y4)
528.196(Hex + HexNAc + Hex)
609.239(y5)
800.436(b7)
937.413(y8)
960.462(b8)
1006.501
1164.547(y10)
1391.635
1408.659(PEP_1)
1595.723
1611.741(PEP+HexNAc(1)_1)
1757.795(PEP+HexNAc(1)Fuc(1)_1)
1814.833(PEP+HexNAc(2)_1)
CID-MS/MS Scan:22172, Noise threshold:1.6
Relative Intensity (%)
350.146(HexNAc + Fuc)
366.141(HexNAc + Hex)
407.168(HexNAc(2)_1)
512.199(HexNAc(1)Hex(1)Fuc(1)_1)
528.196(Hex + HexNAc + Hex)
553.226
933.634
969.649(PEP+HexNAc(6)Hex(5)Fuc(3)_4)
1073.787
1122.134(PEP+HexNAc(5)Hex(4)Fuc(2)_3)
1157.137(PEP+HexNAc(4)Hex(5)Fuc(3)_3)
1206.165
1325.565(PEP+HexNAc(3)Hex(3)Fuc(1)_2)
1427.603
1479.611(PEP+HexNAc(3)Hex(4)Fuc(2)_2)
1500.126(PEP+HexNAc(4)Hex(3)Fuc(2)_2)
1553.656
1582.151
1611.735(PEP+HexNAc(1)_1)
1757.796(PEP+HexNAc(1)Fuc(1)_1)
ETD-MS/MS Scan:22173, Noise threshold:1.9
Relative Intensity (%)
204.087(HexNAc)
138.056(HexNAc − 2H2O − CH2O)
158.067(HexNAc − 2H2O)
366.141(HexNAc + Hex)
512.200(HexNAc(1)Hex(1)Fuc(1)_1)
114.409
155.814
177.832
216.208
240.330
254.012
270.202
392.202
410.016
658.262
988.094
1006.009
1184.565
1342.565
1386.616
1757.213
1918.795
1947.815
1983.819
1991.311
m/z

FPNITNLCPFGE(=PEP)_5_6_4_1_0, 0_None, 0_None,
m/z:1438.25(3+), RT:68.11, Y-score:84.33

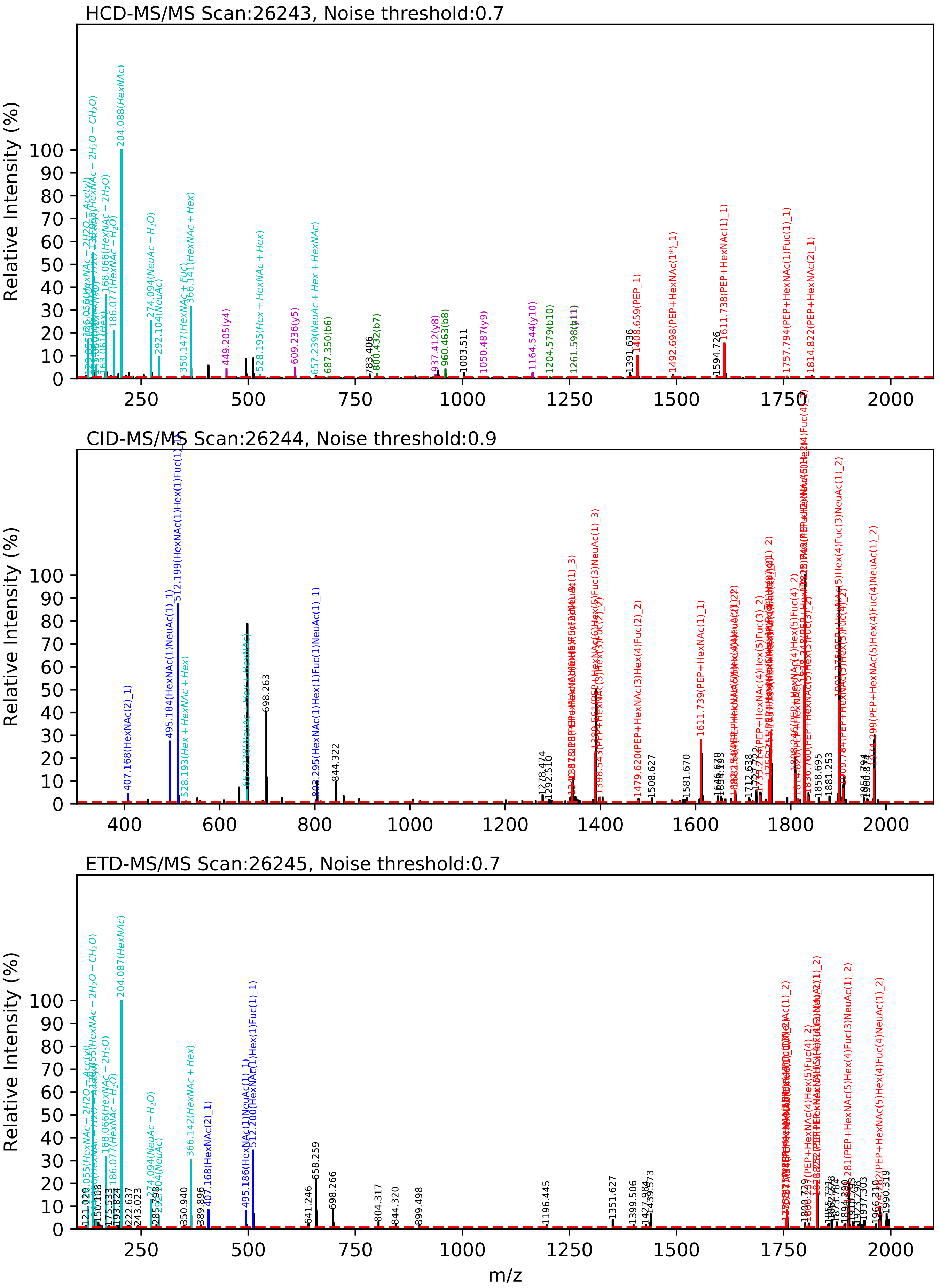

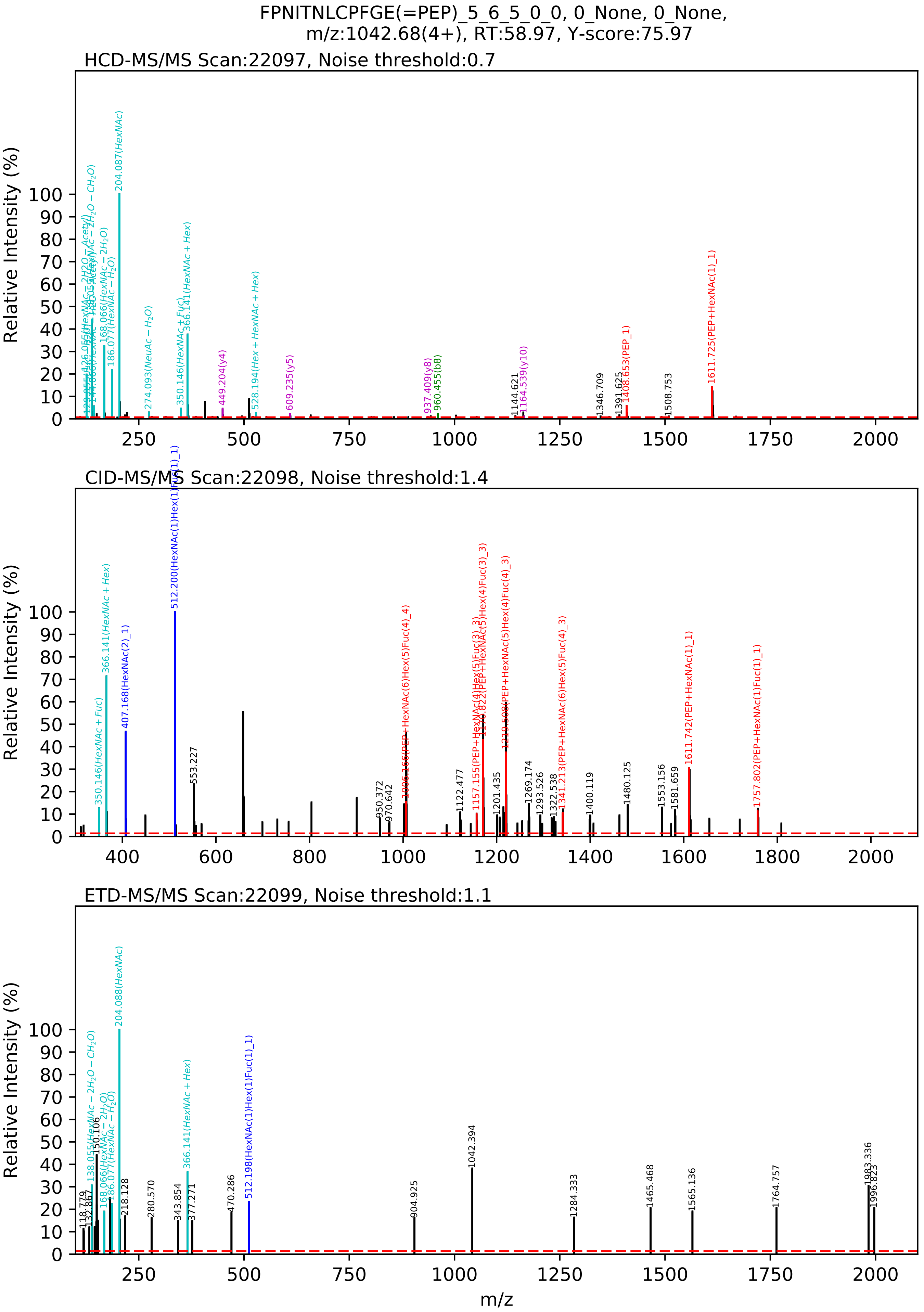
FPNITNLCPFGE(=PEP)_5_6_5_0_0, 0_None, 0_None,
m/z:1042.68(4+), RT:58.97, Y-score:75.97
HCD-MS/MS Scan:22097, Noise threshold:0.7
Relative Intensity (%)
204.087(HexNAc)
366.141(HexNAc+Hex)
350.146(HexNAc+Fuc)
274.093(NeuAc−H2O)
168.066(HexNAc−2H2O)
186.077(HexNAc−H2O)
449.204(y4)
528.194(Hex+HexNAc+Hex)
609.235(y5)
937.409(y8)
960.455(b8)
1144.621
1164.539(y10)
1346.709
1391.625
1408.653(PEP_1)
1508.753
1611.725(PEP+HexNAc(1)_1)
CID-MS/MS Scan:22098, Noise threshold:1.4
Relative Intensity (%)
512.200(HexNAc(1)Hex(1)Fuc(1)_1)
366.141(HexNAc+Hex)
407.168(HexNAc(2)_1)
350.146(HexNAc+Fuc)
553.227
950.372
970.642
1006.166(PEP+HexNAc(6)Hex(5)Fuc(4)_4)
1122.477
1157.155(PEP+HexNAc(4)Hex(5)Fuc(3)_3)
1201.435
1219.508(PEP+HexNAc(5)Hex(4)Fuc(4)_3)
1269.174
1293.526
1322.538
1341.213(PEP+HexNAc(6)Hex(5)Fuc(4)_3)
1400.119
1480.125
1553.156
1581.659
1611.742(PEP+HexNAc(1)_1)
1757.802(PEP+HexNAc(1)Fuc(1)_1)
ETD-MS/MS Scan:22099, Noise threshold:1.1
Relative Intensity (%)
204.088(HexNAc)
138.055(HexNAc−2H2O−CH2O)
150.106
168.066(HexNAc−2H2O)
186.077(HexNAc−H2O)
118.779
132.867
218.128
280.570
343.854
366.141(HexNAc+Hex)
377.271
470.286
512.198(HexNAc(1)Hex(1)Fuc(1)_1)
904.925
1042.394
1284.333
1465.468
1565.136
1764.757
1983.336
1996.823
m/z

FPNITNLCPFGE(=PEP)_5_7_1_0_0, 0_None, 0_None,
m/z:1262.85(3+), RT:59.03, Y-score:70.15

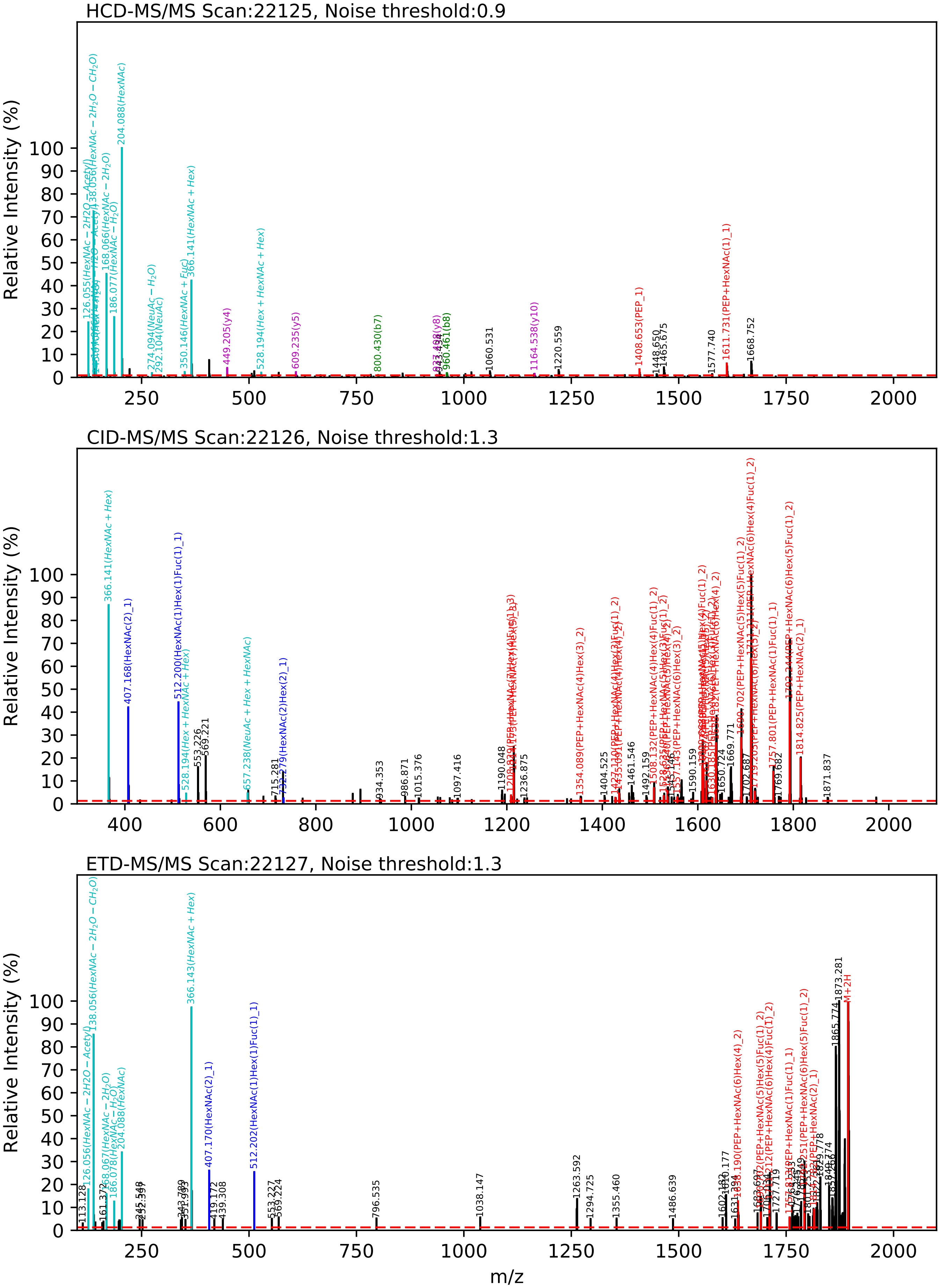

FPNITNLCPFGE(=PEP)_5_7_2_1_0, 0_None, 0_None,
m/z:1408.57(3+), RT:69.27, Y-score:66.29

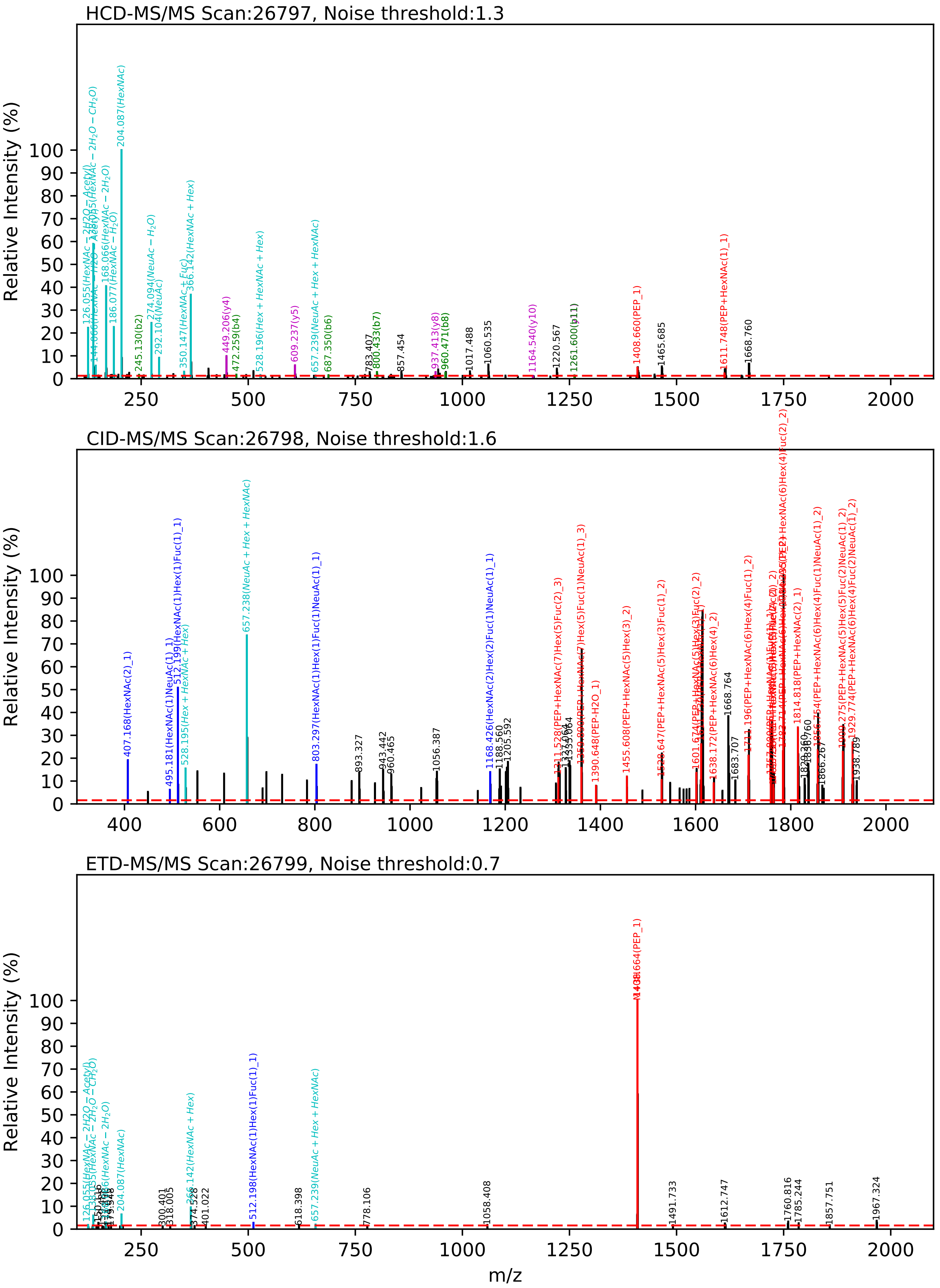

FPNITNLCPFGE(=PEP)_6_2_0_0_0, 0_None, 1_Hex_Phosphorylation,
m/z:956.37(3+), RT:74.36, Y-score:81.61

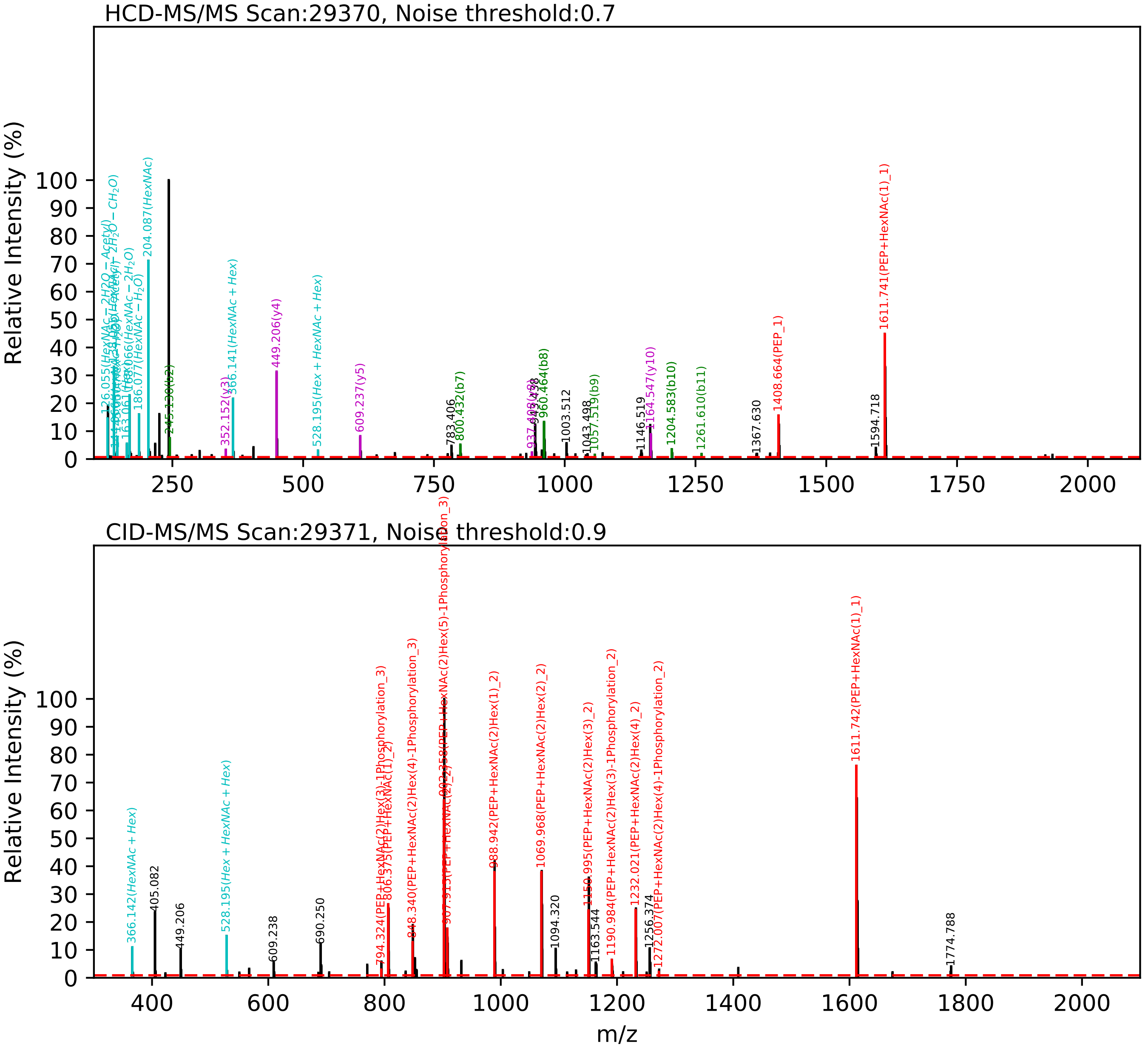

FPNITNLCPFGE(=PEP)_6_3_0_0_0, 0_None, 1_Hex_Phosphorylation, m/z:1024.06(3+), RT:74.26, Y-score:72.86

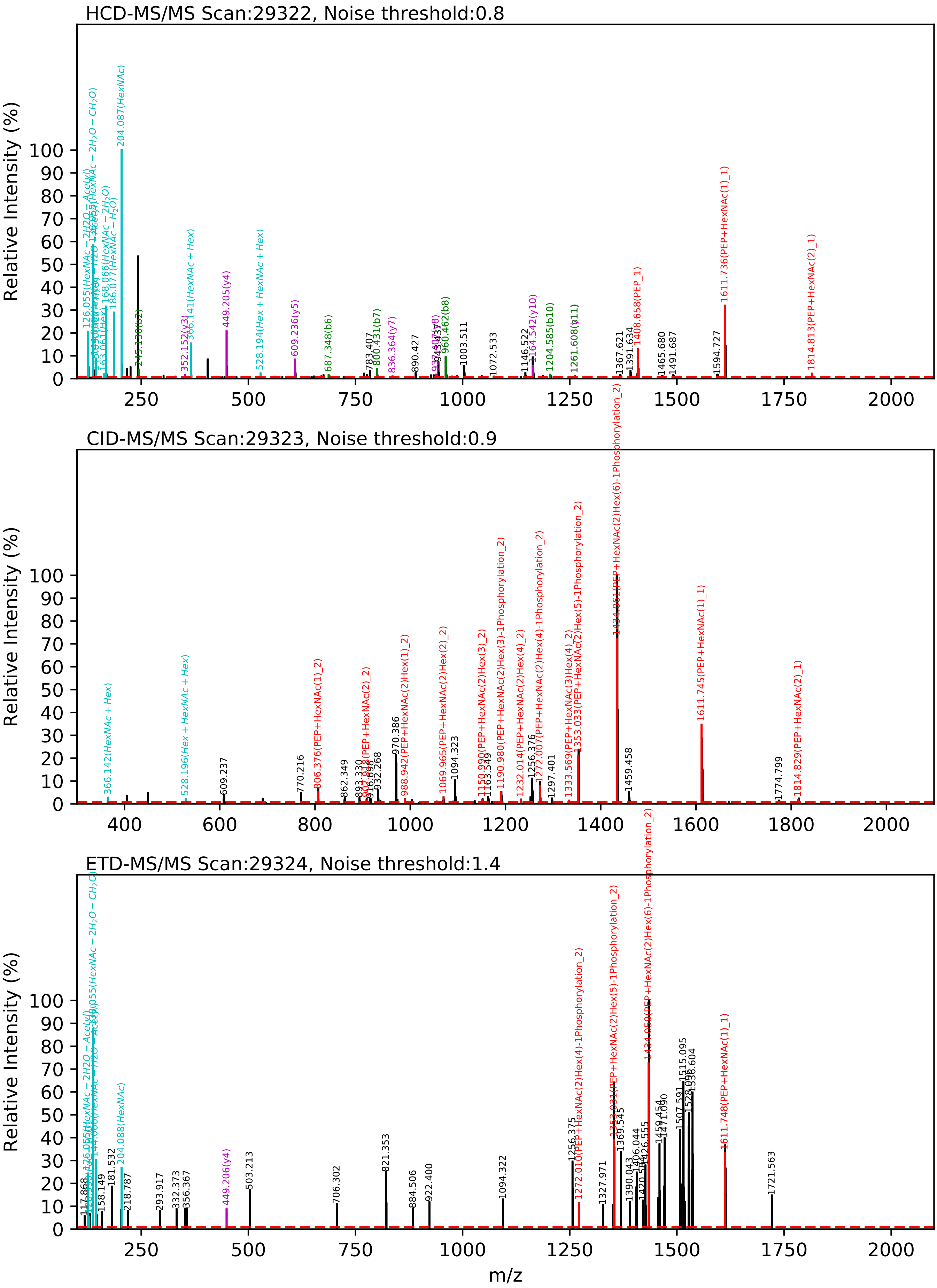

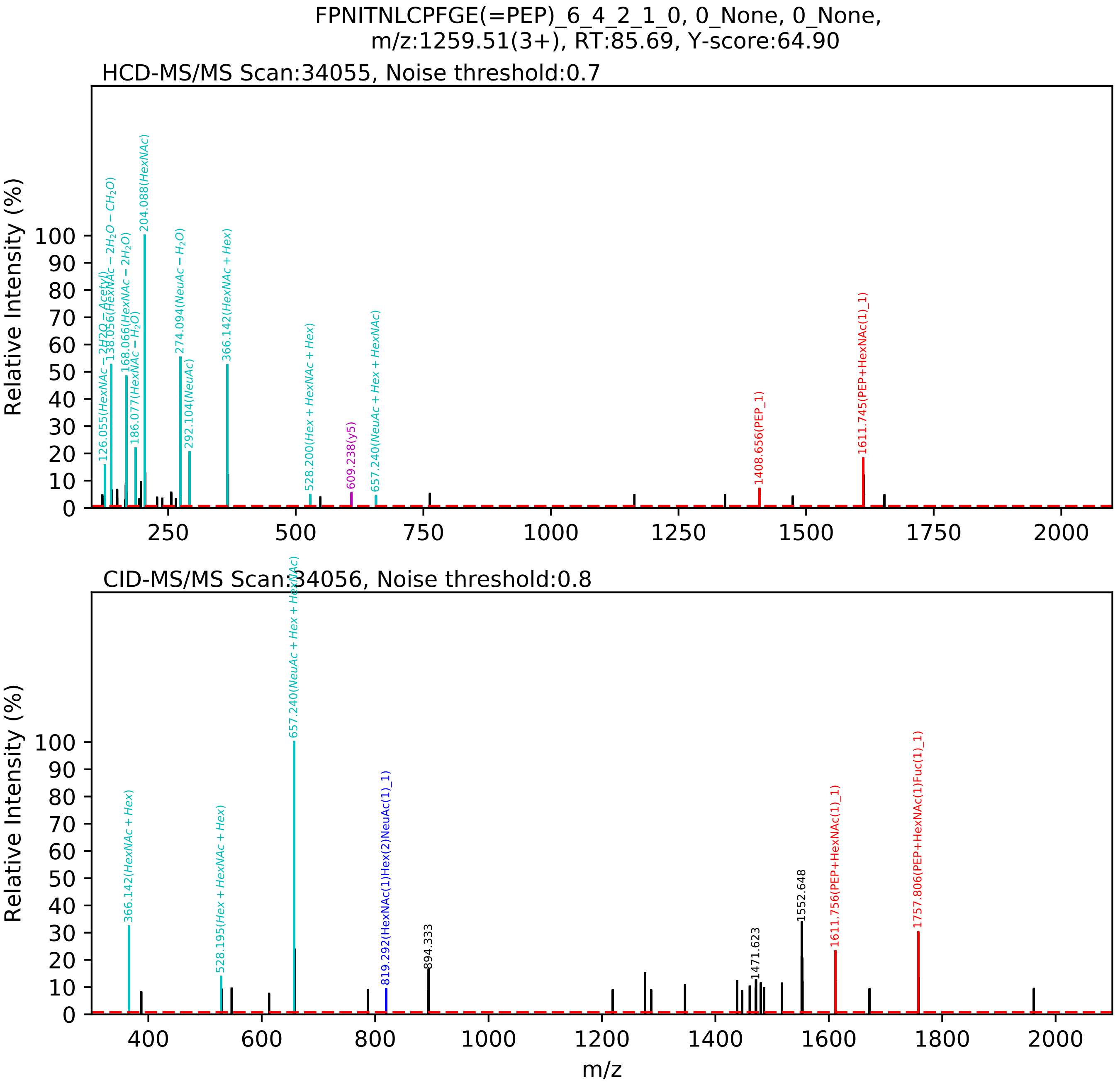

FPNITNLCPFGE(=PEP)_6_4_2_1_0, 0_None, 0_None,
m/z:1259.51(3+), RT:85.69, Y-score:64.90
HCD-MS/MS Scan:34055, Noise threshold:0.7
Relative Intensity (%)
126.055(HexNAc − 2H2O − Acetyl)
138.056(HexNAc − 2H2O − CH2O)
168.066(HexNAc − 2H2O)
186.077(HexNAc − H2O)
204.088(HexNAc)
274.094(NeuAc − H2O)
292.104(NeuAc)
366.142(HexNAc + Hex)
528.200(Hex + HexNAc + Hex)
609.238(y5)
657.240(NeuAc + Hex + HexNAc)
1408.656(PEP_1)
1611.745(PEP+HexNAc(1)_1)
CID-MS/MS Scan:34056, Noise threshold:0.8
366.142(HexNAc + Hex)
528.195(Hex + HexNAc + Hex)
657.240(NeuAc + Hex + HexNAc)
819.292(HexNAc(1)Hex(2)NeuAc(1)_1)
894.333
1471.623
1552.648
1611.756(PEP+HexNAc(1)_1)
1757.806(PEP+HexNAc(1)Fuc(1)_1)
m/z

FPNITNLCPFGE(=PEP)_6_5_1_1_0, 0_None, 0_None,
m/z:1278.51(3+), RT:68.60, Y-score:89.01

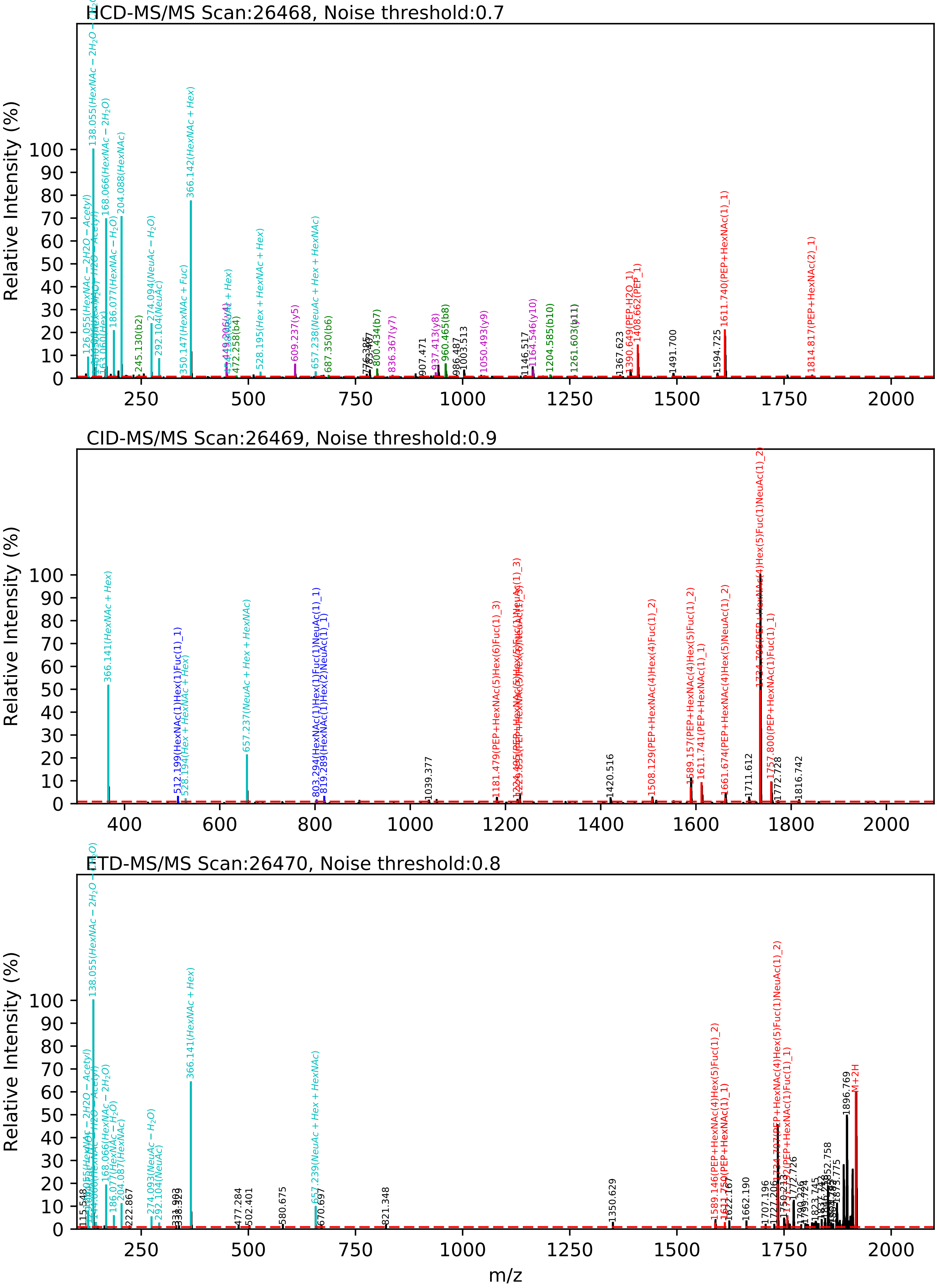

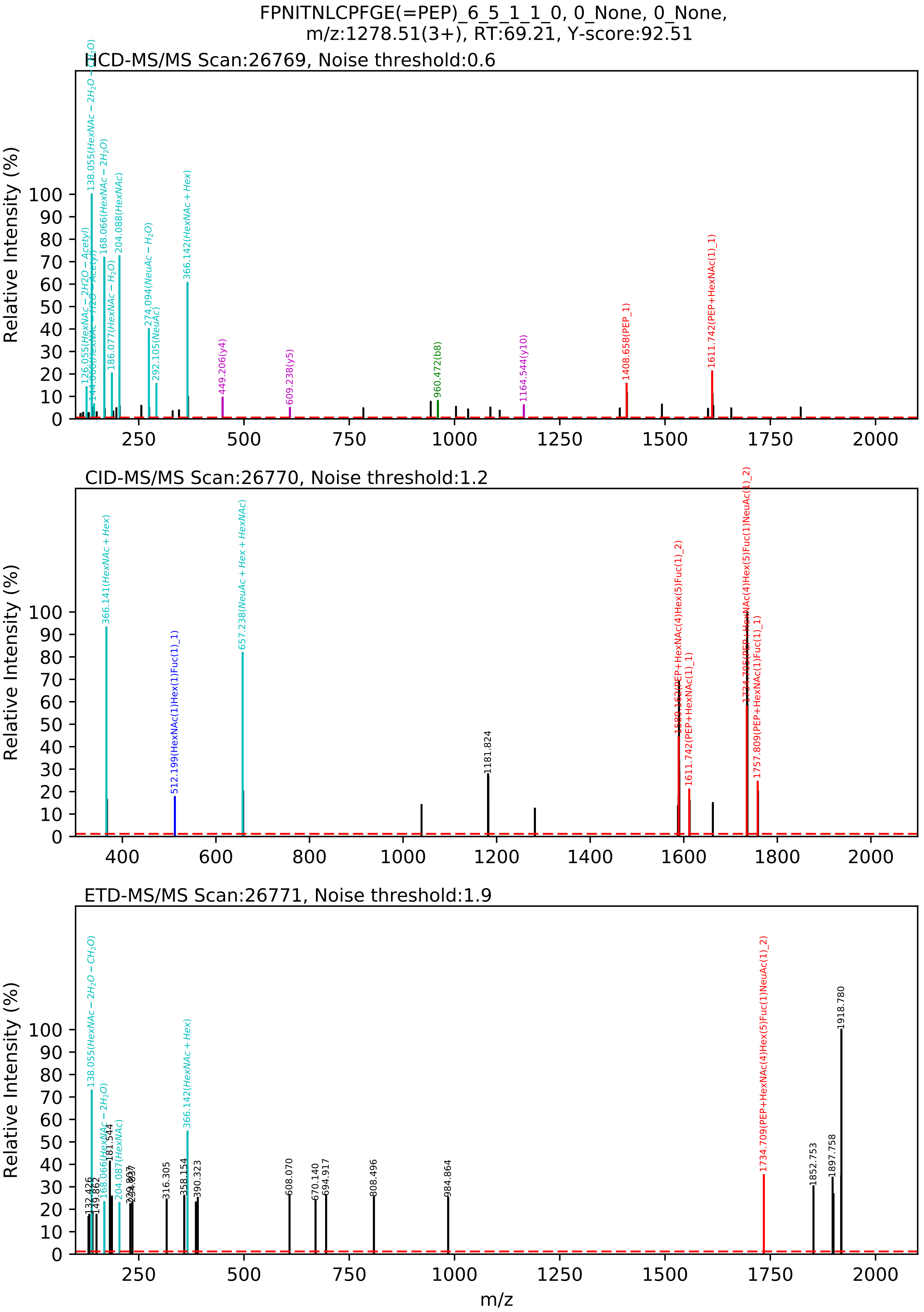

FPNITNLCPFGE(=PEP)_6_5_1_1_0, 0_None, 0_None,
m/z:1278.51(3+), RT:69.21, Y-score:92.51
HCD-MS/MS Scan:26769, Noise threshold:0.6
CID-MS/MS Scan:26770, Noise threshold:1.2
ETD-MS/MS Scan:26771, Noise threshold:1.9
Relative Intensity (%)
m/z
138.055(HexNAc − 2H2O − CH2O)
168.066(HexNAc − 2H2O)
204.088(HexNAc)
186.077(HexNAc − H2O)
274.094(NeuAc − H2O)
292.105(NeuAc)
366.142(HexNAc + Hex)
449.206(y4)
609.238(y5)
960.472(b8)
1164.544(y10)
1408.658(PEP_1)
1611.742(PEP+HexNAc(1)_1)
366.141(HexNAc + Hex)
512.199(HexNAc(1)Hex(1)Fuc(1)_1)
657.238(NeuAc + Hex + HexNAc)
1181.824
1611.742(PEP+HexNAc(1)_1)
1757.809(PEP+HexNAc(1)Fuc(1)_1)
1734.709(PEP+HexNAc(4)Hex(5)Fuc(1)NeuAc(1)_2)
1918.780
1897.758
1852.753
984.864
808.496
694.917
670.140
608.070
390.323
358.154
316.305
181.544
132.426
149.862

FPNITNLCPFGE(=PEP)_6_5_1_1_0, 0_None, 0_None,
m/z:959.14(4+), RT:69.40, Y-score:90.20

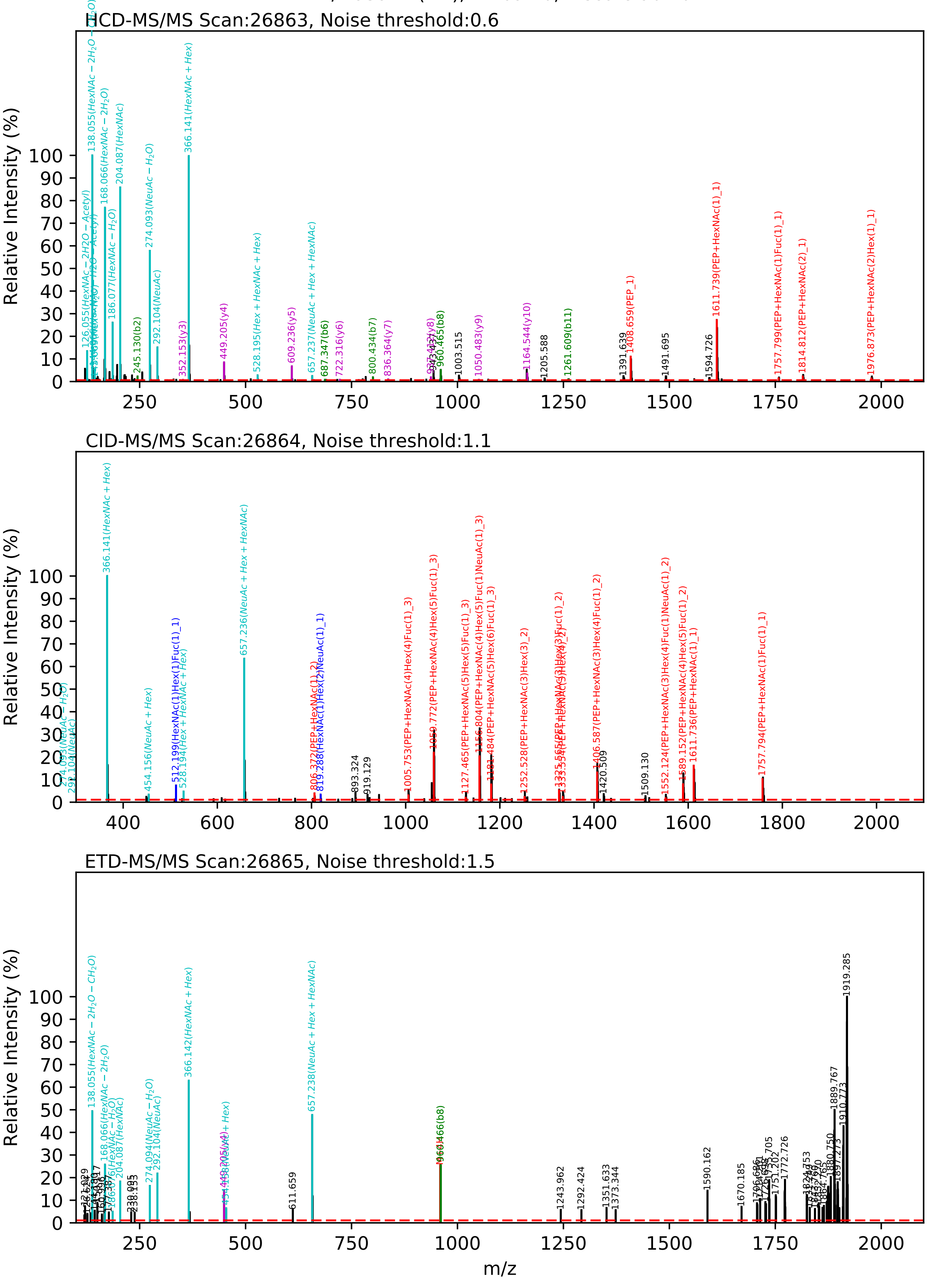

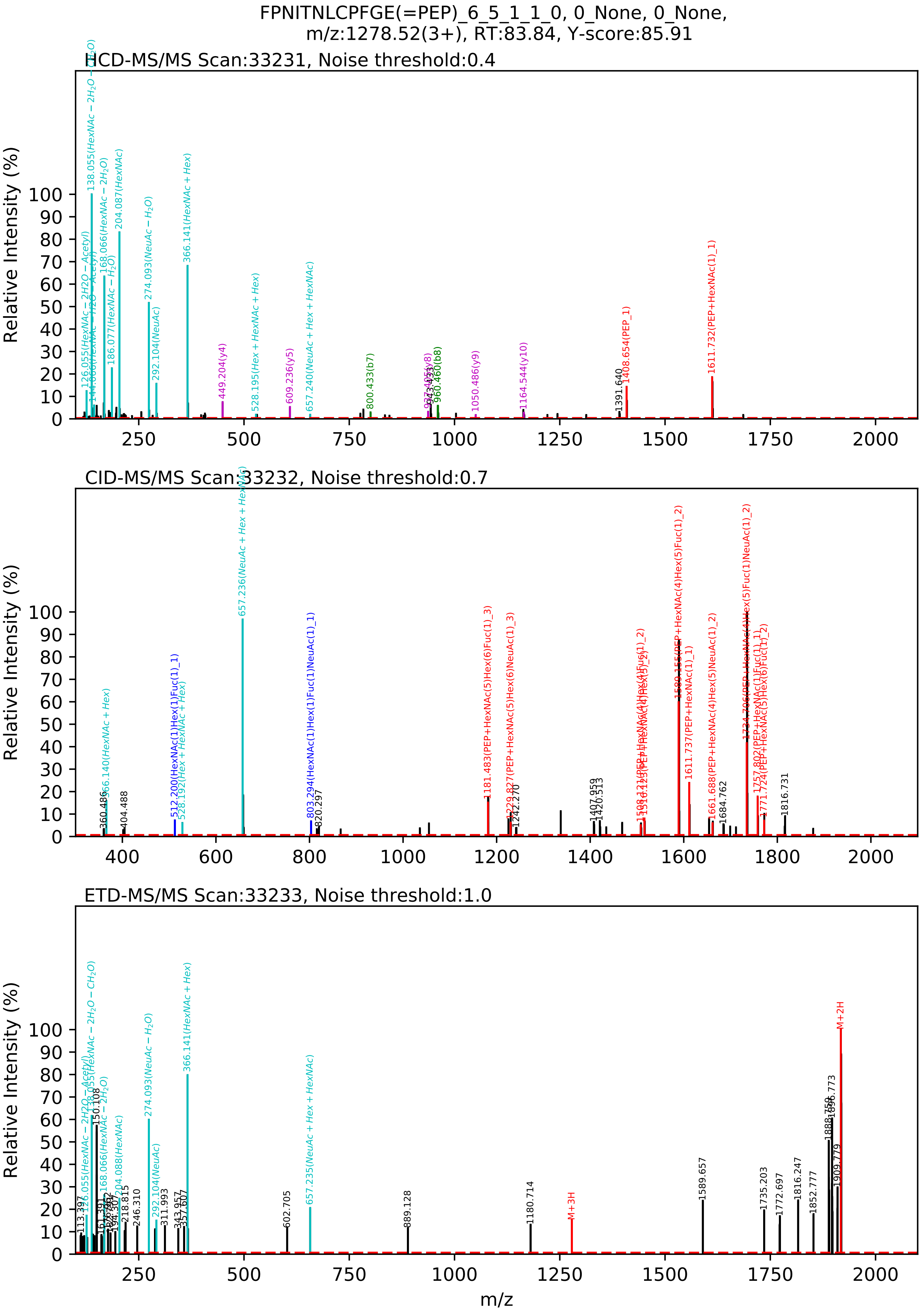

FPNITNLCPFGE(=PEP)_6_5_1_1_0, 0_None, 0_None,
m/z:1278.52(3+), RT:83.84, Y-score:85.91
HCD-MS/MS Scan:33231, Noise threshold:0.4
CID-MS/MS Scan:33232, Noise threshold:0.7
ETD-MS/MS Scan:33233, Noise threshold:1.0
Relative Intensity (%)
m/z

FPNITNLCPFGE(=PEP)_6_5_1_2_0, 0_None, 0_None,
m/z:1031.91(4+), RT:82.52, Y-score:87.84

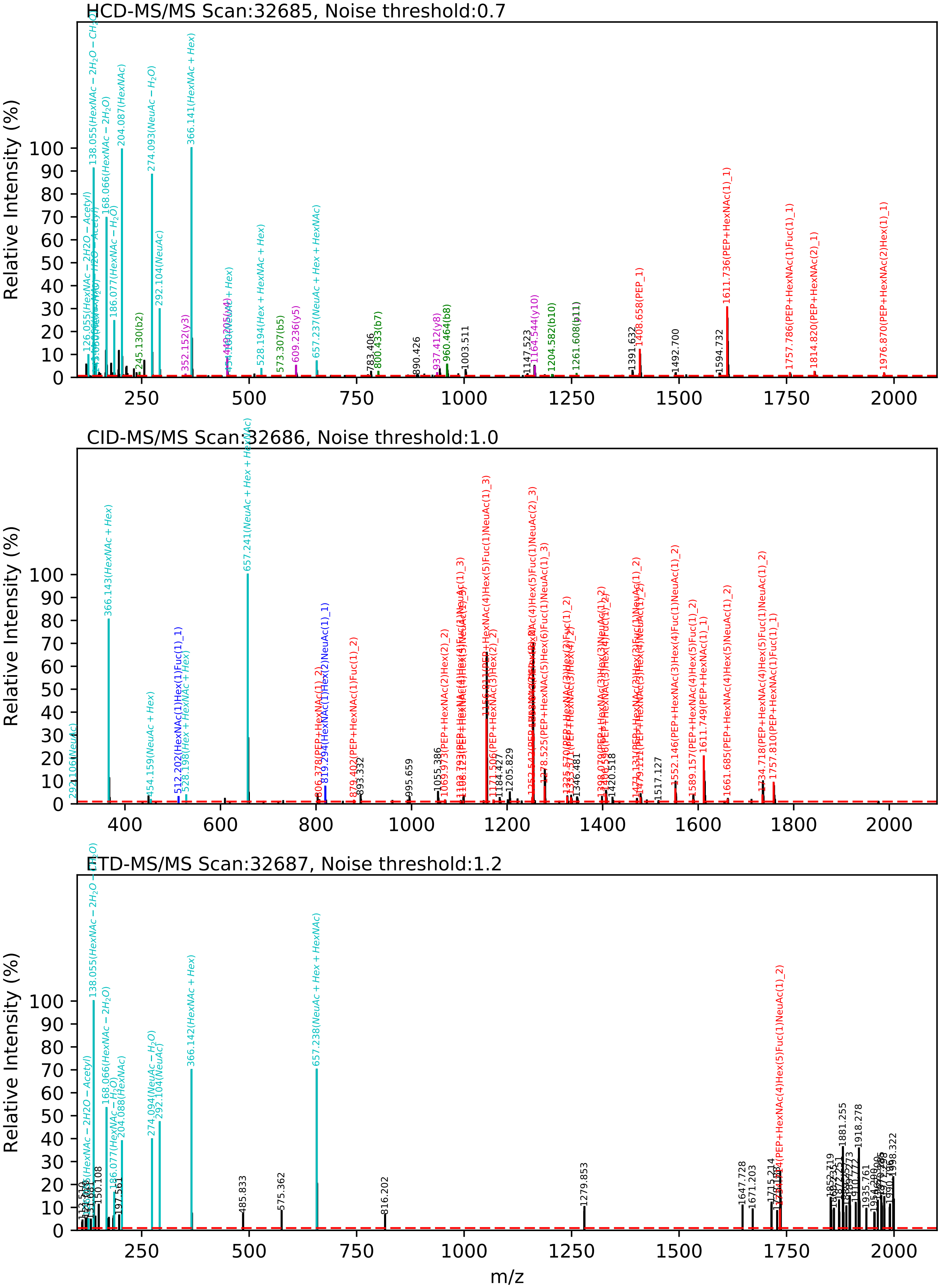

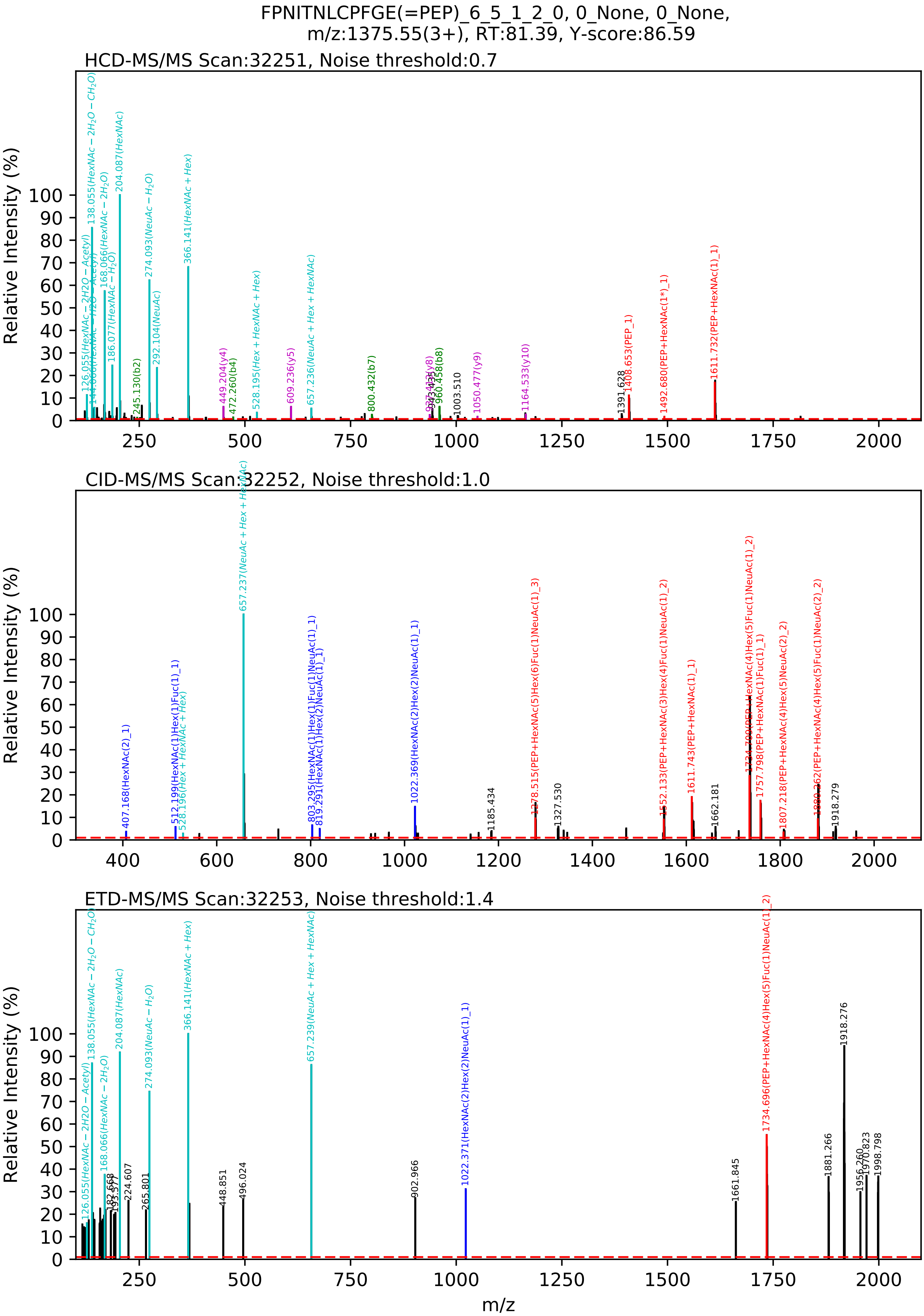

FPNITNLCPFGE(=PEP)_6_5_1_2_0, 0_None, 0_None,
m/z:1375.55(3+), RT:81.39, Y-score:86.59
HCD-MS/MS Scan:32251, Noise threshold:0.7
Relative Intensity (%)
126.055(HexNAc − 2H2O − Acetyl)
138.055(HexNAc − 2H2O − CH2O)
168.066(HexNAc − 2H2O)
186.077(HexNAc − H2O)
204.087(HexNAc)
274.093(NeuAc − H2O)
292.104(NeuAc)
366.141(HexNAc + Hex)
245.130(b2)
449.204(y4)
472.260(b4)
528.195(Hex + HexNAc + Hex)
609.236(y5)
657.236(NeuAc + Hex + HexNAc)
800.432(b7)
960.458(b8)
1003.510
1050.477(y9)
1164.533(y10)
1391.628
1408.653(PEP_1)
1492.680(PEP+HexNAc(1*)_1)
1611.732(PEP+HexNAc(1)_1)
CID-MS/MS Scan:32252, Noise threshold:1.0
407.168(HexNAc(2)_1)
512.199(HexNAc(1)Hex(1)Fuc(1)_1)
528.196(Hex + HexNAc + Hex)
657.237(NeuAc + Hex + HexNAc)
803.295(HexNAc(1)Hex(1)Fuc(1)NeuAc(1)_1)
819.291(HexNAc(1)Hex(2)NeuAc(1)_1)
1022.369(HexNAc(2)Hex(2)NeuAc(1)_1)
1185.434
1278.515(PEP+HexNAc(5)Hex(6)Fuc(1)NeuAc(1)_3)
1327.530
1552.133(PEP+HexNAc(3)Hex(4)Fuc(1)NeuAc(1)_2)
1611.743(PEP+HexNAc(1)_1)
1662.181
1734.700(PEP+HexNAc(4)Hex(5)Fuc(1)NeuAc(1)_2)
1757.798(PEP+HexNAc(1)Fuc(1)_1)
1807.218(PEP+HexNAc(4)Hex(5)NeuAc(2)_2)
1880.262(PEP+HexNAc(4)Hex(5)Fuc(1)NeuAc(2)_2)
1918.279
ETD-MS/MS Scan:32253, Noise threshold:1.4
126.055(HexNAc − 2H2O − Acetyl)
138.055(HexNAc − 2H2O − CH2O)
168.066(HexNAc − 2H2O)
182.668
193.577
204.087(HexNAc)
224.607
265.801
274.093(NeuAc − H2O)
366.141(HexNAc + Hex)
448.851
496.024
657.239(NeuAc + Hex + HexNAc)
902.966
1022.371(HexNAc(2)Hex(2)NeuAc(1)_1)
1661.845
1734.696(PEP+HexNAc(4)Hex(5)Fuc(1)NeuAc(1)_2)
1881.266
1918.276
1956.260
1970.823
1998.798
m/z

FPNITNLCPFGE(=PEP)_6_5_1_2_0, 0_None, 0_None,
m/z:1375.55(3+), RT:82.33, Y-score:89.49

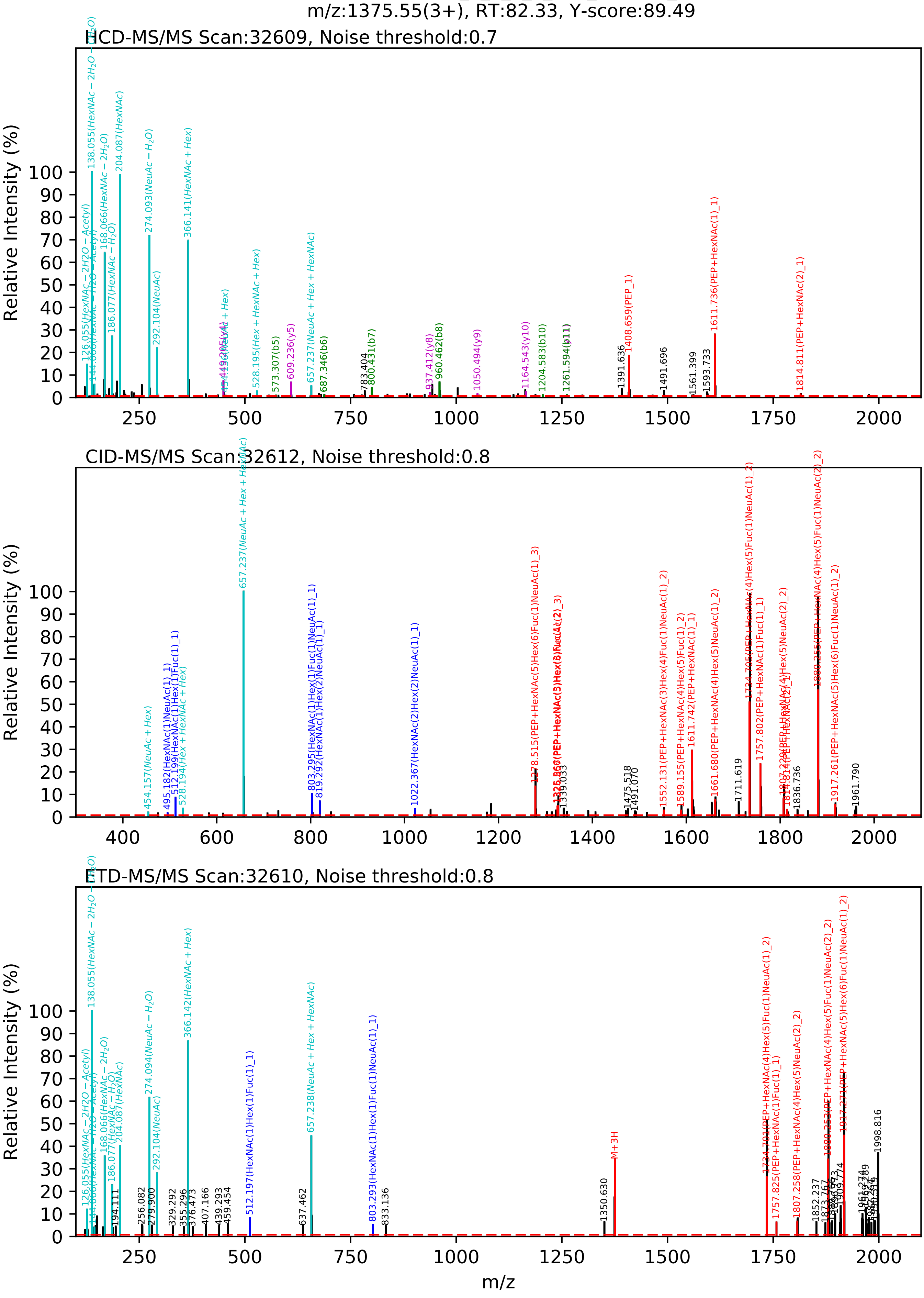

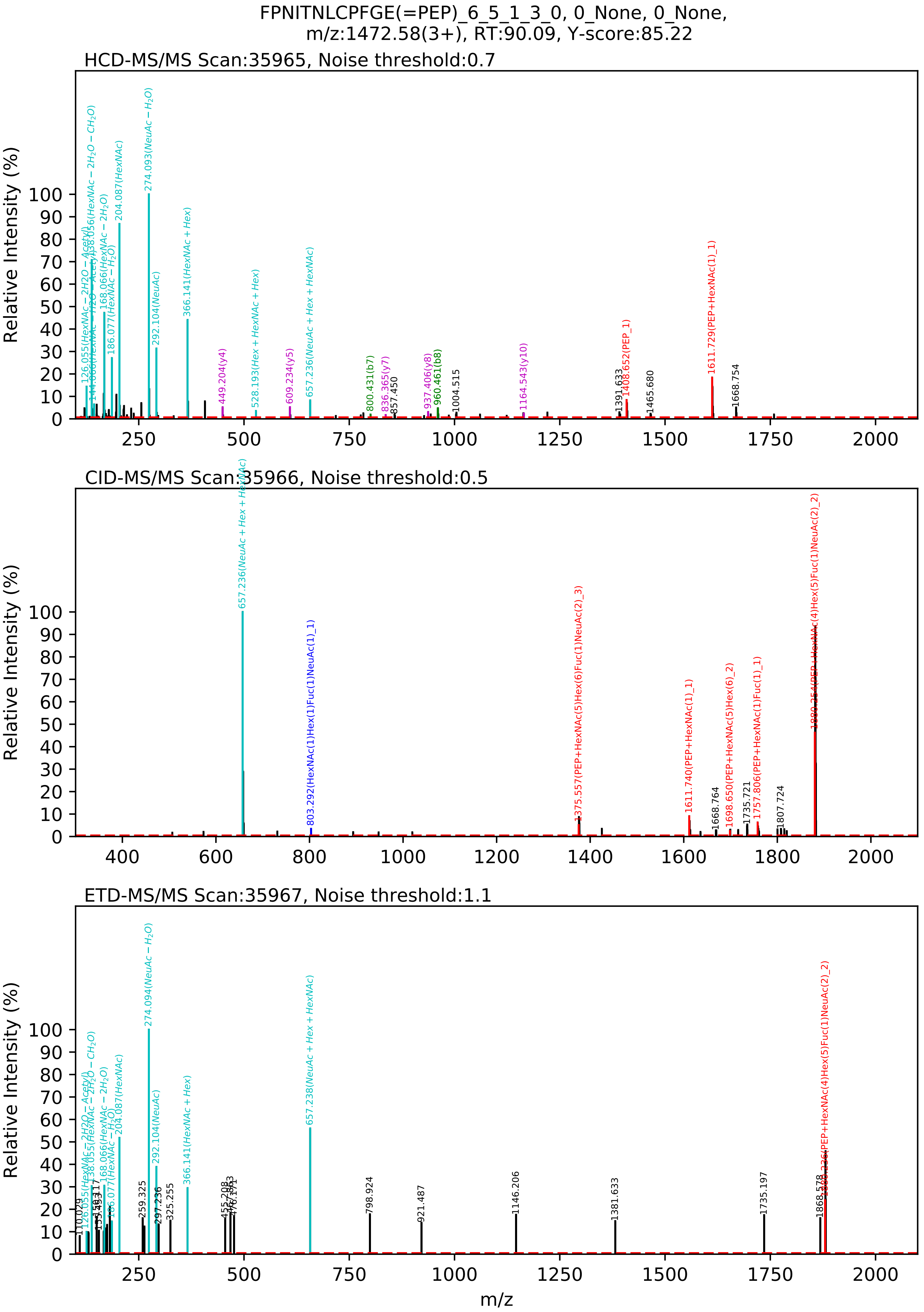

FPNITNLCPFGE(=PEP)_6_5_1_3_0, 0_None, 0_None,
m/z:1472.58(3+), RT:90.09, Y-score:85.22
HCD-MS/MS Scan:35965, Noise threshold:0.7
Relative Intensity (%)
274.093(NeuAc − H2O)
204.087(HexNAc)
366.141(HexNAc + Hex)
292.104(NeuAc)
168.066(HexNAc − 2H2O)
186.077(HexNAc − H2O)
126.055(HexNAc − 2H2O − Acetyl)
449.204(y4)
528.193(Hex + HexNAc + Hex)
609.234(y5)
657.236(NeuAc + Hex + HexNAc)
800.431(b7)
836.365(y7)
857.450
937.406(y8)
960.461(b8)
1004.515
1164.543(y10)
1391.633
1408.652(PEP_1)
1465.680
1611.729(PEP+HexNAc(1)_1)
1668.754
CID-MS/MS Scan:35966, Noise threshold:0.5
657.236(NeuAc + Hex + HexNAc)
803.292(HexNAc(1)Hex(1)Fuc(1)NeuAc(1)_1)
1375.557(PEP+HexNAc(5)Hex(6)Fuc(1)NeuAc(2)_3)
1611.740(PEP+HexNAc(1)_1)
1668.764
1698.650(PEP+HexNAc(5)Hex(6)_2)
1735.721
1757.806(PEP+HexNAc(1)Fuc(1)_1)
1807.724
PEP+HexNAc(4)Hex(5)Fuc(1)NeuAc(2)_2
ETD-MS/MS Scan:35967, Noise threshold:1.1
274.094(NeuAc − H2O)
657.238(NeuAc + Hex + HexNAc)
366.141(HexNAc + Hex)
292.104(NeuAc)
204.087(HexNAc)
168.066(HexNAc − 2H2O)
110.029
259.325
297.236
325.255
798.924
921.487
1146.206
1381.633
1735.197
1868.578
PEP+HexNAc(4)Hex(5)Fuc(1)NeuAc(2)_2
m/z

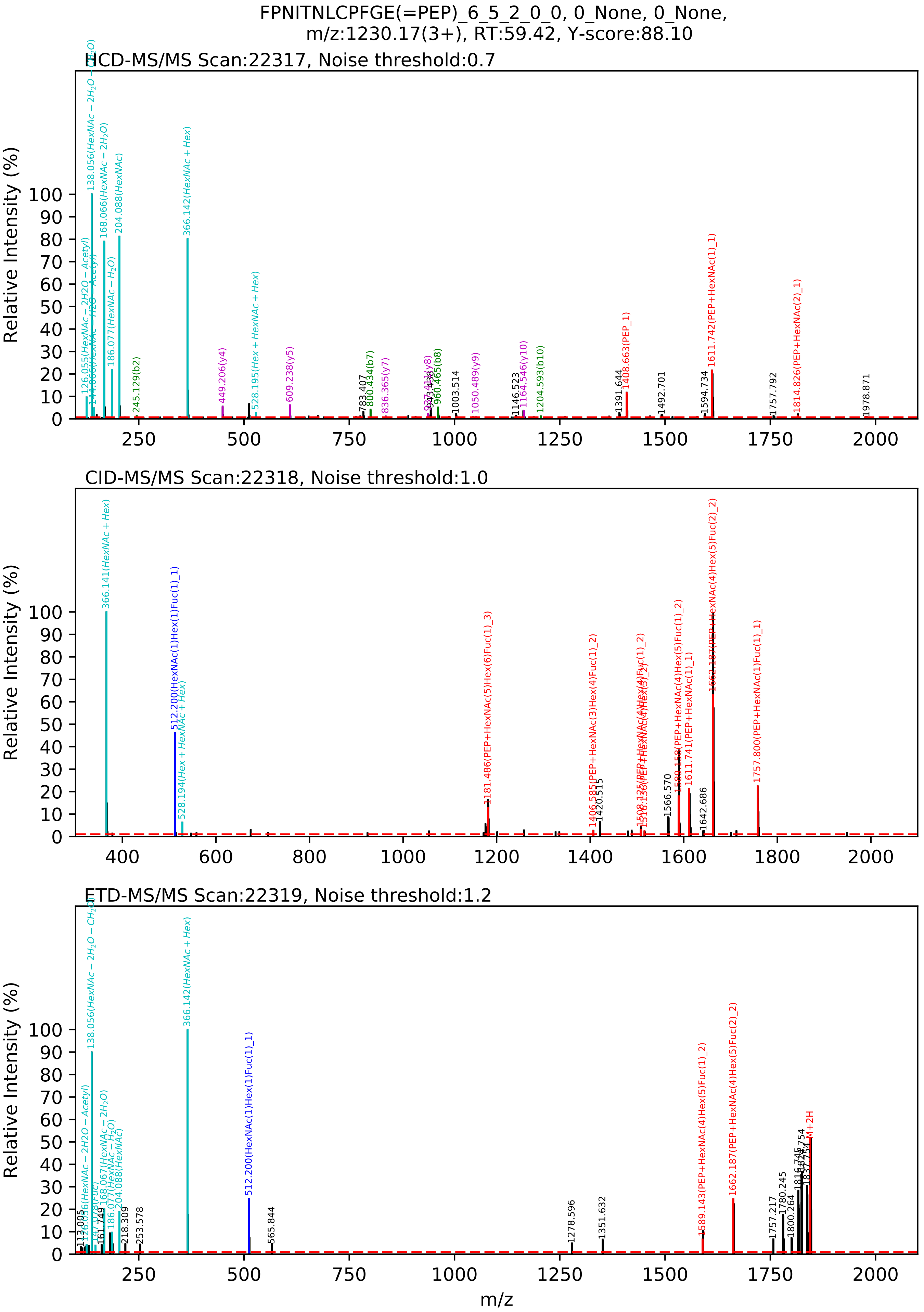

FPNITNLCPFGE(=PEP)_6_5_2_0_0, 0_None, 0_None,
m/z:1230.17(3+), RT:59.42, Y-score:88.10
HCD-MS/MS Scan:22317, Noise threshold:0.7
CID-MS/MS Scan:22318, Noise threshold:1.0
ETD-MS/MS Scan:22319, Noise threshold:1.2
Relative Intensity (%)
m/z
366.142(HexNAc + Hex)
204.088(HexNAc)
1611.742(PEP+HexNAc(1)_1)
1814.826(PEP+HexNAc(2)_1)
1408.663(PEP_1)
366.141(HexNAc + Hex)
512.200(HexNAc(1)Hex(1)Fuc(1)_1)
1181.486(PEP+HexNAc(5)Hex(6)Fuc(1)_3)
1662.187(PEP+HexNAc(4)Hex(5)Fuc(2)_2)
1757.800(PEP+HexNAc(1)Fuc(1)_1)
1589.143(PEP+HexNAc(4)Hex(5)Fuc(1)_2)
M+2H

FPNITNLCPFGE(=PEP)_6_5_2_1_0, 0_None, 0_None,
m/z:1327.20(3+), RT:69.09, Y-score:61.44

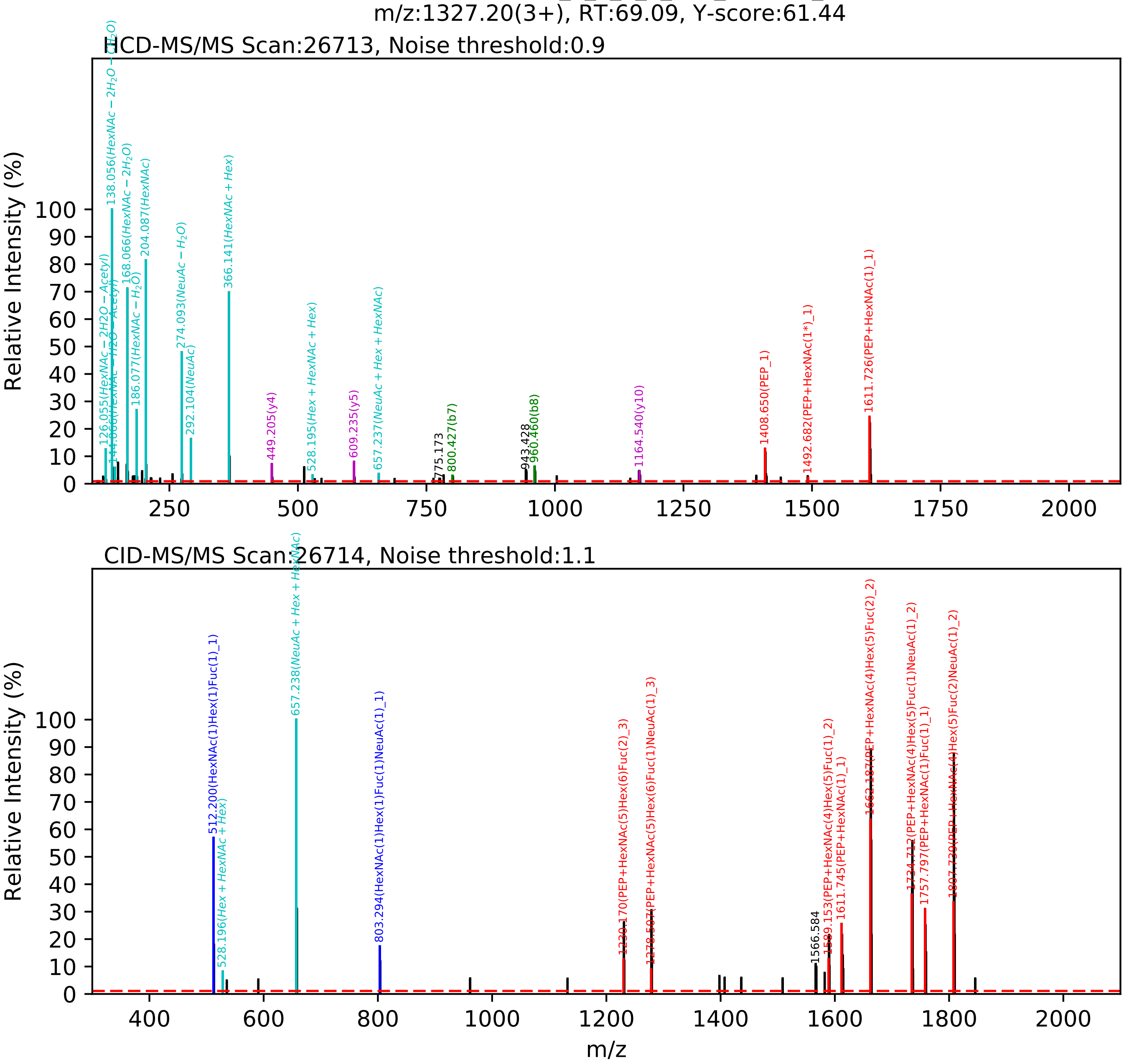

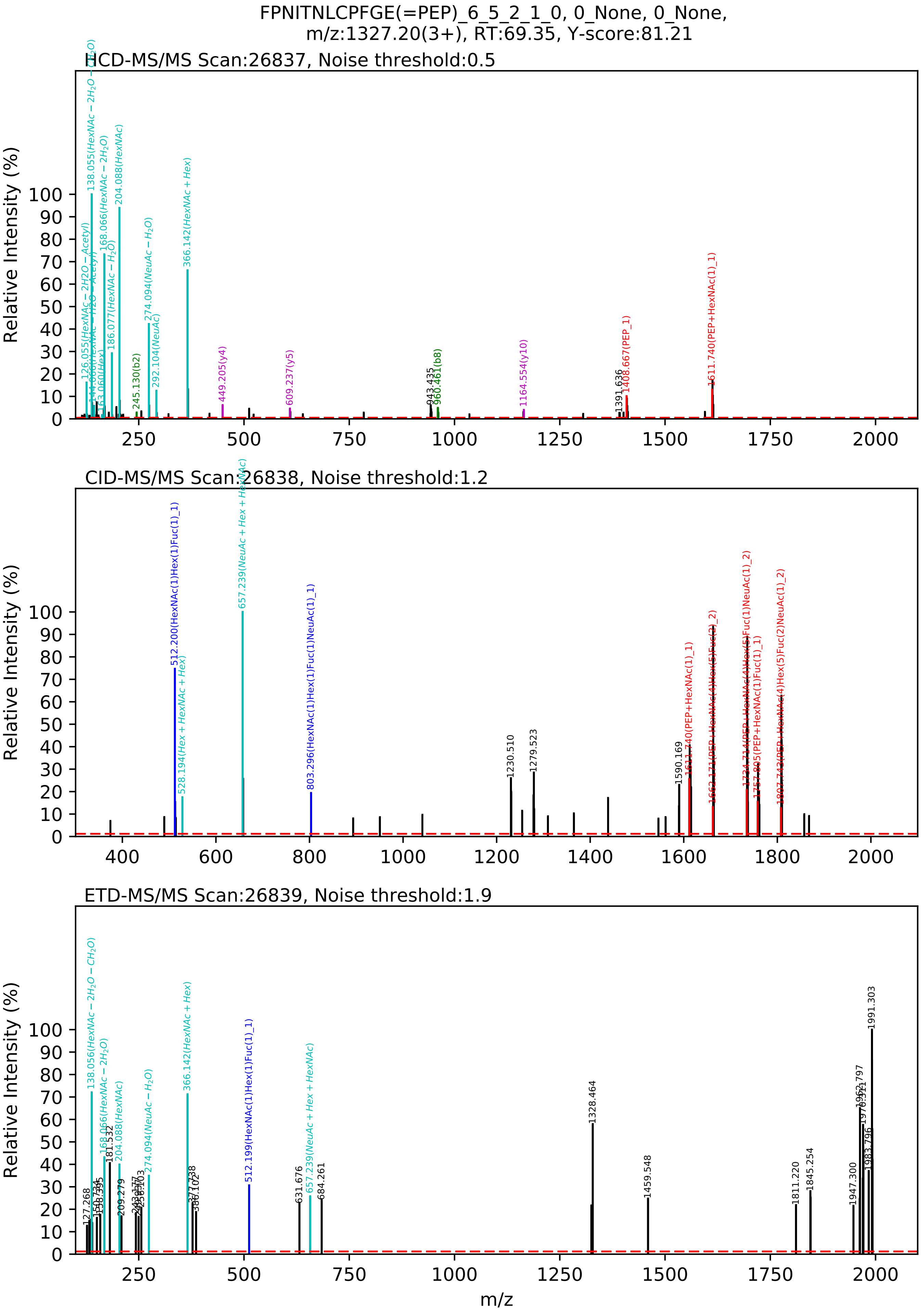
FPNITNLCPFGE(=PEP)_6_5_2_1_0, 0_None, 0_None,
m/z:1327.20(3+), RT:69.35, Y-score:81.21
HCD-MS/MS Scan:26837, Noise threshold:0.5
CID-MS/MS Scan:26838, Noise threshold:1.2
ETD-MS/MS Scan:26839, Noise threshold:1.9
Relative Intensity (%)
m/z

FPNITNLCPFGE(=PEP)_6_5_2_1_0, 0_None, 0_None,
m/z:1327.20(3+), RT:83.66, Y-score:88.79

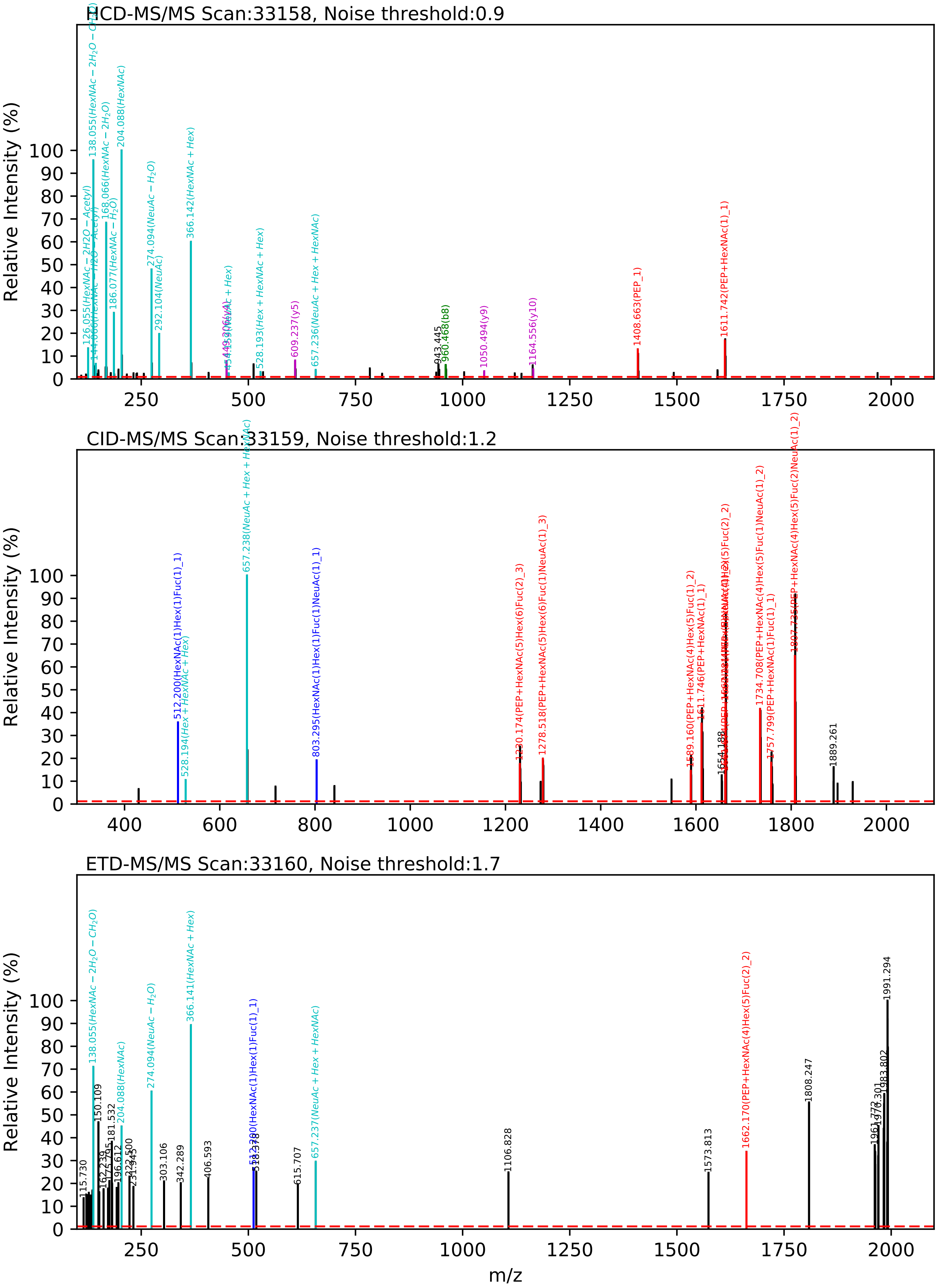

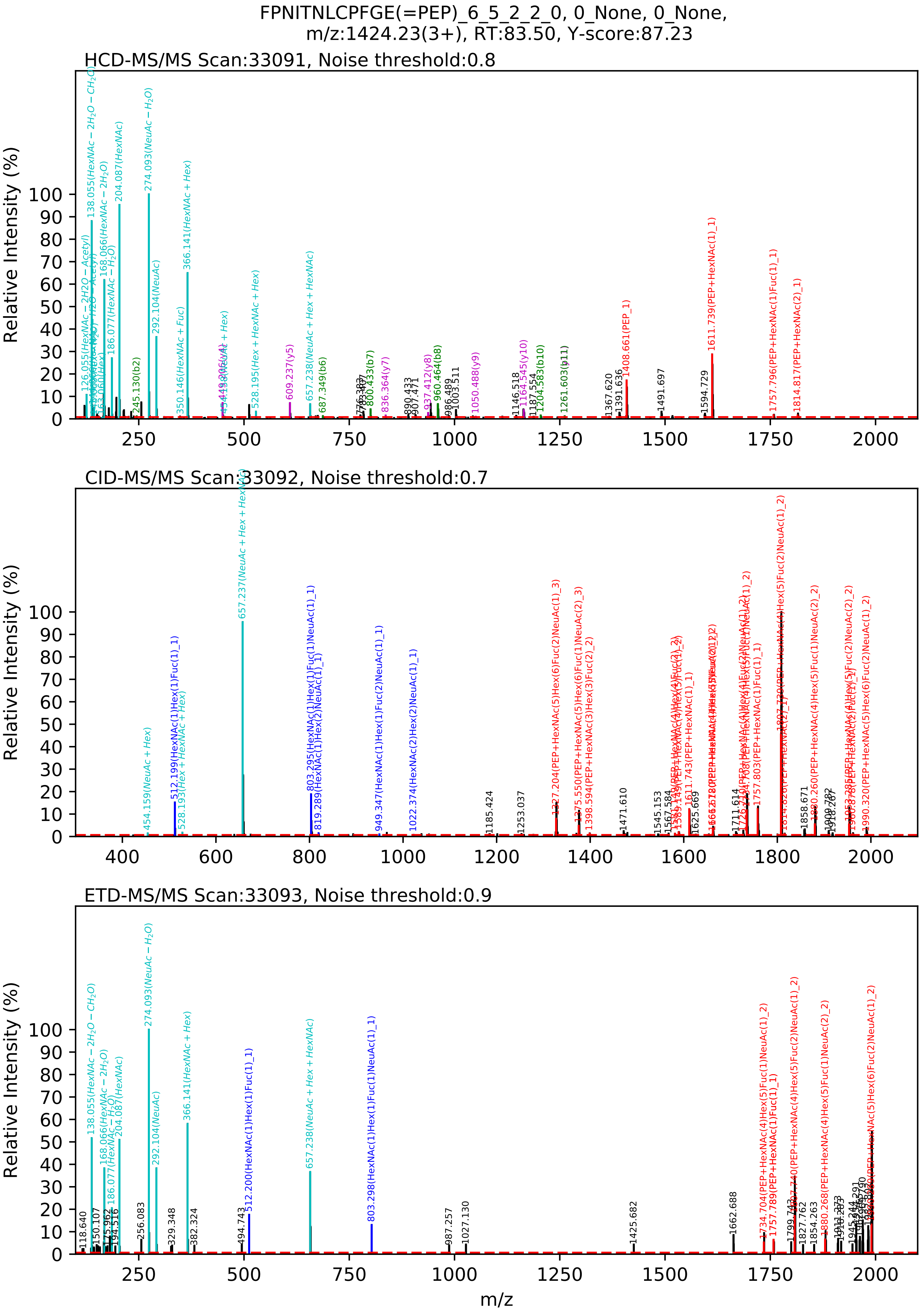
FPNITNLCPFGE(=PEP)_6_5_2_2_0, 0_None, 0_None,
m/z:1424.23(3+), RT:83.50, Y-score:87.23
HCD-MS/MS Scan:33091, Noise threshold:0.8
Relative Intensity (%)
138.055(HexNAc − 2H2O − CH2O)
204.087(HexNAc)
274.093(NeuAc − H2O)
366.141(HexNAc + Hex)
292.104(NeuAc)
168.066(HexNAc − 2H2O)
186.077(HexNAc − H2O)
126.055(HexNAc − 2H2O − Acetyl)
350.146(HexNAc + Fuc)
528.195(Hex + HexNAc + Hex)
657.238(NeuAc + Hex + HexNAc)
609.237(y5)
687.349(b6)
800.433(b7)
836.364(y7)
937.412(y8)
960.464(b8)
1050.488(y9)
1164.545(y10)
1204.583(b10)
1261.603(b11)
245.130(b2)
1408.661(PEP_1)
1611.739(PEP+HexNAc(1)_1)
1757.796(PEP+HexNAc(1)Fuc(1)_1)
1814.817(PEP+HexNAc(2)_1)
1367.620
1391.636
1491.697
1594.729
1146.518
1187.554
986.489
1003.511
890.433
907.471
776.387
CID-MS/MS Scan:33092, Noise threshold:0.7
657.237(NeuAc + Hex + HexNAc)
512.199(HexNAc(1)Hex(1)Fuc(1)_1)
528.193(Hex + HexNAc + Hex)
454.159(NeuAc + Hex)
803.295(HexNAc(1)Hex(1)Fuc(1)NeuAc(1)_1)
819.289(HexNAc(1)Hex(2)NeuAc(1)_1)
949.347(HexNAc(1)Hex(1)Fuc(2)NeuAc(1)_1)
1022.374(HexNAc(2)Hex(2)NeuAc(1)_1)
1185.424
1253.037
1327.204(PEP+HexNAc(5)Hex(6)Fuc(2)NeuAc(1)_3)
1375.550(PEP+HexNAc(5)Hex(6)Fuc(1)NeuAc(2)_3)
1398.594(PEP+HexNAc(3)Hex(3)Fuc(2)_2)
1471.610
1545.153
1567.584
1611.743(PEP+HexNAc(1)_1)
1625.669
1711.614
1757.803(PEP+HexNAc(1)Fuc(1)_1)
1814.826(PEP+HexNAc(2)_1)
1880.260(PEP+HexNAc(4)Hex(5)Fuc(1)NeuAc(2)_2)
1858.671
1990.320(PEP+HexNAc(5)Hex(6)Fuc(2)NeuAc(1)_2)
ETD-MS/MS Scan:33093, Noise threshold:0.9
138.055(HexNAc − 2H2O − CH2O)
168.066(HexNAc − 2H2O)
186.077(HexNAc − H2O)
204.087(HexNAc)
274.093(NeuAc − H2O)
292.104(NeuAc)
366.141(HexNAc + Hex)
512.200(HexNAc(1)Hex(1)Fuc(1)_1)
657.238(NeuAc + Hex + HexNAc)
803.298(HexNAc(1)Hex(1)Fuc(1)NeuAc(1)_1)
118.640
150.107
175.962
194.516
256.083
329.348
382.324
494.743
987.257
1027.130
1425.682
1662.688
1734.704(PEP+HexNAc(4)Hex(5)Fuc(1)NeuAc(1)_2)
1757.789(PEP+HexNAc(1)Fuc(1)_1)
1807.740(PEP+HexNAc(4)Hex(5)Fuc(2)NeuAc(1)_2)
1799.743
1827.762
1854.263
1880.268(PEP+HexNAc(4)Hex(5)Fuc(1)NeuAc(2)_2)
1911.273
m/z

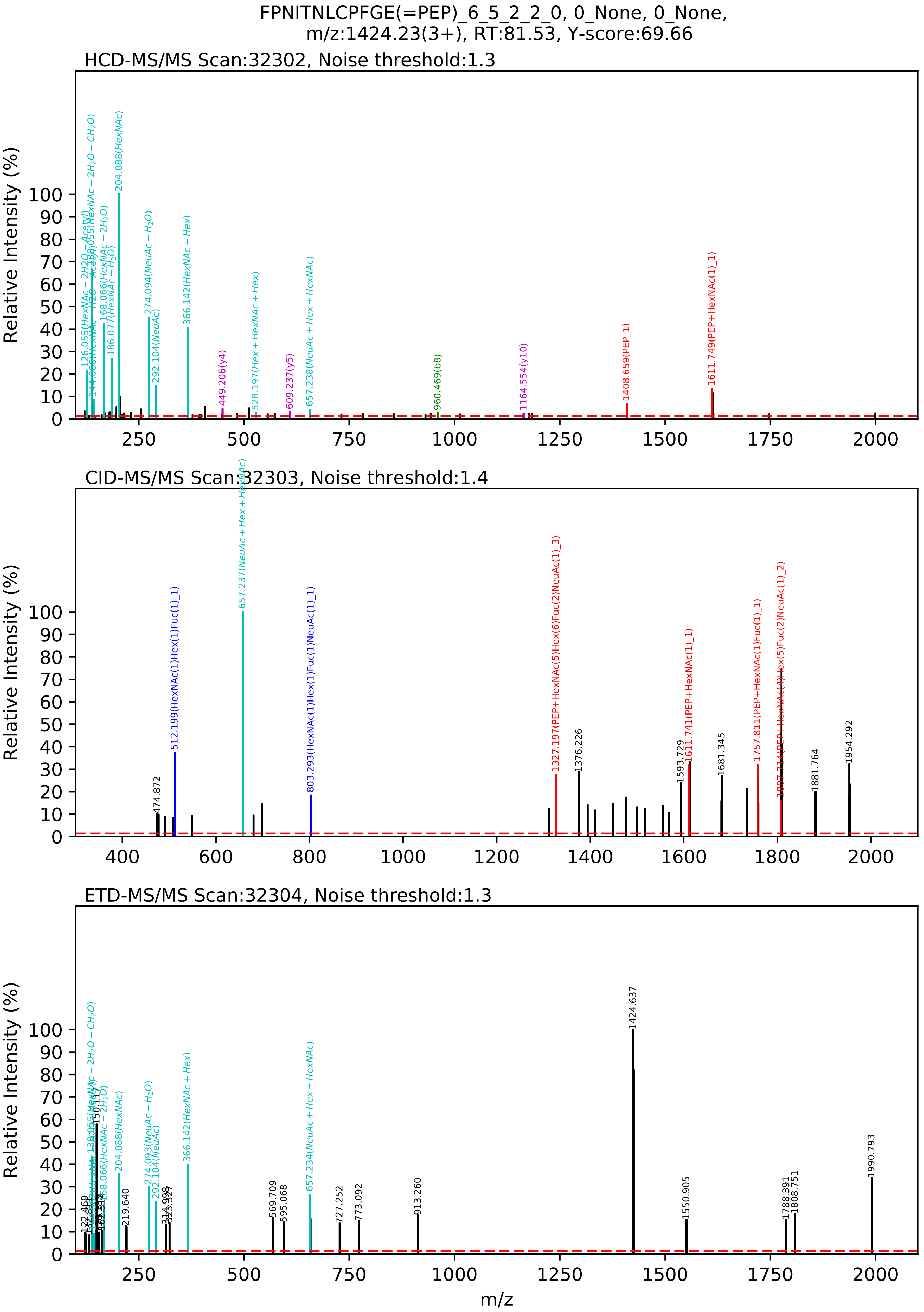

FPNITNLCPFGE(=PEP)_6_5_2_2_0, 0_None, 0_None,
m/z:1424.23(3+), RT:81.53, Y-score:69.66
HCD-MS/MS Scan:32302, Noise threshold:1.3
CID-MS/MS Scan:32303, Noise threshold:1.4
ETD-MS/MS Scan:32304, Noise threshold:1.3
Relative Intensity (%)
m/z

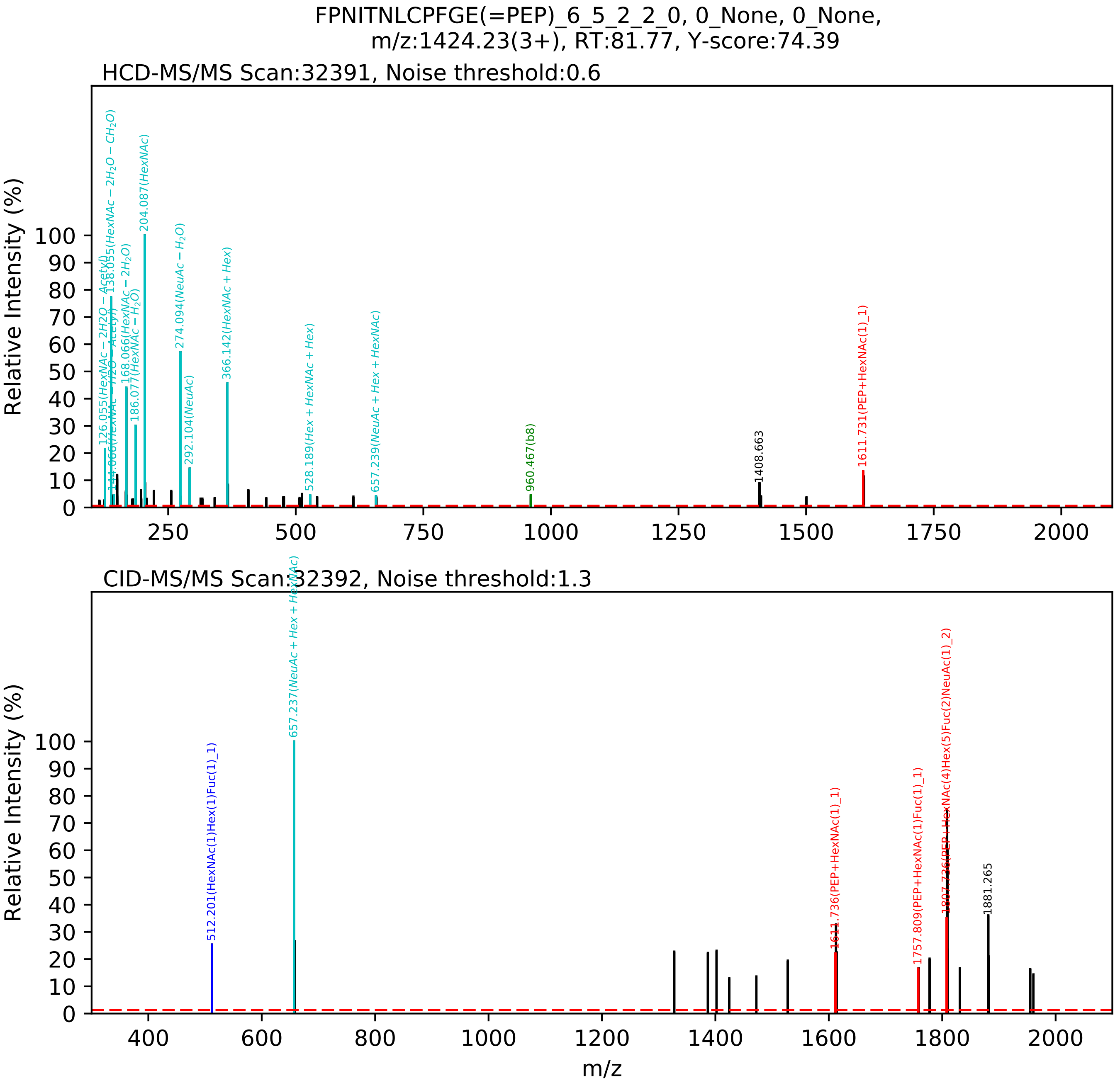
FPNITNLCPFGE(=PEP)_6_5_2_2_0, 0_None, 0_None,
m/z:1424.23(3+), RT:81.77, Y-score:74.39
HCD-MS/MS Scan:32391, Noise threshold:0.6
Relative Intensity (%)
204.087(HexNAc)
274.094(NeuAc − H2O)
292.104(NeuAc)
366.142(HexNAc + Hex)
528.189(Hex + HexNAc + Hex)
657.239(NeuAc + Hex + HexNAc)
960.467(b8)
1408.663
1611.731(PEP+HexNAc(1)_1)
CID-MS/MS Scan:32392, Noise threshold:1.3
512.201(HexNAc(1)Hex(1)Fuc(1)_1)
657.237(NeuAc + Hex + HexNAc)
1611.736(PEP+HexNAc(1)_1)
1757.809(PEP+HexNAc(1)Fuc(1)_1)
1881.265
m/z

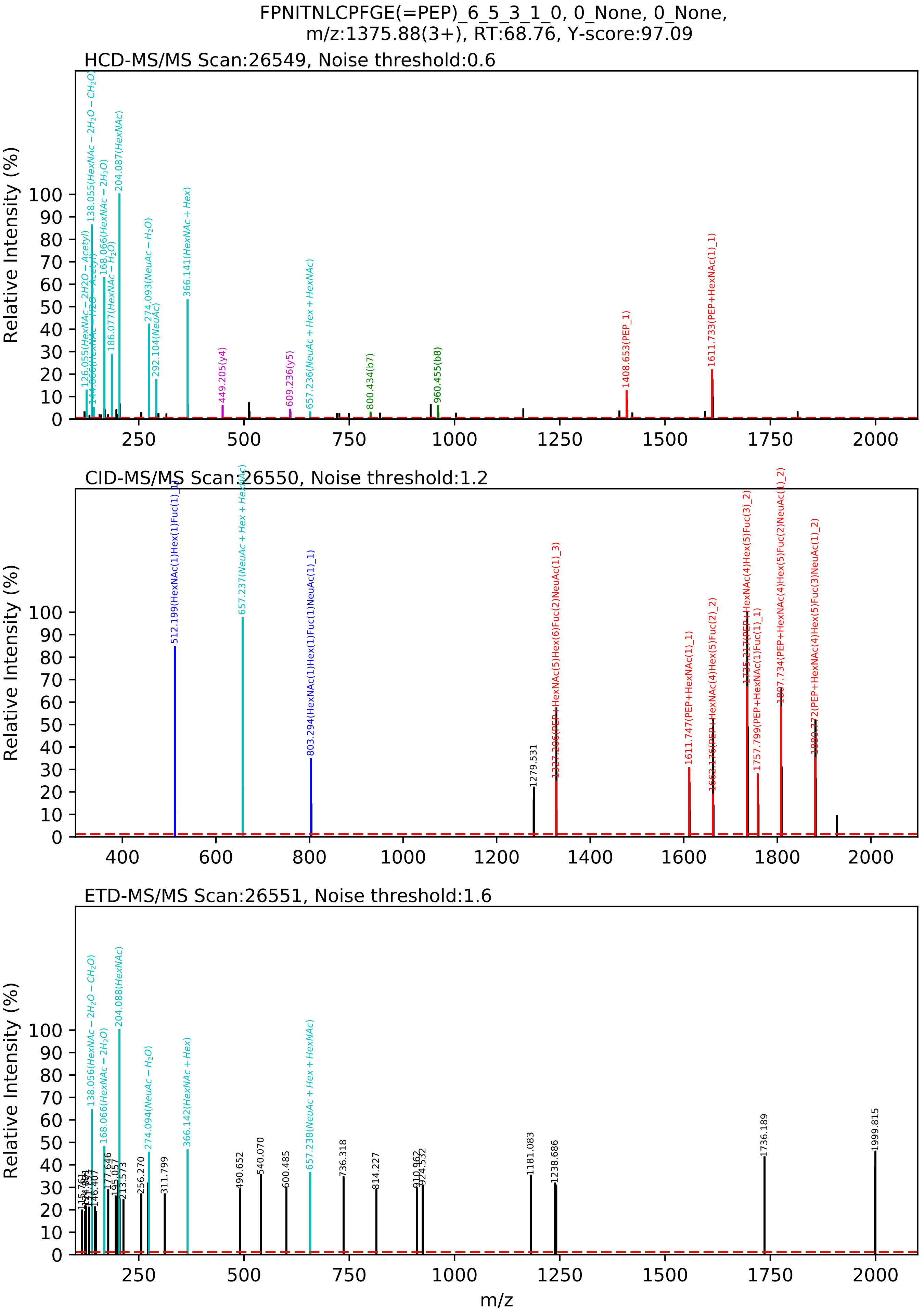

FPNITNLCPFGE(=PEP)_6_5_3_1_0, 0_None, 0_None,
m/z:1375.88(3+), RT:68.76, Y-score:97.09
HCD-MS/MS Scan:26549, Noise threshold:0.6
Relative Intensity (%)
204.087(HexNAc)
138.055(HexNAc − 2H₂O − CH₂O)
168.066(HexNAc − 2H₂O)
186.077(HexNAc − H₂O)
126.055(HexNAc − 2H2O − Acetyl)
274.093(NeuAc − H₂O)
292.104(NeuAc)
366.141(HexNAc + Hex)
449.205(y4)
609.236(y5)
657.236(NeuAc + Hex + HexNAc)
800.434(b7)
960.455(b8)
1408.653(PEP_1)
1611.733(PEP+HexNAc(1)_1)
CID-MS/MS Scan:26550, Noise threshold:1.2
Relative Intensity (%)
512.199(HexNAc(1)Hex(1)Fuc(1)_1)
657.237(NeuAc + Hex + HexNAc)
803.294(HexNAc(1)Hex(1)Fuc(1)NeuAc(1)_1)
1279.531
1611.747(PEP+HexNAc(1)_1)
1757.799(PEP+HexNAc(1)Fuc(1)_1)
ETD-MS/MS Scan:26551, Noise threshold:1.6
Relative Intensity (%)
204.088(HexNAc)
138.056(HexNAc − 2H₂O − CH₂O)
168.066(HexNAc − 2H₂O)
274.094(NeuAc − H₂O)
366.142(HexNAc + Hex)
657.238(NeuAc + Hex + HexNAc)
177.646
195.057
213.573
256.270
311.799
490.652
540.070
600.485
736.318
814.227
910.962
924.532
1181.083
1238.686
1736.189
1999.815
m/z

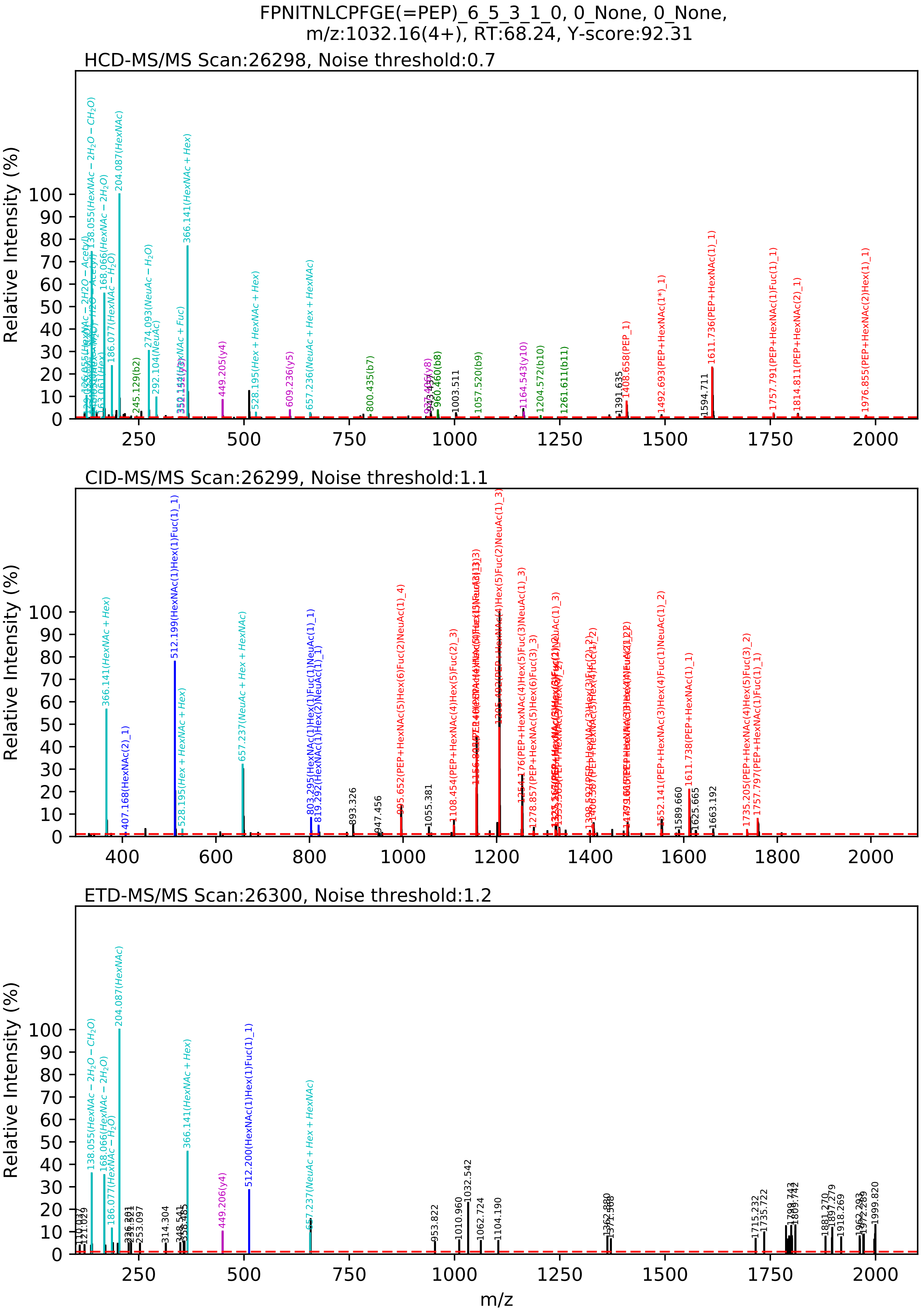
FPNITNLCPFGE(=PEP)_6_5_3_1_0, 0_None, 0_None,
m/z:1032.16(4+), RT:68.24, Y-score:92.31
HCD-MS/MS Scan:26298, Noise threshold:0.7
CID-MS/MS Scan:26299, Noise threshold:1.1
ETD-MS/MS Scan:26300, Noise threshold:1.2
Relative Intensity (%)
m/z

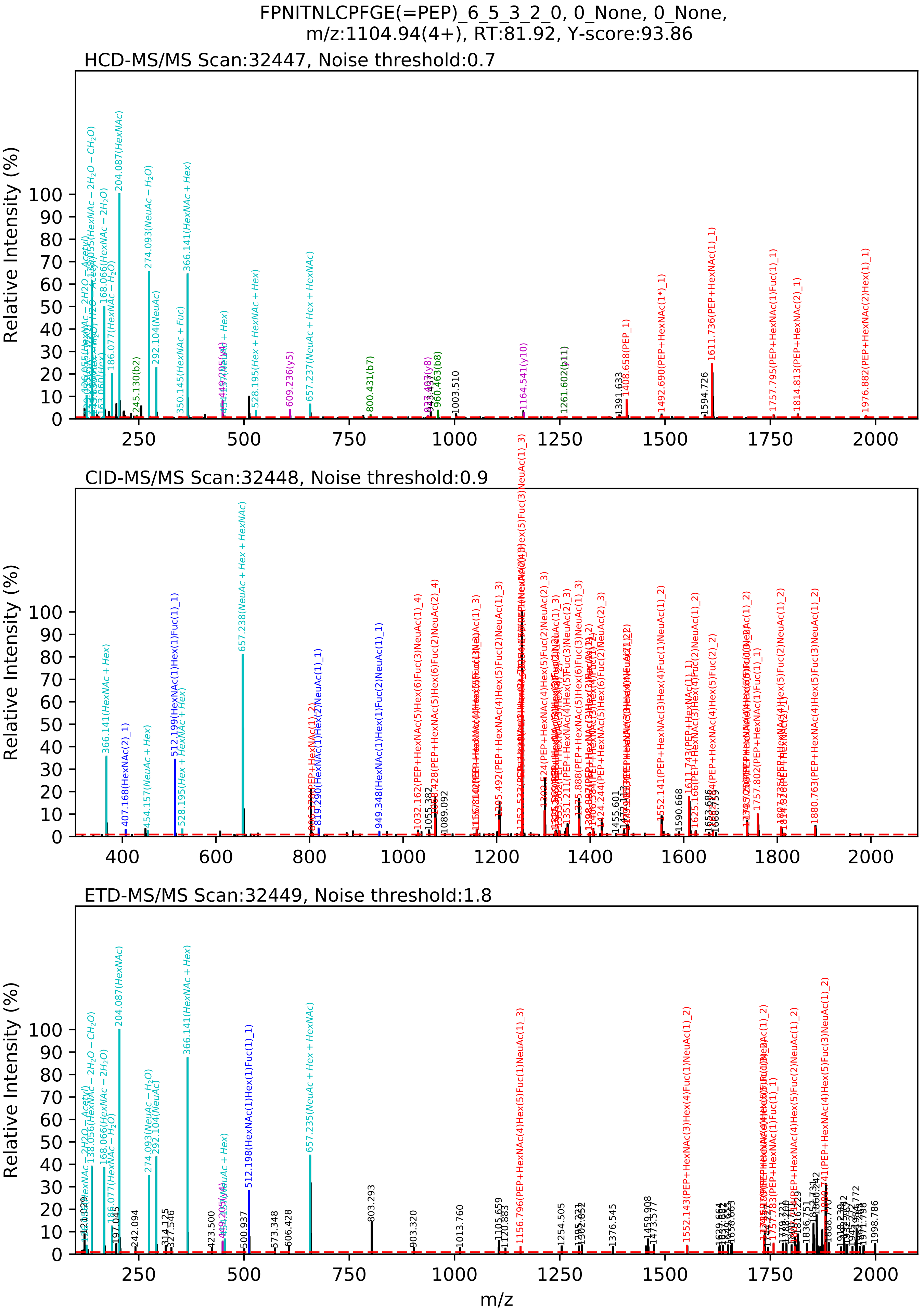
FPNITNLCPFGE(=PEP)_6_5_3_2_0, 0_None, 0_None,
m/z:1104.94(4+), RT:81.92, Y-score:93.86
HCD-MS/MS Scan:32447, Noise threshold:0.7
Relative Intensity (%)
204.087(HexNAc)
274.093(NeuAc − H2O)
292.104(NeuAc)
366.141(HexNAc + Hex)
528.195(Hex + HexNAc + Hex)
609.236(y5)
657.237(NeuAc + Hex + HexNAc)
800.431(b7)
960.463(b8)
1003.510
1164.541(y10)
1261.602(b11)
1391.633
1408.658(PEP_1)
1492.690(PEP+HexNAc(1*)_1)
1594.726
1611.736(PEP+HexNAc(1)_1)
1757.795(PEP+HexNAc(1)Fuc(1)_1)
1814.813(PEP+HexNAc(2)_1)
1976.882(PEP+HexNAc(2)Hex(1)_1)
CID-MS/MS Scan:32448, Noise threshold:0.9
Relative Intensity (%)
366.141(HexNAc + Hex)
407.168(HexNAc(2)_1)
512.199(HexNAc(1)Hex(1)Fuc(1)_1)
657.238(NeuAc + Hex + HexNAc)
949.348(HexNAc(1)Hex(1)Fuc(2)NeuAc(1)_1)
1055.382
1089.092
1295.492(PEP+HexNAc(4)Hex(5)Fuc(2)NeuAc(1)_3)
1552.141(PEP+HexNAc(3)Hex(4)Fuc(1)NeuAc(1)_2)
1590.668
1757.802(PEP+HexNAc(1)Fuc(1)_1)
1880.763(PEP+HexNAc(4)Hex(5)Fuc(3)NeuAc(1)_2)
ETD-MS/MS Scan:32449, Noise threshold:1.8
Relative Intensity (%)
204.087(HexNAc)
366.141(HexNAc + Hex)
512.198(HexNAc(1)Hex(1)Fuc(1)_1)
657.235(NeuAc + Hex + HexNAc)
803.293
903.320
1013.760
1156.796(PEP+HexNAc(4)Hex(5)Fuc(1)NeuAc(1)_3)
1254.505
1376.545
1552.143(PEP+HexNAc(3)Hex(4)Fuc(1)NeuAc(1)_2)
1998.786
m/z

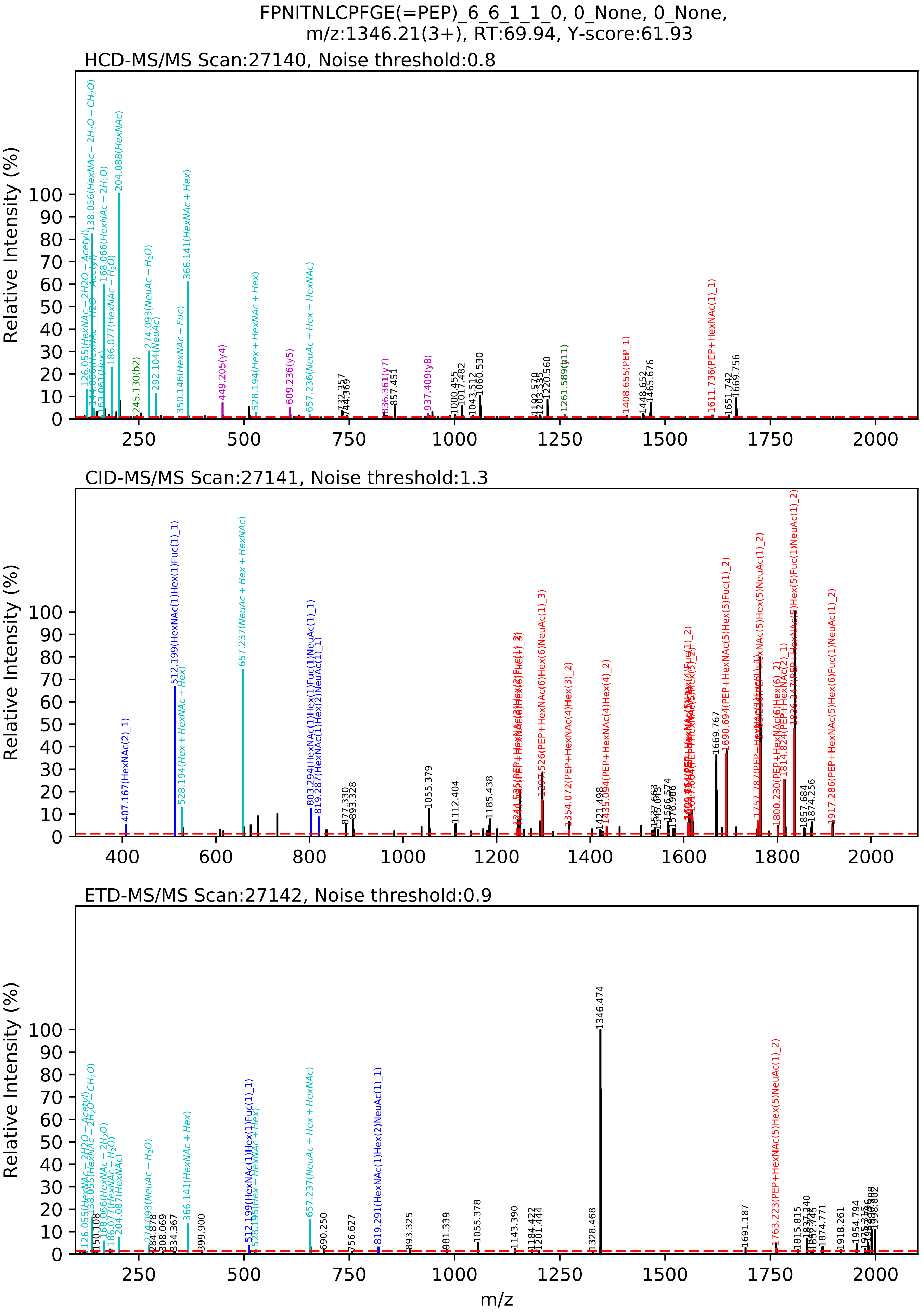
FPNITNLCPFGE(=PEP)_6_6_1_1_0, 0_None, 0_None,
m/z:1346.21(3+), RT:69.94, Y-score:61.93
HCD-MS/MS Scan:27140, Noise threshold:0.8
Relative Intensity (%)
204.088(HexNAc)
138.056(HexNAc − 2H2O − CH2O)
168.066(HexNAc − 2H2O)
186.077(HexNAc − H2O)
126.055(HexNAc − 2H2O − Acetyl)
366.141(HexNAc + Hex)
274.093(NeuAc − H2O)
292.104(NeuAc)
350.146(HexNAc + Fuc)
245.130(b2)
449.205(y4)
528.194(Hex + HexNAc + Hex)
609.236(y5)
657.236(NeuAc + Hex + HexNAc)
732.357
744.369
836.361(y7)
857.451
937.409(y8)
1000.455
1017.482
1043.512
1060.530
1220.560
1261.589(b11)
1408.655(PEP_1)
1448.652
1465.676
1611.736(PEP+HexNAc(1)_1)
1651.742
1669.756
CID-MS/MS Scan:27141, Noise threshold:1.3
Relative Intensity (%)
407.167(HexNAc(2)_1)
512.199(HexNAc(1)Hex(1)Fuc(1)_1)
528.194(Hex + HexNAc + Hex)
657.237(NeuAc + Hex + HexNAc)
803.294(HexNAc(1)Hex(1)Fuc(1)NeuAc(1)_1)
819.287(HexNAc(1)Hex(2)NeuAc(1)_1)
877.330
893.328
1055.379
1112.404
1185.438
1297.526(PEP+HexNAc(6)Hex(6)NeuAc(1)_3)
1354.072(PEP+HexNAc(4)Hex(3)_2)
1435.094(PEP+HexNAc(4)Hex(4)_2)
1421.498
1566.574
1576.986
1669.767
1690.694(PEP+HexNAc(5)Hex(5)Fuc(1)_2)
1757.787
1800.230(PEP+HexNAc(6)Hex(6)_2)
1814.824
1857.684
1874.256
1917.286(PEP+HexNAc(5)Hex(6)Fuc(1)NeuAc(1)_2)
ETD-MS/MS Scan:27142, Noise threshold:0.9
Relative Intensity (%)
1346.474
126.055
150.108
168.066(HexNAc − 2H2O)
186.077(HexNAc − H2O)
204.087(HexNAc)
274.093(NeuAc − H2O)
284.878
308.069
334.367
366.141(HexNAc + Hex)
399.900
512.199(HexNAc(1)Hex(1)Fuc(1)_1)
528.195(Hex + HexNAc + Hex)
657.237(NeuAc + Hex + HexNAc)
690.250
756.627
819.291(HexNAc(1)Hex(2)NeuAc(1)_1)
893.325
981.339
1055.378
1143.390
1184.422
1201.444
1328.468
1691.187
1763.223(PEP+HexNAc(5)Hex(5)NeuAc(1)_2)
1815.815
1852.745
1874.771
1918.261
1954.794
m/z

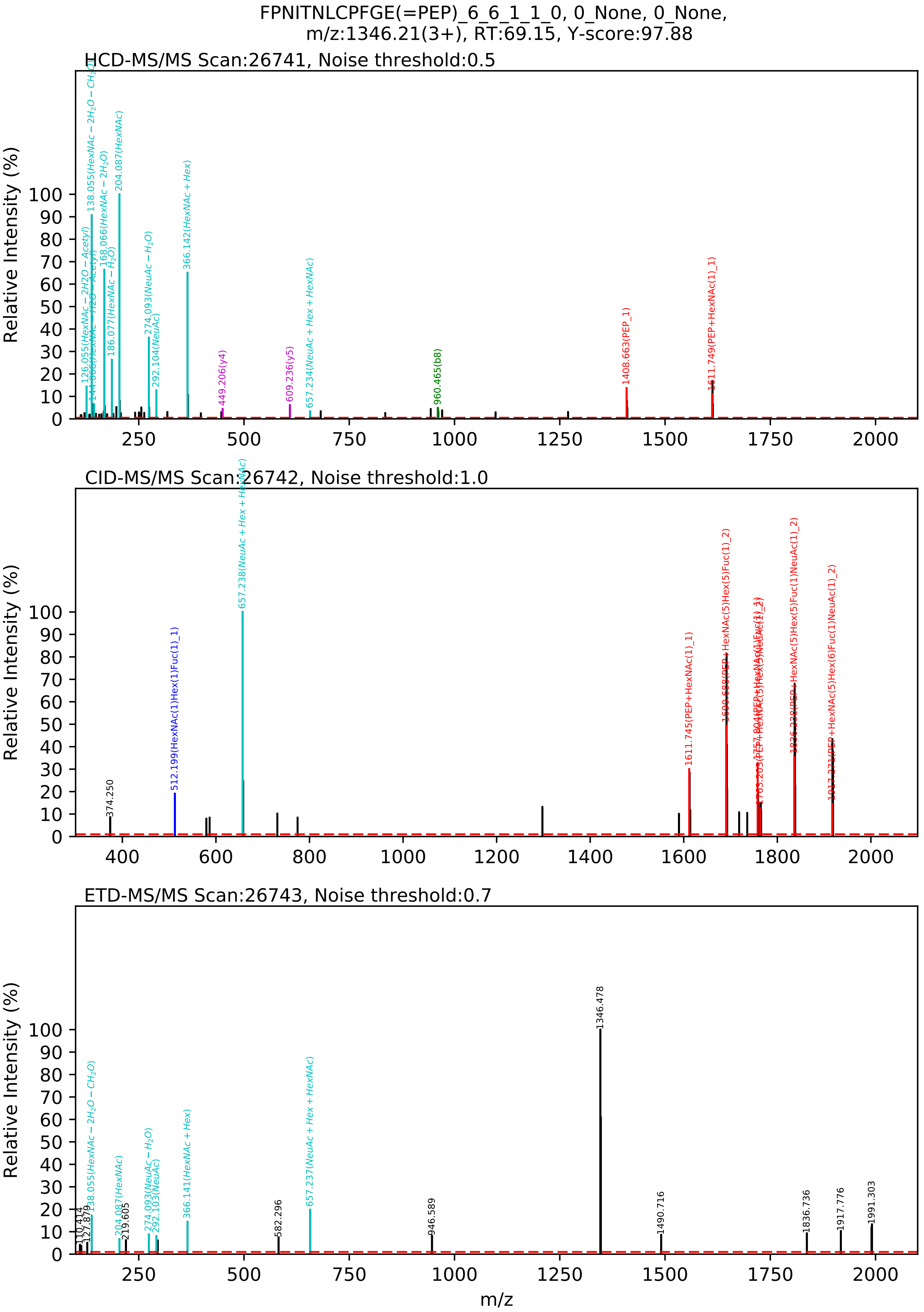

FPNITNLCPFGE(=PEP)_6_6_1_1_0, 0_None, 0_None,
m/z:1346.21(3+), RT:69.15, Y-score:97.88
HCD-MS/MS Scan:26741, Noise threshold:0.5
CID-MS/MS Scan:26742, Noise threshold:1.0
ETD-MS/MS Scan:26743, Noise threshold:0.7
Relative Intensity (%)
m/z
204.087(HexNAc)
366.142(HexNAc+Hex)
1408.663(PEP_1)
1611.749(PEP+HexNAc(1)_1)
449.206(y4)
609.236(y5)
960.465(b8)
657.238(NeuAc+Hex+HexNAc)
512.199(HexNAc(1)Hex(1)Fuc(1)_1)
1611.745(PEP+HexNAc(1)_1)
1346.478

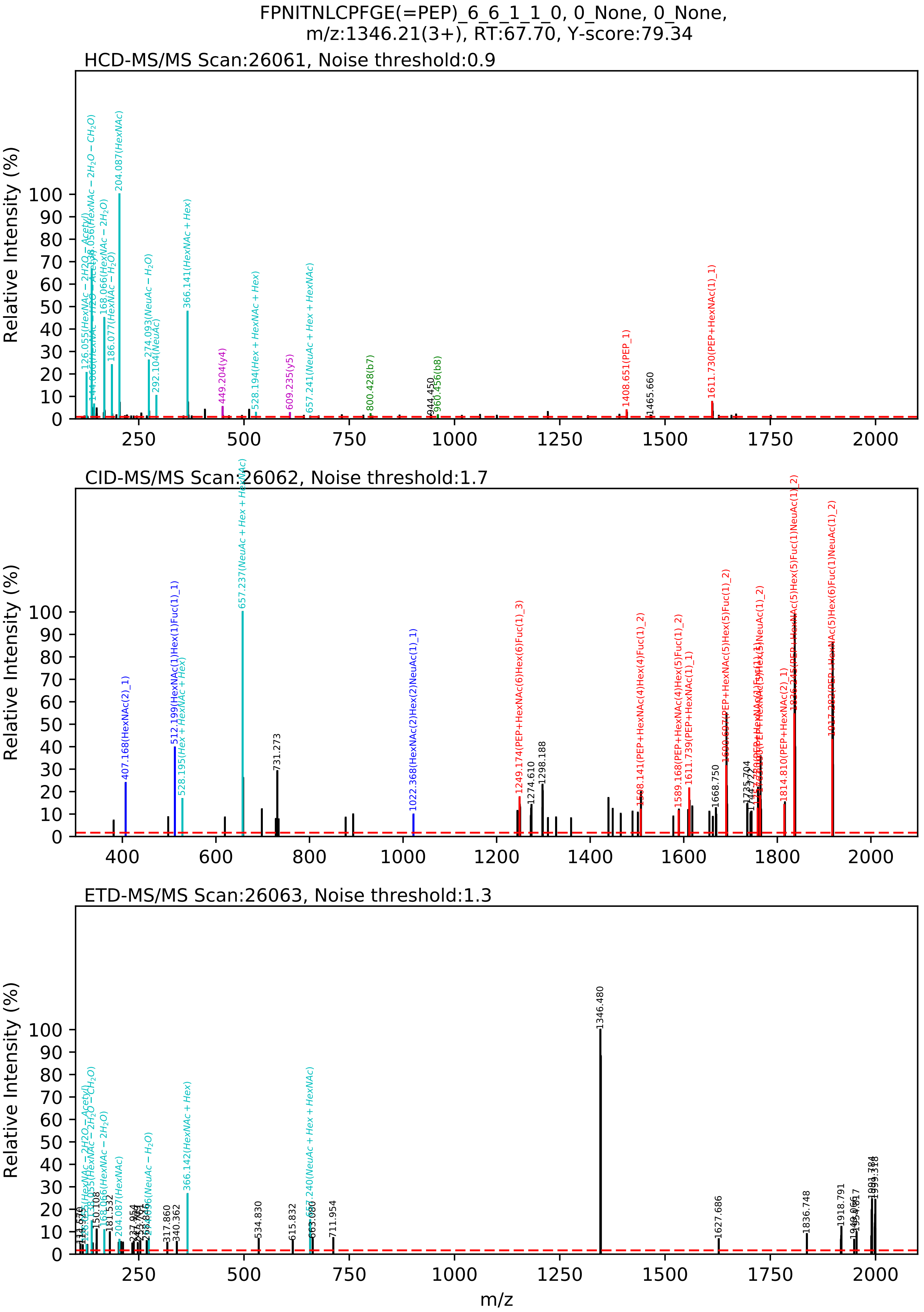

FPNITNLCPFGE(=PEP)_6_6_1_1_0, 0_None, 0_None,
m/z:1346.21(3+), RT:67.70, Y-score:79.34
HCD-MS/MS Scan:26061, Noise threshold:0.9
CID-MS/MS Scan:26062, Noise threshold:1.7
ETD-MS/MS Scan:26063, Noise threshold:1.3
Relative Intensity (%)
m/z
204.087(HexNAc)
366.141(HexNAc + Hex)
1611.730(PEP+HexNAc(1)_1)
1408.651(PEP_1)
657.237(NeuAc + Hex + HexNAc)
512.199(HexNAc(1)Hex(1)Fuc(1)_1)
407.168(HexNAc(2)_1)
1022.368(HexNAc(2)Hex(2)NeuAc(1)_1)
1249.174(PEP+HexNAc(6)Hex(6)Fuc(1)_3)
1346.480

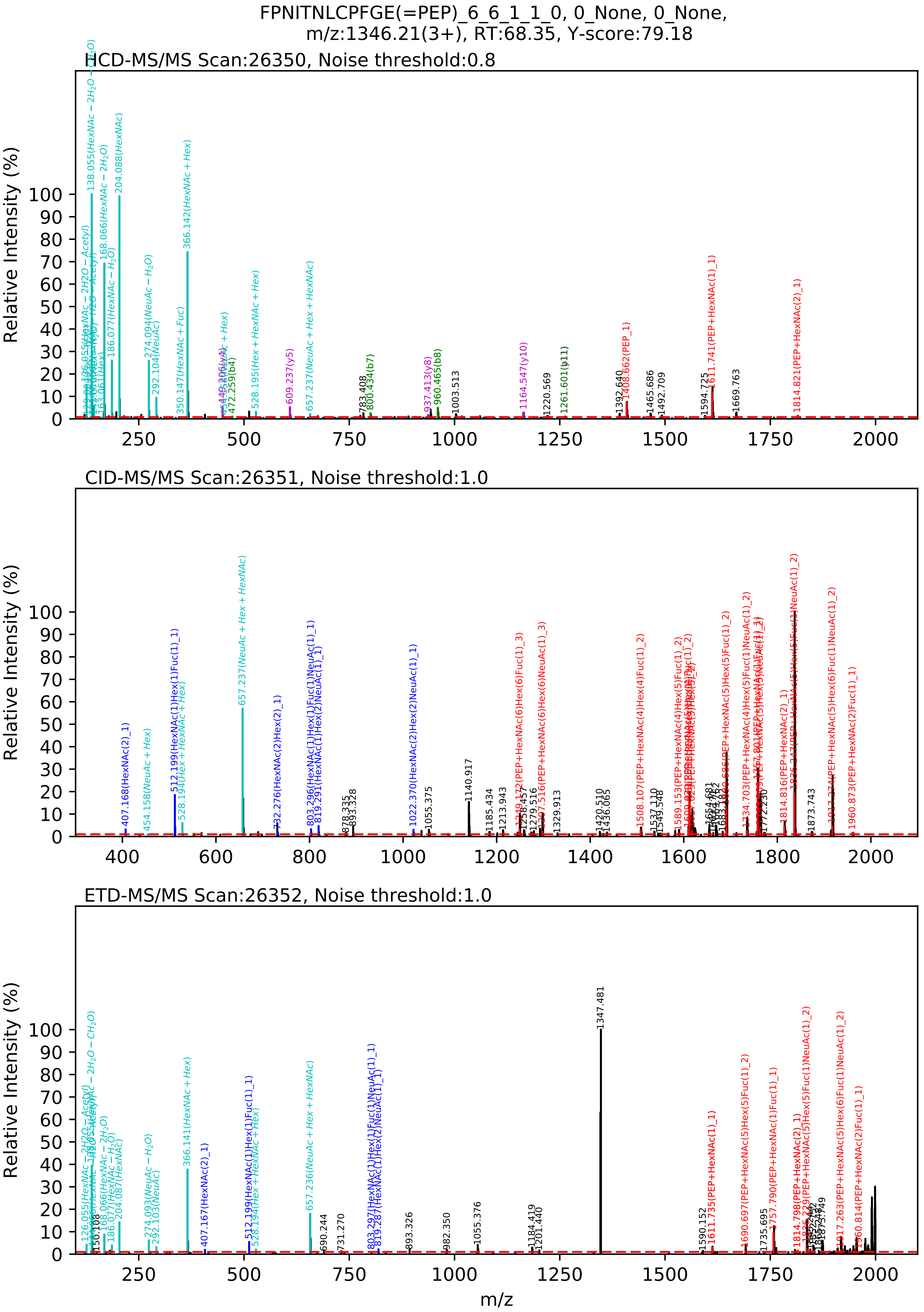
FPNITNLCPFGE(=PEP)_6_6_1_1_0, 0_None, 0_None,
m/z:1346.21(3+), RT:68.35, Y-score:79.18
HCD-MS/MS Scan:26350, Noise threshold:0.8
CID-MS/MS Scan:26351, Noise threshold:1.0
ETD-MS/MS Scan:26352, Noise threshold:1.0
Relative Intensity (%)
m/z

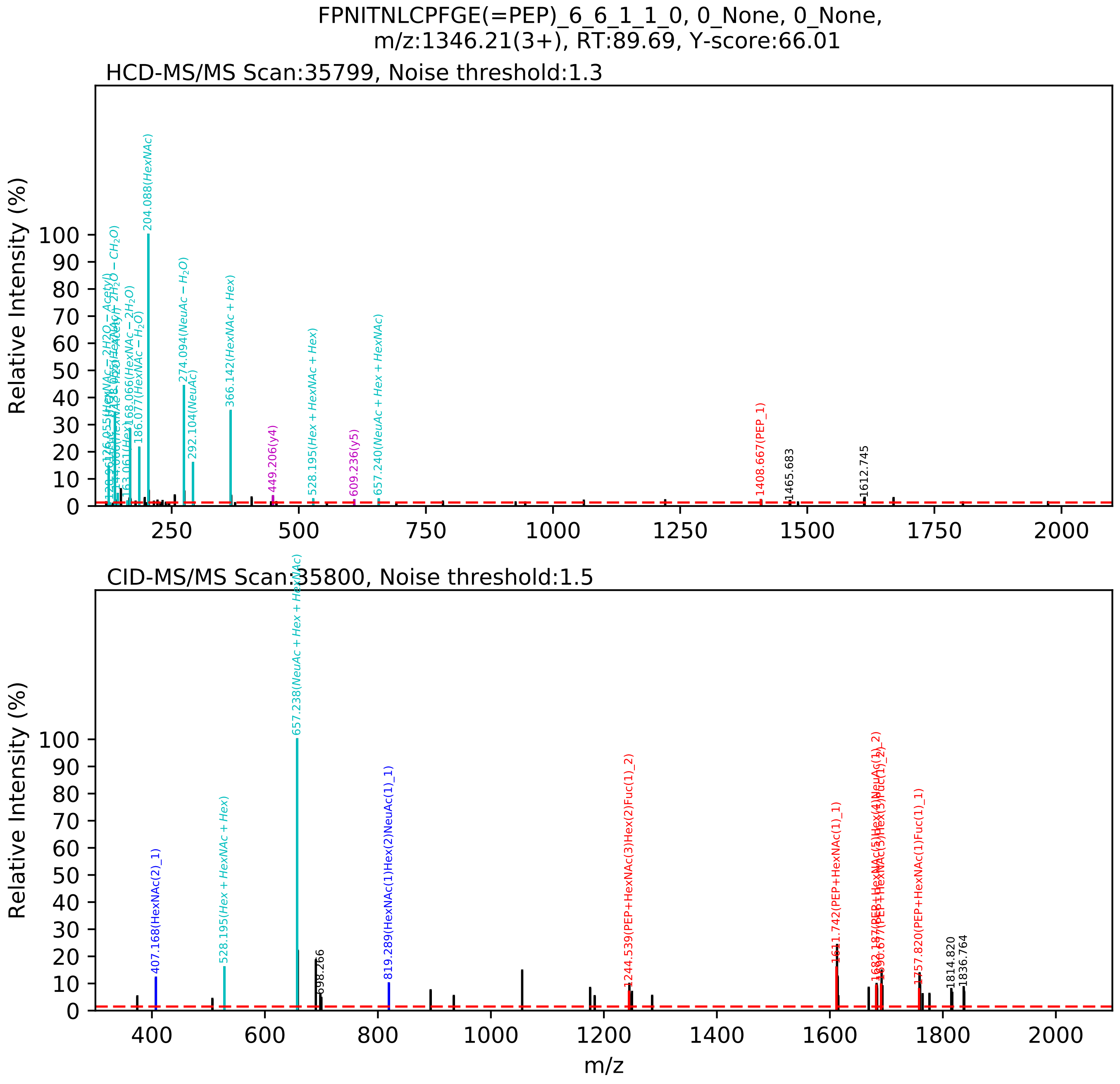

FPNITNLCPFGE(=PEP)_6_6_1_1_0, 0_None, 0_None,
m/z:1346.21(3+), RT:89.69, Y-score:66.01
HCD-MS/MS Scan:35799, Noise threshold:1.3
CID-MS/MS Scan:35800, Noise threshold:1.5
Relative Intensity (%)
m/z

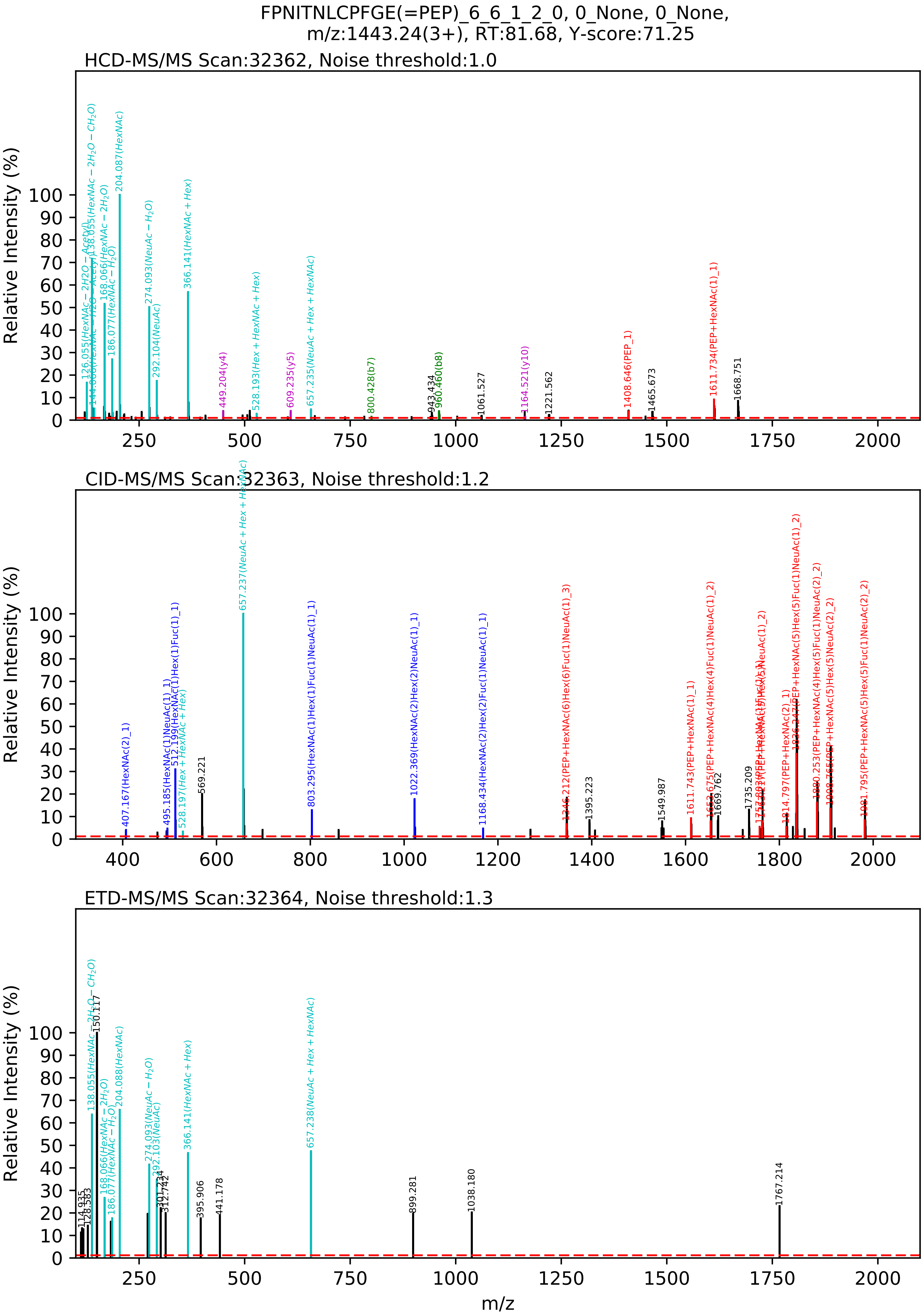

FPNITNLCPFGE(=PEP)_6_6_1_2_0, 0_None, 0_None,
m/z:1443.24(3+), RT:81.68, Y-score:71.25
HCD-MS/MS Scan:32362, Noise threshold:1.0
Relative Intensity (%)
126.055(HexNAc − 2H2O − Acetyl)
138.055(HexNAc − 2H2O − CH2O)
144.066(HexNAc − H2O − Acetyl)
168.066(HexNAc − 2H2O)
186.077(HexNAc − H2O)
204.087(HexNAc)
274.093(NeuAc − H2O)
292.104(NeuAc)
366.141(HexNAc + Hex)
449.204(y4)
528.193(Hex + HexNAc + Hex)
609.235(y5)
657.235(NeuAc + Hex + HexNAc)
800.428(b7)
943.434
960.460(b8)
1061.527
1164.521(y10)
1221.562
1408.646(PEP_1)
1465.673
1611.734(PEP+HexNAc(1)_1)
1668.751
CID-MS/MS Scan:32363, Noise threshold:1.2
407.167(HexNAc(2)_1)
495.185(HexNAc(1)NeuAc(1)_1)
512.199(HexNAc(1)Hex(1)Fuc(1)_1)
528.197(Hex + HexNAc + Hex)
569.221
657.237(NeuAc + Hex + HexNAc)
803.295(HexNAc(1)Hex(1)Fuc(1)NeuAc(1)_1)
1022.369(HexNAc(2)Hex(2)NeuAc(1)_1)
1168.434(HexNAc(2)Hex(2)Fuc(1)NeuAc(1)_1)
1346.212(PEP+HexNAc(6)Hex(6)Fuc(1)NeuAc(1)_3)
1395.223
1549.987
1611.743(PEP+HexNAc(1)_1)
1653.675(PEP+HexNAc(4)Hex(4)Fuc(1)NeuAc(1)_2)
1669.762
1735.209
1814.797(PEP+HexNAc(2)_1)
1836.247(PEP+HexNAc(5)Hex(5)Fuc(1)NeuAc(1)_2)
1880.253(PEP+HexNAc(4)Hex(5)Fuc(1)NeuAc(2)_2)
1908.765(PEP+HexNAc(5)Hex(5)NeuAc(2)_2)
1981.795(PEP+HexNAc(5)Hex(5)Fuc(1)NeuAc(2)_2)
ETD-MS/MS Scan:32364, Noise threshold:1.3
114.935
128.583
138.055(HexNAc − 2H2O − CH2O)
150.117
168.066(HexNAc − 2H2O)
186.077(HexNAc − H2O)
204.088(HexNAc)
274.093(NeuAc − H2O)
292.103(NeuAc)
301.734
312.742
366.141(HexNAc + Hex)
395.906
441.178
657.238(NeuAc + Hex + HexNAc)
899.281
1038.180
1767.214
m/z

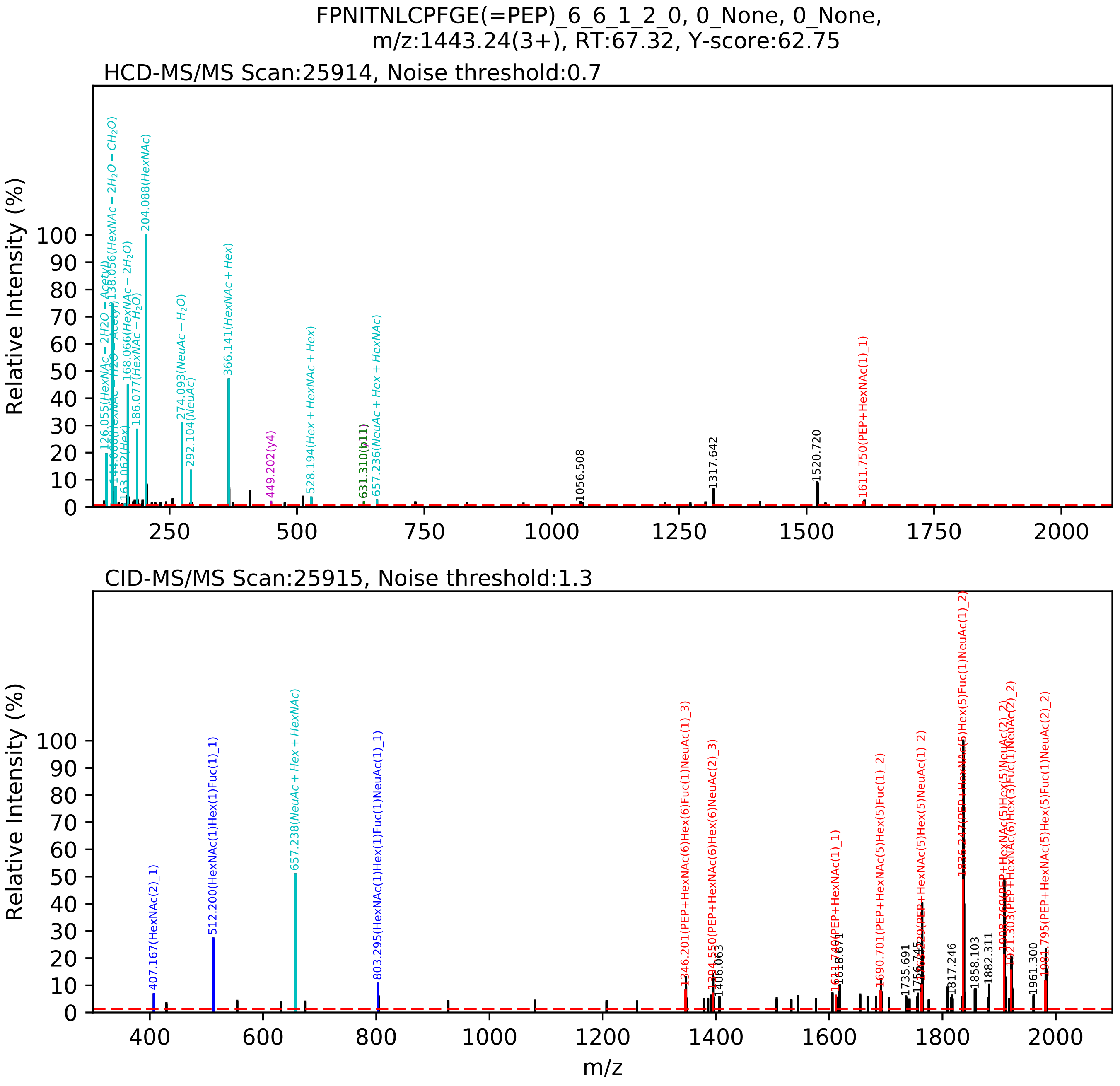
FPNITNLCPFGE(=PEP)_6_6_1_2_0, 0_None, 0_None,
m/z:1443.24(3+), RT:67.32, Y-score:62.75
HCD-MS/MS Scan:25914, Noise threshold:0.7
Relative Intensity (%)
204.088(HexNAc)
366.141(Hex NAc + Hex)
274.093(NeuAc − H2O)
292.104(NeuAc)
186.077(HexNAc − H2O)
168.066(HexNAc − 2H2O)
449.202(y4)
528.194(Hex + HexNAc + Hex)
631.310(b11)
657.236(NeuAc + Hex + HexNAc)
1056.508
1317.642
1520.720
1611.750(PEP+HexNAc(1)_1)
CID-MS/MS Scan:25915, Noise threshold:1.3
Relative Intensity (%)
407.167(HexNAc(2)_1)
512.200(HexNAc(1)Hex(1)Fuc(1)_1)
657.238(NeuAc + Hex + HexNAc)
803.295(HexNAc(1)Hex(1)Fuc(1)NeuAc(1)_1)
1346.201(PEP+HexNAc(6)Hex(6)Fuc(1)NeuAc(1)_3)
1394.550(PEP+HexNAc(6)Hex(6)NeuAc(2)_3)
1406.063
1611.749(PEP+HexNAc(1)_1)
1618.671
1690.701(PEP+HexNAc(5)Hex(5)Fuc(1)_2)
1735.691
1817.246
1858.103
1882.311
1961.300
1981.795(PEP+HexNAc(5)Hex(5)Fuc(1)NeuAc(2)_2)
m/z

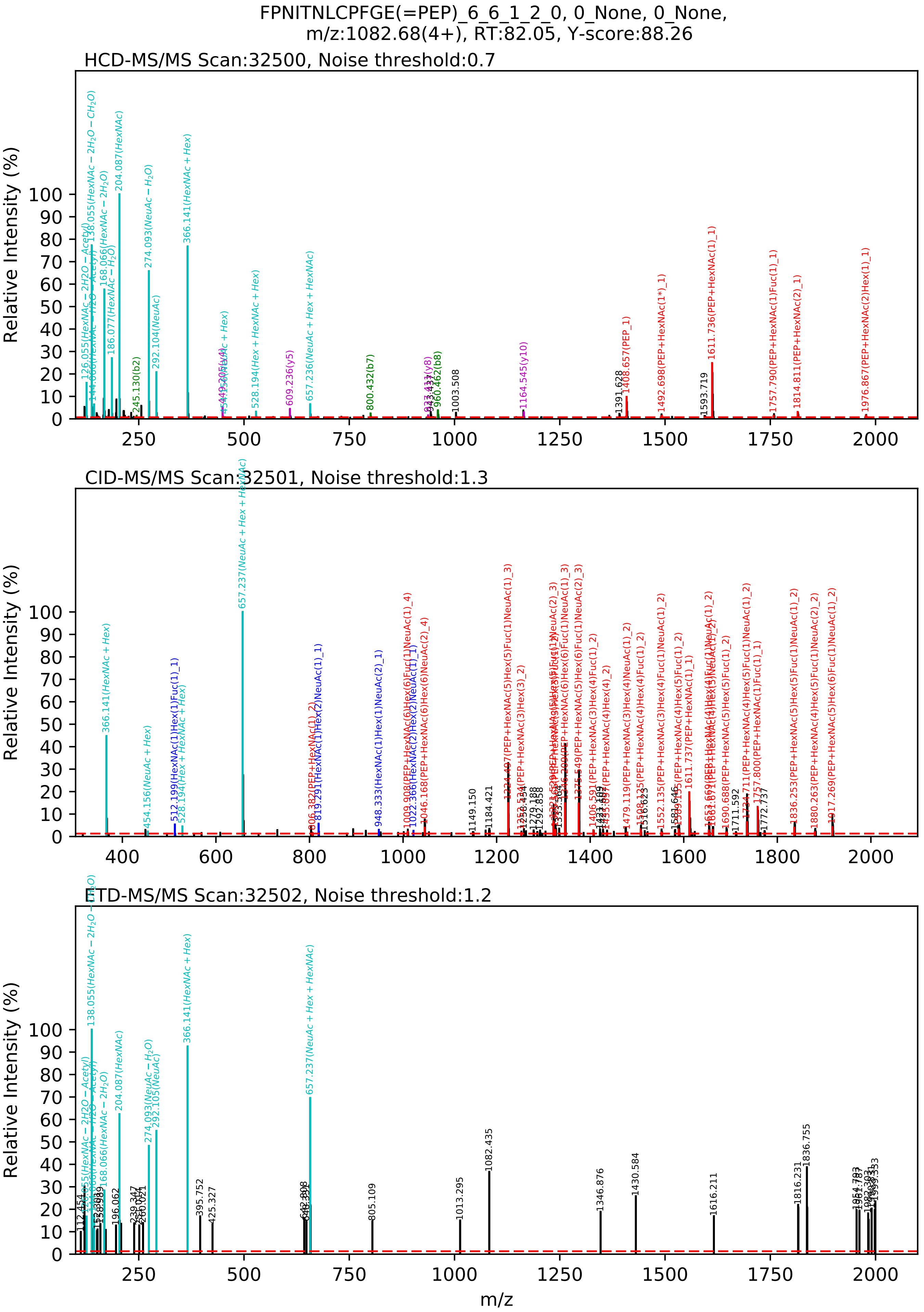

FPNITNLCPFGE(=PEP)_6_6_1_2_0, 0_None, 0_None,
m/z:1082.68(4+), RT:82.05, Y-score:88.26
HCD-MS/MS Scan:32500, Noise threshold:0.7
CID-MS/MS Scan:32501, Noise threshold:1.3
ETD-MS/MS Scan:32502, Noise threshold:1.2
Relative Intensity (%)
m/z
366.141(HexNAc + Hex)
204.087(HexNAc)
274.093(NeuAc − H2O)
292.104(NeuAc)
657.236(NeuAc + Hex + HexNAc)
528.194(Hex + HexNAc + Hex)
609.236(y5)
800.432(b7)
960.462(b8)
1003.508
1164.545(y10)
1391.628
1408.657(PEP_1)
1492.698(PEP+HexNAc(1*)_1)
1593.719
1611.736(PEP+HexNAc(1)_1)
1757.790(PEP+HexNAc(1)Fuc(1)_1)
1814.811(PEP+HexNAc(2)_1)
1976.867(PEP+HexNAc(2)Hex(1)_1)
657.237(NeuAc + Hex + HexNAc)
366.141(HexNAc + Hex)
454.156(NeuAc + Hex)
512.199(HexNAc(1)Hex(1)Fuc(1)_1)
819.291(HexNAc(1)Hex(2)NeuAc(1)_1)
948.333(HexNAc(1)Hex(1)NeuAc(2)_1)
1022.366(HexNAc(2)Hex(2)NeuAc(1)_1)
1046.168(PEP+HexNAc(6)Hex(6)NeuAc(2)_4)
1149.150
1184.421
1224.497(PEP+HexNAc(5)Hex(5)Fuc(1)NeuAc(1)_3)
1375.549(PEP+HexNAc(5)Hex(6)Fuc(1)NeuAc(2)_3)
1406.591(PEP+HexNAc(3)Hex(4)Fuc(1)_2)
1479.119(PEP+HexNAc(3)Hex(4)NeuAc(1)_2)
1552.135(PEP+HexNAc(3)Hex(4)Fuc(1)NeuAc(1)_2)
1611.737(PEP+HexNAc(1)_1)
1690.688(PEP+HexNAc(5)Hex(5)Fuc(1)_2)
1711.592
1757.800(PEP+HexNAc(1)Fuc(1)_1)
1772.737
1836.253(PEP+HexNAc(5)Hex(5)Fuc(1)NeuAc(1)_2)
1880.263(PEP+HexNAc(4)Hex(5)Fuc(1)NeuAc(2)_2)
1917.269(PEP+HexNAc(5)Hex(6)Fuc(1)NeuAc(1)_2)
138.055(HexNAc − 2H2O − CH2O)
168.066(HexNAc − 2H2O)
204.087(HexNAc)
274.093(NeuAc − H2O)
292.105(NeuAc)
366.141(HexNAc + Hex)
657.237(NeuAc + Hex + HexNAc)
196.062
395.752
425.327
805.109
1013.295
1082.435
1346.876
1430.584
1616.211
1816.231
1836.755

FPNITNLCPFGE(=PEP)_6_6_1_2_0, 0_None, 0_None,
m/z:1082.68(4+), RT:82.65, Y-score:72.97

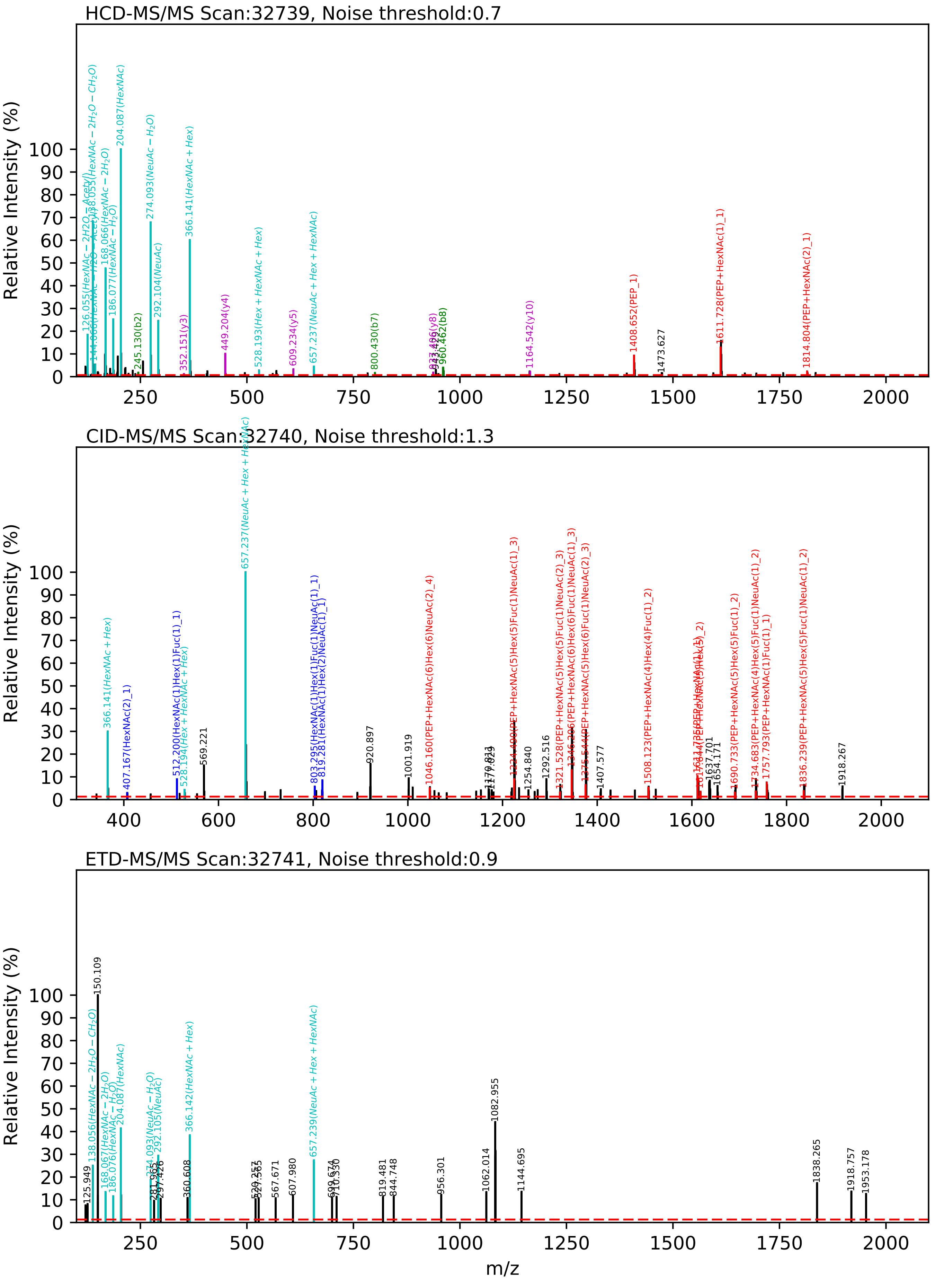

FPNITNLCPFGE(=PEP)_6_6_2_2_0, 0_None, 0_None,
m/z:1491.92(3+), RT:83.10, Y-score:80.07

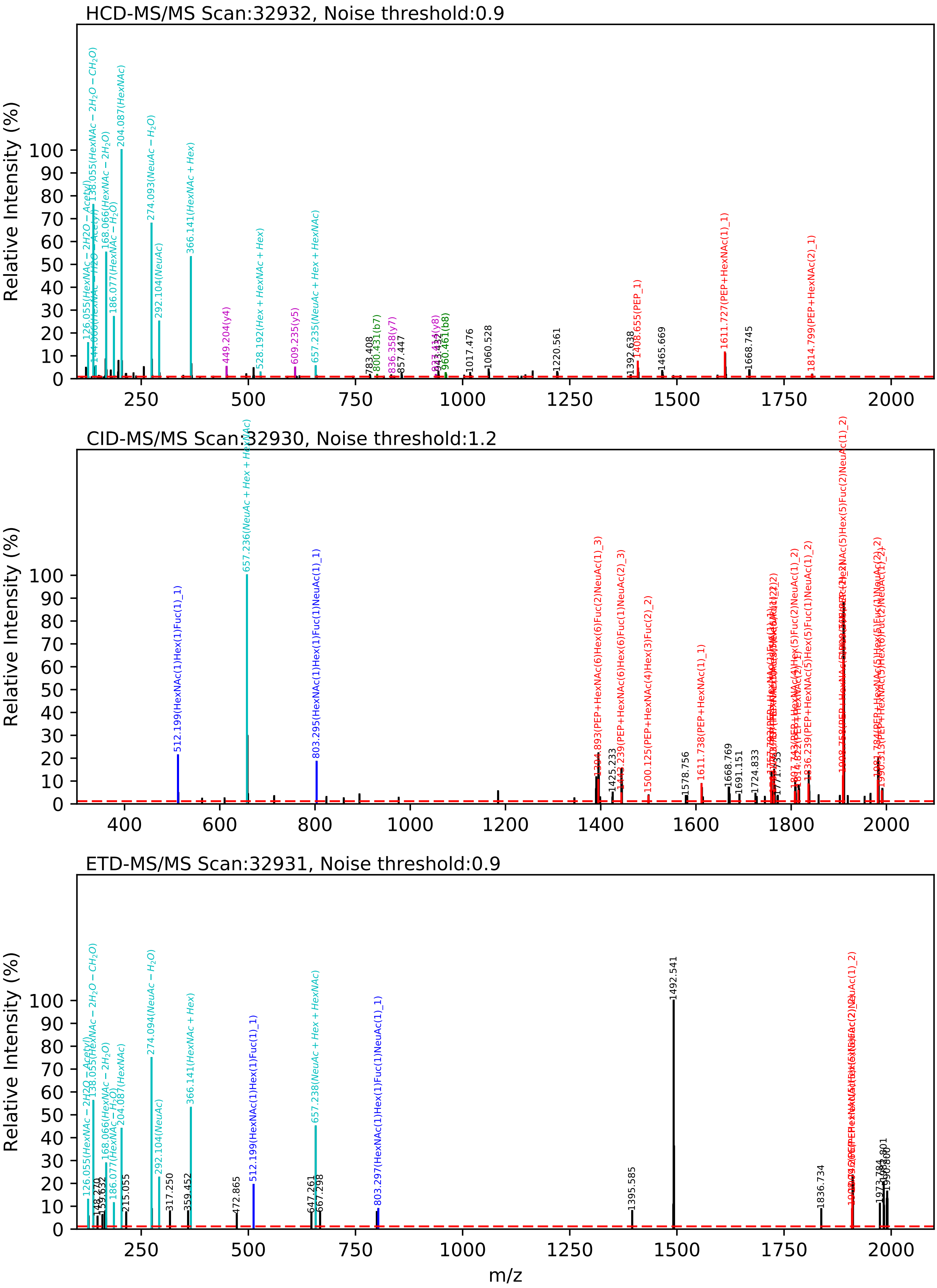

FPNITNLCPFGE(=PEP)_6_6_3_1_0, 0_None, 0_None,
m/z:1443.58(3+), RT:83.29, Y-score:82.80

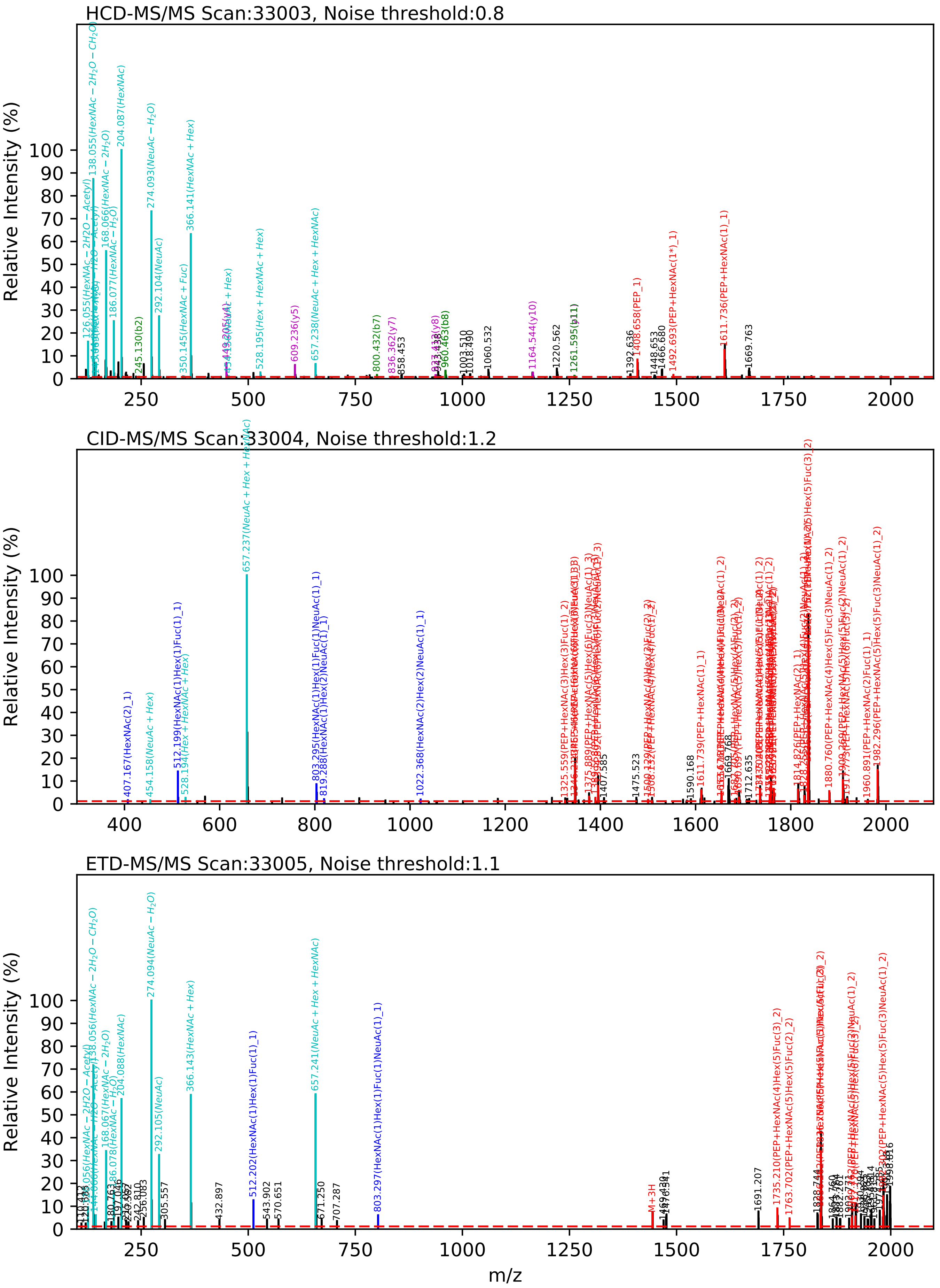

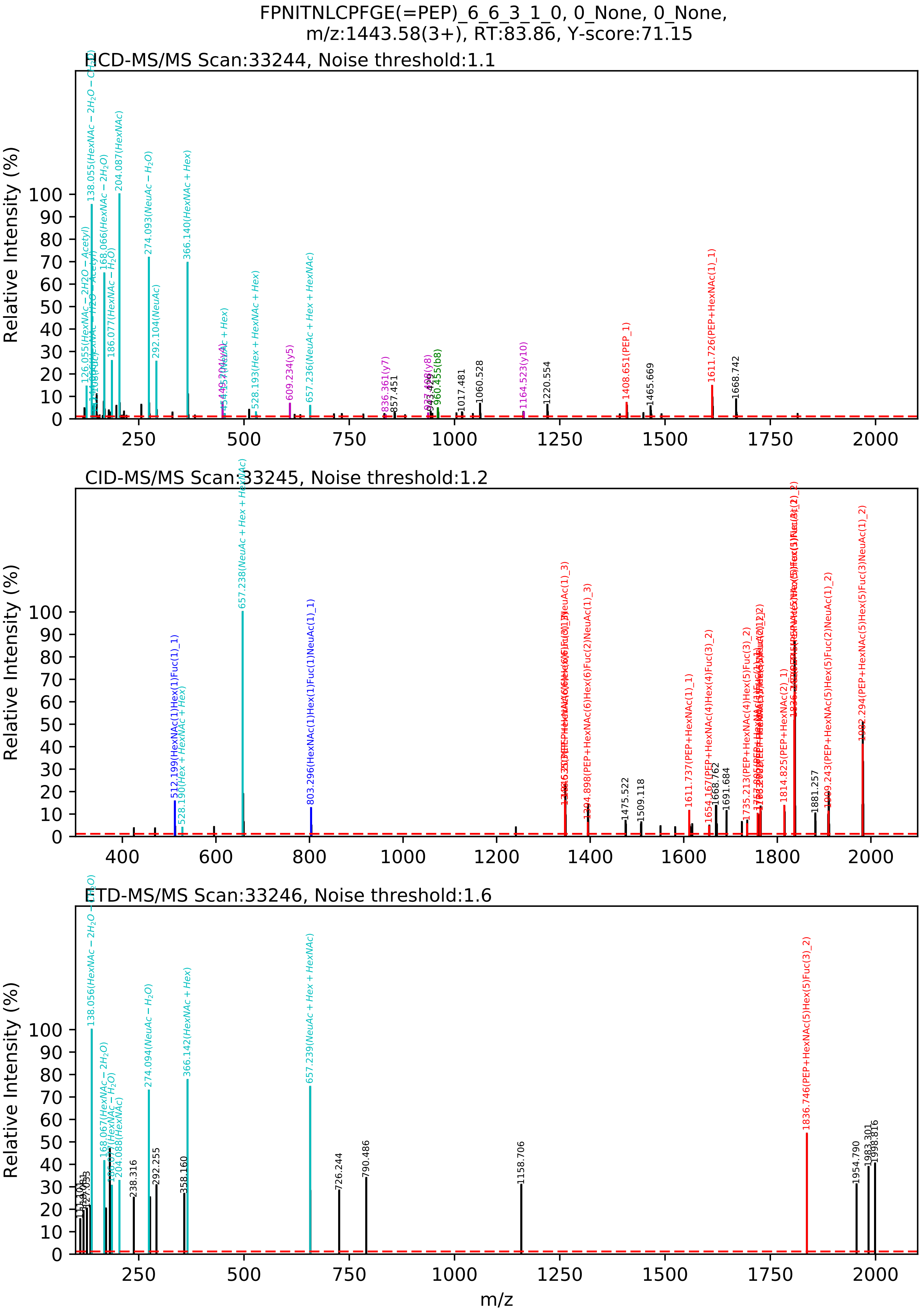
FPNITNLCPFGE(=PEP)_6_6_3_1_0, 0_None, 0_None,
m/z:1443.58(3+), RT:83.86, Y-score:71.15
HCD-MS/MS Scan:33244, Noise threshold:1.1
Relative Intensity (%)
204.087(HexNAc)
138.055(HexNAc − 2H2O − CH2O)
168.066(HexNAc − 2H2O)
186.077(HexNAc − H2O)
274.093(NeuAc − H2O)
292.104(NeuAc)
366.140(HexNAc + Hex)
528.193(Hex + HexNAc + Hex)
609.234(y5)
657.236(NeuAc + Hex + HexNAc)
836.361(y7)
857.451
960.455(b8)
1017.481
1060.528
1164.523(y10)
1220.554
1408.651(PEP_1)
1465.669
1611.726(PEP+HexNAc(1)_1)
1668.742
CID-MS/MS Scan:33245, Noise threshold:1.2
Relative Intensity (%)
657.238(NeuAc + Hex + HexNAc)
512.199(HexNAc(1)Hex(1)Fuc(1)_1)
528.190(Hex + HexNAc + Hex)
803.296(HexNAc(1)Hex(1)Fuc(1)NeuAc(1)_1)
1394.898(PEP+HexNAc(6)Hex(6)Fuc(2)NeuAc(1)_3)
1475.522
1509.118
1611.737(PEP+HexNAc(1)_1)
1654.167(PEP+HexNAc(4)Hex(4)Fuc(3)_2)
1668.762
1691.684
1735.213(PEP+HexNAc(4)Hex(5)Fuc(3)_2)
1814.825(PEP+HexNAc(2)_1)
1881.257
1909.243(PEP+HexNAc(5)Hex(5)Fuc(2)NeuAc(1)_2)
1982.294(PEP+HexNAc(5)Hex(5)Fuc(3)NeuAc(1)_2)
ETD-MS/MS Scan:33246, Noise threshold:1.6
Relative Intensity (%)
138.056(HexNAc − 2H2O − CH2O)
168.067(HexNAc − 2H2O)
186.077(HexNAc − H2O)
204.088(HexNAc)
238.316
274.094(NeuAc − H2O)
292.255
358.160
366.142(HexNAc + Hex)
657.239(NeuAc + Hex + HexNAc)
726.244
790.486
1158.706
1836.746(PEP+HexNAc(5)Hex(5)Fuc(3)_2)
1954.790
1983.301
1998.816
m/z

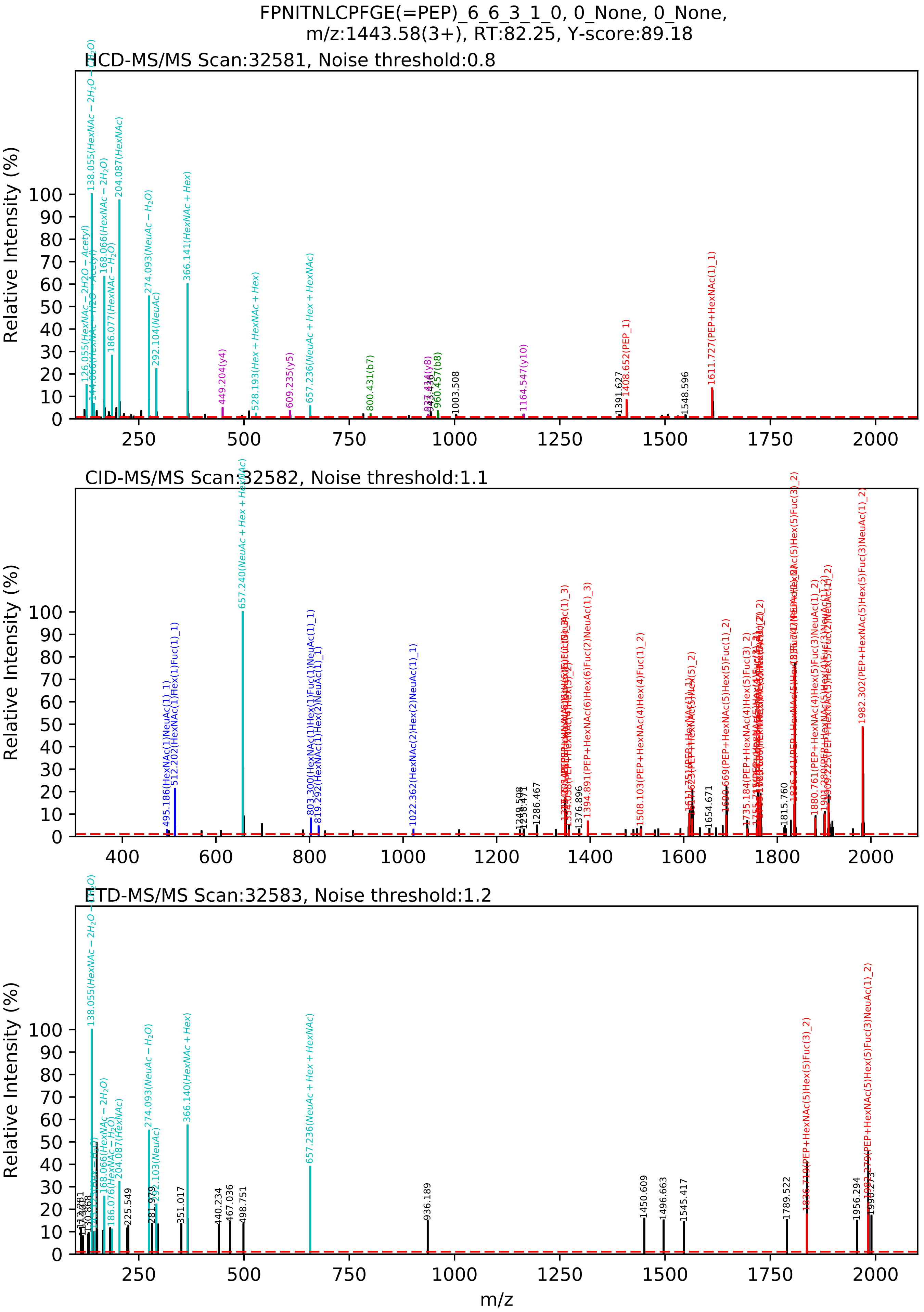
FPNITNLCPFGE(=PEP)_6_6_3_1_0, 0_None, 0_None,
m/z:1443.58(3+), RT:82.25, Y-score:89.18
HCD-MS/MS Scan:32581, Noise threshold:0.8
CID-MS/MS Scan:32582, Noise threshold:1.1
ETD-MS/MS Scan:32583, Noise threshold:1.2
Relative Intensity (%)
m/z
138.055(HexNAc − 2H2O − H2O)
204.087(HexNAc)
366.141(HexNAc + Hex)
274.093(NeuAc − H2O)
292.104(NeuAc)
186.077(HexNAc − H2O)
168.066(HexNAc − 2H2O)
126.055(HexNAc − 2H2O − Acetyl)
449.204(y4)
528.193(Hex + HexNAc + Hex)
609.235(y5)
657.236(NeuAc + Hex + HexNAc)
800.431(b7)
960.457(b8)
1003.508
1164.547(y10)
1391.627
1408.652(PEP_1)
1548.596
1611.727(PEP+HexNAc(1)_1)
657.240(NeuAc + Hex + HexNAc)
495.186(HexNAc(1)NeuAc(1)_1)
512.202(HexNAc(1)Hex(1)Fuc(1)_1)
803.300(HexNAc(1)Hex(1)Fuc(1)NeuAc(1)_1)
819.292(HexNAc(1)Hex(2)NeuAc(1)_1)
1022.362(HexNAc(2)Hex(2)NeuAc(1)_1)
1249.508
1258.471
1286.467
1376.896
1394.891(PEP+HexNAc(6)Hex(6)Fuc(2)NeuAc(1)_3)
1508.103(PEP+HexNAc(4)Hex(4)Fuc(1)_2)
1654.671
1690.669(PEP+HexNAc(5)Hex(5)Fuc(1)_2)
1735.184(PEP+HexNAc(4)Hex(5)Fuc(3)_2)
1815.760
1880.761(PEP+HexNAc(4)Hex(5)Fuc(3)NeuAc(1)_2)
1982.302(PEP+HexNAc(5)Hex(5)Fuc(3)NeuAc(1)_2)
366.140(HexNAc + Hex)
292.103(NeuAc)
186.076(HexNAc − H2O)
225.549
281.979
351.017
440.234
467.036
498.751
936.189
1450.609
1496.663
1545.417
1789.522
1836.710(PEP+HexNAc(5)Hex(5)Fuc(3)_2)
1956.294
1990.273
1982.270(PEP+HexNAc(5)Hex(5)Fuc(3)NeuAc(1)_2)

FPNITNLCPFGE(=PEP)_6_6_4_1_0, 0_None, 0_None,
m/z:1492.26(3+), RT:82.28, Y-score:78.89

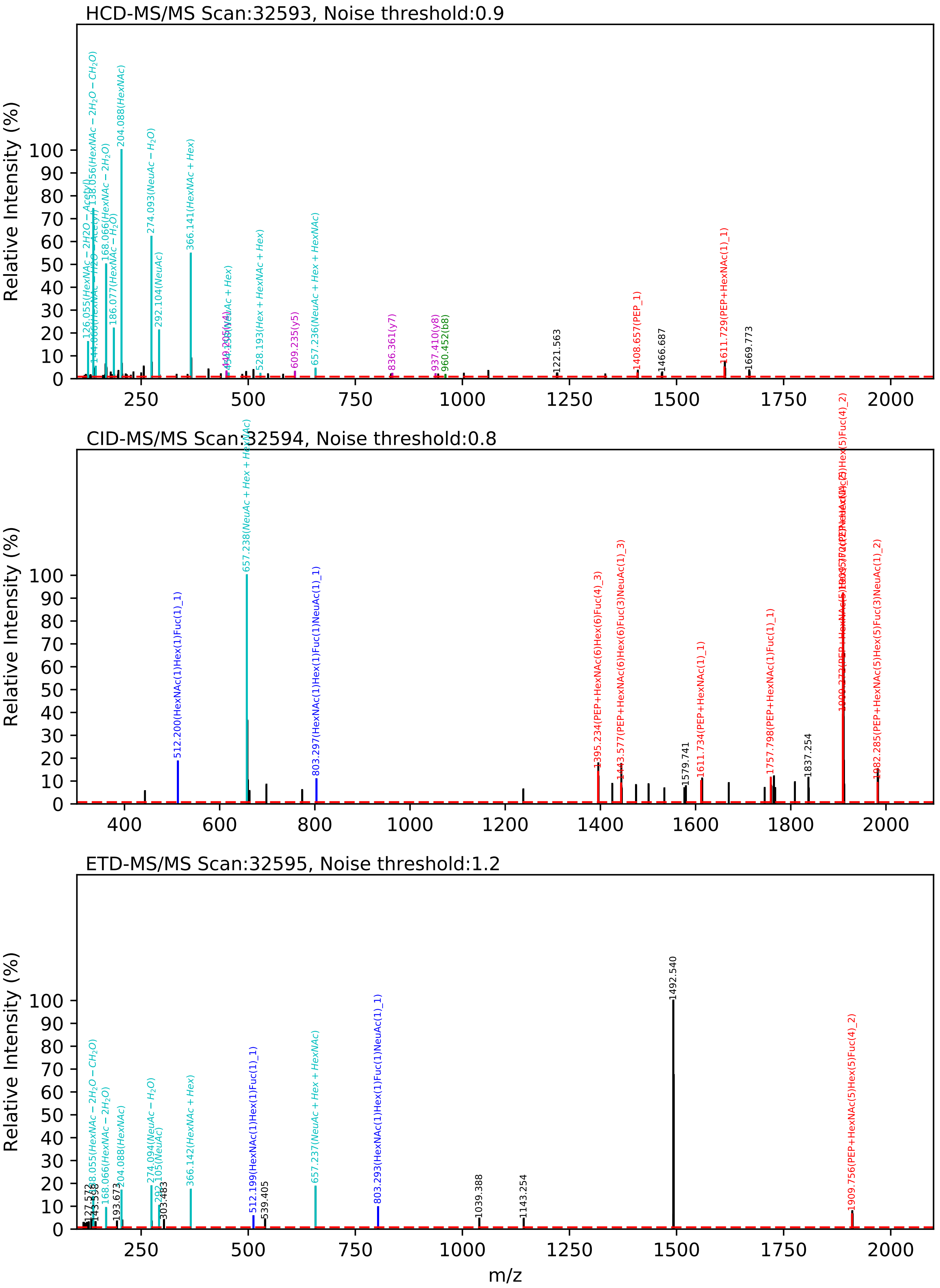

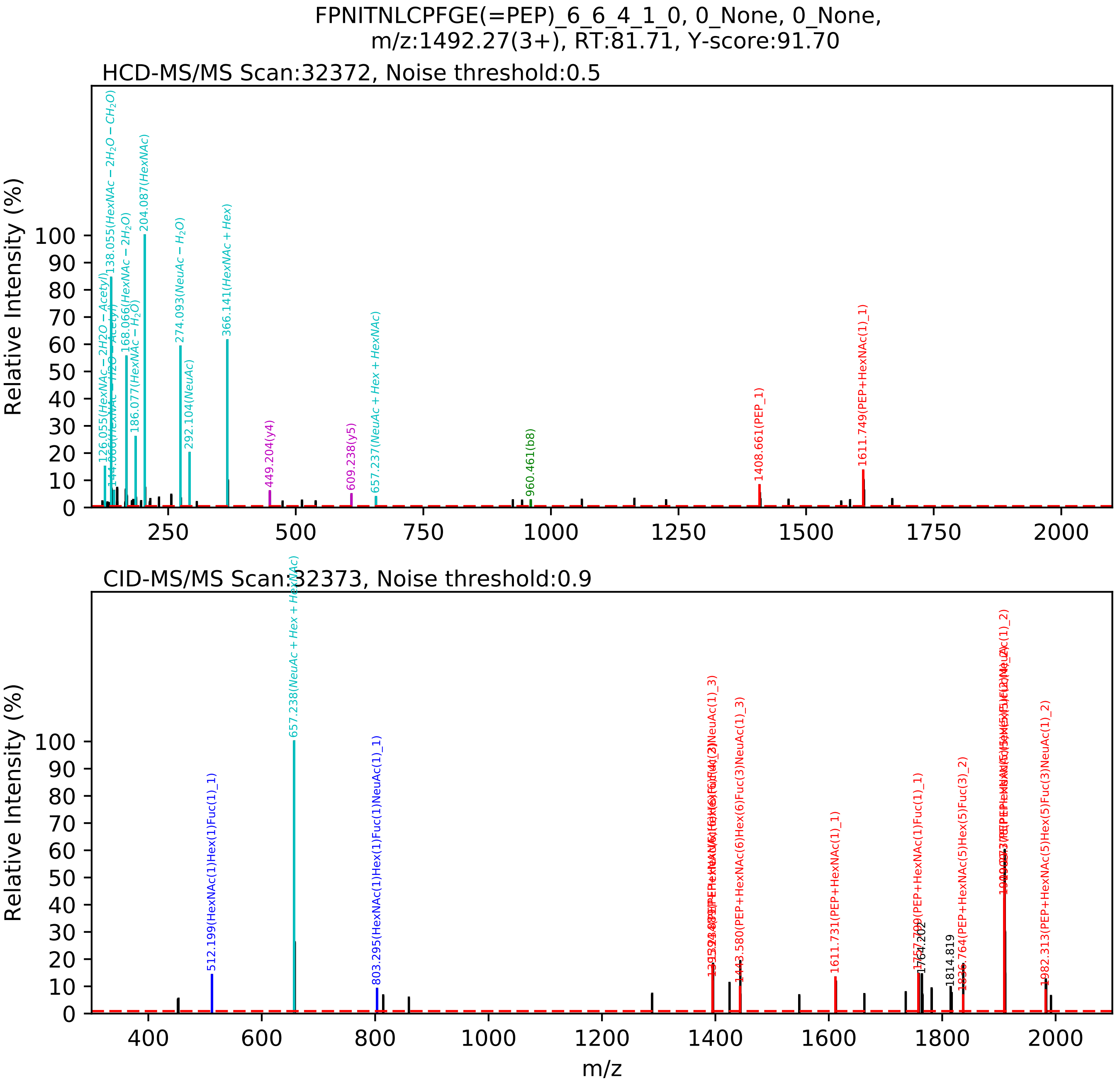

FPNITNLCPFGE(=PEP)_6_6_4_1_0, 0_None, 0_None,
m/z:1492.27(3+), RT:81.71, Y-score:91.70
HCD-MS/MS Scan:32372, Noise threshold:0.5
Relative Intensity (%)
126.055(HexNAc − 2H2O − Acetyl)
138.055(HexNAc − 2H2O − CH2O)
168.066(HexNAc − 2H2O)
186.077(HexNAc − H2O)
204.087(HexNAc)
274.093(NeuAc − H2O)
292.104(NeuAc)
366.141(HexNAc + Hex)
449.204(y4)
609.238(y5)
657.237(NeuAc + Hex + HexNAc)
960.461(b8)
1408.661(PEP_1)
1611.749(PEP+HexNAc(1)_1)
CID-MS/MS Scan:32373, Noise threshold:0.9
512.199(HexNAc(1)Hex(1)Fuc(1)_1)
657.238(NeuAc + Hex + HexNAc)
803.295(HexNAc(1)Hex(1)Fuc(1)NeuAc(1)_1)
1443.580(PEP+HexNAc(6)Hex(6)Fuc(3)NeuAc(1)_3)
1611.731(PEP+HexNAc(1)_1)
1757.799(PEP+HexNAc(1)Fuc(1)_1)
1764.202
1814.819
1836.764(PEP+HexNAc(5)Hex(5)Fuc(3)_2)
1982.313(PEP+HexNAc(5)Hex(5)Fuc(3)NeuAc(1)_2)
m/z

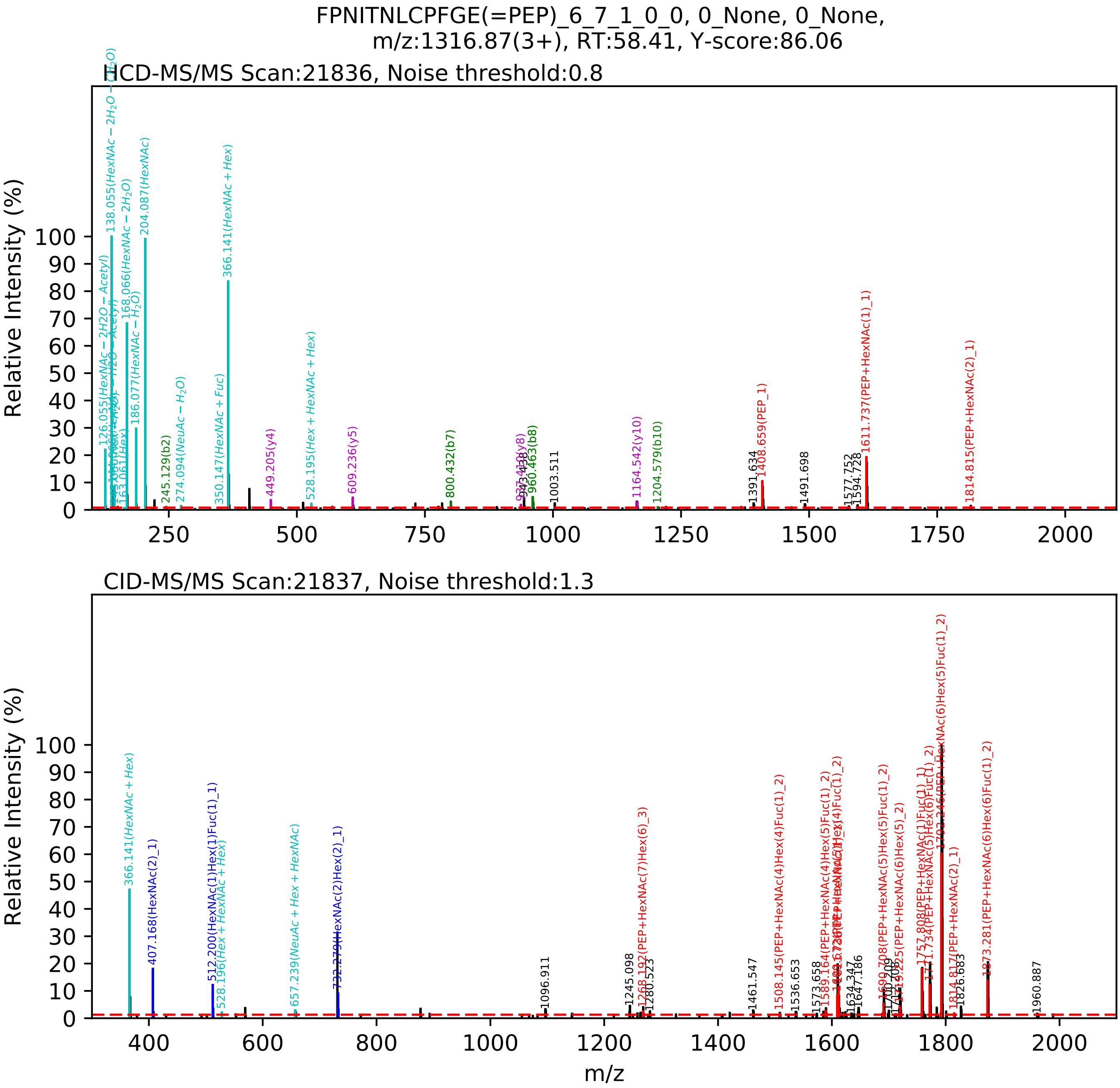
FPNITNLCPFGE(=PEP)_6_7_1_0_0, 0_None, 0_None,
m/z:1316.87(3+), RT:58.41, Y-score:86.06
HCD-MS/MS Scan:21836, Noise threshold:0.8
Relative Intensity (%)
138.055(HexNAc − 2H2O − H2O)
204.087(HexNAc)
366.141(HexNAc + Hex)
168.066(HexNAc − 2H2O)
186.077(HexNAc − H2O)
126.055(HexNAc − 2H2O − Acetyl)
163.061(Hex)
245.129(b2)
274.094(NeuAc − H2O)
350.147(HexNAc + Fuc)
449.205(y4)
528.195(Hex + HexNAc + Hex)
609.236(y5)
800.432(b7)
960.463(b8)
1003.511
1164.542(y10)
1204.579(b10)
1391.634
1408.659(PEP_1)
1491.698
1577.752
1594.728
1611.737(PEP+HexNAc(1)_1)
1814.815(PEP+HexNAc(2)_1)
m/z
CID-MS/MS Scan:21837, Noise threshold:1.3
366.141(HexNAc + Hex)
407.168(HexNAc(2)_1)
512.200(HexNAc(1)Hex(1)Fuc(1)_1)
528.196(Hex + HexNAc + Hex)
657.239(NeuAc + Hex + HexNAc)
732.279(HexNAc(2)Hex(2)_1)
1096.911
1245.098
1268.192(PEP+HexNAc(7)Hex(6)_3)
1280.523
1461.547
1508.145(PEP+HexNAc(4)Hex(4)Fuc(1)_2)
1536.653
1573.658
1589.164(PEP+HexNAc(4)Hex(5)Fuc(1)_2)
1634.347
1647.186
1690.708(PEP+HexNAc(5)Hex(5)Fuc(1)_2)
1757.808(PEP+HexNAc(1)Fuc(1)_1)
1814.817(PEP+HexNAc(2)_1)
1826.683
1873.281(PEP+HexNAc(6)Hex(6)Fuc(1)_2)
1960.887
m/z

FPNITNLCPFGE(=PEP)_6_7_1_0_0, 0_None, 0_None, m/z:1316.87(3+), RT:58.46, Y-score:85.84

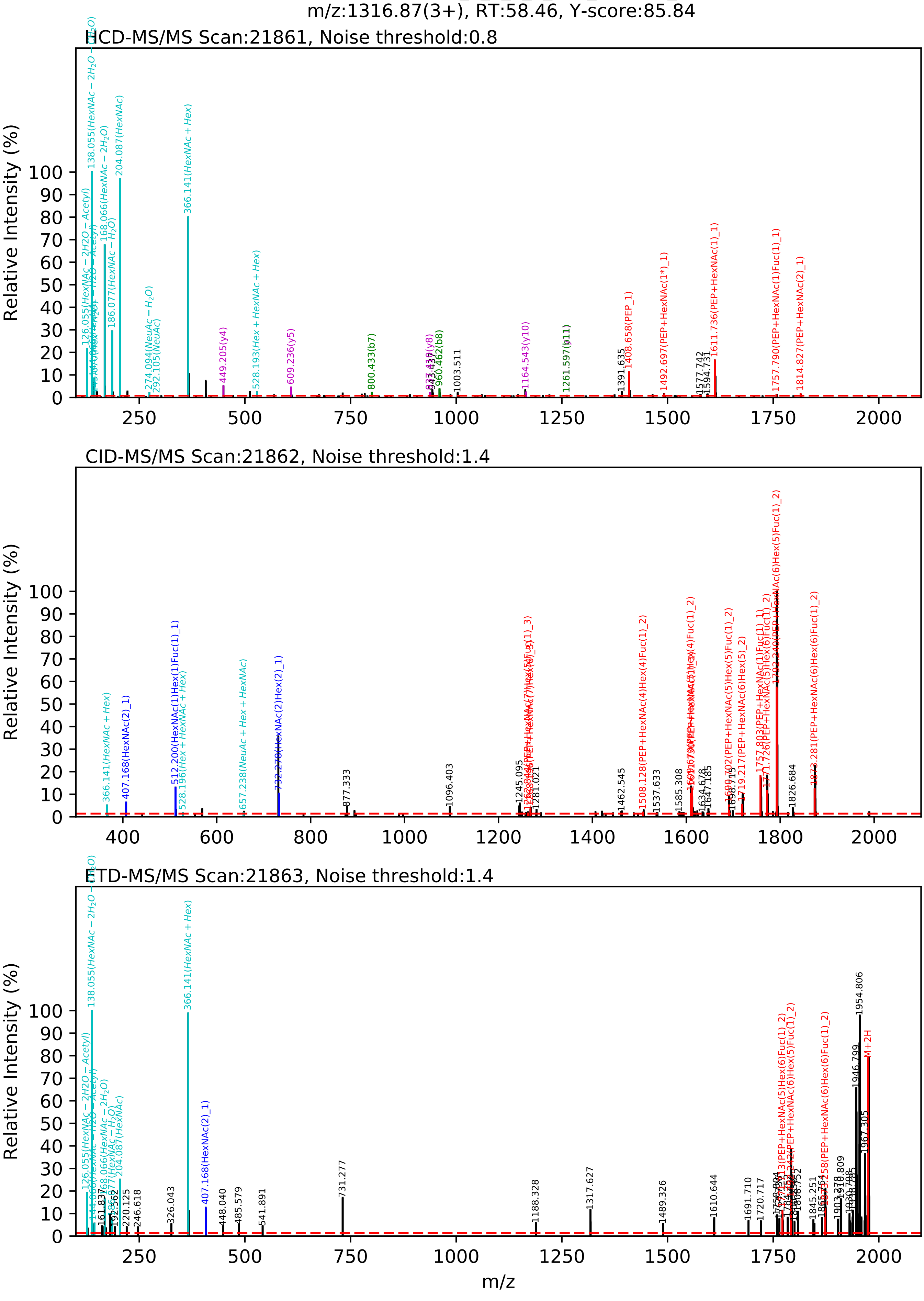

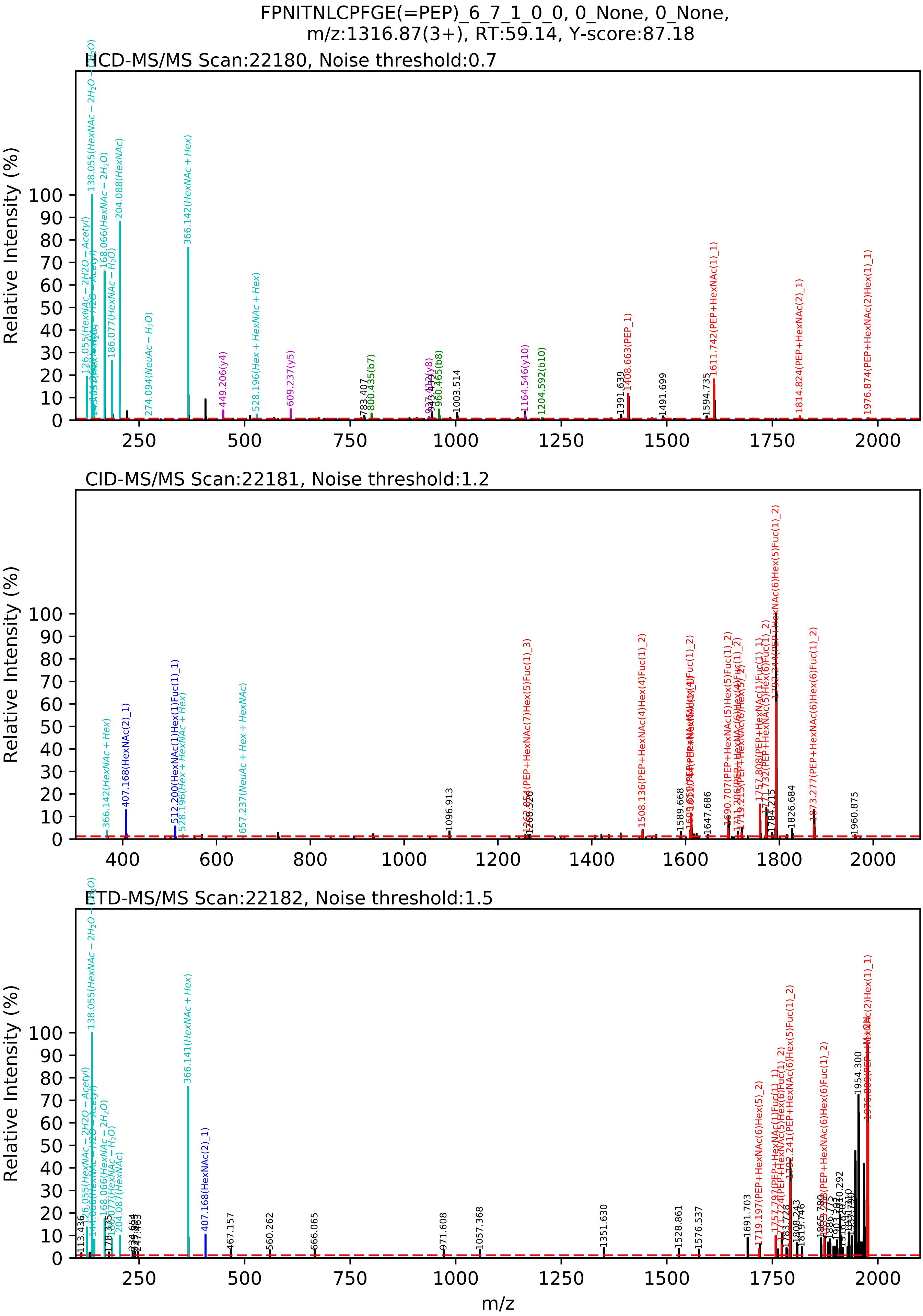

FPNITNLCPFGE(=PEP)_6_7_1_0_0, 0_None, 0_None,
m/z:1316.87(3+), RT:59.14, Y-score:87.18
HCD-MS/MS Scan:22180, Noise threshold:0.7
Relative Intensity (%)
138.055(HexNAc − 2H$_2$O − 2H$_2$O)
204.088(HexNAc)
168.066(HexNAc − 2H$_2$O)
186.077(HexNAc − H$_2$O)
274.094(NeuAc − H$_2$O)
366.142(HexNAc + Hex)
449.206(y4)
528.196(Hex + HexNAc + Hex)
609.237(y5)
783.407
800.435(b7)
960.465(b8)
1003.514
1164.546(y10)
1204.592(b10)
1391.639
1408.663(PEP_1)
1491.699
1594.735
1611.742(PEP+HexNAc(1)_1)
1814.824(PEP+HexNAc(2)_1)
1976.874(PEP+HexNAc(2)Hex(1)_1)
CID-MS/MS Scan:22181, Noise threshold:1.2
Relative Intensity (%)
366.142(HexNAc + Hex)
407.168(HexNAc(2)_1)
512.200(HexNAc(1)Hex(1)Fuc(1)_1)
528.196(Hex + HexNAc + Hex)
657.237(NeuAc + Hex + HexNAc)
1096.913
1268.526
1508.136(PEP+HexNAc(4)Hex(4)Fuc(1)_2)
1589.668
1647.686
1690.707(PEP+HexNAc(5)Hex(5)Fuc(1)_2)
1757.808(PEP+HexNAc(1)Fuc(1)_1)
1784.215
1826.684
1873.277(PEP+HexNAc(6)Hex(6)Fuc(1)_2)
1960.875
ETD-MS/MS Scan:22182, Noise threshold:1.5
Relative Intensity (%)
138.055(HexNAc − 2H$_2$O − 2H$_2$O)
366.141(HexNAc + Hex)
407.168(HexNAc(2)_1)
467.157
560.262
666.065
971.608
1057.368
1351.630
1528.861
1576.537
1691.703
1719.197(PEP+HexNAc(6)Hex(5)_2)
1792.241(PEP+HexNAc(6)Hex(5)Fuc(1)_2)
1954.300
m/z

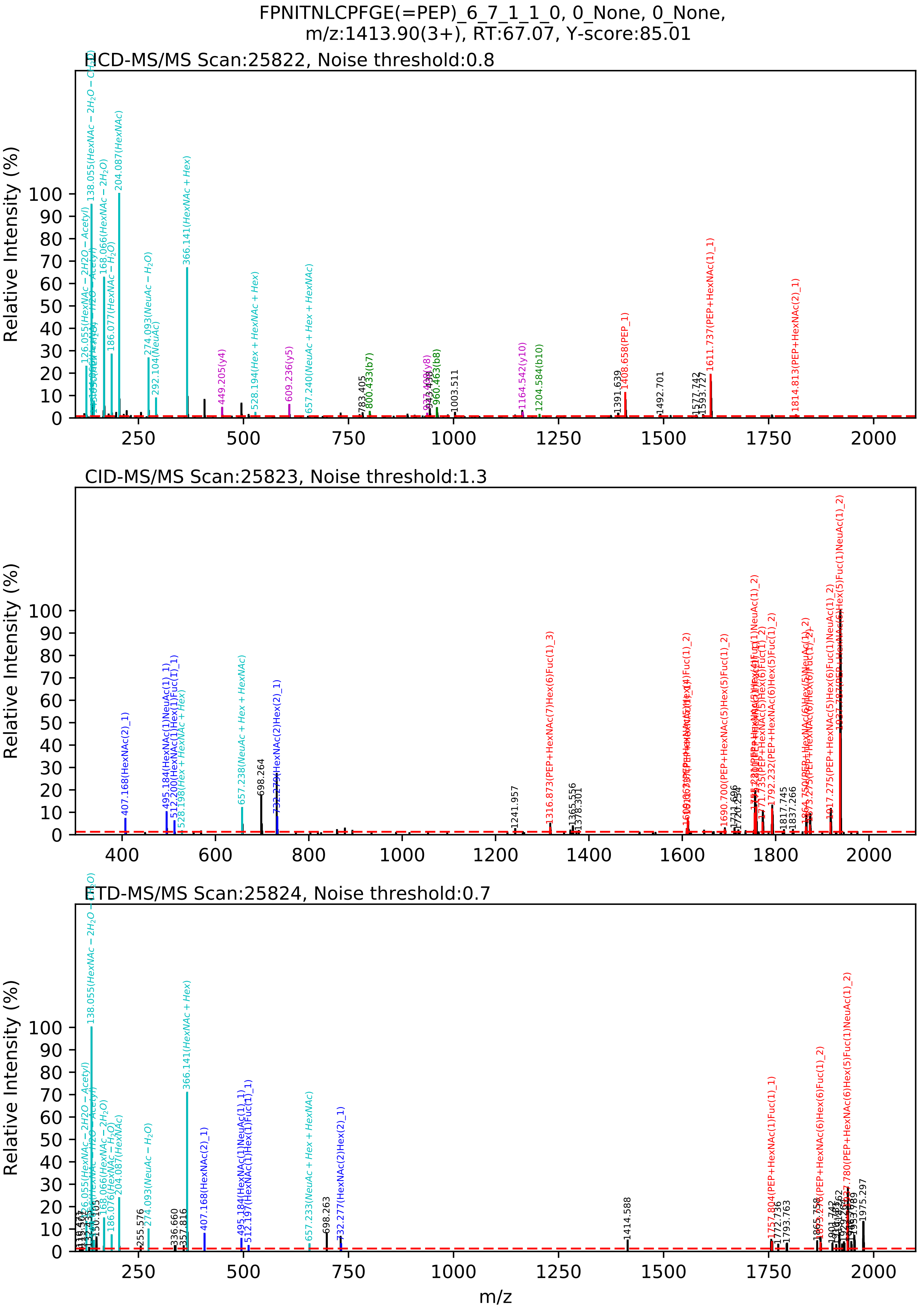

FPNITNLCPFGE(=PEP)_6_7_1_1_0, 0_None, 0_None,
m/z:1413.90(3+), RT:67.07, Y-score:85.01
HCD-MS/MS Scan:25822, Noise threshold:0.8
CID-MS/MS Scan:25823, Noise threshold:1.3
ETD-MS/MS Scan:25824, Noise threshold:0.7
Relative Intensity (%)
m/z

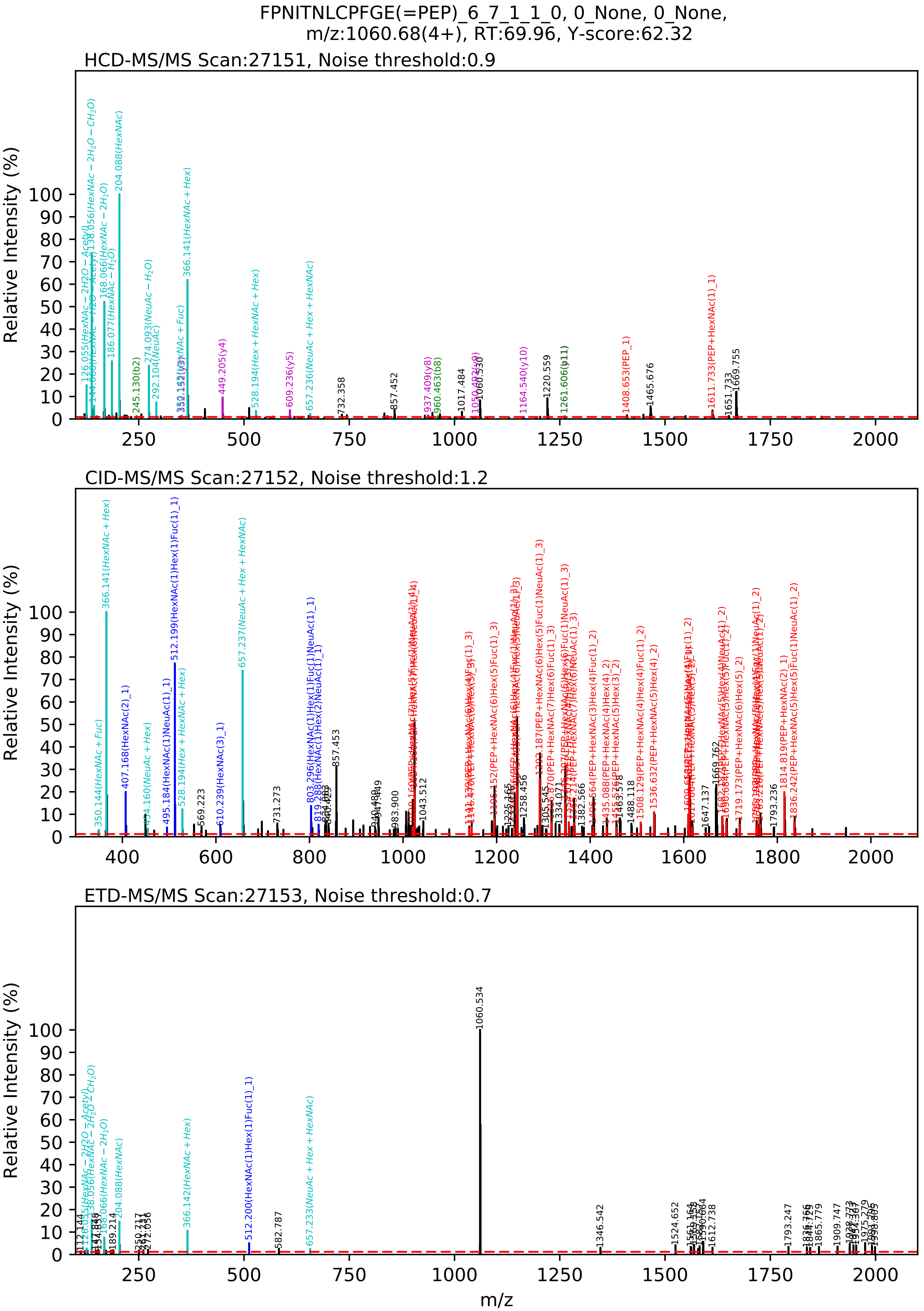
FPNITNLCPFGE(=PEP)_6_7_1_1_0, 0_None, 0_None,
m/z:1060.68(4+), RT:69.96, Y-score:62.32
HCD-MS/MS Scan:27151, Noise threshold:0.9
Relative Intensity (%)
CID-MS/MS Scan:27152, Noise threshold:1.2
Relative Intensity (%)
ETD-MS/MS Scan:27153, Noise threshold:0.7
Relative Intensity (%)
m/z

FPNITNLCPFGE(=PEP)_6_7_1_1_0, 0_None, 0_None,
m/z:1413.90(3+), RT:67.73, Y-score:87.26

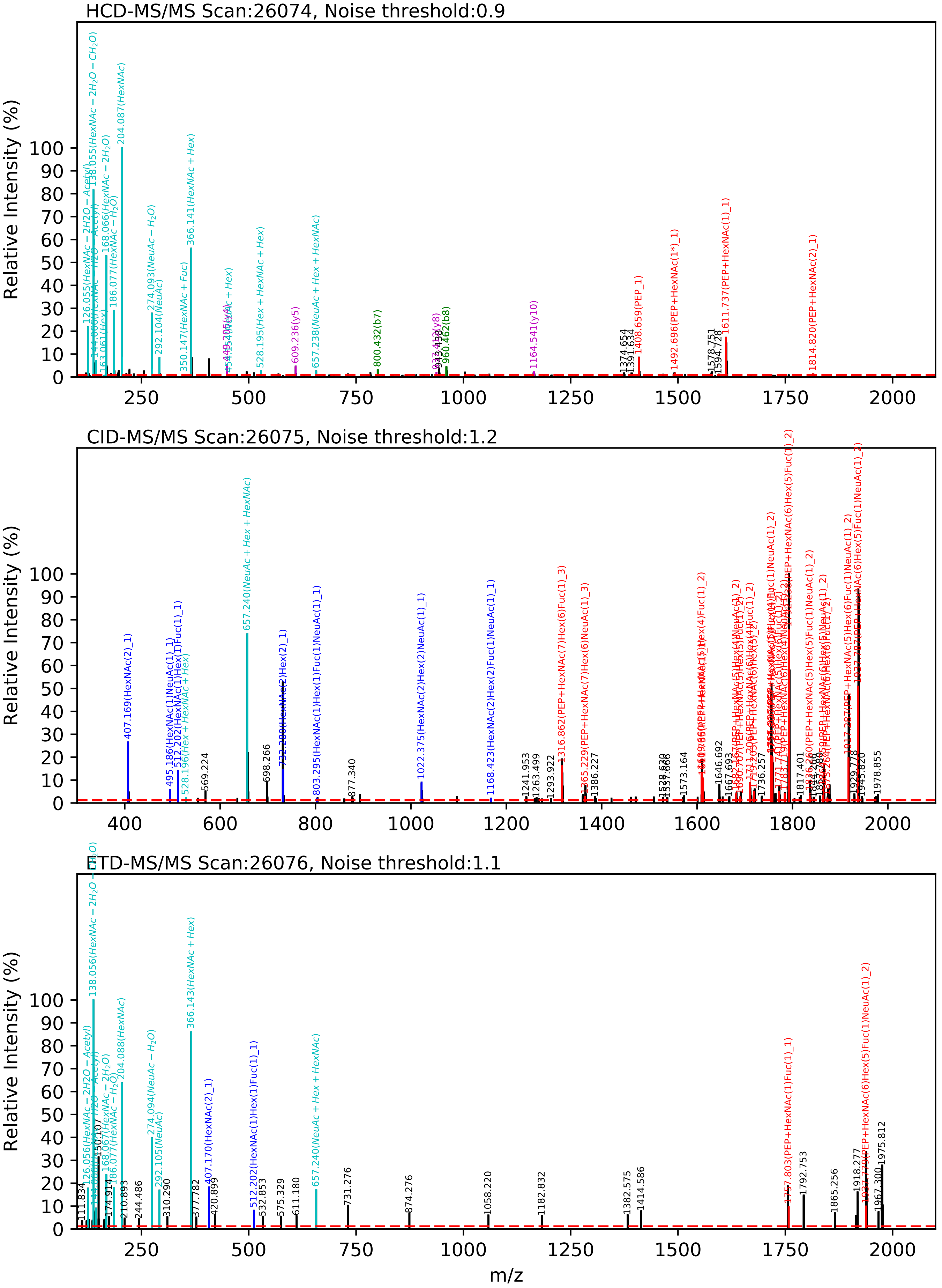

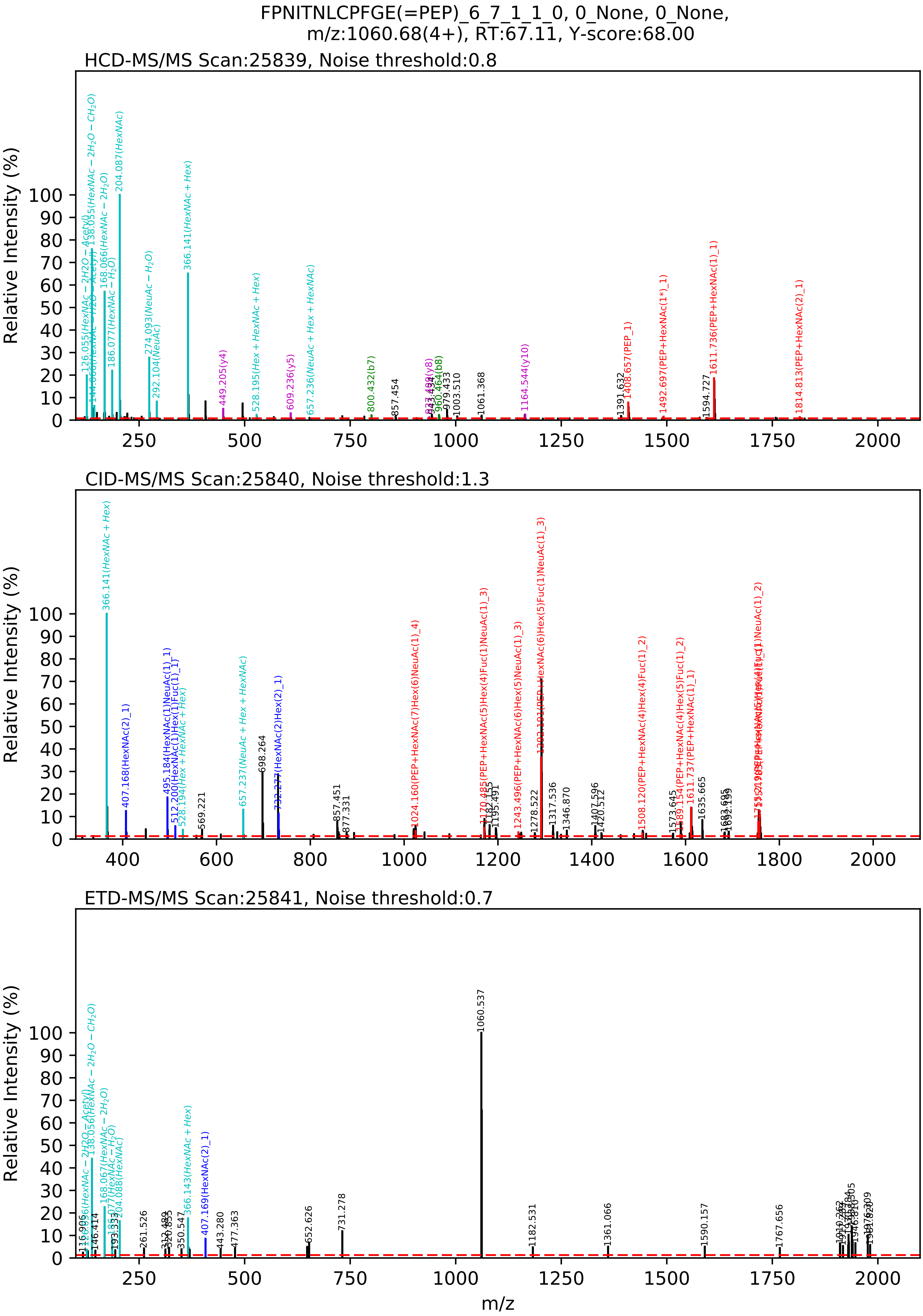

FPNITNLCPFGE(=PEP)_6_7_1_1_0, 0_None, 0_None,
m/z:1060.68(4+), RT:67.11, Y-score:68.00
HCD-MS/MS Scan:25839, Noise threshold:0.8
CID-MS/MS Scan:25840, Noise threshold:1.3
ETD-MS/MS Scan:25841, Noise threshold:0.7
Relative Intensity (%)
m/z

FPNITNLCPFGE(=PEP)_6_7_1_1_0, 0_None, 0_None,
m/z:1413.90(3+), RT:68.31, Y-score:75.40

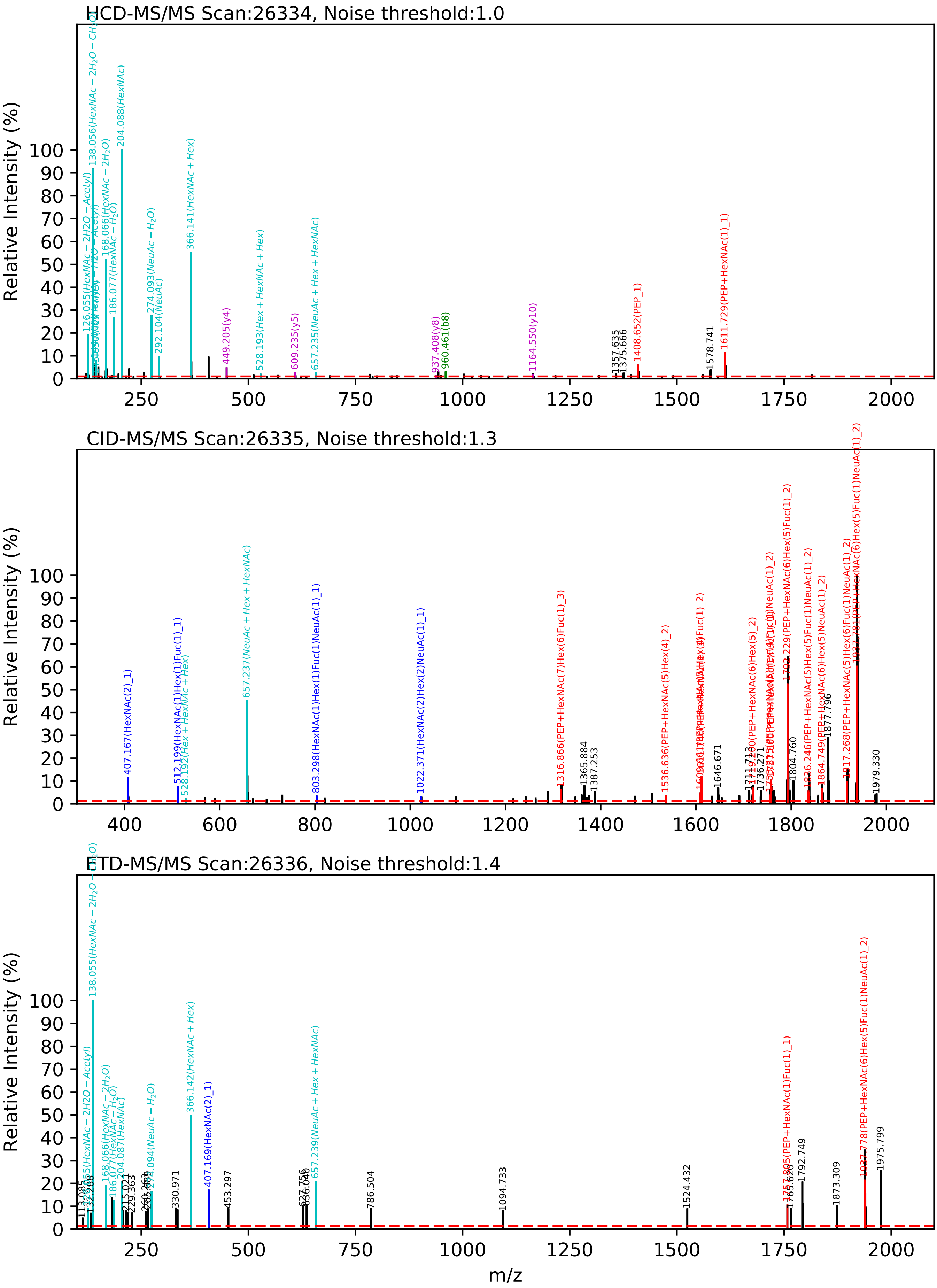

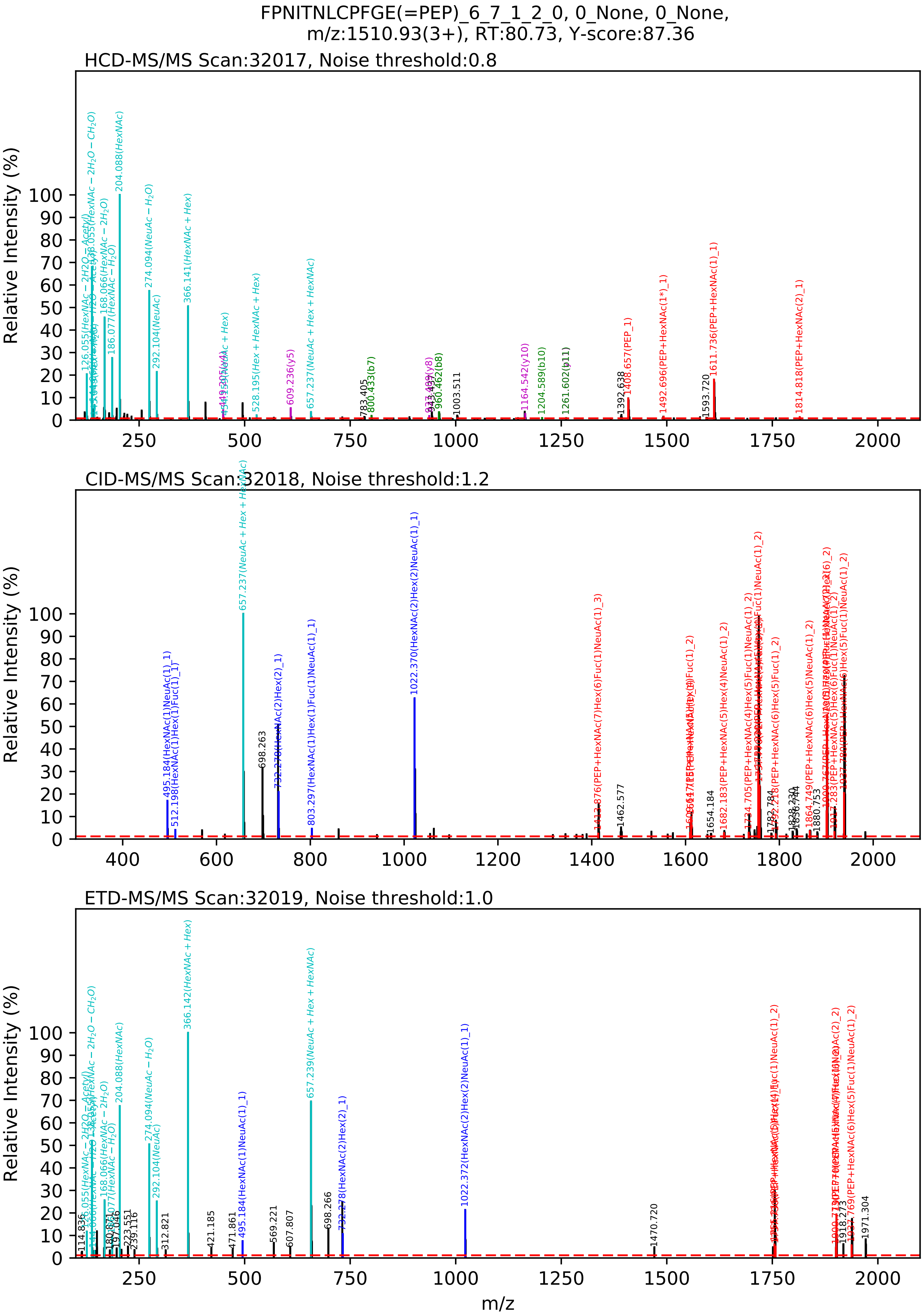
FPNITNLCPFGE(=PEP)_6_7_1_2_0, 0_None, 0_None,
m/z:1510.93(3+), RT:80.73, Y-score:87.36
HCD-MS/MS Scan:32017, Noise threshold:0.8
CID-MS/MS Scan:32018, Noise threshold:1.2
ETD-MS/MS Scan:32019, Noise threshold:1.0
Relative Intensity (%)
m/z

FPNITNLCPFGE(=PEP)_6_7_1_2_0, 0_None, 0_None, m/z:1510.93(3+), RT:82.73, Y-score:87.68

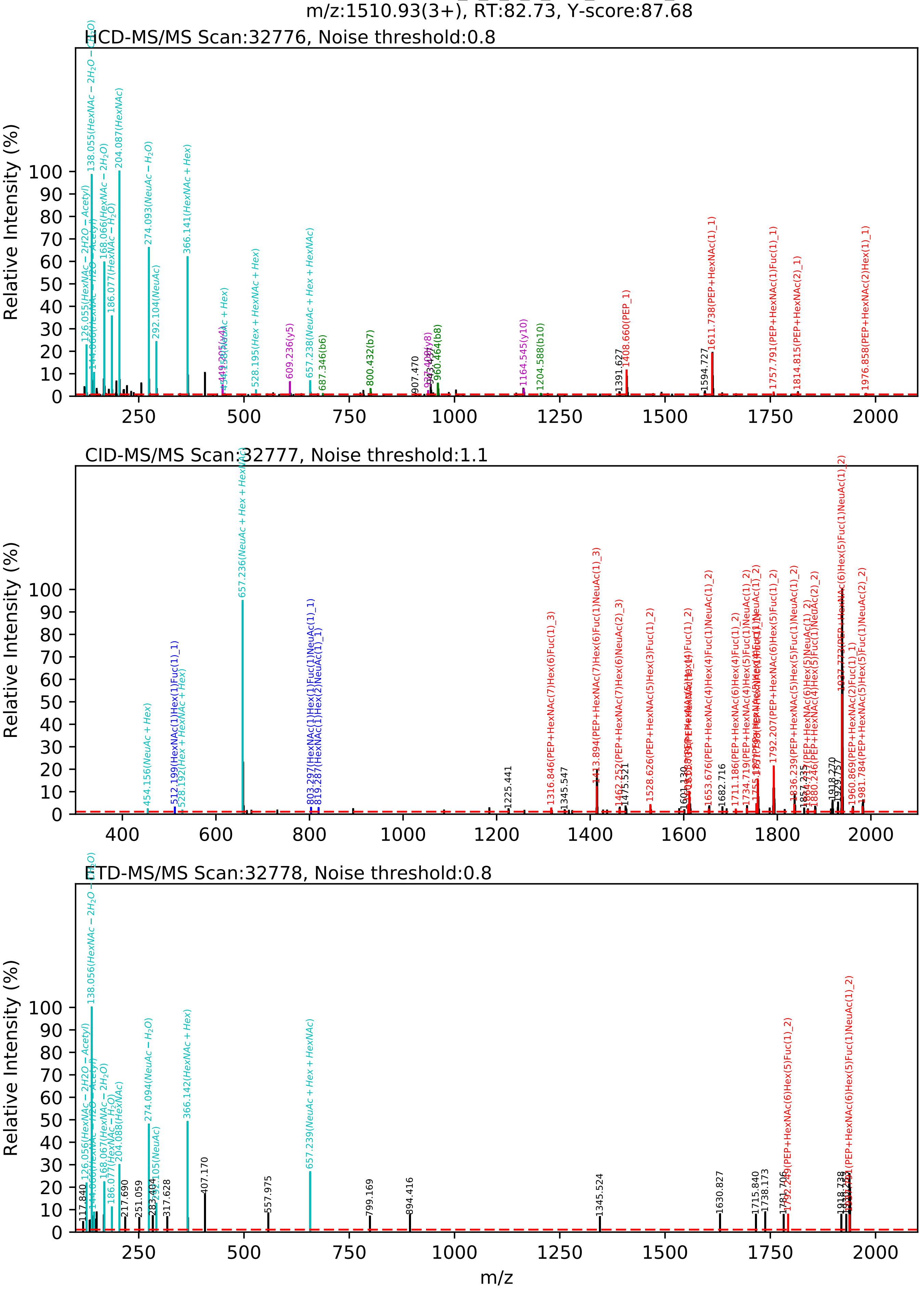

FPNITNLCPFGE(=PEP)_6_7_2_0_0, 0_None, 0_None,
m/z:1365.55(3+), RT:58.73, Y-score:89.24

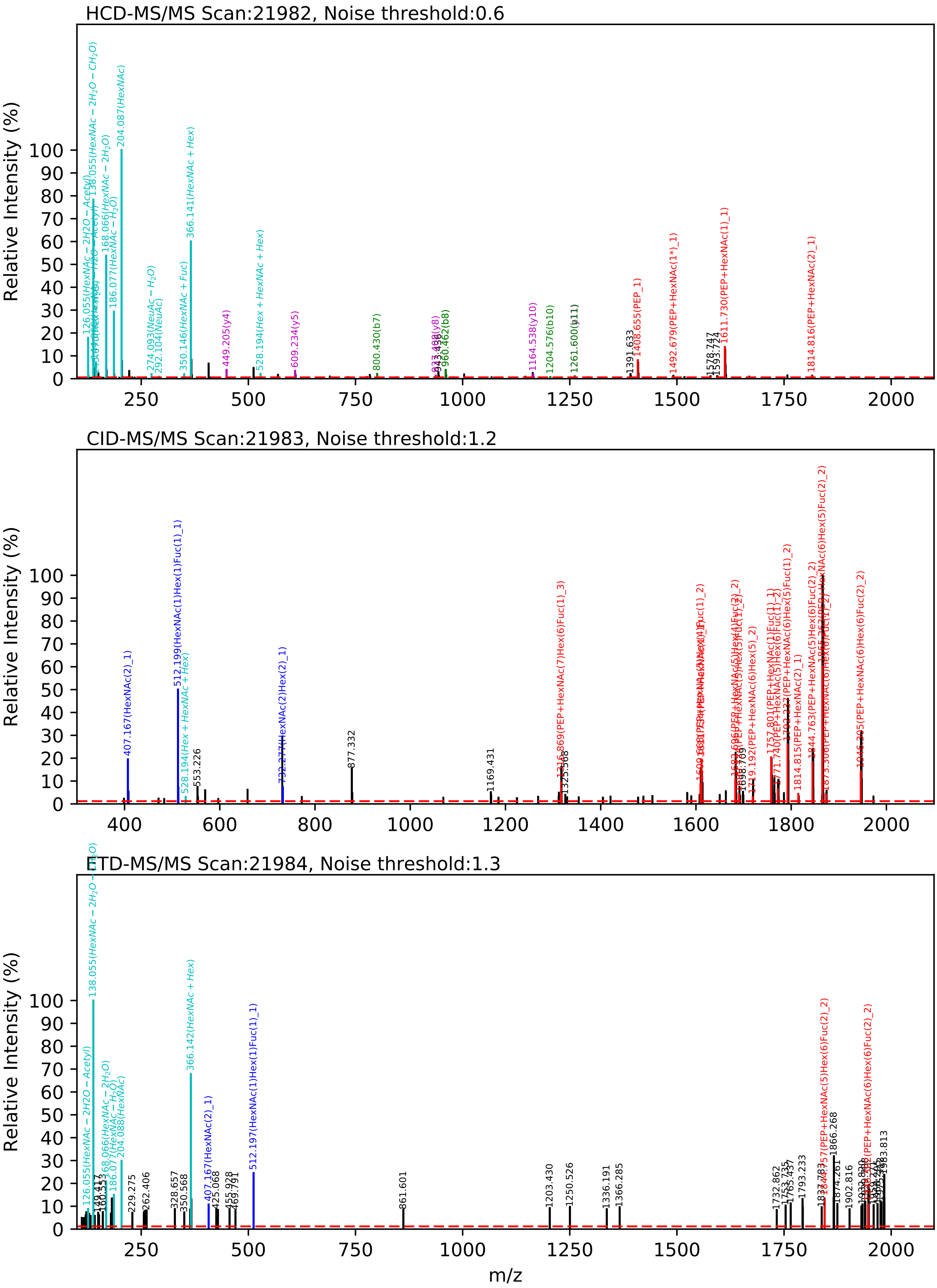

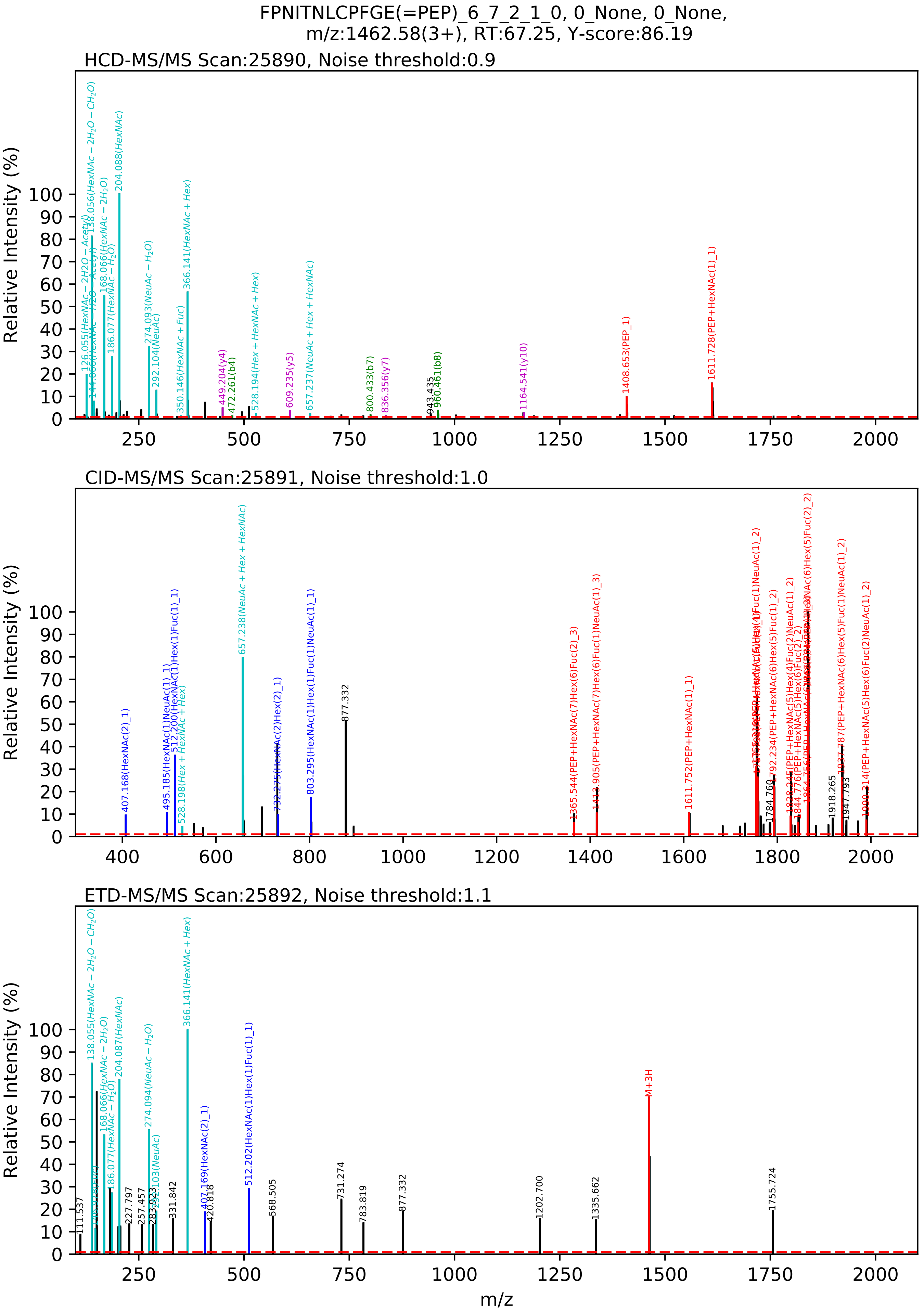

FPNITNLCPFGE(=PEP)_6_7_2_1_0, 0_None, 0_None,
m/z:1462.58(3+), RT:67.25, Y-score:86.19
HCD-MS/MS Scan:25890, Noise threshold:0.9
CID-MS/MS Scan:25891, Noise threshold:1.0
ETD-MS/MS Scan:25892, Noise threshold:1.1
Relative Intensity (%)
m/z
204.088(HexNAc)
366.141(HexNAc + Hex)
1408.653(PEP_1)
1611.728(PEP+HexNAc(1)_1)
657.238(NeuAc + Hex + HexNAc)
877.332
M+3H

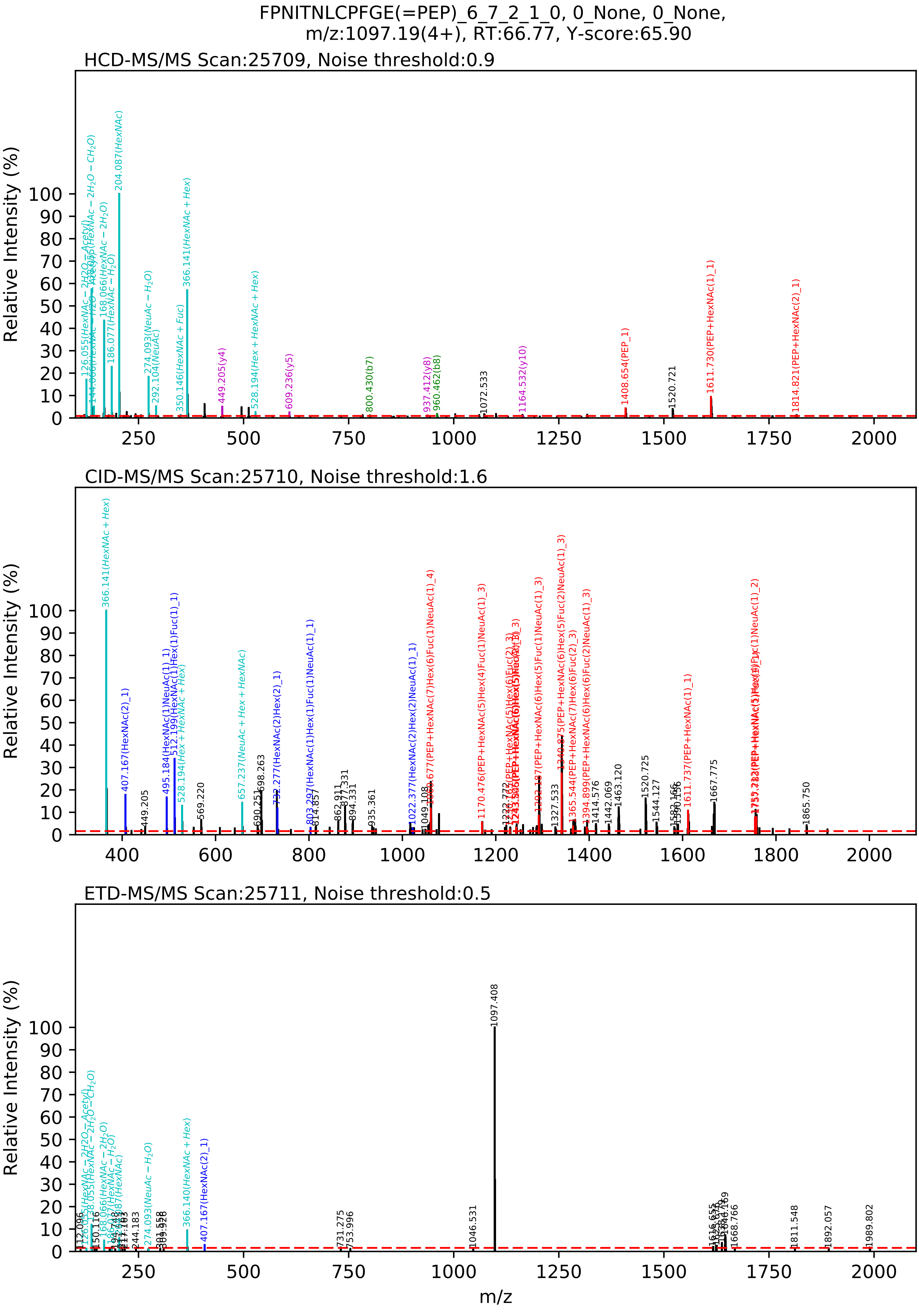

FPNITNLCPFGE(=PEP)_6_7_2_1_0, 0_None, 0_None,
m/z:1097.19(4+), RT:66.77, Y-score:65.90
HCD-MS/MS Scan:25709, Noise threshold:0.9
CID-MS/MS Scan:25710, Noise threshold:1.6
ETD-MS/MS Scan:25711, Noise threshold:0.5
Relative Intensity (%)
m/z

FPNITNLCPFGE(=PEP)_6_7_2_2_0, 0_None, 0_None, m/z:1559.62(3+), RT:79.64, Y-score:78.44

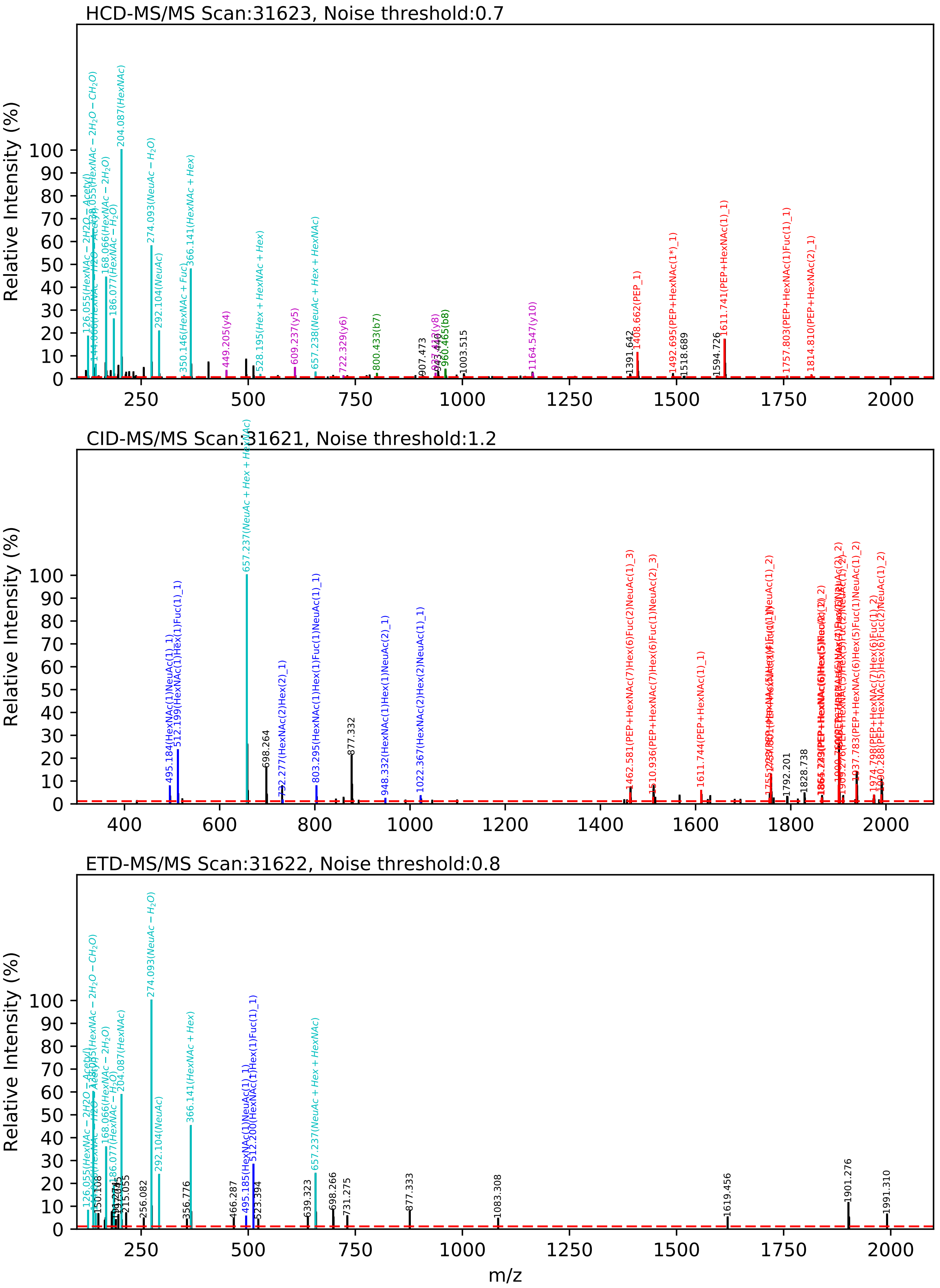

FPNITNLCPFGE(=PEP)_6_7_2_2_0, 0_None, 0_None,
m/z:1169.97(4+), RT:81.69, Y-score:91.43

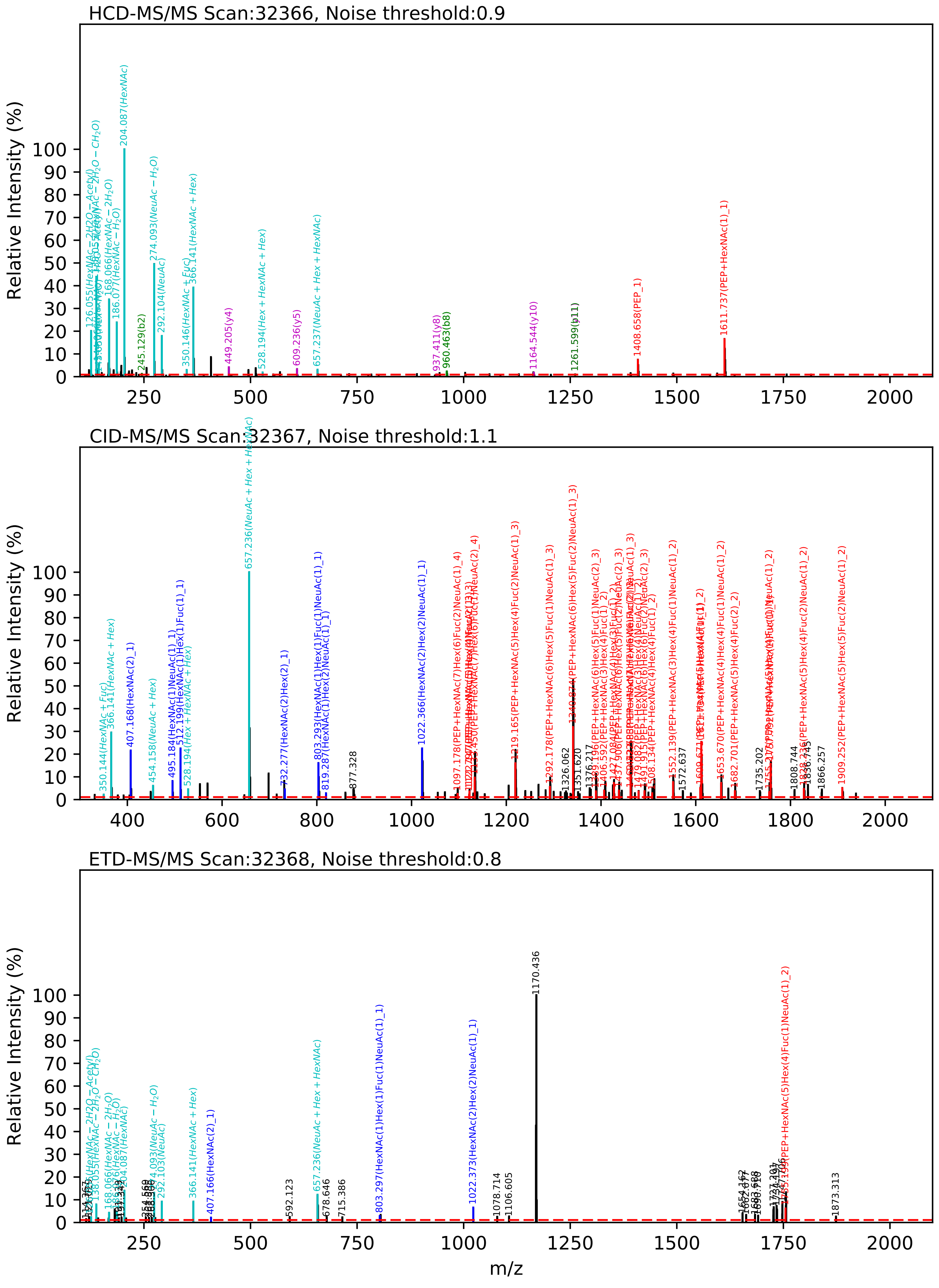

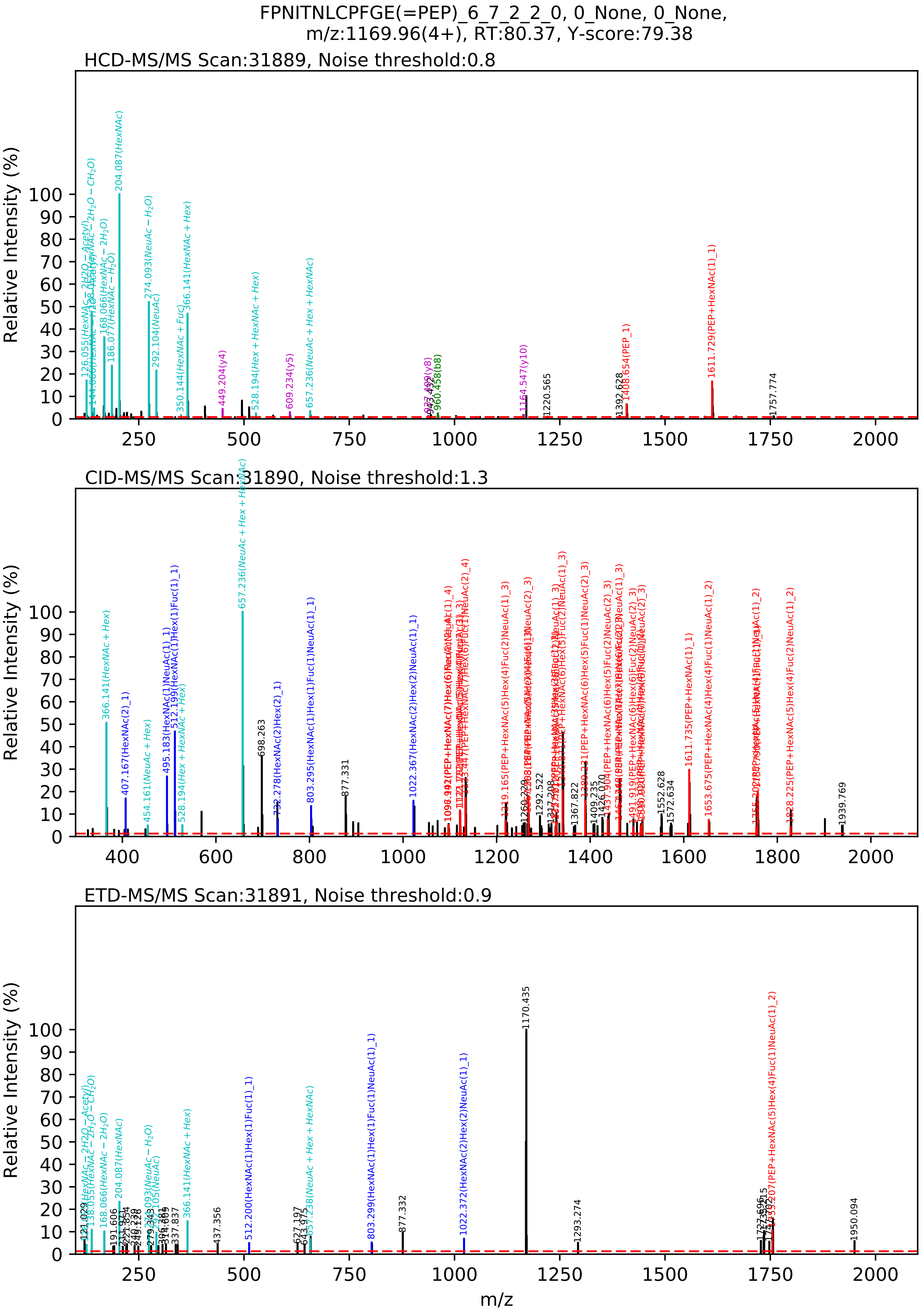
FPNITNLCPFGE(=PEP)_6_7_2_2_0, 0_None, 0_None,
m/z:1169.96(4+), RT:80.37, Y-score:79.38
HCD-MS/MS Scan:31889, Noise threshold:0.8
Relative Intensity (%)
204.087(HexNAc)
274.093(NeuAc − H2O)
366.141(HexNAc + Hex)
292.104(NeuAc)
186.077(HexNAc − H2O)
168.066(HexNAc − 2H2O)
350.144(HexNAc + Fuc)
449.204(y4)
528.194(Hex + HexNAc + Hex)
609.234(y5)
657.236(NeuAc + Hex + HexNAc)
960.458(b8)
1164.547(y10)
1220.565
1392.628
1408.654(PEP_1)
1611.729(PEP+HexNAc(1)_1)
1757.774
CID-MS/MS Scan:31890, Noise threshold:1.3
366.141(HexNAc + Hex)
407.167(HexNAc(2)_1)
454.161(NeuAc + Hex)
495.183(HexNAc(1)NeuAc(1)_1)
512.199(HexNAc(1)Hex(1)Fuc(1)_1)
528.194(Hex + HexNAc + Hex)
657.236(NeuAc + Hex + HexNAc)
698.263
732.278(HexNAc(2)Hex(2)_1)
803.295(HexNAc(1)Hex(1)Fuc(1)NeuAc(1)_1)
877.331
1022.367(HexNAc(2)Hex(2)NeuAc(1)_1)
1260.229
1292.522
1367.822
1409.235
1426.070
1552.628
1572.634
1611.735(PEP+HexNAc(1)_1)
1653.675(PEP+HexNAc(4)Hex(4)Fuc(1)NeuAc(1)_2)
1828.225(PEP+HexNAc(5)Hex(4)Fuc(2)NeuAc(1)_2)
1939.769
ETD-MS/MS Scan:31891, Noise threshold:0.9
121.029
138.055(HexNAc − 2H2O − CH2O)
168.066(HexNAc − 2H2O)
204.087(HexNAc)
191.606
279.343
292.105(NeuAc)
337.837
366.141(HexNAc + Hex)
437.356
512.200(HexNAc(1)Hex(1)Fuc(1)_1)
627.197
657.238(NeuAc + Hex + HexNAc)
803.299(HexNAc(1)Hex(1)Fuc(1)NeuAc(1)_1)
877.332
1022.372(HexNAc(2)Hex(2)NeuAc(1)_1)
1170.435
1293.274
1755.207(PEP+HexNAc(5)Hex(4)Fuc(1)NeuAc(1)_2)
1950.094
m/z

FPNITNLCPFGE(=PEP)_6_7_2_2_0, 0_None, 0_None,
m/z:1559.62(3+), RT:81.18, Y-score:80.26

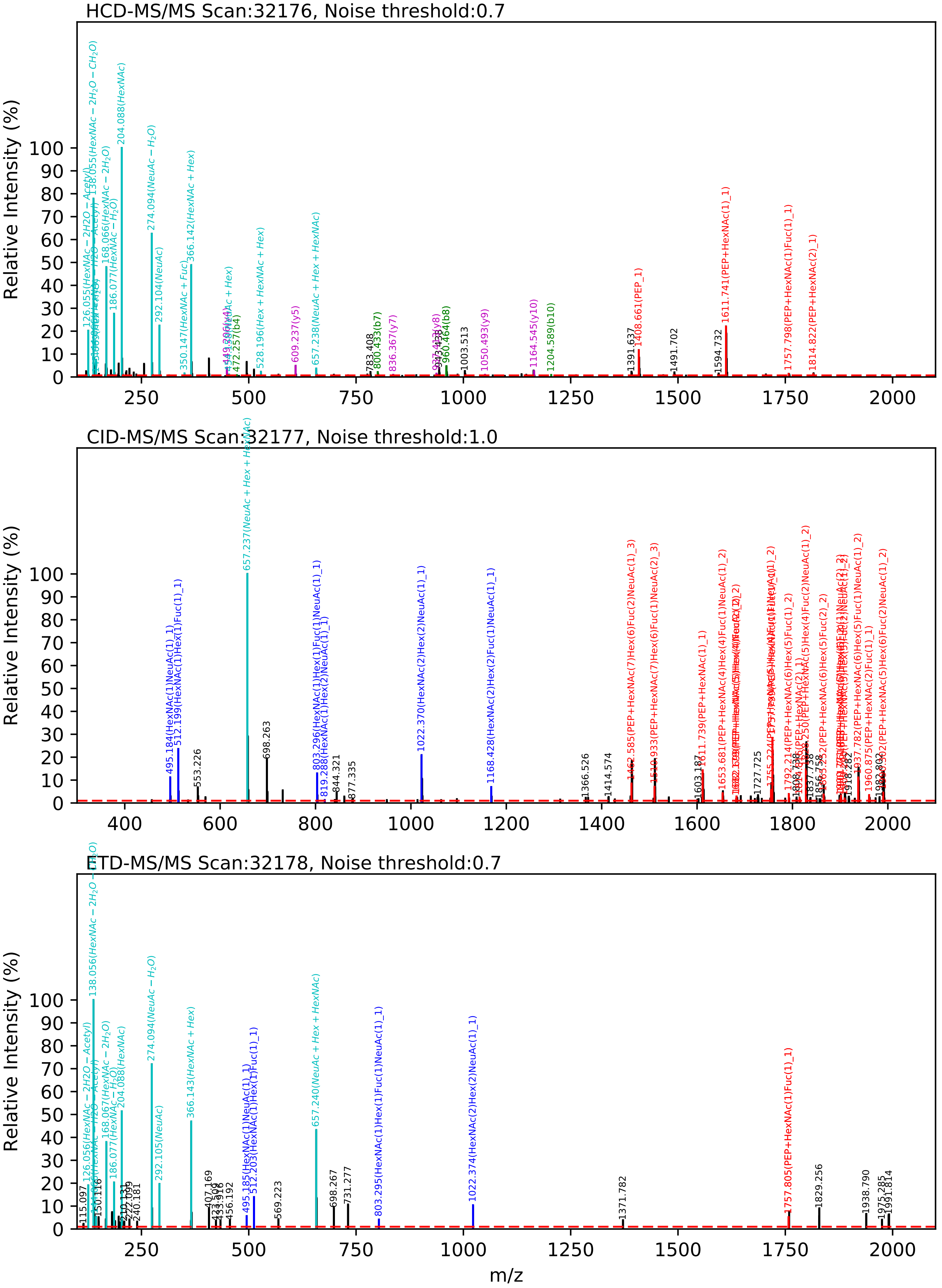

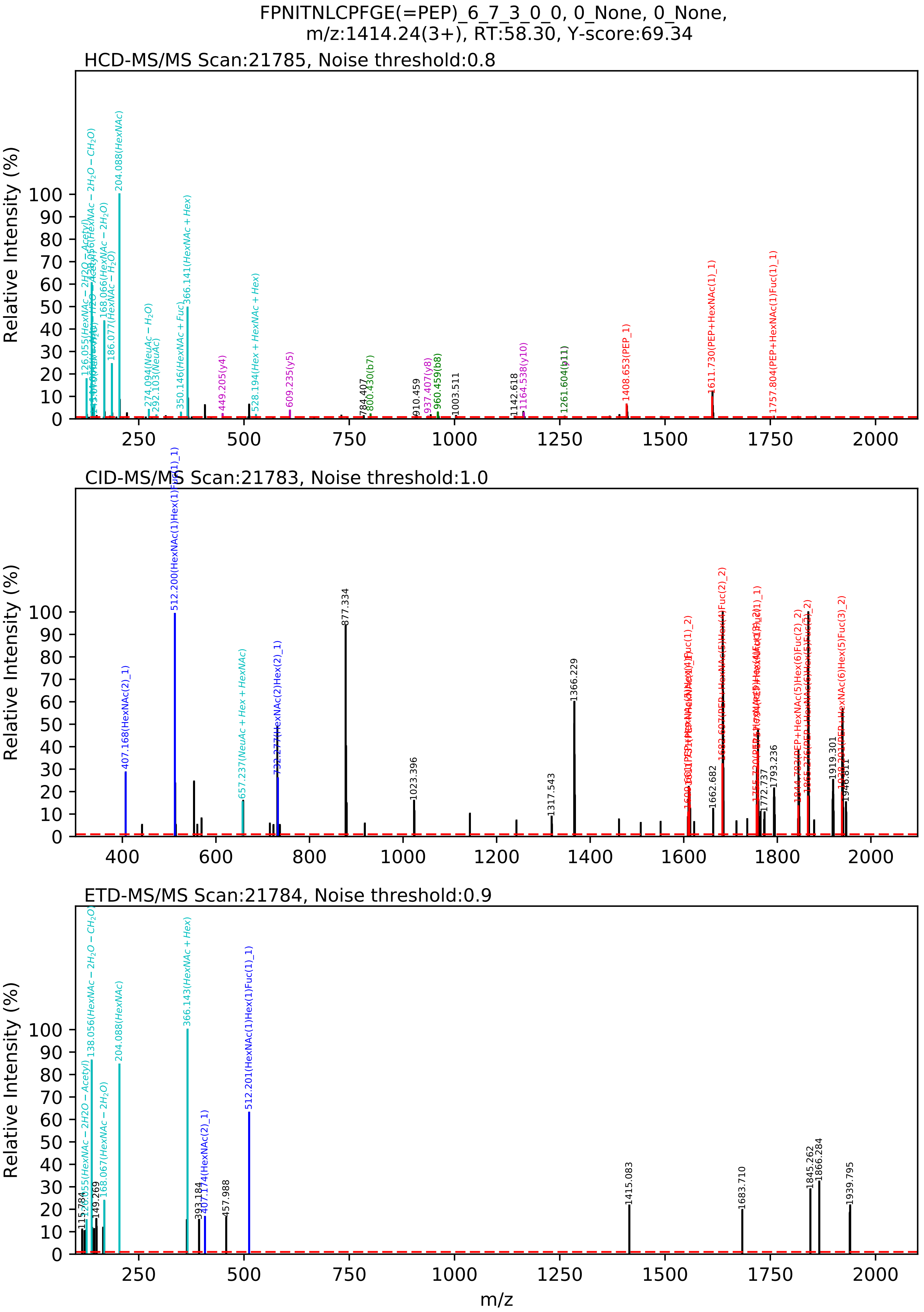

FPNITNLCPFGE(=PEP)_6_7_3_0_0, 0_None, 0_None,
m/z:1414.24(3+), RT:58.30, Y-score:69.34
HCD-MS/MS Scan:21785, Noise threshold:0.8
Relative Intensity (%)
204.088(HexNAc)
168.066(HexNAc − 2H2O)
186.077(HexNAc − H2O)
366.141(HexNAc + Hex)
350.146(HexNAc + Fuc)
274.094(NeuAc − H2O)
292.103(NeuAc)
449.205(y4)
528.194(Hex + HexNAc + Hex)
609.235(y5)
784.407
800.430(b7)
910.459
937.407(y8)
960.459(b8)
1003.511
1142.618
1164.538(y10)
1261.604(b11)
1408.653(PEP_1)
1611.730(PEP+HexNAc(1)_1)
1757.804(PEP+HexNAc(1)Fuc(1)_1)
CID-MS/MS Scan:21783, Noise threshold:1.0
Relative Intensity (%)
407.168(HexNAc(2)_1)
512.200(HexNAc(1)Hex(1)Fuc(1)_1)
657.237(NeuAc + Hex + HexNAc)
732.277(HexNAc(2)Hex(2)_1)
877.334
1023.396
1317.543
1366.229
1662.682
1772.737
1793.236
1919.301
1940.811
ETD-MS/MS Scan:21784, Noise threshold:0.9
Relative Intensity (%)
115.784
138.056(HexNAc − 2H2O − CH2O)
149.269
168.067(HexNAc − 2H2O)
204.088(HexNAc)
366.143(HexNAc + Hex)
393.184
407.174(HexNAc(2)_1)
457.988
512.201(HexNAc(1)Hex(1)Fuc(1)_1)
1415.083
1683.710
1845.262
1866.284
1939.795
m/z

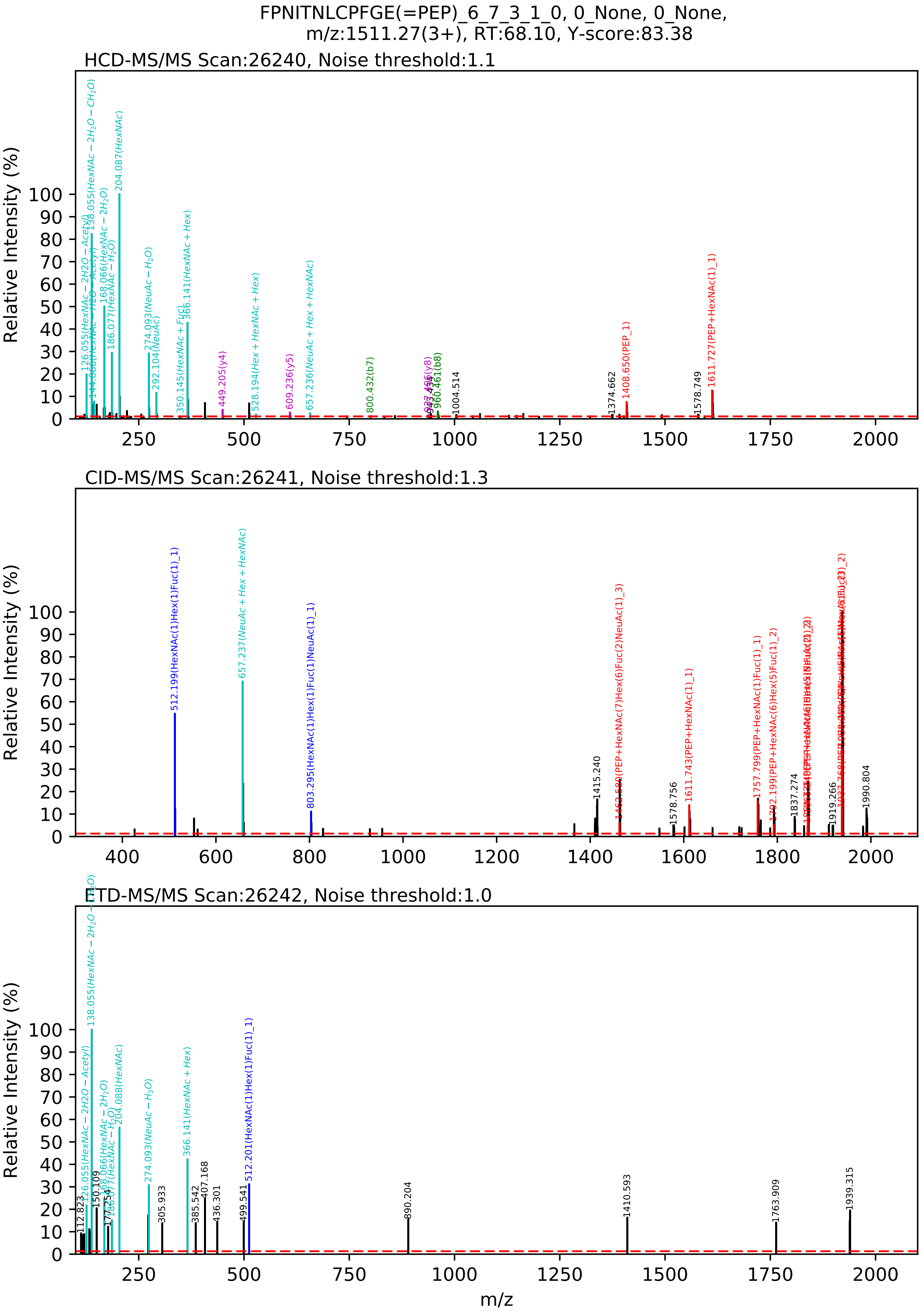

FPNITNLCPFGE(=PEP)_6_7_3_1_0, 0_None, 0_None,
m/z:1511.27(3+), RT:68.10, Y-score:83.38
HCD-MS/MS Scan:26240, Noise threshold:1.1
CID-MS/MS Scan:26241, Noise threshold:1.3
ETD-MS/MS Scan:26242, Noise threshold:1.0
Relative Intensity (%)
m/z
204.087(HexNAc)
138.055(HexNAc − 2H2O − CH2O)
168.066(HexNAc − 2H2O)
186.077(HexNAc − H2O)
274.093(NeuAc − H2O)
292.104(NeuAc)
366.141(HexNAc + Hex)
350.145(HexNAc + Fuc)
449.205(y4)
528.194(Hex + HexNAc + Hex)
609.236(y5)
657.236(NeuAc + Hex + HexNAc)
800.432(b7)
960.461(b8)
1004.514
1374.662
1408.650(PEP_1)
1578.749
1611.727(PEP+HexNAc(1)_1)
512.199(HexNAc(1)Hex(1)Fuc(1)_1)
657.237(NeuAc + Hex + HexNAc)
803.295(HexNAc(1)Hex(1)Fuc(1)NeuAc(1)_1)
1415.240
1462.500(PEP+HexNAc(7)Hex(6)Fuc(2)NeuAc(1)_3)
1578.756
1611.743(PEP+HexNAc(1)_1)
1757.799(PEP+HexNAc(1)Fuc(1)_1)
1792.199(PEP+HexNAc(6)Hex(5)Fuc(1)_2)
1837.274
1919.266
1990.804
112.823
150.109
305.933
385.542
407.168
436.301
499.541
512.201(HexNAc(1)Hex(1)Fuc(1)_1)
890.204
1410.593
1763.909
1939.315

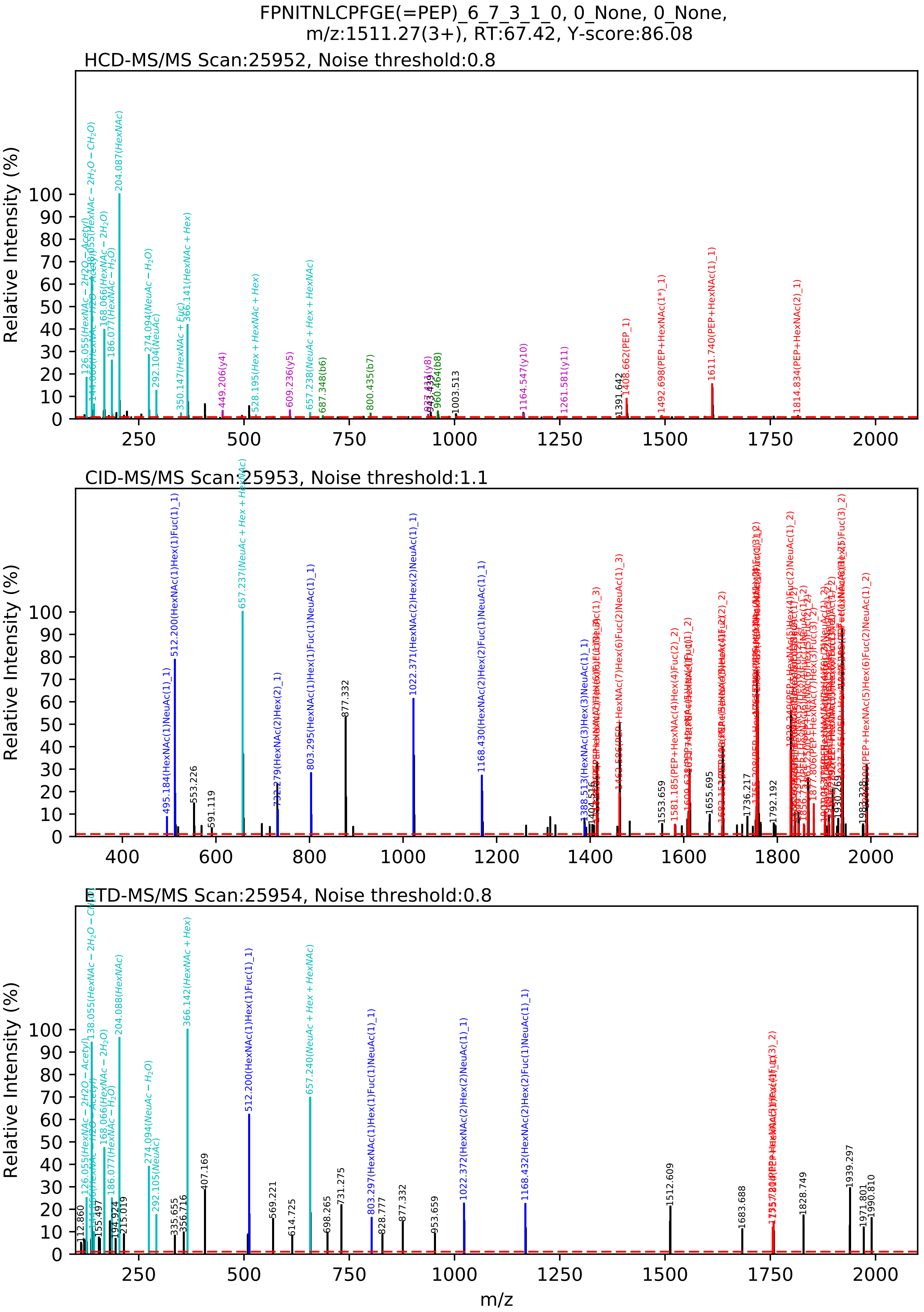

FPNITNLCPFGE(=PEP)_6_7_3_1_0, 0_None, 0_None,
m/z:1511.27(3+), RT:67.42, Y-score:86.08
HCD-MS/MS Scan:25952, Noise threshold:0.8
CID-MS/MS Scan:25953, Noise threshold:1.1
ETD-MS/MS Scan:25954, Noise threshold:0.8
Relative Intensity (%)
m/z

FPNITNLCPFGE(=PEP)_7_6_1_0_0, 0_None, 0_None,
m/z:1303.19(3+), RT:59.16, Y-score:94.31

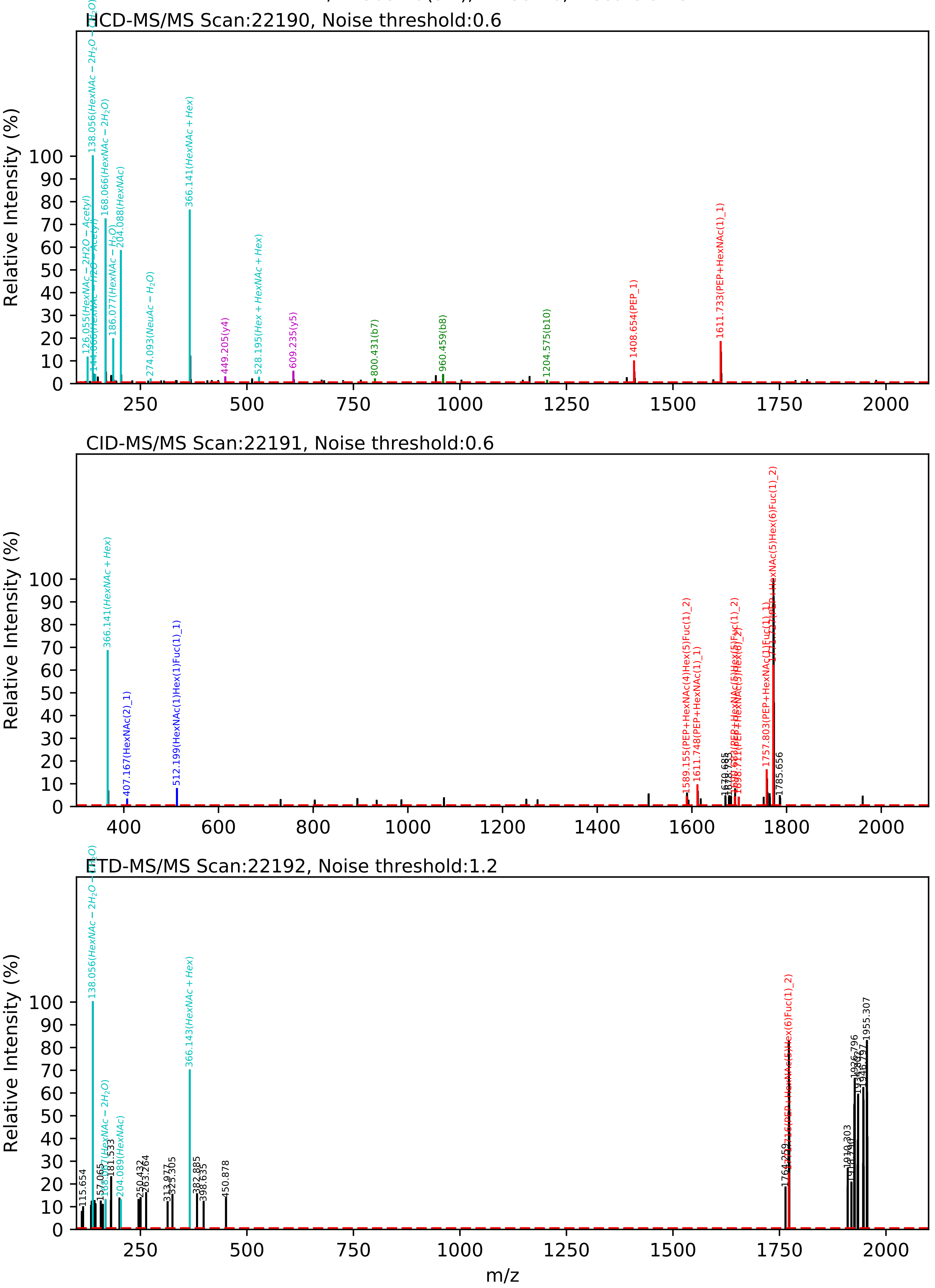

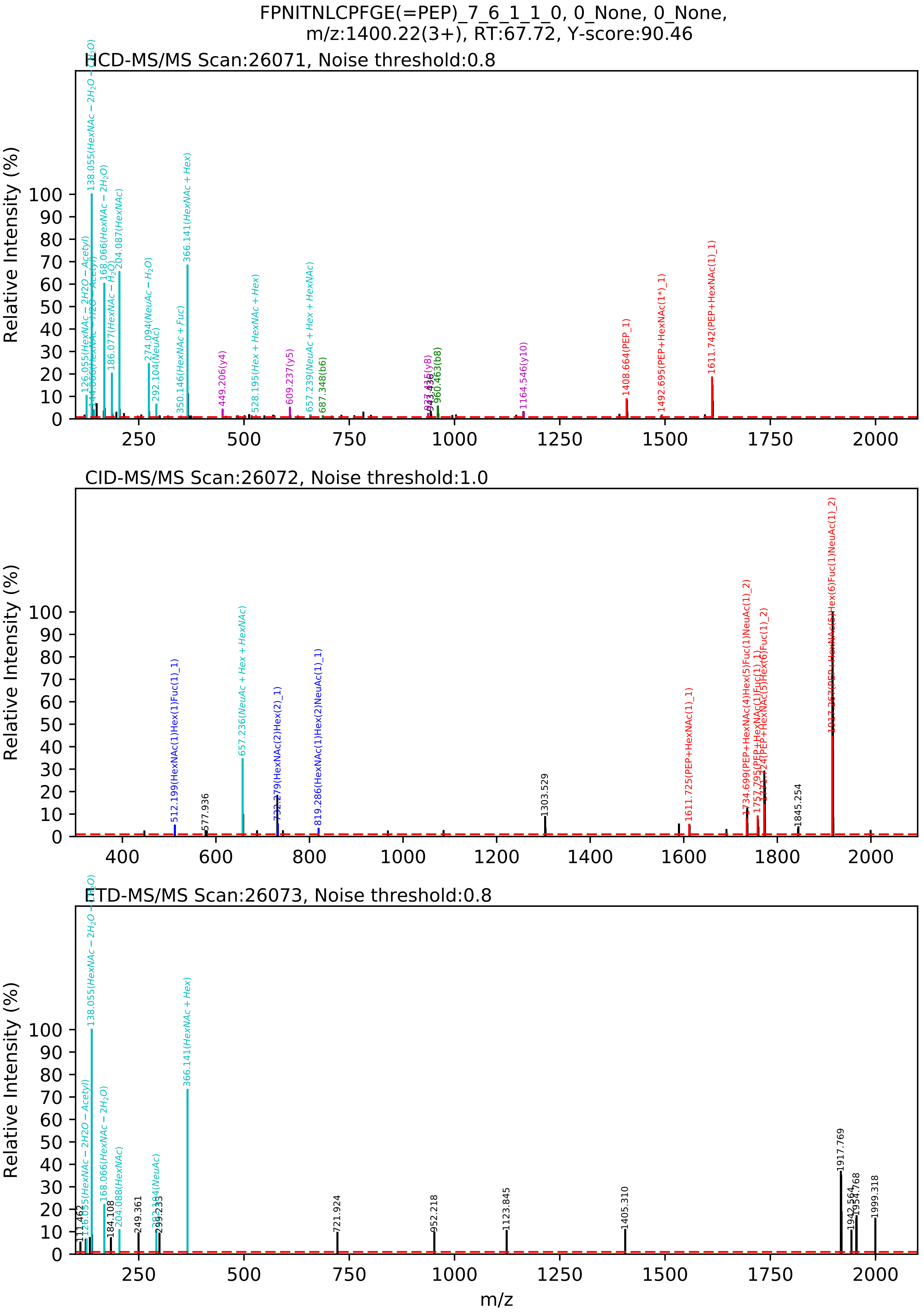
FPNITNLCPFGE(=PEP)_7_6_1_1_0, 0_None, 0_None,
m/z:1400.22(3+), RT:67.72, Y-score:90.46
HCD-MS/MS Scan:26071, Noise threshold:0.8
Relative Intensity (%)
126.055(HexNAc − 2H2O − Acetyl)
144.066(HexNAc − H2O − Acetyl)
138.055(HexNAc − 2H2O − H2O)
168.066(HexNAc − 2H2O)
186.077(HexNAc − H2O)
204.087(HexNAc)
274.094(NeuAc − H2O)
292.104(NeuAc)
350.146(HexNAc + Fuc)
366.141(HexNAc + Hex)
449.206(y4)
528.195(Hex + HexNAc + Hex)
609.237(y5)
657.239(NeuAc + Hex + HexNAc)
687.348(b6)
937.436
960.463(b8)
1164.546(y10)
1408.664(PEP_1)
1492.695(PEP+HexNAc(1*)_1)
1611.742(PEP+HexNAc(1)_1)
CID-MS/MS Scan:26072, Noise threshold:1.0
512.199(HexNAc(1)Hex(1)Fuc(1)_1)
577.936
657.236(NeuAc + Hex + HexNAc)
732.279(HexNAc(2)Hex(2)_1)
819.286(HexNAc(1)Hex(2)NeuAc(1)_1)
1303.529
1611.725(PEP+HexNAc(1)_1)
1734.699(PEP+HexNAc(4)Hex(5)Fuc(1)NeuAc(1)_2)
1845.254
1917.267(PEP+HexNAc(5)Hex(6)Fuc(1)NeuAc(1)_2)
ETD-MS/MS Scan:26073, Noise threshold:0.8
111.462
126.055(HexNAc − 2H2O − Acetyl)
138.055(HexNAc − 2H2O − H2O)
168.066(HexNAc − 2H2O)
184.108
204.088(HexNAc)
249.361
292.104(NeuAc)
299.233
366.141(HexNAc + Hex)
721.924
952.218
1123.845
1405.310
1917.769
1942.564
1954.768
1999.318
m/z

FPNITNLCPFGE(=PEP)_7_6_1_1_0, 0_None, 0_None,
m/z:1400.22(3+), RT:67.00, Y-score:89.49

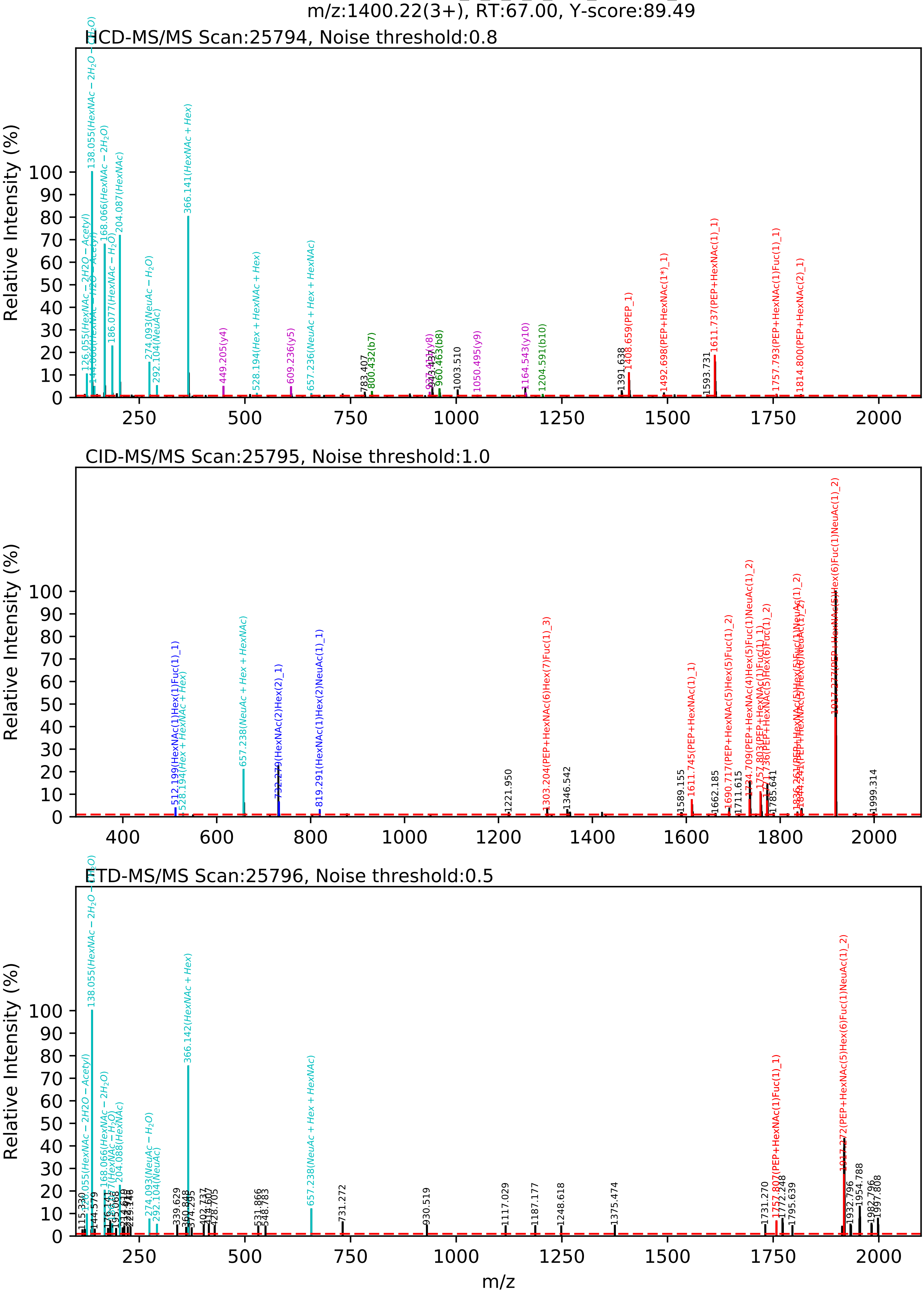

FPNITNLCPFGE(=PEP)_7_6_1_1_0, 0_None, 0_None,
m/z:1400.22(3+), RT:68.31, Y-score:89.08

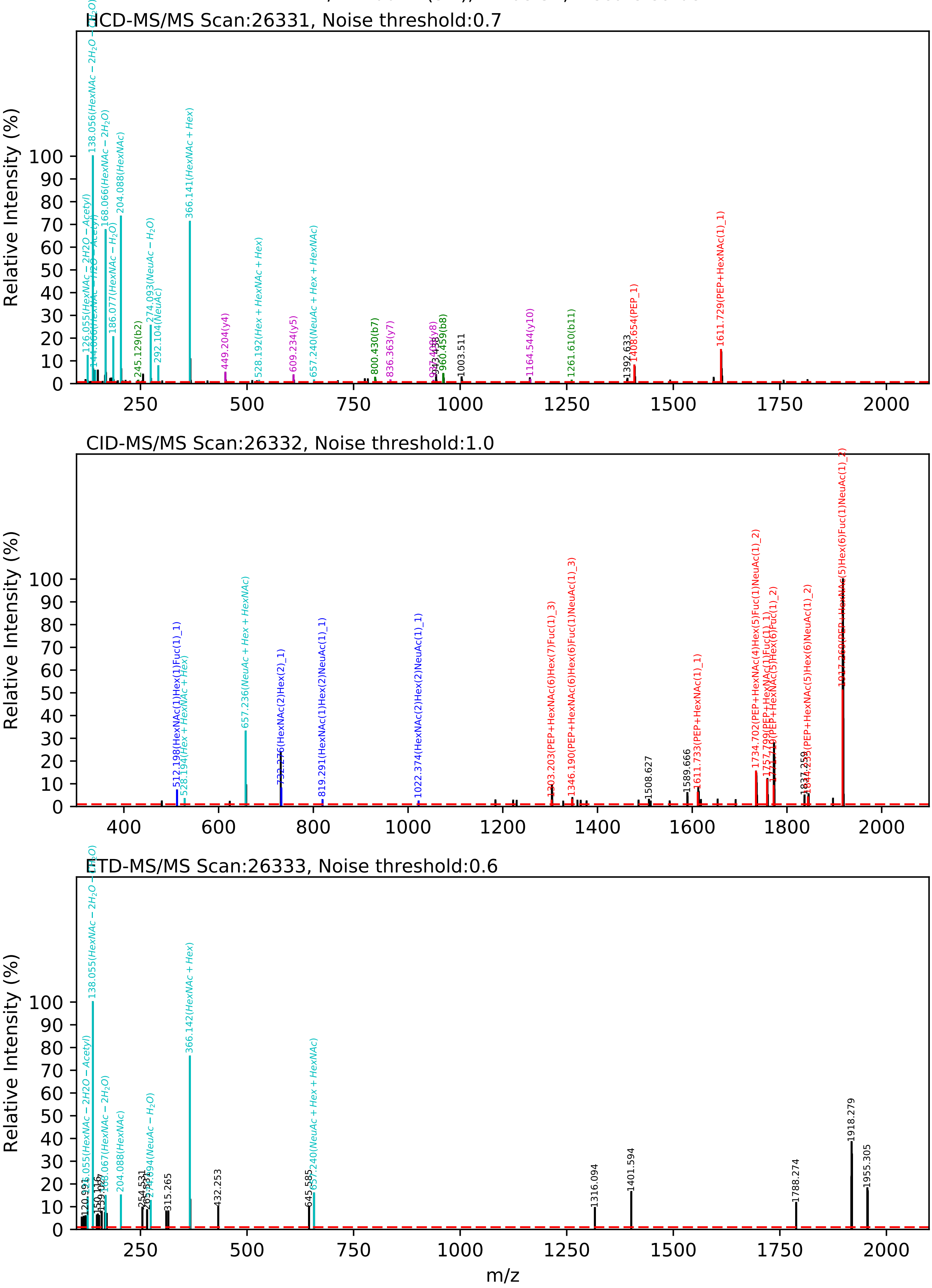

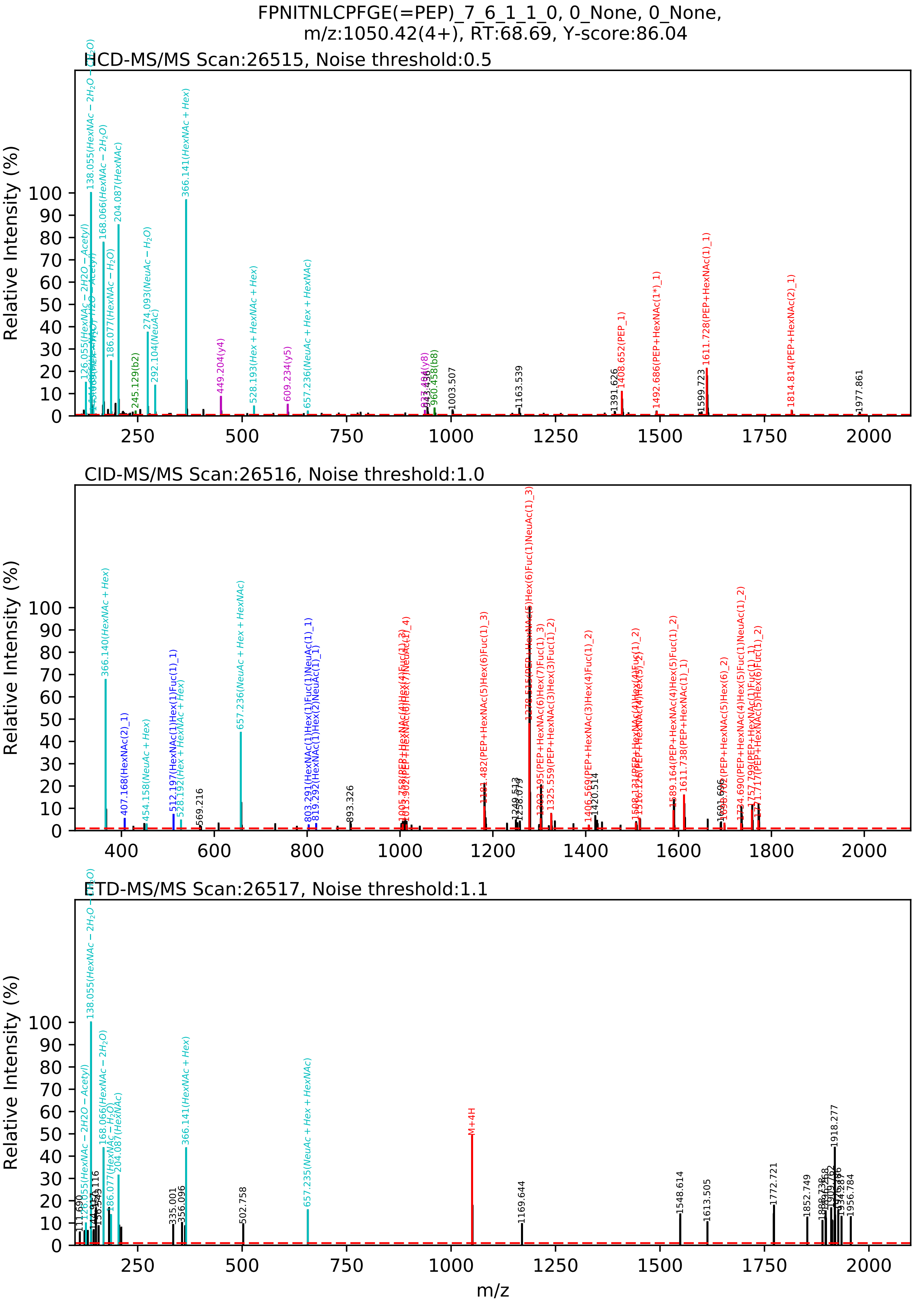
FPNITNLCPFGE(=PEP)_7_6_1_1_0, 0_None, 0_None,
m/z:1050.42(4+), RT:68.69, Y-score:86.04
HCD-MS/MS Scan:26515, Noise threshold:0.5
CID-MS/MS Scan:26516, Noise threshold:1.0
ETD-MS/MS Scan:26517, Noise threshold:1.1
Relative Intensity (%)
m/z
138.055(HexNAc − 2H2O − H2O)
168.066(HexNAc − 2H2O)
204.087(HexNAc)
366.141(HexNAc + Hex)
186.077(HexNAc − H2O)
274.093(NeuAc − H2O)
292.104(NeuAc)
245.129(b2)
449.204(y4)
528.193(Hex + HexNAc + Hex)
609.234(y5)
657.236(NeuAc + Hex + HexNAc)
960.458(b8)
1003.507
1163.539
1391.626
1408.652(PEP_1)
1492.686(PEP+HexNAc(1*)_1)
1599.723
1611.728(PEP+HexNAc(1)_1)
1814.814(PEP+HexNAc(2)_1)
1977.861
366.140(HexNAc + Hex)
407.168(HexNAc(2)_1)
454.158(NeuAc + Hex)
512.197(HexNAc(1)Hex(1)Fuc(1)_1)
569.216
657.236(NeuAc + Hex + HexNAc)
803.291(HexNAc(1)Hex(1)Fuc(1)NeuAc(1)_1)
819.292(HexNAc(1)Hex(2)NeuAc(1)_1)
893.326
1181.482(PEP+HexNAc(5)Hex(6)Fuc(1)_3)
1249.513
1258.079
1303.195(PEP+HexNAc(6)Hex(7)Fuc(1)_3)
1325.559(PEP+HexNAc(3)Hex(3)Fuc(1)_2)
1406.569(PEP+HexNAc(3)Hex(4)Fuc(1)_2)
1420.514
1589.164(PEP+HexNAc(4)Hex(5)Fuc(1)_2)
1611.738(PEP+HexNAc(1)_1)
1691.696
1724.690(PEP+HexNAc(4)Hex(5)Fuc(1)NeuAc(1)_2)
M+4H
335.001
356.096
502.758
657.235(NeuAc + Hex + HexNAc)
1169.644
1548.614
1613.505
1772.721
1852.749
1918.277
1956.784

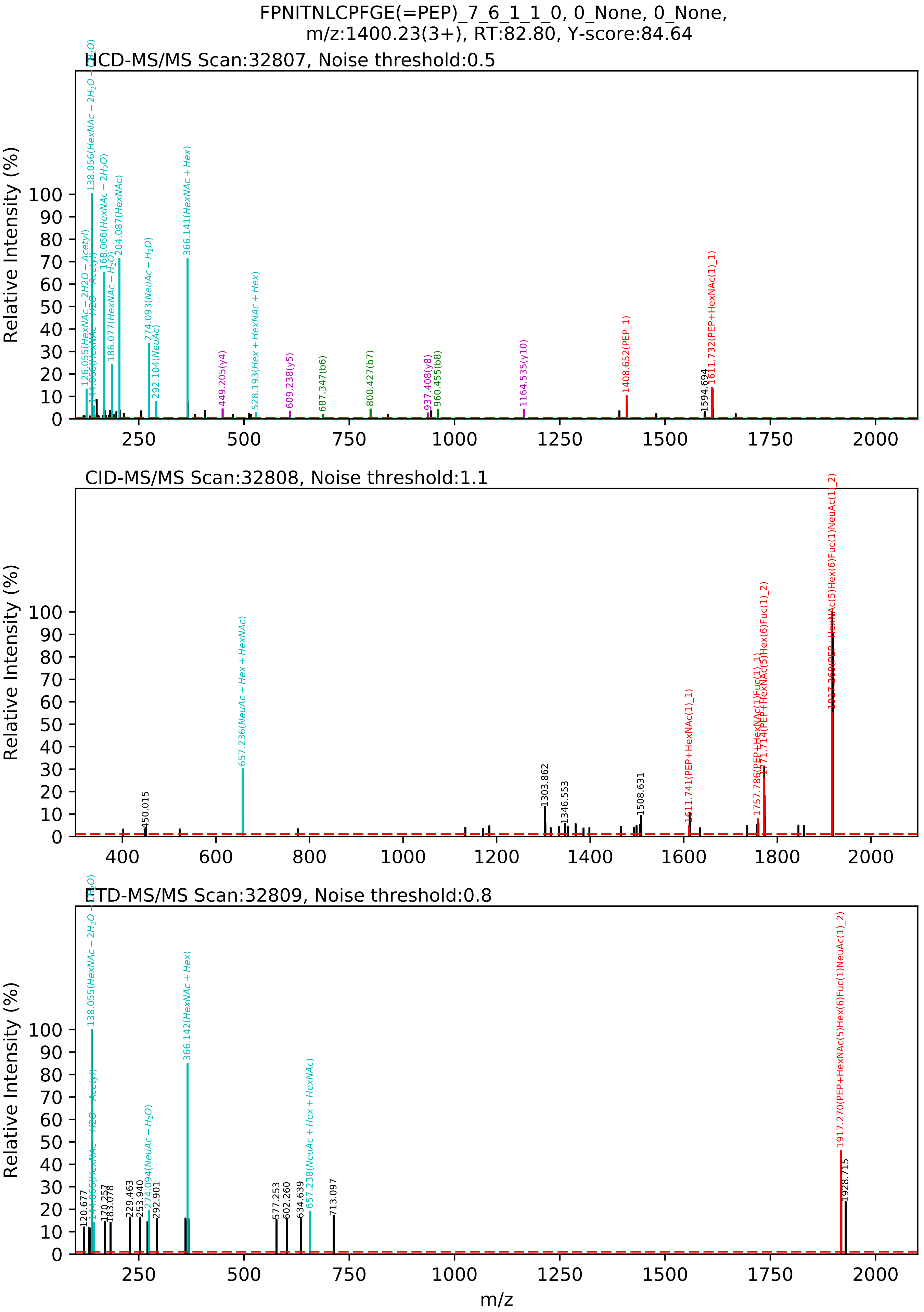
FPNITNLCPFGE(=PEP)_7_6_1_1_0, 0_None, 0_None,
m/z:1400.23(3+), RT:82.80, Y-score:84.64
HCD-MS/MS Scan:32807, Noise threshold:0.5
Relative Intensity (%)
138.056(HexNAc − 2H2O − H2O)
126.055(HexNAc − 2H2O − Acetyl)
144.066(HexNAc − H2O − Acetyl)
168.066(HexNAc − 2H2O)
186.077(HexNAc − H2O)
204.087(HexNAc)
274.093(NeuAc − H2O)
292.104(NeuAc)
366.141(HexNAc + Hex)
449.205(y4)
528.193(Hex + HexNAc + Hex)
609.238(y5)
687.347(b6)
800.427(b7)
937.408(y8)
960.455(b8)
1164.535(y10)
1408.652(PEP_1)
1594.694
1611.732(PEP+HexNAc(1)_1)
CID-MS/MS Scan:32808, Noise threshold:1.1
450.015
657.236(NeuAc + Hex + HexNAc)
1303.862
1346.553
1508.631
1611.741(PEP+HexNAc(1)_1)
1757.786(PEP+HexNAc(1)Fuc(1)_1)
1771.714(PEP+HexNAc(5)Hex(6)Fuc(1)_2)
1917.269(PEP+HexNAc(5)Hex(6)Fuc(1)NeuAc(1)_2)
ETD-MS/MS Scan:32809, Noise threshold:0.8
138.055(HexNAc − 2H2O − H2O)
144.066(HexNAc − H2O − Acetyl)
120.677
170.257
183.078
229.463
253.940
274.094(NeuAc − H2O)
292.901
366.142(HexNAc + Hex)
577.253
602.260
634.639
657.238(NeuAc + Hex + HexNAc)
713.097
1917.270(PEP+HexNAc(5)Hex(6)Fuc(1)NeuAc(1)_2)
1928.715
m/z

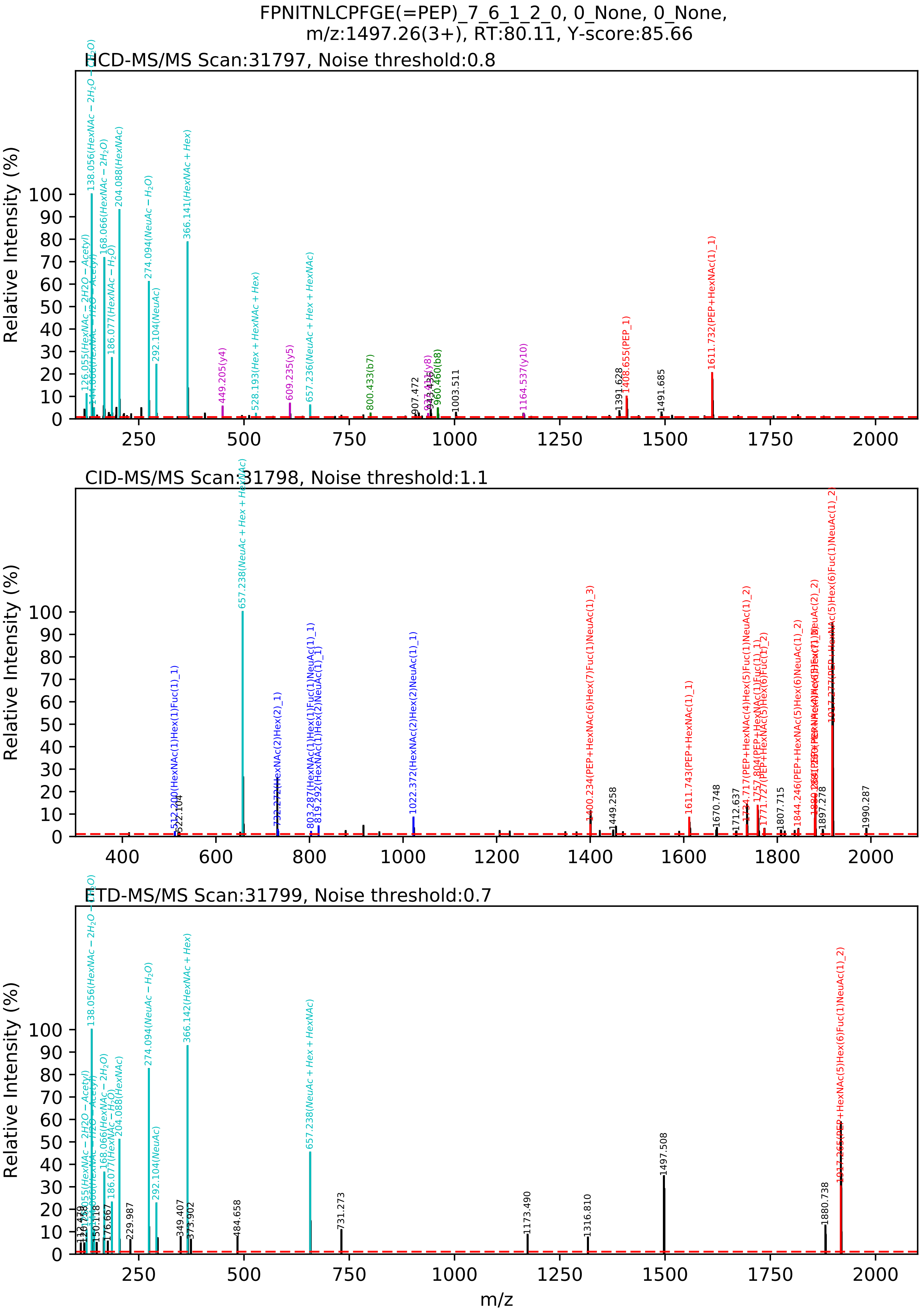

FPNITNLCPFGE(=PEP)_7_6_1_2_0, 0_None, 0_None,
m/z:1497.26(3+), RT:80.11, Y-score:85.66
HCD-MS/MS Scan:31797, Noise threshold:0.8
Relative Intensity (%)
138.056(HexNAc − 2H2O − H2O)
168.066(HexNAc − 2H2O)
204.088(HexNAc)
186.077(HexNAc − H2O)
274.094(NeuAc − H2O)
292.104(NeuAc)
366.141(HexNAc + Hex)
449.205(y4)
528.193(Hex + HexNAc + Hex)
609.235(y5)
657.236(NeuAc + Hex + HexNAc)
800.433(b7)
907.472
960.460(b8)
1003.511
1164.537(y10)
1391.628
1408.655(PEP_1)
1491.685
1611.732(PEP+HexNAc(1)_1)
CID-MS/MS Scan:31798, Noise threshold:1.1
657.238(NeuAc + Hex + HexNAc)
512.200(HexNAc(1)Hex(1)Fuc(1)_1)
522.104
732.272(HexNAc(2)Hex(2)_1)
803.287(HexNAc(1)Hex(1)Fuc(1)NeuAc(1)_1)
819.292(HexNAc(1)Hex(2)NeuAc(1)_1)
1022.372(HexNAc(2)Hex(2)NeuAc(1)_1)
1400.234(PEP+HexNAc(6)Hex(7)Fuc(1)NeuAc(1)_3)
1449.258
1611.743(PEP+HexNAc(1)_1)
1670.748
1712.637
1724.717(PEP+HexNAc(4)Hex(5)Fuc(1)NeuAc(1)_2)
1771.727(PEP+HexNAc(5)Hex(6)Fuc(1)_2)
1807.715
1844.246(PEP+HexNAc(5)Hex(6)NeuAc(1)_2)
1897.278
1917.277(PEP+HexNAc(5)Hex(6)Fuc(1)NeuAc(1)_2)
1990.287
ETD-MS/MS Scan:31799, Noise threshold:0.7
138.056(HexNAc − 2H2O − H2O)
274.094(NeuAc − H2O)
366.142(HexNAc + Hex)
168.066(HexNAc − 2H2O)
204.088(HexNAc)
186.077(HexNAc − H2O)
292.104(NeuAc)
657.238(NeuAc + Hex + HexNAc)
150.118
176.667
229.987
349.407
373.902
484.658
731.273
1173.490
1316.810
1497.508
1880.738
1917.265(PEP+HexNAc(5)Hex(6)Fuc(1)NeuAc(1)_2)
m/z

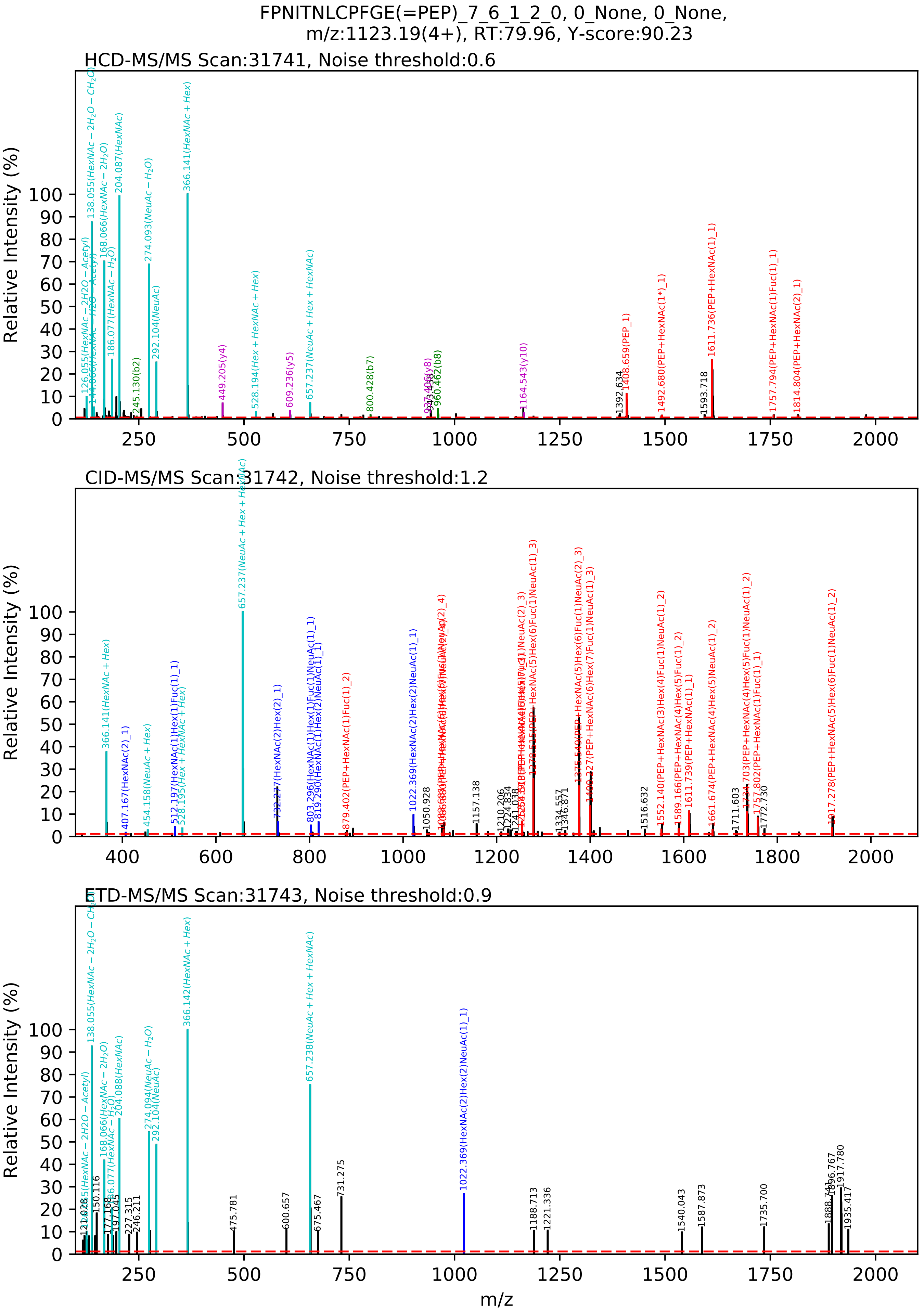

FPNITNLCPFGE(=PEP)_7_6_1_2_0, 0_None, 0_None,
m/z:1123.19(4+), RT:79.96, Y-score:90.23
HCD-MS/MS Scan:31741, Noise threshold:0.6
CID-MS/MS Scan:31742, Noise threshold:1.2
ETD-MS/MS Scan:31743, Noise threshold:0.9
Relative Intensity (%)
m/z

FPNITNLCPFGE(=PEP)_7_6_1_2_0, 0_None, 0_None,
m/z:1497.25(3+), RT:81.85, Y-score:85.58

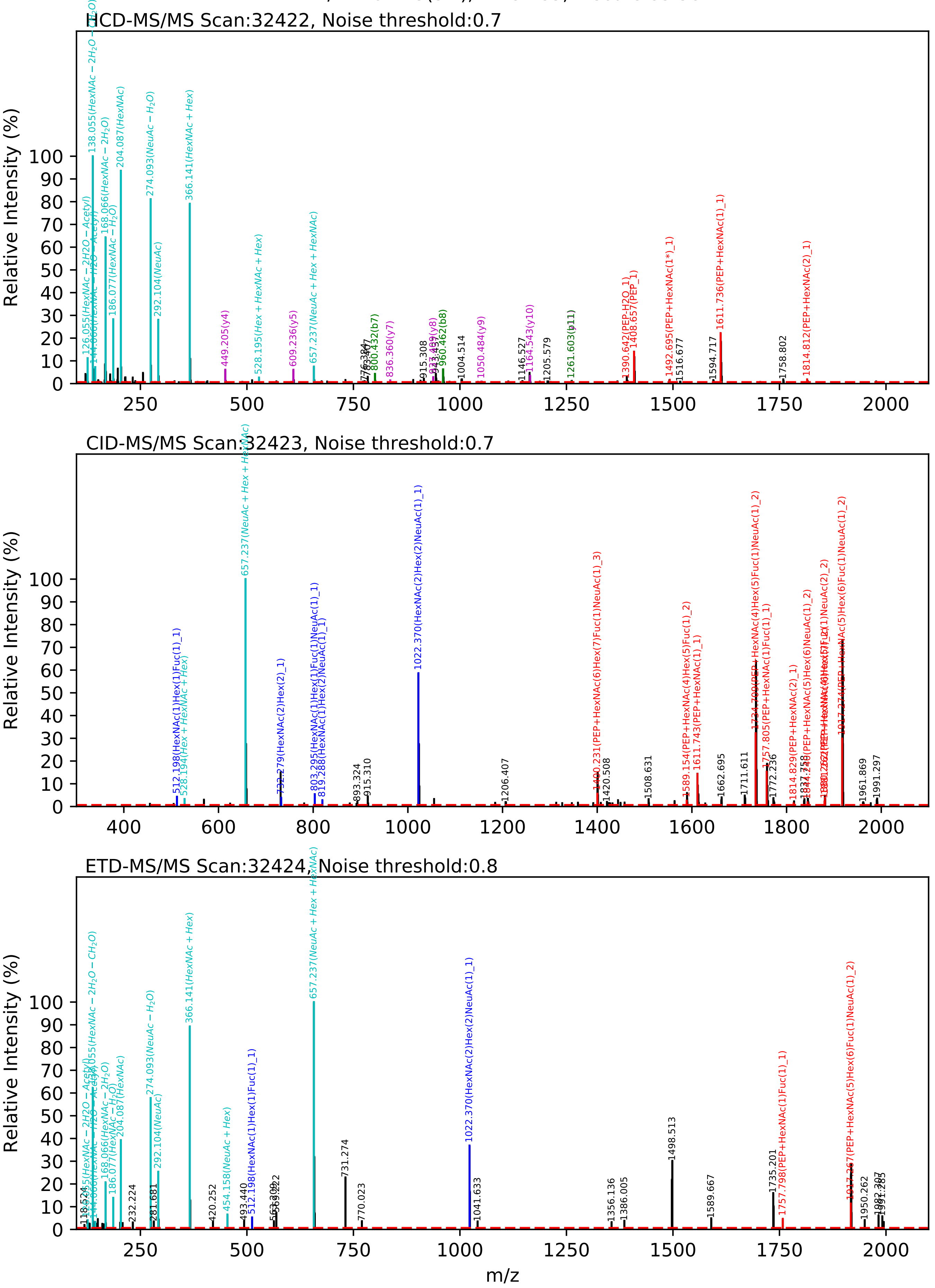

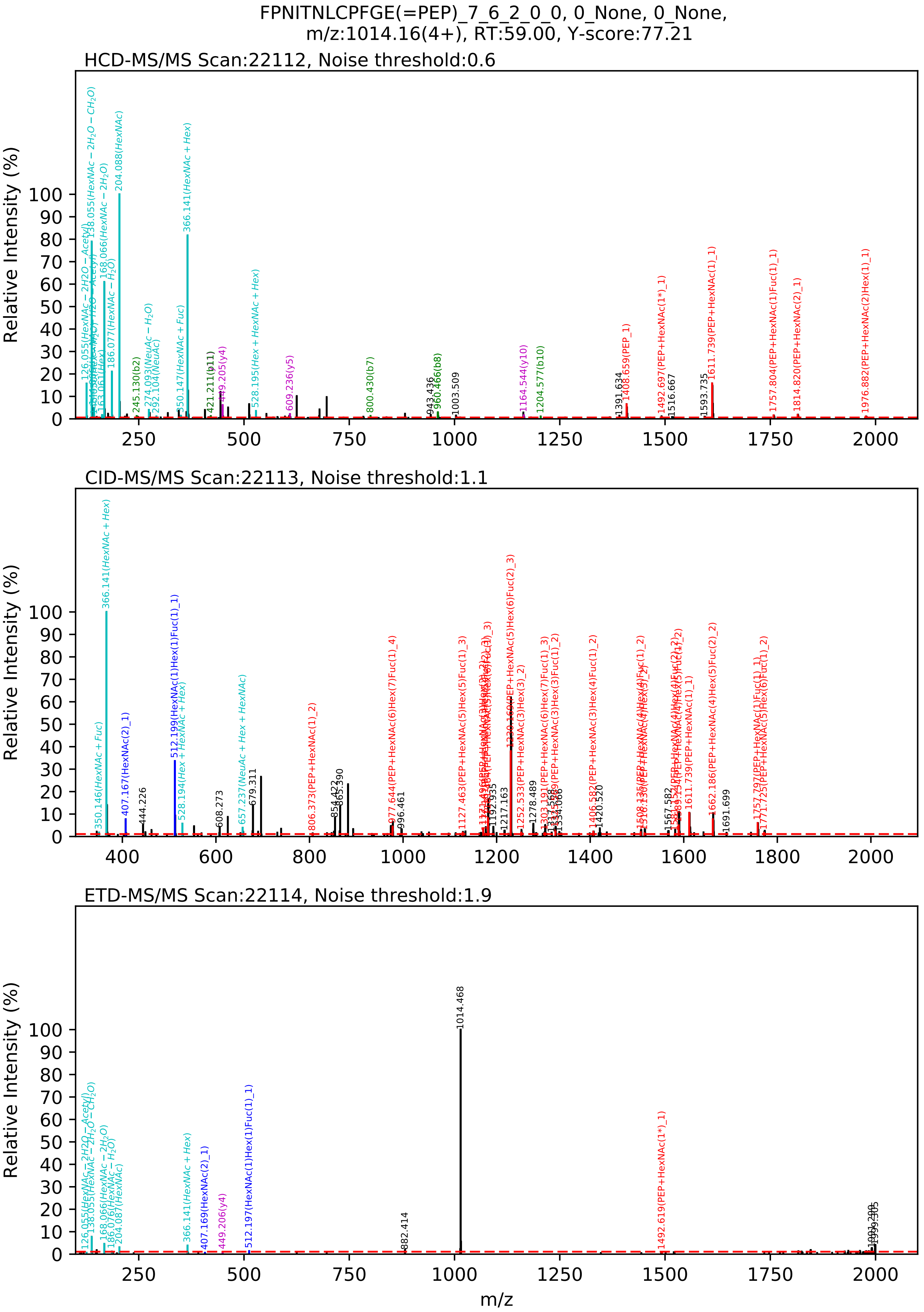

FPNITNLCPFGE(=PEP)_7_6_2_0_0, 0_None, 0_None,
m/z:1014.16(4+), RT:59.00, Y-score:77.21
HCD-MS/MS Scan:22112, Noise threshold:0.6
Relative Intensity (%)
204.088(HexNAc)
366.141(HexNAc + Hex)
1611.739(PEP+HexNAc(1)_1)
1408.659(PEP_1)
CID-MS/MS Scan:22113, Noise threshold:1.1
366.141(HexNAc + Hex)
512.199(HexNAc(1)Hex(1)Fuc(1)_1)
1230.160(PEP+HexNAc(5)Hex(6)Fuc(2)_3)
ETD-MS/MS Scan:22114, Noise threshold:1.9
1014.468
1492.619(PEP+HexNAc(1*)_1)
m/z

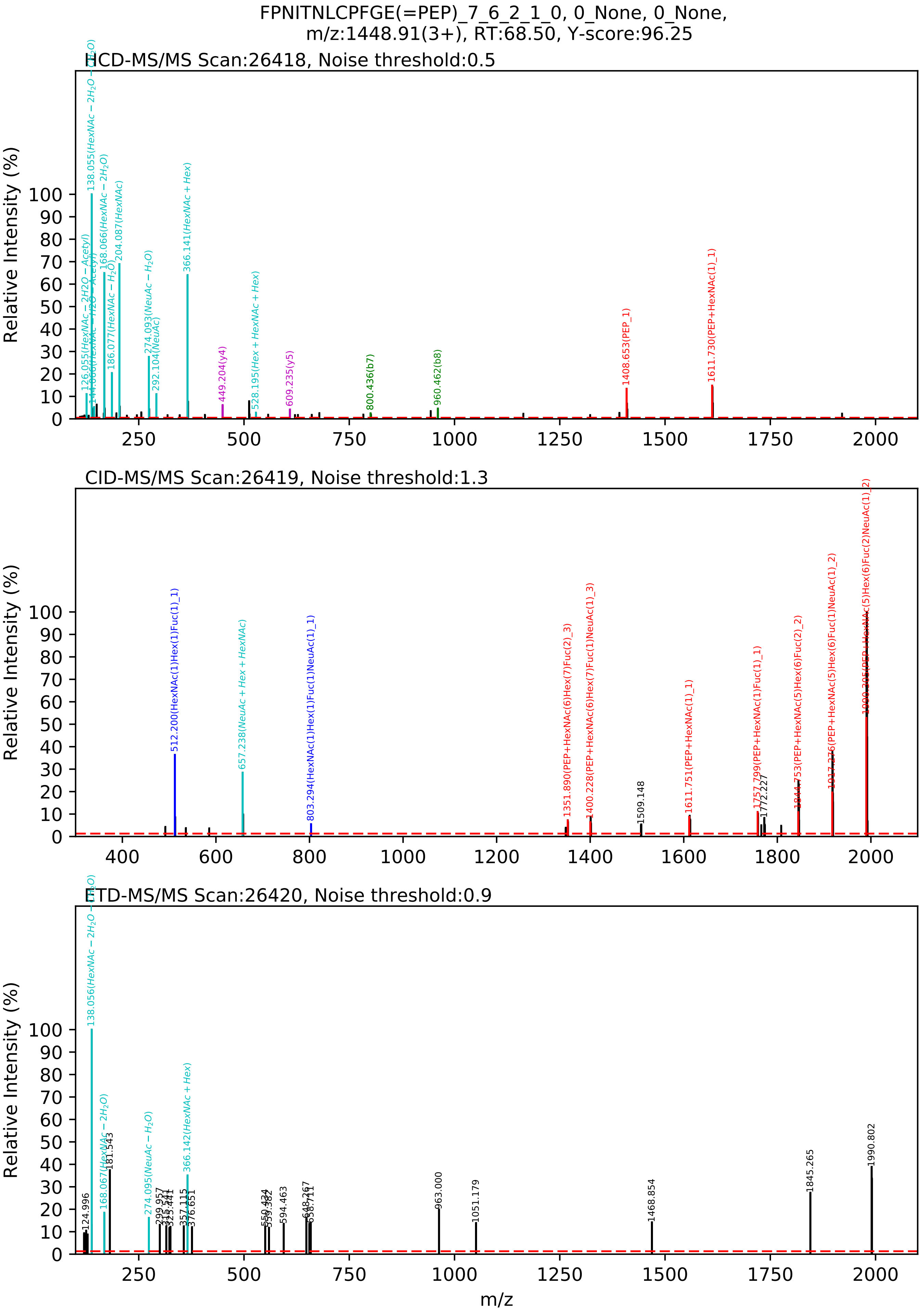

FPNITNLCPFGE(=PEP)_7_6_2_1_0, 0_None, 0_None,
m/z:1448.91(3+), RT:68.50, Y-score:96.25
HCD-MS/MS Scan:26418, Noise threshold:0.5
CID-MS/MS Scan:26419, Noise threshold:1.3
ETD-MS/MS Scan:26420, Noise threshold:0.9
Relative Intensity (%)
m/z

FPNITNLCPFGE(=PEP)_7_6_2_1_0, 0_None, 0_None,
m/z:1086.93(4+), RT:67.78, Y-score:92.07

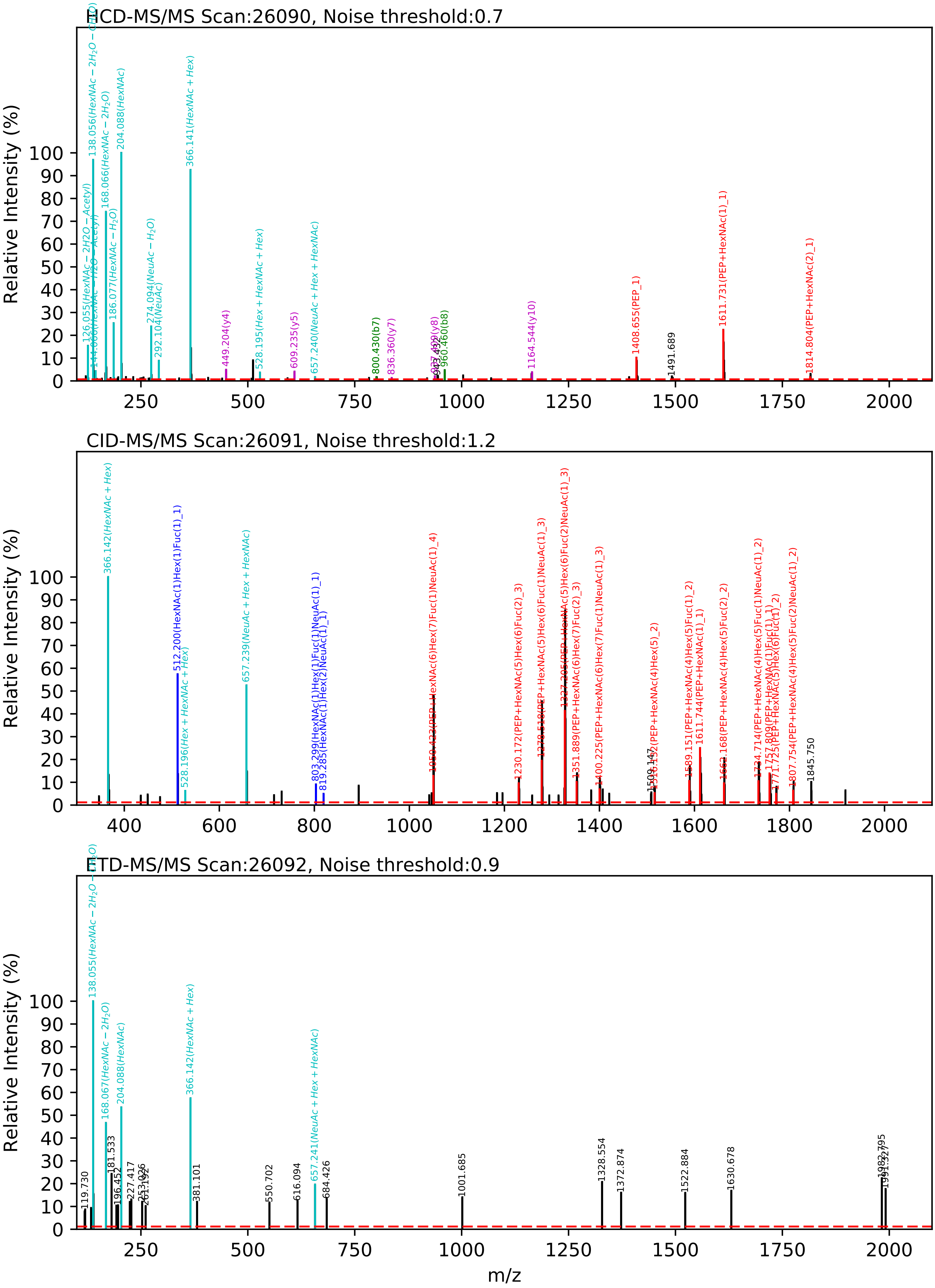

FPNITNLCPFGE(=PEP)_7_6_2_1_0, 0_None, 0_None,
m/z:1448.91(3+), RT:66.80, Y-score:80.72

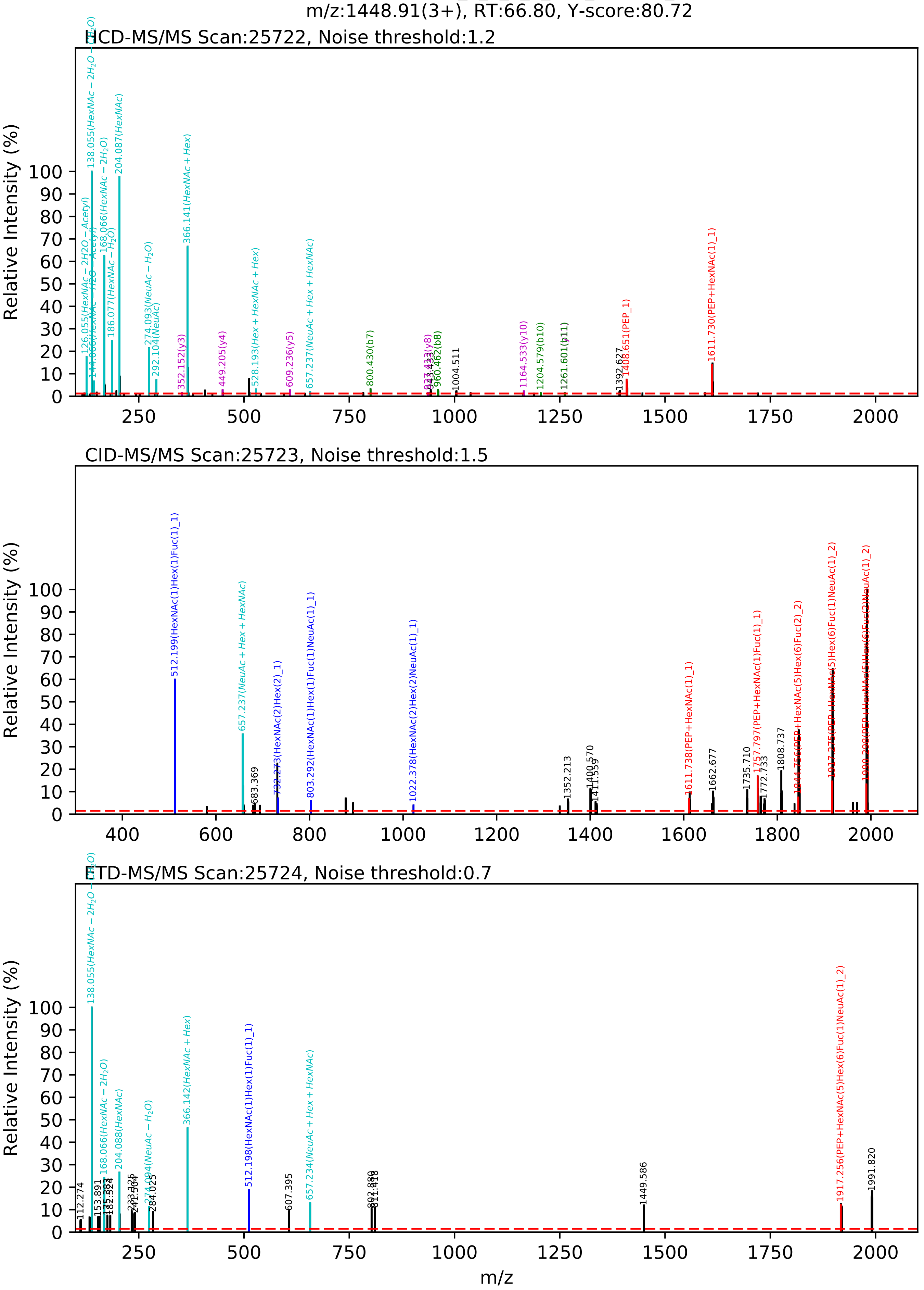

FPNITNLCPFGE(=PEP)_7_6_2_2_0, 0_None, 0_None,
m/z:1545.94(3+), RT:82.14, Y-score:79.94

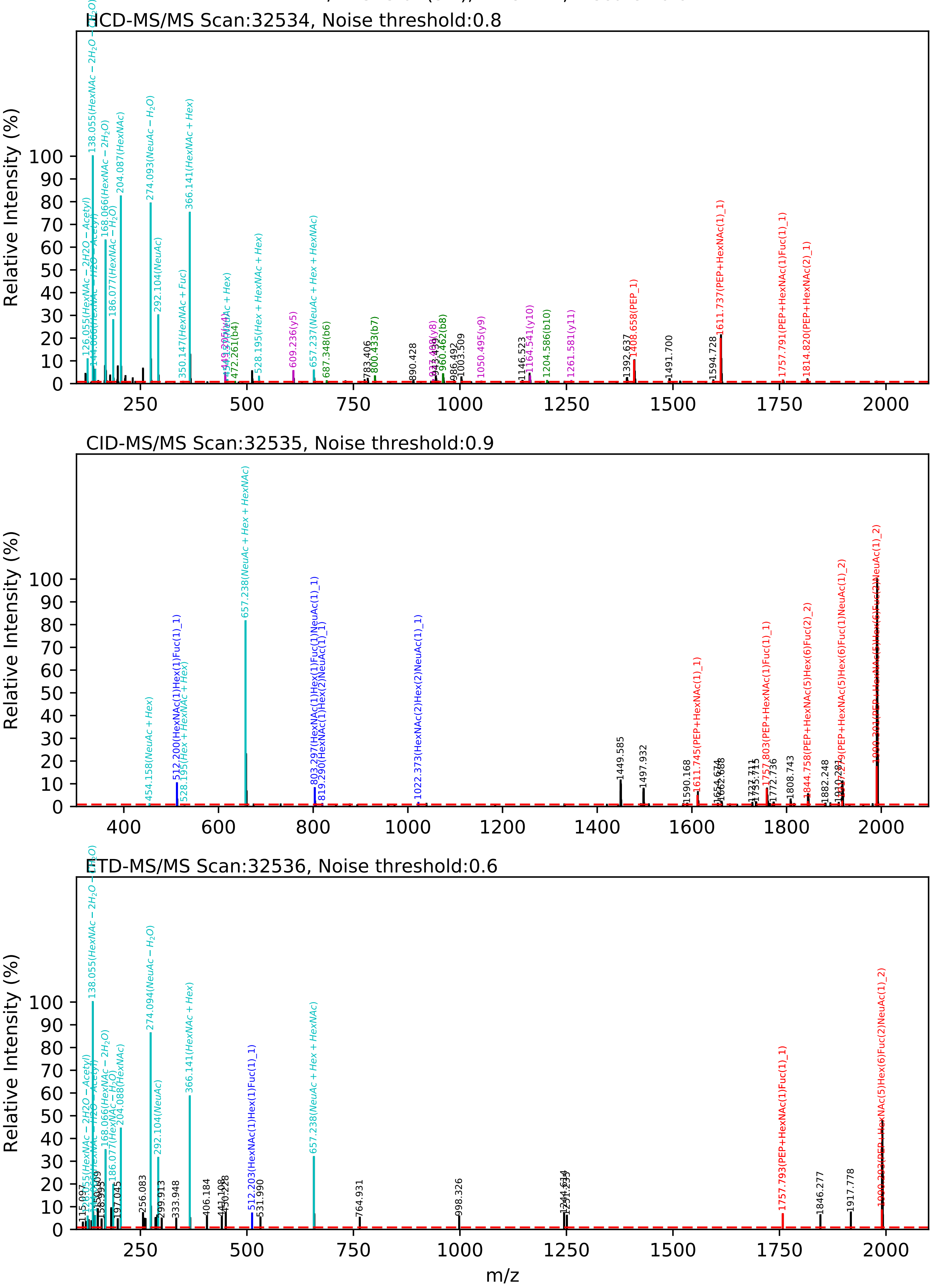

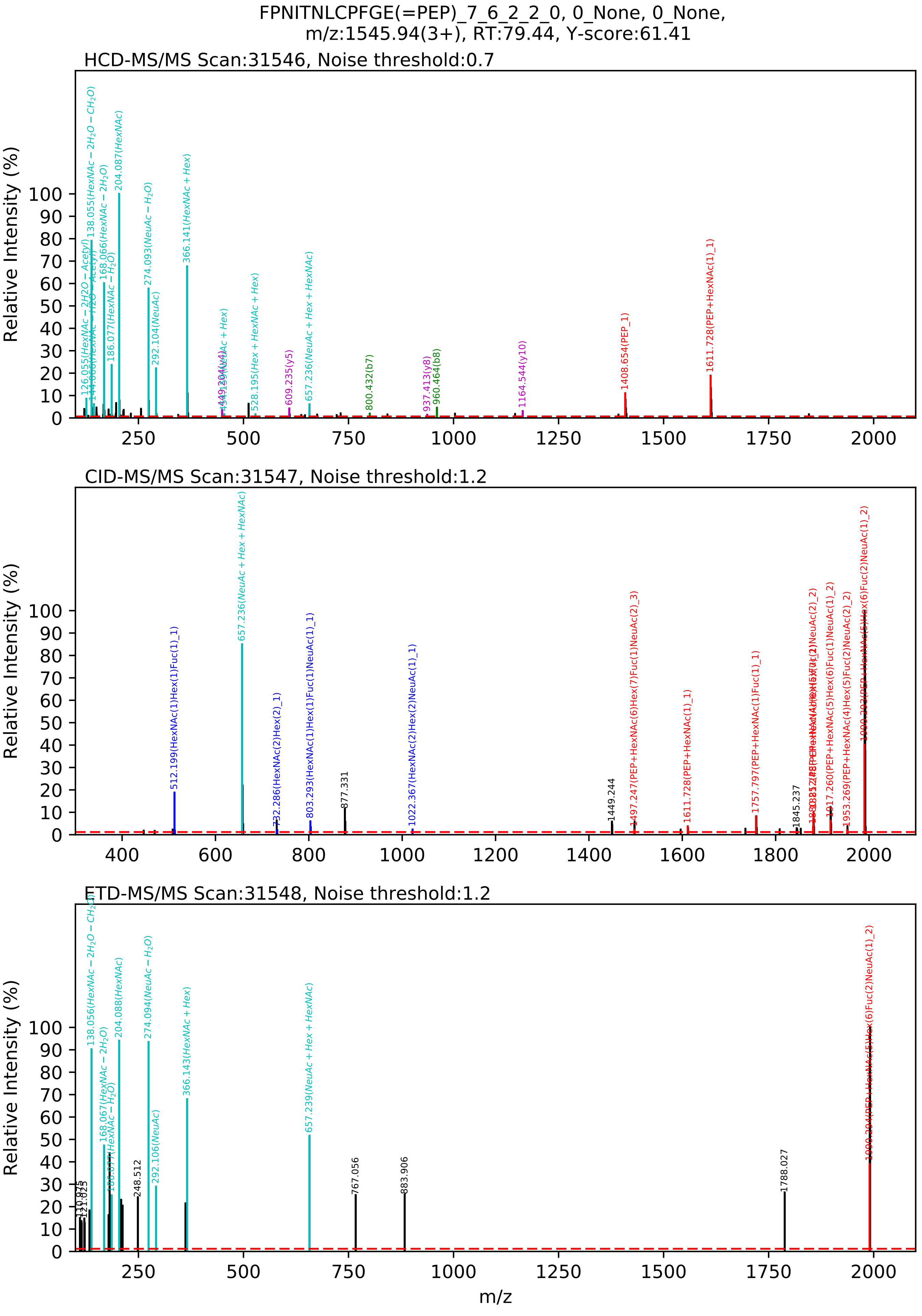

FPNITNLCPFGE(=PEP)_7_6_2_2_0, 0_None, 0_None,
m/z:1545.94(3+), RT:79.44, Y-score:61.41
HCD-MS/MS Scan:31546, Noise threshold:0.7
126.055(HexNAc − 2H2O − Acetyl)
138.055(HexNAc − 2H2O − CH2O)
168.066(HexNAc − 2H2O)
186.077(HexNAc − H2O)
204.087(HexNAc)
274.093(NeuAc − H2O)
292.104(NeuAc)
366.141(HexNAc + Hex)
449.204(y4)
528.195(Hex + HexNAc + Hex)
609.235(y5)
657.236(NeuAc + Hex + HexNAc)
800.432(b7)
937.413(y8)
960.464(b8)
1164.544(y10)
1408.654(PEP_1)
1611.728(PEP+HexNAc(1)_1)
CID-MS/MS Scan:31547, Noise threshold:1.2
512.199(HexNAc(1)Hex(1)Fuc(1)_1)
657.236(NeuAc + Hex + HexNAc)
732.286(HexNAc(2)Hex(2)_1)
803.293(HexNAc(1)Hex(1)Fuc(1)NeuAc(1)_1)
877.331
1022.367(HexNAc(2)Hex(2)NeuAc(1)_1)
1449.244
1497.247(PEP+HexNAc(6)Hex(7)Fuc(1)NeuAc(2)_3)
1611.728(PEP+HexNAc(1)_1)
1757.797(PEP+HexNAc(1)Fuc(1)_1)
1845.237
1917.260(PEP+HexNAc(5)Hex(6)Fuc(1)NeuAc(1)_2)
1953.269(PEP+HexNAc(4)Hex(5)Fuc(2)NeuAc(2)_2)
ETD-MS/MS Scan:31548, Noise threshold:1.2
138.056(HexNAc − 2H2O − CH2O)
168.067(HexNAc − 2H2O)
204.088(HexNAc)
248.512
274.094(NeuAc − H2O)
292.106(NeuAc)
366.143(HexNAc + Hex)
657.239(NeuAc + Hex + HexNAc)
767.056
883.906
1788.027
Relative Intensity (%)
m/z

FPNITNLCPFGE(=PEP)_7_6_2_2_0, 0_None, 0_None,
m/z:1159.71(4+), RT:80.83, Y-score:84.66

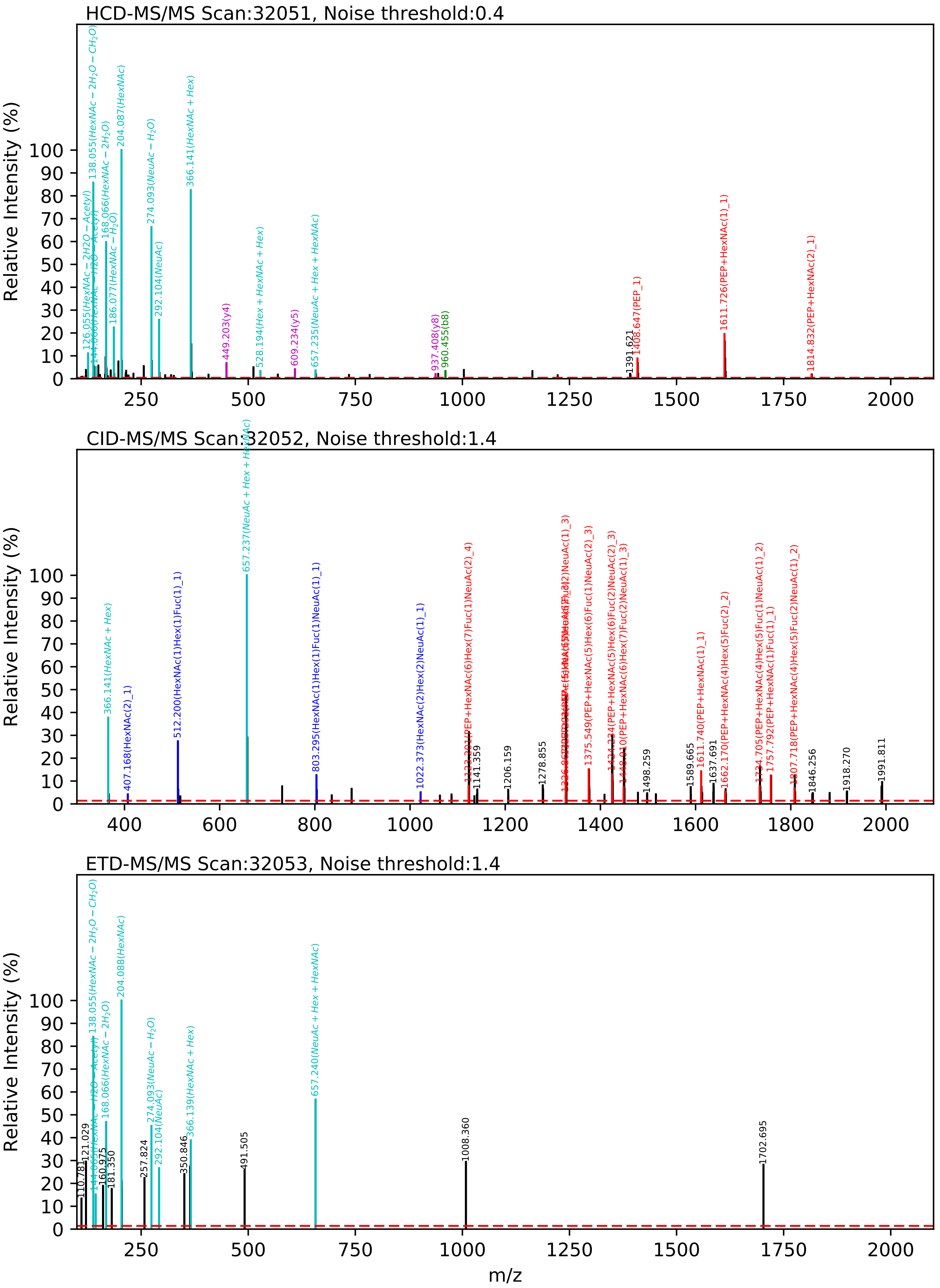

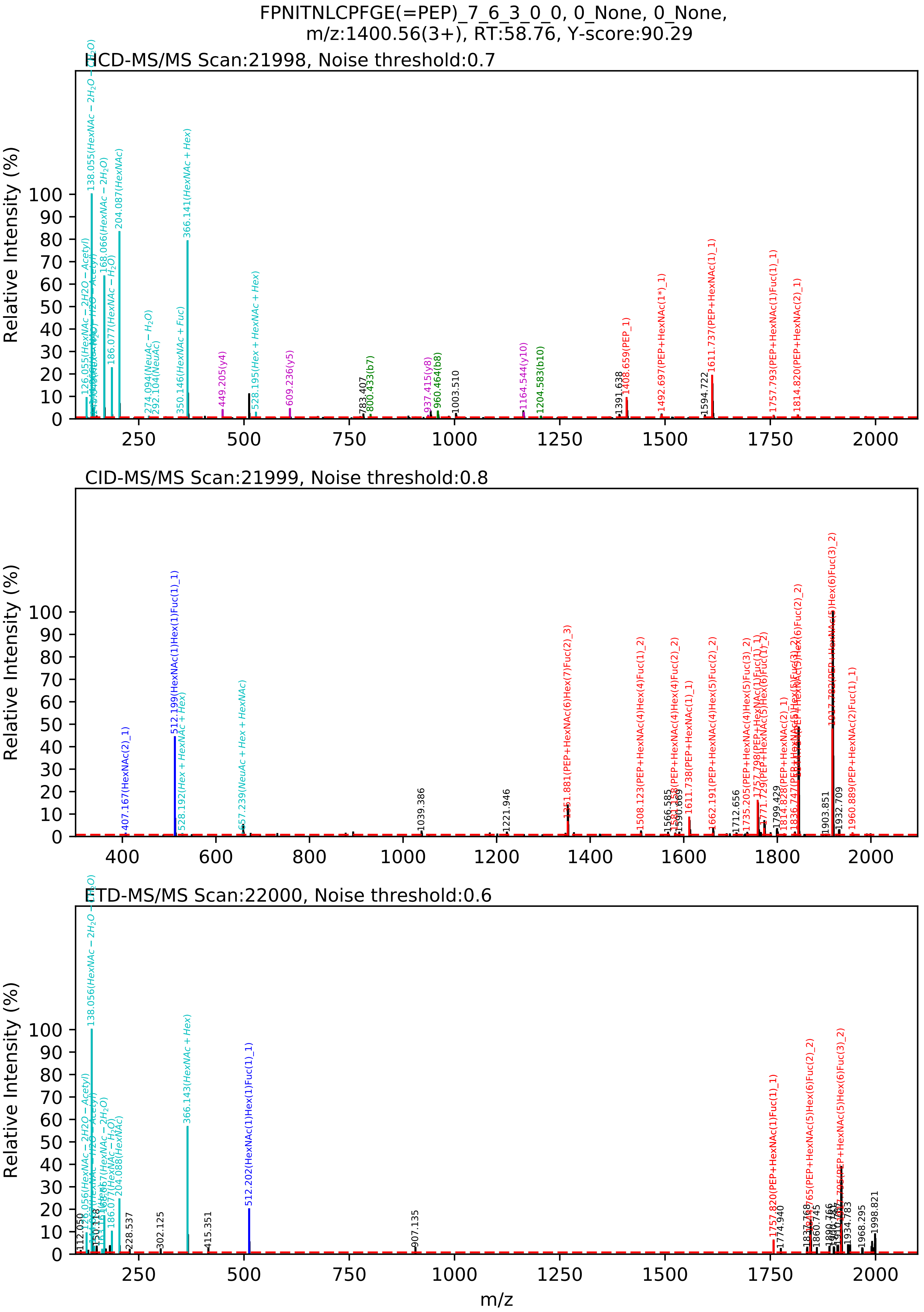
FPNITNLCPFGE(=PEP)_7_6_3_0_0, 0_None, 0_None,
m/z:1400.56(3+), RT:58.76, Y-score:90.29
HCD-MS/MS Scan:21998, Noise threshold:0.7
CID-MS/MS Scan:21999, Noise threshold:0.8
ETD-MS/MS Scan:22000, Noise threshold:0.6
Relative Intensity (%)
m/z

FPNITNLCPFGE(=PEP)_7_6_3_2_0, 0_None, 0_None,
m/z:1594.63(3+), RT:80.85, Y-score:74.35

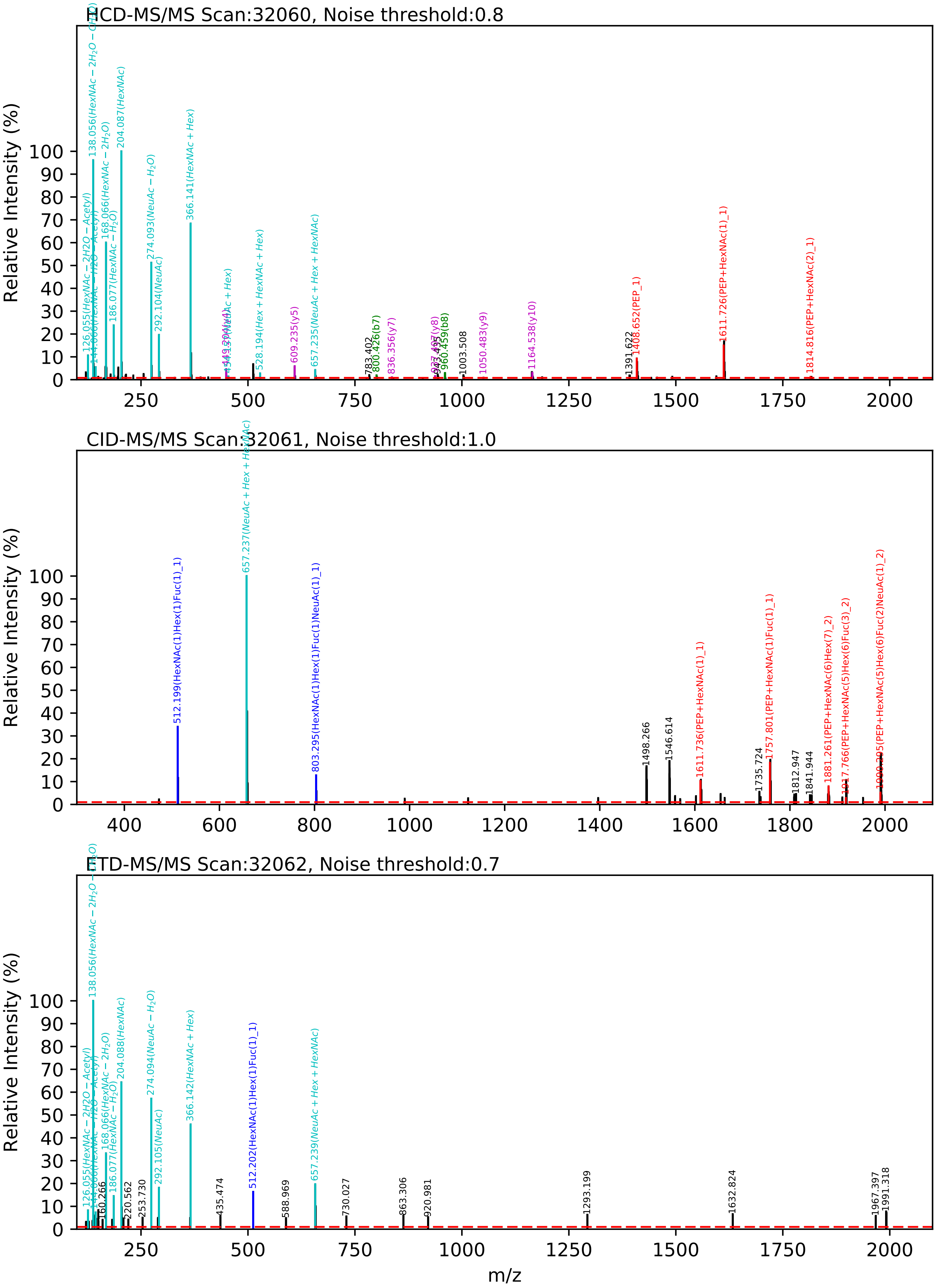

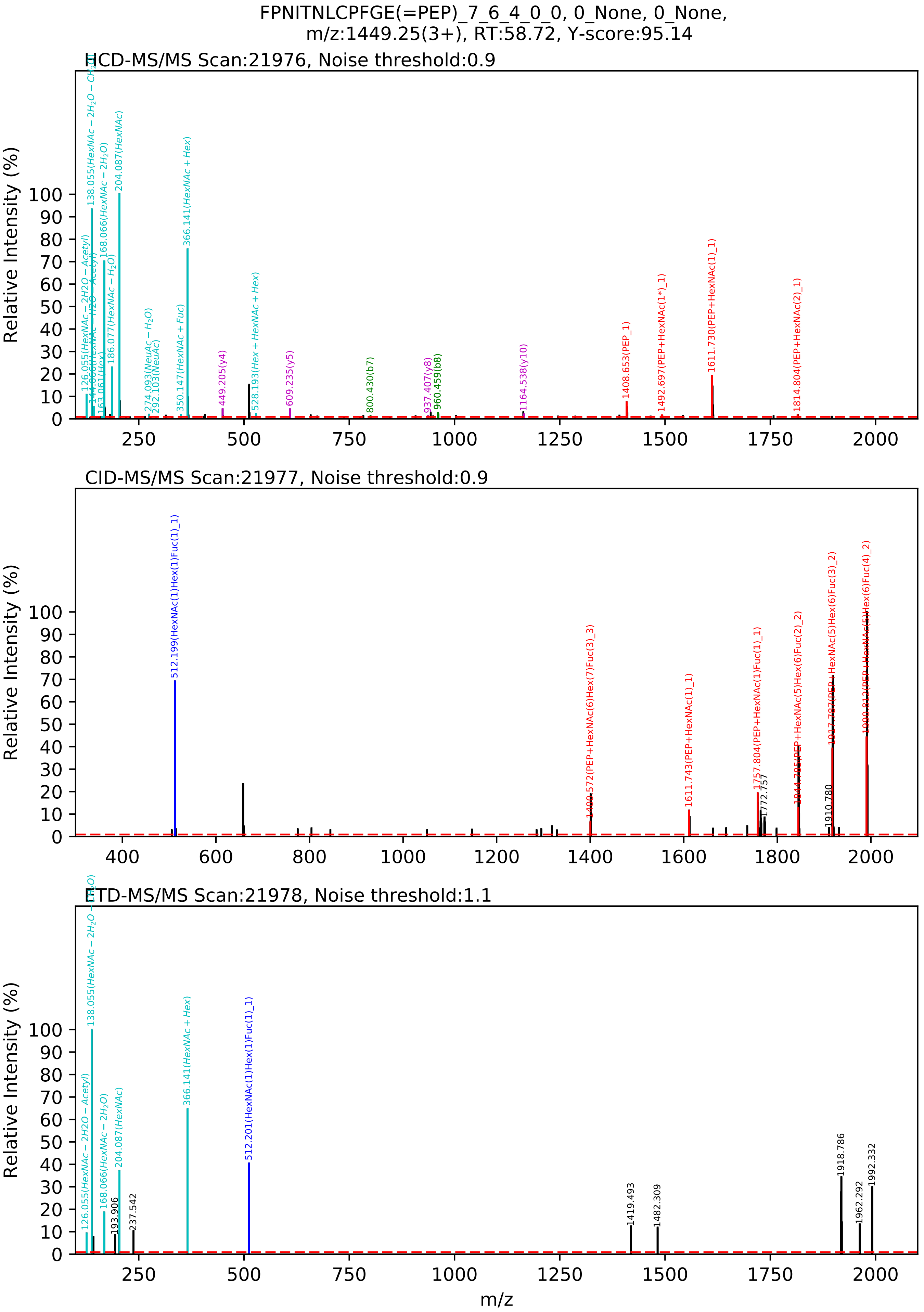

FPNITNLCPFGE(=PEP)_7_6_4_0_0, 0_None, 0_None,
m/z:1449.25(3+), RT:58.72, Y-score:95.14
HCD-MS/MS Scan:21976, Noise threshold:0.9
Relative Intensity (%)
138.055(HexNAc − 2H2O)
204.087(HexNAc)
168.066(HexNAc − 2H2O)
366.141(HexNAc + Hex)
186.077(HexNAc − H2O)
126.055(HexNAc − 2H2O − Acetyl)
163.061(Hex)
274.093(NeuAc − H2O)
292.103(NeuAc)
350.147(HexNAc + Fuc)
449.205(y4)
528.193(Hex + HexNAc + Hex)
609.235(y5)
800.430(b7)
937.407(y8)
960.459(b8)
1164.538(y10)
1408.653(PEP_1)
1492.697(PEP+HexNAc(1*)_1)
1611.730(PEP+HexNAc(1)_1)
1814.804(PEP+HexNAc(2)_1)
CID-MS/MS Scan:21977, Noise threshold:0.9
Relative Intensity (%)
512.199(HexNAc(1)Hex(1)Fuc(1)_1)
1400.572(PEP+HexNAc(6)Hex(7)Fuc(3)_3)
1611.743(PEP+HexNAc(1)_1)
1757.804(PEP+HexNAc(1)Fuc(1)_1)
1772.757
1910.780
ETD-MS/MS Scan:21978, Noise threshold:1.1
Relative Intensity (%)
138.055(HexNAc − 2H2O − CH3O)
126.055(HexNAc − 2H2O − Acetyl)
168.066(HexNAc − 2H2O)
204.087(HexNAc)
193.906
237.542
366.141(HexNAc + Hex)
512.201(HexNAc(1)Hex(1)Fuc(1)_1)
1419.493
1482.309
1918.786
1962.292
1992.332
m/z

FPNITNLCPFGE(=PEP)_7_6_4_1_0, 0_None, 0_None,
m/z:1546.28(3+), RT:68.03, Y-score:87.55

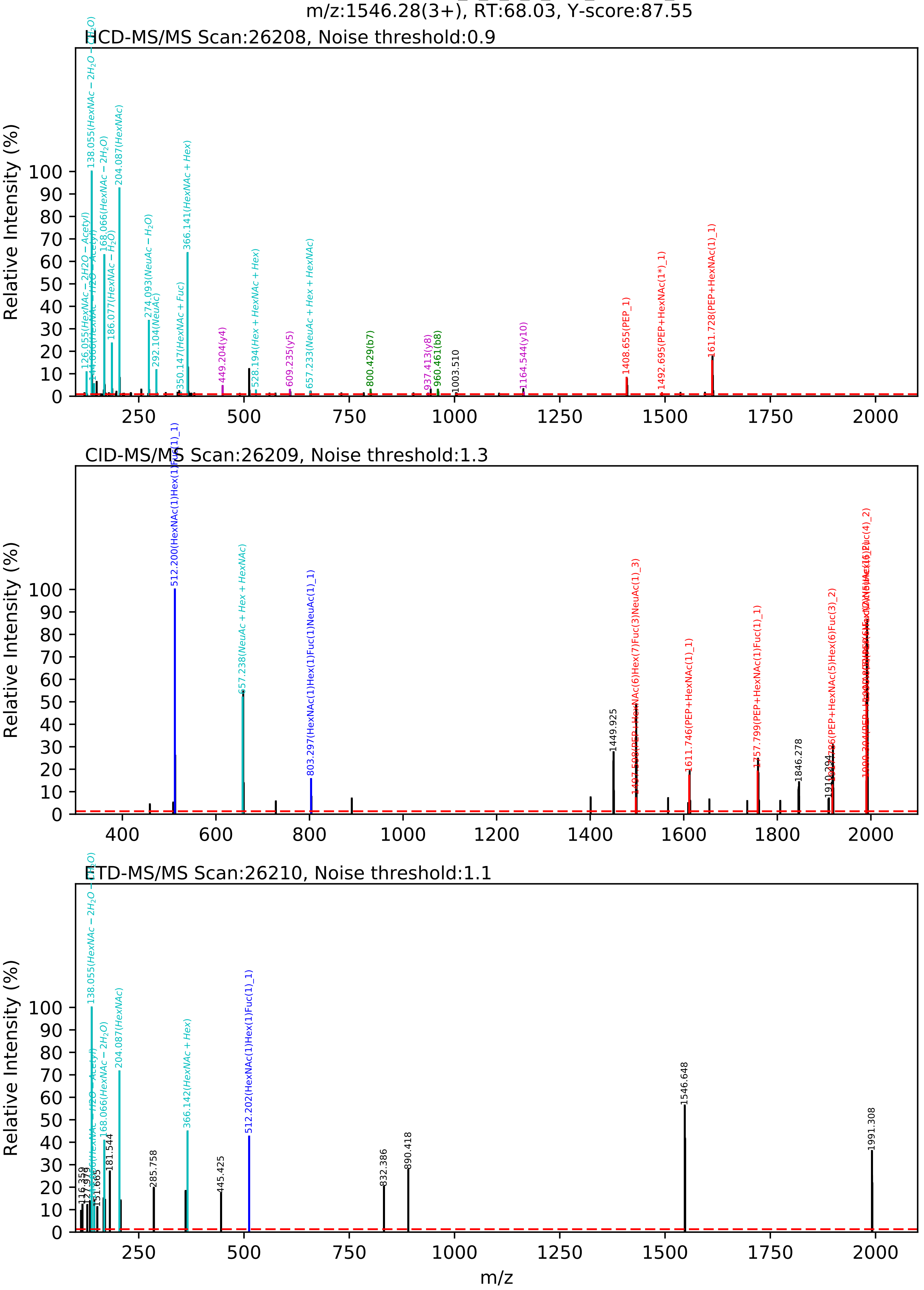

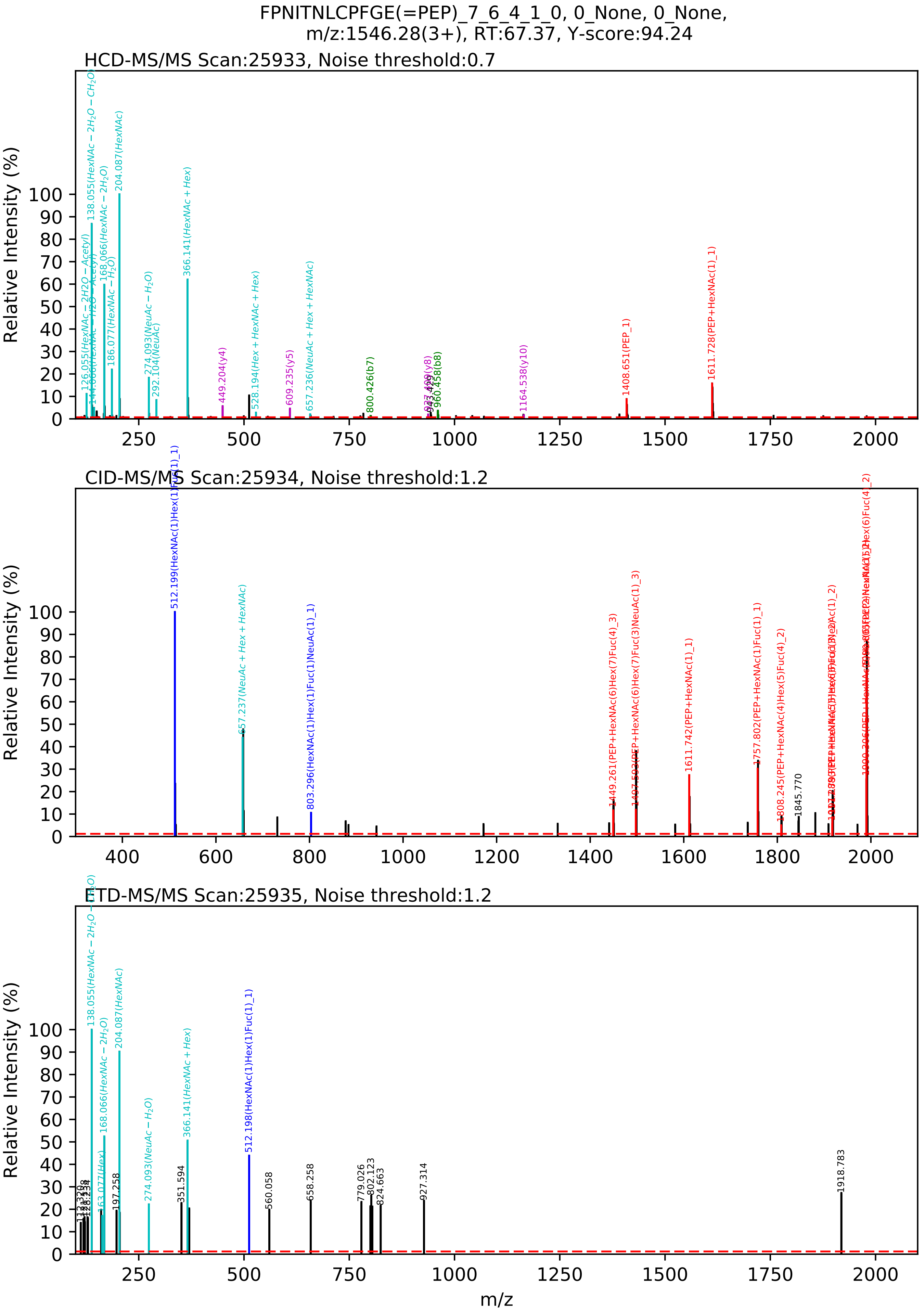

FPNITNLCPFGE(=PEP)_7_6_4_1_0, 0_None, 0_None,
m/z:1546.28(3+), RT:67.37, Y-score:94.24
HCD-MS/MS Scan:25933, Noise threshold:0.7
Relative Intensity (%)
126.055(HexNAc − 2H2O − Acetyl)
138.055(HexNAc − 2H2O − CH2O)
144.066(HexNAc − H2O − Acetyl)
168.066(HexNAc − 2H2O)
186.077(HexNAc − H2O)
204.087(HexNAc)
274.093(NeuAc − H2O)
292.104(NeuAc)
366.141(HexNAc + Hex)
449.204(y4)
528.194(Hex + HexNAc + Hex)
609.235(y5)
657.236(NeuAc + Hex + HexNAc)
800.426(b7)
960.458(b8)
1164.538(y10)
1408.651(PEP_1)
1611.728(PEP+HexNAc(1)_1)
CID-MS/MS Scan:25934, Noise threshold:1.2
512.199(HexNAc(1)Hex(1)Fuc(1)_1)
657.237(NeuAc + Hex + HexNAc)
803.296(HexNAc(1)Hex(1)Fuc(1)NeuAc(1)_1)
1449.261(PEP+HexNAc(6)Hex(7)Fuc(4)_3)
1611.742(PEP+HexNAc(1)_1)
1757.802(PEP+HexNAc(1)Fuc(1)_1)
1808.245(PEP+HexNAc(4)Hex(5)Fuc(4)_2)
1845.770
ETD-MS/MS Scan:25935, Noise threshold:1.2
138.055(HexNAc − 2H2O − CH2O)
168.066(HexNAc − 2H2O)
204.087(HexNAc)
163.077(Hex)
197.258
274.093(NeuAc − H2O)
351.594
366.141(HexNAc + Hex)
512.198(HexNAc(1)Hex(1)Fuc(1)_1)
560.058
658.258
779.026
802.123
824.663
927.314
1918.783
m/z

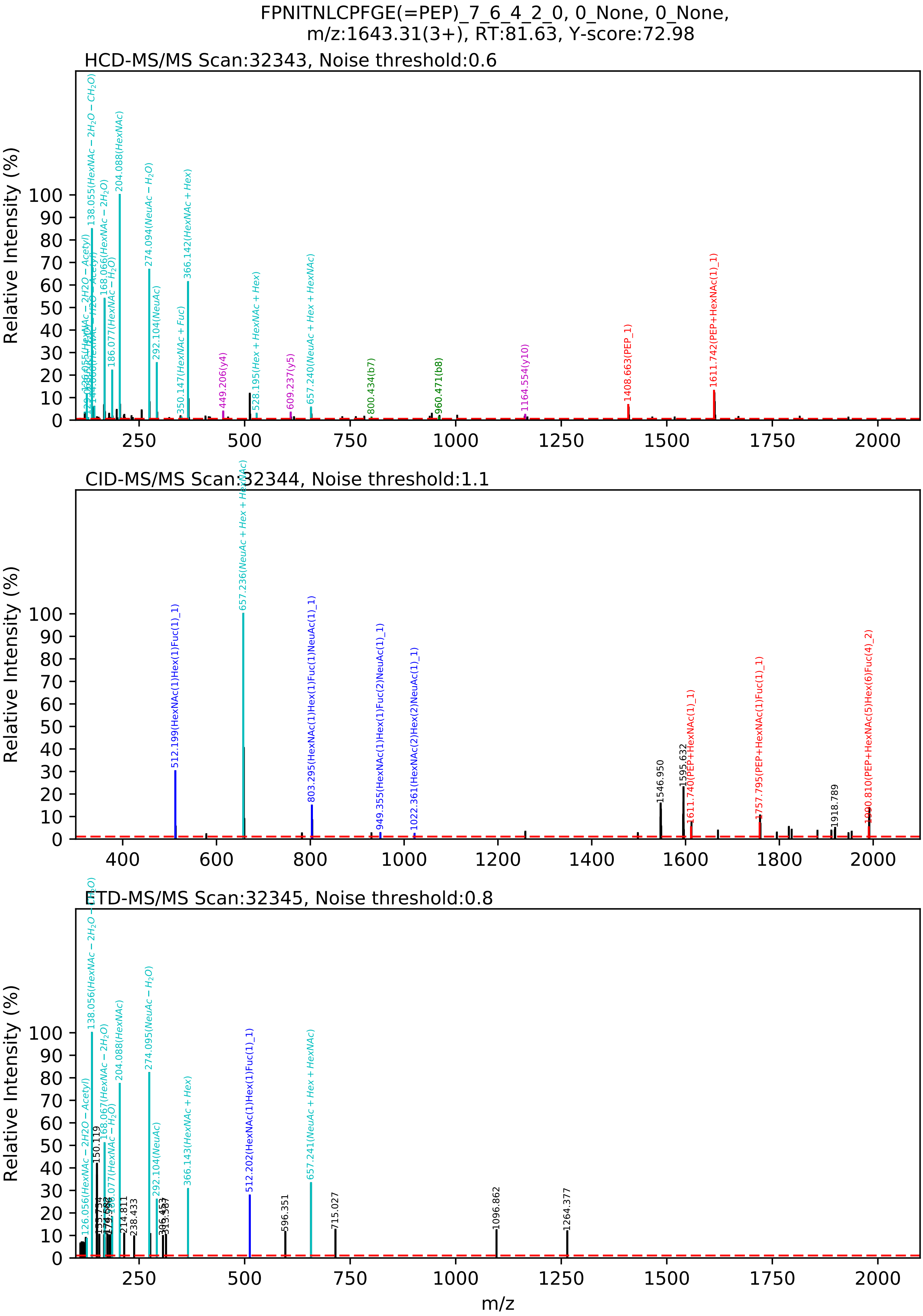

FPNITNLCPFGE(=PEP)_7_6_4_2_0, 0_None, 0_None,
m/z:1643.31(3+), RT:81.63, Y-score:72.98
HCD-MS/MS Scan:32343, Noise threshold:0.6
Relative Intensity (%)
138.055(HexNAc − 2H2O − CH2O)
204.088(HexNAc)
168.066(HexNAc − 2H2O)
186.077(HexNAc − H2O)
274.094(NeuAc − H2O)
292.104(NeuAc)
366.142(HexNAc + Hex)
350.147(HexNAc + Fuc)
449.206(y4)
528.195(Hex + HexNAc + Hex)
609.237(y5)
657.240(NeuAc + Hex + HexNAc)
800.434(b7)
960.471(b8)
1164.554(y10)
1408.663(PEP_1)
1611.742(PEP+HexNAc(1)_1)
CID-MS/MS Scan:32344, Noise threshold:1.1
657.236(NeuAc + Hex + HexNAc)
512.199(HexNAc(1)Hex(1)Fuc(1)_1)
803.295(HexNAc(1)Hex(1)Fuc(1)NeuAc(1)_1)
949.355(HexNAc(1)Hex(1)Fuc(2)NeuAc(1)_1)
1022.361(HexNAc(2)Hex(2)NeuAc(1)_1)
1546.950
1595.632
1611.740(PEP+HexNAc(1)_1)
1757.795(PEP+HexNAc(1)Fuc(1)_1)
1918.789
1990.810(PEP+HexNAc(5)Hex(6)Fuc(4)_2)
ETD-MS/MS Scan:32345, Noise threshold:0.8
138.056(HexNAc − 2H2O − CH2O)
204.088(HexNAc)
274.095(NeuAc − H2O)
168.067(HexNAc − 2H2O)
150.119
126.056(HexNAc − 2H2O − Acetyl)
214.811
238.433
292.104(NeuAc)
366.143(HexNAc + Hex)
512.202(HexNAc(1)Hex(1)Fuc(1)_1)
657.241(NeuAc + Hex + HexNAc)
596.351
715.027
1096.862
1264.377
m/z

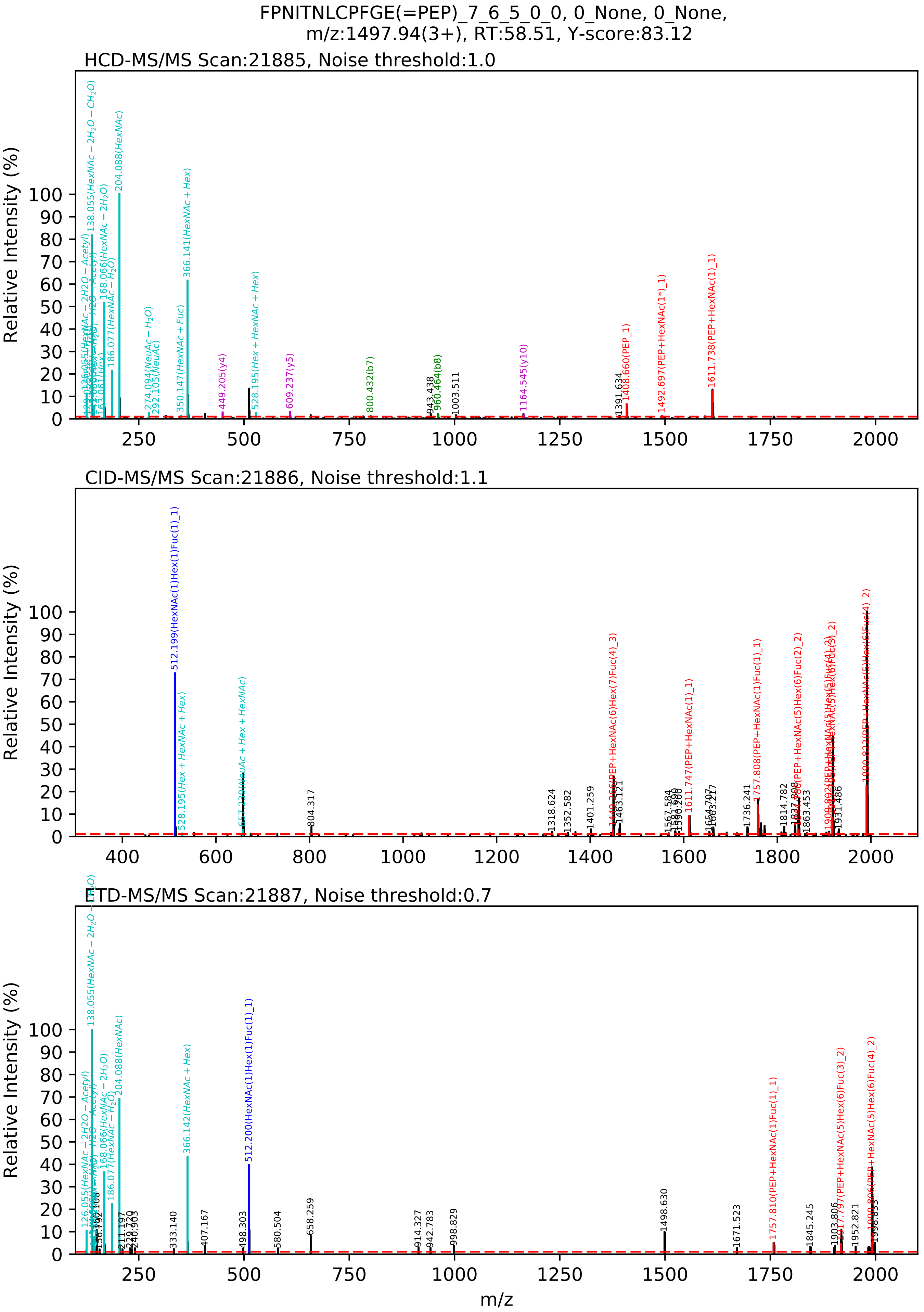

FPNITNLCPFGE(=PEP)_7_6_5_0_0, 0_None, 0_None,
m/z:1497.94(3+), RT:58.51, Y-score:83.12
HCD-MS/MS Scan:21885, Noise threshold:1.0
CID-MS/MS Scan:21886, Noise threshold:1.1
ETD-MS/MS Scan:21887, Noise threshold:0.7
Relative Intensity (%)
m/z
204.088(HexNAc)
366.141(HexNAc + Hex)
168.066(HexNAc − 2H₂O)
186.077(HexNAc − H₂O)
449.205(y4)
528.195(Hex + HexNAc + Hex)
609.237(y5)
800.432(b7)
960.464(b8)
1003.511
1164.545(y10)
1391.634
1408.660(PEP_1)
1492.697(PEP+HexNAc(1*)_1)
1611.738(PEP+HexNAc(1)_1)
512.199(HexNAc(1)Hex(1)Fuc(1)_1)
804.317
1318.624
1352.582
1401.259
1463.121
1611.747(PEP+HexNAc(1)_1)
1736.241
1757.808(PEP+HexNAc(1)Fuc(1)_1)
1814.782
1863.453
1931.486
512.200(HexNAc(1)Hex(1)Fuc(1)_1)
366.142(HexNAc + Hex)
333.140
407.167
498.303
580.504
658.259
914.327
942.783
998.829
1498.630
1671.523
1757.810(PEP+HexNAc(1)Fuc(1)_1)
1845.245
1903.806
1952.821

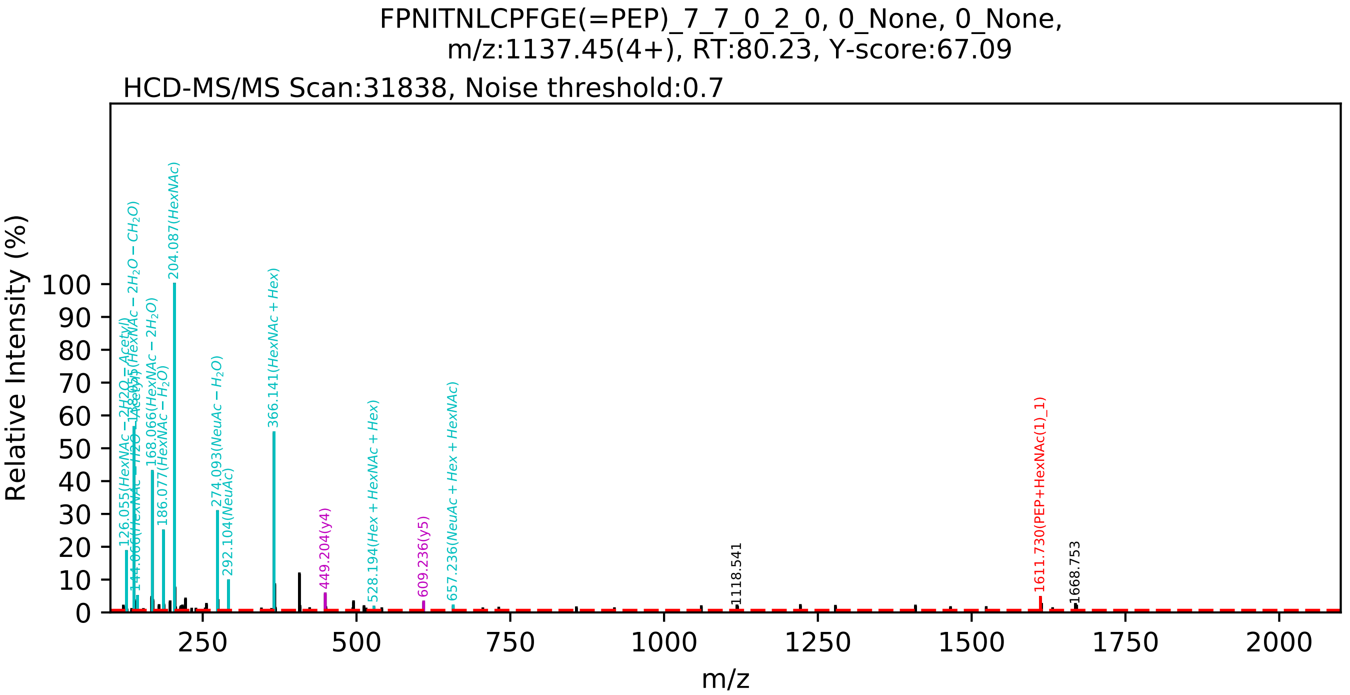

FPNITNLCPFGE(=PEP)_7_7_0_2_0, 0_None, 0_None,
m/z:1137.45(4+), RT:80.23, Y-score:67.09
HCD-MS/MS Scan:31838, Noise threshold:0.7
Relative Intensity (%)
m/z
204.087(HexNAc)
366.141(HexNAc+Hex)
168.066(HexNAc−2H2O)
186.077(HexNAc−H2O)
126.055(HexNAc−2H2O−Acetyl)
144.066(HexNAc−H2O−Acetyl)
274.093(NeuAc−H2O)
292.104(NeuAc)
449.204(y4)
528.194(Hex+HexNAc+Hex)
609.236(y5)
657.236(NeuAc+Hex+HexNAc)
1118.541
1611.730(PEP+HexNAc(1)_1)
1668.753

FPNITNLCPFGE(=PEP)_7_7_0_2_0, 0_None, 0_None,
m/z:1137.45(4+), RT:80.23, Y-score:67.09

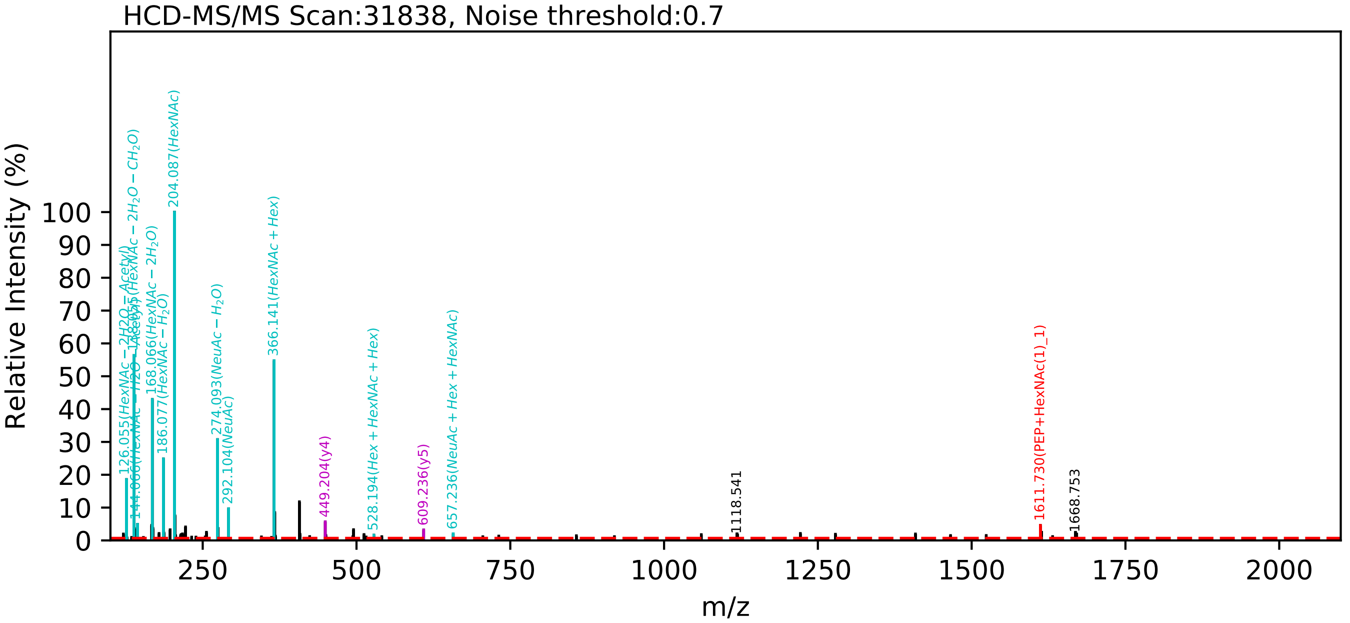

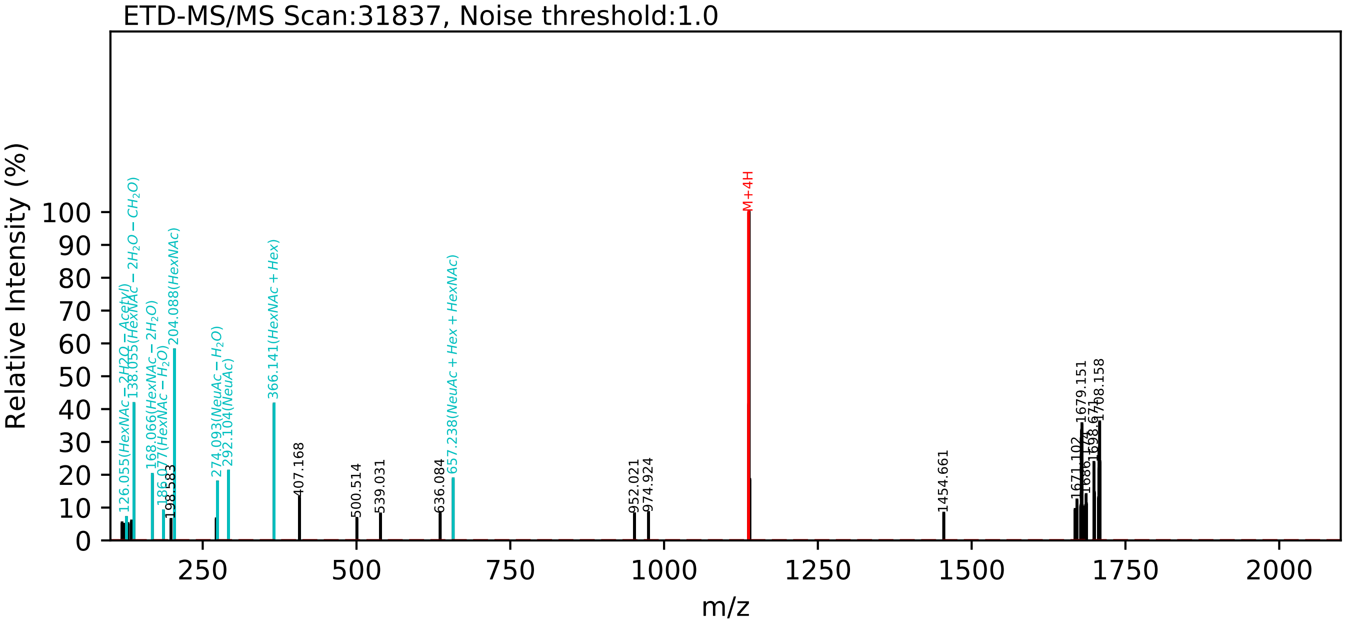

FPNITNLCPFGE(=PEP)_7_7_1_1_0, 0_None, 0_None,
m/z:1101.19(4+), RT:68.27, Y-score:69.14

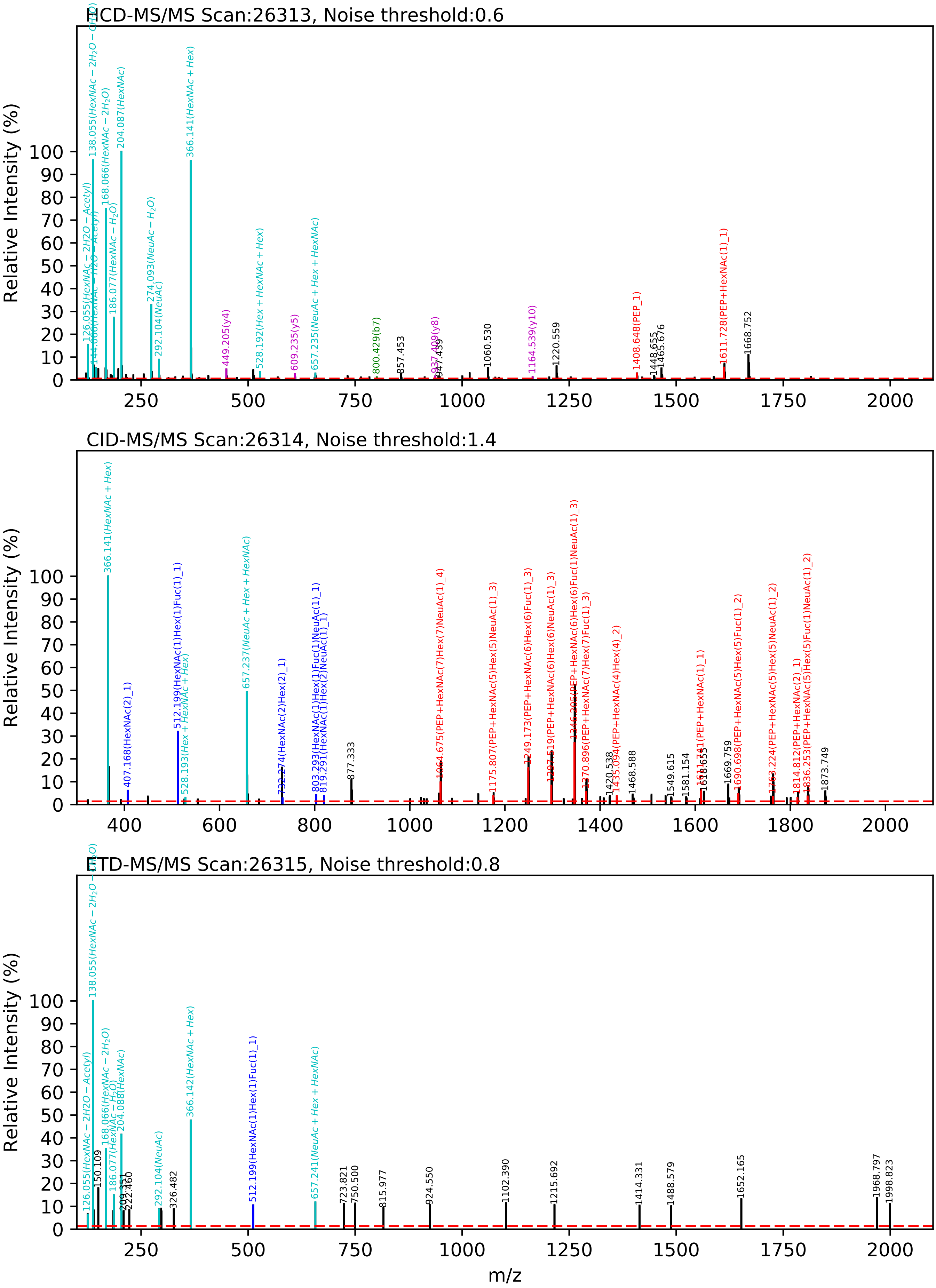

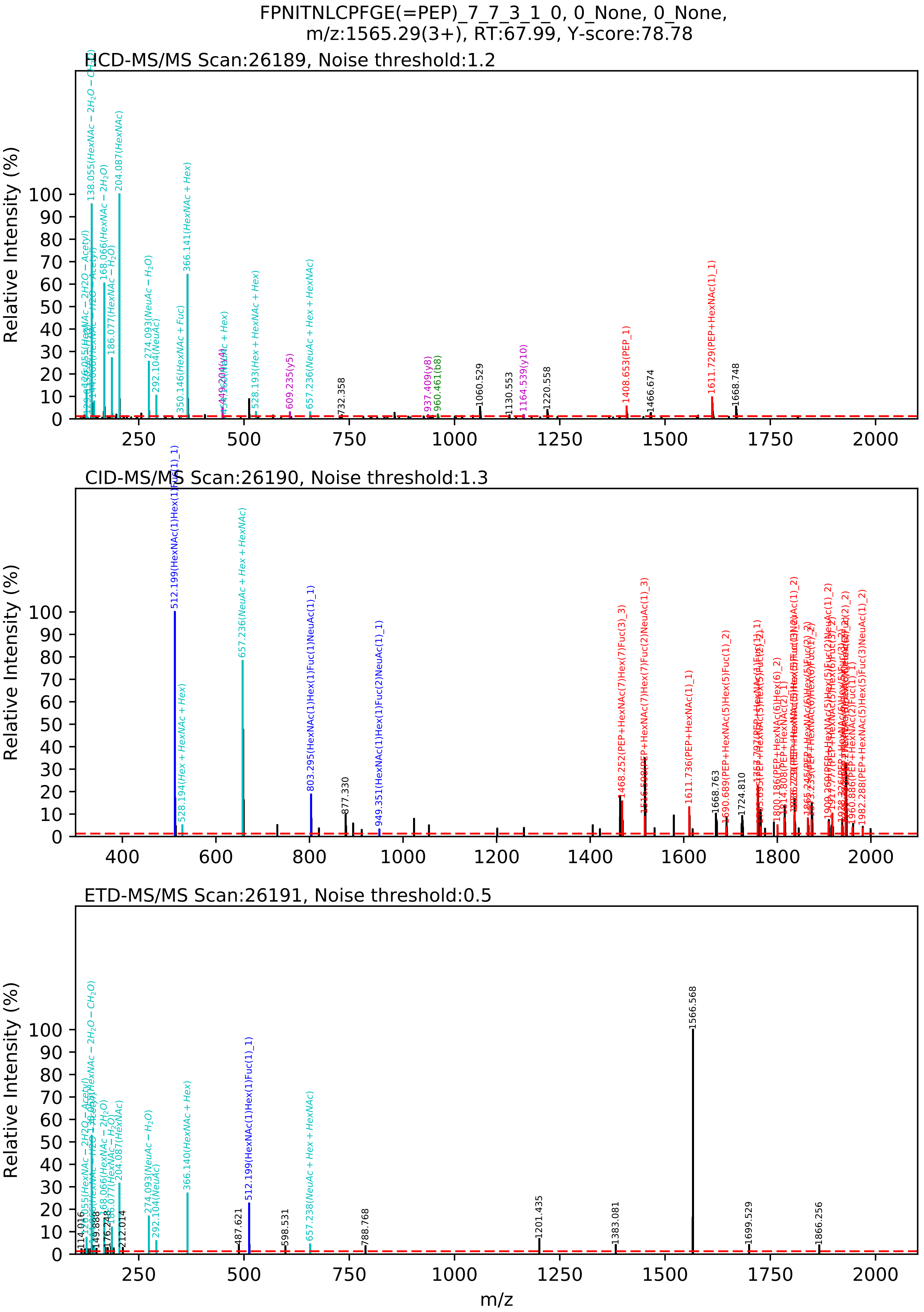

FPNITNLCPFGE(=PEP)_7_7_3_1_0, 0_None, 0_None,
m/z:1565.29(3+), RT:67.99, Y-score:78.78
HCD-MS/MS Scan:26189, Noise threshold:1.2
CID-MS/MS Scan:26190, Noise threshold:1.3
ETD-MS/MS Scan:26191, Noise threshold:0.5
Relative Intensity (%)
m/z
204.087(HexNAc)
138.055(HexNAc − 2H2O − CH2O)
366.141(HexNAc + Hex)
1611.729(PEP+HexNAc(1)_1)
1408.653(PEP_1)
512.199(HexNAc(1)Hex(1)Fuc(1)_1)
657.236(NeuAc + Hex + HexNAc)
803.295(HexNAc(1)Hex(1)Fuc(1)NeuAc(1)_1)
949.351(HexNAc(1)Hex(1)Fuc(2)NeuAc(1)_1)
1468.252(PEP+HexNAc(7)Hex(7)Fuc(3)_3)
1611.736(PEP+HexNAc(1)_1)
1566.568

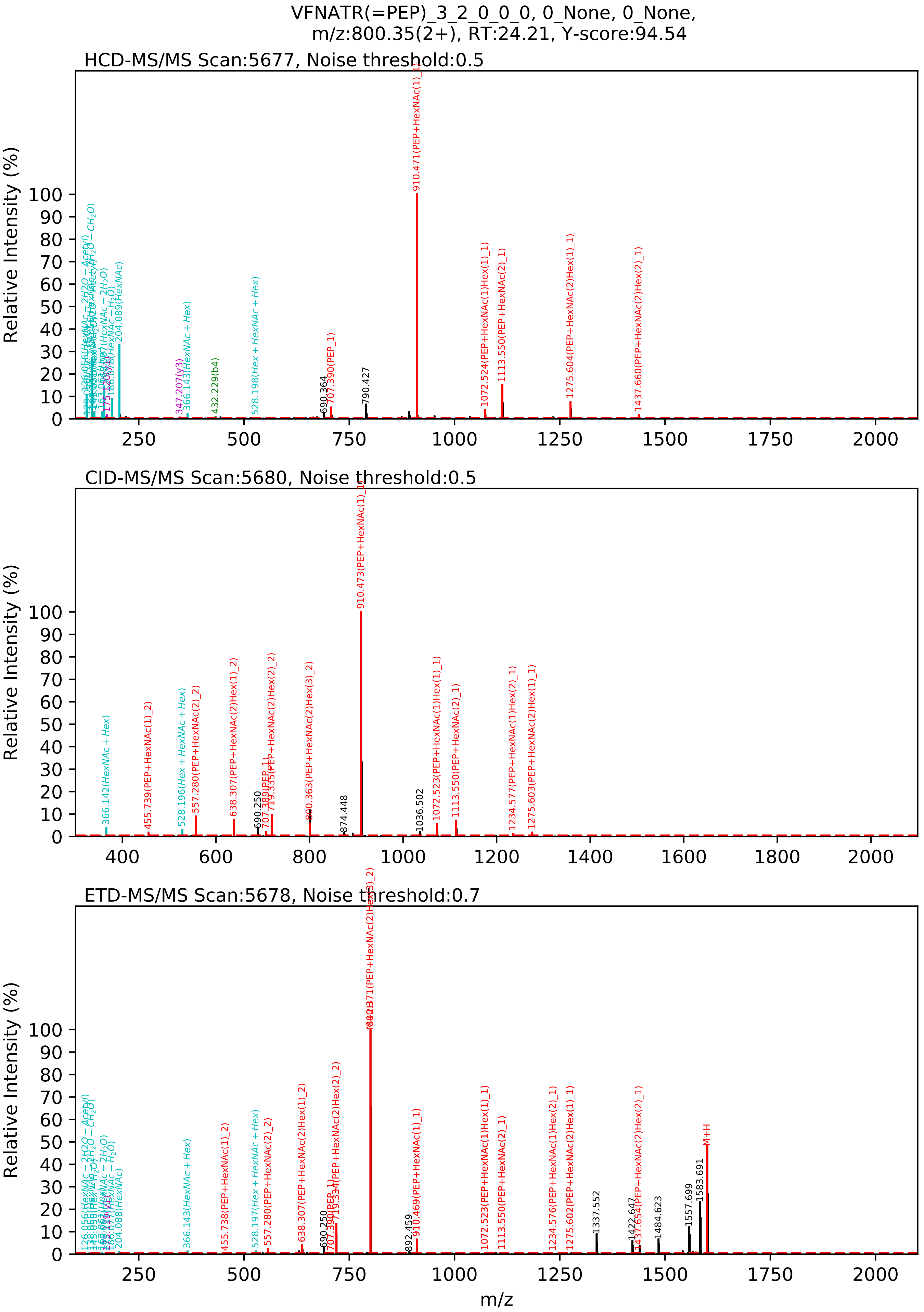

VFNATR(=PEP)_3_2_0_0_0, 0_None, 0_None,
m/z:800.35(2+), RT:24.21, Y-score:94.54
HCD-MS/MS Scan:5677, Noise threshold:0.5
Relative Intensity (%)
910.471(PEP+HexNAc(1)_1)
1072.524(PEP+HexNAc(1)Hex(1)_1)
1113.550(PEP+HexNAc(2)_1)
1275.604(PEP+HexNAc(2)Hex(1)_1)
1437.660(PEP+HexNAc(2)Hex(2)_1)
707.390(PEP_1)
690.364
790.427
528.198(Hex + HexNAc + Hex)
432.229(b4)
347.207(y3)
366.143(HexNAc + Hex)
204.089(HexNAc)
CID-MS/MS Scan:5680, Noise threshold:0.5
910.473(PEP+HexNAc(1)_1)
366.142(HexNAc + Hex)
455.739(PEP+HexNAc(1)_2)
528.196(Hex + HexNAc + Hex)
557.280(PEP+HexNAc(2)_2)
638.307(PEP+HexNAc(2)Hex(1)_2)
690.250
707.389(PEP_1)
719.335(PEP+HexNAc(2)Hex(2)_2)
800.363(PEP+HexNAc(2)Hex(3)_2)
874.448
1036.502
1072.523(PEP+HexNAc(1)Hex(1)_1)
1113.550(PEP+HexNAc(2)_1)
1234.577(PEP+HexNAc(1)Hex(2)_1)
1275.603(PEP+HexNAc(2)Hex(1)_1)
ETD-MS/MS Scan:5678, Noise threshold:0.7
800.371(PEP+HexNAc(2)Hex(3)_2)
366.143(HexNAc + Hex)
455.738(PEP+HexNAc(1)_2)
528.197(Hex + HexNAc + Hex)
557.280(PEP+HexNAc(2)_2)
638.307(PEP+HexNAc(2)Hex(1)_2)
690.250
707.390(PEP_1)
719.334(PEP+HexNAc(2)Hex(2)_2)
892.459
910.469(PEP+HexNAc(1)_1)
1072.523(PEP+HexNAc(1)Hex(1)_1)
1113.550(PEP+HexNAc(2)_1)
1234.576(PEP+HexNAc(1)Hex(2)_1)
1275.602(PEP+HexNAc(2)Hex(1)_1)
1337.552
1422.647
1437.654(PEP+HexNAc(2)Hex(2)_1)
1484.623
1557.699
1583.691
M+H
204.088(HexNAc)
m/z

VFNATR(=PEP)_3_3_1_0_0, 0_None, 0_None,
m/z:974.92(2+), RT:24.46, Y-score:94.48

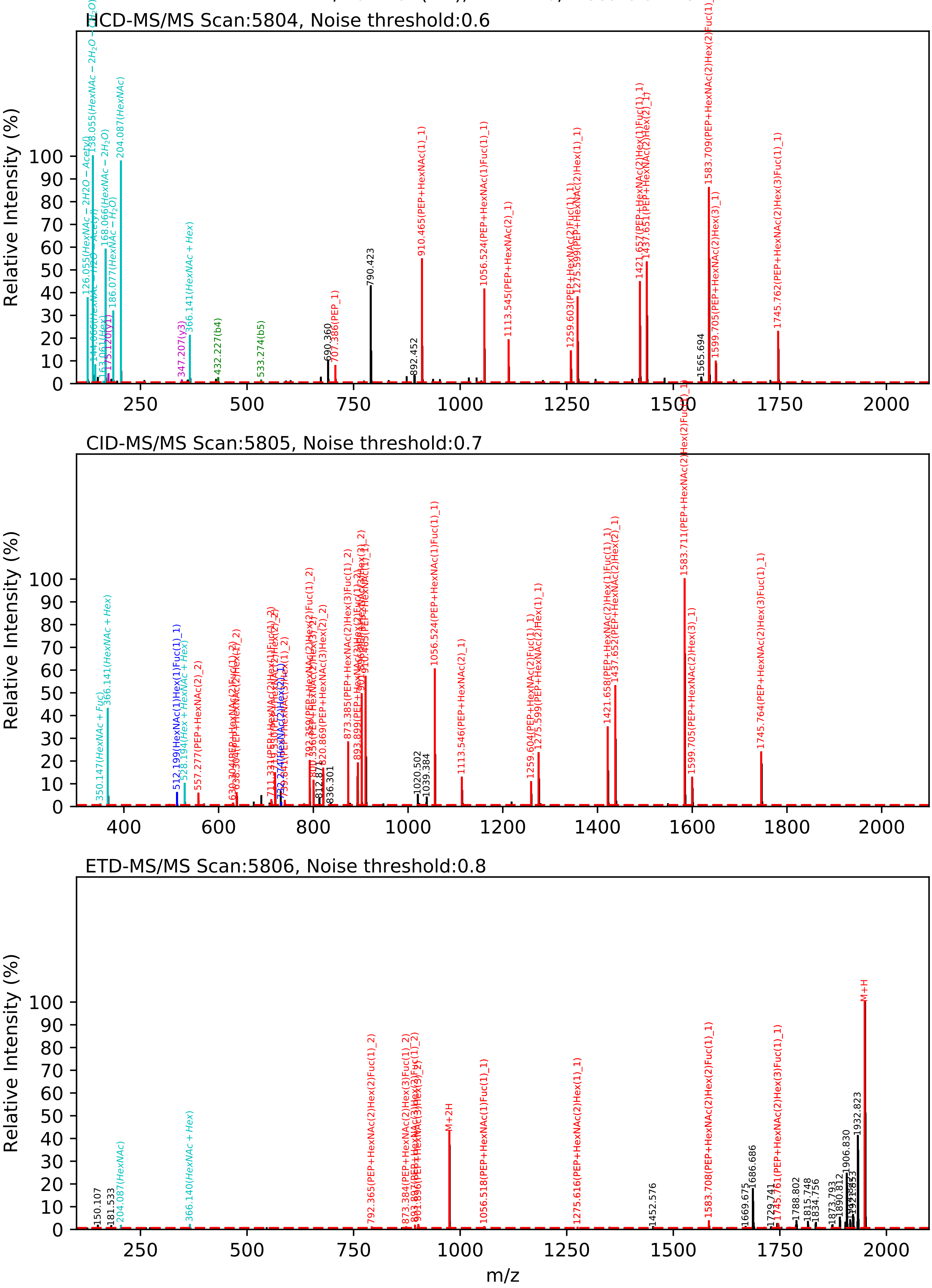

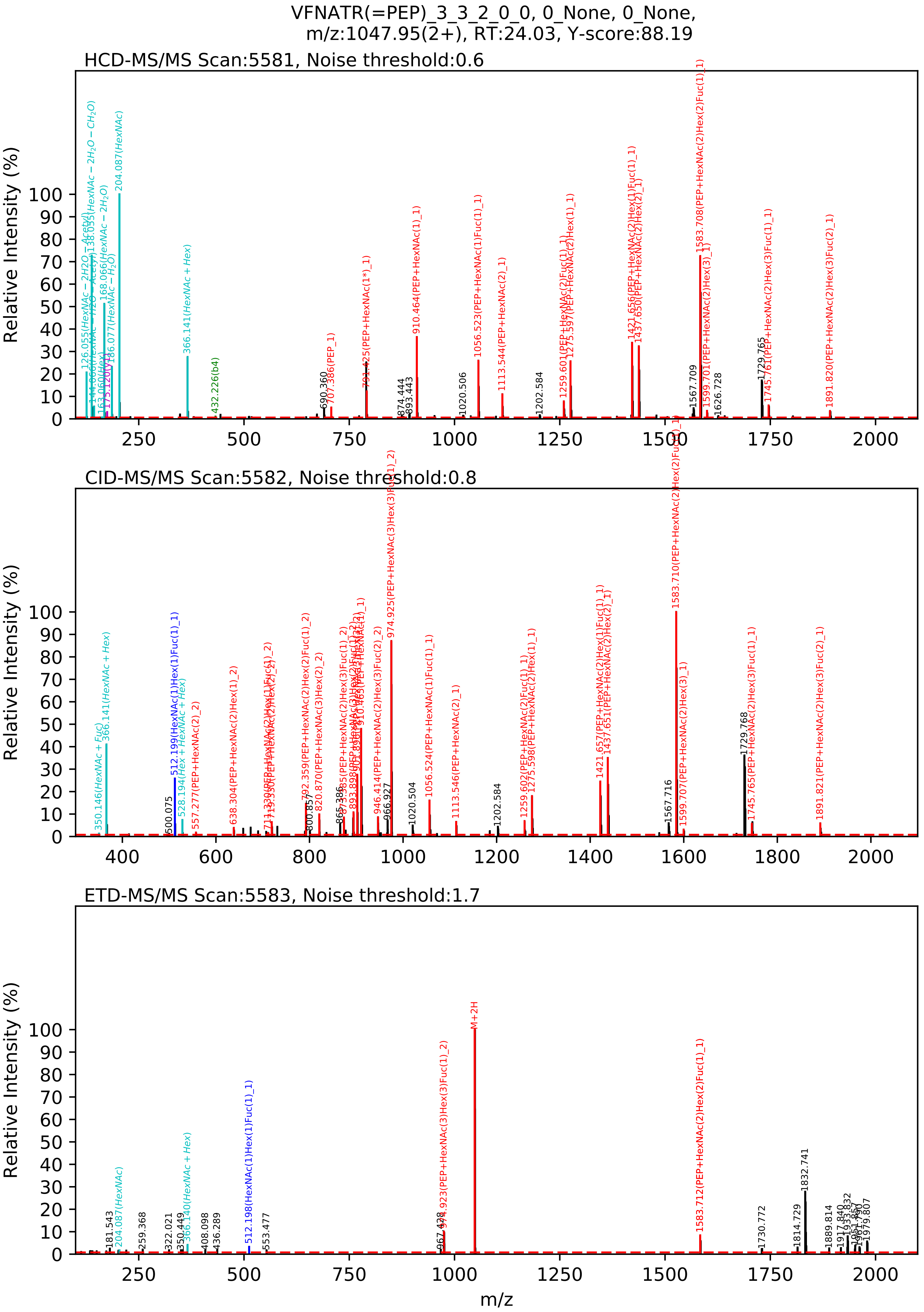

VFNATR(=PEP)_3_3_2_0_0, 0_None, 0_None,
m/z:1047.95(2+), RT:24.03, Y-score:88.19
HCD-MS/MS Scan:5581, Noise threshold:0.6
CID-MS/MS Scan:5582, Noise threshold:0.8
ETD-MS/MS Scan:5583, Noise threshold:1.7
Relative Intensity (%)
m/z

VFNATR(=PEP)_4_2_1_0_0, 0_None, 0_None,
m/z:954.41(2+), RT:24.37, Y-score:88.24

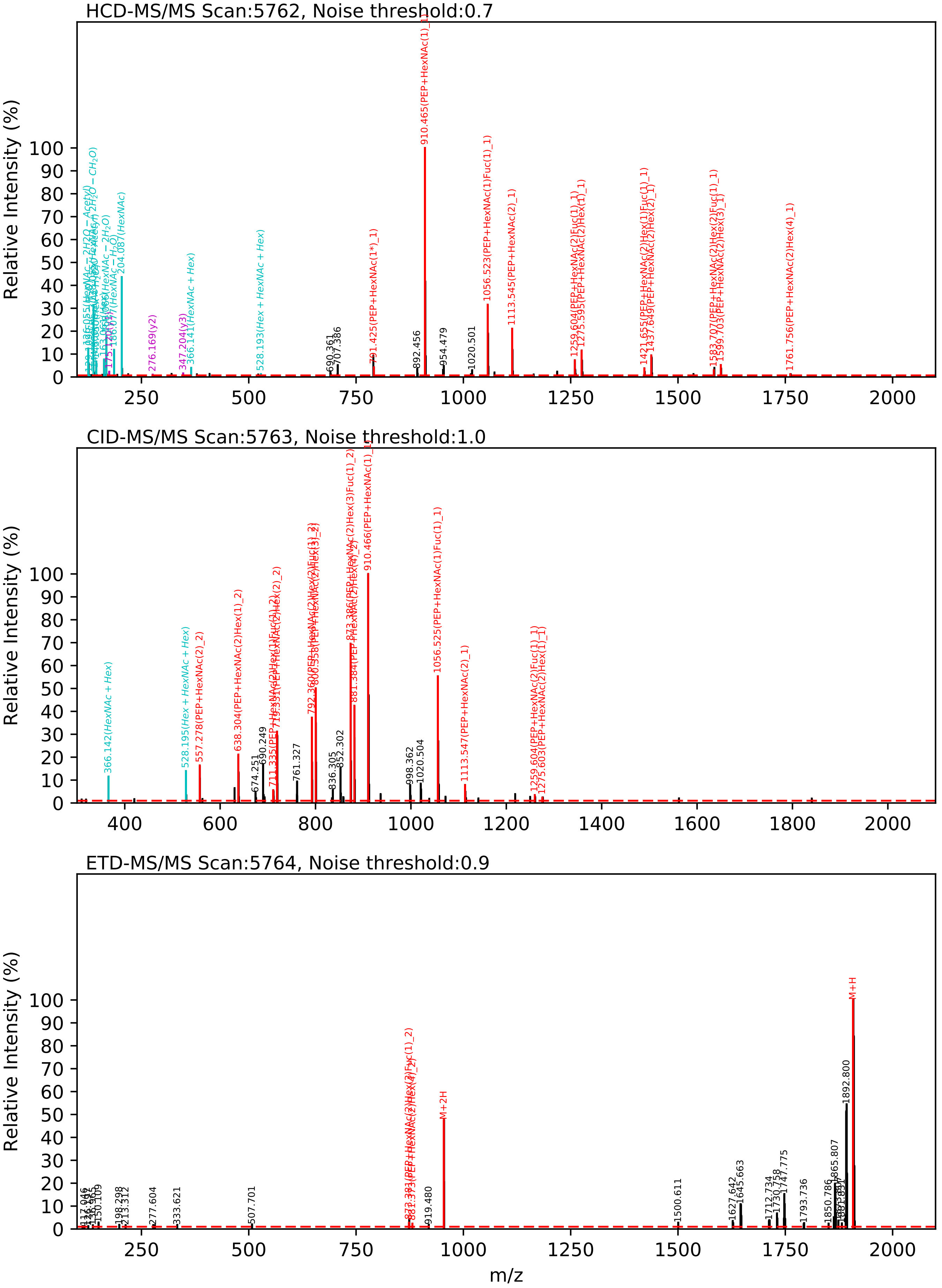

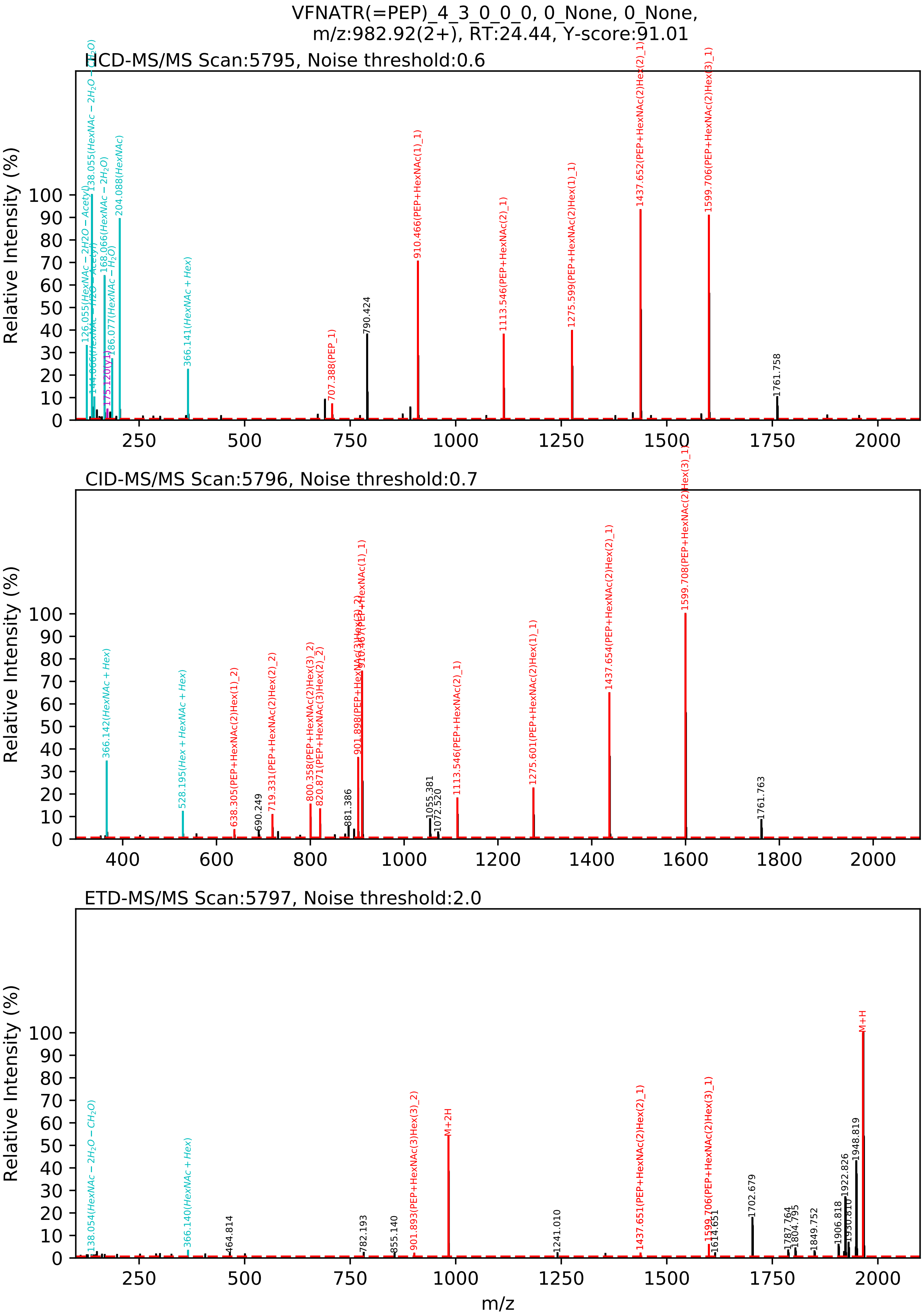
VFNATR(=PEP)_4_3_0_0_0, 0_None, 0_None,
m/z:982.92(2+), RT:24.44, Y-score:91.01
HCD-MS/MS Scan:5795, Noise threshold:0.6
Relative Intensity (%)
126.055(HexNAc − 2H2O − Acetyl)
138.055(HexNAc − 2H2O − CH2O)
144.066(HexNAc − H2O − Acetyl)
168.066(HexNAc − 2H2O)
175.120(y1)
186.077(HexNAc − H2O)
204.088(HexNAc)
366.141(HexNAc + Hex)
707.388(PEP_1)
790.424
910.466(PEP+HexNAc(1)_1)
1113.546(PEP+HexNAc(2)_1)
1275.599(PEP+HexNAc(2)Hex(1)_1)
1437.652(PEP+HexNAc(2)Hex(2)_1)
1599.706(PEP+HexNAc(2)Hex(3)_1)
1761.758
CID-MS/MS Scan:5796, Noise threshold:0.7
366.142(HexNAc + Hex)
528.195(Hex + HexNAc + Hex)
638.305(PEP+HexNAc(2)Hex(1)_2)
690.249
719.331(PEP+HexNAc(2)Hex(2)_2)
800.358(PEP+HexNAc(2)Hex(3)_2)
820.871(PEP+HexNAc(3)Hex(2)_2)
881.386
901.898(PEP+HexNAc(3)Hex(3)_2)
910.467(PEP+HexNAc(1)_1)
1055.381
1072.520
1113.546(PEP+HexNAc(2)_1)
1275.601(PEP+HexNAc(2)Hex(1)_1)
1437.654(PEP+HexNAc(2)Hex(2)_1)
1599.708(PEP+HexNAc(2)Hex(3)_1)
1761.763
ETD-MS/MS Scan:5797, Noise threshold:2.0
138.054(HexNAc − 2H2O − CH2O)
366.140(HexNAc + Hex)
464.814
782.193
855.140
901.893(PEP+HexNAc(3)Hex(3)_2)
M+2H
1241.010
1437.651(PEP+HexNAc(2)Hex(2)_1)
1599.706(PEP+HexNAc(2)Hex(3)_1)
1614.651
1702.679
1787.764
1804.795
1849.752
1906.818
1922.826
1930.810
1948.819
M+H
m/z

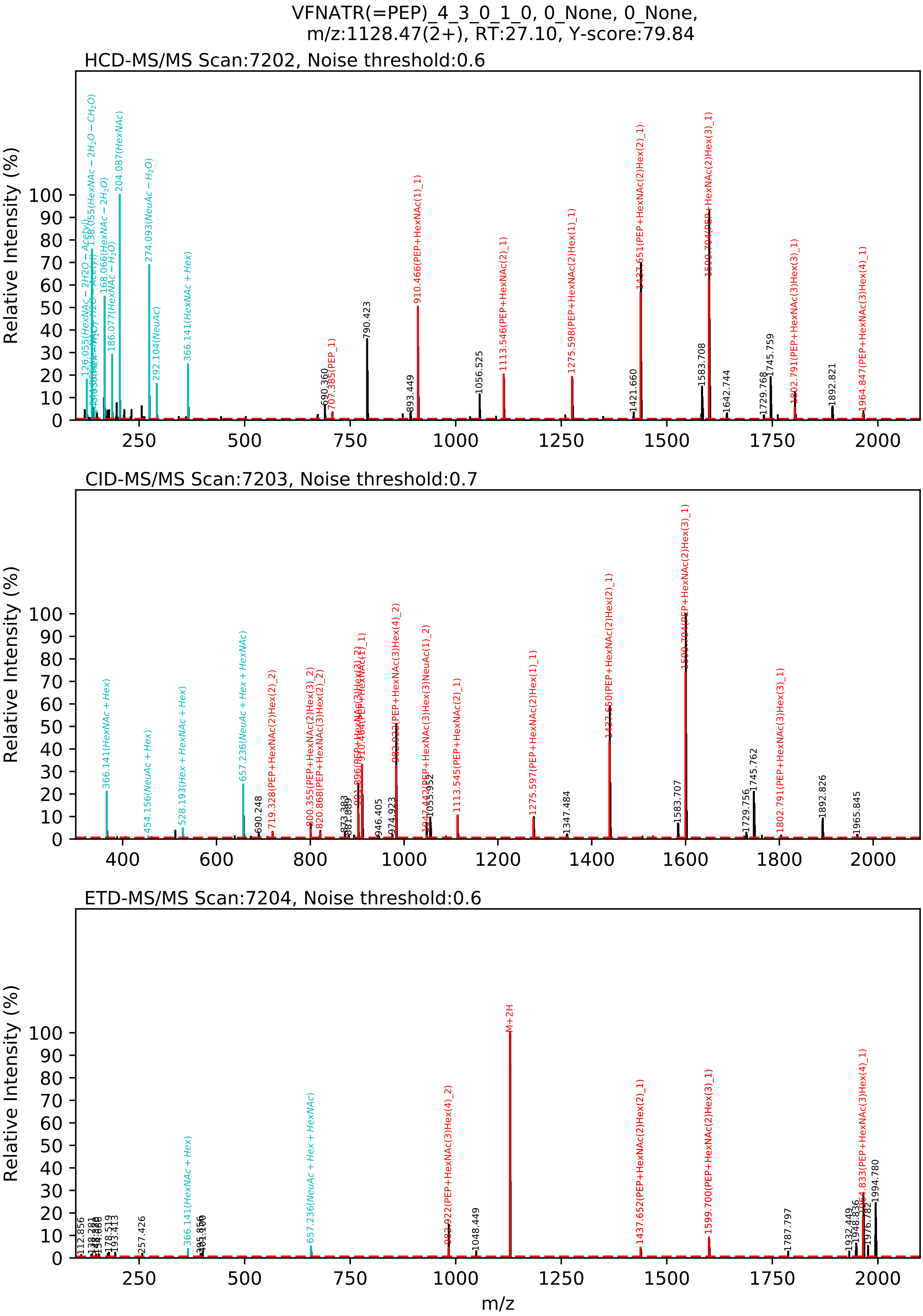
VFNATR(=PEP)_4_3_0_1_0, 0_None, 0_None,
m/z:1128.47(2+), RT:27.10, Y-score:79.84
HCD-MS/MS Scan:7202, Noise threshold:0.6
CID-MS/MS Scan:7203, Noise threshold:0.7
ETD-MS/MS Scan:7204, Noise threshold:0.6
Relative Intensity (%)
m/z
910.466(PEP+HexNAc(1)_1)
1113.546(PEP+HexNAc(2)_1)
1275.598(PEP+HexNAc(2)Hex(1)_1)
1437.651(PEP+HexNAc(2)Hex(2)_1)
1802.791(PEP+HexNAc(3)Hex(3)_1)
1964.847(PEP+HexNAc(3)Hex(4)_1)
204.087(HexNAc)
274.093(NeuAc − H2O)
292.104(NeuAc)
366.141(HexNAc + Hex)
790.423
1056.525
1583.708
1745.759
1892.821
983.922(PEP+HexNAc(3)Hex(4)_2)
1113.545(PEP+HexNAc(2)_1)
1275.597(PEP+HexNAc(2)Hex(1)_1)
1437.650(PEP+HexNAc(2)Hex(2)_1)
1802.791(PEP+HexNAc(3)Hex(3)_1)
657.236(NeuAc + Hex + HexNAc)
528.193(Hex + HexNAc + Hex)
454.156(NeuAc + Hex)
719.328(PEP+HexNAc(2)Hex(2)_2)
1745.762
1892.826
1965.845
M+2H
1437.652(PEP+HexNAc(2)Hex(2)_1)
1599.700(PEP+HexNAc(2)Hex(3)_1)
1994.780
1048.449
1787.797

VFNATR(=PEP)_4_3_0_1_0, 0_None, 0_None,
m/z:1128.47(2+), RT:27.18, Y-score:75.15

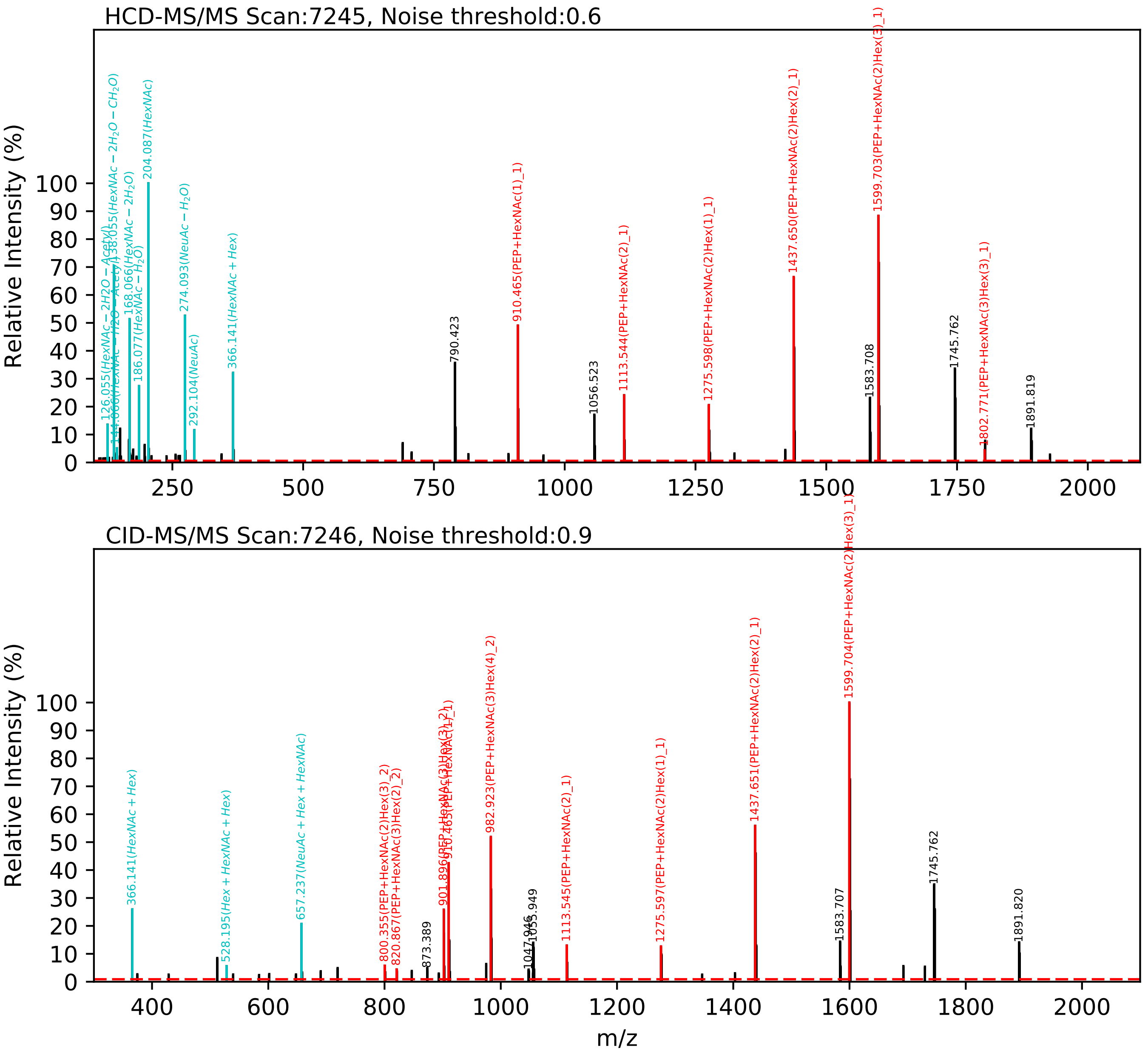

VFNATR(=PEP)_4_3_1_0_0, 0_None, 0_None,
m/z:1055.95(2+), RT:24.19, Y-score:79.10

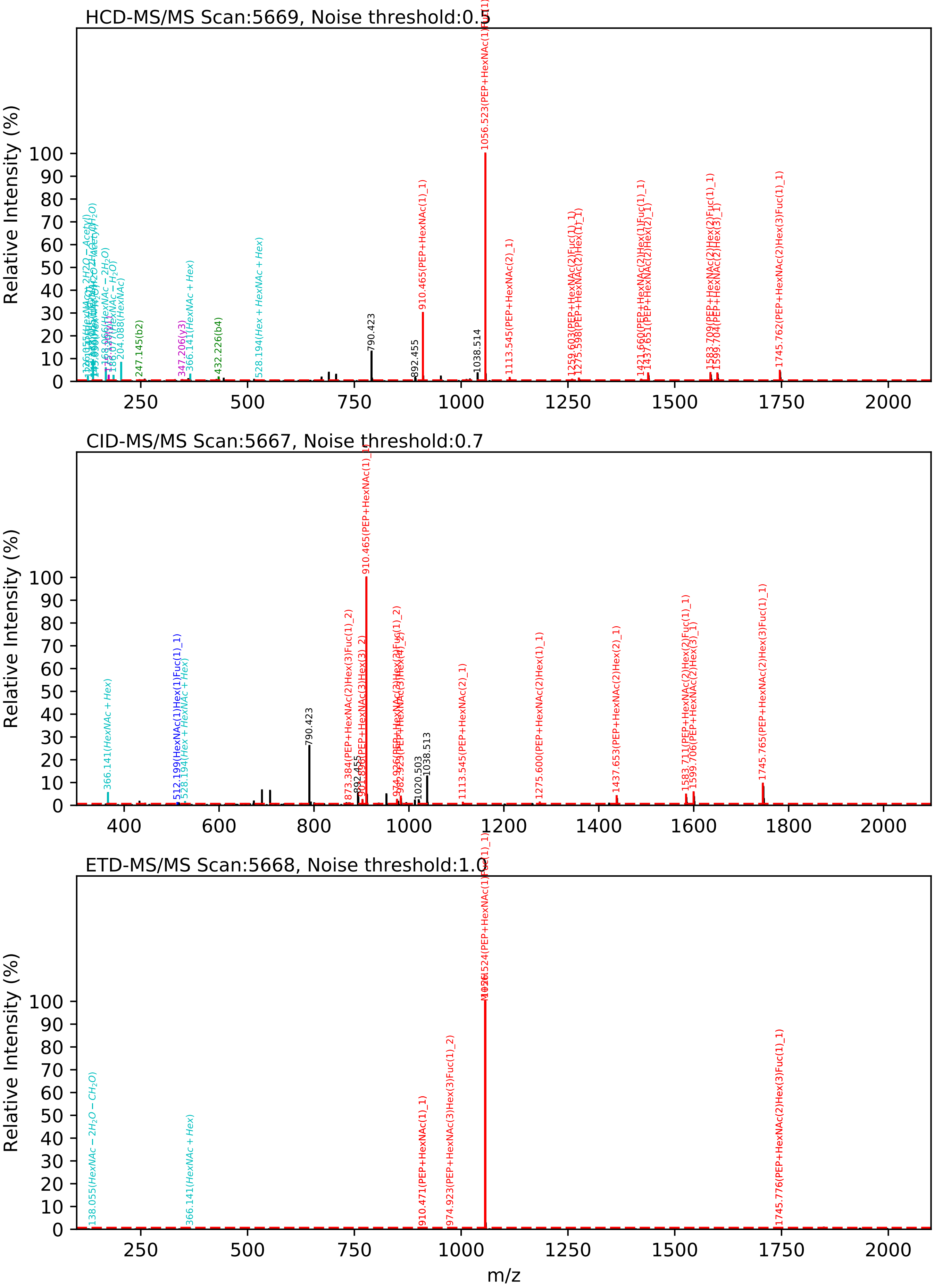

VFNATR(=PEP)_4_3_1_0_0, 0_None, 0_None,
m/z:1055.95(2+), RT:26.65, Y-score:80.83

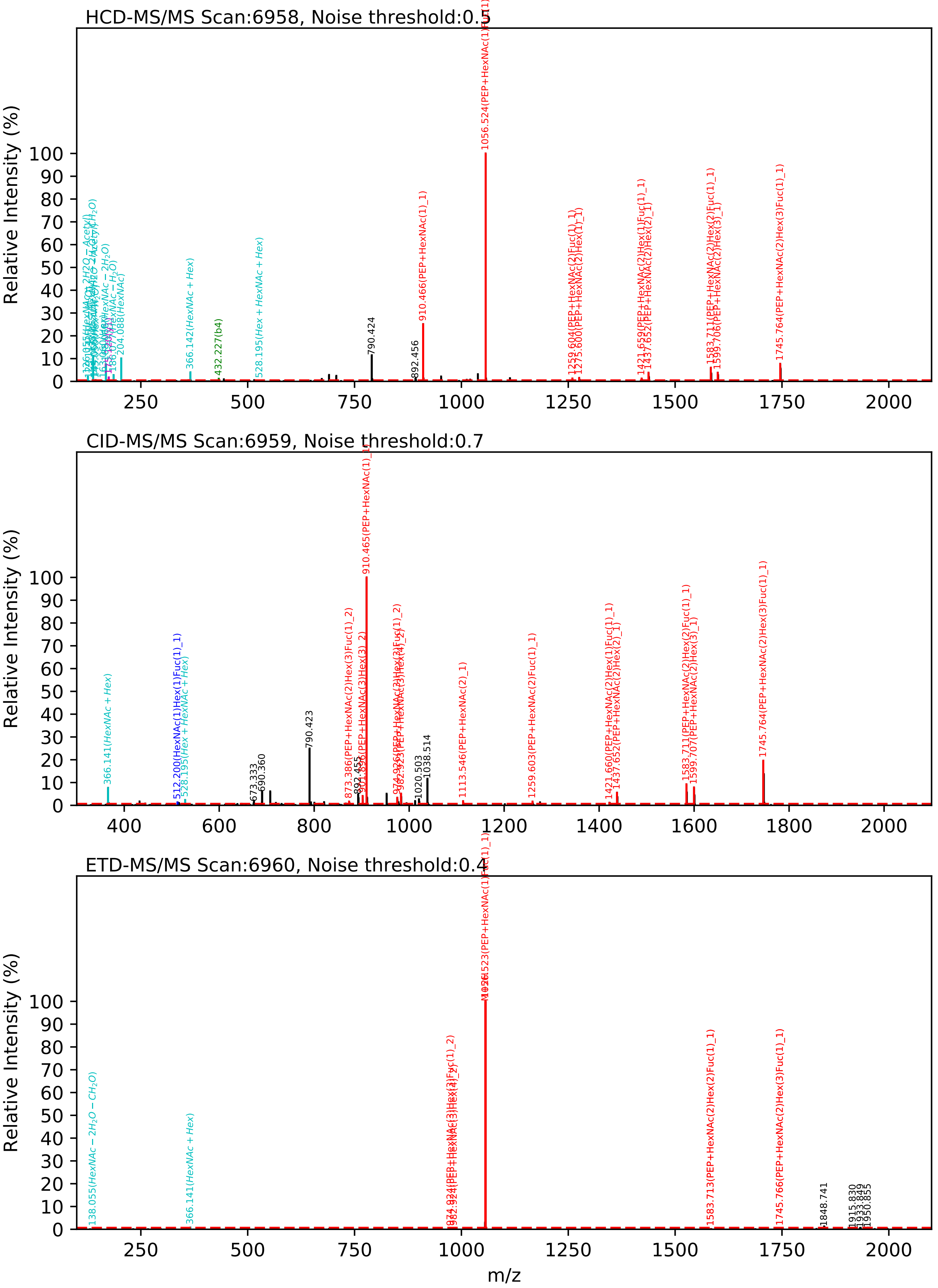

VFNATR(=PEP)_4_3_1_0_0, 0_None, 0_None,
m/z:1055.95(2+), RT:27.27, Y-score:79.41

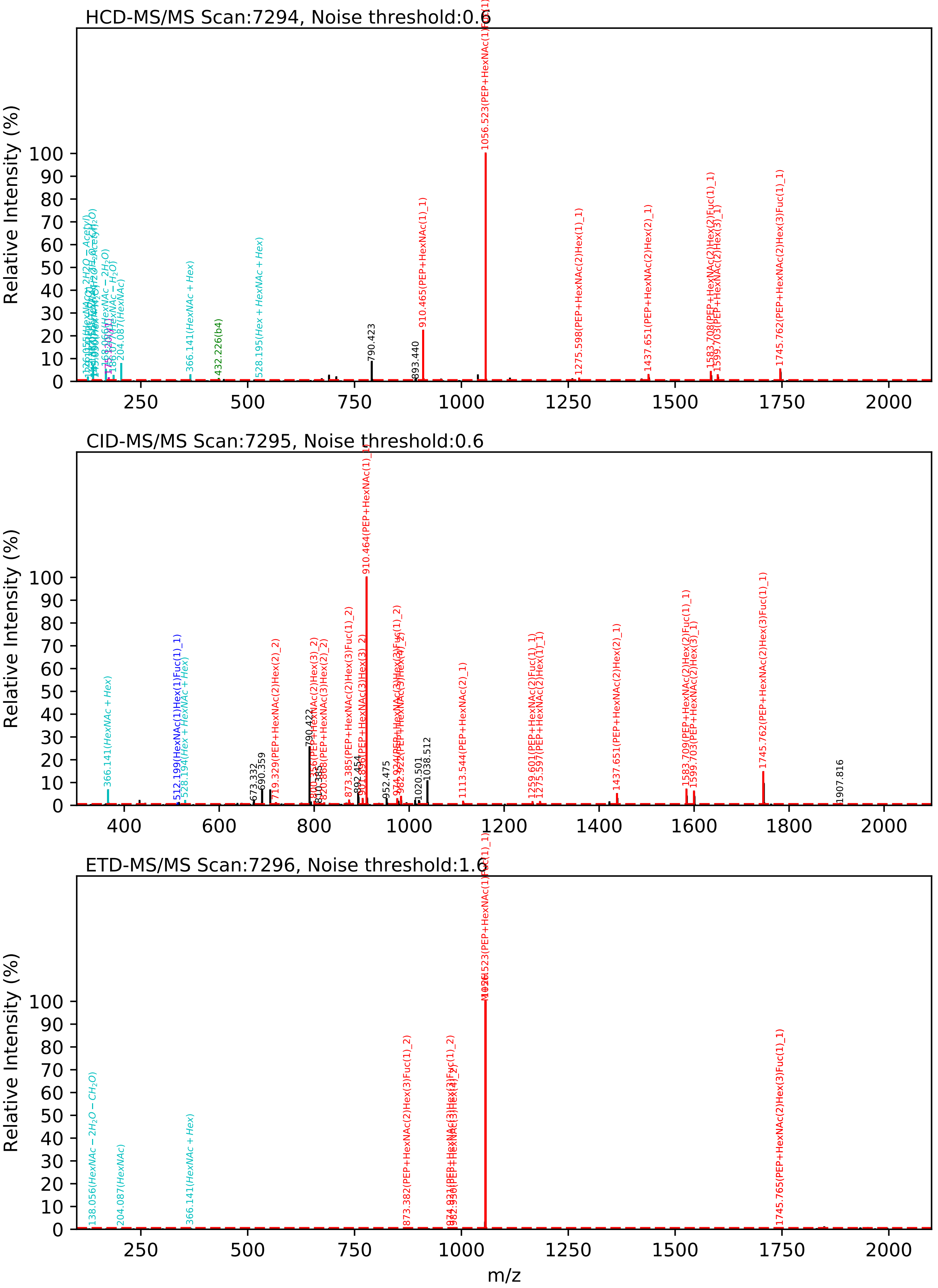

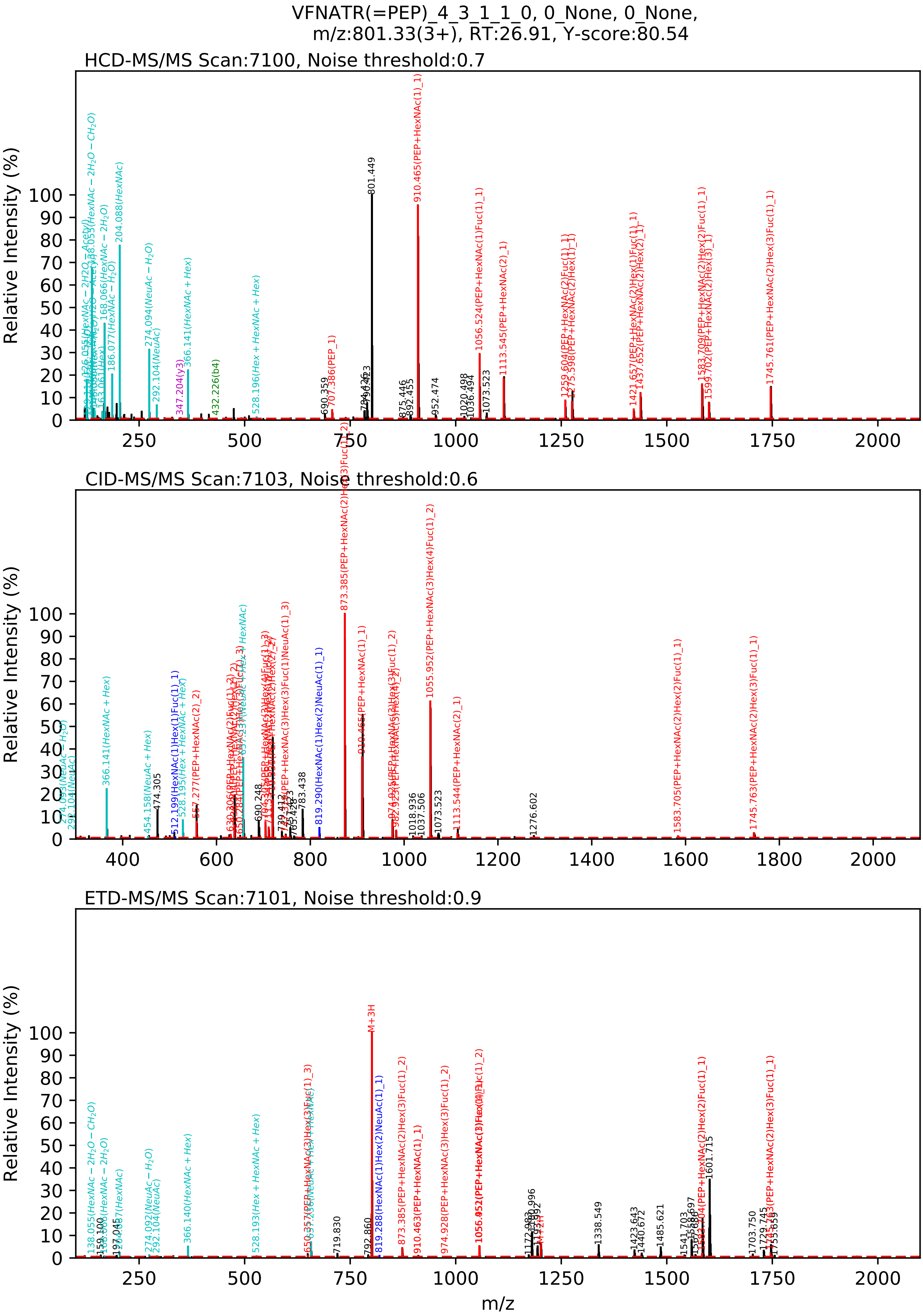

VFNATR(=PEP)_4_3_1_1_0, 0_None, 0_None,
m/z:801.33(3+), RT:26.91, Y-score:80.54
HCD-MS/MS Scan:7100, Noise threshold:0.7
CID-MS/MS Scan:7103, Noise threshold:0.6
ETD-MS/MS Scan:7101, Noise threshold:0.9
Relative Intensity (%)
m/z

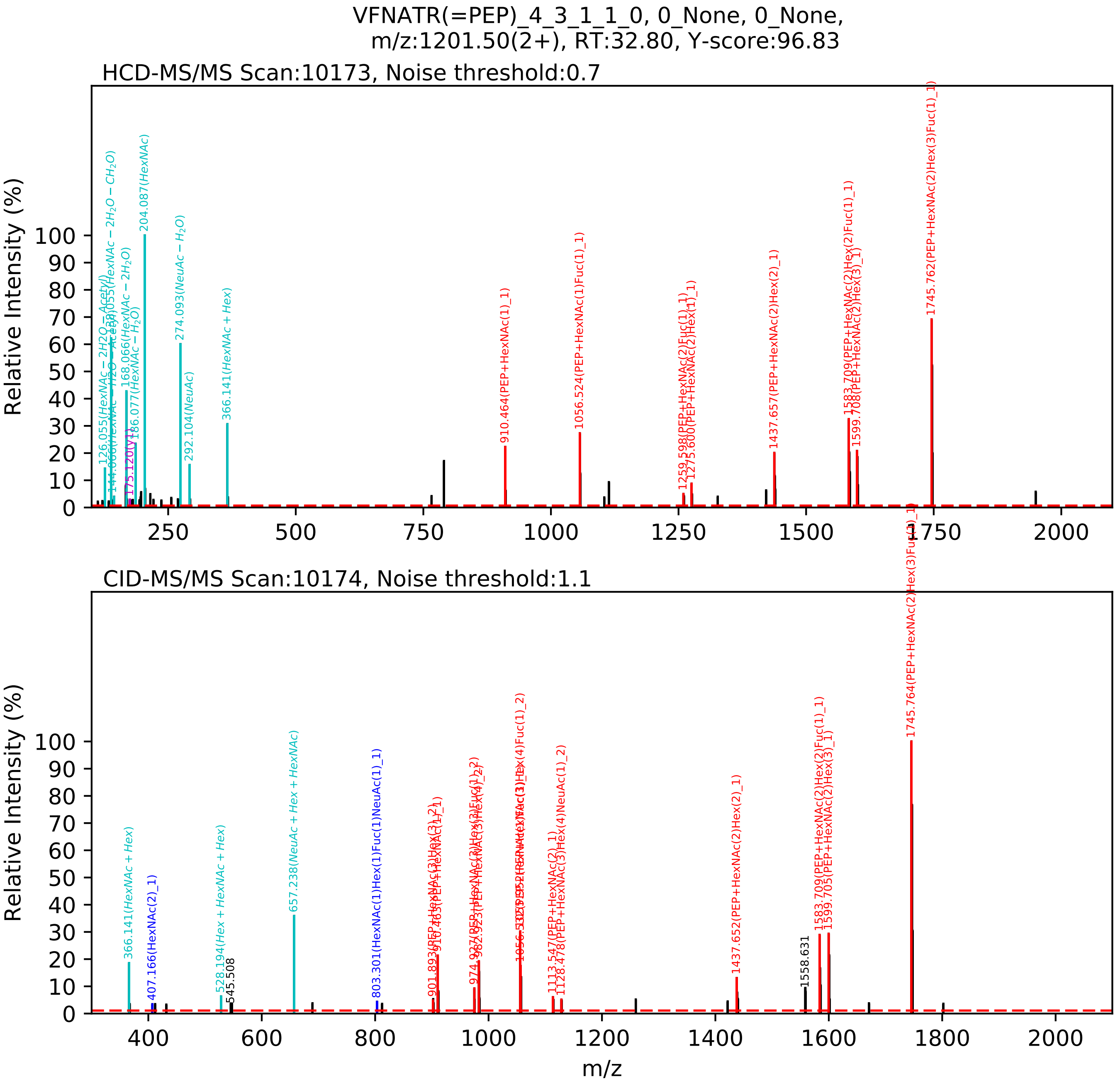

VFNATR(=PEP)_4_3_1_1_0, 0_None, 0_None,
m/z:1201.50(2+), RT:32.80, Y-score:96.83
HCD-MS/MS Scan:10173, Noise threshold:0.7
CID-MS/MS Scan:10174, Noise threshold:1.1
Relative Intensity (%)
m/z
204.087(HexNAc)
274.093(NeuAc − H2O)
292.104(NeuAc)
366.141(HexNAc + Hex)
910.464(PEP+HexNAc(1)_1)
1056.524(PEP+HexNAc(1)Fuc(1)_1)
1437.657(PEP+HexNAc(2)Hex(2)_1)
1745.762(PEP+HexNAc(2)Hex(3)Fuc(1)_1)
657.238(NeuAc + Hex + HexNAc)
803.301(HexNAc(1)Hex(1)Fuc(1)NeuAc(1)_1)
1437.652(PEP+HexNAc(2)Hex(2)_1)
1558.631
1745.764(PEP+HexNAc(2)Hex(3)Fuc(1)_1)

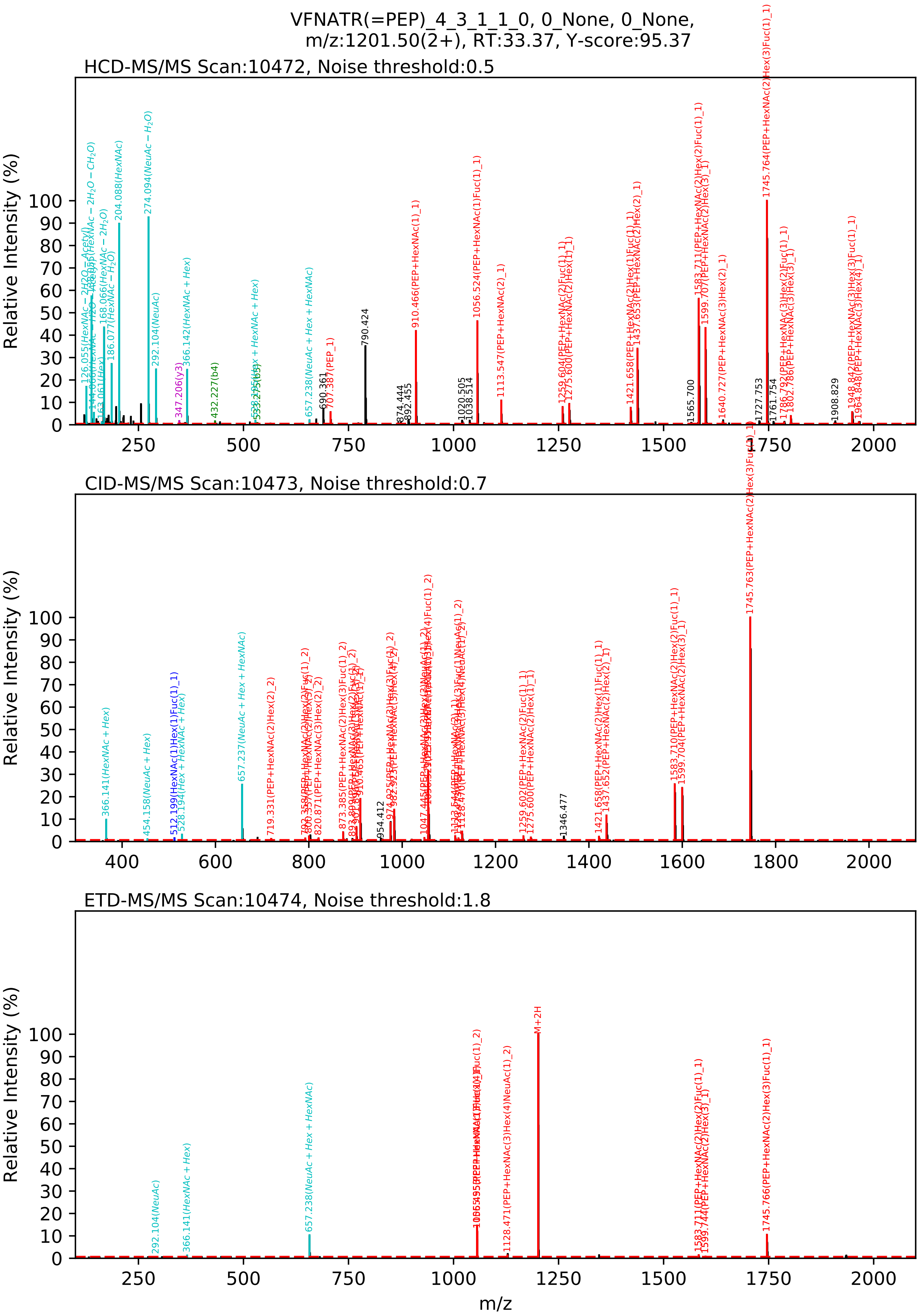
VFNATR(=PEP)_4_3_1_1_0, 0_None, 0_None,
m/z:1201.50(2+), RT:33.37, Y-score:95.37
HCD-MS/MS Scan:10472, Noise threshold:0.5
CID-MS/MS Scan:10473, Noise threshold:0.7
ETD-MS/MS Scan:10474, Noise threshold:1.8
Relative Intensity (%)
m/z
1745.764(PEP+HexNAc(2)Hex(3)Fuc(1)_1)
1583.711(PEP+HexNAc(2)Hex(2)Fuc(1)_1)
1599.707(PEP+HexNAc(2)Hex(3)_1)
1056.524(PEP+HexNAc(1)Fuc(1)_1)
910.466(PEP+HexNAc(1)_1)
1113.547(PEP+HexNAc(2)_1)
1259.604(PEP+HexNAc(2)Fuc(1)_1)
1275.600(PEP+HexNAc(2)Hex(1)_1)
1421.658(PEP+HexNAc(2)Hex(1)Fuc(1)_1)
1437.653(PEP+HexNAc(2)Hex(2)_1)
1640.727(PEP+HexNAc(3)Hex(2)_1)
1948.842(PEP+HexNAc(3)Hex(3)Fuc(1)_1)
1964.848(PEP+HexNAc(3)Hex(4)_1)
204.088(HexNAc)
274.094(NeuAc − H2O)
292.104(NeuAc)
366.142(HexNAc + Hex)
657.238(NeuAc + Hex + HexNAc)
790.424
707.387(PEP_1)
1745.763(PEP+HexNAc(2)Hex(3)Fuc(1)_1)
1583.710(PEP+HexNAc(2)Hex(2)Fuc(1)_1)
1599.704(PEP+HexNAc(2)Hex(3)_1)
719.331(PEP+HexNAc(2)Hex(2)_2)
1346.477
M+2H
1128.471(PEP+HexNAc(3)Hex(4)NeuAc(1)_2)
1745.766(PEP+HexNAc(2)Hex(3)Fuc(1)_1)

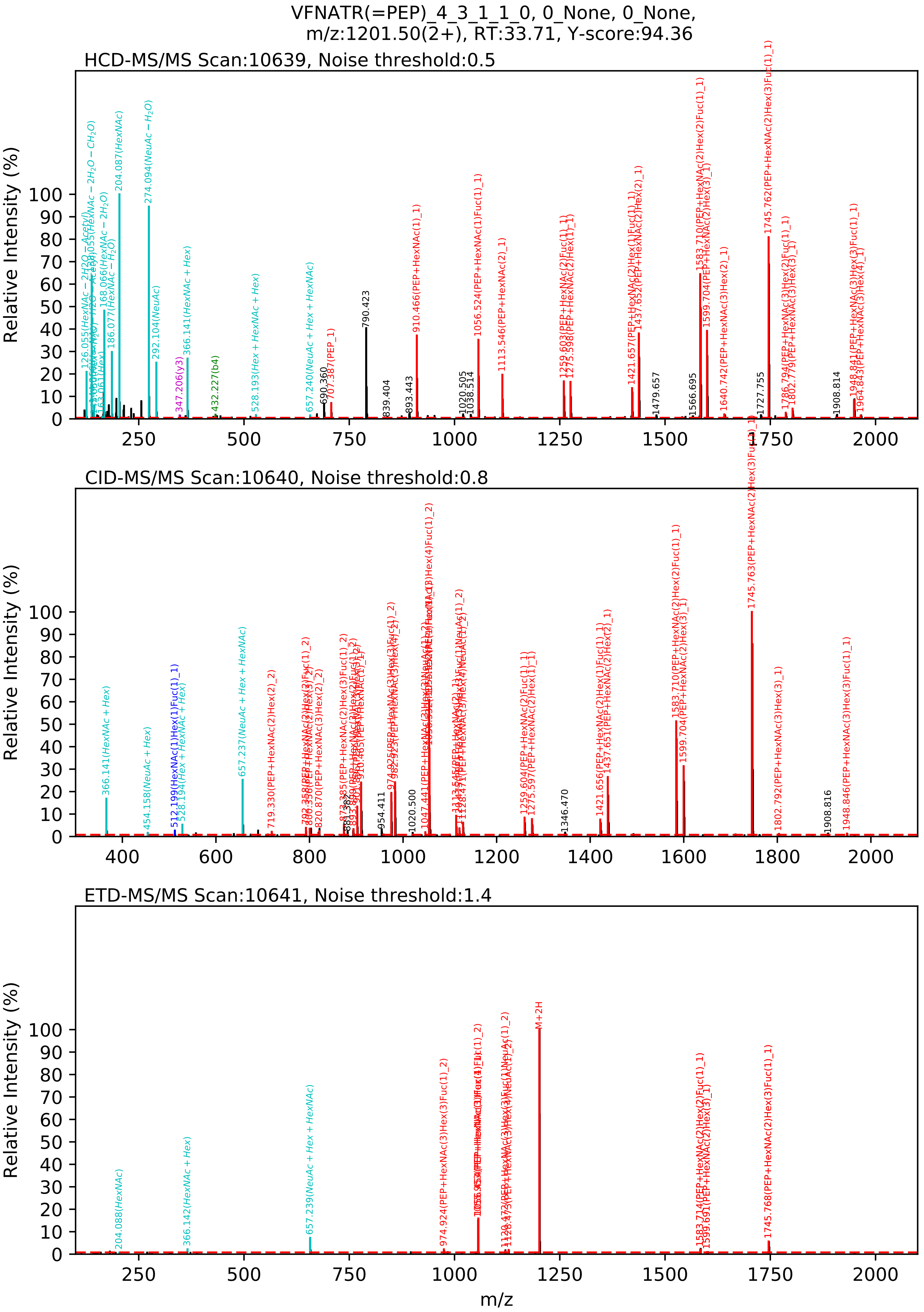
VFNATR(=PEP)_4_3_1_1_0, 0_None, 0_None,
m/z:1201.50(2+), RT:33.71, Y-score:94.36
HCD-MS/MS Scan:10639, Noise threshold:0.5
CID-MS/MS Scan:10640, Noise threshold:0.8
ETD-MS/MS Scan:10641, Noise threshold:1.4
Relative Intensity (%)
m/z
M+2H

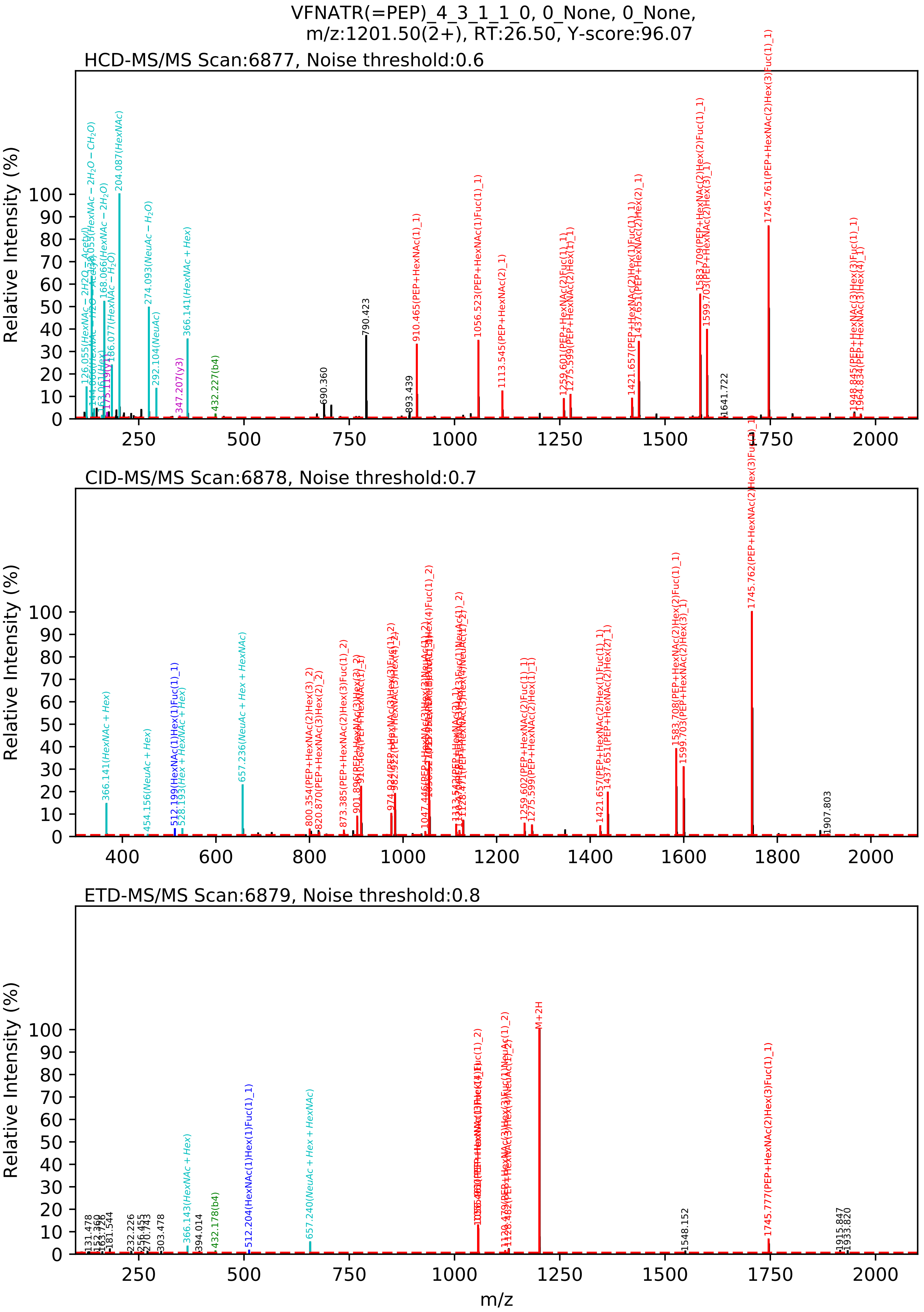
VFNATR(=PEP)_4_3_1_1_0, 0_None, 0_None,
m/z:1201.50(2+), RT:26.50, Y-score:96.07
HCD-MS/MS Scan:6877, Noise threshold:0.6
CID-MS/MS Scan:6878, Noise threshold:0.7
ETD-MS/MS Scan:6879, Noise threshold:0.8
Relative Intensity (%)
m/z

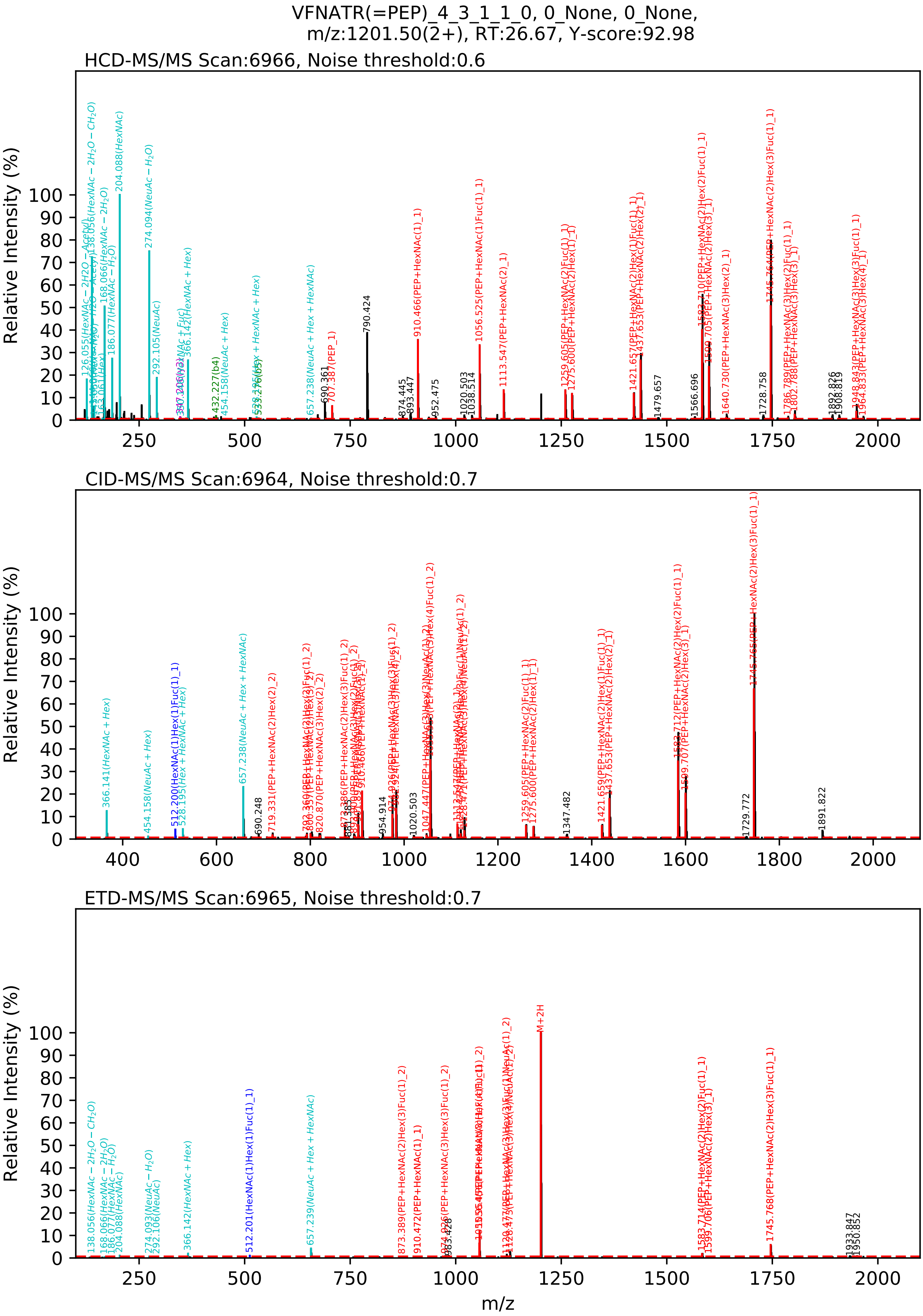

VFNATR(=PEP)_4_3_1_1_0, 0_None, 0_None,
m/z:1201.50(2+), RT:26.67, Y-score:92.98
HCD-MS/MS Scan:6966, Noise threshold:0.6
CID-MS/MS Scan:6964, Noise threshold:0.7
ETD-MS/MS Scan:6965, Noise threshold:0.7
Relative Intensity (%)
m/z

VFNATR(=PEP)_4_3_1_1_0, 0_None, 0_None,
m/z:1201.50(2+), RT:27.12, Y-score:96.46

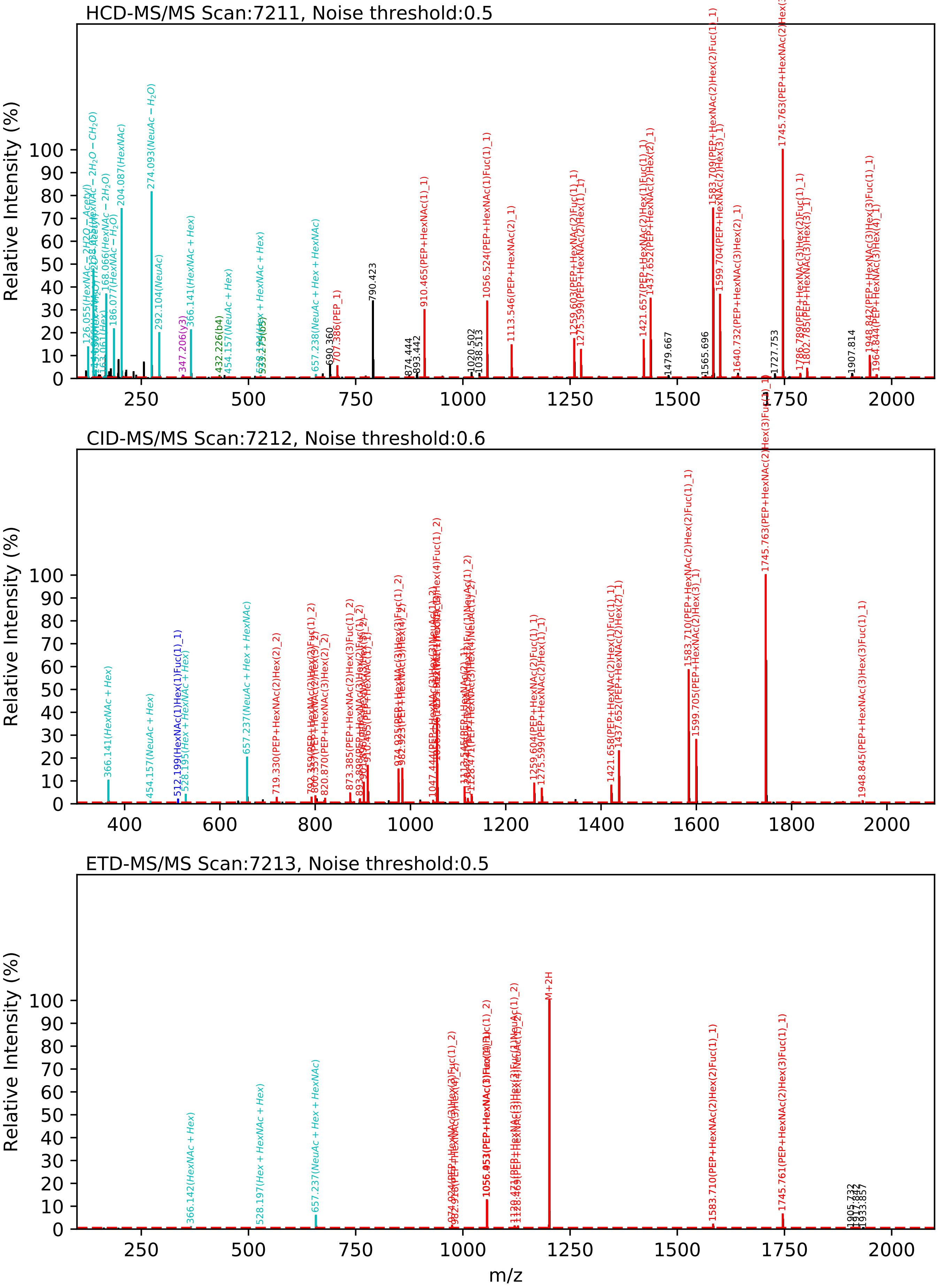

VFNATR(=PEP)_4_3_1_1_0, 0_None, 0_None,
m/z:1201.50(2+), RT:32.18, Y-score:95.38

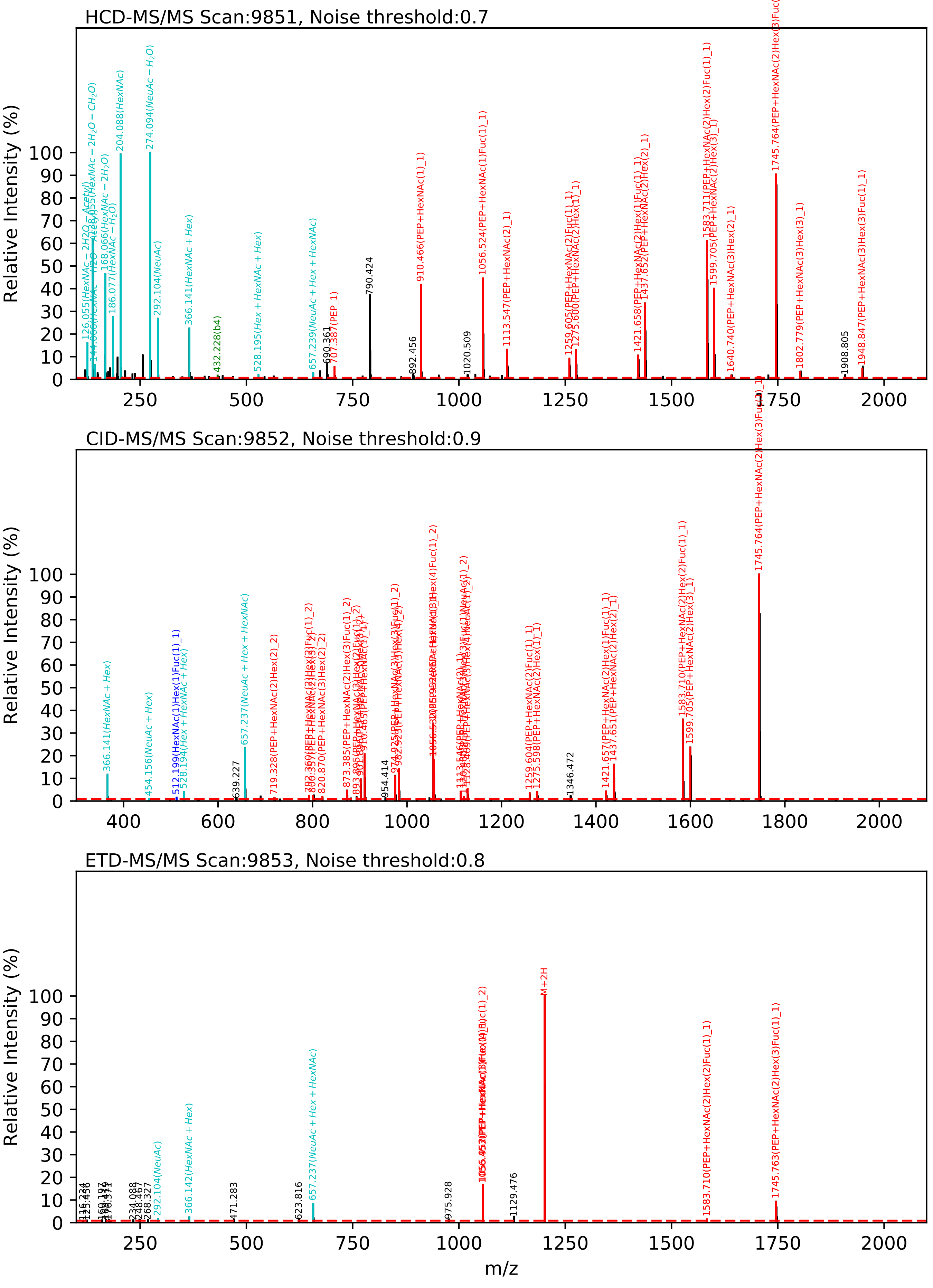

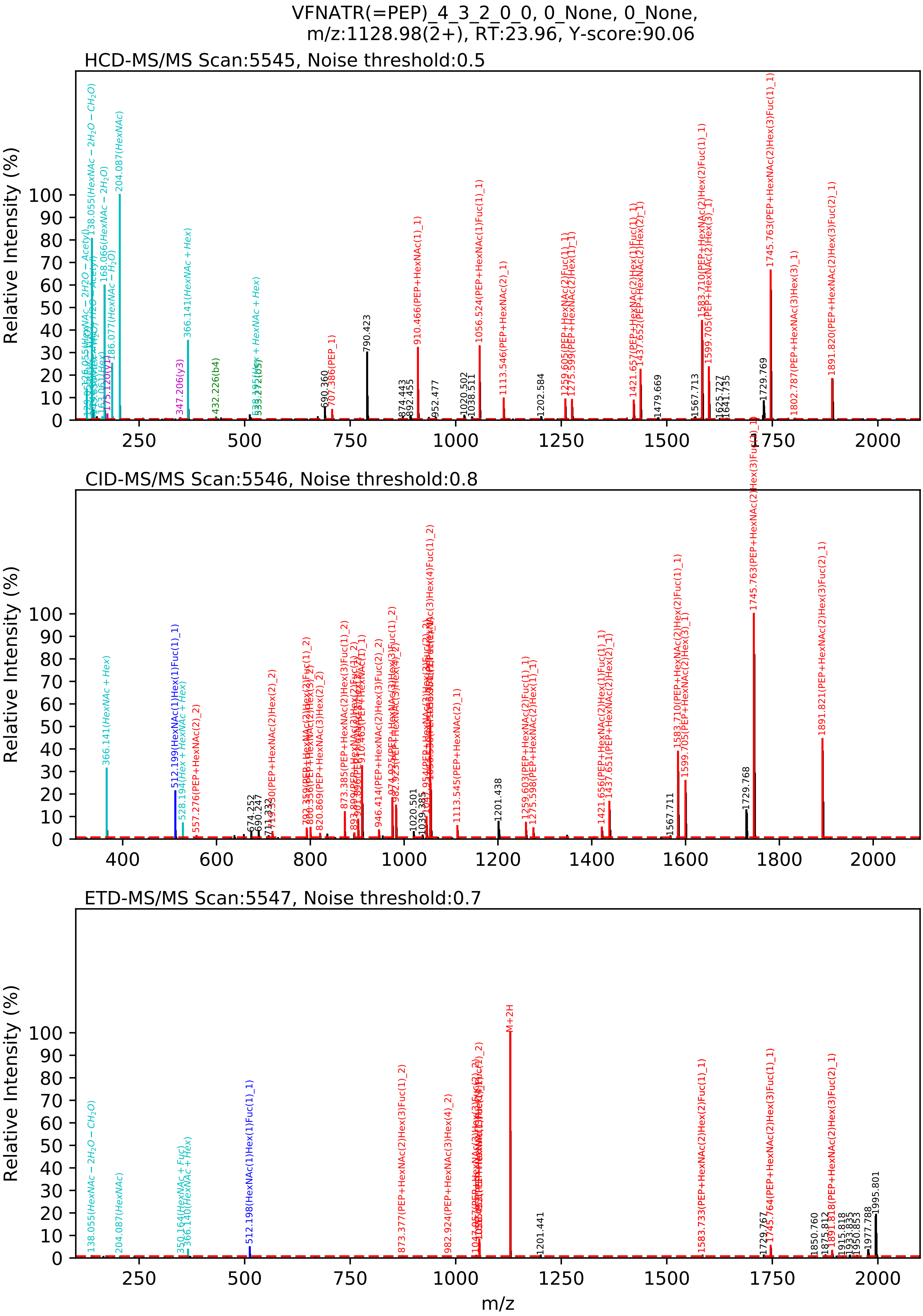
VFNATR(=PEP)_4_3_2_0_0, 0_None, 0_None,
m/z:1128.98(2+), RT:23.96, Y-score:90.06
HCD-MS/MS Scan:5545, Noise threshold:0.5
Relative Intensity (%)
204.087(HexNAc)
366.141(HexNAc + Hex)
347.206(y3)
432.226(b4)
690.360
707.386(PEP_1)
790.423
874.443
892.455
910.466(PEP+HexNAc(1)_1)
952.477
1020.502
1038.511
1056.524(PEP+HexNAc(1)Fuc(1)_1)
1113.546(PEP+HexNAc(2)_1)
1202.584
1421.657(PEP+HexNAc(2)Hex(1)Fuc(1)_1)
1479.669
1567.713
1583.710(PEP+HexNAc(2)Hex(2)Fuc(1)_1)
1625.727
1641.735
1729.769
1745.763(PEP+HexNAc(2)Hex(3)Fuc(1)_1)
1802.787(PEP+HexNAc(3)Hex(3)_1)
1891.820(PEP+HexNAc(2)Hex(3)Fuc(2)_1)
CID-MS/MS Scan:5546, Noise threshold:0.8
Relative Intensity (%)
366.141(HexNAc + Hex)
512.199(HexNAc(1)Hex(1)Fuc(1)_1)
557.276(PEP+HexNAc(2)_2)
674.252
690.247
713.330(PEP+HexNAc(2)Hex(2)_2)
820.869(PEP+HexNAc(3)Hex(2)_2)
873.385(PEP+HexNAc(2)Hex(3)Fuc(1)_2)
946.414(PEP+HexNAc(2)Hex(3)Fuc(2)_2)
1020.501
1039.385
1113.545(PEP+HexNAc(2)_1)
1201.438
1421.656(PEP+HexNAc(2)Hex(1)Fuc(1)_1)
1437.651(PEP+HexNAc(2)Hex(2)_1)
1567.711
1583.710(PEP+HexNAc(2)Hex(2)Fuc(1)_1)
1599.705(PEP+HexNAc(2)Hex(3)_1)
1729.768
1745.763(PEP+HexNAc(2)Hex(3)Fuc(1)_1)
1891.821(PEP+HexNAc(2)Hex(3)Fuc(2)_1)
ETD-MS/MS Scan:5547, Noise threshold:0.7
Relative Intensity (%)
204.087(HexNAc)
512.198(HexNAc(1)Hex(1)Fuc(1)_1)
873.377(PEP+HexNAc(2)Hex(3)Fuc(1)_2)
982.924(PEP+HexNAc(3)Hex(4)_2)
M+2H
1201.441
1583.733(PEP+HexNAc(2)Hex(2)Fuc(1)_1)
1729.767
1745.764(PEP+HexNAc(2)Hex(3)Fuc(1)_1)
1850.760
1995.801
1977.788
m/z

VFNATR(=PEP)_4_4_0_0_0, 0_None, 0_None,
m/z:1084.46(2+), RT:24.53, Y-score:89.73

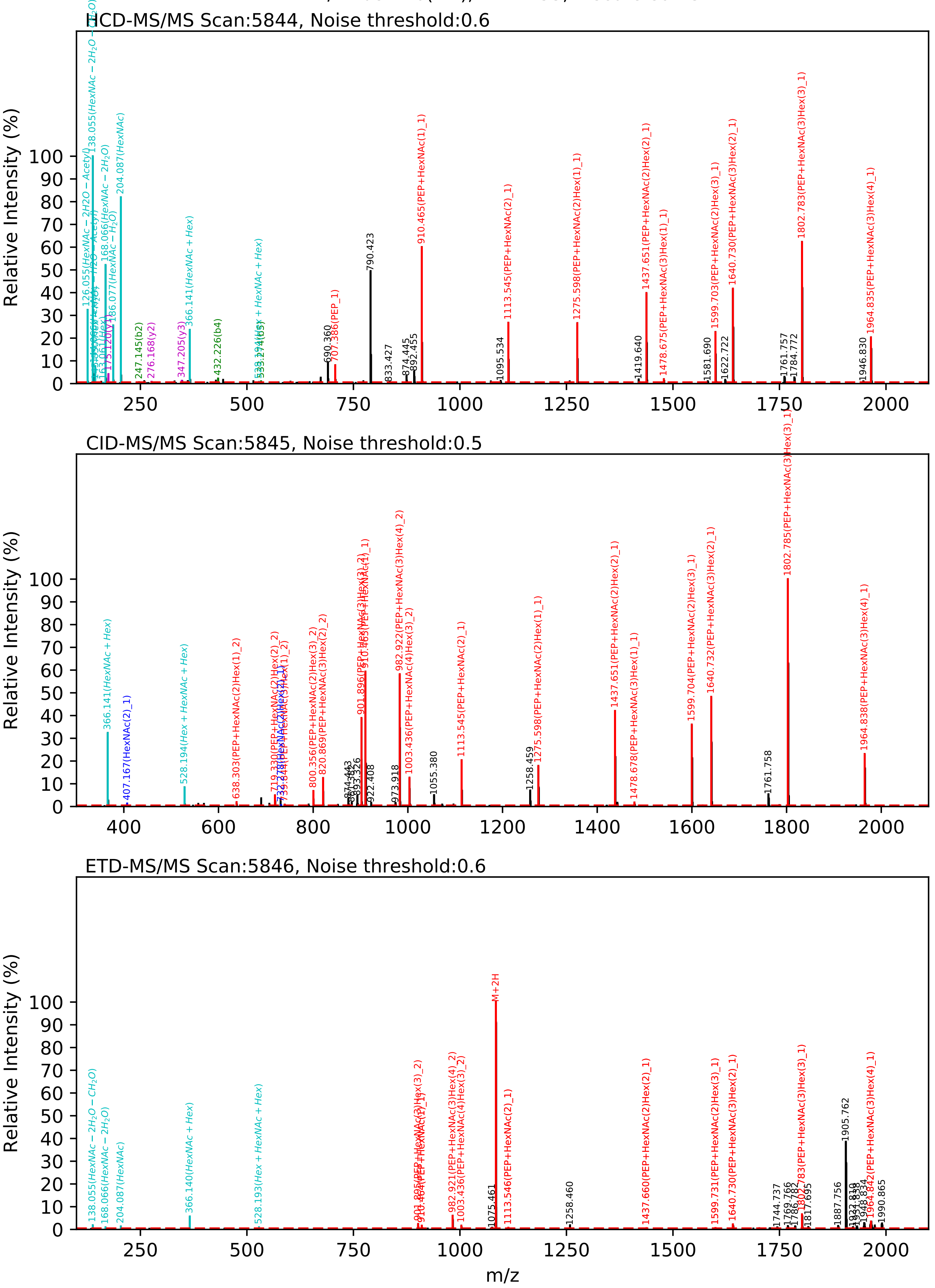

VFNATR(=PEP)_4_4_1_0_0, 0_None, 0_None,
m/z:1157.49(2+), RT:24.65, Y-score:92.71

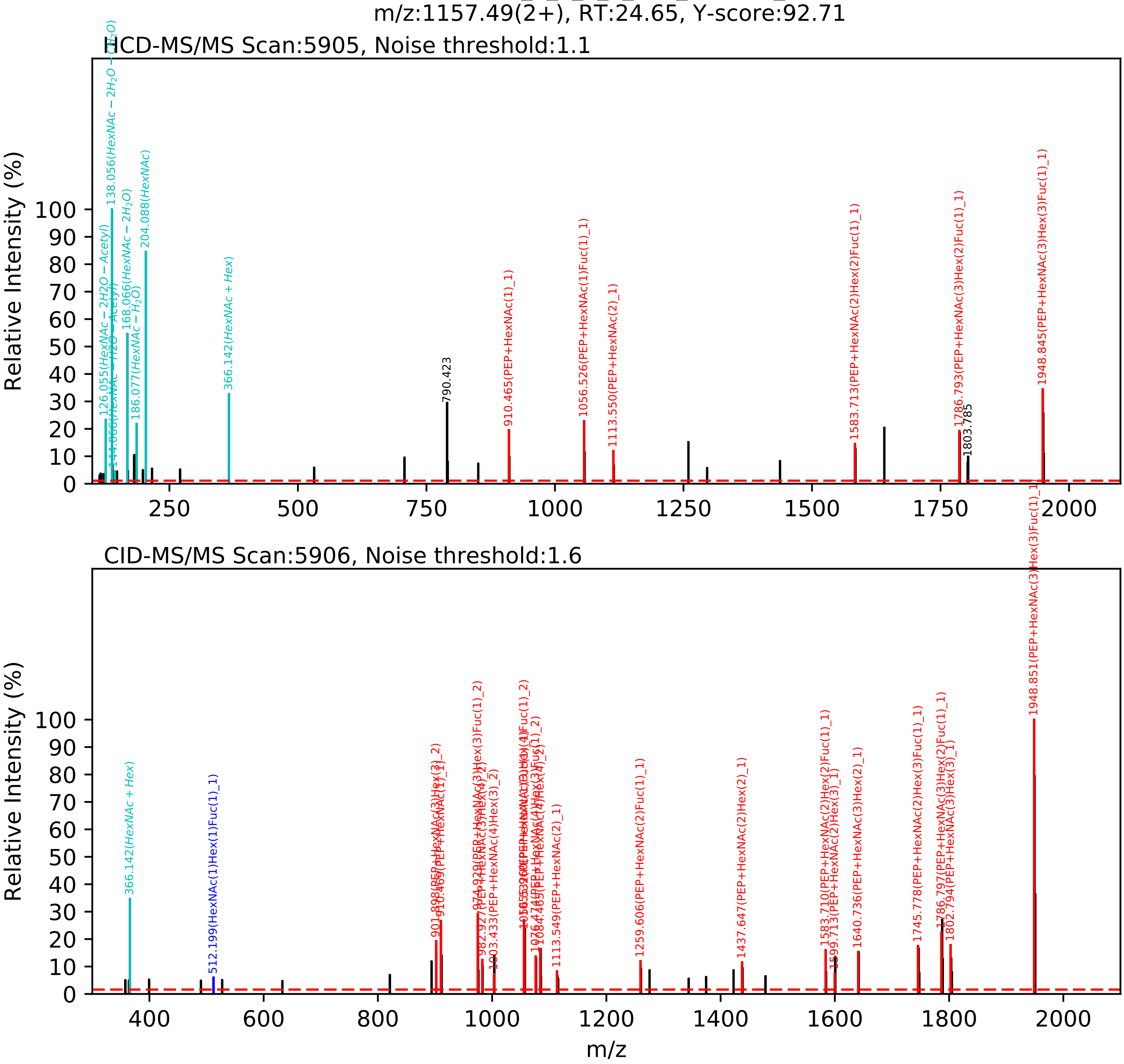

VFNATR(=PEP)_4_4_1_0_0, 0_None, 0_None,
m/z:1157.49(2+), RT:26.76, Y-score:88.41

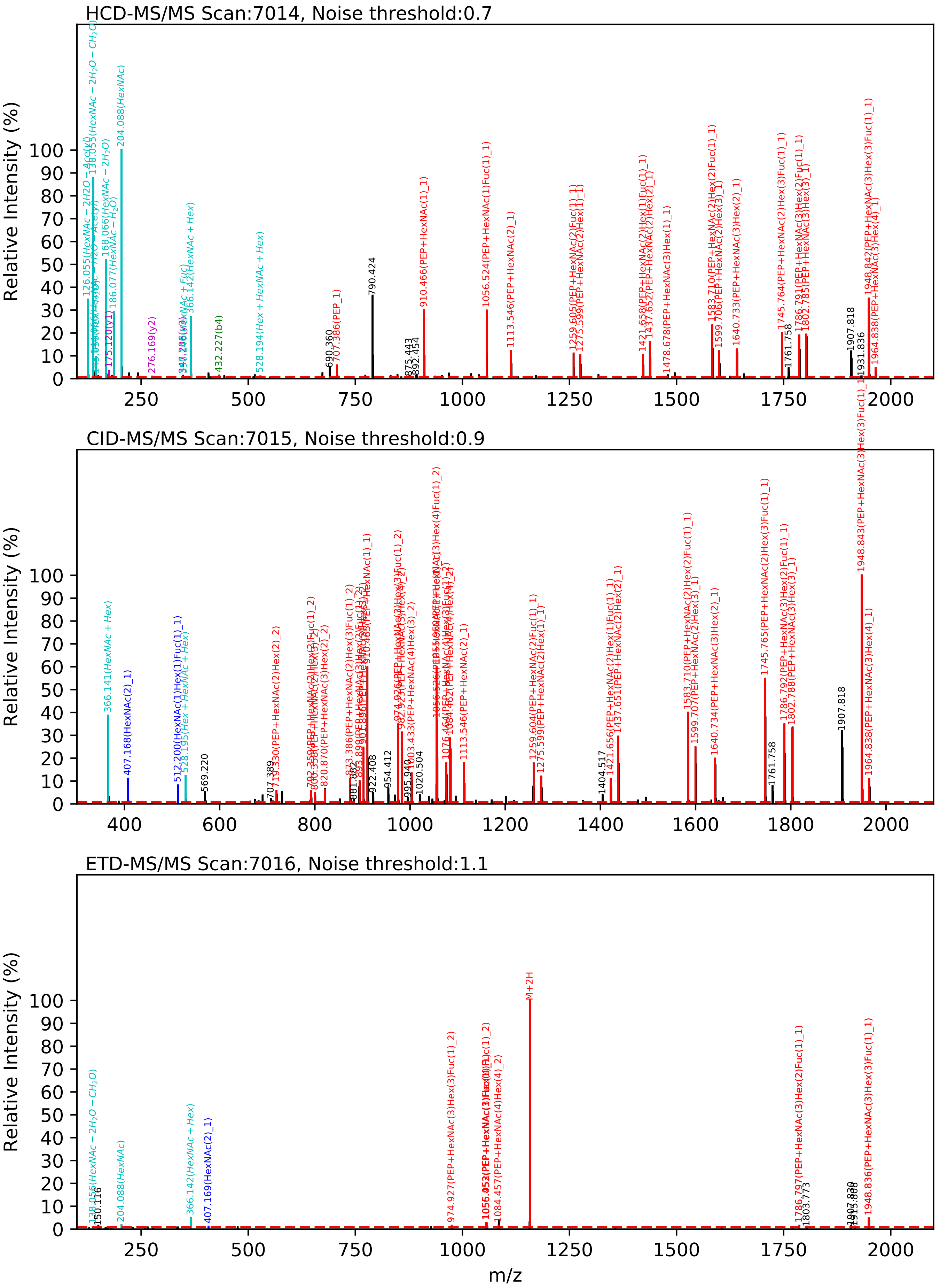

VFNATR(=PEP)_4_4_1_1_0, 0_None, 0_None,
m/z:1303.04(2+), RT:26.78, Y-score:84.80

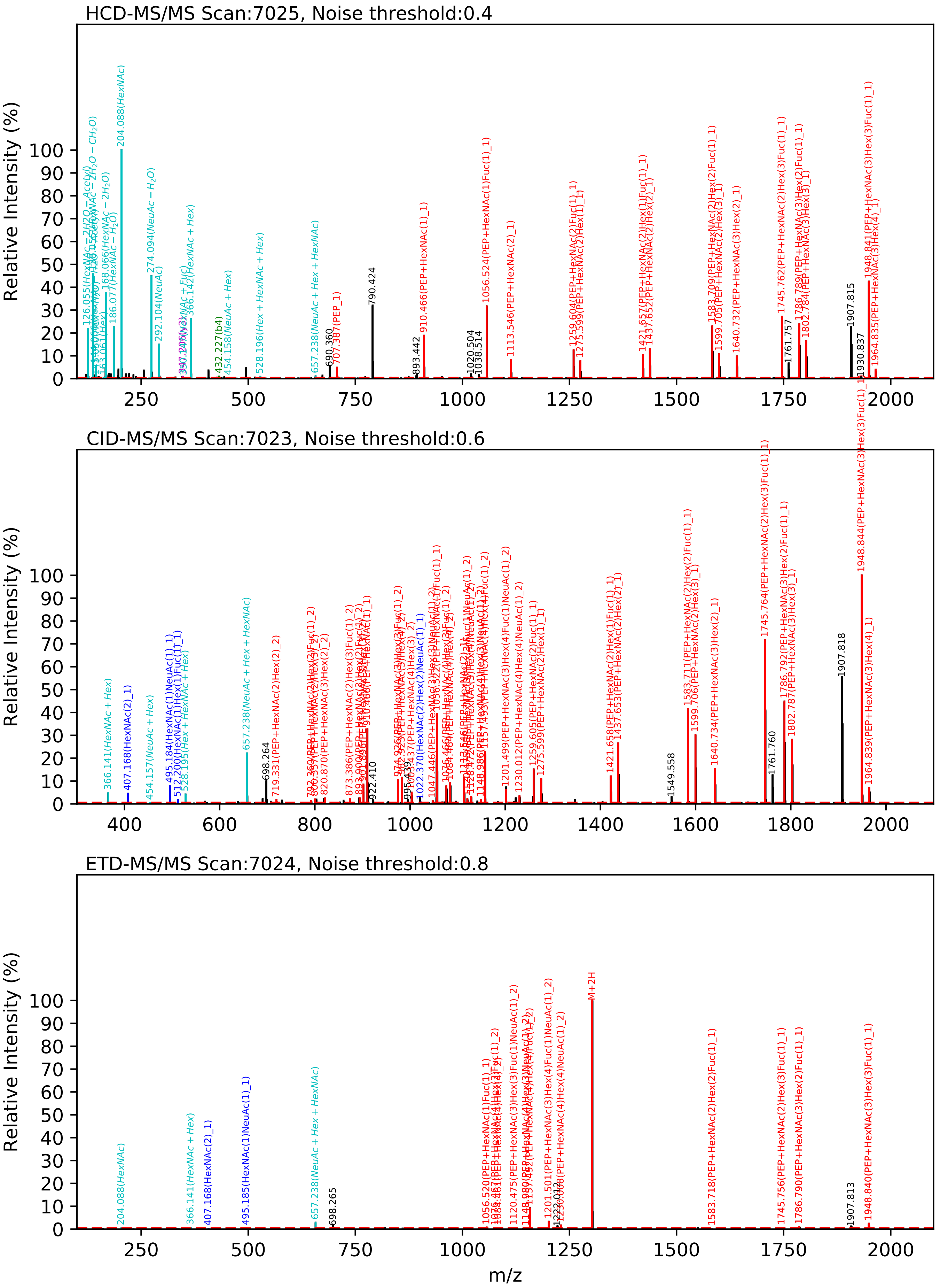

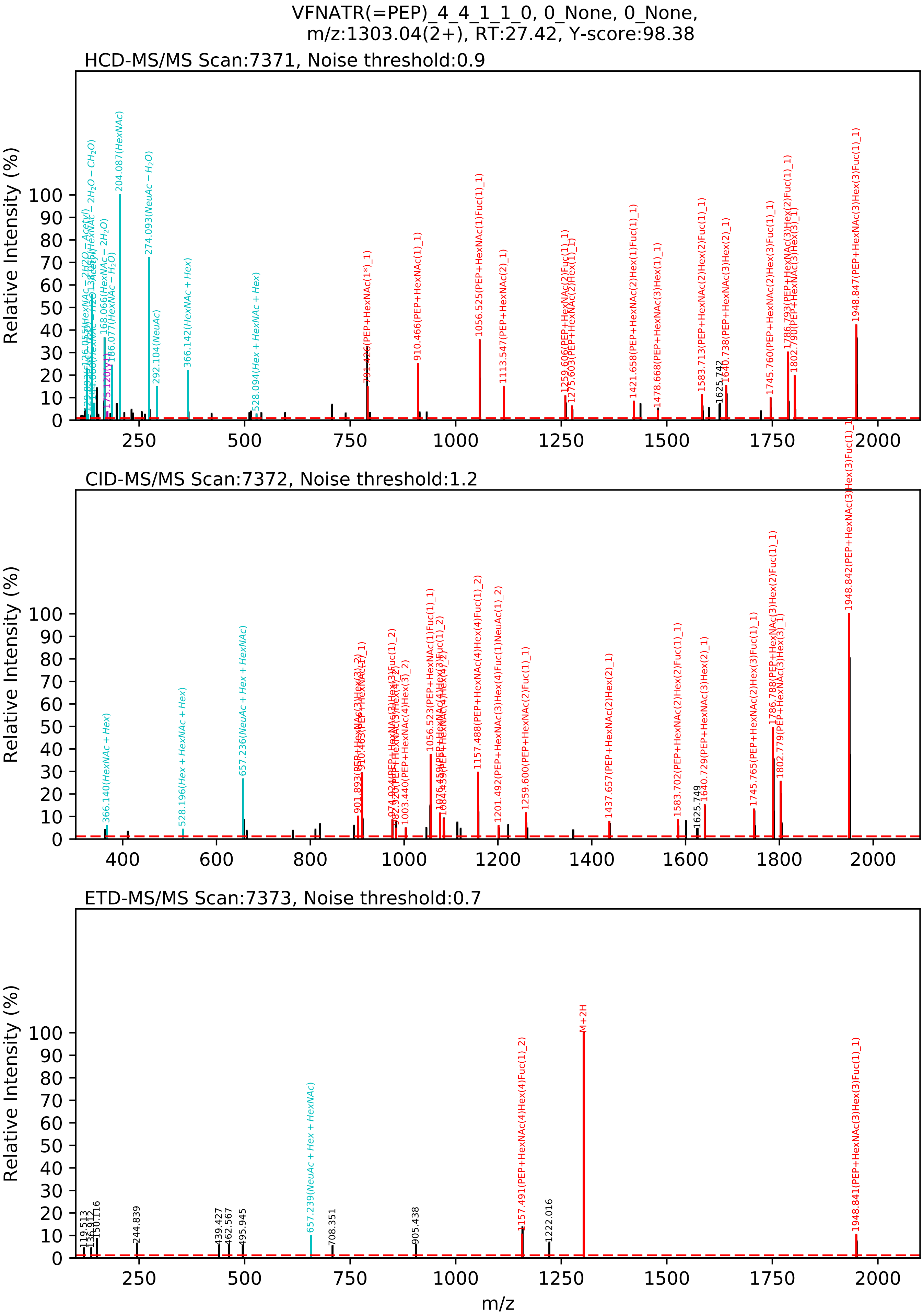

VFNATR(=PEP)_4_4_1_1_0, 0_None, 0_None,
m/z:1303.04(2+), RT:27.42, Y-score:98.38
HCD-MS/MS Scan:7371, Noise threshold:0.9
Relative Intensity (%)
204.087(HexNAc)
274.093(NeuAc − H2O)
292.104(NeuAc)
366.142(HexNAc + Hex)
528.094(Hex + HexNAc + Hex)
791.426(PEP+HexNAc(1*)_1)
910.466(PEP+HexNAc(1)_1)
1056.525(PEP+HexNAc(1)Fuc(1)_1)
1113.547(PEP+HexNAc(2)_1)
1421.658(PEP+HexNAc(2)Hex(1)Fuc(1)_1)
1478.668(PEP+HexNAc(3)Hex(1)_1)
1583.713(PEP+HexNAc(2)Hex(2)Fuc(1)_1)
1625.742
1640.738(PEP+HexNAc(3)Hex(2)_1)
1745.760(PEP+HexNAc(2)Hex(3)Fuc(1)_1)
1802.790(PEP+HexNAc(3)Hex(3)_1)
1948.847(PEP+HexNAc(3)Hex(3)Fuc(1)_1)
CID-MS/MS Scan:7372, Noise threshold:1.2
366.140(HexNAc + Hex)
528.196(Hex + HexNAc + Hex)
657.236(NeuAc + Hex + HexNAc)
910.465(PEP+HexNAc(1)_1)
1003.440(PEP+HexNAc(4)Hex(3)_2)
1056.523(PEP+HexNAc(1)Fuc(1)_1)
1157.488(PEP+HexNAc(4)Hex(4)Fuc(1)_2)
1201.492(PEP+HexNAc(3)Hex(4)Fuc(1)NeuAc(1)_2)
1259.600(PEP+HexNAc(2)Fuc(1)_1)
1437.657(PEP+HexNAc(2)Hex(2)_1)
1583.702(PEP+HexNAc(2)Hex(2)Fuc(1)_1)
1625.749
1640.729(PEP+HexNAc(3)Hex(2)_1)
1745.765(PEP+HexNAc(2)Hex(3)Fuc(1)_1)
1786.788(PEP+HexNAc(3)Hex(2)Fuc(1)_1)
1802.779(PEP+HexNAc(3)Hex(3)_1)
1948.842(PEP+HexNAc(3)Hex(3)Fuc(1)_1)
ETD-MS/MS Scan:7373, Noise threshold:0.7
119.513
150.116
244.839
439.427
462.567
495.945
657.239(NeuAc + Hex + HexNAc)
708.351
905.438
1157.491(PEP+HexNAc(4)Hex(4)Fuc(1)_2)
1222.016
M+2H
1948.841(PEP+HexNAc(3)Hex(3)Fuc(1)_1)
m/z

VFNATR(=PEP)_4_4_1_1_0, 0_None, 0_None,
m/z:1303.04(2+), RT:31.86, Y-score:95.76

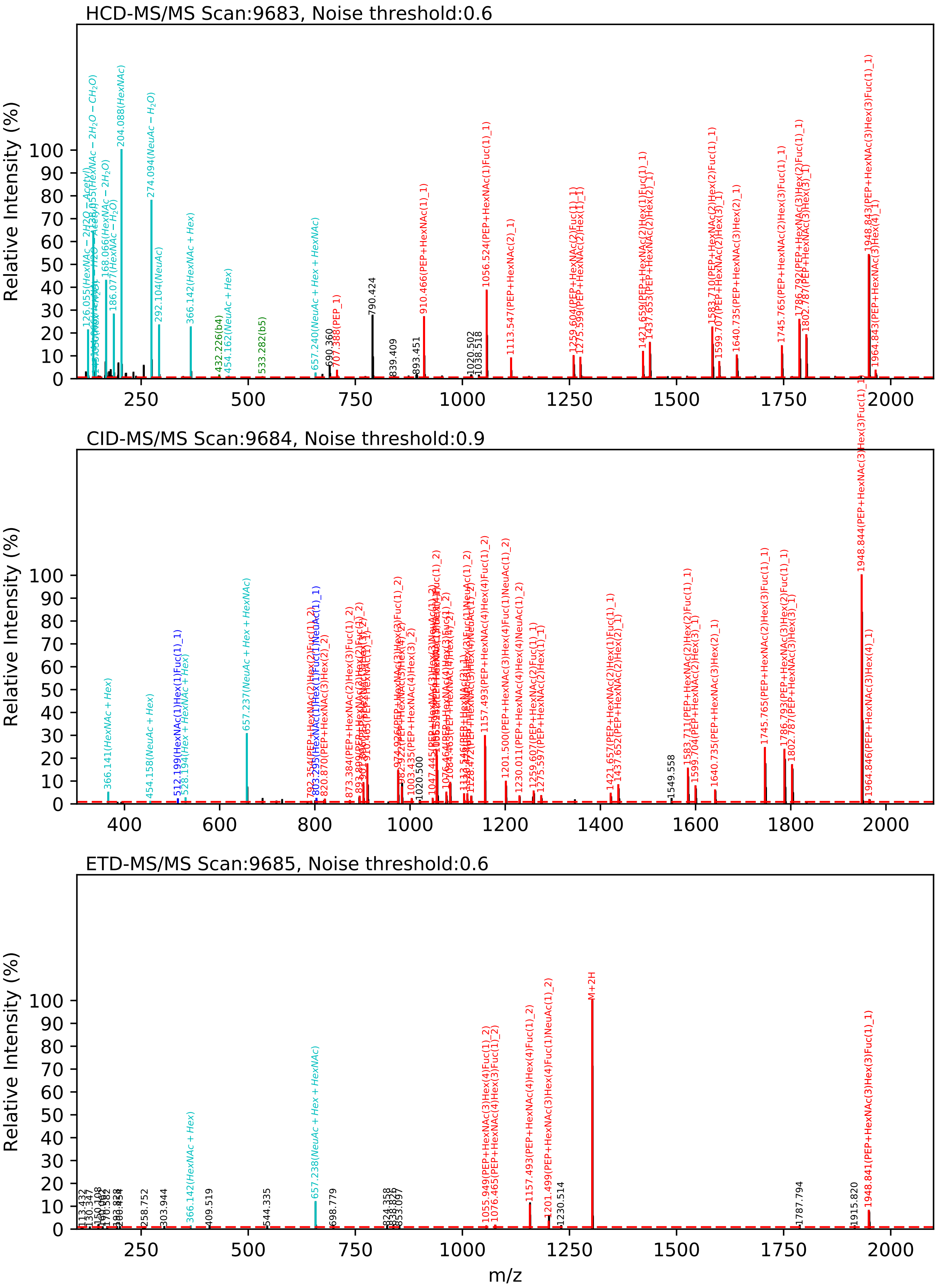

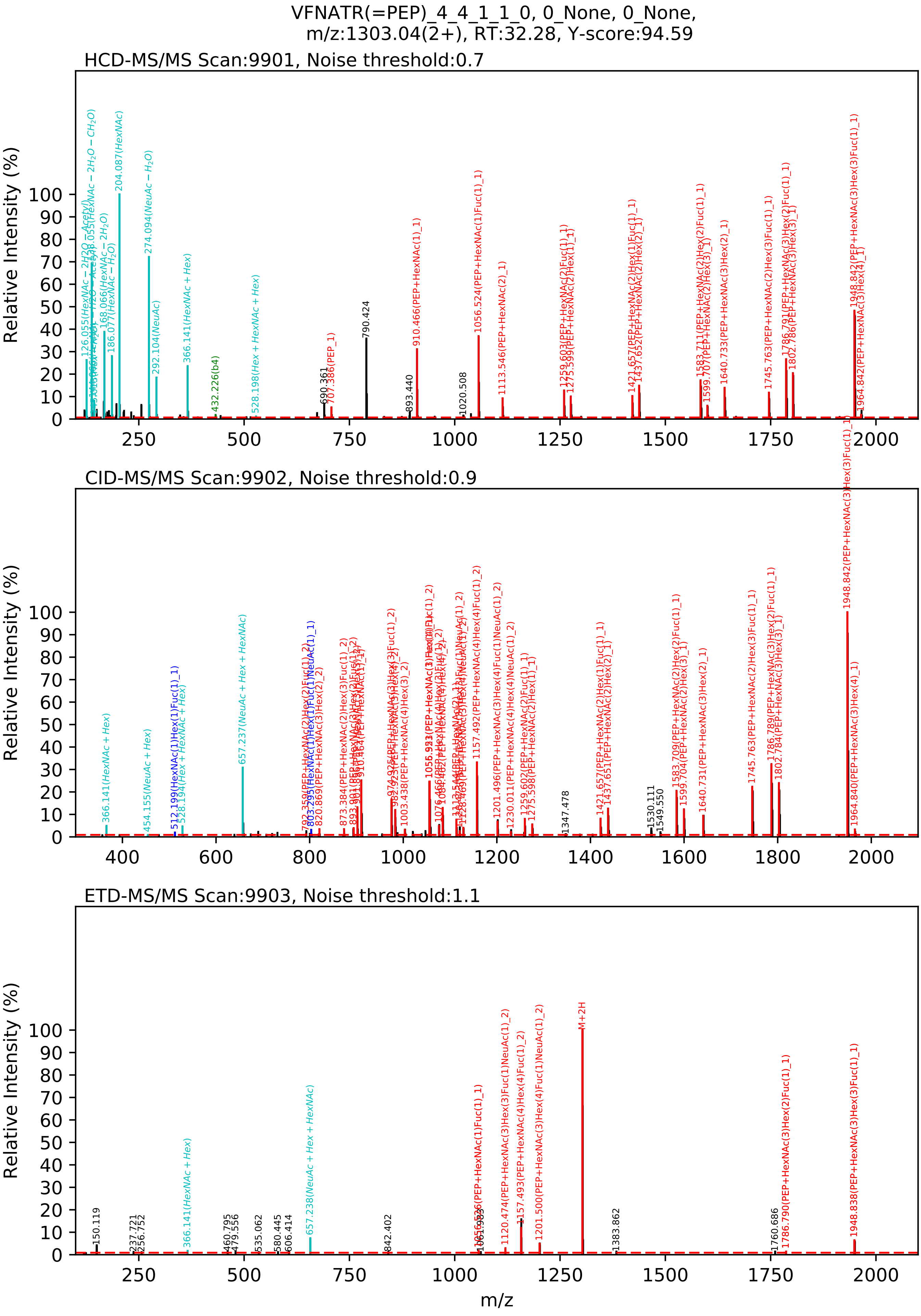

VFNATR(=PEP)_4_4_1_1_0, 0_None, 0_None,
m/z:1303.04(2+), RT:32.28, Y-score:94.59
HCD-MS/MS Scan:9901, Noise threshold:0.7
Relative Intensity (%)
204.087(HexNAc)
274.094(NeuAc − H2O)
292.104(NeuAc)
366.141(HexNAc + Hex)
432.226(b4)
528.198(Hex + HexNAc + Hex)
690.361
707.386(PEP_1)
790.424
893.440
910.466(PEP+HexNAc(1)_1)
1020.508
1056.524(PEP+HexNAc(1)Fuc(1)_1)
1113.546(PEP+HexNAc(2)_1)
1259.603(PEP+HexNAc(2)Fuc(1)_1)
1275.599(PEP+HexNAc(2)Hex(1)_1)
1421.657(PEP+HexNAc(2)Hex(1)Fuc(1)_1)
1437.652(PEP+HexNAc(2)Hex(2)_1)
1583.711(PEP+HexNAc(2)Hex(2)Fuc(1)_1)
1599.707(PEP+HexNAc(2)Hex(3)_1)
1640.733(PEP+HexNAc(3)Hex(2)_1)
1745.763(PEP+HexNAc(2)Hex(3)Fuc(1)_1)
1786.791(PEP+HexNAc(3)Hex(2)Fuc(1)_1)
1802.786(PEP+HexNAc(3)Hex(3)_1)
1948.842(PEP+HexNAc(3)Hex(3)Fuc(1)_1)
1964.842(PEP+HexNAc(3)Hex(4)_1)
CID-MS/MS Scan:9902, Noise threshold:0.9
Relative Intensity (%)
366.141(HexNAc + Hex)
454.155(NeuAc + Hex)
512.199(HexNAc(1)Hex(1)Fuc(1)_1)
528.194(Hex + HexNAc + Hex)
657.237(NeuAc + Hex + HexNAc)
820.869(PEP+HexNAc(3)Hex(2)_2)
873.384(PEP+HexNAc(2)Hex(3)Fuc(1)_2)
910.464(PEP+HexNAc(1)_1)
1003.438(PEP+HexNAc(4)Hex(3)_2)
1157.492(PEP+HexNAc(4)Hex(4)Fuc(1)_2)
1201.496(PEP+HexNAc(3)Hex(4)Fuc(1)NeuAc(1)_2)
1230.011(PEP+HexNAc(4)Hex(4)NeuAc(1)_2)
1259.602(PEP+HexNAc(2)Fuc(1)_1)
1275.598(PEP+HexNAc(2)Hex(1)_1)
1347.478
1421.657(PEP+HexNAc(2)Hex(1)Fuc(1)_1)
1437.651(PEP+HexNAc(2)Hex(2)_1)
1530.111
1549.550
1583.709(PEP+HexNAc(2)Hex(2)Fuc(1)_1)
1599.704(PEP+HexNAc(2)Hex(3)_1)
1640.731(PEP+HexNAc(3)Hex(2)_1)
1745.763(PEP+HexNAc(2)Hex(3)Fuc(1)_1)
1786.789(PEP+HexNAc(3)Hex(2)Fuc(1)_1)
1802.784(PEP+HexNAc(3)Hex(3)_1)
1948.842(PEP+HexNAc(3)Hex(3)Fuc(1)_1)
1964.840(PEP+HexNAc(3)Hex(4)_1)
ETD-MS/MS Scan:9903, Noise threshold:1.1
Relative Intensity (%)
150.119
237.721
256.752
366.141(HexNAc + Hex)
460.795
479.556
535.062
580.445
606.414
657.238(NeuAc + Hex + HexNAc)
842.402
1056.526(PEP+HexNAc(1)Fuc(1)_1)
1120.474(PEP+HexNAc(3)Hex(3)Fuc(1)NeuAc(1)_2)
1157.493(PEP+HexNAc(4)Hex(4)Fuc(1)_2)
1201.500(PEP+HexNAc(3)Hex(4)Fuc(1)NeuAc(1)_2)
M+2H
1383.862
1760.686
1786.790(PEP+HexNAc(3)Hex(2)Fuc(1)_1)
1948.838(PEP+HexNAc(3)Hex(3)Fuc(1)_1)
m/z

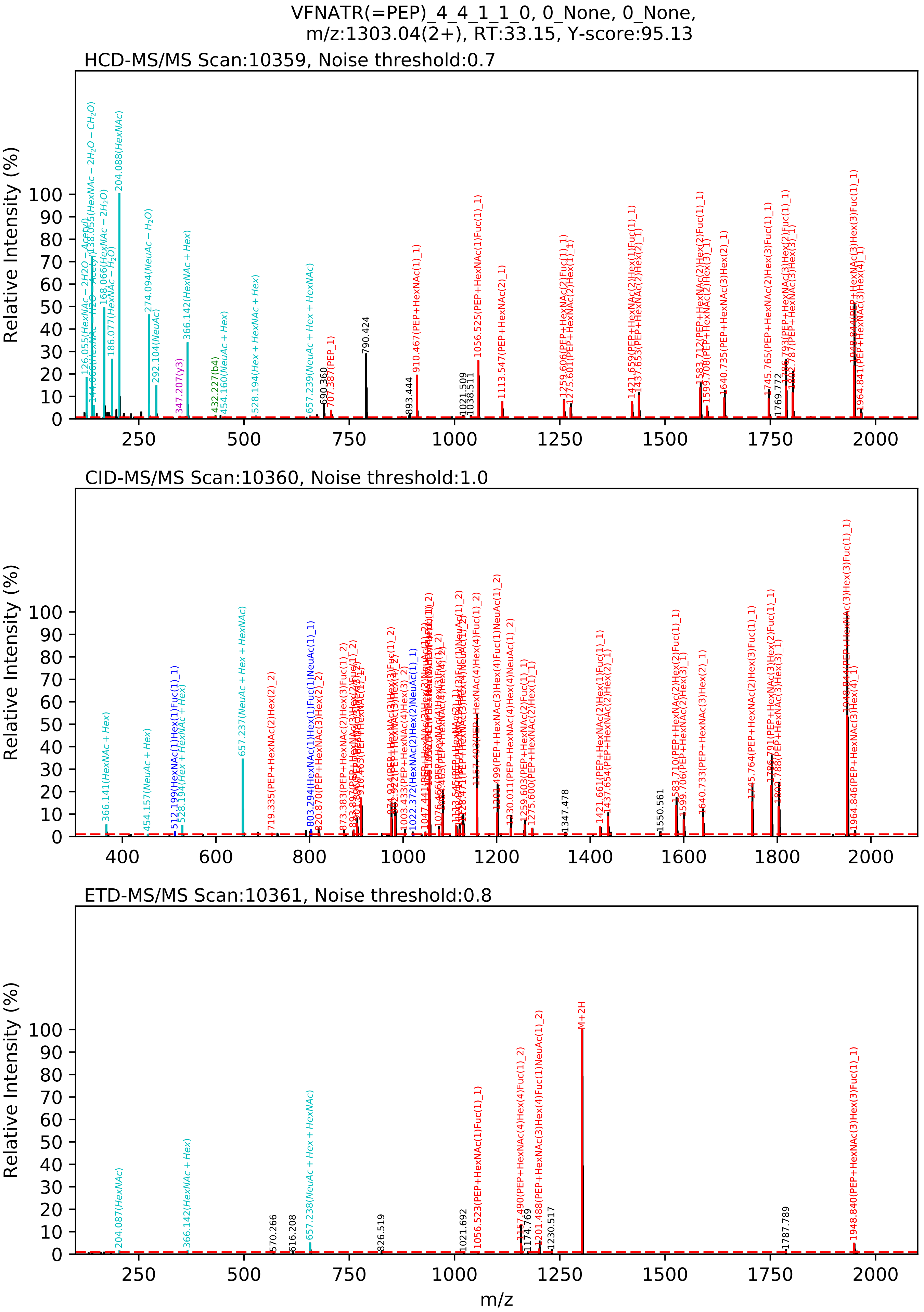

VFNATR(=PEP)_4_4_1_1_0, 0_None, 0_None,
m/z:1303.04(2+), RT:33.15, Y-score:95.13
HCD-MS/MS Scan:10359, Noise threshold:0.7
CID-MS/MS Scan:10360, Noise threshold:1.0
ETD-MS/MS Scan:10361, Noise threshold:0.8
Relative Intensity (%)
m/z
204.088(HexNAc)
274.094(NeuAc − H2O)
292.104(NeuAc)
366.142(HexNAc + Hex)
347.207(y3)
432.227(b4)
454.160(NeuAc + Hex)
528.194(Hex + HexNAc + Hex)
657.239(NeuAc + Hex + HexNAc)
690.360
707.387(PEP_1)
790.424
893.444
910.467(PEP+HexNAc(1)_1)
1021.509
1038.511
1056.525(PEP+HexNAc(1)Fuc(1)_1)
1113.547(PEP+HexNAc(2)_1)
1259.606(PEP+HexNAc(2)Fuc(1)_1)
1275.601(PEP+HexNAc(2)Hex(1)_1)
1421.659(PEP+HexNAc(2)Hex(1)Fuc(1)_1)
1437.653(PEP+HexNAc(2)Hex(2)_1)
1583.712(PEP+HexNAc(2)Hex(2)Fuc(1)_1)
1599.708(PEP+HexNAc(2)Hex(3)_1)
1640.735(PEP+HexNAc(3)Hex(2)_1)
1745.765(PEP+HexNAc(2)Hex(3)Fuc(1)_1)
1769.772
1948.844(PEP+HexNAc(3)Hex(3)Fuc(1)_1)
1964.841(PEP+HexNAc(3)Hex(4)_1)
366.141(HexNAc + Hex)
454.157(NeuAc + Hex)
512.199(HexNAc(1)Hex(1)Fuc(1)_1)
528.194(Hex + HexNAc + Hex)
657.237(NeuAc + Hex + HexNAc)
719.335(PEP+HexNAc(2)Hex(2)_2)
803.294(HexNAc(1)Hex(1)Fuc(1)NeuAc(1)_1)
820.870(PEP+HexNAc(3)Hex(2)_2)
873.383(PEP+HexNAc(2)Hex(3)Fuc(1)_2)
1022.372(HexNAc(2)Hex(2)NeuAc(1)_1)
1003.433(PEP+HexNAc(4)Hex(3)_2)
1157.493(PEP+HexNAc(4)Hex(4)Fuc(1)_2)
1201.499(PEP+HexNAc(3)Hex(4)Fuc(1)NeuAc(1)_2)
1230.011(PEP+HexNAc(4)Hex(4)NeuAc(1)_2)
1259.603(PEP+HexNAc(2)Fuc(1)_1)
1275.600(PEP+HexNAc(2)Hex(1)_1)
1347.478
1421.661(PEP+HexNAc(2)Hex(1)Fuc(1)_1)
1437.654(PEP+HexNAc(2)Hex(2)_1)
1550.561
1583.710(PEP+HexNAc(2)Hex(2)Fuc(1)_1)
1599.706(PEP+HexNAc(2)Hex(3)_1)
1640.733(PEP+HexNAc(3)Hex(2)_1)
1745.764(PEP+HexNAc(2)Hex(3)Fuc(1)_1)
1786.791(PEP+HexNAc(3)Hex(2)Fuc(1)_1)
1802.788(PEP+HexNAc(3)Hex(3)_1)
1948.844(PEP+HexNAc(3)Hex(3)Fuc(1)_1)
1964.846(PEP+HexNAc(3)Hex(4)_1)
204.087(HexNAc)
366.142(HexNAc + Hex)
570.266
616.208
657.238(NeuAc + Hex + HexNAc)
826.519
1021.692
1056.523(PEP+HexNAc(1)Fuc(1)_1)
1157.490(PEP+HexNAc(4)Hex(4)Fuc(1)_2)
1174.769
1201.488(PEP+HexNAc(3)Hex(4)Fuc(1)NeuAc(1)_2)
1230.517
M+2H
1787.789
1948.840(PEP+HexNAc(3)Hex(3)Fuc(1)_1)

VFNATR(=PEP)_4_4_1_1_0, 0_None, 0_None,
m/z:1303.04(2+), RT:33.21, Y-score:96.31

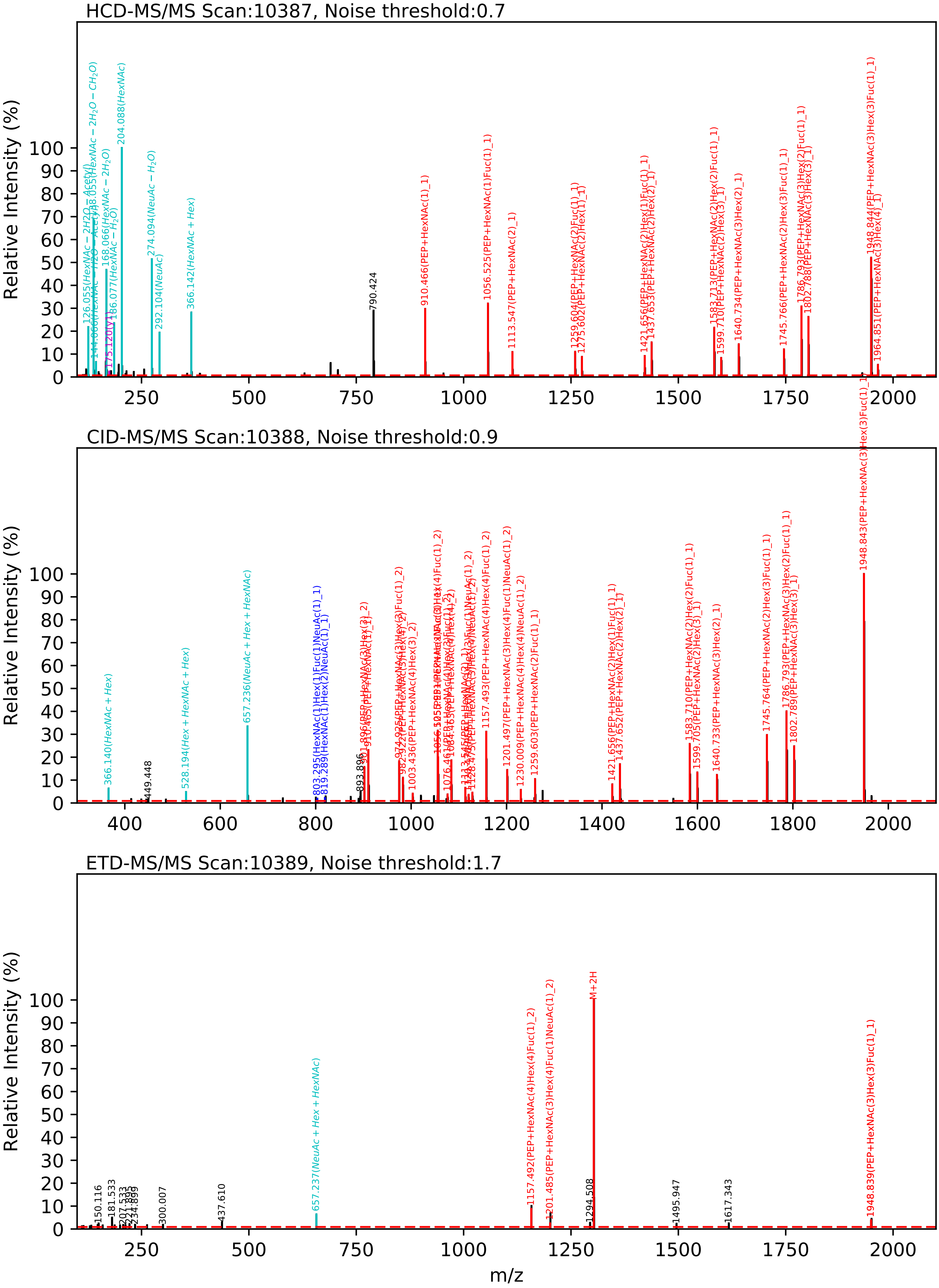

VFNATR(=PEP)_4_4_1_1_0, 0_None, 0_None,
m/z:869.03(3+), RT:26.81, Y-score:92.83

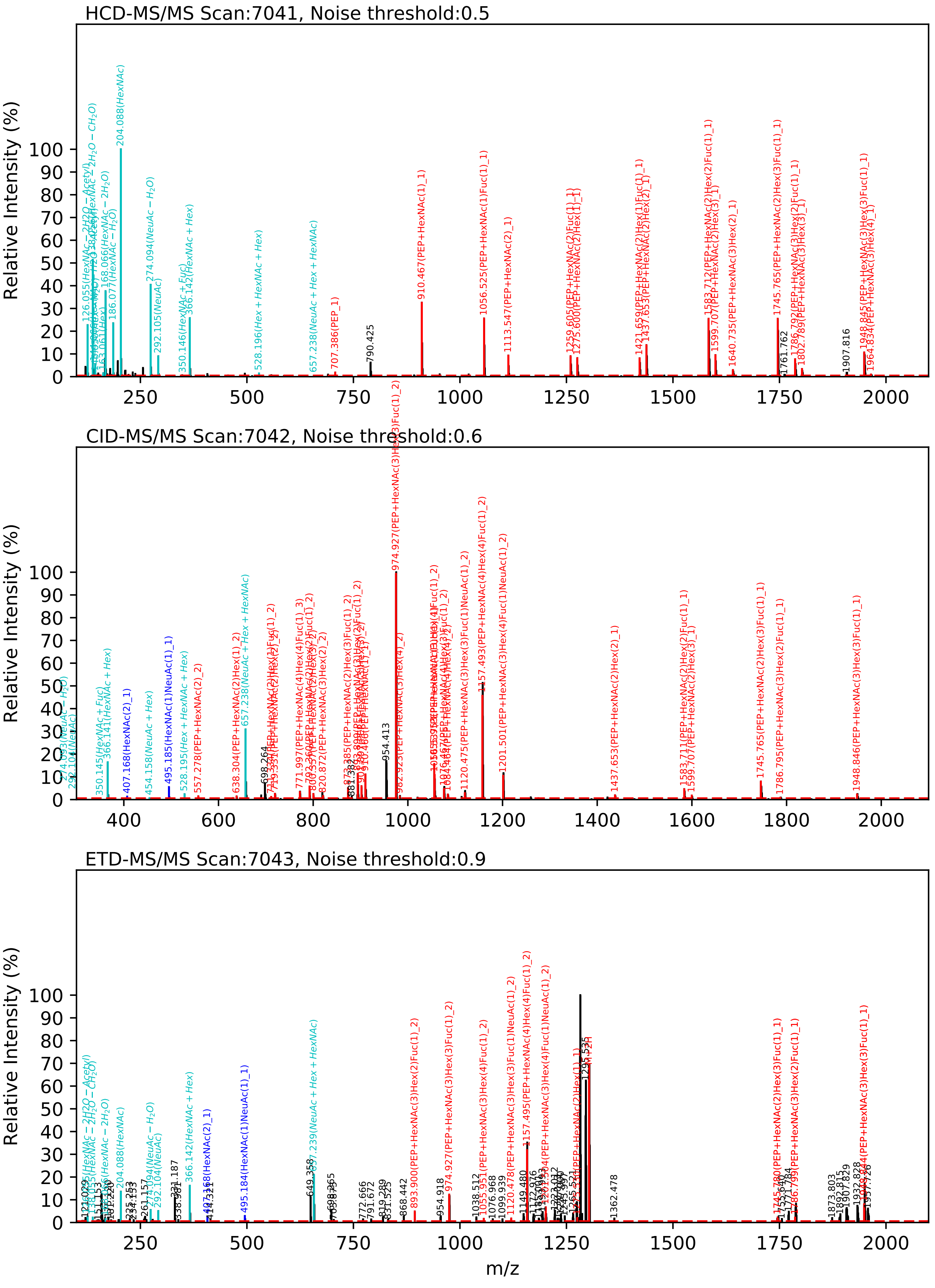

VFNATR(=PEP)_4_4_2_0_0, 0_None, 0_None,
m/z:1230.52(2+), RT:24.08, Y-score:88.34

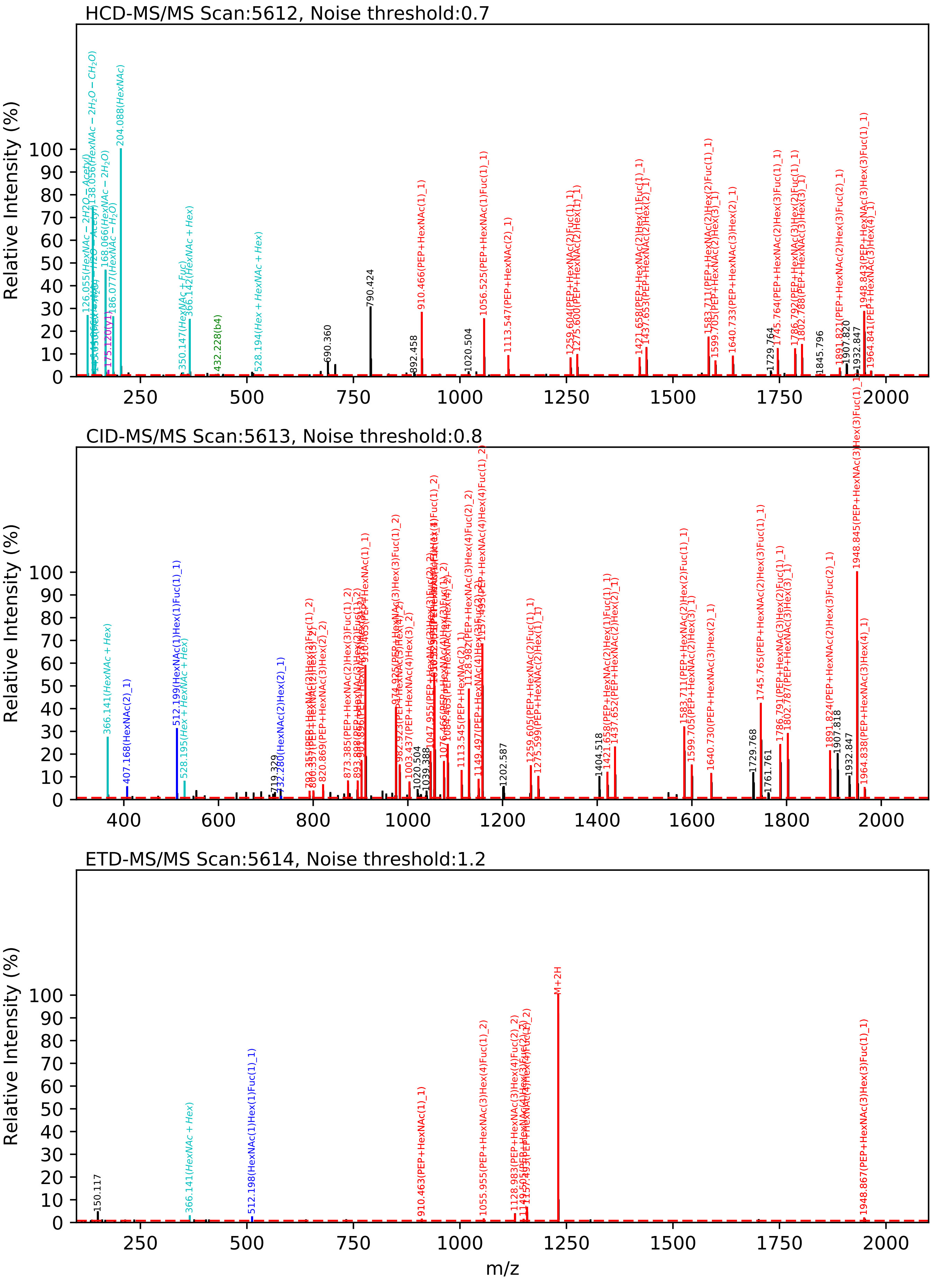

VFNATR(=PEP)_4_5_1_0_0, 0_None, 0_None,
m/z:1259.03(2+), RT:24.82, Y-score:88.61

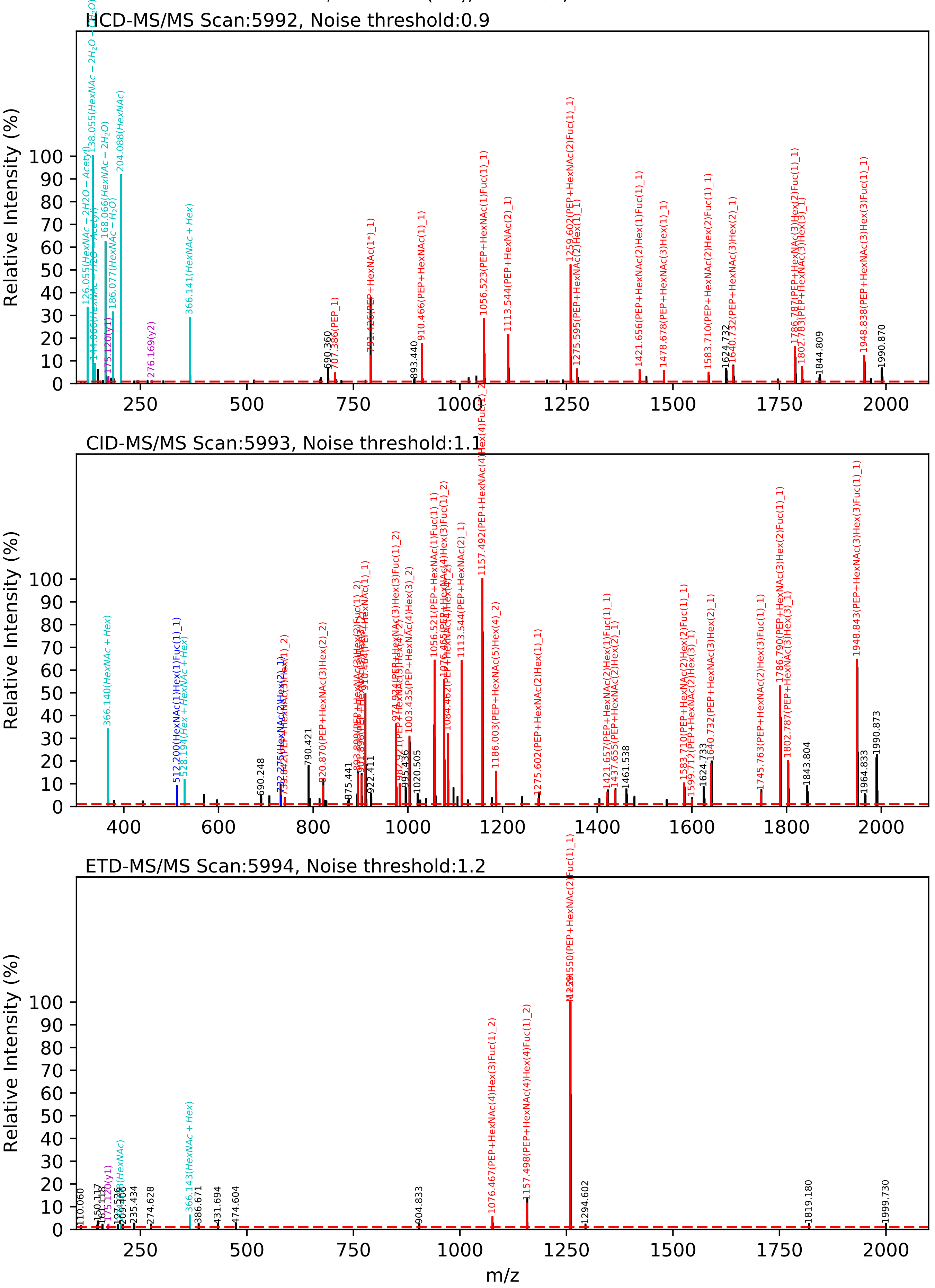

VFNATR(=PEP)_4_5_1_0_0, 0_None, 0_None,
m/z:1259.03(2+), RT:24.26, Y-score:90.27

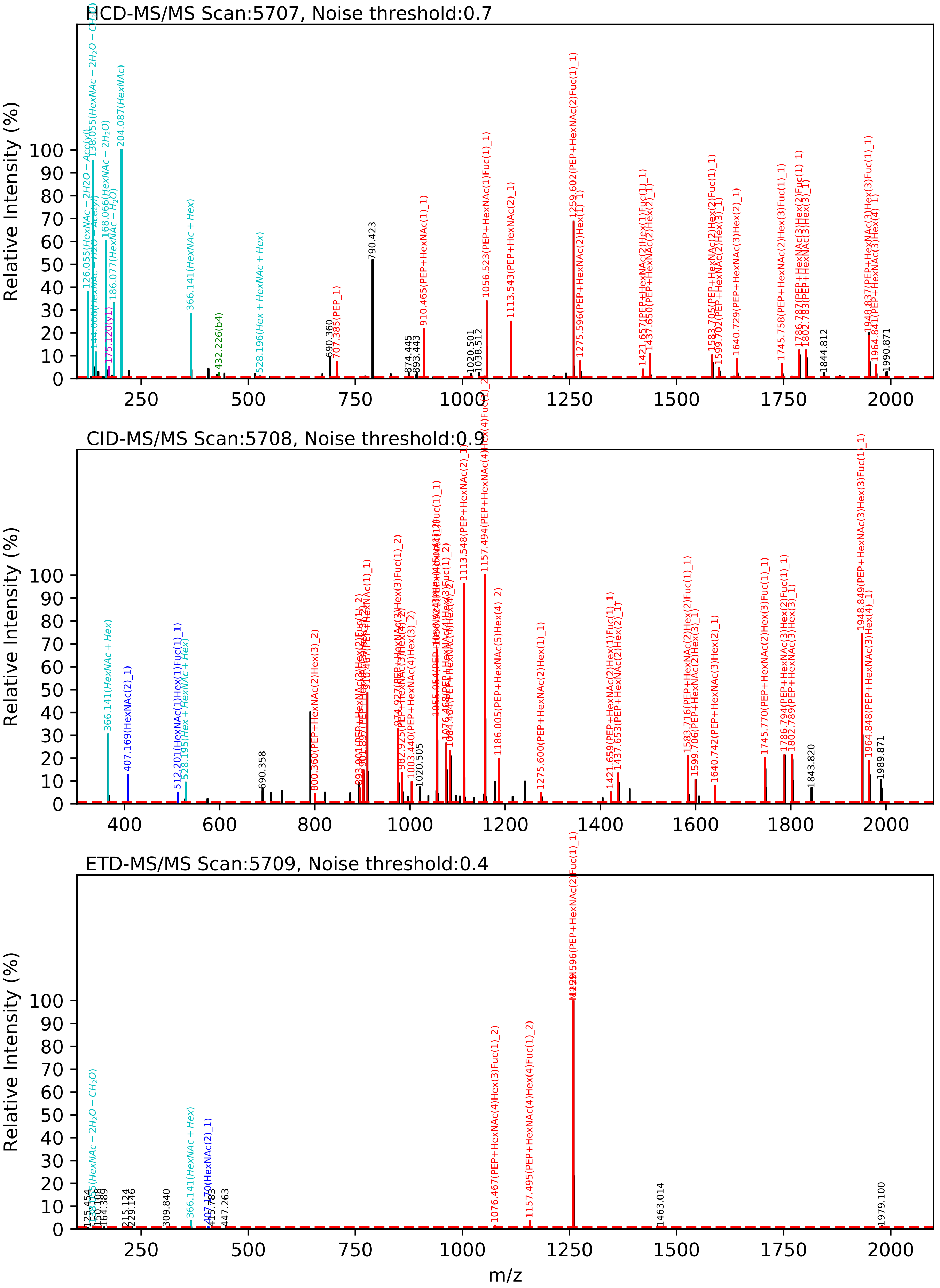

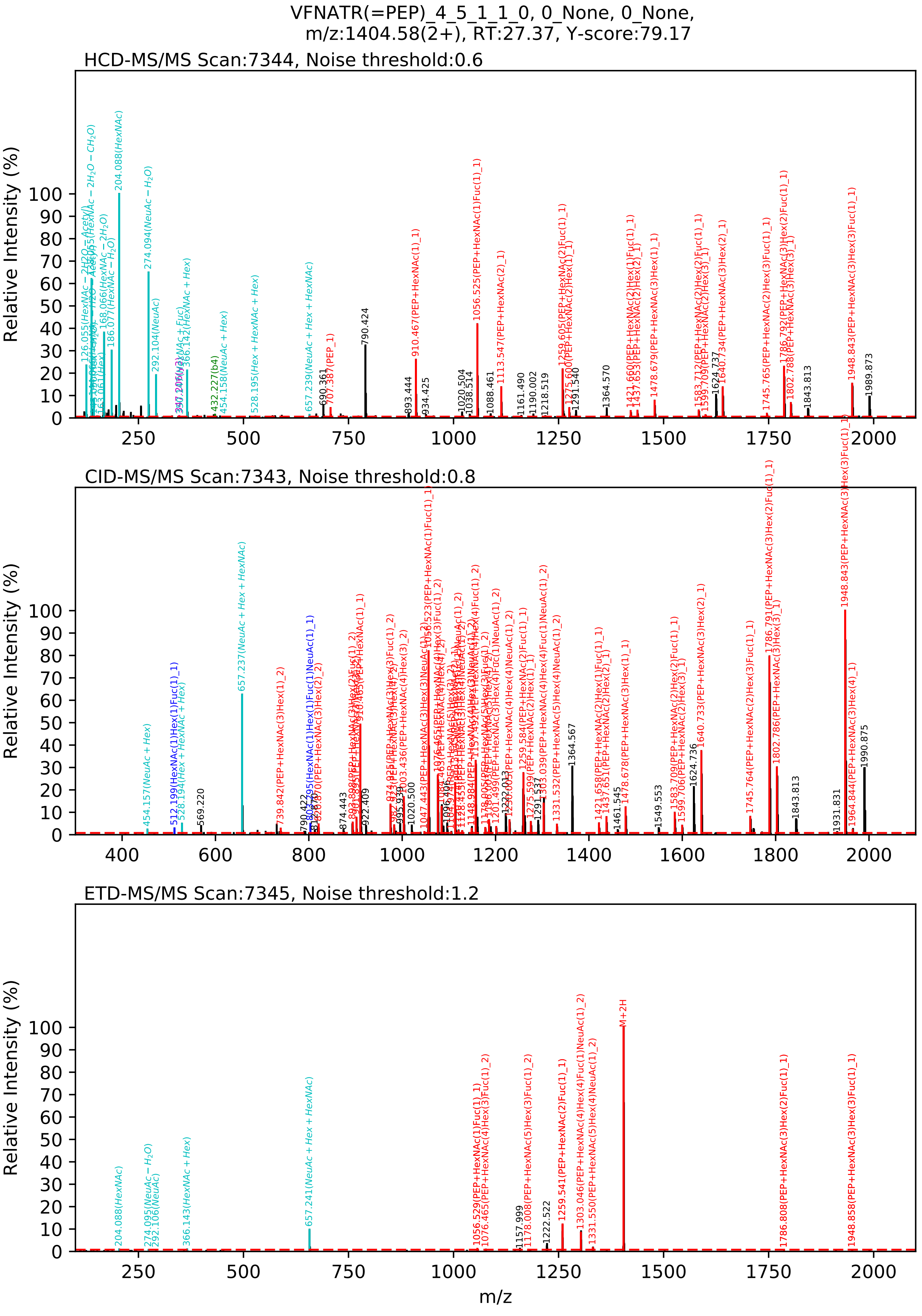
VFNATR(=PEP)_4_5_1_1_0, 0_None, 0_None,
m/z:1404.58(2+), RT:27.37, Y-score:79.17
HCD-MS/MS Scan:7344, Noise threshold:0.6
CID-MS/MS Scan:7343, Noise threshold:0.8
ETD-MS/MS Scan:7345, Noise threshold:1.2
Relative Intensity (%)
m/z
M+2H

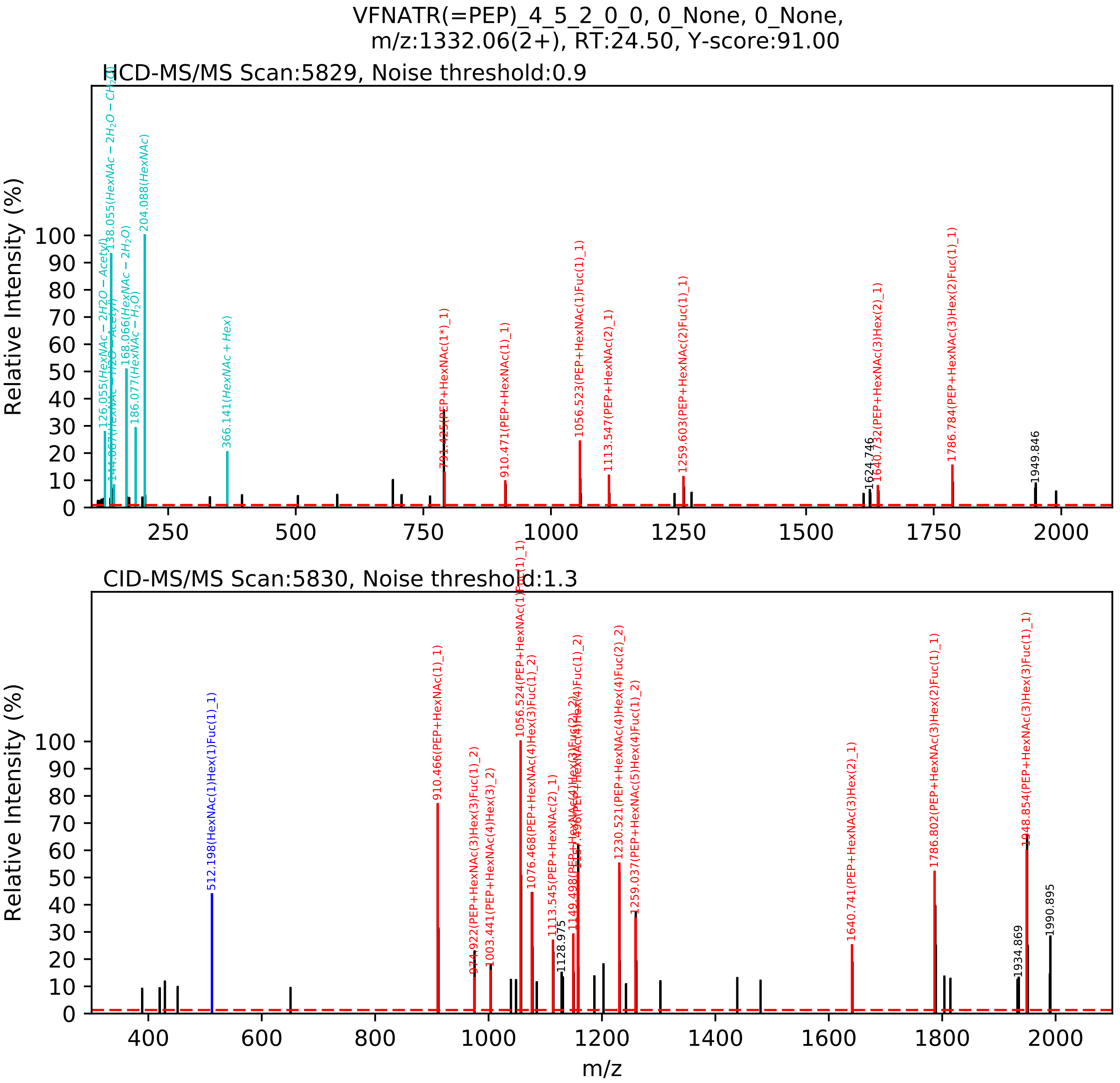

VFNATR(=PEP)_4_5_2_0_0, 0_None, 0_None,
m/z:1332.06(2+), RT:24.50, Y-score:91.00
HCD-MS/MS Scan:5829, Noise threshold:0.9
Relative Intensity (%)
204.088(HexNAc)
366.141(HexNAc + Hex)
791.425(PEP+HexNAc(1*)_1)
910.471(PEP+HexNAc(1)_1)
1056.523(PEP+HexNAc(1)Fuc(1)_1)
1113.547(PEP+HexNAc(2)_1)
1259.603(PEP+HexNAc(2)Fuc(1)_1)
1624.746
1640.732(PEP+HexNAc(3)Hex(2)_1)
1786.784(PEP+HexNAc(3)Hex(2)Fuc(1)_1)
1949.846
CID-MS/MS Scan:5830, Noise threshold:1.3
512.198(HexNAc(1)Hex(1)Fuc(1)_1)
910.466(PEP+HexNAc(1)_1)
974.922(PEP+HexNAc(3)Hex(3)Fuc(1)_2)
1003.441(PEP+HexNAc(4)Hex(3)_2)
1076.468(PEP+HexNAc(4)Hex(3)Fuc(1)_2)
1113.545(PEP+HexNAc(2)_1)
1128.975
1230.521(PEP+HexNAc(4)Hex(4)Fuc(2)_2)
1259.037(PEP+HexNAc(5)Hex(4)Fuc(1)_2)
1640.741(PEP+HexNAc(3)Hex(2)_1)
1786.802(PEP+HexNAc(3)Hex(2)Fuc(1)_1)
1934.869
1948.854(PEP+HexNAc(3)Hex(3)Fuc(1)_1)
1990.895
m/z

VFNATR(=PEP)_4_6_1_0_0, 0_None, 0_None,
m/z:1360.57(2+), RT:24.78, Y-score:77.64

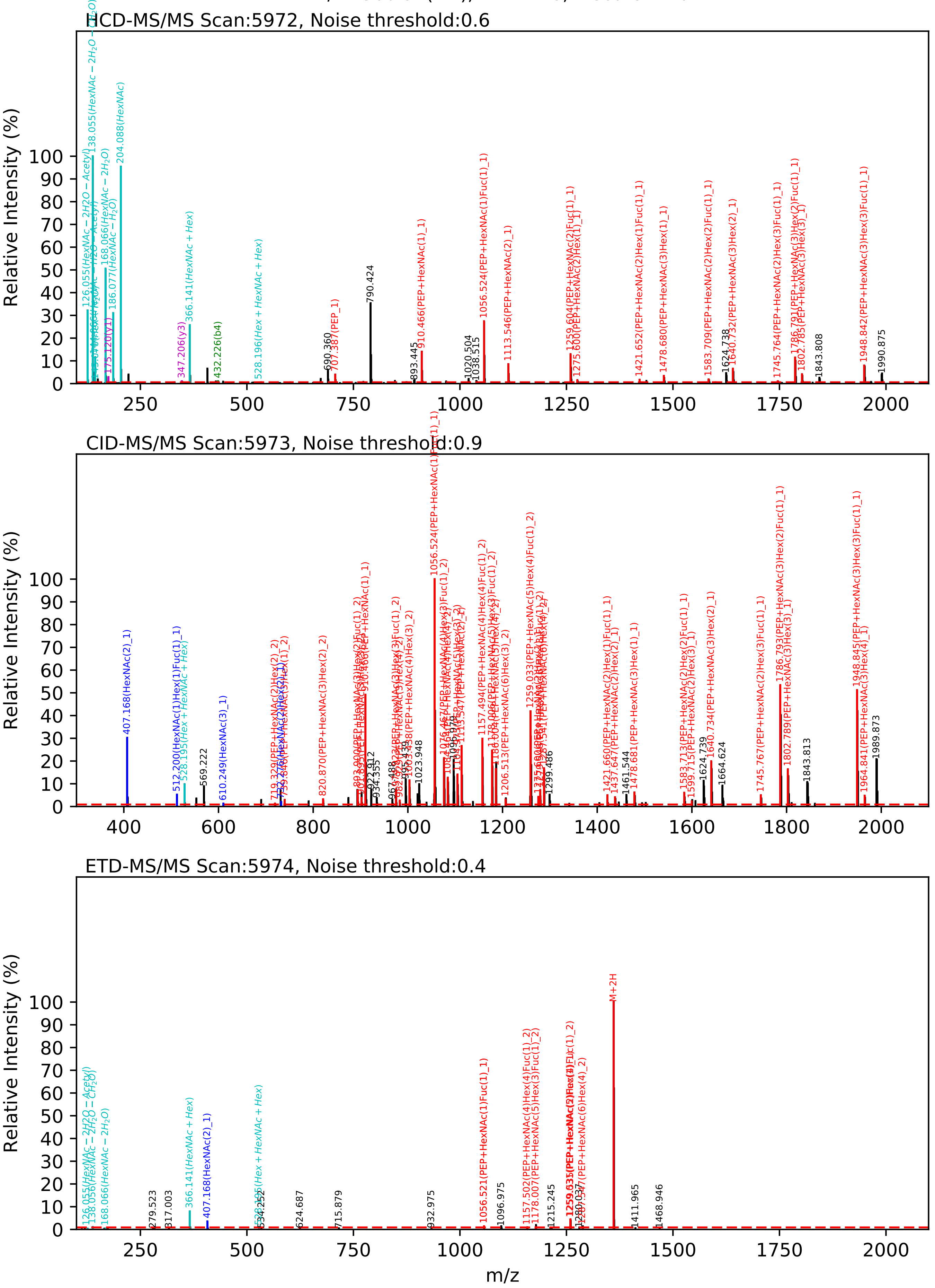

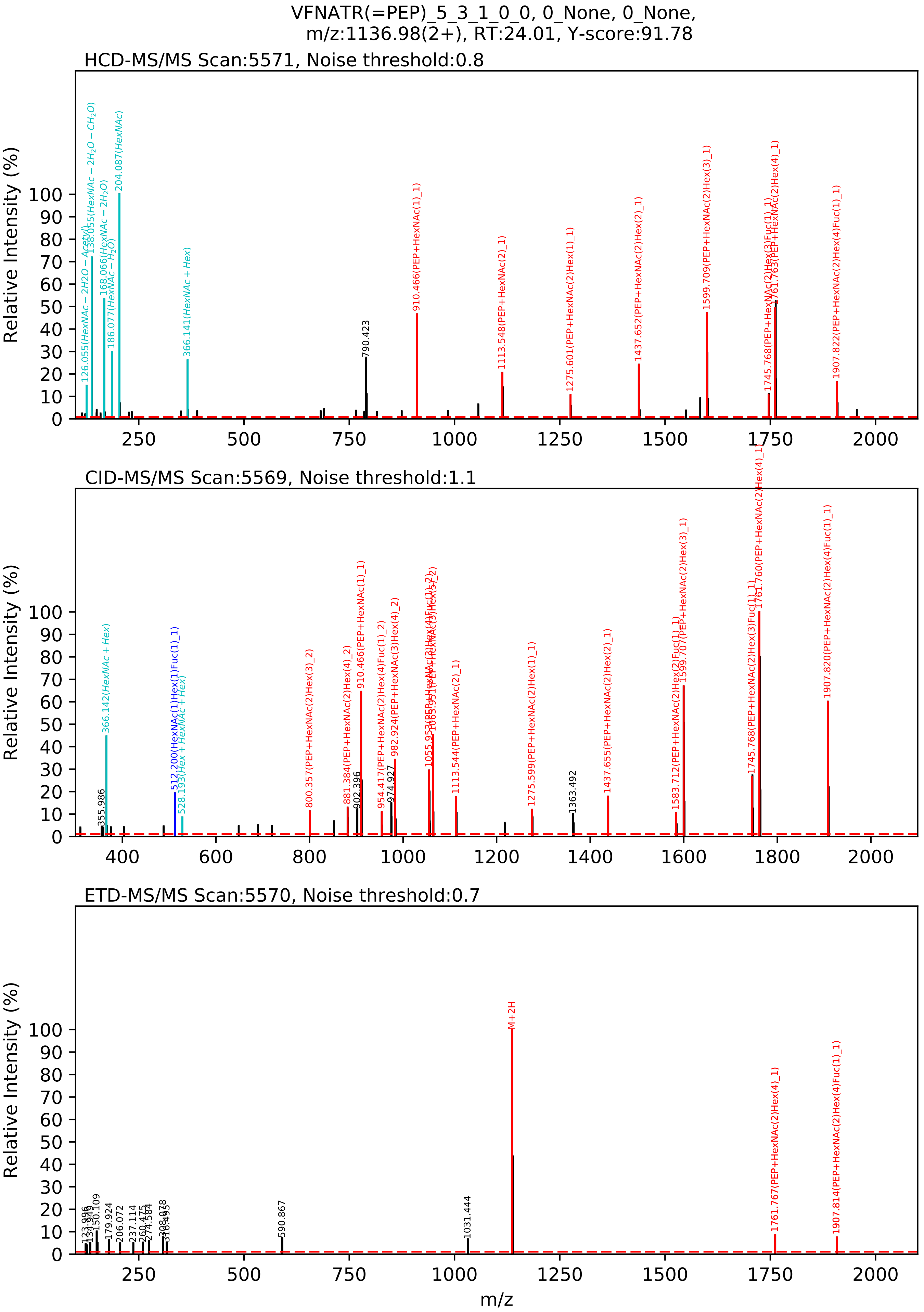
VFNATR(=PEP)_5_3_1_0_0, 0_None, 0_None,
m/z:1136.98(2+), RT:24.01, Y-score:91.78
HCD-MS/MS Scan:5571, Noise threshold:0.8
CID-MS/MS Scan:5569, Noise threshold:1.1
ETD-MS/MS Scan:5570, Noise threshold:0.7
Relative Intensity (%)
m/z
M+2H

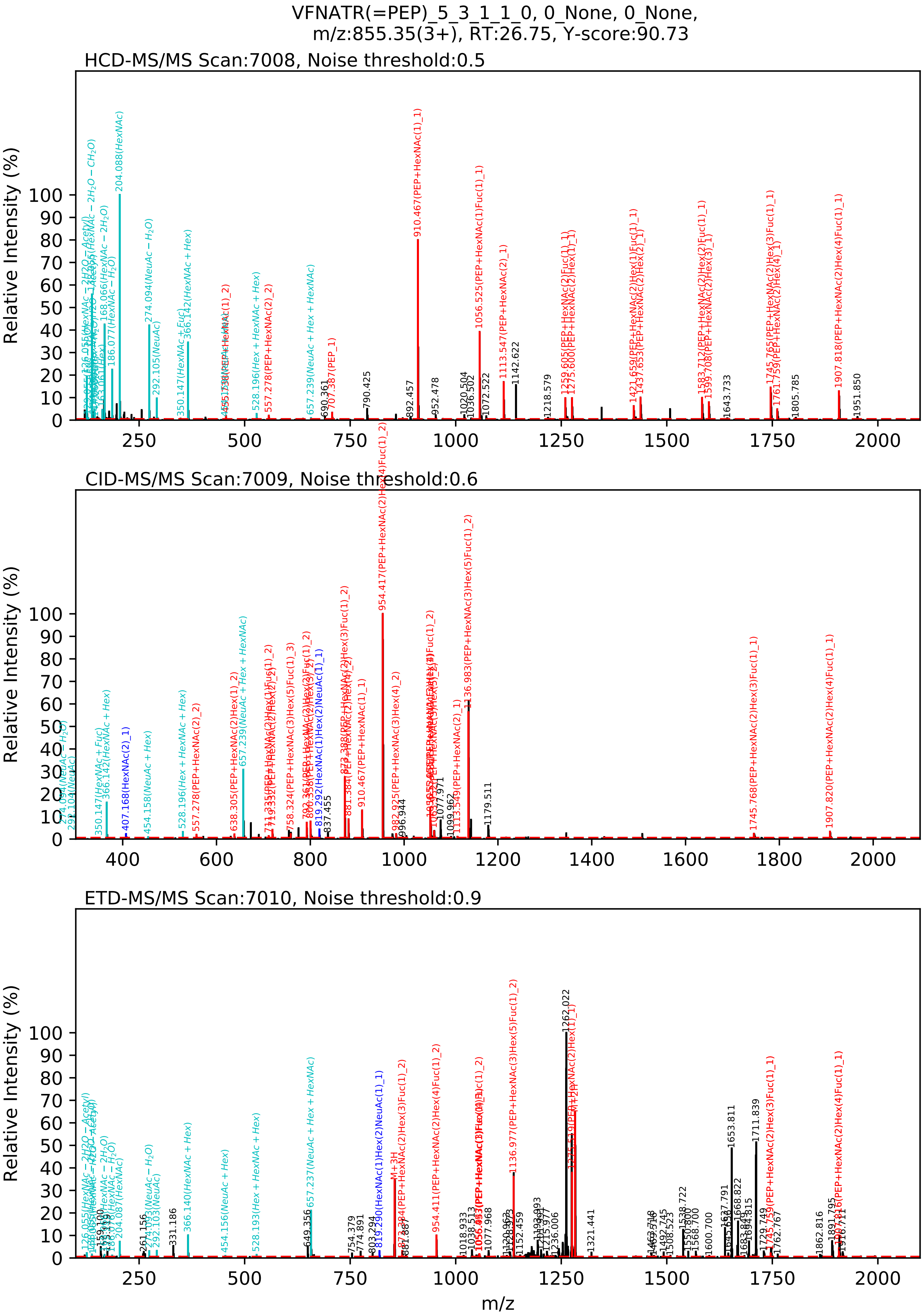

VFNATR(=PEP)_5_3_1_1_0, 0_None, 0_None,
m/z:855.35(3+), RT:26.75, Y-score:90.73
HCD-MS/MS Scan:7008, Noise threshold:0.5
CID-MS/MS Scan:7009, Noise threshold:0.6
ETD-MS/MS Scan:7010, Noise threshold:0.9
Relative Intensity (%)
m/z

VFNATR(=PEP)_5_3_1_1_0, 0_None, 0_None,
m/z:1282.52(2+), RT:26.76, Y-score:92.11

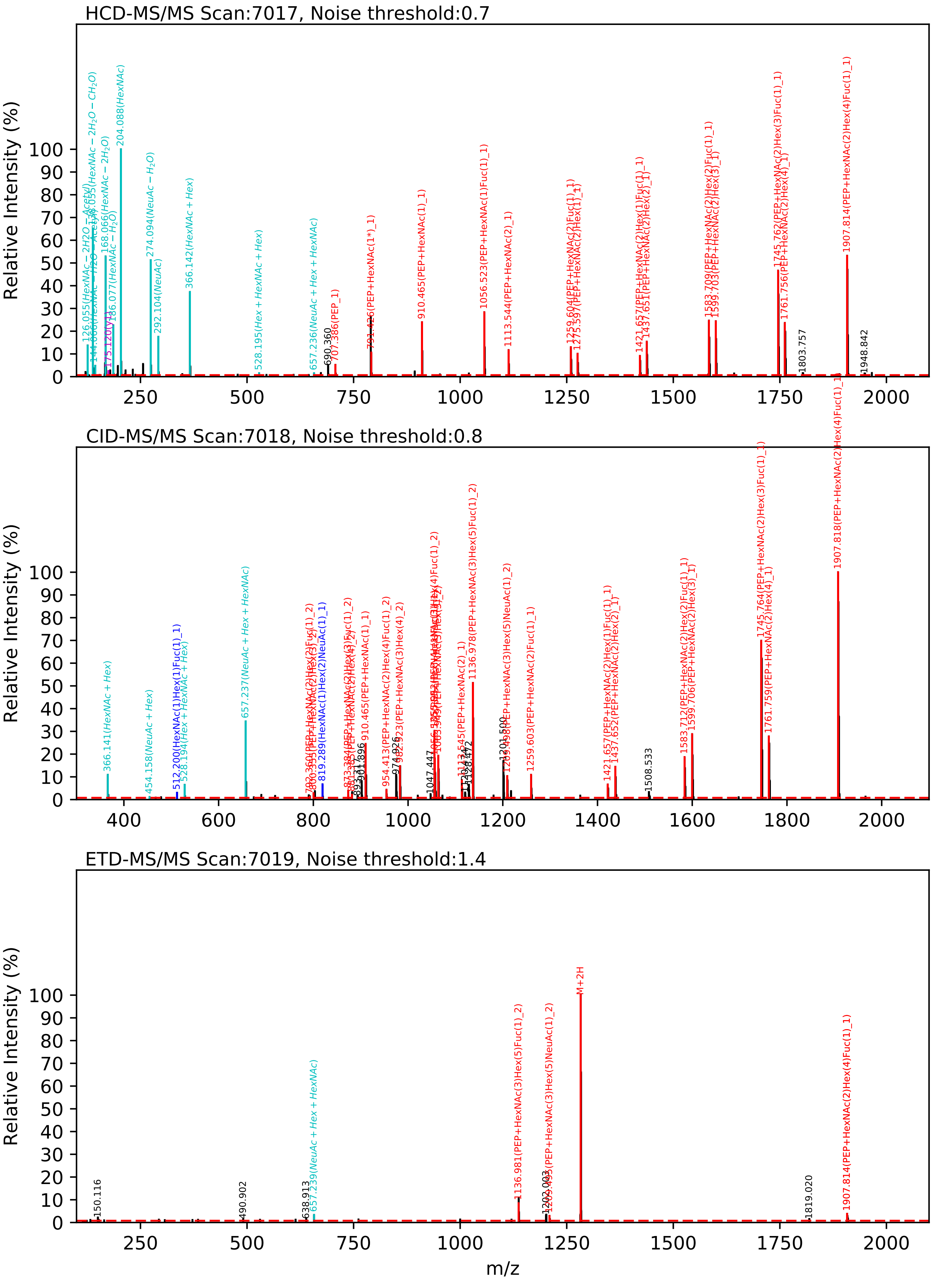

VFNATR(=PEP)_5_4_0_0_0, 0_None, 0_None,
m/z:1165.49(2+), RT:24.23, Y-score:83.56

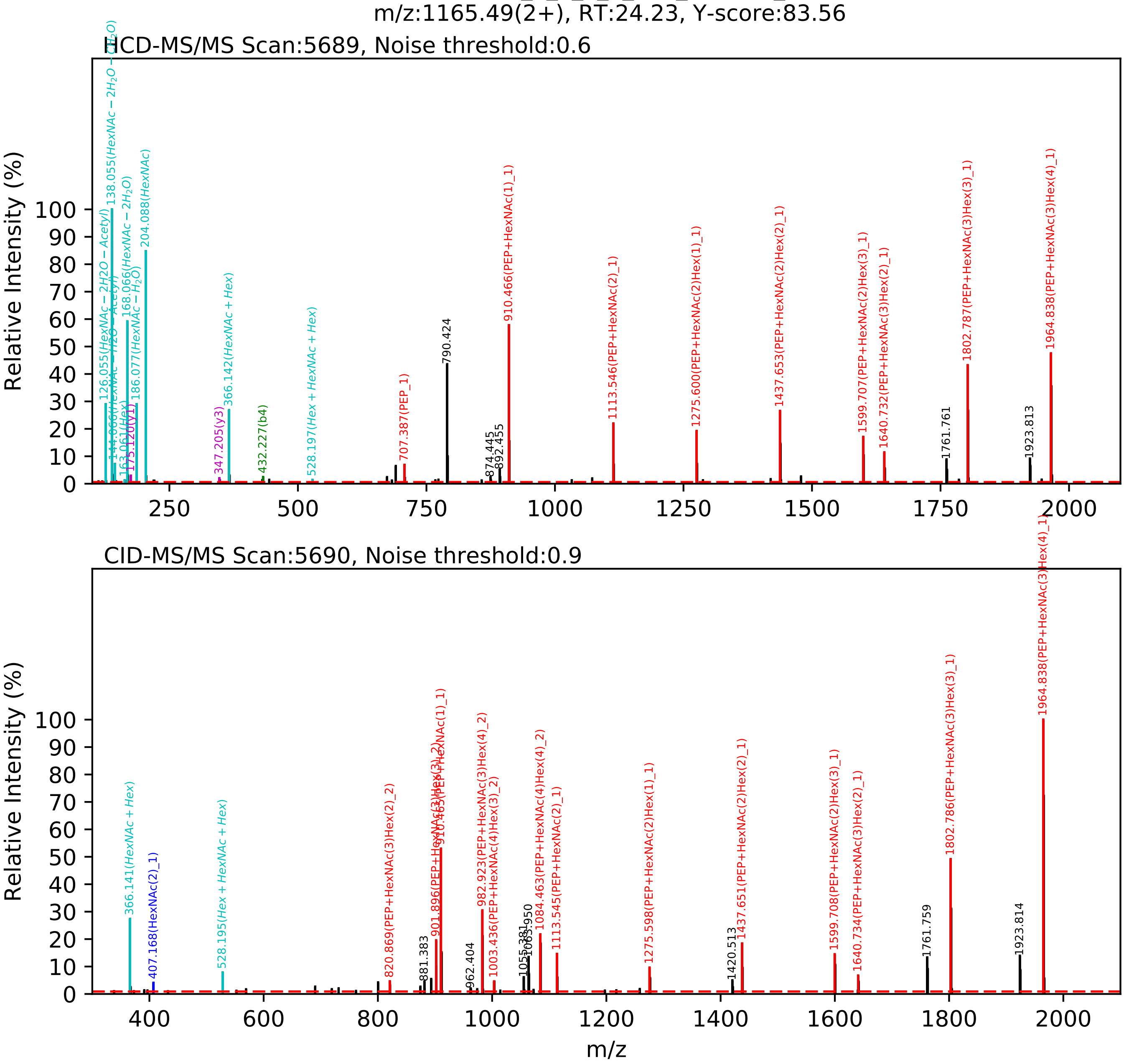

VFNATR(=PEP)_5_4_1_0_0, 0_None, 0_None,
m/z:1238.52(2+), RT:25.10, Y-score:75.25

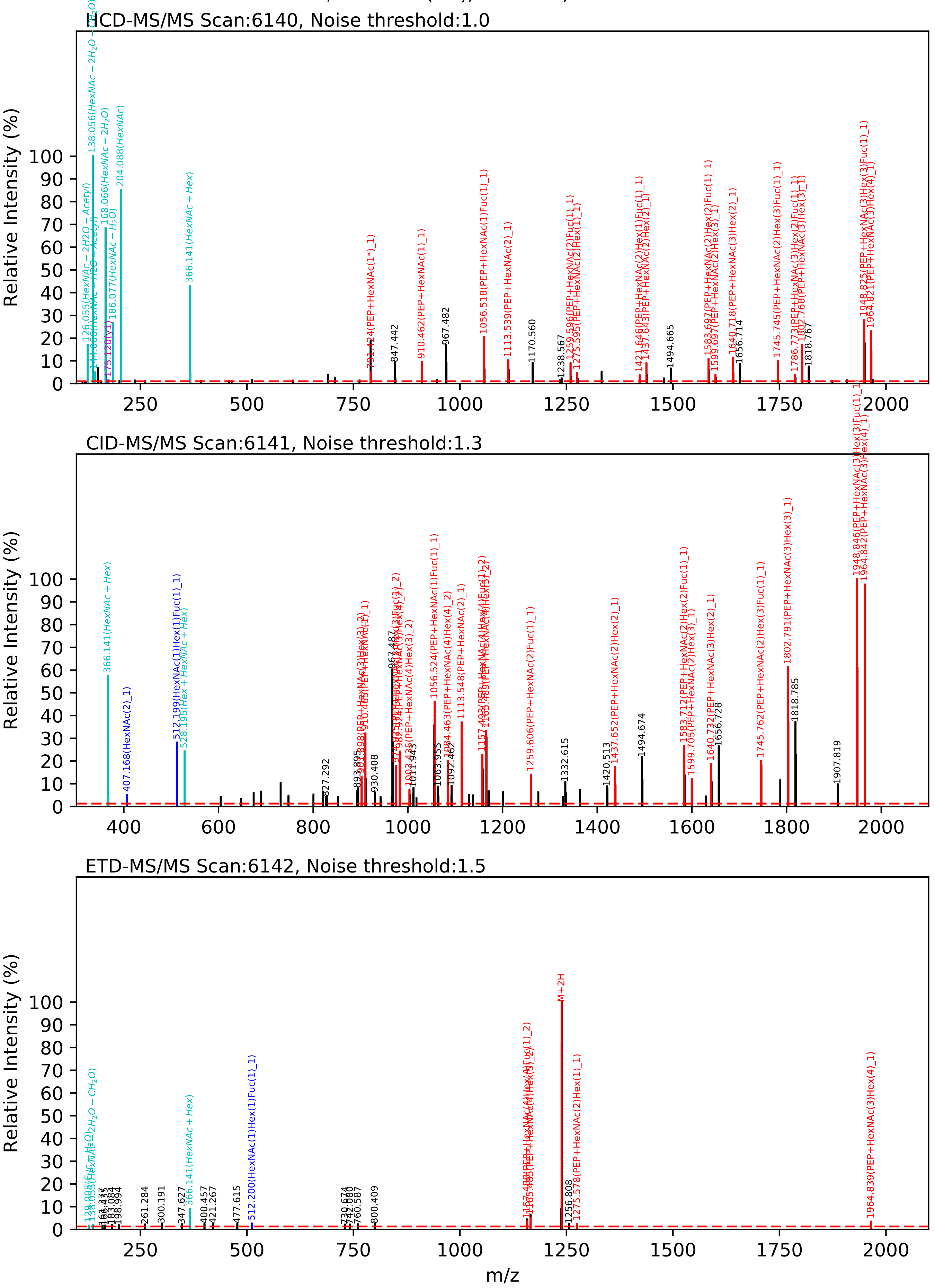

VFNATR(=PEP)_5_4_1_0_0, 0_None, 0_None,
m/z:1238.52(2+), RT:26.37, Y-score:92.08

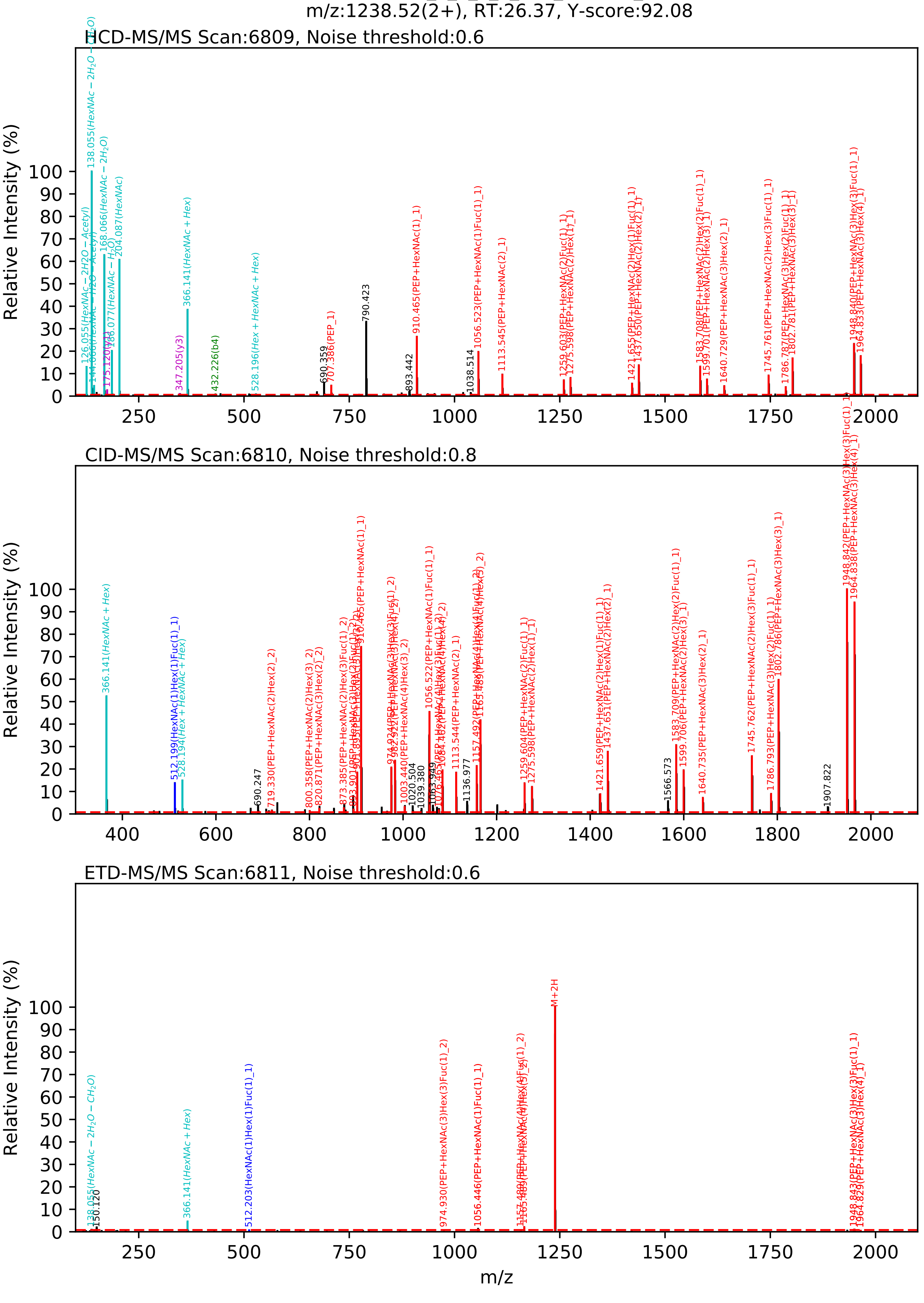

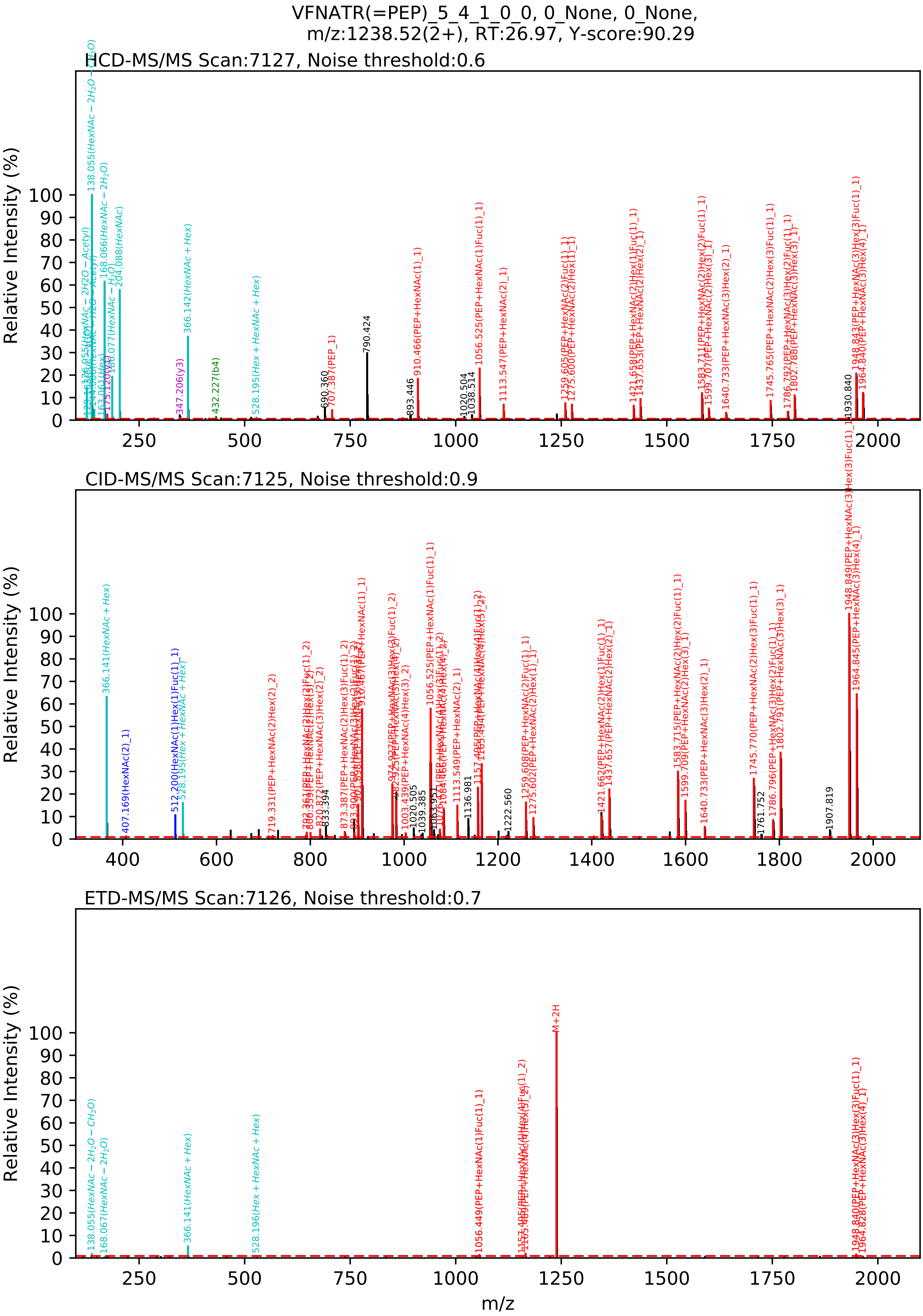
VFNATR(=PEP)_5_4_1_0_0, 0_None, 0_None,
m/z:1238.52(2+), RT:26.97, Y-score:90.29
HCD-MS/MS Scan:7127, Noise threshold:0.6
CID-MS/MS Scan:7125, Noise threshold:0.9
ETD-MS/MS Scan:7126, Noise threshold:0.7
Relative Intensity (%)
m/z

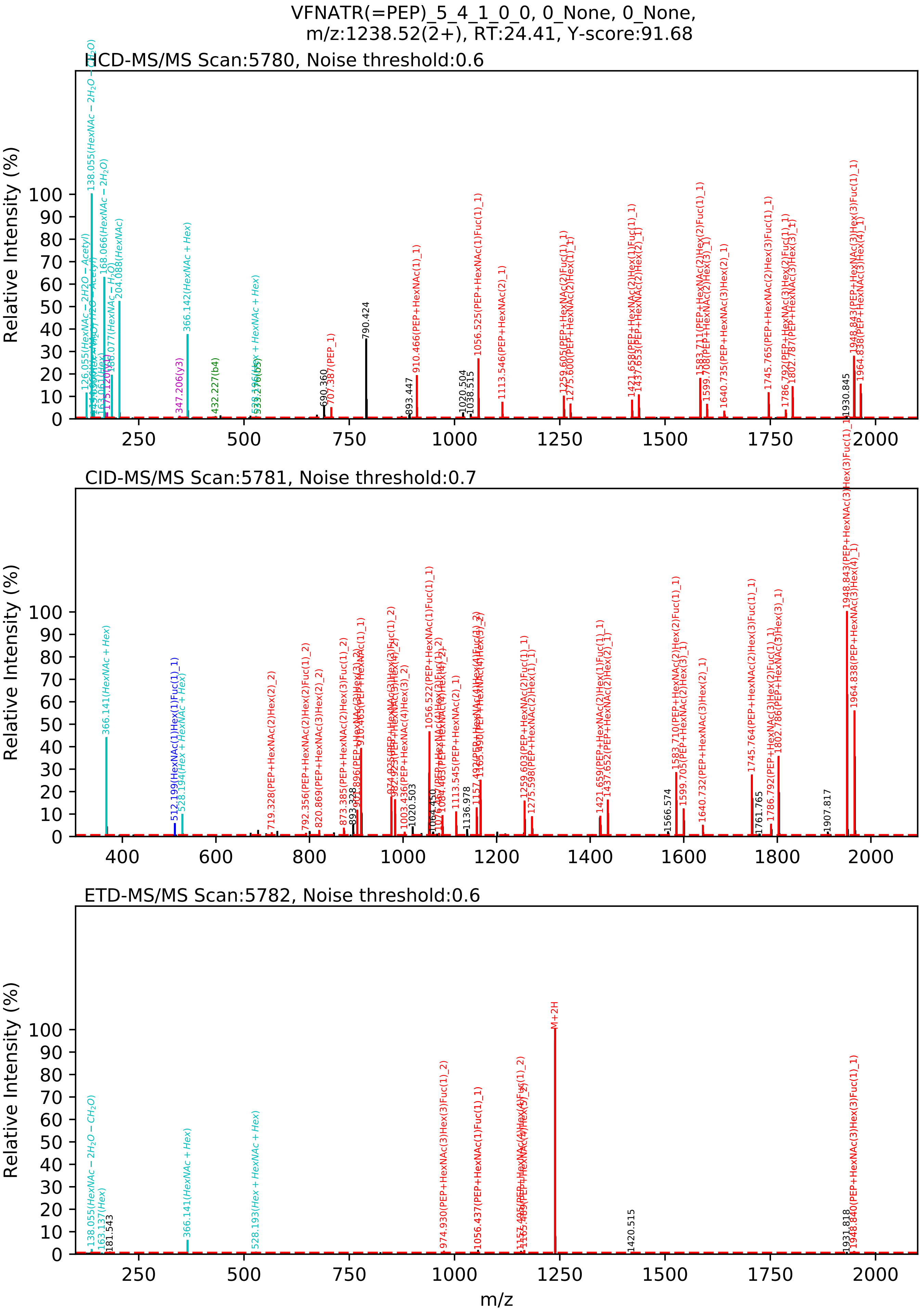
VFNATR(=PEP)_5_4_1_0_0, 0_None, 0_None,
m/z:1238.52(2+), RT:24.41, Y-score:91.68
HCD-MS/MS Scan:5780, Noise threshold:0.6
CID-MS/MS Scan:5781, Noise threshold:0.7
ETD-MS/MS Scan:5782, Noise threshold:0.6
Relative Intensity (%)
m/z

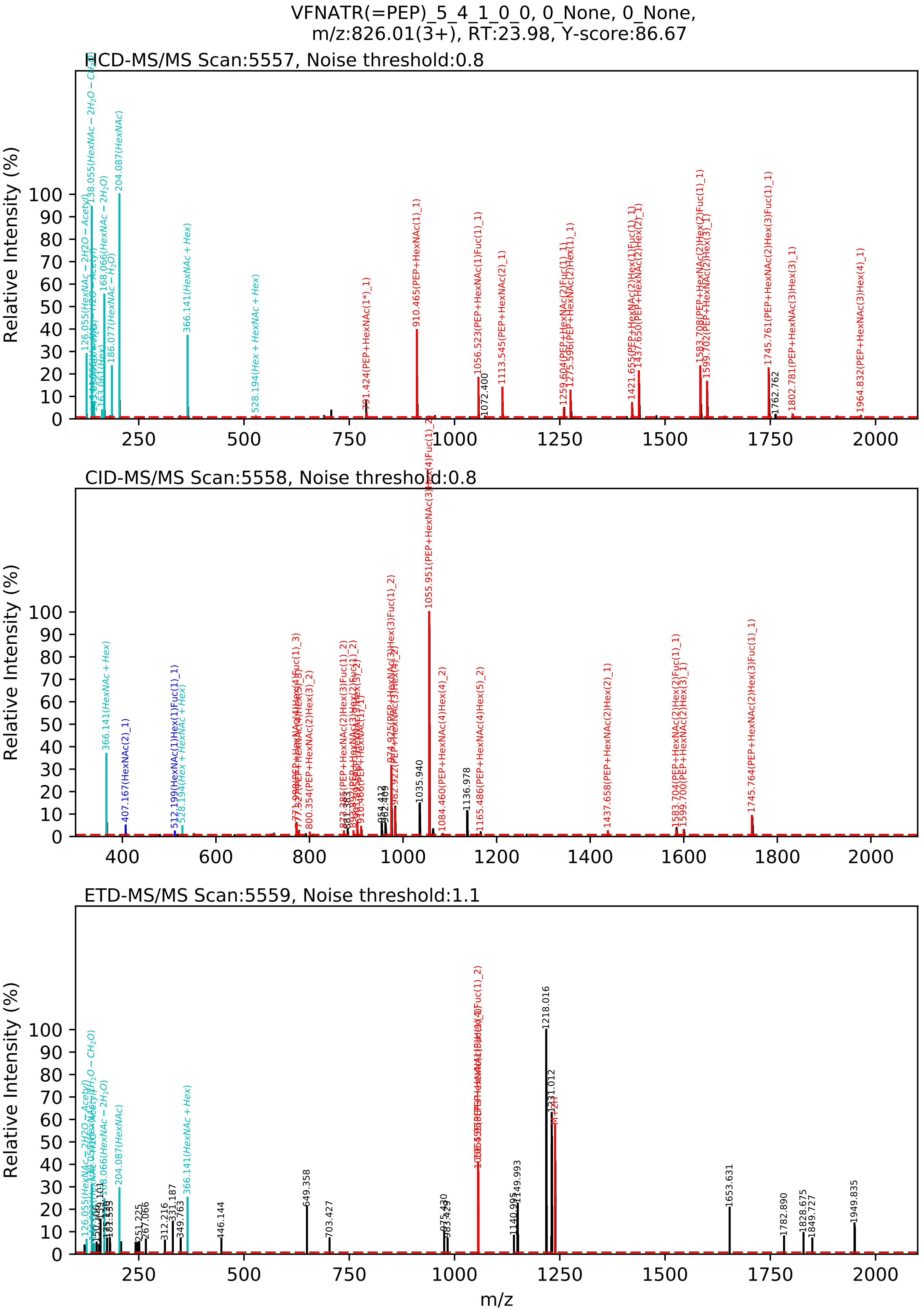
VFNATR(=PEP)_5_4_1_0_0, 0_None, 0_None,
m/z:826.01(3+), RT:23.98, Y-score:86.67
HCD-MS/MS Scan:5557, Noise threshold:0.8
CID-MS/MS Scan:5558, Noise threshold:0.8
ETD-MS/MS Scan:5559, Noise threshold:1.1
Relative Intensity (%)
m/z
910.465(PEP+HexNAc(1)_1)
1056.523(PEP+HexNAc(1)Fuc(1)_1)
1113.545(PEP+HexNAc(2)_1)
1745.761(PEP+HexNAc(2)Hex(3)Fuc(1)_1)
1802.781(PEP+HexNAc(3)Hex(3)_1)
1964.832(PEP+HexNAc(3)Hex(4)_1)
204.087(HexNAc)
366.141(HexNAc + Hex)
1055.951(PEP+HexNAc(3)Hex(4)Fuc(1)_2)
1437.658(PEP+HexNAc(2)Hex(2)_1)
1745.764(PEP+HexNAc(2)Hex(3)Fuc(1)_1)
1218.016
1231.012
1653.631
1949.835

VFNATR(=PEP)_5_4_1_0_0, 0_None, 0_None,
m/z:826.01(3+), RT:24.56, Y-score:92.11

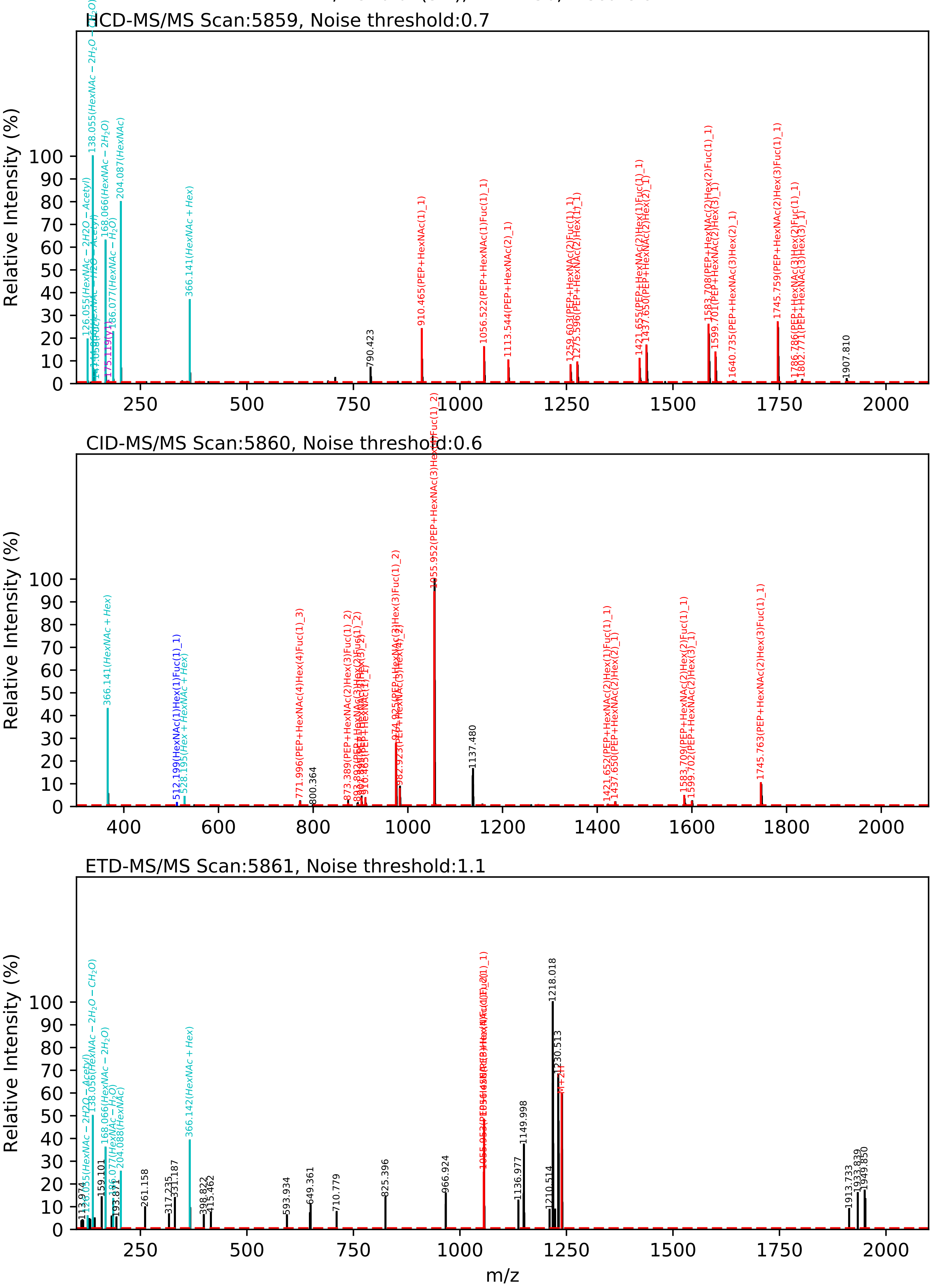

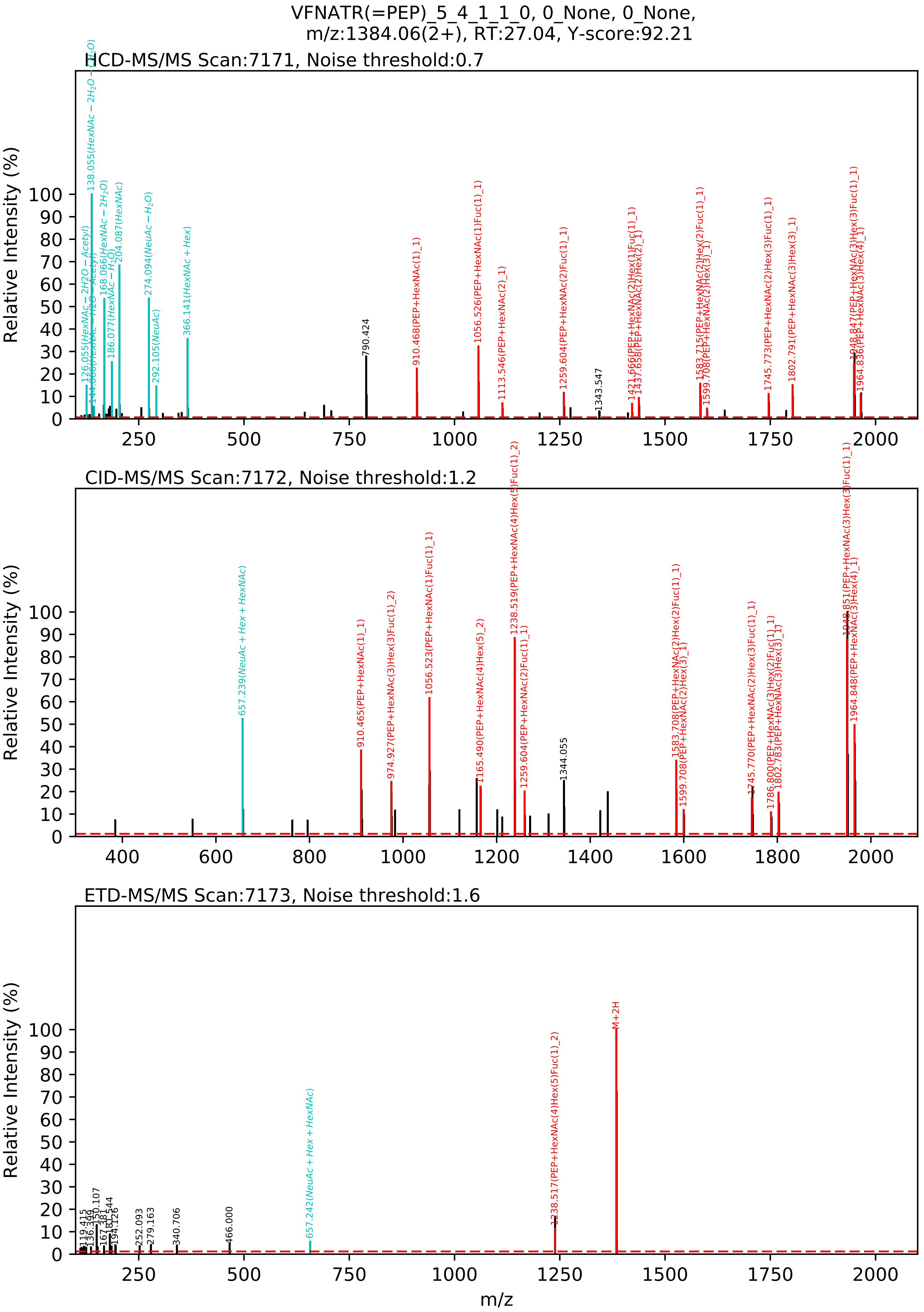
VFNATR(=PEP)_5_4_1_1_0, 0_None, 0_None,
m/z:1384.06(2+), RT:27.04, Y-score:92.21
HCD-MS/MS Scan:7171, Noise threshold:0.7
Relative Intensity (%)
790.424
910.468(PEP+HexNAc(1)_1)
1056.526(PEP+HexNAc(1)Fuc(1)_1)
1113.546(PEP+HexNAc(2)_1)
1259.604(PEP+HexNAc(2)Fuc(1)_1)
1343.547
1745.773(PEP+HexNAc(2)Hex(3)Fuc(1)_1)
1802.791(PEP+HexNAc(3)Hex(3)_1)
CID-MS/MS Scan:7172, Noise threshold:1.2
657.239(NeuAc + Hex + HexNAc)
910.465(PEP+HexNAc(1)_1)
974.927(PEP+HexNAc(3)Hex(3)Fuc(1)_2)
1056.523(PEP+HexNAc(1)Fuc(1)_1)
1165.490(PEP+HexNAc(4)Hex(5)_2)
1238.519(PEP+HexNAc(4)Hex(5)Fuc(1)_2)
1259.604(PEP+HexNAc(2)Fuc(1)_1)
1344.055
1583.708(PEP+HexNAc(2)Hex(2)Fuc(1)_1)
1599.708(PEP+HexNAc(2)Hex(3)_1)
1745.770(PEP+HexNAc(2)Hex(3)Fuc(1)_1)
1786.800(PEP+HexNAc(3)Hex(2)Fuc(1)_1)
1802.783(PEP+HexNAc(3)Hex(3)_1)
1964.848(PEP+HexNAc(3)Hex(4)_1)
ETD-MS/MS Scan:7173, Noise threshold:1.6
657.242(NeuAc + Hex + HexNAc)
1238.517(PEP+HexNAc(4)Hex(5)Fuc(1)_2)
M+2H
252.093
279.163
340.706
466.000
m/z

VFNATR(=PEP)_5_4_1_1_0, 0_None, 0_None,
m/z:923.04(3+), RT:26.63, Y-score:99.60

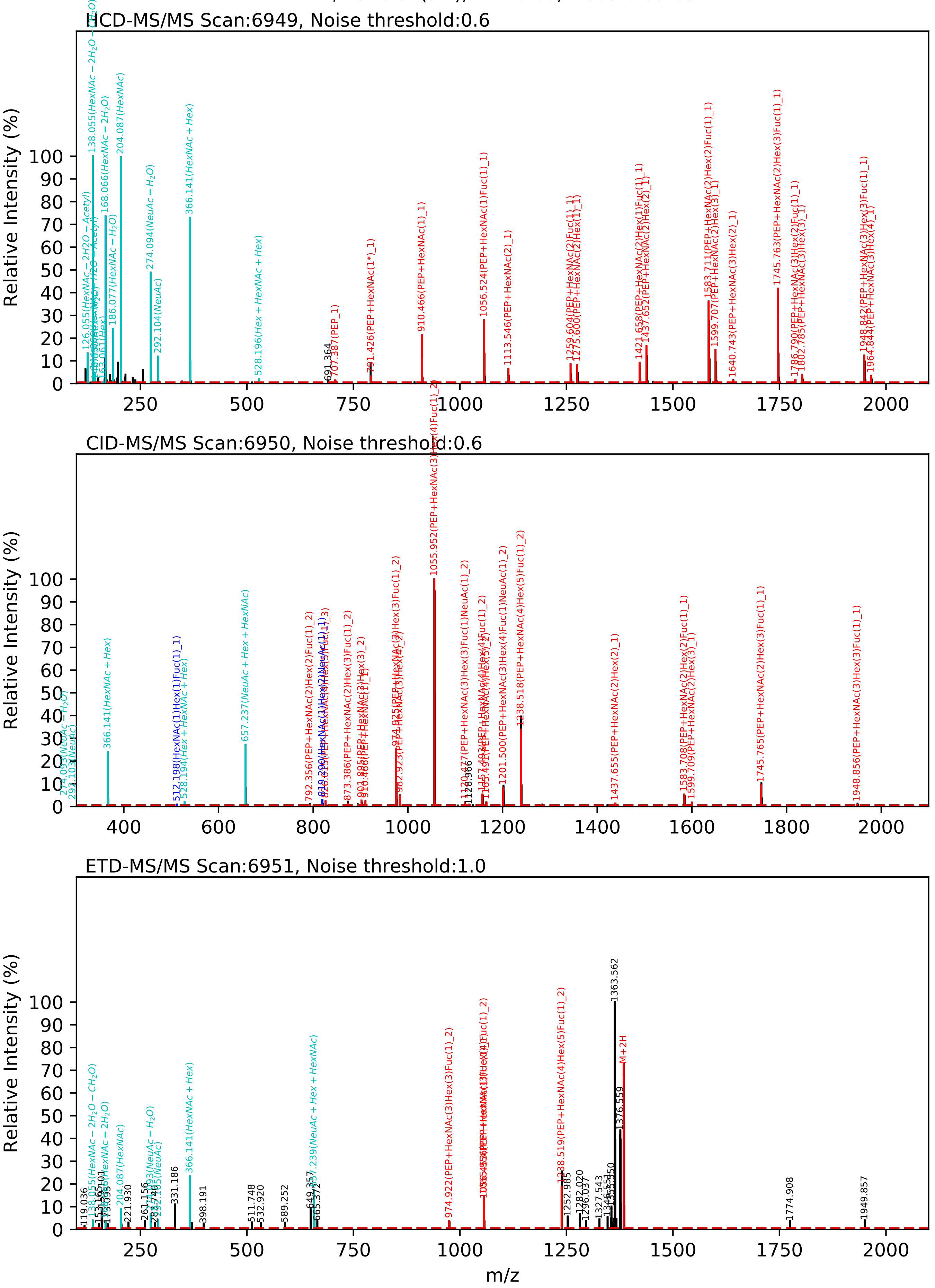

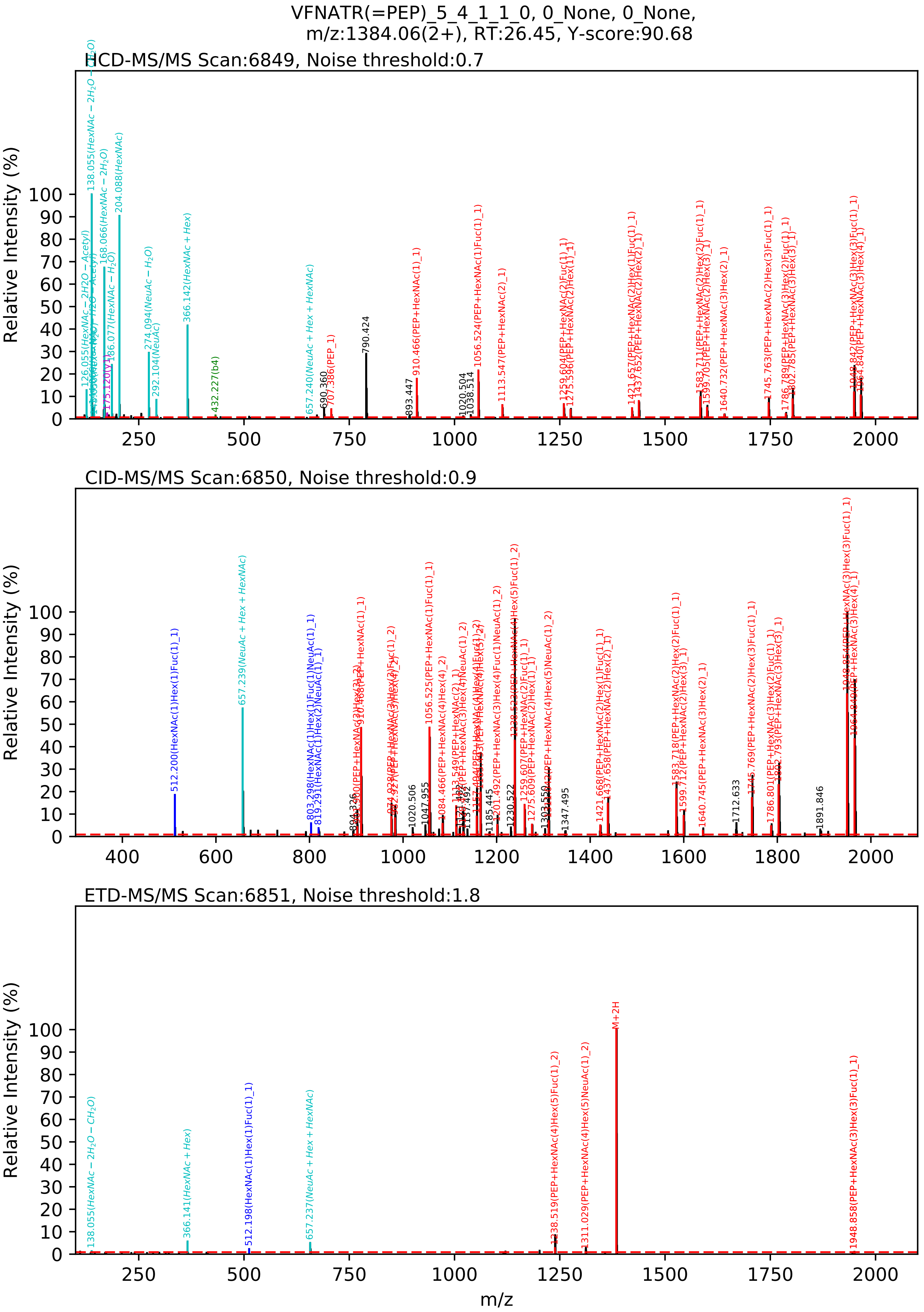
VFNATR(=PEP)_5_4_1_1_0, 0_None, 0_None,
m/z:1384.06(2+), RT:26.45, Y-score:90.68
HCD-MS/MS Scan:6849, Noise threshold:0.7
CID-MS/MS Scan:6850, Noise threshold:0.9
ETD-MS/MS Scan:6851, Noise threshold:1.8
Relative Intensity (%)
m/z

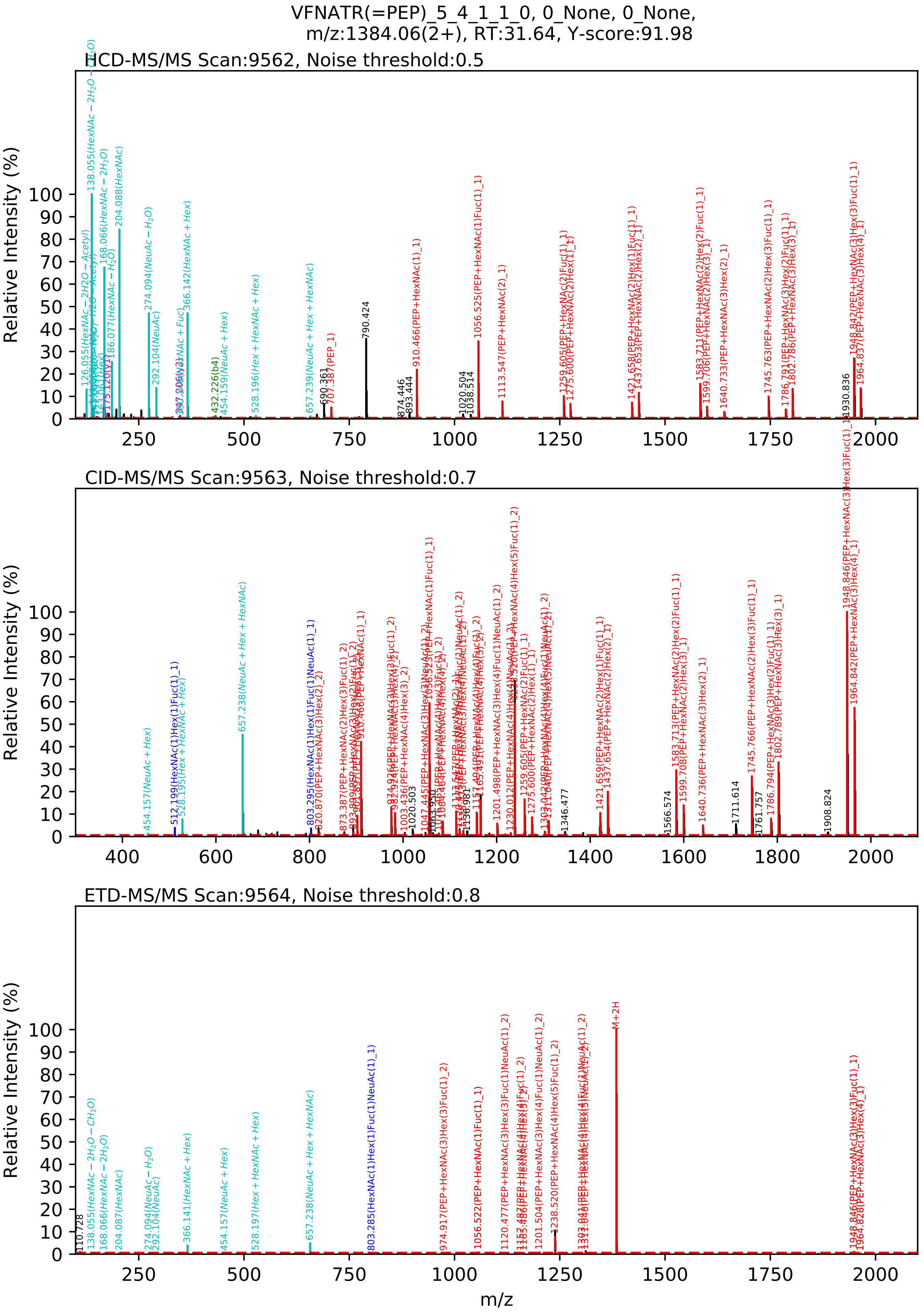
VFNATR(=PEP)_5_4_1_1_0, 0_None, 0_None,
m/z:1384.06(2+), RT:31.64, Y-score:91.98
HCD-MS/MS Scan:9562, Noise threshold:0.5
CID-MS/MS Scan:9563, Noise threshold:0.7
ETD-MS/MS Scan:9564, Noise threshold:0.8
Relative Intensity (%)
m/z
M+2H

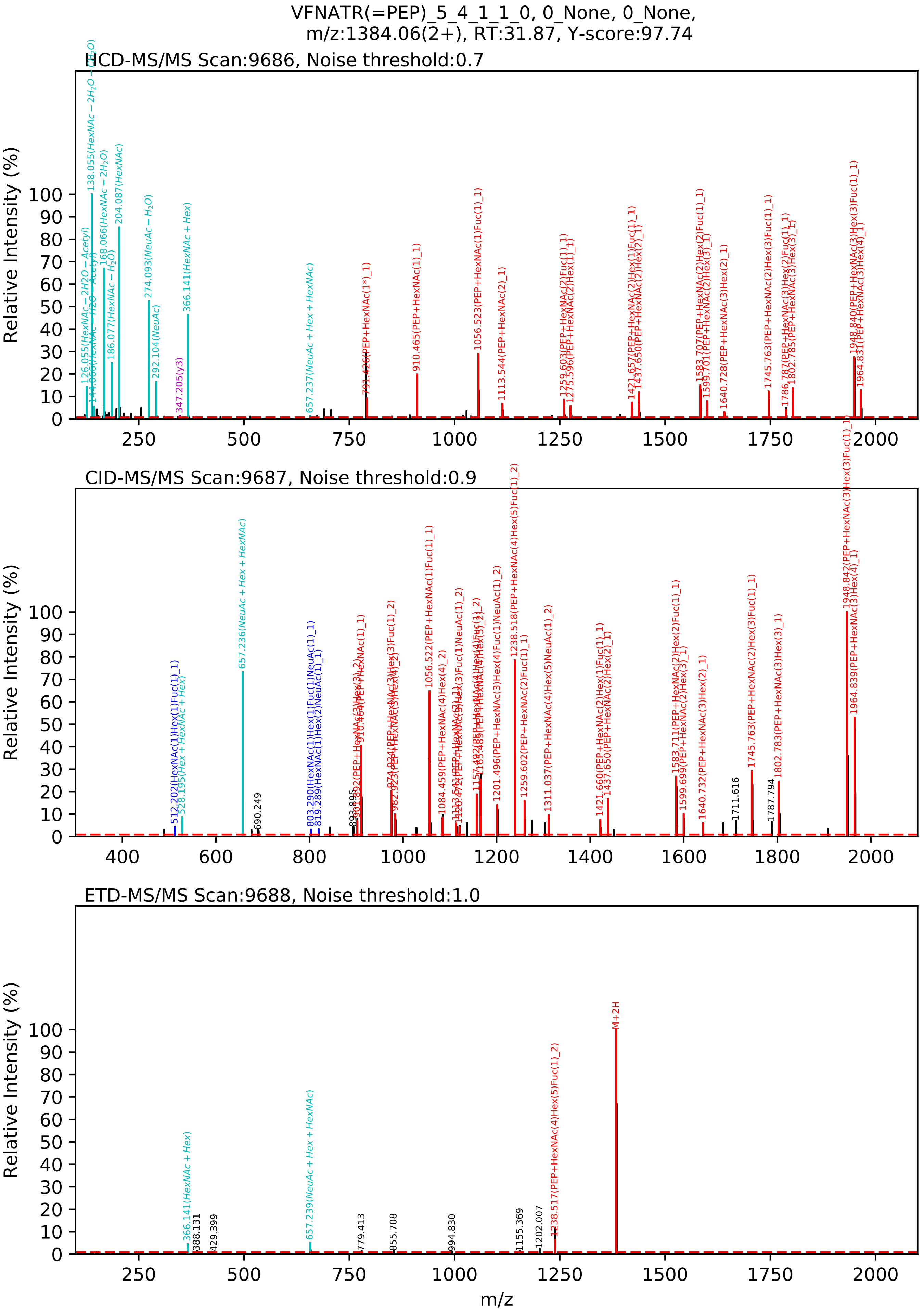
VFNATR(=PEP)_5_4_1_1_0, 0_None, 0_None,
m/z:1384.06(2+), RT:31.87, Y-score:97.74
HCD-MS/MS Scan:9686, Noise threshold:0.7
Relative Intensity (%)
138.055(HexNAc − 2H2O − H2O)
168.066(HexNAc − 2H2O)
186.077(HexNAc − H2O)
204.087(HexNAc)
274.093(NeuAc − H2O)
292.104(NeuAc)
347.205(y3)
366.141(HexNAc + Hex)
657.237(NeuAc + Hex + HexNAc)
791.426(PEP+HexNAc(1*)_1)
910.465(PEP+HexNAc(1)_1)
1056.523(PEP+HexNAc(1)Fuc(1)_1)
1113.544(PEP+HexNAc(2)_1)
1259.603(PEP+HexNAc(2)Fuc(1)_1)
1275.596(PEP+HexNAc(2)Hex(1)_1)
1421.657(PEP+HexNAc(2)Hex(1)Fuc(1)_1)
1437.650(PEP+HexNAc(2)Hex(2)_1)
1583.707(PEP+HexNAc(2)Hex(2)Fuc(1)_1)
1599.701(PEP+HexNAc(2)Hex(3)_1)
1640.728(PEP+HexNAc(3)Hex(2)_1)
1745.763(PEP+HexNAc(2)Hex(3)Fuc(1)_1)
1786.787(PEP+HexNAc(3)Hex(2)Fuc(1)_1)
1802.785(PEP+HexNAc(3)Hex(3)_1)
1948.840(PEP+HexNAc(3)Hex(3)Fuc(1)_1)
1964.831(PEP+HexNAc(3)Hex(4)_1)
CID-MS/MS Scan:9687, Noise threshold:0.9
512.202(HexNAc(1)Hex(1)Fuc(1)_1)
528.195(Hex + HexNAc + Hex)
657.236(NeuAc + Hex + HexNAc)
690.249
803.290(HexNAc(1)Hex(1)Fuc(1)NeuAc(1)_1)
819.289(HexNAc(1)Hex(2)NeuAc(1)_1)
893.895
910.464(PEP+HexNAc(1)_1)
982.923(PEP+HexNAc(3)Hex(4)_2)
1056.522(PEP+HexNAc(1)Fuc(1)_1)
1084.459(PEP+HexNAc(4)Hex(4)_2)
1201.496(PEP+HexNAc(3)Hex(4)Fuc(1)NeuAc(1)_2)
1238.518(PEP+HexNAc(4)Hex(5)Fuc(1)_2)
1259.602(PEP+HexNAc(2)Fuc(1)_1)
1311.037(PEP+HexNAc(4)Hex(5)NeuAc(1)_2)
1421.660(PEP+HexNAc(2)Hex(1)Fuc(1)_1)
1437.650(PEP+HexNAc(2)Hex(2)_1)
1583.711(PEP+HexNAc(2)Hex(2)Fuc(1)_1)
1599.699(PEP+HexNAc(2)Hex(3)_1)
1640.732(PEP+HexNAc(3)Hex(2)_1)
1711.616
1745.763(PEP+HexNAc(2)Hex(3)Fuc(1)_1)
1787.794
1802.783(PEP+HexNAc(3)Hex(3)_1)
1948.842(PEP+HexNAc(3)Hex(3)Fuc(1)_1)
1964.839(PEP+HexNAc(3)Hex(4)_1)
ETD-MS/MS Scan:9688, Noise threshold:1.0
366.141(HexNAc + Hex)
388.131
429.399
657.239(NeuAc + Hex + HexNAc)
779.413
855.708
994.830
1155.369
1202.007
1238.517(PEP+HexNAc(4)Hex(5)Fuc(1)_2)
M+2H
m/z

VFNATR(=PEP)_5_4_1_1_0, 0_None, 0_None,
m/z:1384.06(2+), RT:30.90, Y-score:93.50

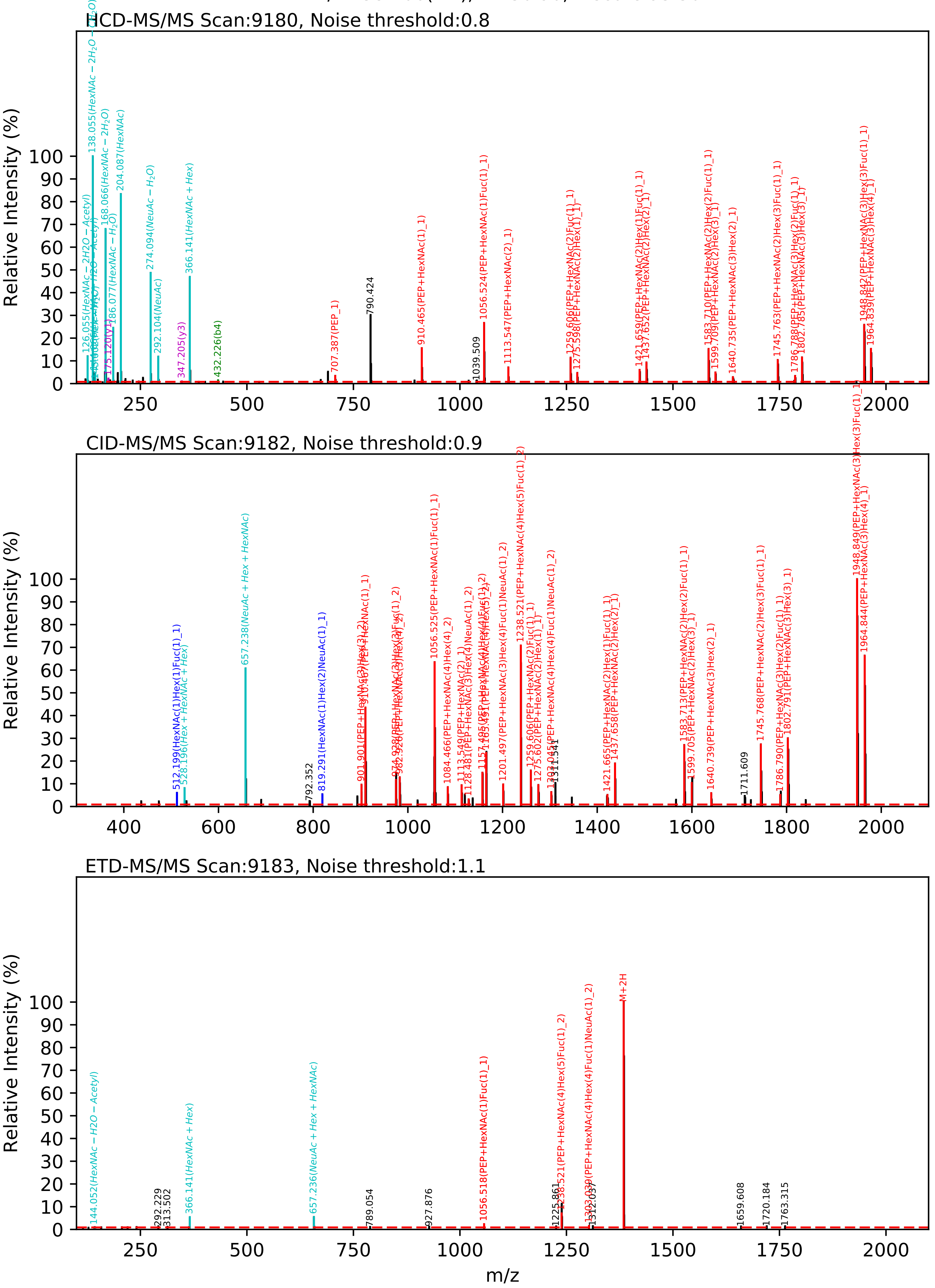

VFNATR(=PEP)_5_4_1_1_0, 0_None, 0_None,
m/z:1384.06(2+), RT:31.42, Y-score:92.42

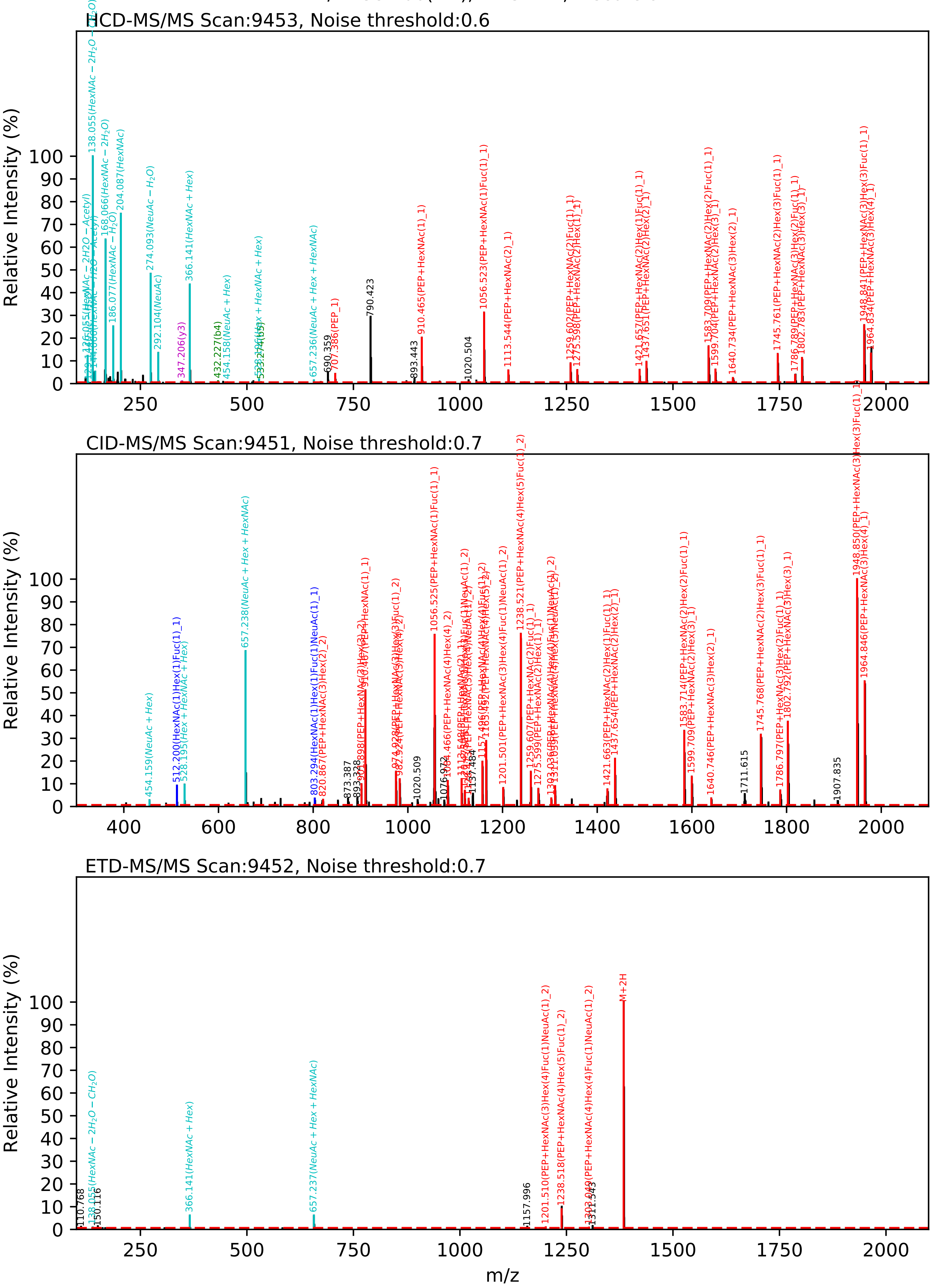

VFNATR(=PEP)_5_4_1_2_0, 0_None, 0_None,
m/z:1529.61(2+), RT:32.12, Y-score:97.11

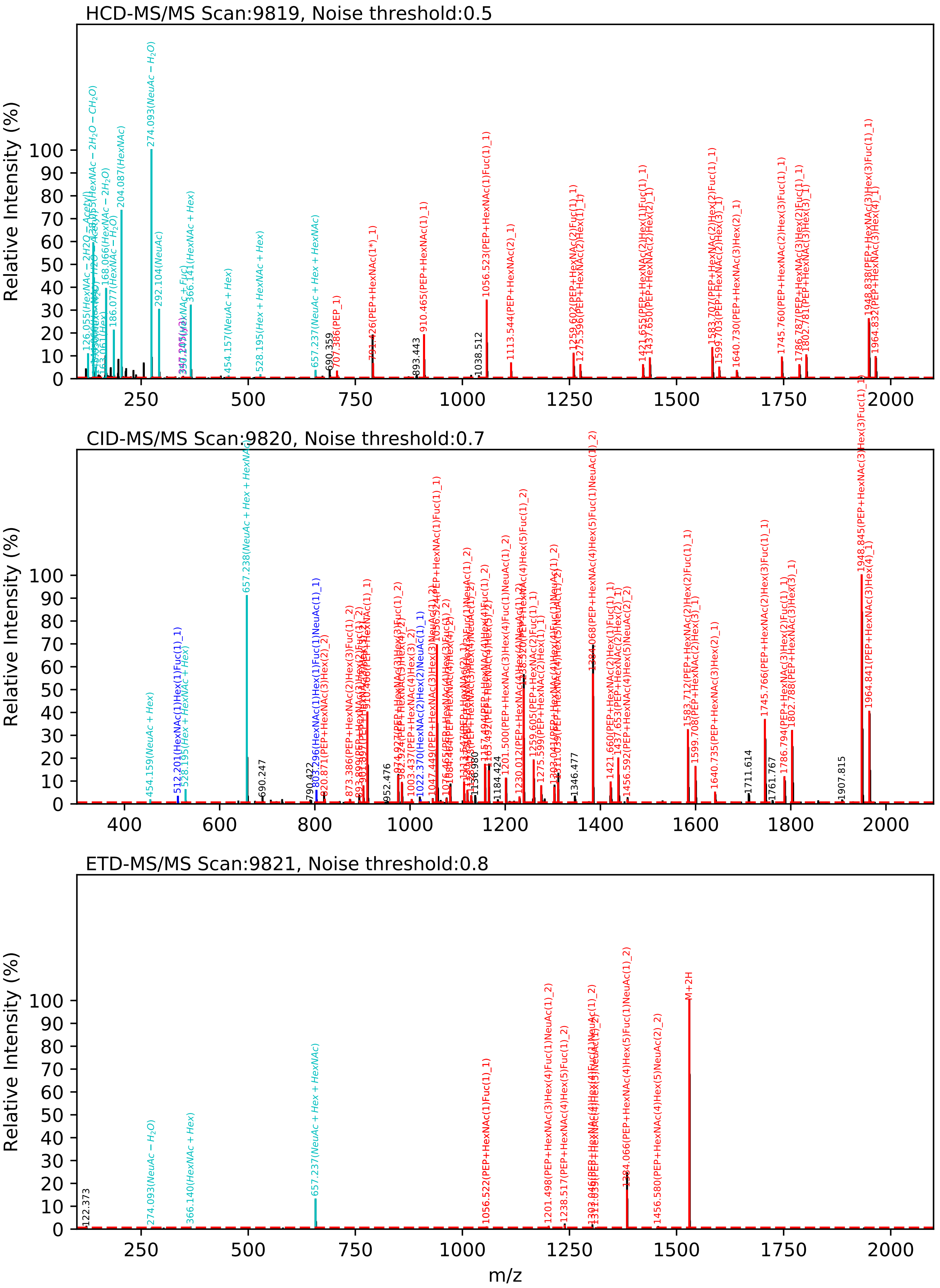

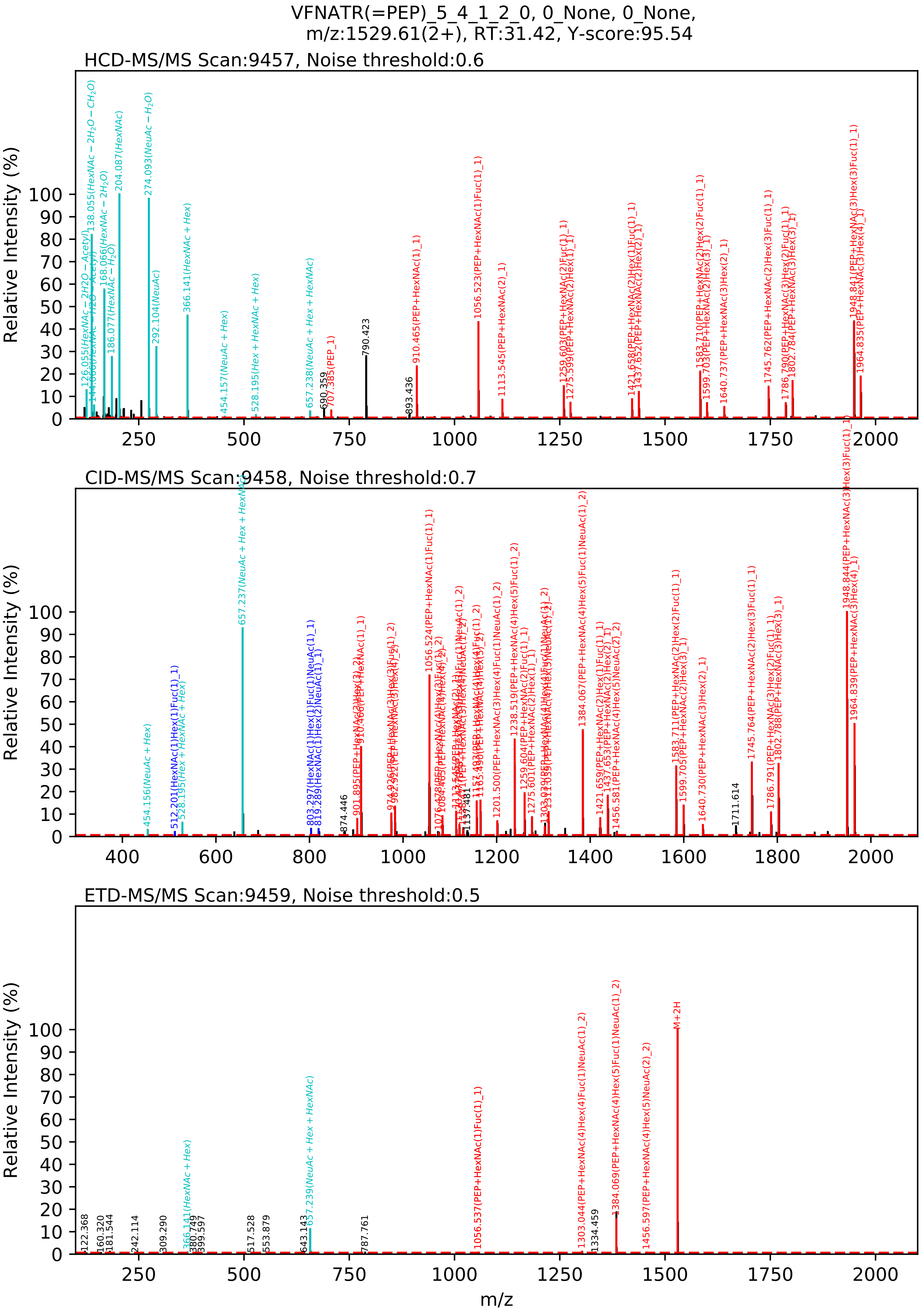
VFNATR(=PEP)_5_4_1_2_0, 0_None, 0_None,
m/z:1529.61(2+), RT:31.42, Y-score:95.54
HCD-MS/MS Scan:9457, Noise threshold:0.6
CID-MS/MS Scan:9458, Noise threshold:0.7
ETD-MS/MS Scan:9459, Noise threshold:0.5
Relative Intensity (%)
m/z
274.093(NeuAc − H2O)
204.087(HexNAc)
366.141(HexNAc + Hex)
292.104(NeuAc)
657.238(NeuAc + Hex + HexNAc)
790.423
910.465(PEP+HexNAc(1)_1)
1056.523(PEP+HexNAc(1)Fuc(1)_1)
1113.545(PEP+HexNAc(2)_1)
1948.841(PEP+HexNAc(3)Hex(3)Fuc(1)_1)
1964.835(PEP+HexNAc(3)Hex(4)_1)
657.237(NeuAc + Hex + HexNAc)
1384.067(PEP+HexNAc(4)Hex(5)Fuc(1)NeuAc(1)_2)
1948.844(PEP+HexNAc(3)Hex(3)Fuc(1)_1)
1964.839(PEP+HexNAc(3)Hex(4)_1)
M+2H
1384.069(PEP+HexNAc(4)Hex(5)Fuc(1)NeuAc(1)_2)
1456.597(PEP+HexNAc(4)Hex(5)NeuAc(2)_2)

VFNATR(=PEP)_5_4_1_2_0, 0_None, 0_None,
m/z:1020.08(3+), RT:31.02, Y-score:97.42

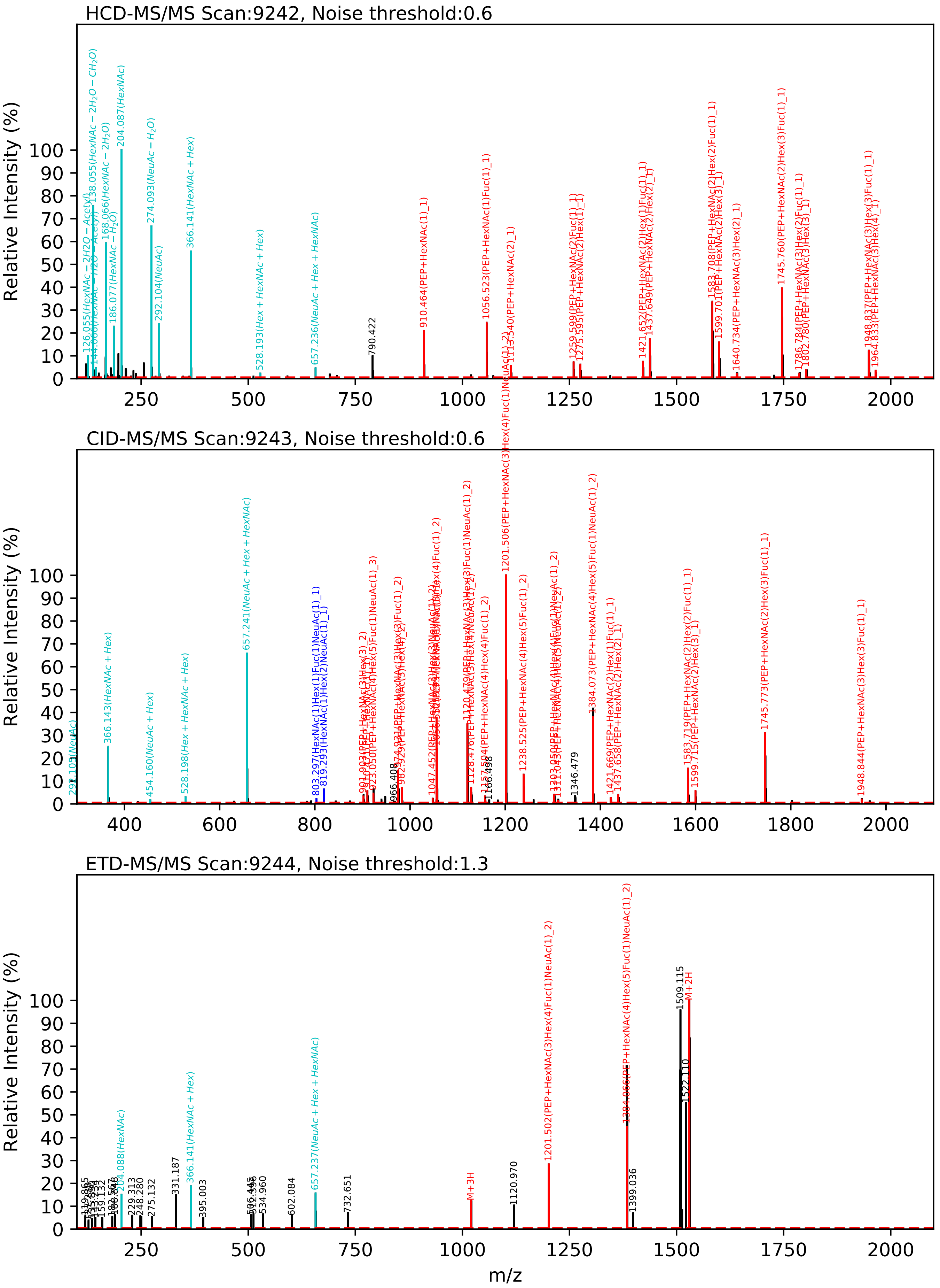

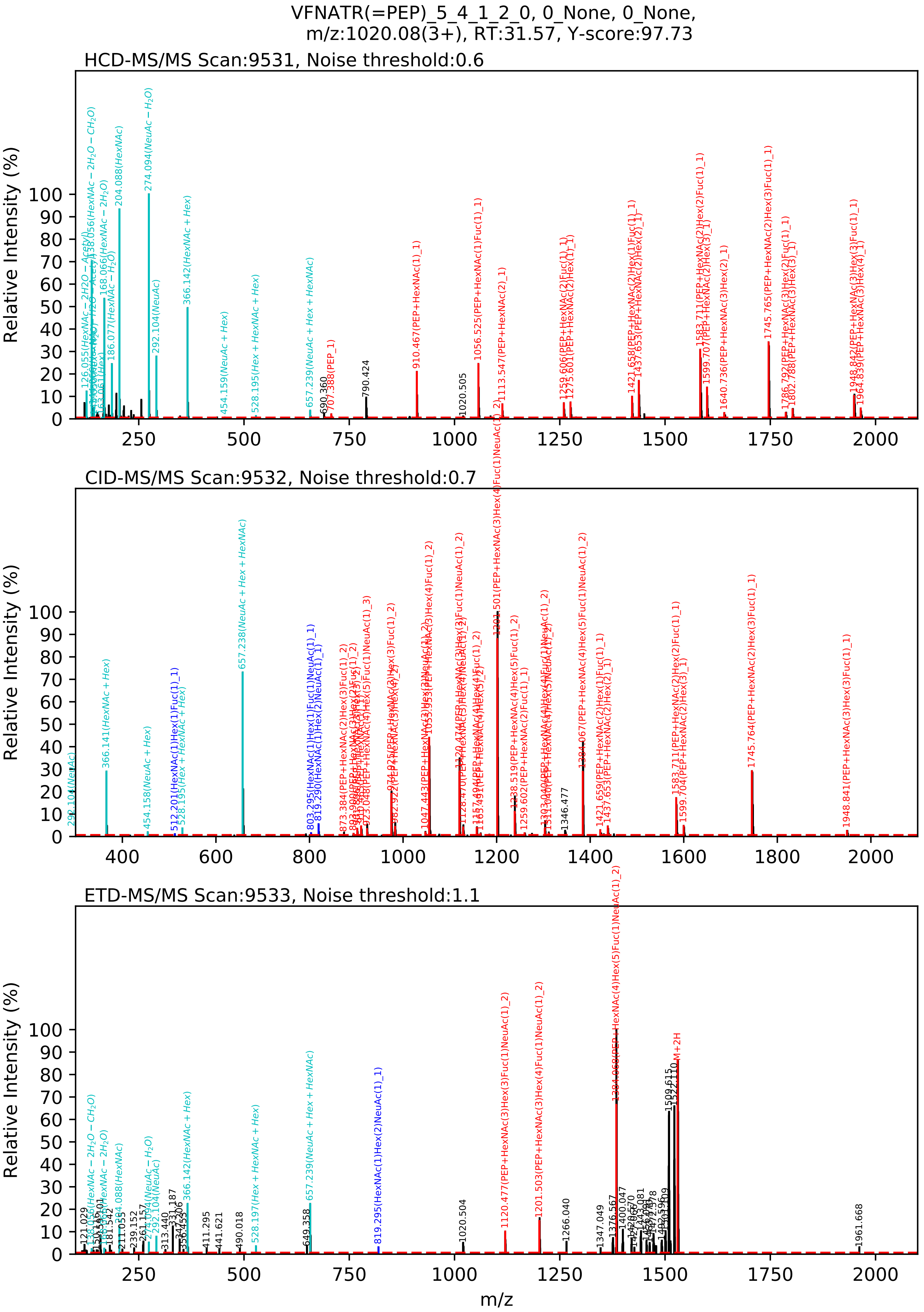

VFNATR(=PEP)_5_4_1_2_0, 0_None, 0_None,
m/z:1020.08(3+), RT:31.57, Y-score:97.73
HCD-MS/MS Scan:9531, Noise threshold:0.6
CID-MS/MS Scan:9532, Noise threshold:0.7
ETD-MS/MS Scan:9533, Noise threshold:1.1
Relative Intensity (%)
m/z

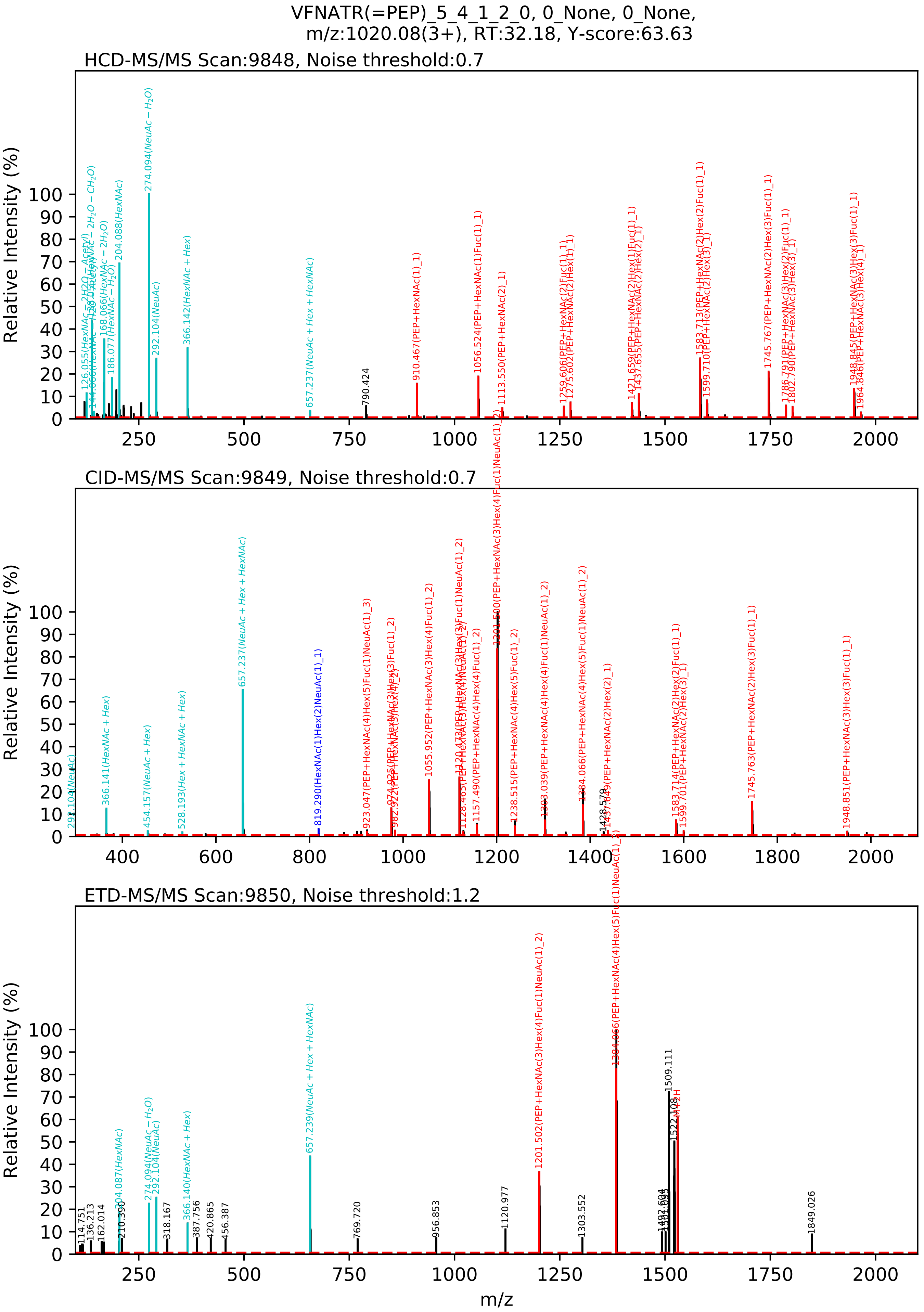

VFNATR(=PEP)_5_4_1_2_0, 0_None, 0_None,
m/z:1020.08(3+), RT:32.18, Y-score:63.63
HCD-MS/MS Scan:9848, Noise threshold:0.7
CID-MS/MS Scan:9849, Noise threshold:0.7
ETD-MS/MS Scan:9850, Noise threshold:1.2
Relative Intensity (%)
m/z

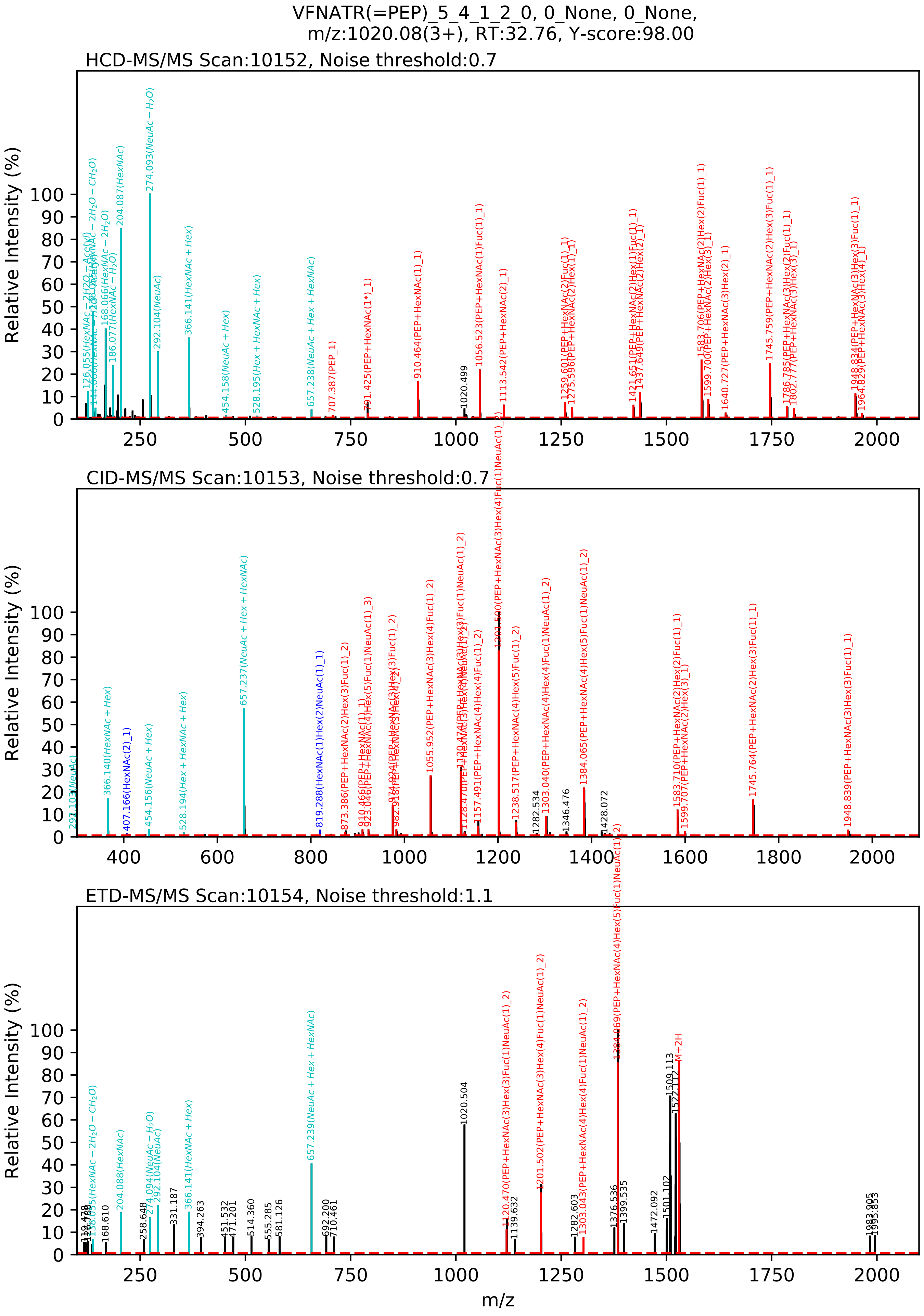
VFNATR(=PEP)_5_4_1_2_0, 0_None, 0_None,
m/z:1020.08(3+), RT:32.76, Y-score:98.00
HCD-MS/MS Scan:10152, Noise threshold:0.7
CID-MS/MS Scan:10153, Noise threshold:0.7
ETD-MS/MS Scan:10154, Noise threshold:1.1
Relative Intensity (%)
m/z

VFNATR(=PEP)_5_4_1_2_0, 0_None, 0_None,
m/z:1529.61(2+), RT:42.70, Y-score:97.83

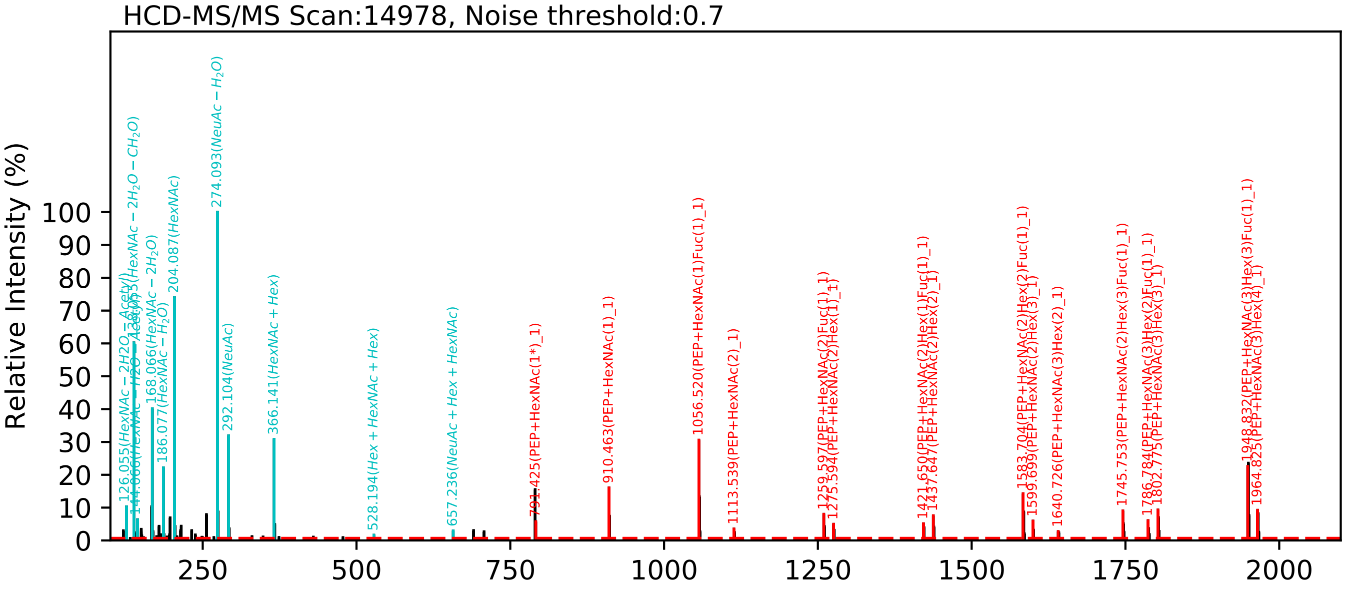

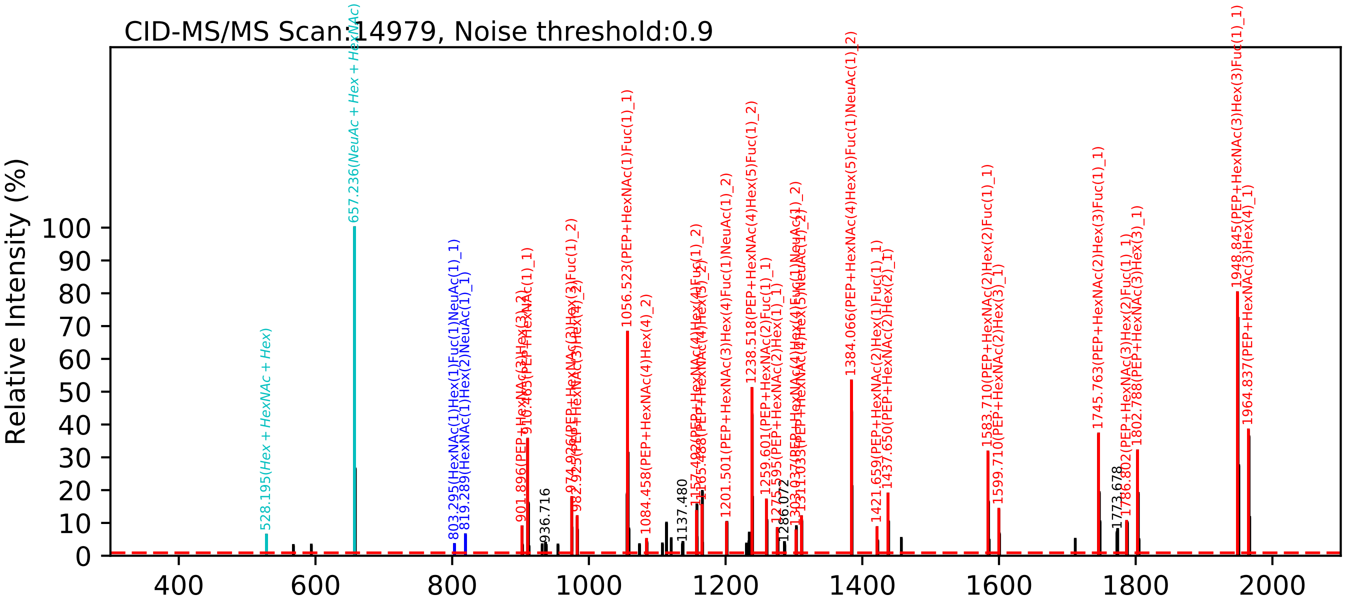

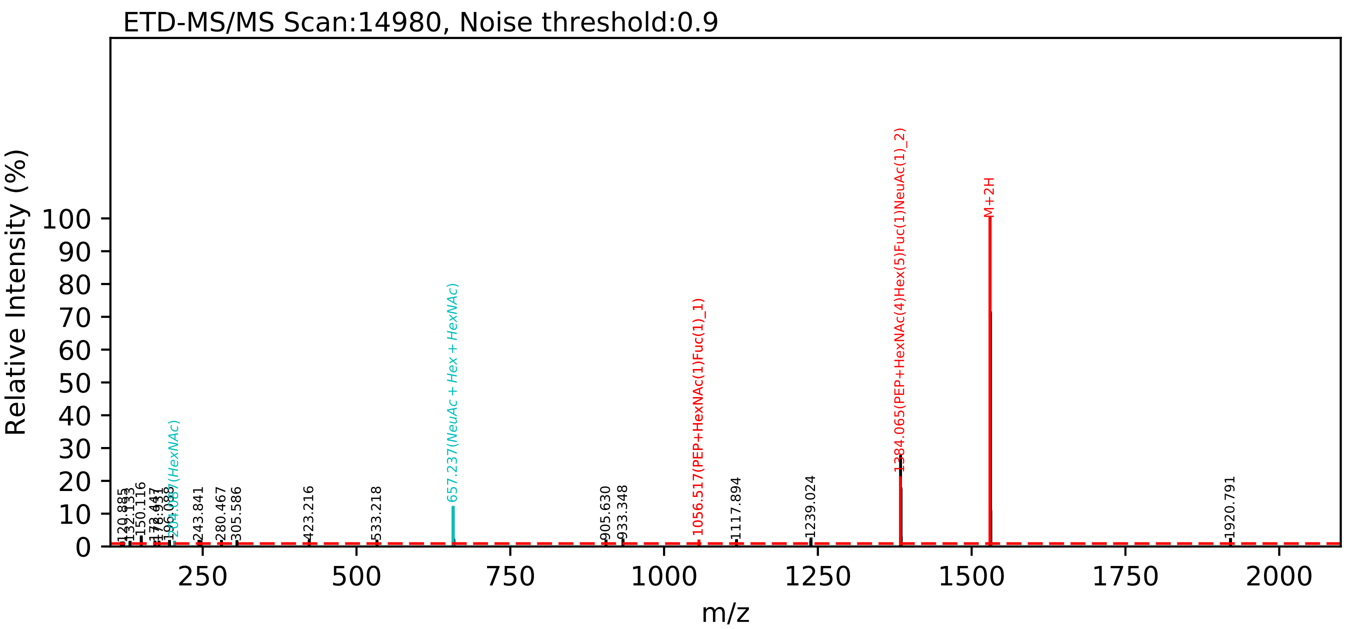

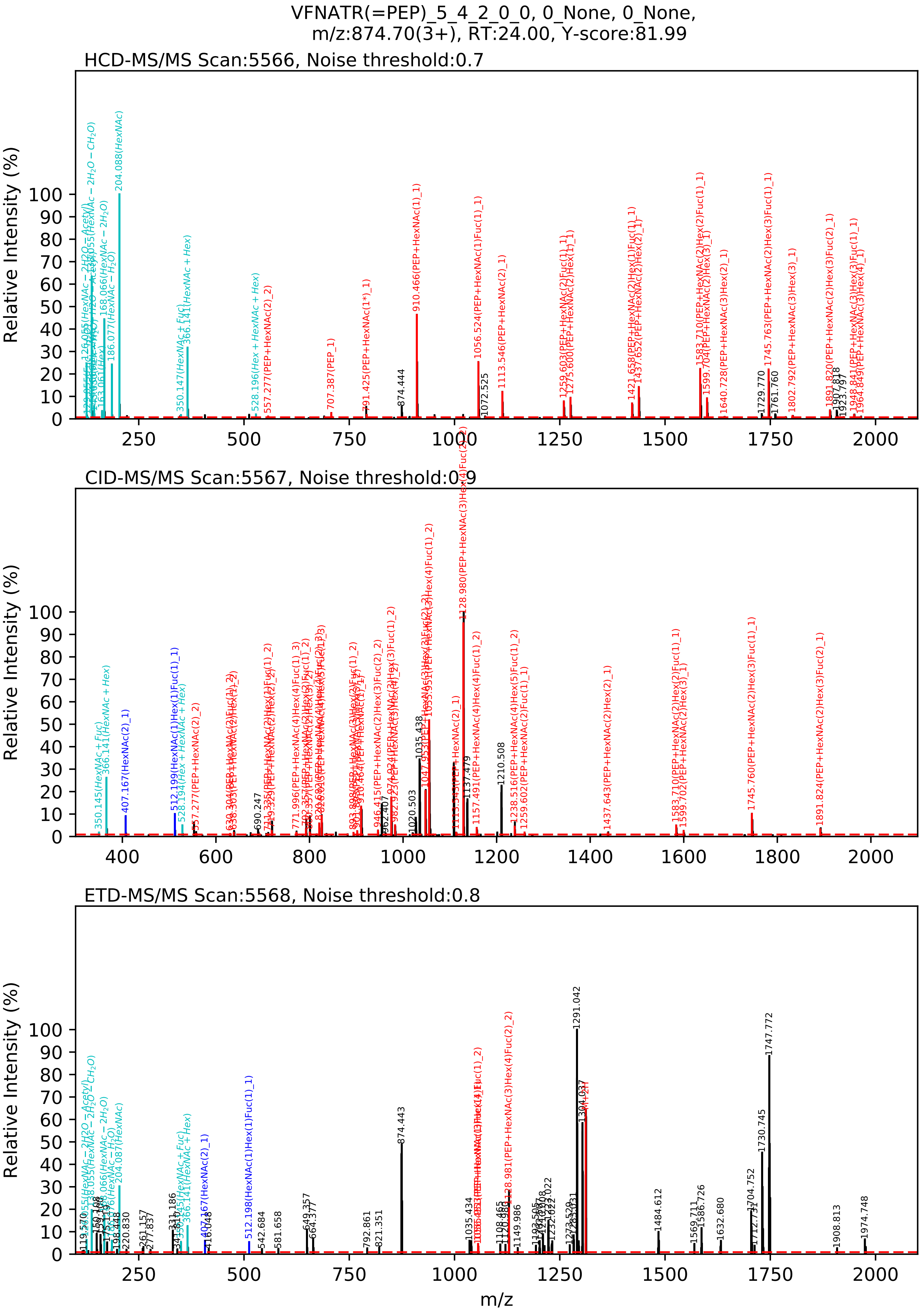
VFNATR(=PEP)_5_4_2_0_0, 0_None, 0_None,
m/z:874.70(3+), RT:24.00, Y-score:81.99
HCD-MS/MS Scan:5566, Noise threshold:0.7
CID-MS/MS Scan:5567, Noise threshold:0.9
ETD-MS/MS Scan:5568, Noise threshold:0.8
Relative Intensity (%)
m/z

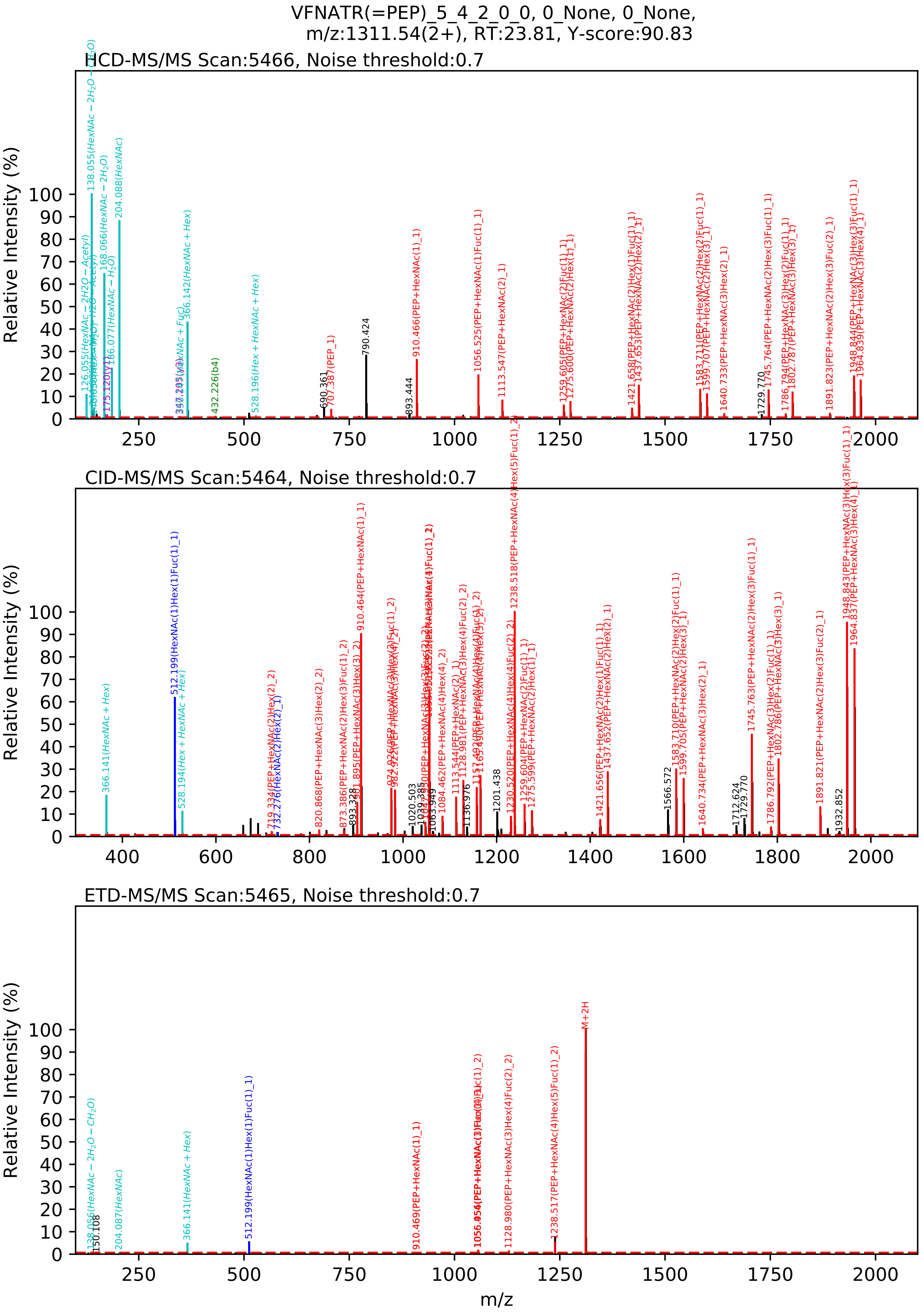
VFNATR(=PEP)_5_4_2_0_0, 0_None, 0_None,
m/z:1311.54(2+), RT:23.81, Y-score:90.83
HCD-MS/MS Scan:5466, Noise threshold:0.7
CID-MS/MS Scan:5464, Noise threshold:0.7
ETD-MS/MS Scan:5465, Noise threshold:0.7
Relative Intensity (%)
m/z
M+2H

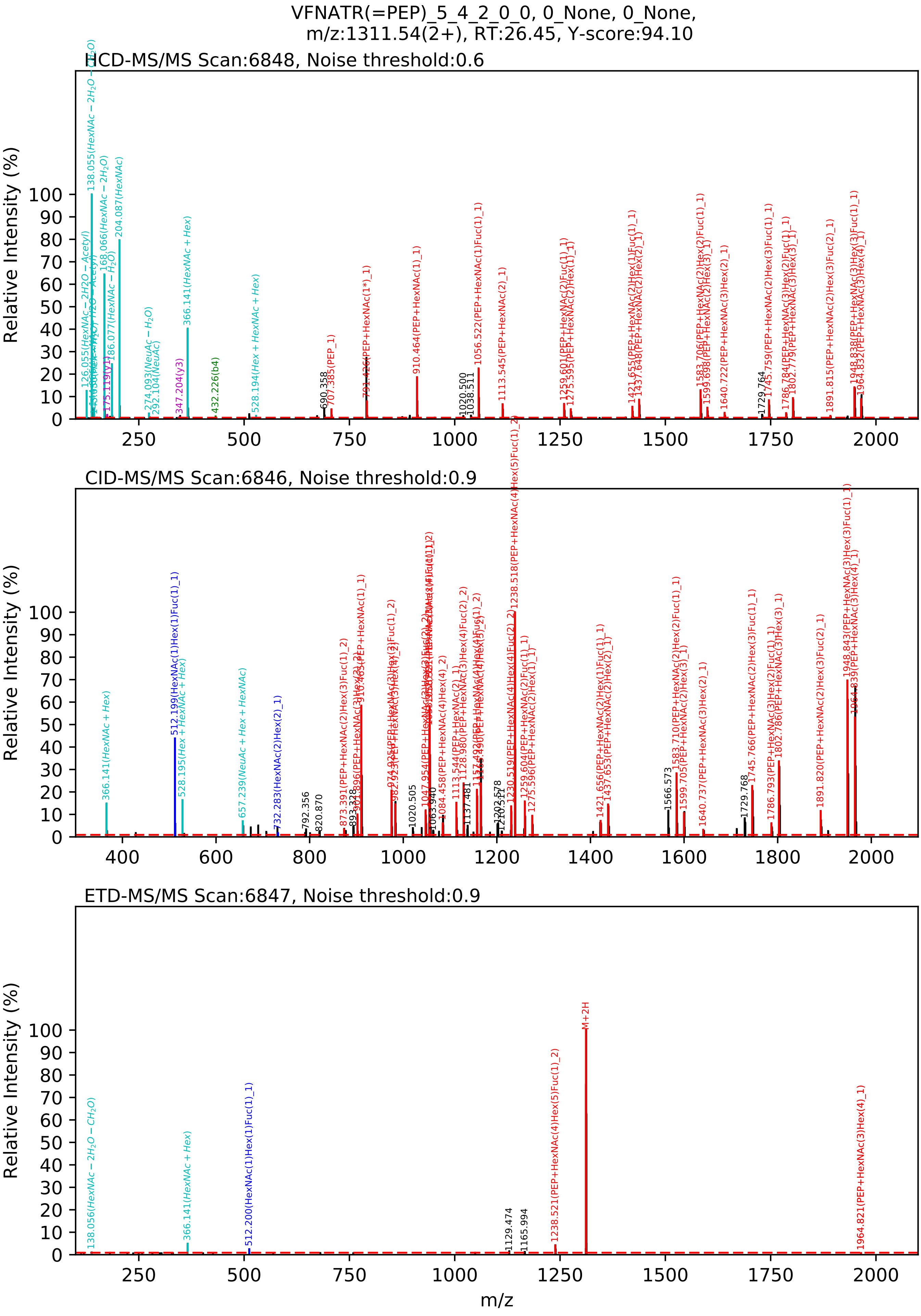
VFNATR(=PEP)_5_4_2_0_0, 0_None, 0_None,
m/z:1311.54(2+), RT:26.45, Y-score:94.10
HCD-MS/MS Scan:6848, Noise threshold:0.6
Relative Intensity (%)
138.055(HexNAc − 2H2O − CH2O)
204.087(HexNAc)
168.066(HexNAc − 2H2O)
186.077(HexNAc − H2O)
366.141(HexNAc + Hex)
274.093(NeuAc − H2O)
292.104(NeuAc)
347.204(y3)
432.226(b4)
528.194(Hex + HexNAc + Hex)
690.358
707.385(PEP_1)
791.426(PEP+HexNAc(1*)_1)
910.464(PEP+HexNAc(1)_1)
1020.500
1038.511
1056.522(PEP+HexNAc(1)Fuc(1)_1)
1113.545(PEP+HexNAc(2)_1)
1259.601(PEP+HexNAc(2)Fuc(1)_1)
1275.595(PEP+HexNAc(2)Hex(1)_1)
1421.655(PEP+HexNAc(2)Hex(1)Fuc(1)_1)
1437.648(PEP+HexNAc(2)Hex(2)_1)
1583.706(PEP+HexNAc(2)Hex(2)Fuc(1)_1)
1640.722(PEP+HexNAc(3)Hex(2)_1)
1729.764
1745.759(PEP+HexNAc(2)Hex(3)Fuc(1)_1)
1802.775(PEP+HexNAc(3)Hex(3)_1)
1891.815(PEP+HexNAc(2)Hex(3)Fuc(2)_1)
1948.838(PEP+HexNAc(3)Hex(3)Fuc(1)_1)
1964.832(PEP+HexNAc(3)Hex(4)_1)
CID-MS/MS Scan:6846, Noise threshold:0.9
366.141(HexNAc + Hex)
512.199(HexNAc(1)Hex(1)Fuc(1)_1)
528.195(Hex + HexNAc + Hex)
657.239(NeuAc + Hex + HexNAc)
732.283(HexNAc(2)Hex(2)_1)
792.356
820.870
873.391(PEP+HexNAc(2)Hex(3)Fuc(1)_2)
893.328
910.463(PEP+HexNAc(1)_1)
974.925(PEP+HexNAc(3)Hex(3)Fuc(1)_2)
1020.505
1047.954(PEP+HexNAc(4)Hex(4)_2)
1063.940
1084.458(PEP+HexNAc(4)Hex(4)_2)
1113.544(PEP+HexNAc(2)_1)
1128.980(PEP+HexNAc(3)Hex(4)Fuc(2)_2)
1137.481
1203.578
1210.511
1230.519(PEP+HexNAc(4)Hex(4)Fuc(2)_2)
1238.518(PEP+HexNAc(4)Hex(5)Fuc(1)_2)
1259.604(PEP+HexNAc(2)Fuc(1)_1)
1275.596(PEP+HexNAc(2)Hex(1)_1)
1421.656(PEP+HexNAc(2)Hex(1)Fuc(1)_1)
1437.653(PEP+HexNAc(2)Hex(2)_1)
1566.573
1583.710(PEP+HexNAc(2)Hex(2)Fuc(1)_1)
1599.705(PEP+HexNAc(2)Hex(3)_1)
1640.737(PEP+HexNAc(3)Hex(2)_1)
1729.768
1745.766(PEP+HexNAc(2)Hex(3)Fuc(1)_1)
1786.793(PEP+HexNAc(3)Hex(2)Fuc(1)_1)
1802.786(PEP+HexNAc(3)Hex(3)_1)
1891.820(PEP+HexNAc(2)Hex(3)Fuc(2)_1)
1948.843(PEP+HexNAc(3)Hex(3)Fuc(1)_1)
1964.839(PEP+HexNAc(3)Hex(4)_1)
ETD-MS/MS Scan:6847, Noise threshold:0.9
138.056(HexNAc − 2H2O − CH2O)
366.141(HexNAc + Hex)
512.200(HexNAc(1)Hex(1)Fuc(1)_1)
1129.474
1165.994
1238.521(PEP+HexNAc(4)Hex(5)Fuc(1)_2)
M+2H
1964.821(PEP+HexNAc(3)Hex(4)_1)
m/z

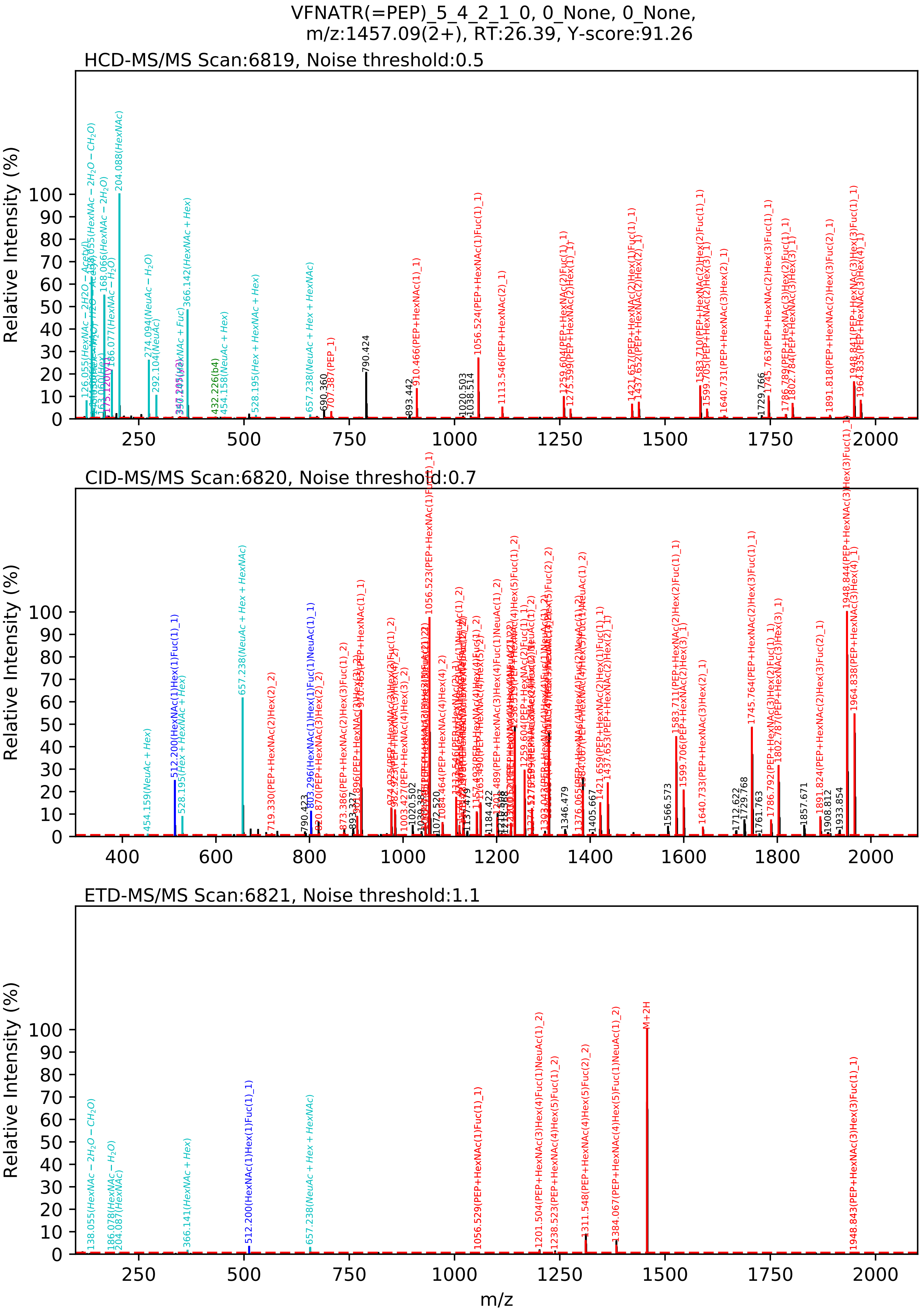

VFNATR(=PEP)_5_4_2_1_0, 0_None, 0_None,
m/z:1457.09(2+), RT:26.39, Y-score:91.26
HCD-MS/MS Scan:6819, Noise threshold:0.5
CID-MS/MS Scan:6820, Noise threshold:0.7
ETD-MS/MS Scan:6821, Noise threshold:1.1
Relative Intensity (%)
m/z

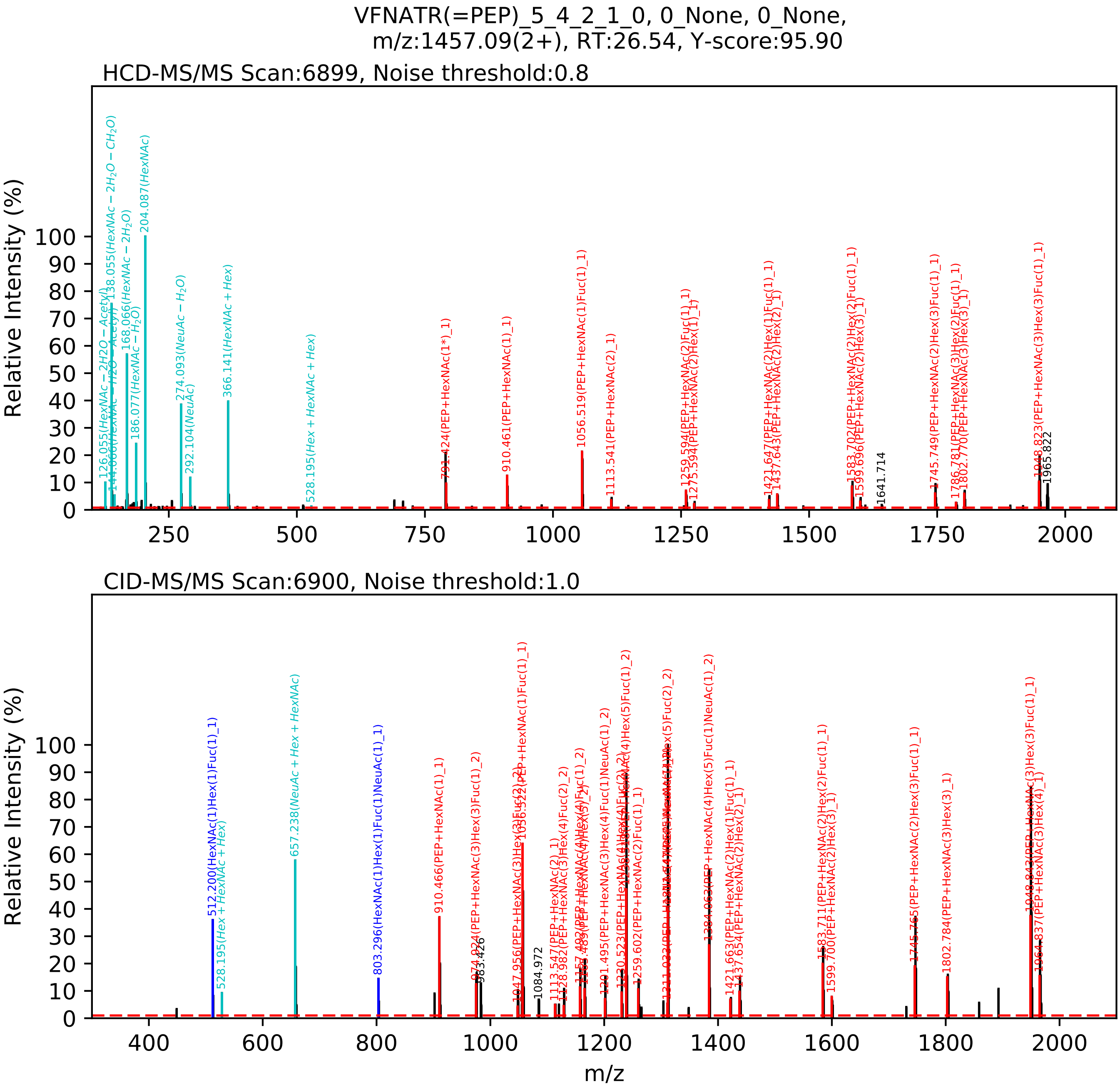

VFNATR(=PEP)_5_4_2_1_0, 0_None, 0_None,
m/z:1457.09(2+), RT:26.54, Y-score:95.90
HCD-MS/MS Scan:6899, Noise threshold:0.8
Relative Intensity (%)
126.055(HexNAc − 2H2O − Acetyl)
138.055(HexNAc − 2H2O − CH2O)
168.066(HexNAc − 2H2O)
186.077(HexNAc − H2O)
204.087(HexNAc)
274.093(NeuAc − H2O)
292.104(NeuAc)
366.141(HexNAc + Hex)
528.195(Hex + HexNAc + Hex)
791.424(PEP+HexNAc(1*)_1)
910.461(PEP+HexNAc(1)_1)
1056.519(PEP+HexNAc(1)Fuc(1)_1)
1113.541(PEP+HexNAc(2)_1)
1259.594(PEP+HexNAc(2)Fuc(1)_1)
1275.594(PEP+HexNAc(2)Hex(1)_1)
1421.647(PEP+HexNAc(2)Hex(1)Fuc(1)_1)
1437.643(PEP+HexNAc(2)Hex(2)_1)
1583.702(PEP+HexNAc(2)Hex(2)Fuc(1)_1)
1599.696(PEP+HexNAc(2)Hex(3)_1)
1641.714
1745.749(PEP+HexNAc(2)Hex(3)Fuc(1)_1)
1786.781(PEP+HexNAc(3)Hex(2)Fuc(1)_1)
1802.770(PEP+HexNAc(3)Hex(3)_1)
1948.823(PEP+HexNAc(3)Hex(3)Fuc(1)_1)
1965.822
CID-MS/MS Scan:6900, Noise threshold:1.0
512.200(HexNAc(1)Hex(1)Fuc(1)_1)
528.195(Hex + HexNAc + Hex)
657.238(NeuAc + Hex + HexNAc)
803.296(HexNAc(1)Hex(1)Fuc(1)NeuAc(1)_1)
910.466(PEP+HexNAc(1)_1)
974.924(PEP+HexNAc(3)Hex(3)Fuc(1)_2)
983.426
1056.522(PEP+HexNAc(1)Fuc(1)_1)
1084.972
1113.547(PEP+HexNAc(2)_1)
1201.495(PEP+HexNAc(3)Hex(4)Fuc(1)NeuAc(1)_2)
1259.602(PEP+HexNAc(2)Fuc(1)_1)
1384.063(PEP+HexNAc(4)Hex(5)Fuc(1)NeuAc(1)_2)
1421.663(PEP+HexNAc(2)Hex(1)Fuc(1)_1)
1437.654(PEP+HexNAc(2)Hex(2)_1)
1583.711(PEP+HexNAc(2)Hex(2)Fuc(1)_1)
1599.700(PEP+HexNAc(2)Hex(3)_1)
1745.765(PEP+HexNAc(2)Hex(3)Fuc(1)_1)
1802.784(PEP+HexNAc(3)Hex(3)_1)
1964.837(PEP+HexNAc(3)Hex(4)_1)
m/z

VFNATR(=PEP)_5_4_2_1_0, 0_None, 0_None,
m/z:1457.09(2+), RT:26.84, Y-score:95.29

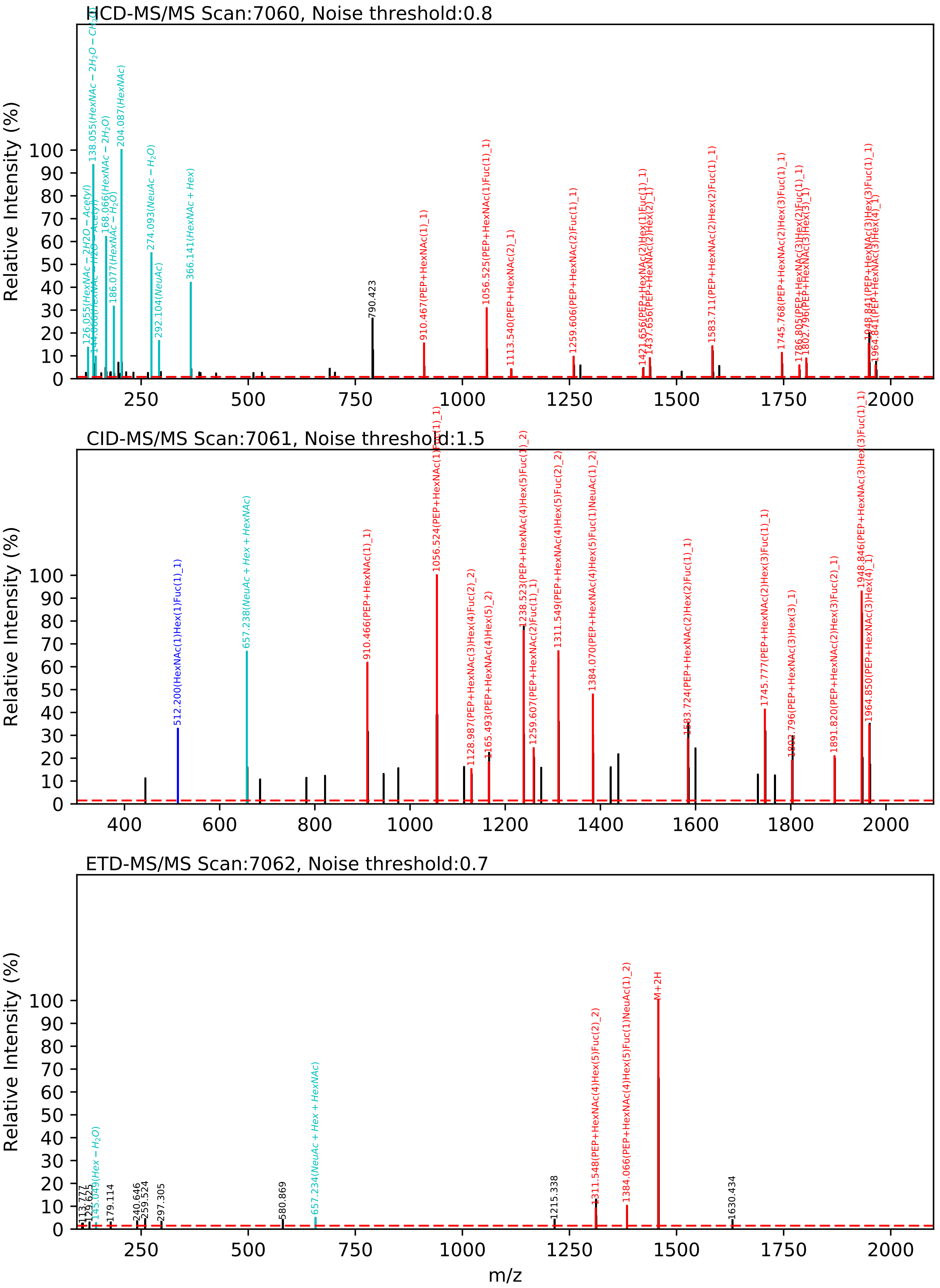

VFNATR(=PEP)_5_4_2_1_0, 0_None, 0_None,
m/z:1457.09(2+), RT:27.03, Y-score:93.61

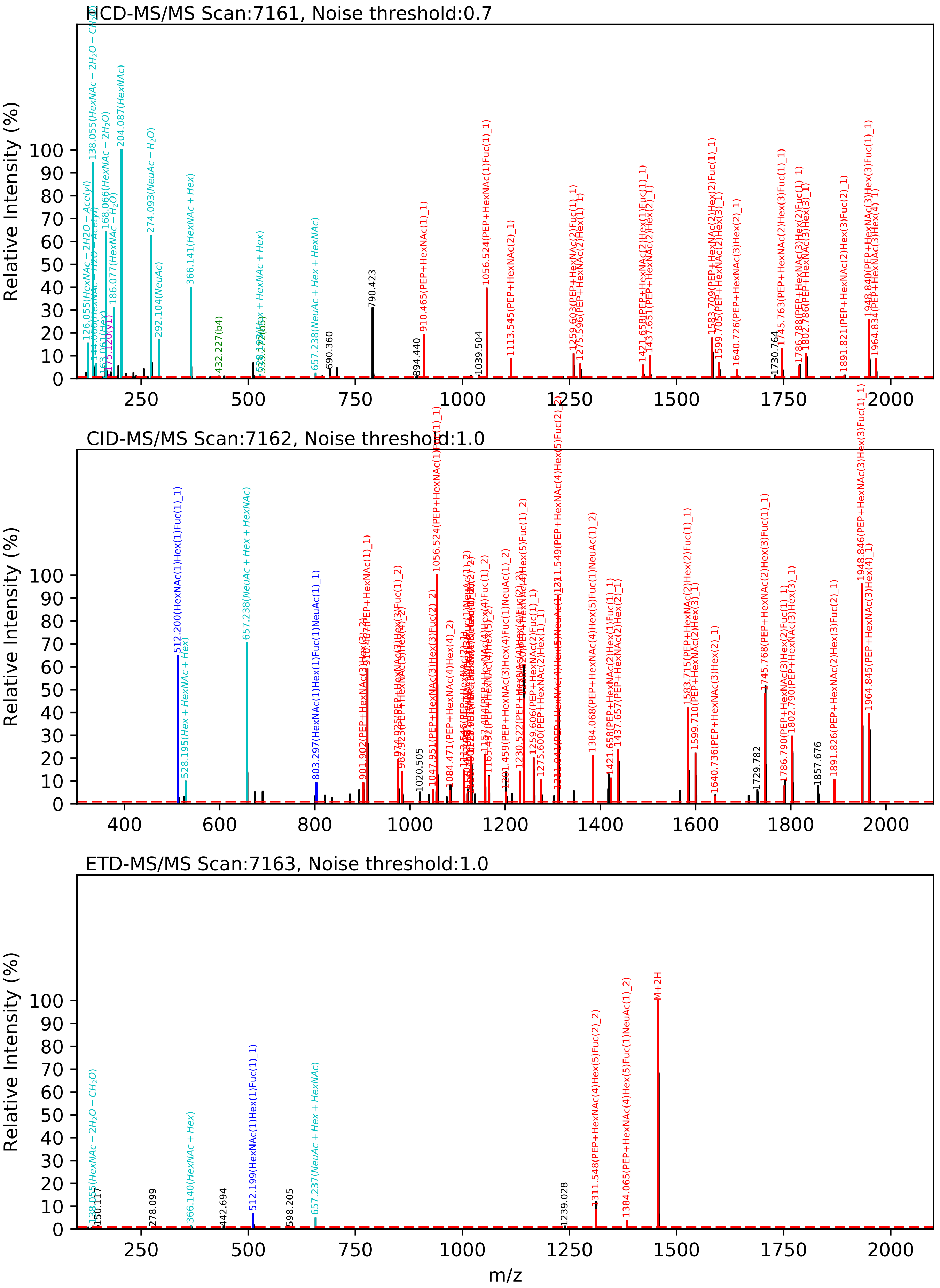

VFNATR(=PEP)_5_4_2_1_0, 0_None, 0_None,
m/z:971.73(3+), RT:26.38, Y-score:98.00

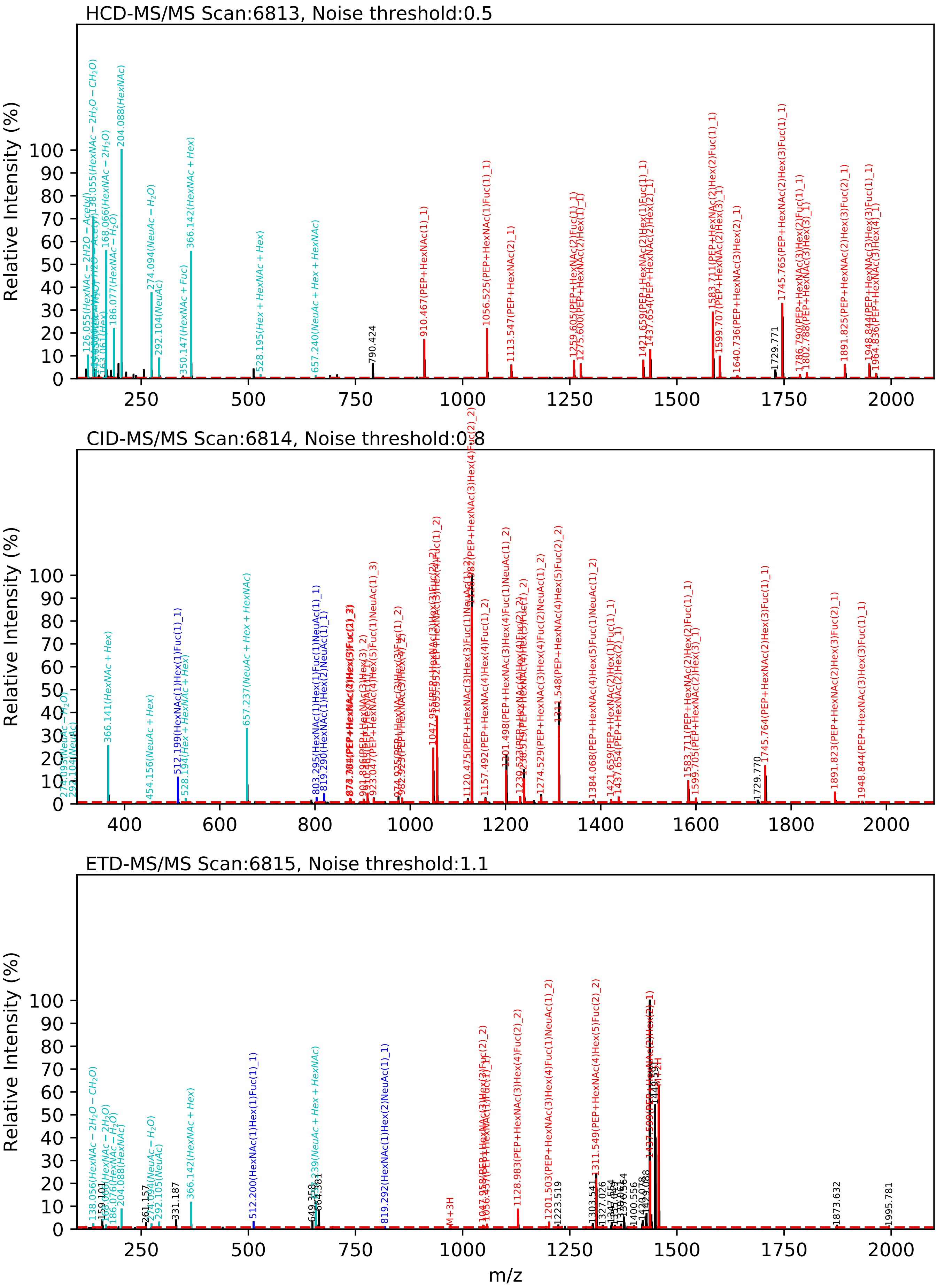

VFNATR(=PEP)_5_4_3_0_0, 0_None, 0_None,
m/z:1384.57(2+), RT:23.80, Y-score:87.42

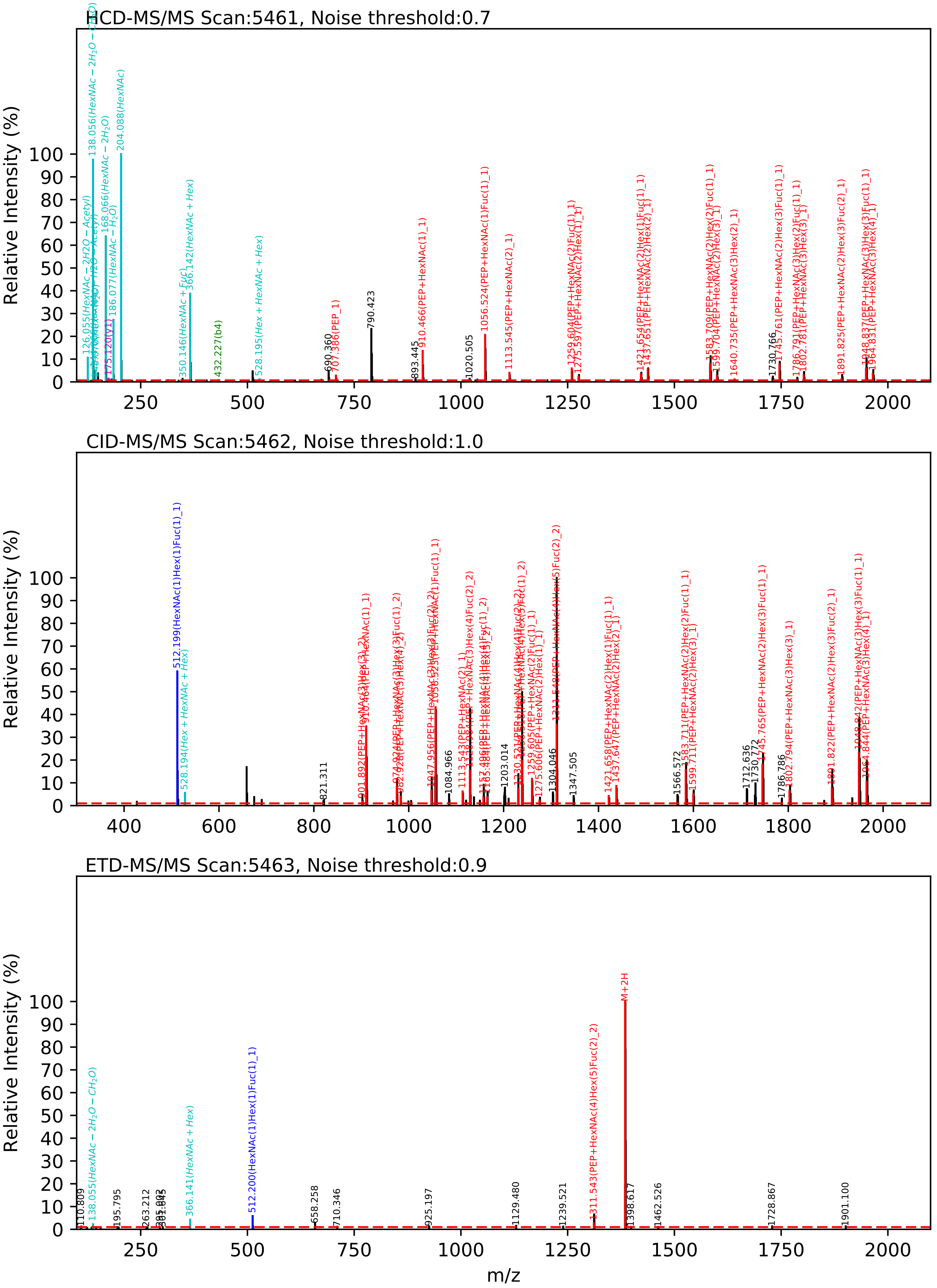

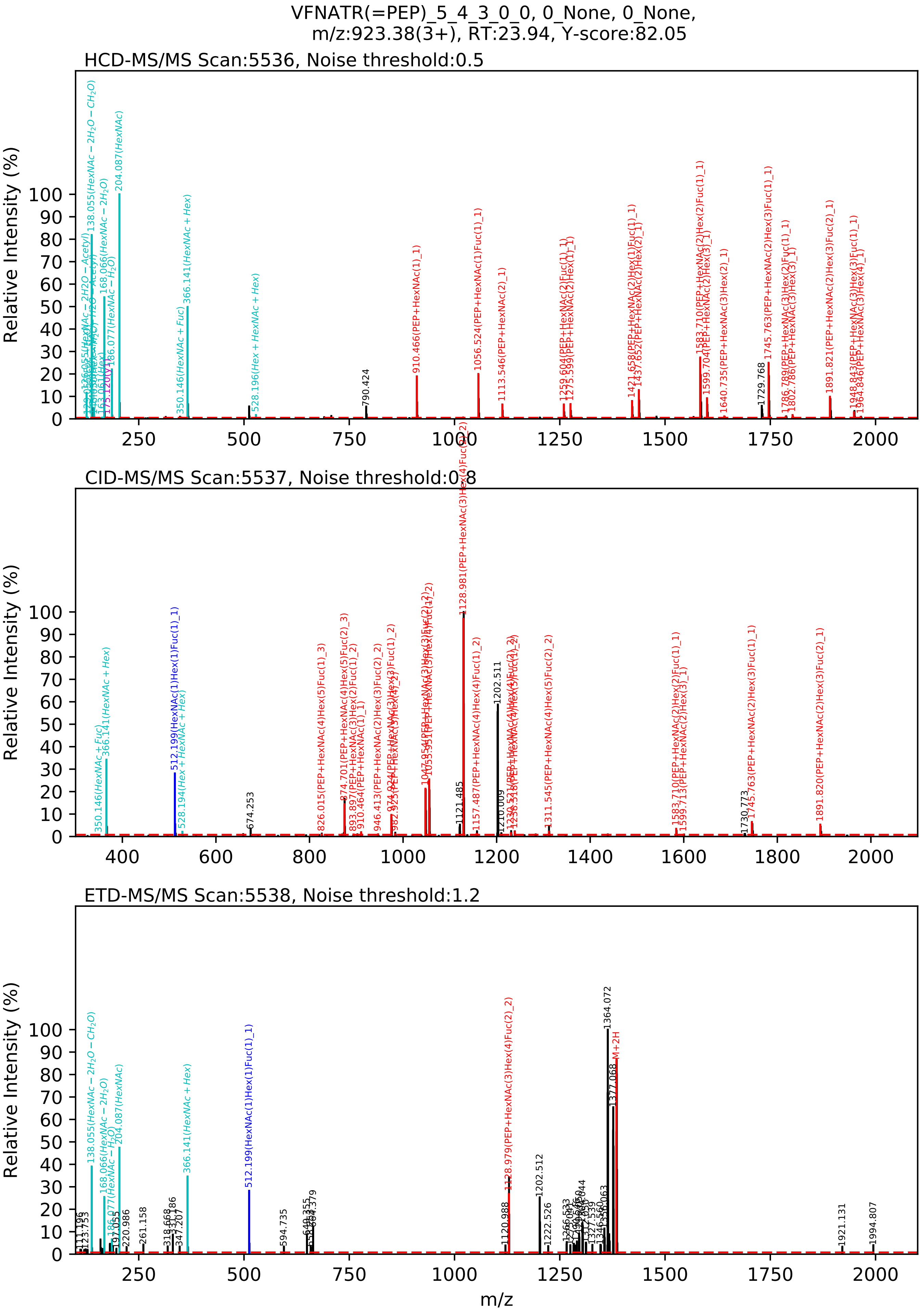

VFNATR(=PEP)_5_4_3_0_0, 0_None, 0_None,
m/z:923.38(3+), RT:23.94, Y-score:82.05
HCD-MS/MS Scan:5536, Noise threshold:0.5
CID-MS/MS Scan:5537, Noise threshold:0.8
ETD-MS/MS Scan:5538, Noise threshold:1.2
Relative Intensity (%)
m/z
204.087(HexNAc)
366.141(HexNAc+Hex)
910.466(PEP+HexNAc(1)_1)
1056.524(PEP+HexNAc(1)Fuc(1)_1)
1113.546(PEP+HexNAc(2)_1)
1745.763(PEP+HexNAc(2)Hex(3)Fuc(1)_1)
1891.821(PEP+HexNAc(2)Hex(3)Fuc(2)_1)
1128.981(PEP+HexNAc(3)Hex(4)Fuc(2)_2)
512.199(HexNAc(1)Hex(1)Fuc(1)_1)
1202.511
1364.072
1377.068
M+2H
1128.979(PEP+HexNAc(3)Hex(4)Fuc(2)_2)
1202.512

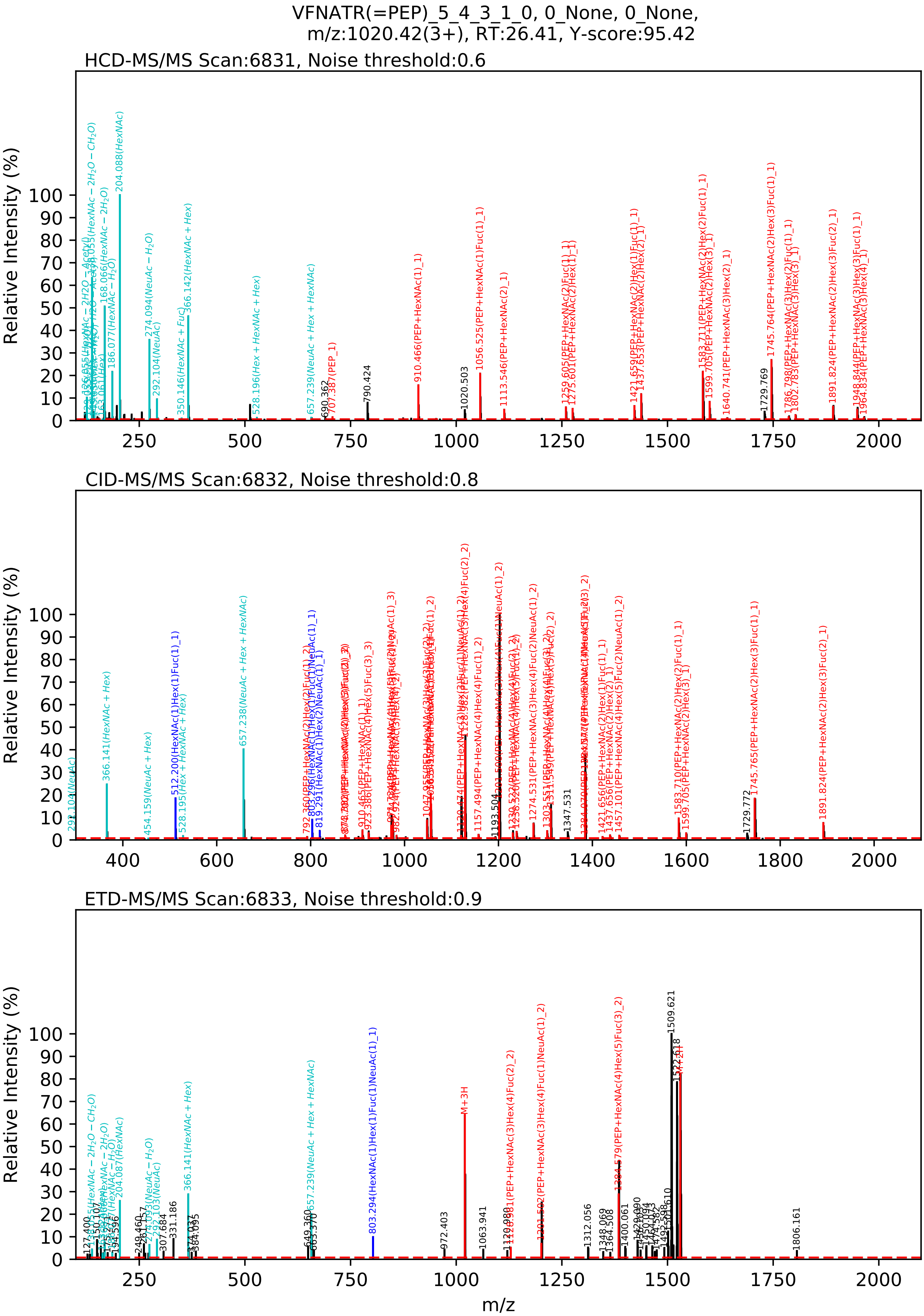
VFNATR(=PEP)_5_4_3_1_0, 0_None, 0_None,
m/z:1020.42(3+), RT:26.41, Y-score:95.42
HCD-MS/MS Scan:6831, Noise threshold:0.6
CID-MS/MS Scan:6832, Noise threshold:0.8
ETD-MS/MS Scan:6833, Noise threshold:0.9
Relative Intensity (%)
m/z
M+3H

VFNATR(=PEP)_5_4_3_1_0, 0_None, 0_None,
m/z:1530.12(2+), RT:26.44, Y-score:94.07

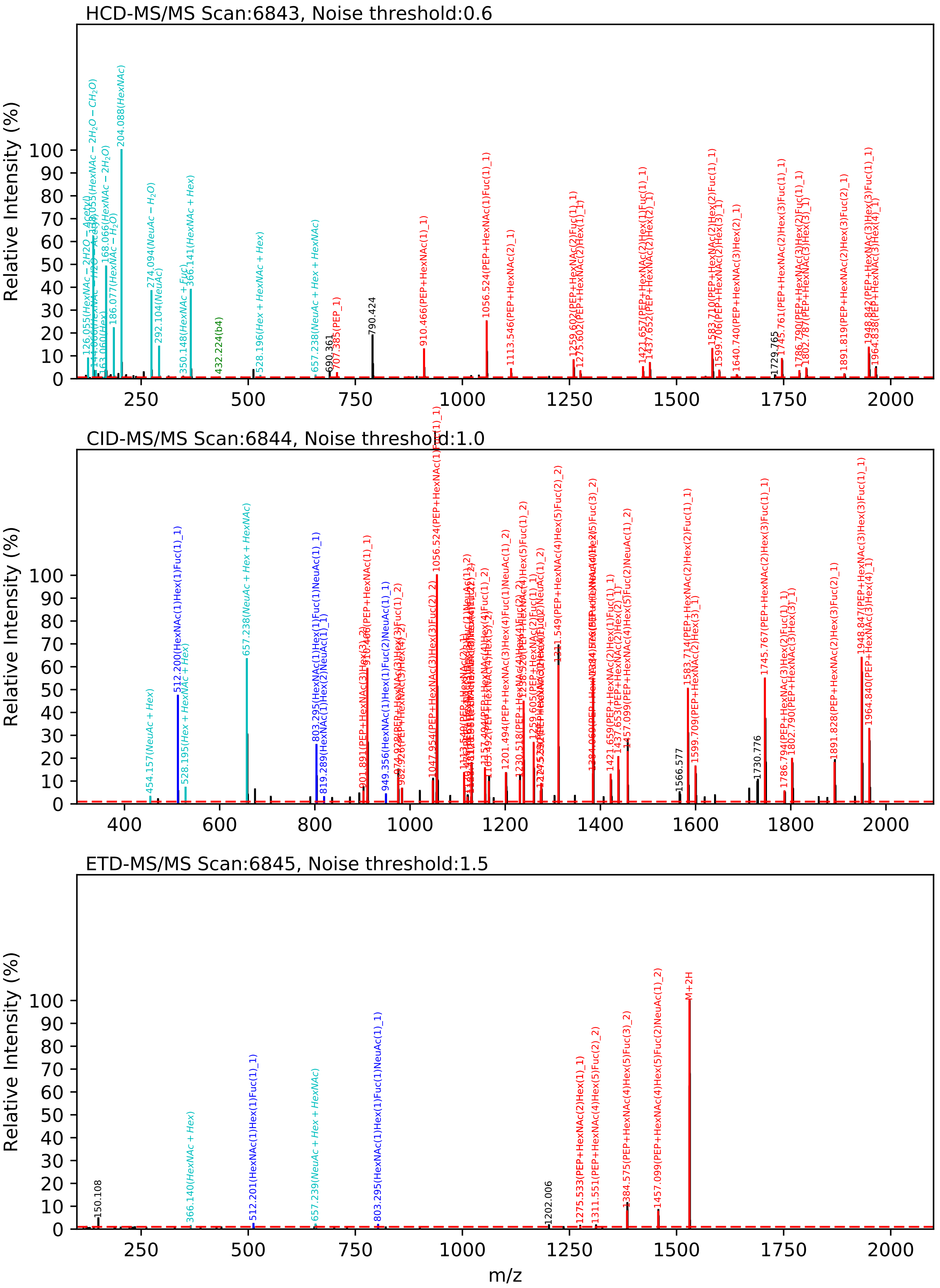

VFNATR(=PEP)_5_5_1_0_0, 0_None, 0_None,
m/z:893.71(3+), RT:24.18, Y-score:95.61

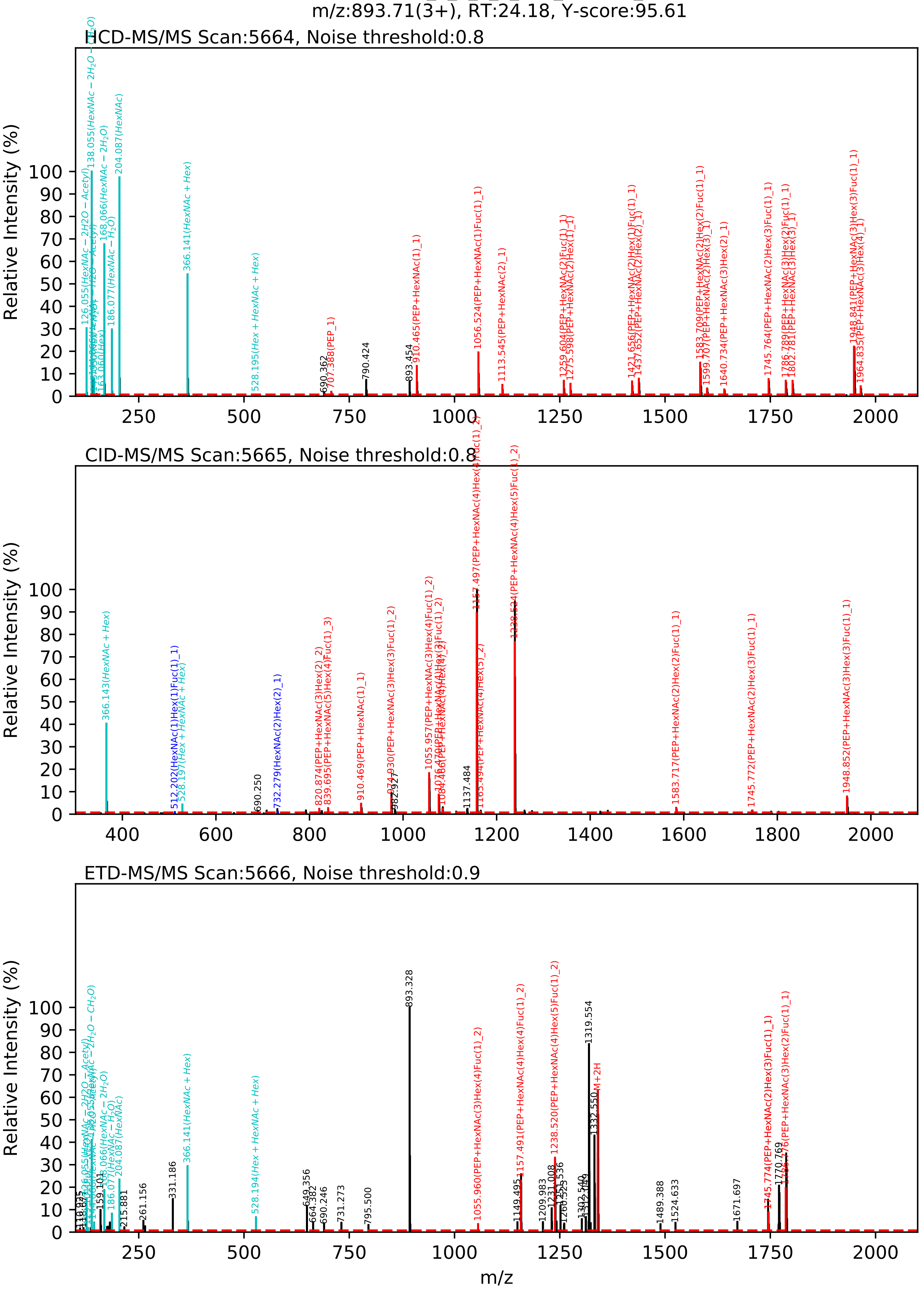

VFNATR(=PEP)_5_5_1_0_0, 0_None, 0_None,
m/z:1340.06(2+), RT:24.16, Y-score:97.71

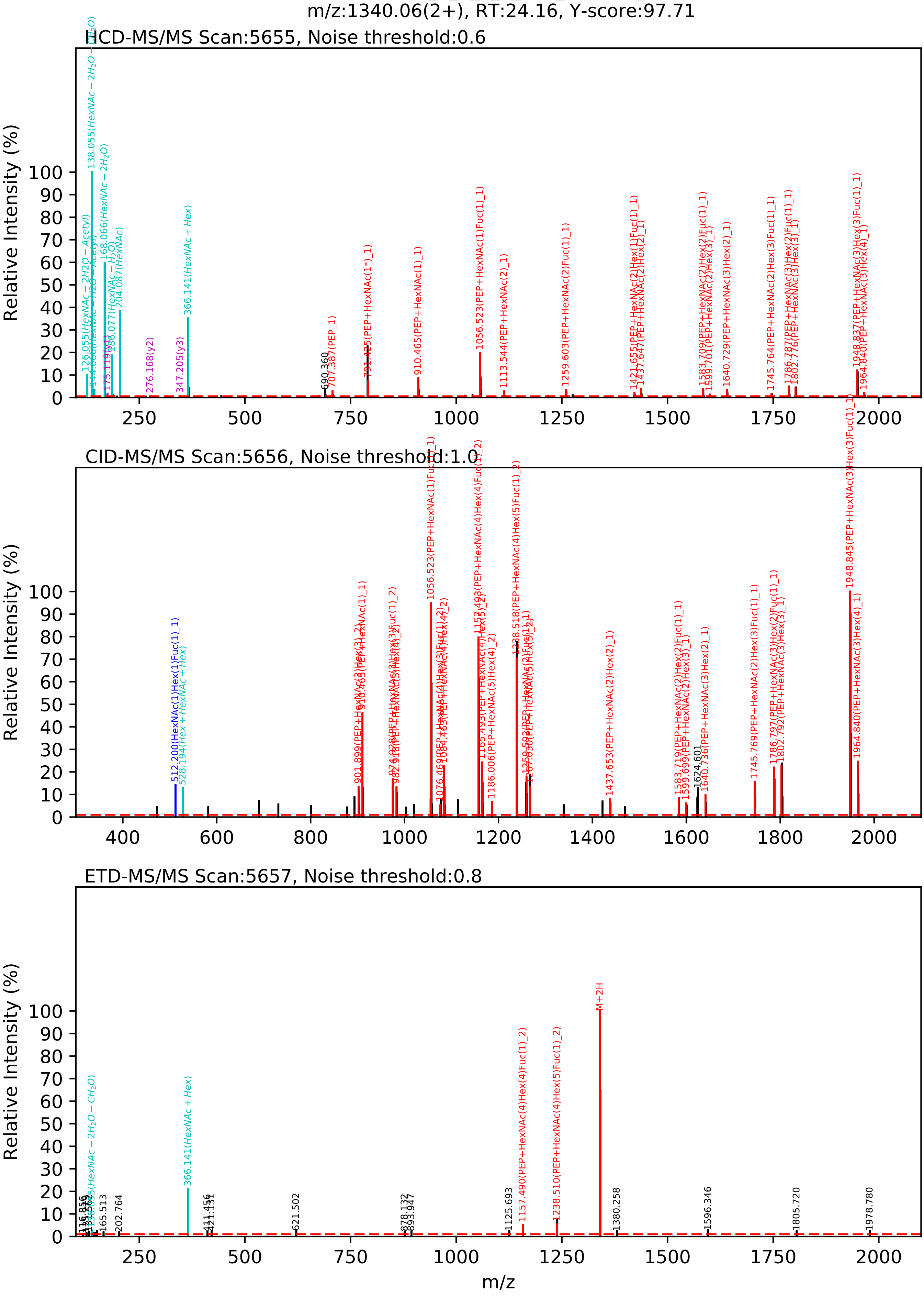

VFNATR(=PEP)_5_5_1_0_0, 0_None, 0_None,
m/z:1340.05(2+), RT:24.51, Y-score:87.91

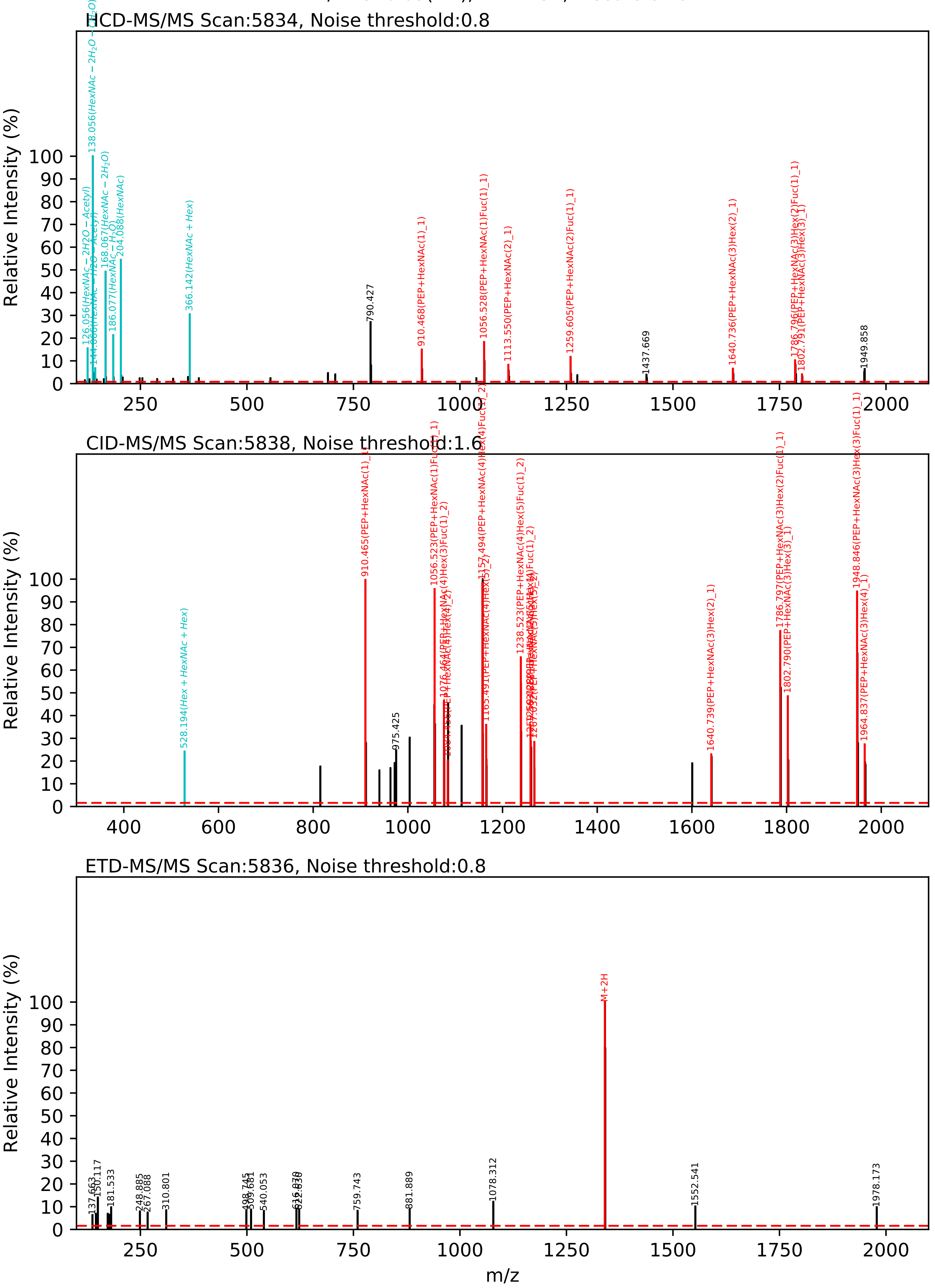

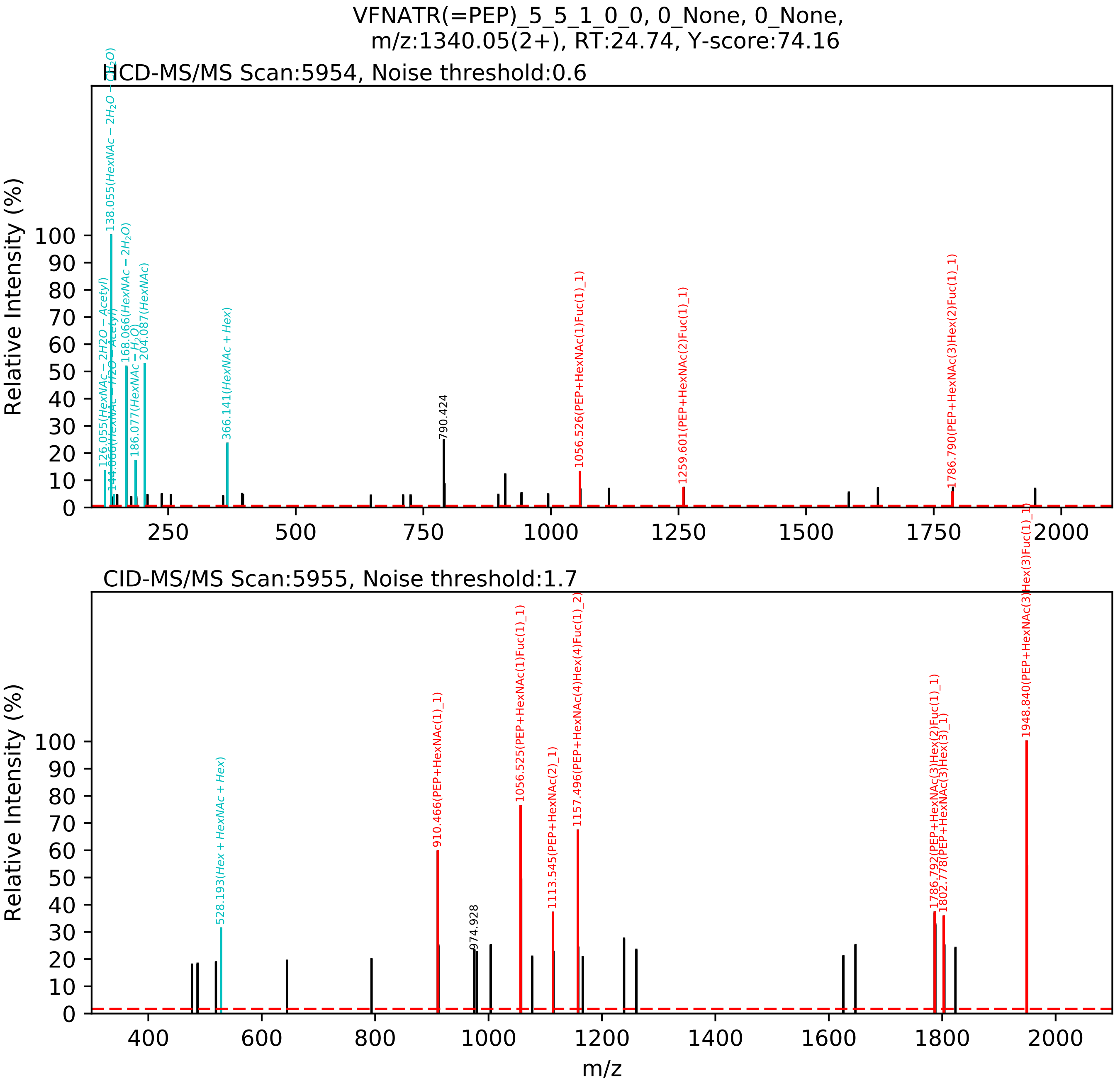

VFNATR(=PEP)_5_5_1_0_0, 0_None, 0_None,
m/z:1340.05(2+), RT:24.74, Y-score:74.16
HCD-MS/MS Scan:5954, Noise threshold:0.6
Relative Intensity (%)
138.055
126.055
168.066
186.077
204.087(HexNAc)
366.141(HexNAc + Hex)
790.424
1056.526(PEP+HexNAc(1)Fuc(1)_1)
1259.601(PEP+HexNAc(2)Fuc(1)_1)
1786.790(PEP+HexNAc(3)Hex(2)Fuc(1)_1)
CID-MS/MS Scan:5955, Noise threshold:1.7
Relative Intensity (%)
528.193(Hex + HexNAc + Hex)
910.466(PEP+HexNAc(1)_1)
974.928
1056.525(PEP+HexNAc(1)Fuc(1)_1)
1113.545(PEP+HexNAc(2)_1)
1157.496(PEP+HexNAc(4)Hex(4)Fuc(1)_2)
1786.792(PEP+HexNAc(3)Hex(2)Fuc(1)_1)
1802.778(PEP+HexNAc(3)Hex(3)_1)
1948.840(PEP+HexNAc(3)Hex(3)Fuc(1)_1)
m/z

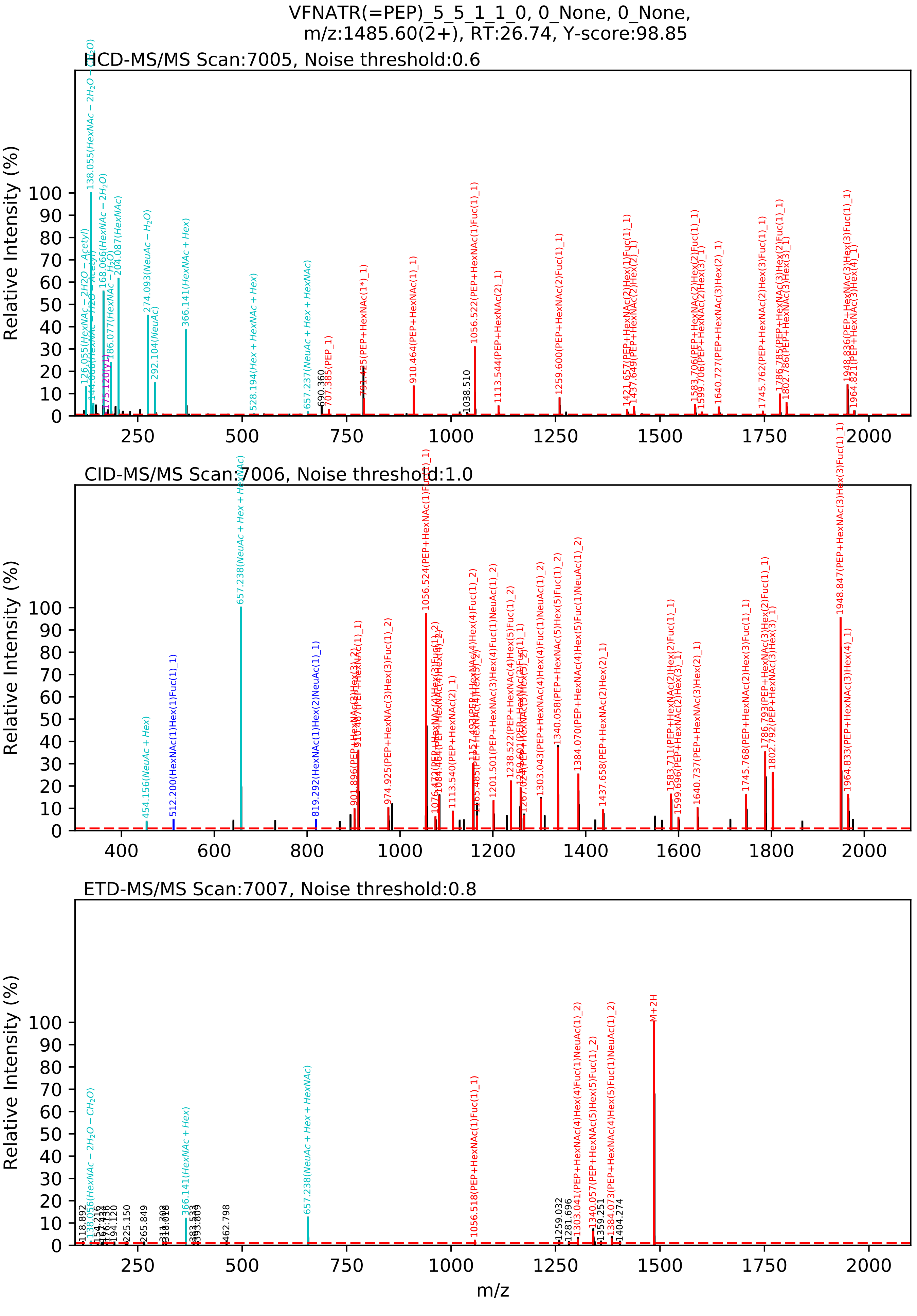

VFNATR(=PEP)_5_5_1_1_0, 0_None, 0_None,
m/z:1485.60(2+), RT:26.74, Y-score:98.85
HCD-MS/MS Scan:7005, Noise threshold:0.6
Relative Intensity (%)
138.055($HexNAc - 2H_2O - CH_2O$)
168.066($HexNAc - 2H_2O$)
204.087($HexNAc$)
274.093($NeuAc - H_2O$)
292.104($NeuAc$)
366.141($HexNAc + Hex$)
528.194($Hex + HexNAc + Hex$)
657.237($NeuAc + Hex + HexNAc$)
690.360
707.385(PEP_1)
791.425(PEP+HexNAc(1*)_1)
910.464(PEP+HexNAc(1)_1)
1038.510
1056.522(PEP+HexNAc(1)Fuc(1)_1)
1113.544(PEP+HexNAc(2)_1)
1259.600(PEP+HexNAc(2)Fuc(1)_1)
1640.727(PEP+HexNAc(3)Hex(2)_1)
1745.762(PEP+HexNAc(2)Hex(3)Fuc(1)_1)
CID-MS/MS Scan:7006, Noise threshold:1.0
454.156($NeuAc + Hex$)
512.200(HexNAc(1)Hex(1)Fuc(1)_1)
657.238($NeuAc + Hex + HexNAc$)
819.292(HexNAc(1)Hex(2)NeuAc(1)_1)
974.925(PEP+HexNAc(3)Hex(3)Fuc(1)_2)
1056.524(PEP+HexNAc(1)Fuc(1)_1)
1113.540(PEP+HexNAc(2)_1)
1201.501(PEP+HexNAc(3)Hex(4)Fuc(1)NeuAc(1)_2)
1303.043(PEP+HexNAc(4)Hex(4)Fuc(1)NeuAc(1)_2)
1340.058(PEP+HexNAc(5)Hex(5)Fuc(1)_2)
1384.070(PEP+HexNAc(4)Hex(5)Fuc(1)NeuAc(1)_2)
1437.658(PEP+HexNAc(2)Hex(2)_1)
1583.711(PEP+HexNAc(2)Hex(2)Fuc(1)_1)
1599.696(PEP+HexNAc(2)Hex(3)_1)
1640.737(PEP+HexNAc(3)Hex(2)_1)
1745.768(PEP+HexNAc(2)Hex(3)Fuc(1)_1)
1786.793(PEP+HexNAc(3)Hex(2)Fuc(1)_1)
1802.792(PEP+HexNAc(3)Hex(3)_1)
1948.847(PEP+HexNAc(3)Hex(3)Fuc(1)_1)
1964.833(PEP+HexNAc(3)Hex(4)_1)
ETD-MS/MS Scan:7007, Noise threshold:0.8
118.892
138.056($HexNAc - 2H_2O - CH_2O$)
194.120
225.150
265.849
366.141($HexNAc + Hex$)
462.798
657.238($NeuAc + Hex + HexNAc$)
1056.518(PEP+HexNAc(1)Fuc(1)_1)
1259.032
1281.696
1303.041(PEP+HexNAc(4)Hex(4)Fuc(1)NeuAc(1)_2)
1340.057(PEP+HexNAc(5)Hex(5)Fuc(1)_2)
1359.251
1384.073(PEP+HexNAc(4)Hex(5)Fuc(1)NeuAc(1)_2)
1404.274
M+2H
m/z

VFNATR(=PEP)_5_5_1_2_0, 0_None, 0_None,
m/z:1631.15(2+), RT:31.84, Y-score:96.26

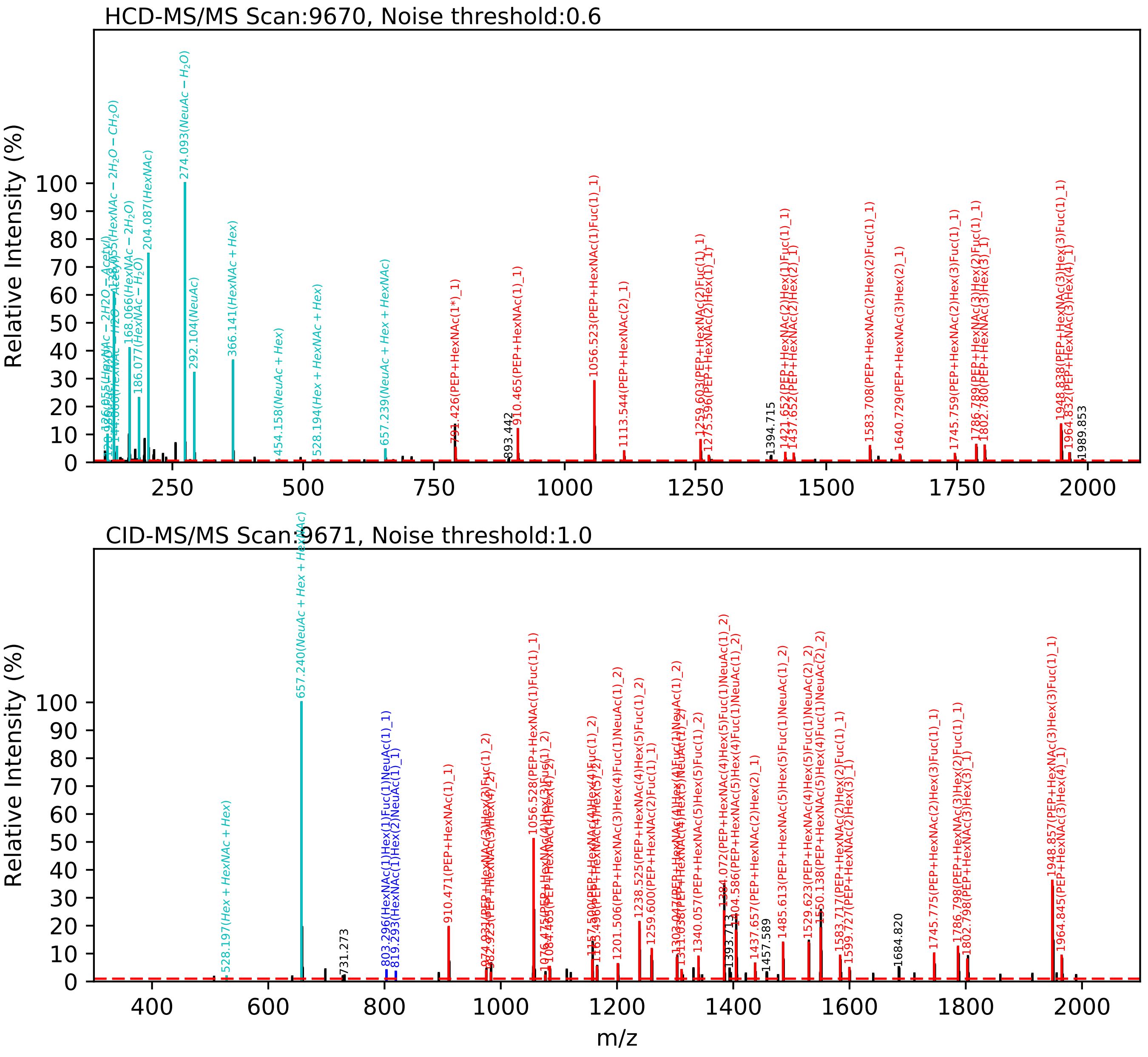

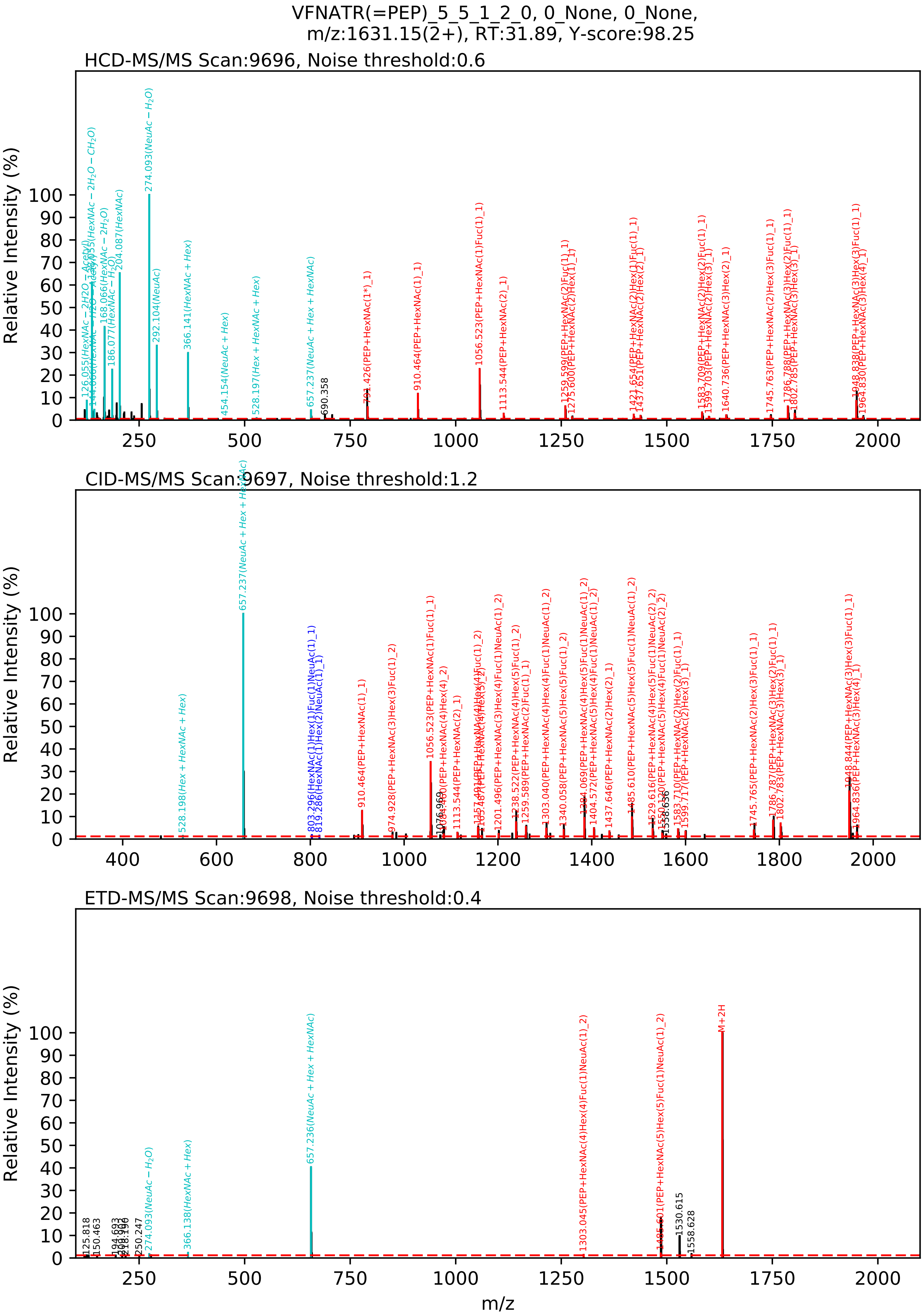

VFNATR(=PEP)_5_5_1_2_0, 0_None, 0_None,
m/z:1631.15(2+), RT:31.89, Y-score:98.25
HCD-MS/MS Scan:9696, Noise threshold:0.6
CID-MS/MS Scan:9697, Noise threshold:1.2
ETD-MS/MS Scan:9698, Noise threshold:0.4
Relative Intensity (%)
m/z

VFNATR(=PEP)_5_5_2_0_0, 0_None, 0_None,
m/z:1413.08(2+), RT:24.01, Y-score:92.58

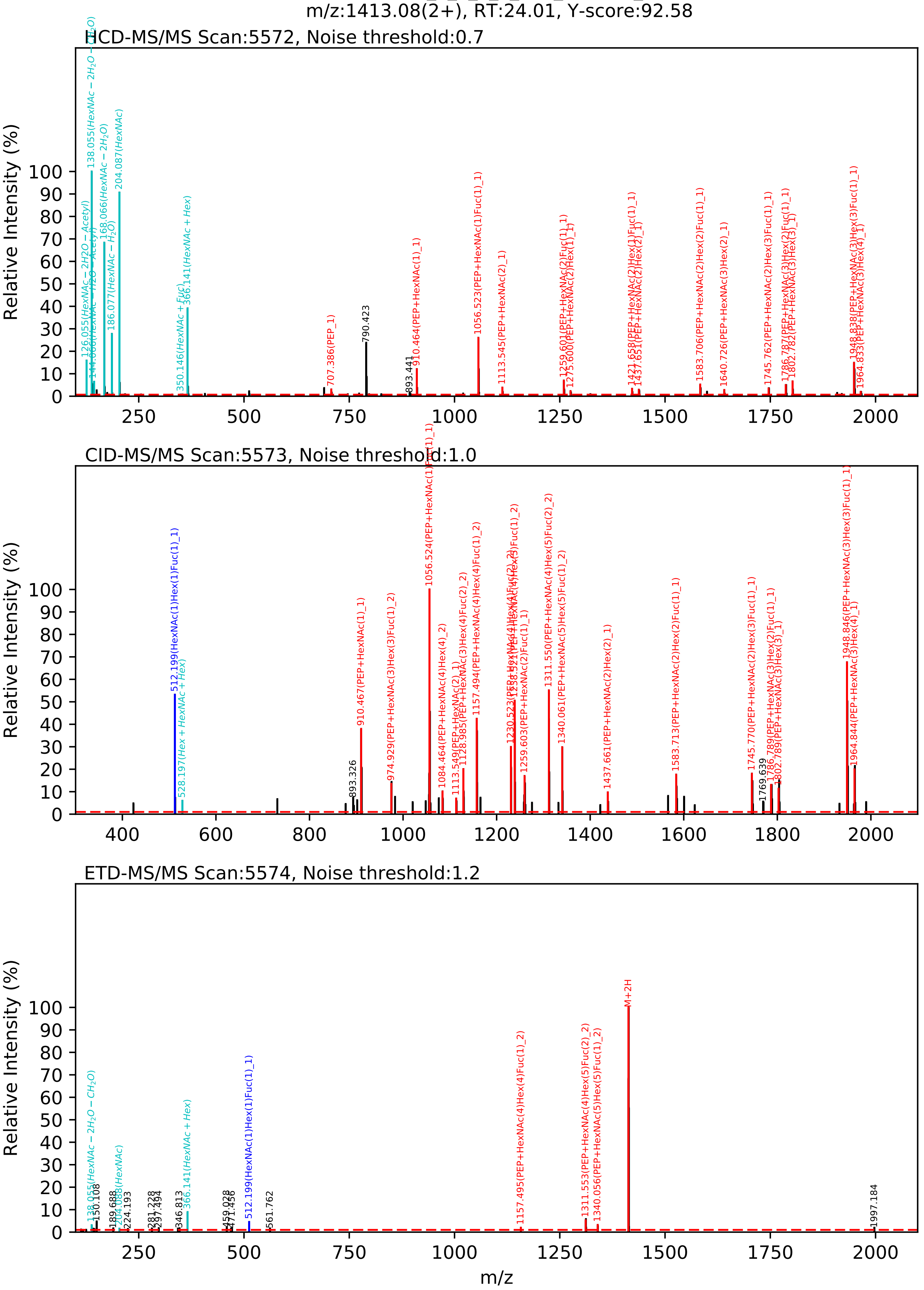

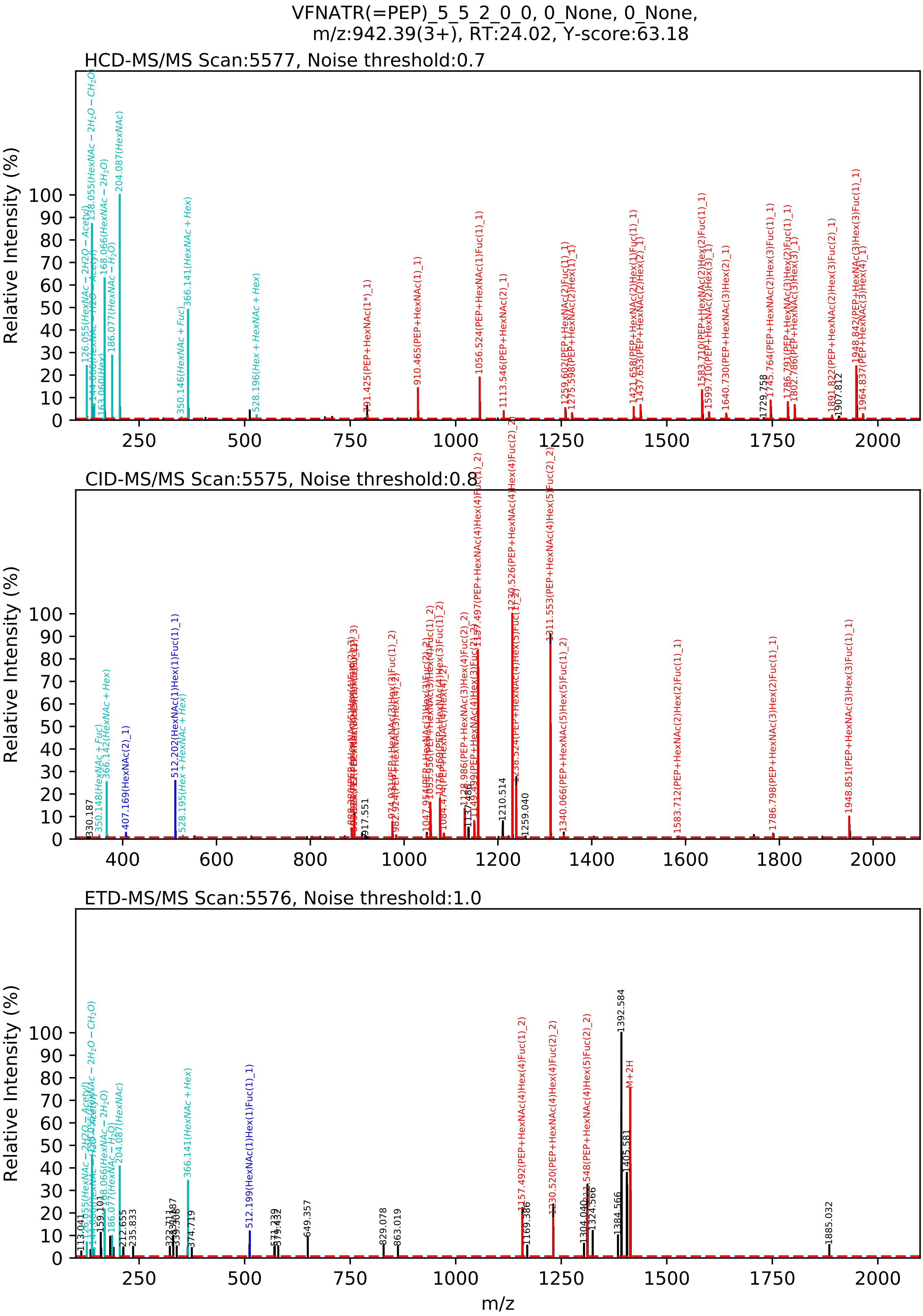

VFNATR(=PEP)_5_5_2_0_0, 0_None, 0_None,
m/z:942.39(3+), RT:24.02, Y-score:63.18
HCD-MS/MS Scan:5577, Noise threshold:0.7
CID-MS/MS Scan:5575, Noise threshold:0.8
ETD-MS/MS Scan:5576, Noise threshold:1.0
Relative Intensity (%)
m/z

VFNATR(=PEP)_5_5_3_0_0, 0_None, 0_None,
m/z:1486.11(2+), RT:23.90, Y-score:85.96

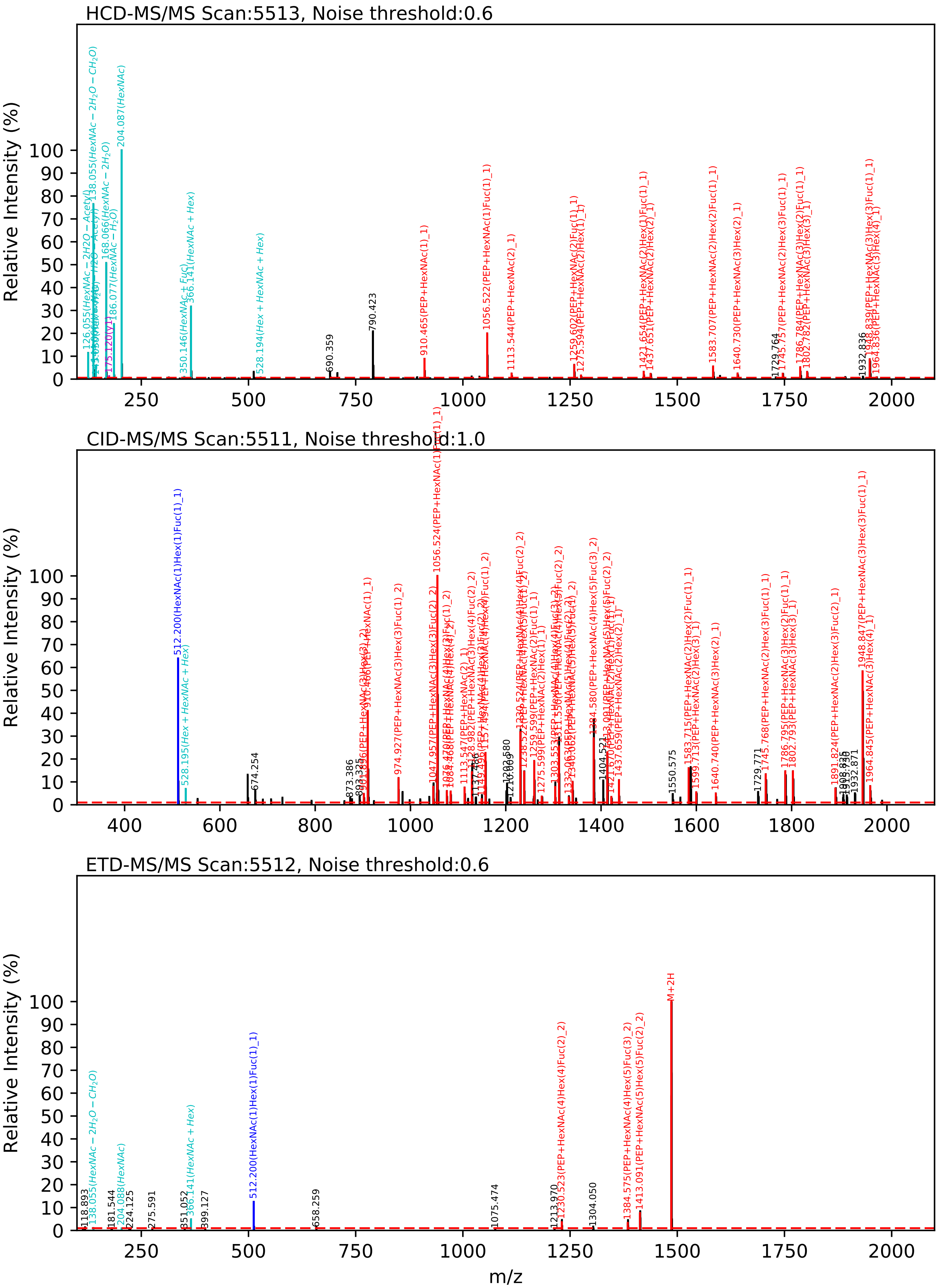

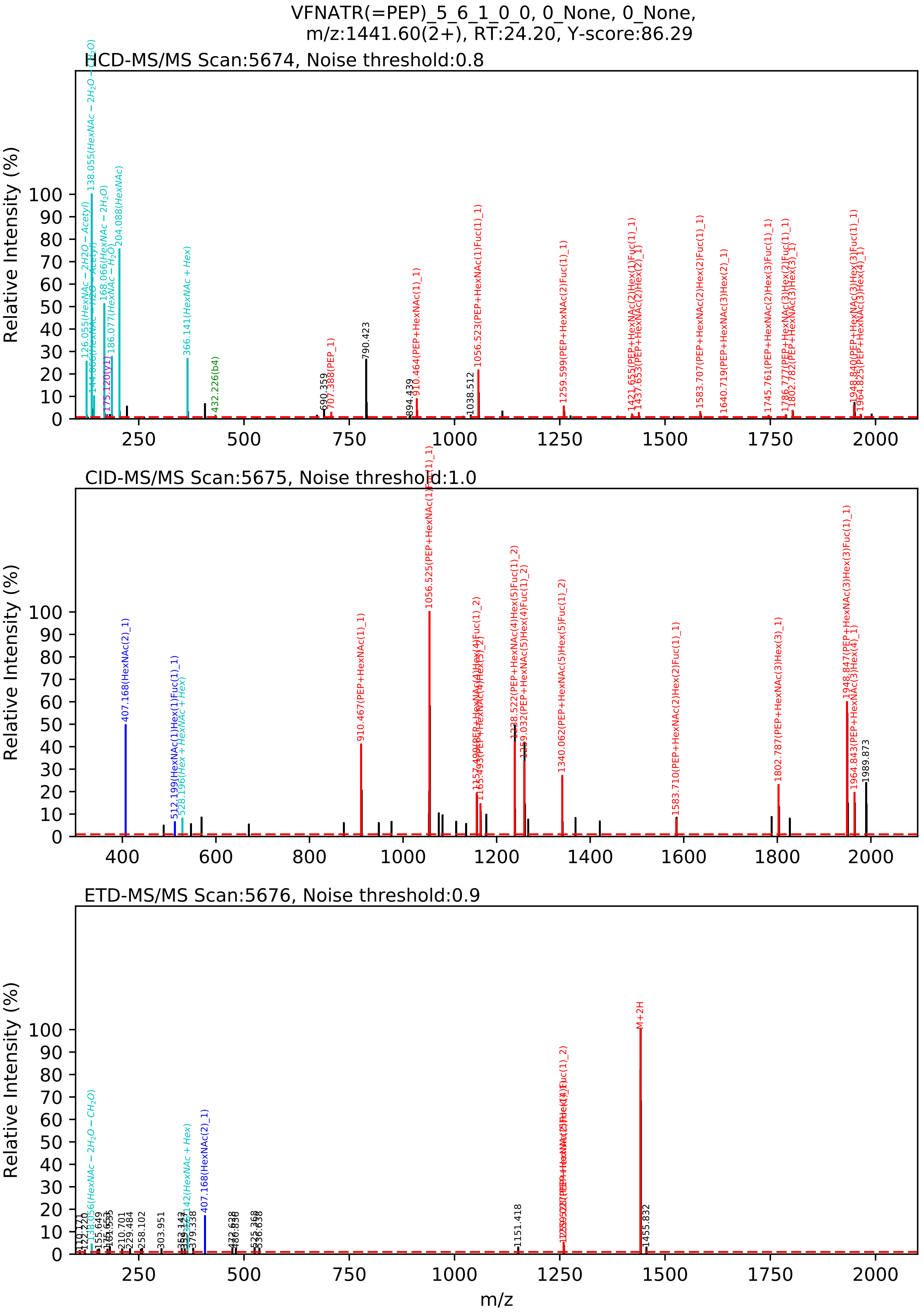
VFNATR(=PEP)_5_6_1_0_0, 0_None, 0_None,
m/z:1441.60(2+), RT:24.20, Y-score:86.29
HCD-MS/MS Scan:5674, Noise threshold:0.8
CID-MS/MS Scan:5675, Noise threshold:1.0
ETD-MS/MS Scan:5676, Noise threshold:0.9
Relative Intensity (%)
m/z
790.423
1056.523(PEP+HexNAc(1)Fuc(1)_1)
1259.599(PEP+HexNAc(2)Fuc(1)_1)
1583.707(PEP+HexNAc(2)Hex(2)Fuc(1)_1)
1640.719(PEP+HexNAc(3)Hex(2)_1)
910.464(PEP+HexNAc(1)_1)
366.141(HexNAc+Hex)
432.226(b4)
204.088(HexNAc)
407.168(HexNAc(2)_1)
910.467(PEP+HexNAc(1)_1)
1238.522(PEP+HexNAc(4)Hex(5)Fuc(1)_2)
1340.062(PEP+HexNAc(5)Hex(5)Fuc(1)_2)
1583.710(PEP+HexNAc(2)Hex(2)Fuc(1)_1)
1802.787(PEP+HexNAc(3)Hex(3)_1)
1989.873
M+2H
1151.418
1455.832
407.168(HexNAc(2)_1)

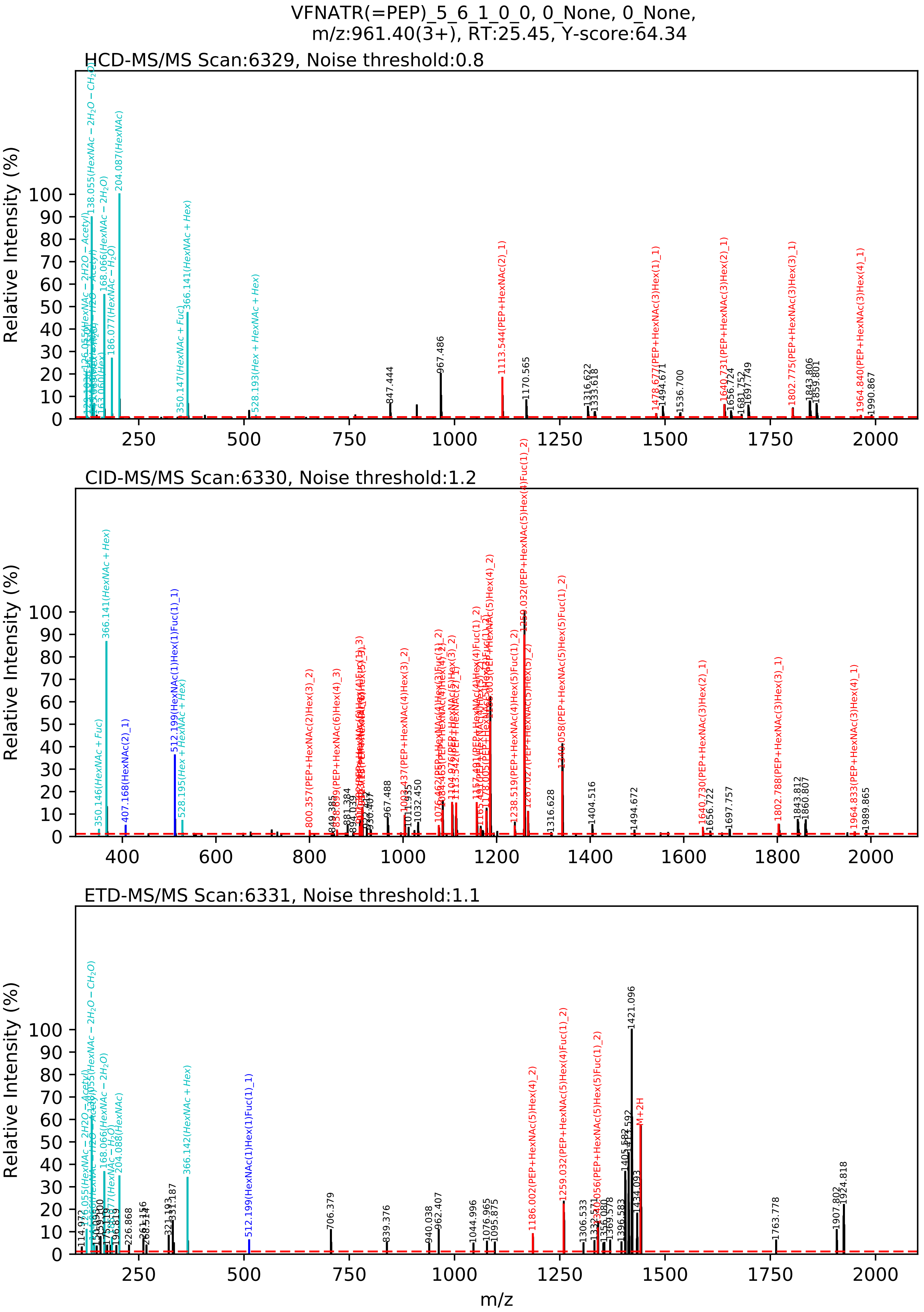

VFNATR(=PEP)_5_6_1_0_0, 0_None, 0_None,
m/z:961.40(3+), RT:25.45, Y-score:64.34
HCD-MS/MS Scan:6329, Noise threshold:0.8
CID-MS/MS Scan:6330, Noise threshold:1.2
ETD-MS/MS Scan:6331, Noise threshold:1.1
Relative Intensity (%)
m/z
204.087(HexNAc)
366.141(HexNAc+Hex)
528.193(Hex+HexNAc+Hex)
1113.544(PEP+HexNAc(2)_1)
1478.677(PEP+HexNAc(3)Hex(1)_1)
1640.731(PEP+HexNAc(3)Hex(2)_1)
1802.775(PEP+HexNAc(3)Hex(3)_1)
1964.840(PEP+HexNAc(3)Hex(4)_1)
512.199(HexNAc(1)Hex(1)Fuc(1)_1)
407.168(HexNAc(2)_1)
1340.058(PEP+HexNAc(5)Hex(5)Fuc(1)_2)
1259.032(PEP+HexNAc(5)Hex(4)Fuc(1)_2)
1186.002(PEP+HexNAc(5)Hex(4)_2)
1421.096
M+2H

VFNATR(=PEP)_5_6_1_1_0, 0_None, 0_None,
m/z:1587.14(2+), RT:26.64, Y-score:92.21

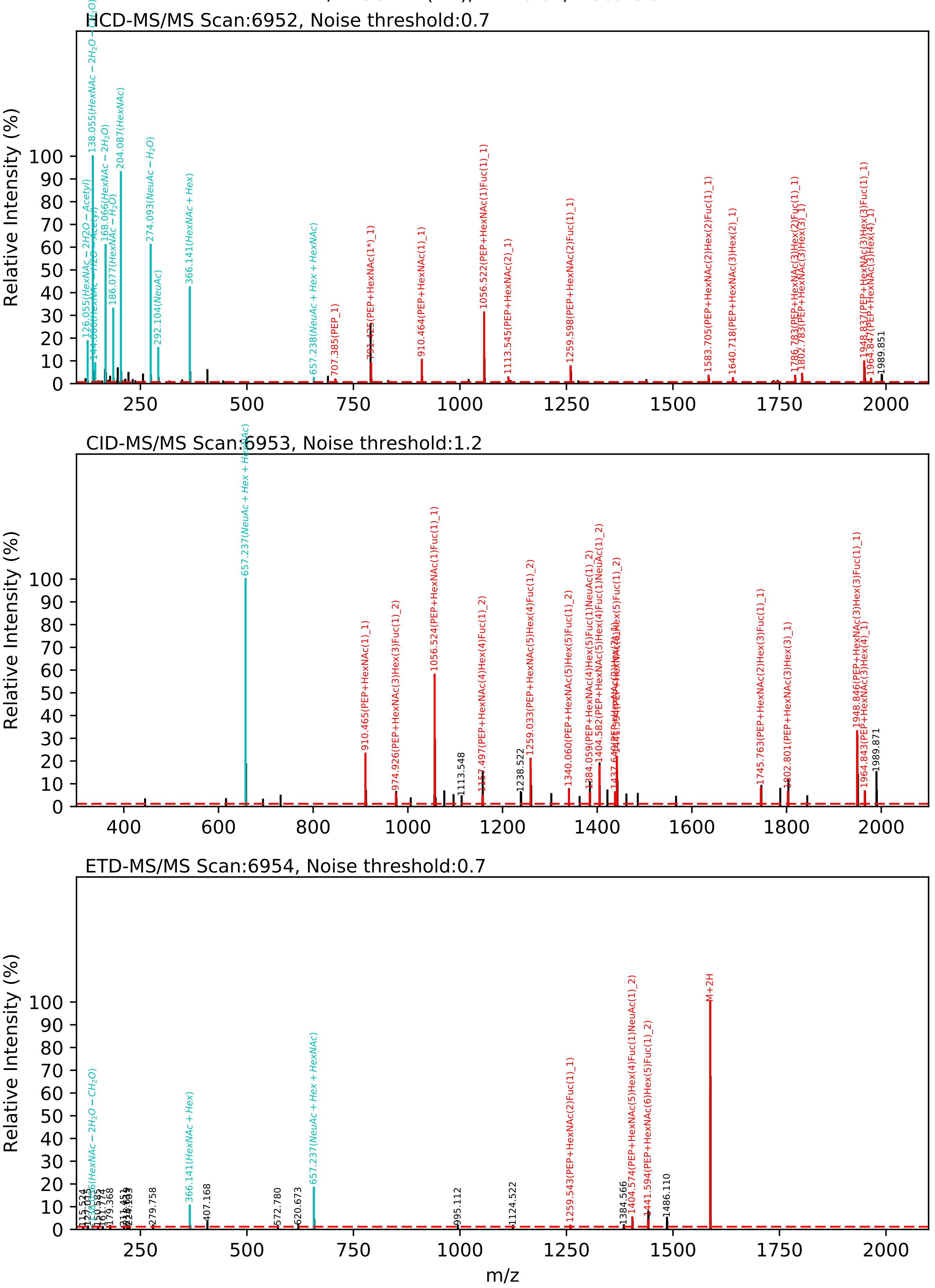

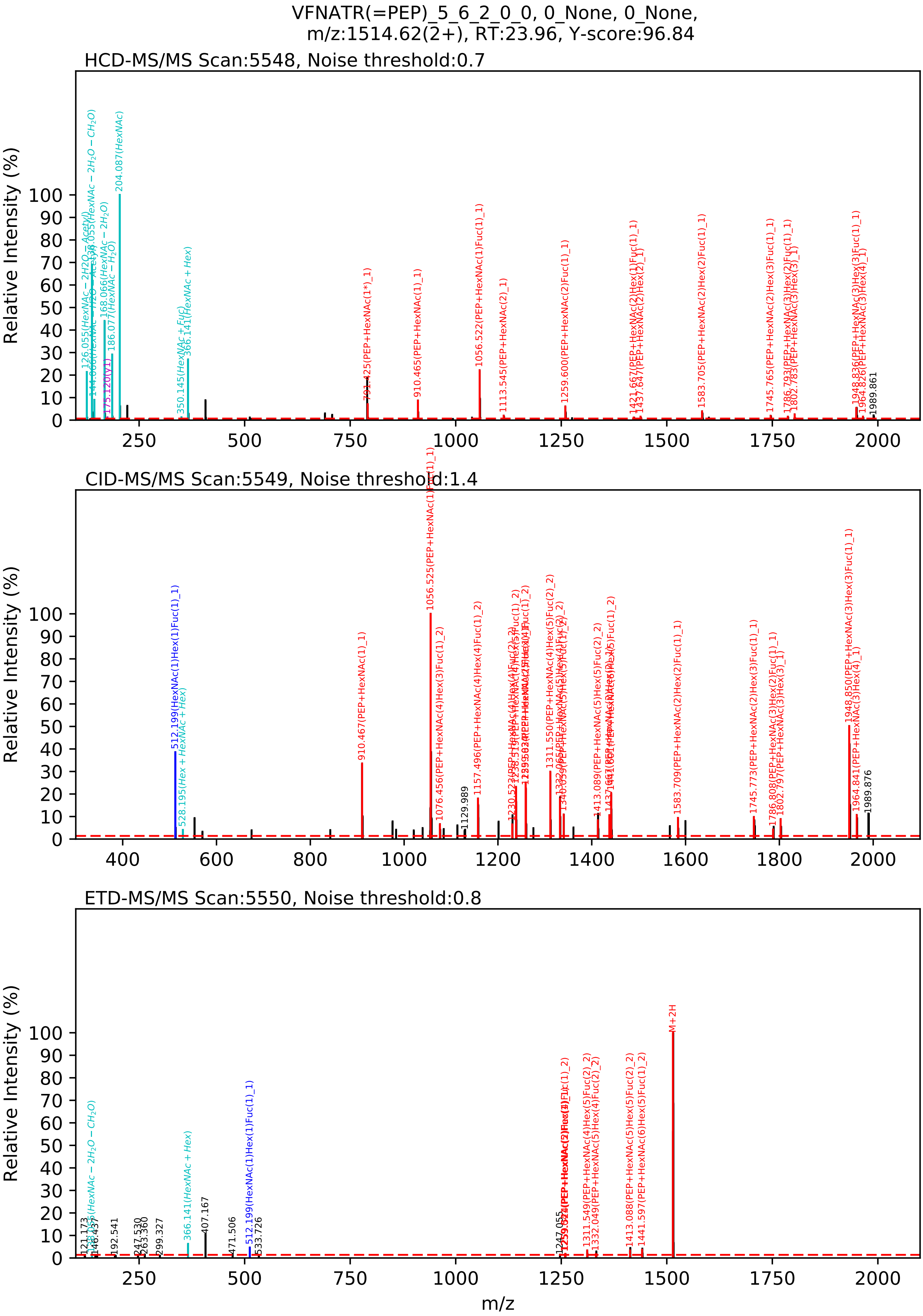

VFNATR(=PEP)_5_6_2_0_0, 0_None, 0_None,
m/z:1514.62(2+), RT:23.96, Y-score:96.84
HCD-MS/MS Scan:5548, Noise threshold:0.7
Relative Intensity (%)
204.087(HexNAc)
168.066(HexNAc − 2H2O)
186.077(HexNAc − H2O)
175.120(y1)
366.141(HexNAc + Hex)
350.145(HexNAc + Fuc)
791.425(PEP+HexNAc(1*)_1)
910.465(PEP+HexNAc(1)_1)
1056.522(PEP+HexNAc(1)Fuc(1)_1)
1113.545(PEP+HexNAc(2)_1)
1259.600(PEP+HexNAc(2)Fuc(1)_1)
1421.667(PEP+HexNAc(2)Hex(1)Fuc(1)_1)
1437.647(PEP+HexNAc(2)Hex(2)_1)
1583.705(PEP+HexNAc(2)Hex(2)Fuc(1)_1)
1745.765(PEP+HexNAc(2)Hex(3)Fuc(1)_1)
1786.793(PEP+HexNAc(3)Hex(2)Fuc(1)_1)
1802.783(PEP+HexNAc(3)Hex(3)_1)
1948.836(PEP+HexNAc(3)Hex(3)Fuc(1)_1)
1964.826(PEP+HexNAc(3)Hex(4)_1)
1989.861
CID-MS/MS Scan:5549, Noise threshold:1.4
Relative Intensity (%)
512.199(HexNAc(1)Hex(1)Fuc(1)_1)
528.195(Hex + HexNAc + Hex)
910.467(PEP+HexNAc(1)_1)
1056.525(PEP+HexNAc(1)Fuc(1)_1)
1076.456(PEP+HexNAc(4)Hex(3)Fuc(1)_2)
1129.989
1157.496(PEP+HexNAc(4)Hex(4)Fuc(1)_2)
1311.550(PEP+HexNAc(4)Hex(5)Fuc(2)_2)
1413.089(PEP+HexNAc(5)Hex(5)Fuc(2)_2)
1583.709(PEP+HexNAc(2)Hex(2)Fuc(1)_1)
1745.773(PEP+HexNAc(2)Hex(3)Fuc(1)_1)
1786.808(PEP+HexNAc(3)Hex(2)Fuc(1)_1)
1802.797(PEP+HexNAc(3)Hex(3)_1)
1948.850(PEP+HexNAc(3)Hex(3)Fuc(1)_1)
1964.841(PEP+HexNAc(3)Hex(4)_1)
1989.876
ETD-MS/MS Scan:5550, Noise threshold:0.8
Relative Intensity (%)
121.173
146.437
192.541
247.530
263.360
299.327
366.141(HexNAc + Hex)
407.167
471.506
512.199(HexNAc(1)Hex(1)Fuc(1)_1)
533.726
1247.055
1311.549(PEP+HexNAc(4)Hex(5)Fuc(2)_2)
1332.049(PEP+HexNAc(5)Hex(4)Fuc(2)_2)
1413.088(PEP+HexNAc(5)Hex(5)Fuc(2)_2)
1441.597(PEP+HexNAc(6)Hex(5)Fuc(1)_2)
M+2H
m/z

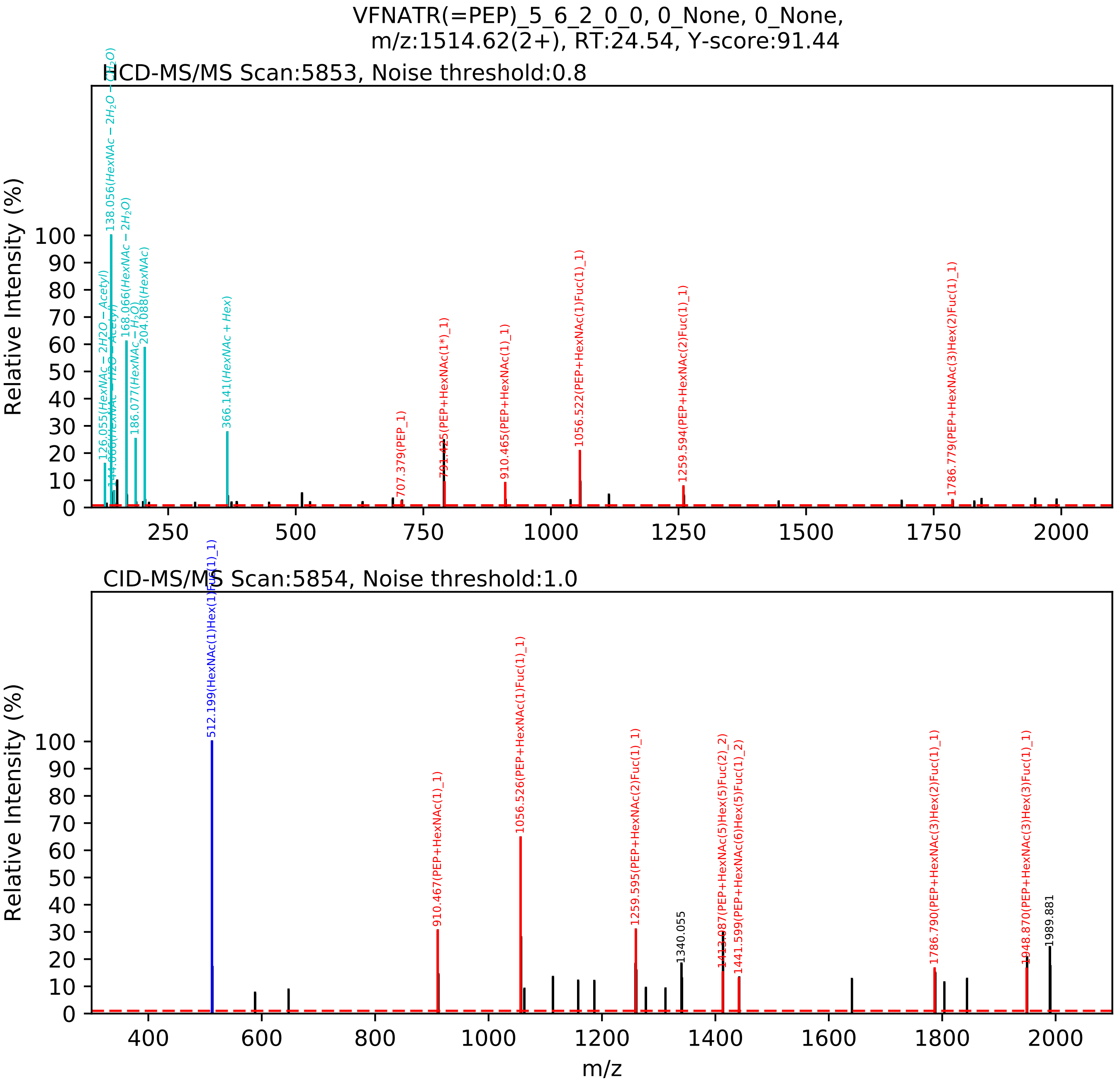

VFNATR(=PEP)_5_6_2_0_0, 0_None, 0_None,
m/z:1514.62(2+), RT:24.54, Y-score:91.44
HCD-MS/MS Scan:5853, Noise threshold:0.8
Relative Intensity (%)
126.055(HexNAc − 2H2O − Acetyl)
138.056(HexNAc − 2H2O − H2O)
144.066(HexNAc − H2O − Acetyl)
168.066(HexNAc − 2H2O)
186.077(HexNAc − H2O)
204.088(HexNAc)
366.141(HexNAc + Hex)
707.379(PEP_1)
791.425(PEP+HexNAc(1*)_1)
910.465(PEP+HexNAc(1)_1)
1056.522(PEP+HexNAc(1)Fuc(1)_1)
1259.594(PEP+HexNAc(2)Fuc(1)_1)
1786.779(PEP+HexNAc(3)Hex(2)Fuc(1)_1)
CID-MS/MS Scan:5854, Noise threshold:1.0
Relative Intensity (%)
512.199(HexNAc(1)Hex(1)Fuc(1)_1)
910.467(PEP+HexNAc(1)_1)
1056.526(PEP+HexNAc(1)Fuc(1)_1)
1259.595(PEP+HexNAc(2)Fuc(1)_1)
1340.055
1412.987(PEP+HexNAc(5)Hex(5)Fuc(2)_2)
1441.599(PEP+HexNAc(6)Hex(5)Fuc(1)_2)
1786.790(PEP+HexNAc(3)Hex(2)Fuc(1)_1)
1948.870(PEP+HexNAc(3)Hex(3)Fuc(1)_1)
1989.881
m/z

VFNATR(=PEP)_5_6_3_0_0, 0_None, 0_None,
m/z:1587.65(2+), RT:23.85, Y-score:81.20

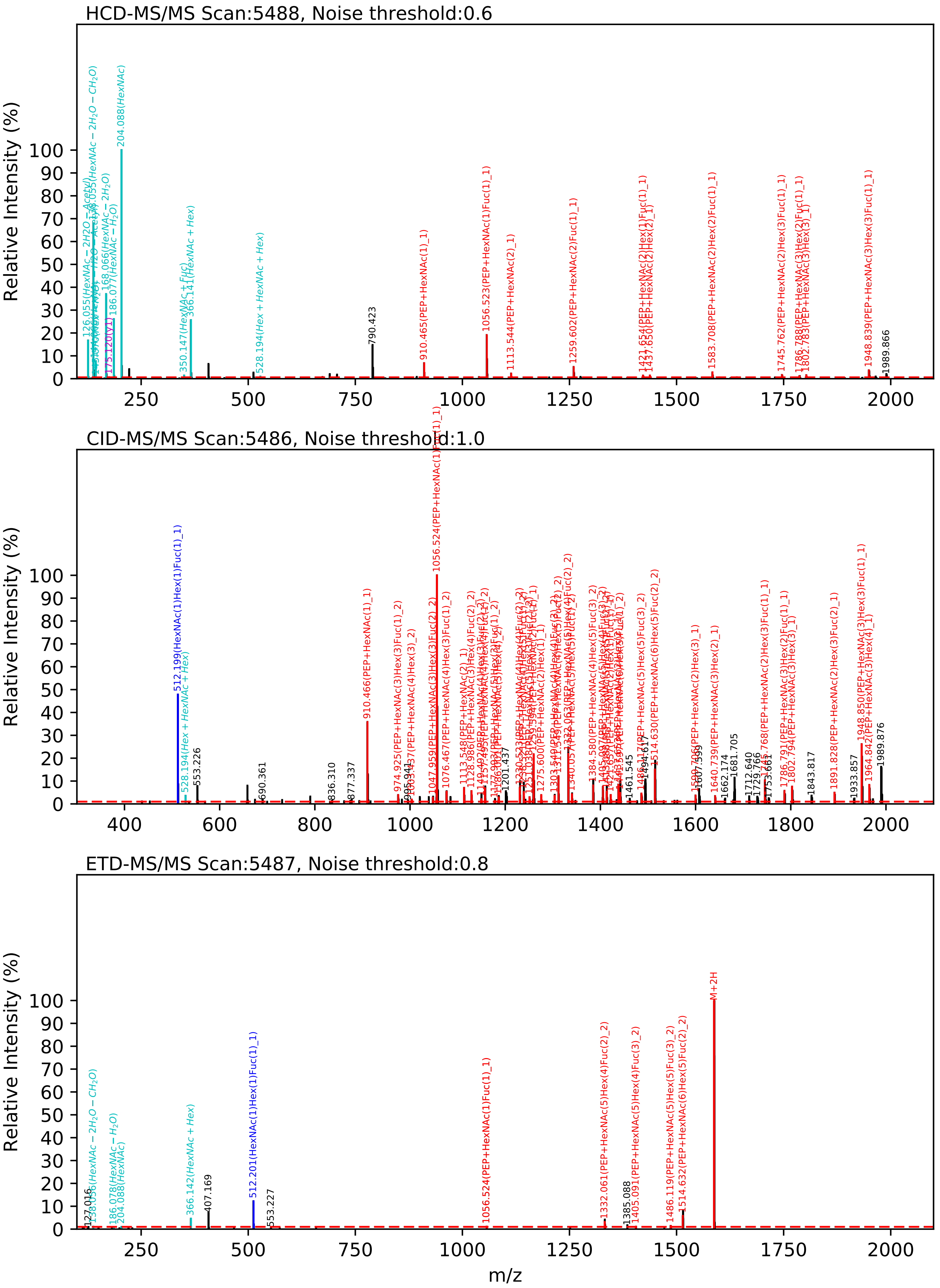

VFNATR(=PEP)_5_6_3_0_0, 0_None, 0_None,
m/z:1058.77(3+), RT:23.89, Y-score:62.37

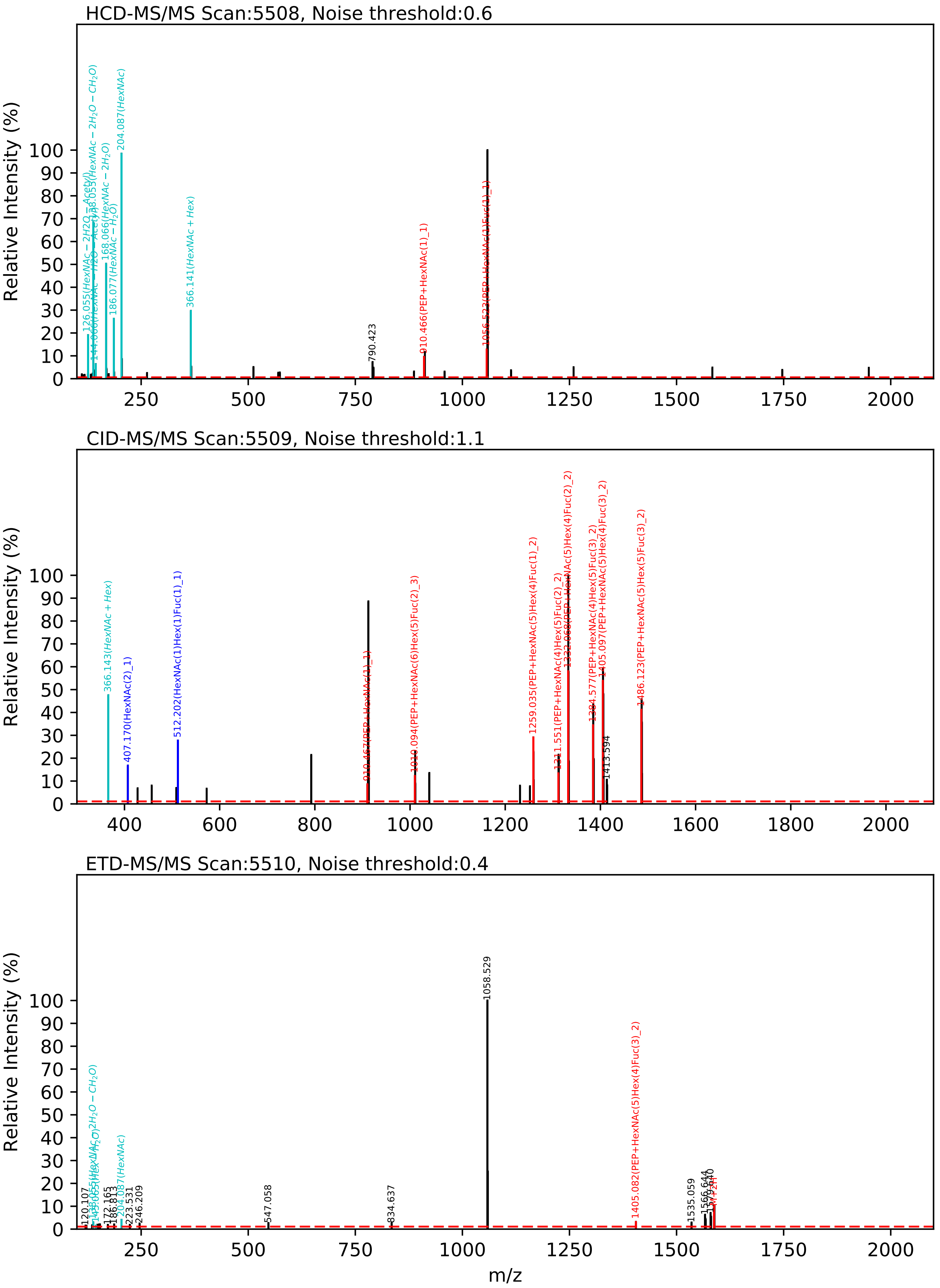

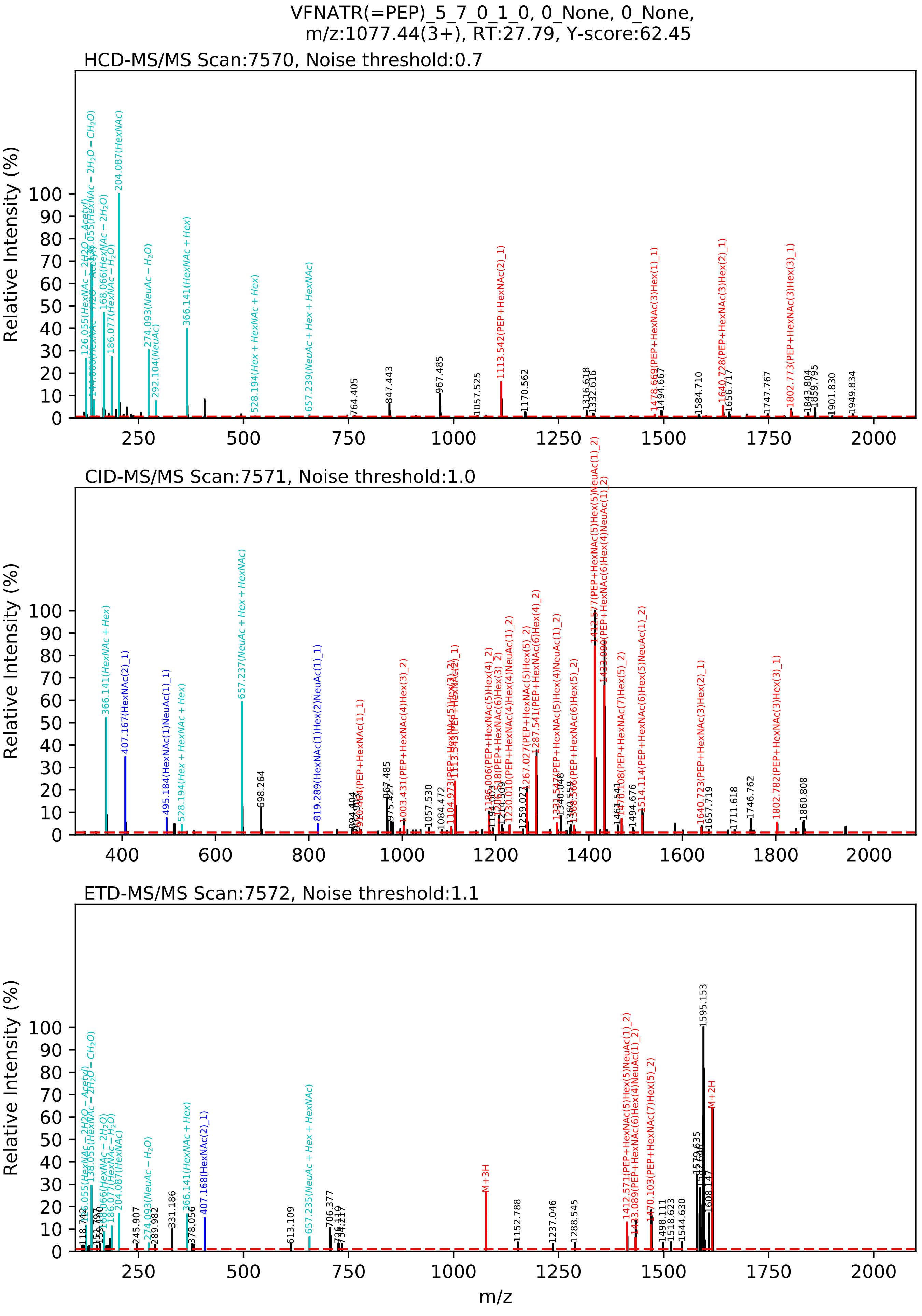

VFNATR(=PEP)_5_7_0_1_0, 0_None, 0_None,
m/z:1077.44(3+), RT:27.79, Y-score:62.45
HCD-MS/MS Scan:7570, Noise threshold:0.7
CID-MS/MS Scan:7571, Noise threshold:1.0
ETD-MS/MS Scan:7572, Noise threshold:1.1
Relative Intensity (%)
m/z

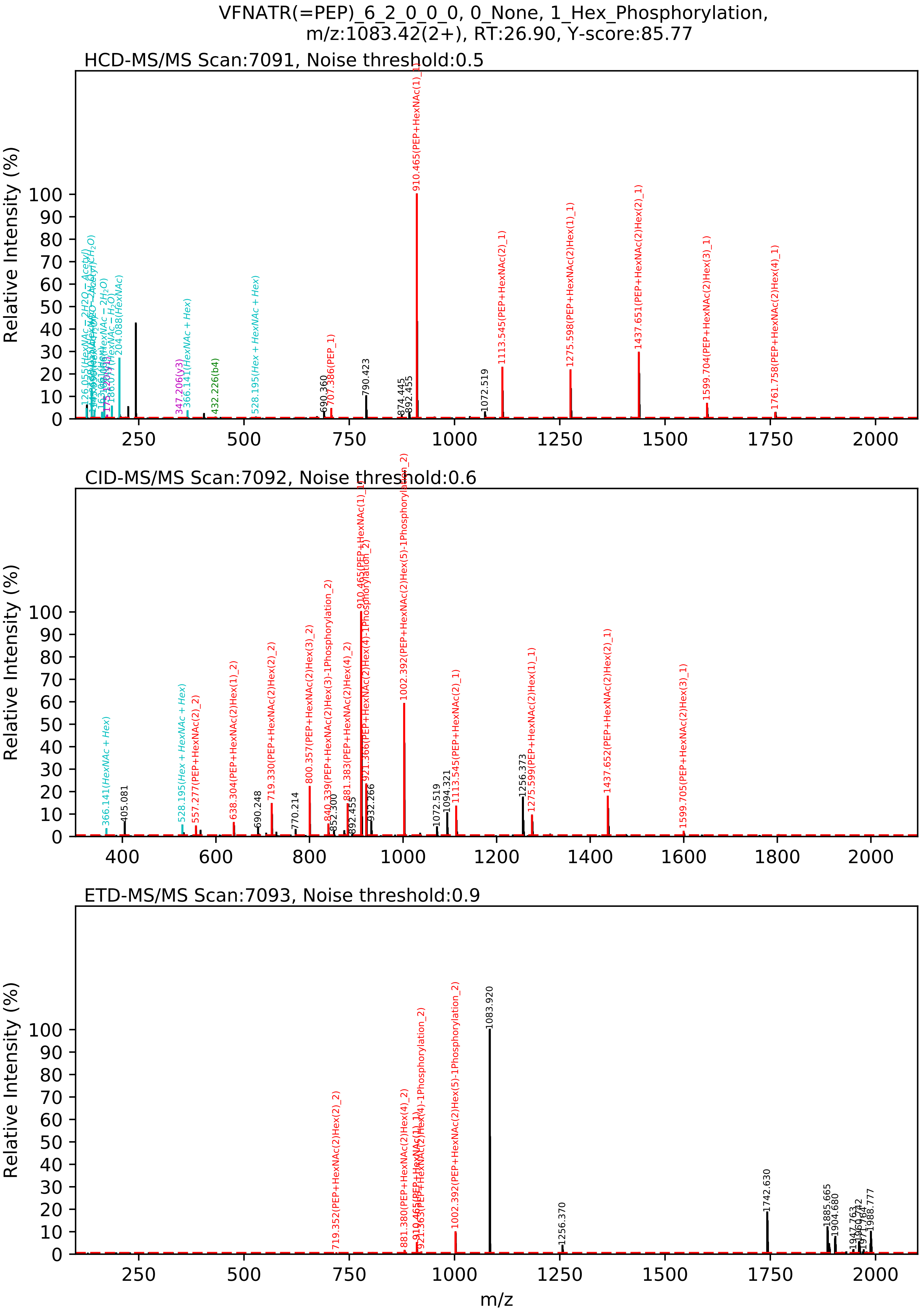

VFNATR(=PEP)_6_2_0_0_0, 0_None, 1_Hex_Phosphorylation,
m/z:1083.42(2+), RT:26.90, Y-score:85.77
HCD-MS/MS Scan:7091, Noise threshold:0.5
CID-MS/MS Scan:7092, Noise threshold:0.6
ETD-MS/MS Scan:7093, Noise threshold:0.9
Relative Intensity (%)
m/z
910.465(PEP+HexNAc(1)_1)
1113.545(PEP+HexNAc(2)_1)
1275.598(PEP+HexNAc(2)Hex(1)_1)
1437.651(PEP+HexNAc(2)Hex(2)_1)
1599.704(PEP+HexNAc(2)Hex(3)_1)
1761.758(PEP+HexNAc(2)Hex(4)_1)
707.386(PEP_1)
790.423
1072.519
1002.392(PEP+HexNAc(2)Hex(5)-1Phosphorylation_2)
1256.373
1083.920
1742.630

VFNATR(=PEP)_6_3_0_0_0, 0_None, 1_Hex_Phosphorylation,
m/z:1184.96(2+), RT:27.00, Y-score:88.01

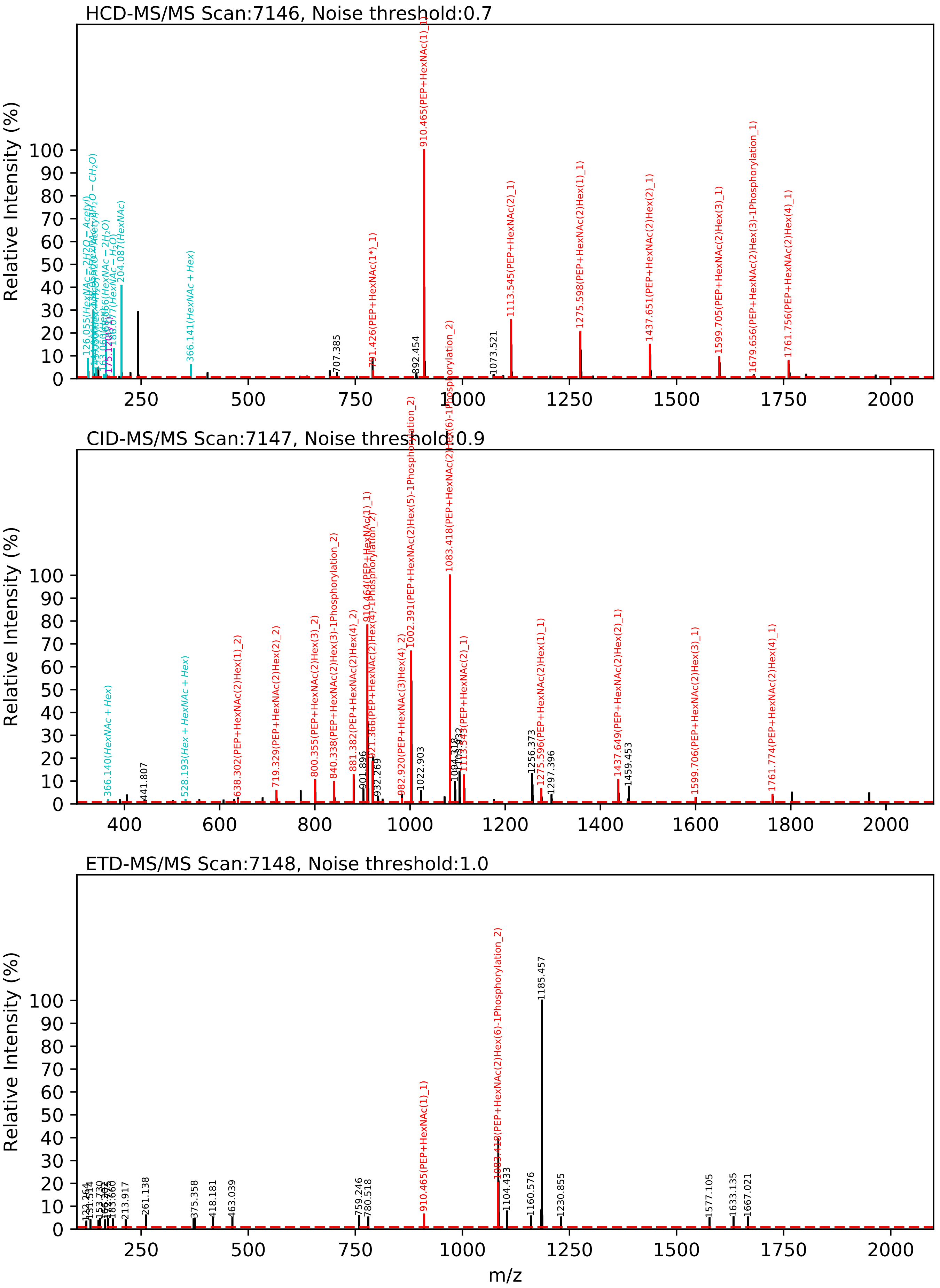

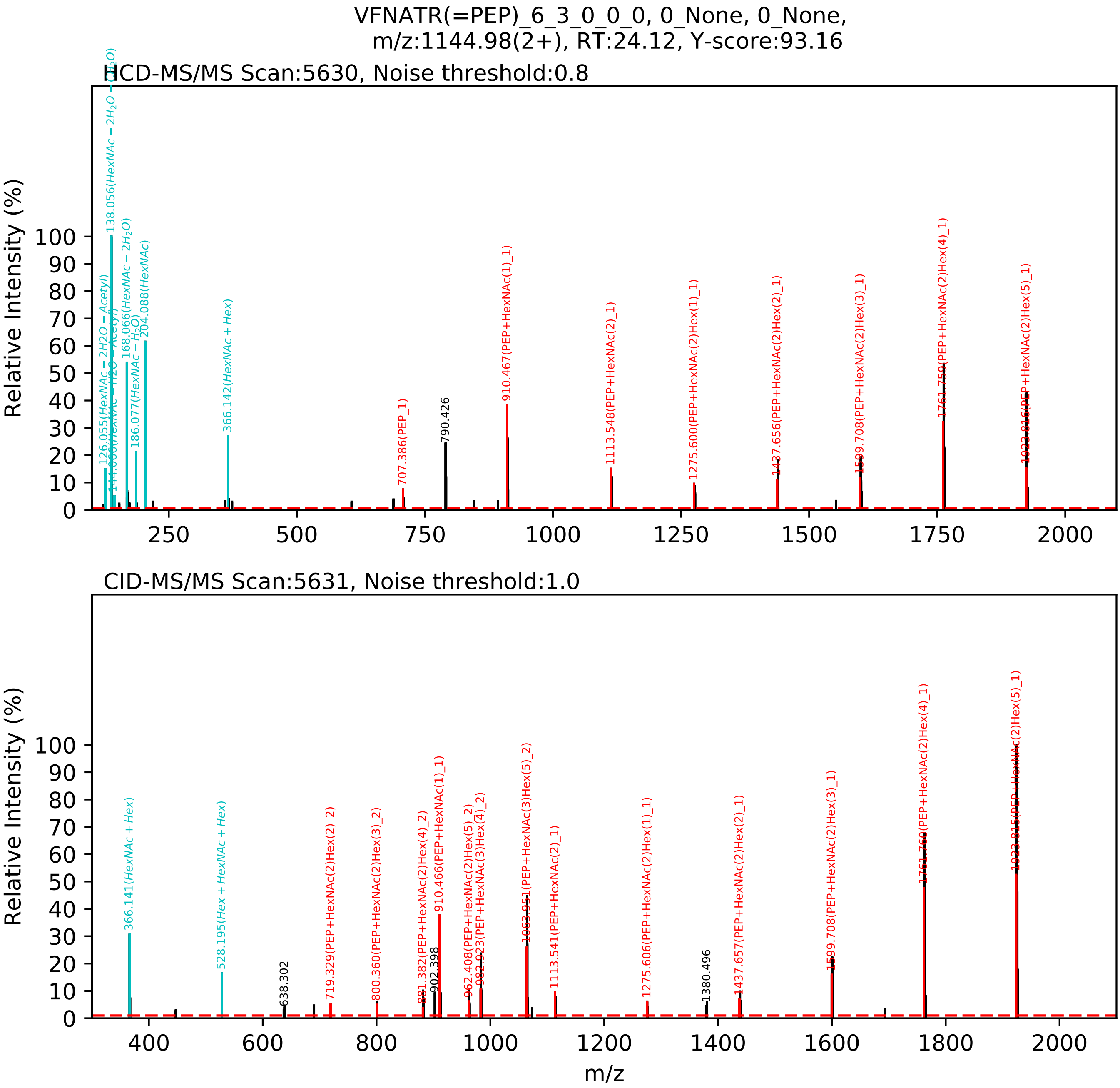
VFNATR(=PEP)_6_3_0_0_0, 0_None, 0_None,
m/z:1144.98(2+), RT:24.12, Y-score:93.16
HCD-MS/MS Scan:5630, Noise threshold:0.8
Relative Intensity (%)
126.055(HexNAc − 2H2O − Acetyl)
144.066(HexNAc − H2O − Acetyl)
138.056(HexNAc − 2H2O − H2O)
168.066(HexNAc − 2H2O)
186.077(HexNAc − H2O)
204.088(HexNAc)
366.142(HexNAc + Hex)
707.386(PEP_1)
790.426
910.467(PEP+HexNAc(1)_1)
1113.548(PEP+HexNAc(2)_1)
1275.600(PEP+HexNAc(2)Hex(1)_1)
1437.656(PEP+HexNAc(2)Hex(2)_1)
1599.708(PEP+HexNAc(2)Hex(3)_1)
1761.759(PEP+HexNAc(2)Hex(4)_1)
1923.816(PEP+HexNAc(2)Hex(5)_1)
CID-MS/MS Scan:5631, Noise threshold:1.0
Relative Intensity (%)
366.141(HexNAc + Hex)
528.195(Hex + HexNAc + Hex)
638.302
719.329(PEP+HexNAc(2)Hex(2)_2)
800.360(PEP+HexNAc(2)Hex(3)_2)
881.382(PEP+HexNAc(2)Hex(4)_2)
902.398
910.466(PEP+HexNAc(1)_1)
962.408(PEP+HexNAc(2)Hex(5)_2)
982.923(PEP+HexNAc(3)Hex(4)_2)
1063.951(PEP+HexNAc(3)Hex(5)_2)
1113.541(PEP+HexNAc(2)_1)
1275.606(PEP+HexNAc(2)Hex(1)_1)
1380.496
1437.657(PEP+HexNAc(2)Hex(2)_1)
1599.708(PEP+HexNAc(2)Hex(3)_1)
1761.760(PEP+HexNAc(2)Hex(4)_1)
1923.815(PEP+HexNAc(2)Hex(5)_1)
m/z

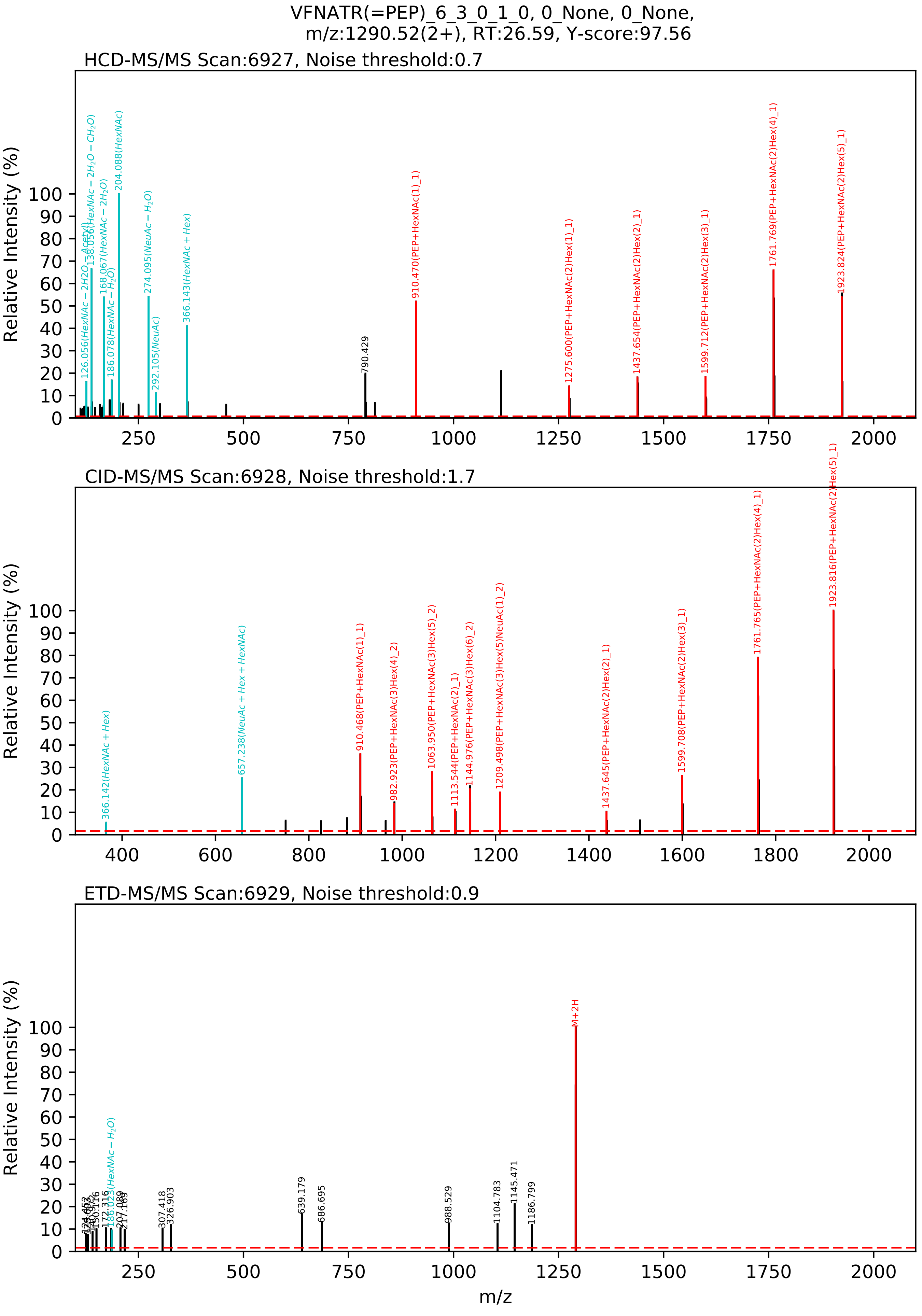
VFNATR(=PEP)_6_3_0_1_0, 0_None, 0_None,
m/z:1290.52(2+), RT:26.59, Y-score:97.56
HCD-MS/MS Scan:6927, Noise threshold:0.7
126.056(HexNAc − 2H2O − Acetyl)
138.056(HexNAc − 2H2O − CH2O)
168.067(HexNAc − 2H2O)
186.078(HexNAc − H2O)
204.088(HexNAc)
274.095(NeuAc − H2O)
292.105(NeuAc)
366.143(HexNAc + Hex)
790.429
910.470(PEP+HexNAc(1)_1)
1275.600(PEP+HexNAc(2)Hex(1)_1)
1437.654(PEP+HexNAc(2)Hex(2)_1)
1599.712(PEP+HexNAc(2)Hex(3)_1)
1761.769(PEP+HexNAc(2)Hex(4)_1)
1923.824(PEP+HexNAc(2)Hex(5)_1)
CID-MS/MS Scan:6928, Noise threshold:1.7
366.142(HexNAc + Hex)
657.238(NeuAc + Hex + HexNAc)
910.468(PEP+HexNAc(1)_1)
982.923(PEP+HexNAc(3)Hex(4)_2)
1063.950(PEP+HexNAc(3)Hex(5)_2)
1113.544(PEP+HexNAc(2)_1)
1144.976(PEP+HexNAc(3)Hex(6)_2)
1209.498(PEP+HexNAc(3)Hex(5)NeuAc(1)_2)
1437.645(PEP+HexNAc(2)Hex(2)_1)
1599.708(PEP+HexNAc(2)Hex(3)_1)
1761.765(PEP+HexNAc(2)Hex(4)_1)
1923.816(PEP+HexNAc(2)Hex(5)_1)
ETD-MS/MS Scan:6929, Noise threshold:0.9
186.023(HexNAc − H2O)
172.316
307.418
326.903
639.179
686.695
988.529
1104.783
1145.471
1186.799
M+2H
Relative Intensity (%)
m/z

VFNATR(=PEP)_6_3_1_0_0, 0_None, 0_None,
m/z:1218.00(2+), RT:23.85, Y-score:91.05

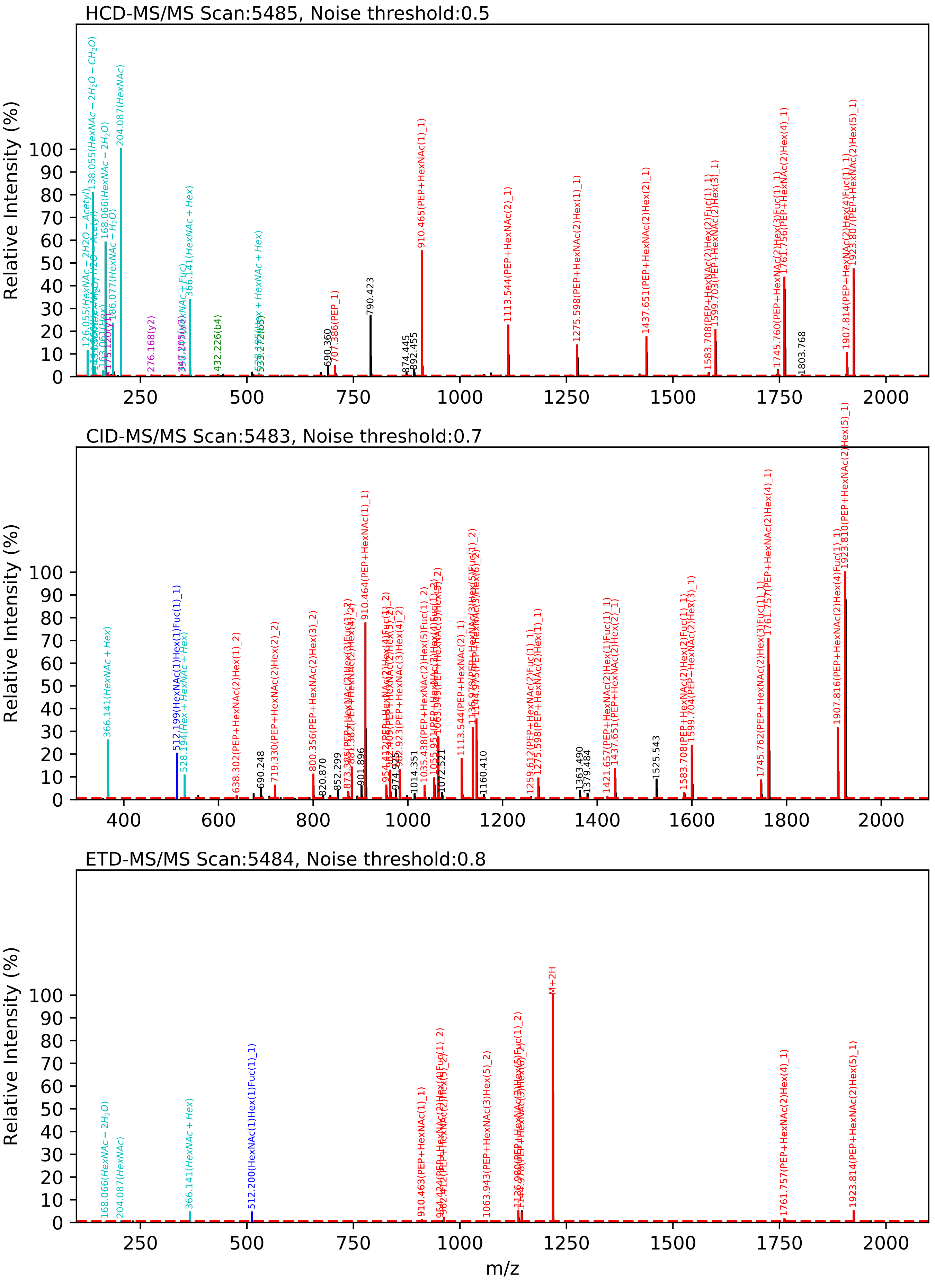

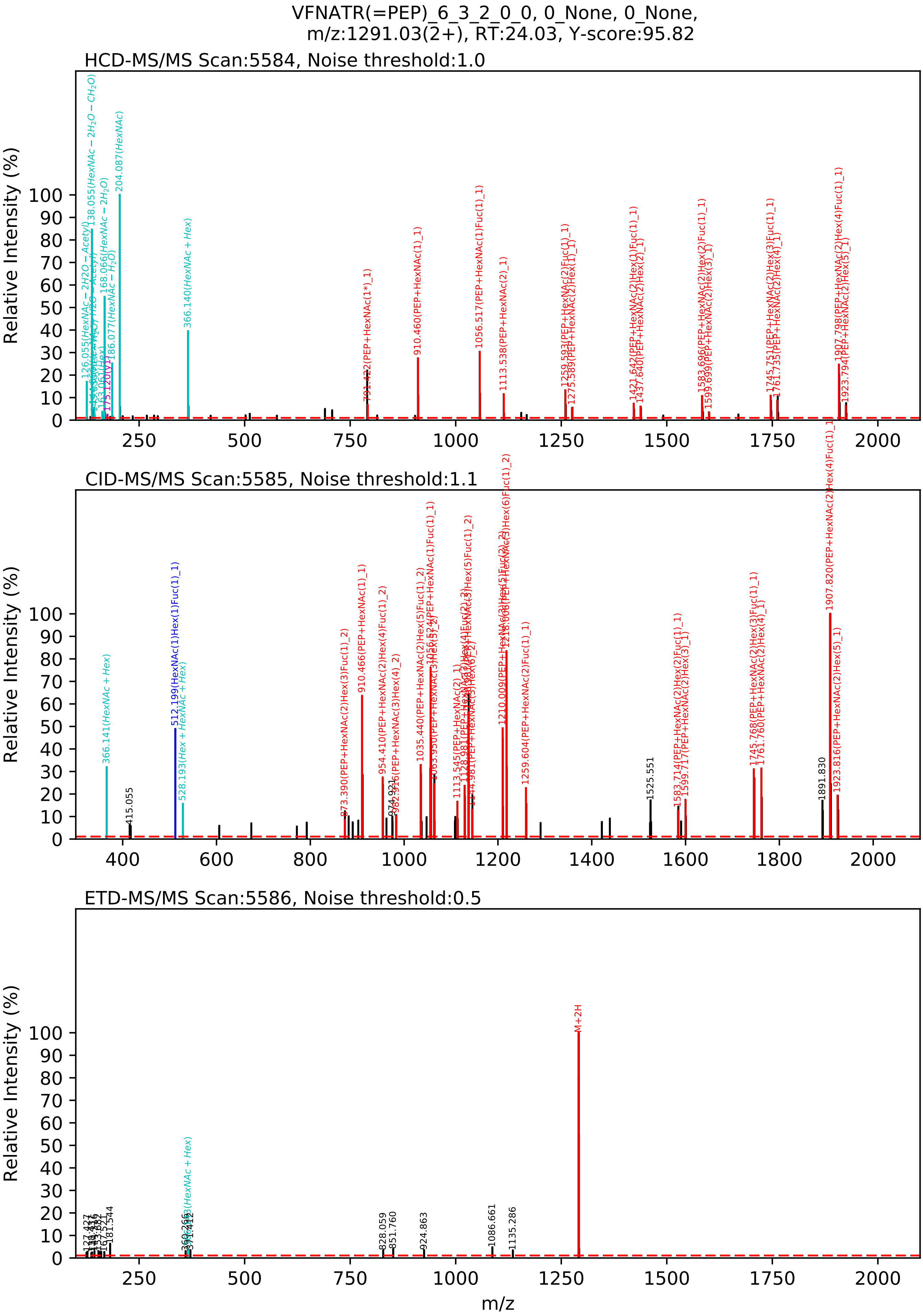
VFNATR(=PEP)_6_3_2_0_0, 0_None, 0_None,
m/z:1291.03(2+), RT:24.03, Y-score:95.82
HCD-MS/MS Scan:5584, Noise threshold:1.0
CID-MS/MS Scan:5585, Noise threshold:1.1
ETD-MS/MS Scan:5586, Noise threshold:0.5
Relative Intensity (%)
m/z
204.087(HexNAc)
366.140(HexNAc + Hex)
910.460(PEP+HexNAc(1)_1)
1056.517(PEP+HexNAc(1)Fuc(1)_1)
1113.538(PEP+HexNAc(2)_1)
1907.798(PEP+HexNAc(2)Hex(4)Fuc(1)_1)
366.141(HexNAc + Hex)
512.199(HexNAc(1)Hex(1)Fuc(1)_1)
415.055
1525.551
1891.830
1907.820(PEP+HexNAc(2)Hex(4)Fuc(1)_1)
1923.816(PEP+HexNAc(2)Hex(5)_1)
1259.604(PEP+HexNAc(2)Fuc(1)_1)
M+2H
828.059
851.760
924.863
1086.661
1135.286

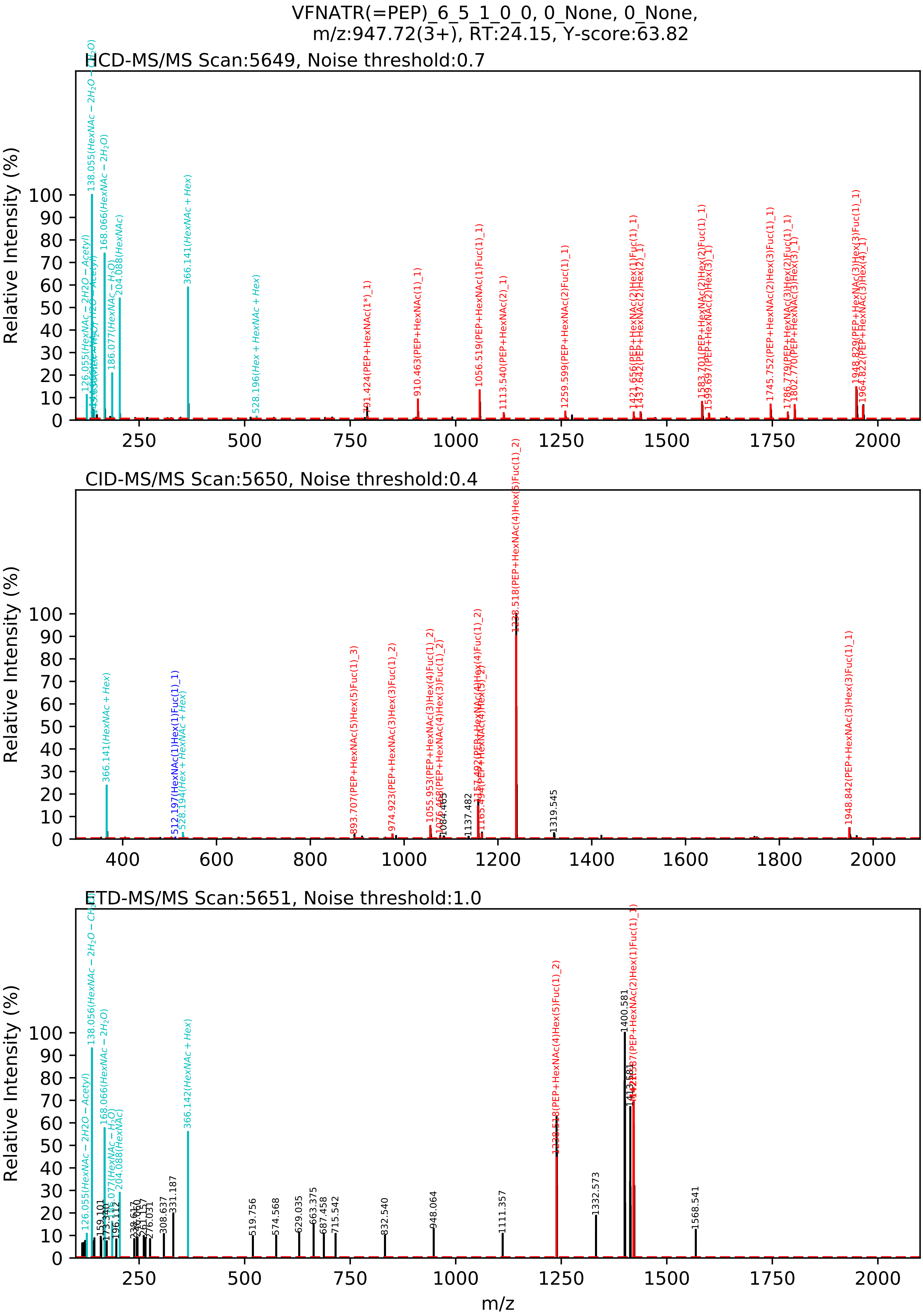

VFNATR(=PEP)_6_5_1_0_0, 0_None, 0_None,
m/z:947.72(3+), RT:24.15, Y-score:63.82
HCD-MS/MS Scan:5649, Noise threshold:0.7
CID-MS/MS Scan:5650, Noise threshold:0.4
ETD-MS/MS Scan:5651, Noise threshold:1.0
Relative Intensity (%)
m/z
366.141(HexNAc + Hex)
910.463(PEP+HexNAc(1)_1)
1056.519(PEP+HexNAc(1)Fuc(1)_1)
1113.540(PEP+HexNAc(2)_1)
1259.599(PEP+HexNAc(2)Fuc(1)_1)
1745.752(PEP+HexNAc(2)Hex(3)Fuc(1)_1)
1238.518(PEP+HexNAc(4)Hex(5)Fuc(1)_2)
893.707(PEP+HexNAc(5)Hex(5)Fuc(1)_3)
974.923(PEP+HexNAc(3)Hex(3)Fuc(1)_2)
1948.842(PEP+HexNAc(3)Hex(3)Fuc(1)_1)
1319.545
1400.581
1332.573
1568.541
1111.357
948.064

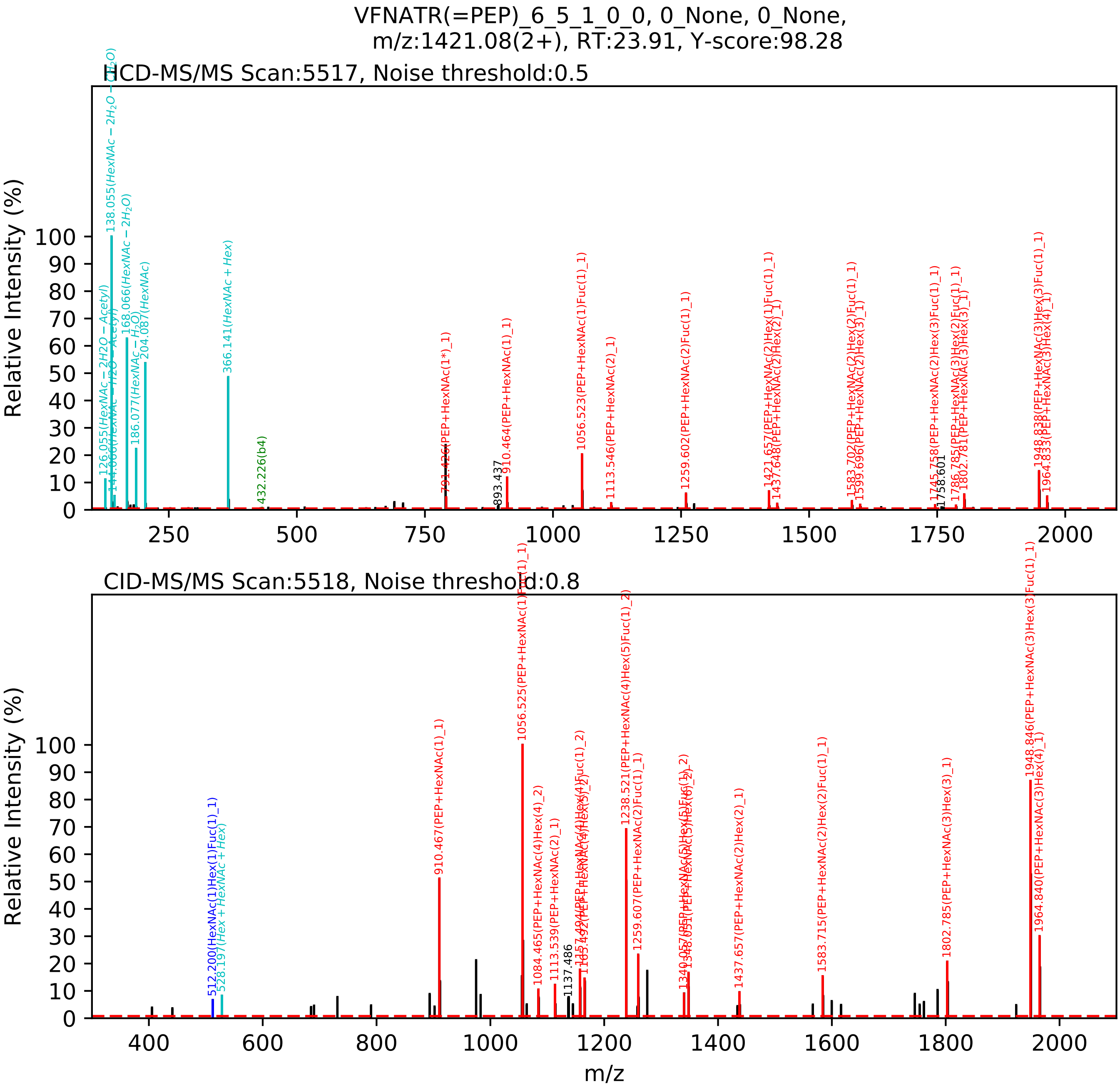

VFNATR(=PEP)_6_5_1_0_0, 0_None, 0_None,
m/z:1421.08(2+), RT:23.91, Y-score:98.28
HCD-MS/MS Scan:5517, Noise threshold:0.5
Relative Intensity (%)
138.055(HexNAc − 2H2O − H2O)
168.066(HexNAc − 2H2O)
204.087(HexNAc)
186.077(HexNAc − H2O)
126.055(HexNAc − 2H2O − Acetyl)
366.141(HexNAc + Hex)
432.226(b4)
791.426(PEP+HexNAc(1*)_1)
893.437
910.464(PEP+HexNAc(1)_1)
1056.523(PEP+HexNAc(1)Fuc(1)_1)
1113.546(PEP+HexNAc(2)_1)
1259.602(PEP+HexNAc(2)Fuc(1)_1)
1421.657(PEP+HexNAc(2)Hex(1)Fuc(1)_1)
1437.648(PEP+HexNAc(2)Hex(2)_1)
1583.702(PEP+HexNAc(2)Hex(2)Fuc(1)_1)
1599.696(PEP+HexNAc(2)Hex(3)_1)
1745.758(PEP+HexNAc(2)Hex(3)Fuc(1)_1)
1758.601
1786.785(PEP+HexNAc(3)Hex(2)Fuc(1)_1)
1802.781(PEP+HexNAc(3)Hex(3)_1)
1948.838(PEP+HexNAc(3)Hex(3)Fuc(1)_1)
1964.833(PEP+HexNAc(3)Hex(4)_1)
CID-MS/MS Scan:5518, Noise threshold:0.8
512.200(HexNAc(1)Hex(1)Fuc(1)_1)
528.197(Hex + HexNAc + Hex)
910.467(PEP+HexNAc(1)_1)
1056.525(PEP+HexNAc(1)Fuc(1)_1)
1084.465(PEP+HexNAc(4)Hex(4)_2)
1113.539(PEP+HexNAc(2)_1)
1137.486
1238.521(PEP+HexNAc(4)Hex(5)Fuc(1)_2)
1259.607(PEP+HexNAc(2)Fuc(1)_1)
1437.657(PEP+HexNAc(2)Hex(2)_1)
1583.715(PEP+HexNAc(2)Hex(2)Fuc(1)_1)
1802.785(PEP+HexNAc(3)Hex(3)_1)
1948.846(PEP+HexNAc(3)Hex(3)Fuc(1)_1)
1964.840(PEP+HexNAc(3)Hex(4)_1)
m/z

VFNATR(=PEP)_6_5_1_1_0, 0_None, 0_None,
m/z:1566.63(2+), RT:26.29, Y-score:95.36

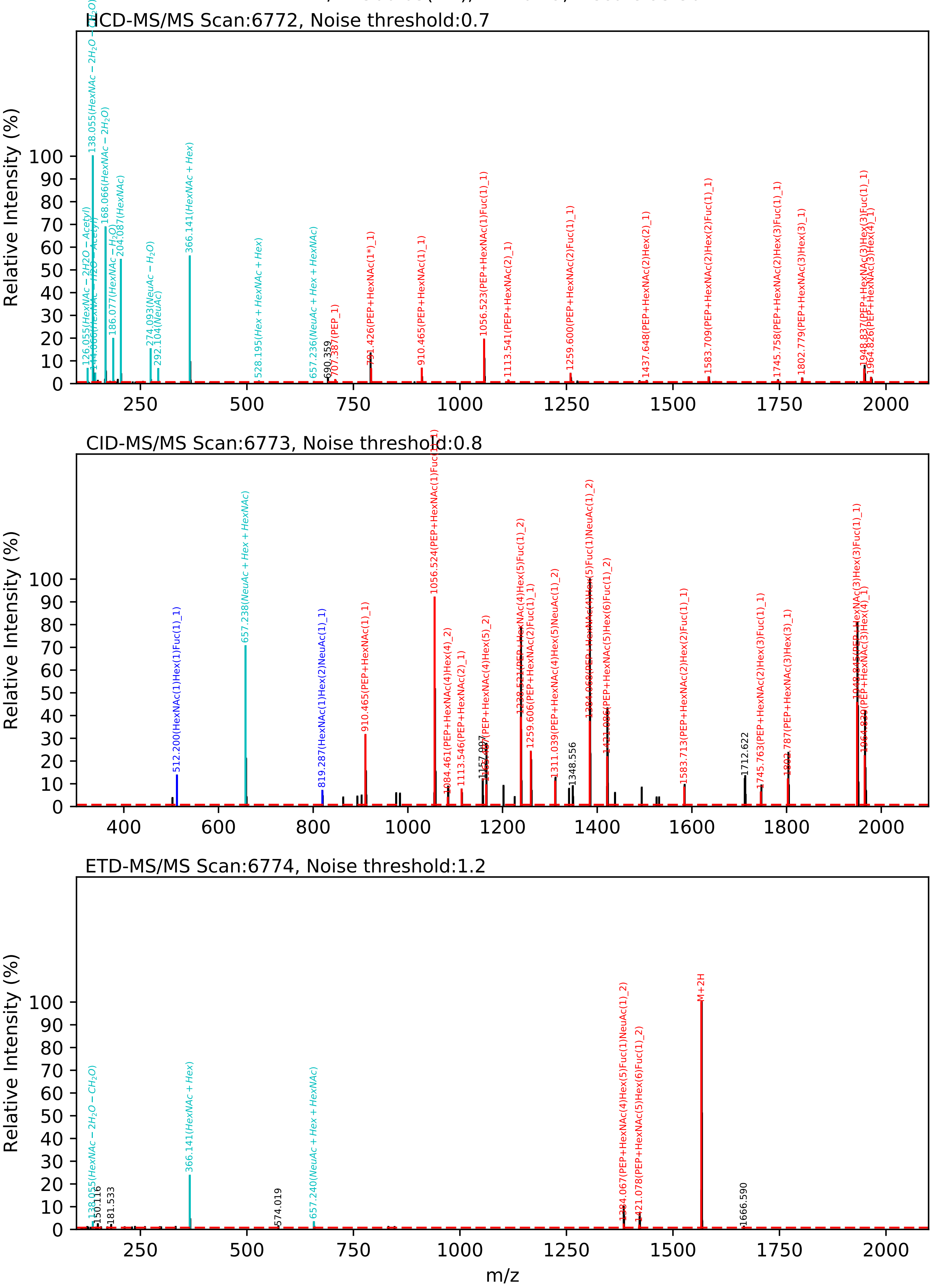

VFNATR(=PEP)_6_5_1_1_0, 0_None, 0_None,
m/z:1044.75(3+), RT:26.27, Y-score:99.14

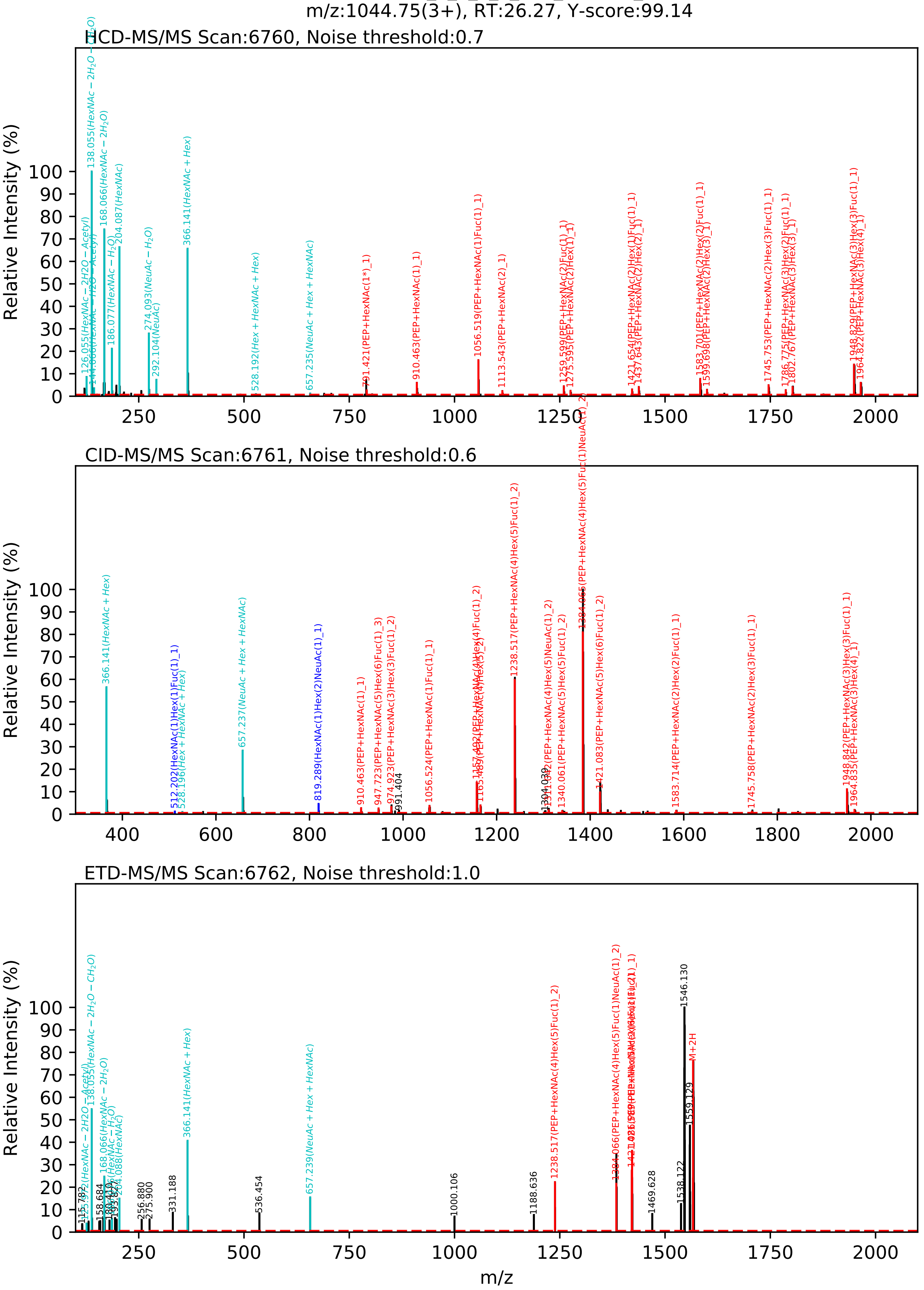

VFNATR(=PEP)_6_5_1_3_0, 0_None, 0_None,
m/z:1238.82(3+), RT:41.71, Y-score:86.03

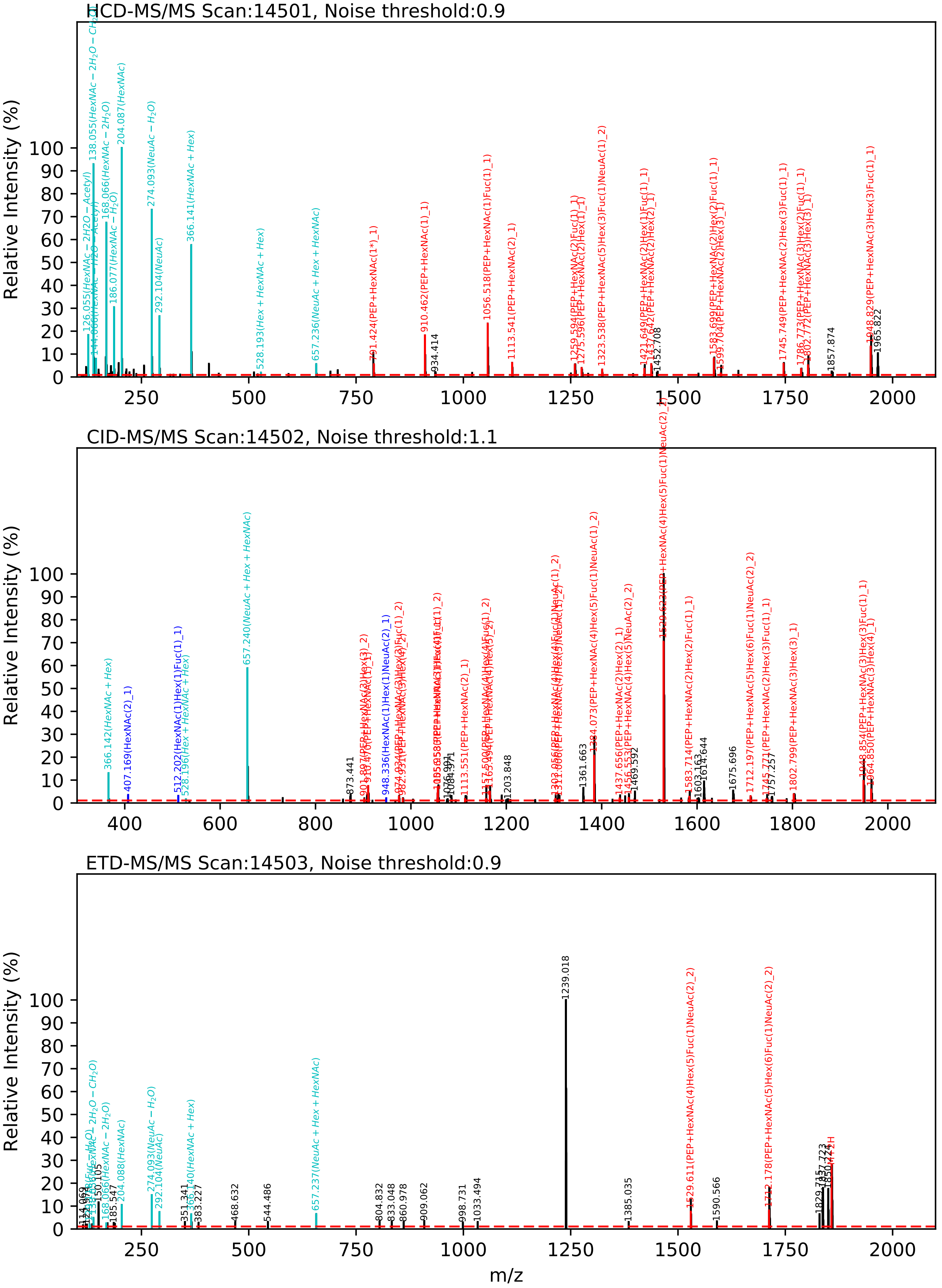

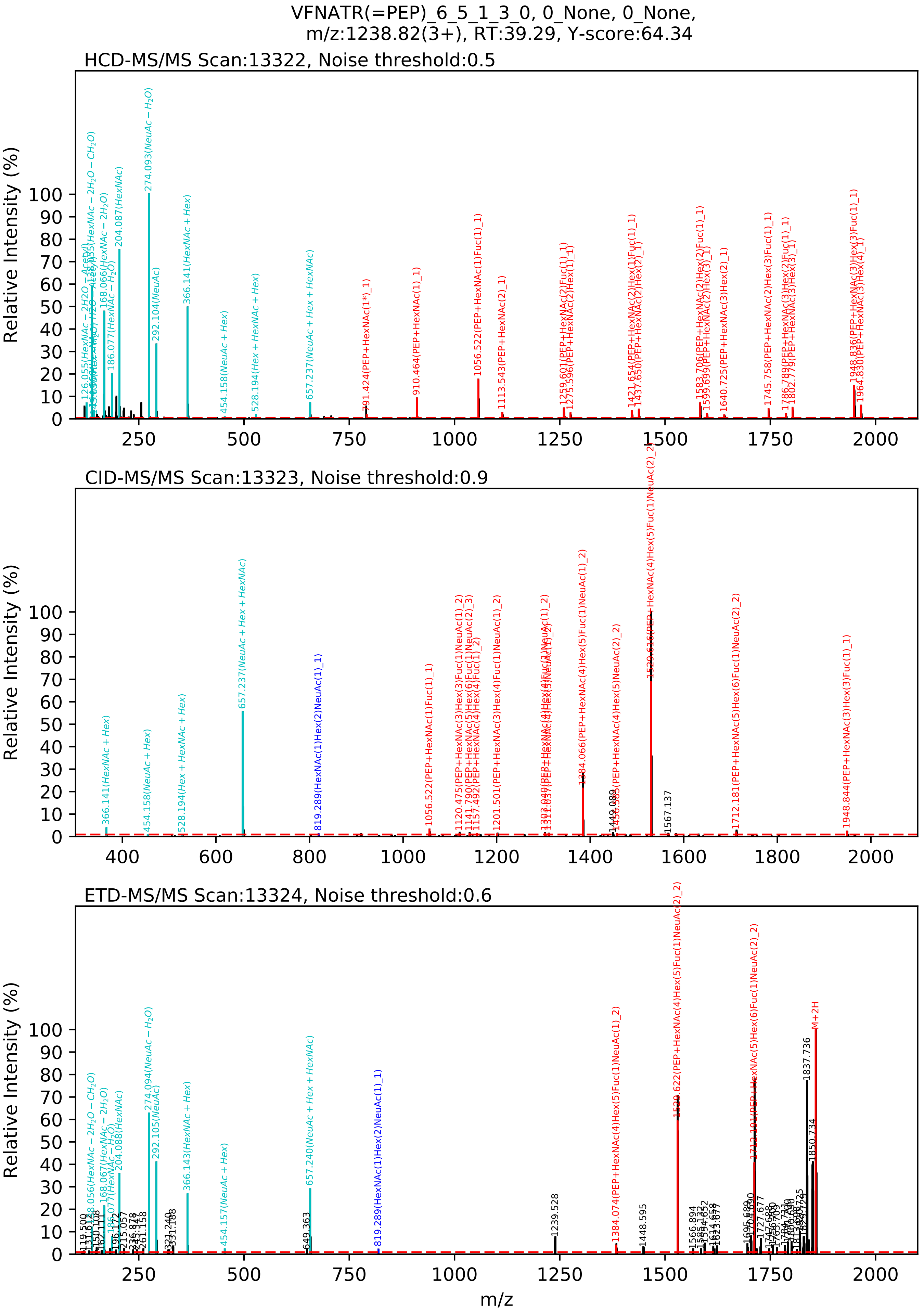

VFNATR(=PEP)_6_5_1_3_0, 0_None, 0_None,
m/z:1238.82(3+), RT:39.29, Y-score:64.34
HCD-MS/MS Scan:13322, Noise threshold:0.5
CID-MS/MS Scan:13323, Noise threshold:0.9
ETD-MS/MS Scan:13324, Noise threshold:0.6
Relative Intensity (%)
m/z
274.093(NeuAc − H2O)
204.087(HexNAc)
366.141(HexNAc + Hex)
292.104(NeuAc)
657.237(NeuAc + Hex + HexNAc)
910.464(PEP+HexNAc(1)_1)
1056.522(PEP+HexNAc(1)Fuc(1)_1)
1948.836(PEP+HexNAc(3)Hex(3)Fuc(1)_1)
1529.616(PEP+HexNAc(4)Hex(5)Fuc(1)NeuAc(2)_2)
1384.066(PEP+HexNAc(4)Hex(5)Fuc(1)NeuAc(1)_2)
819.289(HexNAc(1)Hex(2)NeuAc(1)_1)
1712.181(PEP+HexNAc(5)Hex(6)Fuc(1)NeuAc(2)_2)
1529.622(PEP+HexNAc(4)Hex(5)Fuc(1)NeuAc(2)_2)
1837.736
1850.734
M+2H

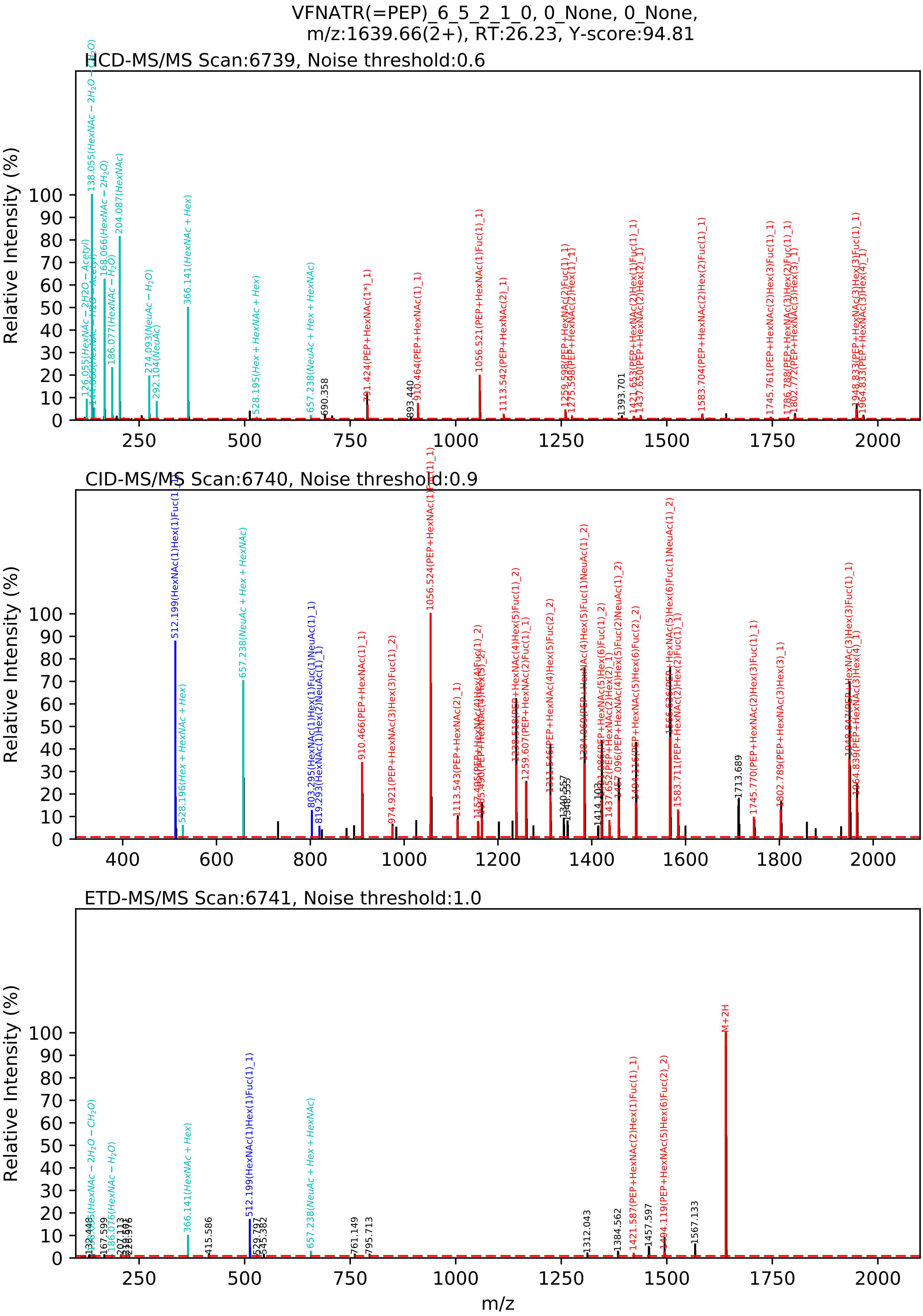
VFNATR(=PEP)_6_5_2_1_0, 0_None, 0_None,
m/z:1639.66(2+), RT:26.23, Y-score:94.81
HCD-MS/MS Scan:6739, Noise threshold:0.6
CID-MS/MS Scan:6740, Noise threshold:0.9
ETD-MS/MS Scan:6741, Noise threshold:1.0
Relative Intensity (%)
m/z
1056.521(PEP+HexNAc(1)Fuc(1)_1)
1056.524(PEP+HexNAc(1)Fuc(1)_1)
512.199(HexNAc(1)Hex(1)Fuc(1)_1)
657.238(NeuAc + Hex + HexNAc)
366.141(HexNAc + Hex)
138.055(HexNAc − 2H2O − CH2O)
204.087(HexNAc)
M+2H
1421.587(PEP+HexNAc(2)Hex(1)Fuc(1)_1)
1494.119(PEP+HexNAc(5)Hex(6)Fuc(2)_2)

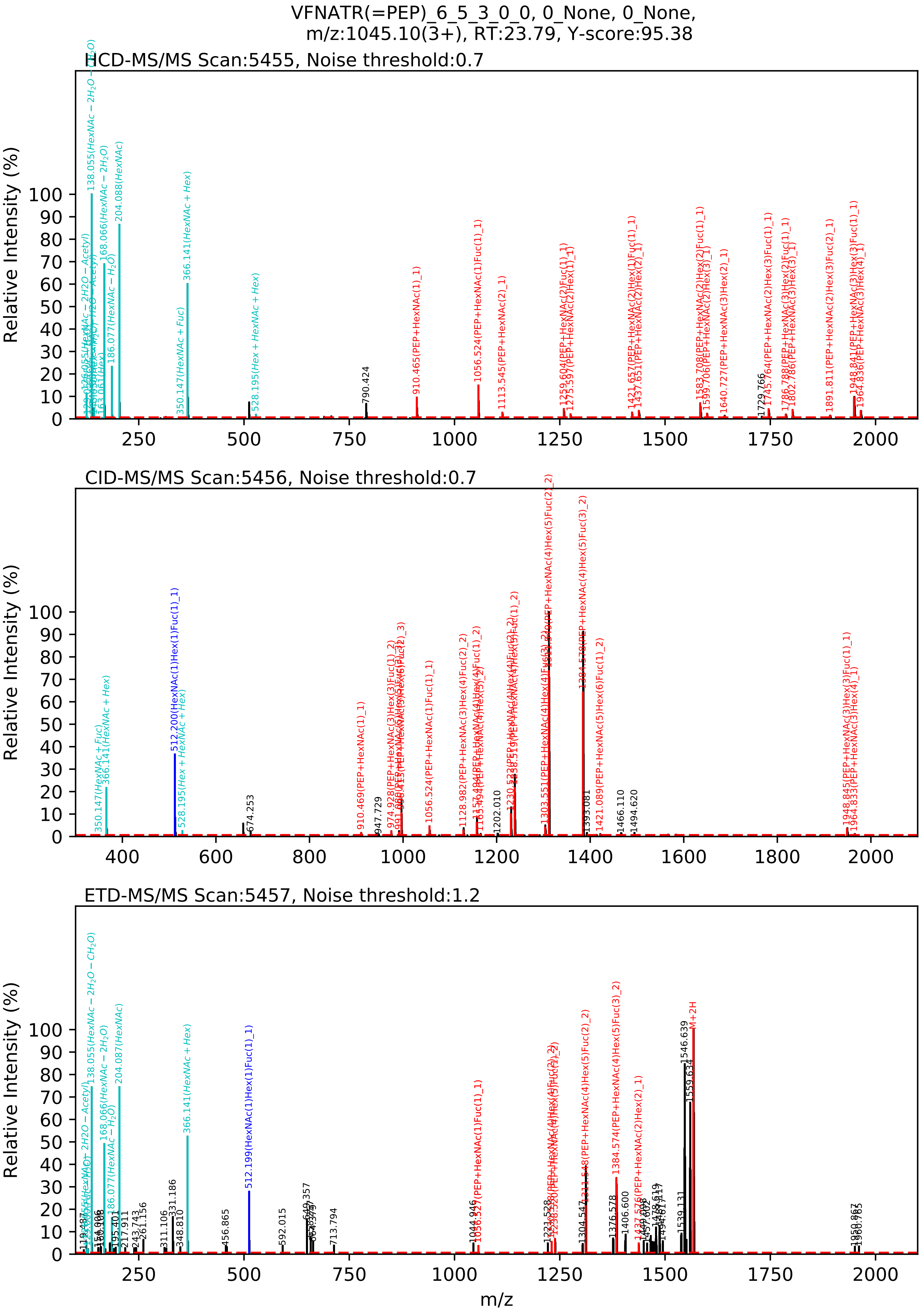

VFNATR(=PEP)_6_5_3_0_0, 0_None, 0_None,
m/z:1045.10(3+), RT:23.79, Y-score:95.38
HCD-MS/MS Scan:5455, Noise threshold:0.7
CID-MS/MS Scan:5456, Noise threshold:0.7
ETD-MS/MS Scan:5457, Noise threshold:1.2
Relative Intensity (%)
m/z

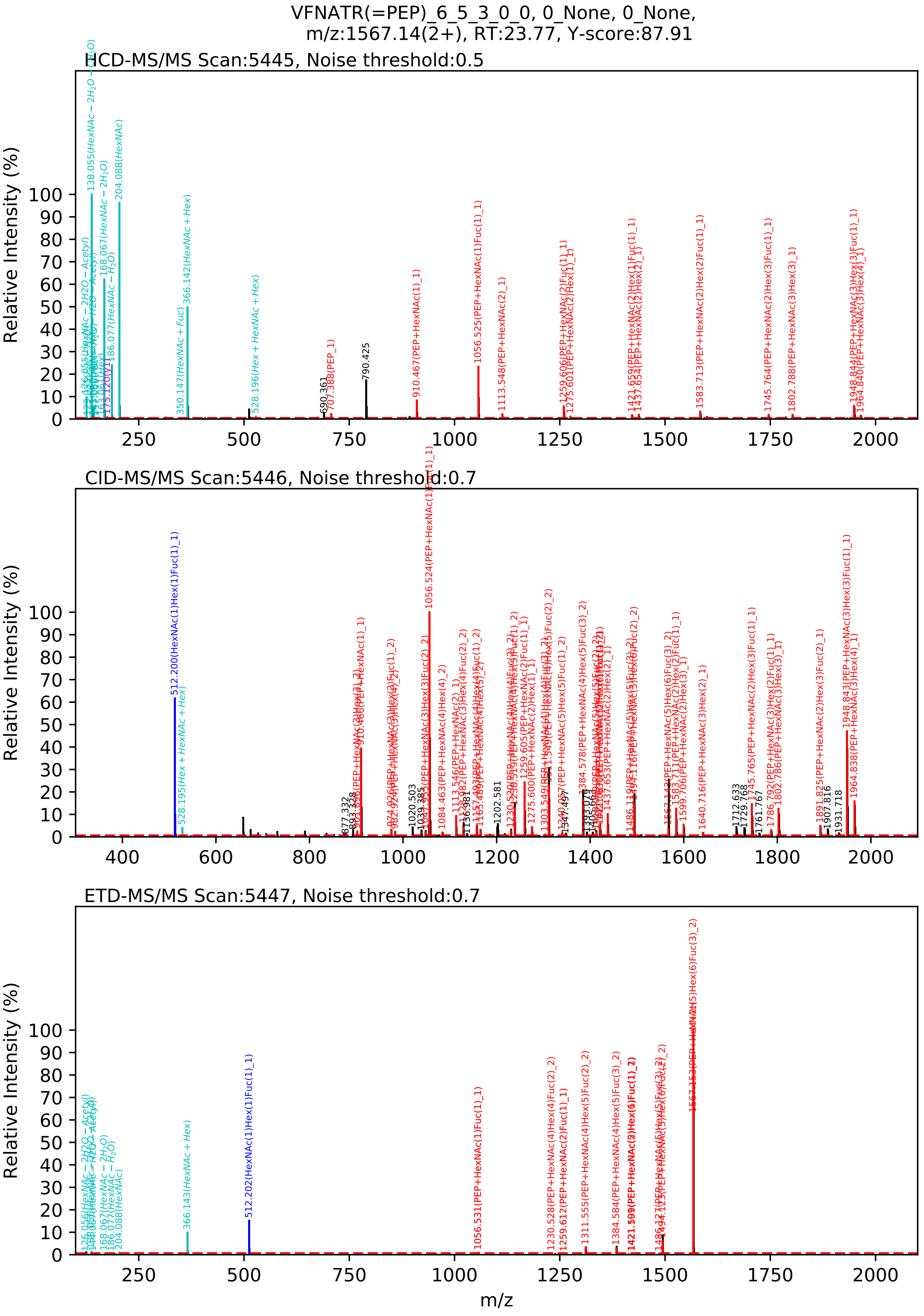
VFNATR(=PEP)_6_5_3_0_0, 0_None, 0_None,
m/z:1567.14(2+), RT:23.77, Y-score:87.91
HCD-MS/MS Scan:5445, Noise threshold:0.5
CID-MS/MS Scan:5446, Noise threshold:0.7
ETD-MS/MS Scan:5447, Noise threshold:0.7
Relative Intensity (%)
m/z

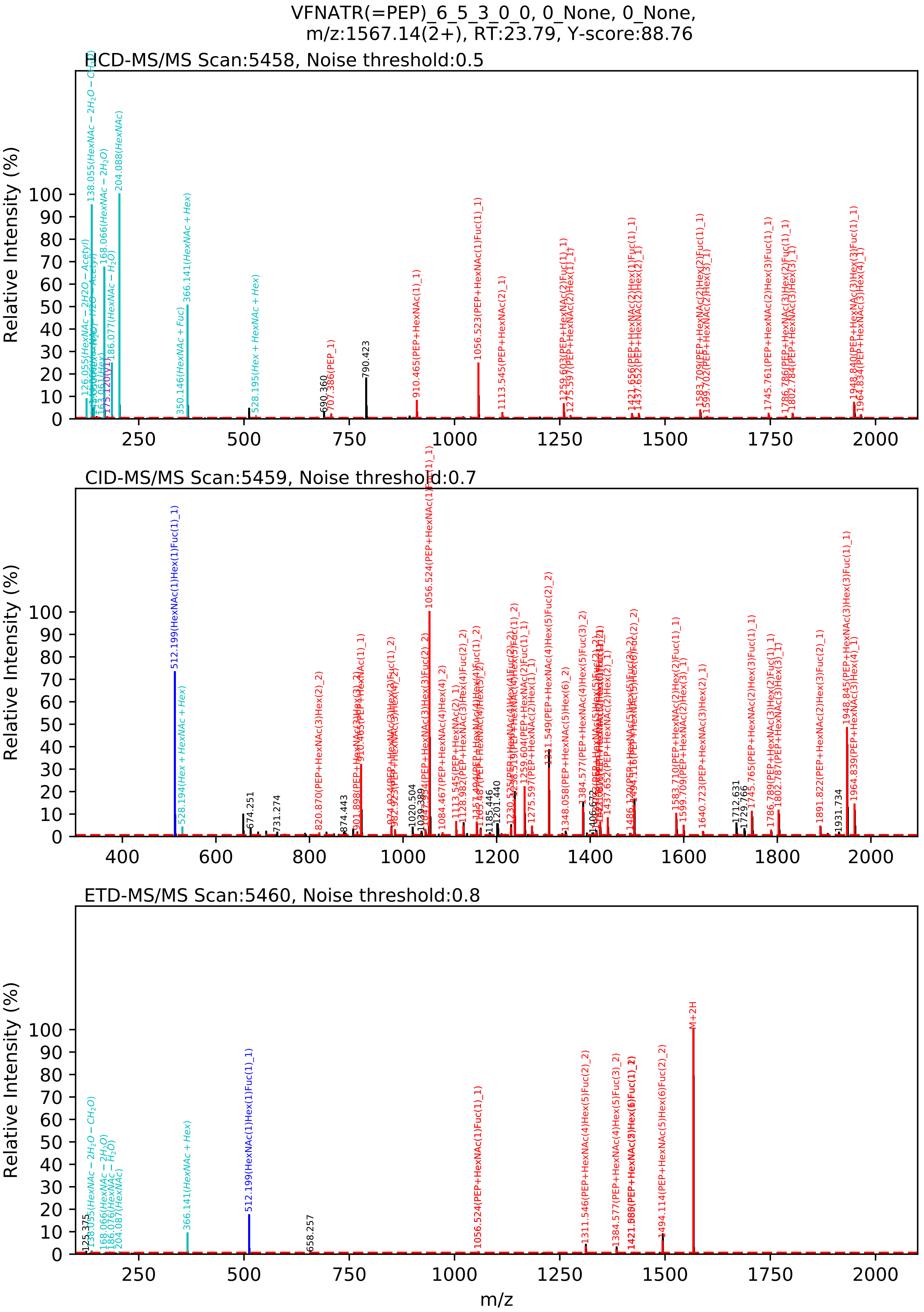

VFNATR(=PEP)_6_5_3_0_0, 0_None, 0_None,
m/z:1567.14(2+), RT:23.79, Y-score:88.76
HCD-MS/MS Scan:5458, Noise threshold:0.5
Relative Intensity (%)
204.088(HexNAc)
366.141(HexNAc + Hex)
528.195(Hex + HexNAc + Hex)
790.423
910.465(PEP+HexNAc(1)_1)
1056.523(PEP+HexNAc(1)Fuc(1)_1)
1113.545(PEP+HexNAc(2)_1)
1745.761(PEP+HexNAc(2)Hex(3)Fuc(1)_1)
CID-MS/MS Scan:5459, Noise threshold:0.7
512.199(HexNAc(1)Hex(1)Fuc(1)_1)
528.194(Hex + HexNAc + Hex)
1056.524(PEP+HexNAc(1)Fuc(1)_1)
1311.549(PEP+HexNAc(4)Hex(5)Fuc(2)_2)
1948.845(PEP+HexNAc(3)Hex(3)Fuc(1)_1)
1964.839(PEP+HexNAc(3)Hex(4)_1)
ETD-MS/MS Scan:5460, Noise threshold:0.8
366.141(HexNAc + Hex)
512.199(HexNAc(1)Hex(1)Fuc(1)_1)
658.257
1056.524(PEP+HexNAc(1)Fuc(1)_1)
1311.546(PEP+HexNAc(4)Hex(5)Fuc(2)_2)
1384.577(PEP+HexNAc(4)Hex(5)Fuc(3)_2)
1494.114(PEP+HexNAc(5)Hex(6)Fuc(2)_2)
M+2H
m/z

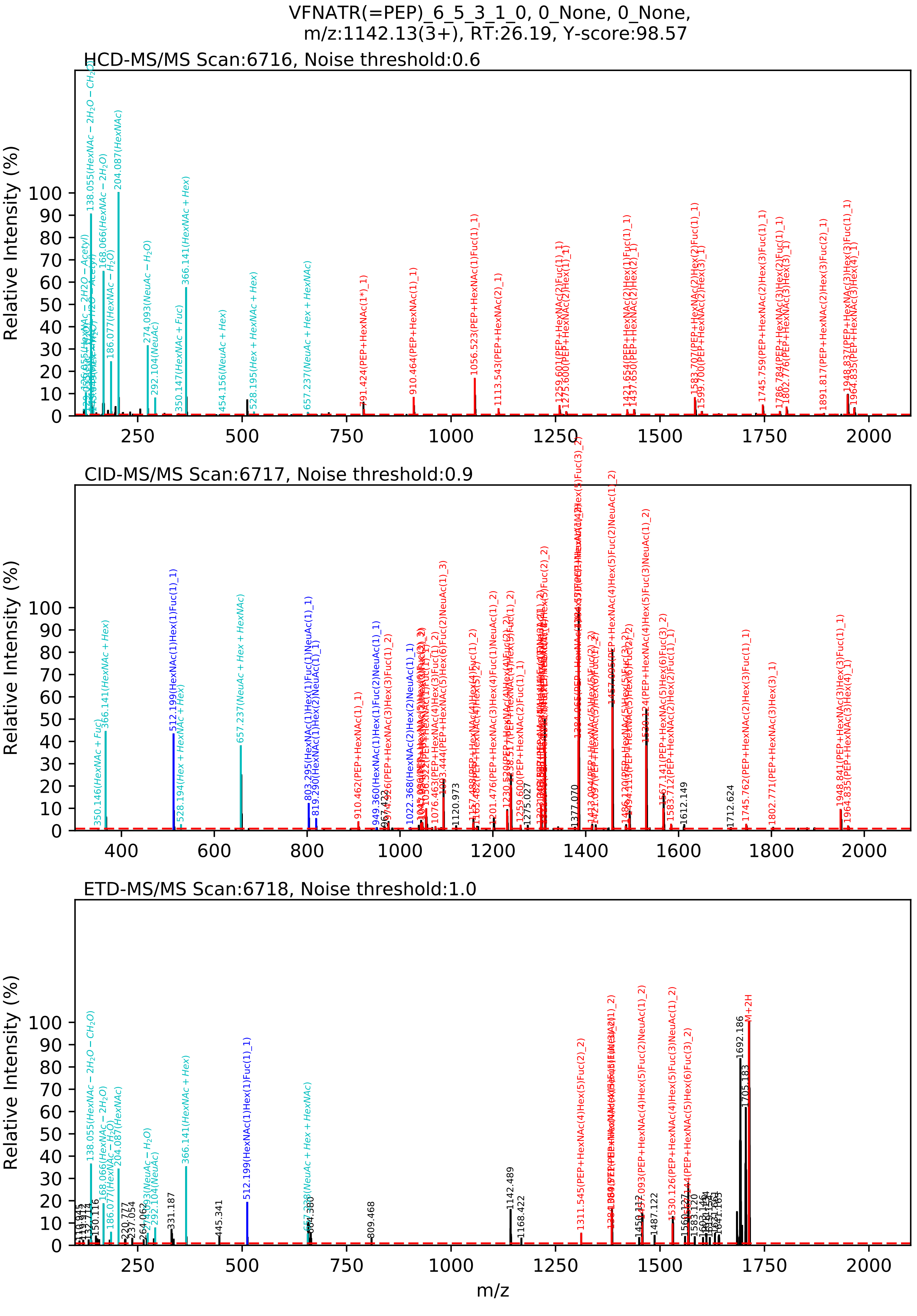

VFNATR(=PEP)_6_5_3_1_0, 0_None, 0_None,
m/z:1142.13(3+), RT:26.19, Y-score:98.57
HCD-MS/MS Scan:6716, Noise threshold:0.6
Relative Intensity (%)
138.055(HexNAc − 2H2O − CH2O)
204.087(HexNAc)
168.066(HexNAc − 2H2O)
186.077(HexNAc − H2O)
274.093(NeuAc − H2O)
292.104(NeuAc)
366.141(HexNAc + Hex)
350.147(HexNAc + Fuc)
454.156(NeuAc + Hex)
528.195(Hex + HexNAc + Hex)
657.237(NeuAc + Hex + HexNAc)
791.424(PEP+HexNAc(1*)_1)
910.464(PEP+HexNAc(1)_1)
1056.523(PEP+HexNAc(1)Fuc(1)_1)
1113.543(PEP+HexNAc(2)_1)
1745.759(PEP+HexNAc(2)Hex(3)Fuc(1)_1)
1891.817(PEP+HexNAc(2)Hex(3)Fuc(2)_1)
1948.837(PEP+HexNAc(3)Hex(3)Fuc(1)_1)
1964.835(PEP+HexNAc(3)Hex(4)_1)
CID-MS/MS Scan:6717, Noise threshold:0.9
366.141(HexNAc + Hex)
350.146(HexNAc + Fuc)
512.199(HexNAc(1)Hex(1)Fuc(1)_1)
528.194(Hex + HexNAc + Hex)
657.237(NeuAc + Hex + HexNAc)
803.295(HexNAc(1)Hex(1)Fuc(1)NeuAc(1)_1)
819.290(HexNAc(1)Hex(2)NeuAc(1)_1)
910.462(PEP+HexNAc(1)_1)
1120.973
1275.027
1377.070
1612.149
1712.624
1745.762(PEP+HexNAc(2)Hex(3)Fuc(1)_1)
1802.771(PEP+HexNAc(3)Hex(3)_1)
1948.841(PEP+HexNAc(3)Hex(3)Fuc(1)_1)
1964.835(PEP+HexNAc(3)Hex(4)_1)
ETD-MS/MS Scan:6718, Noise threshold:1.0
512.199(HexNAc(1)Hex(1)Fuc(1)_1)
445.341
331.187
809.468
1142.489
1168.422
1311.545(PEP+HexNAc(4)Hex(5)Fuc(2)_2)
1487.122
1692.186
1705.183
M+2H
m/z

VFNATR(=PEP)_6_6_2_2_0, 0_None, 0_None,
m/z:1258.17(3+), RT:33.47, Y-score:88.97

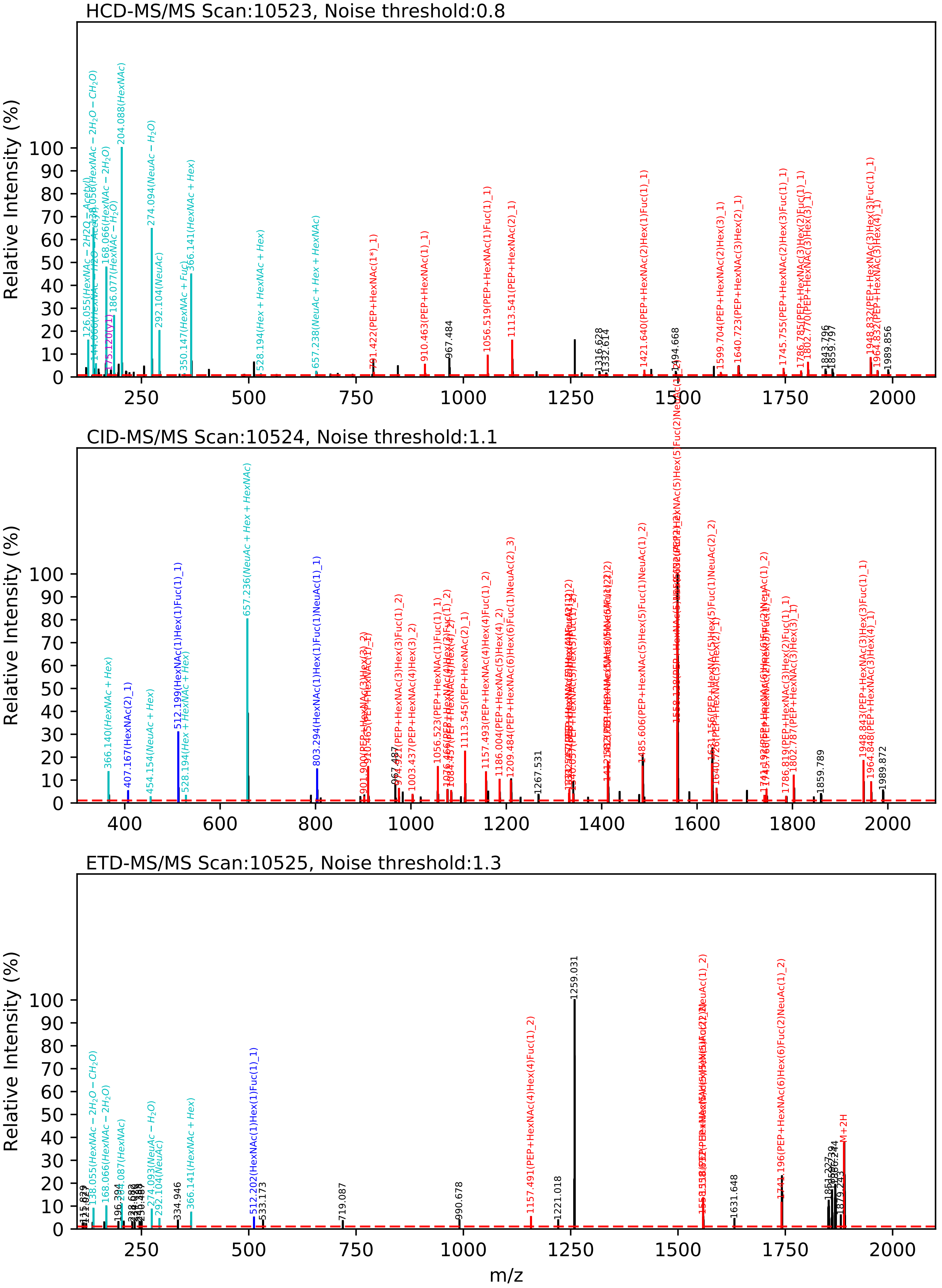

VFNATR(=PEP)_7_2_0_0_0, 0_None, 0_None,
m/z:1124.46(2+), RT:23.87, Y-score:87.31

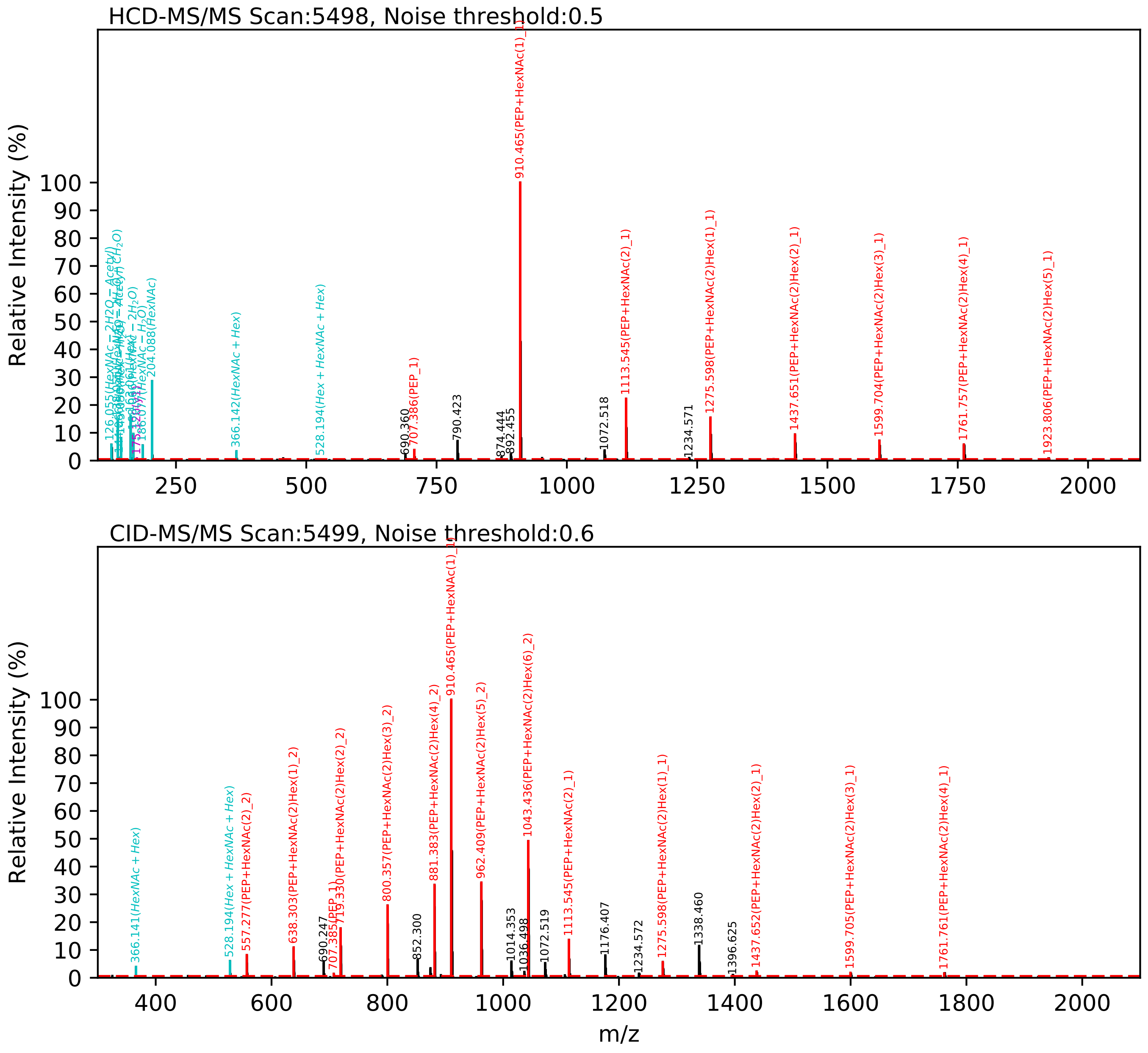

VFNATR(=PEP)_7_4_2_0_0, 0_None, 0_None,
m/z:982.73(3+), RT:23.64, Y-score:90.52

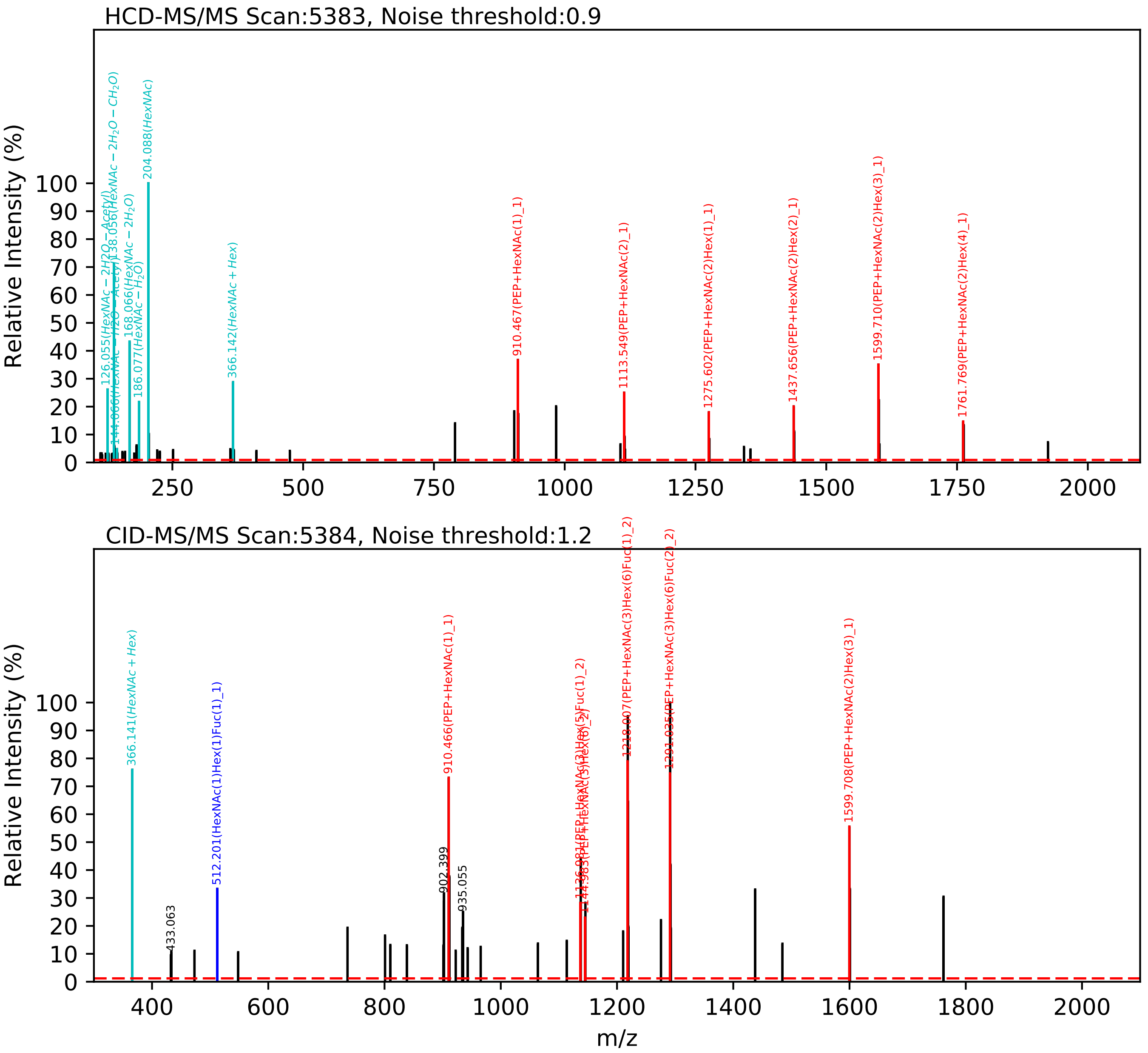

VFNATR(=PEP)_7_7_1_2_0, 0_None, 0_None,
m/z:1331.19(3+), RT:32.88, Y-score:76.63

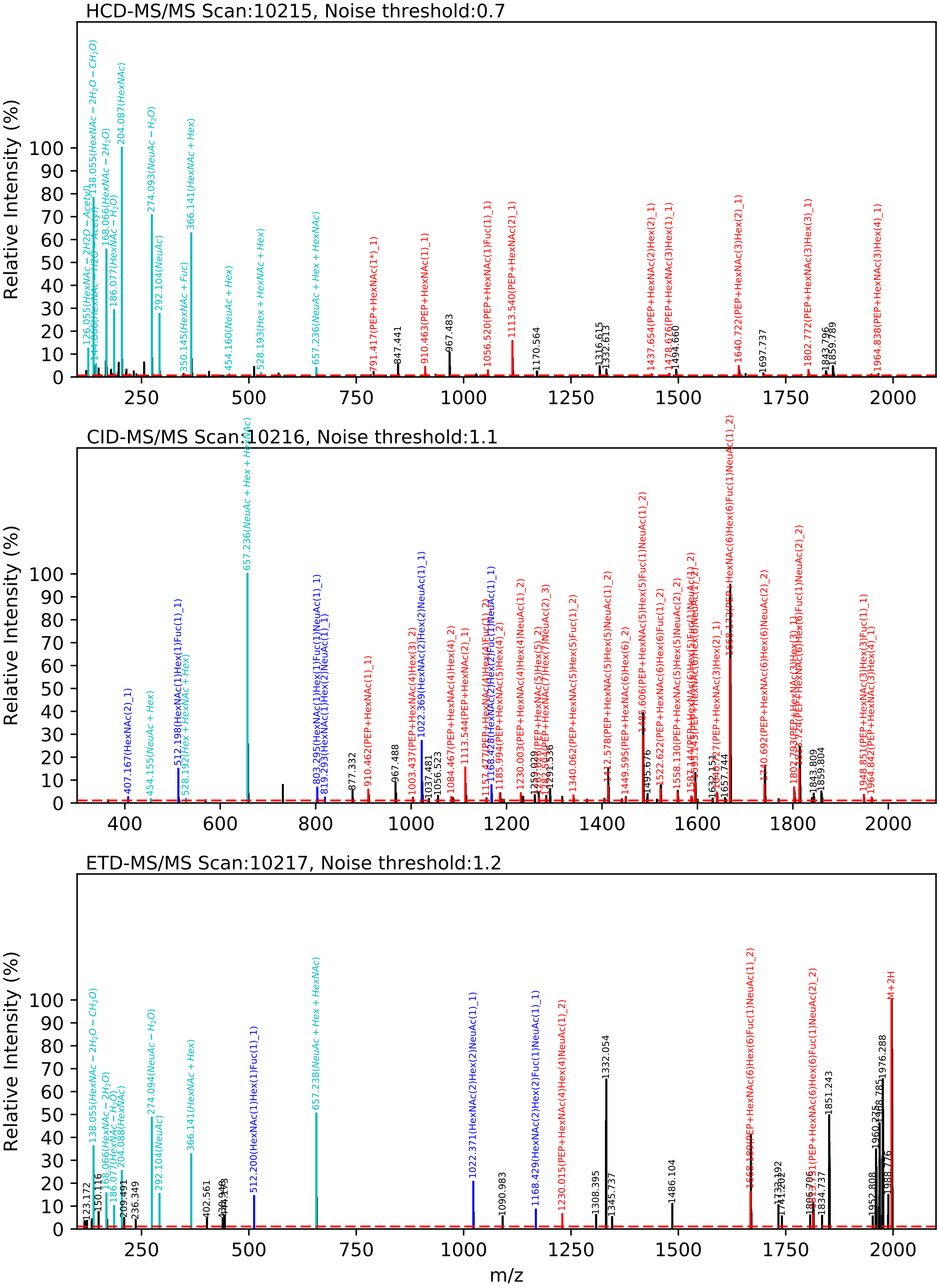

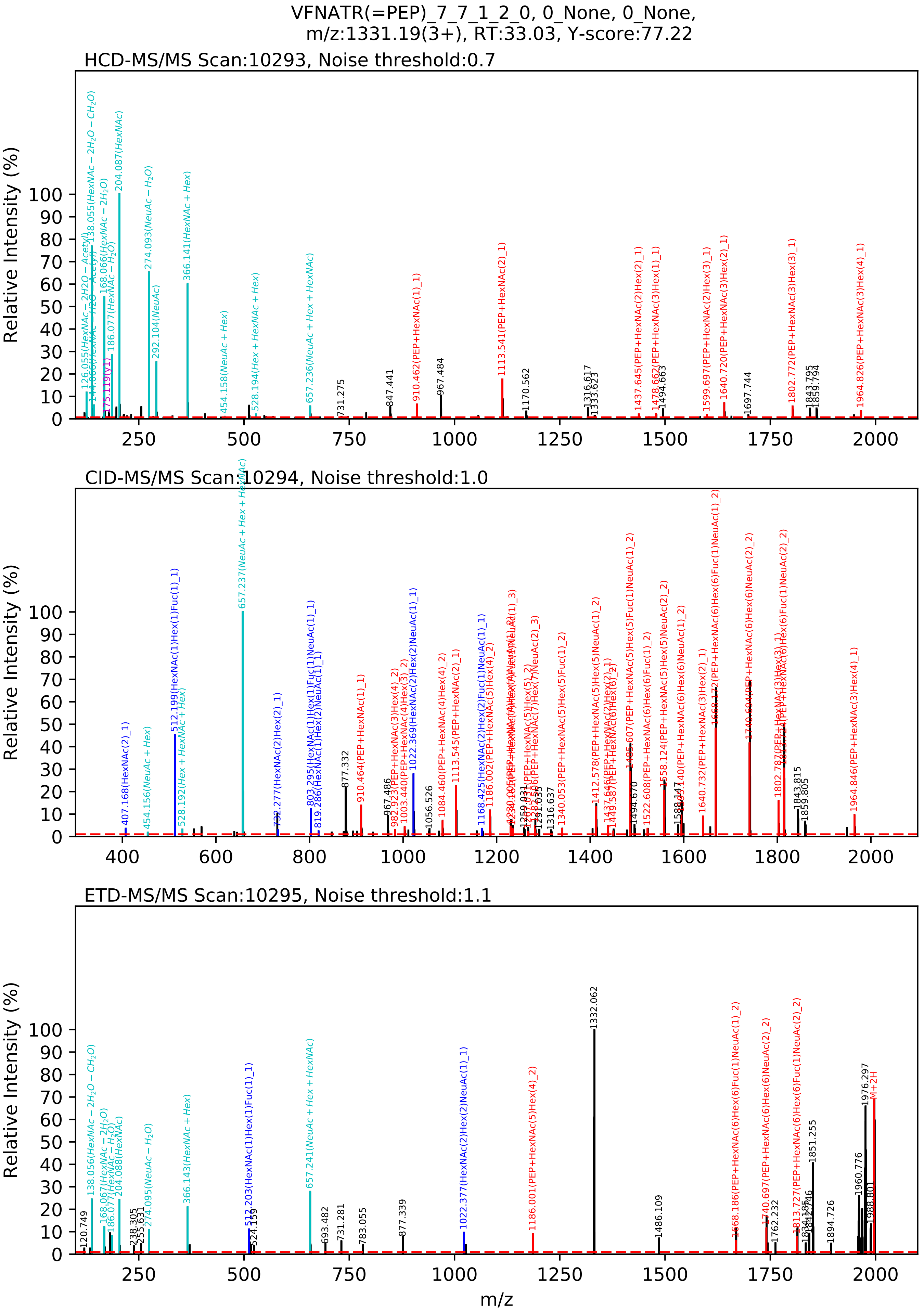

VFNATR(=PEP)_7_7_1_2_0, 0_None, 0_None,
m/z:1331.19(3+), RT:33.03, Y-score:77.22
HCD-MS/MS Scan:10293, Noise threshold:0.7
CID-MS/MS Scan:10294, Noise threshold:1.0
ETD-MS/MS Scan:10295, Noise threshold:1.1
Relative Intensity (%)
m/z
1332.062
M+2H
