# Supplementary material for: Characterization of Site-Specific N- and O-Glycopeptides from Recombinant Spike and ACE2 Glycoproteins Using LC-MS/MS Analysis
Source: Int J Mol Sci. 2024 Dec 20;25(24):13649. doi: 10.3390/ijms252413649 (PMC11678118; doi:10.3390/ijms252413649)

EGVFVSNGTHWFVTQR(=PEP)_8_2_0_0_0, 0_None, 0_None,
m/z:1189.50(2+), RT:64.78, hcd-score:68.98

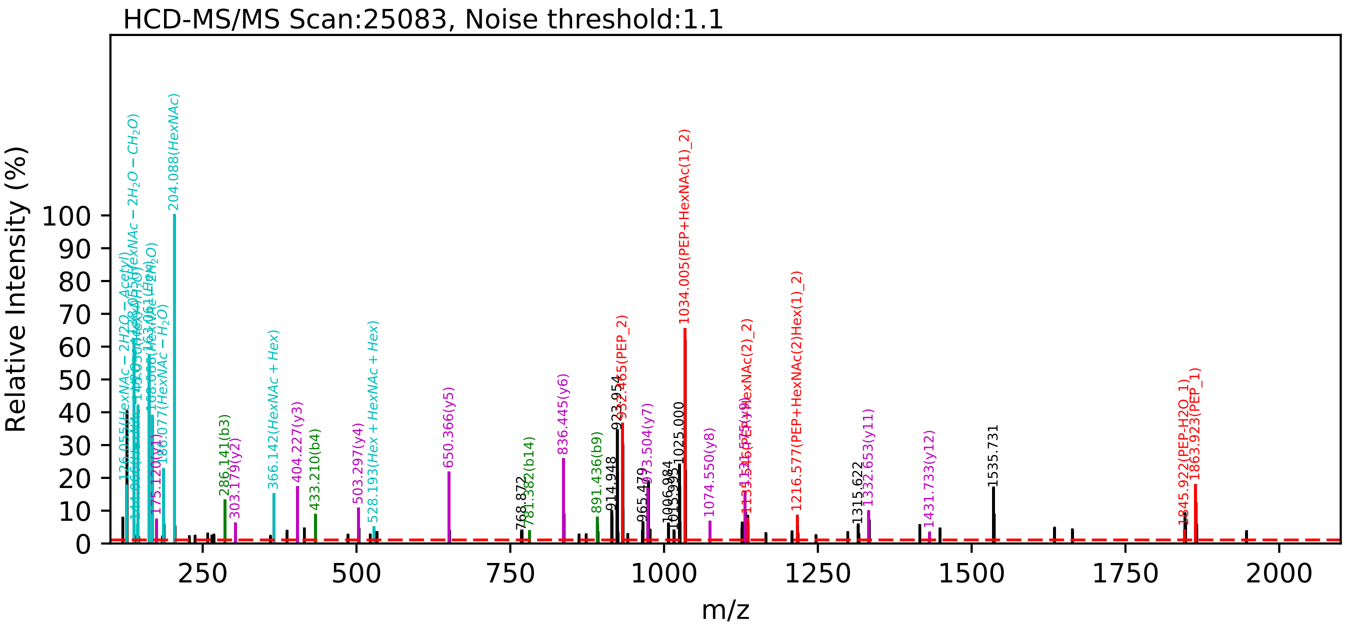

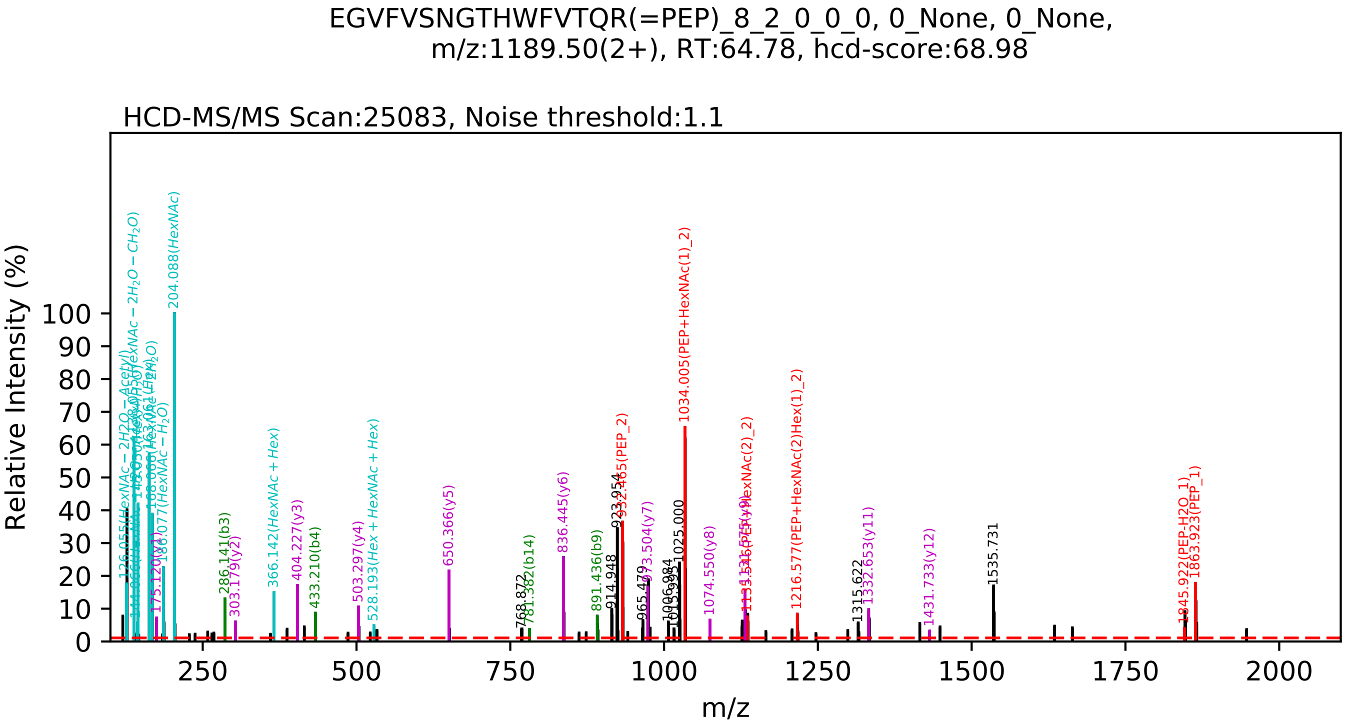

EGVFVSNGTHWFVTQR(=PEP)_8_2_0_0_0, 0_None, 0_None,
m/z:1189.50(2+), RT:64.78, hcd-score:68.98
HCD-MS/MS Scan:25083, Noise threshold:1.1
Relative Intensity (%)
m/z
204.088(HexNAc)
186.077(HexNAc − H2O)
175.120(y1)
286.141(b3)
303.179(y2)
366.142(HexNAc + Hex)
404.227(y3)
433.210(b4)
503.297(y4)
528.193(Hex + HexNAc + Hex)
650.366(y5)
768.872
781.382(b14)
836.445(y6)
891.436(b9)
914.948
923.954
965.479
1006.984
1025.000
1034.005(PEP+HexNAc(1)_2)
1074.550(y8)
1216.577(PEP+HexNAc(2)Hex(1)_2)
1315.622
1332.653(y11)
1431.733(y12)
1535.731
1845.922(PEP-H2O_1)
1863.923(PEP_1)

EGVFVSNGTHWFVTQR(=PEP)_8_2_0_0_0, 0_None, 0_None,
m/z:1189.50(2+), RT:64.88, hcd-score:73.17

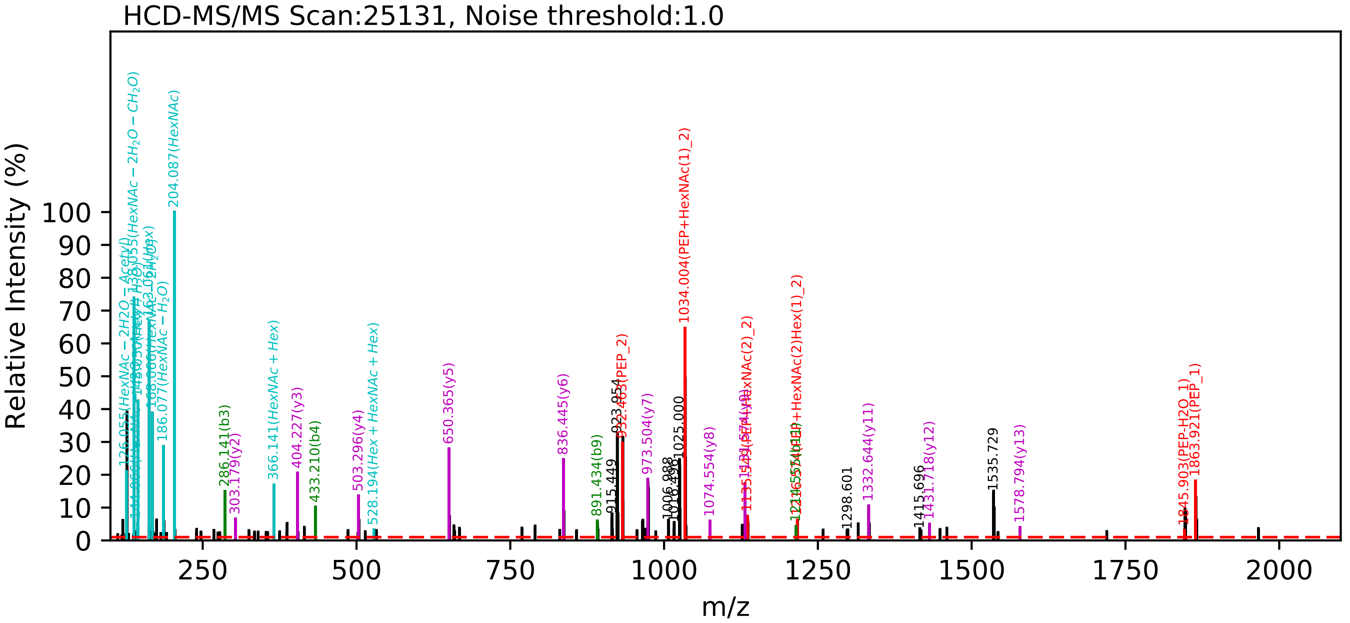

EGVFVSNGTHWFVTQR(=PEP)_8_2_0_0_0, 0_None, 0_None,
m/z:1189.50(2+), RT:64.88, hcd-score:73.17

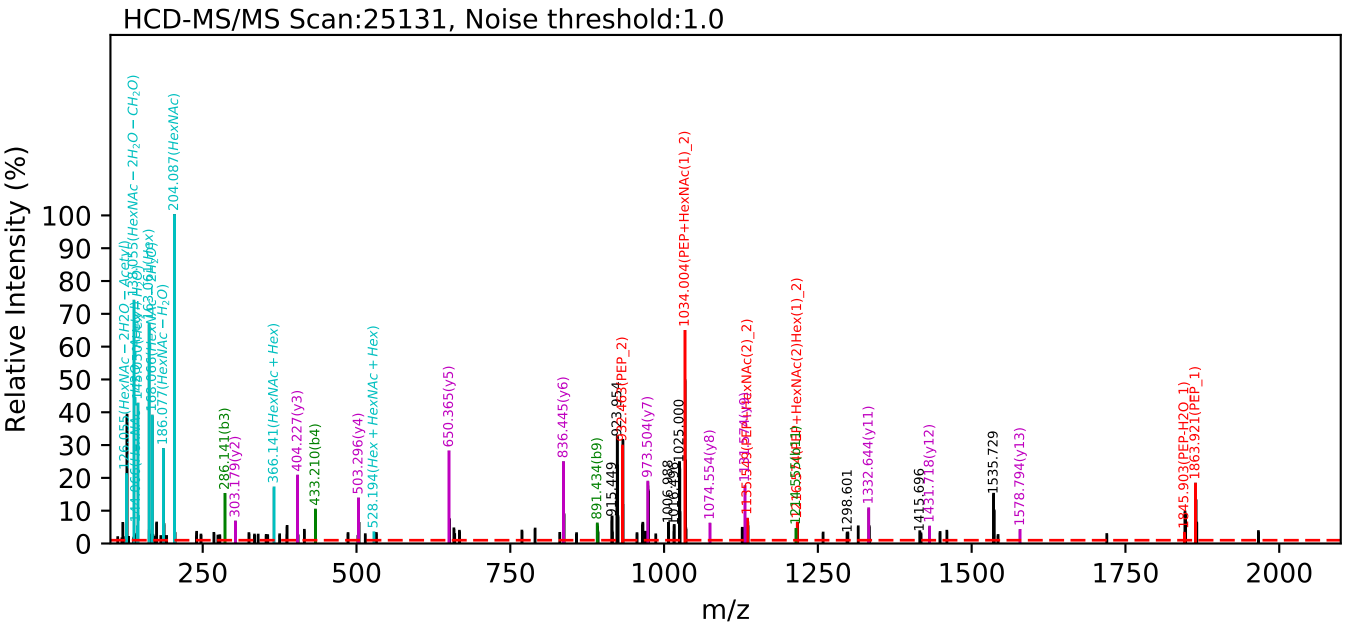

EGVFVSNGTHWFVTQR(=PEP)_8_2_0_0_0, 0_None, 0_None,
m/z:1189.50(2+), RT:65.34, hcd-score:69.62

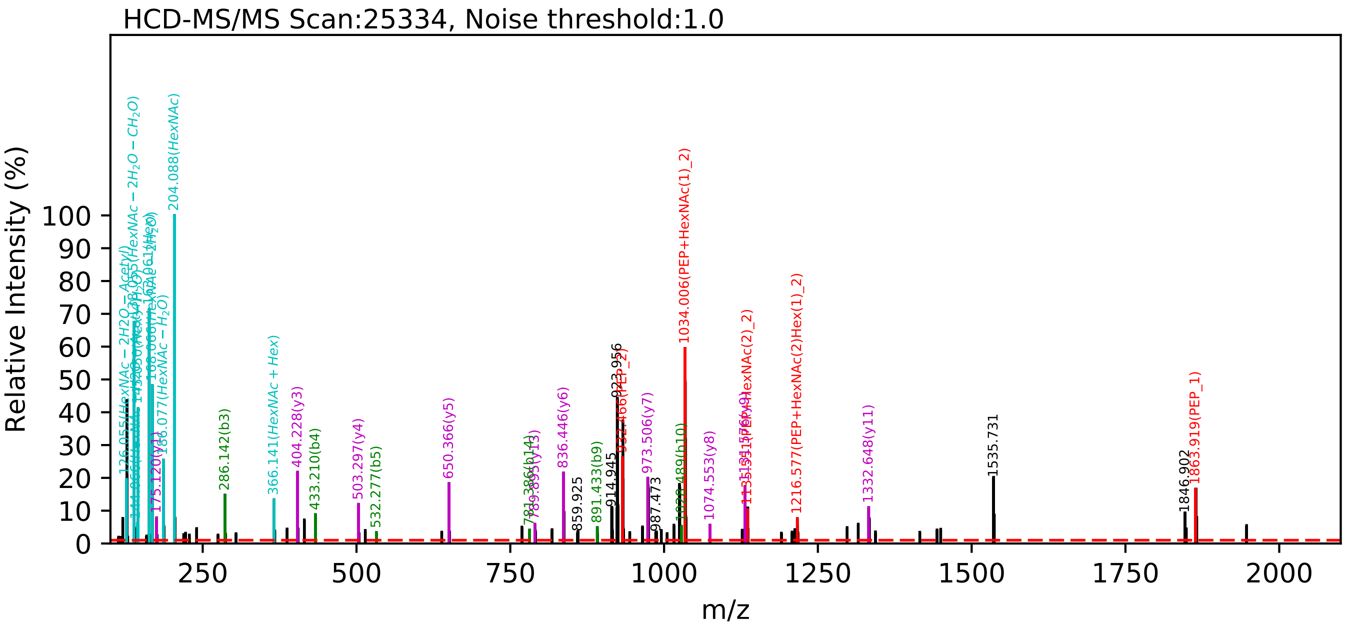

EGVFVSNGTHWFVTQR(=PEP)_8_2_0_0_0, 0_None, 0_None,
m/z:1189.50(2+), RT:65.34, hcd-score:69.62

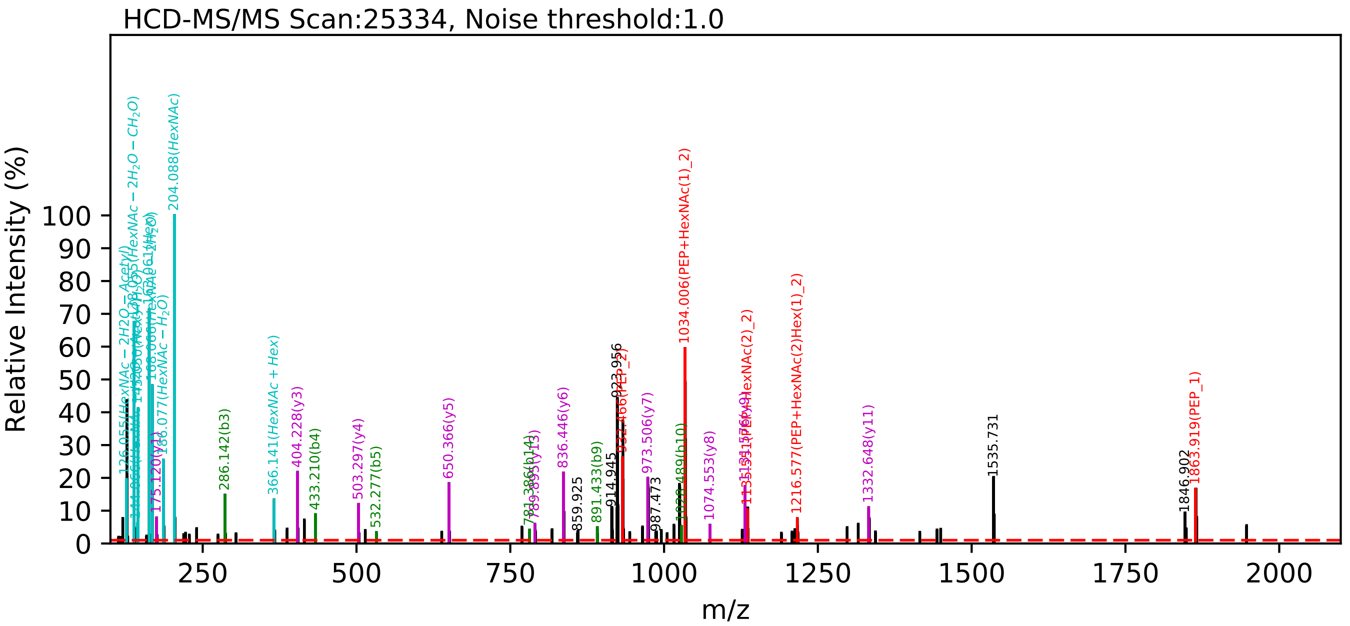

EGVFVSNGTHWFVTQR(=PEP)_8_2_0_0_0, 0_None, 0_None,
m/z:1189.50(2+), RT:65.93, hcd-score:69.67

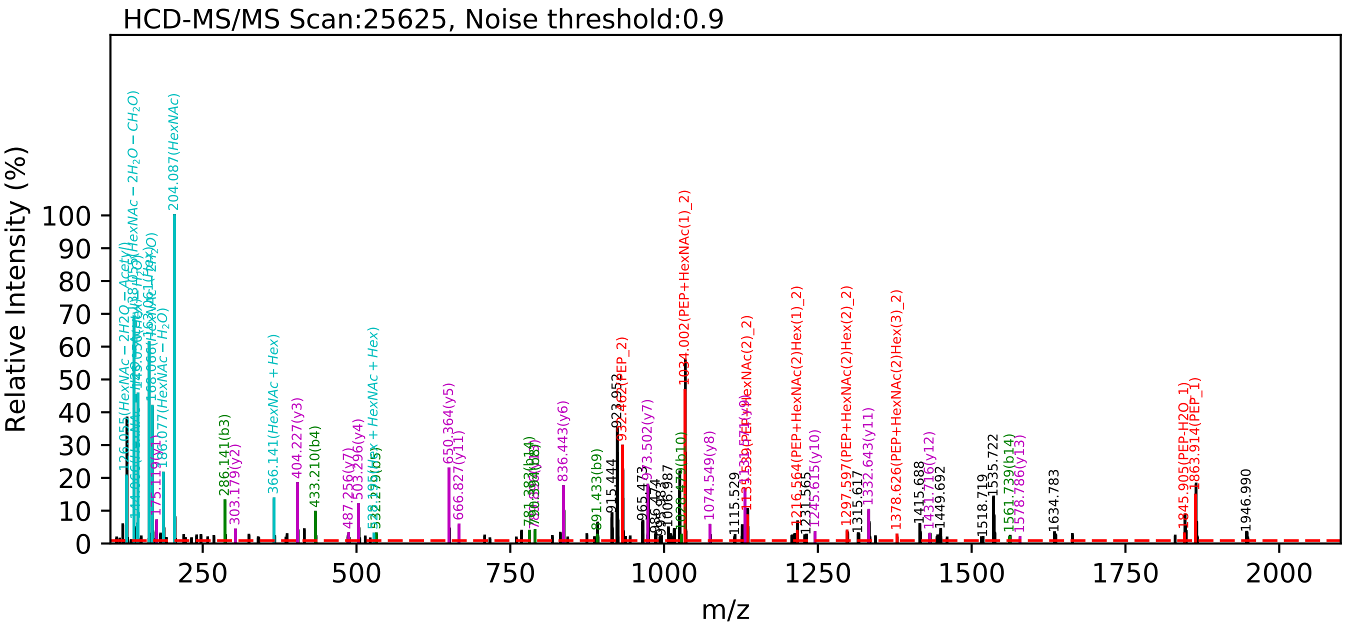

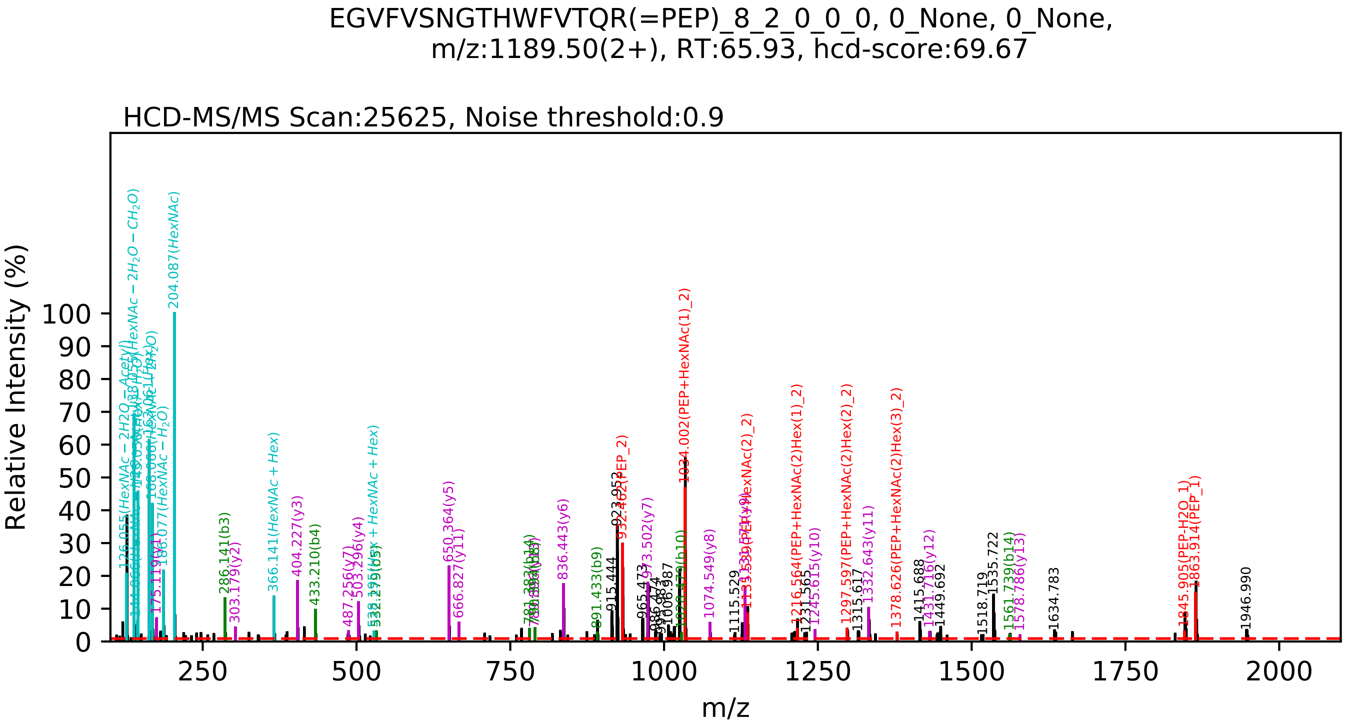
EGVFVSNGTHWFVTQR(=PEP)_8_2_0_0_0, 0_None, 0_None,
m/z:1189.50(2+), RT:65.93, hcd-score:69.67
HCD-MS/MS Scan:25625, Noise threshold:0.9
Relative Intensity (%)
m/z

EGVFVSNGTHWFVTQR(=PEP)_8_2_0_0_0, 0_None, 0_None,
m/z:1189.50(2+), RT:66.48, hcd-score:67.29

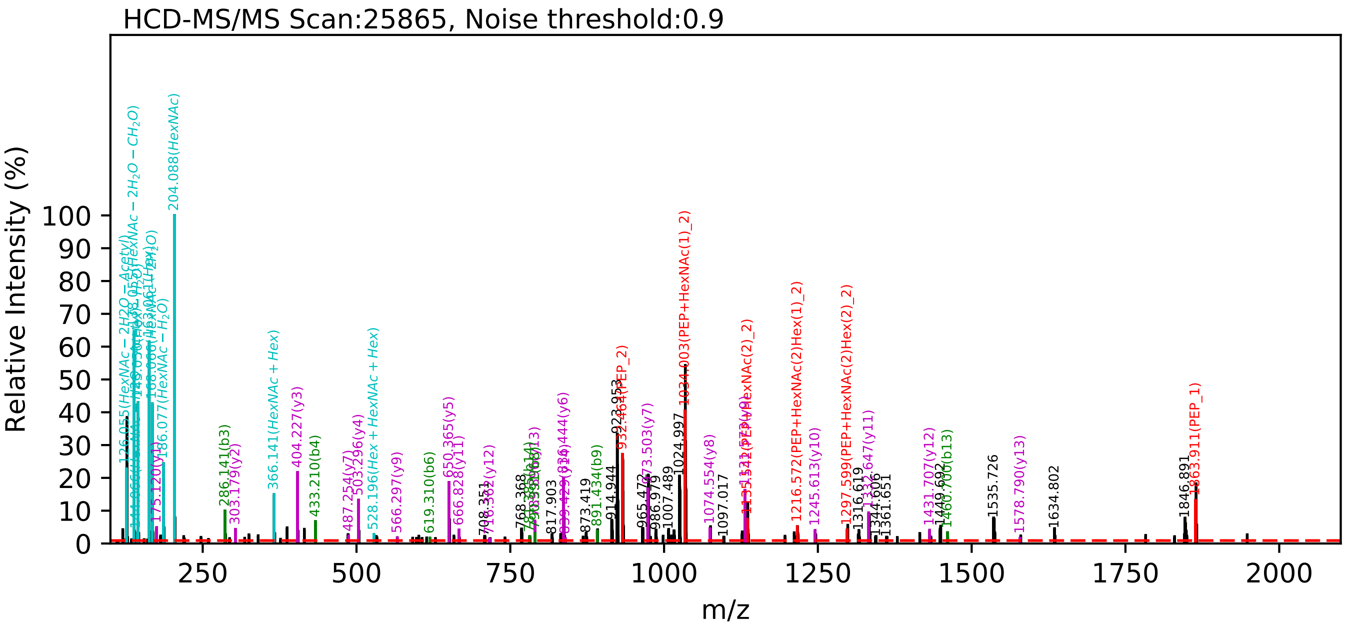

EGVFVSNGTHWFVTQR(=PEP)_8_2_0_0_0, 0_None, 0_None,
m/z:1189.50(2+), RT:66.48, hcd-score:67.29

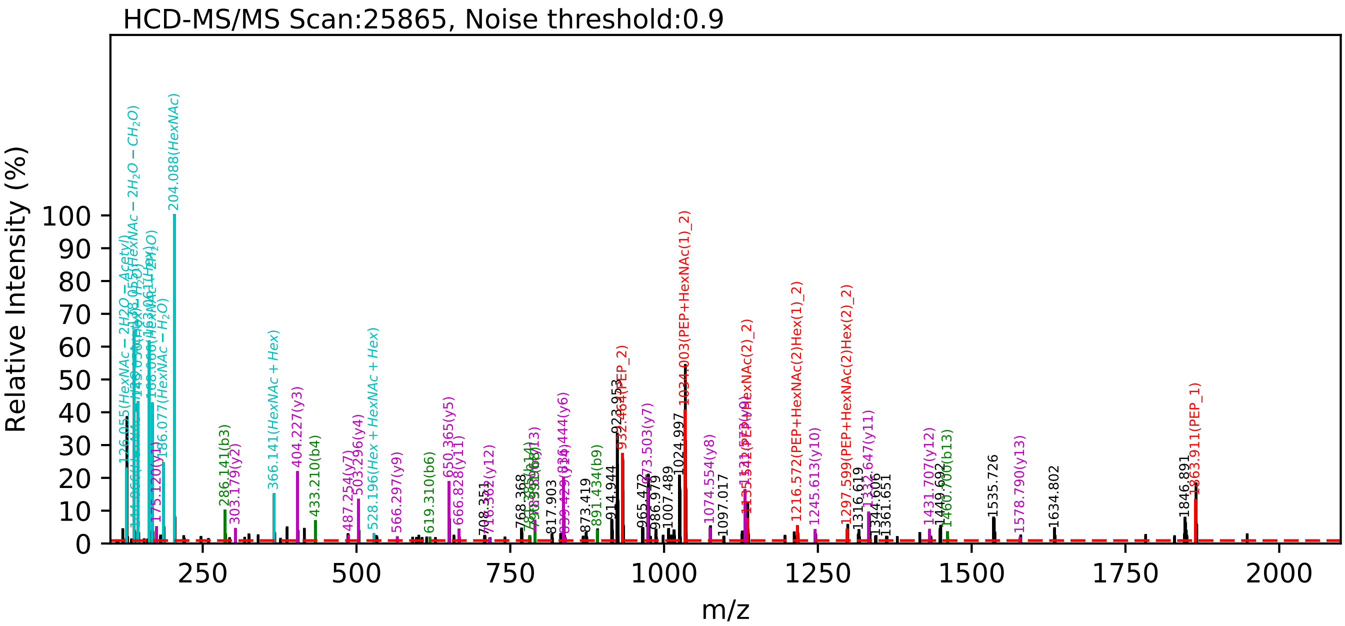

EGVFVSNGTHWFVTQR(=PEP)_8_2_0_0_0, 0_None, 0_None,
m/z:1189.50(2+), RT:67.31, hcd-score:65.49

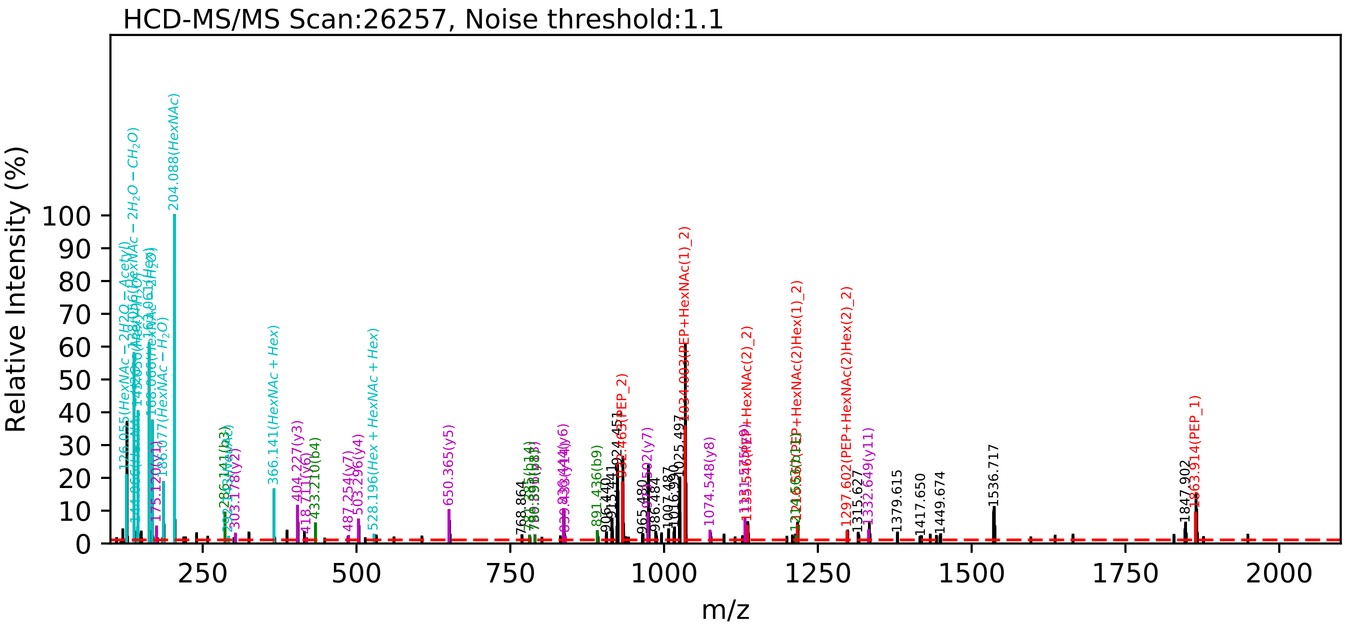

EGVFVSNGTHWFVTQR(=PEP)_8_2_0_0_0, 0_None, 0_None,
m/z:1189.50(2+), RT:67.31, hcd-score:65.49

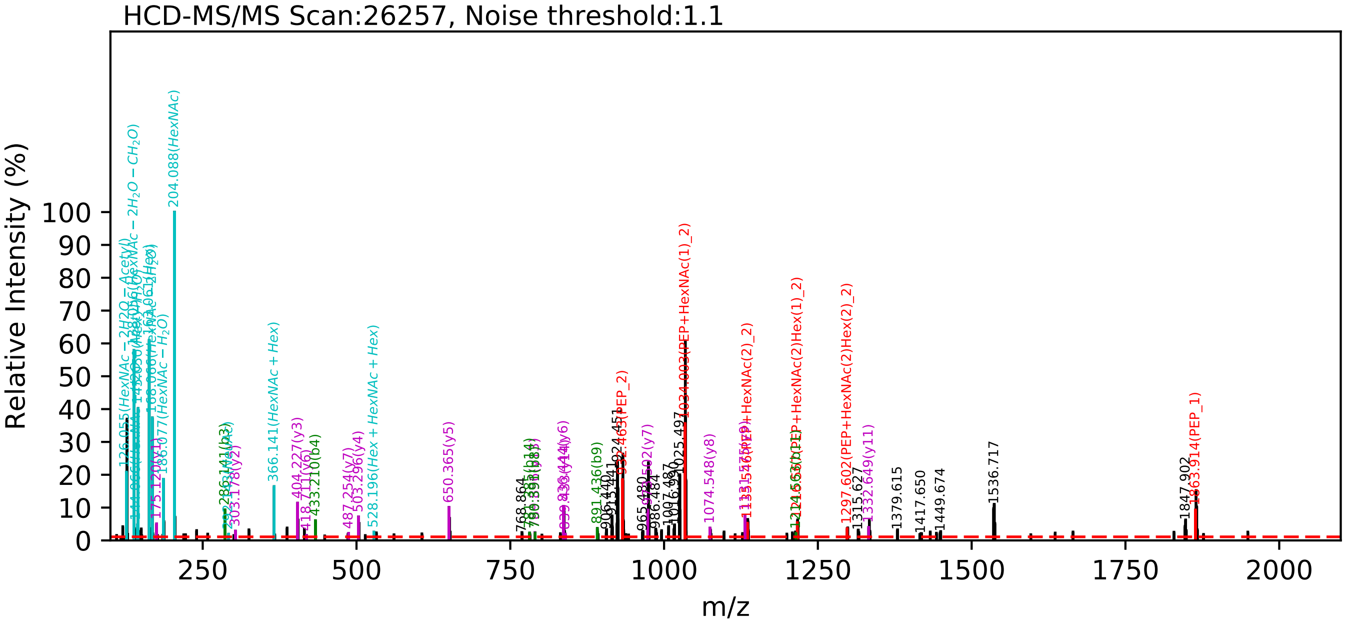

EGVFVSNGTHWFVTQR(=PEP)_8_2_0_0_0, 0_None, 0_None,
m/z:1189.50(2+), RT:68.12, hcd-score:69.03

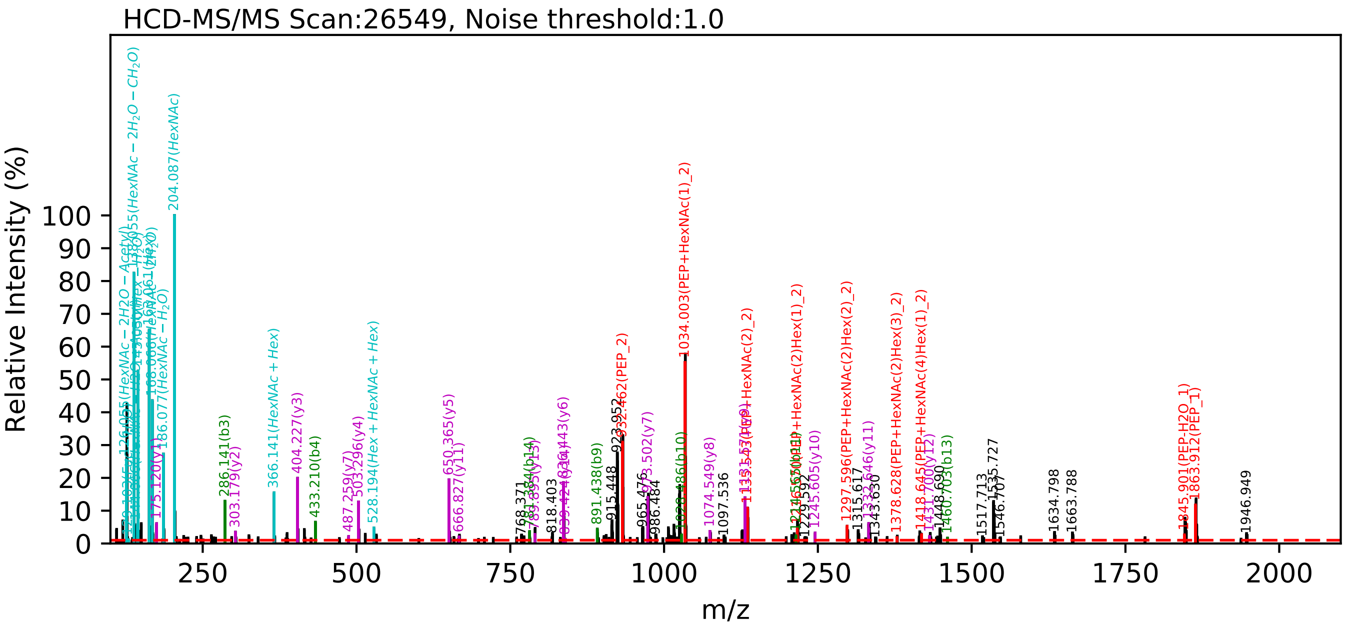

EGVFVSNGTHWFVTQR(=PEP)_8_2_0_0_0, 0_None, 0_None,
m/z:1189.50(2+), RT:68.12, hcd-score:69.03

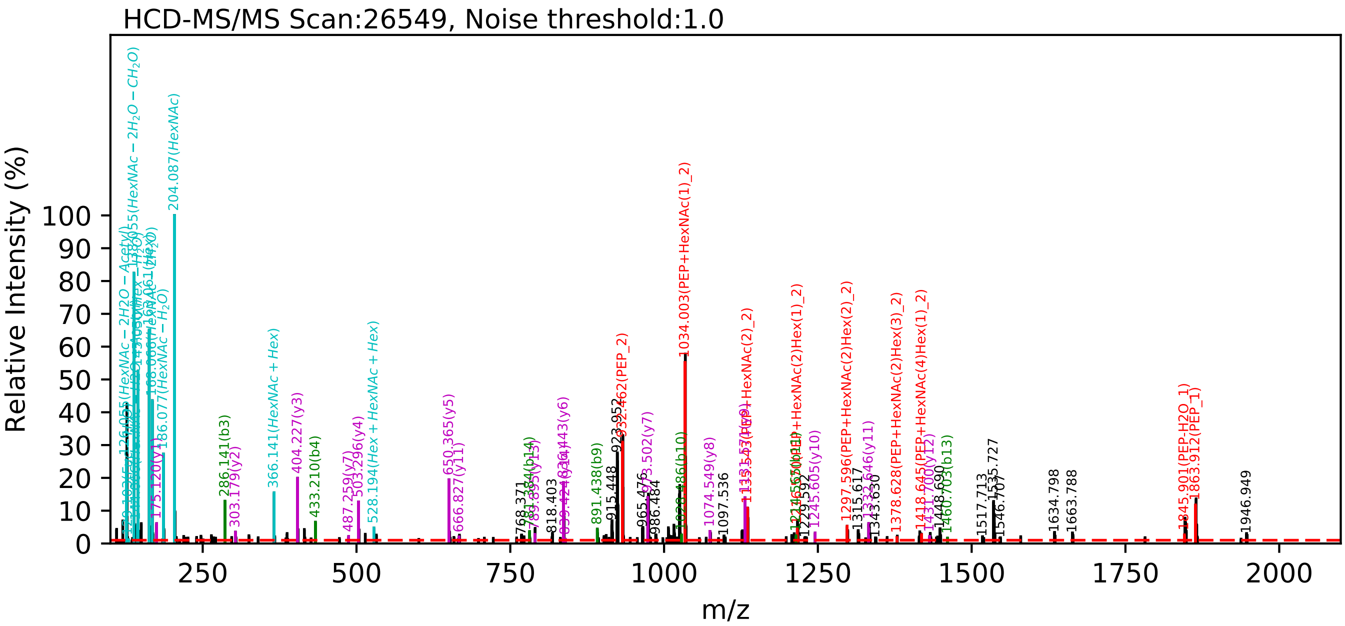

EGVFVSNGTHWFVTQR(=PEP)_8_2_0_0_0, 0_None, 0_None,
m/z:1189.50(2+), RT:68.17, hcd-score:73.95

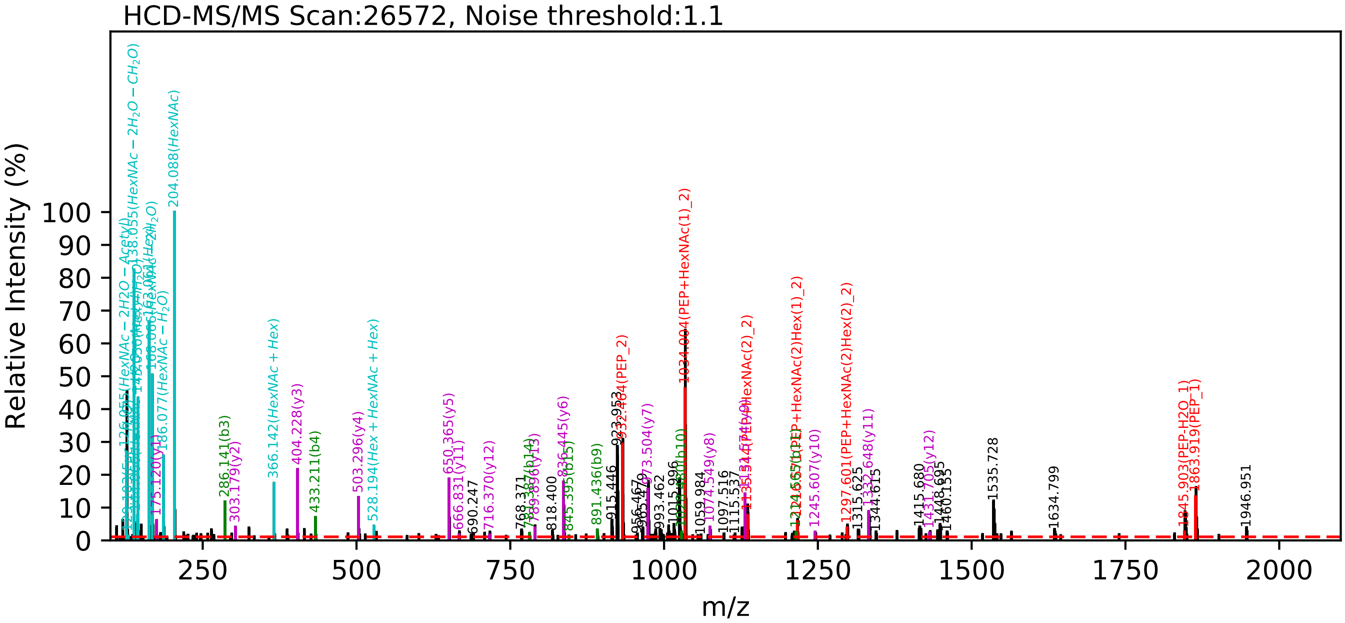

EGVFVSNGTHWFVTQR(=PEP)_8_2_0_0_0, 0_None, 0_None,
m/z:1189.50(2+), RT:68.17, hcd-score:73.95

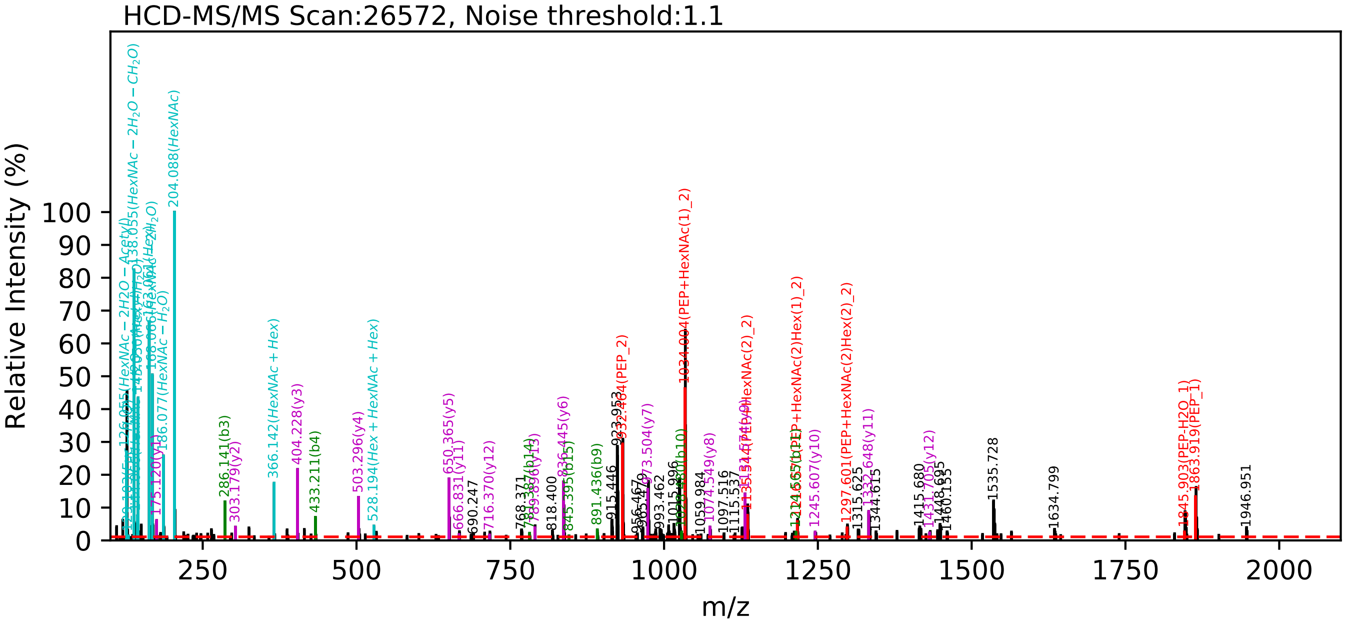

EGVFVSNGTHWFVTQR(=PEP)_8_2_0_0_0, 0_None, 0_None,
m/z:1189.50(2+), RT:68.30, hcd-score:68.16

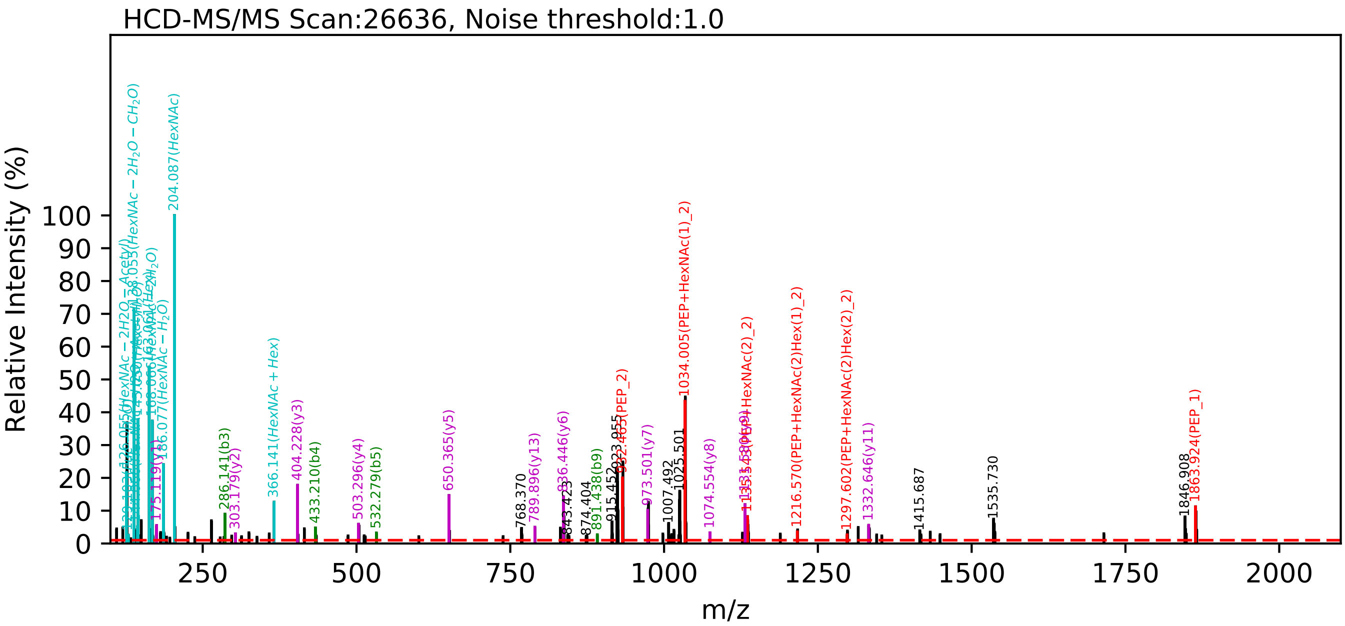

EGVFVSNGTHWFVTQR(=PEP)_8_2_0_0_0, 0_None, 0_None,
m/z:1189.50(2+), RT:68.30, hcd-score:68.16

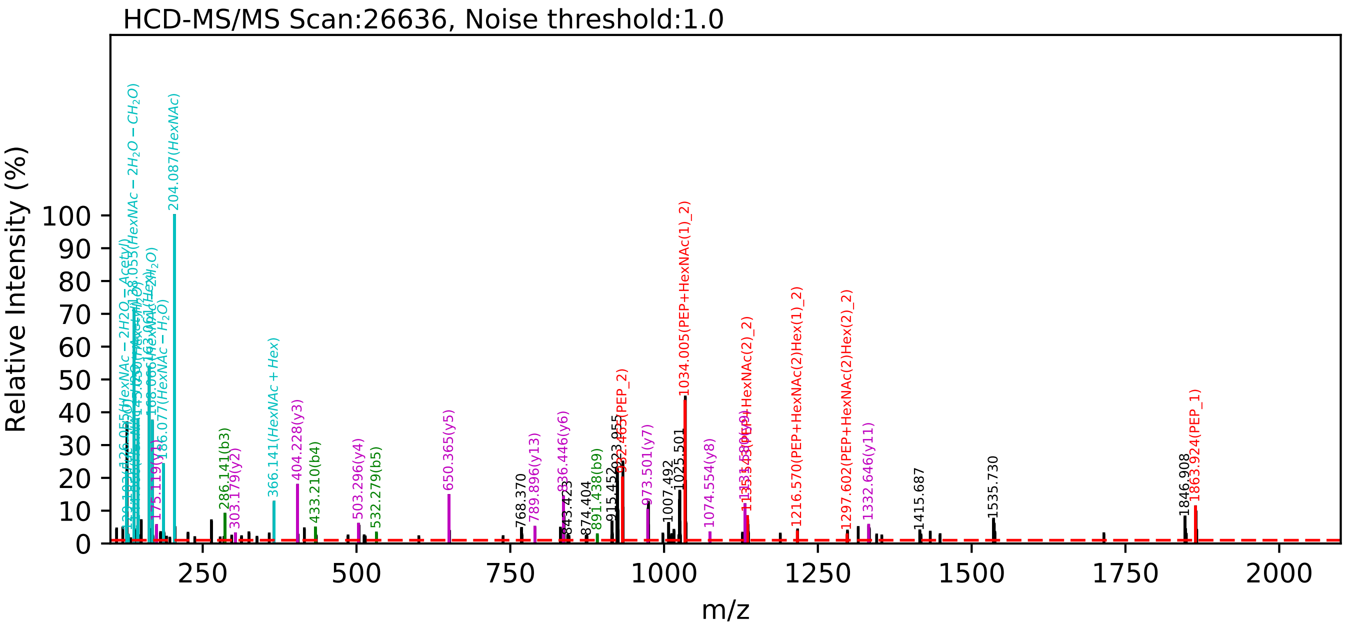

DFGGFNFSQILPDPSKPSKR(=PEP)_10_2_0_0_0, 0_None, 0_None,
m/z:1066.71(4+), RT:88.48, hcd-score:89.65

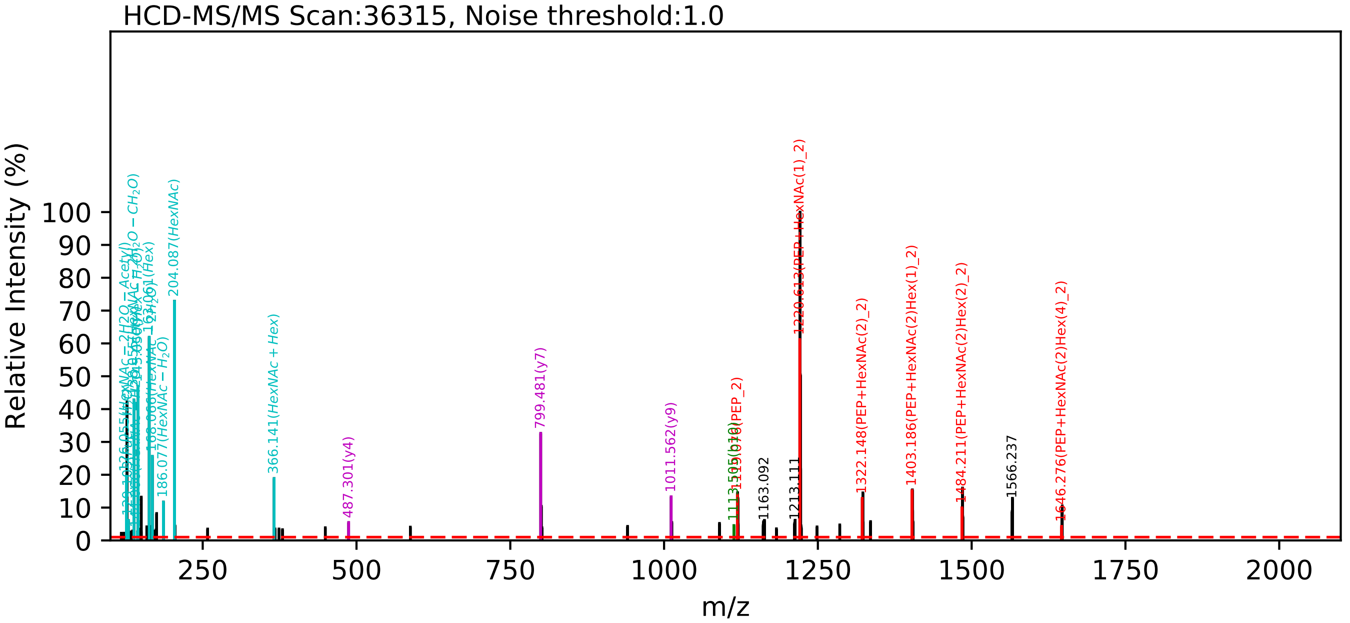

DFGGFNFSQILPDPSKPSKR(=PEP)_10_2_0_0_0, 0_None, 0_None,
m/z:1066.71(4+), RT:88.48, hcd-score:89.65

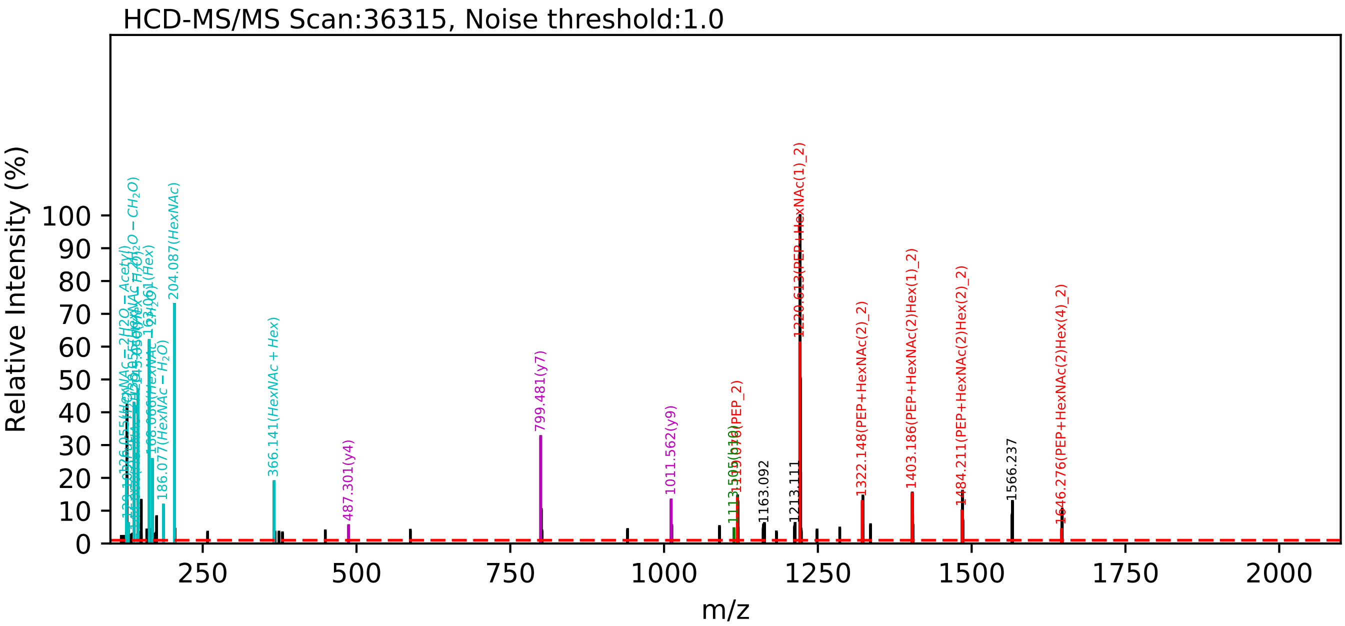

DFGGFNFSQILPDPSKPSK(=PEP)_6_2_0_0_0, 0_None, 0_None,
m/z:1153.84(2+), RT:96.14, hcd-score:87.47

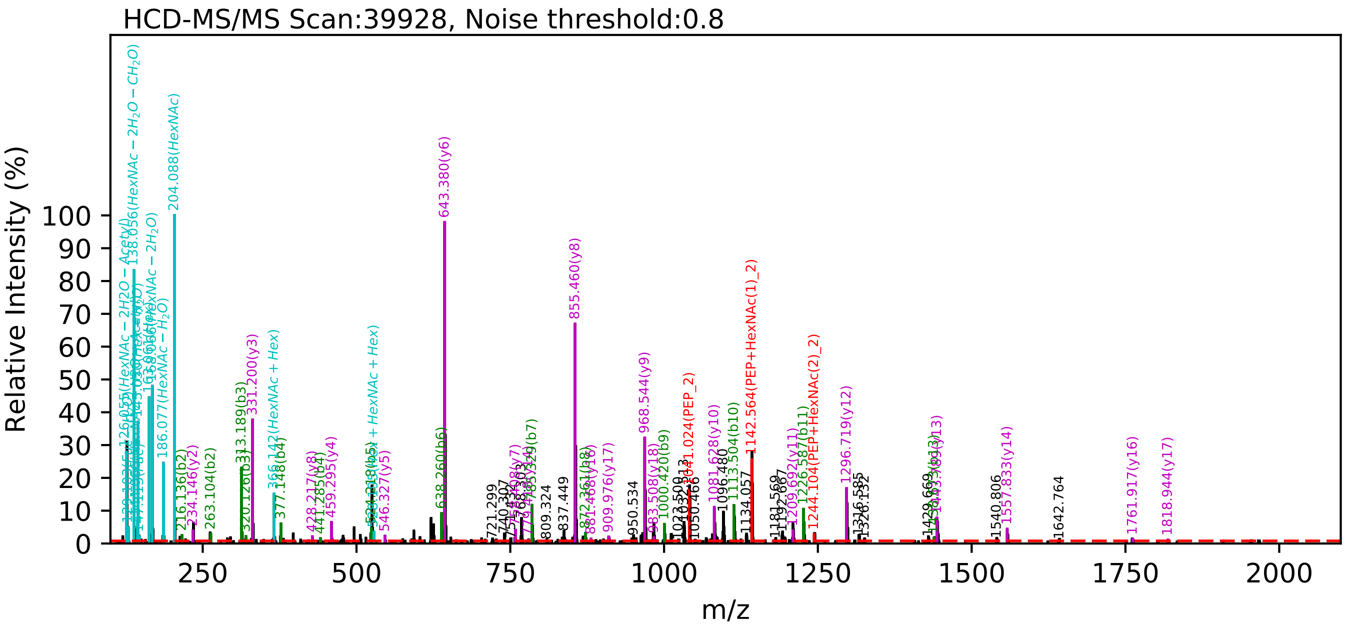

DFGGFNFSQILPDPSKPSK(=PEP)_6_2_0_0_0, 0_None, 0_None,
m/z:1153.84(2+), RT:96.14, hcd-score:87.47

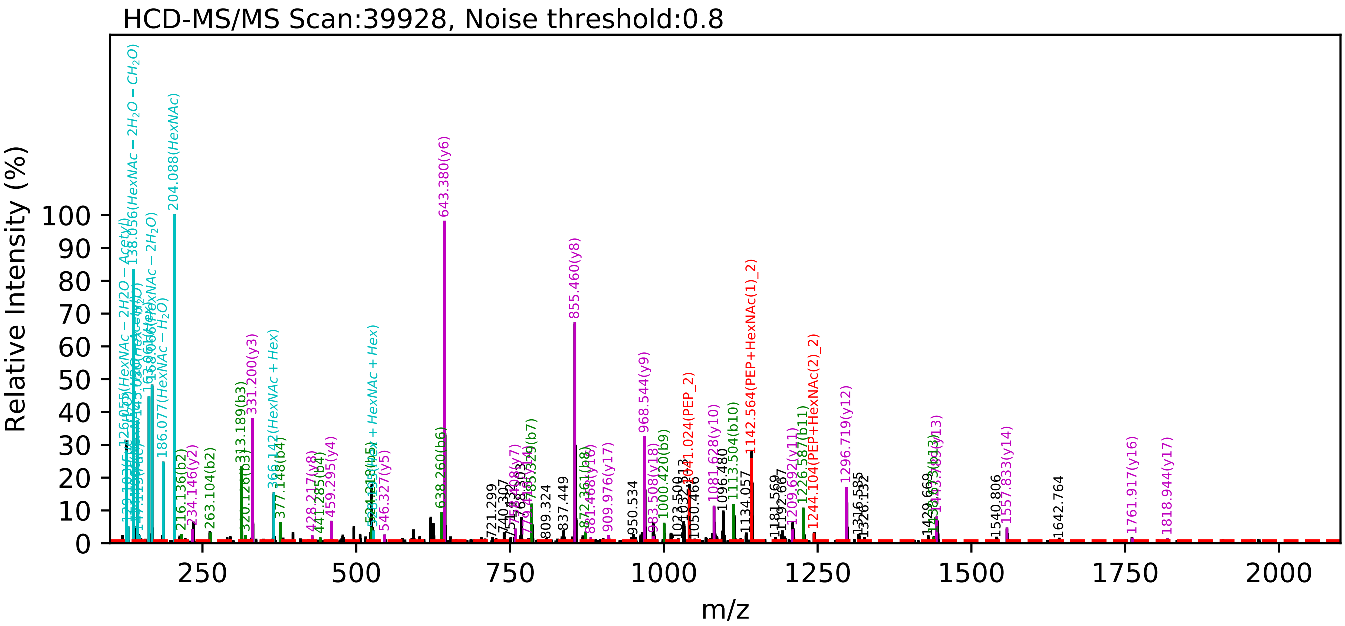

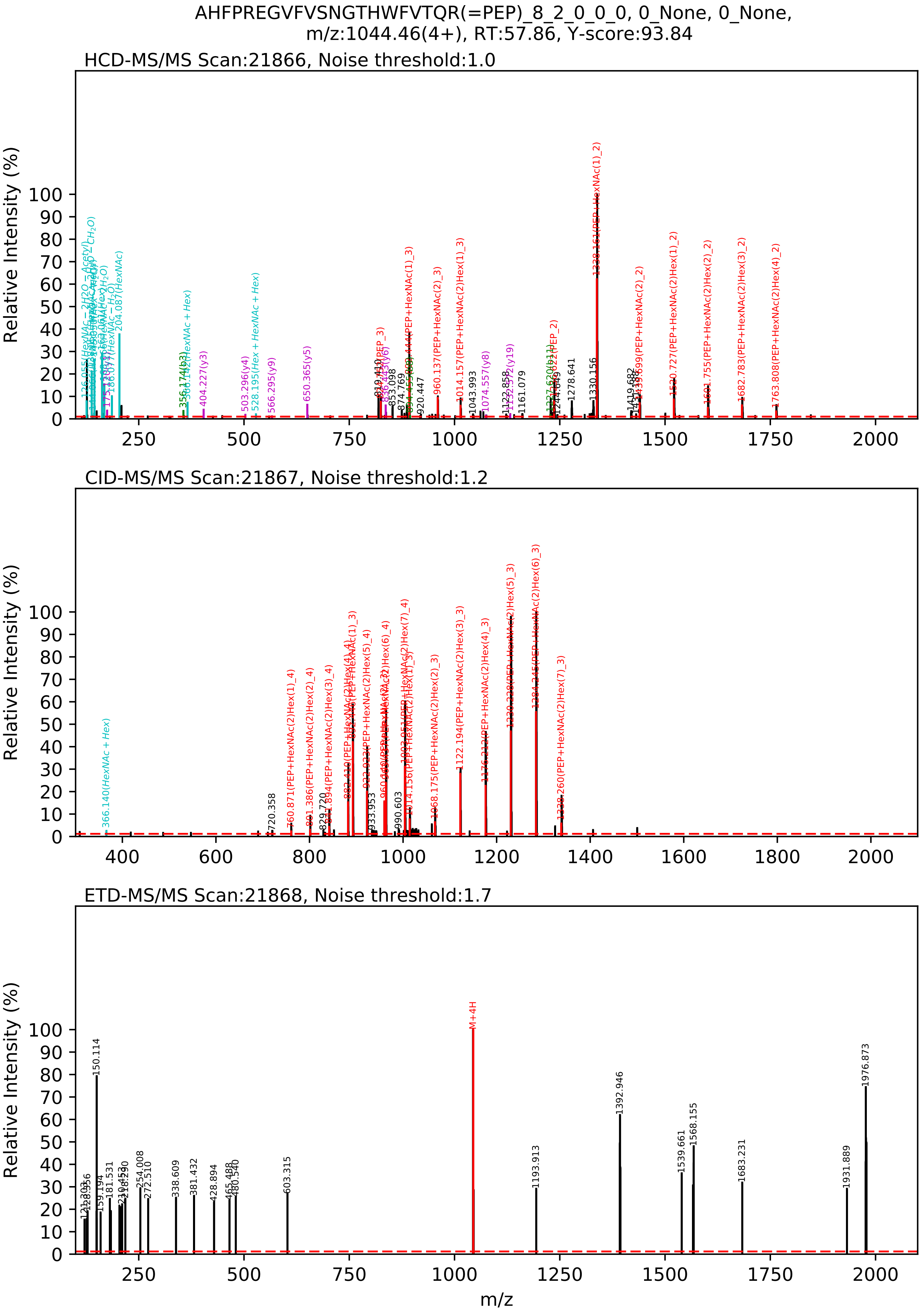

AHFPREGVFVSNGTHWFVTQR(=PEP)_8_2_0_0_0, 0_None, 0_None,
m/z:1044.46(4+), RT:57.86, Y-score:93.84
HCD-MS/MS Scan:21866, Noise threshold:1.0
CID-MS/MS Scan:21867, Noise threshold:1.2
ETD-MS/MS Scan:21868, Noise threshold:1.7
Relative Intensity (%)
m/z
M+4H

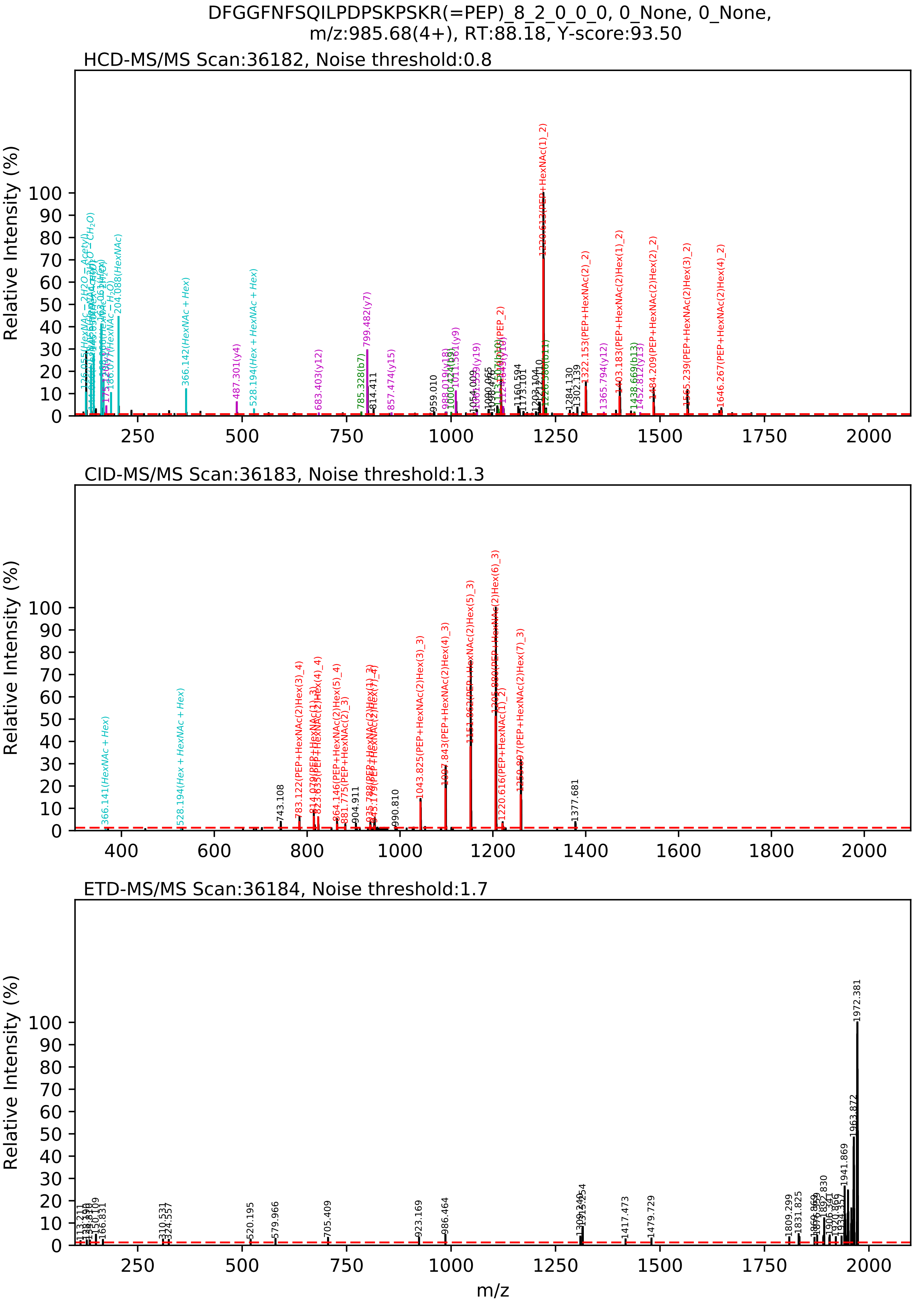

DFGGFNFSQILPDPSKPSKR(=PEP)_8_2_0_0_0, 0_None, 0_None,
m/z:985.68(4+), RT:88.18, Y-score:93.50
HCD-MS/MS Scan:36182, Noise threshold:0.8
CID-MS/MS Scan:36183, Noise threshold:1.3
ETD-MS/MS Scan:36184, Noise threshold:1.7
Relative Intensity (%)
m/z

DFGGFNFSQILPDPSKPSKR(=PEP)_8_2_0_0_0, 0_None, 0_None,
m/z:985.68(4+), RT:88.72, Y-score:95.30

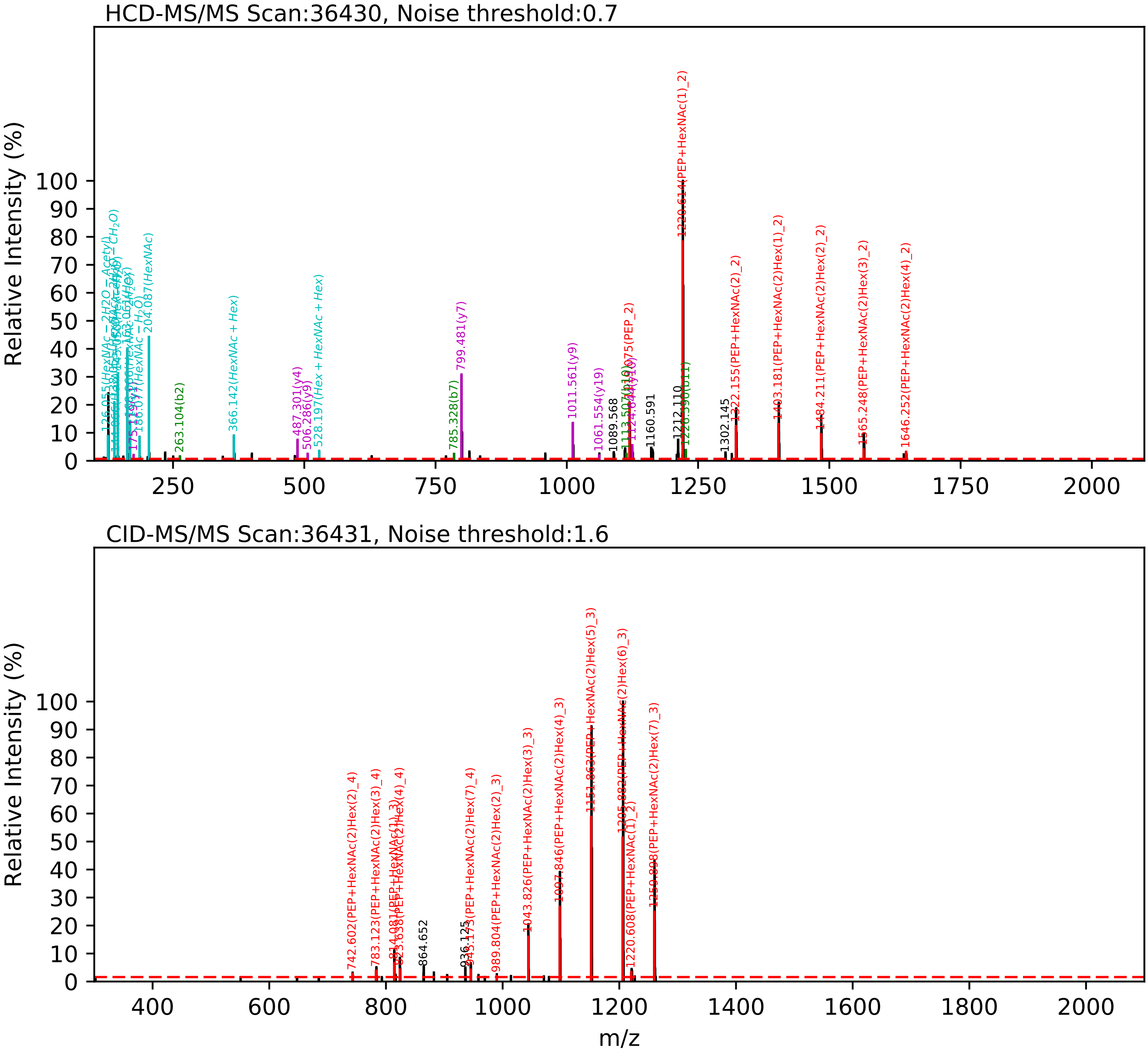

DFGGFNFSQILPDPSKPSKR(=PEP)_8_2_0_0_0, 0_None, 0_None,
m/z:985.68(4+), RT:88.83, Y-score:94.00

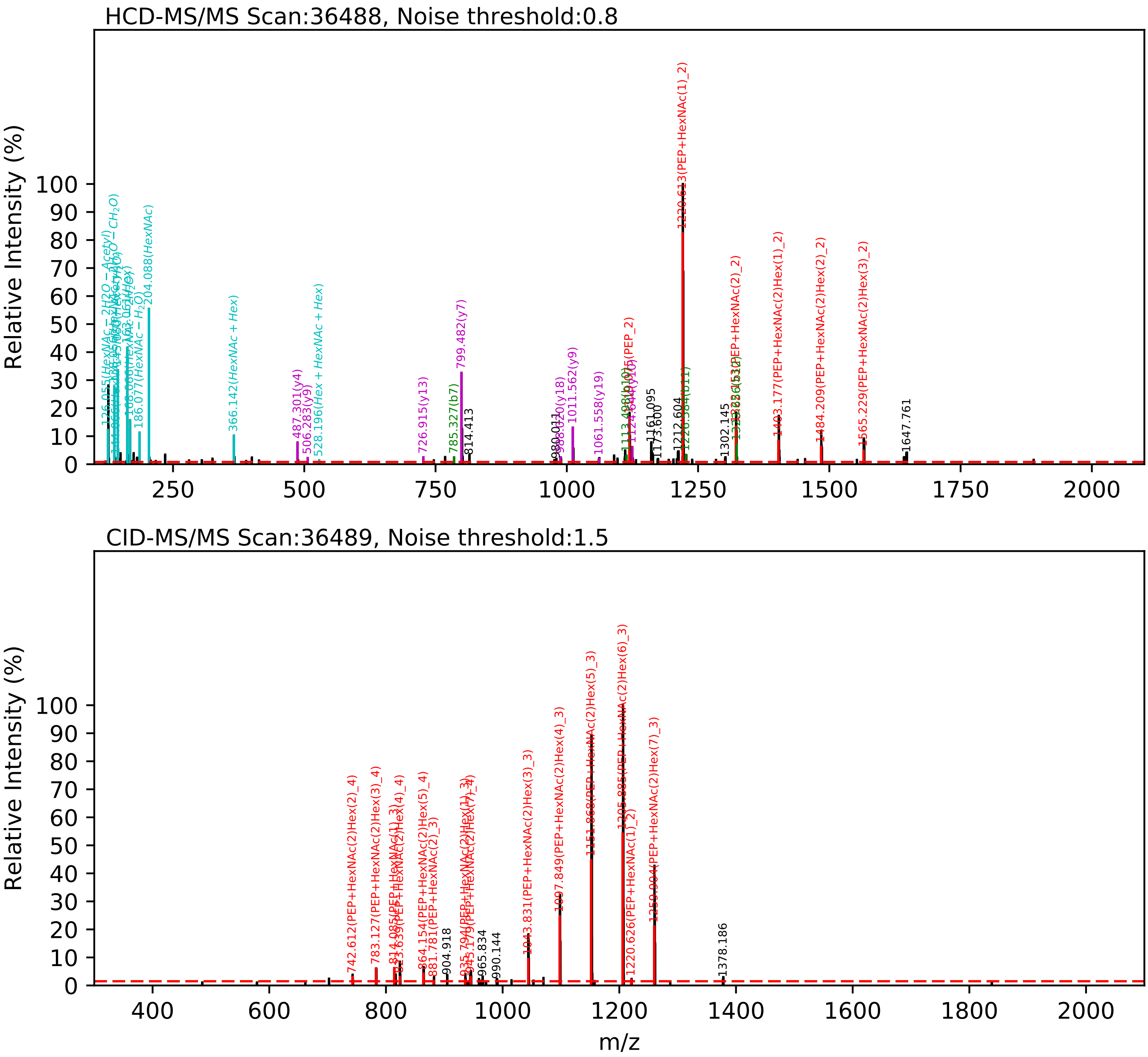

DFGGFNFSQILPDPSKPSKR(=PEP)_8_2_0_0_0, 0_None, 0_None,
m/z:1313.91(3+), RT:88.33, Y-score:91.63

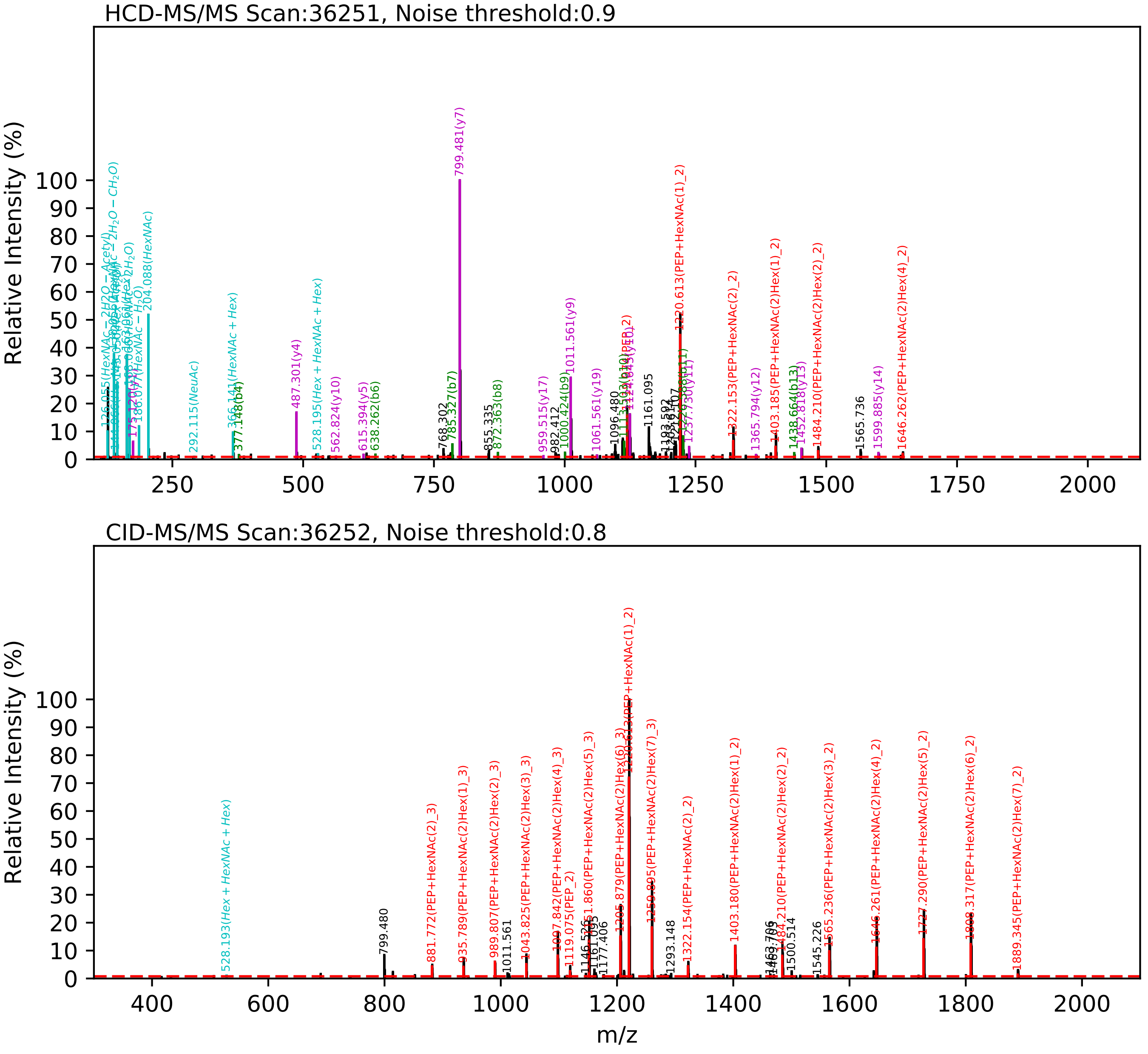

DFGGFNFSQILPDPSKPSKR(=PEP)_8_2_0_0_0, 0_None, 0_None,
m/z:1313.91(3+), RT:88.72, Y-score:92.94

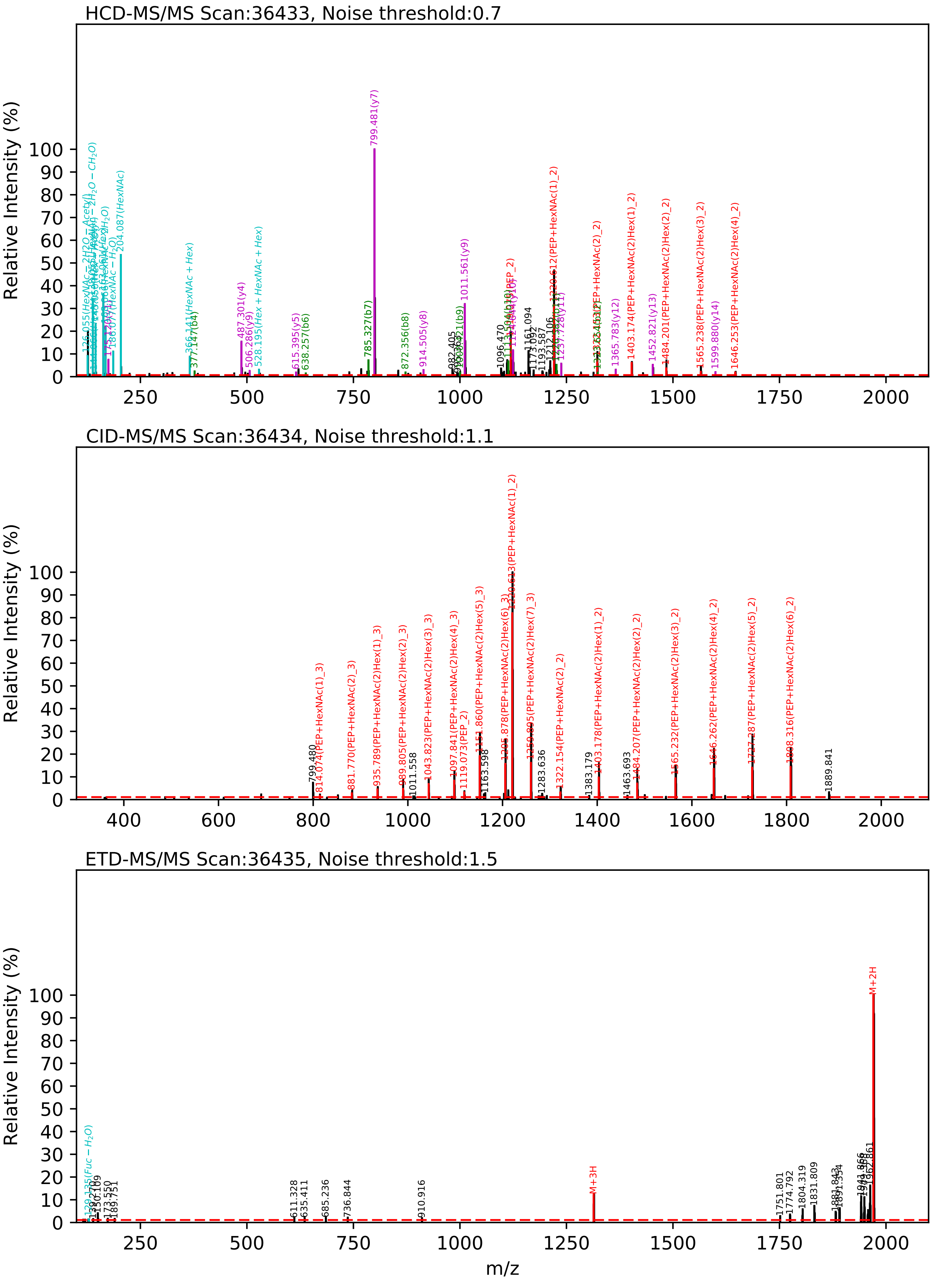

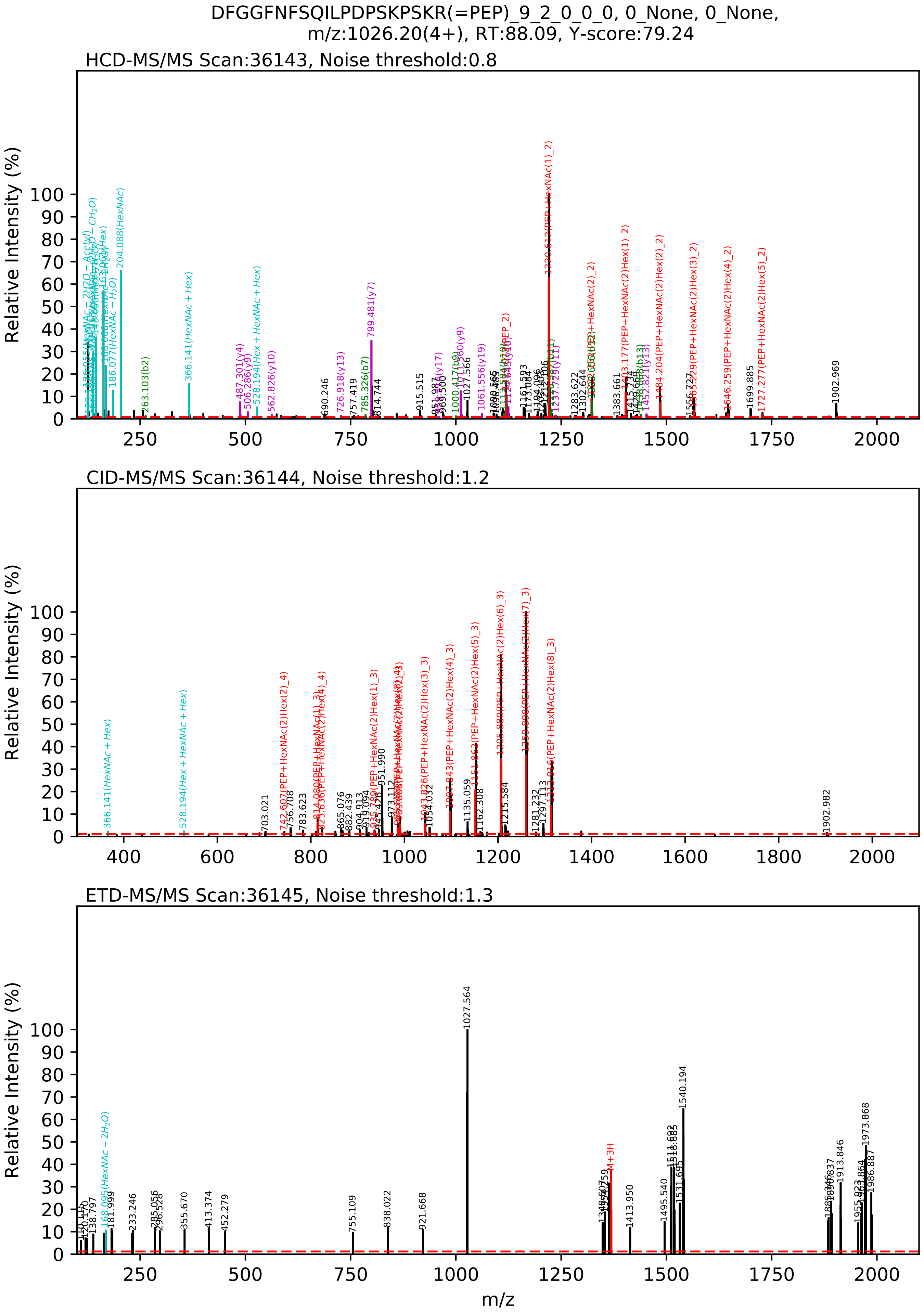

DFGGFNFSQILPDPSKPSKR(=PEP)_9_2_0_0_0, 0_None, 0_None,
m/z:1026.20(4+), RT:88.09, Y-score:79.24
HCD-MS/MS Scan:36143, Noise threshold:0.8
CID-MS/MS Scan:36144, Noise threshold:1.2
ETD-MS/MS Scan:36145, Noise threshold:1.3
Relative Intensity (%)
m/z

DFGGFNFSQILPDPSKPSK(=PEP)_4_2_0_0_0, 0_None, 0_None,
m/z:1045.81(3+), RT:96.17, Y-score:90.44

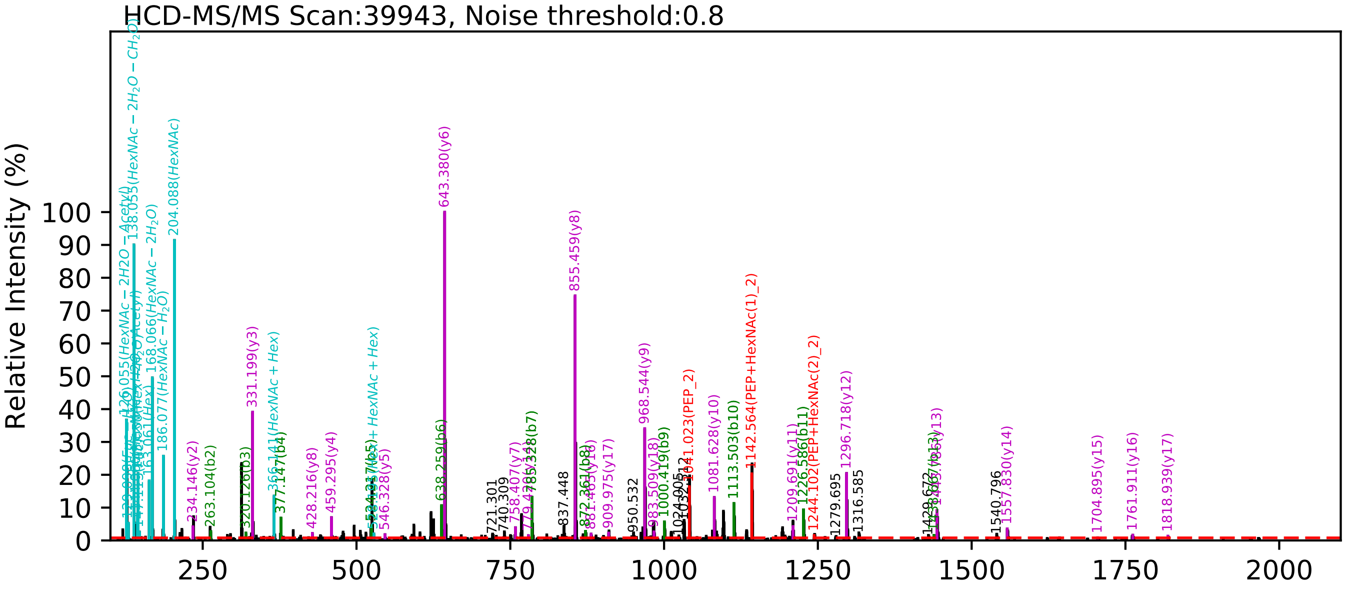

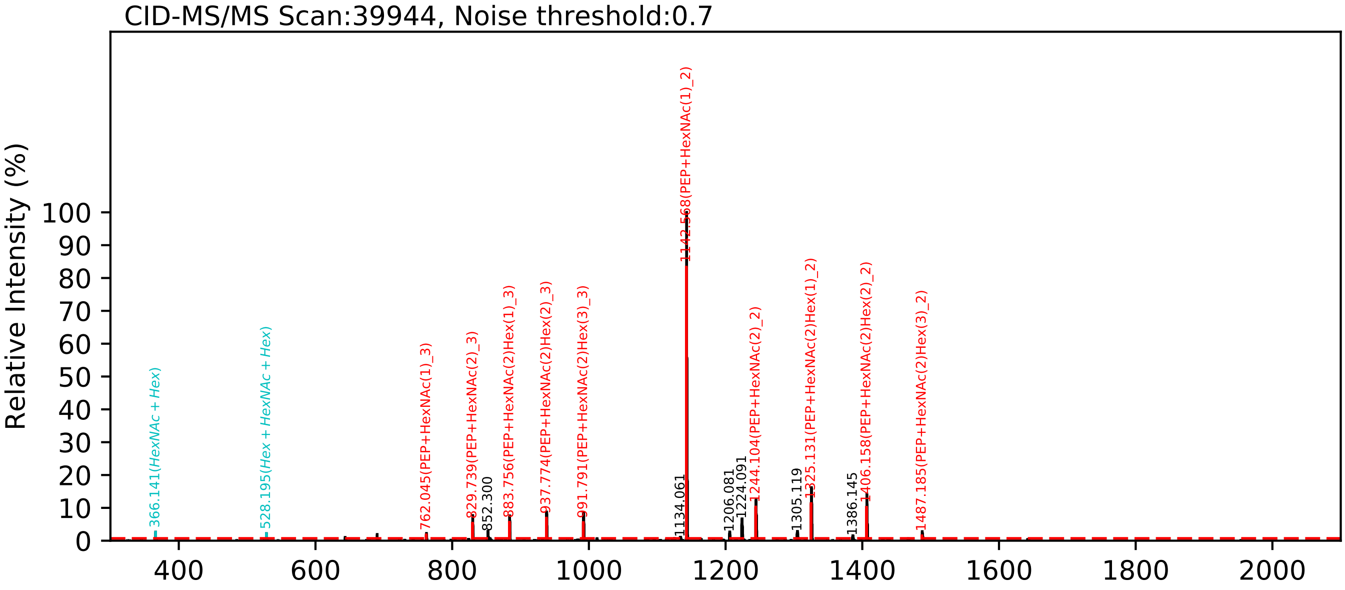

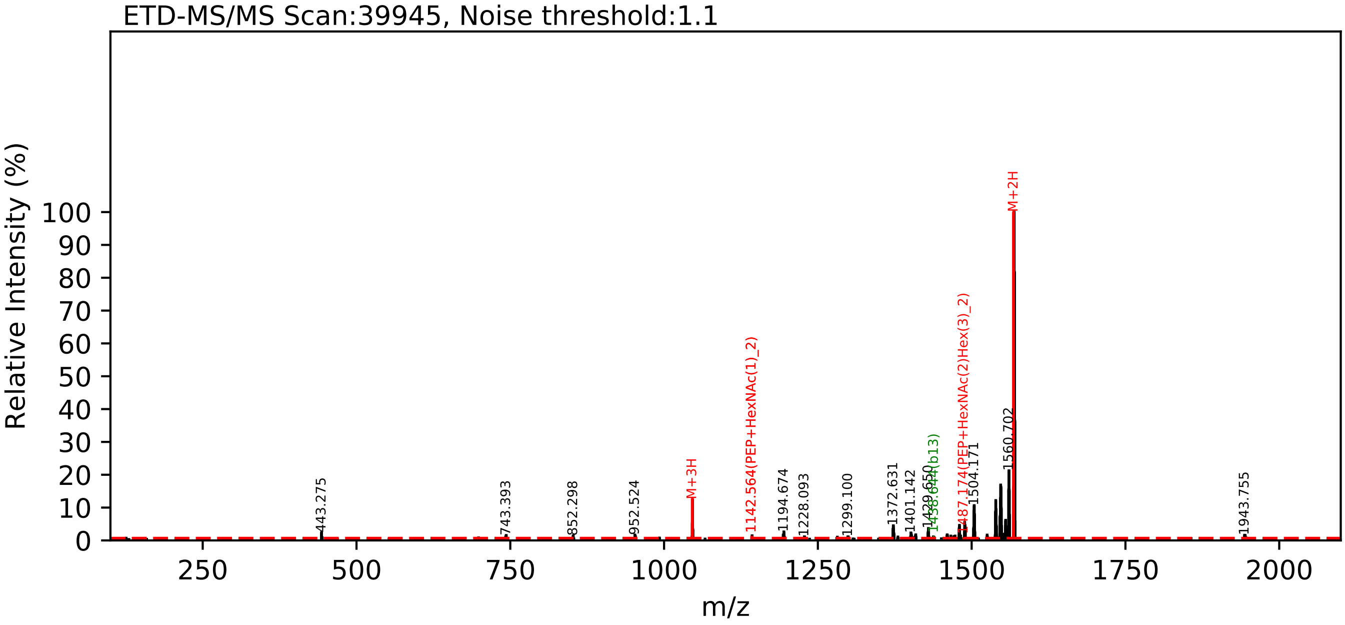

DFGGFNFSQILPDPSKPSK(=PEP)_4_2_0_0_0, 0_None, 0_None,
m/z:1045.81(3+), RT:96.19, Y-score:90.90

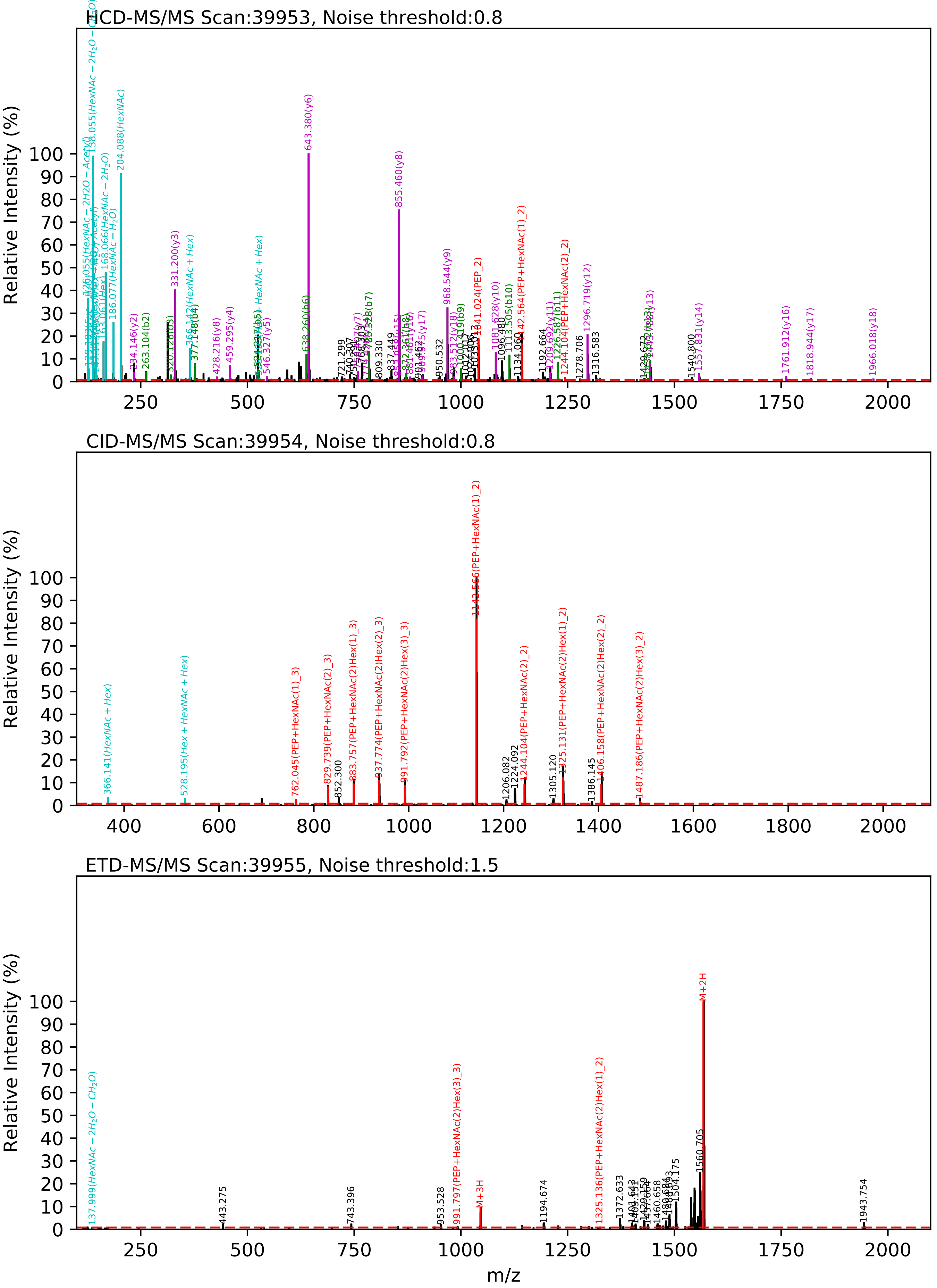

DFGGFNFSQILPDPSKPSK(=PEP)_5_2_0_0_0, 0_None, 0_None,
m/z:1099.82(3+), RT:96.31, Y-score:95.45

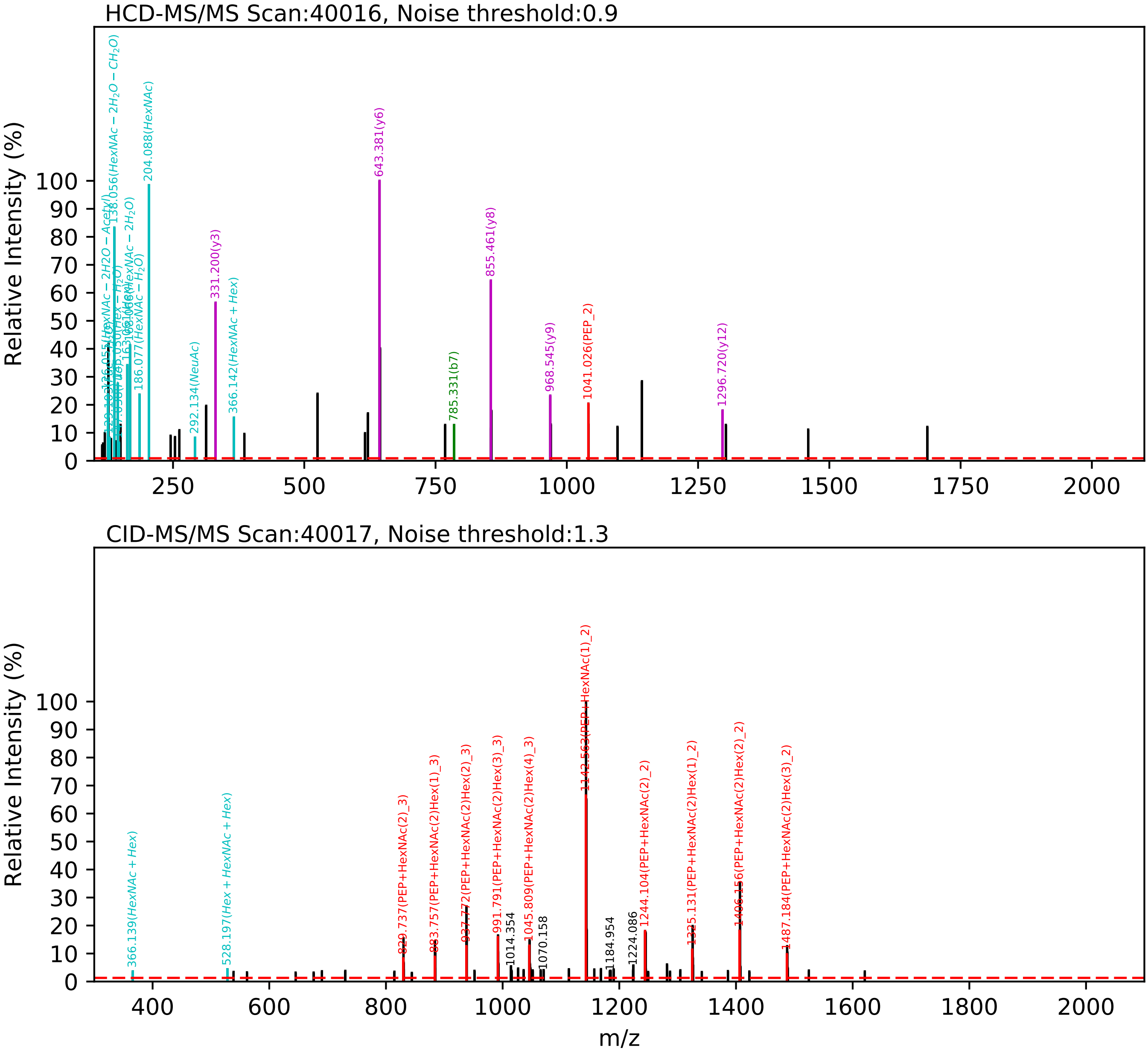

DFGGFNFSQILPDPSKPSK(=PEP)_7_2_0_0_0, 0_None, 0_None,
m/z:1207.86(3+), RT:96.32, Y-score:90.70

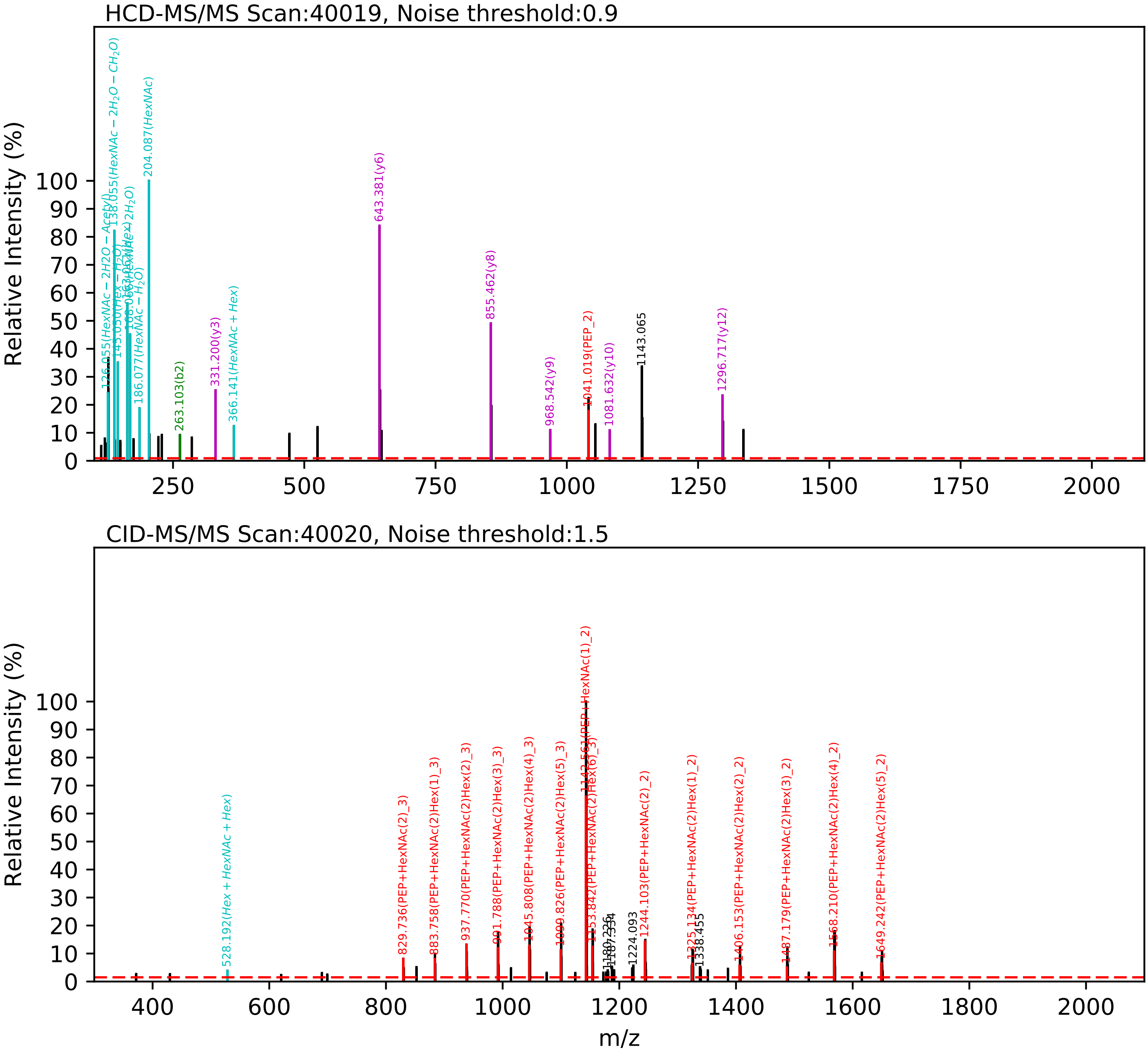

DFGGFNFSQILPDPSKPSK(=PEP)_8_2_0_0_0, 0_None, 0_None,
m/z:946.66(4+), RT:93.40, Y-score:51.62

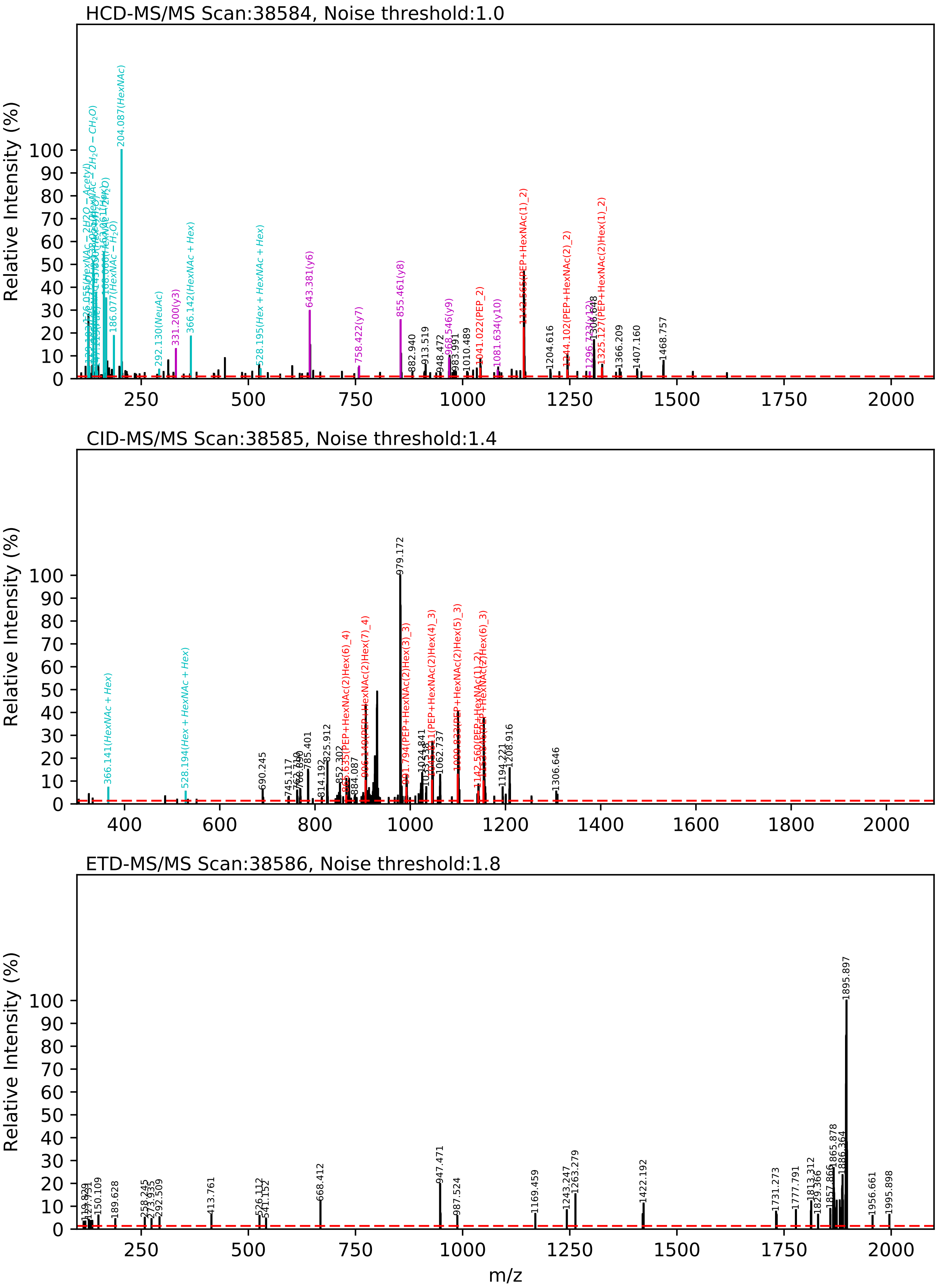

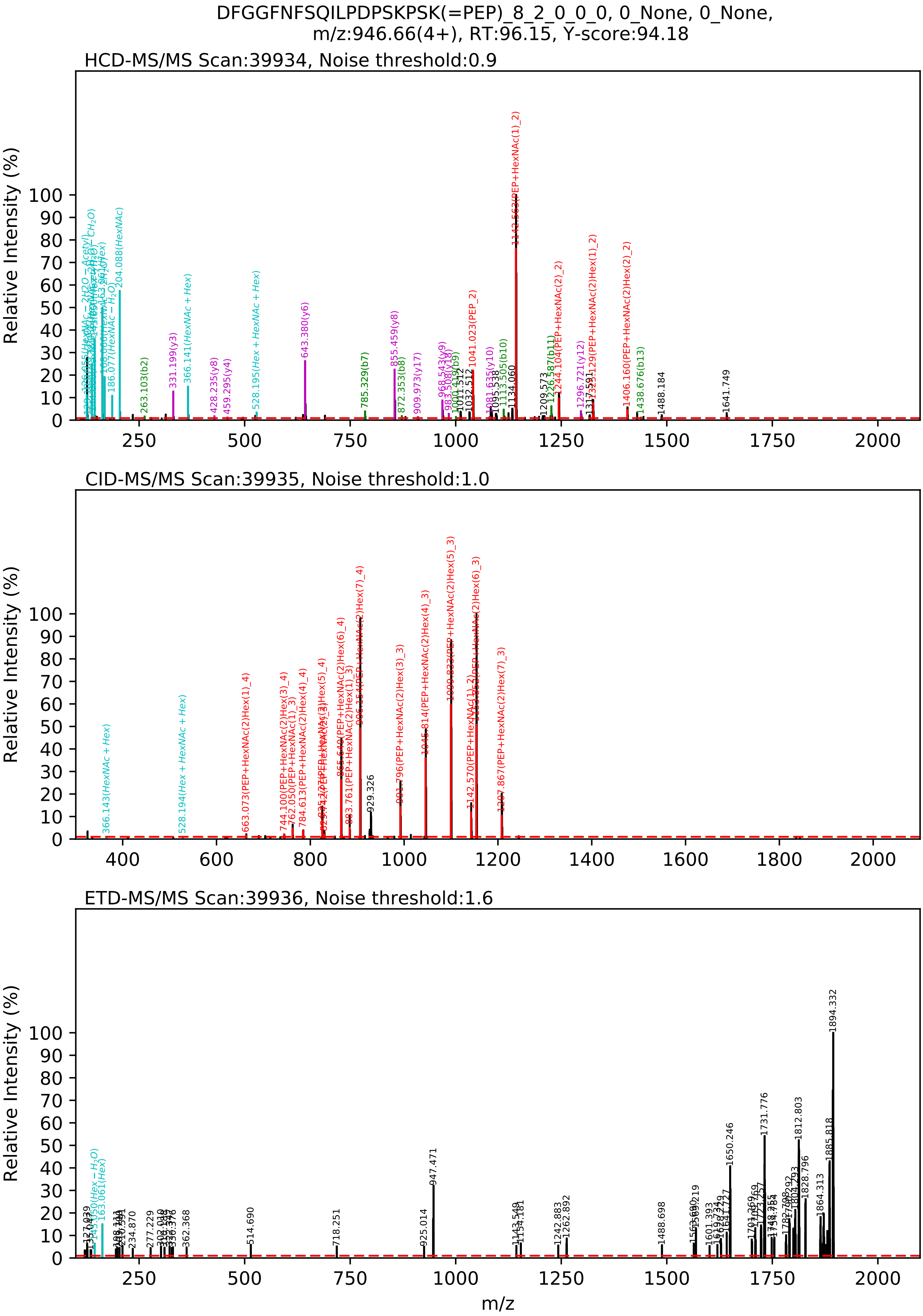
DFGGFNFSQILPDPSKPSK(=PEP)_8_2_0_0_0, 0_None, 0_None,
m/z:946.66(4+), RT:96.15, Y-score:94.18
HCD-MS/MS Scan:39934, Noise threshold:0.9
CID-MS/MS Scan:39935, Noise threshold:1.0
ETD-MS/MS Scan:39936, Noise threshold:1.6
Relative Intensity (%)
m/z

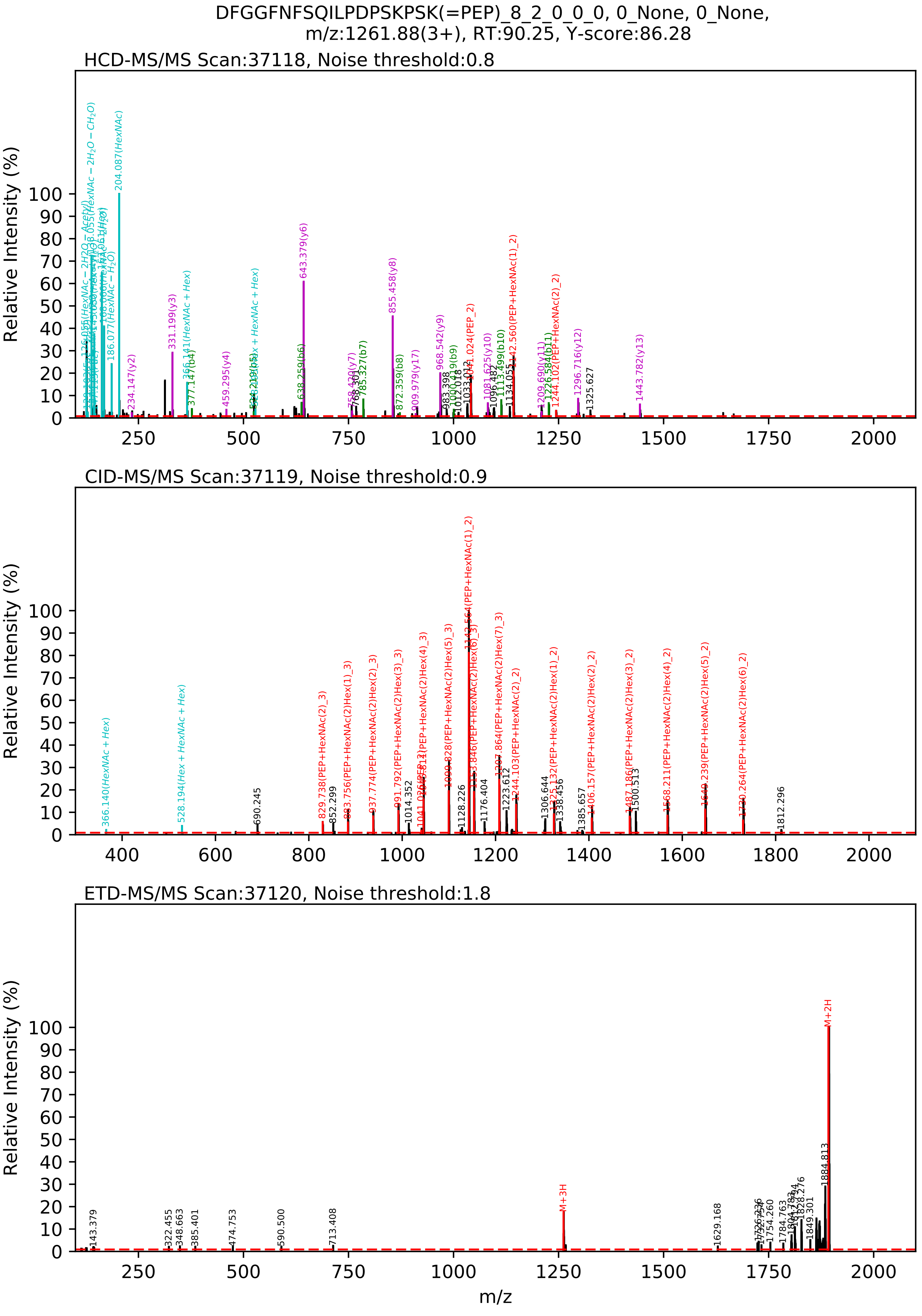

DFGGFNFSQILPDPSKPSK(=PEP)_8_2_0_0_0, 0_None, 0_None,
m/z:1261.88(3+), RT:90.25, Y-score:86.28
HCD-MS/MS Scan:37118, Noise threshold:0.8
Relative Intensity (%)
204.087(HexNAc)
186.077(HexNAc − H2O)
234.147(y2)
331.199(y3)
366.141(HexNAc + Hex)
377.147(b4)
459.295(y4)
643.379(y6)
638.259(b6)
758.420(y7)
768.301
785.327(b7)
855.458(y8)
872.359(b8)
909.979(y17)
968.542(y9)
983.398
1012.018
1033.012
1041.024(PEP_2)
1081.625(y10)
1096.482
1134.055
1142.560(PEP+HexNAc(1)_2)
1209.690(y11)
1244.102(PEP+HexNAc(2)_2)
1296.716(y12)
1325.627
1443.782(y13)
CID-MS/MS Scan:37119, Noise threshold:0.9
366.140(HexNAc + Hex)
528.194(Hex + HexNAc + Hex)
690.245
829.738(PEP+HexNAc(2)_3)
852.299
883.756(PEP+HexNAc(2)Hex(1)_3)
937.774(PEP+HexNAc(2)Hex(2)_3)
991.792(PEP+HexNAc(2)Hex(3)_3)
1014.352
1099.828(PEP+HexNAc(2)Hex(5)_3)
1128.226
1142.564(PEP+HexNAc(1)_2)
1176.404
1207.864(PEP+HexNAc(2)Hex(7)_3)
1223.612
1244.103(PEP+HexNAc(2)_2)
1306.644
1325.132(PEP+HexNAc(2)Hex(1)_2)
1338.456
1385.657
1406.157(PEP+HexNAc(2)Hex(2)_2)
1487.186(PEP+HexNAc(2)Hex(3)_2)
1500.513
1568.211(PEP+HexNAc(2)Hex(4)_2)
1649.239(PEP+HexNAc(2)Hex(5)_2)
1730.264(PEP+HexNAc(2)Hex(6)_2)
1812.296
ETD-MS/MS Scan:37120, Noise threshold:1.8
143.379
322.455
348.663
385.401
474.753
590.500
713.408
M+3H
1629.168
1754.260
1784.763
1828.276
1849.301
1884.813
M+2H
m/z

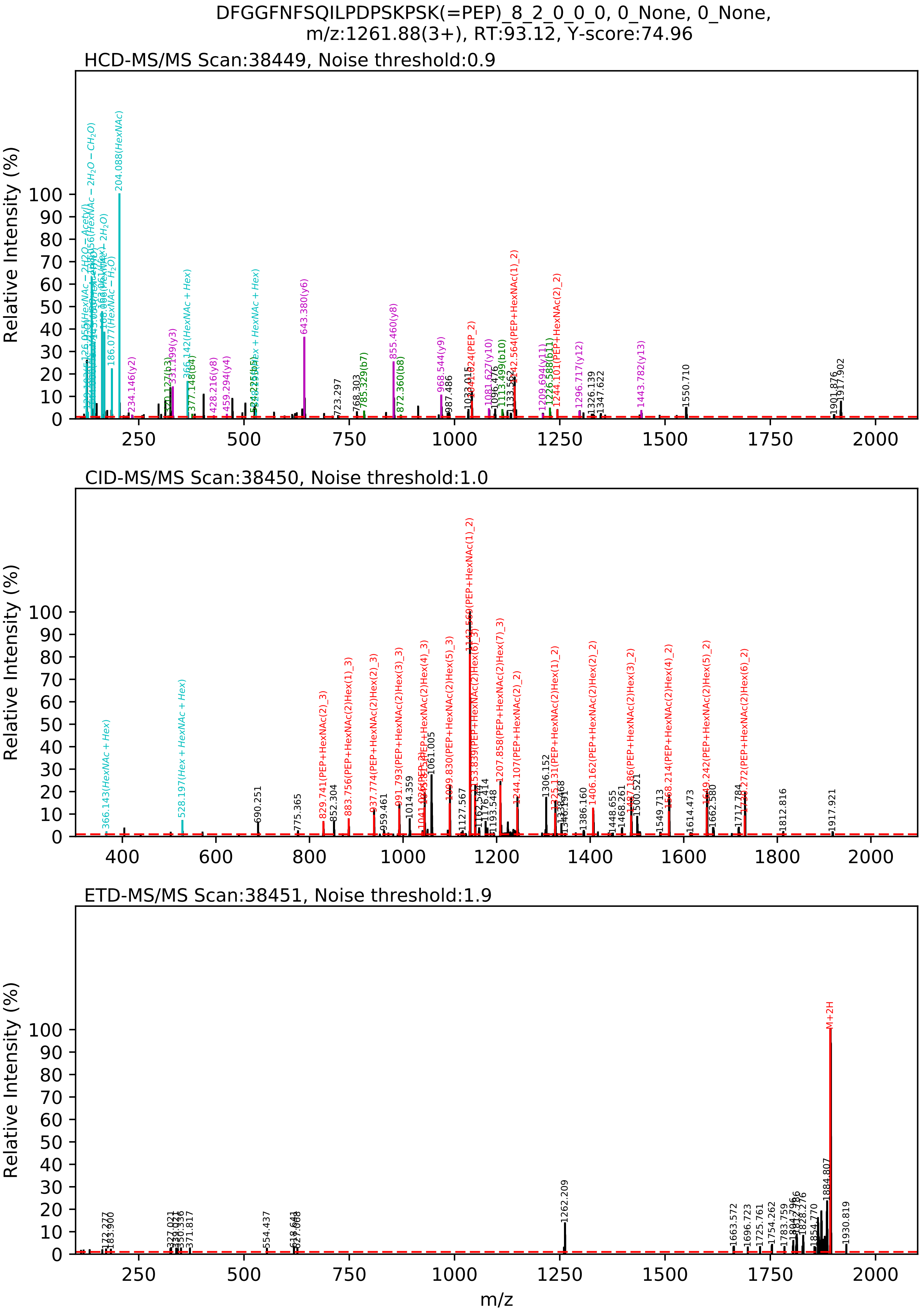
DFGGFNFSQILPDPSKPSK(=PEP)_8_2_0_0_0, 0_None, 0_None,
m/z:1261.88(3+), RT:93.12, Y-score:74.96
HCD-MS/MS Scan:38449, Noise threshold:0.9
Relative Intensity (%)
204.088(HexNAc)
186.077(HexNAc − H2O)
234.146(y2)
331.199(y3)
428.216(y8)
459.294(y4)
643.380(y6)
723.297
768.303
785.329(b7)
855.460(y8)
872.360(b8)
968.544(y9)
987.486
1081.627(y10)
1113.499(b10)
1209.694(y11)
1226.588(b11)
1244.101(PEP+HexNAc(2)_2)
1296.717(y12)
1326.139
1347.622
1443.782(y13)
1550.710
1901.876
1917.902
CID-MS/MS Scan:38450, Noise threshold:1.0
Relative Intensity (%)
366.143(HexNAc + Hex)
528.197(Hex + HexNAc + Hex)
690.251
775.365
829.741(PEP+HexNAc(2)_3)
852.304
883.756(PEP+HexNAc(2)Hex(1)_3)
937.774(PEP+HexNAc(2)Hex(2)_3)
959.461
991.793(PEP+HexNAc(2)Hex(3)_3)
1014.359
1061.005
1099.830(PEP+HexNAc(2)Hex(5)_3)
1127.567
1143.560(PEP+HexNAc(1)_2)
1153.839(PEP+HexNAc(2)Hex(6)_3)
1193.548
1207.858(PEP+HexNAc(2)Hex(7)_3)
1244.107(PEP+HexNAc(2)_2)
1306.152
1325.131(PEP+HexNAc(2)Hex(1)_2)
1386.160
1406.162(PEP+HexNAc(2)Hex(2)_2)
1448.655
1468.261
1487.186(PEP+HexNAc(2)Hex(3)_2)
1500.521
1549.713
1568.214(PEP+HexNAc(2)Hex(4)_2)
1614.473
1649.242(PEP+HexNAc(2)Hex(5)_2)
1662.580
1717.784
1730.272(PEP+HexNAc(2)Hex(6)_2)
1812.816
1917.921
ETD-MS/MS Scan:38451, Noise threshold:1.9
Relative Intensity (%)
M+2H
172.277
183.900
327.021
371.817
554.437
627.068
1262.209
1663.572
1696.723
1725.761
1754.262
1783.759
1828.276
1854.770
1884.807
1930.819
m/z

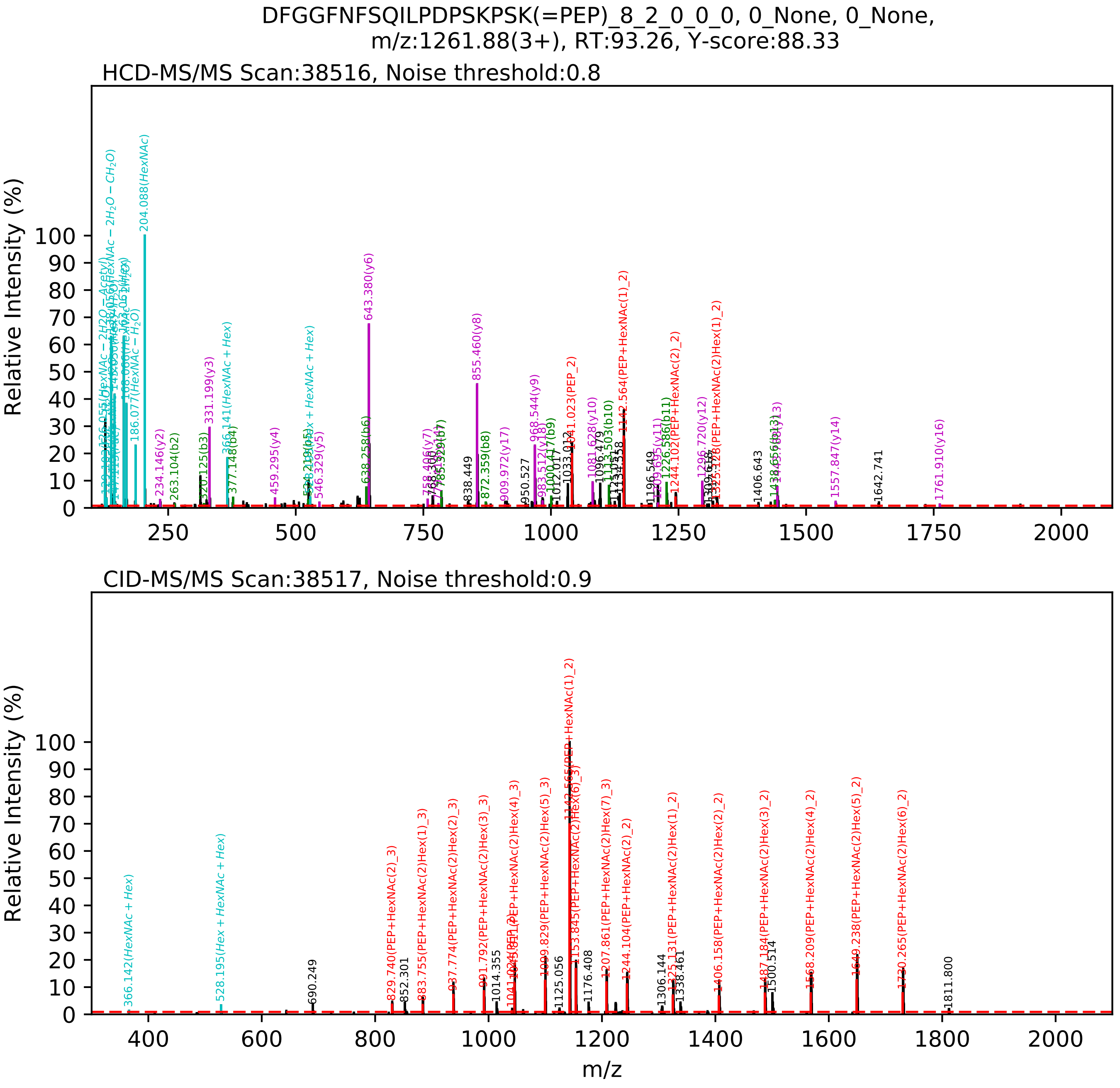
DFGGFNFSQILPDPSKPSK(=PEP)_8_2_0_0_0, 0_None, 0_None,
m/z:1261.88(3+), RT:93.26, Y-score:88.33
HCD-MS/MS Scan:38516, Noise threshold:0.8
Relative Intensity (%)
204.088(HexNAc)
643.380(y6)
855.460(y8)
331.199(y3)
1142.564(PEP+HexNAc(1)_2)
1041.023(PEP_2)
1244.102(PEP+HexNAc(2)_2)
1325.128(PEP+HexNAc(2)Hex(1)_2)
968.544(y9)
1081.628(y10)
1296.720(y12)
1557.847(y14)
1761.910(y16)
1642.741
1406.643
CID-MS/MS Scan:38517, Noise threshold:0.9
Relative Intensity (%)
m/z
366.142(HexNAc + Hex)
528.195(Hex + HexNAc + Hex)
690.249
829.740(PEP+HexNAc(2)_3)
852.301
883.755(PEP+HexNAc(2)Hex(1)_3)
937.774(PEP+HexNAc(2)Hex(2)_3)
991.792(PEP+HexNAc(2)Hex(3)_3)
1014.355
1099.829(PEP+HexNAc(2)Hex(5)_3)
1125.056
1176.408
1207.861(PEP+HexNAc(2)Hex(7)_3)
1244.104(PEP+HexNAc(2)_2)
1306.144
1338.461
1325.131(PEP+HexNAc(2)Hex(1)_2)
1406.158(PEP+HexNAc(2)Hex(2)_2)
1487.184(PEP+HexNAc(2)Hex(3)_2)
1500.514
1568.209(PEP+HexNAc(2)Hex(4)_2)
1649.238(PEP+HexNAc(2)Hex(5)_2)
1730.265(PEP+HexNAc(2)Hex(6)_2)
1811.800

DFGGFNFSQILPDPSKPSK(=PEP)_8_2_0_0_0, 0_None, 0_None,
m/z:1261.88(3+), RT:96.13, Y-score:89.96

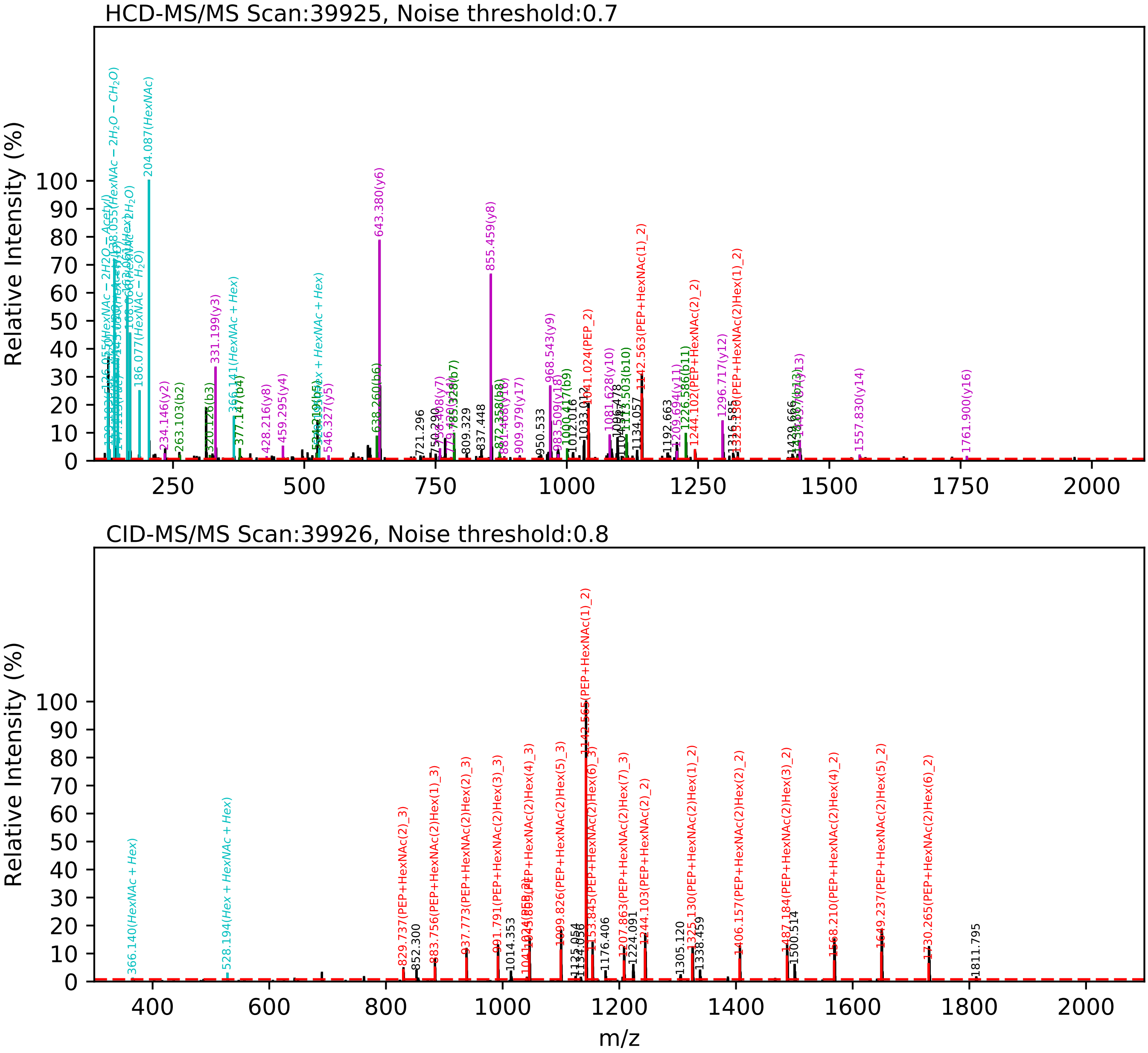

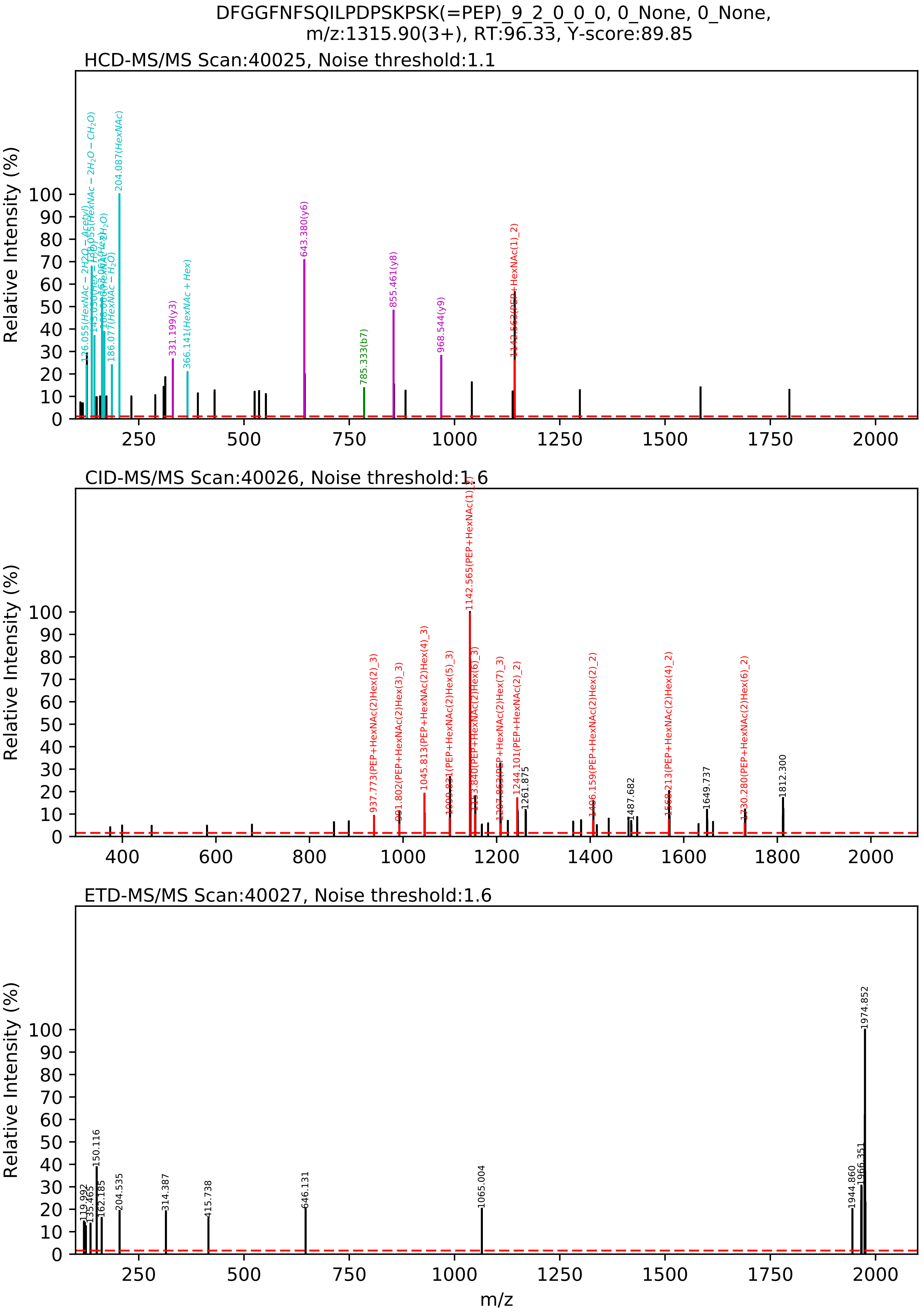
DFGGFNFSQILPDPSKPSK(=PEP)_9_2_0_0_0, 0_None, 0_None,
m/z:1315.90(3+), RT:96.33, Y-score:89.85
HCD-MS/MS Scan:40025, Noise threshold:1.1
Relative Intensity (%)
204.087(HexNAc)
643.380(y6)
855.461(y8)
968.544(y9)
331.199(y3)
366.141(HexNAc+Hex)
785.333(b7)
1142.563(PEP+HexNAc(1)_2)
186.077(HexNAc − H2O)
126.055(HexNAc − 2H2O − Acetyl)
CID-MS/MS Scan:40026, Noise threshold:1.6
1142.565(PEP+HexNAc(1)_2)
937.773(PEP+HexNAc(2)Hex(2)_3)
991.802(PEP+HexNAc(2)Hex(3)_3)
1045.813(PEP+HexNAc(2)Hex(4)_3)
1099.831(PEP+HexNAc(2)Hex(5)_3)
1153.840(PEP+HexNAc(2)Hex(6)_3)
1207.862(PEP+HexNAc(2)Hex(7)_3)
1244.101(PEP+HexNAc(2)_2)
1261.875
1406.159(PEP+HexNAc(2)Hex(2)_2)
1487.682
1568.213(PEP+HexNAc(2)Hex(4)_2)
1649.737
1730.280(PEP+HexNAc(2)Hex(6)_2)
1812.300
ETD-MS/MS Scan:40027, Noise threshold:1.6
1974.852
150.116
119.992
135.465
162.185
204.535
314.387
415.738
646.131
1065.004
1944.860
1966.351
m/z

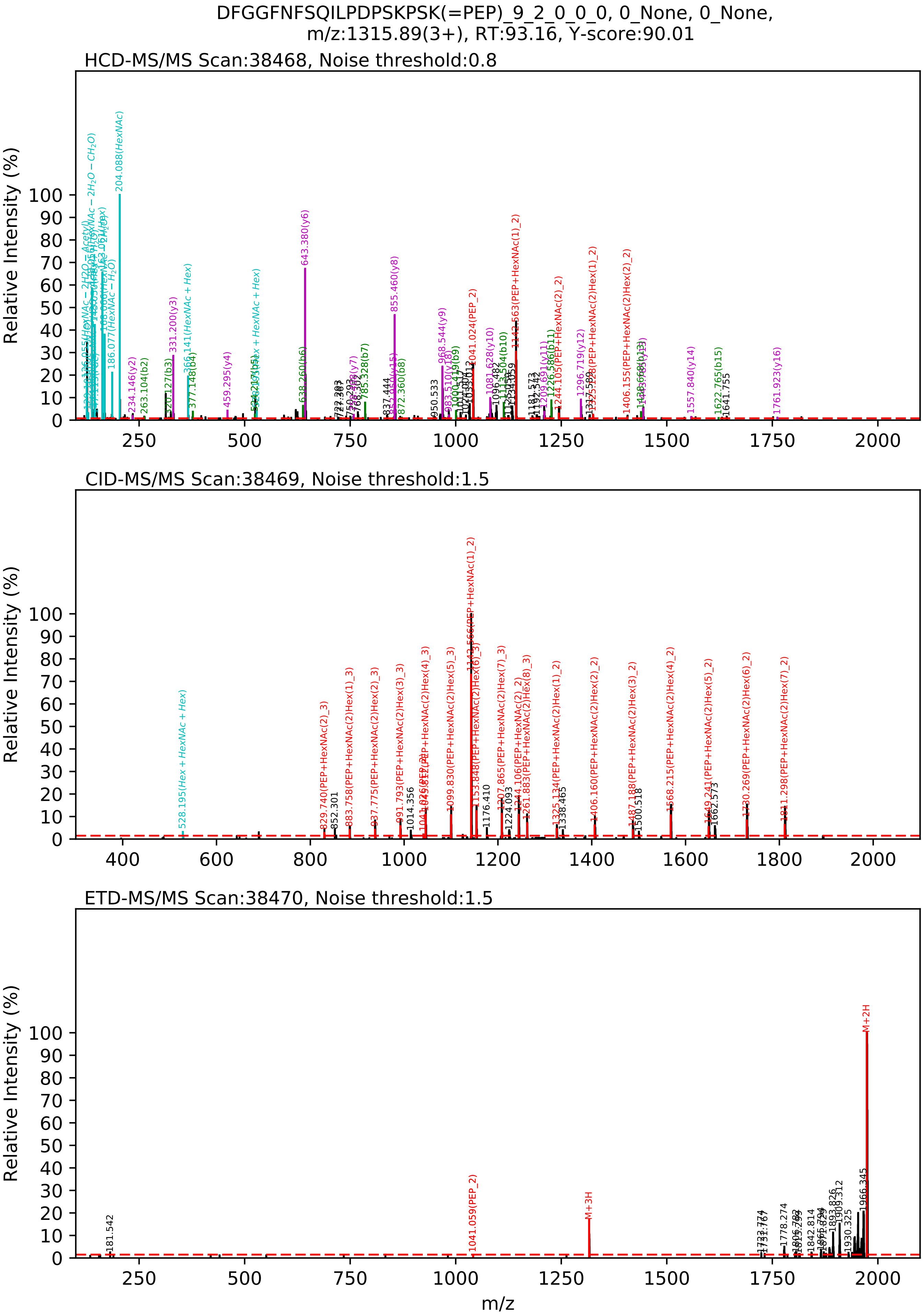

DFGGFNFSQILPDPSKPSK(=PEP)_9_2_0_0_0, 0_None, 0_None,
m/z:1315.89(3+), RT:93.16, Y-score:90.01
HCD-MS/MS Scan:38468, Noise threshold:0.8
CID-MS/MS Scan:38469, Noise threshold:1.5
ETD-MS/MS Scan:38470, Noise threshold:1.5
Relative Intensity (%)
m/z

DFGGFNFSQILPDPSKPSK(=PEP)_9_2_0_0_0, 0_None, 0_None,
m/z:1315.89(3+), RT:96.09, Y-score:89.76

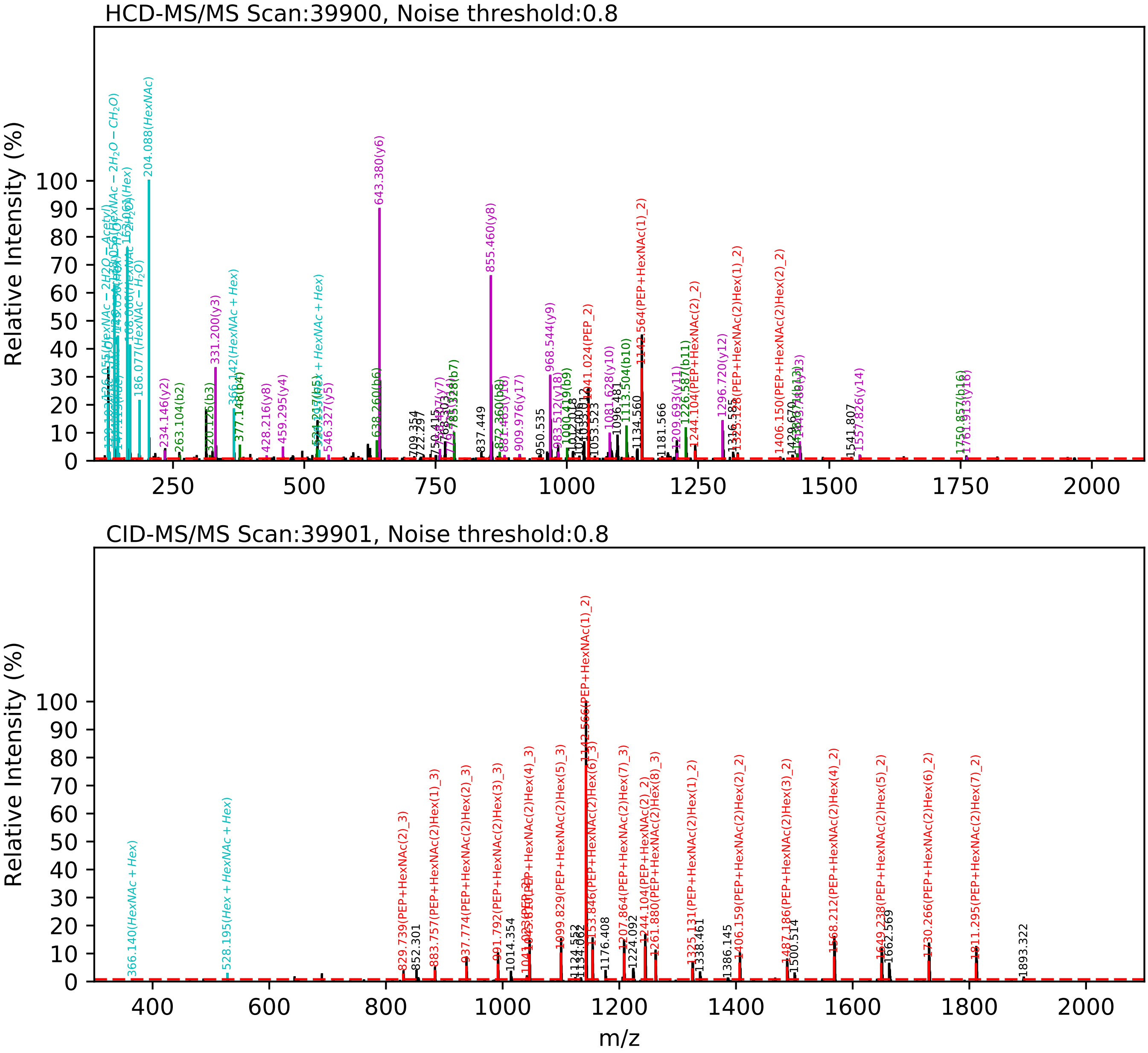

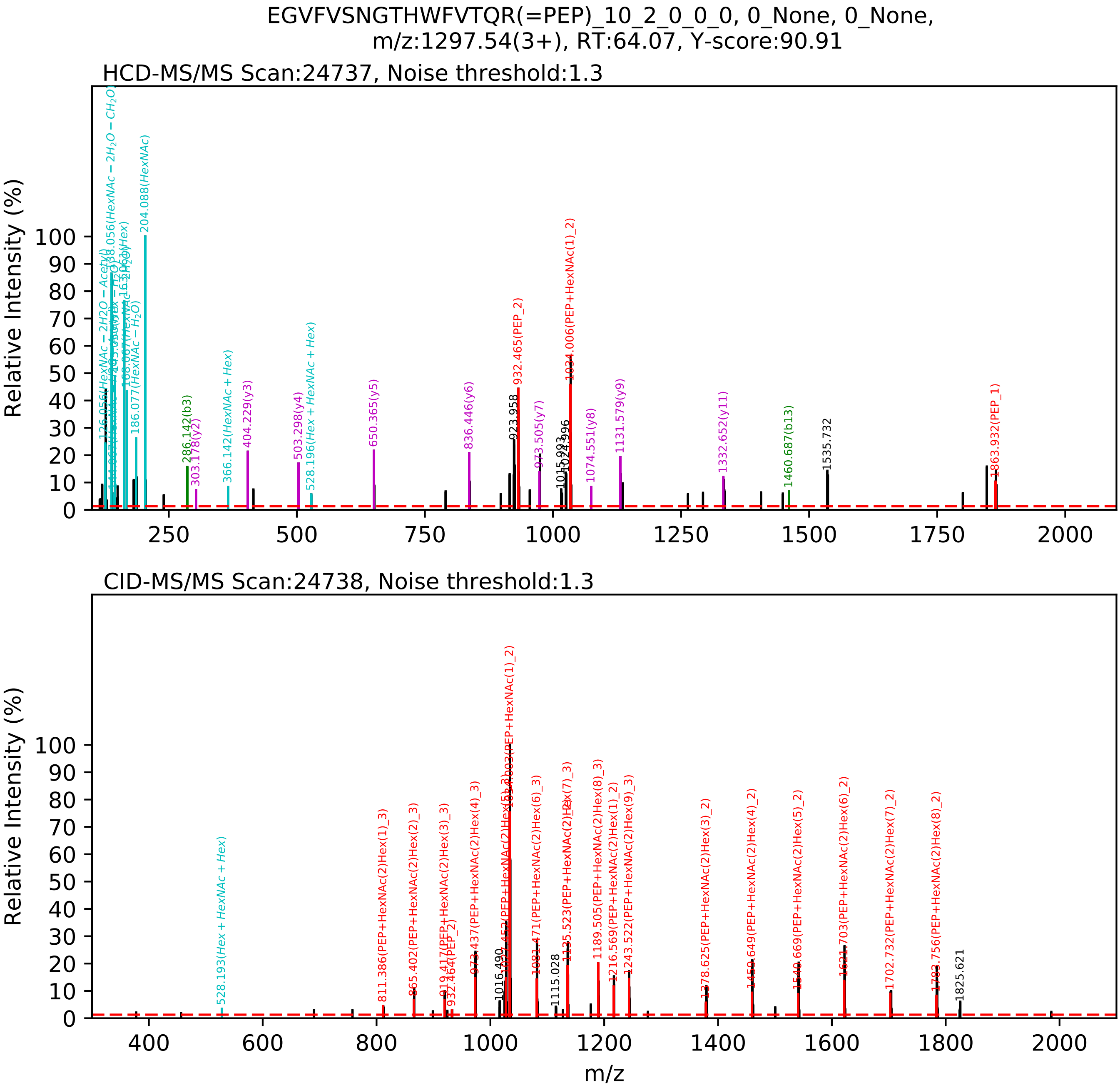

EGVFVSNGTHWFVTQR(=PEP)_10_2_0_0_0, 0_None, 0_None,
m/z:1297.54(3+), RT:64.07, Y-score:90.91
HCD-MS/MS Scan:24737, Noise threshold:1.3
Relative Intensity (%)
204.088(HexNAc)
932.465(PEP_2)
1034.006(PEP+HexNAc(1)_2)
286.142(b3)
303.178(y2)
366.142(HexNAc + Hex)
404.229(y3)
503.298(y4)
528.196(Hex + HexNAc + Hex)
650.365(y5)
836.446(y6)
923.958
973.505(y7)
1074.551(y8)
1131.579(y9)
1332.652(y11)
1460.687(b13)
1535.732
1863.932(PEP_1)
186.077(HexNAc − H2O)
CID-MS/MS Scan:24738, Noise threshold:1.3
Relative Intensity (%)
528.193(Hex + HexNAc + Hex)
811.386(PEP+HexNAc(2)Hex(1)_3)
865.402(PEP+HexNAc(2)Hex(2)_3)
919.417(PEP+HexNAc(2)Hex(3)_3)
932.464(PEP_2)
973.437(PEP+HexNAc(2)Hex(4)_3)
1016.490
1081.471(PEP+HexNAc(2)Hex(6)_3)
1115.028
1135.523(PEP+HexNAc(2)Hex(7)_3)
1189.505(PEP+HexNAc(2)Hex(8)_3)
1216.569(PEP+HexNAc(2)Hex(1)_2)
1243.522(PEP+HexNAc(2)Hex(9)_3)
1378.625(PEP+HexNAc(2)Hex(3)_2)
1459.649(PEP+HexNAc(2)Hex(4)_2)
1540.669(PEP+HexNAc(2)Hex(5)_2)
1621.703(PEP+HexNAc(2)Hex(6)_2)
1702.732(PEP+HexNAc(2)Hex(7)_2)
1783.756(PEP+HexNAc(2)Hex(8)_2)
1825.621
m/z

EGVFVSNGTHWFVTQR(=PEP)_10_2_0_0_0, 0_None, 0_None,
m/z:1297.54(3+), RT:64.36, Y-score:78.12

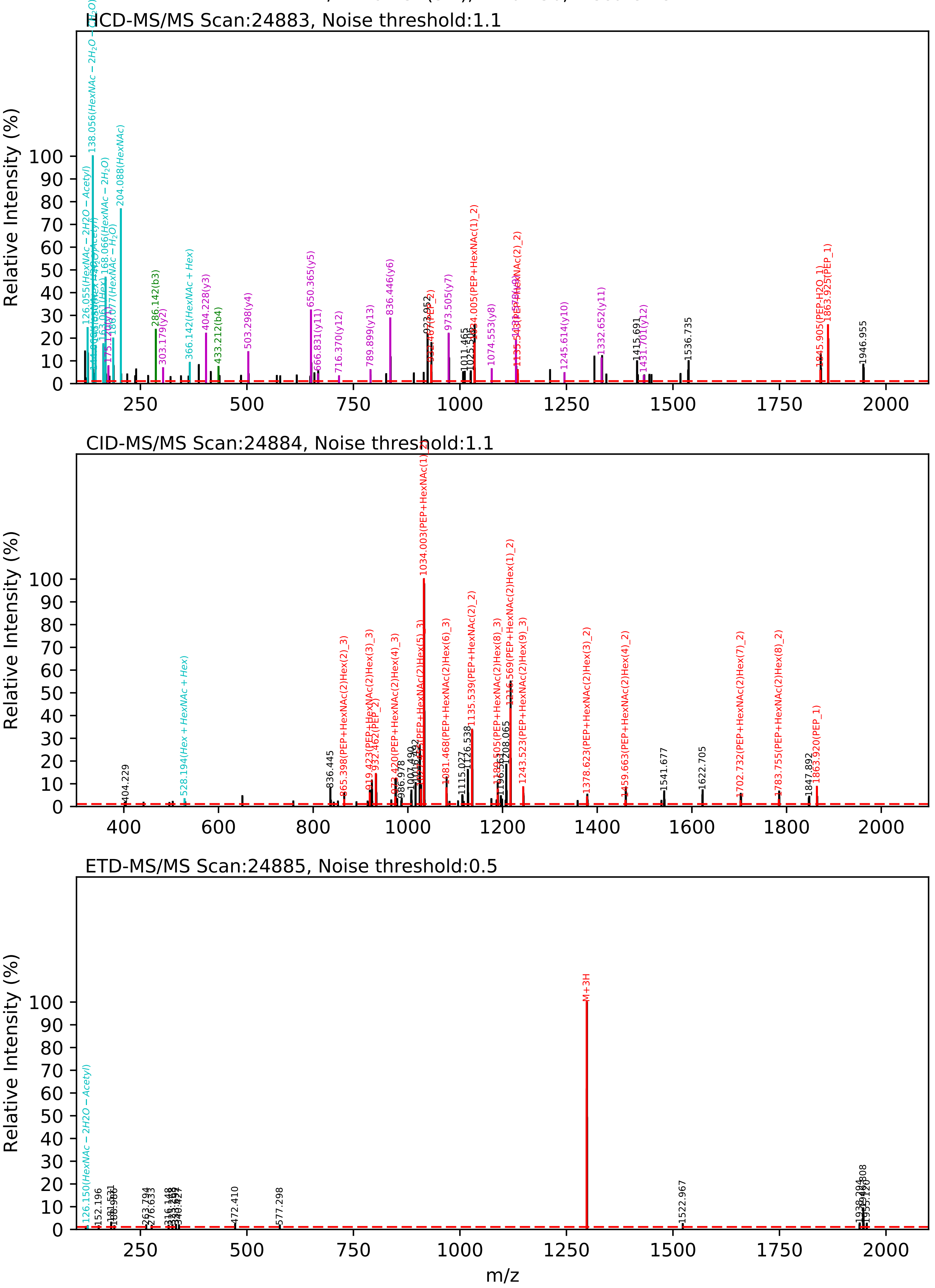

EGVFVSNGTHWFVTQR(=PEP)_11_2_0_0_0, 0_None, 0_None,
m/z:1351.56(3+), RT:63.99, Y-score:88.18

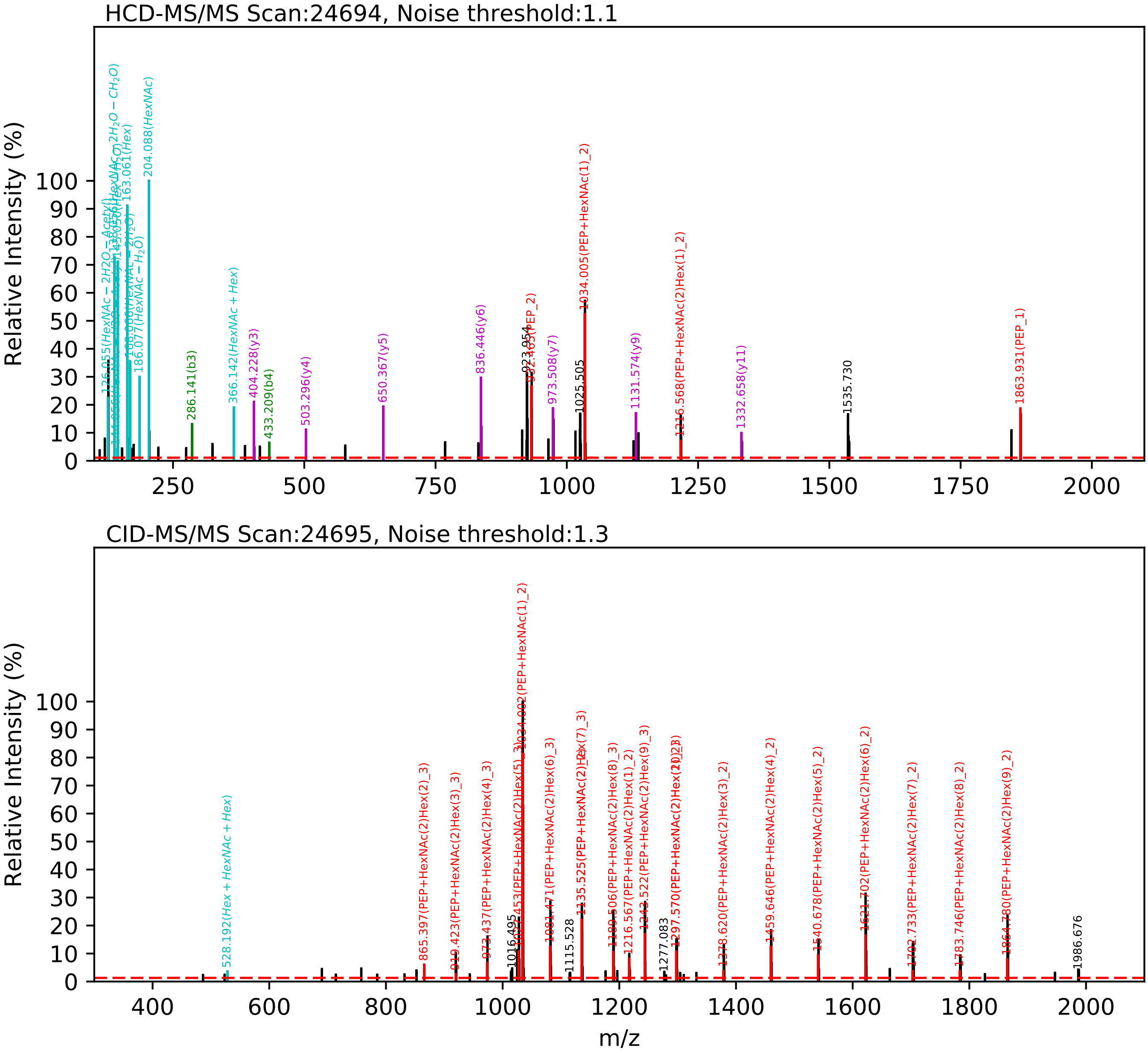

EGVFVSNGTHWFVTQR(=PEP)_11_2_0_0_0, 0_None, 0_None,
m/z:1351.56(3+), RT:63.84, Y-score:84.65

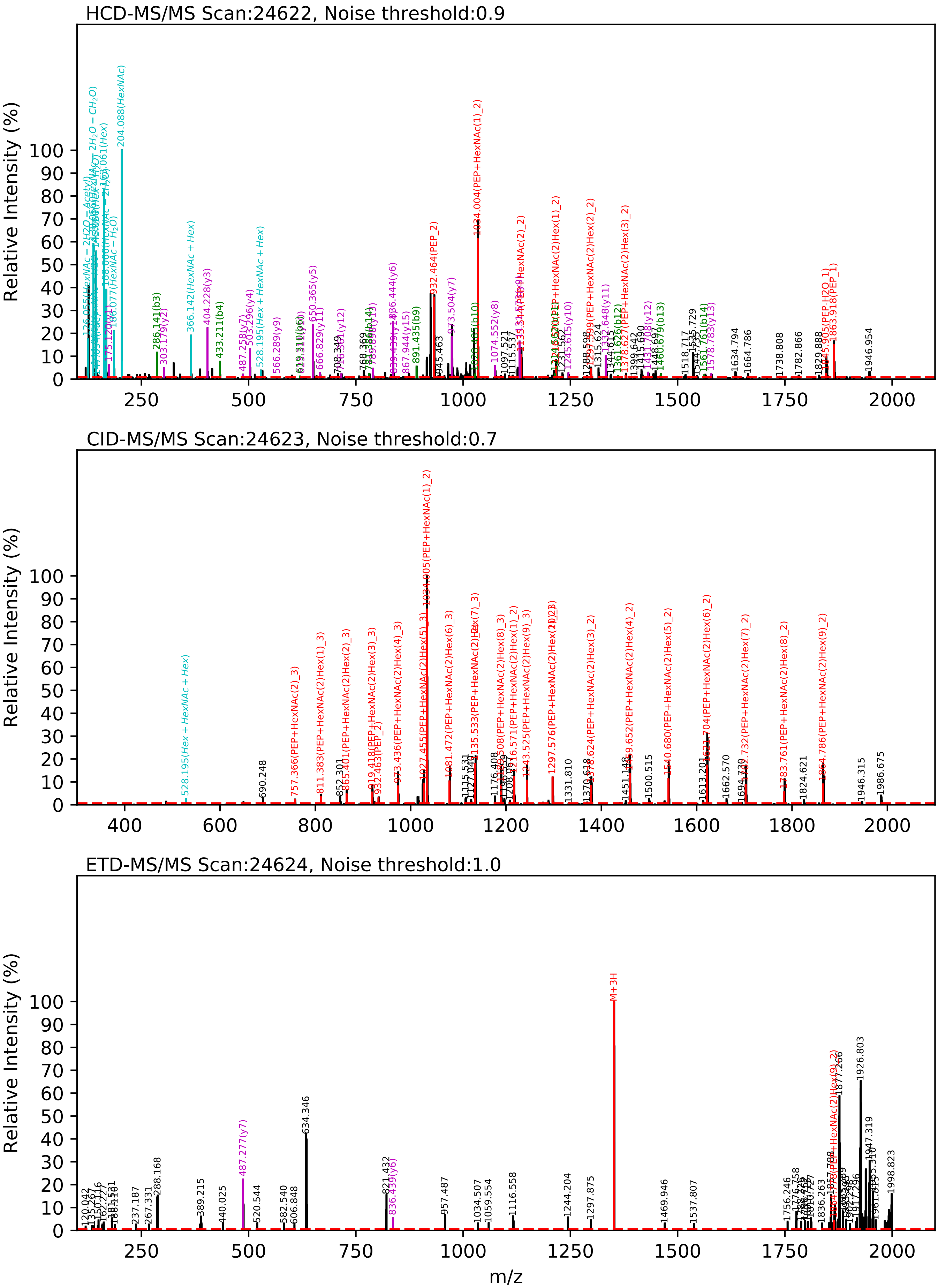

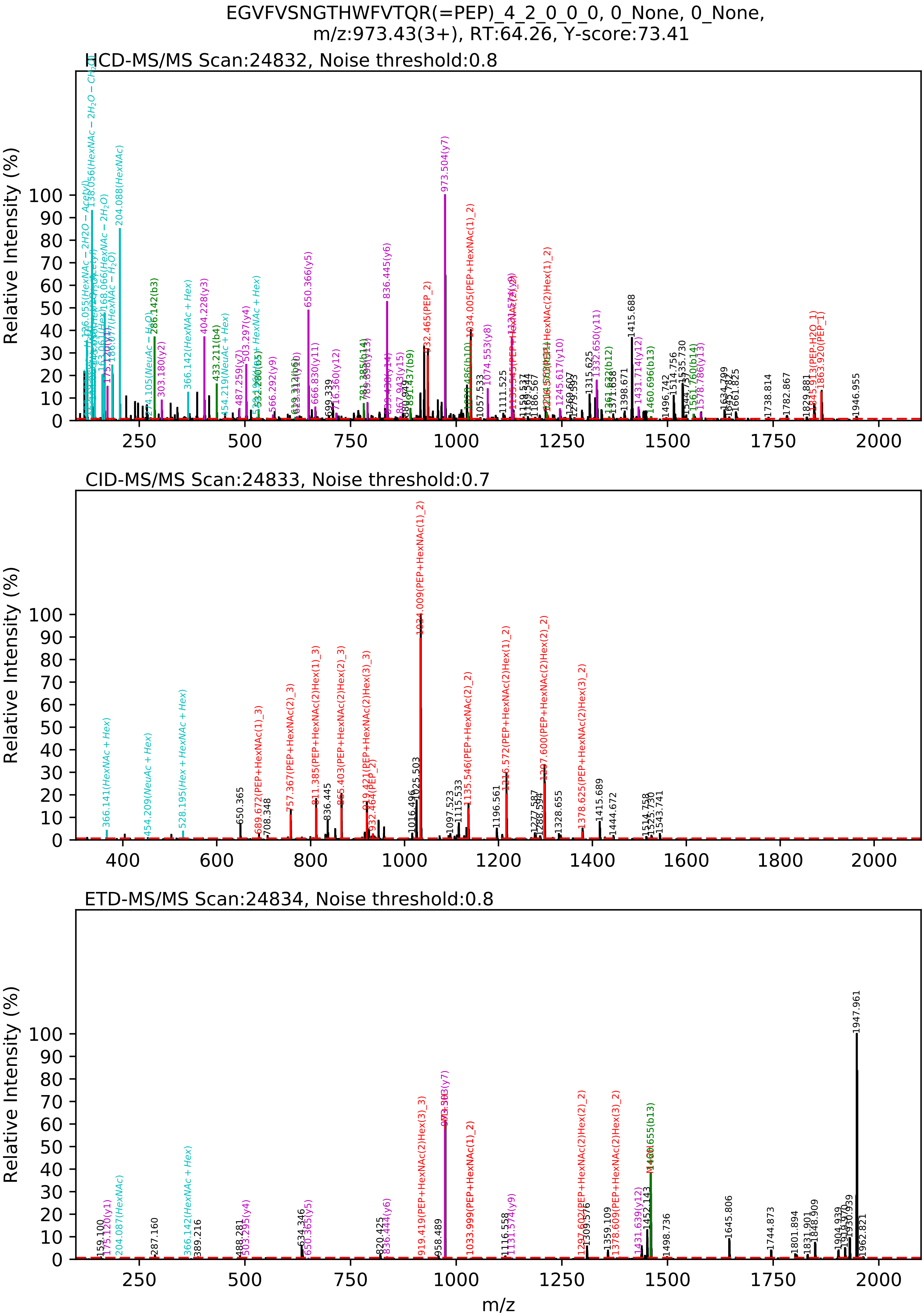

EGVFVSNGTHWFVTQR(=PEP)_4_2_0_0_0, 0_None, 0_None,
m/z:973.43(3+), RT:64.26, Y-score:73.41
HCD-MS/MS Scan:24832, Noise threshold:0.8
Relative Intensity (%)
973.504(y7)
204.088(HexNAc)
836.445(y6)
650.366(y5)
1034.005(PEP+HexNAc(1)_2)
932.465(PEP_2)
1415.688
1863.920(PEP_1)
CID-MS/MS Scan:24833, Noise threshold:0.7
1034.009(PEP+HexNAc(1)_2)
1216.572(PEP+HexNAc(2)Hex(1)_2)
1297.600(PEP+HexNAc(2)Hex(2)_2)
811.385(PEP+HexNAc(2)Hex(1)_3)
865.403(PEP+HexNAc(2)Hex(2)_3)
757.367(PEP+HexNAc(2)_3)
1135.546(PEP+HexNAc(2)_2)
1378.625(PEP+HexNAc(2)Hex(3)_2)
ETD-MS/MS Scan:24834, Noise threshold:0.8
1947.961
973.503(y7)
1460.655(b13)
1645.806
m/z

EGVFVSNGTHWFVTQR(=PEP)_4_2_0_0_0, 0_None, 0_None,
m/z:973.43(3+), RT:64.71, Y-score:74.70

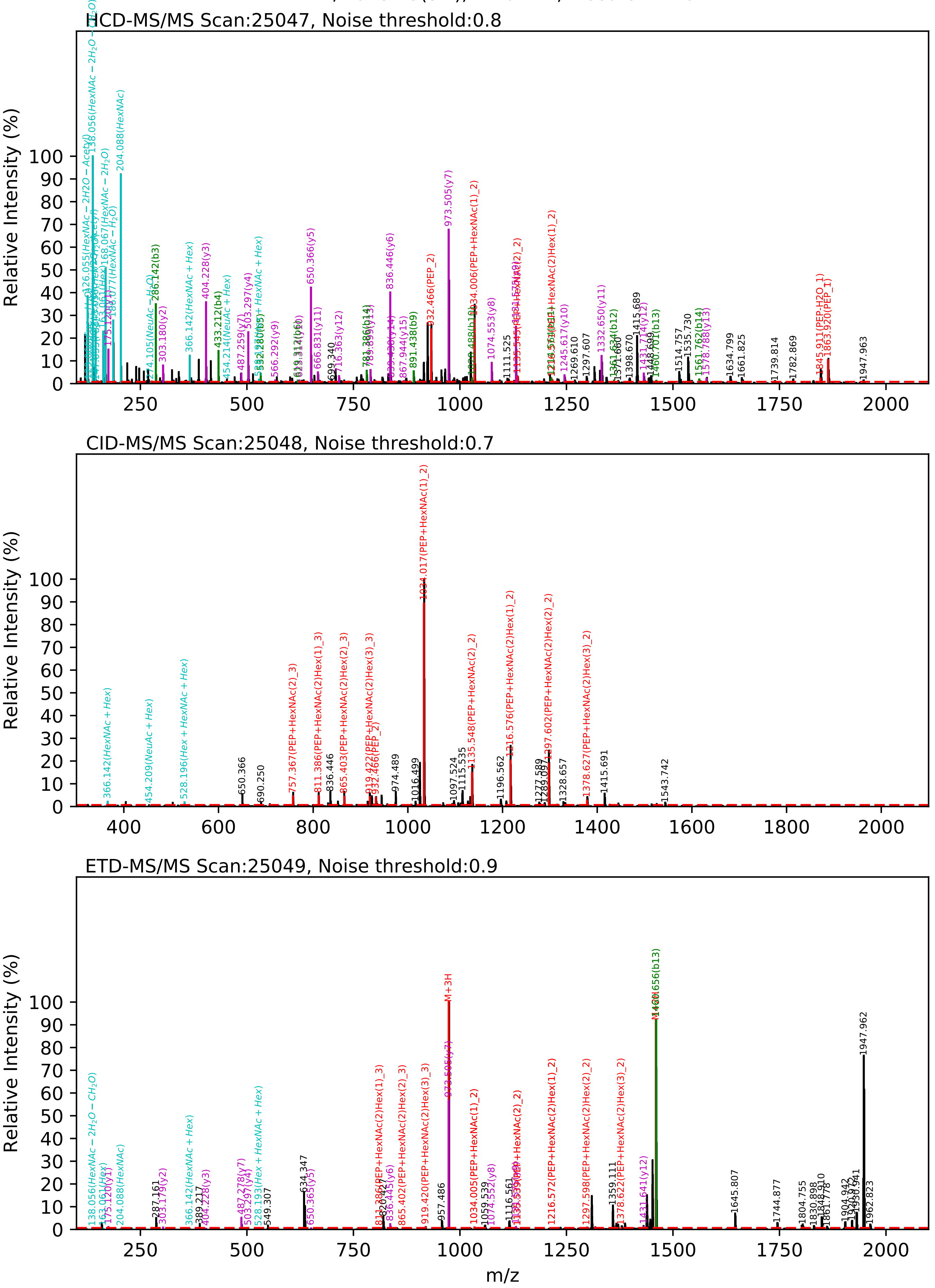

EGVFVSNGTHWFVTQR(=PEP)_4_2_0_0_0, 0_None, 0_None,
m/z:973.43(3+), RT:67.32, Y-score:77.48

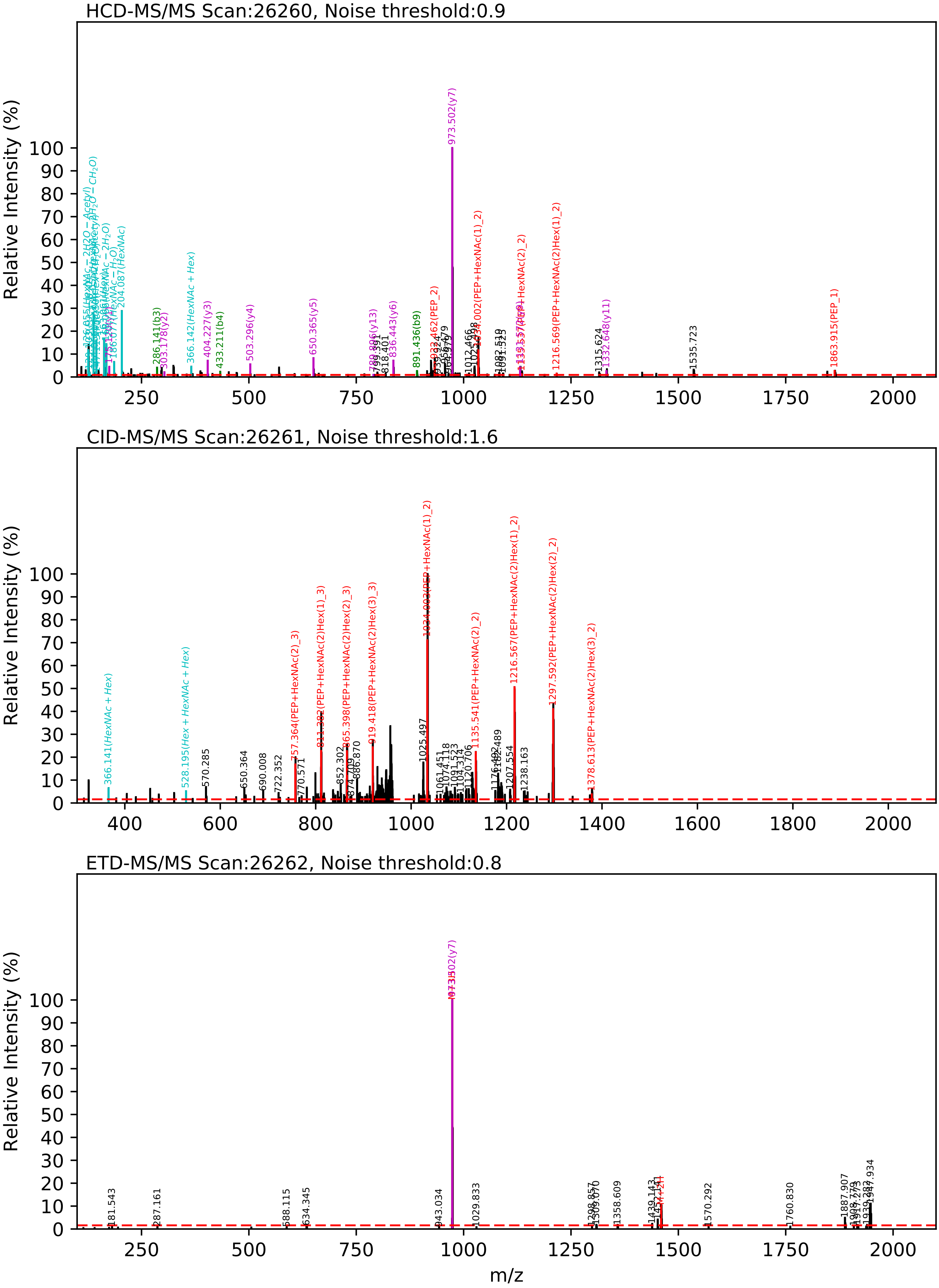

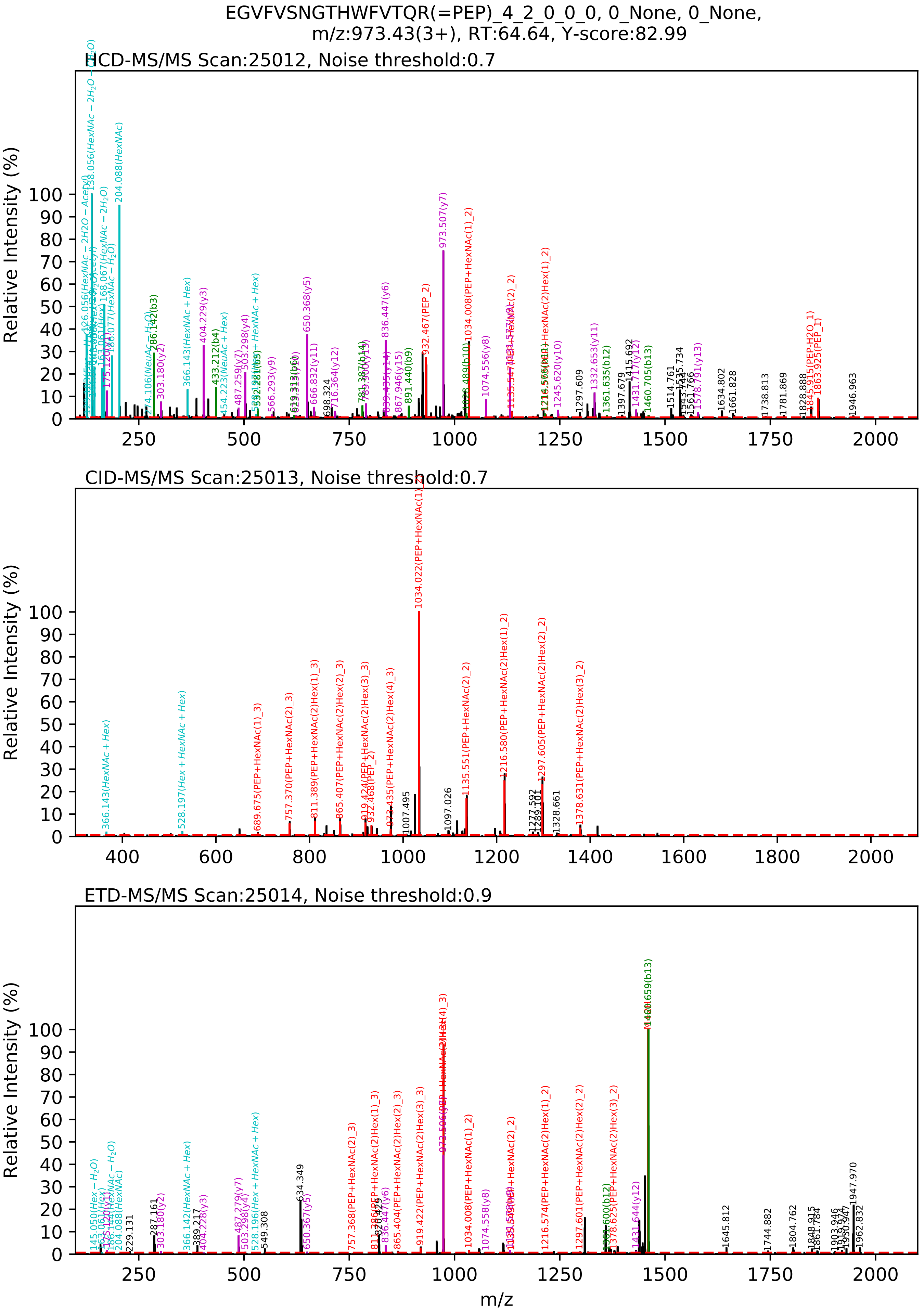
EGVFVSNGTHWFVTQR(=PEP)_4_2_0_0_0, 0_None, 0_None,
m/z:973.43(3+), RT:64.64, Y-score:82.99
HCD-MS/MS Scan:25012, Noise threshold:0.7
CID-MS/MS Scan:25013, Noise threshold:0.7
ETD-MS/MS Scan:25014, Noise threshold:0.9
Relative Intensity (%)
m/z

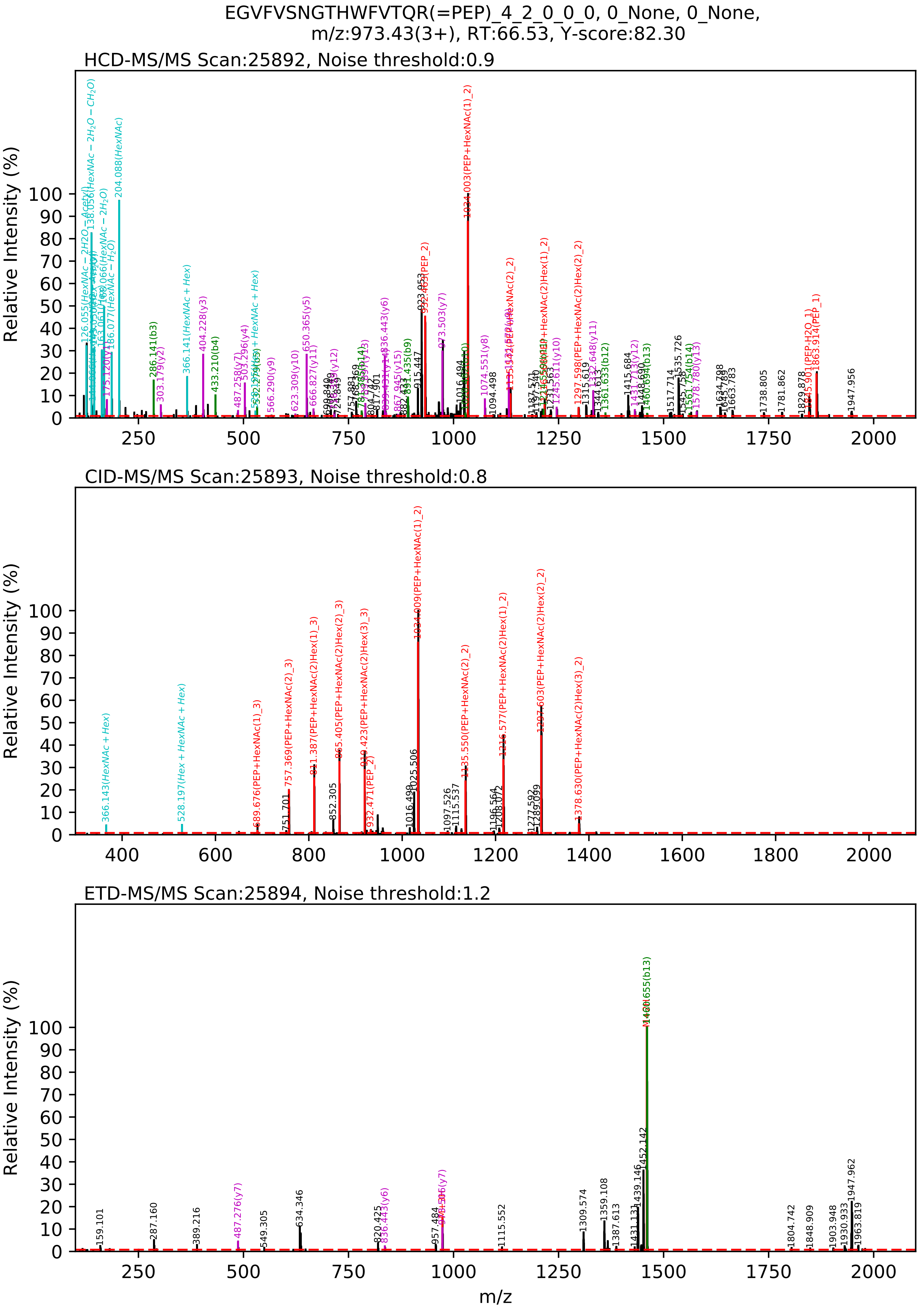
EGVFVSNGTHWFVTQR(=PEP)_4_2_0_0_0, 0_None, 0_None,
m/z:973.43(3+), RT:66.53, Y-score:82.30
HCD-MS/MS Scan:25892, Noise threshold:0.9
CID-MS/MS Scan:25893, Noise threshold:0.8
ETD-MS/MS Scan:25894, Noise threshold:1.2
Relative Intensity (%)
m/z
1034.003(PEP+HexNAc(1)_2)
204.088(HexNAc)
1034.009(PEP+HexNAc(1)_2)
1297.603(PEP+HexNAc(2)Hex(2)_2)
1216.577(PEP+HexNAc(2)Hex(1)_2)
1135.550(PEP+HexNAc(2)_2)
865.405(PEP+HexNAc(2)Hex(2)_3)
811.387(PEP+HexNAc(2)Hex(1)_3)
757.369(PEP+HexNAc(2)_3)
919.423(PEP+HexNAc(2)Hex(3)_3)
689.676(PEP+HexNAc(1)_3)
1378.630(PEP+HexNAc(2)Hex(3)_2)
366.143(HexNAc+Hex)
528.197(Hex+HexNAc+Hex)
1460.655(b13)
1452.142
1947.962

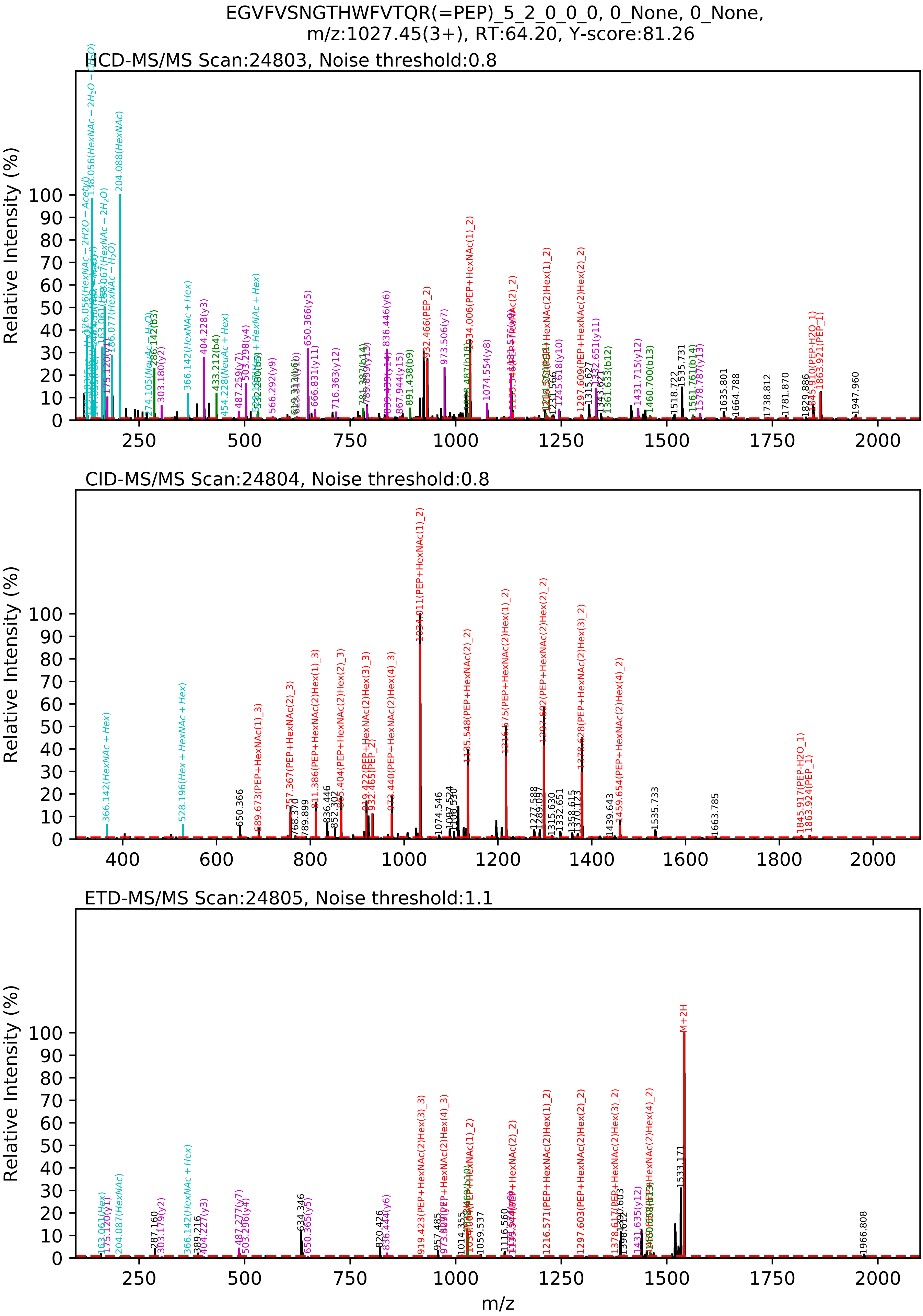

EGVFVSNGTHWFVTQR(=PEP)_5_2_0_0_0, 0_None, 0_None,
m/z:1027.45(3+), RT:64.20, Y-score:81.26
HCD-MS/MS Scan:24803, Noise threshold:0.8
CID-MS/MS Scan:24804, Noise threshold:0.8
ETD-MS/MS Scan:24805, Noise threshold:1.1
Relative Intensity (%)
m/z

EGVFVSNGTHWFVTQR(=PEP)_5_2_0_0_0, 0_None, 0_None,
m/z:1027.45(3+), RT:66.57, Y-score:84.20

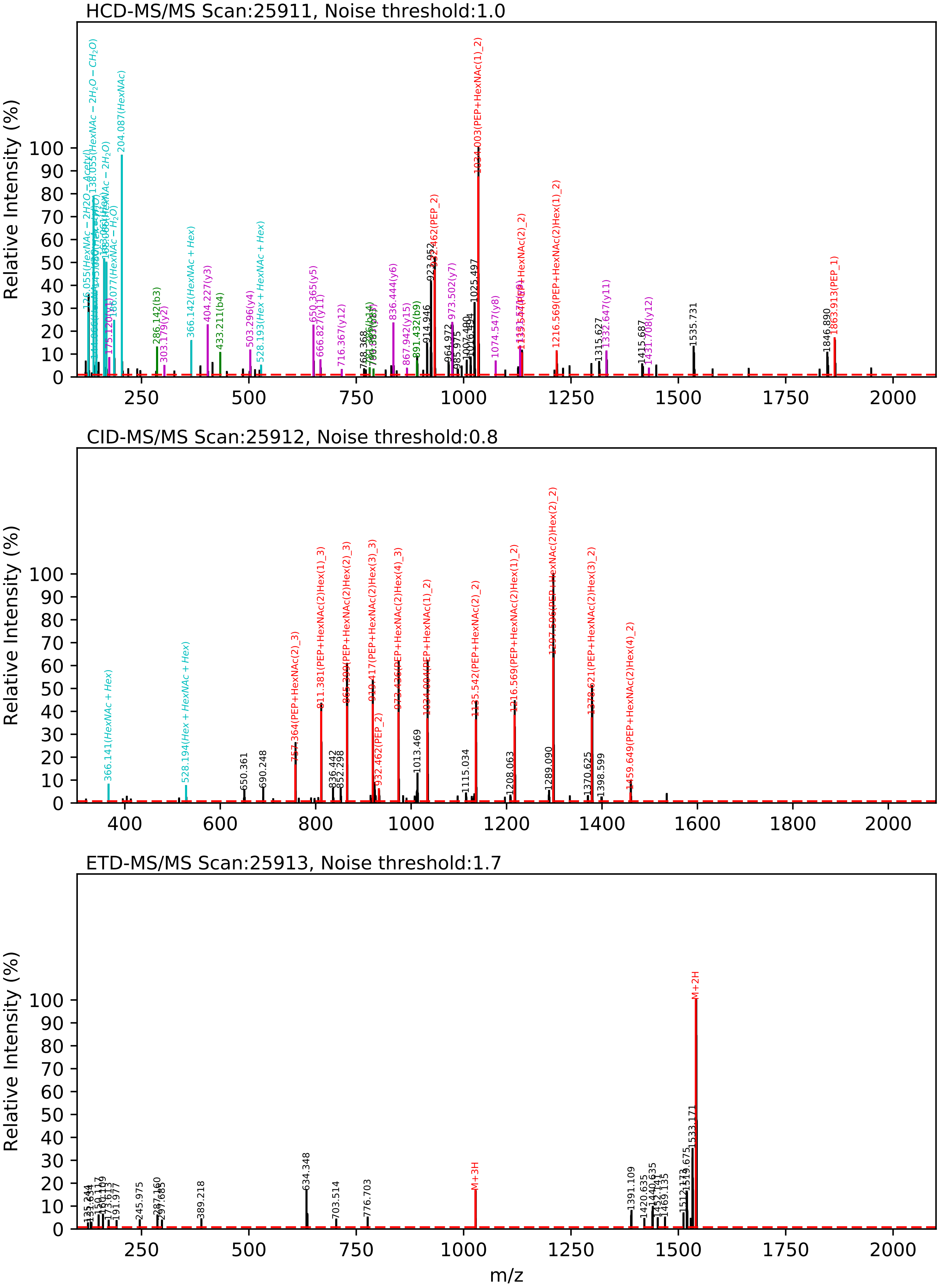

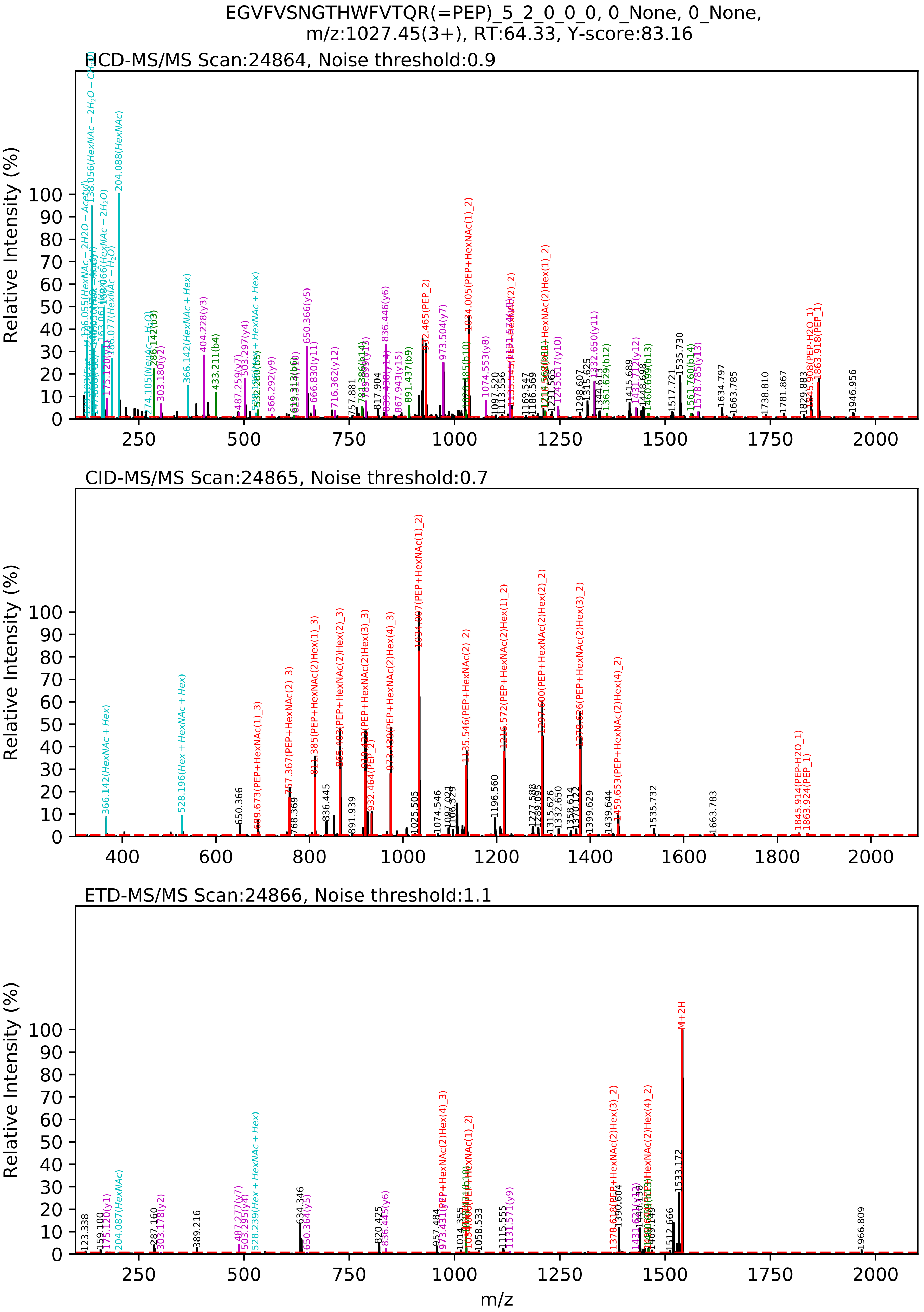
EGVFVSNGTHWFVTQR(=PEP)_5_2_0_0_0, 0_None, 0_None,
m/z:1027.45(3+), RT:64.33, Y-score:83.16
HCD-MS/MS Scan:24864, Noise threshold:0.9
CID-MS/MS Scan:24865, Noise threshold:0.7
ETD-MS/MS Scan:24866, Noise threshold:1.1
Relative Intensity (%)
m/z

EGVFVSNGTHWFVTQR(=PEP)_5_2_0_0_0, 0_None, 0_None,
m/z:1027.45(3+), RT:64.40, Y-score:79.12

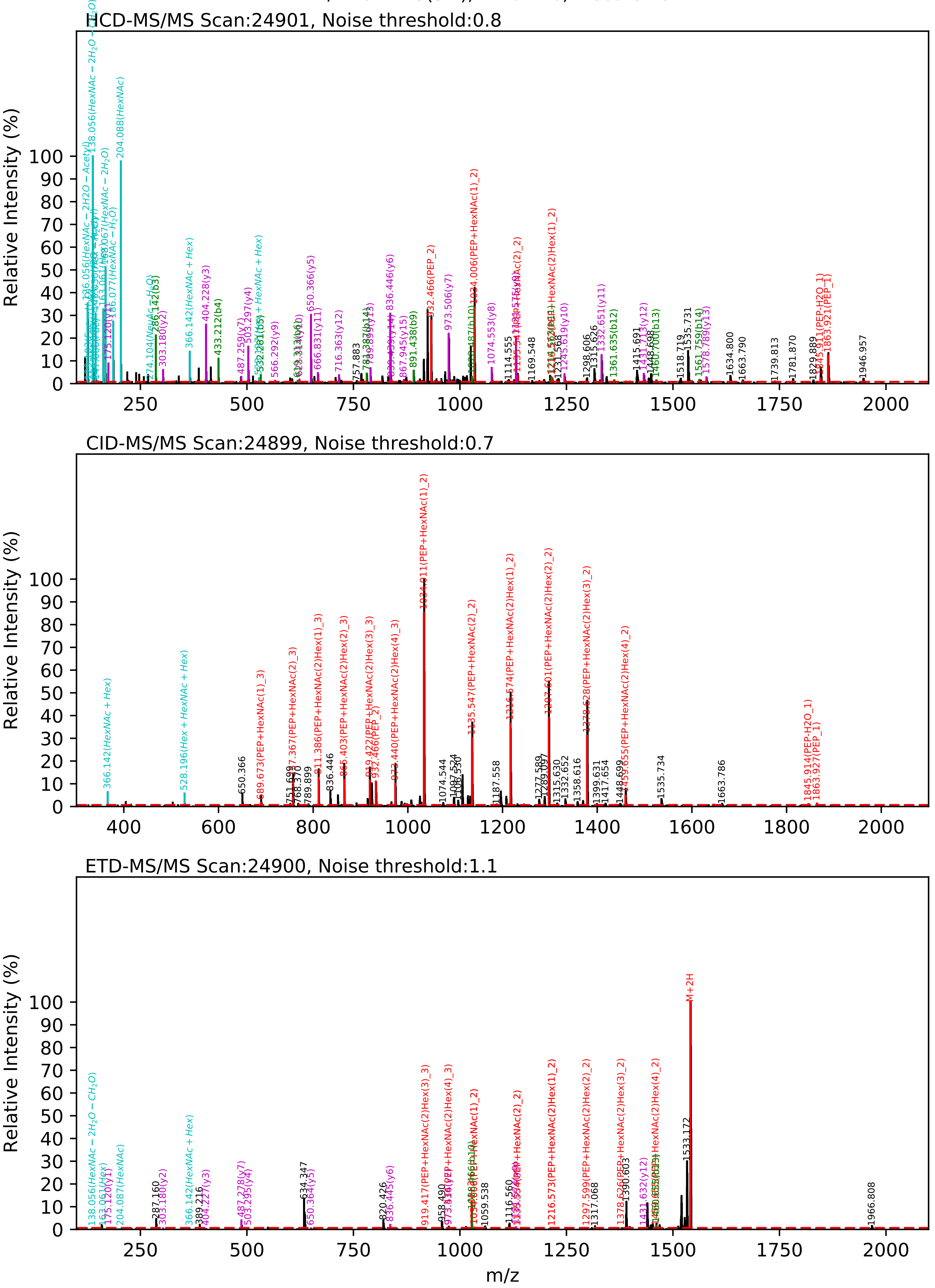

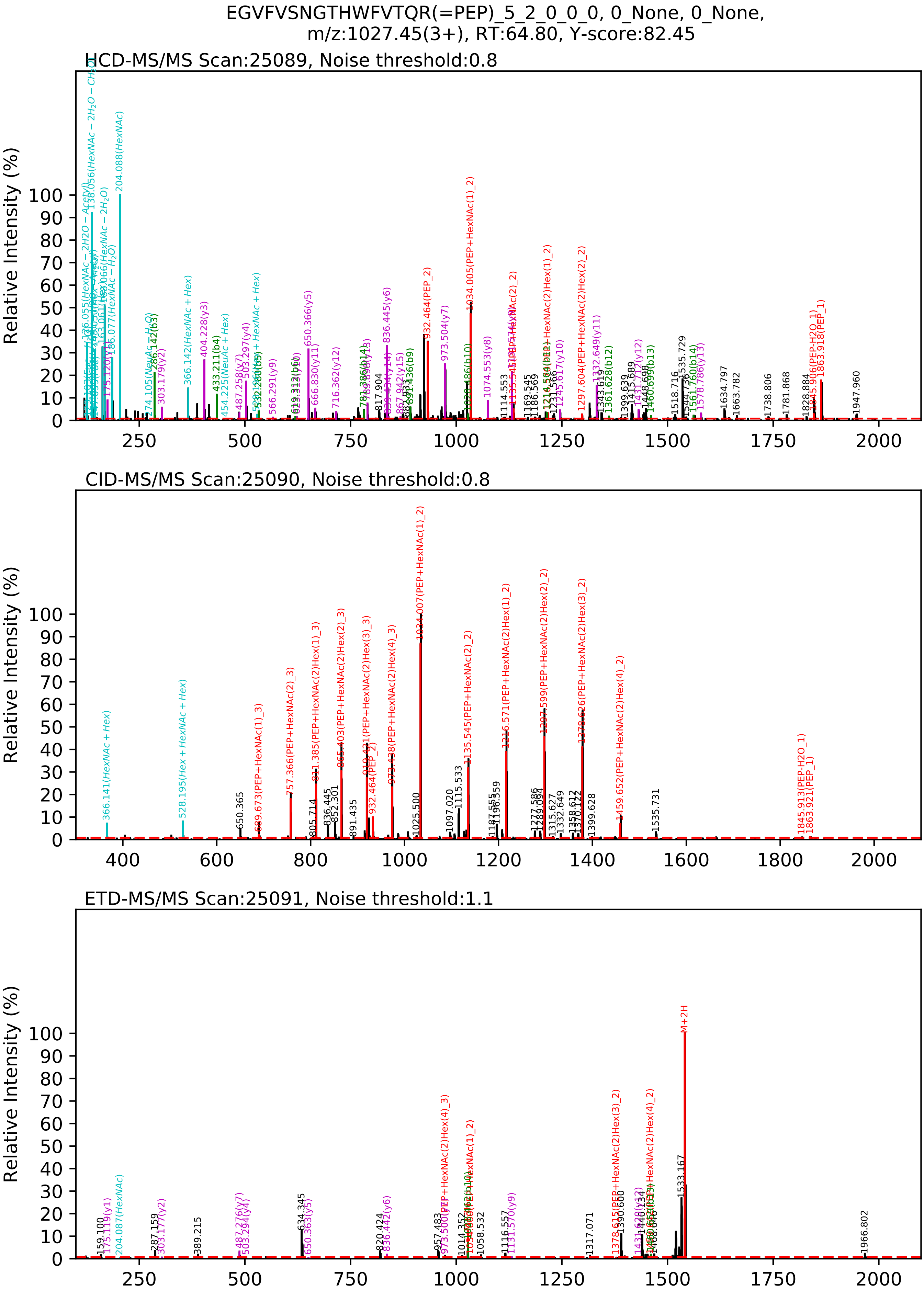
EGVFVSNGTHWFVTQR(=PEP)_5_2_0_0_0, 0_None, 0_None,
m/z:1027.45(3+), RT:64.80, Y-score:82.45
HCD-MS/MS Scan:25089, Noise threshold:0.8
CID-MS/MS Scan:25090, Noise threshold:0.8
ETD-MS/MS Scan:25091, Noise threshold:1.1
Relative Intensity (%)
m/z
204.088(HexNAc)
1034.005(PEP+HexNAc(1)_2)
932.464(PEP_2)
973.504(y7)
836.445(y6)
650.366(y5)
1034.007(PEP+HexNAc(1)_2)
1297.599(PEP+HexNAc(2)Hex(2)_2)
1378.626(PEP+HexNAc(2)Hex(3)_2)
1216.571(PEP+HexNAc(2)Hex(1)_2)
1135.545(PEP+HexNAc(2)_2)
1459.652(PEP+HexNAc(2)Hex(4)_2)
865.403(PEP+HexNAc(2)Hex(2)_3)
919.421(PEP+HexNAc(2)Hex(3)_3)
973.428(PEP+HexNAc(2)Hex(4)_3)
811.385(PEP+HexNAc(2)Hex(1)_3)
757.366(PEP+HexNAc(2)_3)
689.673(PEP+HexNAc(1)_3)
1845.913(PEP-H2O_1)
1863.921(PEP_1)
366.141(HexNAc+Hex)
528.195(Hex+HexNAc+Hex)
M+2H
1533.167
1390.600
634.345
1966.802

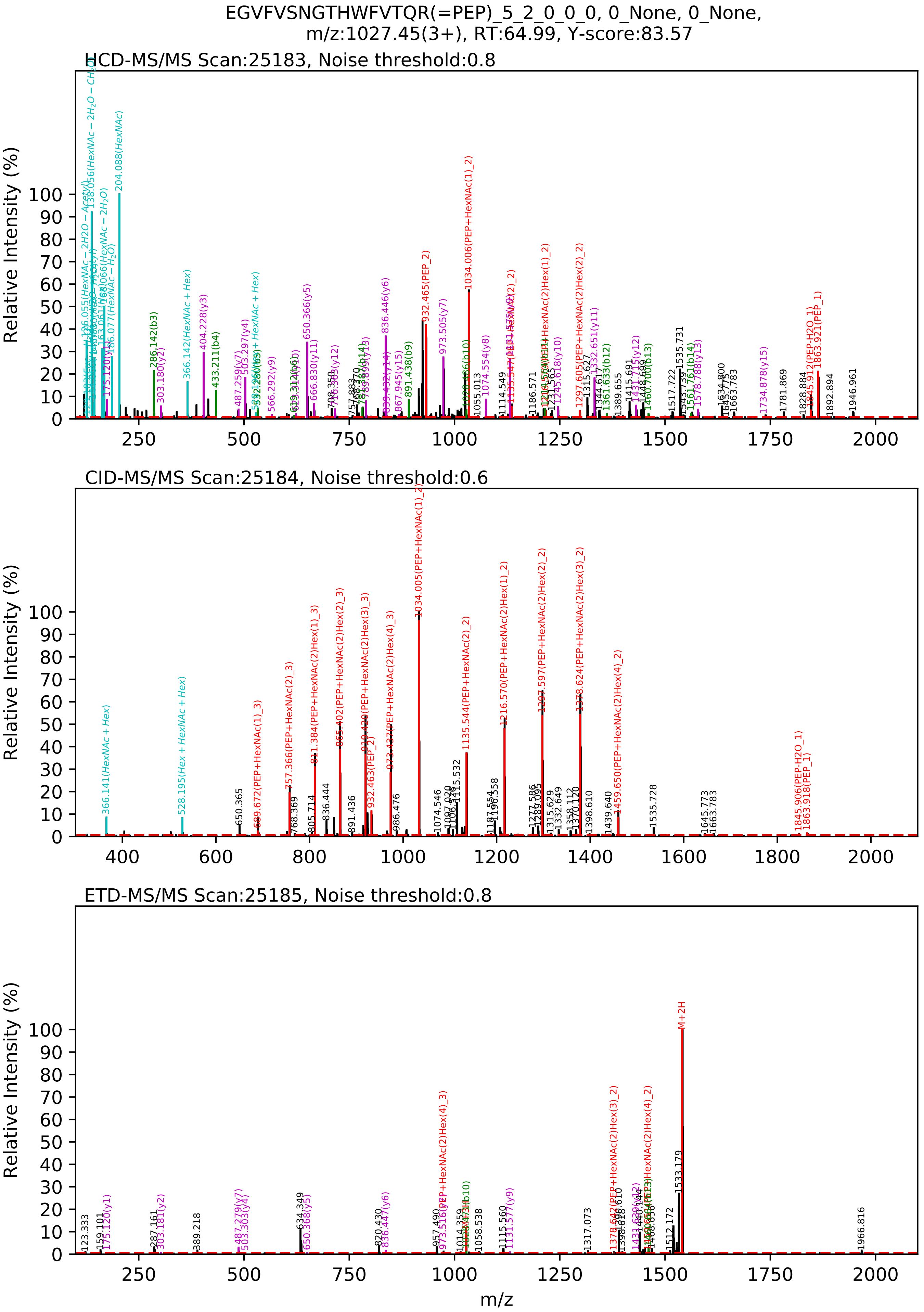

EGVFVSNGTHWFVTQR(=PEP)_5_2_0_0_0, 0_None, 0_None,
m/z:1027.45(3+), RT:64.99, Y-score:83.57
HCD-MS/MS Scan:25183, Noise threshold:0.8
CID-MS/MS Scan:25184, Noise threshold:0.6
ETD-MS/MS Scan:25185, Noise threshold:0.8
Relative Intensity (%)
m/z
204.088(HexNAc)
1034.006(PEP+HexNAc(1)_2)
932.465(PEP_2)
973.505(y7)
836.446(y6)
650.366(y5)
404.228(y3)
366.142(HexNAc + Hex)
286.142(b3)
433.211(b4)
1863.921(PEP_1)
1535.731
1034.005(PEP+HexNAc(1)_2)
1378.624(PEP+HexNAc(2)Hex(3)_2)
1297.597(PEP+HexNAc(2)Hex(2)_2)
1216.570(PEP+HexNAc(2)Hex(1)_2)
1135.544(PEP+HexNAc(2)_2)
865.402(PEP+HexNAc(2)Hex(2)_3)
811.384(PEP+HexNAc(2)Hex(1)_3)
757.366(PEP+HexNAc(2)_3)
1459.650(PEP+HexNAc(2)Hex(4)_2)
1845.906(PEP-H2O_1)
1863.918(PEP_1)
366.141(HexNAc + Hex)
528.195(Hex + HexNAc + Hex)
M+2H
1533.179
634.349
1966.816

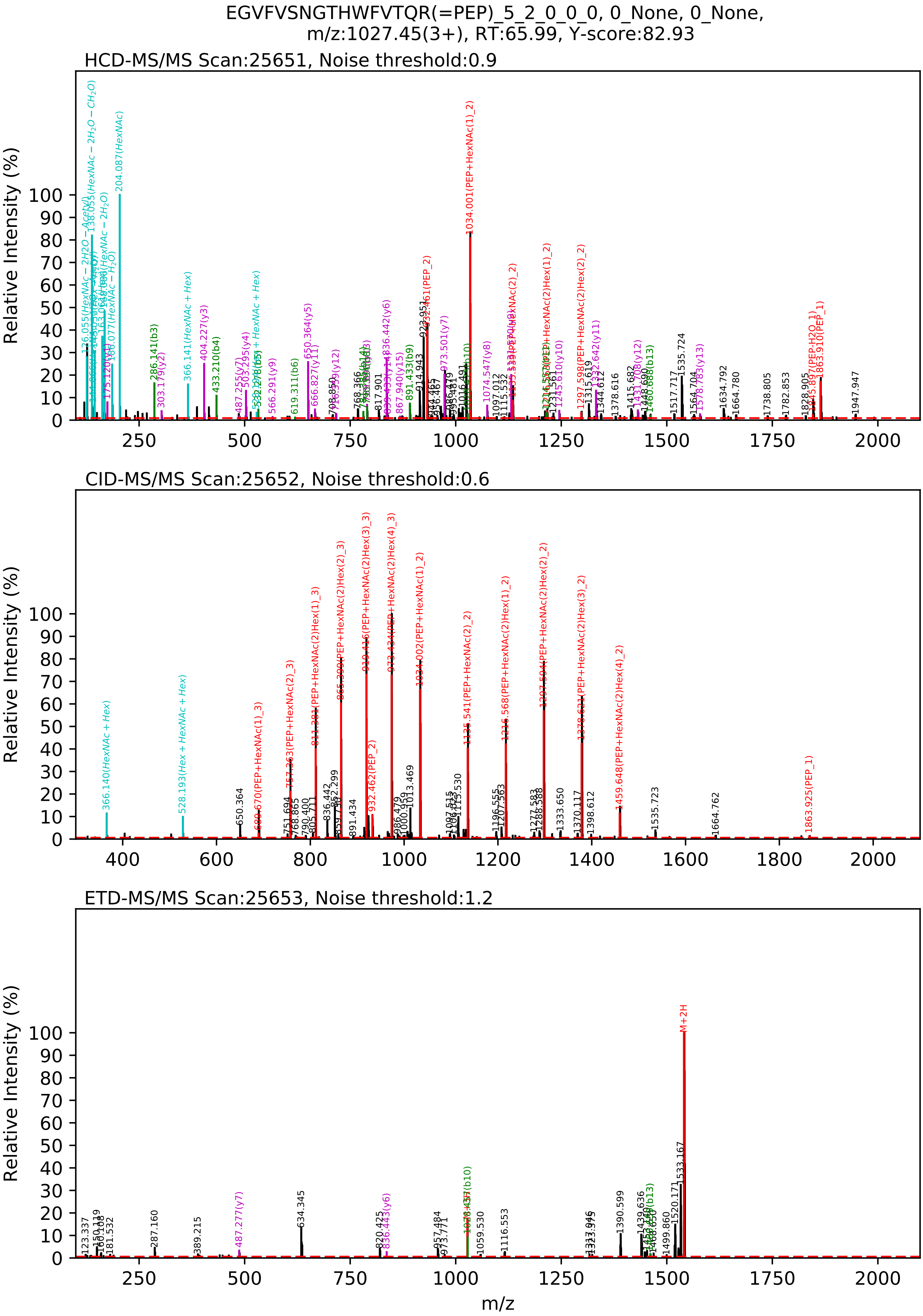

EGVFVSNGTHWFVTQR(=PEP)_5_2_0_0_0, 0_None, 0_None,
m/z:1027.45(3+), RT:65.99, Y-score:82.93
HCD-MS/MS Scan:25651, Noise threshold:0.9
CID-MS/MS Scan:25652, Noise threshold:0.6
ETD-MS/MS Scan:25653, Noise threshold:1.2
Relative Intensity (%)
m/z

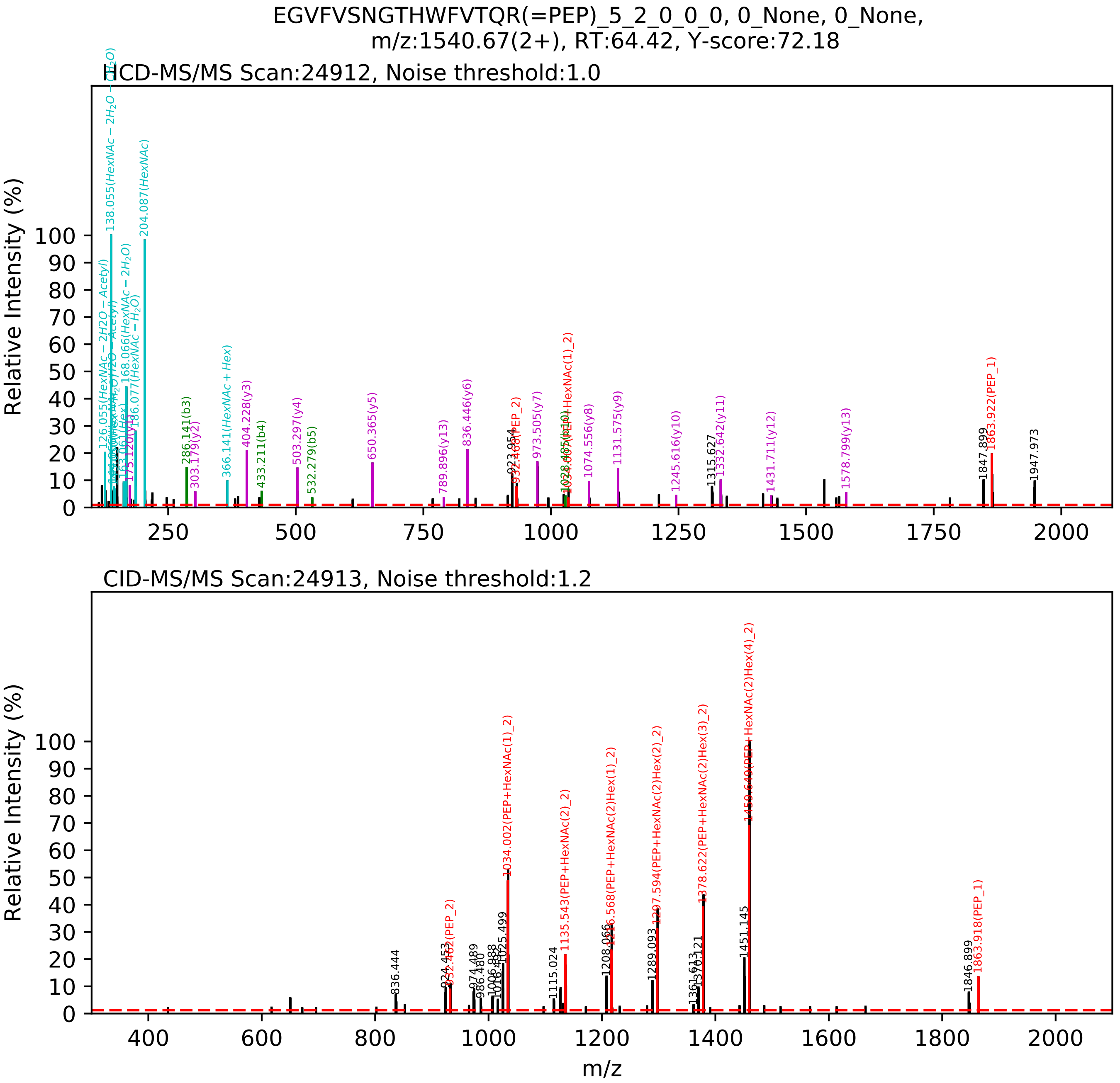
EGVFVSNGTHWFVTQR(=PEP)_5_2_0_0_0, 0_None, 0_None,
m/z:1540.67(2+), RT:64.42, Y-score:72.18
HCD-MS/MS Scan:24912, Noise threshold:1.0
CID-MS/MS Scan:24913, Noise threshold:1.2
Relative Intensity (%)
m/z

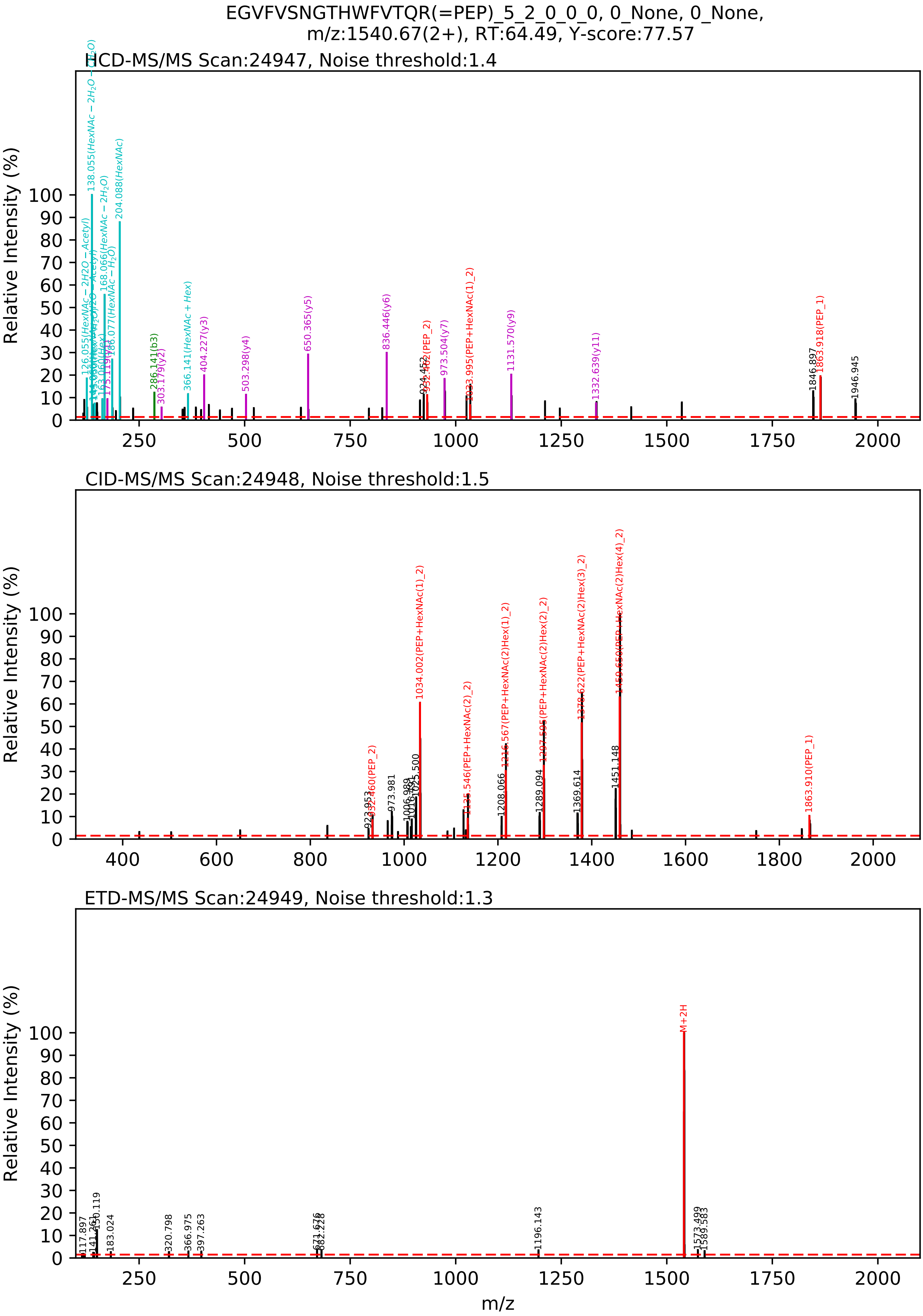

EGVFVSNGTHWFVTQR(=PEP)_5_2_0_0_0, 0_None, 0_None,
m/z:1540.67(2+), RT:64.49, Y-score:77.57
HCD-MS/MS Scan:24947, Noise threshold:1.4
Relative Intensity (%)
138.055(HexNAc)
204.088(HexNAc)
168.066(HexNAc − 2H2O)
186.077(HexNAc − H2O)
126.055(HexNAc − 2H2O − Acetyl)
286.141(b3)
303.179(y2)
366.141(HexNAc + Hex)
404.227(y3)
503.298(y4)
650.365(y5)
836.446(y6)
924.452
932.462(PEP_2)
973.504(y7)
1033.995(PEP+HexNAc(1)_2)
1131.570(y9)
1332.639(y11)
1846.897
1863.918(PEP_1)
1946.945
CID-MS/MS Scan:24948, Noise threshold:1.5
Relative Intensity (%)
923.953
932.460(PEP_2)
973.981
1006.989
1016.491
1025.500
1034.002(PEP+HexNAc(1)_2)
1135.546(PEP+HexNAc(2)_2)
1208.066
1216.567(PEP+HexNAc(2)Hex(1)_2)
1289.094
1297.595(PEP+HexNAc(2)Hex(2)_2)
1369.614
1378.622(PEP+HexNAc(2)Hex(3)_2)
1451.148
1459.650(PEP+HexNAc(2)Hex(4)_2)
1863.910(PEP_1)
ETD-MS/MS Scan:24949, Noise threshold:1.3
Relative Intensity (%)
117.897
141.361
150.119
183.024
320.798
366.975
397.263
671.676
682.228
1196.143
M+2H
1573.499
1589.583
m/z

EGVFVSNGTHWFVTQR(=PEP)_5_2_0_0_0, 0_None, 0_None,
m/z:1540.67(2+), RT:65.01, Y-score:67.56

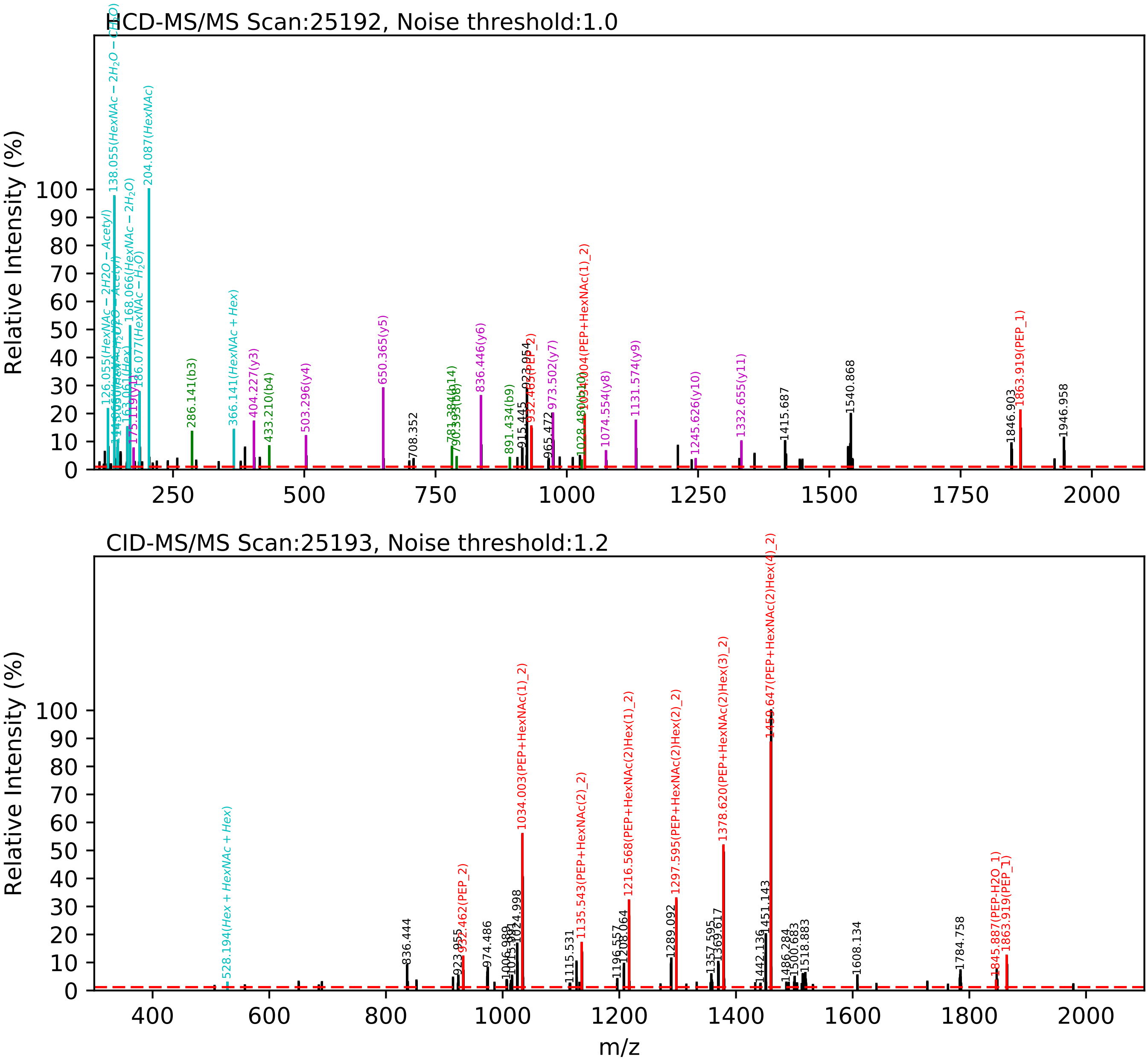

EGVFVSNGTHWFVTQR(=PEP)_5_2_0_0_0, 0_None, 0_None,
m/z:1540.67(2+), RT:66.12, Y-score:68.88

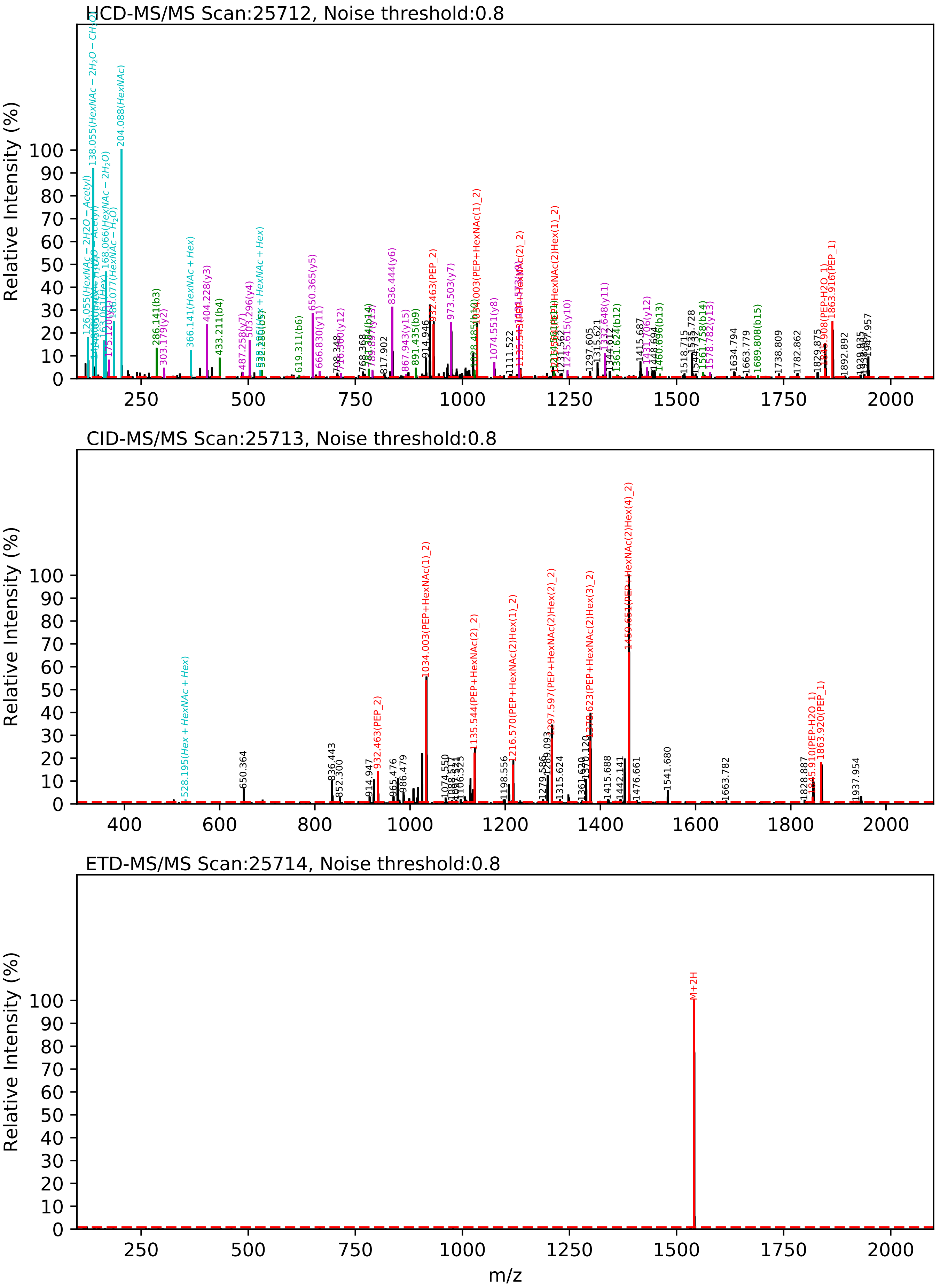

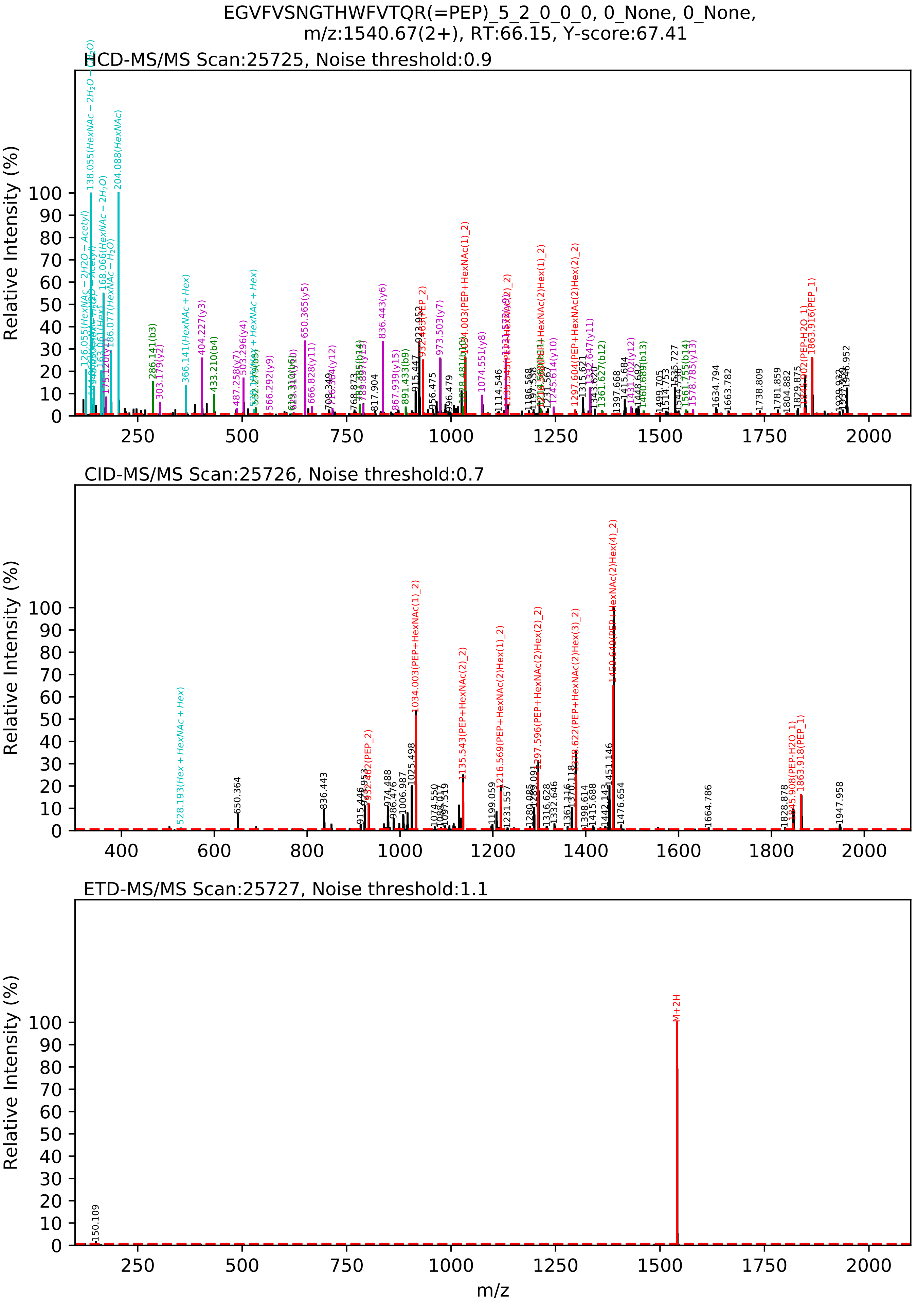

EGVFVSNGTHWFVTQR(=PEP)_5_2_0_0_0, 0_None, 0_None,
m/z:1540.67(2+), RT:66.15, Y-score:67.41
HCD-MS/MS Scan:25725, Noise threshold:0.9
CID-MS/MS Scan:25726, Noise threshold:0.7
ETD-MS/MS Scan:25727, Noise threshold:1.1
Relative Intensity (%)
m/z

EGVFVSNGTHWFVTQR(=PEP)_6_2_0_0_0, 0_None, 0_None,
m/z:1621.70(2+), RT:64.61, Y-score:58.62

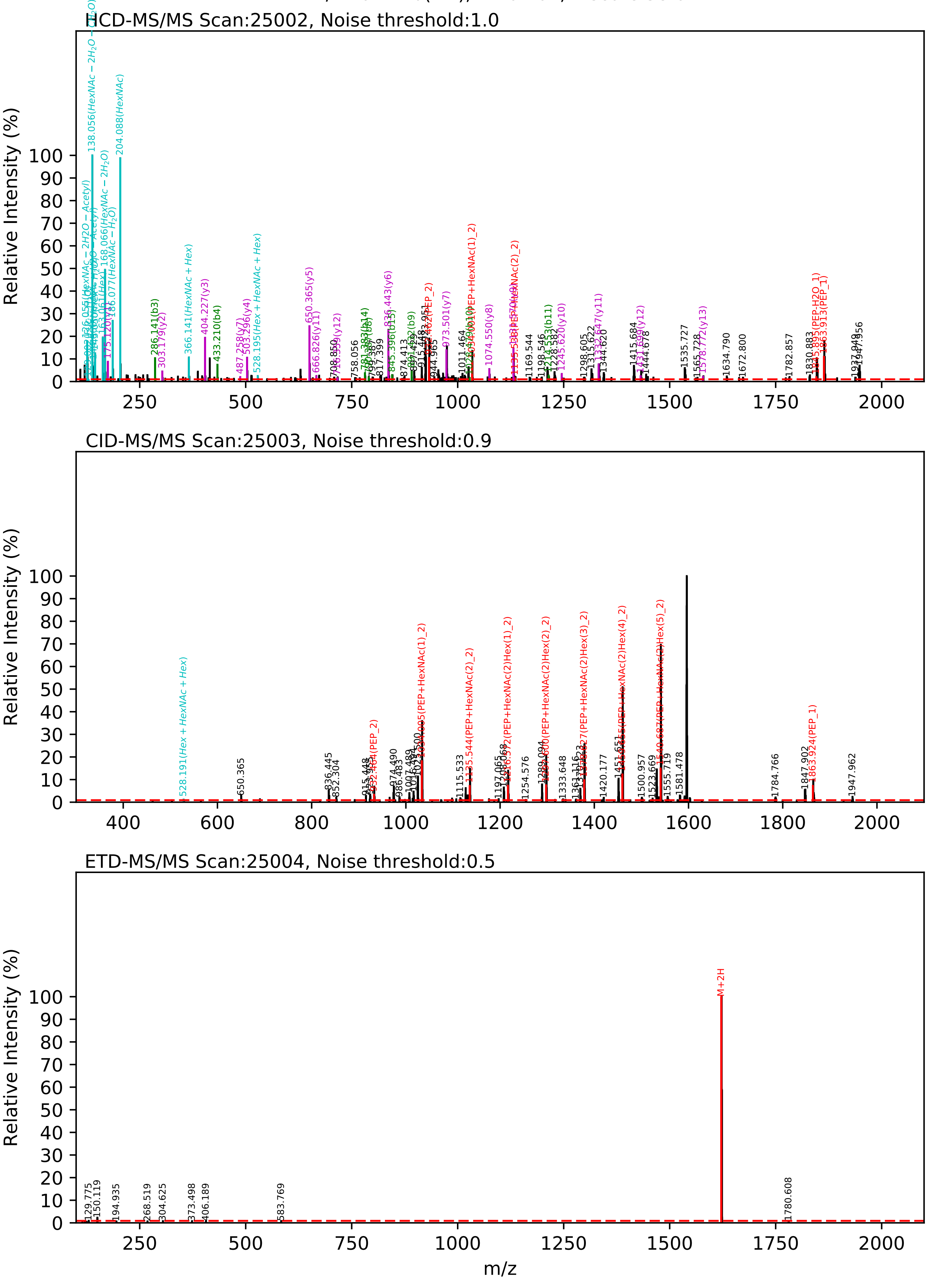

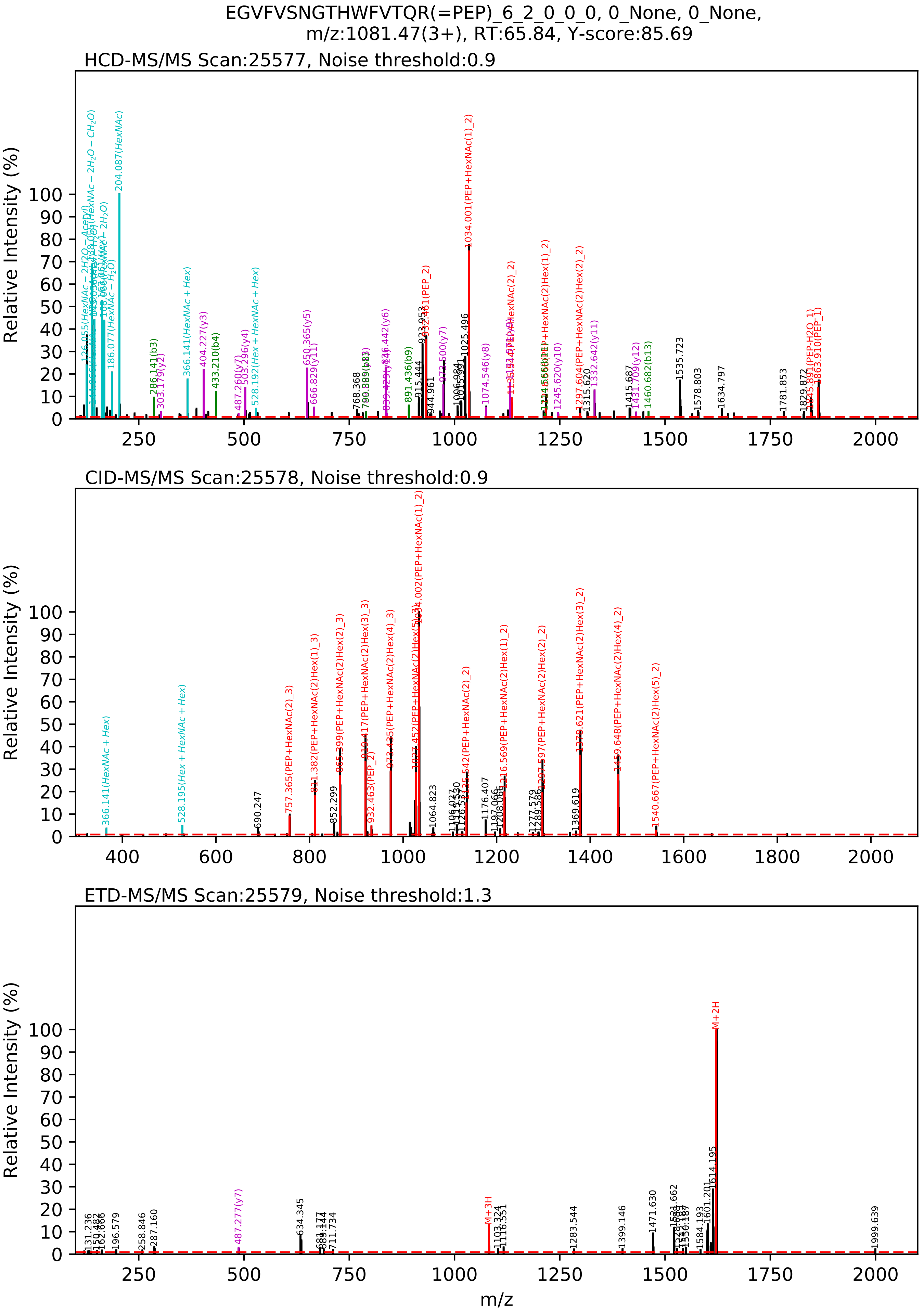

EGVFVSNGTHWFVTQR(=PEP)_6_2_0_0_0, 0_None, 0_None,
m/z:1081.47(3+), RT:65.84, Y-score:85.69
HCD-MS/MS Scan:25577, Noise threshold:0.9
CID-MS/MS Scan:25578, Noise threshold:0.9
ETD-MS/MS Scan:25579, Noise threshold:1.3
Relative Intensity (%)
m/z

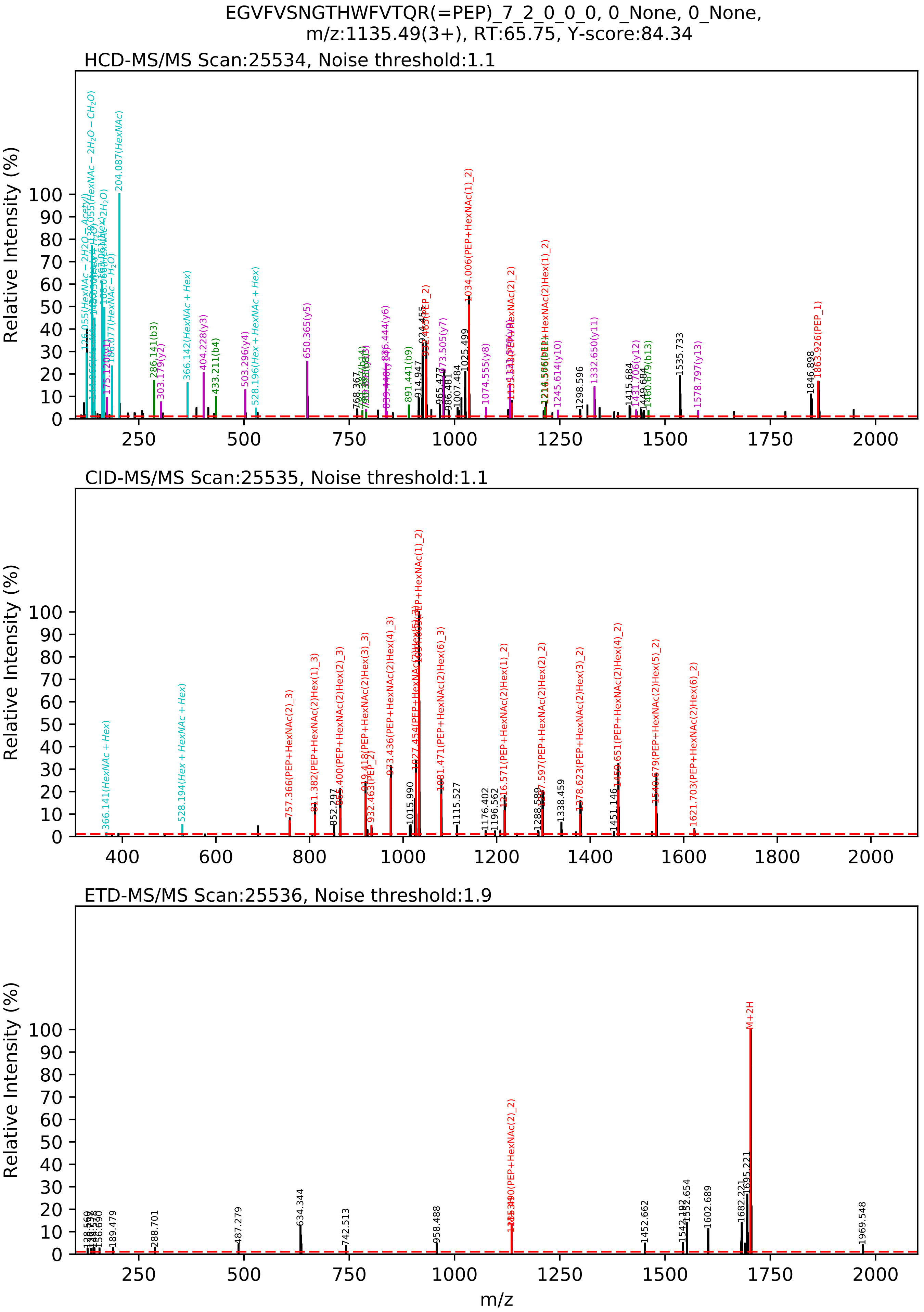

EGVFVSNGTHWFVTQR(=PEP)_7_2_0_0_0, 0_None, 0_None,
m/z:1135.49(3+), RT:65.75, Y-score:84.34
HCD-MS/MS Scan:25534, Noise threshold:1.1
CID-MS/MS Scan:25535, Noise threshold:1.1
ETD-MS/MS Scan:25536, Noise threshold:1.9
Relative Intensity (%)
m/z

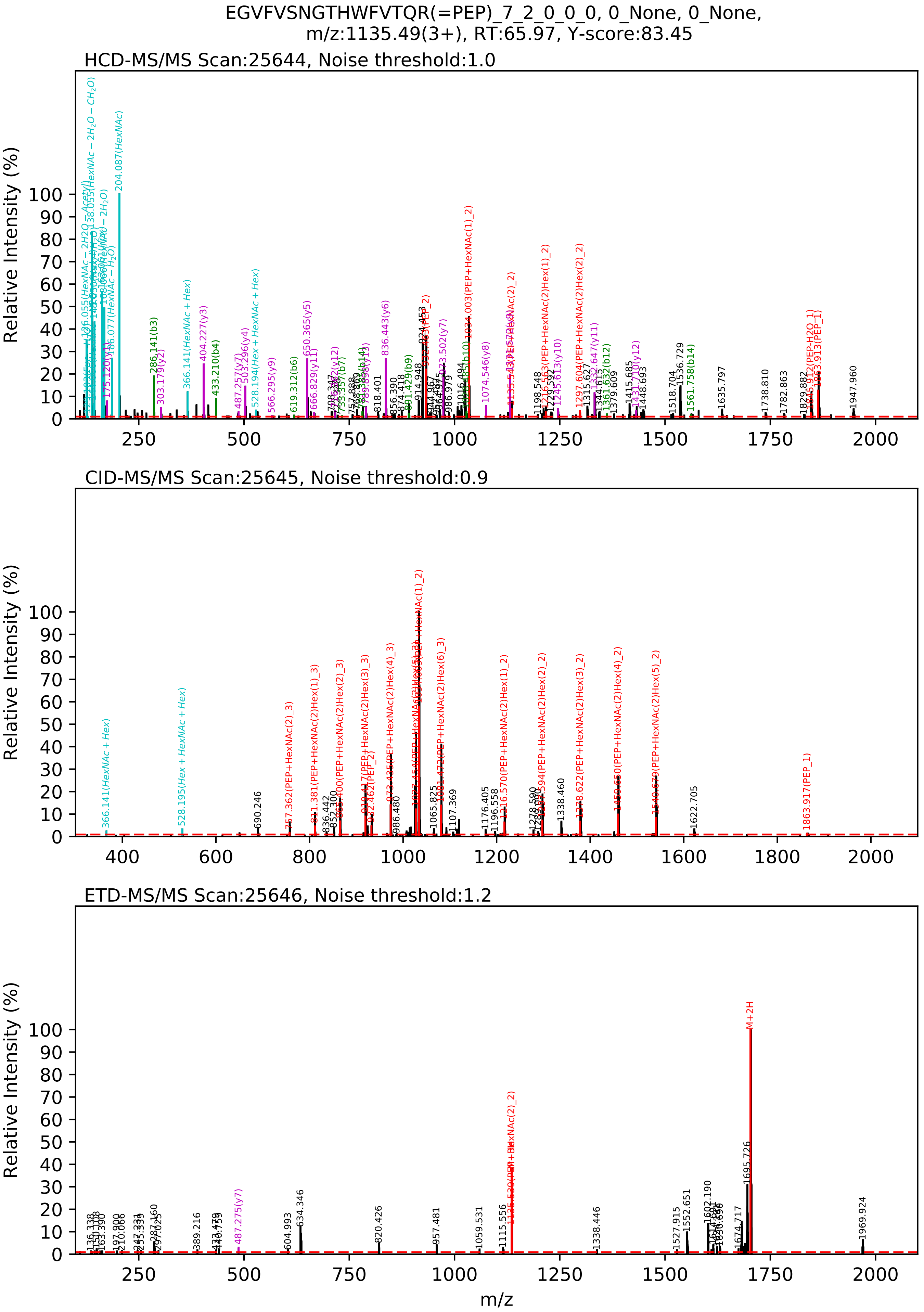
EGVFVSNGTHWFVTQR(=PEP)_7_2_0_0_0, 0_None, 0_None,
m/z:1135.49(3+), RT:65.97, Y-score:83.45
HCD-MS/MS Scan:25644, Noise threshold:1.0
CID-MS/MS Scan:25645, Noise threshold:0.9
ETD-MS/MS Scan:25646, Noise threshold:1.2
Relative Intensity (%)
m/z
M+2H

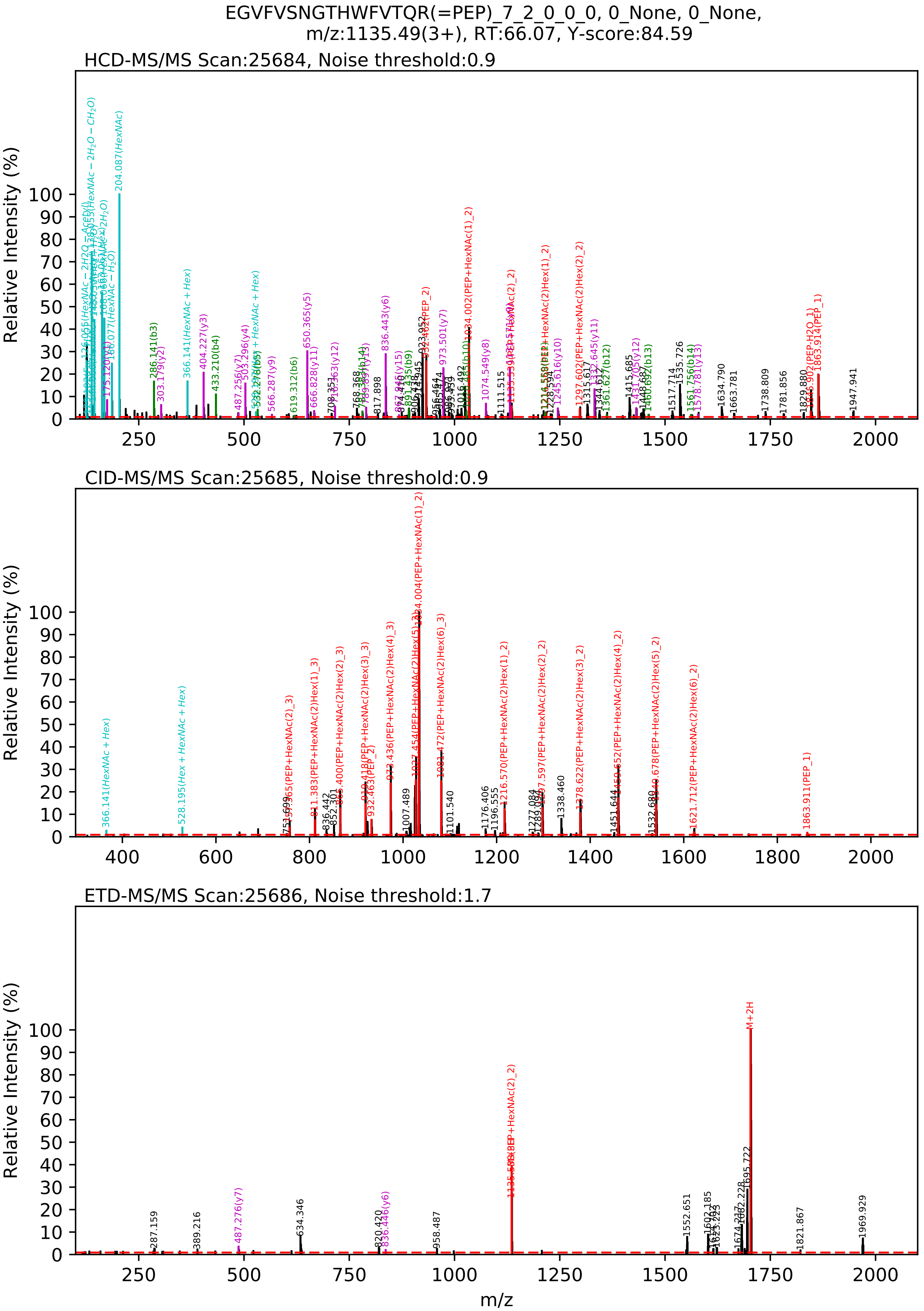
EGVFVSNGTHWFVTQR(=PEP)_7_2_0_0_0, 0_None, 0_None,
m/z:1135.49(3+), RT:66.07, Y-score:84.59
HCD-MS/MS Scan:25684, Noise threshold:0.9
Relative Intensity (%)
204.087(HexNAc)
366.141(HexNAc + Hex)
286.141(b3)
404.227(y3)
433.210(b4)
650.365(y5)
836.443(y6)
973.501(y7)
1034.002(PEP+HexNAc(1)_2)
1074.549(y8)
1297.602(PEP+HexNAc(2)Hex(2)_2)
1863.914(PEP_1)
CID-MS/MS Scan:25685, Noise threshold:0.9
366.141(HexNAc + Hex)
528.195(Hex + HexNAc + Hex)
811.383(PEP+HexNAc(2)Hex(1)_3)
973.436(PEP+HexNAc(2)Hex(4)_3)
1034.004(PEP+HexNAc(1)_2)
1216.570(PEP+HexNAc(2)Hex(1)_2)
1378.622(PEP+HexNAc(2)Hex(3)_2)
1621.712(PEP+HexNAc(2)Hex(6)_2)
1863.911(PEP_1)
ETD-MS/MS Scan:25686, Noise threshold:1.7
M+2H
287.159
389.216
487.276(y7)
634.346
820.420
958.487
1552.651
1695.722
1821.867
1969.929
m/z

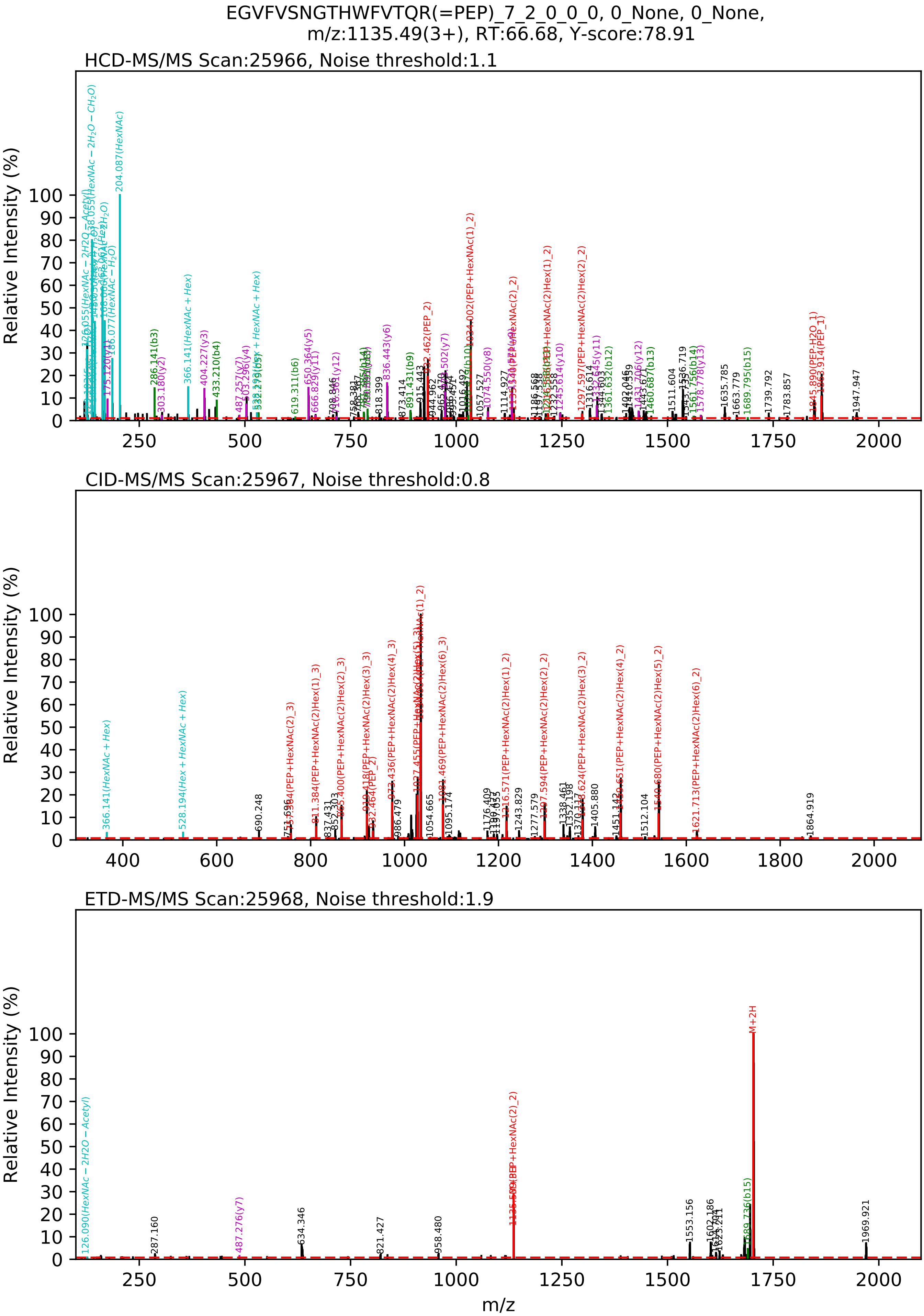

EGVFVSNGTHWFVTQR(=PEP)_7_2_0_0_0, 0_None, 0_None,
m/z:1135.49(3+), RT:66.68, Y-score:78.91
HCD-MS/MS Scan:25966, Noise threshold:1.1
CID-MS/MS Scan:25967, Noise threshold:0.8
ETD-MS/MS Scan:25968, Noise threshold:1.9
Relative Intensity (%)
m/z

EGVFVSNGTHWFVTQR(=PEP)_7_2_0_0_0, 0_None, 0_None,
m/z:1135.49(3+), RT:66.85, Y-score:82.66

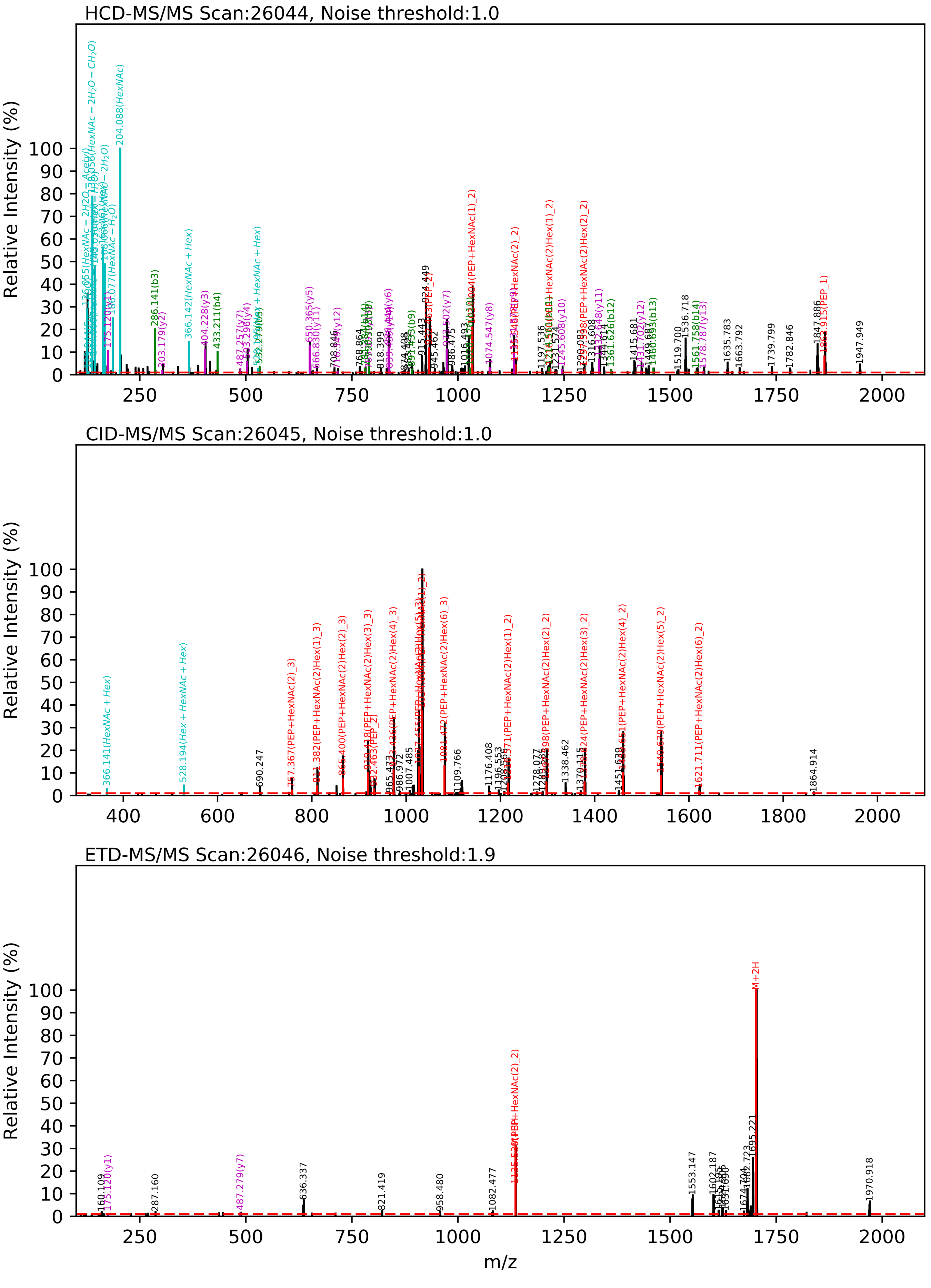

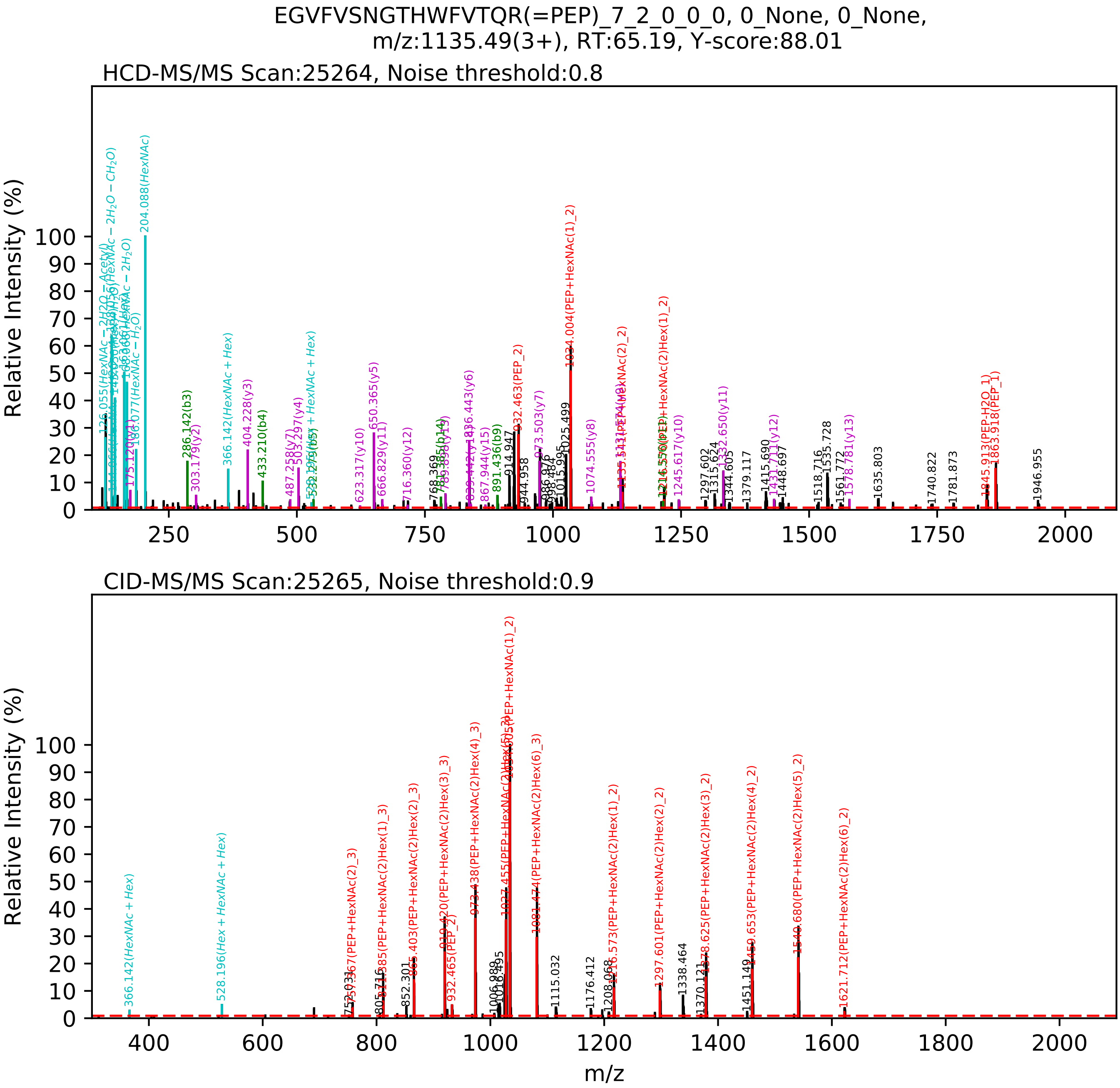
EGVFVSNGTHWFVTQR(=PEP)_7_2_0_0_0, 0_None, 0_None,
m/z:1135.49(3+), RT:65.19, Y-score:88.01
HCD-MS/MS Scan:25264, Noise threshold:0.8
CID-MS/MS Scan:25265, Noise threshold:0.9
Relative Intensity (%)
m/z

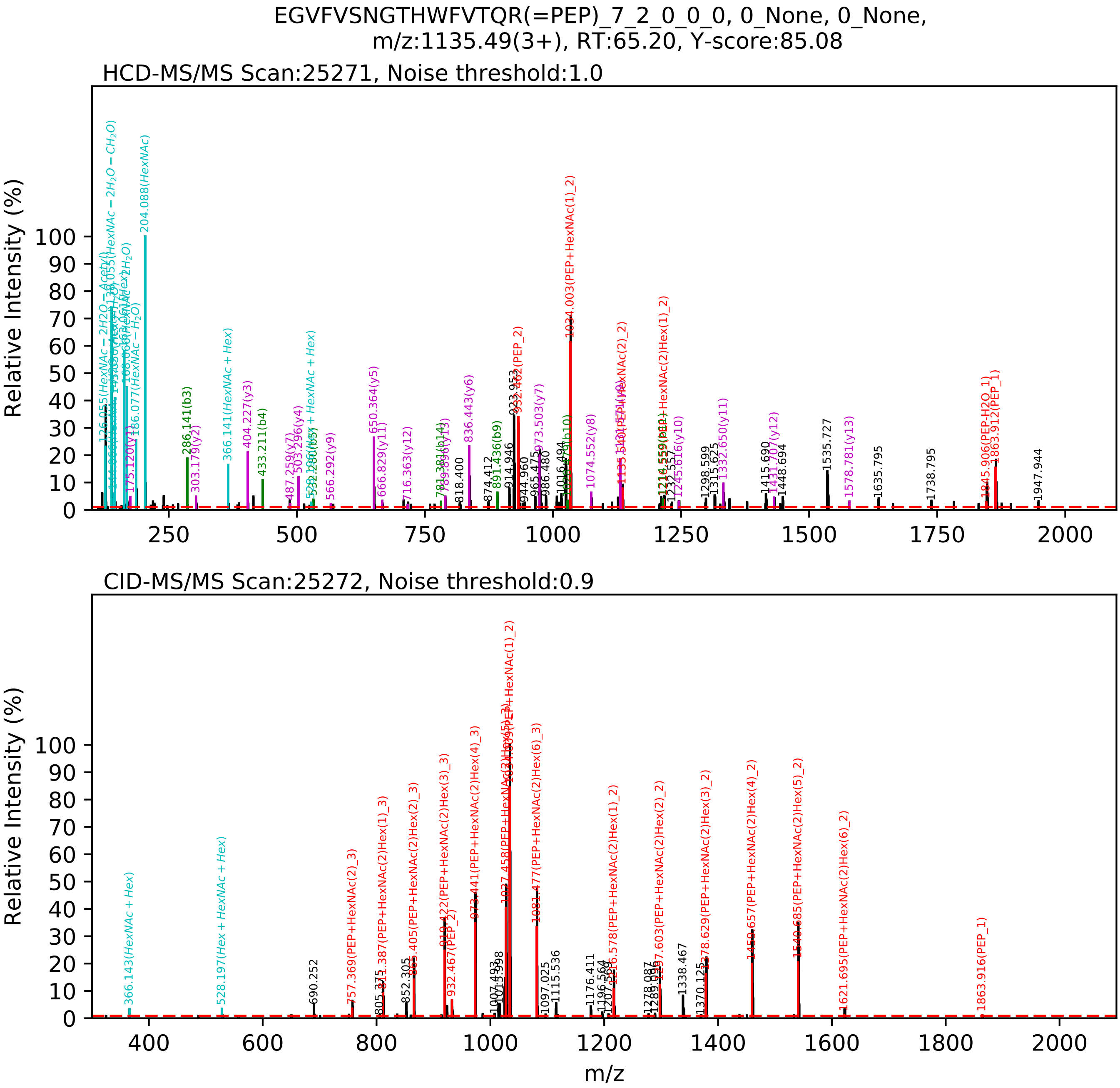

EGVFVSNGTHWFVTQR(=PEP)_7_2_0_0_0, 0_None, 0_None,
m/z:1135.49(3+), RT:65.20, Y-score:85.08
HCD-MS/MS Scan:25271, Noise threshold:1.0
CID-MS/MS Scan:25272, Noise threshold:0.9
Relative Intensity (%)
m/z

EGVFVSNGTHWFVTQR(=PEP)_7_2_0_0_0, 0_None, 0_None,
m/z:1135.49(3+), RT:64.68, Y-score:85.76

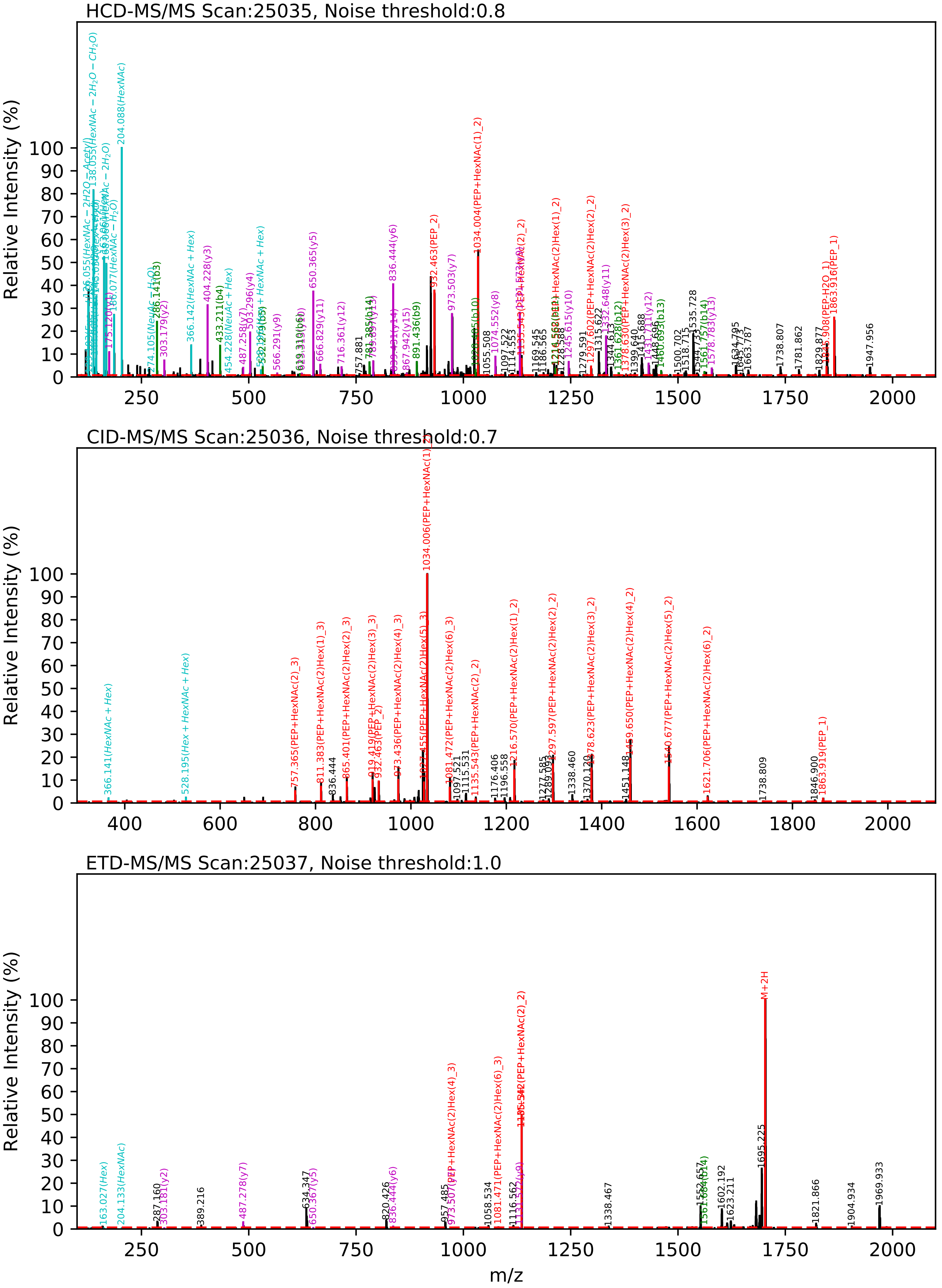

EGVFVSNGTHWFVTQR(=PEP)_8_2_0_0_0, 0_None, 0_None,
m/z:1189.50(3+), RT:67.33, Y-score:84.71

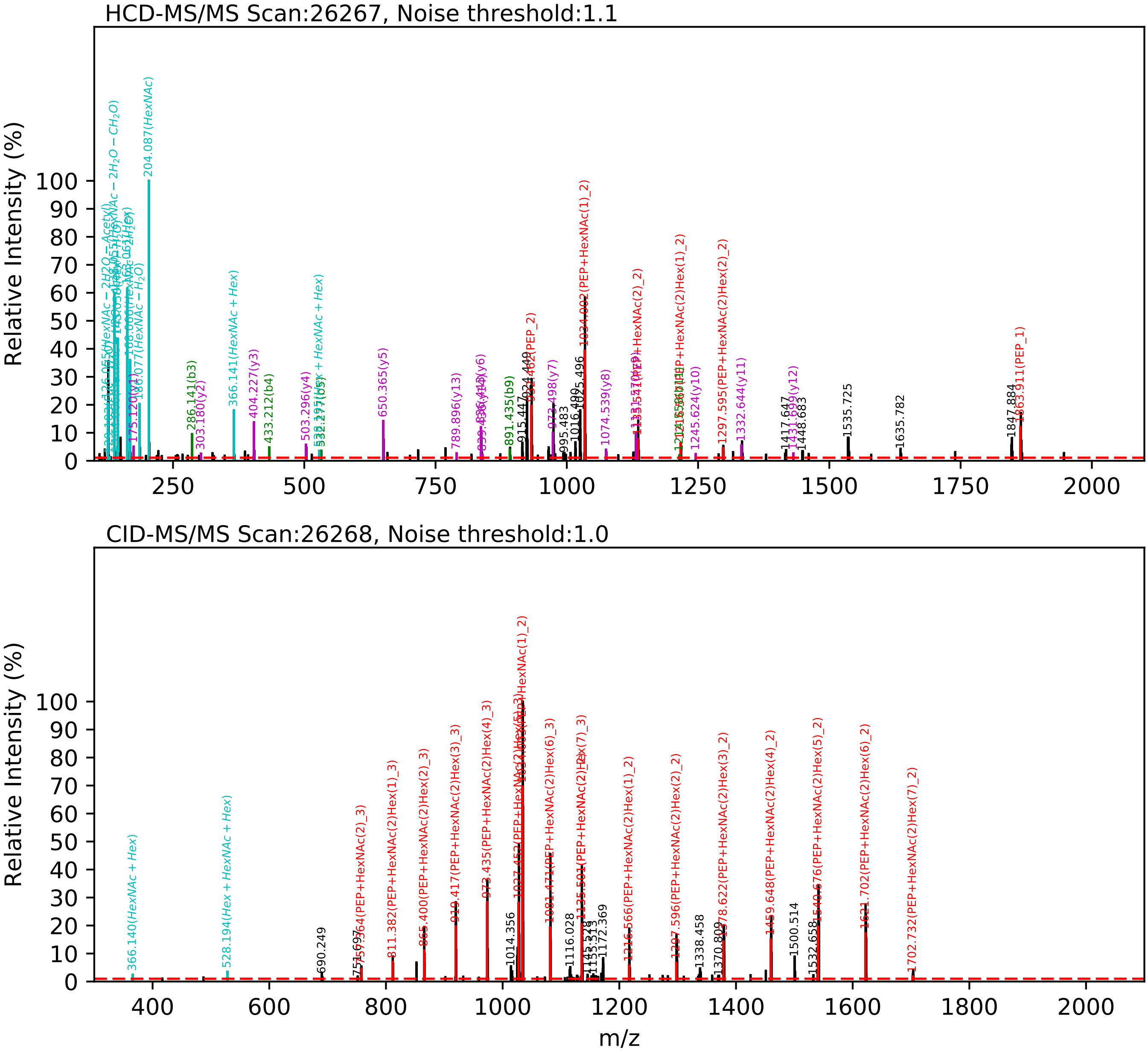

EGVFVSNGTHWFVTQR(=PEP)_8_2_0_0_0, 0_None, 0_None,
m/z:1189.50(3+), RT:68.81, Y-score:75.73

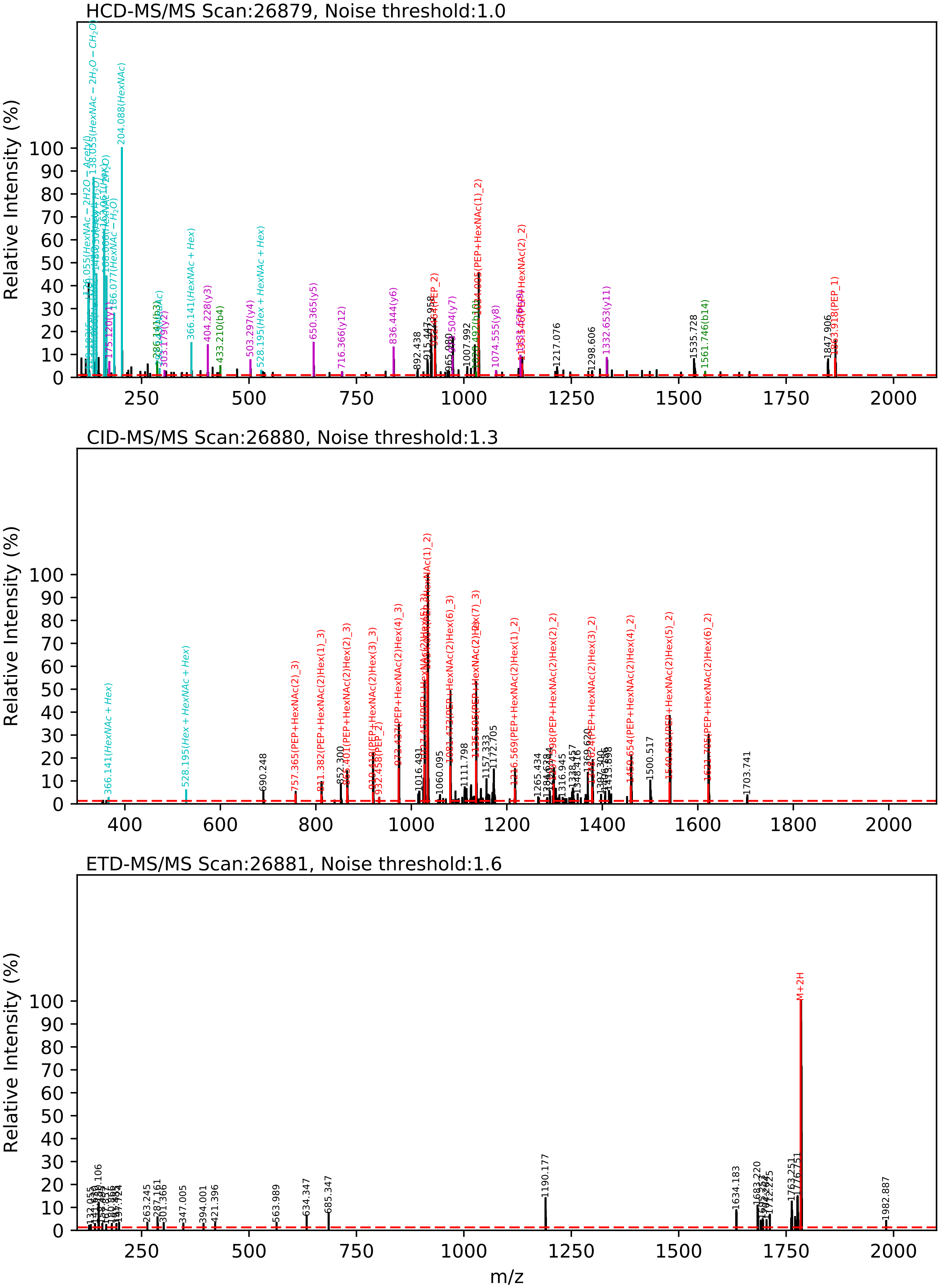

EGVFVSNGTHWFVTQR(=PEP)_8_2_0_0_0, 0_None, 0_None,
m/z:1189.50(3+), RT:73.31, Y-score:79.29

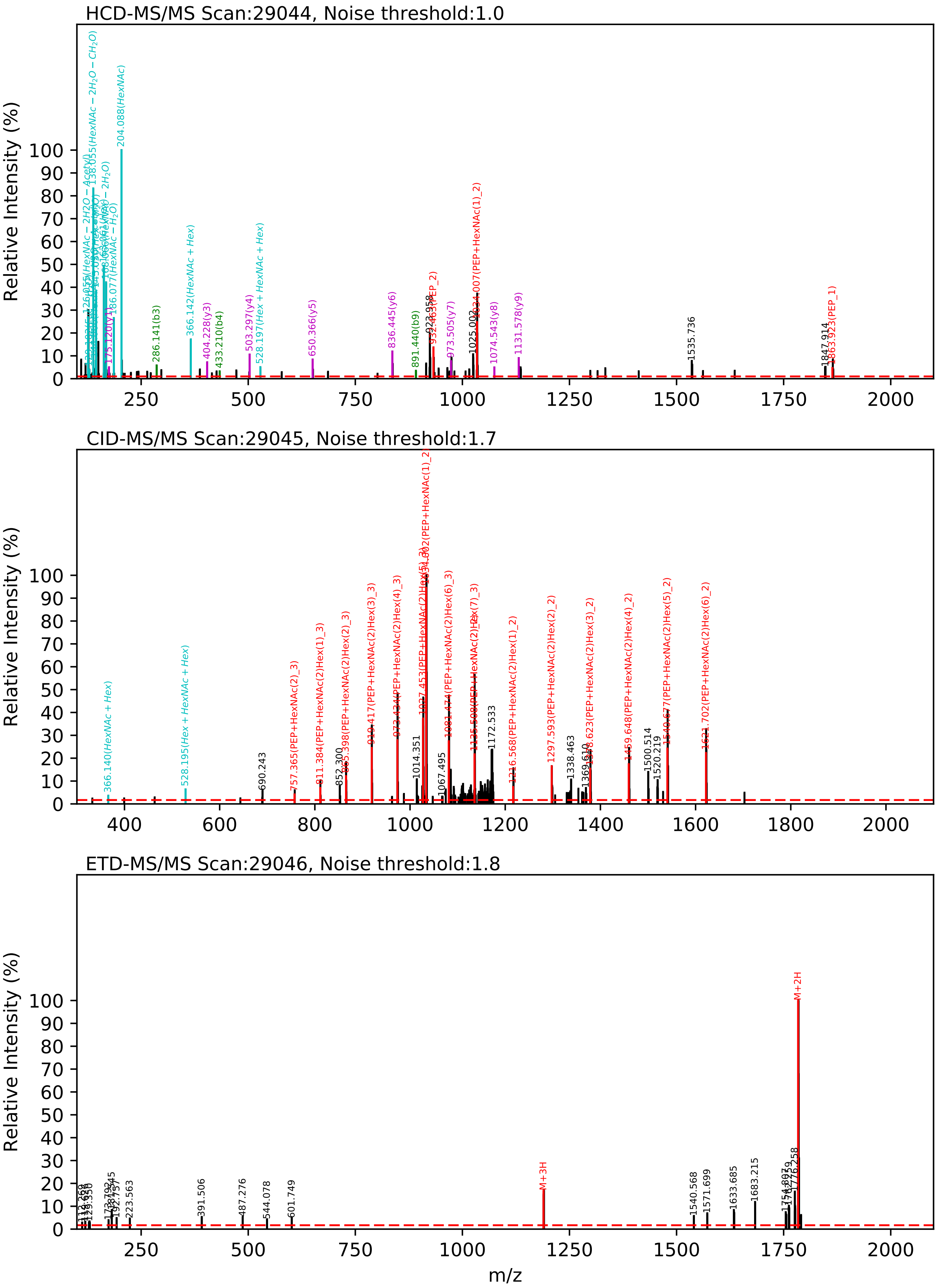

EGVFVSNGTHWFVTQR(=PEP)_8_2_0_0_0, 0_None, 0_None,
m/z:1189.50(3+), RT:74.68, Y-score:83.61

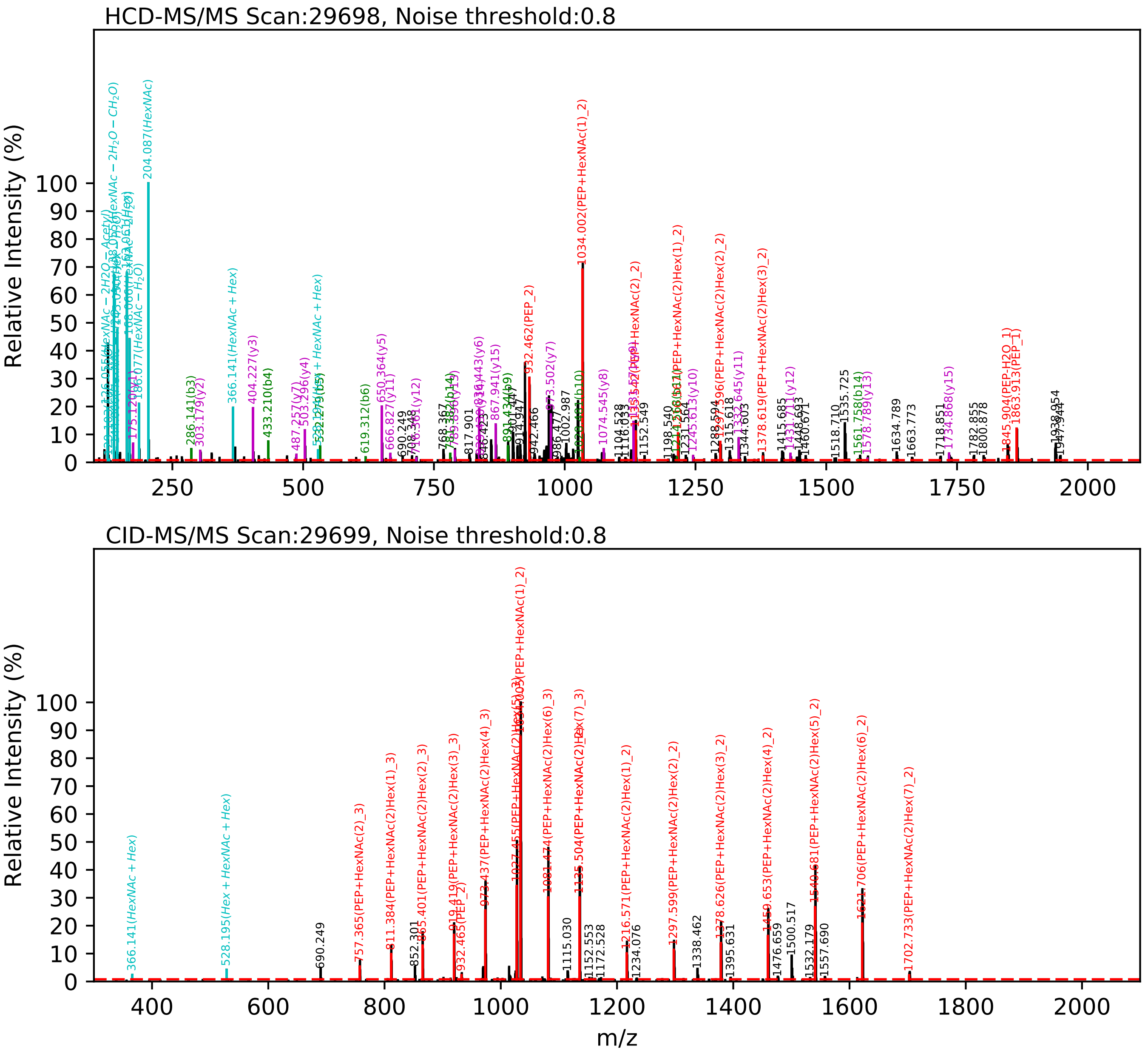

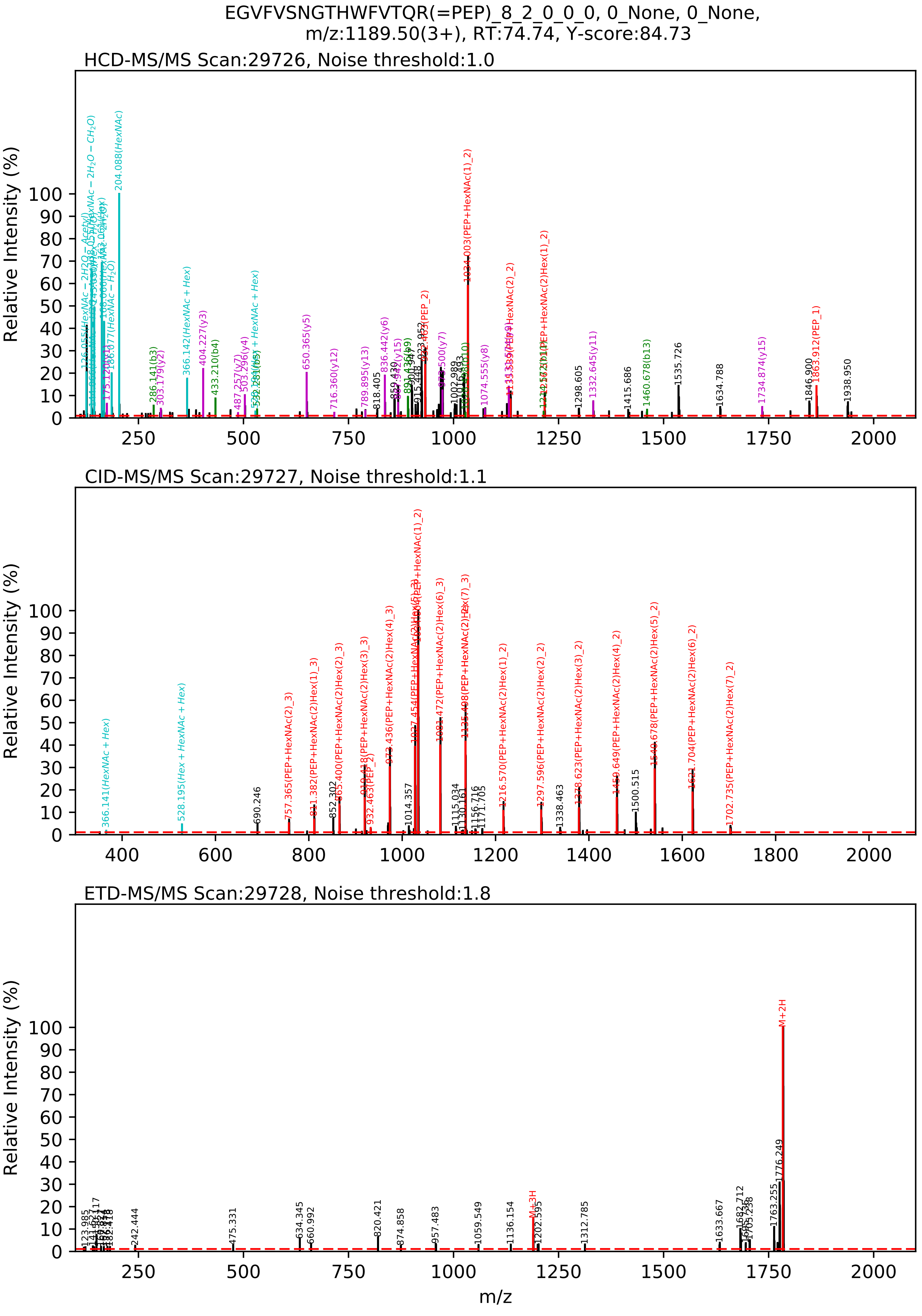
EGVFVSNGTHWFVTQR(=PEP)_8_2_0_0_0, 0_None, 0_None,
m/z:1189.50(3+), RT:74.74, Y-score:84.73
HCD-MS/MS Scan:29726, Noise threshold:1.0
CID-MS/MS Scan:29727, Noise threshold:1.1
ETD-MS/MS Scan:29728, Noise threshold:1.8
Relative Intensity (%)
m/z

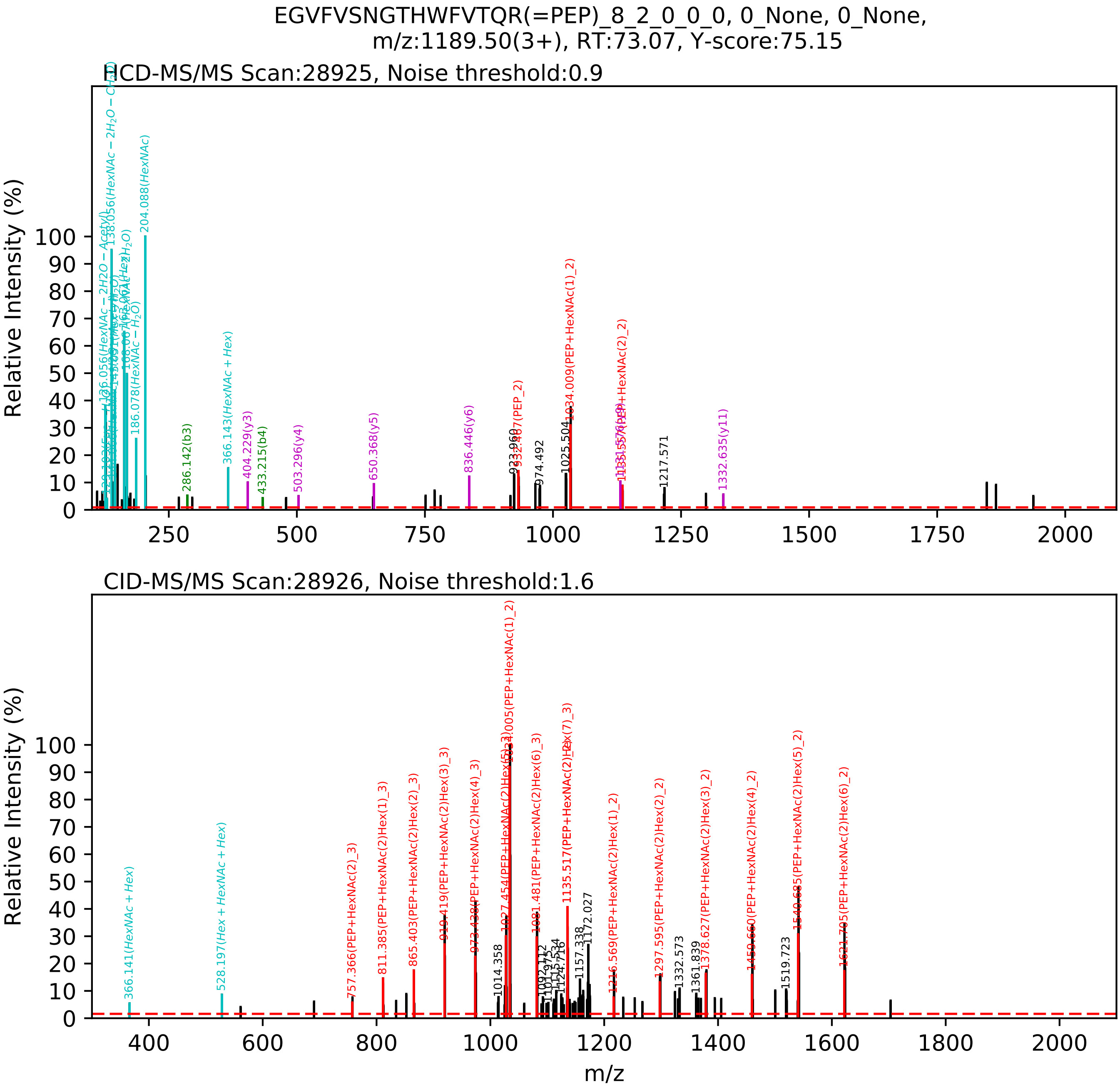

EGVFVSNGTHWFVTQR(=PEP)_8_2_0_0_0, 0_None, 0_None,
m/z:1189.50(3+), RT:73.07, Y-score:75.15
HCD-MS/MS Scan:28925, Noise threshold:0.9
Relative Intensity (%)
204.088(HexNAc)
186.078(HexNAc − H2O)
366.143(HexNAc + Hex)
286.142(b3)
404.229(y3)
433.215(b4)
503.296(y4)
650.368(y5)
836.446(y6)
923.960
932.467(PEP_2)
974.492
1025.504
1034.009(PEP+HexNAc(1)_2)
1217.571
1332.635(y11)
CID-MS/MS Scan:28926, Noise threshold:1.6
366.141(HexNAc + Hex)
528.197(Hex + HexNAc + Hex)
757.366(PEP+HexNAc(2)_3)
811.385(PEP+HexNAc(2)Hex(1)_3)
865.403(PEP+HexNAc(2)Hex(2)_3)
919.419(PEP+HexNAc(2)Hex(3)_3)
973.438(PEP+HexNAc(2)Hex(4)_3)
1014.358
1034.005(PEP+HexNAc(1)_2)
1081.481(PEP+HexNAc(2)Hex(6)_3)
1135.517(PEP+HexNAc(2)Hex(7)_3)
1157.338
1172.027
1216.569(PEP+HexNAc(2)Hex(1)_2)
1297.595(PEP+HexNAc(2)Hex(2)_2)
1332.573
1361.839
1378.627(PEP+HexNAc(2)Hex(3)_2)
1459.660(PEP+HexNAc(2)Hex(4)_2)
1519.723
1540.685(PEP+HexNAc(2)Hex(5)_2)
1621.705(PEP+HexNAc(2)Hex(6)_2)
m/z

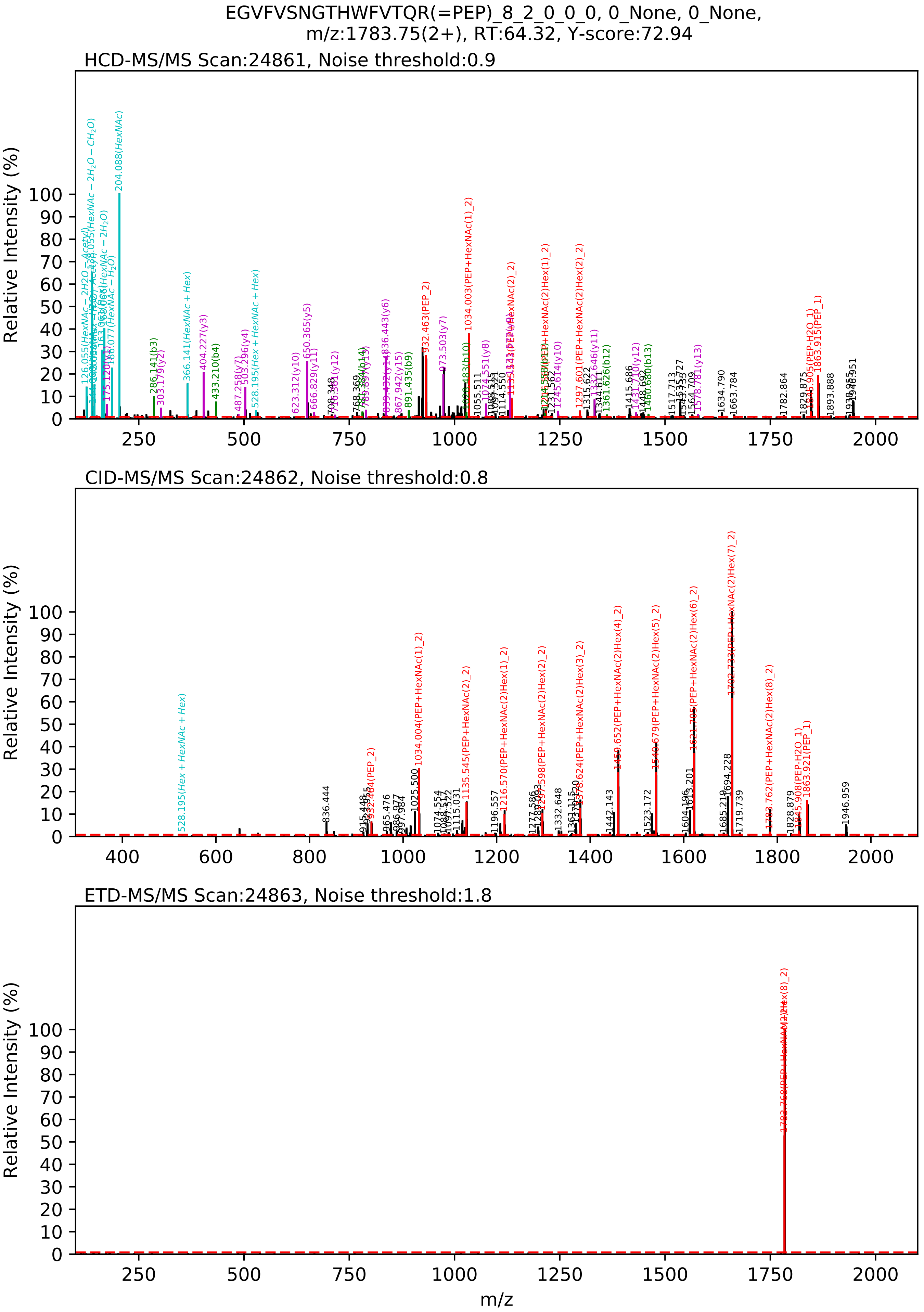

EGVFVSNGTHWFVTQR(=PEP)_8_2_0_0_0, 0_None, 0_None,
m/z:1783.75(2+), RT:64.32, Y-score:72.94
HCD-MS/MS Scan:24861, Noise threshold:0.9
CID-MS/MS Scan:24862, Noise threshold:0.8
ETD-MS/MS Scan:24863, Noise threshold:1.8
Relative Intensity (%)
m/z

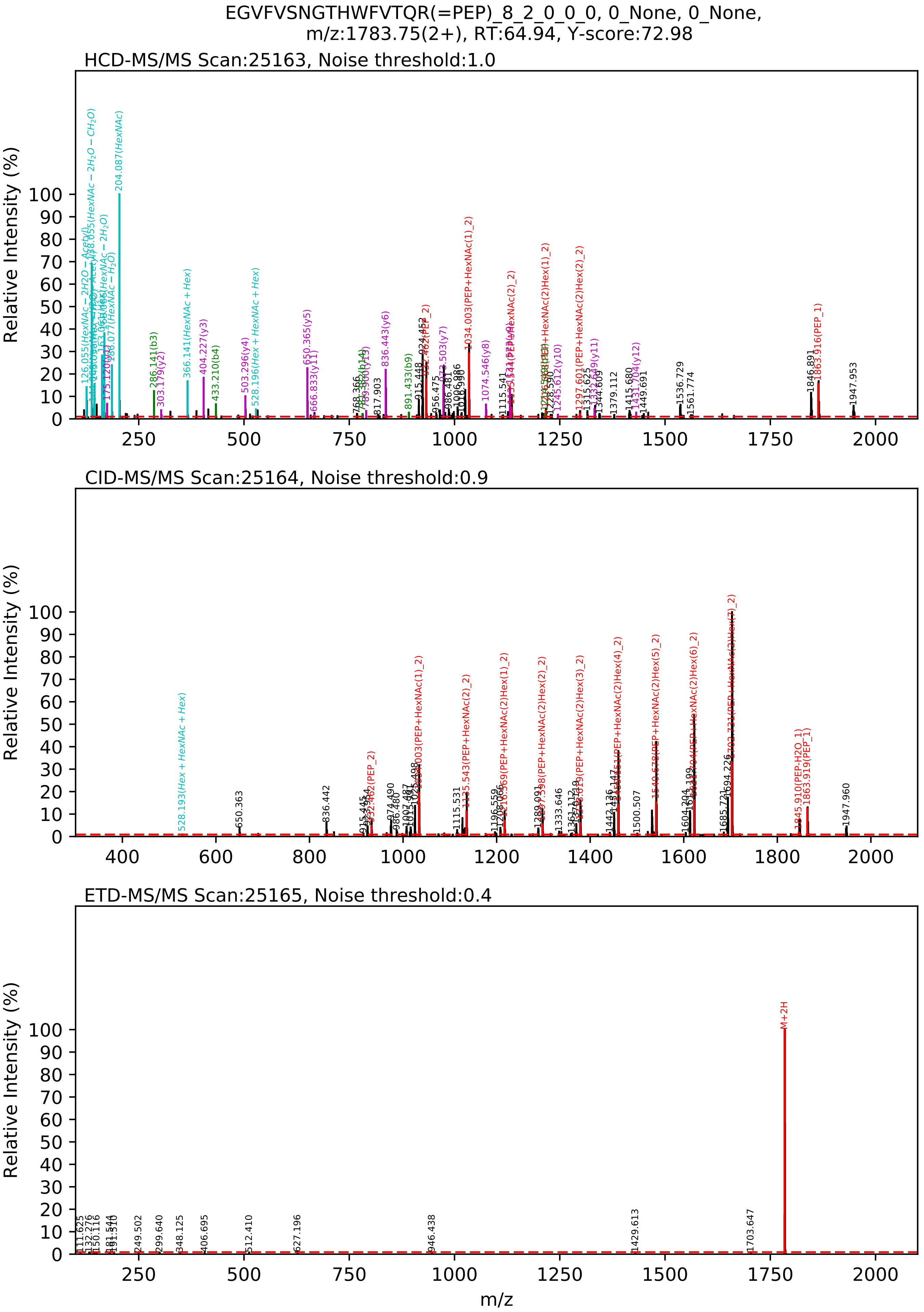
EGVFVSNGTHWFVTQR(=PEP)_8_2_0_0_0, 0_None, 0_None,
m/z:1783.75(2+), RT:64.94, Y-score:72.98
HCD-MS/MS Scan:25163, Noise threshold:1.0
Relative Intensity (%)
204.087(HexNAc)
126.055(HexNAc − 2H2O − Acetyl)
366.141(HexNAc + Hex)
528.196(Hex + HexNAc + Hex)
286.141(b3)
303.179(y2)
404.227(y3)
433.210(b4)
503.296(y4)
650.365(y5)
666.833(y11)
836.443(y6)
891.433(b9)
1034.003(PEP+HexNAc(1)_2)
1074.546(y8)
1243.612(y10)
1297.601(PEP+HexNAc(2)Hex(2)_2)
1863.916(PEP_1)
1846.891
1947.953
1536.729
1561.774
CID-MS/MS Scan:25164, Noise threshold:0.9
528.193(Hex + HexNAc + Hex)
650.363
836.442
1845.910(PEP-H2O_1)
1863.919(PEP_1)
1947.960
1694.226
ETD-MS/MS Scan:25165, Noise threshold:0.4
M+2H
946.438
1429.613
1703.647
m/z

EGVFVSNGTHWFVTQR(=PEP)_8_2_0_0_0, 0_None, 0_None,
m/z:1189.51(3+), RT:61.77, Y-score:83.06

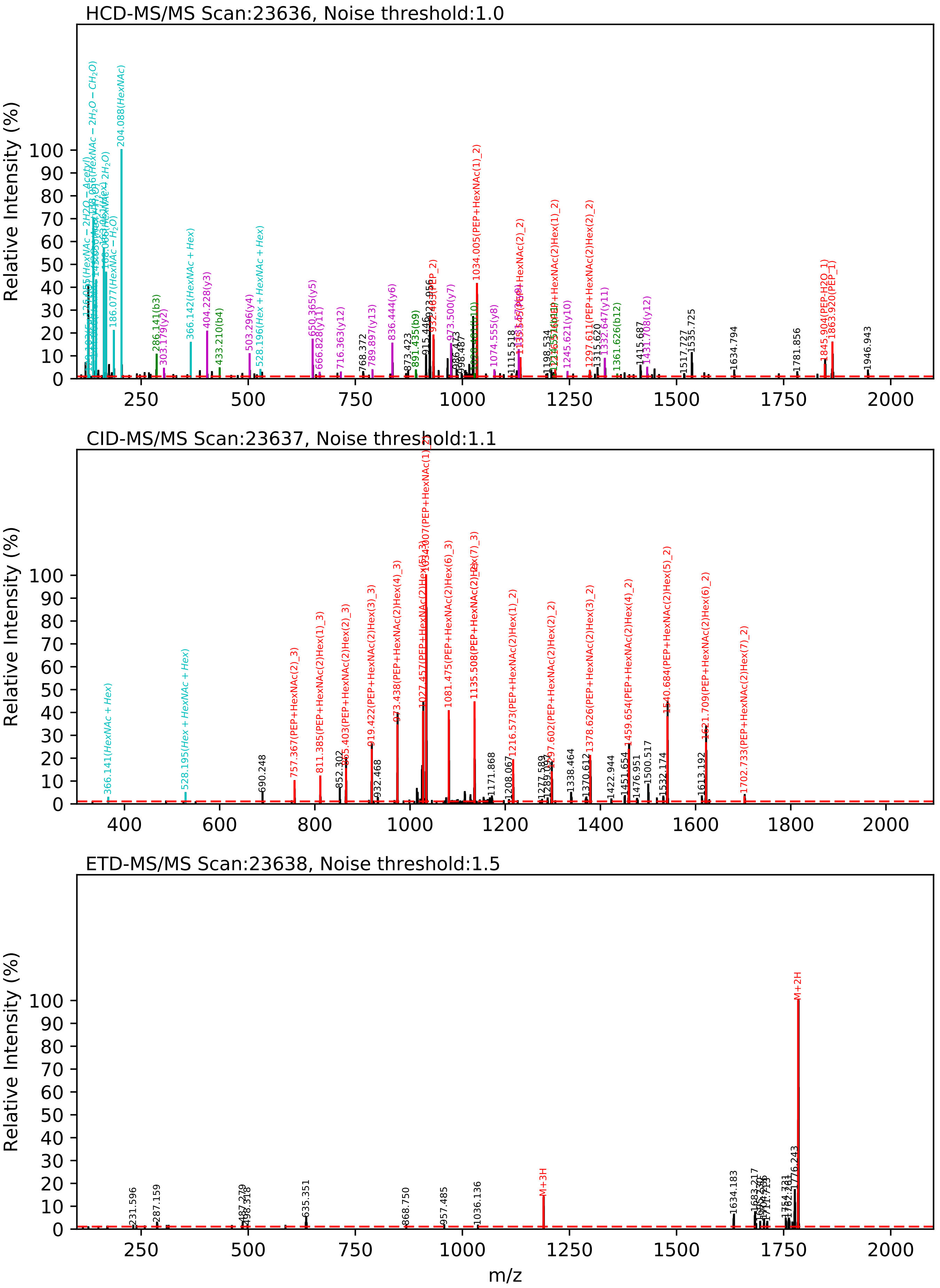

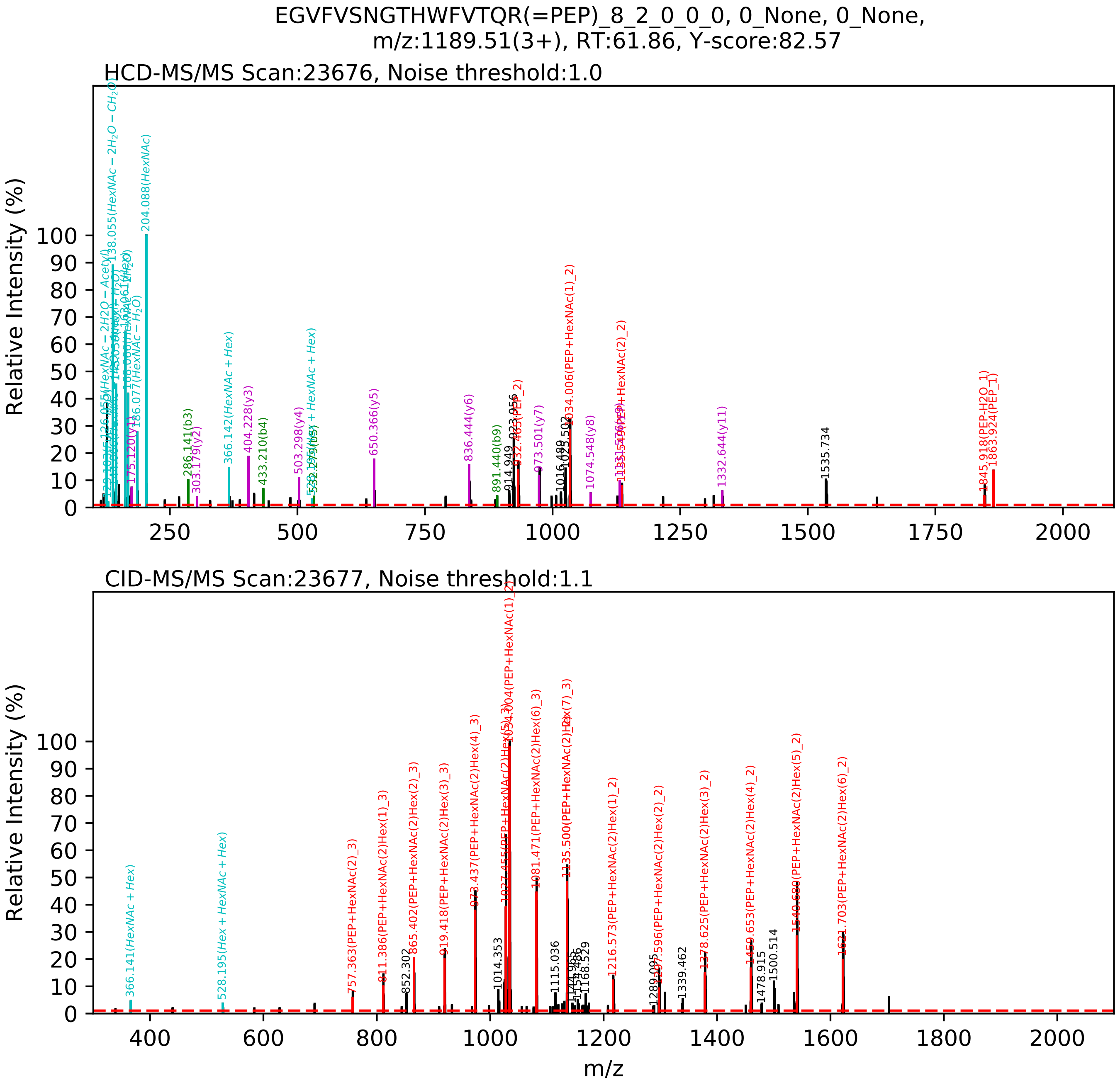
EGVFVSNGTHWFVTQR(=PEP)_8_2_0_0_0, 0_None, 0_None,
m/z:1189.51(3+), RT:61.86, Y-score:82.57
HCD-MS/MS Scan:23676, Noise threshold:1.0
Relative Intensity (%)
204.088(HexNAc)
286.141(b3)
303.179(y2)
366.142(HexNAc + Hex)
404.228(y3)
433.210(b4)
503.298(y4)
650.366(y5)
836.444(y6)
891.440(b9)
914.949
923.956
973.501(y7)
1016.489
1025.503
1034.006(PEP+HexNAc(1)_2)
1074.548(y8)
1332.644(y11)
1535.734
1845.918(PEP-H2O_1)
1863.924(PEP_1)
CID-MS/MS Scan:23677, Noise threshold:1.1
Relative Intensity (%)
366.141(HexNAc + Hex)
528.195(Hex + HexNAc + Hex)
757.363(PEP+HexNAc(2)_3)
811.386(PEP+HexNAc(2)Hex(1)_3)
852.302
865.402(PEP+HexNAc(2)Hex(2)_3)
919.418(PEP+HexNAc(2)Hex(3)_3)
973.437(PEP+HexNAc(2)Hex(4)_3)
1014.353
1081.471(PEP+HexNAc(2)Hex(6)_3)
1115.036
1135.500(PEP+HexNAc(2)Hex(7)_3)
1216.573(PEP+HexNAc(2)Hex(1)_2)
1289.095
1339.462
1378.625(PEP+HexNAc(2)Hex(3)_2)
1459.653(PEP+HexNAc(2)Hex(4)_2)
1478.915
1500.514
1540.680(PEP+HexNAc(2)Hex(5)_2)
1621.703(PEP+HexNAc(2)Hex(6)_2)
m/z

EGVFVSNGTHWFVTQR(=PEP)_8_2_0_0_0, 0_None, 0_None,
m/z:1189.51(3+), RT:62.98, Y-score:84.35

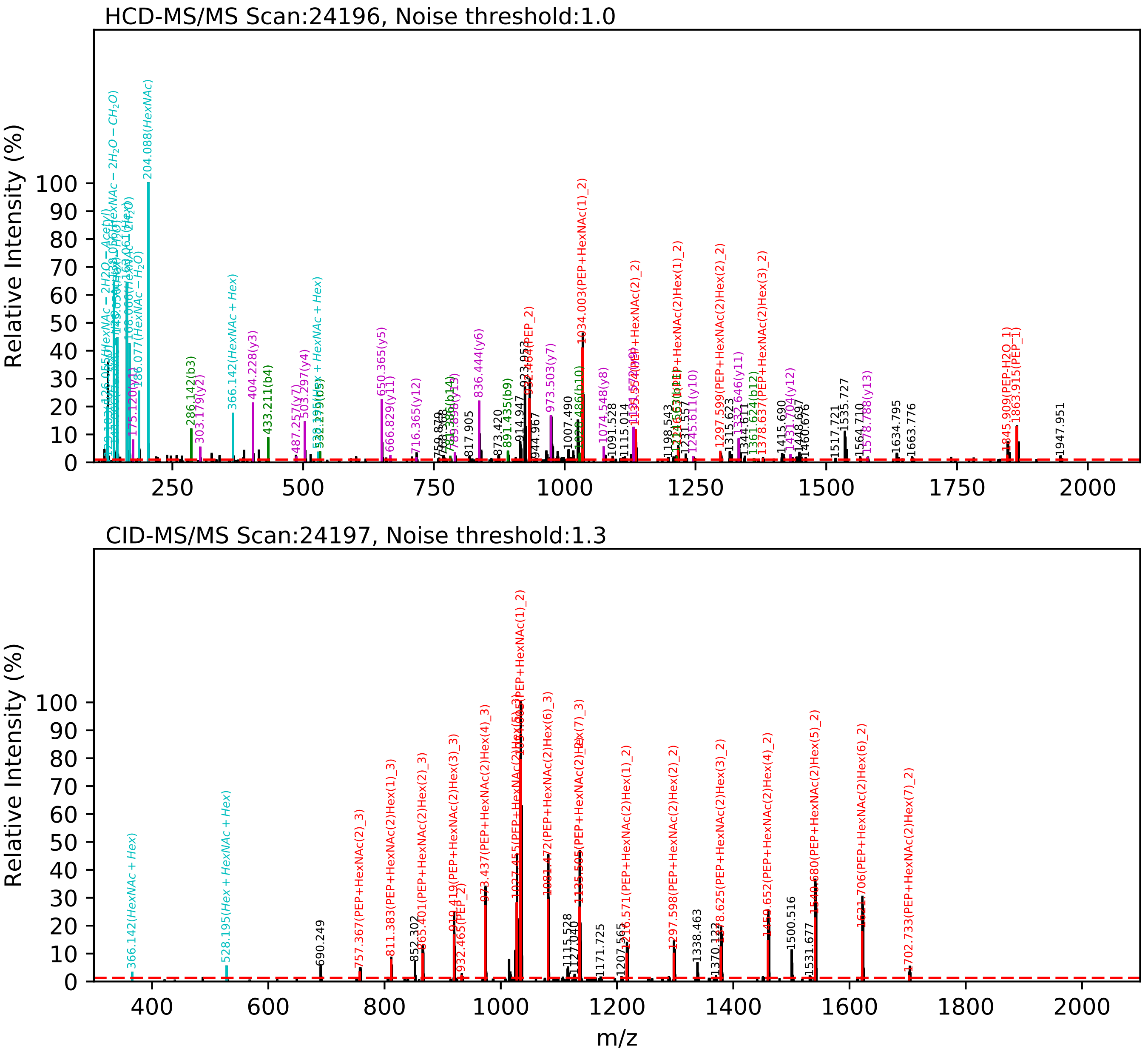

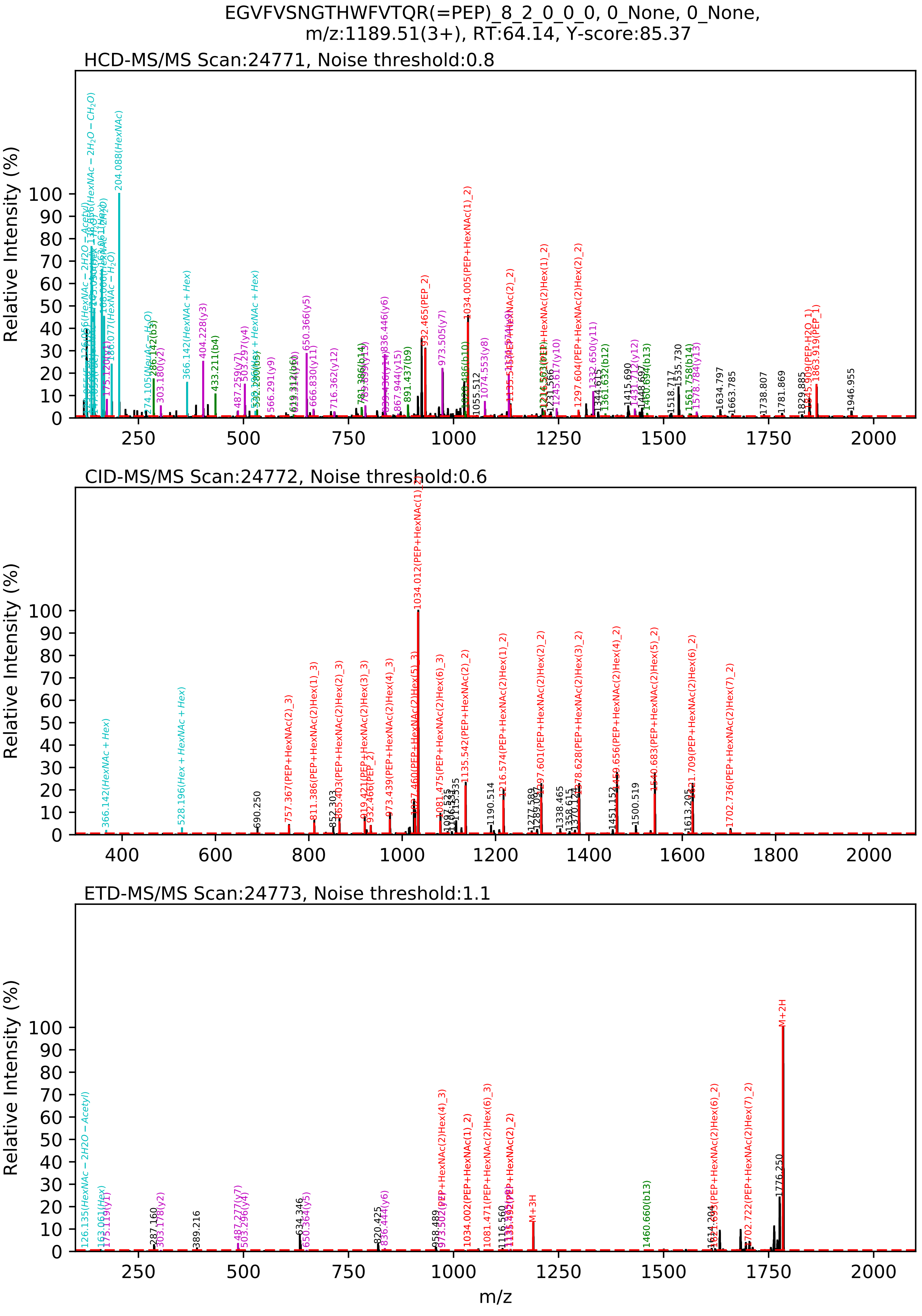
EGVFVSNGTHWFVTQR(=PEP)_8_2_0_0_0, 0_None, 0_None,
m/z:1189.51(3+), RT:64.14, Y-score:85.37
HCD-MS/MS Scan:24771, Noise threshold:0.8
Relative Intensity (%)
204.088(HexNAc)
366.142(HexNAc + Hex)
404.228(y3)
433.211(b4)
303.180(y2)
650.366(y5)
836.446(y6)
932.465(PEP_2)
973.505(y7)
1034.005(PEP+HexNAc(1)_2)
1074.553(y8)
1297.604(PEP+HexNAc(2)Hex(2)_2)
1332.650(y11)
1361.632(b12)
1460.694(b13)
1578.784(y13)
1863.919(PEP_1)
1946.955
CID-MS/MS Scan:24772, Noise threshold:0.6
366.142(HexNAc + Hex)
528.196(Hex + HexNAc + Hex)
690.250
757.367(PEP+HexNAc(2)_3)
811.386(PEP+HexNAc(2)Hex(1)_3)
865.403(PEP+HexNAc(2)Hex(2)_3)
973.439(PEP+HexNAc(2)Hex(4)_3)
1034.012(PEP+HexNAc(1)_2)
1081.475(PEP+HexNAc(2)Hex(6)_3)
1135.542(PEP+HexNAc(2)_2)
1190.514
1216.574(PEP+HexNAc(2)Hex(1)_2)
1338.465
1451.152
1500.519
1540.683(PEP+HexNAc(2)Hex(5)_2)
1613.205
1702.736(PEP+HexNAc(2)Hex(7)_2)
ETD-MS/MS Scan:24773, Noise threshold:1.1
126.135(HexNAc − 2H2O − Acetyl)
287.160
303.178(y2)
389.216
487.277(y7)
503.296(y4)
634.346
650.364(y5)
820.425
836.444(y6)
958.489
1034.002(PEP+HexNAc(1)_2)
1081.471(PEP+HexNAc(2)Hex(6)_3)
1116.560
M+3H
1460.660(b13)
1702.722(PEP+HexNAc(2)Hex(7)_2)
1776.250
M+2H
m/z

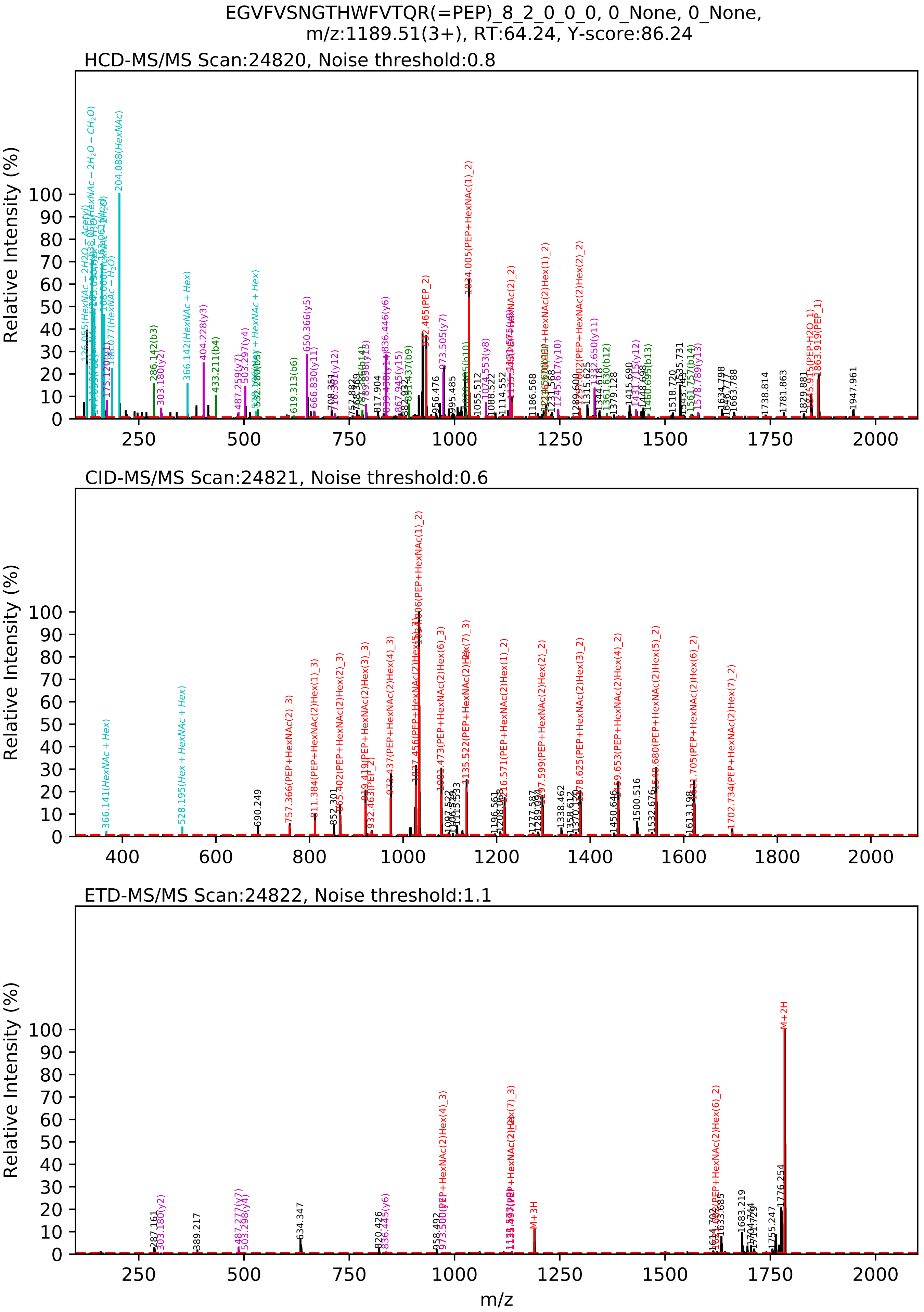

EGVFVSNGTHWFVTQR(=PEP)_8_2_0_0_0, 0_None, 0_None,
m/z:1189.51(3+), RT:64.24, Y-score:86.24
HCD-MS/MS Scan:24820, Noise threshold:0.8
Relative Intensity (%)
204.088(HexNAc)
1034.005(PEP+HexNAc(1)_2)
932.465(PEP_2)
650.366(y5)
404.228(y3)
366.142(HexNAc + Hex)
286.142(b3)
433.211(b4)
973.505(y7)
836.446(y6)
1947.961
1863.919(PEP_1)
CID-MS/MS Scan:24821, Noise threshold:0.6
366.141(HexNAc + Hex)
528.195(Hex + HexNAc + Hex)
690.249
757.366(PEP+HexNAc(2)_3)
811.384(PEP+HexNAc(2)Hex(1)_3)
865.402(PEP+HexNAc(2)Hex(2)_3)
919.419(PEP+HexNAc(2)Hex(3)_3)
973.437(PEP+HexNAc(2)Hex(4)_3)
1081.473(PEP+HexNAc(2)Hex(6)_3)
1135.522(PEP+HexNAc(2)Hex(7)_3)
1216.571(PEP+HexNAc(1)_2)
1297.599(PEP+HexNAc(2)Hex(2)_2)
1378.625(PEP+HexNAc(2)Hex(3)_2)
1459.653(PEP+HexNAc(2)Hex(4)_2)
1540.680(PEP+HexNAc(2)Hex(5)_2)
1621.705(PEP+HexNAc(2)Hex(6)_2)
1702.734(PEP+HexNAc(2)Hex(7)_2)
ETD-MS/MS Scan:24822, Noise threshold:1.1
M+2H
M+3H
287.161
303.180(y2)
389.217
487.277(y7)
503.298(y4)
634.347
820.426
836.445(y6)
958.492
1633.685
1683.219
1755.247
1776.254
m/z

EGVFVSNGTHWFVTQR(=PEP)_9_2_0_0_0, 0_None, 0_None,
m/z:1243.52(3+), RT:64.45, Y-score:90.93

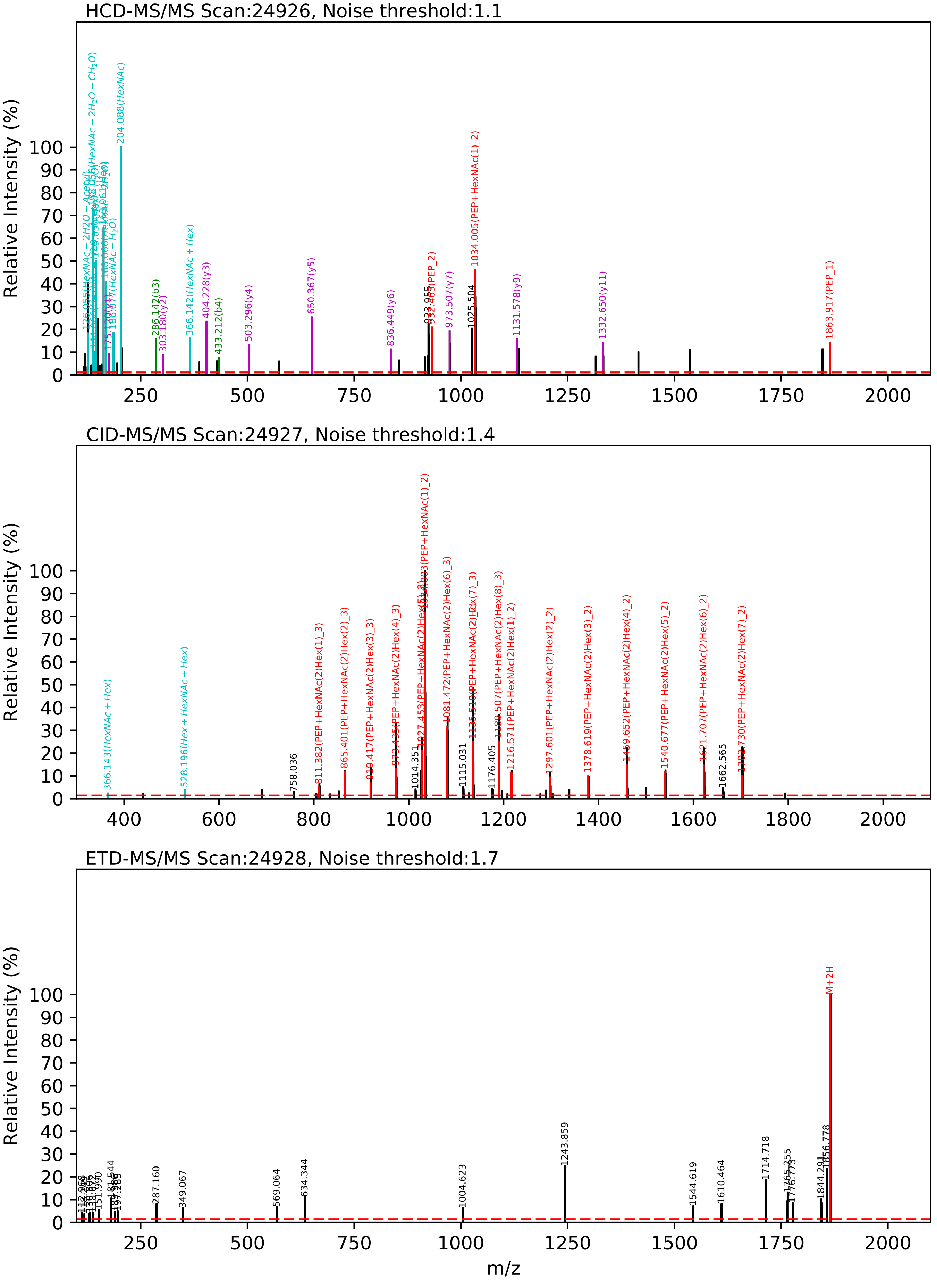

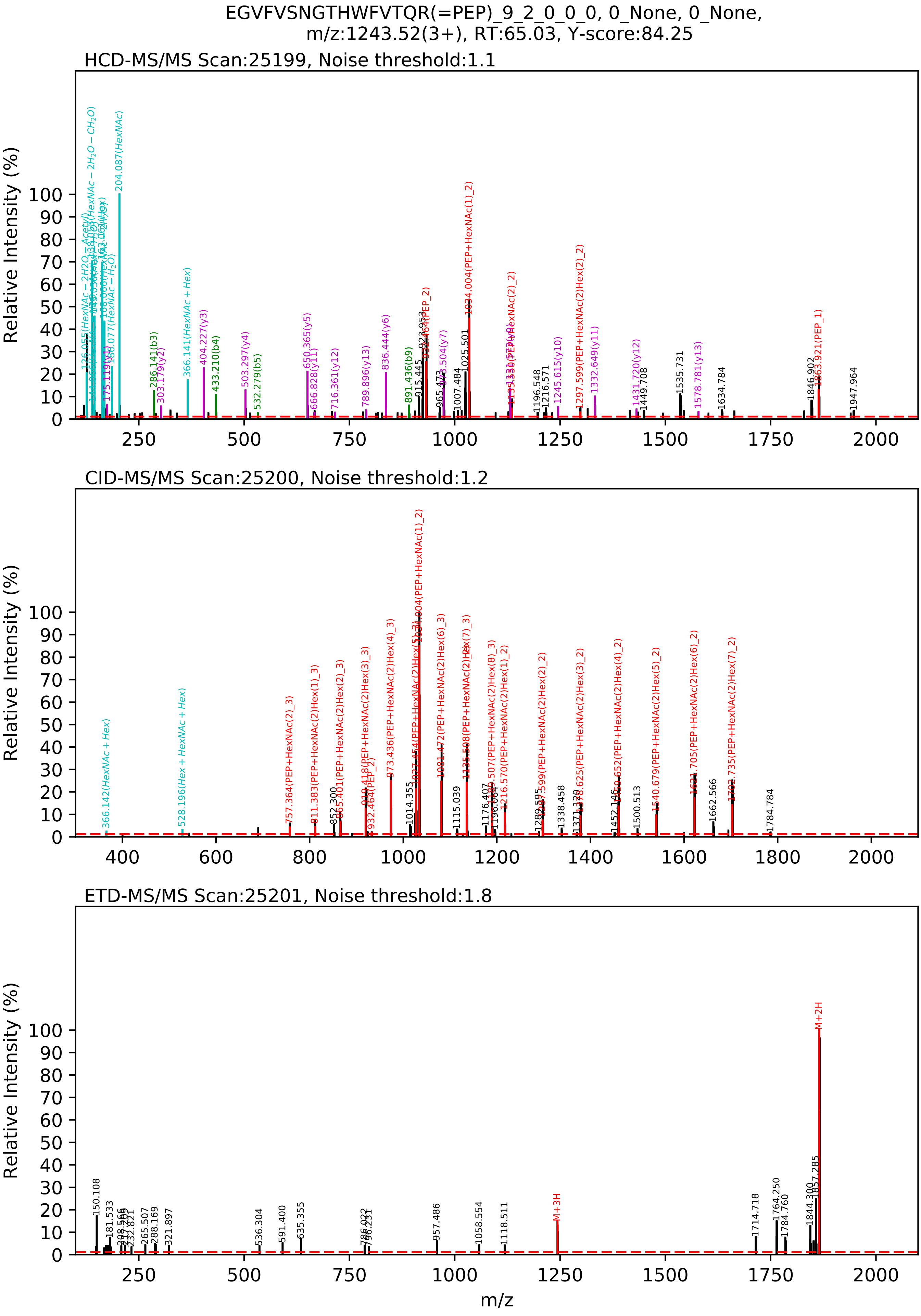

EGVFVSNGTHWFVTQR(=PEP)_9_2_0_0_0, 0_None, 0_None,
m/z:1243.52(3+), RT:65.03, Y-score:84.25
HCD-MS/MS Scan:25199, Noise threshold:1.1
Relative Intensity (%)
204.087(HexNAc)
286.141(b3)
303.179(y2)
366.141(HexNAc + Hex)
404.227(y3)
433.210(b4)
503.297(y4)
532.279(b5)
650.365(y5)
666.828(y11)
716.361(y12)
789.896(y13)
836.444(y6)
891.436(b9)
915.445
923.953
965.473
1007.484
1025.501
1034.004(PEP+HexNAc(1)_2)
1196.548
1216.571
1245.615(y10)
1297.599(PEP+HexNAc(2)Hex(2)_2)
1332.649(y11)
1431.720(y12)
1449.708
1535.731
1578.781(y13)
1634.784
1846.902
1863.921(PEP_1)
1947.964
CID-MS/MS Scan:25200, Noise threshold:1.2
366.142(HexNAc + Hex)
528.196(Hex + HexNAc + Hex)
757.364(PEP+HexNAc(2)_3)
811.383(PEP+HexNAc(2)Hex(1)_3)
865.401(PEP+HexNAc(2)Hex(2)_3)
919.418(PEP+HexNAc(2)Hex(3)_3)
932.464(PEP_2)
973.436(PEP+HexNAc(2)Hex(4)_3)
1014.355
1034.004(PEP+HexNAc(1)_2)
1081.472(PEP+HexNAc(2)Hex(6)_3)
1115.039
1135.508(PEP+HexNAc(2)Hex(7)_3)
1176.407
1216.570(PEP+HexNAc(2)Hex(1)_2)
1289.595
1297.599(PEP+HexNAc(2)Hex(2)_2)
1338.458
1378.625(PEP+HexNAc(2)Hex(3)_2)
1452.146
1459.652(PEP+HexNAc(2)Hex(4)_2)
1500.513
1540.679(PEP+HexNAc(2)Hex(5)_2)
1621.705(PEP+HexNAc(2)Hex(6)_2)
1662.566
1702.735(PEP+HexNAc(2)Hex(7)_2)
1784.784
ETD-MS/MS Scan:25201, Noise threshold:1.8
150.108
181.533
232.821
265.507
288.169
321.897
536.304
591.400
635.355
957.486
1058.554
1118.511
M+3H
1714.718
1764.250
1784.760
1844.300
1857.285
M+2H
m/z

EGVFVSNGTHWFVTQR(=PEP)_9_2_0_0_0, 0_None, 0_None,
m/z:1243.52(3+), RT:65.55, Y-score:84.64

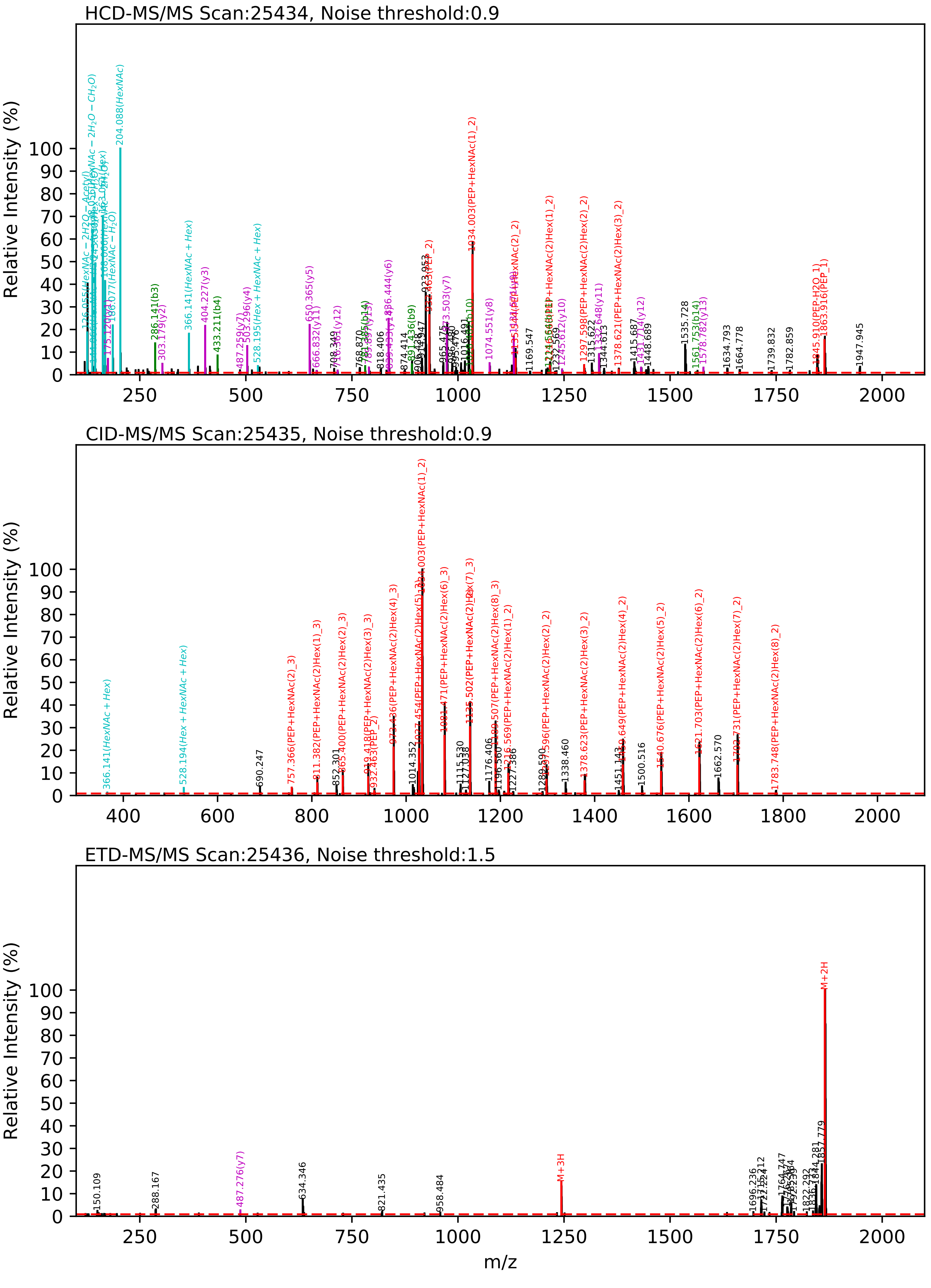

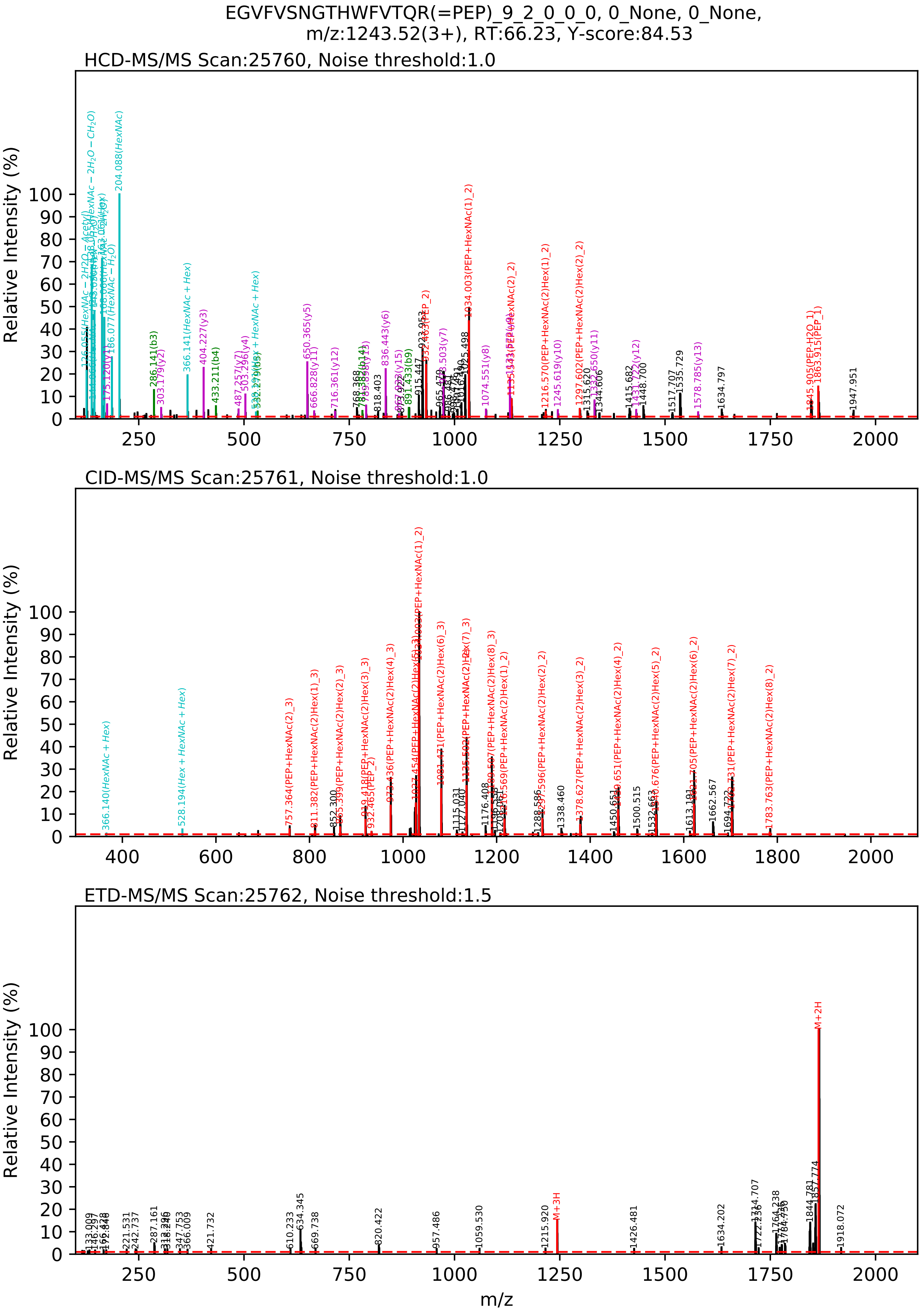

EGVFVSNGTHWFVTQR(=PEP)_9_2_0_0_0, 0_None, 0_None,
m/z:1243.52(3+), RT:66.23, Y-score:84.53
HCD-MS/MS Scan:25760, Noise threshold:1.0
CID-MS/MS Scan:25761, Noise threshold:1.0
ETD-MS/MS Scan:25762, Noise threshold:1.5
Relative Intensity (%)
m/z
204.088(HexNAc)
1034.003(PEP+HexNAc(1)_2)
650.365(y5)
836.443(y6)
404.227(y3)
366.141(HexNAc + Hex)
286.141(b3)
433.211(b4)
1863.915(PEP_1)
1845.905(PEP-H2O_1)
1216.570(PEP+HexNAc(2)Hex(1)_2)
1297.602(PEP+HexNAc(2)Hex(2)_2)
1947.951
757.364(PEP+HexNAc(2)_3)
1783.763(PEP+HexNAc(2)Hex(8)_2)
366.140(HexNAc + Hex)
528.194(Hex + HexNAc + Hex)
M+2H
M+3H
1857.774
1714.707
634.345
820.422

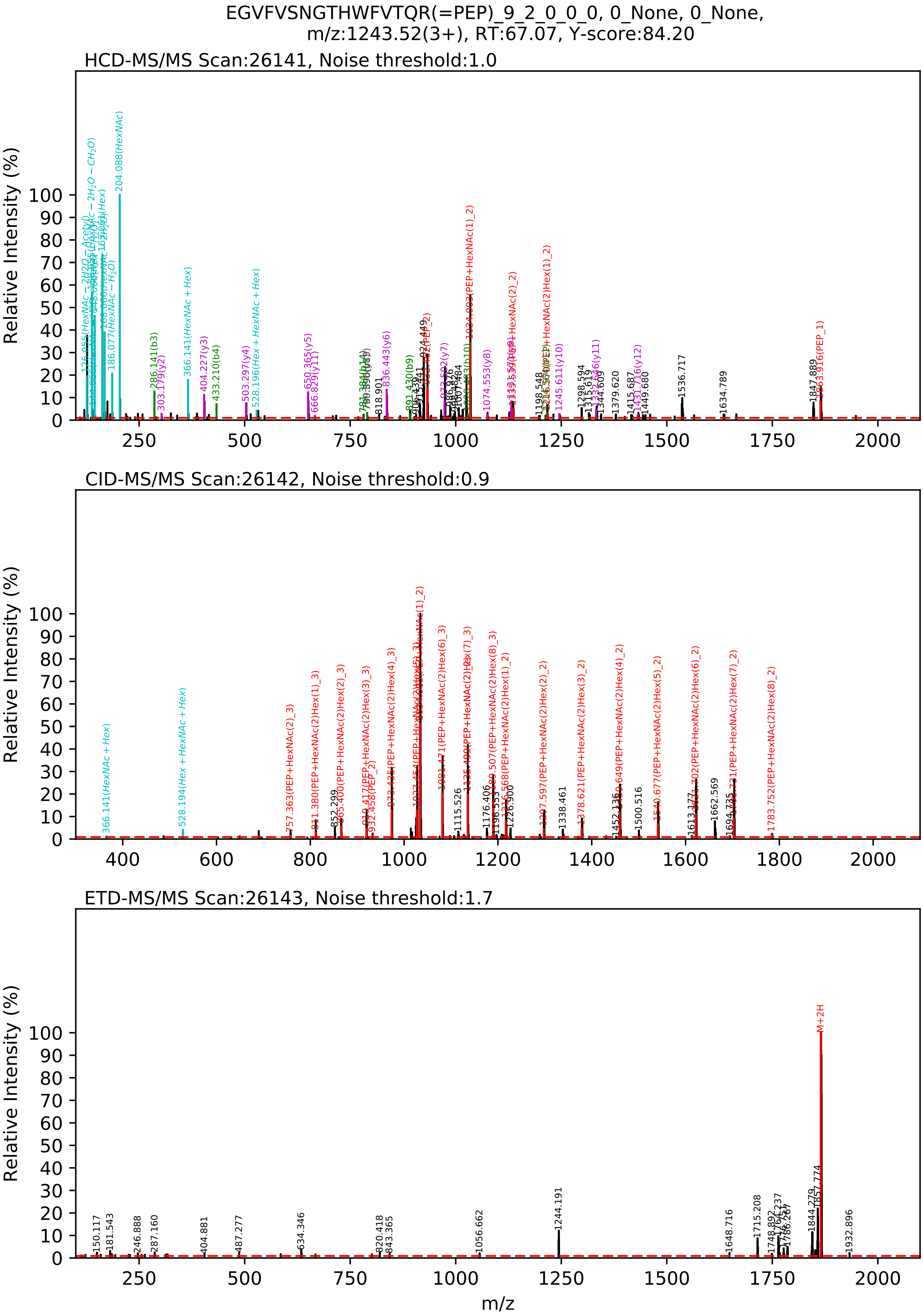
EGVFVSNGTHWFVTQR(=PEP)_9_2_0_0_0, 0_None, 0_None,
m/z:1243.52(3+), RT:67.07, Y-score:84.20
HCD-MS/MS Scan:26141, Noise threshold:1.0
Relative Intensity (%)
204.088(HexNAc)
186.077(HexNAc − H2O)
286.141(b3)
303.179(y2)
366.141(HexNAc + Hex)
404.227(y3)
433.210(b4)
503.297(y4)
528.196(Hex + HexNAc + Hex)
650.365(y5)
666.829(y11)
836.443(y6)
818.901
891.430(b9)
1034.803(PEP+HexNAc(1)_2)
1074.553(y8)
1245.611(y10)
1198.548
1298.594
1344.609
1379.620
1415.687
1449.680
1536.717
1634.789
1847.889
1863.916(PEP_1)
CID-MS/MS Scan:26142, Noise threshold:0.9
366.141(HexNAc + Hex)
528.194(Hex + HexNAc + Hex)
757.363(PEP+HexNAc(2)_3)
811.380(PEP+HexNAc(2)Hex(1)_3)
865.400(PEP+HexNAc(2)Hex(2)_3)
932.458(PEP_2)
852.299
1081.471(PEP+HexNAc(2)Hex(6)_3)
1115.526
1176.406
1196.553
1226.900
1297.597(PEP+HexNAc(2)Hex(2)_2)
1338.461
1378.621(PEP+HexNAc(2)Hex(3)_2)
1452.136
1500.516
1540.677(PEP+HexNAc(2)Hex(5)_2)
1613.177
1662.569
1694.735
1783.752(PEP+HexNAc(2)Hex(8)_2)
ETD-MS/MS Scan:26143, Noise threshold:1.7
M+2H
150.117
181.543
246.888
287.160
404.881
487.277
634.346
820.418
843.365
1056.662
1244.191
1648.716
1715.208
1748.892
1844.279
1857.774
1932.896
m/z

EGVFVSNGTHWFVTQR(=PEP)_9_2_0_0_0, 0_None, 0_None,
m/z:1243.52(3+), RT:67.35, Y-score:81.39

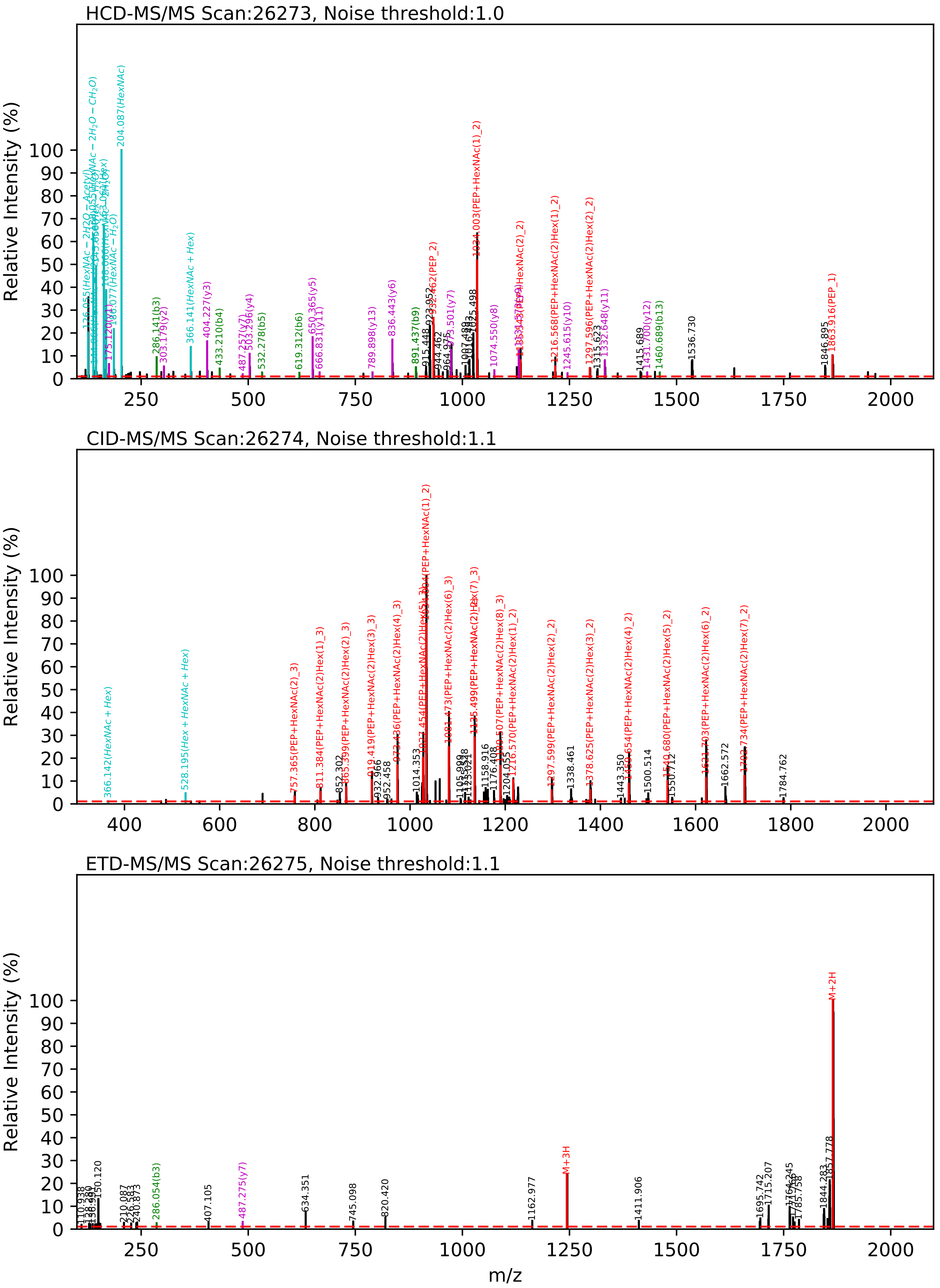

EGVFVSNGTHWFVTQR(=PEP)_9_2_0_0_0, 0_None, 0_None,
m/z:1243.52(3+), RT:68.09, Y-score:79.82

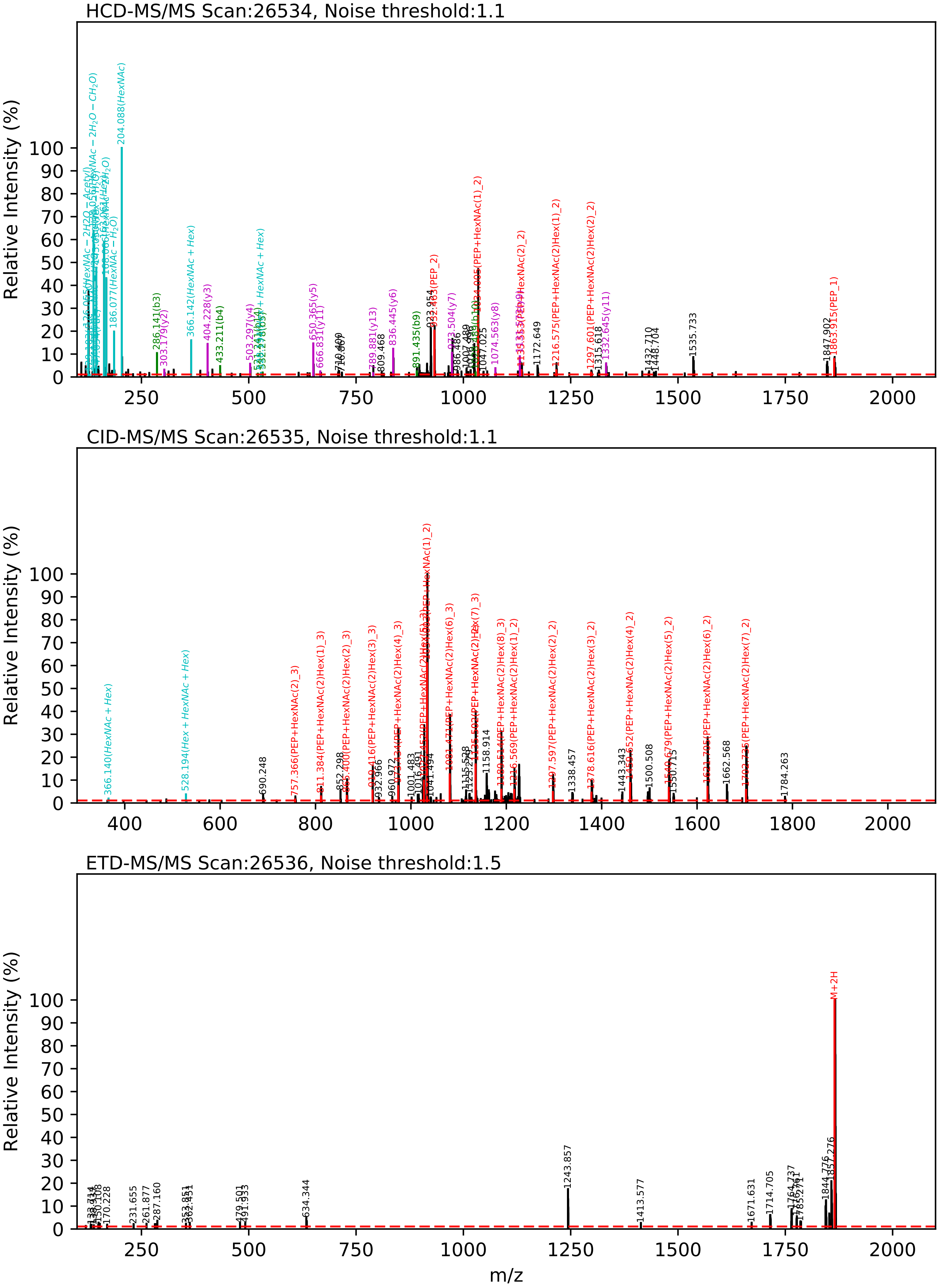

EGVFVSNGTHWFVTQR(=PEP)_9_2_0_0_0, 0_None, 0_None,
m/z:1243.52(3+), RT:68.57, Y-score:85.53

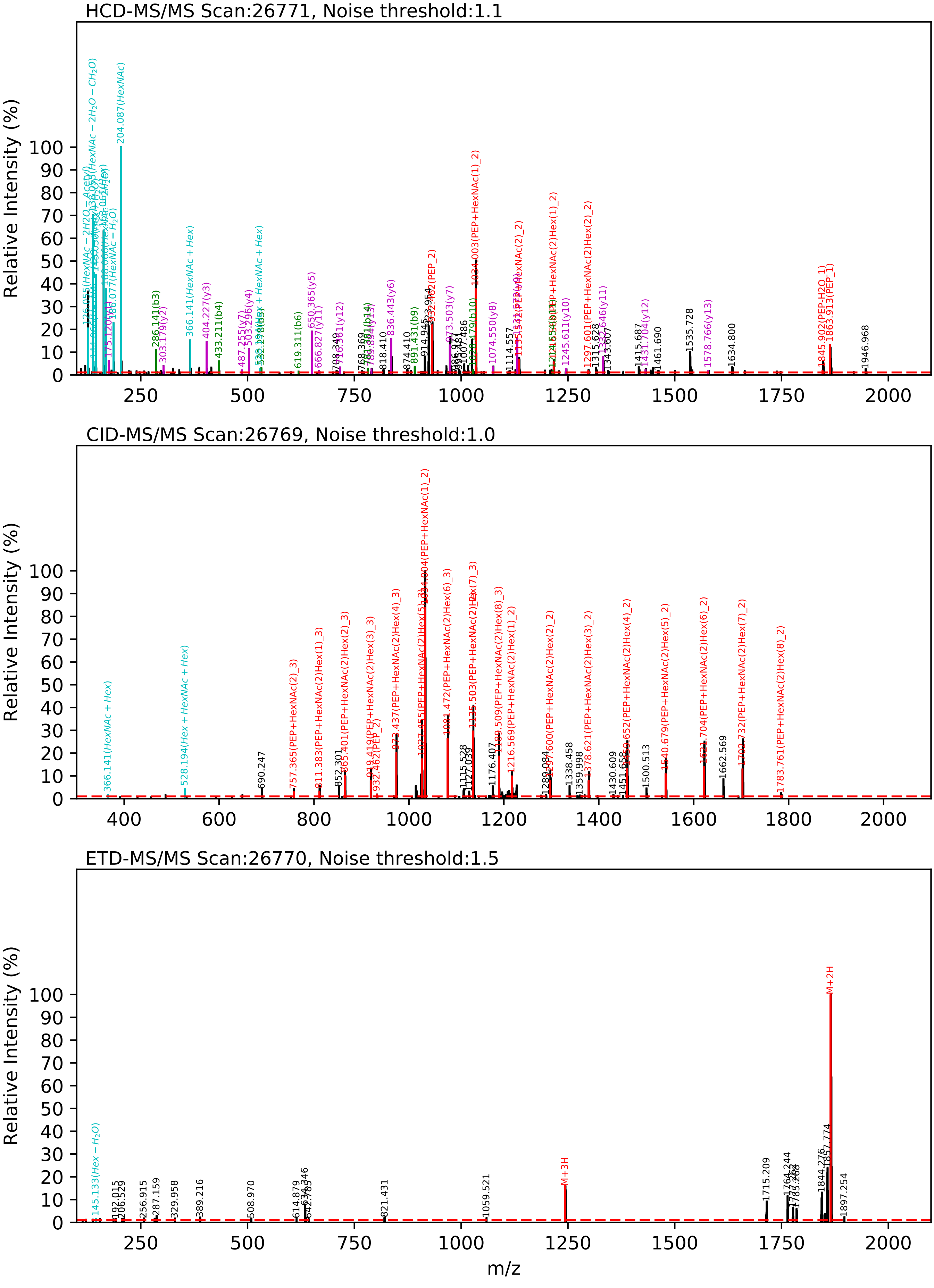

EGVFVSNGTHWFVTQR(=PEP)_9_2_0_0_0, 0_None, 0_None,
m/z:1243.52(3+), RT:74.47, Y-score:85.76

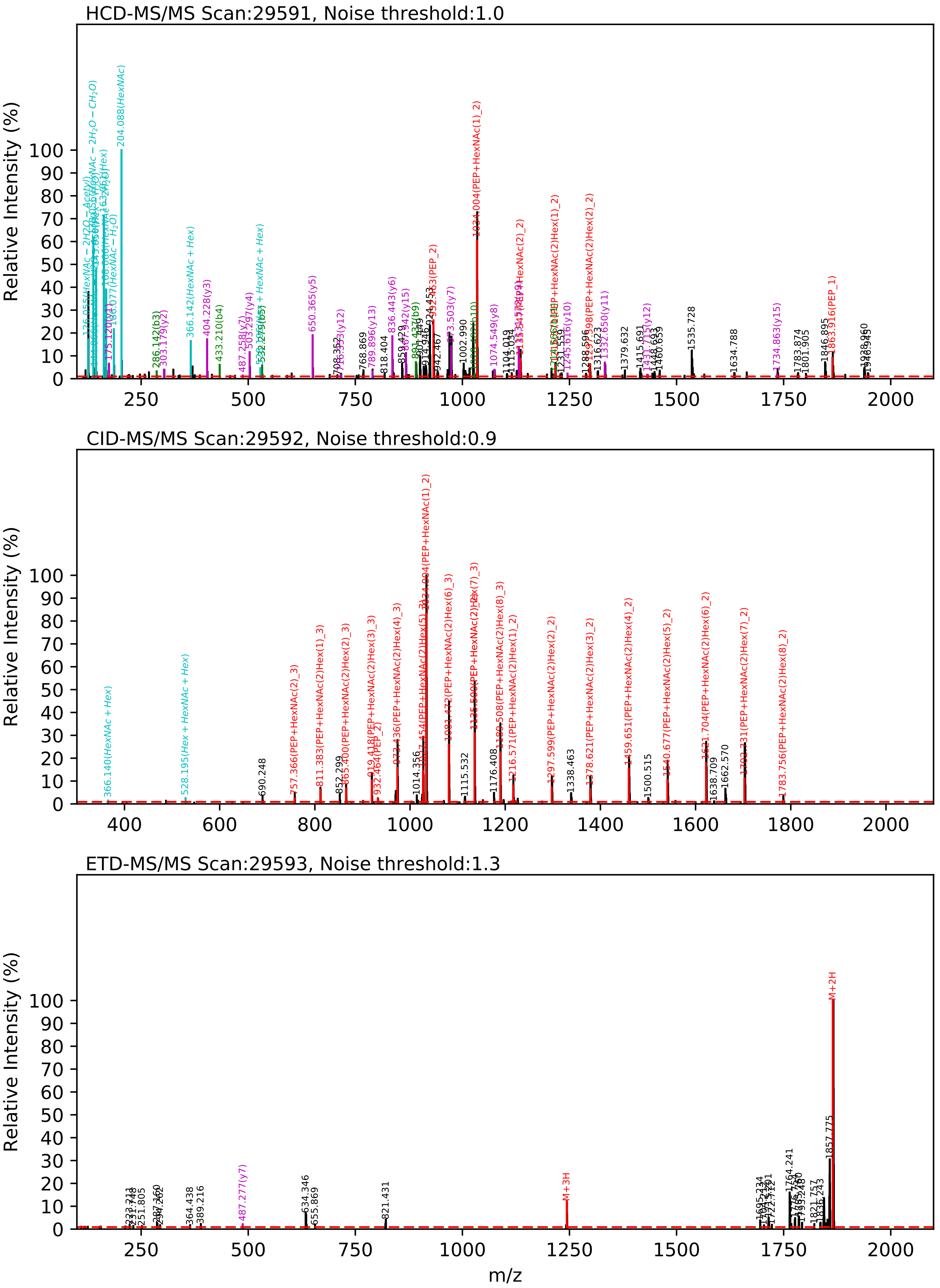

EGVFVSNGTHWFVTQR(=PEP)_9_2_0_0_0, 0_None, 0_None,
m/z:1243.52(3+), RT:62.85, Y-score:84.29

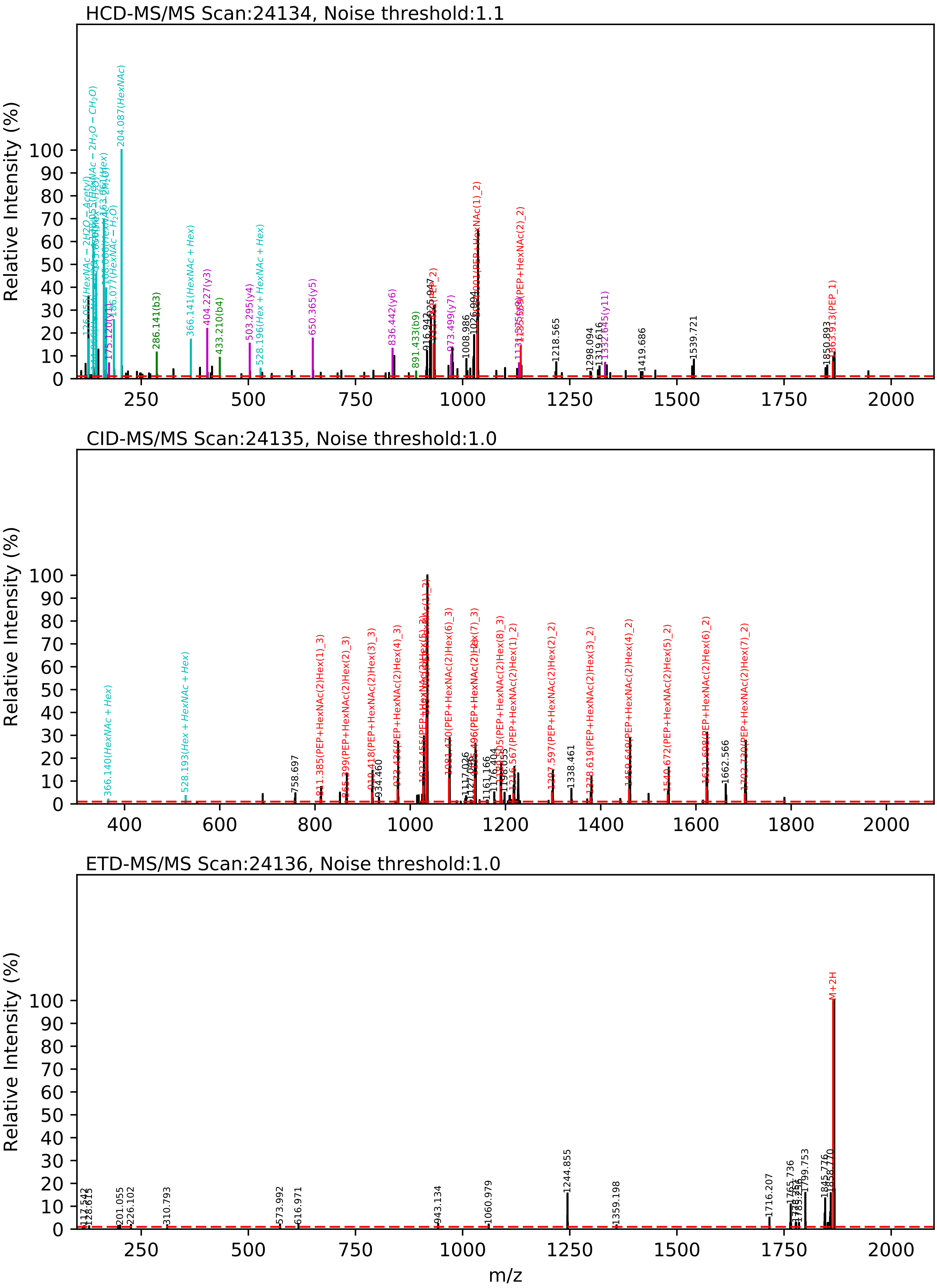

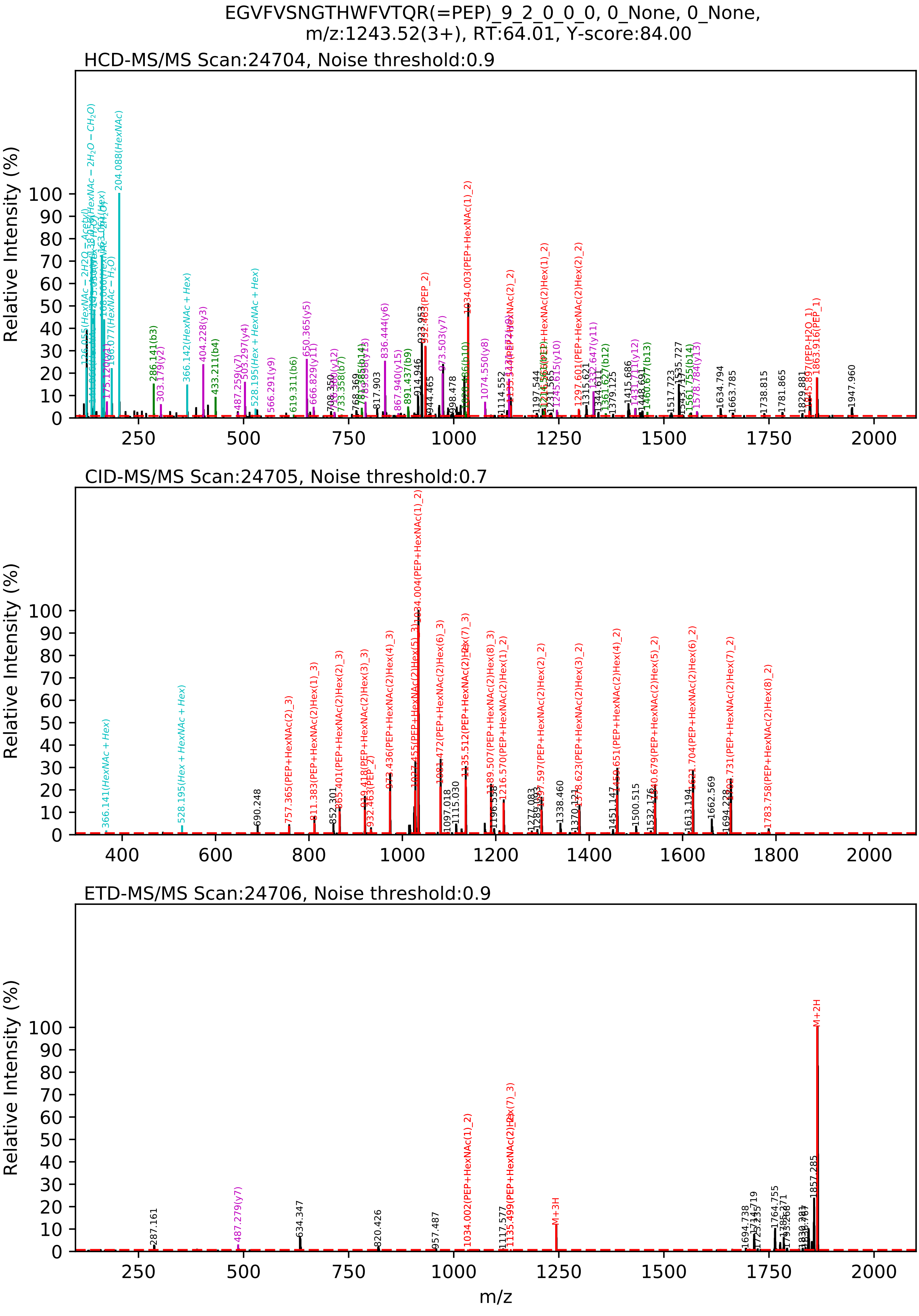

EGVFVSNGTHWFVTQR(=PEP)_9_2_0_0_0, 0_None, 0_None,
m/z:1243.52(3+), RT:64.01, Y-score:84.00
HCD-MS/MS Scan:24704, Noise threshold:0.9
CID-MS/MS Scan:24705, Noise threshold:0.7
ETD-MS/MS Scan:24706, Noise threshold:0.9
Relative Intensity (%)
m/z
204.088(HexNAc)
366.142(HexNAc + Hex)
528.195(Hex + HexNAc + Hex)
1034.003(PEP+HexNAc(1)_2)
932.463(PEP_2)
1863.916(PEP_1)
1034.004(PEP+HexNAc(1)_2)
1783.758(PEP+HexNAc(2)Hex(8)_2)
M+2H
M+3H
1857.285

NFTTAPAICHDGK(=PEP)_6_3_0_0_0, 0_None, 0_None,
m/z:1058.42(3+), RT:27.09, Y-score:48.44

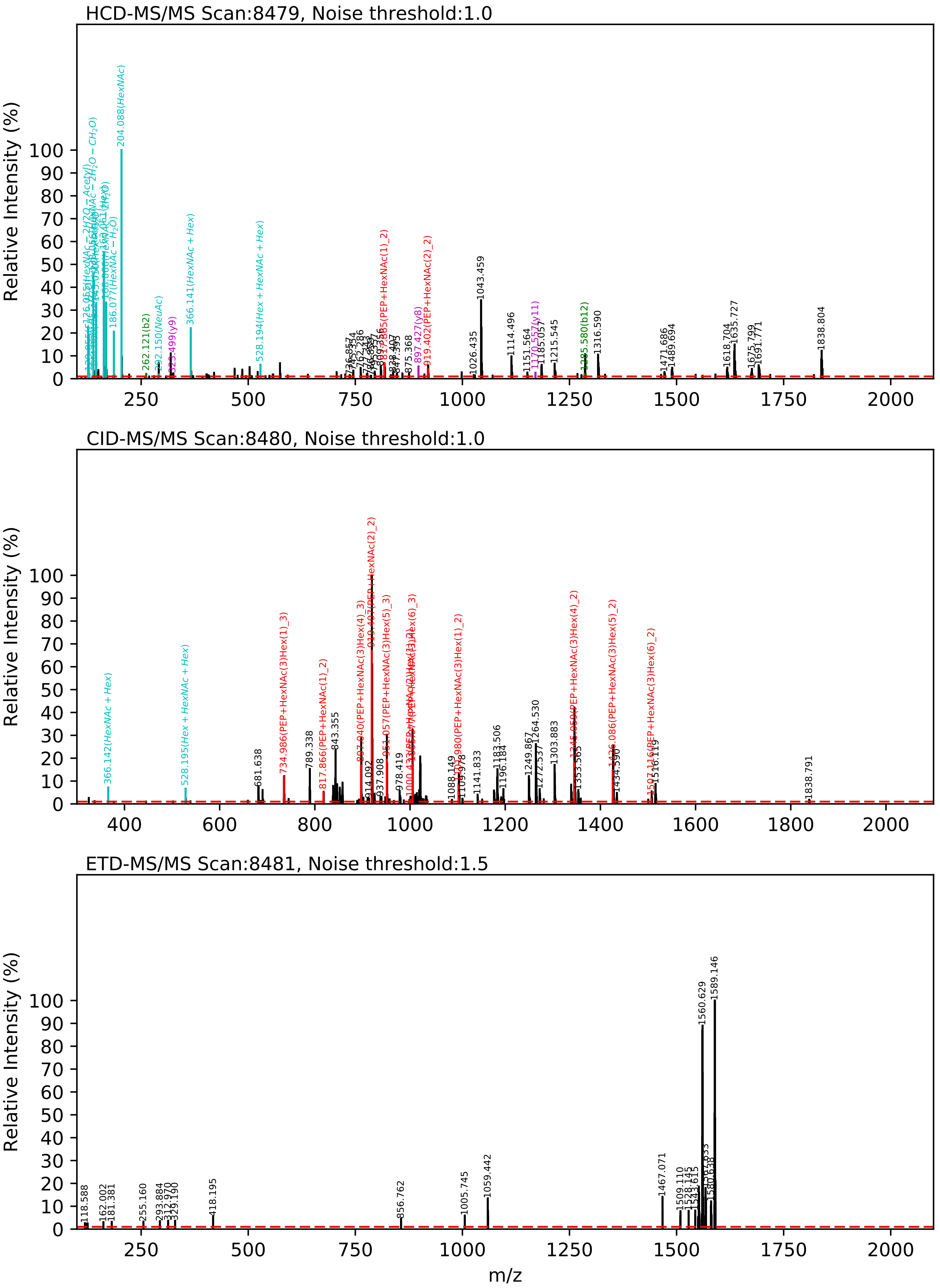

NHTSPDVDLGDISGINASVVNIQK(=PEP)_10_2_0_0_0, 0_None, 0_None,
m/z:1507.32(3+), RT:78.31, Y-score:82.41

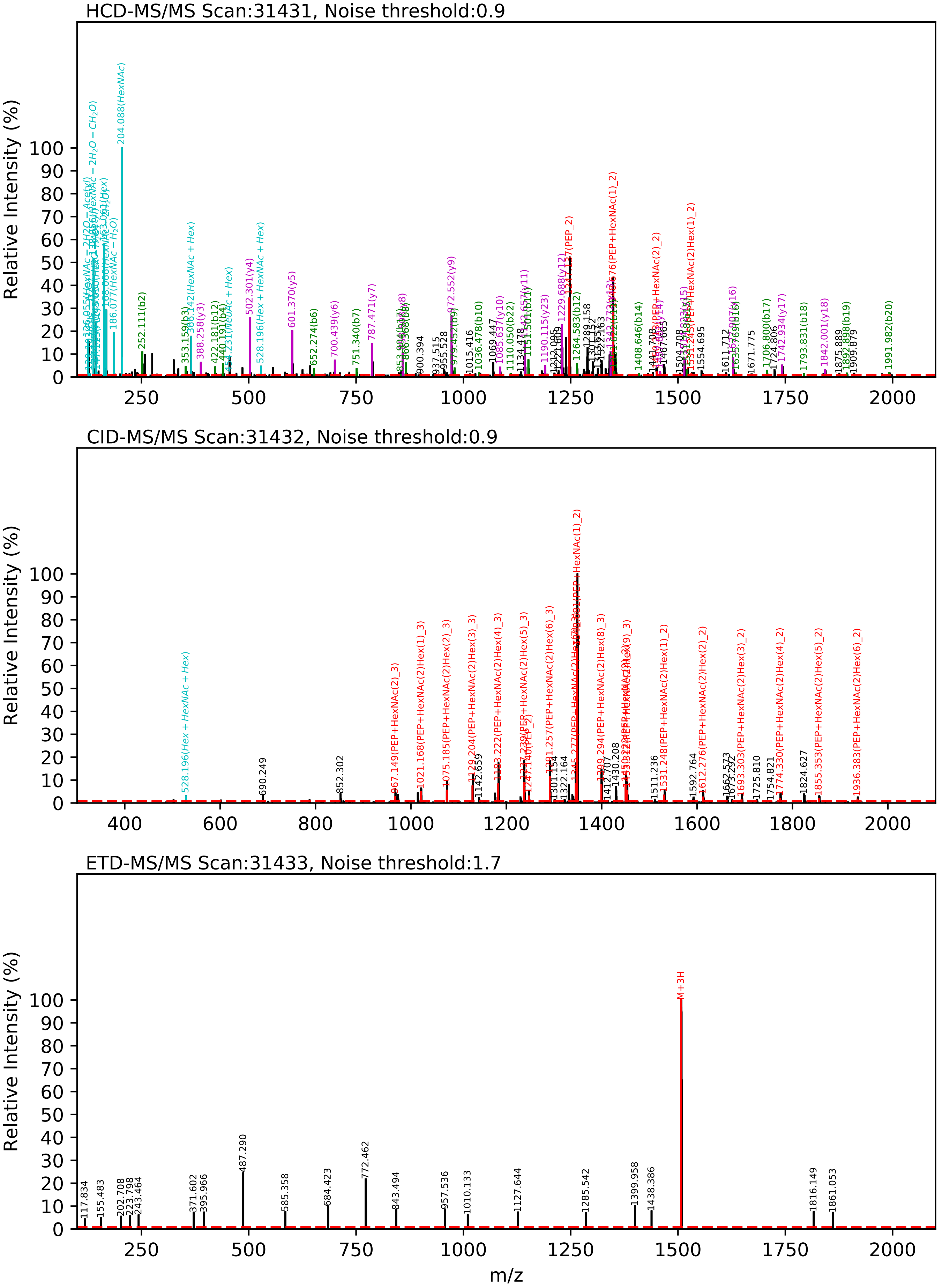

NHTSPDVDLGDISGINASVVNIQK(=PEP)_7_2_0_0_0, 0_None, 0_None,
m/z:1009.20(4+), RT:79.10, Y-score:81.68

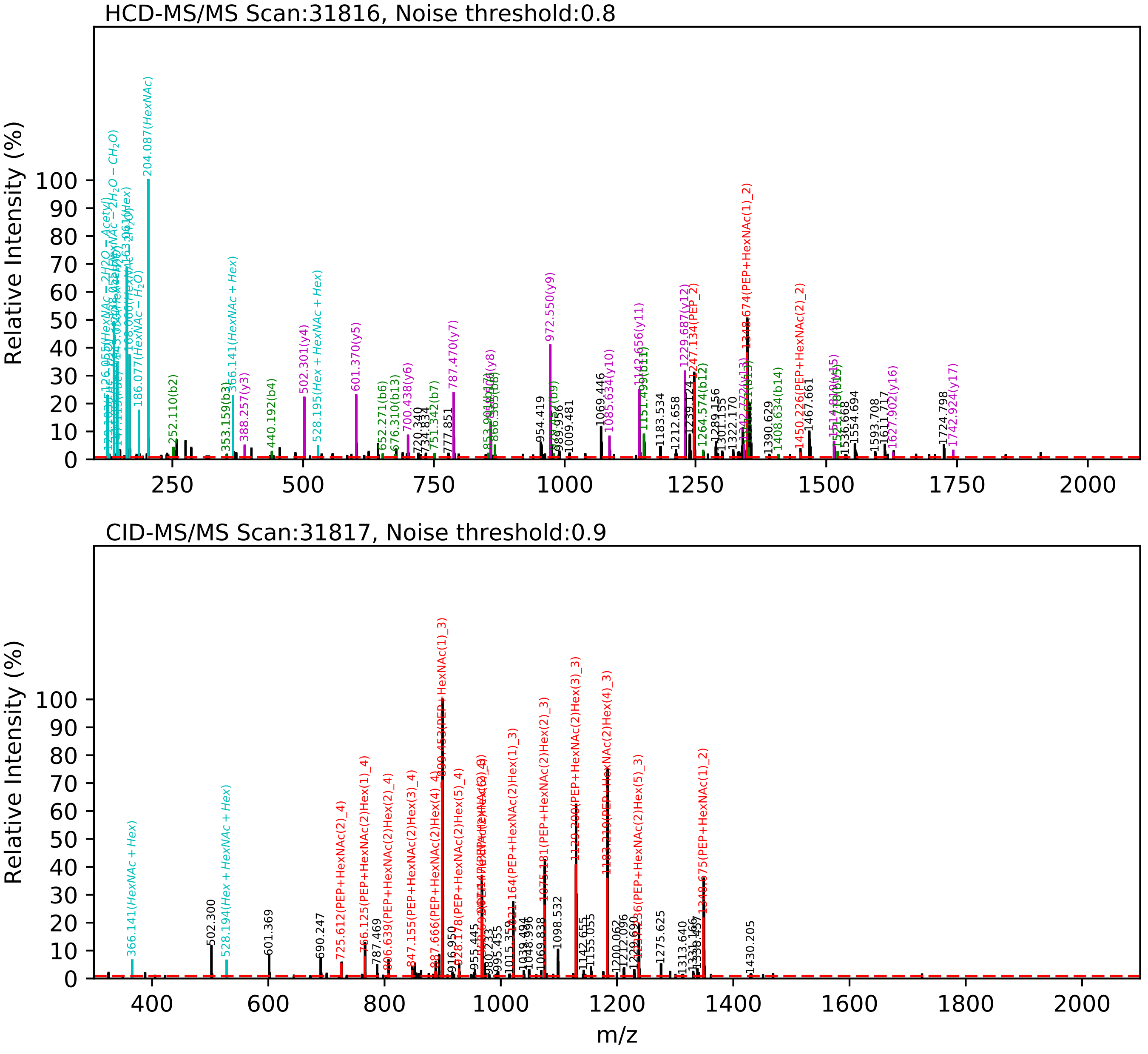

NHTSPDVDLGDISGINASVVNIQK(=PEP)_7_2_0_0_0, 0_None, 0_None,
m/z:1345.27(3+), RT:79.05, Y-score:76.91

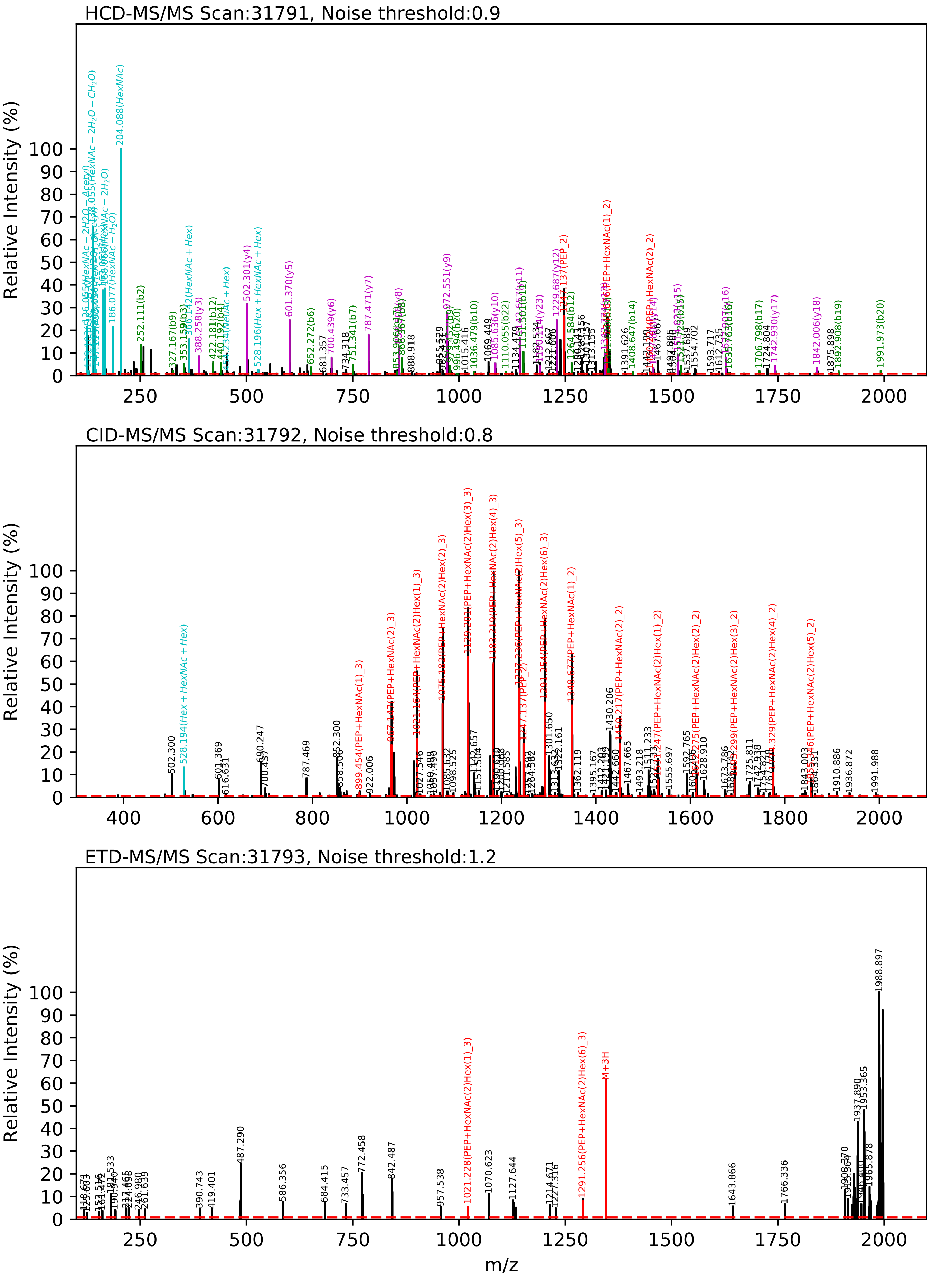

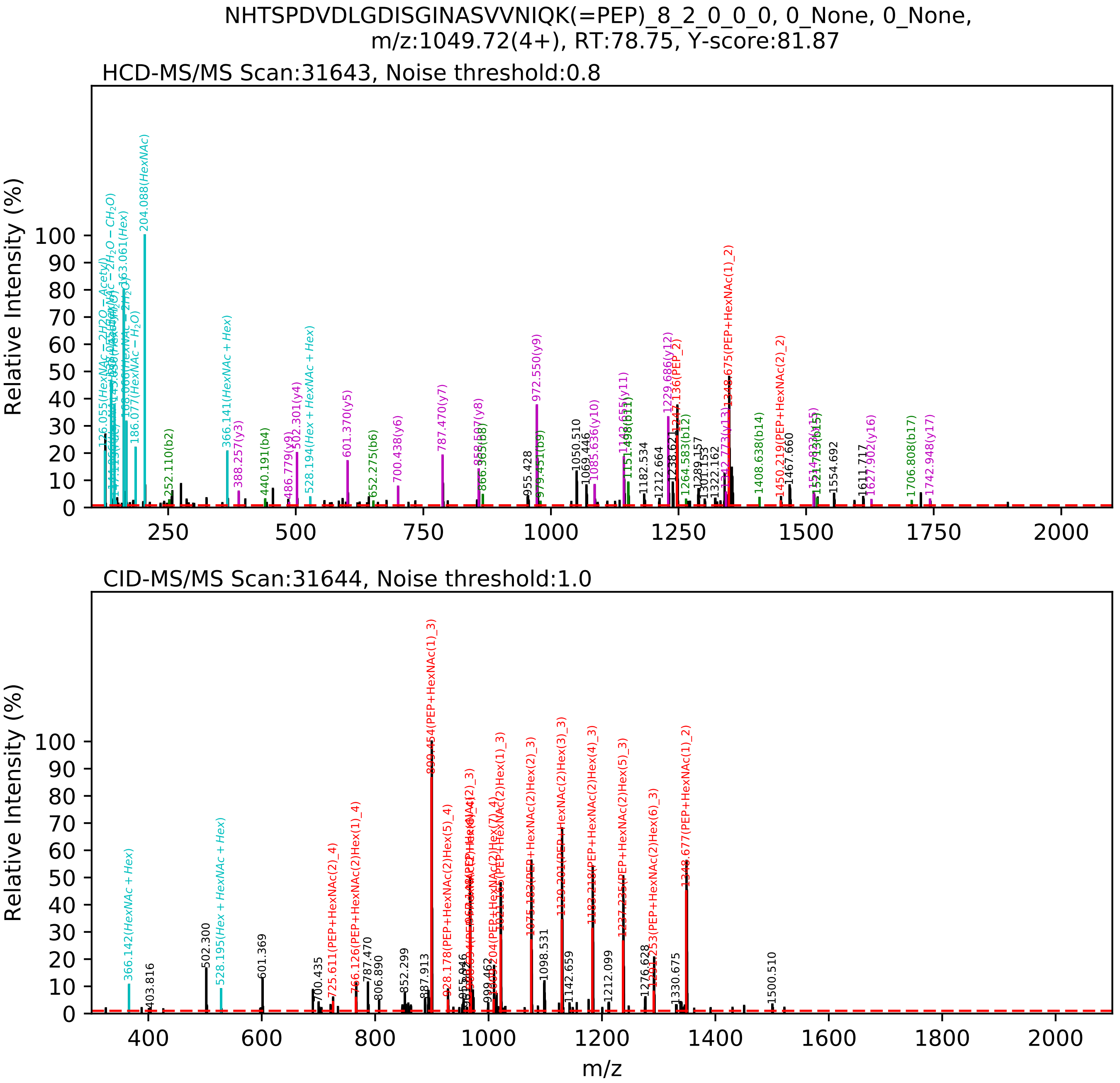
NHTSPDVDLGDISGINASVVNIQK(=PEP)_8_2_0_0_0, 0_None, 0_None,
m/z:1049.72(4+), RT:78.75, Y-score:81.87
HCD-MS/MS Scan:31643, Noise threshold:0.8
CID-MS/MS Scan:31644, Noise threshold:1.0
Relative Intensity (%)
m/z

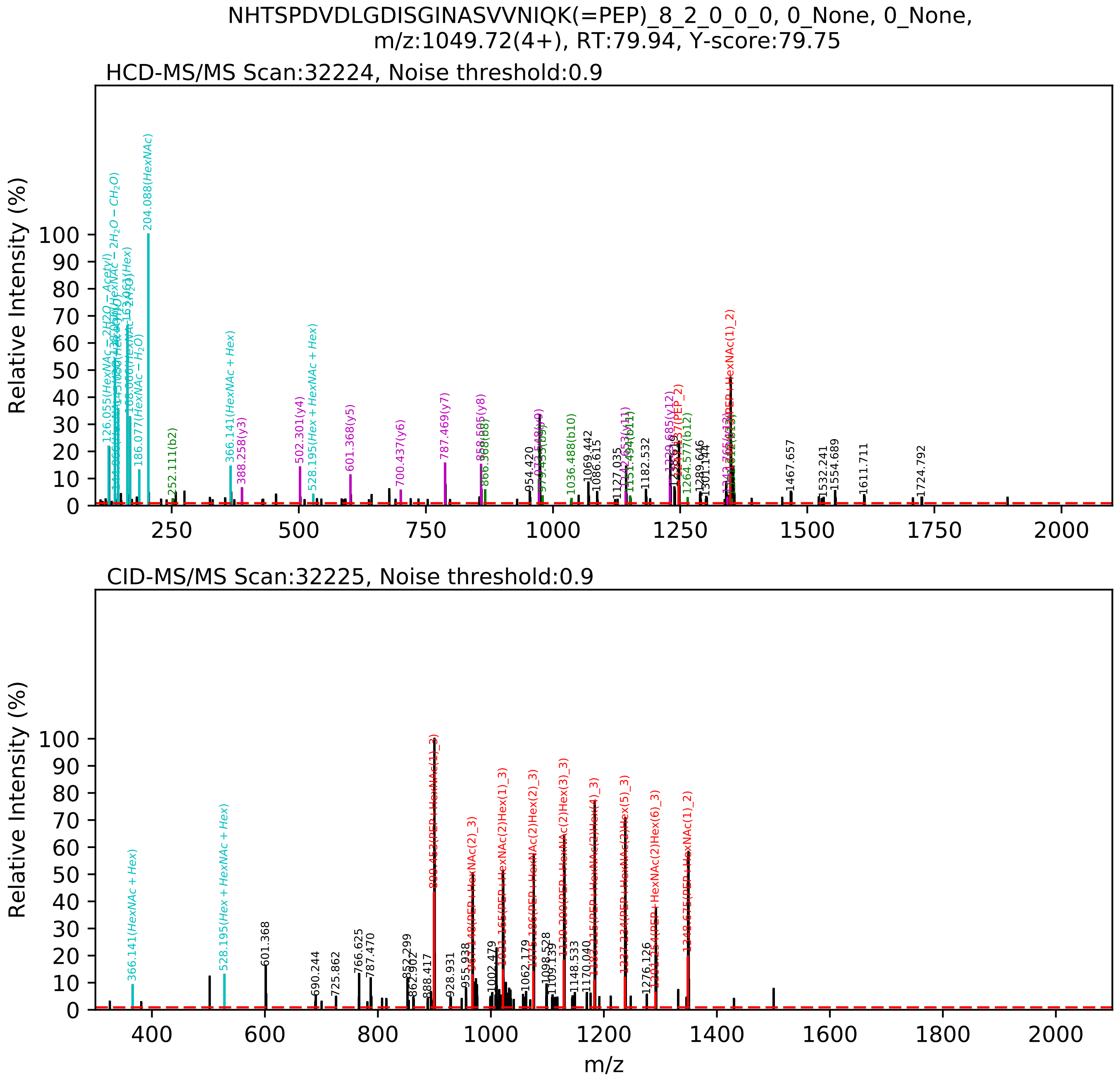
NHTSPDVDLGDISGINASVVNIQK(=PEP)_8_2_0_0_0, 0_None, 0_None,
m/z:1049.72(4+), RT:79.94, Y-score:79.75
HCD-MS/MS Scan:32224, Noise threshold:0.9
Relative Intensity (%)
204.088(HexNAc)
186.077(HexNAc − H2O)
252.111(b2)
366.141(HexNAc + Hex)
388.258(y3)
502.301(y4)
528.195(Hex + HexNAc + Hex)
601.368(y5)
700.437(y6)
787.469(y7)
858.505(y8)
954.420
1036.488(b10)
1069.442
1086.615
1127.035
1182.532
1264.577(b12)
1289.646
1301.144
1467.657
1532.241
1554.689
1611.711
1724.792
CID-MS/MS Scan:32225, Noise threshold:0.9
Relative Intensity (%)
366.141(HexNAc + Hex)
528.195(Hex + HexNAc + Hex)
601.368
690.244
725.862
766.625
787.470
852.299
862.902
888.417
928.931
955.938
1002.479
1062.179
1098.528
1148.533
1170.040
1276.126
m/z

NHTSPDVDLGDISGINASVVNIQK(=PEP)_8_2_0_0_0, 0_None, 0_None,
m/z:1399.29(3+), RT:76.60, Y-score:77.05

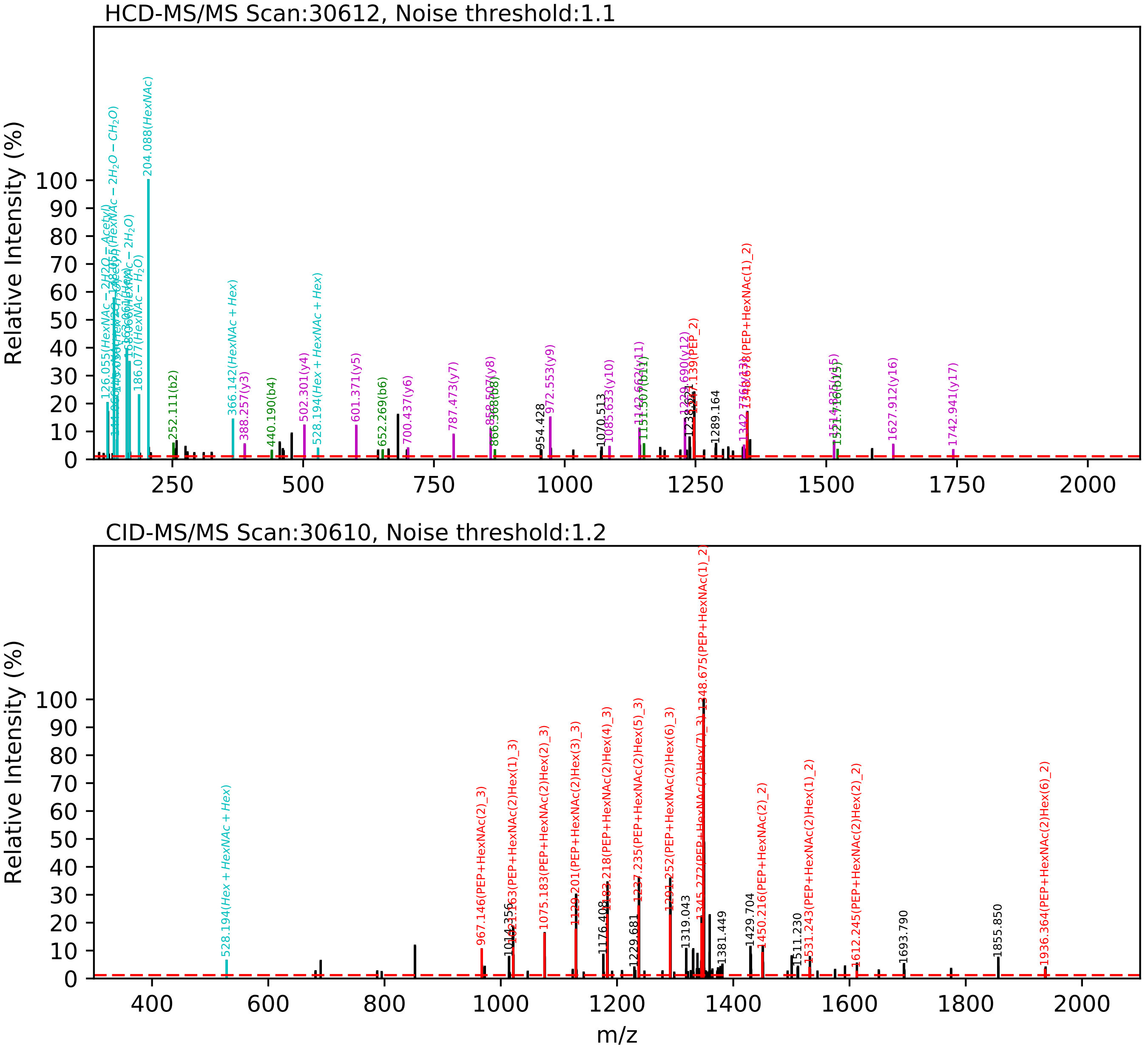

NHTSPDVDLGDISGINASVVNIQK(=PEP)_8_2_0_0_0, 0_None, 0_None,
m/z:1399.29(3+), RT:76.91, Y-score:54.31

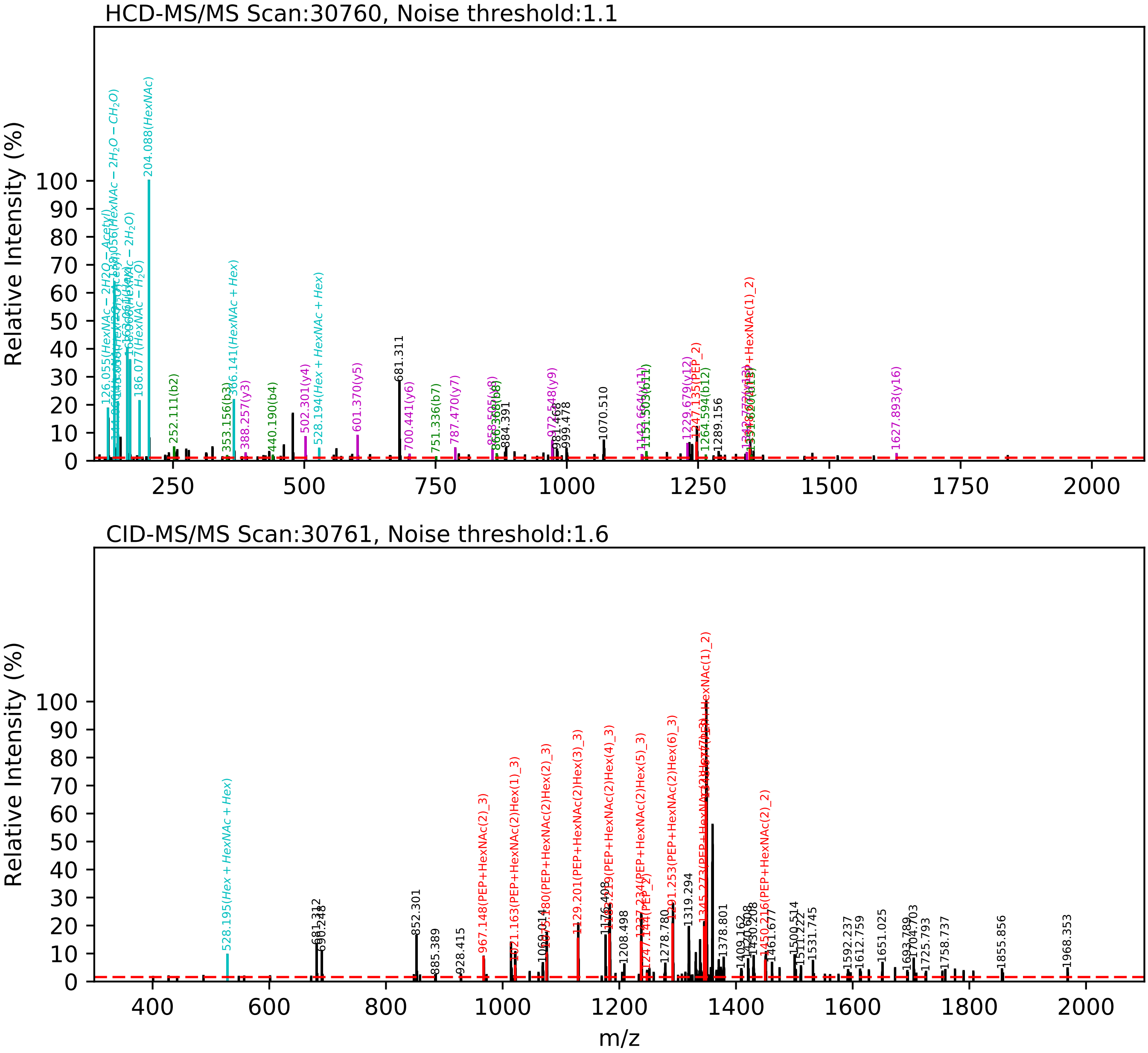

NHTSPDVDLGDISGINASVVNIQK(=PEP)_8_2_0_0_0, 0_None, 0_None,
m/z:1399.29(3+), RT:78.68, Y-score:80.58

HCD-MS/MS Scan:31608, Noise threshold:0.9

CID-MS/MS Scan:31609, Noise threshold:0.8

ETD-MS/MS Scan:31610, Noise threshold:1.6

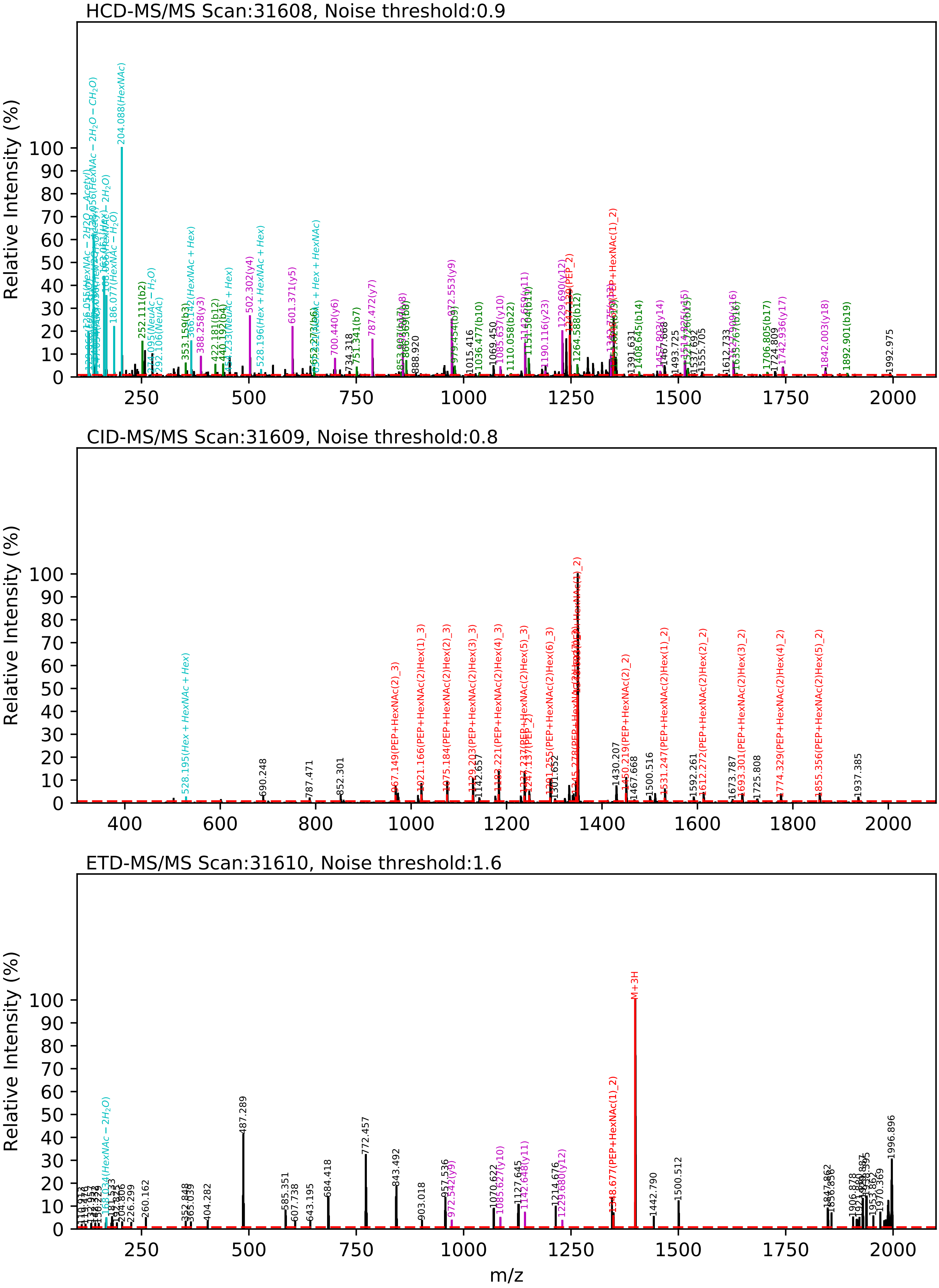

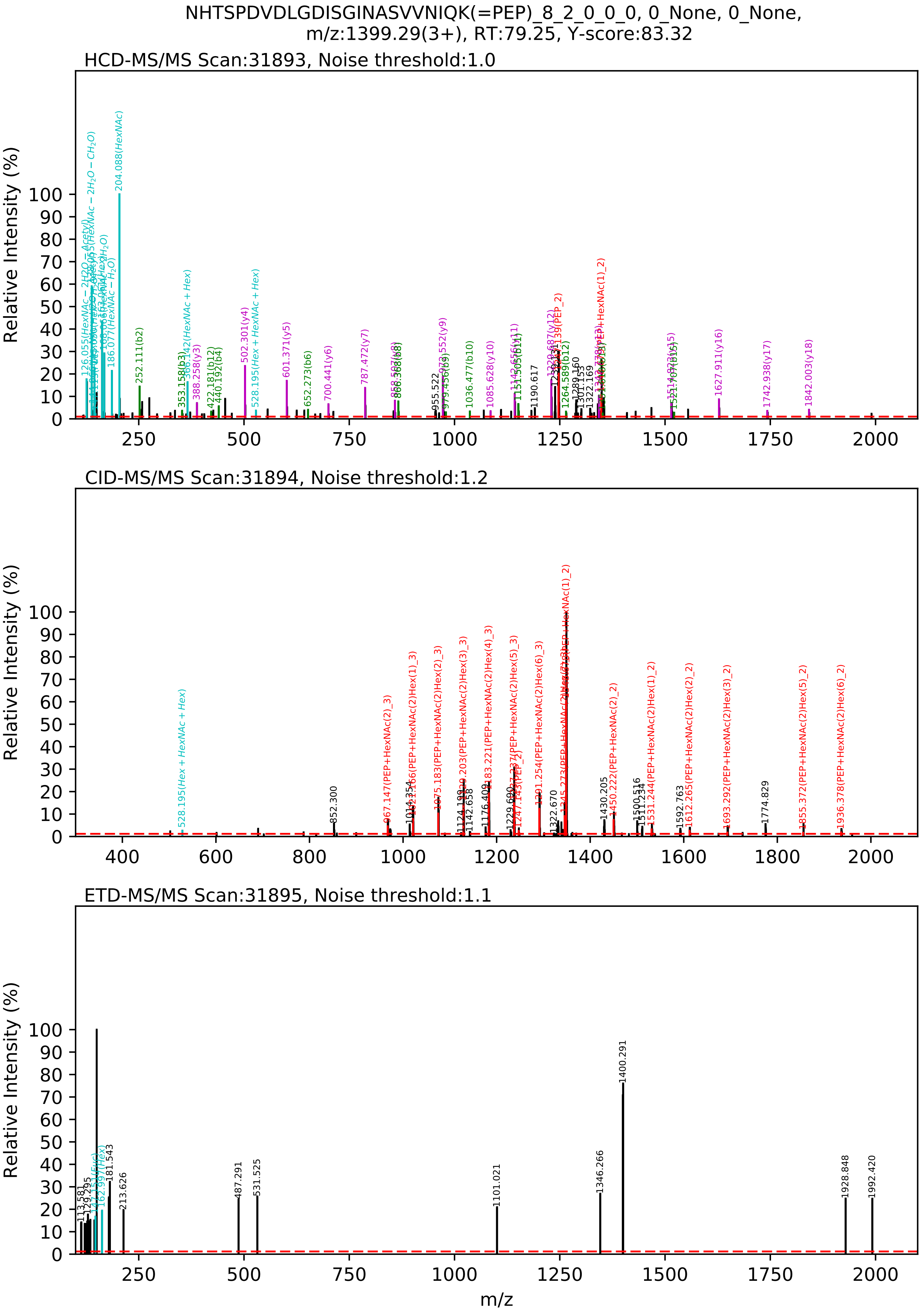
NHTSPDVDLGDISGINASVVNIQK(=PEP)_8_2_0_0_0, 0_None, 0_None,
m/z:1399.29(3+), RT:79.25, Y-score:83.32
HCD-MS/MS Scan:31893, Noise threshold:1.0
Relative Intensity (%)
204.088(HexNAc)
252.111(b2)
353.158(b3)
388.258(y3)
502.301(y4)
528.195(Hex + HexNAc + Hex)
601.371(y5)
652.273(b6)
700.441(y6)
787.472(y7)
955.522
1036.477(b10)
1085.628(y10)
1190.617
1627.911(y16)
1742.938(y17)
1842.003(y18)
CID-MS/MS Scan:31894, Noise threshold:1.2
528.195(Hex + HexNAc + Hex)
852.300
967.147(PEP+HexNAc(2)_3)
1075.183(PEP+HexNAc(2)Hex(2)_3)
1430.205
1450.222(PEP+HexNAc(2)_2)
1531.244(PEP+HexNAc(2)Hex(1)_2)
1612.265(PEP+HexNAc(2)Hex(2)_2)
1693.292(PEP+HexNAc(2)Hex(3)_2)
1774.829
1855.372(PEP+HexNAc(2)Hex(5)_2)
1936.378(PEP+HexNAc(2)Hex(6)_2)
ETD-MS/MS Scan:31895, Noise threshold:1.1
181.543
213.626
487.291
531.525
1101.021
1346.266
1400.291
1928.848
1992.420
m/z

NHTSPDVDLGDISGINASVVNIQK(=PEP)_9_2_0_0_0, 0_None, 0_None,
m/z:1453.30(3+), RT:78.80, Y-score:82.69

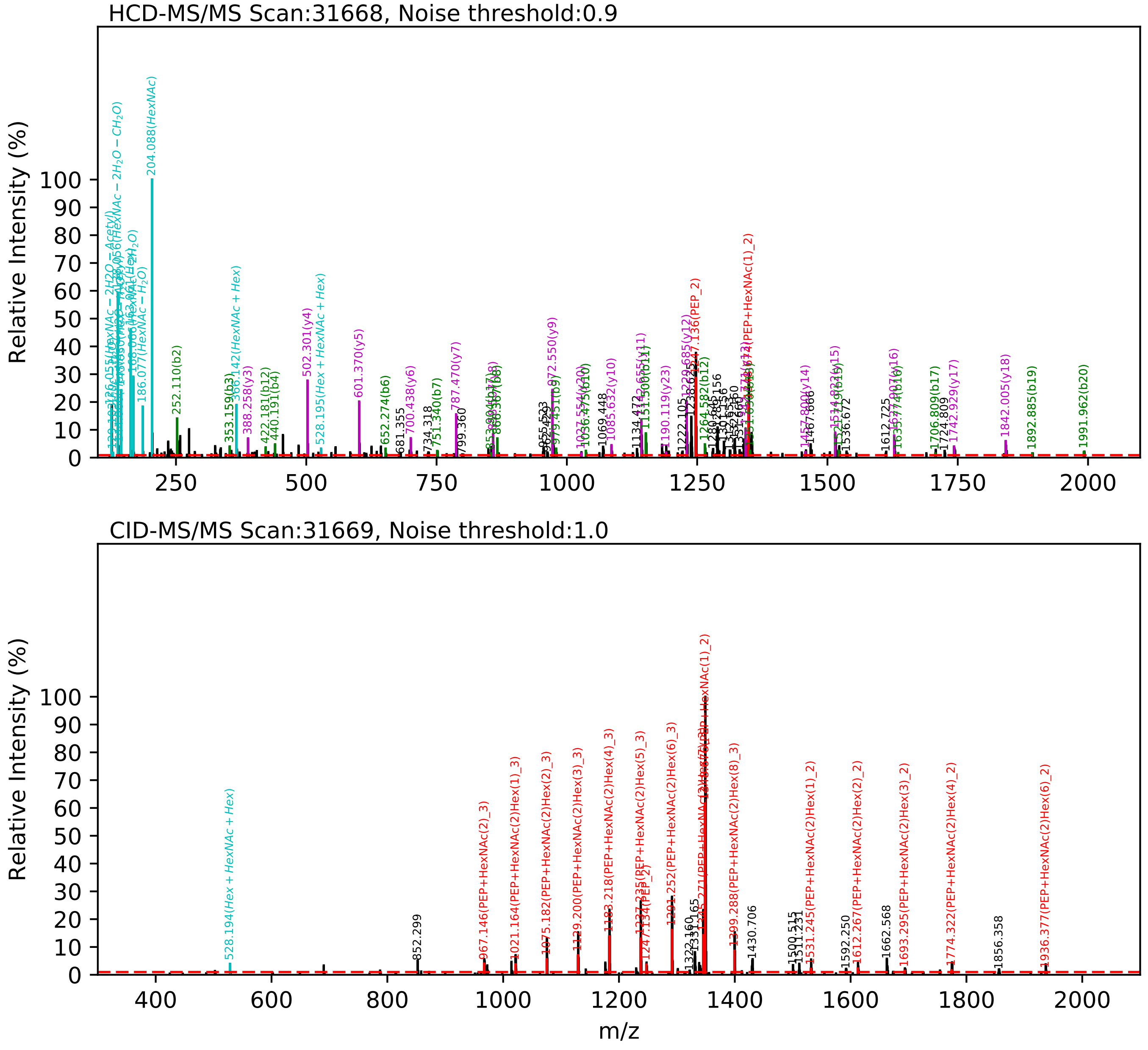

NHTSPDVDLGDISGINASVVNIQK(=PEP)_9_2_0_0_0, 0_None, 0_None,
m/z:1453.30(3+), RT:76.41, Y-score:80.07

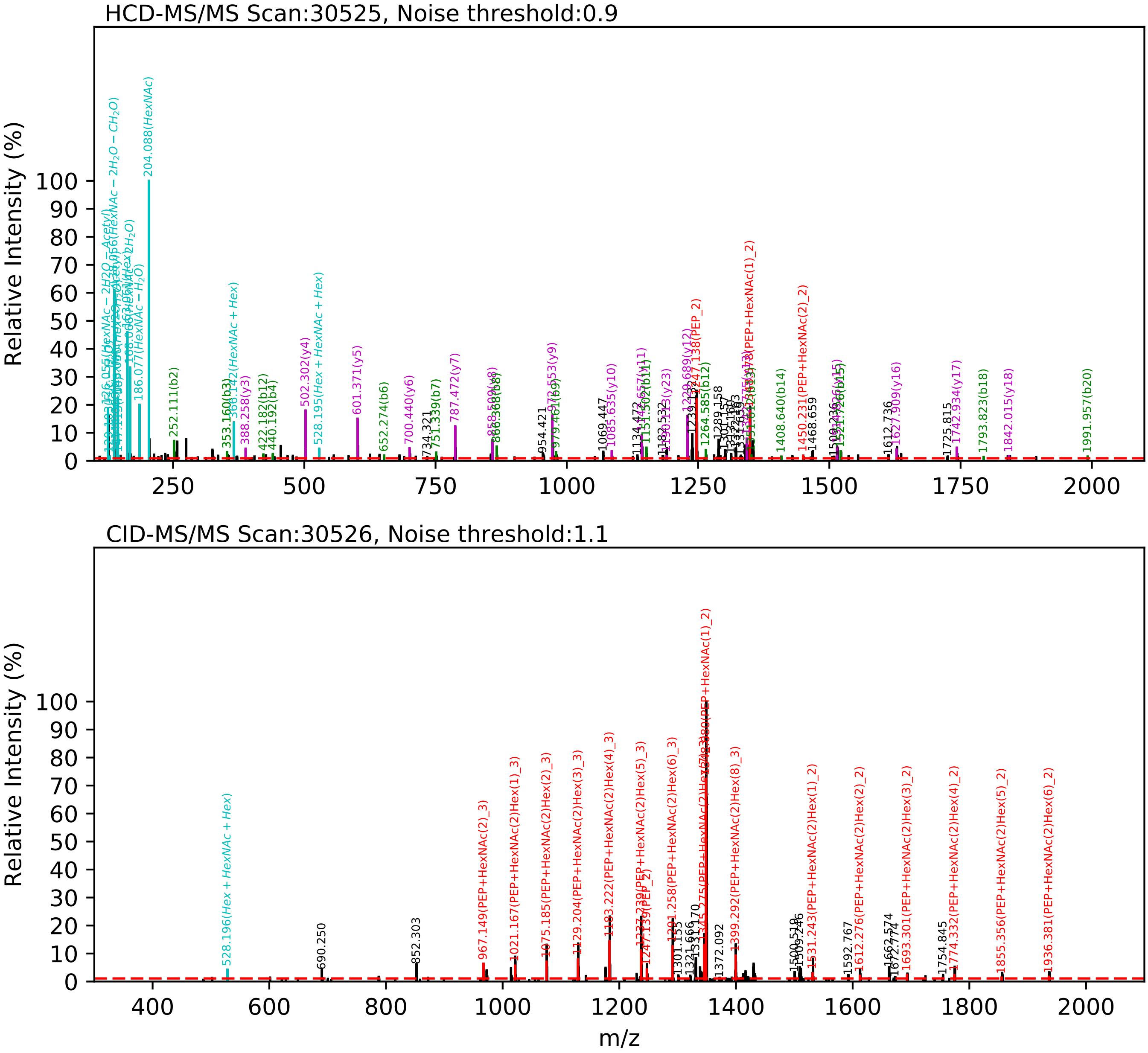

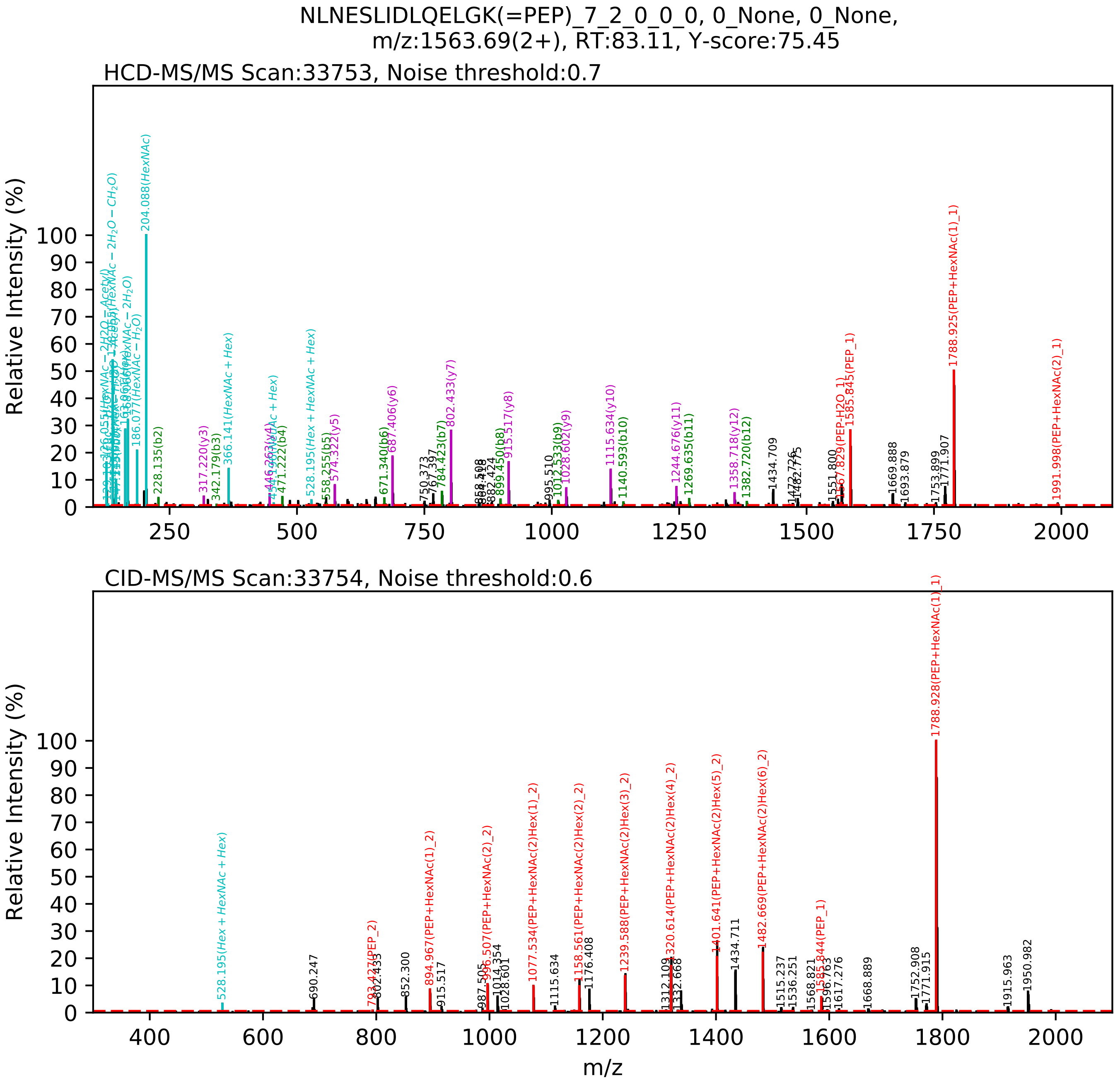
NLNESLIDLQELGK(=PEP)_7_2_0_0_0, 0_None, 0_None,
m/z:1563.69(2+), RT:83.11, Y-score:75.45
HCD-MS/MS Scan:33753, Noise threshold:0.7
Relative Intensity (%)
204.088(HexNAc)
186.077(HexNAc − H2O)
228.135(b2)
317.220(y3)
342.179(b3)
366.141(HexNAc + Hex)
446.263(y4)
471.222(b4)
528.195(Hex + HexNAc + Hex)
558.255(b5)
574.322(y5)
671.340(b6)
687.406(y6)
750.373
767.397
784.423(b7)
802.433(y7)
882.424
899.450(b8)
915.517(y8)
995.510
1012.533(b9)
1028.602(y9)
1115.634(y10)
1140.593(b10)
1244.676(y11)
1269.635(b11)
1358.718(y12)
1382.720(b12)
1434.709
1472.726
1482.775
1551.800
1585.845(PEP_1)
1669.888
1693.879
1753.899
1771.907
1788.925(PEP+HexNAc(1)_1)
1991.998(PEP+HexNAc(2)_1)
CID-MS/MS Scan:33754, Noise threshold:0.6
Relative Intensity (%)
m/z
528.195(Hex + HexNAc + Hex)
690.247
793.427(PEP_2)
802.433
852.300
894.967(PEP+HexNAc(1)_2)
915.517
987.505
996.507(PEP+HexNAc(2)_2)
1014.354
1028.601
1077.534(PEP+HexNAc(2)Hex(1)_2)
1115.634
1158.561(PEP+HexNAc(2)Hex(2)_2)
1176.408
1239.588(PEP+HexNAc(2)Hex(3)_2)
1312.109
1320.614(PEP+HexNAc(2)Hex(4)_2)
1332.668
1401.641(PEP+HexNAc(2)Hex(5)_2)
1434.711
1482.669(PEP+HexNAc(2)Hex(6)_2)
1515.237
1536.251
1568.821
1585.844(PEP_1)
1596.763
1617.276
1668.889
1752.908
1771.915
1788.928(PEP+HexNAc(1)_1)
1915.963
1950.982

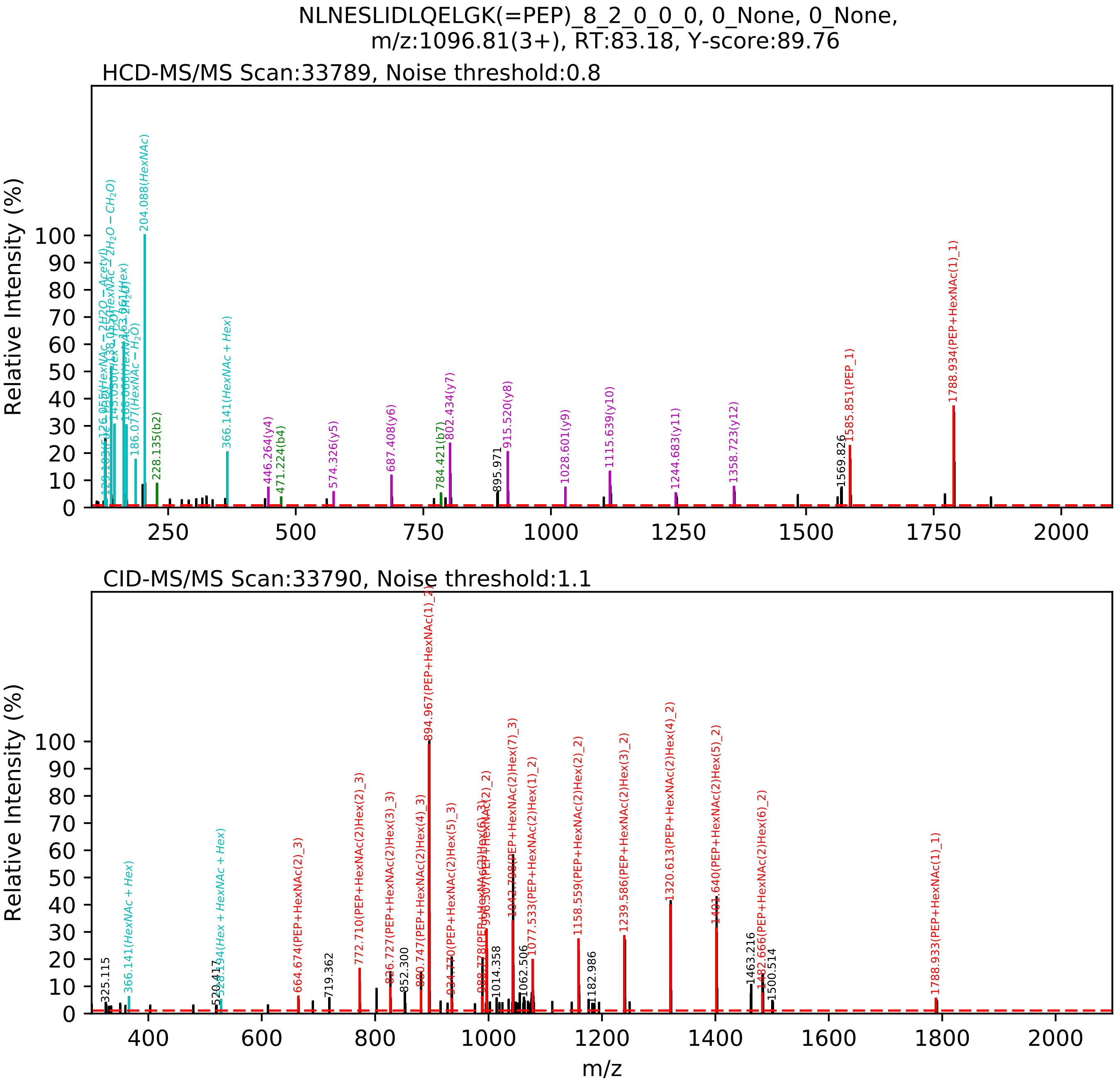
NLNESLIDLQELGK(=PEP)_8_2_0_0_0, 0_None, 0_None,
m/z:1096.81(3+), RT:83.18, Y-score:89.76
HCD-MS/MS Scan:33789, Noise threshold:0.8
Relative Intensity (%)
204.088(HexNAc)
186.077(HexNAc − H2O)
228.135(b2)
366.141(HexNAc + Hex)
446.264(y4)
471.224(b4)
574.326(y5)
687.408(y6)
784.421(b7)
802.434(y7)
895.971
915.520(y8)
1028.601(y9)
1115.639(y10)
1244.683(y11)
1358.723(y12)
1569.826
1585.851(PEP_1)
1788.934(PEP+HexNAc(1)_1)
CID-MS/MS Scan:33790, Noise threshold:1.1
Relative Intensity (%)
325.115
366.141(HexNAc + Hex)
528.194(Hex + HexNAc + Hex)
664.674(PEP+HexNAc(2)_3)
719.362
772.710(PEP+HexNAc(2)Hex(2)_3)
826.727(PEP+HexNAc(2)Hex(3)_3)
852.300
880.747(PEP+HexNAc(2)Hex(4)_3)
894.967(PEP+HexNAc(1)_2)
934.770(PEP+HexNAc(2)Hex(5)_3)
1014.358
1042.798(PEP+HexNAc(2)Hex(7)_3)
1062.506
1077.533(PEP+HexNAc(2)Hex(1)_2)
1158.559(PEP+HexNAc(2)Hex(2)_2)
1182.986
1239.586(PEP+HexNAc(2)Hex(3)_2)
1320.613(PEP+HexNAc(2)Hex(4)_2)
1401.640(PEP+HexNAc(2)Hex(5)_2)
1463.216
1482.666(PEP+HexNAc(2)Hex(6)_2)
1500.514
1788.933(PEP+HexNAc(1)_1)
m/z

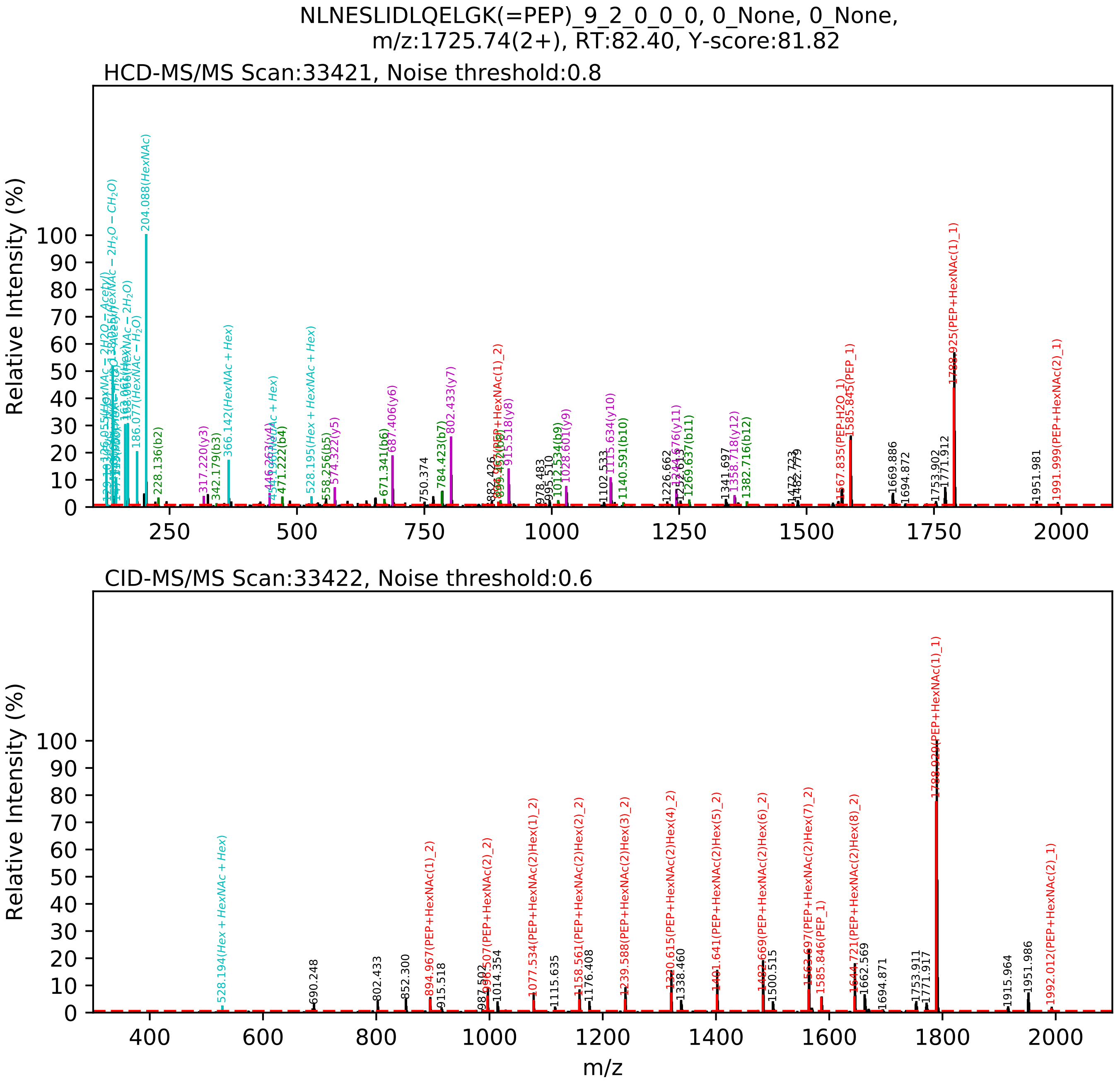

NLNESLIDLQELGK(=PEP)_9_2_0_0_0, 0_None, 0_None,
m/z:1725.74(2+), RT:82.40, Y-score:81.82
HCD-MS/MS Scan:33421, Noise threshold:0.8
Relative Intensity (%)
204.088(HexNAc)
186.077(HexNAc − H2O)
228.136(b2)
317.220(y3)
342.179(b3)
366.142(HexNAc + Hex)
471.222(b4)
528.195(Hex + HexNAc + Hex)
558.256(b5)
574.322(y5)
671.341(b6)
687.406(y6)
750.374
784.423(b7)
802.433(y7)
882.426
915.518(y8)
978.483
995.510
1012.534(b9)
1028.601(y9)
1102.533
1115.634(y10)
1140.591(b10)
1226.662
1269.637(b11)
1341.697
1358.718(y12)
1382.716(b12)
1472.723
1482.779
1567.835(PEP-H2O_1)
1585.845(PEP_1)
1669.886
1694.872
1753.902
1771.912
1788.925(PEP+HexNAc(1)_1)
1951.981
1991.999(PEP+HexNAc(2)_1)
CID-MS/MS Scan:33422, Noise threshold:0.6
Relative Intensity (%)
528.194(Hex + HexNAc + Hex)
690.248
802.433
852.300
894.967(PEP+HexNAc(1)_2)
915.518
987.502
1014.354
1077.534(PEP+HexNAc(2)Hex(1)_2)
1115.635
1158.561(PEP+HexNAc(2)Hex(2)_2)
1176.408
1239.588(PEP+HexNAc(2)Hex(3)_2)
1320.615(PEP+HexNAc(2)Hex(4)_2)
1338.460
1401.641(PEP+HexNAc(2)Hex(5)_2)
1482.669(PEP+HexNAc(2)Hex(6)_2)
1500.515
1585.846(PEP_1)
1644.721(PEP+HexNAc(2)Hex(8)_2)
1662.569
1694.871
1753.911
1771.917
1915.964
1951.986
1992.012(PEP+HexNAc(2)_1)
m/z

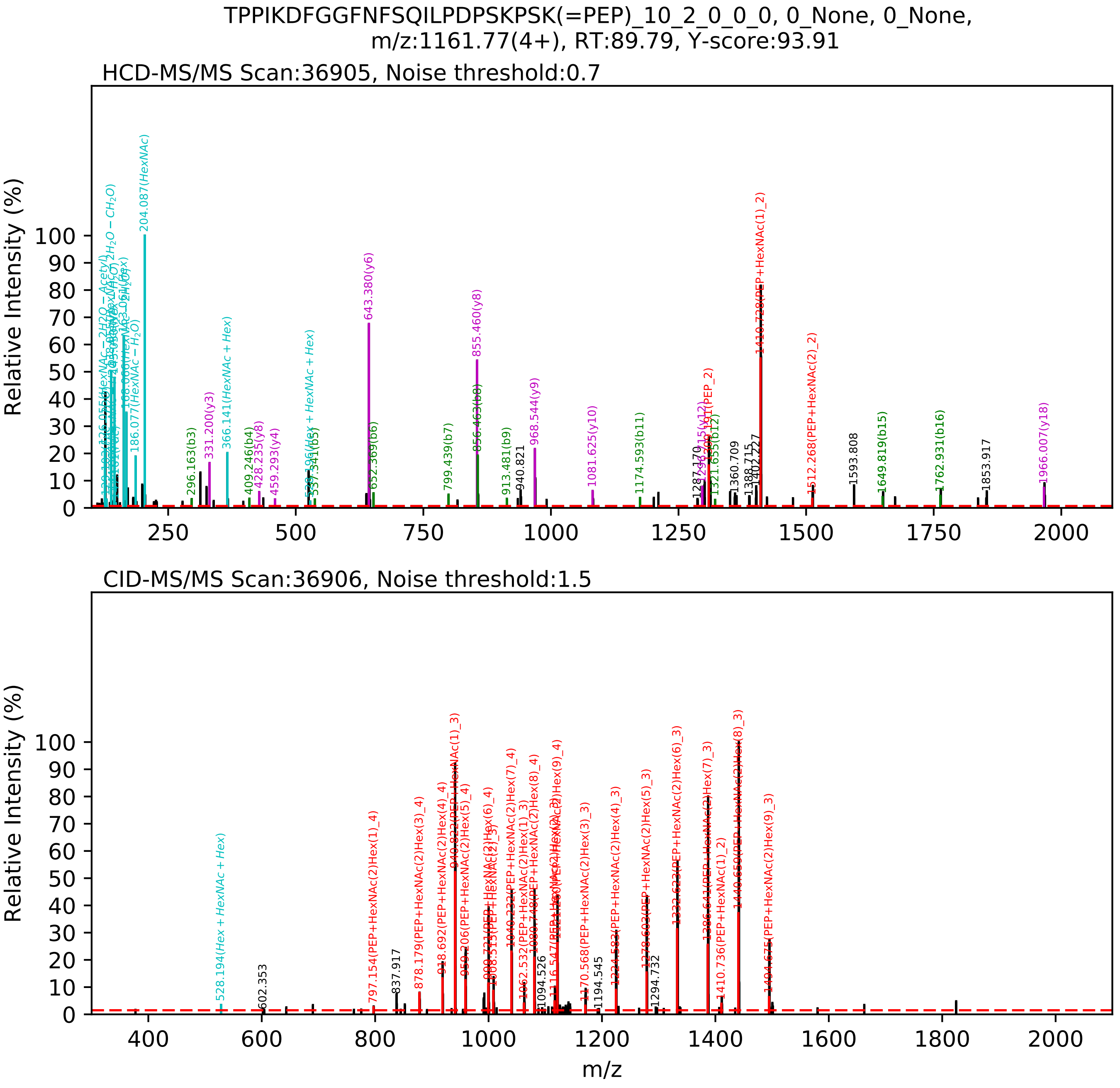
TPPIKDFGGFNFSQILPDPSKPSK(=PEP)_10_2_0_0_0, 0_None, 0_None,
m/z:1161.77(4+), RT:89.79, Y-score:93.91
HCD-MS/MS Scan:36905, Noise threshold:0.7
Relative Intensity (%)
204.087(HexNAc)
186.077(HexNAc − H2O)
296.163(b3)
331.200(y3)
366.141(HexNAc + Hex)
409.246(b4)
428.235(y8)
459.293(y4)
528.196(Hex + HexNAc + Hex)
537.341(b5)
643.380(y6)
652.369(b6)
799.439(b7)
855.460(y8)
856.463(b8)
913.481(b9)
940.821
968.544(y9)
1081.625(y10)
1174.593(b11)
1287.170
1321.655(b12)
1360.709
1388.715
1402.727
1410.728(PEP+HexNAc(1)_2)
1512.268(PEP+HexNAc(2)_2)
1593.808
1649.819(b15)
1762.931(b16)
1853.917
1966.007(y18)
m/z
CID-MS/MS Scan:36906, Noise threshold:1.5
Relative Intensity (%)
528.194(Hex + HexNAc + Hex)
602.353
797.154(PEP+HexNAc(2)Hex(1)_4)
837.917
878.179(PEP+HexNAc(2)Hex(3)_4)
918.692(PEP+HexNAc(2)Hex(4)_4)
959.206(PEP+HexNAc(2)Hex(5)_4)
1040.232(PEP+HexNAc(2)Hex(7)_4)
1062.532(PEP+HexNAc(2)Hex(1)_3)
1094.526
1170.568(PEP+HexNAc(2)Hex(3)_3)
1194.545
1224.583(PEP+HexNAc(2)Hex(4)_3)
1278.602(PEP+HexNAc(2)Hex(5)_3)
1294.732
1332.622(PEP+HexNAc(2)Hex(6)_3)
1410.736(PEP+HexNAc(1)_2)
1494.675(PEP+HexNAc(2)Hex(9)_3)
m/z

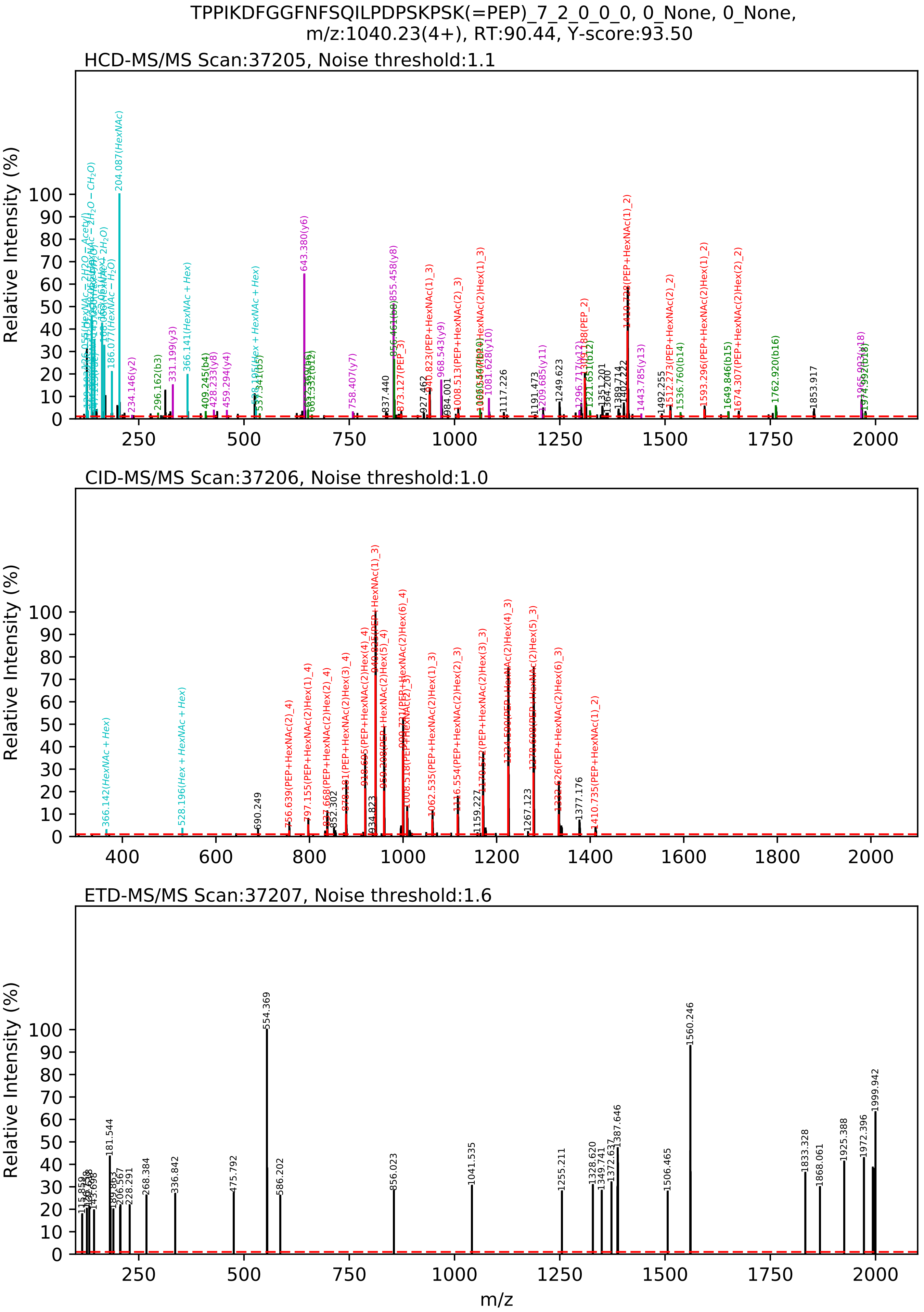
TPPIKDFGGFNFSQILPDPSKPSK(=PEP)_7_2_0_0_0, 0_None, 0_None,
m/z:1040.23(4+), RT:90.44, Y-score:93.50
HCD-MS/MS Scan:37205, Noise threshold:1.1
CID-MS/MS Scan:37206, Noise threshold:1.0
ETD-MS/MS Scan:37207, Noise threshold:1.6
Relative Intensity (%)
m/z

TPPIKDFGGFNFSQILPDPSKPSK(=PEP)_8_2_0_0_0, 0_None, 0_None,
m/z:1080.74(4+), RT:89.88, Y-score:92.82

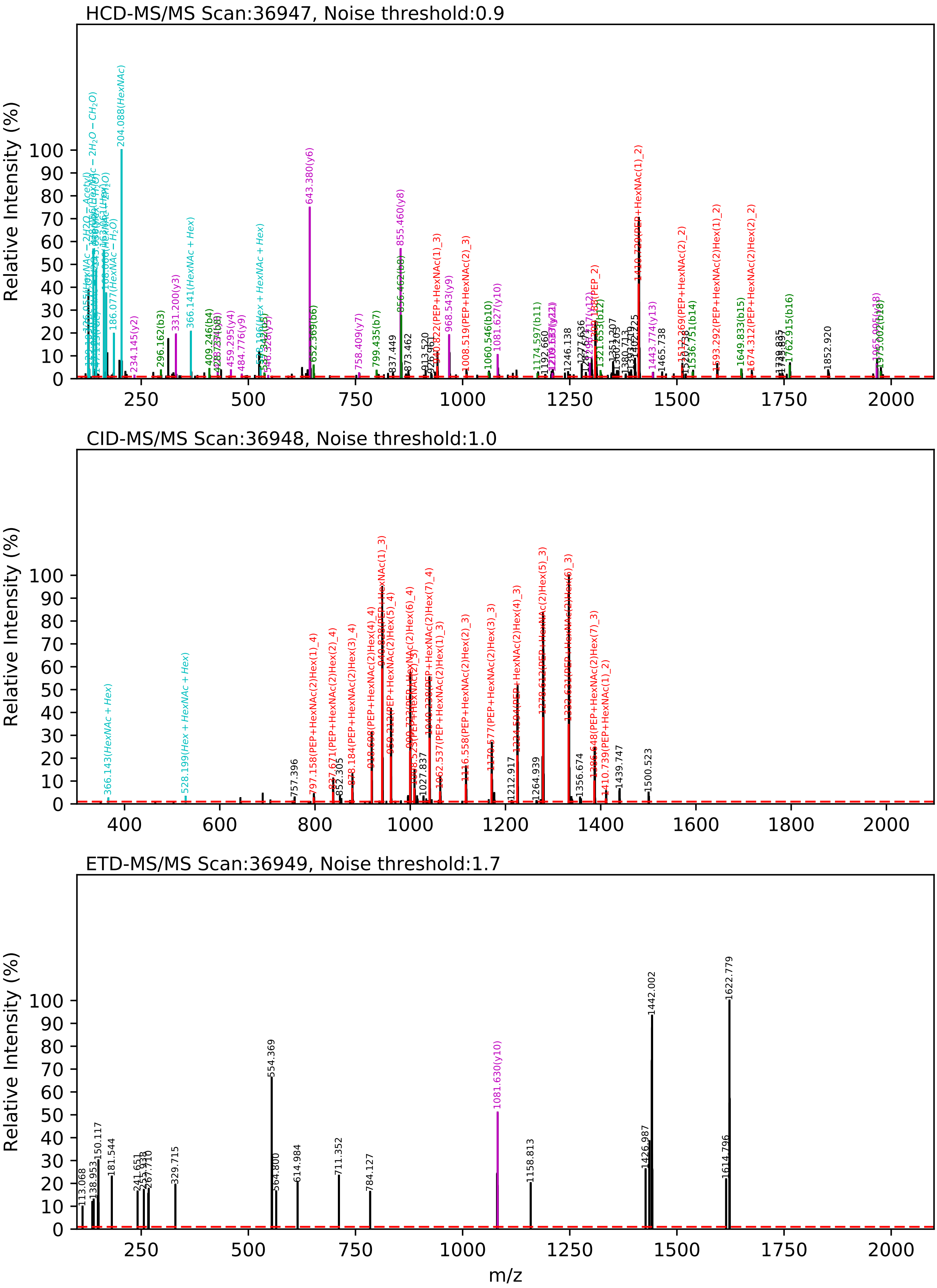

TPPIKDFGGFNFSQILPDPSKPSK(=PEP)_8_2_0_0_0, 0_None, 0_None,
m/z:1080.74(4+), RT:89.97, Y-score:95.62

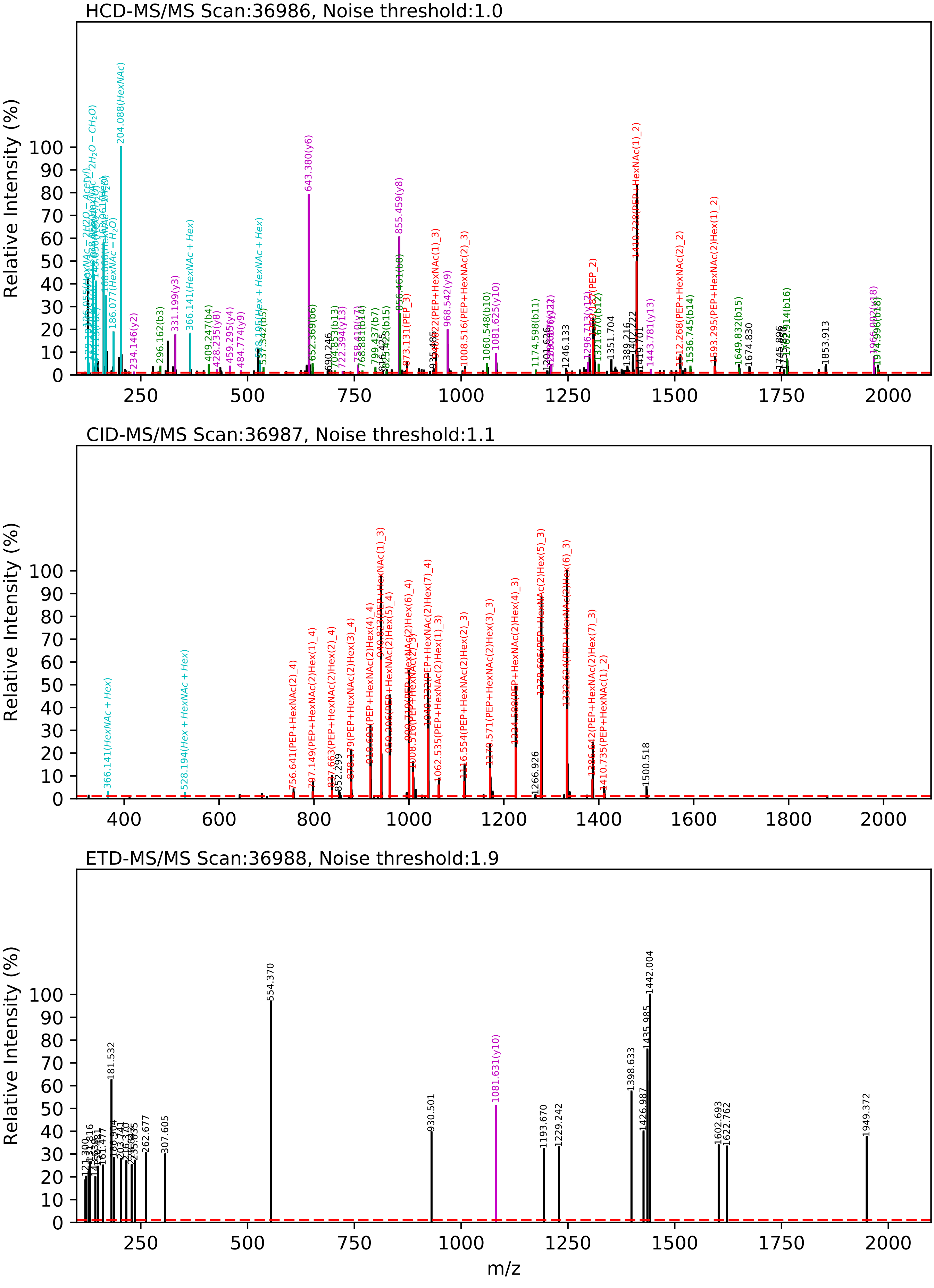

TPPIKDFGGFNFSQILPDPSKPSK(=PEP)_8_2_0_0_0, 0_None, 0_None,
m/z:1080.74(4+), RT:90.59, Y-score:89.53

HCD-MS/MS Scan:37278, Noise threshold:1.2

CID-MS/MS Scan:37279, Noise threshold:1.3

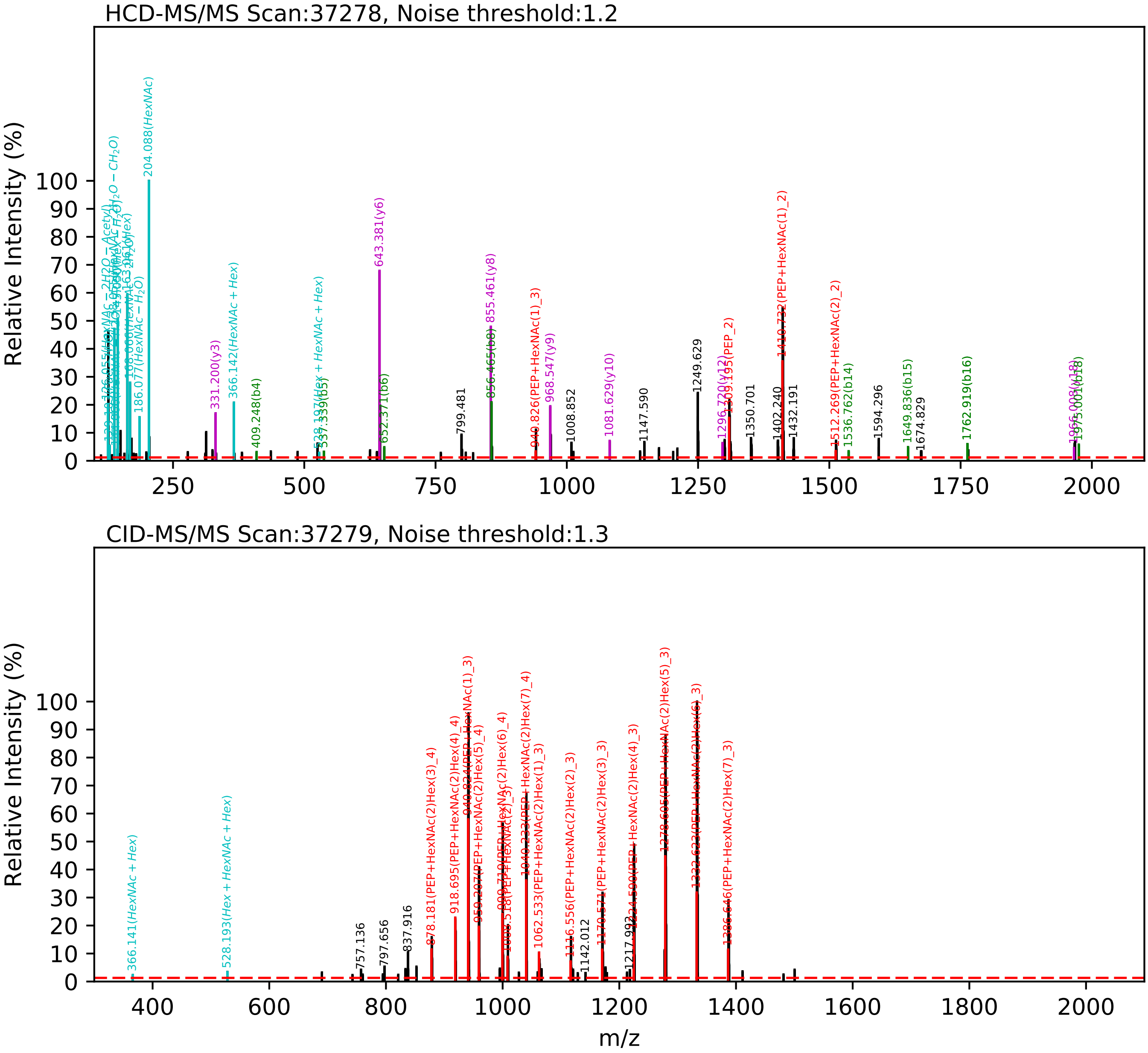

TPPIKDFGGFNFSQILPDPSKPSK(=PEP)_8_2_0_0_0, 0_None, 0_None,
m/z:1080.74(4+), RT:93.36, Y-score:92.92

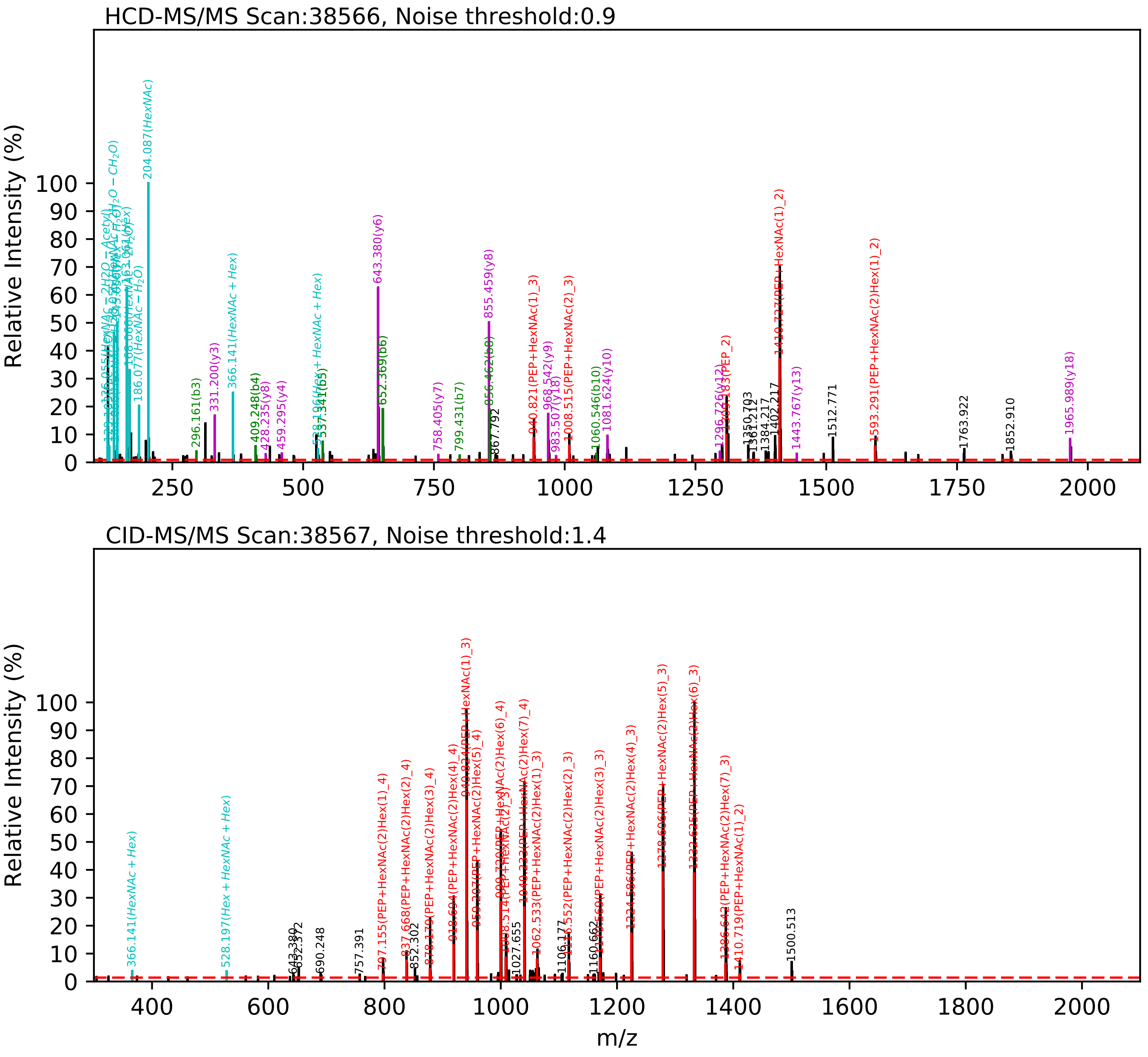

TPPIKDFGGFNFSQILPDPSKPSK(=PEP)_8_2_0_0_0, 0_None, 0_None,
m/z:864.80(5+), RT:90.52, Y-score:95.40

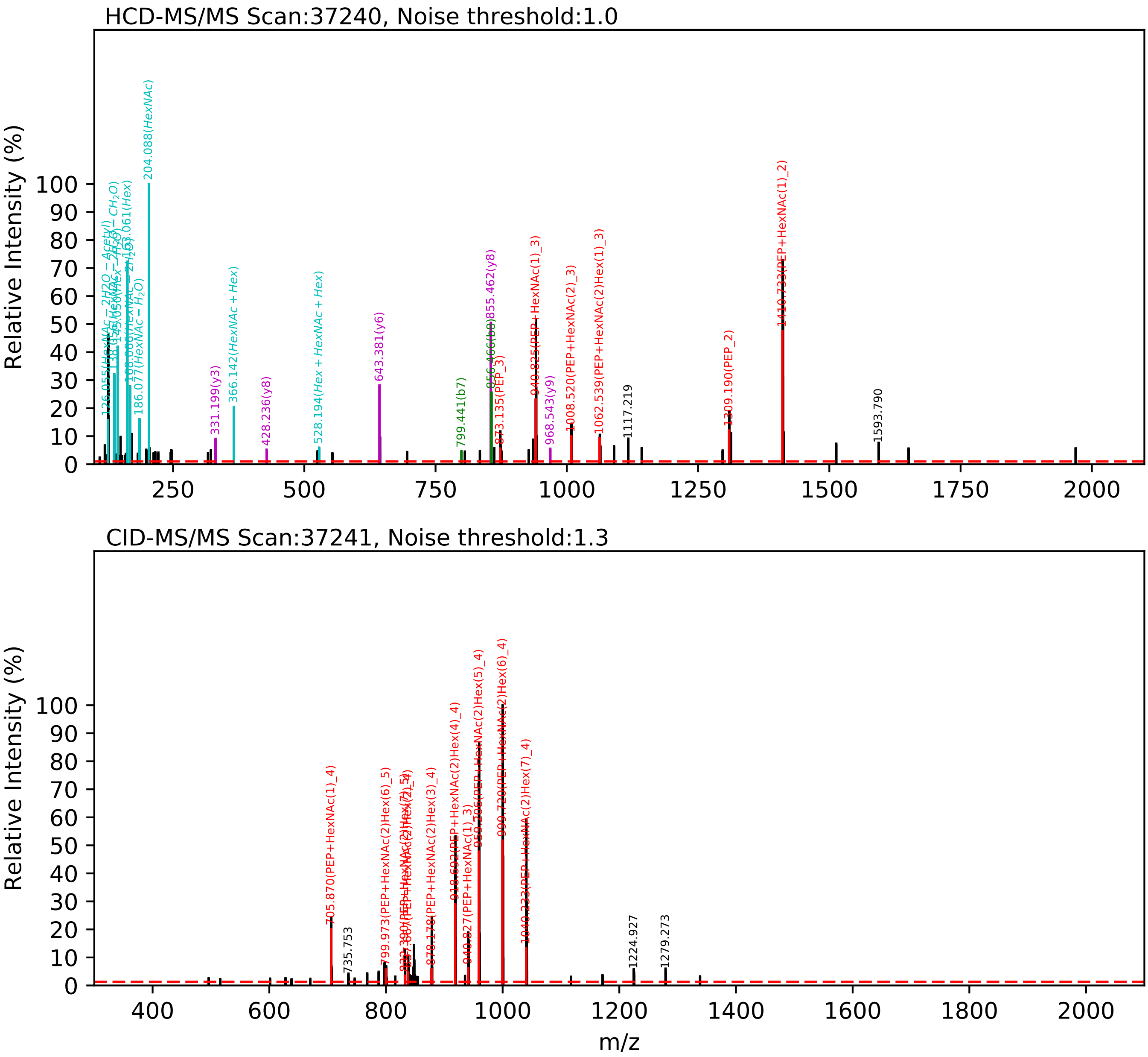

TPPIKDFGGFNFSQILPDPSKPSK(=PEP)_8_2_0_0_0, 0_None, 0_None,
m/z:864.79(5+), RT:90.08, Y-score:92.85

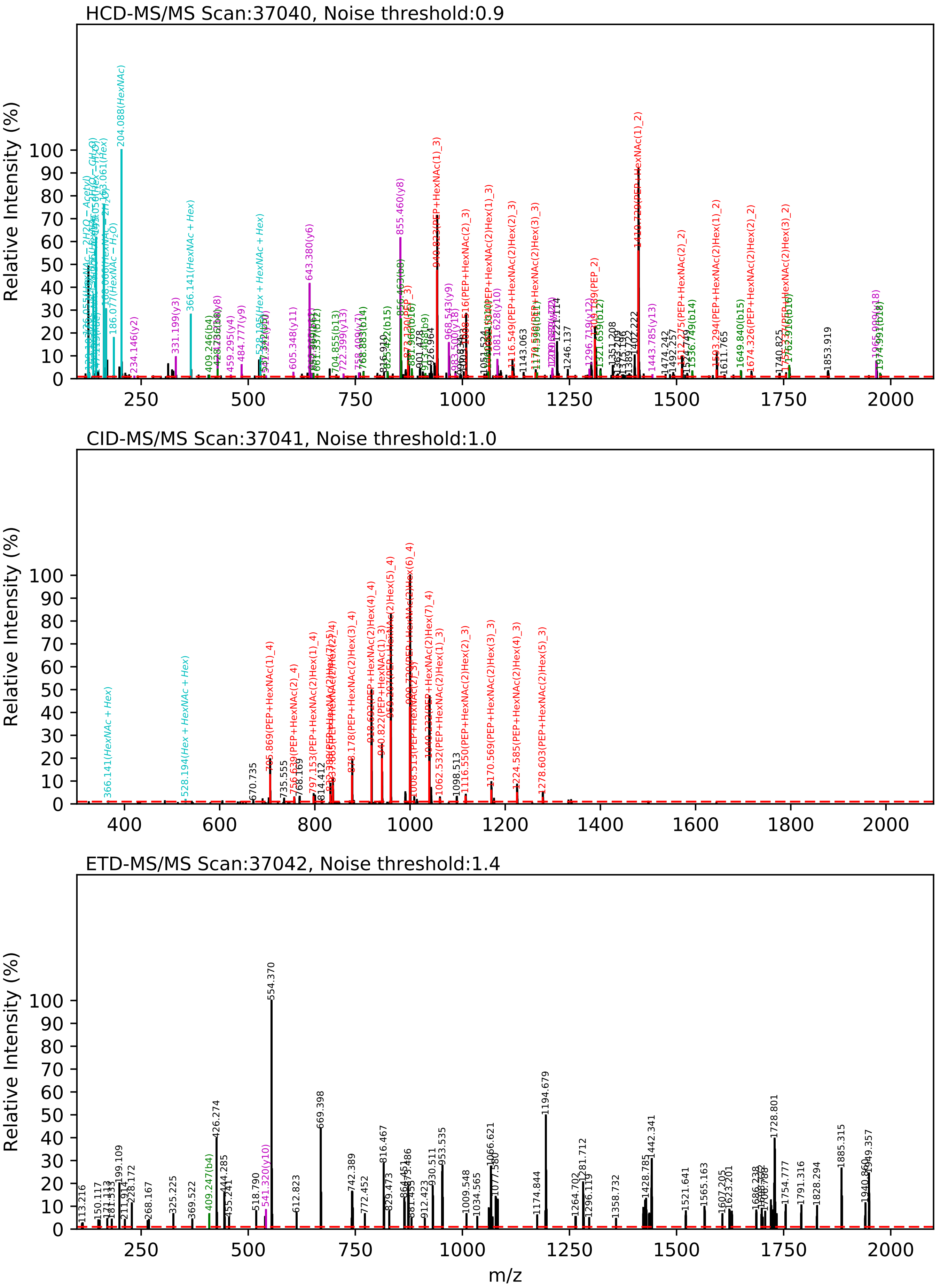

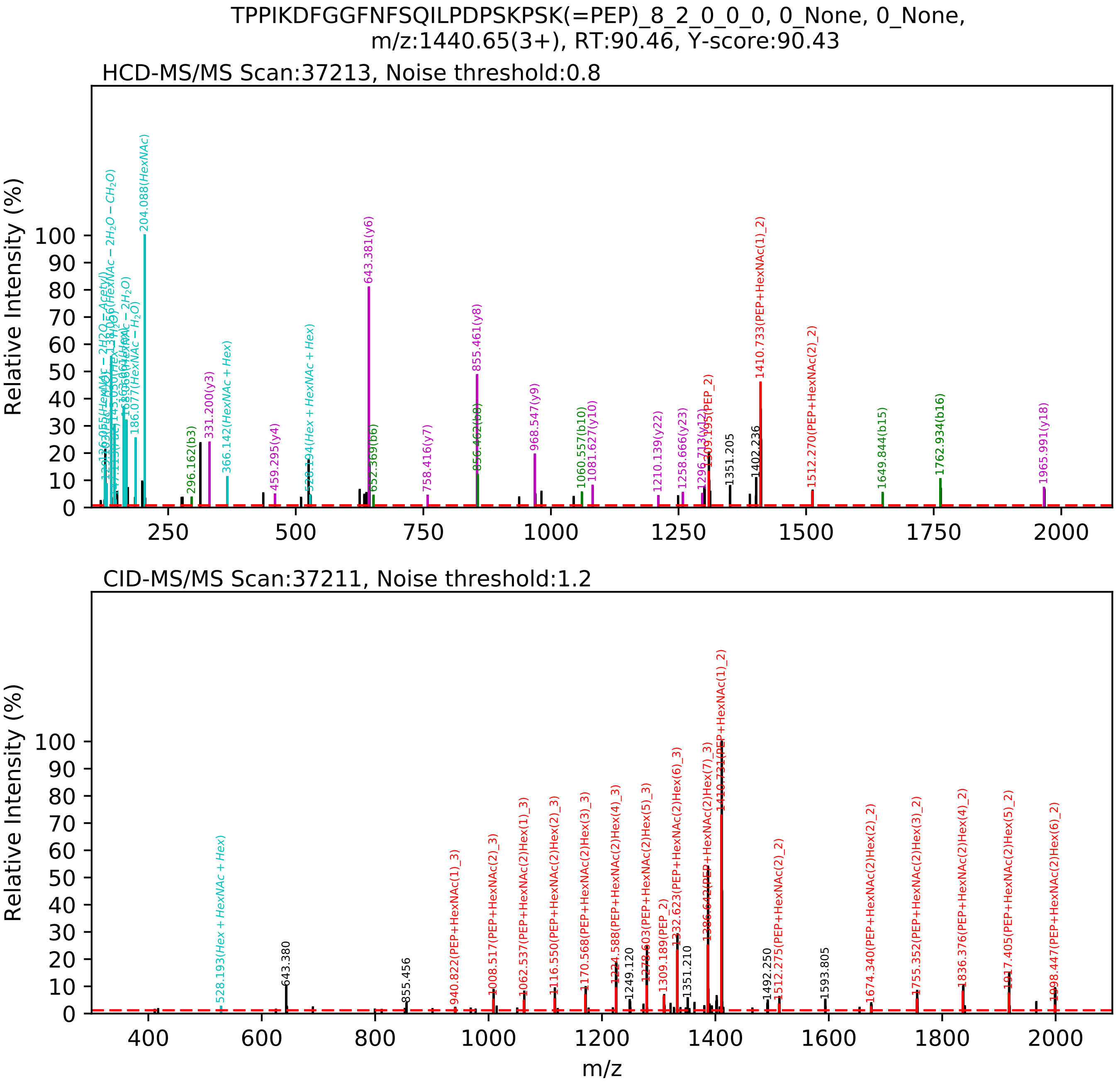
TPPIKDFGGFNFSQILPDPSKPSK(=PEP)_8_2_0_0_0, 0_None, 0_None,
m/z:1440.65(3+), RT:90.46, Y-score:90.43
HCD-MS/MS Scan:37213, Noise threshold:0.8
Relative Intensity (%)
204.088(HexNAc)
643.381(y6)
855.461(y8)
968.547(y9)
1410.733(PEP+HexNAc(1)_2)
1512.270(PEP+HexNAc(2)_2)
1649.844(b15)
1762.934(b16)
1965.991(y18)
296.162(b3)
331.200(y3)
366.142(HexNAc + Hex)
459.295(y4)
652.369(b6)
758.416(y7)
1060.557(b10)
1081.627(y10)
1210.139(y22)
1258.666(y23)
1351.205
1402.236
CID-MS/MS Scan:37211, Noise threshold:1.2
528.193(Hex + HexNAc + Hex)
643.380
855.456
940.822(PEP+HexNAc(1)_3)
1008.517(PEP+HexNAc(2)_3)
1062.537(PEP+HexNAc(2)Hex(1)_3)
1116.550(PEP+HexNAc(2)Hex(2)_3)
1170.568(PEP+HexNAc(2)Hex(3)_3)
1249.120
1309.189(PEP_2)
1351.210
1492.250
1512.275(PEP+HexNAc(2)_2)
1593.805
1674.340(PEP+HexNAc(2)Hex(2)_2)
1755.352(PEP+HexNAc(2)Hex(3)_2)
1836.376(PEP+HexNAc(2)Hex(4)_2)
1998.447(PEP+HexNAc(2)Hex(6)_2)
m/z

TPPIKDFGGFNFSQILPDPSKPSK(=PEP)_9_2_0_0_0, 0_None, 0_None,
m/z:1121.26(4+), RT:90.07, Y-score:94.29

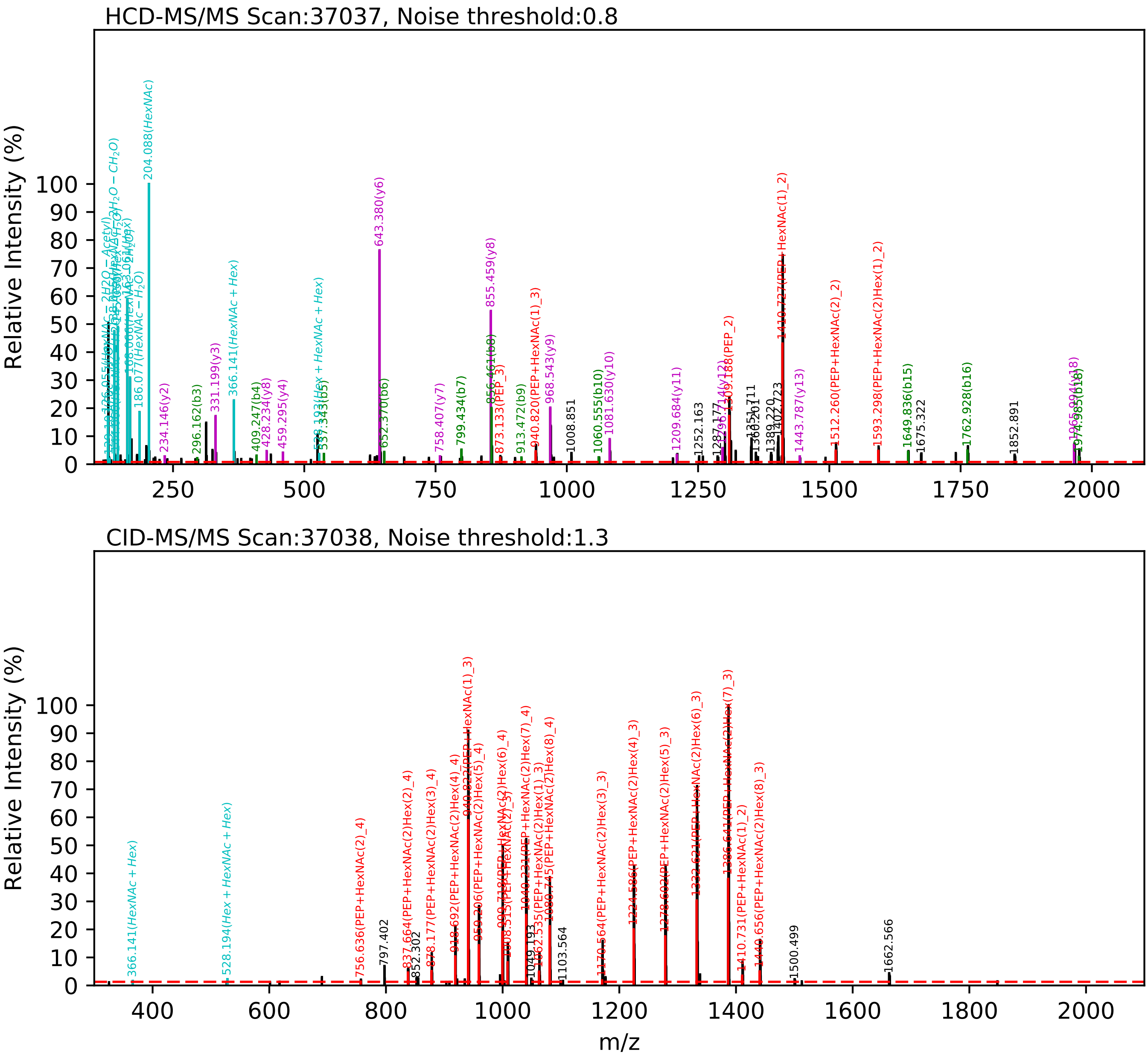

Supplement: Supplementary file 1 [file ijms-25-13649-s001.zip › Supplementary Figure S7(S2_T_N-glycopep_1).pdf]
