# Supplementary material for: Characterization of Site-Specific N- and O-Glycopeptides from Recombinant Spike and ACE2 Glycoproteins Using LC-MS/MS Analysis
Source: Int J Mol Sci. 2024 Dec 20;25(24):13649. doi: 10.3390/ijms252413649 (PMC11678118; doi:10.3390/ijms252413649)

NHTSPDVDLGDISGINASVVNIQK(=PEP)_9_2_0_0_0, 0_None, 0_None,
m/z:1453.30(3+), RT:76.41, hcd-score:78.24

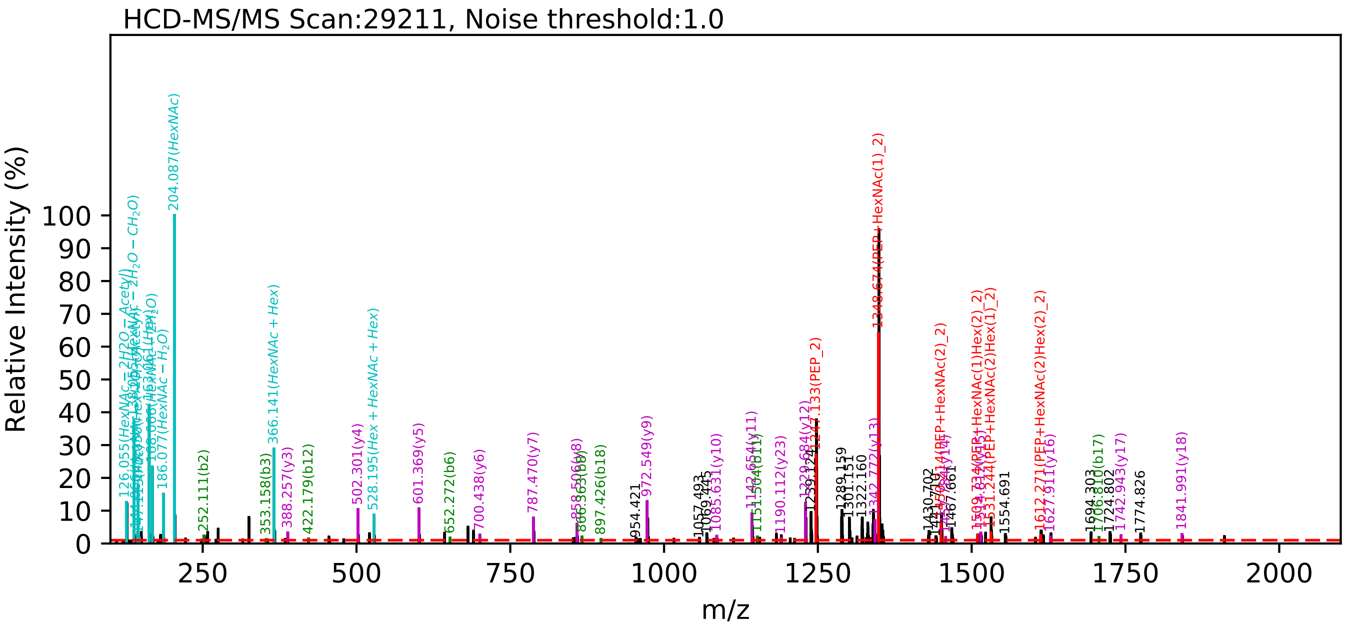

NHTSPDVDLGDISGINASVVNIQK(=PEP)_9_2_0_0_0, 0_None, 0_None,
m/z:1453.30(3+), RT:76.41, hcd-score:78.24

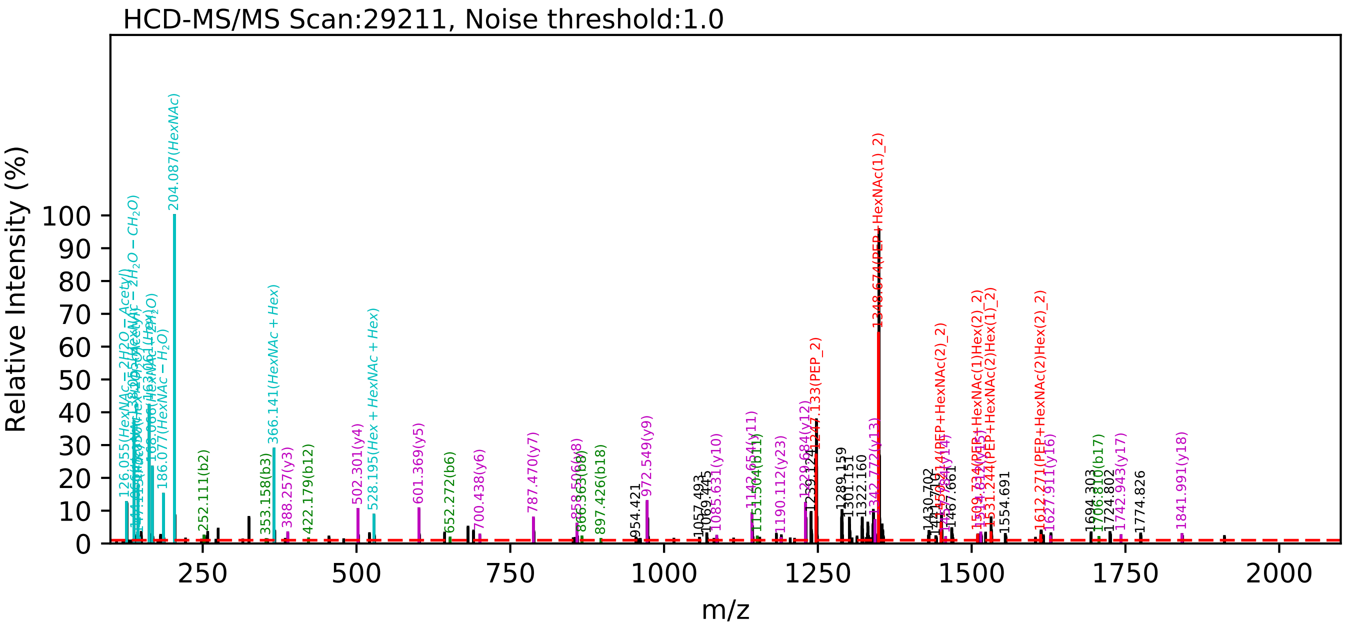

NHTSPDVDLGDISGINASVVNIQK(=PEP)_9_2_0_0_0, 0_None, 0_None,
m/z:1090.23(6+), RT:79.30, hcd-score:88.92

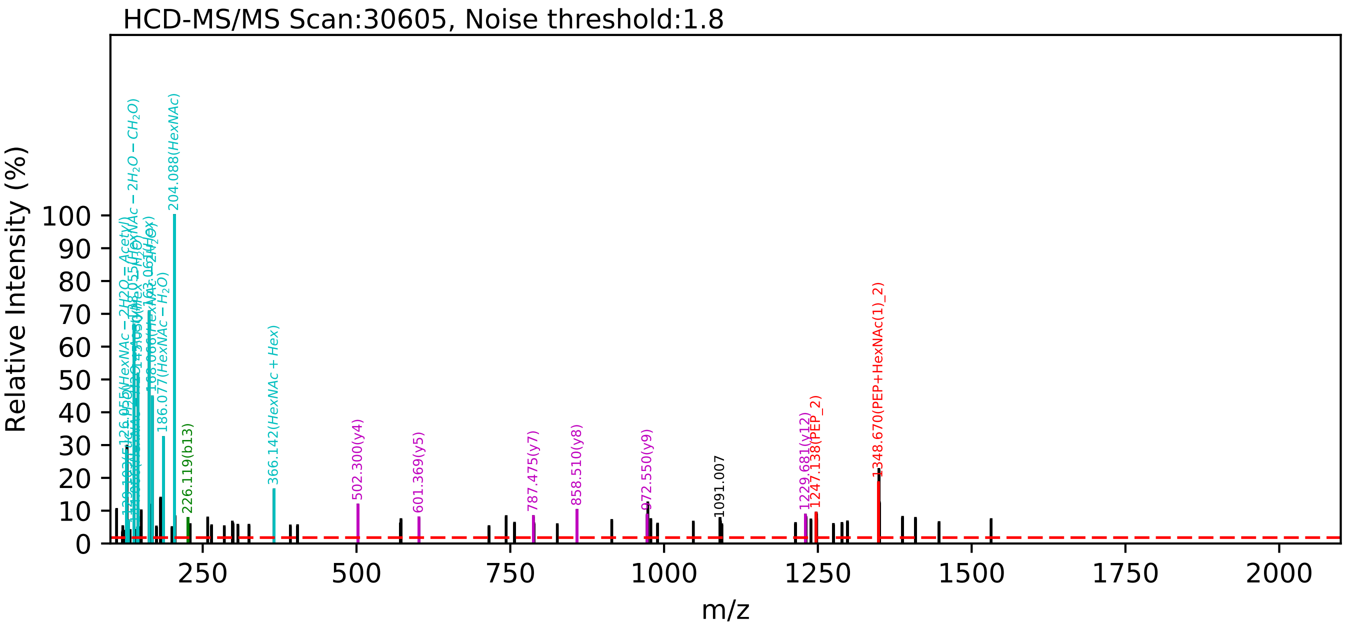

NHTSPDVDLGDISGINASVVNIQK(=PEP)_9_2_0_0_0, 0_None, 0_None,
m/z:1090.23(6+), RT:79.30, hcd-score:88.92

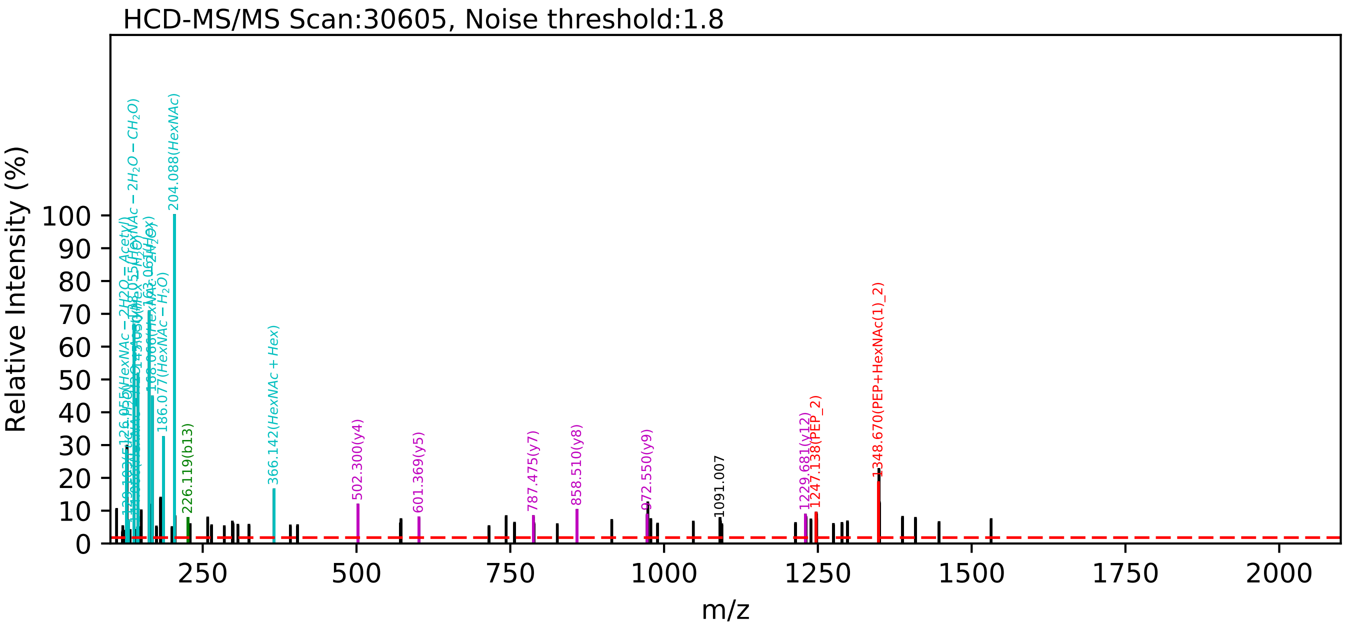

NLNESLIDLQELGK(=PEP)_7_2_0_0_0, 0_None, 0_None,
m/z:1042.80(3+), RT:83.29, hcd-score:88.40

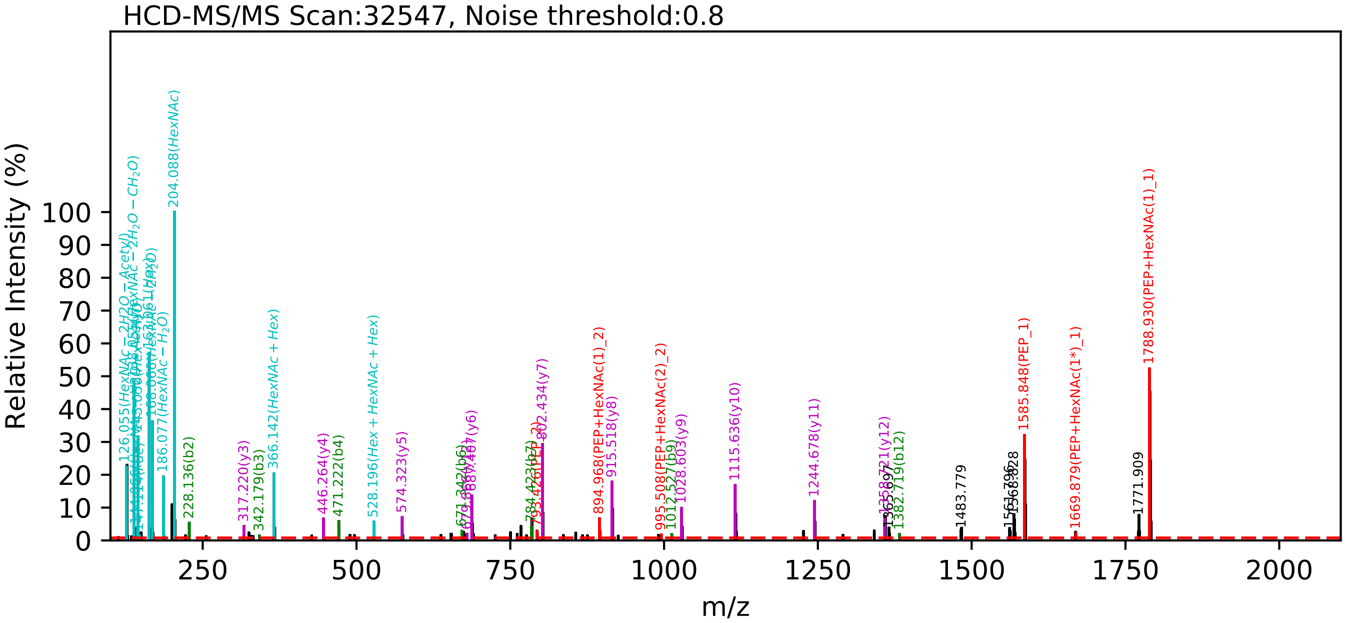

NLNESLIDLQELGK(=PEP)_7_2_0_0_0, 0_None, 0_None,
m/z:1042.80(3+), RT:83.29, hcd-score:88.40

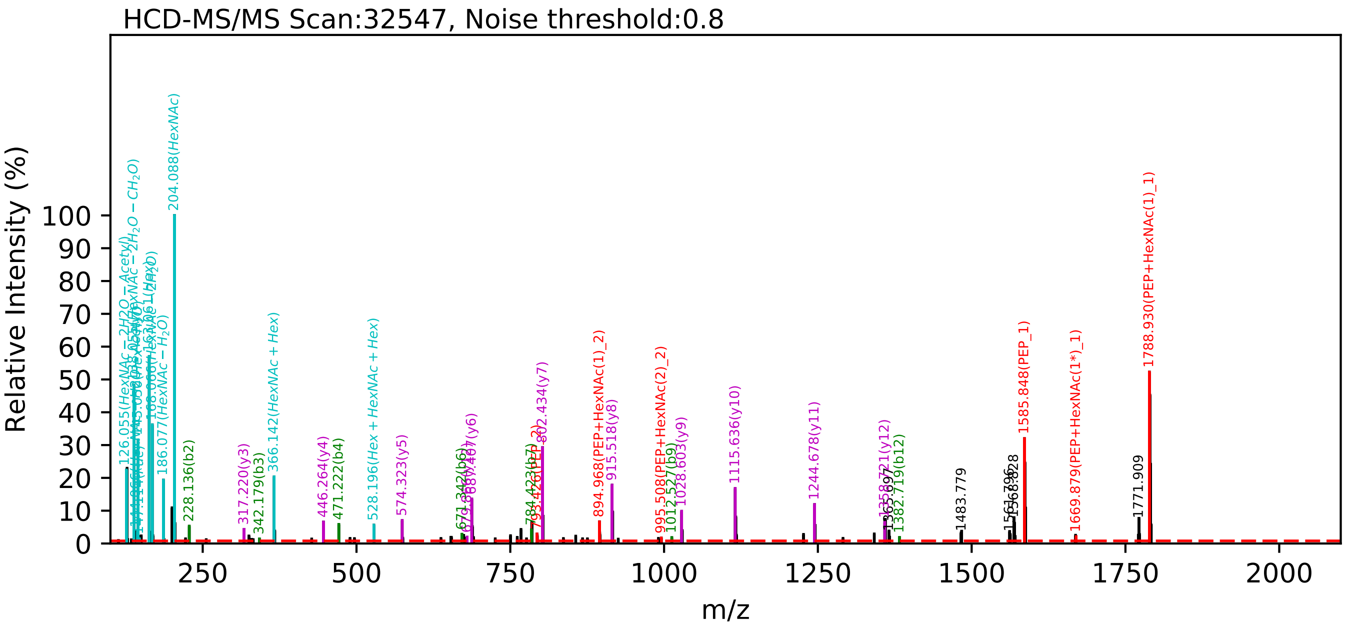

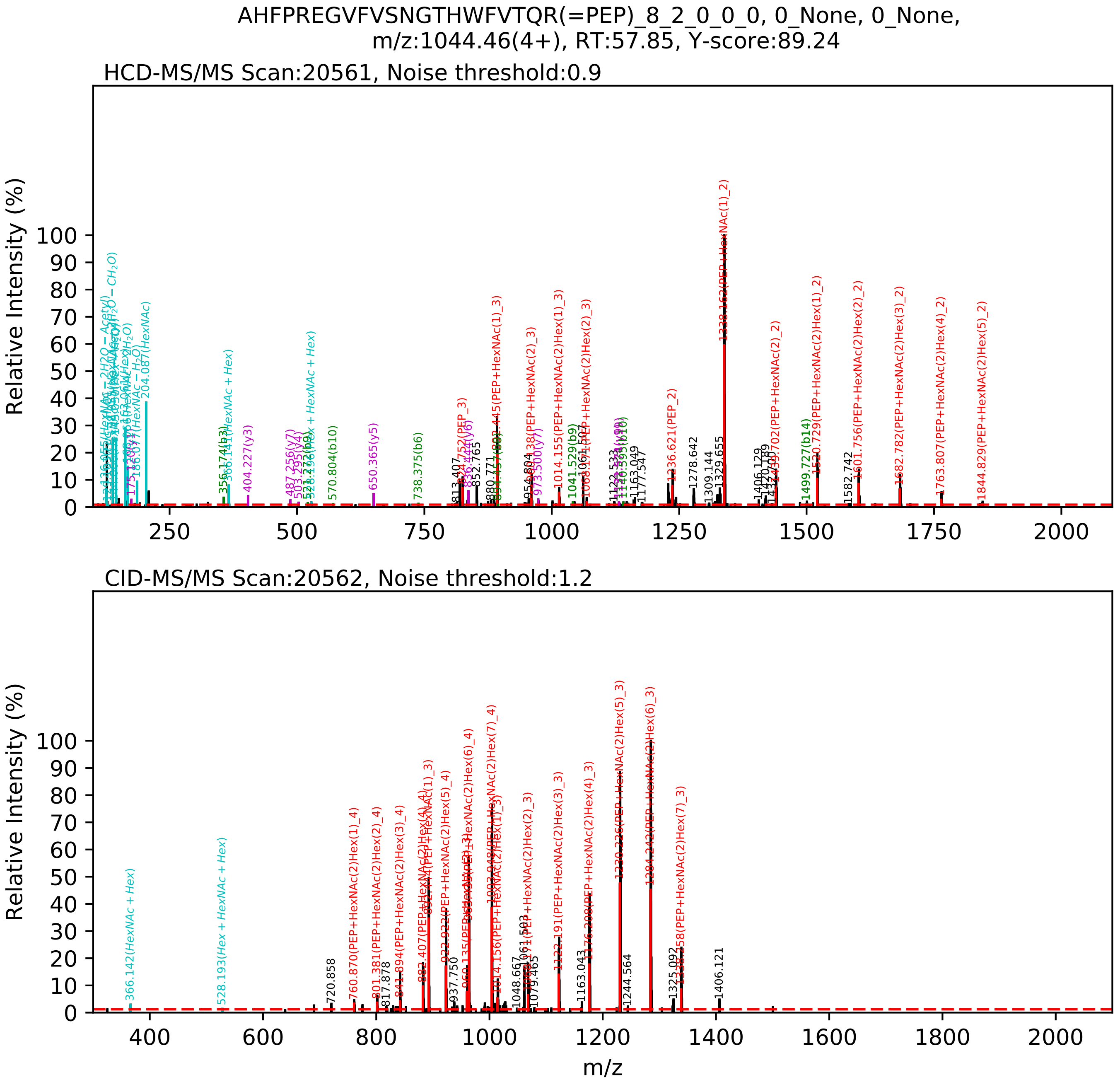
AHFPREGVFVSNGTHWFVTQR(=PEP)_8_2_0_0_0, 0_None, 0_None,
m/z:1044.46(4+), RT:57.85, Y-score:89.24
HCD-MS/MS Scan:20561, Noise threshold:0.9
Relative Intensity (%)
204.087(HexNAc)
356.174(b3)
366.141(HexNAc + Hex)
404.227(y3)
487.256(y7)
528.192(HexNAc + Hex)
570.804(b10)
650.365(y5)
738.375(b6)
852.765
880.771
893.445(PEP+HexNAc(1)_3)
1014.155(PEP+HexNAc(2)Hex(1)_3)
1041.529(b9)
1068.171(PEP+HexNAc(2)Hex(2)_3)
1163.049
1177.547
1236.621(PEP_2)
1278.642
1309.144
1329.655
1339.163(PEP+HexNAc(1)_2)
1406.129
1439.702(PEP+HexNAc(2)_2)
1499.727(b14)
1520.729(PEP+HexNAc(2)Hex(1)_2)
1582.742
1601.756(PEP+HexNAc(2)Hex(2)_2)
1682.782(PEP+HexNAc(2)Hex(3)_2)
1763.807(PEP+HexNAc(2)Hex(4)_2)
1844.829(PEP+HexNAc(2)Hex(5)_2)
CID-MS/MS Scan:20562, Noise threshold:1.2
366.142(HexNAc + Hex)
528.193(Hex + HexNAc + Hex)
720.858
760.870(PEP+HexNAc(2)Hex(1)_4)
801.381(PEP+HexNAc(2)Hex(2)_4)
817.878
841.894(PEP+HexNAc(2)Hex(3)_4)
882.407(PEP+HexNAc(2)Hex(4)_4)
937.750
1002.949(PEP+HexNAc(2)Hex(7)_4)
1014.156(PEP+HexNAc(2)Hex(1)_3)
1048.667
1079.465
1122.191(PEP+HexNAc(2)Hex(3)_3)
1163.043
1176.208(PEP+HexNAc(2)Hex(4)_3)
1230.226(PEP+HexNAc(2)Hex(5)_3)
1244.564
1284.243(PEP+HexNAc(2)Hex(6)_3)
1325.092
1406.121
m/z

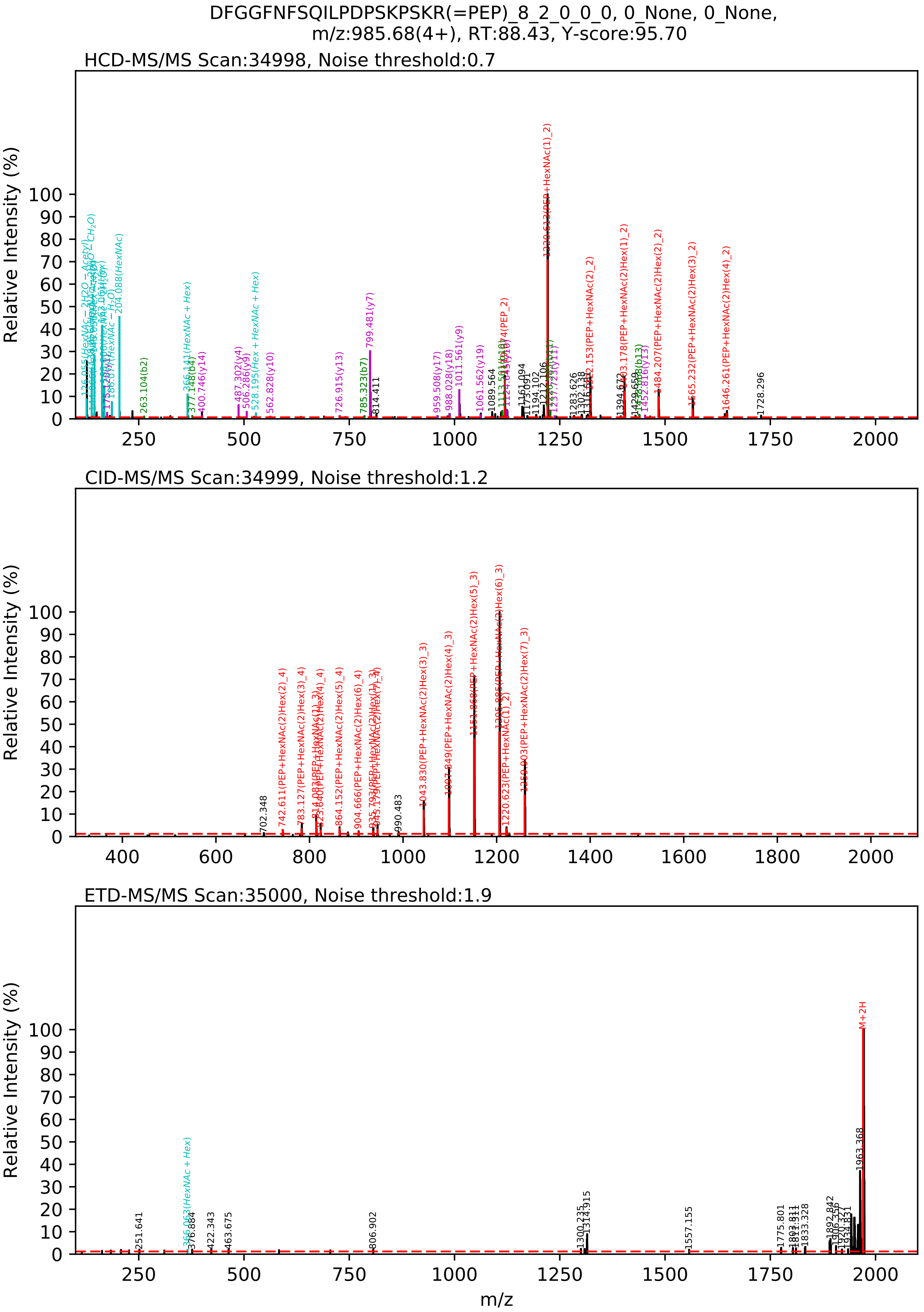
DFGGFNFSQILPDPSKPSKR(=PEP)_8_2_0_0_0, 0_None, 0_None,
m/z:985.68(4+), RT:88.43, Y-score:95.70
HCD-MS/MS Scan:34998, Noise threshold:0.7
Relative Intensity (%)
1220.613(PEP+HexNAc(1)_2)
1322.153(PEP+HexNAc(2)_2)
1403.178(PEP+HexNAc(2)Hex(1)_2)
1484.207(PEP+HexNAc(2)Hex(2)_2)
1565.232(PEP+HexNAc(2)Hex(3)_2)
1646.261(PEP+HexNAc(2)Hex(4)_2)
204.088(HexNAc)
263.104(b2)
366.141(HexNAc + Hex)
400.746(y14)
487.302(y4)
506.286(y9)
528.195(Hex + HexNAc + Hex)
562.828(y10)
726.915(y13)
785.323(b7)
799.481(y7)
814.411
959.508(y17)
988.028(y18)
1011.561(y9)
1061.562(y19)
1089.564
1161.094
1194.102
1212.106
1283.626
1302.138
1394.672
1452.816(y13)
1728.296
m/z
CID-MS/MS Scan:34999, Noise threshold:1.2
Relative Intensity (%)
702.348
742.611(PEP+HexNAc(2)Hex(2)_4)
783.127(PEP+HexNAc(2)Hex(3)_4)
864.152(PEP+HexNAc(2)Hex(5)_4)
904.666(PEP+HexNAc(2)Hex(6)_4)
990.483
1043.830(PEP+HexNAc(2)Hex(3)_3)
1097.849(PEP+HexNAc(2)Hex(4)_3)
1151.868(PEP+HexNAc(2)Hex(5)_3)
1205.885(PEP+HexNAc(2)Hex(6)_3)
1220.623(PEP+HexNAc(1)_2)
1259.903(PEP+HexNAc(2)Hex(7)_3)
ETD-MS/MS Scan:35000, Noise threshold:1.9
Relative Intensity (%)
M+2H
251.641
366.083(HexNAc + Hex)
376.884
422.343
463.675
806.902
1300.235
1314.915
1557.155
1775.801
1833.328
1892.842
1963.368
m/z

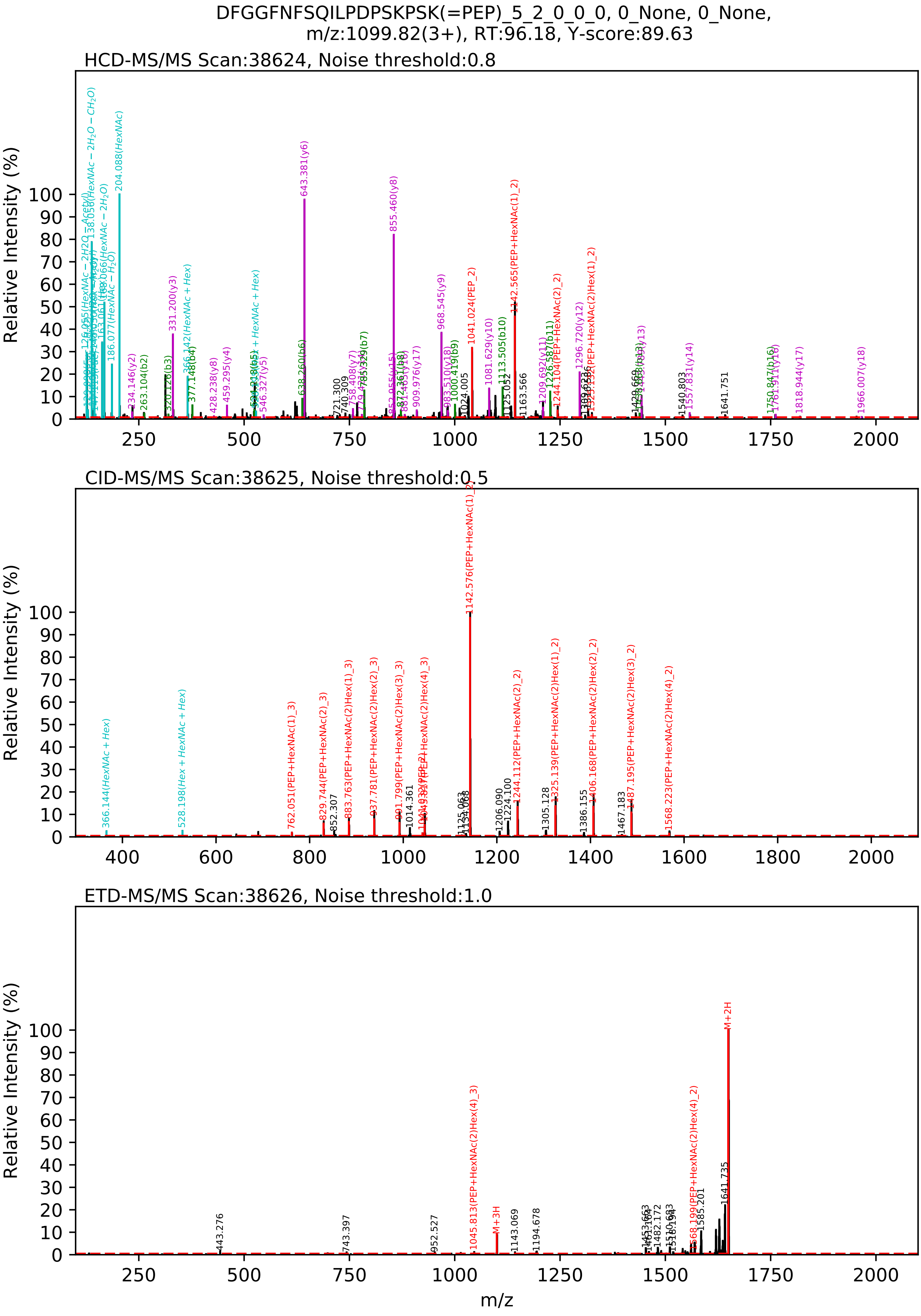

DFGGFNFSQILPDPSKPSK(=PEP)_5_2_0_0_0, 0_None, 0_None,
m/z:1099.82(3+), RT:96.18, Y-score:89.63
HCD-MS/MS Scan:38624, Noise threshold:0.8
Relative Intensity (%)
204.088(HexNAc)
643.381(y6)
855.460(y8)
968.545(y9)
1041.024(PEP_2)
1142.565(PEP+HexNAc(1)_2)
1081.629(y10)
1113.505(b10)
1244.104(PEP+HexNAc(2)_2)
1296.720(y12)
331.200(y3)
263.104(b2)
234.146(y2)
186.077(HexNAc − H2O)
428.238(y8)
459.295(y4)
546.327(y5)
638.260(b6)
785.329(b7)
909.976(y17)
1000.419(b9)
1024.005
1163.566
1209.692(y11)
1429.669
1540.803
1557.831(y14)
1641.751
1750.847(b16)
1761.911(y16)
1818.944(y17)
1966.007(y18)
CID-MS/MS Scan:38625, Noise threshold:0.5
Relative Intensity (%)
1142.576(PEP+HexNAc(1)_2)
366.144(HexNAc + Hex)
528.198(Hex + HexNAc + Hex)
762.051(PEP+HexNAc(1)_3)
829.744(PEP+HexNAc(2)_3)
852.307
883.763(PEP+HexNAc(2)Hex(1)_3)
937.781(PEP+HexNAc(2)Hex(2)_3)
991.799(PEP+HexNAc(2)Hex(3)_3)
1014.361
1125.063
1134.068
1206.090
1224.100
1244.112(PEP+HexNAc(2)_2)
1305.128
1325.139(PEP+HexNAc(2)Hex(1)_2)
1386.155
1406.168(PEP+HexNAc(2)Hex(2)_2)
1467.183
1487.195(PEP+HexNAc(2)Hex(3)_2)
1568.223(PEP+HexNAc(2)Hex(4)_2)
ETD-MS/MS Scan:38626, Noise threshold:1.0
Relative Intensity (%)
M+2H
443.276
743.397
952.527
1045.813(PEP+HexNAc(2)Hex(4)_3)
M+3H
1143.069
1194.678
1453.663
1482.172
1510.683
1518.194
1568.199(PEP+HexNAc(2)Hex(4)_2)
1585.201
1641.735
m/z

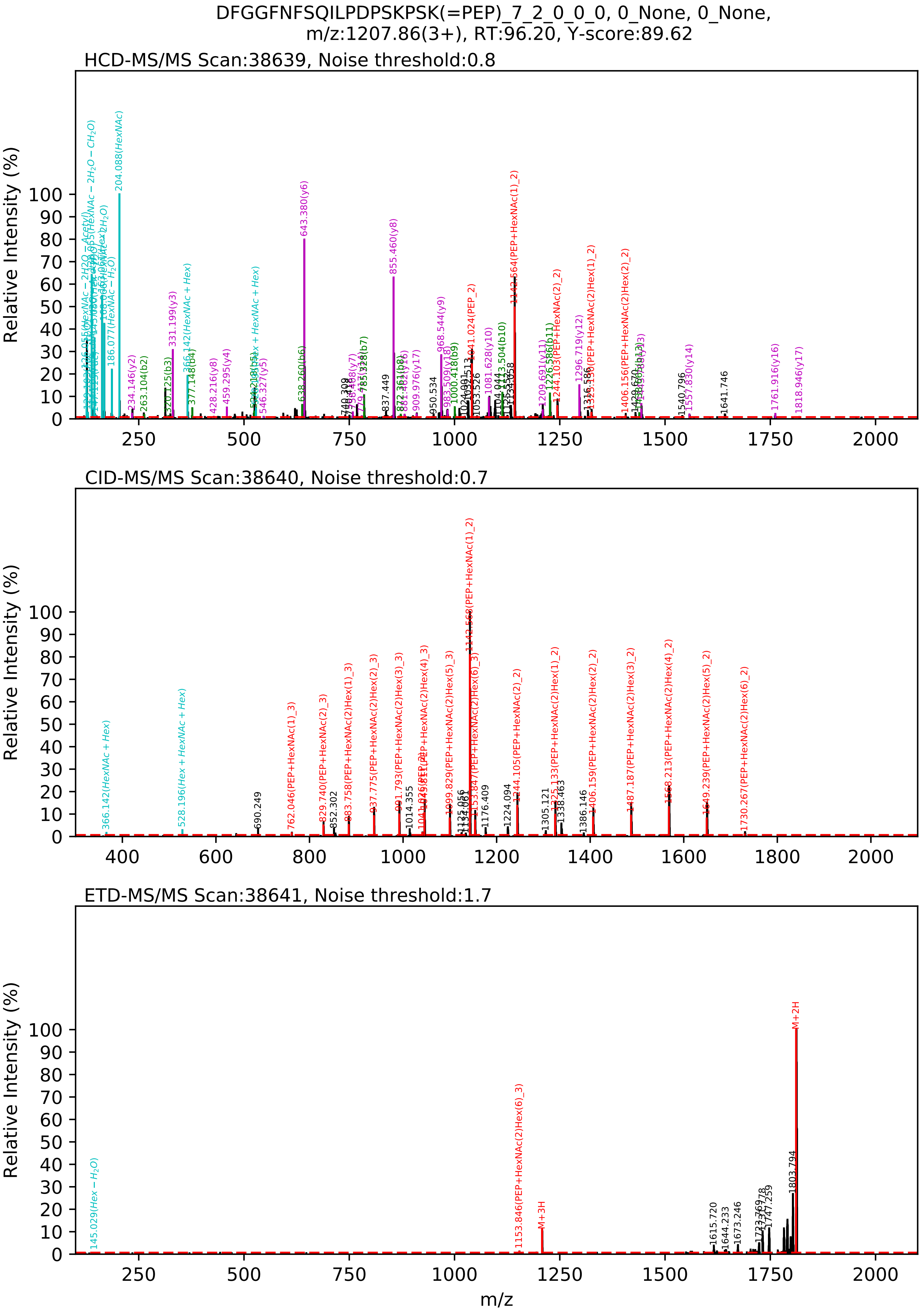
DFGGFNFSQILPDPSKPSK(=PEP)_7_2_0_0_0, 0_None, 0_None,
m/z:1207.86(3+), RT:96.20, Y-score:89.62
HCD-MS/MS Scan:38639, Noise threshold:0.8
Relative Intensity (%)
204.088(HexNAc)
186.077(HexNAc − H2O)
331.199(y3)
643.380(y6)
855.460(y8)
968.544(y9)
1041.024(PEP_2)
1081.628(y10)
1142.564(PEP+HexNAc(1)_2)
1244.103(PEP+HexNAc(2)_2)
1296.719(y12)
1325.136(PEP+HexNAc(2)Hex(1)_2)
1406.156(PEP+HexNAc(2)Hex(2)_2)
1557.830(y14)
1641.746
1761.916(y16)
1818.946(y17)
CID-MS/MS Scan:38640, Noise threshold:0.7
Relative Intensity (%)
366.142(HexNAc + Hex)
528.196(Hex + HexNAc + Hex)
690.249
762.046(PEP+HexNAc(1)_3)
829.740(PEP+HexNAc(2)_3)
852.302
883.758(PEP+HexNAc(2)Hex(1)_3)
937.775(PEP+HexNAc(2)Hex(2)_3)
991.793(PEP+HexNAc(2)Hex(3)_3)
1014.355
1099.829(PEP+HexNAc(2)Hex(5)_3)
1142.568(PEP+HexNAc(1)_2)
1176.409
1224.094
1244.105(PEP+HexNAc(2)_2)
1305.121
1325.133(PEP+HexNAc(2)Hex(1)_2)
1338.463
1386.146
1406.159(PEP+HexNAc(2)Hex(2)_2)
1487.187(PEP+HexNAc(2)Hex(3)_2)
1568.213(PEP+HexNAc(2)Hex(4)_2)
1649.239(PEP+HexNAc(2)Hex(5)_2)
1730.267(PEP+HexNAc(2)Hex(6)_2)
ETD-MS/MS Scan:38641, Noise threshold:1.7
Relative Intensity (%)
145.029(Hex − H2O)
1153.846(PEP+HexNAc(2)Hex(6)_3)
M+3H
1615.720
1644.233
1673.246
1747.259
1803.794
M+2H
m/z

DFGGFNFSQILPDPSKPSK(=PEP)_8_2_0_0_0, 0_None, 0_None,
m/z:1261.88(3+), RT:93.39, Y-score:89.86

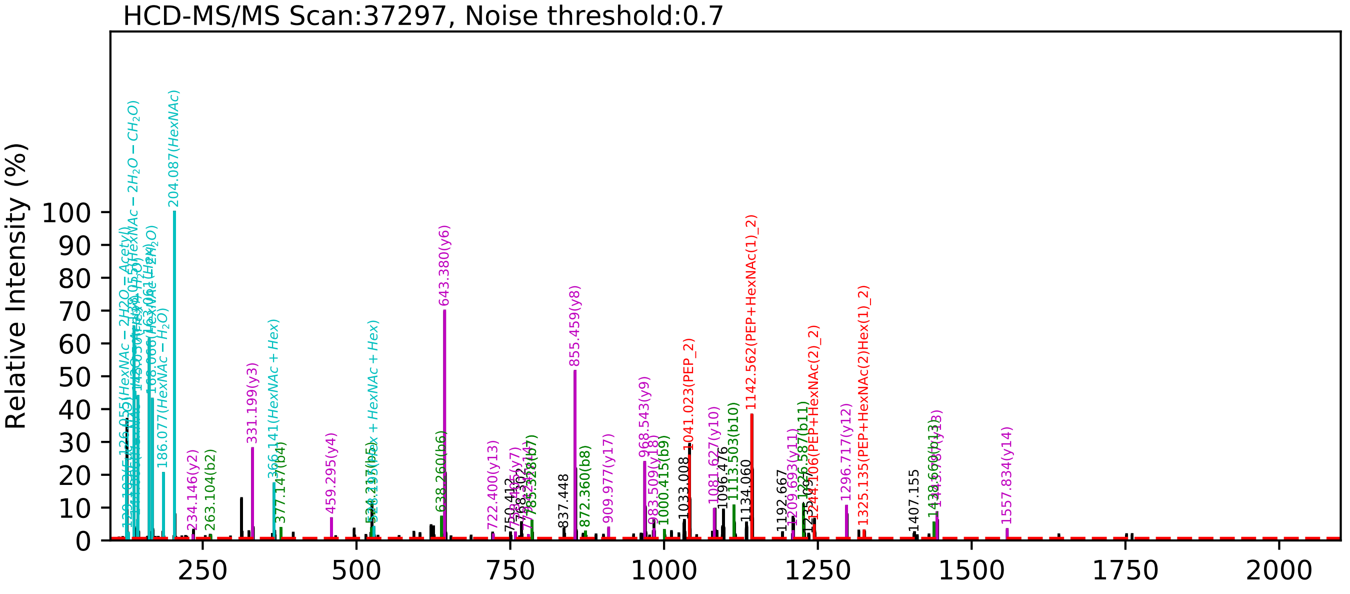

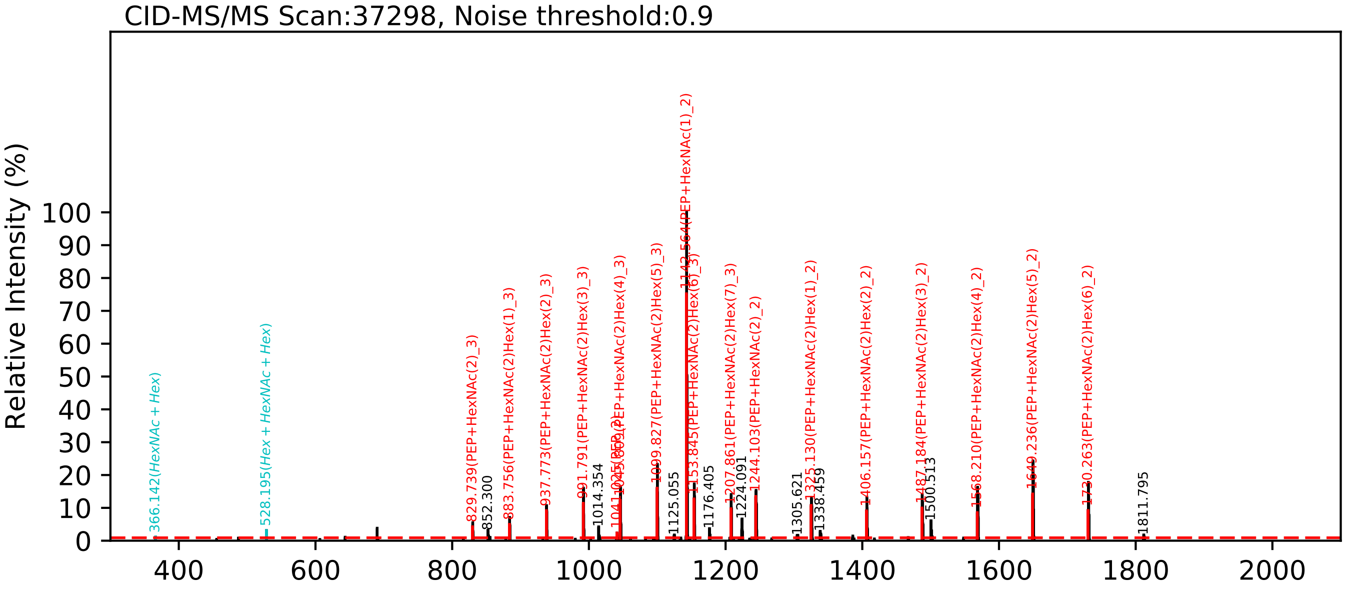

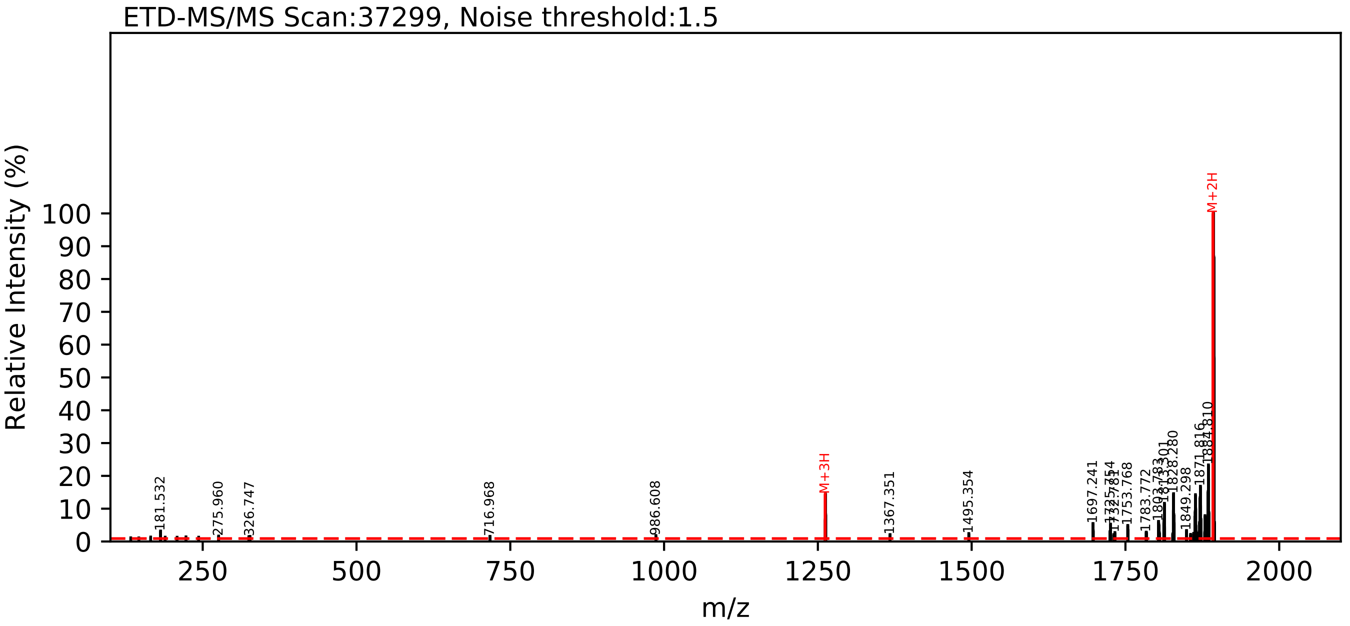

DFGGFNFSQILPDPSKPSK(=PEP)_8_2_0_0_0, 0_None, 0_None,
m/z:1261.88(3+), RT:96.06, Y-score:89.18

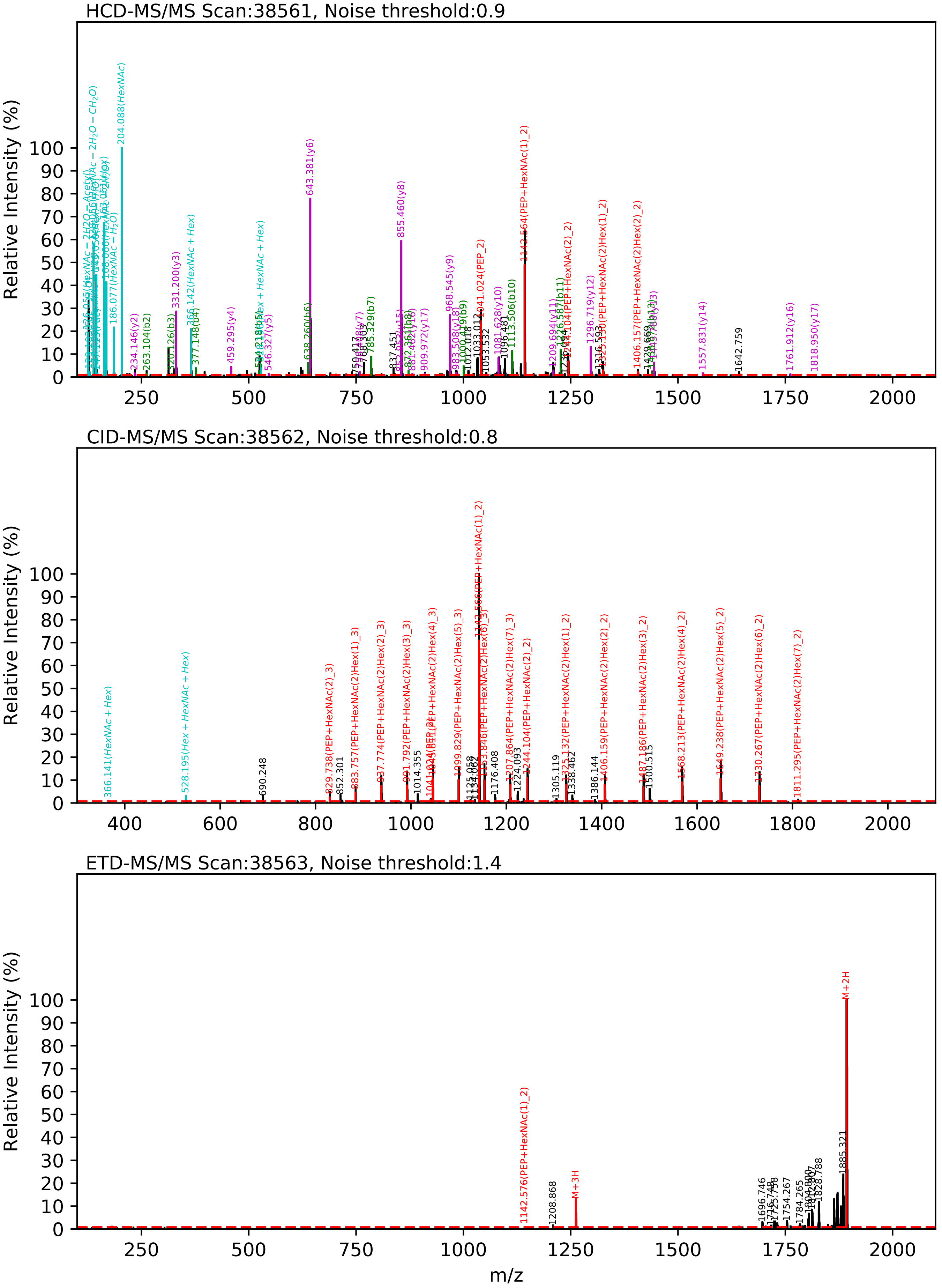

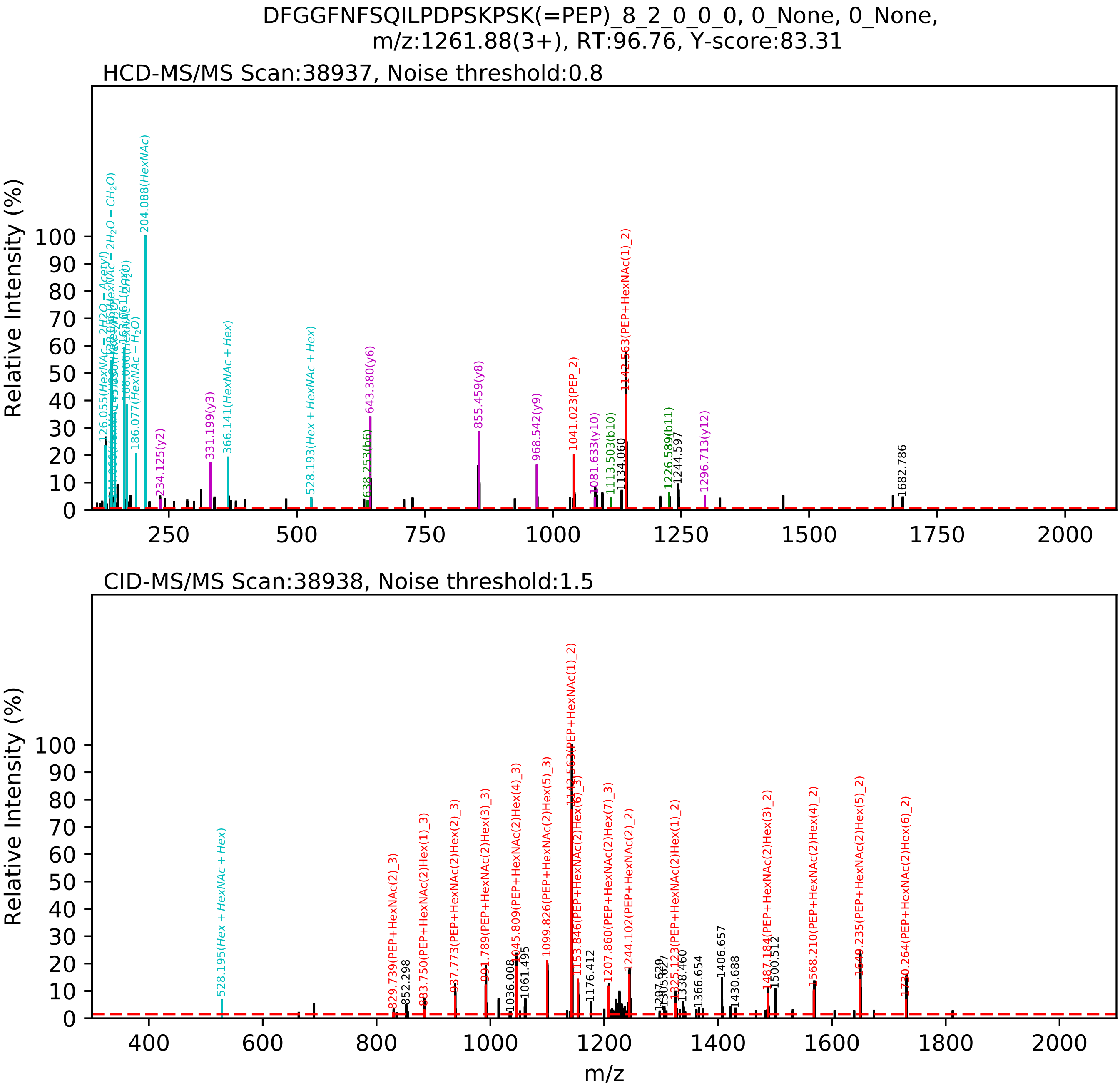
DFGGFNFSQILPDPSKPSK(=PEP)_8_2_0_0_0, 0_None, 0_None,
m/z:1261.88(3+), RT:96.76, Y-score:83.31
HCD-MS/MS Scan:38937, Noise threshold:0.8
Relative Intensity (%)
204.088(HexNAc)
186.077(HexNAc − H2O)
234.125(y2)
331.199(y3)
366.141(HexNAc + Hex)
528.193(Hex + HexNAc + Hex)
638.253(b6)
643.380(y6)
855.459(y8)
968.542(y9)
1041.023(PEP_2)
1081.633(y10)
1113.503(b10)
1134.060
1142.563(PEP+HexNAc(1)_2)
1226.589(b11)
1244.597
1296.713(y12)
1682.786
CID-MS/MS Scan:38938, Noise threshold:1.5
Relative Intensity (%)
528.195(Hex + HexNAc + Hex)
829.739(PEP+HexNAc(2)_3)
852.298
883.750(PEP+HexNAc(2)Hex(1)_3)
937.773(PEP+HexNAc(2)Hex(2)_3)
991.789(PEP+HexNAc(2)Hex(3)_3)
1036.008
1045.809(PEP+HexNAc(2)Hex(4)_3)
1061.495
1099.826(PEP+HexNAc(2)Hex(5)_3)
1142.563(PEP+HexNAc(1)_2)
1153.846(PEP+HexNAc(2)Hex(6)_3)
1176.412
1207.860(PEP+HexNAc(2)Hex(7)_3)
1244.102(PEP+HexNAc(2)_2)
1325.123(PEP+HexNAc(2)Hex(1)_2)
1338.460
1366.654
1406.657
1430.688
1487.184(PEP+HexNAc(2)Hex(3)_2)
1500.512
1568.210(PEP+HexNAc(2)Hex(4)_2)
1649.235(PEP+HexNAc(2)Hex(5)_2)
1730.264(PEP+HexNAc(2)Hex(6)_2)
m/z

DFGGFNFSQILPDPSKPSK(=PEP)_9_2_0_0_0, 0_None, 0_None,
m/z:1315.90(3+), RT:93.30, Y-score:88.51

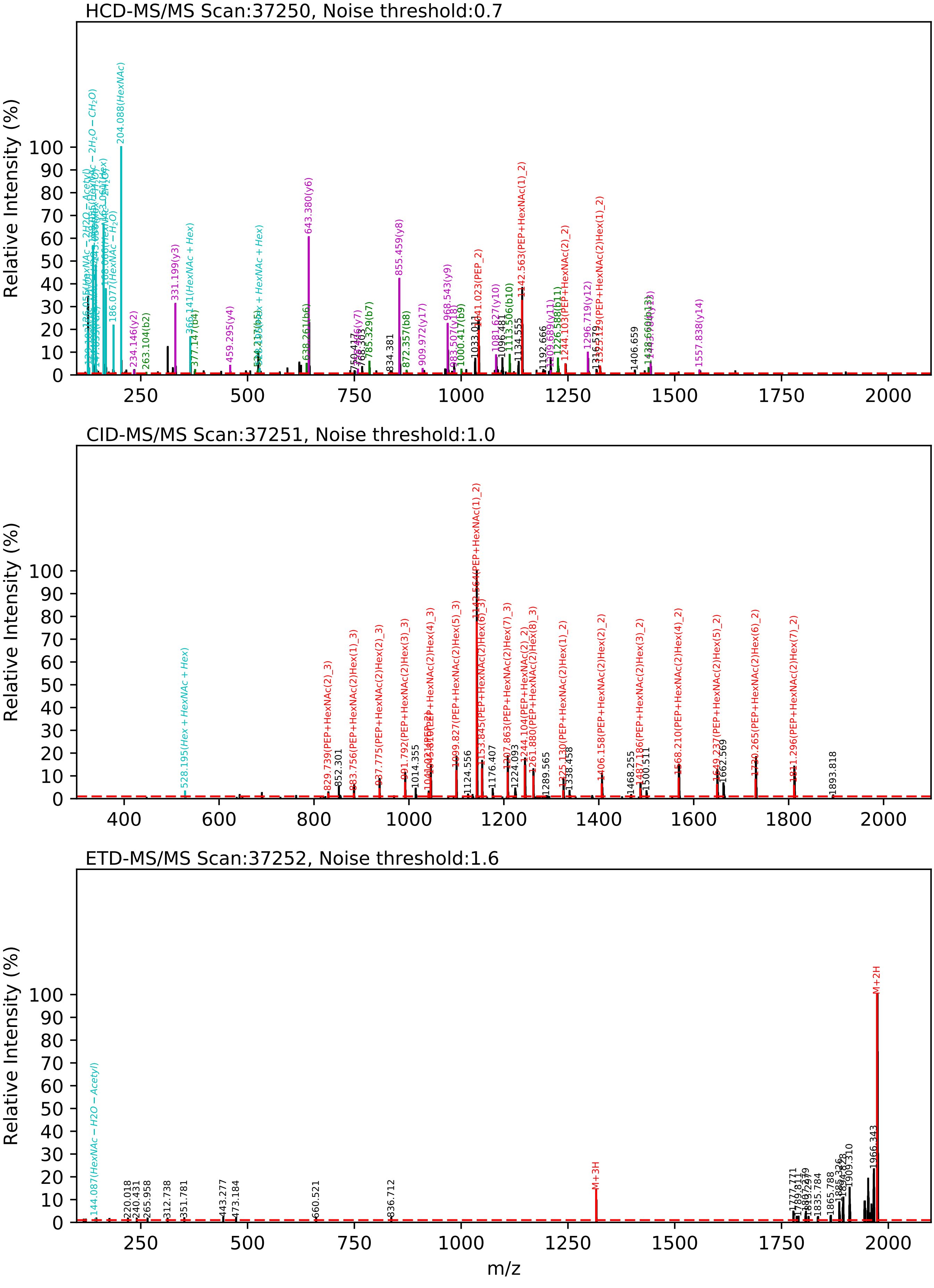

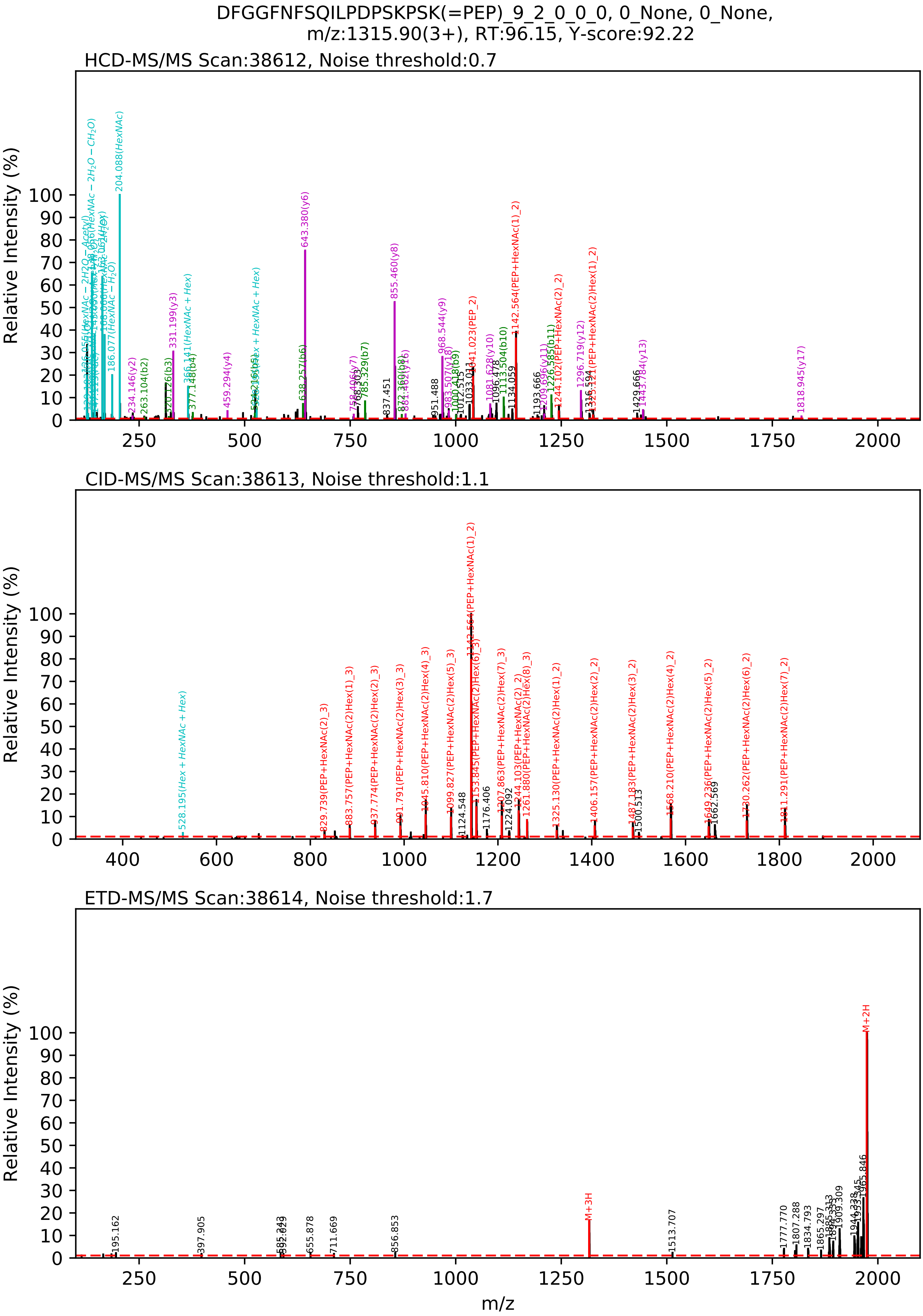
DFGGGFNFSQILPDPSKPSK(=PEP)_9_2_0_0_0, 0_None, 0_None,
m/z:1315.90(3+), RT:96.15, Y-score:92.22
HCD-MS/MS Scan:38612, Noise threshold:0.7
Relative Intensity (%)
204.088(HexNAc)
186.077(HexNAc − H2O)
234.146(y2)
263.104(b2)
320.126(b3)
331.199(y3)
377.148(b4)
459.294(y4)
643.380(y6)
638.257(b6)
758.406(y7)
768.303
785.329(b7)
837.451
855.460(y8)
951.488
968.544(y9)
1012.515
1033.011
1041.023(PEP_2)
1081.628(y10)
1096.478
1113.504(b10)
1134.059
1142.564(PEP+HexNAc(1)_2)
1193.666
1244.102(PEP+HexNAc(2)_2)
1296.719(y12)
1325.131(PEP+HexNAc(2)Hex(1)_2)
1429.666
1443.784(y13)
1818.945(y17)
CID-MS/MS Scan:38613, Noise threshold:1.1
Relative Intensity (%)
528.195(Hex + HexNAc + Hex)
829.739(PEP+HexNAc(2)_3)
883.757(PEP+HexNAc(2)Hex(1)_3)
937.774(PEP+HexNAc(2)Hex(2)_3)
991.791(PEP+HexNAc(2)Hex(3)_3)
1045.810(PEP+HexNAc(2)Hex(4)_3)
1099.827(PEP+HexNAc(2)Hex(5)_3)
1124.548
1142.564(PEP+HexNAc(1)_2)
1153.845(PEP+HexNAc(2)Hex(6)_3)
1176.406
1207.863(PEP+HexNAc(2)Hex(7)_3)
1224.092
1244.103(PEP+HexNAc(2)_2)
1261.880(PEP+HexNAc(2)Hex(8)_3)
1325.130(PEP+HexNAc(2)Hex(1)_2)
1406.157(PEP+HexNAc(2)Hex(2)_2)
1487.183(PEP+HexNAc(2)Hex(3)_2)
1500.513
1568.210(PEP+HexNAc(2)Hex(4)_2)
1649.236(PEP+HexNAc(2)Hex(5)_2)
1662.569
1730.262(PEP+HexNAc(2)Hex(6)_2)
1811.291(PEP+HexNAc(2)Hex(7)_2)
ETD-MS/MS Scan:38614, Noise threshold:1.7
Relative Intensity (%)
195.162
397.905
655.878
711.669
856.853
M+3H
1513.707
1777.770
1807.288
1834.793
1865.297
1909.309
1965.846
M+2H
m/z

DFGGFNFSQILPDPSKPSK(=PEP)_9_2_0_0_0, 0_None, 0_None,
m/z:1315.90(3+), RT:90.51, Y-score:87.50

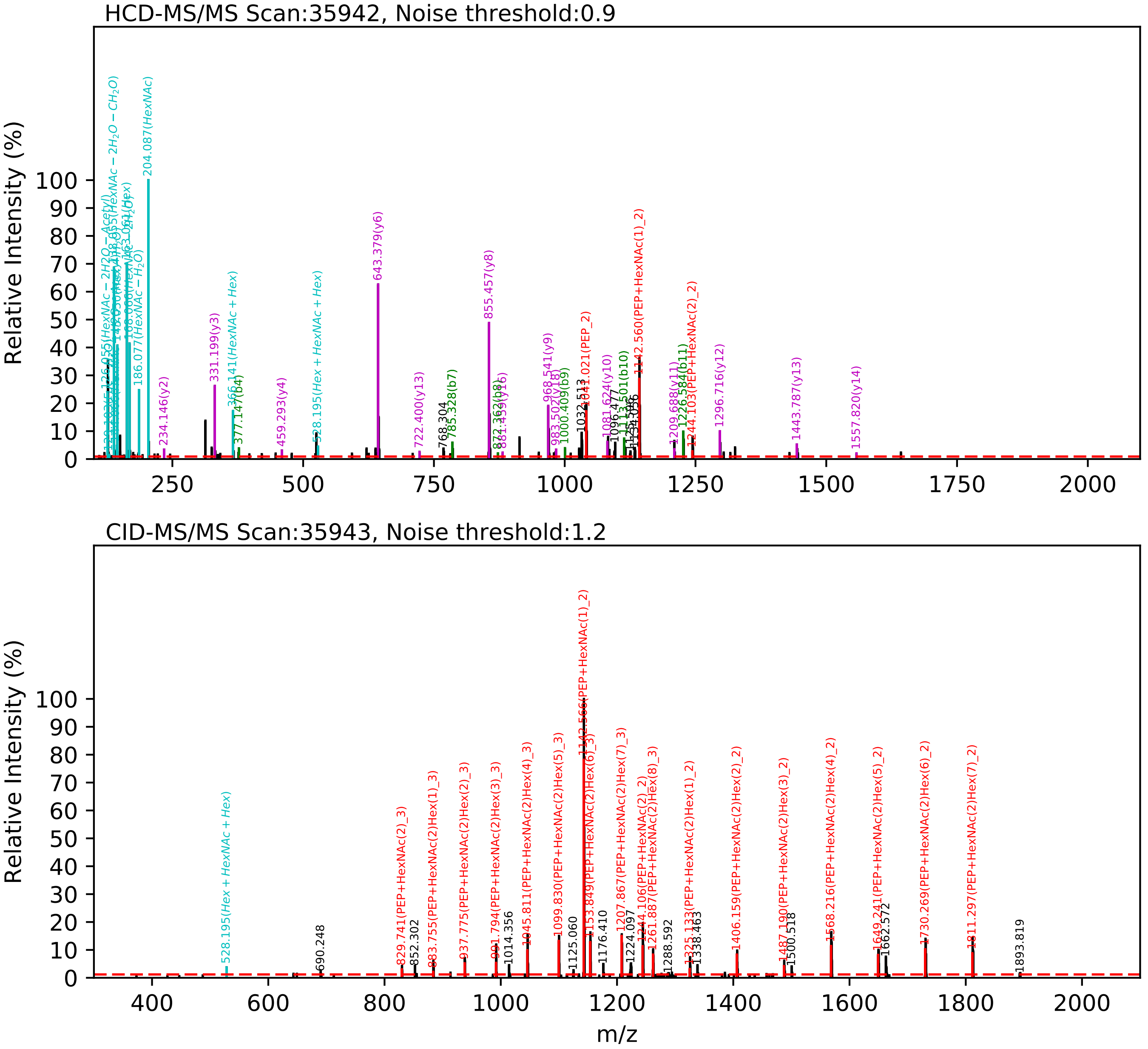

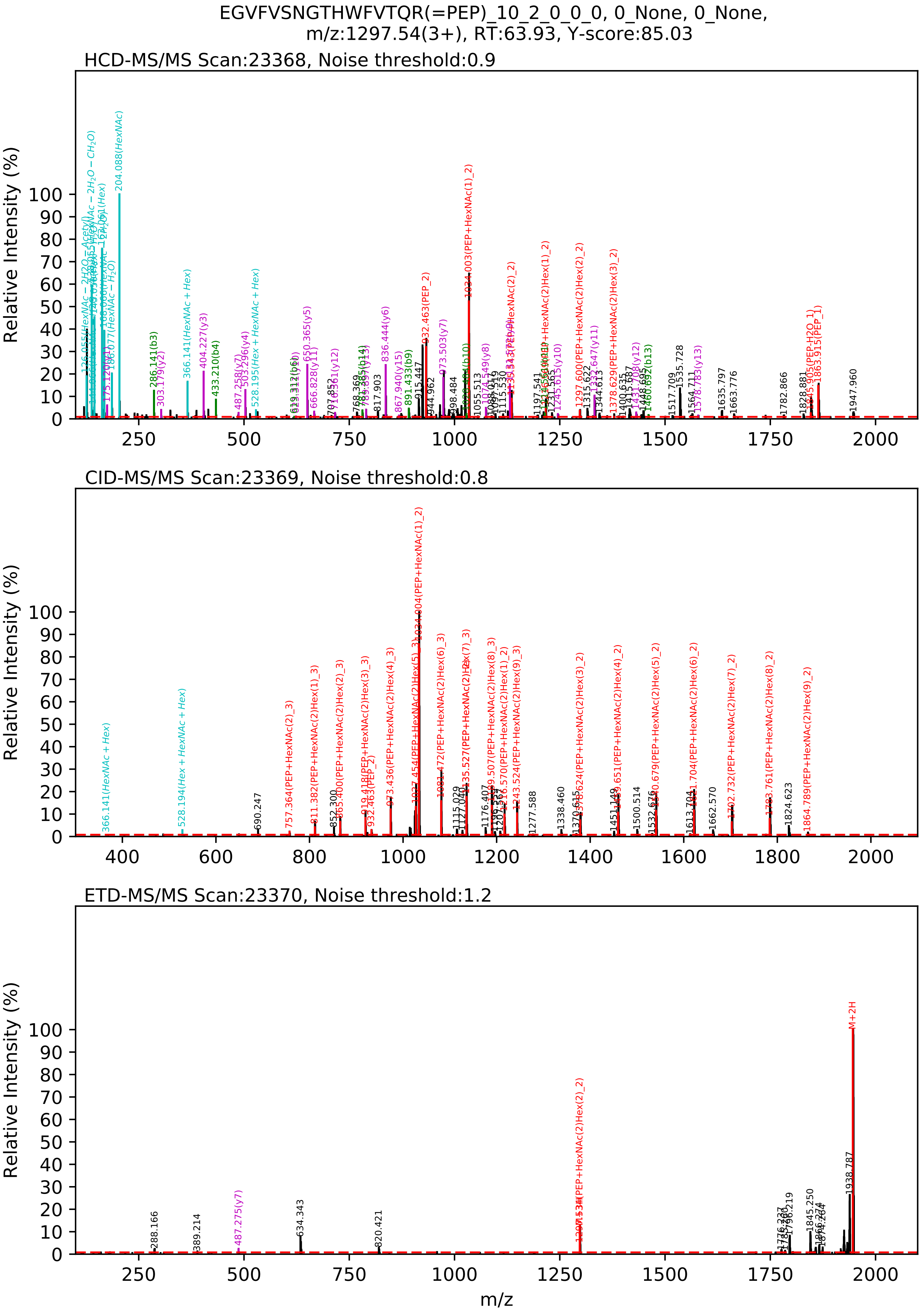
EGVFVSNGTHWFVTQR(=PEP)_10_2_0_0_0, 0_None, 0_None,
m/z:1297.54(3+), RT:63.93, Y-score:85.03
HCD-MS/MS Scan:23368, Noise threshold:0.9
CID-MS/MS Scan:23369, Noise threshold:0.8
ETD-MS/MS Scan:23370, Noise threshold:1.2
Relative Intensity (%)
m/z
204.088(HexNAc)
1034.003(PEP+HexNAc(1)_2)
932.463(PEP_2)
973.503(y7)
1034.004(PEP+HexNAc(1)_2)
M+2H
1938.787

EGVFVSNGTHWFVTQR(=PEP)_10_2_0_0_0, 0_None, 0_None,
m/z:1297.54(3+), RT:64.22, Y-score:69.22

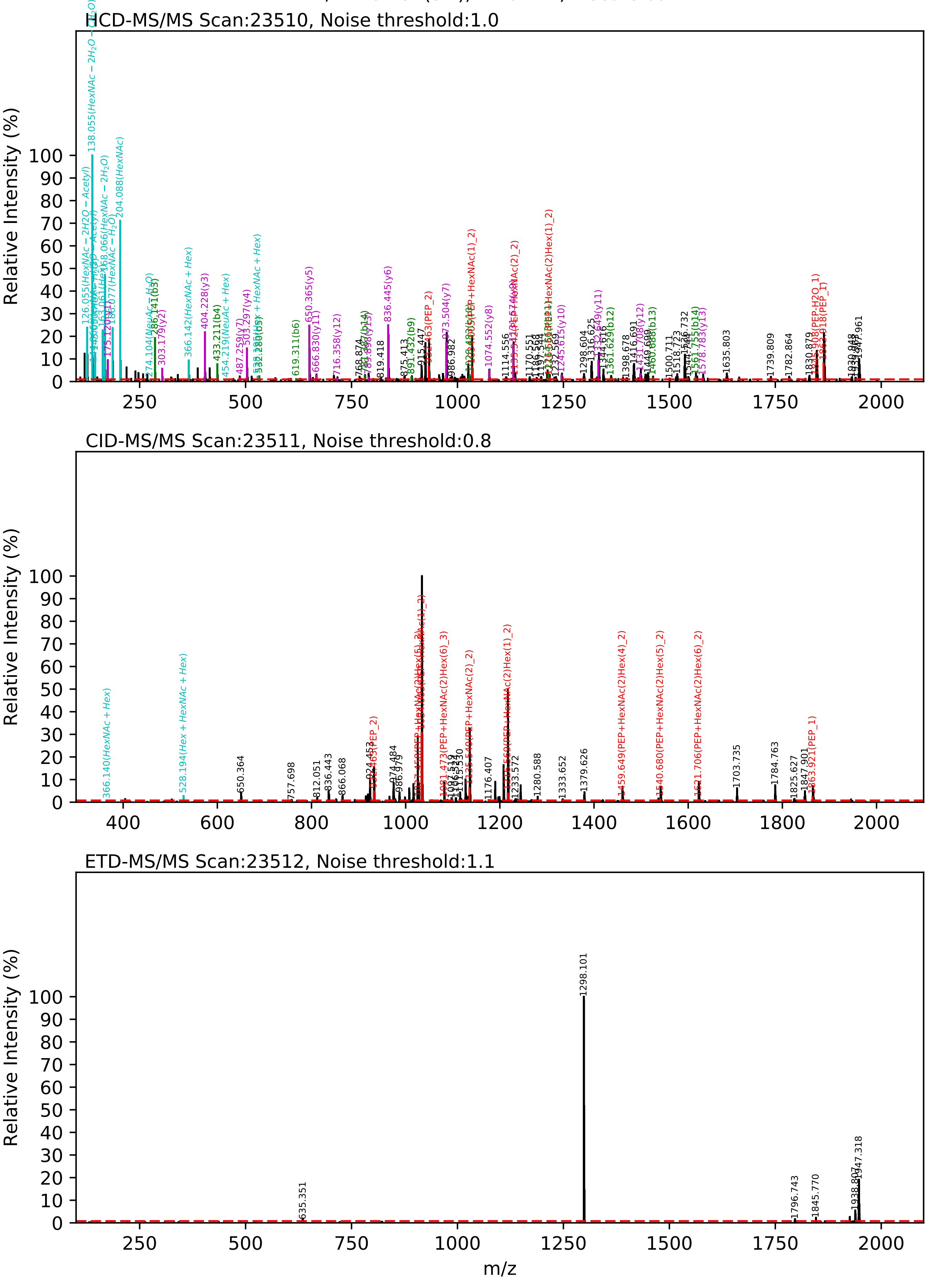

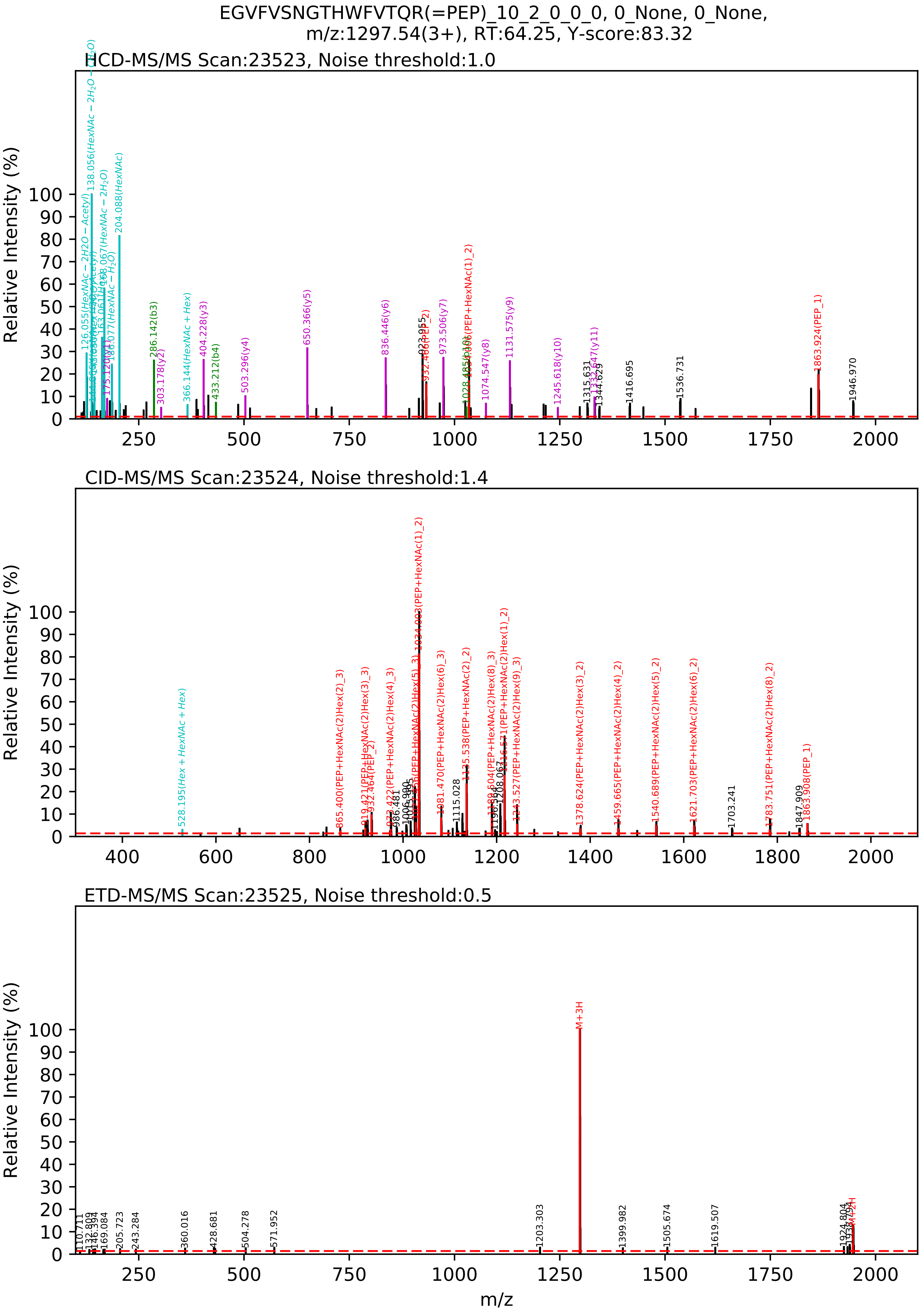
EGVFVSNGTHWFVTQR(=PEP)_10_2_0_0_0, 0_None, 0_None,
m/z:1297.54(3+), RT:64.25, Y-score:83.32
HCD-MS/MS Scan:23523, Noise threshold:1.0
CID-MS/MS Scan:23524, Noise threshold:1.4
ETD-MS/MS Scan:23525, Noise threshold:0.5
Relative Intensity (%)
m/z
138.056(HexNAc − 2H2O − H2O)
204.088(HexNAc)
286.142(b3)
366.144(HexNAc + Hex)
404.228(y3)
433.212(b4)
503.296(y4)
650.366(y5)
836.446(y6)
973.506(y7)
1074.547(y8)
1131.575(y9)
1245.618(y10)
1863.924(PEP_1)
1946.970
1536.731
1416.695
528.195(Hex + HexNAc + Hex)
865.400(PEP+HexNAc(2)Hex(2)_3)
1034.003(PEP+HexNAc(1)_2)
1081.470(PEP+HexNAc(2)Hex(6)_3)
1135.538(PEP+HexNAc(2)Hex(2)_2)
1243.527(PEP+HexNAc(2)Hex(9)_3)
1378.624(PEP+HexNAc(2)Hex(3)_2)
1540.689(PEP+HexNAc(2)Hex(5)_2)
1621.703(PEP+HexNAc(2)Hex(6)_2)
1703.241
1847.909
1863.908(PEP_1)
1115.028
M+3H
1203.303
1399.982
1505.674
1619.507
571.952
504.278
428.681
360.016
243.284
205.723
169.084

EGVFVSNGTHWFVTQR(=PEP)_10_2_0_0_0, 0_None, 0_None,
m/z:1297.54(3+), RT:66.14, Y-score:67.19

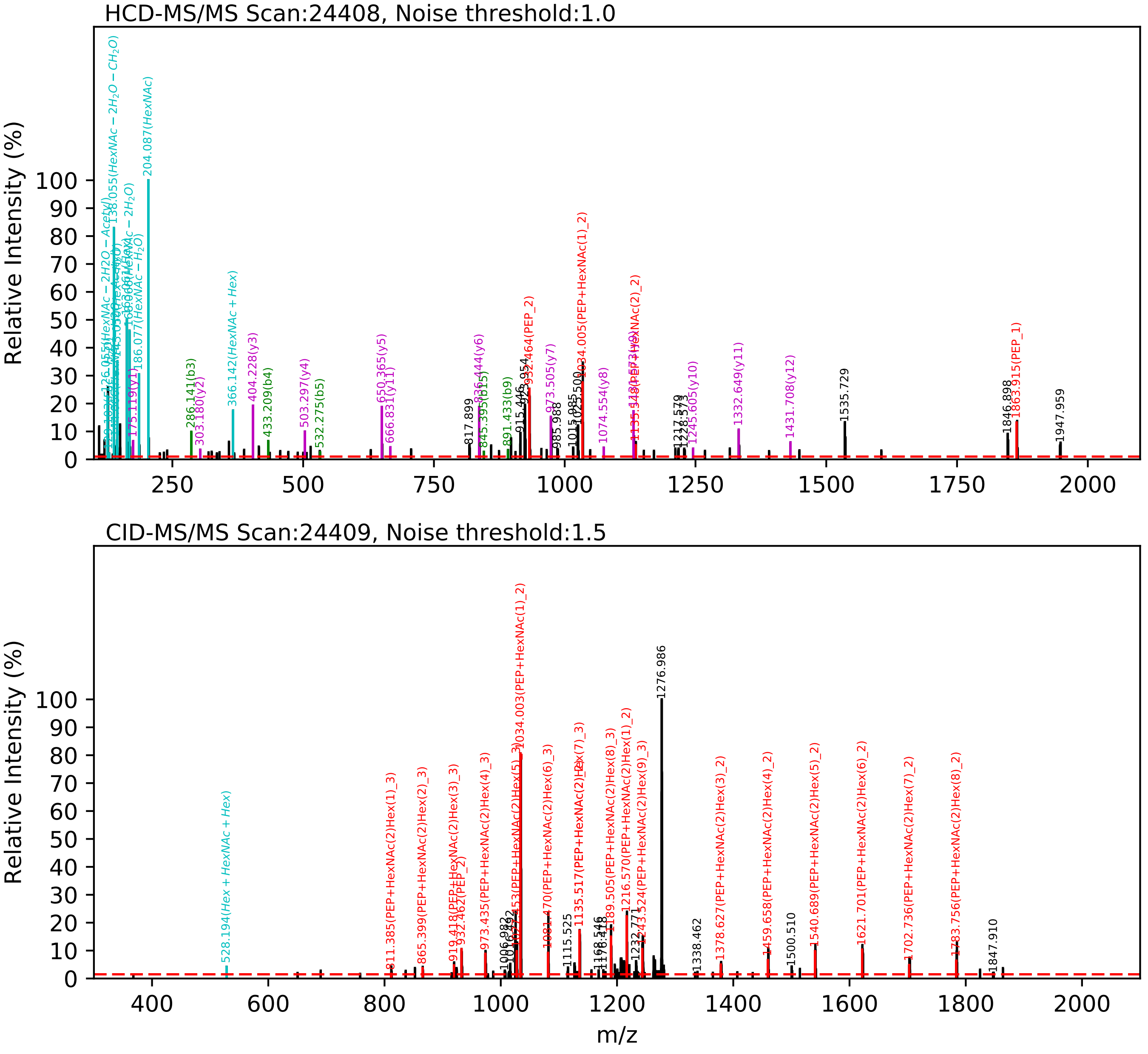

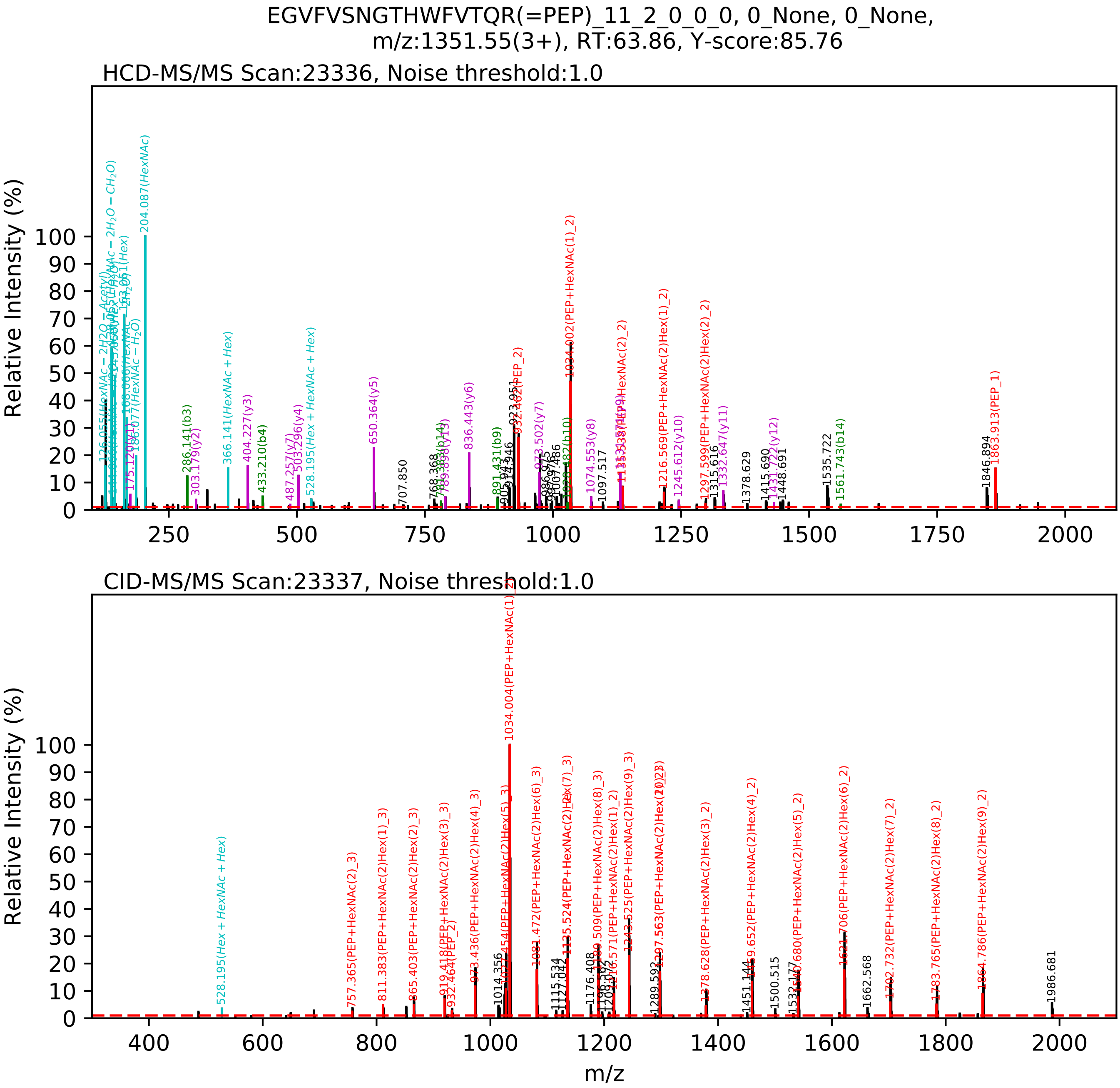
EGVFVSNGTHWFVTQR(=PEP)_11_2_0_0_0, 0_None, 0_None,
m/z:1351.55(3+), RT:63.86, Y-score:85.76
HCD-MS/MS Scan:23336, Noise threshold:1.0
Relative Intensity (%)
204.087(HexNAc)
286.141(b3)
303.179(y2)
366.141(HexNAc + Hex)
404.227(y3)
433.210(b4)
487.257(y7)
503.296(y4)
528.195(Hex + HexNAc + Hex)
650.364(y5)
707.850
768.368
836.443(y6)
891.431(b9)
923.951
932.462(PEP_2)
973.502(y7)
1007.486
1034.002(PEP+HexNAc(1)_2)
1074.553(y8)
1097.517
1135.338(PEP+HexNAc(2)_2)
1216.569(PEP+HexNAc(2)Hex(1)_2)
1245.612(y10)
1297.599(PEP+HexNAc(2)Hex(2)_2)
1315.616
1332.647(y11)
1378.629
1415.690
1431.722(y12)
1448.691
1535.722
1561.743(b14)
1846.894
1863.913(PEP_1)
CID-MS/MS Scan:23337, Noise threshold:1.0
Relative Intensity (%)
528.195(Hex + HexNAc + Hex)
757.365(PEP+HexNAc(2)_3)
811.383(PEP+HexNAc(2)Hex(1)_3)
865.403(PEP+HexNAc(2)Hex(2)_3)
919.418(PEP+HexNAc(2)Hex(3)_3)
932.464(PEP_2)
973.436(PEP+HexNAc(2)Hex(4)_3)
1014.356
1034.004(PEP+HexNAc(1)_2)
1081.472(PEP+HexNAc(2)Hex(6)_3)
1115.534
1135.524(PEP+HexNAc(2)Hex(7)_3)
1176.408
1216.571(PEP+HexNAc(2)Hex(1)_2)
1243.525(PEP+HexNAc(2)Hex(9)_3)
1289.592
1297.563(PEP+HexNAc(2)Hex(2)_2)
1378.628(PEP+HexNAc(2)Hex(3)_2)
1451.144
1459.652(PEP+HexNAc(2)Hex(4)_2)
1500.515
1532.177
1540.680(PEP+HexNAc(2)Hex(5)_2)
1621.706(PEP+HexNAc(2)Hex(6)_2)
1662.568
1702.732(PEP+HexNAc(2)Hex(7)_2)
1783.765(PEP+HexNAc(2)Hex(8)_2)
1864.786(PEP+HexNAc(2)Hex(9)_2)
1986.681
m/z

EGVFVSNGTHWFVTQR(=PEP)_4_2_0_0_0, 0_None, 0_None,
m/z:973.43(3+), RT:64.21, Y-score:82.18

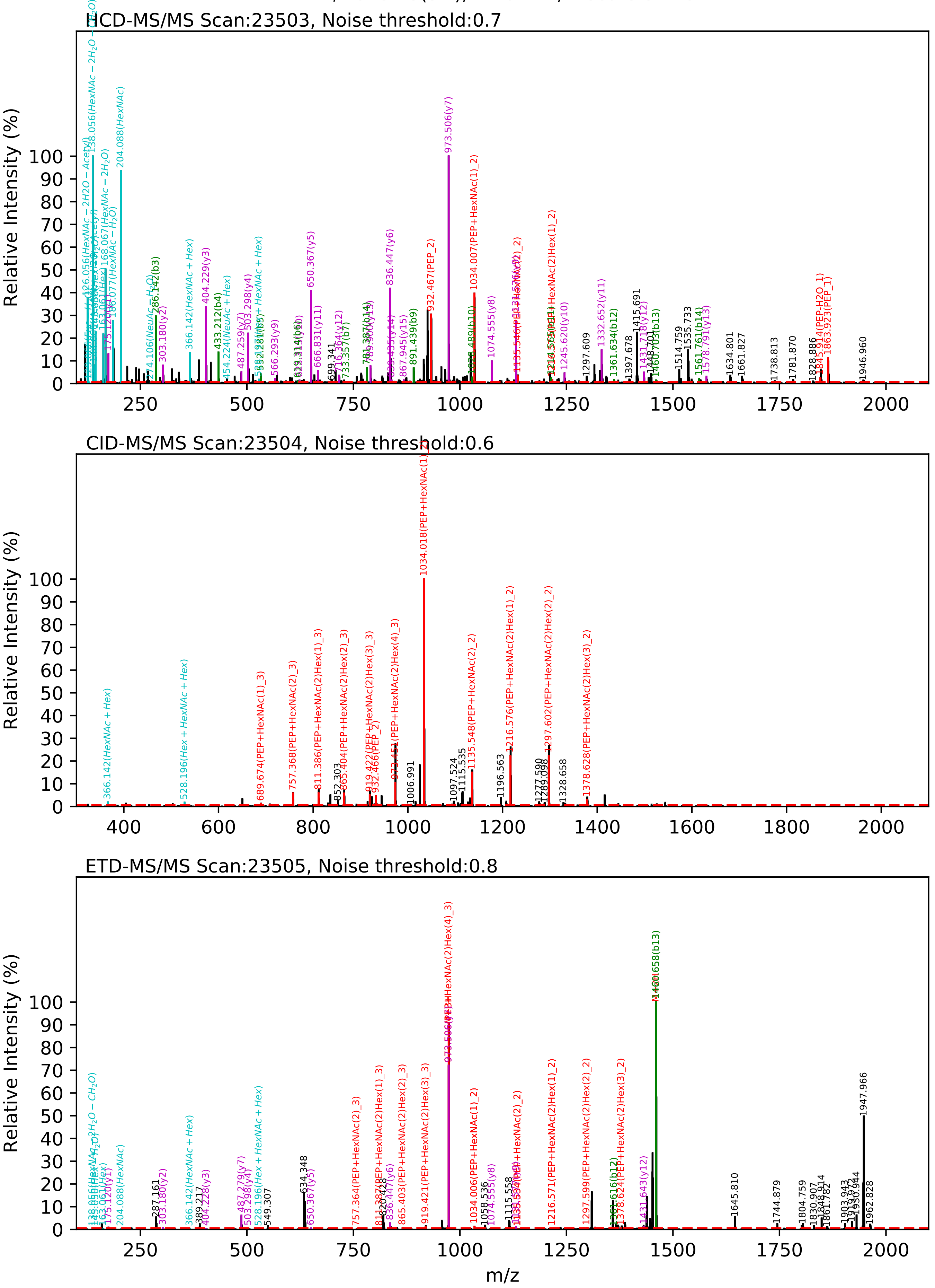

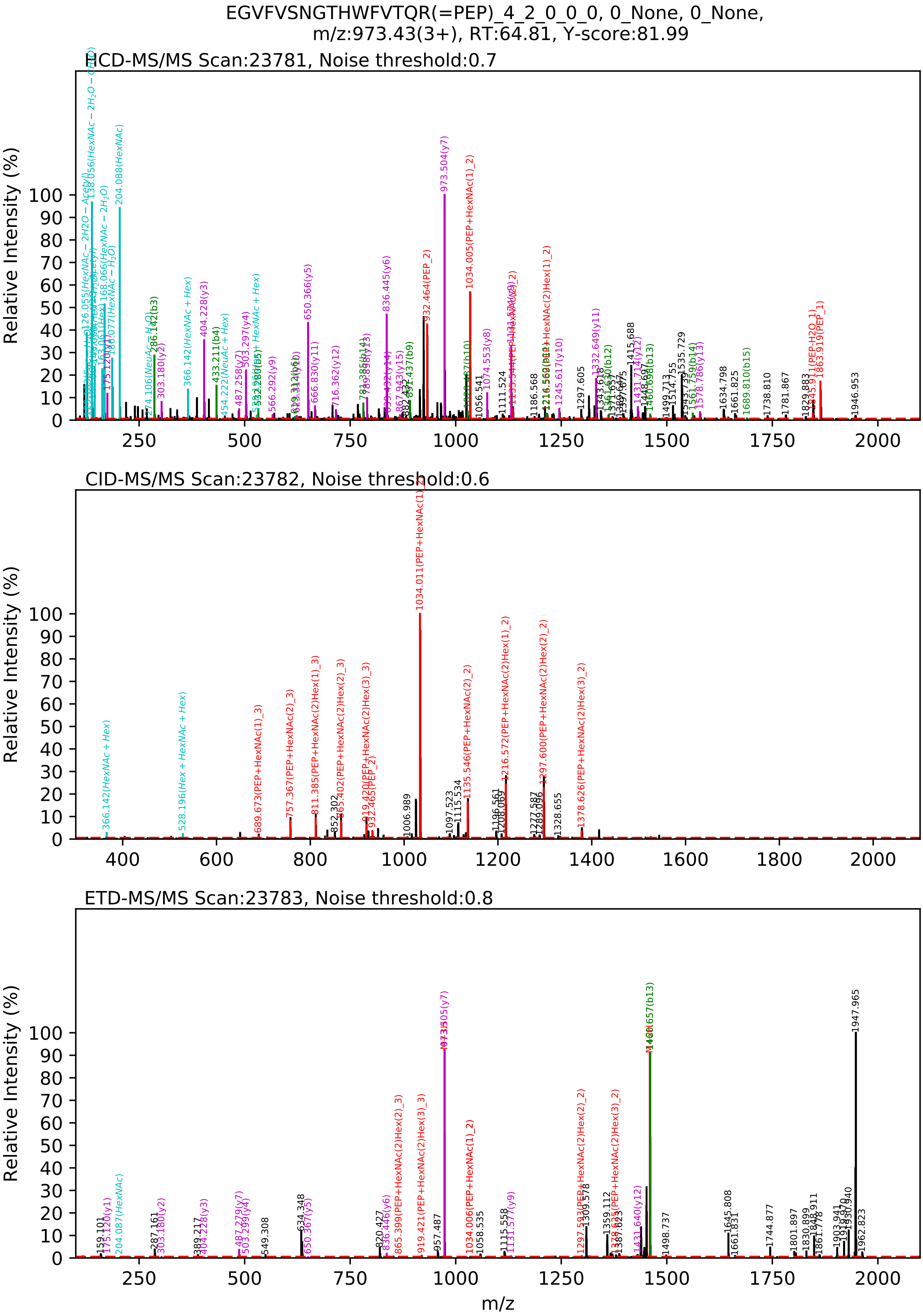
EGVFVSNGTHWFVTQR(=PEP)_4_2_0_0_0, 0_None, 0_None,
m/z:973.43(3+), RT:64.81, Y-score:81.99
HCD-MS/MS Scan:23781, Noise threshold:0.7
CID-MS/MS Scan:23782, Noise threshold:0.6
ETD-MS/MS Scan:23783, Noise threshold:0.8
Relative Intensity (%)
m/z
973.504(y7)
1034.005(PEP+HexNAc(1)_2)
932.464(PEP_2)
836.445(y6)
650.366(y5)
204.088(HexNAc)
404.228(y3)
1034.011(PEP+HexNAc(1)_2)
1216.572(PEP+HexNAc(2)Hex(1)_2)
1297.600(PEP+HexNAc(2)Hex(2)_2)
1378.626(PEP+HexNAc(2)Hex(3)_2)
1135.546(PEP+HexNAc(2)_2)
757.367(PEP+HexNAc(2)_3)
811.385(PEP+HexNAc(2)Hex(1)_3)
689.673(PEP+HexNAc(1)_3)
366.142(HexNAc+Hex)
528.196(Hex+HexNAc+Hex)
973.505(y7)
1947.965
1460.657(b13)
865.399(PEP+HexNAc(2)Hex(2)_3)
919.421(PEP+HexNAc(2)Hex(3)_3)
1034.006(PEP+HexNAc(1)_2)
1645.808

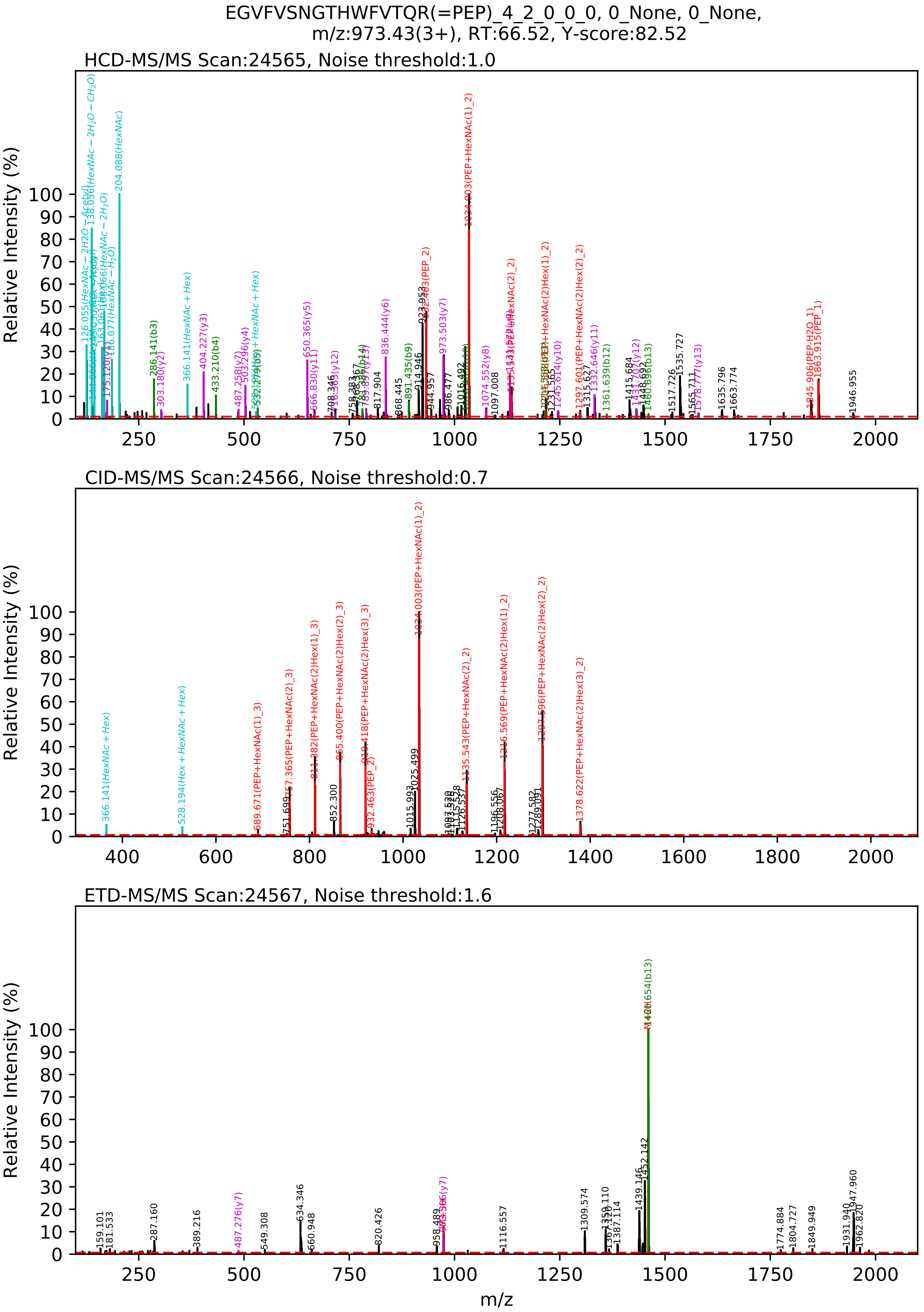
EGVFVSNGTHWFVTQR(=PEP)_4_2_0_0_0, 0_None, 0_None,
m/z:973.43(3+), RT:66.52, Y-score:82.52
HCD-MS/MS Scan:24565, Noise threshold:1.0
Relative Intensity (%)
204.088(HexNAc)
1034.003(PEP+HexNAc(1)_2)
286.141(b3)
303.180(y2)
366.141(HexNAc + Hex)
404.227(y3)
433.210(b4)
487.258(y7)
650.365(y5)
666.830(y11)
708.346
817.904
836.444(y6)
868.445
891.435(b9)
914.946
923.953
944.957
973.503(y7)
986.477
1016.492
1074.552(y8)
1097.008
1231.565
1245.614(y10)
1297.601(PEP+HexNAc(2)Hex(2)_2)
1315.627
1361.639(b12)
1415.684
1517.726
1535.727
1565.711
1578.777(y13)
1635.796
1663.774
1845.906(PEP-H2O_1)
1863.915(PEP_1)
1946.955
CID-MS/MS Scan:24566, Noise threshold:0.7
366.141(HexNAc + Hex)
528.194(Hex + HexNAc + Hex)
689.671(PEP+HexNAc(1)_3)
751.699
757.365(PEP+HexNAc(2)_3)
811.382(PEP+HexNAc(2)Hex(1)_3)
852.300
865.400(PEP+HexNAc(2)Hex(2)_3)
919.418(PEP+HexNAc(2)Hex(3)_3)
932.463(PEP_2)
1015.993
1025.499
1034.003(PEP+HexNAc(1)_2)
1135.543(PEP+HexNAc(2)_2)
1196.556
1208.067
1216.569(PEP+HexNAc(2)Hex(1)_2)
1277.582
1297.596(PEP+HexNAc(2)Hex(2)_2)
1378.622(PEP+HexNAc(2)Hex(3)_2)
ETD-MS/MS Scan:24567, Noise threshold:1.6
159.101
181.533
287.160
389.216
487.276(y7)
549.308
634.346
660.948
820.426
958.489
1116.557
1309.574
1359.110
1367.120
1387.114
1439.146
1452.142
1774.884
1804.727
1849.949
1931.940
1947.960
1962.820
m/z

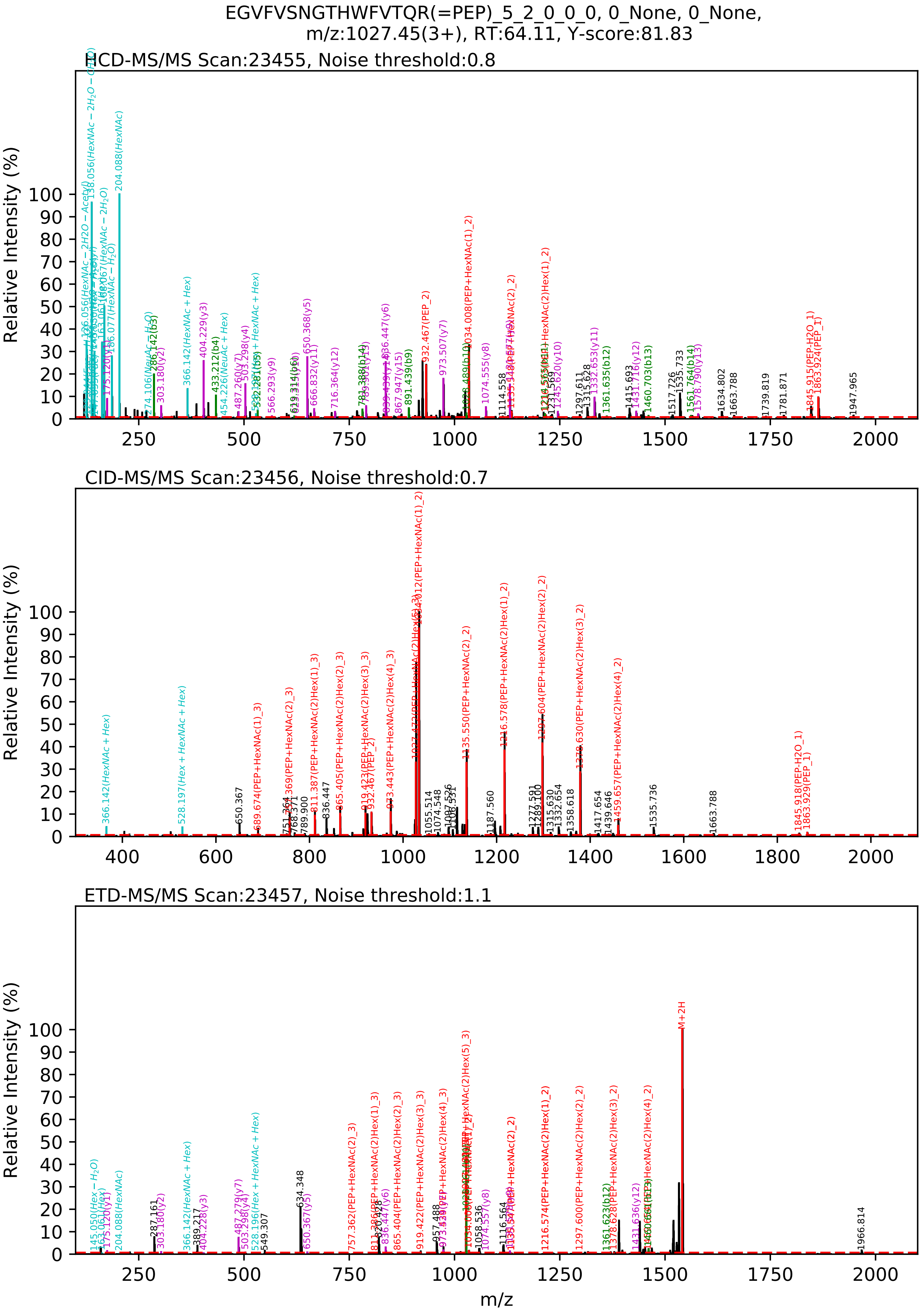

EGVFVSNGTHWFVTQR(=PEP)_5_2_0_0_0, 0_None, 0_None,
m/z:1027.45(3+), RT:64.11, Y-score:81.83
HCD-MS/MS Scan:23455, Noise threshold:0.8
CID-MS/MS Scan:23456, Noise threshold:0.7
ETD-MS/MS Scan:23457, Noise threshold:1.1
Relative Intensity (%)
m/z

EGVFVSNGTHWFVTQR(=PEP)_5_2_0_0_0, 0_None, 0_None,
m/z:1027.45(3+), RT:64.17, Y-score:81.61

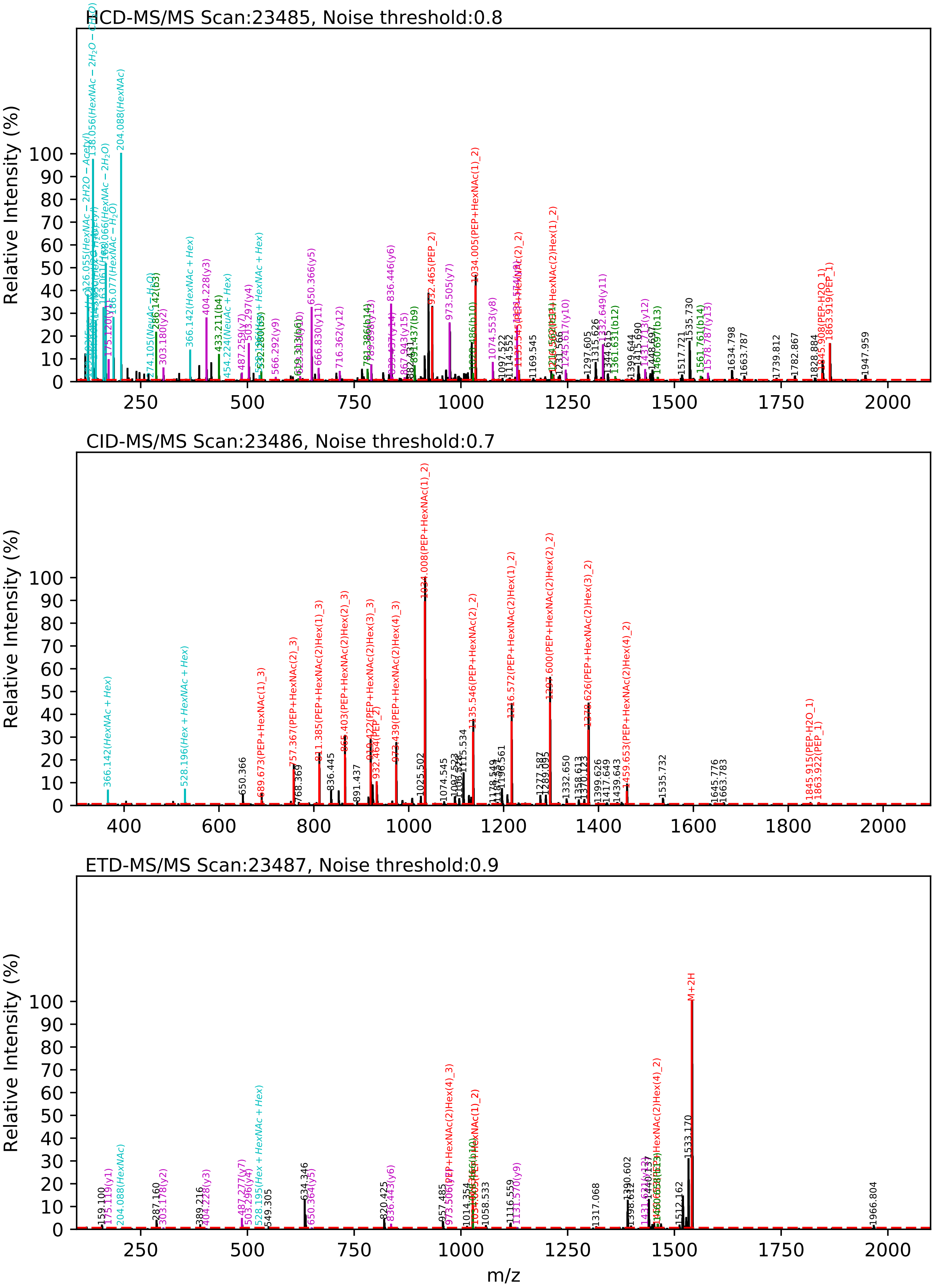

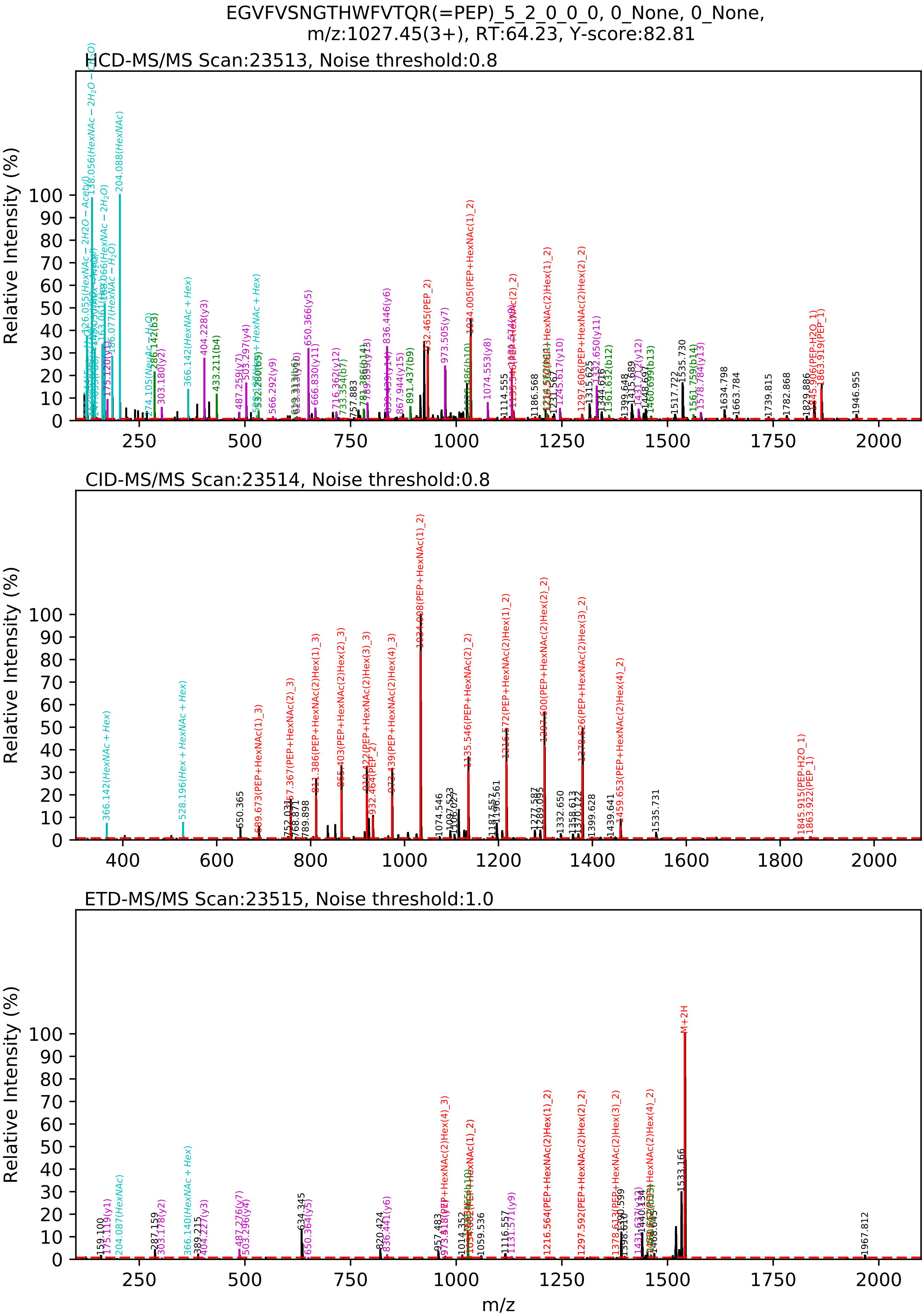
EGVFVSNGTHWFVTQR(=PEP)_5_2_0_0_0, 0_None, 0_None,
m/z:1027.45(3+), RT:64.23, Y-score:82.81
HCD-MS/MS Scan:23513, Noise threshold:0.8
Relative Intensity (%)
204.088(HexNAc)
366.142(HexNAc + Hex)
404.228(y3)
433.211(b4)
650.366(y5)
836.446(y6)
932.465(PEP_2)
973.505(y7)
1034.005(PEP+HexNAc(1)_2)
1074.553(y8)
1297.606(PEP+HexNAc(2)Hex(2)_2)
1535.730
1634.798
1663.784
1739.815
1782.868
1863.919(PEP_1)
1946.955
CID-MS/MS Scan:23514, Noise threshold:0.8
366.142(HexNAc + Hex)
528.196(Hex + HexNAc + Hex)
650.365
689.673(PEP+HexNAc(1)_3)
811.386(PEP+HexNAc(2)Hex(1)_3)
865.403(PEP+HexNAc(2)Hex(2)_3)
972.439(PEP+HexNAc(2)Hex(4)_3)
1034.008(PEP+HexNAc(1)_2)
1135.546(PEP+HexNAc(2)_2)
1216.572(PEP+HexNAc(2)Hex(1)_2)
1297.600(PEP+HexNAc(2)Hex(2)_2)
1378.626(PEP+HexNAc(2)Hex(3)_2)
1459.653(PEP+HexNAc(2)Hex(4)_2)
1535.731
1845.915(PEP-H2O_1)
1863.922(PEP_1)
ETD-MS/MS Scan:23515, Noise threshold:1.0
M+2H
159.100
175.119(y1)
204.087(HexNAc)
287.159
303.178(y2)
366.140(HexNAc + Hex)
389.215
404.227(y3)
487.276(y7)
503.296(y4)
634.345
650.364(y5)
820.424
836.441(y6)
957.483
1014.352
1059.536
1116.557
1131.571(y9)
1216.564(PEP+HexNAc(2)Hex(1)_2)
1297.592(PEP+HexNAc(2)Hex(2)_2)
1533.166
1967.812
m/z

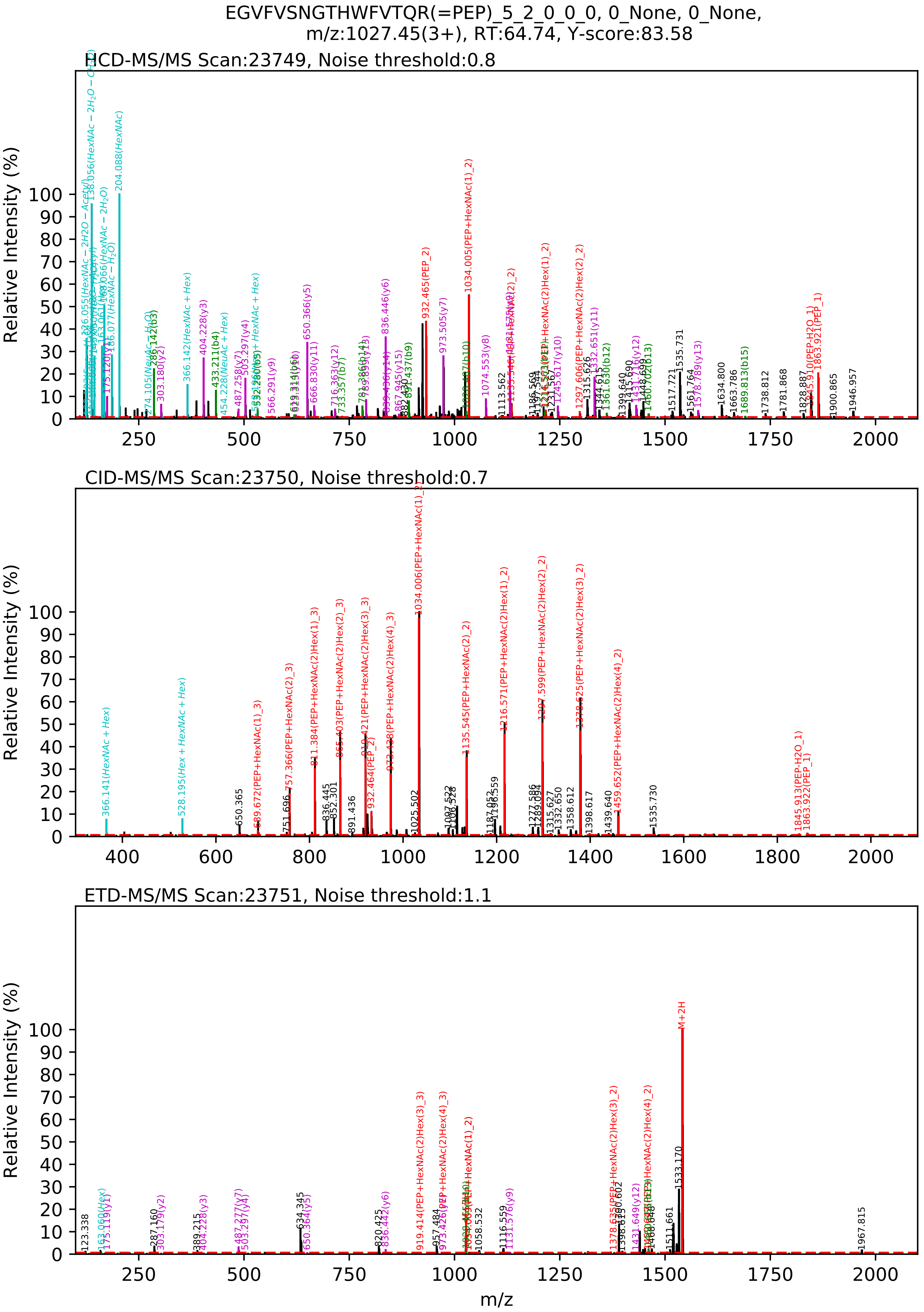
EGVFVSNGTHWFVTQR(=PEP)_5_2_0_0_0, 0_None, 0_None,
m/z:1027.45(3+), RT:64.74, Y-score:83.58
HCD-MS/MS Scan:23749, Noise threshold:0.8
Relative Intensity (%)
204.088(HexNAc)
1034.005(PEP+HexNAc(1)_2)
932.465(PEP_2)
973.505(y7)
836.446(y6)
650.366(y5)
404.228(y3)
366.142(HexNAc + Hex)
CID-MS/MS Scan:23750, Noise threshold:0.7
1034.006(PEP+HexNAc(1)_2)
1378.625(PEP+HexNAc(2)Hex(3)_2)
1297.599(PEP+HexNAc(2)Hex(2)_2)
1216.571(PEP+HexNAc(2)Hex(1)_2)
1135.545(PEP+HexNAc(2)_2)
1459.652(PEP+HexNAc(2)Hex(4)_2)
366.141(HexNAc + Hex)
528.195(Hex + HexNAc + Hex)
ETD-MS/MS Scan:23751, Noise threshold:1.1
M+2H
1533.170
1967.815
m/z

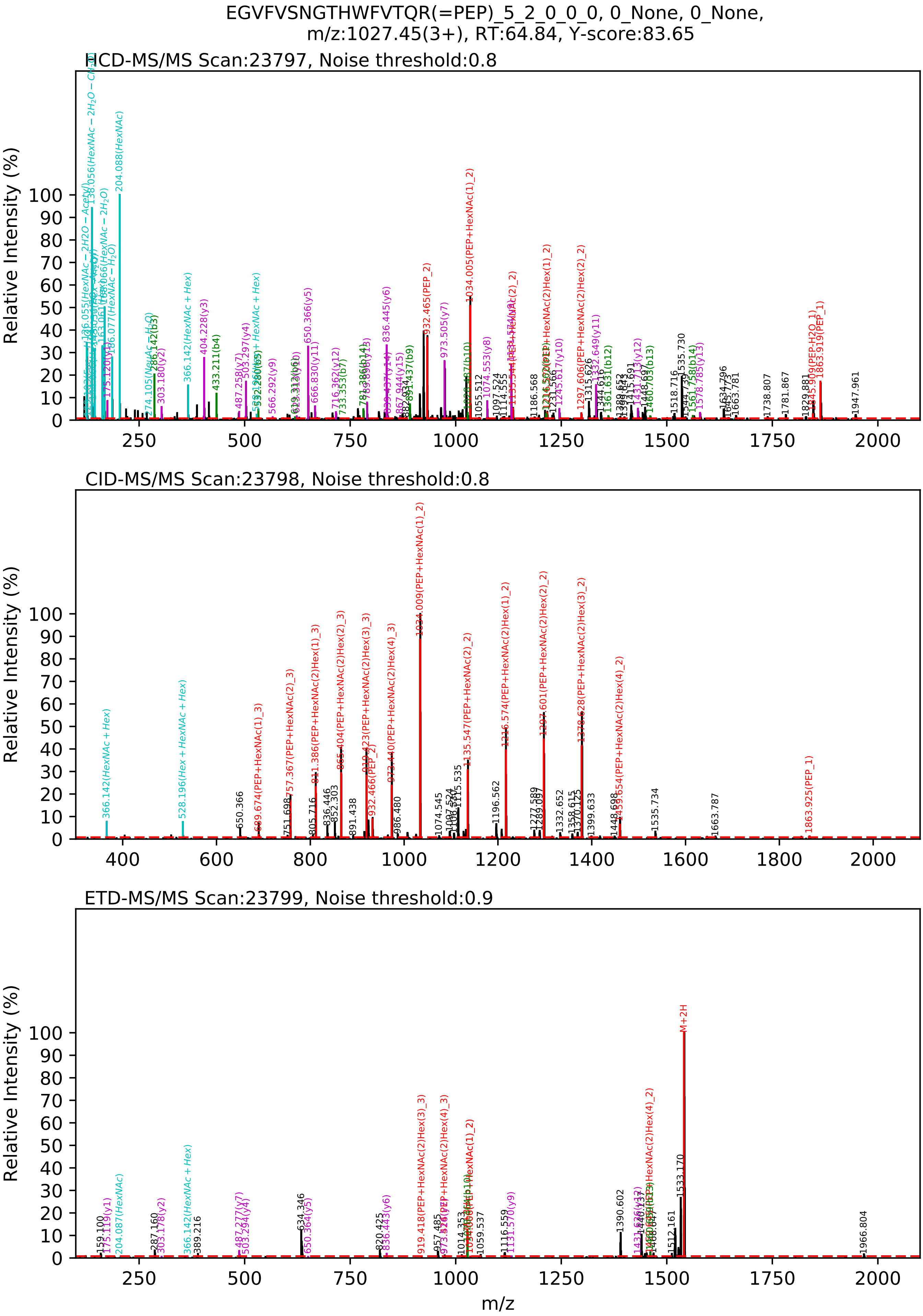
EGVFVSNGTHWFVTQR(=PEP)_5_2_0_0_0, 0_None, 0_None,
m/z:1027.45(3+), RT:64.84, Y-score:83.65
HCD-MS/MS Scan:23797, Noise threshold:0.8
Relative Intensity (%)
204.088(HexNAc)
1034.005(PEP+HexNAc(1)_2)
932.465(PEP_2)
973.505(y7)
836.445(y6)
650.366(y5)
404.228(y3)
366.142(HexNAc + Hex)
433.211(b4)
1863.919(PEP_1)
CID-MS/MS Scan:23798, Noise threshold:0.8
1034.009(PEP+HexNAc(1)_2)
1297.601(PEP+HexNAc(2)Hex(2)_2)
1378.628(PEP+HexNAc(2)Hex(3)_2)
1216.574(PEP+HexNAc(2)Hex(1)_2)
1135.547(PEP+HexNAc(2)_2)
1459.654(PEP+HexNAc(2)Hex(4)_2)
973.440(PEP+HexNAc(2)Hex(4)_3)
865.404(PEP+HexNAc(2)Hex(2)_3)
811.386(PEP+HexNAc(2)Hex(1)_3)
757.367(PEP+HexNAc(2)_3)
689.674(PEP+HexNAc(1)_3)
932.466(PEP_2)
1863.925(PEP_1)
366.142(HexNAc + Hex)
528.196(Hex + HexNAc + Hex)
ETD-MS/MS Scan:23799, Noise threshold:0.9
M+2H
1533.170
1390.602
634.346
650.364(y5)
836.443(y6)
1131.570(y9)
919.418(PEP+HexNAc(2)Hex(3)_3)
1966.804
m/z

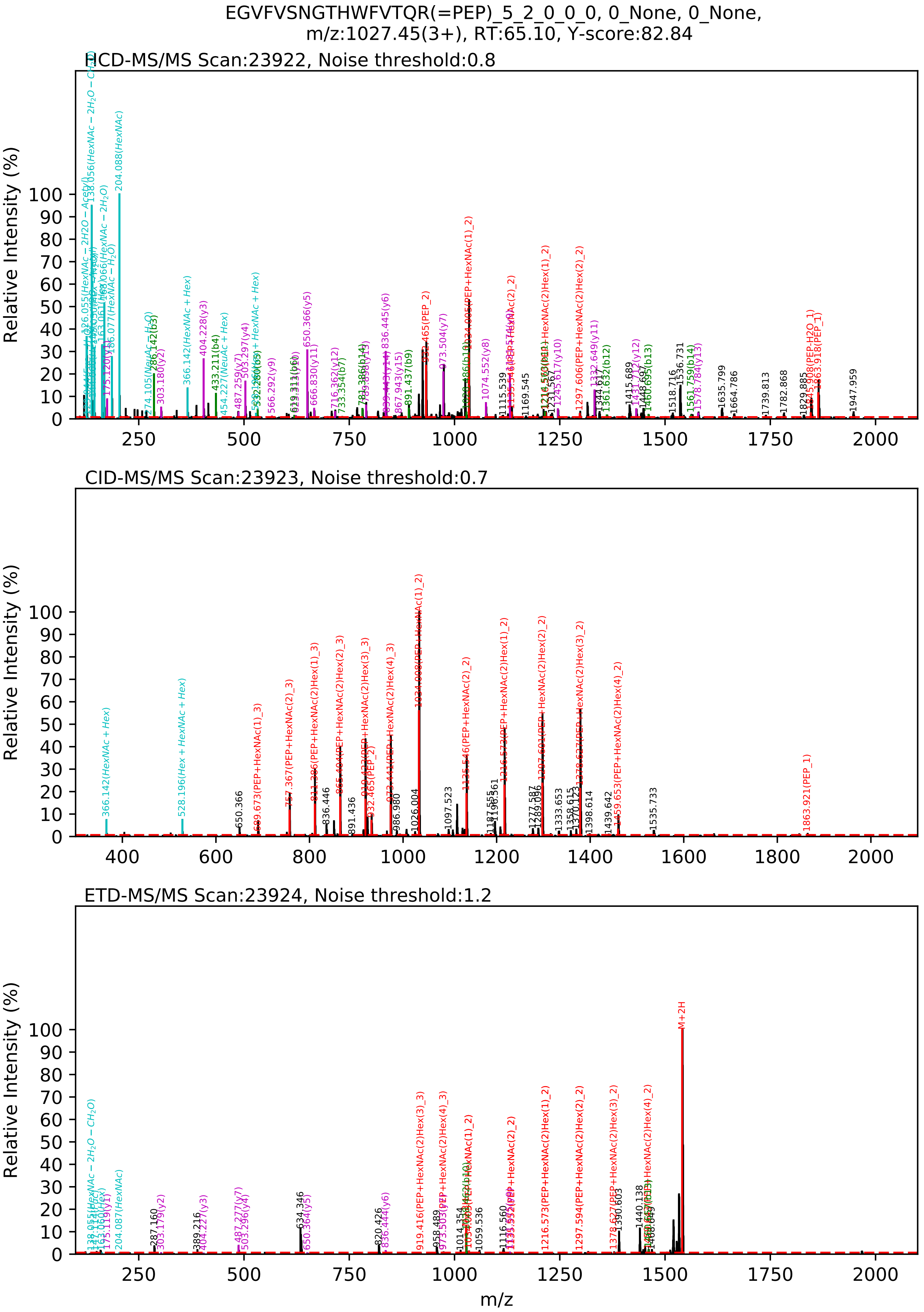

EGVFVSNGTHWFVTQR(=PEP)_5_2_0_0_0, 0_None, 0_None,
m/z:1027.45(3+), RT:65.10, Y-score:82.84
HCD-MS/MS Scan:23922, Noise threshold:0.8
CID-MS/MS Scan:23923, Noise threshold:0.7
ETD-MS/MS Scan:23924, Noise threshold:1.2
Relative Intensity (%)
m/z

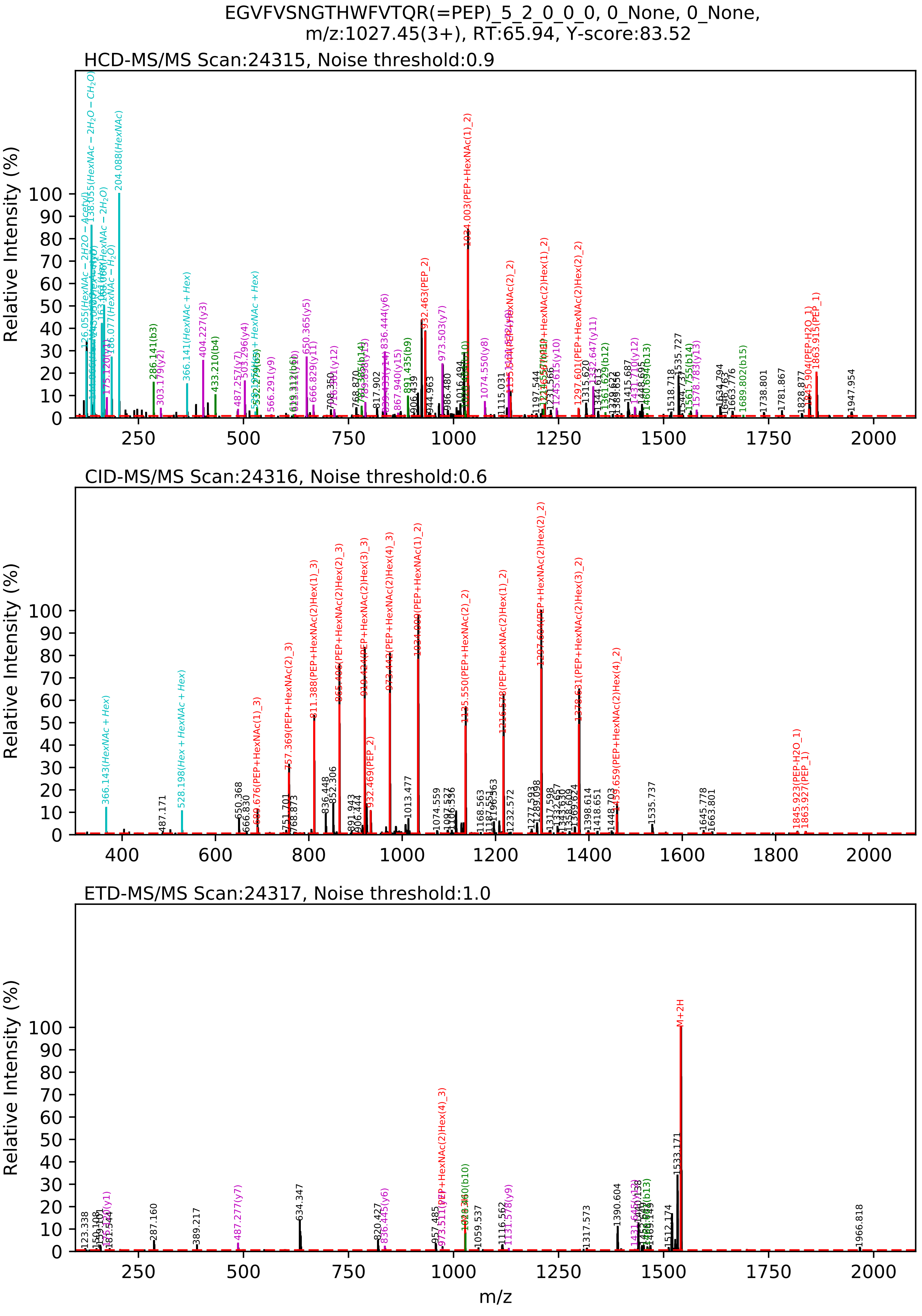
EGVFVSNGTHWFVTQR(=PEP)_5_2_0_0_0, 0_None, 0_None,
m/z:1027.45(3+), RT:65.94, Y-score:83.52
HCD-MS/MS Scan:24315, Noise threshold:0.9
CID-MS/MS Scan:24316, Noise threshold:0.6
ETD-MS/MS Scan:24317, Noise threshold:1.0
Relative Intensity (%)
m/z
1034.003(PEP+HexNAc(1)_2)
932.463(PEP_2)
204.088(HexNAc)
1297.604(PEP+HexNAc(2)Hex(2)_2)
1378.631(PEP+HexNAc(2)Hex(3)_2)
1034.000(PEP+HexNAc(1)_2)
M+2H
1533.171

EGVFVSNGTHWFVTQR(=PEP)_5_2_0_0_0, 0_None, 0_None,
m/z:1027.45(3+), RT:66.47, Y-score:86.77

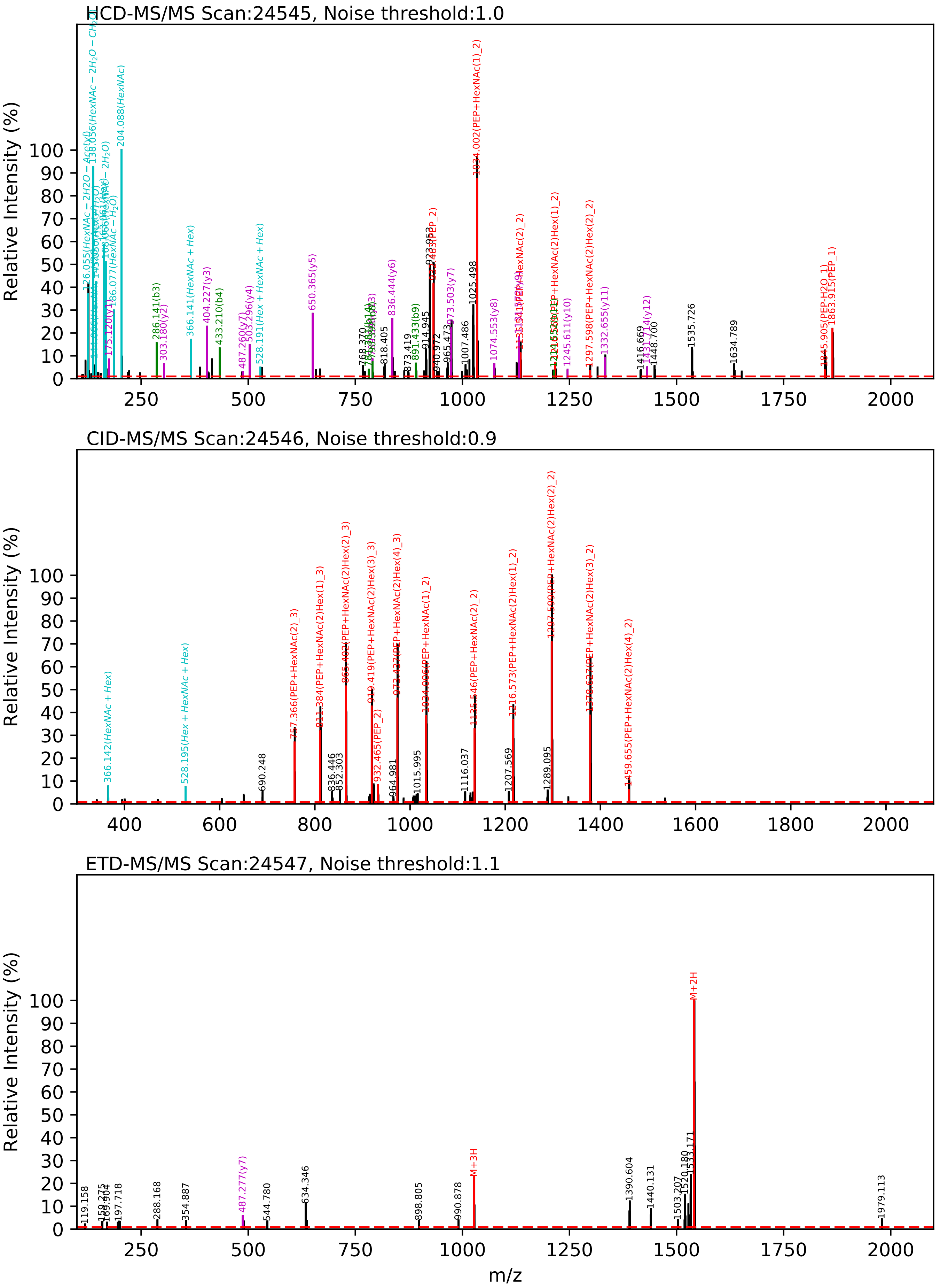

EGVFVSNGTHWFVTQR(=PEP)_6_2_0_0_0, 0_None, 0_None,
m/z:1081.47(3+), RT:68.03, Y-score:81.61

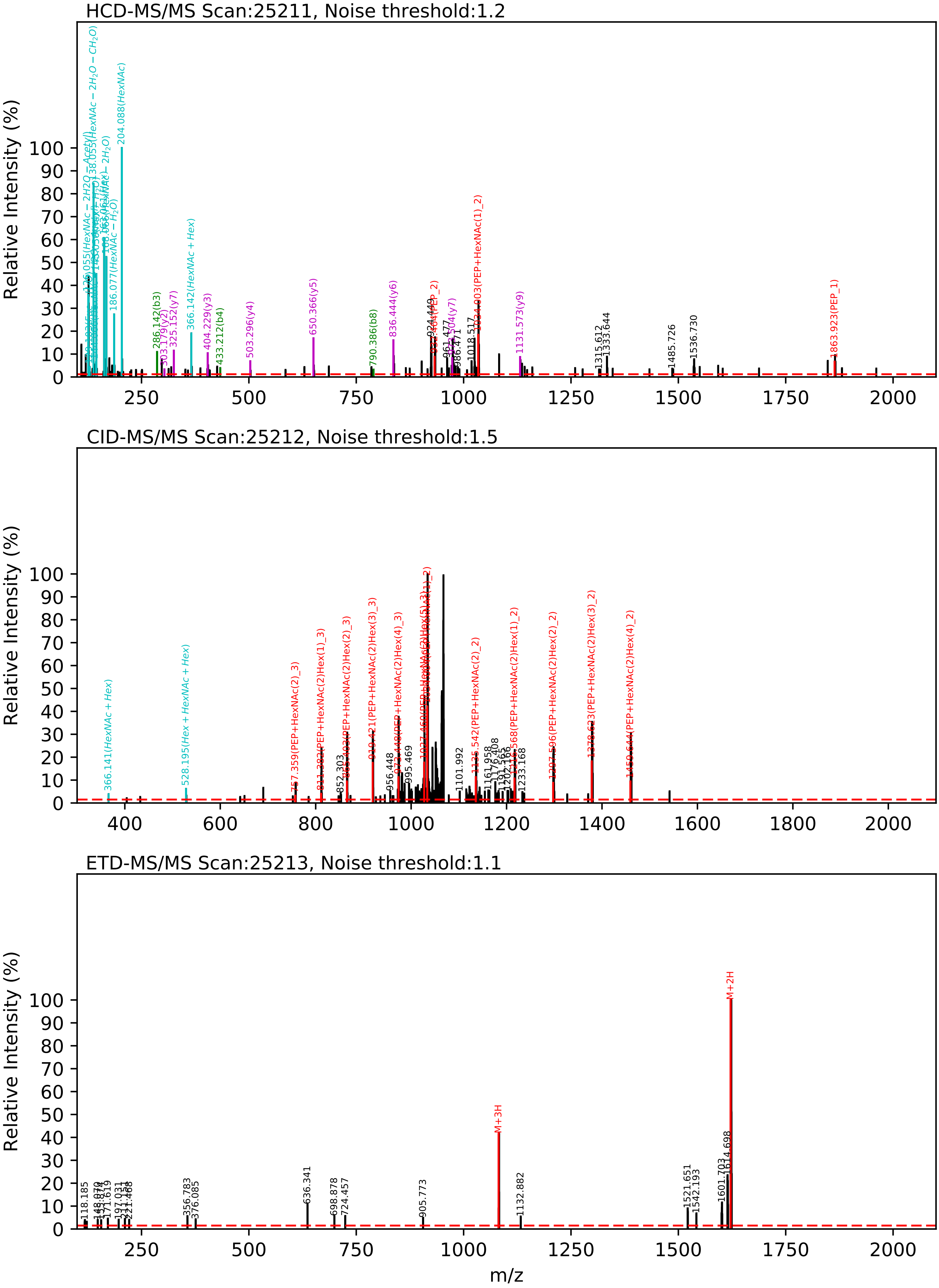

EGVFVSNGTHWFVTQR(=PEP)_6_2_0_0_0, 0_None, 0_None,
m/z:1081.47(3+), RT:65.36, Y-score:79.17

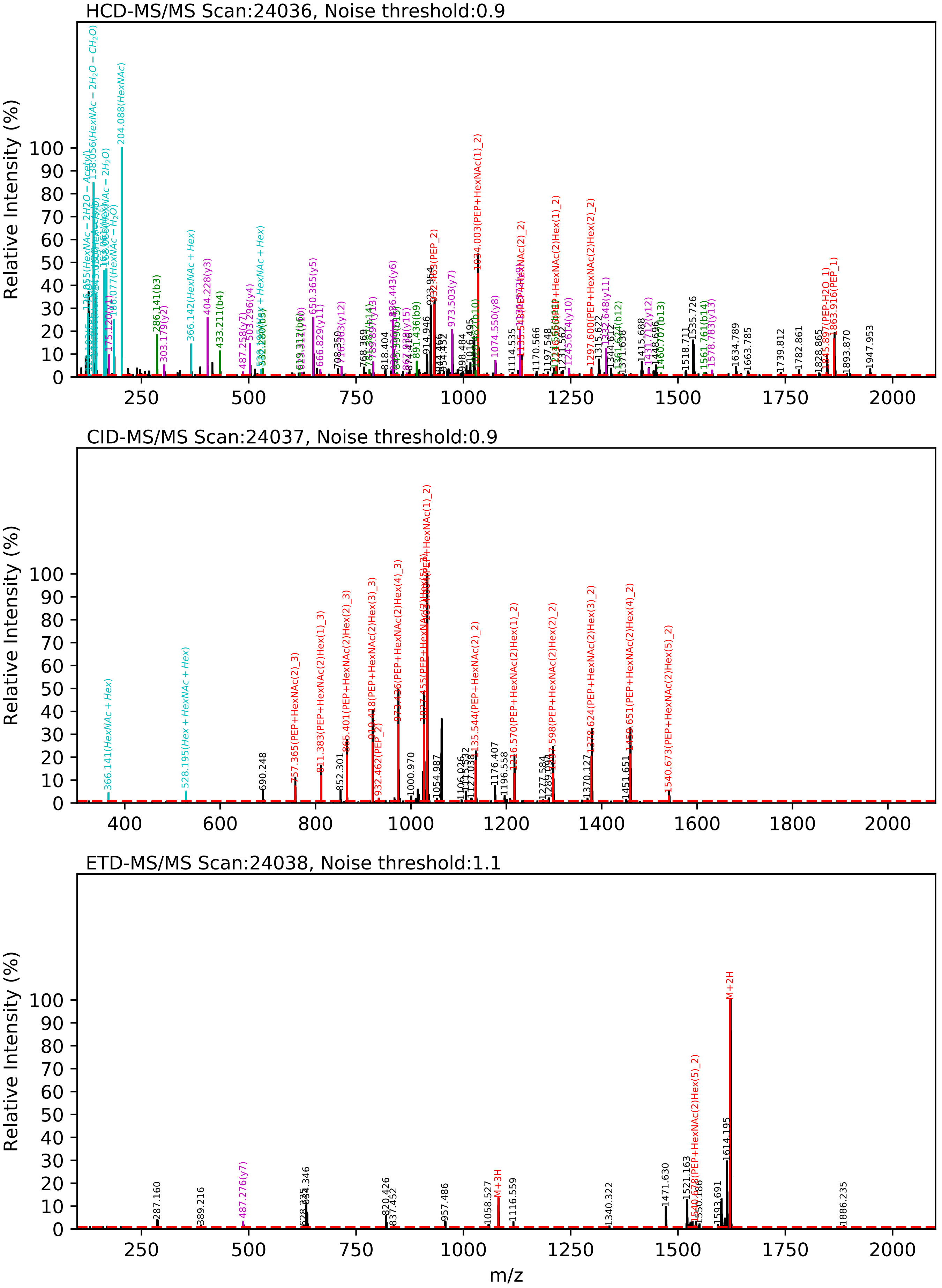

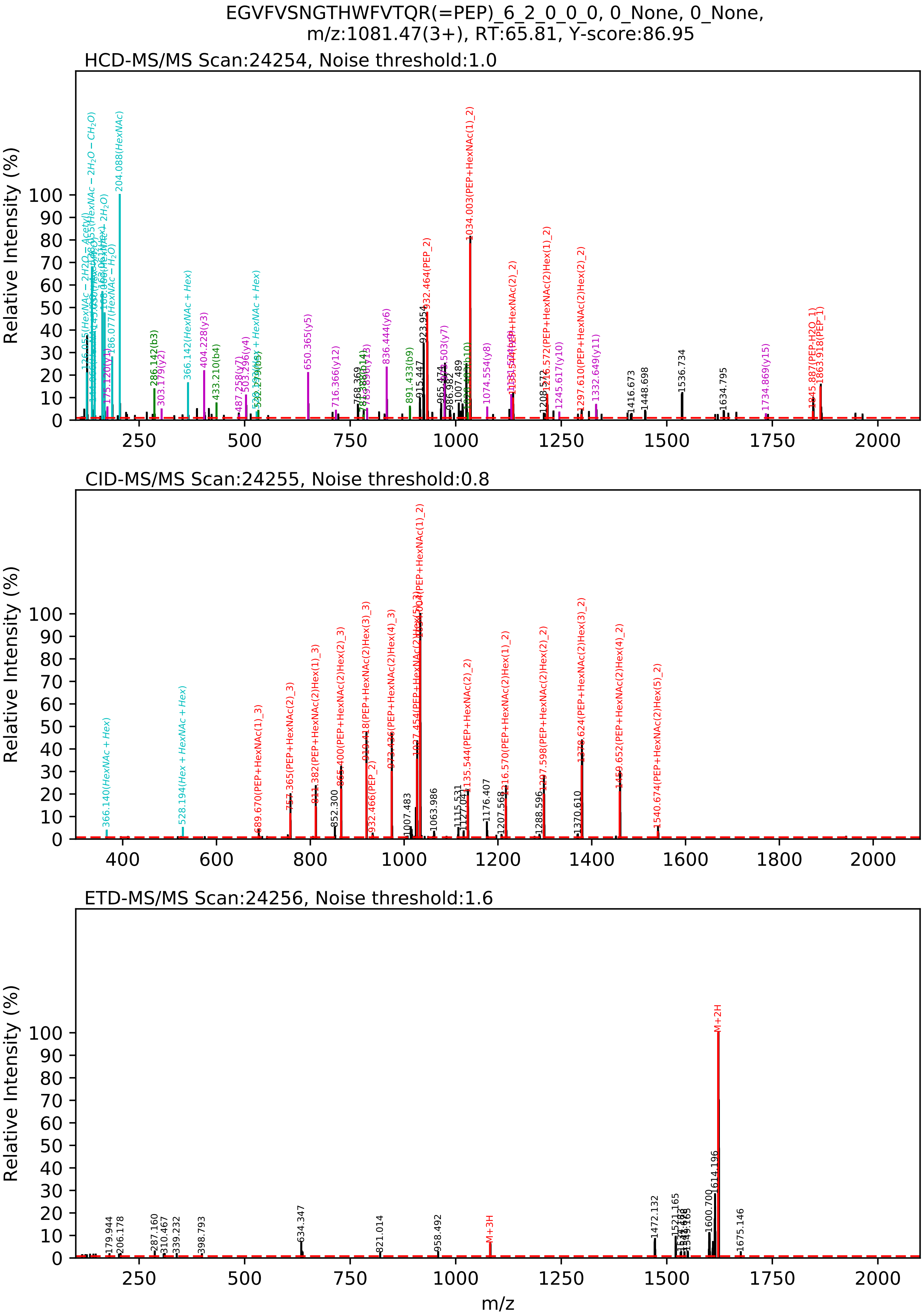
EGVFVSNGTHWFVTQR(=PEP)_6_2_0_0_0, 0_None, 0_None,
m/z:1081.47(3+), RT:65.81, Y-score:86.95
HCD-MS/MS Scan:24254, Noise threshold:1.0
CID-MS/MS Scan:24255, Noise threshold:0.8
ETD-MS/MS Scan:24256, Noise threshold:1.6
Relative Intensity (%)
m/z
204.088(HexNAc)
1034.003(PEP+HexNAc(1)_2)
932.464(PEP_2)
366.142(HexNAc+Hex)
404.228(y3)
650.365(y5)
836.444(y6)
1216.572(PEP+HexNAc(2)Hex(1)_2)
1297.610(PEP+HexNAc(2)Hex(2)_2)
1332.649(y11)
1536.734
1734.869(y15)
1863.918(PEP_1)
1027.454(PEP+HexNAc(2)Hex(5)_3)
1378.624(PEP+HexNAc(2)Hex(3)_2)
1459.652(PEP+HexNAc(2)Hex(4)_2)
1540.674(PEP+HexNAc(2)Hex(5)_2)
M+2H
M+3H
1614.196
1472.132

EGVFVSNGTHWFVTQR(=PEP)_6_2_0_0_0, 0_None, 0_None,
m/z:1081.47(3+), RT:65.93, Y-score:85.12

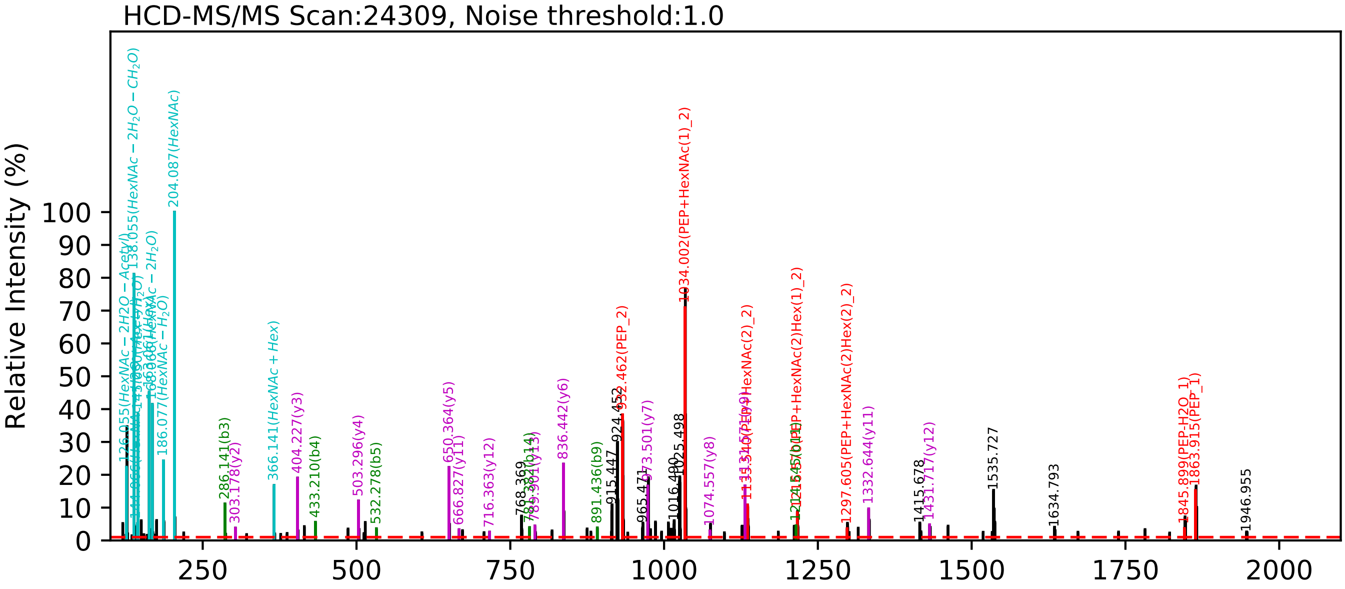

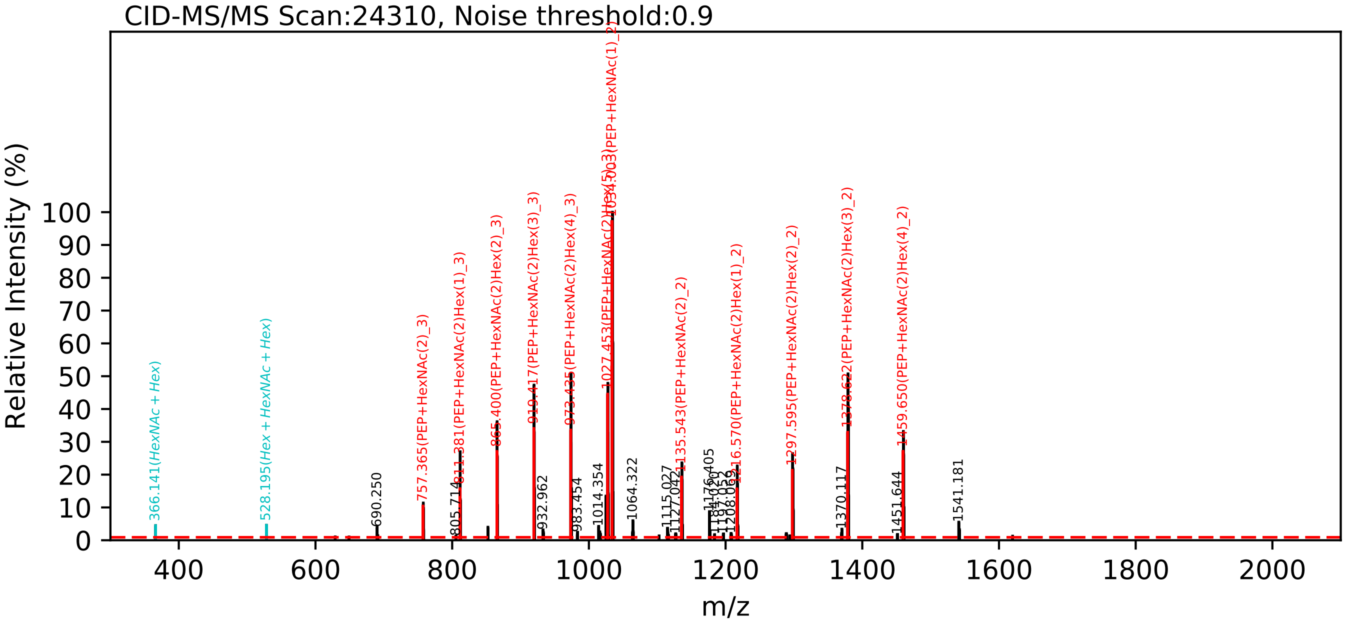

EGVFVSNGTHWFVTQR(=PEP)_6_2_0_0_0, 0_None, 0_None,
m/z:1621.70(2+), RT:64.15, Y-score:67.92

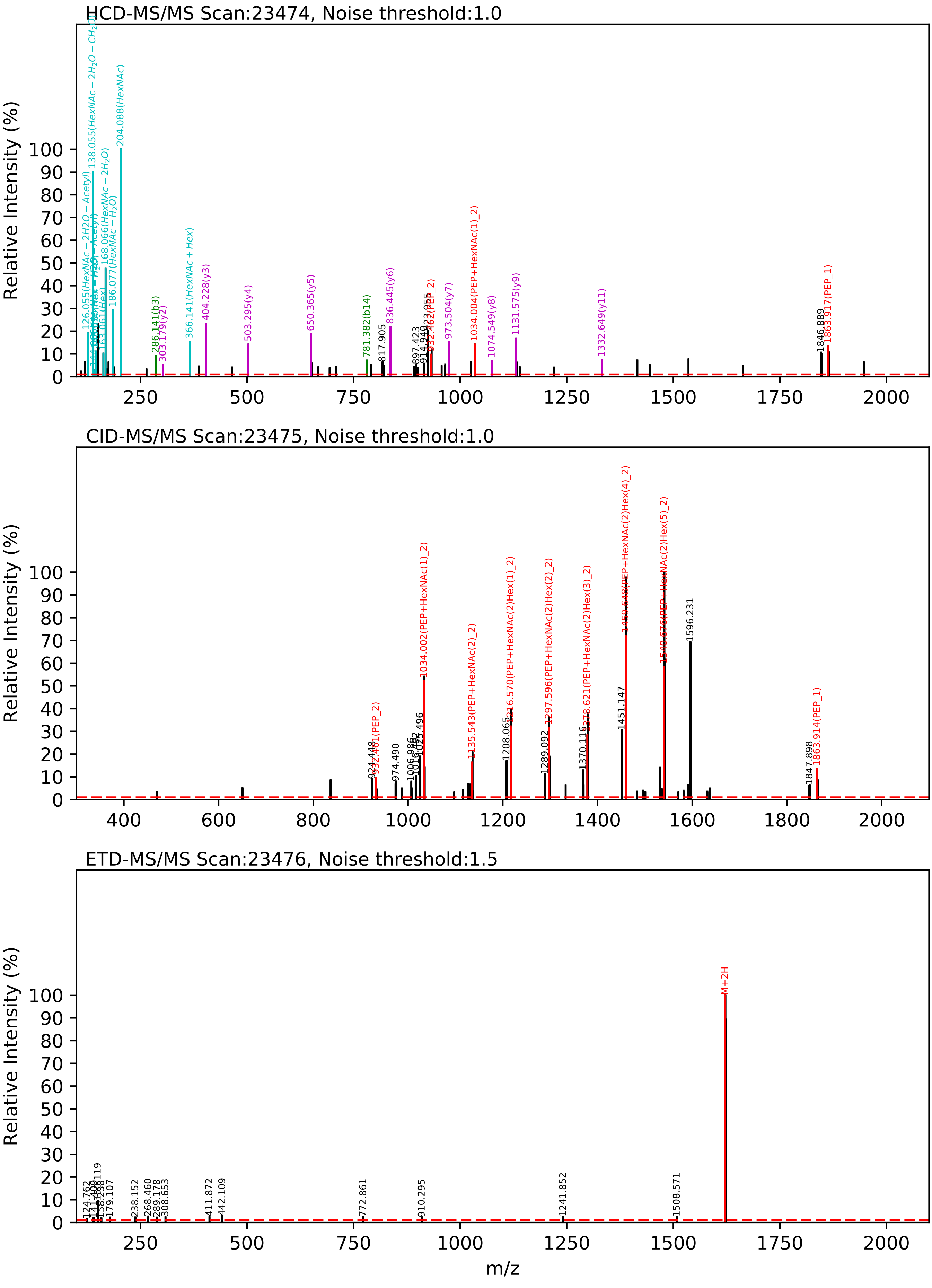

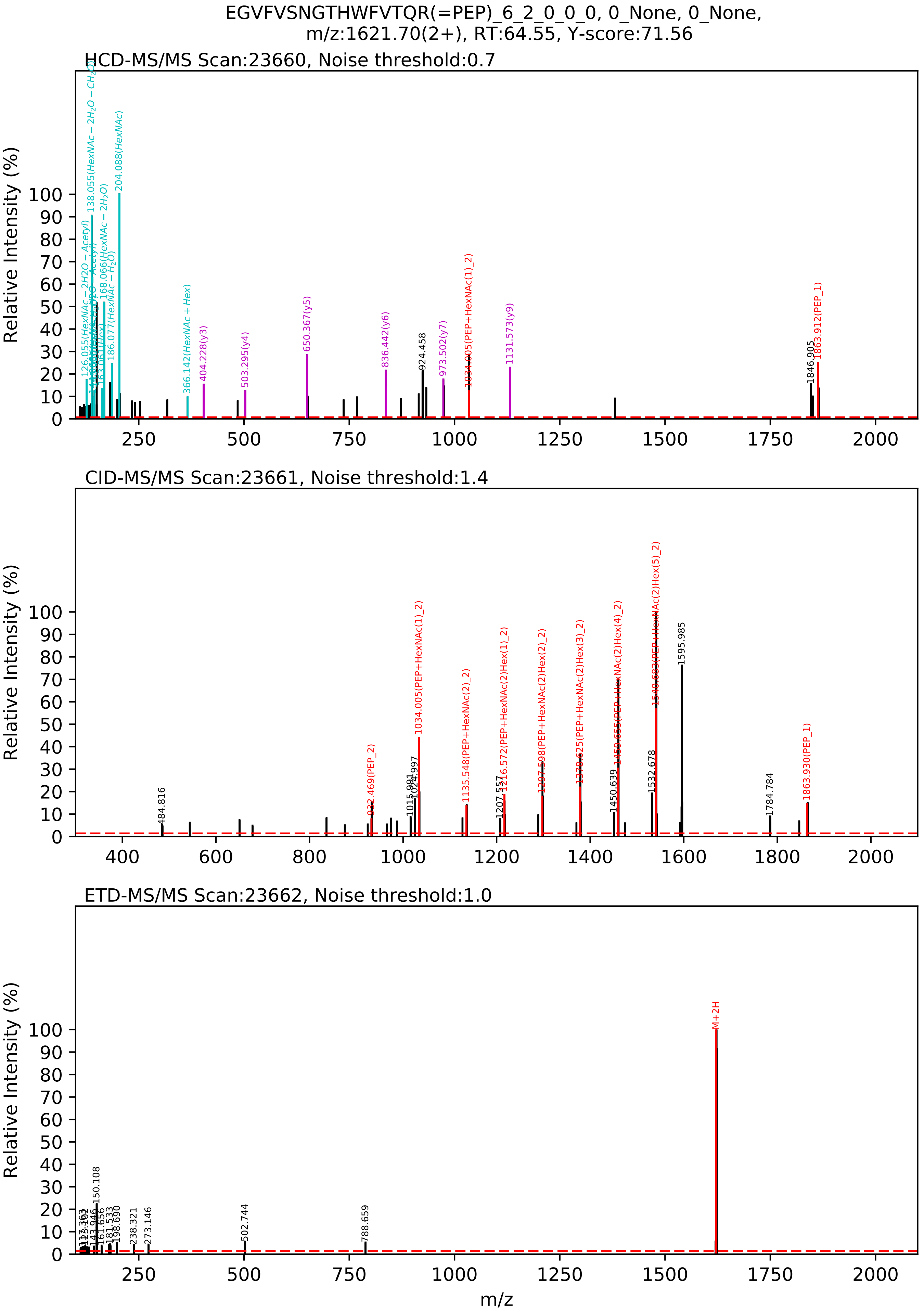
EGVFVSNGTHWFVTQR(=PEP)_6_2_0_0_0, 0_None, 0_None,
m/z:1621.70(2+), RT:64.55, Y-score:71.56
HCD-MS/MS Scan:23660, Noise threshold:0.7
CID-MS/MS Scan:23661, Noise threshold:1.4
ETD-MS/MS Scan:23662, Noise threshold:1.0
Relative Intensity (%)
m/z
204.088(HexNAc)
138.055(HexNAc − 2H2O − CH2O)
168.066(HexNAc − 2H2O)
186.077(HexNAc − H2O)
126.055(HexNAc − 2H2O − Acetyl)
163.061(Hex)
366.142(HexNAc + Hex)
404.228(y3)
503.295(y4)
650.367(y5)
836.442(y6)
924.458
973.502(y7)
1034.005(PEP+HexNAc(1)_2)
1131.573(y9)
1846.905
1863.912(PEP_1)
484.816
932.469(PEP_2)
1015.981
1024.997
1034.005(PEP+HexNAc(1)_2)
1135.548(PEP+HexNAc(2)_2)
1207.557
1216.572(PEP+HexNAc(2)Hex(1)_2)
1297.598(PEP+HexNAc(2)Hex(2)_2)
1378.625(PEP+HexNAc(2)Hex(3)_2)
1450.639
1459.655(PEP+HexNAc(2)Hex(4)_2)
1532.678
1540.682(PEP+HexNAc(2)Hex(5)_2)
1595.985
1784.784
1863.930(PEP_1)
M+2H
150.108
143.946
161.656
181.533
196.690
238.321
273.146
502.744
788.659

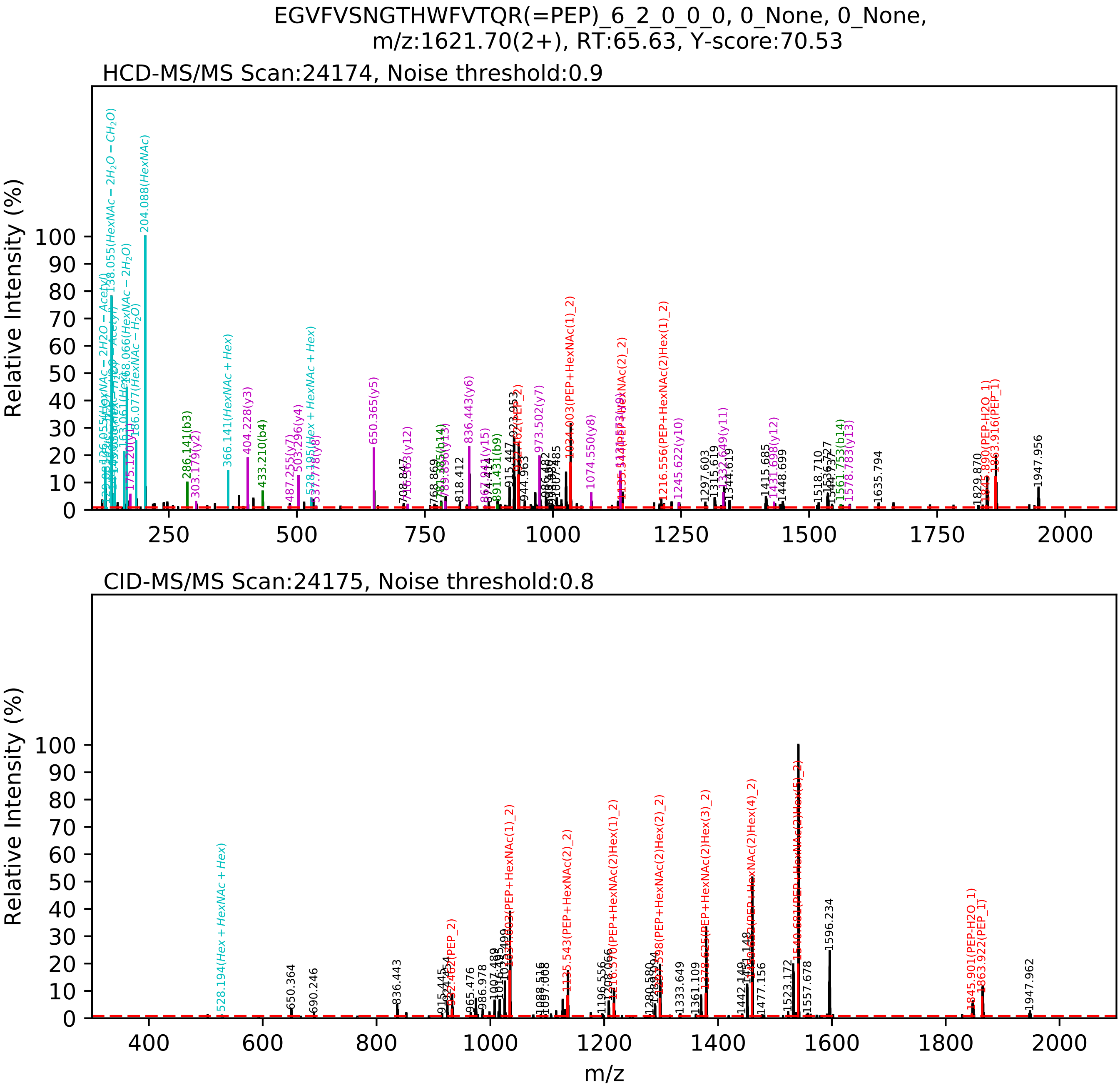
EGVFVSNGTHWFVTQR(=PEP)_6_2_0_0_0, 0_None, 0_None,
m/z:1621.70(2+), RT:65.63, Y-score:70.53
HCD-MS/MS Scan:24174, Noise threshold:0.9
CID-MS/MS Scan:24175, Noise threshold:0.8
Relative Intensity (%)
m/z

EGVFVSNGTHWFVTQR(=PEP)_6_2_0_0_0, 0_None, 0_None,
m/z:1621.70(2+), RT:65.80, Y-score:72.14

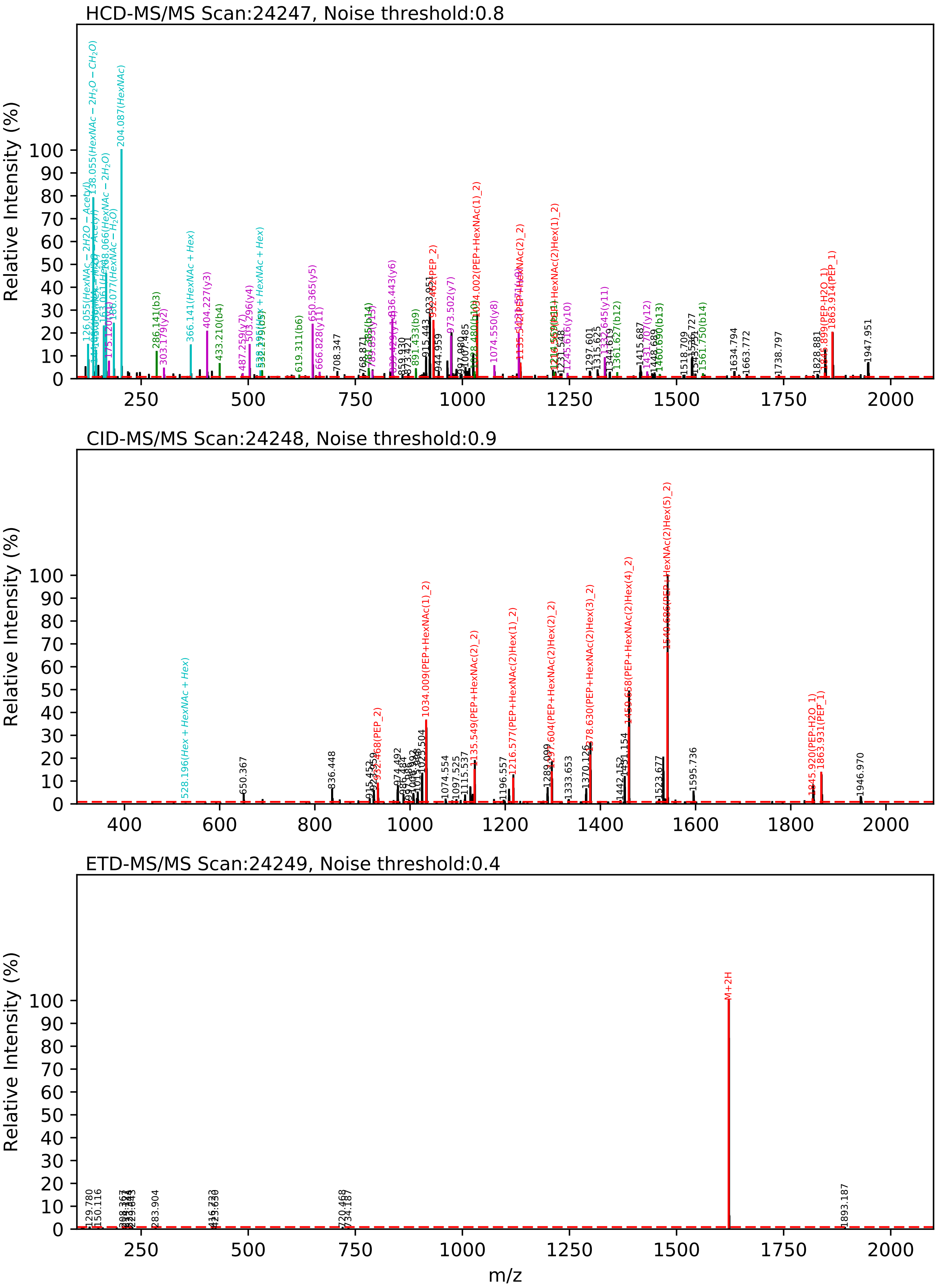

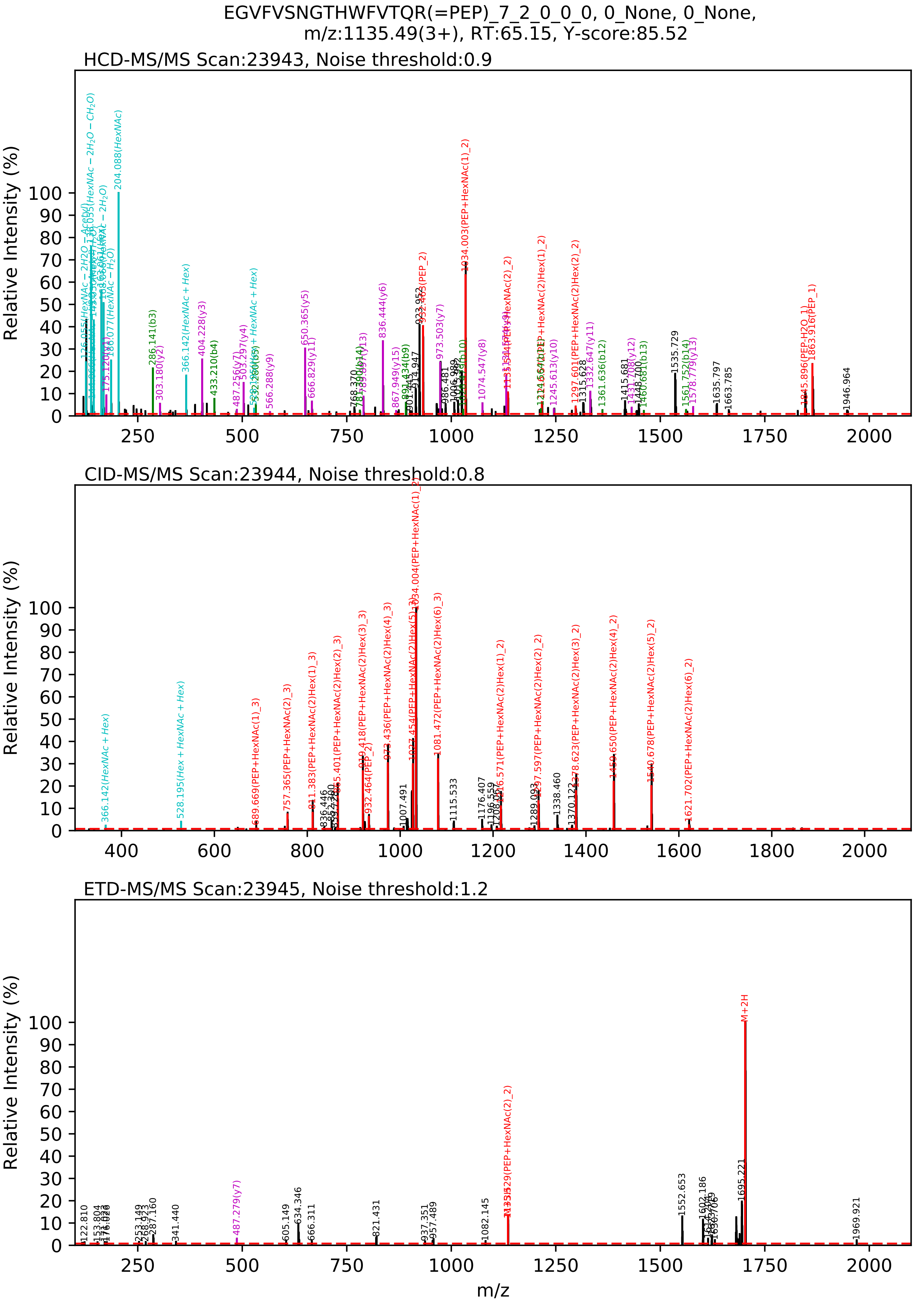
EGVFVSNGTHWFVTQR(=PEP)_7_2_0_0_0, 0_None, 0_None,
m/z:1135.49(3+), RT:65.15, Y-score:85.52
HCD-MS/MS Scan:23943, Noise threshold:0.9
Relative Intensity (%)
204.088(HexNAc)
1034.003(PEP+HexNAc(1)_2)
932.463(PEP_2)
923.952
836.444(y6)
650.365(y5)
973.503(y7)
404.228(y3)
366.142(HexNAc + Hex)
286.141(b3)
433.210(b4)
1535.729
1863.916(PEP_1)
1845.896(PEP-H2O_1)
1946.964
CID-MS/MS Scan:23944, Noise threshold:0.8
1034.004(PEP+HexNAc(1)_2)
1081.472(PEP+HexNAc(2)Hex(6)_3)
973.436(PEP+HexNAc(2)Hex(4)_3)
1459.650(PEP+HexNAc(2)Hex(4)_2)
1540.678(PEP+HexNAc(2)Hex(5)_2)
1621.702(PEP+HexNAc(2)Hex(6)_2)
1378.623(PEP+HexNAc(2)Hex(3)_2)
1297.597(PEP+HexNAc(2)Hex(2)_2)
1216.571(PEP+HexNAc(2)Hex(1)_2)
865.401(PEP+HexNAc(2)Hex(2)_3)
811.383(PEP+HexNAc(2)Hex(1)_3)
757.365(PEP+HexNAc(2)_3)
689.669(PEP+HexNAc(1)_3)
932.464(PEP_2)
1115.533
1338.460
366.142(HexNAc + Hex)
528.195(Hex + HexNAc + Hex)
ETD-MS/MS Scan:23945, Noise threshold:1.2
M+2H
1135.529(PEP+HexNAc(2)_2)
1695.221
1552.653
1602.186
1969.921
487.279(y7)
634.346
821.431
1082.145
m/z

EGVFVSNGTHWFVTQR(=PEP)_7_2_0_0_0, 0_None, 0_None, m/z:1135.49(3+), RT:65.69, Y-score:86.62

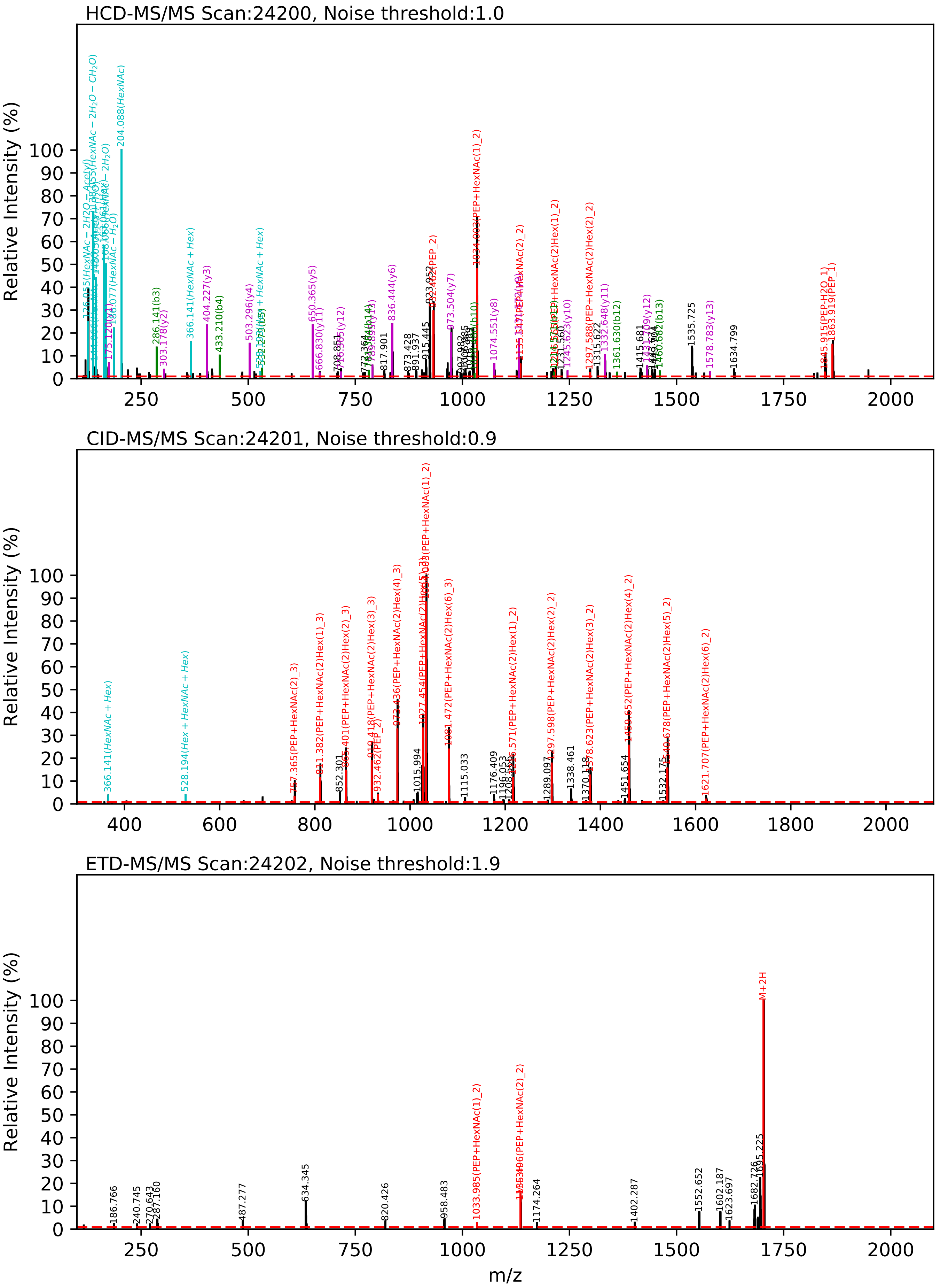

EGVFVSNGTHWFVTQR(=PEP)_7_2_0_0_0, 0_None, 0_None,
m/z:1135.49(3+), RT:66.85, Y-score:78.52

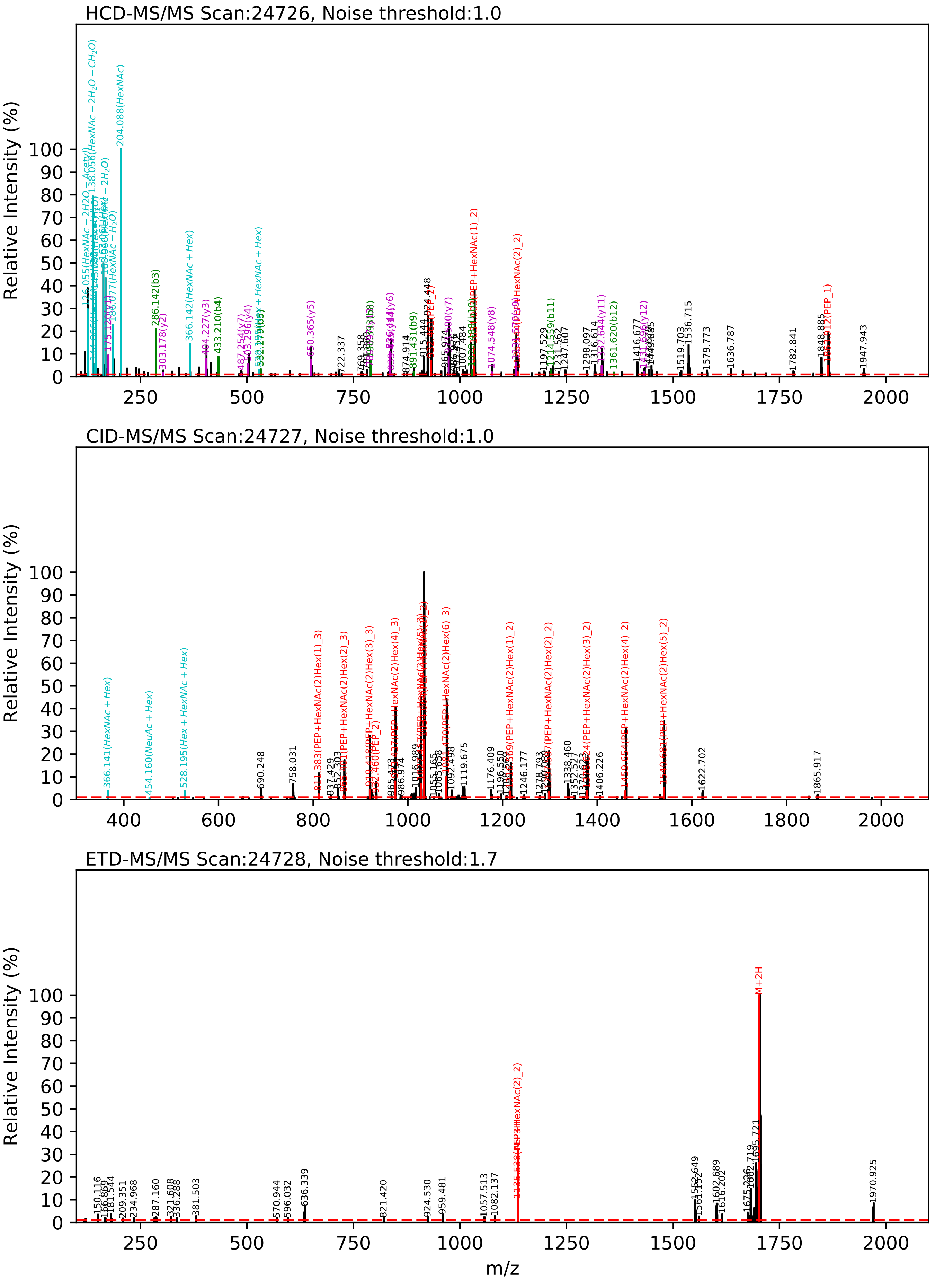

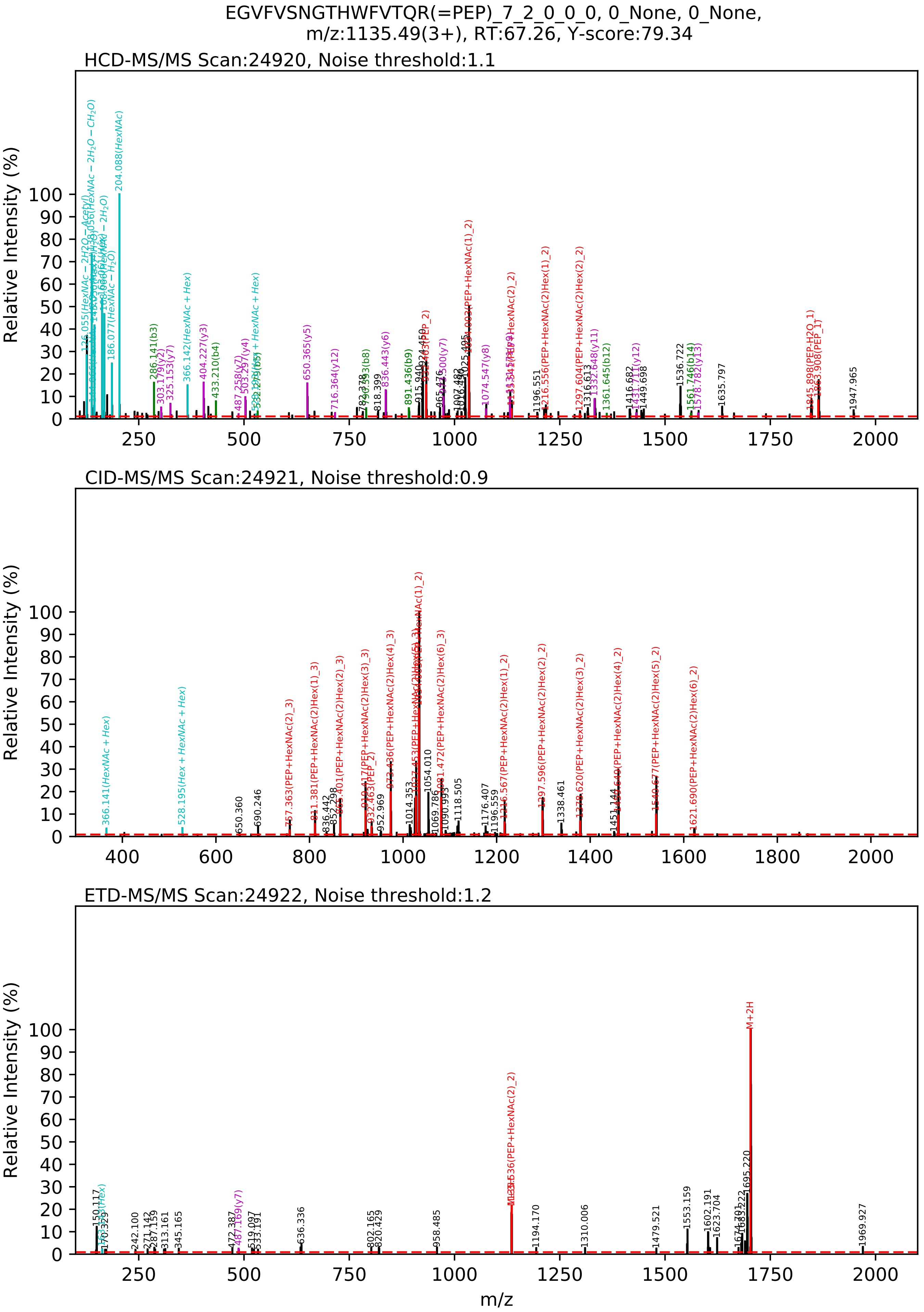
EGVFVSNGTHWFVTQR(=PEP)_7_2_0_0_0, 0_None, 0_None,
m/z:1135.49(3+), RT:67.26, Y-score:79.34
HCD-MS/MS Scan:24920, Noise threshold:1.1
CID-MS/MS Scan:24921, Noise threshold:0.9
ETD-MS/MS Scan:24922, Noise threshold:1.2
Relative Intensity (%)
m/z
204.088(HexNAc)
186.077(HexNAc − H2O)
366.142(HexNAc + Hex)
286.141(b3)
404.227(y3)
433.210(b4)
650.365(y5)
716.364(y12)
836.443(y6)
891.436(b9)
1074.547(y8)
1536.722
1635.797
1845.898(PEP-H2O_1)
1863.908(PEP_1)
1947.965
366.141(HexNAc + Hex)
528.195(Hex + HexNAc + Hex)
757.363(PEP+HexNAc(2)_3)
811.381(PEP+HexNAc(2)Hex(1)_3)
972.436(PEP+HexNAc(2)Hex(4)_3)
1216.567(PEP+HexNAc(2)Hex(1)_2)
1297.596(PEP+HexNAc(2)Hex(2)_2)
1378.620(PEP+HexNAc(2)Hex(3)_2)
1540.677(PEP+HexNAc(2)Hex(5)_2)
1621.690(PEP+HexNAc(2)Hex(6)_2)
M+2H
1695.220
1553.159
1969.927

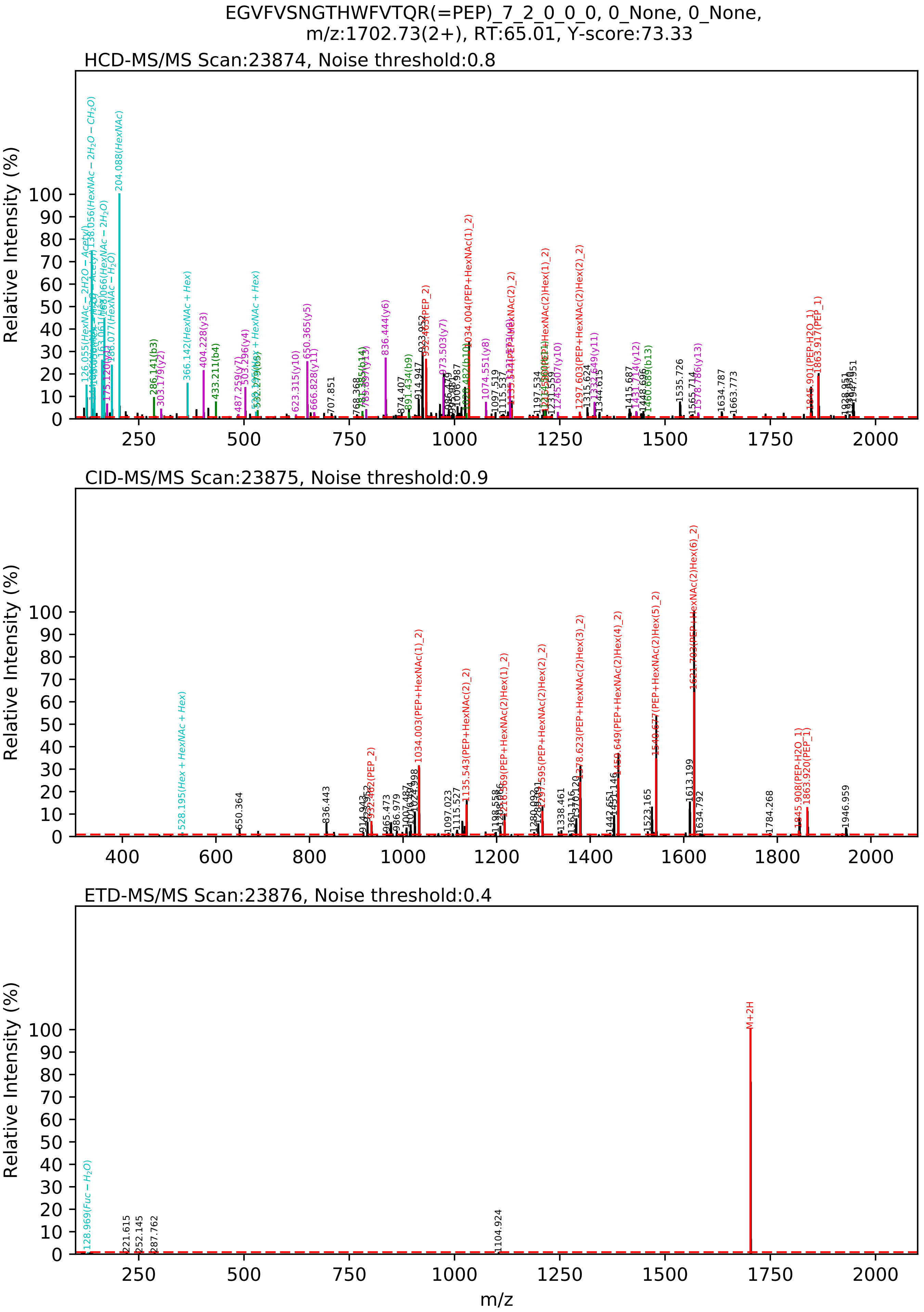
EGVFVSNGTHWFVTQR(=PEP)_7_2_0_0_0, 0_None, 0_None,
m/z:1702.73(2+), RT:65.01, Y-score:73.33
HCD-MS/MS Scan:23874, Noise threshold:0.8
CID-MS/MS Scan:23875, Noise threshold:0.9
ETD-MS/MS Scan:23876, Noise threshold:0.4
Relative Intensity (%)
m/z

EGVFVSNGTHWFVTQR(=PEP)_7_2_0_0_0, 0_None, 0_None,
m/z:1702.73(2+), RT:65.08, Y-score:73.00

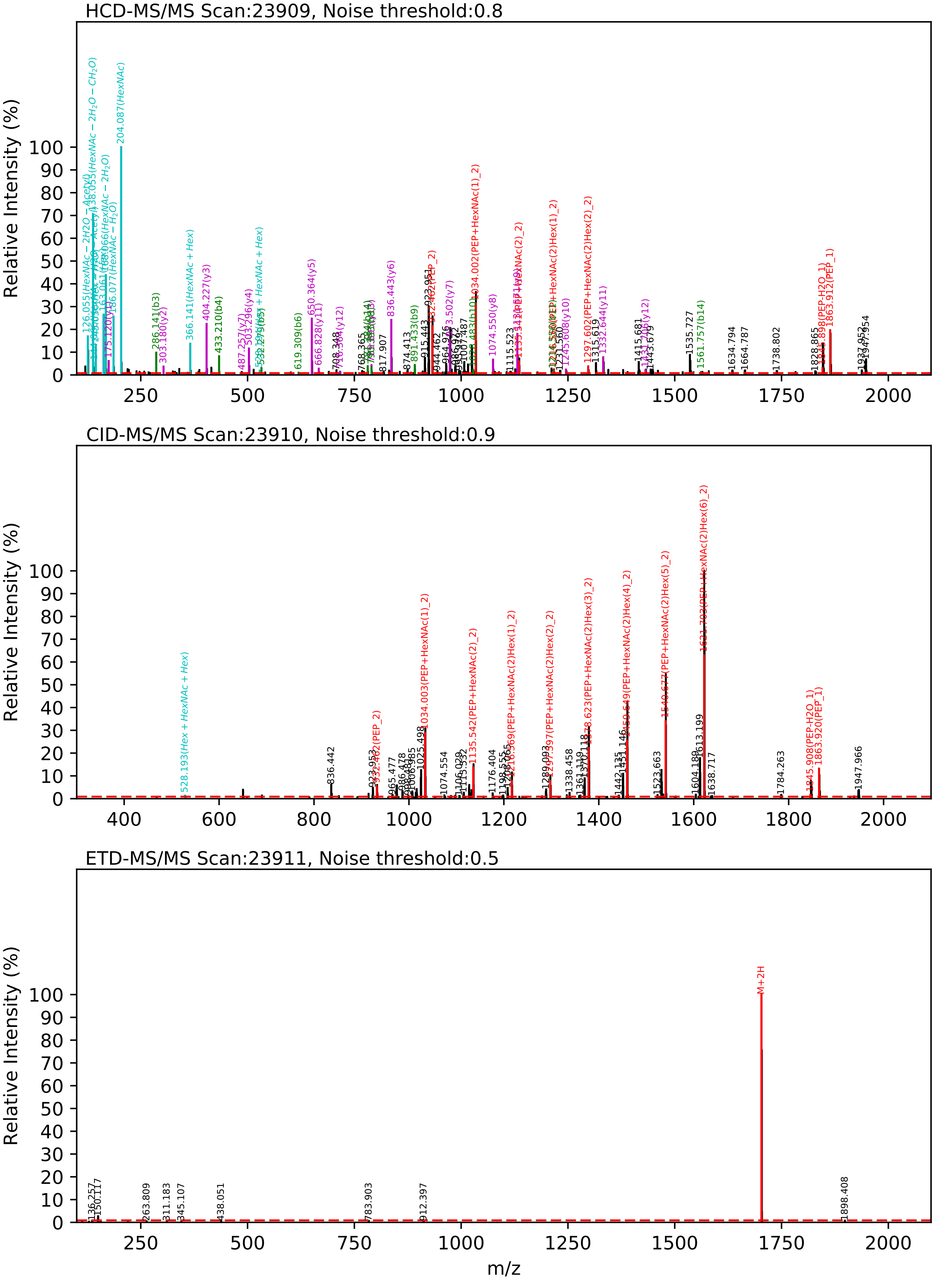

EGVFVSNGTHWFVTQR(=PEP)_7_2_0_0_0, 0_None, 0_None,
m/z:1702.73(2+), RT:65.16, Y-score:71.31

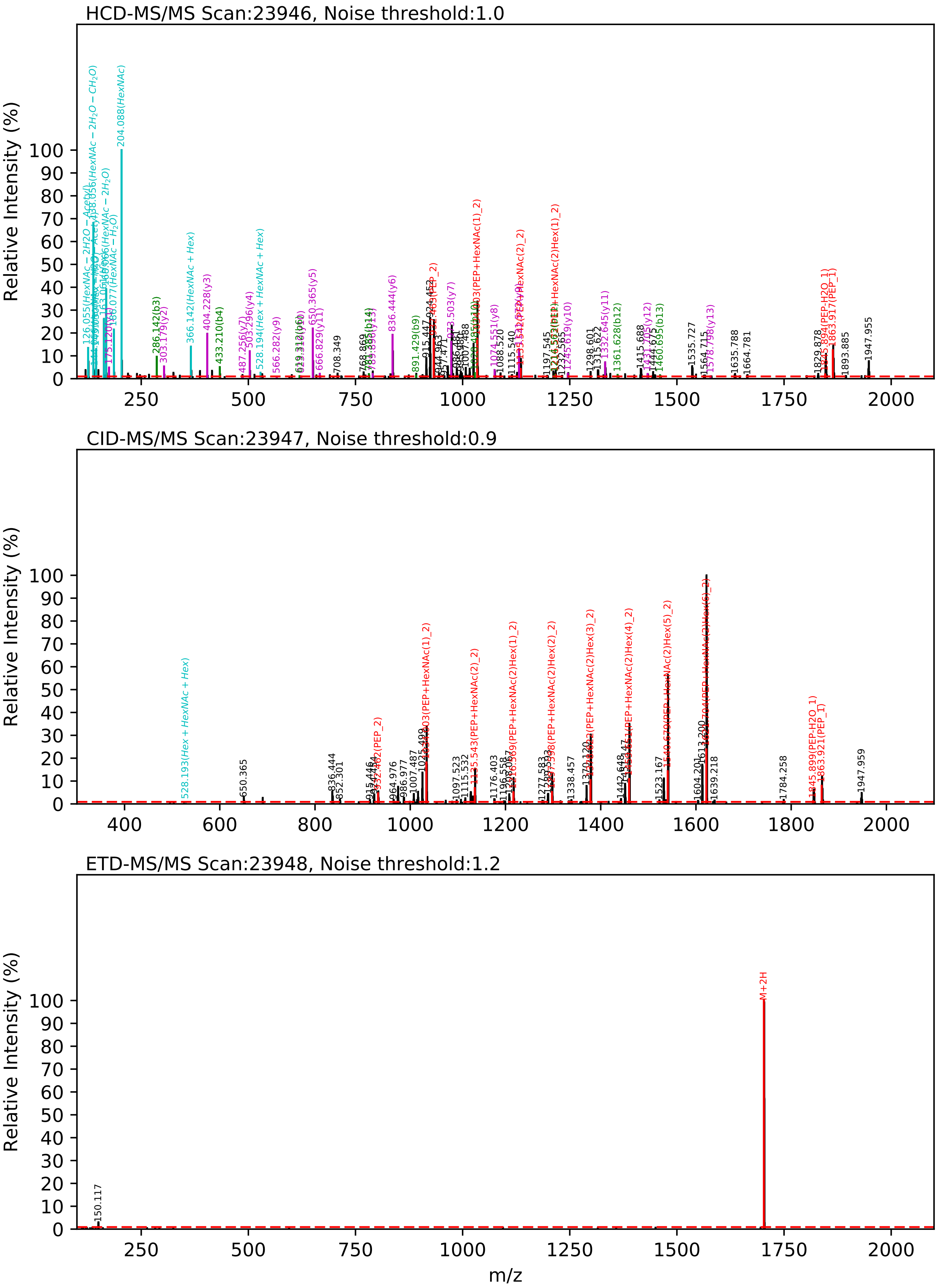

EGVFVSNGTHWFVTQR(=PEP)_8_2_0_0_0, 0_None, 0_None,
m/z:1189.50(3+), RT:64.83, Y-score:85.34

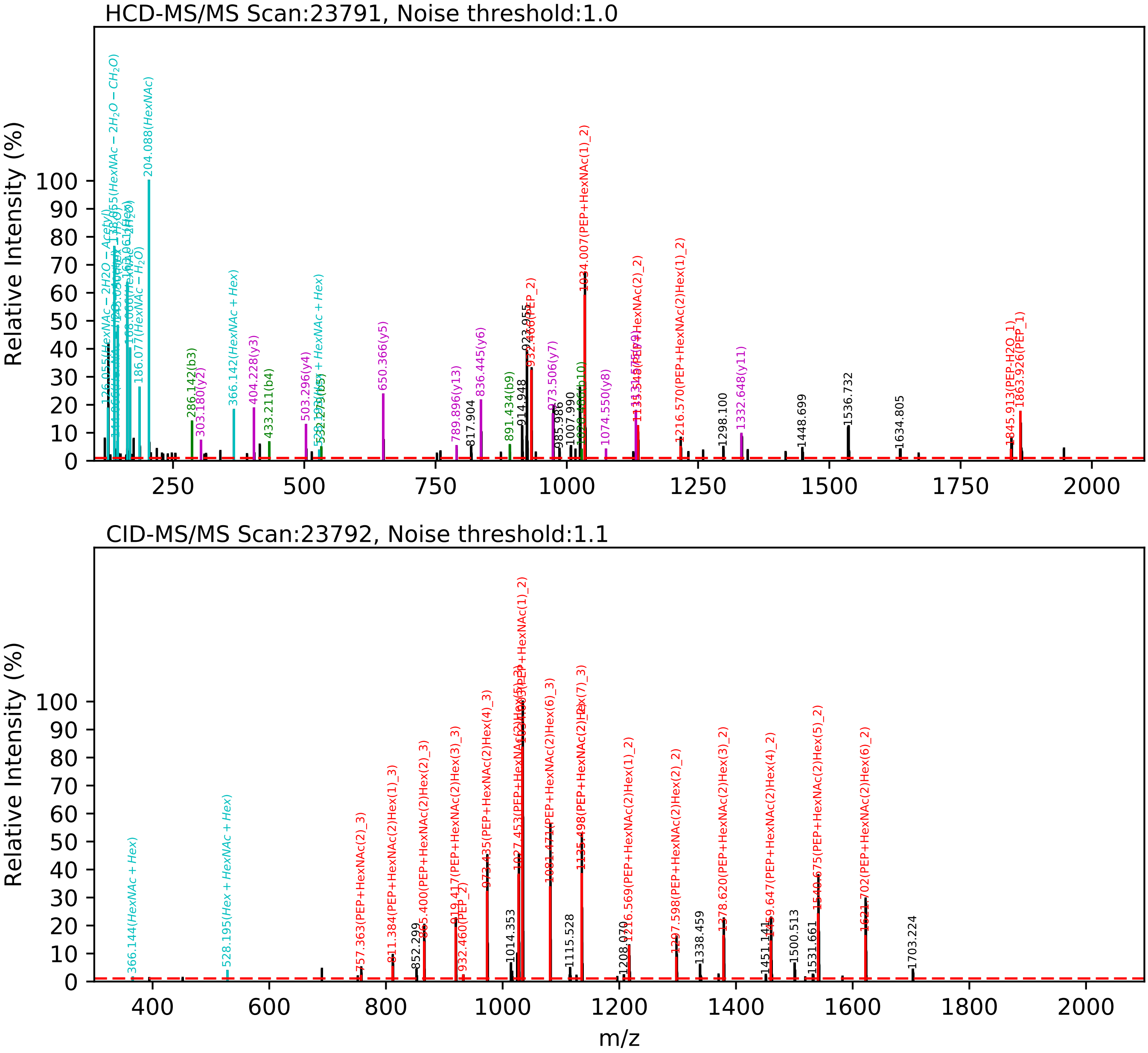

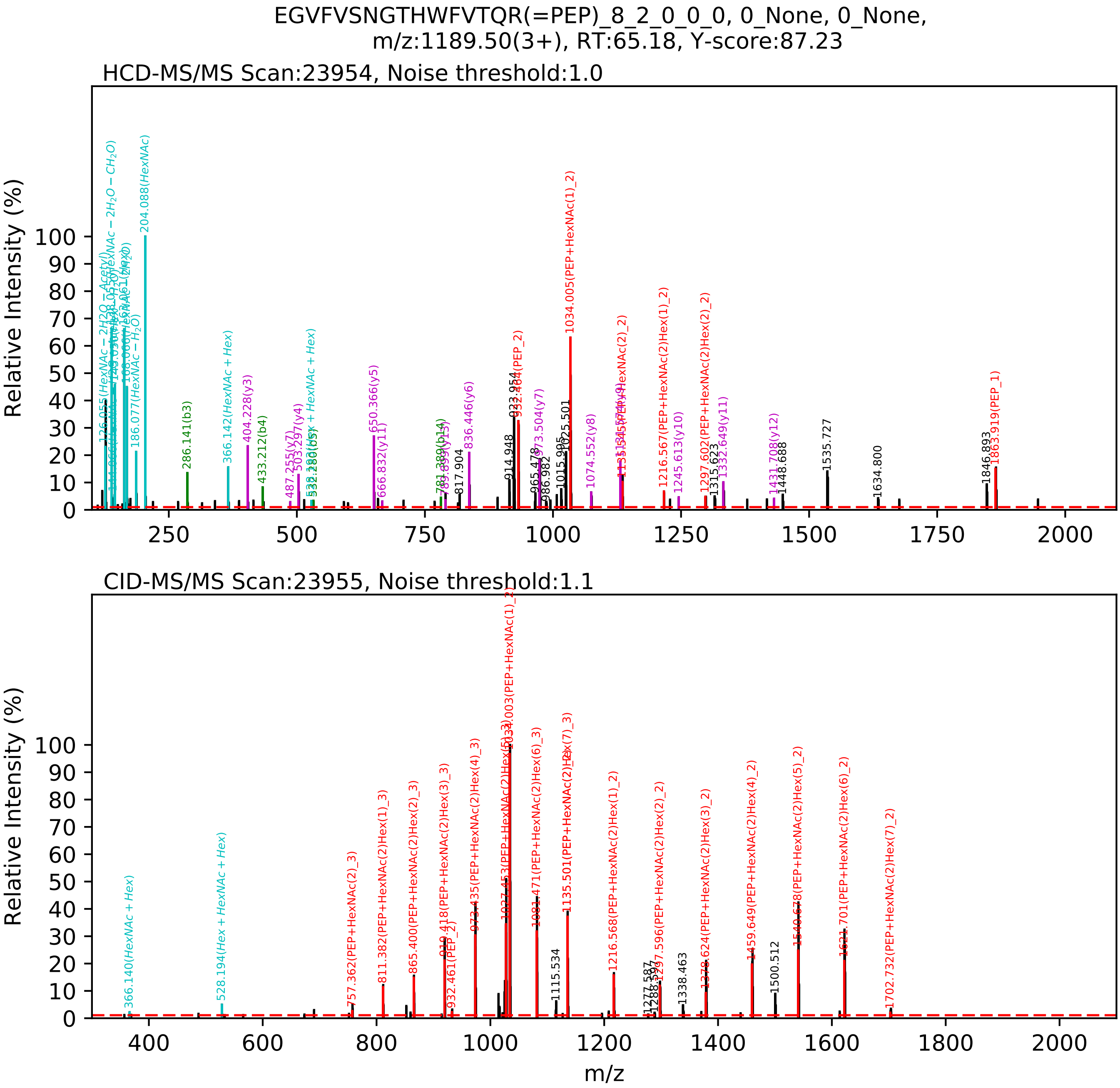
EGVFVSNGTHWFVTQR(=PEP)_8_2_0_0_0, 0_None, 0_None,
m/z:1189.50(3+), RT:65.18, Y-score:87.23
HCD-MS/MS Scan:23954, Noise threshold:1.0
Relative Intensity (%)
204.088(HexNAc)
186.077(HexNAc − H2O)
286.141(b3)
366.142(HexNAc + Hex)
404.228(y3)
433.212(b4)
487.255(y7)
503.297(y4)
650.366(y5)
666.832(y11)
781.389(b14)
817.904
836.446(y6)
914.948
933.954
932.464(PEP_2)
965.478
973.504(y7)
986.982
1015.996
1025.501
1034.005(PEP+HexNAc(1)_2)
1074.552(y8)
1216.567(PEP+HexNAc(2)Hex(1)_2)
1245.613(y10)
1297.602(PEP+HexNAc(2)Hex(2)_2)
1315.623
1332.649(y11)
1431.708(y12)
1448.688
1535.727
1634.800
1846.893
1863.919(PEP_1)
CID-MS/MS Scan:23955, Noise threshold:1.1
Relative Intensity (%)
366.140(HexNAc + Hex)
528.194(Hex + HexNAc + Hex)
757.362(PEP+HexNAc(2)_3)
811.382(PEP+HexNAc(2)Hex(1)_3)
865.400(PEP+HexNAc(2)Hex(2)_3)
919.418(PEP+HexNAc(2)Hex(3)_3)
932.461(PEP_2)
973.435(PEP+HexNAc(2)Hex(4)_3)
1034.003(PEP+HexNAc(1)_2)
1081.471(PEP+HexNAc(2)Hex(6)_3)
1115.534
1135.501(PEP+HexNAc(2)Hex(7)_3)
1216.568(PEP+HexNAc(2)Hex(1)_2)
1277.587
1297.596(PEP+HexNAc(2)Hex(2)_2)
1338.463
1378.624(PEP+HexNAc(2)Hex(3)_2)
1459.649(PEP+HexNAc(2)Hex(4)_2)
1500.512
1540.678(PEP+HexNAc(2)Hex(5)_2)
1621.701(PEP+HexNAc(2)Hex(6)_2)
1702.732(PEP+HexNAc(2)Hex(7)_2)
m/z

EGVFVSNGTHWFVTQR(=PEP)_8_2_0_0_0, 0_None, 0_None,
m/z:1189.50(3+), RT:65.39, Y-score:87.13

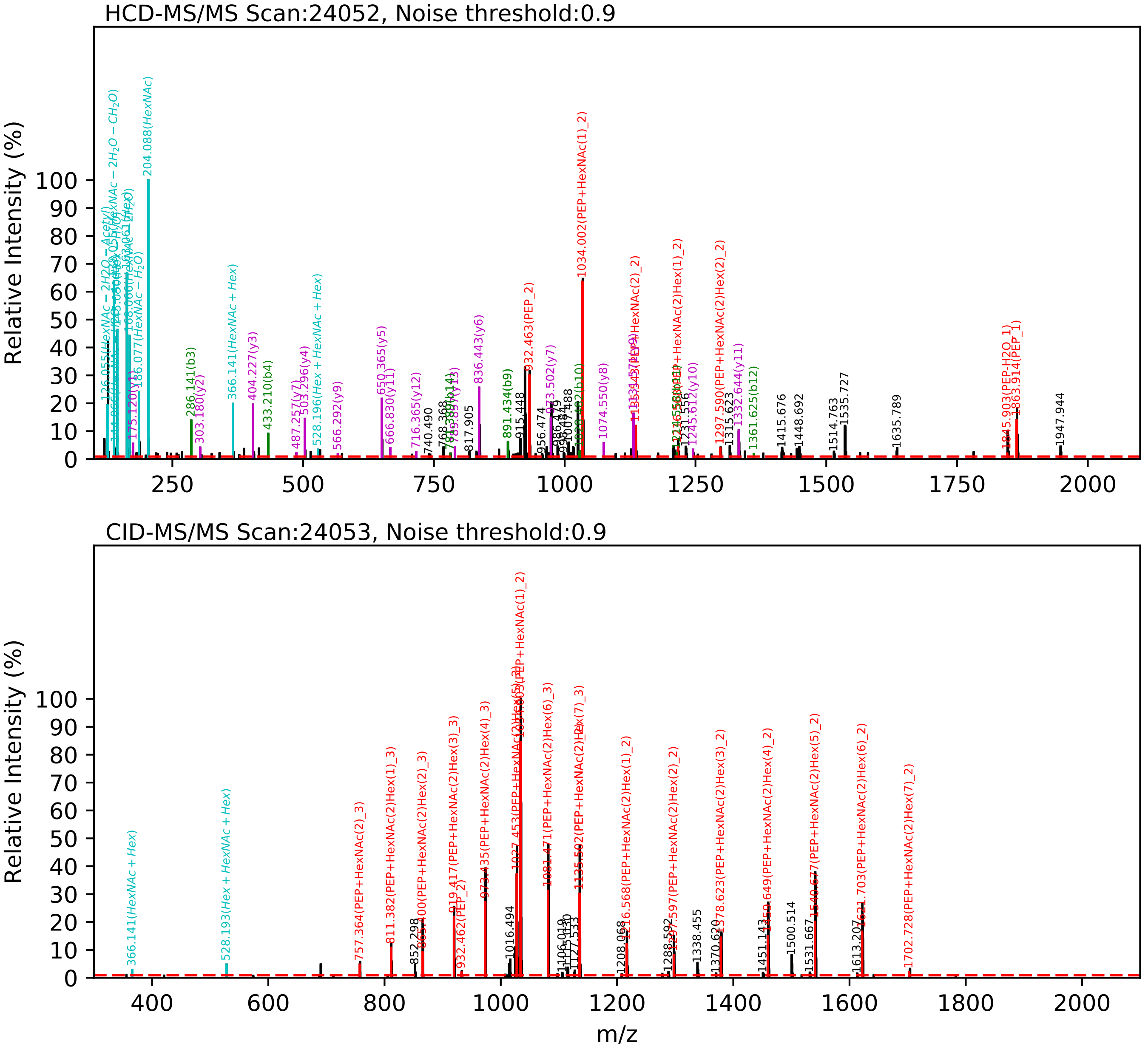

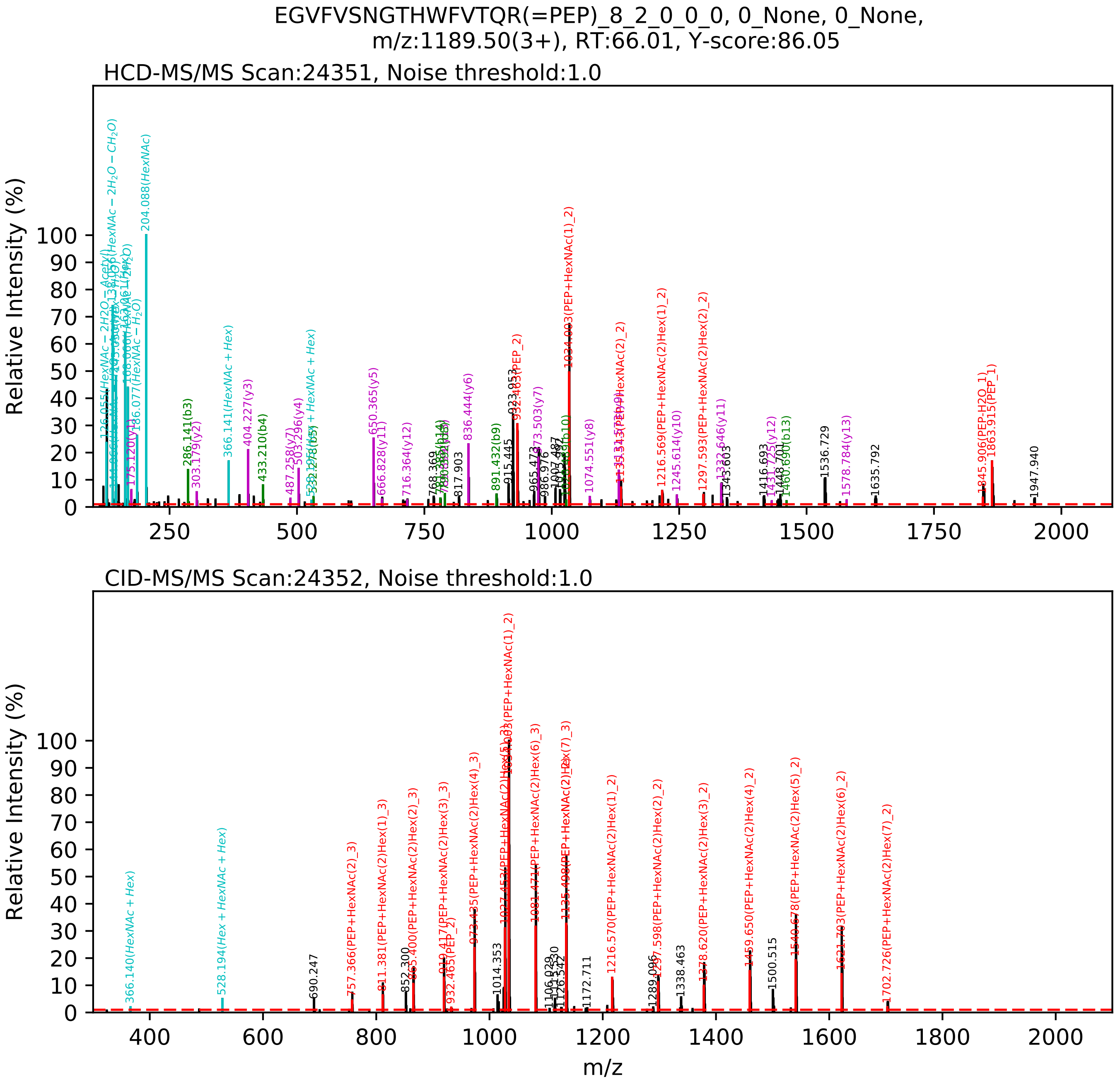
EGVFVSNGTHWFVTQR(=PEP)_8_2_0_0_0, 0_None, 0_None,
m/z:1189.50(3+), RT:66.01, Y-score:86.05
HCD-MS/MS Scan:24351, Noise threshold:1.0
Relative Intensity (%)
204.088(HexNAc)
286.141(b3)
303.179(y2)
366.141(HexNAc + Hex)
404.227(y3)
433.210(b4)
487.258(y7)
503.296(y4)
532.278(b5)
650.365(y5)
666.828(y11)
716.364(y12)
768.369
817.903
836.444(y6)
891.432(b9)
915.445
923.953
932.465(PEP_2)
965.473
973.503(y7)
986.976
1034.003(PEP+HexNAc(1)_2)
1074.551(y8)
1216.569(PEP+HexNAc(2)Hex(1)_2)
1245.614(y10)
1297.593(PEP+HexNAc(2)Hex(2)_2)
1332.646(y11)
1343.603
1416.693
1460.690(b13)
1536.729
1578.784(y13)
1635.792
1845.906(PEP-H2O_1)
1863.915(PEP_1)
1947.940
CID-MS/MS Scan:24352, Noise threshold:1.0
Relative Intensity (%)
366.140(HexNAc + Hex)
528.194(Hex + HexNAc + Hex)
690.247
757.366(PEP+HexNAc(2)_3)
811.381(PEP+HexNAc(2)Hex(1)_3)
852.300
865.400(PEP+HexNAc(2)Hex(2)_3)
919.417(PEP+HexNAc(2)Hex(3)_3)
932.465(PEP_2)
973.435(PEP+HexNAc(2)Hex(4)_3)
1014.353
1081.471(PEP+HexNAc(2)Hex(6)_3)
1135.490(PEP+HexNAc(2)Hex(7)_3)
1172.711
1216.570(PEP+HexNAc(2)Hex(1)_2)
1297.598(PEP+HexNAc(2)Hex(2)_2)
1338.463
1378.620(PEP+HexNAc(2)Hex(3)_2)
1459.650(PEP+HexNAc(2)Hex(4)_2)
1500.515
1540.678(PEP+HexNAc(2)Hex(5)_2)
1621.703(PEP+HexNAc(2)Hex(6)_2)
1702.726(PEP+HexNAc(2)Hex(7)_2)
m/z

EGVFVSNGTHWFVTQR(=PEP)_8_2_0_0_0, 0_None, 0_None,
m/z:1189.50(3+), RT:66.55, Y-score:81.39

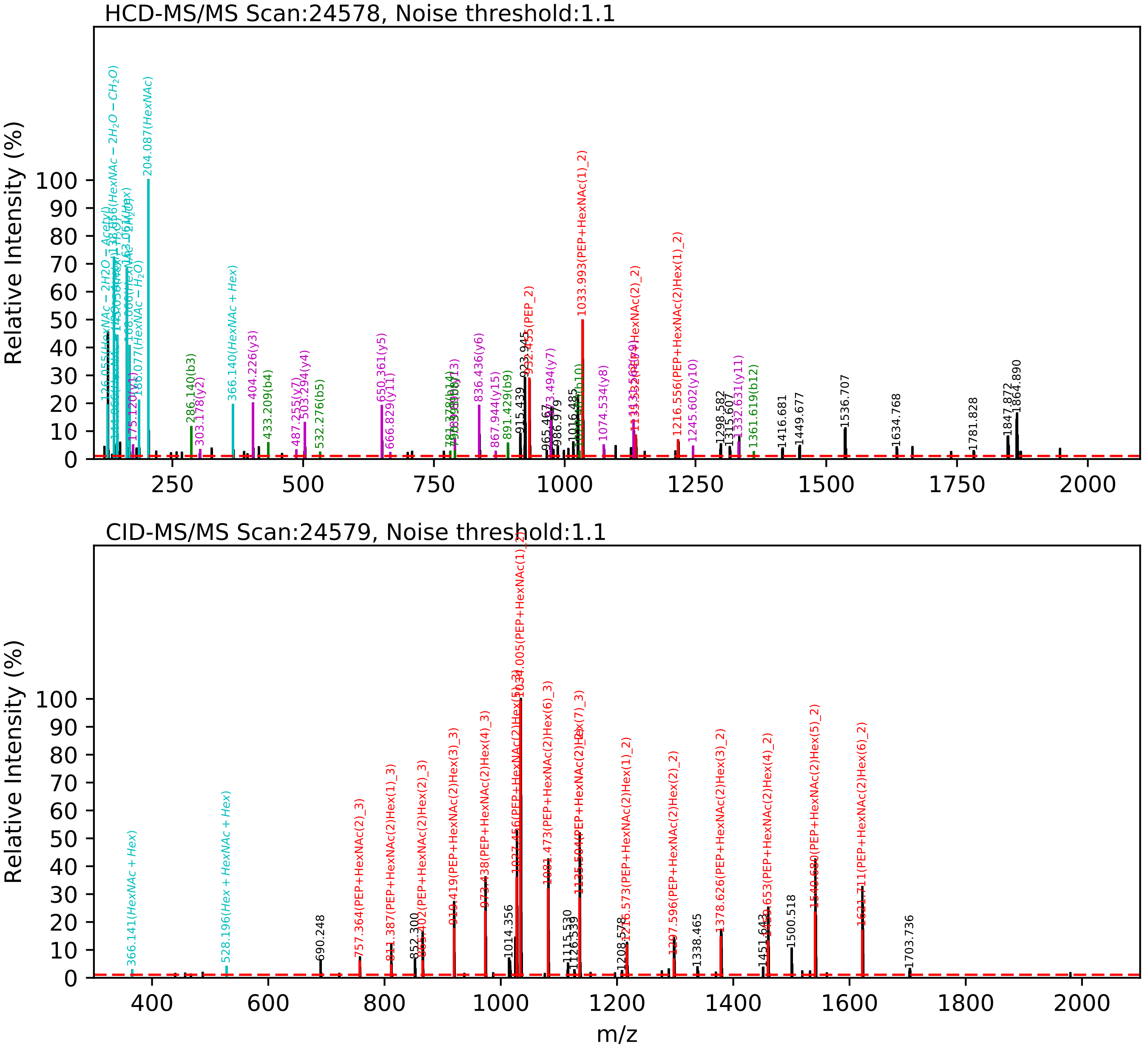

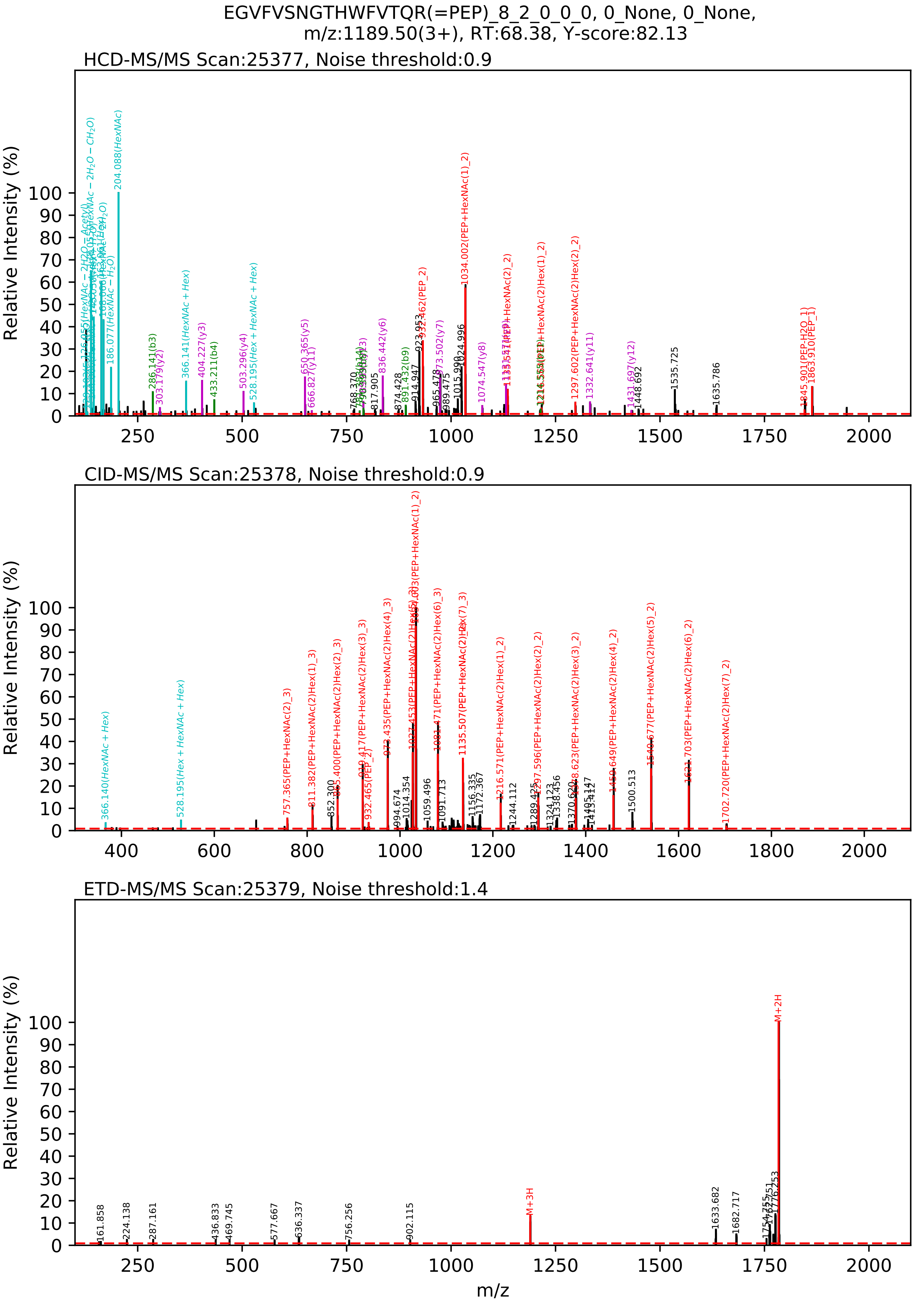
EGVFVSNGTHWFVTQR(=PEP)_8_2_0_0_0, 0_None, 0_None,
m/z:1189.50(3+), RT:68.38, Y-score:82.13
HCD-MS/MS Scan:25377, Noise threshold:0.9
CID-MS/MS Scan:25378, Noise threshold:0.9
ETD-MS/MS Scan:25379, Noise threshold:1.4
Relative Intensity (%)
m/z
M+2H
M+3H

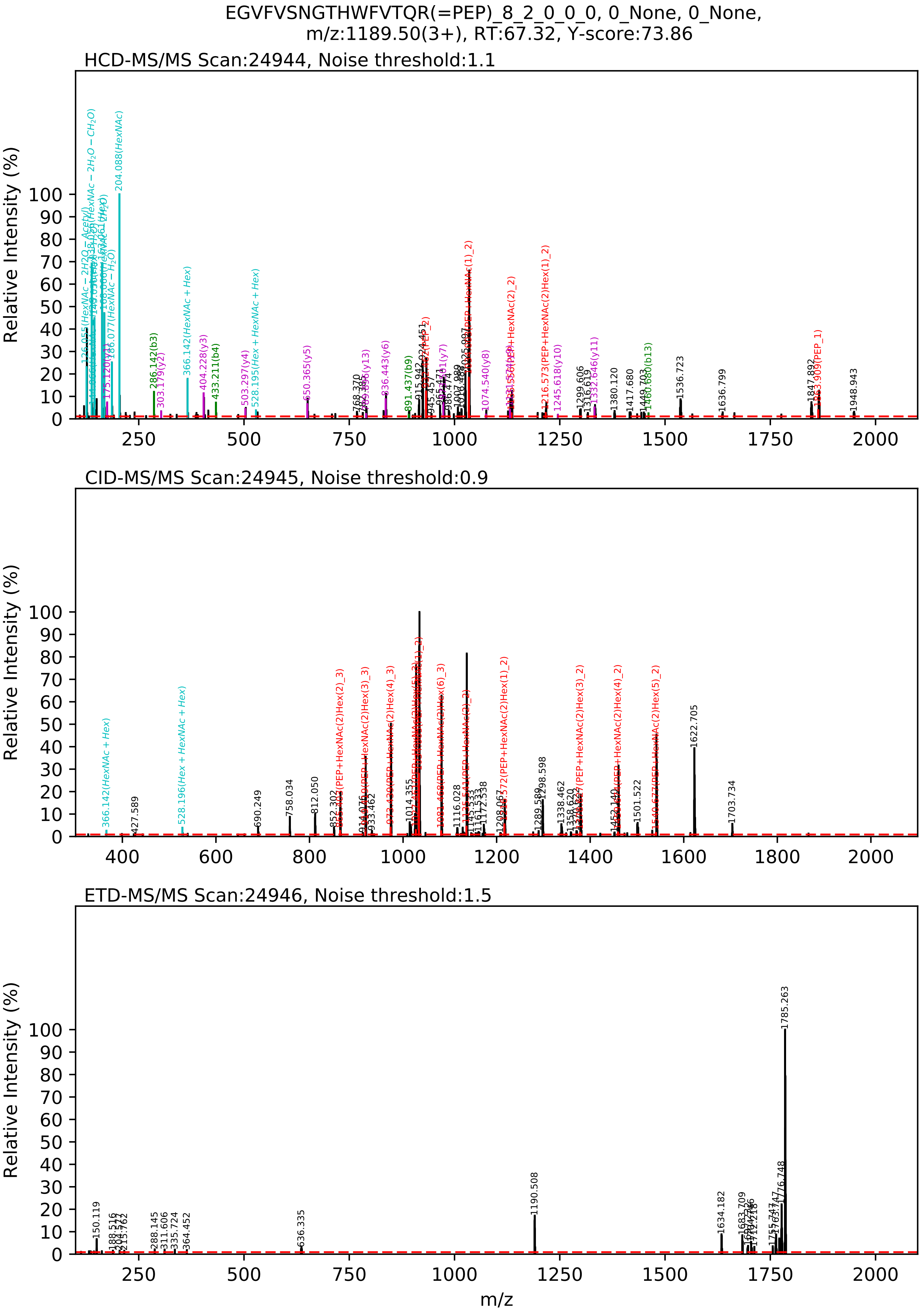
EGVFVSNGTHWFVTQR(=PEP)_8_2_0_0_0, 0_None, 0_None,
m/z:1189.50(3+), RT:67.32, Y-score:73.86
HCD-MS/MS Scan:24944, Noise threshold:1.1
Relative Intensity (%)
204.088(HexNAc)
286.142(b3)
303.179(y2)
366.142(HexNAc+Hex)
404.228(y3)
433.211(b4)
503.297(y4)
528.195(Hex+HexNAc+Hex)
650.365(y5)
768.370
836.443(y6)
891.437(b9)
1074.540(y8)
1216.573(PEP+HexNAc(2)Hex(1)_2)
1245.618(y10)
1299.606
1332.646(y11)
1380.120
1417.680
1449.703
1460.680(b13)
1536.723
1636.799
1847.892
1863.909(PEP_1)
1948.943
CID-MS/MS Scan:24945, Noise threshold:0.9
366.142(HexNAc+Hex)
427.589
528.196(Hex+HexNAc+Hex)
690.249
758.034
812.050
852.302
933.462
1014.355
1116.028
1172.538
1208.067
1289.589
1298.598
1338.462
1358.620
1452.140
1501.522
1622.705
1703.734
ETD-MS/MS Scan:24946, Noise threshold:1.5
150.119
188.516
215.762
288.145
311.606
335.724
364.452
636.335
1190.508
1634.182
1683.709
1712.218
1776.748
1785.263
m/z

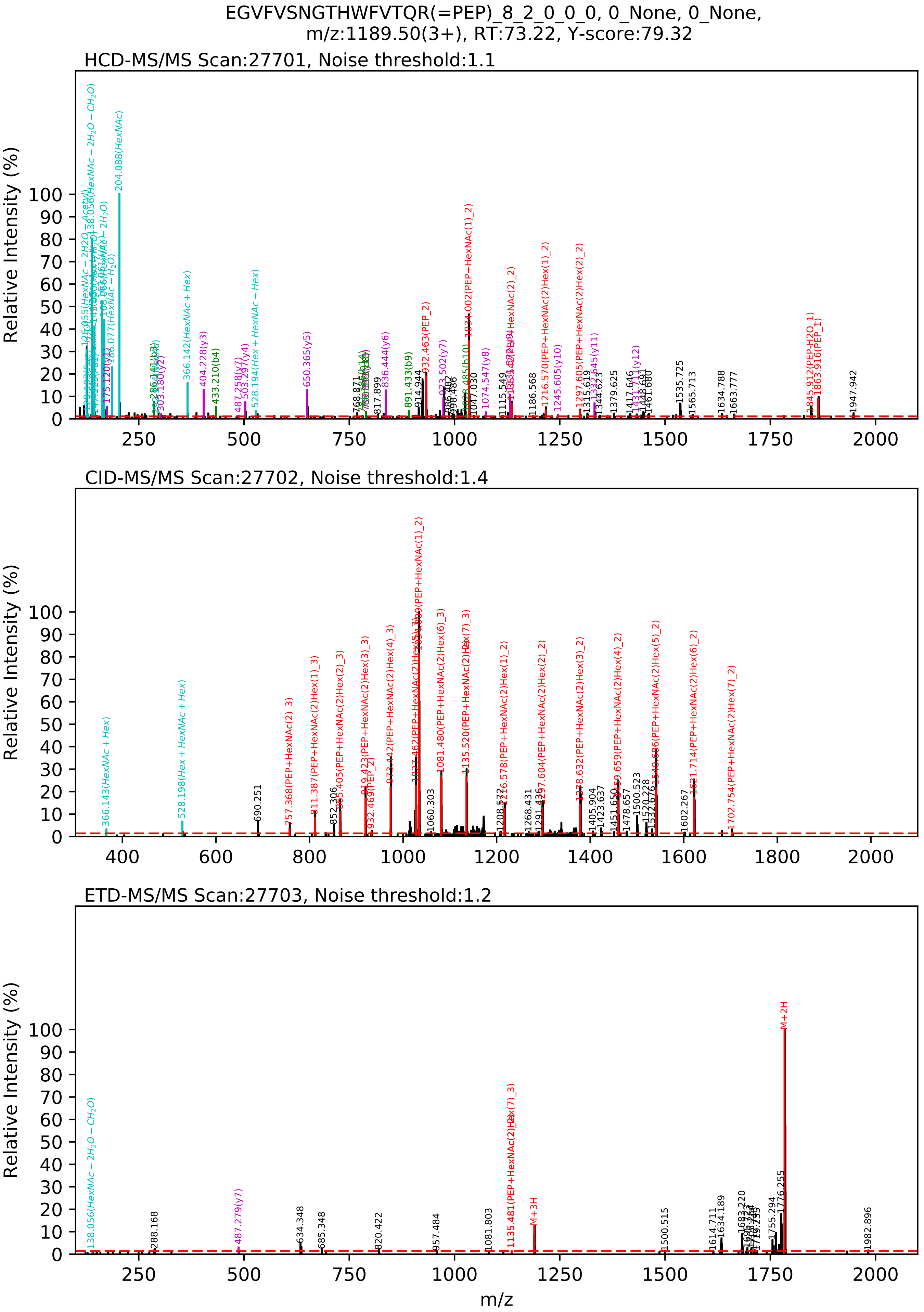

EGVFVSNGTHWFVTQR(=PEP)_8_2_0_0_0, 0_None, 0_None,
m/z:1189.50(3+), RT:73.22, Y-score:79.32
HCD-MS/MS Scan:27701, Noise threshold:1.1
CID-MS/MS Scan:27702, Noise threshold:1.4
ETD-MS/MS Scan:27703, Noise threshold:1.2
Relative Intensity (%)
m/z
204.088(HexNAc)
366.142(Hex + HexNAc)
528.194(Hex + HexNAc + Hex)
404.228(y3)
433.210(b4)
650.365(y5)
836.444(y6)
891.433(b9)
932.463(PEP_2)
1034.002(PEP+HexNAc(1)_2)
1216.570(PEP+HexNAc(2)Hex(1)_2)
1297.605(PEP+HexNAc(2)Hex(2)_2)
1845.912(PEP-H2O_1)
1863.916(PEP_1)
1947.942
757.368(PEP+HexNAc(2)_3)
811.387(PEP+HexNAc(2)Hex(1)_3)
865.405(PEP+HexNAc(2)Hex(2)_3)
919.423(PEP+HexNAc(2)Hex(3)_3)
973.442(PEP+HexNAc(2)Hex(4)_3)
1081.480(PEP+HexNAc(2)Hex(6)_3)
1135.520(PEP+HexNAc(2)Hex(7)_3)
1216.578(PEP+HexNAc(2)Hex(1)_2)
1378.632(PEP+HexNAc(2)Hex(3)_2)
1540.686(PEP+HexNAc(2)Hex(5)_2)
1621.714(PEP+HexNAc(2)Hex(6)_2)
1702.754(PEP+HexNAc(2)Hex(7)_2)
M+2H
M+3H
1776.255
1982.896

EGVFVSNGTHWFVTQR(=PEP)_8_2_0_0_0, 0_None, 0_None,
m/z:1189.50(3+), RT:73.69, Y-score:81.11

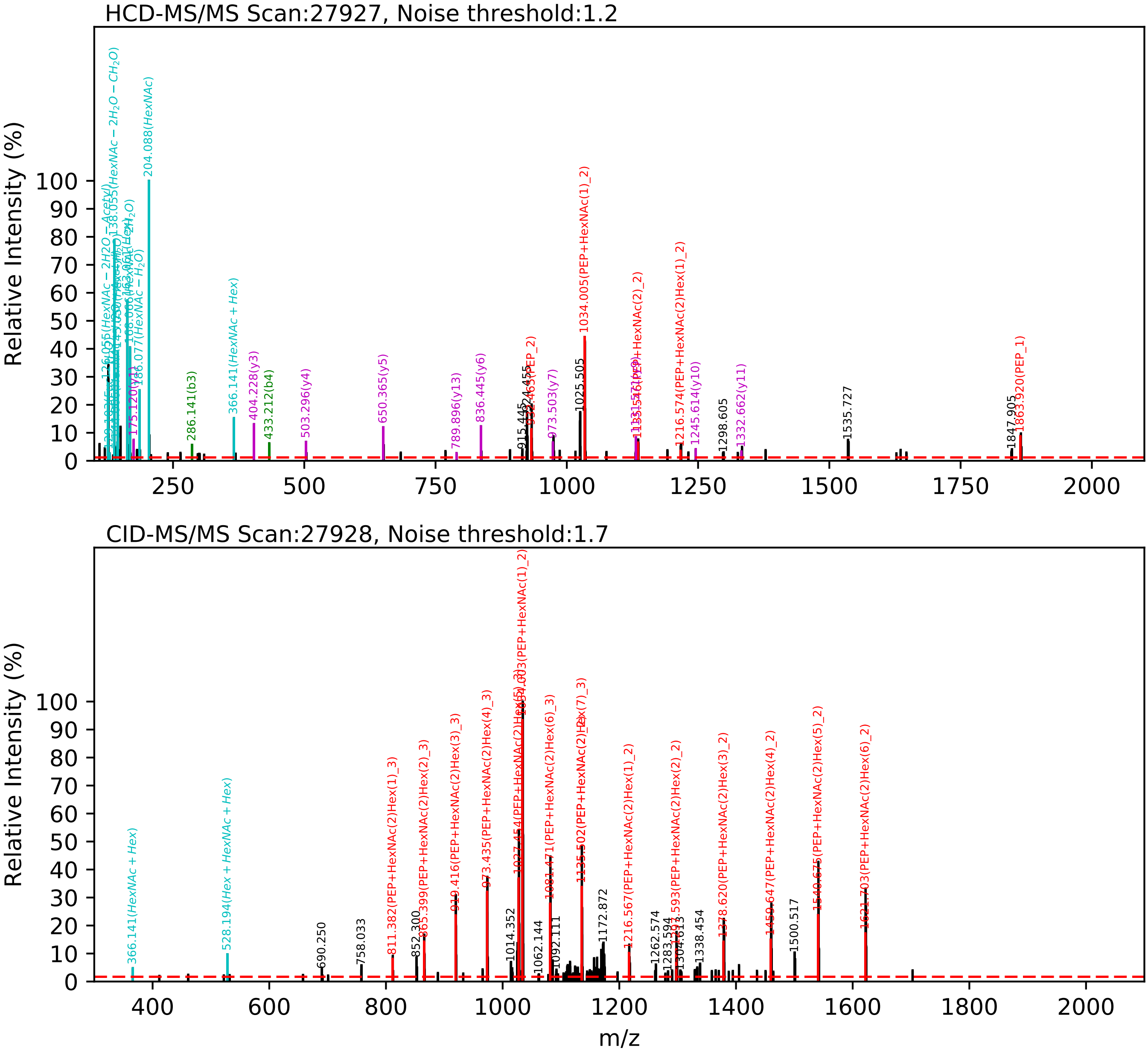

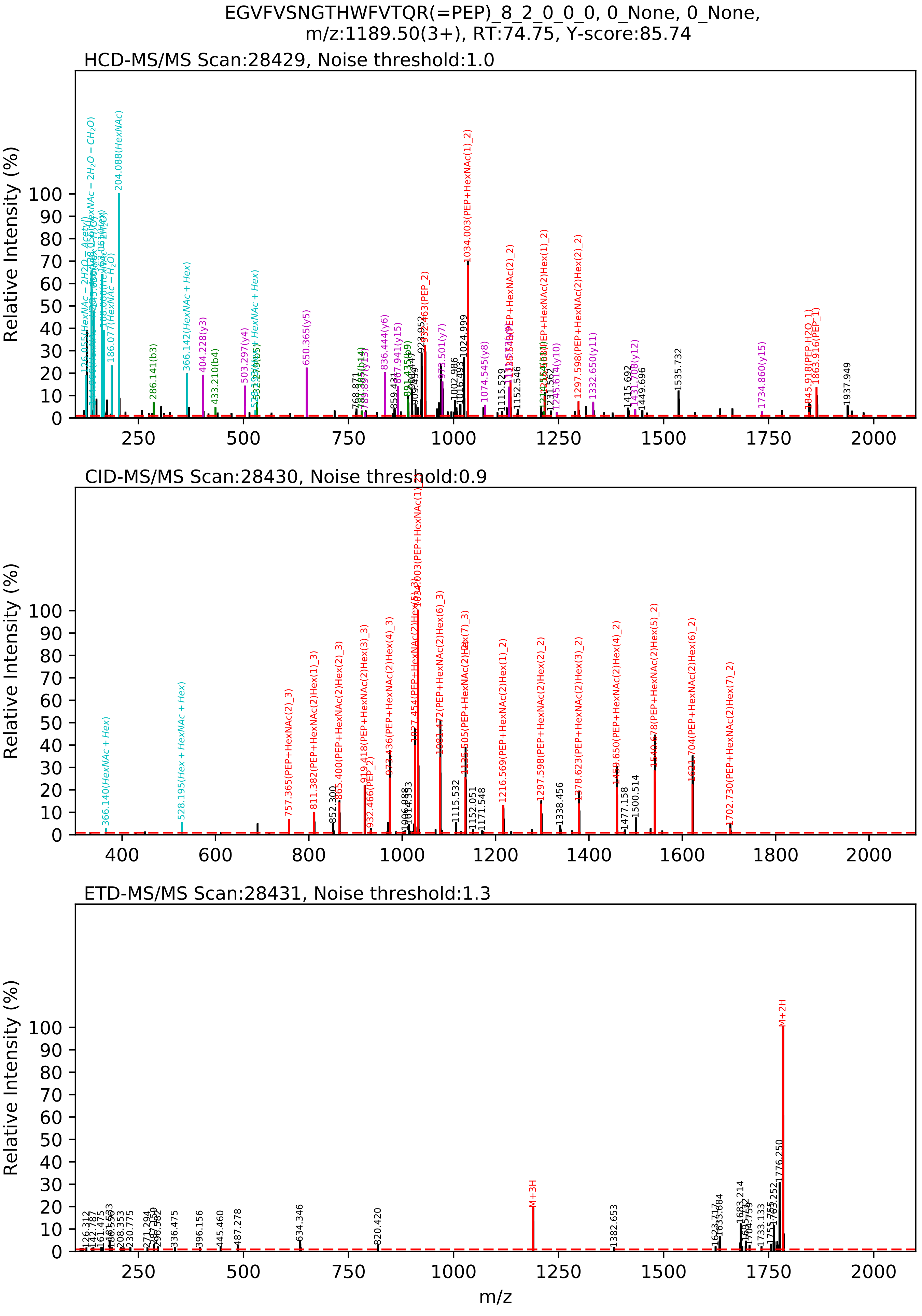
EGVFVSNGTHWFVTQR(=PEP)_8_2_0_0_0, 0_None, 0_None,
m/z:1189.50(3+), RT:74.75, Y-score:85.74
HCD-MS/MS Scan:28429, Noise threshold:1.0
CID-MS/MS Scan:28430, Noise threshold:0.9
ETD-MS/MS Scan:28431, Noise threshold:1.3
Relative Intensity (%)
m/z
204.088(HexNAc)
1034.003(PEP+HexNAc(1)_2)
M+2H
M+3H

EGVFVSNGTHWFVTQR(=PEP)_8_2_0_0_0, 0_None, 0_None,
m/z:1189.50(3+), RT:75.24, Y-score:83.46

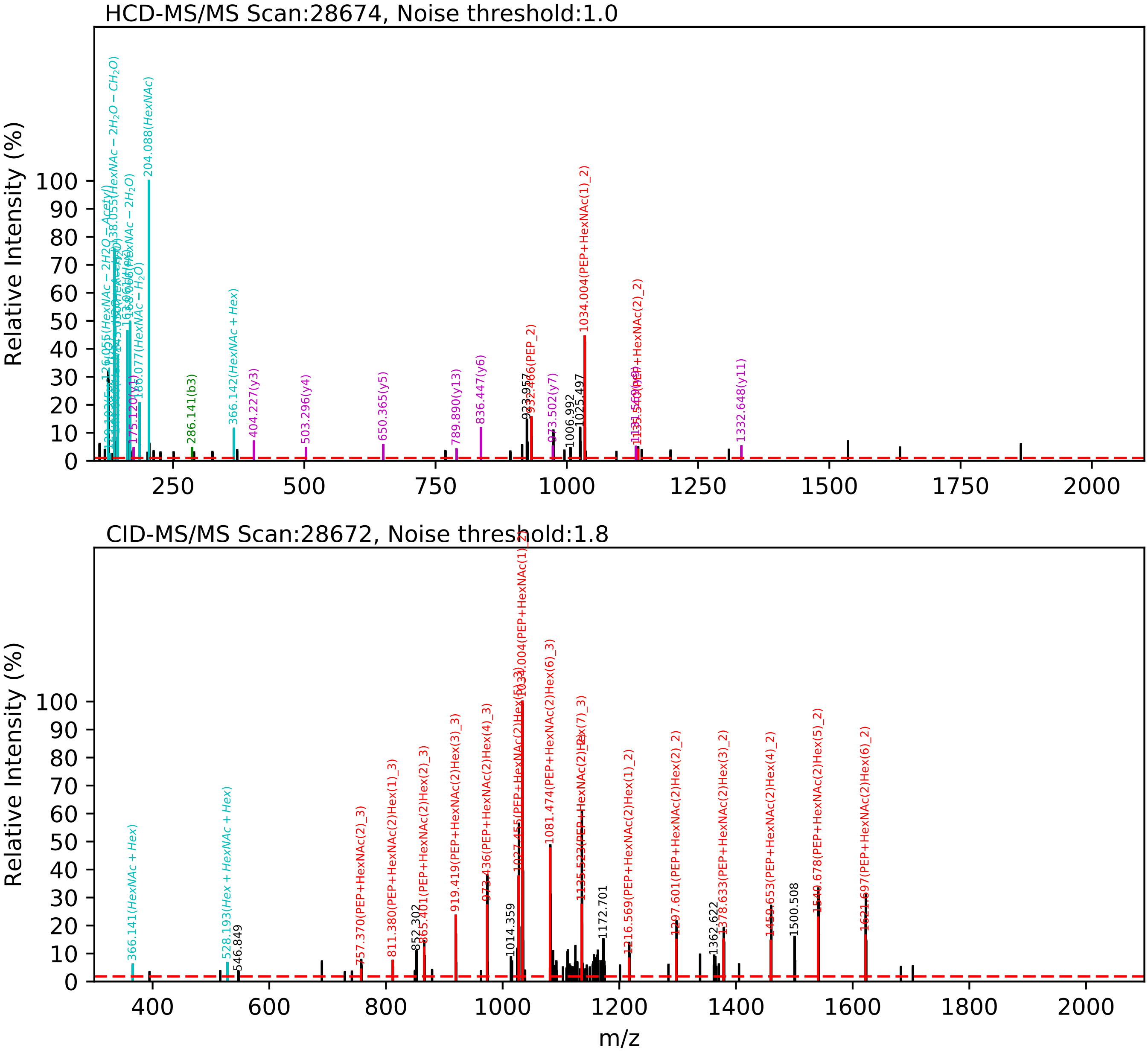

EGVFVSNGTHWFVTQR(=PEP)_8_2_0_0_0, 0_None, 0_None,
m/z:1189.50(3+), RT:71.29, Y-score:77.63

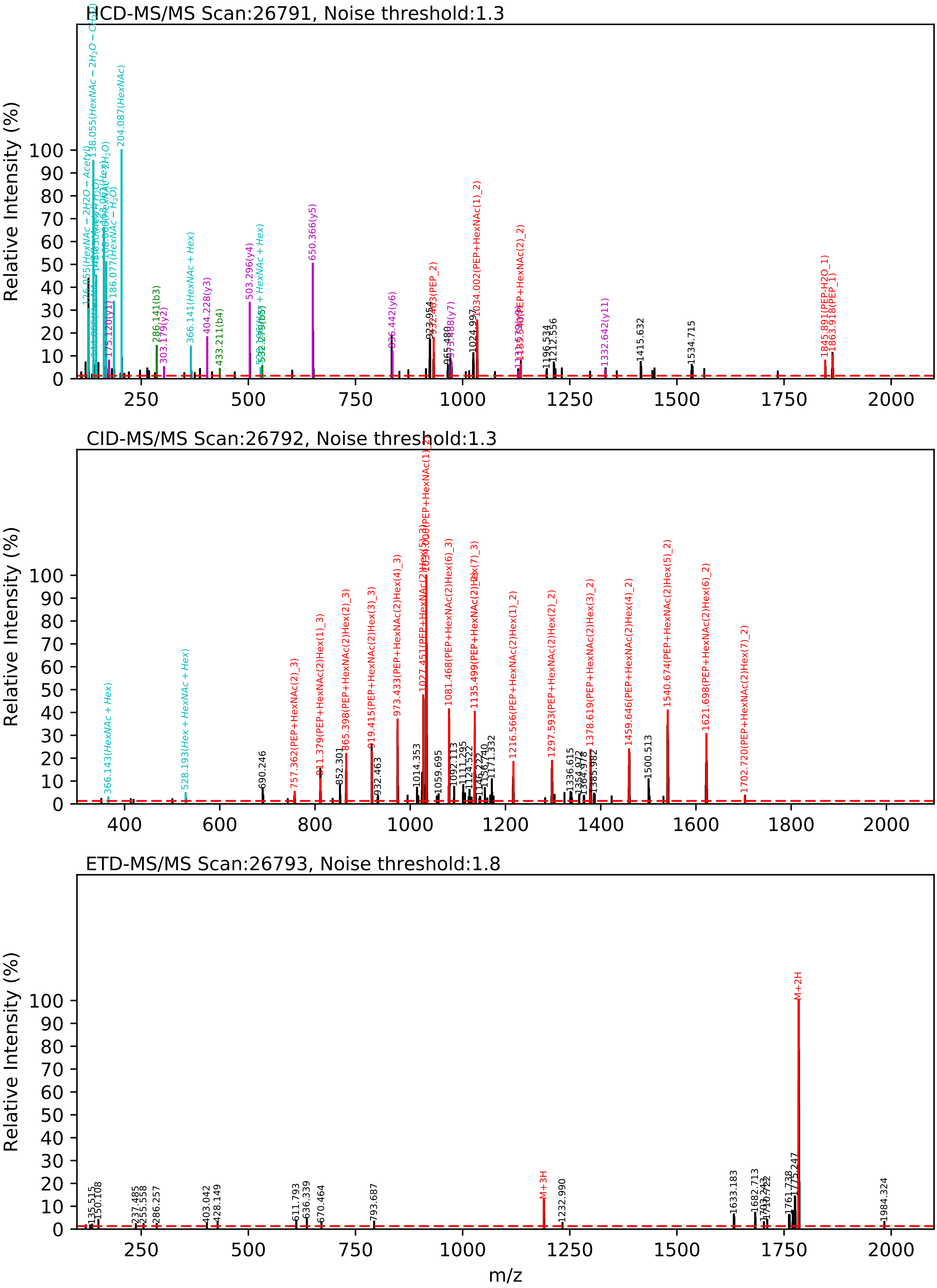

EGVFVSNGTHWFVTQR(=PEP)_8_2_0_0_0, 0_None, 0_None,
m/z:1189.50(3+), RT:71.86, Y-score:79.75

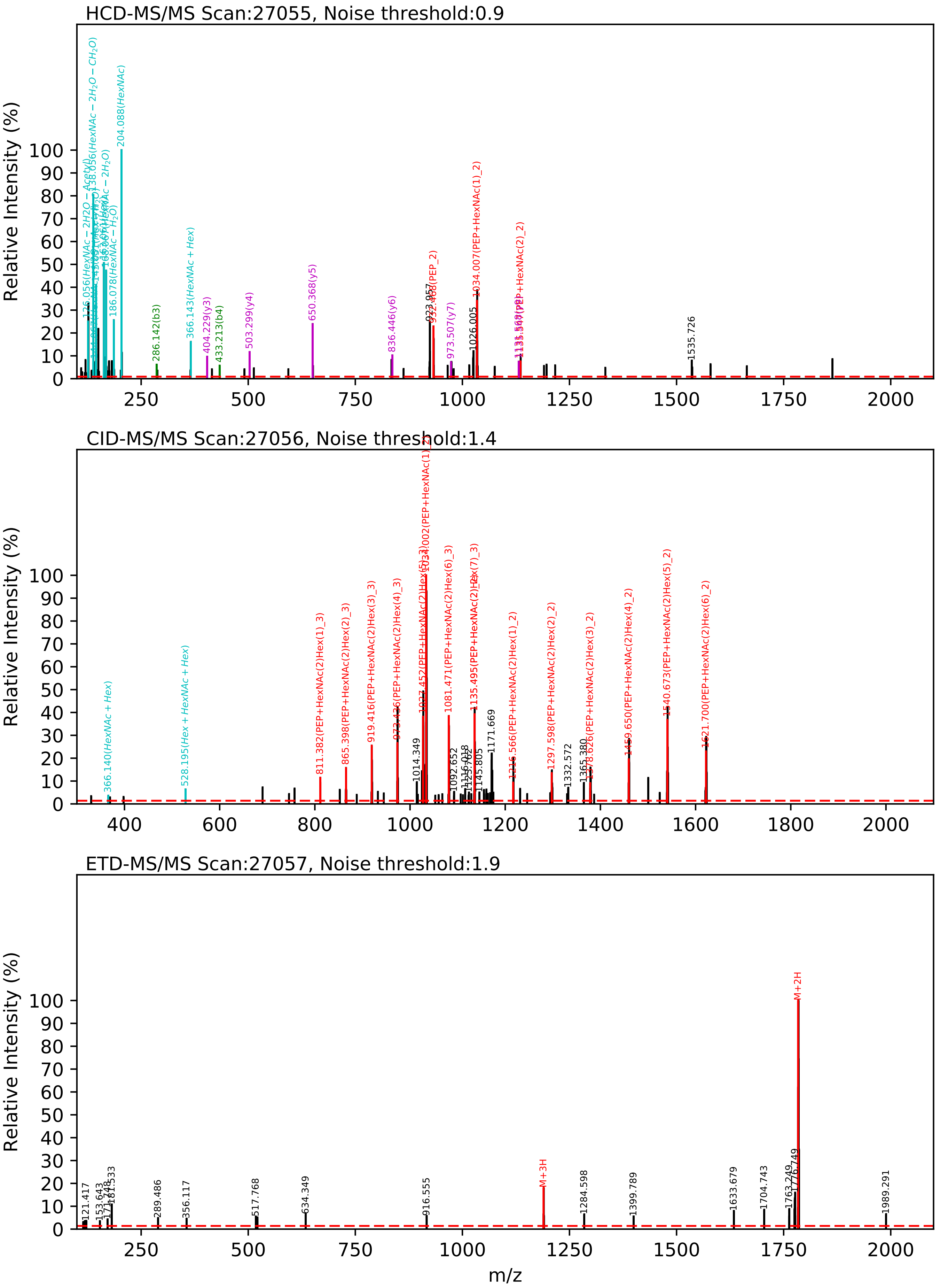

EGVFVSNGTHWFVTQR(=PEP)_8_2_0_0_0, 0_None, 0_None,
m/z:1189.51(3+), RT:62.92, Y-score:84.16

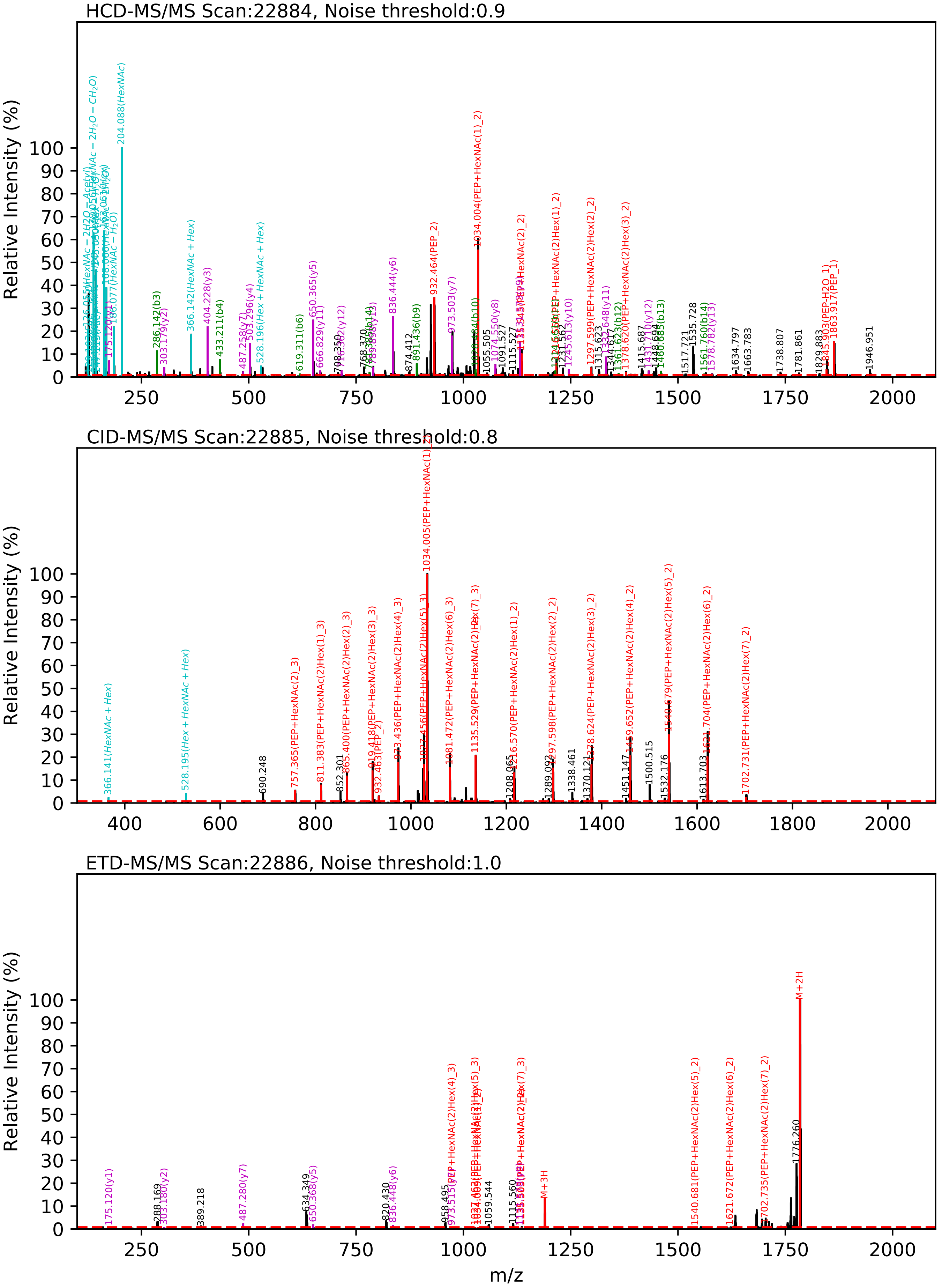

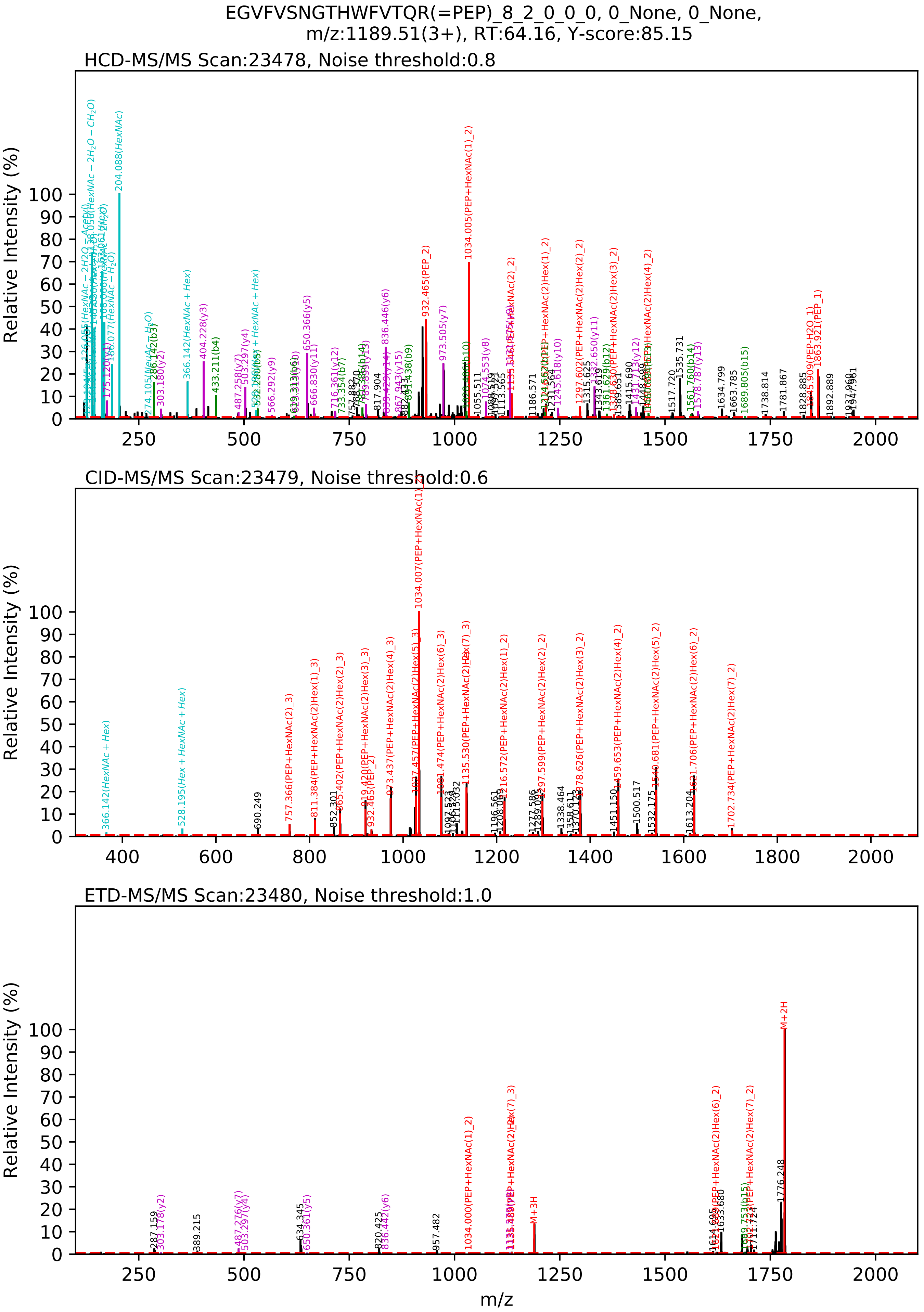
EGVFVSNGTHWFVTQR(=PEP)_8_2_0_0_0, 0_None, 0_None,
m/z:1189.51(3+), RT:64.16, Y-score:85.15
HCD-MS/MS Scan:23478, Noise threshold:0.8
CID-MS/MS Scan:23479, Noise threshold:0.6
ETD-MS/MS Scan:23480, Noise threshold:1.0
Relative Intensity (%)
m/z
1034.005(PEP+HexNAc(1)_2)
204.088(HexNAc)
1034.007(PEP+HexNAc(1)_2)
M+2H
M+3H

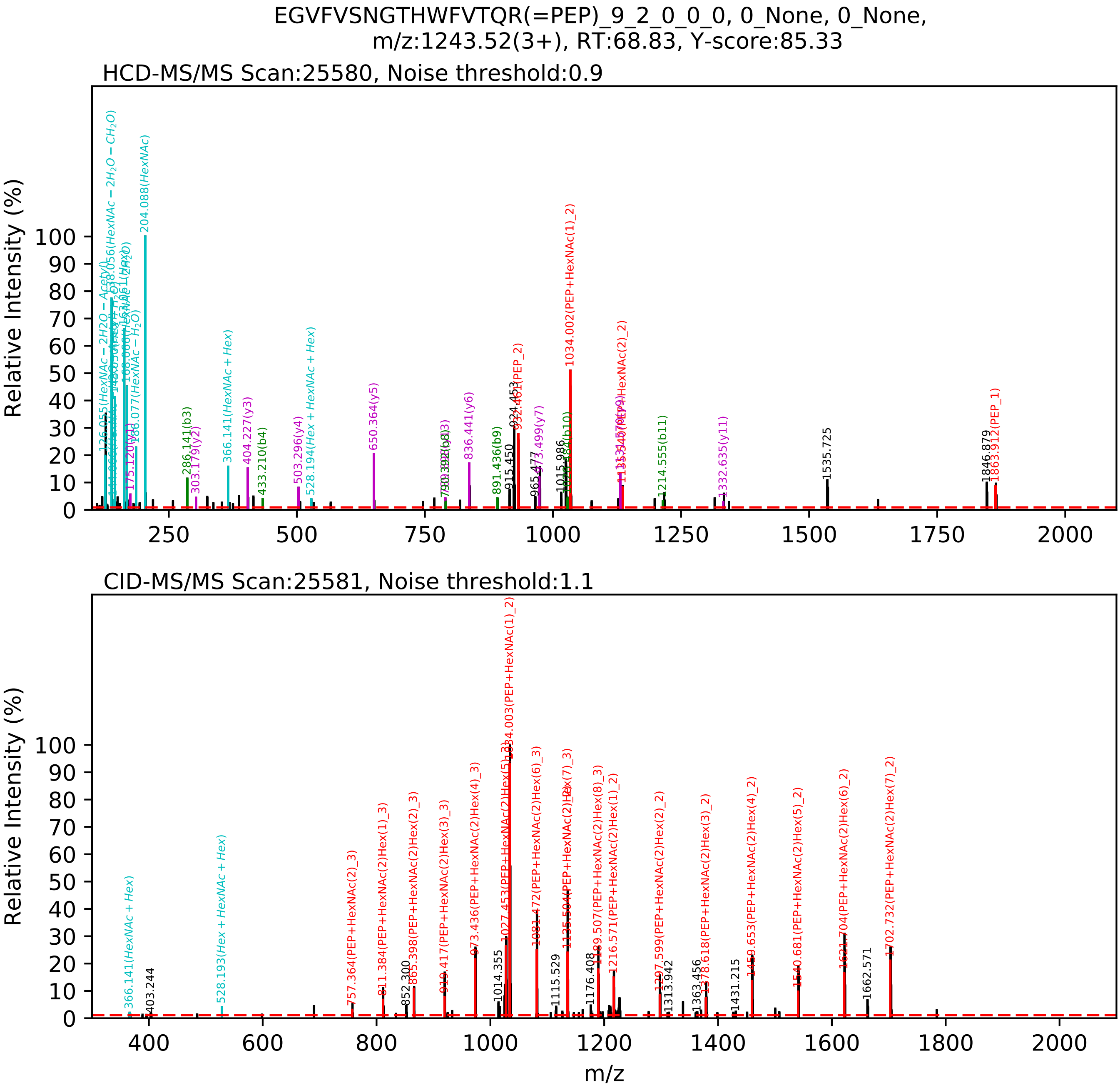
EGVFVSNGTHWFVTQR(=PEP)_9_2_0_0_0, 0_None, 0_None,
m/z:1243.52(3+), RT:68.83, Y-score:85.33
HCD-MS/MS Scan:25580, Noise threshold:0.9
Relative Intensity (%)
204.088(HexNAc)
1034.002(PEP+HexNAc(1)_2)
650.364(y5)
836.441(y6)
1535.725
1846.879
1863.912(PEP_1)
CID-MS/MS Scan:25581, Noise threshold:1.1
Relative Intensity (%)
1034.003(PEP+HexNAc(1)_2)
1702.732(PEP+HexNAc(2)Hex(7)_2)
m/z

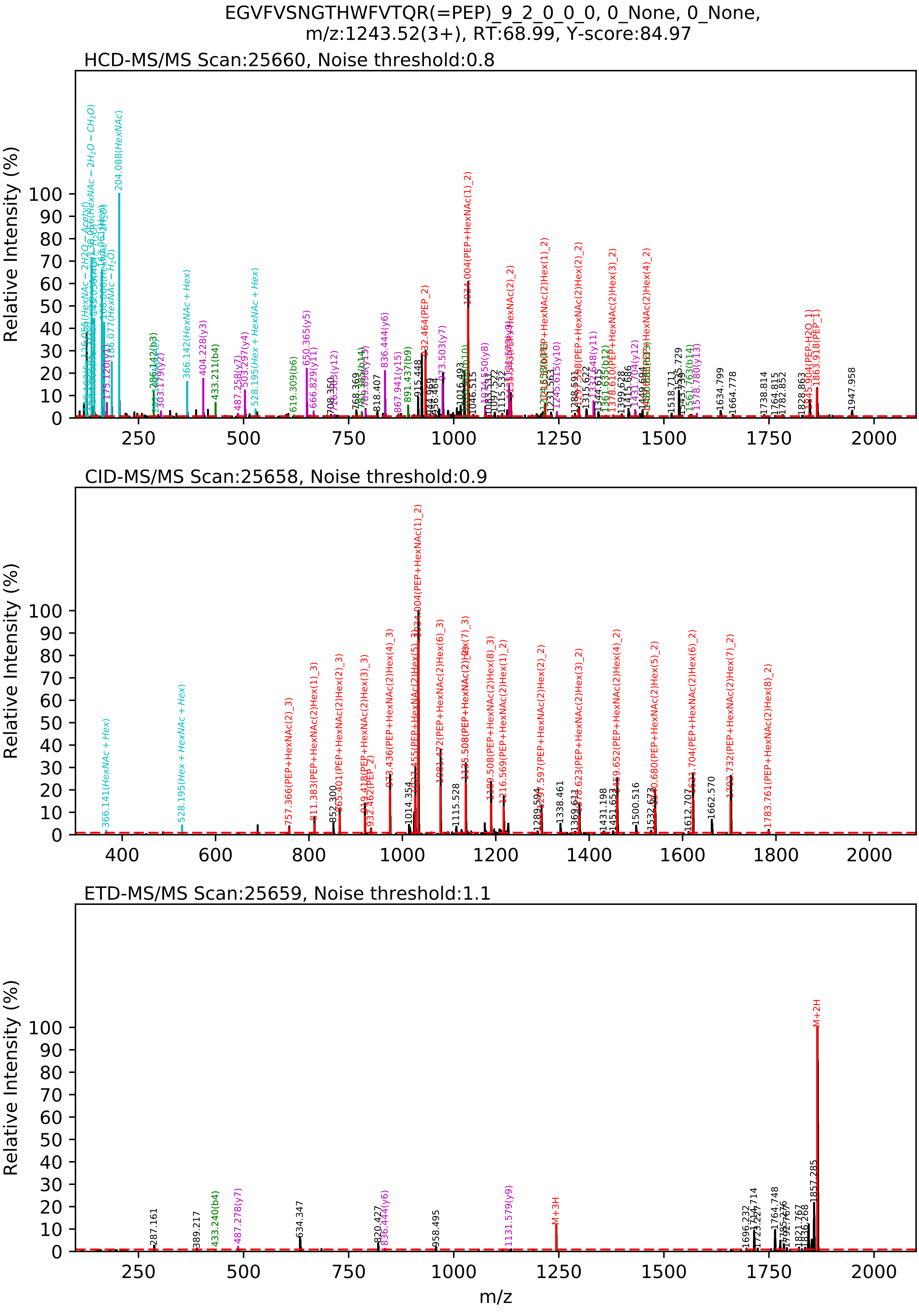
EGVFVSNGTHWFVTQR(=PEP)_9_2_0_0_0, 0_None, 0_None,
m/z:1243.52(3+), RT:68.99, Y-score:84.97
HCD-MS/MS Scan:25660, Noise threshold:0.8
Relative Intensity (%)
204.088(HexNAc)
366.142(HexNAc + Hex)
404.228(y3)
433.211(b4)
528.195(Hex + HexNAc + Hex)
619.309(b6)
650.365(y5)
836.444(y6)
891.437(b9)
1034.004(PEP+HexNAc(1)_2)
1634.799
1664.778
1738.814
1947.958
CID-MS/MS Scan:25658, Noise threshold:0.9
366.141(HexNAc + Hex)
528.195(Hex + HexNAc + Hex)
757.366(PEP+HexNAc(2)_3)
811.383(PEP+HexNAc(2)Hex(1)_3)
1034.004(PEP+HexNAc(1)_2)
1783.761(PEP+HexNAc(2)Hex(8)_2)
ETD-MS/MS Scan:25659, Noise threshold:1.1
287.161
389.217
433.240(b4)
487.278(y7)
634.347
958.495
1131.579(y9)
M+3H
M+2H
1857.285
m/z

EGVFVSNGTHWFVTQR(=PEP)_9_2_0_0_0, 0_None, 0_None,
m/z:1243.52(3+), RT:69.26, Y-score:83.50

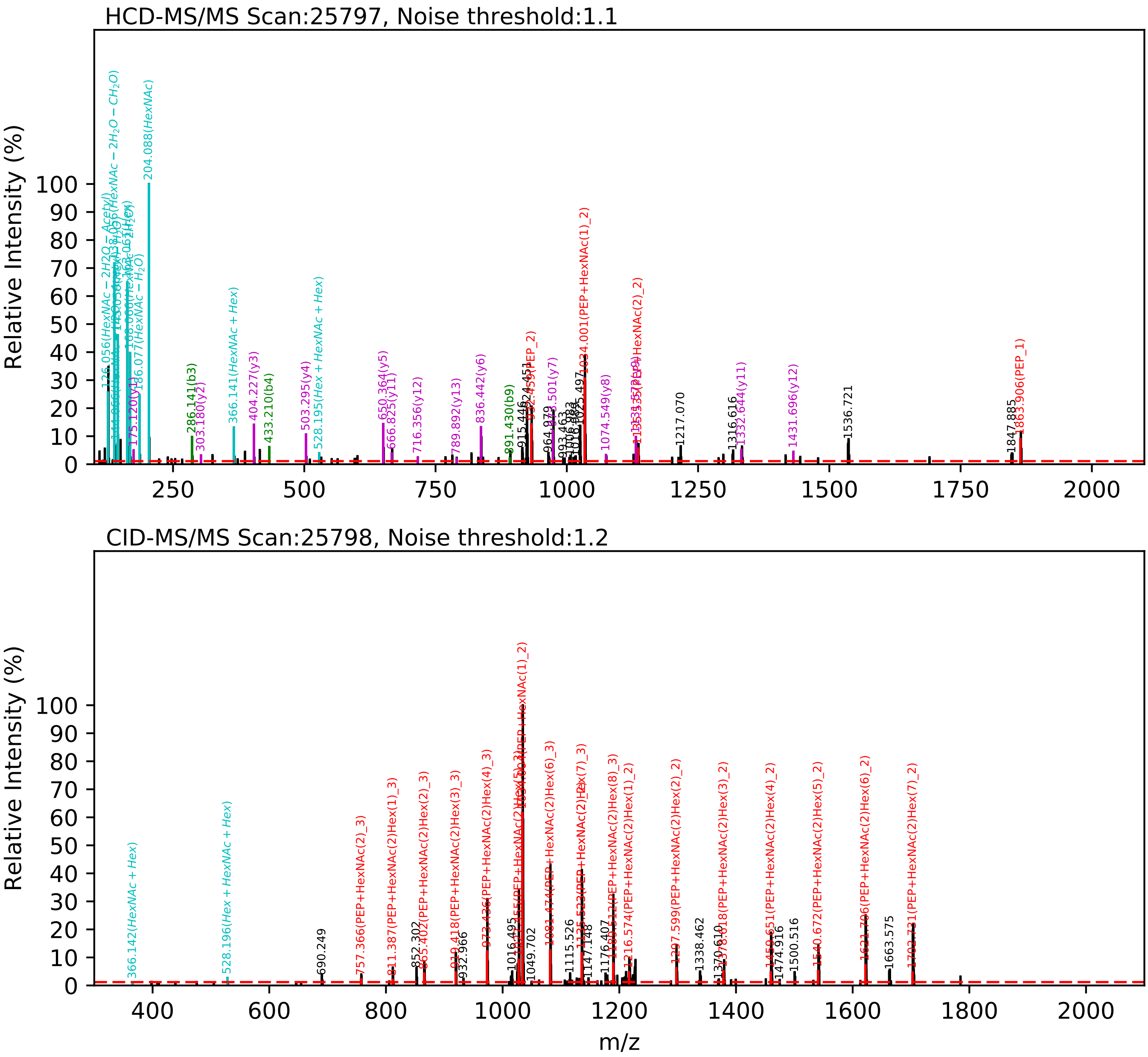

EGVFVSNGTHWFVTQR(=PEP)_9_2_0_0_0, 0_None, 0_None,
m/z:1243.52(3+), RT:69.66, Y-score:82.29

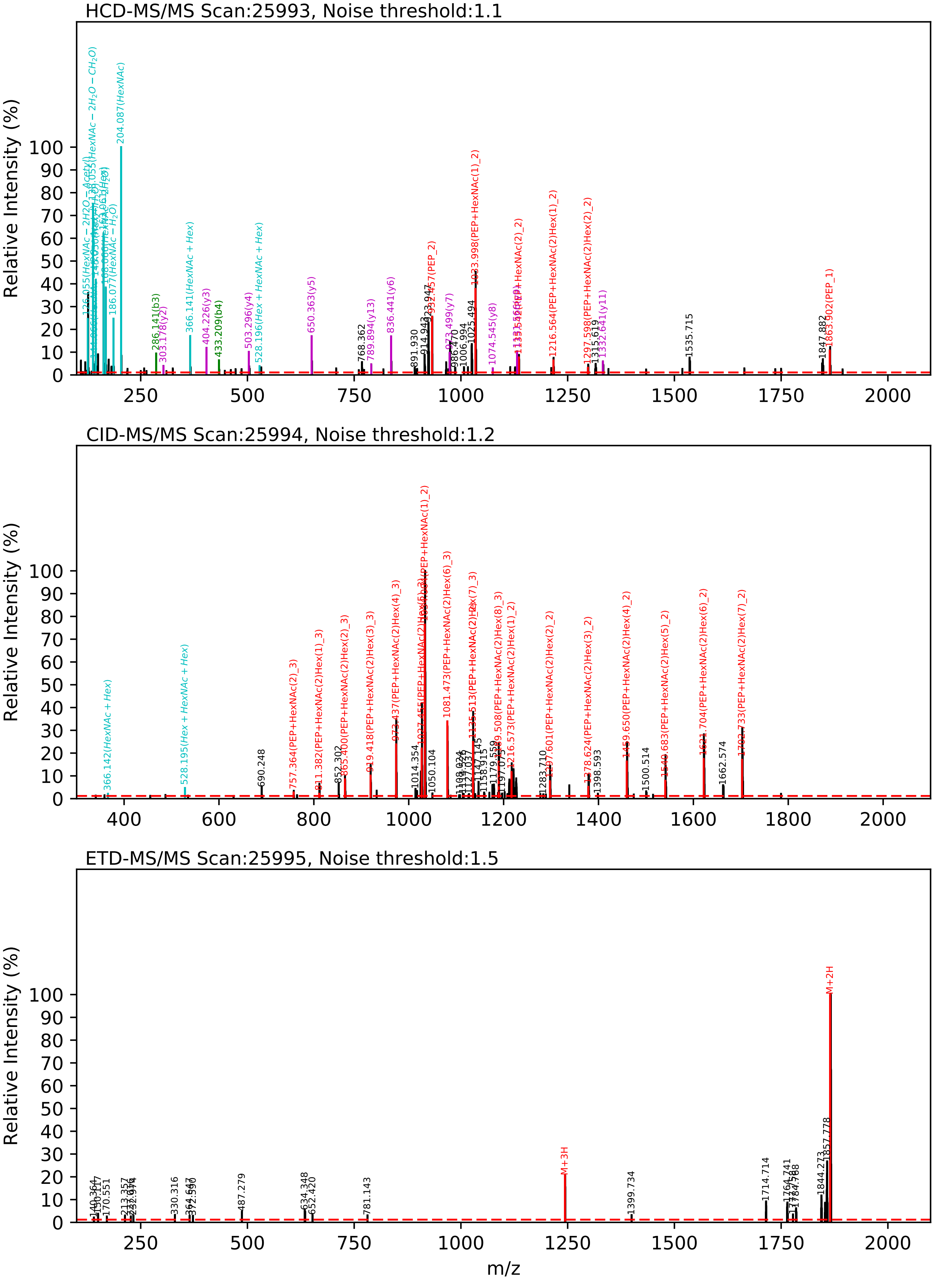

EGVFVSNGTHWFVTQR(=PEP)_9_2_0_0_0, 0_None, 0_None,
m/z:1243.52(3+), RT:69.76, Y-score:84.03

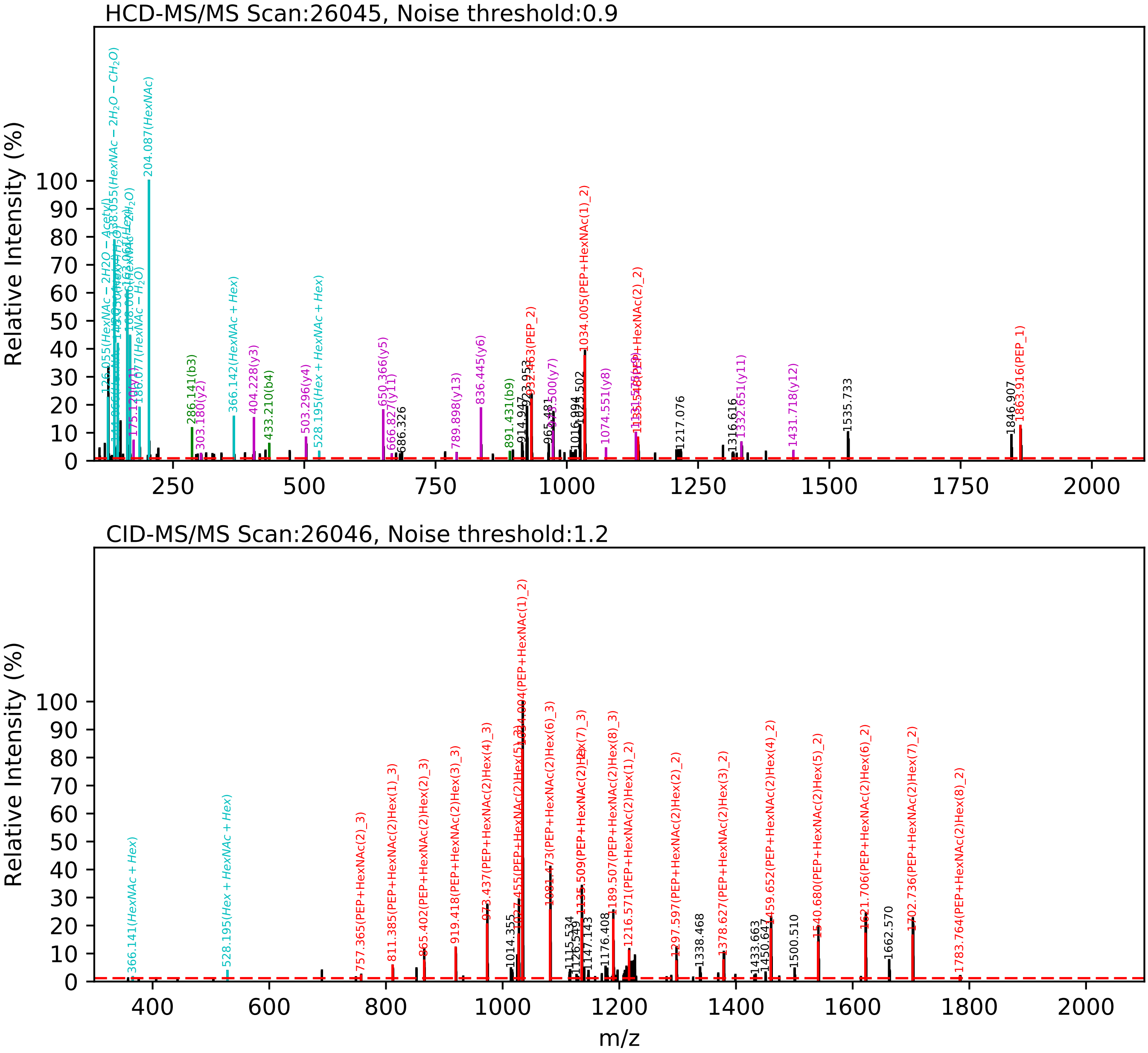

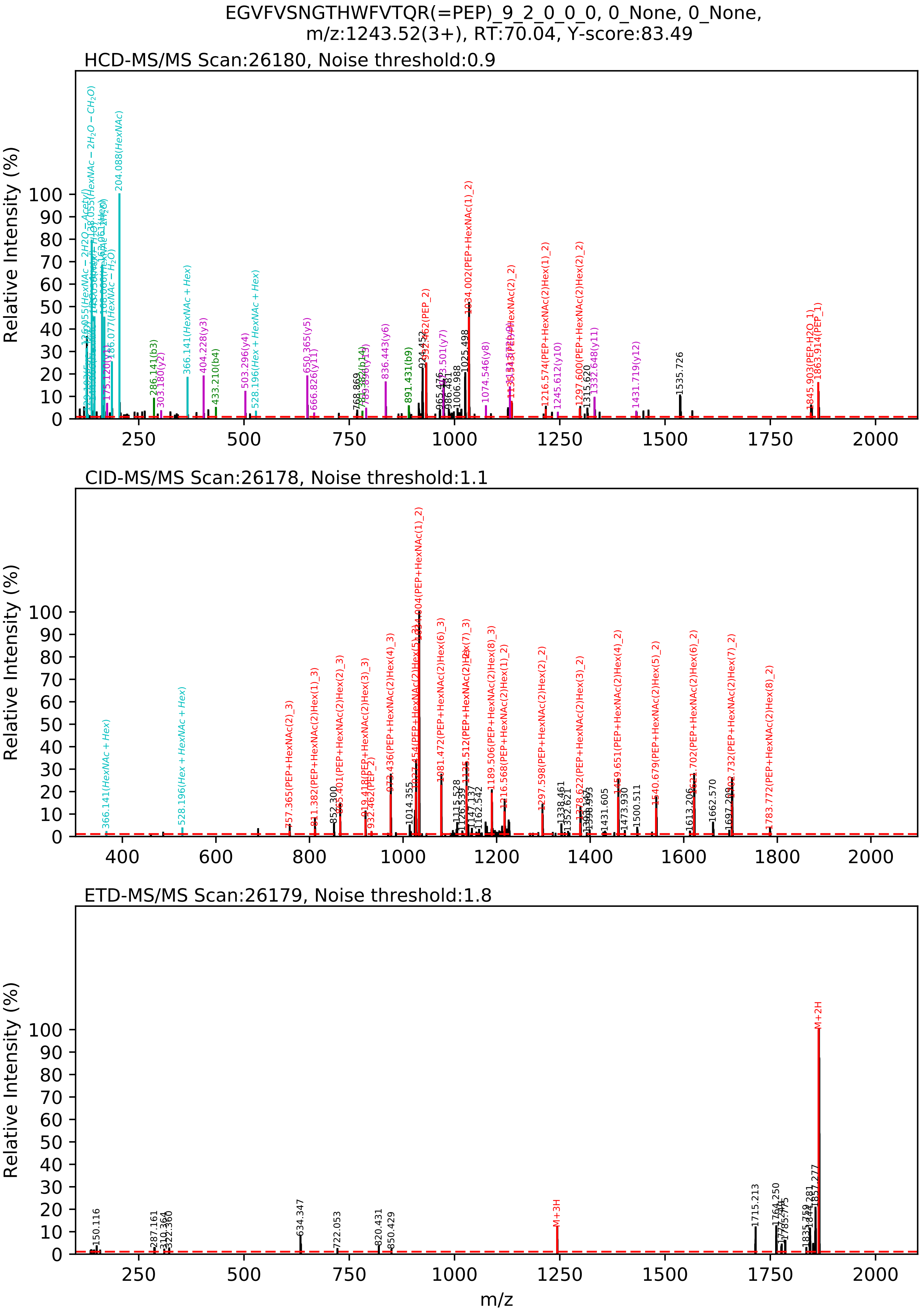
EGVFVSNGTHWFVTQR(=PEP)_9_2_0_0_0, 0_None, 0_None,
m/z:1243.52(3+), RT:70.04, Y-score:83.49
HCD-MS/MS Scan:26180, Noise threshold:0.9
Relative Intensity (%)
1034.002(PEP+HexNAc(1)_2)
204.088(HexNAc)
CID-MS/MS Scan:26178, Noise threshold:1.1
Relative Intensity (%)
1034.004(PEP+HexNAc(1)_2)
ETD-MS/MS Scan:26179, Noise threshold:1.8
Relative Intensity (%)
M+2H
M+3H
m/z

EGVFVSNGTHWFVTQR(=PEP)_9_2_0_0_0, 0_None, 0_None,
m/z:1243.52(3+), RT:72.49, Y-score:85.20

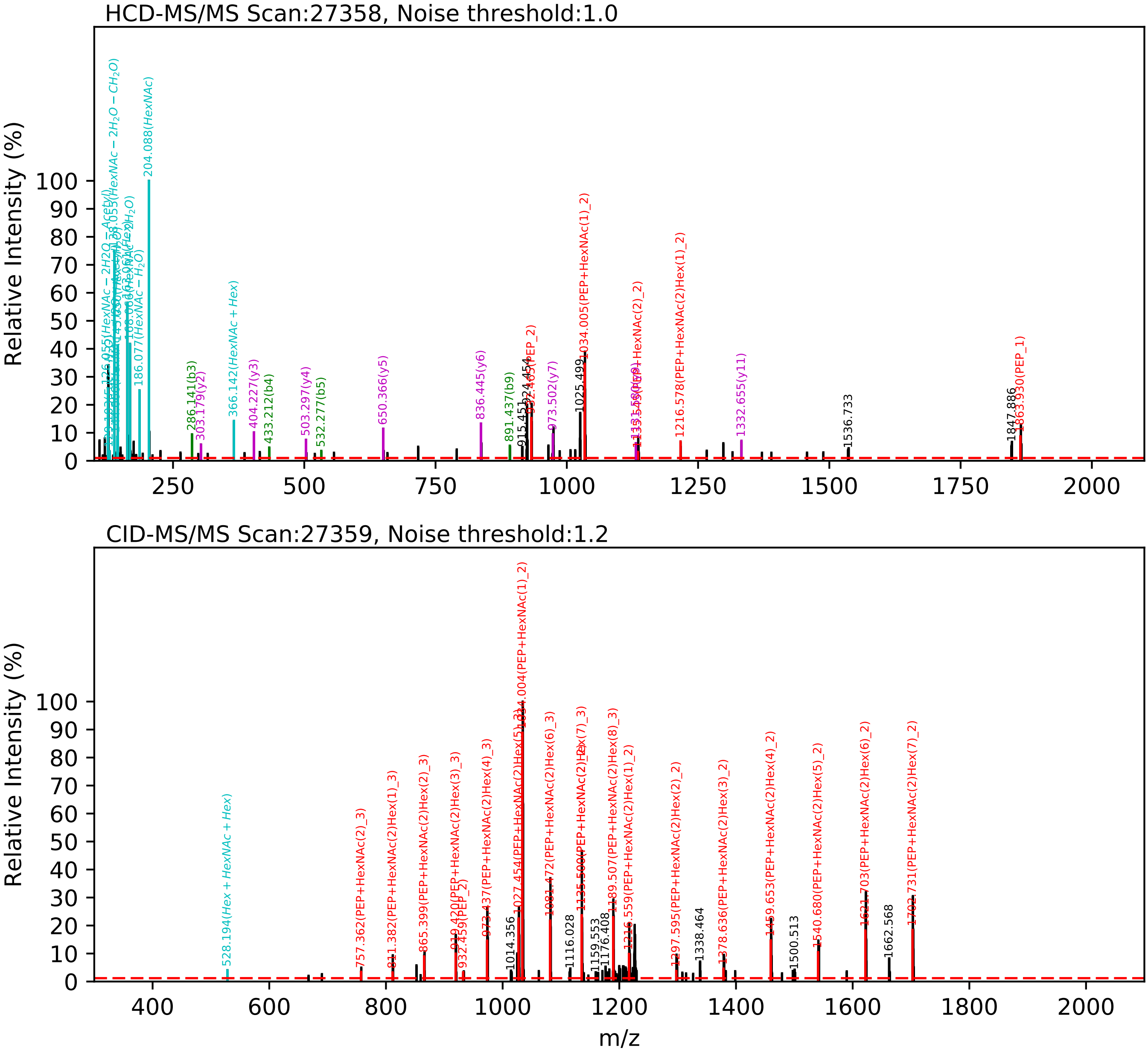

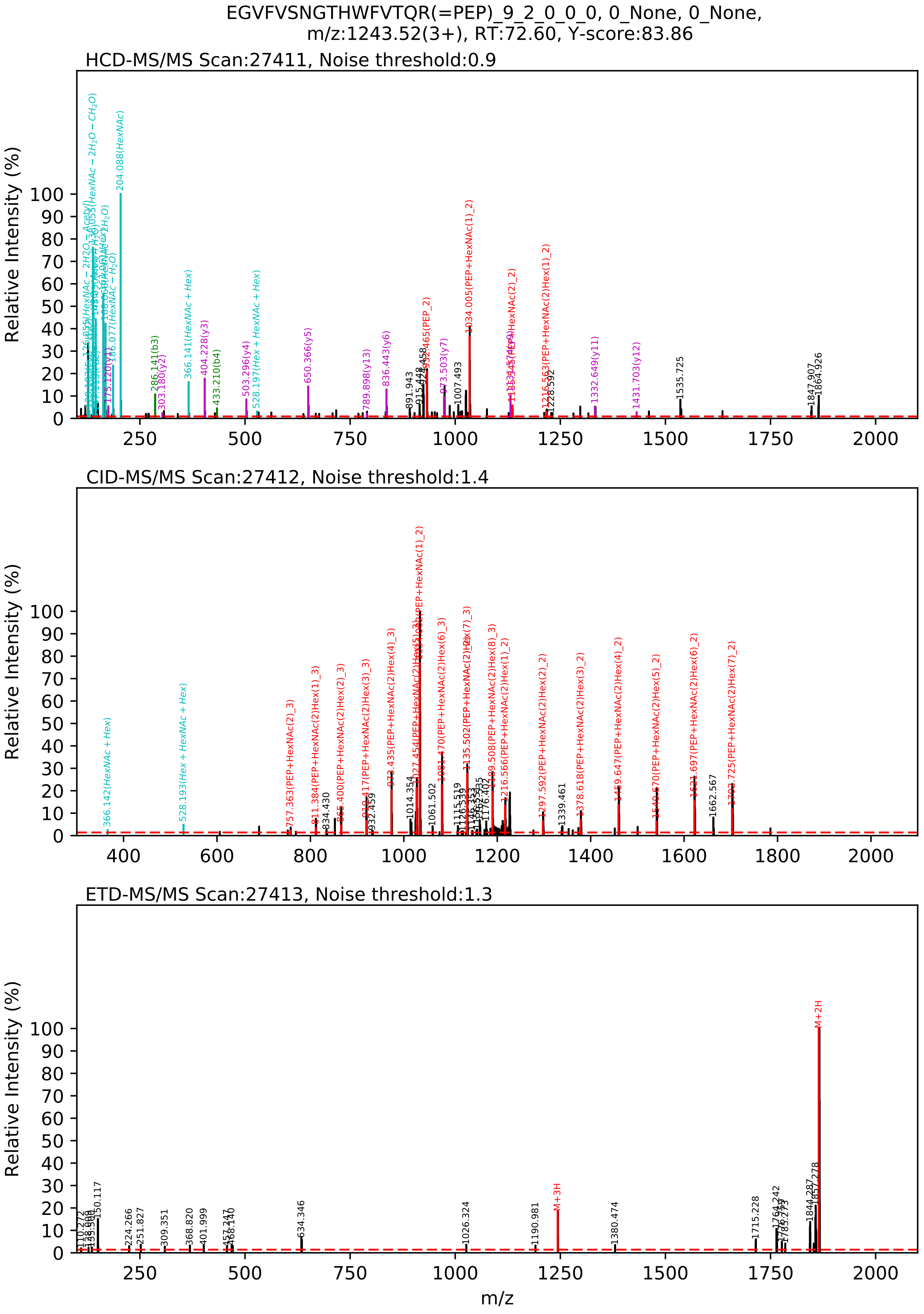
EGVFVSNGTHWFVTQR(=PEP)_9_2_0_0_0, 0_None, 0_None,
m/z:1243.52(3+), RT:72.60, Y-score:83.86
HCD-MS/MS Scan:27411, Noise threshold:0.9
Relative Intensity (%)
204.088(HexNAc)
286.141(b3)
303.180(y2)
366.141(HexNAc + Hex)
404.228(y3)
433.210(b4)
503.296(y4)
528.197(Hex + HexNAc + Hex)
650.366(y5)
789.898(y13)
836.443(y6)
891.943
932.465(PEP_2)
973.503(y7)
1007.493
1034.005(PEP+HexNAc(1)_2)
1216.563(PEP+HexNAc(2)Hex(1)_2)
1228.592
1332.649(y11)
1431.703(y12)
1535.725
1847.907
1864.926
CID-MS/MS Scan:27412, Noise threshold:1.4
366.142(HexNAc + Hex)
528.193(Hex + HexNAc + Hex)
757.363(PEP+HexNAc(2)_3)
811.384(PEP+HexNAc(2)Hex(1)_3)
834.430
865.400(PEP+HexNAc(2)Hex(2)_3)
919.417(PEP+HexNAc(2)Hex(3)_3)
932.459
973.435(PEP+HexNAc(2)Hex(4)_3)
1014.354
1061.502
1081.470(PEP+HexNAc(2)Hex(6)_3)
1135.502(PEP+HexNAc(2)Hex(7)_3)
1216.566(PEP+HexNAc(2)Hex(1)_2)
1297.592(PEP+HexNAc(2)Hex(2)_2)
1339.461
1378.618(PEP+HexNAc(2)Hex(3)_2)
1459.647(PEP+HexNAc(2)Hex(4)_2)
1540.670(PEP+HexNAc(2)Hex(5)_2)
1621.697(PEP+HexNAc(2)Hex(6)_2)
1662.567
1702.725(PEP+HexNAc(2)Hex(7)_2)
ETD-MS/MS Scan:27413, Noise threshold:1.3
150.117
224.266
251.827
309.351
368.820
401.999
468.140
634.346
1026.324
1190.981
M+3H
1380.474
1715.228
1764.242
1783.273
1844.287
1857.278
M+2H
m/z

EGVFVSNGTHWFVTQR(=PEP)_9_2_0_0_0, 0_None, 0_None,
m/z:1243.52(3+), RT:73.60, Y-score:81.81

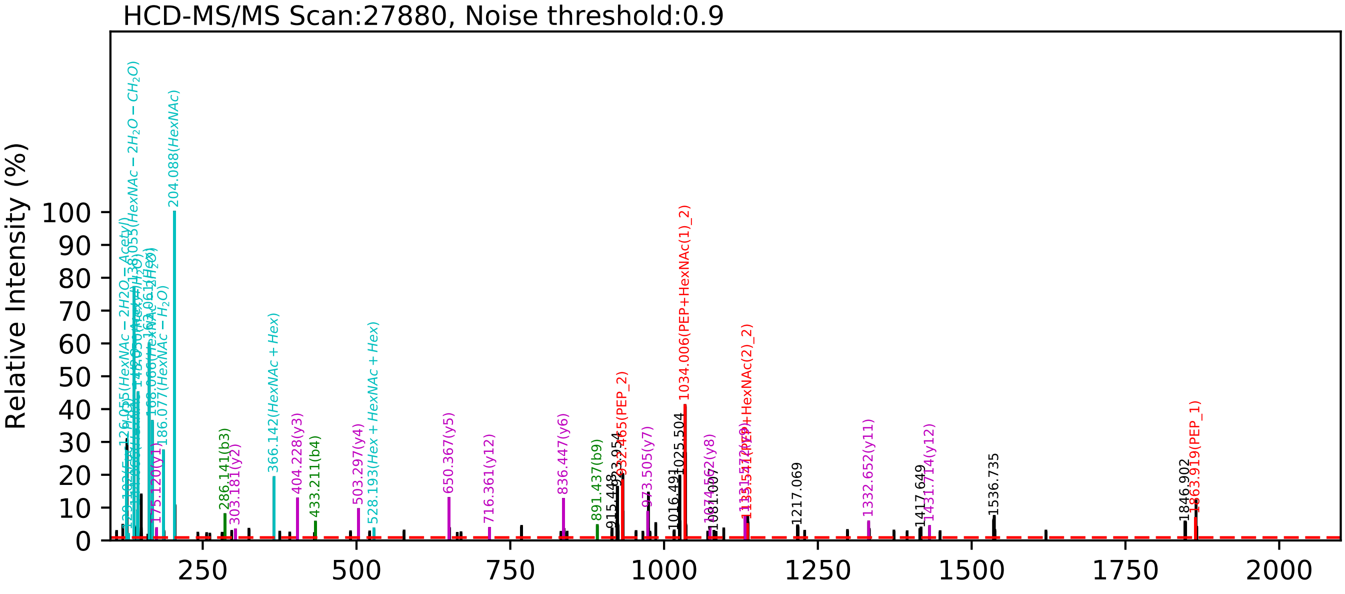

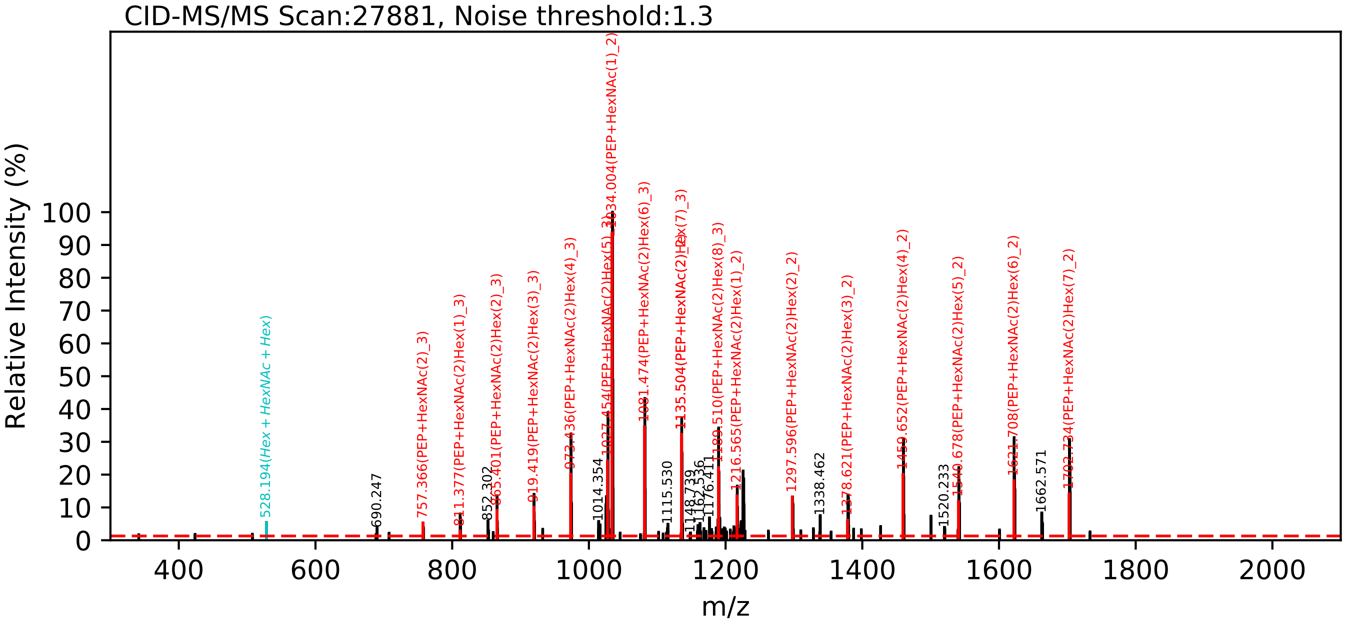

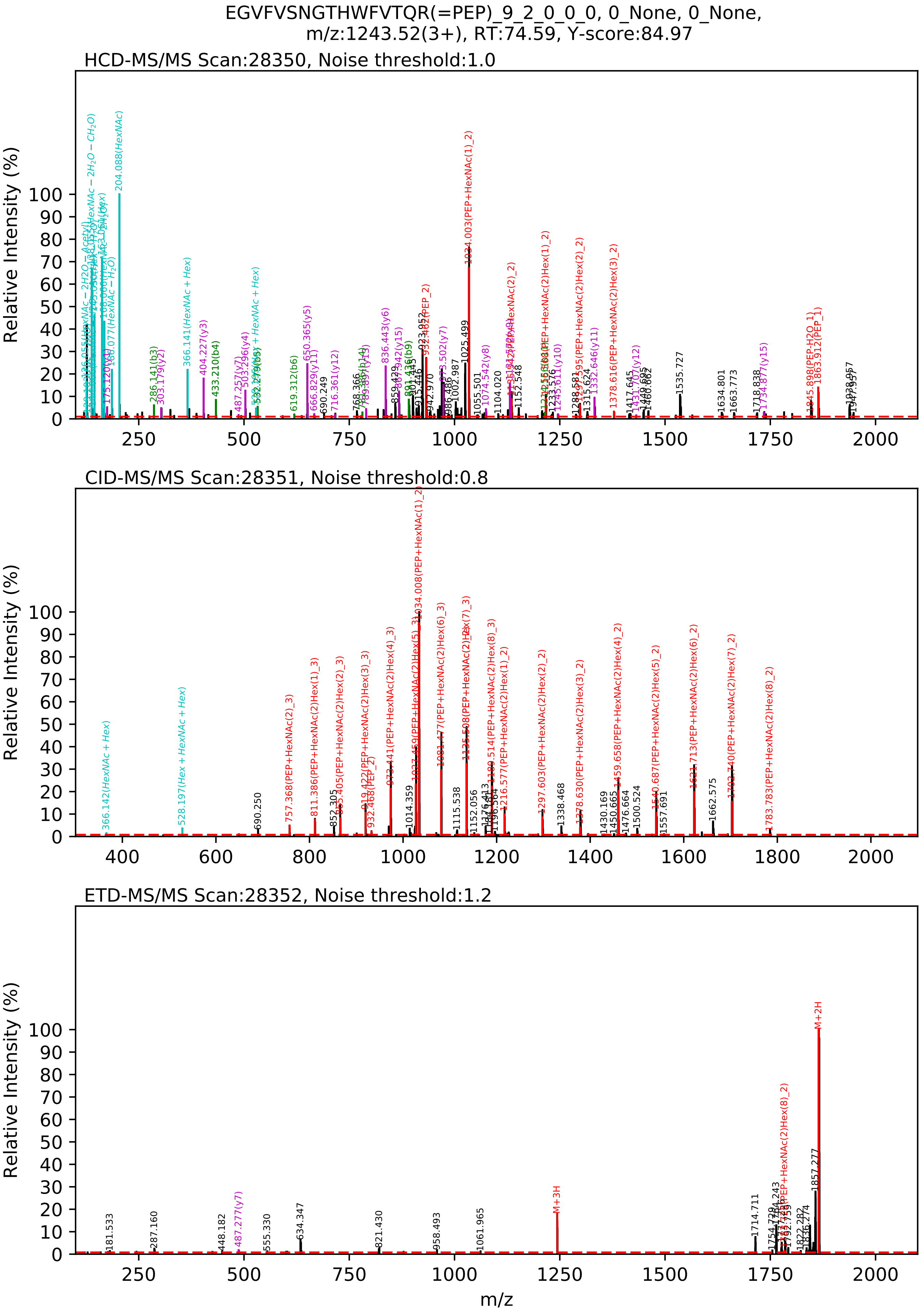
EGVFVSNGTHWFVTQR(=PEP)_9_2_0_0_0, 0_None, 0_None,
m/z:1243.52(3+), RT:74.59, Y-score:84.97
HCD-MS/MS Scan:28350, Noise threshold:1.0
CID-MS/MS Scan:28351, Noise threshold:0.8
ETD-MS/MS Scan:28352, Noise threshold:1.2
Relative Intensity (%)
m/z
204.088(HexNAc)
1034.003(PEP+HexNAc(1)_2)
1034.008(PEP+HexNAc(1)_2)
1863.912(PEP_1)
1845.898(PEP-H2O_1)
M+2H
M+3H
1857.277
1714.711

EGVFVSNGTHWFVTQR(=PEP)_9_2_0_0_0, 0_None, 0_None,
m/z:1243.52(3+), RT:74.78, Y-score:89.11

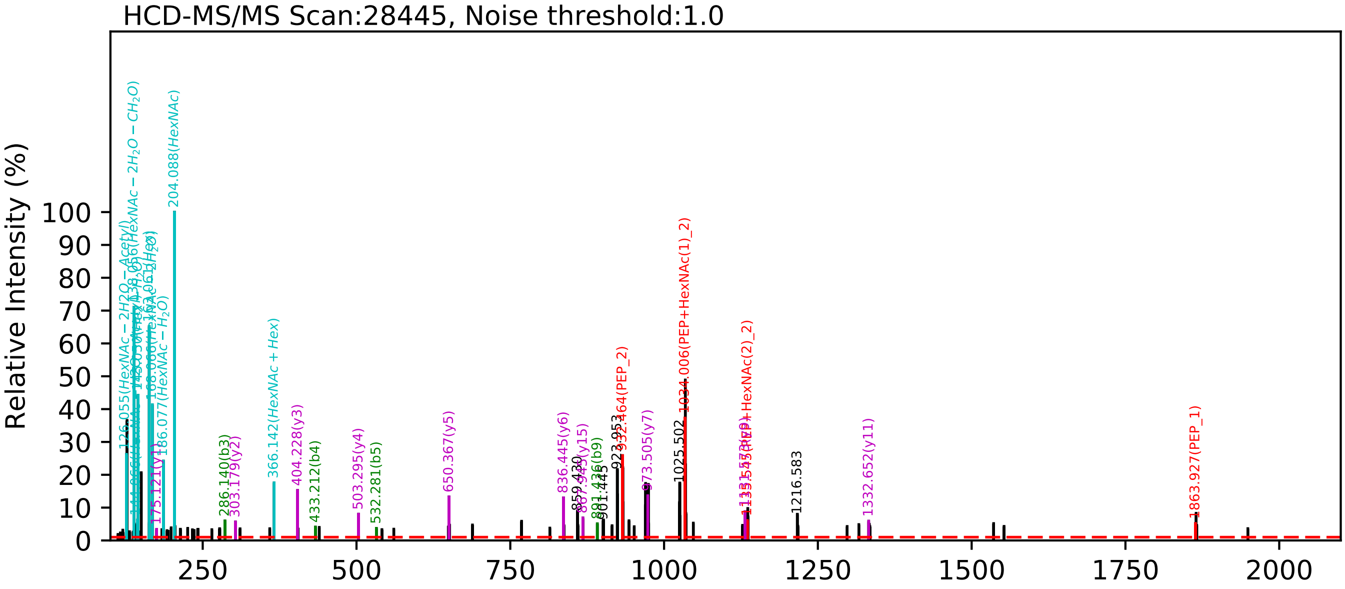

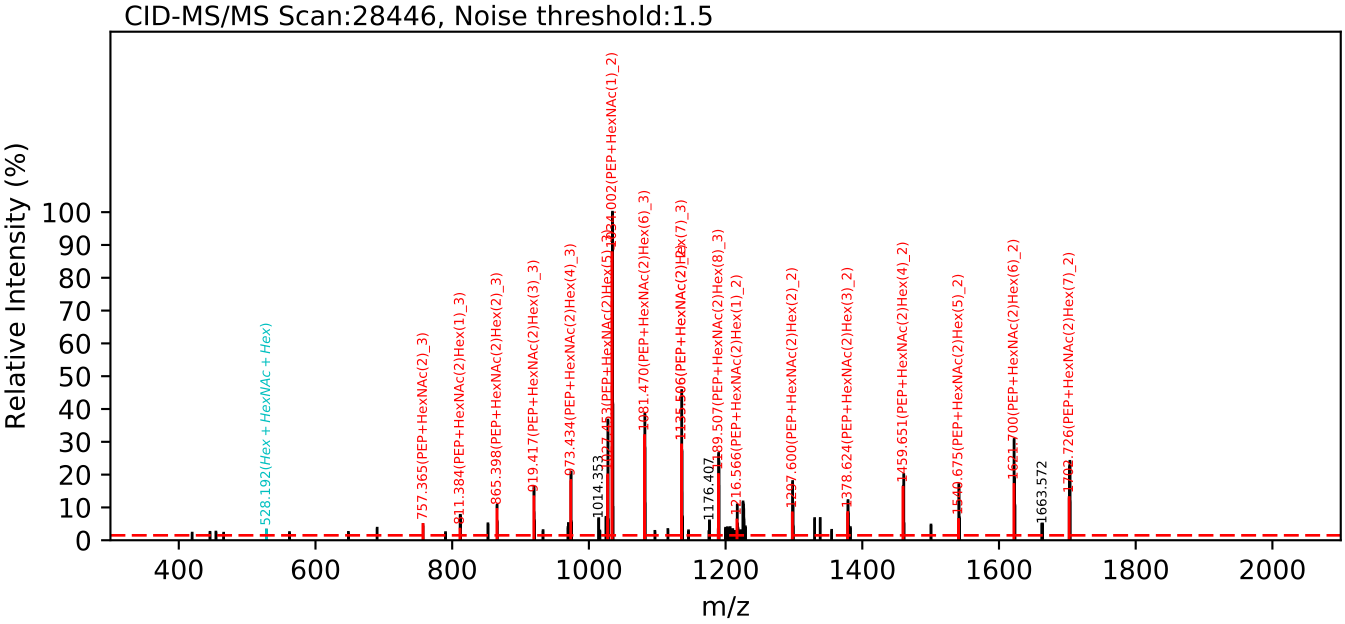

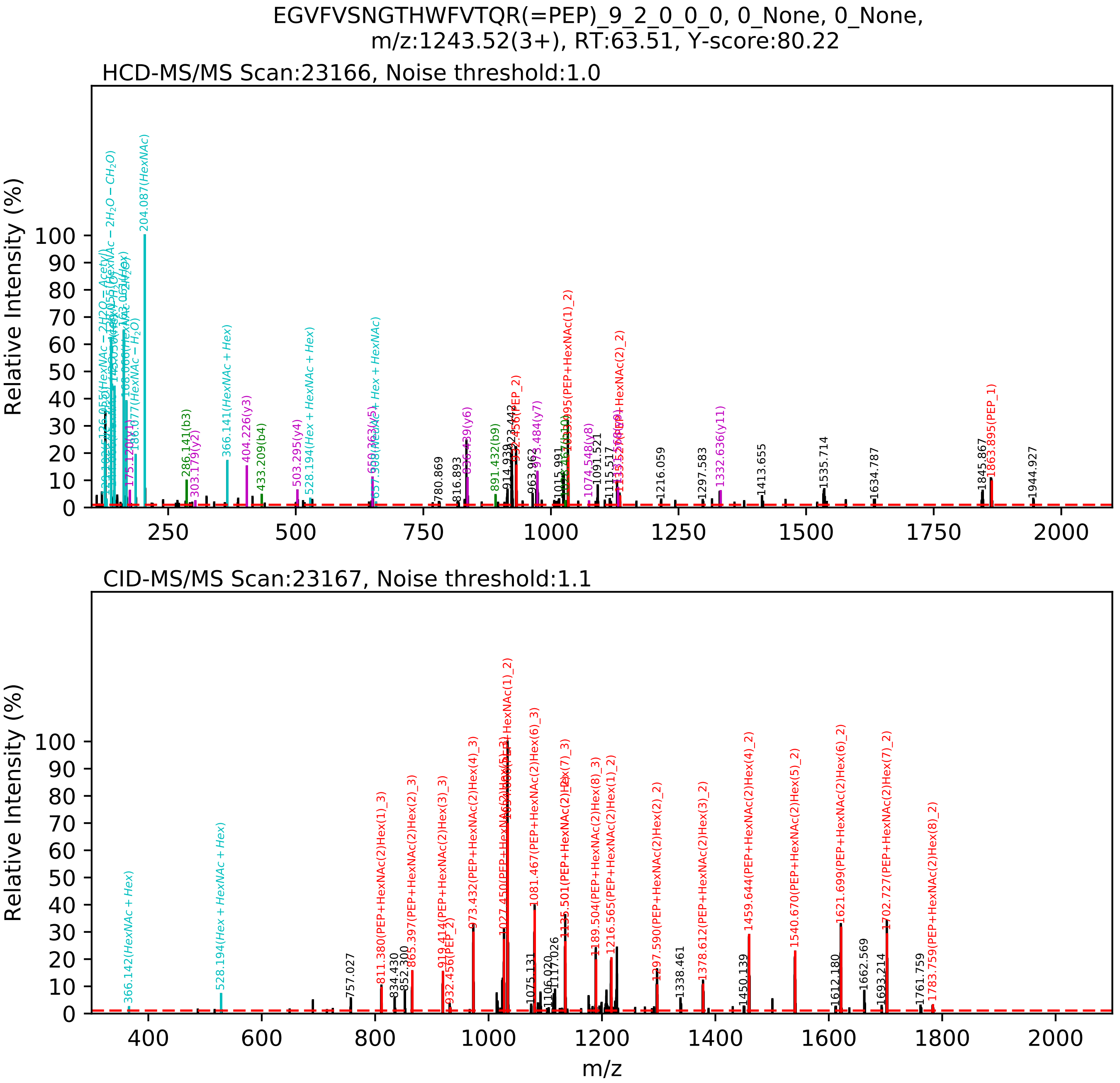

EGVFVSNGTHWFVTQR(=PEP)_9_2_0_0_0, 0_None, 0_None,
m/z:1243.52(3+), RT:63.51, Y-score:80.22
HCD-MS/MS Scan:23166, Noise threshold:1.0
Relative Intensity (%)
204.087(HexNAc)
366.141(HexNAc + Hex)
528.194(Hex + HexNAc + Hex)
286.141(b3)
303.179(y2)
404.226(y3)
433.209(b4)
503.295(y4)
891.432(b9)
973.484(y7)
1074.548(y8)
1332.636(y11)
1863.895(PEP_1)
780.869
816.893
1216.059
1297.583
1413.655
1535.714
1634.787
1845.867
1944.927
CID-MS/MS Scan:23167, Noise threshold:1.1
Relative Intensity (%)
m/z
366.142(HexNAc + Hex)
528.194(Hex + HexNAc + Hex)
757.027
811.380(PEP+HexNAc(2)Hex(1)_3)
834.430
852.300
865.397(PEP+HexNAc(2)Hex(2)_3)
919.414(PEP+HexNAc(2)Hex(3)_3)
932.456(PEP_2)
973.432(PEP+HexNAc(2)Hex(4)_3)
1081.467(PEP+HexNAc(2)Hex(6)_3)
1075.131
1106.020
1117.026
1189.504(PEP+HexNAc(2)Hex(8)_3)
1216.565(PEP+HexNAc(2)Hex(1)_2)
1297.590(PEP+HexNAc(2)Hex(2)_2)
1338.461
1378.612(PEP+HexNAc(2)Hex(3)_2)
1450.139
1459.644(PEP+HexNAc(2)Hex(4)_2)
1540.670(PEP+HexNAc(2)Hex(5)_2)
1612.180
1621.699(PEP+HexNAc(2)Hex(6)_2)
1662.569
1693.214
1702.727(PEP+HexNAc(2)Hex(7)_2)
1761.759
1783.759(PEP+HexNAc(2)Hex(8)_2)

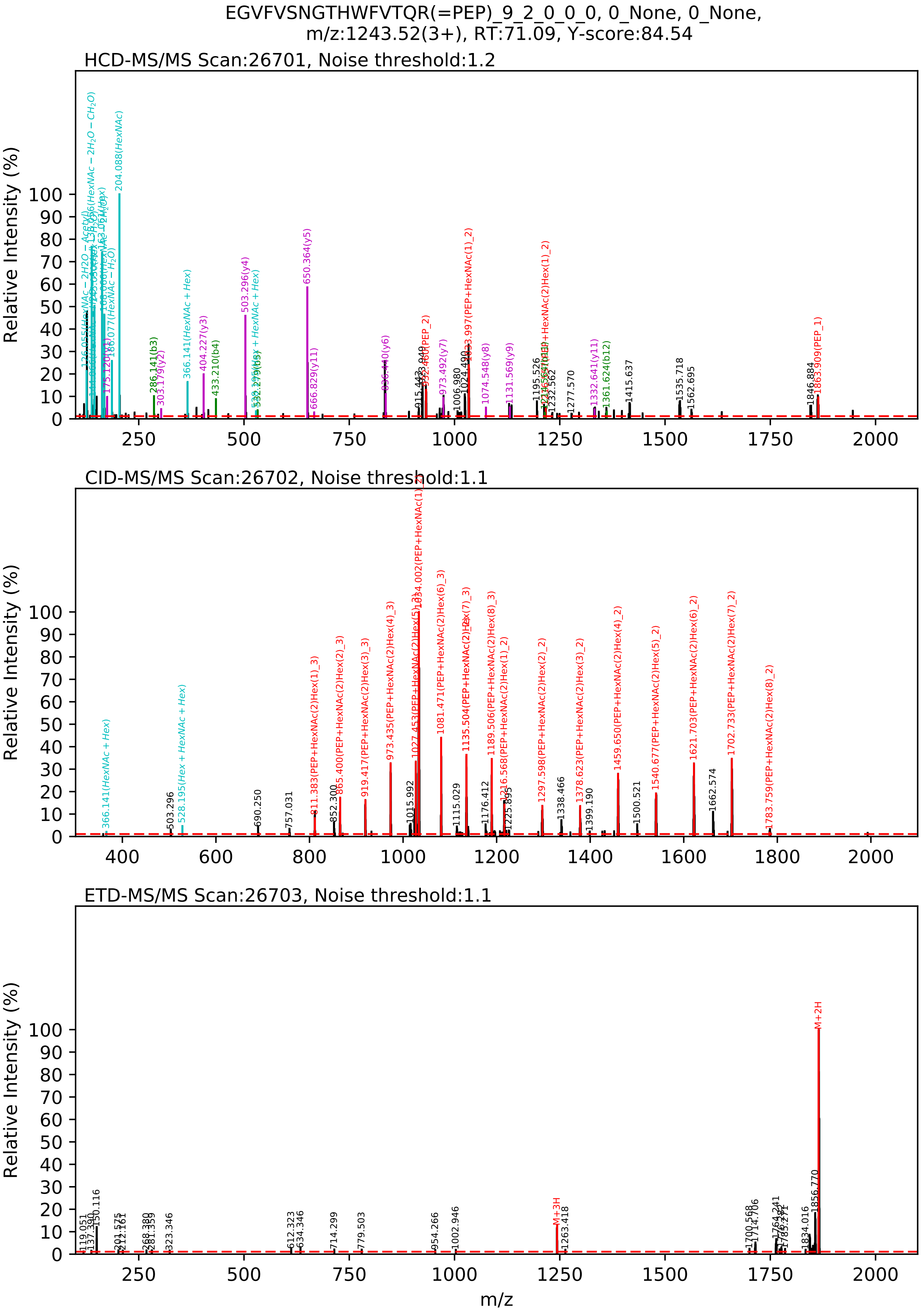

EGVFVSNGTHWFVTQR(=PEP)_9_2_0_0_0, 0_None, 0_None,
m/z:1243.52(3+), RT:71.09, Y-score:84.54
HCD-MS/MS Scan:26701, Noise threshold:1.2
CID-MS/MS Scan:26702, Noise threshold:1.1
ETD-MS/MS Scan:26703, Noise threshold:1.1
Relative Intensity (%)
m/z
204.088(HexNAc)
650.364(y5)
503.296(y4)
366.141(HexNAc + Hex)
404.227(y3)
433.210(b4)
286.141(b3)
303.179(y2)
175.120(y1)
666.829(y11)
836.440(y6)
973.492(y7)
1074.548(y8)
1131.569(y9)
1332.641(y11)
1361.624(b12)
1863.909(PEP_1)
1846.884
1535.718
1562.695
1415.637
1277.570
1232.562
1195.526
1006.980
1024.490
915.443
932.460(PEP_2)
811.383(PEP+HexNAc(2)Hex(1)_3)
865.400(PEP+HexNAc(2)Hex(2)_3)
919.417(PEP+HexNAc(2)Hex(3)_3)
973.435(PEP+HexNAc(2)Hex(4)_3)
1081.471(PEP+HexNAc(2)Hex(6)_3)
1135.504(PEP+HexNAc(2)Hex(7)_3)
1189.506(PEP+HexNAc(2)Hex(8)_3)
1216.568(PEP+HexNAc(2)Hex(1)_2)
1297.598(PEP+HexNAc(2)Hex(2)_2)
1378.623(PEP+HexNAc(2)Hex(3)_2)
1459.650(PEP+HexNAc(2)Hex(4)_2)
1540.677(PEP+HexNAc(2)Hex(5)_2)
1621.703(PEP+HexNAc(2)Hex(6)_2)
1702.733(PEP+HexNAc(2)Hex(7)_2)
1783.759(PEP+HexNAc(2)Hex(8)_2)
366.141(HexNAc + Hex)
528.195(Hex + HexNAc + Hex)
503.296
690.250
757.031
852.300
1015.992
1115.029
1176.412
1225.895
1338.466
1399.190
1500.521
1662.574
M+2H
M+3H
1856.770
1263.418
119.051
137.390
150.116
201.575
212.161
268.380
281.359
323.346
612.323
634.346
714.299
779.503
954.266
1002.946
1700.568
1714.706
1764.241
1783.271
1834.016

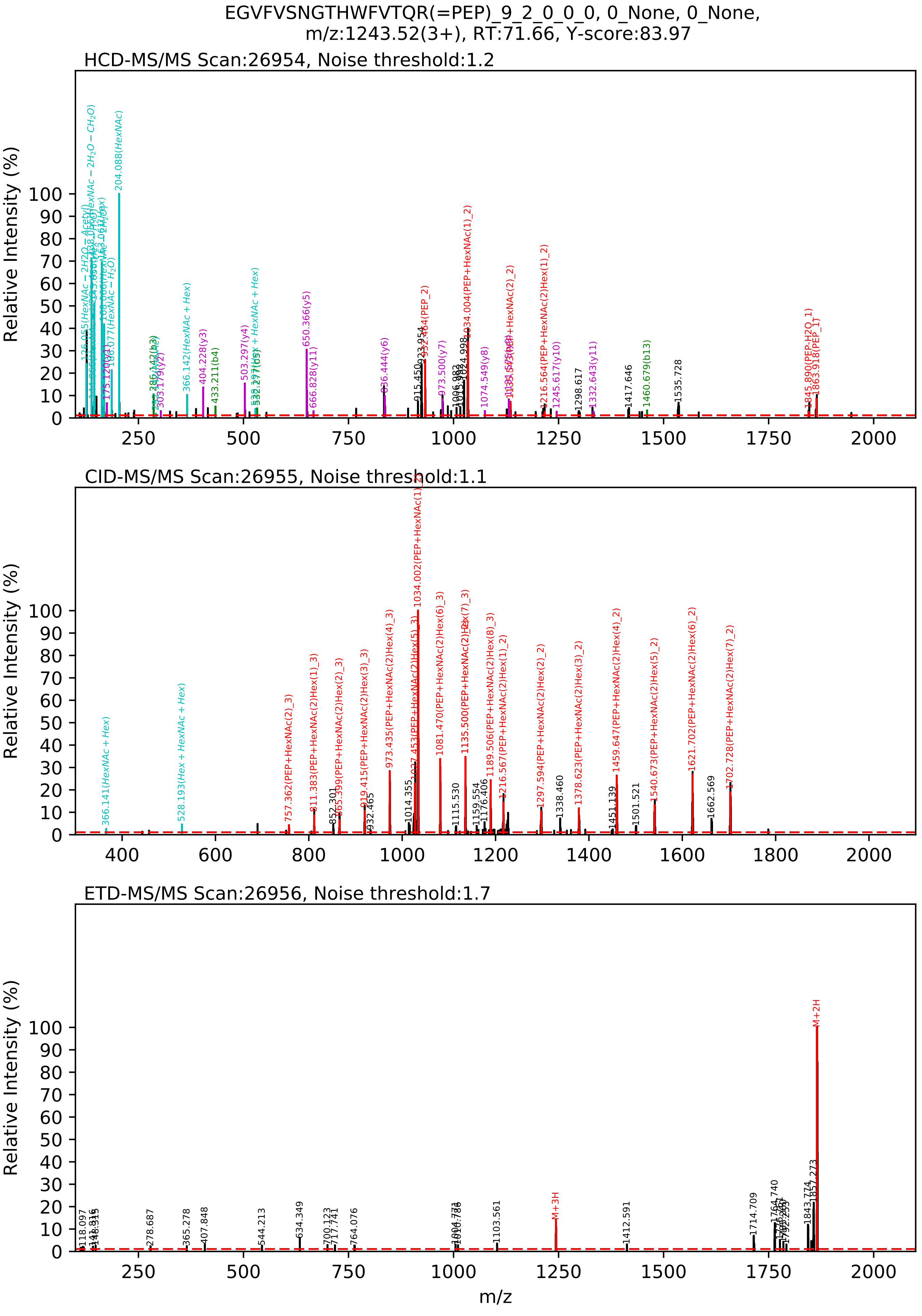
EGVFVSNGTHWFVTQR(=PEP)_9_2_0_0_0, 0_None, 0_None,
m/z:1243.52(3+), RT:71.66, Y-score:83.97
HCD-MS/MS Scan:26954, Noise threshold:1.2
CID-MS/MS Scan:26955, Noise threshold:1.1
ETD-MS/MS Scan:26956, Noise threshold:1.7
Relative Intensity (%)
m/z

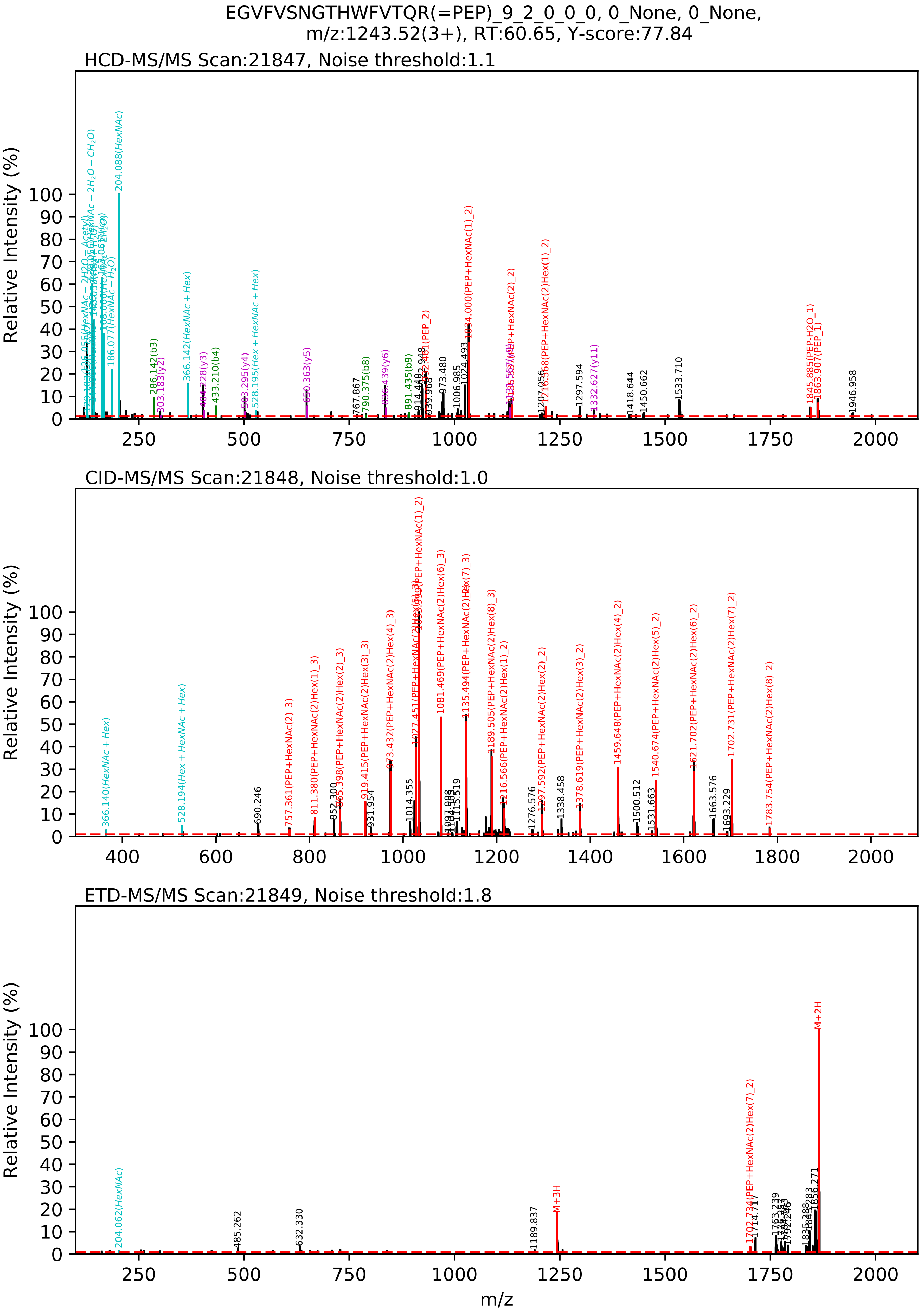
EGVFVSNGTHWFVTQR(=PEP)_9_2_0_0_0, 0_None, 0_None,
m/z:1243.52(3+), RT:60.65, Y-score:77.84
HCD-MS/MS Scan:21847, Noise threshold:1.1
Relative Intensity (%)
204.088(HexNAc)
186.077(HexNAc − H2O)
286.142(b3)
303.183(y2)
366.142(HexNAc + Hex)
433.210(b4)
528.195(Hex + HexNAc + Hex)
650.363(y5)
767.867
790.375(b8)
891.435(b9)
973.480
1006.985
1024.493
1034.000(PEP+HexNAc(1)_2)
1207.056
1297.594
1332.627(y11)
1418.644
1450.662
1533.710
1845.885(PEP-H2O_1)
1863.907(PEP_1)
1946.958
CID-MS/MS Scan:21848, Noise threshold:1.0
366.140(HexNAc + Hex)
528.194(Hex + HexNAc + Hex)
690.246
757.361(PEP+HexNAc(2)_3)
811.380(PEP+HexNAc(2)Hex(1)_3)
852.300
865.398(PEP+HexNAc(2)Hex(2)_3)
919.415(PEP+HexNAc(2)Hex(3)_3)
931.954
973.432(PEP+HexNAc(2)Hex(4)_3)
1014.355
1081.469(PEP+HexNAc(2)Hex(6)_3)
1115.519
1135.494(PEP+HexNAc(2)Hex(7)_3)
1189.505(PEP+HexNAc(2)Hex(8)_3)
1216.566(PEP+HexNAc(2)Hex(1)_2)
1276.576
1297.592(PEP+HexNAc(2)Hex(2)_2)
1338.458
1378.619(PEP+HexNAc(2)Hex(3)_2)
1459.648(PEP+HexNAc(2)Hex(4)_2)
1500.512
1531.663
1540.674(PEP+HexNAc(2)Hex(5)_2)
1621.702(PEP+HexNAc(2)Hex(6)_2)
1663.576
1693.229
1702.731(PEP+HexNAc(2)Hex(7)_2)
1783.754(PEP+HexNAc(2)Hex(8)_2)
ETD-MS/MS Scan:21849, Noise threshold:1.8
204.062(HexNAc)
485.262
632.330
1189.837
M+3H
1702.734(PEP+HexNAc(2)Hex(7)_2)
1714.717
1856.271
M+2H
m/z

EGVFVSNGTHWFVTQR(=PEP)_9_2_0_0_0, 0_None, 0_None,
m/z:1243.52(3+), RT:61.98, Y-score:67.52

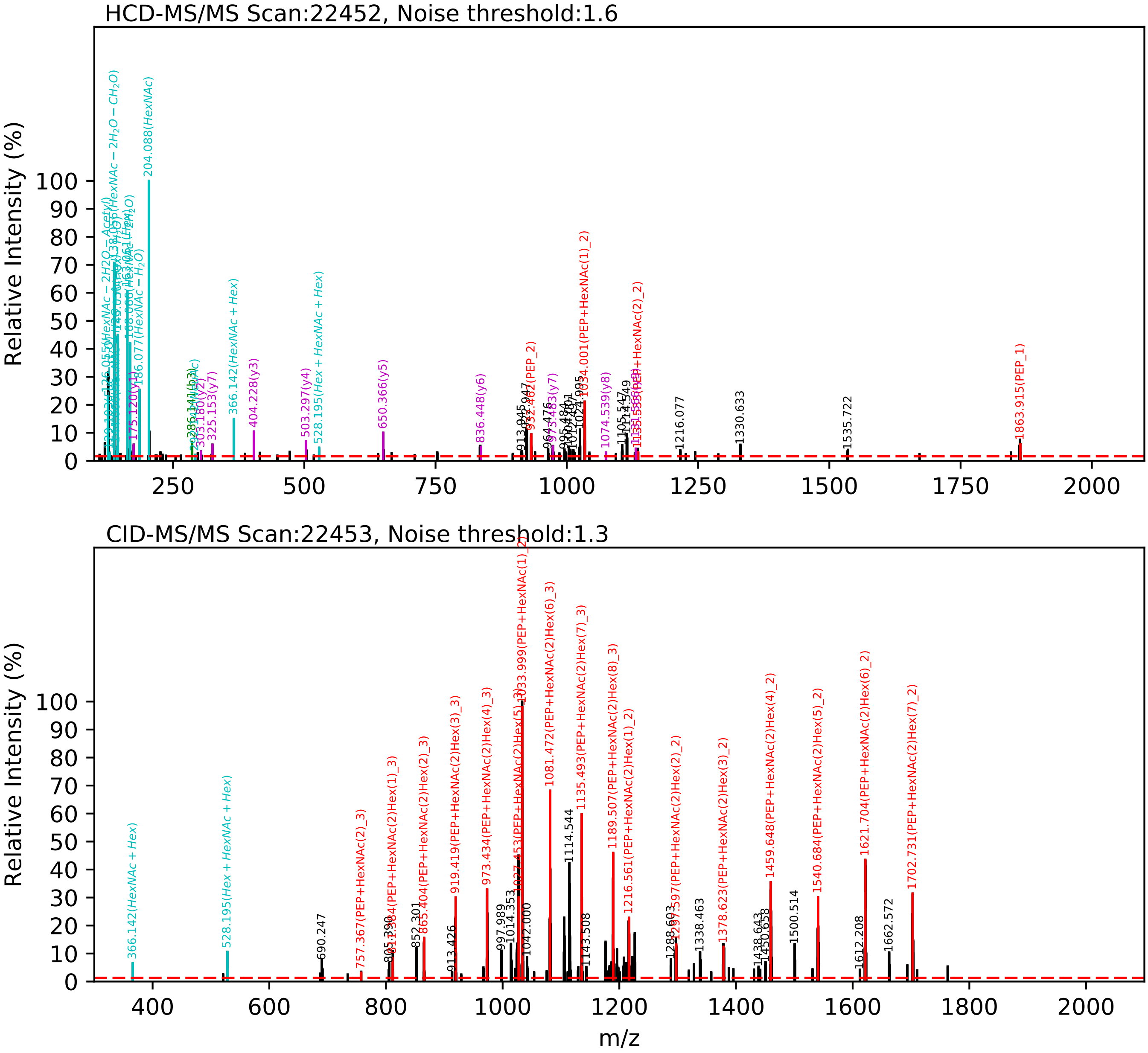

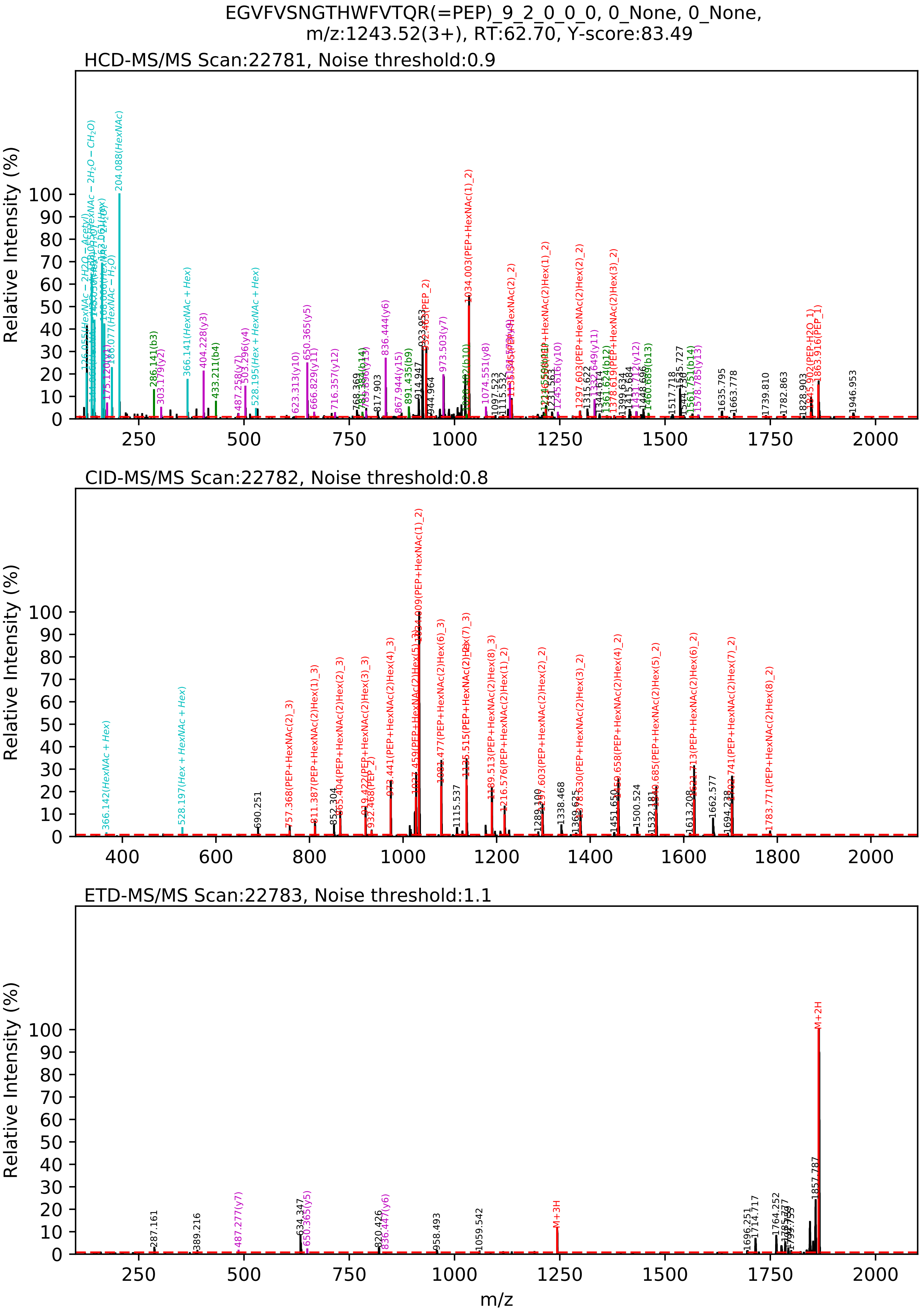
EGVFVSNGTHWFVTQR(=PEP)_9_2_0_0_0, 0_None, 0_None,
m/z:1243.52(3+), RT:62.70, Y-score:83.49
HCD-MS/MS Scan:22781, Noise threshold:0.9
CID-MS/MS Scan:22782, Noise threshold:0.8
ETD-MS/MS Scan:22783, Noise threshold:1.1
Relative Intensity (%)
m/z
1034.003(PEP+HexNAc(1)_2)
204.088(HexNAc)
1034.009(PEP+HexNAc(1)_2)
M+2H
M+3H

EGVFVSNGTHWFVTQR(=PEP)_9_2_0_0_0, 0_None, 0_None,
m/z:1243.52(3+), RT:64.02, Y-score:83.83

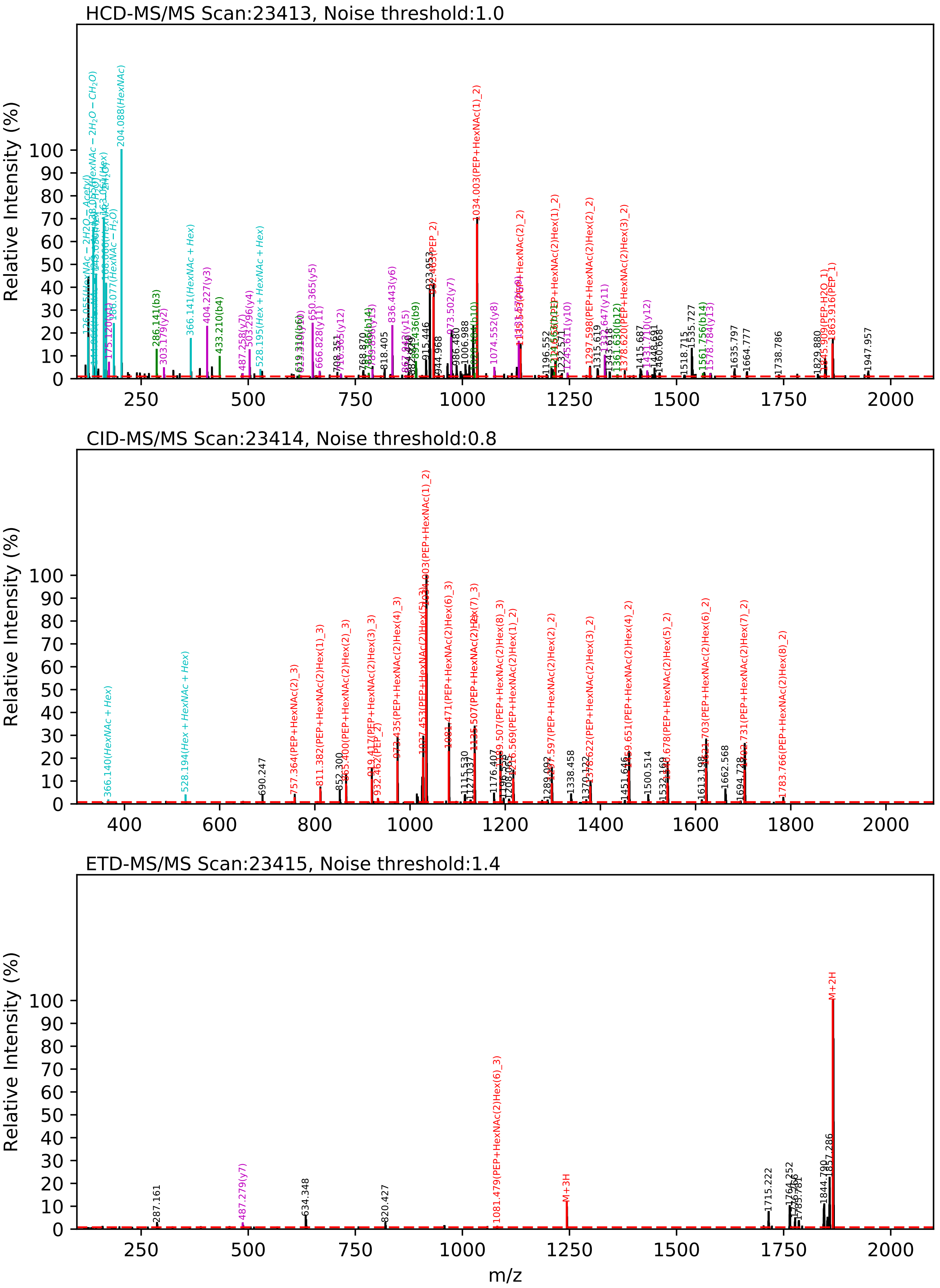

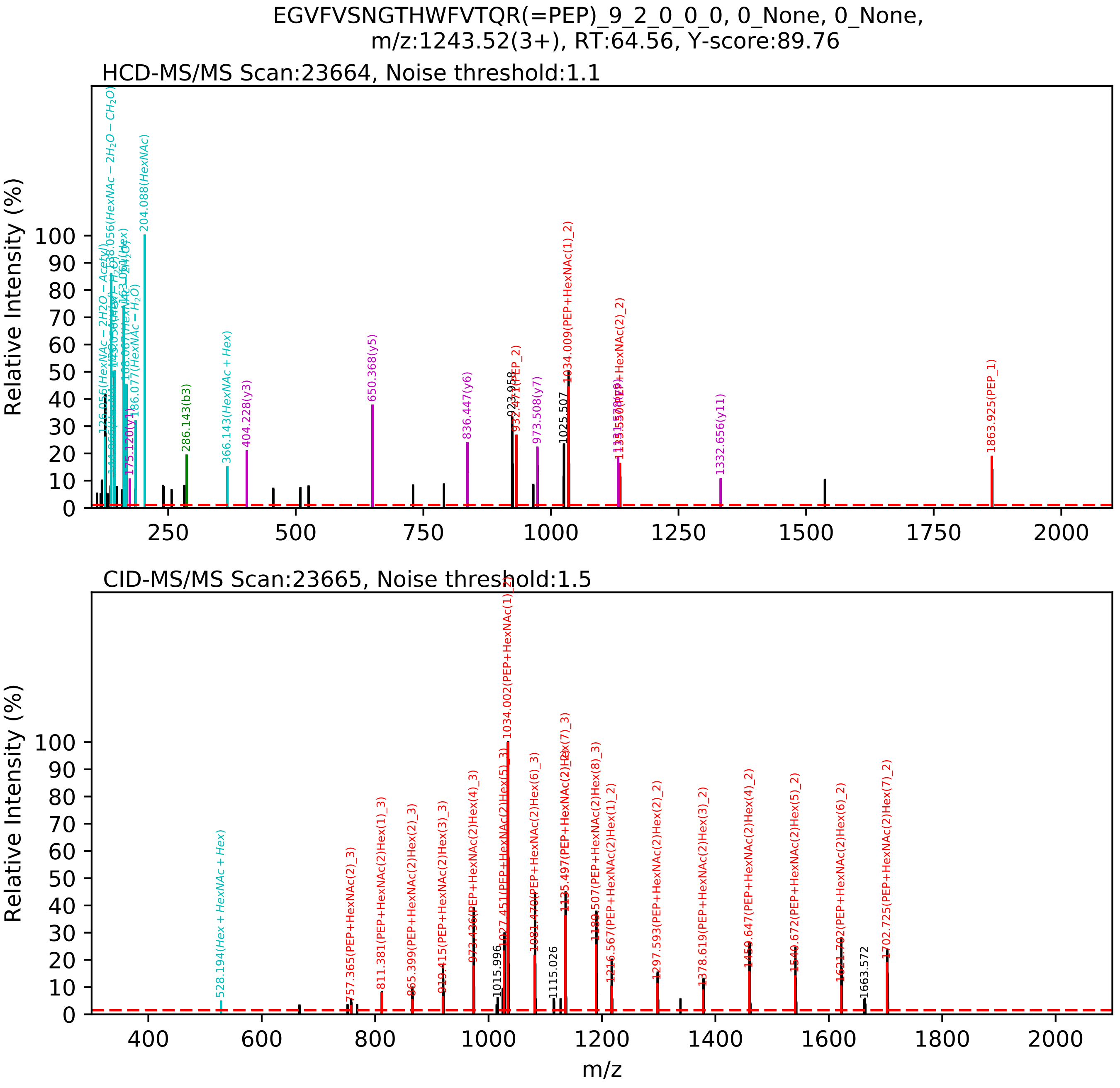
EGVFVSNGTHWFVTQR(=PEP)_9_2_0_0_0, 0_None, 0_None,
m/z:1243.52(3+), RT:64.56, Y-score:89.76
HCD-MS/MS Scan:23664, Noise threshold:1.1
Relative Intensity (%)
204.088(HexNAc)
286.143(b3)
366.143(HexNAc + Hex)
404.228(y3)
650.368(y5)
836.447(y6)
923.958
973.508(y7)
1025.507
1034.009(PEP+HexNAc(1)_2)
1332.656(y11)
1863.925(PEP_1)
175.120(y1)
186.077(HexNAc − H2O)
CID-MS/MS Scan:23665, Noise threshold:1.5
Relative Intensity (%)
528.194(Hex + HexNAc + Hex)
757.365(PEP+HexNAc(2)_3)
811.381(PEP+HexNAc(2)Hex(1)_3)
865.399(PEP+HexNAc(2)Hex(2)_3)
919.415(PEP+HexNAc(2)Hex(3)_3)
973.436(PEP+HexNAc(2)Hex(4)_3)
1015.996
1034.002(PEP+HexNAc(1)_2)
1081.470(PEP+HexNAc(2)Hex(6)_3)
1115.026
1135.497(PEP+HexNAc(2)Hex(7)_3)
1189.507(PEP+HexNAc(2)Hex(8)_3)
1216.567(PEP+HexNAc(2)Hex(1)_2)
1297.593(PEP+HexNAc(2)Hex(2)_2)
1378.619(PEP+HexNAc(2)Hex(3)_2)
1459.647(PEP+HexNAc(2)Hex(4)_2)
1540.672(PEP+HexNAc(2)Hex(5)_2)
1621.702(PEP+HexNAc(2)Hex(6)_2)
1663.572
1702.725(PEP+HexNAc(2)Hex(7)_2)
m/z

EGVFVSNGTHWFVTQR(=PEP)_9_2_0_0_0, 0_None, 0_None,
m/z:1243.52(3+), RT:65.24, Y-score:84.34

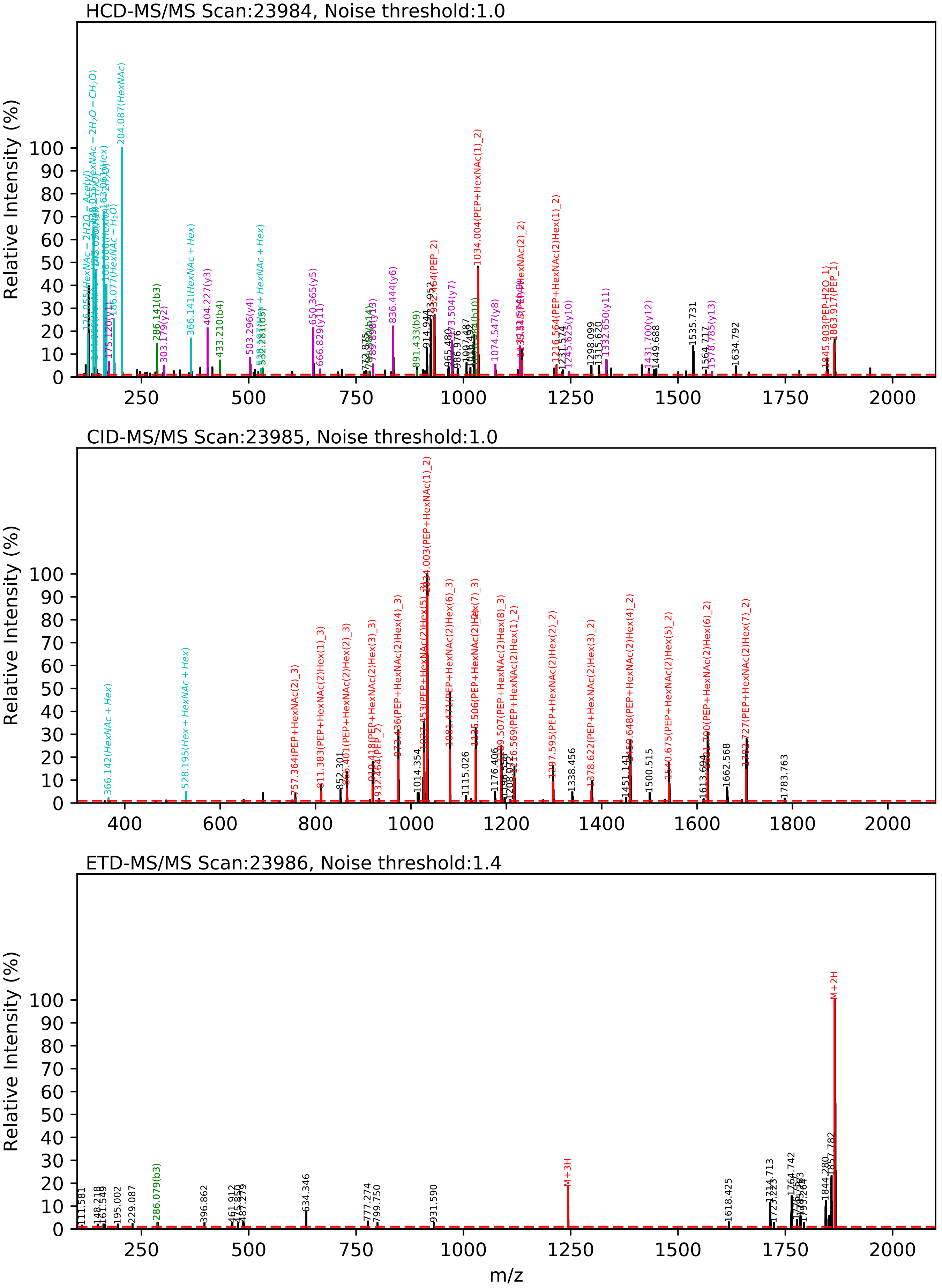

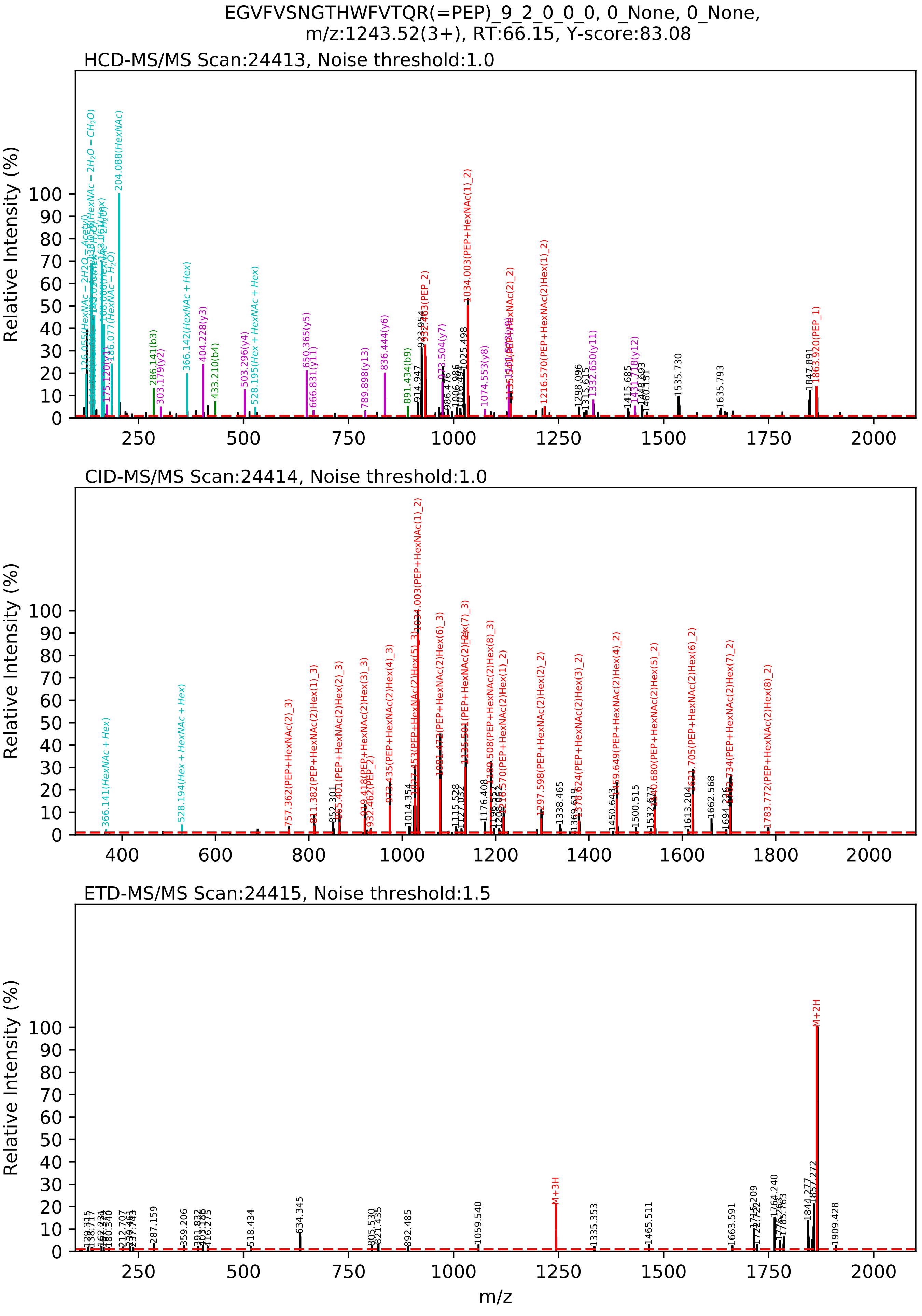
EGVFVSNGTHWFVTQR(=PEP)_9_2_0_0_0, 0_None, 0_None,
m/z:1243.52(3+), RT:66.15, Y-score:83.08
HCD-MS/MS Scan:24413, Noise threshold:1.0
Relative Intensity (%)
204.088(HexNAc)
1034.003(PEP+HexNAc(1)_2)
1216.570(PEP+HexNAc(2)Hex(1)_2)
1863.920(PEP_1)
1847.891
1635.793
1535.730
286.141(b3)
303.179(y2)
366.142(HexNAc+Hex)
404.228(y3)
433.210(b4)
503.296(y4)
528.195(Hex+HexNAc+Hex)
650.365(y5)
666.831(y11)
789.898(y13)
836.444(y6)
891.434(b9)
914.947
923.954
1025.498
1074.553(y8)
1298.096
1332.650(y11)
1415.685
CID-MS/MS Scan:24414, Noise threshold:1.0
366.141(HexNAc+Hex)
528.194(Hex+HexNAc+Hex)
757.362(PEP+HexNAc(2)_3)
811.382(PEP+HexNAc(2)Hex(1)_3)
852.301
865.401(PEP+HexNAc(2)Hex(2)_3)
932.462(PEP_2)
973.435(PEP+HexNAc(2)Hex(4)_3)
1014.354
1034.003(PEP+HexNAc(1)_2)
1081.472(PEP+HexNAc(2)Hex(6)_3)
1115.528
1176.408
1216.570(PEP+HexNAc(2)Hex(1)_2)
1297.598(PEP+HexNAc(2)Hex(2)_2)
1338.465
1369.619
1450.643
1500.515
1532.677
1613.204
1662.568
1694.226
1783.772(PEP+HexNAc(2)Hex(8)_2)
ETD-MS/MS Scan:24415, Noise threshold:1.5
M+2H
M+3H
287.159
359.206
518.434
634.345
892.485
1059.540
1335.353
1465.511
1663.591
1715.209
1764.240
1785.763
1844.277
1857.272
1909.428
m/z

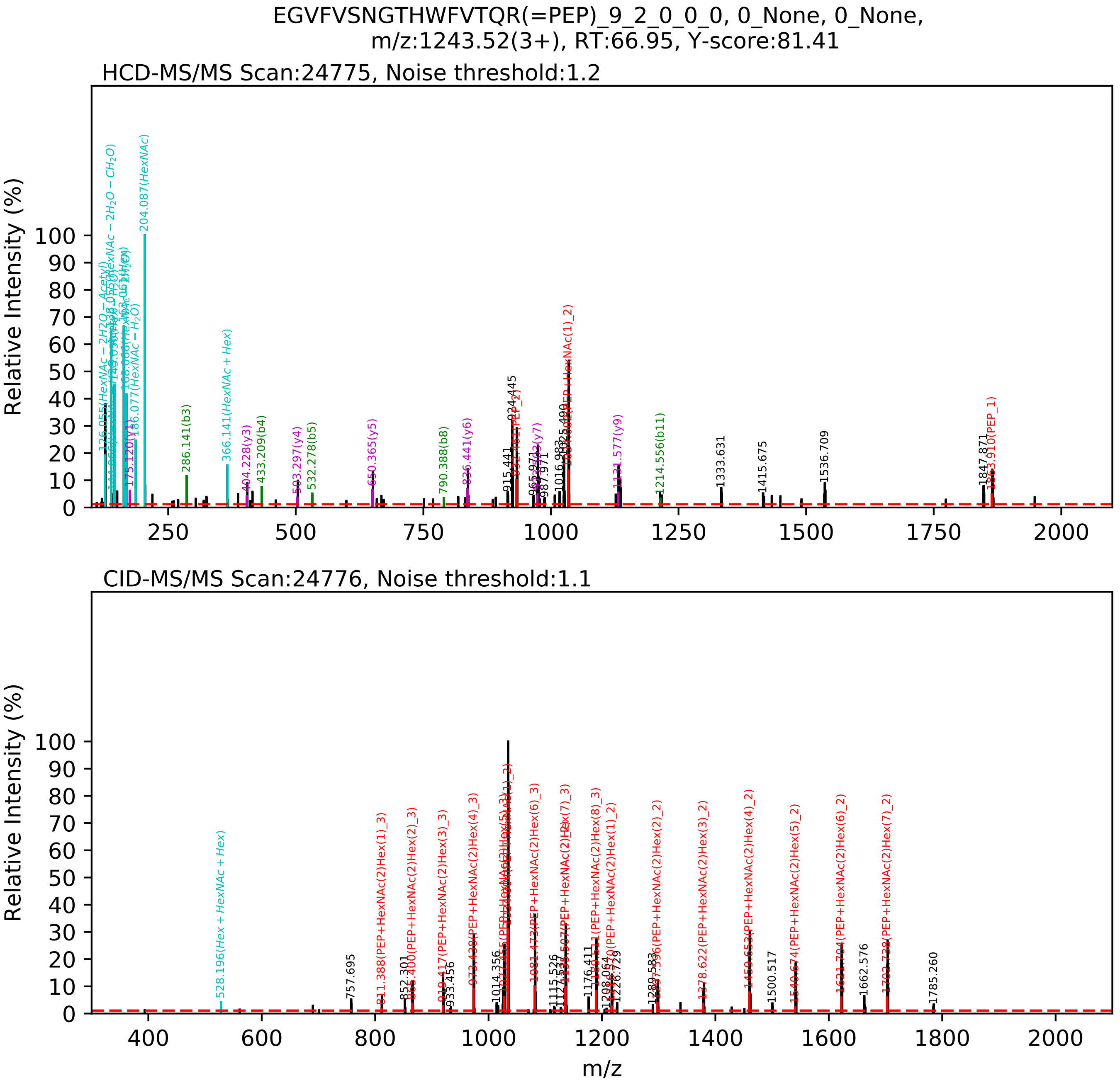
EGVFVSNGTHWFVTQR(=PEP)_9_2_0_0_0, 0_None, 0_None,
m/z:1243.52(3+), RT:66.95, Y-score:81.41
HCD-MS/MS Scan:24775, Noise threshold:1.2
Relative Intensity (%)
CID-MS/MS Scan:24776, Noise threshold:1.1
Relative Intensity (%)
m/z

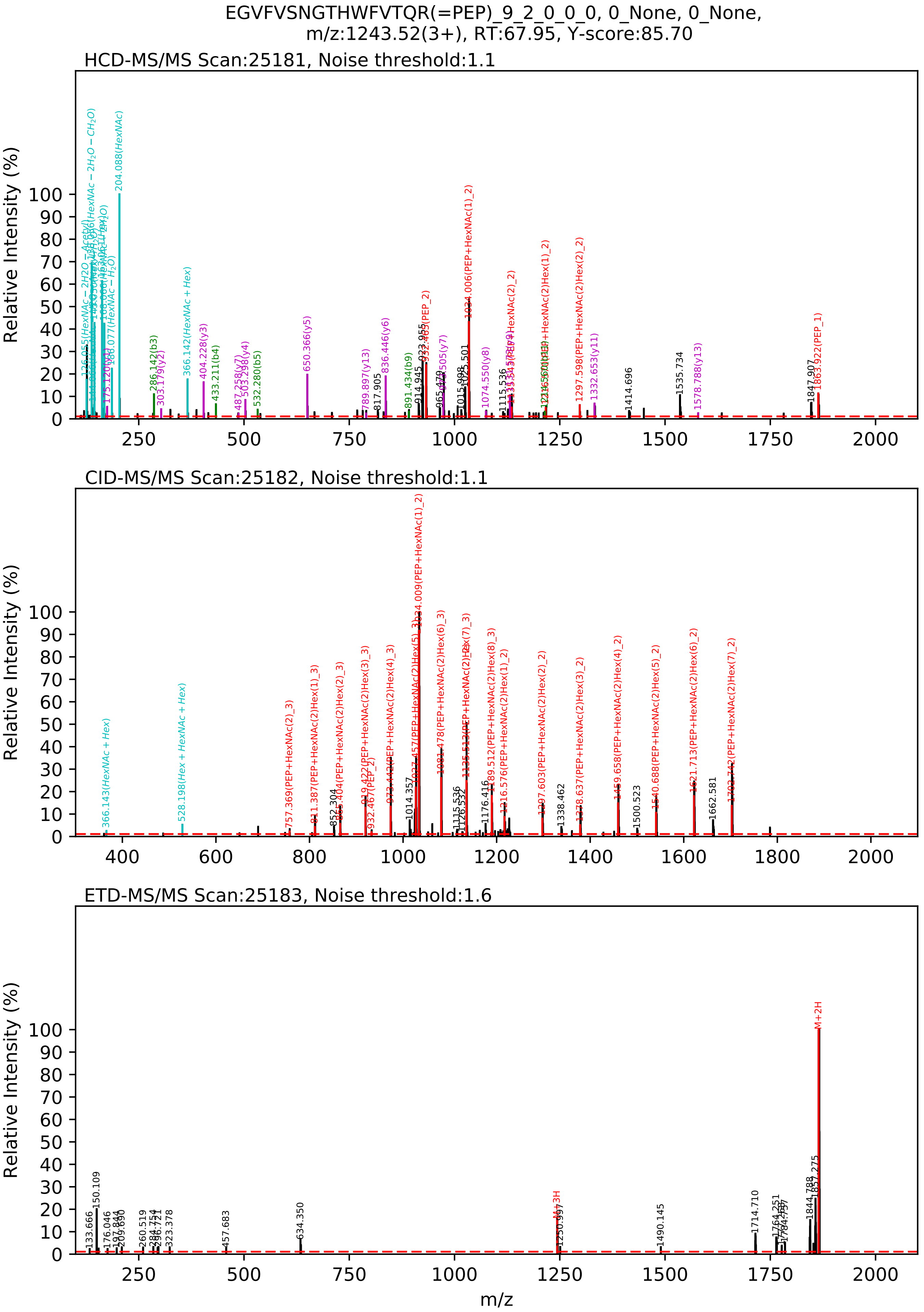
EGVFVSNGTHWFVTQR(=PEP)_9_2_0_0_0, 0_None, 0_None,
m/z:1243.52(3+), RT:67.95, Y-score:85.70
HCD-MS/MS Scan:25181, Noise threshold:1.1
Relative Intensity (%)
204.088(HexNAc)
366.142(HexNAc + Hex)
286.142(b3)
303.179(y2)
404.228(y3)
433.211(b4)
487.258(y7)
503.298(y4)
532.280(b5)
650.366(y5)
789.897(y13)
817.905
836.446(y6)
891.434(b9)
914.945
923.955
932.469(PEP_2)
965.479
1015.998
1025.501
1034.006(PEP+HexNAc(1)_2)
1074.550(y8)
1115.536
1297.598(PEP+HexNAc(2)Hex(2)_2)
1332.653(y11)
1414.696
1535.734
1578.788(y13)
1847.907
1863.922(PEP_1)
CID-MS/MS Scan:25182, Noise threshold:1.1
366.143(HexNAc + Hex)
528.198(Hex + HexNAc + Hex)
757.369(PEP+HexNAc(2)_3)
811.387(PEP+HexNAc(2)Hex(1)_3)
852.304
865.404(PEP+HexNAc(2)Hex(2)_3)
919.422(PEP+HexNAc(2)Hex(3)_3)
932.467(PEP_2)
973.442(PEP+HexNAc(2)Hex(4)_3)
1014.357
1034.009(PEP+HexNAc(1)_2)
1081.478(PEP+HexNAc(2)Hex(6)_3)
1115.536
1176.416
1189.512(PEP+HexNAc(2)Hex(8)_3)
1216.576(PEP+HexNAc(2)Hex(1)_2)
1297.603(PEP+HexNAc(2)Hex(2)_2)
1338.462
1378.637(PEP+HexNAc(2)Hex(3)_2)
1459.658(PEP+HexNAc(2)Hex(4)_2)
1500.523
1540.688(PEP+HexNAc(2)Hex(5)_2)
1621.713(PEP+HexNAc(2)Hex(6)_2)
1662.581
1702.742(PEP+HexNAc(2)Hex(7)_2)
ETD-MS/MS Scan:25183, Noise threshold:1.6
133.666
150.109
176.046
197.844
209.690
260.519
284.754
296.721
323.378
457.683
634.350
M+3H
1250.997
1490.145
1714.710
1764.251
1844.788
1857.275
M+2H
m/z

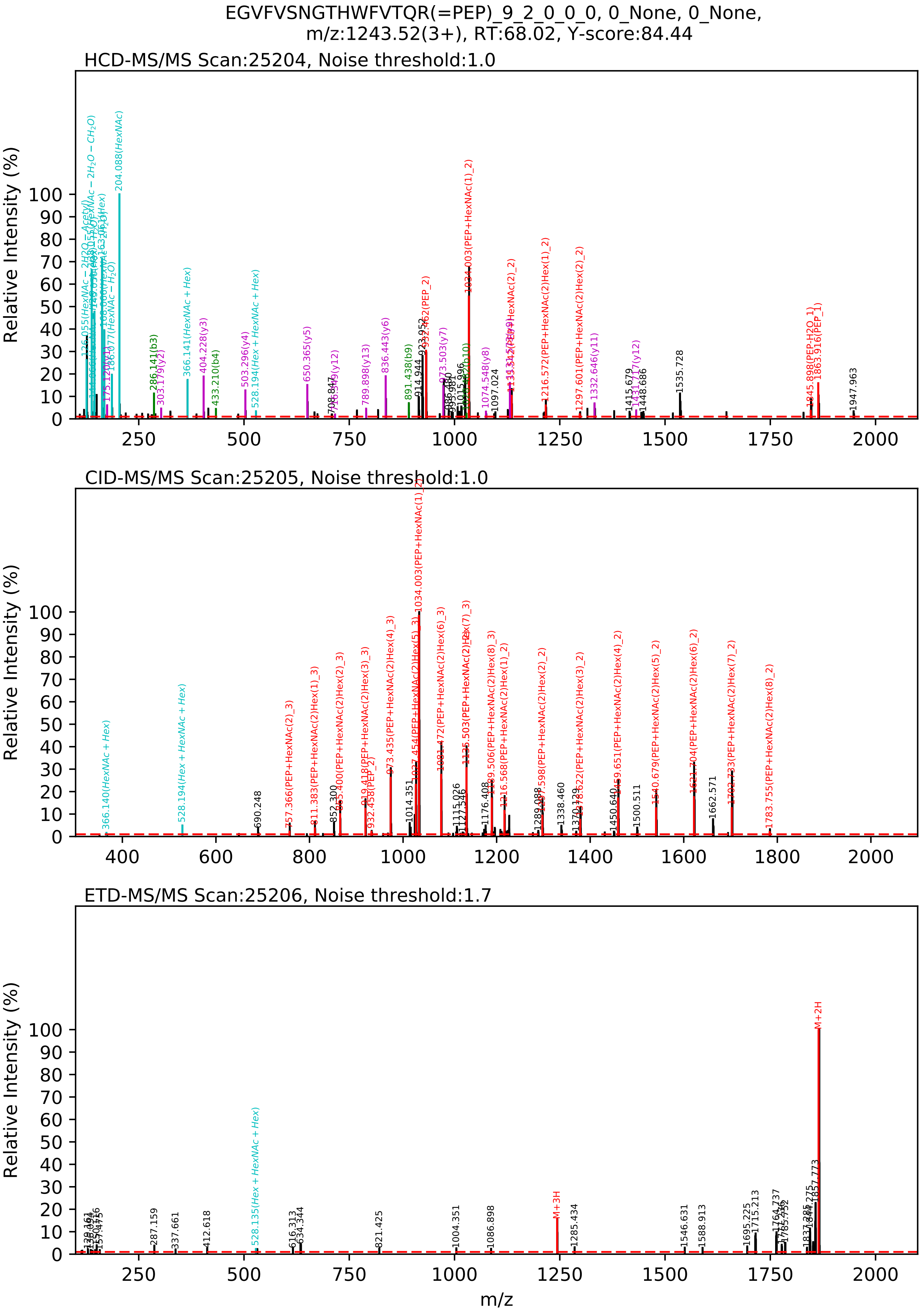
EGVFVSNGTHWFVTQR(=PEP)_9_2_0_0_0, 0_None, 0_None,
m/z:1243.52(3+), RT:68.02, Y-score:84.44
HCD-MS/MS Scan:25204, Noise threshold:1.0
CID-MS/MS Scan:25205, Noise threshold:1.0
ETD-MS/MS Scan:25206, Noise threshold:1.7
Relative Intensity (%)
m/z
204.088(HexNAc)
1034.003(PEP+HexNAc(1)_2)
932.462(PEP_2)
1216.572(PEP+HexNAc(2)Hex(1)_2)
1297.601(PEP+HexNAc(2)Hex(2)_2)
1863.916(PEP_1)
1845.898(PEP-H2O_1)
366.141(HexNAc+Hex)
528.194(Hex+HexNAc+Hex)
286.141(b3)
433.210(b4)
891.438(b9)
404.228(y3)
503.296(y4)
650.365(y5)
836.443(y6)
973.503(y7)
1074.548(y8)
1332.646(y11)
789.898(y13)
1535.728
1947.963
757.366(PEP+HexNAc(2)_3)
811.383(PEP+HexNAc(2)Hex(1)_3)
865.400(PEP+HexNAc(2)Hex(2)_3)
919.418(PEP+HexNAc(2)Hex(3)_3)
973.435(PEP+HexNAc(2)Hex(4)_3)
1081.472(PEP+HexNAc(2)Hex(6)_3)
1783.755(PEP+HexNAc(2)Hex(8)_2)
1378.622(PEP+HexNAc(2)Hex(3)_2)
1540.679(PEP+HexNAc(2)Hex(5)_2)
1621.704(PEP+HexNAc(2)Hex(6)_2)
690.248
1662.571
1338.460
1500.511
M+2H
M+3H
528.135(Hex+HexNAc+Hex)
1857.773
1285.434
1004.351
821.425

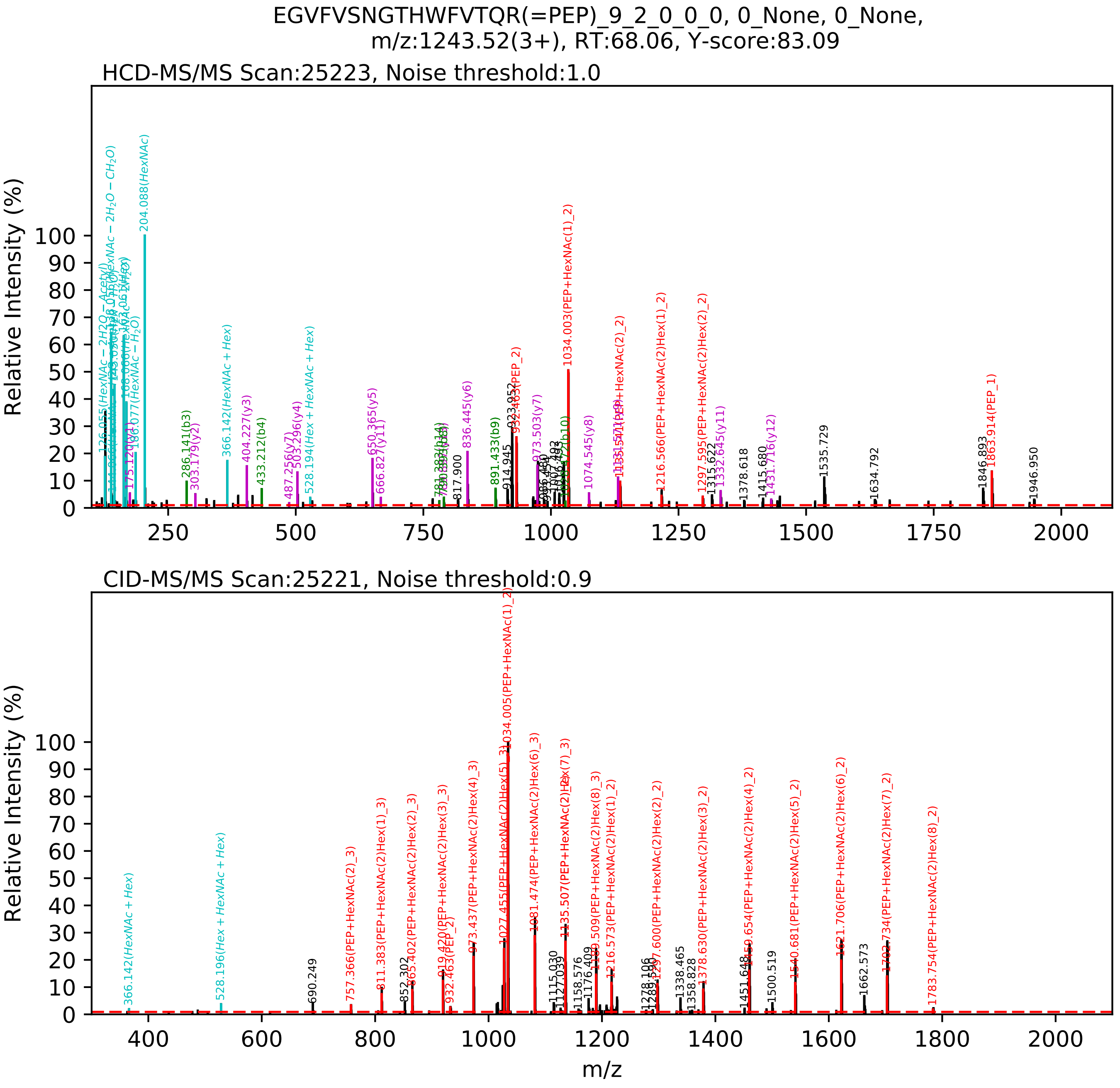
EGVFVSNGTHWFVTQR(=PEP)_9_2_0_0_0, 0_None, 0_None,
m/z:1243.52(3+), RT:68.06, Y-score:83.09
HCD-MS/MS Scan:25223, Noise threshold:1.0
Relative Intensity (%)
204.088(HexNAc)
1034.003(PEP+HexNAc(1)_2)
1216.566(PEP+HexNAc(2)Hex(1)_2)
1297.595(PEP+HexNAc(2)Hex(2)_2)
1863.914(PEP_1)
932.465(PEP_2)
836.445(y6)
650.365(y5)
366.142(HexNAc+Hex)
528.194(Hex+HexNAc+Hex)
1535.729
1634.792
1846.893
1946.950
CID-MS/MS Scan:25221, Noise threshold:0.9
Relative Intensity (%)
m/z
1034.005(PEP+HexNAc(1)_2)
1081.474(PEP+HexNAc(2)Hex(6)_3)
1621.706(PEP+HexNAc(2)Hex(6)_2)
1540.681(PEP+HexNAc(2)Hex(5)_2)
1783.754(PEP+HexNAc(2)Hex(8)_2)
757.366(PEP+HexNAc(2)_3)
690.249
1662.573
1500.519
1338.465

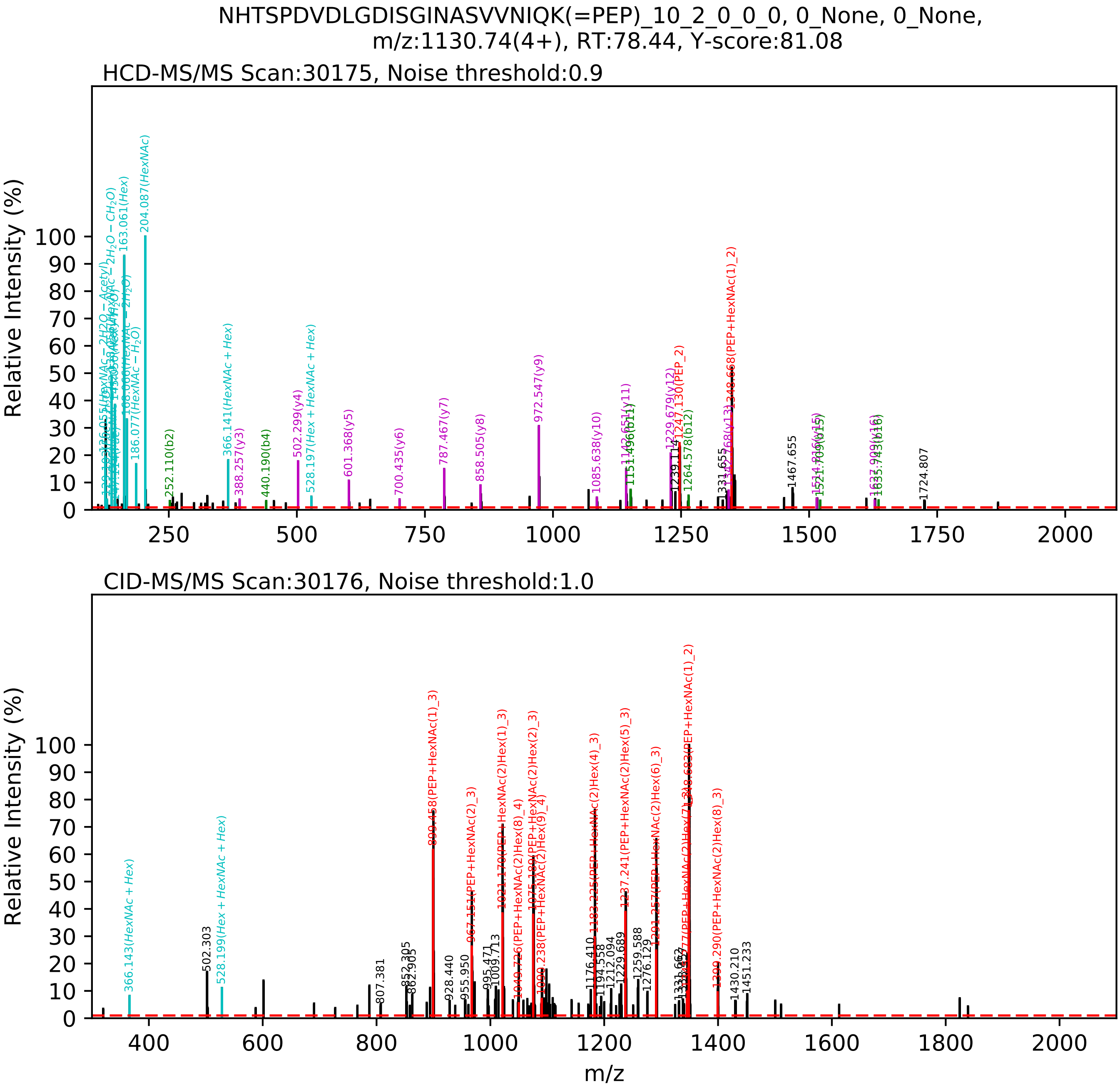
NHTSPDVDLGDISGINASVVNIQK(=PEP)_10_2_0_0_0, 0_None, 0_None,
m/z:1130.74(4+), RT:78.44, Y-score:81.08
HCD-MS/MS Scan:30175, Noise threshold:0.9
Relative Intensity (%)
204.087(HexNAc)
163.061(Hex)
186.077(HexNAc − H2O)
252.110(b2)
366.141(HexNAc + Hex)
388.257(y3)
440.190(b4)
502.299(y4)
528.197(Hex + HexNAc + Hex)
601.368(y5)
700.435(y6)
787.467(y7)
858.505(y8)
972.547(y9)
1085.638(y10)
1264.578(b12)
1331.655
1467.655
1724.807
CID-MS/MS Scan:30176, Noise threshold:1.0
Relative Intensity (%)
366.143(HexNAc + Hex)
502.303
528.199(Hex + HexNAc + Hex)
807.381
852.305
862.905
899.458(PEP+HexNAc(1)_3)
928.440
955.950
967.151(PEP+HexNAc(2)_3)
995.471
1009.713
1237.241(PEP+HexNAc(2)Hex(5)_3)
1259.588
1276.129
1399.290(PEP+HexNAc(2)Hex(8)_3)
1430.210
1451.233
m/z

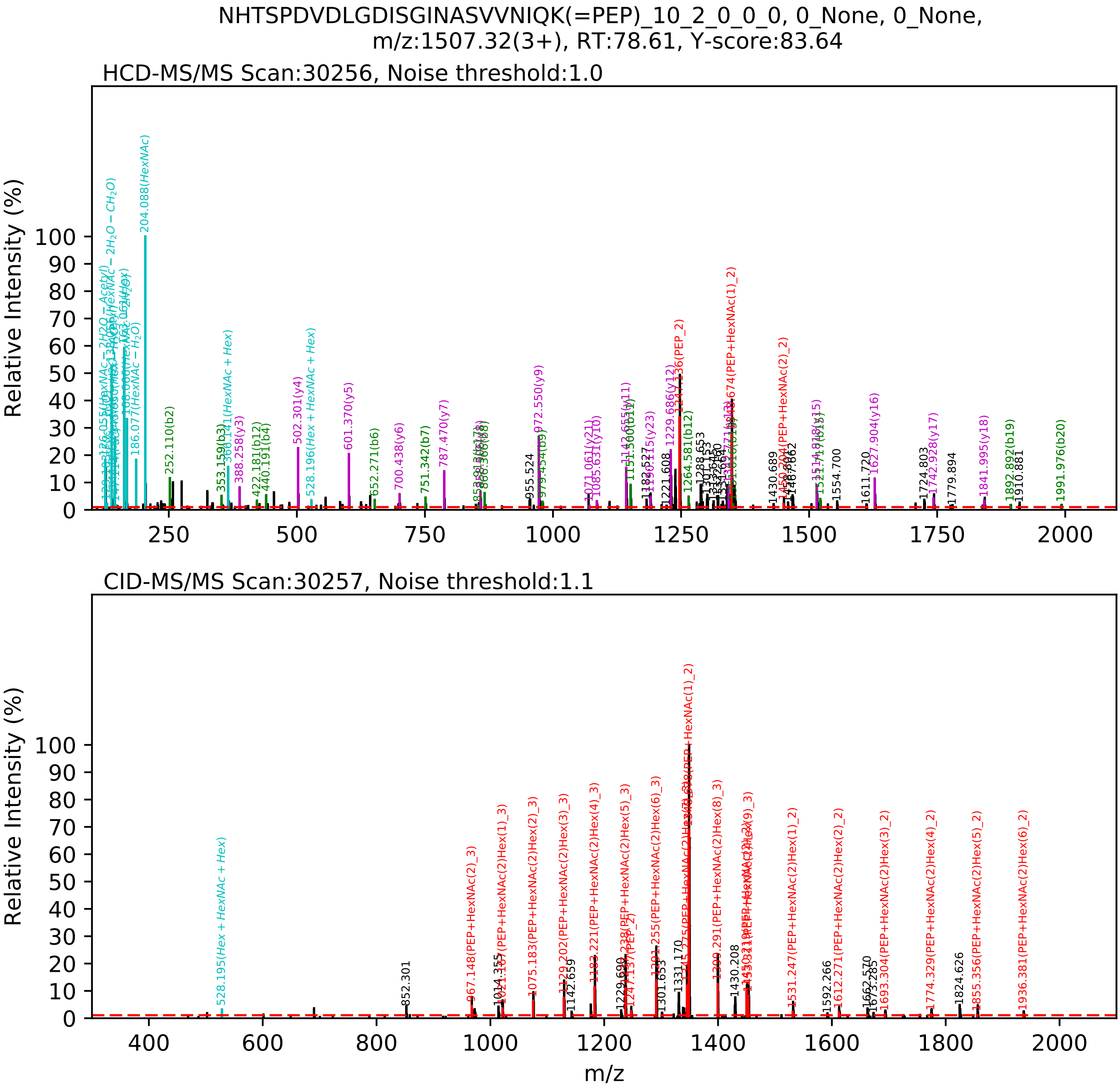

NHTSPDVDLGDISGINASVVNIQK(=PEP)_10_2_0_0_0, 0_None, 0_None,
m/z:1507.32(3+), RT:78.61, Y-score:83.64
HCD-MS/MS Scan:30256, Noise threshold:1.0
Relative Intensity (%)
CID-MS/MS Scan:30257, Noise threshold:1.1
Relative Intensity (%)
m/z

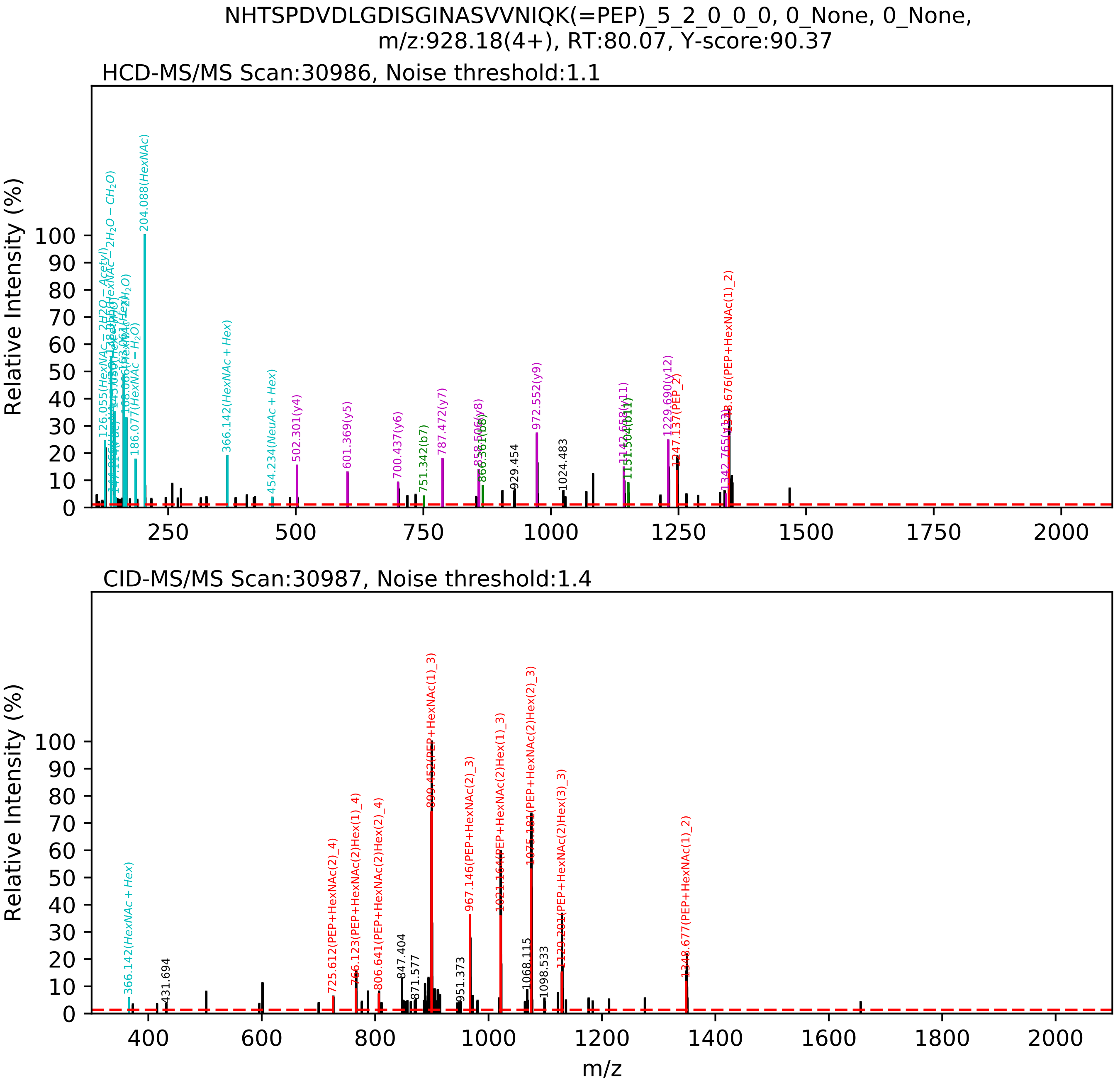
NHTSPDVDLGDISGINASVVNIQK(=PEP)_5_2_0_0_0, 0_None, 0_None,
m/z:928.18(4+), RT:80.07, Y-score:90.37
HCD-MS/MS Scan:30986, Noise threshold:1.1
Relative Intensity (%)
204.088(HexNAc)
126.055(HexNAc-2H2O-Acetyl)
186.077(HexNAc-H2O)
366.142(HexNAc+Hex)
454.234(NeuAc+Hex)
502.301(y4)
601.369(y5)
700.437(y6)
751.342(b7)
787.472(y7)
972.552(y9)
929.454
1024.483
1229.690(y12)
1247.137(PEP_2)
1348.676(PEP+HexNAc(1)_2)
CID-MS/MS Scan:30987, Noise threshold:1.4
Relative Intensity (%)
366.142(HexNAc+Hex)
431.694
725.612(PEP+HexNAc(2)_4)
766.123(PEP+HexNAc(2)Hex(1)_4)
806.641(PEP+HexNAc(2)Hex(2)_4)
847.404
871.577
899.452(PEP+HexNAc(1)_3)
951.373
967.146(PEP+HexNAc(2)_3)
1021.164(PEP+HexNAc(2)Hex(1)_3)
1068.115
1075.181(PEP+HexNAc(2)Hex(2)_3)
1098.533
1129.201(PEP+HexNAc(2)Hex(3)_3)
1348.677(PEP+HexNAc(1)_2)
m/z

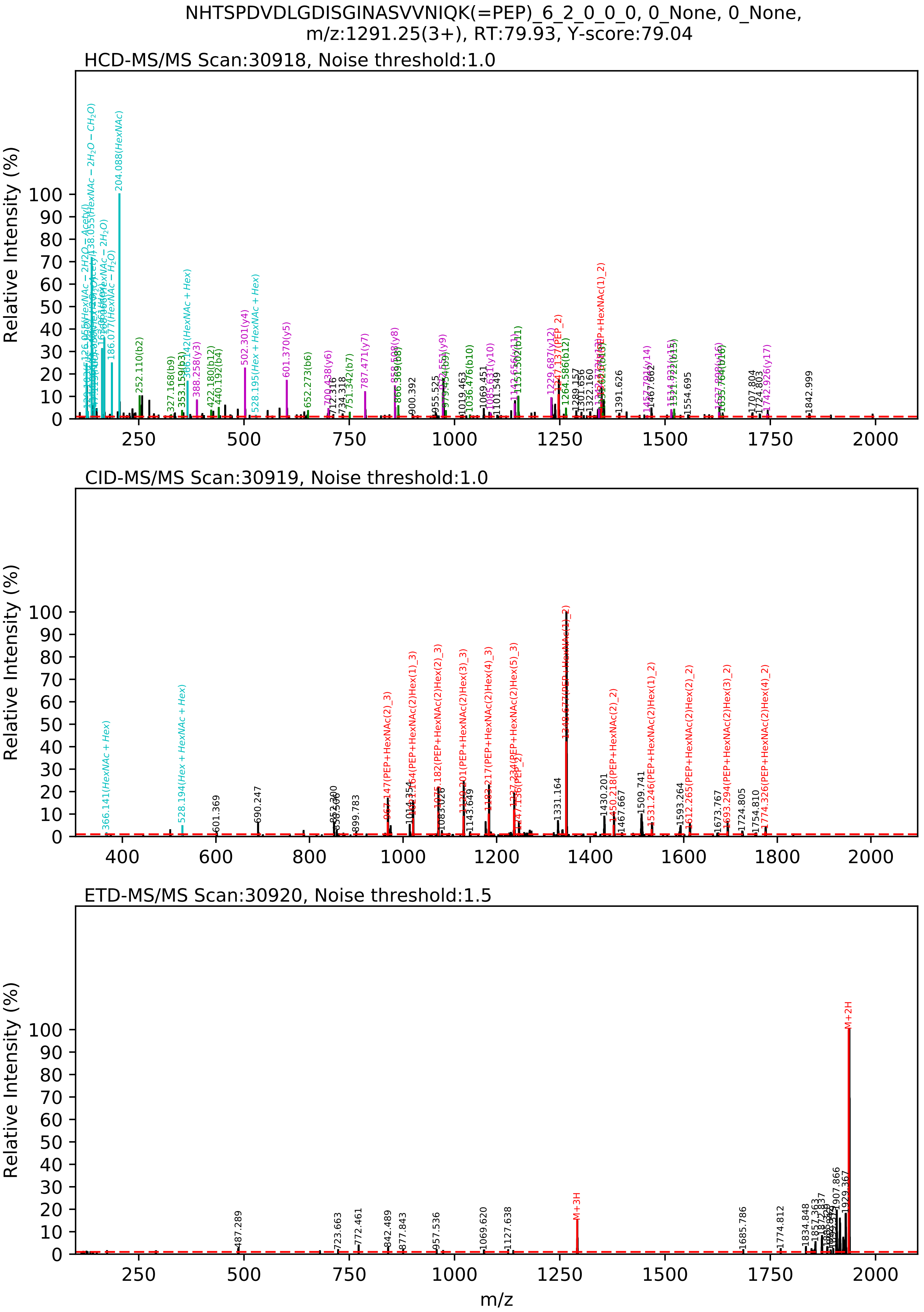
NHTSPDVDLGDISGINASVVNIQK(=PEP)_6_2_0_0_0, 0_None, 0_None,
m/z:1291.25(3+), RT:79.93, Y-score:79.04
HCD-MS/MS Scan:30918, Noise threshold:1.0
CID-MS/MS Scan:30919, Noise threshold:1.0
ETD-MS/MS Scan:30920, Noise threshold:1.5
Relative Intensity (%)
m/z
204.088(HexNAc)
186.077(HexNAc − H2O)
252.110(b2)
327.168(b9)
353.159(b3)
366.142(HexNAc + Hex)
388.258(y3)
422.180(b12)
440.192(b4)
502.301(y4)
528.195(Hex + HexNAc + Hex)
601.370(y5)
652.273(b6)
751.342(b7)
787.471(y7)
858.508(y8)
866.369(b8)
900.392
955.525
979.454(b9)
1019.463
1036.476(b10)
1069.451
1085.631(y10)
1101.549
1151.502(b11)
1229.687(y12)
1247.137(PEP_2)
1264.586(b12)
1289.157
1301.656
1322.160
PEP+HexNAc(1)_2
1391.626
1467.662
1514.831(y15)
1521.722(b15)
1554.695
1627.898(y16)
1635.764(b16)
1707.804
1724.803
1742.926(y17)
1842.999
366.141(HexNAc + Hex)
528.194(Hex + HexNAc + Hex)
601.369
690.247
852.300
899.783
967.147(PEP+HexNAc(2)_3)
1014.354
1075.182(PEP+HexNAc(2)Hex(2)_3)
1083.026
1143.649
1183.217(PEP+HexNAc(2)Hex(4)_3)
1237.234(PEP+HexNAc(2)Hex(5)_3)
1331.164
1430.201
1450.218(PEP+HexNAc(2)_2)
1467.667
1509.741
1531.246(PEP+HexNAc(2)Hex(1)_2)
1593.264
1612.265(PEP+HexNAc(2)Hex(2)_2)
1673.767
1693.294(PEP+HexNAc(2)Hex(3)_2)
1724.805
1754.810
1774.326(PEP+HexNAc(2)Hex(4)_2)
487.289
723.663
772.461
842.489
877.843
957.536
1069.620
1127.638
M+3H
1685.786
1774.812
1834.848
1907.866
1929.367
M+2H

NHTSPDVDLGDISGINASVVNIQK(=PEP)_7_2_0_0_0, 0_None, 0_None,
m/z:1345.27(3+), RT:77.09, Y-score:75.98

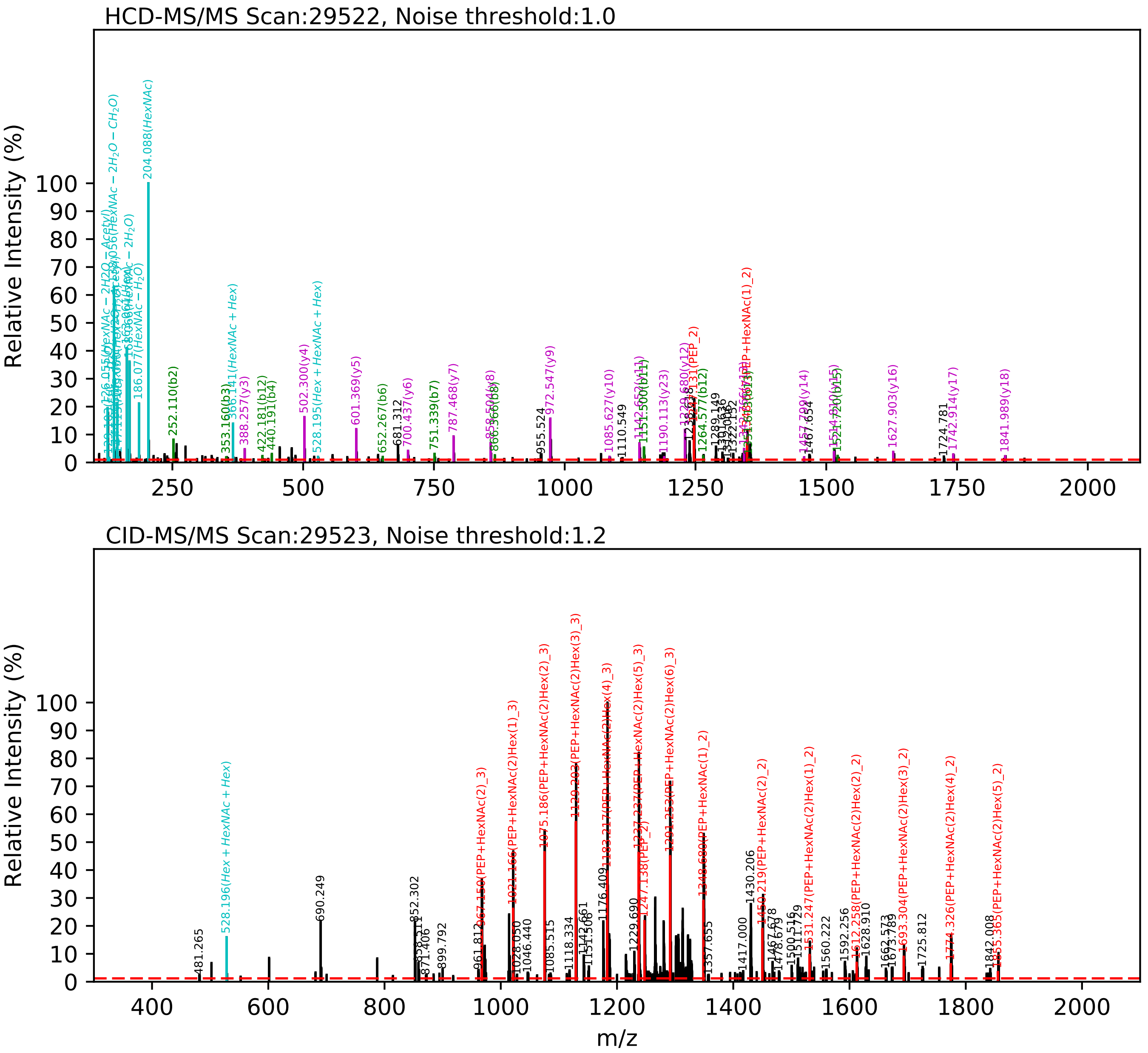

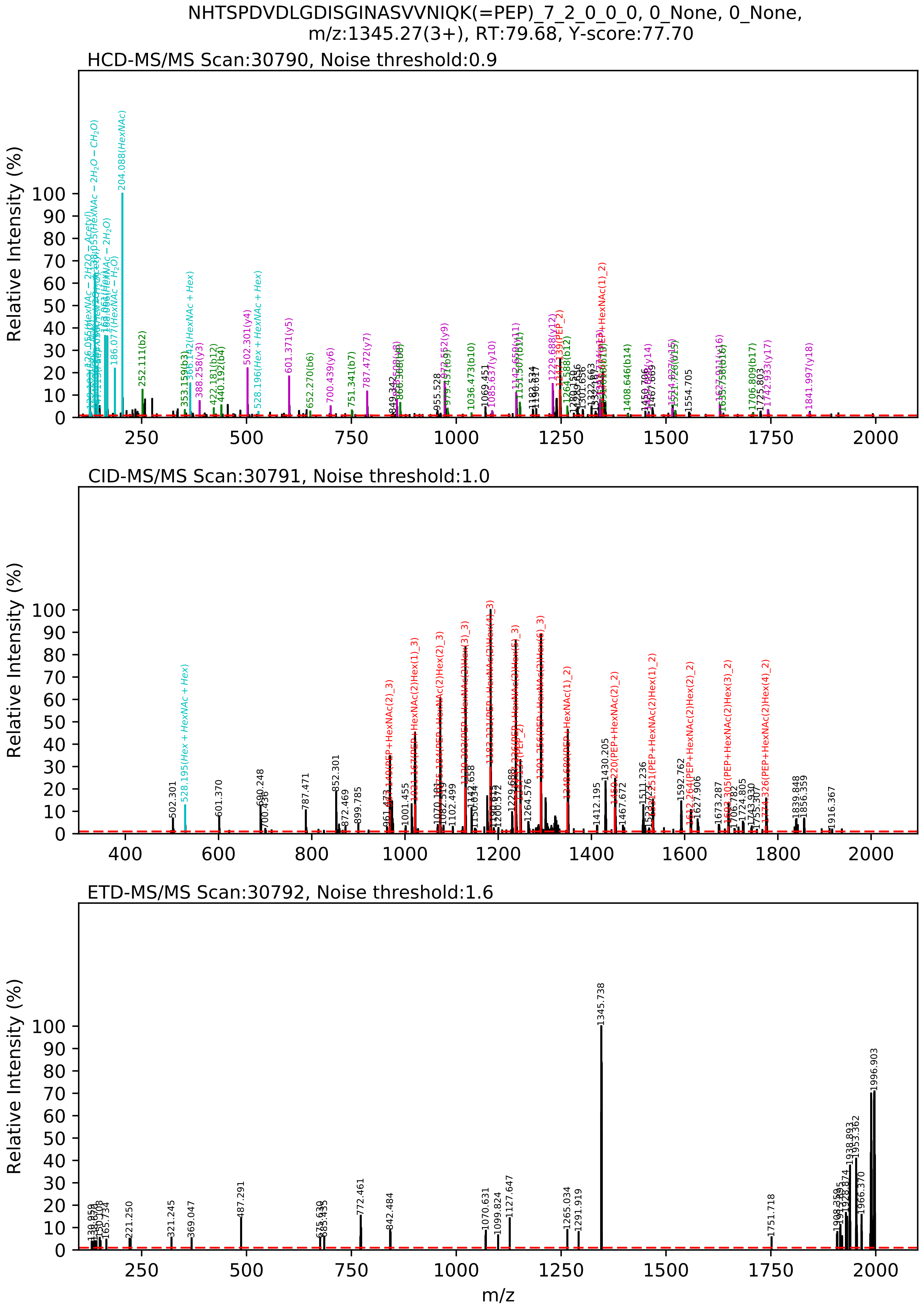
NHTSPDVDLGDISGINASVVNIQK(=PEP)_7_2_0_0_0, 0_None, 0_None,
m/z:1345.27(3+), RT:79.68, Y-score:77.70
HCD-MS/MS Scan:30790, Noise threshold:0.9
Relative Intensity (%)
204.088(HexNAc)
186.077(HexNAc − H2O)
252.111(b2)
353.159(b3)
388.258(y3)
422.181(b12)
440.192(b4)
502.301(y4)
528.196(Hex + HexNAc + Hex)
601.371(y5)
652.270(b6)
700.439(y6)
751.341(b7)
787.472(y7)
849.342
866.368(b8)
955.528
1036.473(b10)
1069.451
1085.637(y10)
1183.534
1190.617
1264.588(b12)
1301.656
1408.646(b14)
1459.706
1467.669
1554.705
1706.809(b17)
1725.803
1742.933(y17)
1841.997(y18)
CID-MS/MS Scan:30791, Noise threshold:1.0
Relative Intensity (%)
502.301
528.195(Hex + HexNAc + Hex)
601.370
690.248
700.436
787.471
852.301
872.469
899.785
1001.455
1070.181
1082.519
1102.499
1142.658
1150.834
1200.572
1229.688
1264.576
1348.690(PEP+HexNAc(1)_2)
1412.195
1430.205
1450.220(PEP+HexNAc(2)_2)
1467.672
1511.236
1531.251(PEP+HexNAc(2)Hex(1)_2)
1592.762
1612.264(PEP+HexNAc(2)Hex(2)_2)
1627.906
1673.287
1693.305(PEP+HexNAc(2)Hex(3)_2)
1706.782
1724.805
1743.930
1753.307
1774.326(PEP+HexNAc(2)Hex(4)_2)
1839.848
1856.359
1916.367
ETD-MS/MS Scan:30792, Noise threshold:1.6
Relative Intensity (%)
138.058
165.734
221.250
321.245
369.047
487.291
675.630
683.435
772.461
842.484
1070.631
1099.824
1127.647
1265.034
1291.919
1345.738
1751.718
1908.359
1928.874
1938.893
1953.362
1966.370
1996.903
m/z

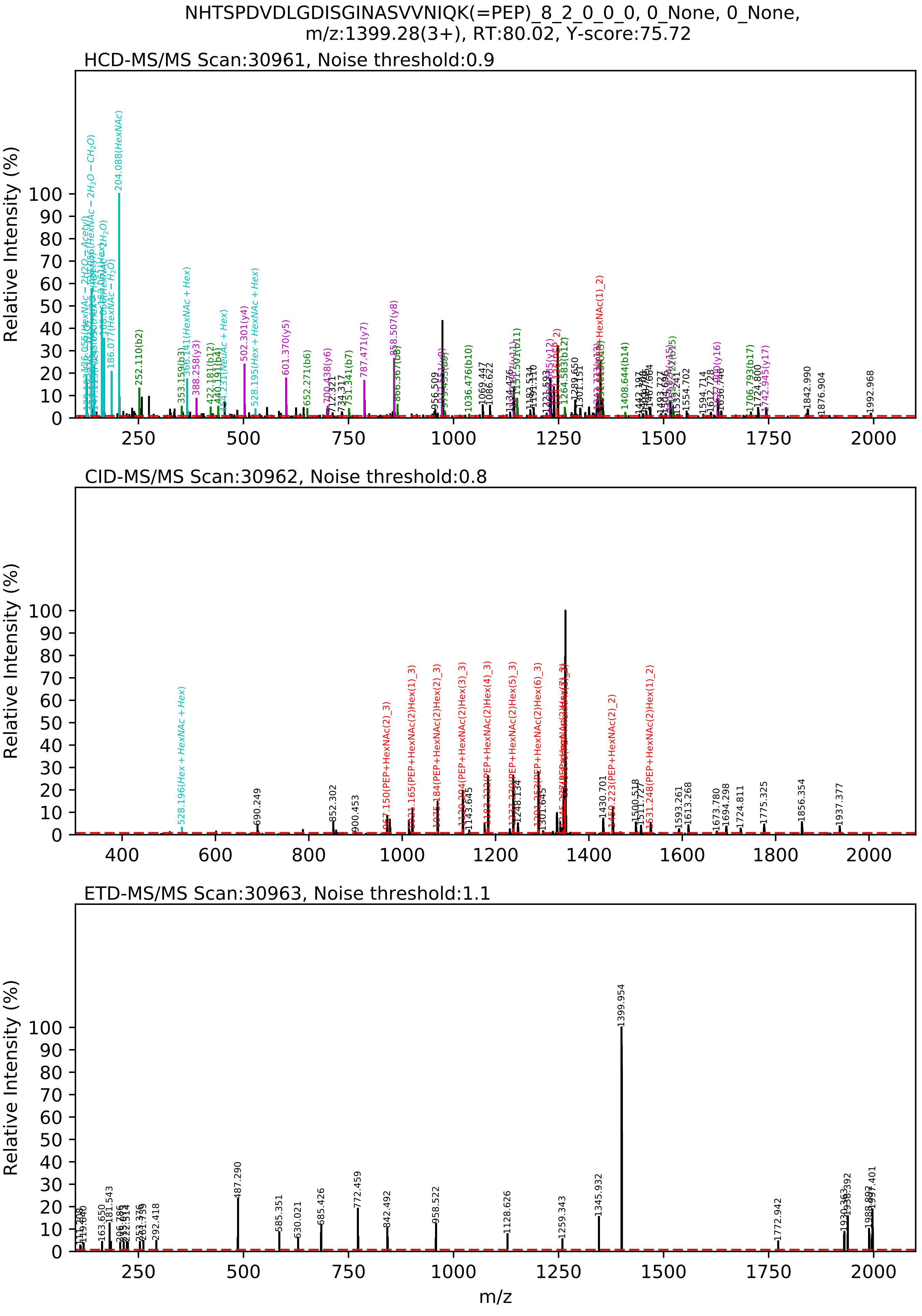
NHTSPDVDLGDISGINASVVNIQK(=PEP)_8_2_0_0_0, 0_None, 0_None,
m/z:1399.28(3+), RT:80.02, Y-score:75.72
HCD-MS/MS Scan:30961, Noise threshold:0.9
CID-MS/MS Scan:30962, Noise threshold:0.8
ETD-MS/MS Scan:30963, Noise threshold:1.1
Relative Intensity (%)
m/z

NHTSPDVDLGDISGINASVVNIQK(=PEP)_8_2_0_0_0, 0_None, 0_None,
m/z:1399.28(3+), RT:77.72, Y-score:63.51

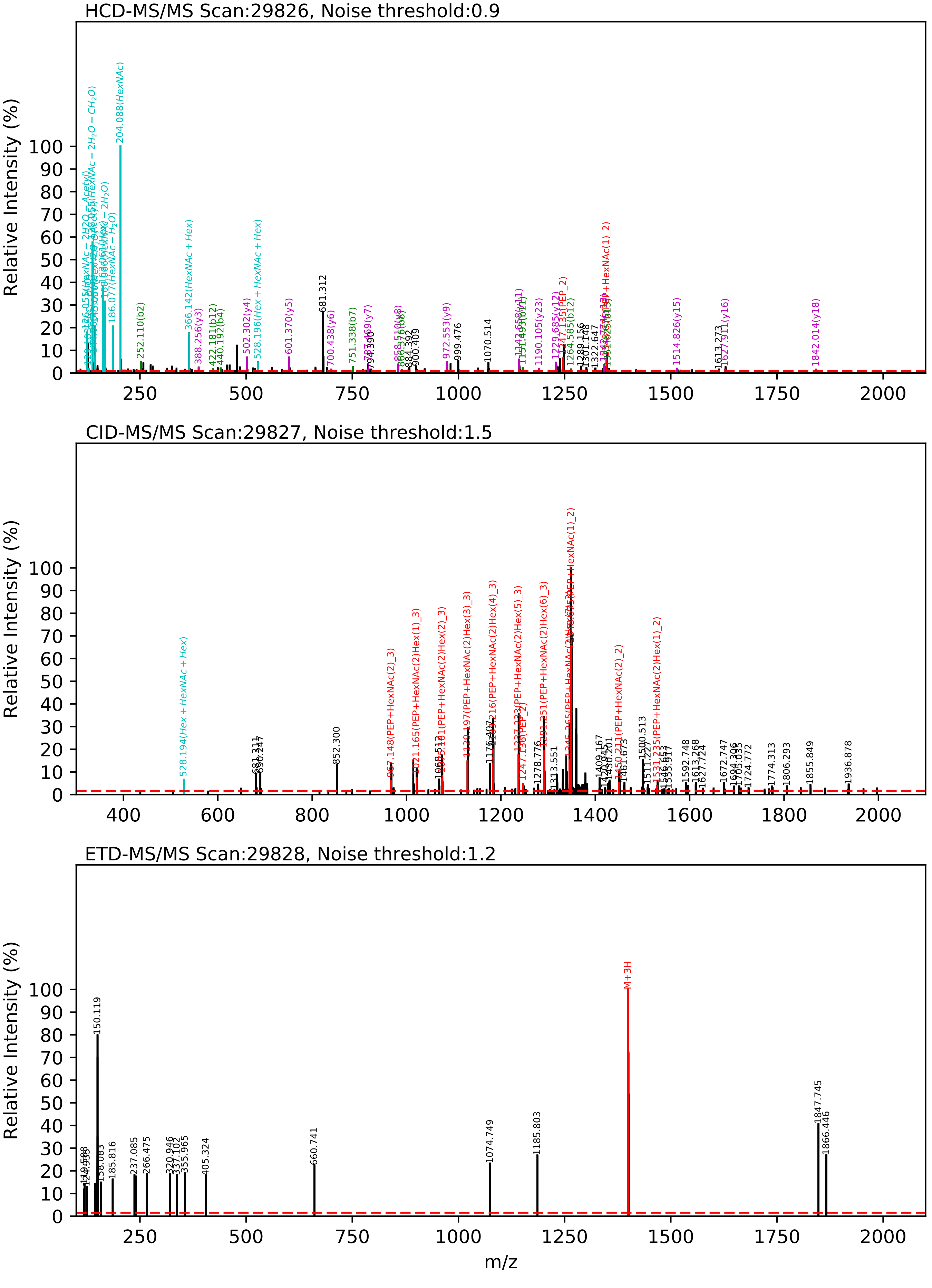

NHTSPDVDLGDISGINASVVNIQK(=PEP)_8_2_0_0_0, 0_None, 0_None,
m/z:1399.28(3+), RT:78.72, Y-score:82.40

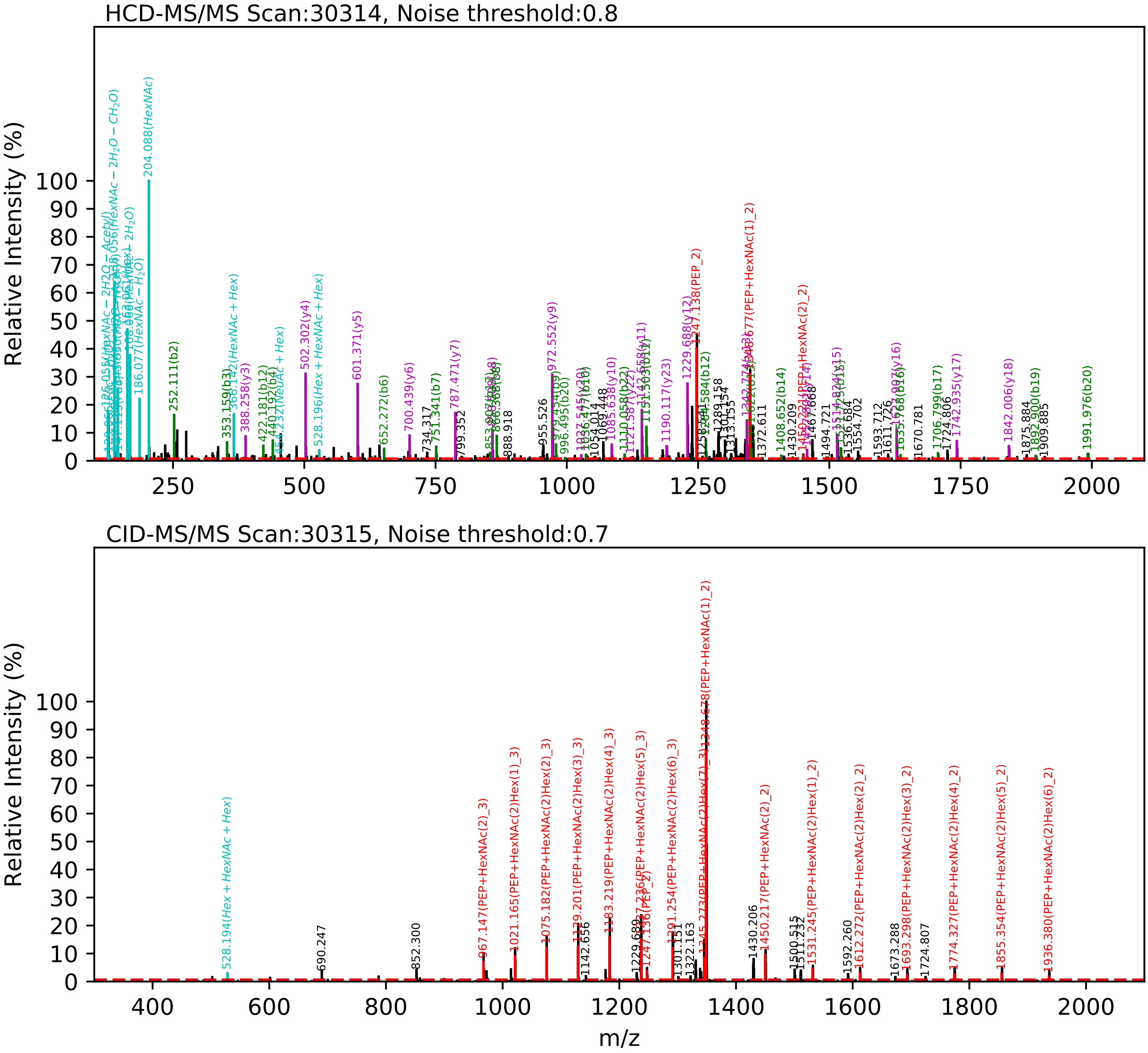

NHTSPDVDLGDISGINASVVNIQK(=PEP)_8_2_0_0_0, 0_None, 0_None,
m/z:1399.28(3+), RT:79.46, Y-score:77.04

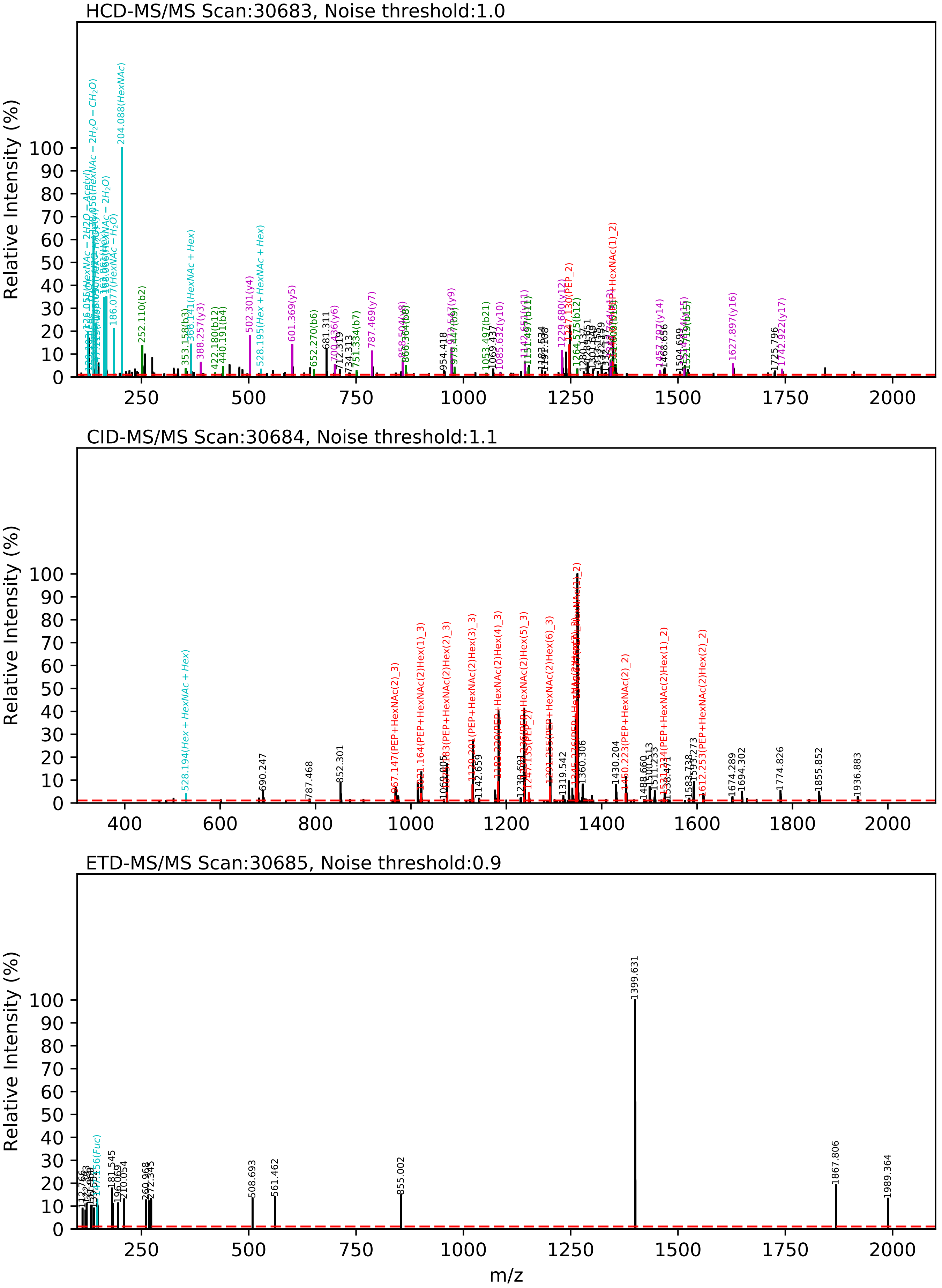

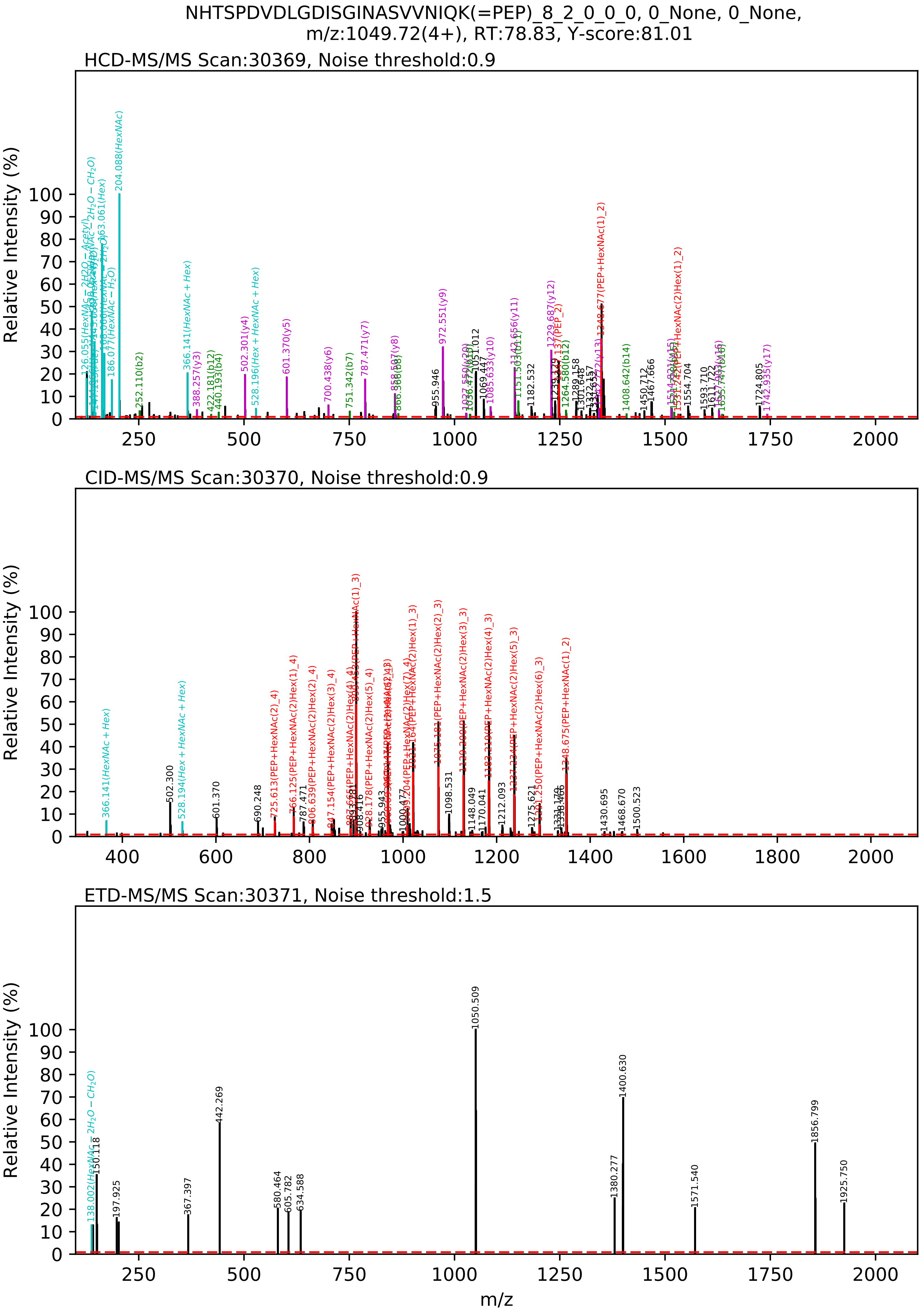
NHTSPDVDLGDISGINASVVNIQK(=PEP)_8_2_0_0_0, 0_None, 0_None,
m/z:1049.72(4+), RT:78.83, Y-score:81.01
HCD-MS/MS Scan:30369, Noise threshold:0.9
Relative Intensity (%)
204.088(HexNAc)
163.061(Hex)
186.077(HexNAc − H2O)
126.055(HexNAc − 2H2O − Acetyl)
252.110(b2)
366.141(HexNAc + Hex)
388.257(y3)
422.181(b12)
440.193(b4)
502.301(y4)
528.196(Hex + HexNAc + Hex)
601.370(y5)
700.438(y6)
751.342(b7)
787.471(y7)
858.587(y8)
866.366(b8)
955.946
972.551(y9)
1051.012
1069.441
1085.633(y10)
1142.656(y11)
1151.503(b11)
1182.532
1229.687(y12)
1239.129
1264.580(b12)
1289.158
1301.648
1348.677(PEP+HexNAc(1)_2)
1408.642(b14)
1450.712
1467.666
1554.704
1593.710
1611.722
1724.805
1742.935(y17)
CID-MS/MS Scan:30370, Noise threshold:0.9
366.141(HexNAc + Hex)
502.300
528.194(Hex + HexNAc + Hex)
601.370
690.248
725.613(PEP+HexNAc(2)_4)
766.125(PEP+HexNAc(2)Hex(1)_4)
787.471
806.639(PEP+HexNAc(2)Hex(2)_4)
847.154(PEP+HexNAc(2)Hex(3)_4)
928.178(PEP+HexNAc(2)Hex(5)_4)
955.943
1000.477
1075.181(PEP+HexNAc(2)Hex(2)_3)
1098.531
1129.200(PEP+HexNAc(2)Hex(3)_3)
1148.049
1170.041
1183.210(PEP+HexNAc(2)Hex(4)_3)
1212.093
1237.224(PEP+HexNAc(2)Hex(5)_3)
1275.621
1291.250(PEP+HexNAc(2)Hex(6)_3)
1331.170
1338.466
1348.675(PEP+HexNAc(1)_2)
1430.695
1468.670
1500.523
ETD-MS/MS Scan:30371, Noise threshold:1.5
138.002(HexNAc − 2H2O − CH2O)
150.118
197.925
367.397
442.269
580.464
605.782
634.588
1050.509
1380.277
1400.630
1571.540
1856.799
1925.750
m/z

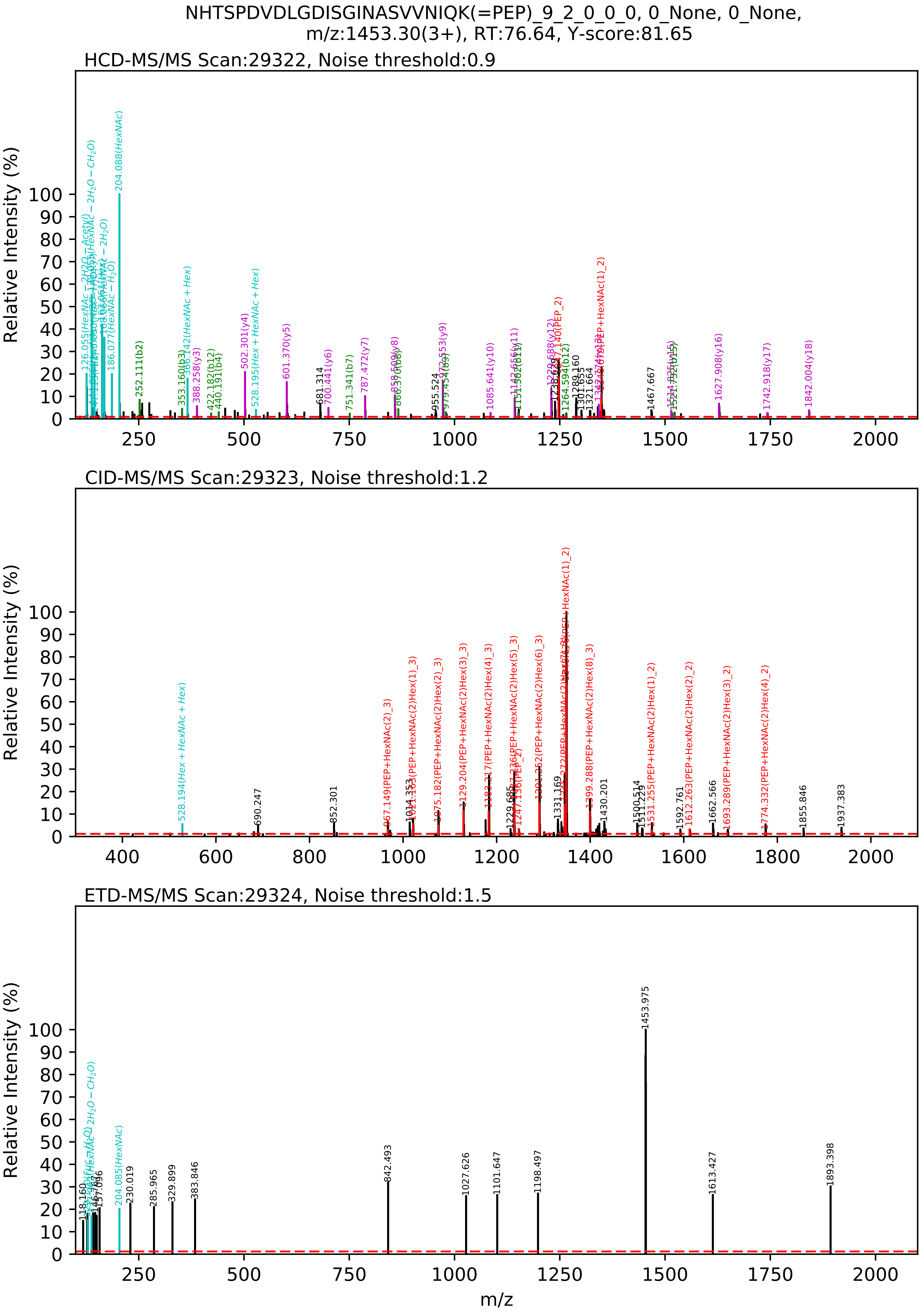
NHTSPDVDLGDISGINASVVNIQK(=PEP)_9_2_0_0_0, 0_None, 0_None,
m/z:1453.30(3+), RT:76.64, Y-score:81.65
HCD-MS/MS Scan:29322, Noise threshold:0.9
CID-MS/MS Scan:29323, Noise threshold:1.2
ETD-MS/MS Scan:29324, Noise threshold:1.5
Relative Intensity (%)
m/z

NHTSPDVDLGDISGINASVVNIQK(=PEP)_9_2_0_0_0, 0_None, 0_None,
m/z:1453.30(3+), RT:79.06, Y-score:82.57

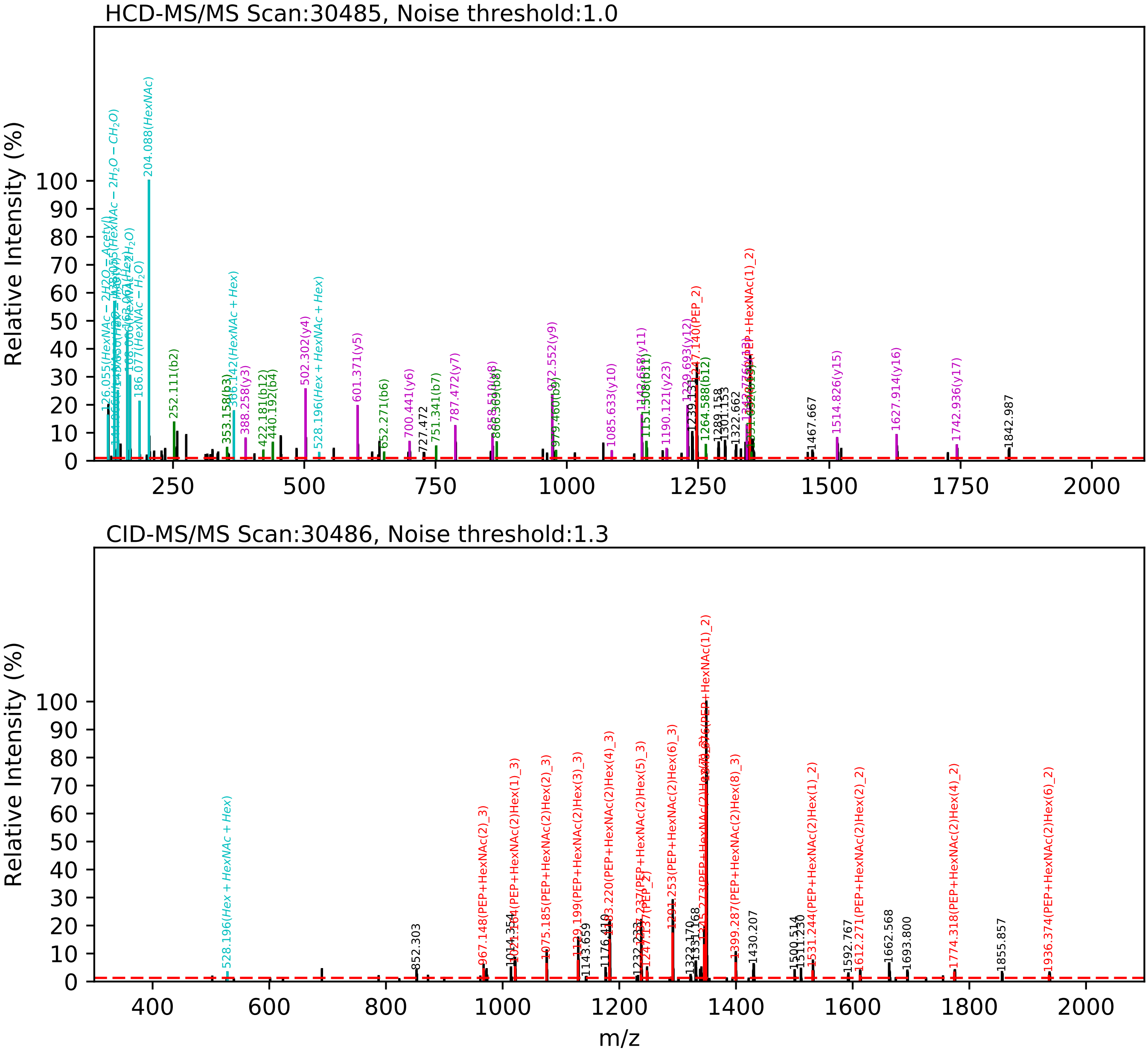

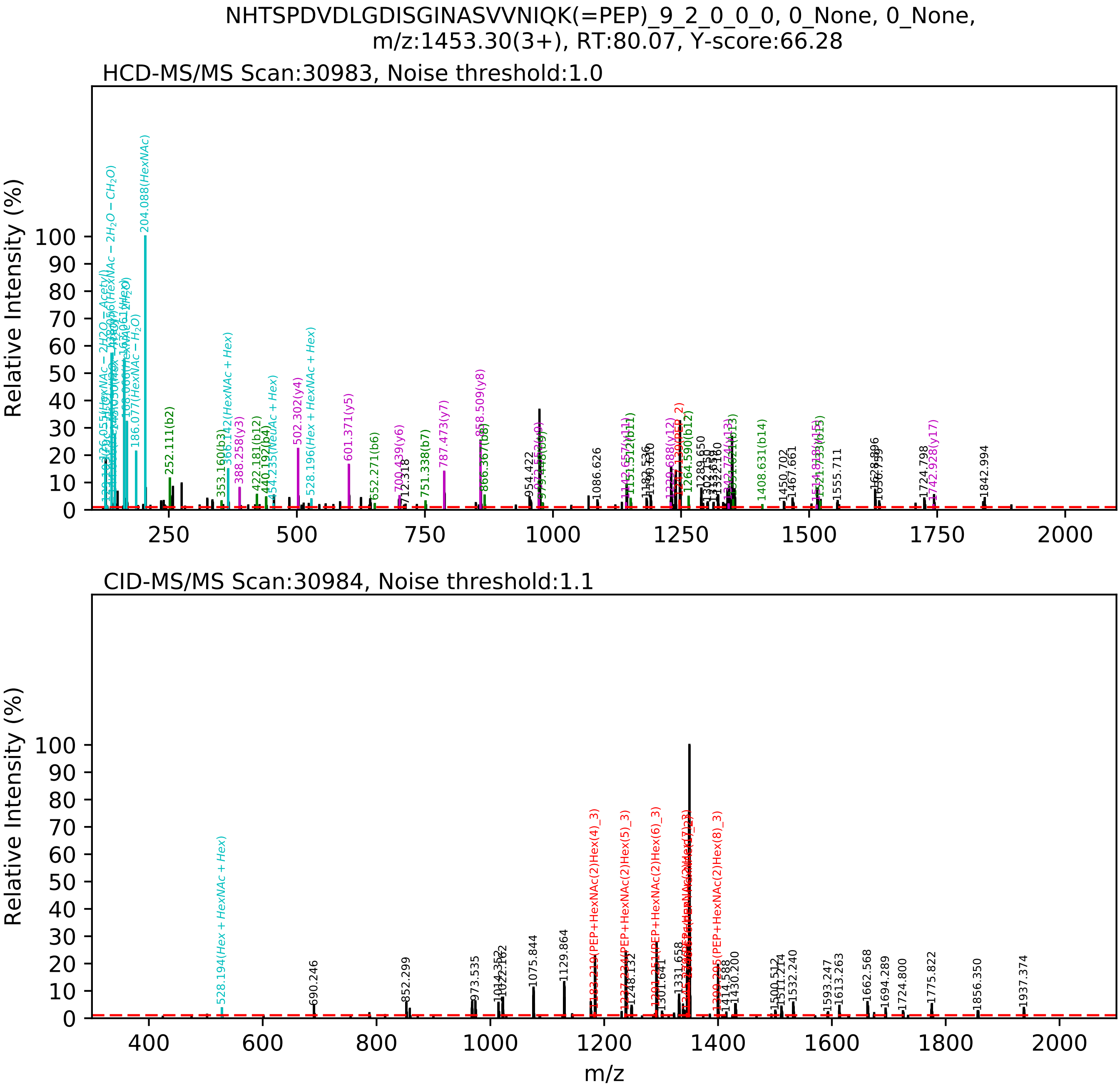
NHTSPDVDLGDISGINASVVNIQK(=PEP)_9_2_0_0_0, 0_None, 0_None,
m/z:1453.30(3+), RT:80.07, Y-score:66.28
HCD-MS/MS Scan:30983, Noise threshold:1.0
Relative Intensity (%)
204.088(HexNAc)
186.077(HexNAc − H2O)
252.111(b2)
353.160(b3)
366.142(HexNAc + Hex)
388.258(y3)
422.181(b12)
440.192(b4)
502.302(y4)
528.196(Hex + HexNAc + Hex)
601.371(y5)
652.271(b6)
700.439(y6)
712.318
751.338(b7)
787.473(y7)
858.509(y8)
866.367(b8)
954.422
1086.626
1182.536
1190.610
1264.590(b12)
1289.650
1408.631(b14)
1450.702
1467.661
1555.711
1628.896
1636.799
1724.798
1742.928(y17)
1842.994
250
500
750
1000
1250
1500
1750
2000
CID-MS/MS Scan:30984, Noise threshold:1.1
Relative Intensity (%)
528.194(Hex + HexNAc + Hex)
690.246
852.299
973.535
1014.352
1022.162
1075.844
1129.864
1182.219(PEP+HexNAc(2)Hex(4)_3)
1237.234(PEP+HexNAc(2)Hex(5)_3)
1248.132
1291.251(PEP+HexNAc(2)Hex(6)_3)
1301.641
1331.658
1399.305(PEP+HexNAc(2)Hex(8)_3)
1430.200
1500.512
1511.124
1532.240
1593.247
1613.263
1662.568
1694.289
1724.800
1775.822
1856.350
1937.374
400
600
800
1000
1200
1400
1600
1800
2000
m/z

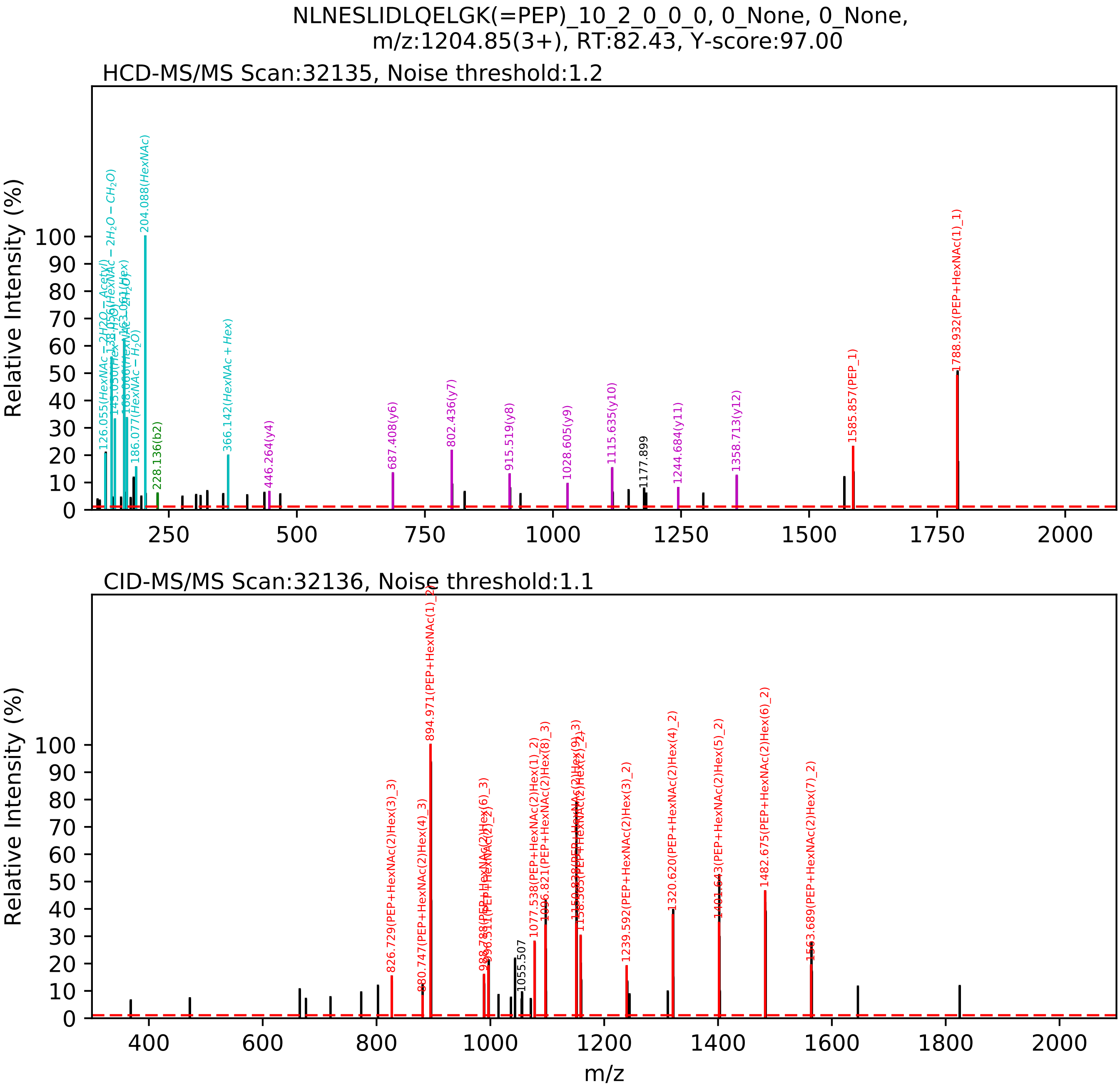

NLNESLIDLQELGK(=PEP)_10_2_0_0_0, 0_None, 0_None,
m/z:1204.85(3+), RT:82.43, Y-score:97.00
HCD-MS/MS Scan:32135, Noise threshold:1.2
Relative Intensity (%)
204.088(HexNAc)
186.077(HexNAc − H2O)
228.136(b2)
366.142(HexNAc + Hex)
446.264(y4)
687.408(y6)
802.436(y7)
915.519(y8)
1028.605(y9)
1115.635(y10)
1177.899
1244.684(y11)
1358.713(y12)
1585.857(PEP_1)
1788.932(PEP+HexNAc(1)_1)
CID-MS/MS Scan:32136, Noise threshold:1.1
826.729(PEP+HexNAc(2)Hex(3)_3)
880.747(PEP+HexNAc(2)Hex(4)_3)
894.971(PEP+HexNAc(1)_2)
1055.507
1077.538(PEP+HexNAc(2)Hex(1)_2)
1096.821(PEP+HexNAc(2)Hex(8)_3)
1239.592(PEP+HexNAc(2)Hex(3)_2)
1320.620(PEP+HexNAc(2)Hex(4)_2)
1401.643(PEP+HexNAc(2)Hex(5)_2)
1482.675(PEP+HexNAc(2)Hex(6)_2)
1563.689(PEP+HexNAc(2)Hex(7)_2)
m/z

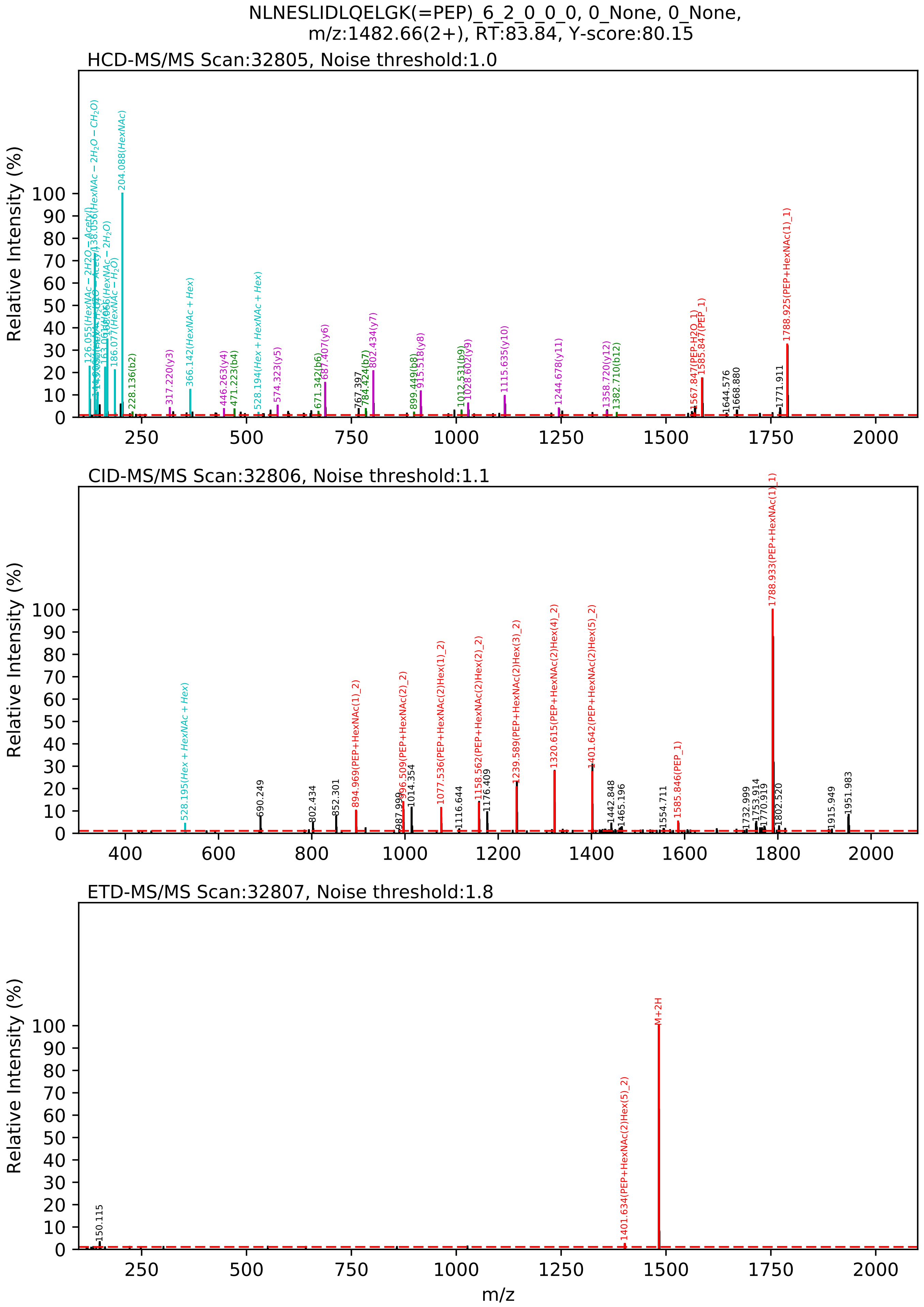

NLNESLIDLQELGK(=PEP)_6_2_0_0_0, 0_None, 0_None,
m/z:1482.66(2+), RT:83.84, Y-score:80.15
HCD-MS/MS Scan:32805, Noise threshold:1.0
Relative Intensity (%)
204.088(HexNAc)
186.077(HexNAc − H2O)
228.136(b2)
317.220(y3)
366.142(HexNAc + Hex)
446.263(y4)
471.223(b4)
528.194(Hex + HexNAc + Hex)
574.323(y5)
671.342(b6)
687.407(y6)
767.397
784.424(b7)
802.434(y7)
899.449(b8)
915.518(y8)
1012.531(b9)
1028.602(y9)
1115.635(y10)
1244.678(y11)
1358.720(y12)
1382.710(b12)
1567.847(PEP-H2O_1)
1585.847(PEP_1)
1644.576
1668.880
1771.911
1788.925(PEP+HexNAc(1)_1)
CID-MS/MS Scan:32806, Noise threshold:1.1
Relative Intensity (%)
528.195(Hex + HexNAc + Hex)
690.249
802.434
852.301
894.969(PEP+HexNAc(1)_2)
987.999
996.509(PEP+HexNAc(2)_2)
1014.354
1077.536(PEP+HexNAc(2)Hex(1)_2)
1116.644
1158.562(PEP+HexNAc(2)Hex(2)_2)
1176.409
1239.589(PEP+HexNAc(2)Hex(3)_2)
1320.615(PEP+HexNAc(2)Hex(4)_2)
1401.642(PEP+HexNAc(2)Hex(5)_2)
1442.848
1465.196
1554.711
1585.846(PEP_1)
1732.999
1753.914
1770.919
1788.933(PEP+HexNAc(1)_1)
1802.520
1915.949
1951.983
ETD-MS/MS Scan:32807, Noise threshold:1.8
Relative Intensity (%)
150.115
1401.634(PEP+HexNAc(2)Hex(5)_2)
M+2H
m/z

NLNESLIDLQELGK(=PEP)_6_2_0_0_0, 0_None, 0_None,
m/z:1482.66(2+), RT:84.01, Y-score:77.94

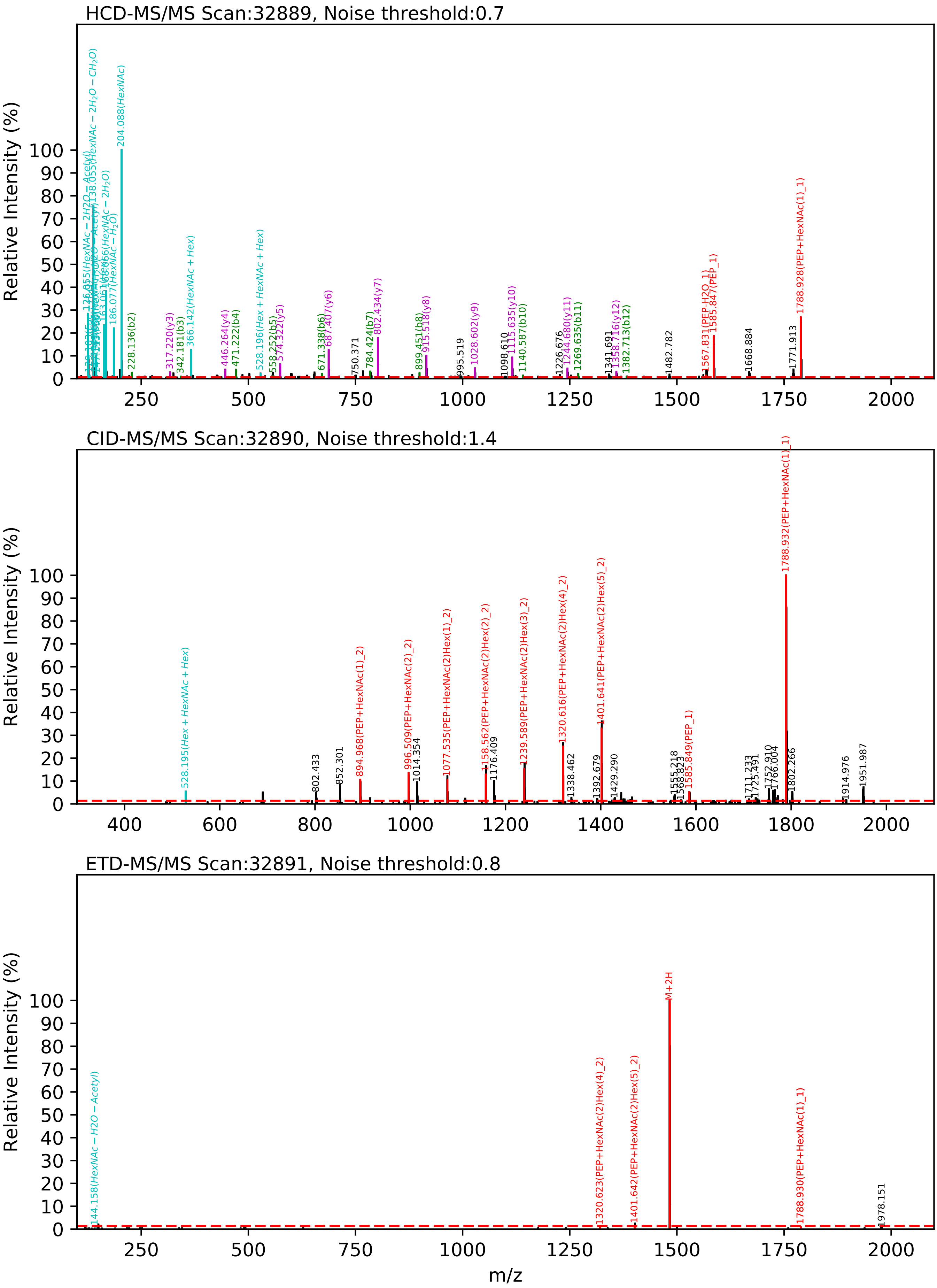

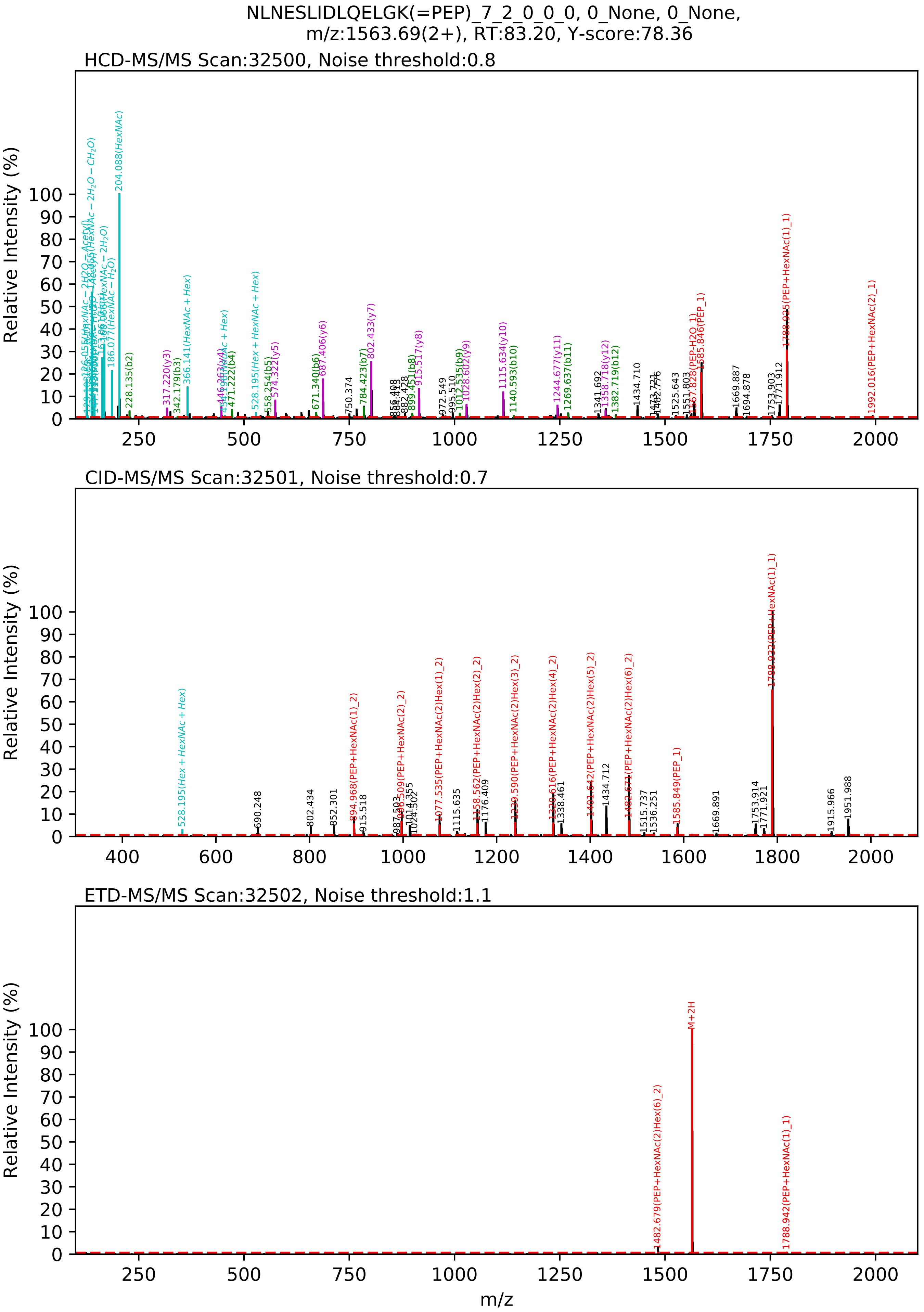
NLNESLIDLQELGK(=PEP)_7_2_0_0_0, 0_None, 0_None,
m/z:1563.69(2+), RT:83.20, Y-score:78.36
HCD-MS/MS Scan:32500, Noise threshold:0.8
CID-MS/MS Scan:32501, Noise threshold:0.7
ETD-MS/MS Scan:32502, Noise threshold:1.1
Relative Intensity (%)
m/z
204.088(HexNAc)
1788.925(PEP+HexNAc(1)_1)
1992.016(PEP+HexNAc(2)_1)
1788.922(PEP+HexNAc(1)_1)
1585.849(PEP_1)
M+2H
1482.679(PEP+HexNAc(2)Hex(6)_2)
1788.942(PEP+HexNAc(1)_1)

NLNESLIDLQELGK(=PEP)_7_2_0_0_0, 0_None, 0_None,
m/z:1563.69(2+), RT:83.81, Y-score:79.56

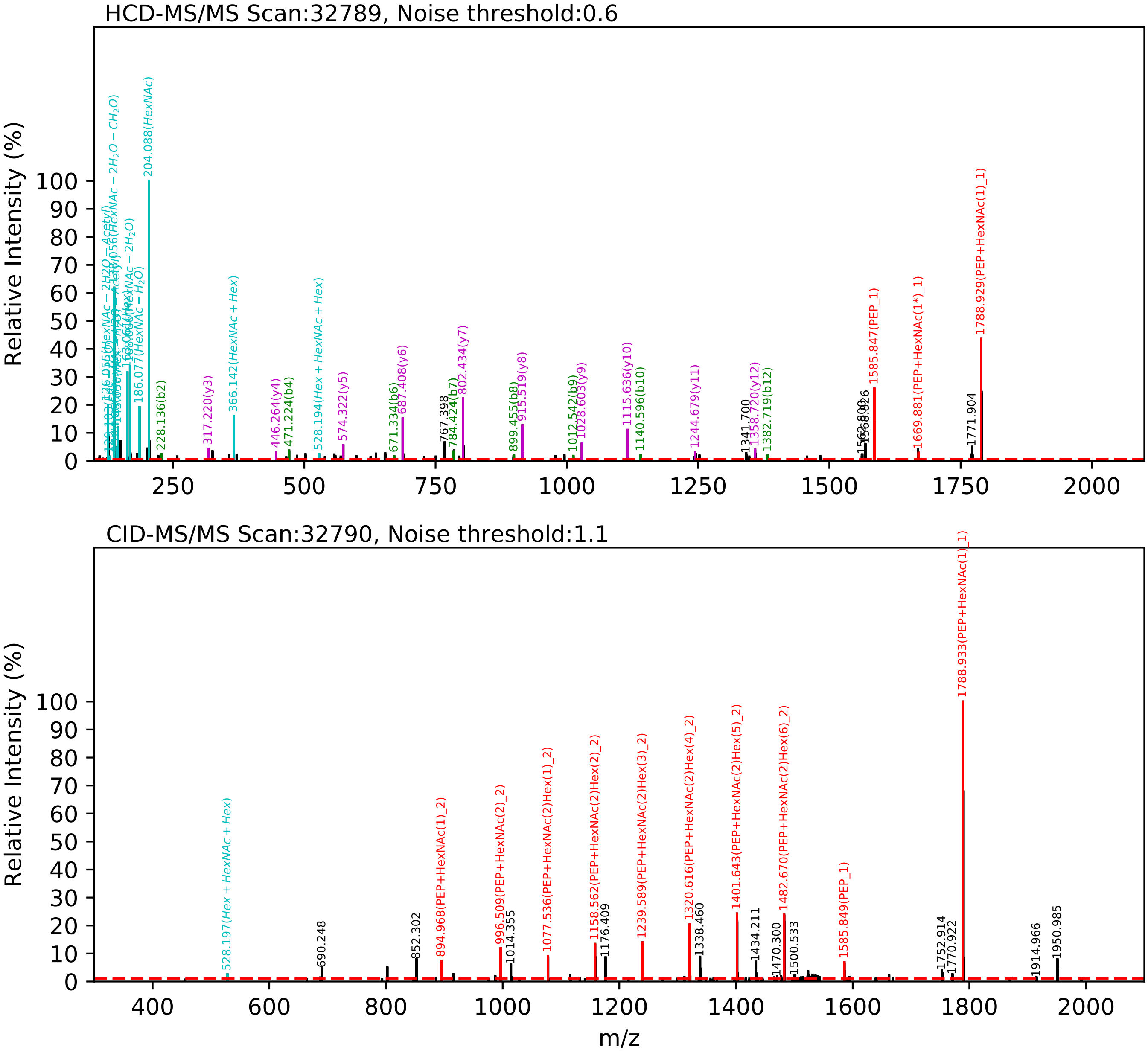

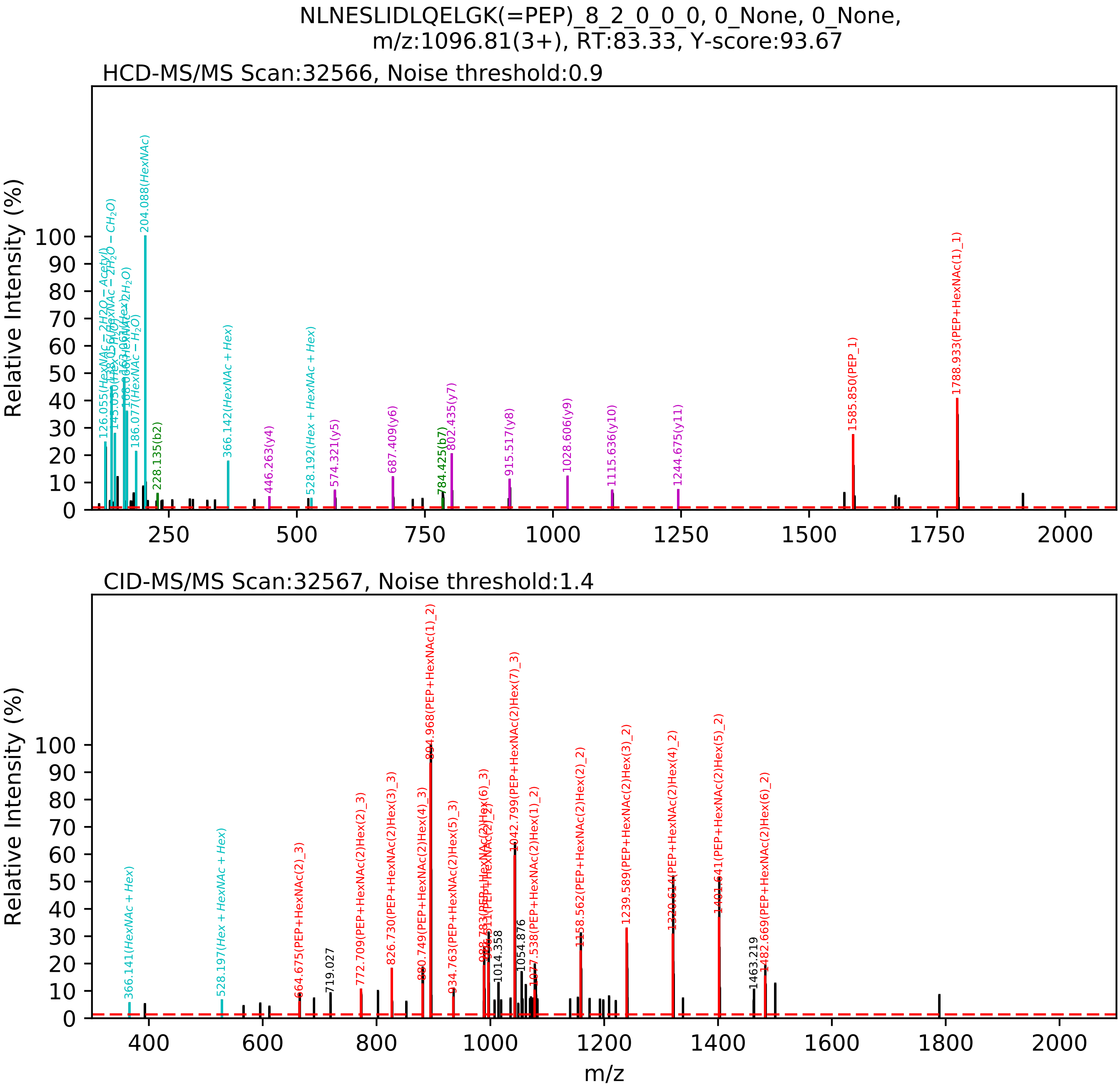

NLNESLIDLQELGK(=PEP)_8_2_0_0_0, 0_None, 0_None,
m/z:1096.81(3+), RT:83.33, Y-score:93.67
HCD-MS/MS Scan:32566, Noise threshold:0.9
Relative Intensity (%)
204.088(HexNAc)
228.135(b2)
366.142(HexNAc+Hex)
446.263(y4)
528.192(Hex+HexNAc+Hex)
574.321(y5)
687.409(y6)
784.425(b7)
802.435(y7)
915.517(y8)
1028.606(y9)
1115.636(y10)
1244.675(y11)
1585.850(PEP_1)
1788.933(PEP+HexNAc(1)_1)
CID-MS/MS Scan:32567, Noise threshold:1.4
366.141(HexNAc+Hex)
528.197(Hex+HexNAc+Hex)
664.675(PEP+HexNAc(2)_3)
719.027
772.709(PEP+HexNAc(2)Hex(2)_3)
826.730(PEP+HexNAc(2)Hex(3)_3)
880.749(PEP+HexNAc(2)Hex(4)_3)
894.968(PEP+HexNAc(1)_2)
934.763(PEP+HexNAc(2)Hex(5)_3)
1014.358
1042.799(PEP+HexNAc(2)Hex(7)_3)
1054.876
1077.538(PEP+HexNAc(2)Hex(1)_2)
1158.562(PEP+HexNAc(2)Hex(2)_2)
1239.589(PEP+HexNAc(2)Hex(3)_2)
1320.614(PEP+HexNAc(2)Hex(4)_2)
1401.641(PEP+HexNAc(2)Hex(5)_2)
1463.219
1482.669(PEP+HexNAc(2)Hex(6)_2)
m/z

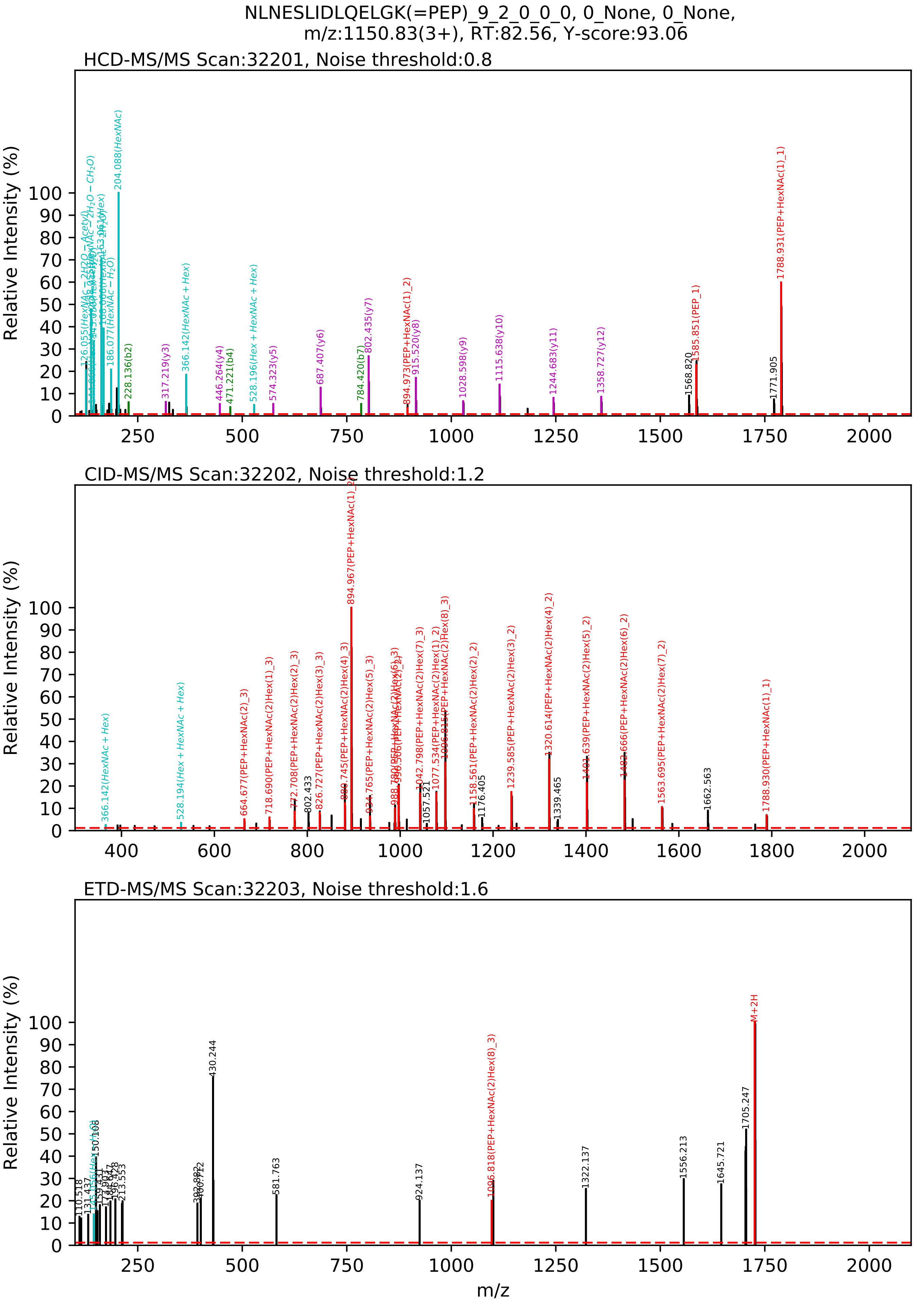

NLNESLIDLQELGK(=PEP)_9_2_0_0_0, 0_None, 0_None,
m/z:1150.83(3+), RT:82.56, Y-score:93.06
HCD-MS/MS Scan:32201, Noise threshold:0.8
Relative Intensity (%)
204.088(HexNAc)
186.077(HexNAc − H2O)
228.136(b2)
317.219(y3)
366.142(HexNAc + Hex)
446.264(y4)
471.221(b4)
528.196(Hex + HexNAc + Hex)
574.323(y5)
687.407(y6)
784.420(b7)
802.435(y7)
894.973(PEP+HexNAc(1)_2)
915.520(y8)
1028.598(y9)
1115.638(y10)
1244.683(y11)
1358.727(y12)
1568.820
1585.851(PEP_1)
1771.905
1788.931(PEP+HexNAc(1)_1)
CID-MS/MS Scan:32202, Noise threshold:1.2
366.142(HexNAc + Hex)
528.194(Hex + HexNAc + Hex)
664.677(PEP+HexNAc(2)_3)
718.690(PEP+HexNAc(2)Hex(1)_3)
772.708(PEP+HexNAc(2)Hex(2)_3)
802.433
826.727(PEP+HexNAc(2)Hex(3)_3)
880.745(PEP+HexNAc(2)Hex(4)_3)
894.967(PEP+HexNAc(1)_2)
934.765(PEP+HexNAc(2)Hex(5)_3)
1042.798(PEP+HexNAc(2)Hex(7)_3)
1057.521
1077.534(PEP+HexNAc(2)Hex(1)_2)
1158.561(PEP+HexNAc(2)Hex(2)_2)
1176.405
1239.585(PEP+HexNAc(2)Hex(3)_2)
1320.614(PEP+HexNAc(2)Hex(4)_2)
1339.465
1401.639(PEP+HexNAc(2)Hex(5)_2)
1482.666(PEP+HexNAc(2)Hex(6)_2)
1563.695(PEP+HexNAc(2)Hex(7)_2)
1662.563
1788.930(PEP+HexNAc(1)_1)
ETD-MS/MS Scan:32203, Noise threshold:1.6
110.518
131.437
150.108
213.553
430.244
581.763
924.137
1096.818(PEP+HexNAc(2)Hex(8)_3)
1322.137
1556.213
1645.721
1705.247
M+2H
m/z

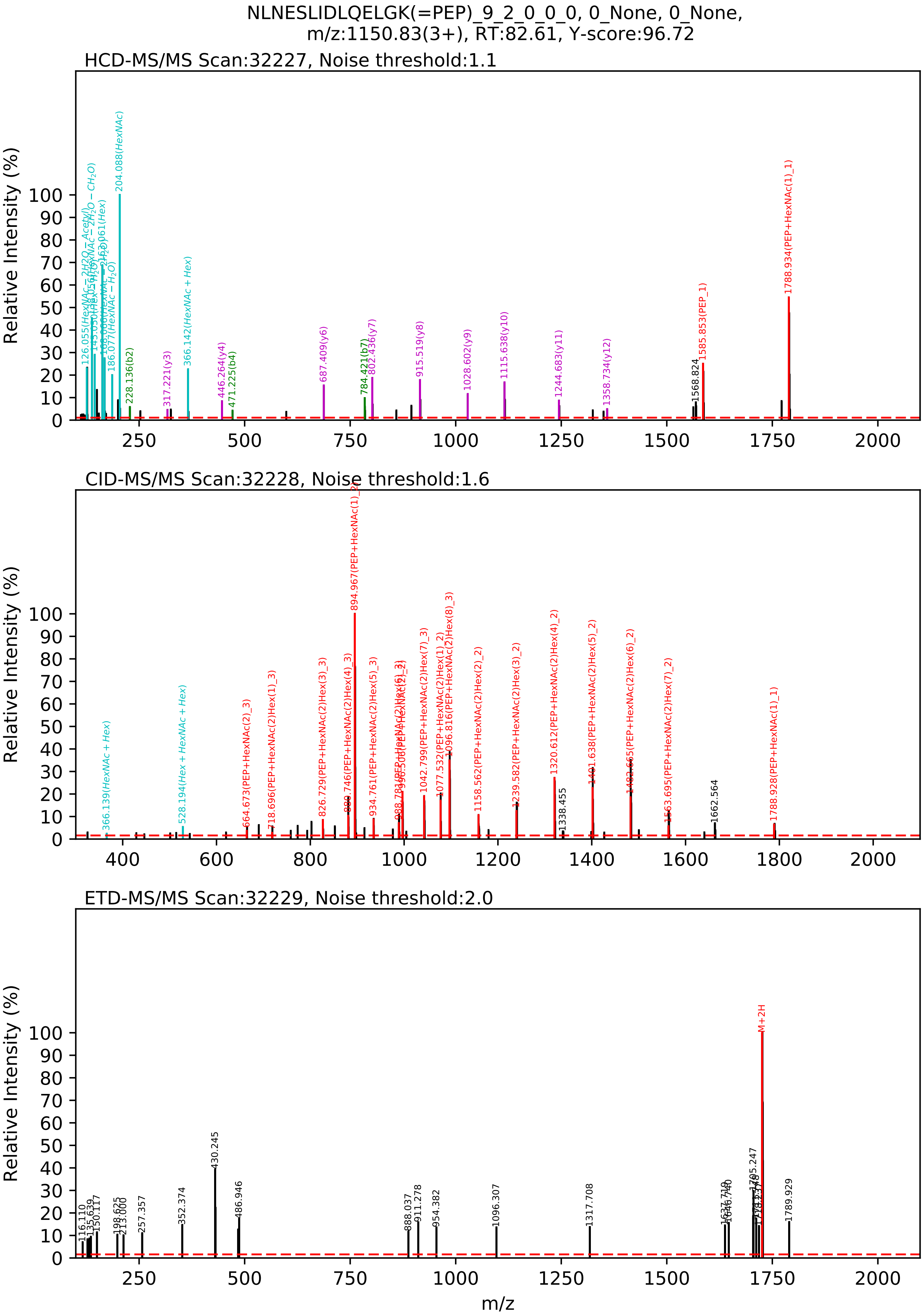
NLNESLIDLQELGK(=PEP)_9_2_0_0_0, 0_None, 0_None,
m/z:1150.83(3+), RT:82.61, Y-score:96.72
HCD-MS/MS Scan:32227, Noise threshold:1.1
204.088(HexNAc)
366.142(HexNAc+Hex)
228.136(b2)
317.221(y3)
446.264(y4)
471.225(b4)
687.409(y6)
784.421(b7)
802.436(y7)
915.519(y8)
1028.602(y9)
1115.638(y10)
1244.683(y11)
1358.734(y12)
1568.824
1585.853(PEP_1)
1788.934(PEP+HexNAc(1)_1)
CID-MS/MS Scan:32228, Noise threshold:1.6
366.139(HexNAc+Hex)
528.194(Hex+HexNAc+Hex)
664.673(PEP+HexNAc(2)_3)
718.696(PEP+HexNAc(2)Hex(1)_3)
826.729(PEP+HexNAc(2)Hex(3)_3)
880.746(PEP+HexNAc(2)Hex(4)_3)
894.967(PEP+HexNAc(1)_2)
934.761(PEP+HexNAc(2)Hex(5)_3)
1042.799(PEP+HexNAc(2)Hex(7)_3)
1077.532(PEP+HexNAc(2)Hex(1)_2)
1096.816(PEP+HexNAc(2)Hex(8)_3)
1158.562(PEP+HexNAc(2)Hex(2)_2)
1239.582(PEP+HexNAc(2)Hex(3)_2)
1320.612(PEP+HexNAc(2)Hex(4)_2)
1338.455
1401.638(PEP+HexNAc(2)Hex(5)_2)
1482.665(PEP+HexNAc(2)Hex(6)_2)
1563.695(PEP+HexNAc(2)Hex(7)_2)
1662.564
1788.928(PEP+HexNAc(1)_1)
ETD-MS/MS Scan:32229, Noise threshold:2.0
M+2H
116.110
150.117
198.625
213.000
257.357
352.374
430.245
486.946
888.037
911.278
954.382
1096.307
1317.708
1646.740
1705.247
1789.929
Relative Intensity (%)
m/z

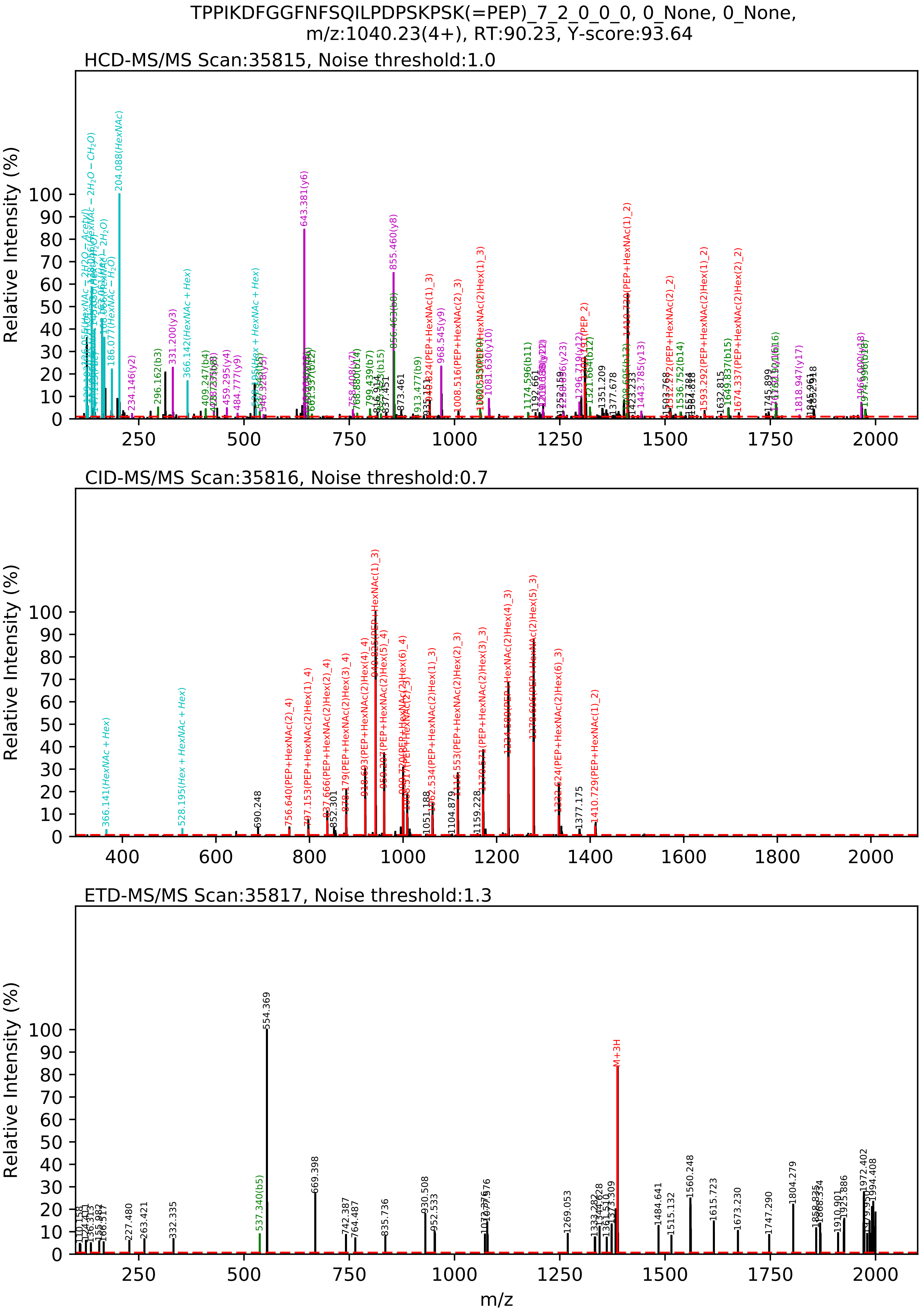
TPPIKDFGGFNFSQILPDPSKPSK(=PEP)_7_2_0_0_0, 0_None, 0_None,
m/z:1040.23(4+), RT:90.23, Y-score:93.64
HCD-MS/MS Scan:35815, Noise threshold:1.0
Relative Intensity (%)
204.088(HexNAc)
643.381(y6)
855.460(y8)
968.545(y9)
1081.630(y10)
1410.730(PEP+HexNAc(1)_2)
1593.292(PEP+HexNAc(2)Hex(1)_2)
1674.337(PEP+HexNAc(2)Hex(2)_2)
1008.516(PEP+HexNAc(2)_3)
366.142(HexNAc+Hex)
331.200(y3)
296.162(b3)
234.146(y2)
186.077(HexNAc−H2O)
409.247(b4)
459.295(y4)
484.777(y9)
873.461
913.477(b9)
1174.596(b11)
1192.661
1252.159
1321.664(b12)
1351.209
1377.678
1443.785(y13)
1503.768
1536.752(b14)
1632.815
1649.837(b15)
1745.899
1818.947(y17)
1845.961
1852.918
CID-MS/MS Scan:35816, Noise threshold:0.7
366.141(HexNAc+Hex)
528.195(Hex+HexNAc+Hex)
690.248
756.640(PEP+HexNAc(2)_4)
797.153(PEP+HexNAc(2)Hex(1)_4)
837.666(PEP+HexNAc(2)Hex(2)_4)
852.301
878.179(PEP+HexNAc(2)Hex(3)_4)
918.693(PEP+HexNAc(2)Hex(4)_4)
940.835(PEP+HexNAc(1)_3)
959.207(PEP+HexNAc(2)Hex(5)_4)
999.720(PEP+HexNAc(2)Hex(6)_4)
1051.188
1062.534(PEP+HexNAc(2)Hex(1)_3)
1104.879
1116.553(PEP+HexNAc(2)Hex(2)_3)
1159.228
1170.571(PEP+HexNAc(2)Hex(3)_3)
1224.590(PEP+HexNAc(2)Hex(4)_3)
1278.606(PEP+HexNAc(2)Hex(5)_3)
1332.624(PEP+HexNAc(2)Hex(6)_3)
1377.175
1410.729(PEP+HexNAc(1)_2)
ETD-MS/MS Scan:35817, Noise threshold:1.3
554.369
M+3H
537.340(b5)
669.398
110.158
227.480
263.421
332.335
742.387
764.487
835.736
930.508
952.533
1269.053
1333.282
1484.641
1515.132
1560.248
1615.723
1673.230
1747.290
1804.279
1910.901
1925.886
1972.402
1994.408
m/z

TPPIKDFGGFNFSQILPDPSKPSK(=PEP)_7_2_0_0_0, 0_None, 0_None,
m/z:1040.23(4+), RT:90.77, Y-score:79.30

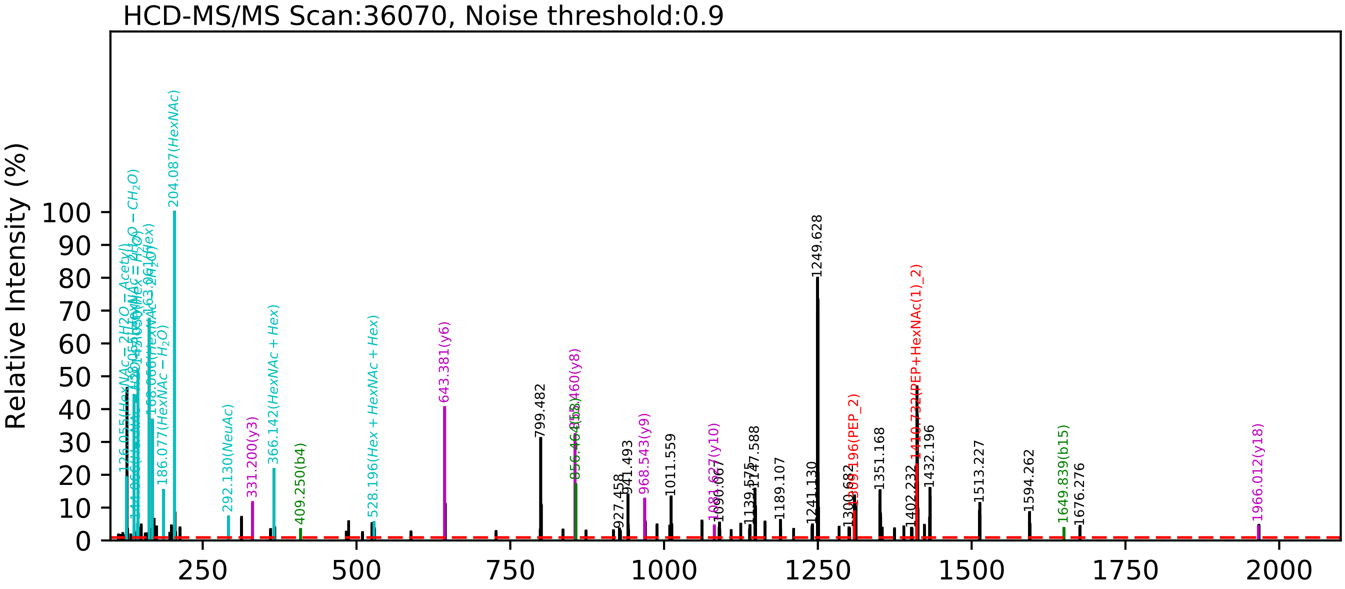

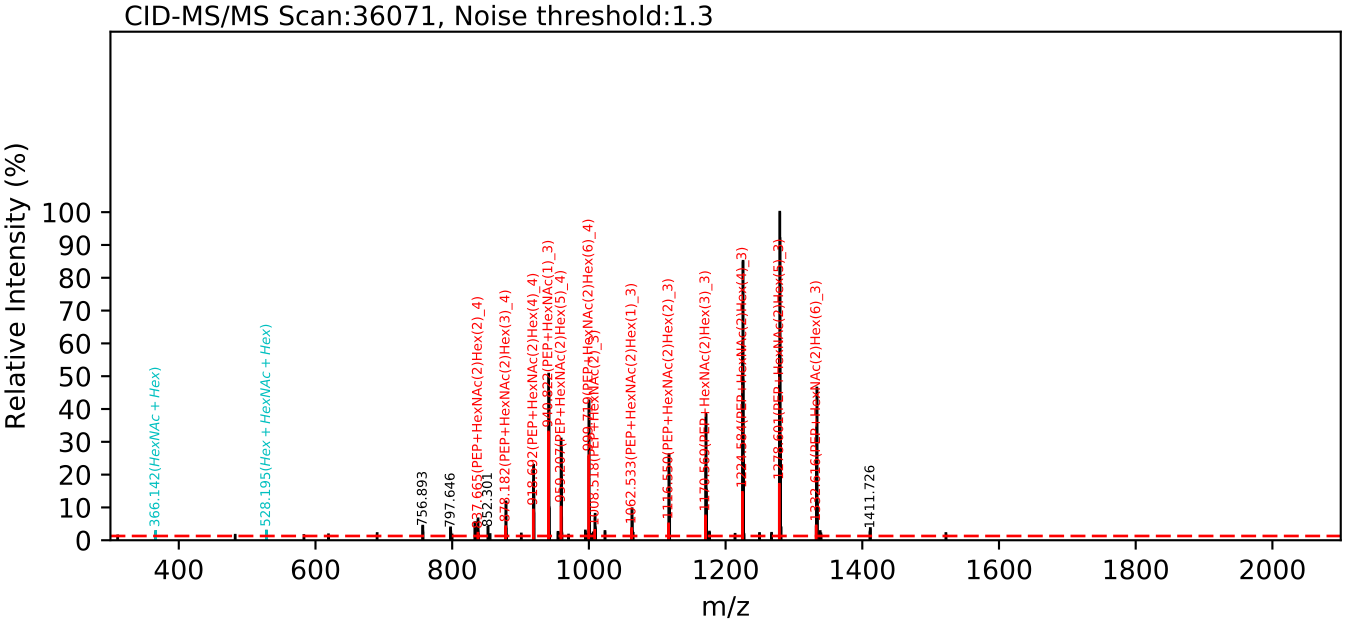

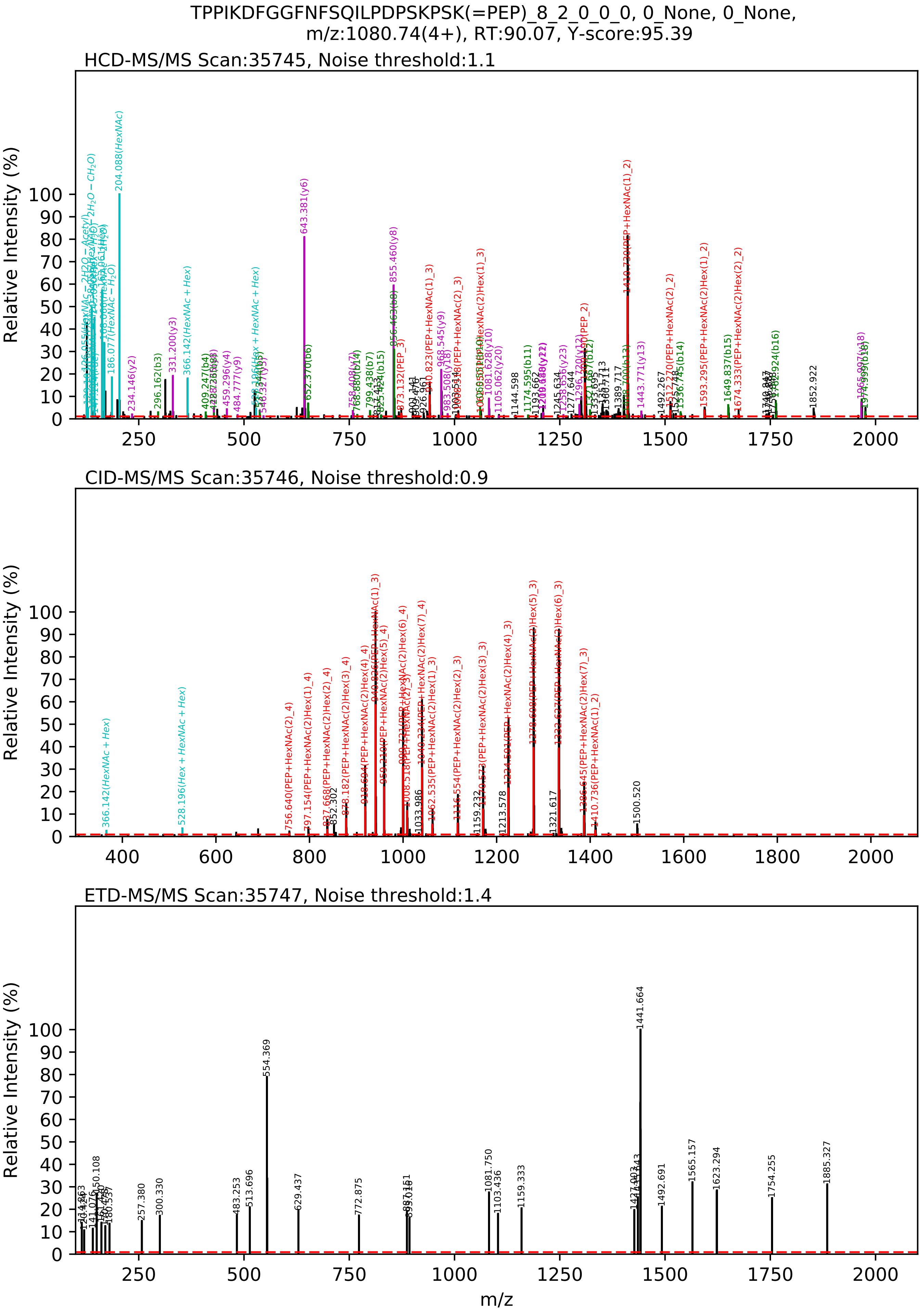
TPPIKDFGGFNFSQILPDPSKPSK(=PEP)_8_2_0_0_0, 0_None, 0_None,
m/z:1080.74(4+), RT:90.07, Y-score:95.39
HCD-MS/MS Scan:35745, Noise threshold:1.1
CID-MS/MS Scan:35746, Noise threshold:0.9
ETD-MS/MS Scan:35747, Noise threshold:1.4
Relative Intensity (%)
m/z
204.088(HexNAc)
643.381(y6)
855.460(y8)
1410.720(PEP+HexNAc(1)_2)
1441.664
554.369

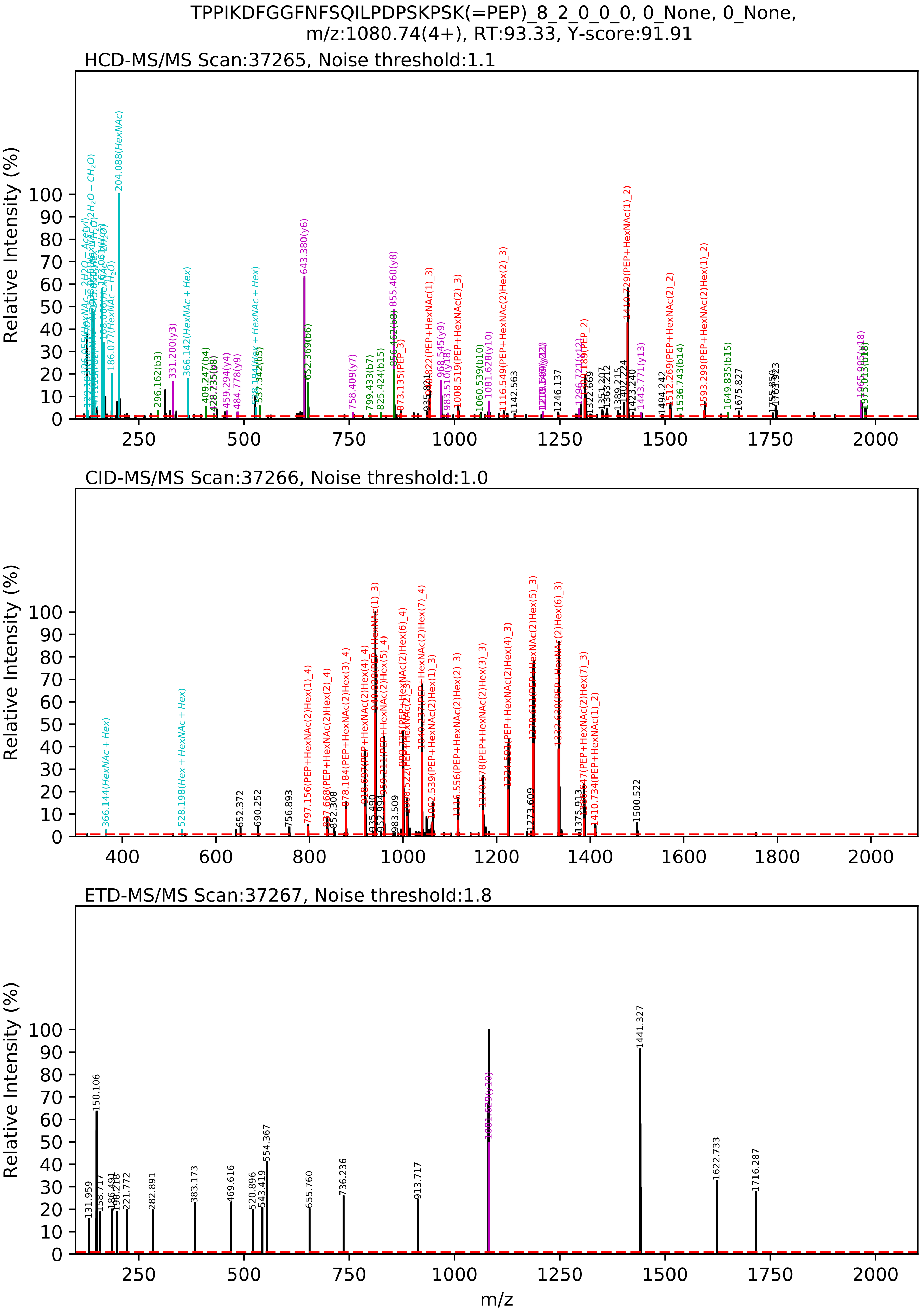
TPPIKDFGGFNFSQILPDPSKPSK(=PEP)_8_2_0_0_0, 0_None, 0_None,
m/z:1080.74(4+), RT:93.33, Y-score:91.91
HCD-MS/MS Scan:37265, Noise threshold:1.1
CID-MS/MS Scan:37266, Noise threshold:1.0
ETD-MS/MS Scan:37267, Noise threshold:1.8
Relative Intensity (%)
m/z

TPPIKDFGGFNFSQILPDPSKPSK(=PEP)_8_2_0_0_0, 0_None, 0_None,
m/z:864.79(5+), RT:90.53, Y-score:95.20

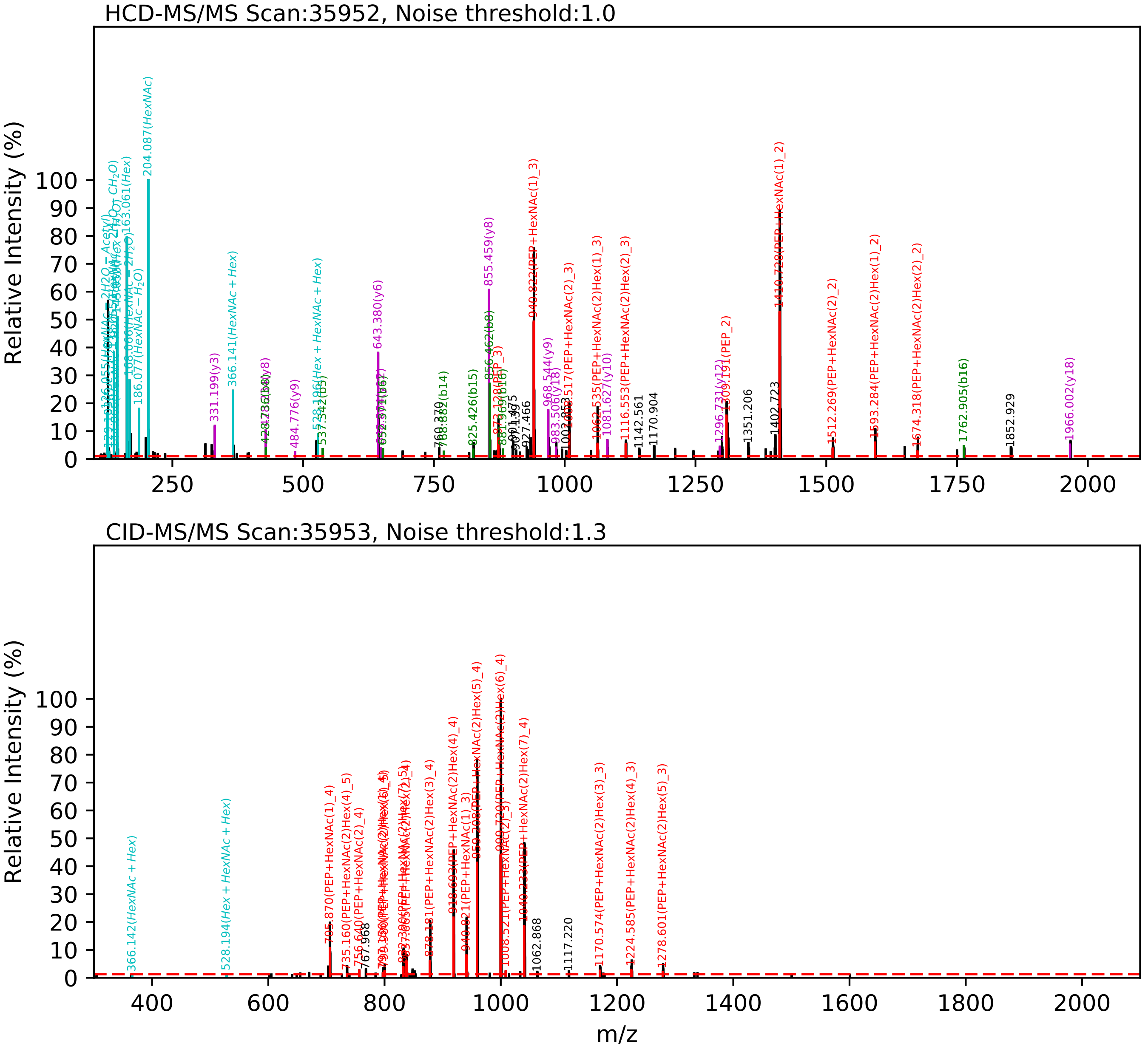

TPPIKDFGGFNFSQILPDPSKPSK(=PEP)_9_2_0_0_0, 0_None, 0_None,
m/z:1121.26(4+), RT:89.95, Y-score:94.27

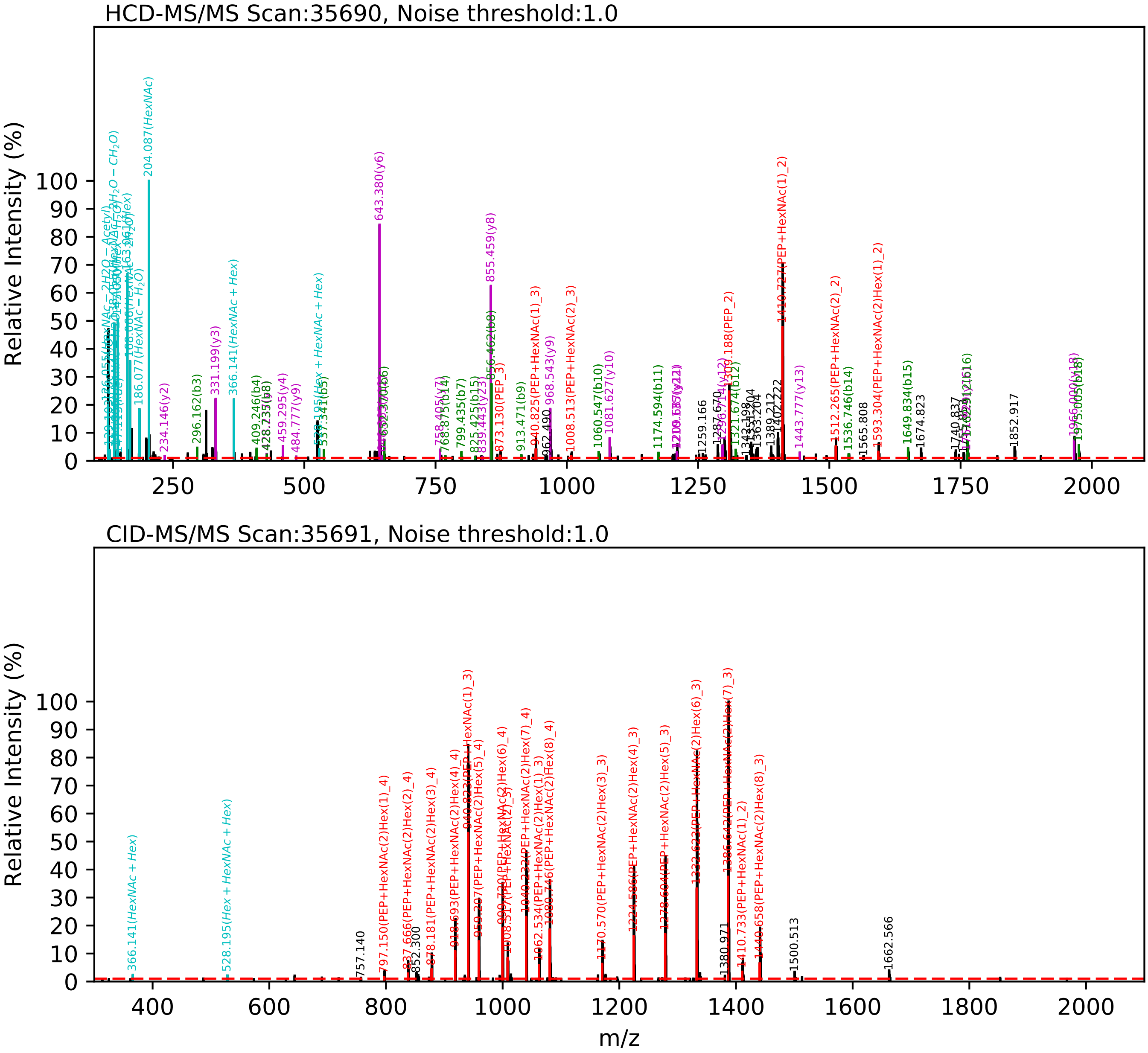

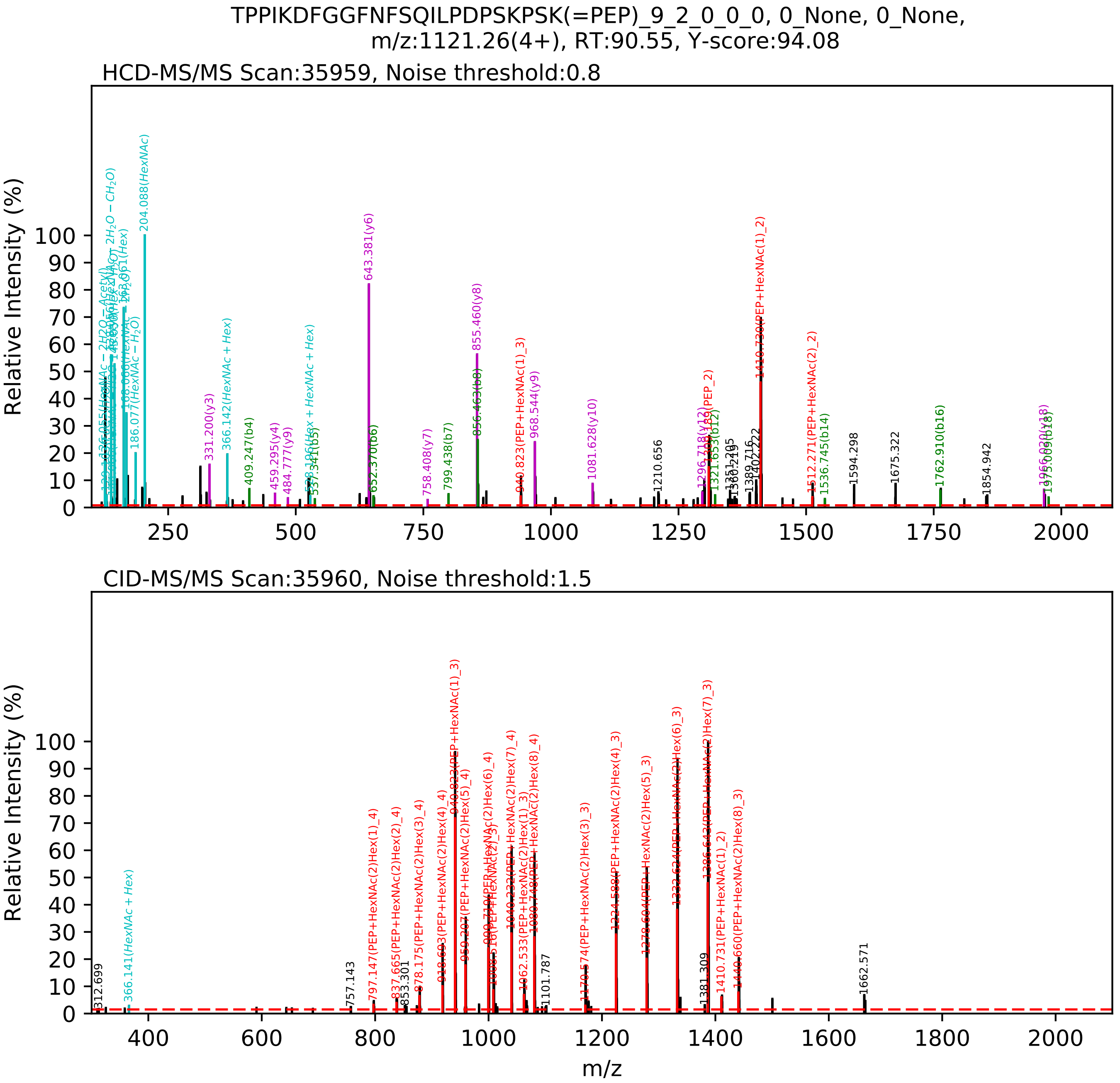
TPPIKDFGGFNFSQILPDPSKPSK(=PEP)_9_2_0_0_0, 0_None, 0_None,
m/z:1121.26(4+), RT:90.55, Y-score:94.08
HCD-MS/MS Scan:35959, Noise threshold:0.8
Relative Intensity (%)
204.088(HexNAc)
186.077(HexNAc − H2O)
168.066(HexNAc − 2H2O)
163.061(Hex)
138.055(HexNAc − 2H2O − CH2O)
126.055(HexNAc − 2H2O − Acetyl)
331.200(y3)
366.142(HexNAc + Hex)
409.247(b4)
459.295(y4)
484.777(y9)
528.196(Hex + HexNAc + Hex)
537.341(b5)
643.381(y6)
652.370(b6)
758.408(y7)
799.438(b7)
855.460(y8)
856.463(b8)
940.823(PEP+HexNAc(1)_3)
968.544(y9)
1081.628(y10)
1210.656
1296.718(y12)
1308.729(PEP_2)
1321.653(b12)
1351.205
1360.219
1389.716
1402.222
1410.730(PEP+HexNAc(1)_2)
1512.271(PEP+HexNAc(2)_2)
1536.745(b14)
1594.298
1675.322
1762.910(b16)
1854.942
1966.030(y18)
1975.009(b18)
CID-MS/MS Scan:35960, Noise threshold:1.5
Relative Intensity (%)
312.699
366.141(HexNAc + Hex)
757.143
797.147(PEP+HexNAc(2)Hex(1)_4)
837.665(PEP+HexNAc(2)Hex(2)_4)
853.301
878.175(PEP+HexNAc(2)Hex(3)_4)
918.693(PEP+HexNAc(2)Hex(4)_4)
940.823(PEP+HexNAc(1)_3)
959.207(PEP+HexNAc(2)Hex(5)_4)
999.719(PEP+HexNAc(2)Hex(6)_4)
1040.232(PEP+HexNAc(2)Hex(7)_4)
1062.533(PEP+HexNAc(2)Hex(1)_3)
1080.748(PEP+HexNAc(2)Hex(8)_4)
1101.787
1170.574(PEP+HexNAc(2)Hex(3)_3)
1224.589(PEP+HexNAc(2)Hex(4)_3)
1278.604(PEP+HexNAc(2)Hex(5)_3)
1332.624(PEP+HexNAc(2)Hex(6)_3)
1381.309
1386.643(PEP+HexNAc(2)Hex(7)_3)
1410.731(PEP+HexNAc(1)_2)
1440.660(PEP+HexNAc(2)Hex(8)_3)
1662.571
m/z

TPPIKDFGGFNFSQILPDPSKPSK(=PEP)_9_2_0_0_0, 0_None, 0_None,
m/z:1121.26(4+), RT:93.44, Y-score:91.64

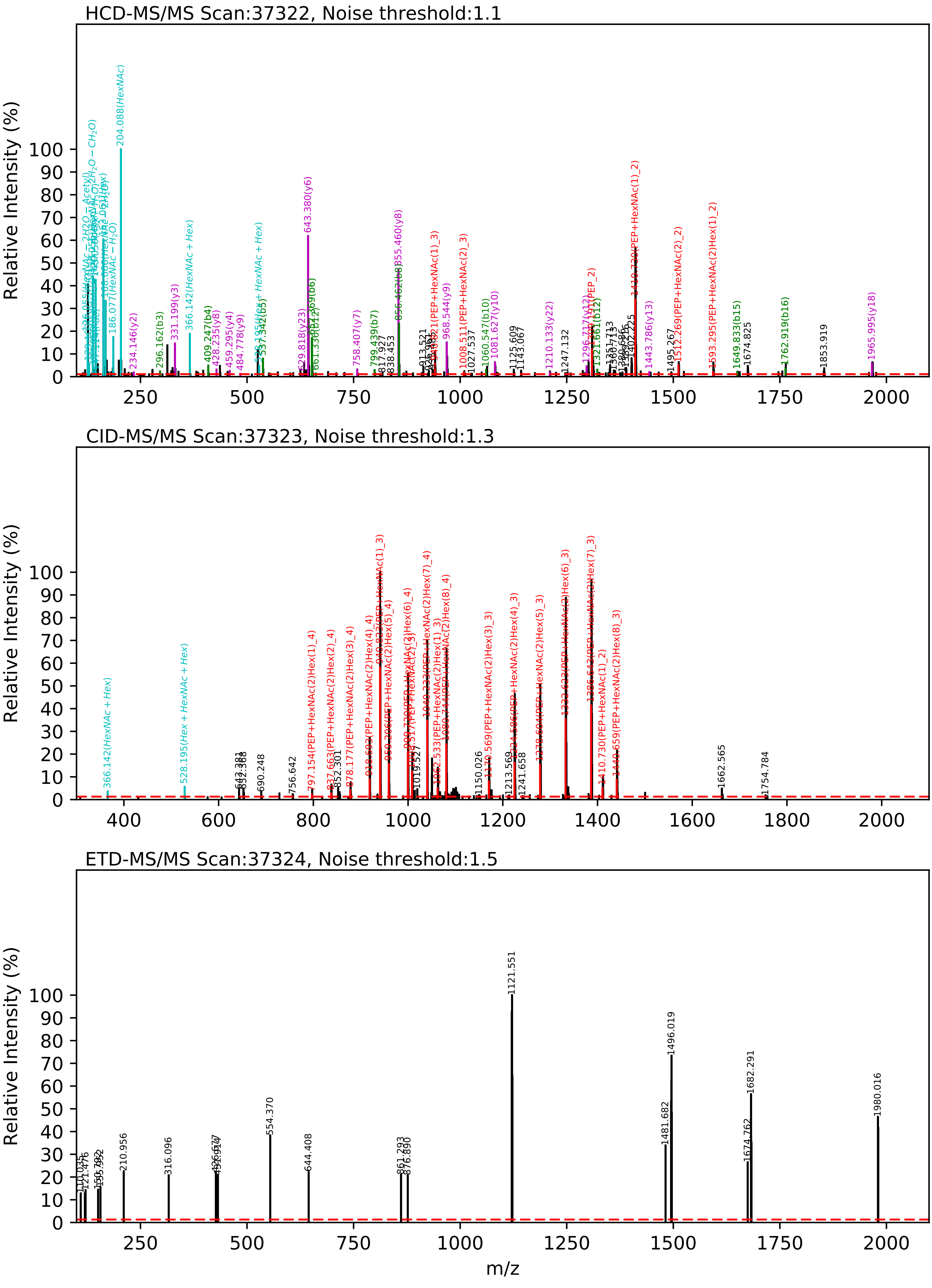

Supplement: Supplementary file 1 [file ijms-25-13649-s001.zip › Supplementary Figure S8(S2_T_N-glycopep_2).pdf]
