# Supplementary material for: Characterization of Site-Specific N- and O-Glycopeptides from Recombinant Spike and ACE2 Glycoproteins Using LC-MS/MS Analysis
Source: Int J Mol Sci. 2024 Dec 20;25(24):13649. doi: 10.3390/ijms252413649 (PMC11678118; doi:10.3390/ijms252413649)
Supplement: Supplementary file 1 [file ijms-25-13649-s001.zip › Supplementary Figure S9(S2_TG_N-glycopep_1).pdf]

EGVFVSNGTHWFVTQR(=PEP)_10_2_0_0_0, 0_None, 0_None,
m/z:1297.54(2+), RT:64.57, hcd-score:68.15

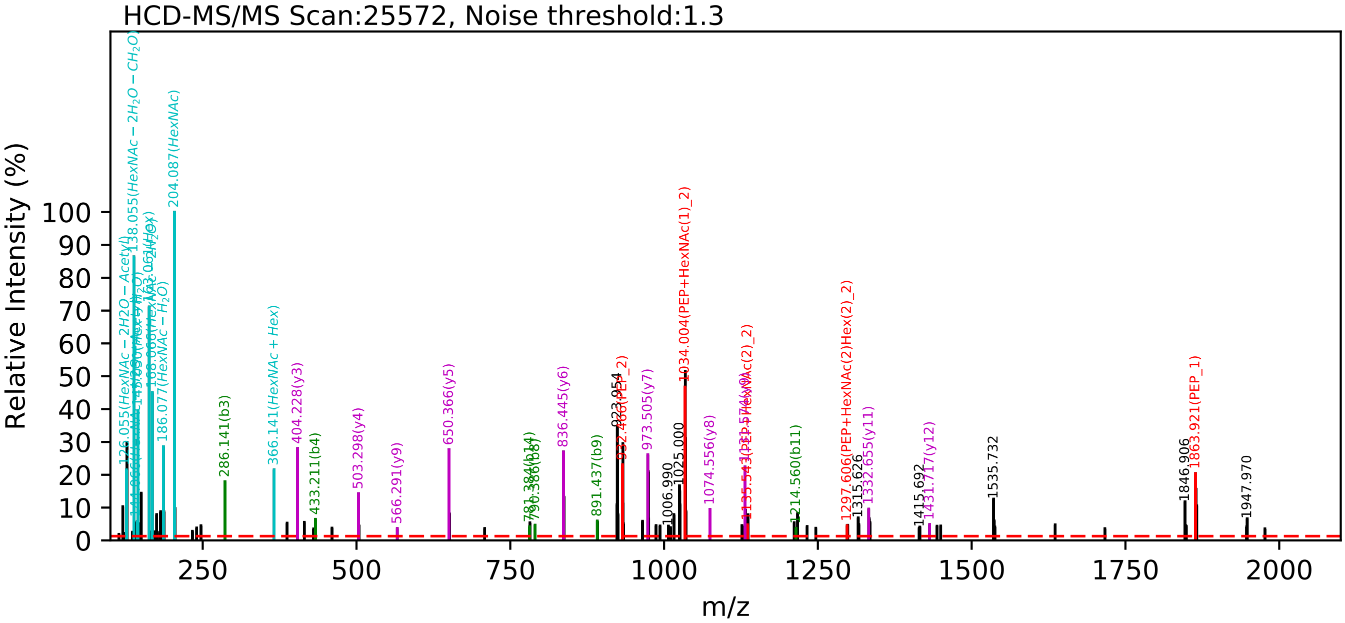

EGVFVSNGTHWFVTQR(=PEP)_10_2_0_0_0, 0_None, 0_None,
m/z:1297.54(2+), RT:64.57, hcd-score:68.15

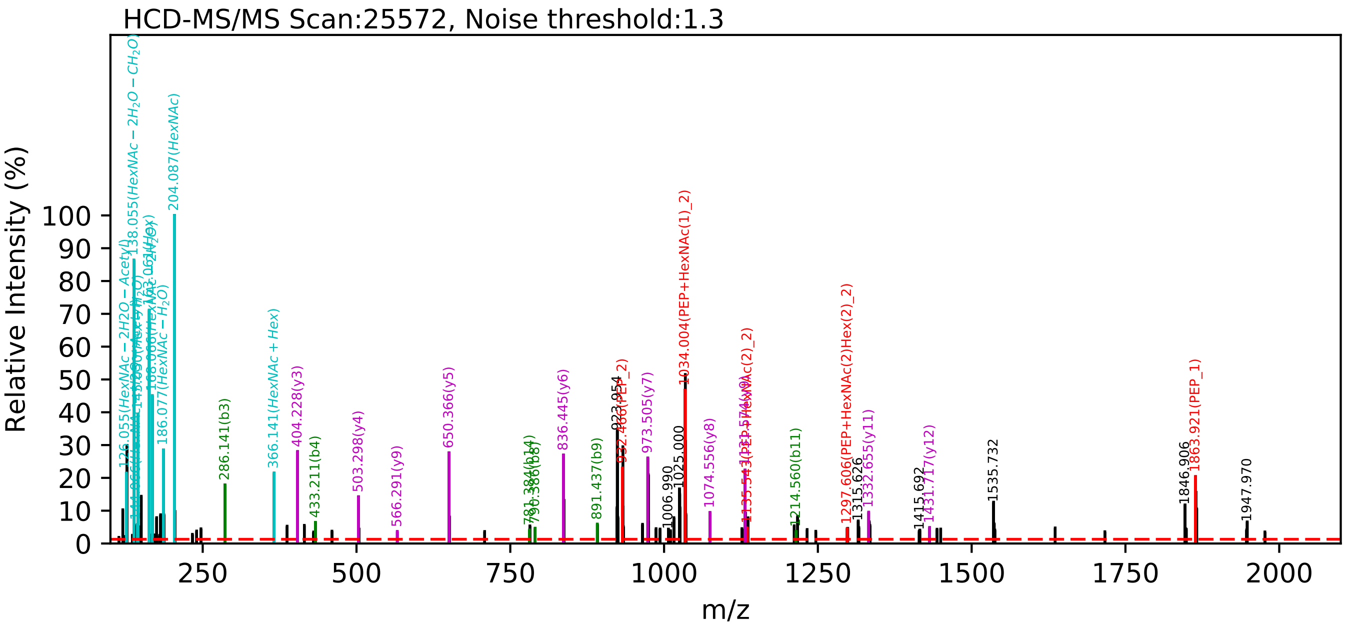

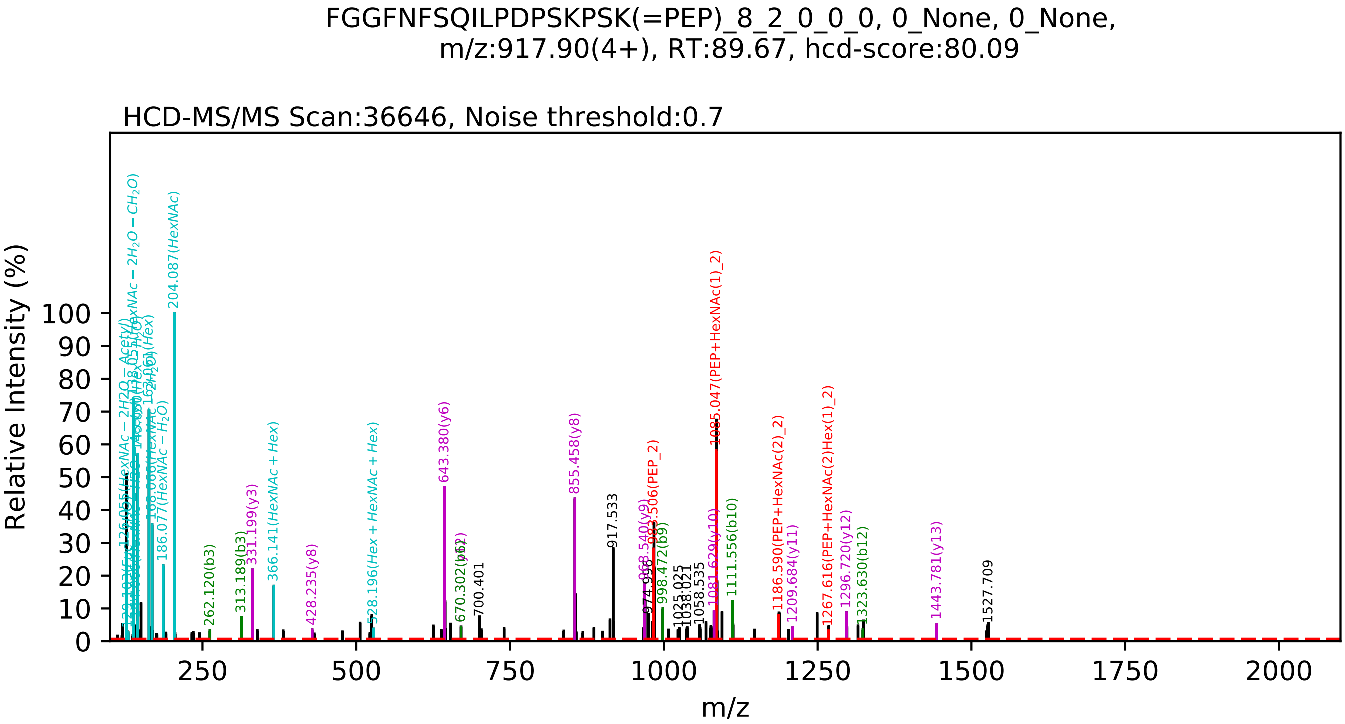

FGGFNFSQILPDPSKPSK(=PEP)_8_2_0_0_0, 0_None, 0_None,
m/z:917.90(4+), RT:89.67, hcd-score:80.09
HCD-MS/MS Scan:36646, Noise threshold:0.7
Relative Intensity (%)
m/z
204.087(HexNAc)
186.077(HexNAc − H2O)
366.141(HexNAc + Hex)
528.196(Hex + HexNAc + Hex)
262.120(b3)
313.189(b3)
331.199(y3)
428.235(y8)
643.380(y6)
670.302(b6)
700.401
855.458(y8)
917.533
998.472(b9)
1025.025
1038.021
1058.535
1085.047(PEP+HexNAc(1)_2)
1081.629(y10)
1111.556(b10)
1186.590(PEP+HexNAc(2)_2)
1209.684(y11)
1267.616(PEP+HexNAc(2)Hex(1)_2)
1296.720(y12)
1323.630(b12)
1443.781(y13)
1527.709

FGGFNFSQILPDPSKPSK(=PEP)_8_2_0_0_0, 0_None, 0_None,
m/z:917.90(4+), RT:89.67, hcd-score:80.09

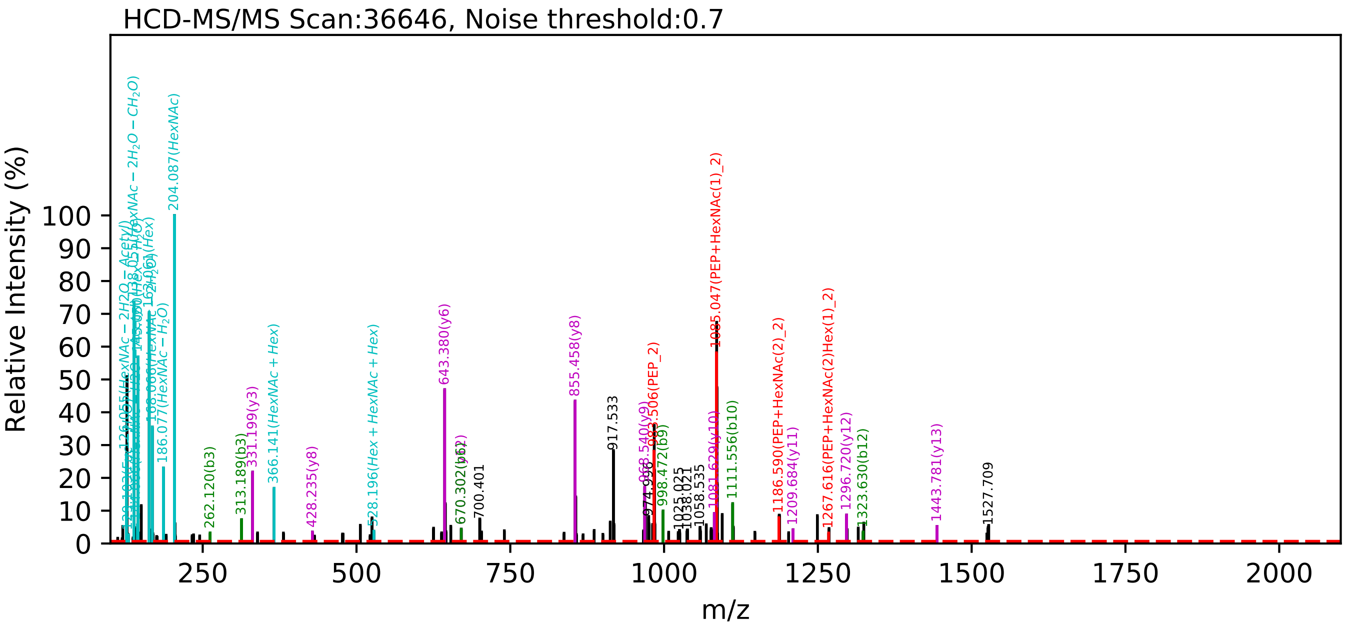

DFGGFNFSQILPD(=PEP)_8_2_0_0_0, 0_None, 0_None,
m/z:1053.76(3+), RT:97.43, Y-score:70.32

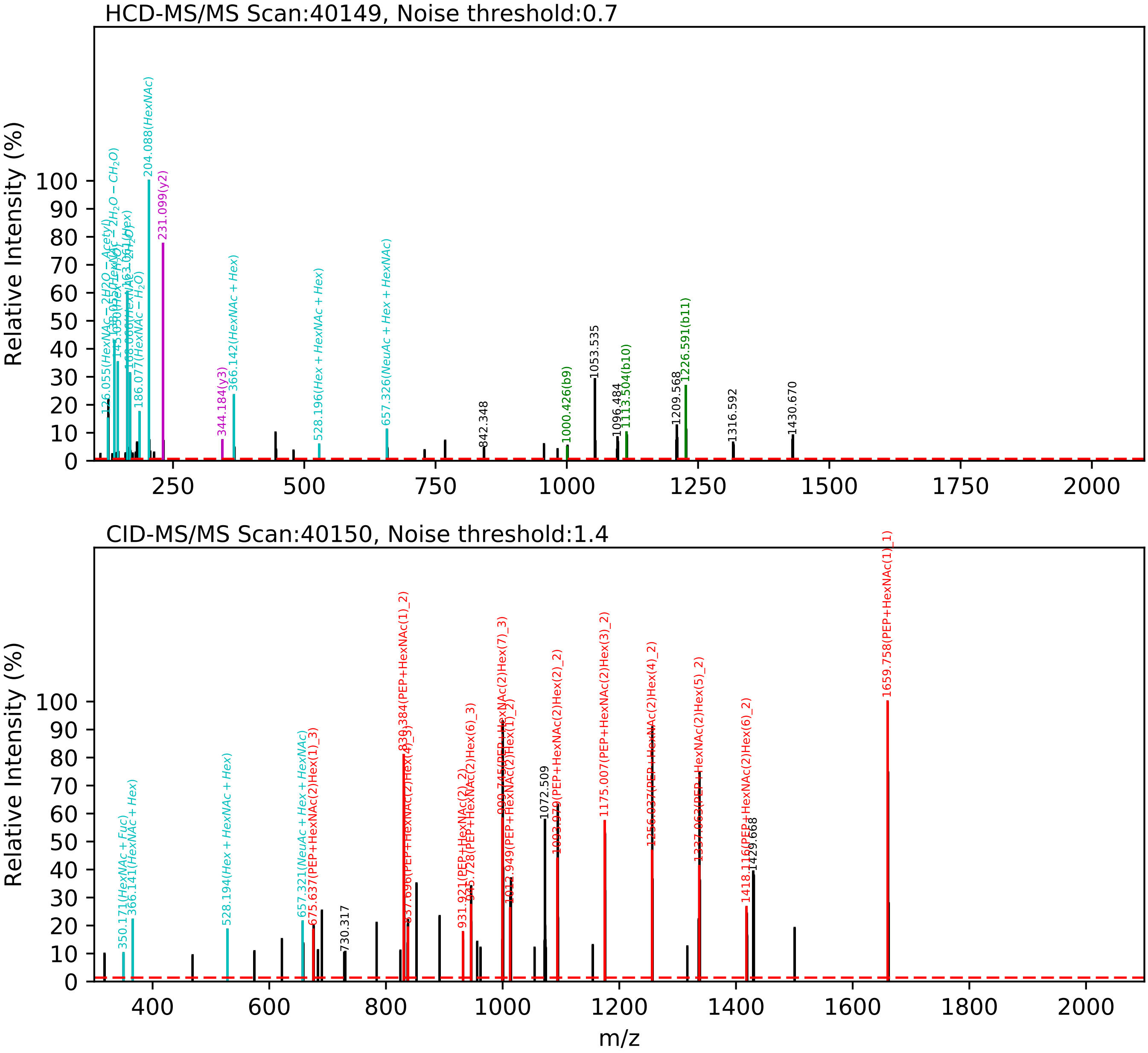

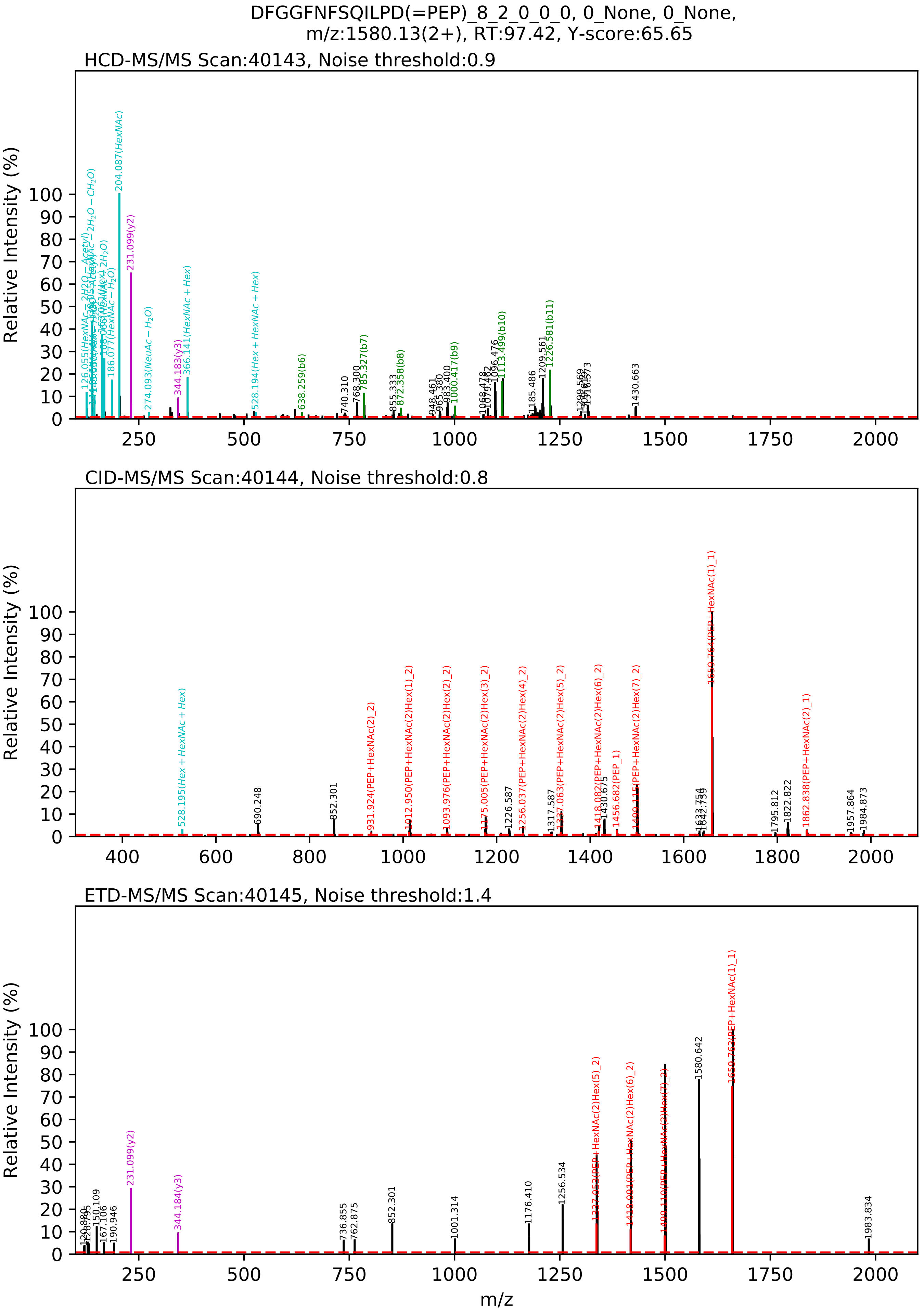

DFGGFNFSQILPD(=PEP)_8_2_0_0_0, 0_None, 0_None,
m/z:1580.13(2+), RT:97.42, Y-score:65.65
HCD-MS/MS Scan:40143, Noise threshold:0.9
CID-MS/MS Scan:40144, Noise threshold:0.8
ETD-MS/MS Scan:40145, Noise threshold:1.4
Relative Intensity (%)
m/z

EGVFVSNGTHWFVTQR(=PEP)_10_2_0_0_0, 0_None, 0_None,
m/z:1297.54(3+), RT:64.62, Y-score:86.64

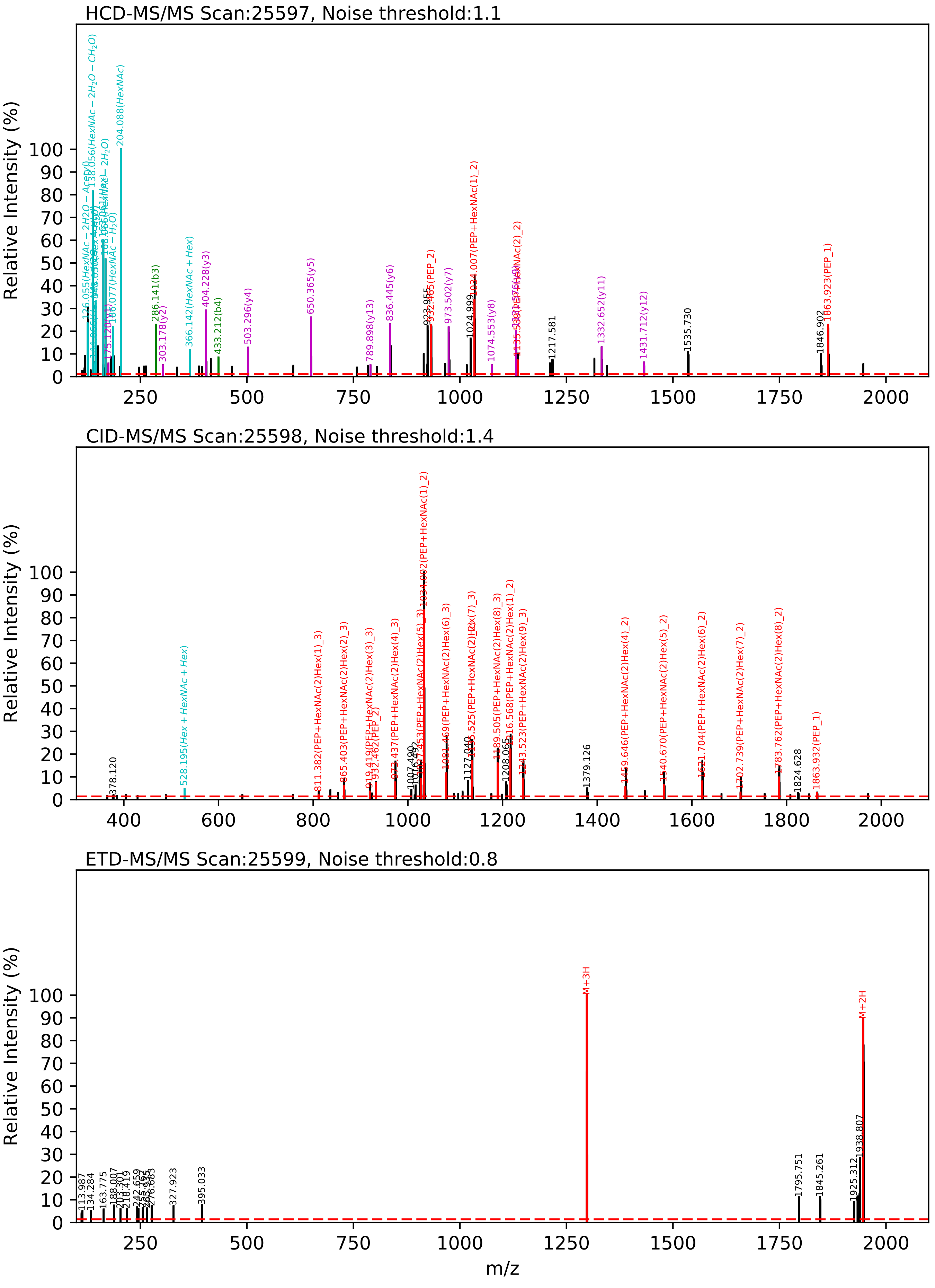

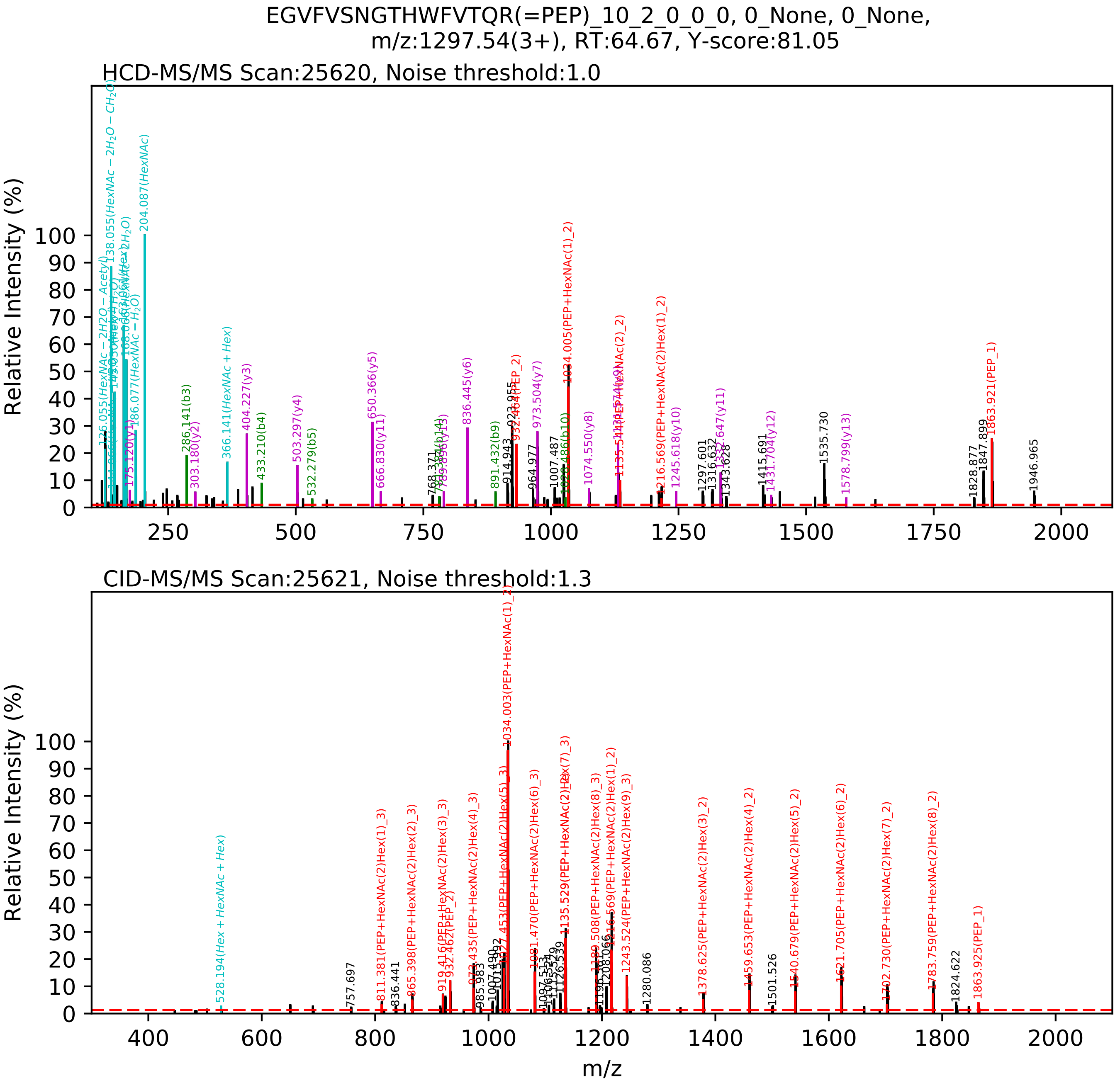

EGVFVSNGTHWFVTQR(=PEP)_10_2_0_0_0, 0_None, 0_None,
m/z:1297.54(3+), RT:64.67, Y-score:81.05
HCD-MS/MS Scan:25620, Noise threshold:1.0
Relative Intensity (%)
204.087(HexNAc)
286.141(b3)
303.180(y2)
366.141(HexNAc + Hex)
404.227(y3)
433.210(b4)
503.297(y4)
532.279(b5)
650.366(y5)
666.830(y11)
836.445(y6)
891.432(b9)
923.955
914.943
964.977
973.504(y7)
1007.487
1034.005(PEP+HexNAc(1)_2)
1074.550(y8)
1216.569(PEP+HexNAc(2)Hex(1)_2)
1245.618(y10)
1297.601
1343.628
1415.691
1431.704(y12)
1535.730
1578.799(y13)
1828.877
1847.899
1863.921(PEP_1)
1946.965
CID-MS/MS Scan:25621, Noise threshold:1.3
Relative Intensity (%)
528.194(Hex + HexNAc + Hex)
757.697
811.381(PEP+HexNAc(2)Hex(1)_3)
836.441
865.398(PEP+HexNAc(2)Hex(2)_3)
932.462(PEP_2)
973.435(PEP+HexNAc(2)Hex(4)_3)
985.983
1034.003(PEP+HexNAc(1)_2)
1081.470(PEP+HexNAc(2)Hex(6)_3)
1135.529(PEP+HexNAc(2)Hex(7)_3)
1189.508(PEP+HexNAc(2)Hex(8)_3)
1216.569(PEP+HexNAc(2)Hex(1)_2)
1243.524(PEP+HexNAc(2)Hex(9)_3)
1280.086
1378.625(PEP+HexNAc(2)Hex(3)_2)
1459.653(PEP+HexNAc(2)Hex(4)_2)
1501.526
1540.679(PEP+HexNAc(2)Hex(5)_2)
1621.705(PEP+HexNAc(2)Hex(6)_2)
1702.730(PEP+HexNAc(2)Hex(7)_2)
1783.759(PEP+HexNAc(2)Hex(8)_2)
1824.622
1863.925(PEP_1)
m/z

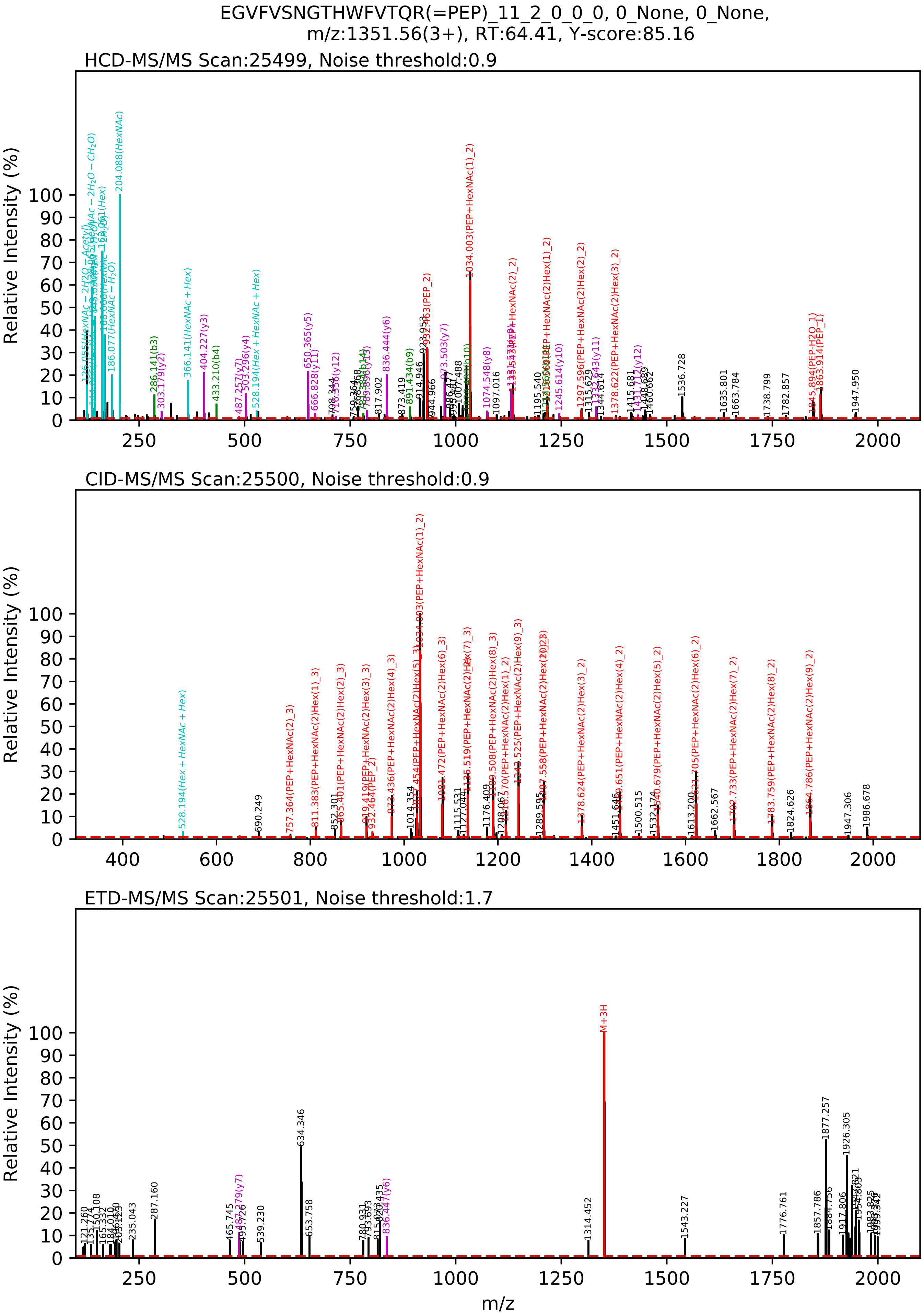

EGVFVSNGTHWFVTQR(=PEP)_11_2_0_0_0, 0_None, 0_None,
m/z:1351.56(3+), RT:64.41, Y-score:85.16
HCD-MS/MS Scan:25499, Noise threshold:0.9
Relative Intensity (%)
204.088(HexNAc)
1034.003(PEP+HexNAc(1)_2)
CID-MS/MS Scan:25500, Noise threshold:0.9
ETD-MS/MS Scan:25501, Noise threshold:1.7
M+3H
m/z

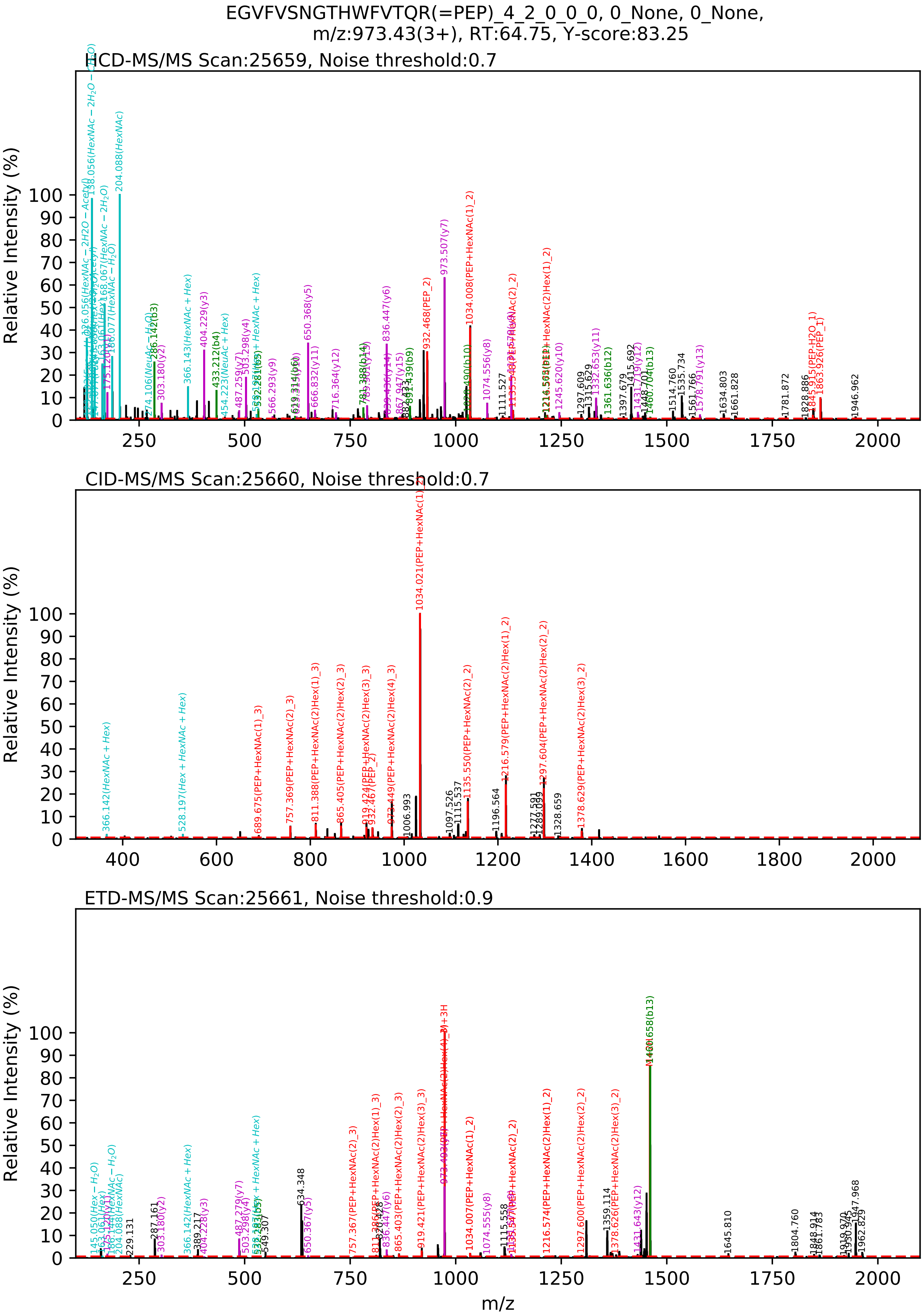

EGVFVSNGTHWFVTQR(=PEP)_4_2_0_0_0, 0_None, 0_None,
m/z:973.43(3+), RT:64.75, Y-score:83.25
HCD-MS/MS Scan:25659, Noise threshold:0.7
CID-MS/MS Scan:25660, Noise threshold:0.7
ETD-MS/MS Scan:25661, Noise threshold:0.9
Relative Intensity (%)
m/z

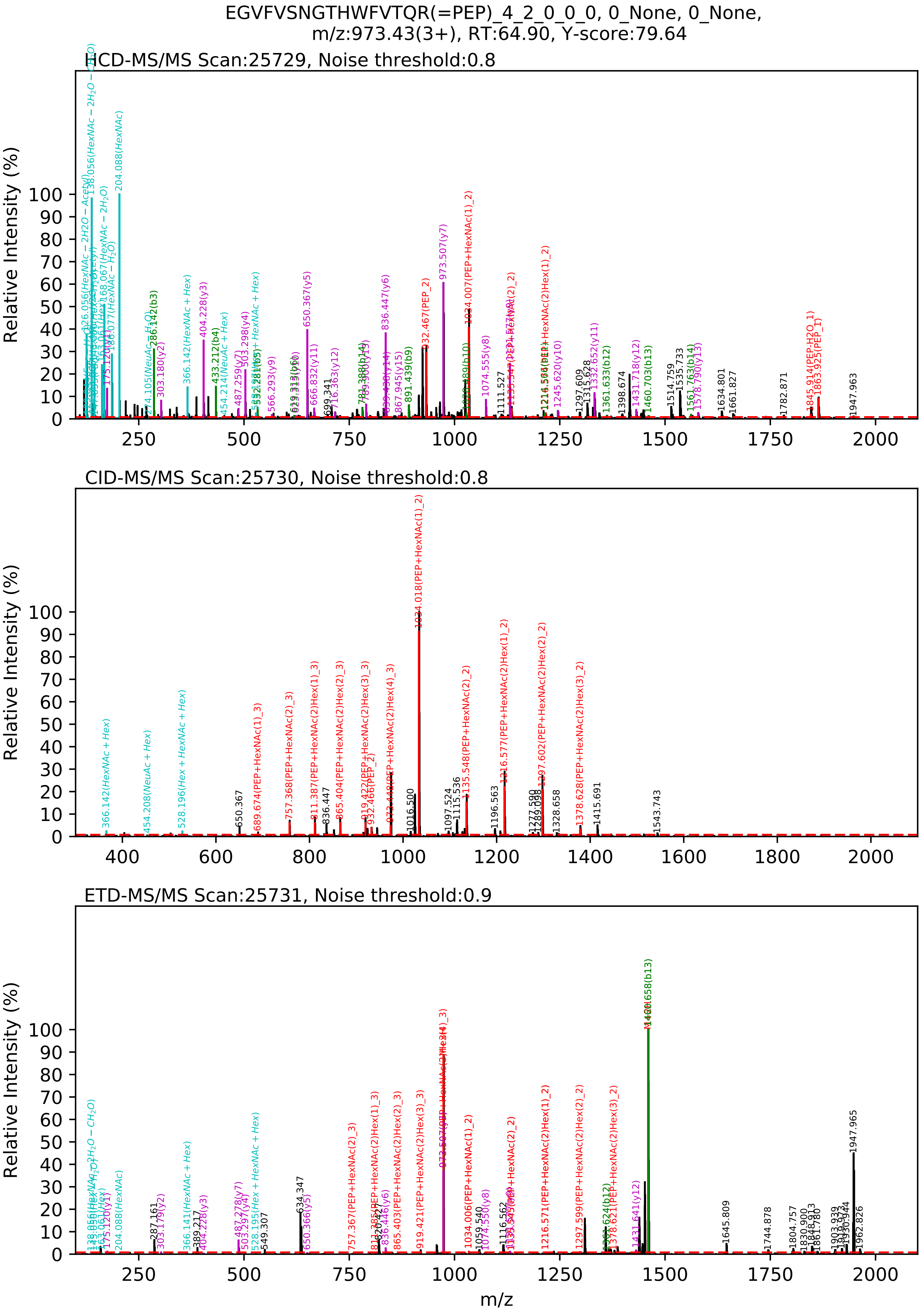
EGVFVSNGTHWFVTQR(=PEP)_4_2_0_0_0, 0_None, 0_None,
m/z:973.43(3+), RT:64.90, Y-score:79.64
HCD-MS/MS Scan:25729, Noise threshold:0.8
Relative Intensity (%)
204.088(HexNAc)
973.507(y7)
1034.007(PEP+HexNAc(1)_2)
932.467(PEP_2)
836.447(y6)
650.367(y5)
404.228(y3)
366.142(HexNAc+Hex)
286.142(b3)
433.212(b4)
503.298(y4)
303.180(y2)
1074.555(y8)
1216.564(PEP+HexNAc(2)Hex(1)_2)
1245.620(y10)
1332.652(y11)
1361.633(b12)
1431.718(y12)
1460.703(b13)
1578.790(y13)
1845.914(PEP-H2O_1)
1863.925(PEP_1)
1297.609
1398.674
1514.759
1535.733
1634.801
1661.827
1782.871
1947.963
1111.527
891.439(b9)
867.945(y15)
566.293(y9)
699.341
CID-MS/MS Scan:25730, Noise threshold:0.8
Relative Intensity (%)
366.142(HexNAc+Hex)
454.208(NeuAc+Hex)
528.196(Hex+HexNAc+Hex)
650.367
689.674(PEP+HexNAc(1)_3)
757.368(PEP+HexNAc(2)_3)
811.387(PEP+HexNAc(2)Hex(1)_3)
836.447
865.404(PEP+HexNAc(2)Hex(2)_3)
919.422(PEP+HexNAc(2)Hex(3)_3)
932.466(PEP_2)
973.448(PEP+HexNAc(2)Hex(4)_3)
1016.500
1034.018(PEP+HexNAc(1)_2)
1097.524
1115.536
1135.548(PEP+HexNAc(2)_2)
1196.563
1216.577(PEP+HexNAc(2)Hex(1)_2)
1297.602(PEP+HexNAc(2)Hex(2)_2)
1328.658
1378.628(PEP+HexNAc(2)Hex(3)_2)
1415.691
1543.743
ETD-MS/MS Scan:25731, Noise threshold:0.9
Relative Intensity (%)
1460.658(b13)
1947.965
204.088(HexNAc)
175.120(y1)
287.161
303.179(y2)
366.141(HexNAc+Hex)
389.217
404.228(y3)
487.278(y7)
503.297(y4)
528.195(Hex+HexNAc+Hex)
549.307
634.347
650.366(y5)
757.367(PEP+HexNAc(2)_3)
811.385(PEP+HexNAc(2)Hex(1)_3)
836.446(y6)
865.403(PEP+HexNAc(2)Hex(2)_3)
919.421(PEP+HexNAc(2)Hex(3)_3)
973.507(y7)
1034.006(PEP+HexNAc(1)_2)
1059.540
1074.550(y8)
1116.562
1216.571(PEP+HexNAc(2)Hex(1)_2)
1297.599(PEP+HexNAc(2)Hex(2)_2)
1361.624(b12)
1378.621(PEP+HexNAc(2)Hex(3)_2)
1431.641(y12)
1645.809
1744.878
1804.757
1830.900
1903.939
1962.826
m/z

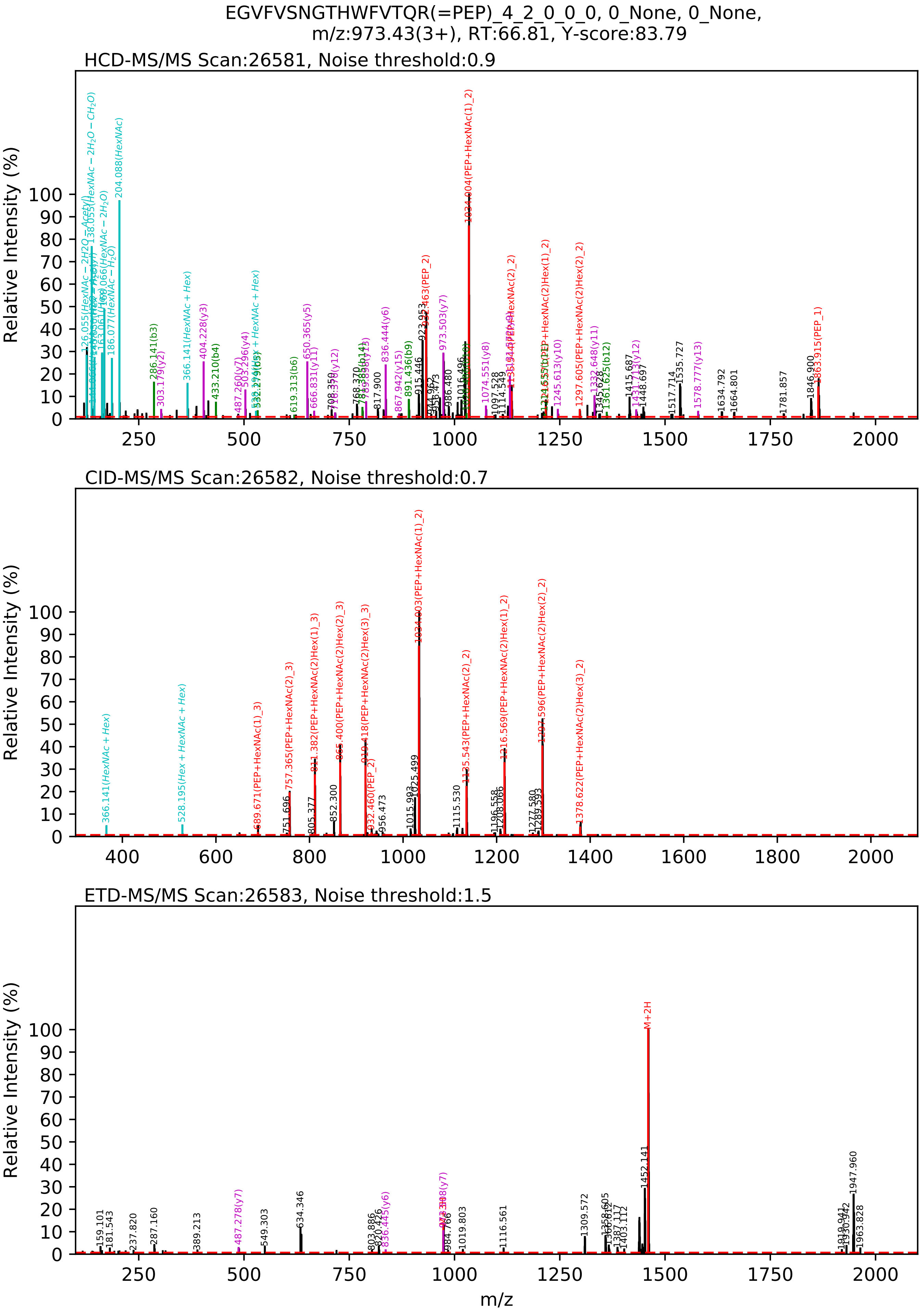
EGVFVSNGTHWFVTQR(=PEP)_4_2_0_0_0, 0_None, 0_None,
m/z:973.43(3+), RT:66.81, Y-score:83.79
HCD-MS/MS Scan:26581, Noise threshold:0.9
CID-MS/MS Scan:26582, Noise threshold:0.7
ETD-MS/MS Scan:26583, Noise threshold:1.5
Relative Intensity (%)
m/z
1034.904(PEP+HexNAc(1)_2)
1034.903(PEP+HexNAc(1)_2)
1297.596(PEP+HexNAc(2)Hex(2)_2)
1216.569(PEP+HexNAc(2)Hex(1)_2)
1378.622(PEP+HexNAc(2)Hex(3)_2)
M+2H
1452.141
1947.960

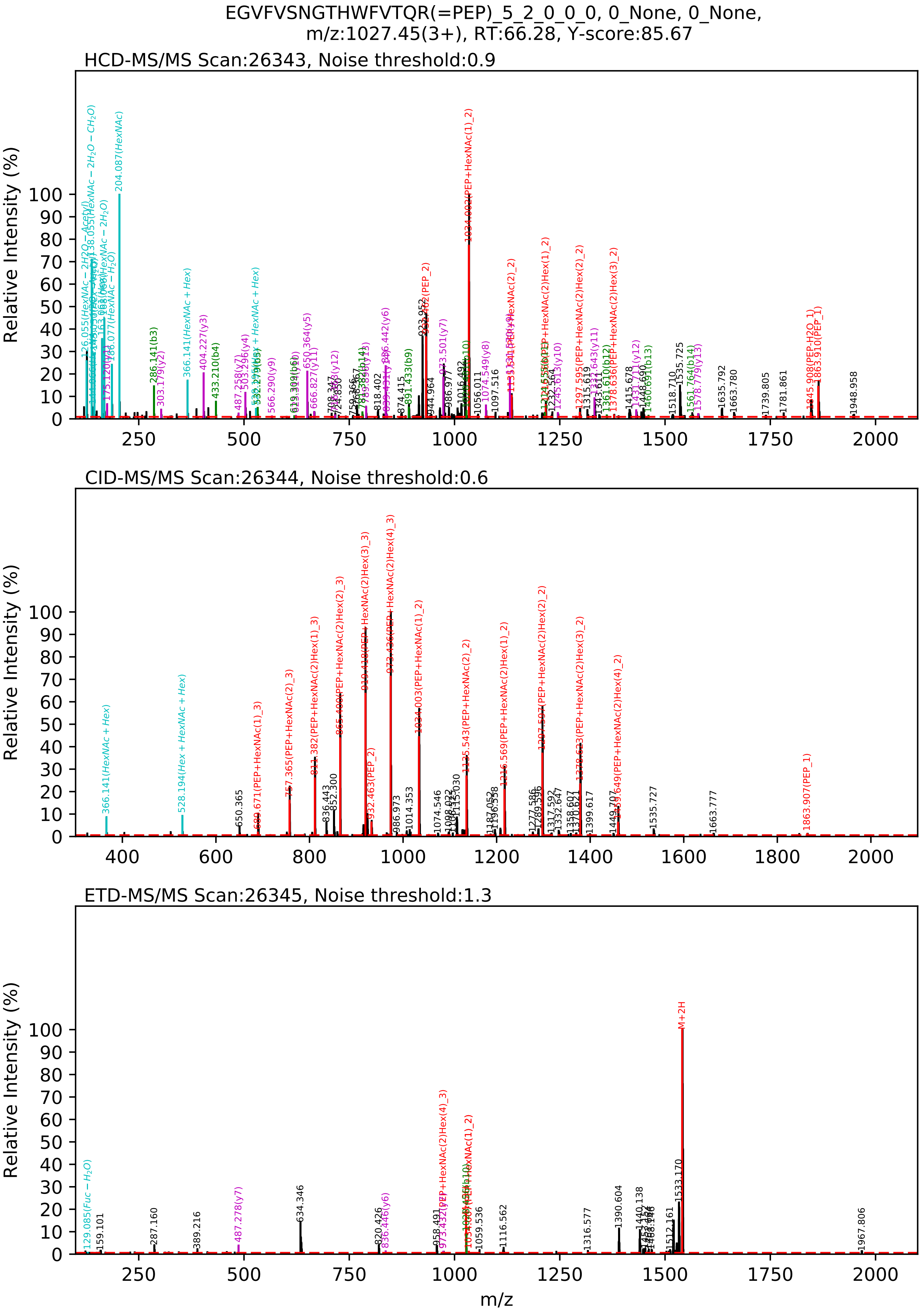

EGVFVSNGTHWFVTQR(=PEP)_5_2_0_0_0, 0_None, 0_None,
m/z:1027.45(3+), RT:66.28, Y-score:85.67
HCD-MS/MS Scan:26343, Noise threshold:0.9
Relative Intensity (%)
204.087(HexNAc)
1034.002(PEP+HexNAc(1)_2)
1863.910(PEP_1)
CID-MS/MS Scan:26344, Noise threshold:0.6
Relative Intensity (%)
973.436(PEP+HexNAc(2)Hex(4)_3)
919.418(PEP+HexNAc(2)Hex(3)_3)
865.400(PEP+HexNAc(2)Hex(2)_3)
811.382(PEP+HexNAc(2)Hex(1)_3)
757.365(PEP+HexNAc(2)_3)
689.671(PEP+HexNAc(1)_3)
1034.003(PEP+HexNAc(1)_2)
1135.543(PEP+HexNAc(2)_2)
1216.569(PEP+HexNAc(2)Hex(1)_2)
1297.597(PEP+HexNAc(2)Hex(2)_2)
1378.623(PEP+HexNAc(2)Hex(3)_2)
1459.649(PEP+HexNAc(2)Hex(4)_2)
1863.907(PEP_1)
366.141(HexNAc + Hex)
528.194(Hex + HexNAc + Hex)
ETD-MS/MS Scan:26345, Noise threshold:1.3
Relative Intensity (%)
M+2H
m/z

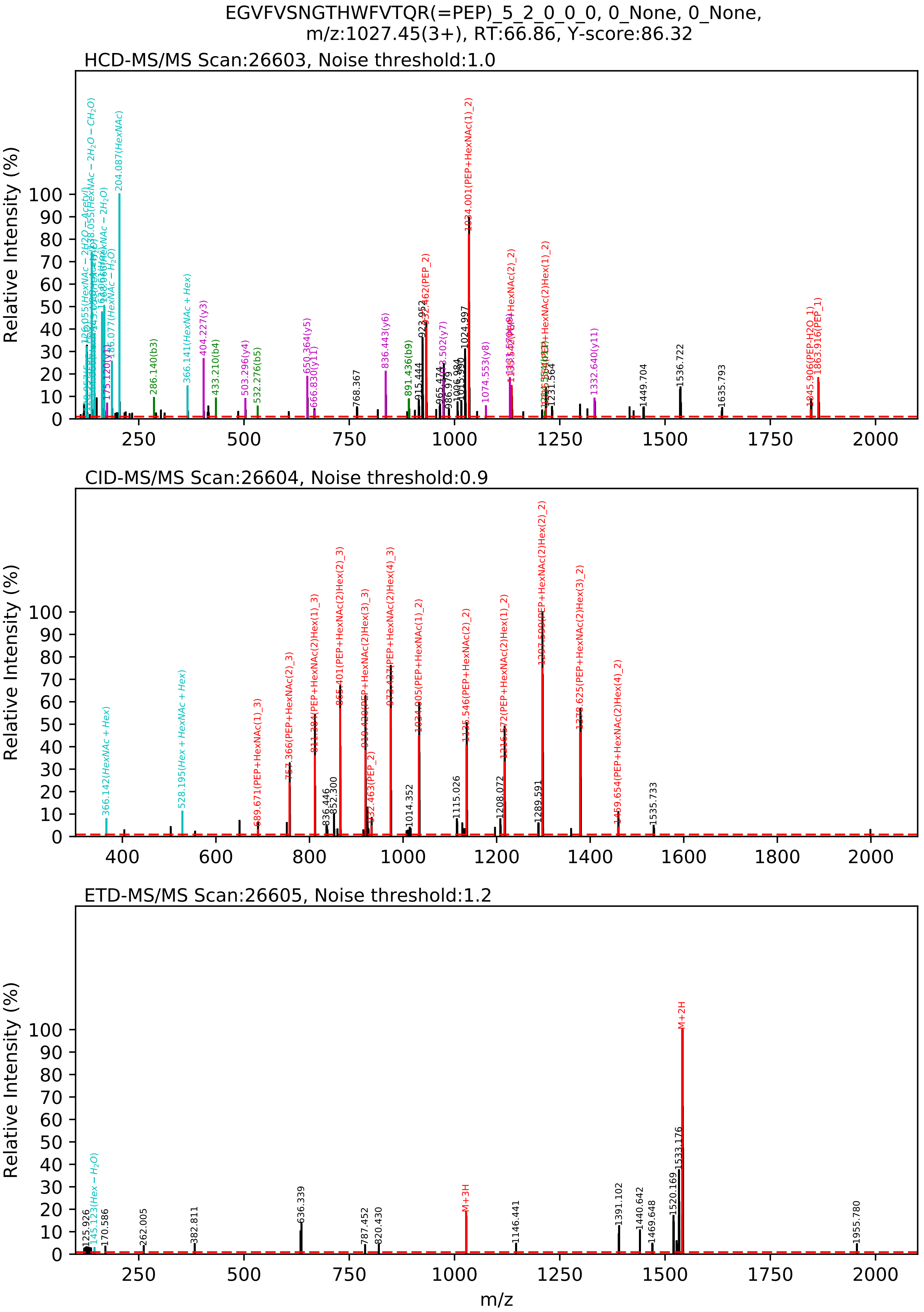
EGVFVSNGTHWFVTQR(=PEP)_5_2_0_0_0, 0_None, 0_None,
m/z:1027.45(3+), RT:66.86, Y-score:86.32
HCD-MS/MS Scan:26603, Noise threshold:1.0
CID-MS/MS Scan:26604, Noise threshold:0.9
ETD-MS/MS Scan:26605, Noise threshold:1.2
Relative Intensity (%)
m/z
204.087(HexNAc)
1034.001(PEP+HexNAc(1)_2)
366.141(HexNAc+Hex)
404.227(y3)
286.140(b3)
433.210(b4)
503.296(y4)
532.276(b5)
650.364(y5)
666.830(y11)
768.367
836.443(y6)
891.436(b9)
915.444
923.952
932.462(PEP_2)
965.474
986.979
1024.997
1074.553(y8)
1231.564
1332.640(y11)
1449.704
1536.722
1635.793
1845.906(PEP-H2O_1)
1863.916(PEP_1)
175.120(y1)
186.077(HexNAc−H2O)
366.142(HexNAc+Hex)
528.195(Hex+HexNAc+Hex)
689.671(PEP+HexNAc(1)_3)
757.366(PEP+HexNAc(2)_3)
811.384(PEP+HexNAc(2)Hex(1)_3)
836.446
852.300
865.401(PEP+HexNAc(2)Hex(2)_3)
919.420(PEP+HexNAc(2)Hex(3)_3)
932.463(PEP_2)
973.437(PEP+HexNAc(2)Hex(4)_3)
1014.352
1034.005(PEP+HexNAc(1)_2)
1115.026
1135.546(PEP+HexNAc(2)_2)
1208.072
1216.572(PEP+HexNAc(2)Hex(1)_2)
1289.591
1297.599(PEP+HexNAc(2)Hex(2)_2)
1378.625(PEP+HexNAc(2)Hex(3)_2)
1459.654(PEP+HexNAc(2)Hex(4)_2)
1535.733
125.926
145.123(Hex−H2O)
170.586
262.005
382.811
636.339
787.452
820.430
M+3H
1146.441
1391.102
1440.642
1469.648
1520.169
1533.176
M+2H
1955.780

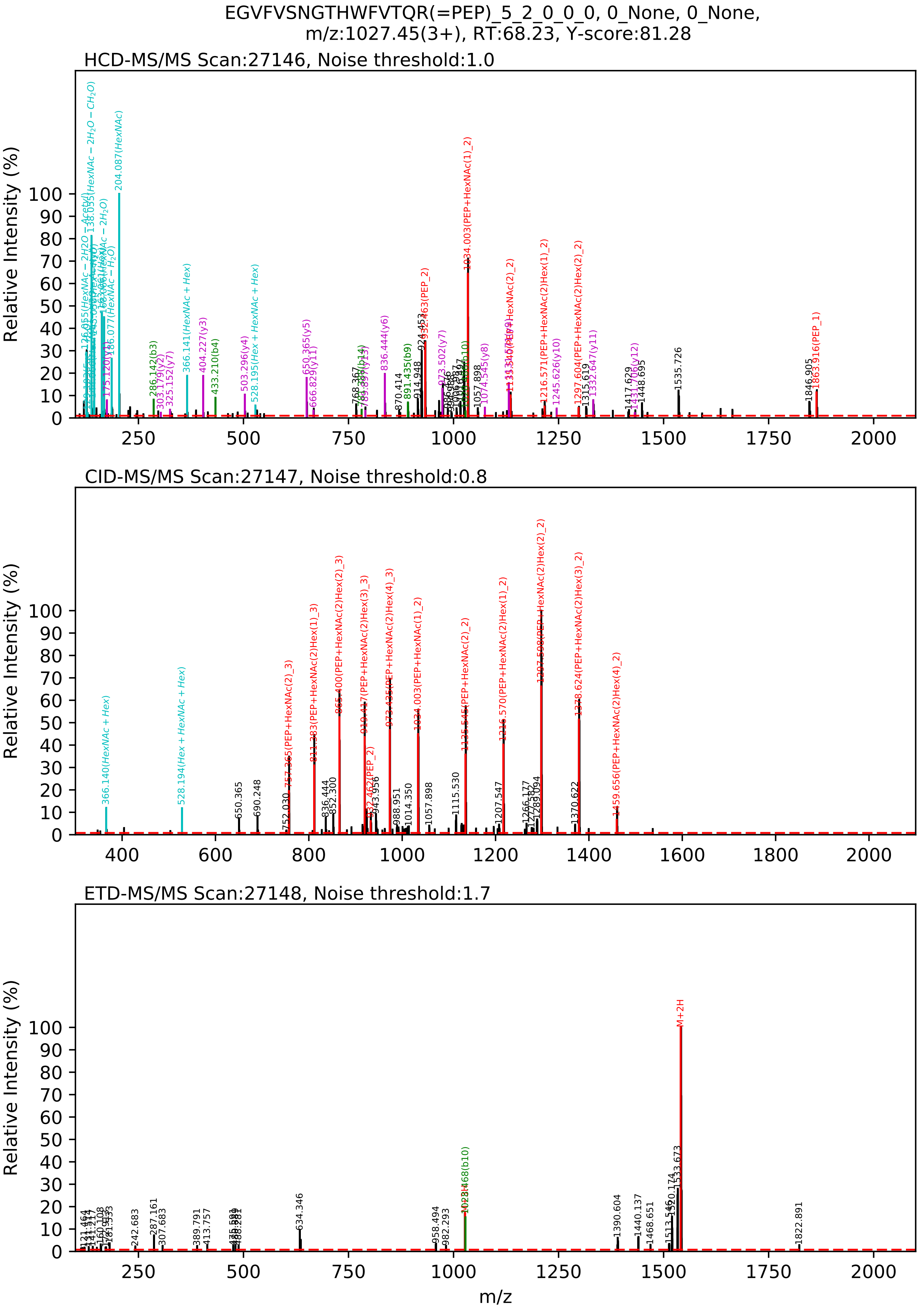

EGVFVSNGTHWFVTQR(=PEP)_5_2_0_0_0, 0_None, 0_None,
m/z:1027.45(3+), RT:68.23, Y-score:81.28
HCD-MS/MS Scan:27146, Noise threshold:1.0
CID-MS/MS Scan:27147, Noise threshold:0.8
ETD-MS/MS Scan:27148, Noise threshold:1.7
Relative Intensity (%)
m/z
204.087(HexNAc)
1034.003(PEP+HexNAc(1)_2)
1297.604(PEP+HexNAc(2)Hex(2)_2)
1378.624(PEP+HexNAc(2)Hex(3)_2)
1459.656(PEP+HexNAc(2)Hex(4)_2)
1863.916(PEP_1)
1535.726
M+2H
1028.468(b10)

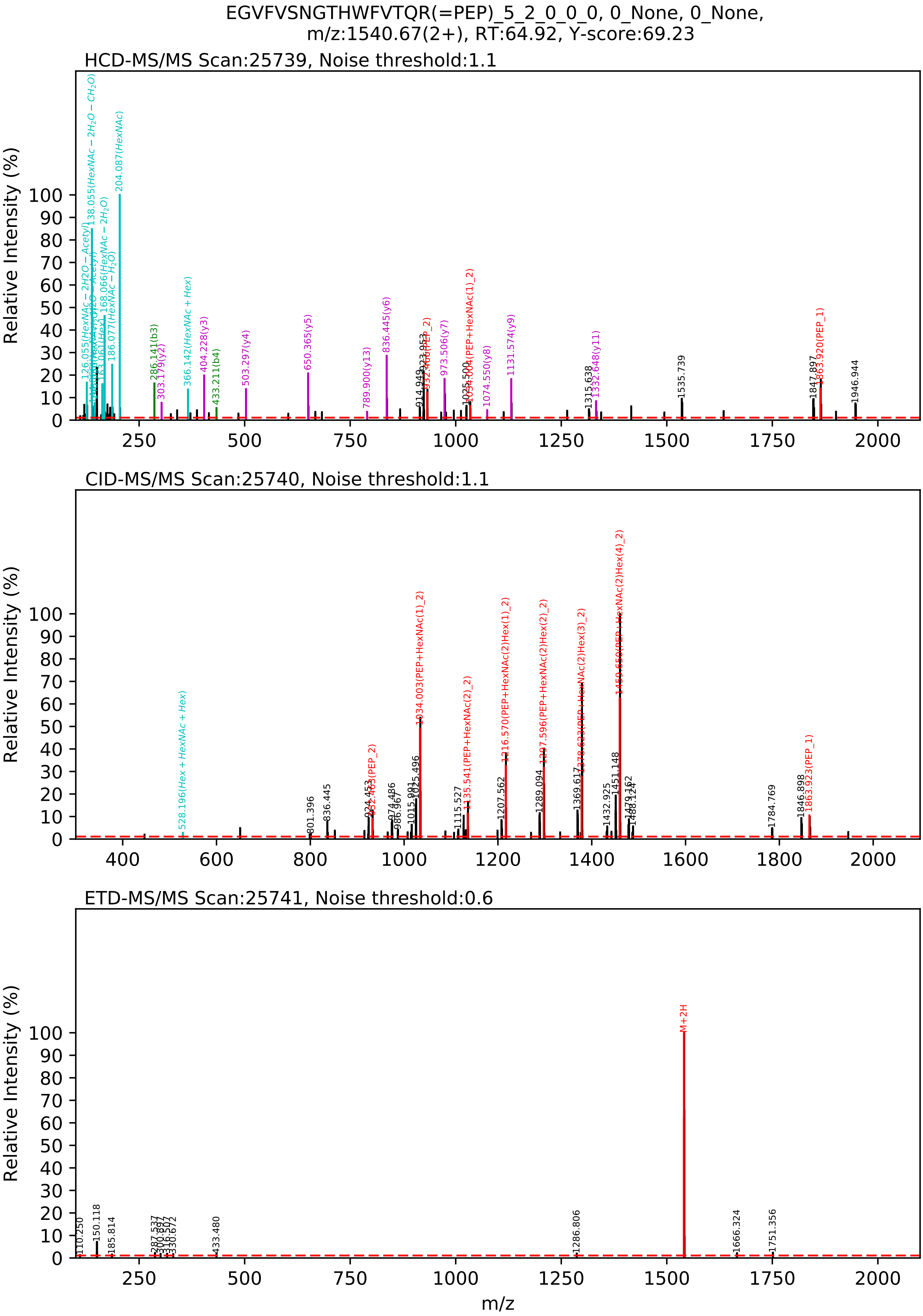

EGVFVSNGTHWFVTQR(=PEP)_5_2_0_0_0, 0_None, 0_None,
m/z:1540.67(2+), RT:64.92, Y-score:69.23
HCD-MS/MS Scan:25739, Noise threshold:1.1
CID-MS/MS Scan:25740, Noise threshold:1.1
ETD-MS/MS Scan:25741, Noise threshold:0.6
Relative Intensity (%)
m/z

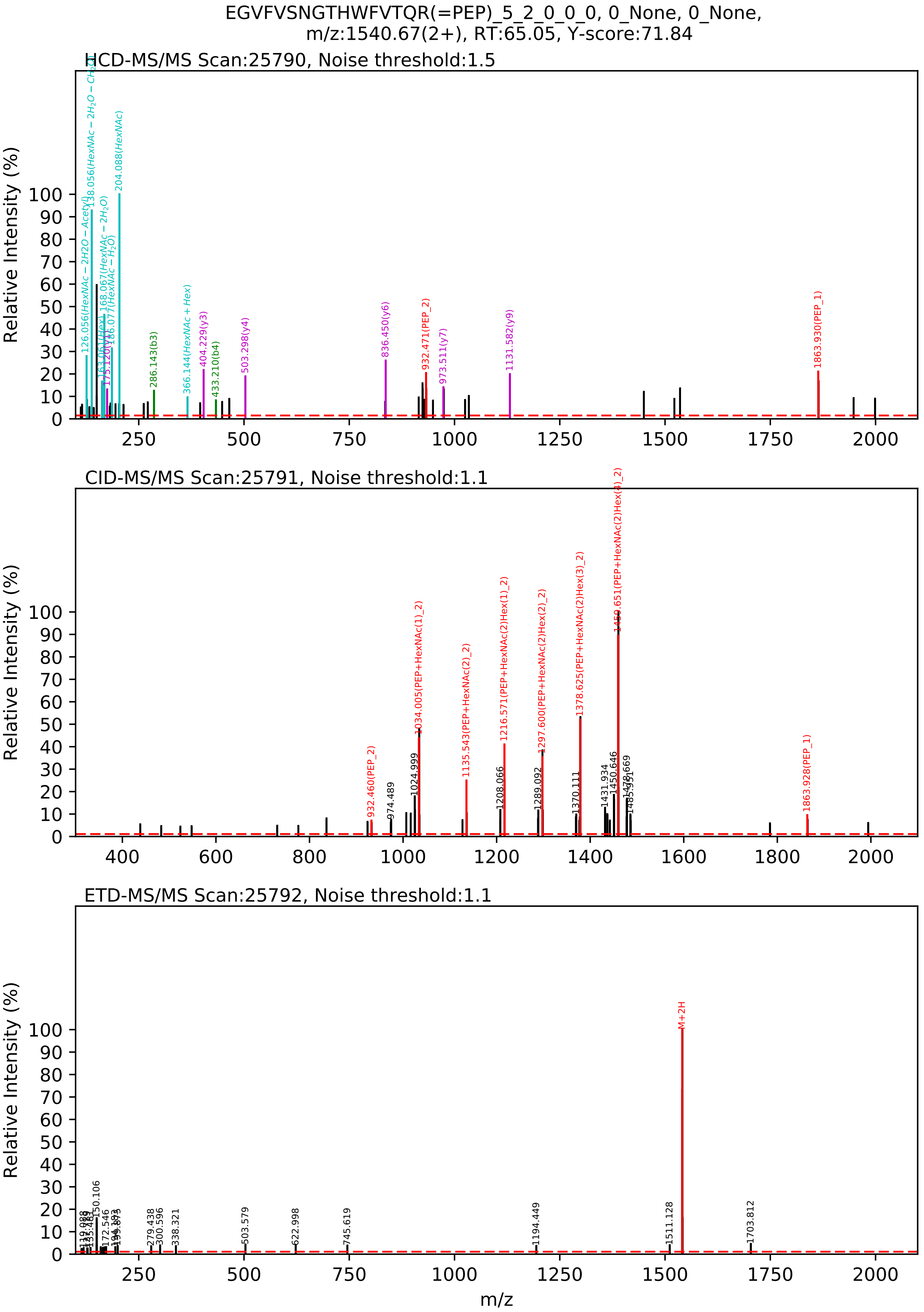
EGVFVSNGTHWFVTQR(=PEP)_5_2_0_0_0, 0_None, 0_None,
m/z:1540.67(2+), RT:65.05, Y-score:71.84
HCD-MS/MS Scan:25790, Noise threshold:1.5
CID-MS/MS Scan:25791, Noise threshold:1.1
ETD-MS/MS Scan:25792, Noise threshold:1.1
Relative Intensity (%)
m/z
204.088(HexNAc)
138.056(HexNAc − 2H2O − CH2O)
126.056(HexNAc − 2H2O − Acetyl)
168.067(HexNAc − 2H2O)
186.077(HexNAc − H2O)
286.143(b3)
366.144(HexNAc + Hex)
404.229(y3)
433.210(b4)
503.298(y4)
836.450(y6)
932.471(PEP_2)
973.511(y7)
1131.582(y9)
1863.930(PEP_1)
932.460(PEP_2)
974.489
1024.999
1034.005(PEP+HexNAc(1)_2)
1135.543(PEP+HexNAc(2)_2)
1208.066
1216.571(PEP+HexNAc(2)Hex(1)_2)
1289.092
1297.600(PEP+HexNAc(2)Hex(2)_2)
1370.111
1378.625(PEP+HexNAc(2)Hex(3)_2)
1431.934
1450.646
1459.651(PEP+HexNAc(2)Hex(4)_2)
1478.669
1863.928(PEP_1)
150.106
172.546
279.438
300.596
338.321
503.579
622.998
745.619
1194.449
1511.128
M+2H
1703.812

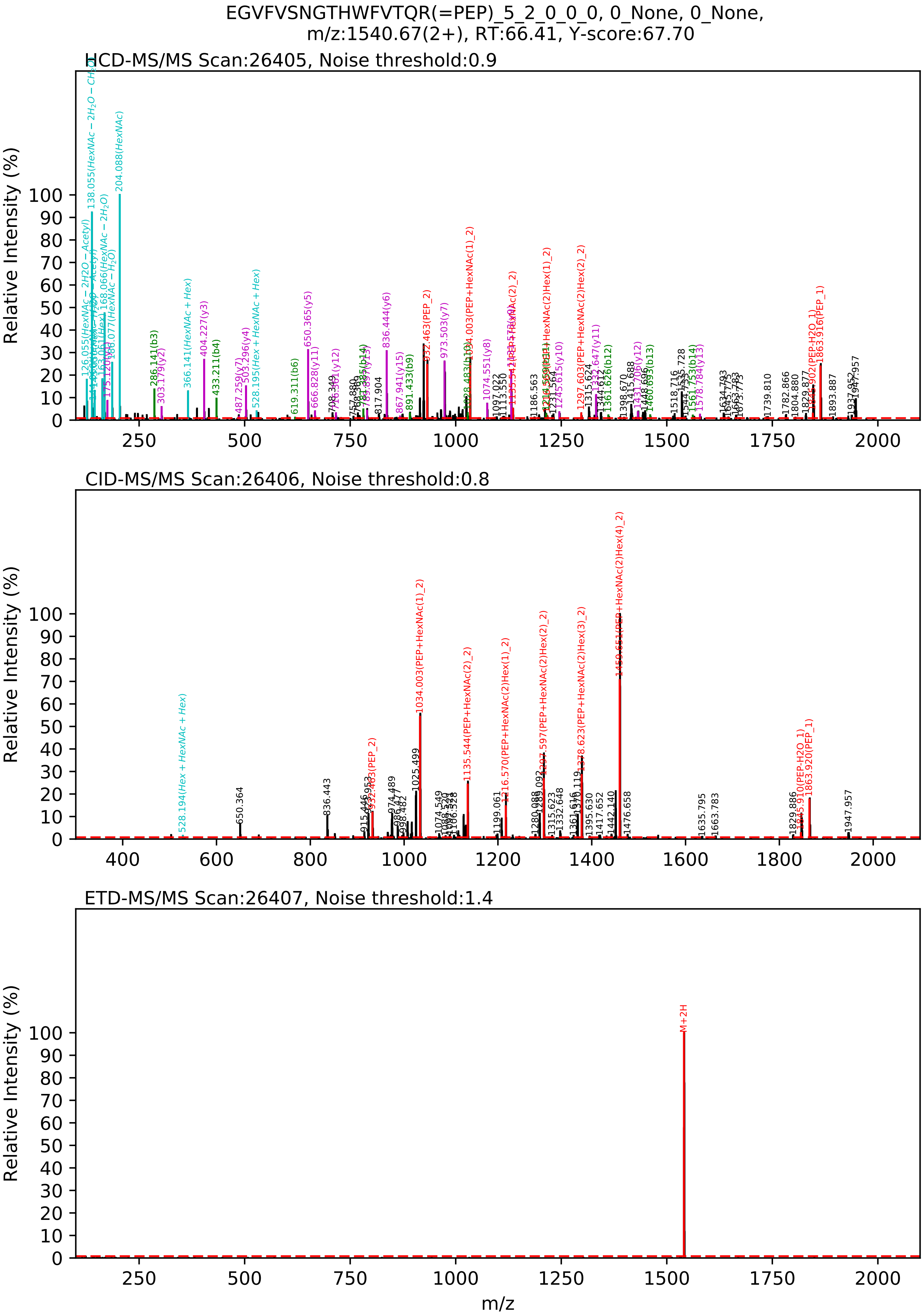
EGVFVSNGTHWFVTQR(=PEP)_5_2_0_0_0, 0_None, 0_None,
m/z:1540.67(2+), RT:66.41, Y-score:67.70
HCD-MS/MS Scan:26405, Noise threshold:0.9
Relative Intensity (%)
204.088(HexNAc)
138.055(HexNAc − 2H2O − CH3)
366.141(HexNAc + Hex)
404.227(y3)
286.141(b3)
303.179(y2)
433.211(b4)
503.296(y4)
528.195(Hex + HexNAc + Hex)
619.311(b6)
650.365(y5)
666.828(y11)
836.444(y6)
867.941(y15)
891.433(b9)
932.463(PEP_2)
973.503(y7)
1034.003(PEP+HexNAc(1)_2)
1074.551(y8)
1245.615(y10)
1297.603(PEP+HexNAc(2)Hex(2)_2)
1863.916(PEP_1)
CID-MS/MS Scan:26406, Noise threshold:0.8
528.194(Hex + HexNAc + Hex)
650.364
836.443
932.463(PEP_2)
1025.499
1034.003(PEP+HexNAc(1)_2)
1135.544(PEP+HexNAc(2)_2)
1216.570(PEP+HexNAc(2)Hex(1)_2)
1297.597(PEP+HexNAc(2)Hex(2)_2)
1378.623(PEP+HexNAc(2)Hex(3)_2)
1459.651(PEP+HexNAc(2)Hex(4)_2)
1845.910(PEP-H2O_1)
1863.920(PEP_1)
1947.957
ETD-MS/MS Scan:26407, Noise threshold:1.4
M+2H
m/z

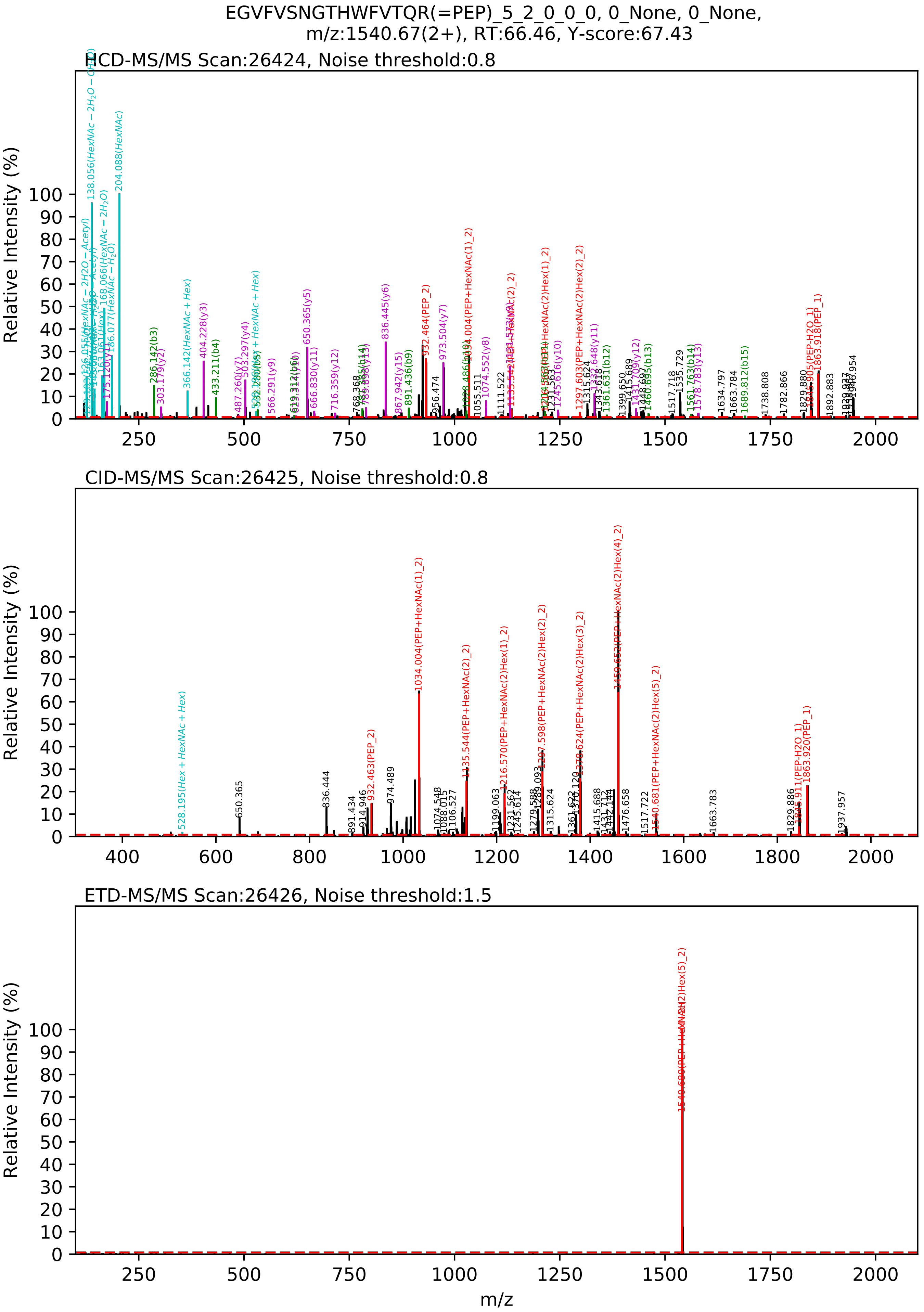
EGVFVSNGTHWFVTQR(=PEP)_5_2_0_0_0, 0_None, 0_None,
m/z:1540.67(2+), RT:66.46, Y-score:67.43
HCD-MS/MS Scan:26424, Noise threshold:0.8
204.088(HexNAc)
138.056(HexNAc − 2H2O − CH4O)
168.066(HexNAc − 2H2O)
186.077(HexNAc − H2O)
366.142(HexNAc + Hex)
528.190(Hex + HexNAc + Hex)
286.142(b3)
303.179(y2)
404.228(y3)
433.211(b4)
487.260(y7)
503.297(y4)
566.291(y9)
650.365(y5)
666.830(y11)
716.359(y12)
836.445(y6)
867.942(y15)
891.436(b9)
932.464(PEP_2)
973.504(y7)
1034.004(PEP+HexNAc(1)_2)
1074.552(y8)
1245.616(y10)
1297.603(PEP+HexNAc(2)Hex(2)_2)
1361.631(b12)
1431.709(y12)
1460.695(b13)
1517.718
1535.729
1561.763(b14)
1578.783(y13)
1634.797
1663.784
1689.812(b15)
1738.808
1782.866
1829.880
1863.918(PEP_1)
1892.883
1937.954
CID-MS/MS Scan:26425, Noise threshold:0.8
528.195(Hex + HexNAc + Hex)
650.365
836.444
891.434
914.946
932.463(PEP_2)
974.489
1034.004(PEP+HexNAc(1)_2)
1135.544(PEP+HexNAc(2)_2)
1216.570(PEP+HexNAc(2)Hex(1)_2)
1297.598(PEP+HexNAc(2)Hex(2)_2)
1378.624(PEP+HexNAc(2)Hex(3)_2)
1459.652(PEP+HexNAc(2)Hex(4)_2)
1540.681(PEP+HexNAc(2)Hex(5)_2)
1845.911(PEP-H2O_1)
1863.920(PEP_1)
1663.783
1829.886
1937.957
ETD-MS/MS Scan:26426, Noise threshold:1.5
1540.680(PEP+HexNAc(2)Hex(5)_2)
Relative Intensity (%)
m/z

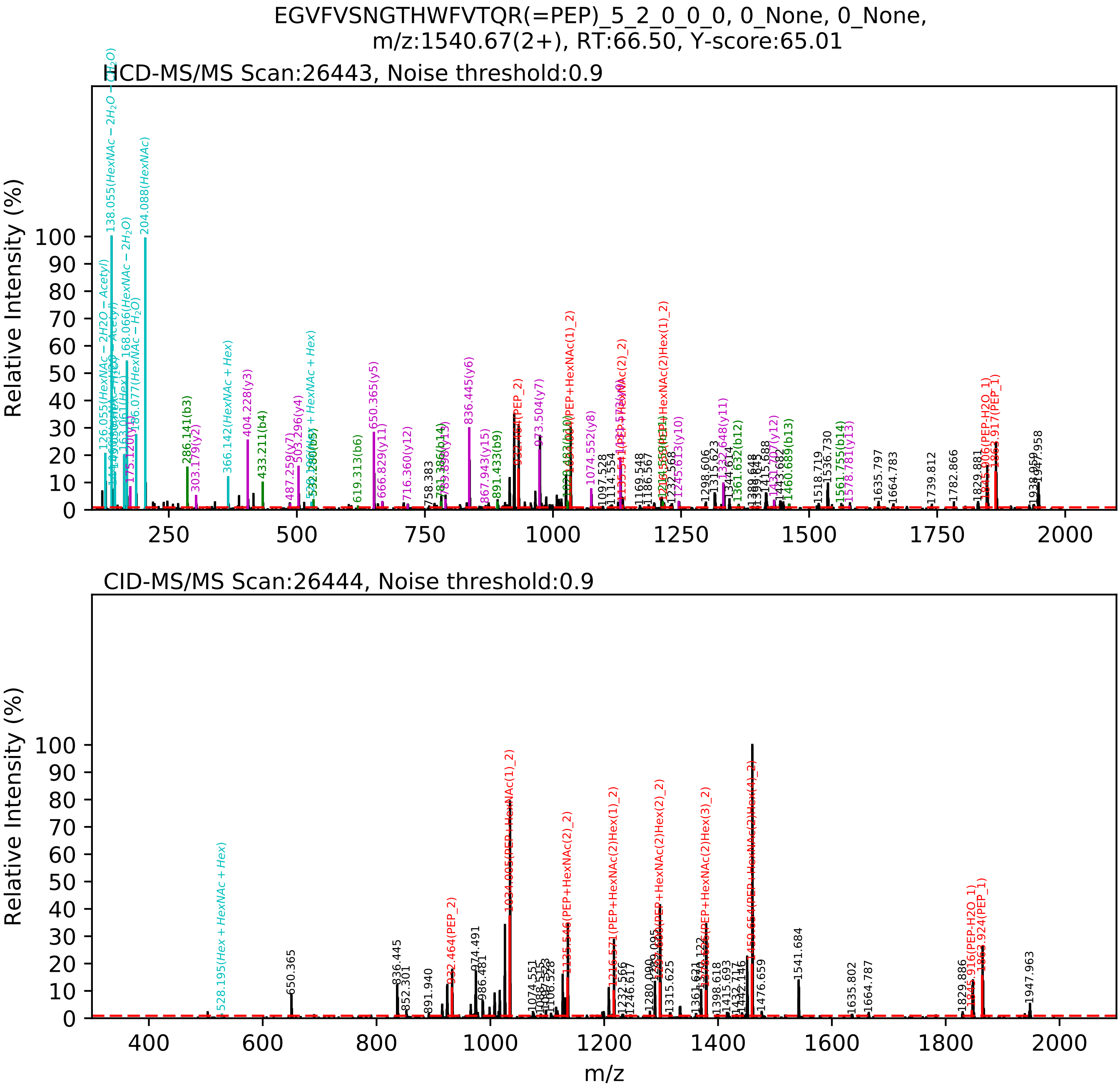
EGVFVSNGTHWFVTQR(=PEP)_5_2_0_0_0, 0_None, 0_None,
m/z:1540.67(2+), RT:66.50, Y-score:65.01
HCD-MS/MS Scan:26443, Noise threshold:0.9
Relative Intensity (%)
CID-MS/MS Scan:26444, Noise threshold:0.9
Relative Intensity (%)
m/z

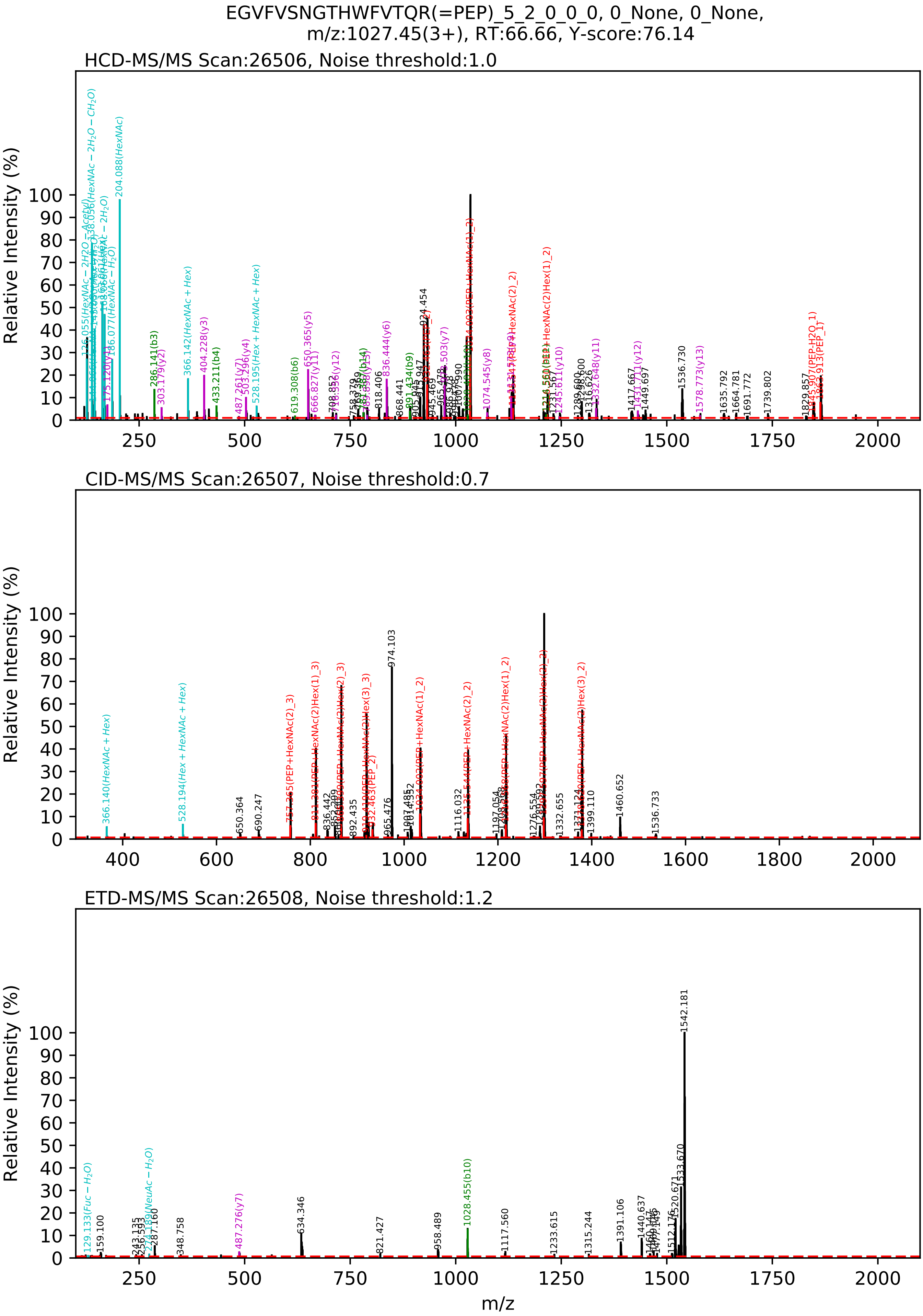
EGVFVSNGTHWFVTQR(=PEP)_5_2_0_0_0, 0_None, 0_None,
m/z:1027.45(3+), RT:66.66, Y-score:76.14
HCD-MS/MS Scan:26506, Noise threshold:1.0
Relative Intensity (%)
204.088(HexNAc)
366.142(HexNAc + Hex)
528.195(Hex + HexNAc + Hex)
404.228(y3)
433.211(b4)
286.141(b3)
303.179(y2)
650.365(y5)
619.308(b6)
836.444(y6)
924.454
1536.730
1578.773(y13)
CID-MS/MS Scan:26507, Noise threshold:0.7
366.140(HexNAc + Hex)
528.194(Hex + HexNAc + Hex)
757.365(PEP+HexNAc(2)_3)
974.103
1460.652
1536.733
ETD-MS/MS Scan:26508, Noise threshold:1.2
129.133(Fuc − H2O)
487.276(y7)
634.346
1028.455(b10)
1542.181
1533.670
m/z

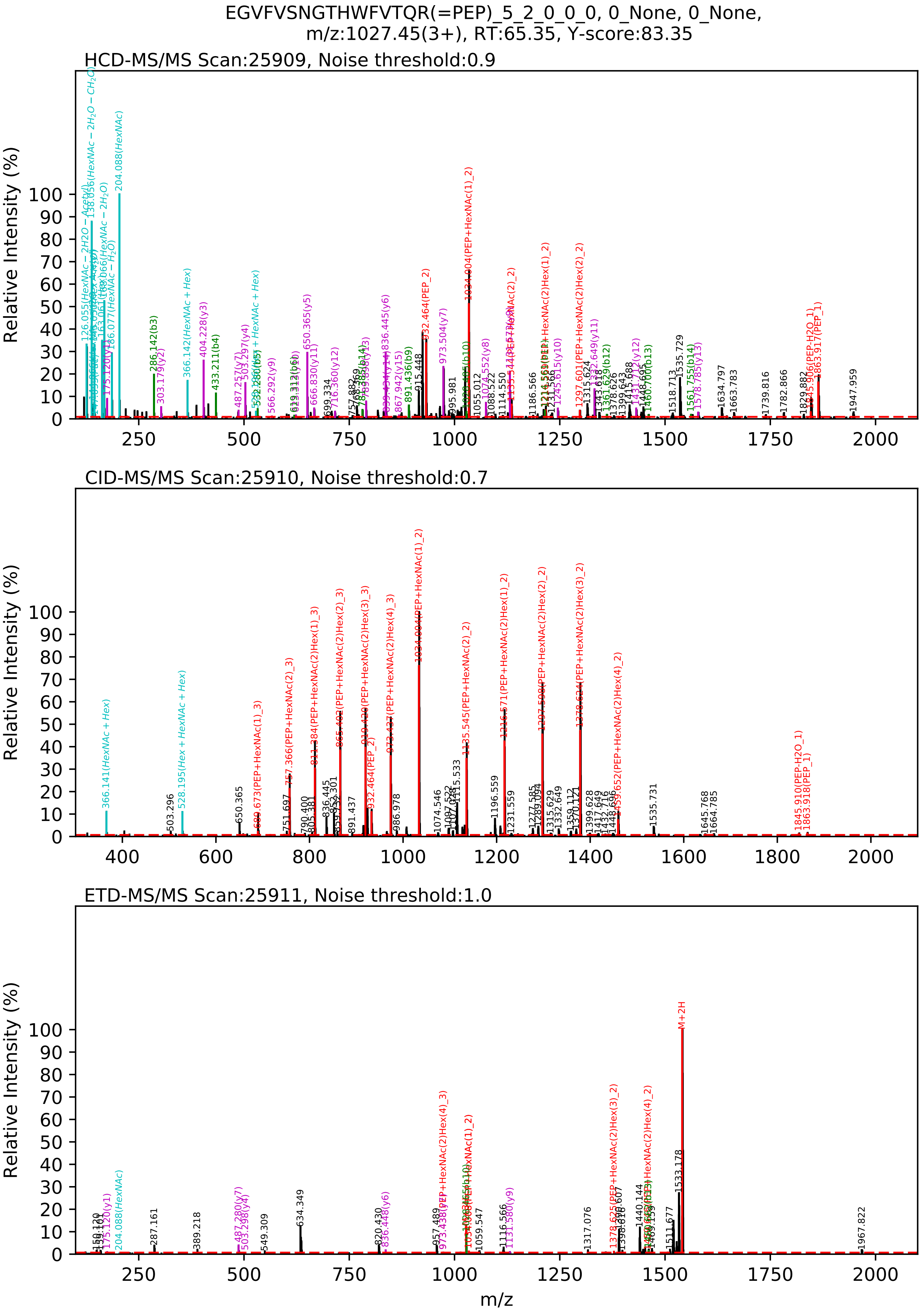
EGVFVSNGTHWFVTQR(=PEP)_5_2_0_0_0, 0_None, 0_None,
m/z:1027.45(3+), RT:65.35, Y-score:83.35
HCD-MS/MS Scan:25909, Noise threshold:0.9
CID-MS/MS Scan:25910, Noise threshold:0.7
ETD-MS/MS Scan:25911, Noise threshold:1.0
Relative Intensity (%)
m/z
M+2H

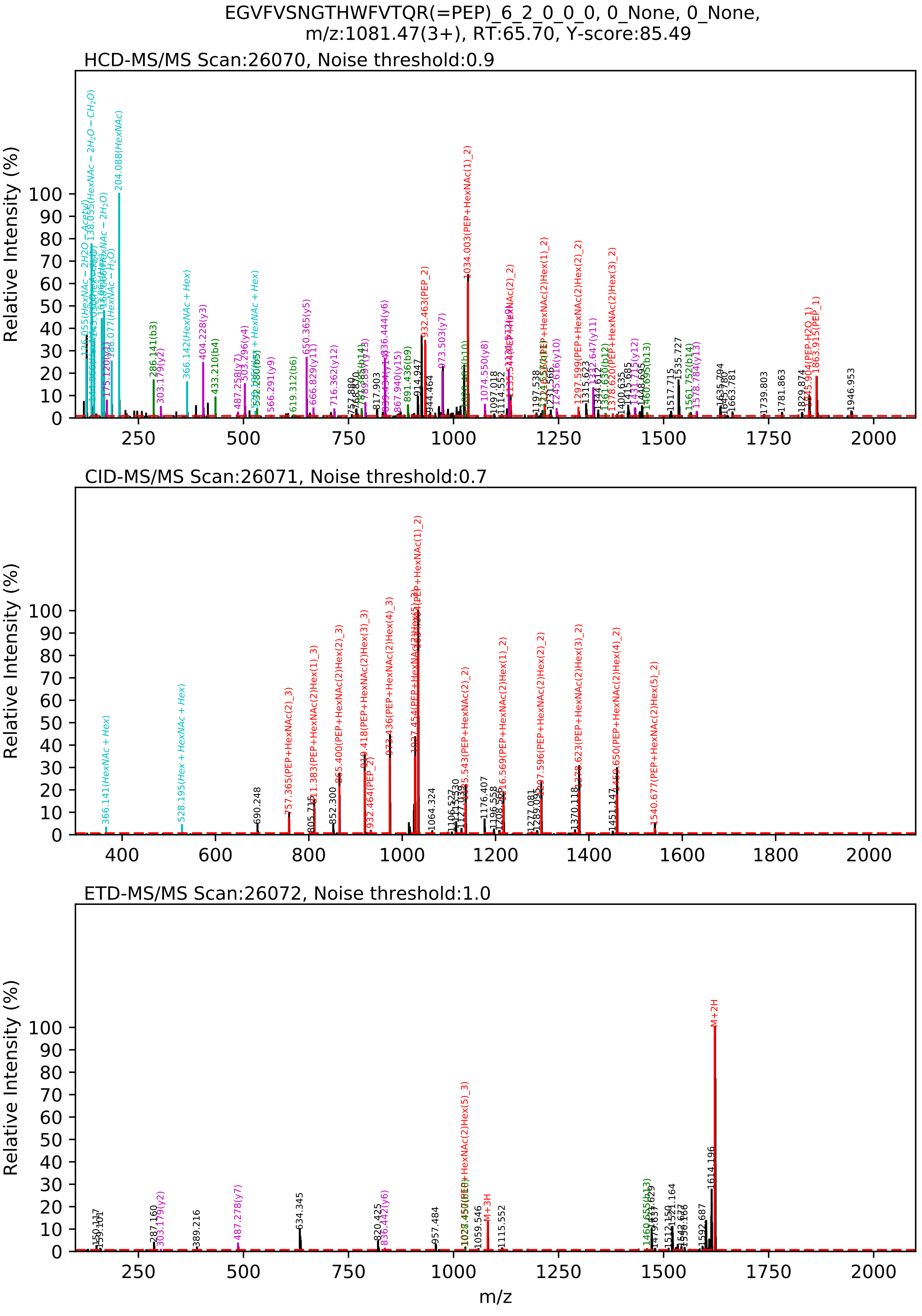
EGVFVSNGTHWFVTQR(=PEP)_6_2_0_0_0, 0_None, 0_None,
m/z:1081.47(3+), RT:65.70, Y-score:85.49
HCD-MS/MS Scan:26070, Noise threshold:0.9
CID-MS/MS Scan:26071, Noise threshold:0.7
ETD-MS/MS Scan:26072, Noise threshold:1.0
Relative Intensity (%)
m/z

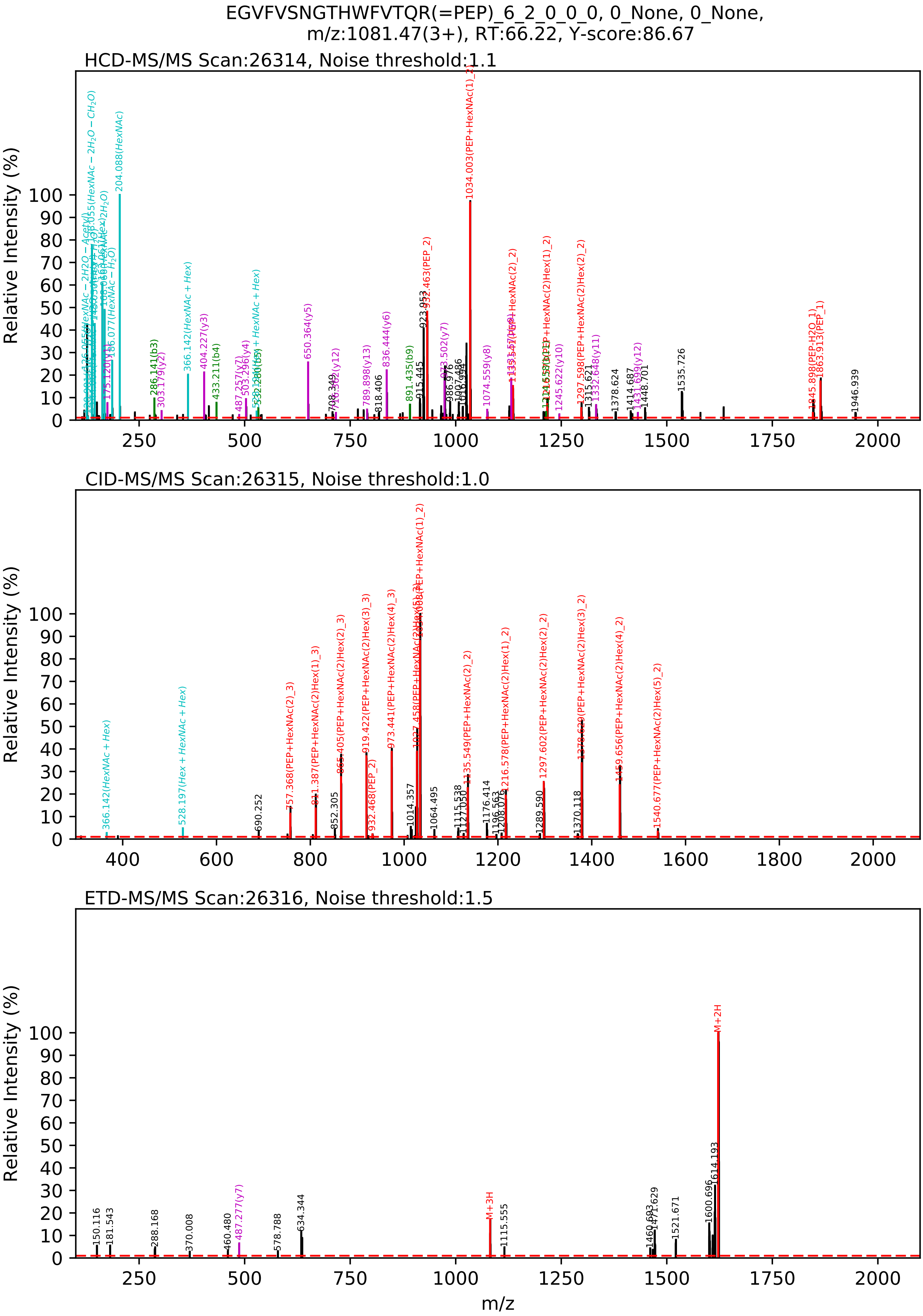
EGVFVSNGTHWFVTQR(=PEP)_6_2_0_0_0, 0_None, 0_None,
m/z:1081.47(3+), RT:66.22, Y-score:86.67
HCD-MS/MS Scan:26314, Noise threshold:1.1
Relative Intensity (%)
204.088(HexNAc)
186.077(HexNAc − H2O)
175.120(y1)
286.141(b3)
303.179(y2)
366.142(HexNAc + Hex)
404.227(y3)
433.211(b4)
487.257(y7)
503.296(y4)
650.364(y5)
708.349
789.898(y13)
818.406
836.444(y6)
891.435(b9)
915.445
923.953
932.463(PEP_2)
973.502(y7)
986.976
1034.003(PEP+HexNAc(1)_2)
1074.559(y8)
1245.622(y10)
1297.598(PEP+HexNAc(2)Hex(2)_2)
1315.621
1378.624
1414.687
1448.701
1535.726
1845.898(PEP-H2O_1)
1863.913(PEP_1)
1946.939
CID-MS/MS Scan:26315, Noise threshold:1.0
366.142(HexNAc + Hex)
528.197(Hex + HexNAc + Hex)
690.252
757.368(PEP+HexNAc(2)_3)
811.387(PEP+HexNAc(2)Hex(1)_3)
852.305
865.405(PEP+HexNAc(2)Hex(2)_3)
919.422(PEP+HexNAc(2)Hex(3)_3)
932.468(PEP_2)
973.441(PEP+HexNAc(2)Hex(4)_3)
1014.357
1064.495
1115.538
1135.549(PEP+HexNAc(2)_2)
1176.414
1216.578(PEP+HexNAc(2)Hex(1)_2)
1289.590
1297.602(PEP+HexNAc(2)Hex(2)_2)
1370.118
1378.629(PEP+HexNAc(2)Hex(3)_2)
1459.656(PEP+HexNAc(2)Hex(4)_2)
1540.677(PEP+HexNAc(2)Hex(5)_2)
ETD-MS/MS Scan:26316, Noise threshold:1.5
150.116
181.543
288.168
370.008
460.480
487.277(y7)
578.788
634.344
M+3H
1115.555
1471.629
1521.671
1600.696
1614.193
M+2H
m/z

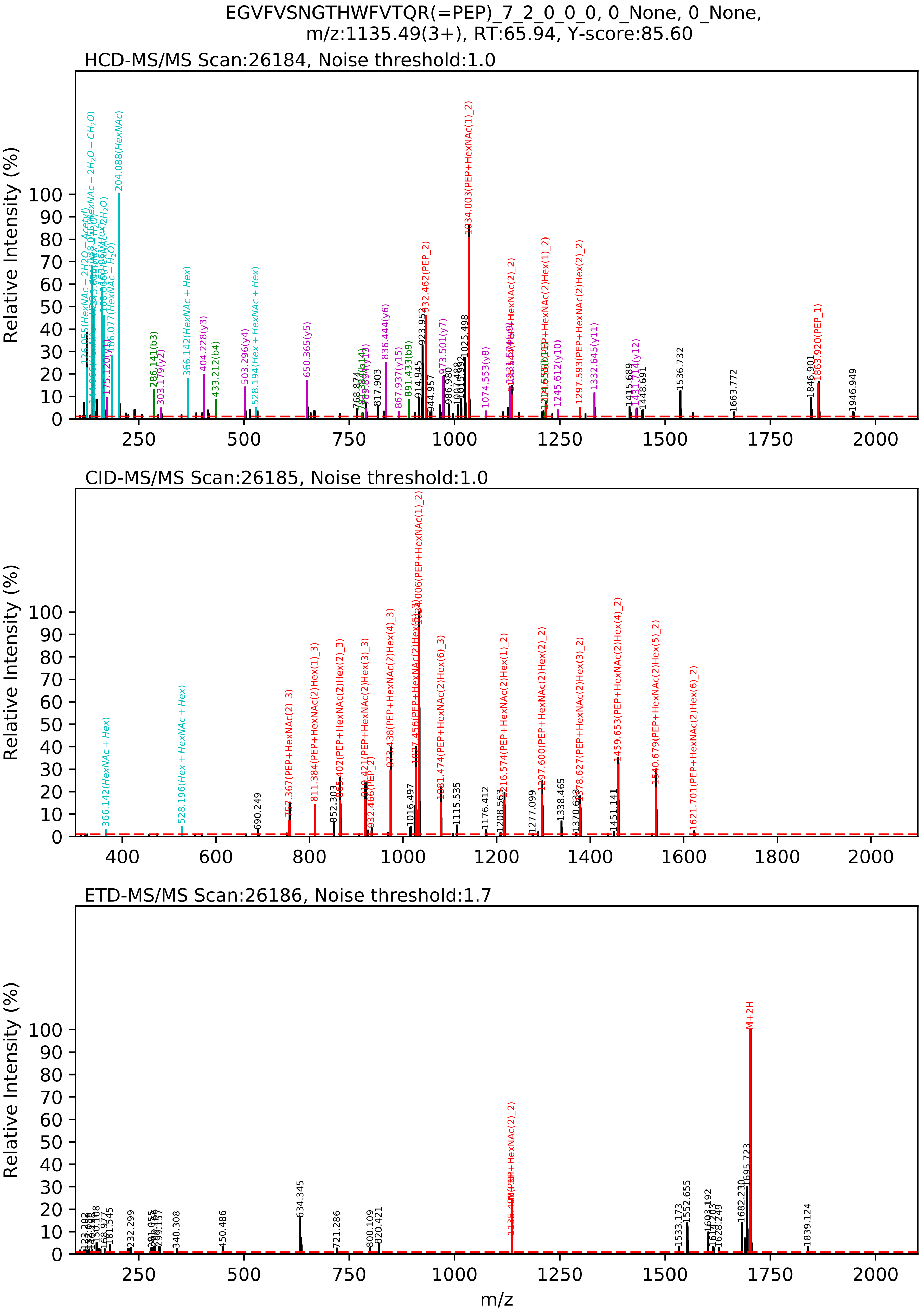
EGVFVSNGTHWFVTQR(=PEP)_7_2_0_0_0, 0_None, 0_None,
m/z:1135.49(3+), RT:65.94, Y-score:85.60
HCD-MS/MS Scan:26184, Noise threshold:1.0
CID-MS/MS Scan:26185, Noise threshold:1.0
ETD-MS/MS Scan:26186, Noise threshold:1.7
Relative Intensity (%)
m/z
204.088(HexNAc)
1034.003(PEP+HexNAc(1)_2)
932.462(PEP_2)
1863.920(PEP_1)
1297.593(PEP+HexNAc(2)Hex(2)_2)
650.365(y5)
836.444(y6)
973.501(y7)
1074.553(y8)
1245.612(y10)
1332.645(y11)
286.141(b3)
433.212(b4)
891.433(b9)
404.228(y3)
303.179(y2)
503.296(y4)
366.142(HexNAc+Hex)
528.194(Hex+HexNAc+Hex)
1536.732
1846.901
1946.949
1663.772
1034.006(PEP+HexNAc(1)_2)
811.384(PEP+HexNAc(2)Hex(1)_3)
757.367(PEP+HexNAc(2)_3)
973.438(PEP+HexNAc(2)Hex(4)_3)
1081.474(PEP+HexNAc(2)Hex(6)_3)
1297.600(PEP+HexNAc(2)Hex(2)_2)
1459.653(PEP+HexNAc(2)Hex(4)_2)
1540.679(PEP+HexNAc(2)Hex(5)_2)
1621.701(PEP+HexNAc(2)Hex(6)_2)
366.142(HexNAc+Hex)
528.196(Hex+HexNAc+Hex)
690.249
852.303
1115.535
1338.465
M+2H
1695.723
1682.230
1552.655
1533.173
1839.124
634.345
721.286
800.109
820.421
450.486
340.308
232.299

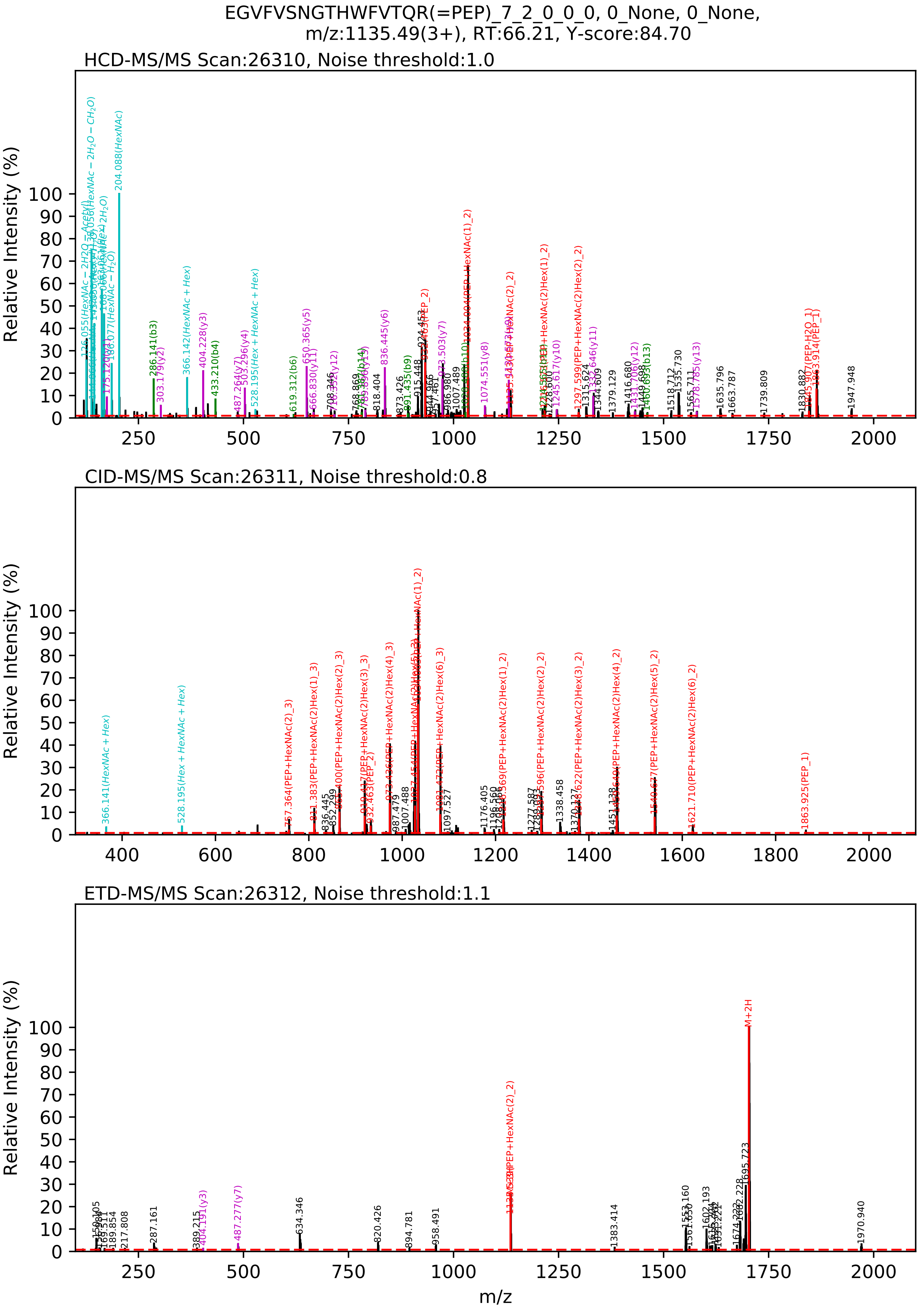

EGVFVSNGTHWFVTQR(=PEP)_7_2_0_0_0, 0_None, 0_None,
m/z:1135.49(3+), RT:66.21, Y-score:84.70
HCD-MS/MS Scan:26310, Noise threshold:1.0
CID-MS/MS Scan:26311, Noise threshold:0.8
ETD-MS/MS Scan:26312, Noise threshold:1.1
Relative Intensity (%)
m/z

EGVFVSNGTHWFVTQR(=PEP)_7_2_0_0_0, 0_None, 0_None,
m/z:1135.49(3+), RT:66.93, Y-score:79.98

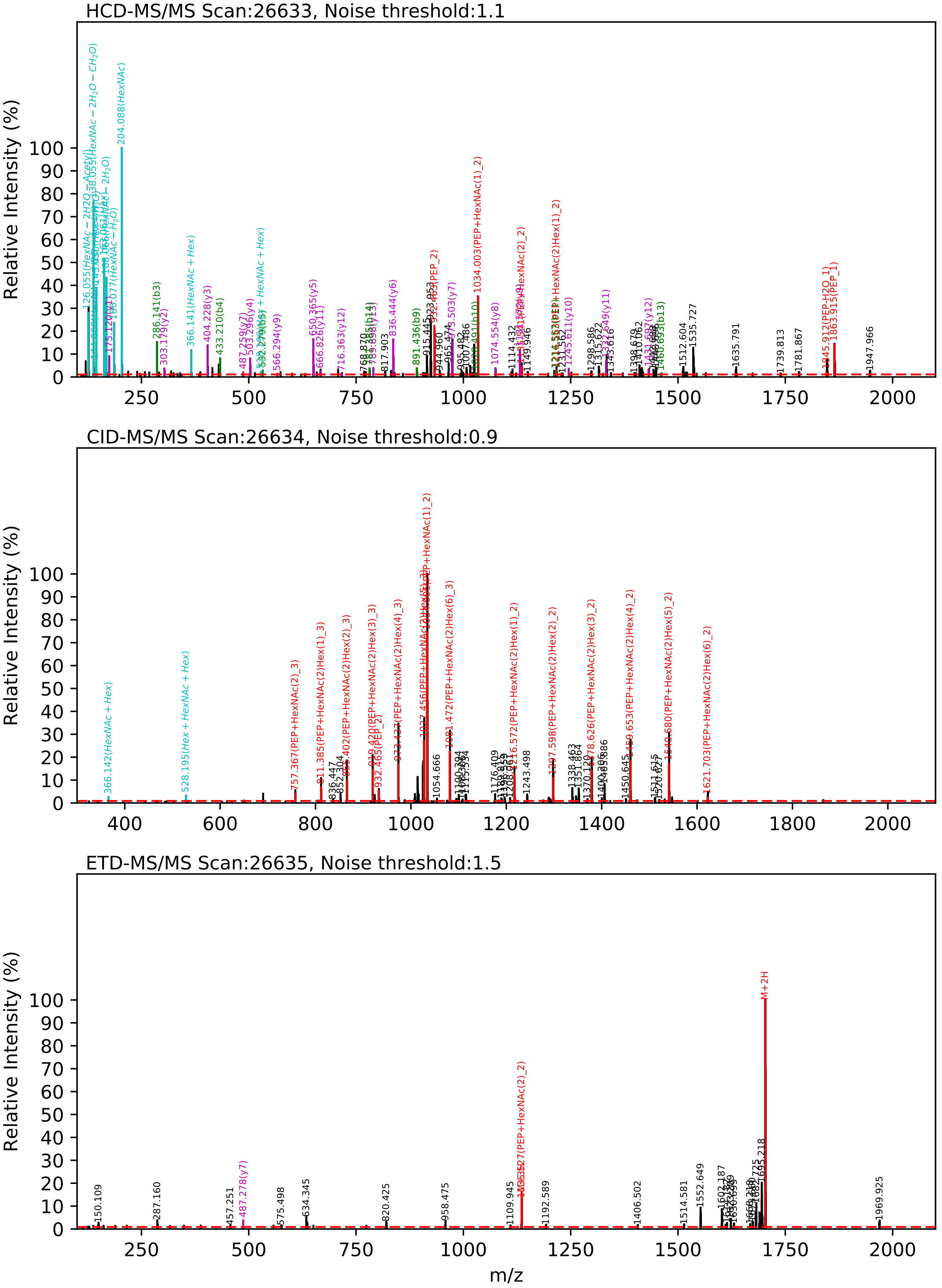

EGVFVSNGTHWFVTQR(=PEP)_7_2_0_0_0, 0_None, 0_None,
m/z:1135.49(3+), RT:65.38, Y-score:84.61

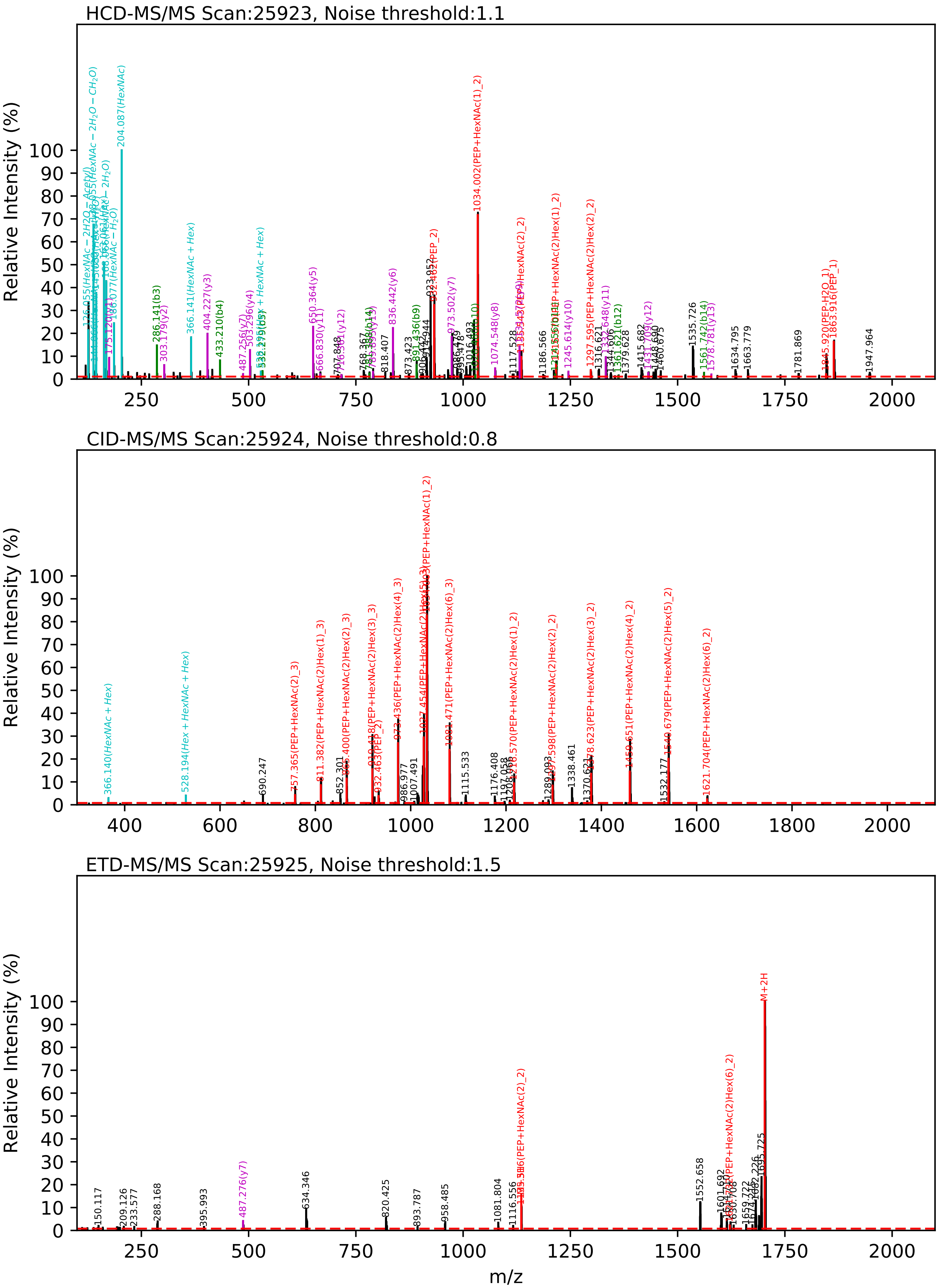

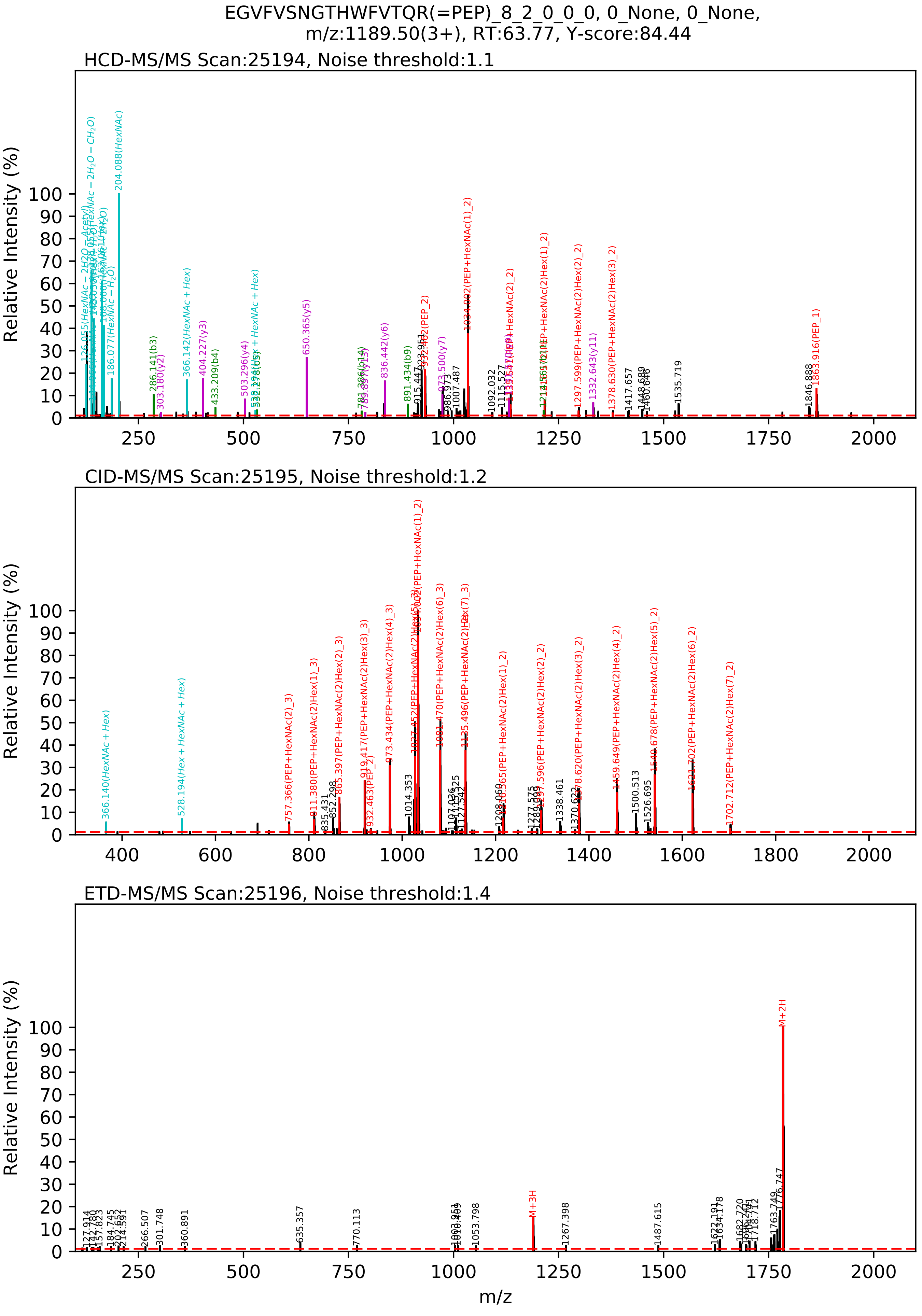
EGVFVSNGTHWFVTQR(=PEP)_8_2_0_0_0, 0_None, 0_None,
m/z:1189.50(3+), RT:63.77, Y-score:84.44
HCD-MS/MS Scan:25194, Noise threshold:1.1
CID-MS/MS Scan:25195, Noise threshold:1.2
ETD-MS/MS Scan:25196, Noise threshold:1.4
Relative Intensity (%)
m/z

EGVFVSNGTHWFVTQR(=PEP)_8_2_0_0_0, 0_None, 0_None,
m/z:1189.50(3+), RT:64.66, Y-score:85.46

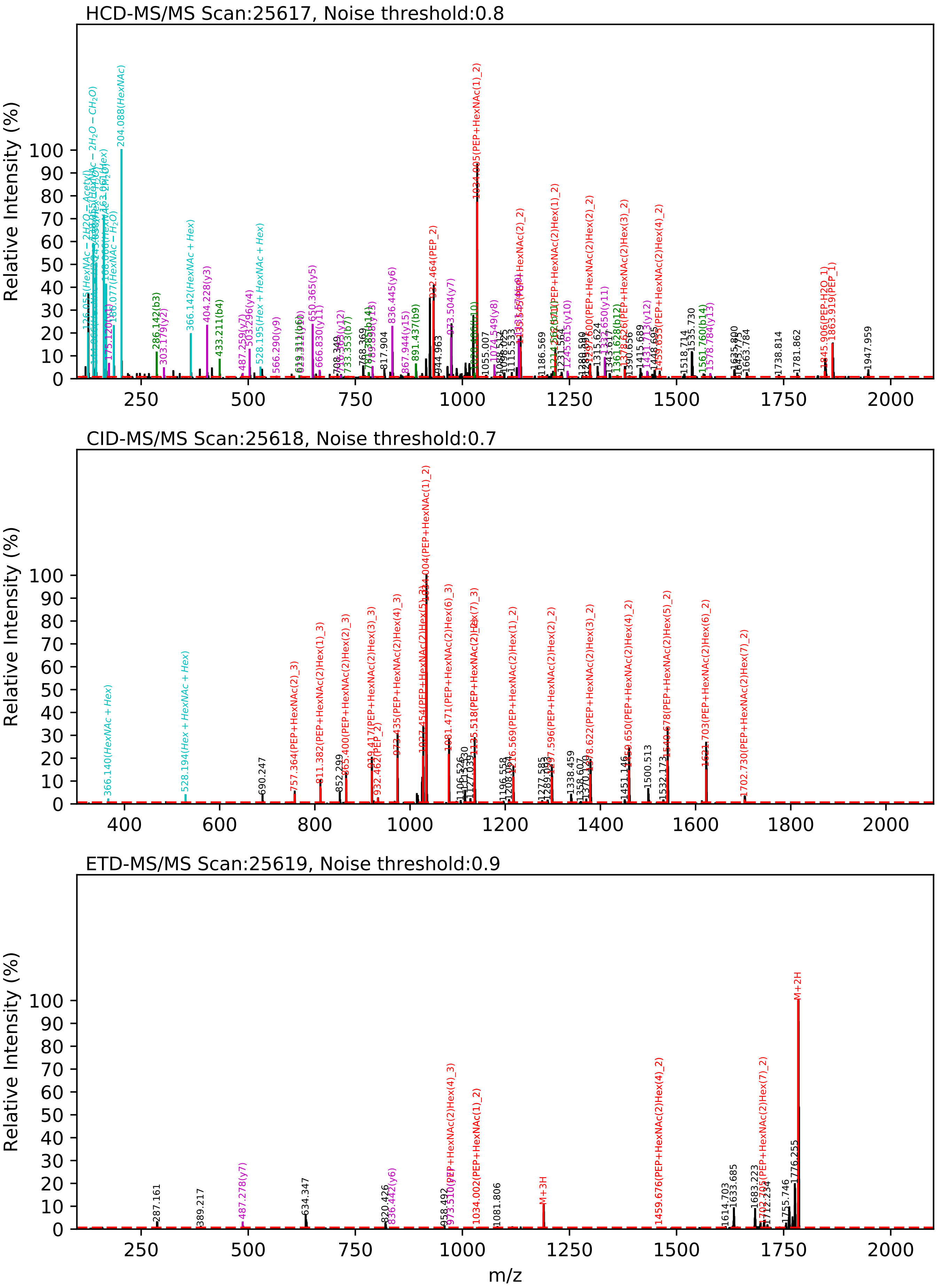

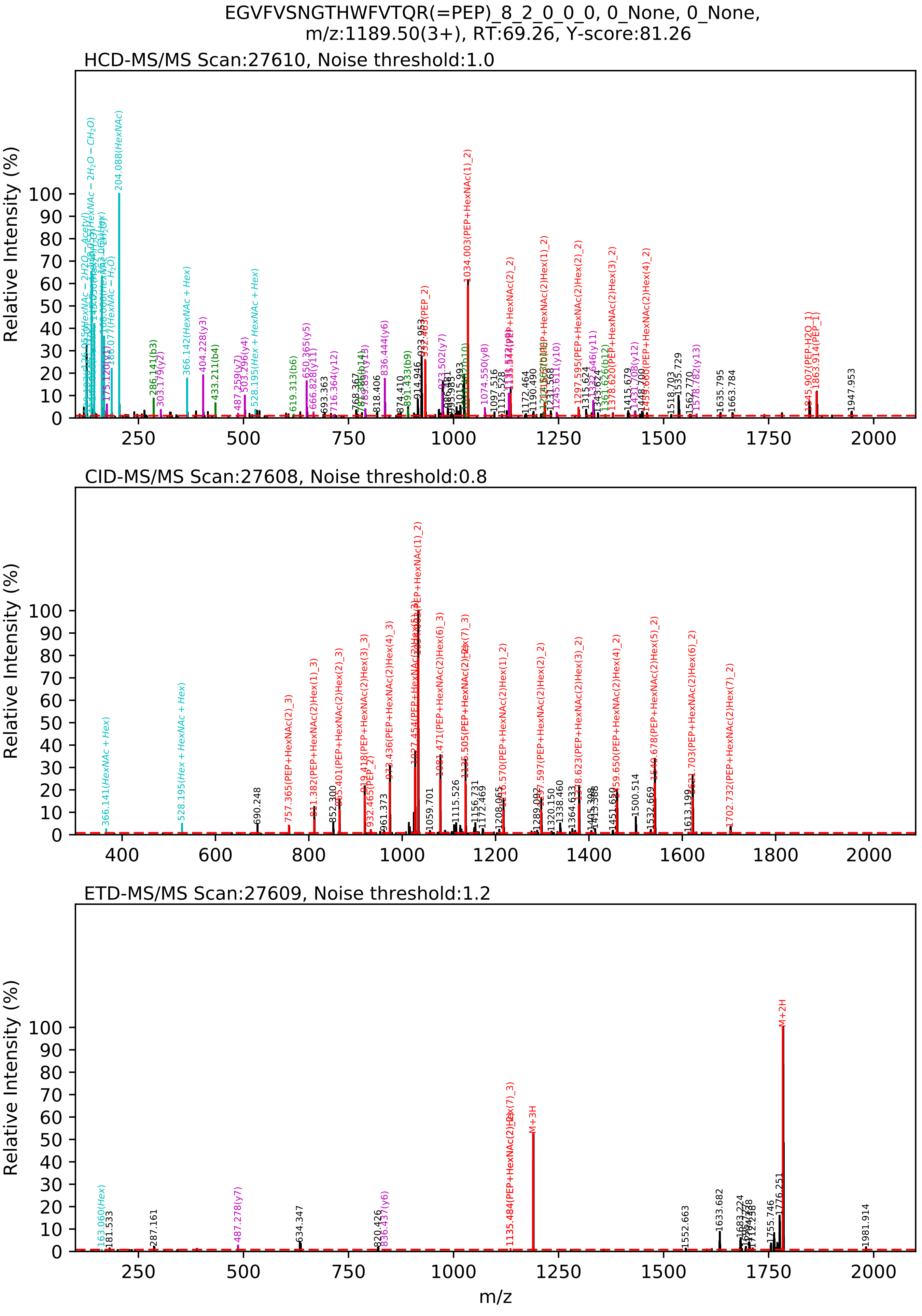

EGVFVSNGTHWFVTQR(=PEP)_8_2_0_0_0, 0_None, 0_None,
m/z:1189.50(3+), RT:69.26, Y-score:81.26
HCD-MS/MS Scan:27610, Noise threshold:1.0
Relative Intensity (%)
204.088(HexNAc)
1034.003(PEP+HexNAc(1)_2)
CID-MS/MS Scan:27608, Noise threshold:0.8
ETD-MS/MS Scan:27609, Noise threshold:1.2
M+2H
M+3H
m/z

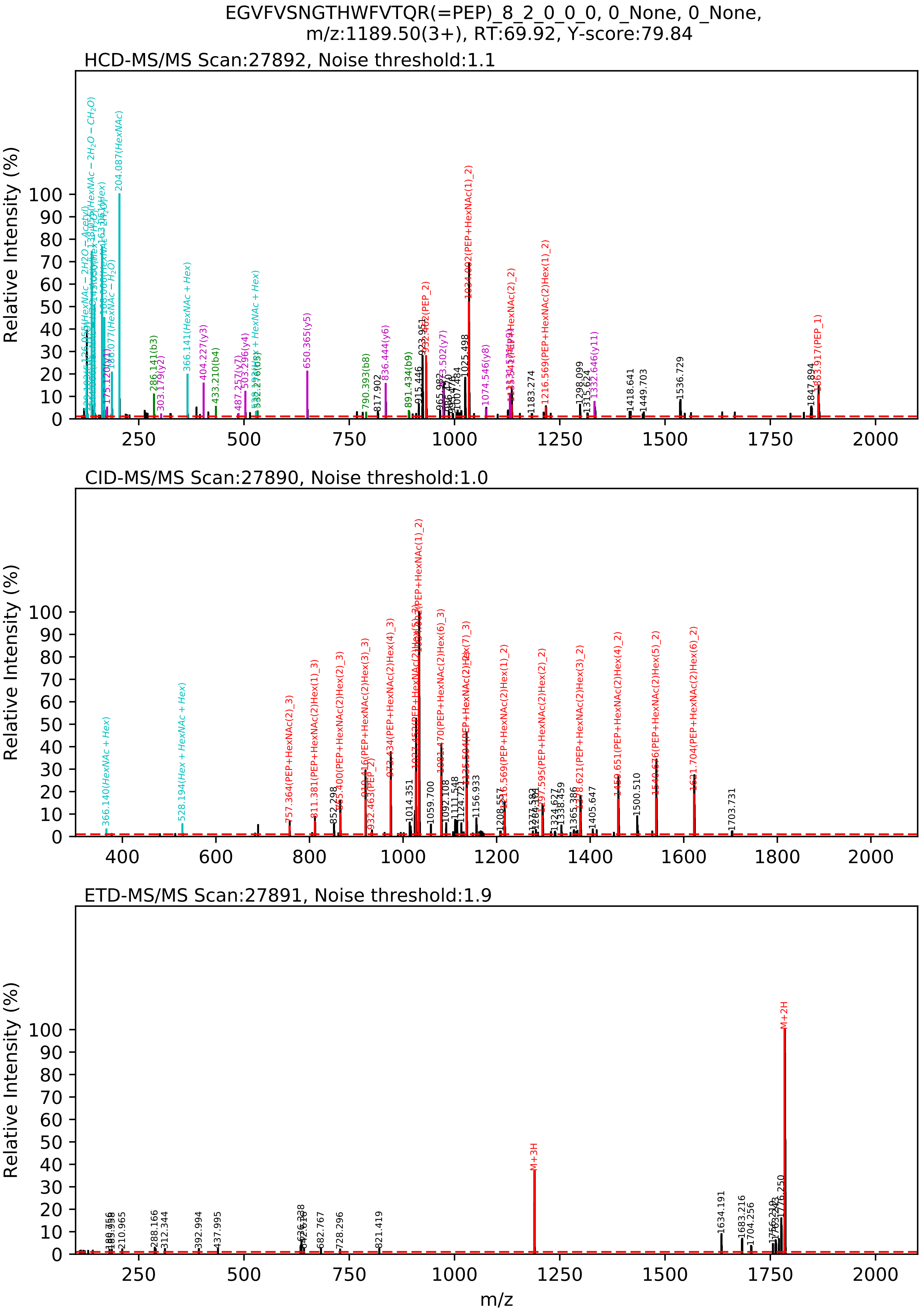

EGVFVSNGTHWFVTQR(=PEP)_8_2_0_0_0, 0_None, 0_None,
m/z:1189.50(3+), RT:69.92, Y-score:79.84
HCD-MS/MS Scan:27892, Noise threshold:1.1
CID-MS/MS Scan:27890, Noise threshold:1.0
ETD-MS/MS Scan:27891, Noise threshold:1.9
Relative Intensity (%)
m/z
204.087(HexNAc)
366.141(HexNAc + Hex)
650.365(y5)
1034.002(PEP+HexNAc(1)_2)
1216.569(PEP+HexNAc(2)Hex(1)_2)
1536.729
1863.917(PEP_1)
1621.704(PEP+HexNAc(2)Hex(6)_2)
1540.676(PEP+HexNAc(2)Hex(5)_2)
1459.651(PEP+HexNAc(2)Hex(4)_2)
M+2H
M+3H
1634.191
1683.216

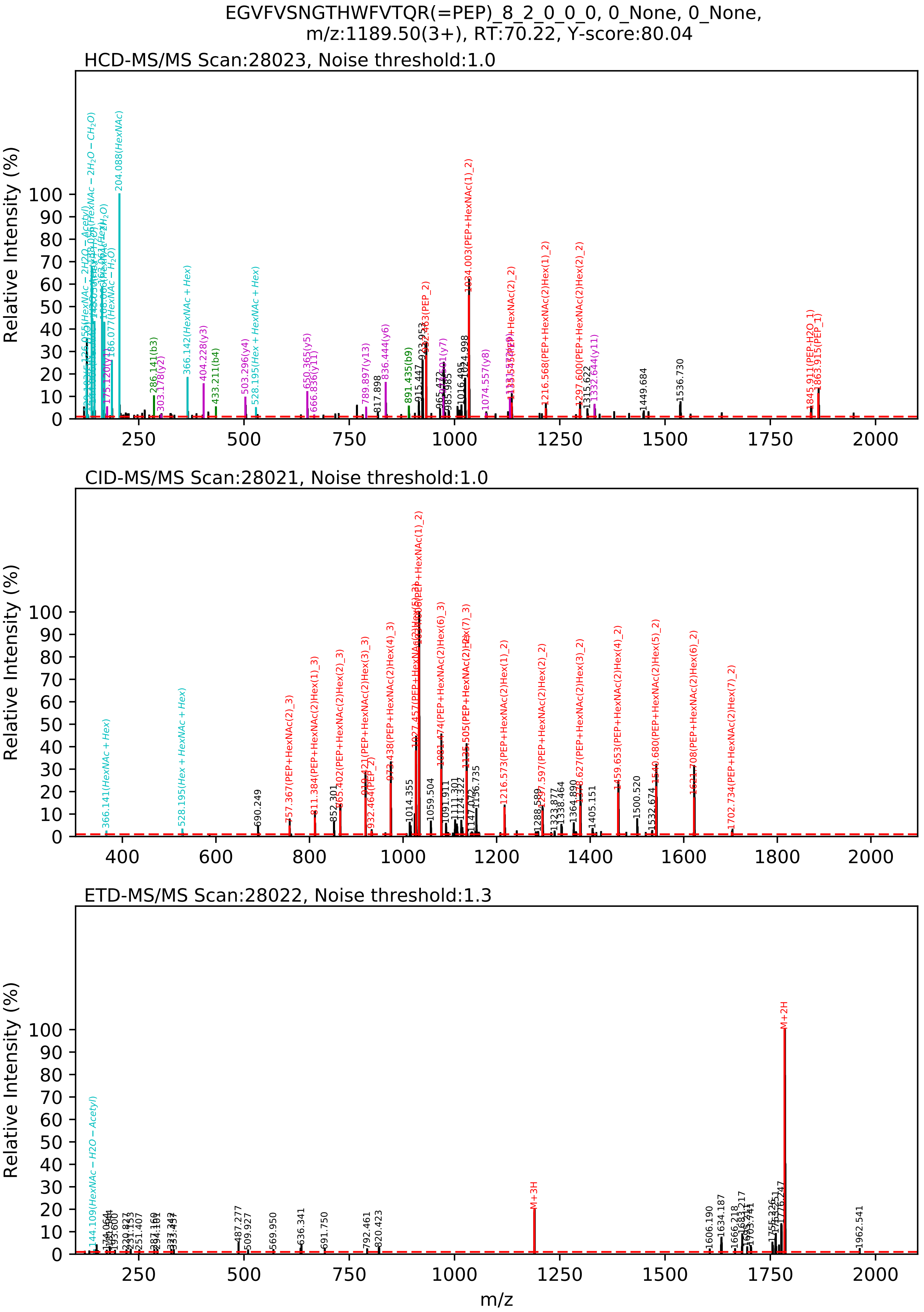

EGVFVSNGTHWFVTQR(=PEP)_8_2_0_0_0, 0_None, 0_None,
m/z:1189.50(3+), RT:70.22, Y-score:80.04
HCD-MS/MS Scan:28023, Noise threshold:1.0
CID-MS/MS Scan:28021, Noise threshold:1.0
ETD-MS/MS Scan:28022, Noise threshold:1.3
Relative Intensity (%)
m/z
M+2H
M+3H

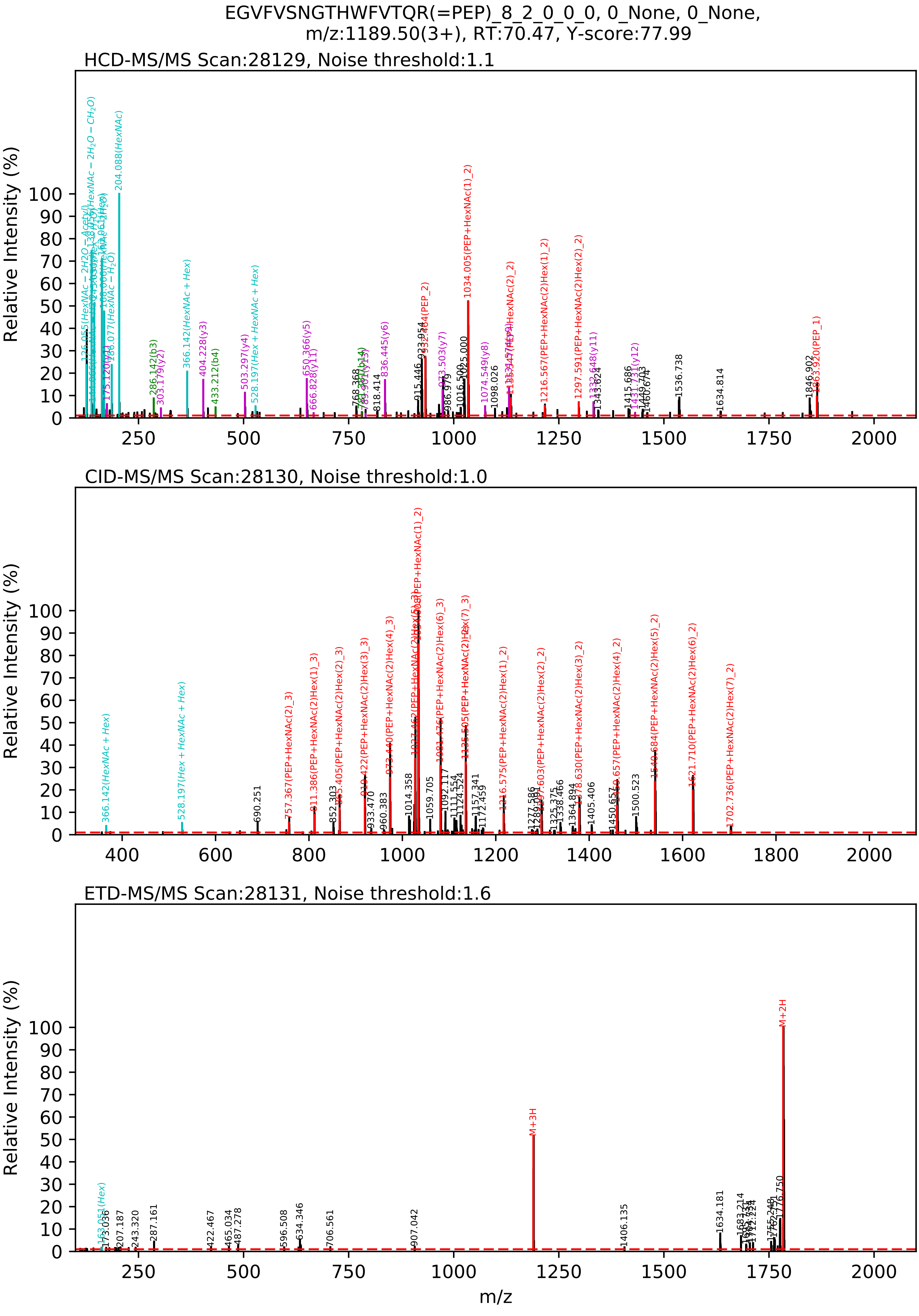

EGVFVSNGTHWFVTQR(=PEP)_8_2_0_0_0, 0_None, 0_None,
m/z:1189.50(3+), RT:70.47, Y-score:77.99
HCD-MS/MS Scan:28129, Noise threshold:1.1
Relative Intensity (%)
204.088(HexNAc)
1034.005(PEP+HexNAc(1)_2)
932.484(PEP_2)
1216.567(PEP+HexNAc(2)Hex(1)_2)
1297.591(PEP+HexNAc(2)Hex(2)_2)
1863.920(PEP_1)
366.142(HexNAc+Hex)
528.197(Hex+HexNAc+Hex)
286.142(b3)
303.179(y2)
404.228(y3)
433.212(b4)
503.297(y4)
650.366(y5)
666.828(y11)
836.445(y6)
973.503(y7)
1074.549(y8)
1332.648(y11)
1431.731(y12)
1536.738
1634.814
1846.902
CID-MS/MS Scan:28130, Noise threshold:1.0
366.142(HexNAc+Hex)
528.197(Hex+HexNAc+Hex)
690.251
757.367(PEP+HexNAc(2)_3)
811.386(PEP+HexNAc(2)Hex(1)_3)
865.405(PEP+HexNAc(2)Hex(2)_3)
919.422(PEP+HexNAc(2)Hex(3)_3)
973.440(PEP+HexNAc(2)Hex(4)_3)
1081.476(PEP+HexNAc(2)Hex(6)_3)
1135.505(PEP+HexNAc(2)Hex(7)_3)
1216.575(PEP+HexNAc(2)Hex(1)_2)
1297.603(PEP+HexNAc(2)Hex(2)_2)
1378.630(PEP+HexNAc(2)Hex(3)_2)
1459.657(PEP+HexNAc(2)Hex(4)_2)
1540.684(PEP+HexNAc(2)Hex(5)_2)
1621.710(PEP+HexNAc(2)Hex(6)_2)
1702.736(PEP+HexNAc(2)Hex(7)_2)
1500.523
1405.406
ETD-MS/MS Scan:28131, Noise threshold:1.6
M+2H
M+3H
163.051(Hex)
207.187
243.320
287.161
422.467
465.034
487.278
596.508
634.346
706.561
907.042
1406.135
1634.181
1683.214
1776.750
m/z

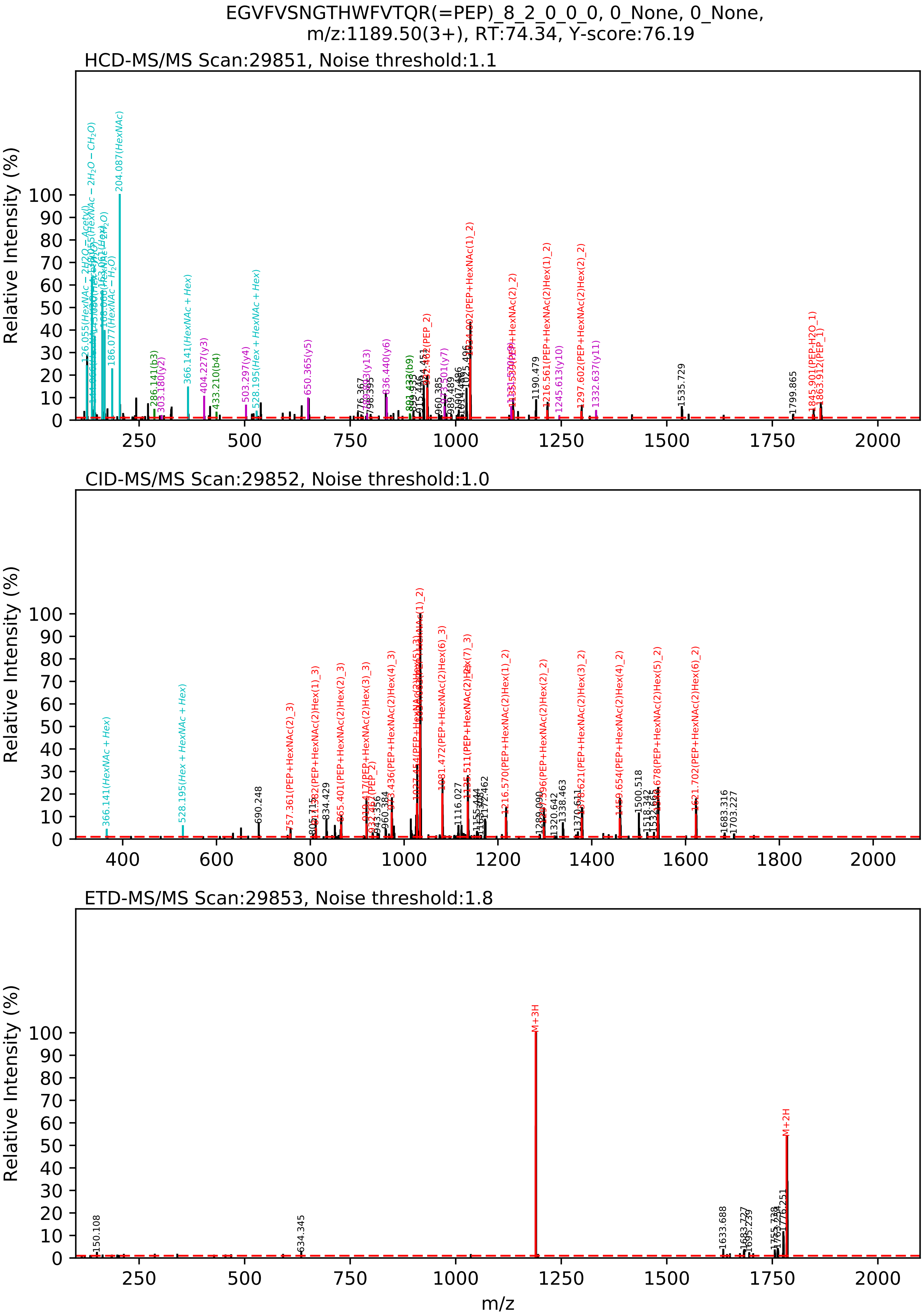
EGVFVSNGTHWFVTQR(=PEP)_8_2_0_0_0, 0_None, 0_None,
m/z:1189.50(3+), RT:74.34, Y-score:76.19
HCD-MS/MS Scan:29851, Noise threshold:1.1
Relative Intensity (%)
204.087(HexNAc)
186.077(HexNAc − H2O)
366.141(HexNAc + Hex)
528.195(Hex + HexNAc + Hex)
286.141(b3)
303.180(y2)
404.227(y3)
433.210(b4)
503.297(y4)
650.365(y5)
836.440(y6)
881.433(b9)
1245.613(y10)
1332.637(y11)
1297.602(PEP+HexNAc(2)Hex(2)_2)
1216.561(PEP+HexNAc(2)Hex(1)_2)
1190.479
1535.729
1799.865
1845.901(PEP-H2O_1)
1863.912(PEP_1)
CID-MS/MS Scan:29852, Noise threshold:1.0
366.141(HexNAc + Hex)
528.195(Hex + HexNAc + Hex)
690.248
757.361(PEP+HexNAc(2)_3)
834.429
865.401(PEP+HexNAc(2)Hex(2)_3)
960.384
1081.472(PEP+HexNAc(2)Hex(6)_3)
1116.027
1172.462
1216.570(PEP+HexNAc(2)Hex(1)_2)
1289.090
1320.642
1338.463
1370.611
1459.654(PEP+HexNAc(2)Hex(4)_2)
1500.518
1621.702(PEP+HexNAc(2)Hex(6)_2)
1683.316
1703.227
ETD-MS/MS Scan:29853, Noise threshold:1.8
M+3H
M+2H
150.108
634.345
1633.688
1683.727
1695.239
1765.738
1778.251
m/z

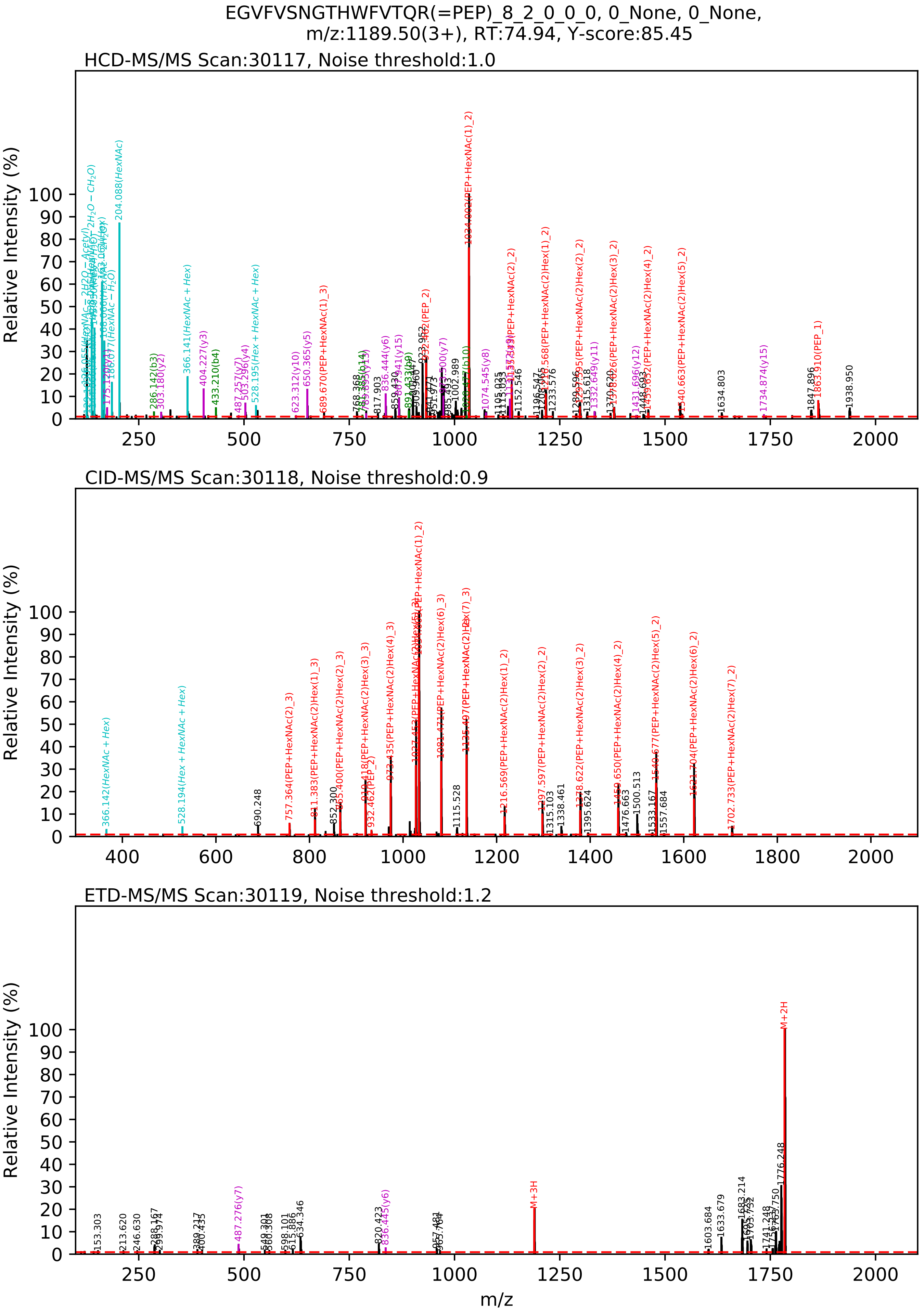
EGVFVSNGTHWFVTQR(=PEP)_8_2_0_0_0, 0_None, 0_None,
m/z:1189.50(3+), RT:74.94, Y-score:85.45
HCD-MS/MS Scan:30117, Noise threshold:1.0
Relative Intensity (%)
CID-MS/MS Scan:30118, Noise threshold:0.9
Relative Intensity (%)
ETD-MS/MS Scan:30119, Noise threshold:1.2
Relative Intensity (%)
m/z

EGVFVSNGTHWFVTQR(=PEP)_8_2_0_0_0, 0_None, 0_None,
m/z:1189.50(3+), RT:75.50, Y-score:75.89

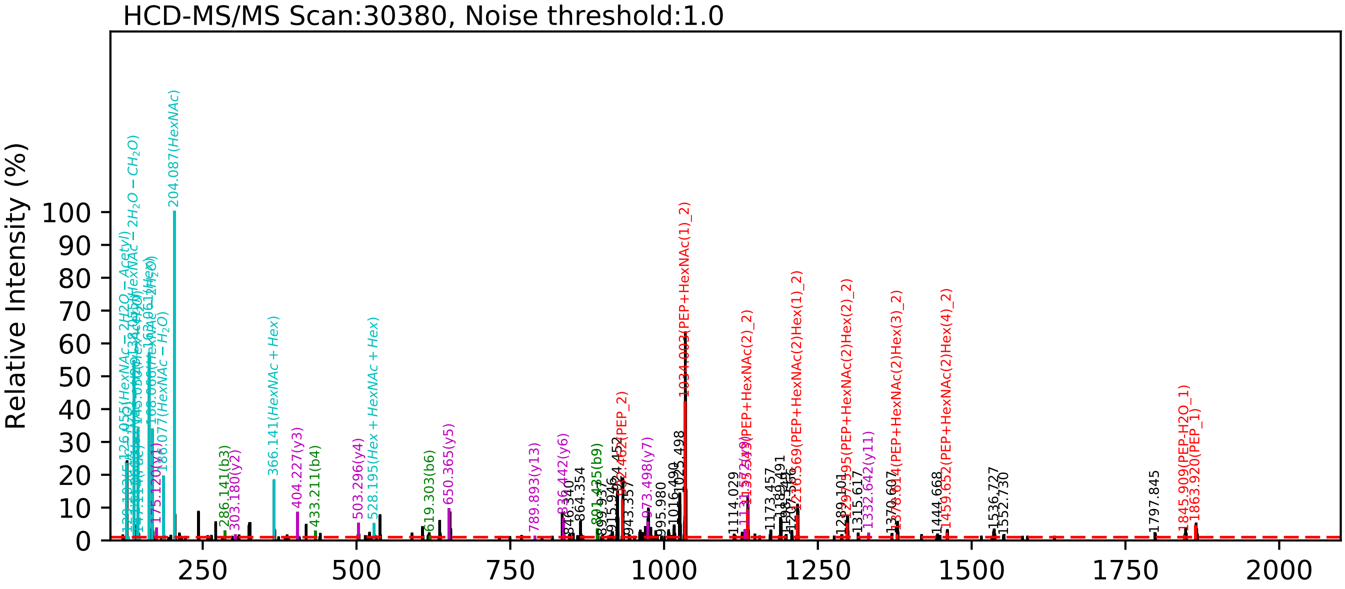

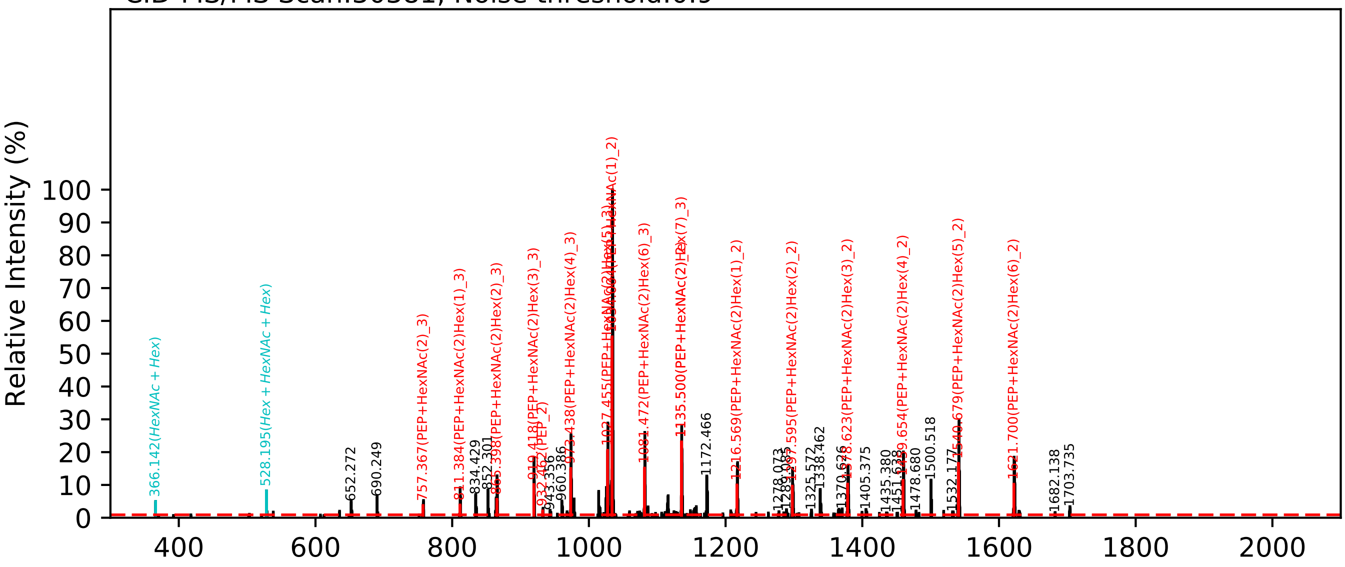

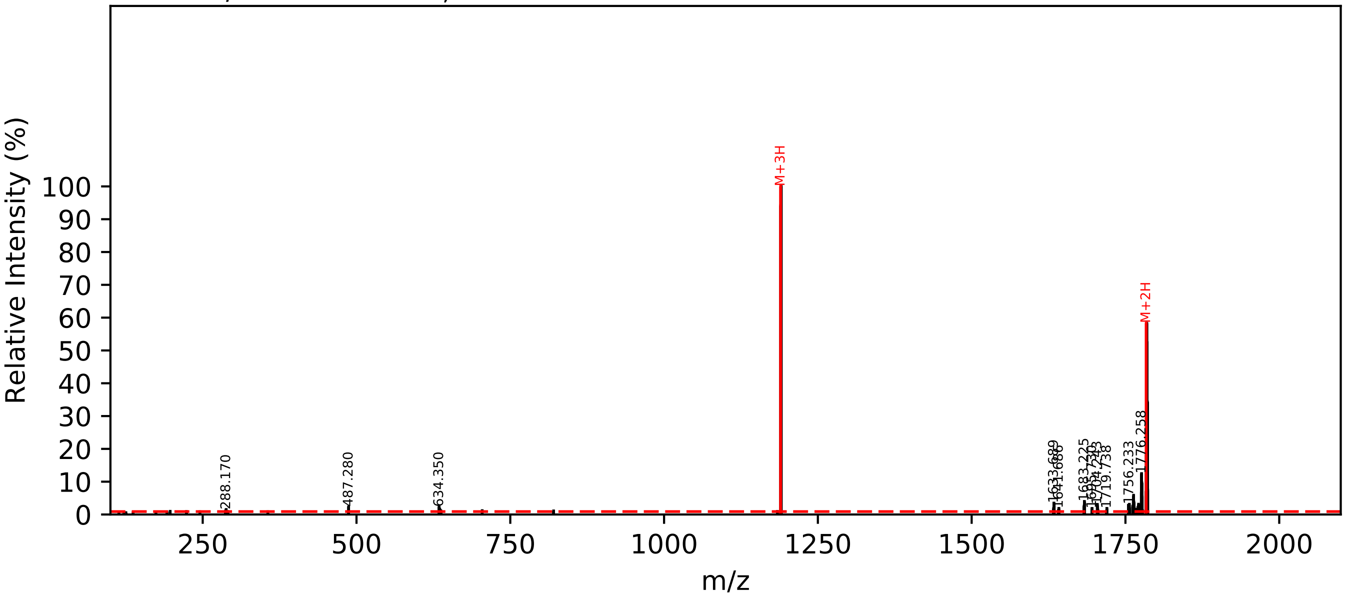

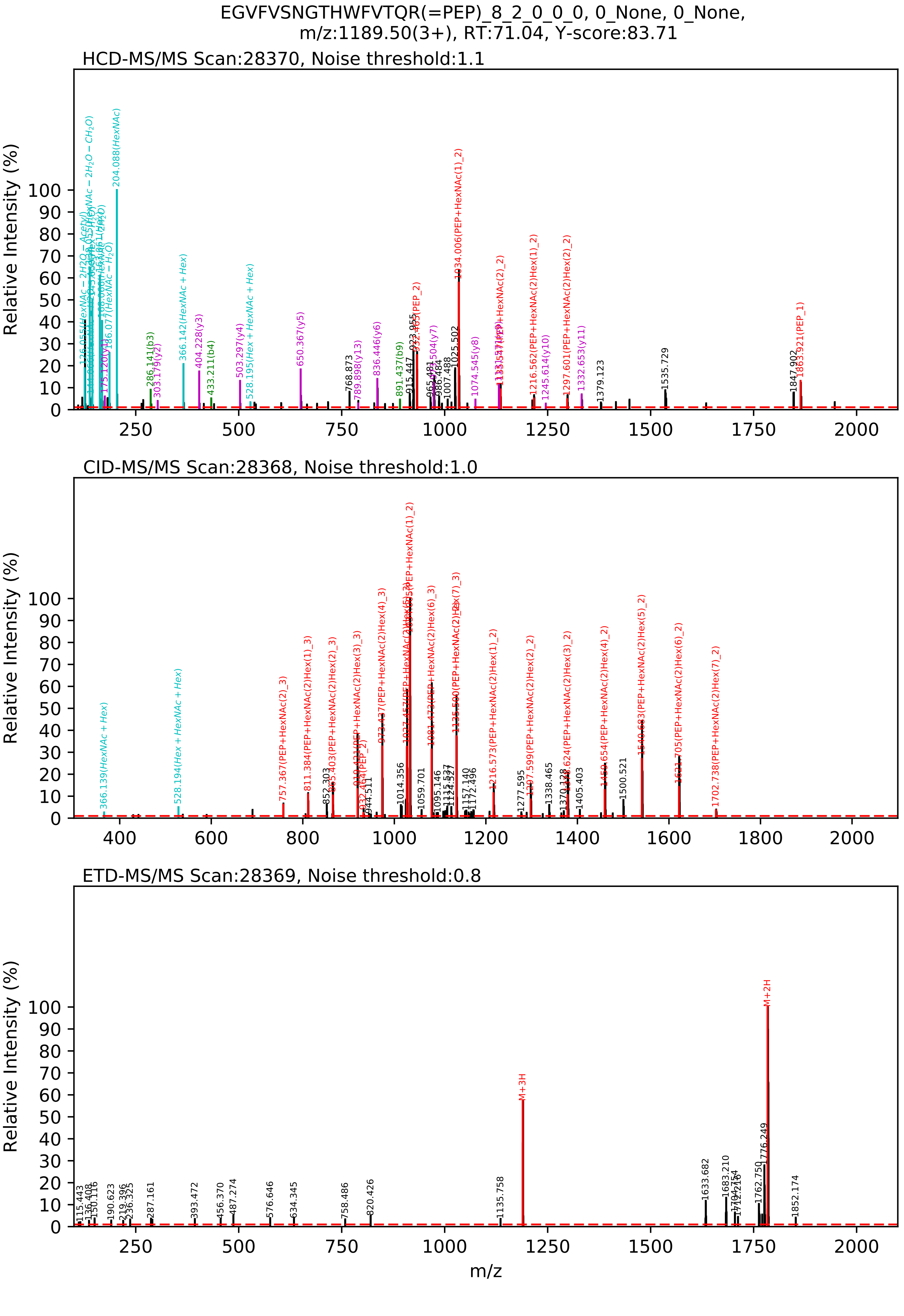

EGVFVSNGTHWFVTQR(=PEP)_8_2_0_0_0, 0_None, 0_None,
m/z:1189.50(3+), RT:71.04, Y-score:83.71
HCD-MS/MS Scan:28370, Noise threshold:1.1
Relative Intensity (%)
204.088(HexNAc)
1034.006(PEP+HexNAc(1)_2)
1863.921(PEP_1)
1535.729
1847.902
CID-MS/MS Scan:28368, Noise threshold:1.0
Relative Intensity (%)
757.367(PEP+HexNAc(2)_3)
811.384(PEP+HexNAc(2)Hex(1)_3)
1216.573(PEP+HexNAc(2)Hex(1)_2)
1540.683(PEP+HexNAc(2)Hex(5)_2)
1621.705(PEP+HexNAc(2)Hex(6)_2)
1702.738(PEP+HexNAc(2)Hex(7)_2)
ETD-MS/MS Scan:28369, Noise threshold:0.8
Relative Intensity (%)
M+3H
M+2H
m/z

EGVFVSNGTHWFVTQR(=PEP)_8_2_0_0_0, 0_None, 0_None,
m/z:1189.50(3+), RT:65.21, Y-score:85.21

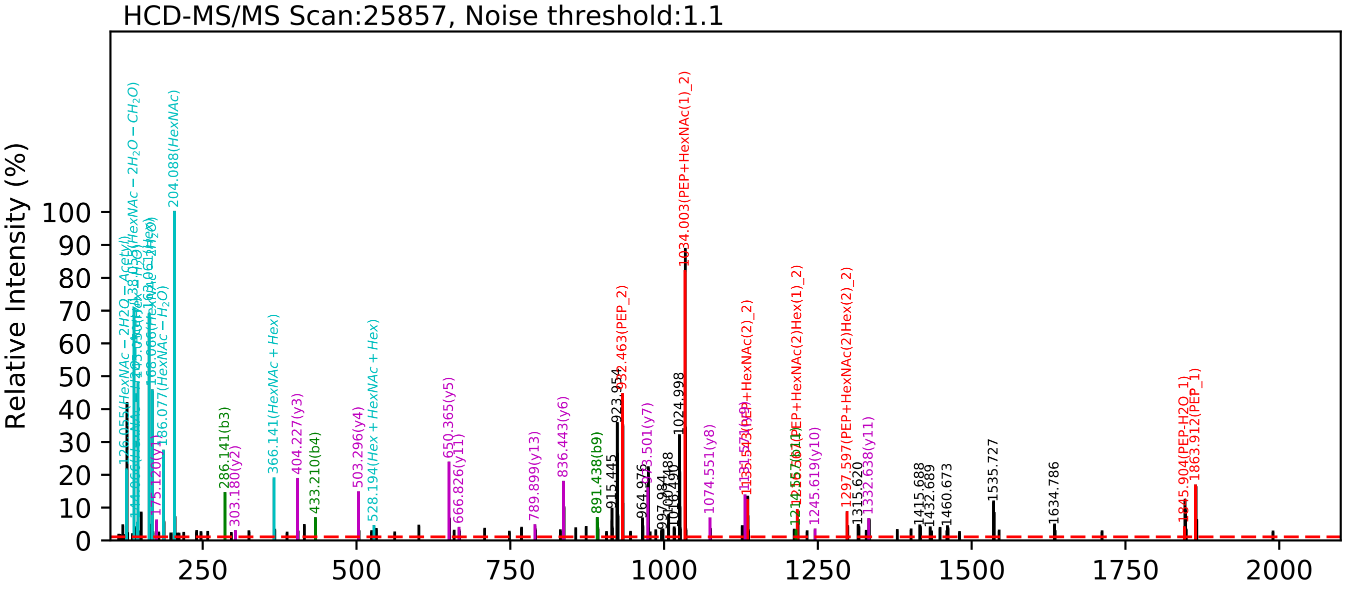

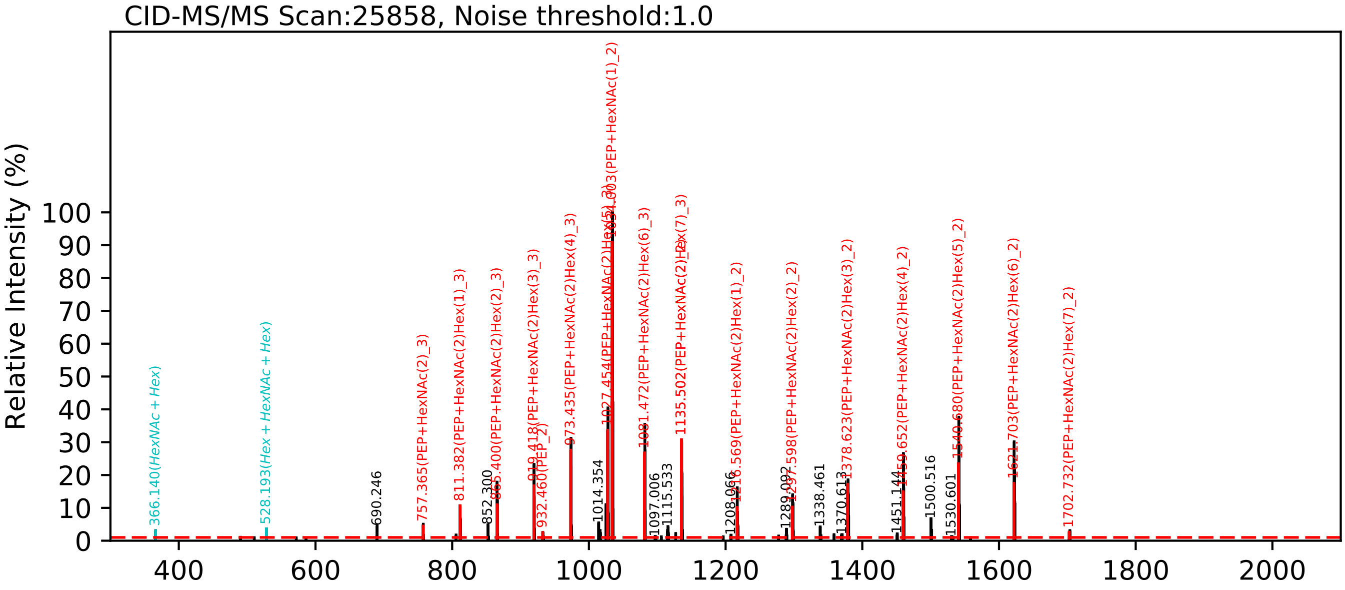

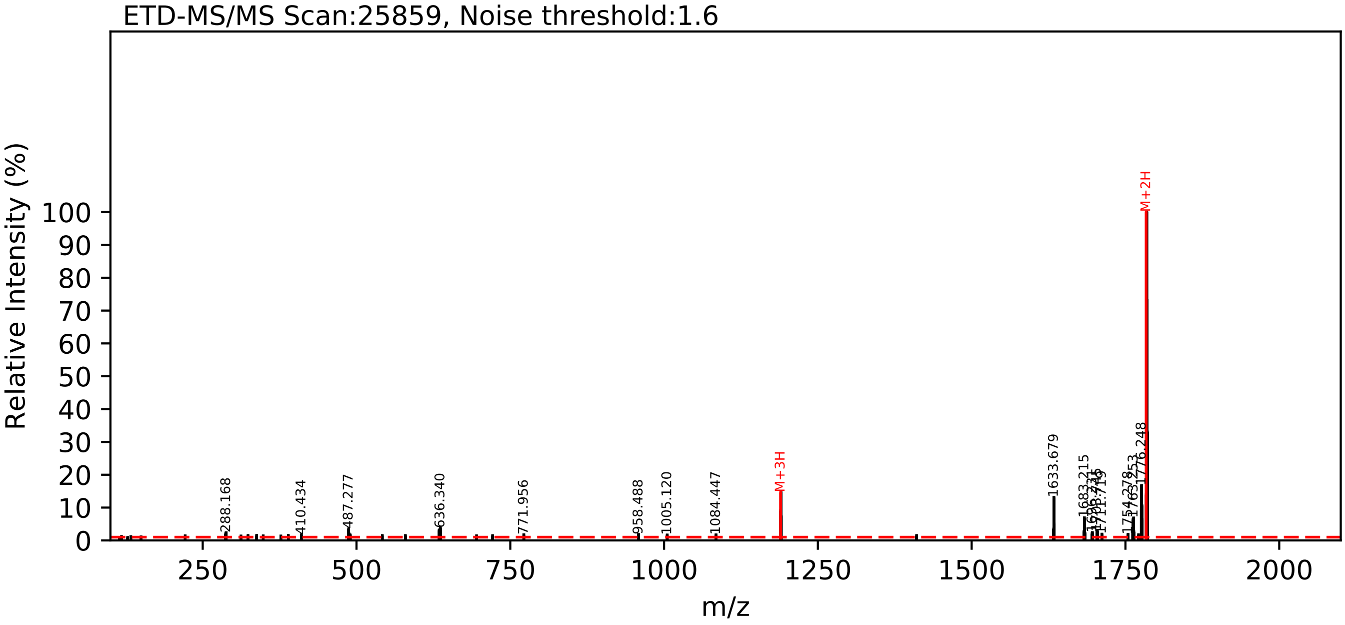

EGVFVSNGTHWFVTQR(=PEP)_8_2_0_0_0, 0_None, 0_None,
m/z:1189.50(3+), RT:65.48, Y-score:87.44

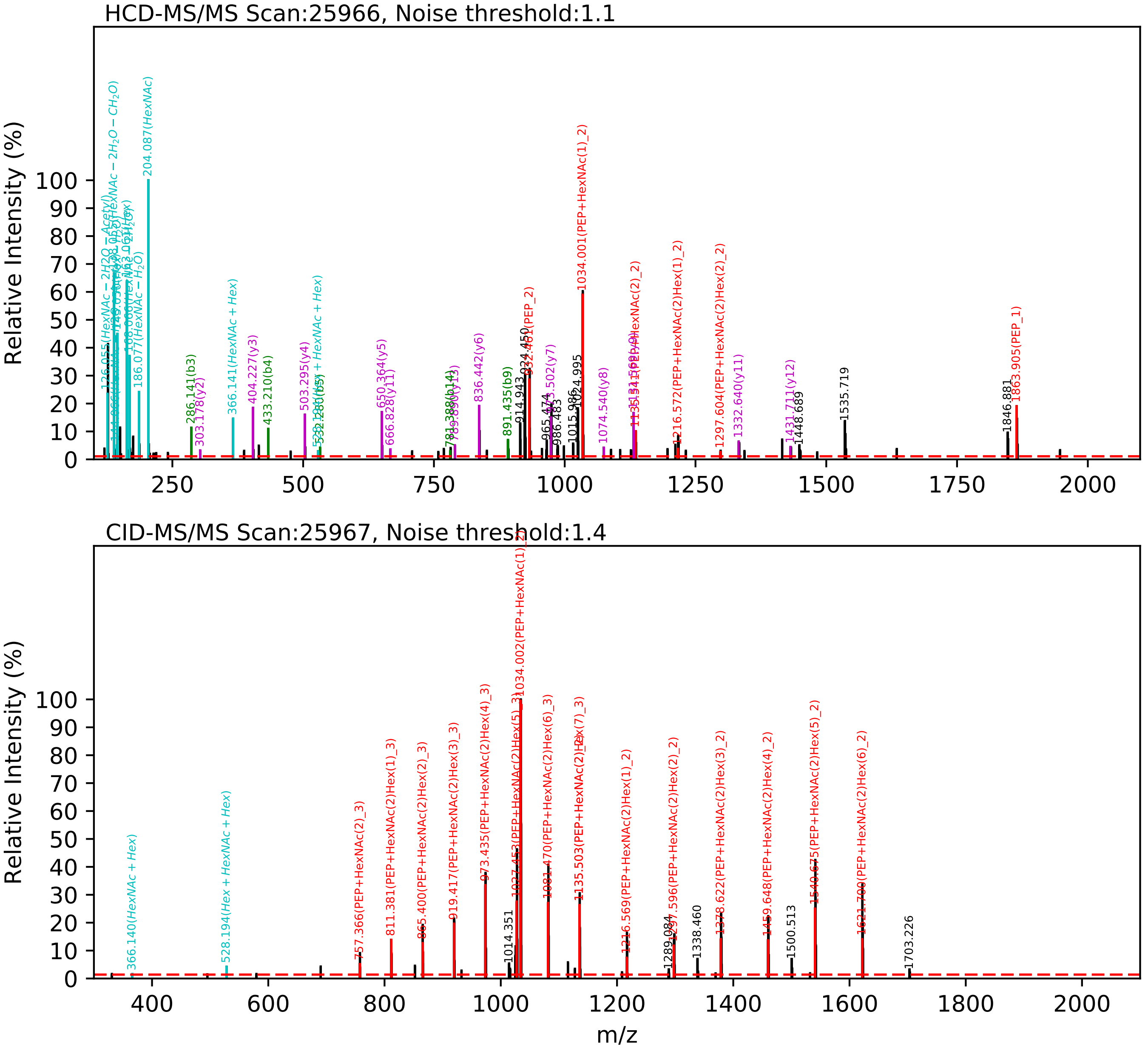

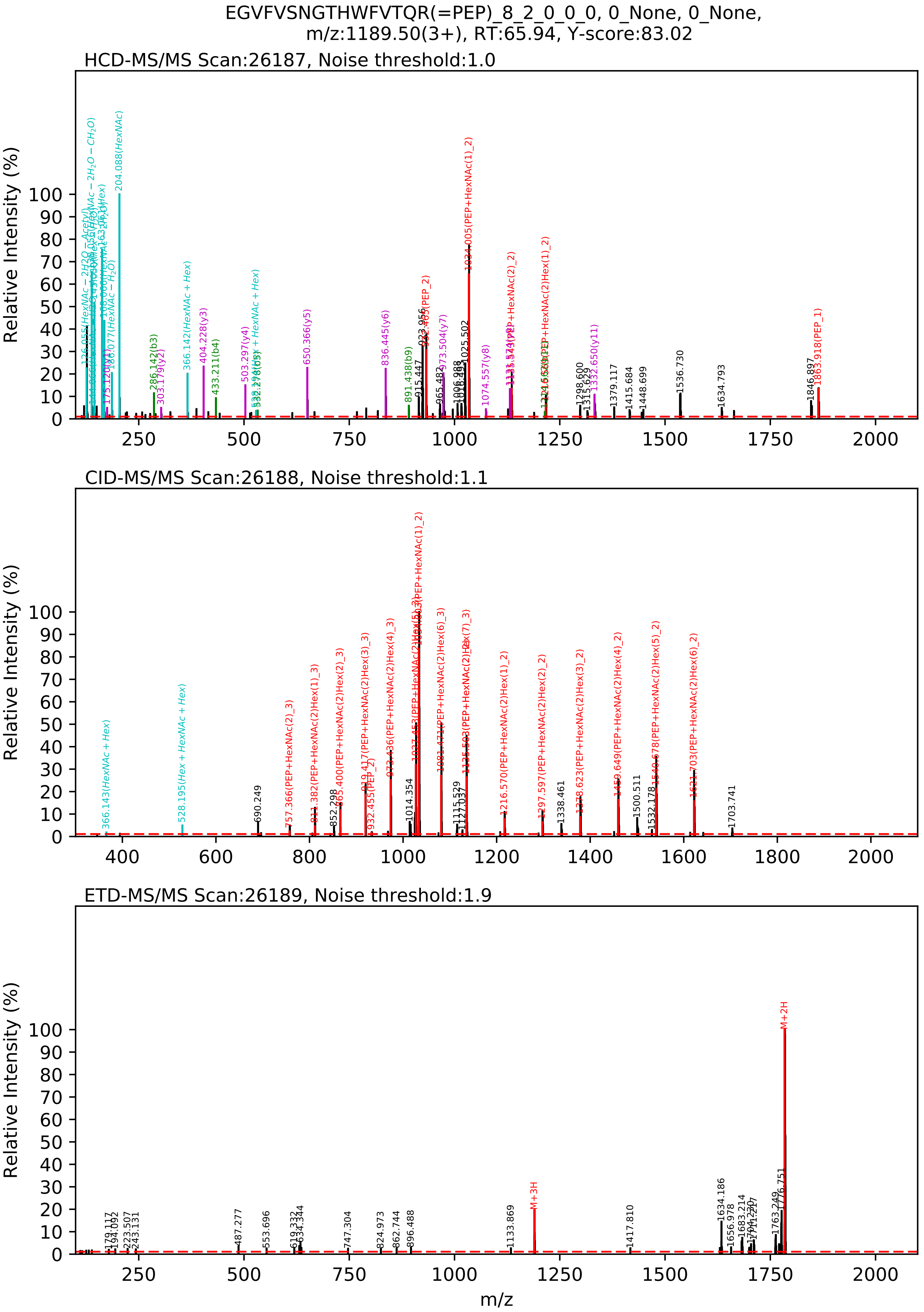
EGVFVSNGTHWFVTQR(=PEP)_8_2_0_0_0, 0_None, 0_None,
m/z:1189.50(3+), RT:65.94, Y-score:83.02
HCD-MS/MS Scan:26187, Noise threshold:1.0
Relative Intensity (%)
204.088(HexNAc)
1034.005(PEP+HexNAc(1)_2)
286.142(b3)
303.179(y2)
366.142(HexNAc + Hex)
404.228(y3)
433.211(b4)
503.297(y4)
650.366(y5)
836.445(y6)
891.438(b9)
915.447
923.956
965.482
973.504(y7)
1006.998
1025.502
1074.557(y8)
1298.600
1313.629
1332.650(y11)
1379.117
1415.684
1448.699
1536.730
1634.793
1846.897
1863.918(PEP_1)
CID-MS/MS Scan:26188, Noise threshold:1.1
366.143(HexNAc + Hex)
528.195(Hex + HexNAc + Hex)
690.249
757.366(PEP+HexNAc(2)_3)
811.382(PEP+HexNAc(2)Hex(1)_3)
852.298
865.400(PEP+HexNAc(2)Hex(2)_3)
919.417(PEP+HexNAc(2)Hex(3)_3)
932.455(PEP_2)
973.436(PEP+HexNAc(2)Hex(4)_3)
1014.354
1081.471(PEP+HexNAc(2)Hex(6)_3)
1115.529
1135.503(PEP+HexNAc(2)Hex(7)_3)
1216.570(PEP+HexNAc(2)Hex(1)_2)
1297.597(PEP+HexNAc(2)Hex(2)_2)
1338.461
1378.623(PEP+HexNAc(2)Hex(3)_2)
1459.649(PEP+HexNAc(2)Hex(4)_2)
1500.511
1532.178
1540.678(PEP+HexNAc(2)Hex(5)_2)
1621.703(PEP+HexNAc(2)Hex(6)_2)
1703.741
ETD-MS/MS Scan:26189, Noise threshold:1.9
M+2H
M+3H
179.117
194.092
223.507
243.131
487.277
553.696
619.332
634.344
747.304
824.973
862.744
896.488
1133.869
1417.810
1634.186
1656.978
1683.214
1763.249
1776.751
m/z

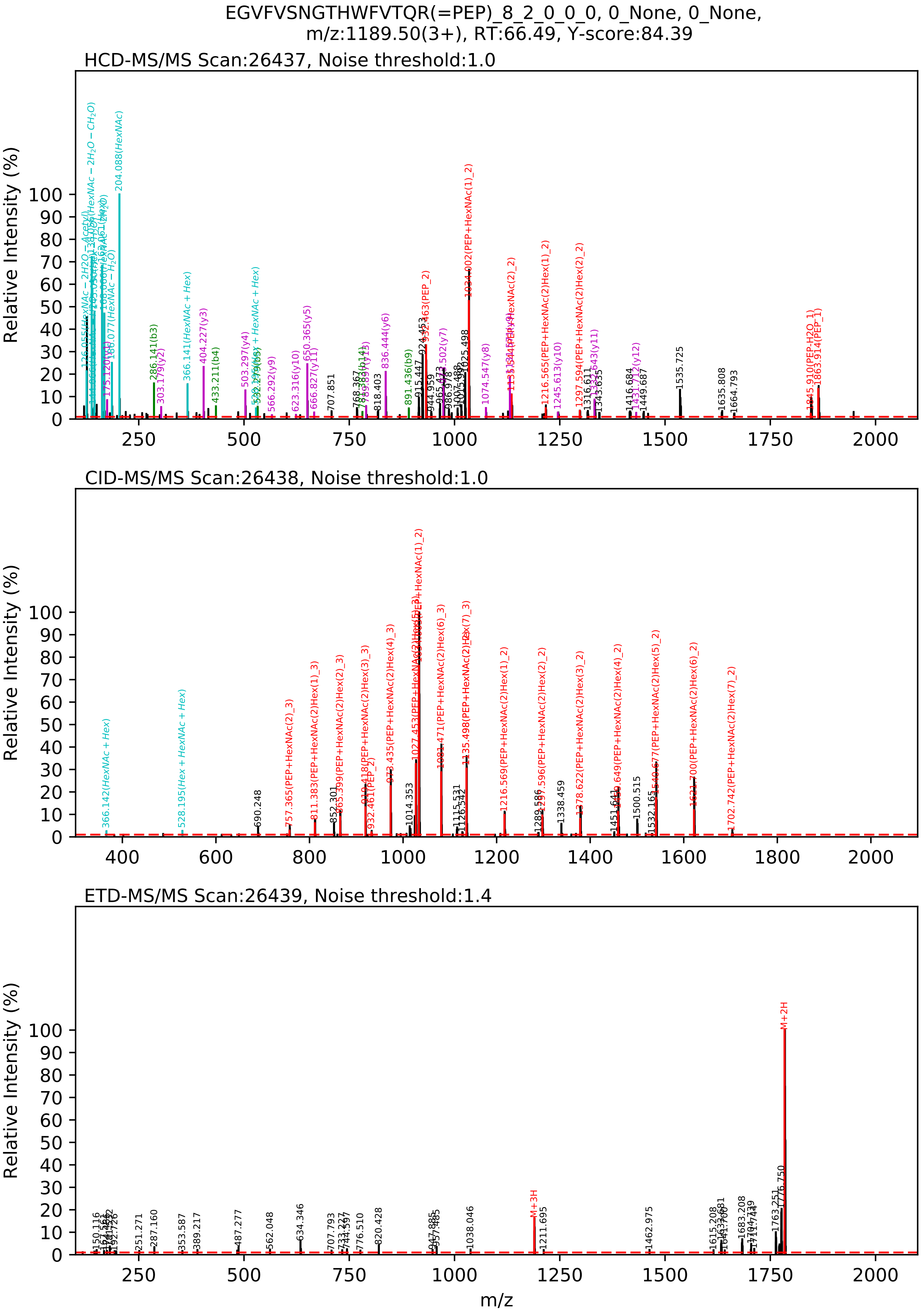
EGVFVSNGTHWFVTQR(=PEP)_8_2_0_0_0, 0_None, 0_None,
m/z:1189.50(3+), RT:66.49, Y-score:84.39
HCD-MS/MS Scan:26437, Noise threshold:1.0
CID-MS/MS Scan:26438, Noise threshold:1.0
ETD-MS/MS Scan:26439, Noise threshold:1.4
Relative Intensity (%)
m/z
M+2H
M+3H

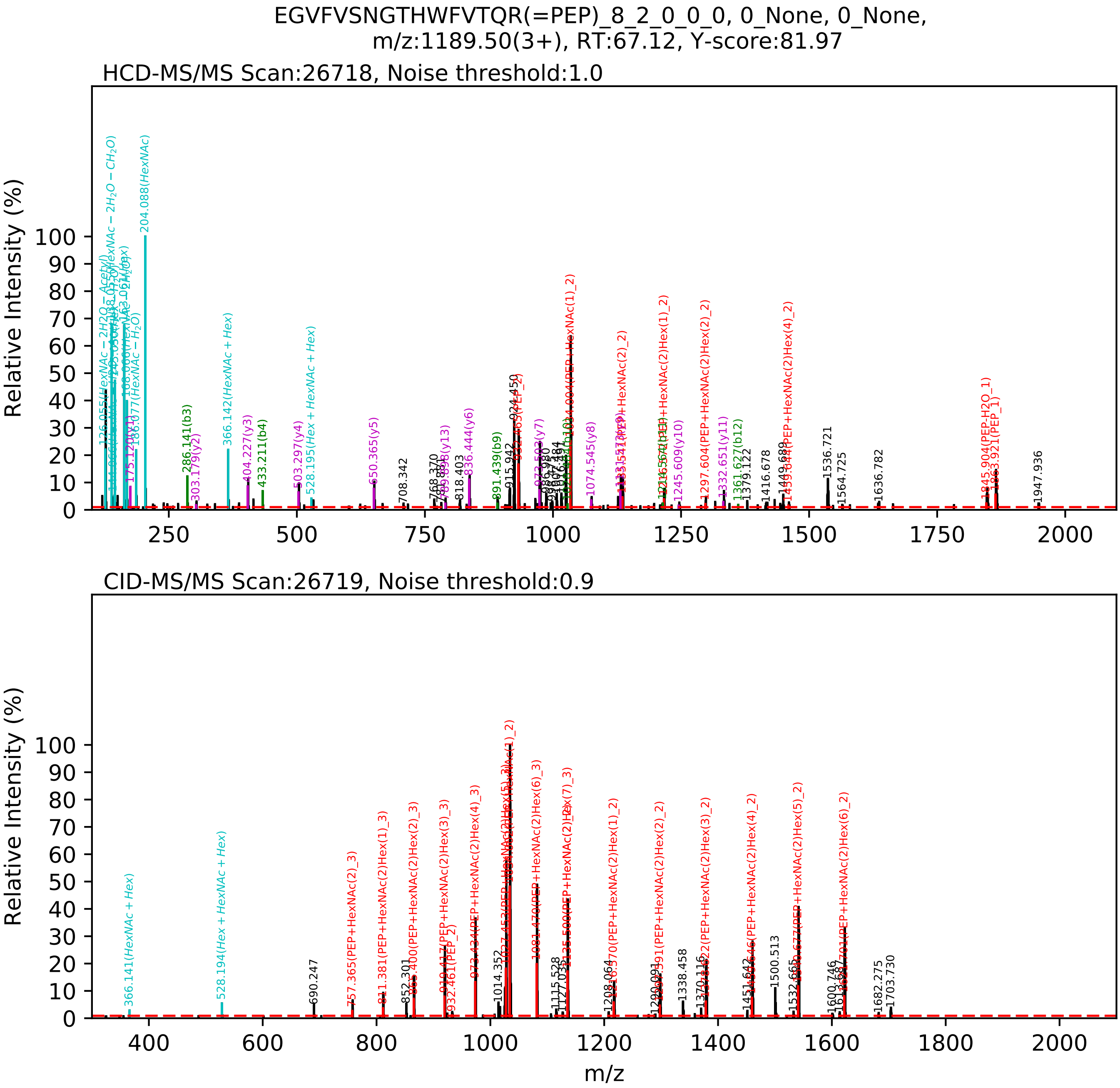
EGVFVSNGTHWFVTQR(=PEP)_8_2_0_0_0, 0_None, 0_None,
m/z:1189.50(3+), RT:67.12, Y-score:81.97
HCD-MS/MS Scan:26718, Noise threshold:1.0
Relative Intensity (%)
CID-MS/MS Scan:26719, Noise threshold:0.9
Relative Intensity (%)
m/z

EGVFVSNGTHWFVTQR(=PEP)_8_2_0_0_0, 0_None, 0_None,
m/z:1189.50(3+), RT:67.55, Y-score:86.55

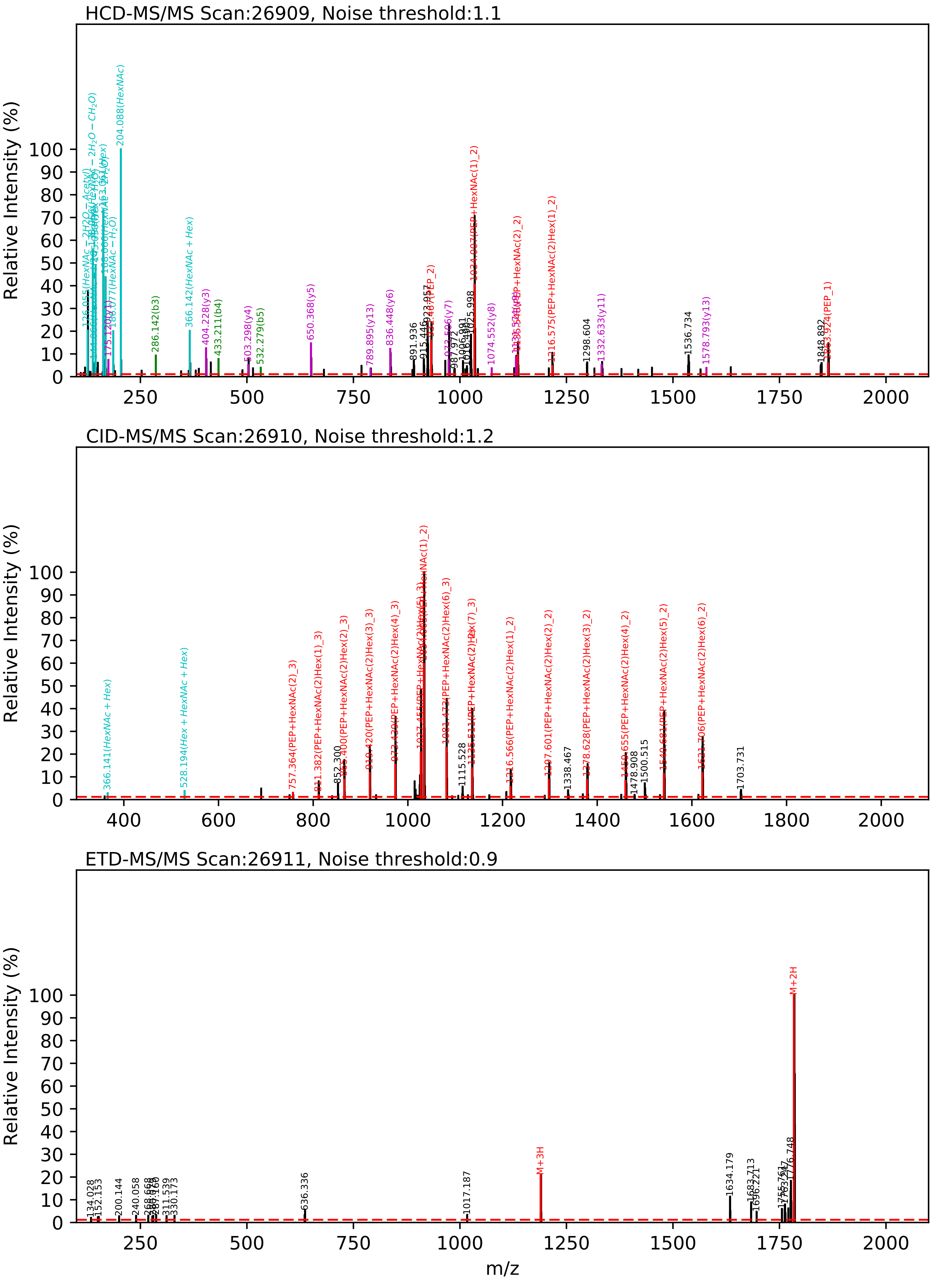

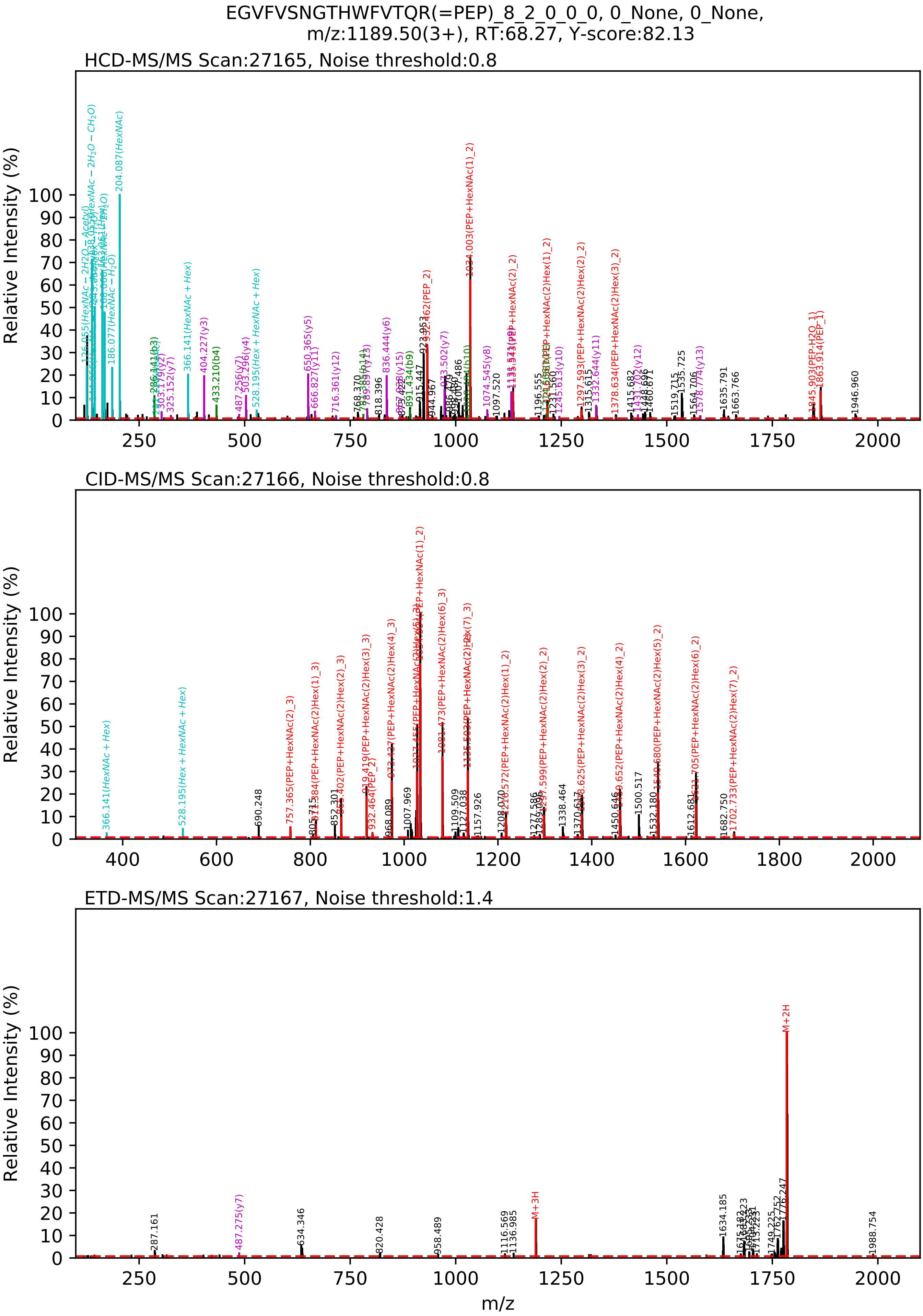

EGVFVSNGTHWFVTQR(=PEP)_8_2_0_0_0, 0_None, 0_None,
m/z:1189.50(3+), RT:68.27, Y-score:82.13
HCD-MS/MS Scan:27165, Noise threshold:0.8
Relative Intensity (%)
204.087(HexNAc)
186.077(HexNAc − H2O)
366.141(HexNAc + Hex)
404.227(y3)
433.210(b4)
528.195(Hex + HexNAc + Hex)
650.365(y5)
666.827(y11)
716.361(y12)
836.444(y6)
923.953
932.462(PEP_2)
1034.003(PEP+HexNAc(1)_2)
1074.545(y8)
1097.520
1135.543(PEP+HexNAc(2)_2)
1297.593(PEP+HexNAc(2)Hex(2)_2)
1378.634(PEP+HexNAc(2)Hex(3)_2)
1332.644(y11)
1535.725
1564.706
1578.774(y13)
1635.791
1663.766
1845.903(PEP-H2O_1)
1863.914(PEP_1)
1946.960
CID-MS/MS Scan:27166, Noise threshold:0.8
Relative Intensity (%)
366.141(HexNAc + Hex)
528.195(Hex + HexNAc + Hex)
690.248
757.365(PEP+HexNAc(2)_3)
852.301
932.464(PEP_2)
968.089
1007.969
1081.473(PEP+HexNAc(2)Hex(6)_3)
1109.509
1127.038
1157.926
1208.070
1338.464
1450.646
1500.517
1532.180
1612.681
1682.750
1702.733(PEP+HexNAc(2)Hex(7)_2)
ETD-MS/MS Scan:27167, Noise threshold:1.4
Relative Intensity (%)
M+2H
M+3H
287.161
487.275(y7)
634.346
820.428
958.489
1116.569
1136.985
1634.185
1988.754
m/z

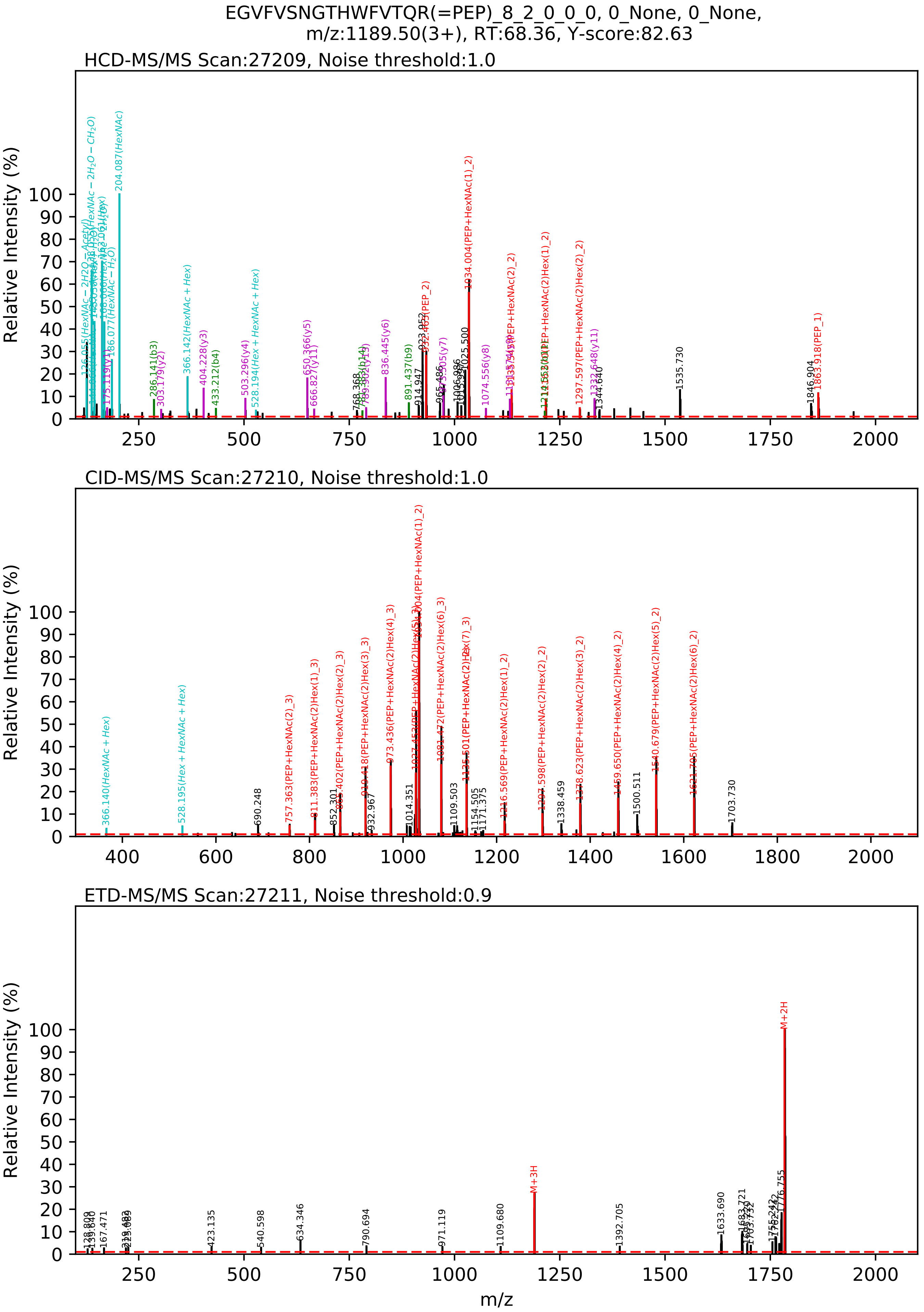
EGVFVSNGTHWFVTQR(=PEP)_8_2_0_0_0, 0_None, 0_None,
m/z:1189.50(3+), RT:68.36, Y-score:82.63
HCD-MS/MS Scan:27209, Noise threshold:1.0
Relative Intensity (%)
204.087(HexNAc)
1034.004(PEP+HexNAc(1)_2)
1863.918(PEP_1)
1535.730
1846.904
1297.597(PEP+HexNAc(2)Hex(2)_2)
1332.648(y11)
1344.640
1074.556(y8)
932.462(PEP_2)
923.952
914.947
891.437(b9)
836.445(y6)
650.366(y5)
666.827(y11)
503.296(y4)
528.194(Hex + HexNAc + Hex)
366.142(HexNAc + Hex)
404.228(y3)
433.212(b4)
286.141(b3)
303.179(y2)
175.119(y1)
186.077(HexNAc − H2O)
CID-MS/MS Scan:27210, Noise threshold:1.0
366.140(HexNAc + Hex)
528.195(Hex + HexNAc + Hex)
690.248
757.363(PEP+HexNAc(2)_3)
811.383(PEP+HexNAc(2)Hex(1)_3)
852.301
932.967
973.436(PEP+HexNAc(2)Hex(4)_3)
1014.351
1109.503
1154.505
1171.375
1216.569(PEP+HexNAc(2)Hex(1)_2)
1297.598(PEP+HexNAc(2)Hex(2)_2)
1338.459
1378.623(PEP+HexNAc(2)Hex(3)_2)
1459.650(PEP+HexNAc(2)Hex(4)_2)
1500.511
1540.679(PEP+HexNAc(2)Hex(5)_2)
1621.705(PEP+HexNAc(2)Hex(6)_2)
1703.730
ETD-MS/MS Scan:27211, Noise threshold:0.9
M+2H
M+3H
128.809
167.471
423.135
540.598
634.346
790.694
971.119
1109.680
1392.705
1633.690
1683.721
1776.755
m/z

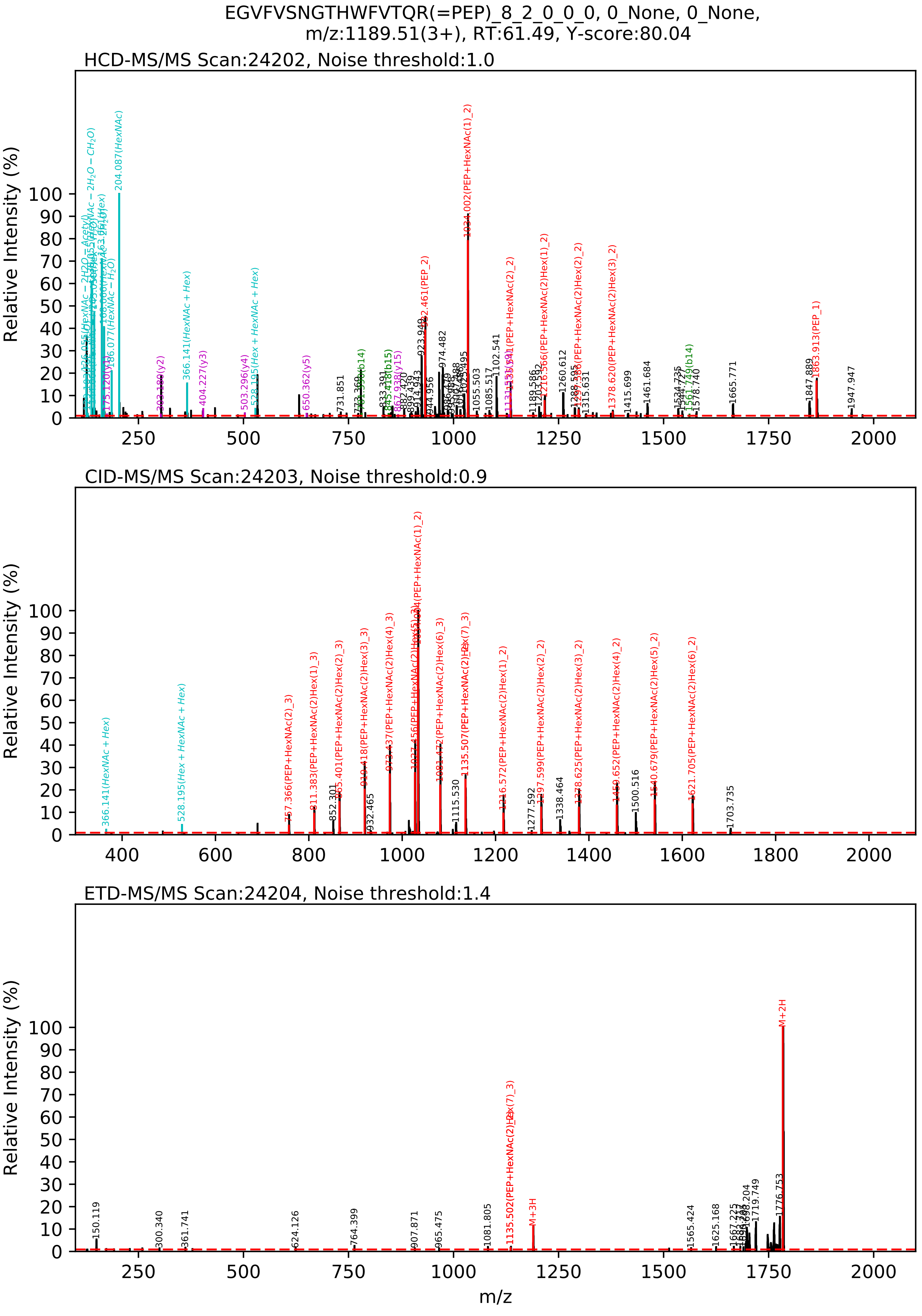
EGVFVSNGTHWFVTQR(=PEP)_8_2_0_0_0, 0_None, 0_None,
m/z:1189.51(3+), RT:61.49, Y-score:80.04
HCD-MS/MS Scan:24202, Noise threshold:1.0
CID-MS/MS Scan:24203, Noise threshold:0.9
ETD-MS/MS Scan:24204, Noise threshold:1.4
Relative Intensity (%)
m/z
204.087(HexNAc)
366.141(HexNAc+Hex)
528.195(Hex+HexNAc+Hex)
1034.002(PEP+HexNAc(1)_2)
1863.913(PEP_1)
M+2H
M+3H

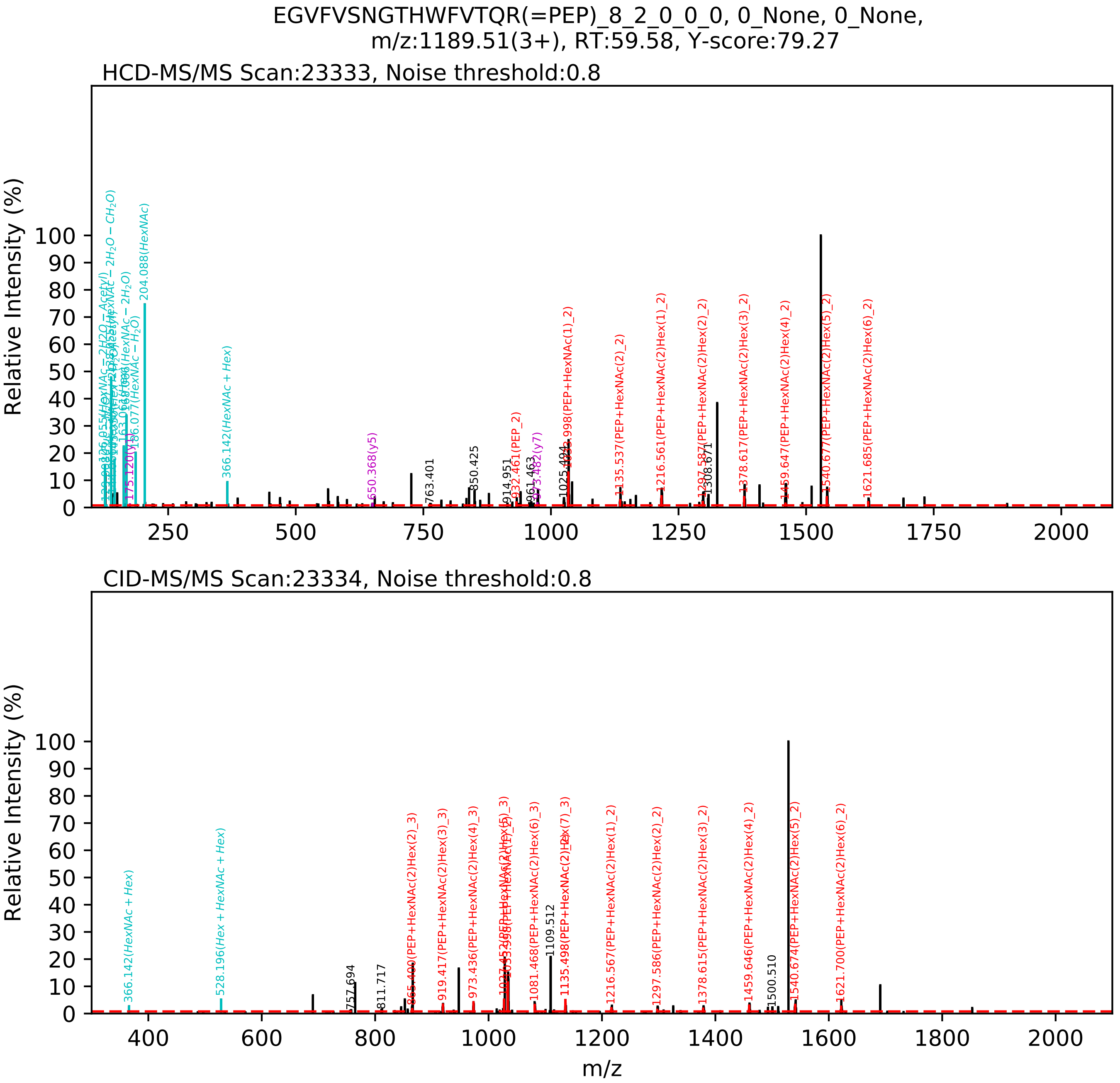
EGVFVSNGTHWFVTQR(=PEP)_8_2_0_0_0, 0_None, 0_None,
m/z:1189.51(3+), RT:59.58, Y-score:79.27
HCD-MS/MS Scan:23333, Noise threshold:0.8
Relative Intensity (%)
204.088(HexNAc)
366.142(HexNAc+Hex)
650.368(y5)
763.401
850.425
914.951
932.461(PEP_2)
961.463
1025.494
1033.998(PEP+HexNAc(1)_2)
1135.537(PEP+HexNAc(2)_2)
1216.561(PEP+HexNAc(2)Hex(1)_2)
1297.587(PEP+HexNAc(2)Hex(2)_2)
1308.671
1378.617(PEP+HexNAc(2)Hex(3)_2)
1459.647(PEP+HexNAc(2)Hex(4)_2)
1540.677(PEP+HexNAc(2)Hex(5)_2)
1621.685(PEP+HexNAc(2)Hex(6)_2)
CID-MS/MS Scan:23334, Noise threshold:0.8
Relative Intensity (%)
366.142(HexNAc+Hex)
528.196(Hex+HexNAc+Hex)
757.694
811.717
919.417(PEP+HexNAc(2)Hex(3)_3)
973.436(PEP+HexNAc(2)Hex(4)_3)
1081.468(PEP+HexNAc(2)Hex(6)_3)
1109.512
1135.498(PEP+HexNAc(2)Hex(7)_3)
1216.567(PEP+HexNAc(2)Hex(1)_2)
1297.586(PEP+HexNAc(2)Hex(2)_2)
1378.615(PEP+HexNAc(2)Hex(3)_2)
1459.646(PEP+HexNAc(2)Hex(4)_2)
1500.510
1540.674(PEP+HexNAc(2)Hex(5)_2)
1621.700(PEP+HexNAc(2)Hex(6)_2)
m/z

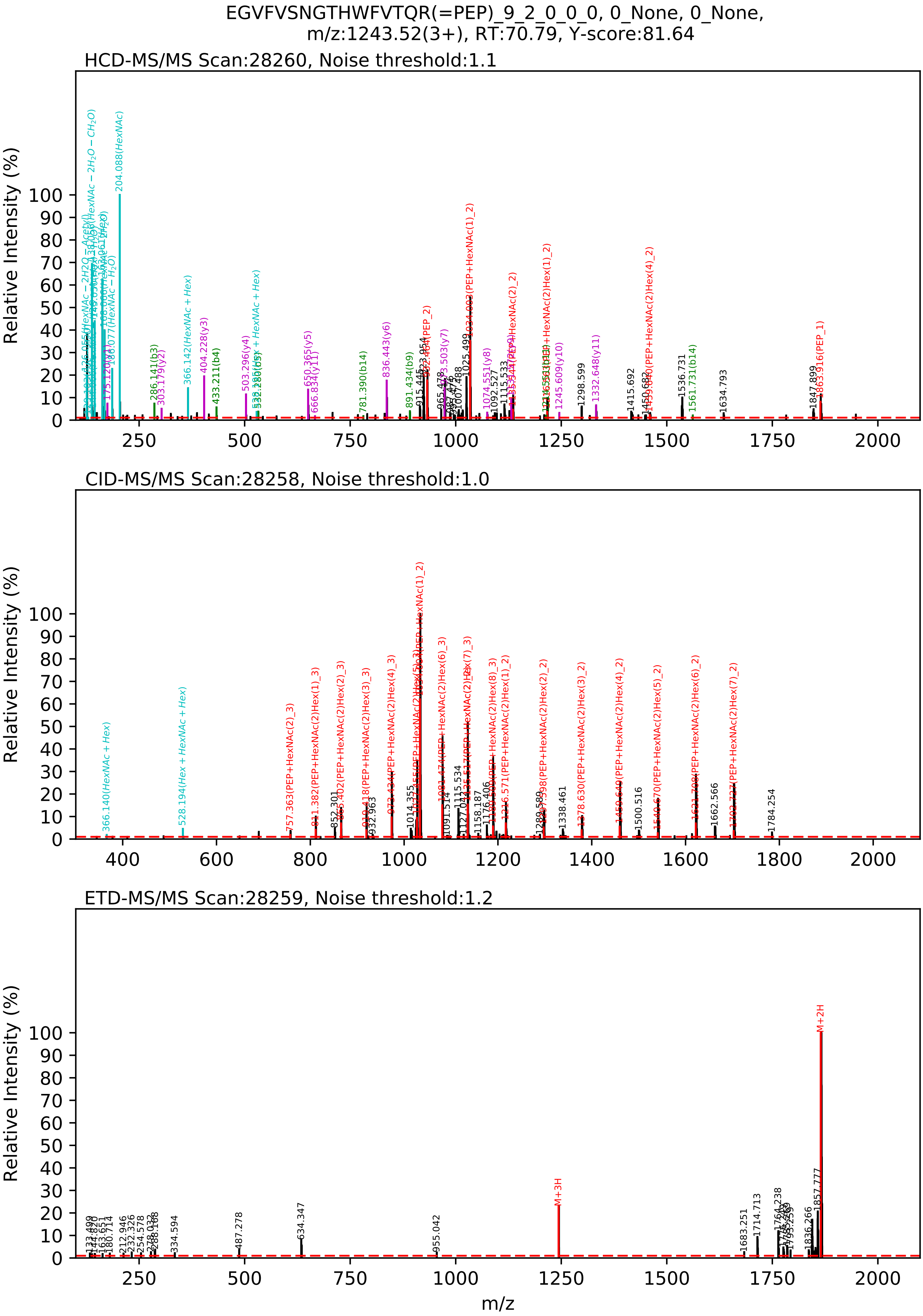
EGVFVSNGTHWFVTQR(=PEP)_9_2_0_0_0, 0_None, 0_None,
m/z:1243.52(3+), RT:70.79, Y-score:81.64
HCD-MS/MS Scan:28260, Noise threshold:1.1
CID-MS/MS Scan:28258, Noise threshold:1.0
ETD-MS/MS Scan:28259, Noise threshold:1.2
Relative Intensity (%)
m/z
204.088(HexNAc)
366.142(HexNAc+Hex)
404.228(y3)
836.443(y6)
1034.003(PEP+HexNAc(1)_2)
1863.916(PEP_1)
M+2H
M+3H

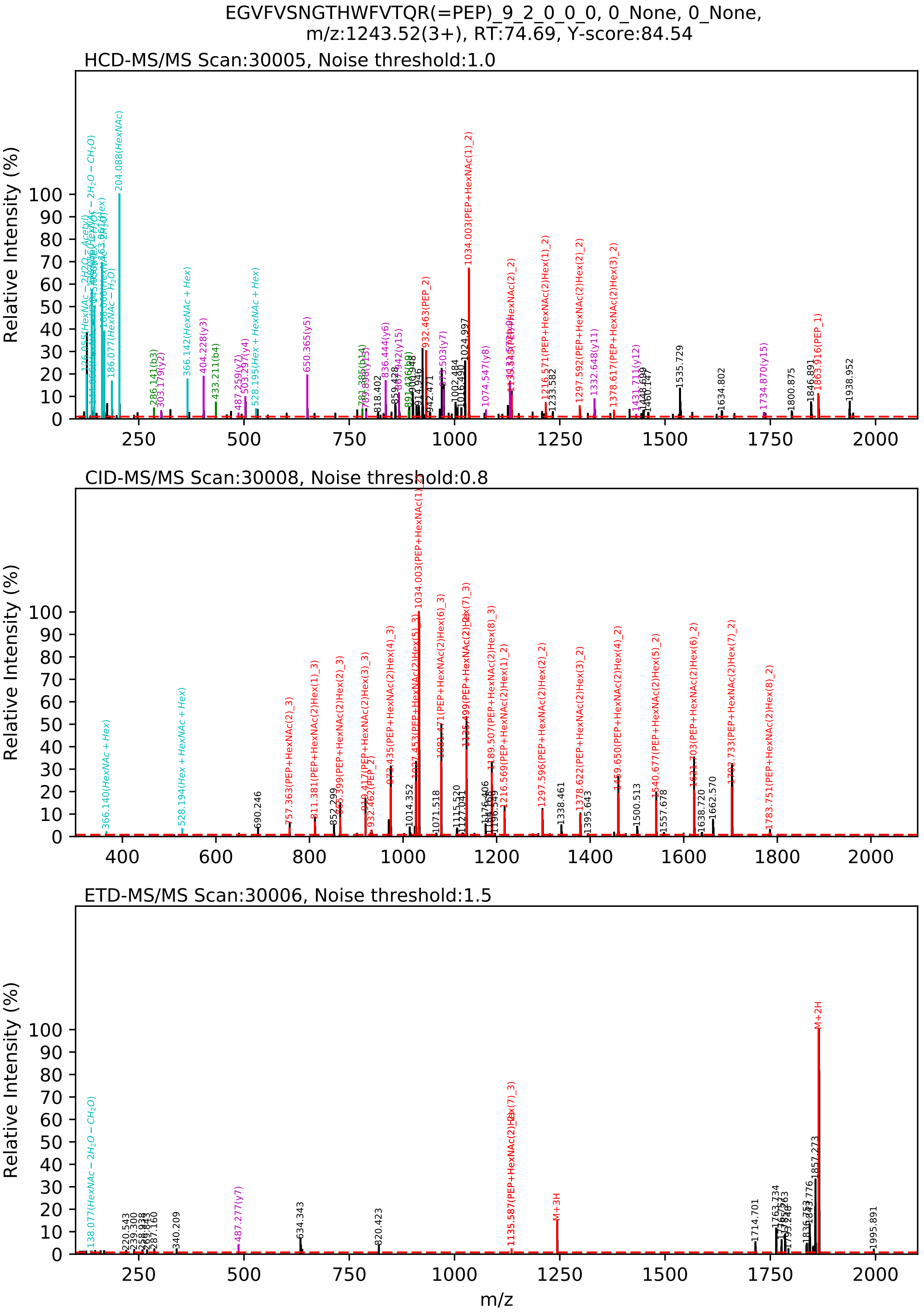
EGVFVSNGTHWFVTQR(=PEP)_9_2_0_0_0, 0_None, 0_None,
m/z:1243.52(3+), RT:74.69, Y-score:84.54
HCD-MS/MS Scan:30005, Noise threshold:1.0
CID-MS/MS Scan:30008, Noise threshold:0.8
ETD-MS/MS Scan:30006, Noise threshold:1.5
Relative Intensity (%)
m/z
204.088(HexNAc)
1034.003(PEP+HexNAc(1)_2)
932.463(PEP_2)
1863.916(PEP_1)
M+2H
M+3H

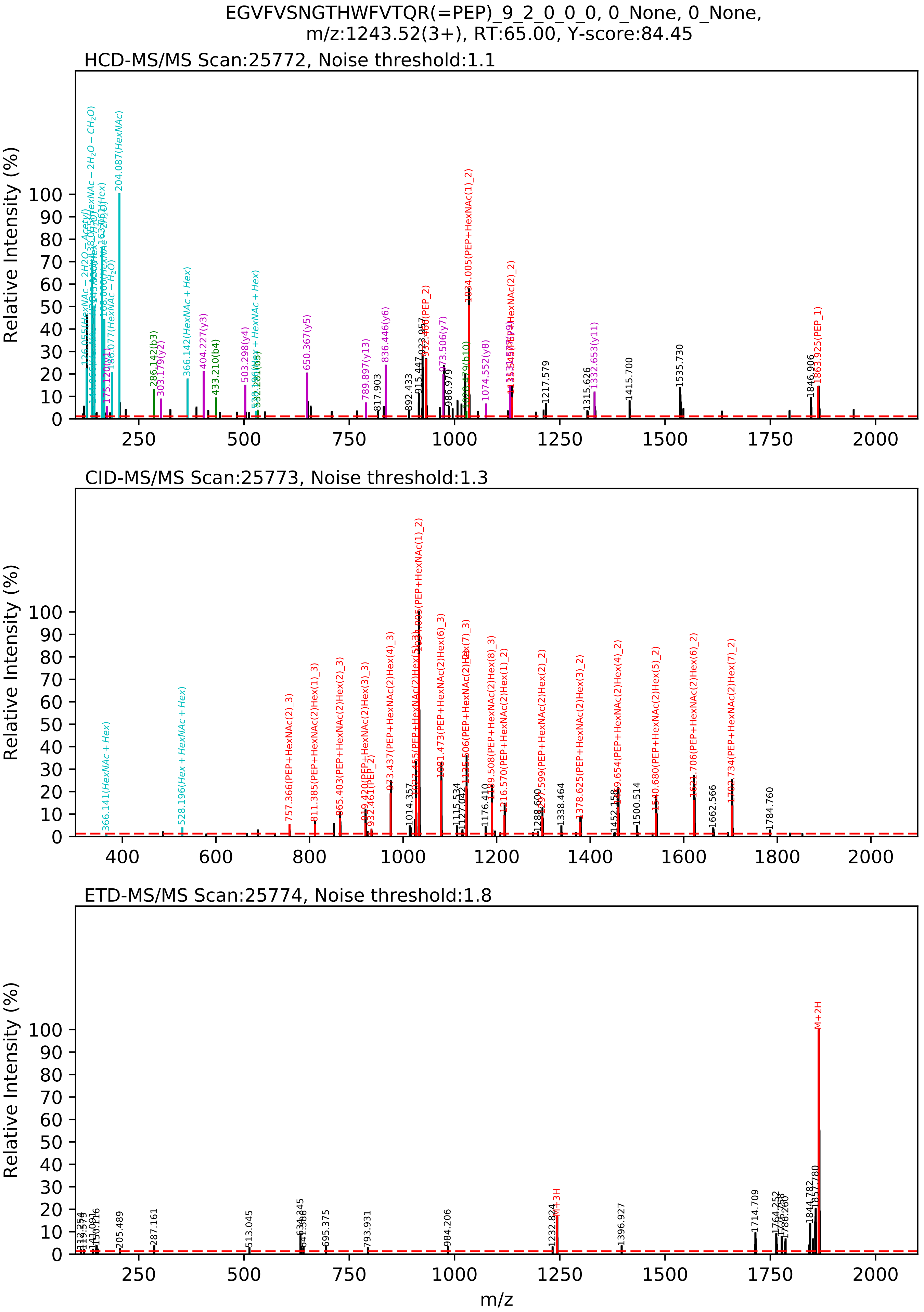

EGVFVSNGTHWFVTQR(=PEP)_9_2_0_0_0, 0_None, 0_None,
m/z:1243.52(3+), RT:65.00, Y-score:84.45
HCD-MS/MS Scan:25772, Noise threshold:1.1
204.087(HexNAc)
1034.005(PEP+HexNAc(1)_2)
1863.925(PEP_1)
650.367(y5)
836.446(y6)
973.506(y7)
1074.552(y8)
1332.653(y11)
404.227(y3)
503.298(y4)
789.897(y13)
286.142(b3)
433.210(b4)
366.142(HexNAc+Hex)
1535.730
1415.700
1846.906
1217.579
1315.626
CID-MS/MS Scan:25773, Noise threshold:1.3
1034.005(PEP+HexNAc(1)_2)
973.437(PEP+HexNAc(2)Hex(4)_3)
1081.473(PEP+HexNAc(2)Hex(6)_3)
1216.570(PEP+HexNAc(2)Hex(1)_2)
1297.599(PEP+HexNAc(2)Hex(2)_2)
1378.625(PEP+HexNAc(2)Hex(3)_2)
1540.680(PEP+HexNAc(2)Hex(5)_2)
1621.706(PEP+HexNAc(2)Hex(6)_2)
1702.734(PEP+HexNAc(2)Hex(7)_2)
757.366(PEP+HexNAc(2)_3)
811.385(PEP+HexNAc(2)Hex(1)_3)
865.403(PEP+HexNAc(2)Hex(2)_3)
932.461(PEP_2)
366.141(HexNAc+Hex)
528.196(Hex+HexNAc+Hex)
1662.566
1784.760
1338.464
1500.514
ETD-MS/MS Scan:25774, Noise threshold:1.8
M+2H
M+3H
1232.824
1396.927
1714.709
984.206
793.931
695.375
513.045
287.161
205.489
1844.782
m/z
Relative Intensity (%)

EGVFVSNGTHWFVTQR(=PEP)_9_2_0_0_0, 0_None, 0_None,
m/z:1243.52(3+), RT:65.56, Y-score:86.41

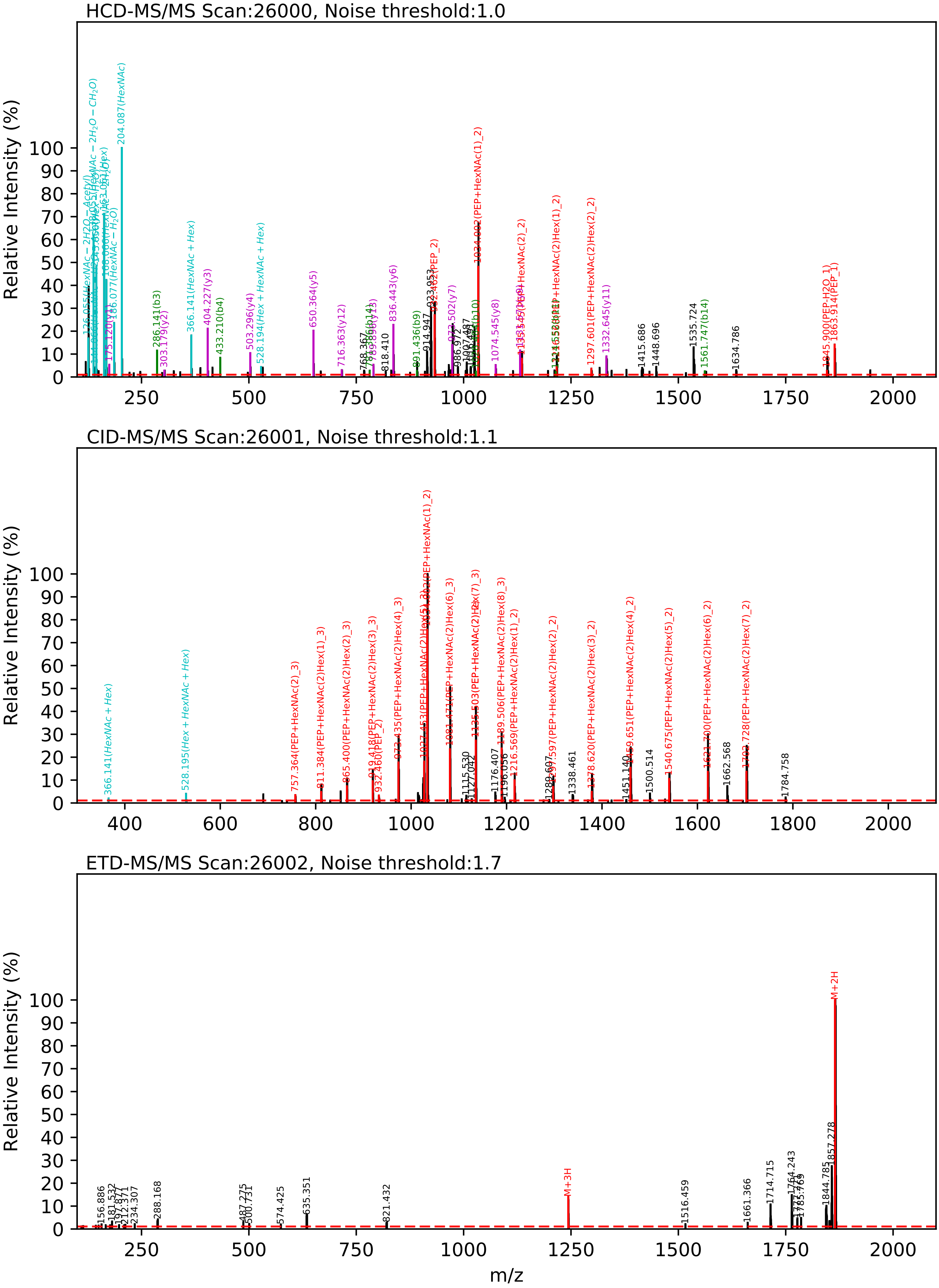

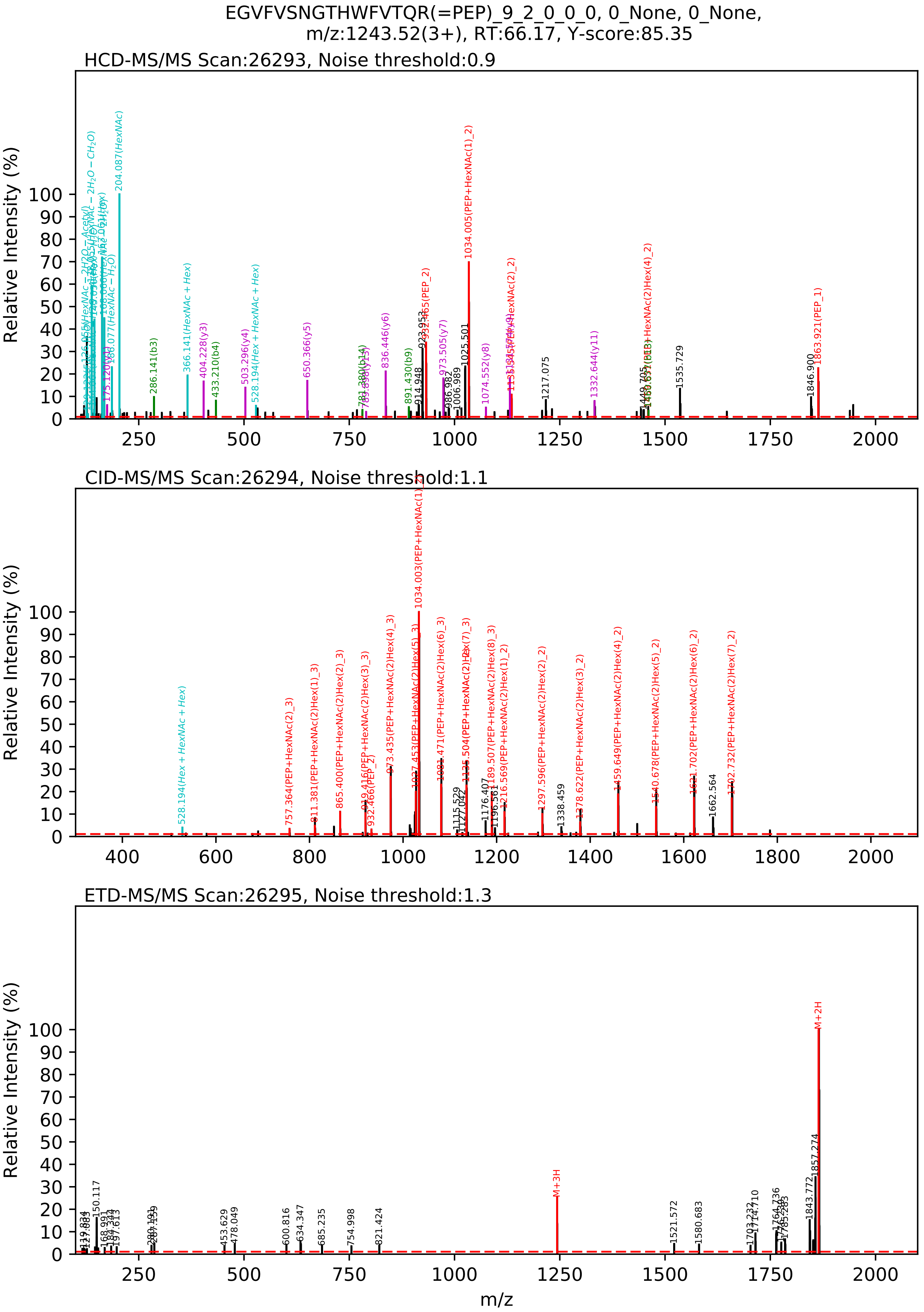
EGVFVSNGTHWFVTQR(=PEP)_9_2_0_0_0, 0_None, 0_None,
m/z:1243.52(3+), RT:66.17, Y-score:85.35
HCD-MS/MS Scan:26293, Noise threshold:0.9
Relative Intensity (%)
204.087(HexNAc)
366.141(HexNAc + Hex)
528.194(Hex + HexNAc + Hex)
286.141(b3)
404.228(y3)
433.210(b4)
503.296(y4)
650.366(y5)
836.446(y6)
891.430(b9)
973.505(y7)
1034.005(PEP+HexNAc(1)_2)
1025.501
1074.552(y8)
1217.075
1332.644(y11)
1535.729
1846.900
1863.921(PEP_1)
CID-MS/MS Scan:26294, Noise threshold:1.1
528.194(Hex + HexNAc + Hex)
757.364(PEP+HexNAc(2)_3)
811.381(PEP+HexNAc(2)Hex(1)_3)
865.400(PEP+HexNAc(2)Hex(2)_3)
973.435(PEP+HexNAc(2)Hex(4)_3)
1034.003(PEP+HexNAc(1)_2)
1081.471(PEP+HexNAc(2)Hex(6)_3)
1297.596(PEP+HexNAc(2)Hex(2)_2)
1338.459
1378.622(PEP+HexNAc(2)Hex(3)_2)
1459.649(PEP+HexNAc(2)Hex(4)_2)
1540.678(PEP+HexNAc(2)Hex(5)_2)
1621.702(PEP+HexNAc(2)Hex(6)_2)
1662.564
1702.732(PEP+HexNAc(2)Hex(7)_2)
ETD-MS/MS Scan:26295, Noise threshold:1.3
150.117
453.629
478.049
600.816
634.347
685.235
754.998
821.424
M+3H
1521.572
1580.683
1714.710
1843.772
1857.274
M+2H
m/z

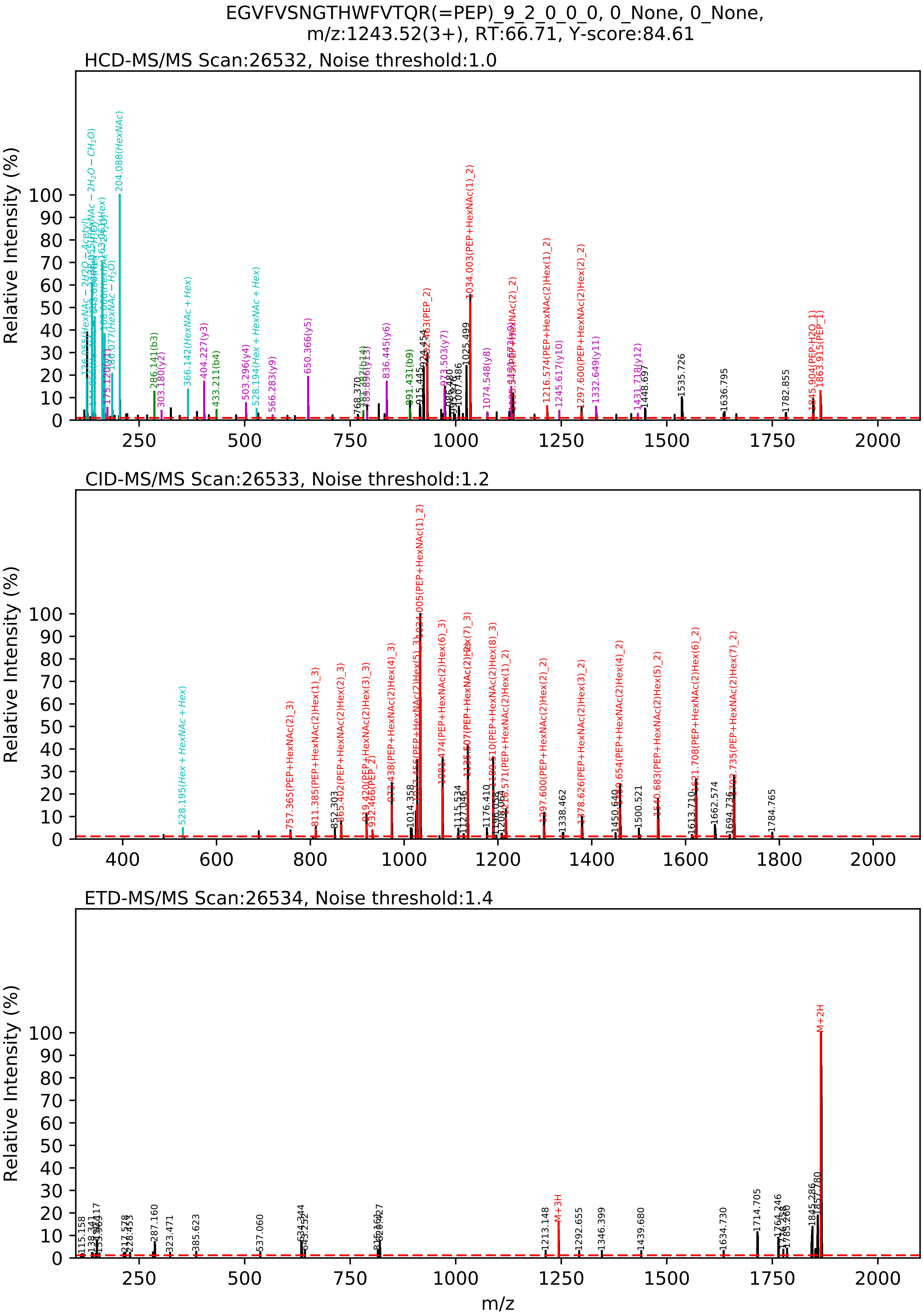
EGVFVSNGTHWFVTQR(=PEP)_9_2_0_0_0, 0_None, 0_None,
m/z:1243.52(3+), RT:66.71, Y-score:84.61
HCD-MS/MS Scan:26532, Noise threshold:1.0
Relative Intensity (%)
204.088(HexNAc)
1034.003(PEP+HexNAc(1)_2)
1216.574(PEP+HexNAc(2)Hex(1)_2)
1297.600(PEP+HexNAc(2)Hex(2)_2)
924.454
1025.499
1535.726
1636.795
1782.855
286.141(b3)
366.142(HexNAc + Hex)
404.227(y3)
433.211(b4)
503.296(y4)
528.194(Hex + HexNAc + Hex)
566.283(y9)
650.366(y5)
836.445(y6)
891.431(b9)
973.503(y7)
1074.548(y8)
1245.617(y10)
1332.649(y11)
1431.718(y12)
1448.697
CID-MS/MS Scan:26533, Noise threshold:1.2
Relative Intensity (%)
528.195(Hex + HexNAc + Hex)
757.365(PEP+HexNAc(2)_3)
811.385(PEP+HexNAc(2)Hex(1)_3)
865.402(PEP+HexNAc(2)Hex(2)_3)
852.303
932.466(PEP_2)
973.438(PEP+HexNAc(2)Hex(4)_3)
1014.358
1034.005(PEP+HexNAc(1)_2)
1081.474(PEP+HexNAc(2)Hex(6)_3)
1115.534
1176.410
1216.571(PEP+HexNAc(2)Hex(1)_2)
1297.600(PEP+HexNAc(2)Hex(2)_2)
1338.462
1378.626(PEP+HexNAc(2)Hex(3)_2)
1450.640
1500.521
1540.683(PEP+HexNAc(2)Hex(5)_2)
1613.710
1662.574
1694.736
1784.765
ETD-MS/MS Scan:26534, Noise threshold:1.4
Relative Intensity (%)
M+2H
M+3H
115.158
287.160
323.471
385.623
537.060
634.344
643.232
1213.148
1292.655
1346.399
1439.680
1634.730
1714.705
1764.246
1785.260
1845.286
m/z

EGVFVSNGTHWFVTQR(=PEP)_9_2_0_0_0, 0_None, 0_None,
m/z:1243.52(3+), RT:67.32, Y-score:84.58

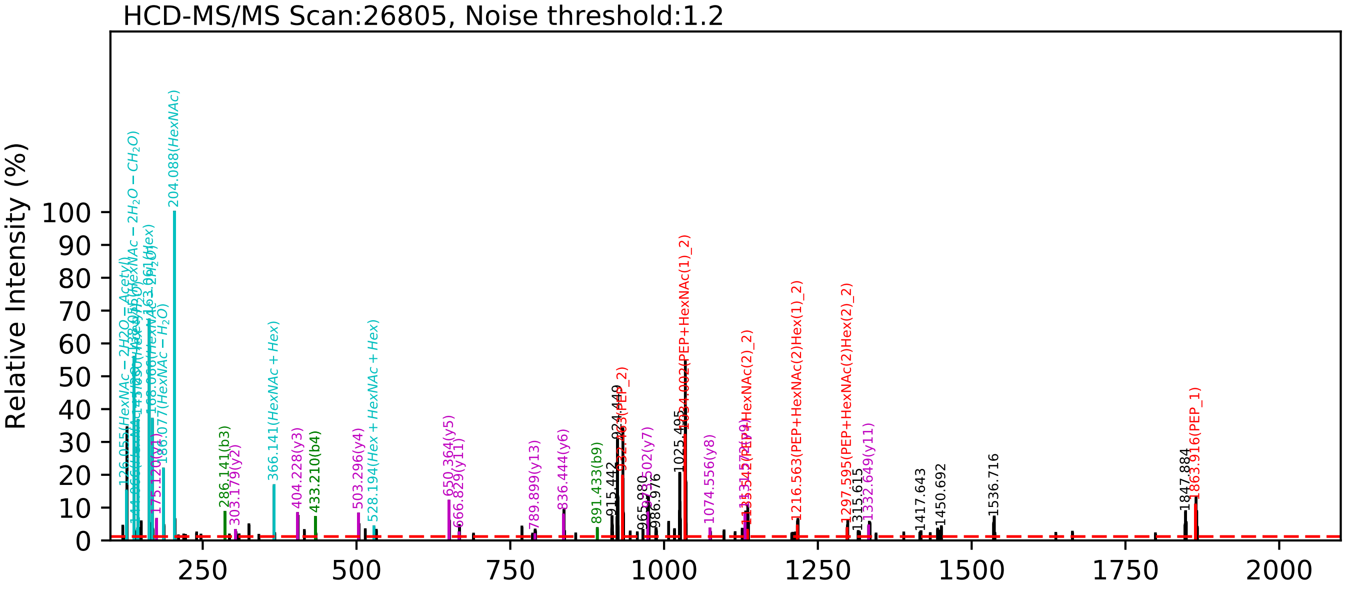

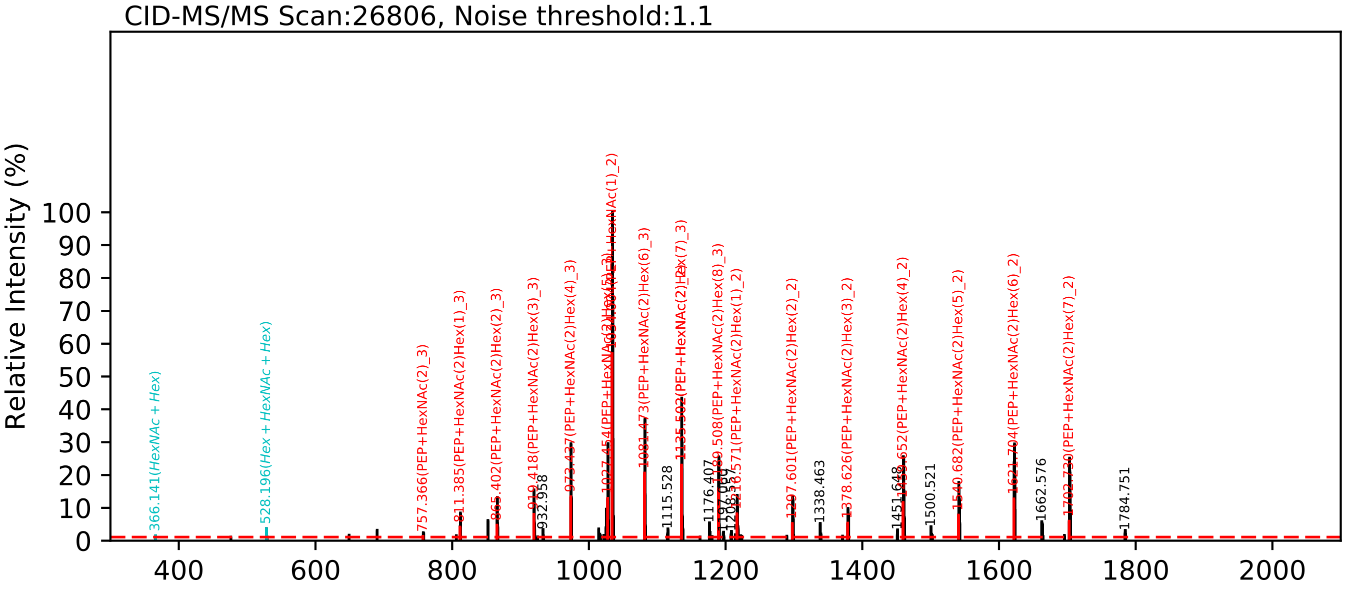

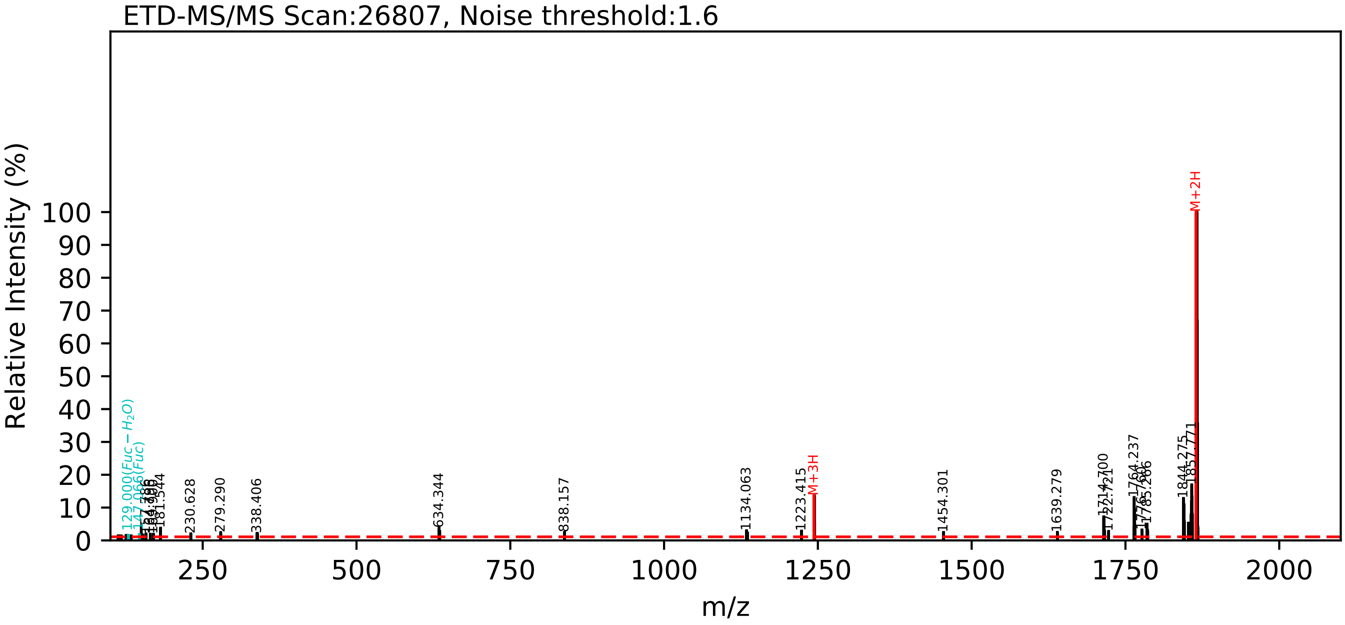

EGVFVSNGTHWFVTQR(=PEP)_9_2_0_0_0, 0_None, 0_None,
m/z:1243.52(3+), RT:68.25, Y-score:86.85

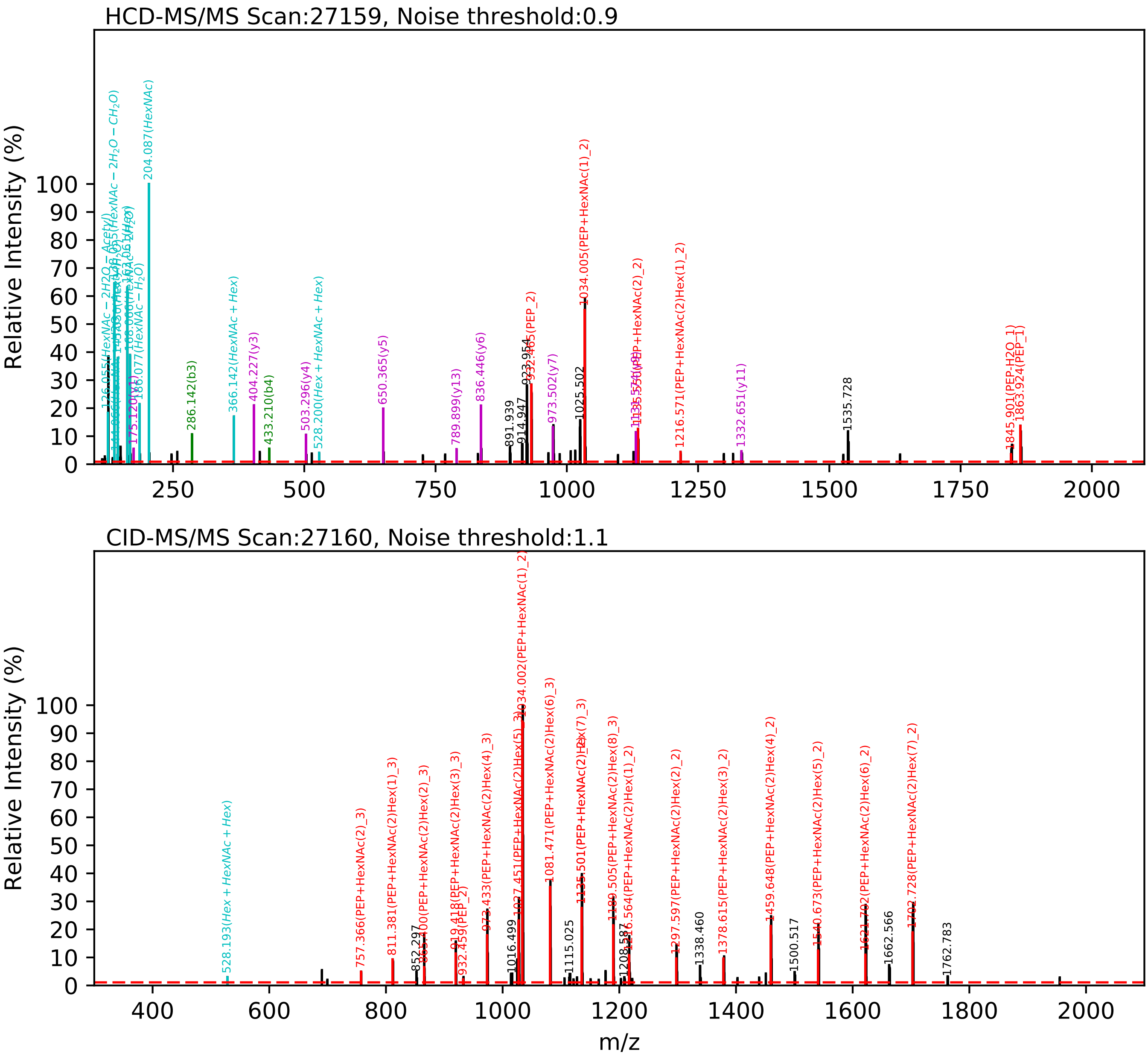

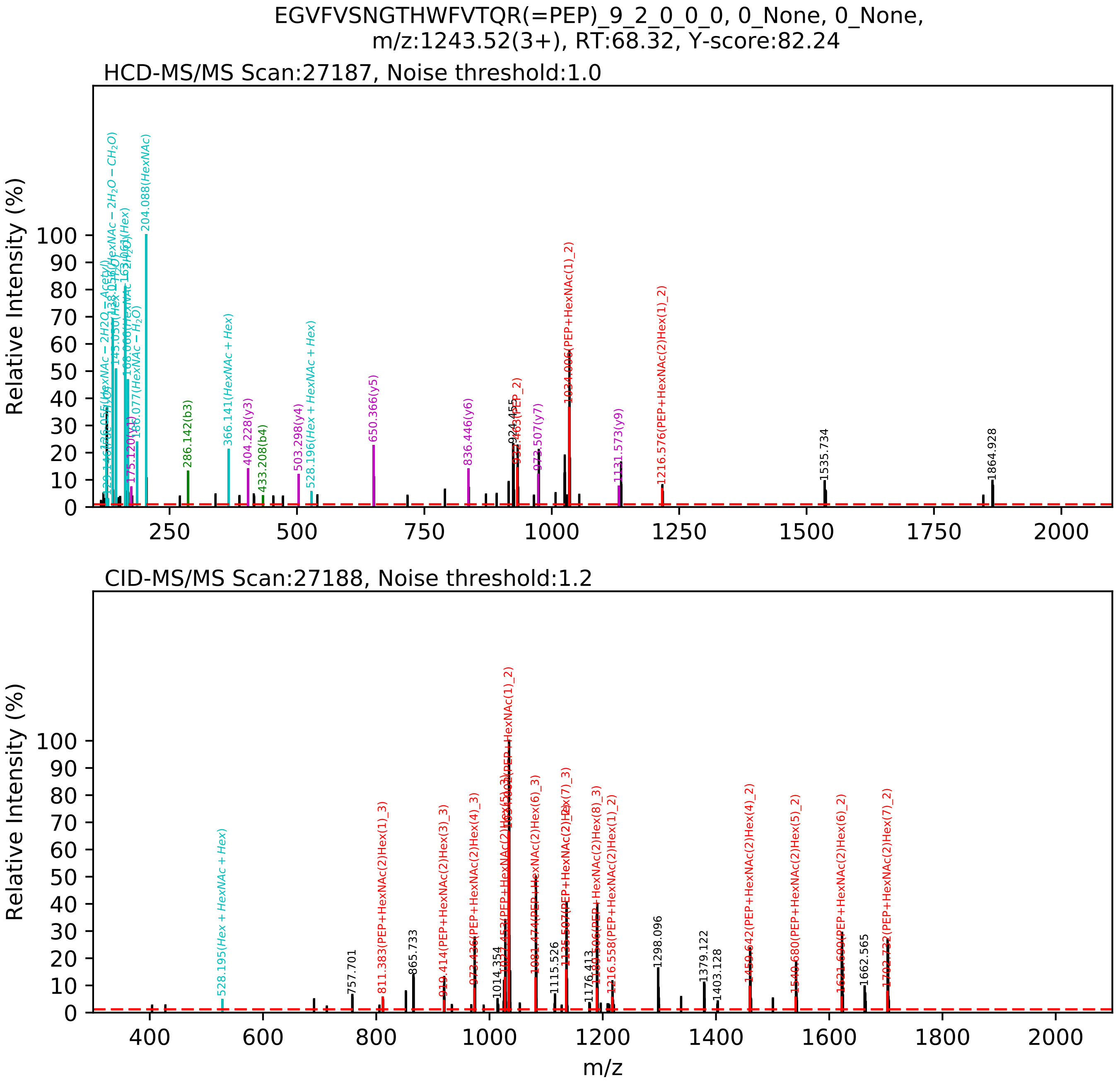

EGVFVSNGTHWFVTQR(=PEP)_9_2_0_0_0, 0_None, 0_None,
m/z:1243.52(3+), RT:68.32, Y-score:82.24
HCD-MS/MS Scan:27187, Noise threshold:1.0
Relative Intensity (%)
204.088(HexNAc)
286.142(b3)
366.141(HexNAc+Hex)
404.228(y3)
433.208(b4)
503.298(y4)
528.196(Hex+HexNAc+Hex)
650.366(y5)
836.446(y6)
1034.096(PEP+HexNAc(1)_2)
1131.573(y9)
1216.576(PEP+HexNAc(2)Hex(1)_2)
1535.734
1864.928
CID-MS/MS Scan:27188, Noise threshold:1.2
Relative Intensity (%)
528.195(Hex+HexNAc+Hex)
757.701
811.383(PEP+HexNAc(2)Hex(1)_3)
865.733
1014.354
1115.526
1176.413
1298.096
1379.122
1403.128
1662.565
m/z

EGVFVSNGTHWFVTQR(=PEP)_9_2_0_0_0, 0_None, 0_None,
m/z:1243.52(3+), RT:68.45, Y-score:82.10

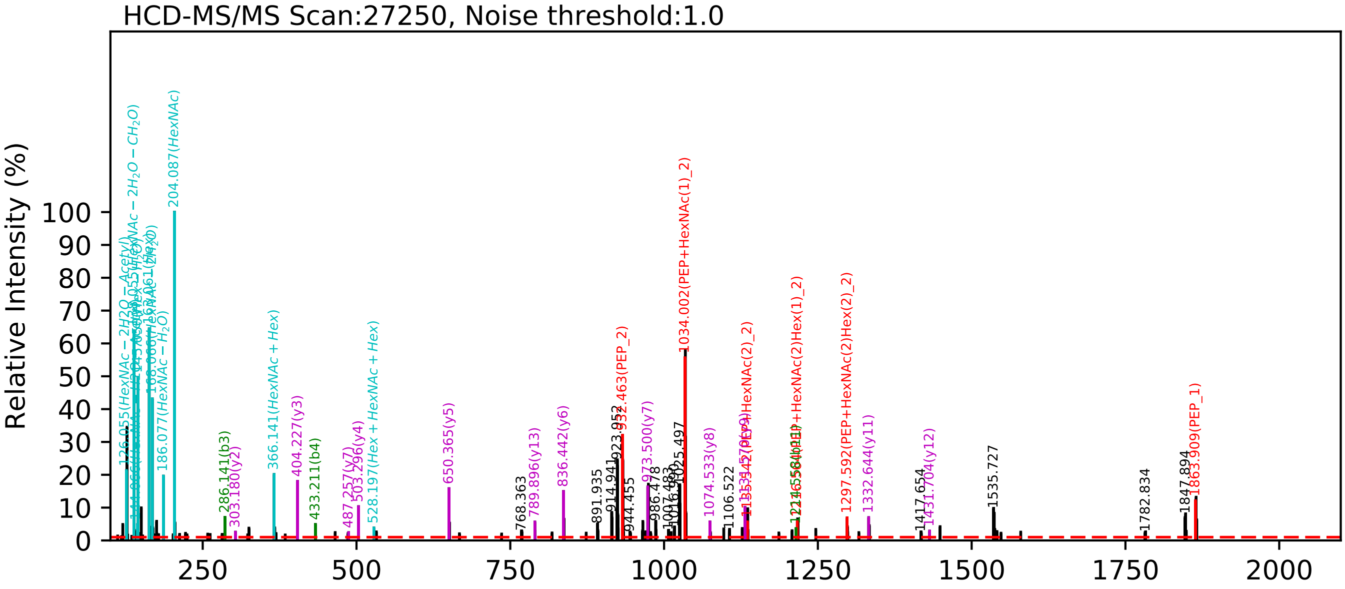

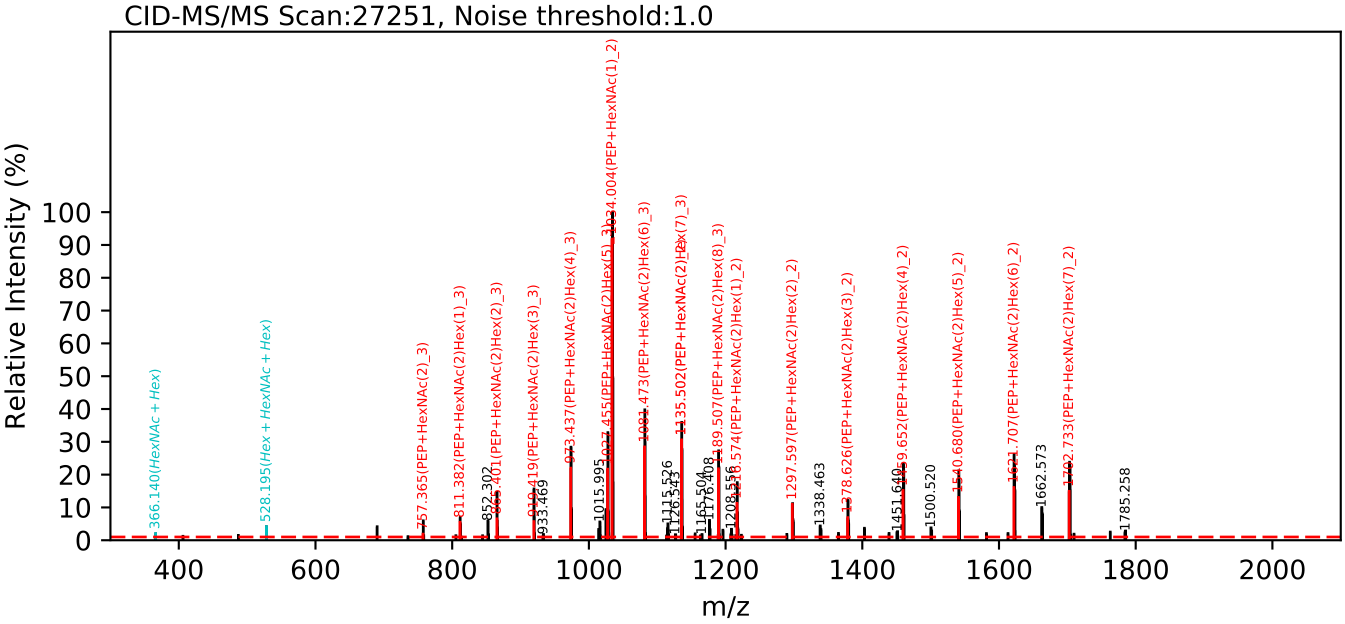

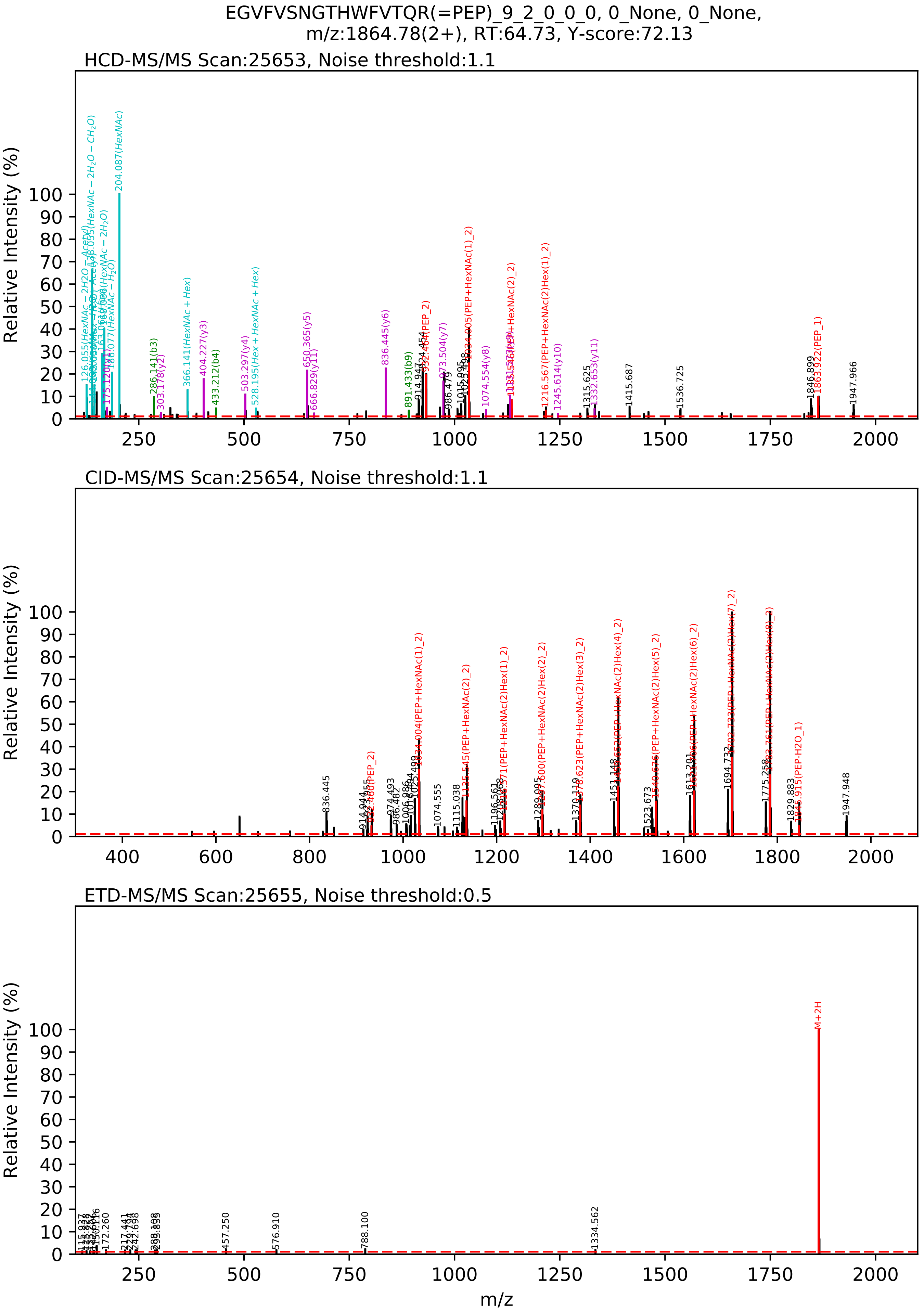

EGVFVSNGTHWFVTQR(=PEP)_9_2_0_0_0, 0_None, 0_None,
m/z:1864.78(2+), RT:64.73, Y-score:72.13
HCD-MS/MS Scan:25653, Noise threshold:1.1
CID-MS/MS Scan:25654, Noise threshold:1.1
ETD-MS/MS Scan:25655, Noise threshold:0.5
Relative Intensity (%)
m/z
204.087(HexNAc)
286.141(b3)
303.178(y2)
366.141(HexNAc + Hex)
404.227(y3)
433.212(b4)
503.297(y4)
528.195(Hex + HexNAc + Hex)
650.365(y5)
666.829(y11)
836.445(y6)
891.433(b9)
932.464(PEP_2)
973.504(y7)
1034.005(PEP+HexNAc(1)_2)
1074.554(y8)
1216.567(PEP+HexNAc(2)Hex(1)_2)
1245.614(y10)
1332.653(y11)
1415.687
1536.725
1846.899
1863.922(PEP_1)
1947.966
836.445
1034.004(PEP+HexNAc(1)_2)
1135.545(PEP+HexNAc(2)_2)
1216.571(PEP+HexNAc(2)Hex(1)_2)
1297.600(PEP+HexNAc(2)Hex(2)_2)
1378.623(PEP+HexNAc(2)Hex(3)_2)
1451.148
1540.676(PEP+HexNAc(2)Hex(5)_2)
1613.201
1694.732
1775.258
1829.883
1845.915(PEP-H2O_1)
1947.948
M+2H
172.260
457.250
576.910
788.100
1334.562

EGVFVSNGTHWFVTQR(=PEP)_9_2_0_0_0, 0_None, 0_None,
m/z:1243.52(3+), RT:61.43, Y-score:80.02

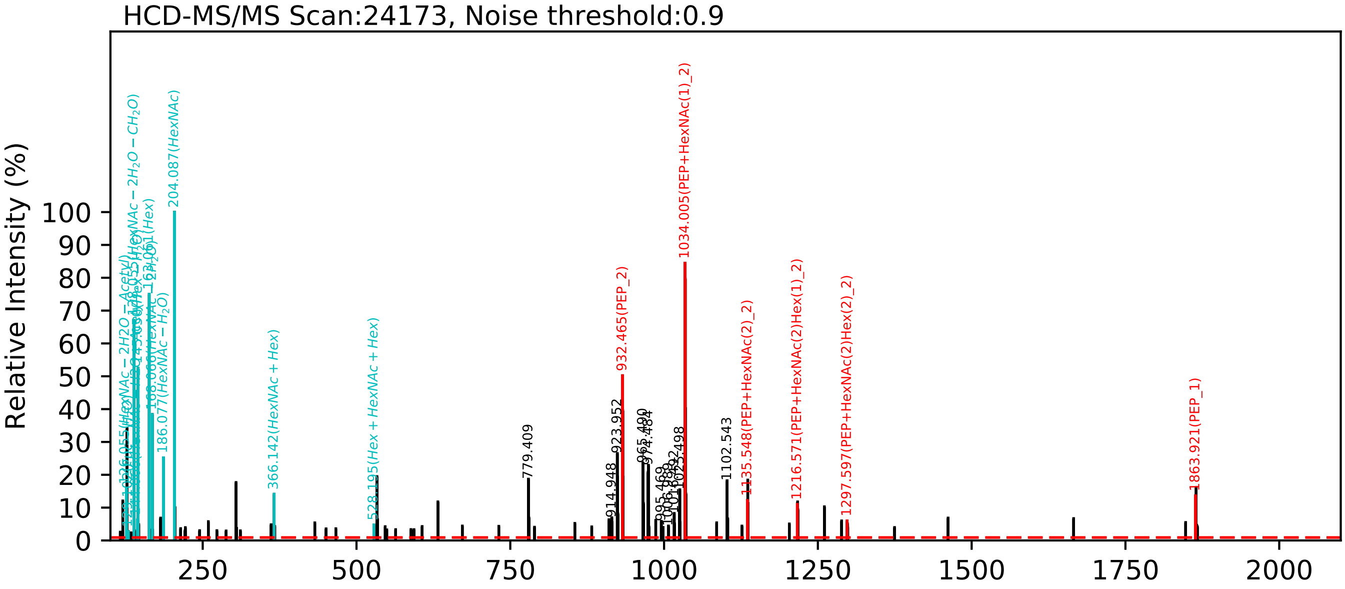

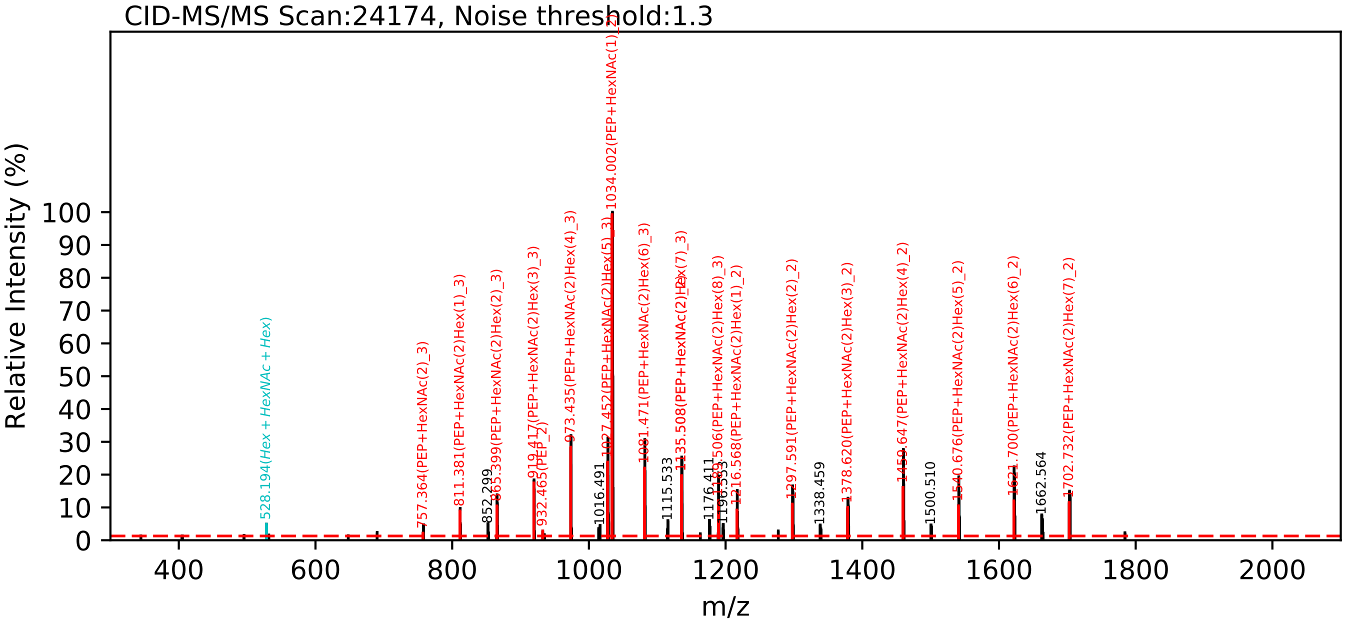

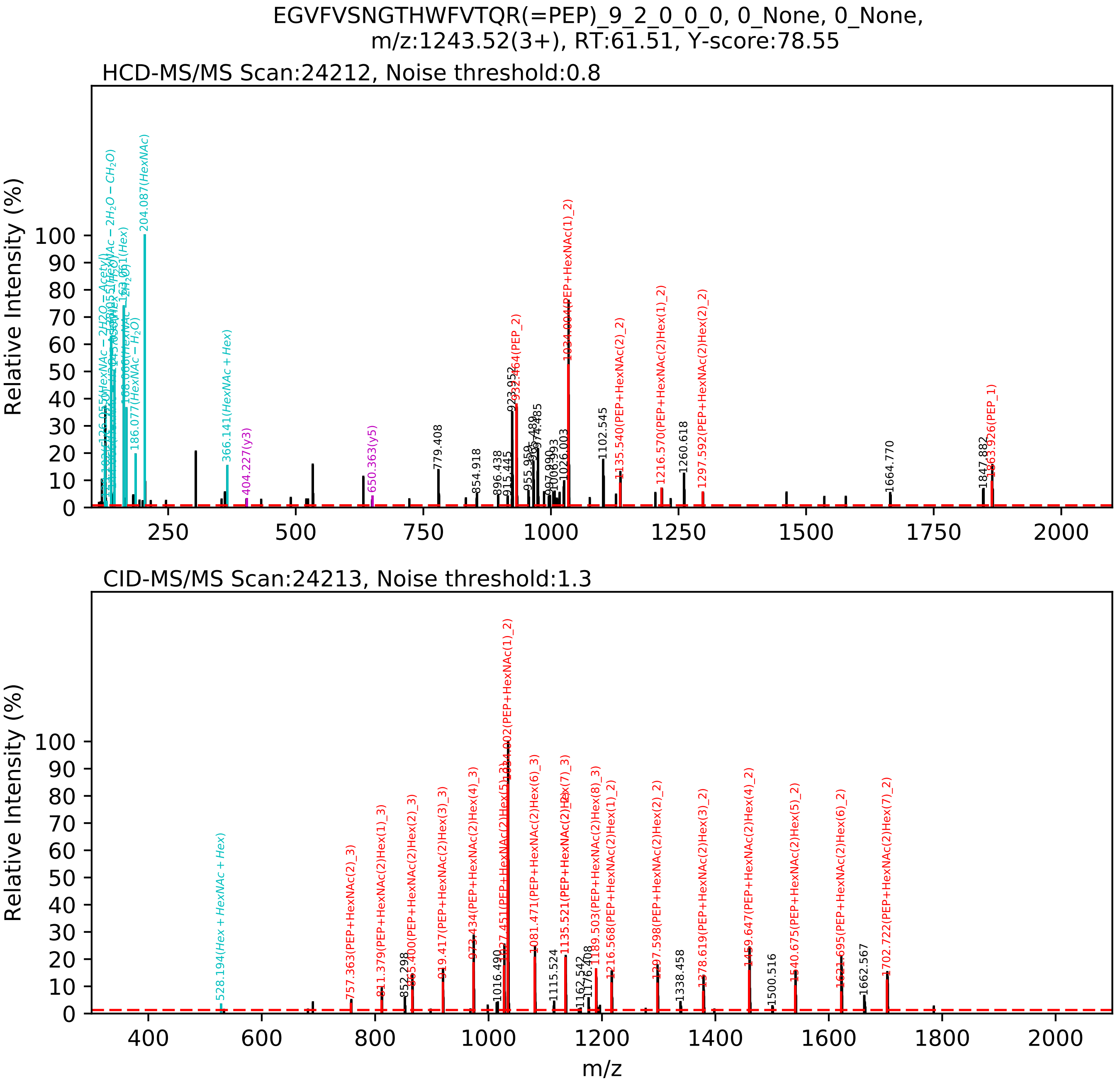
EGVFVSNGTHWFVTQR(=PEP)_9_2_0_0_0, 0_None, 0_None,
m/z:1243.52(3+), RT:61.51, Y-score:78.55
HCD-MS/MS Scan:24212, Noise threshold:0.8
Relative Intensity (%)
204.087(HexNAc)
186.077(HexNAc − H2O)
366.141(HexNAc + Hex)
404.227(y3)
650.363(y5)
779.408
854.918
896.438
915.445
923.952
932.464(PEP_2)
955.959
1026.003
1034.004(PEP+HexNAc(1)_2)
1102.545
1135.540(PEP+HexNAc(2)_2)
1216.570(PEP+HexNAc(2)Hex(1)_2)
1260.618
1297.592(PEP+HexNAc(2)Hex(2)_2)
1664.770
1847.882
1863.926(PEP_1)
CID-MS/MS Scan:24213, Noise threshold:1.3
528.194(Hex + HexNAc + Hex)
757.363(PEP+HexNAc(2)_3)
811.379(PEP+HexNAc(2)Hex(1)_3)
852.298
865.400(PEP+HexNAc(2)Hex(2)_3)
919.417(PEP+HexNAc(2)Hex(3)_3)
973.434(PEP+HexNAc(2)Hex(4)_3)
1016.490
1034.002(PEP+HexNAc(1)_2)
1081.471(PEP+HexNAc(2)Hex(6)_3)
1115.524
1135.521(PEP+HexNAc(2)_2)
1162.542
1176.408
1189.503(PEP+HexNAc(2)Hex(8)_3)
1216.568(PEP+HexNAc(2)Hex(1)_2)
1297.598(PEP+HexNAc(2)Hex(2)_2)
1338.458
1378.619(PEP+HexNAc(2)Hex(3)_2)
1459.647(PEP+HexNAc(2)Hex(4)_2)
1500.516
1540.675(PEP+HexNAc(2)Hex(5)_2)
1621.695(PEP+HexNAc(2)Hex(6)_2)
1662.567
1702.722(PEP+HexNAc(2)Hex(7)_2)
m/z

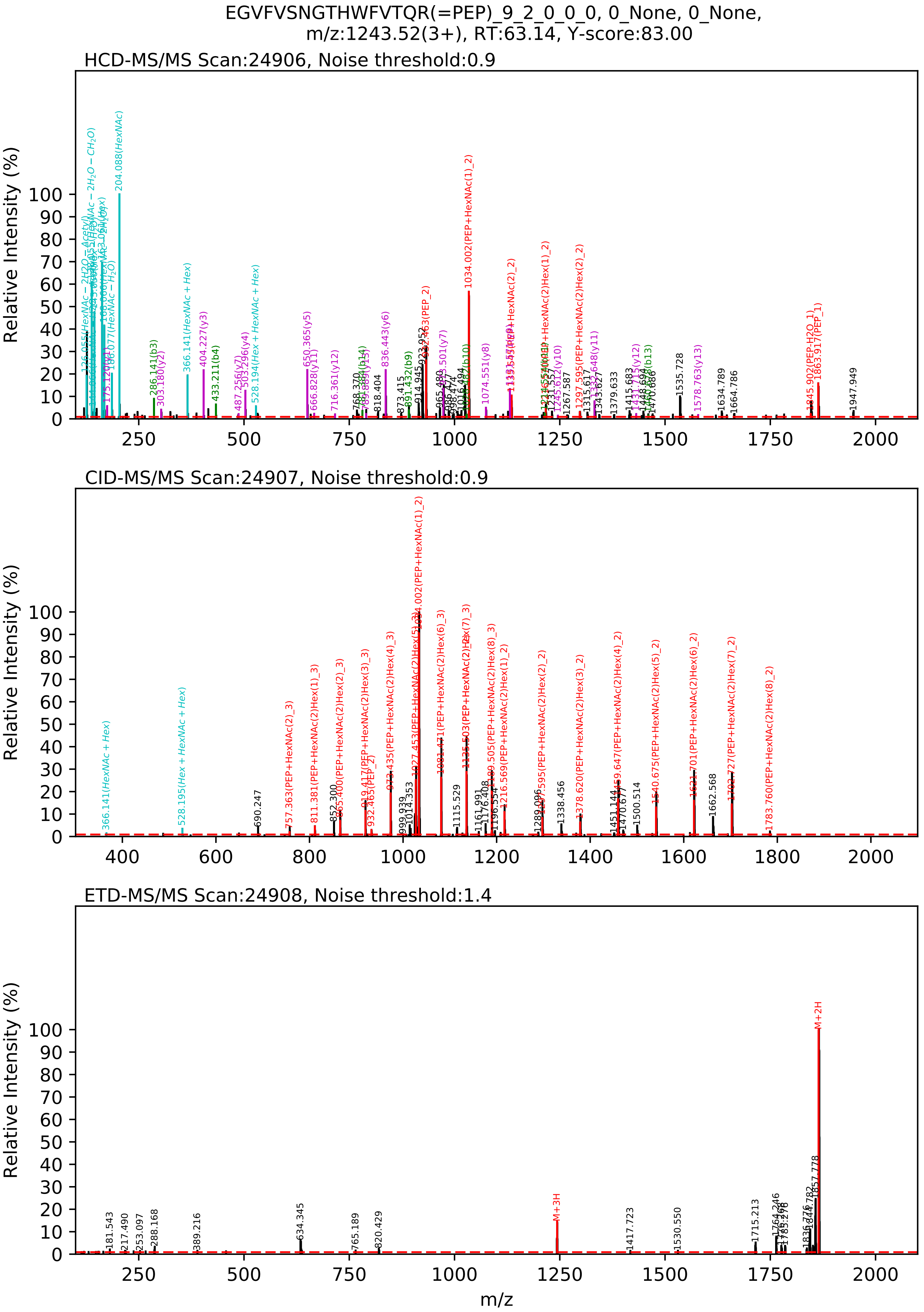
EGVFVSNGTHWFVTQR(=PEP)_9_2_0_0_0, 0_None, 0_None,
m/z:1243.52(3+), RT:63.14, Y-score:83.00
HCD-MS/MS Scan:24906, Noise threshold:0.9
Relative Intensity (%)
204.088(HexNAc)
1034.002(PEP+HexNAc(1)_2)
366.141(HexNAc + Hex)
528.194(Hex + HexNAc + Hex)
CID-MS/MS Scan:24907, Noise threshold:0.9
Relative Intensity (%)
ETD-MS/MS Scan:24908, Noise threshold:1.4
Relative Intensity (%)
M+2H
M+3H
m/z

EGVFVSNGTHWFVTQR(=PEP)_9_2_0_0_0, 0_None, 0_None,
m/z:1243.52(3+), RT:63.68, Y-score:71.71

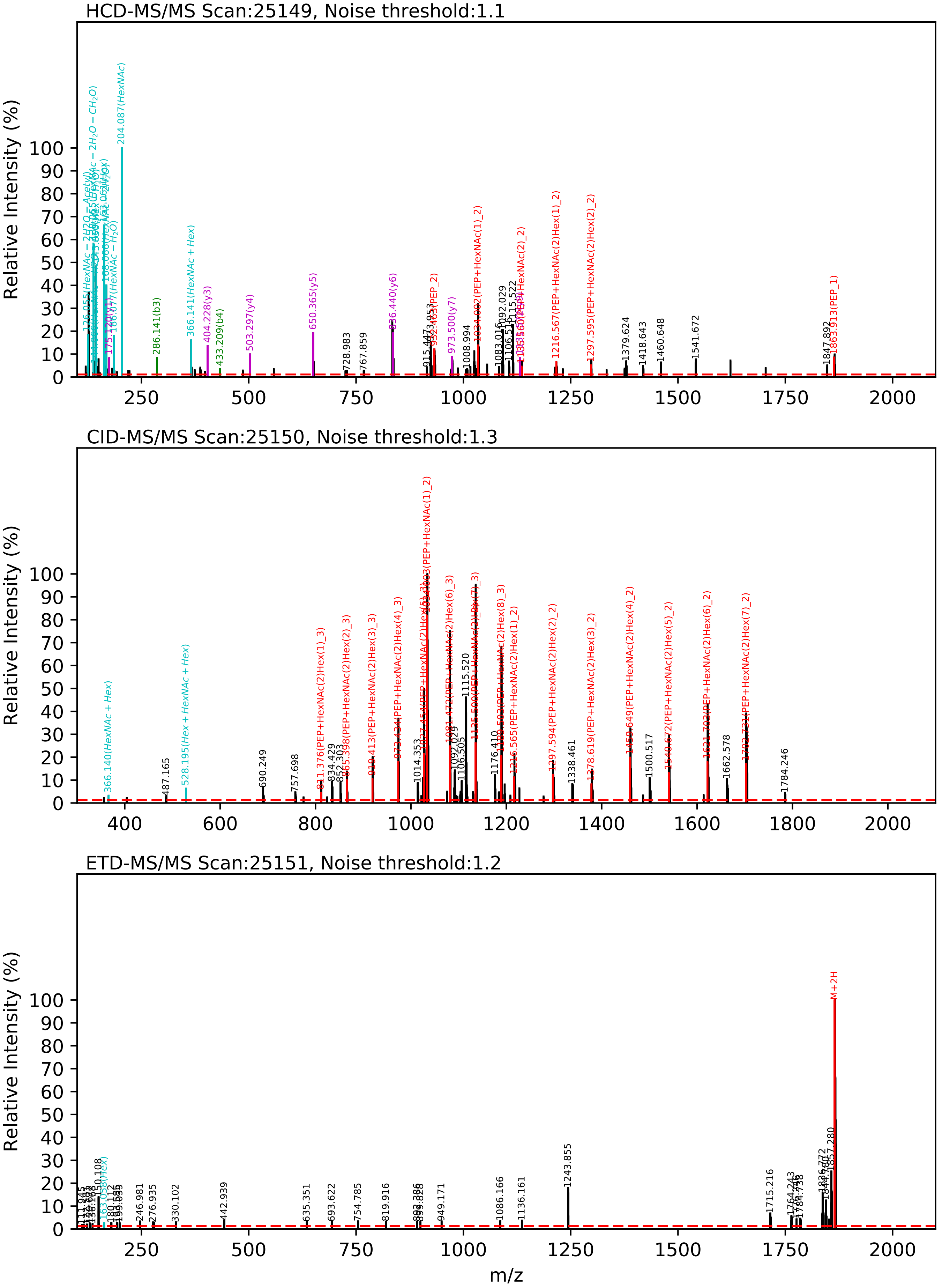

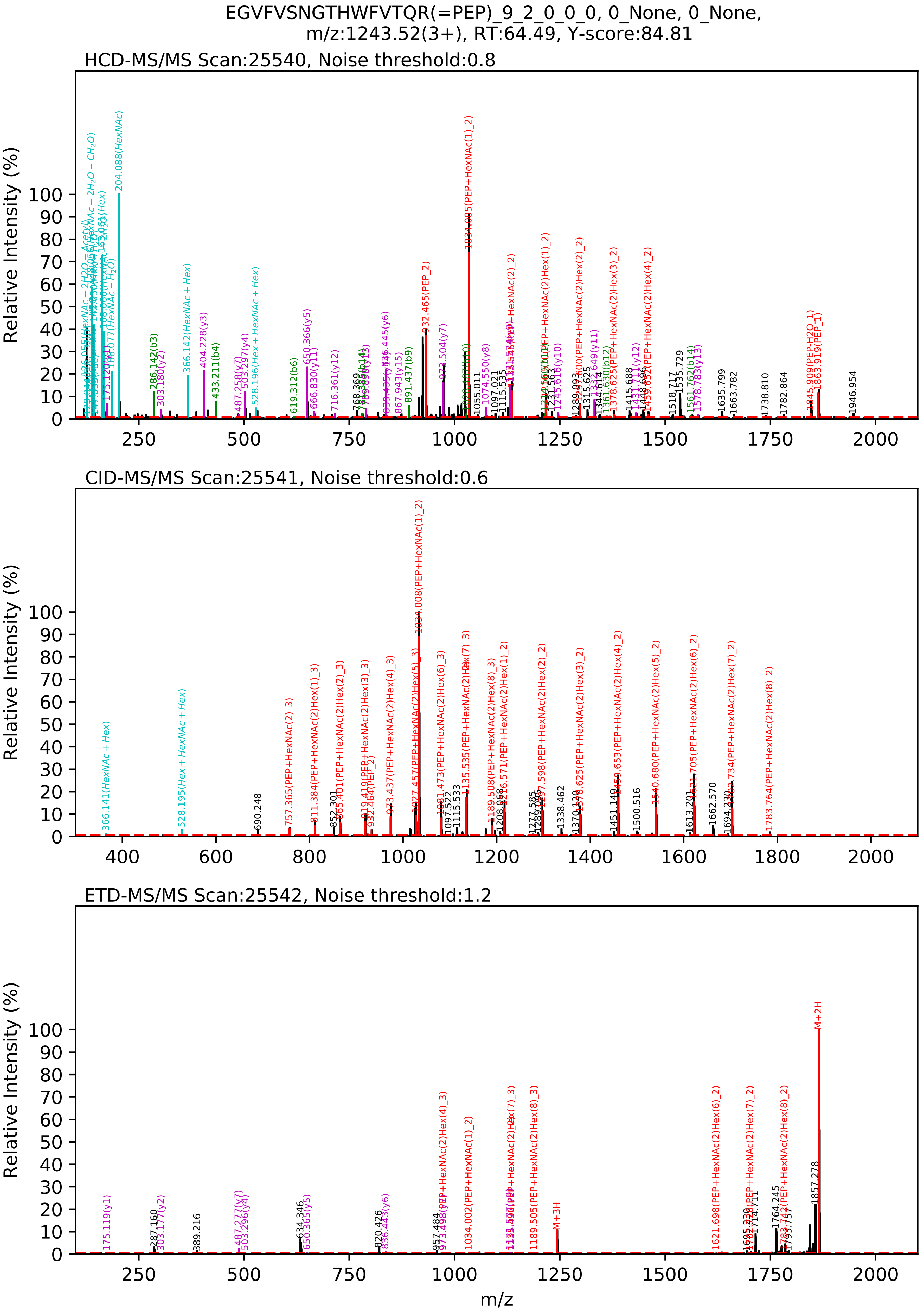
EGVFVSNGTHWFVTQR(=PEP)_9_2_0_0_0, 0_None, 0_None,
m/z:1243.52(3+), RT:64.49, Y-score:84.81
HCD-MS/MS Scan:25540, Noise threshold:0.8
CID-MS/MS Scan:25541, Noise threshold:0.6
ETD-MS/MS Scan:25542, Noise threshold:1.2
Relative Intensity (%)
m/z

EGVFVSNGTHWFVTQR(=PEP)_9_2_0_0_0, 0_None, 0_None,
m/z:1243.52(3+), RT:69.11, Y-score:81.36

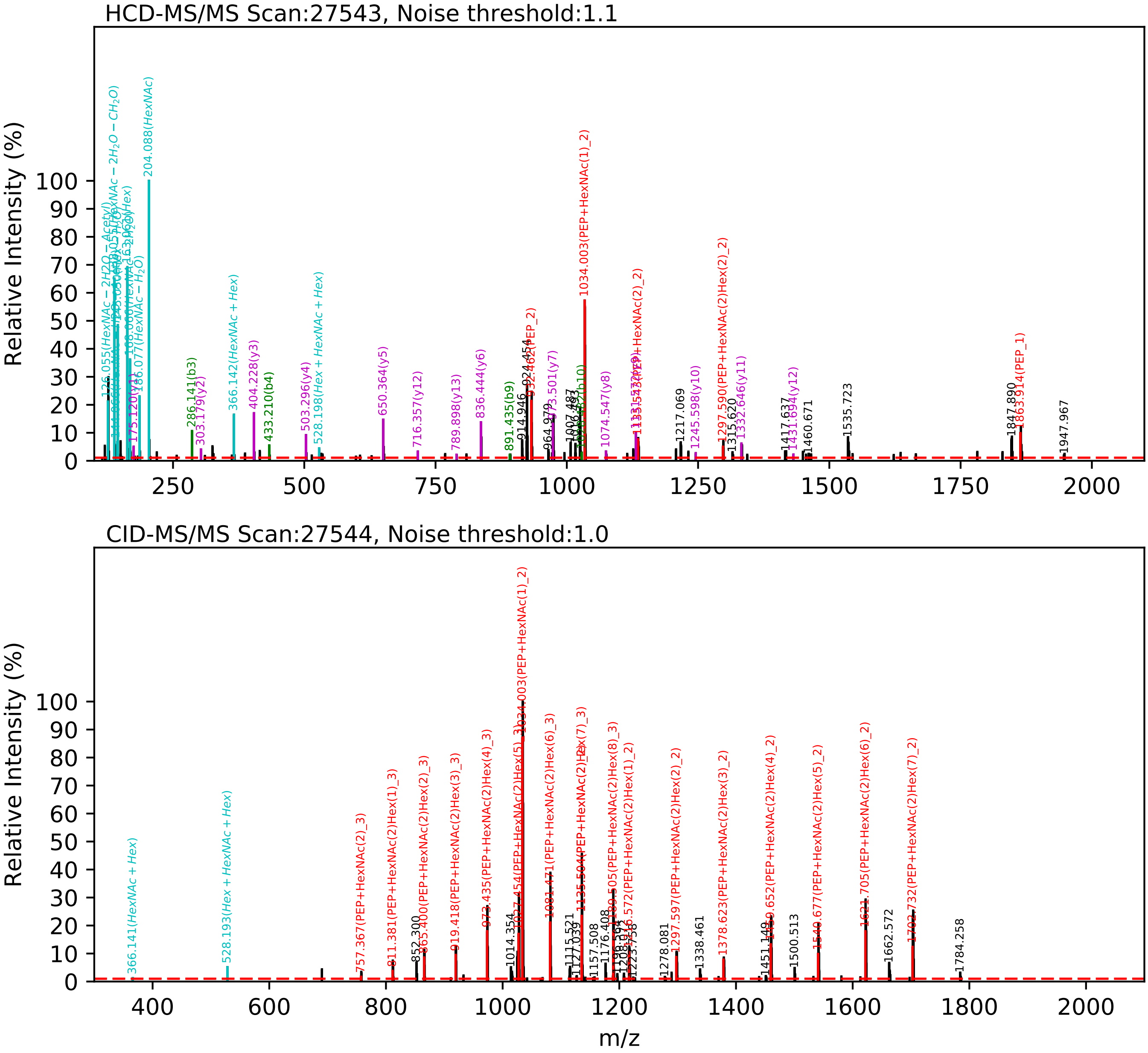

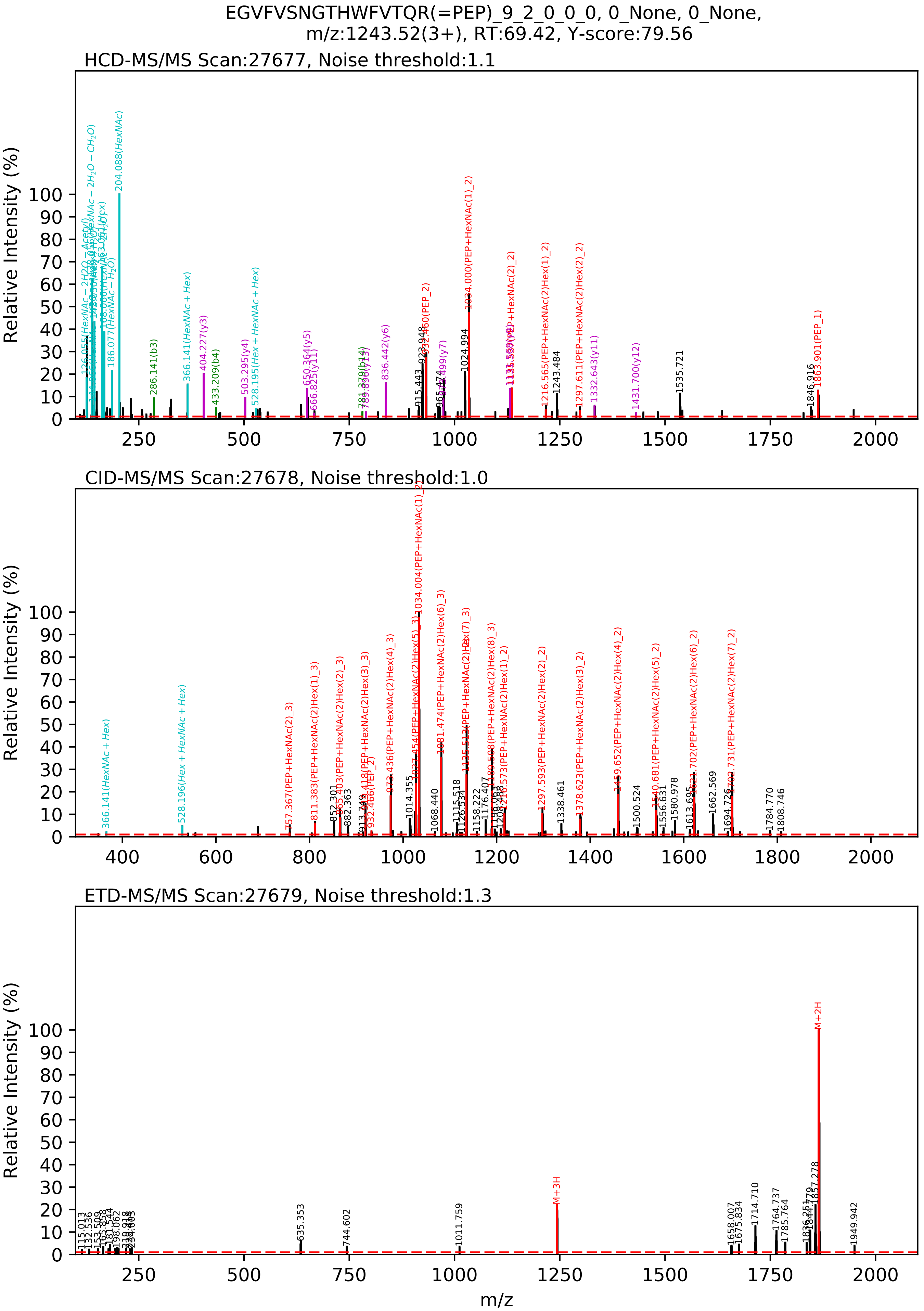
EGVFVSNGTHWFVTQR(=PEP)_9_2_0_0_0, 0_None, 0_None,
m/z:1243.52(3+), RT:69.42, Y-score:79.56
HCD-MS/MS Scan:27677, Noise threshold:1.1
Relative Intensity (%)
204.088(HexNAc)
186.077(HexNAc − H2O)
286.141(b3)
366.141(HexNAc + Hex)
404.227(y3)
433.209(b4)
503.295(y4)
528.195(Hex + HexNAc + Hex)
650.364(y5)
666.825(y11)
781.379(b14)
836.442(y6)
915.443
923.948
932.460(PEP_2)
965.474
1024.994
1034.000(PEP+HexNAc(1)_2)
1135.537(PEP+HexNAc(2)_2)
1216.565(PEP+HexNAc(2)Hex(1)_2)
1243.484
1297.611(PEP+HexNAc(2)Hex(2)_2)
1332.643(y11)
1431.700(y12)
1535.721
1846.916
1863.901(PEP_1)
CID-MS/MS Scan:27678, Noise threshold:1.0
366.141(HexNAc + Hex)
528.196(Hex + HexNAc + Hex)
757.367(PEP+HexNAc(2)_3)
811.383(PEP+HexNAc(2)Hex(1)_3)
852.301
865.403(PEP+HexNAc(2)Hex(2)_3)
882.363
913.749
932.466(PEP_2)
973.436(PEP+HexNAc(2)Hex(4)_3)
1014.355
1034.004(PEP+HexNAc(1)_2)
1068.440
1081.474(PEP+HexNAc(2)Hex(6)_3)
1115.518
1158.222
1176.407
1216.573(PEP+HexNAc(2)Hex(1)_2)
1297.593(PEP+HexNAc(2)Hex(2)_2)
1338.461
1378.623(PEP+HexNAc(2)Hex(3)_2)
1459.652(PEP+HexNAc(2)Hex(4)_2)
1500.524
1540.681(PEP+HexNAc(2)Hex(5)_2)
1556.631
1580.978
1613.695
1662.569
1694.726
1702.731(PEP+HexNAc(2)Hex(7)_2)
1784.770
1808.746
ETD-MS/MS Scan:27679, Noise threshold:1.3
M+2H
M+3H
635.353
744.602
1011.759
1658.007
1675.834
1714.710
1764.737
1785.764
1857.278
1949.942
m/z

EGVFVSNGTHWFVTQR(=PEP)_9_2_0_0_0, 0_None, 0_None,
m/z:1243.52(3+), RT:70.06, Y-score:82.85

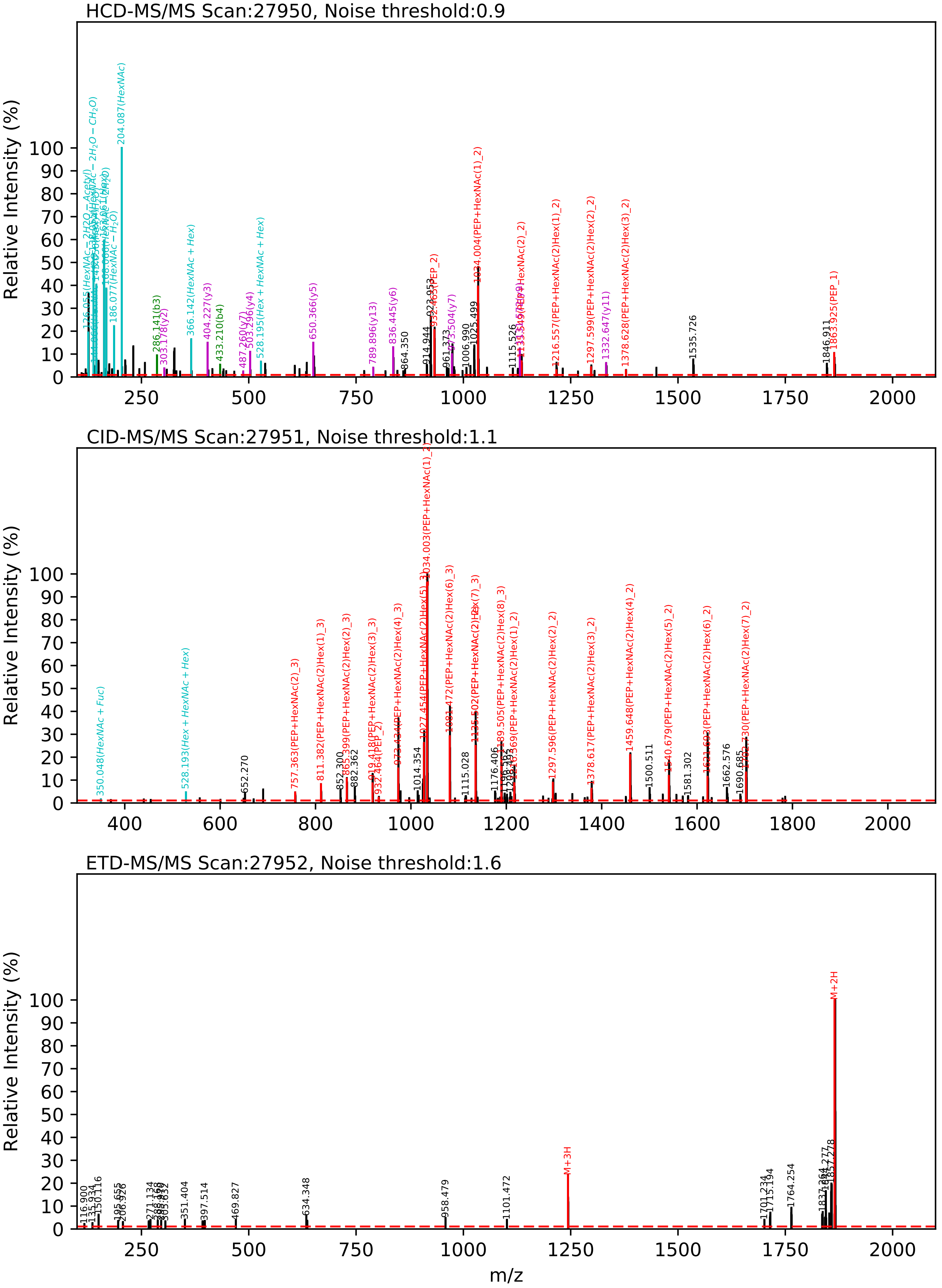

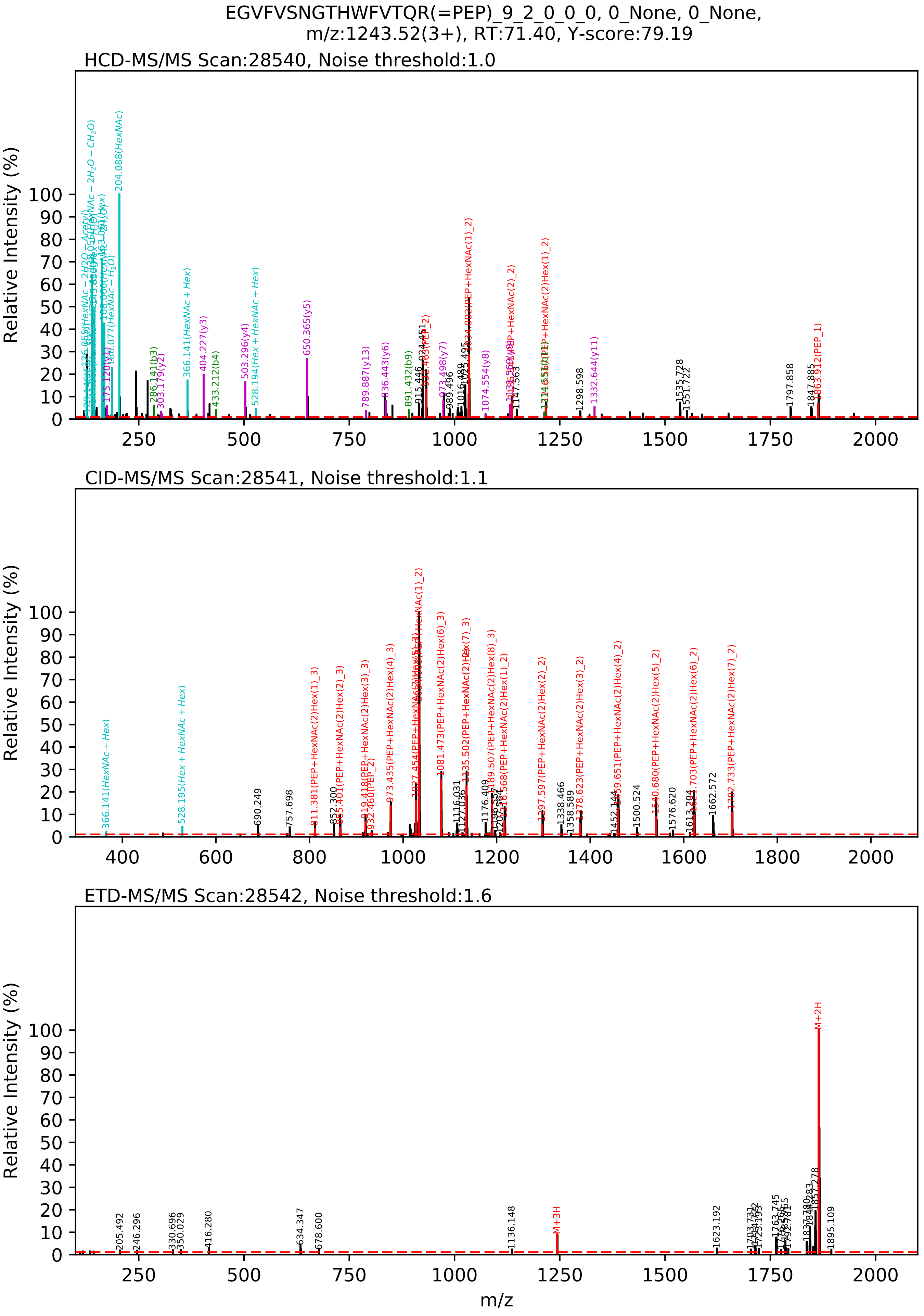
EGVFVSNGTHWFVTQR(=PEP)_9_2_0_0_0, 0_None, 0_None,
m/z:1243.52(3+), RT:71.40, Y-score:79.19
HCD-MS/MS Scan:28540, Noise threshold:1.0
Relative Intensity (%)
204.088(HexNAc)
366.141(HexNAc+Hex)
528.194(Hex+HexNAc+Hex)
175.120(y1)
286.141(b3)
303.179(y2)
404.227(y3)
433.212(b4)
503.296(y4)
650.365(y5)
789.887(y13)
836.443(y6)
891.432(b9)
915.446
973.498(y7)
989.496
1074.554(y8)
1147.563
1298.598
1332.644(y11)
1535.728
1551.722
1797.858
1847.885
1863.912(PEP_1)
CID-MS/MS Scan:28541, Noise threshold:1.1
366.141(HexNAc+Hex)
528.195(Hex+HexNAc+Hex)
690.249
757.698
811.381(PEP+HexNAc(2)Hex(1)_3)
852.300
865.401(PEP+HexNAc(2)Hex(2)_3)
919.418(PEP+HexNAc(2)Hex(3)_3)
932.460(PEP_2)
973.435(PEP+HexNAc(2)Hex(4)_3)
1081.473(PEP+HexNAc(2)Hex(6)_3)
1116.031
1135.502(PEP+HexNAc(2)Hex(7)_3)
1176.409
1189.507(PEP+HexNAc(2)Hex(8)_3)
1216.568(PEP+HexNAc(2)Hex(1)_2)
1297.597(PEP+HexNAc(2)Hex(2)_2)
1338.466
1358.589
1378.623(PEP+HexNAc(2)Hex(3)_2)
1452.144
1459.651(PEP+HexNAc(2)Hex(4)_2)
1500.524
1540.680(PEP+HexNAc(2)Hex(5)_2)
1576.620
1613.294
1621.703(PEP+HexNAc(2)Hex(6)_2)
1662.572
1702.733(PEP+HexNAc(2)Hex(7)_2)
ETD-MS/MS Scan:28542, Noise threshold:1.6
M+2H
M+3H
205.492
246.296
330.696
350.029
416.280
634.347
678.600
1136.148
1623.192
1703.731
1763.745
1857.278
1895.109
m/z

EGVFVSNGTHWFVTQR(=PEP)_9_2_0_0_0, 0_None, 0_None,
m/z:1243.52(3+), RT:72.16, Y-score:84.36

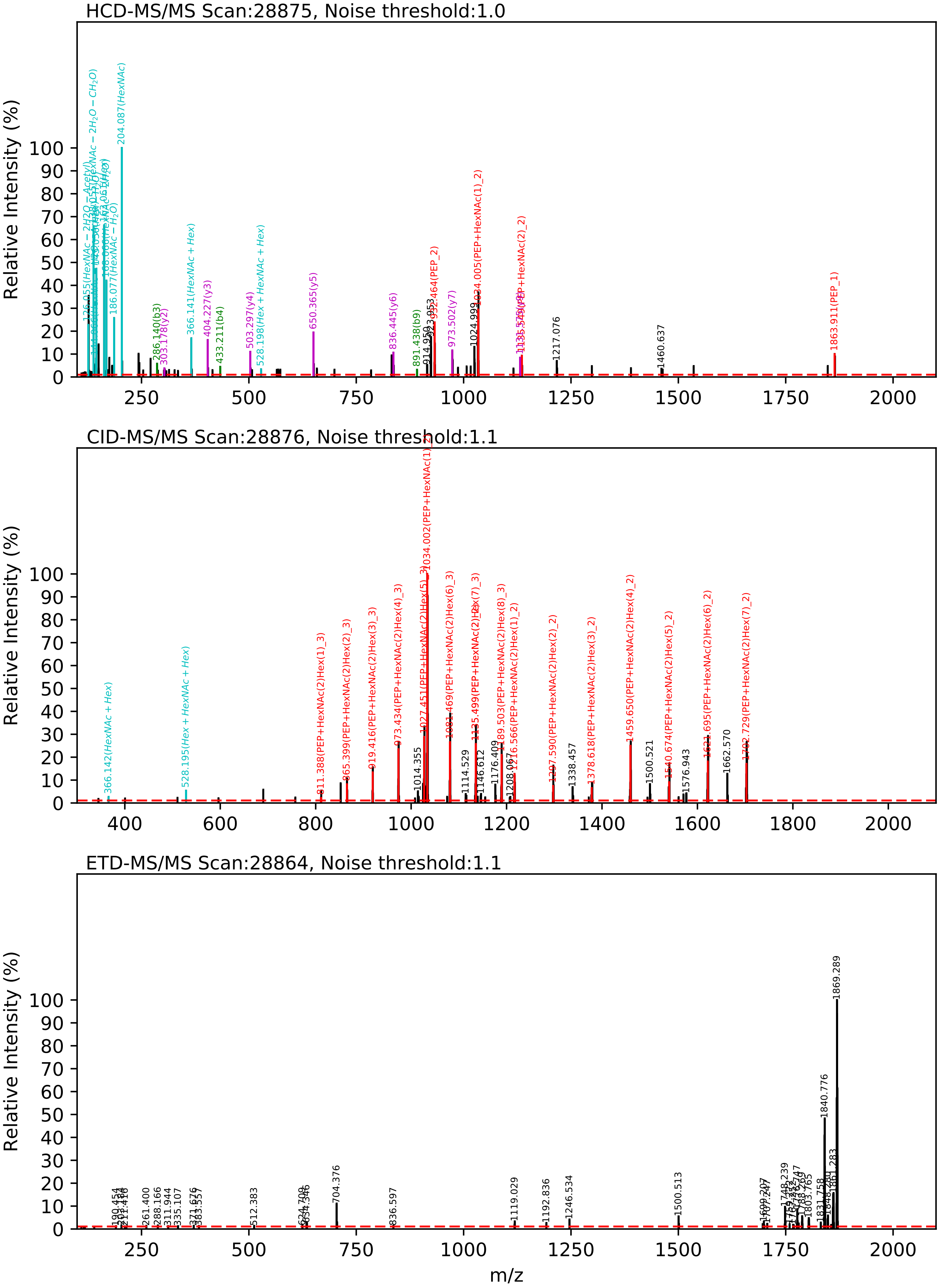

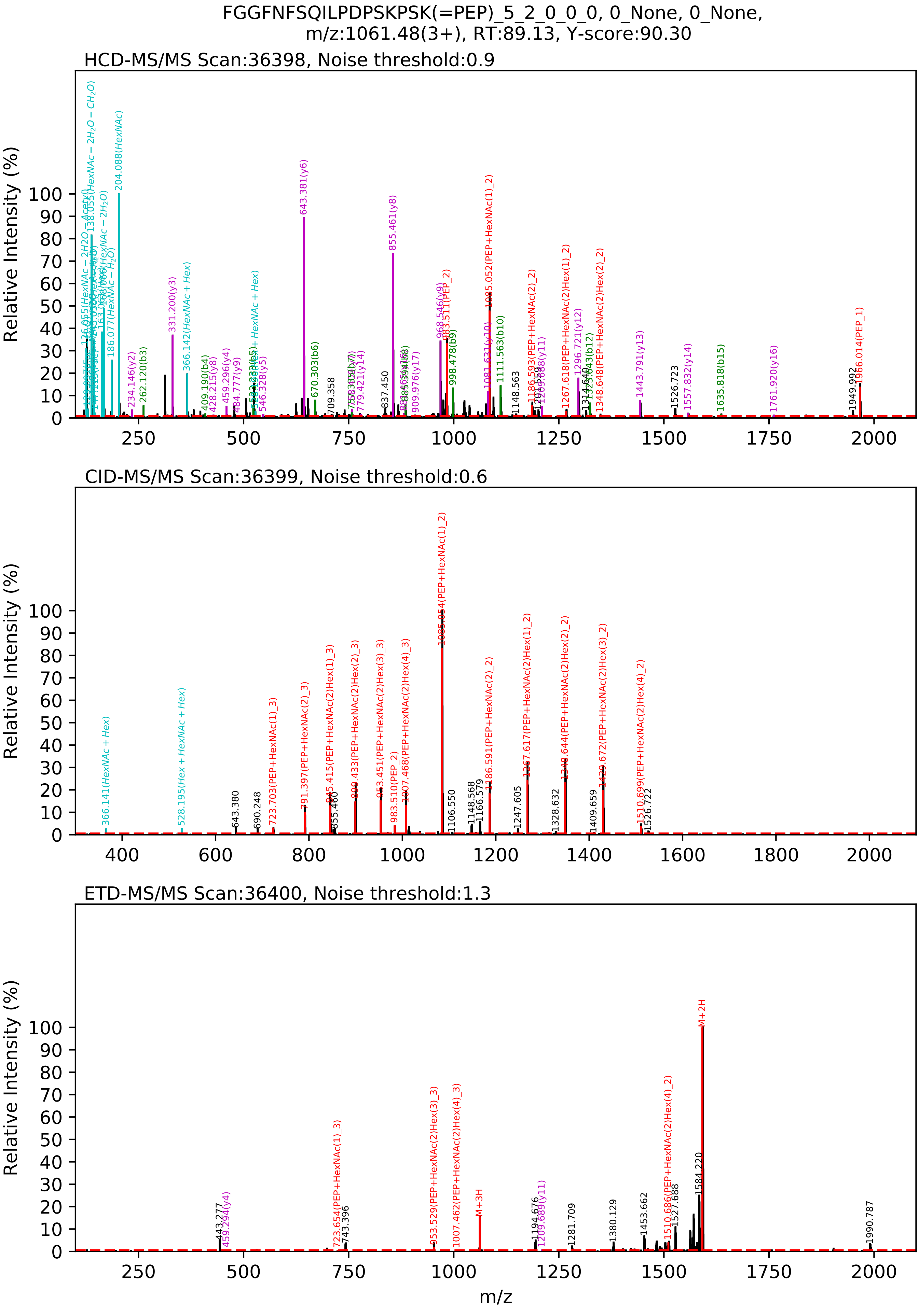

FGGFNFSQILPDPSKPSK(=PEP)_5_2_0_0_0, 0_None, 0_None,
m/z:1061.48(3+), RT:89.13, Y-score:90.30
HCD-MS/MS Scan:36398, Noise threshold:0.9
204.088(HexNAc)
186.077(HexNAc − H2O)
331.200(y3)
366.142(HexNAc + Hex)
234.146(y2)
262.120(b3)
409.190(b4)
428.215(y8)
459.296(y4)
546.328(y5)
643.381(y6)
670.303(b6)
709.358
779.421(y14)
837.450
855.461(y8)
909.976(y17)
968.546(y9)
983.511(PEP_2)
998.478(b9)
1081.631(y10)
1085.052(PEP+HexNAc(1)_2)
1111.563(b10)
1148.563
1186.593(PEP+HexNAc(2)_2)
1267.618(PEP+HexNAc(2)Hex(1)_2)
1296.721(y12)
1348.648(PEP+HexNAc(2)Hex(2)_2)
1443.791(y13)
1526.723
1557.832(y14)
1635.818(b15)
1761.920(y16)
1949.992
1966.014(PEP_1)
CID-MS/MS Scan:36399, Noise threshold:0.6
366.141(HexNAc + Hex)
528.195(Hex + HexNAc + Hex)
643.380
690.248
723.703(PEP+HexNAc(1)_3)
791.397(PEP+HexNAc(2)_3)
845.415(PEP+HexNAc(2)Hex(1)_3)
855.460
899.433(PEP+HexNAc(2)Hex(2)_3)
953.451(PEP+HexNAc(2)Hex(3)_3)
983.510(PEP_2)
1007.468(PEP+HexNAc(2)Hex(4)_3)
1085.054(PEP+HexNAc(1)_2)
1106.550
1148.568
1166.579
1186.591(PEP+HexNAc(2)_2)
1247.605
1267.617(PEP+HexNAc(2)Hex(1)_2)
1328.632
1348.644(PEP+HexNAc(2)Hex(2)_2)
1409.659
1429.672(PEP+HexNAc(2)Hex(3)_2)
1510.699(PEP+HexNAc(2)Hex(4)_2)
1526.722
ETD-MS/MS Scan:36400, Noise threshold:1.3
443.277
459.294(y4)
723.654(PEP+HexNAc(1)_3)
743.396
953.529(PEP+HexNAc(2)Hex(3)_3)
1007.462(PEP+HexNAc(2)Hex(4)_3)
M+3H
1194.676
1209.689(y11)
1281.709
1380.129
1453.662
1510.686(PEP+HexNAc(2)Hex(4)_2)
1527.688
1584.220
M+2H
1990.787
Relative Intensity (%)
m/z

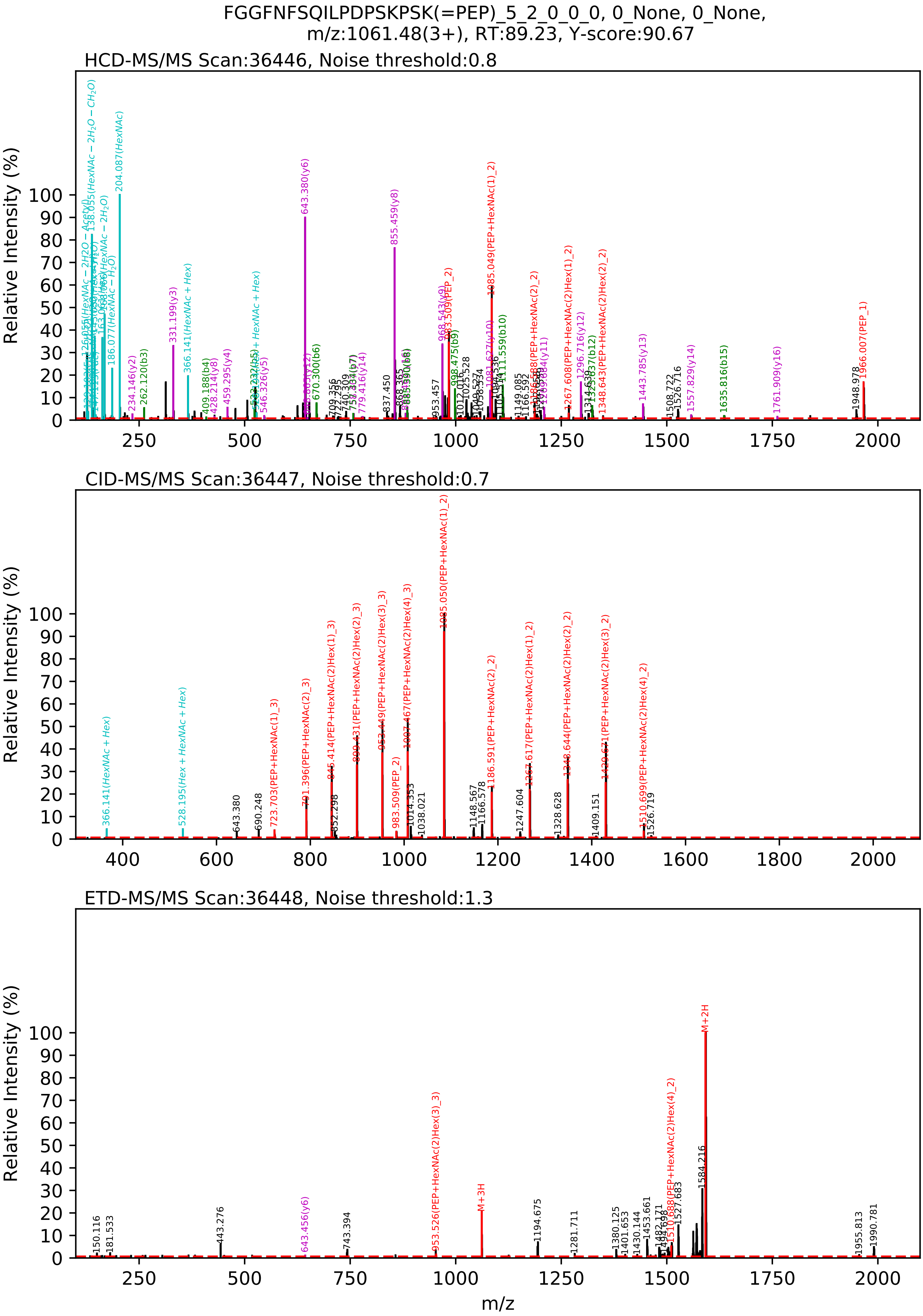
FGGFNFSQILPDPSKPSK(=PEP)_5_2_0_0_0, 0_None, 0_None,
m/z:1061.48(3+), RT:89.23, Y-score:90.67
HCD-MS/MS Scan:36446, Noise threshold:0.8
Relative Intensity (%)
204.087(HexNAc)
186.077(HexNAc − H2O)
234.146(y2)
262.120(b3)
331.199(y3)
366.141(HexNAc + Hex)
409.188(b4)
428.214(y8)
459.295(y4)
546.326(y5)
643.380(y6)
670.300(b6)
779.416(y14)
837.450
855.459(y8)
953.457
968.543(y9)
983.509(PEP_2)
998.475(b9)
1085.049(PEP+HexNAc(1)_2)
1111.559(b10)
1149.085
1166.592
1267.608(PEP+HexNAc(2)Hex(1)_2)
1296.716(y12)
1348.643(PEP+HexNAc(2)Hex(2)_2)
1443.785(y13)
1508.722
1526.716
1557.829(y14)
1635.816(b15)
1761.909(y16)
1948.978
1966.007(PEP_1)
CID-MS/MS Scan:36447, Noise threshold:0.7
Relative Intensity (%)
366.141(HexNAc + Hex)
528.195(Hex + HexNAc + Hex)
643.380
690.248
723.703(PEP+HexNAc(1)_3)
791.396(PEP+HexNAc(2)_3)
845.414(PEP+HexNAc(2)Hex(1)_3)
852.298
899.431(PEP+HexNAc(2)Hex(2)_3)
953.449(PEP+HexNAc(2)Hex(3)_3)
983.509(PEP_2)
1007.467(PEP+HexNAc(2)Hex(4)_3)
1014.353
1038.021
1085.050(PEP+HexNAc(1)_2)
1148.567
1166.578
1186.591(PEP+HexNAc(2)_2)
1247.604
1267.617(PEP+HexNAc(2)Hex(1)_2)
1328.628
1348.644(PEP+HexNAc(2)Hex(2)_2)
1409.151
1429.671(PEP+HexNAc(2)Hex(3)_2)
1510.699(PEP+HexNAc(2)Hex(4)_2)
1526.719
ETD-MS/MS Scan:36448, Noise threshold:1.3
Relative Intensity (%)
150.116
181.533
443.276
643.456(y6)
743.394
953.526(PEP+HexNAc(2)Hex(3)_3)
M+3H
1194.675
1281.711
1380.125
1401.653
1430.144
1453.661
1510.688(PEP+HexNAc(2)Hex(4)_2)
1527.683
1584.216
M+2H
1955.813
1990.781
m/z

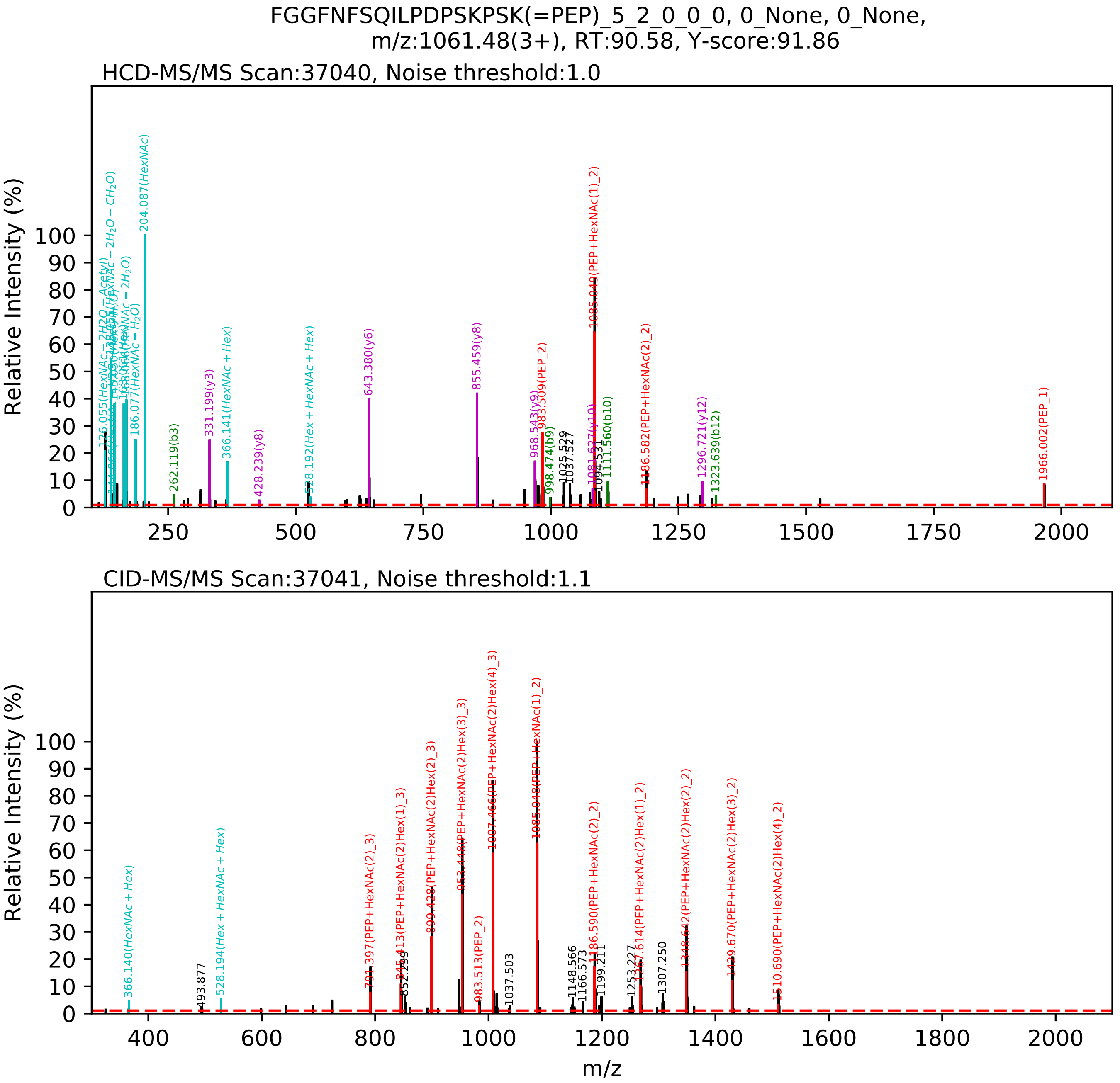
FGGFNFSQILPDPSKPSK(=PEP)_5_2_0_0_0, 0_None, 0_None,
m/z:1061.48(3+), RT:90.58, Y-score:91.86
HCD-MS/MS Scan:37040, Noise threshold:1.0
Relative Intensity (%)
204.087(HexNAc)
186.077(HexNAc − H2O)
262.119(b3)
331.199(y3)
366.141(HexNAc + Hex)
428.239(y8)
528.192(Hex + HexNAc + Hex)
643.380(y6)
855.459(y8)
968.543(y9)
983.509(PEP_2)
998.474(b9)
1025.529
1037.527
1081.627(y10)
1085.049(PEP+HexNAc(1)_2)
1094.535
1111.560(b10)
1186.582(PEP+HexNAc(2)_2)
1296.721(y12)
1323.639(b12)
1966.002(PEP_1)
CID-MS/MS Scan:37041, Noise threshold:1.1
Relative Intensity (%)
366.140(HexNAc + Hex)
493.877
528.194(Hex + HexNAc + Hex)
791.397(PEP+HexNAc(2)_3)
852.299
900.428(PEP+HexNAc(2)Hex(2)_3)
953.448(PEP+HexNAc(2)Hex(3)_3)
983.513(PEP_2)
1007.466(PEP+HexNAc(2)Hex(4)_3)
1037.503
1085.048(PEP+HexNAc(1)_2)
1148.566
1166.573
1186.590(PEP+HexNAc(2)_2)
1199.211
1253.227
1267.614(PEP+HexNAc(2)Hex(1)_2)
1307.250
1348.642(PEP+HexNAc(2)Hex(2)_2)
1429.670(PEP+HexNAc(2)Hex(3)_2)
1510.690(PEP+HexNAc(2)Hex(4)_2)
m/z

FGGFNFSQILPDPSKPSK(=PEP)_6_2_0_0_0, 0_None, 0_None,
m/z:1115.50(3+), RT:90.02, Y-score:90.66

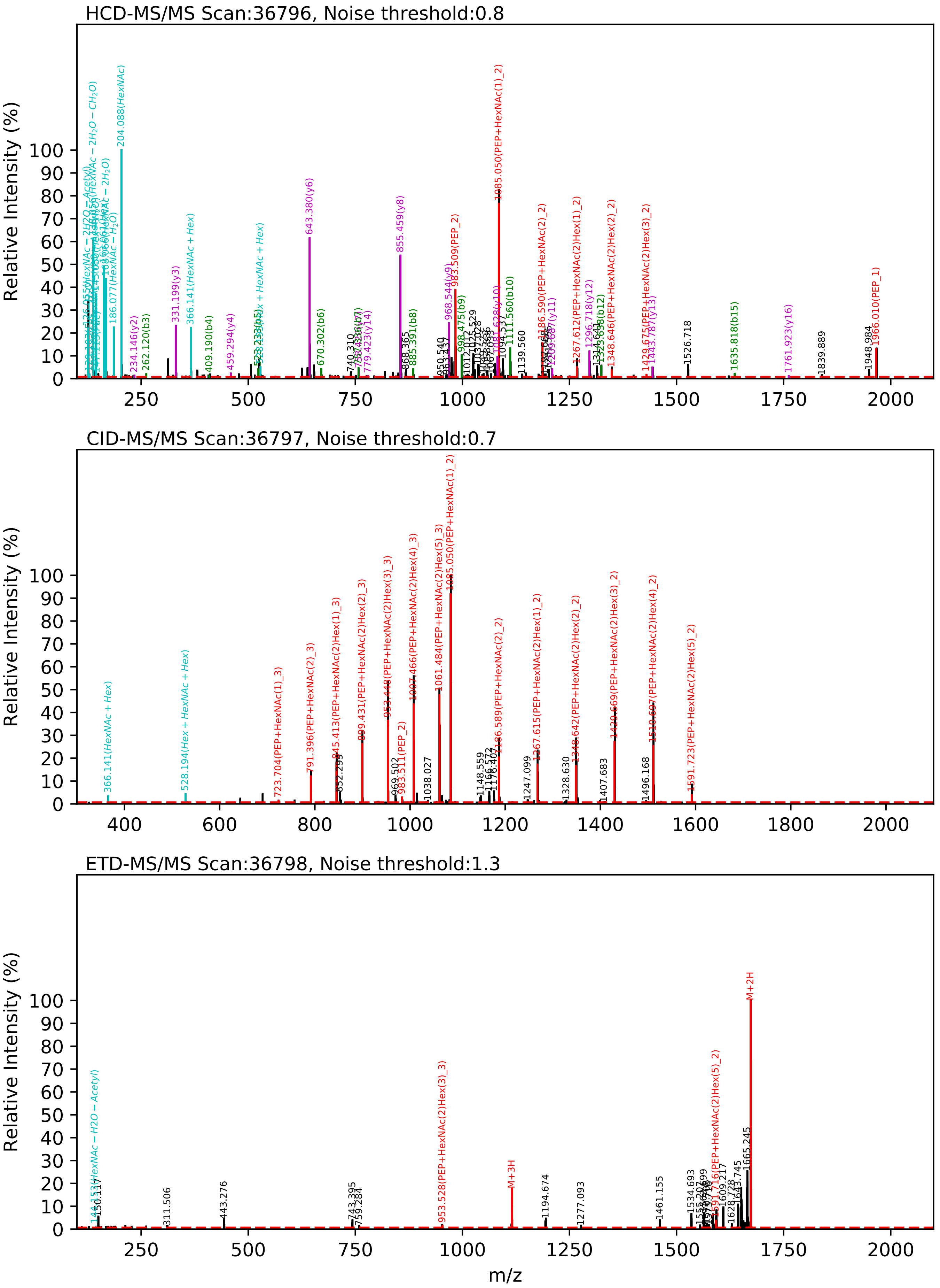

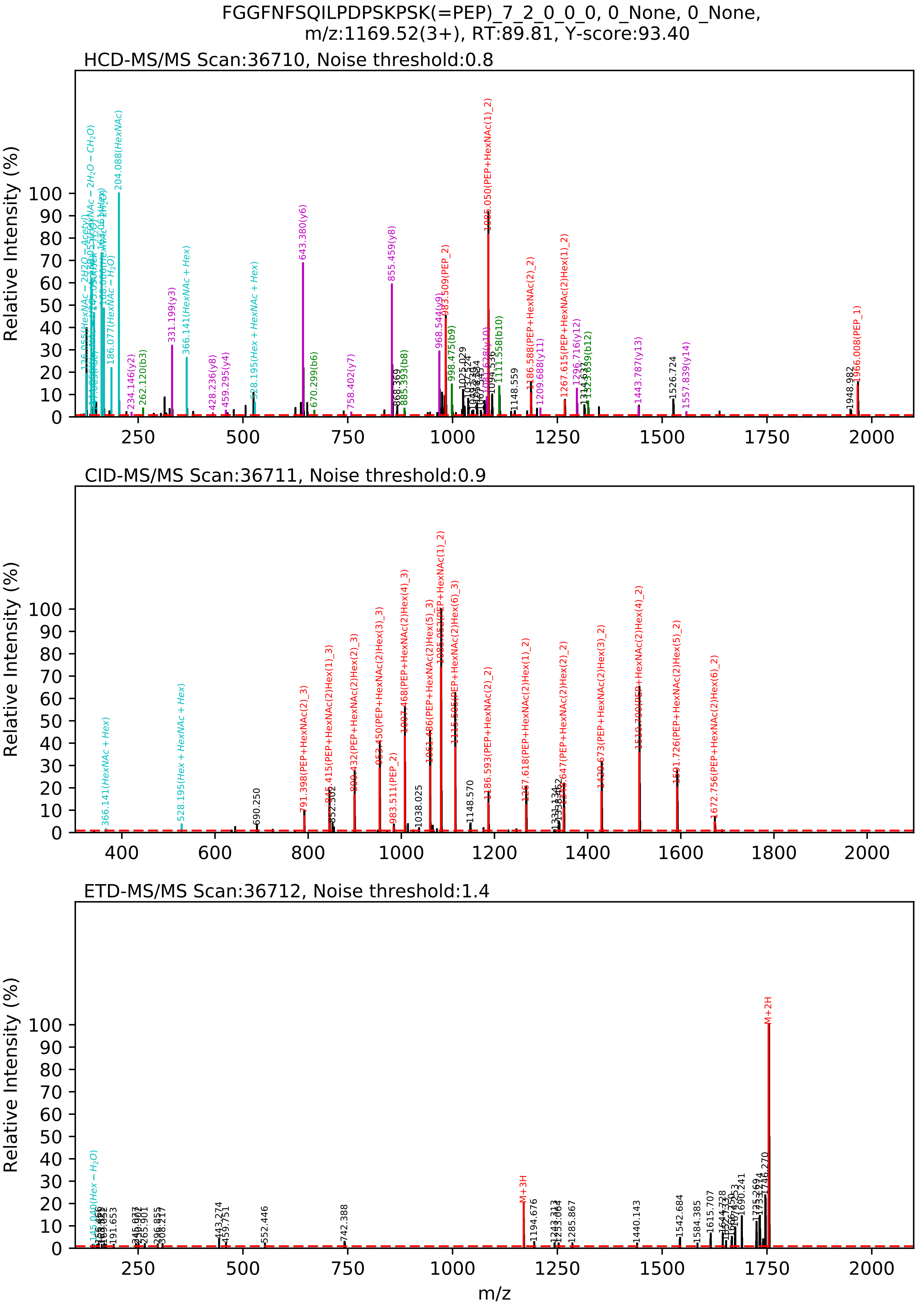

FGGFNFSQILPDPSKPSK(=PEP)_7_2_0_0_0, 0_None, 0_None,
m/z:1169.52(3+), RT:89.81, Y-score:93.40
HCD-MS/MS Scan:36710, Noise threshold:0.8
CID-MS/MS Scan:36711, Noise threshold:0.9
ETD-MS/MS Scan:36712, Noise threshold:1.4
Relative Intensity (%)
m/z

FGGFNFSQILPDPSKPSK(=PEP)_7_2_0_0_0, 0_None, 0_None,
m/z:1169.52(3+), RT:88.92, Y-score:77.28

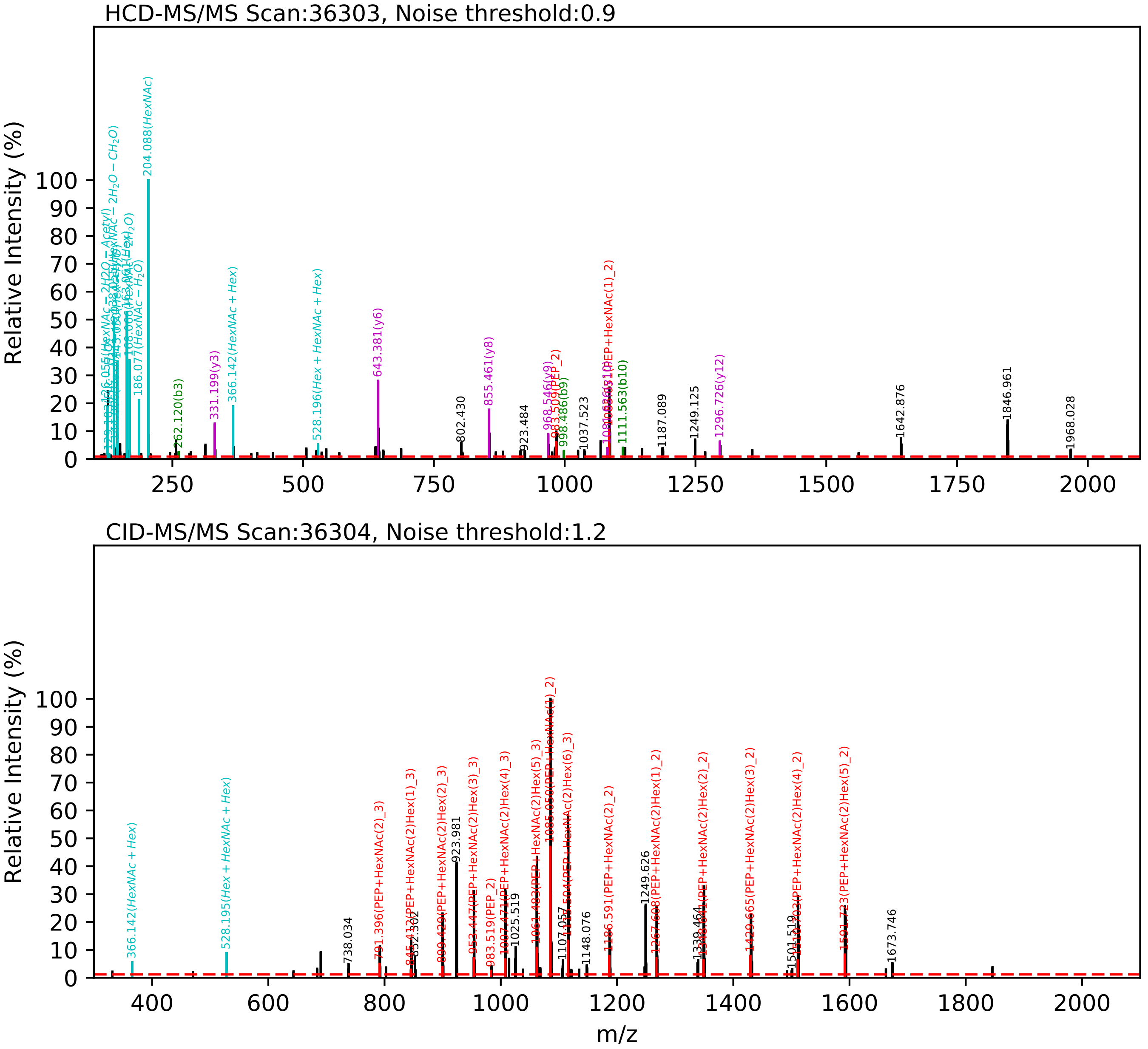

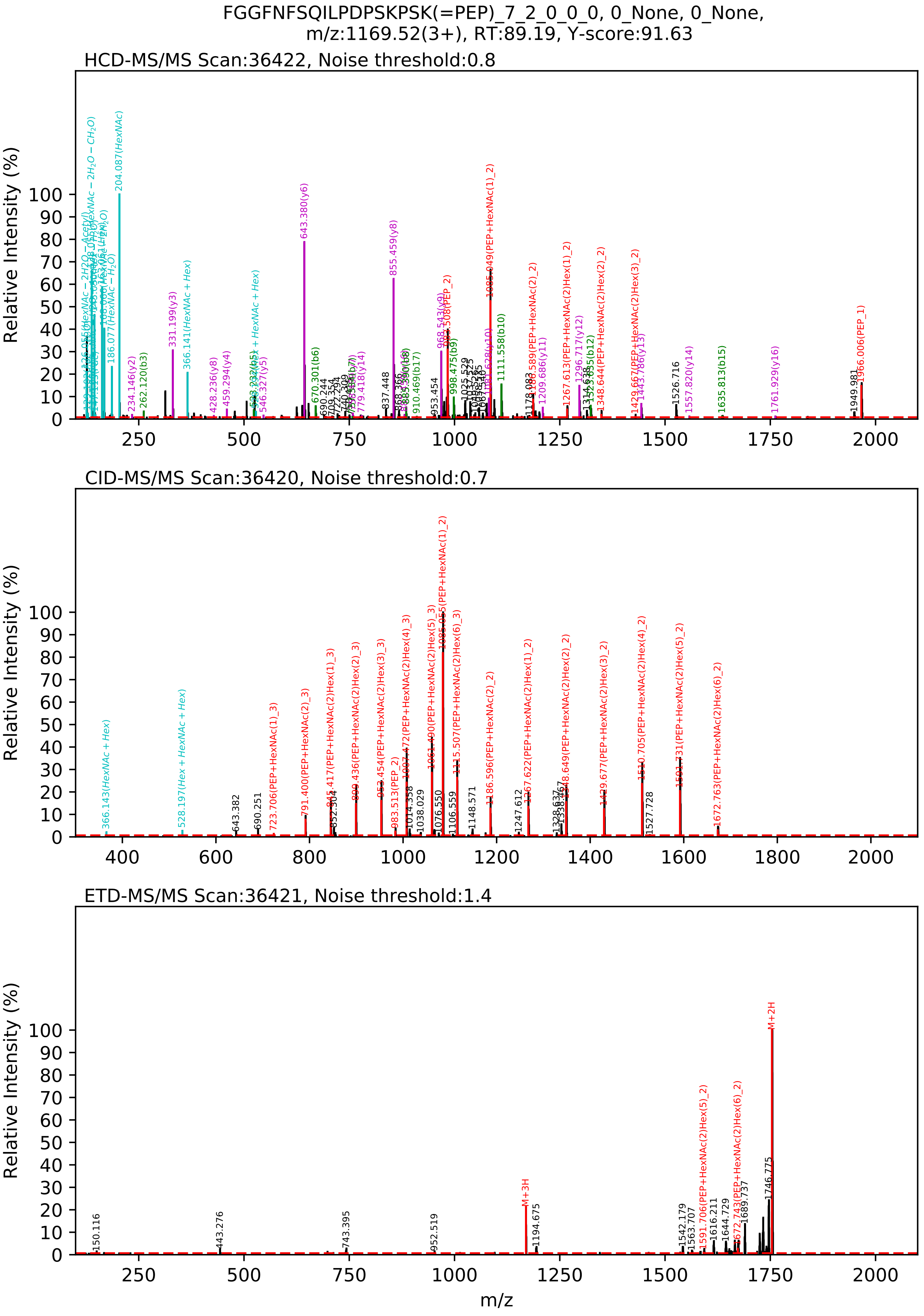
FGGFNFSQILPDPSKPSK(=PEP)_7_2_0_0_0, 0_None, 0_None,
m/z:1169.52(3+), RT:89.19, Y-score:91.63
HCD-MS/MS Scan:36422, Noise threshold:0.8
CID-MS/MS Scan:36420, Noise threshold:0.7
ETD-MS/MS Scan:36421, Noise threshold:1.4
Relative Intensity (%)
m/z
204.087(HexNAc)
643.380(y6)
855.459(y8)
1085.049(PEP+HexNAc(1)_2)
1966.006(PEP_1)
1085.055(PEP+HexNAc(1)_2)
1510.705(PEP+HexNAc(2)Hex(4)_2)
1591.731(PEP+HexNAc(2)Hex(5)_2)
1672.763(PEP+HexNAc(2)Hex(6)_2)
M+2H
M+3H
1746.775

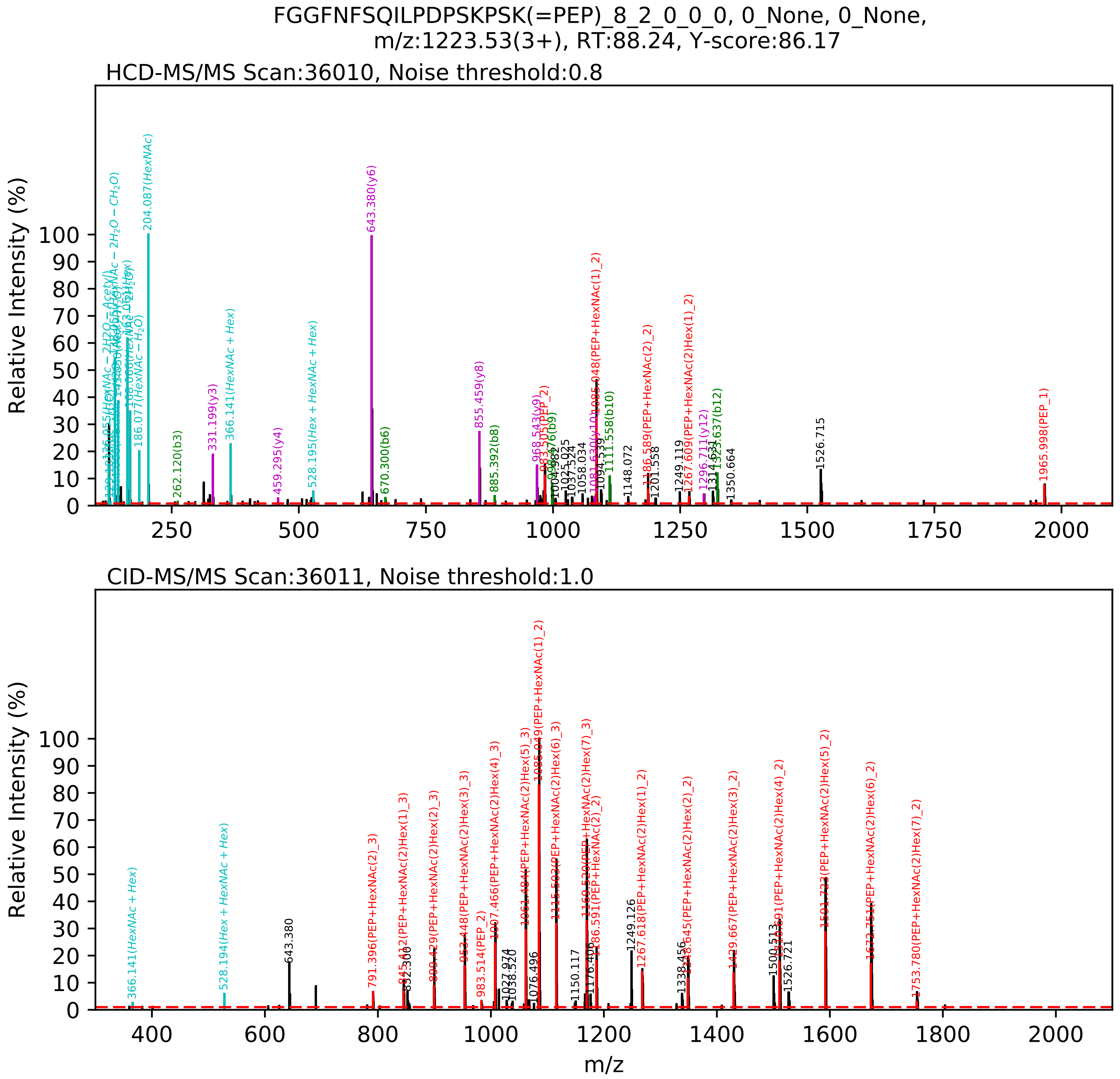
FGGFNFSQILPDPSKPSK(=PEP)_8_2_0_0_0, 0_None, 0_None,
m/z:1223.53(3+), RT:88.24, Y-score:86.17
HCD-MS/MS Scan:36010, Noise threshold:0.8
Relative Intensity (%)
204.087(HexNAc)
643.380(y6)
186.077(HexNAc − H2O)
331.199(y3)
366.141(HexNAc + Hex)
262.120(b3)
459.295(y4)
528.195(Hex + HexNAc + Hex)
670.300(b6)
855.459(y8)
885.392(b8)
968.543(y9)
1085.048(PEP+HexNAc(1)_2)
1111.558(b10)
1148.072
1186.589(PEP+HexNAc(2)_2)
1201.558
1249.119
1267.609(PEP+HexNAc(2)Hex(1)_2)
1296.711(y12)
1350.664
1526.715
1965.998(PEP_1)
CID-MS/MS Scan:36011, Noise threshold:1.0
366.141(HexNAc + Hex)
528.194(Hex + HexNAc + Hex)
643.380
791.396(PEP+HexNAc(2)_3)
852.306
899.429(PEP+HexNAc(2)Hex(2)_3)
953.448(PEP+HexNAc(2)Hex(3)_3)
983.514(PEP_2)
1007.466(PEP+HexNAc(2)Hex(4)_3)
1076.496
1085.049(PEP+HexNAc(1)_2)
1115.503(PEP+HexNAc(2)Hex(6)_3)
1150.117
1249.126
1267.618(PEP+HexNAc(2)Hex(1)_2)
1429.667(PEP+HexNAc(2)Hex(3)_2)
1526.721
1591.722(PEP+HexNAc(2)Hex(5)_2)
1753.780(PEP+HexNAc(2)Hex(7)_2)
m/z

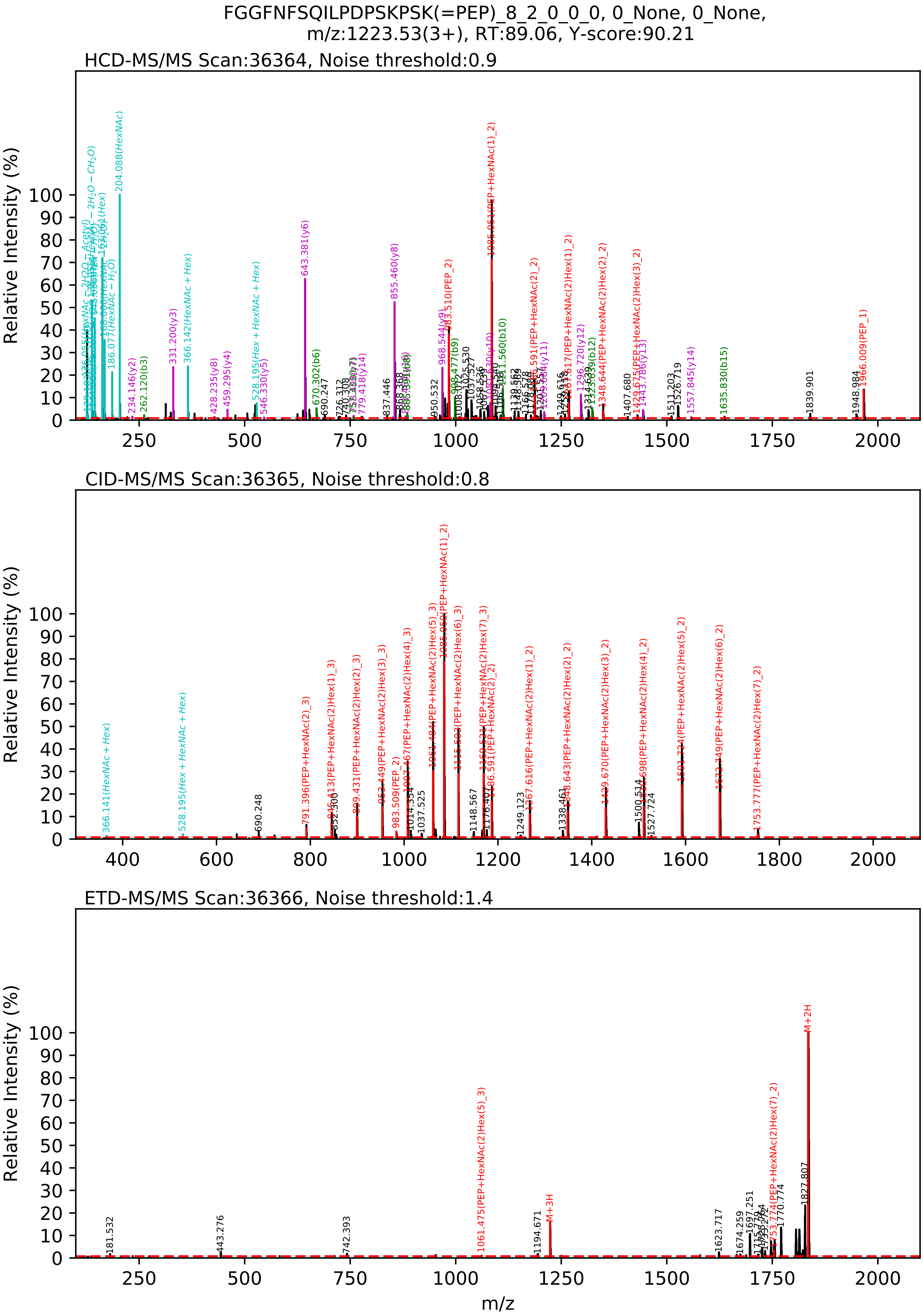
FGGFNFSQILPDPSKPSK(=PEP)_8_2_0_0_0, 0_None, 0_None,
m/z:1223.53(3+), RT:89.06, Y-score:90.21
HCD-MS/MS Scan:36364, Noise threshold:0.9
CID-MS/MS Scan:36365, Noise threshold:0.8
ETD-MS/MS Scan:36366, Noise threshold:1.4
Relative Intensity (%)
m/z
204.088(HexNAc)
643.381(y6)
855.460(y8)
1085.051(PEP+HexNAc(1)_2)
983.510(PEP_2)
1966.009(PEP_1)
331.200(y3)
366.142(HexNAc + Hex)
528.195(Hex + HexNAc + Hex)
1085.050(PEP+HexNAc(1)_2)
1591.724(PEP+HexNAc(2)Hex(5)_2)
1672.749(PEP+HexNAc(2)Hex(6)_2)
1753.777(PEP+HexNAc(2)Hex(7)_2)
M+2H
M+3H
1827.807
1061.475(PEP+HexNAc(2)Hex(5)_3)

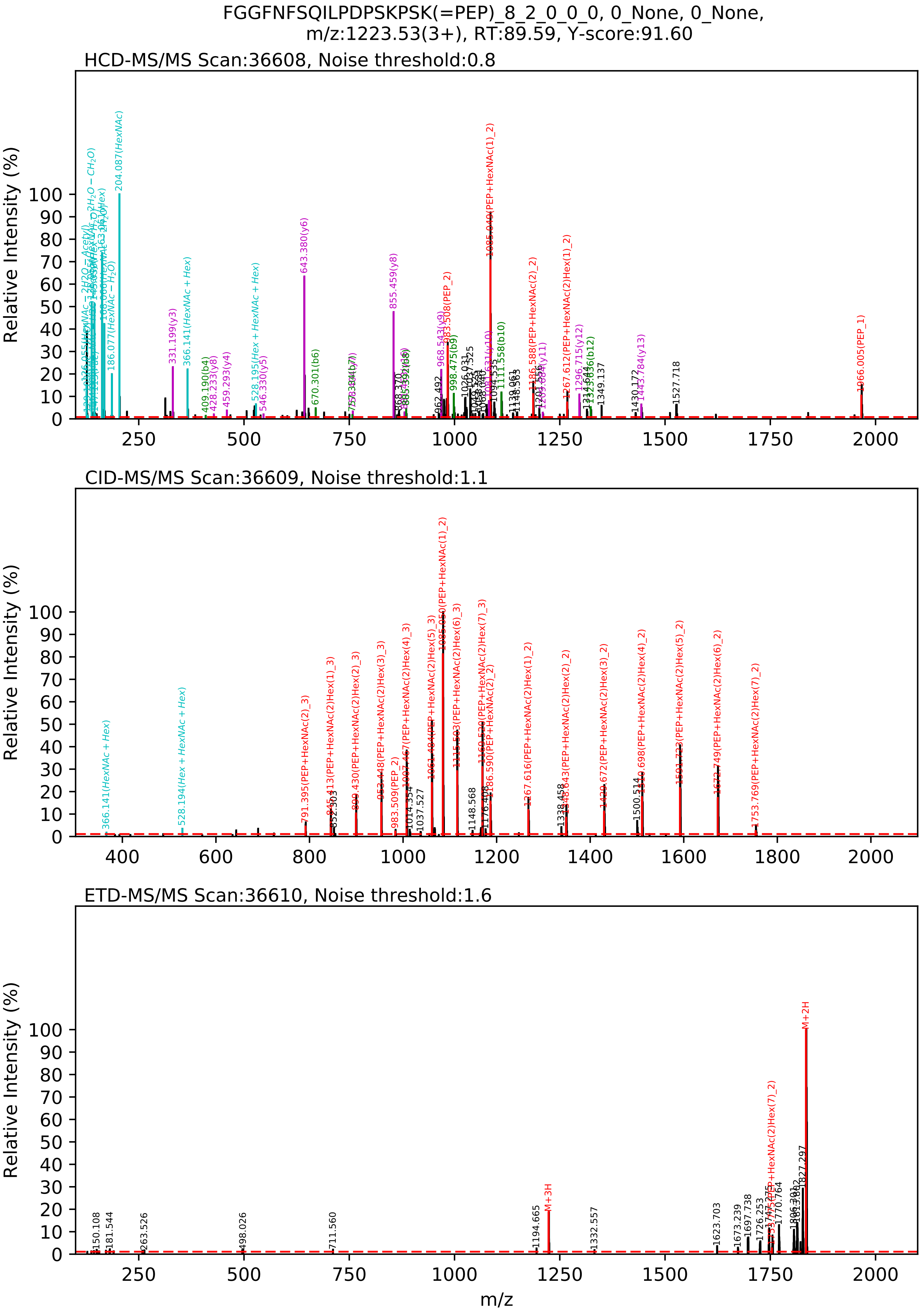
FGGFNFSQILPDPSKPSK(=PEP)_8_2_0_0_0, 0_None, 0_None,
m/z:1223.53(3+), RT:89.59, Y-score:91.60
HCD-MS/MS Scan:36608, Noise threshold:0.8
CID-MS/MS Scan:36609, Noise threshold:1.1
ETD-MS/MS Scan:36610, Noise threshold:1.6
Relative Intensity (%)
m/z

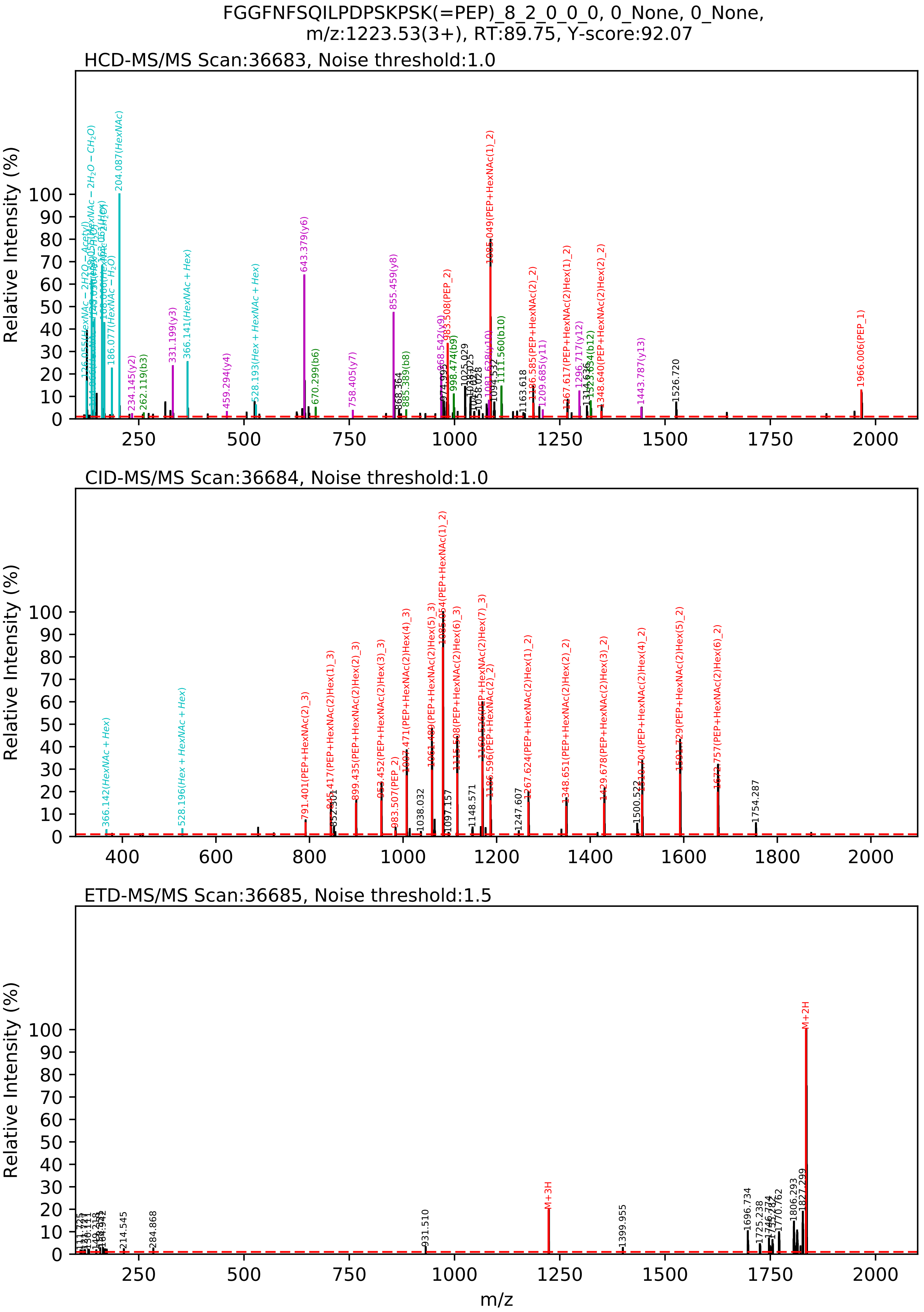
FGGFNFSQILPDPSKPSK(=PEP)_8_2_0_0_0, 0_None, 0_None,
m/z:1223.53(3+), RT:89.75, Y-score:92.07
HCD-MS/MS Scan:36683, Noise threshold:1.0
CID-MS/MS Scan:36684, Noise threshold:1.0
ETD-MS/MS Scan:36685, Noise threshold:1.5
Relative Intensity (%)
m/z
204.087(HexNAc)
643.379(y6)
855.459(y8)
1085.049(PEP+HexNAc(1)_2)
1966.006(PEP_1)
1085.054(PEP+HexNAc(1)_2)
1591.729(PEP+HexNAc(2)Hex(5)_2)
M+2H
M+3H

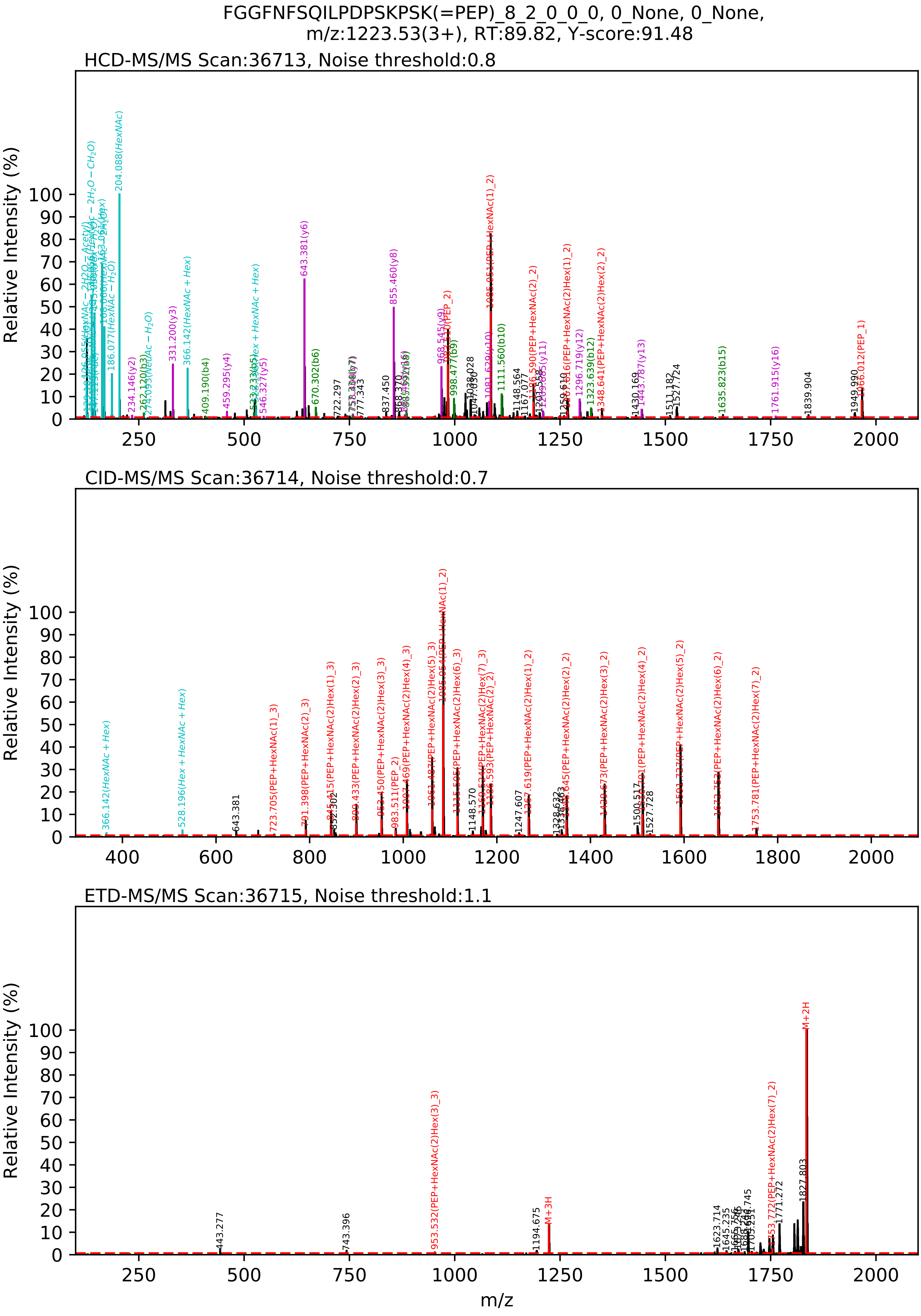

FGGFNFSQILPDPSKPSK(=PEP)_8_2_0_0_0, 0_None, 0_None,
m/z:1223.53(3+), RT:89.82, Y-score:91.48
HCD-MS/MS Scan:36713, Noise threshold:0.8
Relative Intensity (%)
204.088(HexNAc)
186.077(HexNAc − H2O)
643.381(y6)
855.460(y8)
331.200(y3)
366.142(HexNAc + Hex)
234.146(y2)
262.120(b3)
409.190(b4)
459.295(y4)
546.327(y5)
670.302(b6)
722.297
777.343
837.450
968.545(y9)
998.477(b9)
1111.560(b10)
1148.564
1167.077
1296.719(y12)
1323.639(b12)
1348.641(PEP+HexNAc(2)Hex(2)_2)
1430.169
1443.787(y13)
1511.182
1527.724
1635.823(b15)
1761.915(y16)
1839.904
1949.990
1966.012(PEP_1)
CID-MS/MS Scan:36714, Noise threshold:0.7
366.142(HexNAc + Hex)
528.196(Hex + HexNAc + Hex)
643.381
723.705(PEP+HexNAc(1)_3)
791.398(PEP+HexNAc(2)_3)
852.302
899.433(PEP+HexNAc(2)Hex(2)_3)
953.450(PEP+HexNAc(2)Hex(3)_3)
983.511(PEP_2)
1007.469(PEP+HexNAc(2)Hex(4)_3)
1085.054(PEP+HexNAc(1)_2)
1115.505(PEP+HexNAc(2)Hex(6)_3)
1148.570
1186.593(PEP+HexNAc(2)_2)
1247.607
1267.619(PEP+HexNAc(2)Hex(1)_2)
1328.632
1348.645(PEP+HexNAc(2)Hex(2)_2)
1429.673(PEP+HexNAc(2)Hex(3)_2)
1500.517
1527.728
1591.727(PEP+HexNAc(2)Hex(5)_2)
1672.753(PEP+HexNAc(2)Hex(6)_2)
1753.781(PEP+HexNAc(2)Hex(7)_2)
ETD-MS/MS Scan:36715, Noise threshold:1.1
443.277
743.396
953.532(PEP+HexNAc(2)Hex(3)_3)
1194.675
M+3H
1623.714
1645.235
1771.272
1827.803
M+2H
m/z

FGGFNFSQILPDPSKPSK(=PEP)_8_2_0_0_0, 0_None, 0_None,
m/z:1223.53(3+), RT:90.33, Y-score:84.65

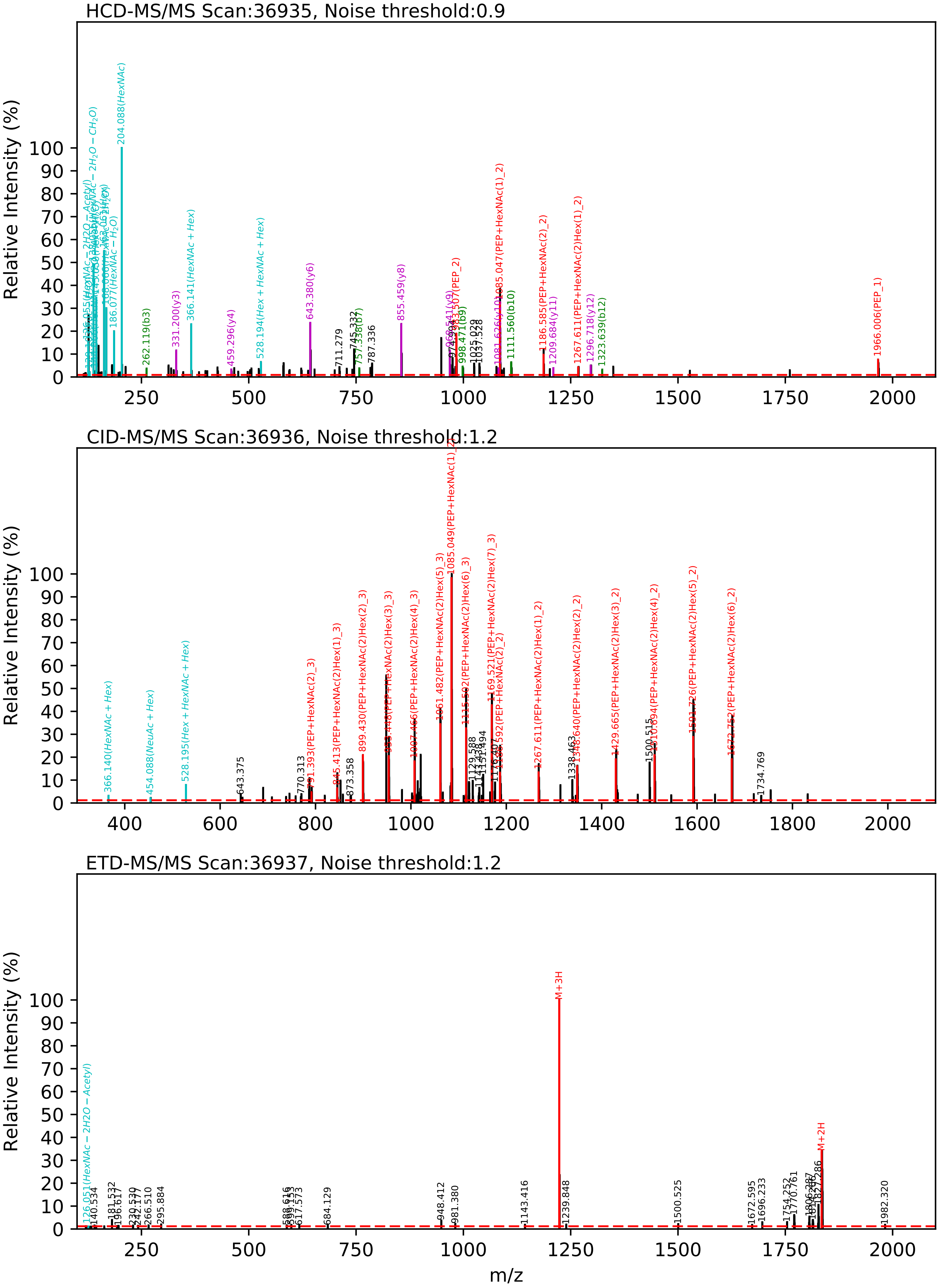

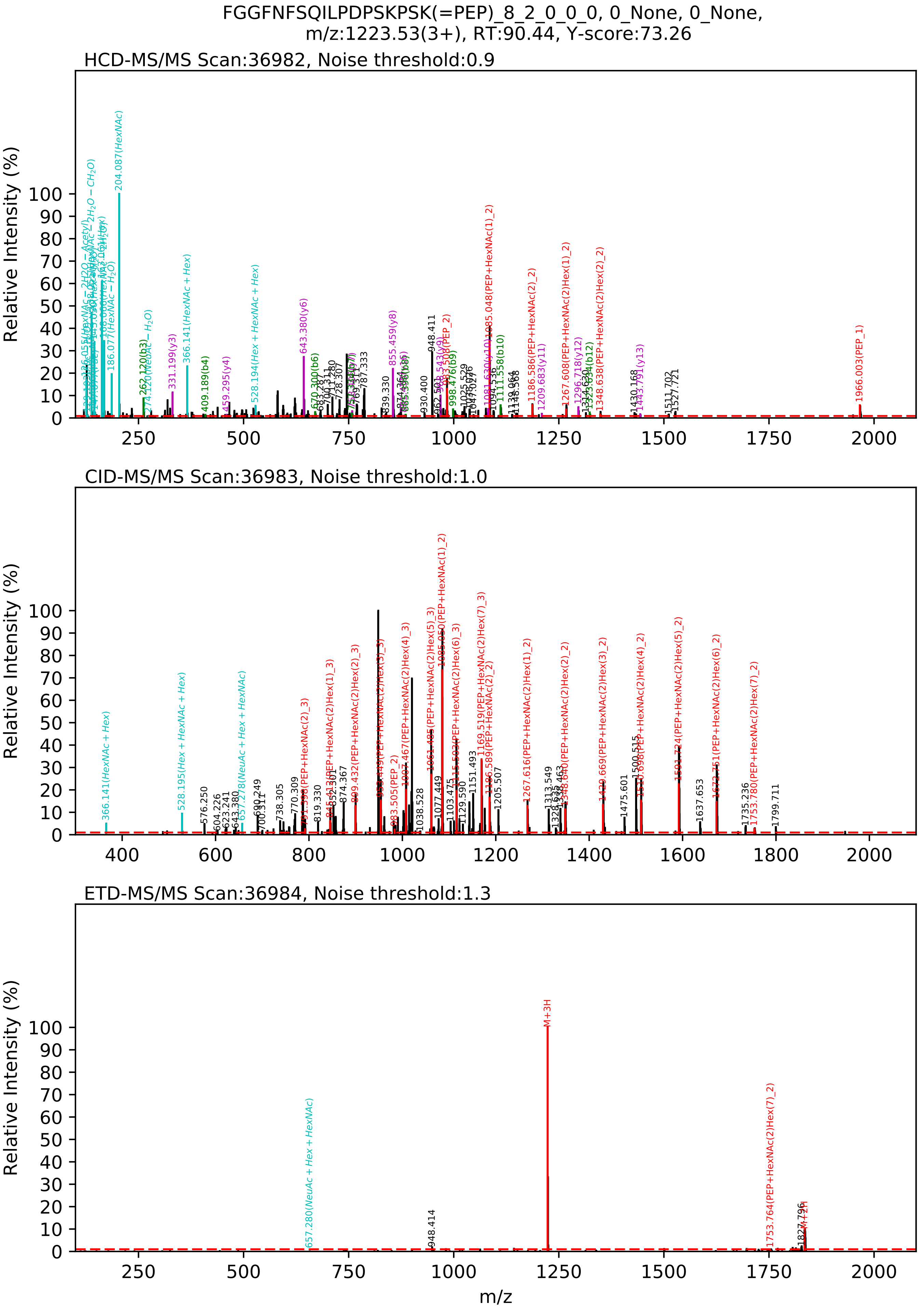

FGGFNFSQILPDPSKPSK(=PEP)_8_2_0_0_0, 0_None, 0_None,
m/z:1223.53(3+), RT:90.44, Y-score:73.26
HCD-MS/MS Scan:36982, Noise threshold:0.9
Relative Intensity (%)
204.087(HexNAc)
186.077(HexNAc − H2O)
262.120(b3)
331.199(y3)
366.141(HexNAc + Hex)
409.189(b4)
459.295(y4)
528.194(Hex + HexNAc + Hex)
643.380(y6)
670.300(b6)
787.333
839.330
855.459(y8)
930.400
948.411
998.476(b9)
1025.529
1085.048(PEP+HexNAc(1)_2)
1111.558(b10)
1186.586(PEP+HexNAc(2)_2)
1209.683(y11)
1267.608(PEP+HexNAc(2)Hex(1)_2)
1296.718(y12)
1348.638(PEP+HexNAc(2)Hex(2)_2)
1430.168
1443.791(y13)
1511.702
1527.721
1966.003(PEP_1)
CID-MS/MS Scan:36983, Noise threshold:1.0
366.141(HexNAc + Hex)
528.195(Hex + HexNAc + Hex)
576.250
604.226
623.241
643.380
690.249
700.311
738.305
770.309
819.330
852.367
874.367
899.432(PEP+HexNAc(2)Hex(2)_3)
983.505(PEP_2)
1007.467(PEP+HexNAc(2)Hex(4)_3)
1038.528
1061.485(PEP+HexNAc(2)Hex(5)_3)
1077.449
1085.050(PEP+HexNAc(1)_2)
1103.475
1129.590
1151.493
1169.519(PEP+HexNAc(2)Hex(7)_3)
1186.589(PEP+HexNAc(2)_2)
1205.507
1267.616(PEP+HexNAc(2)Hex(1)_2)
1313.549
1348.640(PEP+HexNAc(2)Hex(2)_2)
1429.669(PEP+HexNAc(2)Hex(3)_2)
1475.601
1500.515
1591.724(PEP+HexNAc(2)Hex(5)_2)
1637.653
1672.751(PEP+HexNAc(2)Hex(6)_2)
1735.236
1753.780(PEP+HexNAc(2)Hex(7)_2)
1799.711
ETD-MS/MS Scan:36984, Noise threshold:1.3
657.280(NeuAc + Hex + HexNAc)
948.414
M+3H
1753.764(PEP+HexNAc(2)Hex(7)_2)
1827.796
m/z

FGGFNFSQILPDPSKPSK(=PEP)_8_2_0_0_0, 0_None, 0_None,
m/z:1223.54(3+), RT:95.54, Y-score:94.02

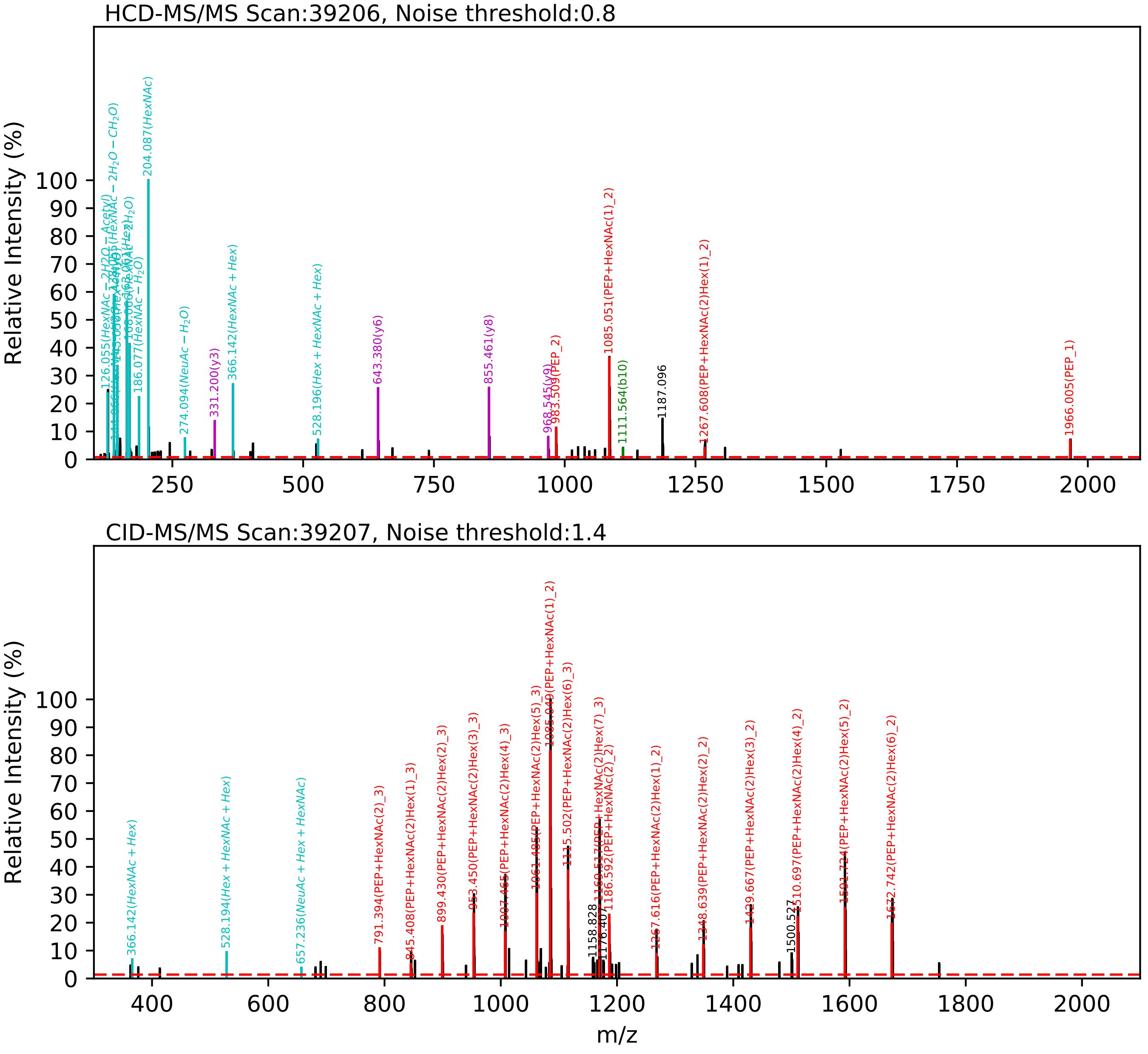

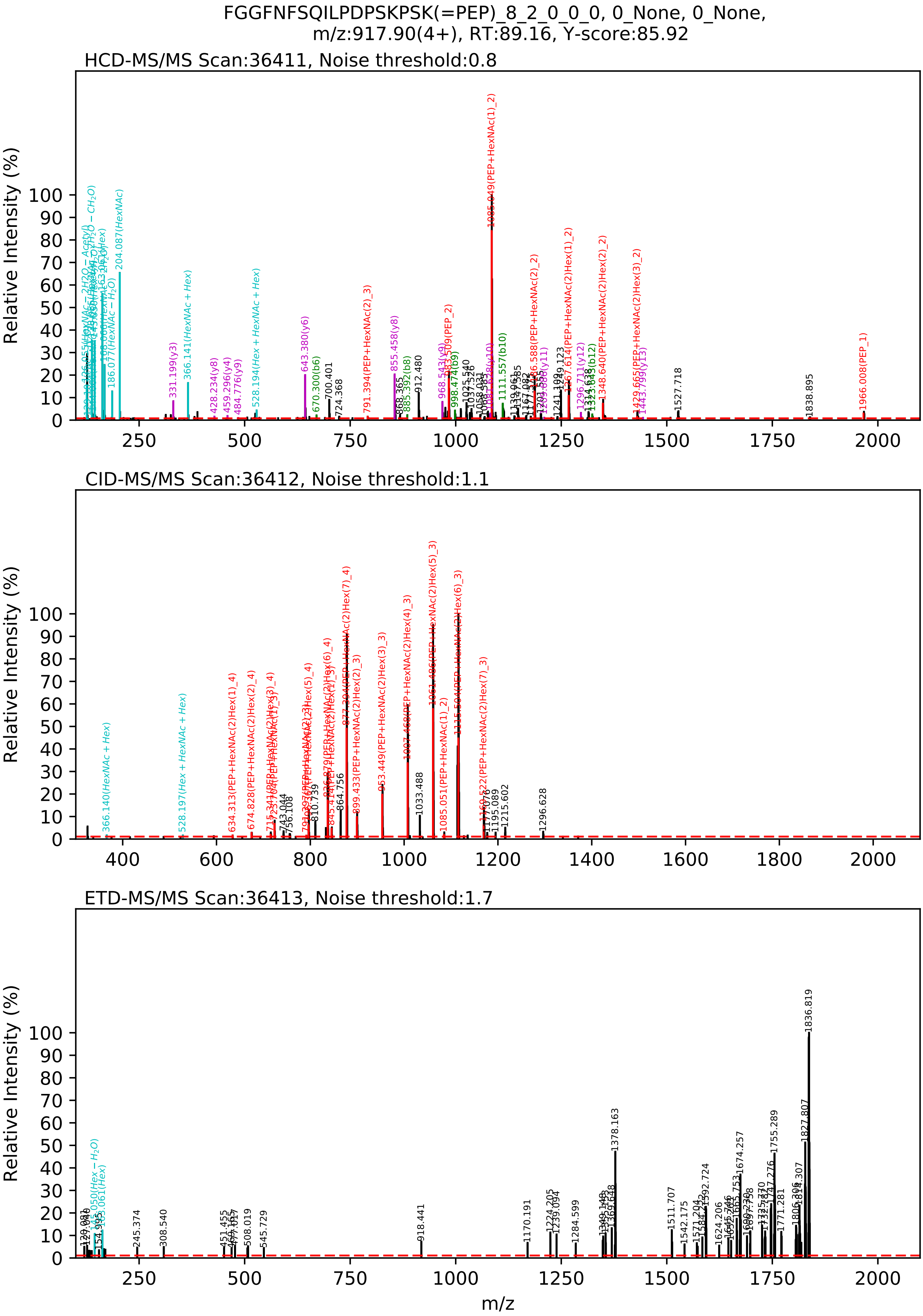
FGGFNFSQILPDPSKPSK(=PEP)_8_2_0_0_0, 0_None, 0_None,
m/z:917.90(4+), RT:89.16, Y-score:85.92
HCD-MS/MS Scan:36411, Noise threshold:0.8
CID-MS/MS Scan:36412, Noise threshold:1.1
ETD-MS/MS Scan:36413, Noise threshold:1.7
Relative Intensity (%)
m/z

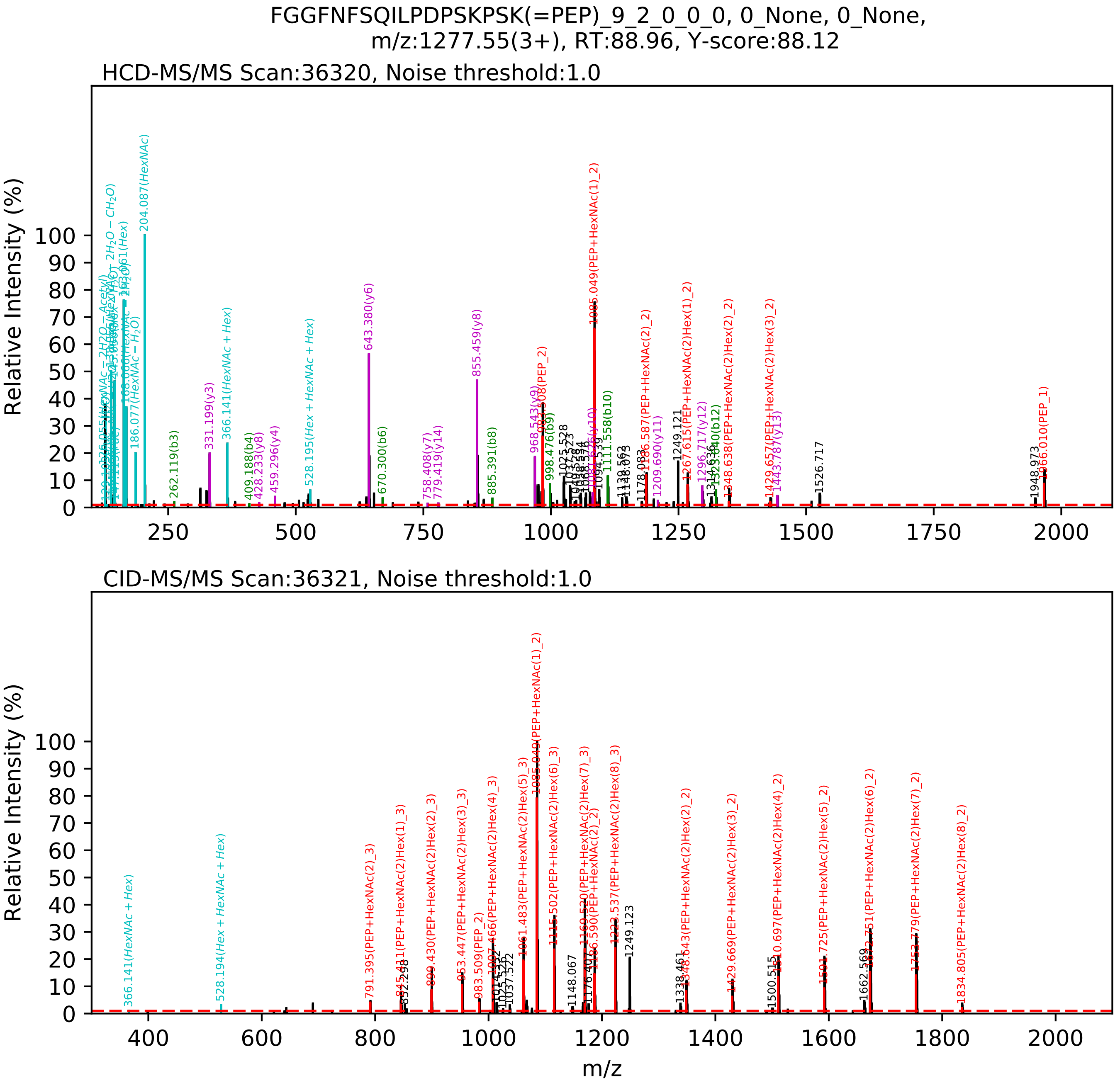
FGGFNFSQILPDPSKPSK(=PEP)_9_2_0_0_0, 0_None, 0_None,
m/z:1277.55(3+), RT:88.96, Y-score:88.12
HCD-MS/MS Scan:36320, Noise threshold:1.0
Relative Intensity (%)
204.087(HexNAc)
643.380(y6)
855.459(y8)
1085.049(PEP+HexNAc(1)_2)
331.199(y3)
366.141(HexNAc + Hex)
528.195(Hex + HexNAc + Hex)
262.119(b3)
409.188(b4)
428.233(y8)
459.296(y4)
670.300(b6)
758.408(y7)
779.419(y14)
885.391(b8)
968.543(y9)
998.476(b9)
1025.528
1111.558(b10)
1186.587(PEP+HexNAc(2)_2)
1209.690(y11)
1249.121
1267.615(PEP+HexNAc(2)Hex(1)_2)
1296.717(y12)
1348.638(PEP+HexNAc(2)Hex(2)_2)
1429.657(PEP+HexNAc(2)Hex(3)_2)
1443.787(y13)
1526.717
1948.973
1966.010(PEP_1)
186.077(HexNAc − H2O)
1178.083
CID-MS/MS Scan:36321, Noise threshold:1.0
366.141(HexNAc + Hex)
528.194(Hex + HexNAc + Hex)
791.395(PEP+HexNAc(2)_3)
845.411(PEP+HexNAc(2)Hex(1)_3)
852.298
899.430(PEP+HexNAc(2)Hex(2)_3)
953.447(PEP+HexNAc(2)Hex(3)_3)
983.509(PEP_2)
1007.466(PEP+HexNAc(2)Hex(4)_3)
1037.522
1061.483(PEP+HexNAc(2)Hex(5)_3)
1085.049(PEP+HexNAc(1)_2)
1115.502(PEP+HexNAc(2)Hex(6)_3)
1148.067
1169.520(PEP+HexNAc(2)Hex(7)_3)
1186.590(PEP+HexNAc(2)_2)
1223.537(PEP+HexNAc(2)Hex(8)_3)
1249.123
1338.461
1348.643(PEP+HexNAc(2)Hex(2)_2)
1429.669(PEP+HexNAc(2)Hex(3)_2)
1500.515
1510.697(PEP+HexNAc(2)Hex(4)_2)
1591.725(PEP+HexNAc(2)Hex(5)_2)
1662.569
1672.751(PEP+HexNAc(2)Hex(6)_2)
1753.779(PEP+HexNAc(2)Hex(7)_2)
1834.805(PEP+HexNAc(2)Hex(8)_2)
m/z

FGGFNFSQILPDPSKPSK(=PEP)_9_2_0_0_0, 0_None, 0_None,
m/z:1277.55(3+), RT:89.21, Y-score:92.48

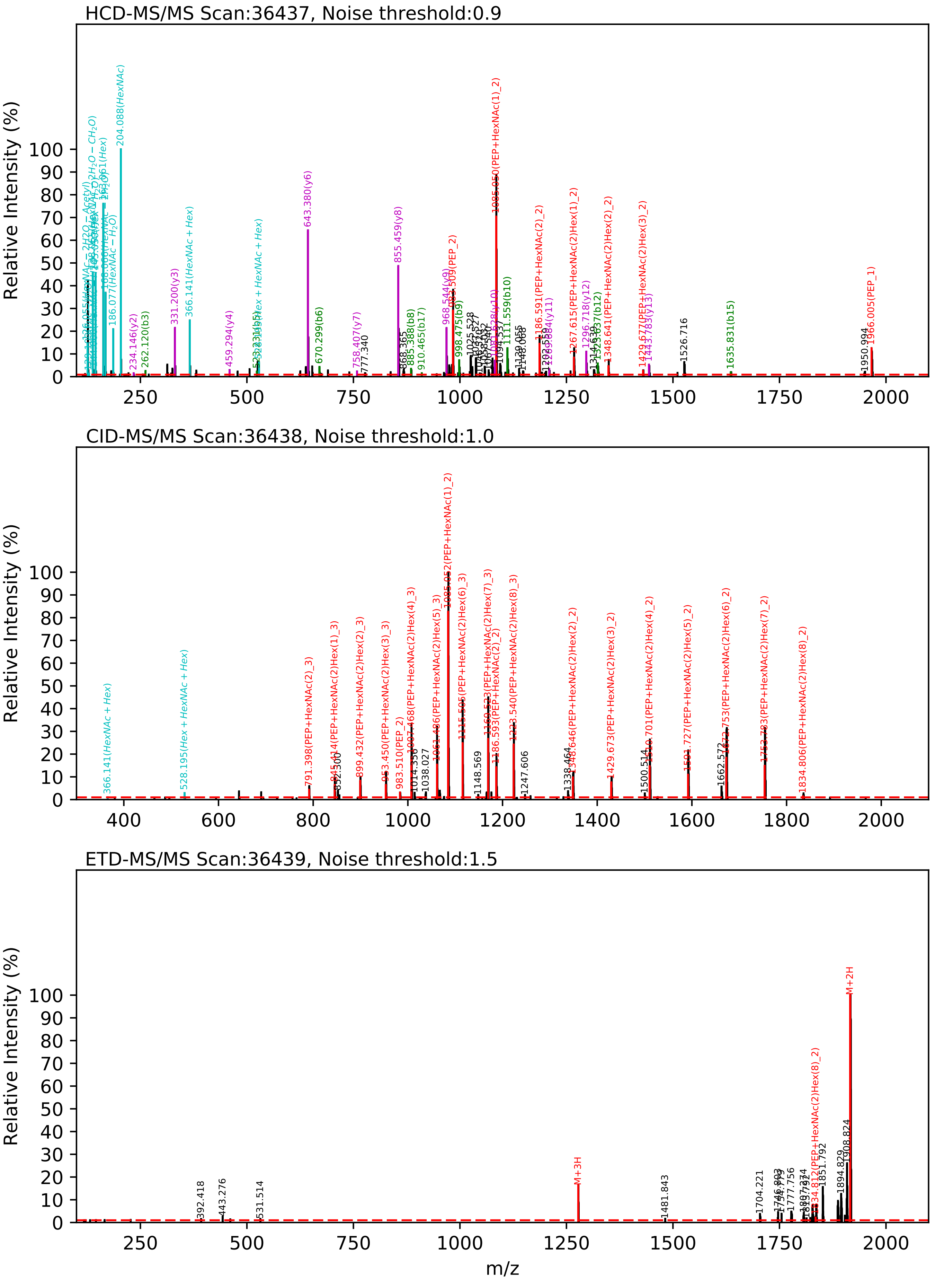

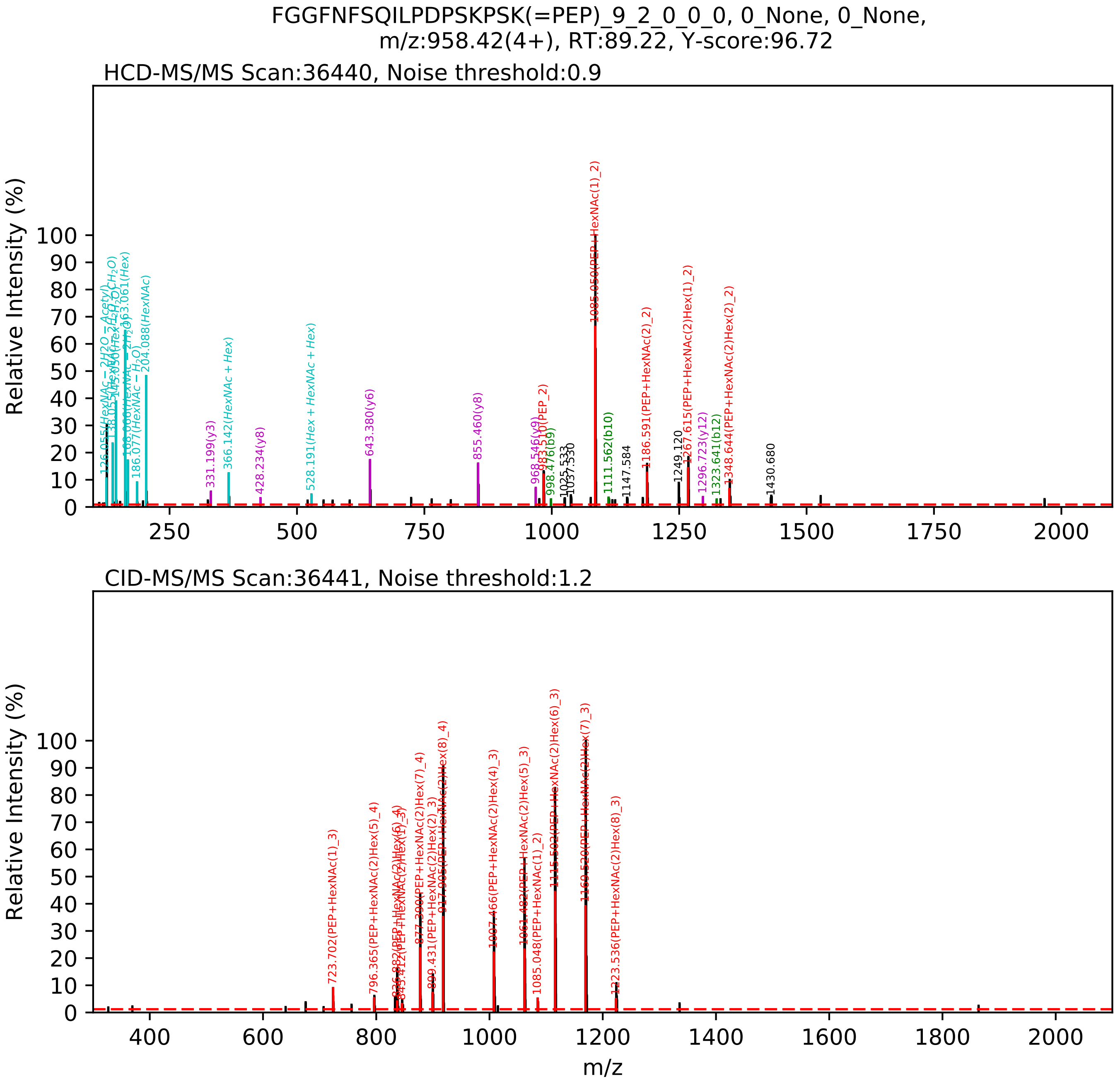

FGGFNFSQILPDPSKPSK(=PEP)_9_2_0_0_0, 0_None, 0_None,
m/z:958.42(4+), RT:89.22, Y-score:96.72
HCD-MS/MS Scan:36440, Noise threshold:0.9
Relative Intensity (%)
204.088(HexNAc)
186.077(HexNAc − H2O)
331.199(y3)
366.142(HexNAc + Hex)
428.234(y8)
528.191(Hex + HexNAc + Hex)
643.380(y6)
855.460(y8)
1025.533
1037.530
1085.050(PEP+HexNAc(1)_2)
1111.562(b10)
1147.584
1186.591(PEP+HexNAc(2)_2)
1249.120
1267.615(PEP+HexNAc(2)Hex(1)_2)
1296.723(y12)
1323.641(b12)
1348.644(PEP+HexNAc(2)Hex(2)_2)
1430.680
CID-MS/MS Scan:36441, Noise threshold:1.2
723.702(PEP+HexNAc(1)_3)
796.365(PEP+HexNAc(2)Hex(5)_4)
877.390(PEP+HexNAc(2)Hex(7)_4)
899.431(PEP+HexNAc(2)Hex(2)_3)
1007.466(PEP+HexNAc(2)Hex(4)_3)
1061.492(PEP+HexNAc(2)Hex(5)_3)
1085.048(PEP+HexNAc(1)_2)
1115.503(PEP+HexNAc(2)Hex(6)_3)
1169.529(PEP+HexNAc(2)Hex(7)_3)
1223.536(PEP+HexNAc(2)Hex(8)_3)
m/z

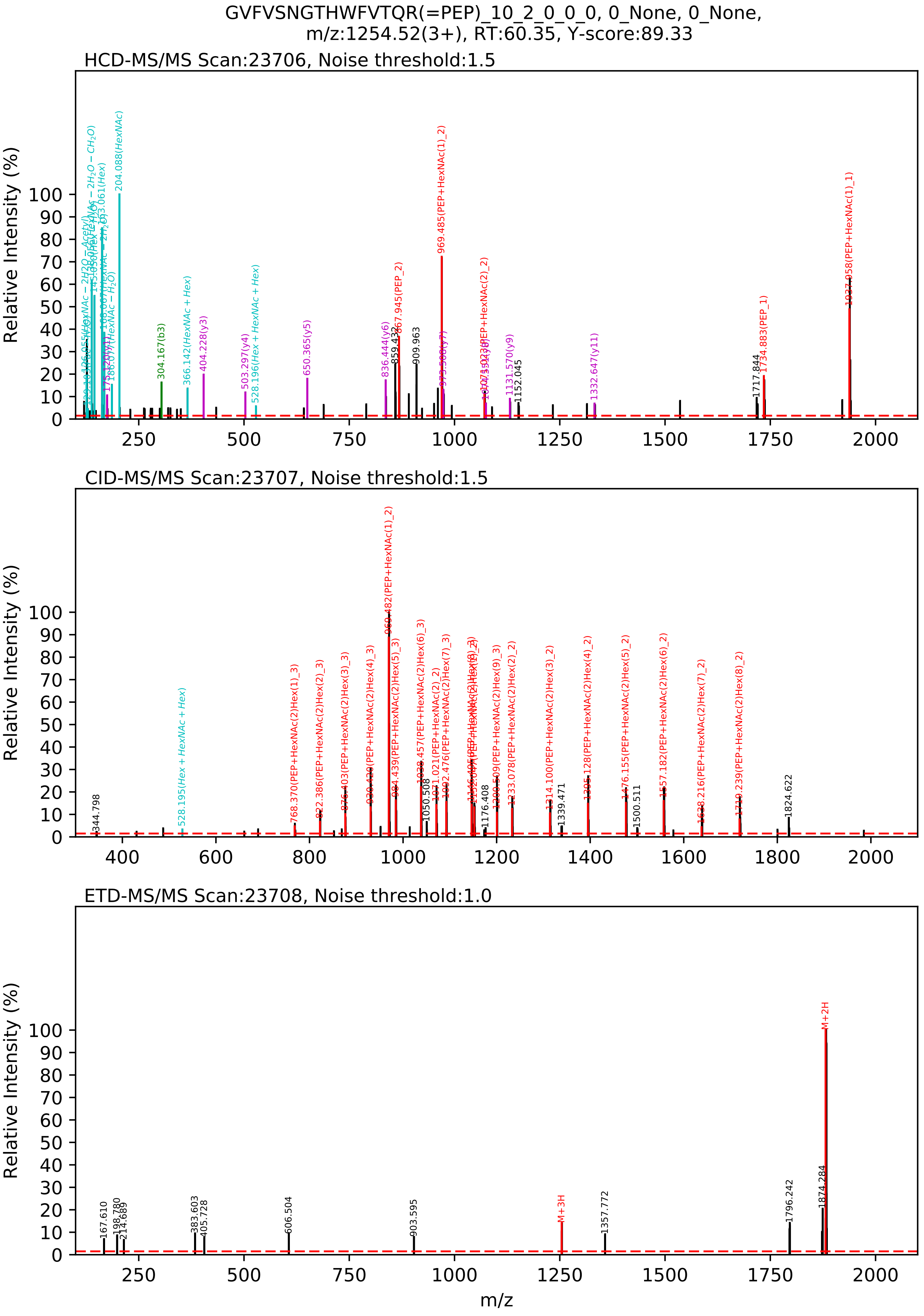

GVFVSNGTHWFVTQR(=PEP)_10_2_0_0_0, 0_None, 0_None,
m/z:1254.52(3+), RT:60.35, Y-score:89.33
HCD-MS/MS Scan:23706, Noise threshold:1.5
Relative Intensity (%)
204.088(HexNAc)
969.485(PEP+HexNAc(1)_2)
1937.958(PEP+HexNAc(1)_1)
867.945(PEP_2)
1734.883(PEP_1)
304.167(b3)
366.142(HexNAc + Hex)
404.228(y3)
503.297(y4)
528.196(Hex + HexNAc + Hex)
650.365(y5)
836.444(y6)
859.432
909.963
1131.570(y9)
1152.045
1332.647(y11)
1717.844
CID-MS/MS Scan:23707, Noise threshold:1.5
344.798
528.195(Hex + HexNAc + Hex)
768.370(PEP+HexNAc(2)Hex(1)_3)
822.386(PEP+HexNAc(2)Hex(2)_3)
876.403(PEP+HexNAc(2)Hex(3)_3)
930.420(PEP+HexNAc(2)Hex(4)_3)
969.482(PEP+HexNAc(1)_2)
984.439(PEP+HexNAc(2)Hex(5)_3)
1038.457(PEP+HexNAc(2)Hex(6)_3)
1050.508
1071.021(PEP+HexNAc(2)_2)
1092.476(PEP+HexNAc(2)Hex(7)_3)
1176.408
1200.509(PEP+HexNAc(2)Hex(9)_3)
1233.078(PEP+HexNAc(2)Hex(2)_2)
1314.100(PEP+HexNAc(2)Hex(3)_2)
1339.471
1395.128(PEP+HexNAc(2)Hex(4)_2)
1476.155(PEP+HexNAc(2)Hex(5)_2)
1500.511
1557.182(PEP+HexNAc(2)Hex(6)_2)
1638.216(PEP+HexNAc(2)Hex(7)_2)
1719.239(PEP+HexNAc(2)Hex(8)_2)
1824.622
ETD-MS/MS Scan:23708, Noise threshold:1.0
167.610
198.780
214.689
383.603
405.728
606.504
903.595
M+3H
1357.772
1796.242
1874.284
M+2H
m/z

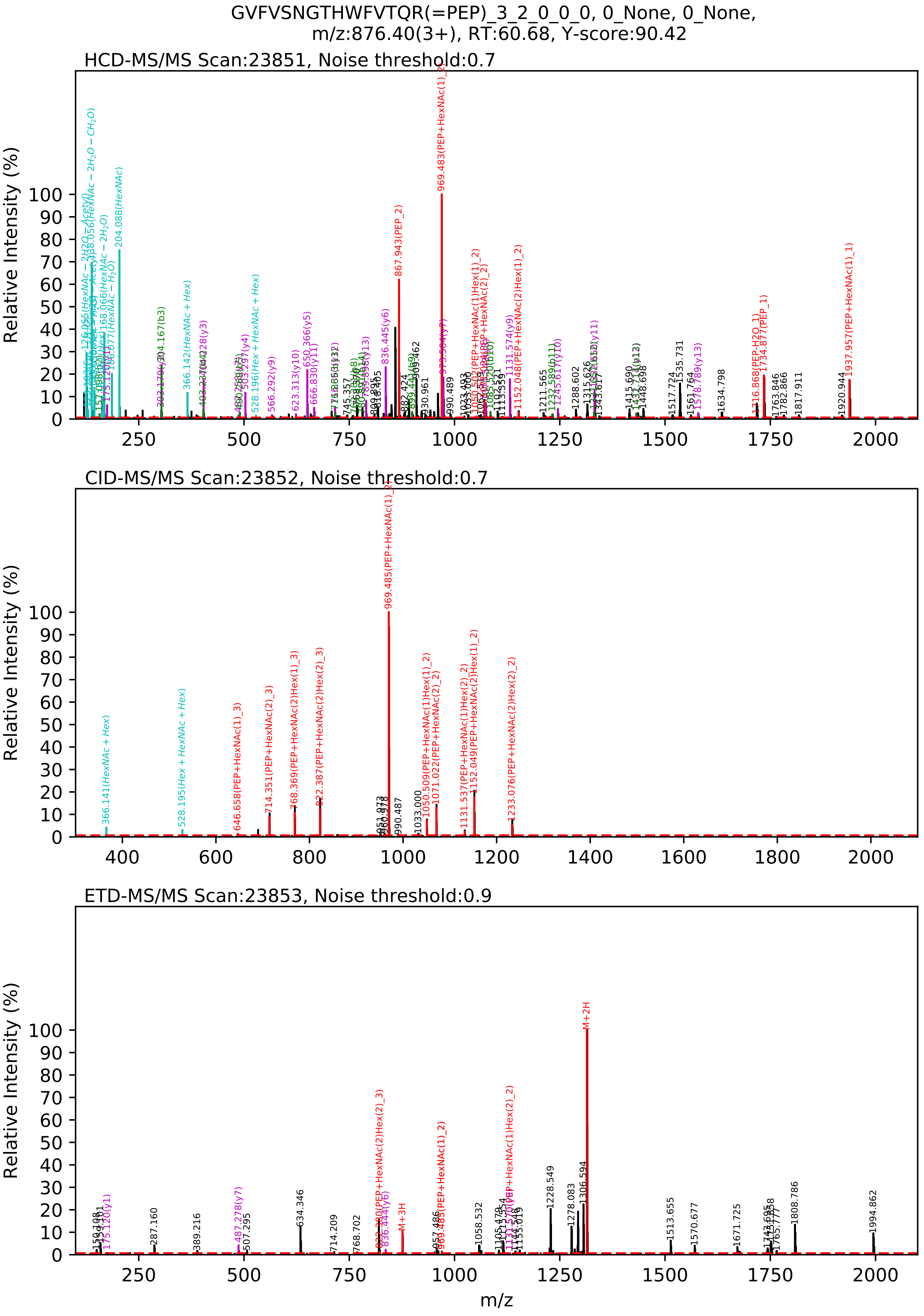
GVFVSNGTHWFVTQR(=PEP)_3_2_0_0_0, 0_None, 0_None,
m/z:876.40(3+), RT:60.68, Y-score:90.42
HCD-MS/MS Scan:23851, Noise threshold:0.7
Relative Intensity (%)
969.483(PEP+HexNAc(1)_2)
867.943(PEP_2)
204.088(HexNAc)
1937.957(PEP+HexNAc(1)_1)
1734.877(PEP_1)
1716.868(PEP-H2O_1)
1152.048(PEP+HexNAc(2)Hex(1)_2)
1131.574(y9)
836.445(y6)
650.366(y5)
366.142(HexNAc + Hex)
528.196(Hex + HexNAc + Hex)
304.167(b3)
CID-MS/MS Scan:23852, Noise threshold:0.7
969.485(PEP+HexNAc(1)_2)
366.141(HexNAc + Hex)
528.195(Hex + HexNAc + Hex)
646.658(PEP+HexNAc(1)_3)
714.351(PEP+HexNAc(2)_3)
768.369(PEP+HexNAc(2)Hex(1)_3)
822.387(PEP+HexNAc(2)Hex(2)_3)
1050.509(PEP+HexNAc(1)Hex(1)_2)
1071.022(PEP+HexNAc(2)_2)
1131.537(PEP+HexNAc(1)Hex(2)_2)
1152.049(PEP+HexNAc(2)Hex(1)_2)
1233.076(PEP+HexNAc(2)Hex(2)_2)
ETD-MS/MS Scan:23853, Noise threshold:0.9
M+2H
M+3H
175.120(y1)
487.278(y7)
836.444(y6)
969.485(PEP+HexNAc(1)_2)
1228.549
1306.594
1808.786
1994.862
m/z

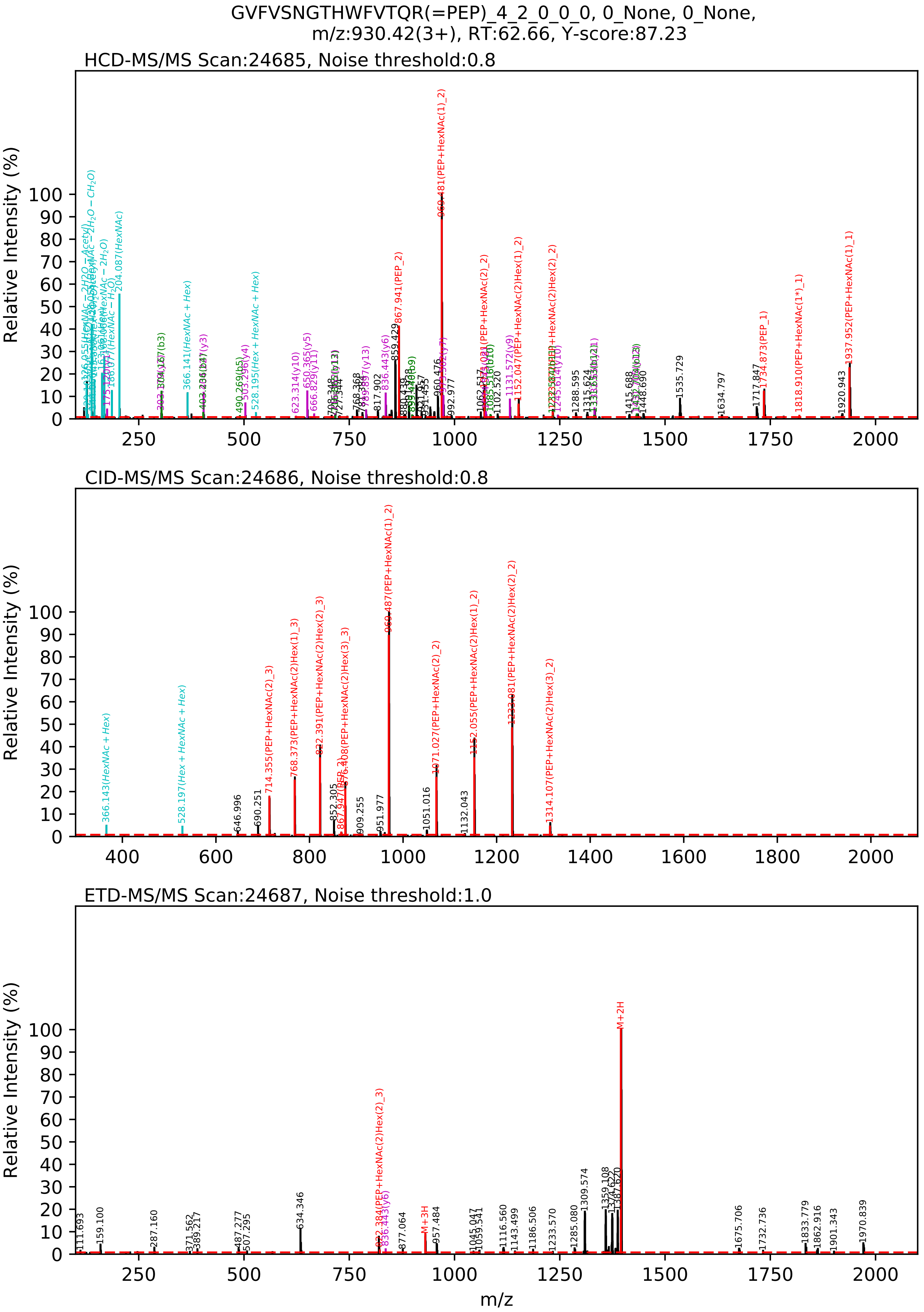

GVFVSNGTHWFVTQR(=PEP)_4_2_0_0_0, 0_None, 0_None,
m/z:930.42(3+), RT:62.66, Y-score:87.23
HCD-MS/MS Scan:24685, Noise threshold:0.8
CID-MS/MS Scan:24686, Noise threshold:0.8
ETD-MS/MS Scan:24687, Noise threshold:1.0
Relative Intensity (%)
m/z
969.481(PEP+HexNAc(1)_2)
867.941(PEP_2)
1937.952(PEP+HexNAc(1)_1)
1734.873(PEP_1)
1818.910(PEP+HexNAc(1*)_1)
969.487(PEP+HexNAc(1)_2)
1233.081(PEP+HexNAc(2)Hex(2)_2)
1152.055(PEP+HexNAc(2)Hex(1)_2)
1071.027(PEP+HexNAc(2)_2)
822.391(PEP+HexNAc(2)Hex(2)_3)
768.373(PEP+HexNAc(2)Hex(1)_3)
714.355(PEP+HexNAc(2)_3)
876.408(PEP+HexNAc(2)Hex(3)_3)
1314.107(PEP+HexNAc(2)Hex(3)_2)
366.143(HexNAc + Hex)
528.197(Hex + HexNAc + Hex)
M+2H
M+3H
822.384(PEP+HexNAc(2)Hex(2)_3)
836.443(y6)

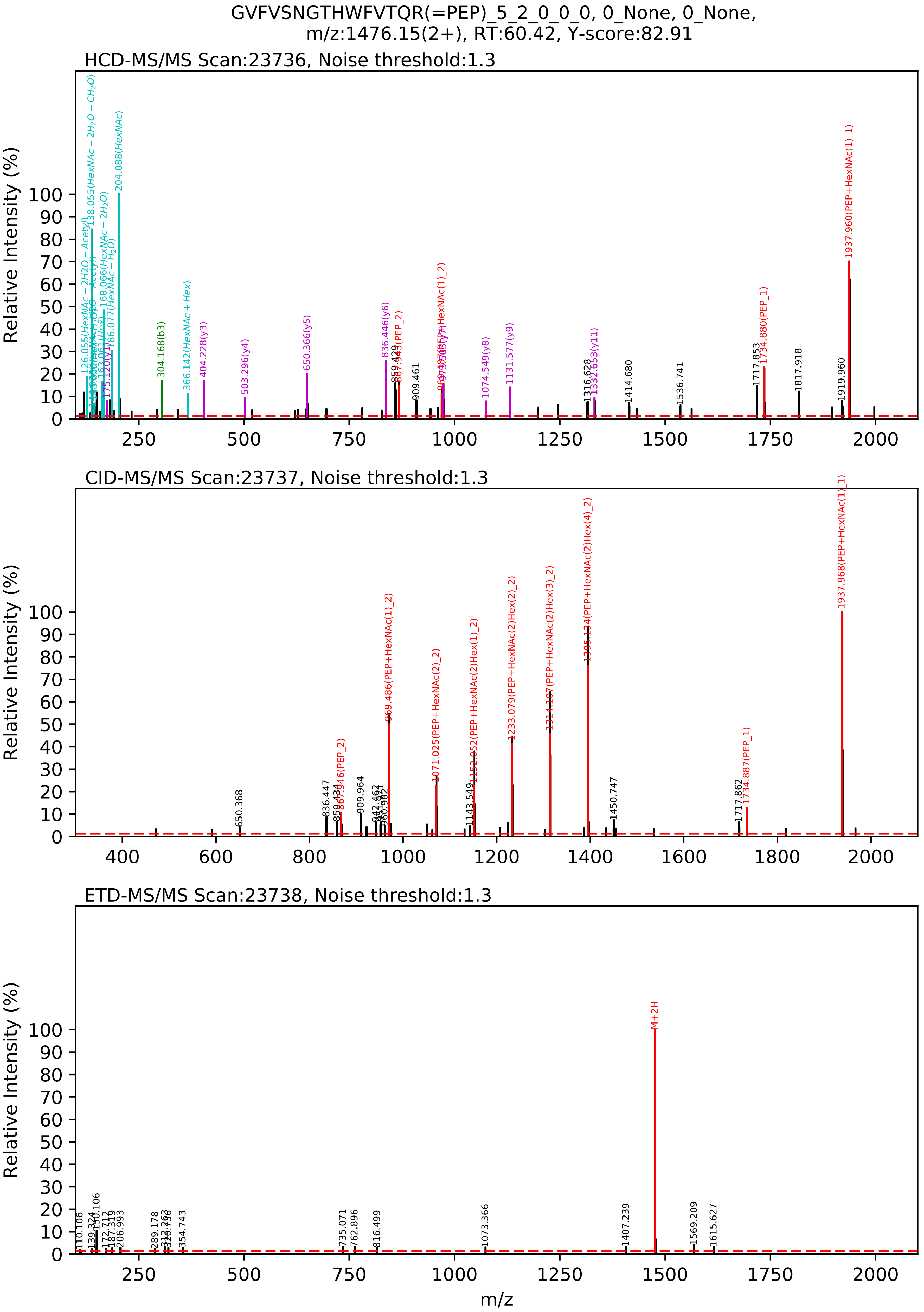

GVFVSNGTHWFVTQR(=PEP)_5_2_0_0_0, 0_None, 0_None,
m/z:1476.15(2+), RT:60.42, Y-score:82.91
HCD-MS/MS Scan:23736, Noise threshold:1.3
Relative Intensity (%)
204.088(HexNAc)
1937.960(PEP+HexNAc(1)_1)
1734.880(PEP_1)
304.168(b3)
366.142(HexNAc + Hex)
404.228(y3)
503.296(y4)
650.366(y5)
836.446(y6)
1074.549(y8)
1131.577(y9)
1316.628
1414.680
1536.741
1717.853
1817.918
1919.960
909.461
CID-MS/MS Scan:23737, Noise threshold:1.3
1937.968(PEP+HexNAc(1)_1)
1395.134(PEP+HexNAc(2)Hex(4)_2)
1314.107(PEP+HexNAc(2)Hex(3)_2)
1233.079(PEP+HexNAc(2)Hex(2)_2)
1152.052(PEP+HexNAc(2)Hex(1)_2)
1071.025(PEP+HexNAc(2)_2)
969.486(PEP+HexNAc(1)_2)
867.946(PEP_2)
1734.887(PEP_1)
650.368
836.447
909.964
1143.549
1450.747
1717.862
ETD-MS/MS Scan:23738, Noise threshold:1.3
M+2H
110.106
139.324
150.106
172.712
187.319
206.993
289.178
354.743
735.071
762.896
816.499
1073.366
1407.239
1569.209
1615.627
m/z

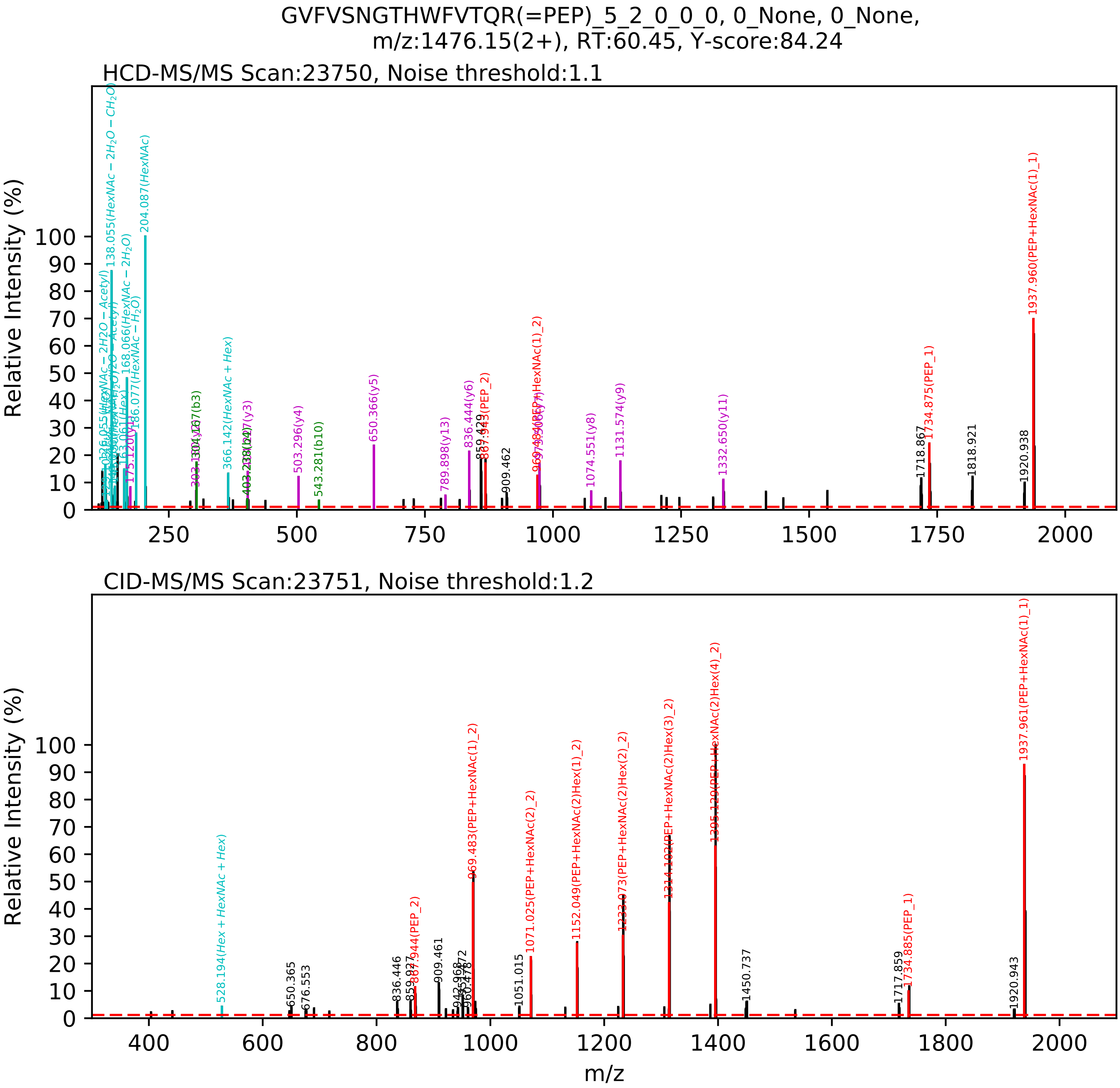
GVFVSNGTHWFVTQR(=PEP)_5_2_0_0_0, 0_None, 0_None,
m/z:1476.15(2+), RT:60.45, Y-score:84.24
HCD-MS/MS Scan:23750, Noise threshold:1.1
Relative Intensity (%)
204.087(HexNAc)
1937.960(PEP+HexNAc(1)_1)
1734.875(PEP_1)
650.366(y5)
836.444(y6)
867.943(PEP_2)
1131.574(y9)
1074.551(y8)
1332.650(y11)
503.296(y4)
543.281(b10)
789.898(y13)
366.142(HexNAc+Hex)
168.066(HexNAc − 2H2O)
186.077(HexNAc − H2O)
1718.867
1818.921
1920.938
909.462
859.429
CID-MS/MS Scan:23751, Noise threshold:1.2
1937.961(PEP+HexNAc(1)_1)
969.483(PEP+HexNAc(1)_2)
1071.025(PEP+HexNAc(2)_2)
1152.049(PEP+HexNAc(2)Hex(1)_2)
1233.073(PEP+HexNAc(2)Hex(2)_2)
1314.102(PEP+HexNAc(2)Hex(3)_2)
1395.129(PEP+HexNAc(2)Hex(4)_2)
867.944(PEP_2)
1734.885(PEP_1)
528.194(Hex + HexNAc + Hex)
650.365
676.553
836.446
909.461
1051.015
1450.737
1717.859
1920.943
m/z

GVFVSNGTHWFVTQR(=PEP)_5_2_0_0_0, 0_None, 0_None,
m/z:1476.15(2+), RT:61.01, Y-score:91.24

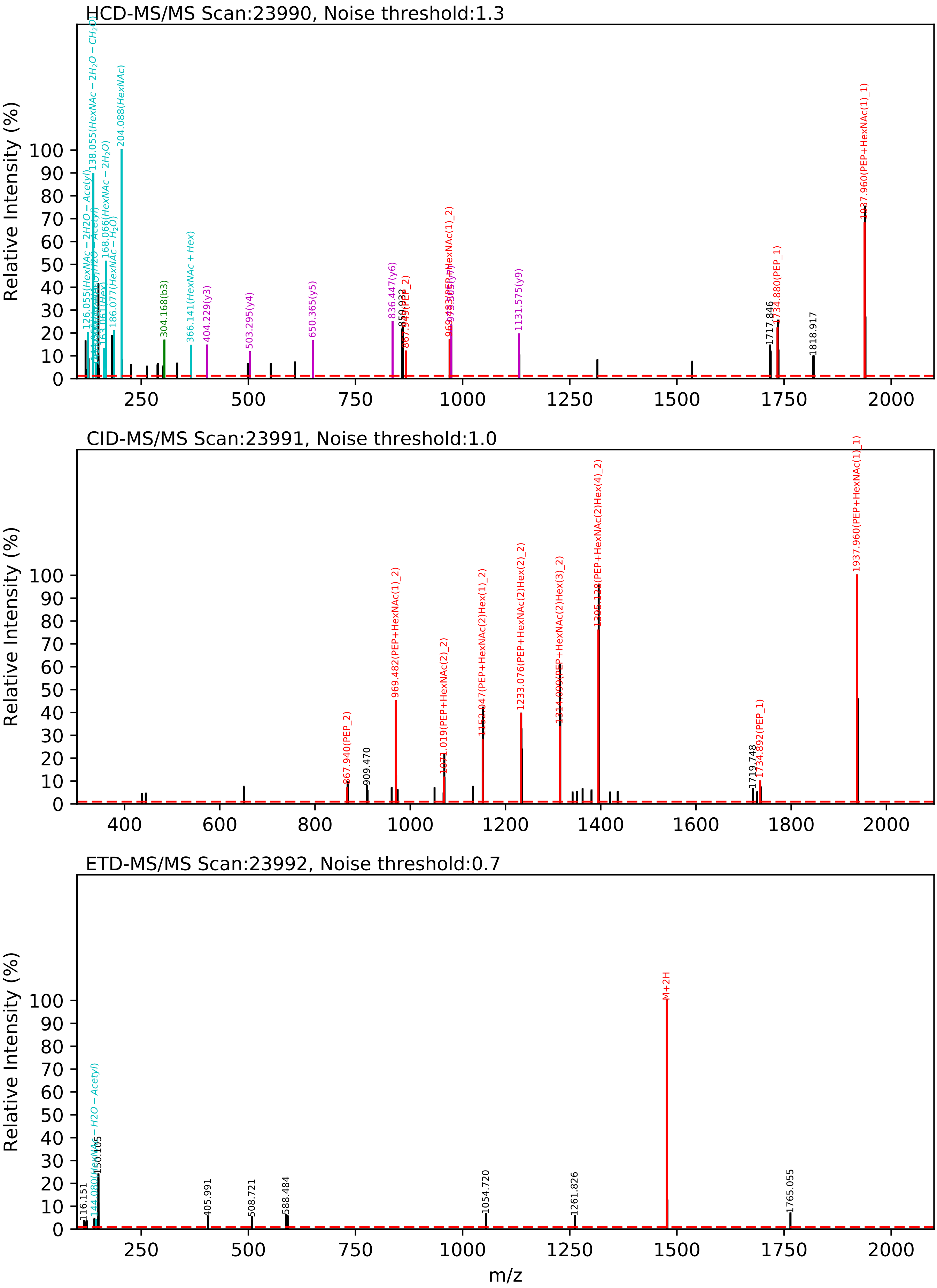

GVFVSNGTHWFVTQR(=PEP)_5_2_0_0_0, 0_None, 0_None,
m/z:1476.15(2+), RT:62.32, Y-score:75.89

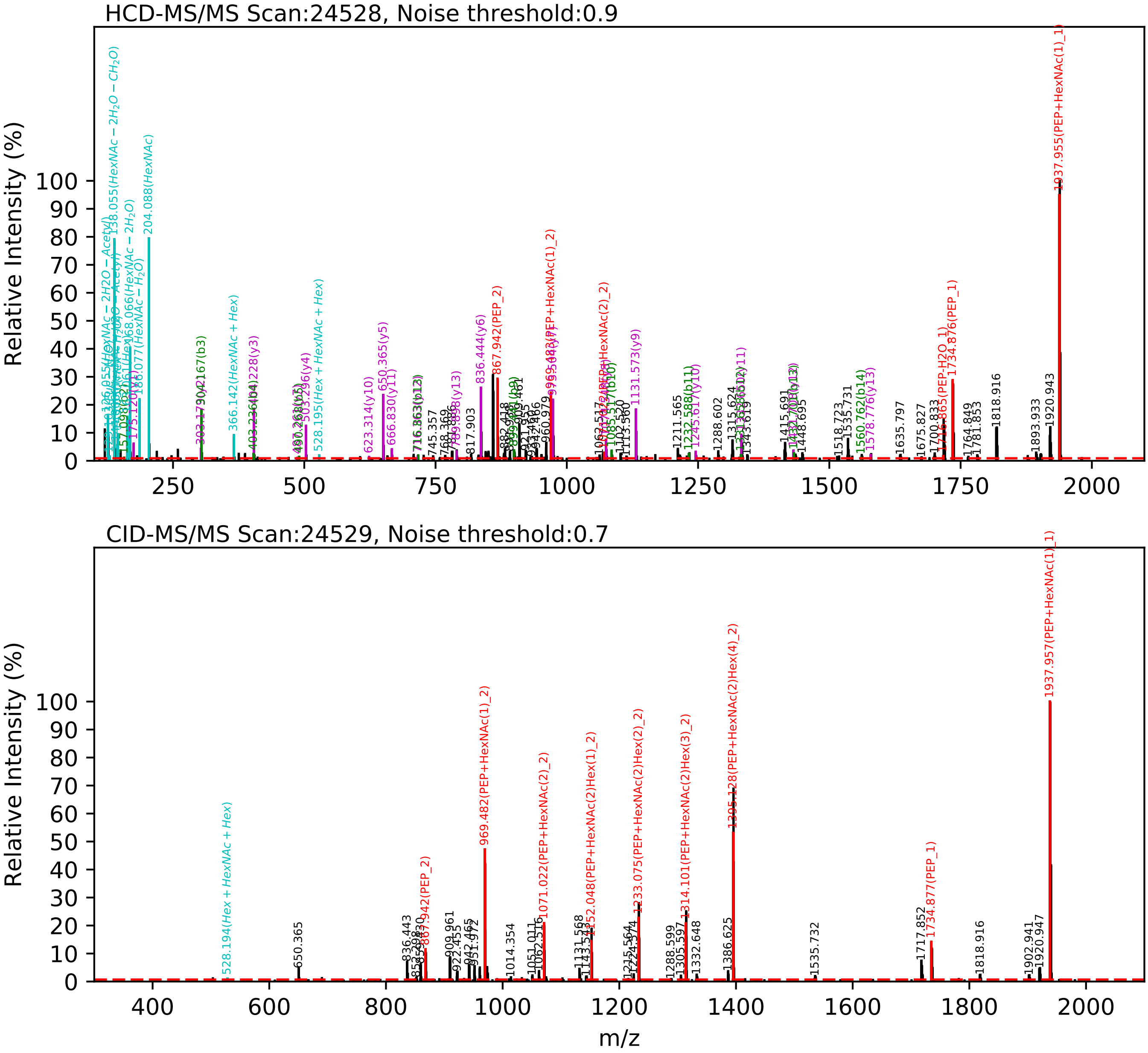

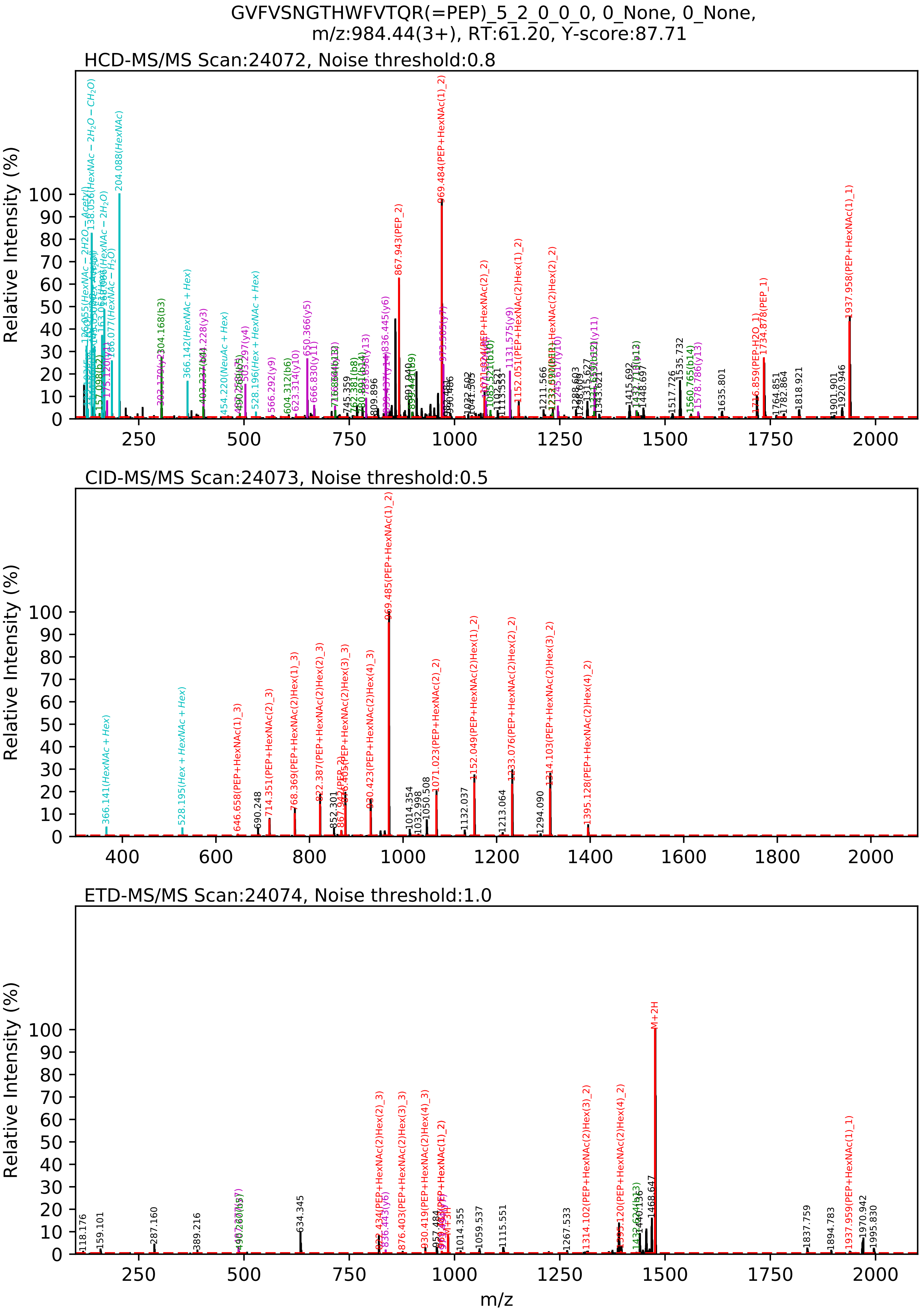
GVFVSNGTHWFVTQR(=PEP)_5_2_0_0_0, 0_None, 0_None,
m/z:984.44(3+), RT:61.20, Y-score:87.71
HCD-MS/MS Scan:24072, Noise threshold:0.8
CID-MS/MS Scan:24073, Noise threshold:0.5
ETD-MS/MS Scan:24074, Noise threshold:1.0
Relative Intensity (%)
m/z
969.484(PEP+HexNAc(1)_2)
867.943(PEP_2)
204.088(HexNAc)
1937.958(PEP+HexNAc(1)_1)
1734.878(PEP_1)
969.485(PEP+HexNAc(1)_2)
1152.049(PEP+HexNAc(2)Hex(1)_2)
1233.076(PEP+HexNAc(2)Hex(2)_2)
1314.103(PEP+HexNAc(2)Hex(3)_2)
1395.128(PEP+HexNAc(2)Hex(4)_2)
M+2H
1937.959(PEP+HexNAc(1)_1)

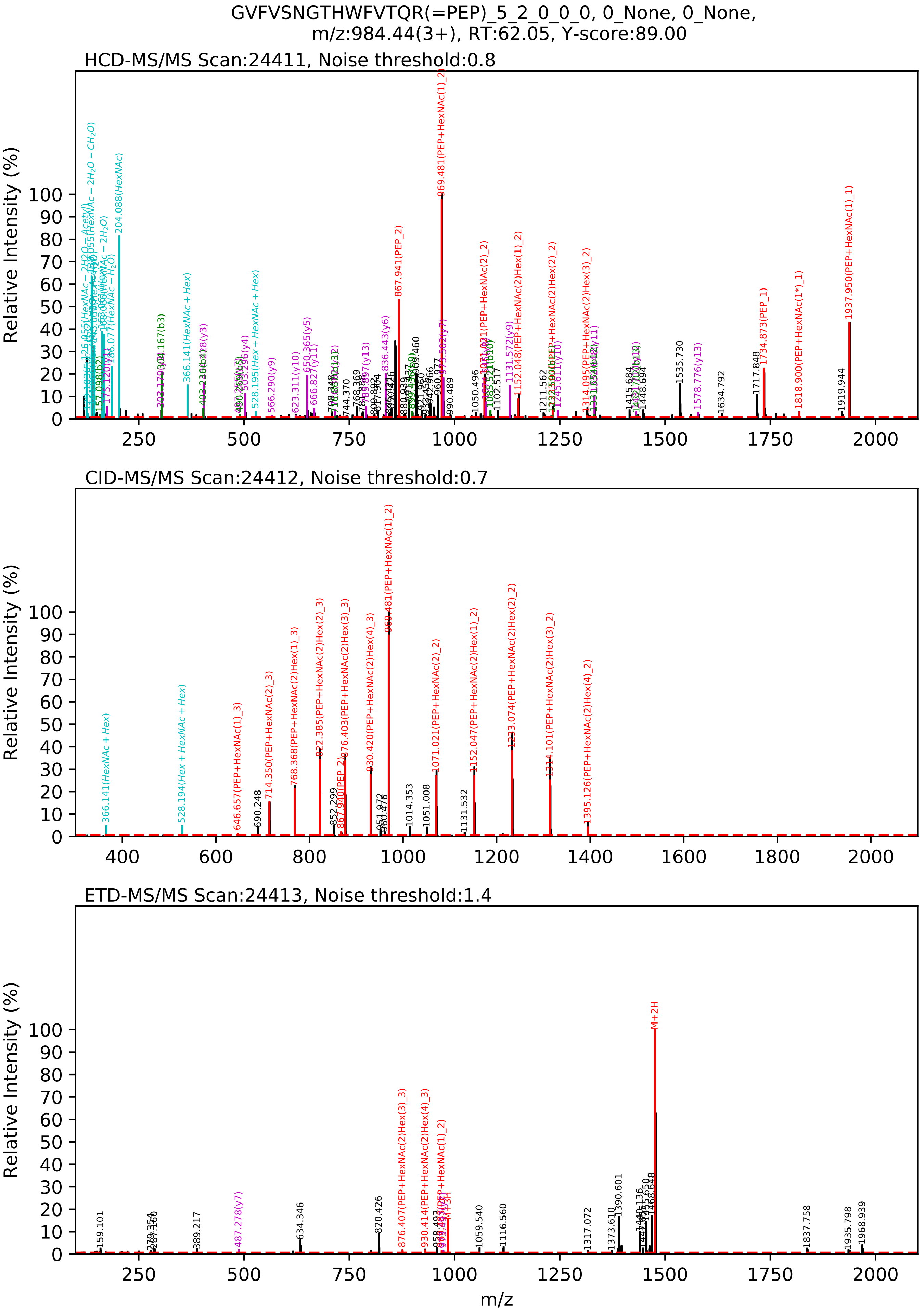
GVFVSNGTHWFVTQR(=PEP)_5_2_0_0_0, 0_None, 0_None,
m/z:984.44(3+), RT:62.05, Y-score:89.00
HCD-MS/MS Scan:24411, Noise threshold:0.8
CID-MS/MS Scan:24412, Noise threshold:0.7
ETD-MS/MS Scan:24413, Noise threshold:1.4
Relative Intensity (%)
m/z
969.481(PEP+HexNAc(1)_2)
1937.950(PEP+HexNAc(1)_1)
867.941(PEP_2)
1734.873(PEP_1)
204.088(HexNAc)
M+2H

GVFVSNGTHWFVTQR(=PEP)_5_2_0_0_0, 0_None, 0_None,
m/z:984.44(3+), RT:62.61, Y-score:92.81

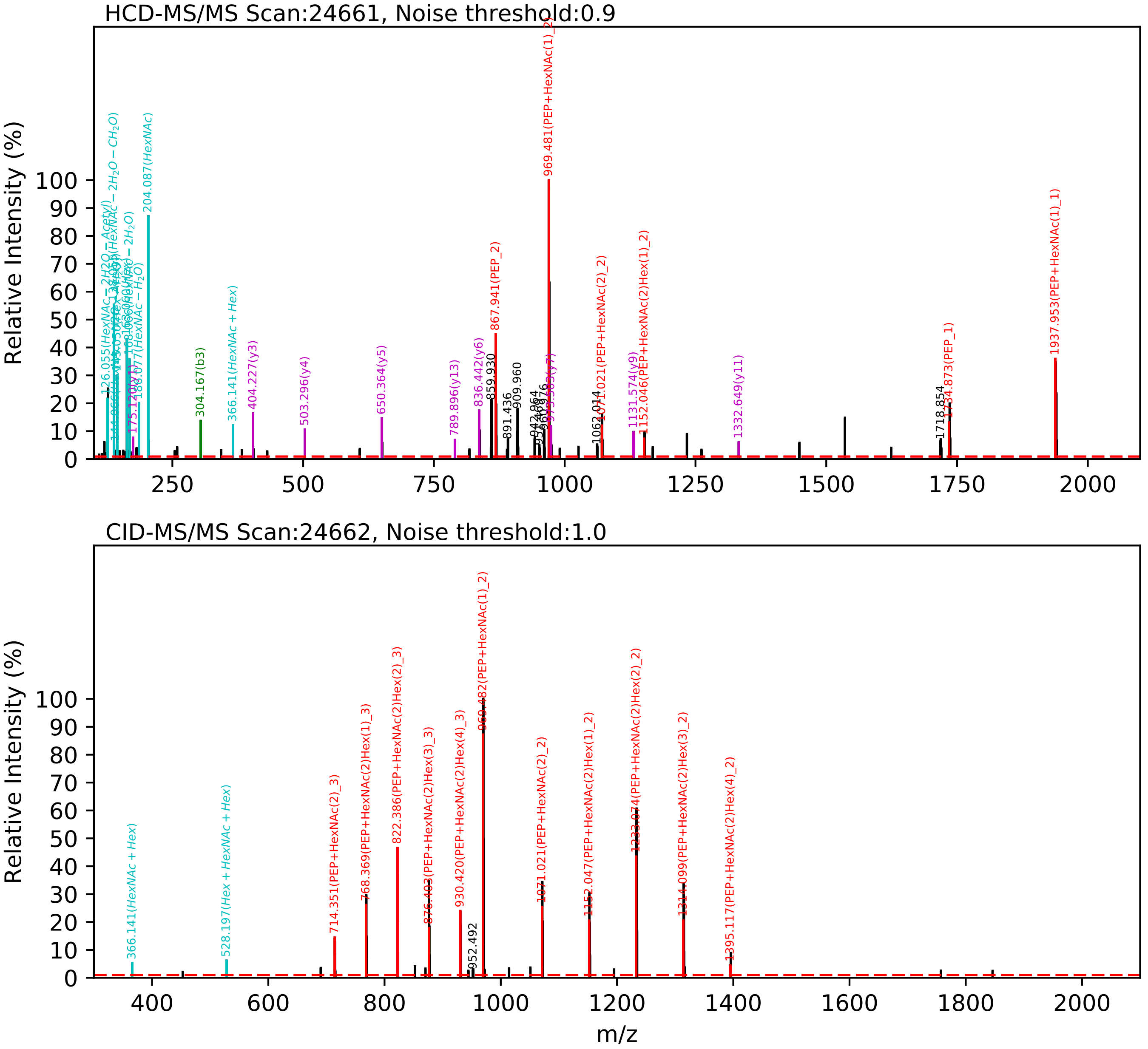

GVFVSNGTHWFVTQR(=PEP)_6_2_0_0_0, 0_None, 0_None,
m/z:1038.45(3+), RT:62.11, Y-score:88.38

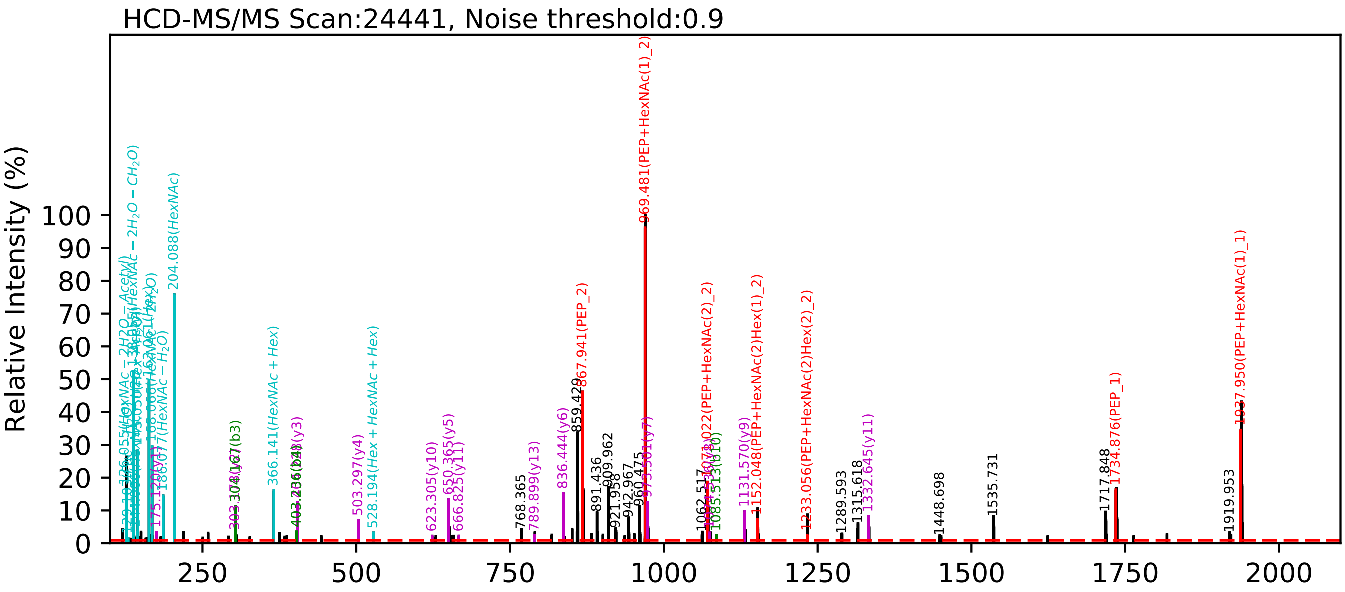

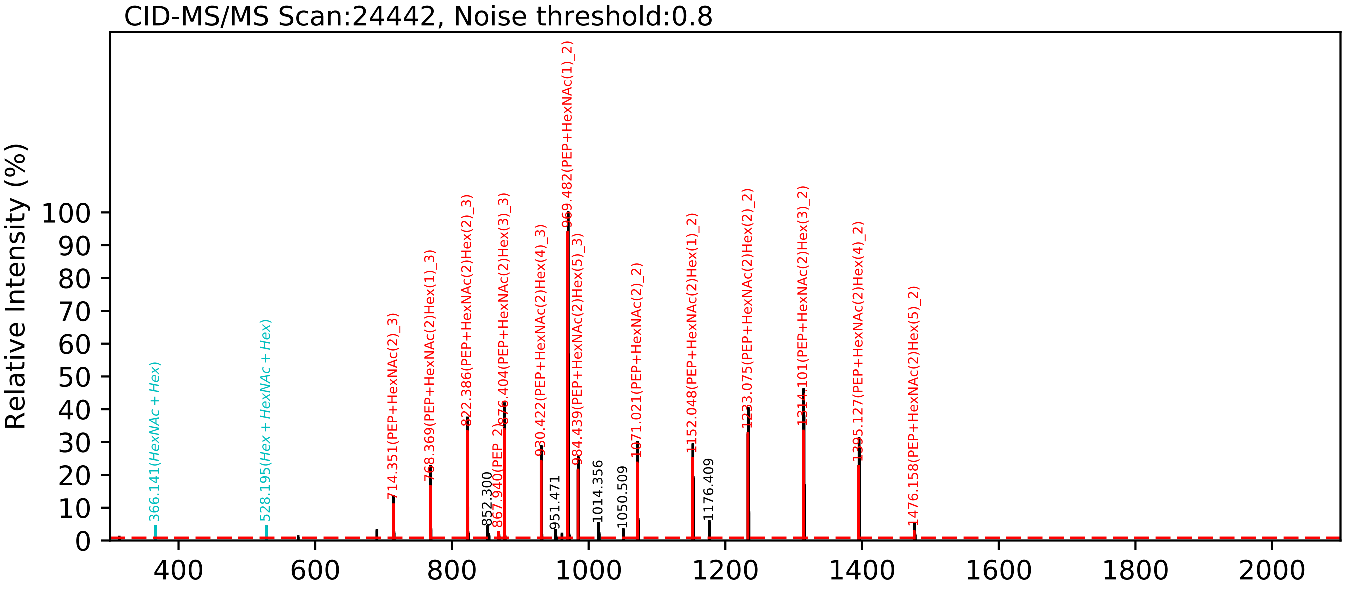

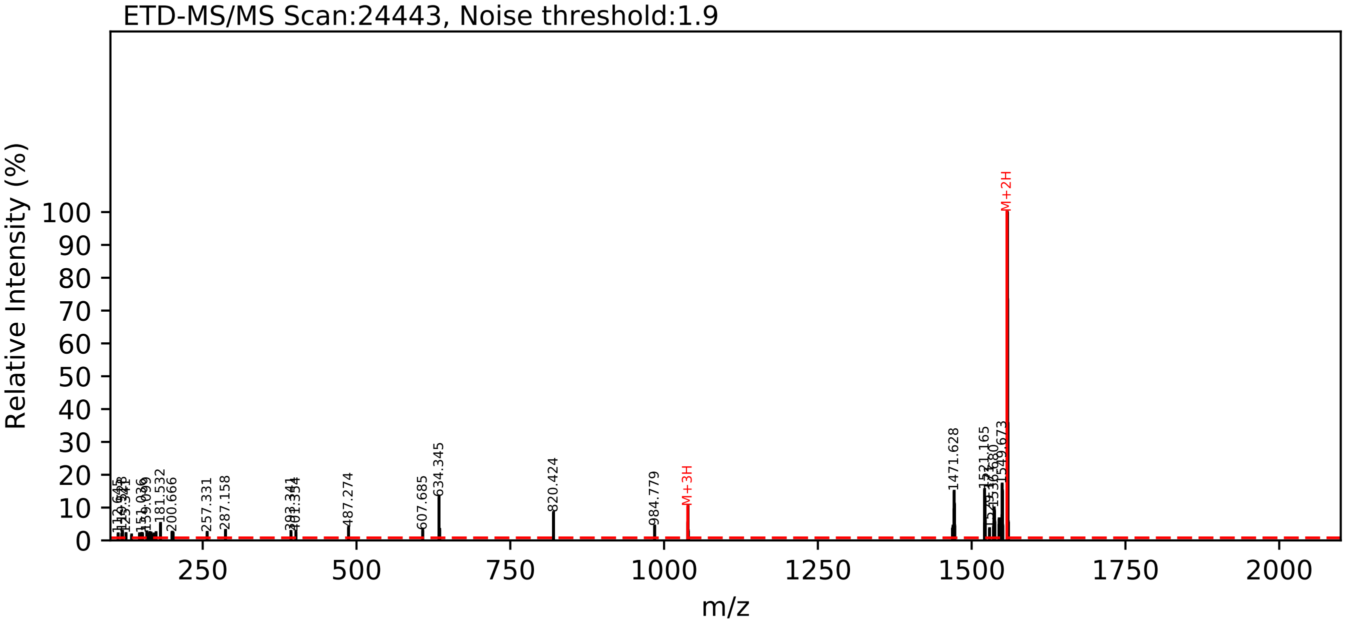

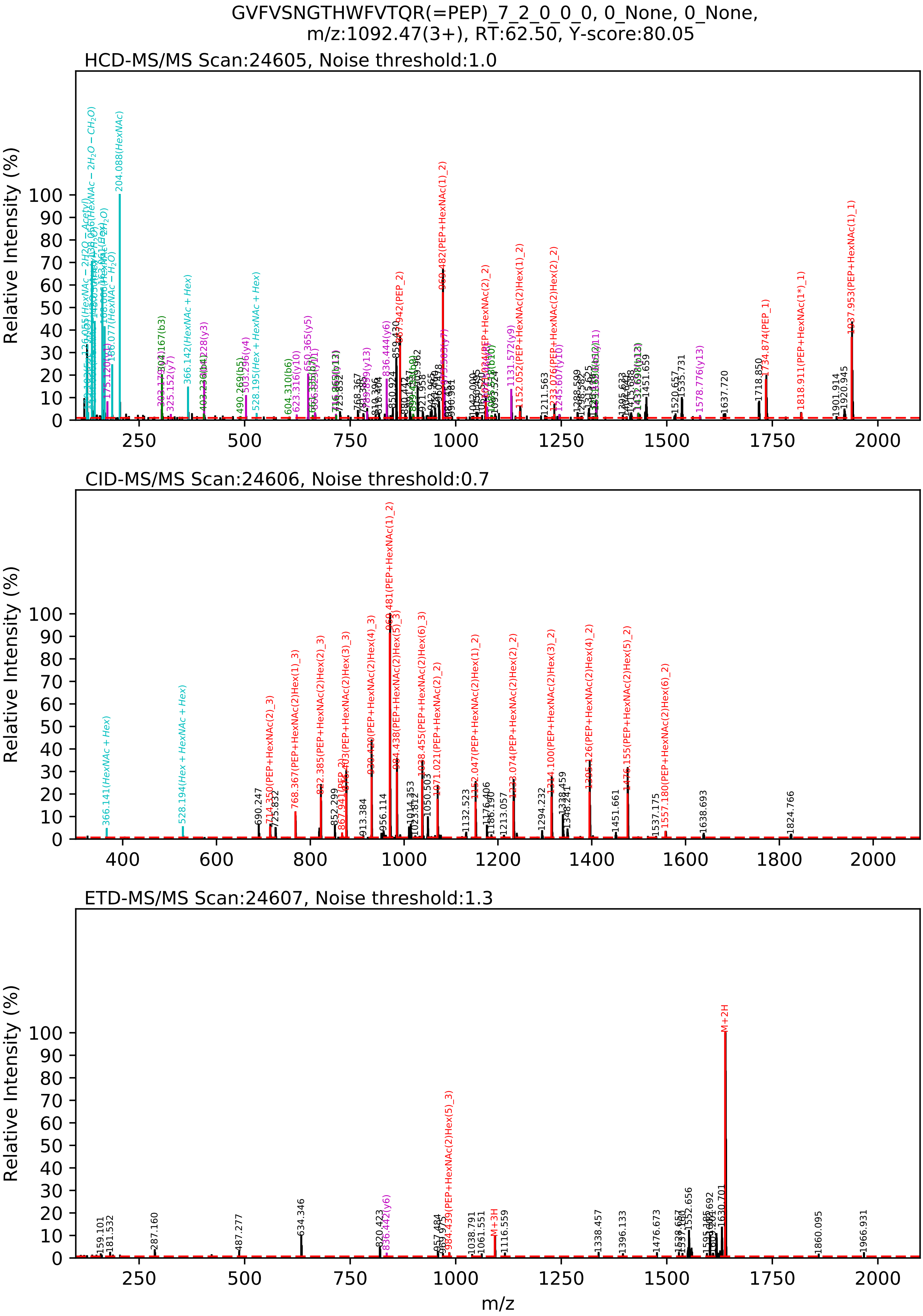
GVFVSNGTHWFVTQR(=PEP)_7_2_0_0_0, 0_None, 0_None,
m/z:1092.47(3+), RT:62.50, Y-score:80.05
HCD-MS/MS Scan:24605, Noise threshold:1.0
Relative Intensity (%)
204.088(HexNAc)
969.482(PEP+HexNAc(1)_2)
867.942(PEP_2)
1937.953(PEP+HexNAc(1)_1)
1734.874(PEP_1)
1818.911(PEP+HexNAc(1*)_1)
1152.052(PEP+HexNAc(2)Hex(1)_2)
1131.572(y9)
836.444(y6)
366.142(HexNAc + Hex)
528.195(Hex + HexNAc + Hex)
1578.776(y13)
CID-MS/MS Scan:24606, Noise threshold:0.7
969.481(PEP+HexNAc(1)_2)
984.438(PEP+HexNAc(2)Hex(5)_3)
930.420(PEP+HexNAc(2)Hex(4)_3)
1038.455(PEP+HexNAc(2)Hex(6)_3)
1071.021(PEP+HexNAc(2)_2)
1152.047(PEP+HexNAc(2)Hex(1)_2)
1233.074(PEP+HexNAc(2)Hex(2)_2)
1314.100(PEP+HexNAc(2)Hex(3)_2)
1395.126(PEP+HexNAc(2)Hex(4)_2)
1476.155(PEP+HexNAc(2)Hex(5)_2)
1557.180(PEP+HexNAc(2)Hex(6)_2)
768.367(PEP+HexNAc(2)Hex(1)_3)
822.385(PEP+HexNAc(2)Hex(2)_3)
876.403(PEP+HexNAc(2)Hex(3)_3)
714.350(PEP+HexNAc(2)_3)
366.141(HexNAc + Hex)
528.194(Hex + HexNAc + Hex)
ETD-MS/MS Scan:24607, Noise threshold:1.3
M+2H
M+3H
984.439(PEP+HexNAc(2)Hex(5)_3)
836.442(y6)
m/z

GVFVSNGTHWFVTQR(=PEP)_7_2_0_0_0, 0_None, 0_None,
m/z:1092.47(3+), RT:60.79, Y-score:87.10

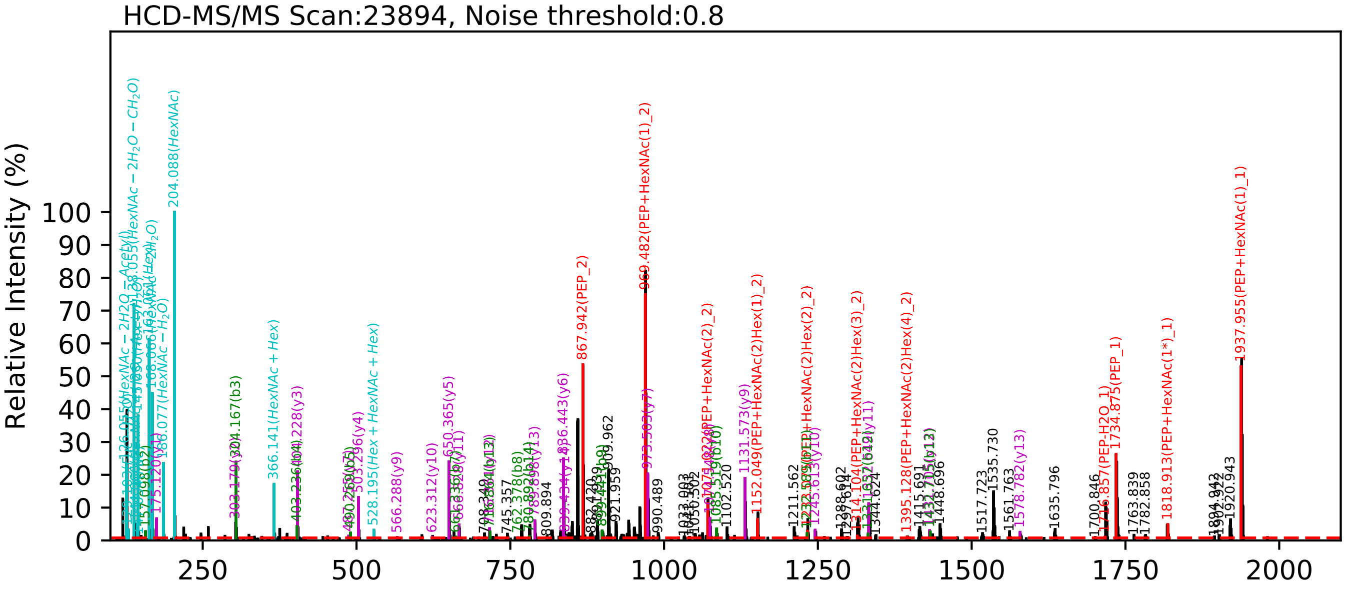

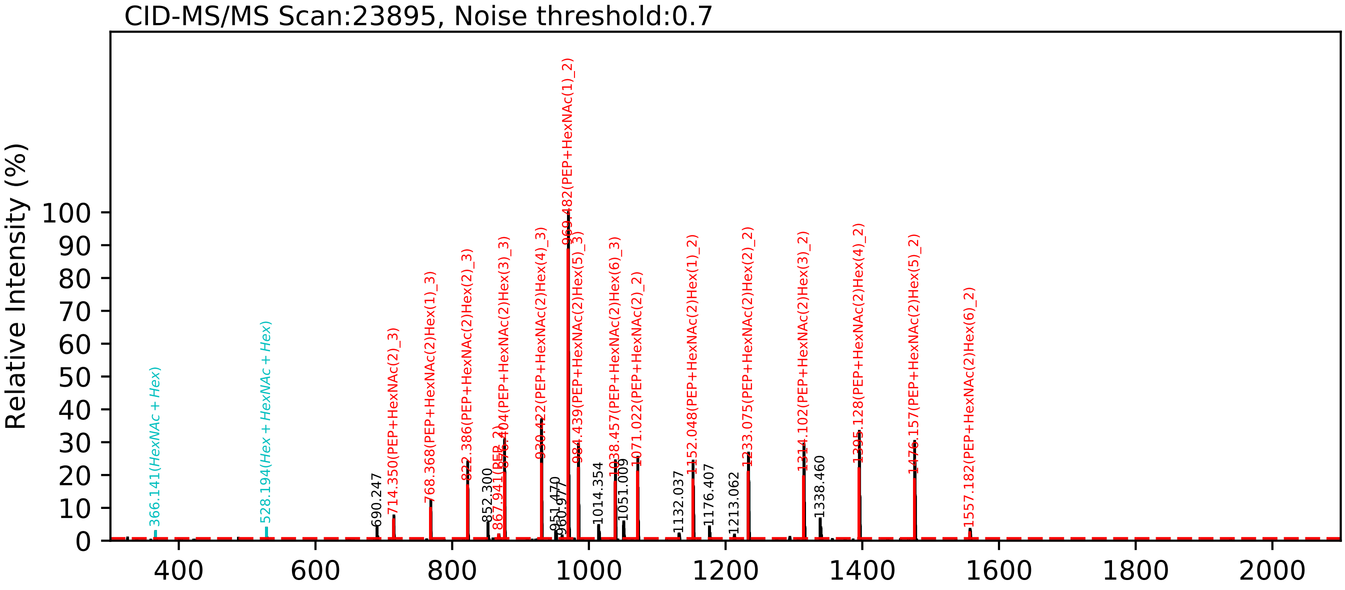

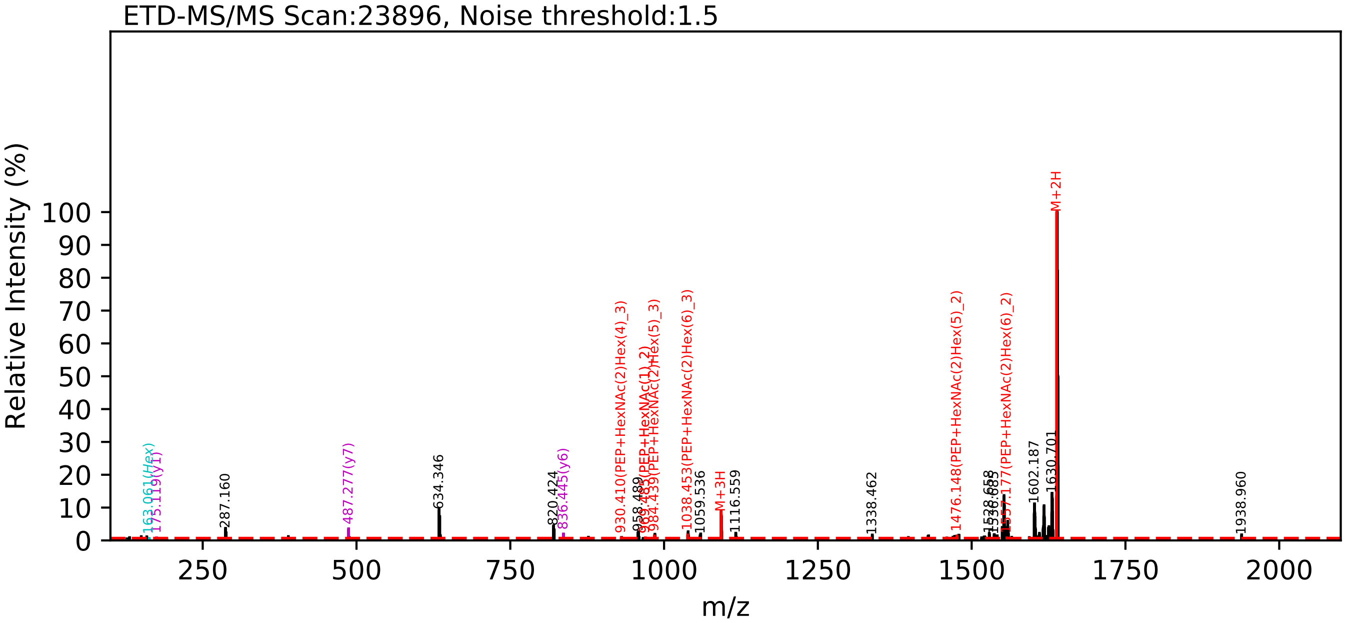

GVFVSNGTHWFVTQR(=PEP)_7_2_0_0_0, 0_None, 0_None,
m/z:1092.47(3+), RT:61.36, Y-score:87.99

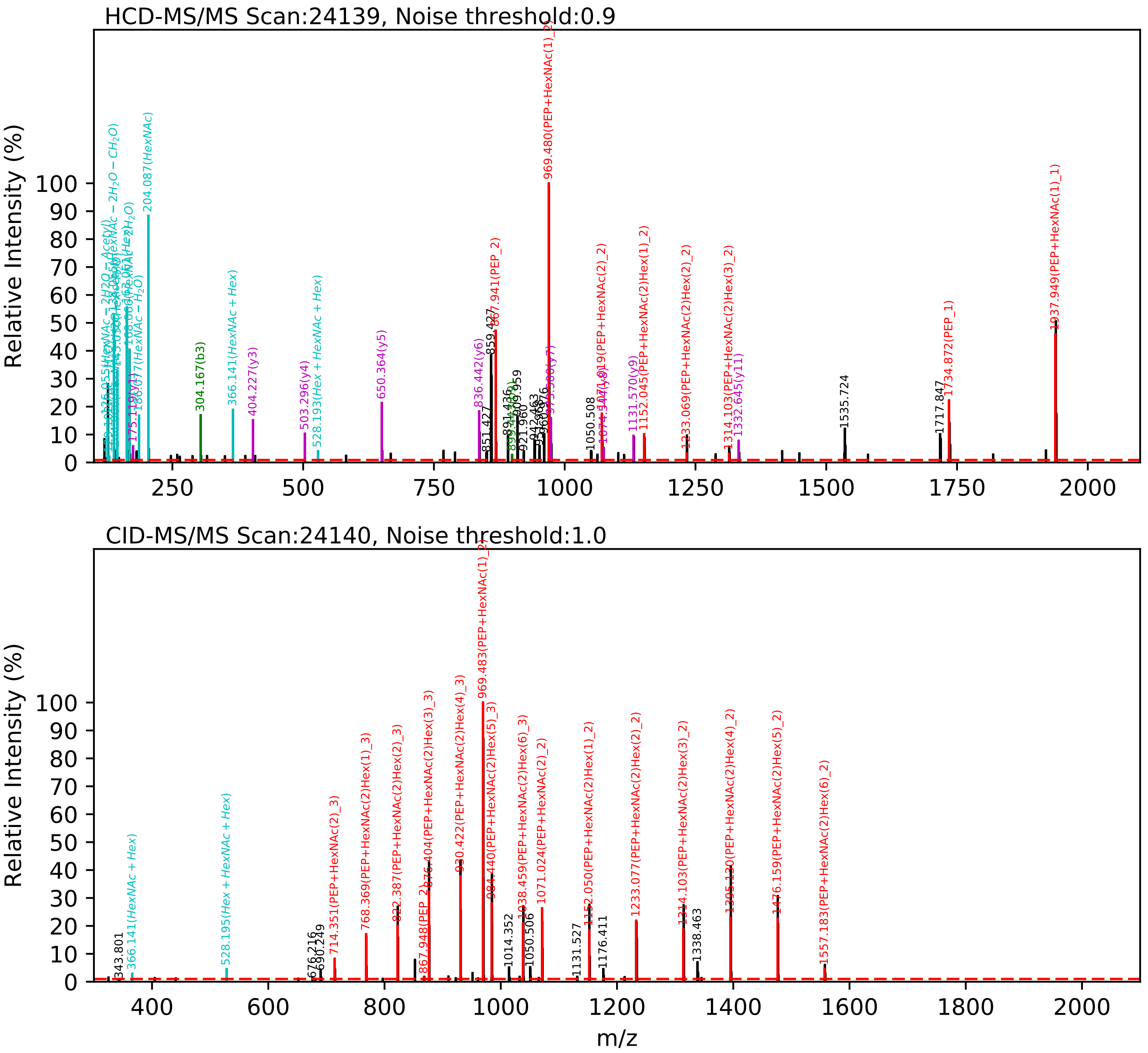

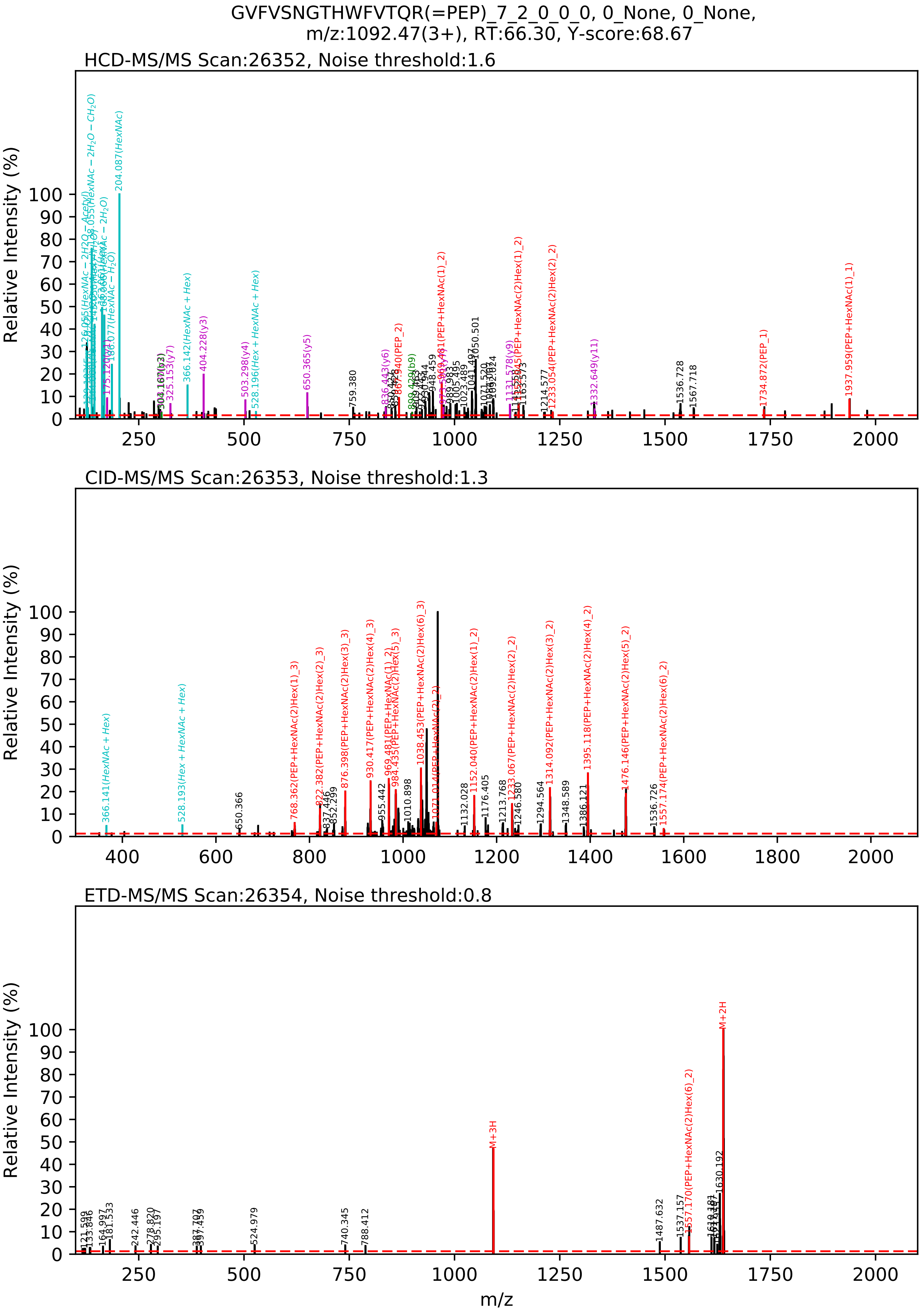
GVFVSNGTHWFVTQR(=PEP)_7_2_0_0_0, 0_None, 0_None,
m/z:1092.47(3+), RT:66.30, Y-score:68.67
HCD-MS/MS Scan:26352, Noise threshold:1.6
Relative Intensity (%)
204.087(HexNAc)
366.142(HexNAc + Hex)
404.228(y3)
325.153(y7)
503.298(y4)
528.196(Hex + HexNAc + Hex)
650.365(y5)
759.380
867.940(PEP_2)
969.481(PEP+HexNAc(1)_2)
1050.501
1131.578(y9)
PEP+HexNAc(2)Hex(1)_2
1233.054(PEP+HexNAc(2)Hex(2)_2)
1214.577
1163.573
1332.649(y11)
1536.728
1567.718
1734.872(PEP_1)
1937.959(PEP+HexNAc(1)_1)
CID-MS/MS Scan:26353, Noise threshold:1.3
Relative Intensity (%)
366.141(HexNAc + Hex)
528.193(Hex + HexNAc + Hex)
650.366
768.362(PEP+HexNAc(2)Hex(1)_3)
822.382(PEP+HexNAc(2)Hex(2)_3)
837.446
852.299
876.398(PEP+HexNAc(2)Hex(3)_3)
930.417(PEP+HexNAc(2)Hex(4)_3)
955.442
969.481(PEP+HexNAc(1)_2)
984.435(PEP+HexNAc(2)Hex(5)_3)
1010.898
1038.453(PEP+HexNAc(2)Hex(6)_3)
1132.028
1152.040(PEP+HexNAc(2)Hex(1)_2)
1176.405
1213.768
1233.067(PEP+HexNAc(2)Hex(2)_2)
1246.580
1294.564
1314.092(PEP+HexNAc(2)Hex(3)_2)
1348.589
1386.121
1395.118(PEP+HexNAc(2)Hex(4)_2)
1476.146(PEP+HexNAc(2)Hex(5)_2)
1536.726
1557.174(PEP+HexNAc(2)Hex(6)_2)
ETD-MS/MS Scan:26354, Noise threshold:0.8
Relative Intensity (%)
121.599
133.846
164.997
181.533
242.446
278.820
295.197
387.792
397.459
524.979
740.345
788.412
M+3H
1487.632
1537.157
1557.170(PEP+HexNAc(2)Hex(6)_2)
1630.192
M+2H
m/z

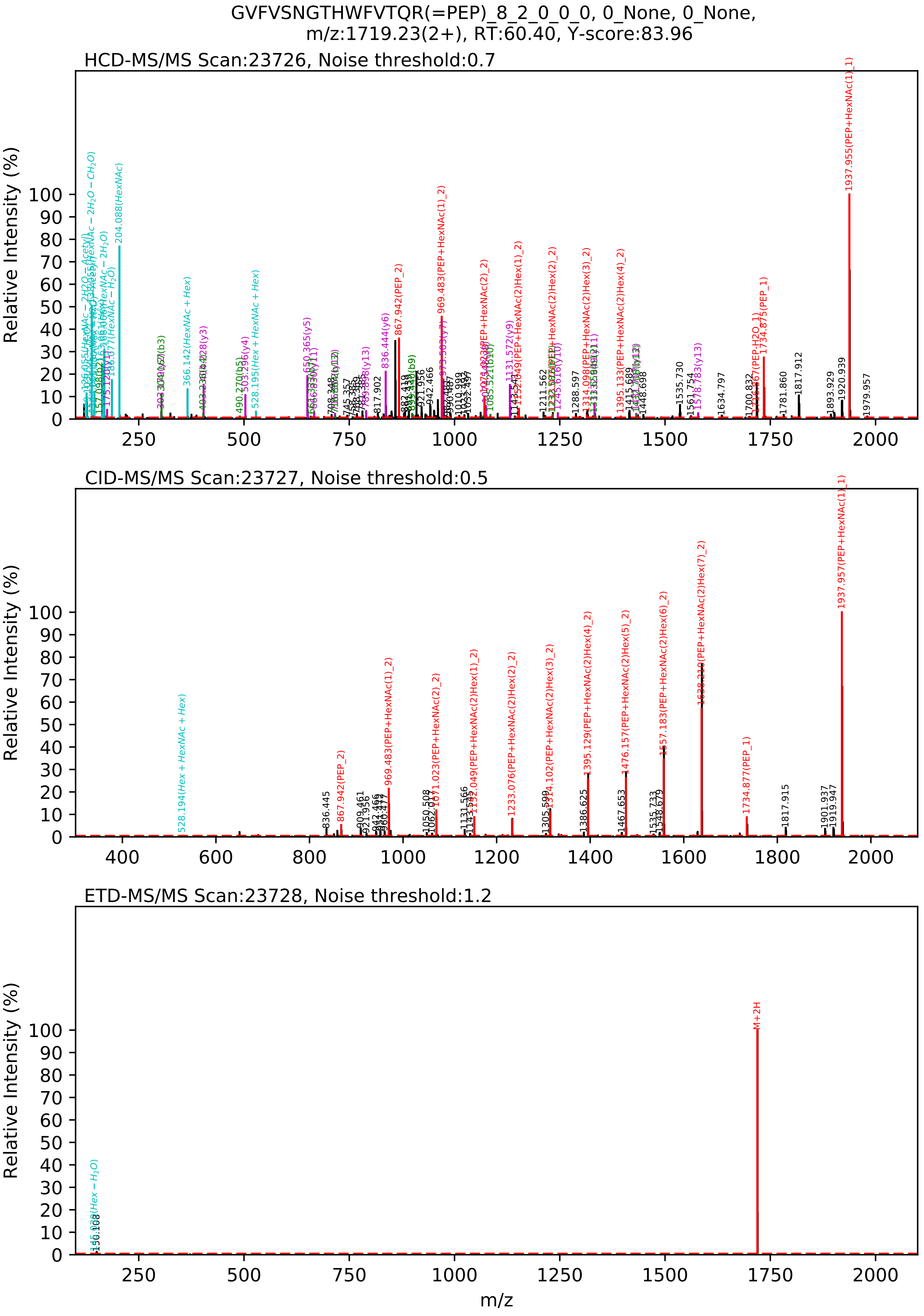
GVFVSNGTHWFVTQR(=PEP)_8_2_0_0_0, 0_None, 0_None,
m/z:1719.23(2+), RT:60.40, Y-score:83.96
HCD-MS/MS Scan:23726, Noise threshold:0.7
Relative Intensity (%)
204.088(HexNAc)
366.142(HexNAc + Hex)
528.195(Hex + HexNAc + Hex)
650.365(y5)
836.444(y6)
867.942(PEP_2)
969.483(PEP+HexNAc(1)_2)
1131.572(y9)
1152.049(PEP+HexNAc(2)Hex(1)_2)
1314.098(PEP+HexNAc(2)Hex(3)_2)
1395.133(PEP+HexNAc(2)Hex(4)_2)
1535.730
1578.783(y13)
1634.797
1734.875(PEP_1)
1781.860
1817.912
1893.929
1920.939
1937.955(PEP+HexNAc(1)_1)
1979.957
CID-MS/MS Scan:23727, Noise threshold:0.5
Relative Intensity (%)
528.194(Hex + HexNAc + Hex)
836.445
867.942(PEP_2)
969.483(PEP+HexNAc(1)_2)
1071.023(PEP+HexNAc(2)_2)
1152.049(PEP+HexNAc(2)Hex(1)_2)
1233.076(PEP+HexNAc(2)Hex(2)_2)
1314.102(PEP+HexNAc(2)Hex(3)_2)
1386.625
1395.129(PEP+HexNAc(2)Hex(4)_2)
1467.653
1476.157(PEP+HexNAc(2)Hex(5)_2)
1557.183(PEP+HexNAc(2)Hex(6)_2)
1638.210(PEP+HexNAc(2)Hex(7)_2)
1734.877(PEP_1)
1817.915
1901.937
1919.947
1937.957(PEP+HexNAc(1)_1)
ETD-MS/MS Scan:23728, Noise threshold:1.2
Relative Intensity (%)
M+2H
m/z

GVFVSNGTHWFVTQR(=PEP)_8_2_0_0_0, 0_None, 0_None,
m/z:1719.23(2+), RT:60.61, Y-score:81.18

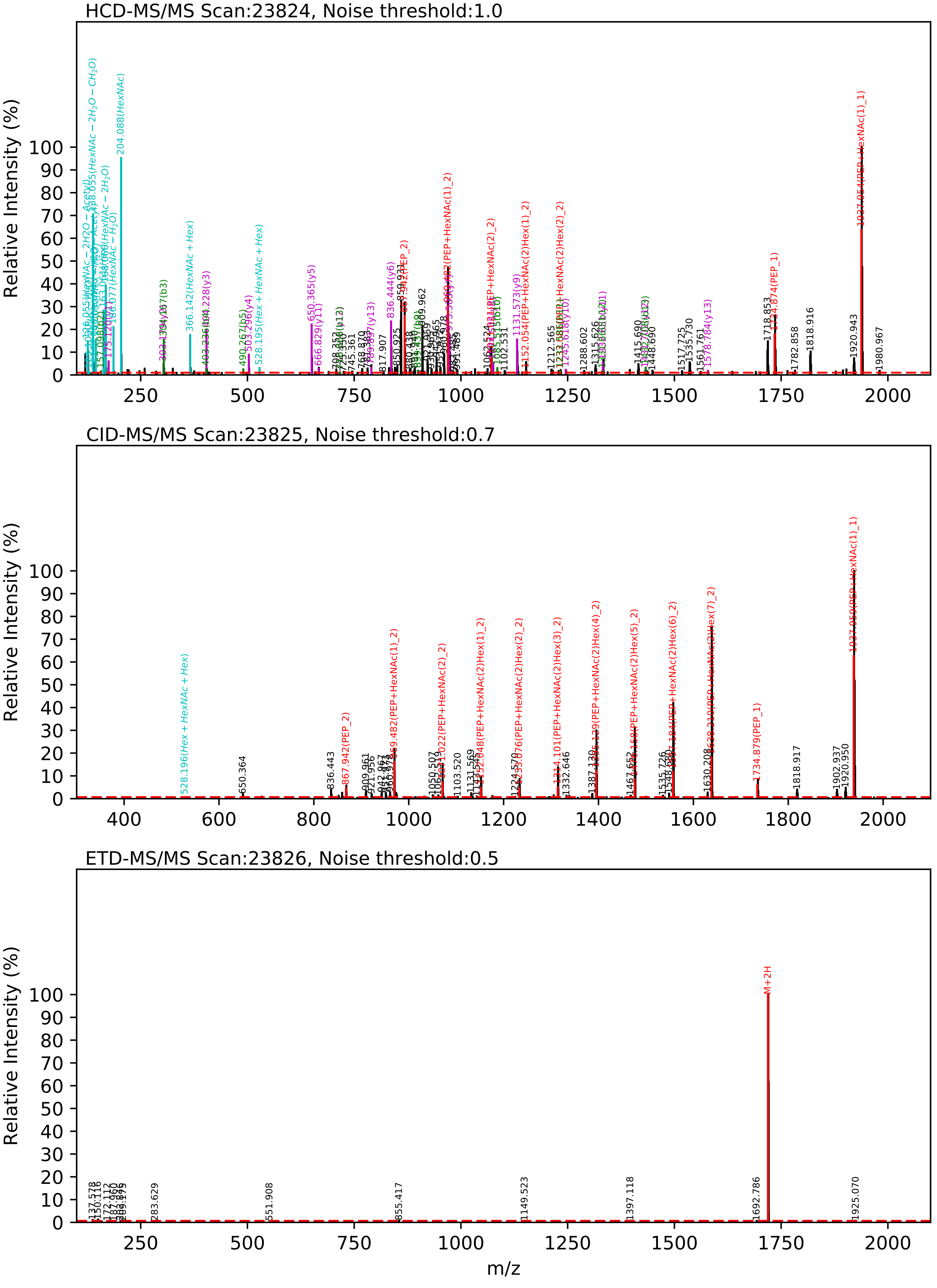

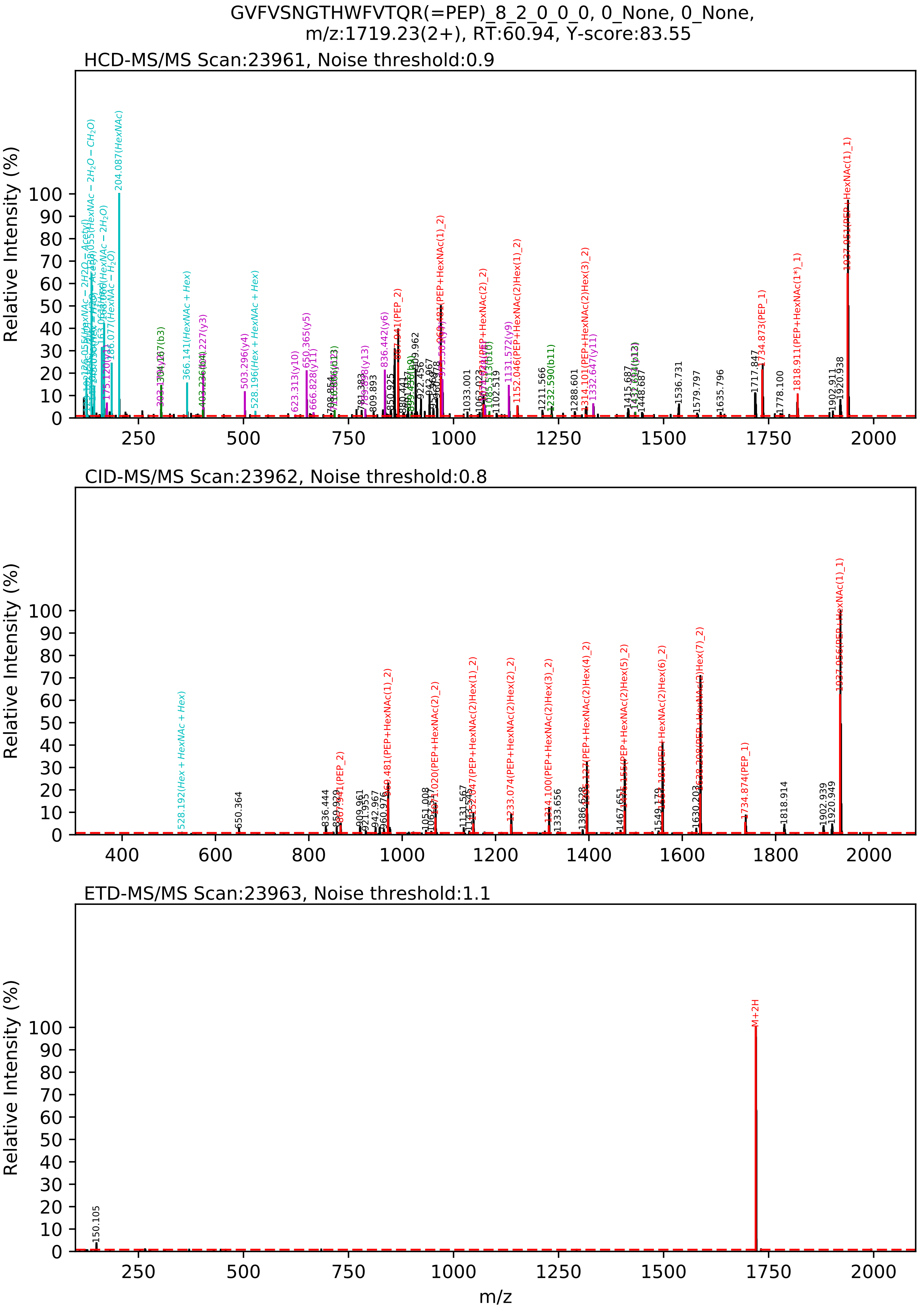

GVFVSNGTHWFVTQR(=PEP)_8_2_0_0_0, 0_None, 0_None,
m/z:1719.23(2+), RT:60.94, Y-score:83.55
HCD-MS/MS Scan:23961, Noise threshold:0.9
CID-MS/MS Scan:23962, Noise threshold:0.8
ETD-MS/MS Scan:23963, Noise threshold:1.1
Relative Intensity (%)
m/z
1937.951(PEP+HexNAc(1)_1)
1937.956(PEP+HexNAc(1)_1)
1734.873(PEP_1)
1734.874(PEP_1)
1818.911(PEP+HexNAc(1*)_1)
M+2H
150.105
204.087(HexNAc)
528.196(Hex + HexNAc + Hex)
528.192(Hex + HexNAc + Hex)
366.141(HexNAc + Hex)

GVFVSNGTHWFVTQR(=PEP)_8_2_0_0_0, 0_None, 0_None,
m/z:1719.23(2+), RT:61.08, Y-score:81.66

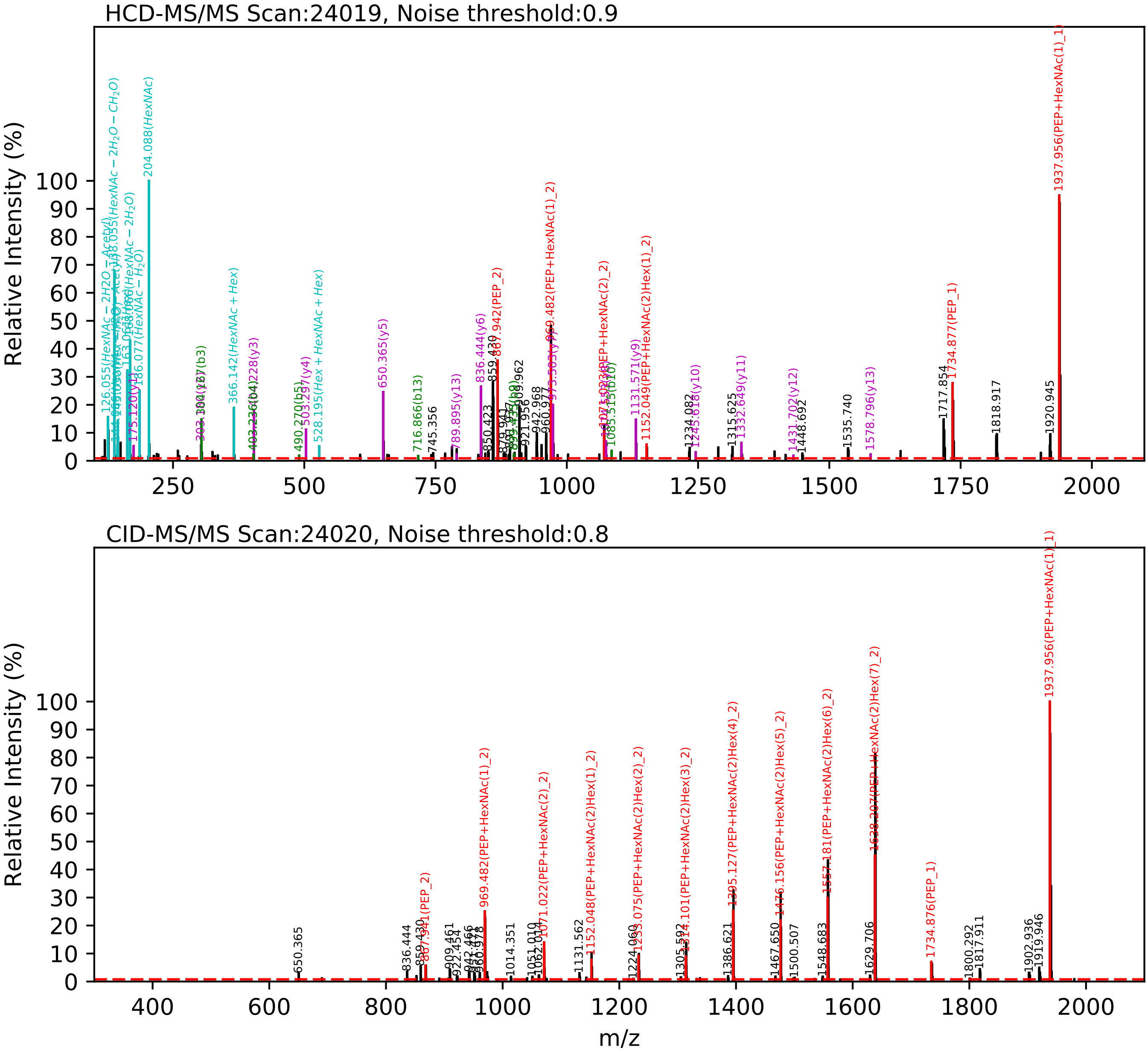

GVFVSNGTHWFVTQR(=PEP)_8_2_0_0_0, 0_None, 0_None,
m/z:1146.49(3+), RT:66.08, Y-score:75.34

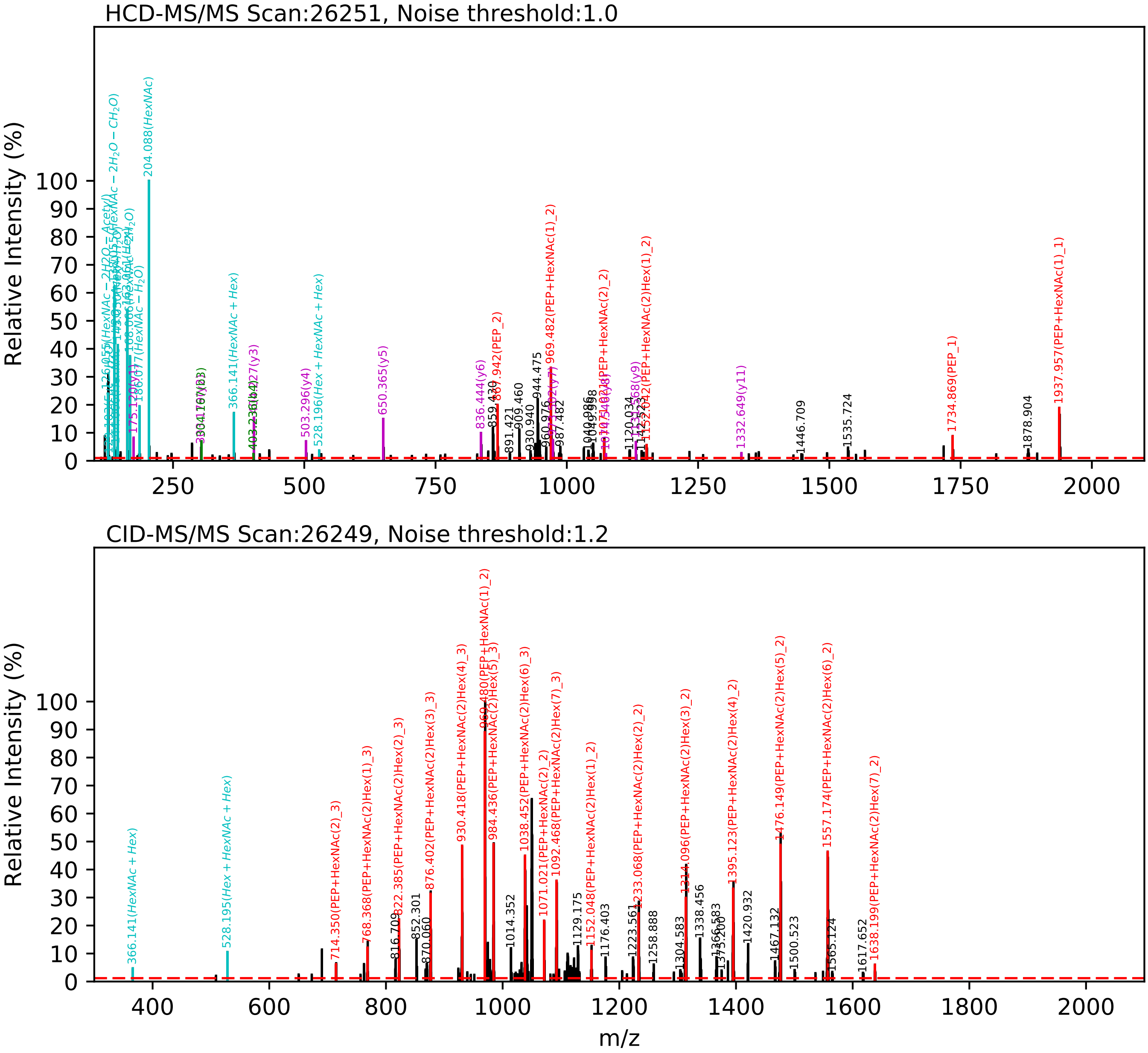

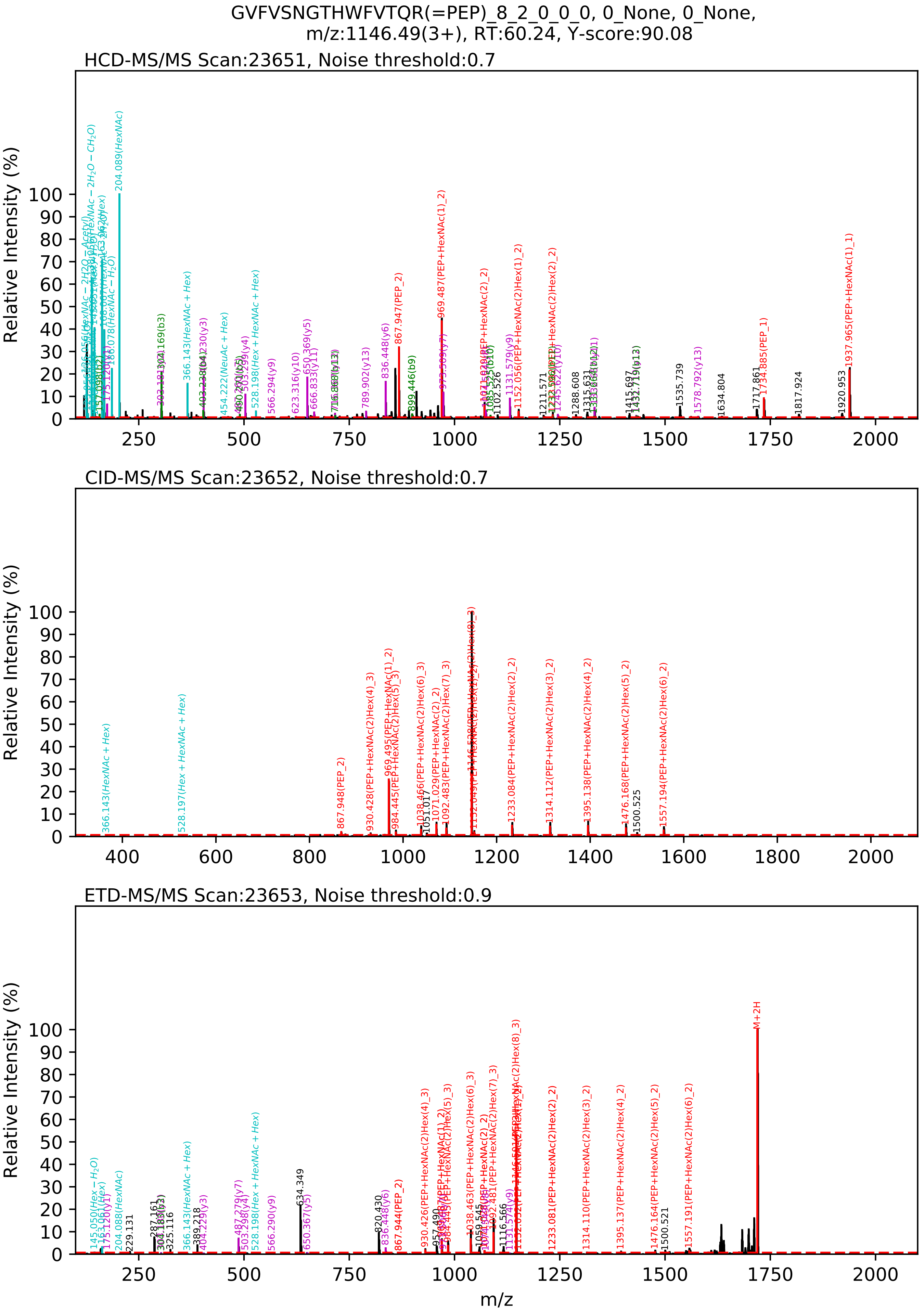
GVFVSNGTHWFVTQR(=PEP)_8_2_0_0_0, 0_None, 0_None,
m/z:1146.49(3+), RT:60.24, Y-score:90.08
HCD-MS/MS Scan:23651, Noise threshold:0.7
CID-MS/MS Scan:23652, Noise threshold:0.7
ETD-MS/MS Scan:23653, Noise threshold:0.9
Relative Intensity (%)
m/z

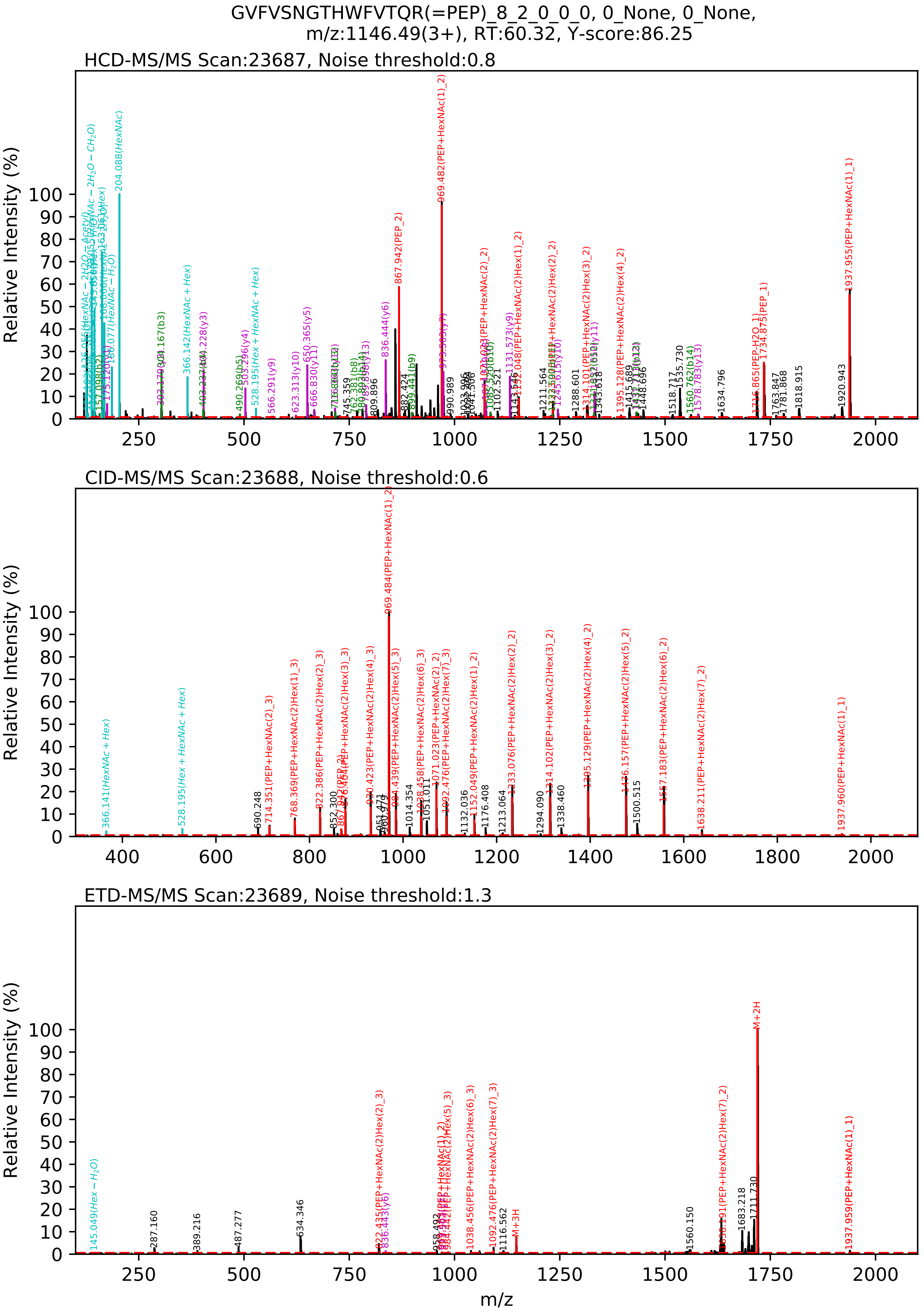
GVFVSNGTHWFVTQR(=PEP)_8_2_0_0_0, 0_None, 0_None,
m/z:1146.49(3+), RT:60.32, Y-score:86.25
HCD-MS/MS Scan:23687, Noise threshold:0.8
Relative Intensity (%)
204.088(HexNAc)
969.482(PEP+HexNAc(1)_2)
867.942(PEP_2)
1937.955(PEP+HexNAc(1)_1)
1152.048(PEP+HexNAc(2)Hex(1)_2)
1233.590(PEP+HexNAc(2)Hex(2)_2)
1314.101(PEP+HexNAc(2)Hex(3)_2)
1395.128(PEP+HexNAc(2)Hex(4)_2)
1734.875(PEP_1)
1716.865(PEP-H2O_1)
836.444(y6)
650.365(y5)
1131.573(y9)
366.142(HexNAc + Hex)
528.195(Hex + HexNAc + Hex)
404.228(y3)
503.296(y4)
566.291(y9)
623.313(y10)
666.830(y11)
899.441(b9)
490.269(b5)
304.167(b3)
1560.762(b14)
1578.783(y13)
1245.615(y10)
186.077(HexNAc − H2O)
175.120(y1)
1920.943
1818.915
1763.847
1781.868
1634.796
1535.730
1518.717
1415.689
1448.696
1343.618
1288.601
1211.564
1143.546
1102.521
990.989
882.424
809.896
745.359
CID-MS/MS Scan:23688, Noise threshold:0.6
Relative Intensity (%)
969.484(PEP+HexNAc(1)_2)
366.141(HexNAc + Hex)
528.195(Hex + HexNAc + Hex)
690.248
714.351(PEP+HexNAc(2)_3)
768.369(PEP+HexNAc(2)Hex(1)_3)
822.386(PEP+HexNAc(2)Hex(2)_3)
876.404(PEP+HexNAc(2)Hex(3)_3)
852.300
930.423(PEP+HexNAc(2)Hex(4)_3)
984.439(PEP+HexNAc(2)Hex(5)_3)
1014.354
1038.458(PEP+HexNAc(2)Hex(6)_3)
1051.011
1071.023(PEP+HexNAc(2)_2)
1092.476(PEP+HexNAc(2)Hex(7)_3)
1132.036
1152.049(PEP+HexNAc(2)Hex(1)_2)
1176.408
1213.064
1233.076(PEP+HexNAc(2)Hex(2)_2)
1294.090
1314.102(PEP+HexNAc(2)Hex(3)_2)
1338.460
1395.129(PEP+HexNAc(2)Hex(4)_2)
1476.157(PEP+HexNAc(2)Hex(5)_2)
1500.515
1557.183(PEP+HexNAc(2)Hex(6)_2)
1638.211(PEP+HexNAc(2)Hex(7)_2)
1937.960(PEP+HexNAc(1)_1)
ETD-MS/MS Scan:23689, Noise threshold:1.3
Relative Intensity (%)
M+2H
M+3H
145.049(Hex − H2O)
287.160
389.216
487.277
634.346
822.435(PEP+HexNAc(2)Hex(2)_3)
836.443(y6)
958.492
1038.456(PEP+HexNAc(2)Hex(6)_3)
1092.476(PEP+HexNAc(2)Hex(7)_3)
1116.562
1560.150
1683.218
1711.730
1937.959(PEP+HexNAc(1)_1)
m/z

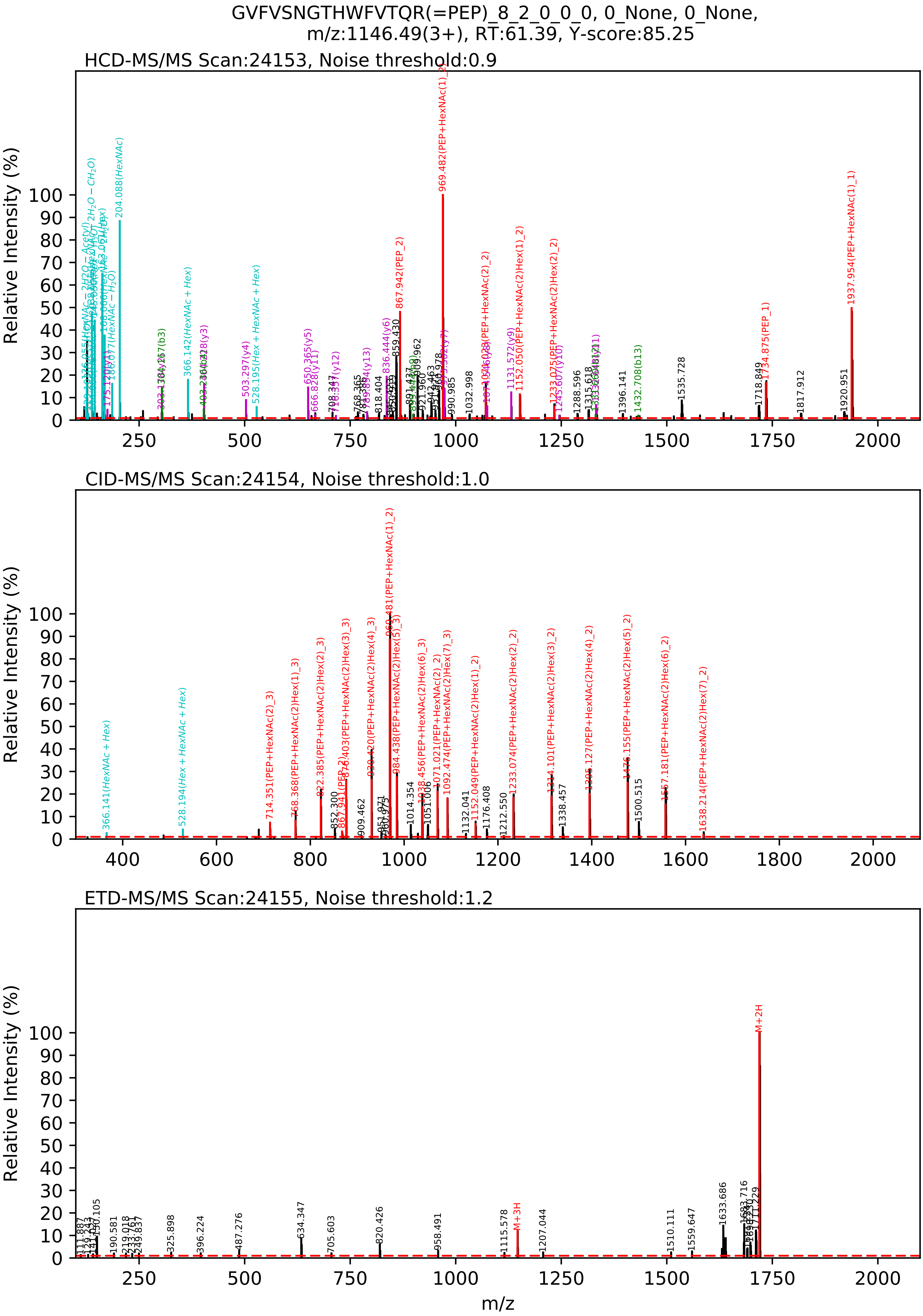
GVFVSNGTHWFVTQR(=PEP)_8_2_0_0_0, 0_None, 0_None,
m/z:1146.49(3+), RT:61.39, Y-score:85.25
HCD-MS/MS Scan:24153, Noise threshold:0.9
Relative Intensity (%)
969.482(PEP+HexNAc(1)_2)
204.088(HexNAc)
1937.954(PEP+HexNAc(1)_1)
867.942(PEP_2)
1074.023(PEP+HexNAc(2)_2)
1152.050(PEP+HexNAc(2)Hex(1)_2)
1233.075(PEP+HexNAc(2)Hex(2)_2)
1734.875(PEP_1)
366.142(HexNAc+Hex)
528.195(Hex+HexNAc+Hex)
503.297(y4)
650.365(y5)
836.444(y6)
1131.572(y9)
1432.708(b13)
1535.728
1718.849
1817.912
1920.951
CID-MS/MS Scan:24154, Noise threshold:1.0
969.481(PEP+HexNAc(1)_2)
366.141(HexNAc+Hex)
528.194(Hex+HexNAc+Hex)
714.351(PEP+HexNAc(2)_3)
768.368(PEP+HexNAc(2)Hex(1)_3)
822.385(PEP+HexNAc(2)Hex(2)_3)
876.403(PEP+HexNAc(2)Hex(3)_3)
930.420(PEP+HexNAc(2)Hex(4)_3)
984.438(PEP+HexNAc(2)Hex(5)_3)
1038.456(PEP+HexNAc(2)Hex(6)_3)
1071.021(PEP+HexNAc(2)_2)
1092.474(PEP+HexNAc(2)Hex(7)_3)
1152.049(PEP+HexNAc(2)Hex(1)_2)
1233.074(PEP+HexNAc(2)Hex(2)_2)
1314.101(PEP+HexNAc(2)Hex(3)_2)
1395.127(PEP+HexNAc(2)Hex(4)_2)
1476.155(PEP+HexNAc(2)Hex(5)_2)
1557.181(PEP+HexNAc(2)Hex(6)_2)
1638.214(PEP+HexNAc(2)Hex(7)_2)
ETD-MS/MS Scan:24155, Noise threshold:1.2
M+2H
M+3H
634.347
820.426
958.491
1115.578
1207.044
1510.111
1559.647
1633.686
1683.716
m/z

GVFVSNGTHWFVTQR(=PEP)_8_2_0_0_0, 0_None, 0_None,
m/z:1146.49(3+), RT:62.04, Y-score:84.95

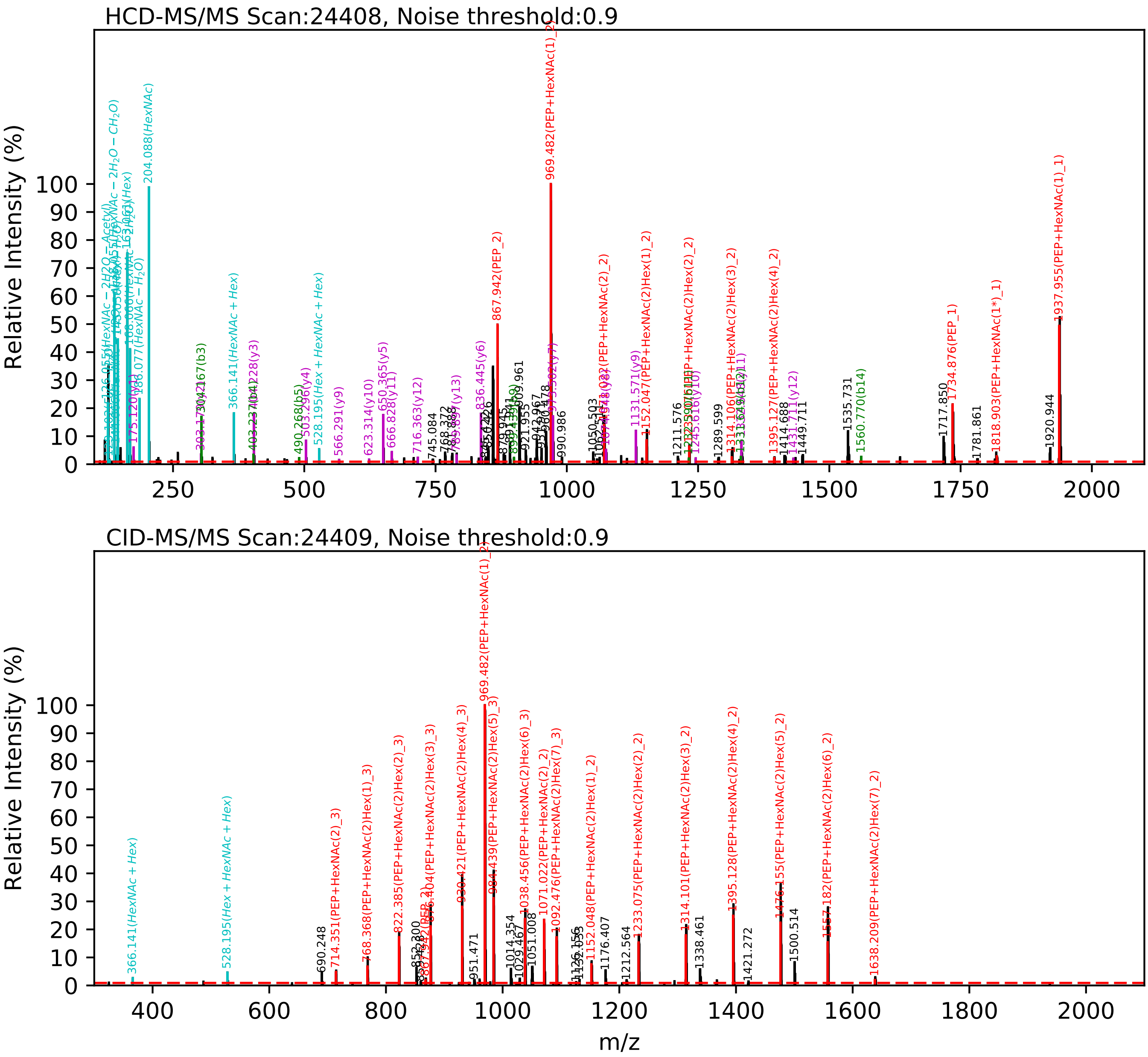

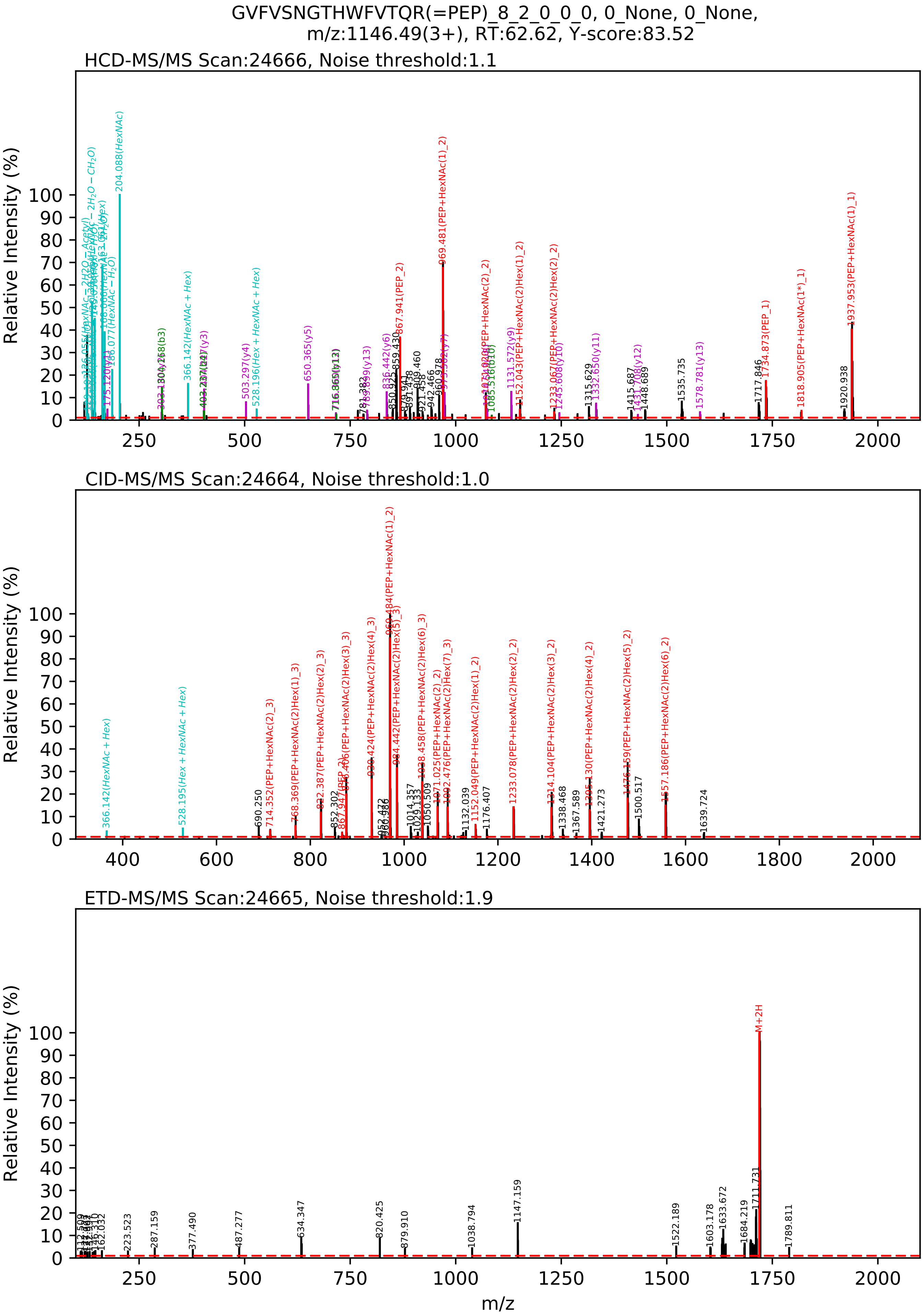

GVFVSNGTHWFVTQR(=PEP)_8_2_0_0_0, 0_None, 0_None,
m/z:1146.49(3+), RT:62.62, Y-score:83.52
HCD-MS/MS Scan:24666, Noise threshold:1.1
Relative Intensity (%)
969.481(PEP+HexNAc(1)_2)
1937.953(PEP+HexNAc(1)_1)
204.088(HexNAc)
CID-MS/MS Scan:24664, Noise threshold:1.0
Relative Intensity (%)
969.484(PEP+HexNAc(1)_2)
ETD-MS/MS Scan:24665, Noise threshold:1.9
Relative Intensity (%)
M+2H
m/z

GVFVSNGTHWFVTQR(=PEP)_8_2_0_0_0, 0_None, 0_None,
m/z:1146.49(3+), RT:63.19, Y-score:86.29

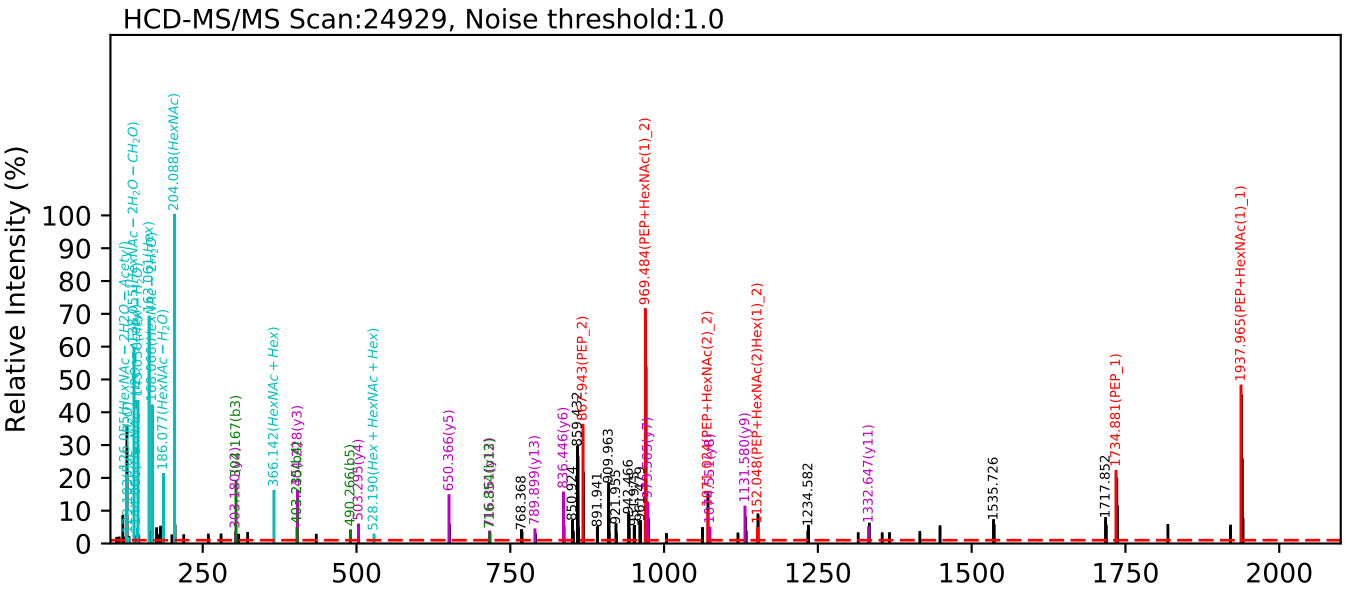

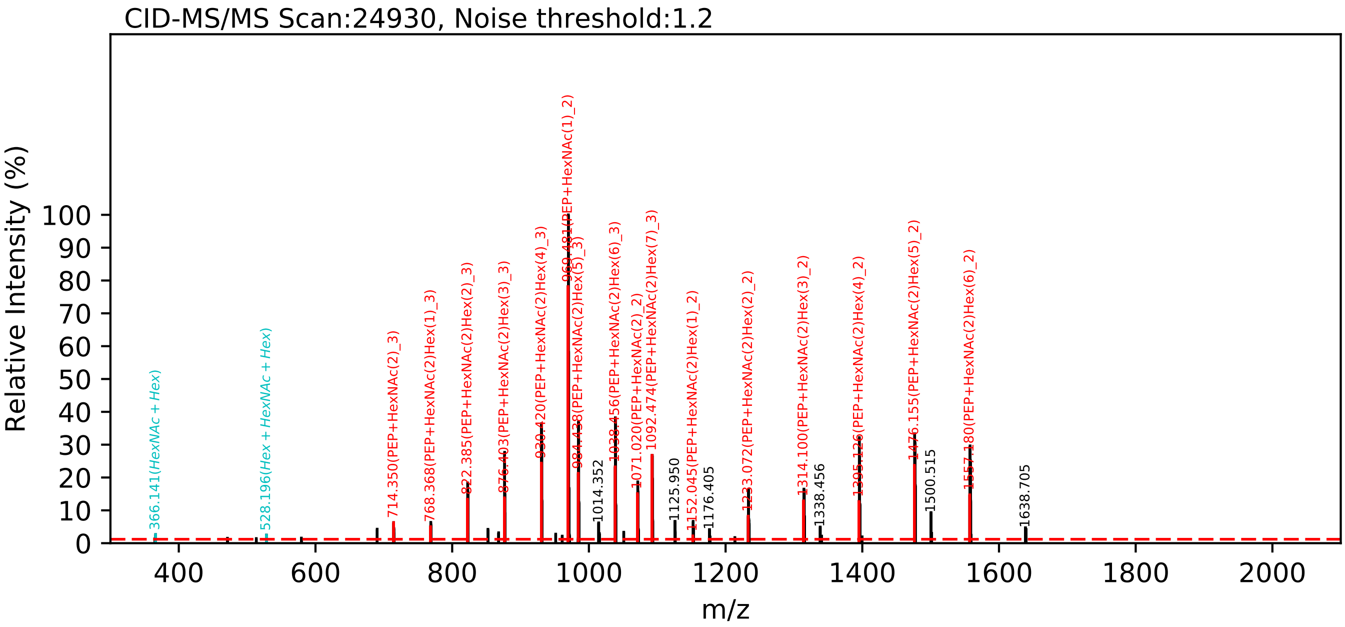

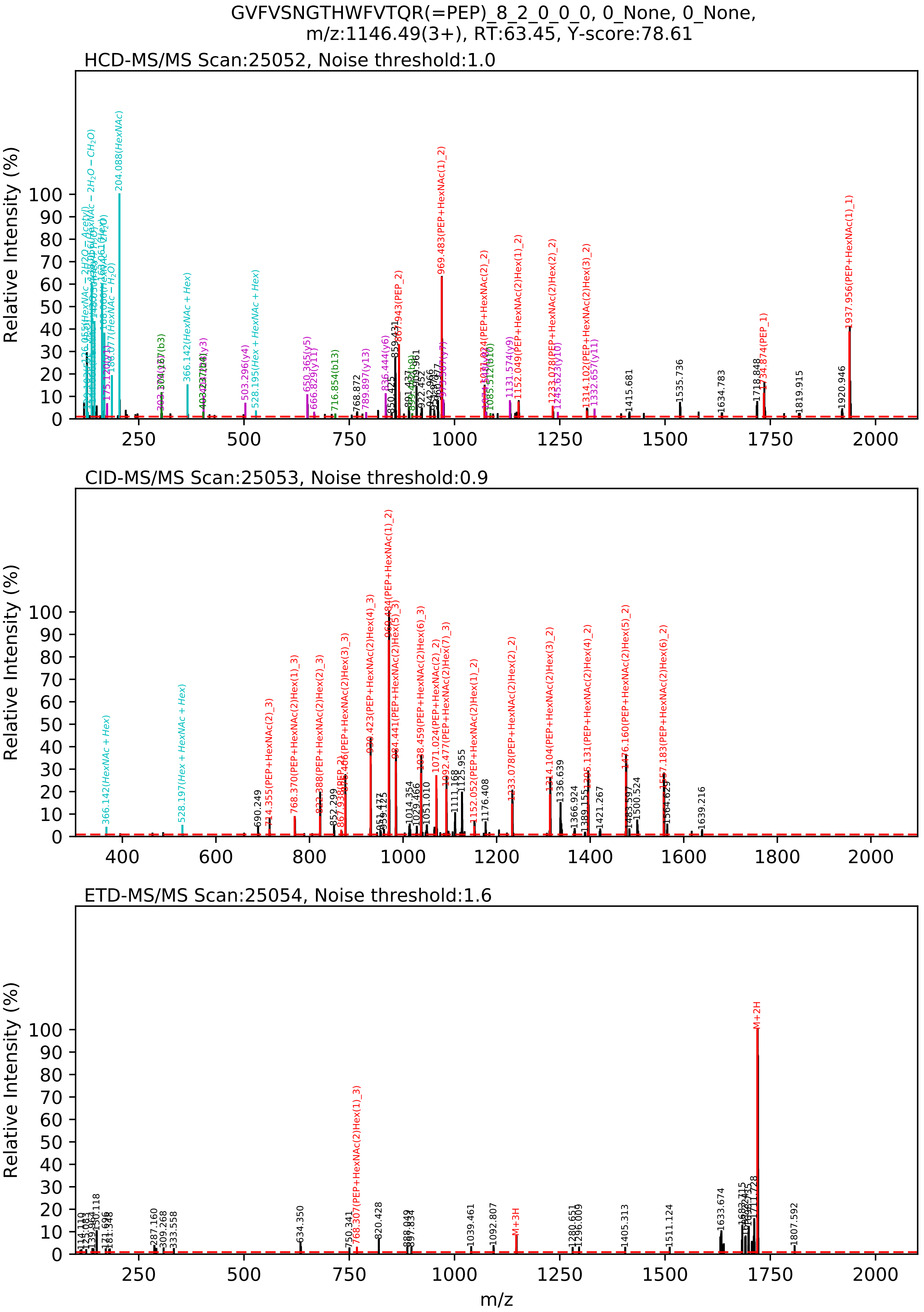
GVFVSNGTHWFVTQR(=PEP)_8_2_0_0_0, 0_None, 0_None,
m/z:1146.49(3+), RT:63.45, Y-score:78.61
HCD-MS/MS Scan:25052, Noise threshold:1.0
CID-MS/MS Scan:25053, Noise threshold:0.9
ETD-MS/MS Scan:25054, Noise threshold:1.6
Relative Intensity (%)
m/z

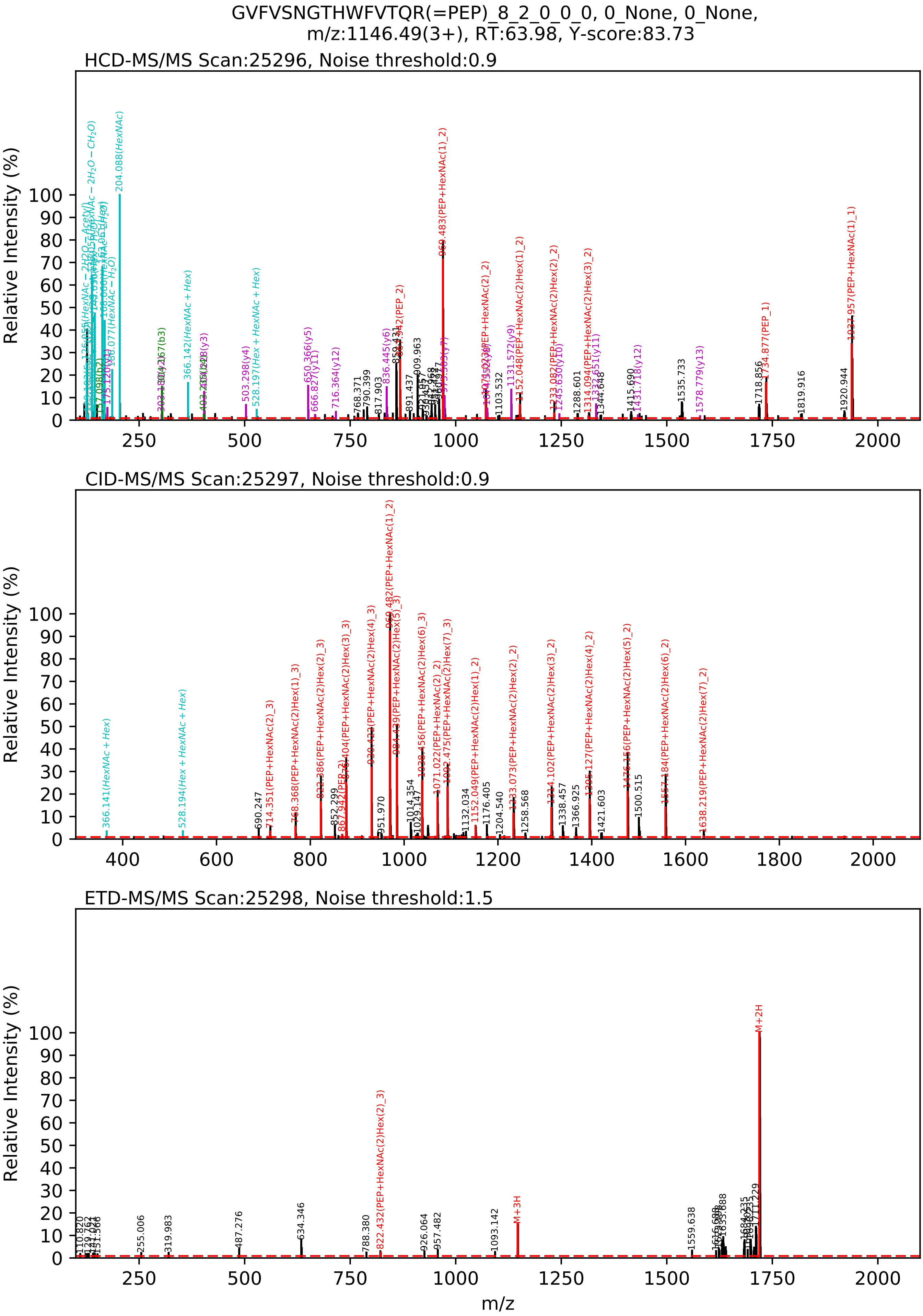

GVFVSNGTHWFVTQR(=PEP)_8_2_0_0_0, 0_None, 0_None,
m/z:1146.49(3+), RT:63.98, Y-score:83.73
HCD-MS/MS Scan:25296, Noise threshold:0.9
CID-MS/MS Scan:25297, Noise threshold:0.9
ETD-MS/MS Scan:25298, Noise threshold:1.5
Relative Intensity (%)
m/z

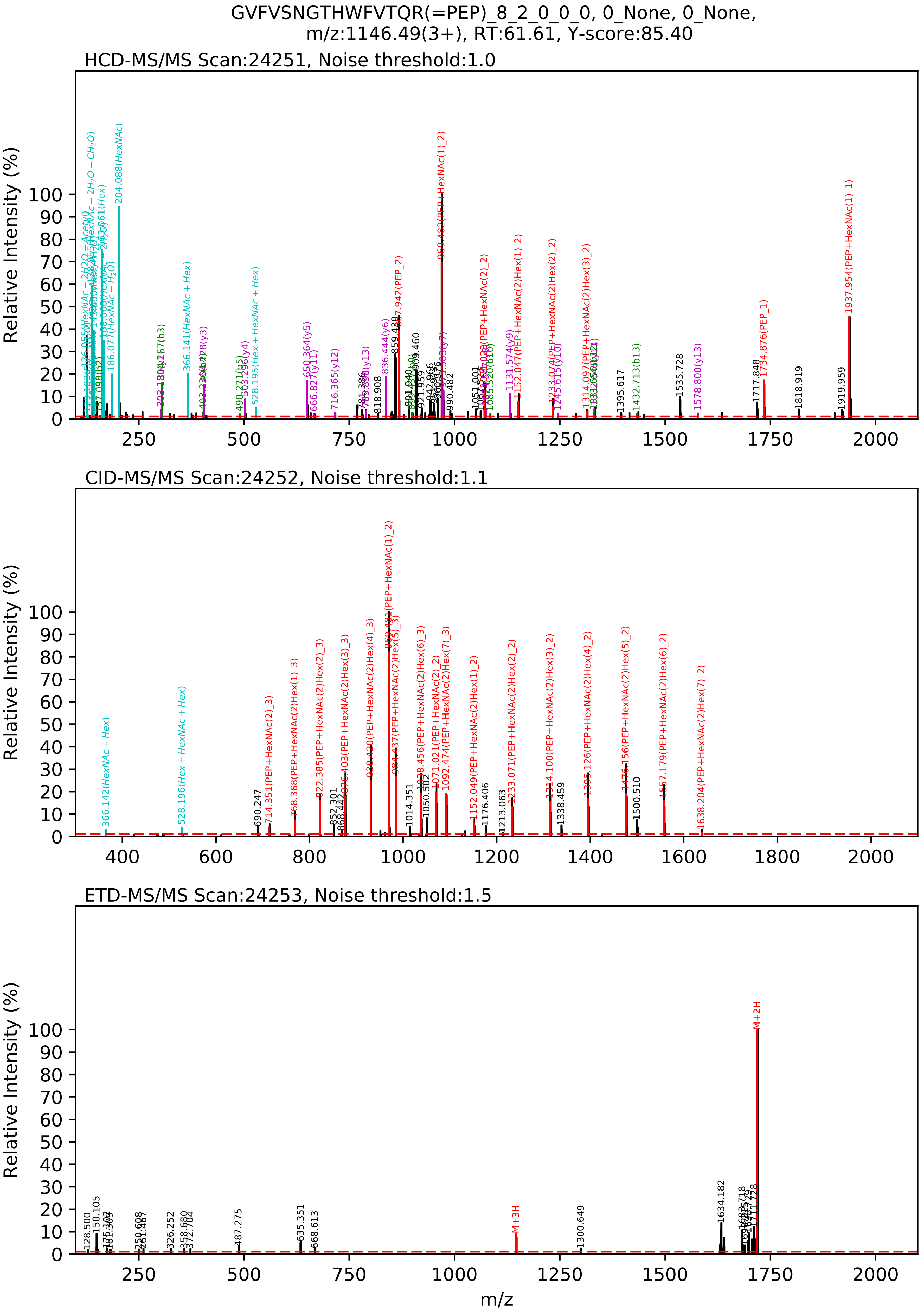

GVFVSNGTHWFVTQR(=PEP)_8_2_0_0_0, 0_None, 0_None,
m/z:1146.49(3+), RT:61.61, Y-score:85.40
HCD-MS/MS Scan:24251, Noise threshold:1.0
CID-MS/MS Scan:24252, Noise threshold:1.1
ETD-MS/MS Scan:24253, Noise threshold:1.5
Relative Intensity (%)
m/z

GVFVSNGTHWFVTQR(=PEP)_8_2_0_0_0, 0_None, 0_None,
m/z:1146.49(3+), RT:60.83, Y-score:89.62

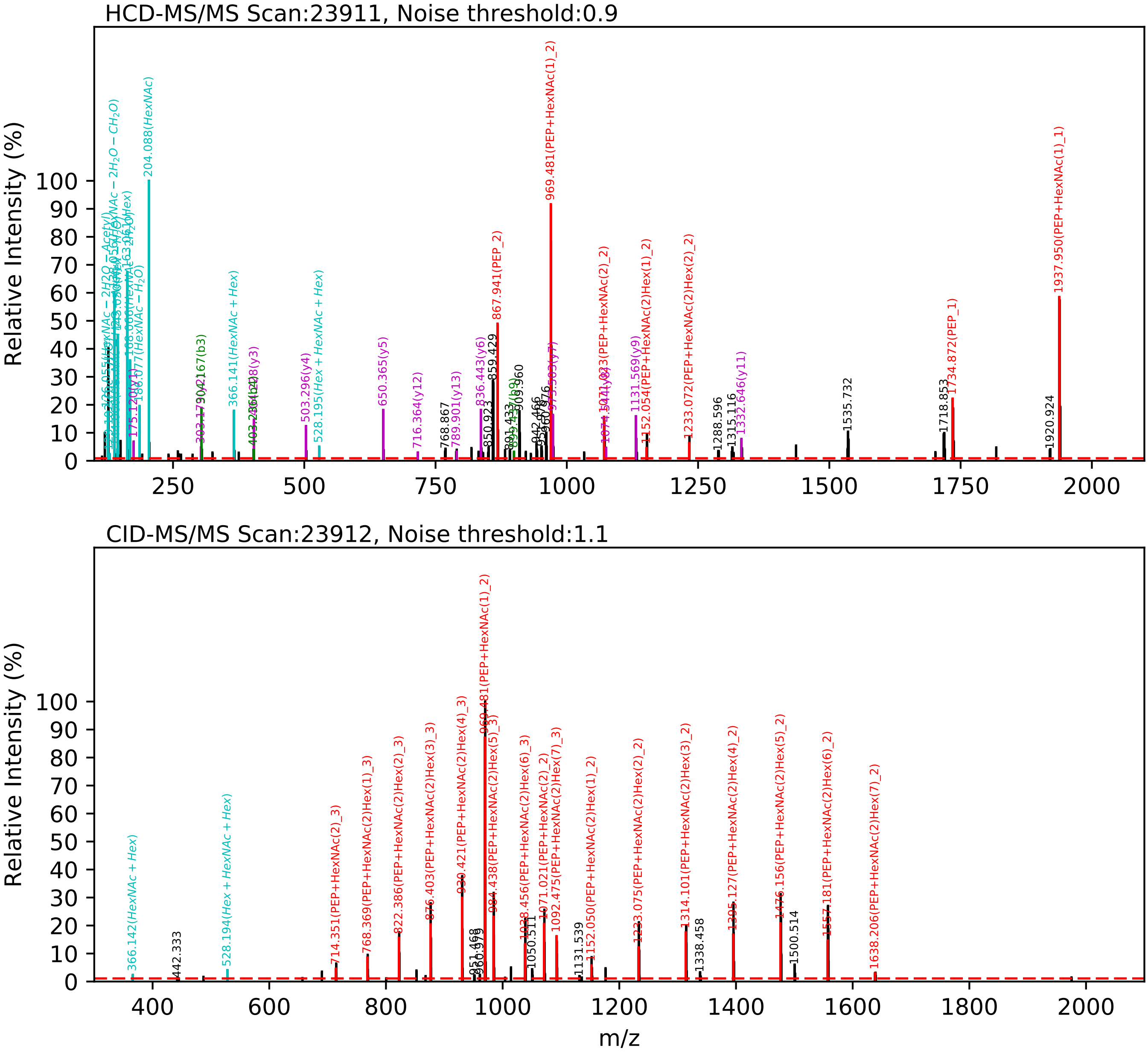

GVFVSNGTHWFVTQR(=PEP)_9_2_0_0_0, 0_None, 0_None,
m/z:1200.51(3+), RT:60.81, Y-score:87.88

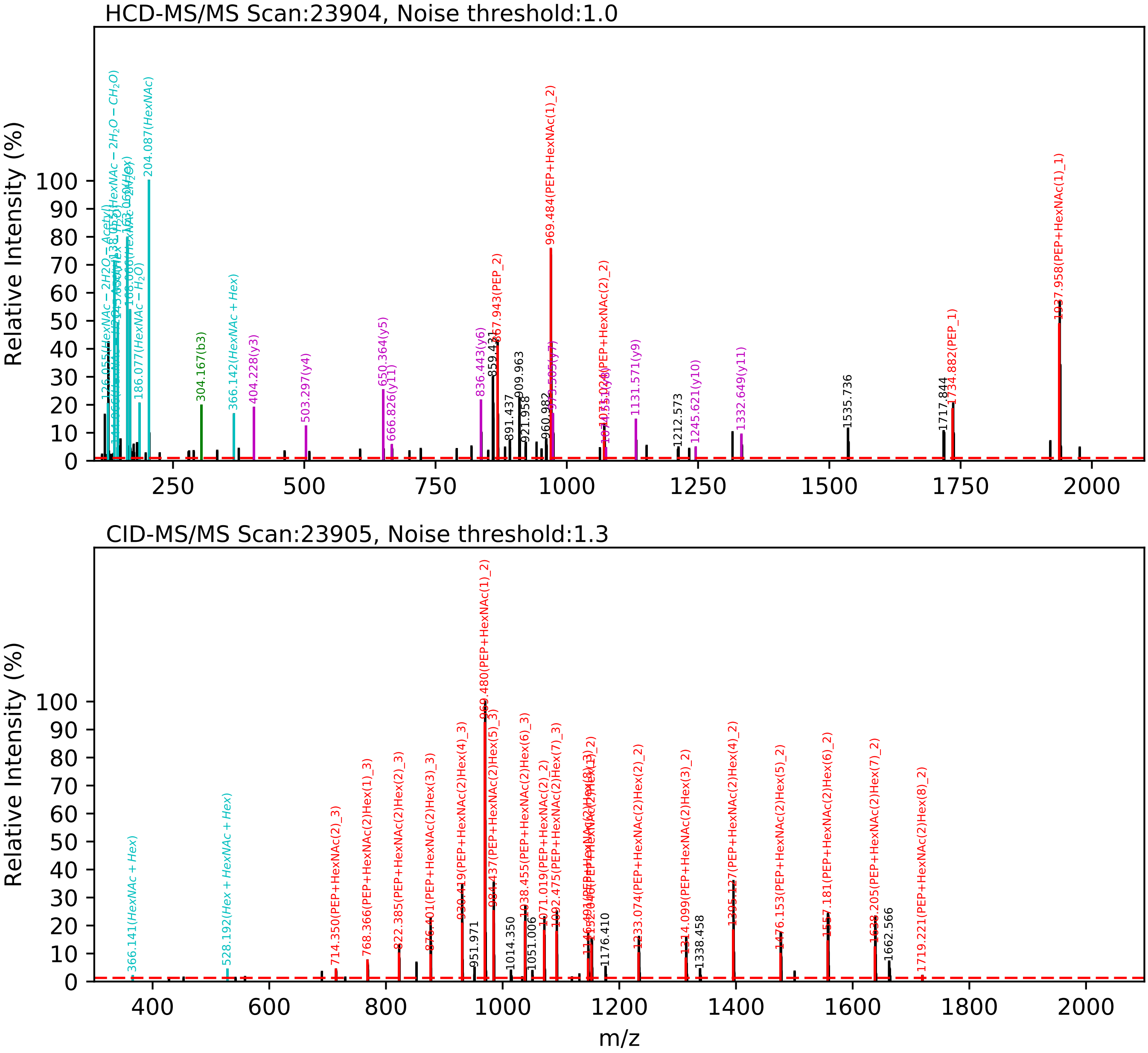

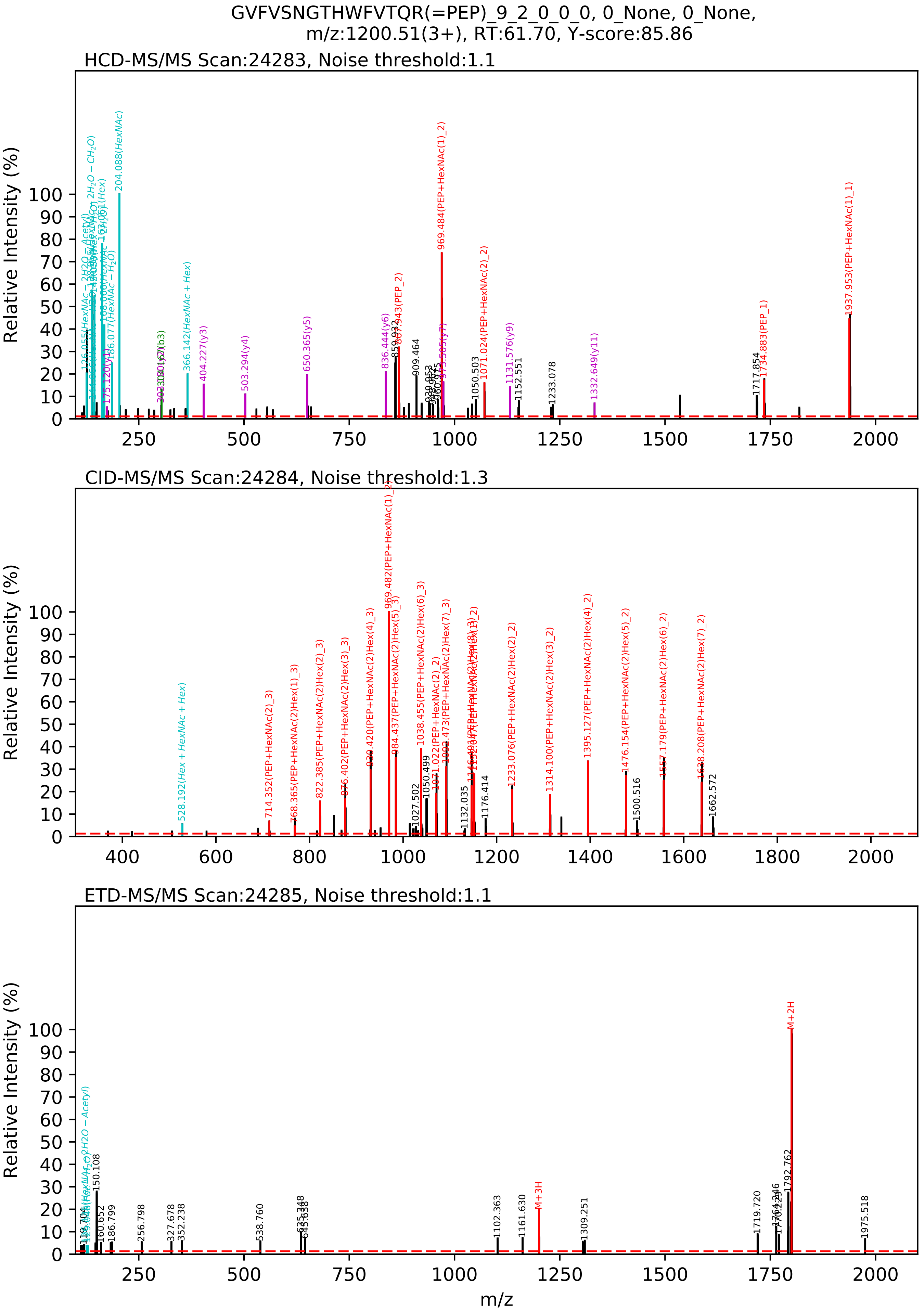

GVFVSNGTHWFVTQR(=PEP)_9_2_0_0_0, 0_None, 0_None,
m/z:1200.51(3+), RT:61.70, Y-score:85.86
HCD-MS/MS Scan:24283, Noise threshold:1.1
CID-MS/MS Scan:24284, Noise threshold:1.3
ETD-MS/MS Scan:24285, Noise threshold:1.1
Relative Intensity (%)
m/z
204.088(HexNAc)
969.484(PEP+HexNAc(1)_2)
1937.953(PEP+HexNAc(1)_1)
1071.024(PEP+HexNAc(2)_2)
1734.883(PEP_1)
867.943(PEP_2)
836.444(y6)
650.365(y5)
503.294(y4)
404.227(y3)
366.142(HexNAc + Hex)
1131.576(y9)
1332.649(y11)
1152.551
1233.078
1717.854
1050.503
909.464
859.932
969.482(PEP+HexNAc(1)_2)
984.437(PEP+HexNAc(2)Hex(5)_3)
1038.455(PEP+HexNAc(2)Hex(6)_3)
1395.127(PEP+HexNAc(2)Hex(4)_2)
1476.154(PEP+HexNAc(2)Hex(5)_2)
1557.179(PEP+HexNAc(2)Hex(6)_2)
1638.208(PEP+HexNAc(2)Hex(7)_2)
1314.100(PEP+HexNAc(2)Hex(3)_2)
1233.076(PEP+HexNAc(2)Hex(2)_2)
1071.022(PEP+HexNAc(2)_2)
930.420(PEP+HexNAc(2)Hex(4)_3)
876.402(PEP+HexNAc(2)Hex(3)_3)
822.385(PEP+HexNAc(2)Hex(2)_3)
768.365(PEP+HexNAc(2)Hex(1)_3)
714.352(PEP+HexNAc(2)_3)
528.192(Hex + HexNAc + Hex)
1662.572
1500.516
1176.414
1132.035
1050.499
1027.502
M+2H
M+3H
1792.762
1975.518
1719.720
1309.251
1161.630
1102.363
538.760
352.238
327.678
256.798
186.799
160.652
150.108

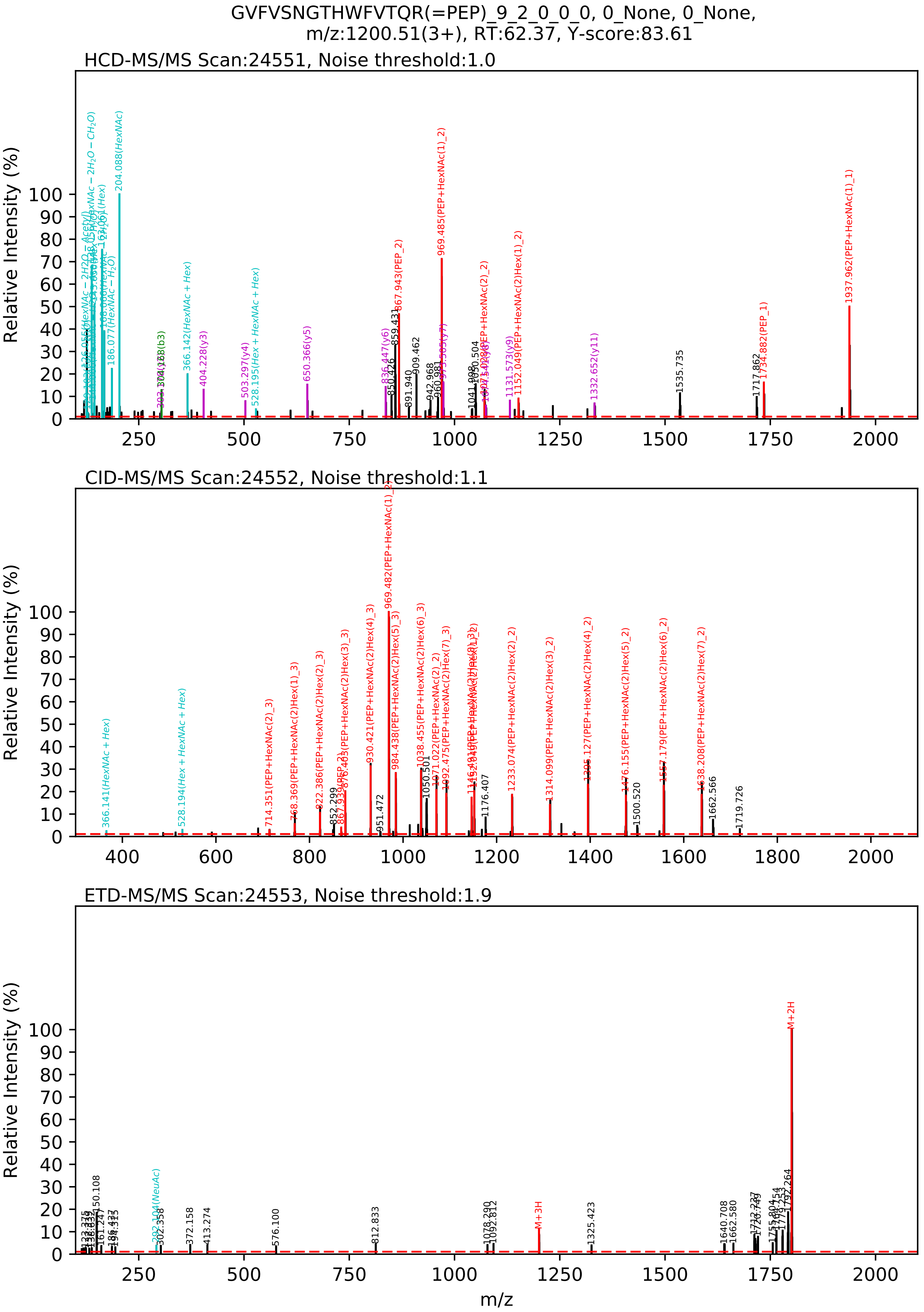

GVFVSNGTHWFVTQR(=PEP)_9_2_0_0_0, 0_None, 0_None,
m/z:1200.51(3+), RT:62.37, Y-score:83.61
HCD-MS/MS Scan:24551, Noise threshold:1.0
Relative Intensity (%)
204.088(HexNAc)
969.485(PEP+HexNAc(1)_2)
867.943(PEP_2)
1937.962(PEP+HexNAc(1)_1)
1734.882(PEP_1)
1152.049(PEP+HexNAc(2)Hex(1)_2)
366.142(HexNAc + Hex)
528.195(Hex + HexNAc + Hex)
650.366(y5)
404.228(y3)
503.297(y4)
1131.573(y9)
1332.652(y11)
1535.735
1717.862
859.431
909.462
CID-MS/MS Scan:24552, Noise threshold:1.1
969.482(PEP+HexNAc(1)_2)
930.421(PEP+HexNAc(2)Hex(4)_3)
984.438(PEP+HexNAc(2)Hex(5)_3)
1038.455(PEP+HexNAc(2)Hex(6)_3)
1092.475(PEP+HexNAc(2)Hex(7)_3)
1233.074(PEP+HexNAc(2)Hex(2)_2)
1314.099(PEP+HexNAc(2)Hex(3)_2)
1395.127(PEP+HexNAc(2)Hex(4)_2)
1476.155(PEP+HexNAc(2)Hex(5)_2)
1557.179(PEP+HexNAc(2)Hex(6)_2)
1638.208(PEP+HexNAc(2)Hex(7)_2)
714.351(PEP+HexNAc(2)_3)
768.369(PEP+HexNAc(2)Hex(1)_3)
822.386(PEP+HexNAc(2)Hex(2)_3)
876.403(PEP+HexNAc(2)Hex(3)_3)
366.141(HexNAc + Hex)
528.194(Hex + HexNAc + Hex)
1662.566
1719.726
1500.520
1176.407
951.472
852.299
ETD-MS/MS Scan:24553, Noise threshold:1.9
M+2H
M+3H
292.104(NeuAc)
302.358
372.158
413.274
576.100
812.833
1078.290
1092.812
1325.423
1640.708
1662.580
1792.264
150.108
161.247
m/z

GVFVSNGTHWFVTQR(=PEP)_9_2_0_0_0, 0_None, 0_None,
m/z:1200.51(3+), RT:62.94, Y-score:85.55

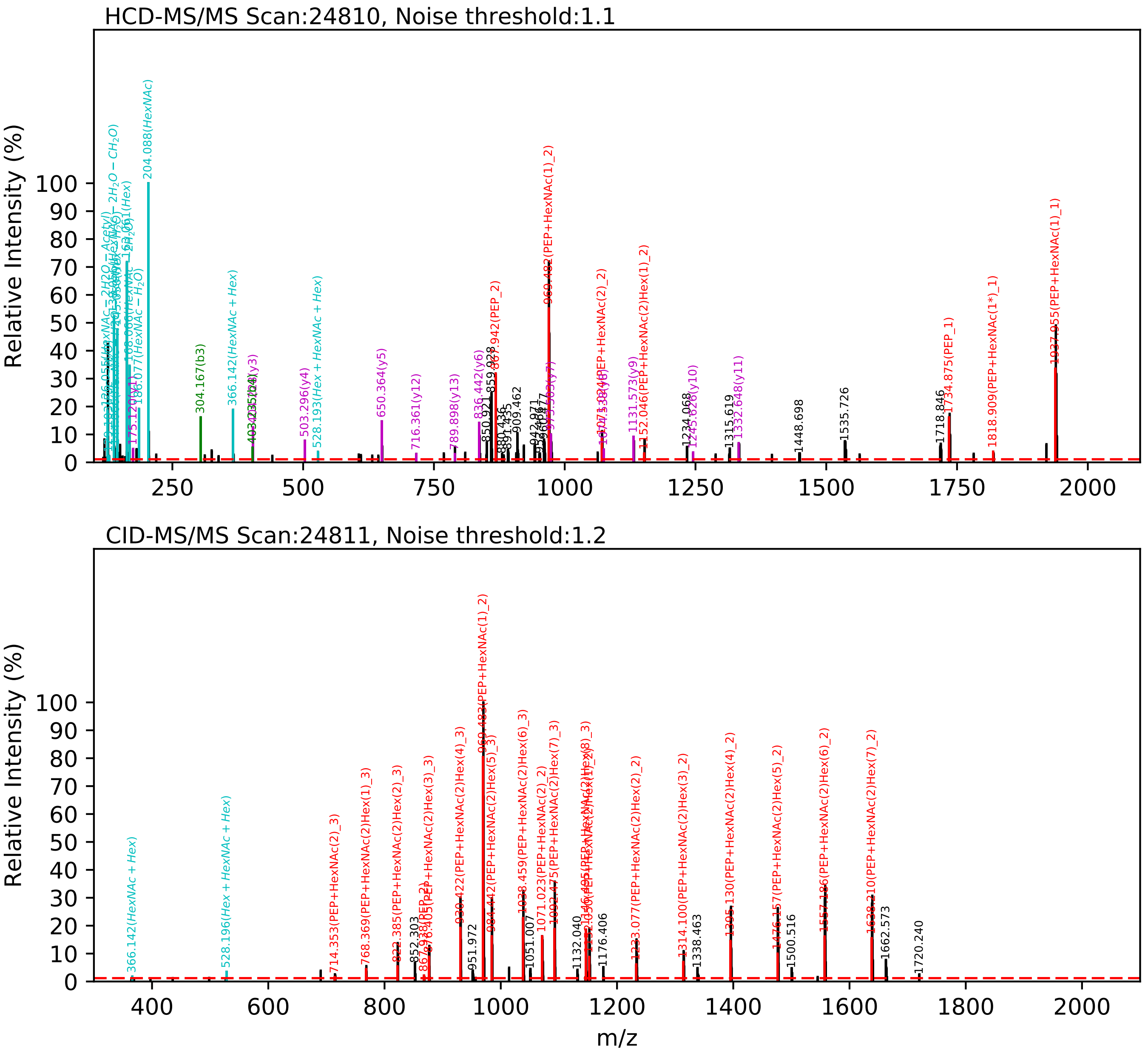

GVFVSNGTHWFVTQR(=PEP)_9_2_0_0_0, 0_None, 0_None,
m/z:1200.51(3+), RT:63.78, Y-score:81.23

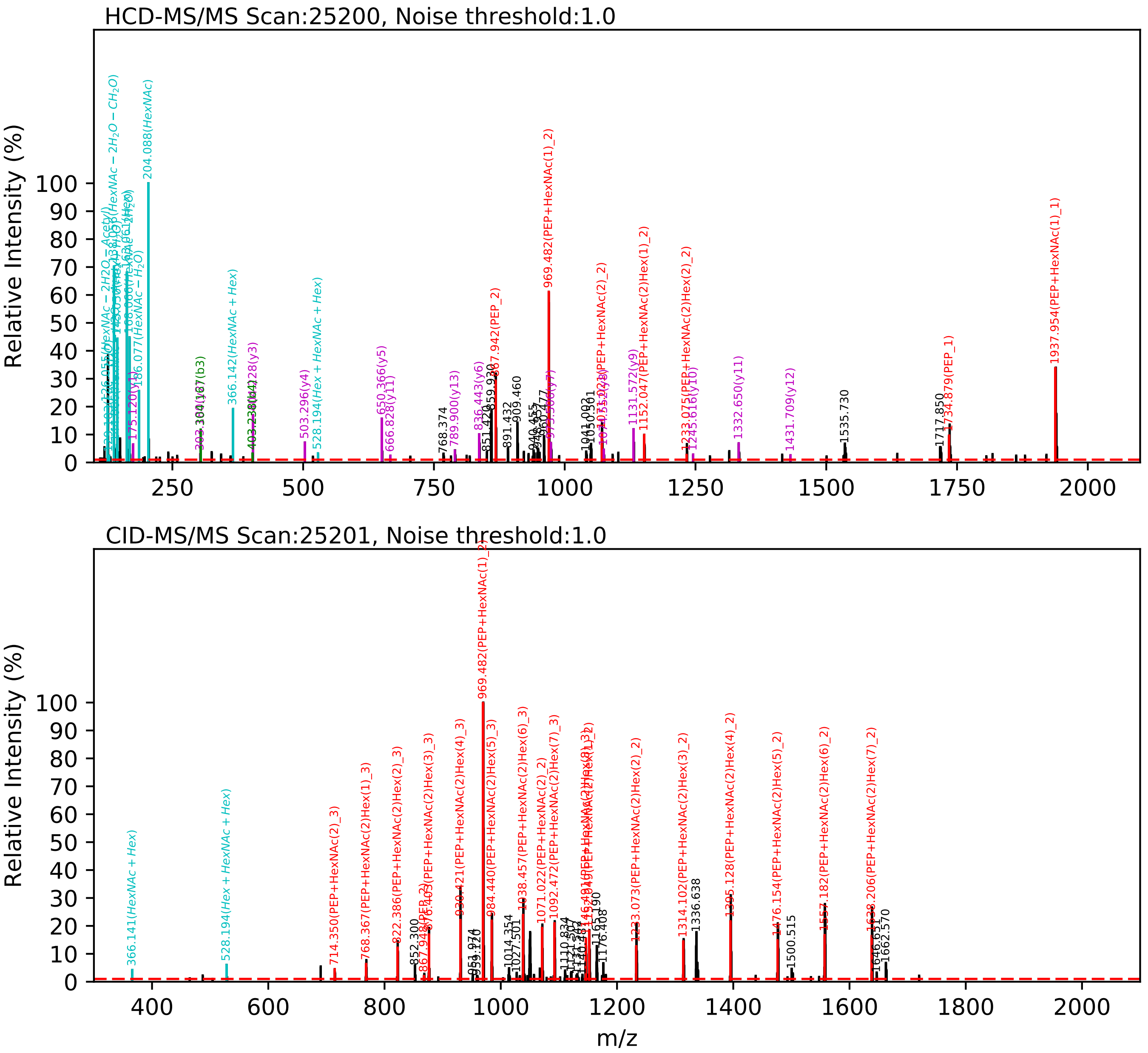

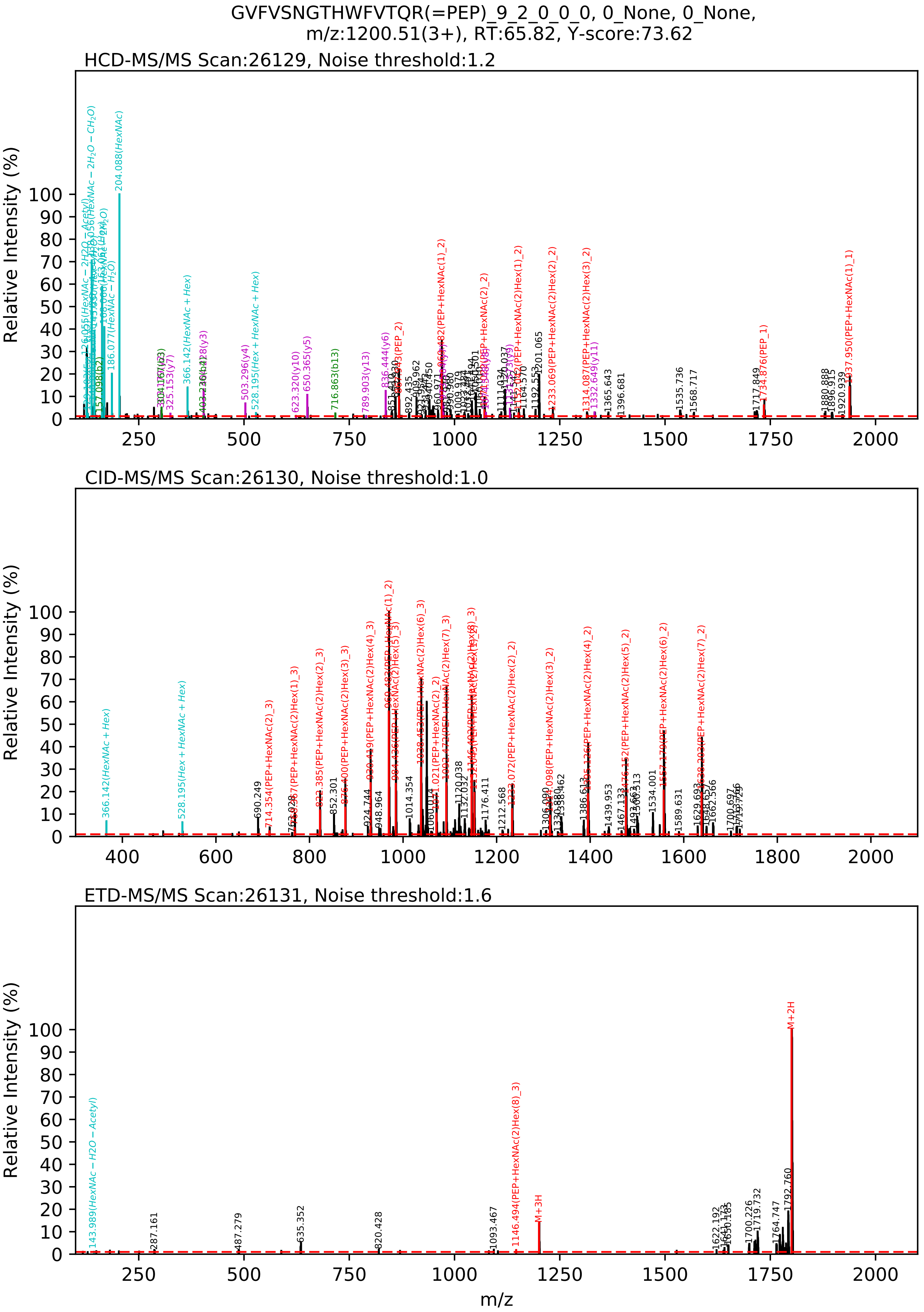
GVFVSNGTHWFVTQR(=PEP)_9_2_0_0_0, 0_None, 0_None,
m/z:1200.51(3+), RT:65.82, Y-score:73.62
HCD-MS/MS Scan:26129, Noise threshold:1.2
CID-MS/MS Scan:26130, Noise threshold:1.0
ETD-MS/MS Scan:26131, Noise threshold:1.6
Relative Intensity (%)
m/z
204.088(HexNAc)
366.142(HexNAc + Hex)
528.195(Hex + HexNAc + Hex)
1937.950(PEP+HexNAc(1)_1)
1734.876(PEP_1)
960.482(PEP+HexNAc(1)_2)
1146.494(PEP+HexNAc(2)Hex(8)_3)
M+3H
M+2H
143.989(HexNAc − H2O − Acetyl)

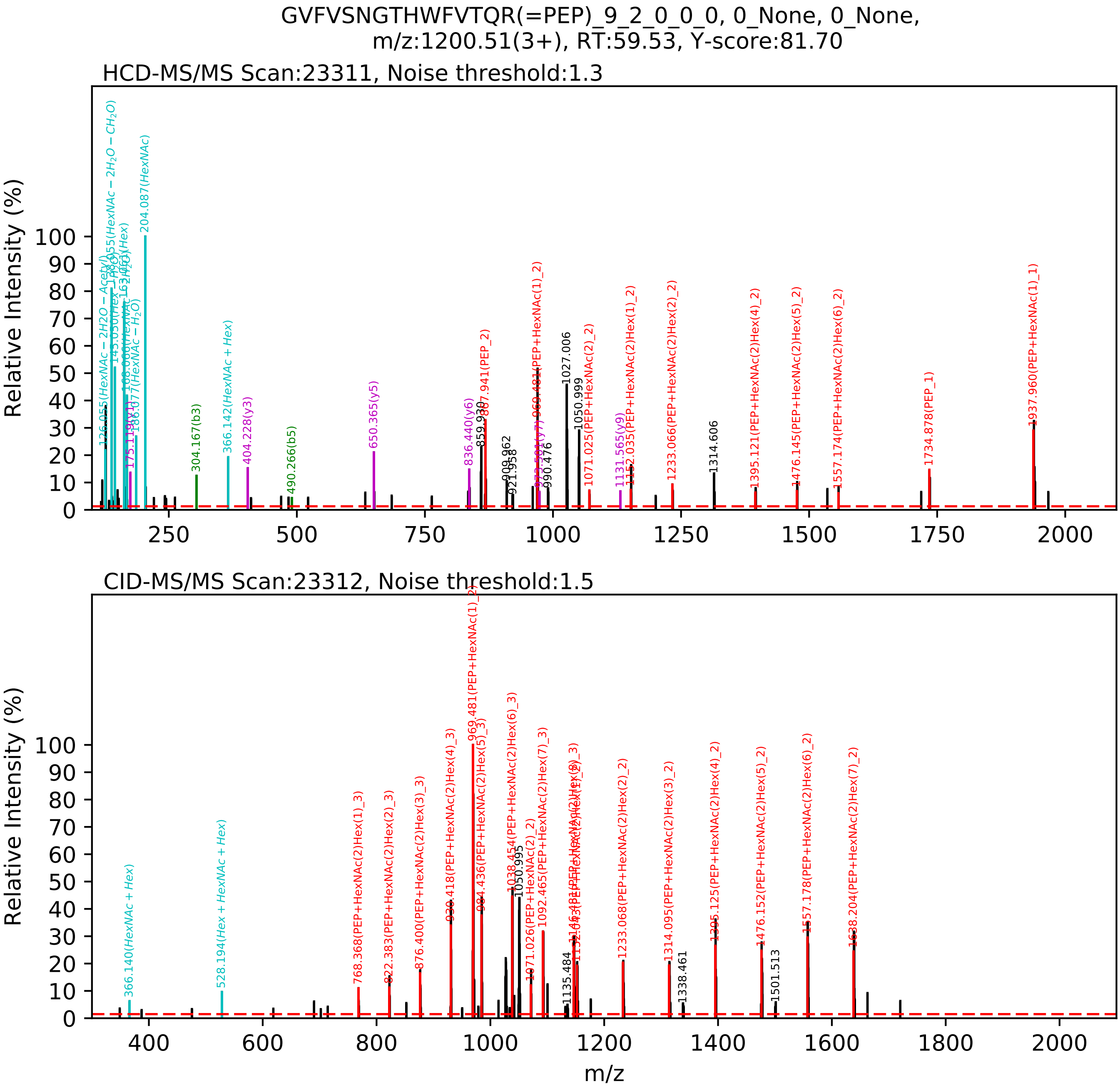
GVFVSNGTHWFVTQR(=PEP)_9_2_0_0_0, 0_None, 0_None,
m/z:1200.51(3+), RT:59.53, Y-score:81.70
HCD-MS/MS Scan:23311, Noise threshold:1.3
CID-MS/MS Scan:23312, Noise threshold:1.5
Relative Intensity (%)
m/z
204.087(HexNAc)
304.167(b3)
366.142(HexNAc + Hex)
404.228(y3)
490.266(b5)
650.365(y5)
836.440(y6)
867.941(PEP_2)
1027.006
1050.999
1071.025(PEP+HexNAc(2)_2)
1131.565(y9)
1152.035(PEP+HexNAc(2)Hex(1)_2)
1233.066(PEP+HexNAc(2)Hex(2)_2)
1314.606
1395.121(PEP+HexNAc(2)Hex(4)_2)
1476.145(PEP+HexNAc(2)Hex(5)_2)
1557.174(PEP+HexNAc(2)Hex(6)_2)
1734.878(PEP_1)
1937.960(PEP+HexNAc(1)_1)
366.140(HexNAc + Hex)
528.194(Hex + HexNAc + Hex)
768.368(PEP+HexNAc(2)Hex(1)_3)
822.383(PEP+HexNAc(2)Hex(2)_3)
876.400(PEP+HexNAc(2)Hex(3)_3)
930.418(PEP+HexNAc(2)Hex(4)_3)
969.481(PEP+HexNAc(1)_2)
984.436(PEP+HexNAc(2)Hex(5)_3)
1038.454(PEP+HexNAc(2)Hex(6)_3)
1071.026(PEP+HexNAc(2)_2)
1092.465(PEP+HexNAc(2)Hex(7)_3)
1135.484
1233.068(PEP+HexNAc(2)Hex(2)_2)
1314.095(PEP+HexNAc(2)Hex(3)_2)
1338.461
1476.152(PEP+HexNAc(2)Hex(5)_2)
1501.513
1557.178(PEP+HexNAc(2)Hex(6)_2)
1638.204(PEP+HexNAc(2)Hex(7)_2)

GVFVSNGTHWFVTQR(=PEP)_9_2_0_0_0, 0_None, 0_None,
m/z:1200.51(3+), RT:59.67, Y-score:80.04

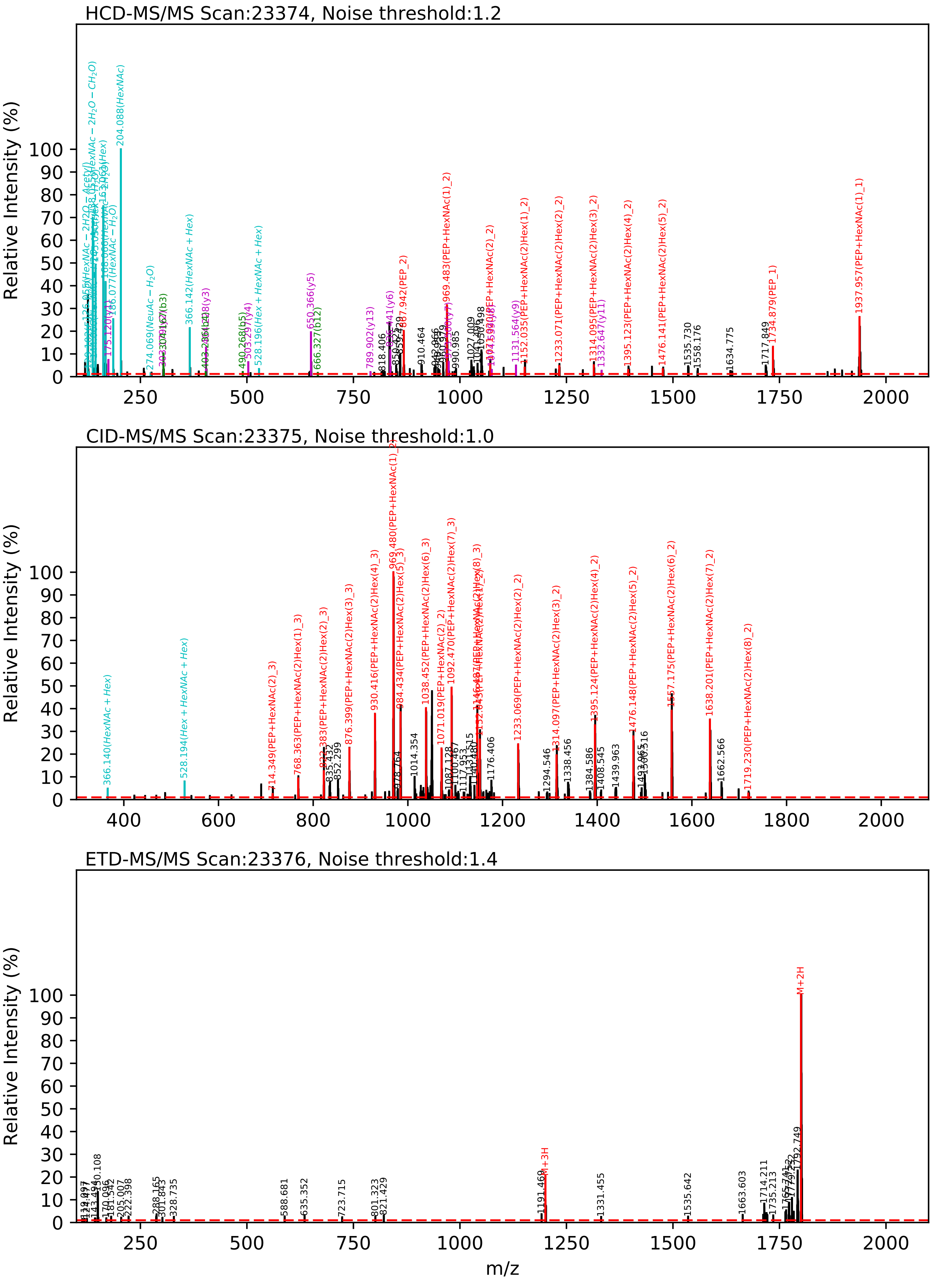

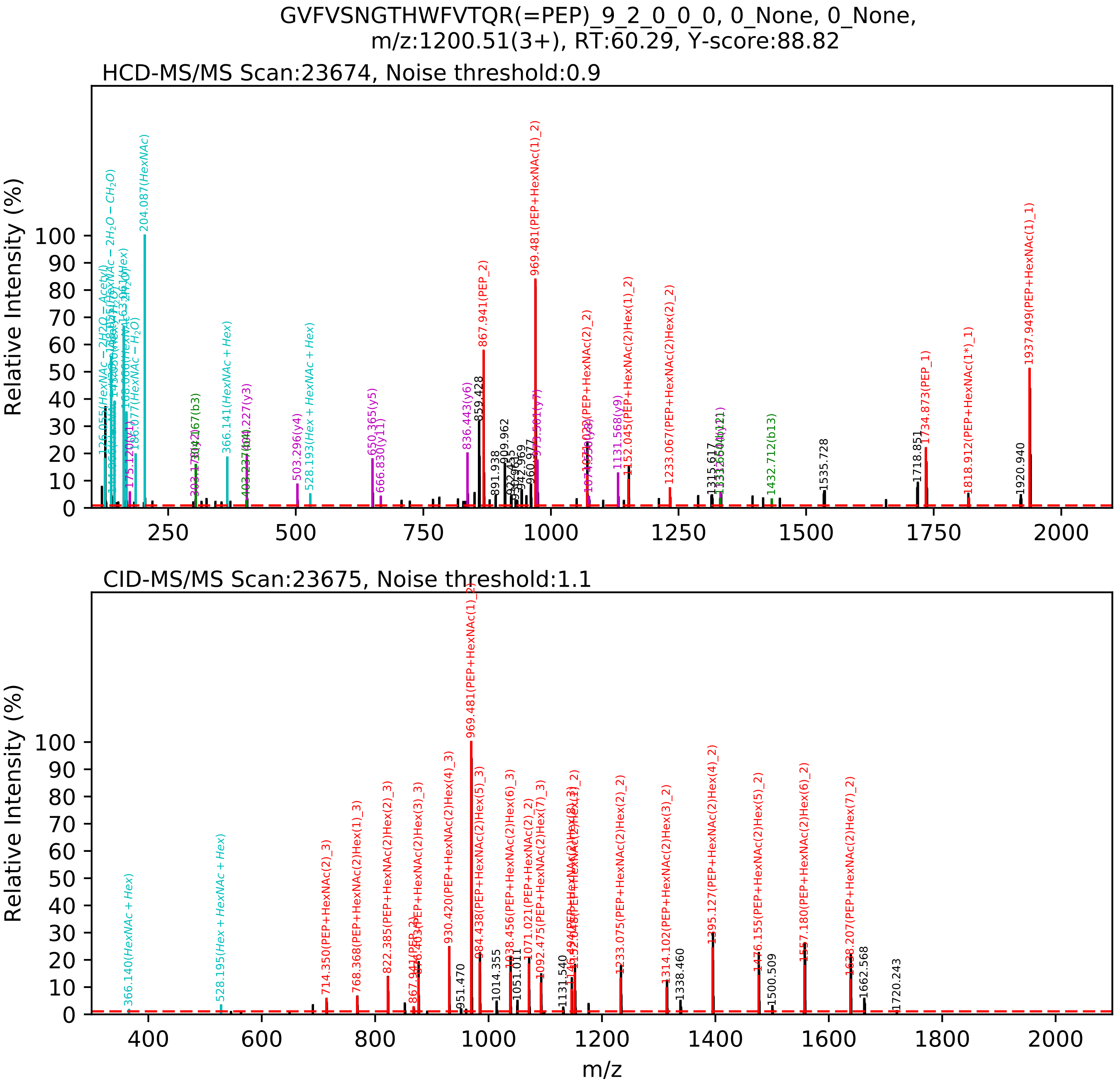

GVFVSNGTHWFVTQR(=PEP)_9_2_0_0_0, 0_None, 0_None,
m/z:1200.51(3+), RT:60.29, Y-score:88.82
HCD-MS/MS Scan:23674, Noise threshold:0.9
Relative Intensity (%)
CID-MS/MS Scan:23675, Noise threshold:1.1
Relative Intensity (%)
m/z

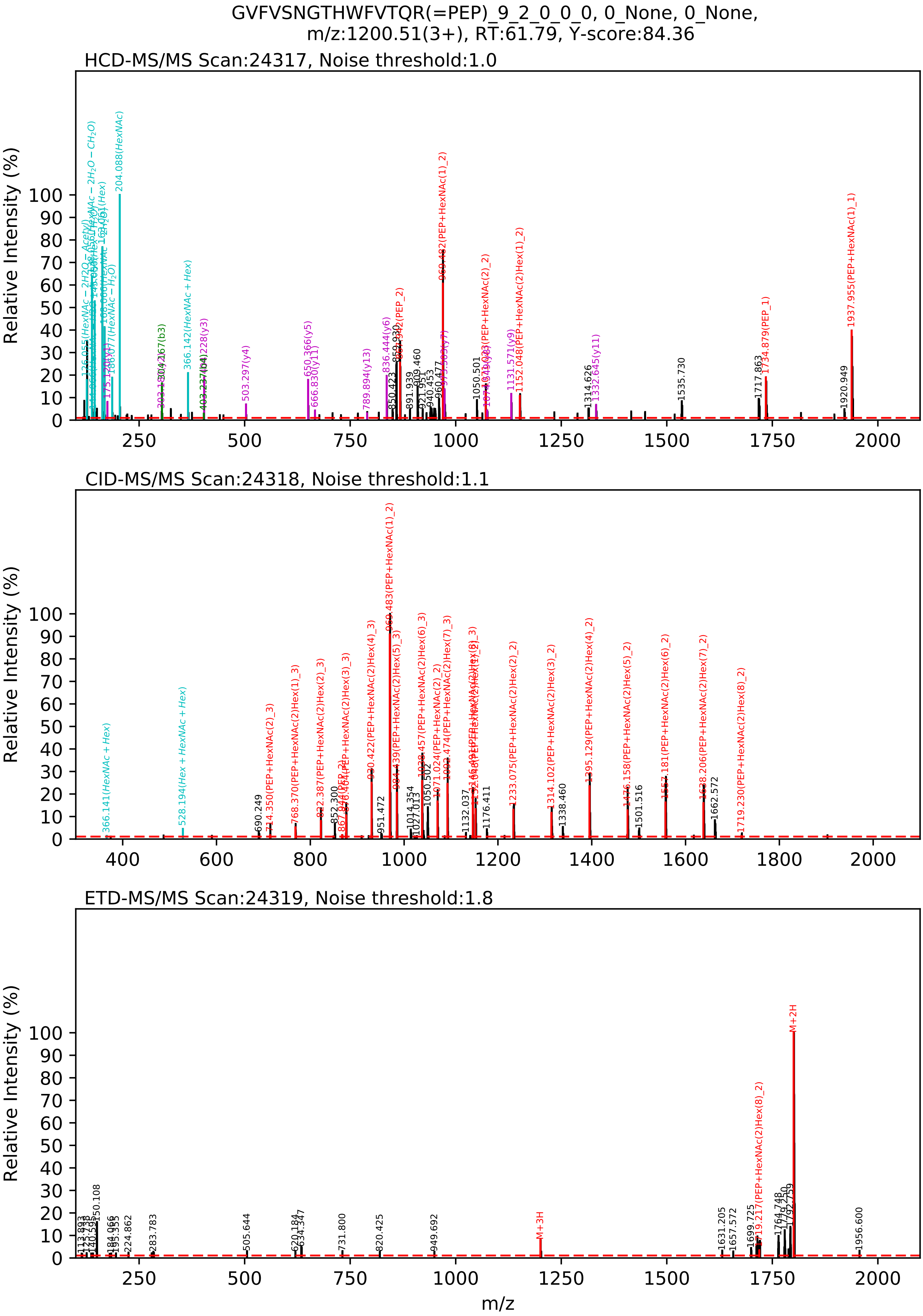
GVFVSNGTHWFVTQR(=PEP)_9_2_0_0_0, 0_None, 0_None,
m/z:1200.51(3+), RT:61.79, Y-score:84.36
HCD-MS/MS Scan:24317, Noise threshold:1.0
CID-MS/MS Scan:24318, Noise threshold:1.1
ETD-MS/MS Scan:24319, Noise threshold:1.8
Relative Intensity (%)
m/z
204.088(HexNAc)
366.142(HexNAc + Hex)
969.482(PEP+HexNAc(1)_2)
1152.048(PEP+HexNAc(2)Hex(1)_2)
1734.879(PEP_1)
1937.955(PEP+HexNAc(1)_1)
969.483(PEP+HexNAc(1)_2)
528.194(Hex + HexNAc + Hex)
1395.129(PEP+HexNAc(2)Hex(4)_2)
1557.181(PEP+HexNAc(2)Hex(6)_2)
1638.206(PEP+HexNAc(2)Hex(7)_2)
1719.230(PEP+HexNAc(2)Hex(8)_2)
M+2H
M+3H
1719.217(PEP+HexNAc(2)Hex(8)_2)

GVFVSNGTHWFVTQR(=PEP)_9_2_0_0_0, 0_None, 0_None,
m/z:1200.51(3+), RT:61.25, Y-score:77.58

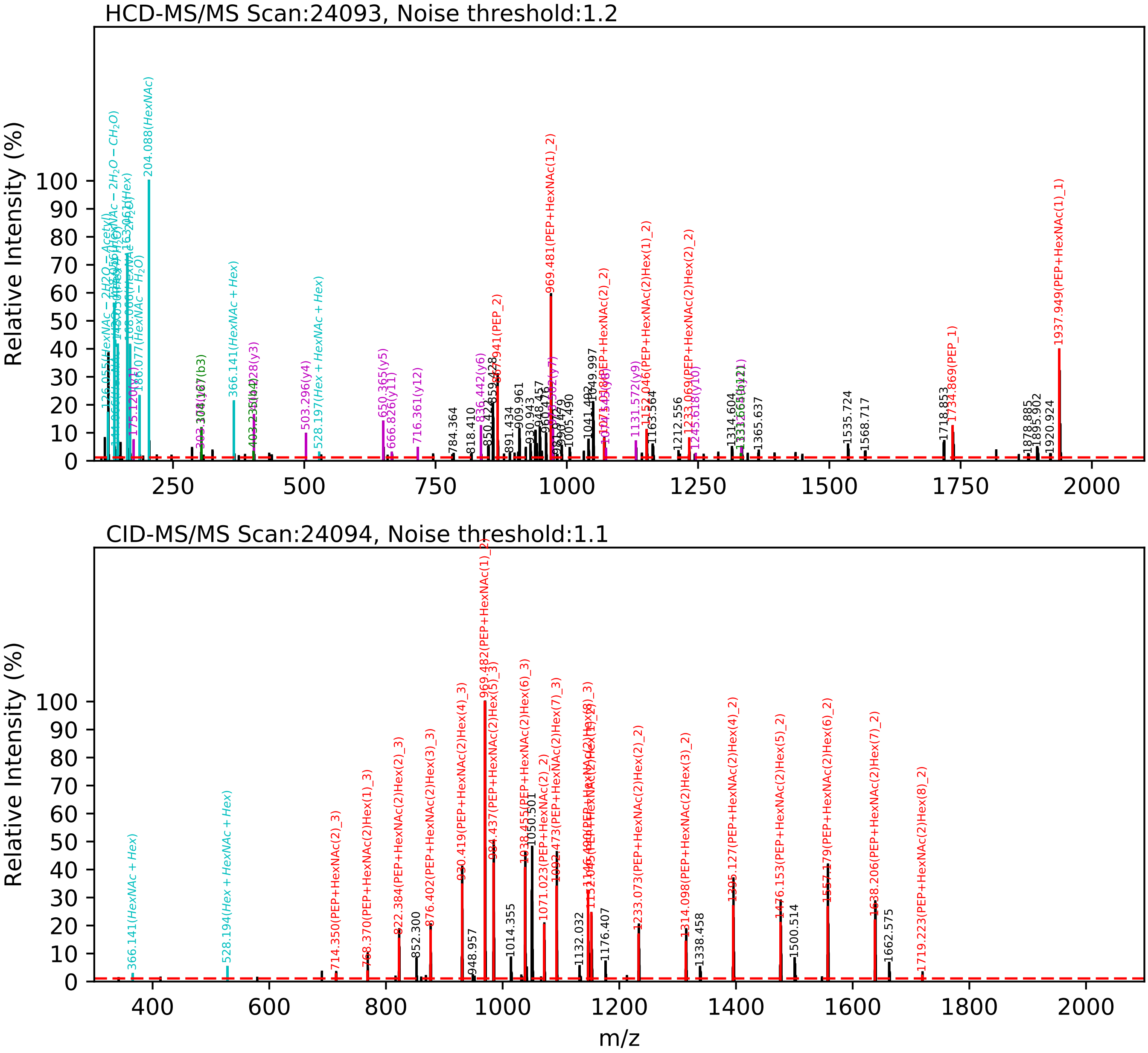

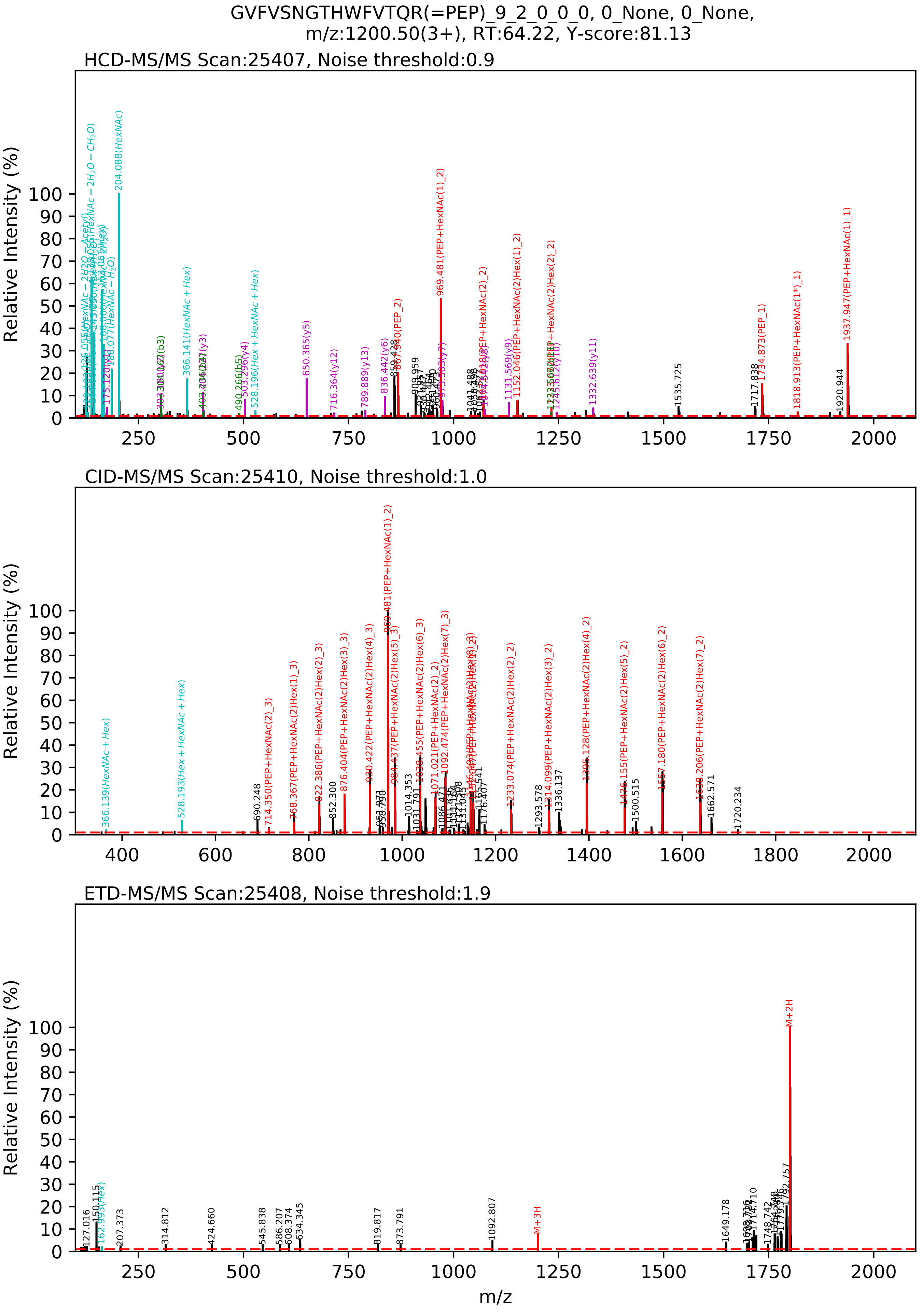

GVFVSNGTHWFVTQR(=PEP)_9_2_0_0_0, 0_None, 0_None,
m/z:1200.50(3+), RT:64.22, Y-score:81.13
HCD-MS/MS Scan:25407, Noise threshold:0.9
204.088(HexNAc)
366.141(HexNAc + Hex)
528.196(Hex + HexNAc + Hex)
650.365(y5)
716.364(y12)
789.889(y13)
836.442(y6)
859.428
867.940(PEP_2)
909.959
969.481(PEP+HexNAc(1)_2)
1131.569(y9)
1152.046(PEP+HexNAc(2)Hex(1)_2)
1332.639(y11)
1535.725
1717.838
1734.873(PEP_1)
1818.913(PEP+HexNAc(1*)_1)
1920.944
1937.947(PEP+HexNAc(1)_1)
CID-MS/MS Scan:25410, Noise threshold:1.0
366.139(HexNAc + Hex)
528.193(Hex + HexNAc + Hex)
690.248
714.350(PEP+HexNAc(2)_3)
768.367(PEP+HexNAc(2)Hex(1)_3)
822.386(PEP+HexNAc(2)Hex(2)_3)
852.300
876.404(PEP+HexNAc(2)Hex(3)_3)
930.422(PEP+HexNAc(2)Hex(4)_3)
969.481(PEP+HexNAc(1)_2)
984.437(PEP+HexNAc(2)Hex(5)_3)
1014.353
1038.455(PEP+HexNAc(2)Hex(6)_3)
1071.021(PEP+HexNAc(2)_2)
1092.474(PEP+HexNAc(2)Hex(7)_3)
1165.541
1176.407
1233.074(PEP+HexNAc(2)Hex(2)_2)
1293.578
1314.099(PEP+HexNAc(2)Hex(3)_2)
1336.137
1395.128(PEP+HexNAc(2)Hex(4)_2)
1476.155(PEP+HexNAc(2)Hex(5)_2)
1500.515
1557.180(PEP+HexNAc(2)Hex(6)_2)
1638.206(PEP+HexNAc(2)Hex(7)_2)
1662.571
1720.234
ETD-MS/MS Scan:25408, Noise threshold:1.9
127.016
150.115
162.993(Hex)
207.373
314.812
424.660
545.838
586.207
608.374
634.345
819.817
873.791
1092.807
M+3H
1649.178
1714.710
1748.742
1792.757
M+2H
Relative Intensity (%)
m/z

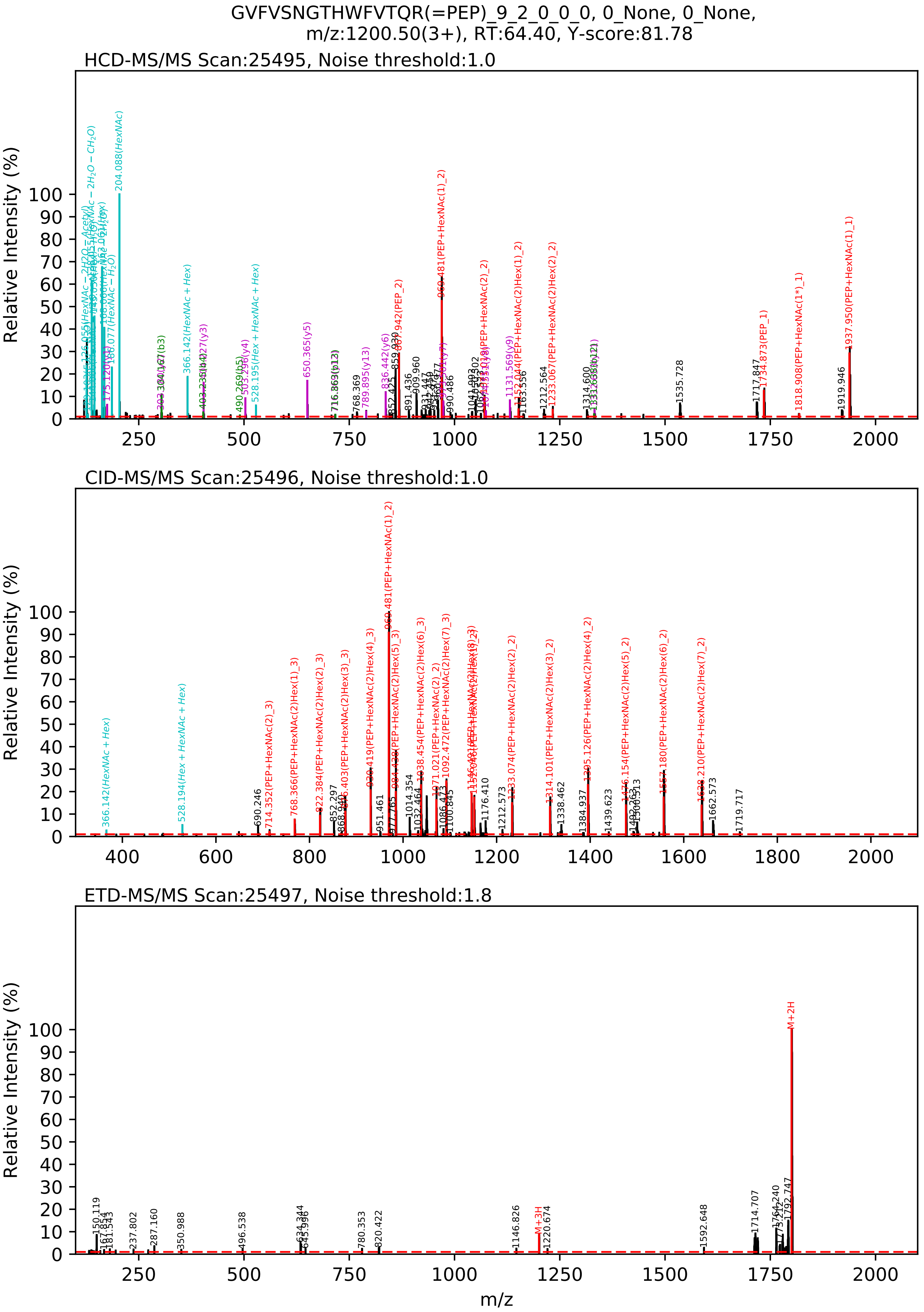
GVFVSNGTHWFVTQR(=PEP)_9_2_0_0_0, 0_None, 0_None,
m/z:1200.50(3+), RT:64.40, Y-score:81.78
HCD-MS/MS Scan:25495, Noise threshold:1.0
CID-MS/MS Scan:25496, Noise threshold:1.0
ETD-MS/MS Scan:25497, Noise threshold:1.8
Relative Intensity (%)
m/z

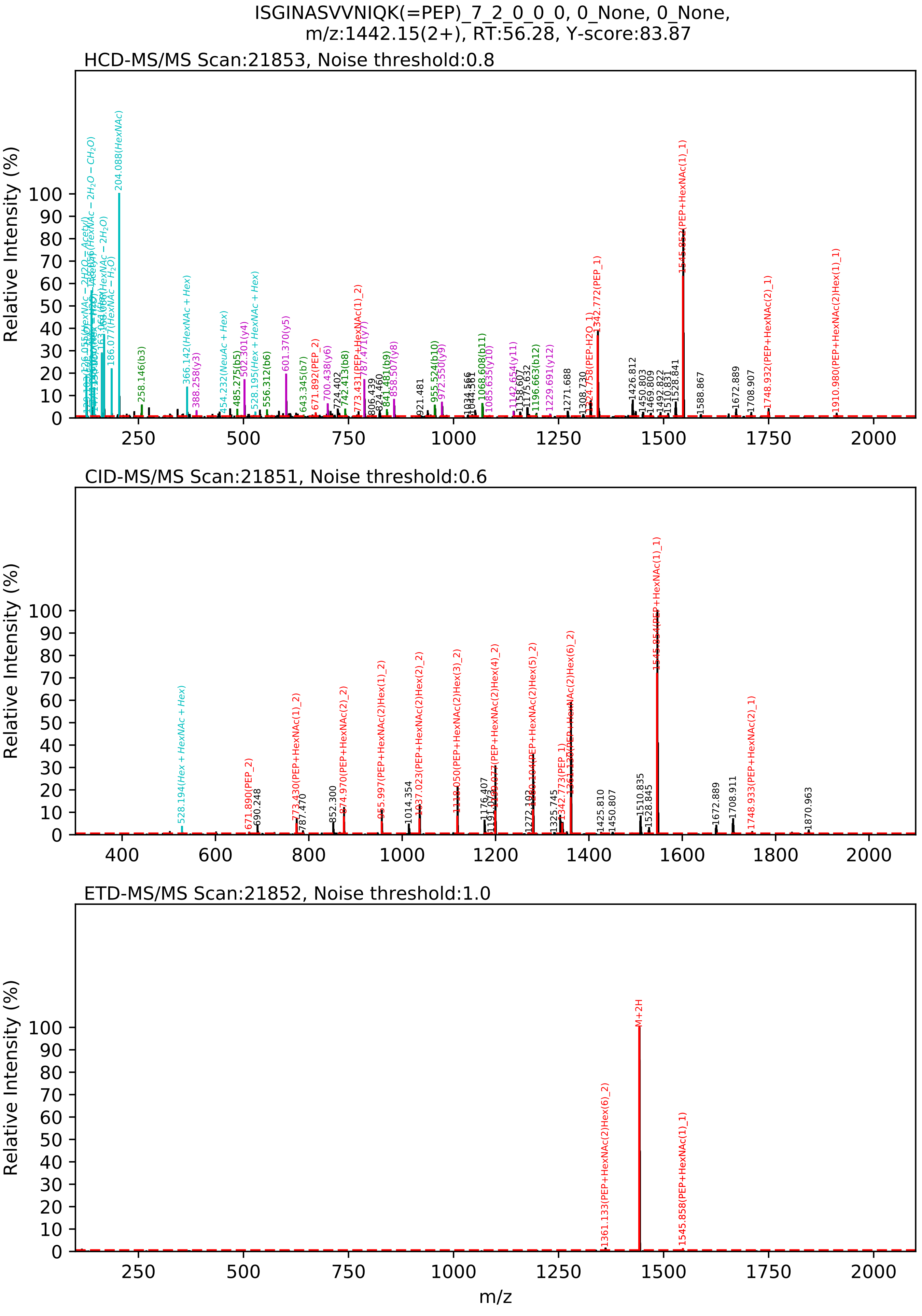
ISGINASVVNIQK(=PEP)_7_2_0_0_0, 0_None, 0_None,
m/z:1442.15(2+), RT:56.28, Y-score:83.87
HCD-MS/MS Scan:21853, Noise threshold:0.8
Relative Intensity (%)
204.088(HexNAc)
186.077(HexNAc − H2O)
258.146(b3)
366.142(HexNAc + Hex)
388.258(y3)
454.232(NeuAc + Hex)
485.275(b5)
502.301(y4)
528.195(Hex + HexNAc + Hex)
556.312(b6)
601.370(y5)
643.345(b7)
671.892(PEP_2)
700.438(y6)
724.402
742.413(b8)
773.431(PEP+HexNAc(1)_2)
787.471(y7)
806.439
824.460
841.481(b9)
858.507(y8)
921.481
955.524(b10)
972.550(y9)
1068.608(b11)
1085.635(y10)
1142.654(y11)
1175.632
1196.663(b12)
1229.691(y12)
1271.688
1308.730
1342.772(PEP_1)
1426.812
1450.803
1469.809
1510.831
1528.841
1545.853(PEP+HexNAc(1)_1)
1588.867
1672.889
1708.907
1748.932(PEP+HexNAc(2)_1)
1910.980(PEP+HexNAc(2)Hex(1)_1)
CID-MS/MS Scan:21851, Noise threshold:0.6
528.194(Hex + HexNAc + Hex)
671.890(PEP_2)
690.248
773.430(PEP+HexNAc(1)_2)
787.470
852.300
874.970(PEP+HexNAc(2)_2)
955.997(PEP+HexNAc(2)Hex(1)_2)
1014.354
1037.023(PEP+HexNAc(2)Hex(2)_2)
1118.050(PEP+HexNAc(2)Hex(3)_2)
1176.407
1199.077(PEP+HexNAc(2)Hex(4)_2)
1272.102
1280.104(PEP+HexNAc(2)Hex(5)_2)
1325.745
1342.773(PEP_1)
1425.810
1450.807
1510.835
1528.845
1545.854(PEP+HexNAc(1)_1)
1672.889
1708.911
1748.933(PEP+HexNAc(2)_1)
1870.963
ETD-MS/MS Scan:21852, Noise threshold:1.0
M+2H
1361.133(PEP+HexNAc(2)Hex(6)_2)
1545.858(PEP+HexNAc(1)_1)
m/z

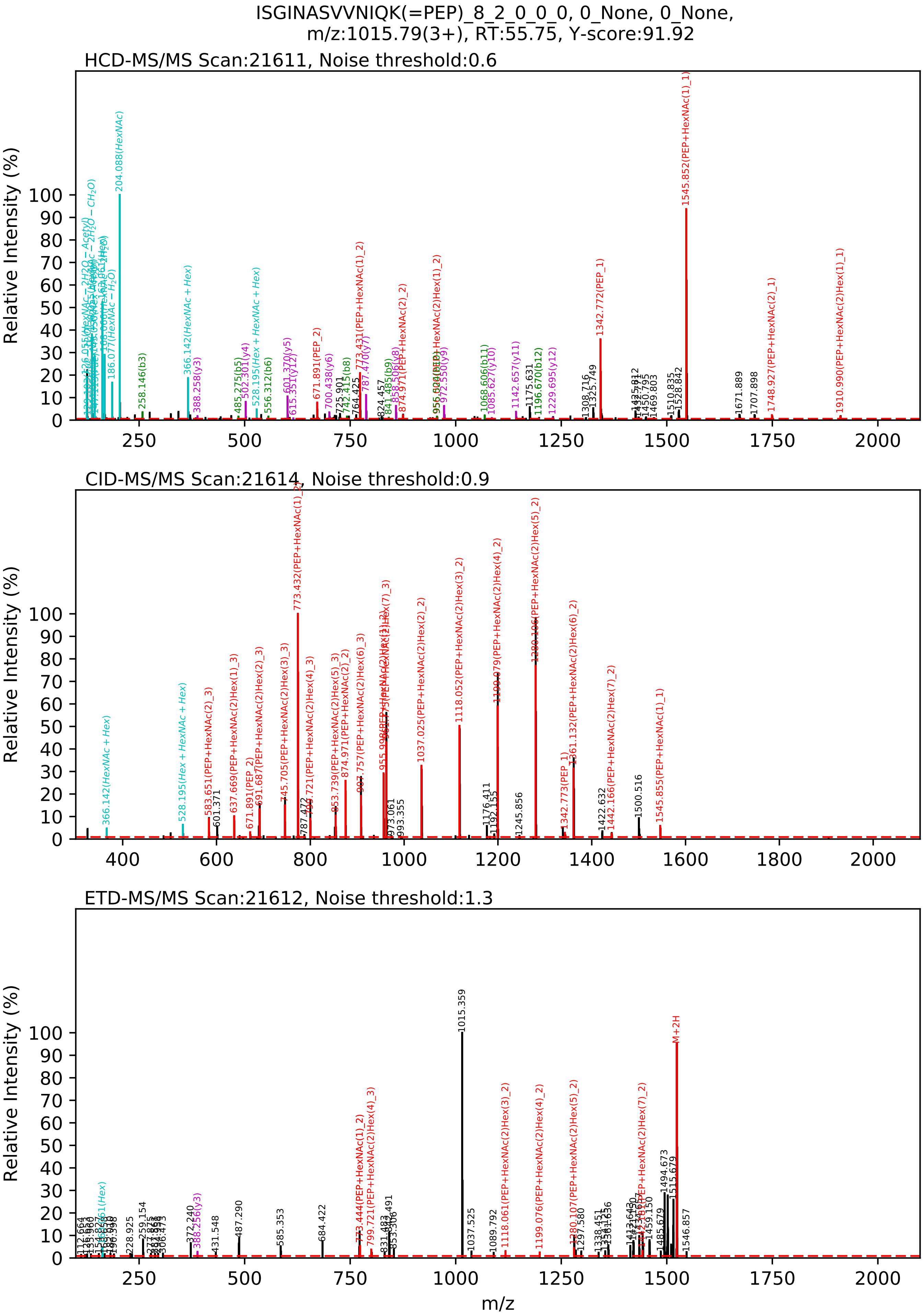

ISGINASVVNIQK(=PEP)_8_2_0_0_0, 0_None, 0_None,
m/z:1015.79(3+), RT:55.75, Y-score:91.92
HCD-MS/MS Scan:21611, Noise threshold:0.6
CID-MS/MS Scan:21614, Noise threshold:0.9
ETD-MS/MS Scan:21612, Noise threshold:1.3
Relative Intensity (%)
m/z
204.088(HexNAc)
1545.852(PEP+HexNAc(1)_1)
1342.772(PEP_1)
773.432(PEP+HexNAc(1)_2)
1280.106(PEP+HexNAc(2)Hex(5)_2)
1199.979(PEP+HexNAc(2)Hex(4)_2)
1118.052(PEP+HexNAc(2)Hex(3)_2)
1015.359
M+2H

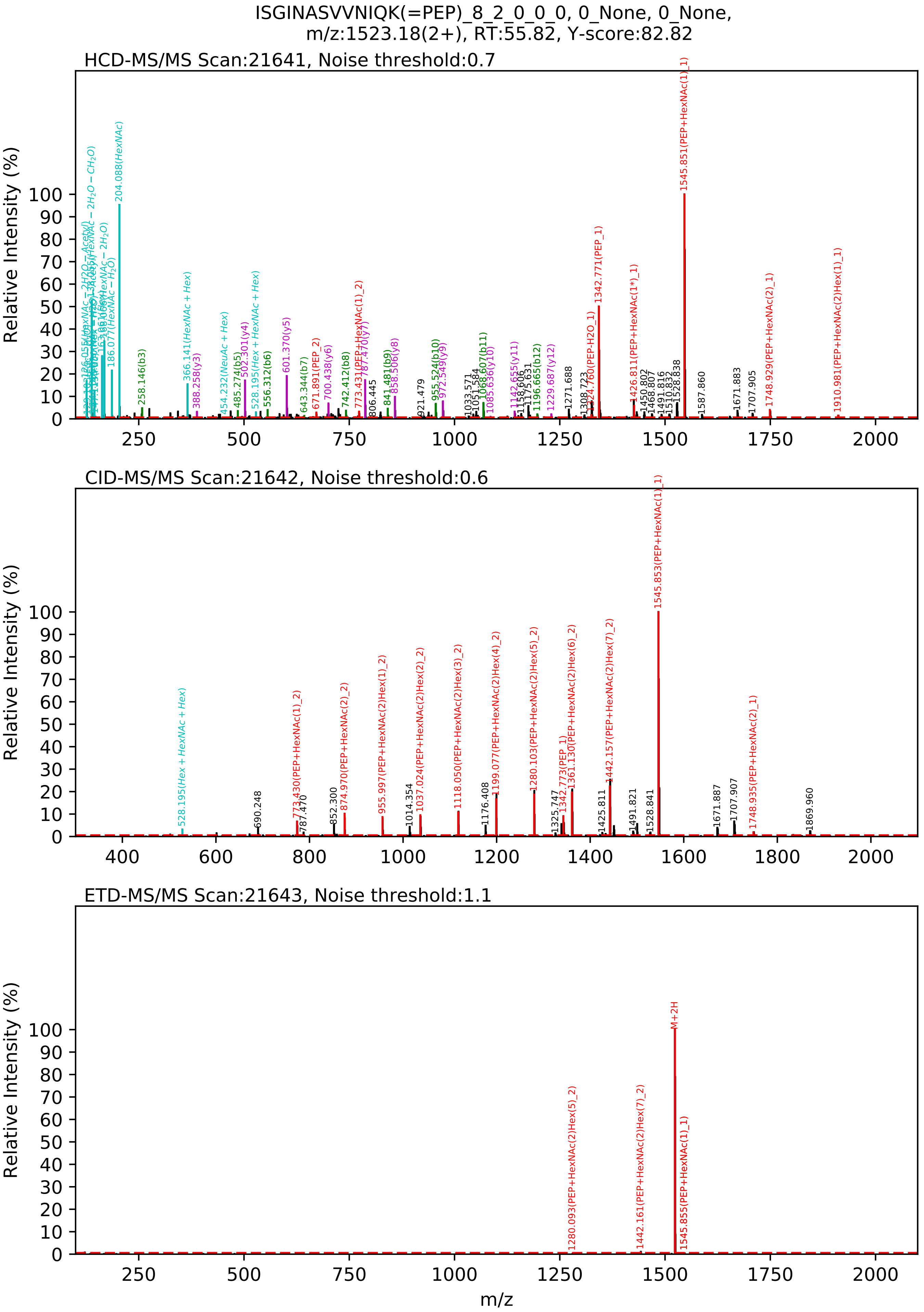
ISGINASVVNIQK(=PEP)_8_2_0_0_0, 0_None, 0_None,
m/z:1523.18(2+), RT:55.82, Y-score:82.82
HCD-MS/MS Scan:21641, Noise threshold:0.7
CID-MS/MS Scan:21642, Noise threshold:0.6
ETD-MS/MS Scan:21643, Noise threshold:1.1
Relative Intensity (%)
m/z
1545.851(PEP+HexNAc(1)_1)
1342.771(PEP_1)
204.088(HexNAc)
1545.853(PEP+HexNAc(1)_1)
M+2H
1545.855(PEP+HexNAc(1)_1)
1280.093(PEP+HexNAc(2)Hex(5)_2)
1442.161(PEP+HexNAc(2)Hex(7)_2)

ISGINASVVNIQK(=PEP)_9_2_0_0_0, 0_None, 0_None,
m/z:1604.21(2+), RT:55.68, Y-score:84.95

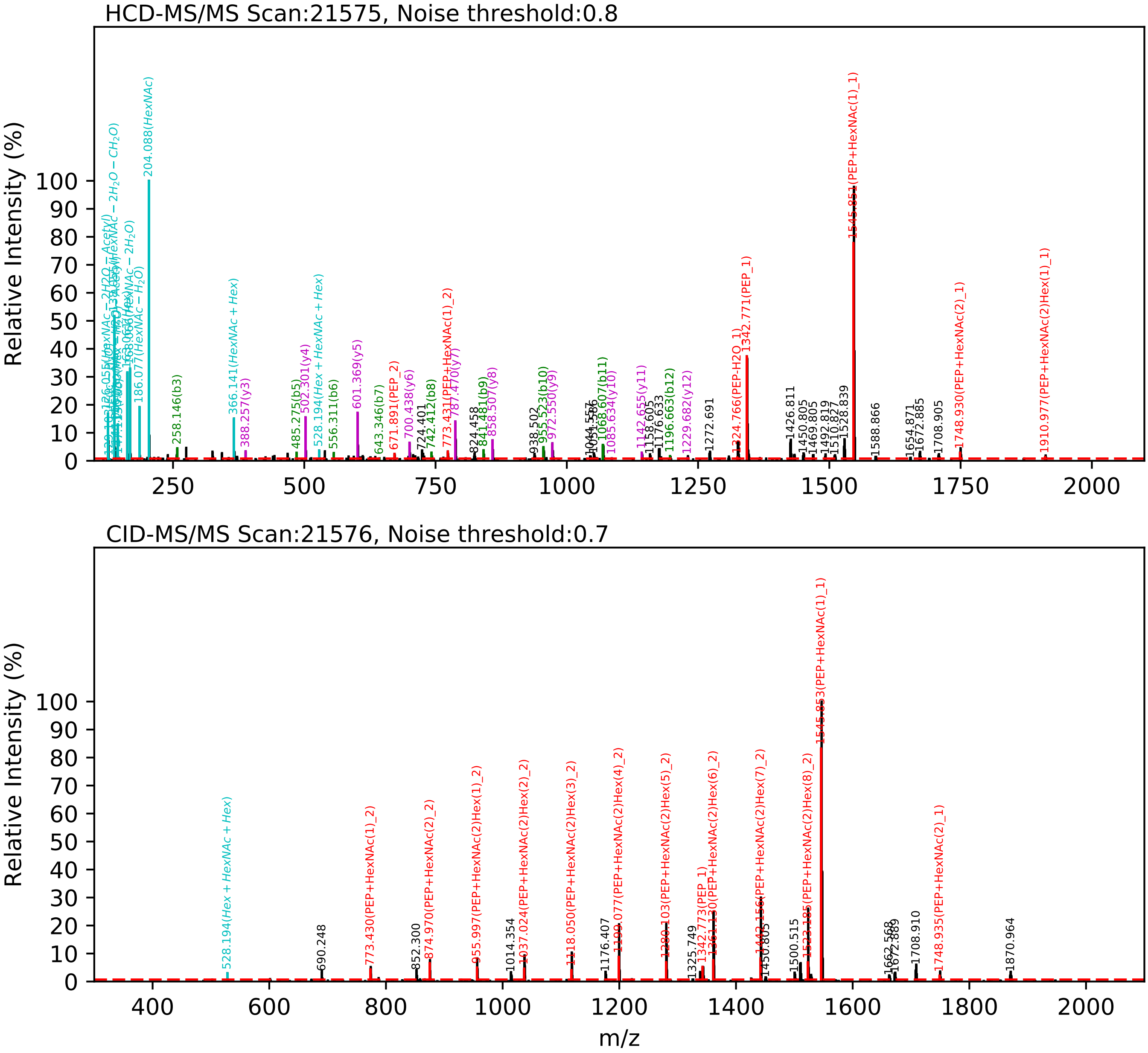

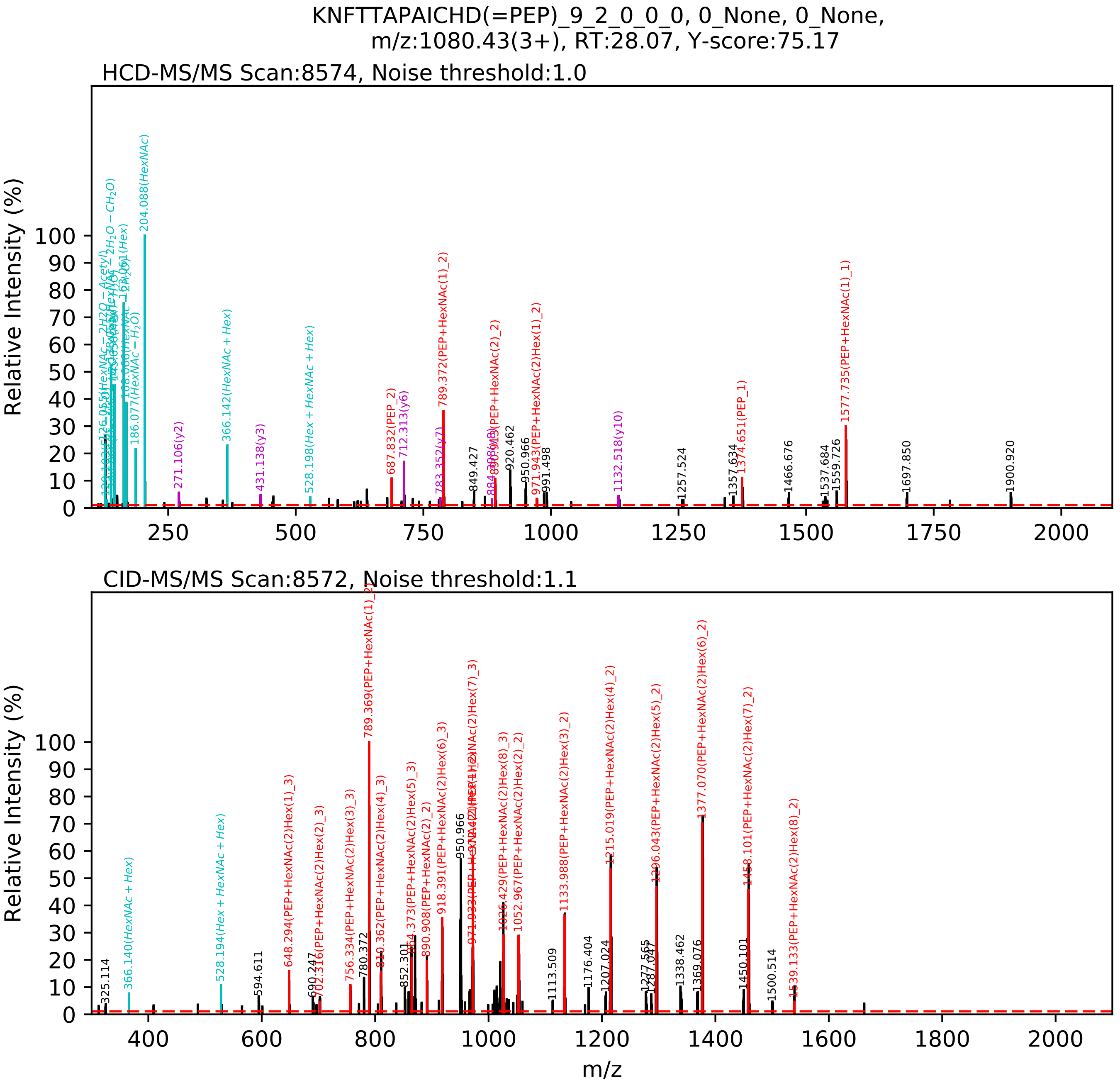

KNFTTAPAICHD(=PEP)_9_2_0_0_0, 0_None, 0_None,
m/z:1080.43(3+), RT:28.07, Y-score:75.17
HCD-MS/MS Scan:8574, Noise threshold:1.0
Relative Intensity (%)
204.088(HexNAc)
366.142(HexNAc + Hex)
528.198(Hex + HexNAc + Hex)
186.077(HexNAc − H2O)
271.106(y2)
431.138(y3)
687.832(PEP_2)
712.313(y6)
783.352(y7)
789.372(PEP+HexNAc(1)_2)
849.427
920.462
950.966
971.943(PEP+HexNAc(2)Hex(1)_2)
991.498
1132.518(y10)
1257.524
1357.634
1374.651(PEP_1)
1466.676
1537.684
1559.726
1577.735(PEP+HexNAc(1)_1)
1697.850
1900.920
CID-MS/MS Scan:8572, Noise threshold:1.1
325.114
366.140(HexNAc + Hex)
528.194(Hex + HexNAc + Hex)
594.611
648.294(PEP+HexNAc(2)Hex(1)_3)
690.247
702.316(PEP+HexNAc(2)Hex(2)_3)
756.334(PEP+HexNAc(2)Hex(3)_3)
780.372
789.369(PEP+HexNAc(1)_2)
810.362(PEP+HexNAc(2)Hex(4)_3)
852.301
864.373(PEP+HexNAc(2)Hex(5)_3)
890.908(PEP+HexNAc(2)_2)
918.391(PEP+HexNAc(2)Hex(6)_3)
950.966
972.433(PEP+HexNAc(2)Hex(7)_3)
1026.429(PEP+HexNAc(2)Hex(8)_3)
1052.967(PEP+HexNAc(2)Hex(2)_2)
1113.509
1133.988(PEP+HexNAc(2)Hex(3)_2)
1176.404
1207.024
1215.019(PEP+HexNAc(2)Hex(4)_2)
1277.565
1296.043(PEP+HexNAc(2)Hex(5)_2)
1338.462
1369.076
1377.070(PEP+HexNAc(2)Hex(6)_2)
1450.101
1458.101(PEP+HexNAc(2)Hex(7)_2)
1500.514
1539.133(PEP+HexNAc(2)Hex(8)_2)
m/z

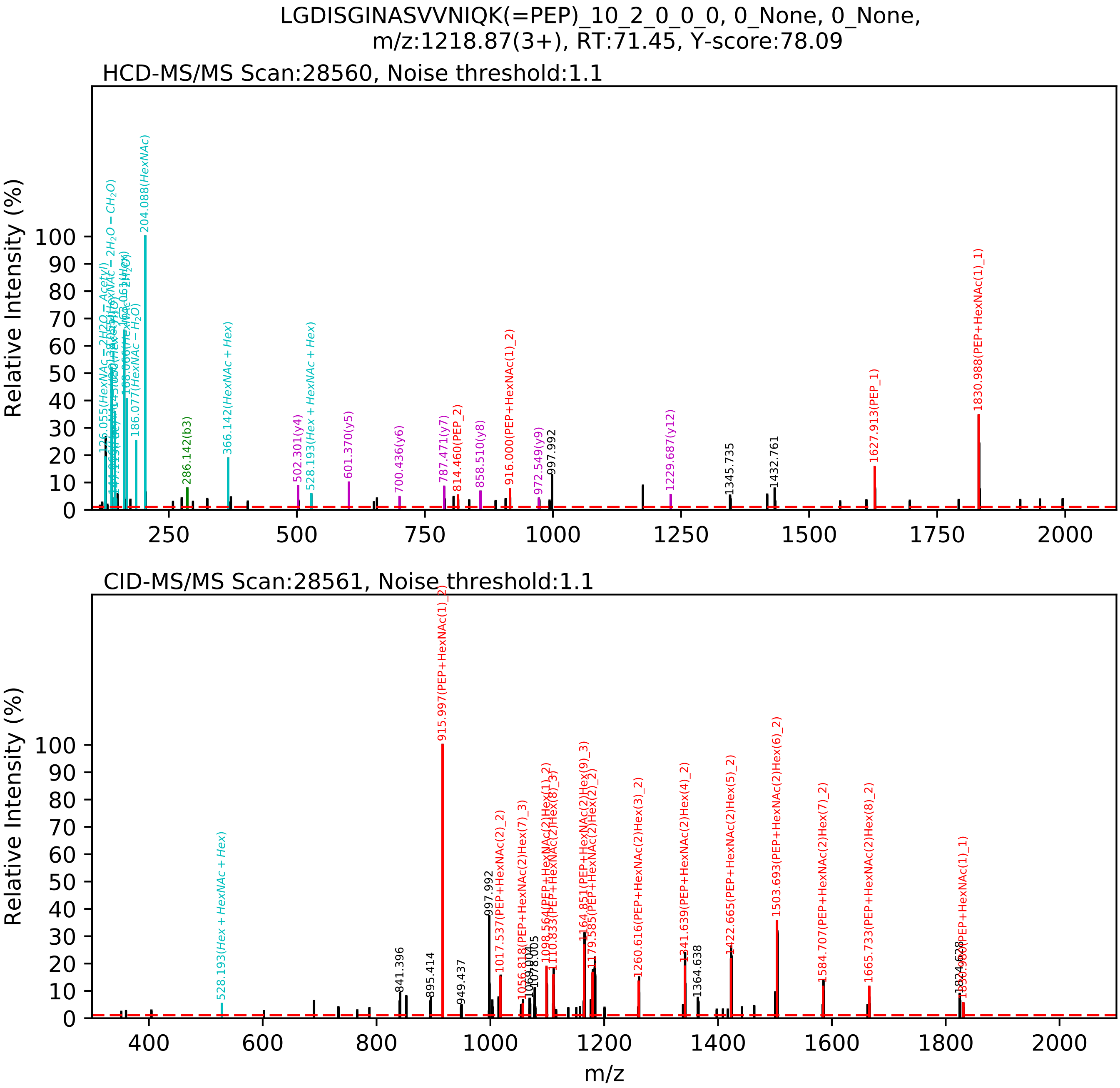

LGDISGINASVVNIQK(=PEP)_10_2_0_0_0, 0_None, 0_None,
m/z:1218.87(3+), RT:71.45, Y-score:78.09
HCD-MS/MS Scan:28560, Noise threshold:1.1
Relative Intensity (%)
204.088(HexNAc)
186.077(HexNAc − H2O)
286.142(b3)
366.142(HexNAc + Hex)
502.301(y4)
528.193(Hex + HexNAc + Hex)
601.370(y5)
700.436(y6)
787.471(y7)
814.460(PEP_2)
858.510(y8)
916.000(PEP+HexNAc(1)_2)
972.549(y9)
997.992
1229.687(y12)
1345.735
1432.761
1627.913(PEP_1)
1830.988(PEP+HexNAc(1)_1)
CID-MS/MS Scan:28561, Noise threshold:1.1
915.997(PEP+HexNAc(1)_2)
528.193(Hex + HexNAc + Hex)
841.396
895.414
949.437
997.992
1017.537(PEP+HexNAc(2)_2)
1260.616(PEP+HexNAc(2)Hex(3)_2)
1341.639(PEP+HexNAc(2)Hex(4)_2)
1364.638
1422.665(PEP+HexNAc(2)Hex(5)_2)
1503.693(PEP+HexNAc(2)Hex(6)_2)
1584.707(PEP+HexNAc(2)Hex(7)_2)
1665.733(PEP+HexNAc(2)Hex(8)_2)
m/z

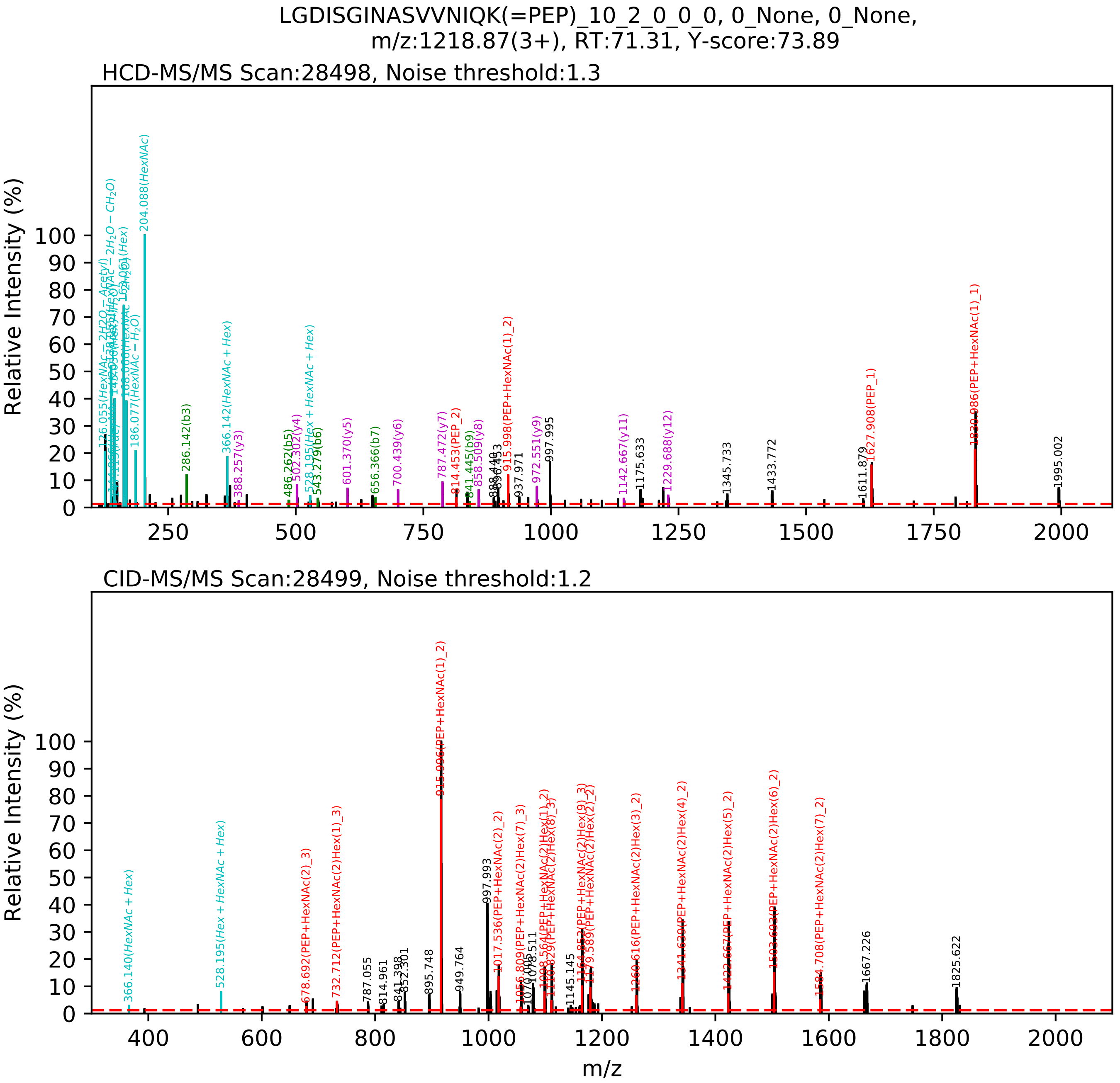

LGDISGINASVVNIQK(=PEP)_10_2_0_0_0, 0_None, 0_None,
m/z:1218.87(3+), RT:71.31, Y-score:73.89
HCD-MS/MS Scan:28498, Noise threshold:1.3
Relative Intensity (%)
204.088(HexNAc)
186.077(HexNAc − H2O)
286.142(b3)
366.142(HexNAc + Hex)
388.257(y3)
486.262(b5)
502.302(y4)
528.195(Hex + HexNAc + Hex)
543.279(b6)
601.370(y5)
656.366(b7)
700.439(y6)
787.472(y7)
814.453(PEP_2)
841.445(b9)
858.509(y8)
915.998(PEP+HexNAc(1)_2)
937.971
972.551(y9)
997.995
1142.667(y11)
1175.633
1229.688(y12)
1345.733
1433.772
1611.879
1627.908(PEP_1)
1830.986(PEP+HexNAc(1)_1)
1995.002
CID-MS/MS Scan:28499, Noise threshold:1.2
Relative Intensity (%)
366.140(HexNAc + Hex)
528.195(Hex + HexNAc + Hex)
678.692(PEP+HexNAc(2)_3)
732.712(PEP+HexNAc(2)Hex(1)_3)
787.055
814.961
841.398
852.301
895.748
915.996(PEP+HexNAc(1)_2)
949.764
997.993
1017.536(PEP+HexNAc(2)_2)
1056.809(PEP+HexNAc(2)Hex(7)_3)
1078.511
1145.145
1260.616(PEP+HexNAc(2)Hex(3)_2)
1341.630(PEP+HexNAc(2)Hex(4)_2)
1422.667(PEP+HexNAc(2)Hex(5)_2)
1503.603(PEP+HexNAc(2)Hex(6)_2)
1584.708(PEP+HexNAc(2)Hex(7)_2)
1667.226
1825.622
m/z

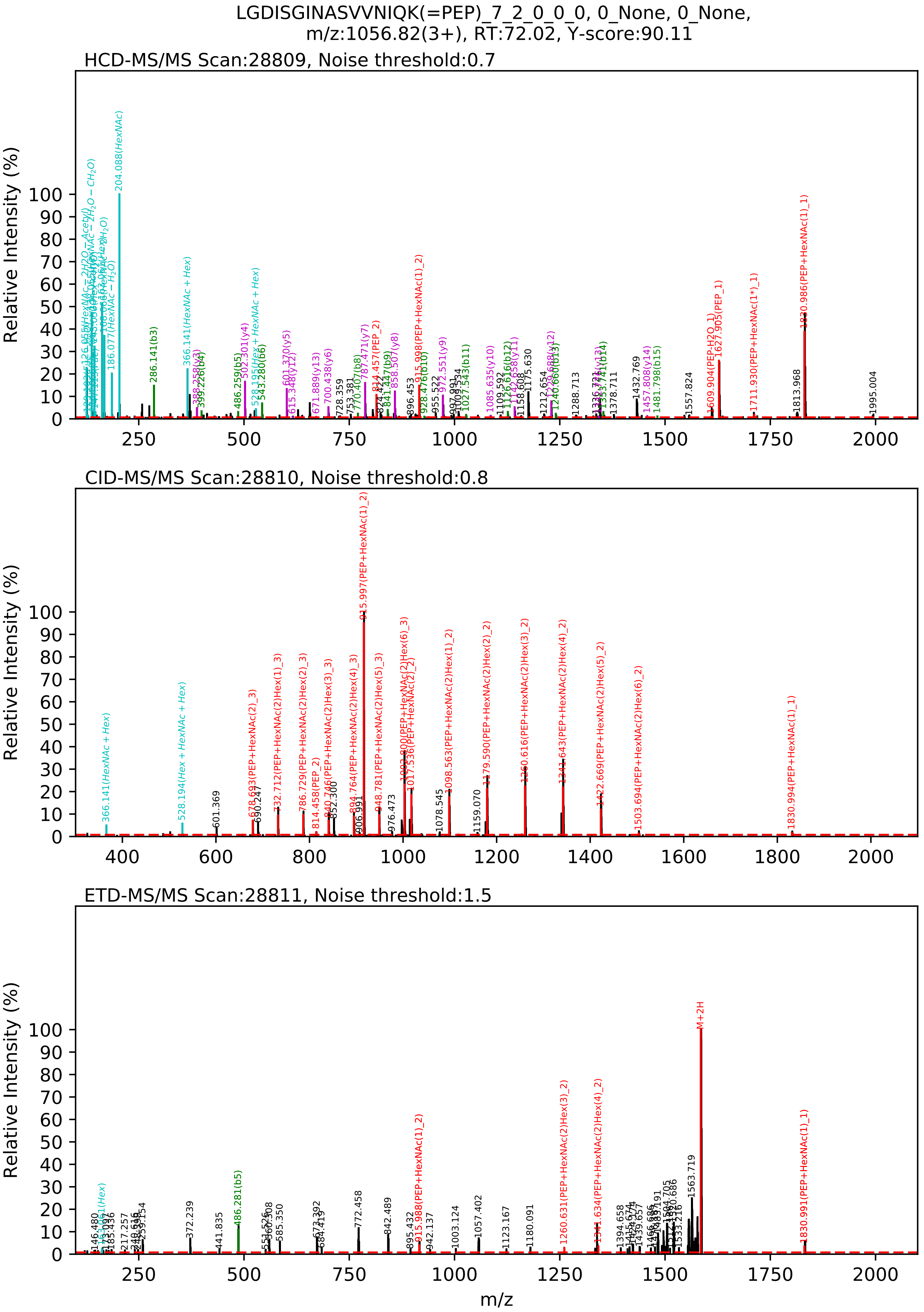

LGDISGINASVVNIQK(=PEP)_7_2_0_0_0, 0_None, 0_None,
m/z:1056.82(3+), RT:72.02, Y-score:90.11
HCD-MS/MS Scan:28809, Noise threshold:0.7
Relative Intensity (%)
204.088(HexNAc)
186.077(HexNAc − H2O)
286.141(b3)
366.141(HexNAc + Hex)
399.226(b4)
486.259(b5)
502.301(y4)
528.195(Hex + HexNAc + Hex)
543.280(b6)
601.370(y5)
615.348(y12)
671.889(y13)
700.438(y6)
728.359
753.381
770.407(b8)
787.471(y7)
814.457(PEP_2)
841.447(b9)
858.507(y8)
896.453
915.998(PEP+HexNAc(1)_2)
928.476(b10)
955.522
972.551(y9)
997.991
1009.534
1027.543(b11)
1085.635(y10)
1109.592
1126.616(b12)
1158.608
1175.630
1212.654
1240.660(b13)
1288.713
1353.741(b14)
1378.711
1432.769
1457.808(y14)
1481.798(b15)
1557.824
1609.904(PEP-H2O_1)
1627.905(PEP_1)
1711.930(PEP+HexNAc(1*)_1)
1813.968
1830.986(PEP+HexNAc(1)_1)
1995.004
CID-MS/MS Scan:28810, Noise threshold:0.8
Relative Intensity (%)
366.141(HexNAc + Hex)
528.194(Hex + HexNAc + Hex)
601.369
678.693(PEP+HexNAc(2)_3)
690.247
732.712(PEP+HexNAc(2)Hex(1)_3)
786.729(PEP+HexNAc(2)Hex(2)_3)
814.458(PEP_2)
840.746(PEP+HexNAc(2)Hex(3)_3)
852.300
894.764(PEP+HexNAc(2)Hex(4)_3)
906.991
915.997(PEP+HexNAc(1)_2)
948.781(PEP+HexNAc(2)Hex(5)_3)
976.473
1002.800(PEP+HexNAc(2)Hex(6)_3)
1017.536(PEP+HexNAc(2)_2)
1078.545
1098.563(PEP+HexNAc(2)Hex(1)_2)
1159.070
1179.590(PEP+HexNAc(2)Hex(2)_2)
1260.616(PEP+HexNAc(2)Hex(3)_2)
1341.643(PEP+HexNAc(2)Hex(4)_2)
1422.669(PEP+HexNAc(2)Hex(5)_2)
1503.694(PEP+HexNAc(2)Hex(6)_2)
1830.994(PEP+HexNAc(1)_1)
ETD-MS/MS Scan:28811, Noise threshold:1.5
Relative Intensity (%)
146.480
185.436
217.257
259.154
372.239
441.835
486.281(b5)
551.526
560.308
585.350
673.392
684.419
772.458
842.489
895.432
915.988(PEP+HexNAc(1)_2)
942.137
1003.124
1057.402
1123.167
1180.091
1260.631(PEP+HexNAc(2)Hex(3)_2)
1341.634(PEP+HexNAc(2)Hex(4)_2)
1394.658
1439.657
1466.686
1563.719
M+2H
1830.991(PEP+HexNAc(1)_1)
m/z

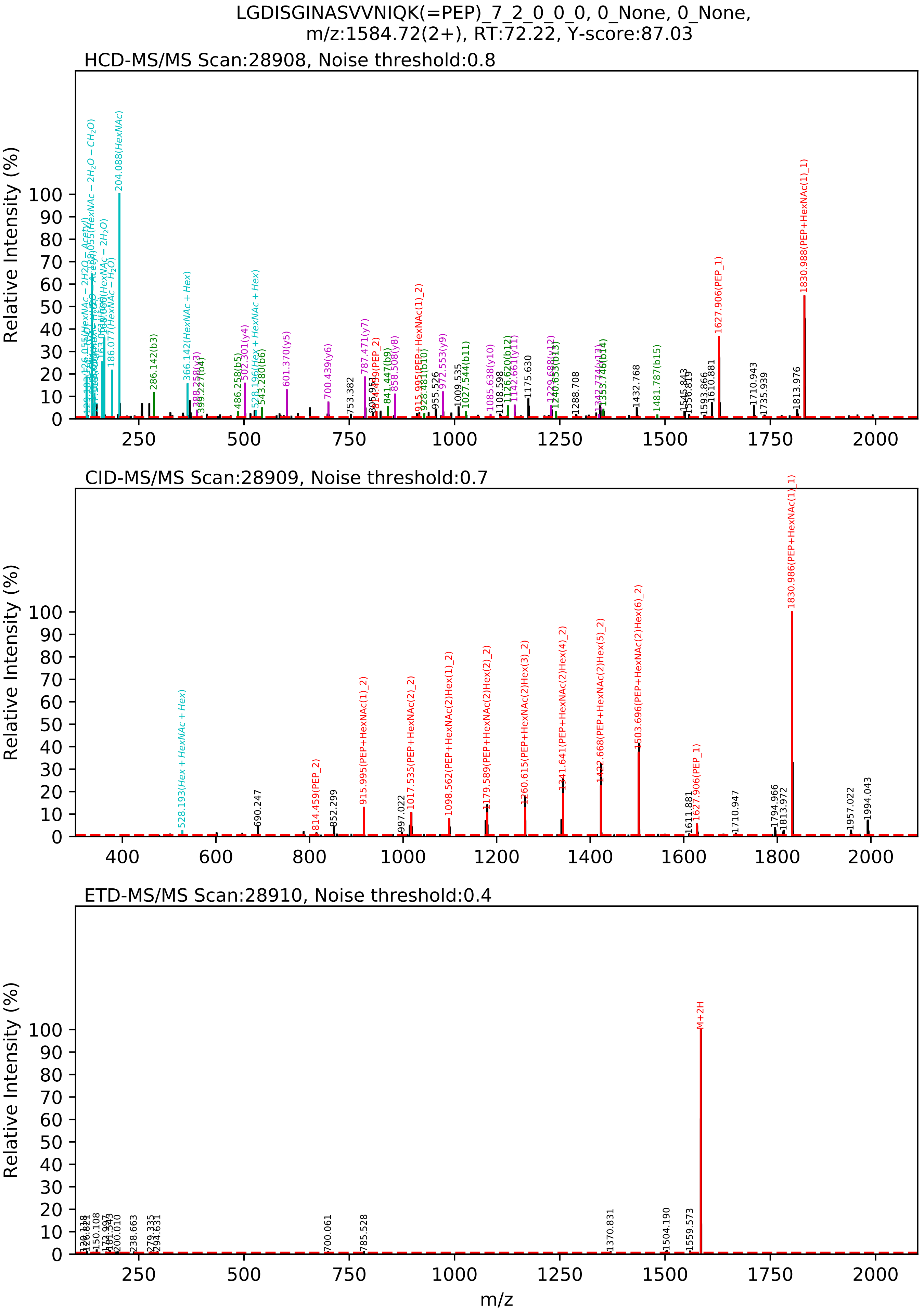
LGDISGINASVVNIQK(=PEP)_7_2_0_0_0, 0_None, 0_None,
m/z:1584.72(2+), RT:72.22, Y-score:87.03
HCD-MS/MS Scan:28908, Noise threshold:0.8
Relative Intensity (%)
204.088(HexNAc)
186.077(HexNAc − H2O)
286.142(b3)
366.142(HexNAc + Hex)
502.301(y4)
528.196(Hex + HexNAc + Hex)
543.280(b6)
601.370(y5)
700.439(y6)
753.382
787.471(y7)
841.447(b9)
858.508(y8)
915.995(PEP+HexNAc(1)_2)
928.481(b10)
955.526
972.553(y9)
1009.535
1027.544(b11)
1085.638(y10)
1108.598
1126.620(b12)
1142.661(y11)
1175.630
1288.708
1432.768
1481.787(b15)
1593.866
1610.881
1627.906(PEP_1)
1710.943
1735.939
1813.976
1830.988(PEP+HexNAc(1)_1)
CID-MS/MS Scan:28909, Noise threshold:0.7
528.193(Hex + HexNAc + Hex)
690.247
814.459(PEP_2)
852.299
915.995(PEP+HexNAc(1)_2)
997.022
1017.535(PEP+HexNAc(2)_2)
1098.562(PEP+HexNAc(2)Hex(1)_2)
1179.589(PEP+HexNAc(2)Hex(2)_2)
1260.615(PEP+HexNAc(2)Hex(3)_2)
1341.641(PEP+HexNAc(2)Hex(4)_2)
1422.668(PEP+HexNAc(2)Hex(5)_2)
1503.696(PEP+HexNAc(2)Hex(6)_2)
1611.881
1627.906(PEP_1)
1710.947
1794.966
1813.972
1830.986(PEP+HexNAc(1)_1)
1957.022
1994.043
ETD-MS/MS Scan:28910, Noise threshold:0.4
M+2H
150.108
200.010
238.663
279.335
294.631
700.061
785.528
1370.831
1504.190
1559.573
m/z

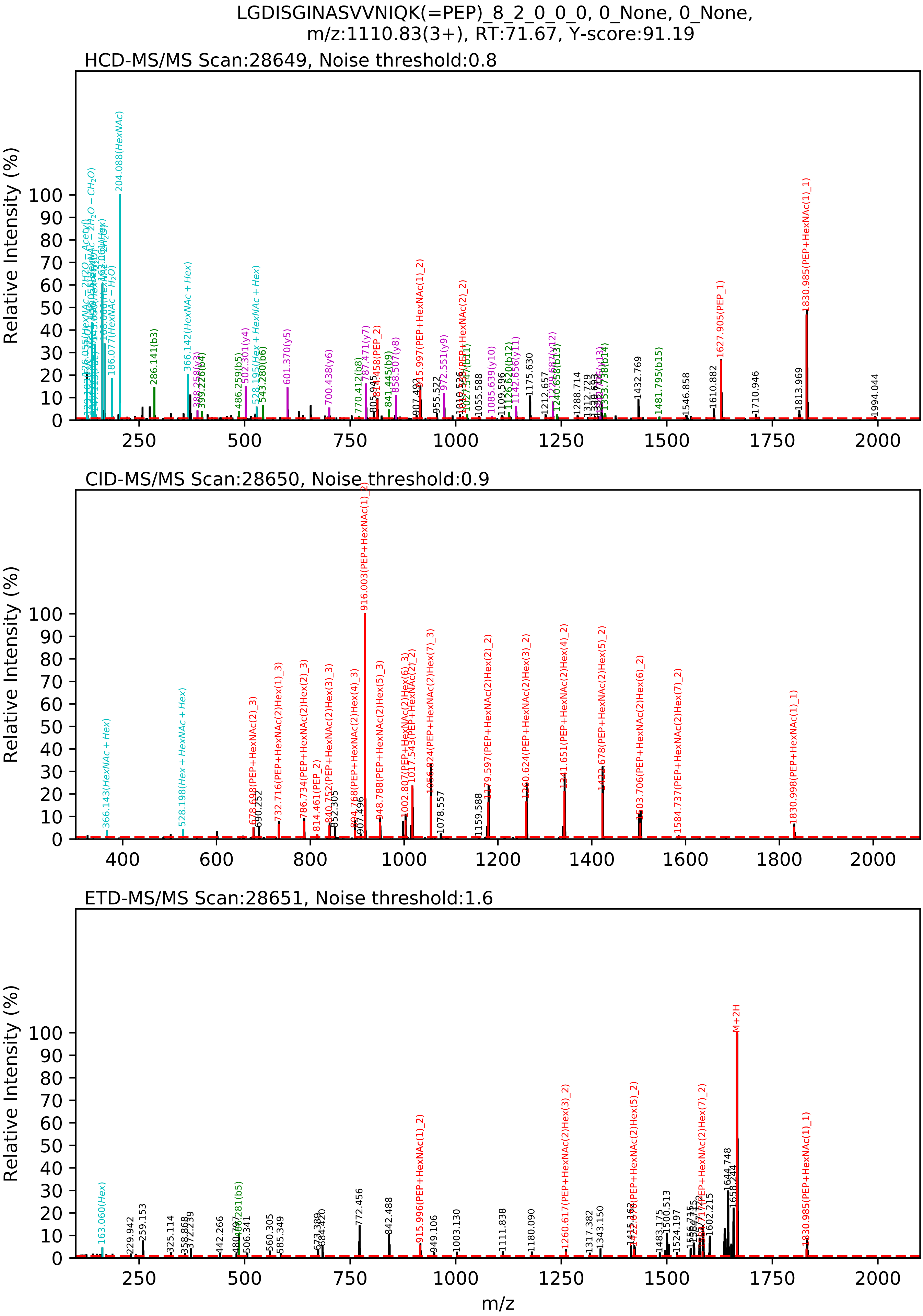

LGDISGINASVVNIQK(=PEP)_8_2_0_0_0, 0_None, 0_None,
m/z:1110.83(3+), RT:71.67, Y-score:91.19
HCD-MS/MS Scan:28649, Noise threshold:0.8
Relative Intensity (%)
204.088(HexNAc)
186.077(HexNAc − H2O)
286.141(b3)
366.142(HexNAc + Hex)
399.226(b4)
486.259(b5)
502.301(y4)
528.195(Hex + HexNAc + Hex)
543.280(b6)
601.370(y5)
700.438(y6)
770.412(b8)
787.471(y7)
841.445(b9)
858.507(y8)
907.492
915.997(PEP+HexNAc(1)_2)
955.522
972.551(y9)
1027.547(b11)
1055.588
1085.639(y10)
1109.596
1126.620(b12)
1142.656(y11)
1175.630
1212.657
1288.714
1432.769
1481.795(b15)
1546.858
1610.882
1627.905(PEP_1)
1710.946
1813.969
1830.985(PEP+HexNAc(1)_1)
1994.044
CID-MS/MS Scan:28650, Noise threshold:0.9
Relative Intensity (%)
366.143(HexNAc + Hex)
528.198(Hex + HexNAc + Hex)
678.698(PEP+HexNAc(2)_3)
690.252
732.716(PEP+HexNAc(2)Hex(1)_3)
786.734(PEP+HexNAc(2)Hex(2)_3)
814.461(PEP_2)
840.752(PEP+HexNAc(2)Hex(3)_3)
852.305
894.768(PEP+HexNAc(2)Hex(4)_3)
907.496
916.003(PEP+HexNAc(1)_2)
948.788(PEP+HexNAc(2)Hex(5)_3)
1017.543(PEP+HexNAc(2)_2)
1056.824(PEP+HexNAc(2)Hex(7)_3)
1078.557
1159.588
1260.624(PEP+HexNAc(2)Hex(3)_2)
1341.651(PEP+HexNAc(2)Hex(4)_2)
1422.678(PEP+HexNAc(2)Hex(5)_2)
1503.706(PEP+HexNAc(2)Hex(6)_2)
1584.737(PEP+HexNAc(2)Hex(7)_2)
1830.998(PEP+HexNAc(1)_1)
ETD-MS/MS Scan:28651, Noise threshold:1.6
Relative Intensity (%)
163.060(Hex)
229.942
259.153
325.114
442.266
480.281(b5)
506.341
560.305
585.349
772.456
842.488
915.996(PEP+HexNAc(1)_2)
949.106
1003.130
1111.838
1180.090
1260.617(PEP+HexNAc(2)Hex(3)_2)
1317.382
1343.150
1422.676(PEP+HexNAc(2)Hex(5)_2)
1483.175
1500.513
1524.197
1584.7(PEP+HexNAc(2)Hex(7)_2)
1602.215
1644.748
1658.244
M+2H
1830.985(PEP+HexNAc(1)_1)
m/z

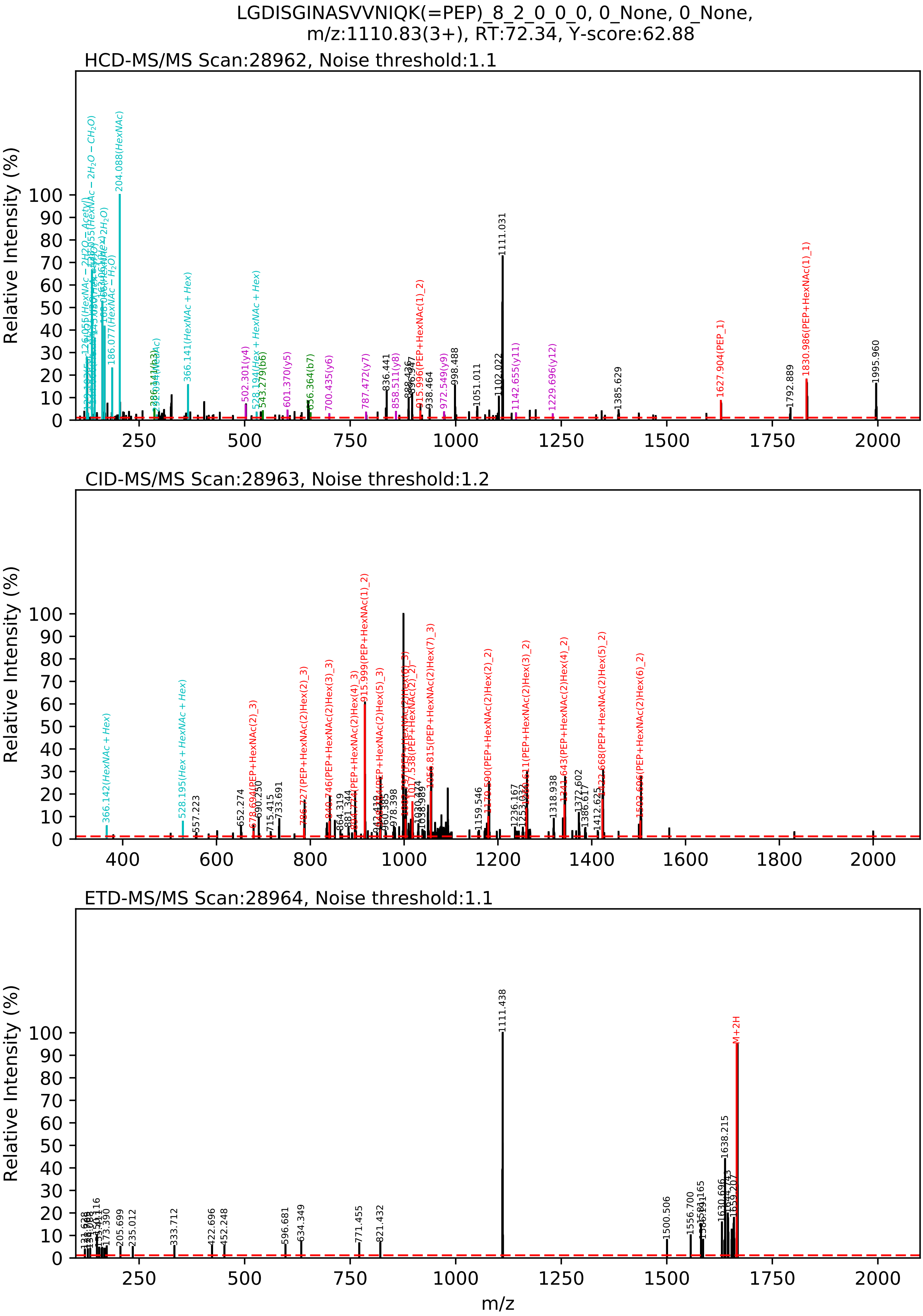
LGDISGINASVVNIQK(=PEP)_8_2_0_0_0, 0_None, 0_None,
m/z:1110.83(3+), RT:72.34, Y-score:62.88
HCD-MS/MS Scan:28962, Noise threshold:1.1
Relative Intensity (%)
204.088(HexNAc)
186.077(HexNAc − H2O)
366.141(HexNAc + Hex)
528.194(Hex + HexNAc + Hex)
543.279(b6)
656.364(b7)
502.301(y4)
601.370(y5)
700.435(y6)
787.472(y7)
858.511(y8)
915.996(PEP+HexNAc(1)_2)
972.549(y9)
998.488
836.441
938.464
1051.011
1102.022
1111.031
1142.655(y11)
1229.696(y12)
1385.629
1627.904(PEP_1)
1792.889
1830.986(PEP+HexNAc(1)_1)
1995.960
CID-MS/MS Scan:28963, Noise threshold:1.2
366.142(HexNAc + Hex)
528.195(Hex + HexNAc + Hex)
557.223
652.274
690.250
715.415
733.691
786.727(PEP+HexNAc(2)Hex(2)_3)
840.746(PEP+HexNAc(2)Hex(3)_3)
864.319
881.344
915.999(PEP+HexNAc(1)_2)
942.438
960.385
978.398
1056.815(PEP+HexNAc(2)Hex(7)_3)
1159.546
1179.590(PEP+HexNAc(2)Hex(2)_2)
1236.167
1318.938
1372.602
1386.617
1412.625
1341.643(PEP+HexNAc(2)Hex(4)_2)
1422.668(PEP+HexNAc(2)Hex(5)_2)
1503.696(PEP+HexNAc(2)Hex(6)_2)
ETD-MS/MS Scan:28964, Noise threshold:1.1
1111.438
M+2H
205.699
235.012
333.712
422.696
452.248
596.681
634.349
771.455
821.432
1500.506
1556.700
1638.215
1659.207
m/z

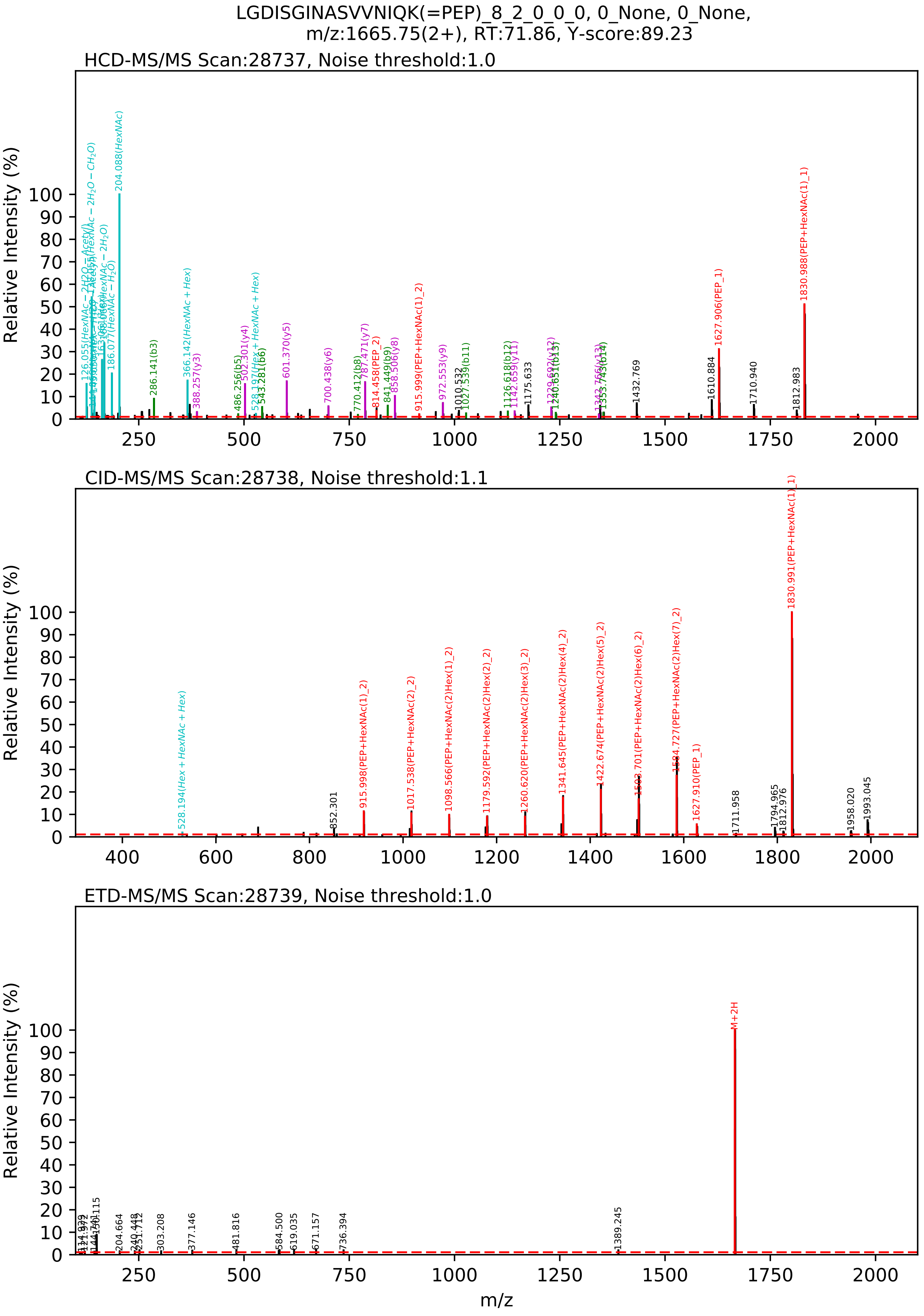
LGDISGINASVVNIQK(=PEP)_8_2_0_0_0, 0_None, 0_None,
m/z:1665.75(2+), RT:71.86, Y-score:89.23
HCD-MS/MS Scan:28737, Noise threshold:1.0
Relative Intensity (%)
204.088(HexNAc)
186.077(HexNAc − H2O)
126.055(HexNAc − 2H2O − CH2O)
286.141(b3)
366.142(HexNAc + Hex)
388.257(y3)
486.256(b5)
502.301(y4)
528.197(Hex + HexNAc + Hex)
543.281(b6)
601.370(y5)
700.438(y6)
770.412(b8)
787.471(y7)
814.458(PEP_2)
841.449(b9)
858.506(y8)
915.999(PEP+HexNAc(1)_2)
972.553(y9)
1010.532
1027.539(b11)
1126.618(b12)
1142.659(y11)
1175.633
1432.769
1610.884
1627.906(PEP_1)
1710.940
1812.983
1830.988(PEP+HexNAc(1)_1)
CID-MS/MS Scan:28738, Noise threshold:1.1
Relative Intensity (%)
528.194(Hex + HexNAc + Hex)
852.301
915.998(PEP+HexNAc(1)_2)
1017.538(PEP+HexNAc(2)_2)
1098.566(PEP+HexNAc(2)Hex(1)_2)
1179.592(PEP+HexNAc(2)Hex(2)_2)
1260.620(PEP+HexNAc(2)Hex(3)_2)
1341.645(PEP+HexNAc(2)Hex(4)_2)
1422.674(PEP+HexNAc(2)Hex(5)_2)
1503.701(PEP+HexNAc(2)Hex(6)_2)
1584.727(PEP+HexNAc(2)Hex(7)_2)
1627.910(PEP_1)
1711.958
1794.965
1812.976
1830.991(PEP+HexNAc(1)_1)
1958.020
1993.045
ETD-MS/MS Scan:28739, Noise threshold:1.0
Relative Intensity (%)
M+2H
204.664
240.448
251.712
303.208
377.146
481.816
584.500
619.035
671.157
736.394
1389.245
m/z

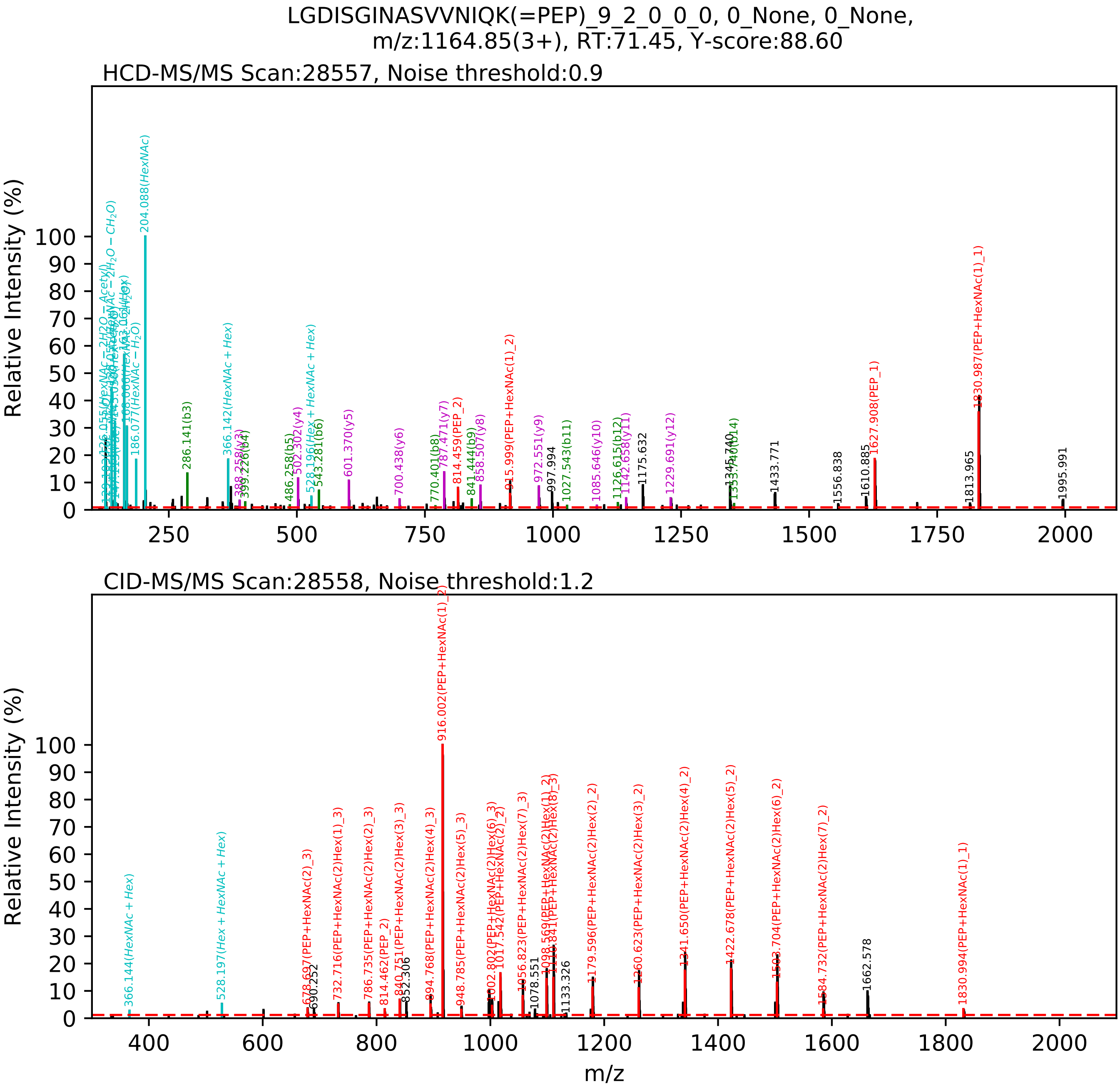
LGDISGINASVVNIQK(=PEP)_9_2_0_0_0, 0_None, 0_None,
m/z:1164.85(3+), RT:71.45, Y-score:88.60
HCD-MS/MS Scan:28557, Noise threshold:0.9
Relative Intensity (%)
204.088(HexNAc)
286.141(b3)
366.142(HexNAc + Hex)
601.370(y5)
700.438(y6)
787.471(y7)
814.459(PEP_2)
915.999(PEP+HexNAc(1)_2)
972.551(y9)
997.994
1027.543(b11)
1085.646(y10)
1175.632
1229.691(y12)
1433.771
1556.838
1610.885
1627.908(PEP_1)
1813.965
1830.987(PEP+HexNAc(1)_1)
1995.991
CID-MS/MS Scan:28558, Noise threshold:1.2
916.002(PEP+HexNAc(1)_2)
366.144(HexNAc + Hex)
528.197(Hex + HexNAc + Hex)
678.697(PEP+HexNAc(2)_3)
732.716(PEP+HexNAc(2)Hex(1)_3)
786.735(PEP+HexNAc(2)Hex(2)_3)
814.462(PEP_2)
840.751(PEP+HexNAc(2)Hex(3)_3)
852.306
894.768(PEP+HexNAc(2)Hex(4)_3)
948.785(PEP+HexNAc(2)Hex(5)_3)
1017.542(PEP+HexNAc(2)_2)
1056.823(PEP+HexNAc(2)Hex(7)_3)
1078.551
1133.326
1179.596(PEP+HexNAc(2)Hex(2)_2)
1260.623(PEP+HexNAc(2)Hex(3)_2)
1341.650(PEP+HexNAc(2)Hex(4)_2)
1422.678(PEP+HexNAc(2)Hex(5)_2)
1503.704(PEP+HexNAc(2)Hex(6)_2)
1584.732(PEP+HexNAc(2)Hex(7)_2)
1662.578
1830.994(PEP+HexNAc(1)_1)
m/z

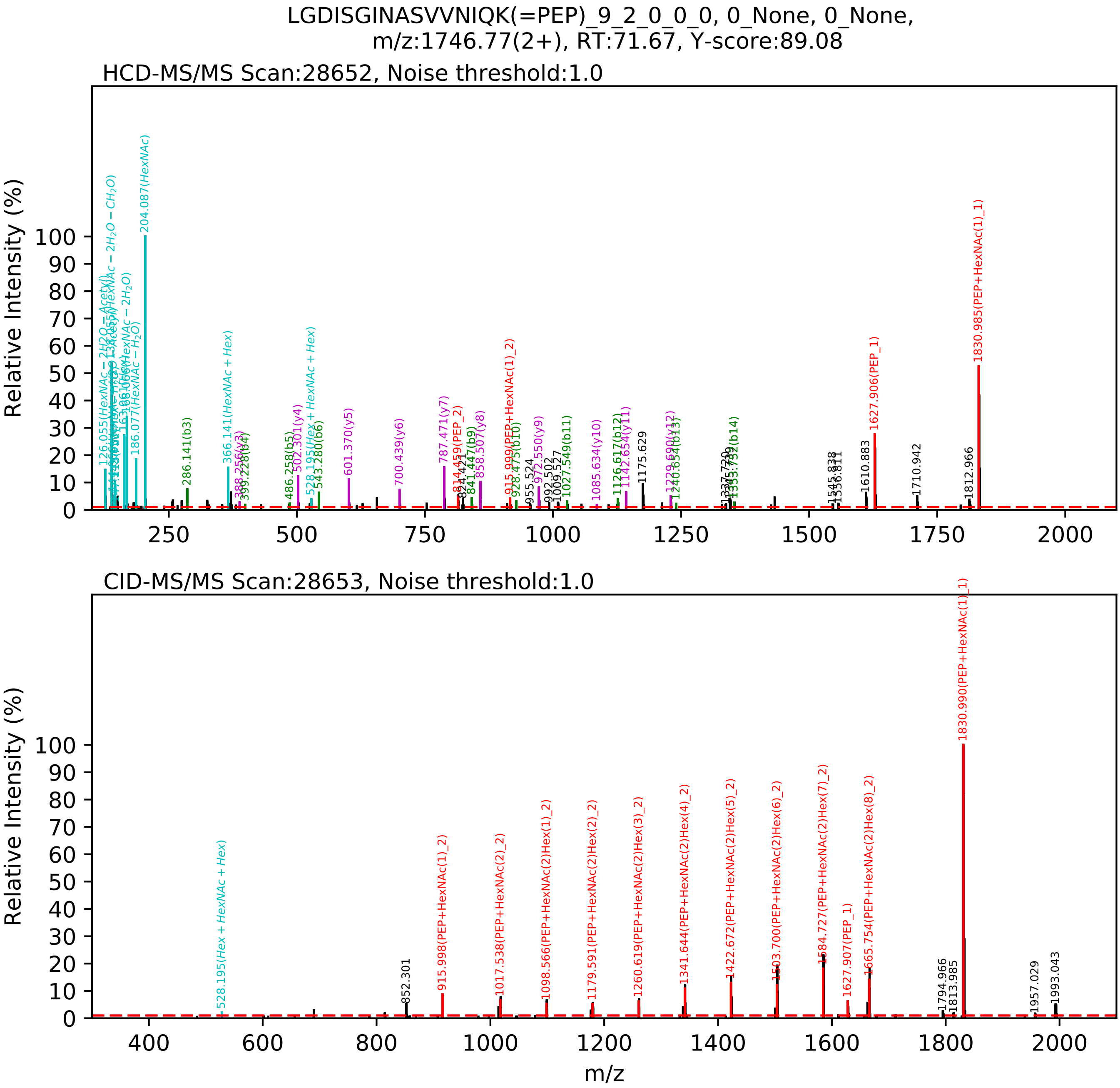

LGDISGINASVVNIQK(=PEP)_9_2_0_0_0, 0_None, 0_None,
m/z:1746.77(2+), RT:71.67, Y-score:89.08
HCD-MS/MS Scan:28652, Noise threshold:1.0
Relative Intensity (%)
204.087(HexNAc)
186.077(HexNAc − H2O)
286.141(b3)
366.141(HexNAc + Hex)
486.258(b5)
502.301(y4)
528.195(Hex + HexNAc + Hex)
543.280(b6)
601.370(y5)
700.439(y6)
787.471(y7)
858.507(y8)
915.999(PEP+HexNAc(1)_2)
928.475(b10)
955.524
972.550(y9)
992.502
1009.527
1027.549(b11)
1085.634(y10)
1126.617(b12)
1142.654(y11)
1175.629
1240.634(b13)
1353.732(b14)
1545.838
1556.811
1610.883
1627.906(PEP_1)
1710.942
1812.966
1830.985(PEP+HexNAc(1)_1)
CID-MS/MS Scan:28653, Noise threshold:1.0
528.195(Hex + HexNAc + Hex)
852.301
915.998(PEP+HexNAc(1)_2)
1017.538(PEP+HexNAc(2)_2)
1098.566(PEP+HexNAc(2)Hex(1)_2)
1179.591(PEP+HexNAc(2)Hex(2)_2)
1260.619(PEP+HexNAc(2)Hex(3)_2)
1341.644(PEP+HexNAc(2)Hex(4)_2)
1422.672(PEP+HexNAc(2)Hex(5)_2)
1503.700(PEP+HexNAc(2)Hex(6)_2)
1584.727(PEP+HexNAc(2)Hex(7)_2)
1627.907(PEP_1)
1665.754(PEP+HexNAc(2)Hex(8)_2)
1794.966
1813.985
1830.990(PEP+HexNAc(1)_1)
1957.029
1993.043
m/z

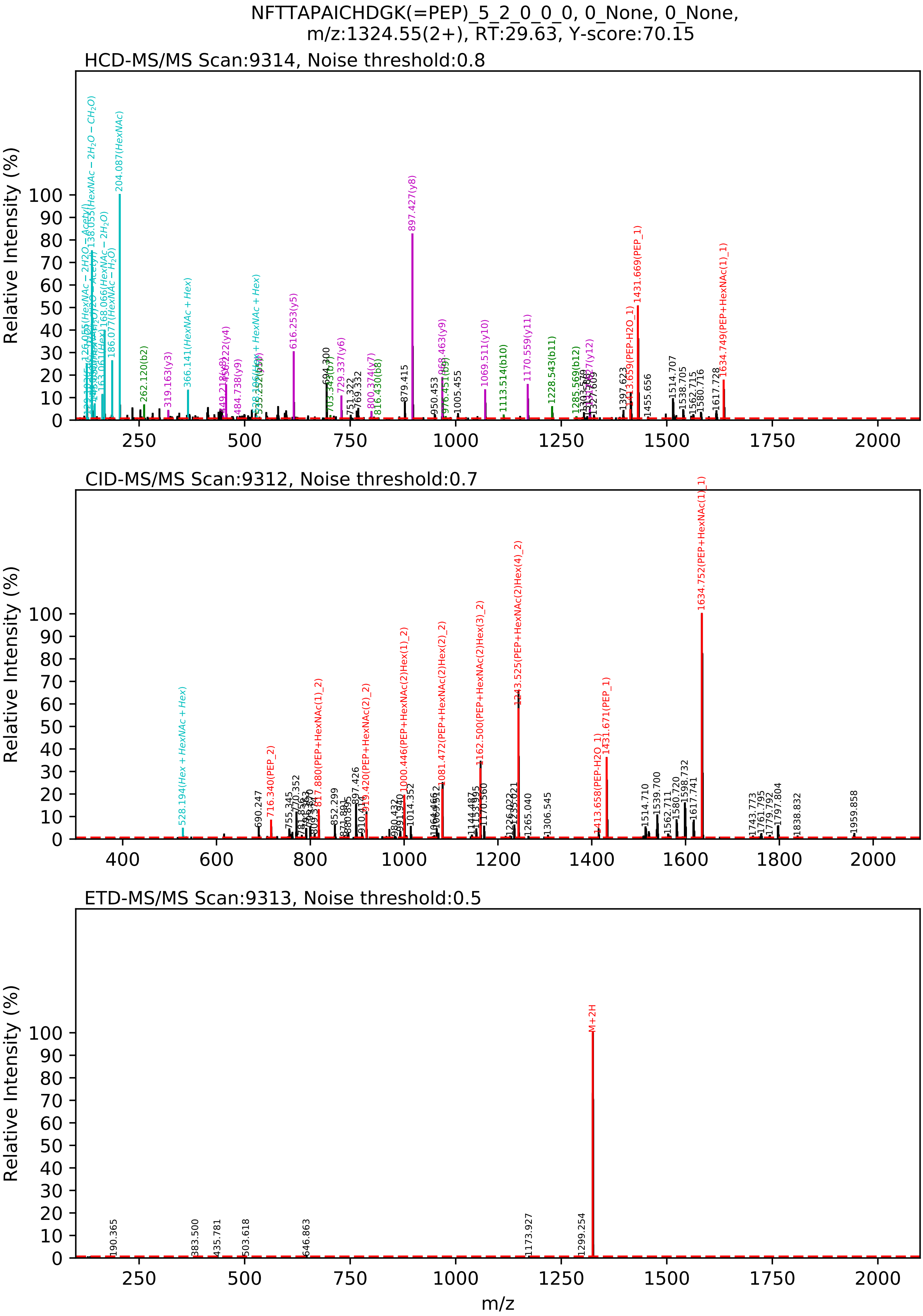

NFTTAPAICHDGK(=PEP)_5_2_0_0_0, 0_None, 0_None,
m/z:1324.55(2+), RT:29.63, Y-score:70.15
HCD-MS/MS Scan:9314, Noise threshold:0.8
Relative Intensity (%)
204.087(HexNAc)
897.427(y8)
1431.669(PEP_1)
1634.749(PEP+HexNAc(1)_1)
616.253(y5)
1170.559(y11)
1069.511(y10)
366.141(HexNAc+Hex)
262.120(b2)
1228.543(b11)
1113.514(b10)
CID-MS/MS Scan:9312, Noise threshold:0.7
1634.752(PEP+HexNAc(1)_1)
1243.525(PEP+HexNAc(2)Hex(4)_2)
1162.500(PEP+HexNAc(2)Hex(3)_2)
1081.472(PEP+HexNAc(2)Hex(2)_2)
1000.446(PEP+HexNAc(2)Hex(1)_2)
919.420(PEP+HexNAc(2)_2)
817.880(PEP+HexNAc(1)_2)
716.340(PEP_2)
528.194(Hex+HexNAc+Hex)
1431.671(PEP_1)
1413.658(PEP-H2O_1)
ETD-MS/MS Scan:9313, Noise threshold:0.5
M+2H
m/z

NFTTAPAICHDGK(=PEP)_5_2_0_0_0, 0_None, 0_None,
m/z:1324.55(2+), RT:29.71, Y-score:73.32

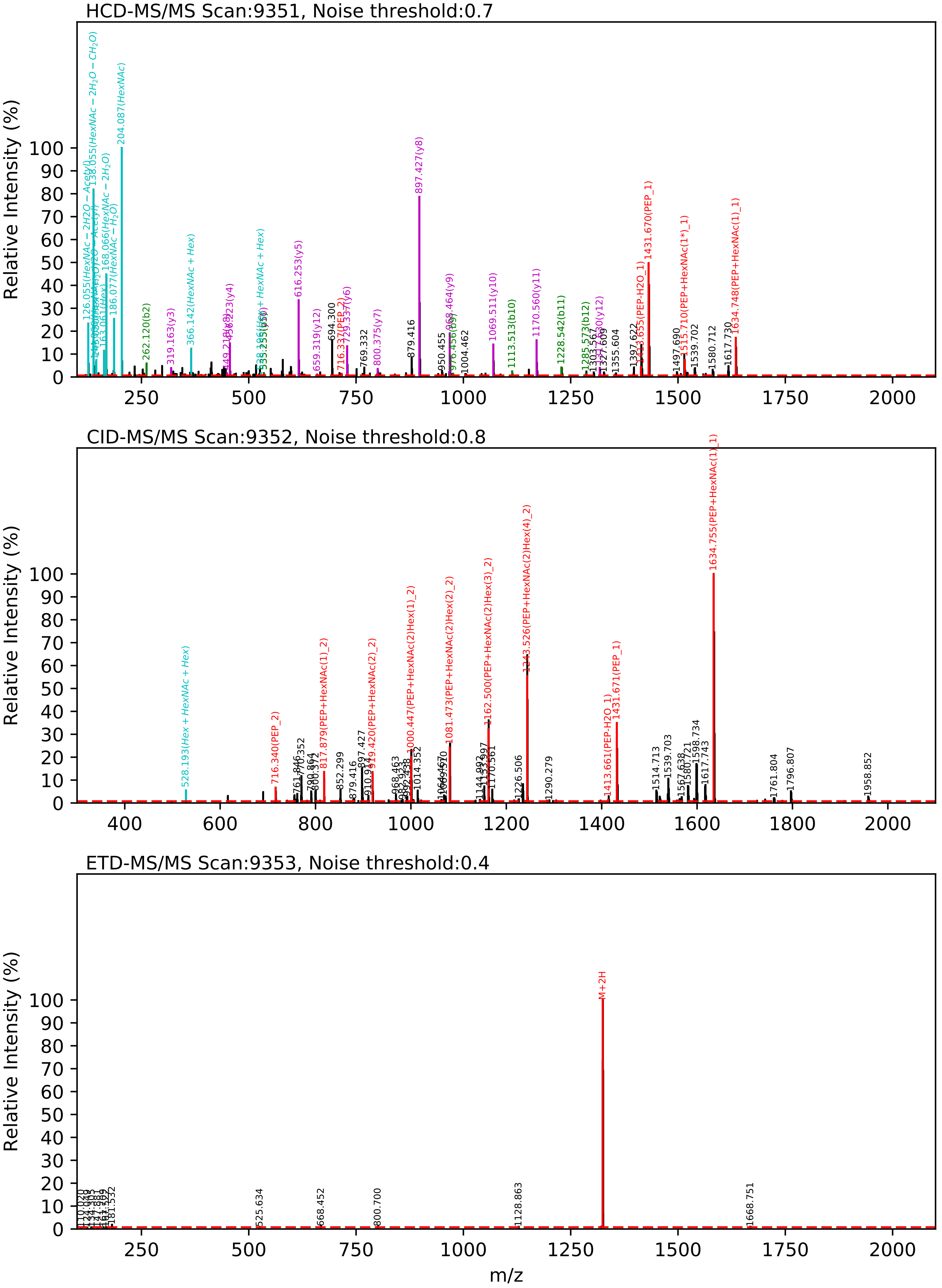

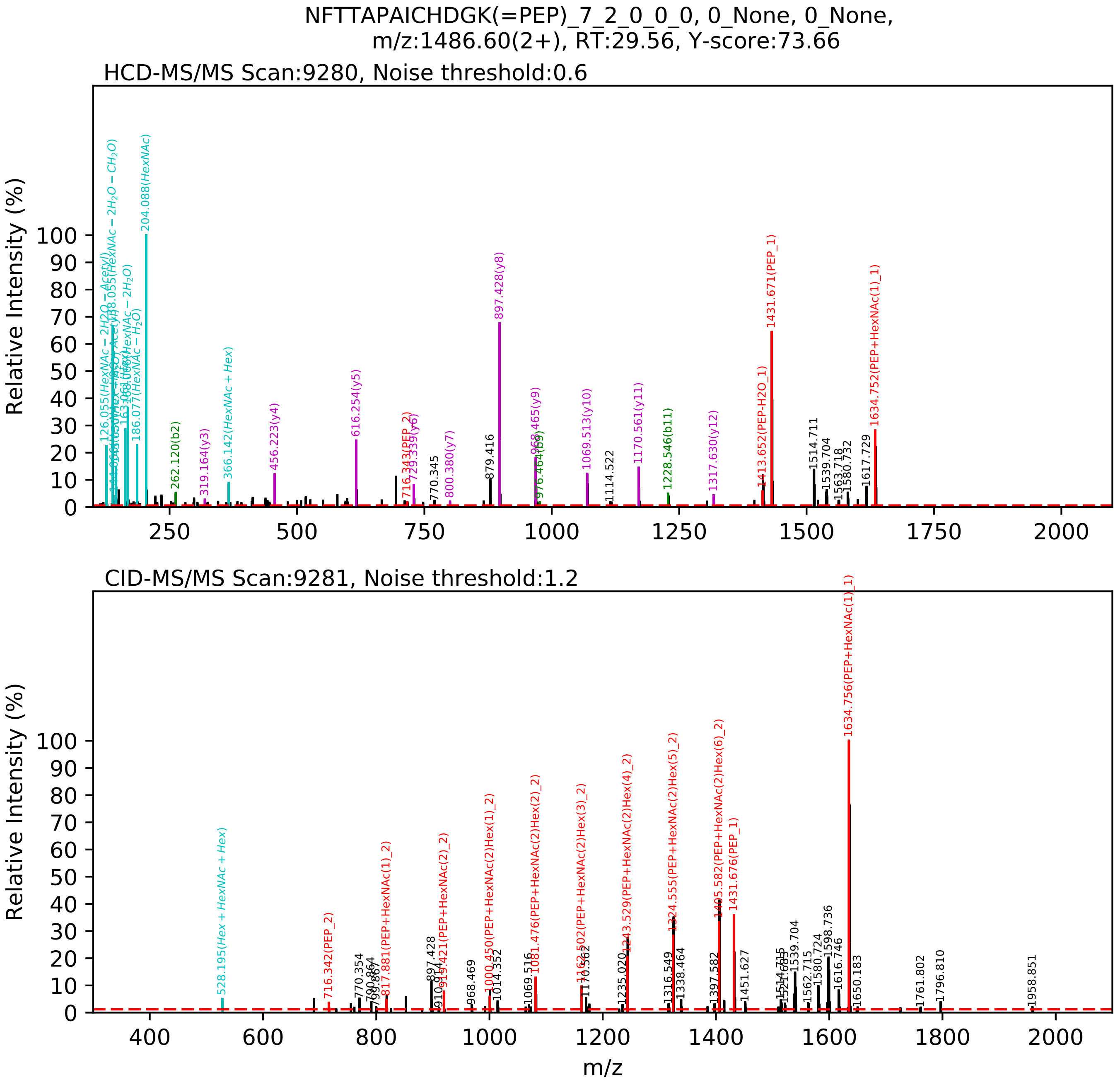
NFTTAPAICHDGK(=PEP)_7_2_0_0_0, 0_None, 0_None,
m/z:1486.60(2+), RT:29.56, Y-score:73.66
HCD-MS/MS Scan:9280, Noise threshold:0.6
Relative Intensity (%)
204.088(HexNAc)
186.077(HexNAc − H2O)
126.055(HexNAc − 2H2O − CH2O)
262.120(b2)
319.164(y3)
366.142(HexNAc + Hex)
456.223(y4)
616.254(y5)
716.343(PEP_2)
729.339(y6)
770.345
800.380(y7)
879.416
897.428(y8)
968.465(y9)
976.464(b9)
1069.513(y10)
1114.522
1170.561(y11)
1228.546(b11)
1317.630(y12)
1413.652(PEP-H2O_1)
1431.671(PEP_1)
1514.711
1539.704
1563.718
1580.732
1617.729
1634.752(PEP+HexNAc(1)_1)
CID-MS/MS Scan:9281, Noise threshold:1.2
Relative Intensity (%)
528.195(Hex + HexNAc + Hex)
716.342(PEP_2)
770.354
790.864
799.867
817.881(PEP+HexNAc(1)_2)
897.428
910.914
919.421(PEP+HexNAc(2)_2)
968.469
1000.450(PEP+HexNAc(2)Hex(1)_2)
1014.352
1069.516
1081.476(PEP+HexNAc(2)Hex(2)_2)
1162.502(PEP+HexNAc(2)Hex(3)_2)
1170.562
1235.020
1243.529(PEP+HexNAc(2)Hex(4)_2)
1316.549
1324.555(PEP+HexNAc(2)Hex(5)_2)
1338.464
1397.582
1405.582(PEP+HexNAc(2)Hex(6)_2)
1431.676(PEP_1)
1451.627
1514.715
1539.704
1562.715
1580.724
1598.736
1616.746
1634.756(PEP+HexNAc(1)_1)
1650.183
1761.802
1796.810
1958.851
m/z

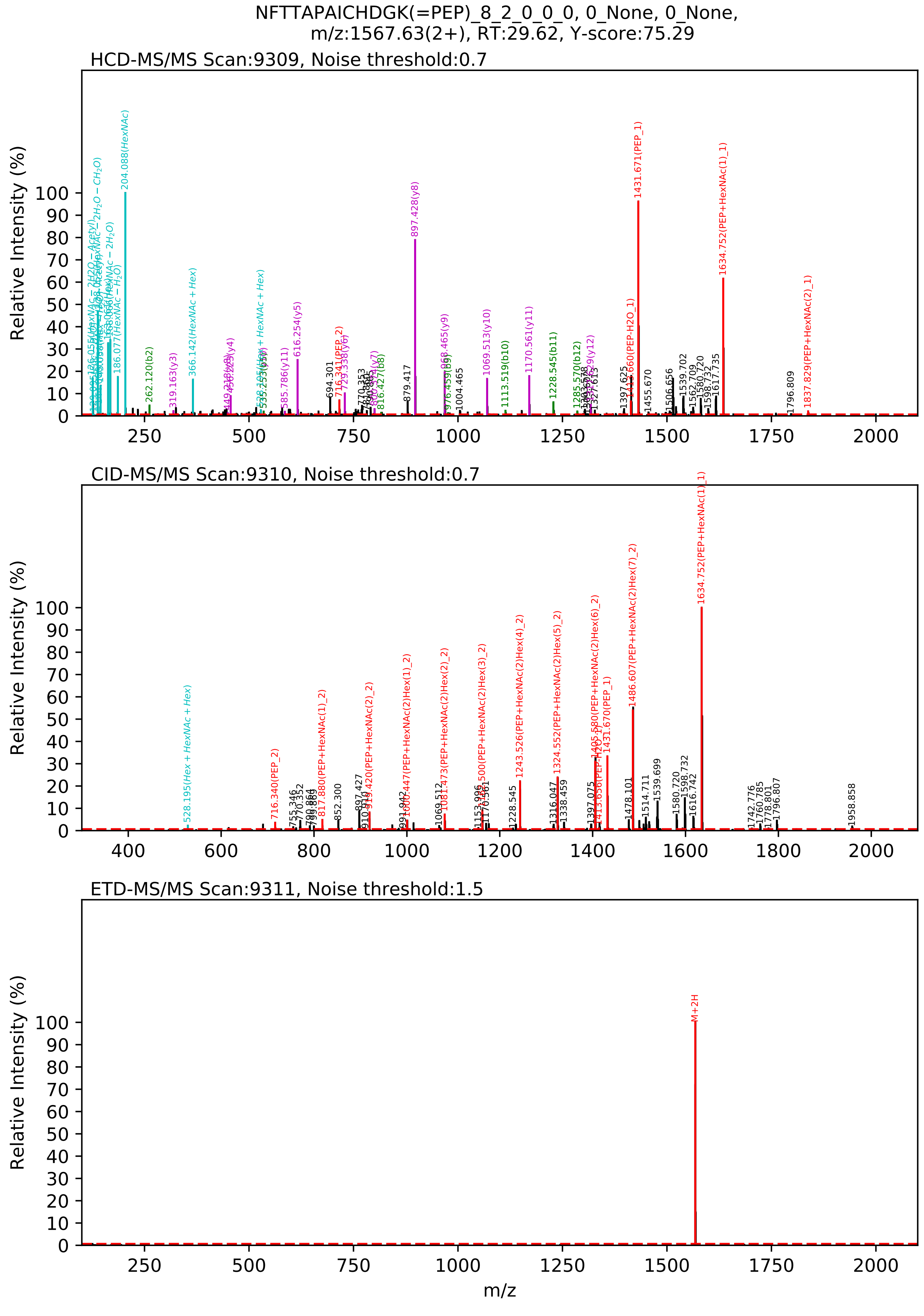
NFTTAPAICHDGK(=PEP)_8_2_0_0_0, 0_None, 0_None,
m/z:1567.63(2+), RT:29.62, Y-score:75.29
HCD-MS/MS Scan:9309, Noise threshold:0.7
204.088(HexNAc)
897.428(y8)
1431.671(PEP_1)
1634.752(PEP+HexNAc(1)_1)
1837.829(PEP+HexNAc(2)_1)
366.142(HexNAc + Hex)
262.120(b2)
319.163(y3)
616.254(y5)
1069.513(y10)
1170.561(y11)
1228.545(b11)
1113.519(b10)
1285.570(b12)
186.077(HexNAc − H2O)
CID-MS/MS Scan:9310, Noise threshold:0.7
528.195(Hex + HexNAc + Hex)
716.340(PEP_2)
817.880(PEP+HexNAc(1)_2)
919.420(PEP+HexNAc(2)_2)
1000.447(PEP+HexNAc(2)Hex(1)_2)
1081.473(PEP+HexNAc(2)Hex(2)_2)
1162.500(PEP+HexNAc(2)Hex(3)_2)
1243.526(PEP+HexNAc(2)Hex(4)_2)
1324.552(PEP+HexNAc(2)Hex(5)_2)
1405.580(PEP+HexNAc(2)Hex(6)_2)
1486.607(PEP+HexNAc(2)Hex(7)_2)
1431.670(PEP_1)
1634.752(PEP+HexNAc(1)_1)
ETD-MS/MS Scan:9311, Noise threshold:1.5
M+2H
Relative Intensity (%)
m/z

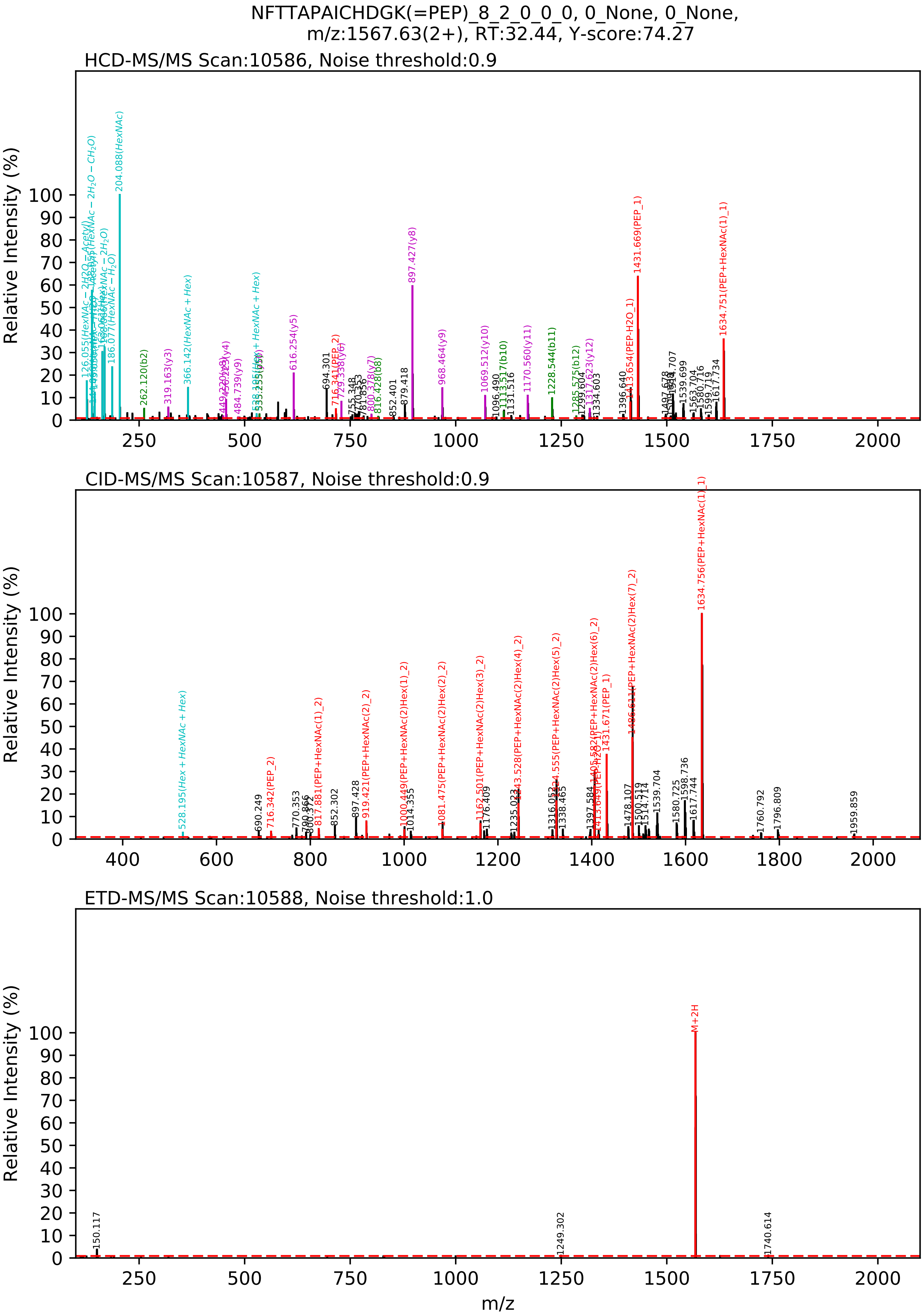

NFTTAPAICHDGK(=PEP)_8_2_0_0_0, 0_None, 0_None,
m/z:1567.63(2+), RT:32.44, Y-score:74.27
HCD-MS/MS Scan:10586, Noise threshold:0.9
Relative Intensity (%)
204.088(HexNAc)
186.077(HexNAc − H2O)
262.120(b2)
319.163(y3)
366.142(HexNAc + Hex)
484.739(y9)
616.254(y5)
694.301
755.348
852.401
879.418
897.427(y8)
968.464(y9)
1069.512(y10)
1096.490
1131.516
1170.560(y11)
1228.544(b11)
1285.575(b12)
1299.604
1334.603
1396.640
1413.654(PEP-H2O_1)
1431.669(PEP_1)
1539.699
1617.734
1634.751(PEP+HexNAc(1)_1)
CID-MS/MS Scan:10587, Noise threshold:0.9
528.195(Hex + HexNAc + Hex)
690.249
716.342(PEP_2)
770.353
817.881(PEP+HexNAc(1)_2)
852.302
897.428
919.421(PEP+HexNAc(2)_2)
1000.449(PEP+HexNAc(2)Hex(1)_2)
1014.355
1081.475(PEP+HexNAc(2)Hex(2)_2)
1162.501(PEP+HexNAc(2)Hex(3)_2)
1176.409
1235.023
1243.528(PEP+HexNAc(2)Hex(4)_2)
1316.053
1324.555(PEP+HexNAc(2)Hex(5)_2)
1338.465
1397.584
1405.582(PEP+HexNAc(2)Hex(6)_2)
1413.649(PEP-H2O_1)
1431.671(PEP_1)
1478.107
1486.611(PEP+HexNAc(2)Hex(7)_2)
1500.519
1514.714
1539.704
1580.725
1598.736
1617.744
1634.756(PEP+HexNAc(1)_1)
1760.792
1796.809
1959.859
ETD-MS/MS Scan:10588, Noise threshold:1.0
150.117
1249.302
M+2H
1740.614
m/z

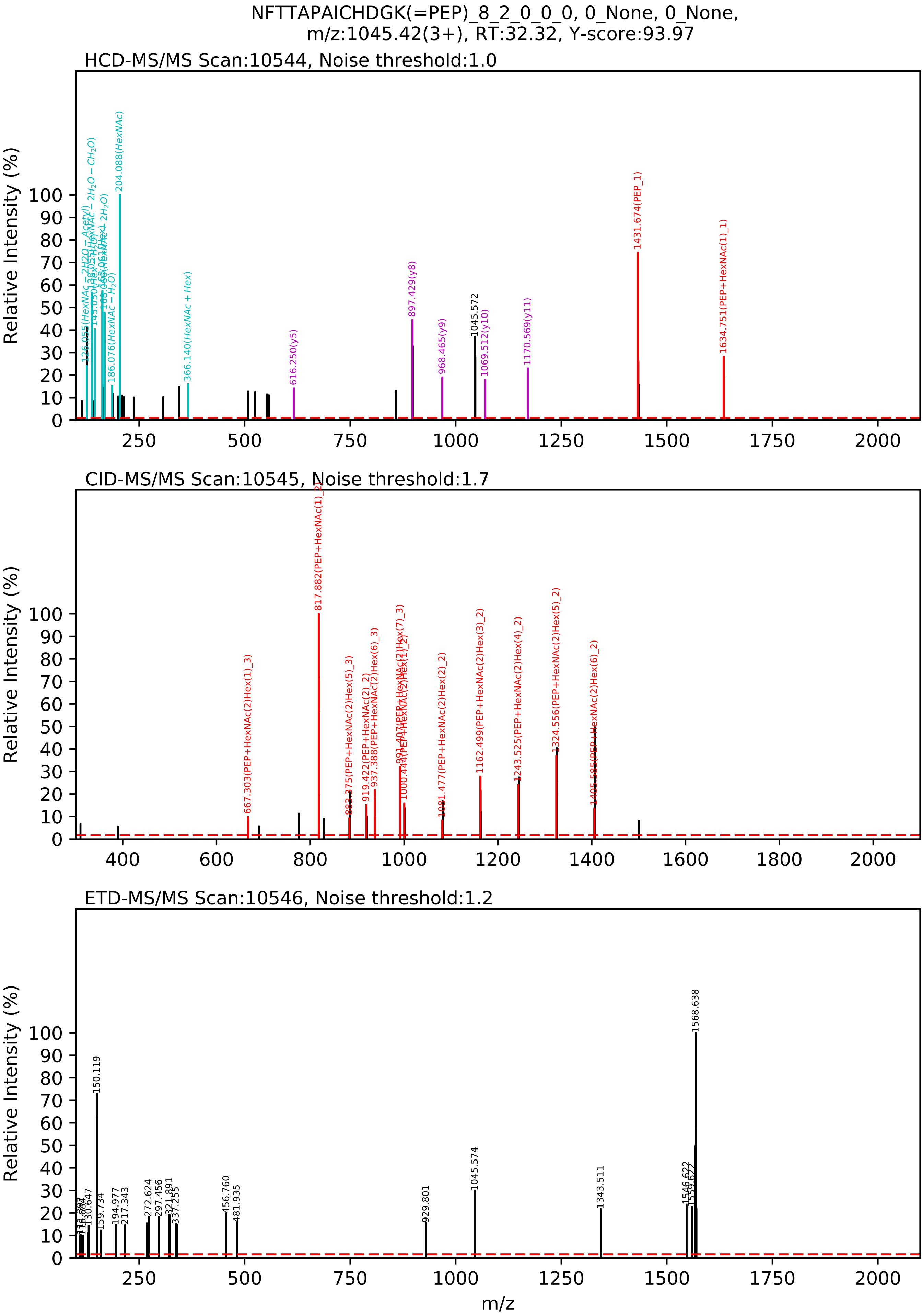
NFTTAPAICHDGK(=PEP)_8_2_0_0_0, 0_None, 0_None,
m/z:1045.42(3+), RT:32.32, Y-score:93.97
HCD-MS/MS Scan:10544, Noise threshold:1.0
204.088(HexNAc)
1431.674(PEP_1)
1634.751(PEP+HexNAc(1)_1)
897.429(y8)
968.465(y9)
1045.572
1069.512(y10)
1170.569(y11)
616.250(y5)
366.140(HexNAc + Hex)
186.076(HexNAc − H2O)
CID-MS/MS Scan:10545, Noise threshold:1.7
817.882(PEP+HexNAc(1)_2)
667.303(PEP+HexNAc(2)Hex(1)_3)
883.275(PEP+HexNAc(2)Hex(5)_3)
919.422(PEP+HexNAc(2)_2)
937.388(PEP+HexNAc(2)Hex(6)_3)
991.407(PEP+HexNAc(2)Hex(7)_3)
1081.477(PEP+HexNAc(2)Hex(2)_2)
1162.499(PEP+HexNAc(2)Hex(3)_2)
1243.525(PEP+HexNAc(2)Hex(4)_2)
1324.556(PEP+HexNAc(2)Hex(5)_2)
1405.585(PEP+HexNAc(2)Hex(6)_2)
ETD-MS/MS Scan:10546, Noise threshold:1.2
1568.638
150.119
1045.574
1343.511
1546.622
929.801
456.760
481.935
272.624
297.456
321.891
337.255
194.977
217.343
159.734
130.047
Relative Intensity (%)
m/z

NFTTAPAICHDGK(=PEP)_9_2_0_0_0, 0_None, 0_None,
m/z:1099.44(3+), RT:27.84, Y-score:82.05

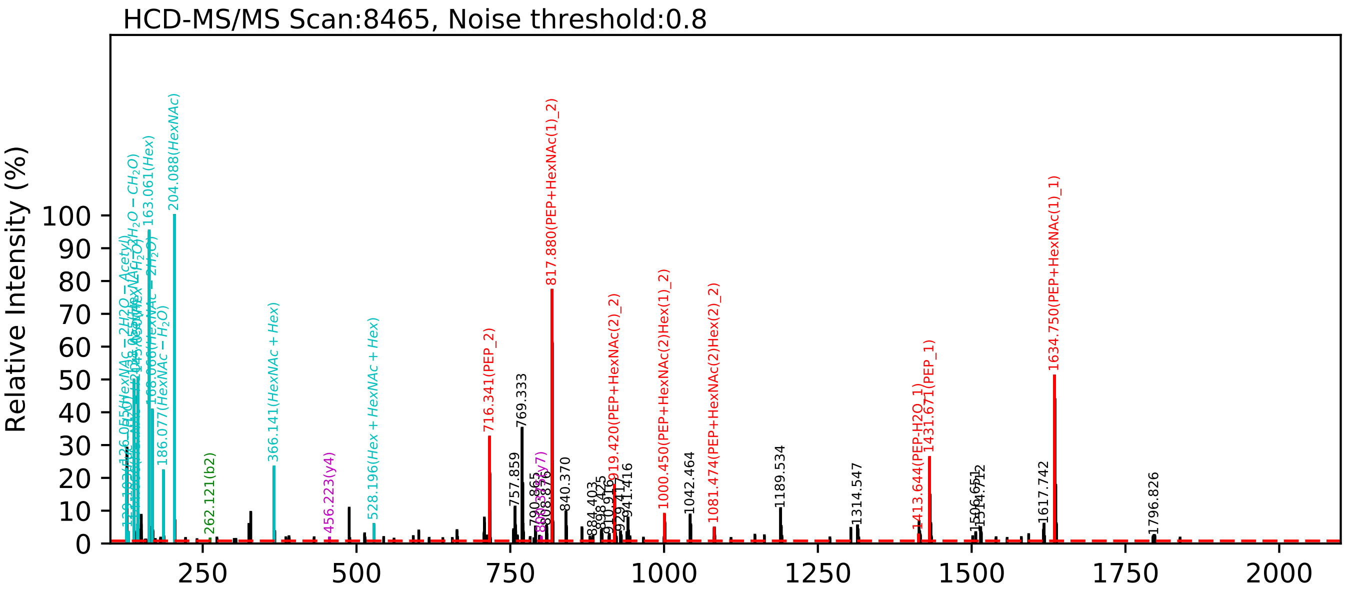

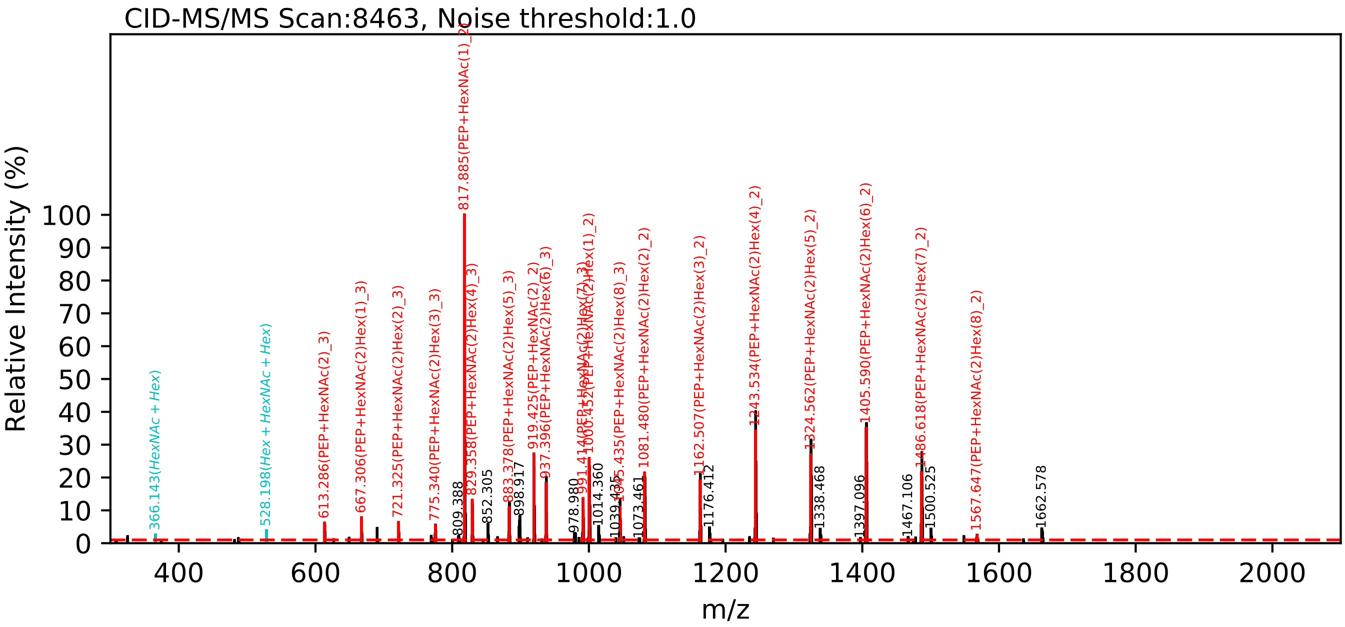

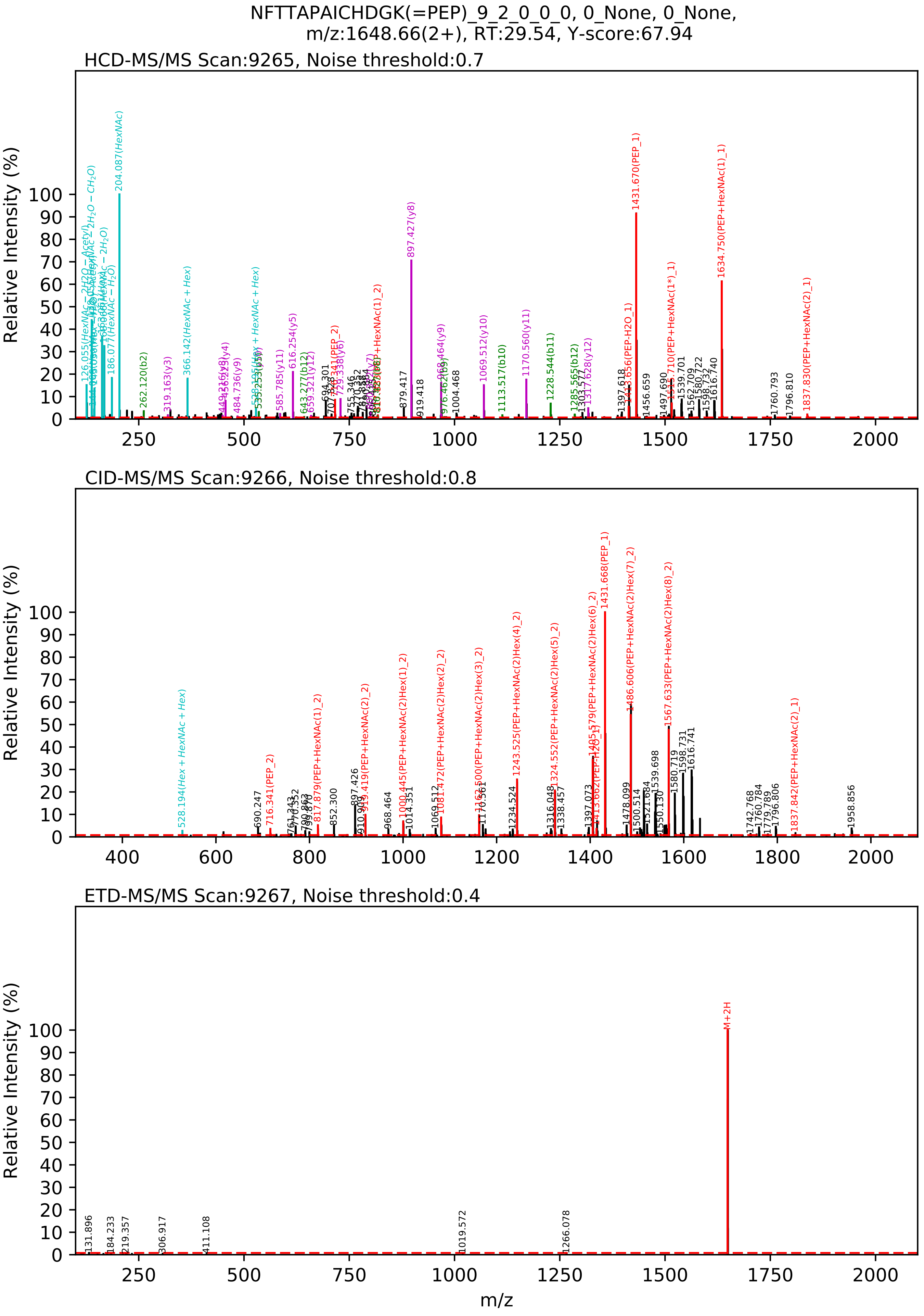

NFTTAPAICHDGK(=PEP)_9_2_0_0_0, 0_None, 0_None,
m/z:1648.66(2+), RT:29.54, Y-score:67.94
HCD-MS/MS Scan:9265, Noise threshold:0.7
Relative Intensity (%)
204.087(HexNAc)
366.142(HexNAc+Hex)
262.120(b2)
319.163(y3)
616.254(y5)
585.785(y11)
643.277(b12)
659.321(y12)
897.427(y8)
968.464(y9)
1004.468
1069.512(y10)
1113.517(b10)
1170.560(y11)
1228.544(b11)
1285.565(b12)
1317.628(y12)
1397.618
1431.670(PEP_1)
1456.659
1497.690
1539.701
1616.740
1634.750(PEP+HexNAc(1)_1)
1760.793
1796.810
1837.830(PEP+HexNAc(2)_1)
879.417
919.418
CID-MS/MS Scan:9266, Noise threshold:0.8
528.194(Hex+HexNAc+Hex)
690.247
716.341(PEP_2)
817.879(PEP+HexNAc(1)_2)
852.300
897.426
919.419(PEP+HexNAc(2)_2)
968.464
1000.445(PEP+HexNAc(2)Hex(1)_2)
1014.351
1069.512
1081.472(PEP+HexNAc(2)Hex(2)_2)
1170.561
1234.524
1243.525(PEP+HexNAc(2)Hex(4)_2)
1316.048
1324.552(PEP+HexNAc(2)Hex(5)_2)
1338.457
1397.073
1405.579(PEP+HexNAc(2)Hex(6)_2)
1431.668(PEP_1)
1478.099
1486.606(PEP+HexNAc(2)Hex(7)_2)
1500.514
1521.684
1539.698
1550.130
1567.633(PEP+HexNAc(2)Hex(8)_2)
1580.719
1598.731
1616.741
1742.768
1760.784
1779.789
1796.806
1837.842(PEP+HexNAc(2)_1)
1958.856
ETD-MS/MS Scan:9267, Noise threshold:0.4
M+2H
131.896
184.233
219.357
306.917
411.108
1019.572
1266.078
m/z

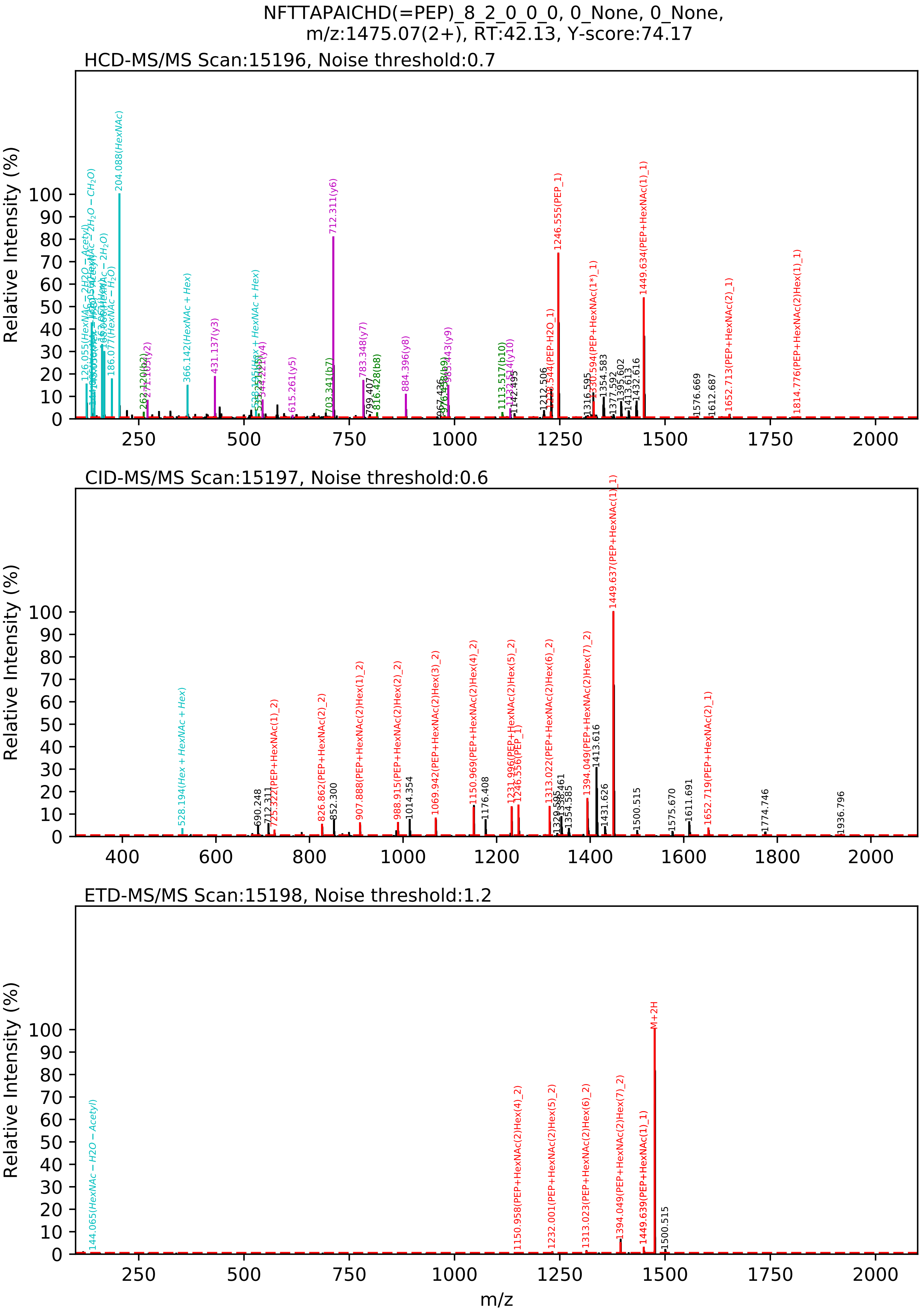
NFTTAPAICHD(=PEP)_8_2_0_0_0, 0_None, 0_None,
m/z:1475.07(2+), RT:42.13, Y-score:74.17
HCD-MS/MS Scan:15196, Noise threshold:0.7
Relative Intensity (%)
204.088(HexNAc)
186.077(HexNAc − H₂O)
126.055(HexNAc − 2H2O − Acetyl)
366.142(HexNAc + Hex)
431.137(y3)
712.311(y6)
783.348(y7)
816.428(b8)
884.396(y8)
985.443(y9)
1113.517(b10)
1142.495
1212.506
1246.555(PEP_1)
1228.544(PEP-H2O_1)
1330.594(PEP+HexNAc(1*)_1)
1316.595
1354.583
1377.592
1395.602
1413.613
1432.616
1449.634(PEP+HexNAc(1)_1)
1576.669
1612.687
1652.713(PEP+HexNAc(2)_1)
1814.776(PEP+HexNAc(2)Hex(1)_1)
CID-MS/MS Scan:15197, Noise threshold:0.6
Relative Intensity (%)
528.194(Hex + HexNAc + Hex)
690.248
712.311
725.322(PEP+HexNAc(1)_2)
826.862(PEP+HexNAc(2)_2)
852.300
907.888(PEP+HexNAc(2)Hex(1)_2)
988.915(PEP+HexNAc(2)Hex(2)_2)
1014.354
1069.942(PEP+HexNAc(2)Hex(3)_2)
1150.969(PEP+HexNAc(2)Hex(4)_2)
1176.408
1231.996(PEP+HexNAc(2)Hex(5)_2)
1246.556(PEP_1)
1313.022(PEP+HexNAc(2)Hex(6)_2)
1329.595
1338.461
1354.585
1394.049(PEP+HexNAc(2)Hex(7)_2)
1413.616
1431.626
1449.637(PEP+HexNAc(1)_1)
1500.515
1575.670
1611.691
1652.719(PEP+HexNAc(2)_1)
1774.746
1936.796
ETD-MS/MS Scan:15198, Noise threshold:1.2
Relative Intensity (%)
144.065(HexNAc − H2O − Acetyl)
1150.958(PEP+HexNAc(2)Hex(4)_2)
1232.001(PEP+HexNAc(2)Hex(5)_2)
1313.023(PEP+HexNAc(2)Hex(6)_2)
1394.049(PEP+HexNAc(2)Hex(7)_2)
1449.639(PEP+HexNAc(1)_1)
M+2H
1500.515
m/z

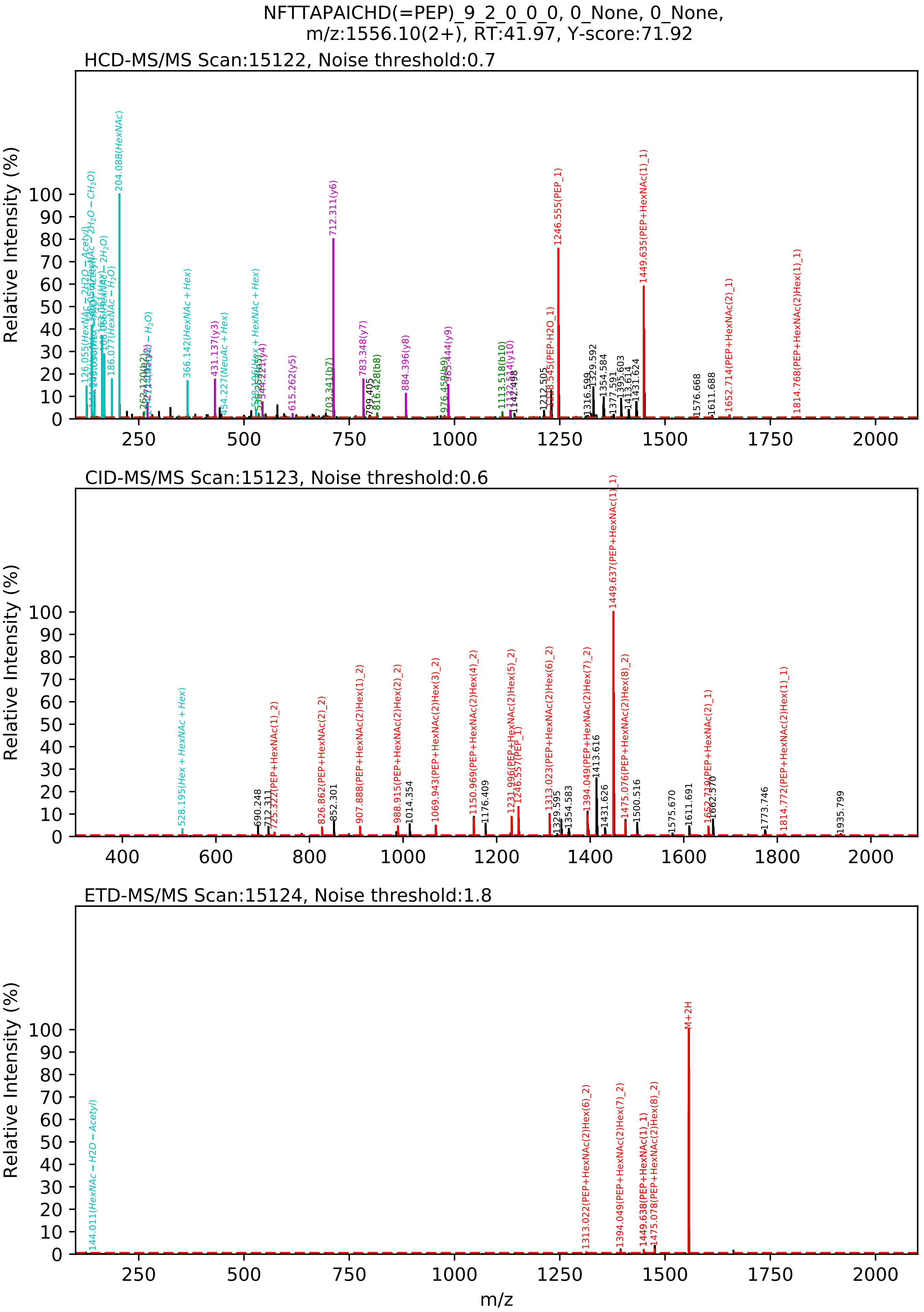

NFTTAPAICHD(=PEP)_9_2_0_0_0, 0_None, 0_None,
m/z:1556.10(2+), RT:41.97, Y-score:71.92
HCD-MS/MS Scan:15122, Noise threshold:0.7
CID-MS/MS Scan:15123, Noise threshold:0.6
ETD-MS/MS Scan:15124, Noise threshold:1.8
Relative Intensity (%)
m/z
204.088(HexNAc)
712.311(y6)
1246.555(PEP_1)
1449.635(PEP+HexNAc(1)_1)
1652.714(PEP+HexNAc(2)_1)
1814.768(PEP+HexNAc(2)Hex(1)_1)
366.142(HexNAc+Hex)
431.137(y3)
783.348(y7)
884.396(y8)
985.444(y9)
1449.637(PEP+HexNAc(1)_1)
1394.049(PEP+HexNAc(2)Hex(7)_2)
1313.023(PEP+HexNAc(2)Hex(6)_2)
1475.076(PEP+HexNAc(2)Hex(8)_2)
M+2H
144.011(HexNAc−H2O−Acetyl)

NHTSPDVD(=PEP)_7_2_0_0_0, 0_None, 0_None,
m/z:1212.95(2+), RT:13.83, Y-score:91.97

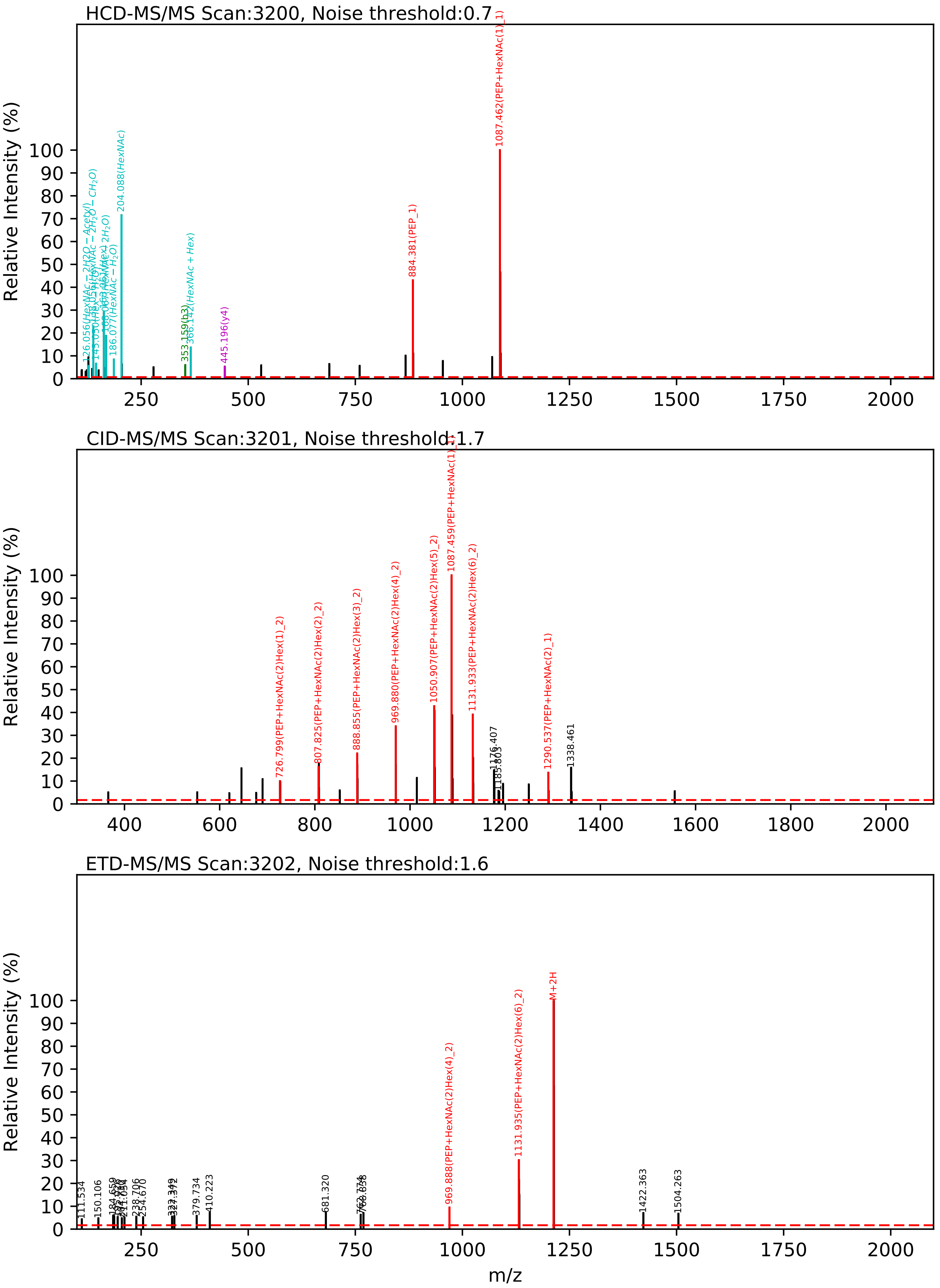

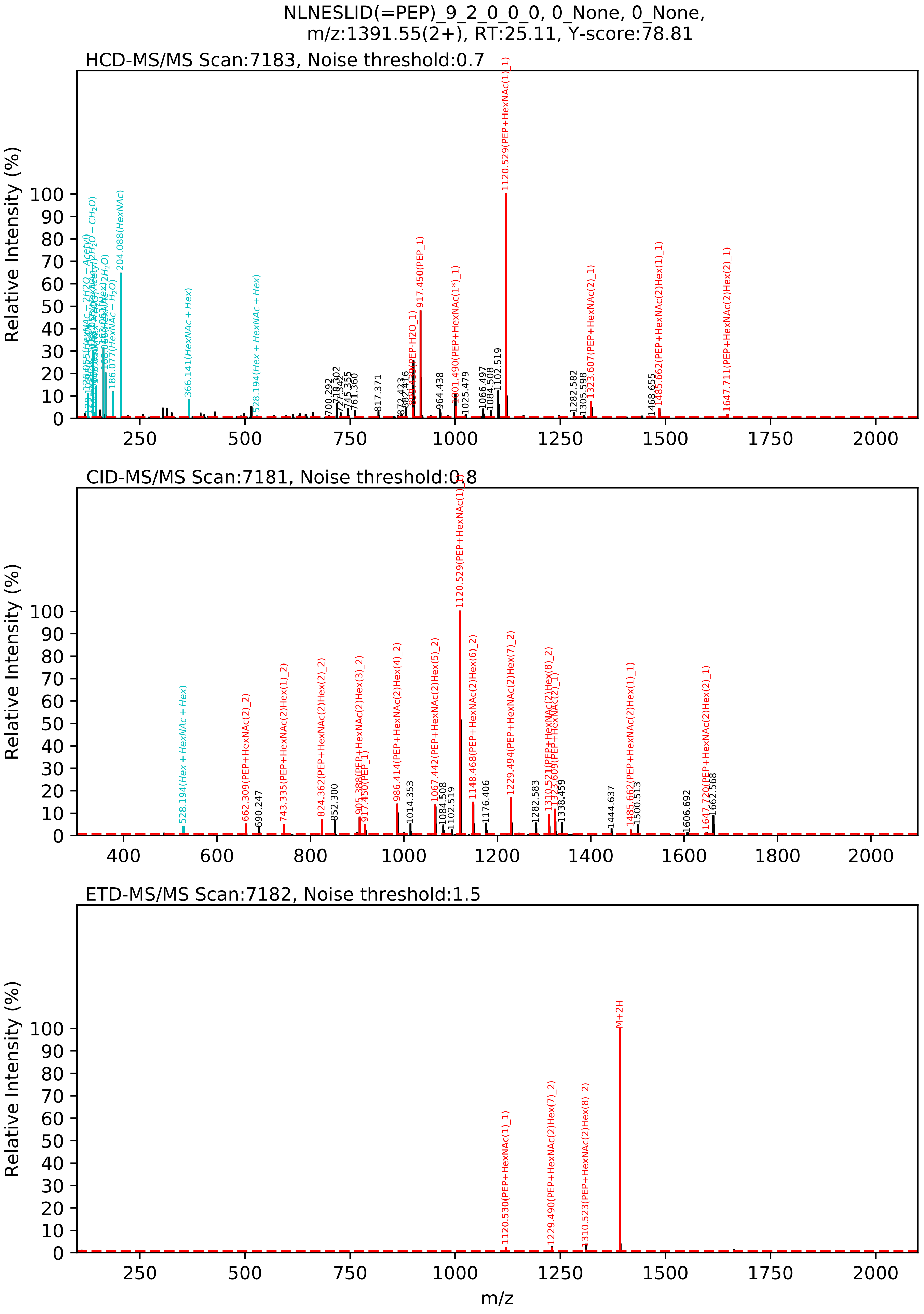
NLNESLID(=PEP)_9_2_0_0_0, 0_None, 0_None,
m/z:1391.55(2+), RT:25.11, Y-score:78.81
HCD-MS/MS Scan:7183, Noise threshold:0.7
Relative Intensity (%)
366.141(HexNAc + Hex)
528.194(Hex + HexNAc + Hex)
204.088(HexNAc)
186.077(HexNAc − H2O)
700.292
718.302
745.355
761.360
817.371
964.438
1025.479
1066.497
1084.508
1102.519
1120.529(PEP+HexNAc(1)_1)
917.450(PEP_1)
1001.490(PEP+HexNAc(1*)_1)
1282.582
1305.598
1323.607(PEP+HexNAc(2)_1)
1468.655
1485.662(PEP+HexNAc(2)Hex(1)_1)
1647.711(PEP+HexNAc(2)Hex(2)_1)
CID-MS/MS Scan:7181, Noise threshold:0.8
528.194(Hex + HexNAc + Hex)
662.309(PEP+HexNAc(2)_2)
690.247
743.335(PEP+HexNAc(2)Hex(1)_2)
824.362(PEP+HexNAc(2)Hex(2)_2)
852.300
905.388(PEP+HexNAc(2)Hex(3)_2)
917.450(PEP_1)
986.414(PEP+HexNAc(2)Hex(4)_2)
1014.353
1067.442(PEP+HexNAc(2)Hex(5)_2)
1084.508
1102.519
1120.529(PEP+HexNAc(1)_1)
1148.468(PEP+HexNAc(2)Hex(6)_2)
1176.406
1229.494(PEP+HexNAc(2)Hex(7)_2)
1282.583
1310.521(PEP+HexNAc(2)Hex(8)_2)
1323.609(PEP+HexNAc(2)_1)
1338.459
1444.637
1485.662(PEP+HexNAc(2)Hex(1)_1)
1500.513
1606.692
1647.720(PEP+HexNAc(2)Hex(2)_1)
1662.568
ETD-MS/MS Scan:7182, Noise threshold:1.5
1120.530(PEP+HexNAc(1)_1)
1229.490(PEP+HexNAc(2)Hex(7)_2)
1310.523(PEP+HexNAc(2)Hex(8)_2)
M+2H
m/z
